# Supplementary material for: Comparative Fitting of Mathematical Models to Carvedilol Release Profiles Obtained from Hypromellose Matrix Tablets
Source: Pharmaceutics. 2024 Apr 4;16(4):498. doi: 10.3390/pharmaceutics16040498 (PMC11053526; doi:10.3390/pharmaceutics16040498)

Supplementary materials – Criteria for visual assessment of model fit

1) Progression of drug release

A mathematical model, which predicts a maximum of drug released before the last experimentally tested time point in the data set and afterwards demonstrates a significantly lower fraction of drug released in further successive time points, is inferior to a model with a similar RSS result, which predicts a progressively higher fraction of drug released throughout the drug release profile from t = 0 to t = max in the studied dissolution data range.

Example: the model fit to the left is considered inferior to the model fit to the right due to the above–stated rule (the example is taken from the model fitting to the entire relevant carvedilol release profile for tested tablet No. 2 of the Polyglykol® 4000 P formulation; the Quadratic model fit is shown on the left chart and the Weibull_2 model fit is shown on the right chart)

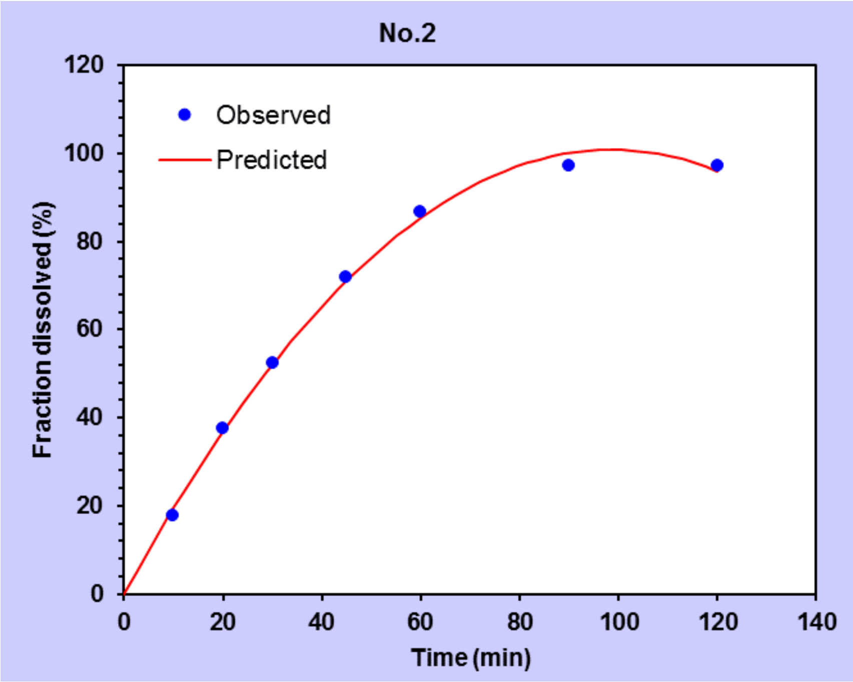

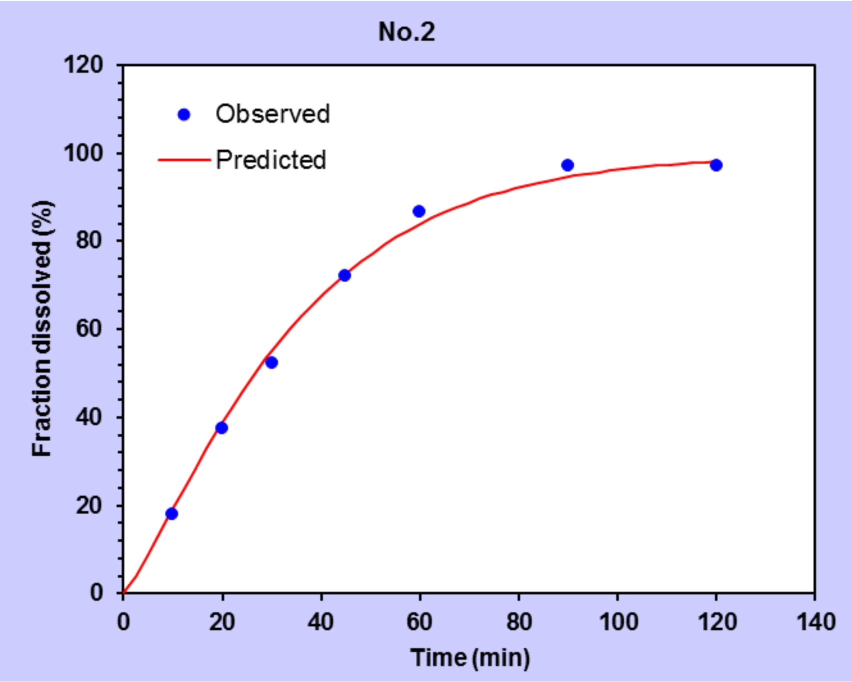

2) The ability of the mathematical model to reproduce a sigmoid shape of a drug release profile from the experimental dissolution data

If the experimental dissolution data clearly demonstrates a sigmoid shape of a dissolution profile, a mathematical model, which is capable of reproducing/following this sigmoid shape, is superior to a model with a similar RSS result, which is not able to demonstrate a sigmoid shape.

Example: the model fit to the left is considered superior to the model fit to the right due to the above–stated rule (the example is taken from the model fitting to the entire relevant carvedilol release profile for tested tablet No. 1 of the POLYOX™ WSR N-80 LEO NF formulation; the Weibull_2 model fit is shown on the left chart and the Hopfenberg with T_{lag} model fit is shown on the right chart)

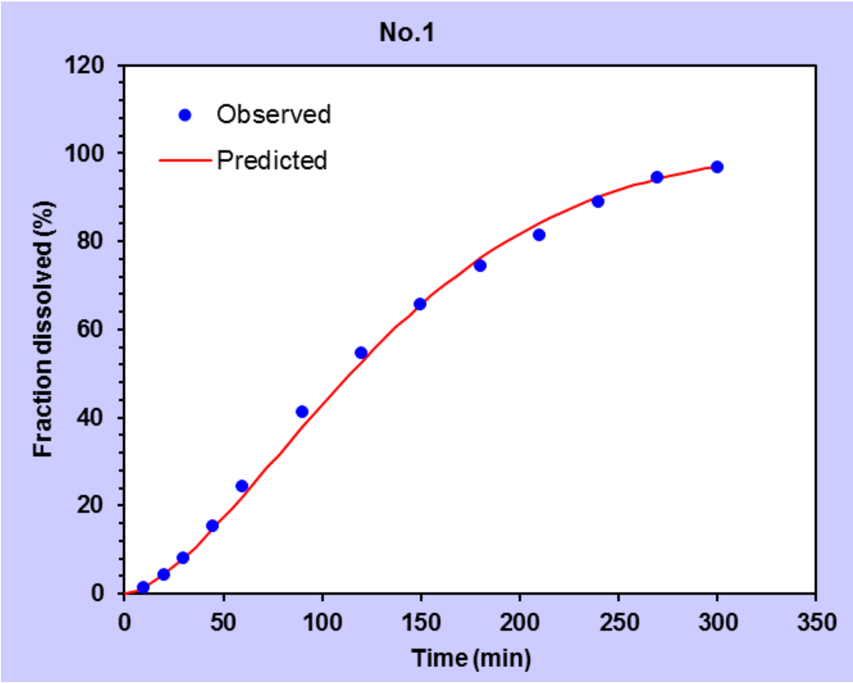

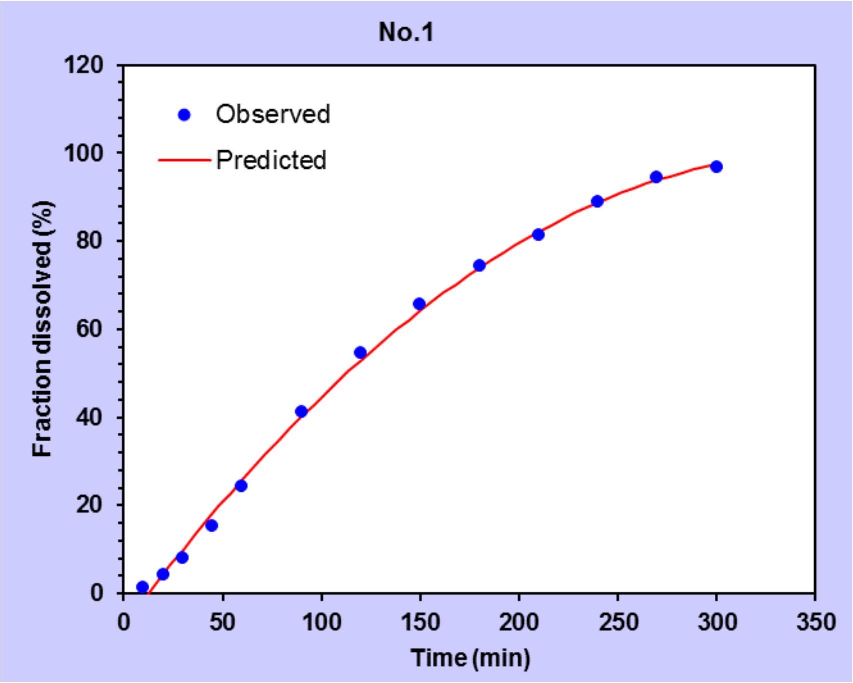

3) Matching indications of burst release or lag time (mathematical model vs. experimental dissolution data)

If the experimental dissolution data indicates a possible burst release or lag time, this should be matched by the fitted mathematical model's indication of the same two phenomena. The model, which fails to do so is considered inferior to the model, which matches the experimental dissolution data up to t = 60 min more accurately, where both phenomena can be observed.

See below for practical examples…

3.1) If two mathematical models demonstrate a similar overall fit to experimental dissolution data and one of the models indicates a positive F_0 or negative T_{lag}, in both cases an indication of burst release, which is not clearly visible in experimental dissolution data, the model which does not indicate burst release or better fits the initial part of the dissolution profile up to t = 60 min, is considered superior.

Example: model 'A' shows a similar overall fit to dissolution data as model 'B', but model 'A' also indicates burst release, which is not clearly visible in experimental dissolution data, and model 'B' does not, which makes model 'B' superior to model 'A' (the example is taken from model fitting to the entire relevant carvedilol release profile for tested tablet No. 1 of the AVICEL PH 102 formulation; the Hopfenberg with T_{lag} model fit is shown as the model 'A' and the Makoid–Banakar model fit is shown as the model 'B')

a) Model 'A' (the original figure is shown on the left and a zoomed–in image is shown on the right to demonstrate the deviation of the model from the experimental data points in the initial stage of carvedilol release)

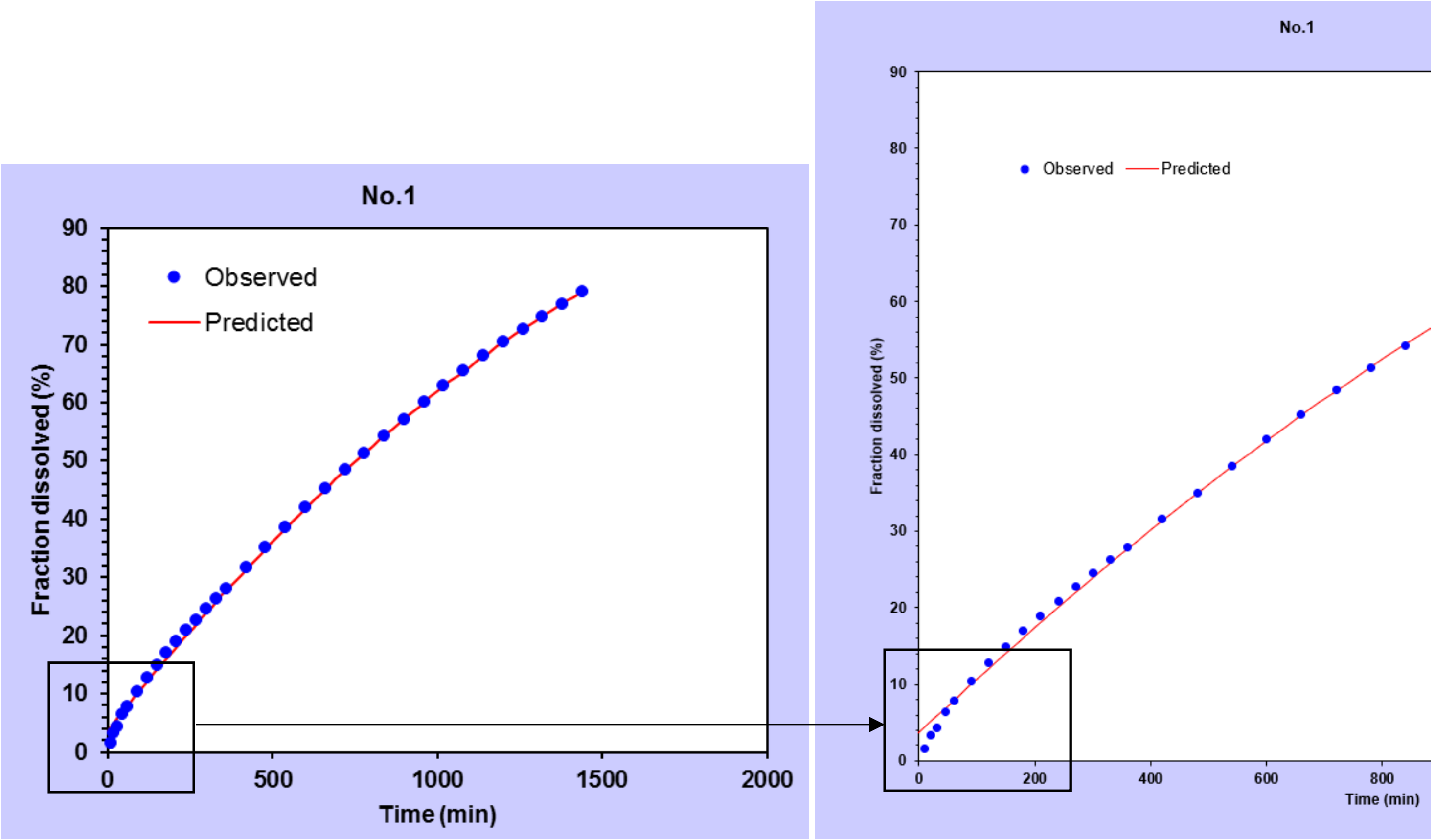

b) Model 'B' (the original figure is shown on the left and a zoomed–in image is shown on the right to demonstrate the model fit matching the experimental data points in the initial stage of carvedilol release)

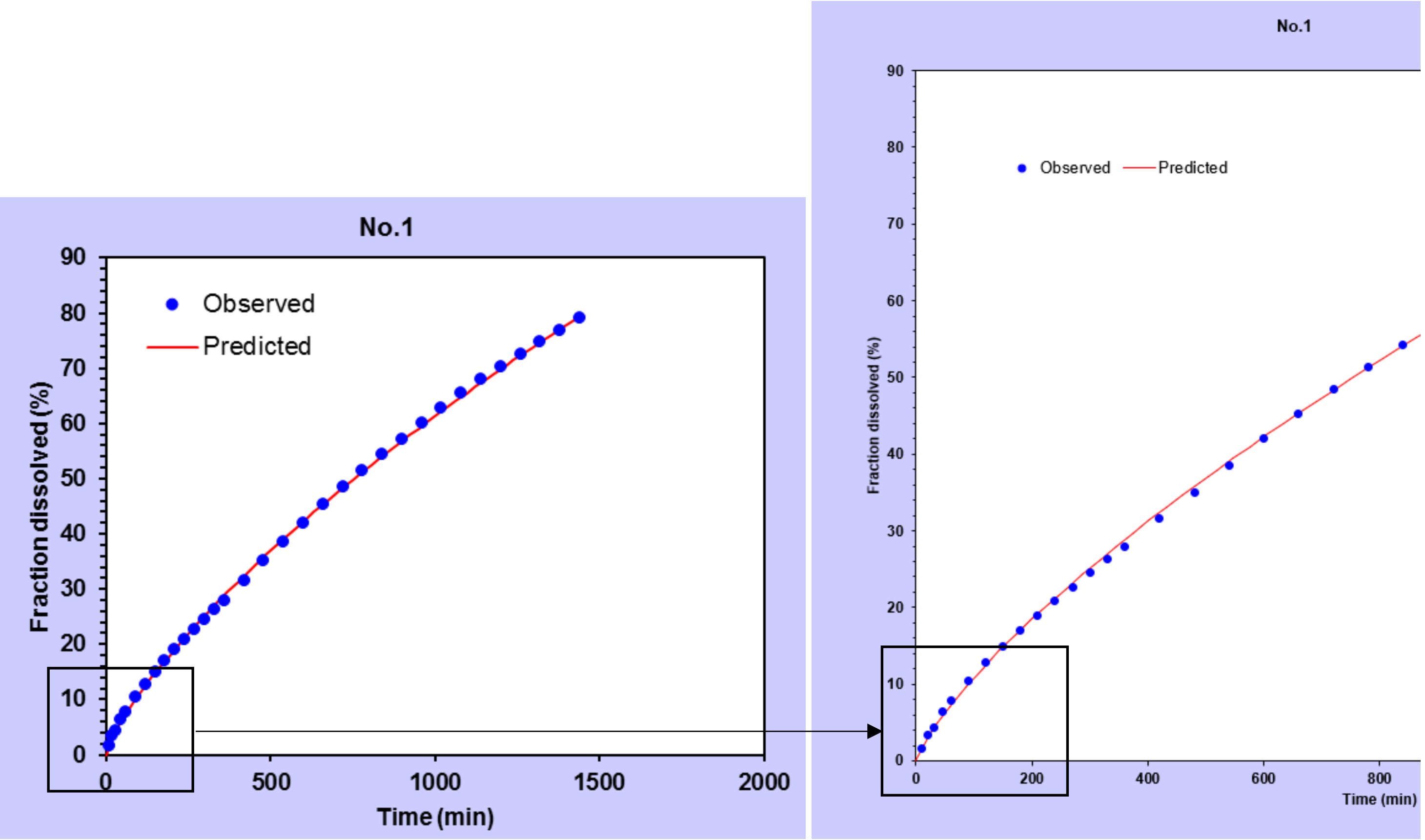

3.2) If two mathematical models demonstrate a similar overall fit to experimental dissolution data and one of the models indicates a negative F_0 or positive T_{lag}, in both cases an indication of lag time, which is not clearly visible in experimental dissolution data, the model which does not indicate lag time or better fits the initial part of the dissolution profile up to t = 60 min, is considered superior.

Example: the model fit to the left is considered superior to the model fit to the right due to the above–stated rule (the example is taken from the model fitting to the entire relevant carvedilol release profile for tested tablet No. 2 of the Polyglykol® 4000 P formulation; the Weibull_2 model fit is shown on the left chart and the Weibull_4 model fit is shown on the right chart)

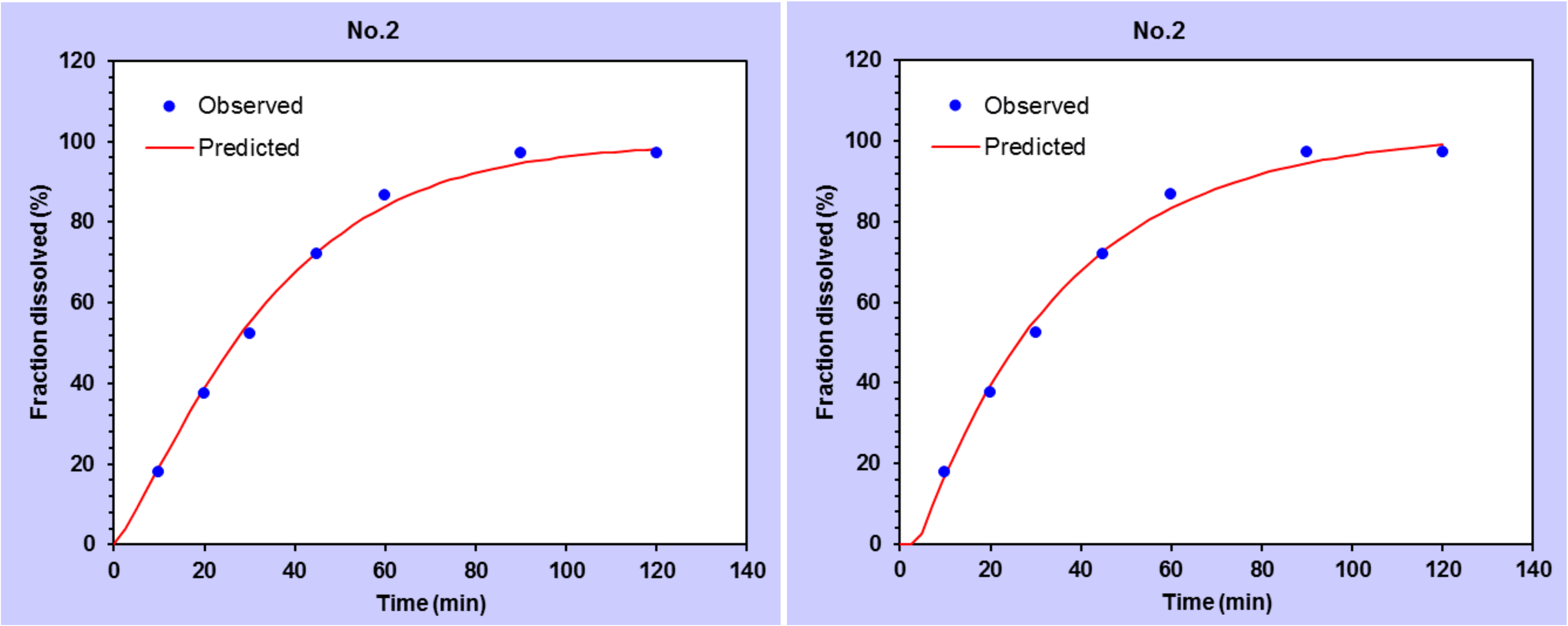

3.3) If two mathematical models demonstrate a similar overall fit to experimental dissolution data and one of the models indicates a positive F_0 or negative T_{lag}, in both cases an indication of burst release, which is clearly visible in experimental dissolution data, and the other model does not, the model which indicates burst release or better fits the initial part of the dissolution profile up to t = 60 min, is considered superior.

Example: the model fit to the left is considered inferior to the model fit to the right due to the above–stated rule (the example is taken from model fitting to the entire relevant carvedilol release profile for tested tablet No. 1 of the Tablettose® 70 formulation; the Makoid–Banakar model fit is shown on the left chart and the Higuchi with F_0 model fit is shown on the right chart)

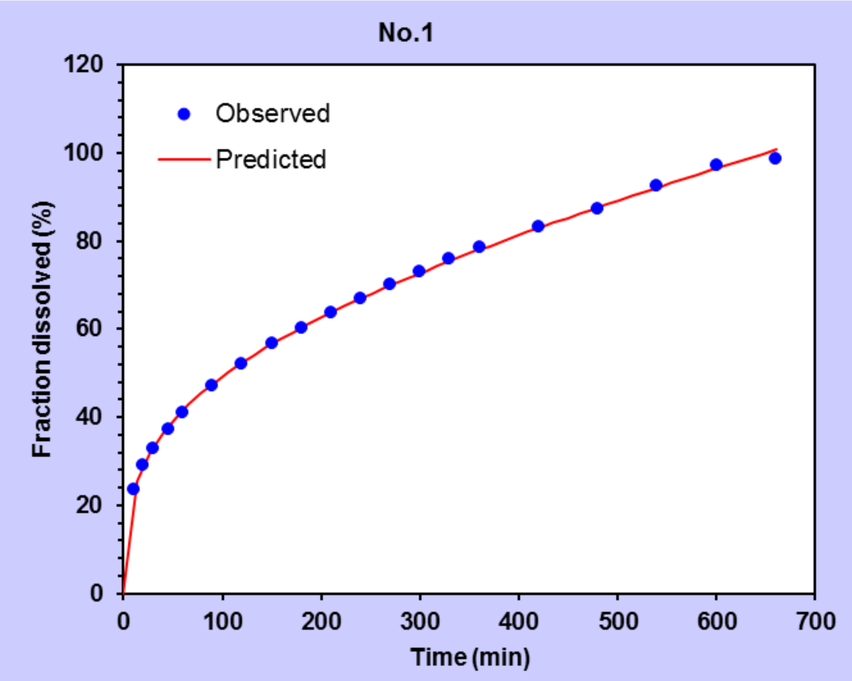

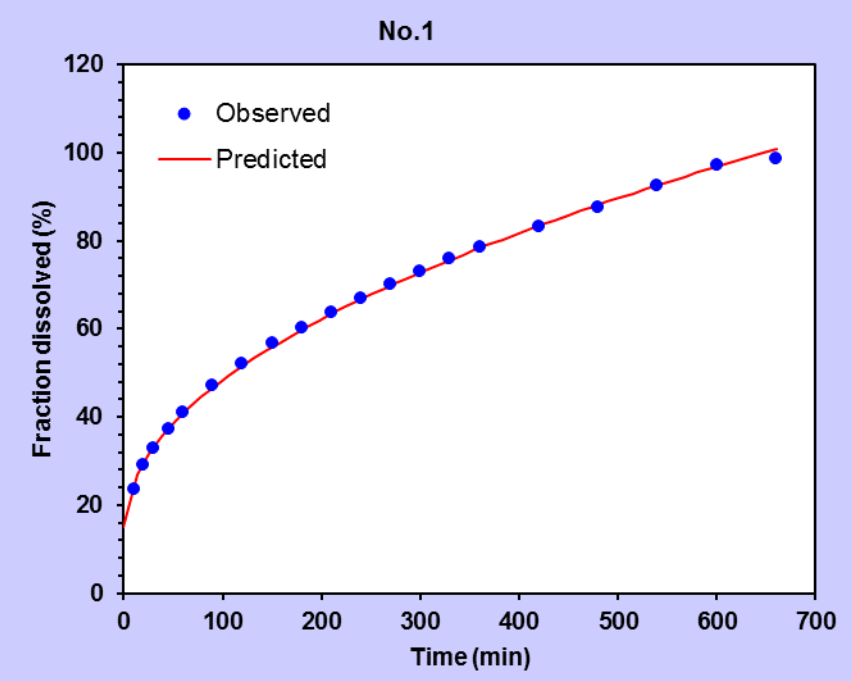

3.4) If two mathematical models demonstrate a similar overall fit to experimental dissolution data and one of the models indicates a negative F_0 or positive T_{lag}, in both cases an indication of lag time, which is clearly visible in experimental dissolution data, and the other model does not, the model which indicates lag time or better fits the initial part of the dissolution profile up to t = 60 min, is considered superior

Example: model 'A' shows a similar overall fit to dissolution data as model 'B', but model 'B' also indicates lag time, which is clearly visible in experimental dissolution data, and model 'A' does not, which makes model 'B' superior to model 'A' (the example is taken from model fitting to the entire relevant carvedilol release profile for tested tablet No. 3 of the Parteck® M 100 formulation; the Hopfenberg model fit is shown as the model 'A' and the Hopfenberg with T_{lag} model fit is shown as the model 'B')

a) Model 'A' (the original figure is shown on the left and a zoomed–in image is shown on the right)

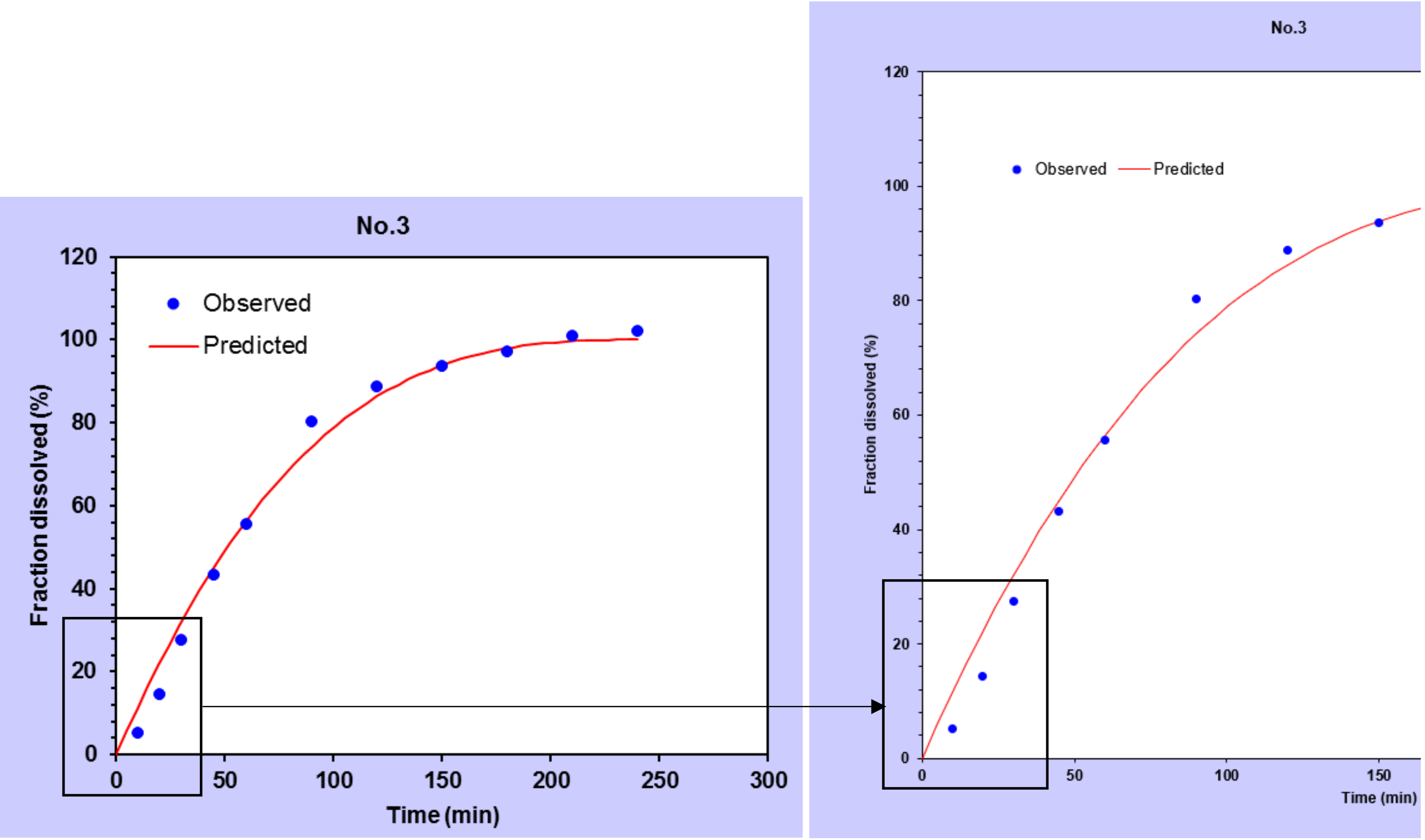

b) Model 'B' (the original figure is shown on the left and a zoomed–in image is shown on the right)

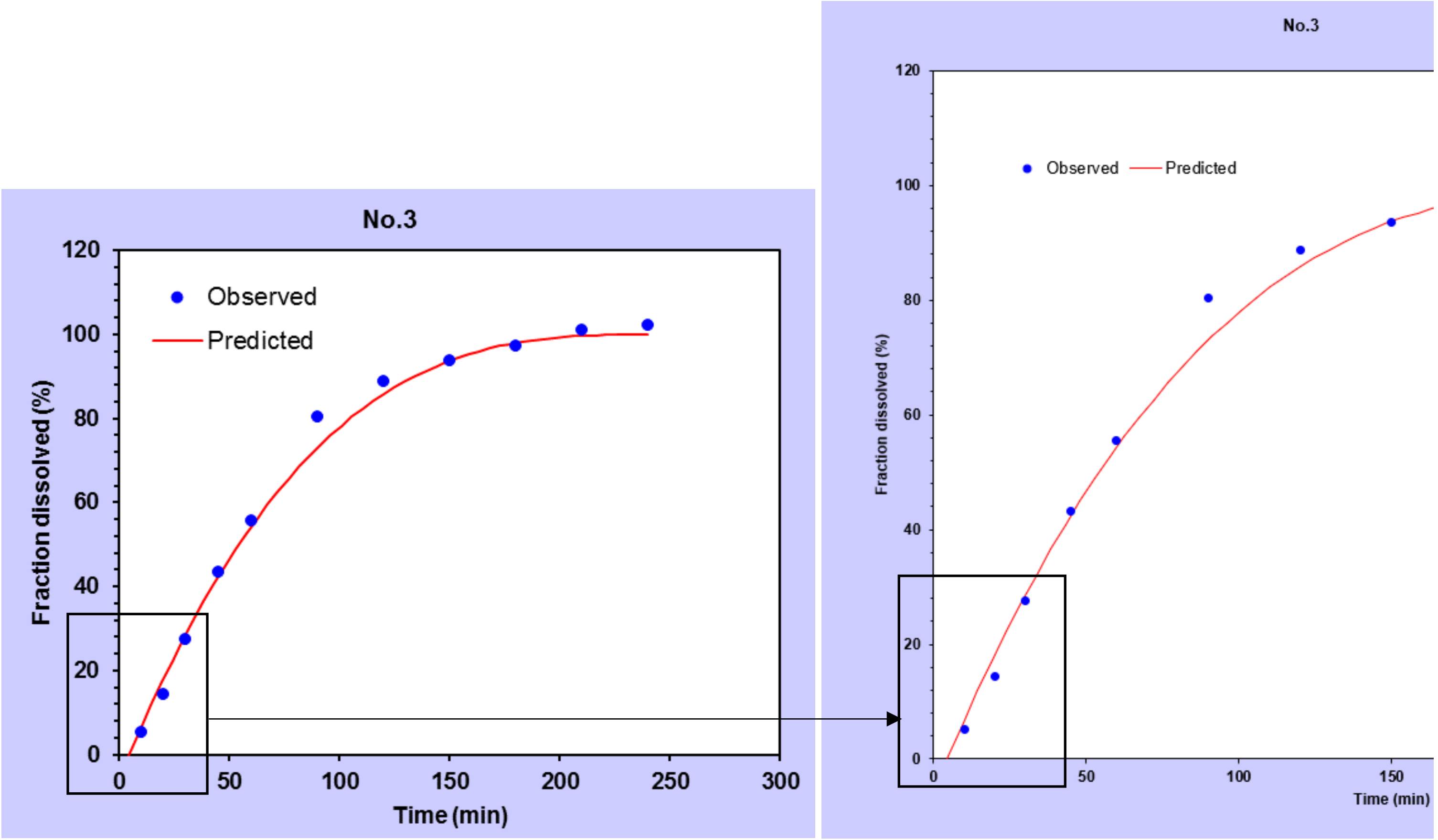

4. Uniformity of model fit

If two mathematical models demonstrate a similar overall fit to experimental dissolution data i.e. RSS result and the first one demonstrates a more uniform fit throughout the entire drug release profile than the second one (for example the second model demonstrates a similar fit to the first one throughout the majority of the drug release profile except at the beginning etc.), the model, which demonstrates a more uniform fit throughout the entire drug release profile is considered superior.

Example: The fit of the model on the left is more uniform throughout the entire drug release profile than the model fit to the right, therefore exhibiting a more uniform distribution of residuals; the model fit to the right underestimates the drug release at the beginning of the drug release profile whereas the model fit to the left does not (the example is taken from model fitting to the entire relevant carvedilol release profile for tested tablet No. 2 of the SuperTab® 11SD formulation; the Higuchi with F_0 model fit is shown on the left chart and the Korsmeyer–Peppas with F0 model fit is shown on the right chart)

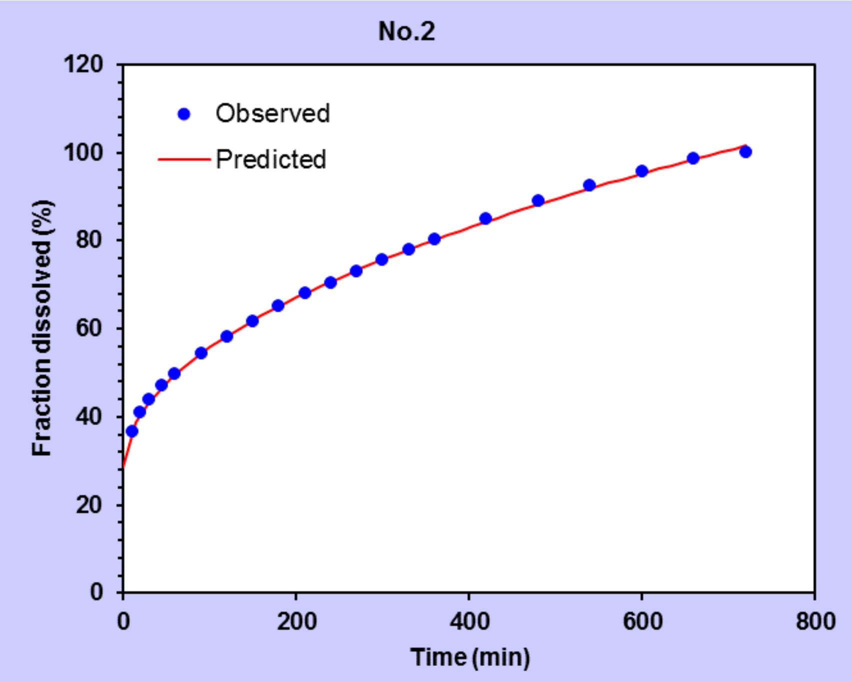

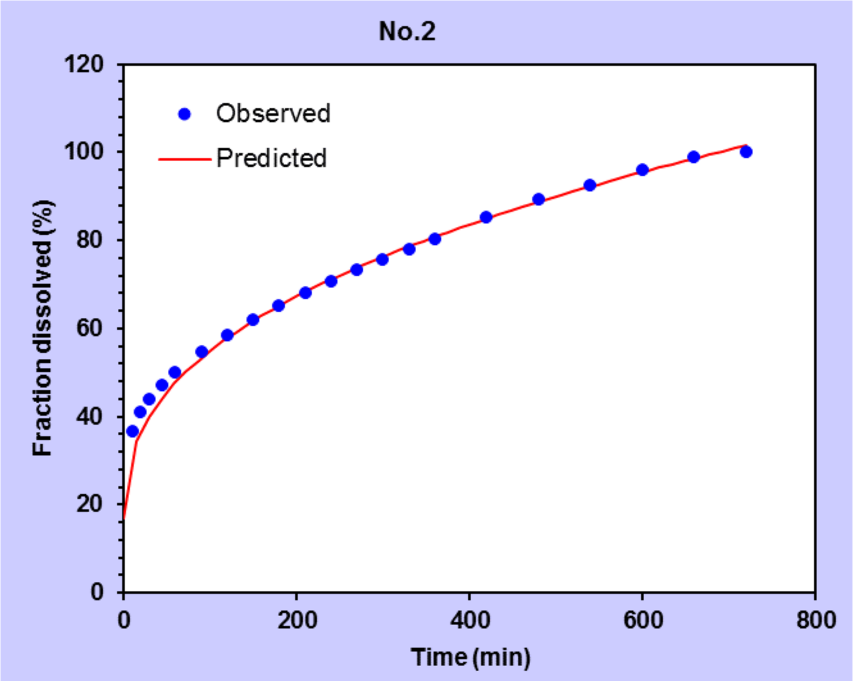

Supplement: Supplementary file 1 [file pharmaceutics-16-00498-s001.zip › Supplementary materials_Criteria for visual assessment of model fit.pdf]
