# Supplementary material for: Comparative Fitting of Mathematical Models to Carvedilol Release Profiles Obtained from Hypromellose Matrix Tablets
Source: Pharmaceutics. 2024 Apr 4;16(4):498. doi: 10.3390/pharmaceutics16040498 (PMC11053526; doi:10.3390/pharmaceutics16040498)
Supplement: Supplementary file 1 [file pharmaceutics-16-00498-s001.zip › Supplementary materials_Introduction to model fitting summaries.pdf]

Introduction to the Model fitting summaries

The Model fitting summaries, which are a part of the supplements/attachments of the article, are all structured in the same manner. The following information is important for guiding oneself through these Model fitting summaries:

1) Formulation's name in the file name of each Model fitting summary

The formulations are named after the filler used in each formulation as this is the only difference in composition among the formulations (also see section '2.1. Materials' of the article):

- Polyglykol® 4000 P
- Polyglykol® 8000 P
- Polyox™ WSR N-80
- Kollidon® 25
- Kollidon® 90 F
- C*Pharm Mannidex 16700
- Pearlitol® 160C
- Parteck® M 100
- Parteck® M 200
- Lactochem® Crystals
- Lactochem® Fine Powder
- SuperTab® 11SD
- FlowLac® 100
- Tablettose® 70
- Granulated sugar N°1 600
- Glucidex® 19
- Di-Cafos® A12
- Emcompress® Anhydrous
- Avicel® PH-102
- Avicel® PH-200
- Ethocel™ Standard 20 Premium
- Starch 1500® sample with smaller particle size
- Starch 1500® sample with larger particle size

The formulation name is contained in the file name of each Model fitting summary to guide an interested reader, which file to open if he or she wants to inspect the Model fitting summary of a chosen formulation.

2) Information in the heading of each Model fitting summary

Once a file of a chosen Model fitting summary is opened, there are two important pieces of information available in the heading of each document:

1. **Name of the formulation**

   The formulation name to which the opened file of a chosen Model fitting summary belongs to is visible in the heading of the document on all pages of the document, just as in the name of the file.

2. **Section of the experimental dissolution data the model was applied to**

   The heading of each page of the chosen Model fitting summary contains information about the section of the experimental dissolution data used in fitting a model. This information is included in the heading of the document so the reader can know at any point within the document to what section of the experimental dissolution data any chosen model was applied.

   The first section of each Model fitting summary contains a summary of modelling the entire relevant dissolution data as determined by the paired t–test (also see section '2.2.3. 2.2.3. Determination of the approximate end–point of carvedilol release using a paired t–test' and section '2.2.4. Fitting of mathematical models to carvedilol release data using DDSolver and overall comparison of model fit' in the article).

   The second section of each Model fitting summary contains a summary of modelling experimental dissolution data up to app. 60% of carvedilol released.

   The third section is only available for the Polyglykol® 4000 P, the Polyglykol® 8000 P, and the Parteck® M 100 formulations. These three formulations released carvedilol so fast, that an app. average of 60 % of carvedilol released was achieved in just 45 minutes, yielding only four dissolution data points to be considered for modelling. Four dissolution data points were not enough to fit all the available models in DDSolver to experimental dissolution data. For this reason, **an additional dissolution data point at t = 60 min was included in the mentioned formulations, yielding the necessary minimum of five dissolution data points to fit all the models**. **This resulted in utilizing dissolution data up to app. 75 % of carvedilol released in the case of the Polyglykol® 4000 P and the Polyglykol® 8000 P formulations, and up to app. 70 % of carvedilol released in the case of the Parteck® M 100 formulation.**

3) The order of mathematical models applied to dissolution data of each formulation in each Model fitting summary

The models fitted to all formulations are presented in the same order in each Model fitting summary, and independently for each section of the experimental dissolution data the models were applied to (modelling the entire relevant dissolution data as determined by the paired t–test, modelling experimental dissolution data up to app. 60% of carvedilol released). When an interested reader finds himself or herself on a chosen page within a chosen Model fitting summary, belonging to a chosen model applied to a chosen section of the experimental dissolution profile, he or she can always determine if he or she needs to scroll forward or backwards within the document to find the information about fitting a certain chosen model to the chosen section of the experimental dissolution data by considering the order in which the models are summarized in the document.

The order in which the models are summarized for each section of the experimental dissolution data is the following (also see Table 1 in the article)**:**

the Zero–order model
the Zero–order with T_{lag} model
the Zero–order with F_0 model
the First–order model
the First–order with T_{lag} model
the First–order with F_{max} model
the First–order with T_{lag} and F_{max} model
the Higuchi model
the Higuchi with T_{lag} model
the Higuchi with F_0 model
the Korsmeyer–Peppas model
the Korsmeyer–Peppas with T_{lag} model
the Korsmeyer–Peppas with F_0 model
the Hixson–Crowell model
the Hixson–Crowell with T_{lag} model
the Hopfenberg model
the Hopfenberg with T_{lag} model
the Baker–Lonsdale model
the Baker–Lonsdale with T_{lag} model
the Makoid–Banakar model
the Makoid–Banakar with T_{lag} model
the Peppas–Sahlin_1 model
the Peppas–Sahlin_1 with T_{lag} model
the Peppas–Sahlin_2 model
the Peppas–Sahlin_2 with T_{lag} model
the Quadratic model
the Quadratic with T_{lag} model
the Weibull_1 model
the Weibull_2 model
the Weibull_3 model
the Weibull_4 model
the Logistic_1 model
the Logistic_2 model
the Logistic_3 model
the Gompertz_1 model
the Gompertz_2 model
the Gompertz_3 model
the Gompertz_4 model
the Probit_1 model
the Probit_2 model

4) The structure of provided information for each applied mathematical model in each Model fitting summary

A model fitting summary for each fitted model and each independent section of experimental dissolution data consists of two pages.

On the first page, the following information is provided:

1. The name of the model (for example 'the Zero–order model')

2. The model equation (also see Table 1 in the article for the explanation of model parameters)

3. A table summarizing the fitted model parameters

   The model parameters were fitted to experimental dissolution data for four independently analysed tablets per formulation; symbols No.1, No.2, No.3, and No.4 are used for tablet 1, tablet 2, tablet 3, and tablet 4, respectively. In addition, the mean, the standard deviation (SD), and the relative standard deviation % (RSD%) are presented. All the presented model parameter values and their statistics are rounded and presented for illustration purposes only. More precise estimations are available at request from the authors or can be obtained using DDSolver (freely available MS Excel plugin; see the supplements/attachment of the article by Zhang, et al., 2010) and the experimental dissolution data provided in Tables S1–S8.

4. A table summarizing the number of dissolution data points used for model fitting (N), the degrees of freedom (df), and selected goodness of fit criteria – Pearson correlation coefficient (R), coefficient of determination (R^2), adjusted coefficient of determination ($R^2_{adjusted}$), and residual sum of squares (RSS)

5. Graphical abstract of model fit presented as mean ± 1 standard deviation (SD) of the fraction % of released carvedilol:

   A chart of the experimentally determined fraction of carvedilol released as a function of time represented as mean carvedilol release (blue dots) ± 1 SD (blue error bars), and the model fit (red line) obtained with mean values of model parameters. The chart is titled 'Mean'. Note to the reader – a good average model fit does not mean the model fit was of the same or similar quality for each of the four independently tested tablets per formulation (always check the quality of model fit for each of the four independently tested tablets per formulation).

On the second page, the following information is provided:

1. Graphical abstract of model fit presented as the fraction % of released carvedilol per tested tablet

   Four charts are presented titled 'No.1', 'No.2', 'No.3', and 'No.4'. They represent experimental dissolution data (blue dots) i.e. the fraction of carvedilol released at tested time points, and the model fit (red line) for each of the four independently tested tablets per formulation. By inspecting these four charts, an interested reader can get insight into how well is each individual model able to adapt and model the different experimentally obtained carvedilol dissolution profiles within the same formulation i.e. how well is each individual model able to model intertablet carvedilol release variability.

Example of a Model fitting summary with marked information elements described above: see the next two pages

Suppleme[illegible]l fitting summary – FlowLac® 100 (modelling the entire relevant dissolution data)

The name of the model

Model: **Korsmeyer–Peppas with F_0**

Name of the formulation

Section of the experimental dissolution data the model was applied to

Model equation: $F = F_0 + k_{KP} \cdot t^n$

The model equation

Fitted model parameters per tested tablet (N = 4) with statistics – mean, standard deviation (SD), and relative standard deviation expressed in % (RSD%) (output from DDSolver):

Parameter	No.1	No.2	No.3	No.4	Mean	SD	RSD(%)
k_{KP}	0.896	1.038	1.532	0.922	1.097	0.296	27.023
n	0.690	0.665	0.612	0.690	0.664	0.037	5.528
F_0	3.120	3.319	4.519	2.960	3.479	0.709	20.363

A table summarizing the fitted model parameters

Number of dissolution data points (N), degrees of freedom (df), and selected goodness of fit criteria – Pearson correlation coefficient (R), coefficient of determination (R^2), adjusted coefficient of determination ($R^2_{adjusted}$), and residual sum of squares (RSS) (manual calculation in MS Excel):

Parameter	No.1	No.2	No.3	No.4
N	23	23	23	23
df	20	20	20	20
R	0.99921626	0.99883013	0.995805792	0.999218773
R^2	0.998433134	0.997661629	0.991629176	0.998438155
$R^2_{adjusted}$	0.998276447	0.997427792	0.990792093	0.998281971
RSS	55.07203956	68.16830056	217.1200689	41.60340454

A table summarizing the number of dissolution data points used for model fitting (N), the degrees of freedom (df), and selected goodness of fit criteria – Pearson correlation coefficient (R), coefficient of determination (R^2), adjusted coefficient of determination ($R^2_{adjusted}$), and residual sum of squares (RSS)

Graphical abstract of model fit presented as mean ± 1 SD of the fraction % of released carvedilol:

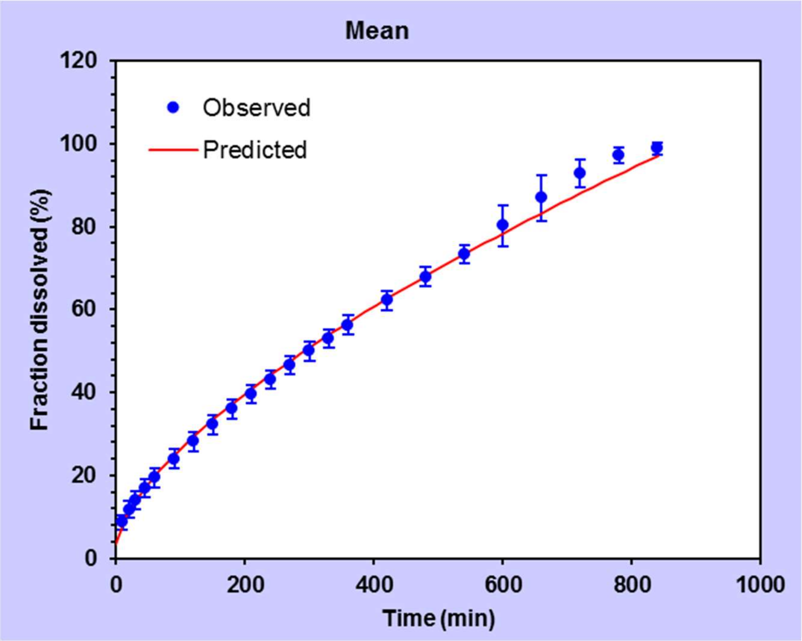

Graphical abstract of model fit presented as mean ± 1 standard deviation (SD) of the fraction % of released carvedilol

Page 1 of 2 of a chosen fitted model (in this case the Korsmeyer–Peppas with F_0 model) and a chosen section of experimental dissolution data

Note: this example excerpt of two pages from the Model fitting summary of the FlowLac® 100 formulation is concerned with the Korsmeyer–Peppas with F_0 model and the 'entire relevant dissolution data' section of dissolution data; according to the order in which the models are summarized for each section of the experimental dissolution data, the next model presented in the document for the chosen section of dissolution data will be the Hixson–Crowell model and the previous model will be the Korsmeyer–Peppas with T_{lag} model; since we are located in the 'modelling the entire relevant dissolution data' section of dissolution data, the section of dissolution data 'modelling up to app. 60 % of carvedilol released' will be located further down in the document

Supplementary materials – Model fitting summary – FlowLac® 100 (modelling the entire relevant dissolution data)

Graphical abstract of model fit presented as the fraction % of released carvedilol per tested tablet:

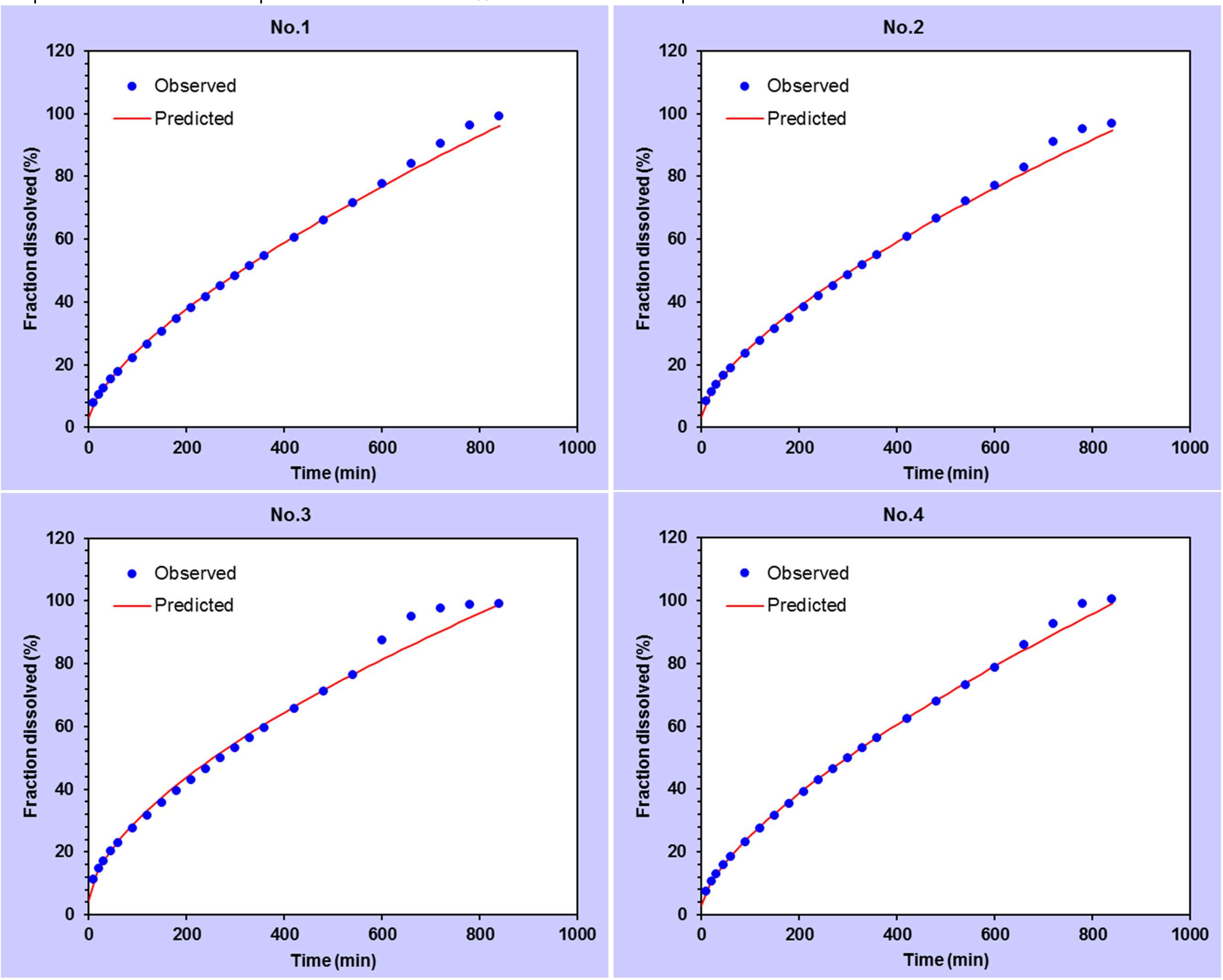

Page 2 of 2 of a chosen fitted model (in this case the Korsmeyer–Peppas with F_0 model) and a chosen section of experimental dissolution data (in this case the entire relevant dissolution data)

Graphical abstract of model fit presented as the fraction % of released carvedilol per tested tablet
