# Supplementary material for: Comparative Fitting of Mathematical Models to Carvedilol Release Profiles Obtained from Hypromellose Matrix Tablets
Source: Pharmaceutics. 2024 Apr 4;16(4):498. doi: 10.3390/pharmaceutics16040498 (PMC11053526; doi:10.3390/pharmaceutics16040498)

Supplementary materials – Model fitting summary – Avicel® PH-102 (modelling the entire relevant dissolution data)

Model: **Zero–order**

Model equation: $F = k_0 \cdot t$

Fitted model parameters per tested tablet (N = 4) with statistics – mean, standard deviation (SD), and relative standard deviation expressed in % (RSD%) (output from DDSolver):

Parameter	No.1	No.2	No.3	No.4	Mean	SD	RSD(%)
k_0	0.061	0.059	0.064	0.061	0.061	0.002	3.237

Number of dissolution data points (N), degrees of freedom (df), and selected goodness of fit criteria – Pearson correlation coefficient (R), coefficient of determination (R^2), adjusted coefficient of determination ($R^2_{adjusted}$), and residual sum of squares (RSS) (manual calculation in MS Excel):

Parameter	No.1	No.2	No.3	No.4
N	33	33	33	33
df	32	32	32	32
R	0.994420743	0.99290383	0.993463989	0.993642748
R^2	0.988872615	0.985858015	0.986970697	0.987325911
$R^2_{adjusted}$	0.988872615	0.985858015	0.986970697	0.987325911
RSS	792.7955997	804.3422243	1205.320708	921.7118693

Graphical abstract of model fit presented as mean ± 1 SD of the fraction % of released carvedilol:

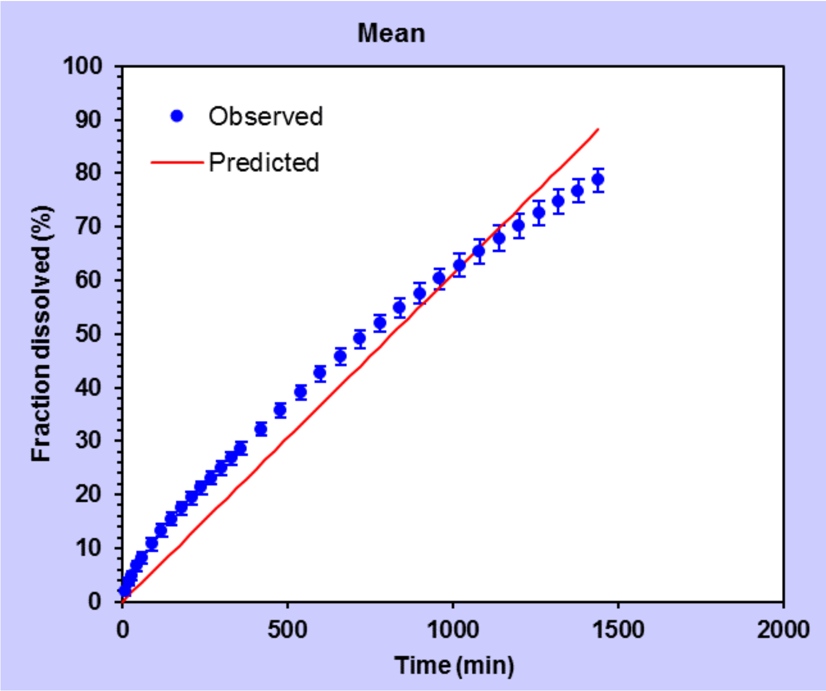

Supplementary materials – Model fitting summary – Avicel® PH-102 (modelling the entire relevant dissolution data)

Graphical abstract of model fit presented as the fraction % of released carvedilol per tested tablet:

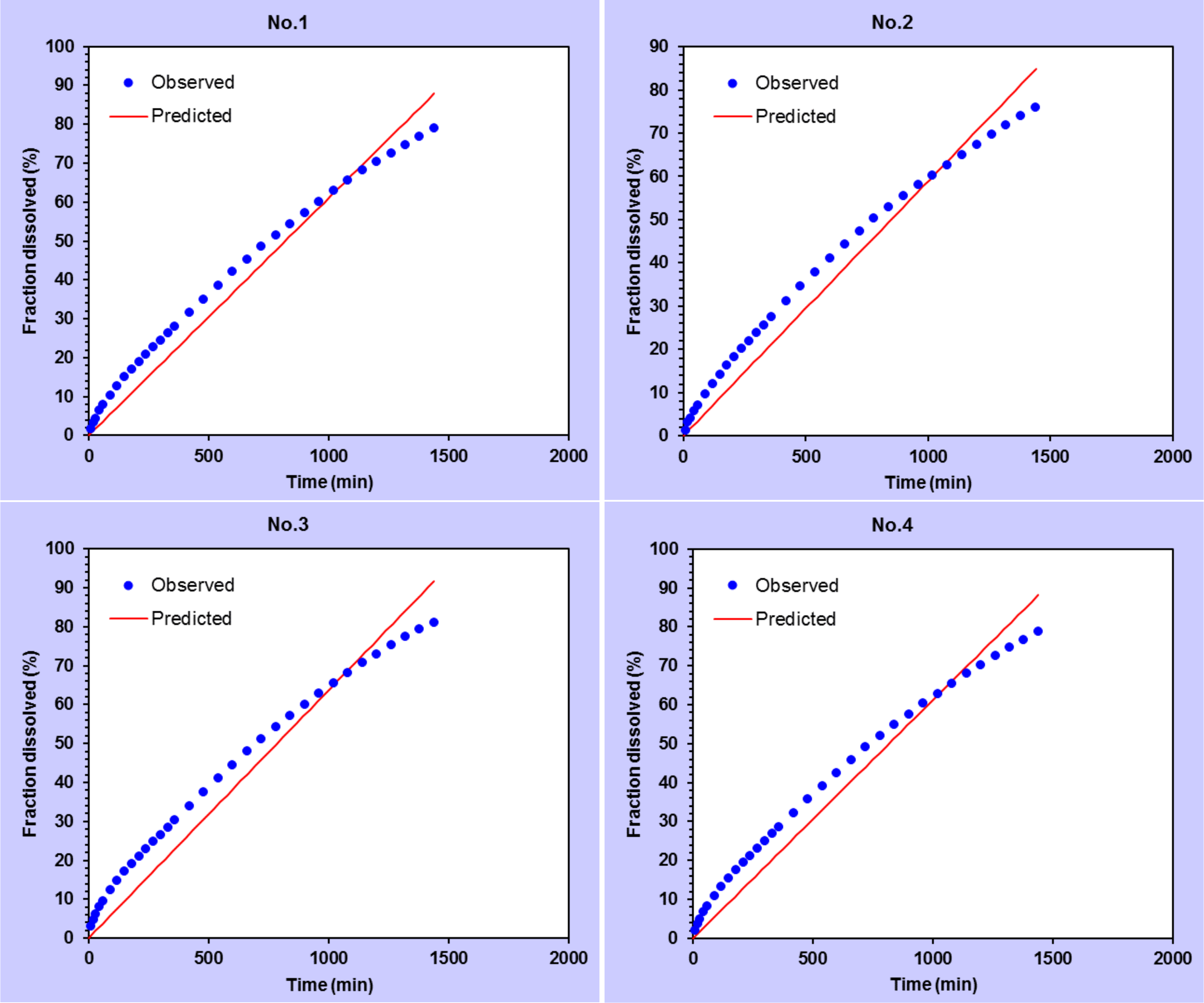

Supplementary materials – Model fitting summary – Avicel® PH-102 (modelling the entire relevant dissolution data)

Model: **Zero–order with T_{lag}**

Model equation: $F = k_0 \cdot (t - T_{lag})$

Fitted model parameters per tested tablet (N = 4) with statistics – mean, standard deviation (SD), and relative standard deviation expressed in % (RSD%) (output from DDSolver):

Parameter	No.1	No.2	No.3	No.4	Mean	SD	RSD(%)
k_0	0.054	0.052	0.054	0.053	0.053	0.001	2.094
T_{lag}	-125.715	-127.240	-159.331	-137.641	-137.482	15.500	-11.274

Number of dissolution data points (N), degrees of freedom (df), and selected goodness of fit criteria – Pearson correlation coefficient (R), coefficient of determination (R^2), adjusted coefficient of determination ($R^2_{adjusted}$), and residual sum of squares (RSS) (manual calculation in MS Excel):

Parameter	No.1	No.2	No.3	No.4
N	33	33	33	33
df	31	31	31	31
R	0.994420743	0.99290383	0.993463989	0.993642748
R^2	0.988872615	0.985858015	0.986970697	0.987325911
$R^2_{adjusted}$	0.988513667	0.985401822	0.986550397	0.986917069
RSS	220.8759027	261.0884291	265.1823666	247.7154417

Graphical abstract of model fit presented as mean ± 1 SD of the fraction % of released carvedilol:

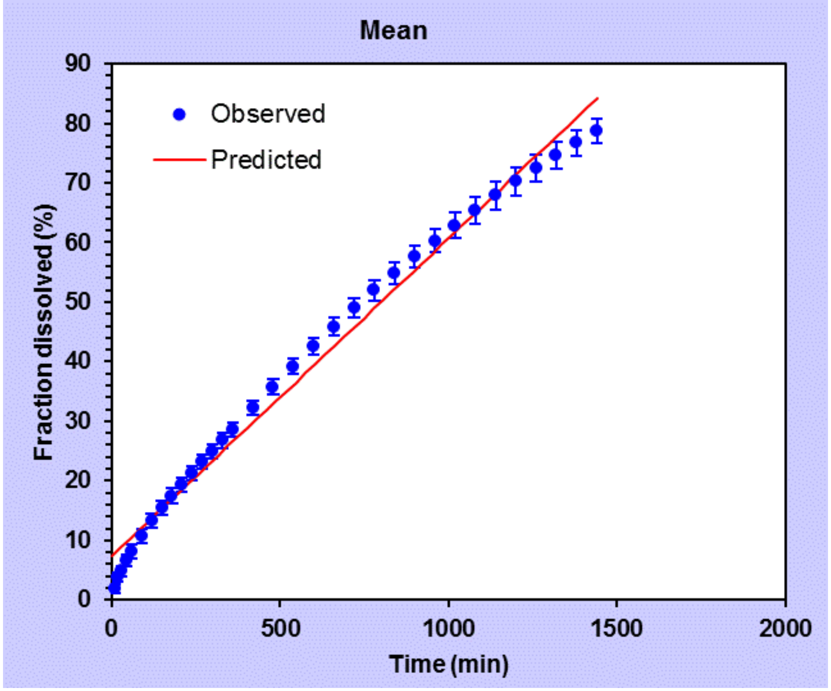

Supplementary materials – Model fitting summary – Avicel® PH-102 (modelling the entire relevant dissolution data)

Graphical abstract of model fit presented as the fraction % of released carvedilol per tested tablet:

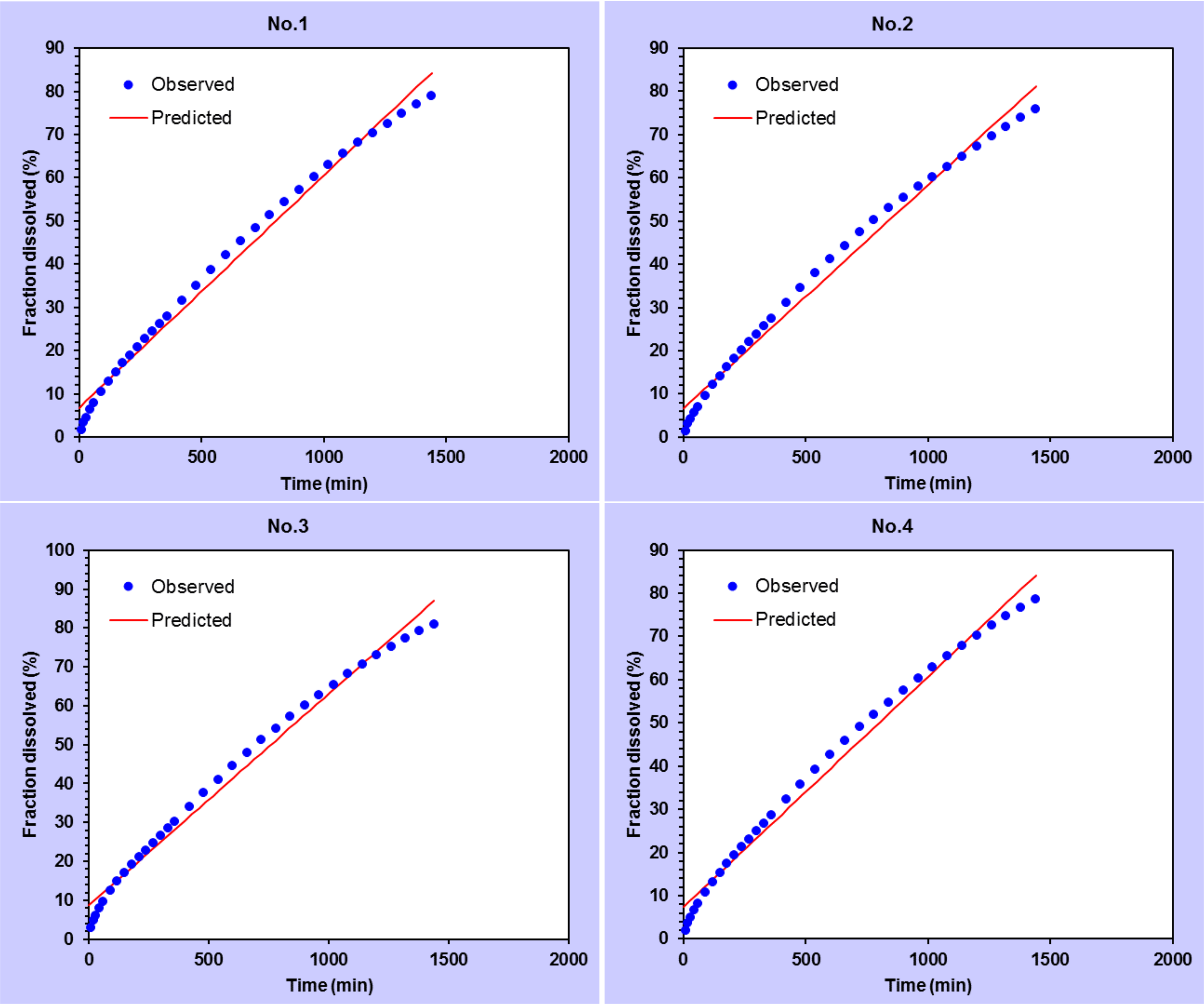

Supplementary materials – Model fitting summary – Avicel® PH-102 (modelling the entire relevant dissolution data)

Model: **Zero–order with F_0**

Model equation: $F = F_0 + k_0 \cdot t$

Fitted model parameters per tested tablet (N = 4) with statistics – mean, standard deviation (SD), and relative standard deviation expressed in % (RSD%) (output from DDSolver):

Parameter	No.1	No.2	No.3	No.4	Mean	SD	RSD(%)
k_0	0.054	0.052	0.054	0.053	0.053	0.001	2.094
F_0	6.764	6.592	8.672	7.342	7.342	0.943	12.837

Number of dissolution data points (N), degrees of freedom (df), and selected goodness of fit criteria – Pearson correlation coefficient (R), coefficient of determination (R^2), adjusted coefficient of determination ($R^2_{adjusted}$), and residual sum of squares (RSS) (manual calculation in MS Excel):

Parameter	No.1	No.2	No.3	No.4
N	33	33	33	33
df	31	31	31	31
R	0.994420743	0.99290383	0.993463989	0.993642748
R^2	0.988872615	0.985858015	0.986970697	0.987325911
$R^2_{adjusted}$	0.988513667	0.985401822	0.986550397	0.986917069
RSS	220.8759027	261.0884291	265.1823666	247.7154417

Graphical abstract of model fit presented as mean ± 1 SD of the fraction % of released carvedilol:

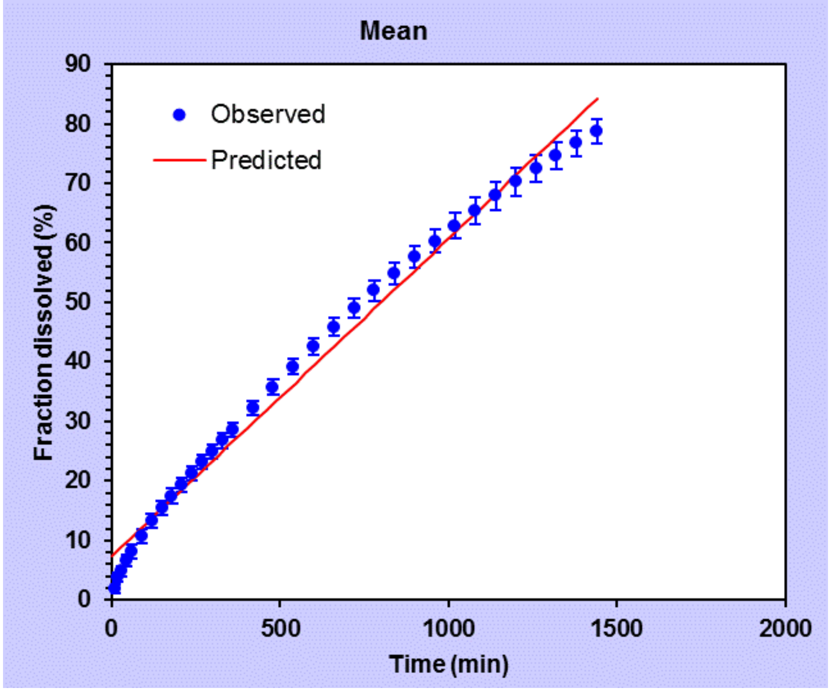

Supplementary materials – Model fitting summary – Avicel® PH-102 (modelling the entire relevant dissolution data)

Graphical abstract of model fit presented as the fraction % of released carvedilol per tested tablet:

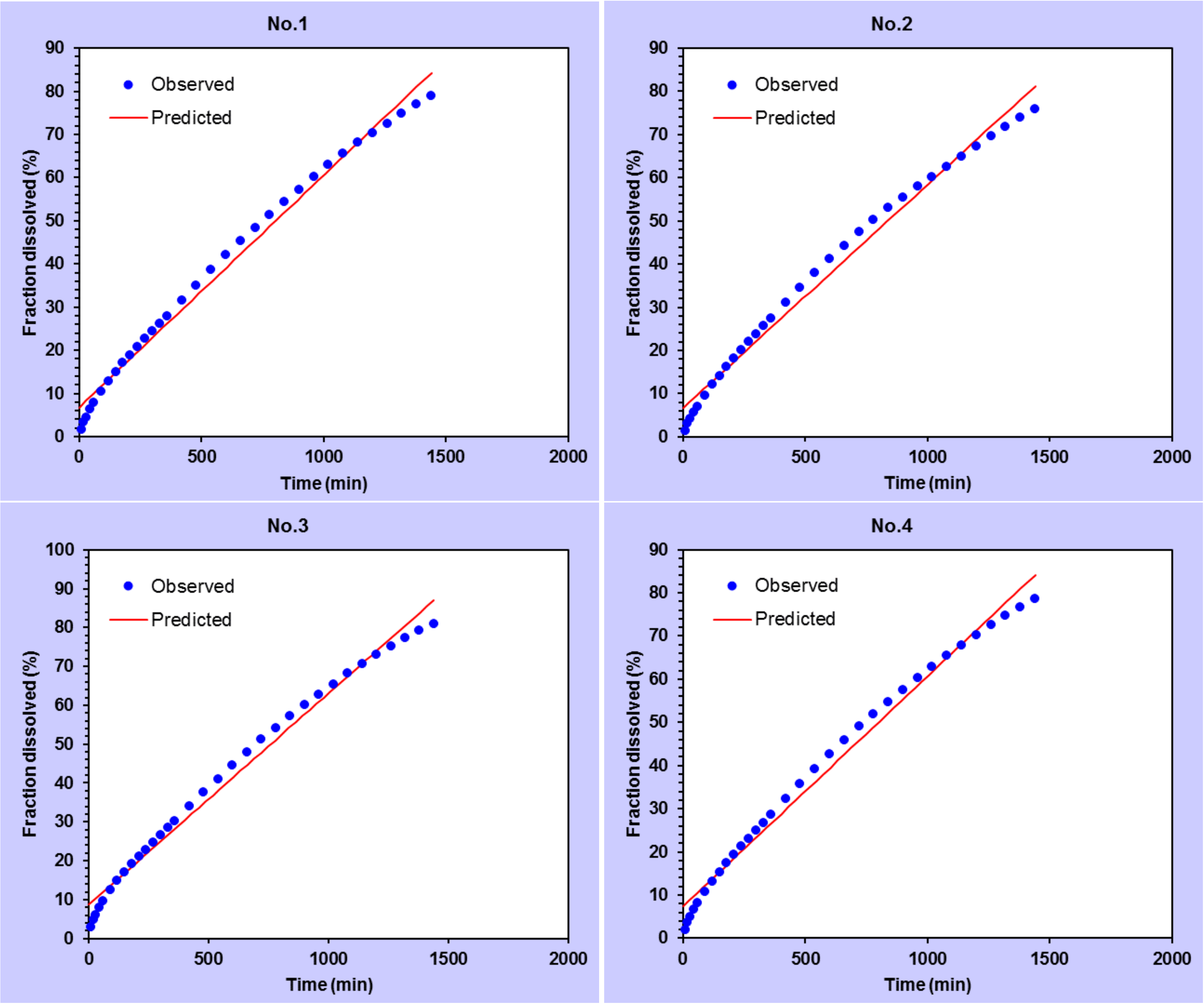

Supplementary materials – Model fitting summary – Avicel® PH-102 (modelling the entire relevant dissolution data)

Model: **First–order**

Model equation: $F = 100 \cdot (1 - e^{-k_1 \cdot t})$

Fitted model parameters per tested tablet (N = 4) with statistics – mean, standard deviation (SD), and relative standard deviation expressed in % (RSD%) (output from DDSolver):

Parameter	No.1	No.2	No.3	No.4	Mean	SD	RSD(%)
k_1	0.001	0.001	0.001	0.001	0.001	0.000	5.881

Number of dissolution data points (N), degrees of freedom (df), and selected goodness of fit criteria – Pearson correlation coefficient (R), coefficient of determination (R^2), adjusted coefficient of determination ($R^2_{adjusted}$), and residual sum of squares (RSS) (manual calculation in MS Excel):

Parameter	No.1	No.2	No.3	No.4
N	33	33	33	33
df	32	32	32	32
R	0.997235262	0.998822999	0.996891424	0.997773552
R^2	0.994478168	0.997647383	0.993792512	0.995552061
$R^2_{adjusted}$	0.994478168	0.997647383	0.993792512	0.995552061
RSS	122.6872776	49.43678329	156.2034625	99.67492562

Graphical abstract of model fit presented as mean ± 1 SD of the fraction % of released carvedilol:

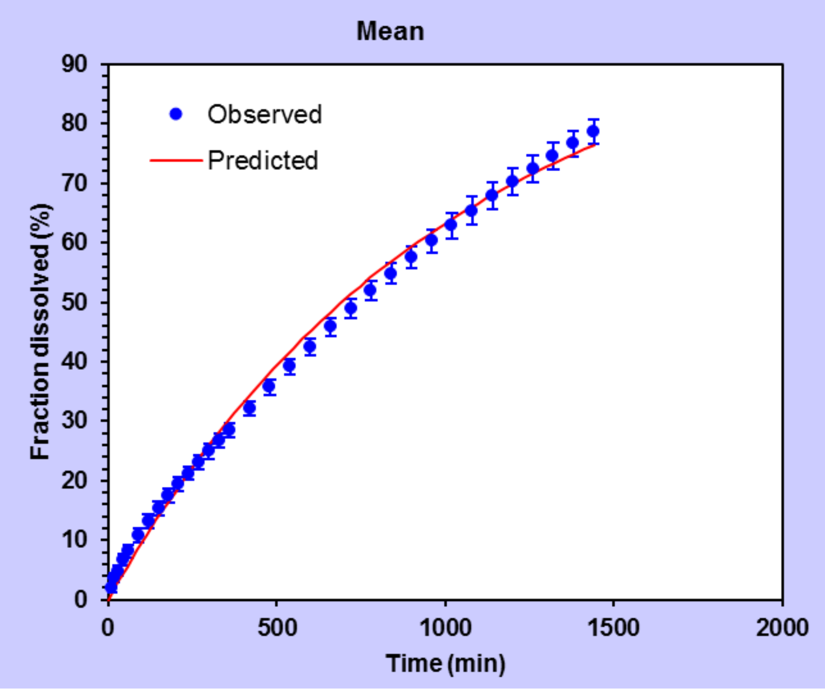

Supplementary materials – Model fitting summary – Avicel® PH-102 (modelling the entire relevant dissolution data)

Graphical abstract of model fit presented as the fraction % of released carvedilol per tested tablet:

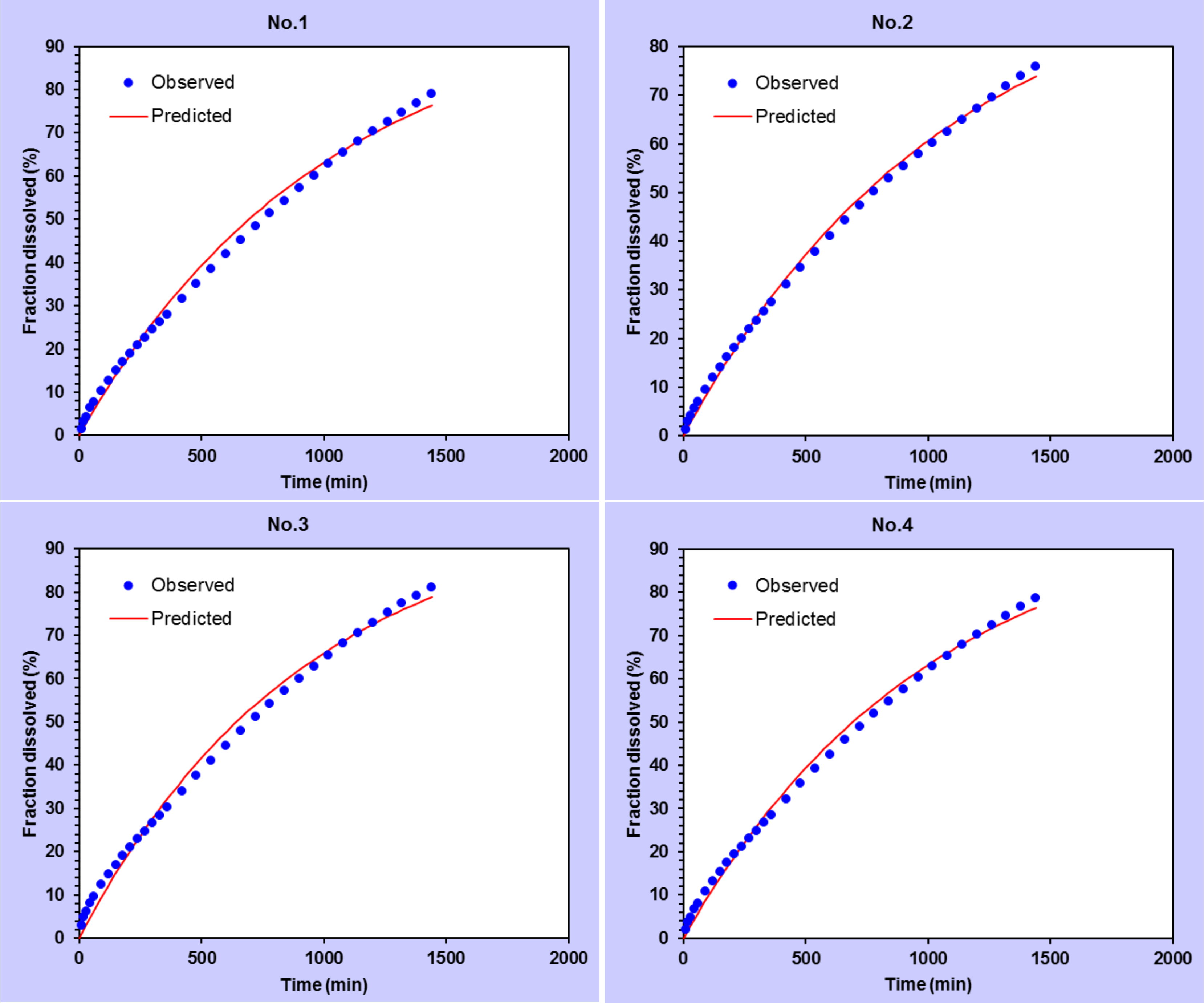

Supplementary materials – Model fitting summary – Avicel® PH-102 (modelling the entire relevant dissolution data)

Model: **First–order with T_{lag}**

Model equation: $F = 100 \cdot \left[1 - e^{-k_1 \cdot (t - T_{lag})}\right]$

Fitted model parameters per tested tablet (N = 4) with statistics – mean, standard deviation (SD), and relative standard deviation expressed in % (RSD%) (output from DDSolver):

Parameter	No.1	No.2	No.3	No.4	Mean	SD	RSD(%)
k_1	0.001	0.001	0.001	0.001	0.001	0.000	5.862
T_{lag}	17.020	5.394	5.293	8.688	9.099	5.511	60.570

Number of dissolution data points (N), degrees of freedom (df), and selected goodness of fit criteria – Pearson correlation coefficient (R), coefficient of determination (R^2), adjusted coefficient of determination ($R^2_{adjusted}$), and residual sum of squares (RSS) (manual calculation in MS Excel):

Parameter	No.1	No.2	No.3	No.4
N	33	33	33	33
df	31	31	31	31
R	0.99701658	0.998782876	0.996816525	0.997675421
R^2	0.994042061	0.997567234	0.993643185	0.995356246
$R^2_{adjusted}$	0.993849869	0.997488758	0.993438126	0.995206447
RSS	155.9840718	57.21482693	174.881437	119.2579953

Graphical abstract of model fit presented as mean ± 1 SD of the fraction % of released carvedilol:

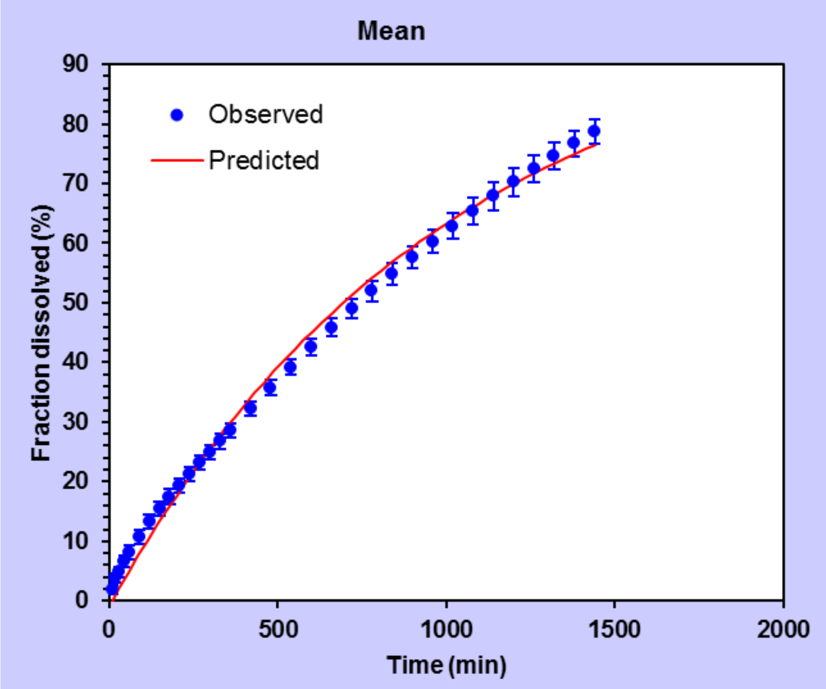

Supplementary materials – Model fitting summary – Avicel® PH-102 (modelling the entire relevant dissolution data)

Graphical abstract of model fit presented as the fraction % of released carvedilol per tested tablet:

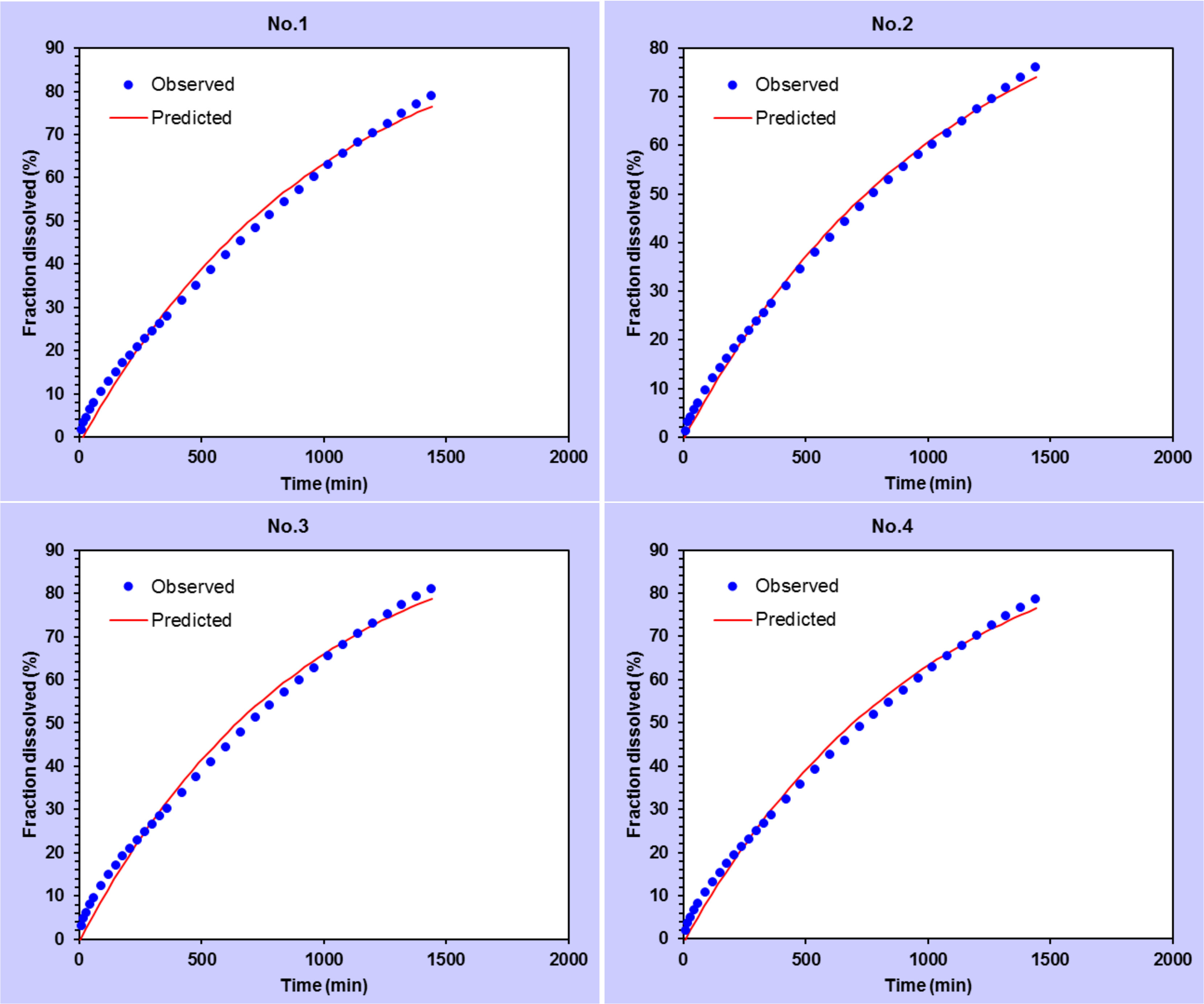

Supplementary materials – Model fitting summary – Avicel® PH-102 (modelling the entire relevant dissolution data)

Model: **First–order with F_{max}**

Model equation: $F = F_{max} \cdot (1 - e^{-k_1 \cdot t})$

Fitted model parameters per tested tablet (N = 4) with statistics – mean, standard deviation (SD), and relative standard deviation expressed in % (RSD%) (output from DDSolver):

Parameter	No.1	No.2	No.3	No.4	Mean	SD	RSD(%)
k_1	0.002	0.002	0.002	0.002	0.002	0.000	1.497
F_{max}	82.955	79.779	85.073	82.602	82.602	2.175	2.634

Number of dissolution data points (N), degrees of freedom (df), and selected goodness of fit criteria – Pearson correlation coefficient (R), coefficient of determination (R^2), adjusted coefficient of determination ($R^2_{adjusted}$), and residual sum of squares (RSS) (manual calculation in MS Excel):

Parameter	No.1	No.2	No.3	No.4
N	33	33	33	33
df	31	31	31	31
R	0.987044456	0.989405666	0.987107777	0.987879002
R^2	0.974256758	0.978923572	0.974381763	0.975904923
$R^2_{adjusted}$	0.973426331	0.978243687	0.973555368	0.975127662
RSS	882.681236	705.5762546	799.3458618	788.260125

Graphical abstract of model fit presented as mean ± 1 SD of the fraction % of released carvedilol:

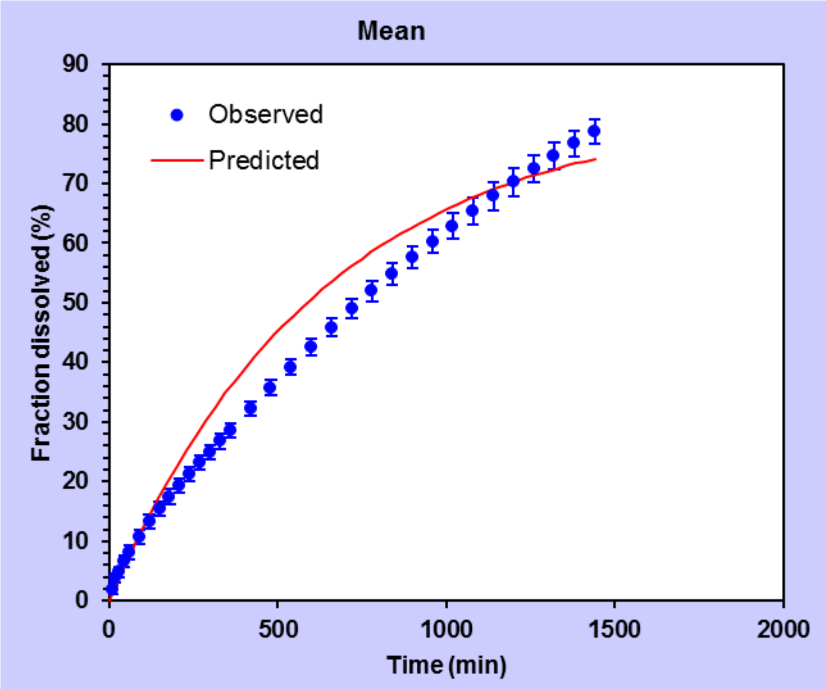

Supplementary materials – Model fitting summary – Avicel® PH-102 (modelling the entire relevant dissolution data)

Graphical abstract of model fit presented as the fraction % of released carvedilol per tested tablet:

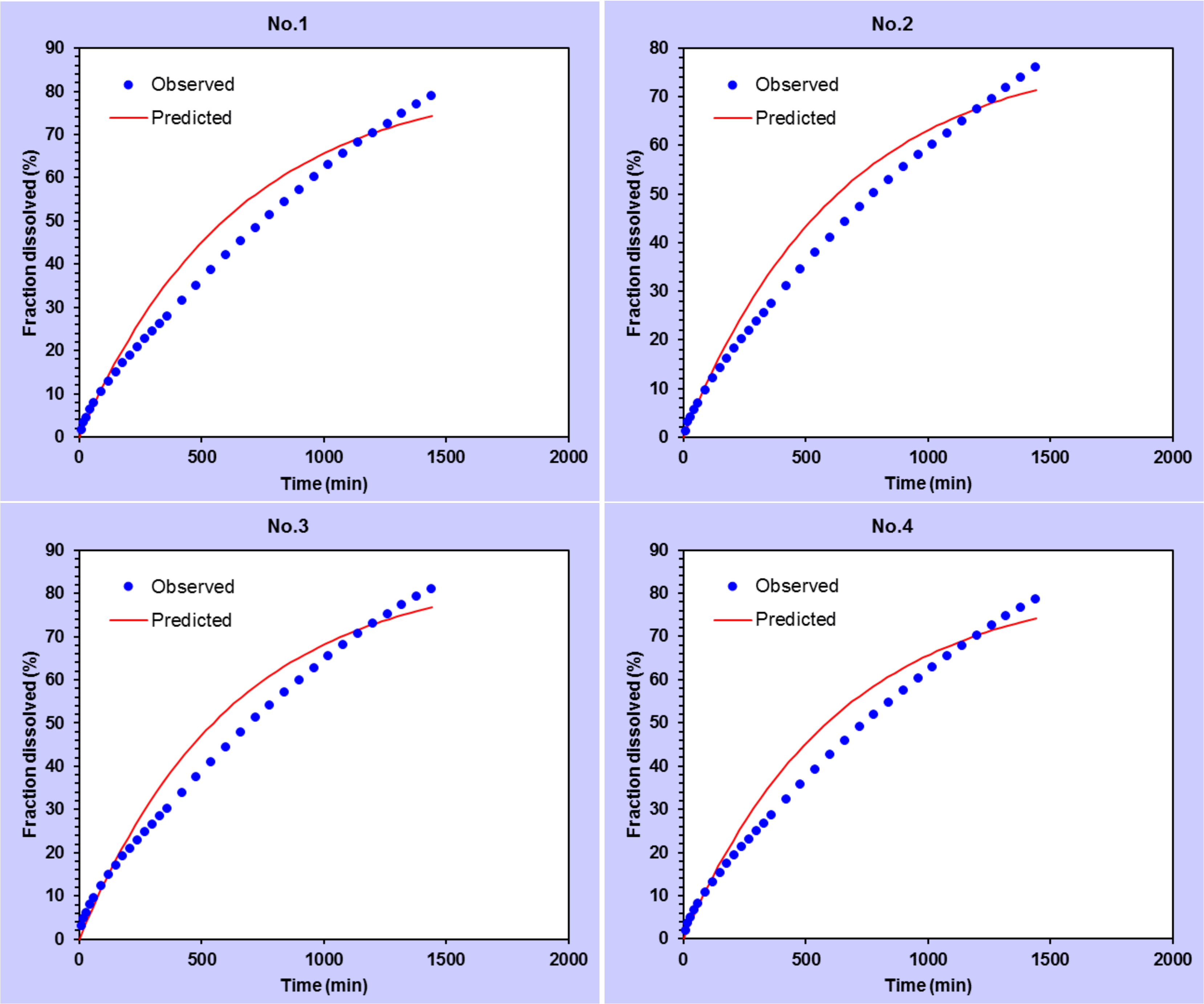

Supplementary materials – Model fitting summary – Avicel® PH-102 (modelling the entire relevant dissolution data)

Model: **First–order with T_{lag} and F_{max}**

Model equation: $F = F_{max} \cdot \left[1 - e^{-k_1 \cdot (t - T_{lag})}\right]$

Fitted model parameters per tested tablet (N = 4) with statistics – mean, standard deviation (SD), and relative standard deviation expressed in % (RSD%) (output from DDSolver):

Parameter	No.1	No.2	No.3	No.4	Mean	SD	RSD(%)
k_1	0.002	0.002	0.002	0.002	0.002	0.000	0.913
T_{lag}	84.789	81.914	72.623	79.647	79.743	5.192	6.511
F_{max}	82.955	79.779	85.073	82.602	82.602	2.175	2.634

Number of dissolution data points (N), degrees of freedom (df), and selected goodness of fit criteria – Pearson correlation coefficient (R), coefficient of determination (R^2), adjusted coefficient of determination ($R^2_{adjusted}$), and residual sum of squares (RSS) (manual calculation in MS Excel):

Parameter	No.1	No.2	No.3	No.4
N	33	33	33	33
df	30	30	30	30
R	0.983129969	0.985969522	0.983756501	0.984312747
R^2	0.966544535	0.972135899	0.967776852	0.968871584
$R^2_{adjusted}$	0.964314171	0.970278292	0.965628642	0.966796356
RSS	1440.888296	1141.795425	1428.286342	1331.570074

Graphical abstract of model fit presented as mean ± 1 SD of the fraction % of released carvedilol:

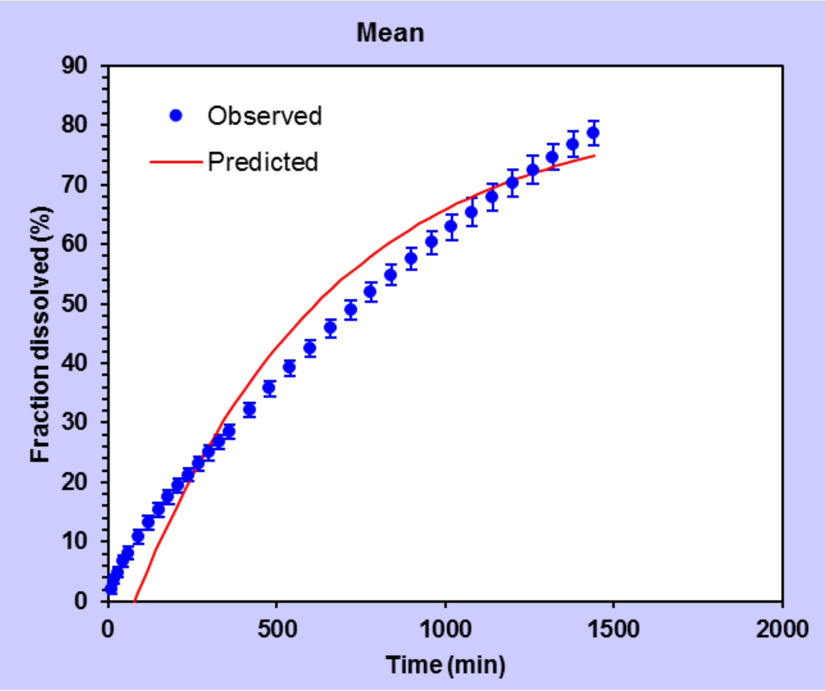

Supplementary materials – Model fitting summary – Avicel® PH-102 (modelling the entire relevant dissolution data)

Graphical abstract of model fit presented as the fraction % of released carvedilol per tested tablet:

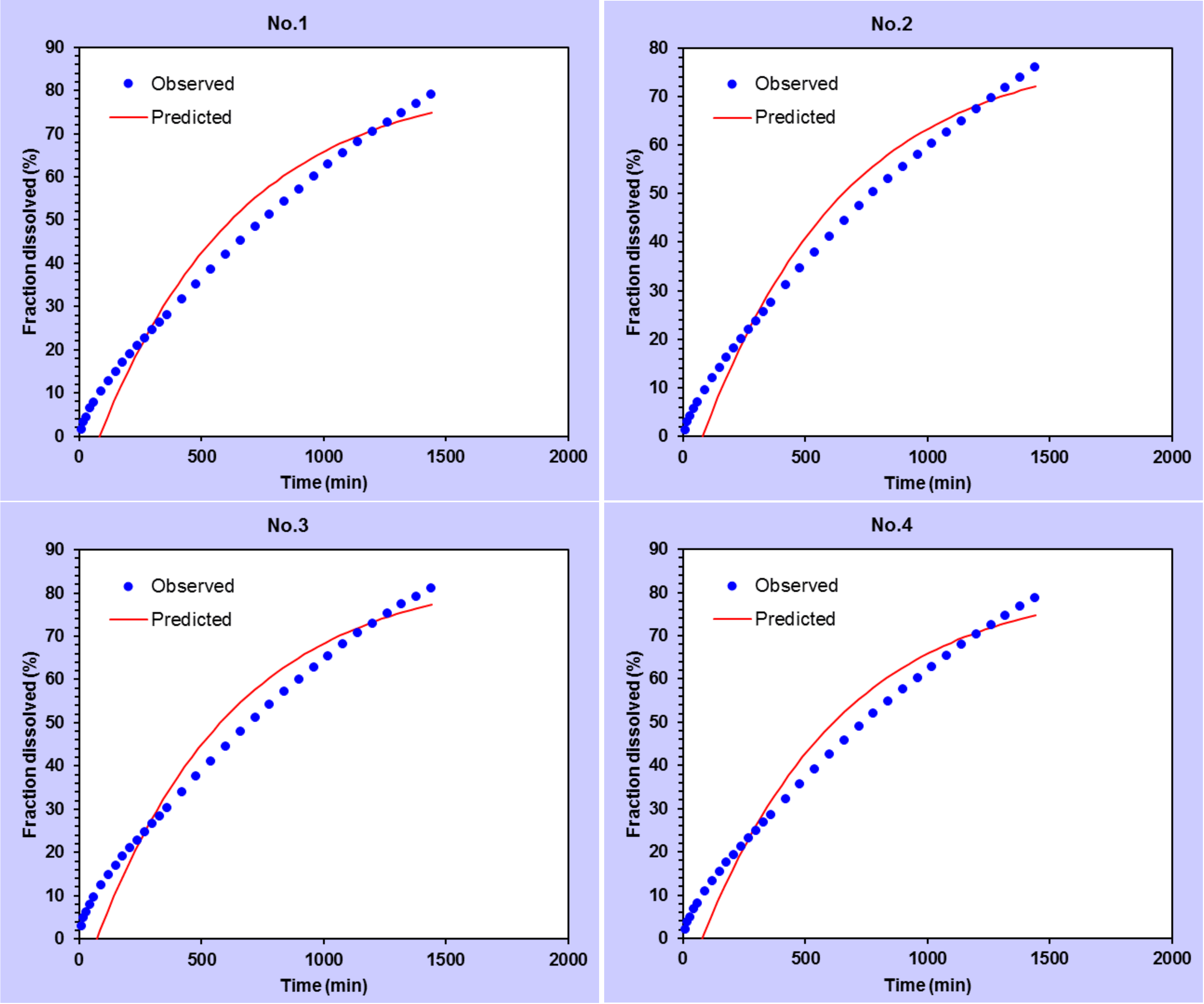

Supplementary materials – Model fitting summary – Avicel® PH-102 (modelling the entire relevant dissolution data)

Model: **Higuchi**

Model equation: $F = k_H \cdot t^{0.5}$

Fitted model parameters per tested tablet (N = 4) with statistics – mean, standard deviation (SD), and relative standard deviation expressed in % (RSD%) (output from DDSolver):

Parameter	No.1	No.2	No.3	No.4	Mean	SD	RSD(%)
k_H	1.860	1.795	1.951	1.869	1.869	0.064	3.412

Number of dissolution data points (N), degrees of freedom (df), and selected goodness of fit criteria – Pearson correlation coefficient (R), coefficient of determination (R^2), adjusted coefficient of determination ($R^2_{adjusted}$), and residual sum of squares (RSS) (manual calculation in MS Excel):

Parameter	No.1	No.2	No.3	No.4
N	33	33	33	33
df	32	32	32	32
R	0.993328218	0.994597873	0.99403975	0.994020403
R^2	0.986700949	0.989224929	0.988115026	0.988076562
$R^2_{adjusted}$	0.986700949	0.989224929	0.988115026	0.988076562
RSS	1199.258639	1074.558402	947.4061901	1068.9252

Graphical abstract of model fit presented as mean ± 1 SD of the fraction % of released carvedilol:

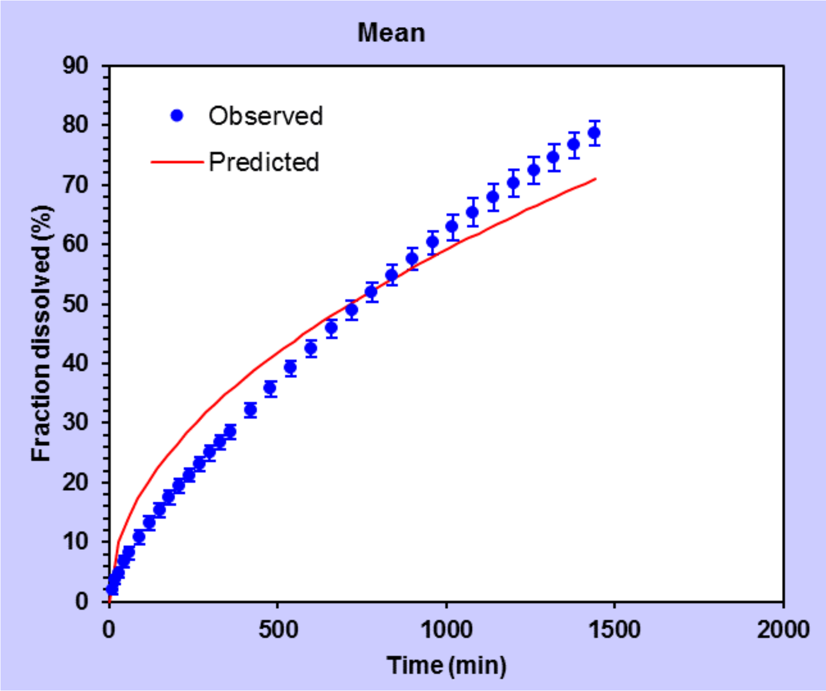

Supplementary materials – Model fitting summary – Avicel® PH-102 (modelling the entire relevant dissolution data)

Graphical abstract of model fit presented as the fraction % of released carvedilol per tested tablet:

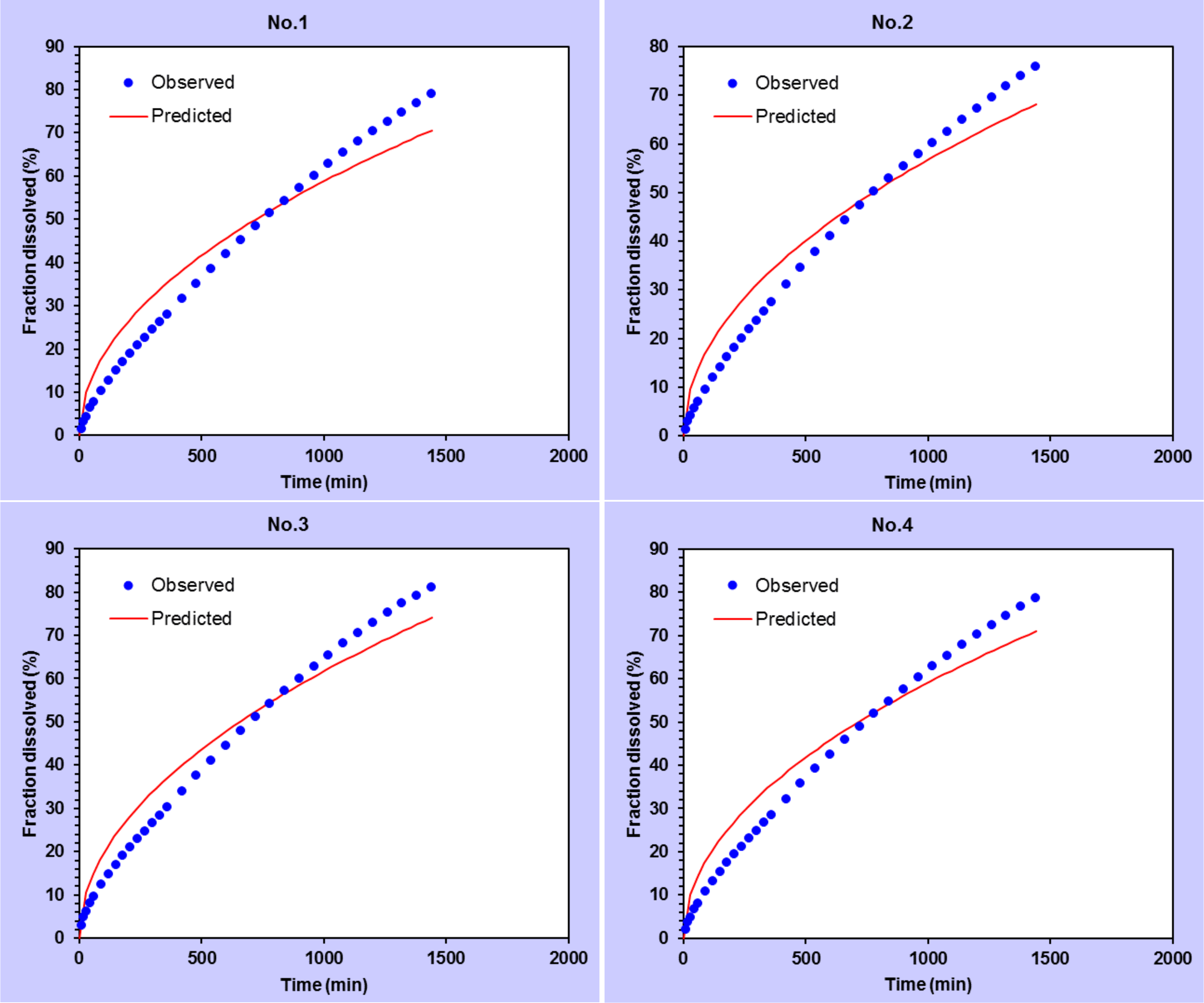

Supplementary materials – Model fitting summary – Avicel® PH-102 (modelling the entire relevant dissolution data)

Model: **Higuchi with T_{lag}**

Model equation: $F = k_H \cdot (t - T_{lag})^{0.5}$

Fitted model parameters per tested tablet (N = 4) with statistics – mean, standard deviation (SD), and relative standard deviation expressed in % (RSD%) (output from DDSolver):

Parameter	No.1	No.2	No.3	No.4	Mean	SD	RSD(%)
k_H	2.099	2.016	2.165	2.094	2.093	0.061	2.917
T_{lag}	116.218	112.122	103.182	110.524	110.512	5.443	4.925

Number of dissolution data points (N), degrees of freedom (df), and selected goodness of fit criteria – Pearson correlation coefficient (R), coefficient of determination (R^2), adjusted coefficient of determination ($R^2_{adjusted}$), and residual sum of squares (RSS) (manual calculation in MS Excel):

Parameter	No.1	No.2	No.3	No.4
N	33	33	33	33
df	31	31	31	31
R	0.98817004	0.990257475	0.988275248	0.989052948
R^2	0.976480027	0.980609866	0.976687965	0.978225734
$R^2_{adjusted}$	0.975721319	0.979984378	0.975935964	0.977523338
RSS	551.9071237	416.3394694	604.8710451	512.1775992

Graphical abstract of model fit presented as mean ± 1 SD of the fraction % of released carvedilol:

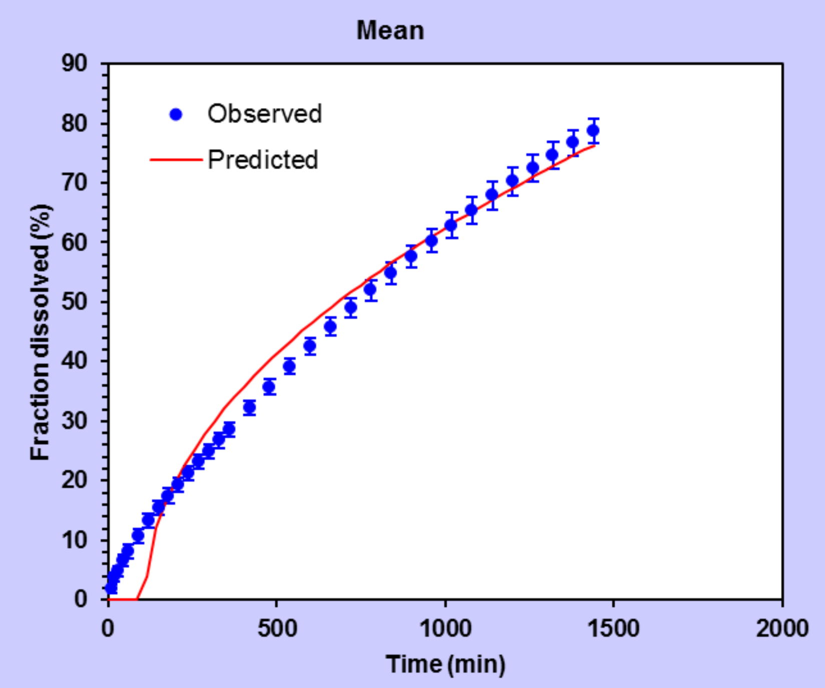

Supplementary materials – Model fitting summary – Avicel® PH-102 (modelling the entire relevant dissolution data)

Graphical abstract of model fit presented as the fraction % of released carvedilol per tested tablet:

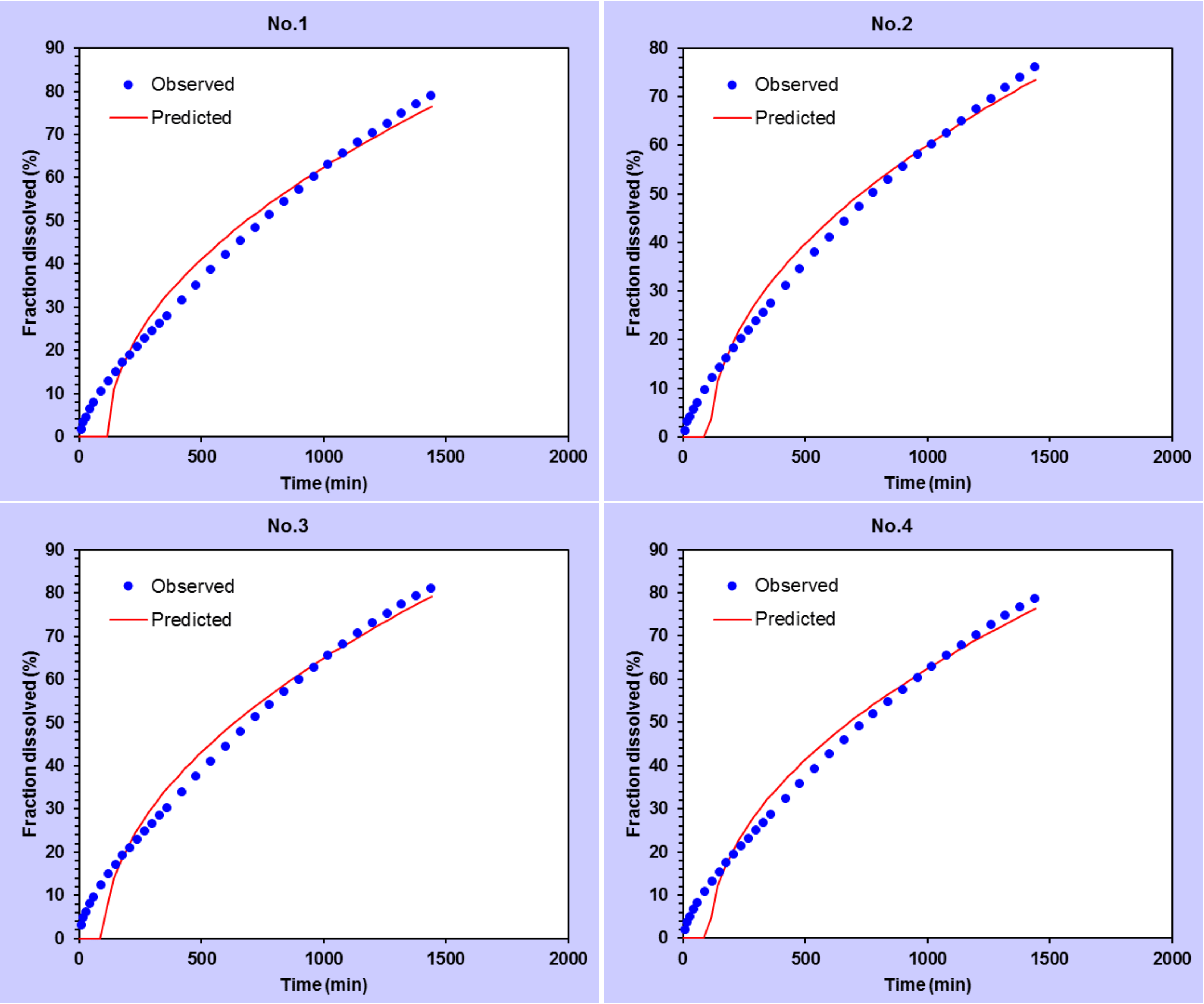

Supplementary materials – Model fitting summary – Avicel® PH-102 (modelling the entire relevant dissolution data)

Model: **Higuchi with F_0**

Model equation: $F = F_0 + k_H \cdot t^{0.5}$

Fitted model parameters per tested tablet (N = 4) with statistics – mean, standard deviation (SD), and relative standard deviation expressed in % (RSD%) (output from DDSolver):

Parameter	No.1	No.2	No.3	No.4	Mean	SD	RSD(%)
k_H	2.315	2.236	2.346	2.299	2.299	0.047	2.023
F_0	-12.190	-11.795	-10.587	-11.524	-11.524	0.682	-5.916

Number of dissolution data points (N), degrees of freedom (df), and selected goodness of fit criteria – Pearson correlation coefficient (R), coefficient of determination (R^2), adjusted coefficient of determination ($R^2_{adjusted}$), and residual sum of squares (RSS) (manual calculation in MS Excel):

Parameter	No.1	No.2	No.3	No.4
N	33	33	33	33
df	31	31	31	31
R	0.993328218	0.994597873	0.99403975	0.994020403
R^2	0.986700949	0.989224929	0.988115026	0.988076562
$R^2_{adjusted}$	0.986271947	0.988877346	0.987731639	0.987691935
RSS	263.9829504	198.9286806	241.8921134	233.0439394

Graphical abstract of model fit presented as mean ± 1 SD of the fraction % of released carvedilol:

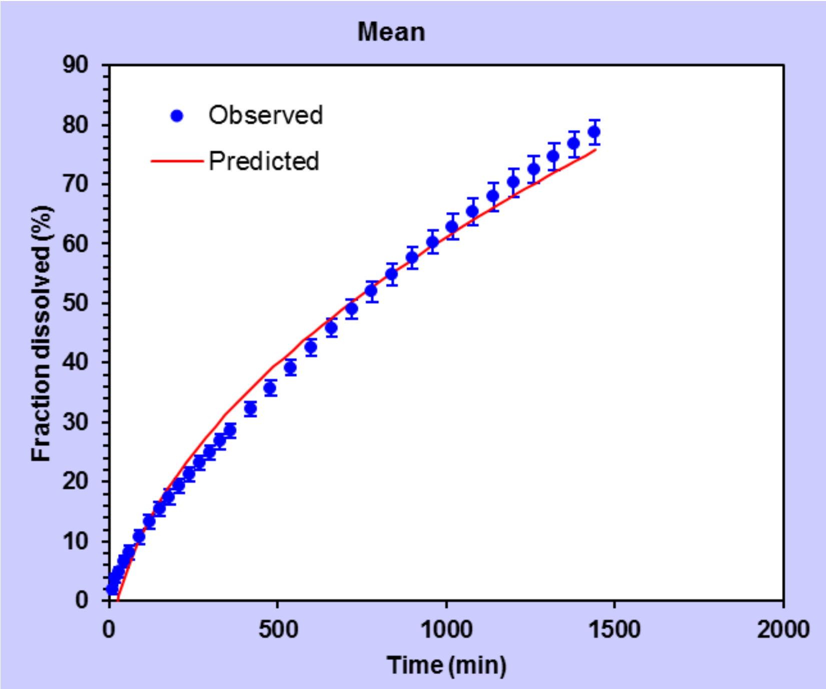

Supplementary materials – Model fitting summary – Avicel® PH-102 (modelling the entire relevant dissolution data)

Graphical abstract of model fit presented as the fraction % of released carvedilol per tested tablet:

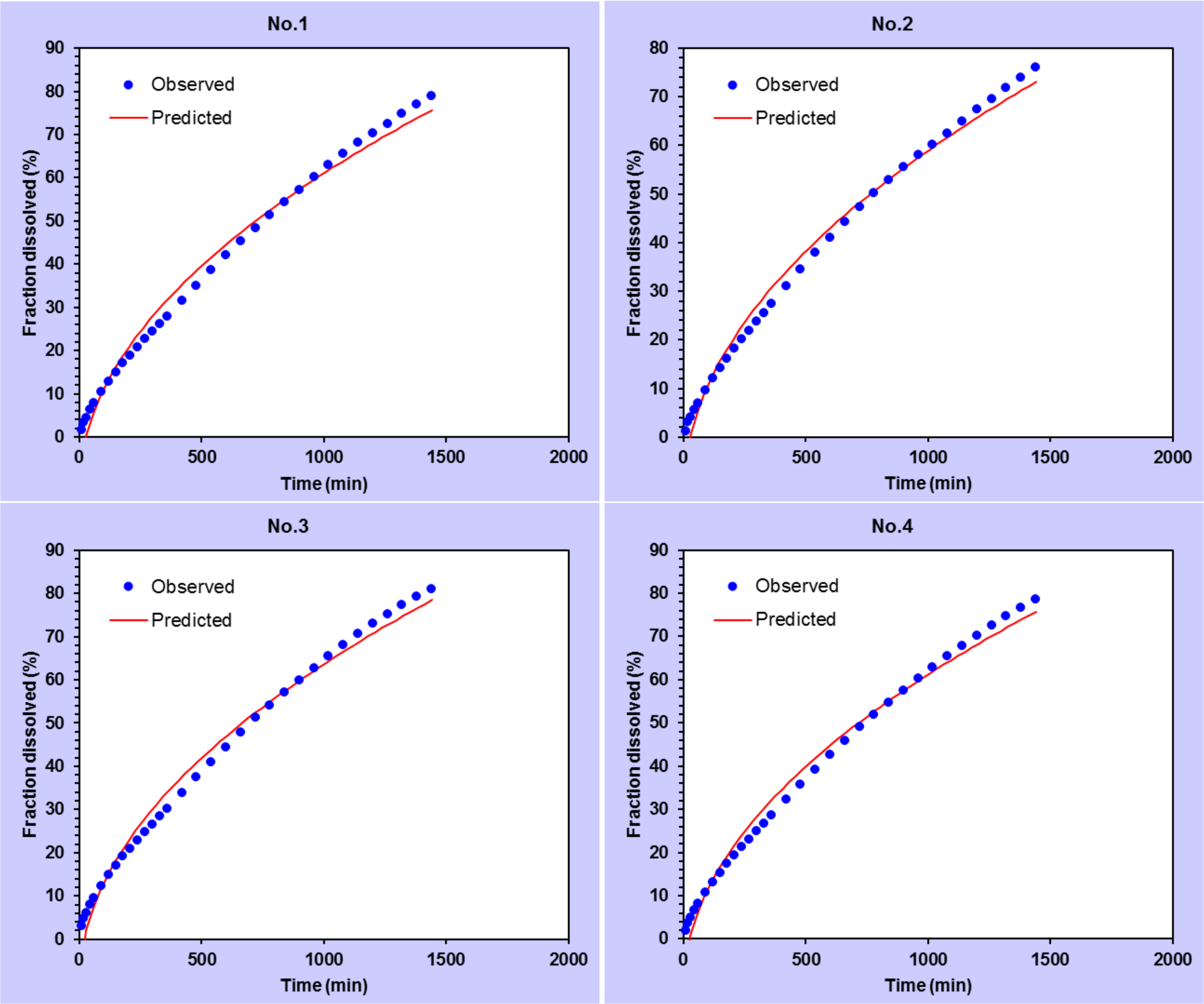

Supplementary materials – Model fitting summary – Avicel® PH-102 (modelling the entire relevant dissolution data)

Model: **Korsmeyer–Peppas**

Model equation: $F = k_{KP} \cdot t^n$

Fitted model parameters per tested tablet (N = 4) with statistics – mean, standard deviation (SD), and relative standard deviation expressed in % (RSD%) (output from DDSolver):

Parameter	No.1	No.2	No.3	No.4	Mean	SD	RSD(%)
k_{KP}	0.331	0.320	0.607	0.400	0.414	0.133	32.114
n	0.757	0.754	0.672	0.729	0.728	0.040	5.428

Number of dissolution data points (N), degrees of freedom (df), and selected goodness of fit criteria – Pearson correlation coefficient (R), coefficient of determination (R^2), adjusted coefficient of determination ($R^2_{adjusted}$), and residual sum of squares (RSS) (manual calculation in MS Excel):

Parameter	No.1	No.2	No.3	No.4
N	33	33	33	33
df	31	31	31	31
R	0.999767514	0.999570531	0.999452269	0.999776342
R^2	0.999535082	0.999141247	0.998904839	0.999552735
$R^2_{adjusted}$	0.999520085	0.999113546	0.998869511	0.999538307
RSS	13.206762	21.96224082	45.80772103	8.742042704

Graphical abstract of model fit presented as mean ± 1 SD of the fraction % of released carvedilol:

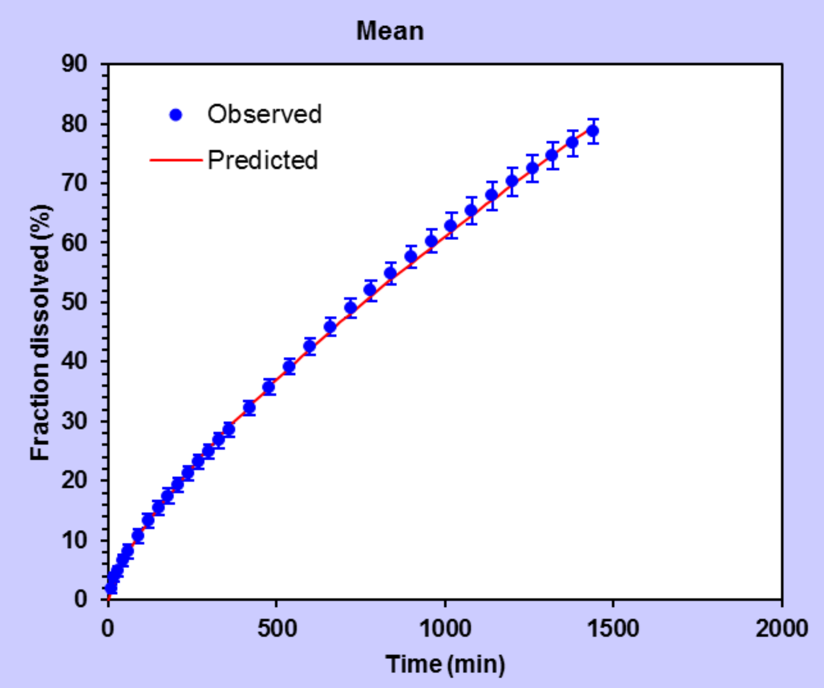

Supplementary materials – Model fitting summary – Avicel® PH-102 (modelling the entire relevant dissolution data)

Graphical abstract of model fit presented as the fraction % of released carvedilol per tested tablet:

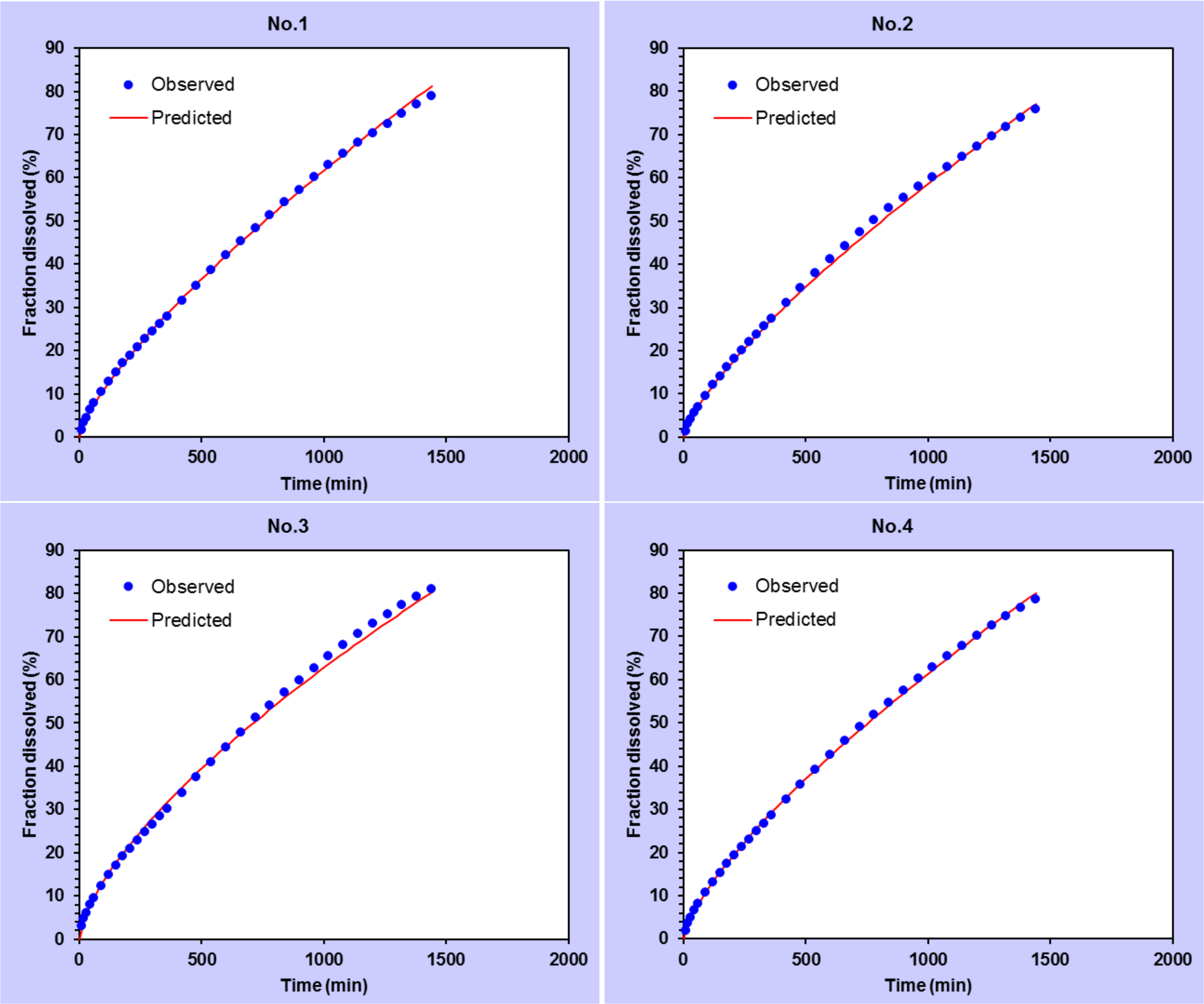

Supplementary materials – Model fitting summary – Avicel® PH-102 (modelling the entire relevant dissolution data)

Model: **Korsmeyer–Peppas with T_{lag}**

Model equation: $F = k_{KP} \cdot (t - T_{lag})^n$

Fitted model parameters per tested tablet (N = 4) with statistics – mean, standard deviation (SD), and relative standard deviation expressed in % (RSD%) (output from DDSolver):

Parameter	No.1	No.2	No.3	No.4	Mean	SD	RSD(%)
k_{KP}	0.431	0.366	0.774	0.517	0.522	0.179	34.266
n	0.716	0.736	0.634	0.689	0.694	0.044	6.382
T_{lag}	4.000	4.000	4.000	4.000	4.000	0.000	0.000

Number of dissolution data points (N), degrees of freedom (df), and selected goodness of fit criteria – Pearson correlation coefficient (R), coefficient of determination (R^2), adjusted coefficient of determination ($R^2_{adjusted}$), and residual sum of squares (RSS) (manual calculation in MS Excel):

Parameter	No.1	No.2	No.3	No.4
N	33	33	33	33
df	30	30	30	30
R	0.999721946	0.999708158	0.99874027	0.999608778
R^2	0.999443969	0.9994164	0.997482127	0.99921771
$R^2_{adjusted}$	0.9994069	0.999377494	0.997314269	0.999165557
RSS	21.60464987	10.84409208	147.4957093	41.65767663

Graphical abstract of model fit presented as mean ± 1 SD of the fraction % of released carvedilol:

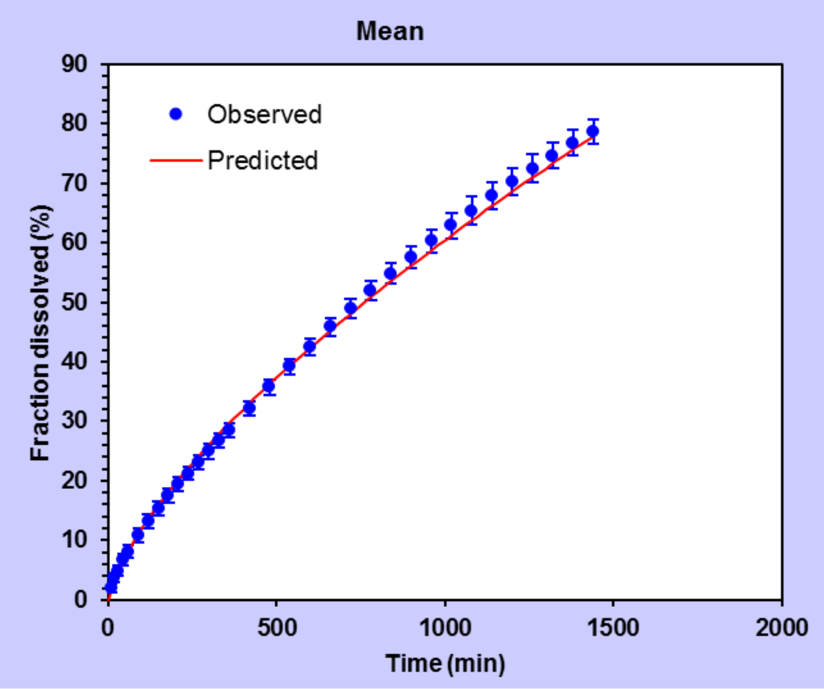

Supplementary materials – Model fitting summary – Avicel® PH-102 (modelling the entire relevant dissolution data)

Graphical abstract of model fit presented as the fraction % of released carvedilol per tested tablet:

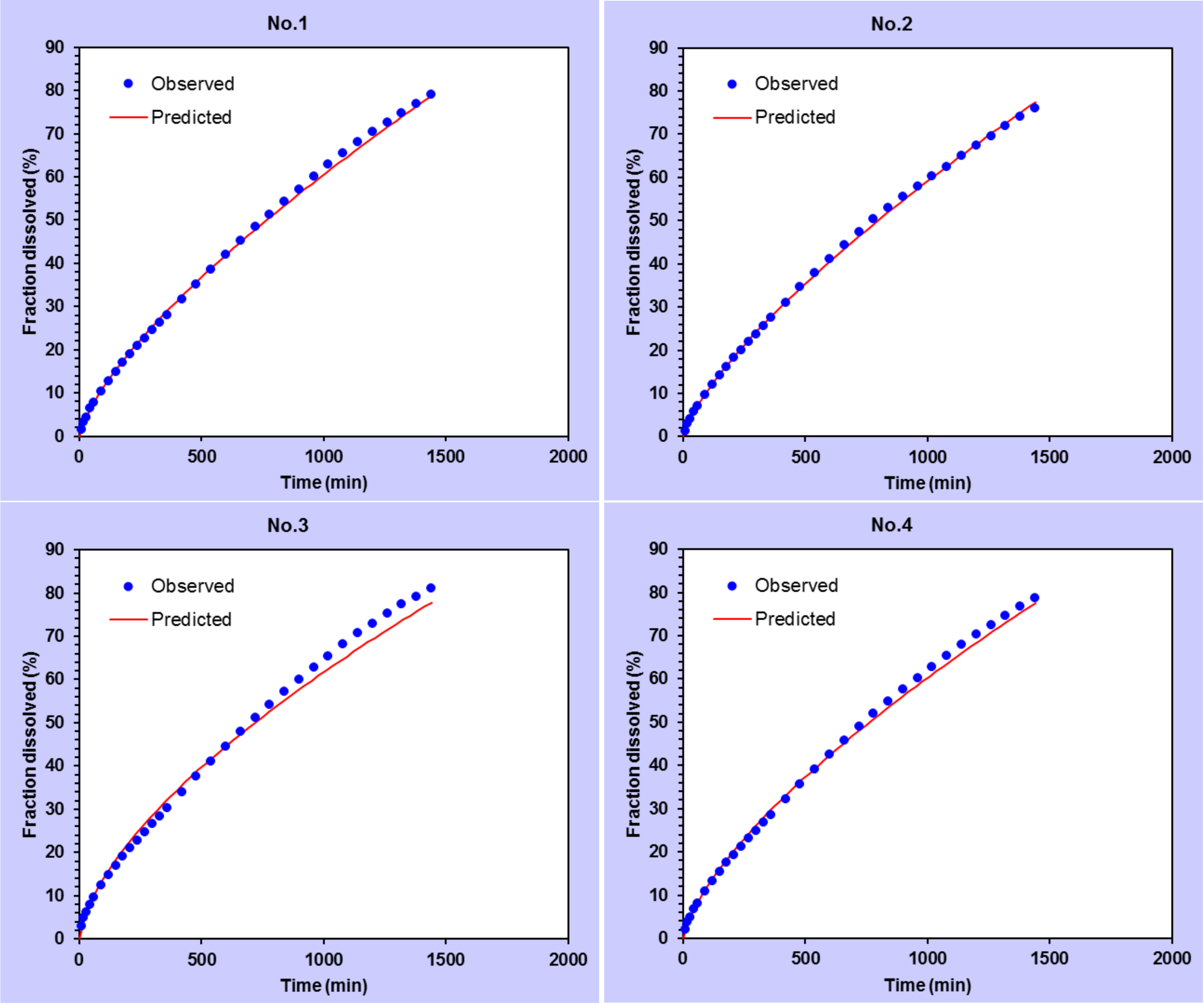

Supplementary materials – Model fitting summary – Avicel® PH-102 (modelling the entire relevant dissolution data)

Model: **Korsmeyer–Peppas with F_0**

Model equation: $F = F_0 + k_{KP} \cdot t^n$

Fitted model parameters per tested tablet (N = 4) with statistics – mean, standard deviation (SD), and relative standard deviation expressed in % (RSD%) (output from DDSolver):

Parameter	No.1	No.2	No.3	No.4	Mean	SD	RSD(%)
k_{KP}	0.226	0.196	0.384	0.288	0.274	0.083	30.380
n	0.813	0.831	0.738	0.772	0.789	0.042	5.293
F_0	0.640	0.520	1.200	0.960	0.830	0.309	37.200

Number of dissolution data points (N), degrees of freedom (df), and selected goodness of fit criteria – Pearson correlation coefficient (R), coefficient of determination (R^2), adjusted coefficient of determination ($R^2_{adjusted}$), and residual sum of squares (RSS) (manual calculation in MS Excel):

Parameter	No.1	No.2	No.3	No.4
N	33	33	33	33
df	30	30	30	30
R	0.999288944	0.998401003	0.999665252	0.999546174
R^2	0.998578394	0.996804563	0.999330616	0.999092553
$R^2_{adjusted}$	0.998483621	0.996591534	0.99928599	0.999032057
RSS	104.6270991	202.4531919	16.39963905	29.50352956

Graphical abstract of model fit presented as mean ± 1 SD of the fraction % of released carvedilol:

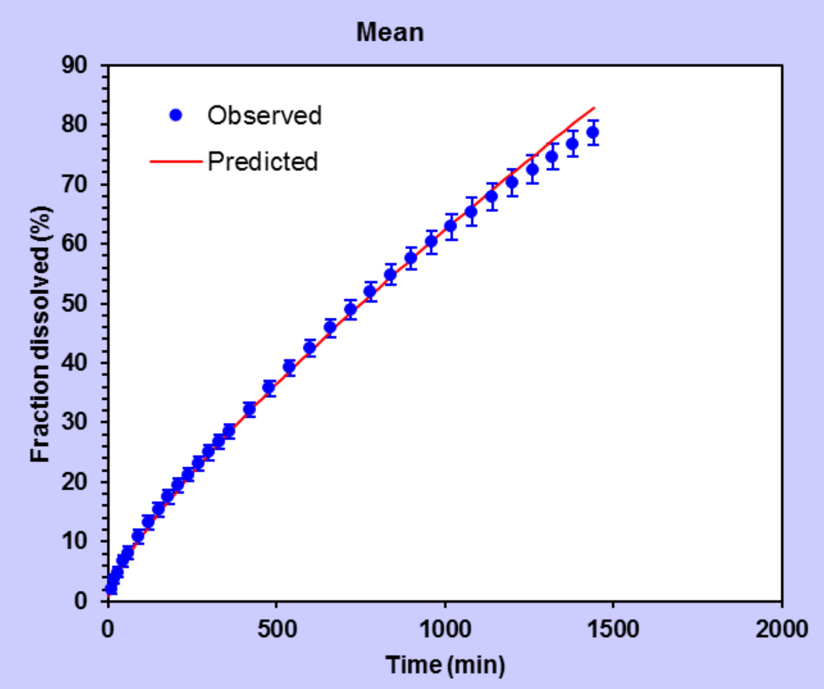

Supplementary materials – Model fitting summary – Avicel® PH-102 (modelling the entire relevant dissolution data)

Graphical abstract of model fit presented as the fraction % of released carvedilol per tested tablet:

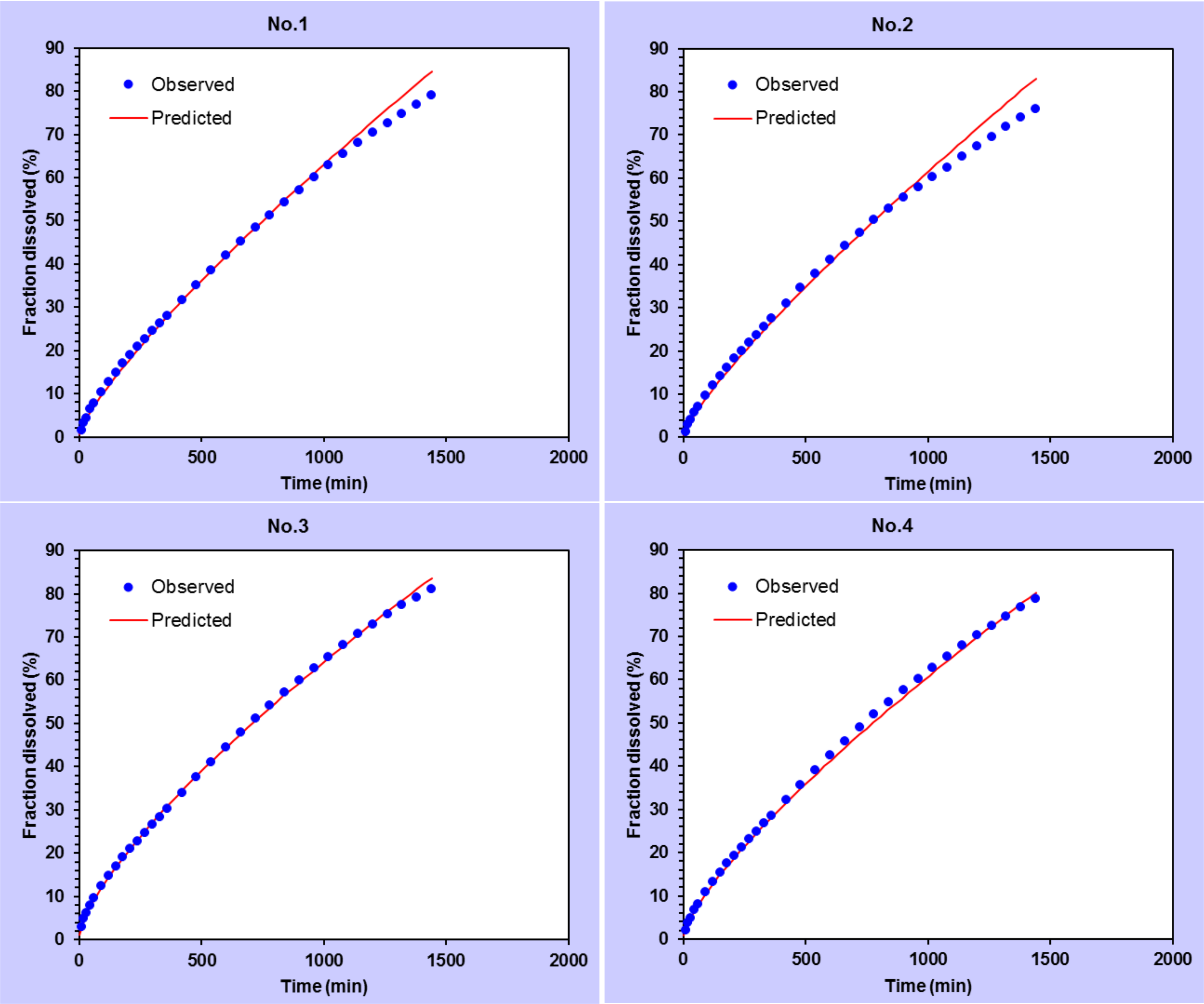

Supplementary materials – Model fitting summary – Avicel® PH-102 (modelling the entire relevant dissolution data)

Model: **Hixson–Crowell**

Model equation: $F = 100 \cdot [1 - (1 - k_{HC} \cdot t)^3]$

Fitted model parameters per tested tablet (N = 4) with statistics – mean, standard deviation (SD), and relative standard deviation expressed in % (RSD%) (output from DDSolver):

Parameter	No.1	No.2	No.3	No.4	Mean	SD	RSD(%)
k_{HC}	0.0003	0.0003	0.0003	0.0003	0.0003	0.0000	4.8223

Number of dissolution data points (N), degrees of freedom (df), and selected goodness of fit criteria – Pearson correlation coefficient (R), coefficient of determination (R^2), adjusted coefficient of determination ($R^2_{adjusted}$), and residual sum of squares (RSS) (manual calculation in MS Excel):

Parameter	No.1	No.2	No.3	No.4
N	33	33	33	33
df	32	32	32	32
R	0.999507425	0.999726782	0.999461248	0.999644287
R^2	0.999015093	0.999453638	0.998922786	0.999288701
$R^2_{adjusted}$	0.999015093	0.999453638	0.998922786	0.999288701
RSS	77.7113861	80.40200041	177.9961136	104.9465027

Graphical abstract of model fit presented as mean ± 1 SD of the fraction % of released carvedilol:

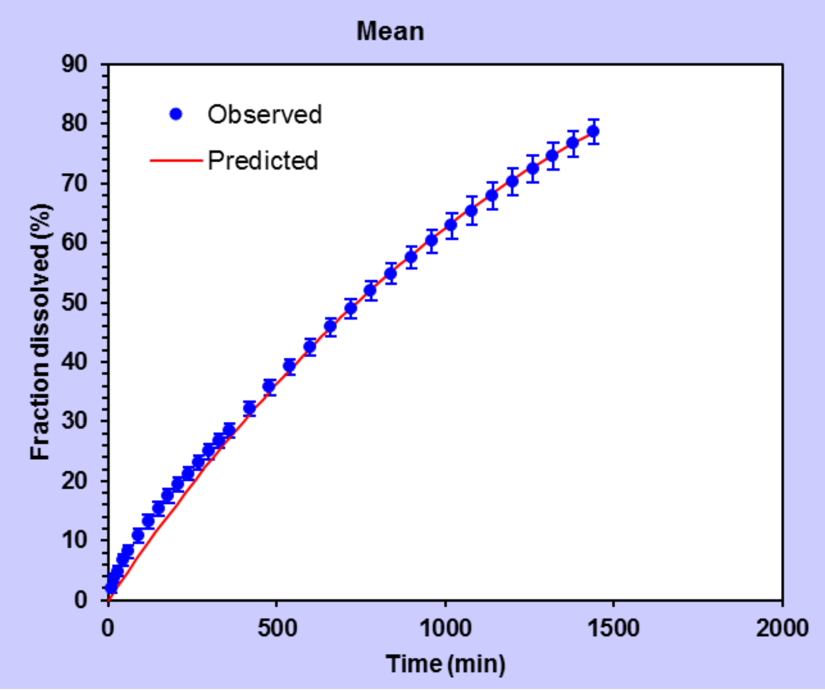

Supplementary materials – Model fitting summary – Avicel® PH-102 (modelling the entire relevant dissolution data)

Graphical abstract of model fit presented as the fraction % of released carvedilol per tested tablet:

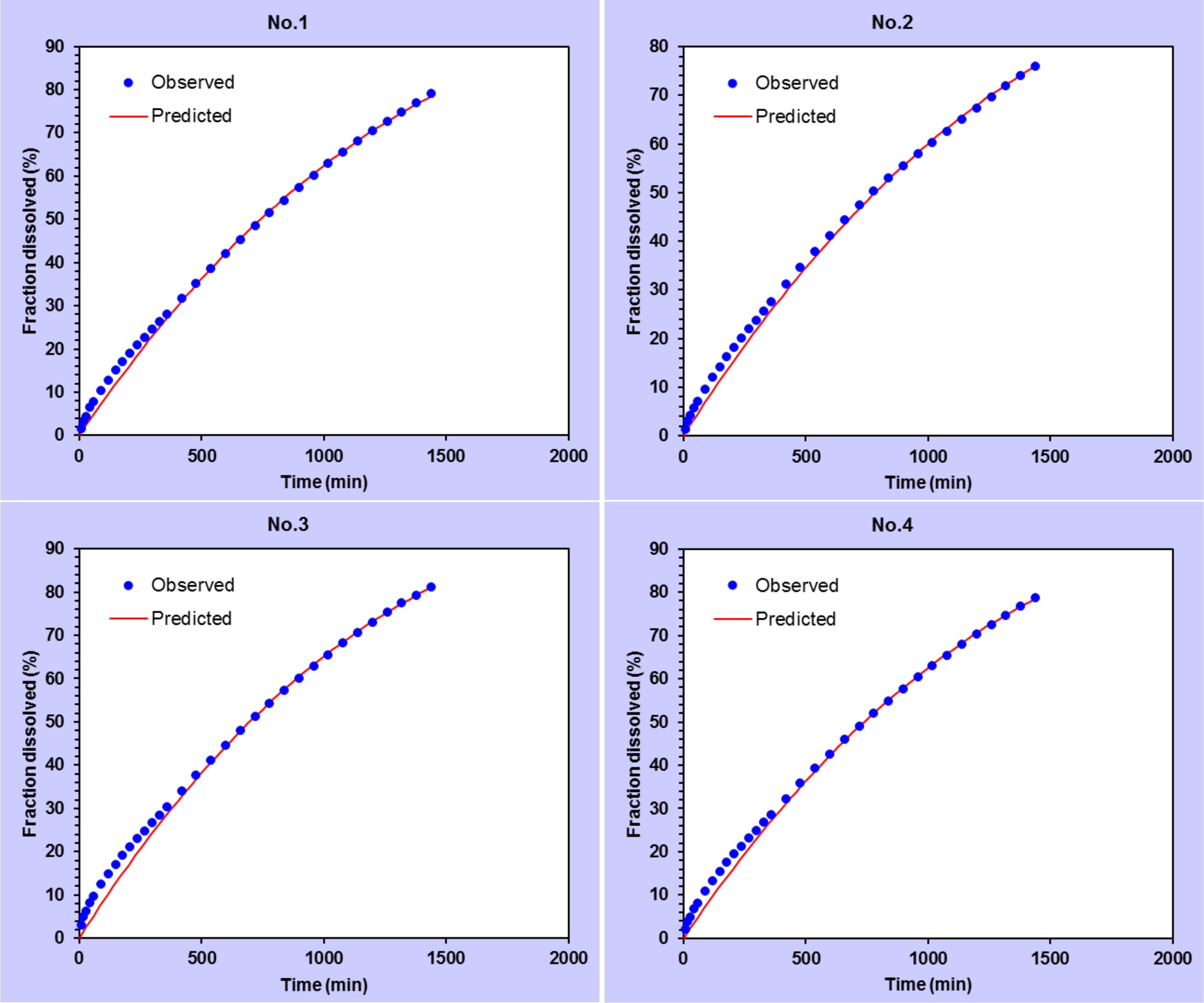

Supplementary materials – Model fitting summary – Avicel® PH-102 (modelling the entire relevant dissolution data)

Model: **Hixson–Crowell with T_{lag}**

Model equation: $F = 100 \cdot \left\{1 - \left[1 - k_{HC} \cdot \left(t - T_{lag}\right)\right]^3\right\}$

Fitted model parameters per tested tablet (N = 4) with statistics – mean, standard deviation (SD), and relative standard deviation expressed in % (RSD%) (output from DDSolver):

Parameter	No.1	No.2	No.3	No.4	Mean	SD	RSD(%)
k_{HC}	0.0003	0.0003	0.0003	0.0003	0.0003	0.0000	4.4354
T_{lag}	-27.2735	-35.9850	-45.2353	-36.6349	-36.2822	7.3377	-20.2240

Number of dissolution data points (N), degrees of freedom (df), and selected goodness of fit criteria – Pearson correlation coefficient (R), coefficient of determination (R^2), adjusted coefficient of determination ($R^2_{adjusted}$), and residual sum of squares (RSS) (manual calculation in MS Excel):

Parameter	No.1	No.2	No.3	No.4
N	33	33	33	33
df	31	31	31	31
R	0.99955836	0.999705285	0.999546888	0.999680568
R^2	0.999116916	0.999410658	0.999093982	0.999361238
$R^2_{adjusted}$	0.999088429	0.999391646	0.999064756	0.999340633
RSS	18.40173468	10.92480268	19.23406584	12.72663803

Graphical abstract of model fit presented as mean ± 1 SD of the fraction % of released carvedilol:

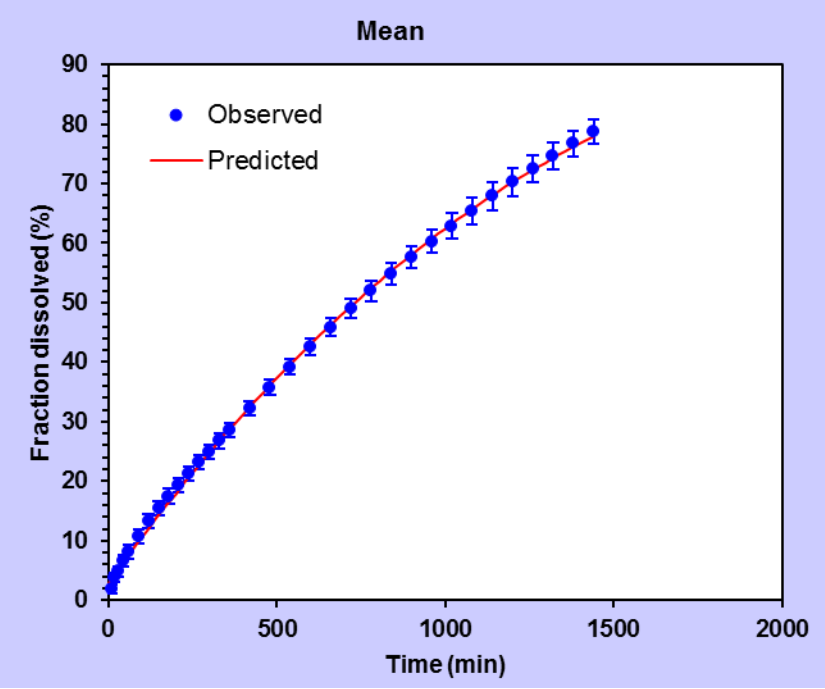

Supplementary materials – Model fitting summary – Avicel® PH-102 (modelling the entire relevant dissolution data)

Graphical abstract of model fit presented as the fraction % of released carvedilol per tested tablet:

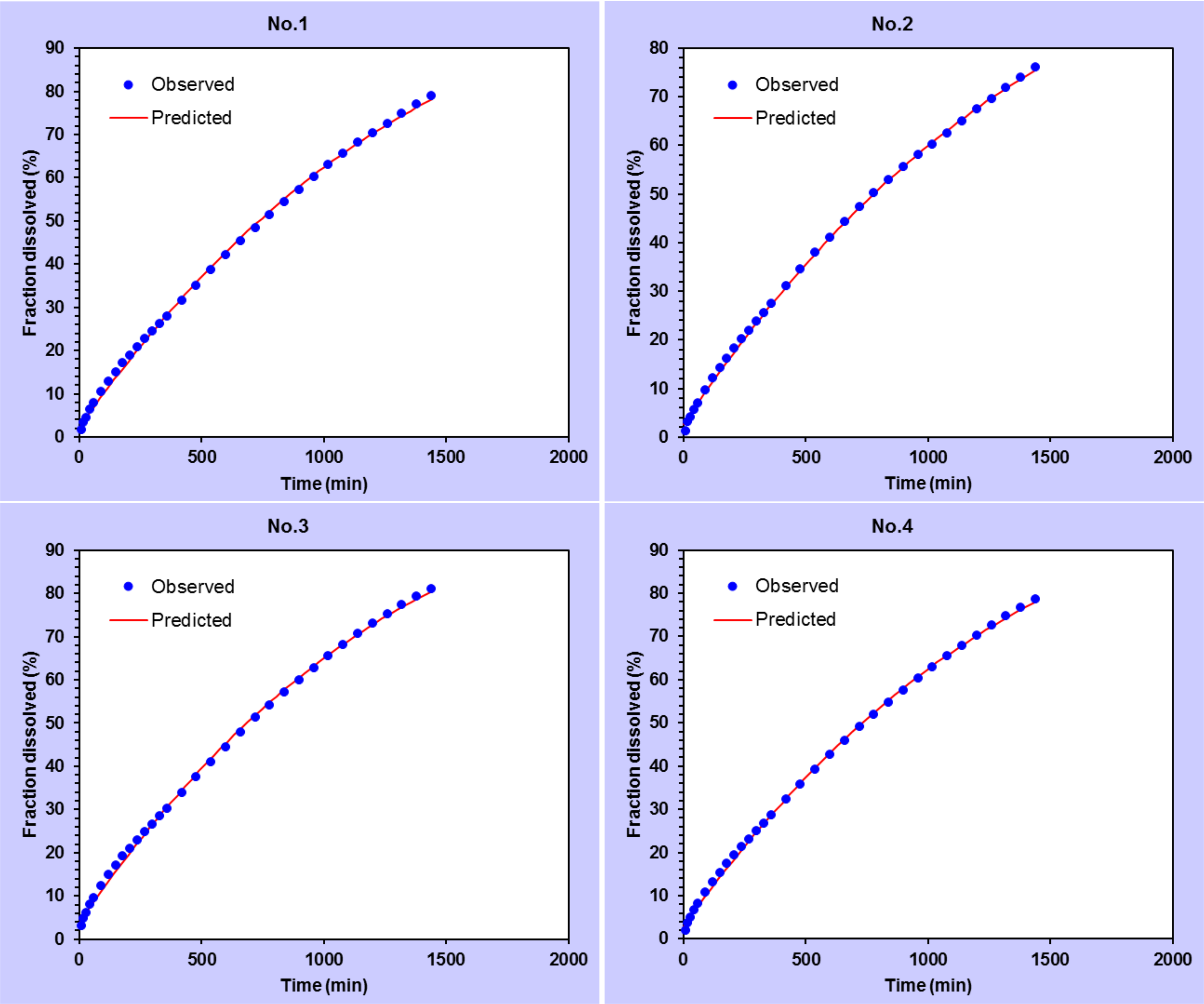

Supplementary materials – Model fitting summary – Avicel® PH-102 (modelling the entire relevant dissolution data)

Model: **Hopfenberg**

Model equation: $F = 100 \cdot [1 - (1 - k_{HB} \cdot t)^n]$

Fitted model parameters per tested tablet (N = 4) with statistics – mean, standard deviation (SD), and relative standard deviation expressed in % (RSD%) (output from DDSolver):

Parameter	No.1	No.2	No.3	No.4	Mean	SD	RSD(%)
k_{HB}	0.0002	0.0003	0.0003	0.0003	0.0003	0.0000	10.5245
n	3.7207	3.0000	3.0000	3.0000	3.1802	0.3604	11.3312

Number of dissolution data points (N), degrees of freedom (df), and selected goodness of fit criteria – Pearson correlation coefficient (R), coefficient of determination (R^2), adjusted coefficient of determination ($R^2_{adjusted}$), and residual sum of squares (RSS) (manual calculation in MS Excel):

Parameter	No.1	No.2	No.3	No.4
N	33	33	33	33
df	31	31	31	31
R	0.999316992	0.999726782	0.999461248	0.999644287
R^2	0.998634451	0.999453638	0.998922786	0.999288701
$R^2_{adjusted}$	0.998590401	0.999436013	0.998888037	0.999265756
RSS	69.55664421	80.40200041	177.9961136	104.9465027

Graphical abstract of model fit presented as mean ± 1 SD of the fraction % of released carvedilol:

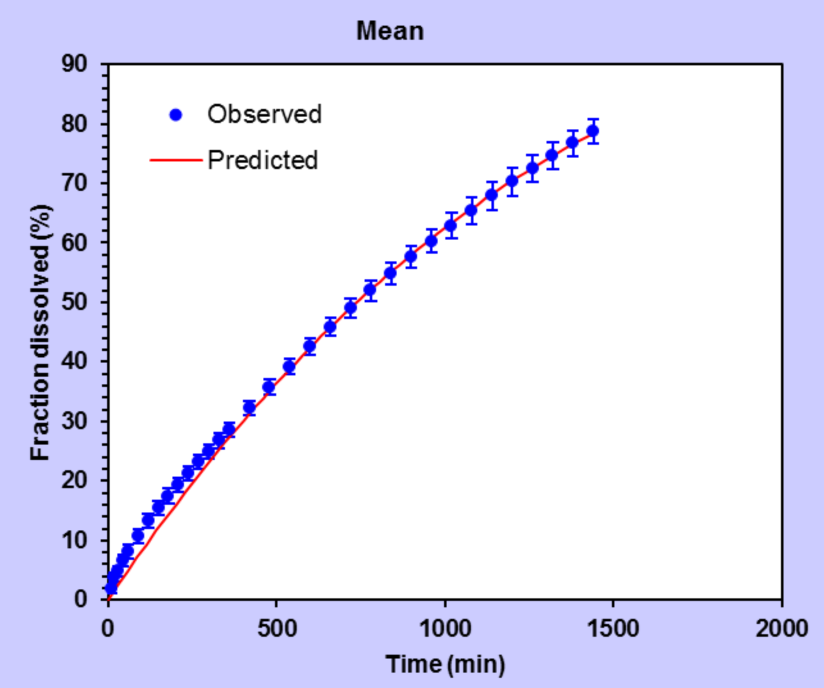

Supplementary materials – Model fitting summary – Avicel® PH-102 (modelling the entire relevant dissolution data)

Graphical abstract of model fit presented as the fraction % of released carvedilol per tested tablet:

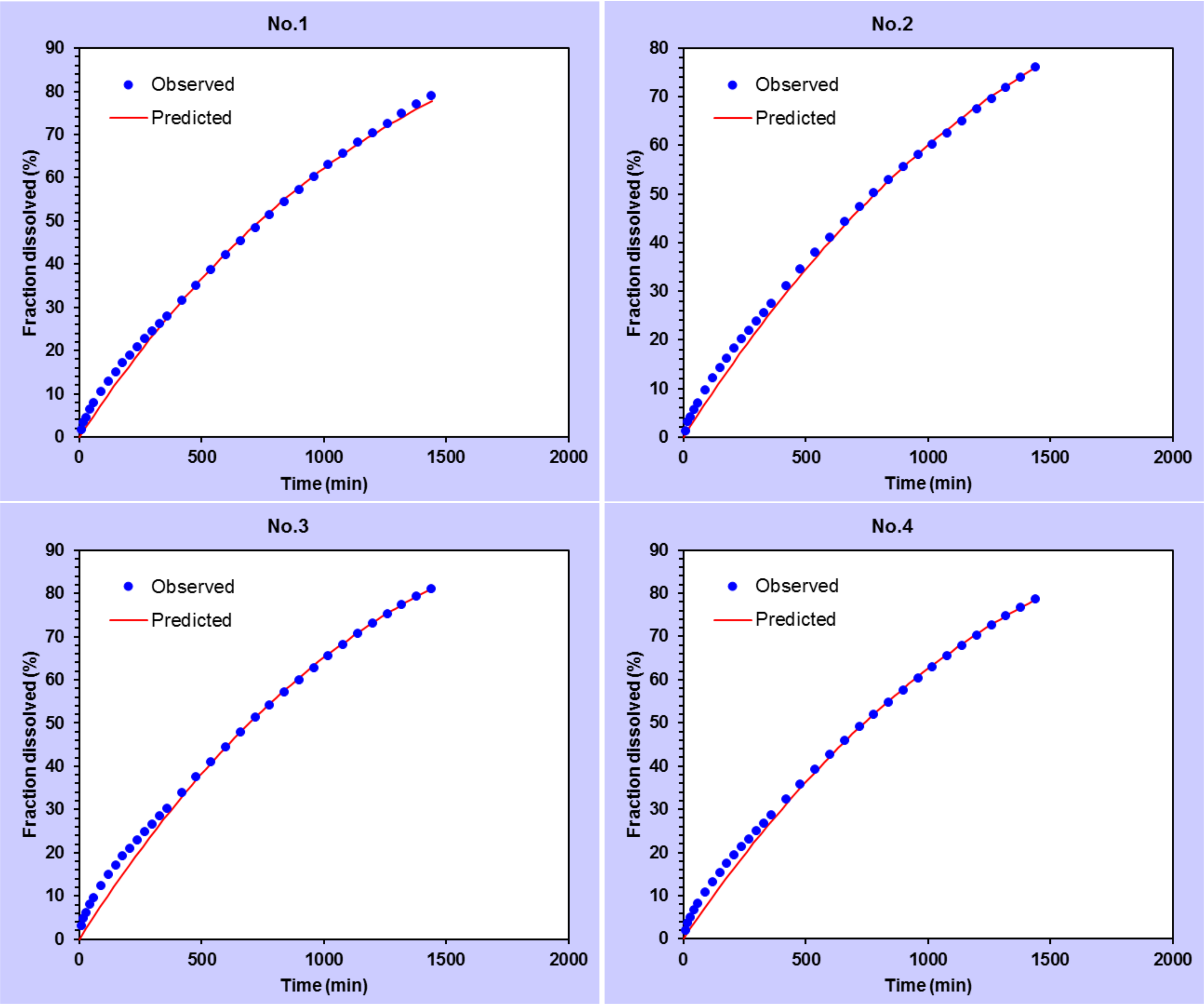

Supplementary materials – Model fitting summary – Avicel® PH-102 (modelling the entire relevant dissolution data)

Model: **Hopfenberg with T_{lag}**

Model equation: $F = 100 \cdot \{1 - [1 - k_{HB} \cdot (t - T_{lag})]^n\}$

Fitted model parameters per tested tablet (N = 4) with statistics – mean, standard deviation (SD), and relative standard deviation expressed in % (RSD%) (output from DDSolver):

Parameter	No.1	No.2	No.3	No.4	Mean	SD	RSD(%)
k_{HB}	0.0004	0.0003	0.0003	0.0003	0.0003	0.0000	16.9578
n	2.0000	3.0000	3.0000	3.0000	2.7500	0.5000	18.1818
T_{lag}	-50.7044	-35.9850	-45.2353	-36.6349	-42.1399	7.0974	-16.8425

Number of dissolution data points (N), degrees of freedom (df), and selected goodness of fit criteria – Pearson correlation coefficient (R), coefficient of determination (R^2), adjusted coefficient of determination ($R^2_{adjusted}$), and residual sum of squares (RSS) (manual calculation in MS Excel):

Parameter	No.1	No.2	No.3	No.4
N	33	33	33	33
df	30	30	30	30
R	0.999551032	0.999705285	0.999546888	0.999680568
R^2	0.999102266	0.999410658	0.999093982	0.999361238
$R^2_{adjusted}$	0.999042417	0.999371368	0.999033581	0.999318654
RSS	18.01254945	10.92480268	19.23406584	12.72663803

Graphical abstract of model fit presented as mean ± 1 SD of the fraction % of released carvedilol:

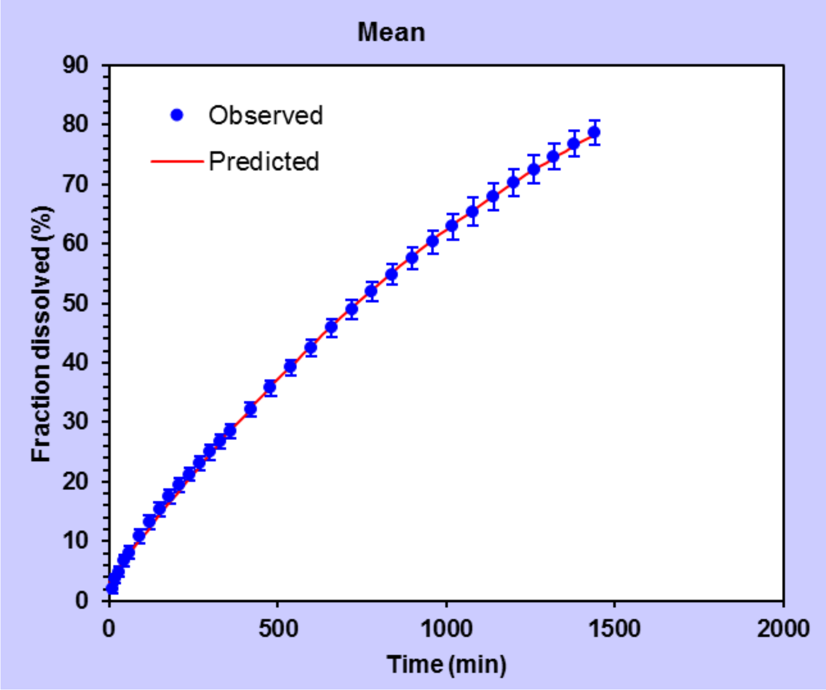

Supplementary materials – Model fitting summary – Avicel® PH-102 (modelling the entire relevant dissolution data)

Graphical abstract of model fit presented as the fraction % of released carvedilol per tested tablet:

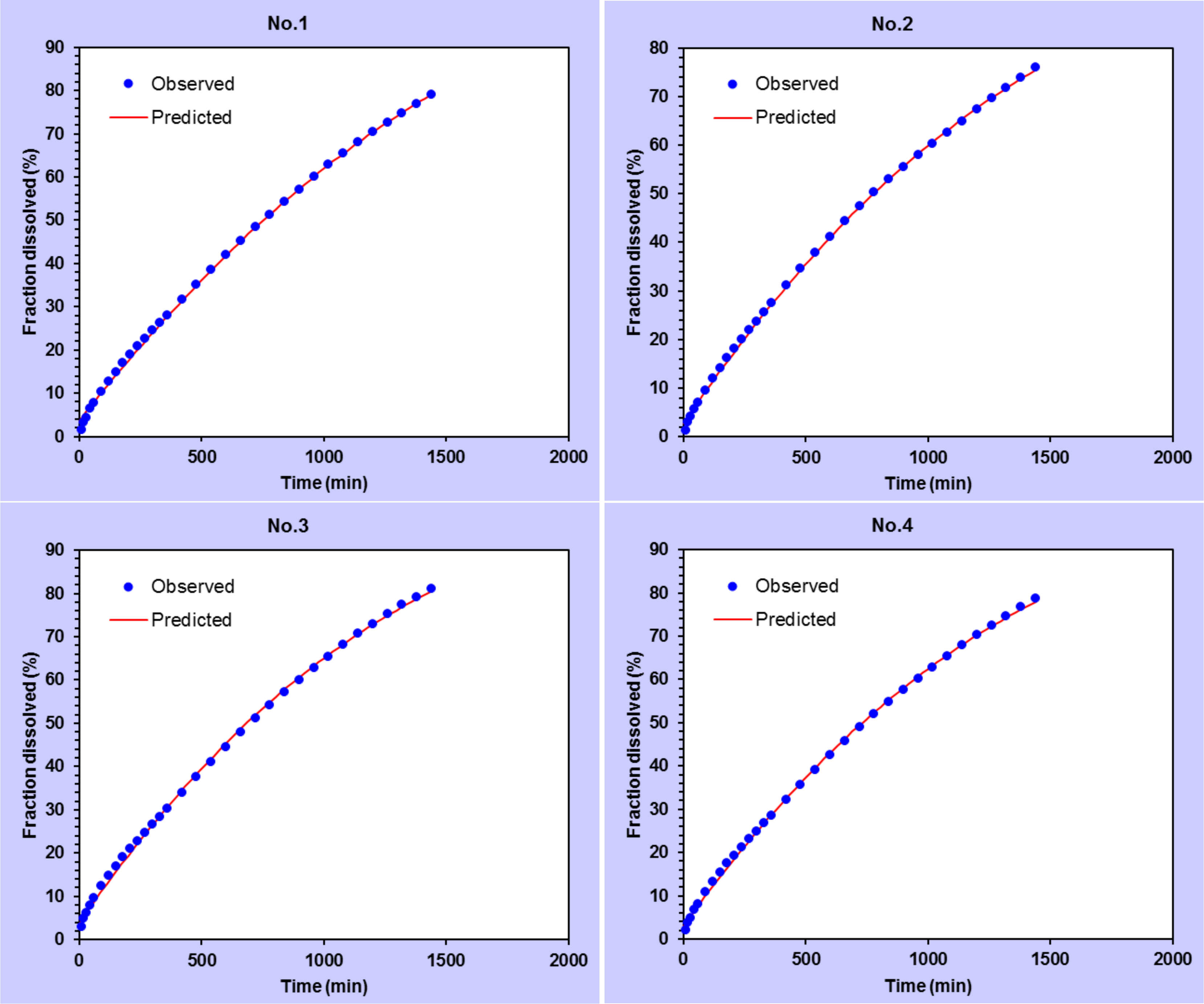

Supplementary materials – Model fitting summary – Avicel® PH-102 (modelling the entire relevant dissolution data)

Model: **Baker–Lonsdale**

Model equation: $\frac{3}{2}\cdot\left[1-\left(1-\frac{F}{100}\right)^{\frac{2}{3}}\right]-\frac{F}{100}=k_{BL}\cdot t$

Fitted model parameters per tested tablet (N = 4) with statistics – mean, standard deviation (SD), and relative standard deviation expressed in % (RSD%) (output from DDSolver):

Parameter	No.1	No.2	No.3	No.4	Mean	SD	RSD(%)
k_{BL}	0.0001	0.0001	0.0001	0.0001	0.0001	0.0000	8.2336

Number of dissolution data points (N), degrees of freedom (df), and selected goodness of fit criteria – Pearson correlation coefficient (R), coefficient of determination (R^2), adjusted coefficient of determination ($R^2_{adjusted}$), and residual sum of squares (RSS) (manual calculation in MS Excel):

Parameter	No.1	No.2	No.3	No.4
N	33	33	33	33
df	32	32	32	32
R	0.981549609	0.984858883	0.981873283	0.982837935
R^2	0.963439634	0.96994702	0.964075145	0.965970406
$R^2_{adjusted}$	0.963439634	0.96994702	0.964075145	0.965970406
RSS	4635.132475	3939.087029	4254.340646	4259.585619

Graphical abstract of model fit presented as mean ± 1 SD of the fraction % of released carvedilol:

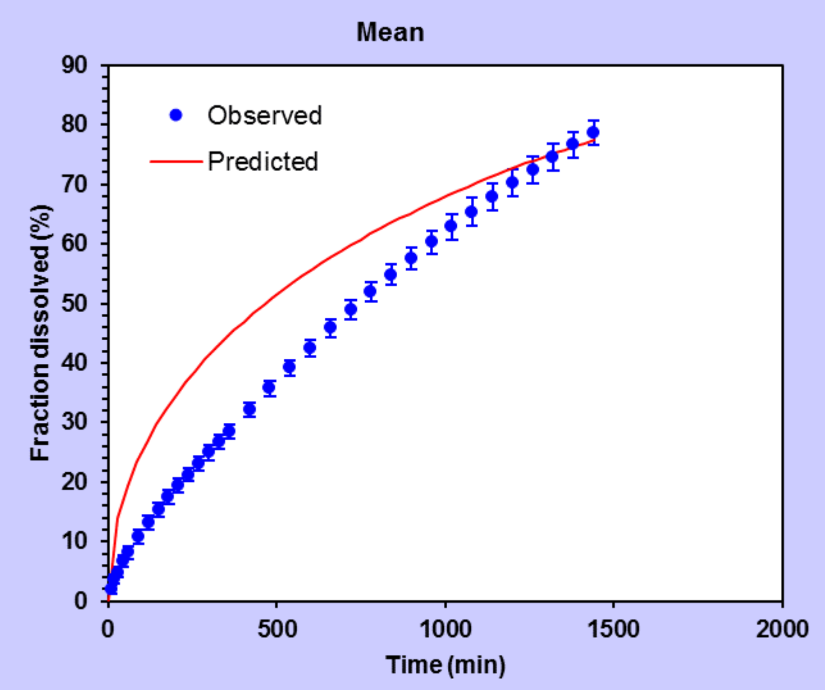

Supplementary materials – Model fitting summary – Avicel® PH-102 (modelling the entire relevant dissolution data)

Graphical abstract of model fit presented as the fraction % of released carvedilol per tested tablet:

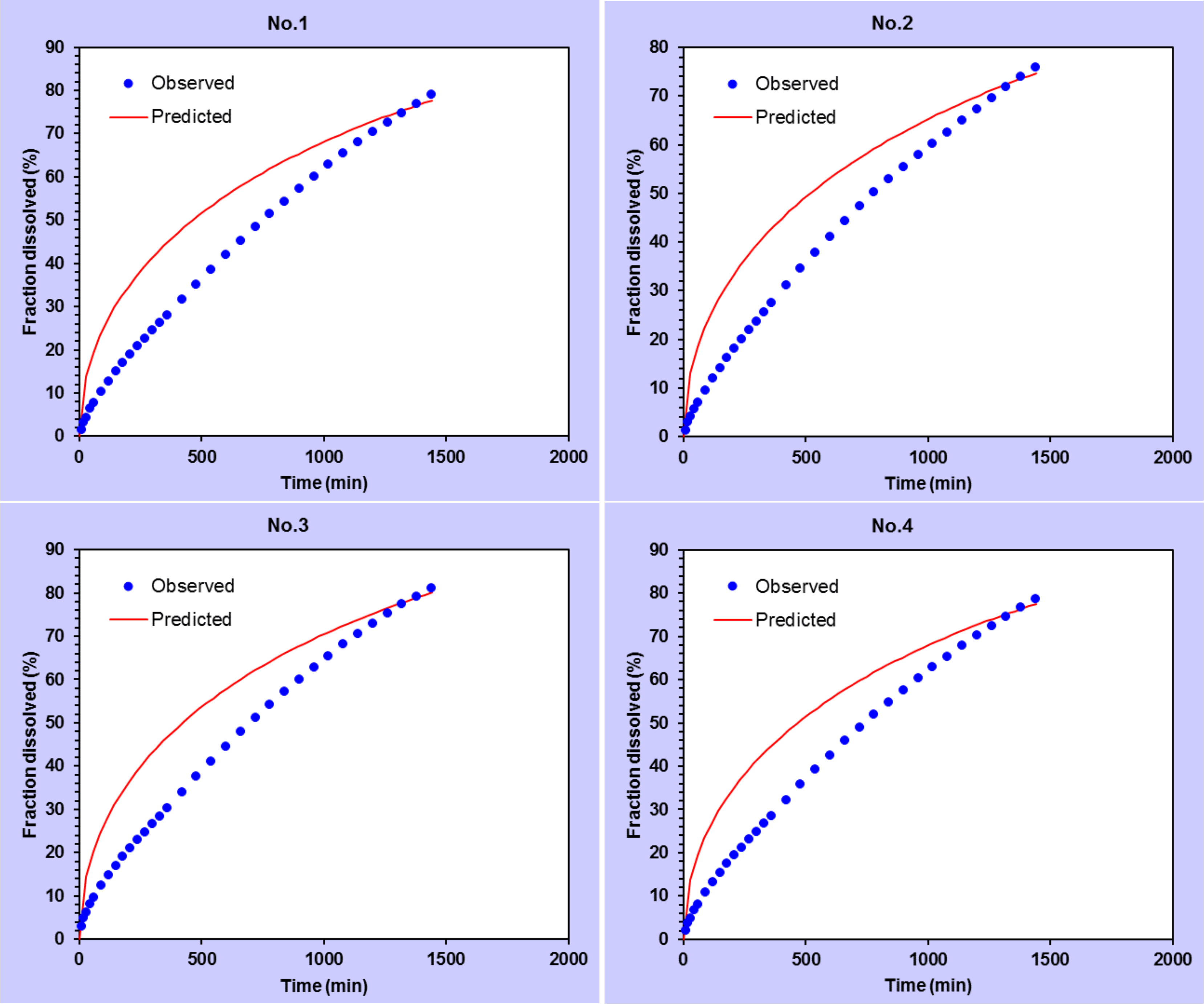

Supplementary materials – Model fitting summary – Avicel® PH-102 (modelling the entire relevant dissolution data)

Model: **Baker–Lonsdale with T_{lag}**

Model equation: $\frac{3}{2} \cdot \left[1 - \left(1 - \frac{F}{100}\right)^{\frac{2}{3}}\right] - \frac{F}{100} = k_{BL} \cdot (t - T_{lag})$

Fitted model parameters per tested tablet (N = 4) with statistics – mean, standard deviation (SD), and relative standard deviation expressed in % (RSD%) (output from DDSolver):

Parameter	No.1	No.2	No.3	No.4	Mean	SD	RSD(%)
k_{BL}	0.0001	0.0001	0.0001	0.0001	0.0001	0.0000	8.2336
T_{lag}	155.1442	148.7736	147.1455	150.2672	150.3326	3.4518	2.2961

Number of dissolution data points (N), degrees of freedom (df), and selected goodness of fit criteria – Pearson correlation coefficient (R), coefficient of determination (R^2), adjusted coefficient of determination ($R^2_{adjusted}$), and residual sum of squares (RSS) (manual calculation in MS Excel):

Parameter	No.1	No.2	No.3	No.4
N	33	33	33	33
df	31	31	31	31
R	0.976315167	0.980525808	0.976879043	0.976847668
R^2	0.953191305	0.96143086	0.954292665	0.954231367
$R^2_{adjusted}$	0.951681347	0.960186695	0.952818235	0.95275496
RSS	1202.988564	903.1742502	1284.009932	1186.285918

Graphical abstract of model fit presented as mean ± 1 SD of the fraction % of released carvedilol:

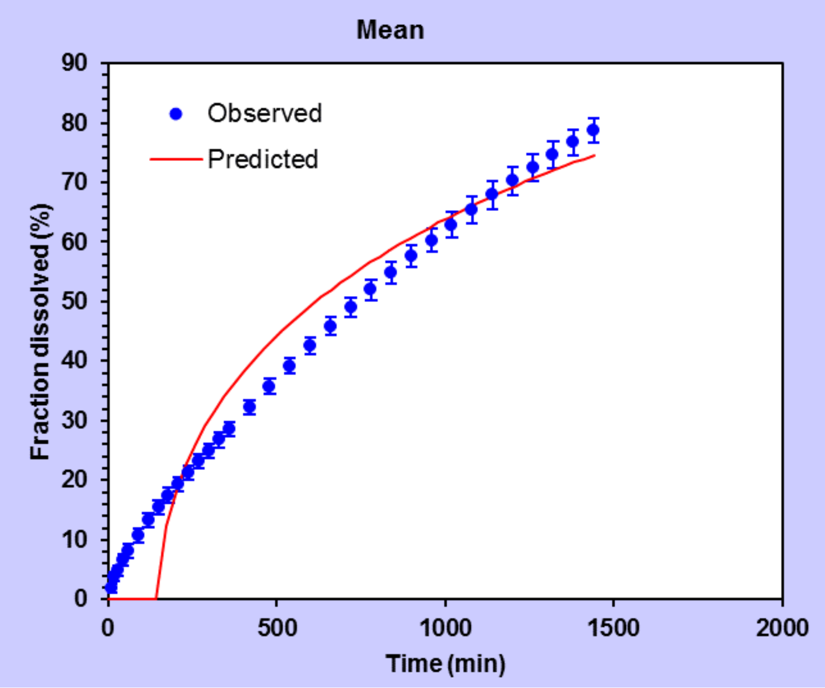

Supplementary materials – Model fitting summary – Avicel® PH-102 (modelling the entire relevant dissolution data)

Graphical abstract of model fit presented as the fraction % of released carvedilol per tested tablet:

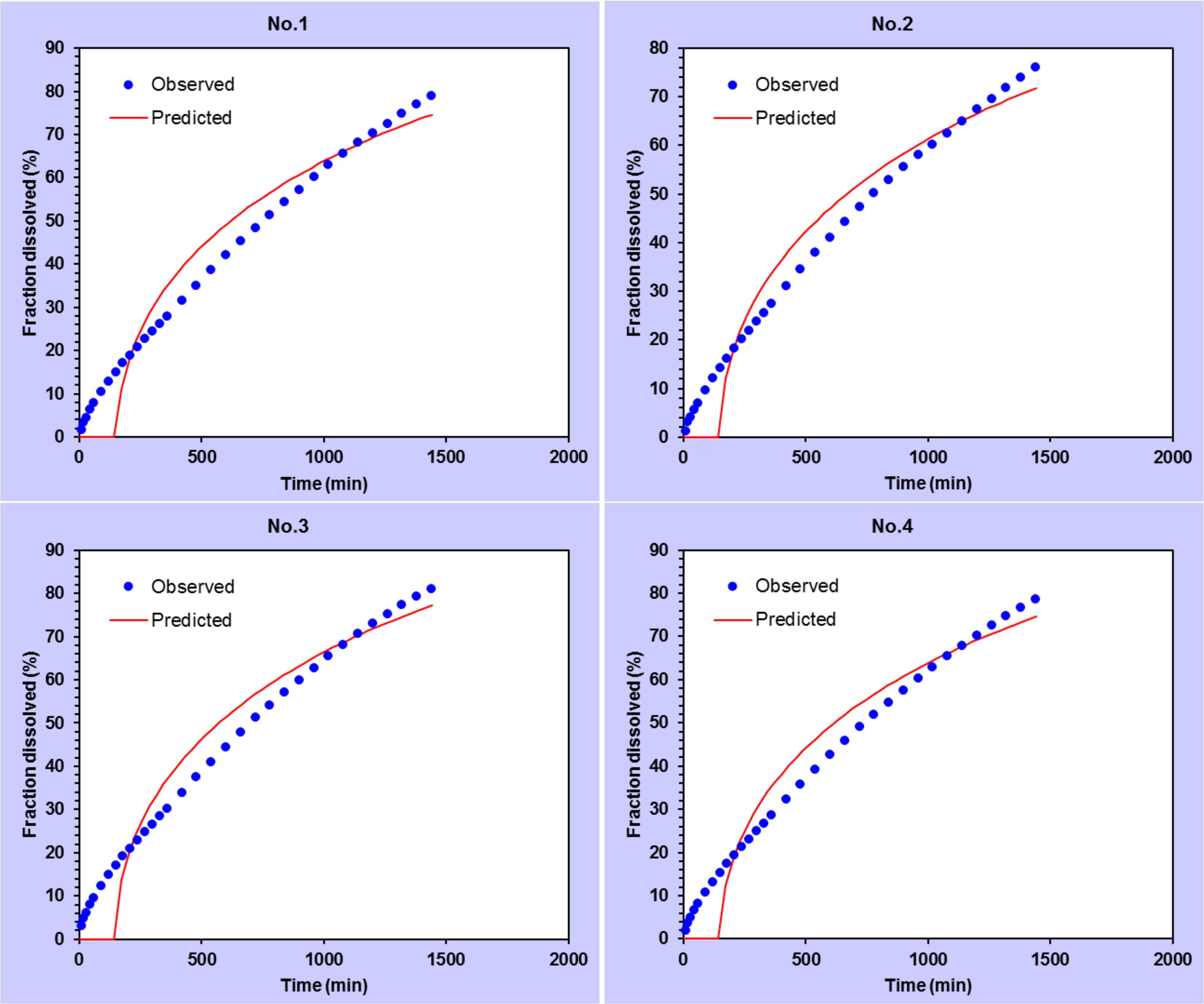

Supplementary materials – Model fitting summary – Avicel® PH-102 (modelling the entire relevant dissolution data)

Model: **Makoid–Banakar**

Model equation: $F = k_{MB} \cdot t^n \cdot e^{-k \cdot t}$

Fitted model parameters per tested tablet (N = 4) with statistics – mean, standard deviation (SD), and relative standard deviation expressed in % (RSD%) (output from DDSolver):

Parameter	No.1	No.2	No.3	No.4	Mean	SD	RSD(%)
k_{MB}	0.309816	0.238540	0.697454	0.399097	0.411227	0.201807	49.074329
n	0.773653	0.818460	0.635886	0.729111	0.739278	0.077984	10.548728
k	0.000056	0.000135	-0.000118	0.000001	0.000019	0.000107	575.229331

Number of dissolution data points (N), degrees of freedom (df), and selected goodness of fit criteria – Pearson correlation coefficient (R), coefficient of determination (R^2), adjusted coefficient of determination ($R^2_{adjusted}$), and residual sum of squares (RSS) (manual calculation in MS Excel):

Parameter	No.1	No.2	No.3	No.4
N	33	33	33	33
df	30	30	30	30
R	0.999834124	0.99990598	0.999206185	0.999780589
R^2	0.999668276	0.999811969	0.998413001	0.999561227
$R^2_{adjusted}$	0.999646161	0.999799434	0.998307201	0.999531975
RSS	6.911176964	3.787365236	32.69178568	8.604118666

Graphical abstract of model fit presented as mean ± 1 SD of the fraction % of released carvedilol:

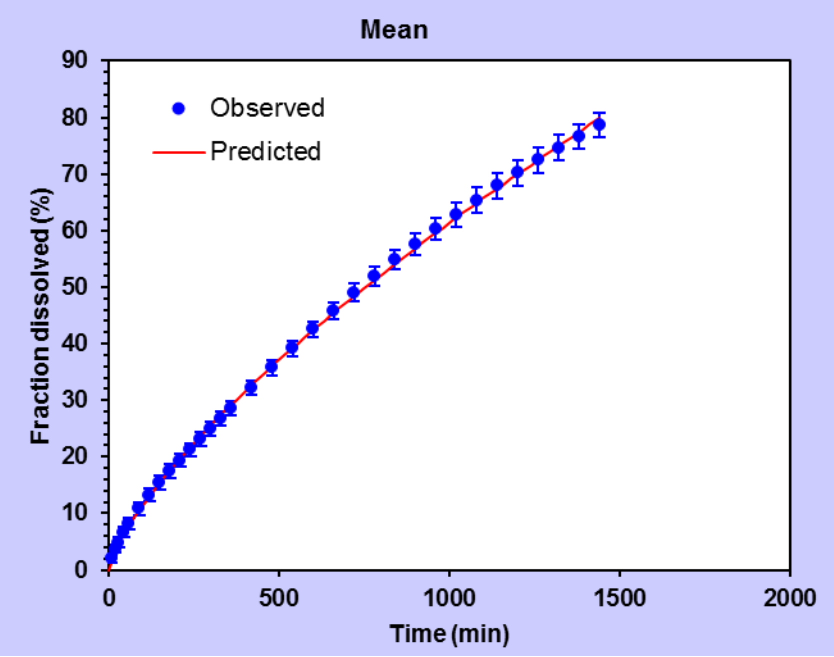

Supplementary materials – Model fitting summary – Avicel® PH-102 (modelling the entire relevant dissolution data)

Graphical abstract of model fit presented as the fraction % of released carvedilol per tested tablet:

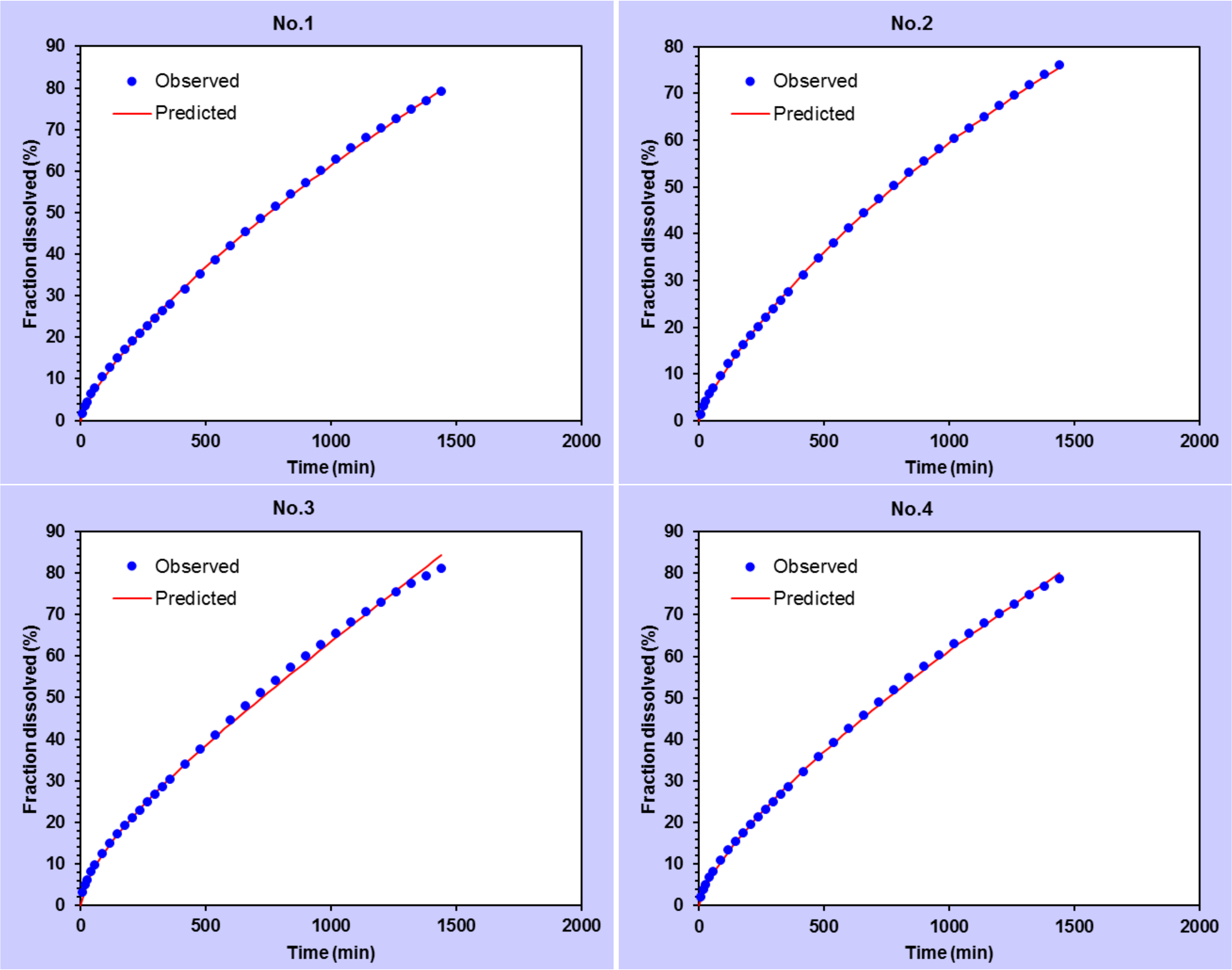

Supplementary materials – Model fitting summary – Avicel® PH-102 (modelling the entire relevant dissolution data)

Model: **Makoid–Banakar with T_{lag}**

Model equation: $F = k_{MB} \cdot (t - T_{lag})^n \cdot e^{-k \cdot (t - T_{lag})}$

Fitted model parameters per tested tablet (N = 4) with statistics – mean, standard deviation (SD), and relative standard deviation expressed in % (RSD%) (output from DDSolver):

Parameter	No.1	No.2	No.3	No.4	Mean	SD	RSD(%)
k_{MB}	0.466243	0.366879	0.986108	0.589520	0.602188	0.271665	45.113073
n	0.695207	0.735847	0.569132	0.654081	0.663567	0.071259	10.738743
k	-0.000075	-0.000002	-0.000232	-0.000125	-0.000108	0.000096	-88.950437
T_{lag}	4.000000	4.000000	4.000000	4.000000	4.000000	0.000000	0.000000

Number of dissolution data points (N), degrees of freedom (df), and selected goodness of fit criteria – Pearson correlation coefficient (R), coefficient of determination (R^2), adjusted coefficient of determination ($R^2_{adjusted}$), and residual sum of squares (RSS) (manual calculation in MS Excel):

Parameter	No.1	No.2	No.3	No.4
N	33	33	33	33
df	29	29	29	29
R	0.999556299	0.999696215	0.998154599	0.999132403
R^2	0.999112795	0.999392523	0.996312604	0.998265559
$R^2_{adjusted}$	0.999021016	0.99932968	0.995931149	0.998086134
RSS	17.7868606	11.33796642	78.23087922	34.93918571

Graphical abstract of model fit presented as mean ± 1 SD of the fraction % of released carvedilol:

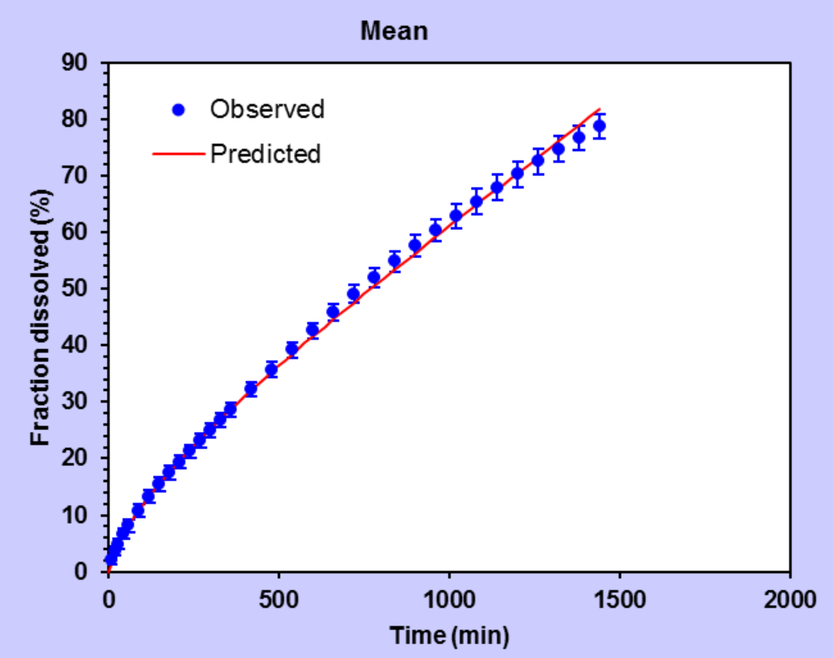

Supplementary materials – Model fitting summary – Avicel® PH-102 (modelling the entire relevant dissolution data)

Graphical abstract of model fit presented as the fraction % of released carvedilol per tested tablet:

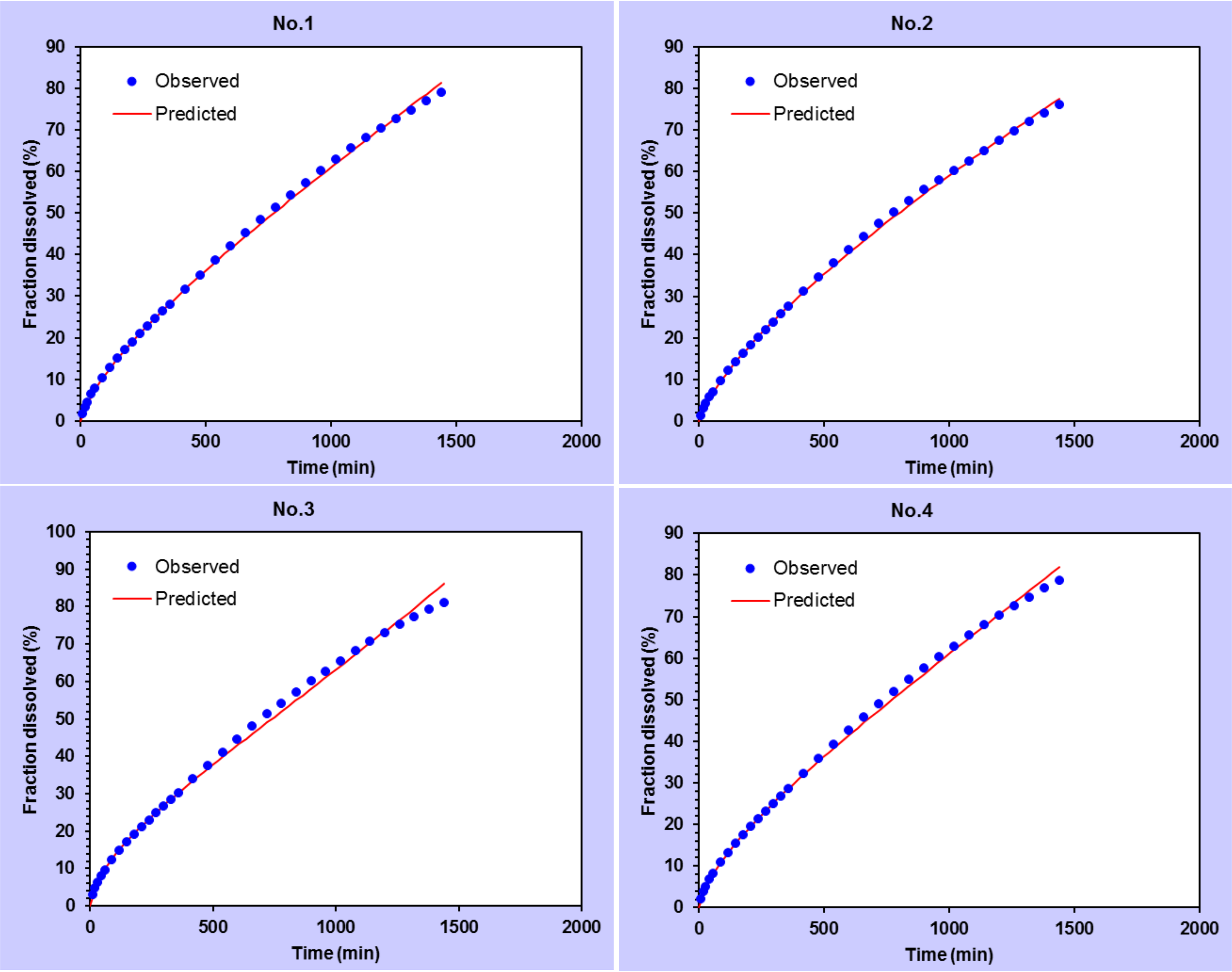

Supplementary materials – Model fitting summary – Avicel® PH-102 (modelling the entire relevant dissolution data)

Model: **Peppas–Sahlin_1**

Model equation: $F = k_1 \cdot t^m + k_2 \cdot t^{2m}$

Fitted model parameters per tested tablet (N = 4) with statistics – mean, standard deviation (SD), and relative standard deviation expressed in % (RSD%) (output from DDSolver):

Parameter	No.1	No.2	No.3	No.4	Mean	SD	RSD(%)
k_1	0.767	0.775	1.045	0.862	0.862	0.129	15.002
k_2	0.087	0.083	0.080	0.083	0.083	0.003	3.582
m	0.450	0.450	0.450	0.450	0.450	0.000	0.000

Number of dissolution data points (N), degrees of freedom (df), and selected goodness of fit criteria – Pearson correlation coefficient (R), coefficient of determination (R^2), adjusted coefficient of determination ($R^2_{adjusted}$), and residual sum of squares (RSS) (manual calculation in MS Excel):

Parameter	No.1	No.2	No.3	No.4
N	33	33	33	33
df	30	30	30	30
R	0.999511098	0.999148992	0.999503874	0.999444787
R^2	0.999022434	0.998298708	0.999007993	0.998889883
$R^2_{adjusted}$	0.998957263	0.998185288	0.99894186	0.998815875
RSS	20.86465337	34.55603638	20.77515397	23.26860042

Graphical abstract of model fit presented as mean ± 1 SD of the fraction % of released carvedilol:

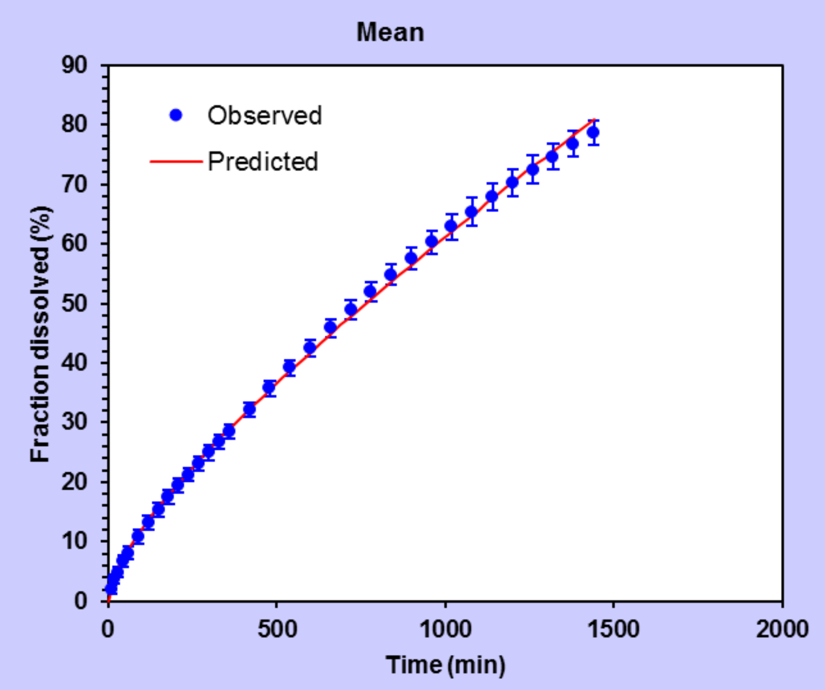

Supplementary materials – Model fitting summary – Avicel® PH-102 (modelling the entire relevant dissolution data)

Graphical abstract of model fit presented as the fraction % of released carvedilol per tested tablet:

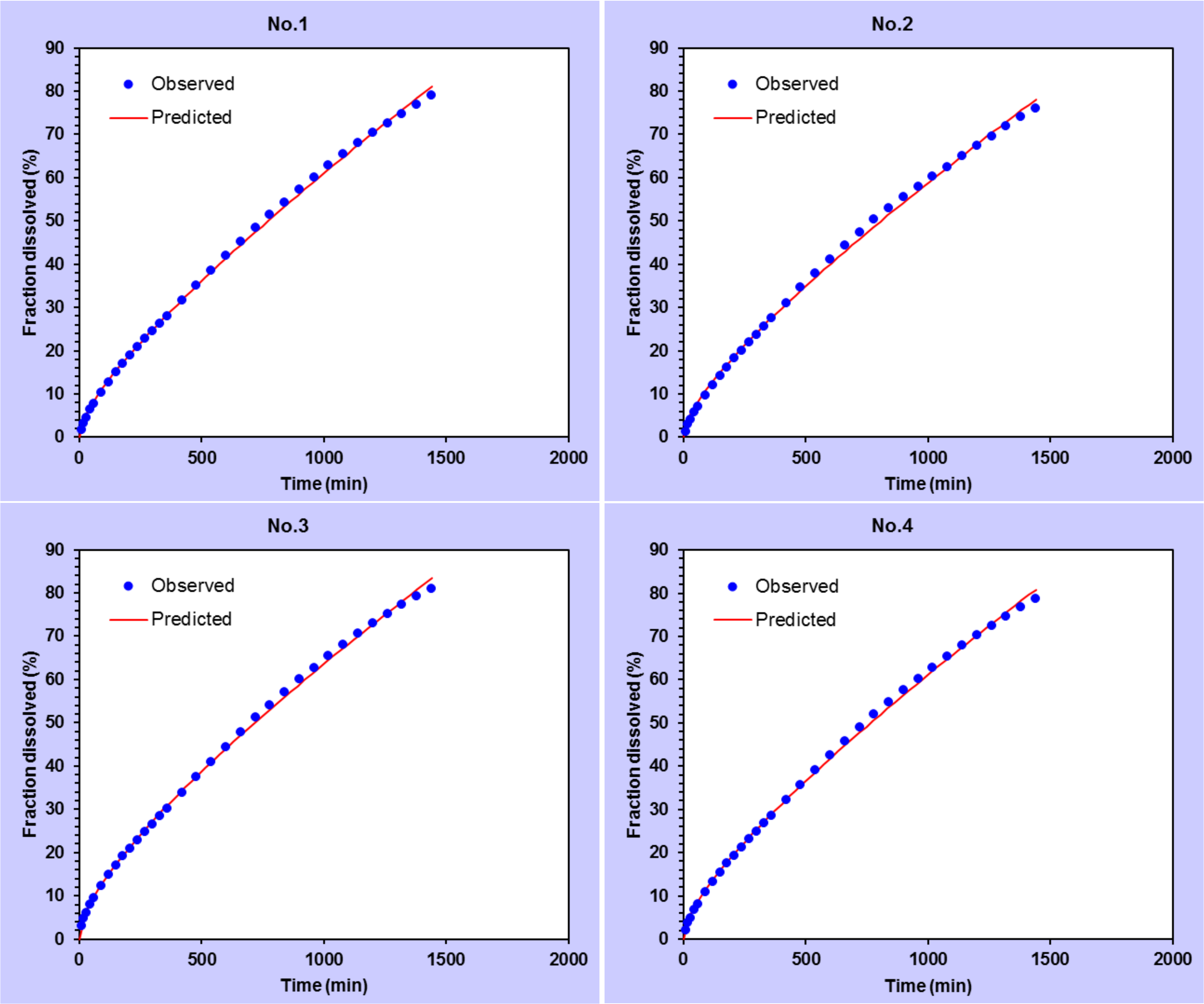

Supplementary materials – Model fitting summary – Avicel® PH-102 (modelling the entire relevant dissolution data)

Model: **Peppas–Sahlin_1 with T_{lag}**

Model equation: $F = k_1 \cdot (t - T_{lag})^m + k_2 \cdot (t - T_{lag})^{2m}$

Fitted model parameters per tested tablet (N = 4) with statistics – mean, standard deviation (SD), and relative standard deviation expressed in % (RSD%) (output from DDSolver):

Parameter	No.1	No.2	No.3	No.4	Mean	SD	RSD(%)
k_1	0.813	0.820	1.094	0.909	0.909	0.131	14.367
k_2	0.086	0.081	0.078	0.082	0.082	0.003	3.704
m	0.450	0.450	0.450	0.450	0.450	0.000	0.000
T_{lag}	6.000	6.000	4.000	6.000	5.500	1.000	18.182

Number of dissolution data points (N), degrees of freedom (df), and selected goodness of fit criteria – Pearson correlation coefficient (R), coefficient of determination (R^2), adjusted coefficient of determination ($R^2_{adjusted}$), and residual sum of squares (RSS) (manual calculation in MS Excel):

Parameter	No.1	No.2	No.3	No.4
N	33	33	33	33
df	29	29	29	29
R	0.999605203	0.99930096	0.999538678	0.999537629
R^2	0.999210563	0.99860241	0.999077568	0.999075472
$R^2_{adjusted}$	0.999128897	0.998457831	0.998982144	0.998979831
RSS	15.99229622	26.8058568	18.90231145	18.3897341

Graphical abstract of model fit presented as mean ± 1 SD of the fraction % of released carvedilol:

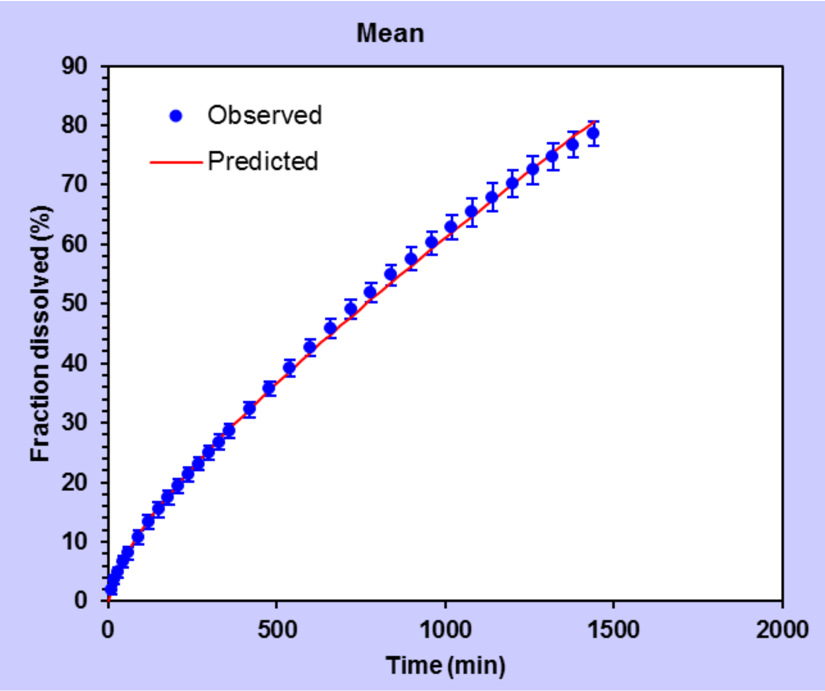

Supplementary materials – Model fitting summary – Avicel® PH-102 (modelling the entire relevant dissolution data)

Graphical abstract of model fit presented as the fraction % of released carvedilol per tested tablet:

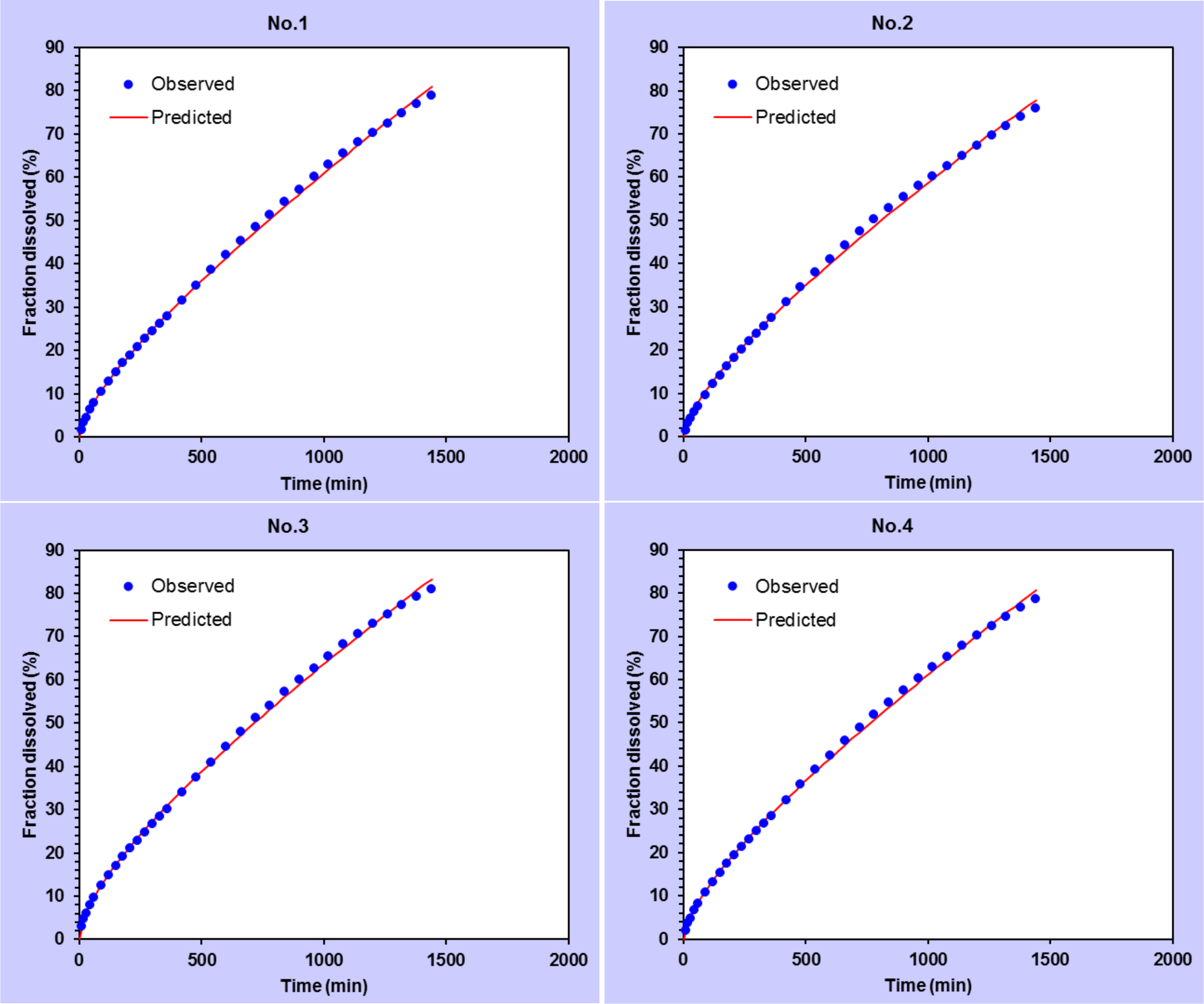

Supplementary materials – Model fitting summary – Avicel® PH-102 (modelling the entire relevant dissolution data)

Model: **Peppas–Sahlin_2**

Model equation: $F = k_1 \cdot t^{0.5} + k_2 \cdot t$

Fitted model parameters per tested tablet (N = 4) with statistics – mean, standard deviation (SD), and relative standard deviation expressed in % (RSD%) (output from DDSolver):

Parameter	No.1	No.2	No.3	No.4	Mean	SD	RSD(%)
k_1	0.845	0.843	1.049	0.912	0.912	0.097	10.640
k_2	0.034	0.032	0.030	0.032	0.032	0.002	4.879

Number of dissolution data points (N), degrees of freedom (df), and selected goodness of fit criteria – Pearson correlation coefficient (R), coefficient of determination (R^2), adjusted coefficient of determination ($R^2_{adjusted}$), and residual sum of squares (RSS) (manual calculation in MS Excel):

Parameter	No.1	No.2	No.3	No.4
N	33	33	33	33
df	31	31	31	31
R	0.999370443	0.998975992	0.99942472	0.999318324
R^2	0.998741282	0.997953034	0.99884977	0.998637113
$R^2_{adjusted}$	0.998700678	0.997887002	0.998812666	0.998593149
RSS	27.96152106	43.36106593	24.59169851	29.62921444

Graphical abstract of model fit presented as mean ± 1 SD of the fraction % of released carvedilol:

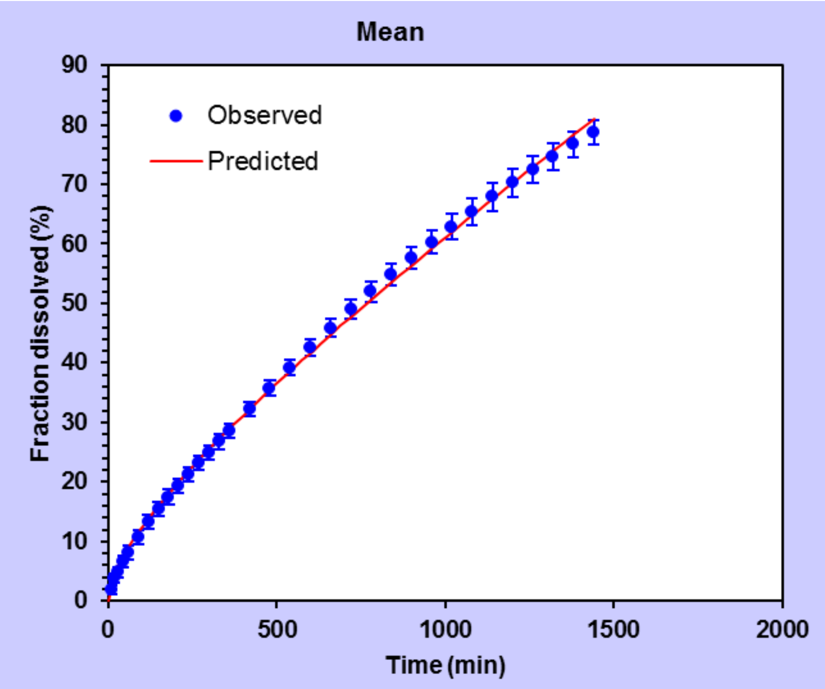

Supplementary materials – Model fitting summary – Avicel® PH-102 (modelling the entire relevant dissolution data)

Graphical abstract of model fit presented as the fraction % of released carvedilol per tested tablet:

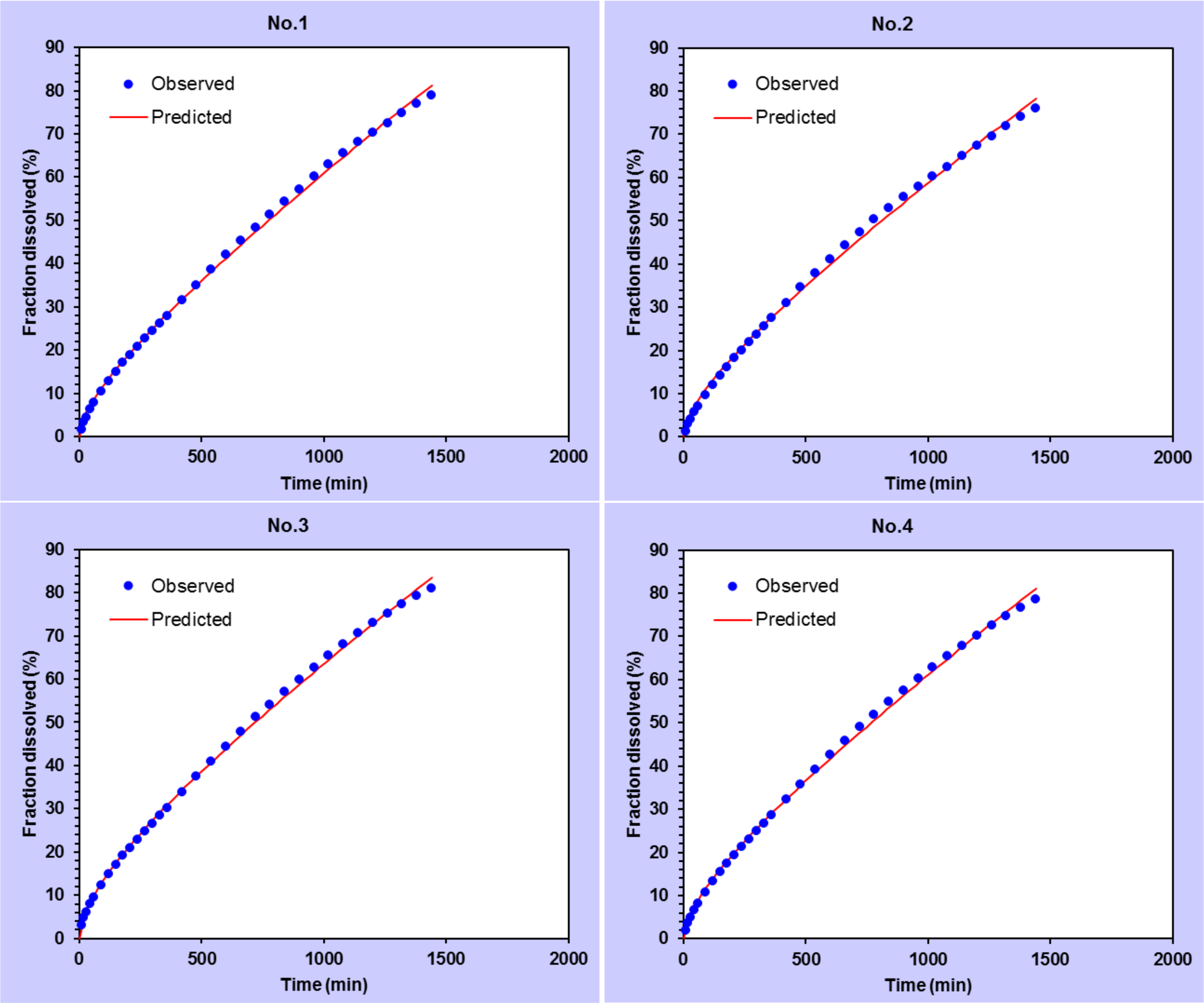

Supplementary materials – Model fitting summary – Avicel® PH-102 (modelling the entire relevant dissolution data)

Model: **Peppas–Sahlin_2 with T_{lag}**

Model equation: $F = k_1 \cdot (t - T_{lag})^{0.5} + k_2 \cdot (t - T_{lag})$

Fitted model parameters per tested tablet (N = 4) with statistics – mean, standard deviation (SD), and relative standard deviation expressed in % (RSD%) (output from DDSolver):

Parameter	No.1	No.2	No.3	No.4	Mean	SD	RSD(%)
k_1	0.876	0.874	1.083	0.944	0.944	0.098	10.355
k_2	0.033	0.031	0.030	0.031	0.031	0.002	5.062
T_{lag}	6.000	6.000	4.000	6.000	5.500	1.000	18.182

Number of dissolution data points (N), degrees of freedom (df), and selected goodness of fit criteria – Pearson correlation coefficient (R), coefficient of determination (R^2), adjusted coefficient of determination ($R^2_{adjusted}$), and residual sum of squares (RSS) (manual calculation in MS Excel):

Parameter	No.1	No.2	No.3	No.4
N	33	33	33	33
df	30	30	30	30
R	0.999490596	0.999155094	0.999469173	0.999431267
R^2	0.998981452	0.998310902	0.998938627	0.998862858
$R^2_{adjusted}$	0.998913549	0.998198296	0.998867869	0.998787048
RSS	20.96836568	33.33081299	21.95171904	22.90836328

Graphical abstract of model fit presented as mean ± 1 SD of the fraction % of released carvedilol:

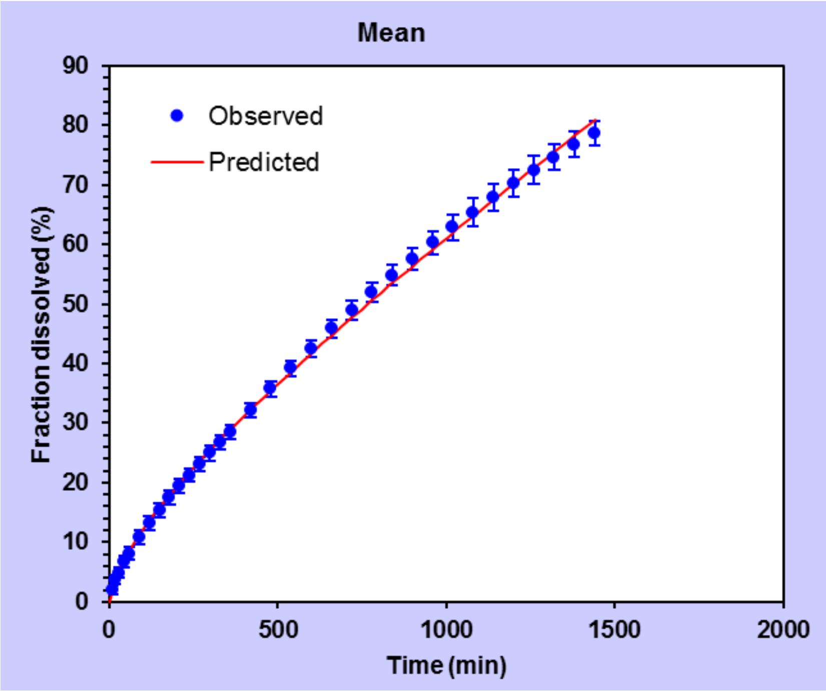

Supplementary materials – Model fitting summary – Avicel® PH-102 (modelling the entire relevant dissolution data)

Graphical abstract of model fit presented as the fraction % of released carvedilol per tested tablet:

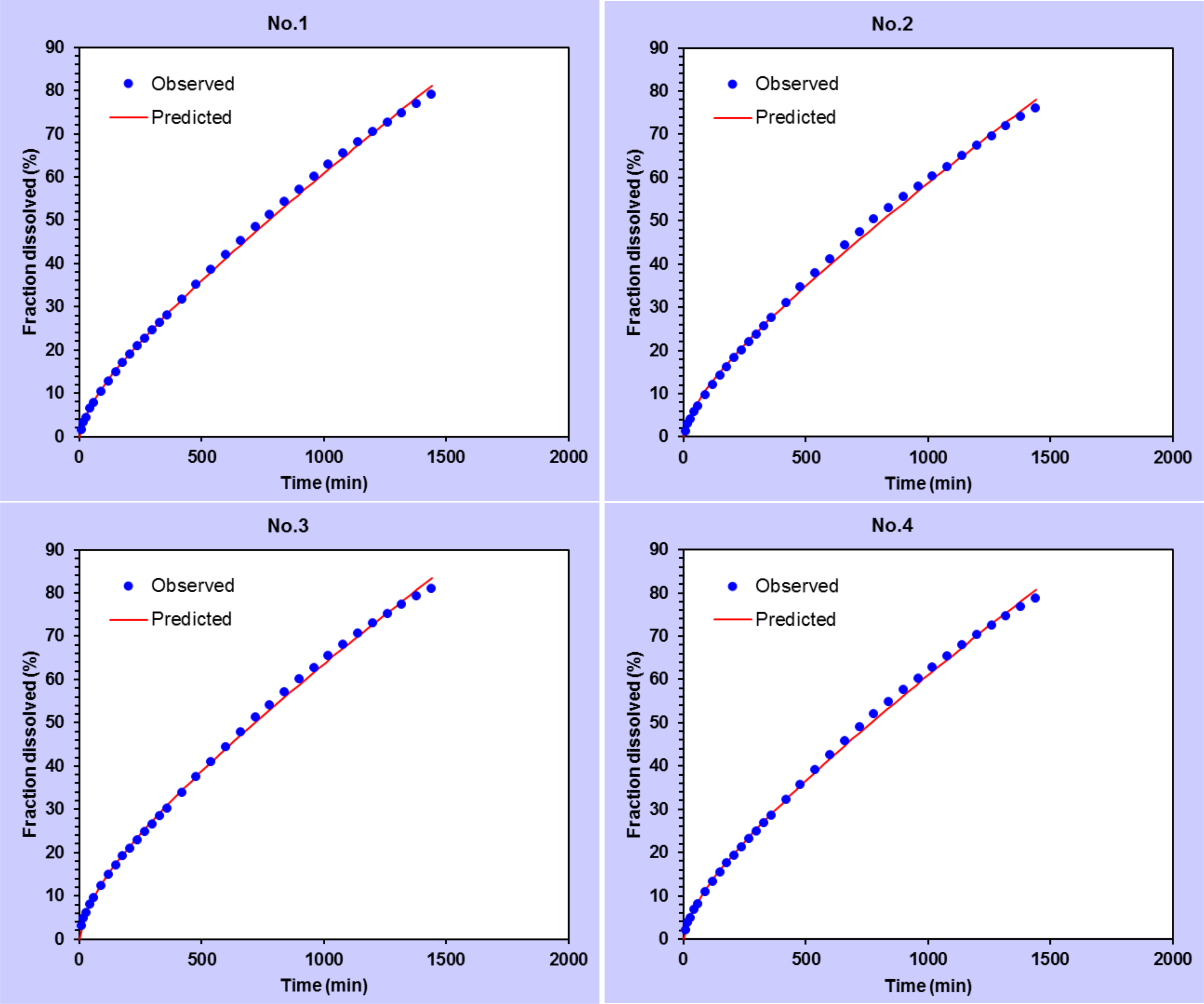

Supplementary materials – Model fitting summary – Avicel® PH-102 (modelling the entire relevant dissolution data)

Model: **Quadratic**

Model equation: $F = 100 \cdot (k_1 \cdot t^2 + k_2 \cdot t)$

Fitted model parameters per tested tablet (N = 4) with statistics – mean, standard deviation (SD), and relative standard deviation expressed in % (RSD%) (output from DDSolver):

Parameter	No.1	No.2	No.3	No.4	Mean	SD	RSD(%)
k_1	-0.0000002	-0.0000002	-0.0000003	-0.0000002	-0.0000002	0.0000000	-8.6289329
k_2	0.0008394	0.0008230	0.0009137	0.0008587	0.0008587	0.0000394	4.5932501

Number of dissolution data points (N), degrees of freedom (df), and selected goodness of fit criteria – Pearson correlation coefficient (R), coefficient of determination (R^2), adjusted coefficient of determination ($R^2_{adjusted}$), and residual sum of squares (RSS) (manual calculation in MS Excel):

Parameter	No.1	No.2	No.3	No.4
N	33	33	33	33
df	31	31	31	31
R	0.99904202	0.999210064	0.998387813	0.998928633
R^2	0.998084958	0.998420751	0.996778225	0.997858413
$R^2_{adjusted}$	0.998023183	0.998369808	0.996674296	0.99778933
RSS	84.21440661	61.71676585	171.3449501	99.44024278

Graphical abstract of model fit presented as mean ± 1 SD of the fraction % of released carvedilol:

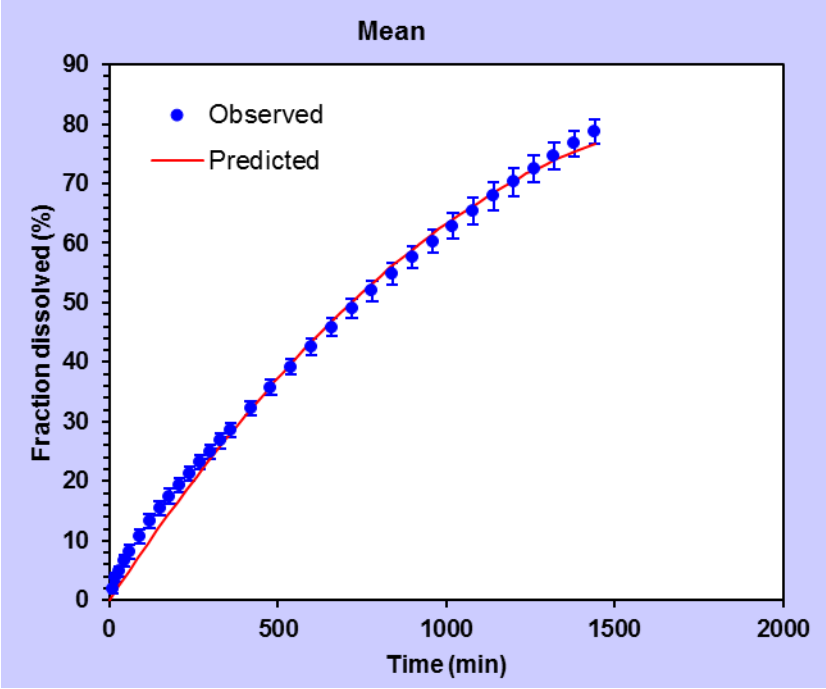

Supplementary materials – Model fitting summary – Avicel® PH-102 (modelling the entire relevant dissolution data)

Graphical abstract of model fit presented as the fraction % of released carvedilol per tested tablet:

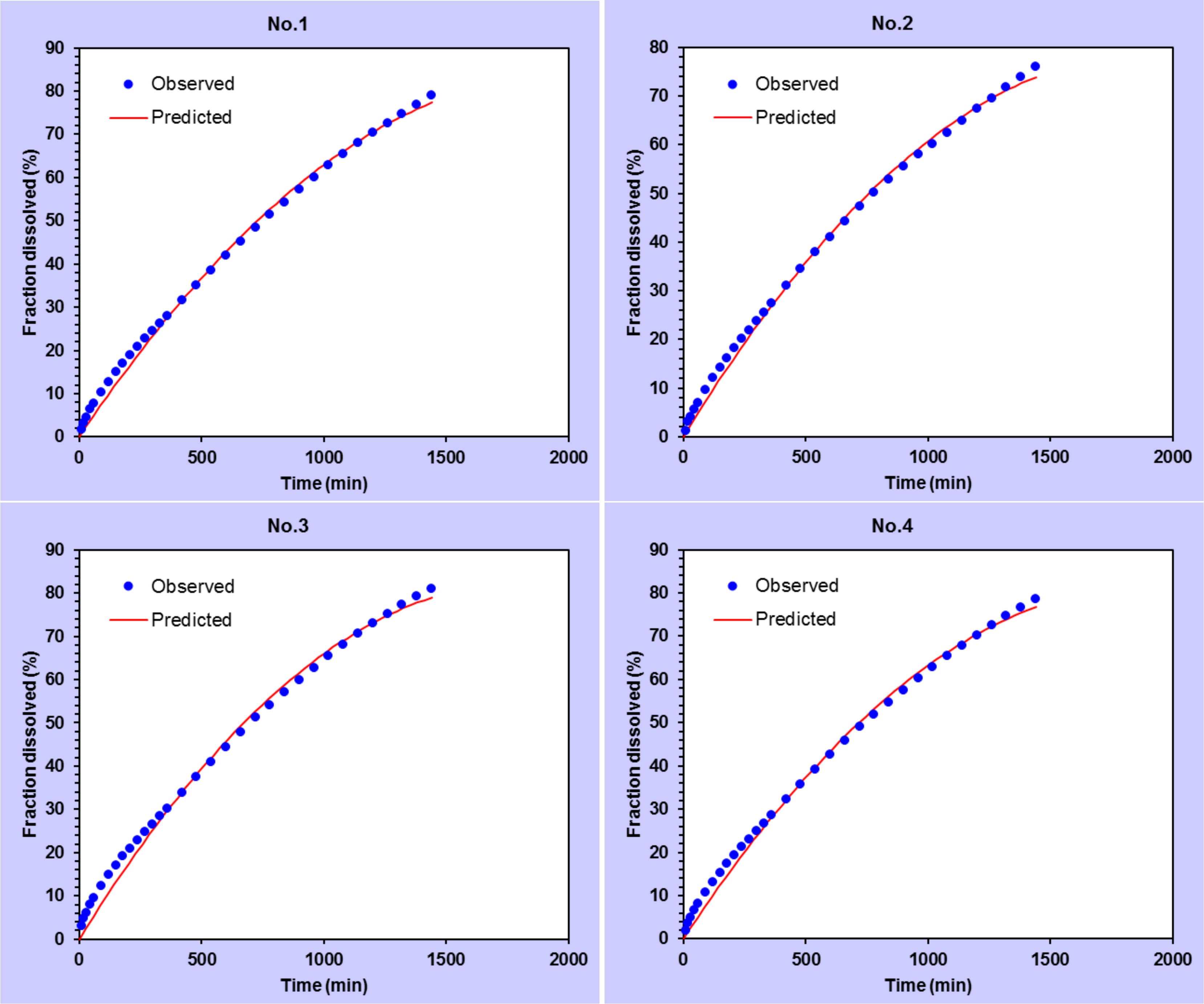

Supplementary materials – Model fitting summary – Avicel® PH-102 (modelling the entire relevant dissolution data)

Model: **Quadratic with T_{lag}**

Model equation: $F = 100 \cdot \left[k_1 \cdot (t - T_{lag})^2 + k_2 \cdot (t - T_{lag})\right]$

Fitted model parameters per tested tablet (N = 4) with statistics – mean, standard deviation (SD), and relative standard deviation expressed in % (RSD%) (output from DDSolver):

Parameter	No.1	No.2	No.3	No.4	Mean	SD	RSD(%)
k_1	-0.0000002	-0.0000002	-0.0000003	-0.0000002	-0.0000002	0.0000000	-8.4694390
k_2	0.0008473	0.0008309	0.0009218	0.0008667	0.0008667	0.0000396	4.5667695
T_{lag}	4.0000000	4.0000000	4.0000000	4.0000000	4.0000000	0.0000000	0.0000000

Number of dissolution data points (N), degrees of freedom (df), and selected goodness of fit criteria – Pearson correlation coefficient (R), coefficient of determination (R^2), adjusted coefficient of determination ($R^2_{adjusted}$), and residual sum of squares (RSS) (manual calculation in MS Excel):

Parameter	No.1	No.2	No.3	No.4
N	33	33	33	33
df	30	30	30	30
R	0.998932213	0.999112451	0.998237713	0.998808156
R^2	0.997865566	0.99822569	0.996478531	0.997617732
$R^2_{adjusted}$	0.997723271	0.998107403	0.996243767	0.997458914
RSS	99.96373718	74.72720902	196.5746647	117.2398701

Graphical abstract of model fit presented as mean ± 1 SD of the fraction % of released carvedilol:

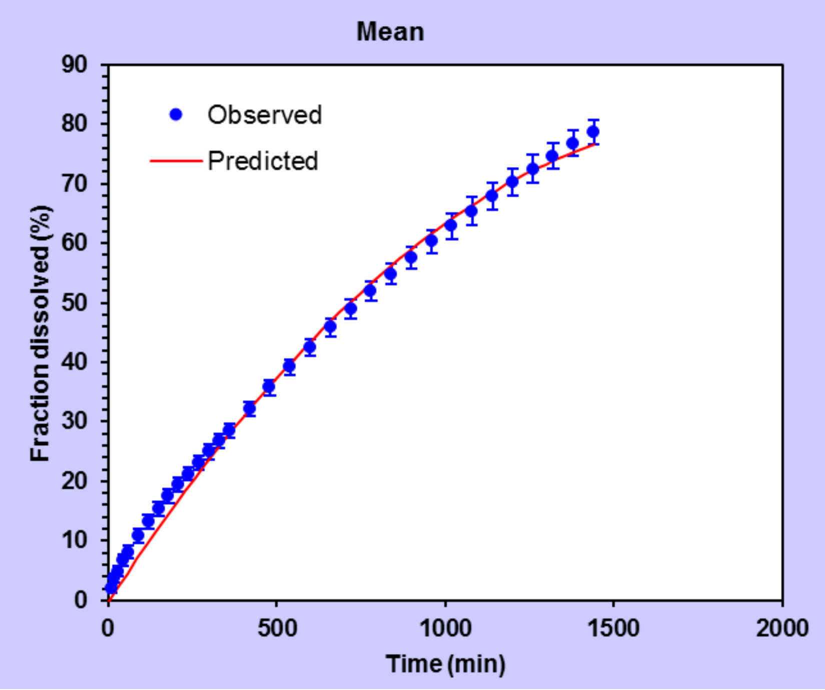

Supplementary materials – Model fitting summary – Avicel® PH-102 (modelling the entire relevant dissolution data)

Graphical abstract of model fit presented as the fraction % of released carvedilol per tested tablet:

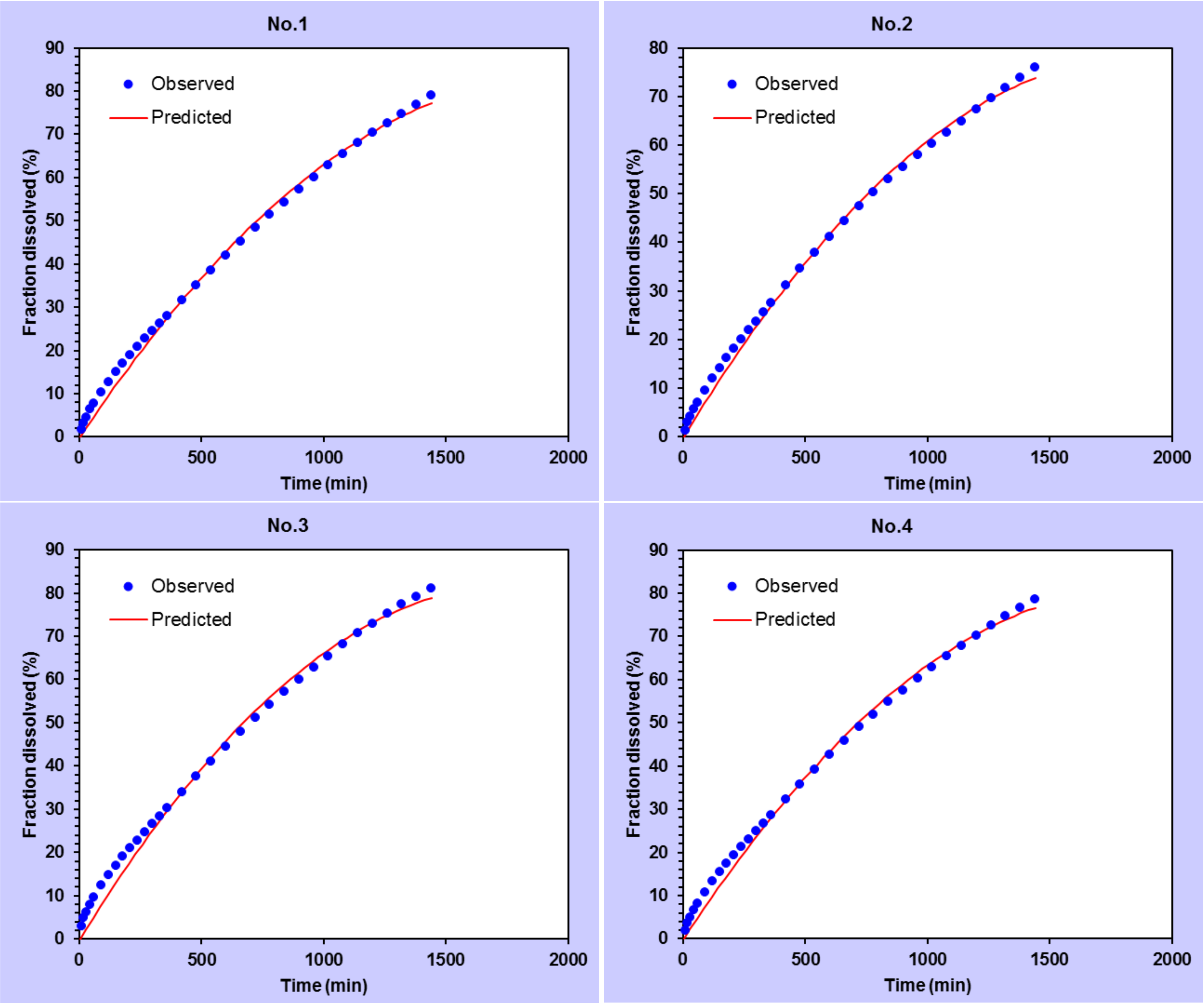

Supplementary materials – Model fitting summary – Avicel® PH-102 (modelling the entire relevant dissolution data)

Model: **Weibull_1**

Model equation: $F = 100 \cdot \left[1 - e^{-\frac{(t-T_i)^\beta}{\alpha}}\right]$

Fitted model parameters per tested tablet (N = 4) with statistics – mean, standard deviation (SD), and relative standard deviation expressed in % (RSD%) (output from DDSolver):

Parameter	No.1	No.2	No.3	No.4	Mean	SD	RSD(%)
α	365.958	418.704	207.310	303.549	323.880	90.855	28.052
β	0.841	0.854	0.766	0.814	0.819	0.039	4.771
T_i	6.000	6.000	6.000	6.000	6.000	0.000	0.000

Number of dissolution data points (N), degrees of freedom (df), and selected goodness of fit criteria – Pearson correlation coefficient (R), coefficient of determination (R^2), adjusted coefficient of determination ($R^2_{adjusted}$), and residual sum of squares (RSS) (manual calculation in MS Excel):

Parameter	No.1	No.2	No.3	No.4
N	33	33	33	33
df	30	30	30	30
R	0.993744886	0.996342541	0.990063927	0.993451511
R^2	0.987528899	0.992698459	0.98022658	0.986945906
$R^2_{adjusted}$	0.986697492	0.992211689	0.978908351	0.986075633
RSS	375.6575696	217.6917716	607.9496946	401.4520122

Graphical abstract of model fit presented as mean ± 1 SD of the fraction % of released carvedilol:

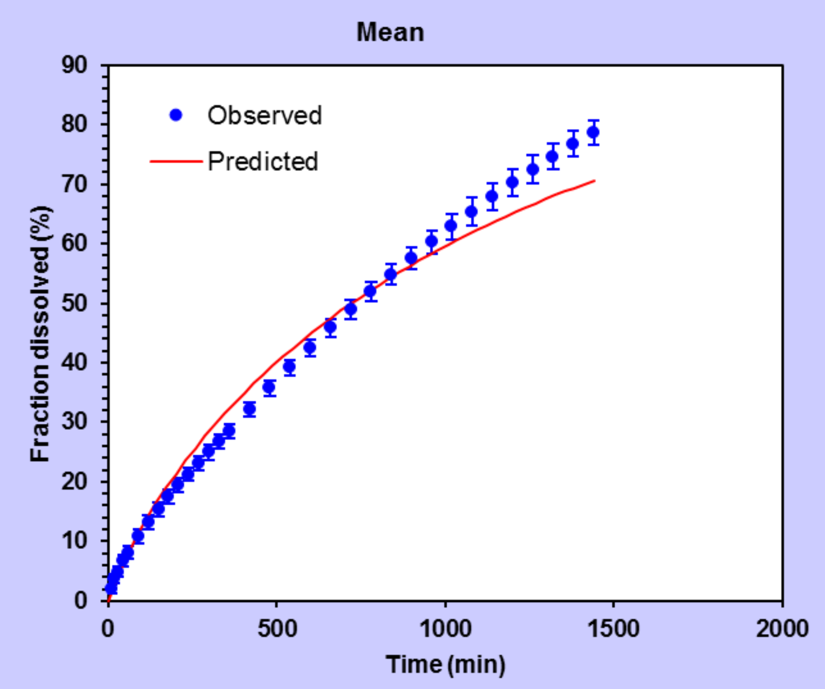

Supplementary materials – Model fitting summary – Avicel® PH-102 (modelling the entire relevant dissolution data)

Graphical abstract of model fit presented as the fraction % of released carvedilol per tested tablet:

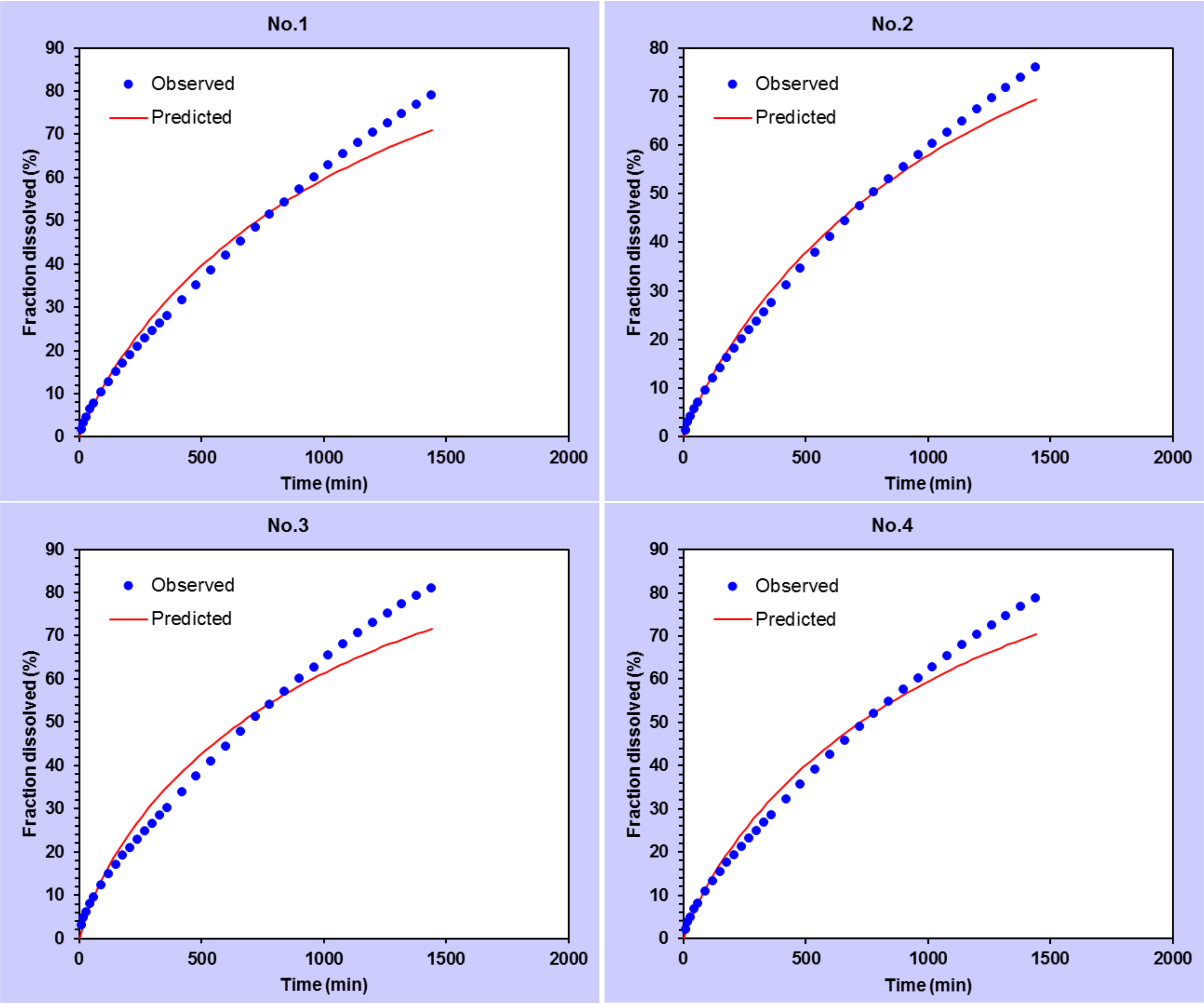

Supplementary materials – Model fitting summary – Avicel® PH-102 (modelling the entire relevant dissolution data)

Model: **Weibull_2**

Model equation: $F = 100 \cdot \left(1 - e^{-\frac{t^{\beta}}{\alpha}}\right)$

Fitted model parameters per tested tablet (N = 4) with statistics – mean, standard deviation (SD), and relative standard deviation expressed in % (RSD%) (output from DDSolver):

Parameter	No.1	No.2	No.3	No.4	Mean	SD	RSD(%)
α	506.575	579.881	281.828	417.321	446.401	128.281	28.737
β	0.891	0.904	0.813	0.863	0.868	0.040	4.640

Number of dissolution data points (N), degrees of freedom (df), and selected goodness of fit criteria – Pearson correlation coefficient (R), coefficient of determination (R^2), adjusted coefficient of determination ($R^2_{adjusted}$), and residual sum of squares (RSS) (manual calculation in MS Excel):

Parameter	No.1	No.2	No.3	No.4
N	33	33	33	33
df	31	31	31	31
R	0.995437647	0.997612446	0.992527067	0.99529533
R^2	0.990896109	0.995230592	0.985109979	0.990612794
$R^2_{adjusted}$	0.990602435	0.99507674	0.984629655	0.990309981
RSS	249.4198368	124.0462498	437.489476	266.6764235

Graphical abstract of model fit presented as mean ± 1 SD of the fraction % of released carvedilol:

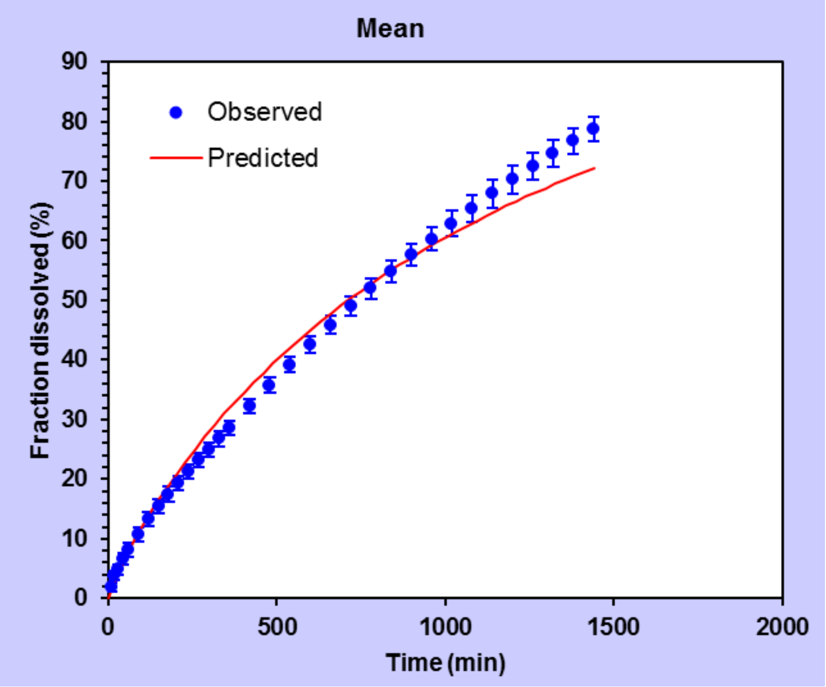

Supplementary materials – Model fitting summary – Avicel® PH-102 (modelling the entire relevant dissolution data)

Graphical abstract of model fit presented as the fraction % of released carvedilol per tested tablet:

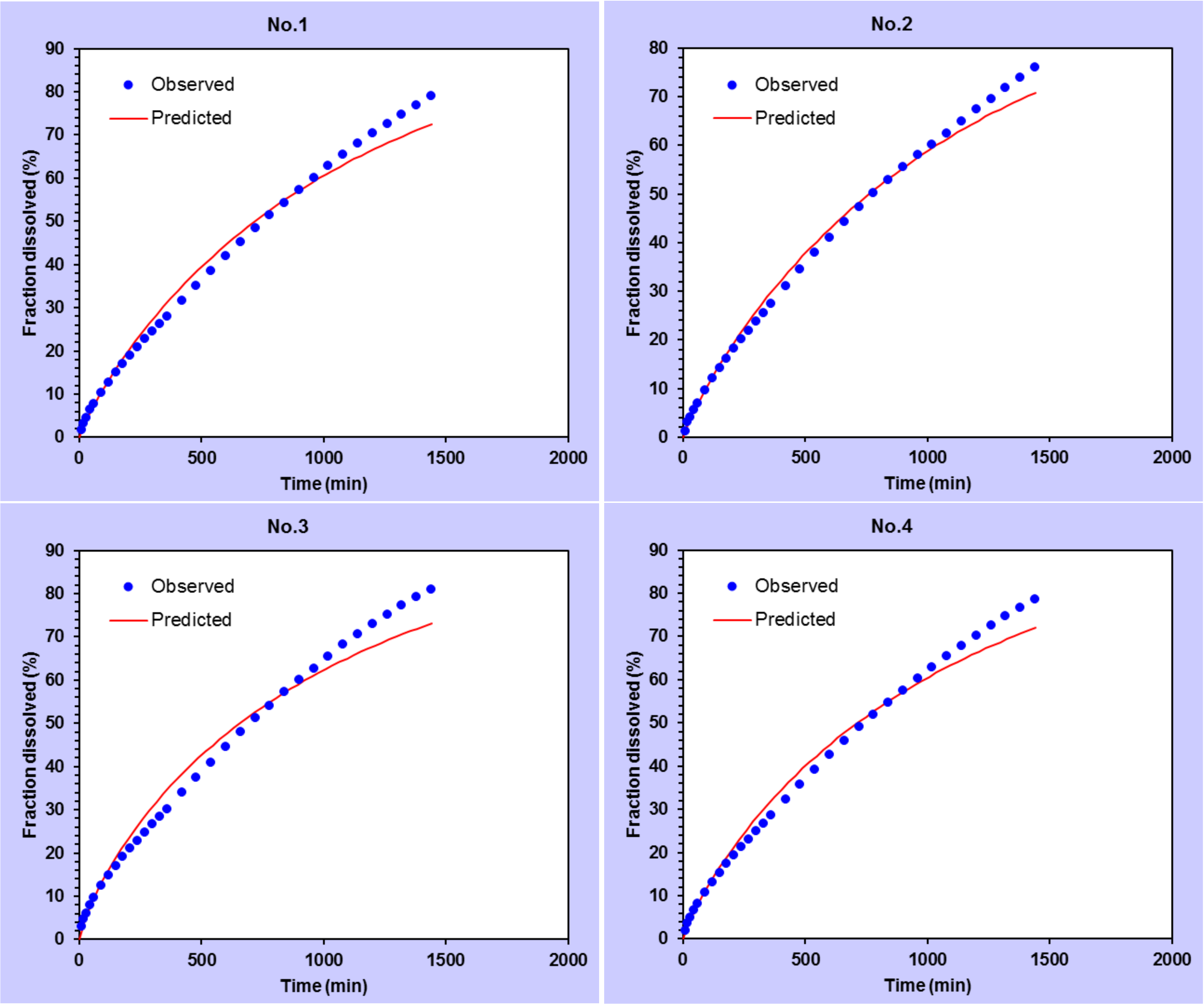

Supplementary materials – Model fitting summary – Avicel® PH-102 (modelling the entire relevant dissolution data)

Model: **Weibull_3**

Model equation: $F = F_{max} \cdot \left(1 - e^{-\frac{t^{\beta}}{\alpha}}\right)$

Fitted model parameters per tested tablet (N = 4) with statistics – mean, standard deviation (SD), and relative standard deviation expressed in % (RSD%) (output from DDSolver):

Parameter	No.1	No.2	No.3	No.4	Mean	SD	RSD(%)
α	552.986	707.567	309.663	456.448	506.666	167.171	32.994
β	0.957	0.959	0.874	0.930	0.930	0.039	4.219
F_{max}	82.955	94.345	85.073	82.602	86.244	5.510	6.389

Number of dissolution data points (N), degrees of freedom (df), and selected goodness of fit criteria – Pearson correlation coefficient (R), coefficient of determination (R^2), adjusted coefficient of determination ($R^2_{adjusted}$), and residual sum of squares (RSS) (manual calculation in MS Excel):

Parameter	No.1	No.2	No.3	No.4
N	33	33	33	33
df	30	30	30	30
R	0.990241305	0.996796656	0.987130948	0.989982596
R^2	0.980577842	0.993603573	0.974427508	0.980065541
$R^2_{adjusted}$	0.979283032	0.993177145	0.972722675	0.978736577
RSS	465.5402955	194.62223	646.9550437	480.3896164

Graphical abstract of model fit presented as mean ± 1 SD of the fraction % of released carvedilol:

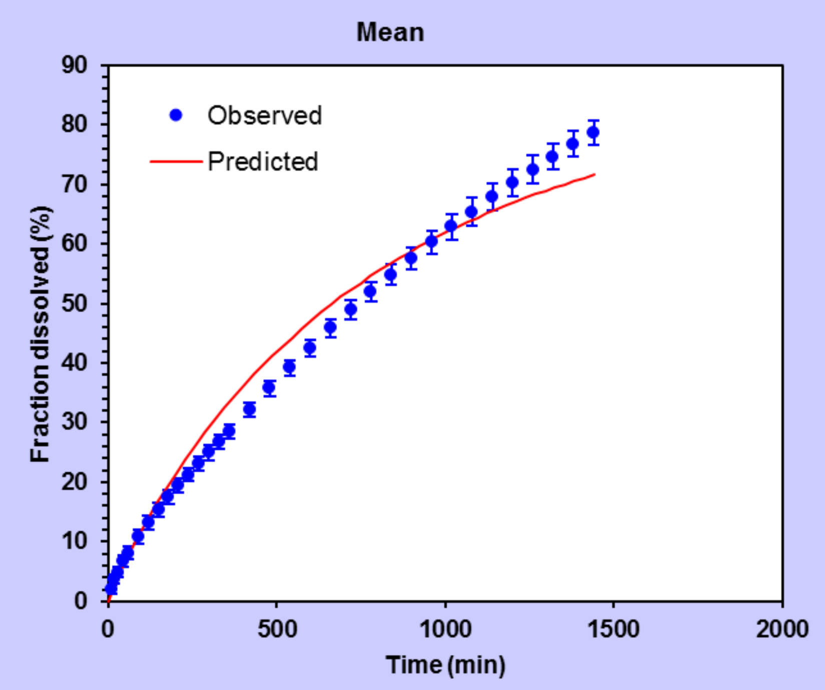

Supplementary materials – Model fitting summary – Avicel® PH-102 (modelling the entire relevant dissolution data)

Graphical abstract of model fit presented as the fraction % of released carvedilol per tested tablet:

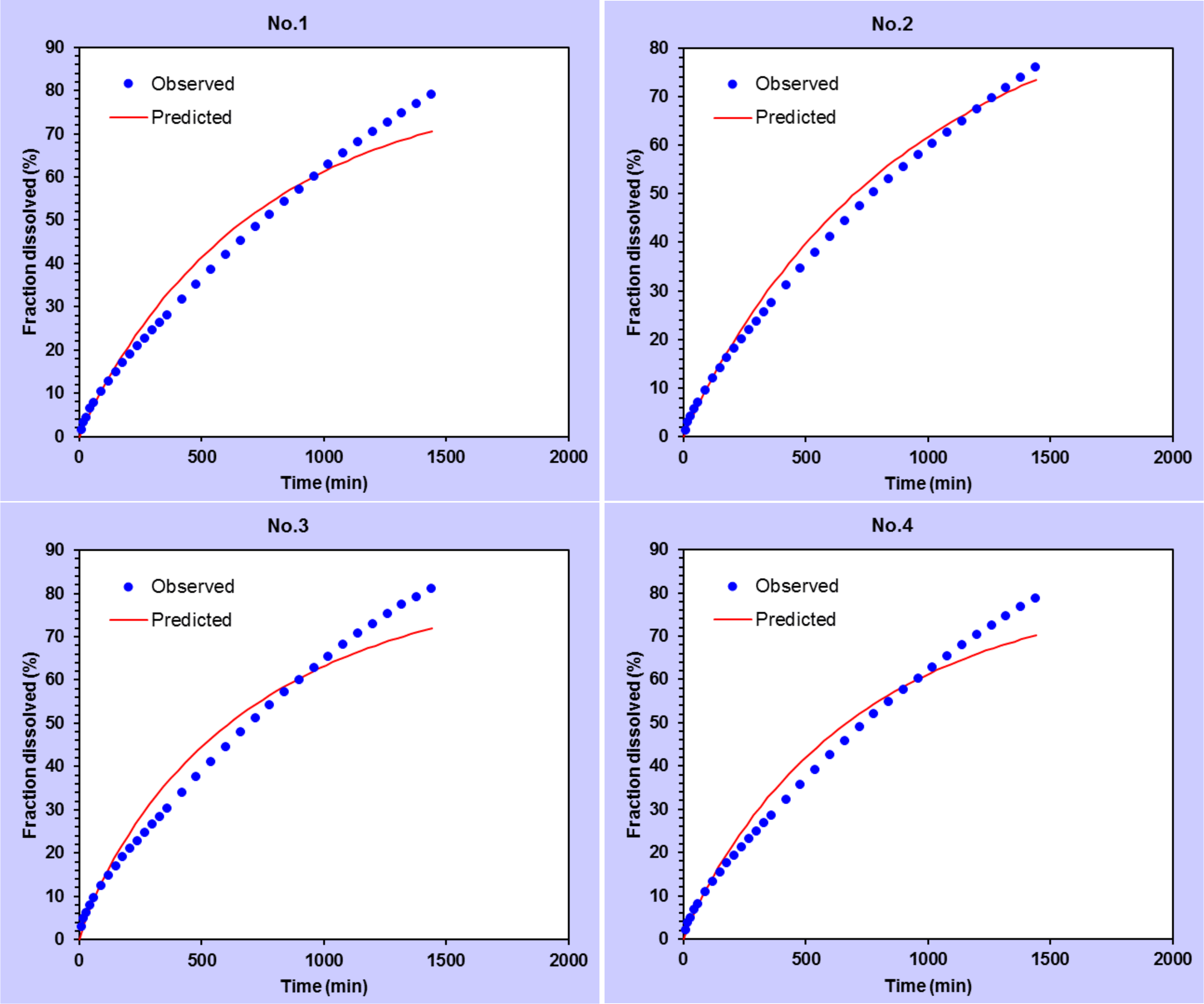

Supplementary materials – Model fitting summary – Avicel® PH-102 (modelling the entire relevant dissolution data)

Model: **Weibull_4**

Model equation: $F = F_{max} \cdot \left[1 - e^{-\frac{(t-T_i)^{\beta}}{\alpha}}\right]$

Fitted model parameters per tested tablet (N = 4) with statistics – mean, standard deviation (SD), and relative standard deviation expressed in % (RSD%) (output from DDSolver):

Parameter	No.1	No.2	No.3	No.4	Mean	SD	RSD(%)
α	387.244	474.957	221.246	321.590	351.259	107.051	30.476
β	0.902	0.909	0.822	0.876	0.877	0.039	4.468
T_i	6.000	4.557	6.000	6.000	5.639	0.721	12.792
F_{max}	82.955	92.192	85.073	82.602	85.705	4.460	5.204

Number of dissolution data points (N), degrees of freedom (df), and selected goodness of fit criteria – Pearson correlation coefficient (R), coefficient of determination (R^2), adjusted coefficient of determination ($R^2_{adjusted}$), and residual sum of squares (RSS) (manual calculation in MS Excel):

Parameter	No.1	No.2	No.3	No.4
N	33	33	33	33
df	29	29	29	29
R	0.988226265	0.994383731	0.984389254	0.987793934
R^2	0.976591151	0.988799004	0.969022204	0.975736856
$R^2_{adjusted}$	0.974169546	0.98764028	0.965817605	0.973226876
RSS	585.0410026	375.4321995	802.6663465	606.8164646

Graphical abstract of model fit presented as mean ± 1 SD of the fraction % of released carvedilol:

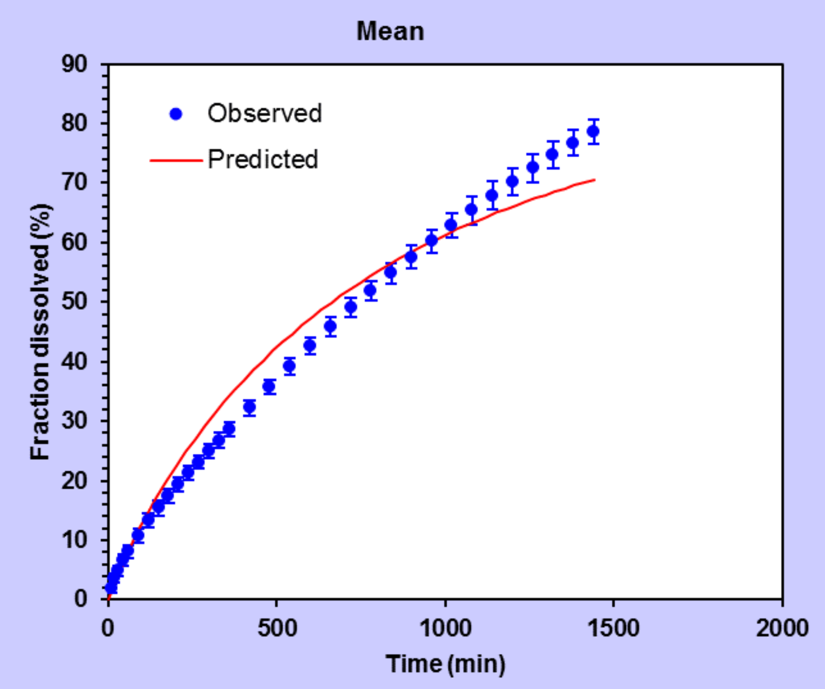

Supplementary materials – Model fitting summary – Avicel® PH-102 (modelling the entire relevant dissolution data)

Graphical abstract of model fit presented as the fraction % of released carvedilol per tested tablet:

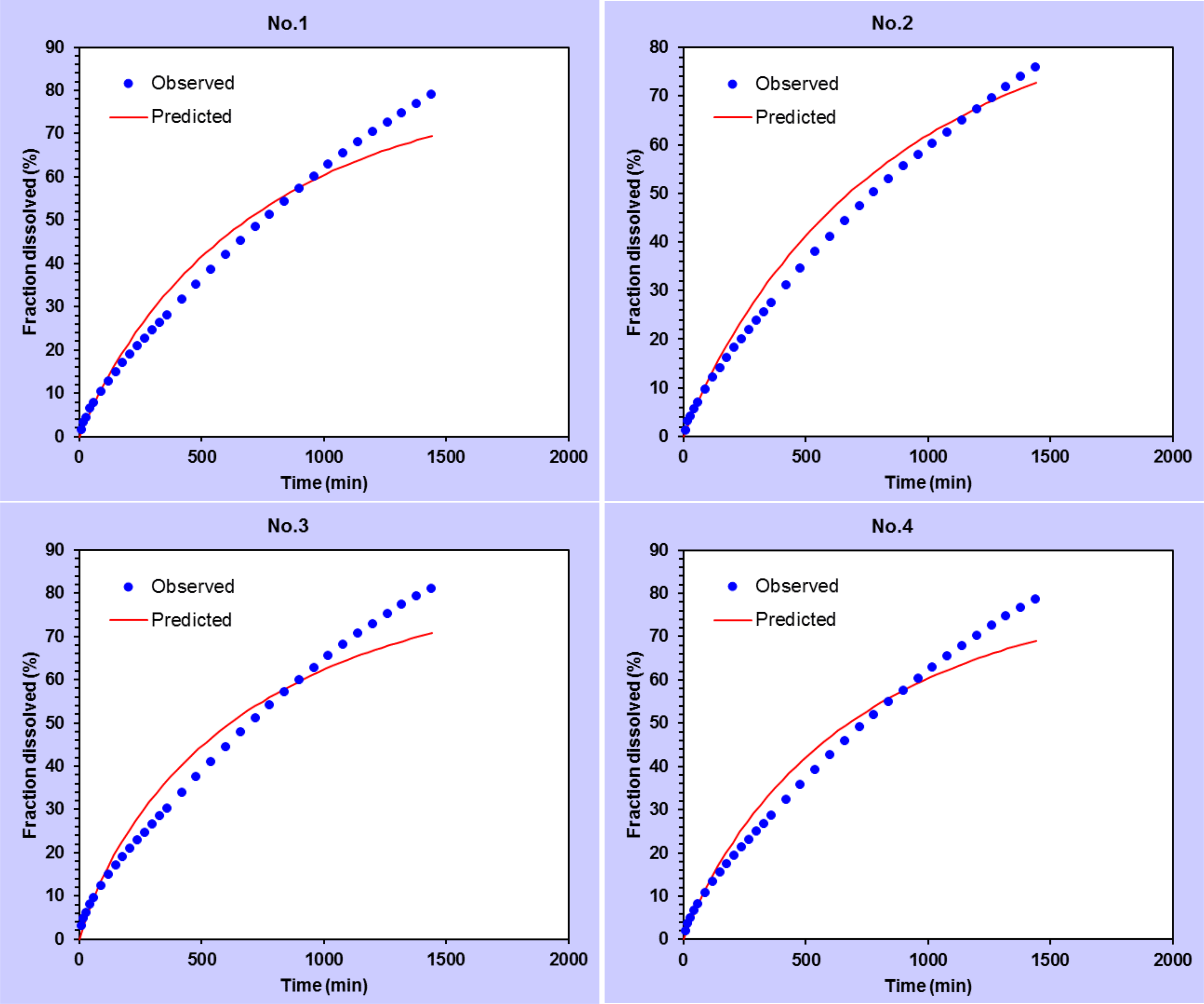

Supplementary materials – Model fitting summary – Avicel® PH-102 (modelling the entire relevant dissolution data)

Model: **Logistic_1**

Model equation: $F = 100 \cdot \frac{e^{\alpha+\beta \cdot \log(t)}}{1+e^{\alpha+\beta \cdot \log(t)}}$

Fitted model parameters per tested tablet (N = 4) with statistics – mean, standard deviation (SD), and relative standard deviation expressed in % (RSD%) (output from DDSolver):

Parameter	No.1	No.2	No.3	No.4	Mean	SD	RSD(%)
α	-6.876	-7.522	-7.039	-6.676	-7.028	0.361	-5.137
β	2.431	2.664	2.470	2.364	2.482	0.129	5.182

Number of dissolution data points (N), degrees of freedom (df), and selected goodness of fit criteria – Pearson correlation coefficient (R), coefficient of determination (R^2), adjusted coefficient of determination ($R^2_{adjusted}$), and residual sum of squares (RSS) (manual calculation in MS Excel):

Parameter	No.1	No.2	No.3	No.4
N	33	33	33	33
df	31	31	31	31
R	0.985835009	0.991073124	0.989029563	0.985718902
R^2	0.971870665	0.982225936	0.978179476	0.971641753
$R^2_{adjusted}$	0.970963268	0.981652579	0.977475588	0.970726971
RSS	689.4693133	425.3320513	905.5714788	695.2173268

Graphical abstract of model fit presented as mean ± 1 SD of the fraction % of released carvedilol:

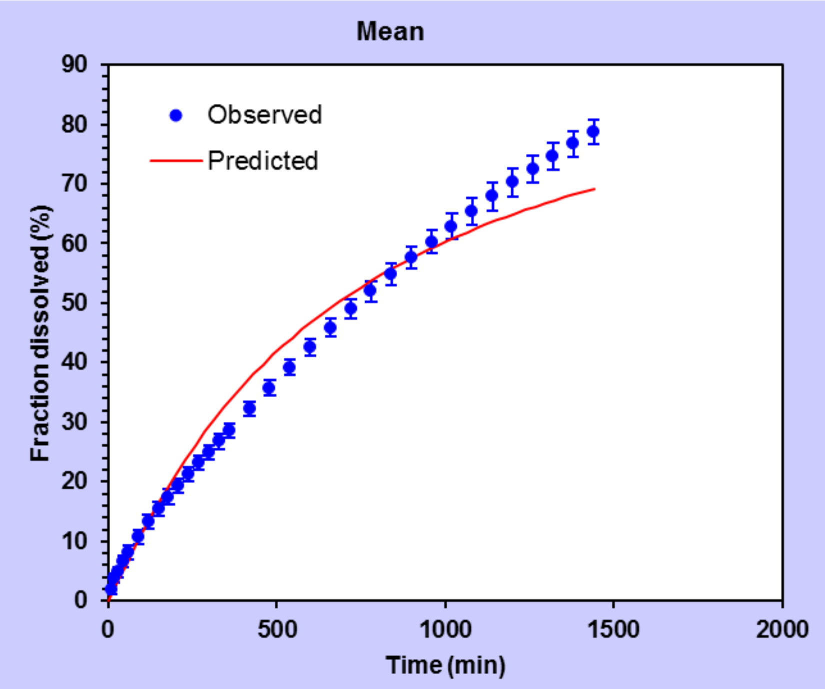

Supplementary materials – Model fitting summary – Avicel® PH-102 (modelling the entire relevant dissolution data)

Graphical abstract of model fit presented as the fraction % of released carvedilol per tested tablet:

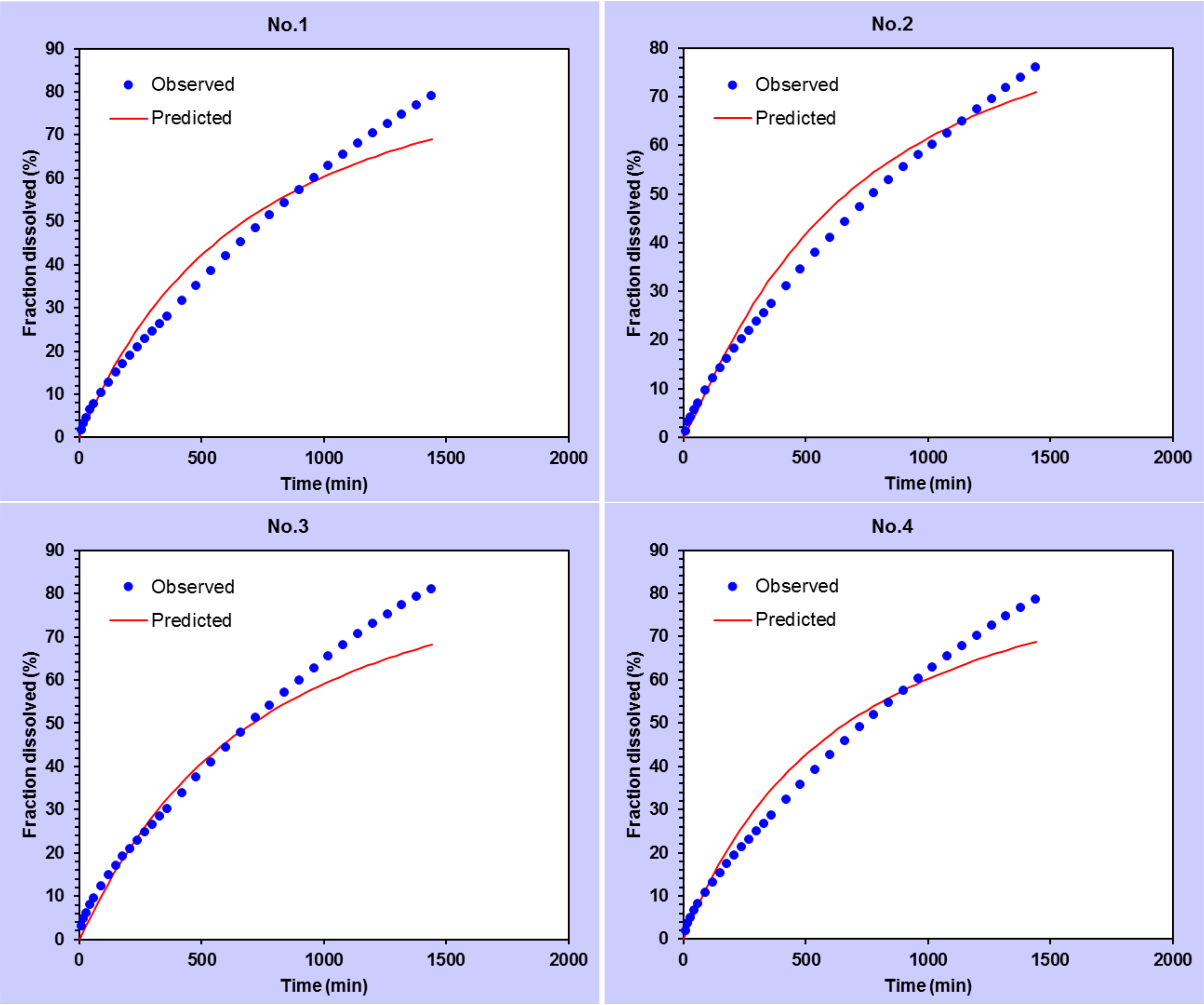

Supplementary materials – Model fitting summary – Avicel® PH-102 (modelling the entire relevant dissolution data)

Model: **Logistic_2**

Model equation: $F = F_{max} \cdot \frac{e^{\alpha+\beta\cdot\log(t)}}{1+e^{\alpha+\beta\cdot\log(t)}}$

Fitted model parameters per tested tablet (N = 4) with statistics – mean, standard deviation (SD), and relative standard deviation expressed in % (RSD%) (output from DDSolver):

Parameter	No.1	No.2	No.3	No.4	Mean	SD	RSD(%)
α	-8.561	-8.712	-7.968	-8.348	-8.397	0.323	-3.846
β	3.008	3.060	2.844	2.948	2.965	0.093	3.135
F_{max}	92.647	89.100	92.285	92.253	91.571	1.657	1.810

Number of dissolution data points (N), degrees of freedom (df), and selected goodness of fit criteria – Pearson correlation coefficient (R), coefficient of determination (R^2), adjusted coefficient of determination ($R^2_{adjusted}$), and residual sum of squares (RSS) (manual calculation in MS Excel):

Parameter	No.1	No.2	No.3	No.4
N	33	33	33	33
df	30	30	30	30
R	0.993398803	0.995390125	0.990393821	0.993095171
R^2	0.986841181	0.9908015	0.98087992	0.986238019
$R^2_{adjusted}$	0.985963927	0.990188267	0.979605248	0.985320554
RSS	861.286168	771.156623	1042.383473	774.5841701

Graphical abstract of model fit presented as mean ± 1 SD of the fraction % of released carvedilol:

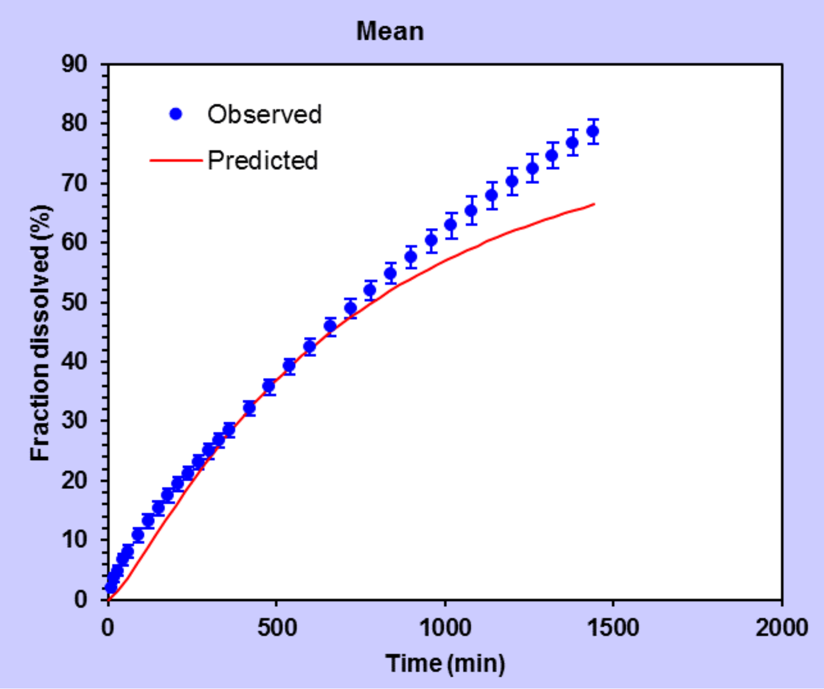

Supplementary materials – Model fitting summary – Avicel® PH-102 (modelling the entire relevant dissolution data)

Graphical abstract of model fit presented as the fraction % of released carvedilol per tested tablet:

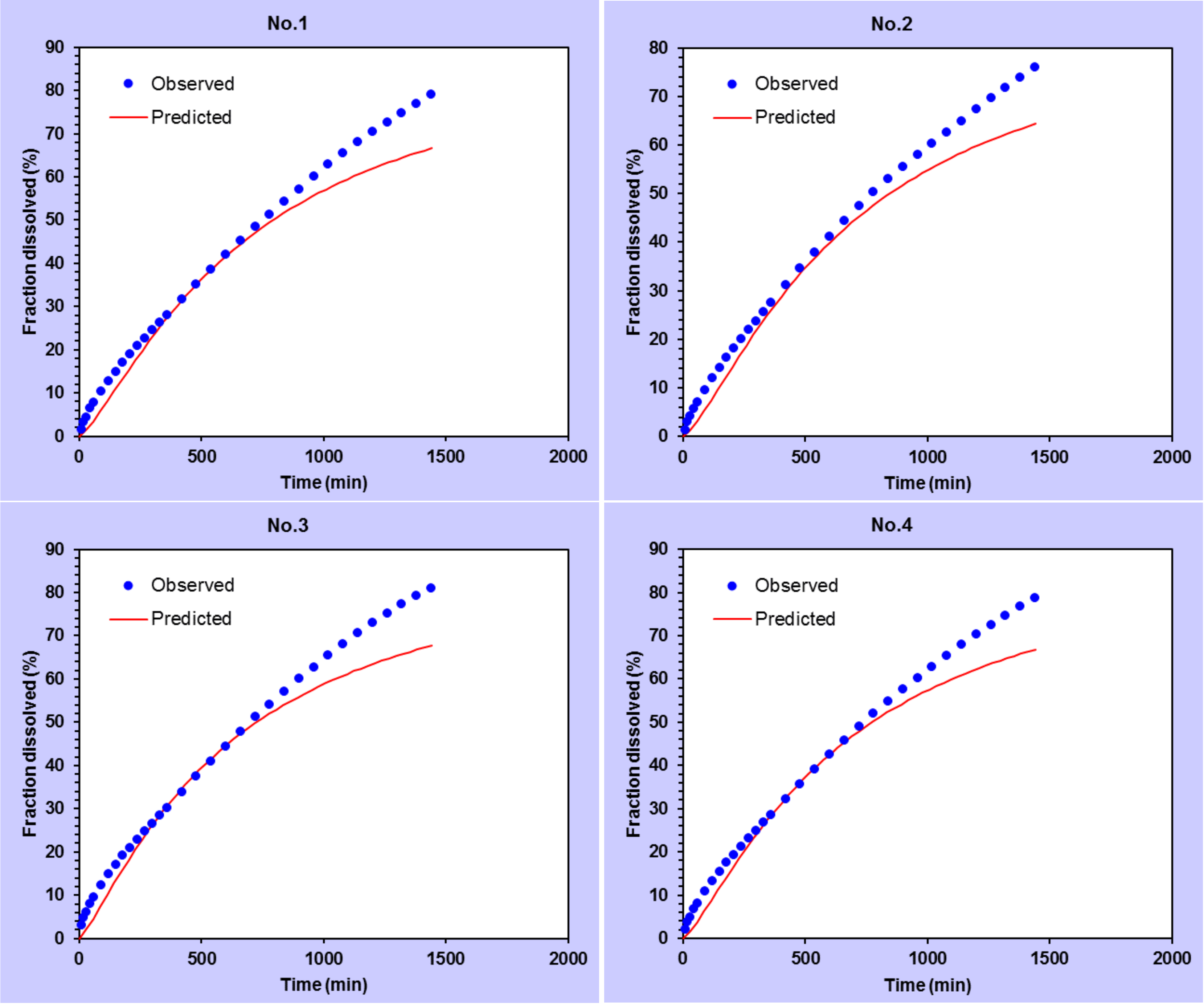

Supplementary materials – Model fitting summary – Avicel® PH-102 (modelling the entire relevant dissolution data)

Model: **Logistic_3**

Model equation: $F = F_{max} \cdot \frac{1}{1+e^{-k\cdot(t-\gamma)}}$

Fitted model parameters per tested tablet (N = 4) with statistics – mean, standard deviation (SD), and relative standard deviation expressed in % (RSD%) (output from DDSolver):

Parameter	No.1	No.2	No.3	No.4	Mean	SD	RSD(%)
k	0.003	0.003	0.003	0.003	0.003	0.000	4.071
γ	559.590	560.533	623.361	550.091	573.394	33.644	5.867
F_{max}	81.034	77.932	85.073	80.690	81.182	2.942	3.624

Number of dissolution data points (N), degrees of freedom (df), and selected goodness of fit criteria – Pearson correlation coefficient (R), coefficient of determination (R^2), adjusted coefficient of determination ($R^2_{adjusted}$), and residual sum of squares (RSS) (manual calculation in MS Excel):

Parameter	No.1	No.2	No.3	No.4
N	33	33	33	33
df	30	30	30	30
R	0.994670776	0.993767693	0.991370787	0.994743104
R^2	0.989369952	0.987574227	0.982816037	0.989513843
$R^2_{adjusted}$	0.988661283	0.986745842	0.98167044	0.988814766
RSS	447.766576	417.7750684	421.1648453	448.2583775

Graphical abstract of model fit presented as mean ± 1 SD of the fraction % of released carvedilol:

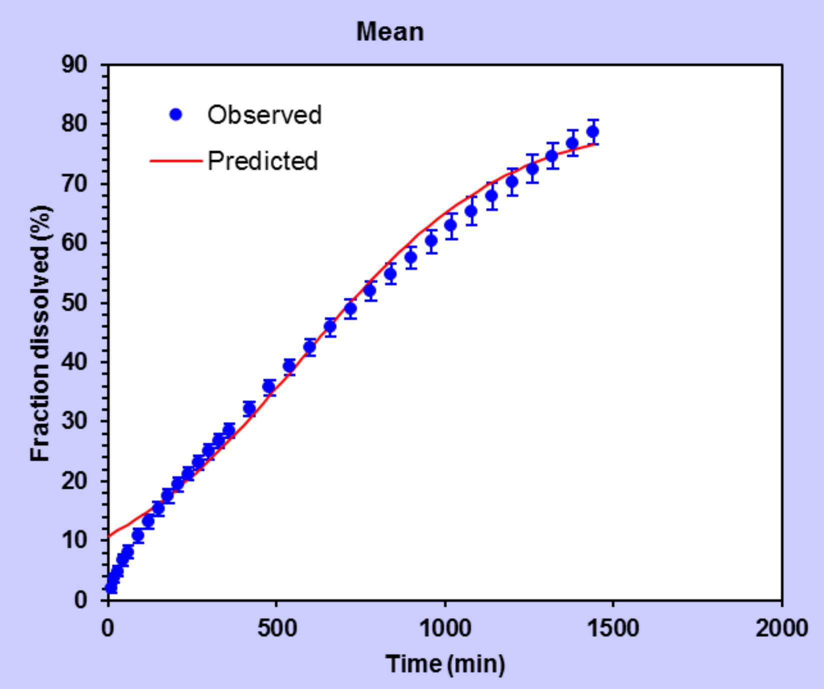

Supplementary materials – Model fitting summary – Avicel® PH-102 (modelling the entire relevant dissolution data)

Graphical abstract of model fit presented as the fraction % of released carvedilol per tested tablet:

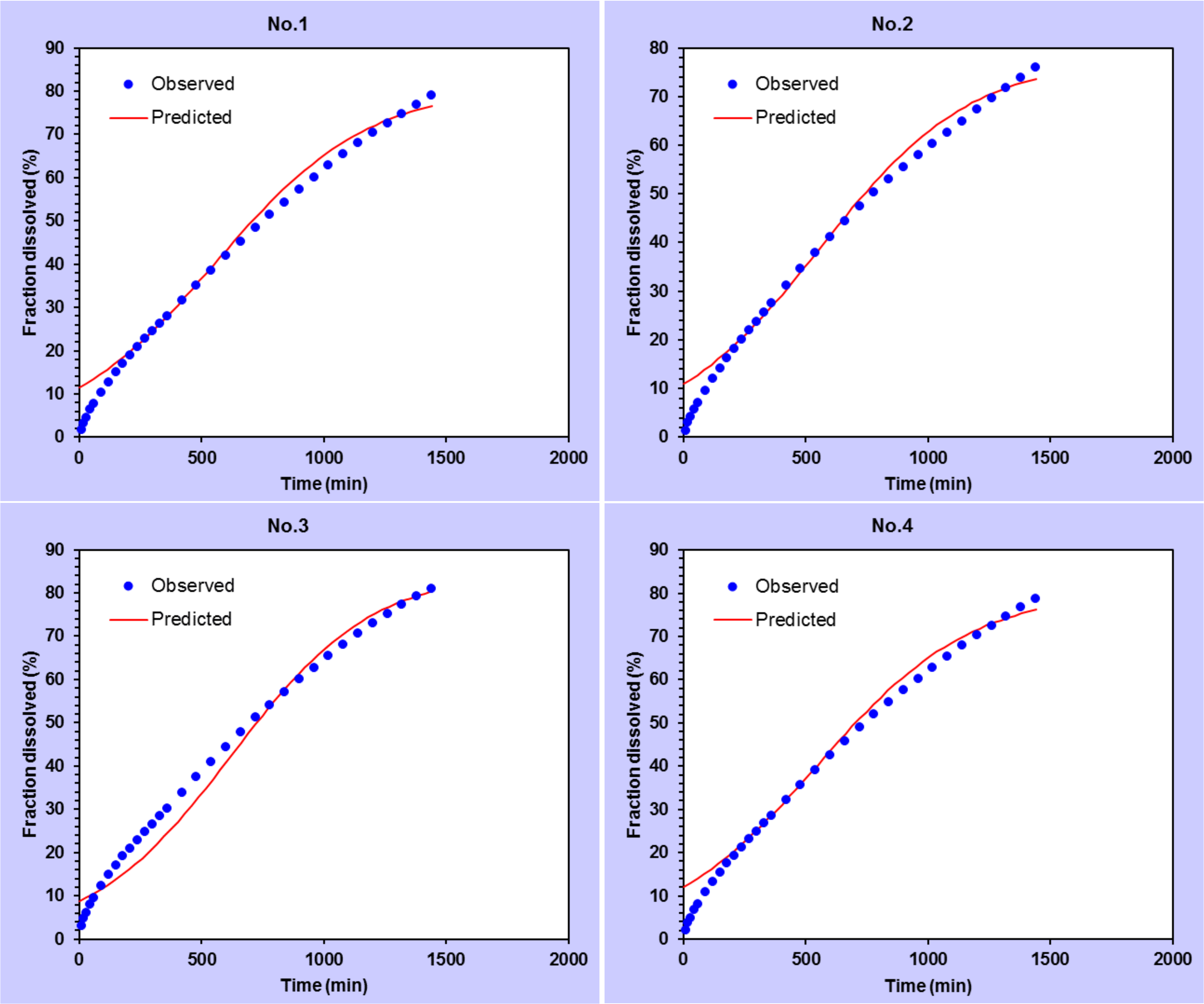

Supplementary materials – Model fitting summary – Avicel® PH-102 (modelling the entire relevant dissolution data)

Model: **Gompertz_1**

Model equation: $F = 100 \cdot e^{-\alpha \cdot e^{-\beta \cdot \log(t)}}$

Fitted model parameters per tested tablet (N = 4) with statistics – mean, standard deviation (SD), and relative standard deviation expressed in % (RSD%) (output from DDSolver):

Parameter	No.1	No.2	No.3	No.4	Mean	SD	RSD(%)
α	27.038	33.435	24.448	25.436	27.589	4.040	14.645
β	1.316	1.316	1.310	1.296	1.310	0.009	0.714

Number of dissolution data points (N), degrees of freedom (df), and selected goodness of fit criteria – Pearson correlation coefficient (R), coefficient of determination (R^2), adjusted coefficient of determination ($R^2_{adjusted}$), and residual sum of squares (RSS) (manual calculation in MS Excel):

Parameter	No.1	No.2	No.3	No.4
N	33	33	33	33
df	31	31	31	31
R	0.958009926	0.977670045	0.952229531	0.958333822
R^2	0.917783018	0.955838718	0.906741081	0.918403714
$R^2_{adjusted}$	0.915130858	0.95441416	0.903732728	0.915771575
RSS	1915.526497	1482.054598	2177.951814	1867.399676

Graphical abstract of model fit presented as mean ± 1 SD of the fraction % of released carvedilol:

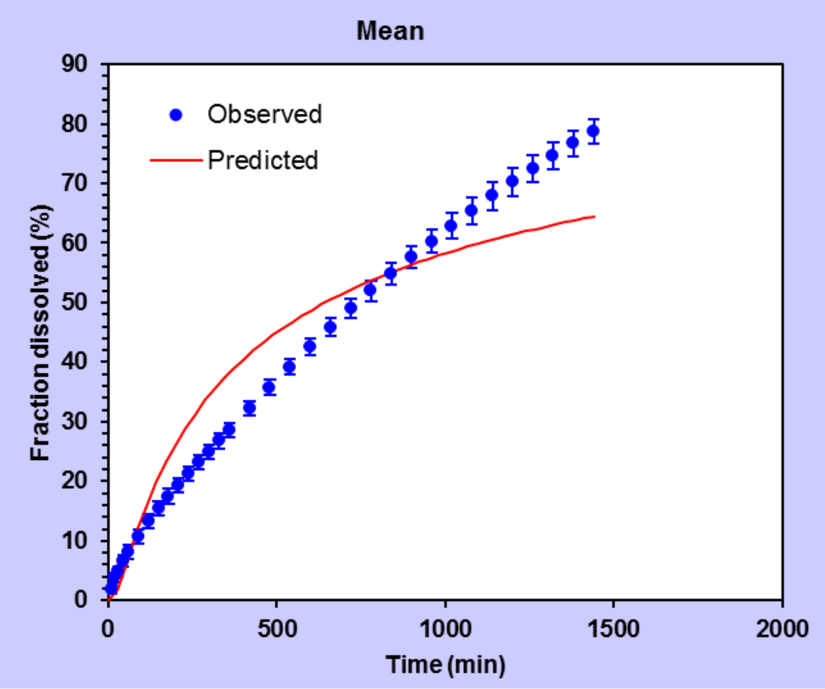

Supplementary materials – Model fitting summary – Avicel® PH-102 (modelling the entire relevant dissolution data)

Graphical abstract of model fit presented as the fraction % of released carvedilol per tested tablet:

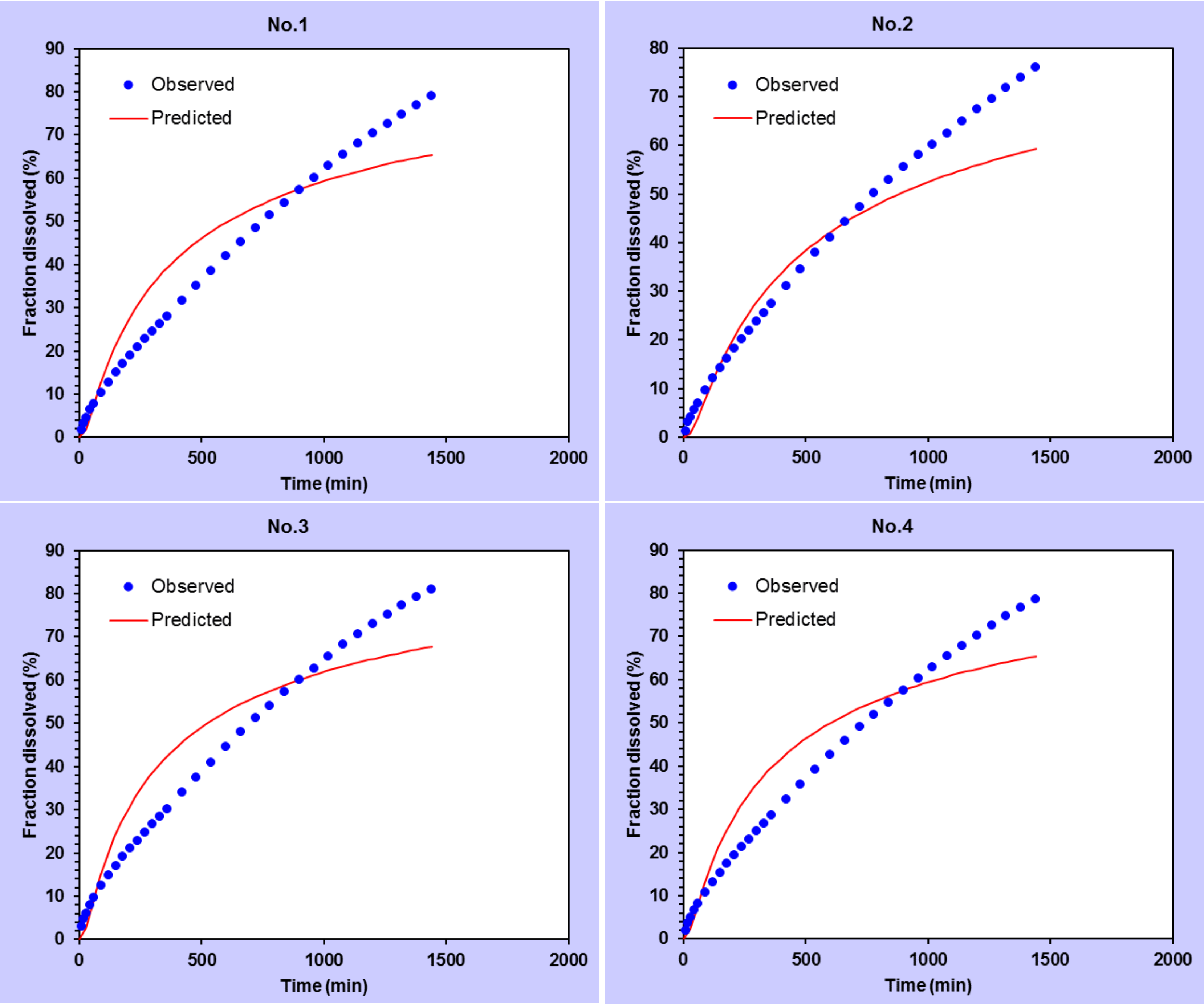

Supplementary materials – Model fitting summary – Avicel® PH-102 (modelling the entire relevant dissolution data)

Model: **Gompertz_2**

Model equation: $F = F_{max} \cdot e^{-\alpha \cdot e^{-\beta \cdot \log(t)}}$

Fitted model parameters per tested tablet (N = 4) with statistics – mean, standard deviation (SD), and relative standard deviation expressed in % (RSD%) (output from DDSolver):

Parameter	No.1	No.2	No.3	No.4	Mean	SD	RSD(%)
α	69.856	72.483	74.484	66.771	70.898	3.341	4.713
β	1.626	1.640	1.723	1.618	1.652	0.048	2.925
F_{max}	94.188	90.582	85.073	93.788	90.908	4.211	4.633

Number of dissolution data points (N), degrees of freedom (df), and selected goodness of fit criteria – Pearson correlation coefficient (R), coefficient of determination (R^2), adjusted coefficient of determination ($R^2_{adjusted}$), and residual sum of squares (RSS) (manual calculation in MS Excel):

Parameter	No.1	No.2	No.3	No.4
N	33	33	33	33
df	30	30	30	30
R	0.977217146	0.980772343	0.968114858	0.97719132
R^2	0.954953351	0.961914389	0.937246379	0.954902877
$R^2_{adjusted}$	0.951950241	0.959375348	0.933062804	0.951896402
RSS	1489.344563	1288.498569	2260.00918	1418.101323

Graphical abstract of model fit presented as mean ± 1 SD of the fraction % of released carvedilol:

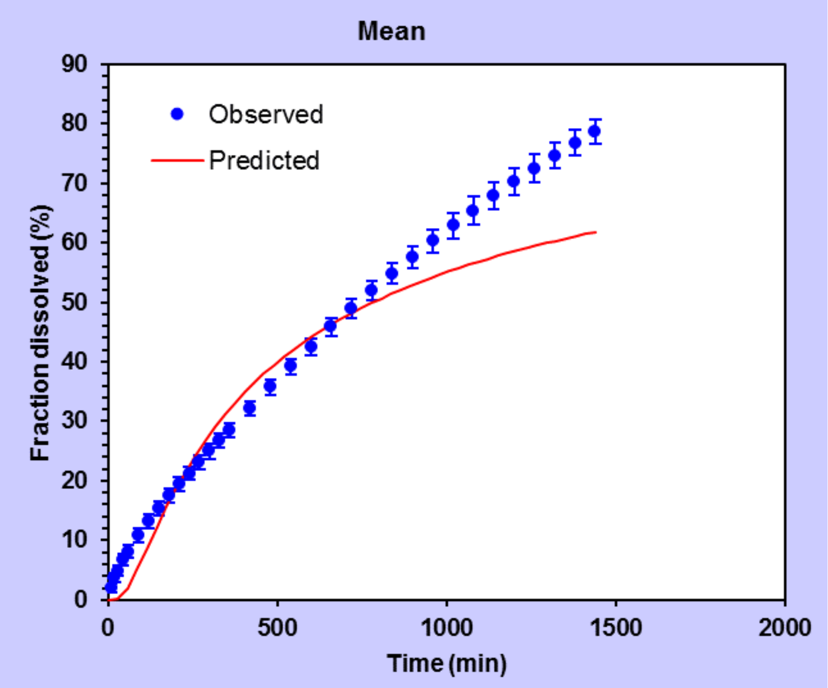

Supplementary materials – Model fitting summary – Avicel® PH-102 (modelling the entire relevant dissolution data)

Graphical abstract of model fit presented as the fraction % of released carvedilol per tested tablet:

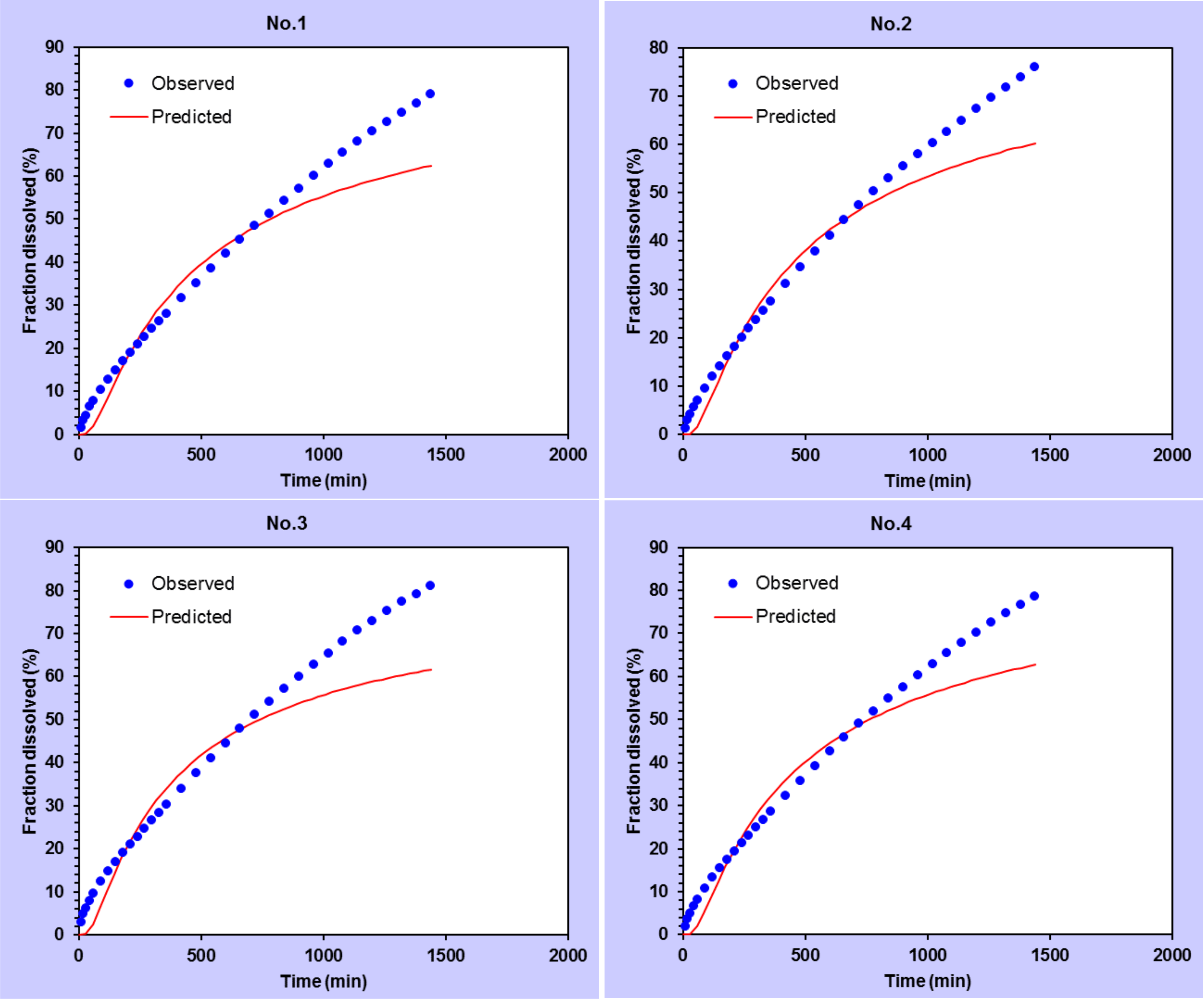

Supplementary materials – Model fitting summary – Avicel® PH-102 (modelling the entire relevant dissolution data)

Model: **Gompertz_3**

Model equation: $F = F_{max} \cdot e^{-e^{-k \cdot (t-\gamma)}}$

Fitted model parameters per tested tablet (N = 4) with statistics – mean, standard deviation (SD), and relative standard deviation expressed in % (RSD%) (output from DDSolver):

Parameter	No.1	No.2	No.3	No.4	Mean	SD	RSD(%)
k	0.002	0.002	0.002	0.002	0.002	0.000	0.417
γ	426.494	426.454	398.677	416.623	417.062	13.107	3.143
F_{max}	82.955	79.779	85.073	82.602	82.602	2.175	2.634

Number of dissolution data points (N), degrees of freedom (df), and selected goodness of fit criteria – Pearson correlation coefficient (R), coefficient of determination (R^2), adjusted coefficient of determination ($R^2_{adjusted}$), and residual sum of squares (RSS) (manual calculation in MS Excel):

Parameter	No.1	No.2	No.3	No.4
N	33	33	33	33
df	30	30	30	30
R	0.995567834	0.995628448	0.996062946	0.99583929
R^2	0.991155311	0.991276007	0.992141392	0.991695892
$R^2_{adjusted}$	0.990565665	0.990694408	0.991617485	0.991142285
RSS	217.3695837	197.5969195	196.1720118	199.2719059

Graphical abstract of model fit presented as mean ± 1 SD of the fraction % of released carvedilol:

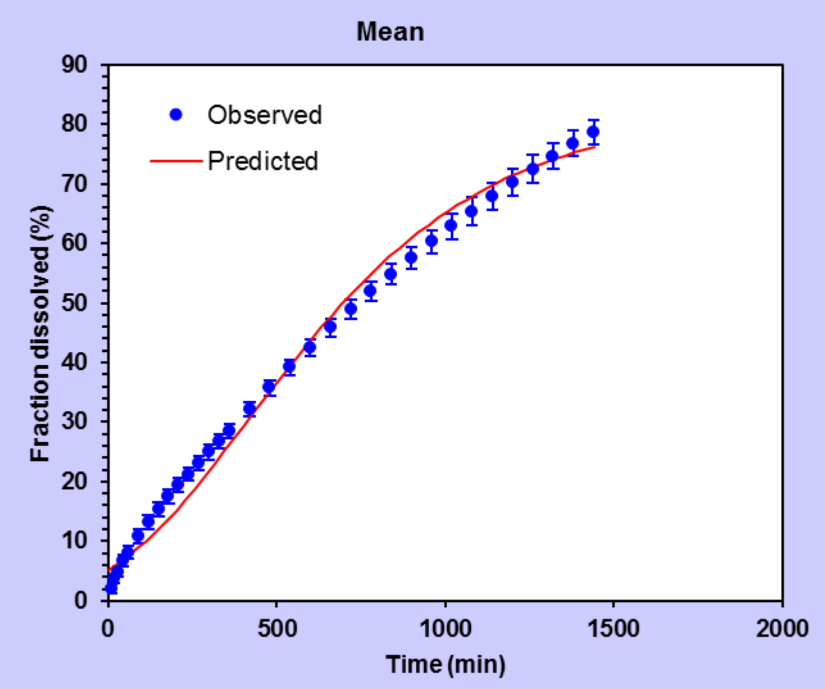

Supplementary materials – Model fitting summary – Avicel® PH-102 (modelling the entire relevant dissolution data)

Graphical abstract of model fit presented as the fraction % of released carvedilol per tested tablet:

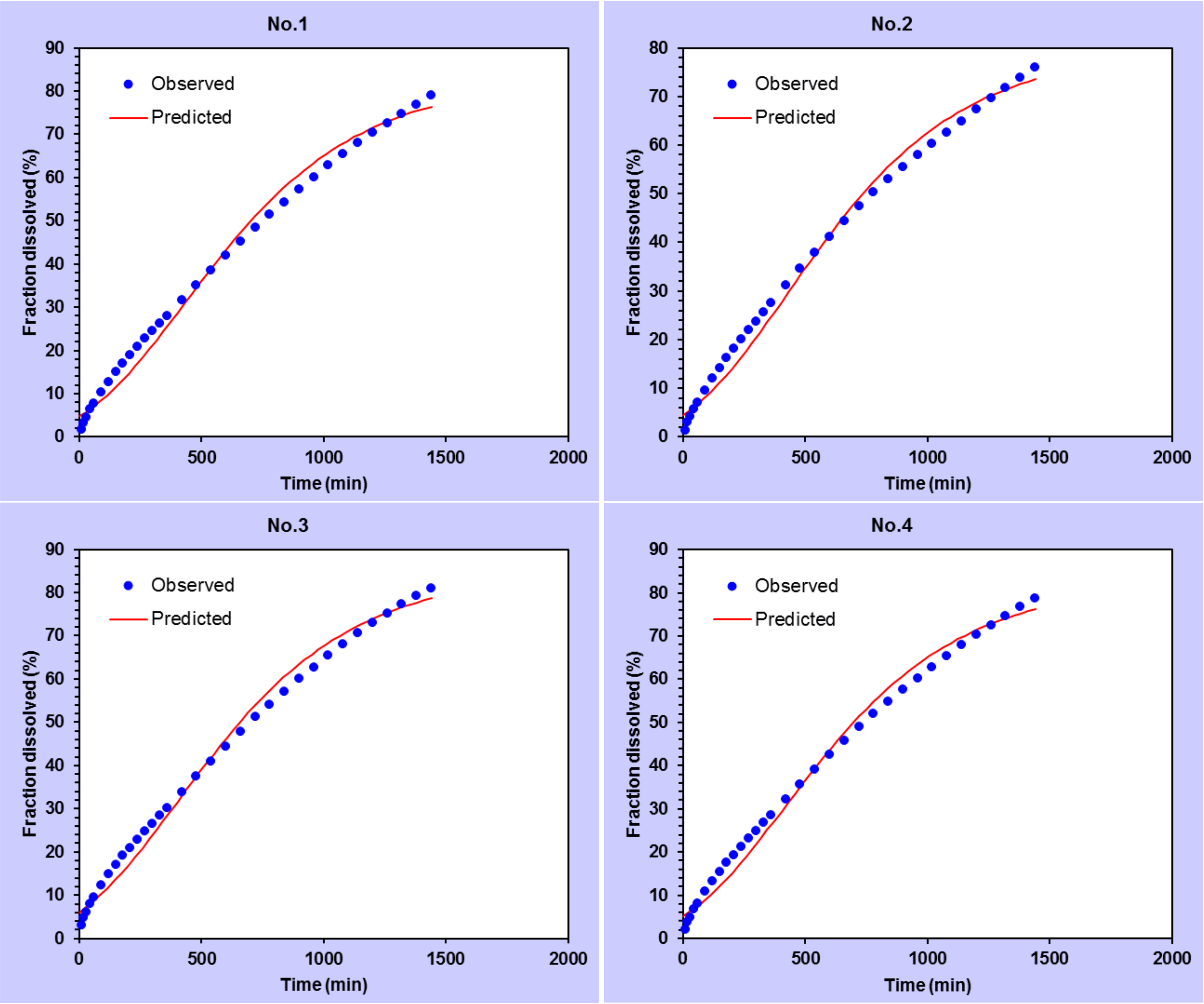

Supplementary materials – Model fitting summary – Avicel® PH-102 (modelling the entire relevant dissolution data)

Model: **Gompertz_4**

Model equation: $F = F_{max} \cdot e^{-\beta \cdot e^{-k \cdot t}}$

Fitted model parameters per tested tablet (N = 4) with statistics – mean, standard deviation (SD), and relative standard deviation expressed in % (RSD%) (output from DDSolver):

Parameter	No.1	No.2	No.3	No.4	Mean	SD	RSD(%)
k	0.002	0.002	0.002	0.002	0.002	0.000	0.417
β	2.862	2.866	2.651	2.784	2.791	0.100	3.595
F_{max}	82.955	79.779	85.073	82.602	82.602	2.175	2.634

Number of dissolution data points (N), degrees of freedom (df), and selected goodness of fit criteria – Pearson correlation coefficient (R), coefficient of determination (R^2), adjusted coefficient of determination ($R^2_{adjusted}$), and residual sum of squares (RSS) (manual calculation in MS Excel):

Parameter	No.1	No.2	No.3	No.4
N	33	33	33	33
df	30	30	30	30
R	0.995567834	0.995628448	0.996062946	0.99583929
R^2	0.991155311	0.991276007	0.992141392	0.991695892
$R^2_{adjusted}$	0.990565665	0.990694408	0.991617485	0.991142285
RSS	217.3695837	197.5969195	196.1720118	199.2719059

Graphical abstract of model fit presented as mean ± 1 SD of the fraction % of released carvedilol:

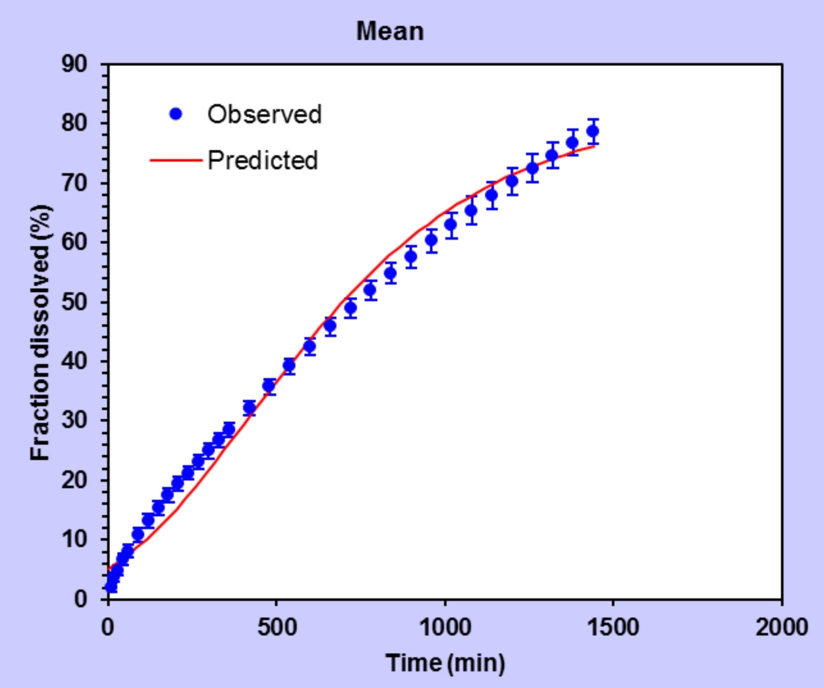

Supplementary materials – Model fitting summary – Avicel® PH-102 (modelling the entire relevant dissolution data)

Graphical abstract of model fit presented as the fraction % of released carvedilol per tested tablet:

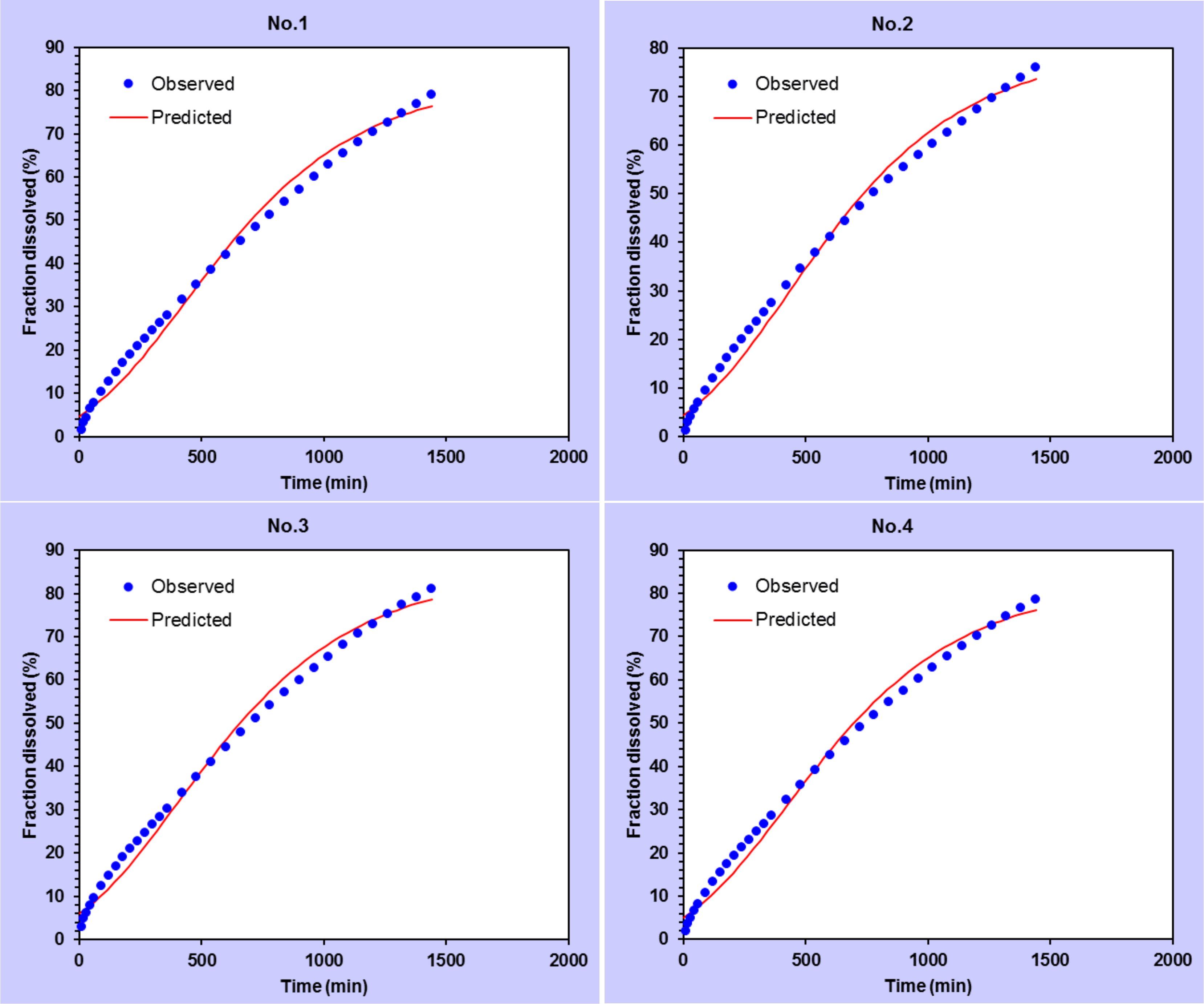

Supplementary materials – Model fitting summary – Avicel® PH-102 (modelling the entire relevant dissolution data)

Model: **Probit_1**

Model equation: $F = 100 \cdot \phi[\alpha + \beta \cdot \log(t)]$

Fitted model parameters per tested tablet (N = 4) with statistics – mean, standard deviation (SD), and relative standard deviation expressed in % (RSD%) (output from DDSolver):

Parameter	No.1	No.2	No.3	No.4	Mean	SD	RSD(%)
α	-3.871	-3.888	-3.641	-3.783	-3.796	0.113	-2.975
β	1.368	1.357	1.312	1.339	1.344	0.025	1.835

Number of dissolution data points (N), degrees of freedom (df), and selected goodness of fit criteria – Pearson correlation coefficient (R), coefficient of determination (R^2), adjusted coefficient of determination ($R^2_{adjusted}$), and residual sum of squares (RSS) (manual calculation in MS Excel):

Parameter	No.1	No.2	No.3	No.4
N	33	33	33	33
df	31	31	31	31
R	0.979255554	0.9839951	0.974694509	0.979288182
R^2	0.95894144	0.968246356	0.950029387	0.959005343
$R^2_{adjusted}$	0.95761697	0.967222045	0.948417431	0.957682934
RSS	1027.547556	767.0148954	1247.533862	1014.457279

Graphical abstract of model fit presented as mean ± 1 SD of the fraction % of released carvedilol:

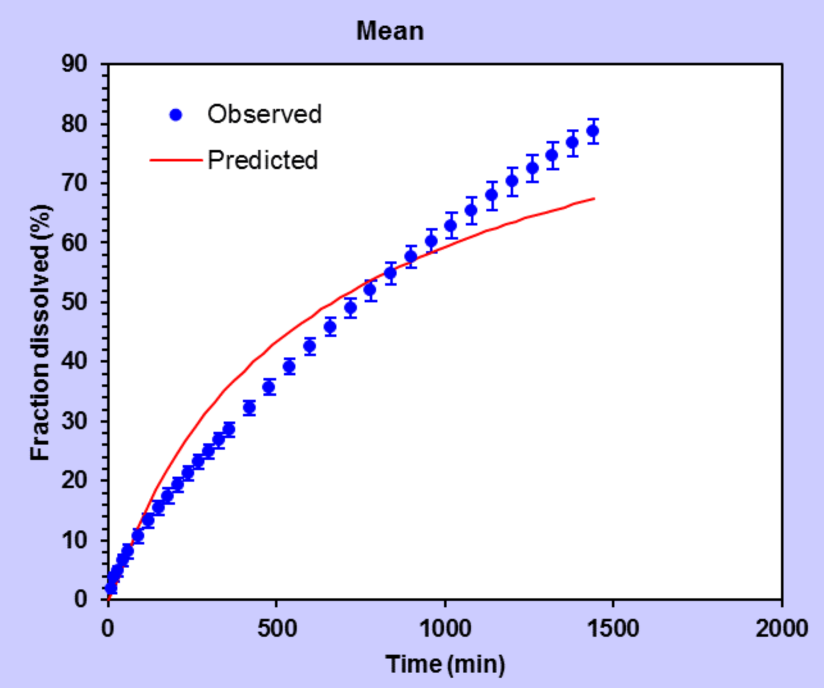

Supplementary materials – Model fitting summary – Avicel® PH-102 (modelling the entire relevant dissolution data)

Graphical abstract of model fit presented as the fraction % of released carvedilol per tested tablet:

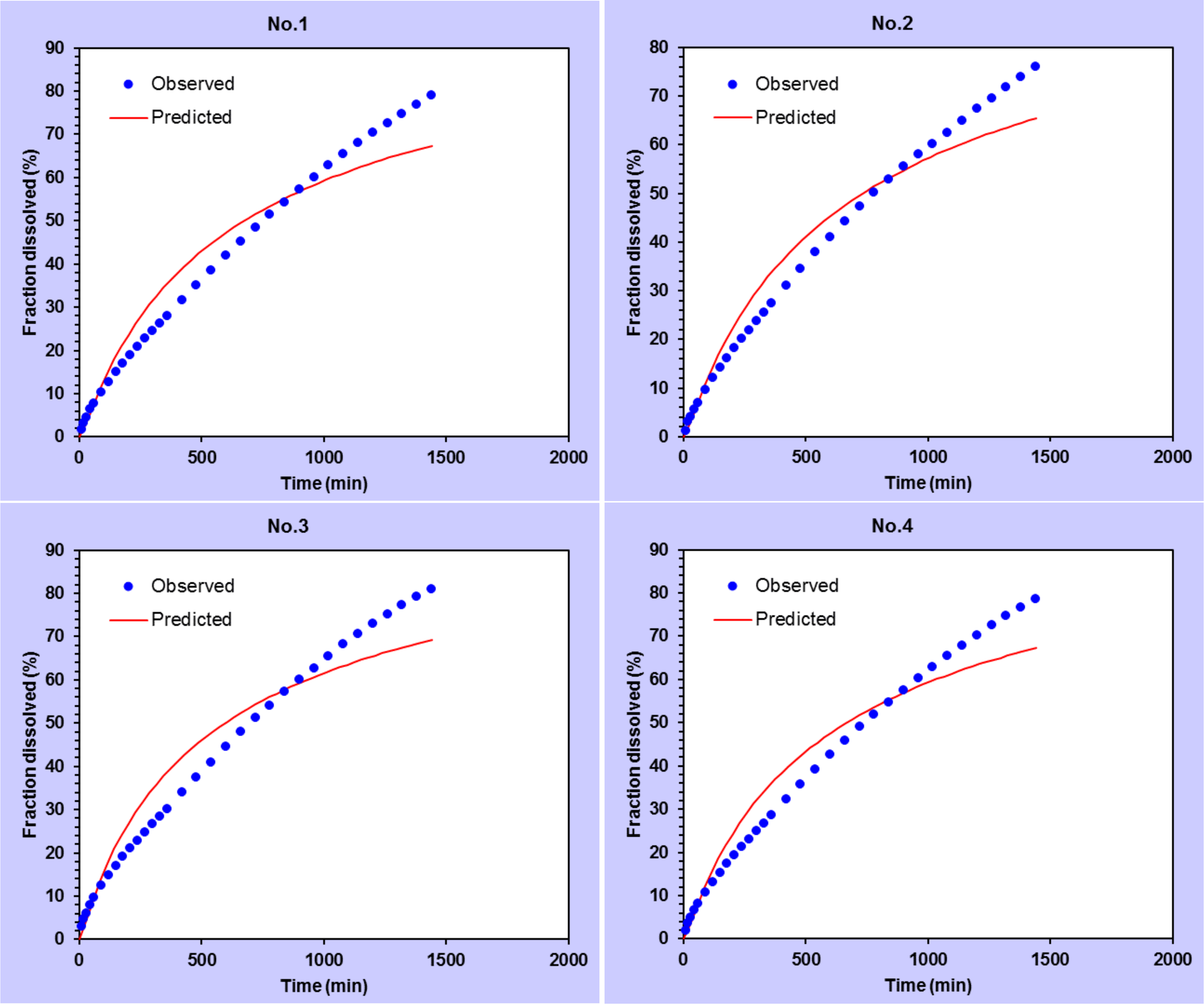

Supplementary materials – Model fitting summary – Avicel® PH-102 (modelling the entire relevant dissolution data)

Model: **Probit_2**

Model equation: $F = F_{max} \cdot \phi[\alpha + \beta \cdot \log(t)]$

Fitted model parameters per tested tablet (N = 4) with statistics – mean, standard deviation (SD), and relative standard deviation expressed in % (RSD%) (output from DDSolver):

Parameter	No.1	No.2	No.3	No.4	Mean	SD	RSD(%)
α	-4.861	-4.929	-4.597	-4.767	-4.789	0.144	-3.006
β	1.709	1.734	1.641	1.685	1.692	0.040	2.342
F_{max}	92.647	89.100	92.285	92.253	91.571	1.657	1.810

Number of dissolution data points (N), degrees of freedom (df), and selected goodness of fit criteria – Pearson correlation coefficient (R), coefficient of determination (R^2), adjusted coefficient of determination ($R^2_{adjusted}$), and residual sum of squares (RSS) (manual calculation in MS Excel):

Parameter	No.1	No.2	No.3	No.4
N	33	33	33	33
df	30	30	30	30
R	0.989753167	0.992286031	0.986721644	0.989527454
R^2	0.979611332	0.984631567	0.973619603	0.979164583
$R^2_{adjusted}$	0.978252088	0.983607005	0.97186091	0.977775555
RSS	1032.133344	897.4827372	1267.313658	949.554759

Graphical abstract of model fit presented as mean ± 1 SD of the fraction % of released carvedilol:

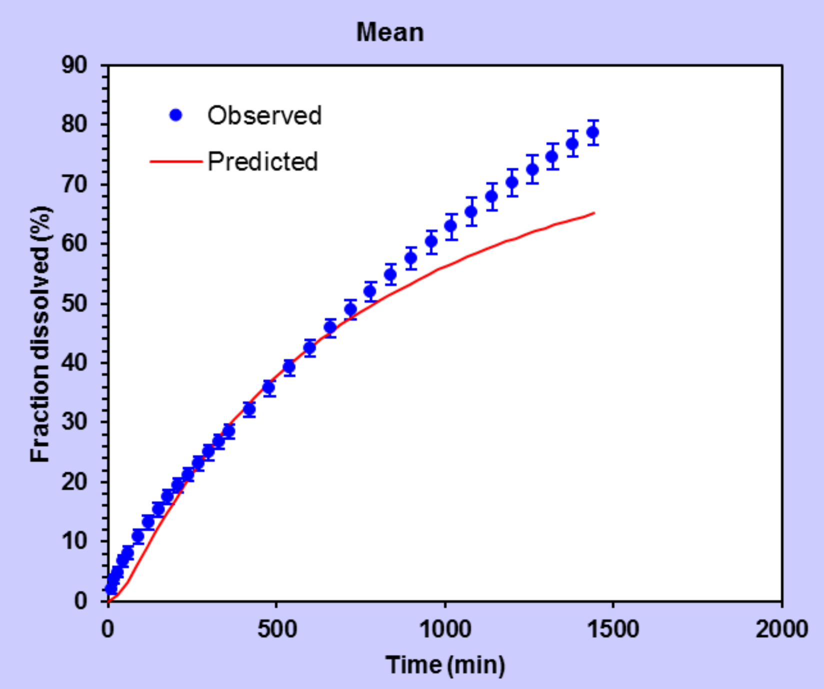

Supplementary materials – Model fitting summary – Avicel® PH-102 (modelling the entire relevant dissolution data)

Graphical abstract of model fit presented as the fraction % of released carvedilol per tested tablet:

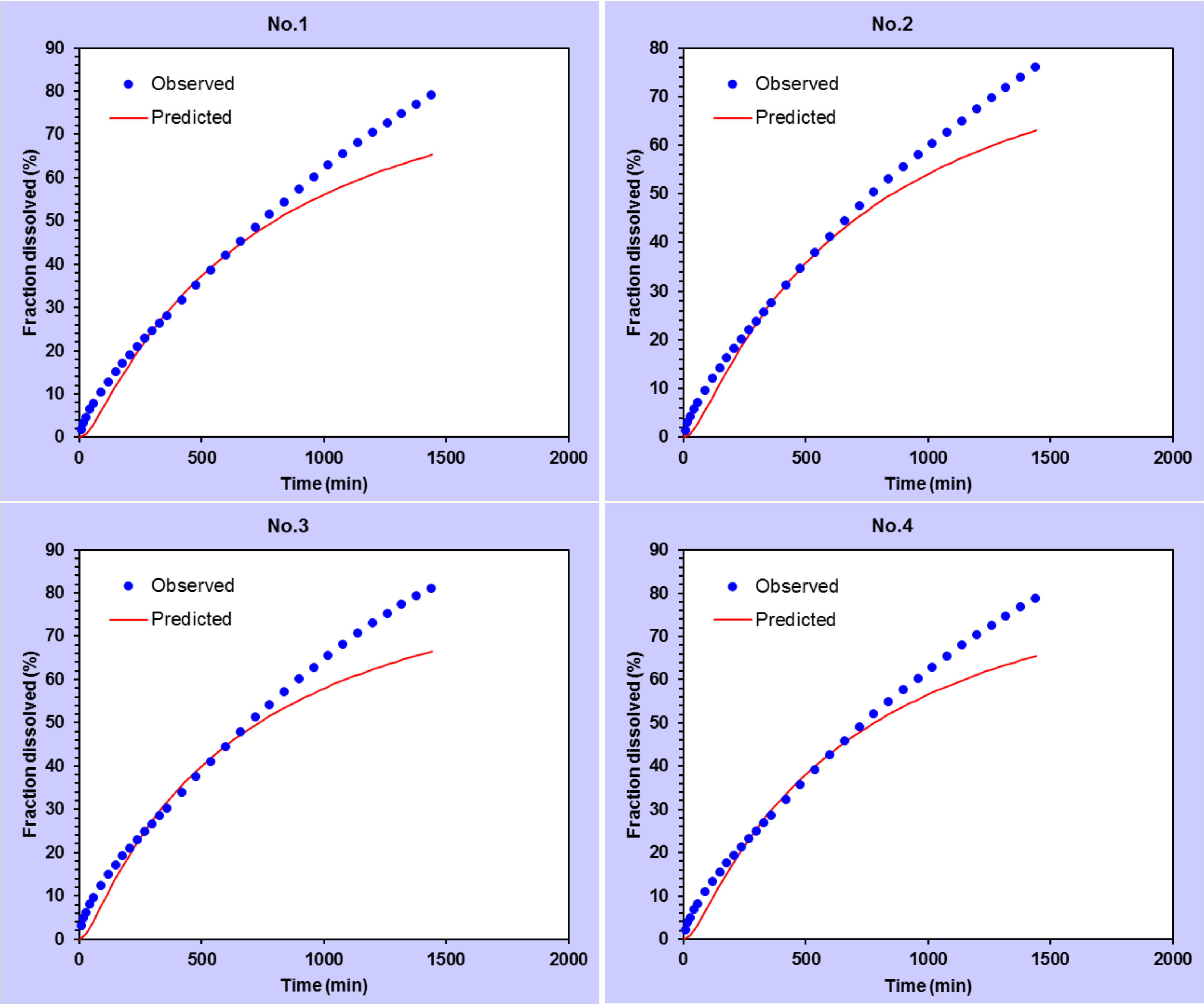

Supplementary materials – Model fitting summary – Avicel® PH-102 (modelling up to app. 60 % of carvedilol released)

Model: **Zero–order**

Model equation: $F = k_0 \cdot t$

Fitted model parameters per tested tablet (N = 4) with statistics – mean, standard deviation (SD), and relative standard deviation expressed in % (RSD%) (output from DDSolver):

Parameter	No.1	No.2	No.3	No.4	Mean	SD	RSD(%)
k_0	0.068	0.067	0.072	0.069	0.069	0.002	3.566

Number of dissolution data points (N), degrees of freedom (df), and selected goodness of fit criteria – Pearson correlation coefficient (R), coefficient of determination (R^2), adjusted coefficient of determination ($R^2_{adjusted}$), and residual sum of squares (RSS) (manual calculation in MS Excel):

Parameter	No.1	No.2	No.3	No.4
N	25	25	25	25
df	24	24	24	24
R	0.995910926	0.995288144	0.995563642	0.995617822
R^2	0.991838573	0.990598489	0.991146964	0.991254847
$R^2_{adjusted}$	0.991838573	0.990598489	0.991146964	0.991254847
RSS	298.0517256	268.0129342	497.0680472	345.7900375

Graphical abstract of model fit presented as mean ± 1 SD of the fraction % of released carvedilol:

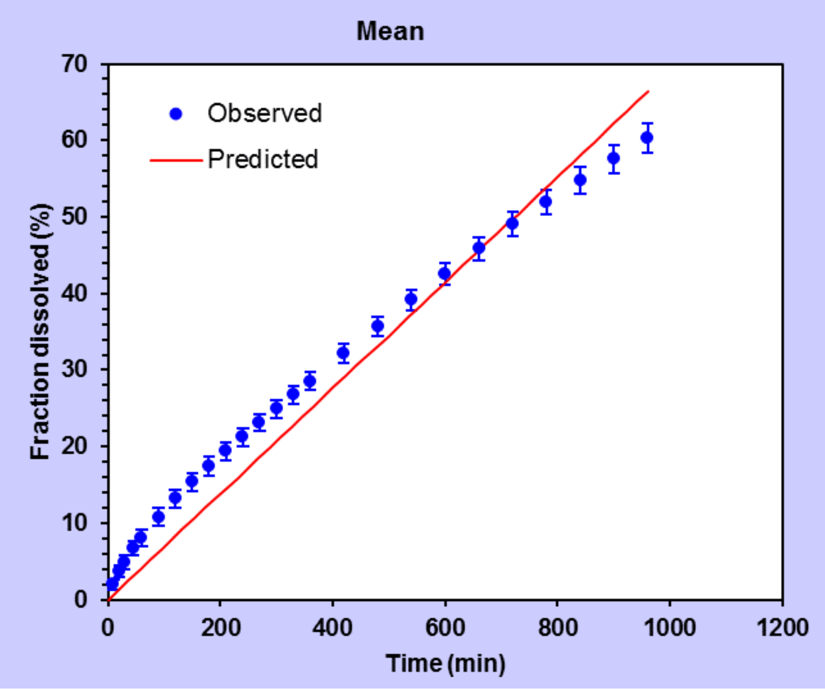

Supplementary materials – Model fitting summary – Avicel® PH-102 (modelling up to app. 60 % of carvedilol released)

Graphical abstract of model fit presented as the fraction % of released carvedilol per tested tablet:

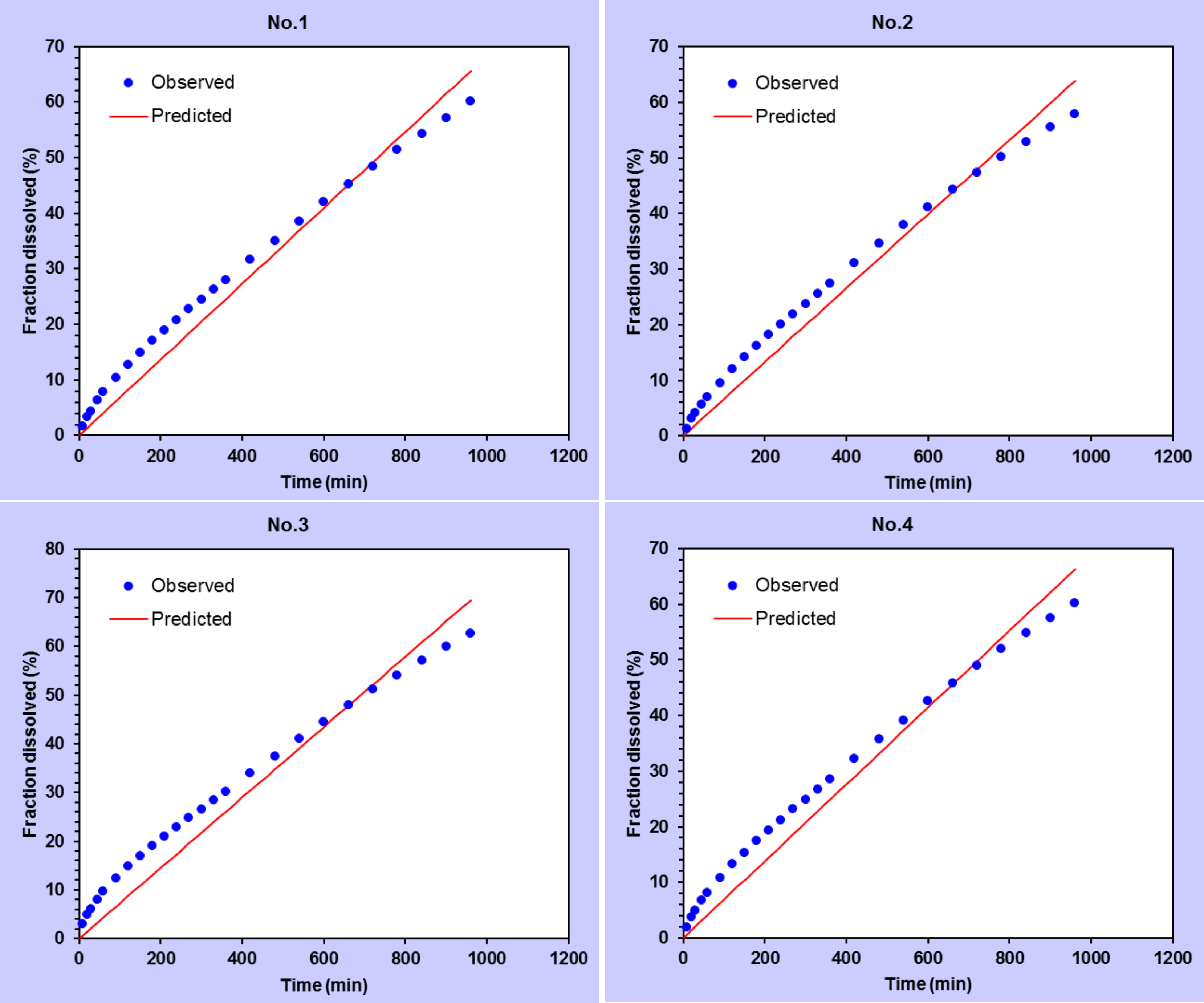

Supplementary materials – Model fitting summary – Avicel® PH-102 (modelling up to app. 60 % of carvedilol released)

Model: **Zero–order with T_{lag}**

Model equation: $F = k_0 \cdot (t - T_{lag})$

Fitted model parameters per tested tablet (N = 4) with statistics – mean, standard deviation (SD), and relative standard deviation expressed in % (RSD%) (output from DDSolver):

Parameter	No.1	No.2	No.3	No.4	Mean	SD	RSD(%)
k_0	0.060	0.059	0.061	0.060	0.060	0.001	1.612
T_{lag}	-81.762	-76.281	-107.847	-88.842	-88.683	13.772	-15.529

Number of dissolution data points (N), degrees of freedom (df), and selected goodness of fit criteria – Pearson correlation coefficient (R), coefficient of determination (R^2), adjusted coefficient of determination ($R^2_{adjusted}$), and residual sum of squares (RSS) (manual calculation in MS Excel):

Parameter	No.1	No.2	No.3	No.4
N	25	25	25	25
df	23	23	23	23
R	0.995910926	0.995288144	0.995563642	0.995617822
R^2	0.991838573	0.990598489	0.991146964	0.991254847
$R^2_{adjusted}$	0.991483728	0.990189728	0.99076205	0.990874623
RSS	64.82804686	72.14299384	73.46212631	69.69076284

Graphical abstract of model fit presented as mean ± 1 SD of the fraction % of released carvedilol:

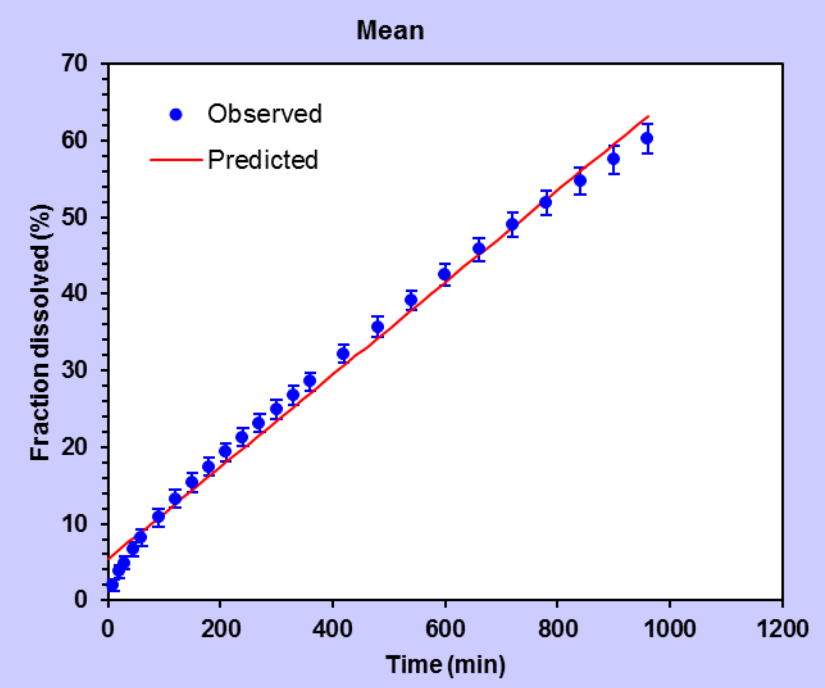

Supplementary materials – Model fitting summary – Avicel® PH-102 (modelling up to app. 60 % of carvedilol released)

Graphical abstract of model fit presented as the fraction % of released carvedilol per tested tablet:

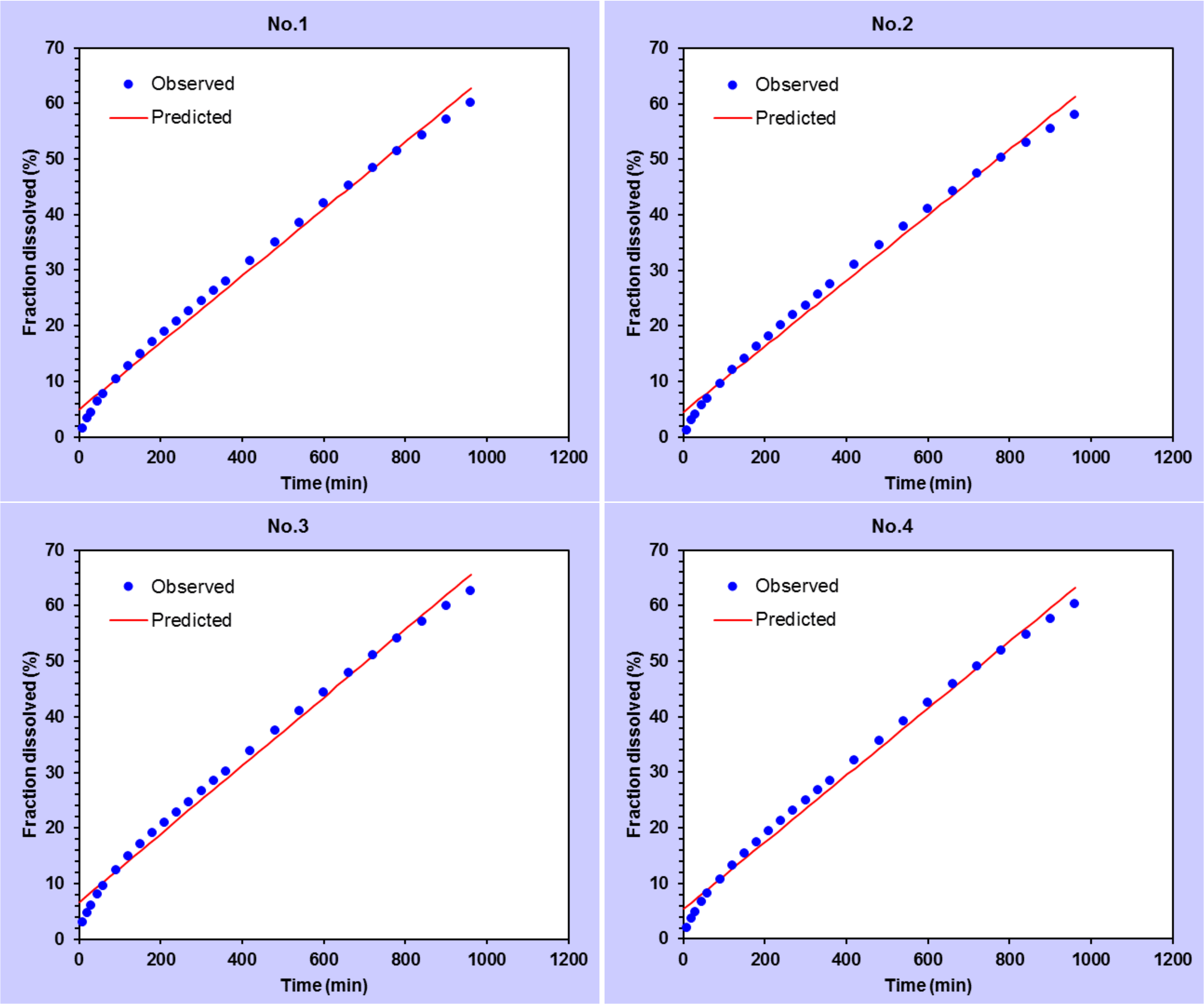

Supplementary materials – Model fitting summary – Avicel® PH-102 (modelling up to app. 60 % of carvedilol released)

Model: **Zero–order with F_0**

Model equation: $F = F_0 + k_0 \cdot t$

Fitted model parameters per tested tablet (N = 4) with statistics – mean, standard deviation (SD), and relative standard deviation expressed in % (RSD%) (output from DDSolver):

Parameter	No.1	No.2	No.3	No.4	Mean	SD	RSD(%)
k_0	0.060	0.059	0.061	0.060	0.060	0.001	1.612
F_0	4.921	4.510	6.632	5.354	5.354	0.919	17.164

Number of dissolution data points (N), degrees of freedom (df), and selected goodness of fit criteria – Pearson correlation coefficient (R), coefficient of determination (R^2), adjusted coefficient of determination ($R^2_{adjusted}$), and residual sum of squares (RSS) (manual calculation in MS Excel):

Parameter	No.1	No.2	No.3	No.4
N	25	25	25	25
df	23	23	23	23
R	0.995910926	0.995288144	0.995563642	0.995617822
R^2	0.991838573	0.990598489	0.991146964	0.991254847
$R^2_{adjusted}$	0.991483728	0.990189728	0.99076205	0.990874623
RSS	64.82804686	72.14299384	73.46212631	69.69076284

Graphical abstract of model fit presented as mean ± 1 SD of the fraction % of released carvedilol:

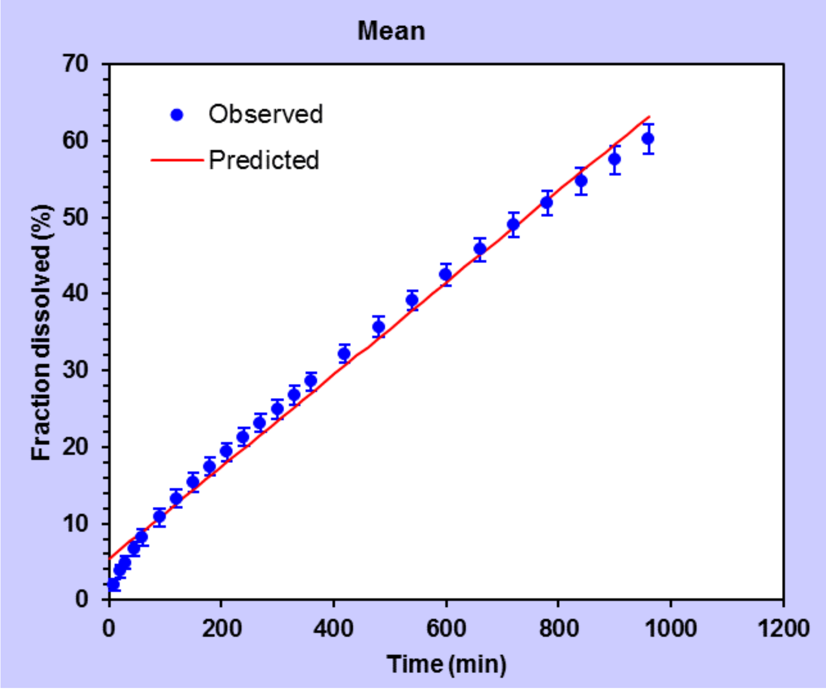

Supplementary materials – Model fitting summary – Avicel® PH-102 (modelling up to app. 60 % of carvedilol released)

Graphical abstract of model fit presented as the fraction % of released carvedilol per tested tablet:

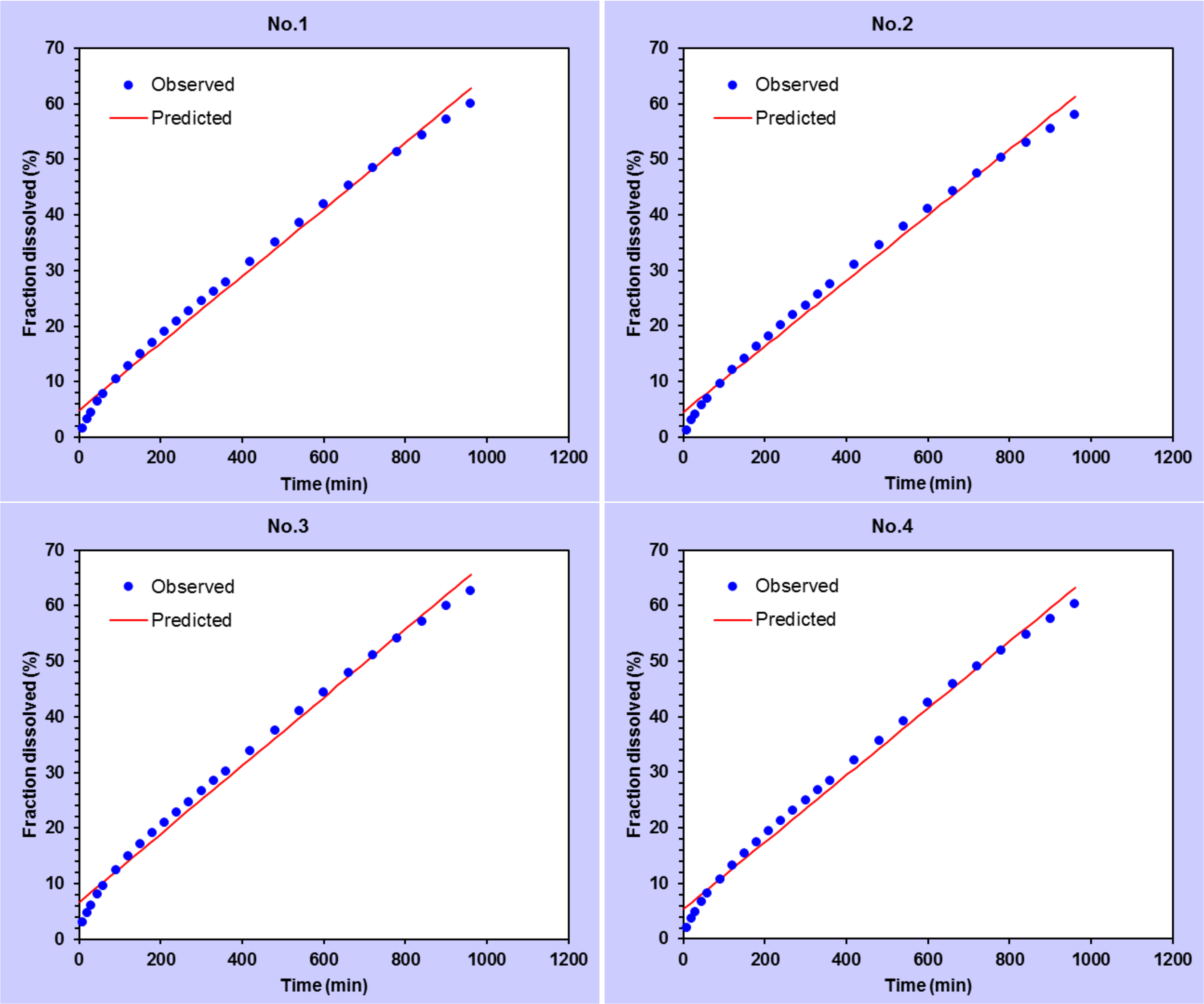

Supplementary materials – Model fitting summary – Avicel® PH-102 (modelling up to app. 60 % of carvedilol released)

Model: **First–order**

Model equation: $F = 100 \cdot (1 - e^{-k_1 \cdot t})$

Fitted model parameters per tested tablet (N = 4) with statistics – mean, standard deviation (SD), and relative standard deviation expressed in % (RSD%) (output from DDSolver):

Parameter	No.1	No.2	No.3	No.4	Mean	SD	RSD(%)
k_1	0.001	0.001	0.001	0.001	0.001	0.000	4.999

Number of dissolution data points (N), degrees of freedom (df), and selected goodness of fit criteria – Pearson correlation coefficient (R), coefficient of determination (R^2), adjusted coefficient of determination ($R^2_{adjusted}$), and residual sum of squares (RSS) (manual calculation in MS Excel):

Parameter	No.1	No.2	No.3	No.4
N	25	25	25	25
df	24	24	24	24
R	0.998956514	0.999562937	0.998635749	0.999106247
R^2	0.997914117	0.999126065	0.997273359	0.998213294
$R^2_{adjusted}$	0.997914117	0.999126065	0.997273359	0.998213294
RSS	40.30120617	23.69099274	98.84494132	49.3785832

Graphical abstract of model fit presented as mean ± 1 SD of the fraction % of released carvedilol:

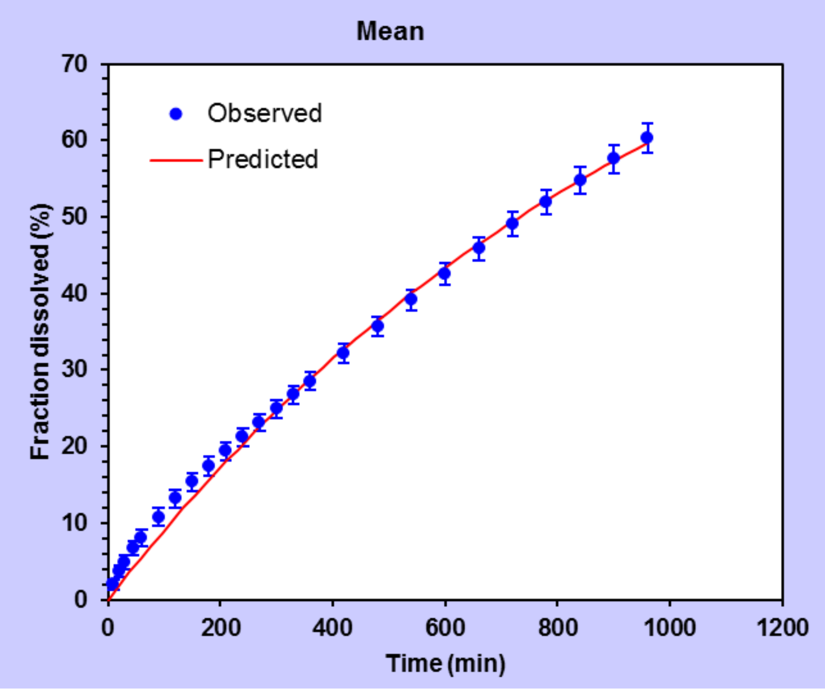

Supplementary materials – Model fitting summary – Avicel® PH-102 (modelling up to app. 60 % of carvedilol released)

Graphical abstract of model fit presented as the fraction % of released carvedilol per tested tablet:

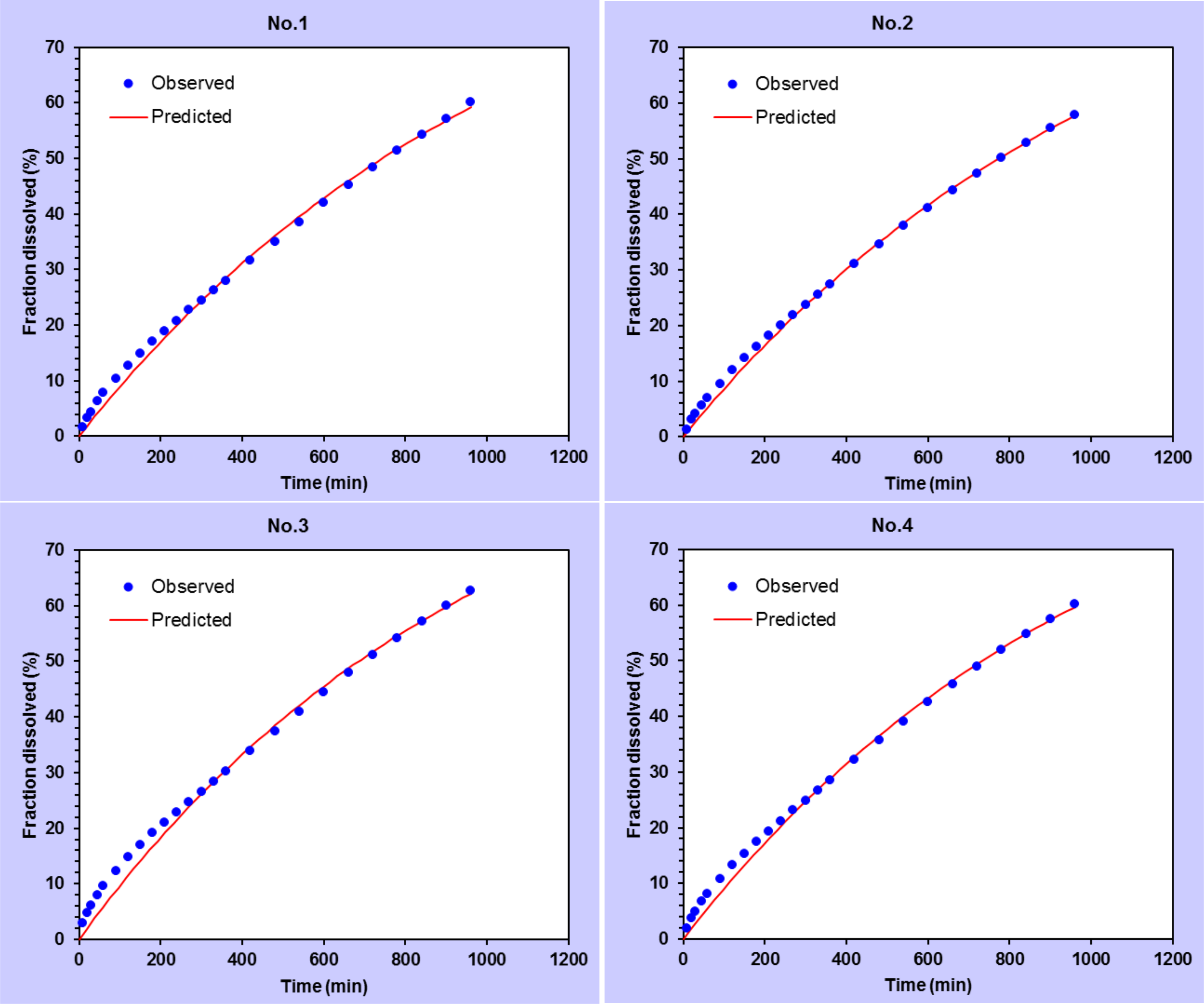

Supplementary materials – Model fitting summary – Avicel® PH-102 (modelling up to app. 60 % of carvedilol released)

Model: **First–order with T_{lag}**

Model equation: $F = 100 \cdot \left[1 - e^{-k_1 \cdot (t - T_{lag})}\right]$

Fitted model parameters per tested tablet (N = 4) with statistics – mean, standard deviation (SD), and relative standard deviation expressed in % (RSD%) (output from DDSolver):

Parameter	No.1	No.2	No.3	No.4	Mean	SD	RSD(%)
k_1	0.001	0.001	0.001	0.001	0.001	0.000	3.955
T_{lag}	-16.725	-15.835	-30.438	-21.356	-21.088	6.686	-31.706

Number of dissolution data points (N), degrees of freedom (df), and selected goodness of fit criteria – Pearson correlation coefficient (R), coefficient of determination (R^2), adjusted coefficient of determination ($R^2_{adjusted}$), and residual sum of squares (RSS) (manual calculation in MS Excel):

Parameter	No.1	No.2	No.3	No.4
N	25	25	25	25
df	23	23	23	23
R	0.99904083	0.999603483	0.998827154	0.999203925
R^2	0.99808258	0.999207124	0.997655683	0.998408484
$R^2_{adjusted}$	0.997999214	0.999172651	0.997553756	0.998339288
RSS	16.12611651	6.288589031	20.78143523	13.37740209

Graphical abstract of model fit presented as mean ± 1 SD of the fraction % of released carvedilol:

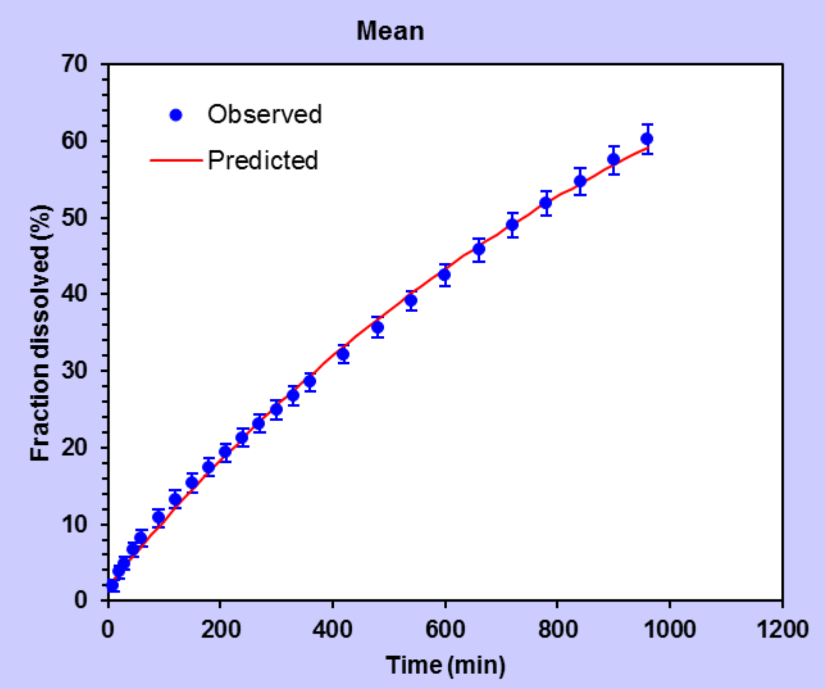

Supplementary materials – Model fitting summary – Avicel® PH-102 (modelling up to app. 60 % of carvedilol released)

Graphical abstract of model fit presented as the fraction % of released carvedilol per tested tablet:

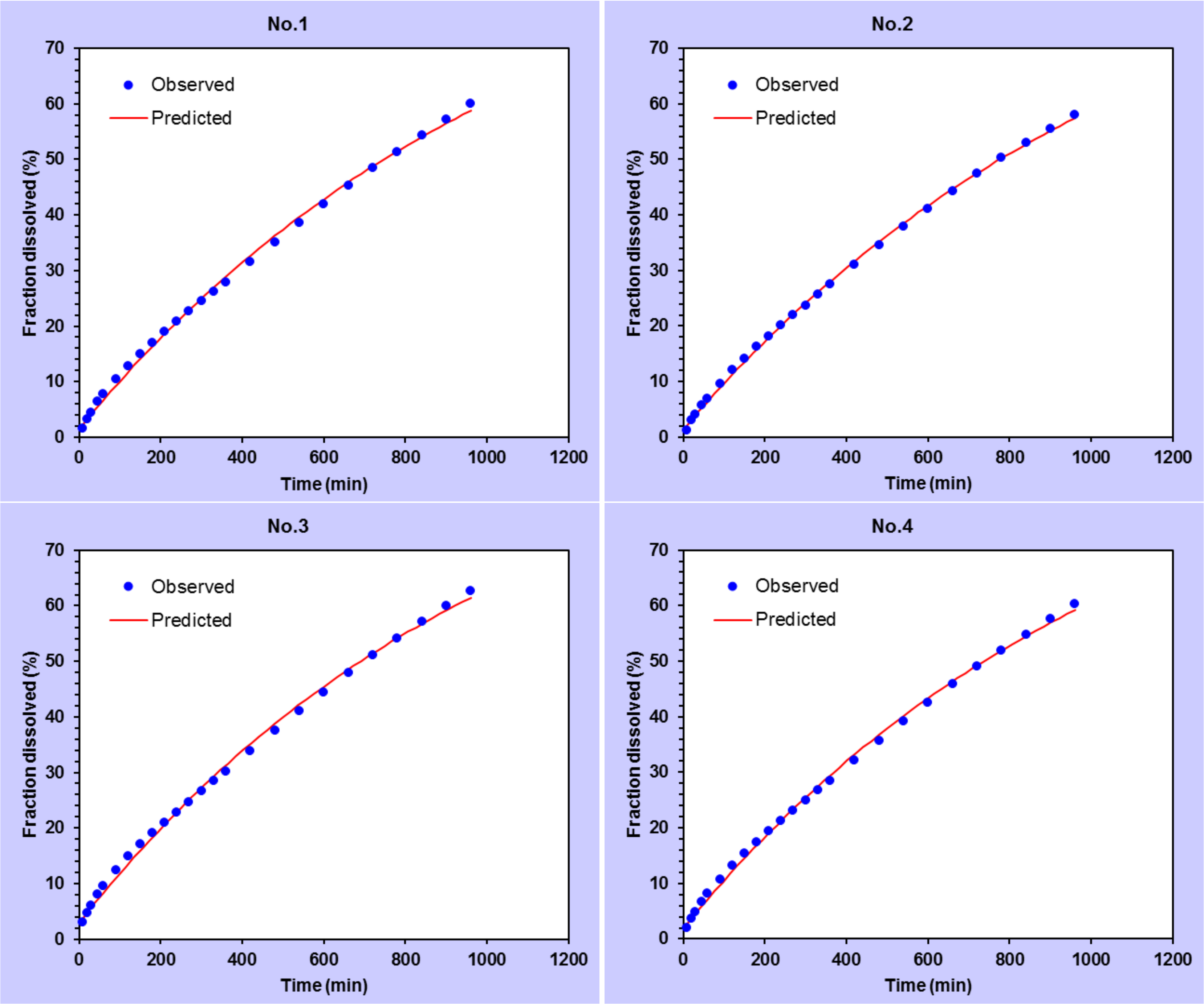

Supplementary materials – Model fitting summary – Avicel® PH-102 (modelling up to app. 60 % of carvedilol released)

Model: **First–order with F_{max}**

Model equation: $F = F_{max} \cdot (1 - e^{-k_1 \cdot t})$

Fitted model parameters per tested tablet (N = 4) with statistics – mean, standard deviation (SD), and relative standard deviation expressed in % (RSD%) (output from DDSolver):

Parameter	No.1	No.2	No.3	No.4	Mean	SD	RSD(%)
k_1	0.002	0.002	0.002	0.002	0.002	0.000	1.079
F_{max}	63.101	60.881	65.867	63.283	63.283	2.039	3.223

Number of dissolution data points (N), degrees of freedom (df), and selected goodness of fit criteria – Pearson correlation coefficient (R), coefficient of determination (R^2), adjusted coefficient of determination ($R^2_{adjusted}$), and residual sum of squares (RSS) (manual calculation in MS Excel):

Parameter	No.1	No.2	No.3	No.4
N	25	25	25	25
df	23	23	23	23
R	0.983551843	0.984391013	0.982949959	0.983651495
R^2	0.967374228	0.969025667	0.966190623	0.967570263
$R^2_{adjusted}$	0.965955717	0.967678957	0.96472065	0.966160274
RSS	460.2226922	449.6569995	436.588363	443.8498338

Graphical abstract of model fit presented as mean ± 1 SD of the fraction % of released carvedilol:

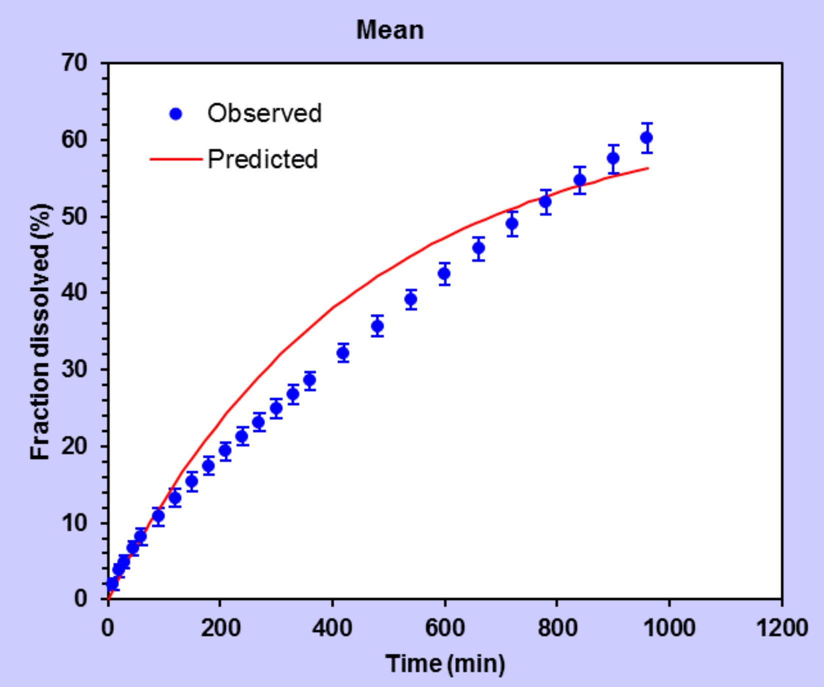

Supplementary materials – Model fitting summary – Avicel® PH-102 (modelling up to app. 60 % of carvedilol released)

Graphical abstract of model fit presented as the fraction % of released carvedilol per tested tablet:

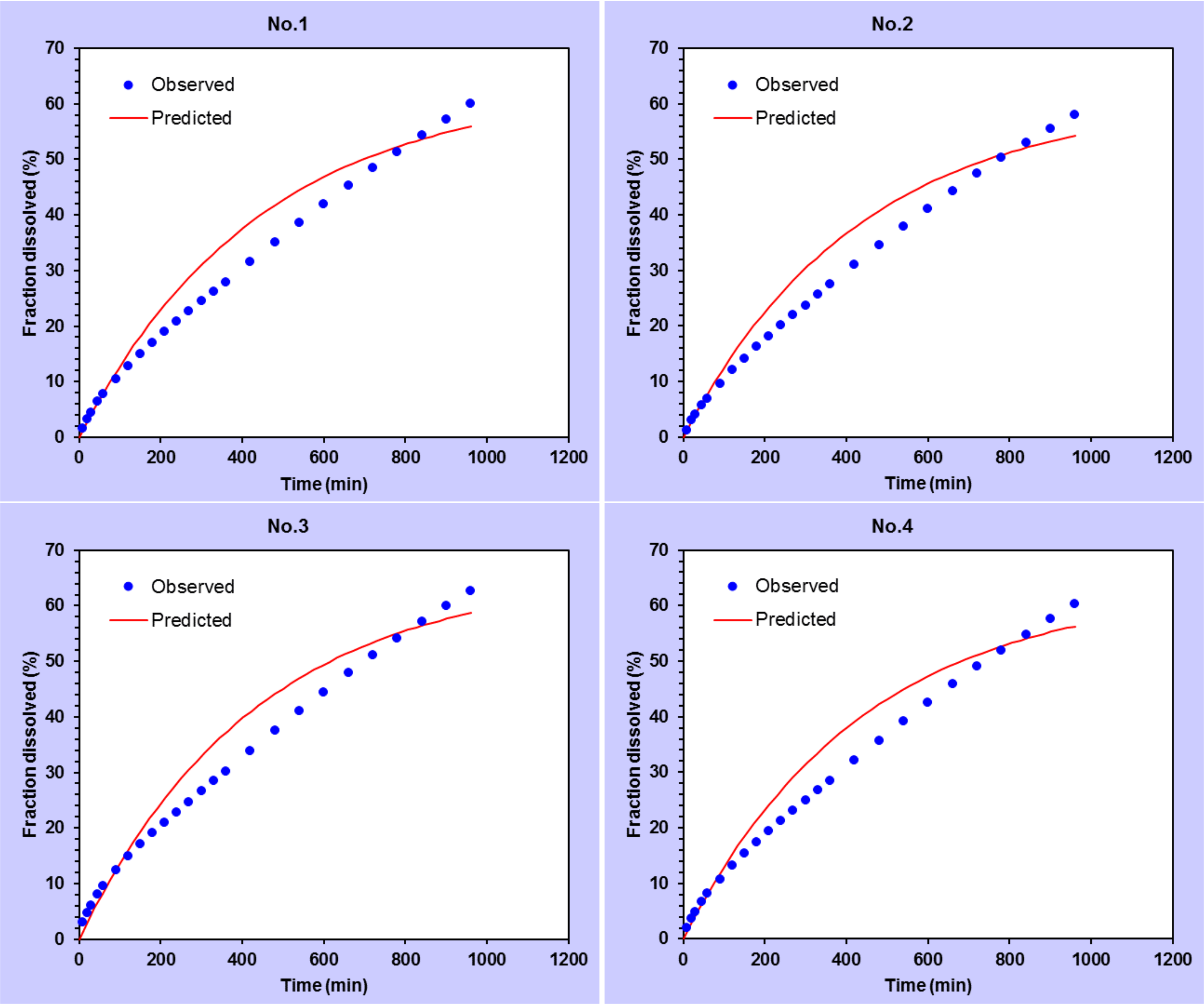

Supplementary materials – Model fitting summary – Avicel® PH-102 (modelling up to app. 60 % of carvedilol released)

Model: **First–order with T_{lag} and F_{max}**

Model equation: $F = F_{max} \cdot \left[1 - e^{-k_1 \cdot (t - T_{lag})}\right]$

Fitted model parameters per tested tablet (N = 4) with statistics – mean, standard deviation (SD), and relative standard deviation expressed in % (RSD%) (output from DDSolver):

Parameter	No.1	No.2	No.3	No.4	Mean	SD	RSD(%)
k_1	0.003	0.003	0.003	0.003	0.003	0.000	1.159
T_{lag}	61.386	63.983	51.566	58.868	58.951	5.348	9.072
F_{max}	63.101	60.881	65.867	63.283	63.283	2.039	3.223

Number of dissolution data points (N), degrees of freedom (df), and selected goodness of fit criteria – Pearson correlation coefficient (R), coefficient of determination (R^2), adjusted coefficient of determination ($R^2_{adjusted}$), and residual sum of squares (RSS) (manual calculation in MS Excel):

Parameter	No.1	No.2	No.3	No.4
N	25	25	25	25
df	22	22	22	22
R	0.978585748	0.979128626	0.978758697	0.978857306
R^2	0.957630066	0.958692867	0.957968588	0.958161625
$R^2_{adjusted}$	0.953778254	0.954937673	0.95414755	0.954358136
RSS	750.0253262	710.7832369	773.926856	743.8576485

Graphical abstract of model fit presented as mean ± 1 SD of the fraction % of released carvedilol:

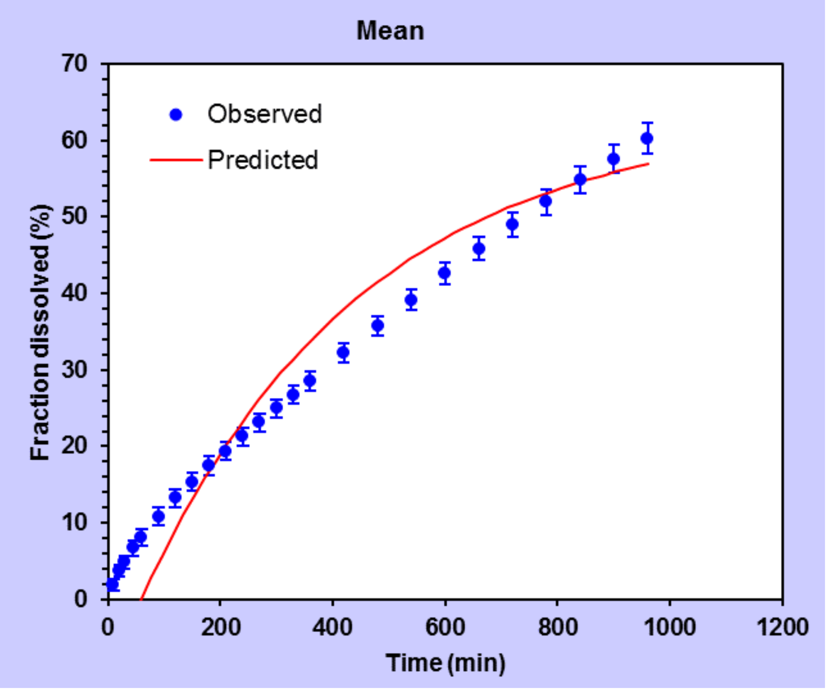

Supplementary materials – Model fitting summary – Avicel® PH-102 (modelling up to app. 60 % of carvedilol released)

Graphical abstract of model fit presented as the fraction % of released carvedilol per tested tablet:

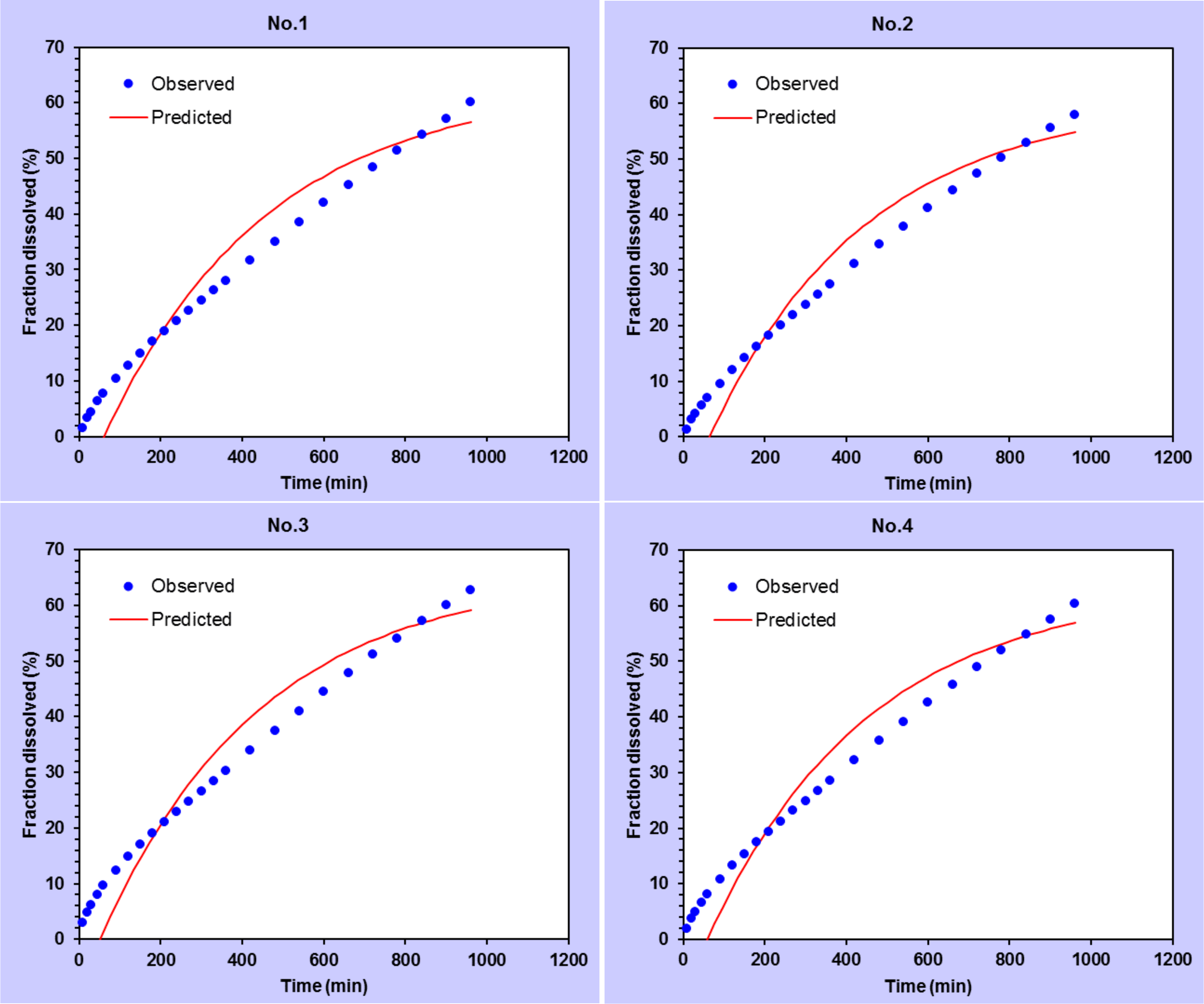

Supplementary materials – Model fitting summary – Avicel® PH-102 (modelling up to app. 60 % of carvedilol released)

Model: **Higuchi**

Model equation: $F = k_H \cdot t^{0.5}$

Fitted model parameters per tested tablet (N = 4) with statistics – mean, standard deviation (SD), and relative standard deviation expressed in % (RSD%) (output from DDSolver):

Parameter	No.1	No.2	No.3	No.4	Mean	SD	RSD(%)
k_H	1.673	1.629	1.785	1.696	1.696	0.066	3.868

Number of dissolution data points (N), degrees of freedom (df), and selected goodness of fit criteria – Pearson correlation coefficient (R), coefficient of determination (R^2), adjusted coefficient of determination ($R^2_{adjusted}$), and residual sum of squares (RSS) (manual calculation in MS Excel):

Parameter	No.1	No.2	No.3	No.4
N	25	25	25	25
df	24	24	24	24
R	0.991645763	0.992273745	0.992032985	0.992011491
R^2	0.98336132	0.984607186	0.984129443	0.984086797
$R^2_{adjusted}$	0.98336132	0.984607186	0.984129443	0.984086797
RSS	554.2995042	558.3092289	440.8718738	515.0366457

Graphical abstract of model fit presented as mean ± 1 SD of the fraction % of released carvedilol:

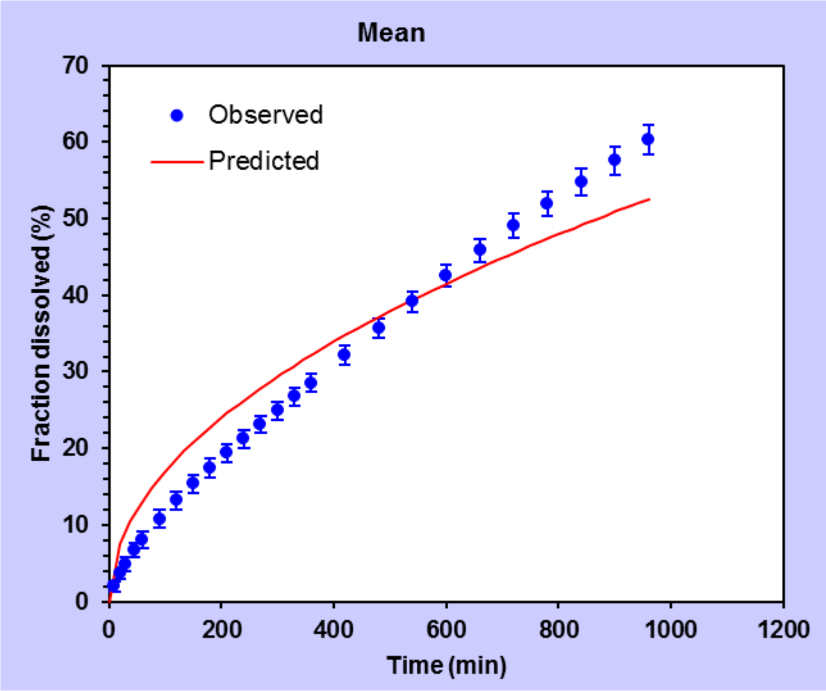

Supplementary materials – Model fitting summary – Avicel® PH-102 (modelling up to app. 60 % of carvedilol released)

Graphical abstract of model fit presented as the fraction % of released carvedilol per tested tablet:

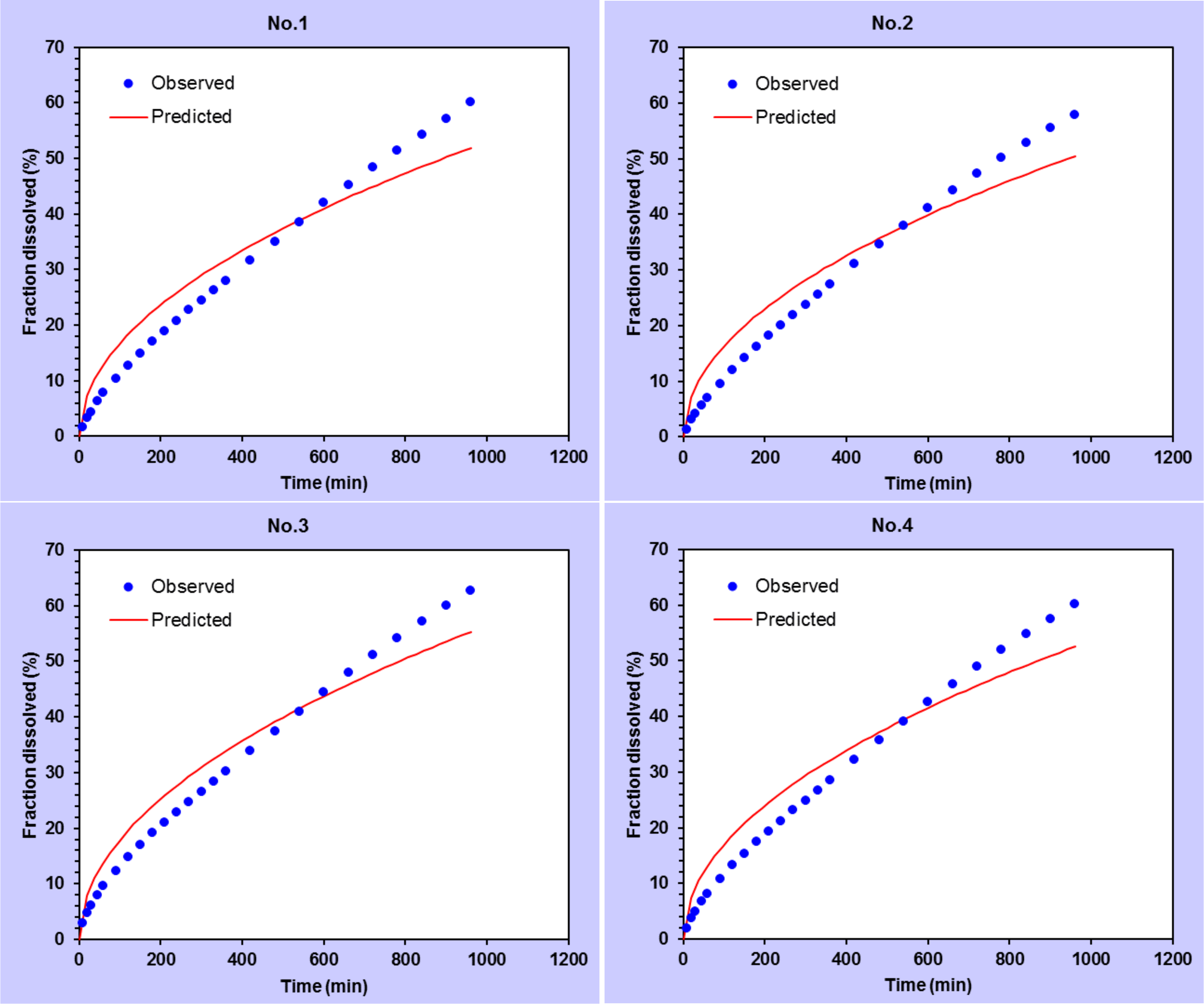

Supplementary materials – Model fitting summary – Avicel® PH-102 (modelling up to app. 60 % of carvedilol released)

Model: **Higuchi with T_{lag}**

Model equation: $F = k_H \cdot (t - T_{lag})^{0.5}$

Fitted model parameters per tested tablet (N = 4) with statistics – mean, standard deviation (SD), and relative standard deviation expressed in % (RSD%) (output from DDSolver):

Parameter	No.1	No.2	No.3	No.4	Mean	SD	RSD(%)
k_H	1.925	1.876	2.015	1.939	1.939	0.057	2.965
T_{lag}	85.072	85.359	75.878	82.108	82.104	4.403	5.363

Number of dissolution data points (N), degrees of freedom (df), and selected goodness of fit criteria – Pearson correlation coefficient (R), coefficient of determination (R^2), adjusted coefficient of determination ($R^2_{adjusted}$), and residual sum of squares (RSS) (manual calculation in MS Excel):

Parameter	No.1	No.2	No.3	No.4
N	25	25	25	25
df	23	23	23	23
R	0.984606874	0.986112969	0.984017098	0.985074149
R^2	0.969450695	0.972418788	0.968289648	0.970371078
$R^2_{adjusted}$	0.968122465	0.971219605	0.966910937	0.969082865
RSS	300.9345607	254.9477694	363.8459144	299.9228879

Graphical abstract of model fit presented as mean ± 1 SD of the fraction % of released carvedilol:

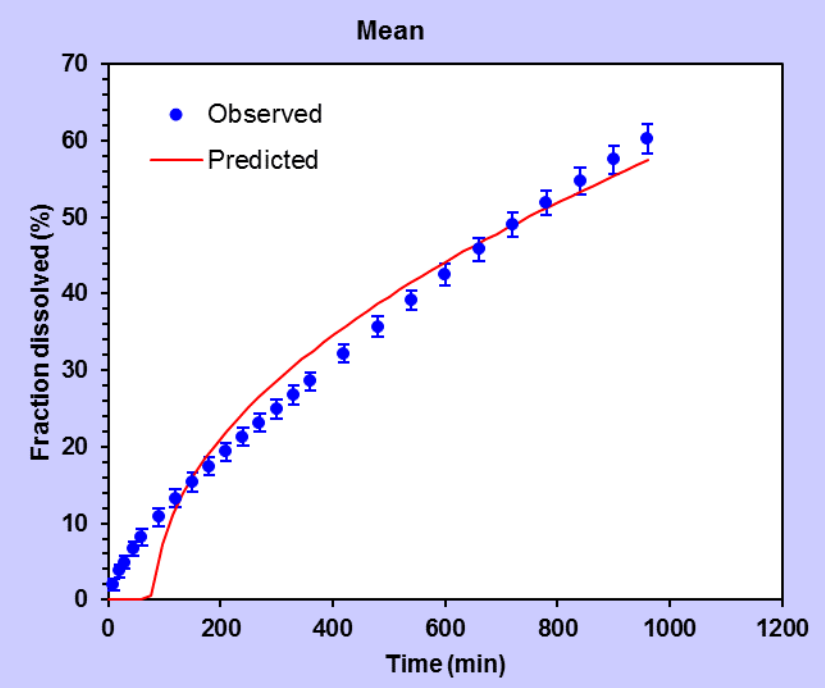

Supplementary materials – Model fitting summary – Avicel® PH-102 (modelling up to app. 60 % of carvedilol released)

Graphical abstract of model fit presented as the fraction % of released carvedilol per tested tablet:

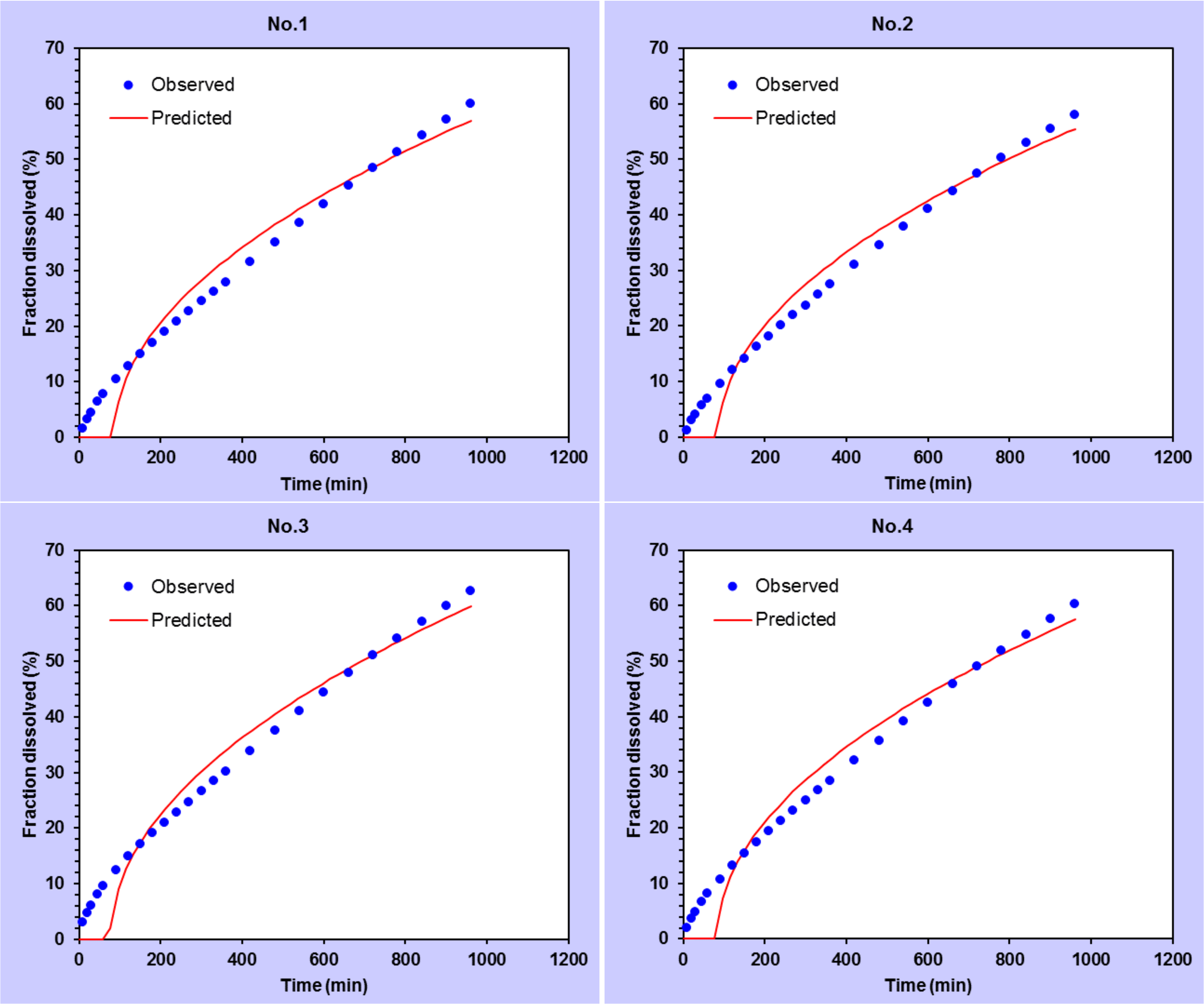

Supplementary materials – Model fitting summary – Avicel® PH-102 (modelling up to app. 60 % of carvedilol released)

Model: **Higuchi with F_0**

Model equation: $F = F_0 + k_H \cdot t^{0.5}$

Fitted model parameters per tested tablet (N = 4) with statistics – mean, standard deviation (SD), and relative standard deviation expressed in % (RSD%) (output from DDSolver):

Parameter	No.1	No.2	No.3	No.4	Mean	SD	RSD(%)
k_H	2.117	2.082	2.165	2.121	2.121	0.034	1.595
F_0	-9.501	-9.702	-8.131	-9.111	-9.111	0.698	-7.661

Number of dissolution data points (N), degrees of freedom (df), and selected goodness of fit criteria – Pearson correlation coefficient (R), coefficient of determination (R^2), adjusted coefficient of determination ($R^2_{adjusted}$), and residual sum of squares (RSS) (manual calculation in MS Excel):

Parameter	No.1	No.2	No.3	No.4
N	25	25	25	25
df	23	23	23	23
R	0.991645763	0.992273745	0.992032985	0.992011491
R^2	0.98336132	0.984607186	0.984129443	0.984086797
$R^2_{adjusted}$	0.982637899	0.983937933	0.983439419	0.983394919
RSS	132.1647688	118.1175836	131.6932288	126.8134776

Graphical abstract of model fit presented as mean ± 1 SD of the fraction % of released carvedilol:

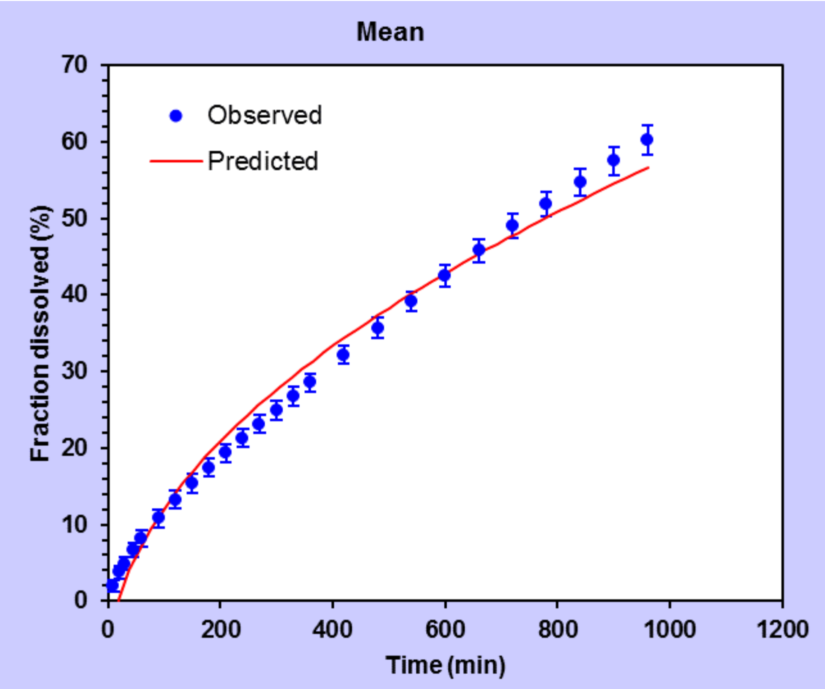

Supplementary materials – Model fitting summary – Avicel® PH-102 (modelling up to app. 60 % of carvedilol released)

Graphical abstract of model fit presented as the fraction % of released carvedilol per tested tablet:

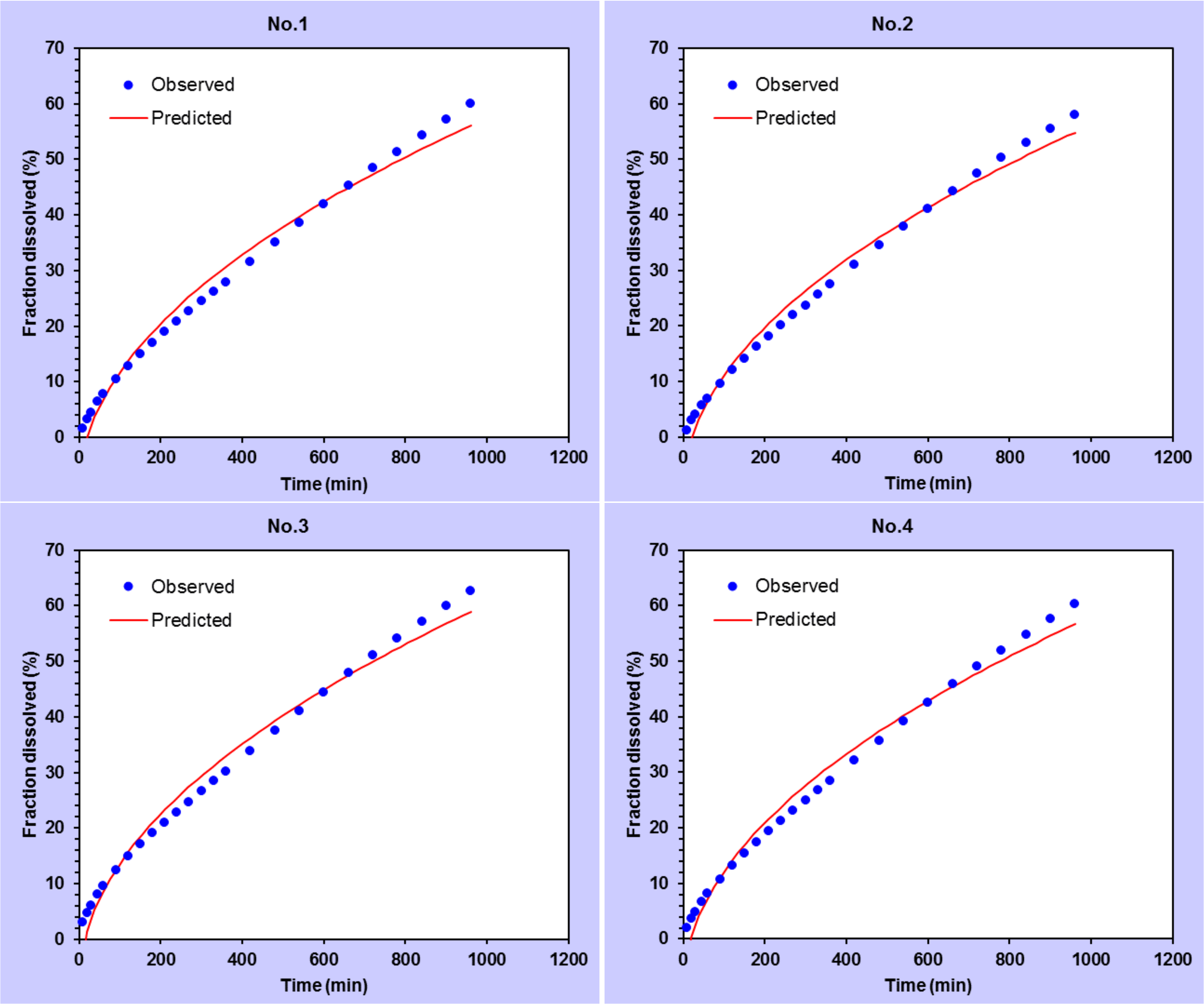

Supplementary materials – Model fitting summary – Avicel® PH-102 (modelling up to app. 60 % of carvedilol released)

Model: **Korsmeyer–Peppas**

Model equation: $F = k_{KP} \cdot t^n$

Fitted model parameters per tested tablet (N = 4) with statistics – mean, standard deviation (SD), and relative standard deviation expressed in % (RSD%) (output from DDSolver):

Parameter	No.1	No.2	No.3	No.4	Mean	SD	RSD(%)
k_{KP}	0.325	0.299	0.629	0.398	0.413	0.150	36.407
n	0.761	0.767	0.664	0.730	0.730	0.047	6.473

Number of dissolution data points (N), degrees of freedom (df), and selected goodness of fit criteria – Pearson correlation coefficient (R), coefficient of determination (R^2), adjusted coefficient of determination ($R^2_{adjusted}$), and residual sum of squares (RSS) (manual calculation in MS Excel):

Parameter	No.1	No.2	No.3	No.4
N	25	25	25	25
df	23	23	23	23
R	0.999870437	0.999906568	0.998786599	0.99976981
R^2	0.999740892	0.999813145	0.99757467	0.999539673
$R^2_{adjusted}$	0.999729626	0.999805021	0.99746922	0.999519659
RSS	2.888422149	6.146014288	36.07285394	4.443595236

Graphical abstract of model fit presented as mean ± 1 SD of the fraction % of released carvedilol:

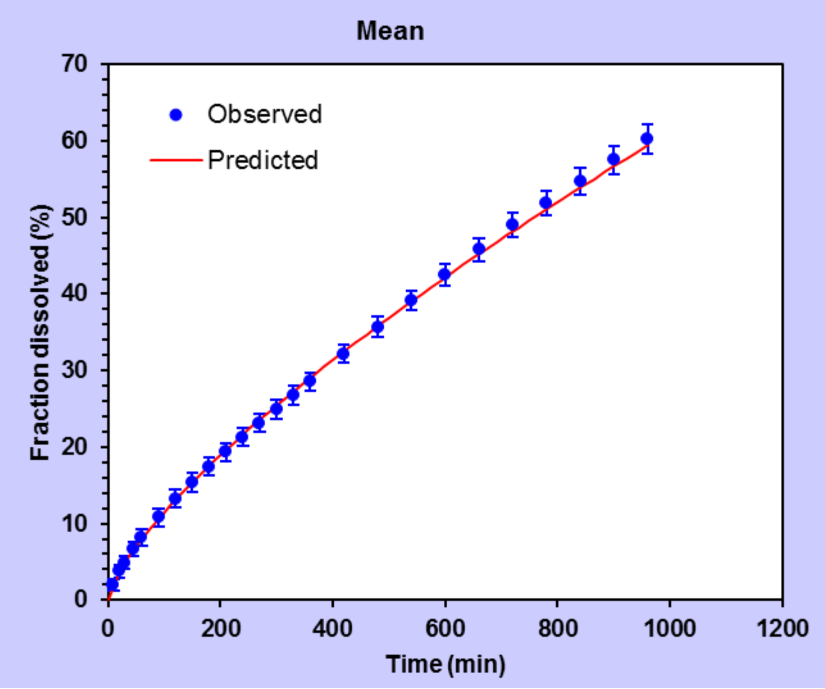

Supplementary materials – Model fitting summary – Avicel® PH-102 (modelling up to app. 60 % of carvedilol released)

Graphical abstract of model fit presented as the fraction % of released carvedilol per tested tablet:

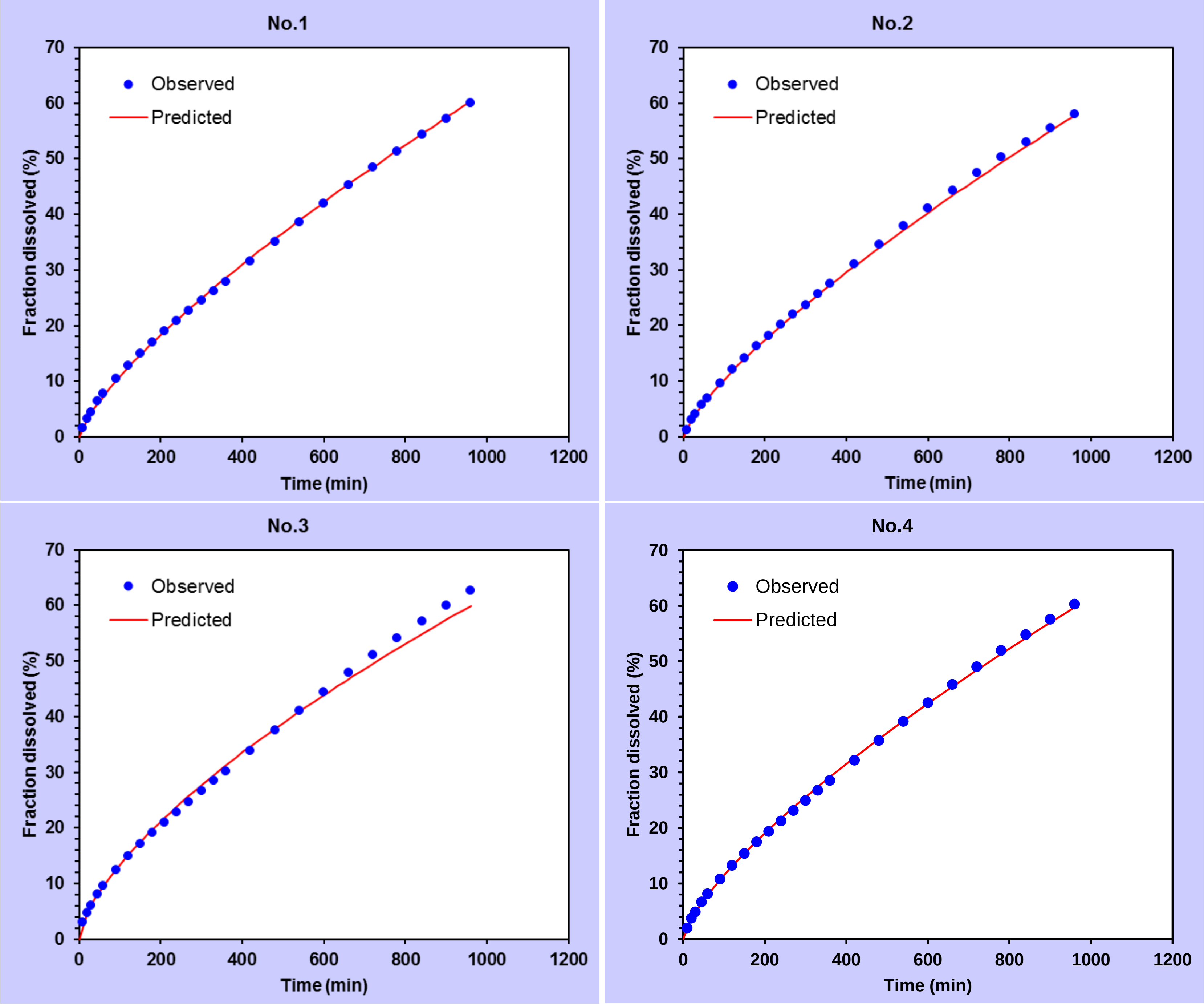

Supplementary materials – Model fitting summary – Avicel® PH-102 (modelling up to app. 60 % of carvedilol released)

Model: **Korsmeyer–Peppas with T_{lag}**

Model equation: $F = k_{KP} \cdot (t - T_{lag})^n$

Fitted model parameters per tested tablet (N = 4) with statistics – mean, standard deviation (SD), and relative standard deviation expressed in % (RSD%) (output from DDSolver):

Parameter	No.1	No.2	No.3	No.4	Mean	SD	RSD(%)
k_{KP}	0.440	0.363	0.827	0.534	0.541	0.203	37.569
n	0.711	0.739	0.619	0.682	0.688	0.051	7.474
T_{lag}	4.000	4.000	4.000	4.000	4.000	0.000	0.000

Number of dissolution data points (N), degrees of freedom (df), and selected goodness of fit criteria – Pearson correlation coefficient (R), coefficient of determination (R^2), adjusted coefficient of determination ($R^2_{adjusted}$), and residual sum of squares (RSS) (manual calculation in MS Excel):

Parameter	No.1	No.2	No.3	No.4
N	25	25	25	25
df	22	22	22	22
R	0.99942108	0.999830823	0.997261627	0.999034969
R^2	0.998842496	0.999661674	0.994530753	0.99807087
$R^2_{adjusted}$	0.998737268	0.999630917	0.994033549	0.997895494
RSS	17.17347643	4.474388553	100.3253252	35.51000563

Graphical abstract of model fit presented as mean ± 1 SD of the fraction % of released carvedilol:

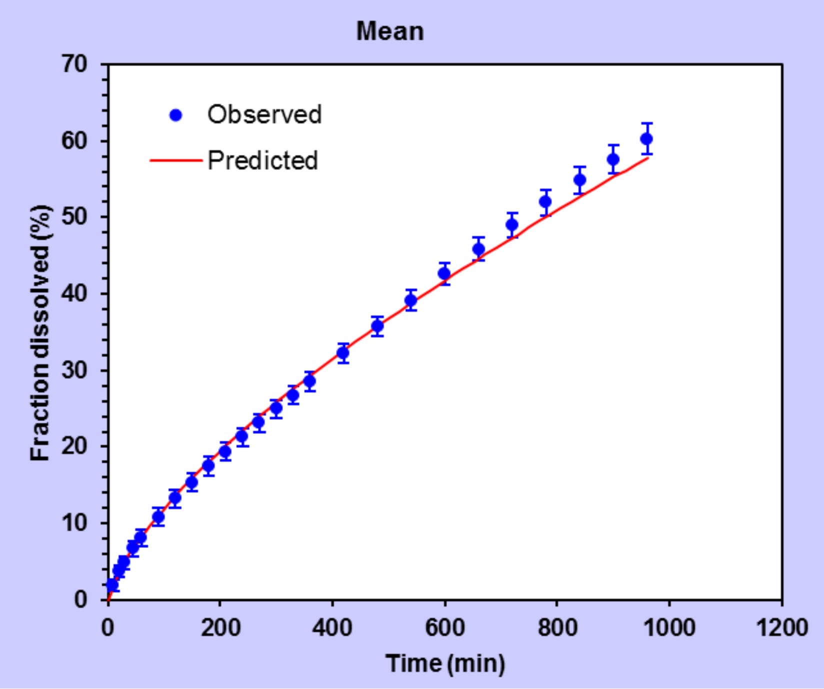

Supplementary materials – Model fitting summary – Avicel® PH-102 (modelling up to app. 60 % of carvedilol released)

Graphical abstract of model fit presented as the fraction % of released carvedilol per tested tablet:

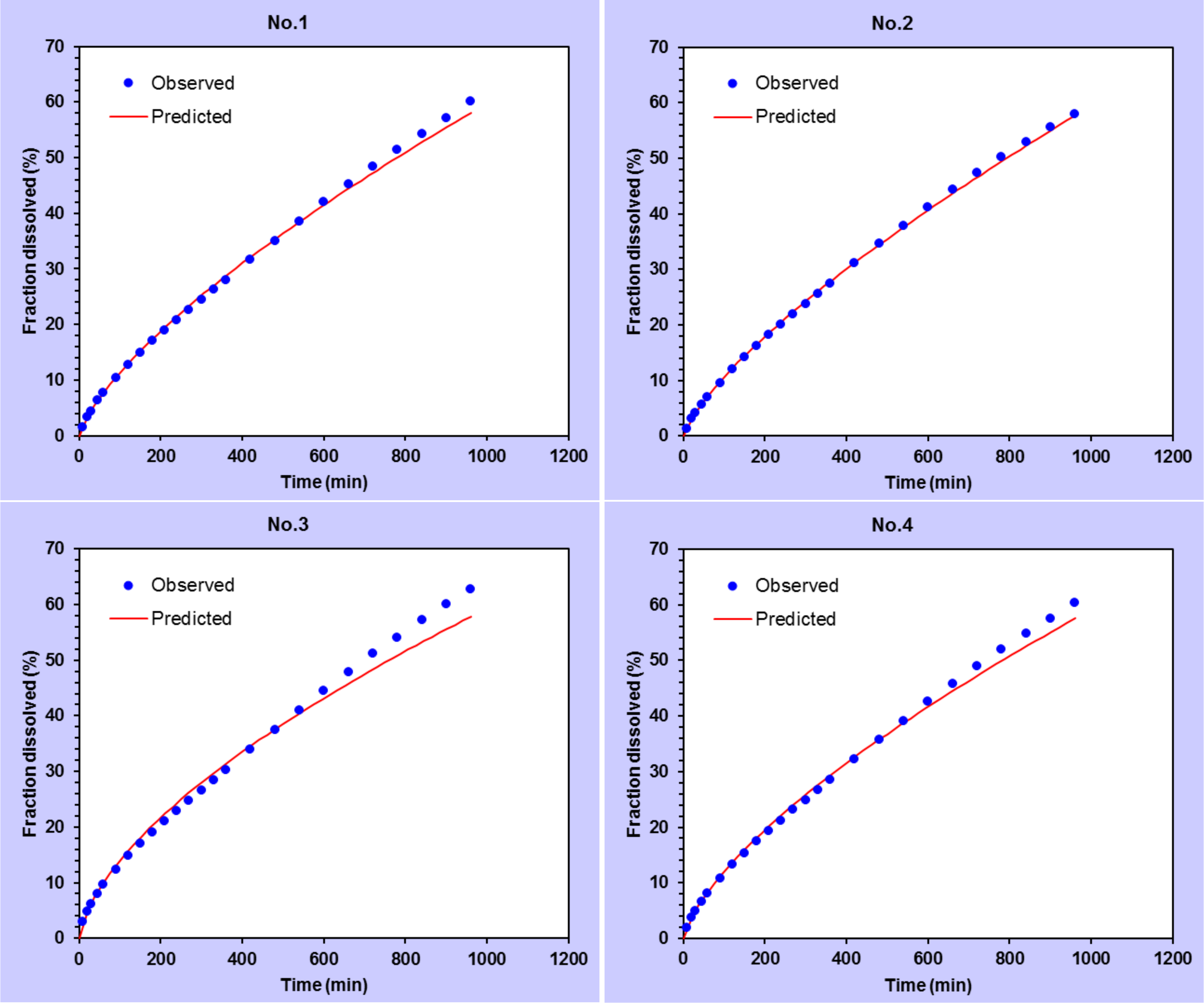

Supplementary materials – Model fitting summary – Avicel® PH-102 (modelling up to app. 60 % of carvedilol released)

Model: **Korsmeyer–Peppas with F_0**

Model equation: $F = F_0 + k_{KP} \cdot t^n$

Fitted model parameters per tested tablet (N = 4) with statistics – mean, standard deviation (SD), and relative standard deviation expressed in % (RSD%) (output from DDSolver):

Parameter	No.1	No.2	No.3	No.4	Mean	SD	RSD(%)
k_{KP}	0.211	0.177	0.379	0.270	0.259	0.088	34.154
n	0.829	0.854	0.741	0.784	0.802	0.050	6.195
F_0	0.640	0.520	1.200	0.964	0.831	0.309	37.224

Number of dissolution data points (N), degrees of freedom (df), and selected goodness of fit criteria – Pearson correlation coefficient (R), coefficient of determination (R^2), adjusted coefficient of determination ($R^2_{adjusted}$), and residual sum of squares (RSS) (manual calculation in MS Excel):

Parameter	No.1	No.2	No.3	No.4
N	25	25	25	25
df	22	22	22	22
R	0.999628122	0.999128691	0.999774445	0.999875603
R^2	0.999256382	0.998258141	0.999548941	0.999751222
$R^2_{adjusted}$	0.99918878	0.998099791	0.999507935	0.999728606
RSS	38.65146118	65.36494255	3.981988908	10.75697171

Graphical abstract of model fit presented as mean ± 1 SD of the fraction % of released carvedilol:

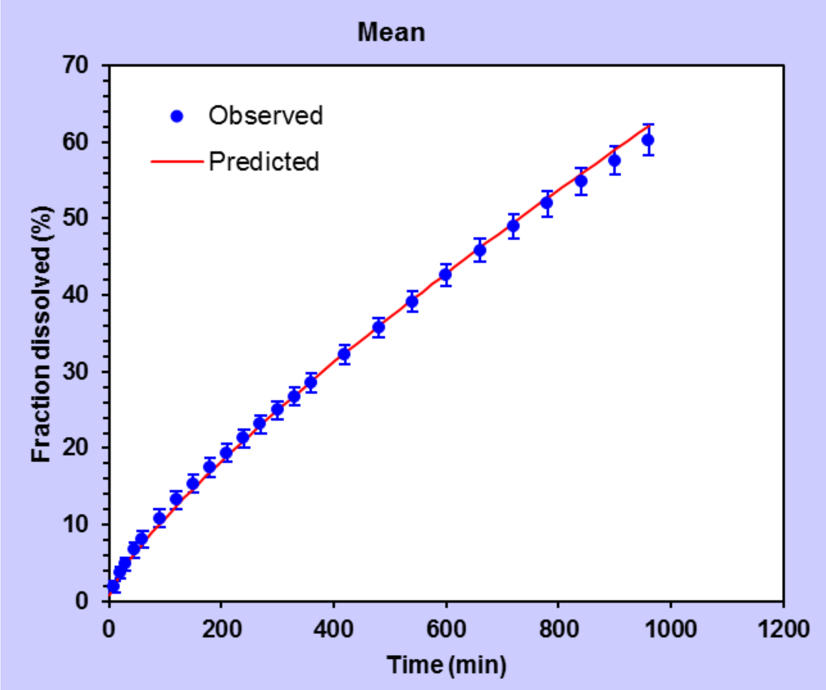

Supplementary materials – Model fitting summary – Avicel® PH-102 (modelling up to app. 60 % of carvedilol released)

Graphical abstract of model fit presented as the fraction % of released carvedilol per tested tablet:

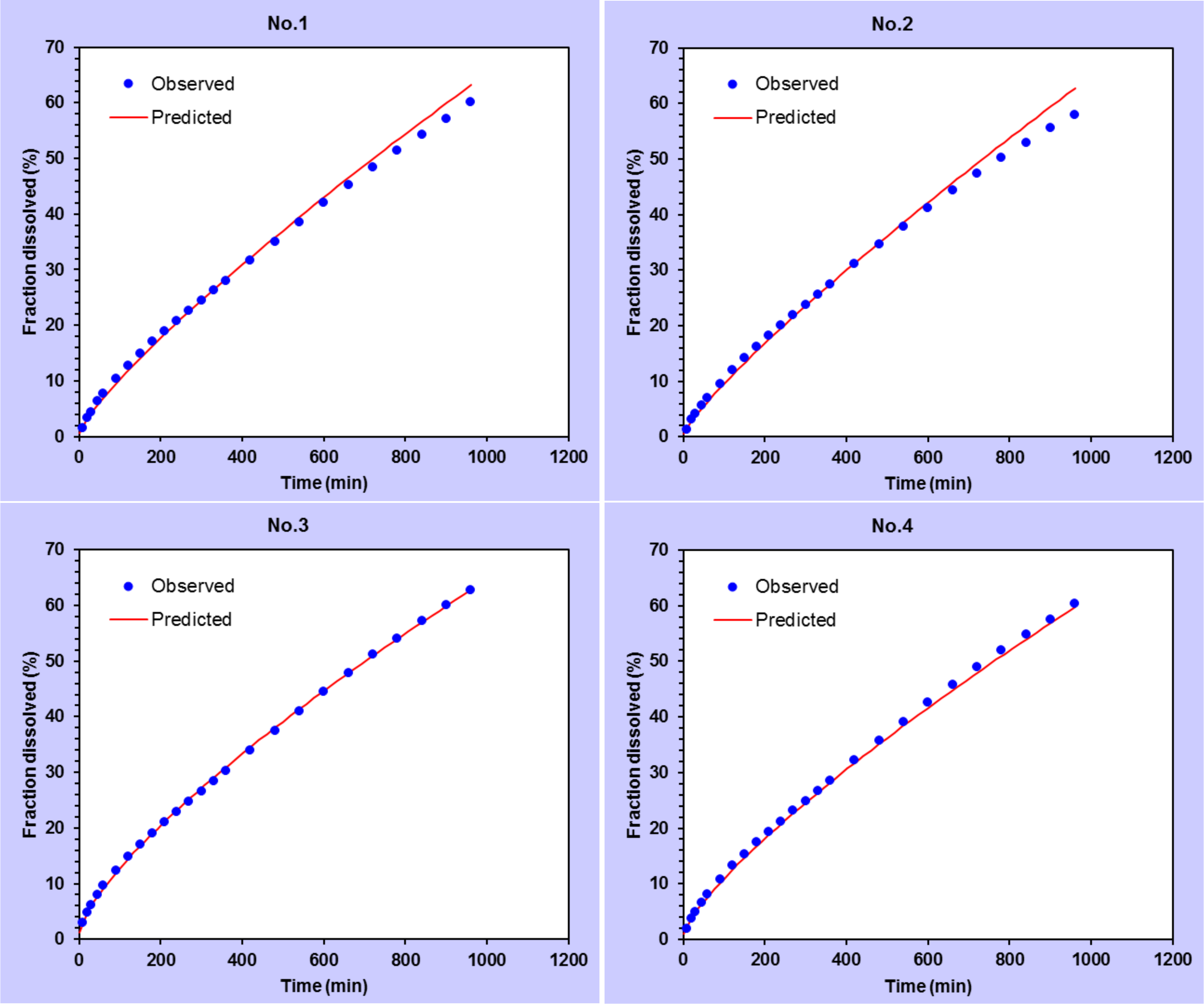

Supplementary materials – Model fitting summary – Avicel® PH-102 (modelling up to app. 60 % of carvedilol released)

Model: **Hixson–Crowell**

Model equation: $F = 100 \cdot [1 - (1 - k_{HC} \cdot t)^3]$

Fitted model parameters per tested tablet (N = 4) with statistics – mean, standard deviation (SD), and relative standard deviation expressed in % (RSD%) (output from DDSolver):

Parameter	No.1	No.2	No.3	No.4	Mean	SD	RSD(%)
k_{HC}	0.0003	0.0003	0.0003	0.0003	0.0003	0.0000	4.4646

Number of dissolution data points (N), degrees of freedom (df), and selected goodness of fit criteria – Pearson correlation coefficient (R), coefficient of determination (R^2), adjusted coefficient of determination ($R^2_{adjusted}$), and residual sum of squares (RSS) (manual calculation in MS Excel):

Parameter	No.1	No.2	No.3	No.4
N	25	25	25	25
df	24	24	24	24
R	0.999334708	0.999496786	0.999234513	0.999398786
R^2	0.998669859	0.998993825	0.998469613	0.998797934
$R^2_{adjusted}$	0.998669859	0.998993825	0.998469613	0.998797934
RSS	77.10673598	60.33327041	168.122128	96.20044249

Graphical abstract of model fit presented as mean ± 1 SD of the fraction % of released carvedilol:

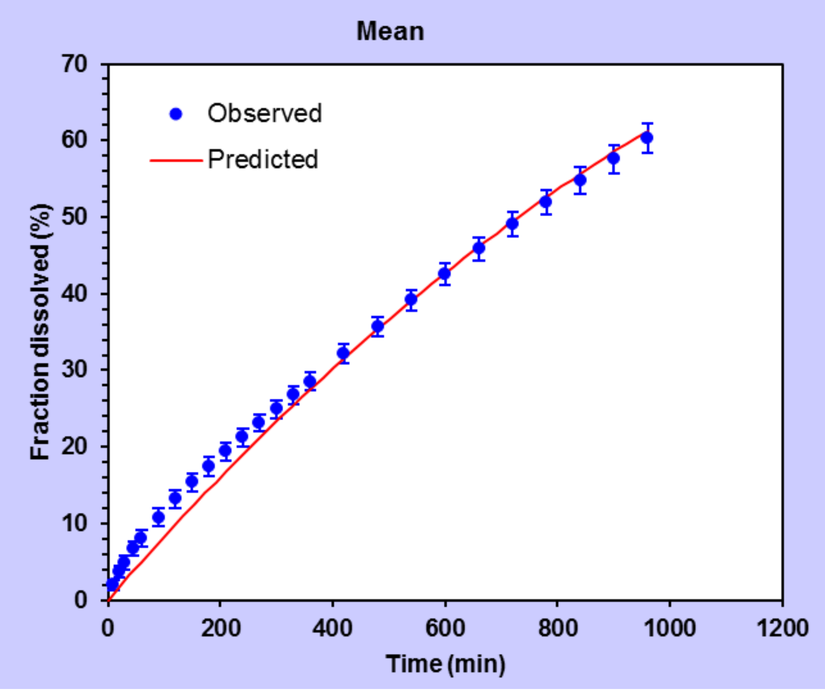

Supplementary materials – Model fitting summary – Avicel® PH-102 (modelling up to app. 60 % of carvedilol released)

Graphical abstract of model fit presented as the fraction % of released carvedilol per tested tablet:

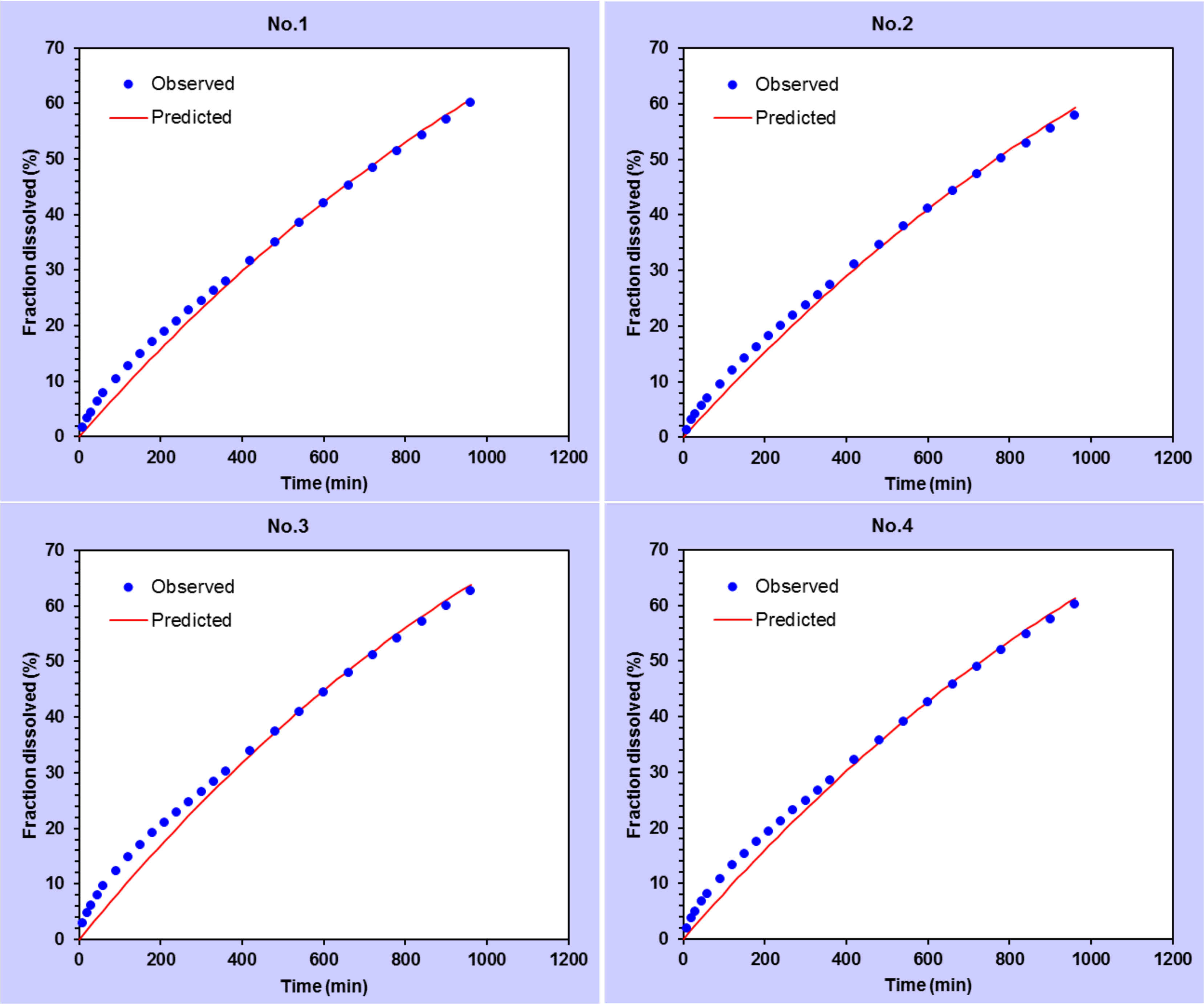

Supplementary materials – Model fitting summary – Avicel® PH-102 (modelling up to app. 60 % of carvedilol released)

Model: **Hixson–Crowell with T_{lag}**

Model equation: $F = 100 \cdot \left\{1 - \left[1 - k_{HC} \cdot \left(t - T_{lag}\right)\right]^3\right\}$

Fitted model parameters per tested tablet (N = 4) with statistics – mean, standard deviation (SD), and relative standard deviation expressed in % (RSD%) (output from DDSolver):

Parameter	No.1	No.2	No.3	No.4	Mean	SD	RSD(%)
k_{HC}	0.0003	0.0003	0.0003	0.0003	0.0003	0.0000	3.1262
T_{lag}	-37.3154	-35.0238	-54.7205	-42.6748	-42.4336	8.7963	-20.7296

Number of dissolution data points (N), degrees of freedom (df), and selected goodness of fit criteria – Pearson correlation coefficient (R), coefficient of determination (R^2), adjusted coefficient of determination ($R^2_{adjusted}$), and residual sum of squares (RSS) (manual calculation in MS Excel):

Parameter	No.1	No.2	No.3	No.4
N	25	25	25	25
df	23	23	23	23
R	0.999307198	0.999420976	0.999210261	0.999350169
R^2	0.998614877	0.998842287	0.998421145	0.99870076
$R^2_{adjusted}$	0.998554654	0.998791951	0.998352499	0.998644271
RSS	11.01760661	9.031334194	13.1110305	10.3975905

Graphical abstract of model fit presented as mean ± 1 SD of the fraction % of released carvedilol:

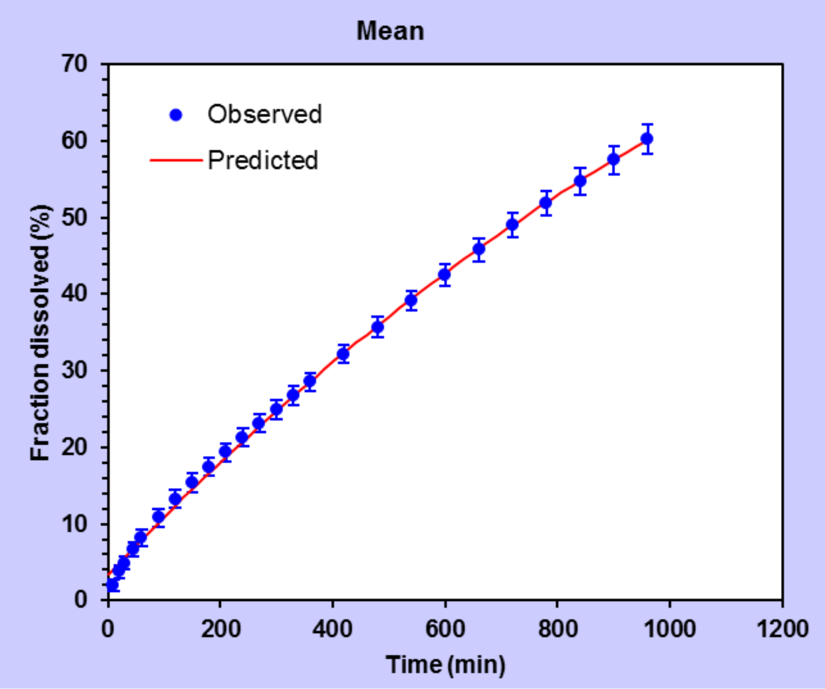

Supplementary materials – Model fitting summary – Avicel® PH-102 (modelling up to app. 60 % of carvedilol released)

Graphical abstract of model fit presented as the fraction % of released carvedilol per tested tablet:

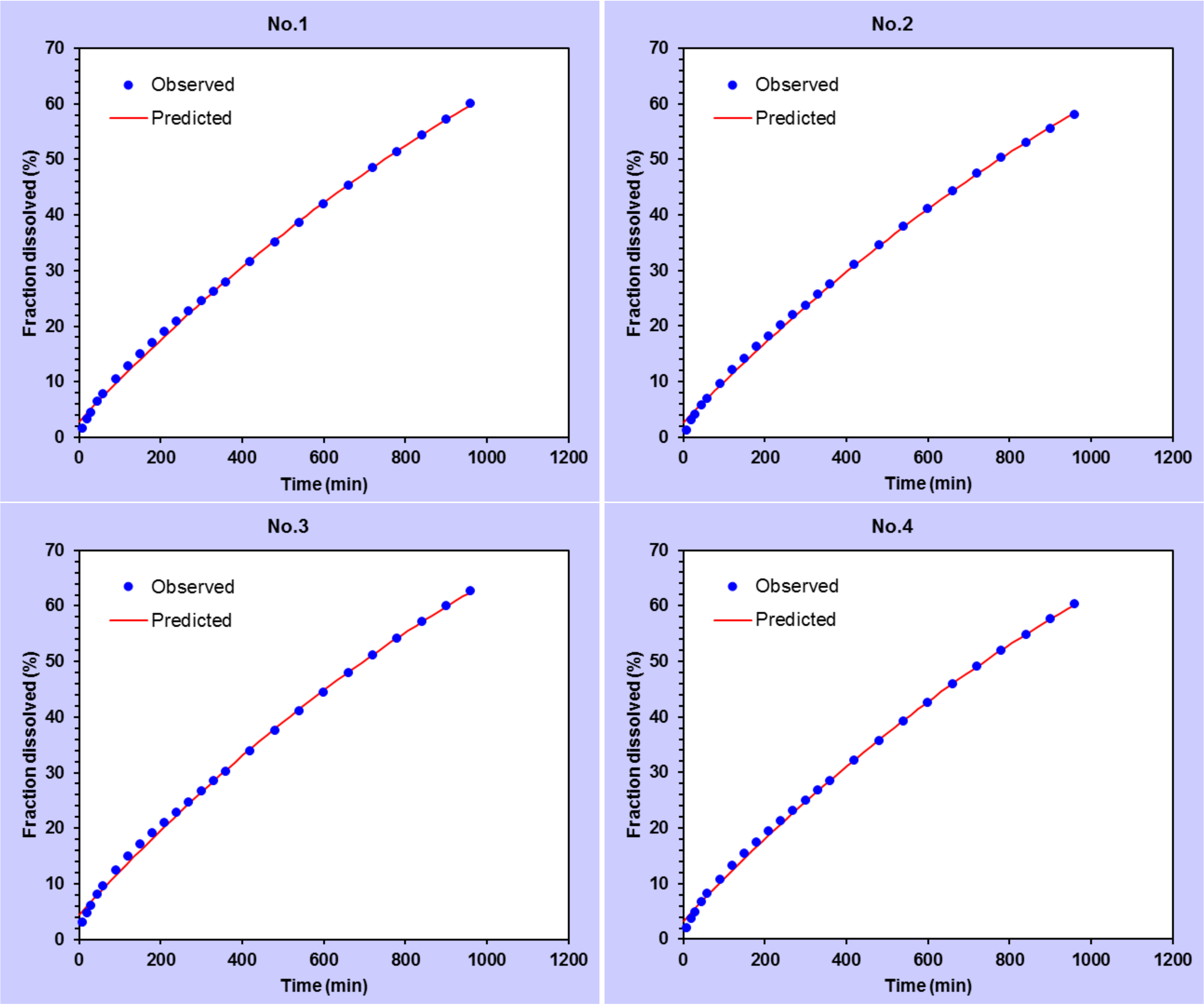

Supplementary materials – Model fitting summary – Avicel® PH-102 (modelling up to app. 60 % of carvedilol released)

Model: **Hopfenberg**

Model equation: $F = 100 \cdot [1 - (1 - k_{HB} \cdot t)^n]$

Fitted model parameters per tested tablet (N = 4) with statistics – mean, standard deviation (SD), and relative standard deviation expressed in % (RSD%) (output from DDSolver):

Parameter	No.1	No.2	No.3	No.4	Mean	SD	RSD(%)
k_{HB}	0.0003	0.0003	0.0003	0.0003	0.0003	0.0000	4.4646
n	3.0000	3.0000	3.0000	3.0000	3.0000	0.0000	0.0000

Number of dissolution data points (N), degrees of freedom (df), and selected goodness of fit criteria – Pearson correlation coefficient (R), coefficient of determination (R^2), adjusted coefficient of determination ($R^2_{adjusted}$), and residual sum of squares (RSS) (manual calculation in MS Excel):

Parameter	No.1	No.2	No.3	No.4
N	25	25	25	25
df	23	23	23	23
R	0.999334708	0.999496786	0.999234513	0.999398786
R^2	0.998669859	0.998993825	0.998469613	0.998797934
$R^2_{adjusted}$	0.998612026	0.998950078	0.998403074	0.99874567
RSS	77.10673598	60.33327041	168.122128	96.20044249

Graphical abstract of model fit presented as mean ± 1 SD of the fraction % of released carvedilol:

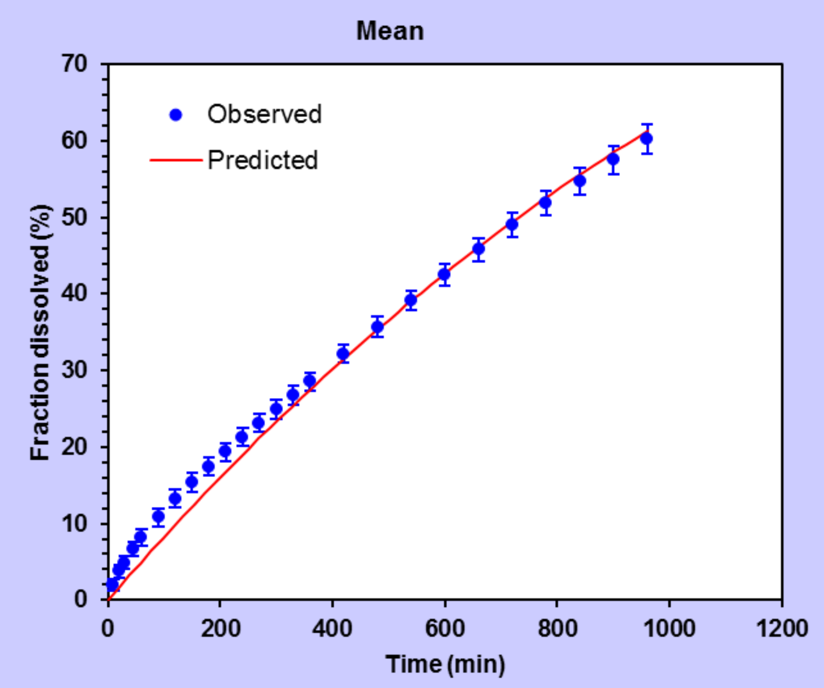

Supplementary materials – Model fitting summary – Avicel® PH-102 (modelling up to app. 60 % of carvedilol released)

Graphical abstract of model fit presented as the fraction % of released carvedilol per tested tablet:

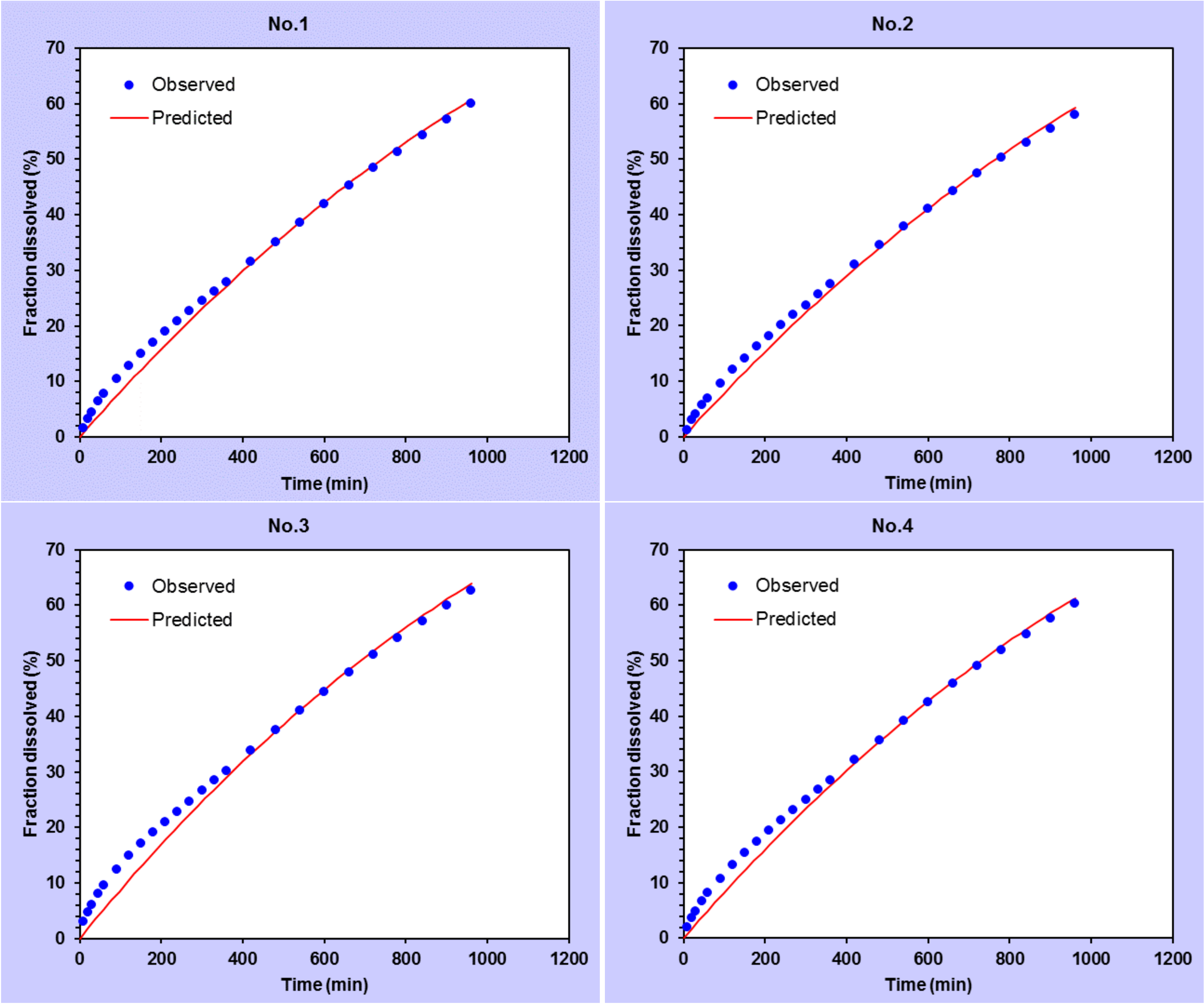

Supplementary materials – Model fitting summary – Avicel® PH-102 (modelling up to app. 60 % of carvedilol released)

Model: **Hopfenberg with T_{lag}**

Model equation: $F = 100 \cdot \{1 - [1 - k_{HB} \cdot (t - T_{lag})]^n\}$

Fitted model parameters per tested tablet (N = 4) with statistics – mean, standard deviation (SD), and relative standard deviation expressed in % (RSD%) (output from DDSolver):

Parameter	No.1	No.2	No.3	No.4	Mean	SD	RSD(%)
k_{HB}	0.0003	0.0003	0.0003	0.0003	0.0003	0.0000	3.1262
n	3.0000	3.0000	3.0000	3.0000	3.0000	0.0000	0.0000
T_{lag}	-37.3154	-35.0238	-54.7205	-42.6748	-42.4336	8.7963	-20.7296

Number of dissolution data points (N), degrees of freedom (df), and selected goodness of fit criteria – Pearson correlation coefficient (R), coefficient of determination (R^2), adjusted coefficient of determination ($R^2_{adjusted}$), and residual sum of squares (RSS) (manual calculation in MS Excel):

Parameter	No.1	No.2	No.3	No.4
N	25	25	25	25
df	22	22	22	22
R	0.999307198	0.999420976	0.999210261	0.999350169
R^2	0.998614877	0.998842287	0.998421145	0.99870076
$R^2_{adjusted}$	0.998488956	0.99873704	0.998277613	0.998582647
RSS	11.01760661	9.031334194	13.1110305	10.3975905

Graphical abstract of model fit presented as mean ± 1 SD of the fraction % of released carvedilol:

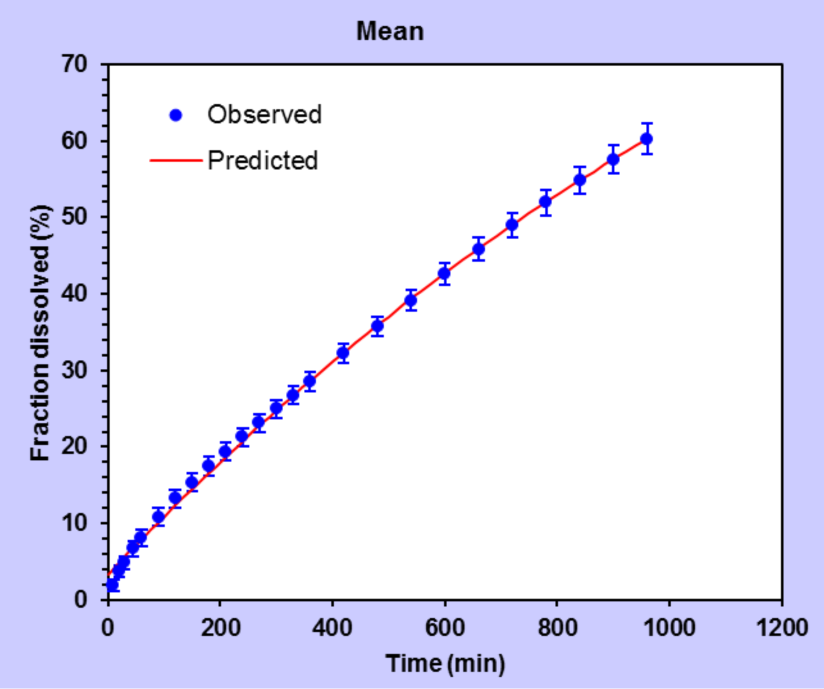

Supplementary materials – Model fitting summary – Avicel® PH-102 (modelling up to app. 60 % of carvedilol released)

Graphical abstract of model fit presented as the fraction % of released carvedilol per tested tablet:

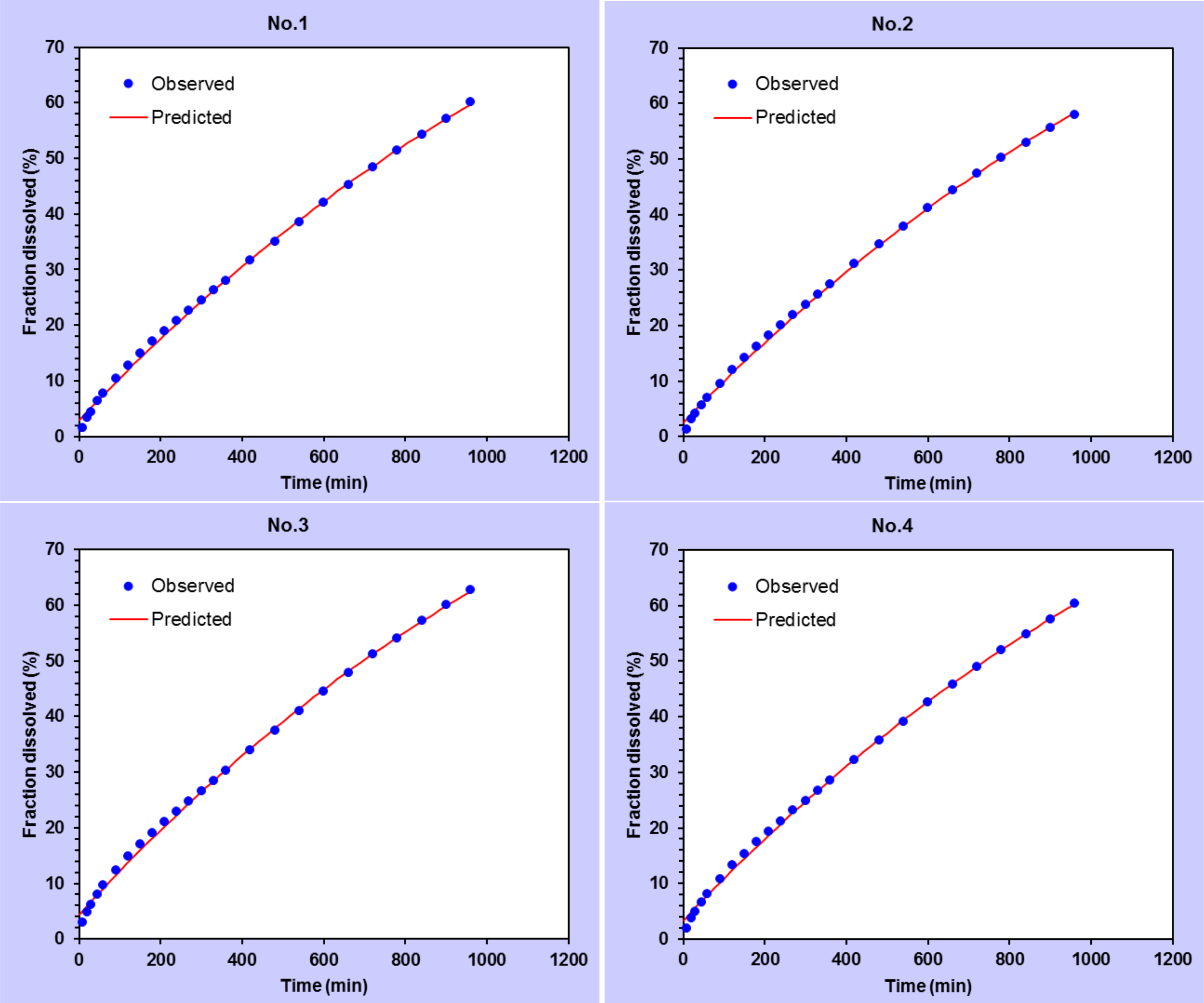

Supplementary materials – Model fitting summary – Avicel® PH-102 (modelling up to app. 60 % of carvedilol released)

Model: **Baker–Lonsdale**

Model equation: $\frac{3}{2}\cdot\left[1-\left(1-\frac{F}{100}\right)^{\frac{2}{3}}\right]-\frac{F}{100}=k_{BL}\cdot t$

Fitted model parameters per tested tablet (N = 4) with statistics – mean, standard deviation (SD), and relative standard deviation expressed in % (RSD%) (output from DDSolver):

Parameter	No.1	No.2	No.3	No.4	Mean	SD	RSD(%)
k_{BL}	0.00008	0.00008	0.00005	0.00004	0.00006	0.00002	33.61678

Number of dissolution data points (N), degrees of freedom (df), and selected goodness of fit criteria – Pearson correlation coefficient (R), coefficient of determination (R^2), adjusted coefficient of determination ($R^2_{adjusted}$), and residual sum of squares (RSS) (manual calculation in MS Excel):

Parameter	No.1	No.2	No.3	No.4
N	25	25	25	25
df	24	24	24	24
R	0.984240494	0.985475034	0.987190385	0.987458304
R^2	0.968729351	0.971161042	0.974544857	0.975073903
$R^2_{adjusted}$	0.968729351	0.971161042	0.974544857	0.975073903
RSS	1808.974756	1731.654421	1189.983742	1155.099365

Graphical abstract of model fit presented as mean ± 1 SD of the fraction % of released carvedilol:

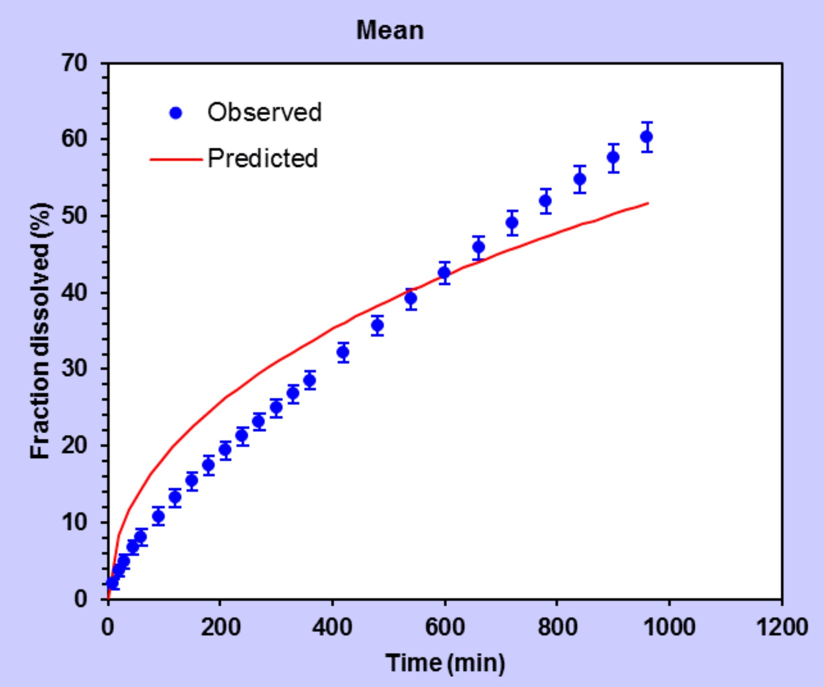

Supplementary materials – Model fitting summary – Avicel® PH-102 (modelling up to app. 60 % of carvedilol released)

Graphical abstract of model fit presented as the fraction % of released carvedilol per tested tablet:

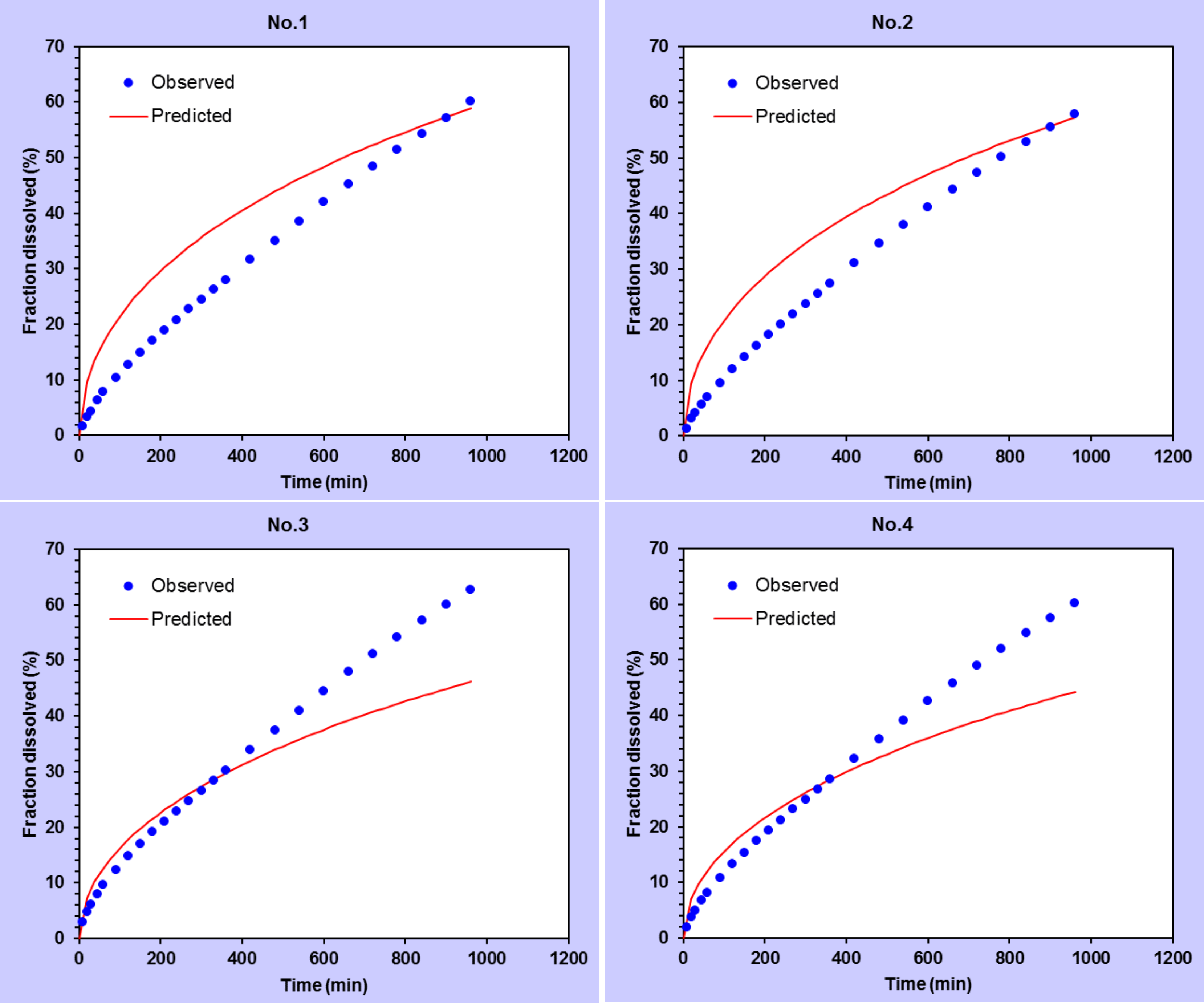

Supplementary materials – Model fitting summary – Avicel® PH-102 (modelling up to app. 60 % of carvedilol released)

Model: **Baker–Lonsdale with T_{lag}**

Model equation: $\frac{3}{2}\cdot\left[1-\left(1-\frac{F}{100}\right)^{\frac{2}{3}}\right]-\frac{F}{100}=k_{BL}\cdot\left(t-T_{lag}\right)$

Fitted model parameters per tested tablet (N = 4) with statistics – mean, standard deviation (SD), and relative standard deviation expressed in % (RSD%) (output from DDSolver):

Parameter	No.1	No.2	No.3	No.4	Mean	SD	RSD(%)
k_{BL}	0.00008	0.00008	0.00010	0.00009	0.00009	0.00001	7.49126
T_{lag}	102.34644	101.50916	95.89090	99.87225	99.90469	2.86636	2.86909

Number of dissolution data points (N), degrees of freedom (df), and selected goodness of fit criteria – Pearson correlation coefficient (R), coefficient of determination (R^2), adjusted coefficient of determination ($R^2_{adjusted}$), and residual sum of squares (RSS) (manual calculation in MS Excel):

Parameter	No.1	No.2	No.3	No.4
N	25	25	25	25
df	23	23	23	23
R	0.97638404	0.978584056	0.974218329	0.976439163
R^2	0.953325794	0.957626755	0.949101353	0.95343344
$R^2_{adjusted}$	0.951296481	0.95578444	0.946888368	0.951408807
RSS	479.7020437	408.8769663	607.963961	492.2680906

Graphical abstract of model fit presented as mean ± 1 SD of the fraction % of released carvedilol:

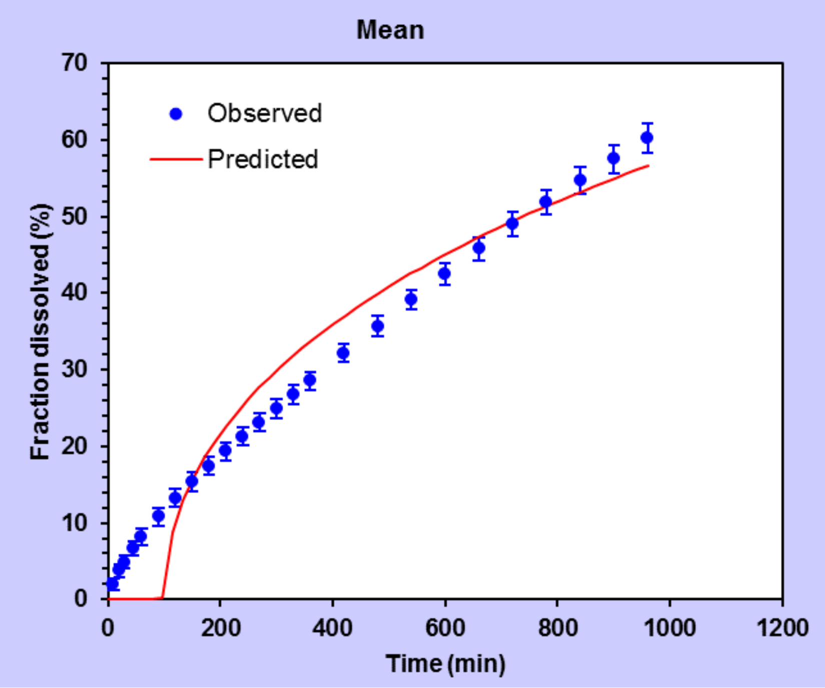

Supplementary materials – Model fitting summary – Avicel® PH-102 (modelling up to app. 60 % of carvedilol released)

Graphical abstract of model fit presented as the fraction % of released carvedilol per tested tablet:

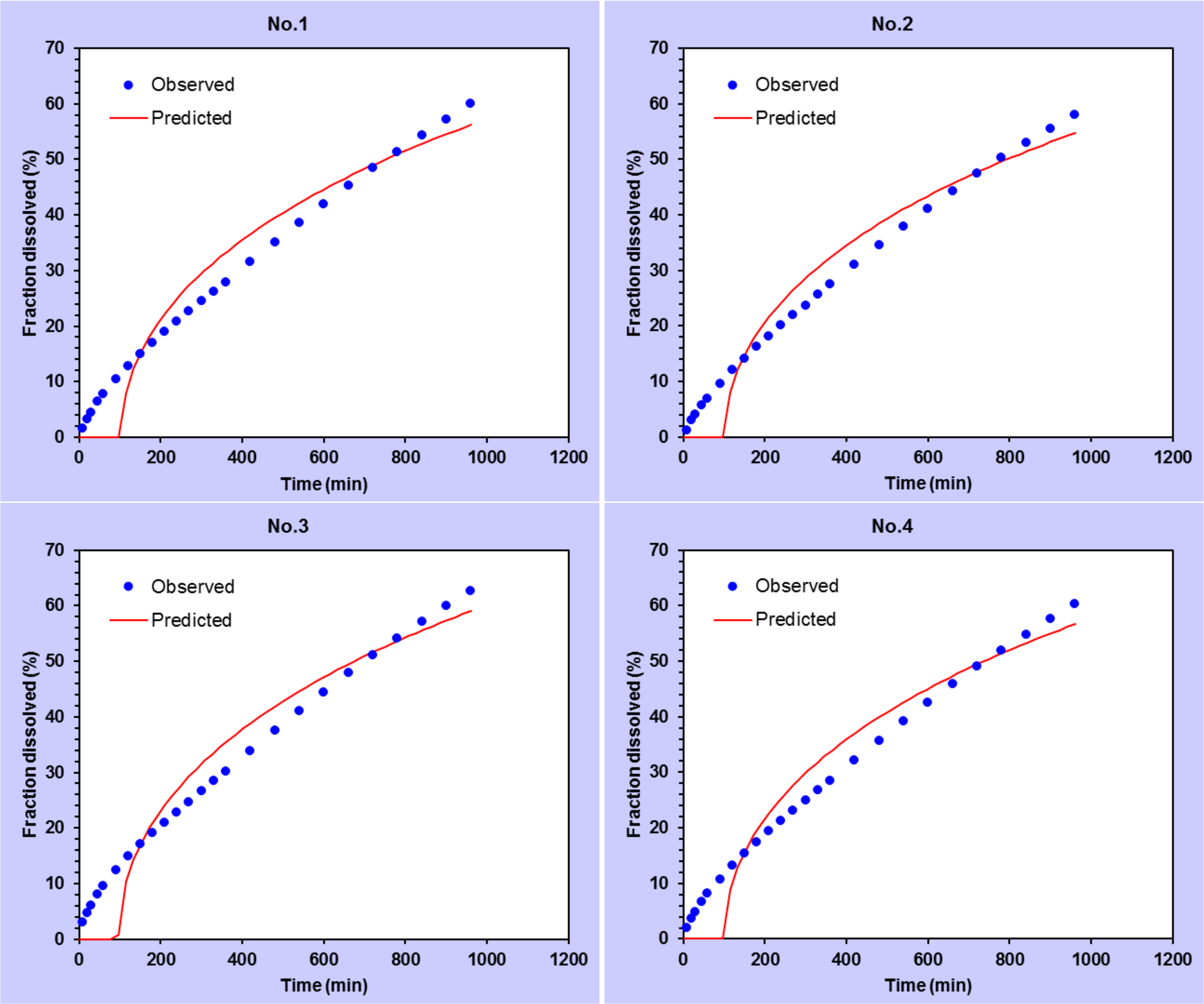

Supplementary materials – Model fitting summary – Avicel® PH-102 (modelling up to app. 60 % of carvedilol released)

Model: **Makoid–Banakar**

Model equation: $F = k_{MB} \cdot t^n \cdot e^{-k \cdot t}$

Fitted model parameters per tested tablet (N = 4) with statistics – mean, standard deviation (SD), and relative standard deviation expressed in % (RSD%) (output from DDSolver):

Parameter	No.1	No.2	No.3	No.4	Mean	SD	RSD(%)
k_{MB}	0.30580	0.23507	0.73577	0.40628	0.42073	0.22146	52.63824
n	0.77760	0.82278	0.62024	0.72393	0.73614	0.08719	11.84471
k	0.00008	0.00016	-0.00021	-0.00003	0.00000	0.00016	8759.55354

Number of dissolution data points (N), degrees of freedom (df), and selected goodness of fit criteria – Pearson correlation coefficient (R), coefficient of determination (R^2), adjusted coefficient of determination ($R^2_{adjusted}$), and residual sum of squares (RSS) (manual calculation in MS Excel):

Parameter	No.1	No.2	No.3	No.4
N	25	25	25	25
df	22	22	22	22
R	0.999555501	0.999698376	0.999848259	0.999854155
R^2	0.9991112	0.999396843	0.99969654	0.999708332
$R^2_{adjusted}$	0.9990304	0.999342011	0.999668953	0.999681816
RSS	7.550559059	5.038976926	2.52033621	2.464209652

Graphical abstract of model fit presented as mean ± 1 SD of the fraction % of released carvedilol:

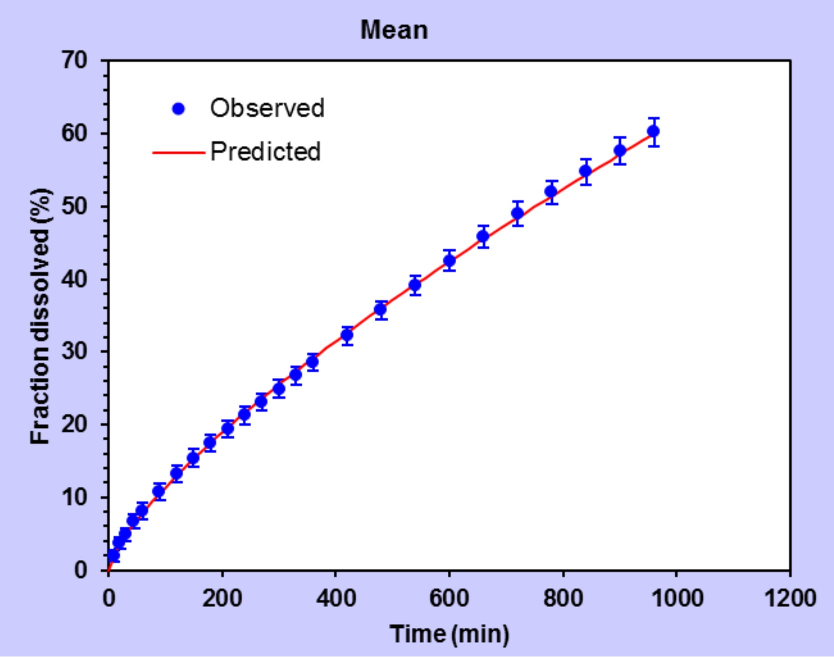

Supplementary materials – Model fitting summary – Avicel® PH-102 (modelling up to app. 60 % of carvedilol released)

Graphical abstract of model fit presented as the fraction % of released carvedilol per tested tablet:

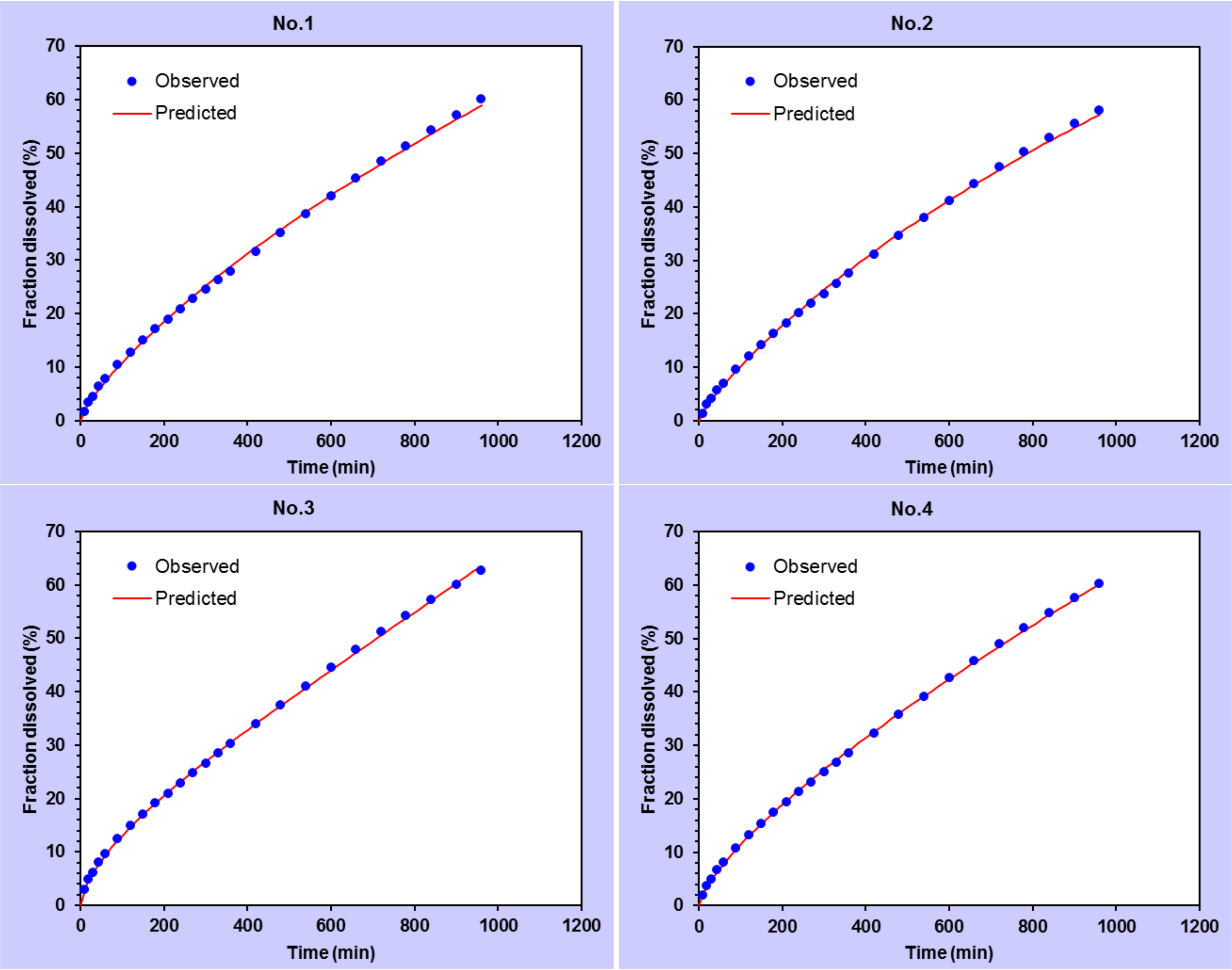

Supplementary materials – Model fitting summary – Avicel® PH-102 (modelling up to app. 60 % of carvedilol released)

Model: **Makoid–Banakar with T_{lag}**

Model equation: $F = k_{MB} \cdot (t - T_{lag})^n \cdot e^{-k \cdot (t - T_{lag})}$

Fitted model parameters per tested tablet (N = 4) with statistics – mean, standard deviation (SD), and relative standard deviation expressed in % (RSD%) (output from DDSolver):

Parameter	No.1	No.2	No.3	No.4	Mean	SD	RSD(%)
k_{MB}	0.48040	0.37822	1.06546	0.62162	0.63643	0.30293	47.59904
n	0.68587	0.72623	0.54481	0.63741	0.64858	0.07813	12.04665
k	-0.00013	-0.00007	-0.00039	-0.00023	-0.00020	0.00014	-68.49598
T_{lag}	4.00000	4.00000	4.00000	4.00000	4.00000	0.00000	0.00000

Number of dissolution data points (N), degrees of freedom (df), and selected goodness of fit criteria – Pearson correlation coefficient (R), coefficient of determination (R^2), adjusted coefficient of determination ($R^2_{adjusted}$), and residual sum of squares (RSS) (manual calculation in MS Excel):

Parameter	No.1	No.2	No.3	No.4
N	25	25	25	25
df	21	21	21	21
R	0.999920759	0.999865103	0.999436299	0.999753485
R^2	0.999841525	0.999730225	0.998872915	0.99950703
$R^2_{adjusted}$	0.999818886	0.999691686	0.998711903	0.999436606
RSS	1.275186473	2.073173808	9.750508176	4.002148602

Graphical abstract of model fit presented as mean ± 1 SD of the fraction % of released carvedilol:

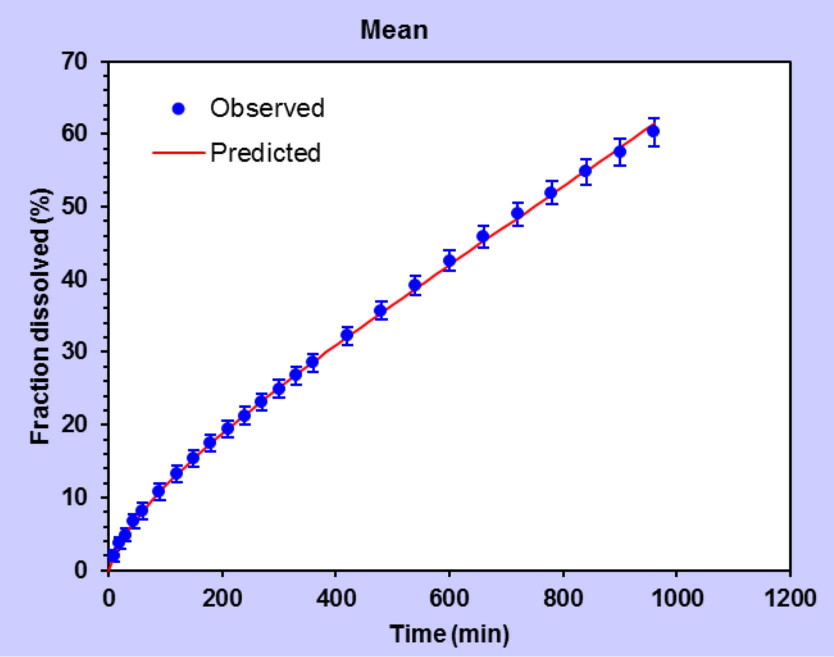

Supplementary materials – Model fitting summary – Avicel® PH-102 (modelling up to app. 60 % of carvedilol released)

Graphical abstract of model fit presented as the fraction % of released carvedilol per tested tablet:

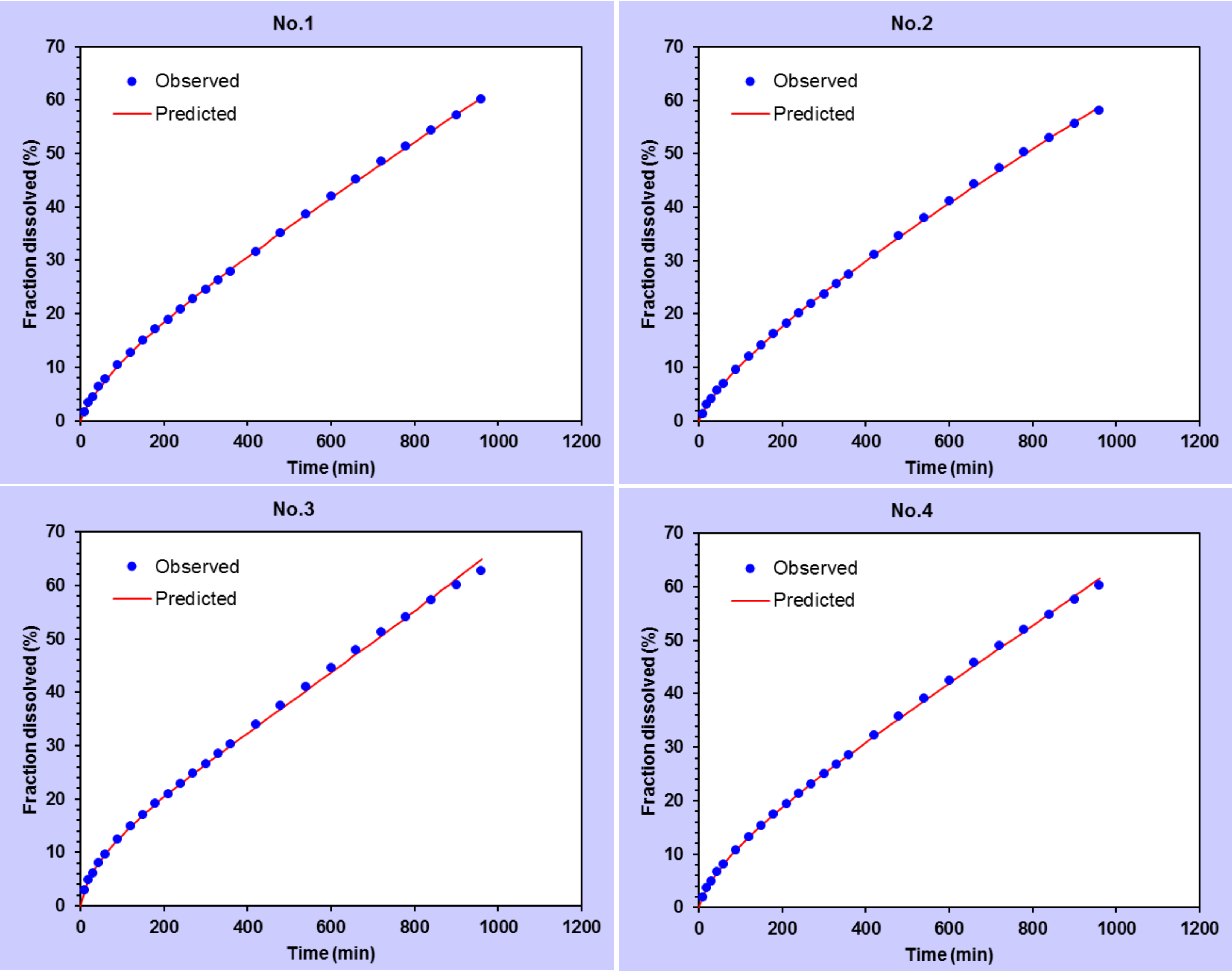

Supplementary materials – Model fitting summary – Avicel® PH-102 (modelling up to app. 60 % of carvedilol released)

Model: **Peppas–Sahlin_1**

Model equation: $F = k_1 \cdot t^m + k_2 \cdot t^{2m}$

Fitted model parameters per tested tablet (N = 4) with statistics – mean, standard deviation (SD), and relative standard deviation expressed in % (RSD%) (output from DDSolver):

Parameter	No.1	No.2	No.3	No.4	Mean	SD	RSD(%)
k_1	0.621	0.565	0.905	0.697	0.697	0.149	21.330
k_2	0.097	0.097	0.089	0.094	0.094	0.004	3.743
m	0.450	0.450	0.450	0.450	0.450	0.000	0.000

Number of dissolution data points (N), degrees of freedom (df), and selected goodness of fit criteria – Pearson correlation coefficient (R), coefficient of determination (R^2), adjusted coefficient of determination ($R^2_{adjusted}$), and residual sum of squares (RSS) (manual calculation in MS Excel):

Parameter	No.1	No.2	No.3	No.4
N	25	25	25	25
df	22	22	22	22
R	0.999873437	0.999692903	0.999891326	0.999888026
R^2	0.99974689	0.999385901	0.999782664	0.999776064
$R^2_{adjusted}$	0.99972388	0.999330073	0.999762906	0.999755706
RSS	2.17977456	5.186041271	1.812620938	1.90051543

Graphical abstract of model fit presented as mean ± 1 SD of the fraction % of released carvedilol:

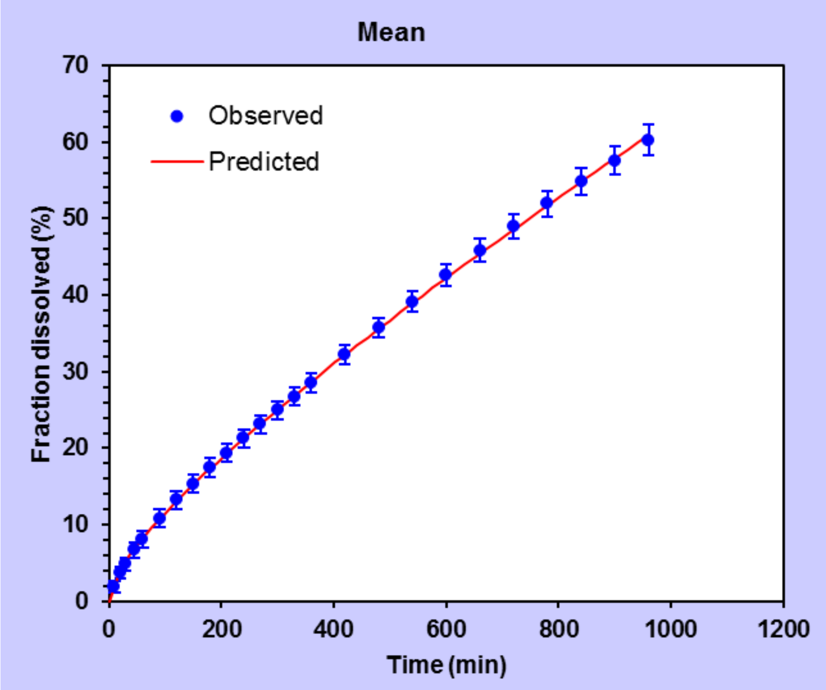

Supplementary materials – Model fitting summary – Avicel® PH-102 (modelling up to app. 60 % of carvedilol released)

Graphical abstract of model fit presented as the fraction % of released carvedilol per tested tablet:

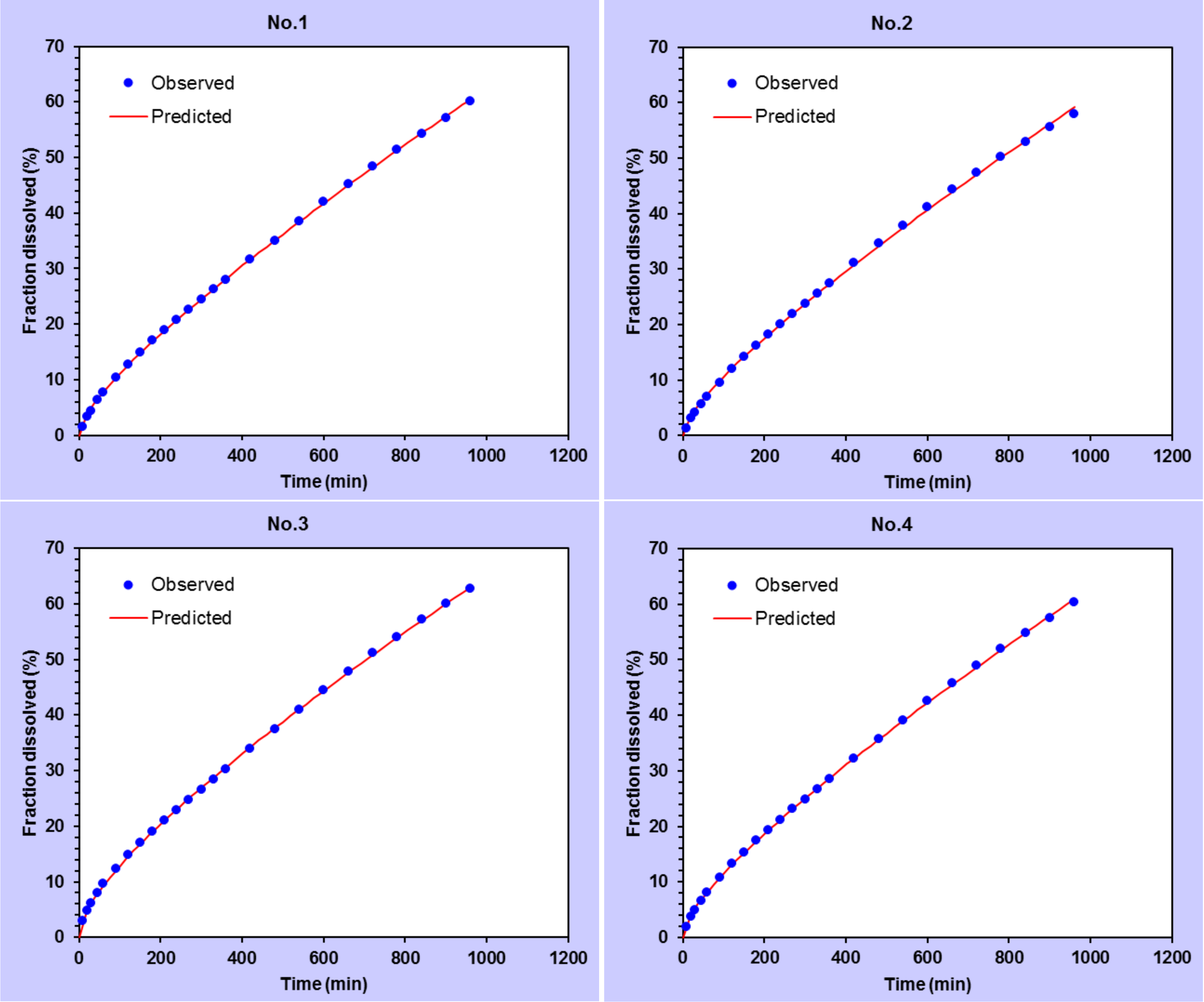

Supplementary materials – Model fitting summary – Avicel® PH-102 (modelling up to app. 60 % of carvedilol released)

Model: **Peppas–Sahlin_1 with T_{lag}**

Model equation: $F = k_1 \cdot (t - T_{lag})^m + k_2 \cdot (t - T_{lag})^{2m}$

Fitted model parameters per tested tablet (N = 4) with statistics – mean, standard deviation (SD), and relative standard deviation expressed in % (RSD%) (output from DDSolver):

Parameter	No.1	No.2	No.3	No.4	Mean	SD	RSD(%)
k_1	0.680	0.623	0.967	0.756	0.756	0.151	19.923
k_2	0.094	0.094	0.087	0.092	0.092	0.004	3.960
m	0.450	0.450	0.450	0.450	0.450	0.000	0.000
T_{lag}	4.000	6.000	4.000	4.000	4.500	1.000	22.222

Number of dissolution data points (N), degrees of freedom (df), and selected goodness of fit criteria – Pearson correlation coefficient (R), coefficient of determination (R^2), adjusted coefficient of determination ($R^2_{adjusted}$), and residual sum of squares (RSS) (manual calculation in MS Excel):

Parameter	No.1	No.2	No.3	No.4
N	25	25	25	25
df	21	21	21	21
R	0.999939056	0.999818445	0.99984691	0.99993442
R^2	0.999878115	0.999636922	0.999693844	0.999868845
$R^2_{adjusted}$	0.999860703	0.999585054	0.999650107	0.999850109
RSS	0.99138523	3.028443333	2.759560203	1.048116787

Graphical abstract of model fit presented as mean ± 1 SD of the fraction % of released carvedilol:

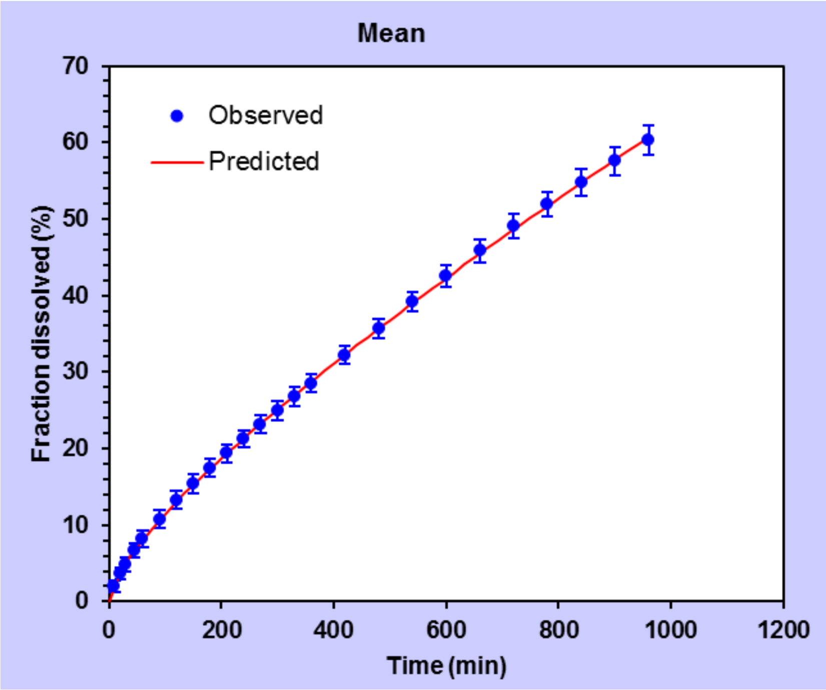

Supplementary materials – Model fitting summary – Avicel® PH-102 (modelling up to app. 60 % of carvedilol released)

Graphical abstract of model fit presented as the fraction % of released carvedilol per tested tablet:

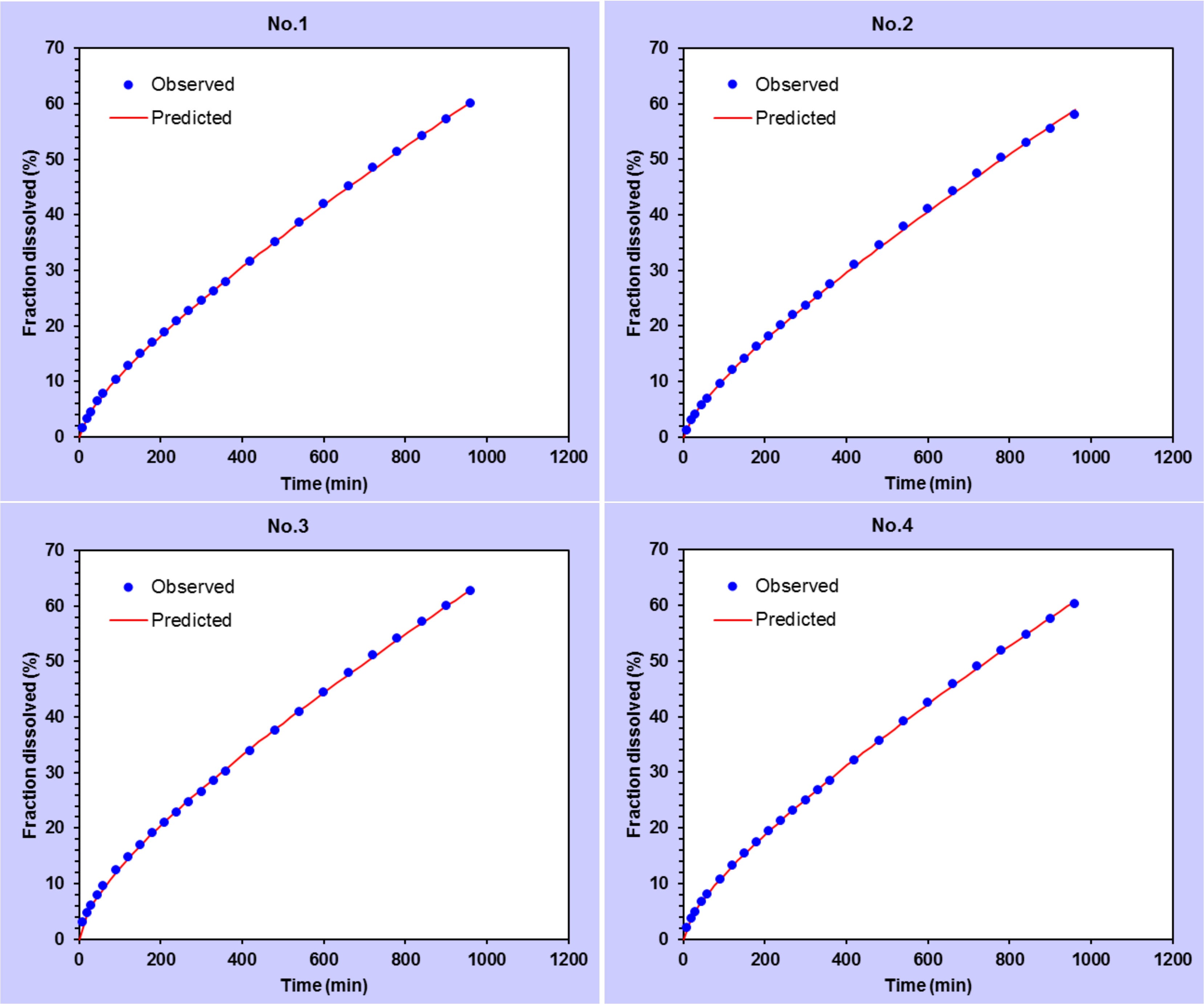

Supplementary materials – Model fitting summary – Avicel® PH-102 (modelling up to app. 60 % of carvedilol released)

Model: **Peppas–Sahlin_2**

Model equation: $F = k_1 \cdot t^{0.5} + k_2 \cdot t$

Fitted model parameters per tested tablet (N = 4) with statistics – mean, standard deviation (SD), and relative standard deviation expressed in % (RSD%) (output from DDSolver):

Parameter	No.1	No.2	No.3	No.4	Mean	SD	RSD(%)
k_1	0.720	0.676	0.933	0.776	0.776	0.112	14.483
k_2	0.040	0.040	0.036	0.039	0.039	0.002	5.217

Number of dissolution data points (N), degrees of freedom (df), and selected goodness of fit criteria – Pearson correlation coefficient (R), coefficient of determination (R^2), adjusted coefficient of determination ($R^2_{adjusted}$), and residual sum of squares (RSS) (manual calculation in MS Excel):

Parameter	No.1	No.2	No.3	No.4
N	25	25	25	25
df	23	23	23	23
R	0.99981441	0.999542409	0.999904352	0.999834896
R^2	0.999628855	0.999085027	0.999808713	0.99966982
$R^2_{adjusted}$	0.999612718	0.999045245	0.999800396	0.999655464
RSS	3.429019296	8.111654647	1.587404129	2.979559334

Graphical abstract of model fit presented as mean ± 1 SD of the fraction % of released carvedilol:

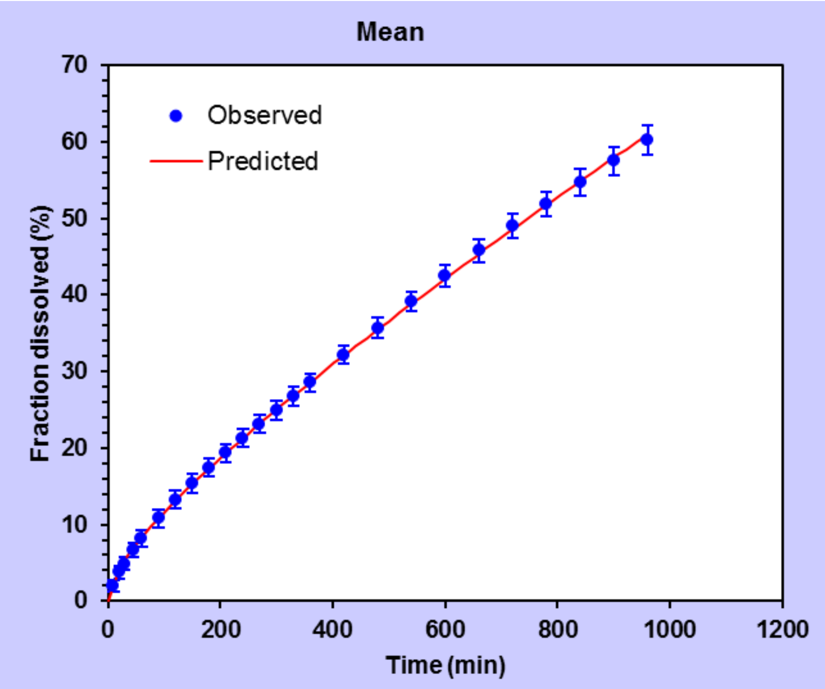

Supplementary materials – Model fitting summary – Avicel® PH-102 (modelling up to app. 60 % of carvedilol released)

Graphical abstract of model fit presented as the fraction % of released carvedilol per tested tablet:

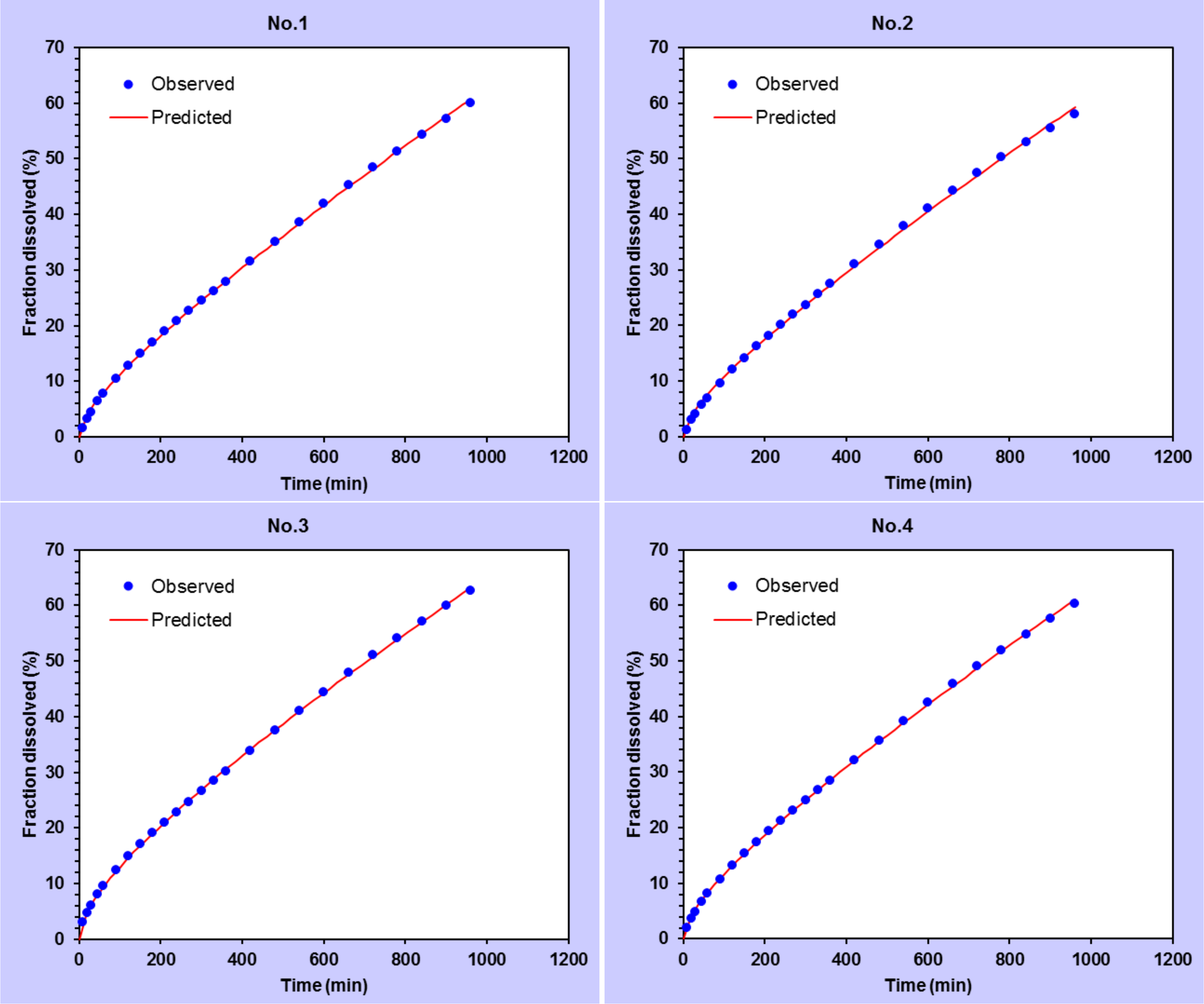

Supplementary materials – Model fitting summary – Avicel® PH-102 (modelling up to app. 60 % of carvedilol released)

Model: **Peppas–Sahlin_2 with T_{lag}**

Model equation: $F = k_1 \cdot (t - T_{lag})^{0.5} + k_2 \cdot (t - T_{lag})$

Fitted model parameters per tested tablet (N = 4) with statistics – mean, standard deviation (SD), and relative standard deviation expressed in % (RSD%) (output from DDSolver):

Parameter	No.1	No.2	No.3	No.4	Mean	SD	RSD(%)
k_1	0.761	0.717	0.977	0.818	0.818	0.114	13.895
k_2	0.039	0.039	0.034	0.037	0.037	0.002	5.515
T_{lag}	6.000	6.000	4.000	4.000	5.000	1.155	23.094

Number of dissolution data points (N), degrees of freedom (df), and selected goodness of fit criteria – Pearson correlation coefficient (R), coefficient of determination (R^2), adjusted coefficient of determination ($R^2_{adjusted}$), and residual sum of squares (RSS) (manual calculation in MS Excel):

Parameter	No.1	No.2	No.3	No.4
N	25	25	25	25
df	22	22	22	22
R	0.999933741	0.999718769	0.999874707	0.999913084
R^2	0.999867486	0.999437616	0.99974943	0.999826176
$R^2_{adjusted}$	0.999855439	0.999386491	0.999726651	0.999810374
RSS	1.347410941	4.579722061	2.273897309	1.441429409

Graphical abstract of model fit presented as mean ± 1 SD of the fraction % of released carvedilol:

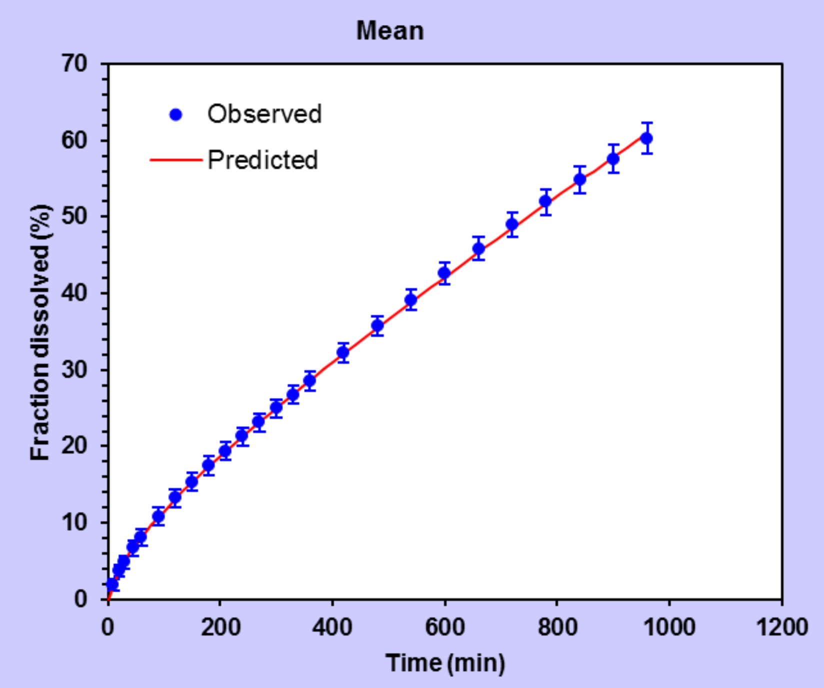

Supplementary materials – Model fitting summary – Avicel® PH-102 (modelling up to app. 60 % of carvedilol released)

Graphical abstract of model fit presented as the fraction % of released carvedilol per tested tablet:

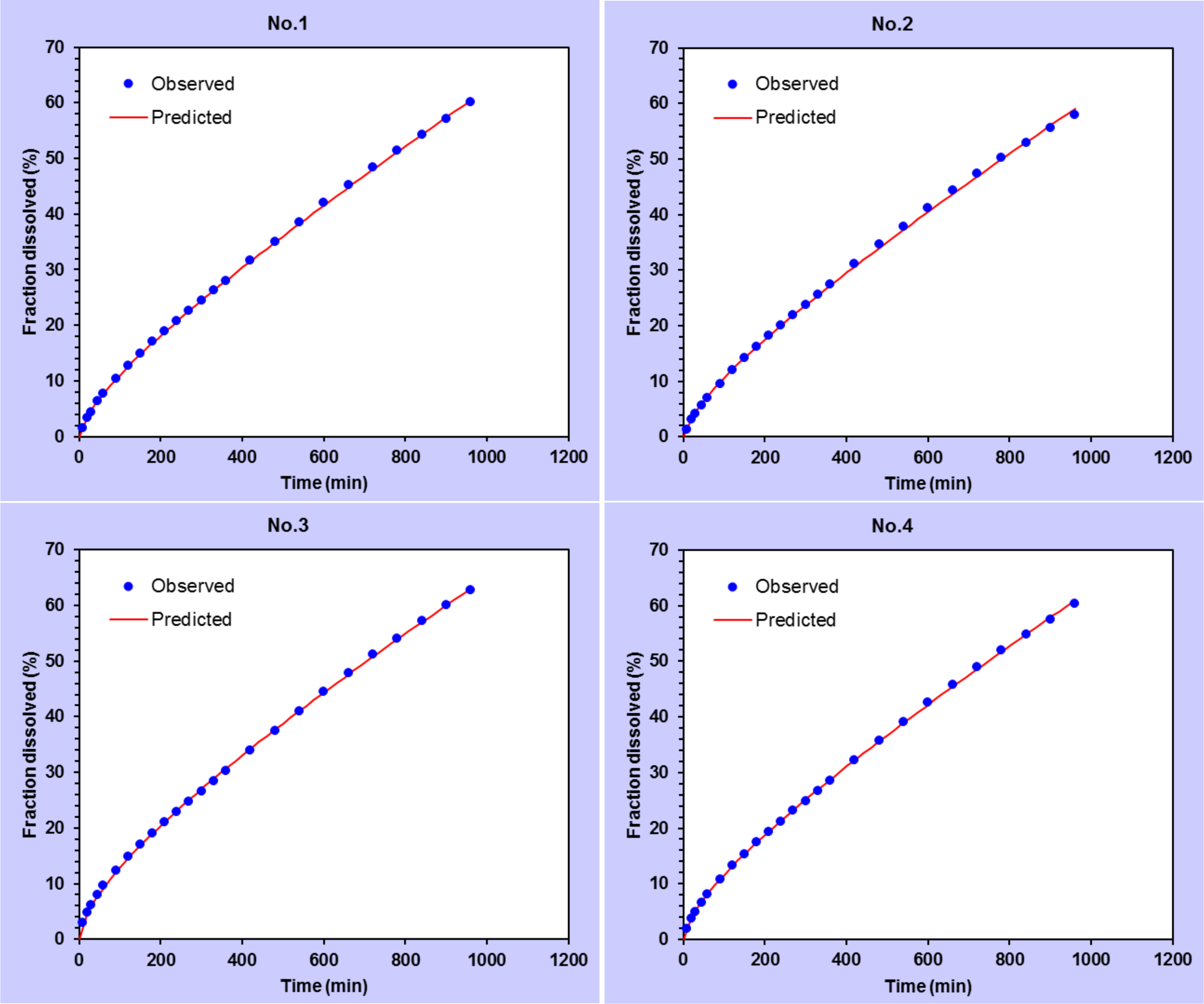

Supplementary materials – Model fitting summary – Avicel® PH-102 (modelling up to app. 60 % of carvedilol released)

Model: **Quadratic**

Model equation: $F = 100 \cdot (k_1 \cdot t^2 + k_2 \cdot t)$

Fitted model parameters per tested tablet (N = 4) with statistics – mean, standard deviation (SD), and relative standard deviation expressed in % (RSD%) (output from DDSolver):

Parameter	No.1	No.2	No.3	No.4	Mean	SD	RSD(%)
k_1	-0.0000003	-0.0000003	-0.0000004	-0.0000003	-0.0000003	0.0000000	-11.2603005
k_2	0.0009077	0.0008864	0.0010048	0.0009330	0.0009330	0.0000515	5.5229309

Number of dissolution data points (N), degrees of freedom (df), and selected goodness of fit criteria – Pearson correlation coefficient (R), coefficient of determination (R^2), adjusted coefficient of determination ($R^2_{adjusted}$), and residual sum of squares (RSS) (manual calculation in MS Excel):

Parameter	No.1	No.2	No.3	No.4
N	25	25	25	25
df	23	23	23	23
R	0.998390642	0.999063131	0.997151647	0.998284174
R^2	0.996783874	0.99812714	0.994311406	0.996571293
$R^2_{adjusted}$	0.996644042	0.998045712	0.994064076	0.996422218
RSS	50.65625516	28.9959496	111.5551239	58.78727802

Graphical abstract of model fit presented as mean ± 1 SD of the fraction % of released carvedilol:

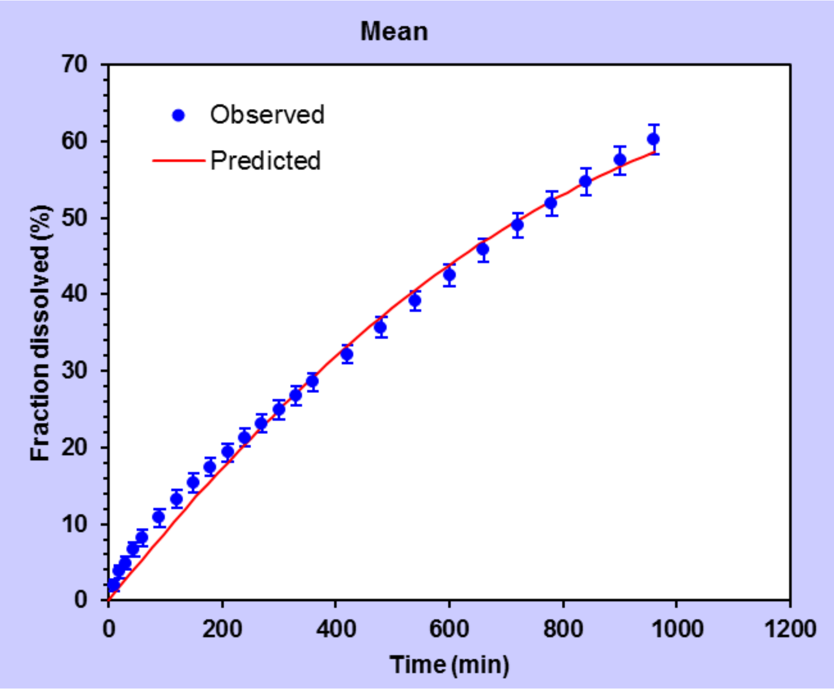

Supplementary materials – Model fitting summary – Avicel® PH-102 (modelling up to app. 60 % of carvedilol released)

Graphical abstract of model fit presented as the fraction % of released carvedilol per tested tablet:

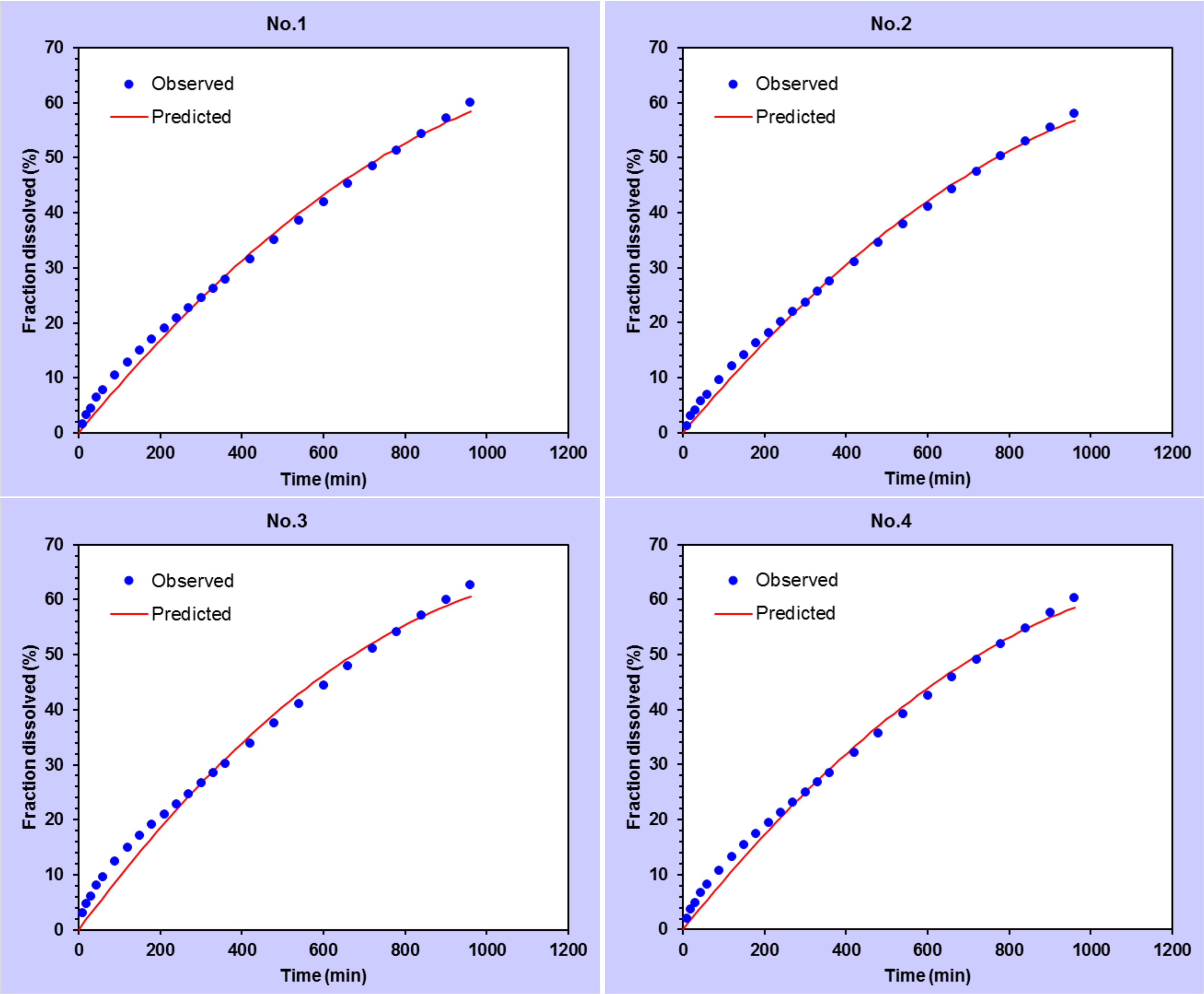

Supplementary materials – Model fitting summary – Avicel® PH-102 (modelling up to app. 60 % of carvedilol released)

Model: **Quadratic with T_{lag}**

Model equation: $F = 100 \cdot \left[k_1 \cdot (t - T_{lag})^2 + k_2 \cdot (t - T_{lag})\right]$

Fitted model parameters per tested tablet (N = 4) with statistics – mean, standard deviation (SD), and relative standard deviation expressed in % (RSD%) (output from DDSolver):

Parameter	No.1	No.2	No.3	No.4	Mean	SD	RSD(%)
k_1	-0.0000003	-0.0000003	-0.0000004	-0.0000003	-0.0000003	0.0000000	-10.9034029
k_2	0.0009208	0.0008995	0.0010184	0.0009462	0.0009462	0.0000517	5.4685340
T_{lag}	4.0000000	4.0000000	4.0000000	4.0000000	4.0000000	0.0000000	0.0000000

Number of dissolution data points (N), degrees of freedom (df), and selected goodness of fit criteria – Pearson correlation coefficient (R), coefficient of determination (R^2), adjusted coefficient of determination ($R^2_{adjusted}$), and residual sum of squares (RSS) (manual calculation in MS Excel):

Parameter	No.1	No.2	No.3	No.4
N	25	25	25	25
df	22	22	22	22
R	0.998184418	0.998895016	0.996861412	0.998059167
R^2	0.996372132	0.997791252	0.993732676	0.9961221
$R^2_{adjusted}$	0.996042326	0.997590457	0.993162919	0.995769564
RSS	61.92624366	37.53845071	130.8574228	71.62055398

Graphical abstract of model fit presented as mean ± 1 SD of the fraction % of released carvedilol:

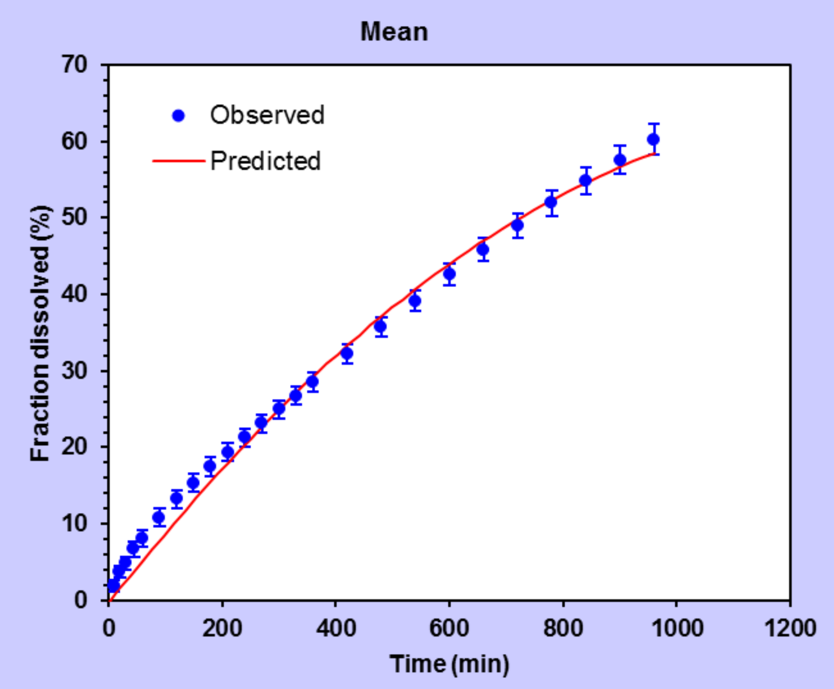

Supplementary materials – Model fitting summary – Avicel® PH-102 (modelling up to app. 60 % of carvedilol released)

Graphical abstract of model fit presented as the fraction % of released carvedilol per tested tablet:

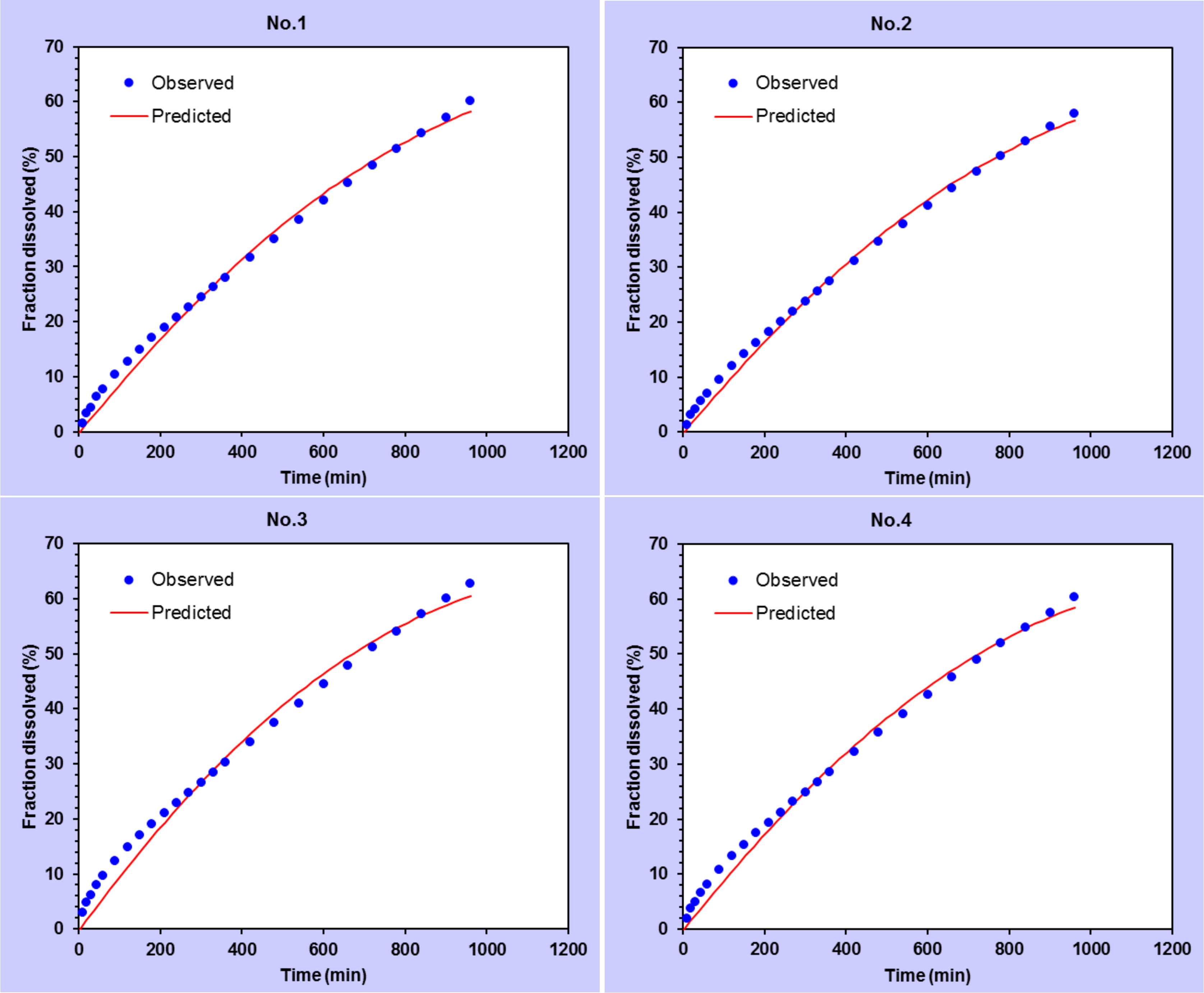

Supplementary materials – Model fitting summary – Avicel® PH-102 (modelling up to app. 60 % of carvedilol released)

Model: **Weibull_1**

Model equation: $F = 100 \cdot \left[1 - e^{-\frac{(t-T_i)^\beta}{\alpha}}\right]$

Fitted model parameters per tested tablet (N = 4) with statistics – mean, standard deviation (SD), and relative standard deviation expressed in % (RSD%) (output from DDSolver):

Parameter	No.1	No.2	No.3	No.4	Mean	SD	RSD(%)
α	324.861	356.700	158.315	243.515	270.848	88.879	32.815
β	0.819	0.817	0.704	0.763	0.776	0.054	7.020
T_i	4.733	6.000	6.000	6.000	5.683	0.634	11.150

Number of dissolution data points (N), degrees of freedom (df), and selected goodness of fit criteria – Pearson correlation coefficient (R), coefficient of determination (R^2), adjusted coefficient of determination ($R^2_{adjusted}$), and residual sum of squares (RSS) (manual calculation in MS Excel):

Parameter	No.1	No.2	No.3	No.4
N	25	25	25	25
df	22	22	22	22
R	0.995217627	0.996822923	0.990310278	0.994054895
R^2	0.990458126	0.99365594	0.980714446	0.988145135
$R^2_{adjusted}$	0.989590683	0.993079208	0.978961214	0.98706742
RSS	109.4367698	80.85124459	247.6485514	152.798623

Graphical abstract of model fit presented as mean ± 1 SD of the fraction % of released carvedilol:

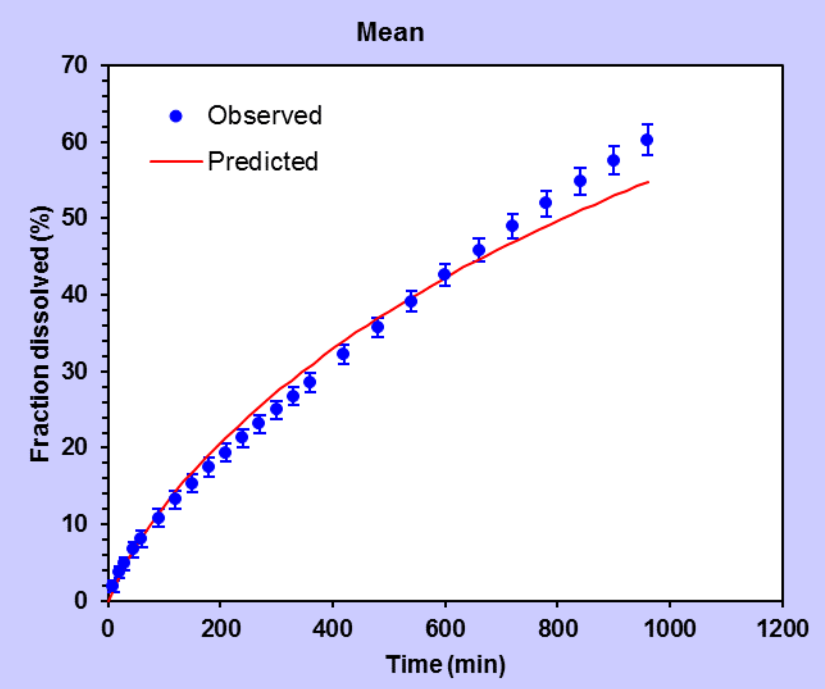

Supplementary materials – Model fitting summary – Avicel® PH-102 (modelling up to app. 60 % of carvedilol released)

Graphical abstract of model fit presented as the fraction % of released carvedilol per tested tablet:

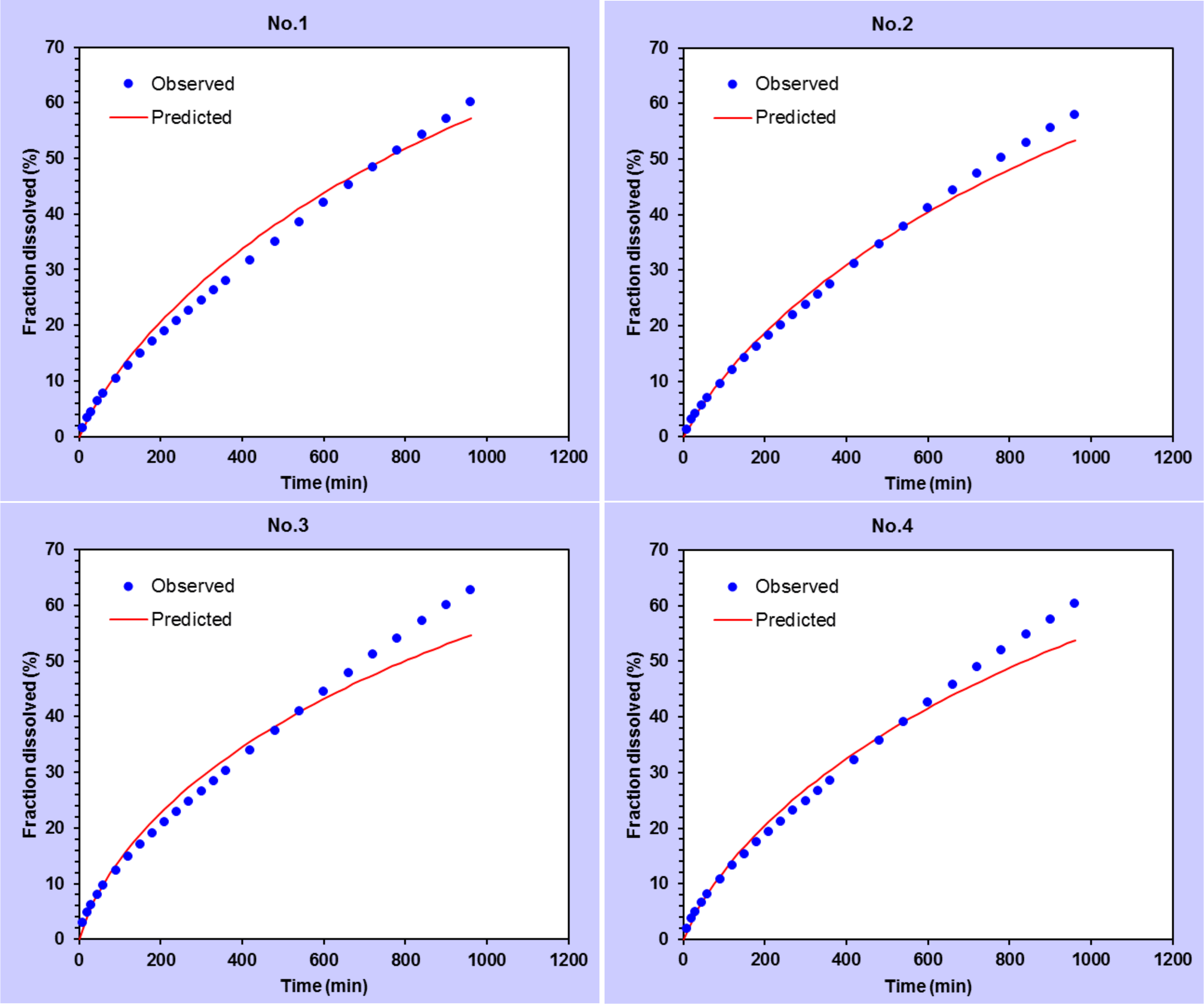

Supplementary materials – Model fitting summary – Avicel® PH-102 (modelling up to app. 60 % of carvedilol released)

Model: **Weibull_2**

Model equation: $F = 100 \cdot \left(1 - e^{-\frac{t^{\beta}}{\alpha}}\right)$

Fitted model parameters per tested tablet (N = 4) with statistics – mean, standard deviation (SD), and relative standard deviation expressed in % (RSD%) (output from DDSolver):

Parameter	No.1	No.2	No.3	No.4	Mean	SD	RSD(%)
α	485.158	508.600	217.910	341.669	388.334	135.476	34.886
β	0.880	0.875	0.756	0.818	0.832	0.058	6.963

Number of dissolution data points (N), degrees of freedom (df), and selected goodness of fit criteria – Pearson correlation coefficient (R), coefficient of determination (R^2), adjusted coefficient of determination ($R^2_{adjusted}$), and residual sum of squares (RSS) (manual calculation in MS Excel):

Parameter	No.1	No.2	No.3	No.4
N	25	25	25	25
df	23	23	23	23
R	0.997193559	0.998328007	0.99344048	0.996300072
R^2	0.994394995	0.996658809	0.986923988	0.992613833
$R^2_{adjusted}$	0.994151299	0.99651354	0.986355465	0.992292695
RSS	60.09044288	34.73198623	159.3343202	85.44454675

Graphical abstract of model fit presented as mean ± 1 SD of the fraction % of released carvedilol:

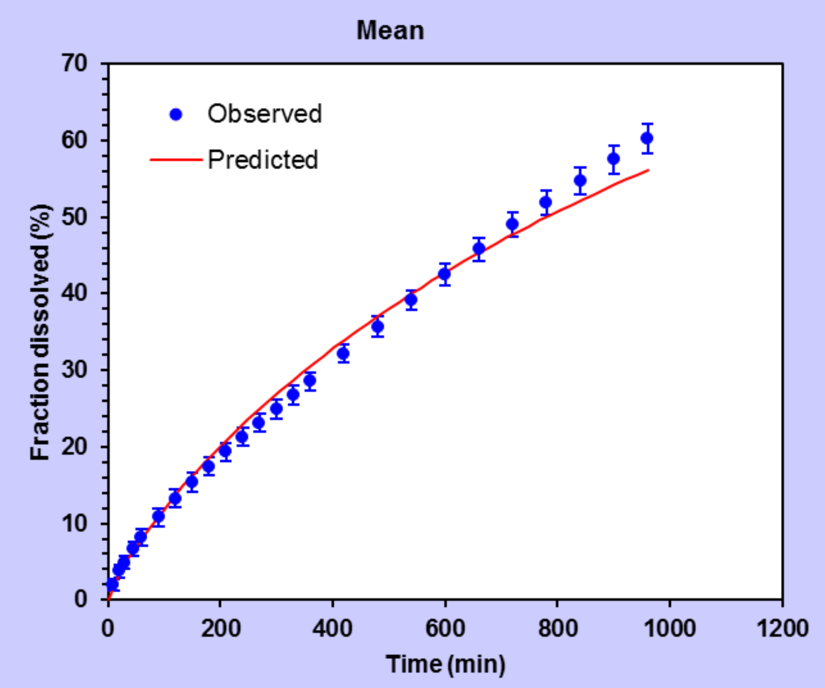

Supplementary materials – Model fitting summary – Avicel® PH-102 (modelling up to app. 60 % of carvedilol released)

Graphical abstract of model fit presented as the fraction % of released carvedilol per tested tablet:

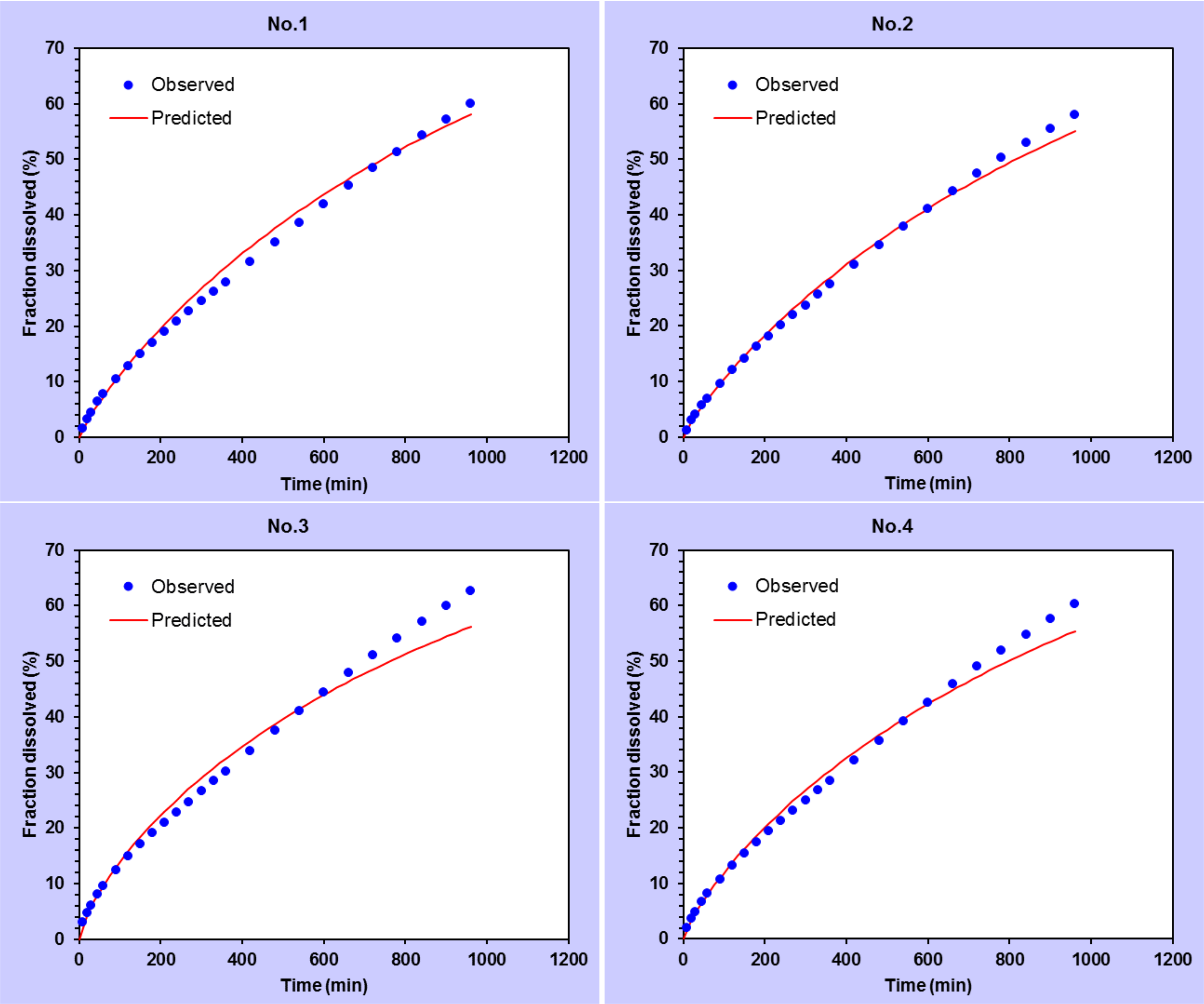

Supplementary materials – Model fitting summary – Avicel® PH-102 (modelling up to app. 60 % of carvedilol released)

Model: **Weibull_3**

Model equation: $F = F_{max} \cdot \left(1 - e^{-\frac{t^\beta}{\alpha}}\right)$

Fitted model parameters per tested tablet (N = 4) with statistics – mean, standard deviation (SD), and relative standard deviation expressed in % (RSD%) (output from DDSolver):

Parameter	No.1	No.2	No.3	No.4	Mean	SD	RSD(%)
α	403.053	487.925	215.215	331.675	359.467	115.444	32.115
β	0.959	0.993	0.863	0.930	0.936	0.055	5.923
F_{max}	63.101	60.881	65.867	63.283	63.283	2.039	3.223

Number of dissolution data points (N), degrees of freedom (df), and selected goodness of fit criteria – Pearson correlation coefficient (R), coefficient of determination (R^2), adjusted coefficient of determination ($R^2_{adjusted}$), and residual sum of squares (RSS) (manual calculation in MS Excel):

Parameter	No.1	No.2	No.3	No.4
N	25	25	25	25
df	22	22	22	22
R	0.987808918	0.989847055	0.9833047	0.986899845
R^2	0.975766458	0.979797192	0.966888133	0.973971304
$R^2_{adjusted}$	0.973563409	0.977960573	0.963877964	0.971605059
RSS	221.8776913	180.601965	323.3165044	243.6488996

Graphical abstract of model fit presented as mean ± 1 SD of the fraction % of released carvedilol:

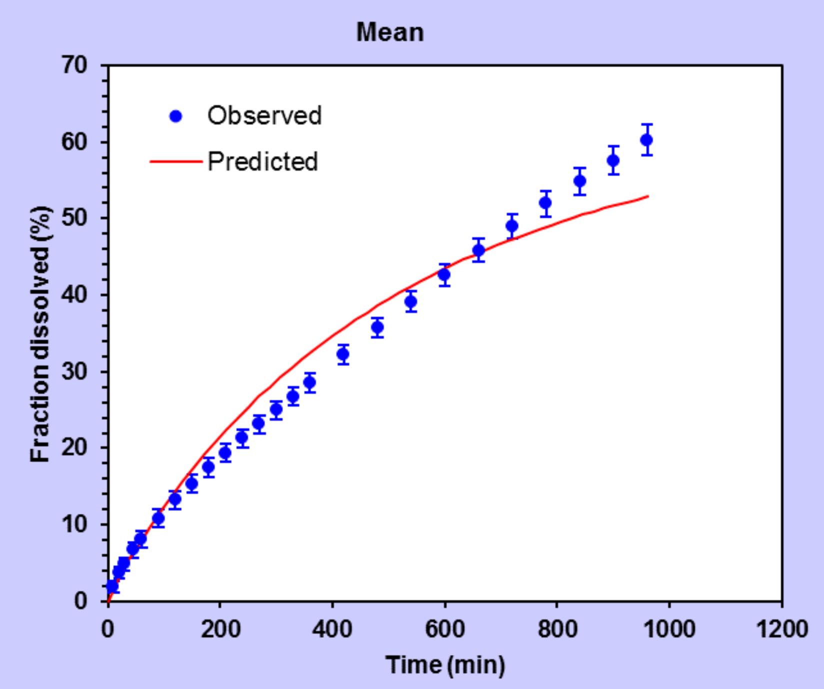

Graphical abstract of model fit presented as the fraction % of released carvedilol per tested tablet:

Supplementary materials – Model fitting summary – Avicel® PH-102 (modelling up to app. 60 % of carvedilol released)

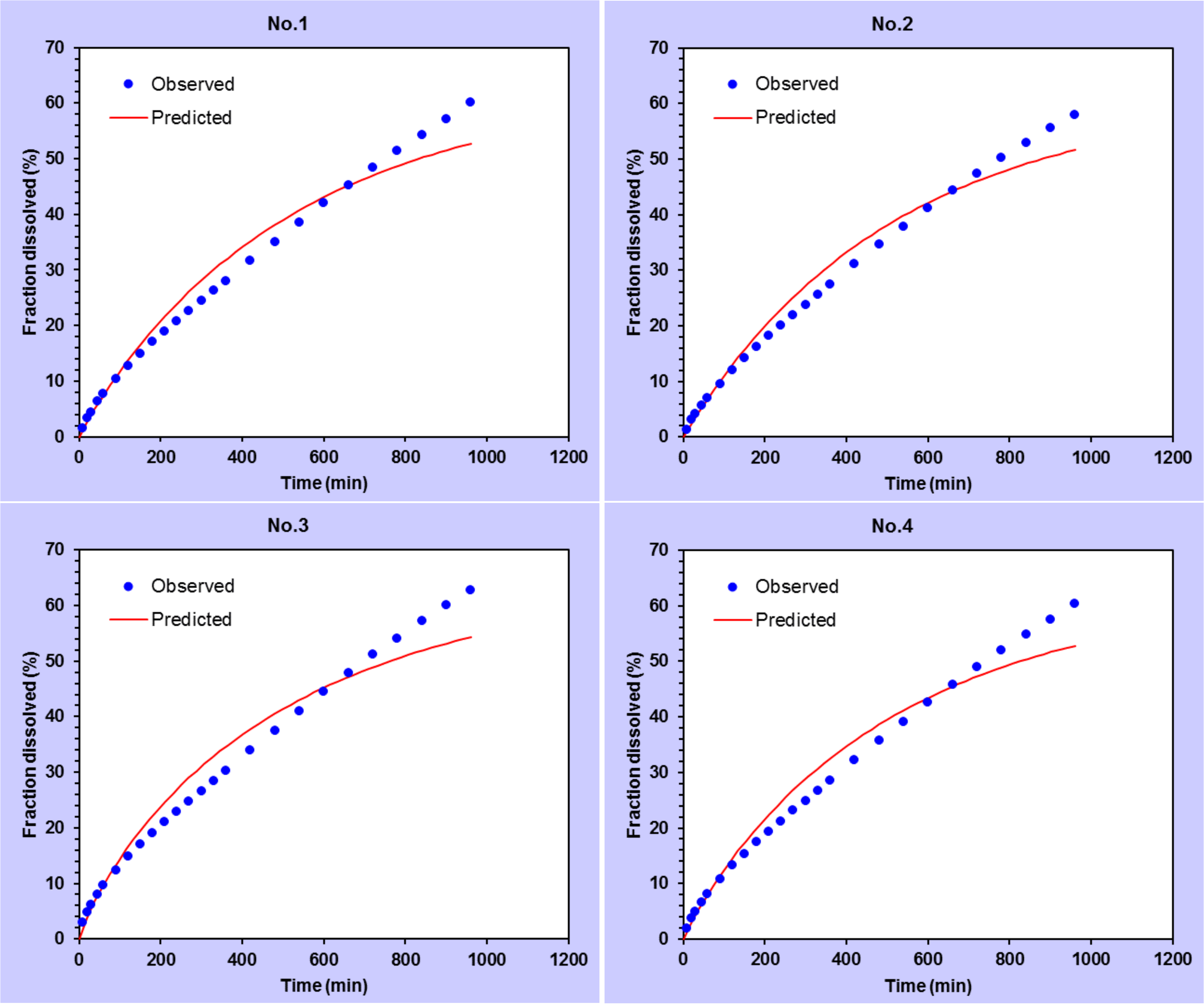

Supplementary materials – Model fitting summary – Avicel® PH-102 (modelling up to app. 60 % of carvedilol released)

Model: **Weibull_4**

Model equation: $F = F_{max} \cdot \left[1 - e^{-\frac{(t-T_i)^\beta}{\alpha}}\right]$

Fitted model parameters per tested tablet (N = 4) with statistics – mean, standard deviation (SD), and relative standard deviation expressed in % (RSD%) (output from DDSolver):

Parameter	No.1	No.2	No.3	No.4	Mean	SD	RSD(%)
α	268.853	321.599	147.857	222.920	240.307	73.649	30.648
β	0.893	0.925	0.801	0.865	0.871	0.053	6.055
T_i	6.000	6.000	6.000	6.000	6.000	0.000	0.000
F_{max}	63.101	60.881	65.867	63.283	63.283	2.039	3.223

Number of dissolution data points (N), degrees of freedom (df), and selected goodness of fit criteria – Pearson correlation coefficient (R), coefficient of determination (R^2), adjusted coefficient of determination ($R^2_{adjusted}$), and residual sum of squares (RSS) (manual calculation in MS Excel):

Parameter	No.1	No.2	No.3	No.4
N	25	25	25	25
df	21	21	21	21
R	0.985070606	0.987357921	0.979494391	0.983838736
R^2	0.970364099	0.974875664	0.959409262	0.967938659
$R^2_{adjusted}$	0.966130399	0.971286474	0.953610585	0.963358467
RSS	279.7970143	232.4335633	401.4808691	307.7289107

Graphical abstract of model fit presented as mean ± 1 SD of the fraction % of released carvedilol:

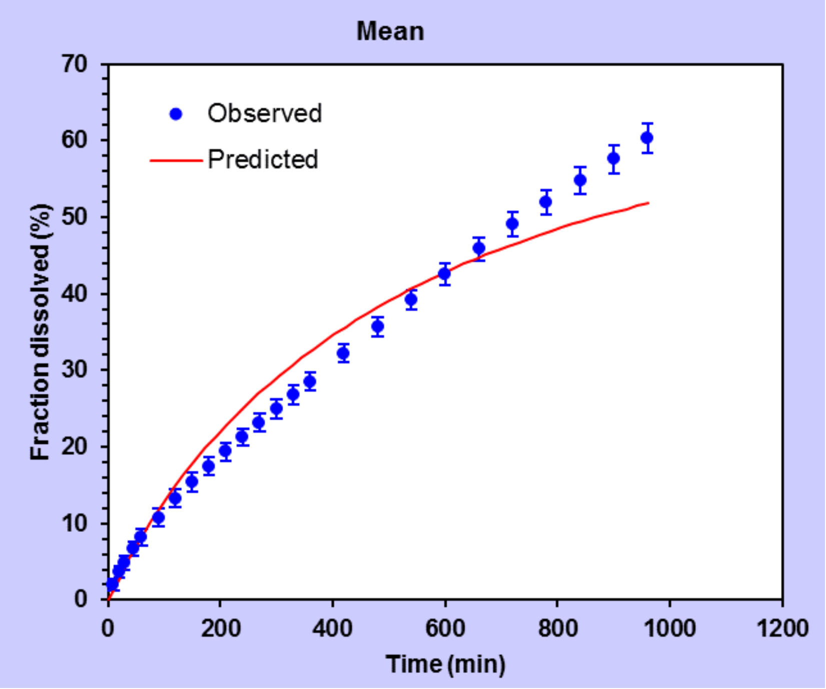

Supplementary materials – Model fitting summary – Avicel® PH-102 (modelling up to app. 60 % of carvedilol released)

Graphical abstract of model fit presented as the fraction % of released carvedilol per tested tablet:

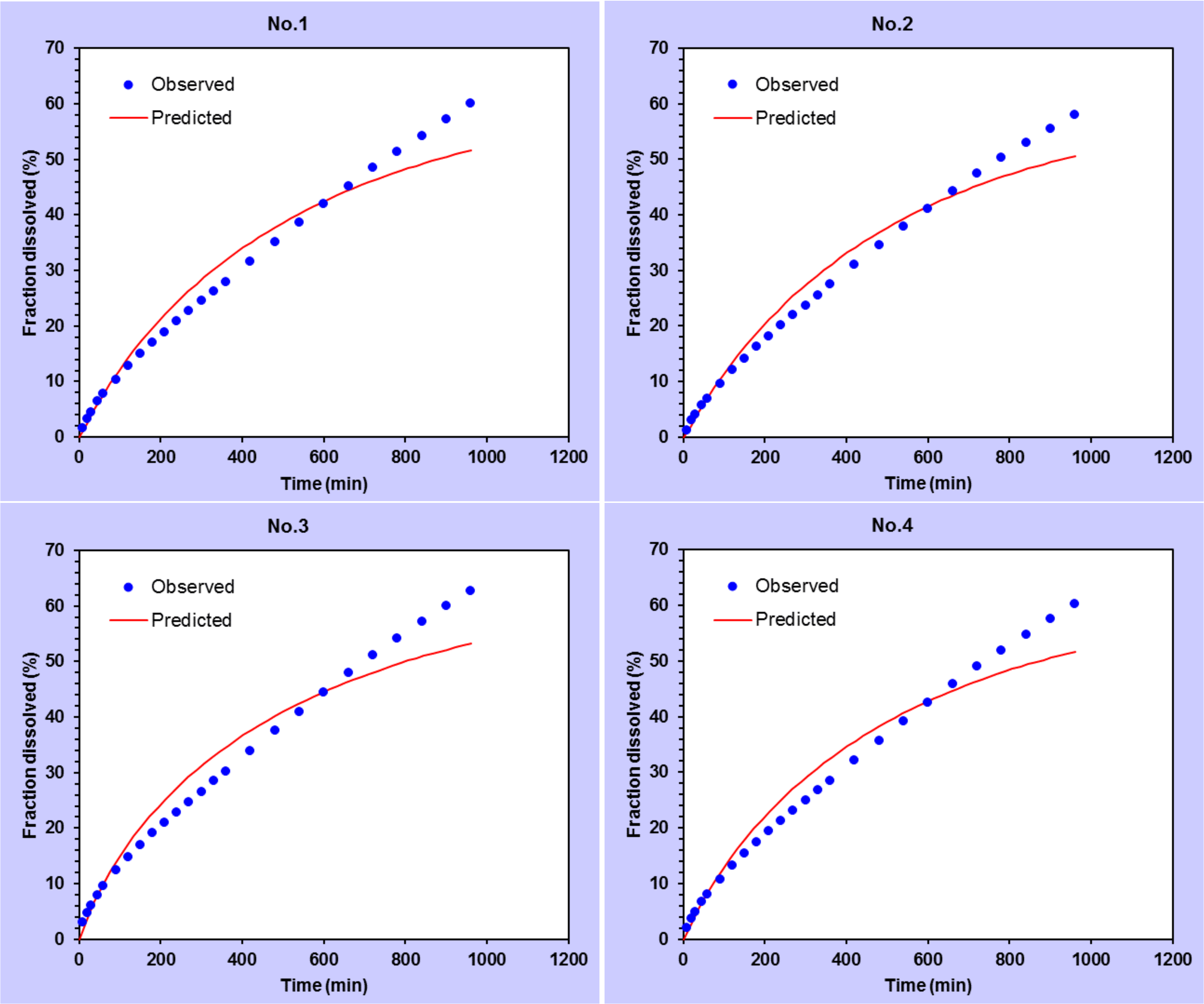

Supplementary materials – Model fitting summary – Avicel® PH-102 (modelling up to app. 60 % of carvedilol released)

Model: **Logistic_1**

Model equation: $F = 100 \cdot \frac{e^{\alpha+\beta\cdot\log(t)}}{1+e^{\alpha+\beta\cdot\log(t)}}$

Fitted model parameters per tested tablet (N = 4) with statistics – mean, standard deviation (SD), and relative standard deviation expressed in % (RSD%) (output from DDSolver):

Parameter	No.1	No.2	No.3	No.4	Mean	SD	RSD(%)
α	-6.916	-7.117	-5.755	-6.189	-6.494	0.634	-9.764
β	2.356	2.414	1.986	2.117	2.218	0.201	9.071

Number of dissolution data points (N), degrees of freedom (df), and selected goodness of fit criteria – Pearson correlation coefficient (R), coefficient of determination (R^2), adjusted coefficient of determination ($R^2_{adjusted}$), and residual sum of squares (RSS) (manual calculation in MS Excel):

Parameter	No.1	No.2	No.3	No.4
N	25	25	25	25
df	23	23	23	23
R	0.994481379	0.996614776	0.986198318	0.990485893
R^2	0.988993213	0.993241012	0.972587123	0.981062304
$R^2_{adjusted}$	0.988514657	0.992947143	0.971395258	0.980238925
RSS	161.4484088	123.2672968	295.3772277	199.868164

Graphical abstract of model fit presented as mean ± 1 SD of the fraction % of released carvedilol:

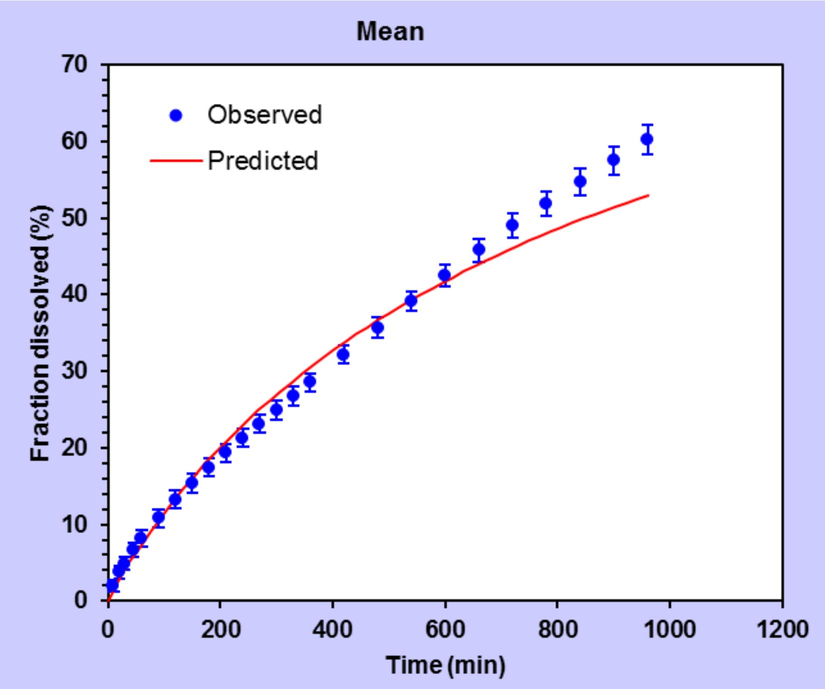

Supplementary materials – Model fitting summary – Avicel® PH-102 (modelling up to app. 60 % of carvedilol released)

Graphical abstract of model fit presented as the fraction % of released carvedilol per tested tablet:

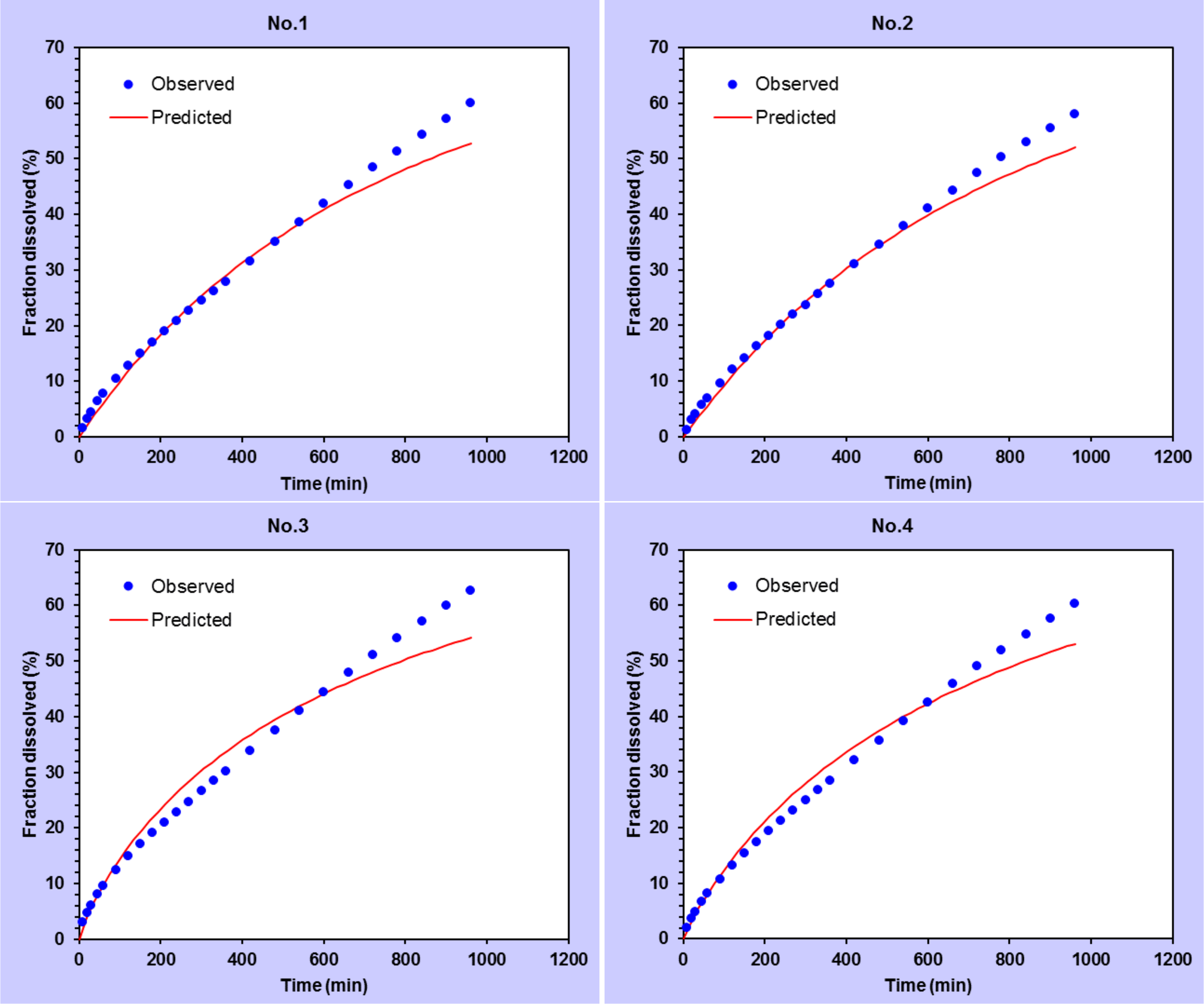

Supplementary materials – Model fitting summary – Avicel® PH-102 (modelling up to app. 60 % of carvedilol released)

Model: **Logistic_2**

Model equation: $F = F_{max} \cdot \frac{e^{\alpha+\beta \cdot \log(t)}}{1+e^{\alpha+\beta \cdot \log(t)}}$

Fitted model parameters per tested tablet (N = 4) with statistics – mean, standard deviation (SD), and relative standard deviation expressed in % (RSD%) (output from DDSolver):

Parameter	No.1	No.2	No.3	No.4	Mean	SD	RSD(%)
α	-7.439	-8.360	-7.402	-7.246	-7.612	0.506	-6.642
β	2.732	3.111	2.804	2.676	2.831	0.194	6.858
F_{max}	75.215	67.994	72.122	75.431	72.691	3.477	4.783

Number of dissolution data points (N), degrees of freedom (df), and selected goodness of fit criteria – Pearson correlation coefficient (R), coefficient of determination (R^2), adjusted coefficient of determination ($R^2_{adjusted}$), and residual sum of squares (RSS) (manual calculation in MS Excel):

Parameter	No.1	No.2	No.3	No.4
N	25	25	25	25
df	22	22	22	22
R	0.990060503	0.992976497	0.985404233	0.989034598
R^2	0.980219799	0.986002323	0.971021503	0.978189436
$R^2_{adjusted}$	0.978421599	0.984729807	0.968387094	0.976206657
RSS	291.9145801	346.1043119	397.9474141	277.5106328

Graphical abstract of model fit presented as mean ± 1 SD of the fraction % of released carvedilol:

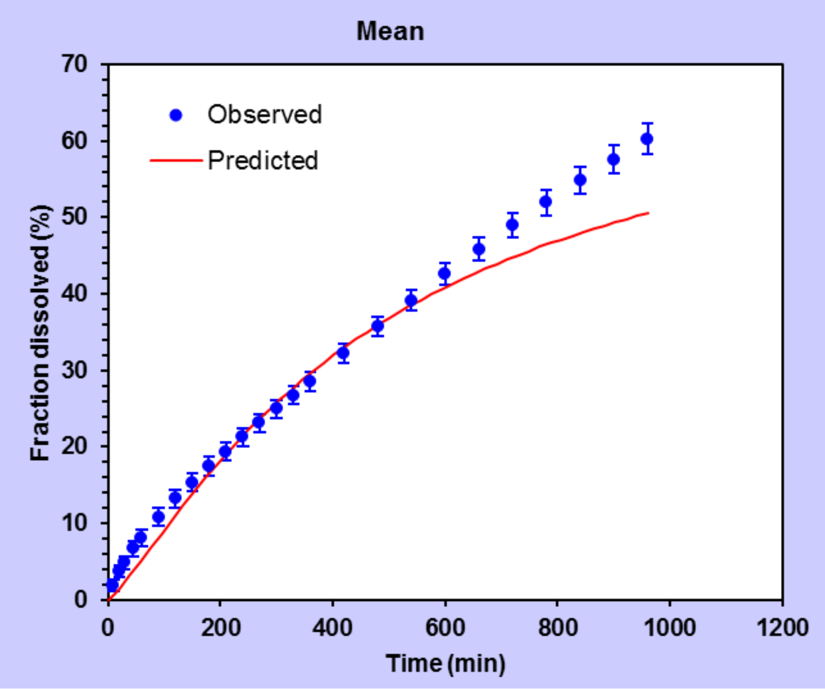

Supplementary materials – Model fitting summary – Avicel® PH-102 (modelling up to app. 60 % of carvedilol released)

Graphical abstract of model fit presented as the fraction % of released carvedilol per tested tablet:

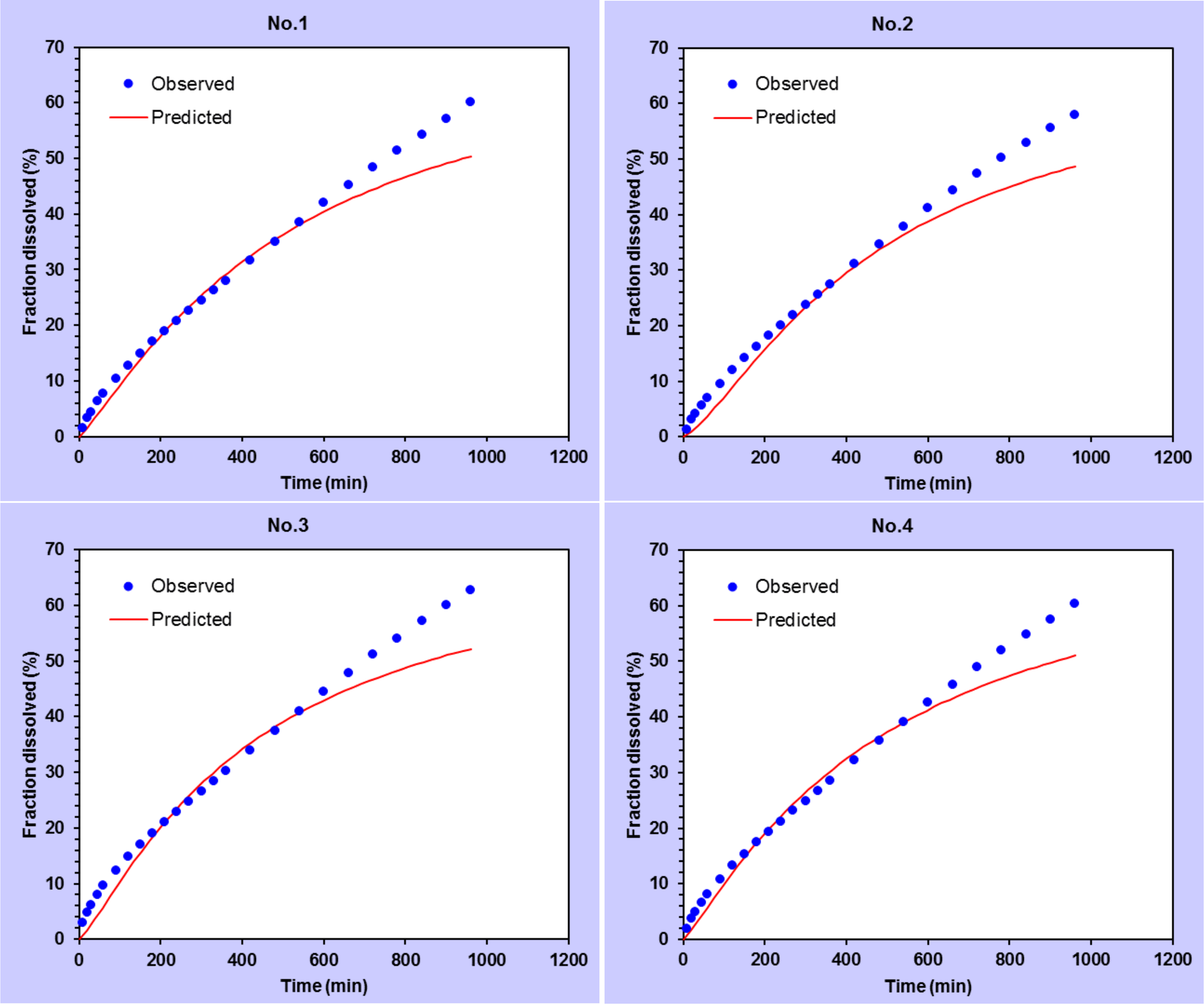

Supplementary materials – Model fitting summary – Avicel® PH-102 (modelling up to app. 60 % of carvedilol released)

Model: **Logistic_3**

Model equation: $F = F_{max} \cdot \frac{1}{1+e^{-k\cdot(t-\gamma)}}$

Fitted model parameters per tested tablet (N = 4) with statistics – mean, standard deviation (SD), and relative standard deviation expressed in % (RSD%) (output from DDSolver):

Parameter	No.1	No.2	No.3	No.4	Mean	SD	RSD(%)
k	0.005	0.004	0.005	0.005	0.005	0.001	14.521
γ	444.715	504.754	424.156	436.553	452.545	35.818	7.915
F_{max}	63.101	68.914	65.867	63.283	65.291	2.726	4.175

Number of dissolution data points (N), degrees of freedom (df), and selected goodness of fit criteria – Pearson correlation coefficient (R), coefficient of determination (R^2), adjusted coefficient of determination ($R^2_{adjusted}$), and residual sum of squares (RSS) (manual calculation in MS Excel):

Parameter	No.1	No.2	No.3	No.4
N	25	25	25	25
df	22	22	22	22
R	0.988510441	0.988789708	0.990601764	0.989411397
R^2	0.977152892	0.977705086	0.981291855	0.978934912
$R^2_{adjusted}$	0.975075882	0.975678276	0.979591114	0.977019904
RSS	230.4758077	208.5447148	183.707818	207.666214

Graphical abstract of model fit presented as mean ± 1 SD of the fraction % of released carvedilol:

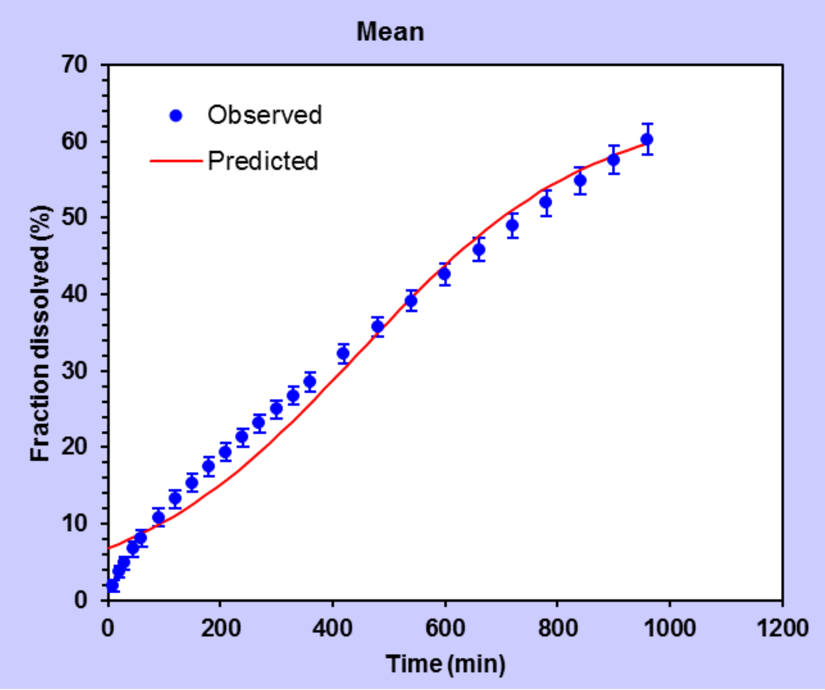

Supplementary materials – Model fitting summary – Avicel® PH-102 (modelling up to app. 60 % of carvedilol released)

Graphical abstract of model fit presented as the fraction % of released carvedilol per tested tablet:

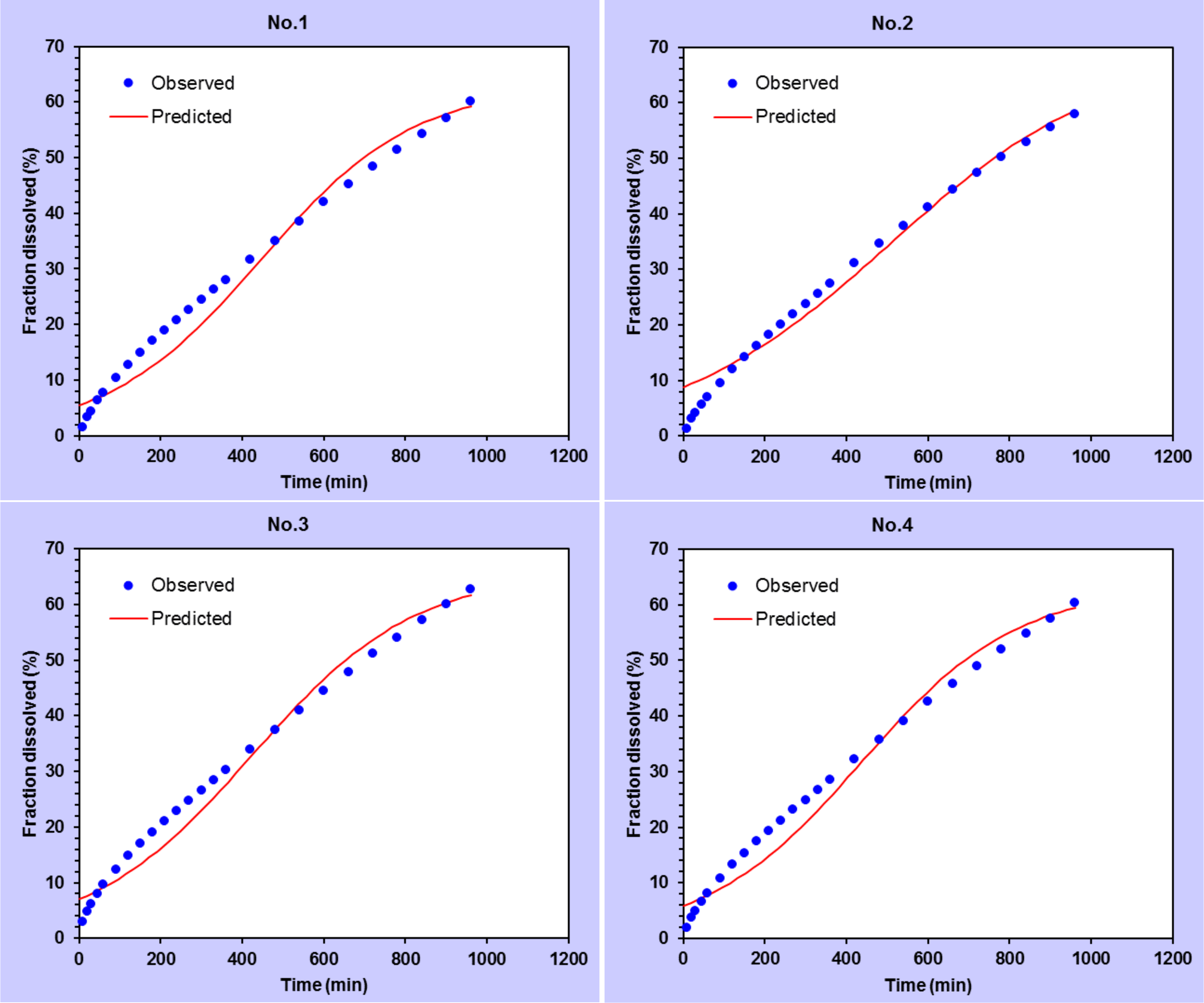

Supplementary materials – Model fitting summary – Avicel® PH-102 (modelling up to app. 60 % of carvedilol released)

Model: **Gompertz_1**

Model equation: $F = 100 \cdot e^{-\alpha \cdot e^{-\beta \cdot \log(t)}}$

Fitted model parameters per tested tablet (N = 4) with statistics – mean, standard deviation (SD), and relative standard deviation expressed in % (RSD%) (output from DDSolver):

Parameter	No.1	No.2	No.3	No.4	Mean	SD	RSD(%)
α	15.519	16.003	13.446	14.788	14.939	1.114	7.454
β	1.033	1.034	1.006	1.020	1.023	0.013	1.285

Number of dissolution data points (N), degrees of freedom (df), and selected goodness of fit criteria – Pearson correlation coefficient (R), coefficient of determination (R^2), adjusted coefficient of determination ($R^2_{adjusted}$), and residual sum of squares (RSS) (manual calculation in MS Excel):

Parameter	No.1	No.2	No.3	No.4
N	25	25	25	25
df	23	23	23	23
R	0.968121576	0.971902157	0.961757047	0.967100247
R^2	0.937259385	0.944593803	0.924976617	0.935282888
$R^2_{adjusted}$	0.934531532	0.942184837	0.921714731	0.932469101
RSS	589.3367483	515.8577354	710.7433937	607.3646303

Graphical abstract of model fit presented as mean ± 1 SD of the fraction % of released carvedilol:

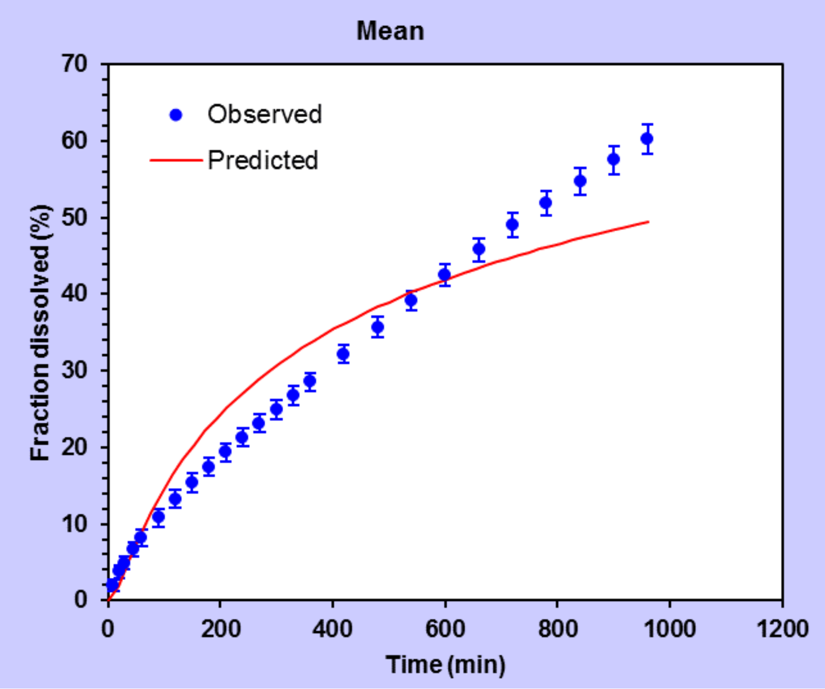

Supplementary materials – Model fitting summary – Avicel® PH-102 (modelling up to app. 60 % of carvedilol released)

Graphical abstract of model fit presented as the fraction % of released carvedilol per tested tablet:

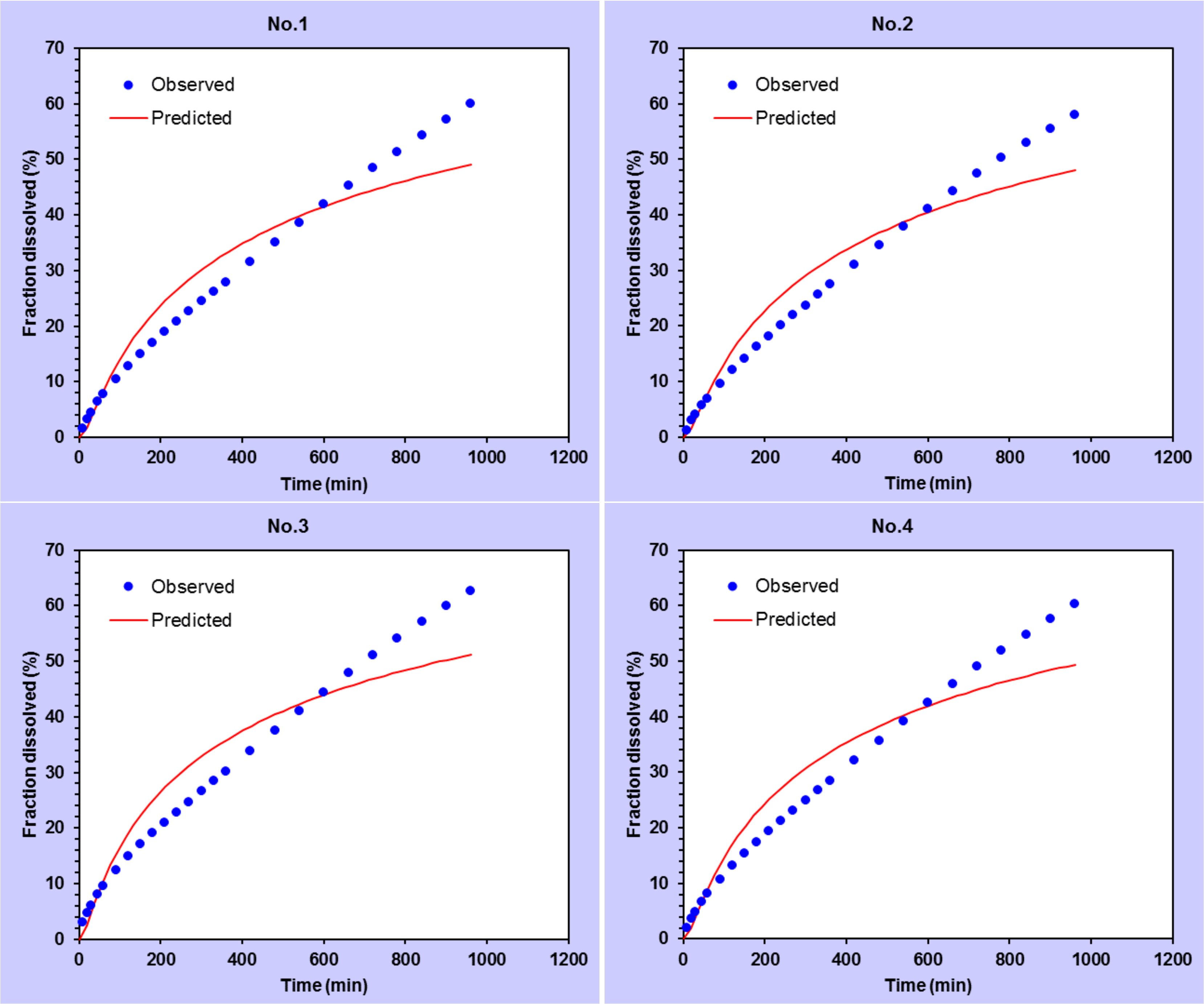

Supplementary materials – Model fitting summary – Avicel® PH-102 (modelling up to app. 60 % of carvedilol released)

Model: **Gompertz_2**

Model equation: $F = F_{max} \cdot e^{-\alpha \cdot e^{-\beta \cdot \log(t)}}$

Fitted model parameters per tested tablet (N = 4) with statistics – mean, standard deviation (SD), and relative standard deviation expressed in % (RSD%) (output from DDSolver):

Parameter	No.1	No.2	No.3	No.4	Mean	SD	RSD(%)
α	68.374	61.958	56.550	65.732	63.154	5.130	8.122
β	1.752	1.673	1.697	1.747	1.717	0.039	2.255
F_{max}	63.101	69.126	65.867	63.283	65.344	2.820	4.315

Number of dissolution data points (N), degrees of freedom (df), and selected goodness of fit criteria – Pearson correlation coefficient (R), coefficient of determination (R^2), adjusted coefficient of determination ($R^2_{adjusted}$), and residual sum of squares (RSS) (manual calculation in MS Excel):

Parameter	No.1	No.2	No.3	No.4
N	25	25	25	25
df	22	22	22	22
R	0.968606724	0.975975747	0.96400058	0.96763189
R^2	0.938198986	0.952528659	0.929297119	0.936311474
$R^2_{adjusted}$	0.932580713	0.948213082	0.922869584	0.930521608
RSS	980.774243	605.4057764	1093.769835	981.257361

Graphical abstract of model fit presented as mean ± 1 SD of the fraction % of released carvedilol:

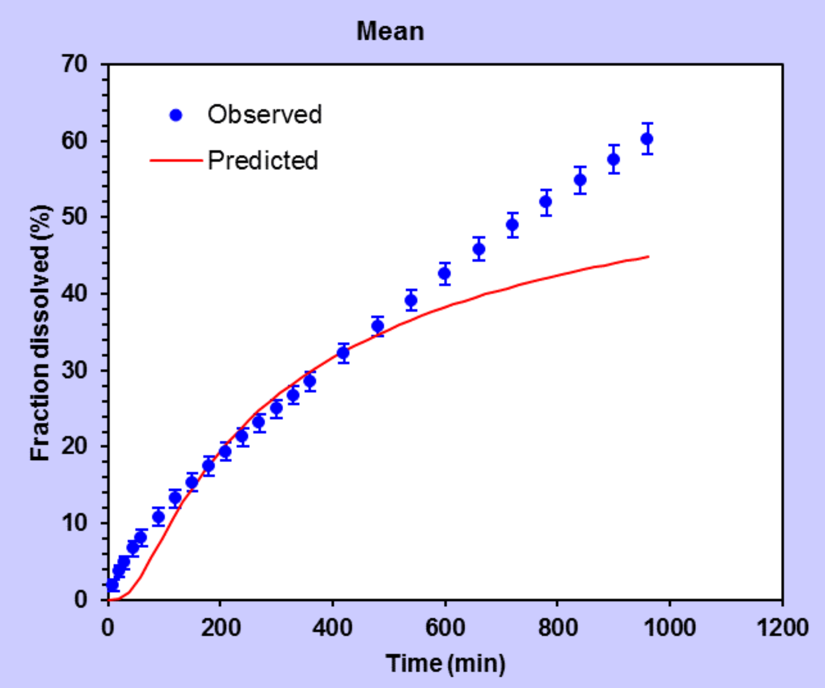

Supplementary materials – Model fitting summary – Avicel® PH-102 (modelling up to app. 60 % of carvedilol released)

Graphical abstract of model fit presented as the fraction % of released carvedilol per tested tablet:

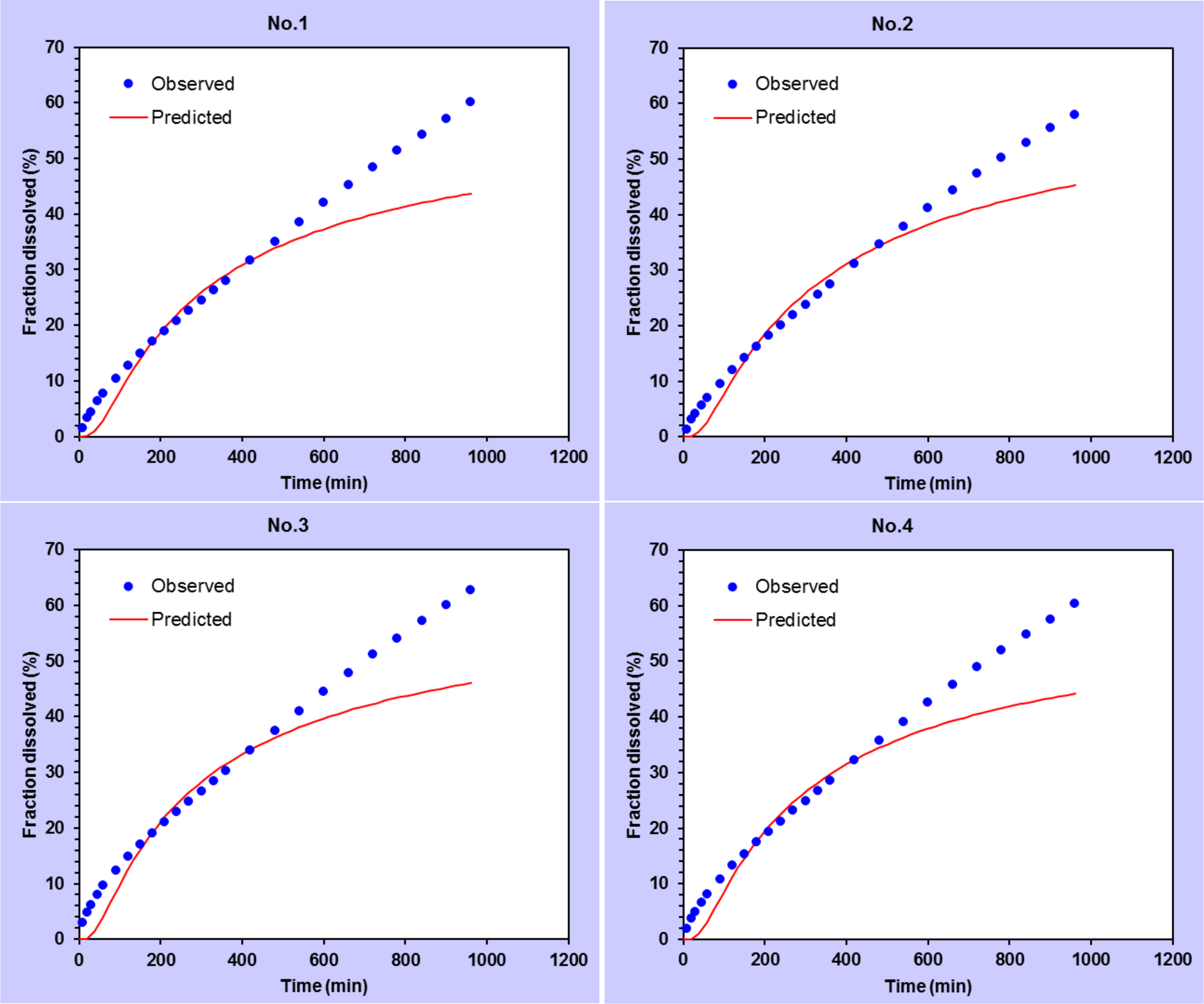

Supplementary materials – Model fitting summary – Avicel® PH-102 (modelling up to app. 60 % of carvedilol released)

Model: **Gompertz_3**

Model equation: $F = F_{max} \cdot e^{-e^{-k \cdot (t-\gamma)}}$

Fitted model parameters per tested tablet (N = 4) with statistics – mean, standard deviation (SD), and relative standard deviation expressed in % (RSD%) (output from DDSolver):

Parameter	No.1	No.2	No.3	No.4	Mean	SD	RSD(%)
k	0.004	0.004	0.004	0.004	0.004	0.000	1.924
γ	292.605	291.501	273.877	285.581	285.891	8.583	3.002
F_{max}	63.101	60.881	65.867	63.283	63.283	2.039	3.223

Number of dissolution data points (N), degrees of freedom (df), and selected goodness of fit criteria – Pearson correlation coefficient (R), coefficient of determination (R^2), adjusted coefficient of determination ($R^2_{adjusted}$), and residual sum of squares (RSS) (manual calculation in MS Excel):

Parameter	No.1	No.2	No.3	No.4
N	25	25	25	25
df	22	22	22	22
R	0.994045581	0.994961452	0.994261156	0.994479556
R^2	0.988126618	0.989948291	0.988555246	0.988989588
$R^2_{adjusted}$	0.987047219	0.9890345	0.987514814	0.987988642
RSS	117.9034926	96.3790435	118.8797848	109.5110888

Graphical abstract of model fit presented as mean ± 1 SD of the fraction % of released carvedilol:

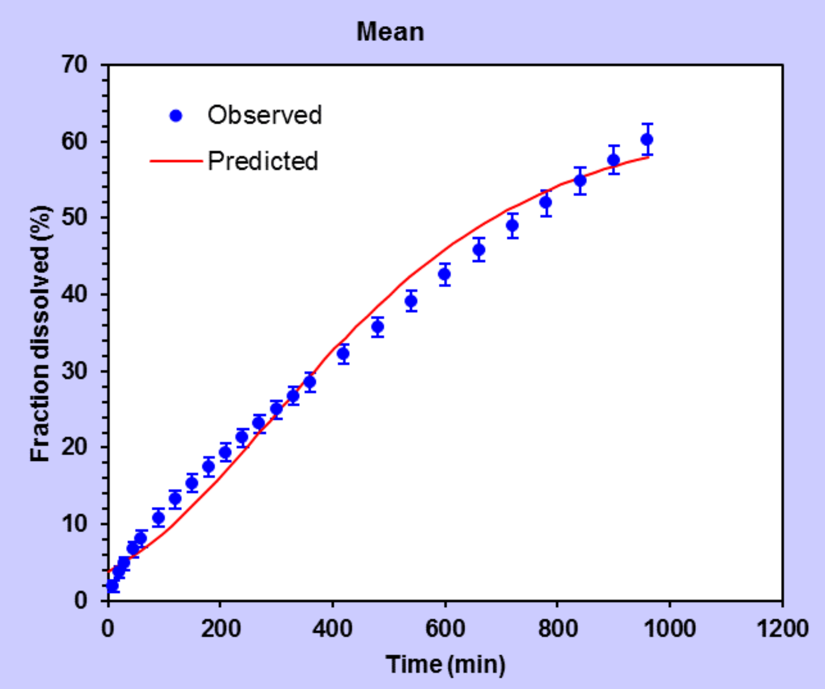

Supplementary materials – Model fitting summary – Avicel® PH-102 (modelling up to app. 60 % of carvedilol released)

Graphical abstract of model fit presented as the fraction % of released carvedilol per tested tablet:

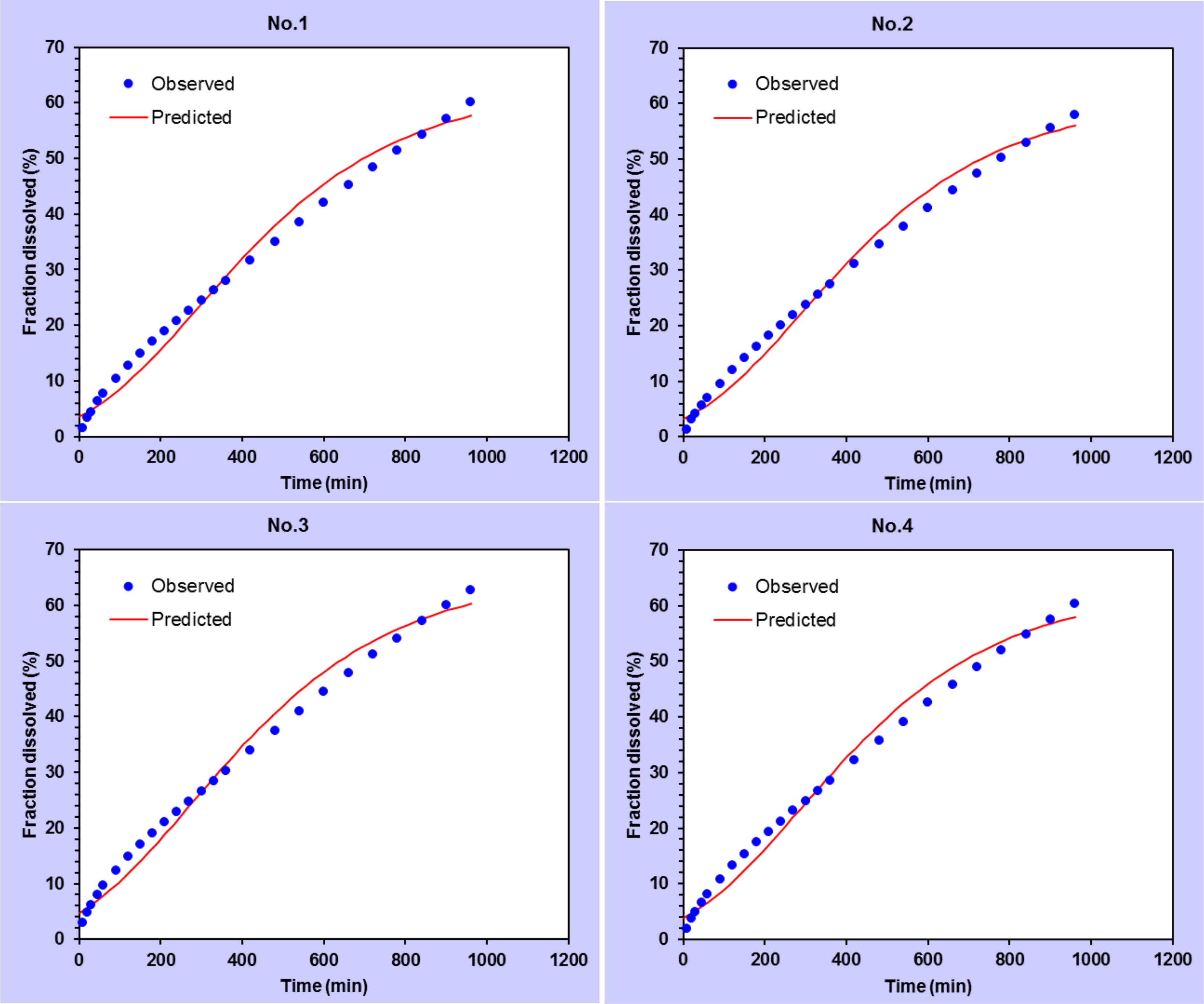

Supplementary materials – Model fitting summary – Avicel® PH-102 (modelling up to app. 60 % of carvedilol released)

Model: **Gompertz_4**

Model equation: $F = F_{max} \cdot e^{-\beta \cdot e^{-k \cdot t}}$

Fitted model parameters per tested tablet (N = 4) with statistics – mean, standard deviation (SD), and relative standard deviation expressed in % (RSD%) (output from DDSolver):

Parameter	No.1	No.2	No.3	No.4	Mean	SD	RSD(%)
k	0.004	0.004	0.004	0.004	0.004	0.000	1.924
β	2.874	2.945	2.633	2.804	2.814	0.133	4.742
F_{max}	63.101	60.881	65.867	63.283	63.283	2.039	3.223

Number of dissolution data points (N), degrees of freedom (df), and selected goodness of fit criteria – Pearson correlation coefficient (R), coefficient of determination (R^2), adjusted coefficient of determination ($R^2_{adjusted}$), and residual sum of squares (RSS) (manual calculation in MS Excel):

Parameter	No.1	No.2	No.3	No.4
N	25	25	25	25
df	22	22	22	22
R	0.994045581	0.994961452	0.994261156	0.994479556
R^2	0.988126618	0.989948291	0.988555246	0.988989588
$R^2_{adjusted}$	0.987047219	0.9890345	0.987514814	0.987988642
RSS	117.9034926	96.3790435	118.8797848	109.5110888

Graphical abstract of model fit presented as mean ± 1 SD of the fraction % of released carvedilol:

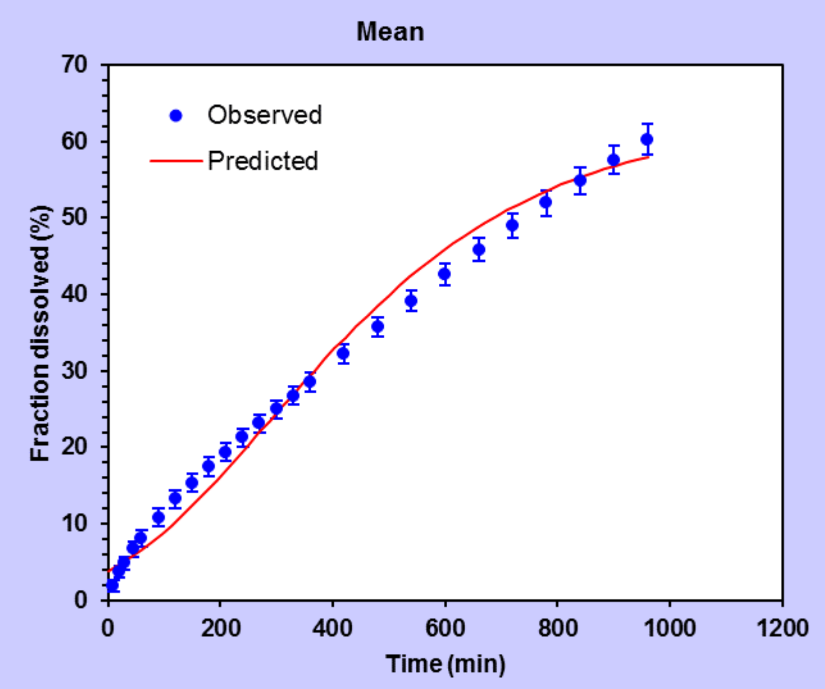

Supplementary materials – Model fitting summary – Avicel® PH-102 (modelling up to app. 60 % of carvedilol released)

Graphical abstract of model fit presented as the fraction % of released carvedilol per tested tablet:

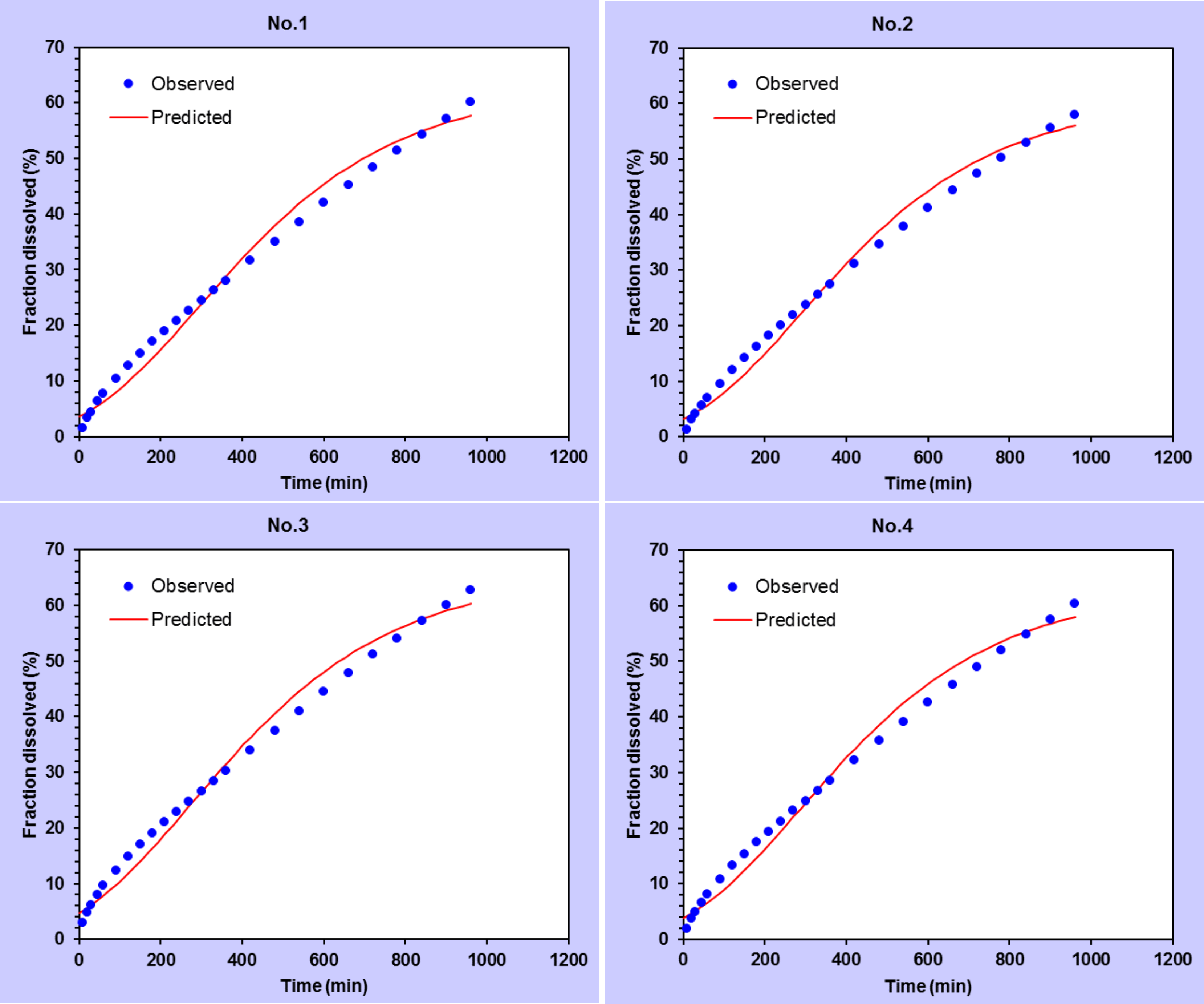

Supplementary materials – Model fitting summary – Avicel® PH-102 (modelling up to app. 60 % of carvedilol released)

Model: **Probit_1**

Model equation: $F = 100 \cdot \phi[\alpha + \beta \cdot \log(t)]$

Fitted model parameters per tested tablet (N = 4) with statistics – mean, standard deviation (SD), and relative standard deviation expressed in % (RSD%) (output from DDSolver):

Parameter	No.1	No.2	No.3	No.4	Mean	SD	RSD(%)
α	-3.516	-3.588	-3.252	-3.434	-3.447	0.145	-4.203
β	1.188	1.204	1.113	1.162	1.167	0.040	3.396

Number of dissolution data points (N), degrees of freedom (df), and selected goodness of fit criteria – Pearson correlation coefficient (R), coefficient of determination (R^2), adjusted coefficient of determination ($R^2_{adjusted}$), and residual sum of squares (RSS) (manual calculation in MS Excel):

Parameter	No.1	No.2	No.3	No.4
N	25	25	25	25
df	23	23	23	23
R	0.984029008	0.986981138	0.978700352	0.983123185
R^2	0.968313089	0.974131767	0.957854379	0.966531198
$R^2_{adjusted}$	0.966935397	0.973007061	0.956021961	0.965076032
RSS	327.9198587	269.0401204	441.2079628	349.0531048

Graphical abstract of model fit presented as mean ± 1 SD of the fraction % of released carvedilol:

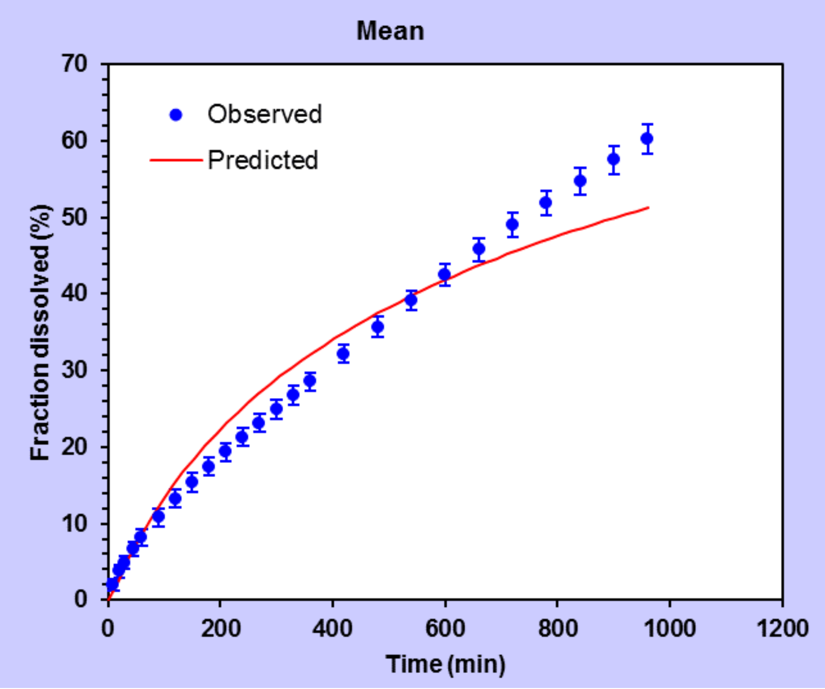

Supplementary materials – Model fitting summary – Avicel® PH-102 (modelling up to app. 60 % of carvedilol released)

Graphical abstract of model fit presented as the fraction % of released carvedilol per tested tablet:

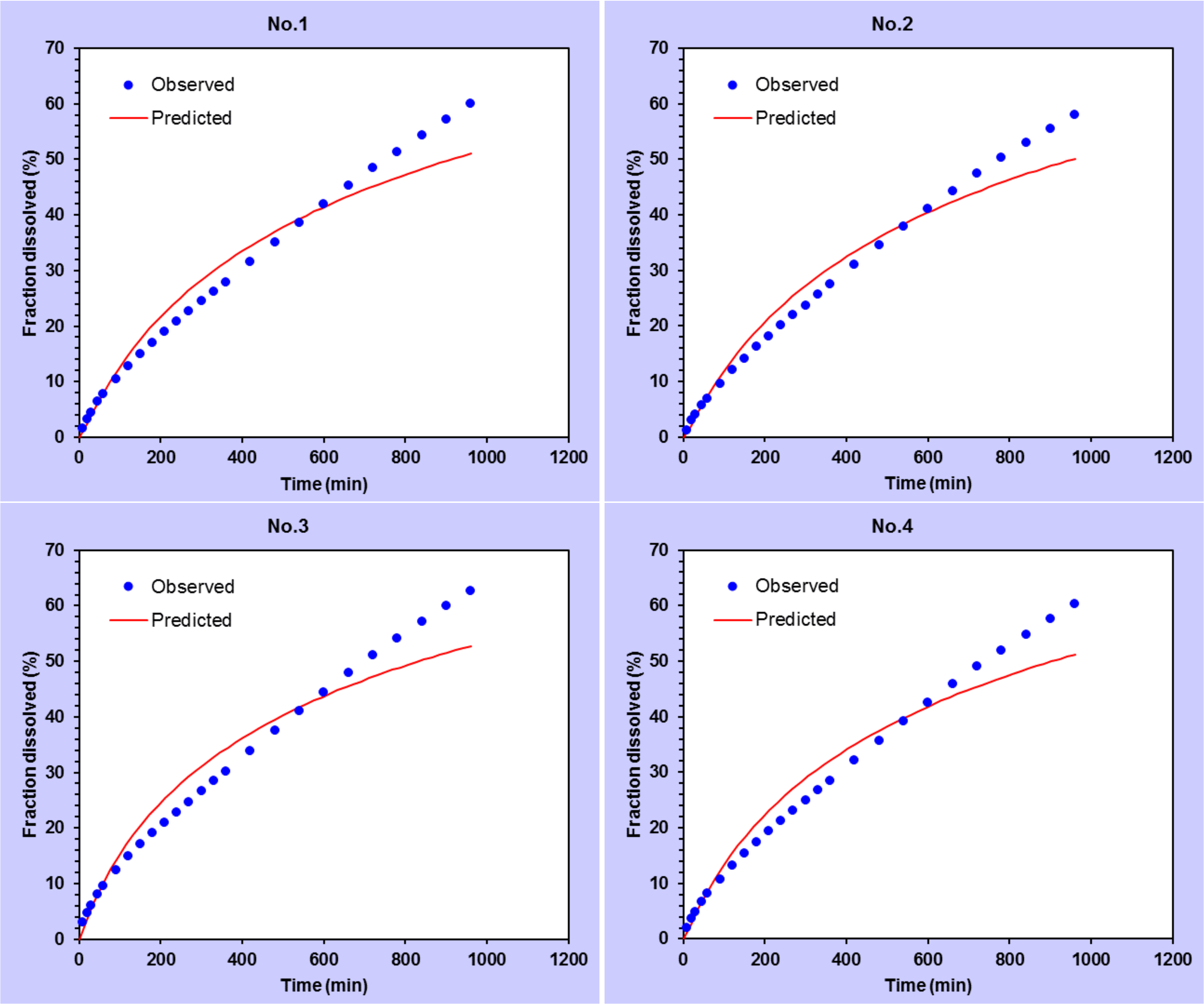

Supplementary materials – Model fitting summary – Avicel® PH-102 (modelling up to app. 60 % of carvedilol released)

Model: **Probit_2**

Model equation: $F = F_{max} \cdot \phi[\alpha + \beta \cdot \log(t)]$

Fitted model parameters per tested tablet (N = 4) with statistics – mean, standard deviation (SD), and relative standard deviation expressed in % (RSD%) (output from DDSolver):

Parameter	No.1	No.2	No.3	No.4	Mean	SD	RSD(%)
α	-4.633	-4.359	-4.292	-4.155	-4.360	0.201	-4.603
β	1.729	1.604	1.624	1.534	1.623	0.081	4.968
F_{max}	69.094	72.569	72.122	75.431	72.304	2.594	3.588

Number of dissolution data points (N), degrees of freedom (df), and selected goodness of fit criteria – Pearson correlation coefficient (R), coefficient of determination (R^2), adjusted coefficient of determination ($R^2_{adjusted}$), and residual sum of squares (RSS) (manual calculation in MS Excel):

Parameter	No.1	No.2	No.3	No.4
N	25	25	25	25
df	22	22	22	22
R	0.986133723	0.987398131	0.981185878	0.983911002
R^2	0.972459719	0.974955069	0.962725728	0.968080861
$R^2_{adjusted}$	0.969956057	0.972678257	0.959337158	0.965179121
RSS	482.9804975	332.4941213	493.6337093	364.4278608

Graphical abstract of model fit presented as mean ± 1 SD of the fraction % of released carvedilol:

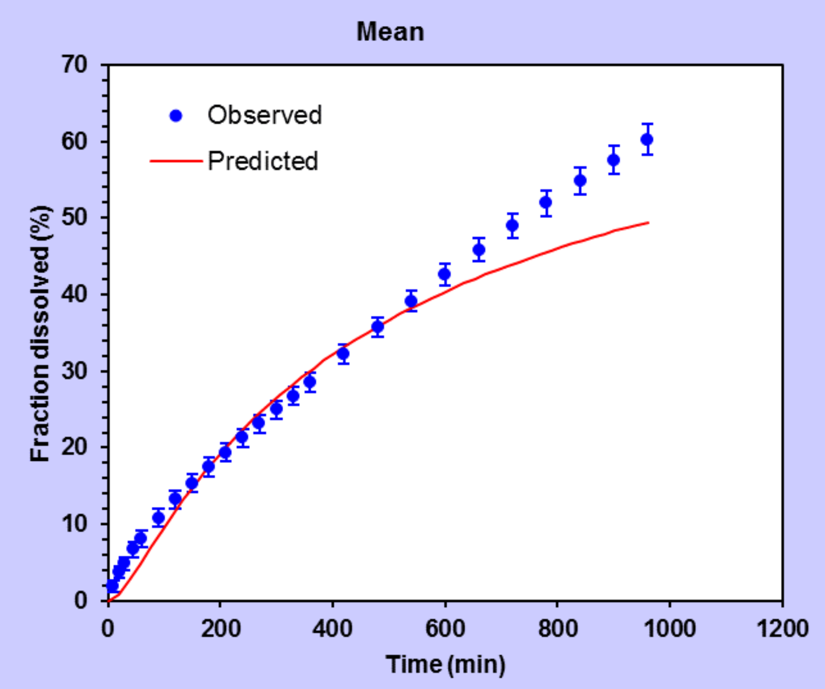

Supplementary materials – Model fitting summary – Avicel® PH-102 (modelling up to app. 60 % of carvedilol released)

Graphical abstract of model fit presented as the fraction % of released carvedilol per tested tablet:

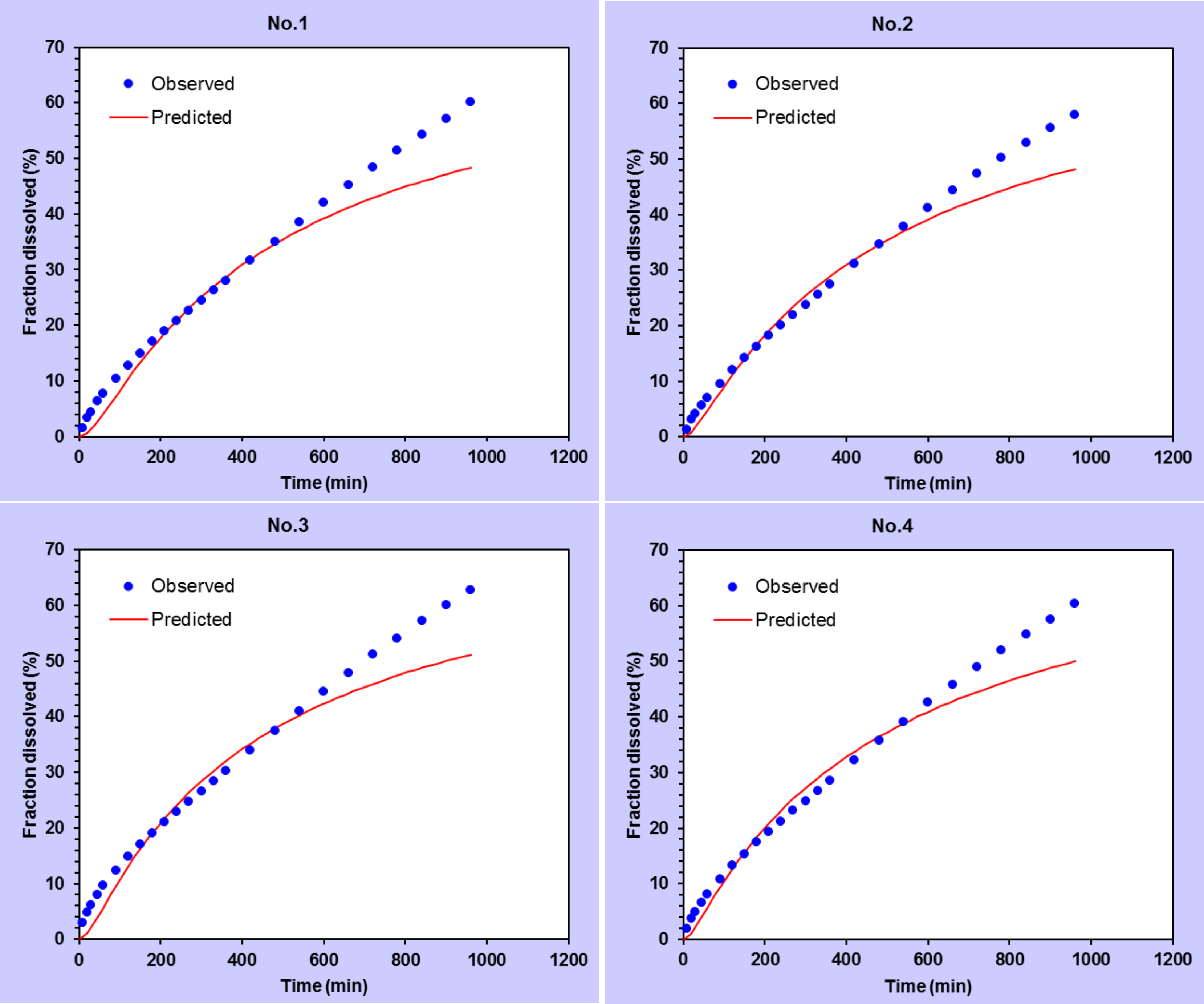

Supplement: Supplementary file 1 [file pharmaceutics-16-00498-s001.zip › Supplementary materials_Model fitting summary_Avicel® PH-102.pdf]
