# Supplementary material for: Comparative Fitting of Mathematical Models to Carvedilol Release Profiles Obtained from Hypromellose Matrix Tablets
Source: Pharmaceutics. 2024 Apr 4;16(4):498. doi: 10.3390/pharmaceutics16040498 (PMC11053526; doi:10.3390/pharmaceutics16040498)

Supplementary materials – Model fitting summary – Avicel® PH-200 (modelling the entire relevant dissolution data)

Model: **Zero–order**

Model equation: $F = k_0 \cdot t$

Fitted model parameters per tested tablet (N = 4) with statistics – mean, standard deviation (SD), and relative standard deviation expressed in % (RSD%) (output from DDSolver):

Parameter	No.1	No.2	No.3	No.4	Mean	SD	RSD(%)
k_0	0.066	0.069	0.067	0.063	0.066	0.003	3.831

Number of dissolution data points (N), degrees of freedom (df), and selected goodness of fit criteria – Pearson correlation coefficient (R), coefficient of determination (R^2), adjusted coefficient of determination ($R^2_{adjusted}$), and residual sum of squares (RSS) (manual calculation in MS Excel):

Parameter	No.1	No.2	No.3	No.4
N	33	33	33	33
df	32	32	32	32
R	0.993042228	0.991824852	0.991895946	0.99096662
R^2	0.986132866	0.983716536	0.983857567	0.982014842
$R^2_{adjusted}$	0.986132866	0.983716536	0.983857567	0.982014842
RSS	1360.440387	1276.445604	1273.491538	1182.496409

Graphical abstract of model fit presented as mean ± 1 SD of the fraction % of released carvedilol:

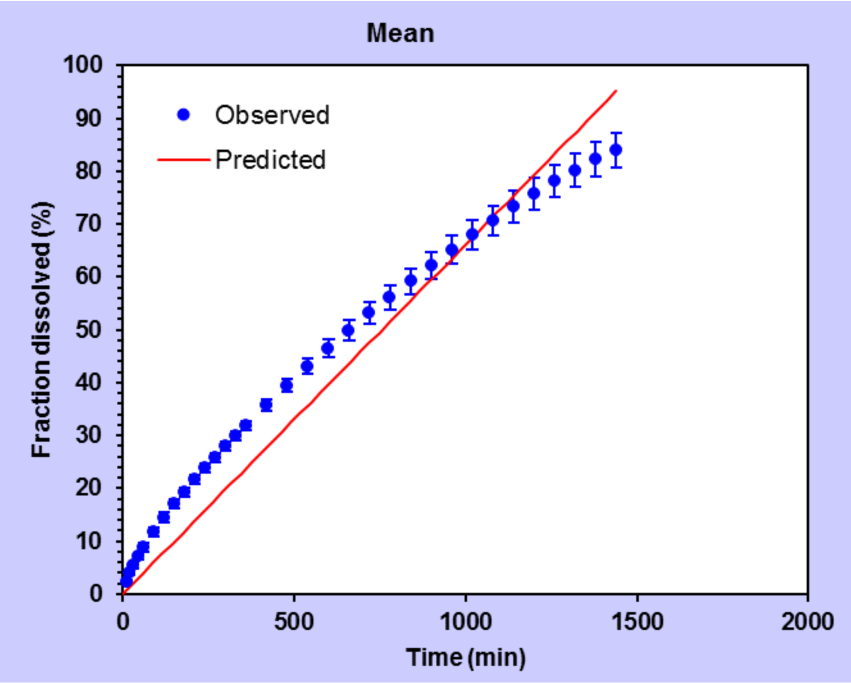

Supplementary materials – Model fitting summary – Avicel® PH-200 (modelling the entire relevant dissolution data)

Graphical abstract of model fit presented as the fraction % of released carvedilol per tested tablet:

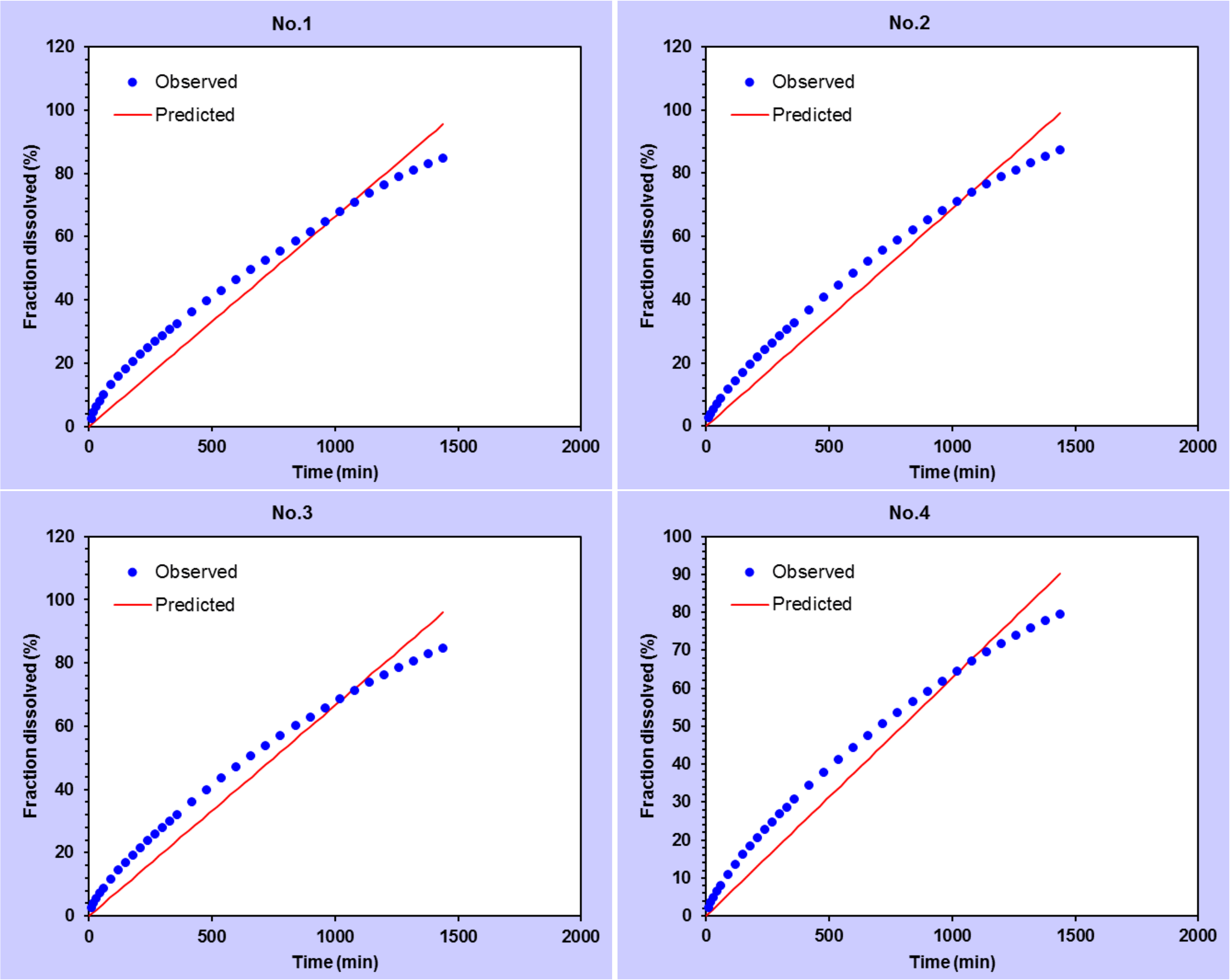

Supplementary materials – Model fitting summary – Avicel® PH-200 (modelling the entire relevant dissolution data)

Model: **Zero–order with T_{lag}**

Model equation: $F = k_0 \cdot (t - T_{lag})$

Fitted model parameters per tested tablet (N = 4) with statistics – mean, standard deviation (SD), and relative standard deviation expressed in % (RSD%) (output from DDSolver):

Parameter	No.1	No.2	No.3	No.4	Mean	SD	RSD(%)
k_0	0.057	0.060	0.058	0.054	0.057	0.002	4.235
T_{lag}	-162.580	-139.735	-147.942	-149.910	-150.042	9.449	-6.298

Number of dissolution data points (N), degrees of freedom (df), and selected goodness of fit criteria – Pearson correlation coefficient (R), coefficient of determination (R^2), adjusted coefficient of determination ($R^2_{adjusted}$), and residual sum of squares (RSS) (manual calculation in MS Excel):

Parameter	No.1	No.2	No.3	No.4
N	33	33	33	33
df	31	31	31	31
R	0.993042228	0.991824852	0.991895946	0.99096662
R^2	0.986132866	0.983716536	0.983857567	0.982014842
$R^2_{adjusted}$	0.985685539	0.983191263	0.983336843	0.981434676
RSS	304.666507	402.0693889	368.1388524	362.4851702

Graphical abstract of model fit presented as mean ± 1 SD of the fraction % of released carvedilol:

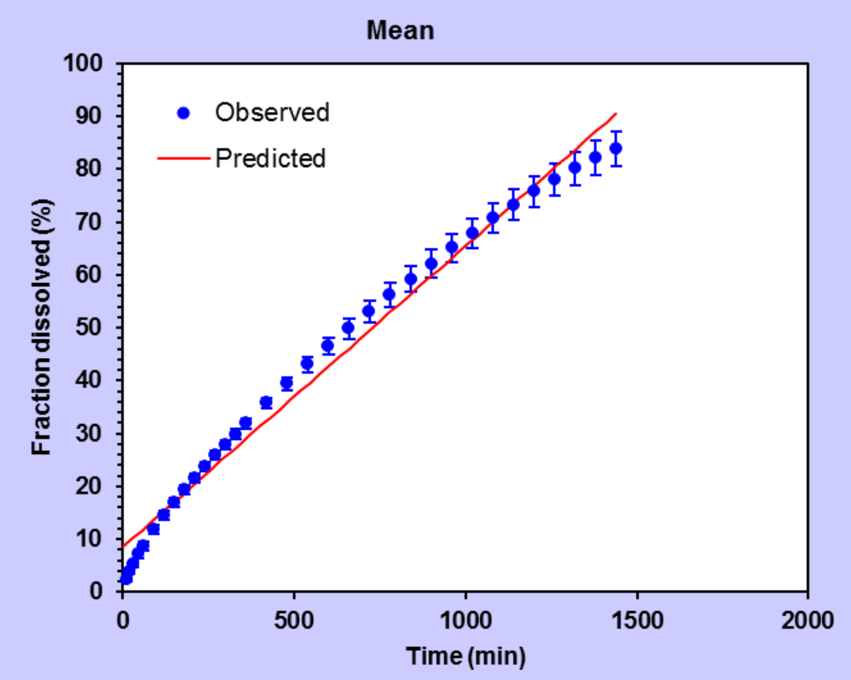

Supplementary materials – Model fitting summary – Avicel® PH-200 (modelling the entire relevant dissolution data)

Graphical abstract of model fit presented as the fraction % of released carvedilol per tested tablet:

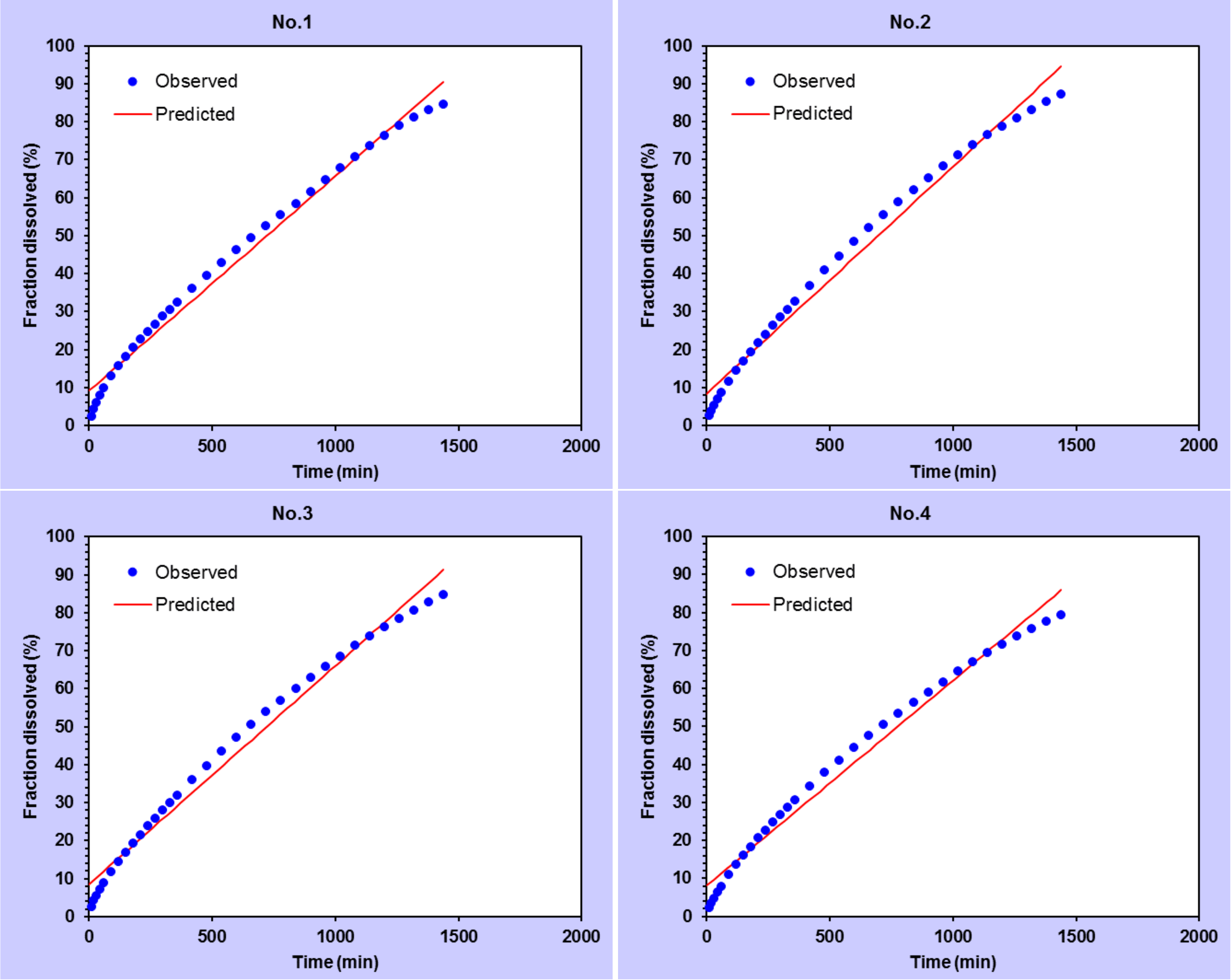

Supplementary materials – Model fitting summary – Avicel® PH-200 (modelling the entire relevant dissolution data)

Model: **Zero–order with F_0**

Model equation: $F = F_0 + k_0 \cdot t$

Fitted model parameters per tested tablet (N = 4) with statistics – mean, standard deviation (SD), and relative standard deviation expressed in % (RSD%) (output from DDSolver):

Parameter	No.1	No.2	No.3	No.4	Mean	SD	RSD(%)
k_0	0.057	0.060	0.058	0.054	0.057	0.002	4.235
F_0	9.190	8.363	8.510	8.099	8.540	0.465	5.446

Number of dissolution data points (N), degrees of freedom (df), and selected goodness of fit criteria – Pearson correlation coefficient (R), coefficient of determination (R^2), adjusted coefficient of determination ($R^2_{adjusted}$), and residual sum of squares (RSS) (manual calculation in MS Excel):

Parameter	No.1	No.2	No.3	No.4
N	33	33	33	33
df	31	31	31	31
R	0.993042228	0.991824852	0.991895946	0.99096662
R^2	0.986132866	0.983716536	0.983857567	0.982014842
$R^2_{adjusted}$	0.985685539	0.983191263	0.983336843	0.981434676
RSS	304.666507	402.0693889	368.1388524	362.4851702

Graphical abstract of model fit presented as mean ± 1 SD of the fraction % of released carvedilol:

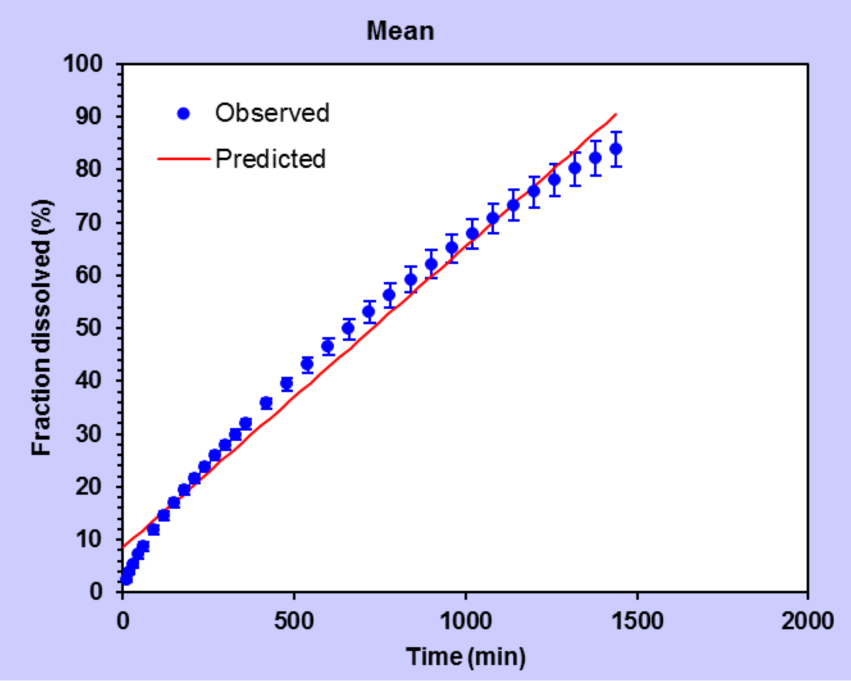

Supplementary materials – Model fitting summary – Avicel® PH-200 (modelling the entire relevant dissolution data)

Graphical abstract of model fit presented as the fraction % of released carvedilol per tested tablet:

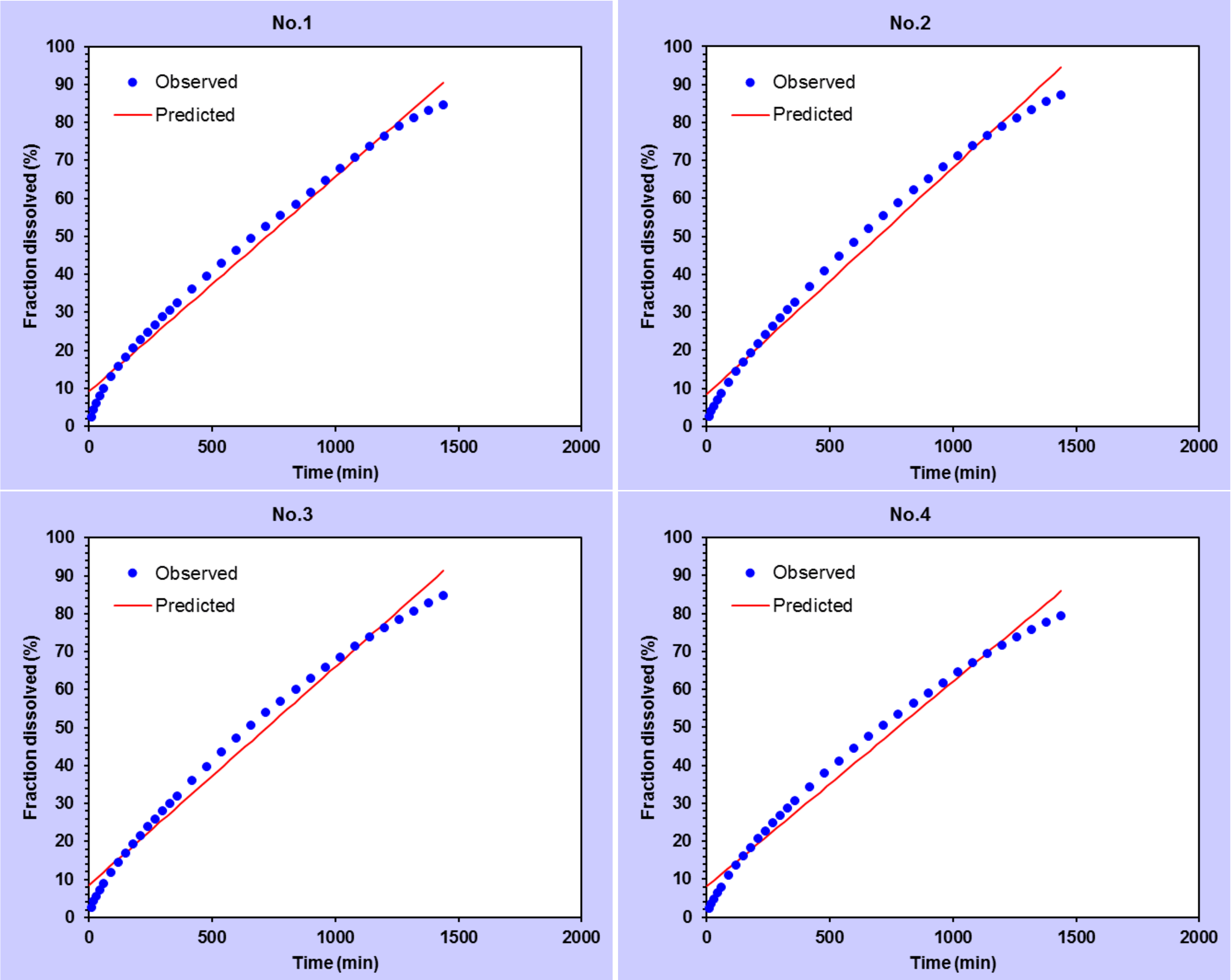

Supplementary materials – Model fitting summary – Avicel® PH-200 (modelling the entire relevant dissolution data)

Model: **First–order**

Model equation: $F = 100 \cdot (1 - e^{-k_1 \cdot t})$

Fitted model parameters per tested tablet (N = 4) with statistics – mean, standard deviation (SD), and relative standard deviation expressed in % (RSD%) (output from DDSolver):

Parameter	No.1	No.2	No.3	No.4	Mean	SD	RSD(%)
k_1	0.001	0.001	0.001	0.001	0.001	0.000	8.112

Number of dissolution data points (N), degrees of freedom (df), and selected goodness of fit criteria – Pearson correlation coefficient (R), coefficient of determination (R^2), adjusted coefficient of determination ($R^2_{adjusted}$), and residual sum of squares (RSS) (manual calculation in MS Excel):

Parameter	No.1	No.2	No.3	No.4
N	33	33	33	33
df	32	32	32	32
R	0.995224065	0.995773582	0.996907251	0.998625455
R^2	0.990470939	0.991565027	0.993824067	0.9972528
$R^2_{adjusted}$	0.990470939	0.991565027	0.993824067	0.9972528
RSS	248.8397296	272.8636365	166.3213731	68.87749053

Graphical abstract of model fit presented as mean ± 1 SD of the fraction % of released carvedilol:

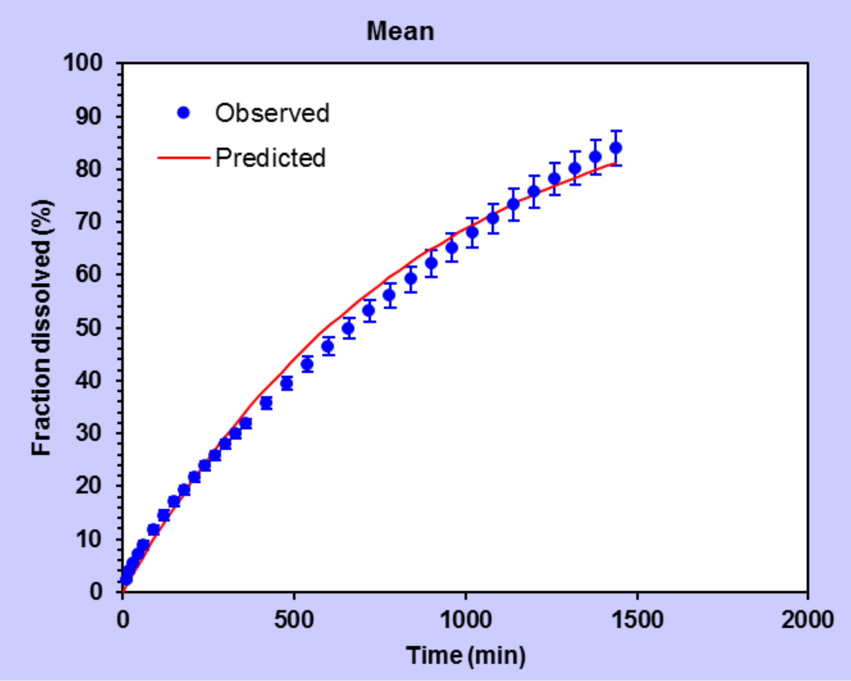

Supplementary materials – Model fitting summary – Avicel® PH-200 (modelling the entire relevant dissolution data)

Graphical abstract of model fit presented as the fraction % of released carvedilol per tested tablet:

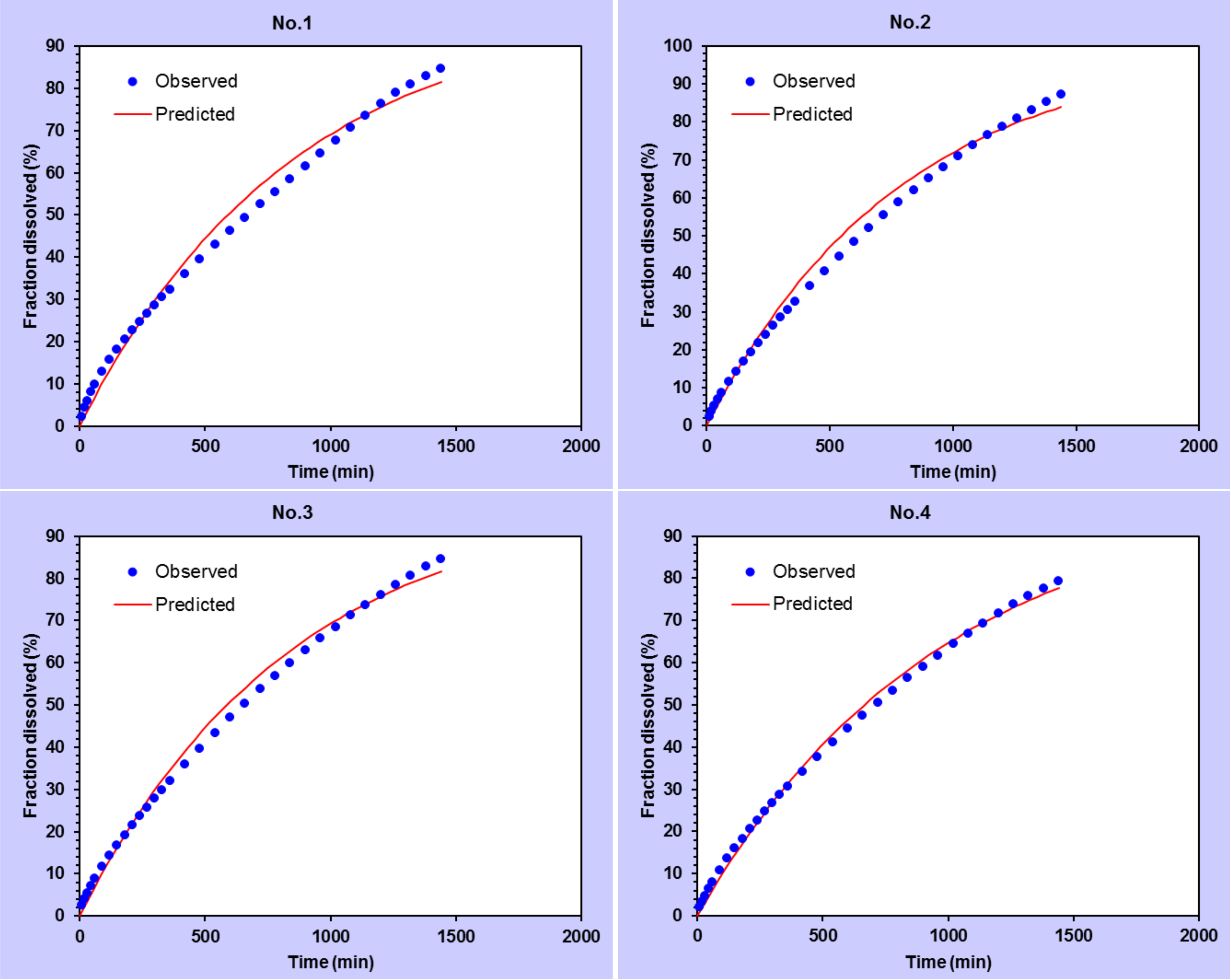

Supplementary materials – Model fitting summary – Avicel® PH-200 (modelling the entire relevant dissolution data)

Model: **First–order with T_{lag}**

Model equation: $F = 100 \cdot \left[1 - e^{-k_1 \cdot (t - T_{lag})}\right]$

Fitted model parameters per tested tablet (N = 4) with statistics – mean, standard deviation (SD), and relative standard deviation expressed in % (RSD%) (output from DDSolver):

Parameter	No.1	No.2	No.3	No.4	Mean	SD	RSD(%)
k_1	0.001	0.001	0.001	0.001	0.001	0.000	9.606
T_{lag}	17.698	37.199	22.522	2.846	20.066	14.162	70.577

Number of dissolution data points (N), degrees of freedom (df), and selected goodness of fit criteria – Pearson correlation coefficient (R), coefficient of determination (R^2), adjusted coefficient of determination ($R^2_{adjusted}$), and residual sum of squares (RSS) (manual calculation in MS Excel):

Parameter	No.1	No.2	No.3	No.4
N	33	33	33	33
df	31	31	31	31
R	0.994902939	0.994988237	0.996541215	0.998601238
R^2	0.989831859	0.990001592	0.993094393	0.997204432
$R^2_{adjusted}$	0.989503854	0.989679062	0.992871631	0.997114252
RSS	328.1576734	390.9210514	235.1313809	75.30906692

Graphical abstract of model fit presented as mean ± 1 SD of the fraction % of released carvedilol:

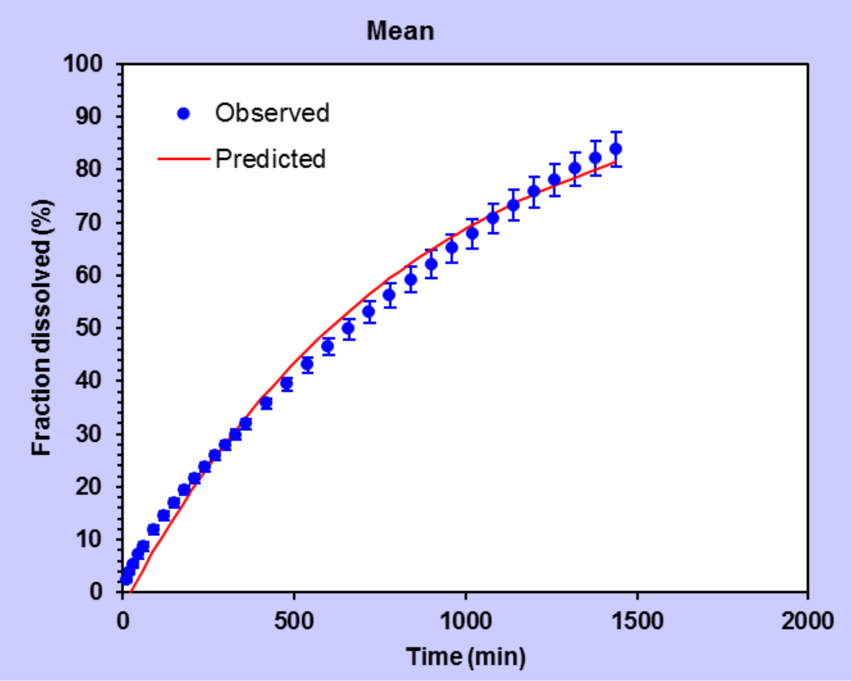

Supplementary materials – Model fitting summary – Avicel® PH-200 (modelling the entire relevant dissolution data)

Graphical abstract of model fit presented as the fraction % of released carvedilol per tested tablet:

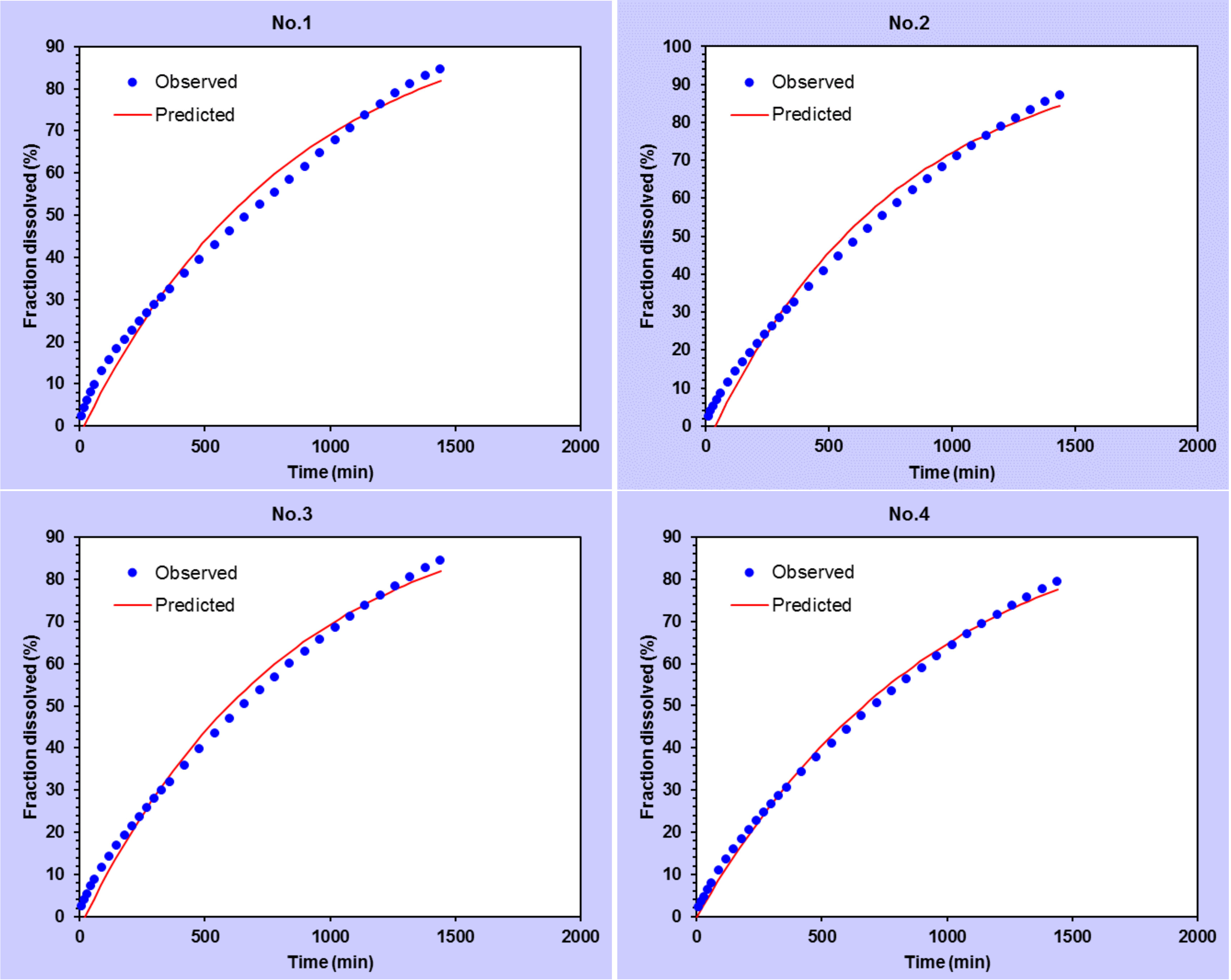

Supplementary materials – Model fitting summary – Avicel® PH-200 (modelling the entire relevant dissolution data)

Model: **First–order with F_{max}**

Model equation: $F = F_{max} \cdot (1 - e^{-k_1 \cdot t})$

Fitted model parameters per tested tablet (N = 4) with statistics – mean, standard deviation (SD), and relative standard deviation expressed in % (RSD%) (output from DDSolver):

Parameter	No.1	No.2	No.3	No.4	Mean	SD	RSD(%)
k_1	0.002	0.002	0.002	0.002	0.002	0.000	0.335
F_{max}	88.807	91.579	88.795	83.377	88.139	3.434	3.896

Number of dissolution data points (N), degrees of freedom (df), and selected goodness of fit criteria – Pearson correlation coefficient (R), coefficient of determination (R^2), adjusted coefficient of determination ($R^2_{adjusted}$), and residual sum of squares (RSS) (manual calculation in MS Excel):

Parameter	No.1	No.2	No.3	No.4
N	33	33	33	33
df	31	31	31	31
R	0.987076445	0.98929608	0.989415321	0.990288489
R^2	0.974319909	0.978706733	0.978942677	0.980671291
$R^2_{adjusted}$	0.973491519	0.978019853	0.978263408	0.980047784
RSS	876.5460167	916.3718775	806.9293165	666.0307912

Graphical abstract of model fit presented as mean ± 1 SD of the fraction % of released carvedilol:

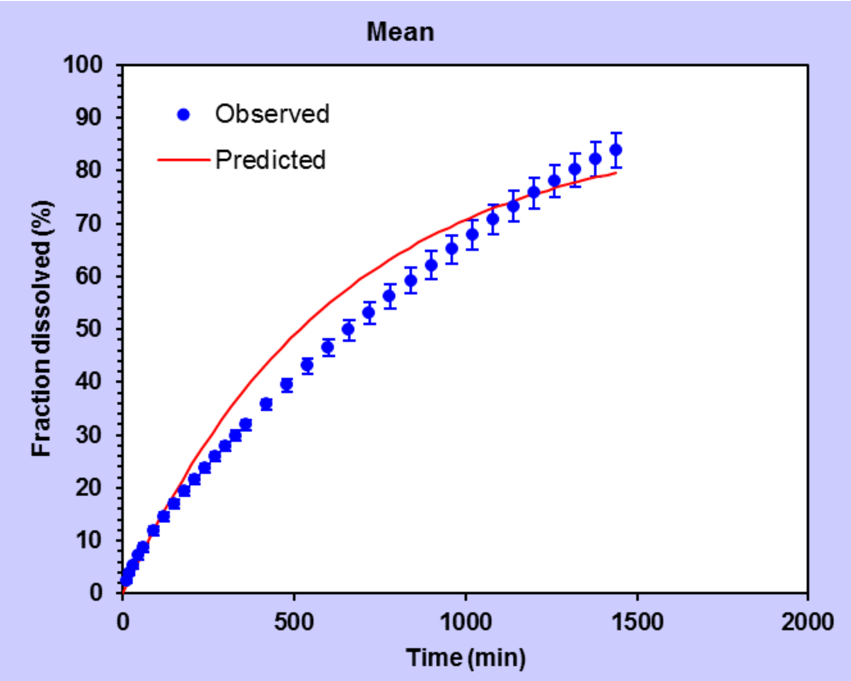

Supplementary materials – Model fitting summary – Avicel® PH-200 (modelling the entire relevant dissolution data)

Graphical abstract of model fit presented as the fraction % of released carvedilol per tested tablet:

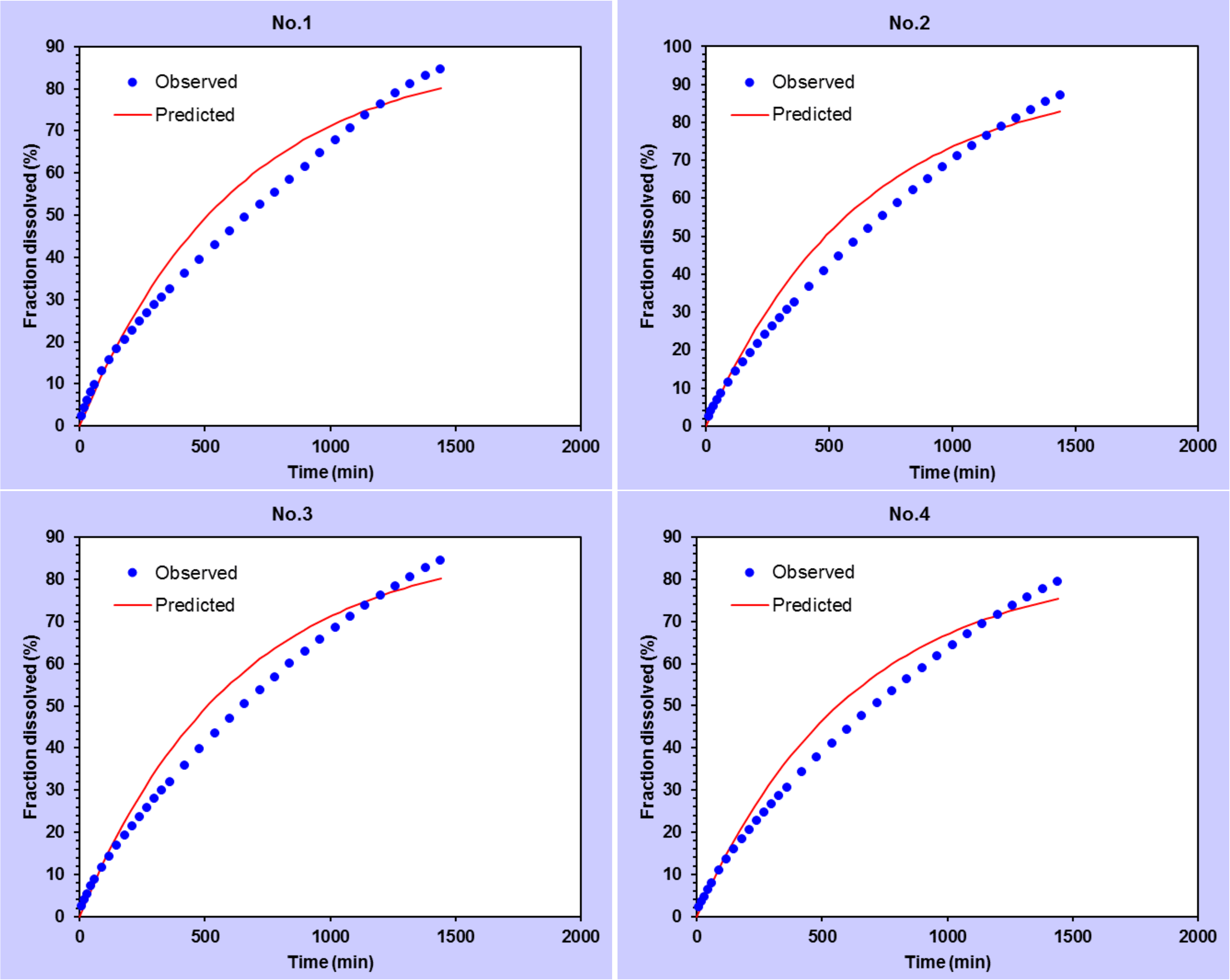

Supplementary materials – Model fitting summary – Avicel® PH-200 (modelling the entire relevant dissolution data)

Model: **First–order with T_{lag} and F_{max}**

Model equation: $F = F_{max} \cdot \left[1 - e^{-k_1 \cdot (t - T_{lag})}\right]$

Fitted model parameters per tested tablet (N = 4) with statistics – mean, standard deviation (SD), and relative standard deviation expressed in % (RSD%) (output from DDSolver):

Parameter	No.1	No.2	No.3	No.4	Mean	SD	RSD(%)
k_1	0.002	0.002	0.002	0.002	0.002	0.000	0.575
T_{lag}	73.226	78.710	75.436	74.139	75.378	2.399	3.183
F_{max}	88.807	91.579	88.795	83.377	88.139	3.434	3.896

Number of dissolution data points (N), degrees of freedom (df), and selected goodness of fit criteria – Pearson correlation coefficient (R), coefficient of determination (R^2), adjusted coefficient of determination ($R^2_{adjusted}$), and residual sum of squares (RSS) (manual calculation in MS Excel):

Parameter	No.1	No.2	No.3	No.4
N	33	33	33	33
df	30	30	30	30
R	0.983822763	0.985871348	0.986202006	0.987291449
R^2	0.96790723	0.971942315	0.972594396	0.974744405
$R^2_{adjusted}$	0.965767711	0.970071802	0.970767355	0.973060699
RSS	1598.231221	1531.99916	1398.749718	1159.06538

Graphical abstract of model fit presented as mean ± 1 SD of the fraction % of released carvedilol:

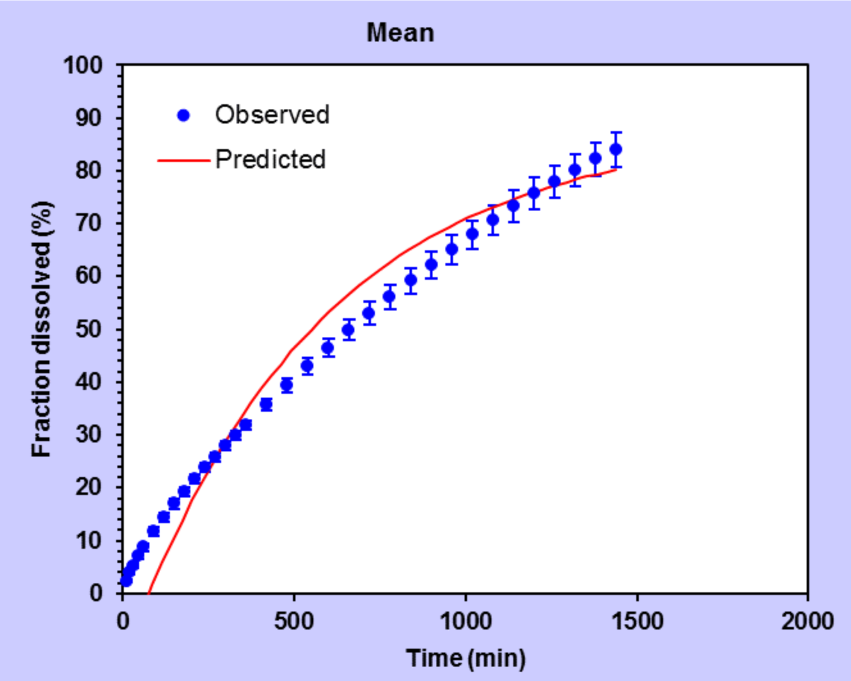

Supplementary materials – Model fitting summary – Avicel® PH-200 (modelling the entire relevant dissolution data)

Graphical abstract of model fit presented as the fraction % of released carvedilol per tested tablet:

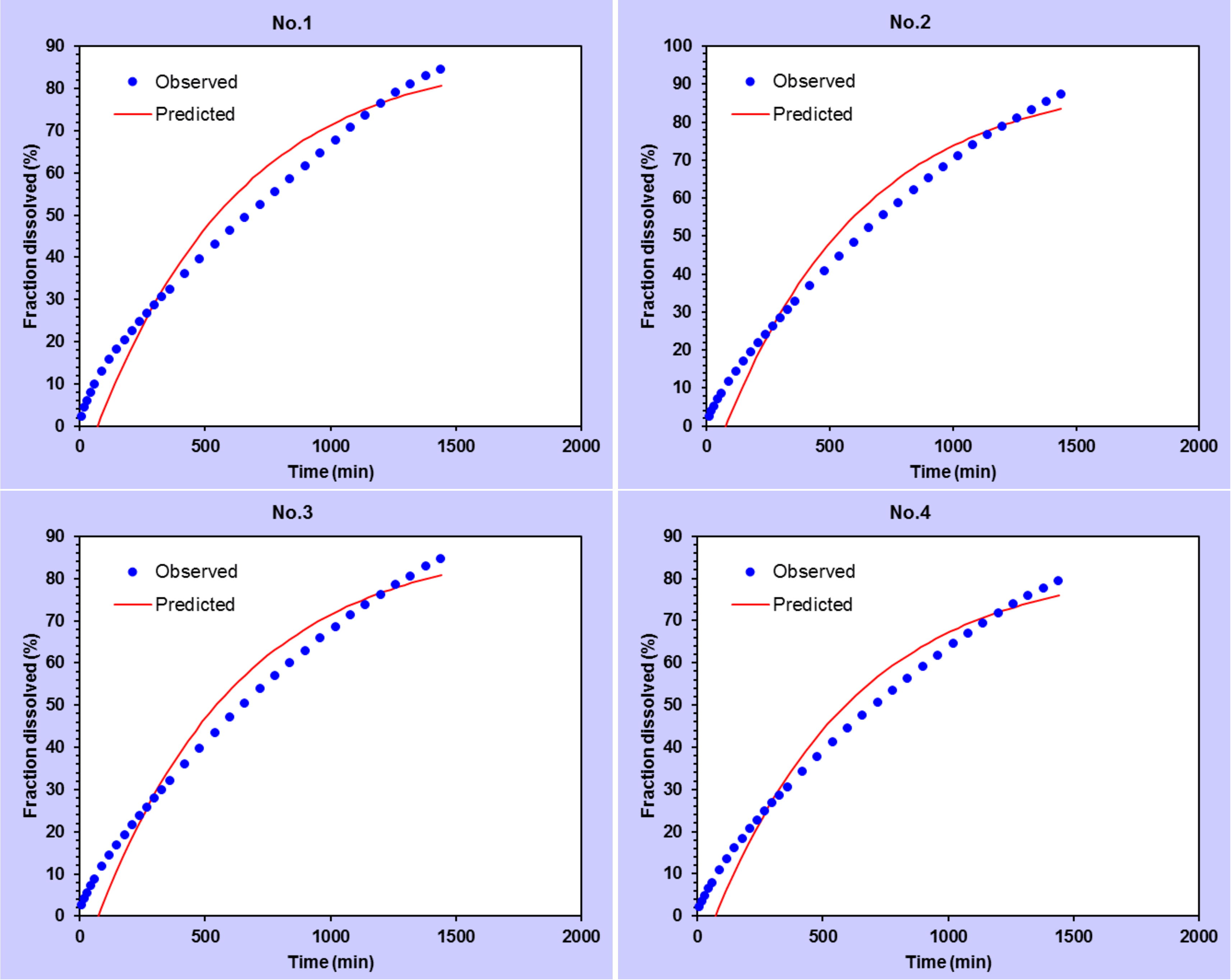

Supplementary materials – Model fitting summary – Avicel® PH-200 (modelling the entire relevant dissolution data)

Model: **Higuchi**

Model equation: $F = k_H \cdot t^{0.5}$

Fitted model parameters per tested tablet (N = 4) with statistics – mean, standard deviation (SD), and relative standard deviation expressed in % (RSD%) (output from DDSolver):

Parameter	No.1	No.2	No.3	No.4	Mean	SD	RSD(%)
k_H	2.034	2.103	2.039	1.920	2.024	0.076	3.766

Number of dissolution data points (N), degrees of freedom (df), and selected goodness of fit criteria – Pearson correlation coefficient (R), coefficient of determination (R^2), adjusted coefficient of determination ($R^2_{adjusted}$), and residual sum of squares (RSS) (manual calculation in MS Excel):

Parameter	No.1	No.2	No.3	No.4
N	33	33	33	33
df	32	32	32	32
R	0.995047736	0.994989566	0.995258838	0.996105558
R^2	0.990119997	0.990004235	0.990540154	0.992226283
$R^2_{adjusted}$	0.990119997	0.990004235	0.990540154	0.992226283
RSS	963.1839536	1304.126992	1121.422206	953.3454421

Graphical abstract of model fit presented as mean ± 1 SD of the fraction % of released carvedilol:

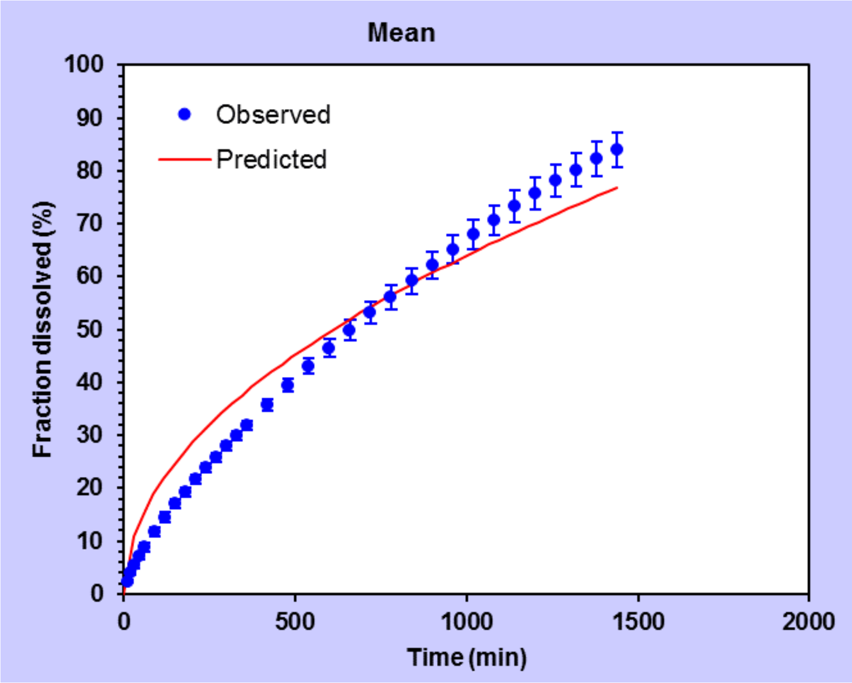

Supplementary materials – Model fitting summary – Avicel® PH-200 (modelling the entire relevant dissolution data)

Graphical abstract of model fit presented as the fraction % of released carvedilol per tested tablet:

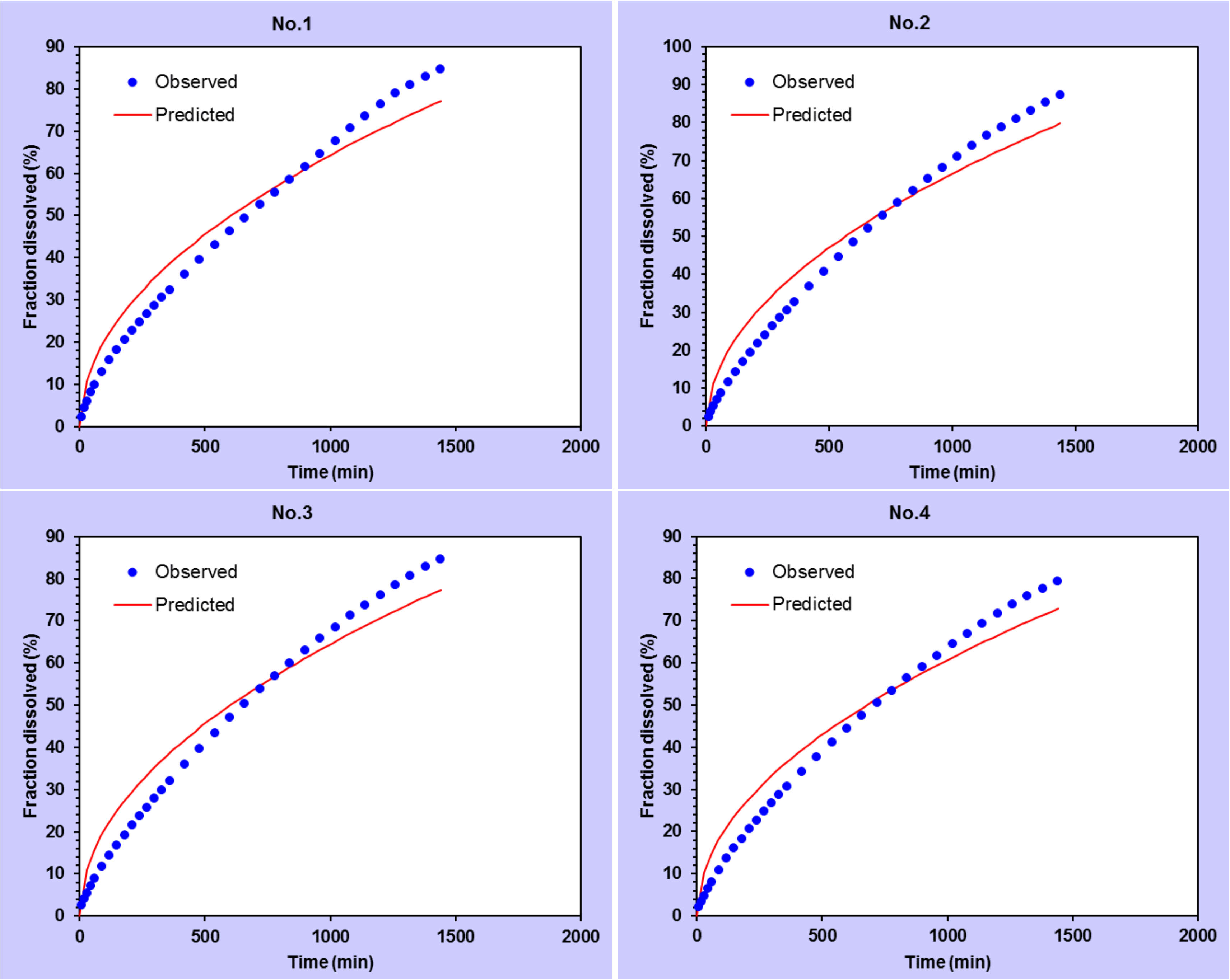

Supplementary materials – Model fitting summary – Avicel® PH-200 (modelling the entire relevant dissolution data)

Model: **Higuchi with T_{lag}**

Model equation: $F = k_H \cdot (t - T_{lag})^{0.5}$

Fitted model parameters per tested tablet (N = 4) with statistics – mean, standard deviation (SD), and relative standard deviation expressed in % (RSD%) (output from DDSolver):

Parameter	No.1	No.2	No.3	No.4	Mean	SD	RSD(%)
k_H	2.253	2.343	2.265	2.126	2.247	0.090	3.997
T_{lag}	101.994	105.837	103.354	100.984	103.042	2.101	2.039

Number of dissolution data points (N), degrees of freedom (df), and selected goodness of fit criteria – Pearson correlation coefficient (R), coefficient of determination (R^2), adjusted coefficient of determination ($R^2_{adjusted}$), and residual sum of squares (RSS) (manual calculation in MS Excel):

Parameter	No.1	No.2	No.3	No.4
N	33	33	33	33
df	31	31	31	31
R	0.988980946	0.990725979	0.990602222	0.991503494
R^2	0.978083312	0.981537966	0.981292763	0.983079178
$R^2_{adjusted}$	0.977376322	0.980942416	0.980689304	0.982533345
RSS	625.508973	539.7024198	519.3817791	415.6946601

Graphical abstract of model fit presented as mean ± 1 SD of the fraction % of released carvedilol:

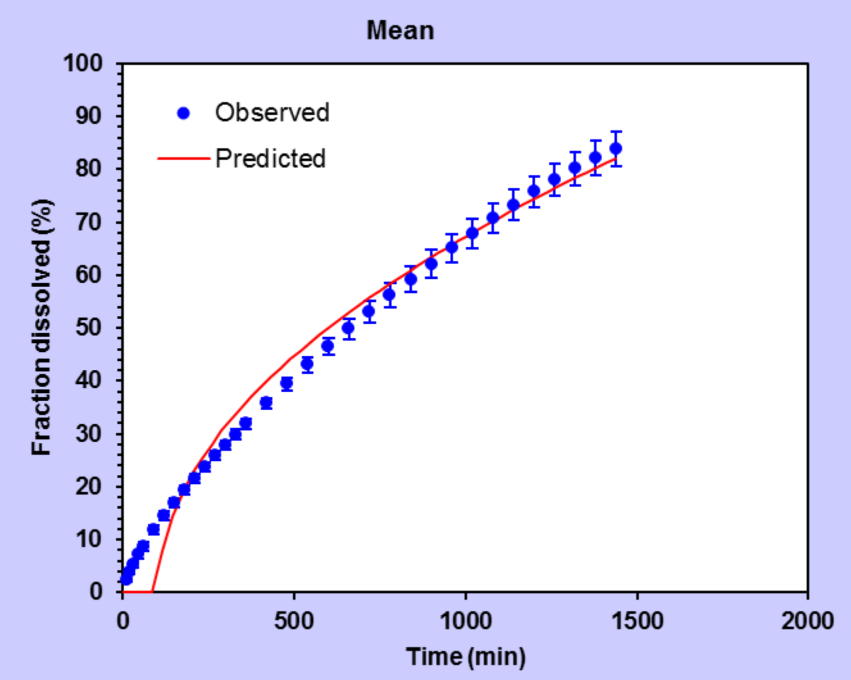

Supplementary materials – Model fitting summary – Avicel® PH-200 (modelling the entire relevant dissolution data)

Graphical abstract of model fit presented as the fraction % of released carvedilol per tested tablet:

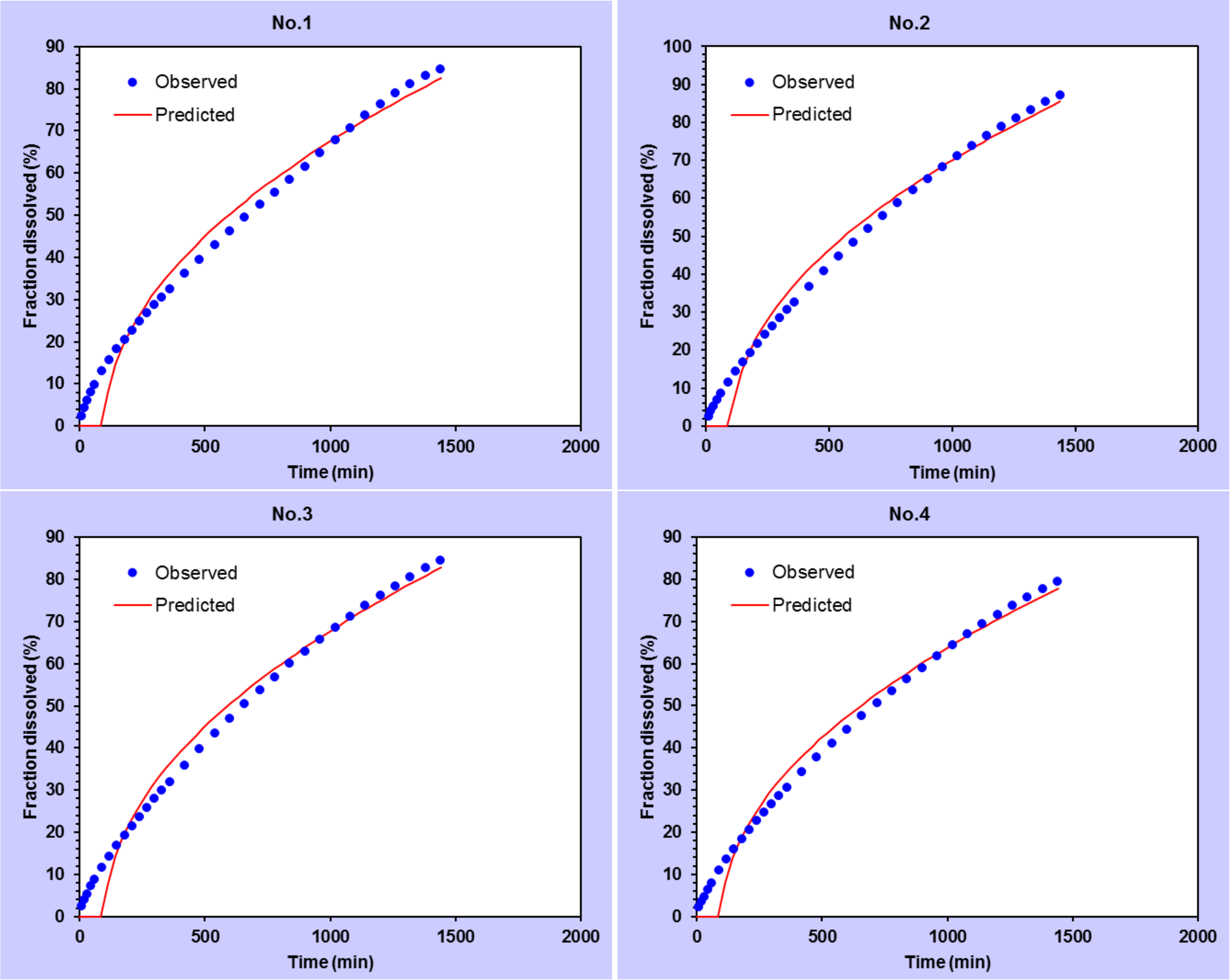

Supplementary materials – Model fitting summary – Avicel® PH-200 (modelling the entire relevant dissolution data)

Model: **Higuchi with F_0**

Model equation: $F = F_0 + k_H \cdot t^{0.5}$

Fitted model parameters per tested tablet (N = 4) with statistics – mean, standard deviation (SD), and relative standard deviation expressed in % (RSD%) (output from DDSolver):

Parameter	No.1	No.2	No.3	No.4	Mean	SD	RSD(%)
k_H	2.440	2.587	2.487	2.340	2.463	0.103	4.167
F_0	-10.888	-12.961	-11.996	-11.251	-11.774	0.916	-7.780

Number of dissolution data points (N), degrees of freedom (df), and selected goodness of fit criteria – Pearson correlation coefficient (R), coefficient of determination (R^2), adjusted coefficient of determination ($R^2_{adjusted}$), and residual sum of squares (RSS) (manual calculation in MS Excel):

Parameter	No.1	No.2	No.3	No.4
N	33	33	33	33
df	31	31	31	31
R	0.995047736	0.994989566	0.995258838	0.996105558
R^2	0.990119997	0.990004235	0.990540154	0.992226283
$R^2_{adjusted}$	0.989801287	0.989681791	0.990234998	0.991975518
RSS	217.067639	246.8142551	215.7380336	156.676811

Graphical abstract of model fit presented as mean ± 1 SD of the fraction % of released carvedilol:

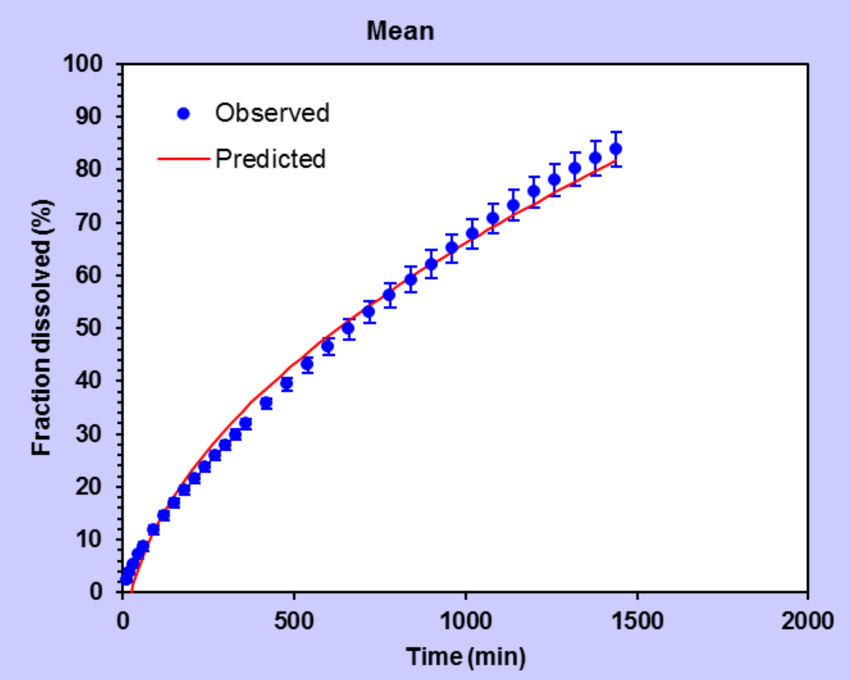

Supplementary materials – Model fitting summary – Avicel® PH-200 (modelling the entire relevant dissolution data)

Graphical abstract of model fit presented as the fraction % of released carvedilol per tested tablet:

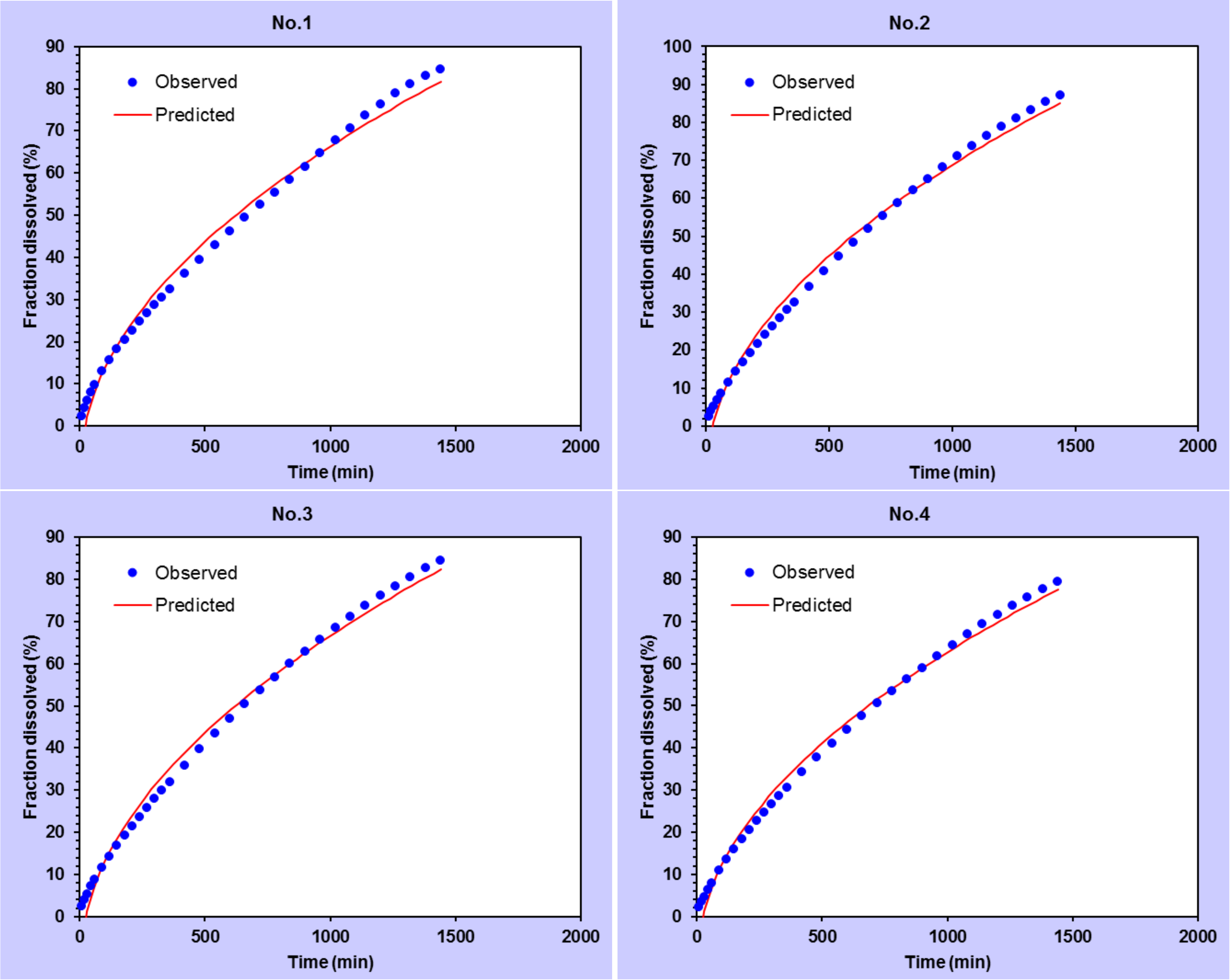

Supplementary materials – Model fitting summary – Avicel® PH-200 (modelling the entire relevant dissolution data)

Model: **Korsmeyer–Peppas**

Model equation: $F = k_{KP} \cdot t^n$

Fitted model parameters per tested tablet (N = 4) with statistics – mean, standard deviation (SD), and relative standard deviation expressed in % (RSD%) (output from DDSolver):

Parameter	No.1	No.2	No.3	No.4	Mean	SD	RSD(%)
k_{KP}	0.534	0.435	0.468	0.443	0.470	0.045	9.541
n	0.699	0.734	0.718	0.715	0.717	0.014	1.982

Number of dissolution data points (N), degrees of freedom (df), and selected goodness of fit criteria – Pearson correlation coefficient (R), coefficient of determination (R^2), adjusted coefficient of determination ($R^2_{adjusted}$), and residual sum of squares (RSS) (manual calculation in MS Excel):

Parameter	No.1	No.2	No.3	No.4
N	33	33	33	33
df	31	31	31	31
R	0.999857148	0.999416991	0.999644247	0.999627536
R^2	0.999714317	0.998834321	0.99928862	0.999255211
$R^2_{adjusted}$	0.999705101	0.998796719	0.999265673	0.999231185
RSS	7.951268502	29.54625551	16.33610616	30.04698357

Graphical abstract of model fit presented as mean ± 1 SD of the fraction % of released carvedilol:

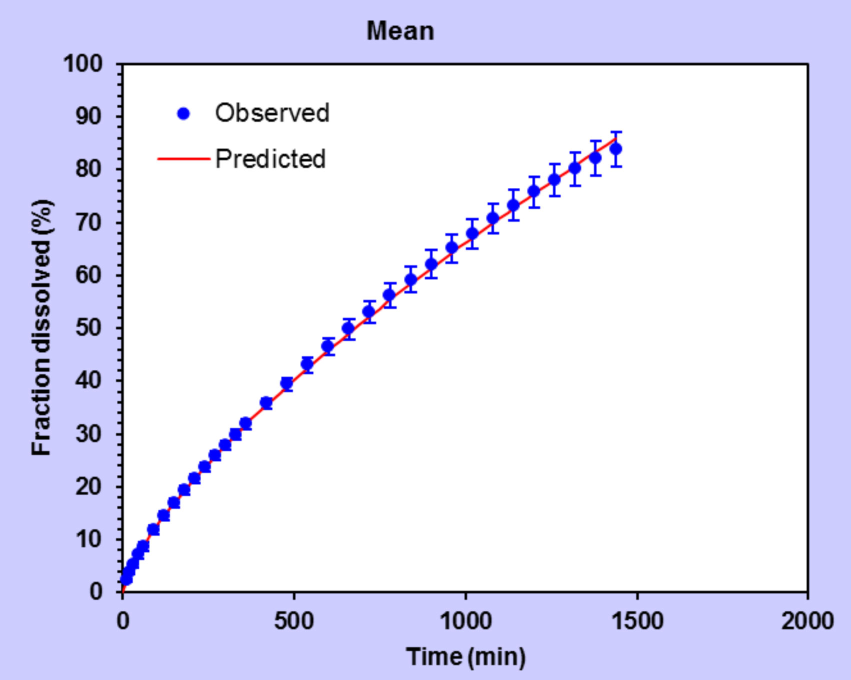

Supplementary materials – Model fitting summary – Avicel® PH-200 (modelling the entire relevant dissolution data)

Graphical abstract of model fit presented as the fraction % of released carvedilol per tested tablet:

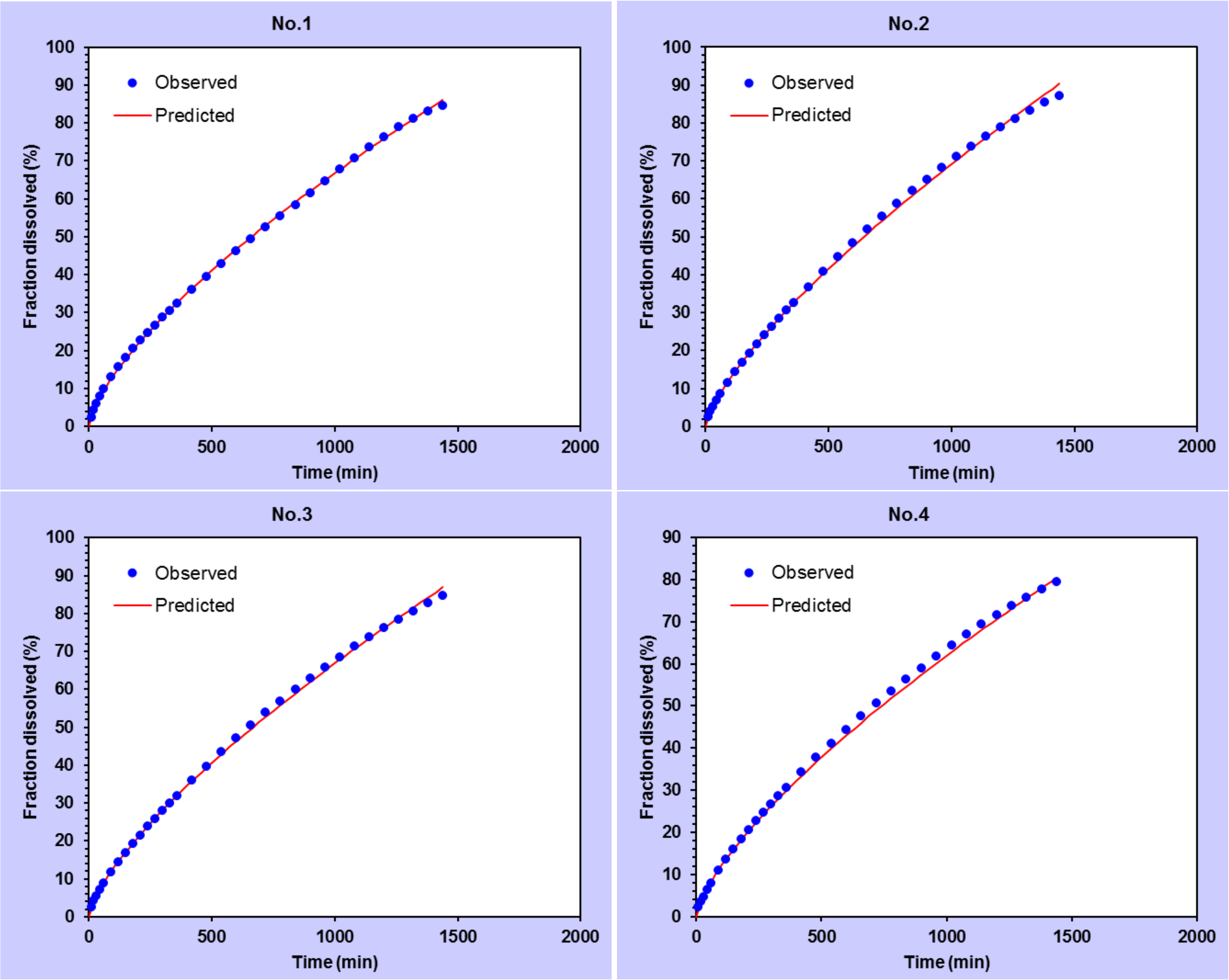

Supplementary materials – Model fitting summary – Avicel® PH-200 (modelling the entire relevant dissolution data)

Model: **Korsmeyer–Peppas with T_{lag}**

Model equation: $F = k_{KP} \cdot (t - T_{lag})^n$

Fitted model parameters per tested tablet (N = 4) with statistics – mean, standard deviation (SD), and relative standard deviation expressed in % (RSD%) (output from DDSolver):

Parameter	No.1	No.2	No.3	No.4	Mean	SD	RSD(%)
k_{KP}	0.681	0.566	0.605	0.511	0.591	0.072	12.151
n	0.662	0.693	0.679	0.696	0.683	0.016	2.321
T_{lag}	4.000	4.000	4.000	4.000	4.000	0.000	0.000

Number of dissolution data points (N), degrees of freedom (df), and selected goodness of fit criteria – Pearson correlation coefficient (R), coefficient of determination (R^2), adjusted coefficient of determination ($R^2_{adjusted}$), and residual sum of squares (RSS) (manual calculation in MS Excel):

Parameter	No.1	No.2	No.3	No.4
N	33	33	33	33
df	30	30	30	30
R	0.999610797	0.999539909	0.999646012	0.999756273
R^2	0.999221746	0.99908003	0.99929215	0.999512606
$R^2_{adjusted}$	0.999169862	0.999018699	0.99924496	0.999480113
RSS	32.09151286	51.4680371	46.24307308	12.78757395

Graphical abstract of model fit presented as mean ± 1 SD of the fraction % of released carvedilol:

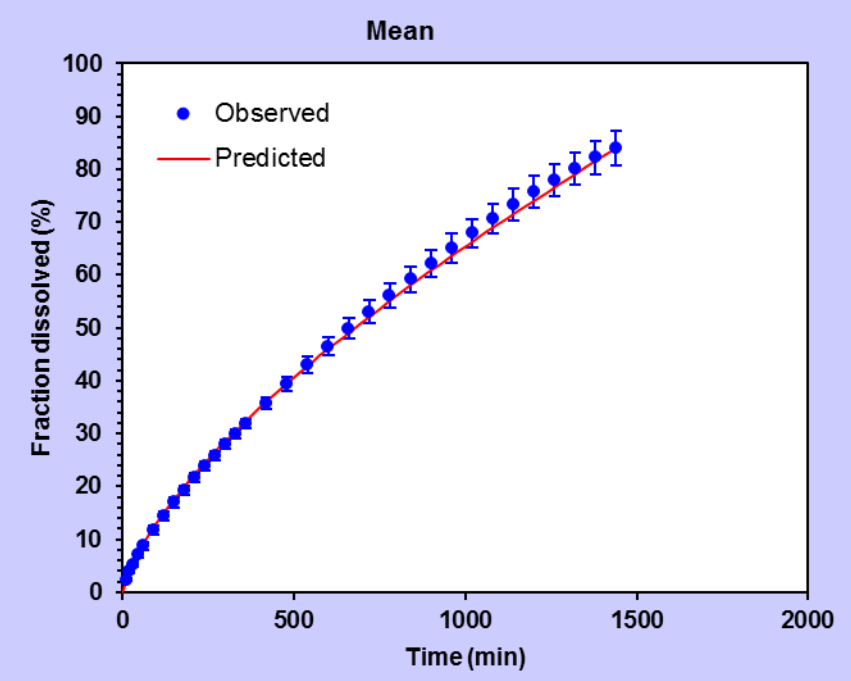

Supplementary materials – Model fitting summary – Avicel® PH-200 (modelling the entire relevant dissolution data)

Graphical abstract of model fit presented as the fraction % of released carvedilol per tested tablet:

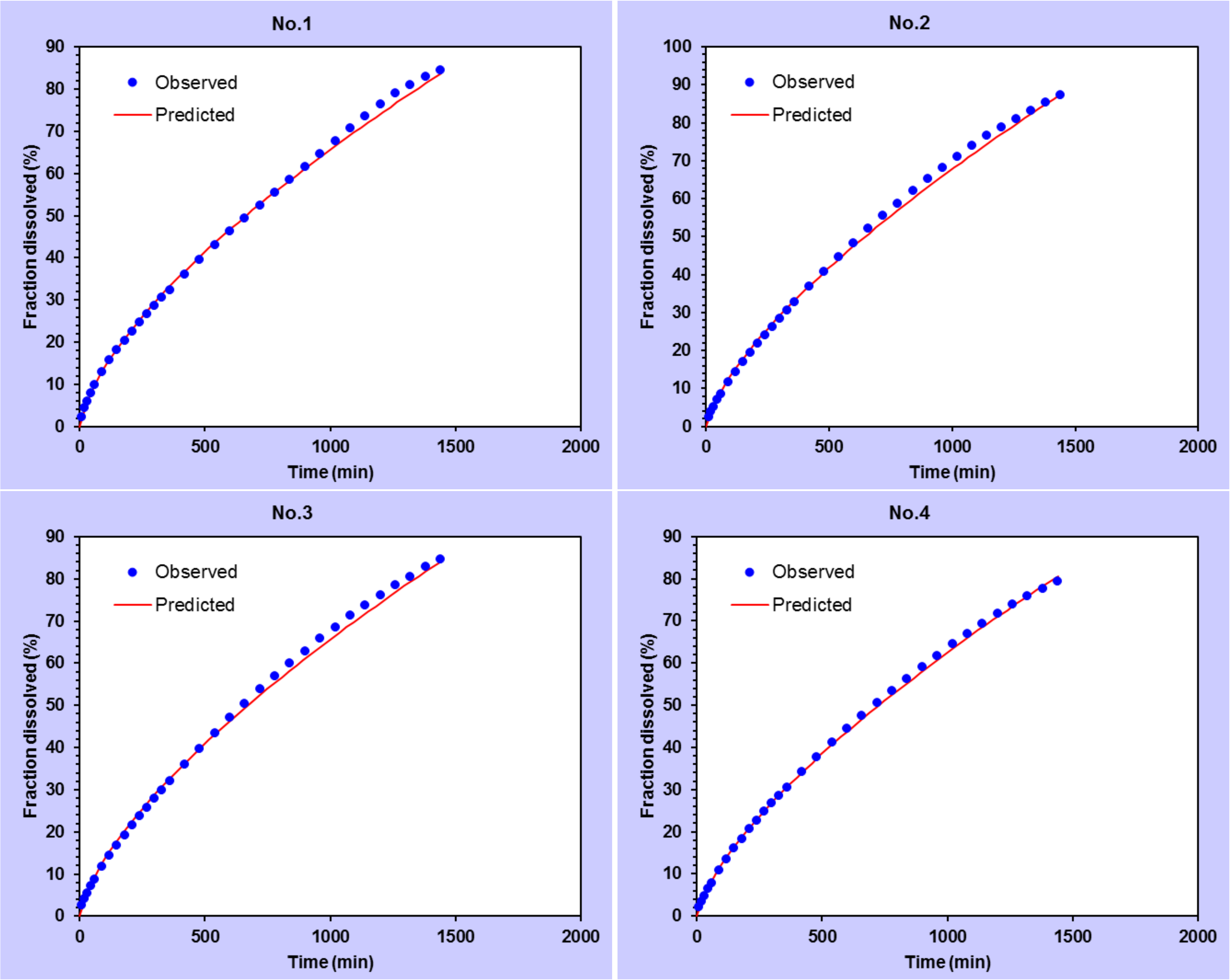

Supplementary materials – Model fitting summary – Avicel® PH-200 (modelling the entire relevant dissolution data)

Model: **Korsmeyer–Peppas with F_0**

Model equation: $F = F_0 + k_{KP} \cdot t^n$

Fitted model parameters per tested tablet (N = 4) with statistics – mean, standard deviation (SD), and relative standard deviation expressed in % (RSD%) (output from DDSolver):

Parameter	No.1	No.2	No.3	No.4	Mean	SD	RSD(%)
k_{KP}	0.383	0.300	0.326	0.256	0.316	0.053	16.815
n	0.742	0.783	0.766	0.800	0.773	0.025	3.204
F_0	1.128	1.221	1.221	0.840	1.102	0.180	16.367

Number of dissolution data points (N), degrees of freedom (df), and selected goodness of fit criteria – Pearson correlation coefficient (R), coefficient of determination (R^2), adjusted coefficient of determination ($R^2_{adjusted}$), and residual sum of squares (RSS) (manual calculation in MS Excel):

Parameter	No.1	No.2	No.3	No.4
N	33	33	33	33
df	30	30	30	30
R	0.999729974	0.998814661	0.999179205	0.998274261
R^2	0.999460021	0.997630728	0.998359084	0.996551501
$R^2_{adjusted}$	0.999424022	0.997472776	0.99824969	0.996321601
RSS	35.02195602	86.93549676	59.96080429	196.079837

Graphical abstract of model fit presented as mean ± 1 SD of the fraction % of released carvedilol:

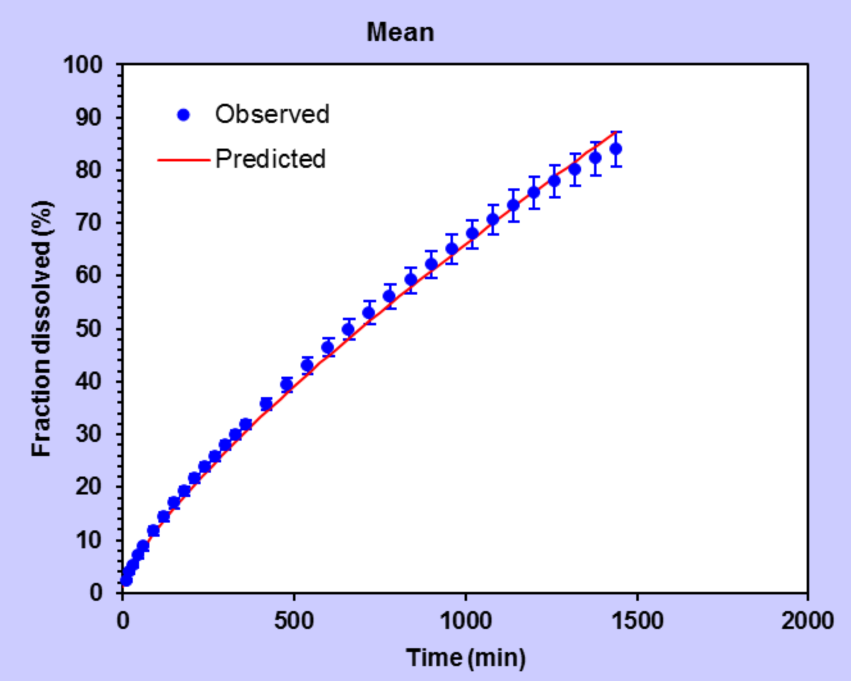

Supplementary materials – Model fitting summary – Avicel® PH-200 (modelling the entire relevant dissolution data)

Graphical abstract of model fit presented as the fraction % of released carvedilol per tested tablet:

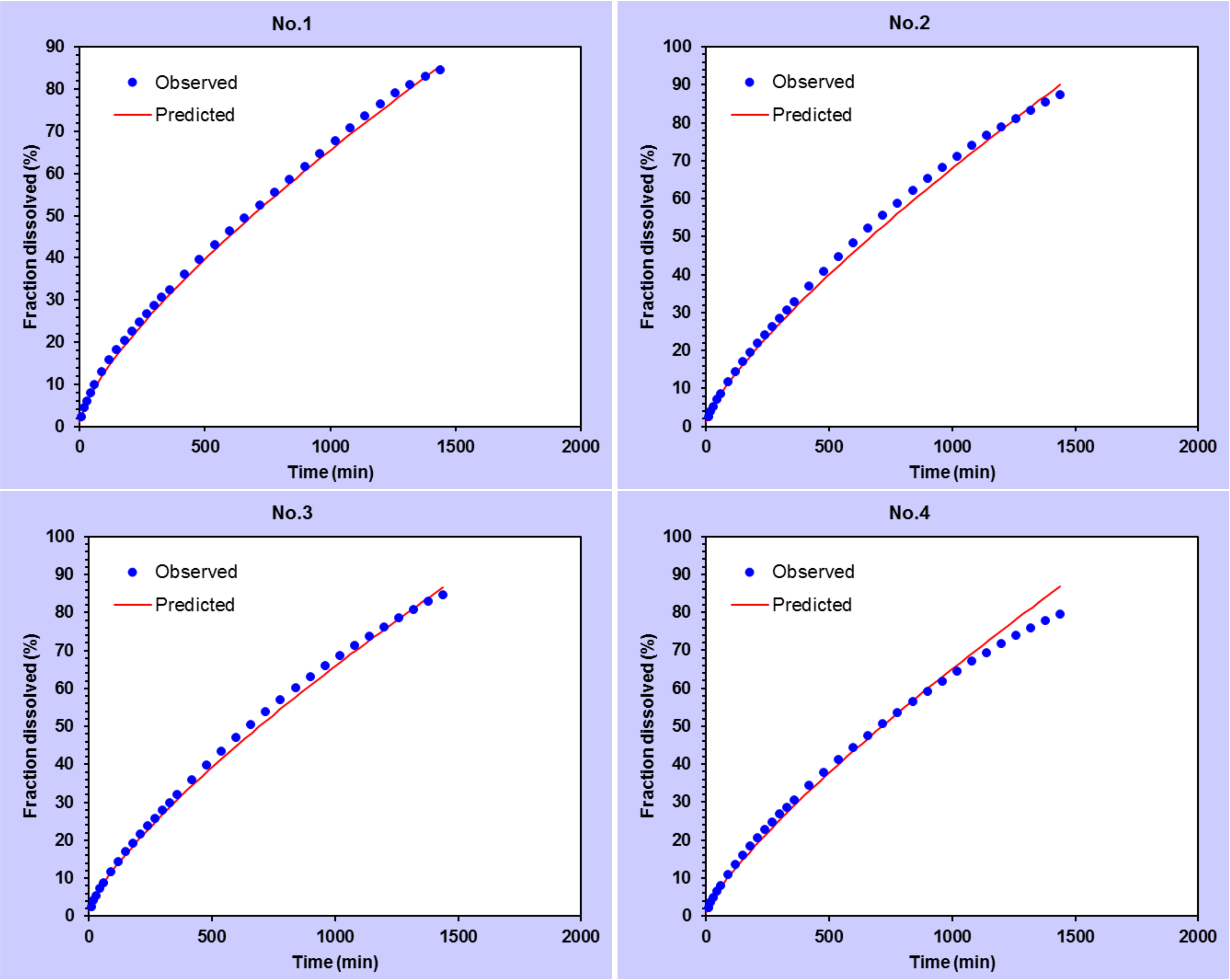

Supplementary materials – Model fitting summary – Avicel® PH-200 (modelling the entire relevant dissolution data)

Model: **Hixson–Crowell**

Model equation: $F = 100 \cdot [1 - (1 - k_{HC} \cdot t)^3]$

Fitted model parameters per tested tablet (N = 4) with statistics – mean, standard deviation (SD), and relative standard deviation expressed in % (RSD%) (output from DDSolver):

Parameter	No.1	No.2	No.3	No.4	Mean	SD	RSD(%)
k_{HC}	0.0003	0.0003	0.0003	0.0003	0.0003	0.0000	6.3379

Number of dissolution data points (N), degrees of freedom (df), and selected goodness of fit criteria – Pearson correlation coefficient (R), coefficient of determination (R^2), adjusted coefficient of determination ($R^2_{adjusted}$), and residual sum of squares (RSS) (manual calculation in MS Excel):

Parameter	No.1	No.2	No.3	No.4
N	33	33	33	33
df	32	32	32	32
R	0.998533946	0.999518408	0.999627924	0.999575865
R^2	0.997070042	0.999037047	0.999255986	0.999151909
$R^2_{adjusted}$	0.997070042	0.999037047	0.999255986	0.999151909
RSS	212.9675006	70.43210485	104.1321678	138.7805403

Graphical abstract of model fit presented as mean ± 1 SD of the fraction % of released carvedilol:

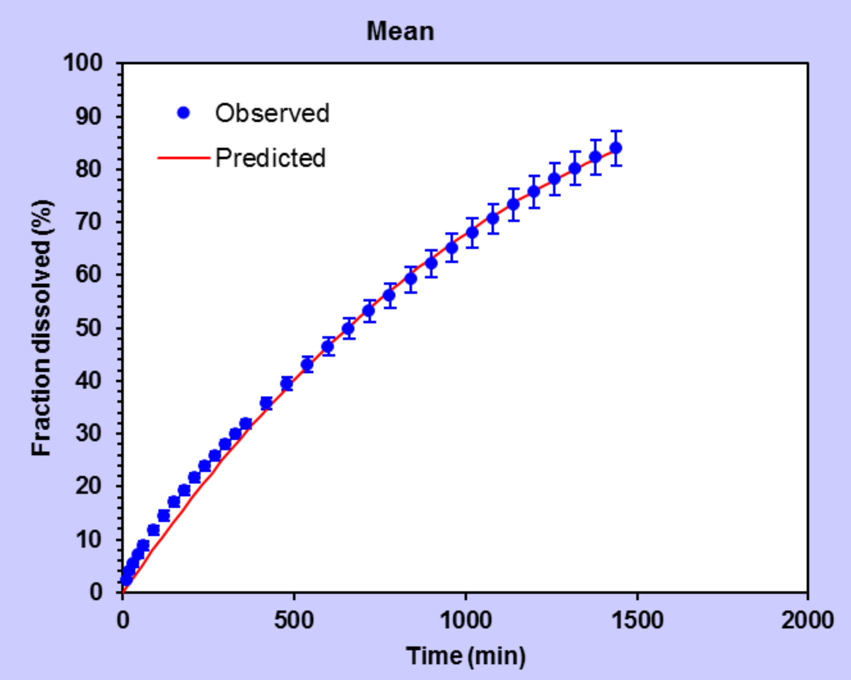

Supplementary materials – Model fitting summary – Avicel® PH-200 (modelling the entire relevant dissolution data)

Graphical abstract of model fit presented as the fraction % of released carvedilol per tested tablet:

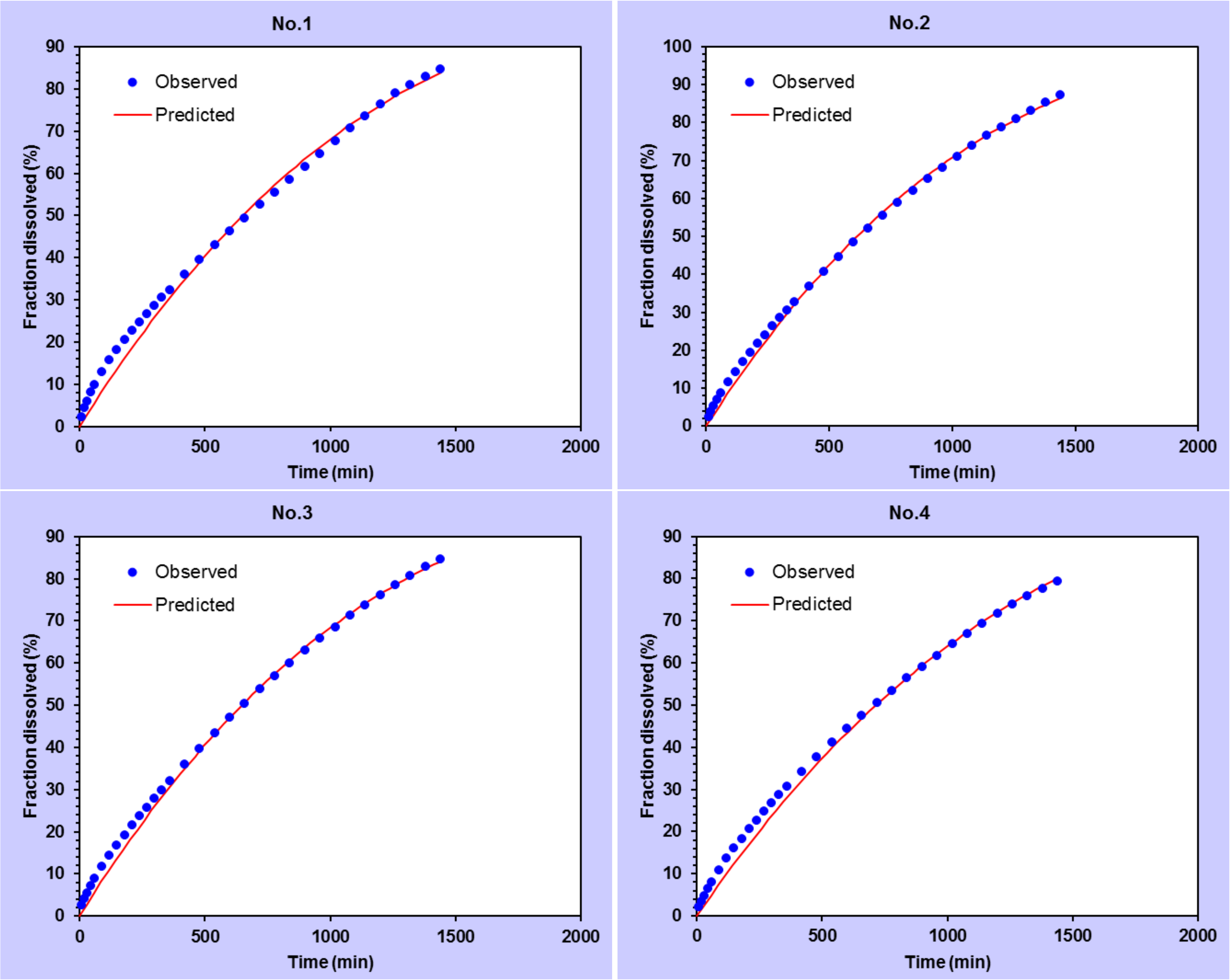

Supplementary materials – Model fitting summary – Avicel® PH-200 (modelling the entire relevant dissolution data)

Model: **Hixson–Crowell with T_{lag}**

Model equation: $F = 100 \cdot \left\{1 - \left[1 - k_{HC} \cdot \left(t - T_{lag}\right)\right]^3\right\}$

Fitted model parameters per tested tablet (N = 4) with statistics – mean, standard deviation (SD), and relative standard deviation expressed in % (RSD%) (output from DDSolver):

Parameter	No.1	No.2	No.3	No.4	Mean	SD	RSD(%)
k_{HC}	0.0003	0.0003	0.0003	0.0003	0.0003	0.0000	7.4652
T_{lag}	-37.5534	-17.2103	-29.9255	-44.4133	-32.2756	11.6571	-36.1175

Number of dissolution data points (N), degrees of freedom (df), and selected goodness of fit criteria – Pearson correlation coefficient (R), coefficient of determination (R^2), adjusted coefficient of determination ($R^2_{adjusted}$), and residual sum of squares (RSS) (manual calculation in MS Excel):

Parameter	No.1	No.2	No.3	No.4
N	33	33	33	33
df	31	31	31	31
R	0.998665309	0.999573653	0.999681701	0.999534592
R^2	0.9973324	0.999147488	0.999363503	0.9990694
$R^2_{adjusted}$	0.997246348	0.999119988	0.999342971	0.999039381
RSS	63.18307865	24.90540004	15.68364576	18.89623284

Graphical abstract of model fit presented as mean ± 1 SD of the fraction % of released carvedilol:

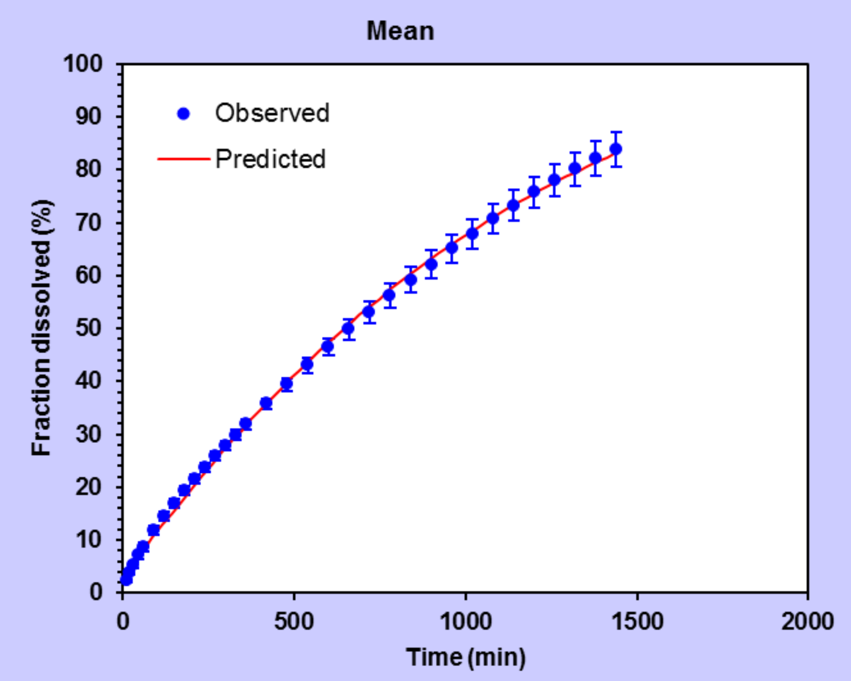

Supplementary materials – Model fitting summary – Avicel® PH-200 (modelling the entire relevant dissolution data)

Graphical abstract of model fit presented as the fraction % of released carvedilol per tested tablet:

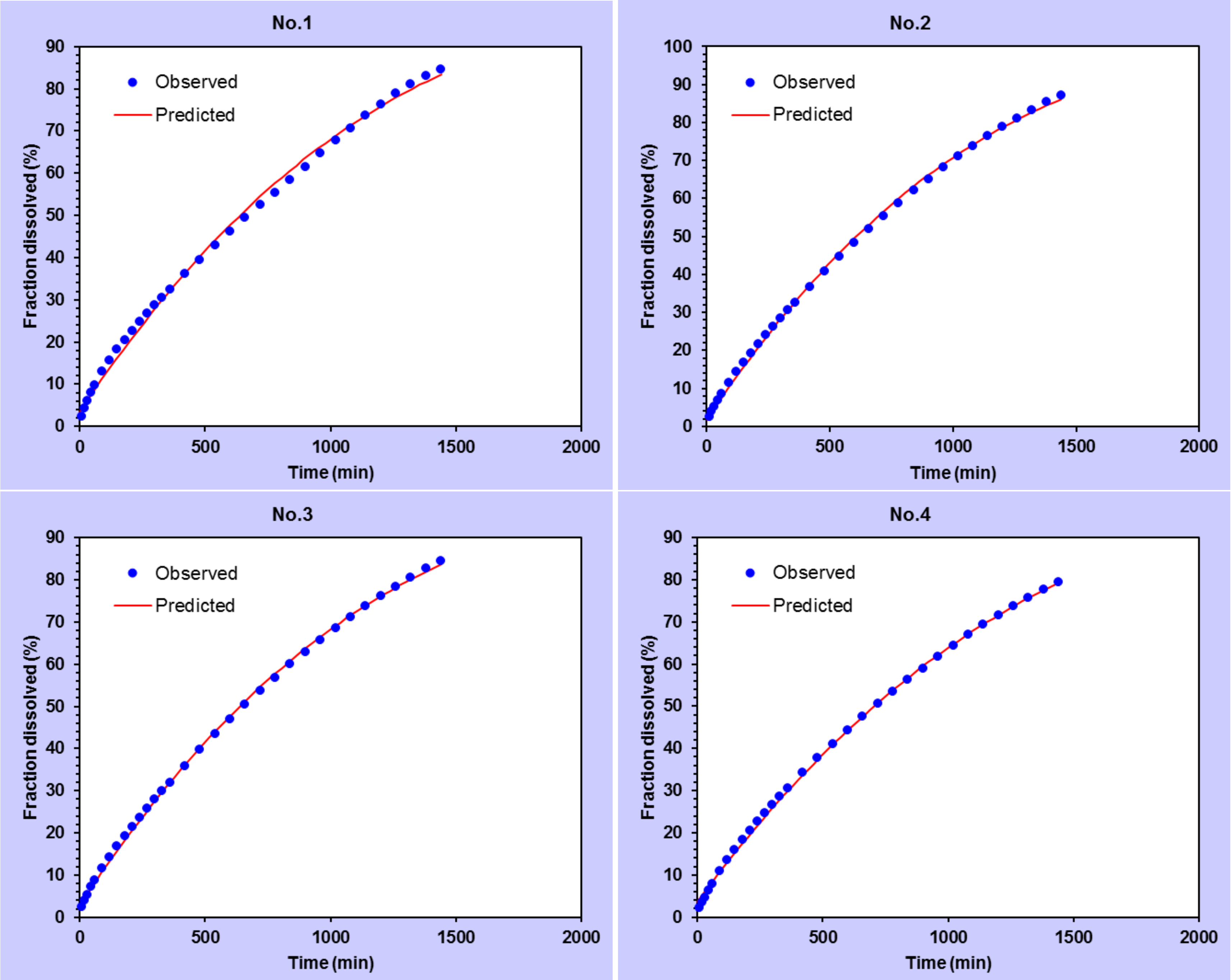

Supplementary materials – Model fitting summary – Avicel® PH-200 (modelling the entire relevant dissolution data)

Model: **Hopfenberg**

Model equation: $F = 100 \cdot [1 - (1 - k_{HB} \cdot t)^n]$

Fitted model parameters per tested tablet (N = 4) with statistics – mean, standard deviation (SD), and relative standard deviation expressed in % (RSD%) (output from DDSolver):

Parameter	No.1	No.2	No.3	No.4	Mean	SD	RSD(%)
k_{HB}	0.0003	0.0003	0.0003	0.0003	0.0003	0.0000	6.7466
n	3.0000	3.7207	3.0000	3.0000	3.1802	0.3604	11.3312

Number of dissolution data points (N), degrees of freedom (df), and selected goodness of fit criteria – Pearson correlation coefficient (R), coefficient of determination (R^2), adjusted coefficient of determination ($R^2_{adjusted}$), and residual sum of squares (RSS) (manual calculation in MS Excel):

Parameter	No.1	No.2	No.3	No.4
N	33	33	33	33
df	31	31	31	31
R	0.998533946	0.999252121	0.999627924	0.999575865
R^2	0.997070042	0.998504802	0.999255986	0.999151909
$R^2_{adjusted}$	0.996975527	0.998456569	0.999231986	0.999124551
RSS	212.9675006	68.54301899	104.1321678	138.7805403

Graphical abstract of model fit presented as mean ± 1 SD of the fraction % of released carvedilol:

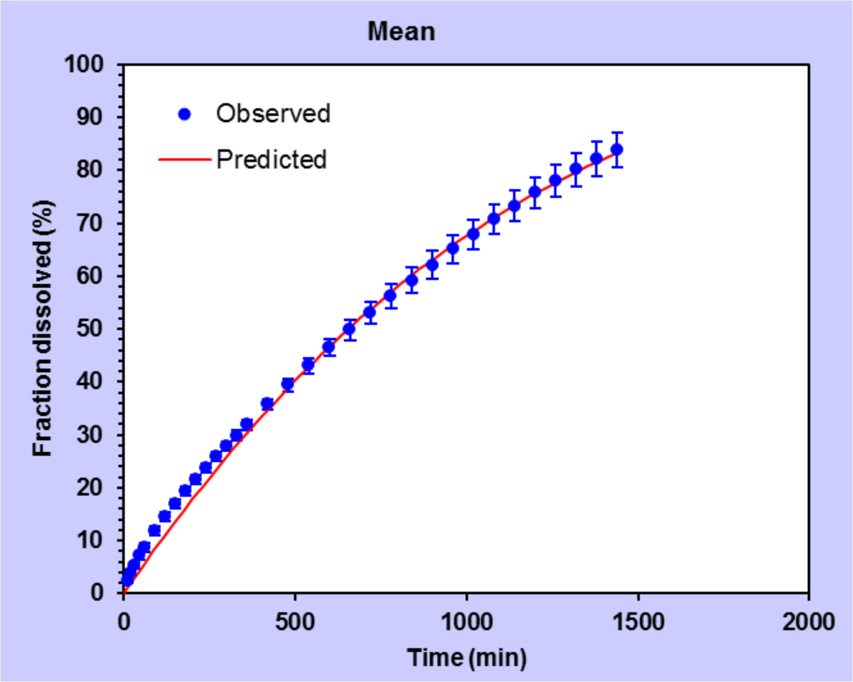

Supplementary materials – Model fitting summary – Avicel® PH-200 (modelling the entire relevant dissolution data)

Graphical abstract of model fit presented as the fraction % of released carvedilol per tested tablet:

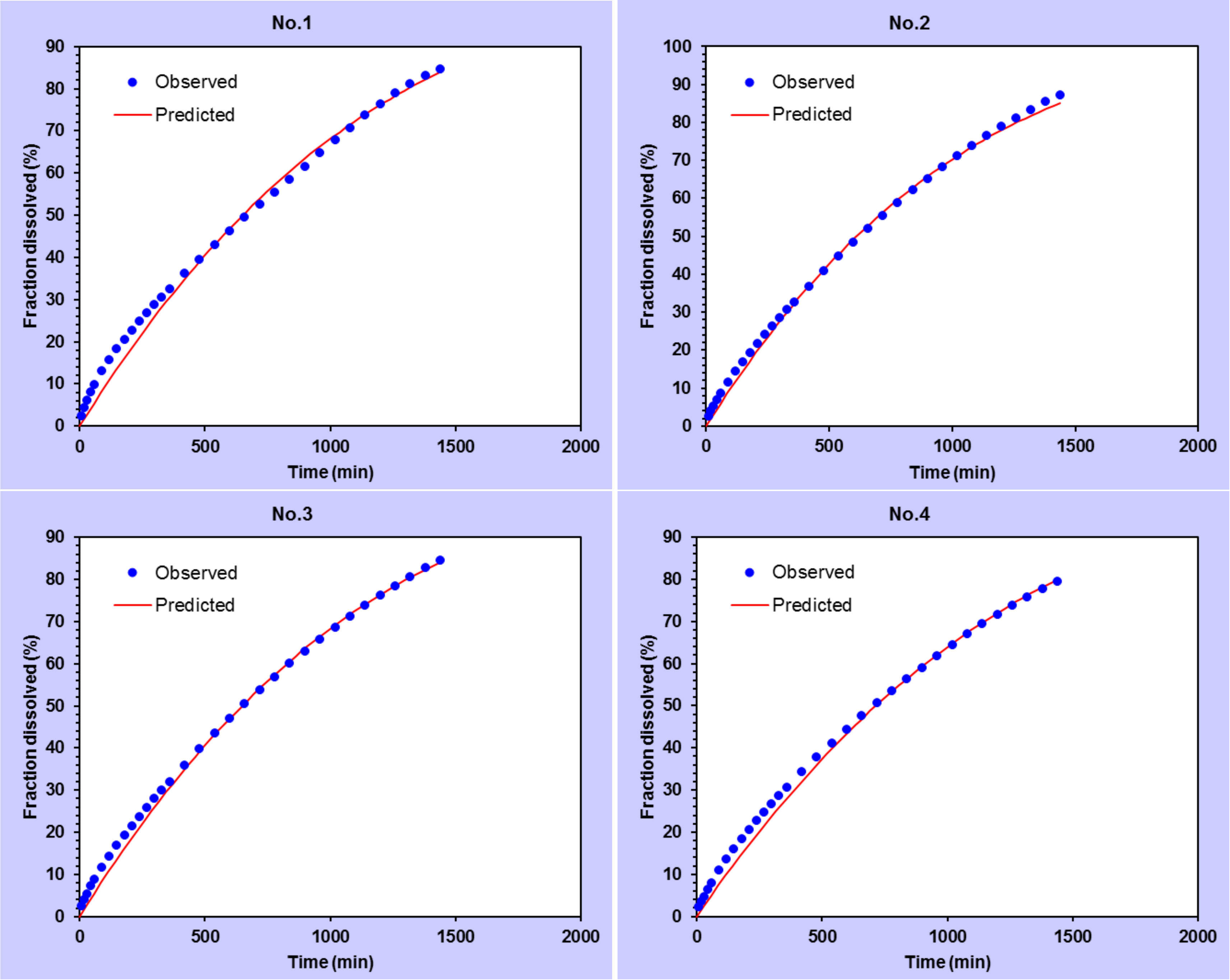

Supplementary materials – Model fitting summary – Avicel® PH-200 (modelling the entire relevant dissolution data)

Model: **Hopfenberg with T_{lag}**

Model equation: $F = 100 \cdot \{1 - [1 - k_{HB} \cdot (t - T_{lag})]^n\}$

Fitted model parameters per tested tablet (N = 4) with statistics – mean, standard deviation (SD), and relative standard deviation expressed in % (RSD%) (output from DDSolver):

Parameter	No.1	No.2	No.3	No.4	Mean	SD	RSD(%)
k_{HB}	0.0004	0.0004	0.0003	0.0003	0.0004	0.0001	21.1493
n	2.0000	2.0000	3.0000	3.0000	2.5000	0.5774	23.0940
T_{lag}	-67.0905	-46.2527	-29.9255	-44.4133	-46.9205	15.3013	-32.6112

Number of dissolution data points (N), degrees of freedom (df), and selected goodness of fit criteria – Pearson correlation coefficient (R), coefficient of determination (R^2), adjusted coefficient of determination ($R^2_{adjusted}$), and residual sum of squares (RSS) (manual calculation in MS Excel):

Parameter	No.1	No.2	No.3	No.4
N	33	33	33	33
df	30	30	30	30
R	0.998871507	0.999696627	0.999681701	0.999534592
R^2	0.997744288	0.999393347	0.999363503	0.9990694
$R^2_{adjusted}$	0.997593907	0.999352903	0.99932107	0.99900736
RSS	49.56044665	15.34167087	15.68364576	18.89623284

Graphical abstract of model fit presented as mean ± 1 SD of the fraction % of released carvedilol:

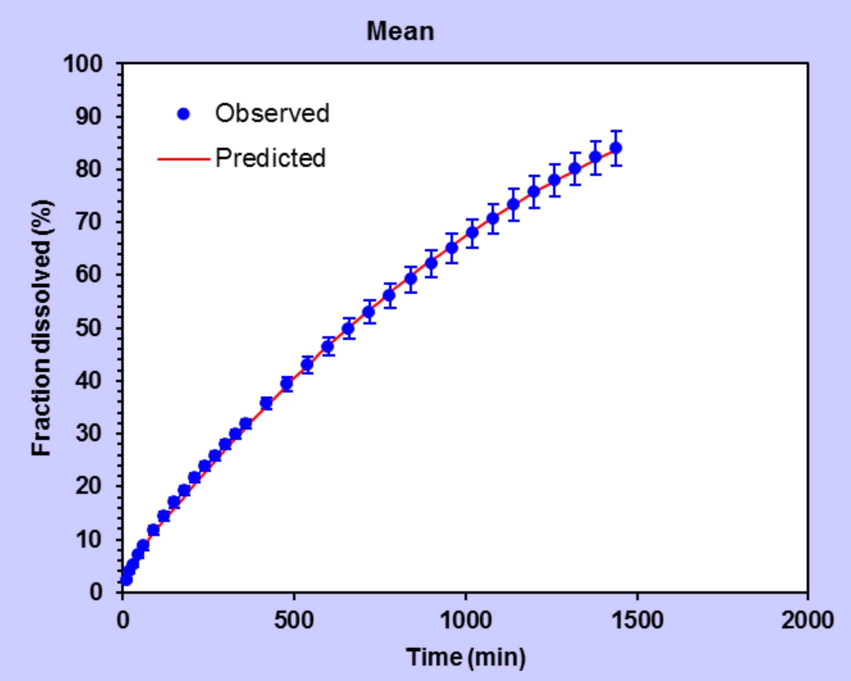

Supplementary materials – Model fitting summary – Avicel® PH-200 (modelling the entire relevant dissolution data)

Graphical abstract of model fit presented as the fraction % of released carvedilol per tested tablet:

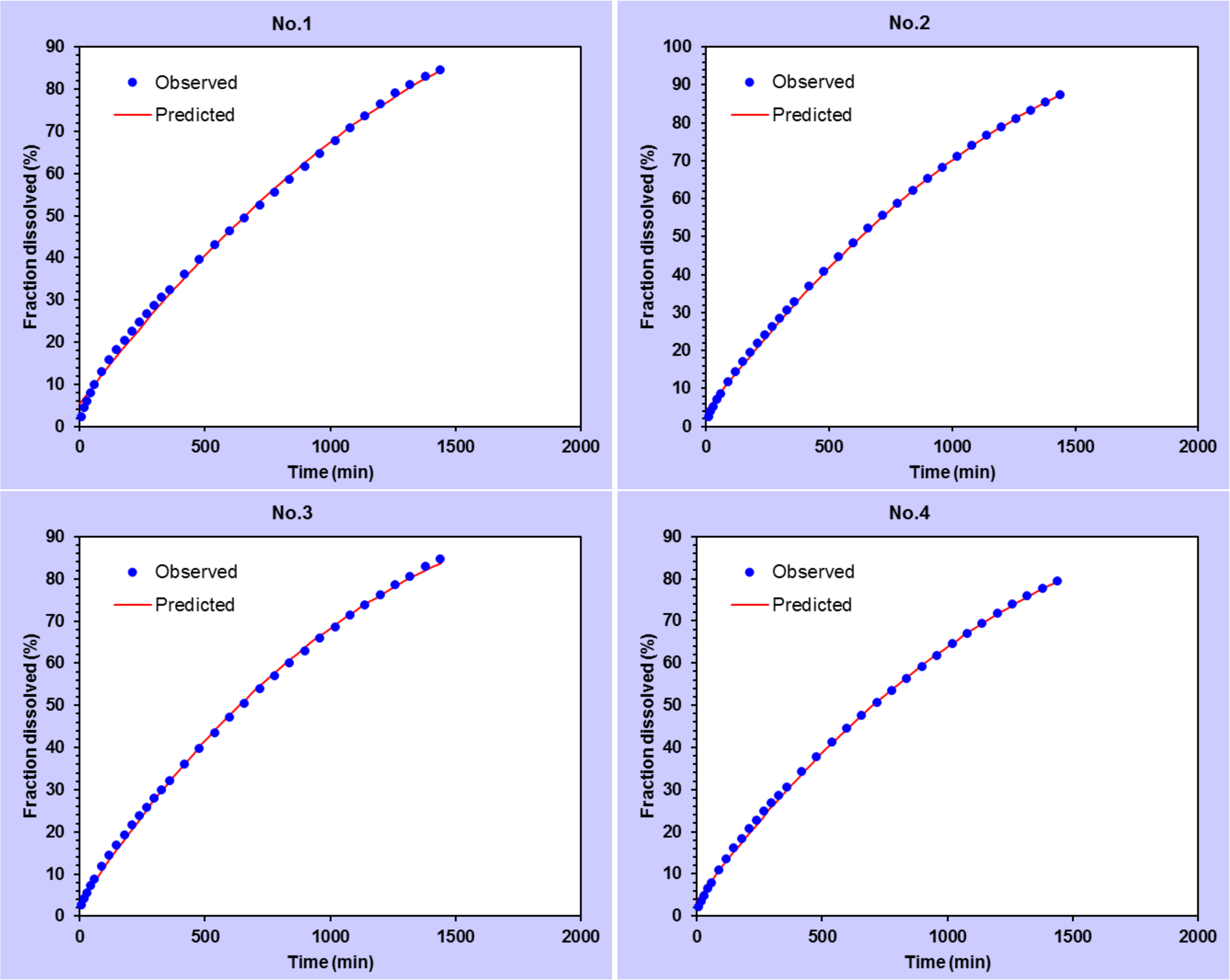

Supplementary materials – Model fitting summary – Avicel® PH-200 (modelling the entire relevant dissolution data)

Model: **Baker–Lonsdale**

Model equation: $\frac{3}{2}\cdot\left[1-\left(1-\frac{F}{100}\right)^{\frac{2}{3}}\right]-\frac{F}{100}=k_{BL}\cdot t$

Fitted model parameters per tested tablet (N = 4) with statistics – mean, standard deviation (SD), and relative standard deviation expressed in % (RSD%) (output from DDSolver):

Parameter	No.1	No.2	No.3	No.4	Mean	SD	RSD(%)
k_{BL}	0.0001	0.0002	0.0001	0.0001	0.0001	0.0000	11.4726

Number of dissolution data points (N), degrees of freedom (df), and selected goodness of fit criteria – Pearson correlation coefficient (R), coefficient of determination (R^2), adjusted coefficient of determination ($R^2_{adjusted}$), and residual sum of squares (RSS) (manual calculation in MS Excel):

Parameter	No.1	No.2	No.3	No.4
N	33	33	33	33
df	32	32	32	32
R	0.981926148	0.980909788	0.982663554	0.986226589
R^2	0.964178959	0.962184013	0.965627661	0.972642884
$R^2_{adjusted}$	0.964178959	0.962184013	0.965627661	0.972642884
RSS	4814.873321	5958.692502	5075.45778	3987.004934

Graphical abstract of model fit presented as mean ± 1 SD of the fraction % of released carvedilol:

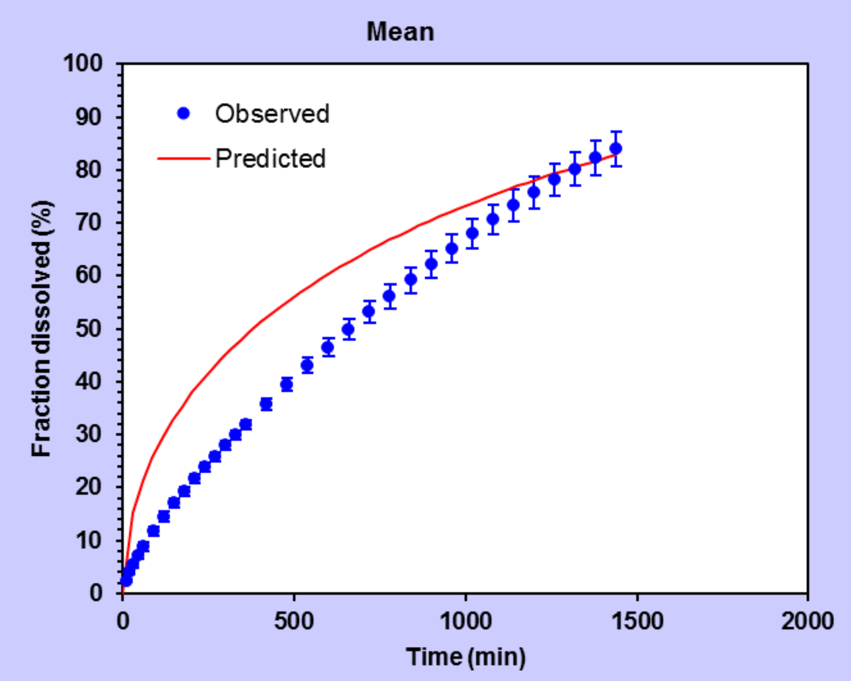

Supplementary materials – Model fitting summary – Avicel® PH-200 (modelling the entire relevant dissolution data)

Graphical abstract of model fit presented as the fraction % of released carvedilol per tested tablet:

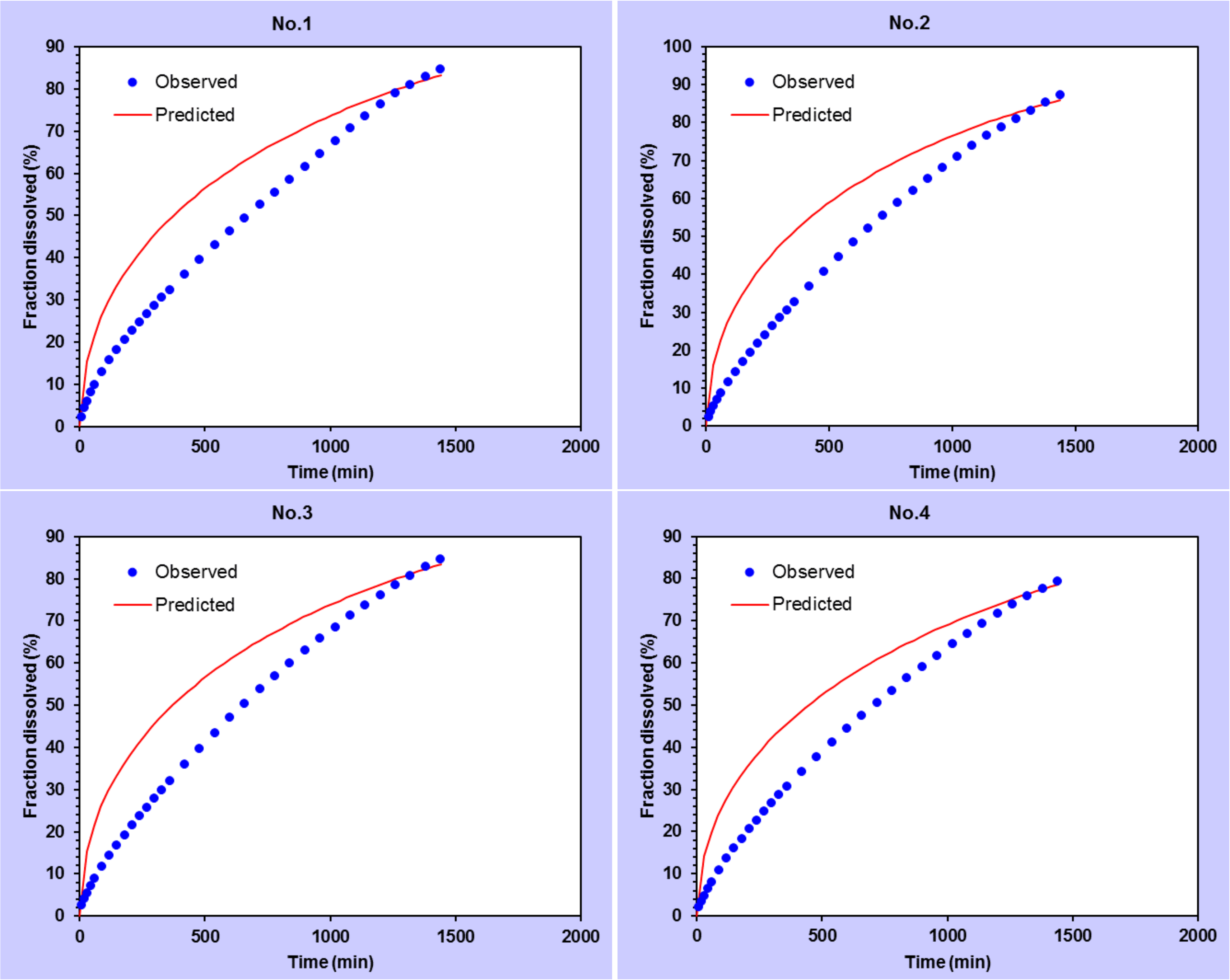

Supplementary materials – Model fitting summary – Avicel® PH-200 (modelling the entire relevant dissolution data)

Model: **Baker–Lonsdale with T_{lag}**

Model equation: $\frac{3}{2}\cdot\left[1-\left(1-\frac{F}{100}\right)^{\frac{2}{3}}\right]-\frac{F}{100}=k_{BL}\cdot\left(t-T_{lag}\right)$

Fitted model parameters per tested tablet (N = 4) with statistics – mean, standard deviation (SD), and relative standard deviation expressed in % (RSD%) (output from DDSolver):

Parameter	No.1	No.2	No.3	No.4	Mean	SD	RSD(%)
k_{BL}	0.0001	0.0002	0.0001	0.0001	0.0001	0.0000	11.4726
T_{lag}	151.2385	154.9549	150.3544	143.2421	149.9475	4.8945	3.2642

Number of dissolution data points (N), degrees of freedom (df), and selected goodness of fit criteria – Pearson correlation coefficient (R), coefficient of determination (R^2), adjusted coefficient of determination ($R^2_{adjusted}$), and residual sum of squares (RSS) (manual calculation in MS Excel):

Parameter	No.1	No.2	No.3	No.4
N	33	33	33	33
df	31	31	31	31
R	0.974369671	0.977417411	0.977655778	0.982097701
R^2	0.949396255	0.955344795	0.955810821	0.964515893
$R^2_{adjusted}$	0.947763877	0.953904305	0.954385364	0.963371245
RSS	1599.2542	1477.429482	1375.698872	943.5476332

Graphical abstract of model fit presented as mean ± 1 SD of the fraction % of released carvedilol:

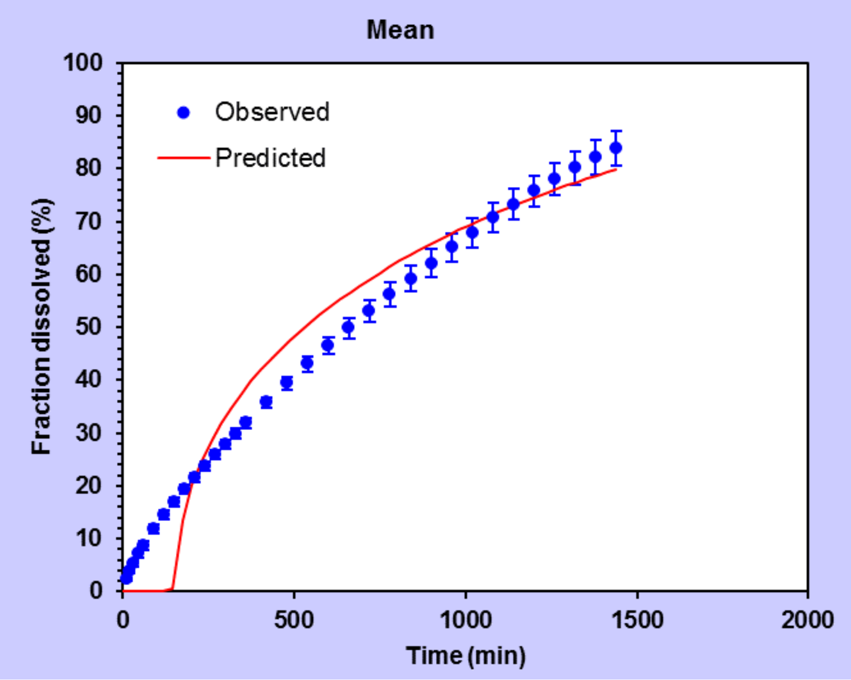

Supplementary materials – Model fitting summary – Avicel® PH-200 (modelling the entire relevant dissolution data)

Graphical abstract of model fit presented as the fraction % of released carvedilol per tested tablet:

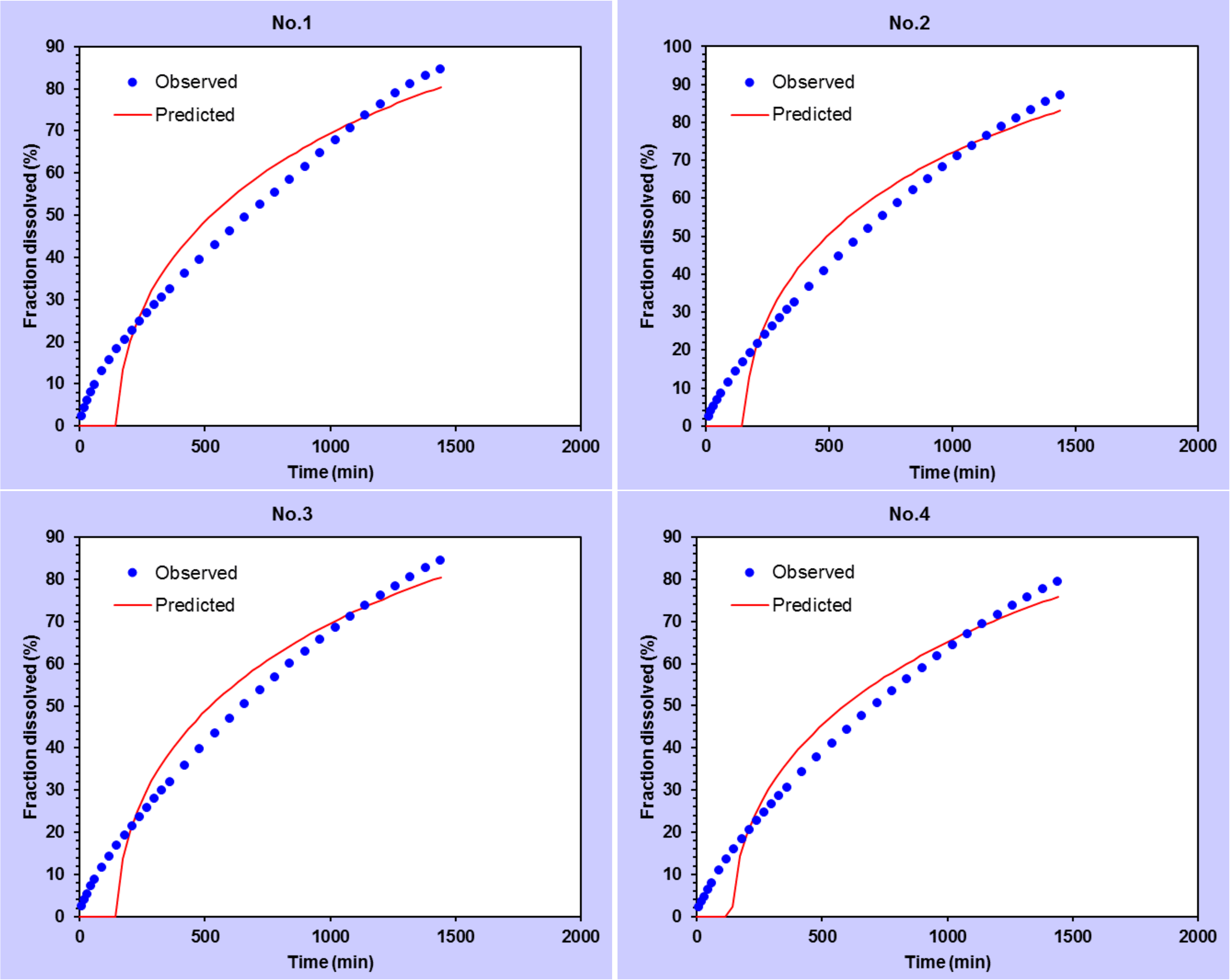

Supplementary materials – Model fitting summary – Avicel® PH-200 (modelling the entire relevant dissolution data)

Model: **Makoid–Banakar**

Model equation: $F = k_{MB} \cdot t^{n} \cdot e^{-k \cdot t}$

Fitted model parameters per tested tablet (N = 4) with statistics – mean, standard deviation (SD), and relative standard deviation expressed in % (RSD%) (output from DDSolver):

Parameter	No.1	No.2	No.3	No.4	Mean	SD	RSD(%)
k_{MB}	0.507953	0.434335	0.471098	0.357679	0.442766	0.064195	14.498606
n	0.711793	0.734085	0.716544	0.760688	0.730777	0.022125	3.027643
k	0.000042	0.000001	-0.000006	0.000080	0.000029	0.000040	136.035229

Number of dissolution data points (N), degrees of freedom (df), and selected goodness of fit criteria – Pearson correlation coefficient (R), coefficient of determination (R^2), adjusted coefficient of determination ($R^2_{adjusted}$), and residual sum of squares (RSS) (manual calculation in MS Excel):

Parameter	No.1	No.2	No.3	No.4
N	33	33	33	33
df	30	30	30	30
R	0.999708164	0.999428945	0.999603608	0.999937818
R^2	0.999416413	0.998858217	0.999207372	0.99987564
$R^2_{adjusted}$	0.999377507	0.998782098	0.99915453	0.999867349
RSS	13.37394514	28.77784263	18.41008359	2.532064262

Graphical abstract of model fit presented as mean ± 1 SD of the fraction % of released carvedilol:

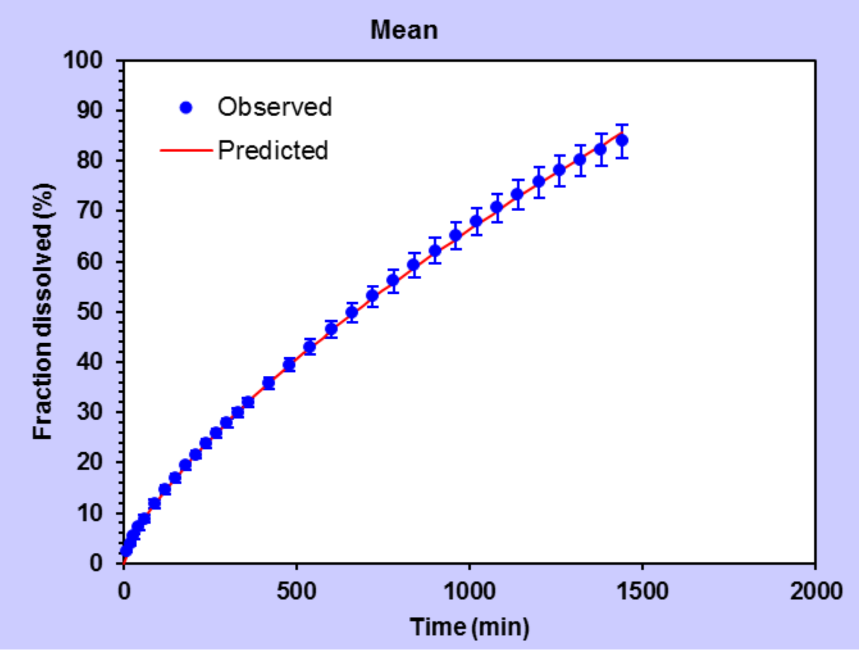

Supplementary materials – Model fitting summary – Avicel® PH-200 (modelling the entire relevant dissolution data)

Graphical abstract of model fit presented as the fraction % of released carvedilol per tested tablet:

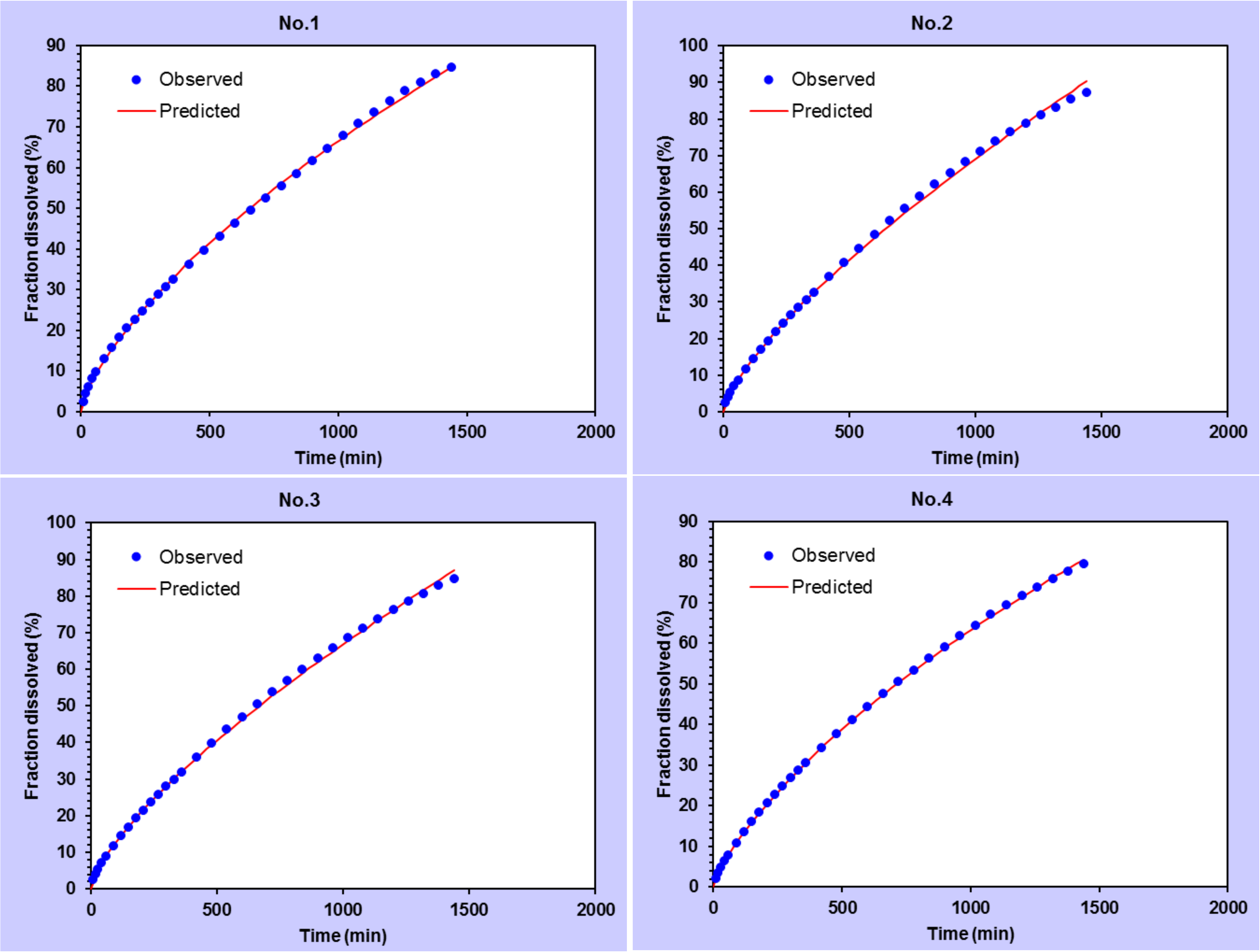

Supplementary materials – Model fitting summary – Avicel® PH-200 (modelling the entire relevant dissolution data)

Model: **Makoid–Banakar with T_{lag}**

Model equation: $F = k_{MB} \cdot (t - T_{lag})^n \cdot e^{-k \cdot (t - T_{lag})}$

Fitted model parameters per tested tablet (N = 4) with statistics – mean, standard deviation (SD), and relative standard deviation expressed in % (RSD%) (output from DDSolver):

Parameter	No.1	No.2	No.3	No.4	Mean	SD	RSD(%)
k_{MB}	0.73946	0.65086	0.69711	0.54147	0.65723	0.08523	12.96828
n	0.63975	0.65580	0.64082	0.68054	0.65423	0.01901	2.90578
k	-0.00008	-0.00013	-0.00013	-0.00006	-0.00010	0.00004	-39.39160
T_{lag}	4.00000	4.00000	4.00000	4.00000	4.00000	0.00000	0.00000

Number of dissolution data points (N), degrees of freedom (df), and selected goodness of fit criteria – Pearson correlation coefficient (R), coefficient of determination (R^2), adjusted coefficient of determination ($R^2_{adjusted}$), and residual sum of squares (RSS) (manual calculation in MS Excel):

Parameter	No.1	No.2	No.3	No.4
N	33	33	33	33
df	29	29	29	29
R	0.999829276	0.998174983	0.998554183	0.999295439
R^2	0.99965858	0.996353296	0.997110457	0.998591375
$R^2_{adjusted}$	0.999623261	0.995976051	0.996811539	0.998445655
RSS	7.50465369	95.49741824	69.4813751	30.08056695

Graphical abstract of model fit presented as mean ± 1 SD of the fraction % of released carvedilol:

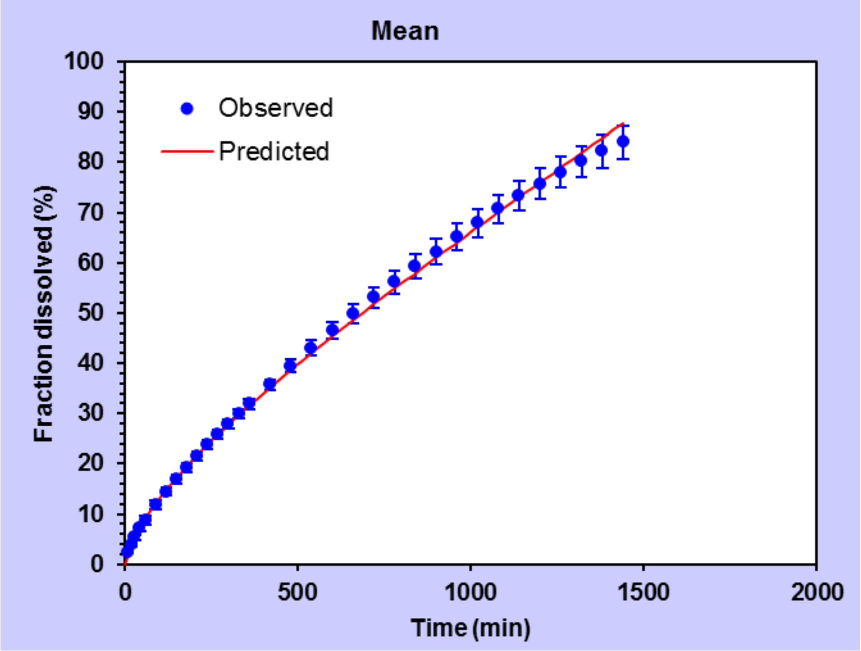

Supplementary materials – Model fitting summary – Avicel® PH-200 (modelling the entire relevant dissolution data)

Graphical abstract of model fit presented as the fraction % of released carvedilol per tested tablet:

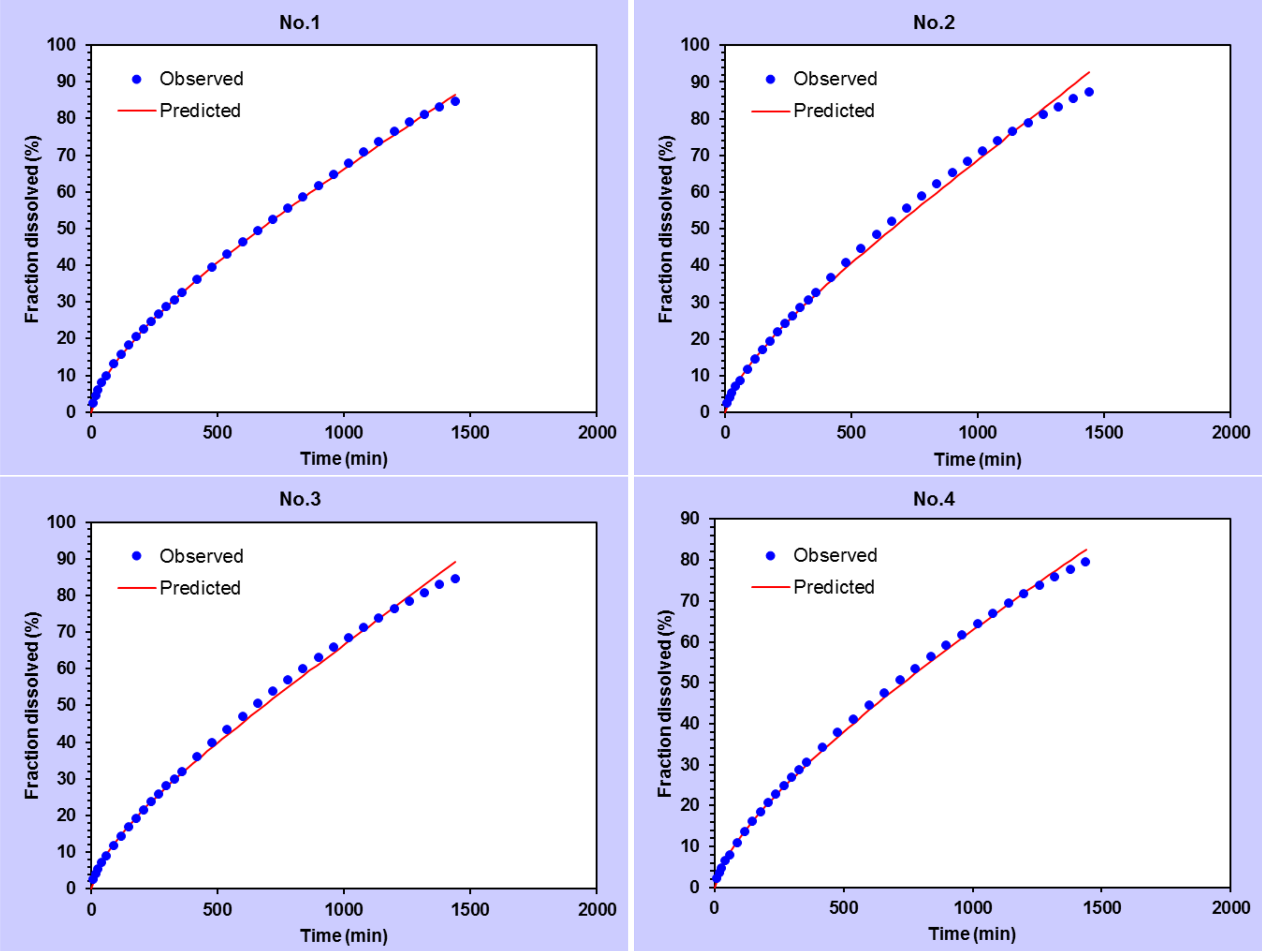

Supplementary materials – Model fitting summary – Avicel® PH-200 (modelling the entire relevant dissolution data)

Model: **Peppas–Sahlin_1**

Model equation: $F = k_1 \cdot t^m + k_2 \cdot t^{2m}$

Fitted model parameters per tested tablet (N = 4) with statistics – mean, standard deviation (SD), and relative standard deviation expressed in % (RSD%) (output from DDSolver):

Parameter	No.1	No.2	No.3	No.4	Mean	SD	RSD(%)
k_1	1.128	1.013	1.042	1.011	1.048	0.055	5.249
k_2	0.082	0.092	0.086	0.080	0.085	0.005	6.329
m	0.450	0.450	0.450	0.450	0.450	0.000	0.000

Number of dissolution data points (N), degrees of freedom (df), and selected goodness of fit criteria – Pearson correlation coefficient (R), coefficient of determination (R^2), adjusted coefficient of determination ($R^2_{adjusted}$), and residual sum of squares (RSS) (manual calculation in MS Excel):

Parameter	No.1	No.2	No.3	No.4
N	33	33	33	33
df	30	30	30	30
R	0.999727518	0.998877434	0.999128508	0.999035812
R^2	0.99945511	0.997756129	0.998257776	0.998072554
$R^2_{adjusted}$	0.999418784	0.997606537	0.998141627	0.997944057
RSS	13.26729145	59.78528744	43.18516052	43.513248

Graphical abstract of model fit presented as mean ± 1 SD of the fraction % of released carvedilol:

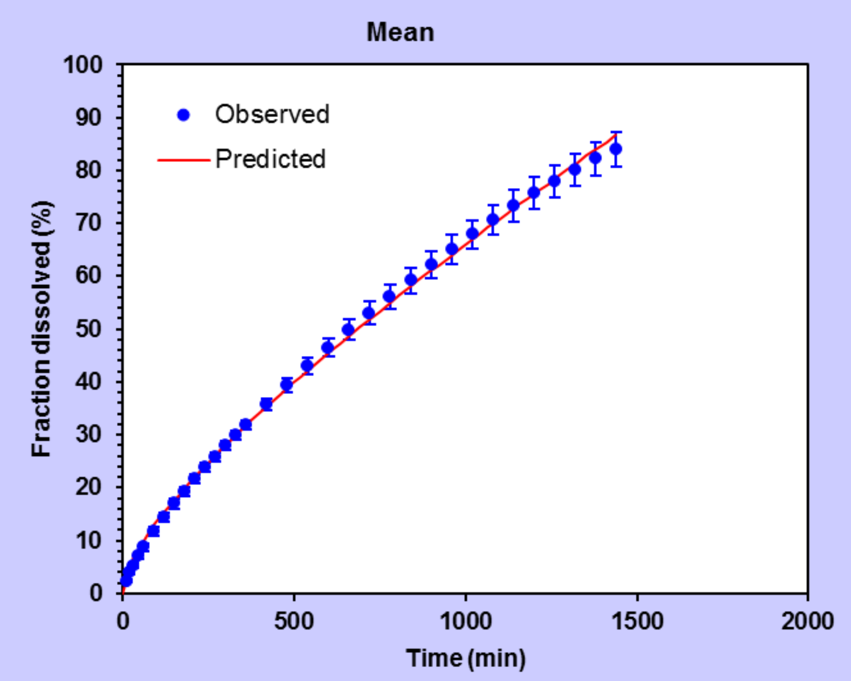

Supplementary materials – Model fitting summary – Avicel® PH-200 (modelling the entire relevant dissolution data)

Graphical abstract of model fit presented as the fraction % of released carvedilol per tested tablet:

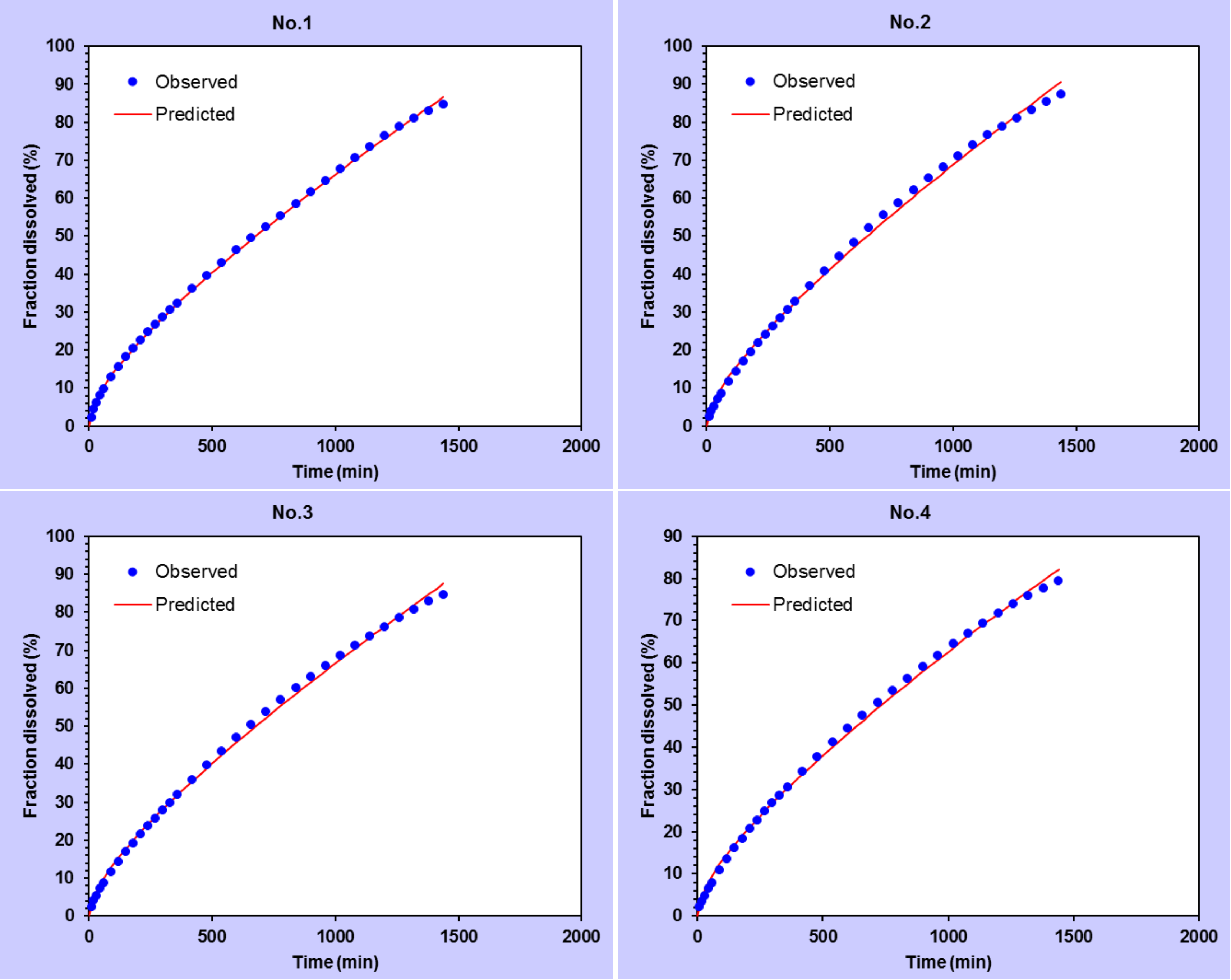

Supplementary materials – Model fitting summary – Avicel® PH-200 (modelling the entire relevant dissolution data)

Model: **Peppas–Sahlin_1 with T_{lag}**

Model equation: $F = k_1 \cdot (t - T_{lag})^m + k_2 \cdot (t - T_{lag})^{2m}$

Fitted model parameters per tested tablet (N = 4) with statistics – mean, standard deviation (SD), and relative standard deviation expressed in % (RSD%) (output from DDSolver):

Parameter	No.1	No.2	No.3	No.4	Mean	SD	RSD(%)
k_1	1.180	1.066	1.094	1.061	1.100	0.055	5.016
k_2	0.080	0.090	0.084	0.078	0.083	0.005	6.429
m	0.450	0.450	0.450	0.450	0.450	0.000	0.000
T_{lag}	6.000	6.000	6.000	6.000	6.000	0.000	0.000

Number of dissolution data points (N), degrees of freedom (df), and selected goodness of fit criteria – Pearson correlation coefficient (R), coefficient of determination (R^2), adjusted coefficient of determination ($R^2_{adjusted}$), and residual sum of squares (RSS) (manual calculation in MS Excel):

Parameter	No.1	No.2	No.3	No.4
N	33	33	33	33
df	29	29	29	29
R	0.999832225	0.999014648	0.999257427	0.999220173
R^2	0.999664479	0.998030266	0.998515406	0.998440953
$R^2_{adjusted}$	0.99962977	0.997826501	0.998361828	0.998279673
RSS	7.6581521	49.9209794	34.71638333	32.96022213

Graphical abstract of model fit presented as mean ± 1 SD of the fraction % of released carvedilol:

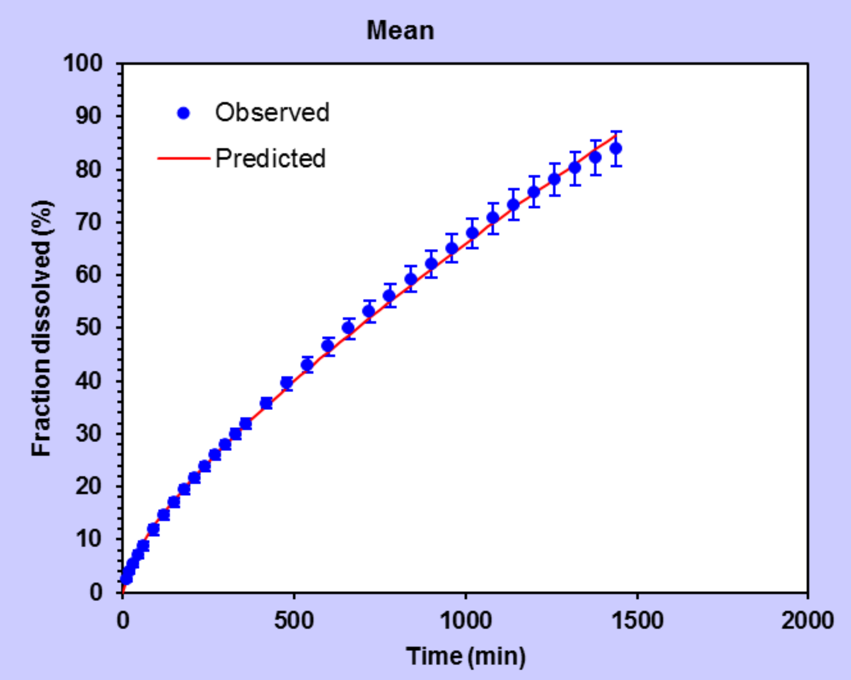

Supplementary materials – Model fitting summary – Avicel® PH-200 (modelling the entire relevant dissolution data)

Graphical abstract of model fit presented as the fraction % of released carvedilol per tested tablet:

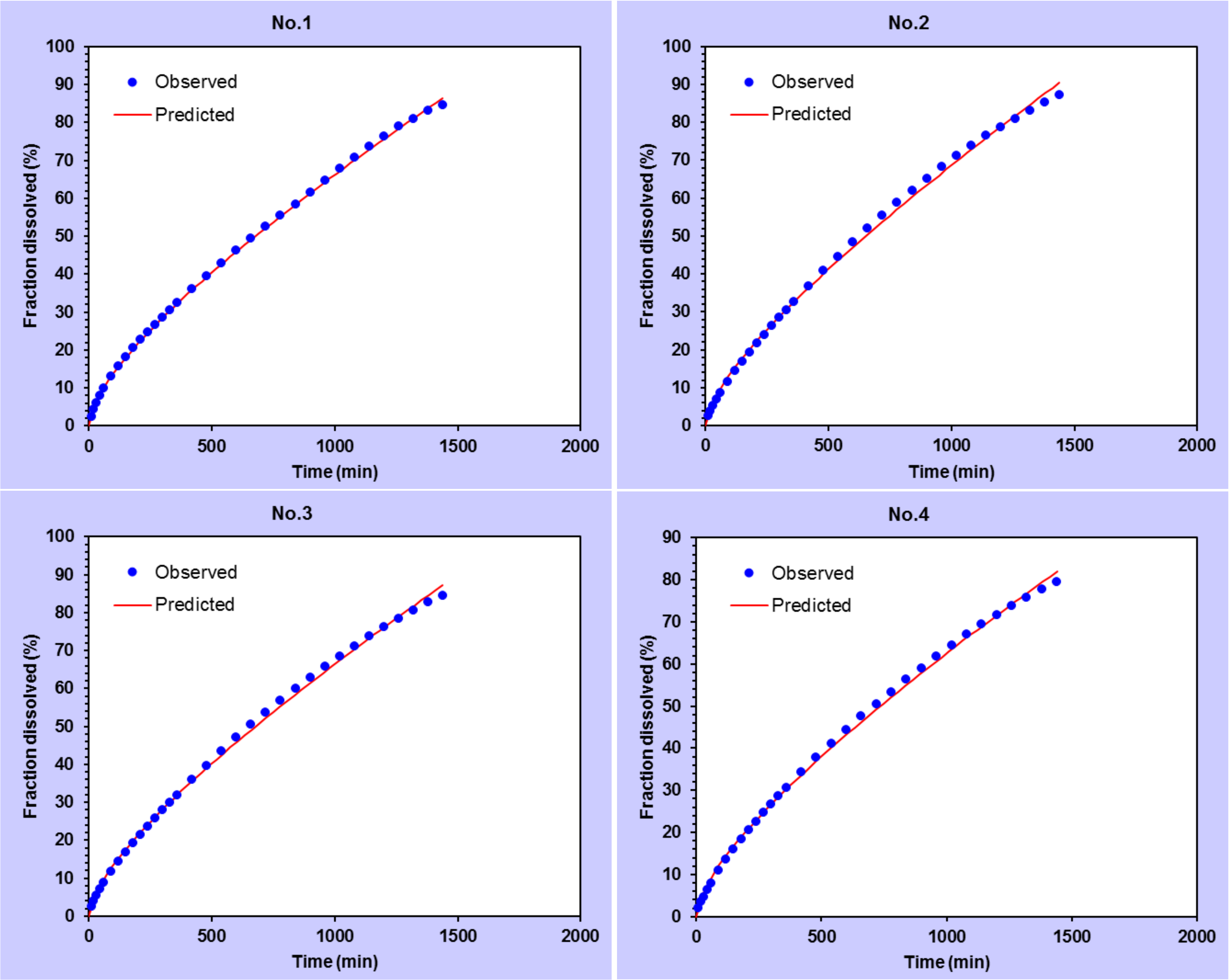

Supplementary materials – Model fitting summary – Avicel® PH-200 (modelling the entire relevant dissolution data)

Model: **Peppas–Sahlin_2**

Model equation: $F = k_1 \cdot t^{0.5} + k_2 \cdot t$

Fitted model parameters per tested tablet (N = 4) with statistics – mean, standard deviation (SD), and relative standard deviation expressed in % (RSD%) (output from DDSolver):

Parameter	No.1	No.2	No.3	No.4	Mean	SD	RSD(%)
k_1	1.120	1.061	1.068	1.028	1.069	0.038	3.576
k_2	0.031	0.035	0.033	0.030	0.032	0.002	7.045

Number of dissolution data points (N), degrees of freedom (df), and selected goodness of fit criteria – Pearson correlation coefficient (R), coefficient of determination (R^2), adjusted coefficient of determination ($R^2_{adjusted}$), and residual sum of squares (RSS) (manual calculation in MS Excel):

Parameter	No.1	No.2	No.3	No.4
N	33	33	33	33
df	31	31	31	31
R	0.999695555	0.998721373	0.999008232	0.998936861
R^2	0.999391202	0.997444381	0.998017447	0.997874852
$R^2_{adjusted}$	0.999371563	0.997361941	0.997953494	0.997806299
RSS	15.45149373	70.60192835	50.9965263	50.14541137

Graphical abstract of model fit presented as mean ± 1 SD of the fraction % of released carvedilol:

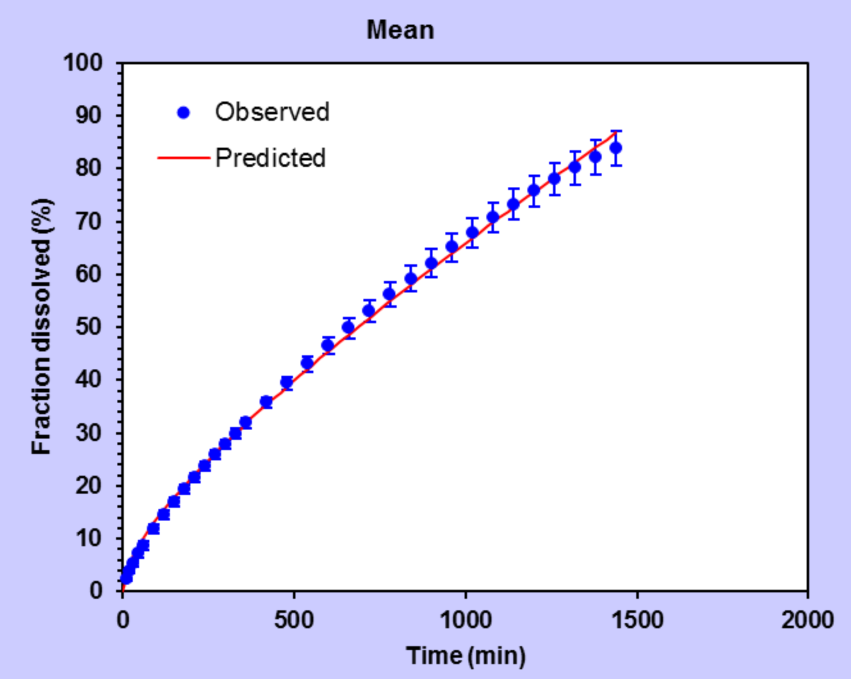

Supplementary materials – Model fitting summary – Avicel® PH-200 (modelling the entire relevant dissolution data)

Graphical abstract of model fit presented as the fraction % of released carvedilol per tested tablet:

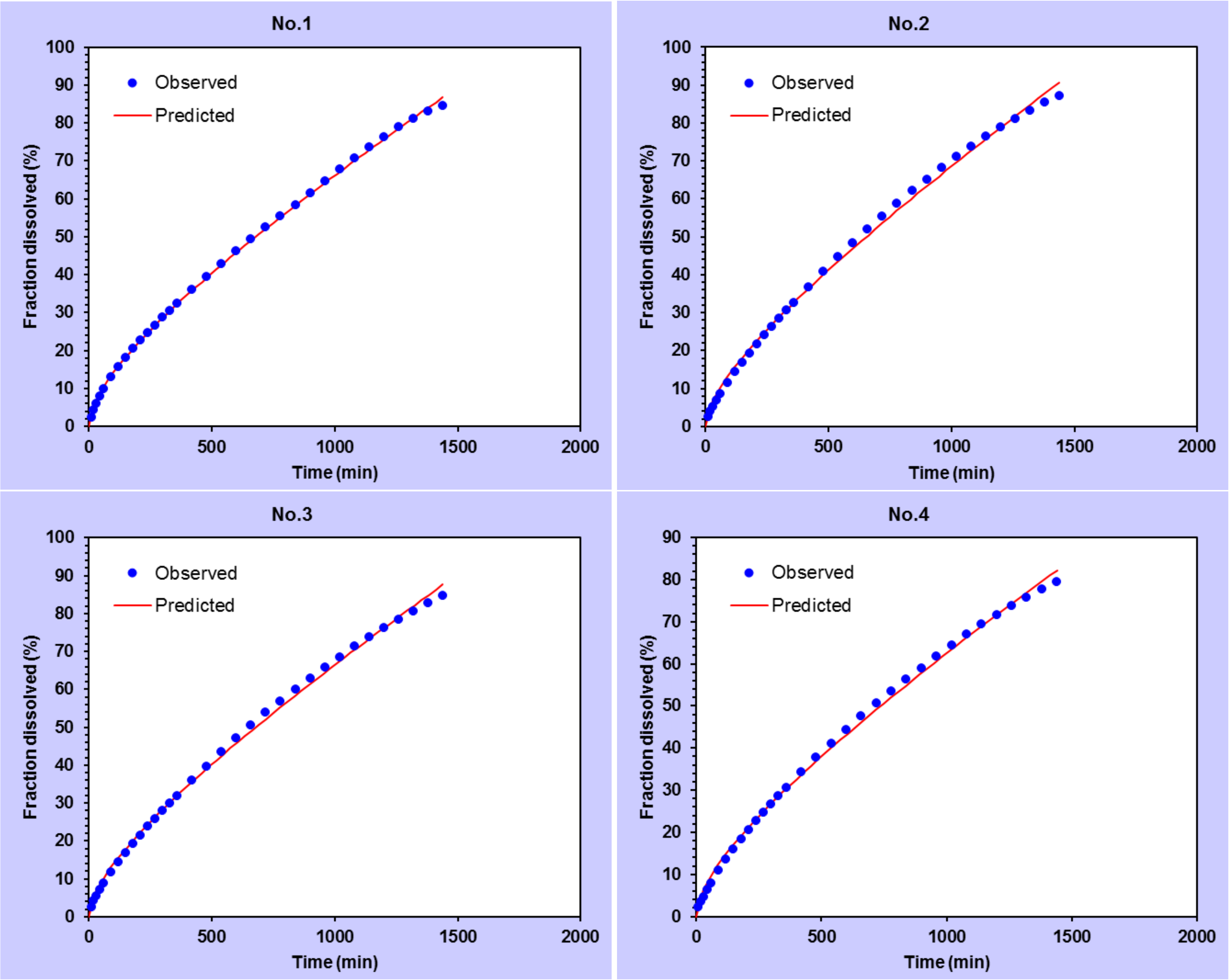

Supplementary materials – Model fitting summary – Avicel® PH-200 (modelling the entire relevant dissolution data)

Model: **Peppas–Sahlin_2 with T_{lag}**

Model equation: $F = k_1 \cdot (t - T_{lag})^{0.5} + k_2 \cdot (t - T_{lag})$

Fitted model parameters per tested tablet (N = 4) with statistics – mean, standard deviation (SD), and relative standard deviation expressed in % (RSD%) (output from DDSolver):

Parameter	No.1	No.2	No.3	No.4	Mean	SD	RSD(%)
k_1	1.156	1.097	1.104	1.062	1.105	0.039	3.489
k_2	0.030	0.034	0.032	0.029	0.031	0.002	7.185
T_{lag}	6.000	6.000	6.000	6.000	6.000	0.000	0.000

Number of dissolution data points (N), degrees of freedom (df), and selected goodness of fit criteria – Pearson correlation coefficient (R), coefficient of determination (R^2), adjusted coefficient of determination ($R^2_{adjusted}$), and residual sum of squares (RSS) (manual calculation in MS Excel):

Parameter	No.1	No.2	No.3	No.4
N	33	33	33	33
df	30	30	30	30
R	0.999807753	0.99887814	0.999152196	0.999136216
R^2	0.999615542	0.997757539	0.99830511	0.998273179
$R^2_{adjusted}$	0.999589912	0.997608041	0.998192118	0.998158057
RSS	8.768267892	58.02726996	40.38338416	37.54828583

Graphical abstract of model fit presented as mean ± 1 SD of the fraction % of released carvedilol:

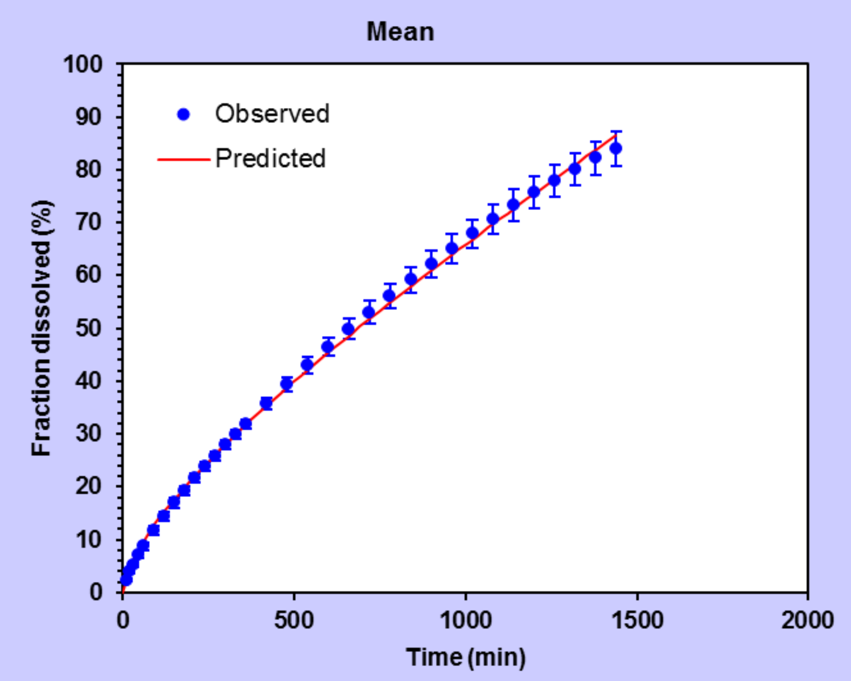

Supplementary materials – Model fitting summary – Avicel® PH-200 (modelling the entire relevant dissolution data)

Graphical abstract of model fit presented as the fraction % of released carvedilol per tested tablet:

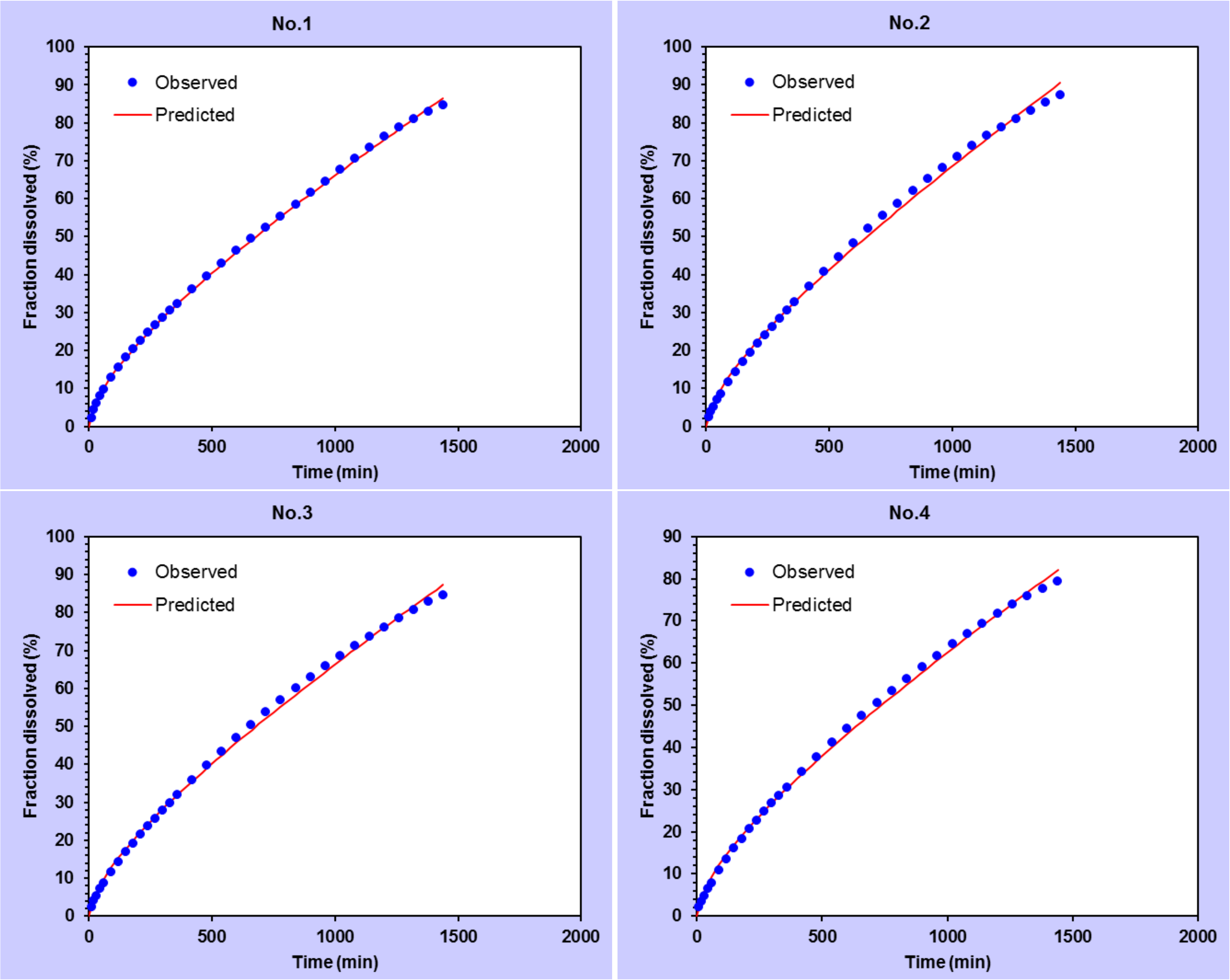

Supplementary materials – Model fitting summary – Avicel® PH-200 (modelling the entire relevant dissolution data)

Model: **Quadratic**

Model equation: $F = 100 \cdot (k_1 \cdot t^2 + k_2 \cdot t)$

Fitted model parameters per tested tablet (N = 4) with statistics – mean, standard deviation (SD), and relative standard deviation expressed in % (RSD%) (output from DDSolver):

Parameter	No.1	No.2	No.3	No.4	Mean	SD	RSD(%)
k_1	-0.0000003	-0.0000003	-0.0000003	-0.0000003	-0.0000003	0.0000000	-2.0614419
k_2	0.0009521	0.0009841	0.0009590	0.0009091	0.0009511	0.0000311	3.2752728

Number of dissolution data points (N), degrees of freedom (df), and selected goodness of fit criteria – Pearson correlation coefficient (R), coefficient of determination (R^2), adjusted coefficient of determination ($R^2_{adjusted}$), and residual sum of squares (RSS) (manual calculation in MS Excel):

Parameter	No.1	No.2	No.3	No.4
N	33	33	33	33
df	31	31	31	31
R	0.997536934	0.999253717	0.998973485	0.998798205
R^2	0.995079935	0.99850799	0.997948024	0.997597854
$R^2_{adjusted}$	0.994921224	0.998459861	0.997881831	0.997520365
RSS	235.7001757	91.66416402	115.3807085	106.010553

Graphical abstract of model fit presented as mean ± 1 SD of the fraction % of released carvedilol:

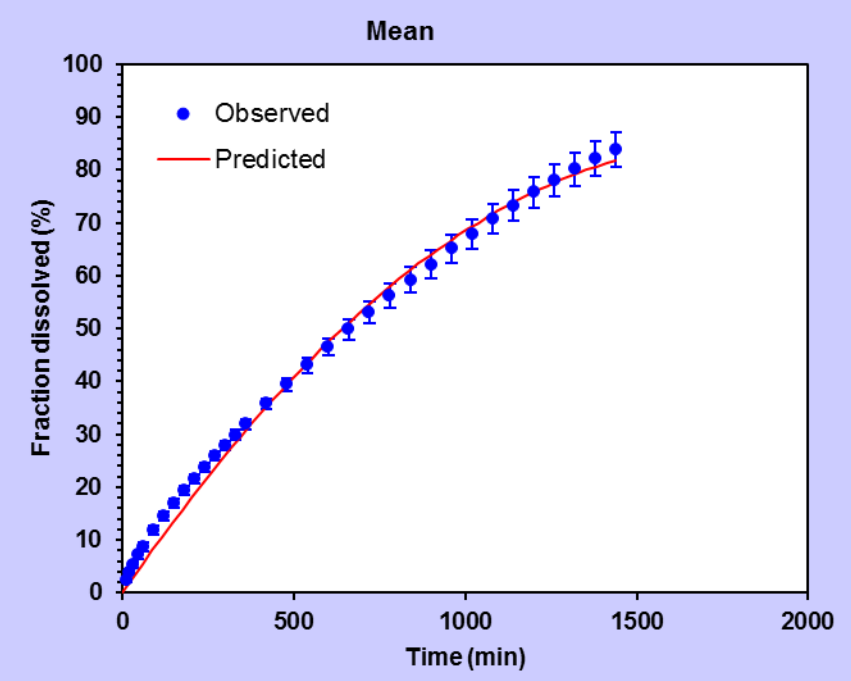

Supplementary materials – Model fitting summary – Avicel® PH-200 (modelling the entire relevant dissolution data)

Graphical abstract of model fit presented as the fraction % of released carvedilol per tested tablet:

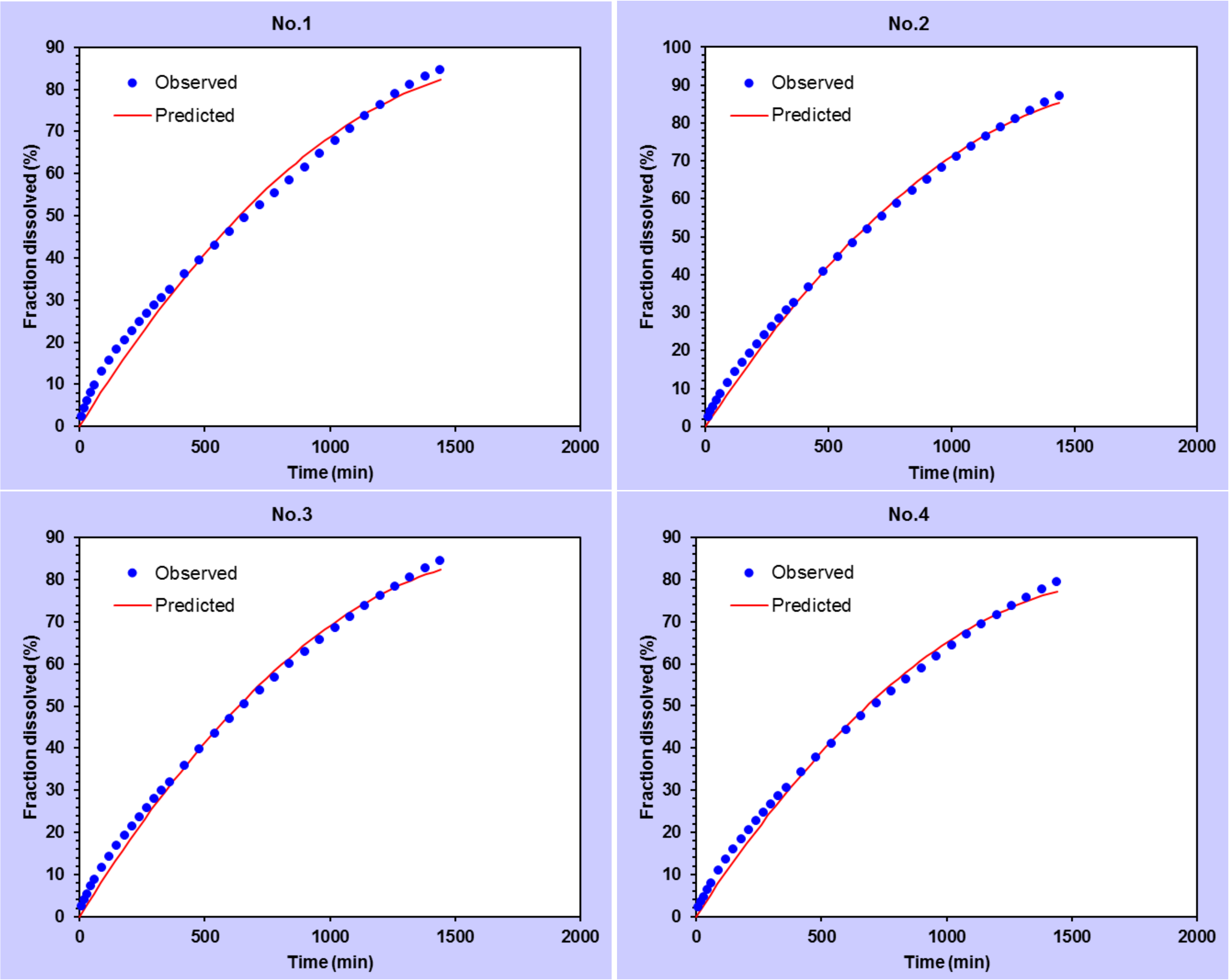

Supplementary materials – Model fitting summary – Avicel® PH-200 (modelling the entire relevant dissolution data)

Model: **Quadratic with T_{lag}**

Model equation: $F = 100 \cdot \left[k_1 \cdot \left(t - T_{lag}\right)^2 + k_2 \cdot \left(t - T_{lag}\right)\right]$

Fitted model parameters per tested tablet (N = 4) with statistics – mean, standard deviation (SD), and relative standard deviation expressed in % (RSD%) (output from DDSolver):

Parameter	No.1	No.2	No.3	No.4	Mean	SD	RSD(%)
k_1	-0.0000003	-0.0000003	-0.0000003	-0.0000003	-0.0000003	0.0000000	-2.1049533
k_2	0.0009605	0.0009933	0.0009679	0.0009175	0.0009598	0.0000315	3.2806687
T_{lag}	4.0000000	4.0000000	4.0000000	4.0000000	4.0000000	0.0000000	0.0000000

Number of dissolution data points (N), degrees of freedom (df), and selected goodness of fit criteria – Pearson correlation coefficient (R), coefficient of determination (R^2), adjusted coefficient of determination ($R^2_{adjusted}$), and residual sum of squares (RSS) (manual calculation in MS Excel):

Parameter	No.1	No.2	No.3	No.4
N	33	33	33	33
df	30	30	30	30
R	0.997380327	0.999146936	0.998852817	0.998680316
R^2	0.994767517	0.998294599	0.99770695	0.997362374
$R^2_{adjusted}$	0.994418685	0.998180906	0.99755408	0.997186532
RSS	264.5158544	111.6147089	136.9282205	124.7173946

Graphical abstract of model fit presented as mean ± 1 SD of the fraction % of released carvedilol:

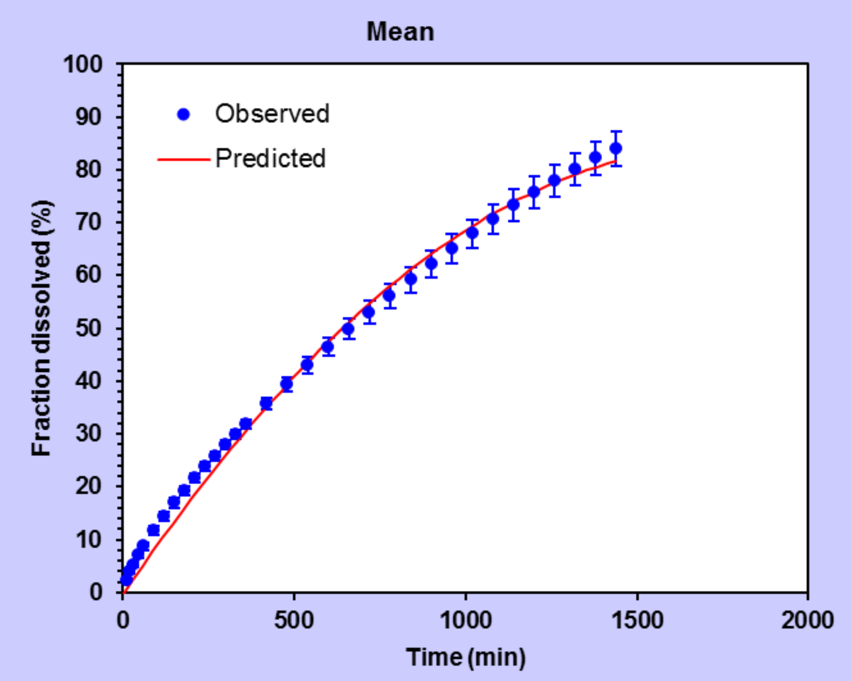

Supplementary materials – Model fitting summary – Avicel® PH-200 (modelling the entire relevant dissolution data)

Graphical abstract of model fit presented as the fraction % of released carvedilol per tested tablet:

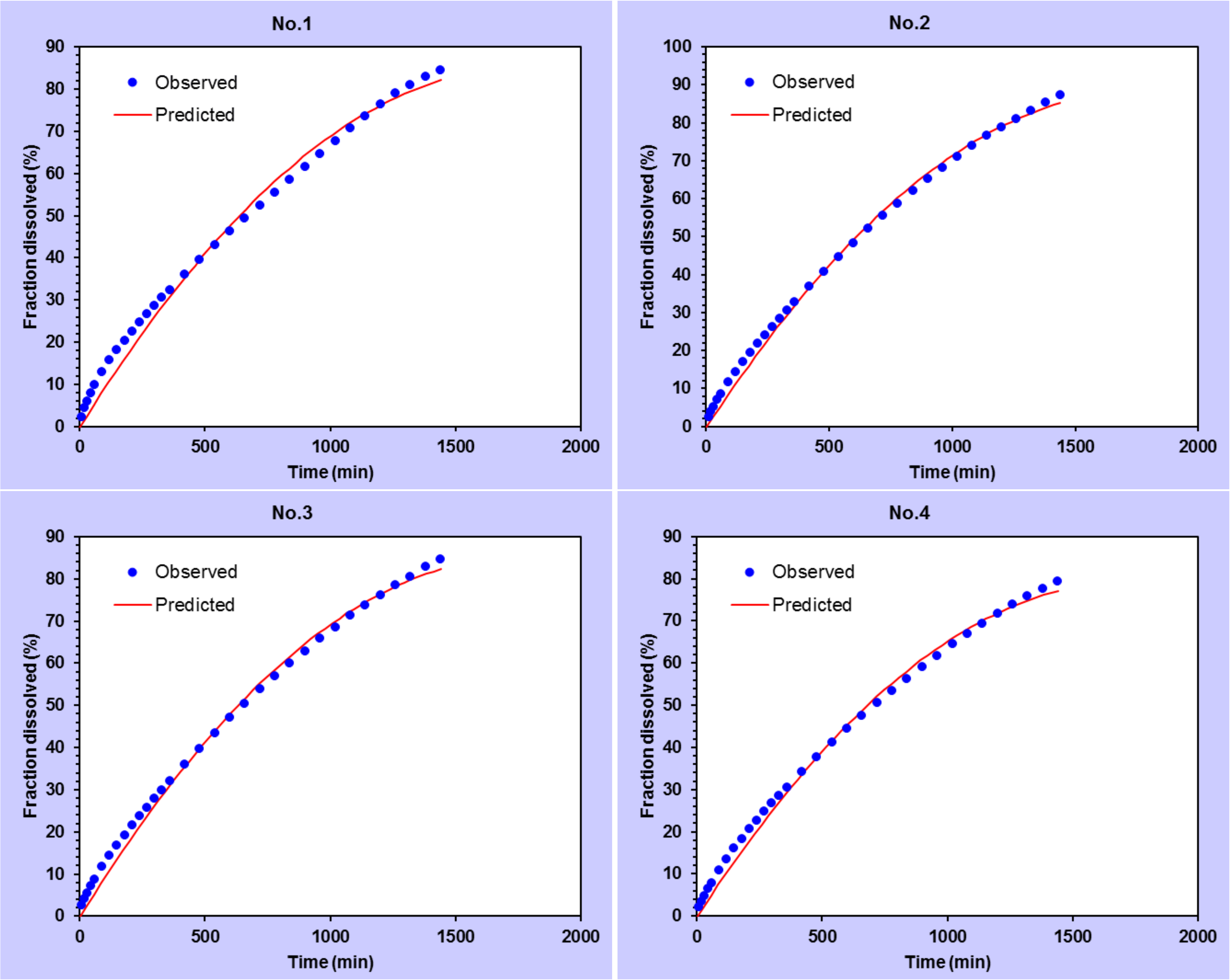

Supplementary materials – Model fitting summary – Avicel® PH-200 (modelling the entire relevant dissolution data)

Model: **Weibull_1**

Model equation: $F = 100 \cdot \left[1 - e^{-\frac{(t-T_i)^{\beta}}{\alpha}}\right]$

Fitted model parameters per tested tablet (N = 4) with statistics – mean, standard deviation (SD), and relative standard deviation expressed in % (RSD%) (output from DDSolver):

Parameter	No.1	No.2	No.3	No.4	Mean	SD	RSD(%)
α	245.764	312.668	279.912	312.339	287.671	31.884	11.084
β	0.804	0.848	0.823	0.825	0.825	0.018	2.191
T_i	6.000	6.000	6.000	6.000	6.000	0.000	0.000

Number of dissolution data points (N), degrees of freedom (df), and selected goodness of fit criteria – Pearson correlation coefficient (R), coefficient of determination (R^2), adjusted coefficient of determination ($R^2_{adjusted}$), and residual sum of squares (RSS) (manual calculation in MS Excel):

Parameter	No.1	No.2	No.3	No.4
N	33	33	33	33
df	30	30	30	30
R	0.990634952	0.992712506	0.992934283	0.995522961
R^2	0.981357608	0.98547812	0.98591849	0.991065966
$R^2_{adjusted}$	0.980114782	0.984509995	0.984979723	0.990470364
RSS	538.3731023	545.4118402	492.7752446	289.2499829

Graphical abstract of model fit presented as mean ± 1 SD of the fraction % of released carvedilol:

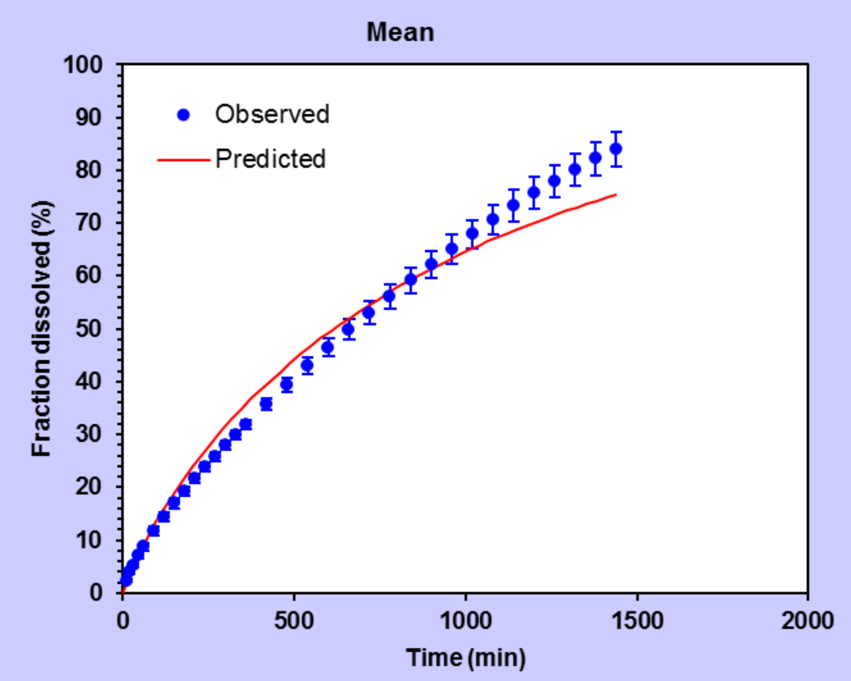

Supplementary materials – Model fitting summary – Avicel® PH-200 (modelling the entire relevant dissolution data)

Graphical abstract of model fit presented as the fraction % of released carvedilol per tested tablet:

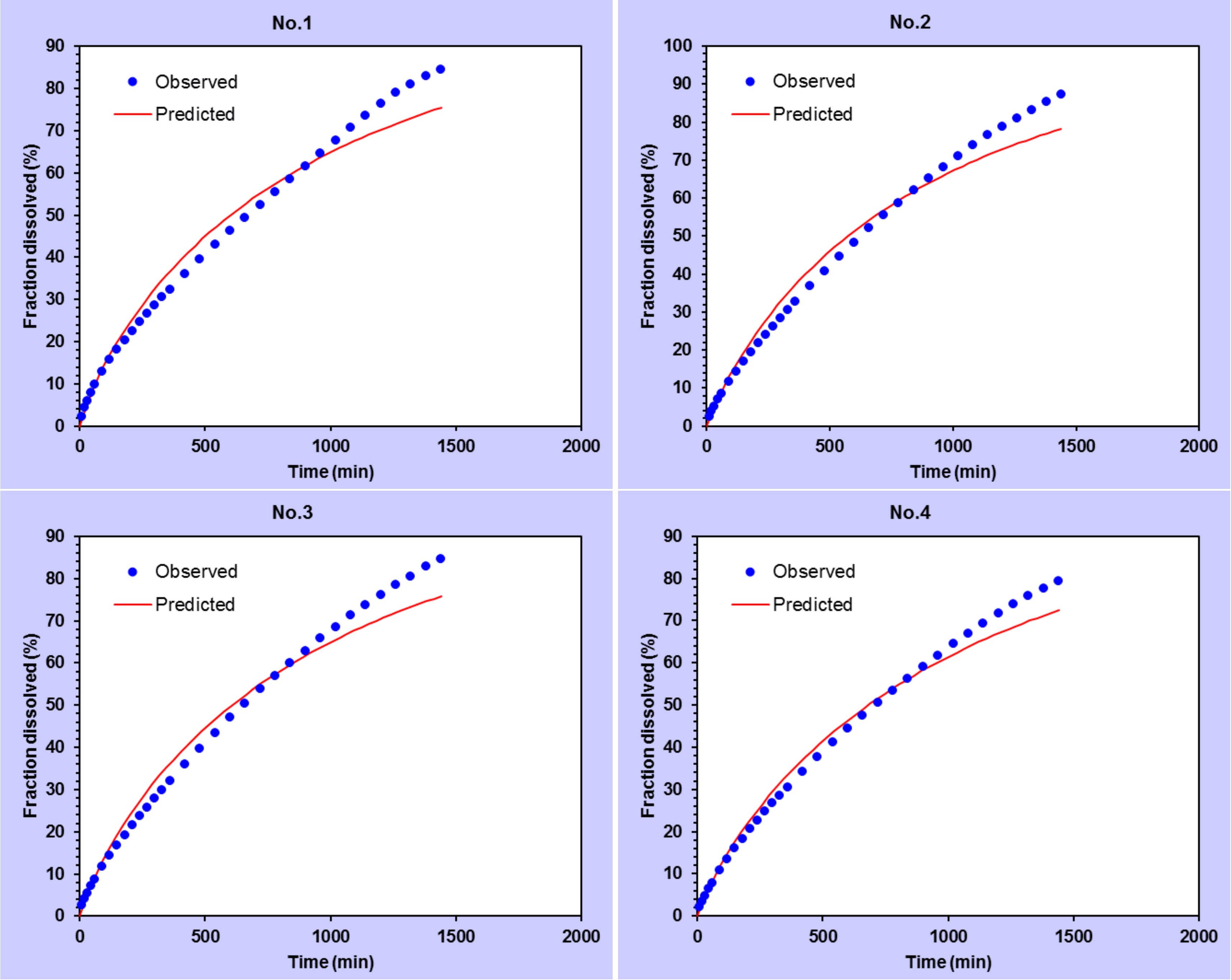

Supplementary materials – Model fitting summary – Avicel® PH-200 (modelling the entire relevant dissolution data)

Model: **Weibull_2**

Model equation: $F = 100 \cdot \left(1 - e^{-\frac{t^{\beta}}{\alpha}}\right)$

Fitted model parameters per tested tablet (N = 4) with statistics – mean, standard deviation (SD), and relative standard deviation expressed in % (RSD%) (output from DDSolver):

Parameter	No.1	No.2	No.3	No.4	Mean	SD	RSD(%)
α	336.264	439.190	388.524	431.692	398.918	47.362	11.873
β	0.852	0.901	0.874	0.875	0.876	0.020	2.260

Number of dissolution data points (N), degrees of freedom (df), and selected goodness of fit criteria – Pearson correlation coefficient (R), coefficient of determination (R^2), adjusted coefficient of determination ($R^2_{adjusted}$), and residual sum of squares (RSS) (manual calculation in MS Excel):

Parameter	No.1	No.2	No.3	No.4
N	33	33	33	33
df	31	31	31	31
R	0.992614332	0.994611675	0.994850599	0.996980328
R^2	0.985283211	0.989252384	0.989727714	0.993969775
$R^2_{adjusted}$	0.984808476	0.988905687	0.98939635	0.993775252
RSS	399.716223	378.7258952	337.0611248	175.8285777

Graphical abstract of model fit presented as mean ± 1 SD of the fraction % of released carvedilol:

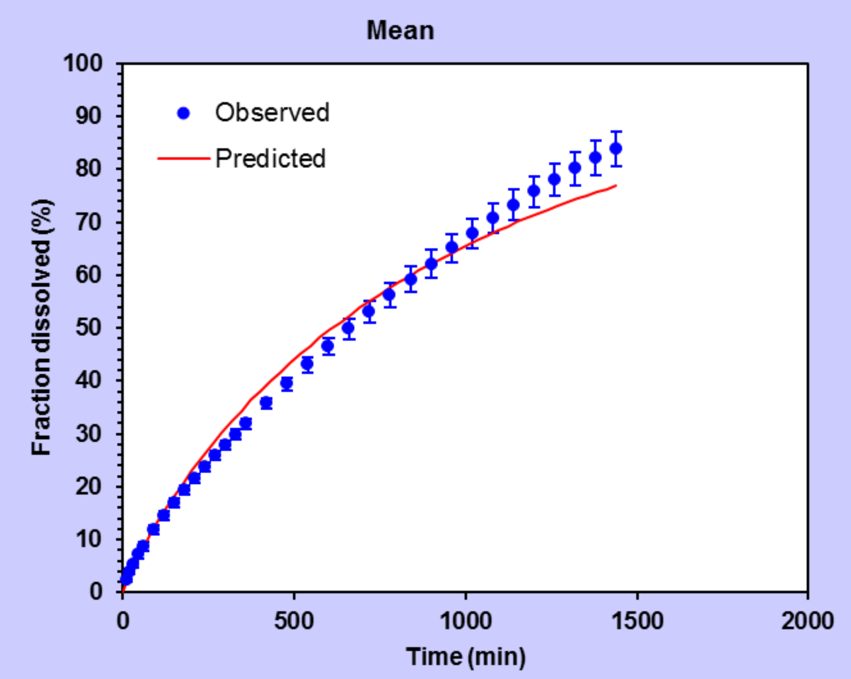

Supplementary materials – Model fitting summary – Avicel® PH-200 (modelling the entire relevant dissolution data)

Graphical abstract of model fit presented as the fraction % of released carvedilol per tested tablet:

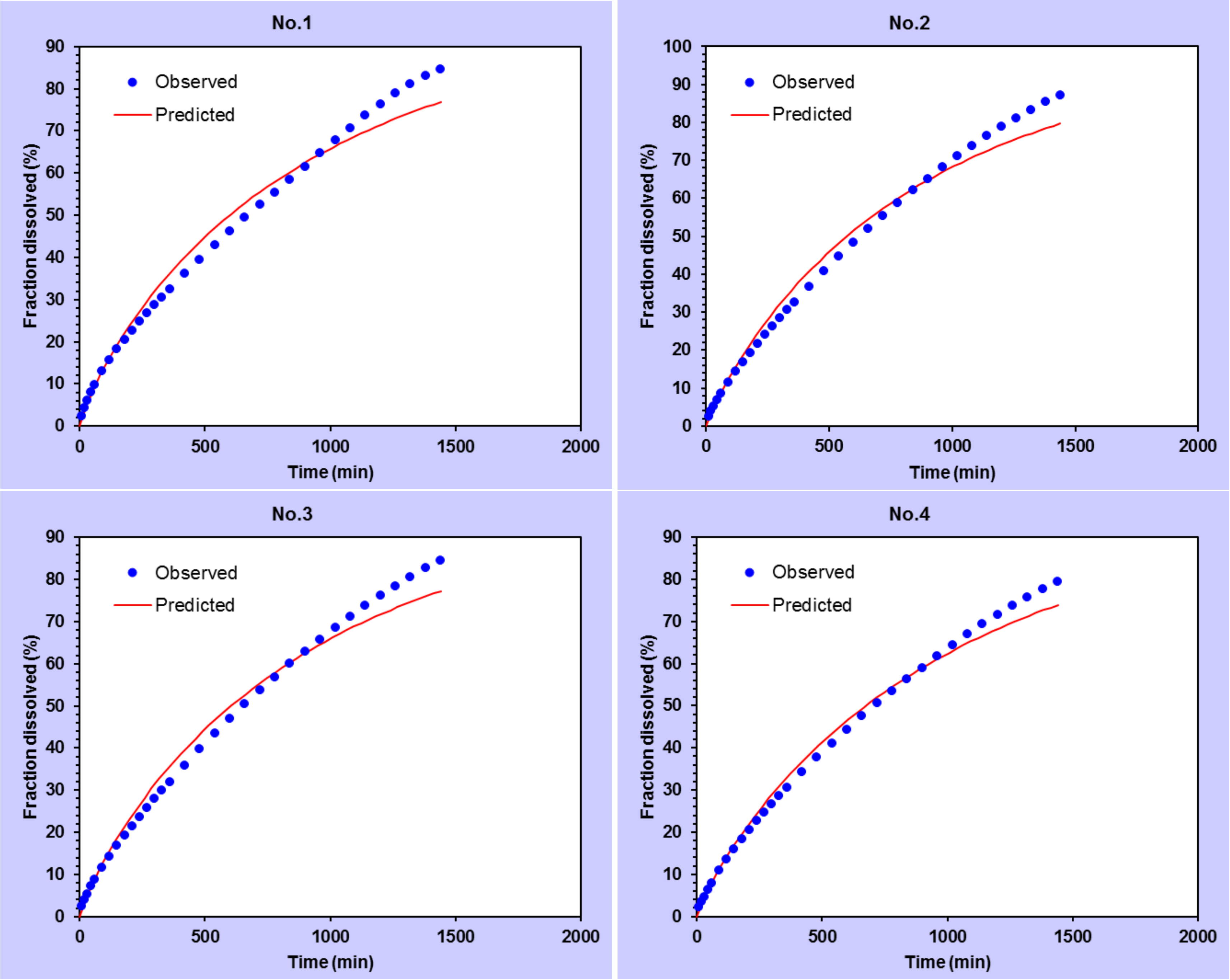

Supplementary materials – Model fitting summary – Avicel® PH-200 (modelling the entire relevant dissolution data)

Model: **Weibull_3**

Model equation: $F = F_{max} \cdot \left(1 - e^{-\frac{t^\beta}{\alpha}}\right)$

Fitted model parameters per tested tablet (N = 4) with statistics – mean, standard deviation (SD), and relative standard deviation expressed in % (RSD%) (output from DDSolver):

Parameter	No.1	No.2	No.3	No.4	Mean	SD	RSD(%)
α	367.275	475.908	424.933	476.370	436.122	51.858	11.891
β	0.902	0.941	0.923	0.942	0.927	0.019	2.039
F_{max}	88.807	91.579	88.795	83.377	88.139	3.434	3.896

Number of dissolution data points (N), degrees of freedom (df), and selected goodness of fit criteria – Pearson correlation coefficient (R), coefficient of determination (R^2), adjusted coefficient of determination ($R^2_{adjusted}$), and residual sum of squares (RSS) (manual calculation in MS Excel):

Parameter	No.1	No.2	No.3	No.4
N	33	33	33	33
df	30	30	30	30
R	0.988239859	0.99163959	0.991072024	0.992313026
R^2	0.976618018	0.983349077	0.982223757	0.984685142
$R^2_{adjusted}$	0.975059219	0.982239015	0.981038674	0.983664152
RSS	595.7293212	525.9575174	511.0731017	377.8205993

Graphical abstract of model fit presented as mean ± 1 SD of the fraction % of released carvedilol:

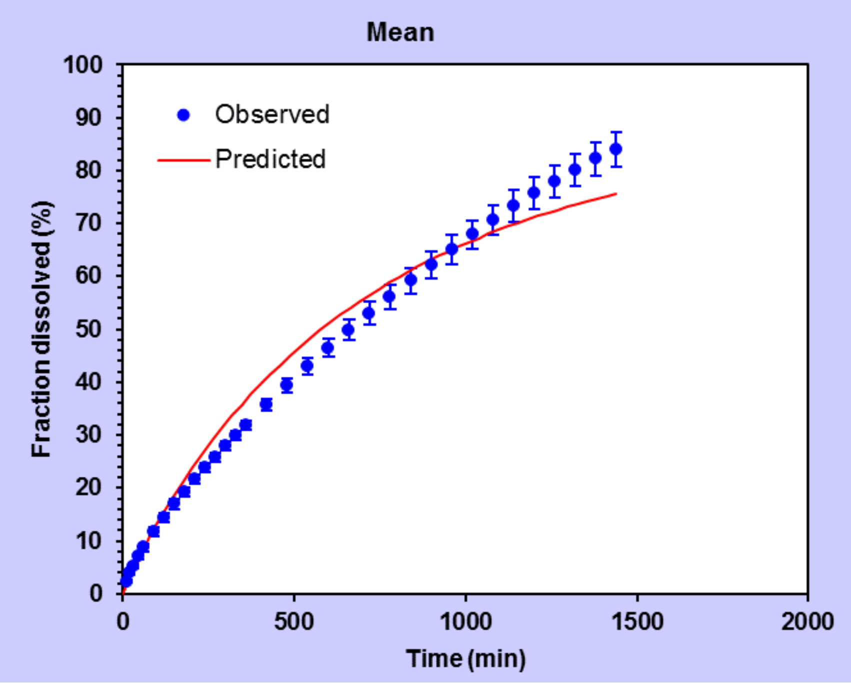

Supplementary materials – Model fitting summary – Avicel® PH-200 (modelling the entire relevant dissolution data)

Graphical abstract of model fit presented as the fraction % of released carvedilol per tested tablet:

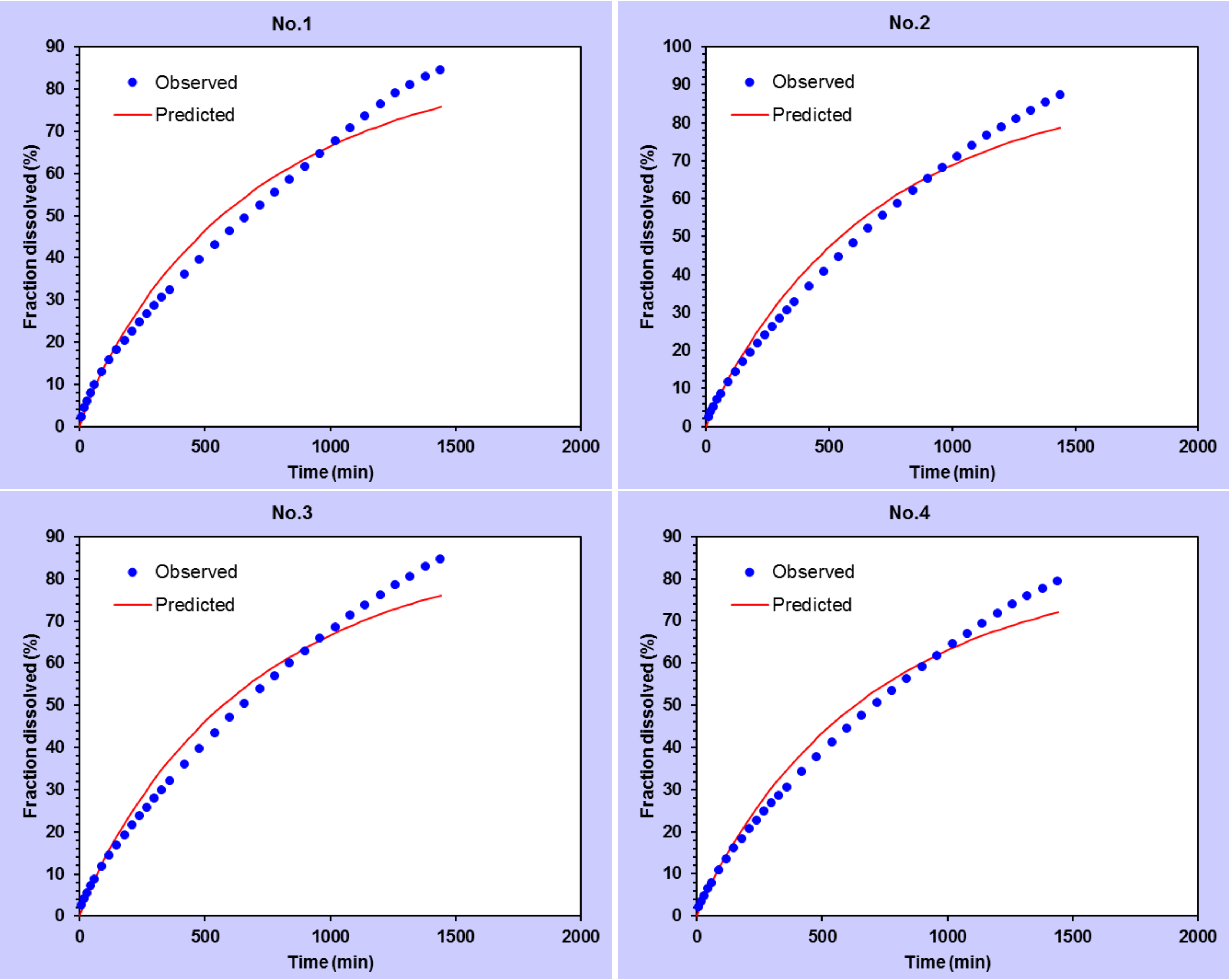

Supplementary materials – Model fitting summary – Avicel® PH-200 (modelling the entire relevant dissolution data)

Model: **Weibull_4**

Model equation: $F = F_{max} \cdot \left[1 - e^{-\frac{(t-T_i)^\beta}{\alpha}}\right]$

Fitted model parameters per tested tablet (N = 4) with statistics – mean, standard deviation (SD), and relative standard deviation expressed in % (RSD%) (output from DDSolver):

Parameter	No.1	No.2	No.3	No.4	Mean	SD	RSD(%)
α	262.201	332.426	298.998	333.873	306.874	33.860	11.034
β	0.850	0.885	0.869	0.887	0.873	0.017	1.998
T_i	6.000	6.000	6.000	6.000	6.000	0.000	0.000
F_{max}	88.807	91.579	88.795	83.377	88.139	3.434	3.896

Number of dissolution data points (N), degrees of freedom (df), and selected goodness of fit criteria – Pearson correlation coefficient (R), coefficient of determination (R^2), adjusted coefficient of determination ($R^2_{adjusted}$), and residual sum of squares (RSS) (manual calculation in MS Excel):

Parameter	No.1	No.2	No.3	No.4
N	33	33	33	33
df	29	29	29	29
R	0.986065756	0.989564884	0.988919923	0.990478863
R^2	0.972325676	0.97923866	0.977962615	0.981048378
$R^2_{adjusted}$	0.969462815	0.977090935	0.975682886	0.979087865
RSS	725.2052659	682.8280939	657.126744	488.3729211

Graphical abstract of model fit presented as mean ± 1 SD of the fraction % of released carvedilol:

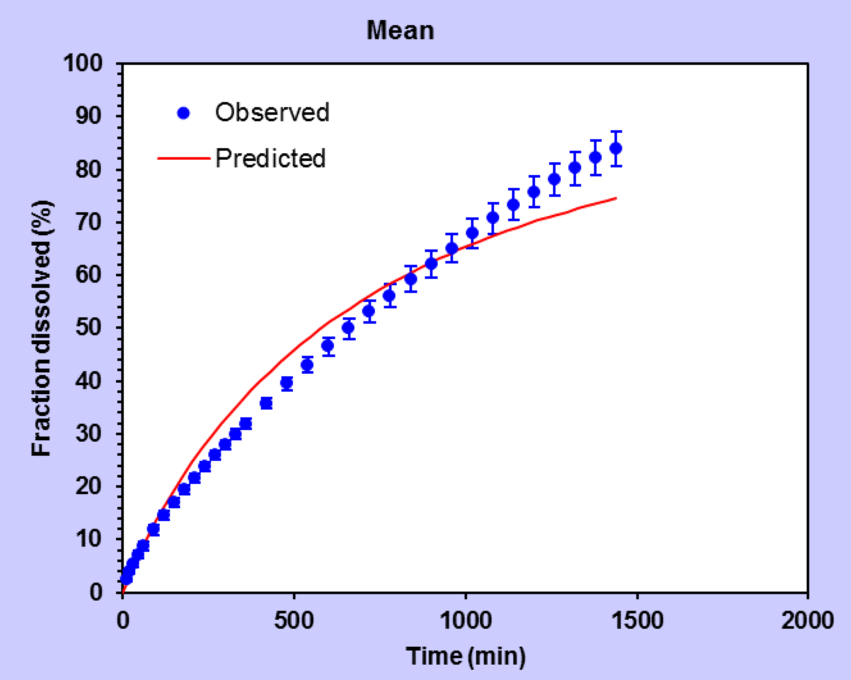

Supplementary materials – Model fitting summary – Avicel® PH-200 (modelling the entire relevant dissolution data)

Graphical abstract of model fit presented as the fraction % of released carvedilol per tested tablet:

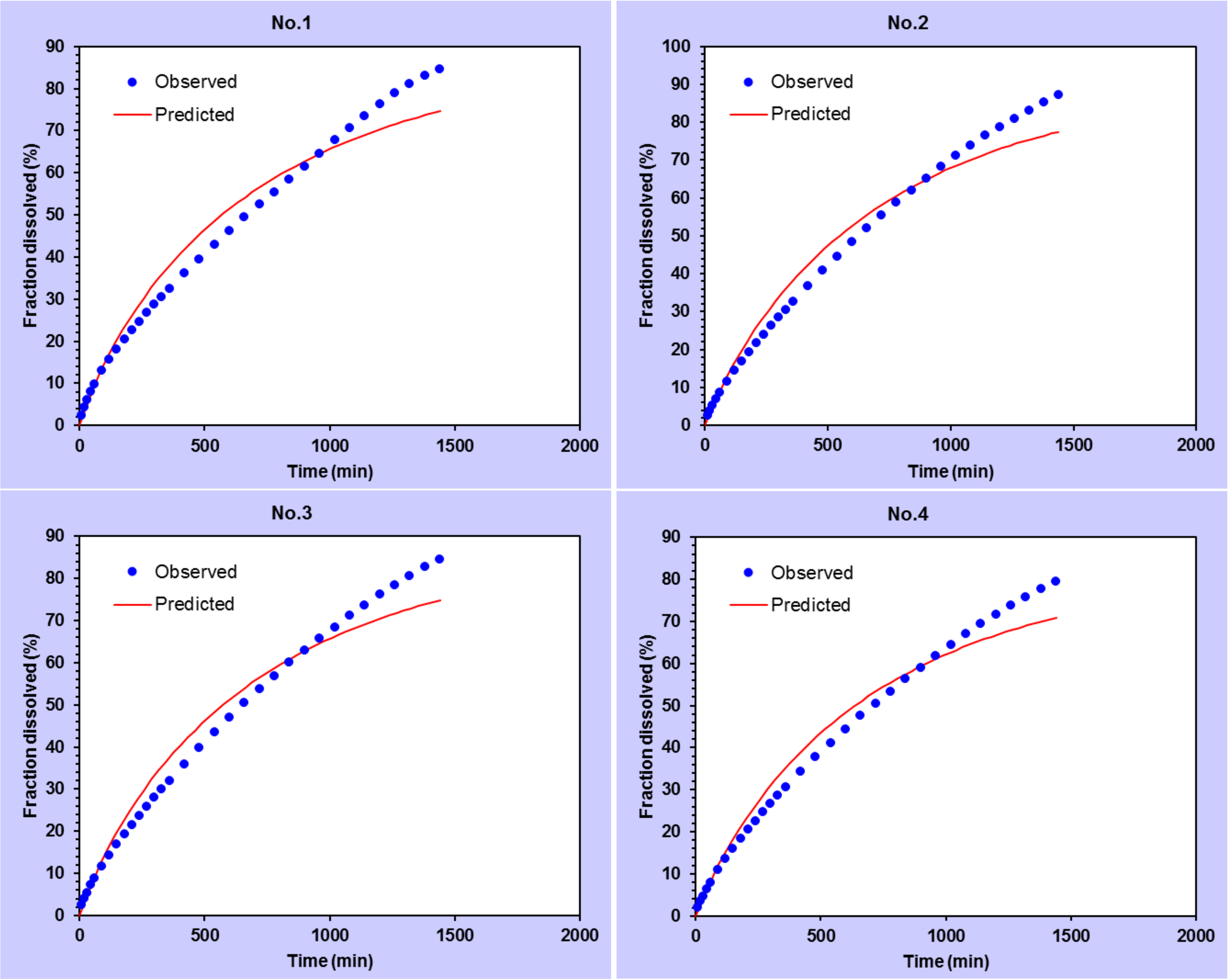

Supplementary materials – Model fitting summary – Avicel® PH-200 (modelling the entire relevant dissolution data)

Model: **Logistic_1**

Model equation: $F = 100 \cdot \frac{e^{\alpha+\beta\cdot\log(t)}}{1+e^{\alpha+\beta\cdot\log(t)}}$

Fitted model parameters per tested tablet (N = 4) with statistics – mean, standard deviation (SD), and relative standard deviation expressed in % (RSD%) (output from DDSolver):

Parameter	No.1	No.2	No.3	No.4	Mean	SD	RSD(%)
α	-7.324	-6.947	-7.501	-6.736	-7.127	0.348	-4.885
β	2.615	2.572	2.675	2.409	2.568	0.114	4.435

Number of dissolution data points (N), degrees of freedom (df), and selected goodness of fit criteria – Pearson correlation coefficient (R), coefficient of determination (R^2), adjusted coefficient of determination ($R^2_{adjusted}$), and residual sum of squares (RSS) (manual calculation in MS Excel):

Parameter	No.1	No.2	No.3	No.4
N	33	33	33	33
df	31	31	31	31
R	0.987949423	0.982035757	0.991088015	0.988383836
R^2	0.976044062	0.964394228	0.982255454	0.976902608
$R^2_{adjusted}$	0.975271289	0.963245654	0.981683049	0.976157531
RSS	849.1543936	1075.932486	793.7272598	583.7520789

Graphical abstract of model fit presented as mean ± 1 SD of the fraction % of released carvedilol:

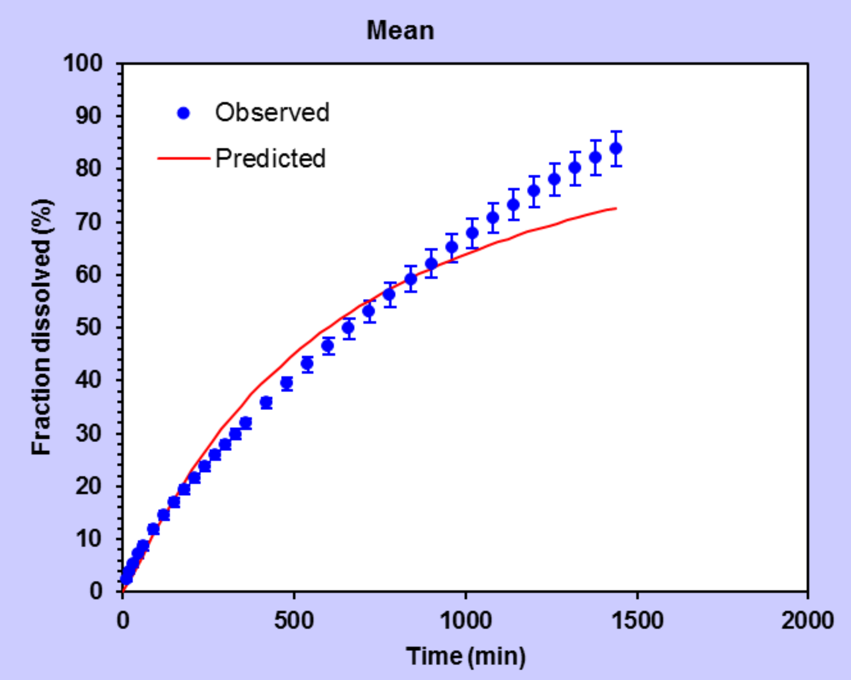

Supplementary materials – Model fitting summary – Avicel® PH-200 (modelling the entire relevant dissolution data)

Graphical abstract of model fit presented as the fraction % of released carvedilol per tested tablet:

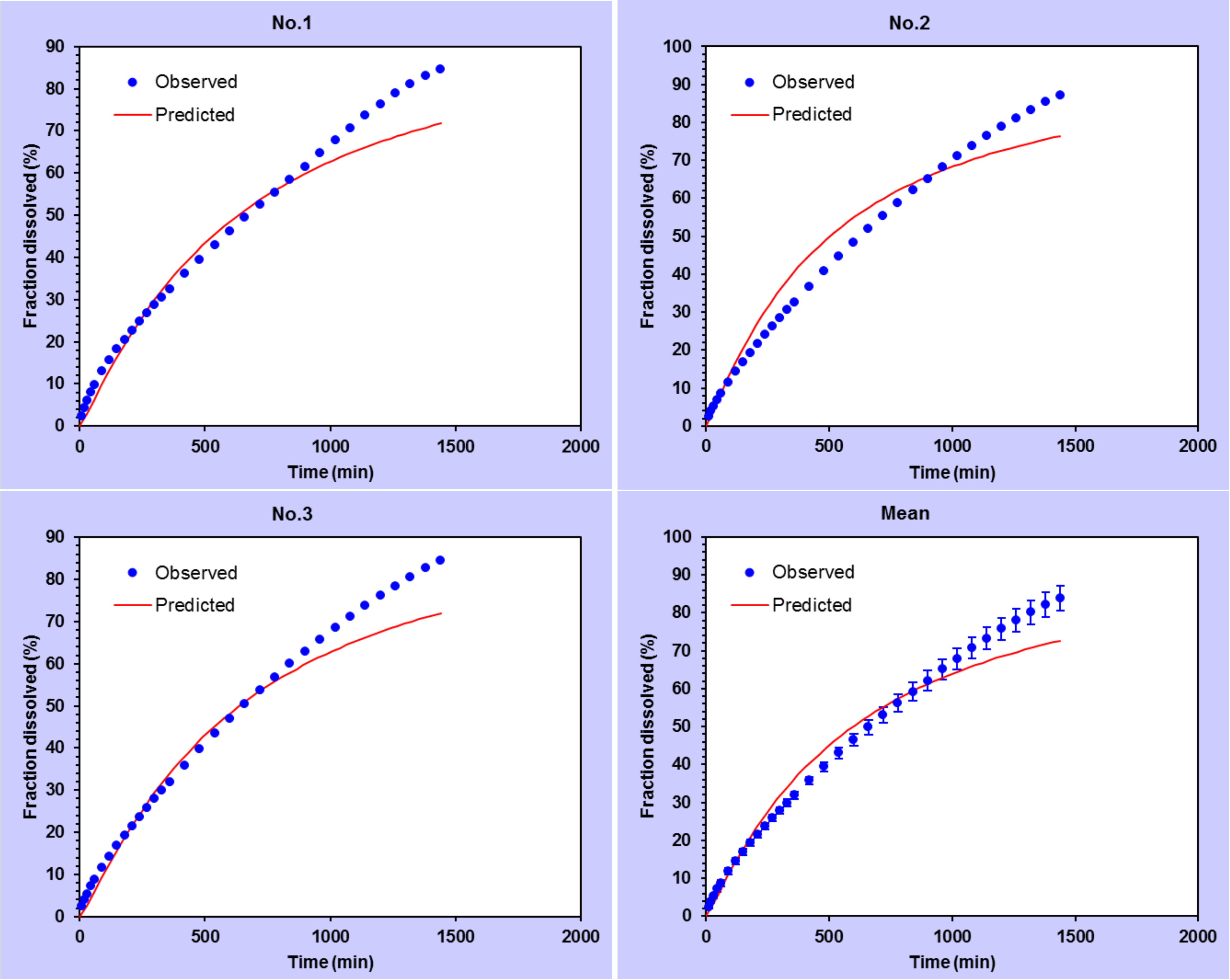

Supplementary materials – Model fitting summary – Avicel® PH-200 (modelling the entire relevant dissolution data)

Model: **Logistic_2**

Model equation: $F = F_{max} \cdot \frac{e^{\alpha+\beta\cdot\log(t)}}{1+e^{\alpha+\beta\cdot\log(t)}}$

Fitted model parameters per tested tablet (N = 4) with statistics – mean, standard deviation (SD), and relative standard deviation expressed in % (RSD%) (output from DDSolver):

Parameter	No.1	No.2	No.3	No.4	Mean	SD	RSD(%)
α	-8.166	-8.441	-8.297	-8.433	-8.334	0.130	-1.565
β	2.911	2.997	2.950	2.999	2.964	0.042	1.423
F_{max}	96.336	102.278	99.169	93.118	97.725	3.914	4.006

Number of dissolution data points (N), degrees of freedom (df), and selected goodness of fit criteria – Pearson correlation coefficient (R), coefficient of determination (R^2), adjusted coefficient of determination ($R^2_{adjusted}$), and residual sum of squares (RSS) (manual calculation in MS Excel):

Parameter	No.1	No.2	No.3	No.4
N	33	33	33	33
df	30	30	30	30
R	0.990769729	0.994528991	0.99391184	0.994707352
R^2	0.981624656	0.989087914	0.987860746	0.989442716
$R^2_{adjusted}$	0.980399633	0.988360441	0.987051462	0.988738897
RSS	1135.398561	850.3165295	778.2716277	676.1609559

Graphical abstract of model fit presented as mean ± 1 SD of the fraction % of released carvedilol:

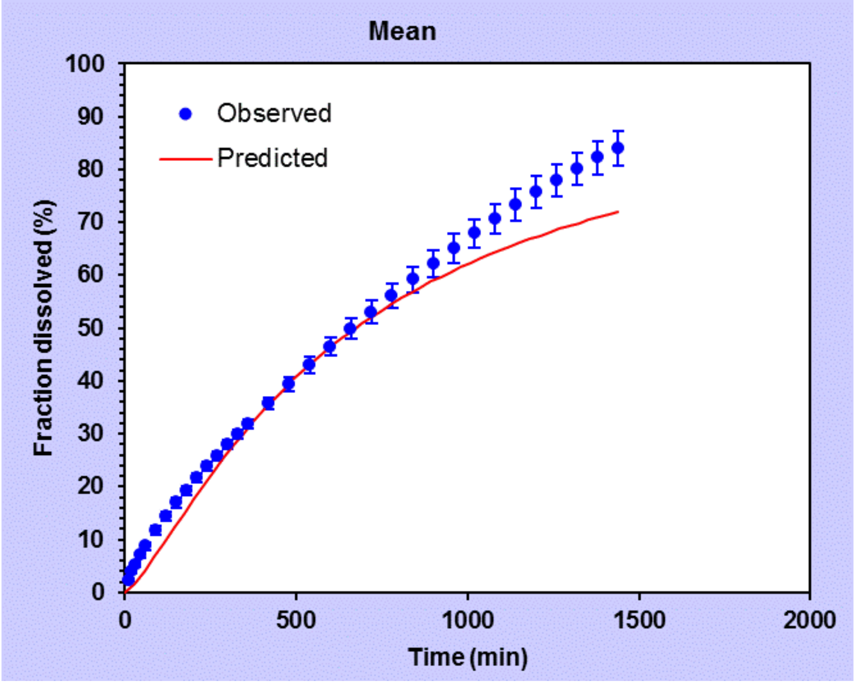

Supplementary materials – Model fitting summary – Avicel® PH-200 (modelling the entire relevant dissolution data)

Graphical abstract of model fit presented as the fraction % of released carvedilol per tested tablet:

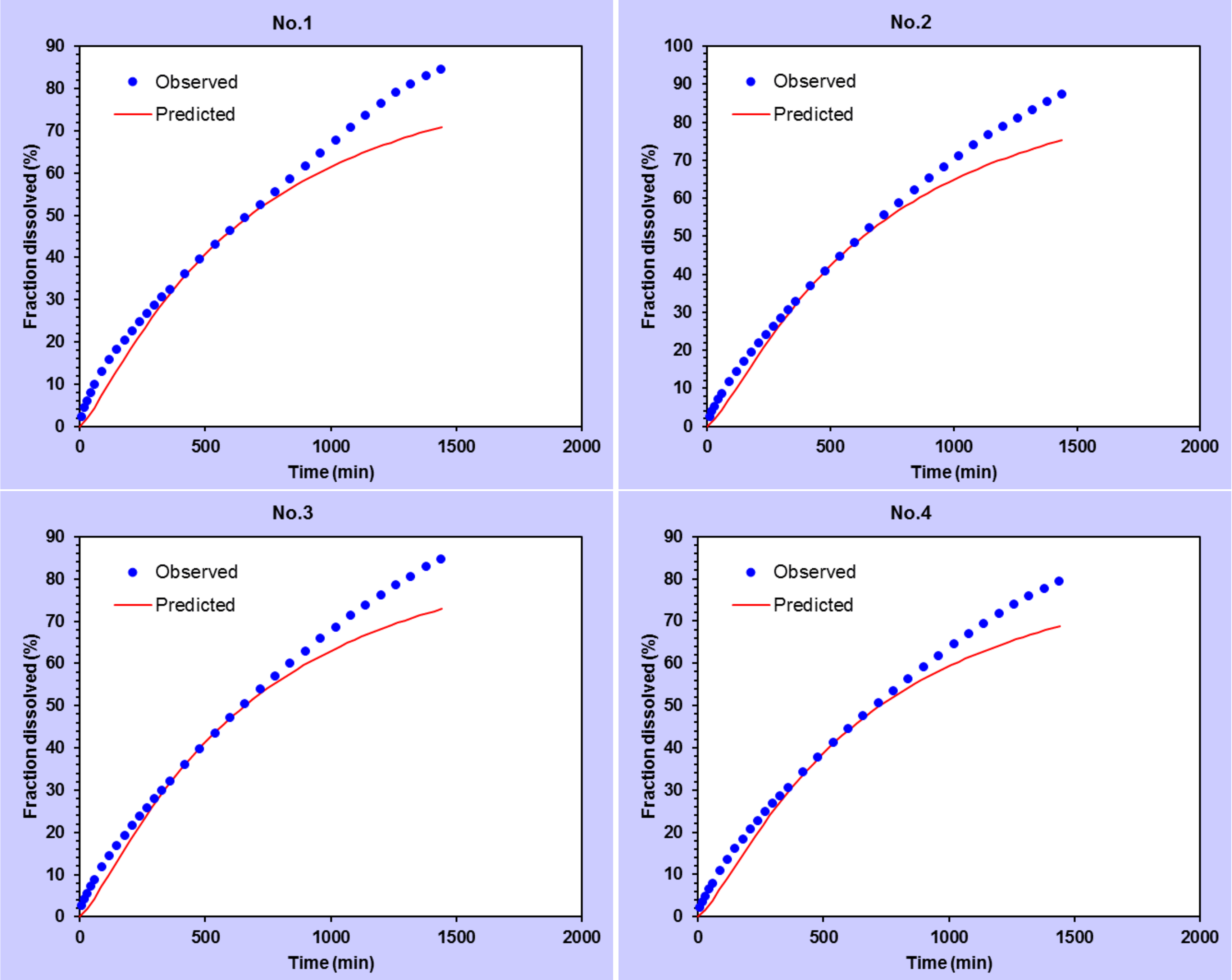

Supplementary materials – Model fitting summary – Avicel® PH-200 (modelling the entire relevant dissolution data)

Model: **Logistic_3**

Model equation: $F = F_{max} \cdot \frac{1}{1+e^{-k\cdot(t-\gamma)}}$

Fitted model parameters per tested tablet (N = 4) with statistics – mean, standard deviation (SD), and relative standard deviation expressed in % (RSD%) (output from DDSolver):

Parameter	No.1	No.2	No.3	No.4	Mean	SD	RSD(%)
k	0.003	0.003	0.003	0.003	0.003	0.000	1.470
γ	534.753	540.789	538.423	538.675	538.160	2.507	0.466
F_{max}	86.751	89.459	86.740	81.447	86.099	3.355	3.896

Number of dissolution data points (N), degrees of freedom (df), and selected goodness of fit criteria – Pearson correlation coefficient (R), coefficient of determination (R^2), adjusted coefficient of determination ($R^2_{adjusted}$), and residual sum of squares (RSS) (manual calculation in MS Excel):

Parameter	No.1	No.2	No.3	No.4
N	33	33	33	33
df	30	30	30	30
R	0.993047115	0.994626561	0.994345003	0.993205821
R^2	0.986142573	0.989281996	0.988721986	0.986457803
$R^2_{adjusted}$	0.985218745	0.988567463	0.987970118	0.98555499
RSS	569.9912786	547.773027	526.8839095	480.7161909

Graphical abstract of model fit presented as mean ± 1 SD of the fraction % of released carvedilol:

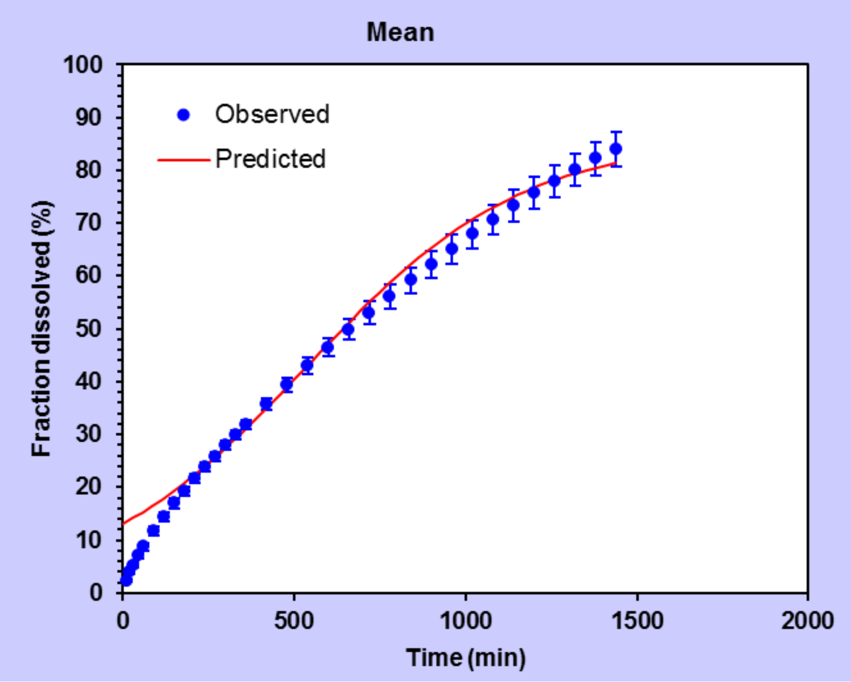

Supplementary materials – Model fitting summary – Avicel® PH-200 (modelling the entire relevant dissolution data)

Graphical abstract of model fit presented as the fraction % of released carvedilol per tested tablet:

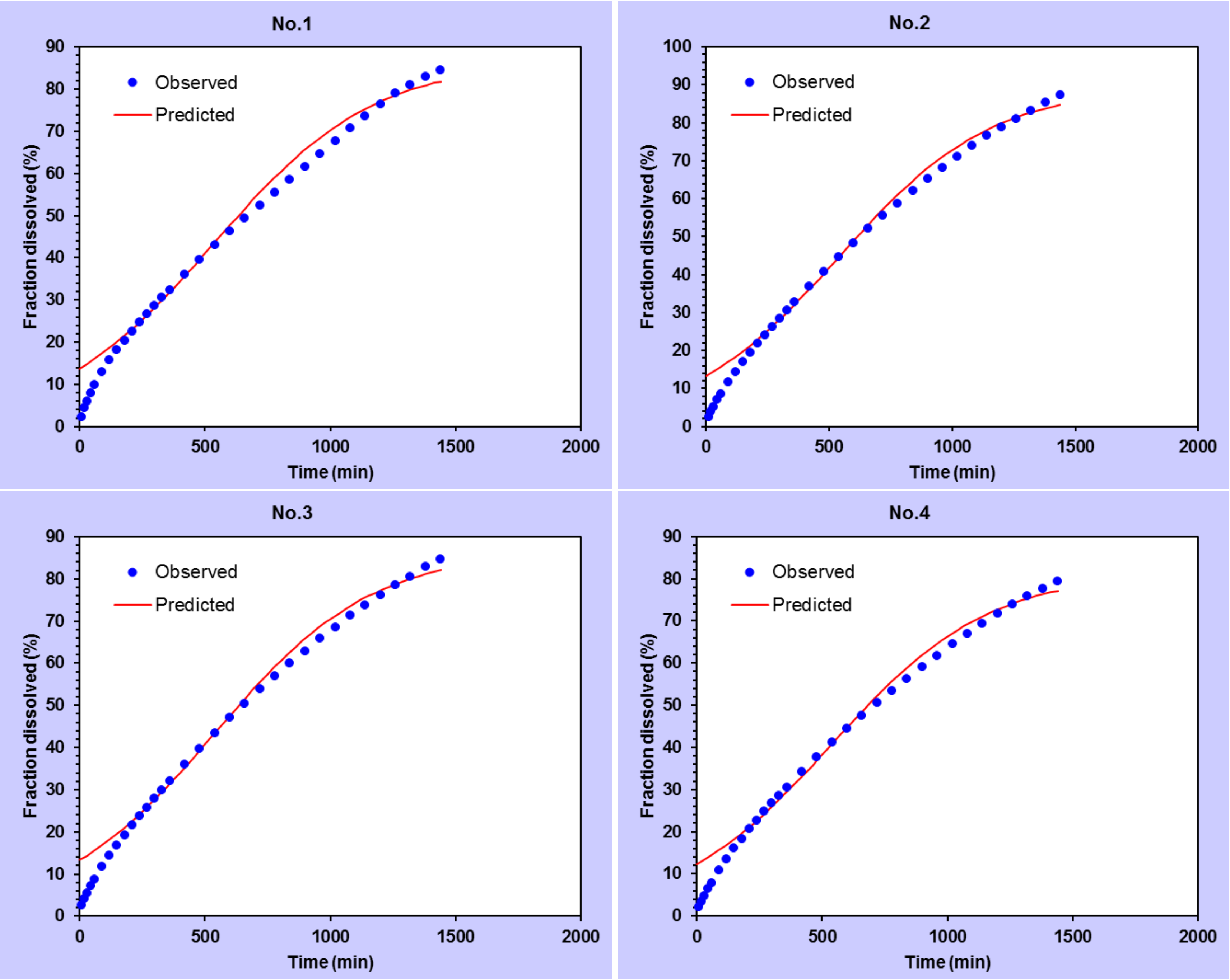

Supplementary materials – Model fitting summary – Avicel® PH-200 (modelling the entire relevant dissolution data)

Model: **Gompertz_1**

Model equation: $F = 100 \cdot e^{-\alpha \cdot e^{-\beta \cdot \log(t)}}$

Fitted model parameters per tested tablet (N = 4) with statistics – mean, standard deviation (SD), and relative standard deviation expressed in % (RSD%) (output from DDSolver):

Parameter	No.1	No.2	No.3	No.4	Mean	SD	RSD(%)
α	28.824	36.317	30.996	26.792	30.733	4.100	13.341
β	1.401	1.511	1.428	1.331	1.418	0.074	5.248

Number of dissolution data points (N), degrees of freedom (df), and selected goodness of fit criteria – Pearson correlation coefficient (R), coefficient of determination (R^2), adjusted coefficient of determination ($R^2_{adjusted}$), and residual sum of squares (RSS) (manual calculation in MS Excel):

Parameter	No.1	No.2	No.3	No.4
N	33	33	33	33
df	31	31	31	31
R	0.951689666	0.95223295	0.954849647	0.962890025
R^2	0.905713221	0.906747591	0.911737849	0.9271572
$R^2_{adjusted}$	0.902671712	0.903739449	0.908890683	0.924807432
RSS	2375.725423	2729.082302	2361.487805	1735.518769

Graphical abstract of model fit presented as mean ± 1 SD of the fraction % of released carvedilol:

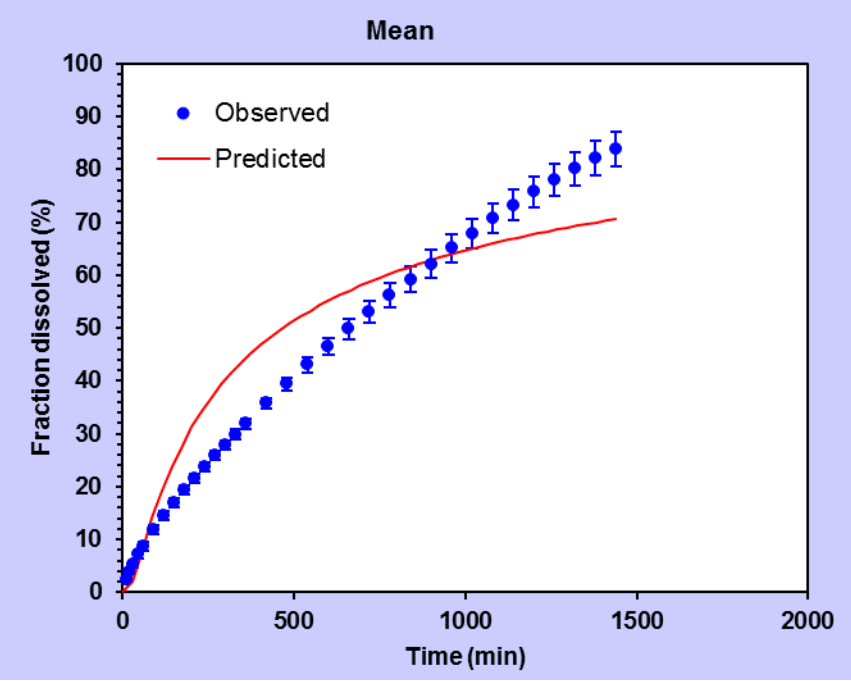

Supplementary materials – Model fitting summary – Avicel® PH-200 (modelling the entire relevant dissolution data)

Graphical abstract of model fit presented as the fraction % of released carvedilol per tested tablet:

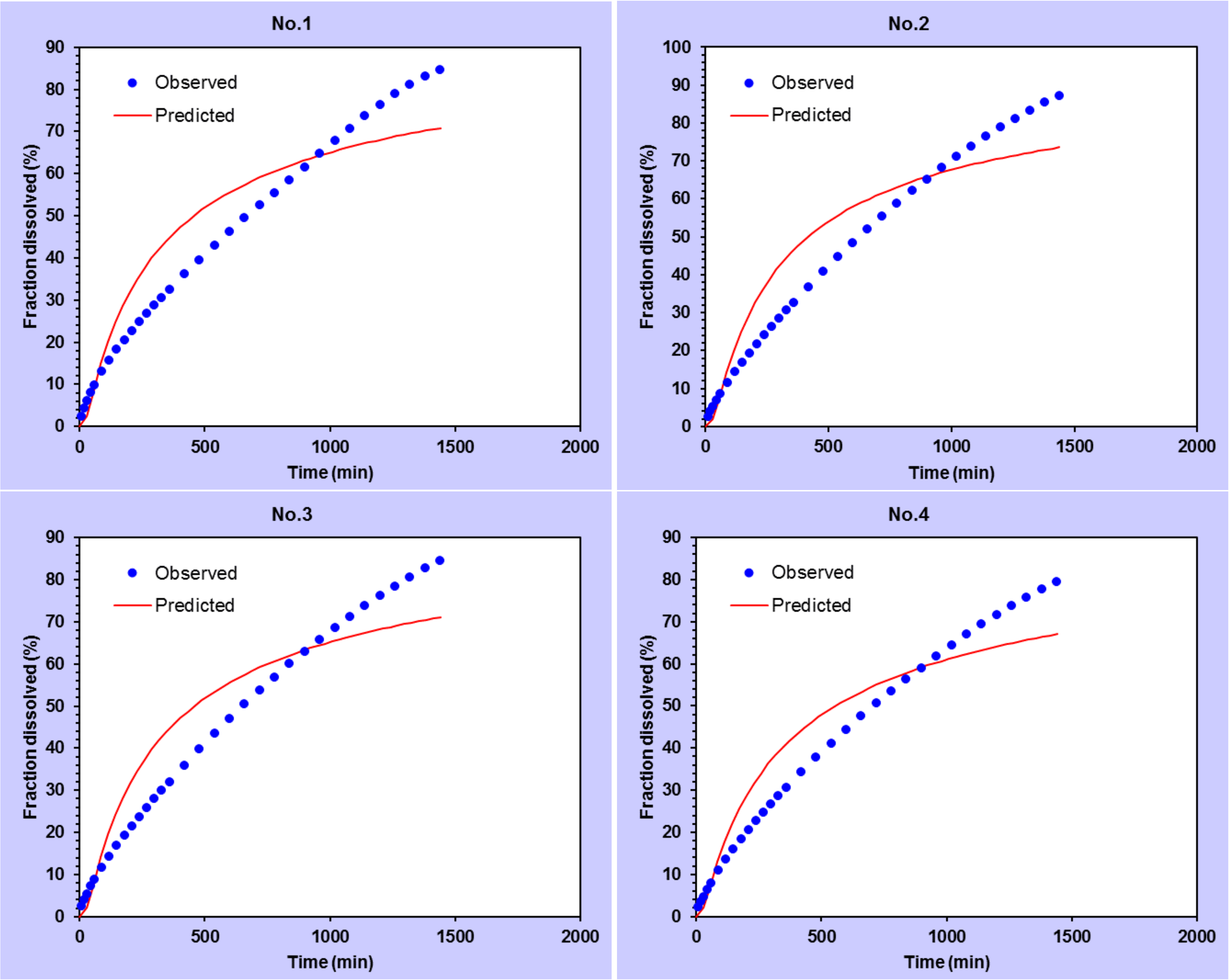

Supplementary materials – Model fitting summary – Avicel® PH-200 (modelling the entire relevant dissolution data)

Model: **Gompertz_2**

Model equation: $F = F_{max} \cdot e^{-\alpha \cdot e^{-\beta \cdot \log(t)}}$

Fitted model parameters per tested tablet (N = 4) with statistics – mean, standard deviation (SD), and relative standard deviation expressed in % (RSD%) (output from DDSolver):

Parameter	No.1	No.2	No.3	No.4	Mean	SD	RSD(%)
α	63.198	71.875	68.132	70.717	68.481	3.853	5.627
β	1.612	1.656	1.638	1.652	1.640	0.020	1.222
F_{max}	100.833	103.980	100.819	94.668	100.075	3.899	3.896

Number of dissolution data points (N), degrees of freedom (df), and selected goodness of fit criteria – Pearson correlation coefficient (R), coefficient of determination (R^2), adjusted coefficient of determination ($R^2_{adjusted}$), and residual sum of squares (RSS) (manual calculation in MS Excel):

Parameter	No.1	No.2	No.3	No.4
N	33	33	33	33
df	30	30	30	30
R	0.975121289	0.979648398	0.979013811	0.980772552
R^2	0.950861527	0.959710984	0.958468042	0.961914798
$R^2_{adjusted}$	0.947585629	0.95702505	0.955699245	0.959375785
RSS	1561.53317	1596.211156	1496.574171	1259.405767

Graphical abstract of model fit presented as mean ± 1 SD of the fraction % of released carvedilol:

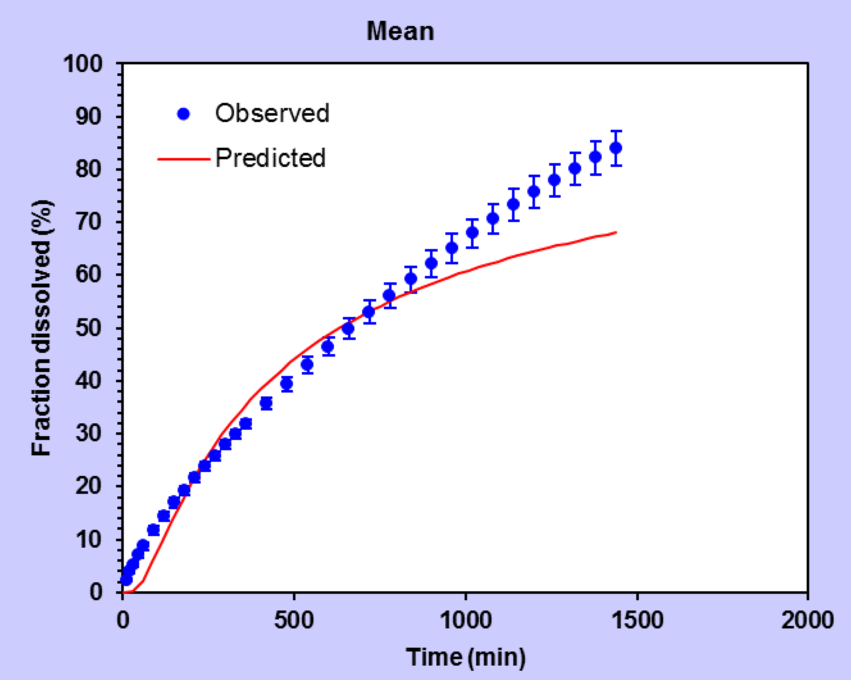

Supplementary materials – Model fitting summary – Avicel® PH-200 (modelling the entire relevant dissolution data)

Graphical abstract of model fit presented as the fraction % of released carvedilol per tested tablet:

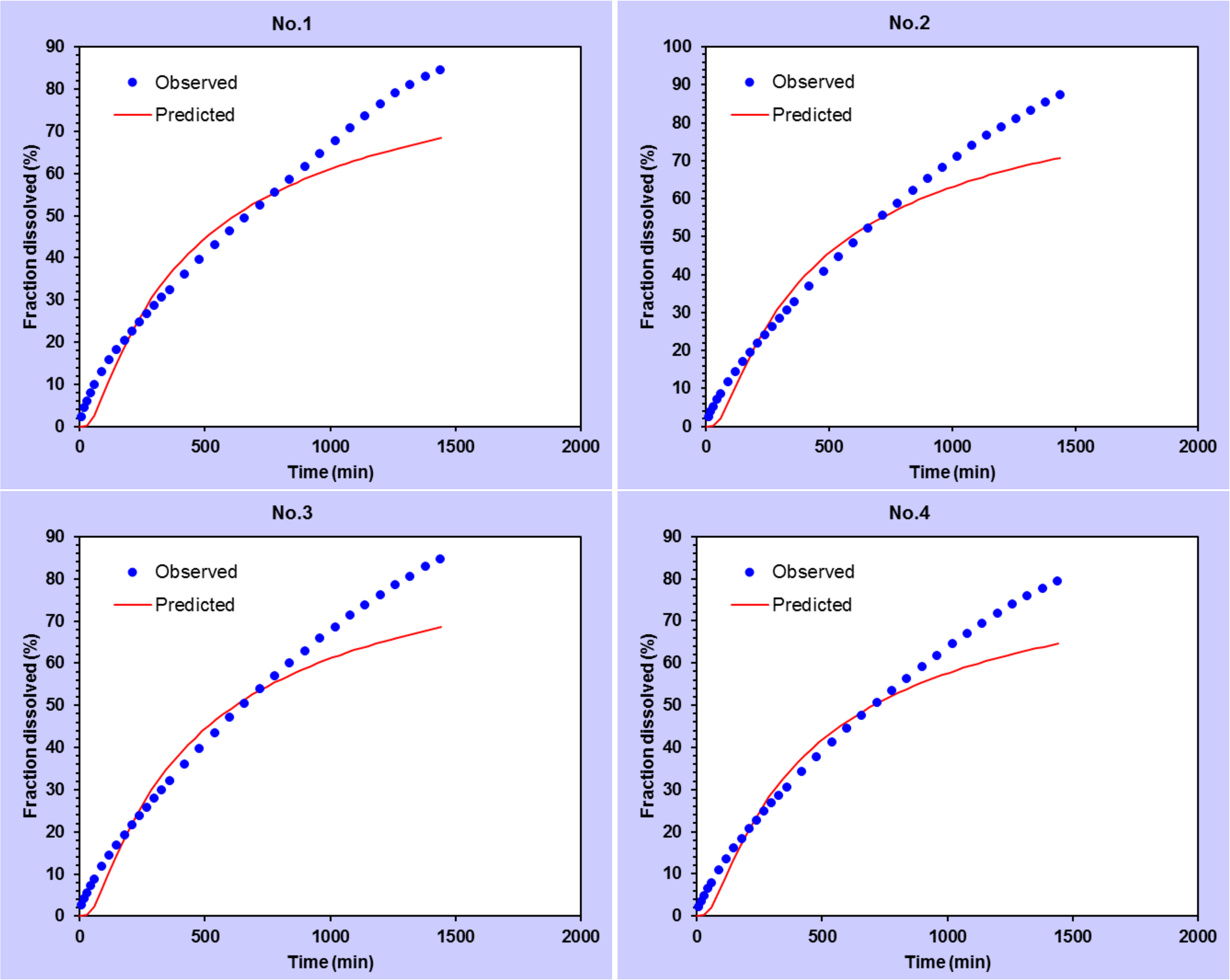

Supplementary materials – Model fitting summary – Avicel® PH-200 (modelling the entire relevant dissolution data)

Model: **Gompertz_3**

Model equation: $F = F_{max} \cdot e^{-e^{-k\cdot(t-\gamma)}}$

Fitted model parameters per tested tablet (N = 4) with statistics – mean, standard deviation (SD), and relative standard deviation expressed in % (RSD%) (output from DDSolver):

Parameter	No.1	No.2	No.3	No.4	Mean	SD	RSD(%)
k	0.002	0.003	0.002	0.002	0.002	0.000	0.979
γ	400.673	408.098	405.042	404.625	404.610	3.047	0.753
F_{max}	88.807	91.579	88.795	83.377	88.139	3.434	3.896

Number of dissolution data points (N), degrees of freedom (df), and selected goodness of fit criteria – Pearson correlation coefficient (R), coefficient of determination (R^2), adjusted coefficient of determination ($R^2_{adjusted}$), and residual sum of squares (RSS) (manual calculation in MS Excel):

Parameter	No.1	No.2	No.3	No.4
N	33	33	33	33
df	30	30	30	30
R	0.99382209	0.996235998	0.995958895	0.995198681
R^2	0.987682347	0.992486164	0.991934121	0.990420414
$R^2_{adjusted}$	0.98686117	0.991985241	0.991396396	0.989781775
RSS	329.8490835	222.7636828	221.2390596	229.4994907

Graphical abstract of model fit presented as mean ± 1 SD of the fraction % of released carvedilol:

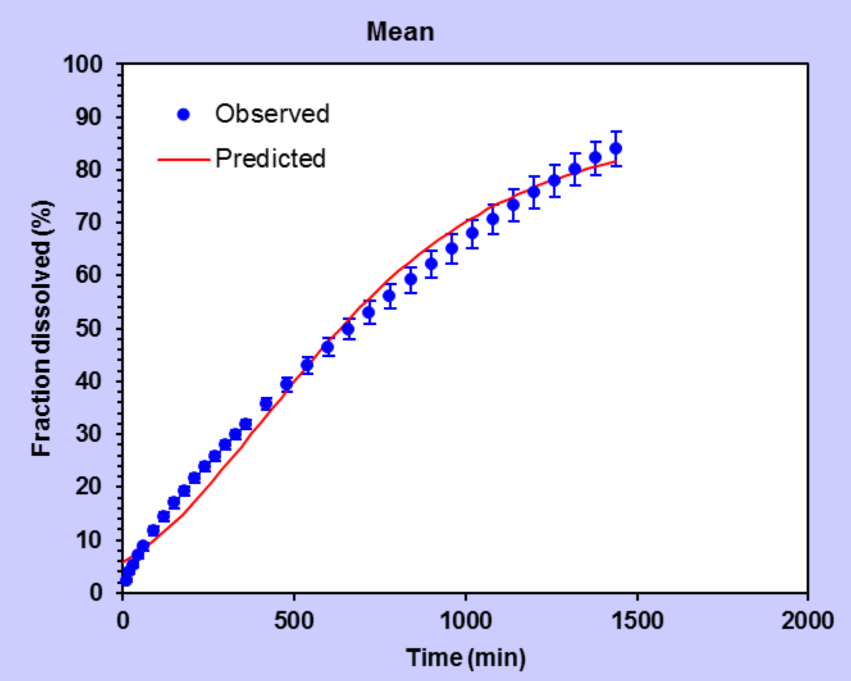

Supplementary materials – Model fitting summary – Avicel® PH-200 (modelling the entire relevant dissolution data)

Graphical abstract of model fit presented as the fraction % of released carvedilol per tested tablet:

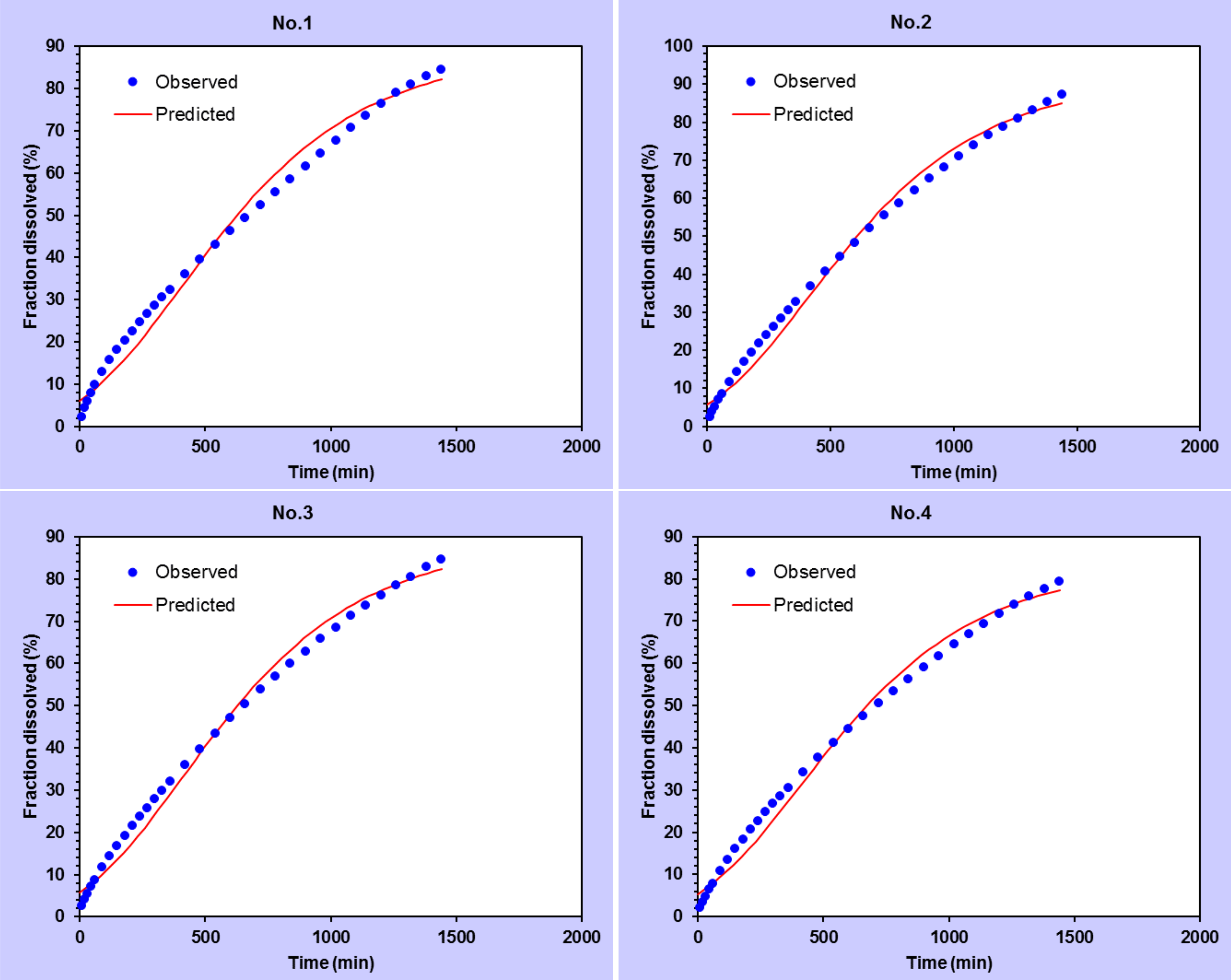

Supplementary materials – Model fitting summary – Avicel® PH-200 (modelling the entire relevant dissolution data)

Model: **Gompertz_4**

Model equation: $F = F_{max} \cdot e^{-\beta \cdot e^{-k \cdot t}}$

Fitted model parameters per tested tablet (N = 4) with statistics – mean, standard deviation (SD), and relative standard deviation expressed in % (RSD%) (output from DDSolver):

Parameter	No.1	No.2	No.3	No.4	Mean	SD	RSD(%)
k	0.002	0.003	0.002	0.002	0.002	0.000	0.979
β	2.671	2.786	2.735	2.741	2.733	0.047	1.725
F_{max}	88.807	91.579	88.795	83.377	88.139	3.434	3.896

Number of dissolution data points (N), degrees of freedom (df), and selected goodness of fit criteria – Pearson correlation coefficient (R), coefficient of determination (R^2), adjusted coefficient of determination ($R^2_{adjusted}$), and residual sum of squares (RSS) (manual calculation in MS Excel):

Parameter	No.1	No.2	No.3	No.4
N	33	33	33	33
df	30	30	30	30
R	0.99382209	0.996235998	0.995958895	0.995198681
R^2	0.987682347	0.992486164	0.991934121	0.990420414
$R^2_{adjusted}$	0.98686117	0.991985241	0.991396396	0.989781775
RSS	329.8490835	222.7636828	221.2390596	229.4994907

Graphical abstract of model fit presented as mean ± 1 SD of the fraction % of released carvedilol:

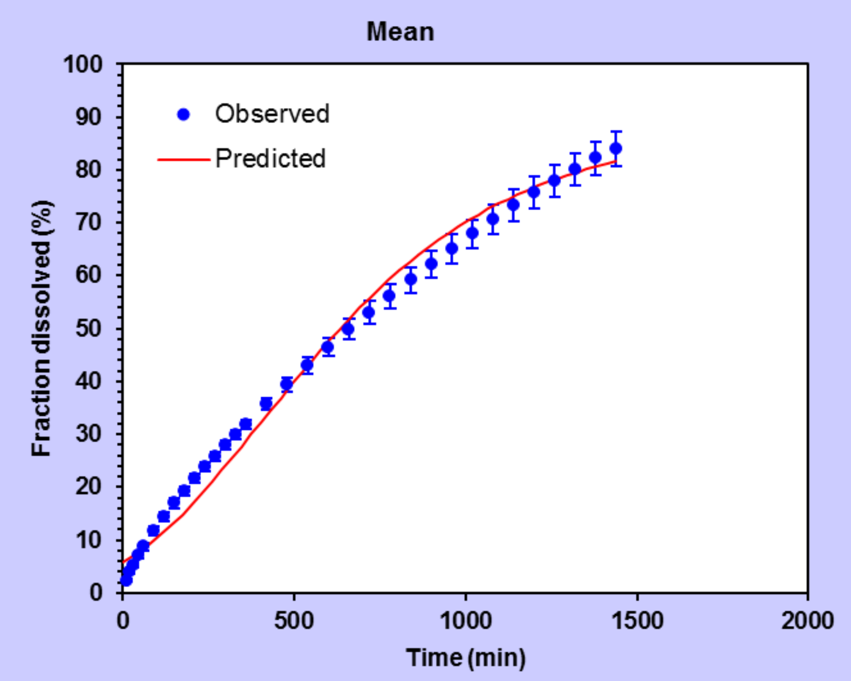

Supplementary materials – Model fitting summary – Avicel® PH-200 (modelling the entire relevant dissolution data)

Graphical abstract of model fit presented as the fraction % of released carvedilol per tested tablet:

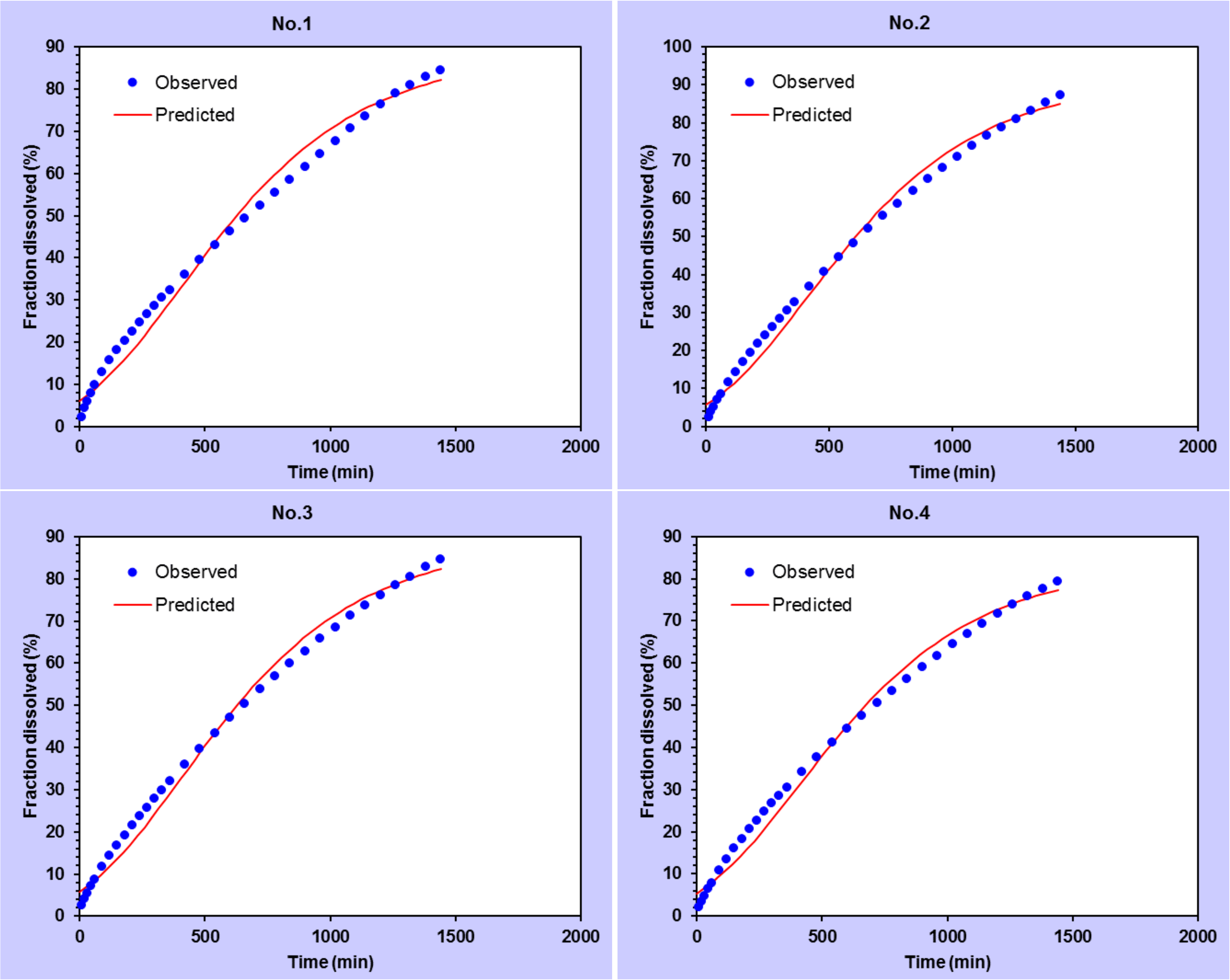

Supplementary materials – Model fitting summary – Avicel® PH-200 (modelling the entire relevant dissolution data)

Model: **Probit_1**

Model equation: $F = 100 \cdot \phi[\alpha + \beta \cdot \log(t)]$

Fitted model parameters per tested tablet (N = 4) with statistics – mean, standard deviation (SD), and relative standard deviation expressed in % (RSD%) (output from DDSolver):

Parameter	No.1	No.2	No.3	No.4	Mean	SD	RSD(%)
α	-3.770	-4.559	-3.857	-3.819	-4.001	0.374	-9.337
β	1.383	1.628	1.413	1.367	1.448	0.122	8.407

Number of dissolution data points (N), degrees of freedom (df), and selected goodness of fit criteria – Pearson correlation coefficient (R), coefficient of determination (R^2), adjusted coefficient of determination ($R^2_{adjusted}$), and residual sum of squares (RSS) (manual calculation in MS Excel):

Parameter	No.1	No.2	No.3	No.4
N	33	33	33	33
df	31	31	31	31
R	0.974788103	0.988907603	0.977844954	0.982808357
R^2	0.950211845	0.977938248	0.956180755	0.965912267
$R^2_{adjusted}$	0.948605776	0.977226578	0.954767231	0.964812663
RSS	1292.84556	1341.512085	1239.852792	877.1914029

Graphical abstract of model fit presented as mean ± 1 SD of the fraction % of released carvedilol:

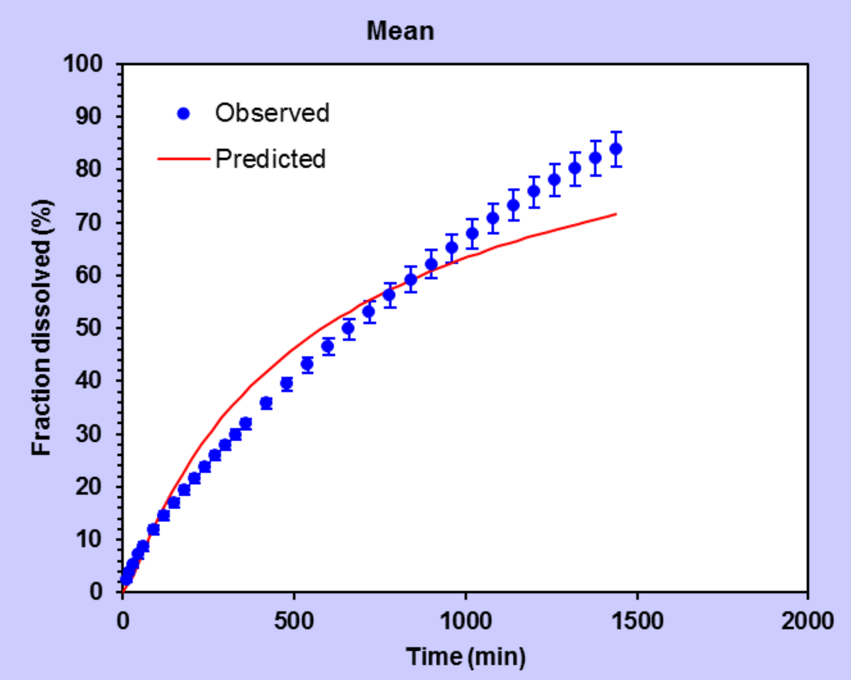

Supplementary materials – Model fitting summary – Avicel® PH-200 (modelling the entire relevant dissolution data)

Graphical abstract of model fit presented as the fraction % of released carvedilol per tested tablet:

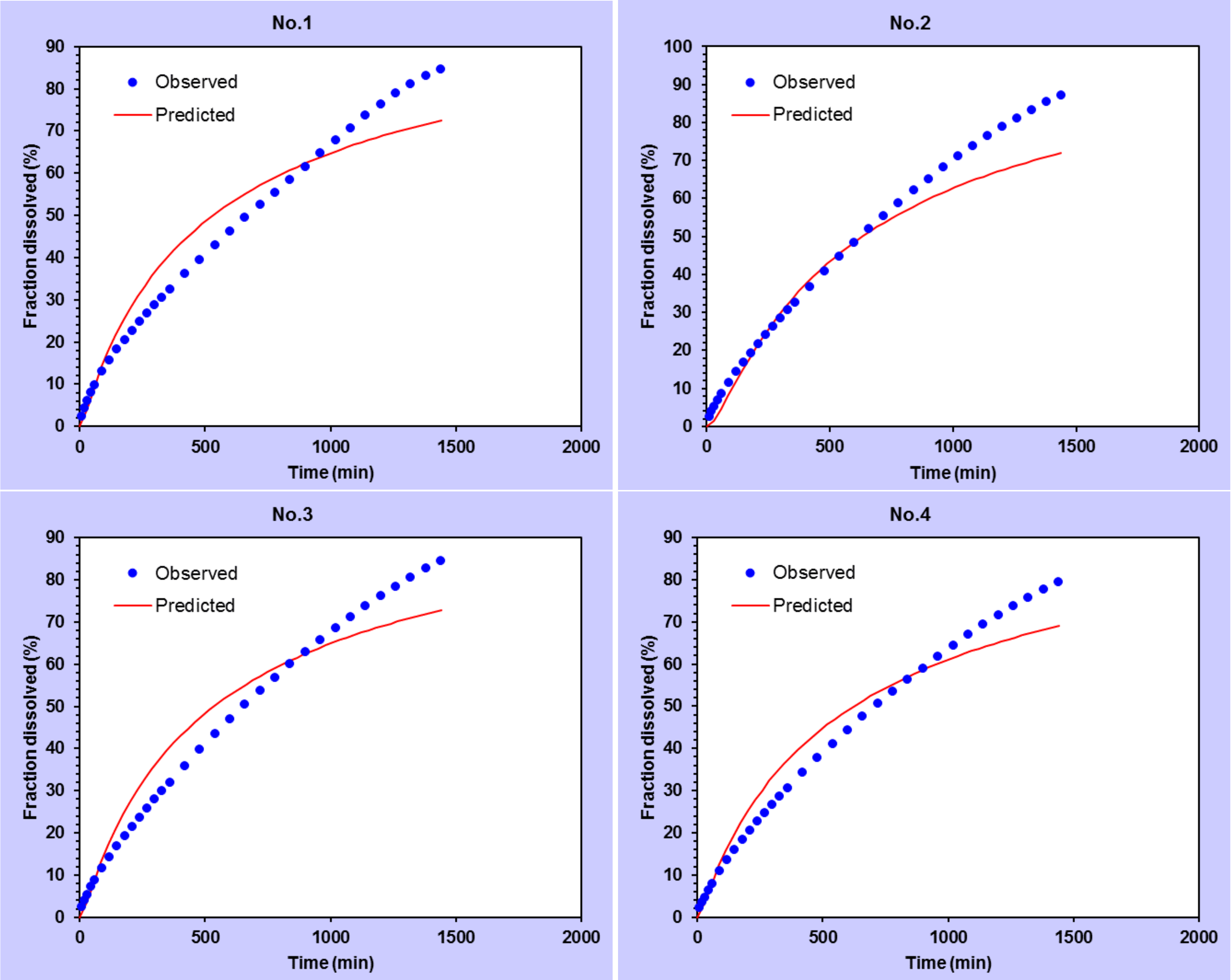

Supplementary materials – Model fitting summary – Avicel® PH-200 (modelling the entire relevant dissolution data)

Model: **Probit_2**

Model equation: $F = F_{max} \cdot \phi[\alpha + \beta \cdot \log(t)]$

Fitted model parameters per tested tablet (N = 4) with statistics – mean, standard deviation (SD), and relative standard deviation expressed in % (RSD%) (output from DDSolver):

Parameter	No.1	No.2	No.3	No.4	Mean	SD	RSD(%)
α	-4.679	-4.823	-4.751	-4.812	-4.766	0.066	-1.391
β	1.668	1.714	1.690	1.713	1.696	0.022	1.271
F_{max}	96.336	102.278	99.169	93.118	97.725	3.914	4.006

Number of dissolution data points (N), degrees of freedom (df), and selected goodness of fit criteria – Pearson correlation coefficient (R), coefficient of determination (R^2), adjusted coefficient of determination ($R^2_{adjusted}$), and residual sum of squares (RSS) (manual calculation in MS Excel):

Parameter	No.1	No.2	No.3	No.4
N	33	33	33	33
df	30	30	30	30
R	0.987590488	0.991411517	0.990758554	0.991966786
R^2	0.975334972	0.982896796	0.981602512	0.983998104
$R^2_{adjusted}$	0.973690636	0.981756583	0.980376013	0.982931311
RSS	1345.014787	1054.083995	970.1107458	819.6406253

Graphical abstract of model fit presented as mean ± 1 SD of the fraction % of released carvedilol:

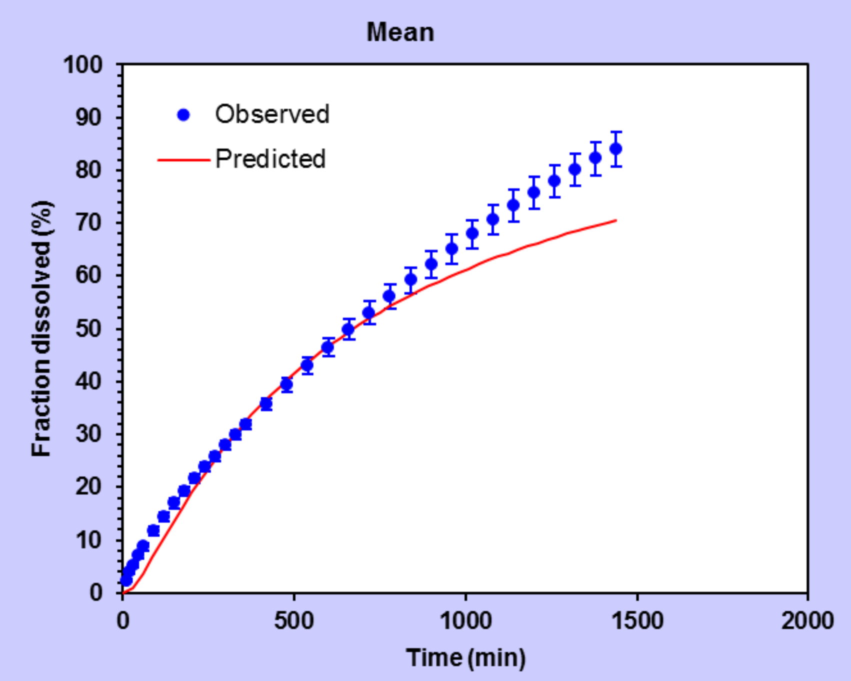

Supplementary materials – Model fitting summary – Avicel® PH-200 (modelling the entire relevant dissolution data)

Graphical abstract of model fit presented as the fraction % of released carvedilol per tested tablet:

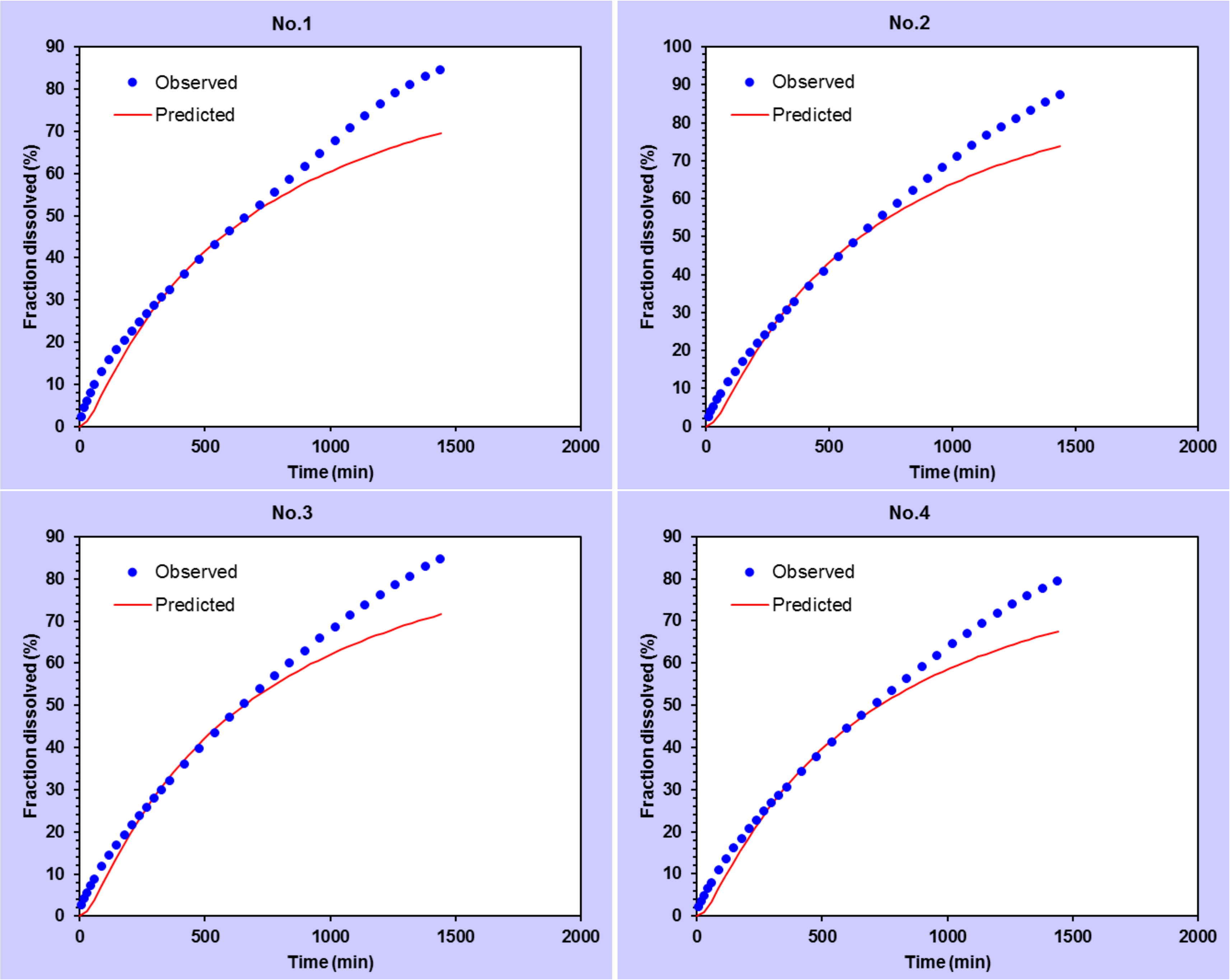

Supplementary materials – Model fitting summary – Avicel® PH-200 (modelling up to app. 60 % of carvedilol released)

Model: **Zero–order**

Model equation: $F = k_0 \cdot t$

Fitted model parameters per tested tablet (N = 4) with statistics – mean, standard deviation (SD), and relative standard deviation expressed in % (RSD%) (output from DDSolver):

Parameter	No.1	No.2	No.3	No.4	Mean	SD	RSD(%)
k_0	0.078	0.081	0.079	0.075	0.078	0.003	3.515

Number of dissolution data points (N), degrees of freedom (df), and selected goodness of fit criteria – Pearson correlation coefficient (R), coefficient of determination (R^2), adjusted coefficient of determination ($R^2_{adjusted}$), and residual sum of squares (RSS) (manual calculation in MS Excel):

Parameter	No.1	No.2	No.3	No.4
N	23	23	23	23
df	22	22	22	22
R	0.991400504	0.995560267	0.995003294	0.993278134
R^2	0.982874959	0.991140244	0.990031555	0.986601452
$R^2_{adjusted}$	0.982874959	0.991140244	0.990031555	0.986601452
RSS	517.1085183	309.9314252	339.6648027	323.5540509

Graphical abstract of model fit presented as mean ± 1 SD of the fraction % of released carvedilol:

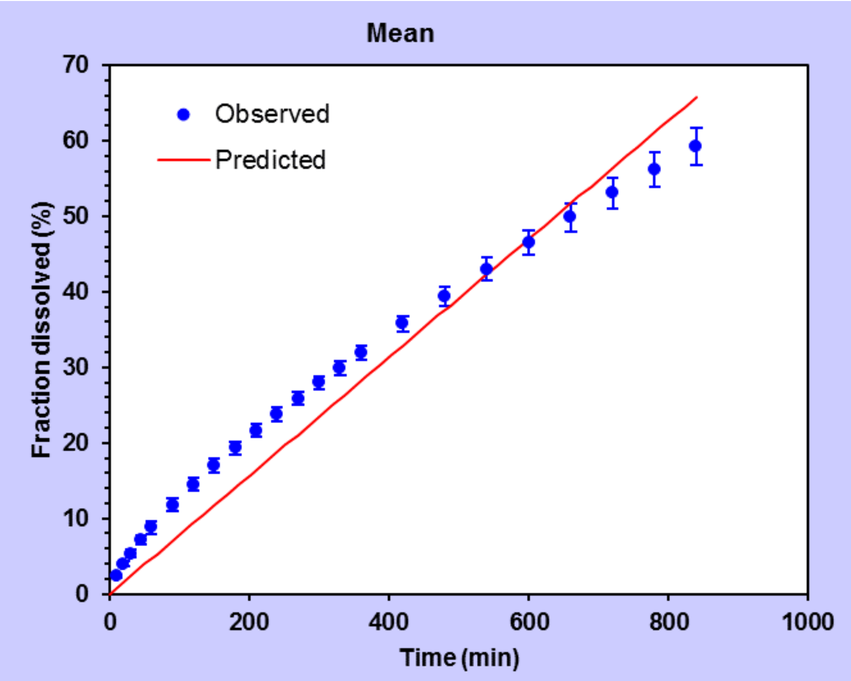

Supplementary materials – Model fitting summary – Avicel® PH-200 (modelling up to app. 60 % of carvedilol released)

Graphical abstract of model fit presented as the fraction % of released carvedilol per tested tablet:

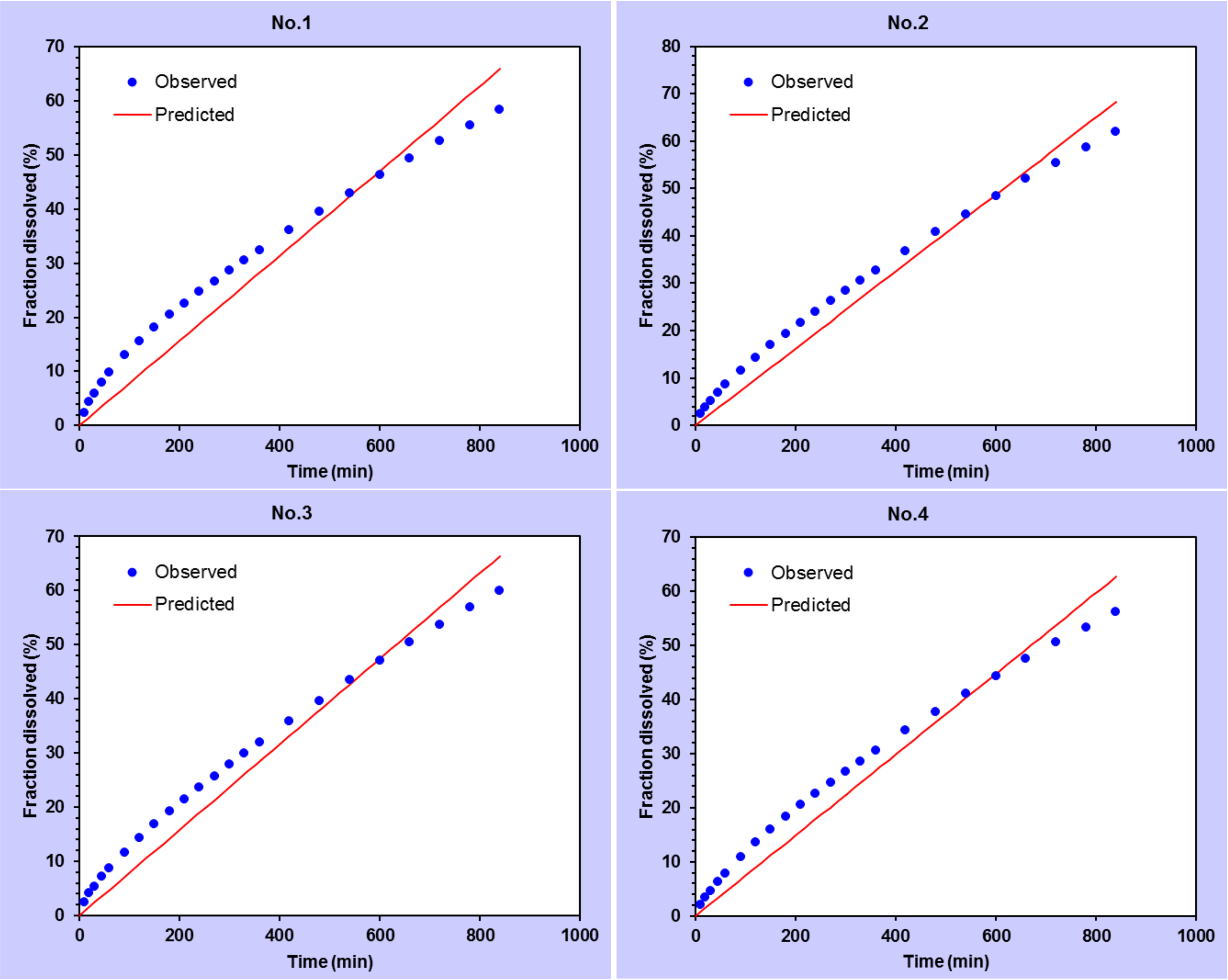

Supplementary materials – Model fitting summary – Avicel® PH-200 (modelling up to app. 60 % of carvedilol released)

Model: **Zero–order with T_{lag}**

Model equation: $F = k_0 \cdot (t - T_{lag})$

Fitted model parameters per tested tablet (N = 4) with statistics – mean, standard deviation (SD), and relative standard deviation expressed in % (RSD%) (output from DDSolver):

Parameter	No.1	No.2	No.3	No.4	Mean	SD	RSD(%)
k_0	0.065	0.071	0.069	0.065	0.067	0.003	4.469
T_{lag}	-103.724	-73.545	-80.665	-80.386	-84.580	13.181	-15.584

Number of dissolution data points (N), degrees of freedom (df), and selected goodness of fit criteria – Pearson correlation coefficient (R), coefficient of determination (R^2), adjusted coefficient of determination ($R^2_{adjusted}$), and residual sum of squares (RSS) (manual calculation in MS Excel):

Parameter	No.1	No.2	No.3	No.4
N	23	23	23	23
df	21	21	21	21
R	0.991400504	0.995560267	0.995003294	0.993278134
R^2	0.982874959	0.991140244	0.990031555	0.986601452
$R^2_{adjusted}$	0.982059481	0.990718351	0.989556867	0.985963426
RSS	111.7193355	68.03065885	70.79872141	85.2325952

Graphical abstract of model fit presented as mean ± 1 SD of the fraction % of released carvedilol:

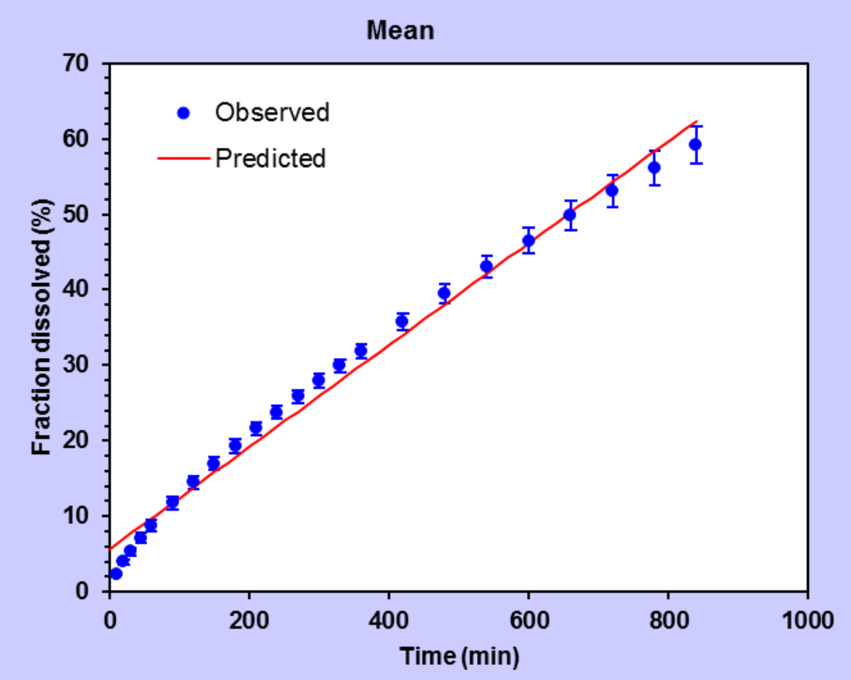

Supplementary materials – Model fitting summary – Avicel® PH-200 (modelling up to app. 60 % of carvedilol released)

Graphical abstract of model fit presented as the fraction % of released carvedilol per tested tablet:

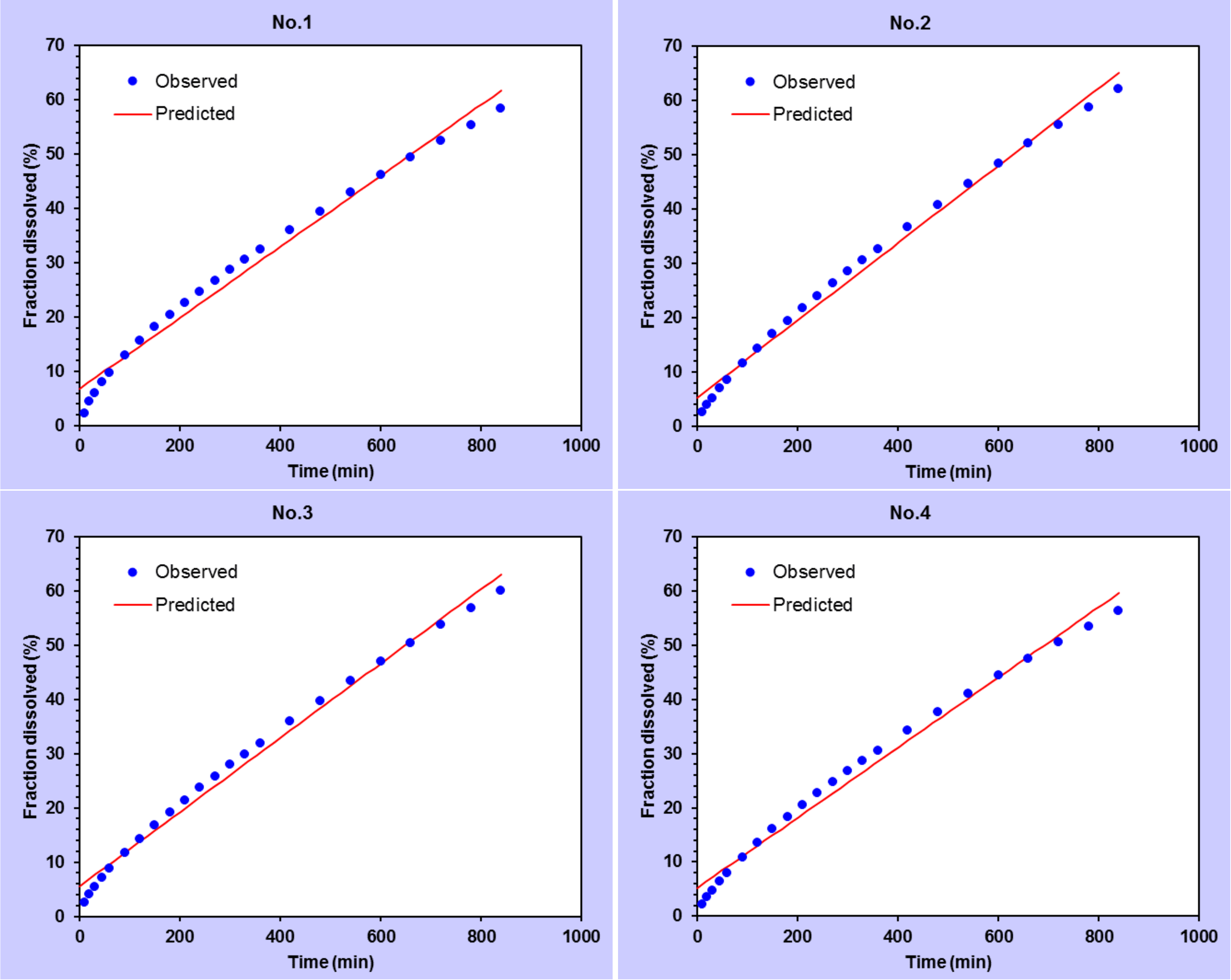

Supplementary materials – Model fitting summary – Avicel® PH-200 (modelling up to app. 60 % of carvedilol released)

Model: **Zero–order with F_0**

Model equation: $F = F_0 + k_0 \cdot t$

Fitted model parameters per tested tablet (N = 4) with statistics – mean, standard deviation (SD), and relative standard deviation expressed in % (RSD%) (output from DDSolver):

Parameter	No.1	No.2	No.3	No.4	Mean	SD	RSD(%)
k_0	0.065	0.071	0.069	0.065	0.067	0.003	4.469
F_0	6.787	5.243	5.527	5.204	5.690	0.745	13.097

Number of dissolution data points (N), degrees of freedom (df), and selected goodness of fit criteria – Pearson correlation coefficient (R), coefficient of determination (R^2), adjusted coefficient of determination ($R^2_{adjusted}$), and residual sum of squares (RSS) (manual calculation in MS Excel):

Parameter	No.1	No.2	No.3	No.4
N	23	23	23	23
df	21	21	21	21
R	0.991400504	0.995560267	0.995003294	0.993278134
R^2	0.982874959	0.991140244	0.990031555	0.986601452
$R^2_{adjusted}$	0.982059481	0.990718351	0.989556867	0.985963426
RSS	111.7193355	68.03065885	70.79872141	85.2325952

Graphical abstract of model fit presented as mean ± 1 SD of the fraction % of released carvedilol:

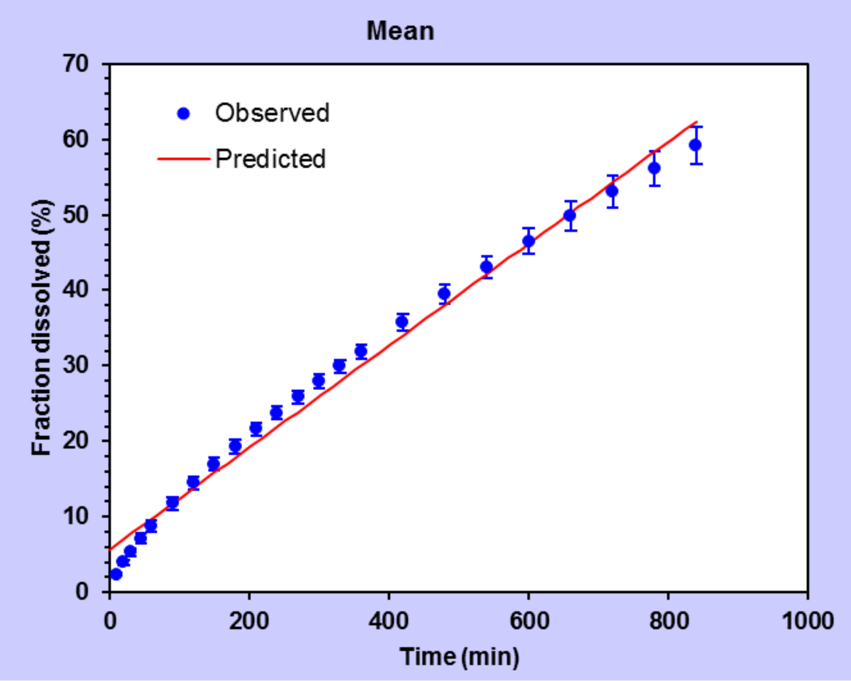

Supplementary materials – Model fitting summary – Avicel® PH-200 (modelling up to app. 60 % of carvedilol released)

Graphical abstract of model fit presented as the fraction % of released carvedilol per tested tablet:

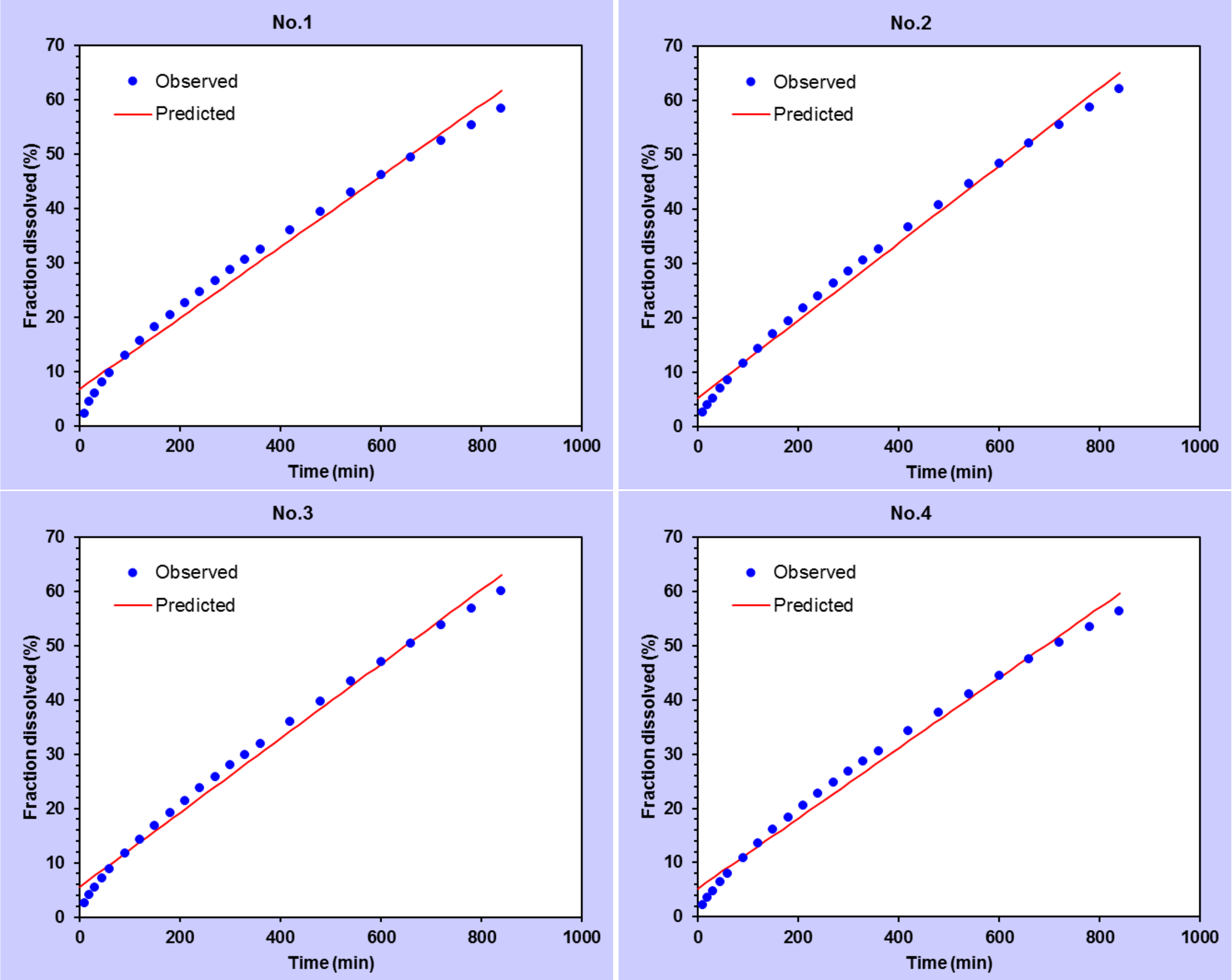

Supplementary materials – Model fitting summary – Avicel® PH-200 (modelling up to app. 60 % of carvedilol released)

Model: **First–order**

Model equation: $F = 100 \cdot (1 - e^{-k_1 \cdot t})$

Fitted model parameters per tested tablet (N = 4) with statistics – mean, standard deviation (SD), and relative standard deviation expressed in % (RSD%) (output from DDSolver):

Parameter	No.1	No.2	No.3	No.4	Mean	SD	RSD(%)
k_1	0.001	0.001	0.001	0.001	0.001	0.000	5.145

Number of dissolution data points (N), degrees of freedom (df), and selected goodness of fit criteria – Pearson correlation coefficient (R), coefficient of determination (R^2), adjusted coefficient of determination ($R^2_{adjusted}$), and residual sum of squares (RSS) (manual calculation in MS Excel):

Parameter	No.1	No.2	No.3	No.4
N	23	23	23	23
df	22	22	22	22
R	0.998903243	0.999234393	0.999431002	0.999647053
R^2	0.997807689	0.998469373	0.998862327	0.999294232
$R^2_{adjusted}$	0.997807689	0.998469373	0.998862327	0.999294232
RSS	113.1643411	32.19294329	44.51441621	43.92463581

Graphical abstract of model fit presented as mean ± 1 SD of the fraction % of released carvedilol:

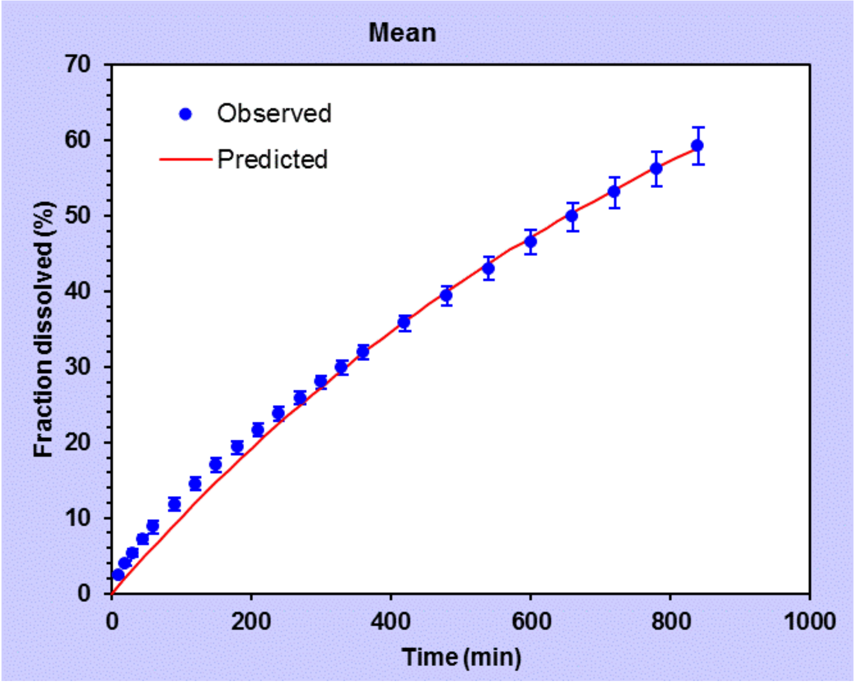

Supplementary materials – Model fitting summary – Avicel® PH-200 (modelling up to app. 60 % of carvedilol released)

Graphical abstract of model fit presented as the fraction % of released carvedilol per tested tablet:

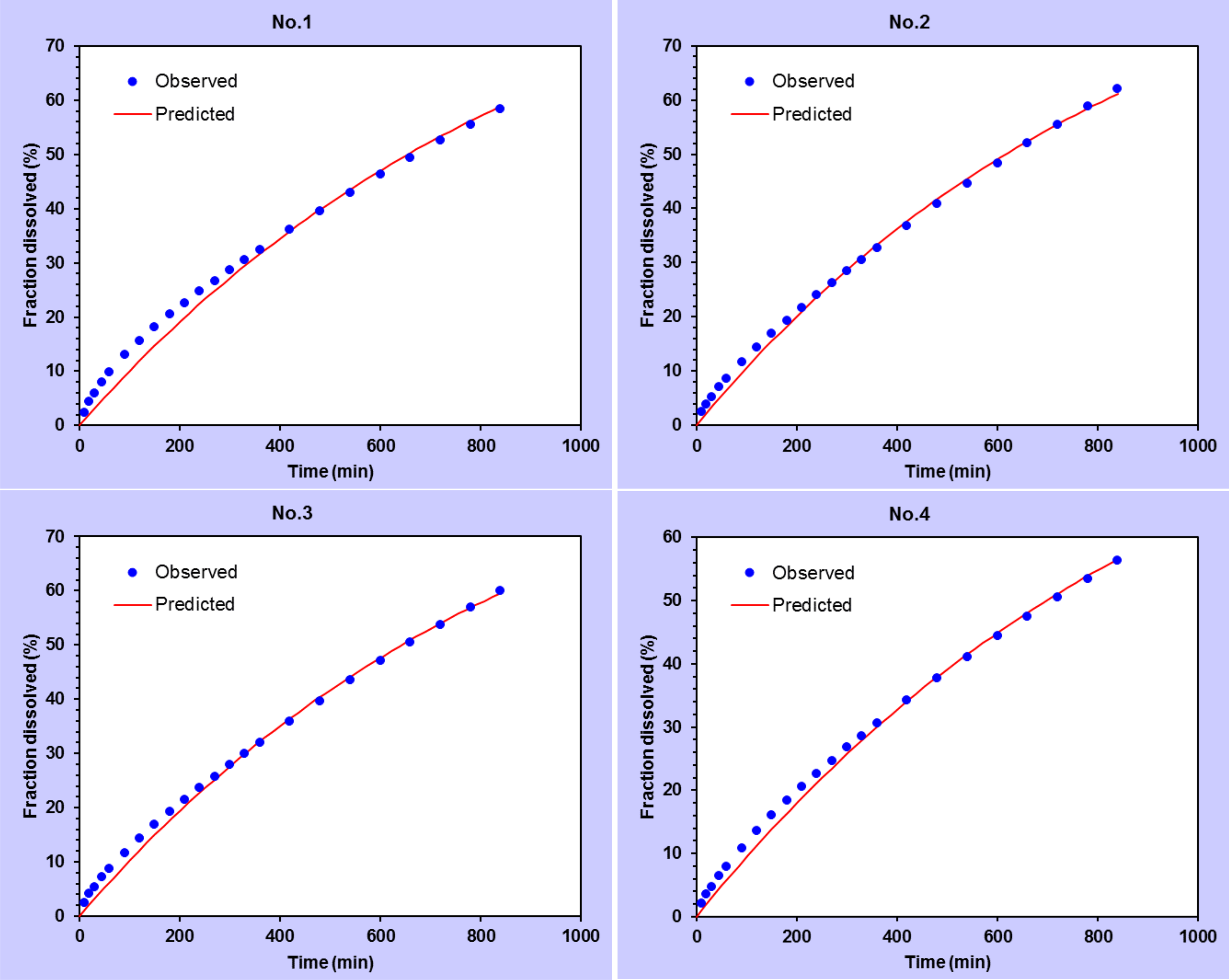

Supplementary materials – Model fitting summary – Avicel® PH-200 (modelling up to app. 60 % of carvedilol released)

Model: **First–order with T_{lag}**

Model equation: $F = 100 \cdot \left[1 - e^{-k_1 \cdot (t - T_{lag})}\right]$

Fitted model parameters per tested tablet (N = 4) with statistics – mean, standard deviation (SD), and relative standard deviation expressed in % (RSD%) (output from DDSolver):

Parameter	No.1	No.2	No.3	No.4	Mean	SD	RSD(%)
k_1	0.001	0.001	0.001	0.001	0.001	0.000	6.522
T_{lag}	-40.590	-13.008	-20.879	-26.192	-25.167	11.621	-46.175

Number of dissolution data points (N), degrees of freedom (df), and selected goodness of fit criteria – Pearson correlation coefficient (R), coefficient of determination (R^2), adjusted coefficient of determination ($R^2_{adjusted}$), and residual sum of squares (RSS) (manual calculation in MS Excel):

Parameter	No.1	No.2	No.3	No.4
N	23	23	23	23
df	21	21	21	21
R	0.998800473	0.999320839	0.999514109	0.999610384
R^2	0.997602385	0.99864214	0.999028453	0.99922092
$R^2_{adjusted}$	0.997488213	0.99857748	0.998982189	0.999183821
RSS	15.71479817	11.5136666	7.261605061	4.977430556

Graphical abstract of model fit presented as mean ± 1 SD of the fraction % of released carvedilol:

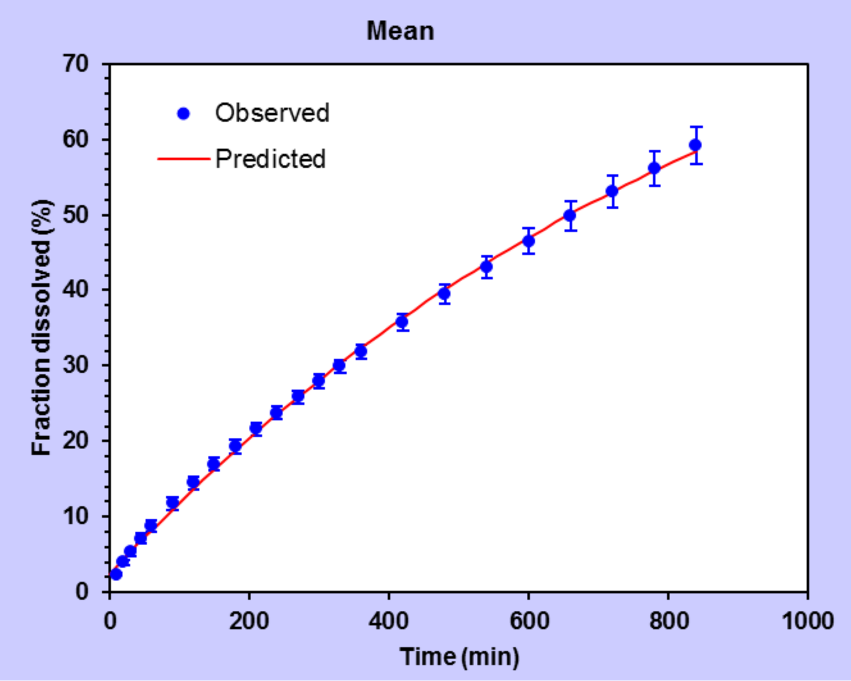

Supplementary materials – Model fitting summary – Avicel® PH-200 (modelling up to app. 60 % of carvedilol released)

Graphical abstract of model fit presented as the fraction % of released carvedilol per tested tablet:

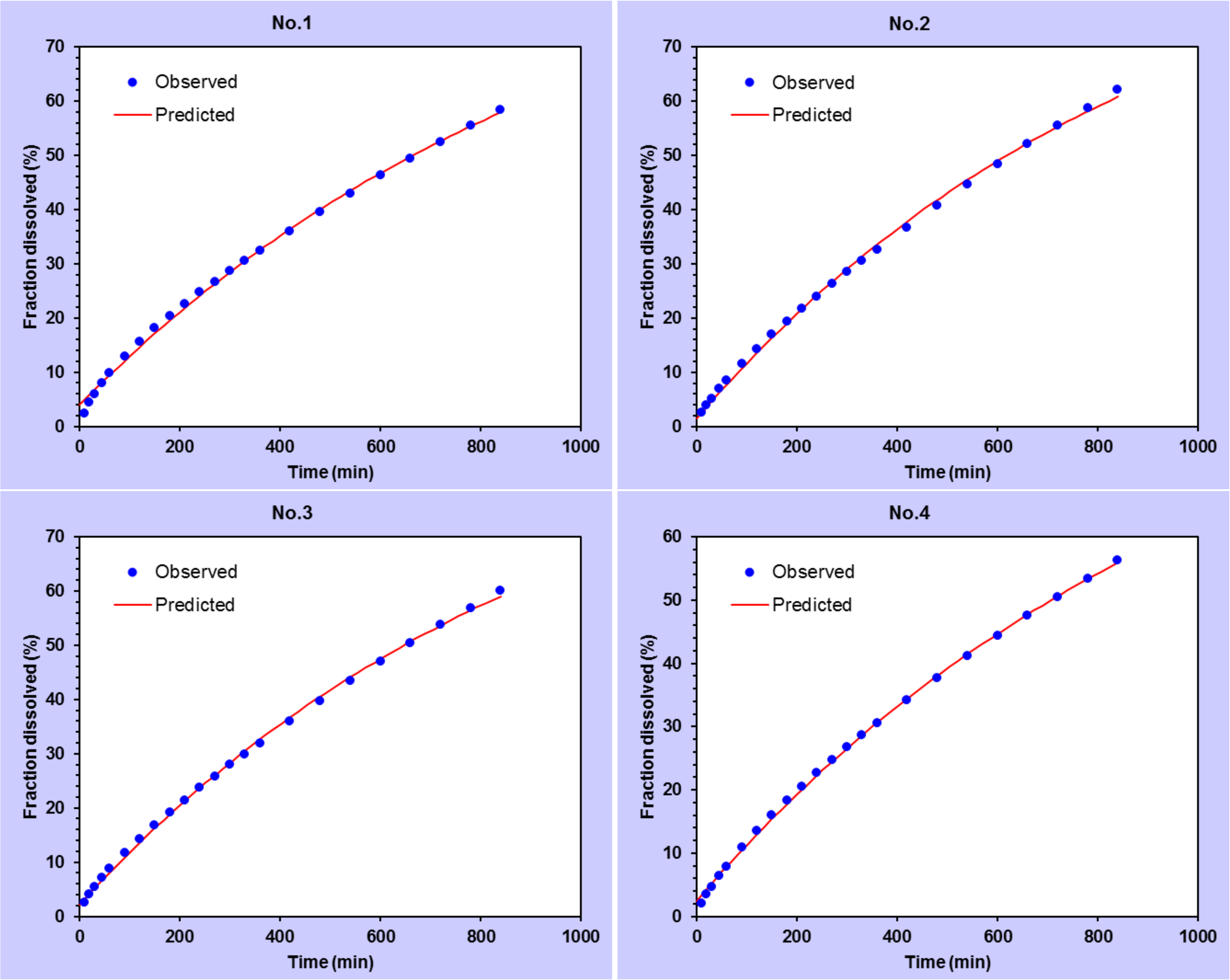

Supplementary materials – Model fitting summary – Avicel® PH-200 (modelling up to app. 60 % of carvedilol released)

Model: **First–order with F_{max}**

Model equation: $F = F_{max} \cdot (1 - e^{-k_1 \cdot t})$

Fitted model parameters per tested tablet (N = 4) with statistics – mean, standard deviation (SD), and relative standard deviation expressed in % (RSD%) (output from DDSolver):

Parameter	No.1	No.2	No.3	No.4	Mean	SD	RSD(%)
k_1	0.003	0.003	0.003	0.003	0.003	0.000	1.188
F_{max}	61.354	65.189	63.003	59.117	62.166	2.569	4.132

Number of dissolution data points (N), degrees of freedom (df), and selected goodness of fit criteria – Pearson correlation coefficient (R), coefficient of determination (R^2), adjusted coefficient of determination ($R^2_{adjusted}$), and residual sum of squares (RSS) (manual calculation in MS Excel):

Parameter	No.1	No.2	No.3	No.4
N	23	23	23	23
df	21	21	21	21
R	0.987611121	0.983740214	0.984262267	0.986762646
R^2	0.975375726	0.967744809	0.968772209	0.973700519
$R^2_{adjusted}$	0.974203141	0.966208847	0.967285172	0.972448163
RSS	258.2164687	452.1208794	389.9144204	308.7684415

Graphical abstract of model fit presented as mean ± 1 SD of the fraction % of released carvedilol:

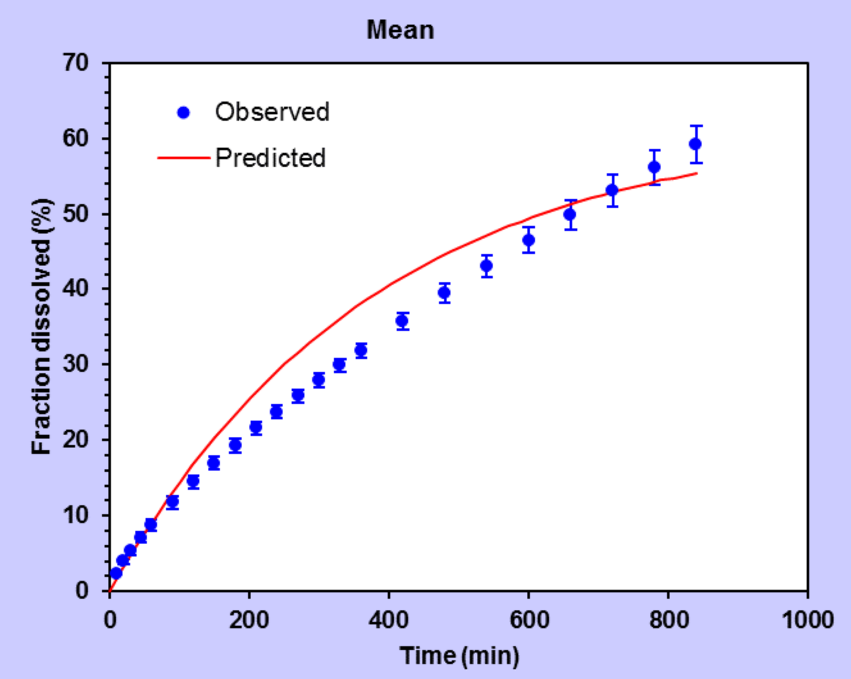

Supplementary materials – Model fitting summary – Avicel® PH-200 (modelling up to app. 60 % of carvedilol released)

Graphical abstract of model fit presented as the fraction % of released carvedilol per tested tablet:

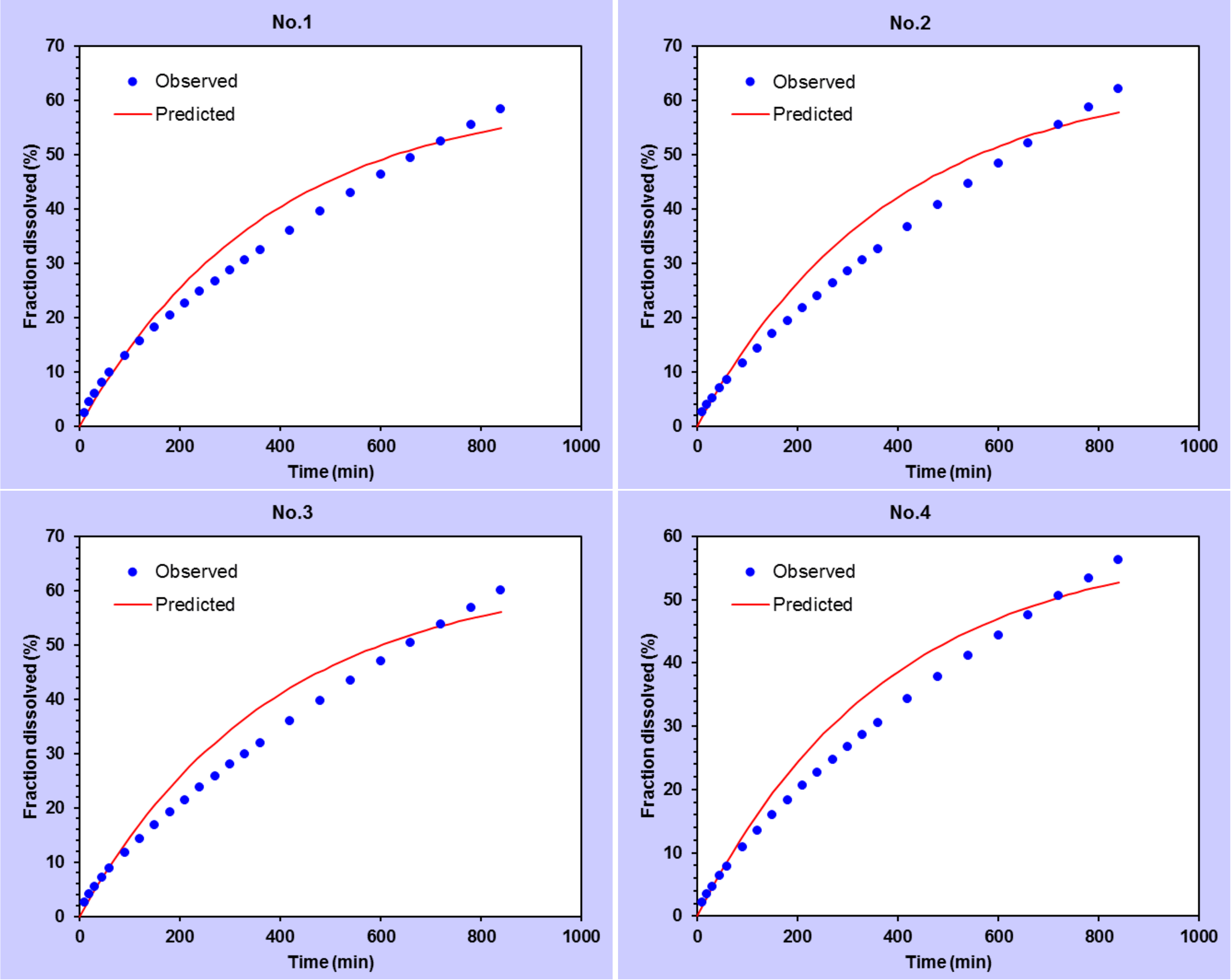

Supplementary materials – Model fitting summary – Avicel® PH-200 (modelling up to app. 60 % of carvedilol released)

Model: **First–order with T_{lag} and F_{max}**

Model equation: $F = F_{max} \cdot \left[1 - e^{-k_1 \cdot (t - T_{lag})}\right]$

Fitted model parameters per tested tablet (N = 4) with statistics – mean, standard deviation (SD), and relative standard deviation expressed in % (RSD%) (output from DDSolver):

Parameter	No.1	No.2	No.3	No.4	Mean	SD	RSD(%)
k_1	0.003	0.003	0.003	0.003	0.003	0.000	0.431
T_{lag}	40.941	54.756	51.759	50.859	49.579	5.995	12.091
F_{max}	61.354	65.189	63.003	59.117	62.166	2.569	4.132

Number of dissolution data points (N), degrees of freedom (df), and selected goodness of fit criteria – Pearson correlation coefficient (R), coefficient of determination (R^2), adjusted coefficient of determination ($R^2_{adjusted}$), and residual sum of squares (RSS) (manual calculation in MS Excel):

Parameter	No.1	No.2	No.3	No.4
N	23	23	23	23
df	20	20	20	20
R	0.984365123	0.978452045	0.97937136	0.98231892
R^2	0.968974696	0.957368405	0.95916826	0.964950461
$R^2_{adjusted}$	0.965872166	0.953105246	0.955085086	0.961445507
RSS	482.6681794	758.1637889	673.7891085	528.1626999

Graphical abstract of model fit presented as mean ± 1 SD of the fraction % of released carvedilol:

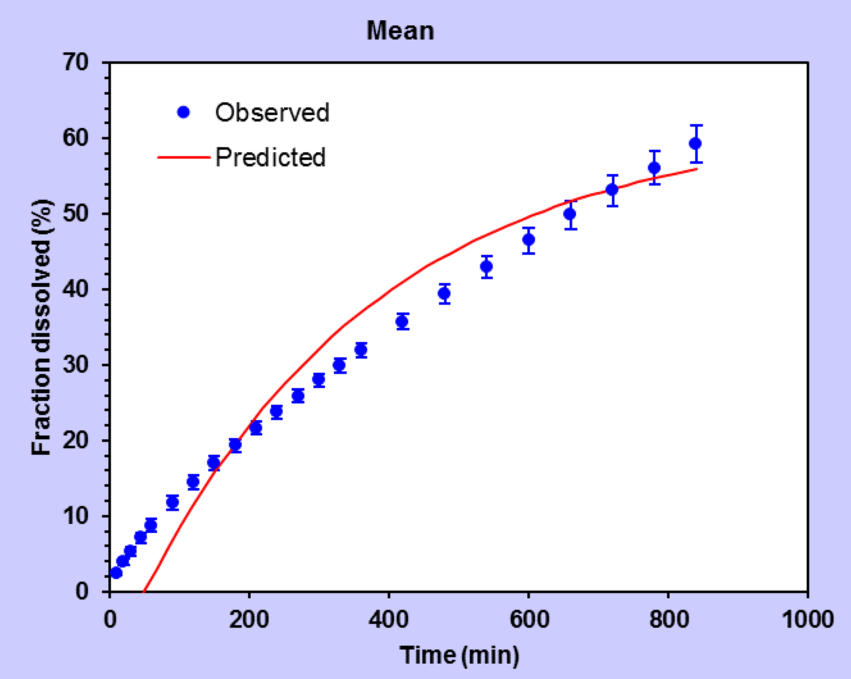

Supplementary materials – Model fitting summary – Avicel® PH-200 (modelling up to app. 60 % of carvedilol released)

Graphical abstract of model fit presented as the fraction % of released carvedilol per tested tablet:

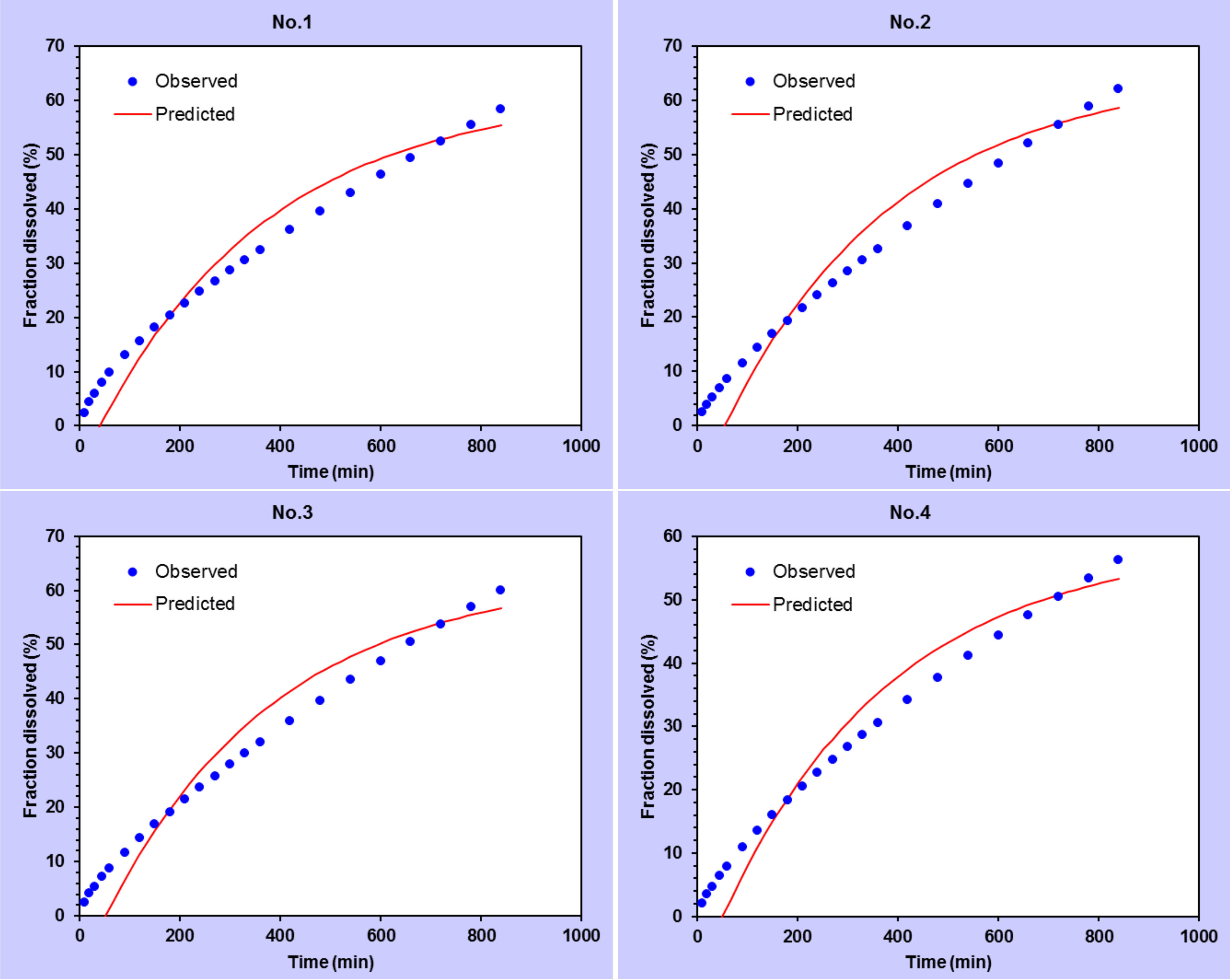

Supplementary materials – Model fitting summary – Avicel® PH-200 (modelling up to app. 60 % of carvedilol released)

Model: **Higuchi**

Model equation: $F = k_H \cdot t^{0.5}$

Fitted model parameters per tested tablet (N = 4) with statistics – mean, standard deviation (SD), and relative standard deviation expressed in % (RSD%) (output from DDSolver):

Parameter	No.1	No.2	No.3	No.4	Mean	SD	RSD(%)
k_H	1.804	1.854	1.807	1.708	1.793	0.061	3.425

Number of dissolution data points (N), degrees of freedom (df), and selected goodness of fit criteria – Pearson correlation coefficient (R), coefficient of determination (R^2), adjusted coefficient of determination ($R^2_{adjusted}$), and residual sum of squares (RSS) (manual calculation in MS Excel):

Parameter	No.1	No.2	No.3	No.4
N	23	23	23	23
df	22	22	22	22
R	0.99610179	0.992031191	0.992760982	0.994451871
R^2	0.992218776	0.984125884	0.985574368	0.988934524
$R^2_{adjusted}$	0.992218776	0.984125884	0.985574368	0.988934524
RSS	279.9363025	532.4499228	447.3350332	388.2346869

Graphical abstract of model fit presented as mean ± 1 SD of the fraction % of released carvedilol:

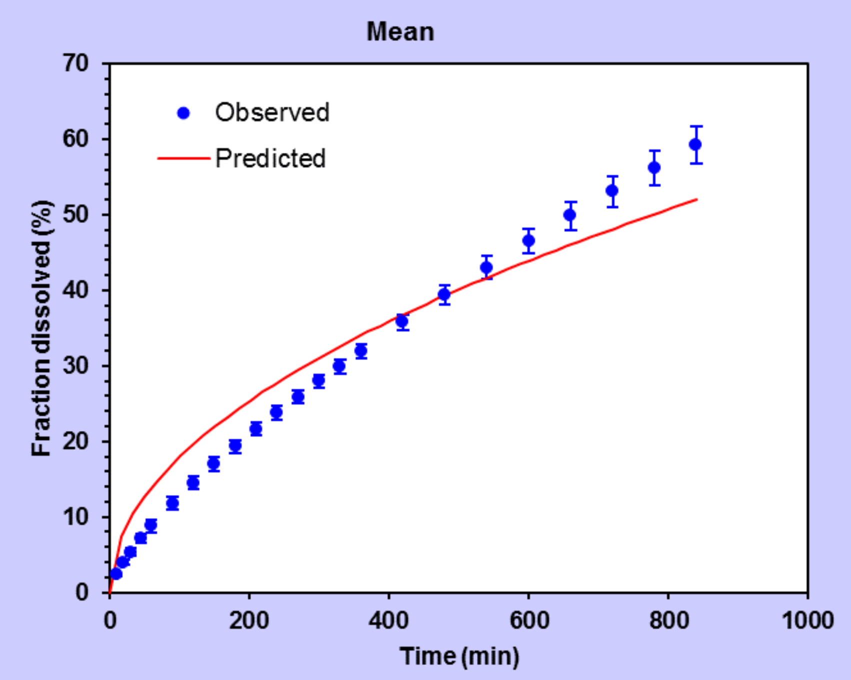

Supplementary materials – Model fitting summary – Avicel® PH-200 (modelling up to app. 60 % of carvedilol released)

Graphical abstract of model fit presented as the fraction % of released carvedilol per tested tablet:

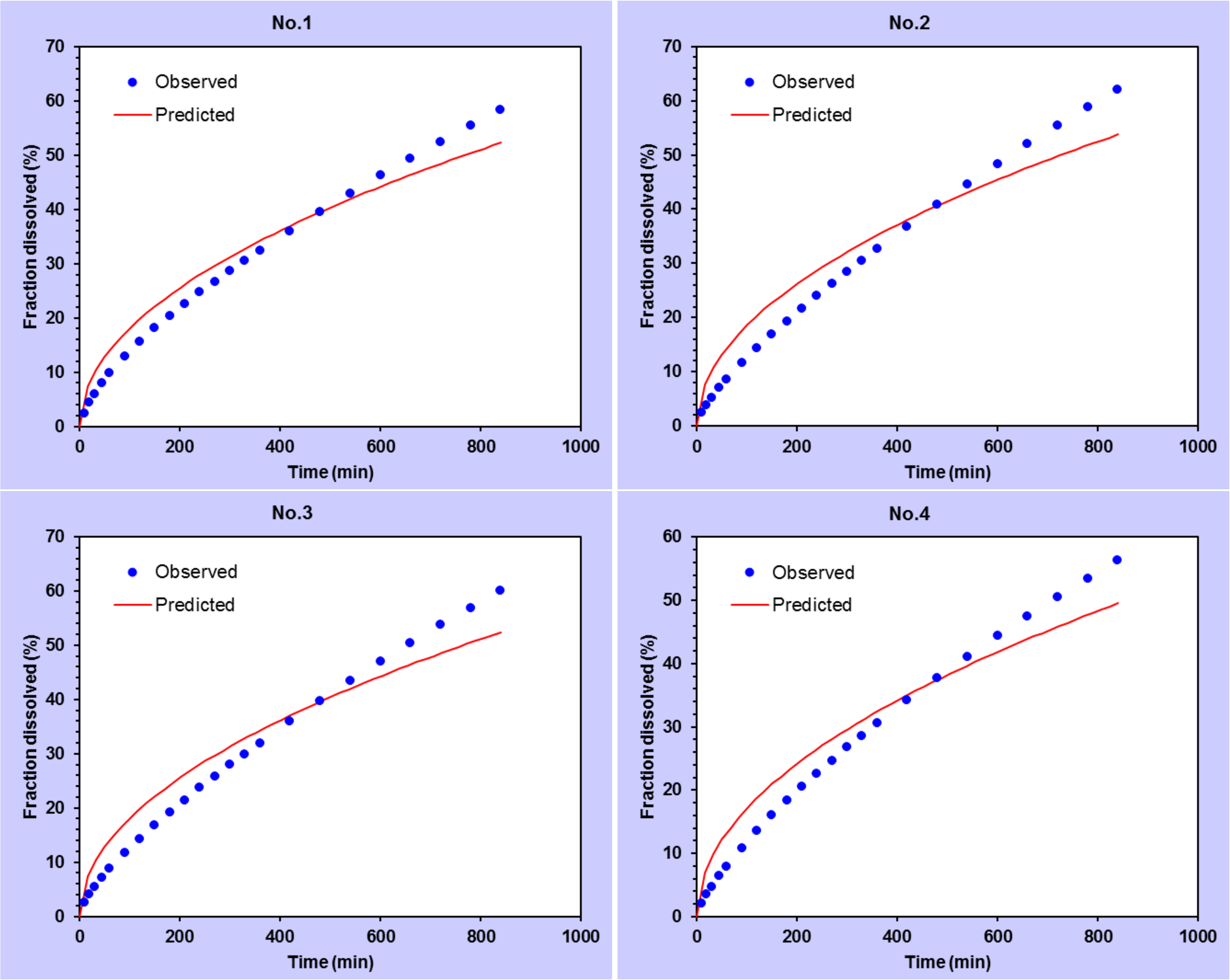

Supplementary materials – Model fitting summary – Avicel® PH-200 (modelling up to app. 60 % of carvedilol released)

Model: **Higuchi with T_{lag}**

Model equation: $F = k_H \cdot (t - T_{lag})^{0.5}$

Fitted model parameters per tested tablet (N = 4) with statistics – mean, standard deviation (SD), and relative standard deviation expressed in % (RSD%) (output from DDSolver):

Parameter	No.1	No.2	No.3	No.4	Mean	SD	RSD(%)
k_H	2.008	2.133	2.064	1.942	2.037	0.082	4.007
T_{lag}	59.280	74.167	71.066	68.845	68.339	6.422	9.397

Number of dissolution data points (N), degrees of freedom (df), and selected goodness of fit criteria – Pearson correlation coefficient (R), coefficient of determination (R^2), adjusted coefficient of determination ($R^2_{adjusted}$), and residual sum of squares (RSS) (manual calculation in MS Excel):

Parameter	No.1	No.2	No.3	No.4
N	23	23	23	23
df	21	21	21	21
R	0.987327089	0.985032587	0.984965672	0.987197296
R^2	0.974814781	0.970289198	0.970157375	0.974558501
$R^2_{adjusted}$	0.973615485	0.968874398	0.968736298	0.973347002
RSS	247.3445659	293.3462617	282.5250438	214.2828365

Graphical abstract of model fit presented as mean ± 1 SD of the fraction % of released carvedilol:

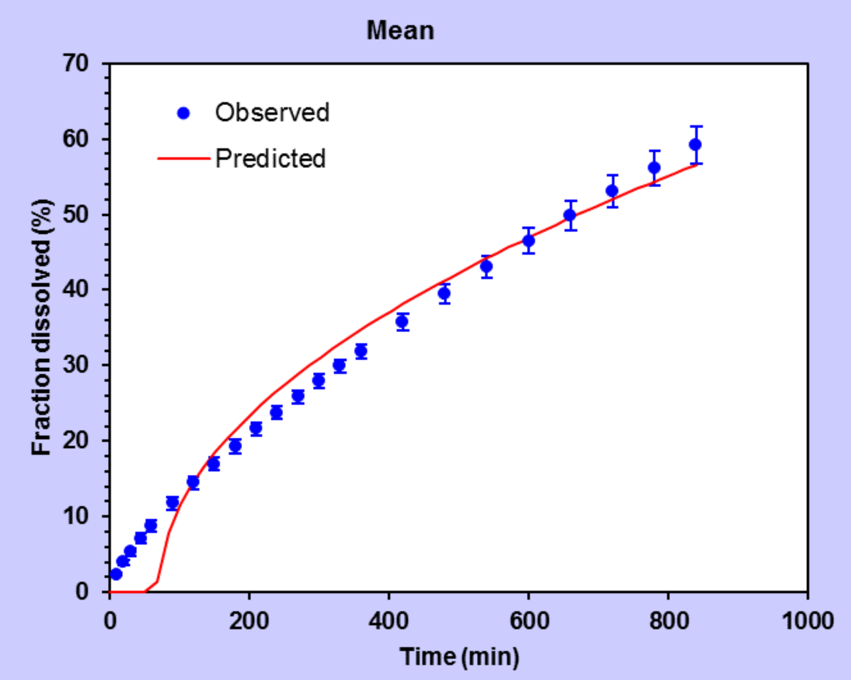

Supplementary materials – Model fitting summary – Avicel® PH-200 (modelling up to app. 60 % of carvedilol released)

Graphical abstract of model fit presented as the fraction % of released carvedilol per tested tablet:

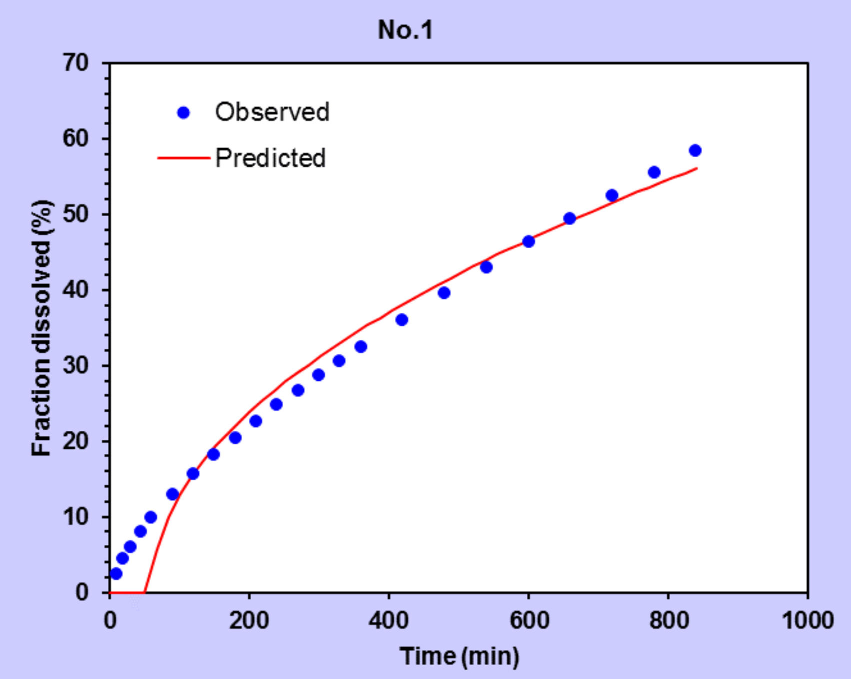

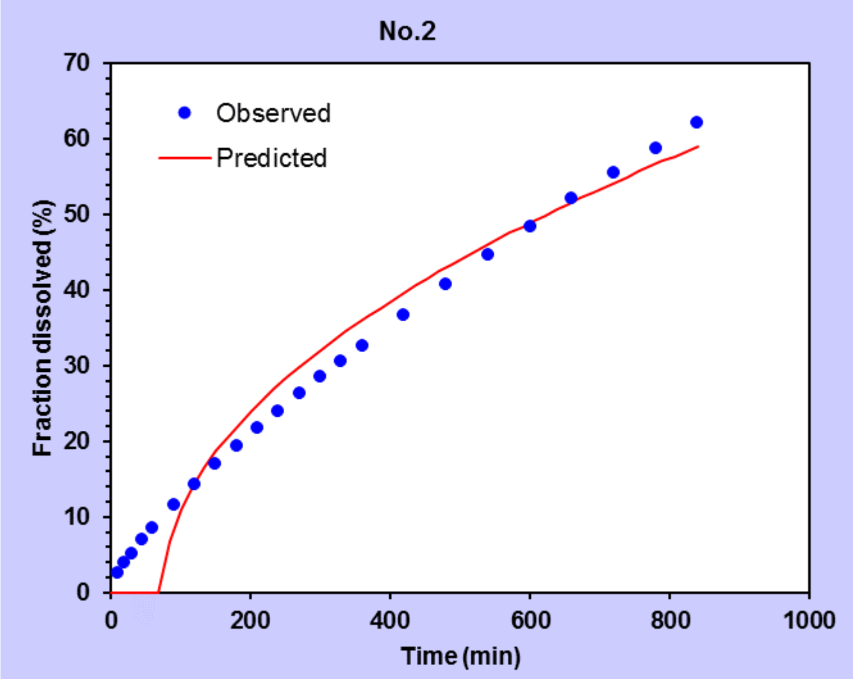

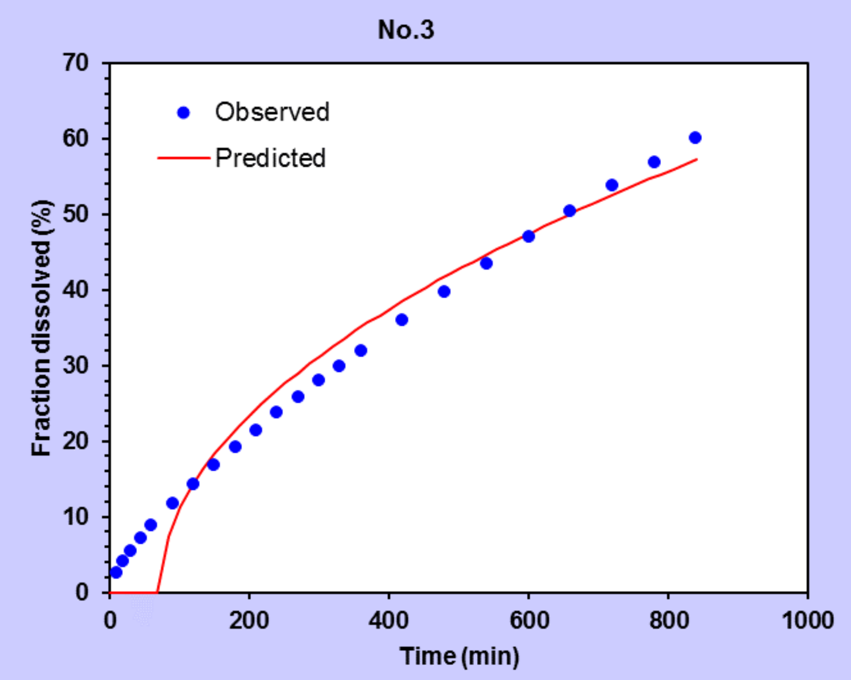

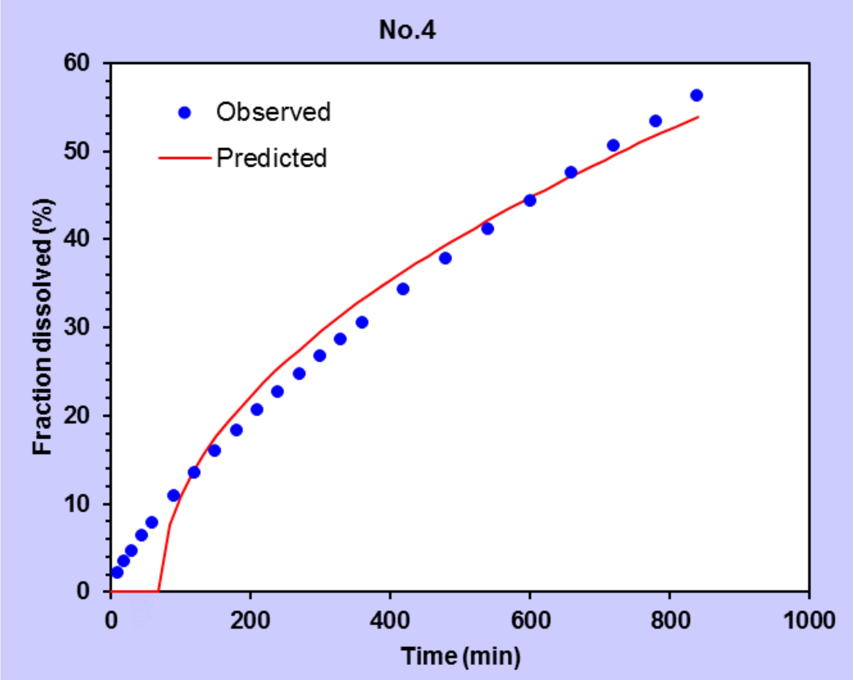

Supplementary materials – Model fitting summary – Avicel® PH-200 (modelling up to app. 60 % of carvedilol released)

Model: **Higuchi with F_0**

Model equation: $F = F_0 + k_H \cdot t^{0.5}$

Fitted model parameters per tested tablet (N = 4) with statistics – mean, standard deviation (SD), and relative standard deviation expressed in % (RSD%) (output from DDSolver):

Parameter	No.1	No.2	No.3	No.4	Mean	SD	RSD(%)
k_H	2.174	2.349	2.261	2.143	2.232	0.093	4.151
F_0	-7.364	-9.857	-9.034	-8.673	-8.732	1.038	-11.884

Number of dissolution data points (N), degrees of freedom (df), and selected goodness of fit criteria – Pearson correlation coefficient (R), coefficient of determination (R^2), adjusted coefficient of determination ($R^2_{adjusted}$), and residual sum of squares (RSS) (manual calculation in MS Excel):

Parameter	No.1	No.2	No.3	No.4
N	23	23	23	23
df	21	21	21	21
R	0.99610179	0.992031191	0.992760982	0.994451871
R^2	0.992218776	0.984125884	0.985574368	0.988934524
$R^2_{adjusted}$	0.991848241	0.983369974	0.984887433	0.988407596
RSS	50.76269374	121.8912326	102.454921	70.39115362

Graphical abstract of model fit presented as mean ± 1 SD of the fraction % of released carvedilol:

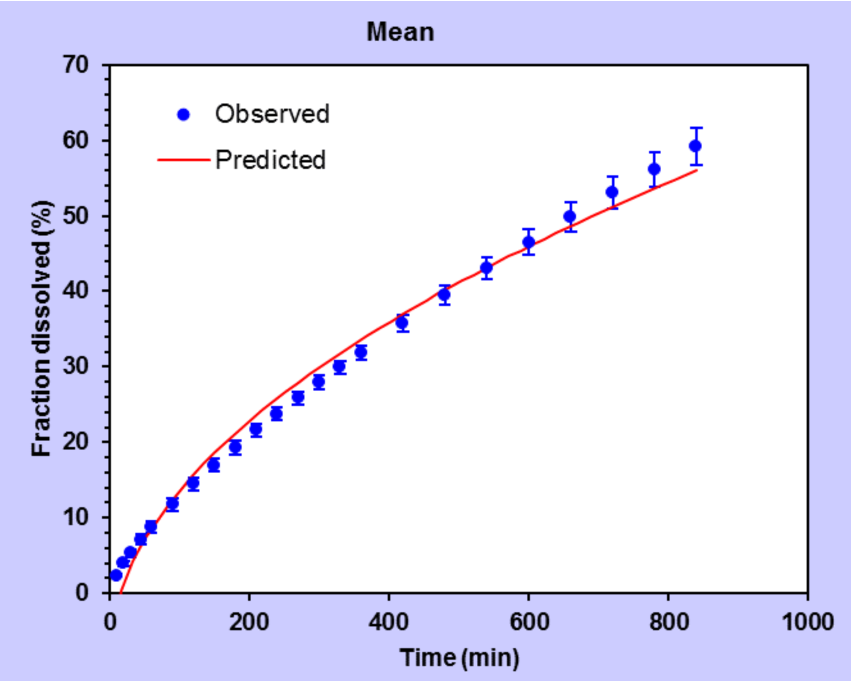

Supplementary materials – Model fitting summary – Avicel® PH-200 (modelling up to app. 60 % of carvedilol released)

Graphical abstract of model fit presented as the fraction % of released carvedilol per tested tablet:

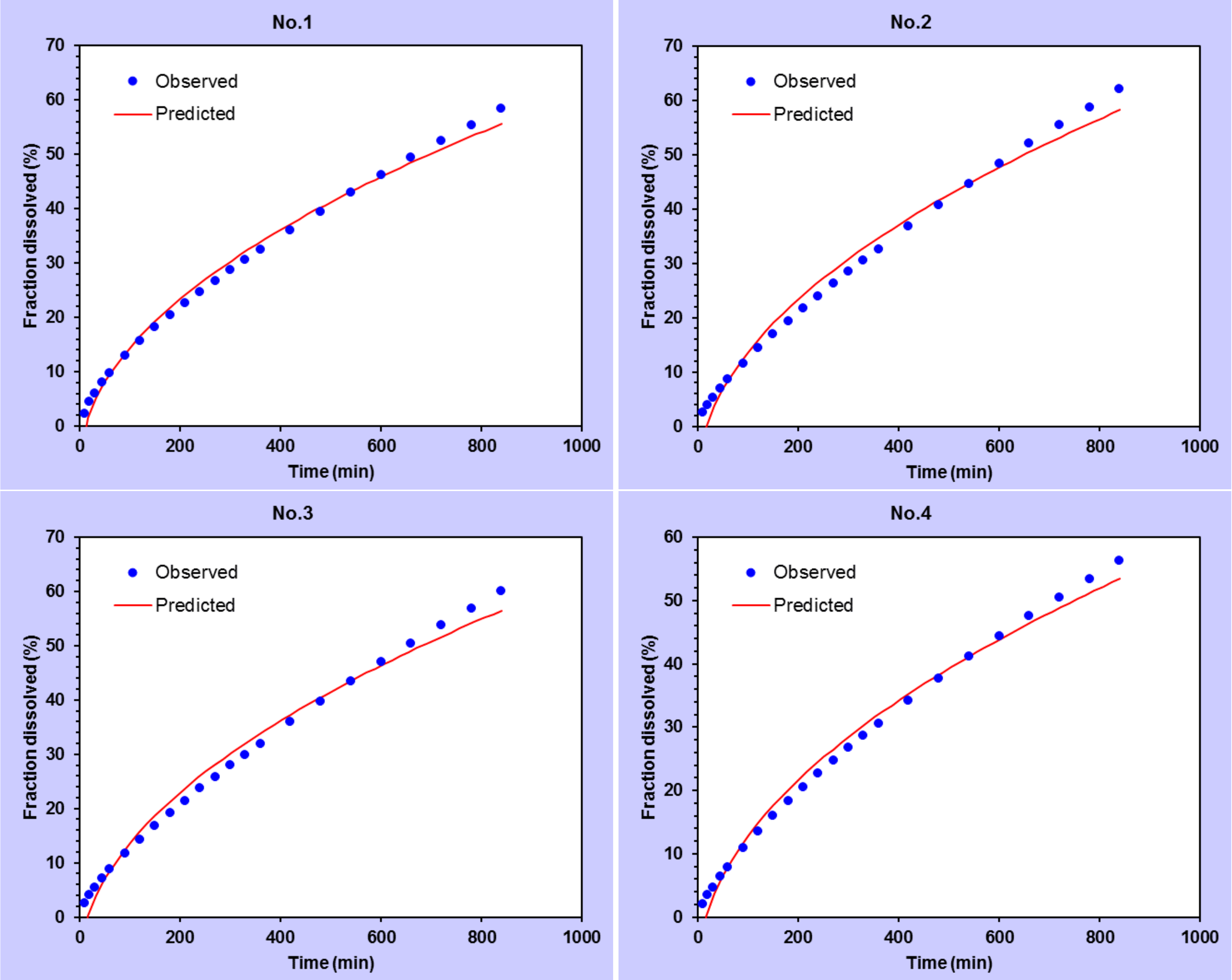

Supplementary materials – Model fitting summary – Avicel® PH-200 (modelling up to app. 60 % of carvedilol released)

Model: **Korsmeyer–Peppas**

Model equation: $F = k_{KP} \cdot t^n$

Fitted model parameters per tested tablet (N = 4) with statistics – mean, standard deviation (SD), and relative standard deviation expressed in % (RSD%) (output from DDSolver):

Parameter	No.1	No.2	No.3	No.4	Mean	SD	RSD(%)
k_{KP}	0.531	0.377	0.465	0.376	0.437	0.075	17.147
n	0.700	0.758	0.720	0.746	0.731	0.026	3.562

Number of dissolution data points (N), degrees of freedom (df), and selected goodness of fit criteria – Pearson correlation coefficient (R), coefficient of determination (R^2), adjusted coefficient of determination ($R^2_{adjusted}$), and residual sum of squares (RSS) (manual calculation in MS Excel):

Parameter	No.1	No.2	No.3	No.4
N	23	23	23	23
df	21	21	21	21
R	0.99990011	0.999980127	0.99986934	0.999905365
R^2	0.999800229	0.999960254	0.999738696	0.99981074
$R^2_{adjusted}$	0.999790716	0.999958361	0.999726253	0.999801727
RSS	5.036944276	0.669581734	4.182760173	2.012009349

Graphical abstract of model fit presented as mean ± 1 SD of the fraction % of released carvedilol:

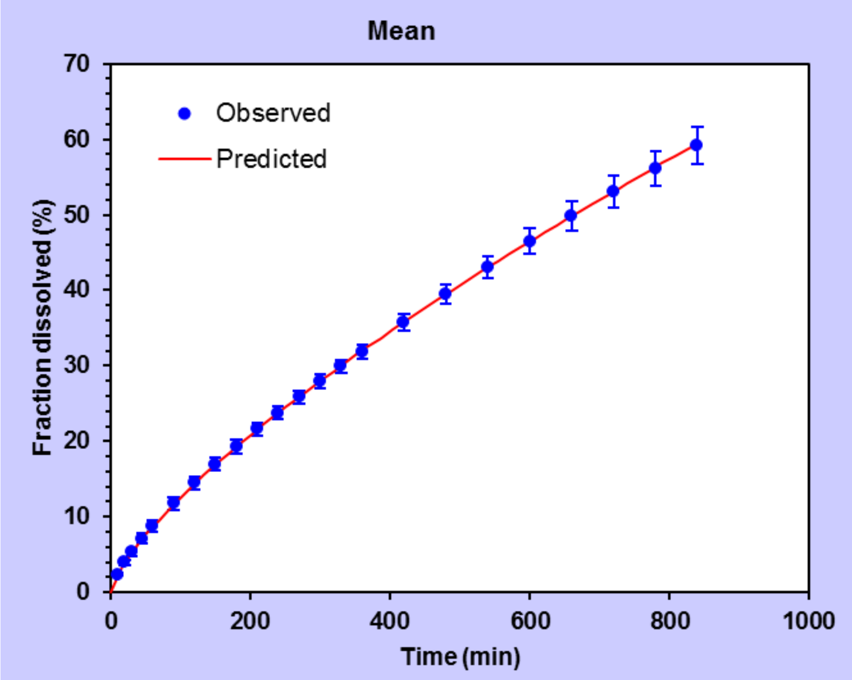

Supplementary materials – Model fitting summary – Avicel® PH-200 (modelling up to app. 60 % of carvedilol released)

Graphical abstract of model fit presented as the fraction % of released carvedilol per tested tablet:

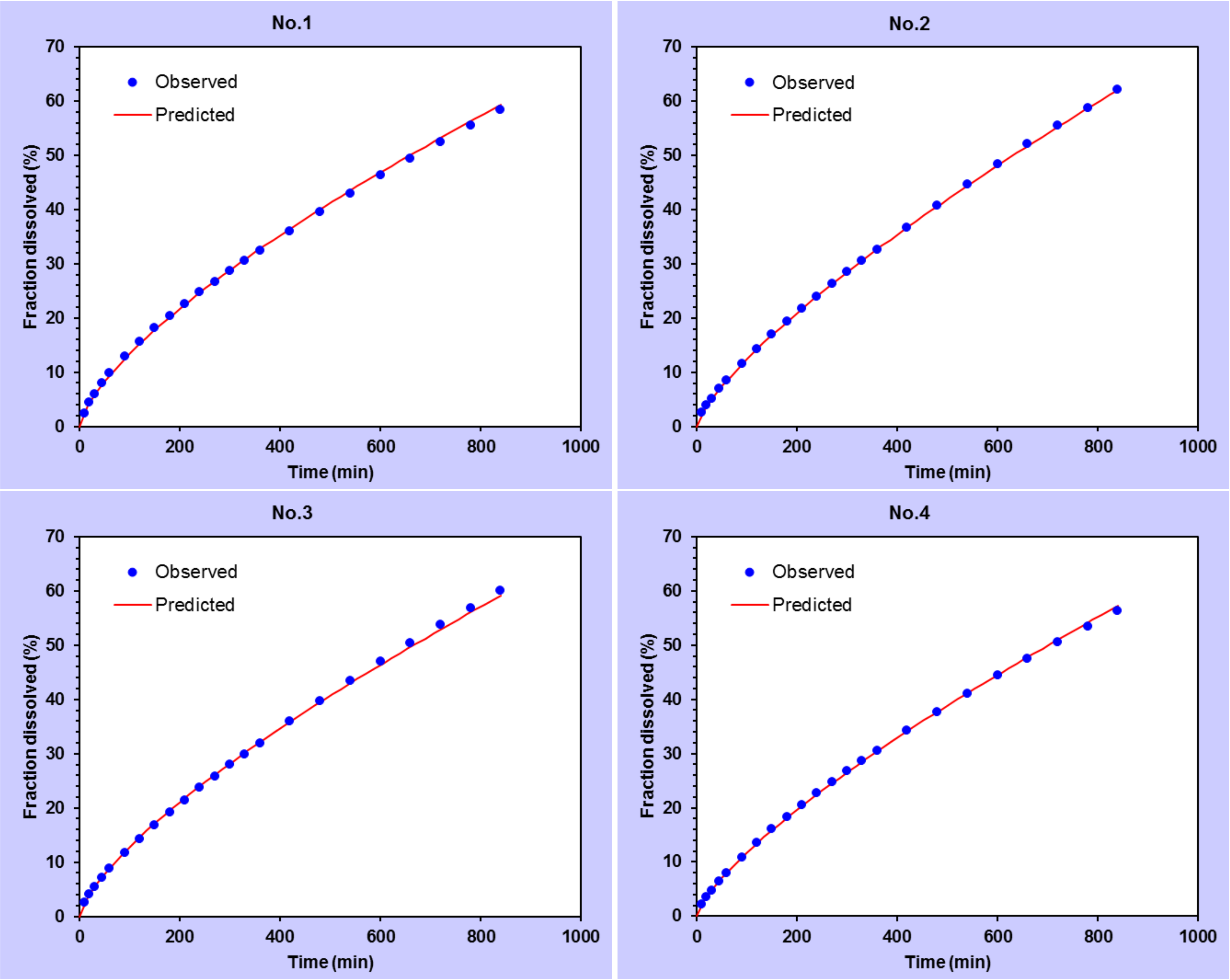

Supplementary materials – Model fitting summary – Avicel® PH-200 (modelling up to app. 60 % of carvedilol released)

Model: **Korsmeyer–Peppas with T_{lag}**

Model equation: $F = k_{KP} \cdot (t - T_{lag})^n$

Fitted model parameters per tested tablet (N = 4) with statistics – mean, standard deviation (SD), and relative standard deviation expressed in % (RSD%) (output from DDSolver):

Parameter	No.1	No.2	No.3	No.4	Mean	SD	RSD(%)
k_{KP}	0.710	0.590	0.632	0.521	0.613	0.079	12.898
n	0.652	0.684	0.668	0.696	0.675	0.019	2.816
T_{lag}	4.000	4.000	4.000	4.975	4.244	0.488	11.490

Number of dissolution data points (N), degrees of freedom (df), and selected goodness of fit criteria – Pearson correlation coefficient (R), coefficient of determination (R^2), adjusted coefficient of determination ($R^2_{adjusted}$), and residual sum of squares (RSS) (manual calculation in MS Excel):

Parameter	No.1	No.2	No.3	No.4
N	23	23	23	23
df	20	20	20	20
R	0.999775009	0.999106516	0.999043718	0.999839285
R^2	0.999550068	0.998213829	0.99808835	0.999678596
$R^2_{adjusted}$	0.999505075	0.998035212	0.997897185	0.999646456
RSS	4.71650012	44.68697237	39.67646555	4.984418371

Graphical abstract of model fit presented as mean ± 1 SD of the fraction % of released carvedilol:

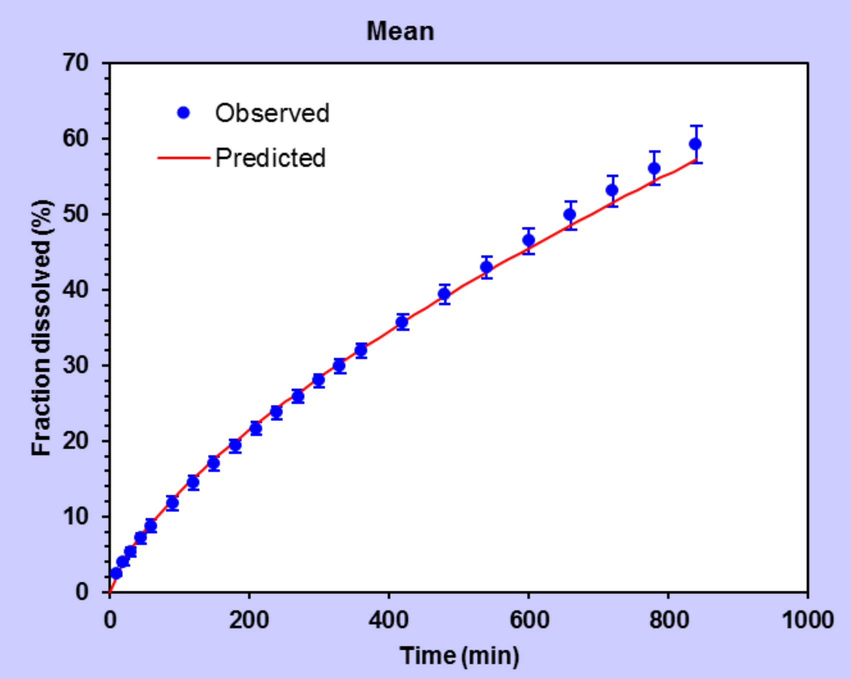

Supplementary materials – Model fitting summary – Avicel® PH-200 (modelling up to app. 60 % of carvedilol released)

Graphical abstract of model fit presented as the fraction % of released carvedilol per tested tablet:

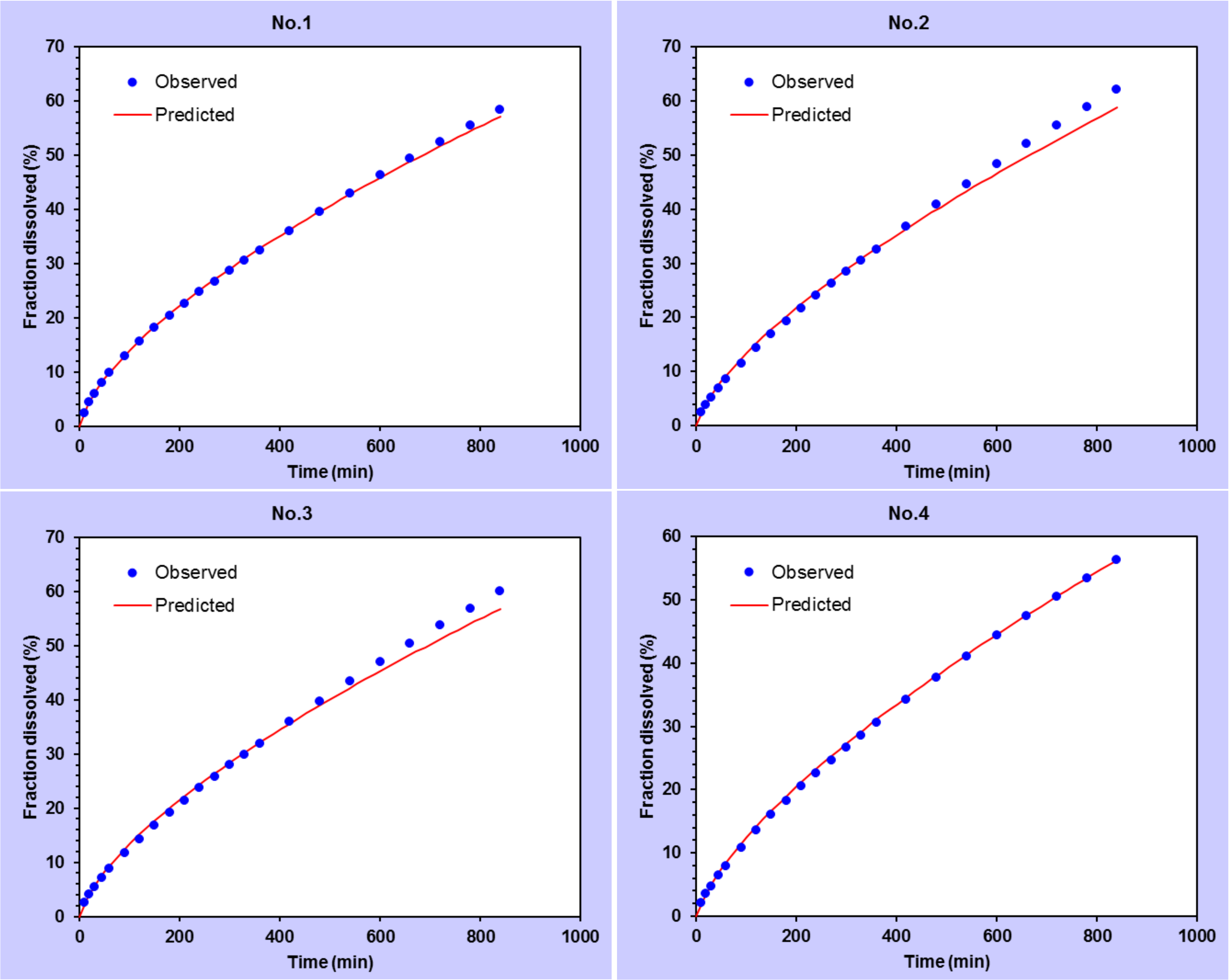

Supplementary materials – Model fitting summary – Avicel® PH-200 (modelling up to app. 60 % of carvedilol released)

Model: **Korsmeyer–Peppas with F_0**

Model equation: $F = F_0 + k_{KP} \cdot t^n$

Fitted model parameters per tested tablet (N = 4) with statistics – mean, standard deviation (SD), and relative standard deviation expressed in % (RSD%) (output from DDSolver):

Parameter	No.1	No.2	No.3	No.4	Mean	SD	RSD(%)
k_{KP}	0.322	0.272	0.297	0.227	0.280	0.041	14.505
n	0.769	0.802	0.784	0.826	0.795	0.025	3.136
F_0	1.168	1.226	1.226	0.840	1.115	0.185	16.634

Number of dissolution data points (N), degrees of freedom (df), and selected goodness of fit criteria – Pearson correlation coefficient (R), coefficient of determination (R^2), adjusted coefficient of determination ($R^2_{adjusted}$), and residual sum of squares (RSS) (manual calculation in MS Excel):

Parameter	No.1	No.2	No.3	No.4
N	23	23	23	23
df	20	20	20	20
R	0.999200327	0.999871492	0.999896426	0.998877886
R^2	0.998401294	0.999743	0.999792863	0.99775703
$R^2_{adjusted}$	0.998241424	0.9997173	0.999772149	0.997532733
RSS	39.39975056	12.55275936	8.988596726	45.21993295

Graphical abstract of model fit presented as mean ± 1 SD of the fraction % of released carvedilol:

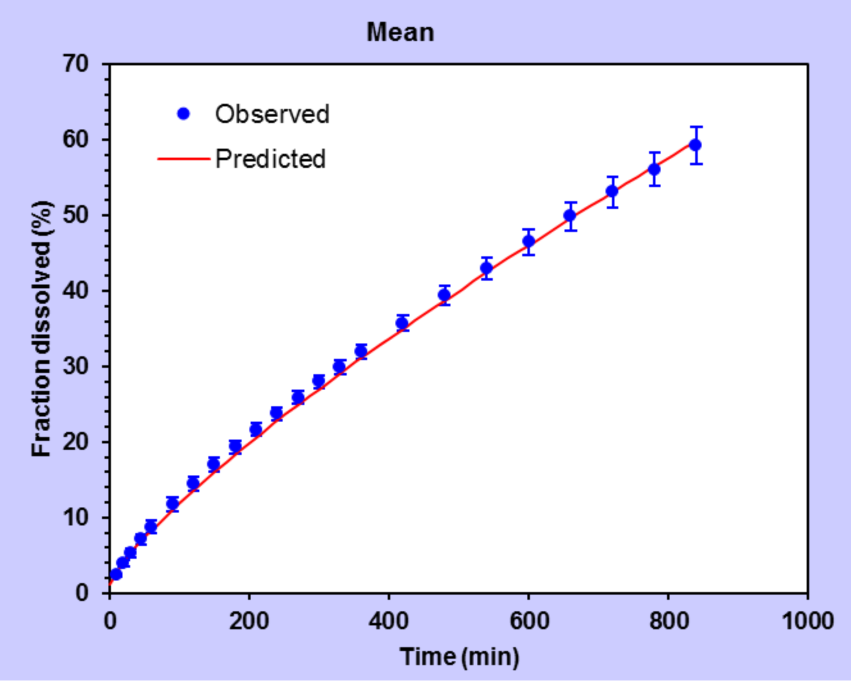

Supplementary materials – Model fitting summary – Avicel® PH-200 (modelling up to app. 60 % of carvedilol released)

Graphical abstract of model fit presented as the fraction % of released carvedilol per tested tablet:

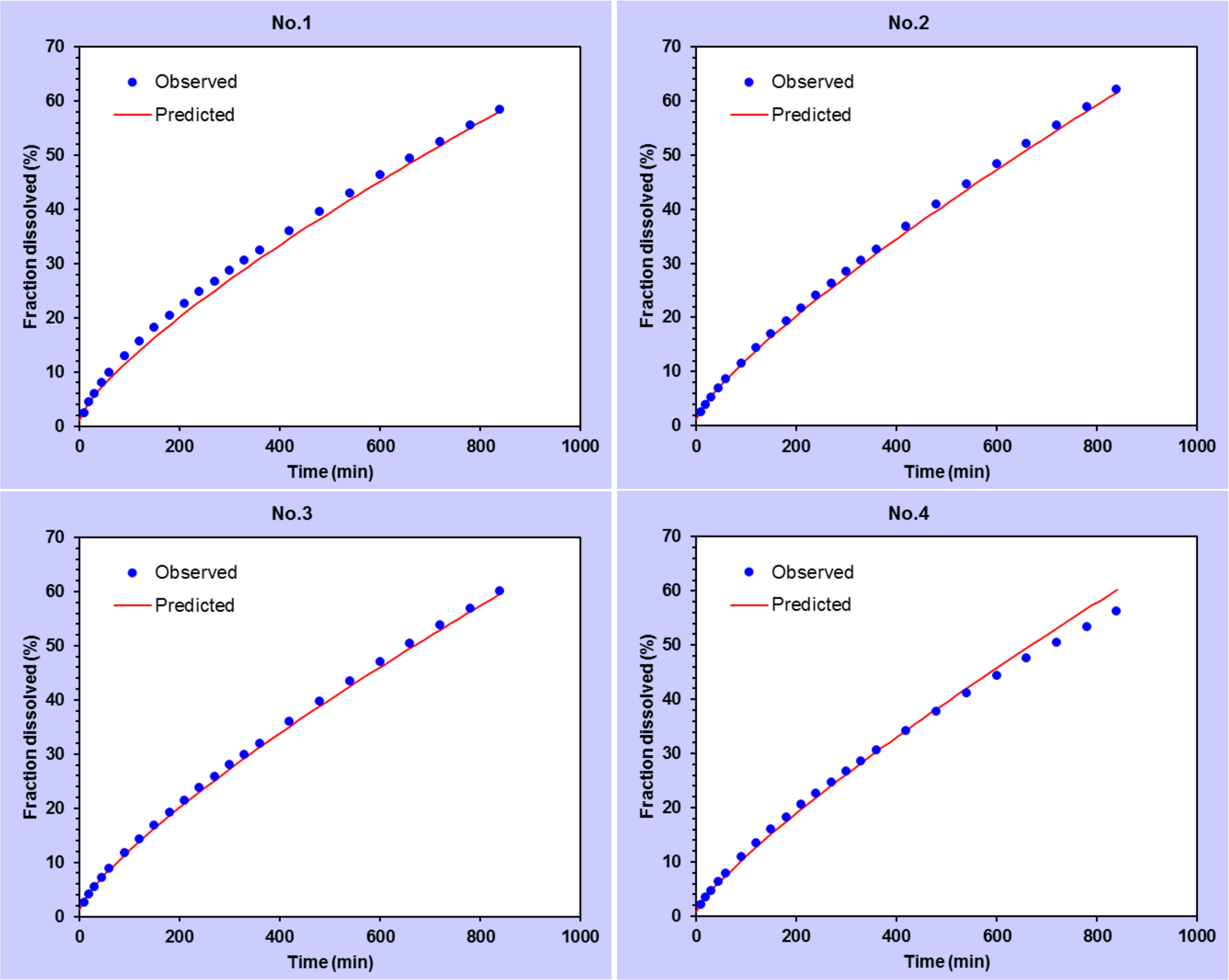

Supplementary materials – Model fitting summary – Avicel® PH-200 (modelling up to app. 60 % of carvedilol released)

Model: **Hixson–Crowell**

Model equation: $F = 100 \cdot [1 - (1 - k_{HC} \cdot t)^3]$

Fitted model parameters per tested tablet (N = 4) with statistics – mean, standard deviation (SD), and relative standard deviation expressed in % (RSD%) (output from DDSolver):

Parameter	No.1	No.2	No.3	No.4	Mean	SD	RSD(%)
k_{HC}	0.0003	0.0003	0.0003	0.0003	0.0003	0.0000	4.5347

Number of dissolution data points (N), degrees of freedom (df), and selected goodness of fit criteria – Pearson correlation coefficient (R), coefficient of determination (R^2), adjusted coefficient of determination ($R^2_{adjusted}$), and residual sum of squares (RSS) (manual calculation in MS Excel):

Parameter	No.1	No.2	No.3	No.4
N	23	23	23	23
df	22	22	22	22
R	0.997884116	0.999657641	0.999488852	0.99887057
R^2	0.995772709	0.9993154	0.998977965	0.997742415
$R^2_{adjusted}$	0.995772709	0.9993154	0.998977965	0.997742415
RSS	200.2712127	68.88025006	93.6775615	98.41099489

Graphical abstract of model fit presented as mean ± 1 SD of the fraction % of released carvedilol:

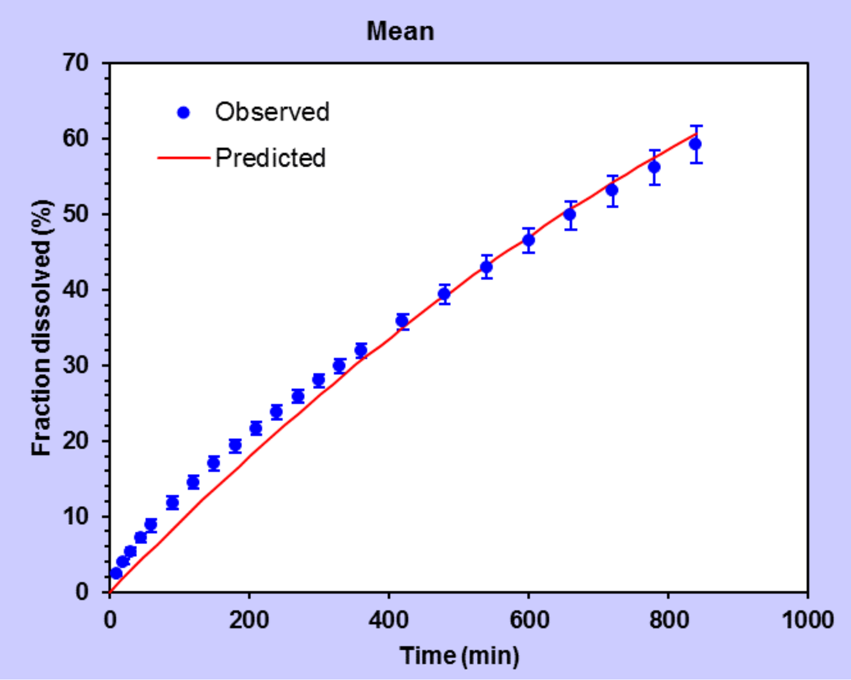

Supplementary materials – Model fitting summary – Avicel® PH-200 (modelling up to app. 60 % of carvedilol released)

Graphical abstract of model fit presented as the fraction % of released carvedilol per tested tablet:

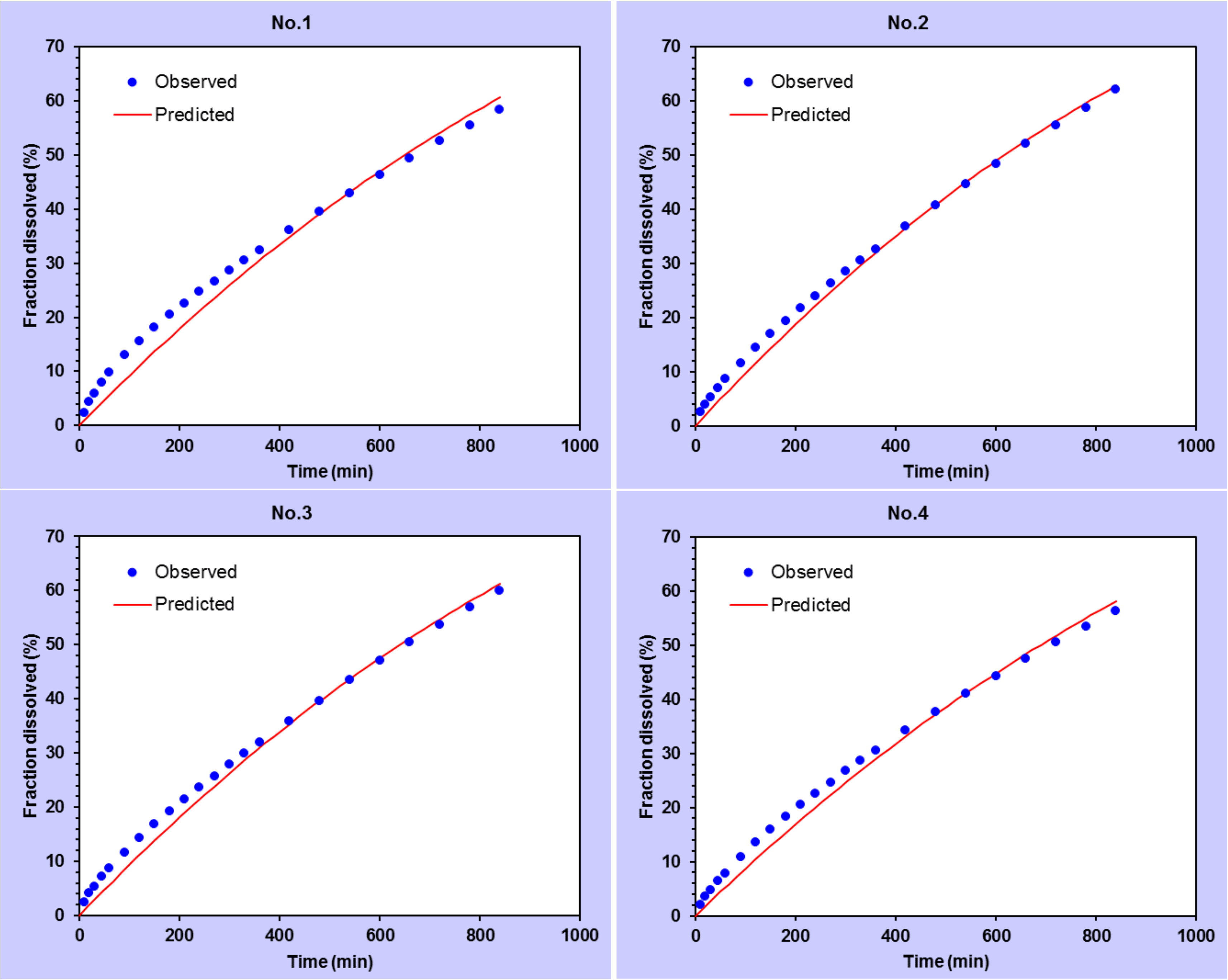

Supplementary materials – Model fitting summary – Avicel® PH-200 (modelling up to app. 60 % of carvedilol released)

Model: **Hixson–Crowell with T_{lag}**

Model equation: $F = 100 \cdot \left\{1 - \left[1 - k_{HC} \cdot \left(t - T_{lag}\right)\right]^3\right\}$

Fitted model parameters per tested tablet (N = 4) with statistics – mean, standard deviation (SD), and relative standard deviation expressed in % (RSD%) (output from DDSolver):

Parameter	No.1	No.2	No.3	No.4	Mean	SD	RSD(%)
k_{HC}	0.0003	0.0003	0.0003	0.0003	0.0003	0.0000	5.7650
T_{lag}	-60.5459	-32.1442	-39.7878	-43.4226	-43.9751	12.0055	-27.3008

Number of dissolution data points (N), degrees of freedom (df), and selected goodness of fit criteria – Pearson correlation coefficient (R), coefficient of determination (R^2), adjusted coefficient of determination ($R^2_{adjusted}$), and residual sum of squares (RSS) (manual calculation in MS Excel):

Parameter	No.1	No.2	No.3	No.4
N	23	23	23	23
df	21	21	21	21
R	0.997530438	0.999621144	0.999397345	0.998654796
R^2	0.995066974	0.999242432	0.998795052	0.997311402
$R^2_{adjusted}$	0.994832068	0.999206357	0.998737674	0.997183373
RSS	32.8473457	5.846537204	8.674647982	17.56530286

Graphical abstract of model fit presented as mean ± 1 SD of the fraction % of released carvedilol:

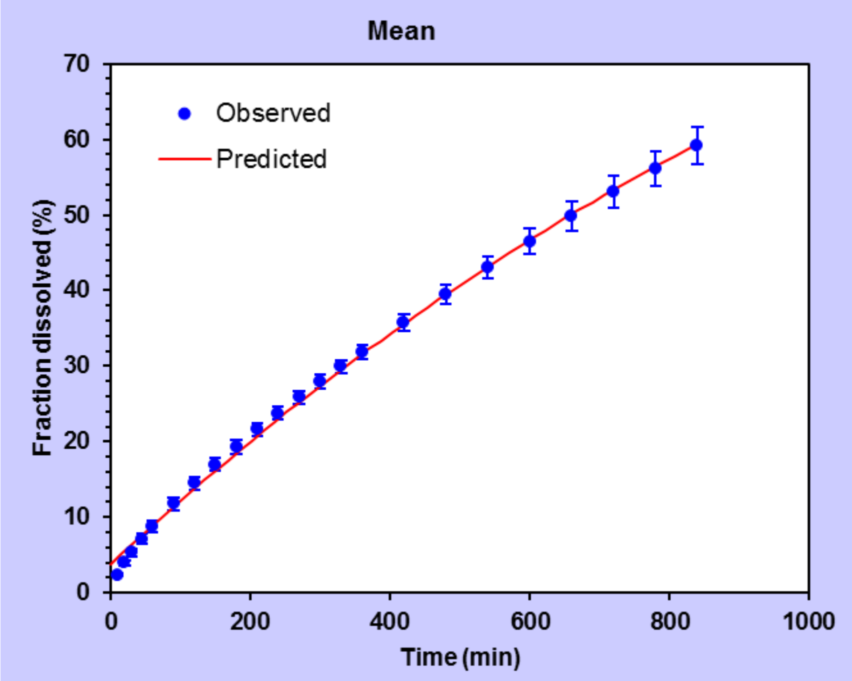

Supplementary materials – Model fitting summary – Avicel® PH-200 (modelling up to app. 60 % of carvedilol released)

Graphical abstract of model fit presented as the fraction % of released carvedilol per tested tablet:

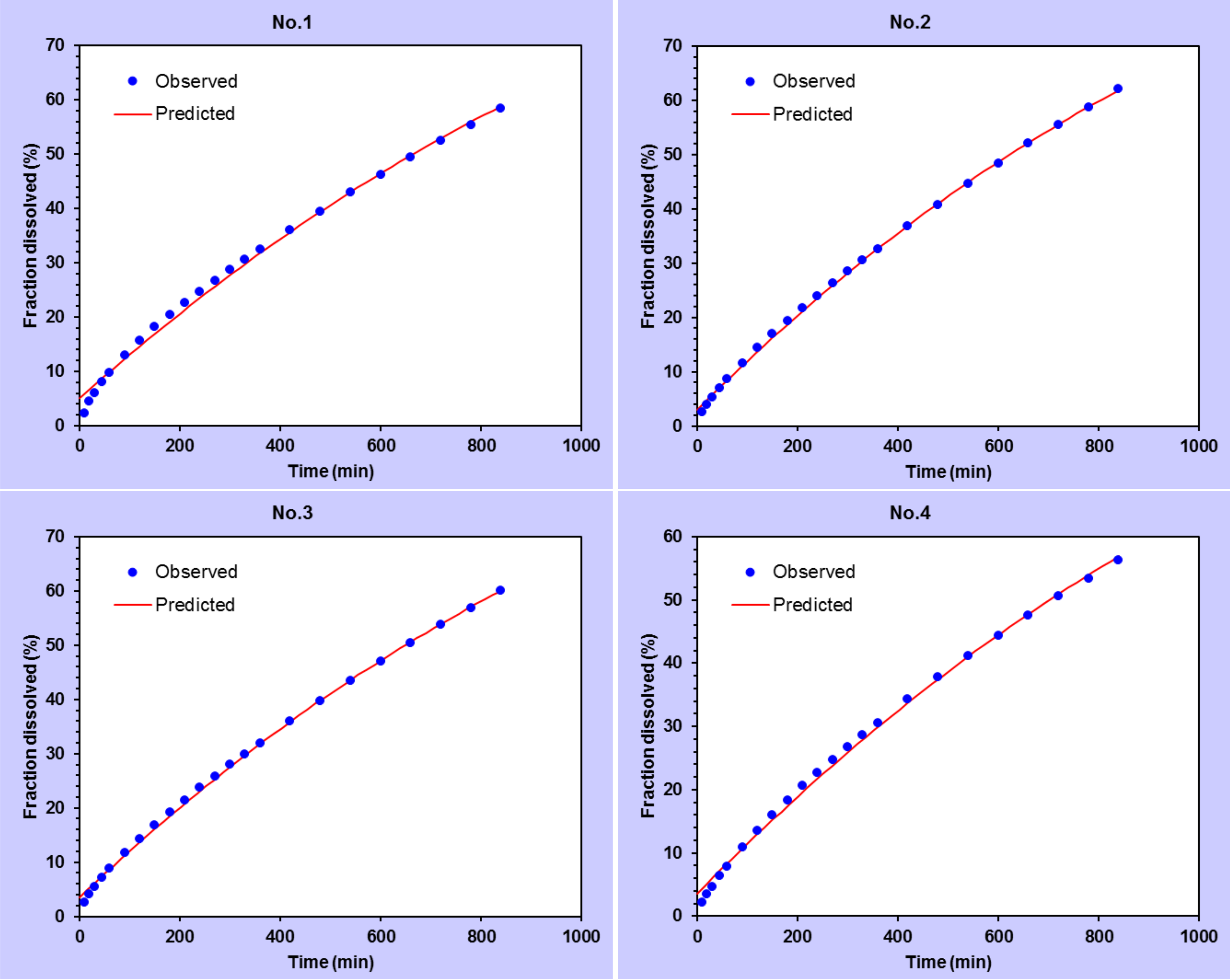

Supplementary materials – Model fitting summary – Avicel® PH-200 (modelling up to app. 60 % of carvedilol released)

Model: **Hopfenberg**

Model equation: $F = 100 \cdot [1 - (1 - k_{HB} \cdot t)^n]$

Fitted model parameters per tested tablet (N = 4) with statistics – mean, standard deviation (SD), and relative standard deviation expressed in % (RSD%) (output from DDSolver):

Parameter	No.1	No.2	No.3	No.4	Mean	SD	RSD(%)
k_{HB}	0.0003	0.0003	0.0003	0.0003	0.0003	0.0000	4.5347
n	3.0000	3.0000	3.0000	3.0000	3.0000	0.0000	0.0000

Number of dissolution data points (N), degrees of freedom (df), and selected goodness of fit criteria – Pearson correlation coefficient (R), coefficient of determination (R^2), adjusted coefficient of determination ($R^2_{adjusted}$), and residual sum of squares (RSS) (manual calculation in MS Excel):

Parameter	No.1	No.2	No.3	No.4
N	23	23	23	23
df	21	21	21	21
R	0.997884116	0.999657641	0.999488852	0.99887057
R^2	0.995772709	0.9993154	0.998977965	0.997742415
$R^2_{adjusted}$	0.995571409	0.9992828	0.998929297	0.997634911
RSS	200.2712127	68.88025006	93.6775615	98.41099489

Graphical abstract of model fit presented as mean ± 1 SD of the fraction % of released carvedilol:

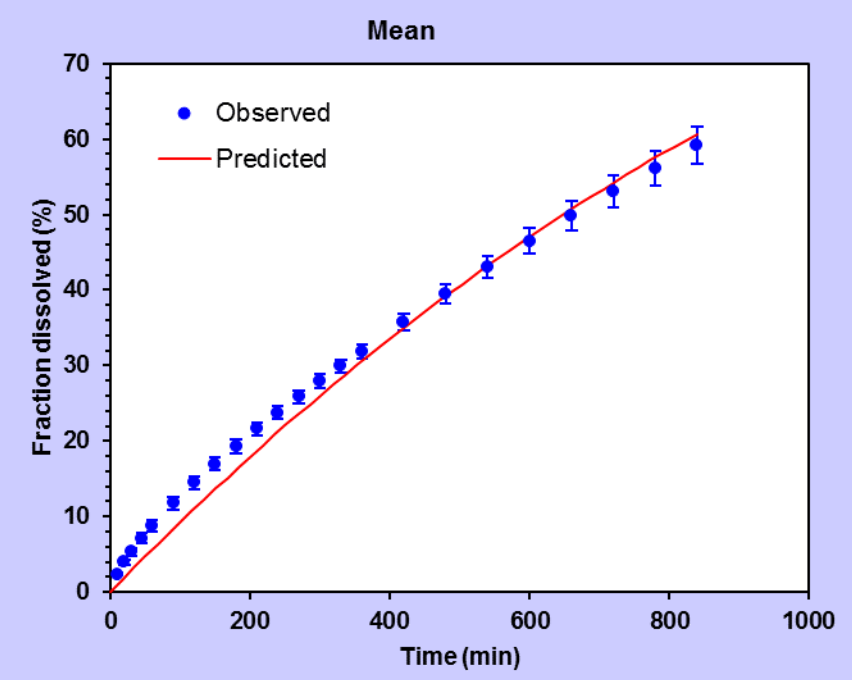

Supplementary materials – Model fitting summary – Avicel® PH-200 (modelling up to app. 60 % of carvedilol released)

Graphical abstract of model fit presented as the fraction % of released carvedilol per tested tablet:

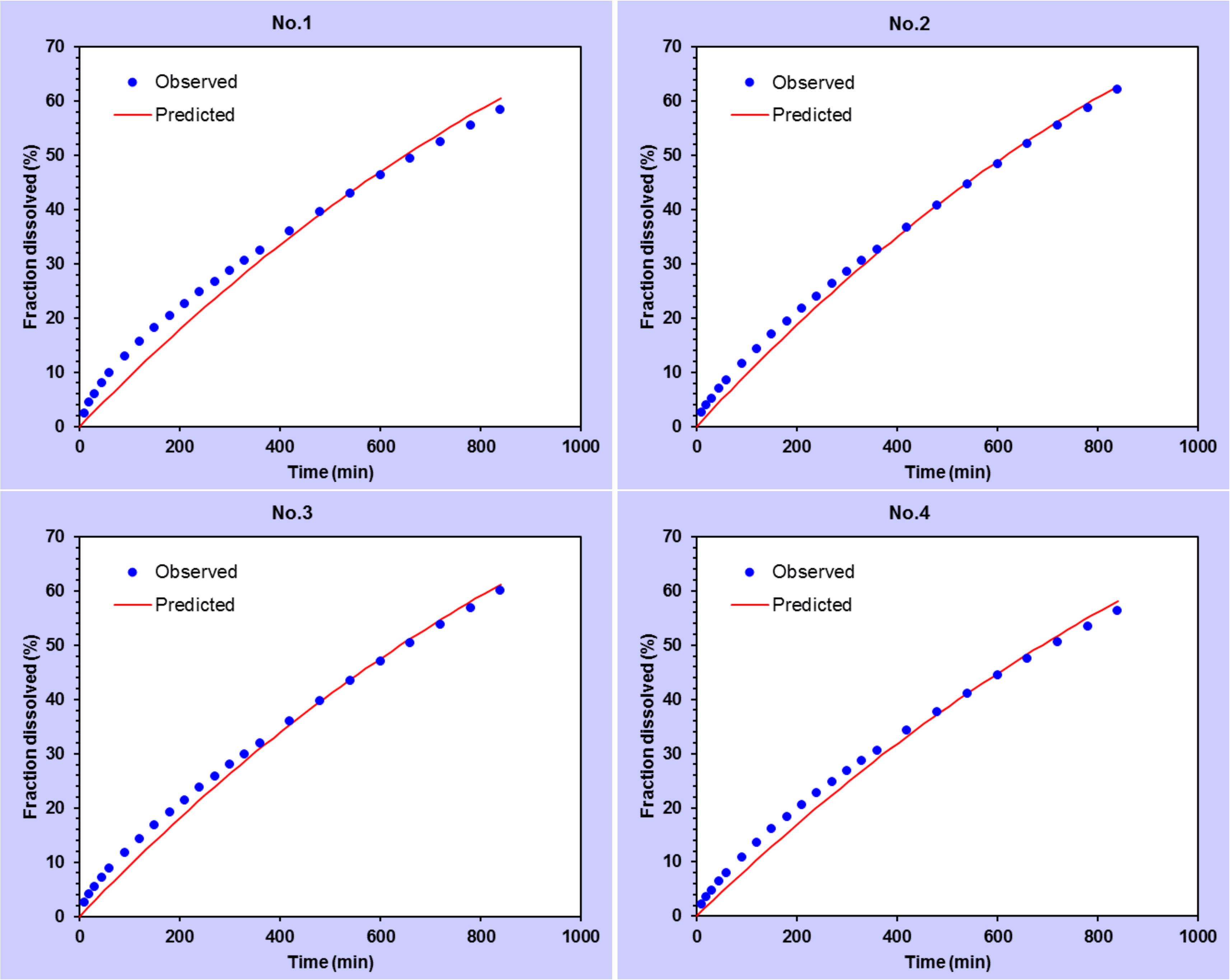

Supplementary materials – Model fitting summary – Avicel® PH-200 (modelling up to app. 60 % of carvedilol released)

Model: **Hopfenberg with T_{lag}**

Model equation: $F = 100 \cdot \{1 - [1 - k_{HB} \cdot (t - T_{lag})]^n\}$

Fitted model parameters per tested tablet (N = 4) with statistics – mean, standard deviation (SD), and relative standard deviation expressed in % (RSD%) (output from DDSolver):

Parameter	No.1	No.2	No.3	No.4	Mean	SD	RSD(%)
k_{HB}	0.0003	0.0003	0.0003	0.0003	0.0003	0.0000	5.7650
n	3.0000	3.0000	3.0000	3.0000	3.0000	0.0000	0.0000
T_{lag}	-60.5459	-32.1442	-39.7878	-43.4226	-43.9751	12.0055	-27.3008

Number of dissolution data points (N), degrees of freedom (df), and selected goodness of fit criteria – Pearson correlation coefficient (R), coefficient of determination (R^2), adjusted coefficient of determination ($R^2_{adjusted}$), and residual sum of squares (RSS) (manual calculation in MS Excel):

Parameter	No.1	No.2	No.3	No.4
N	23	23	23	23
df	20	20	20	20
R	0.997530438	0.999621144	0.999397345	0.998654796
R^2	0.995066974	0.999242432	0.998795052	0.997311402
$R^2_{adjusted}$	0.994573672	0.999166675	0.998674557	0.997042542
RSS	32.8473457	5.846537204	8.674647982	17.56530286

Graphical abstract of model fit presented as mean ± 1 SD of the fraction % of released carvedilol:

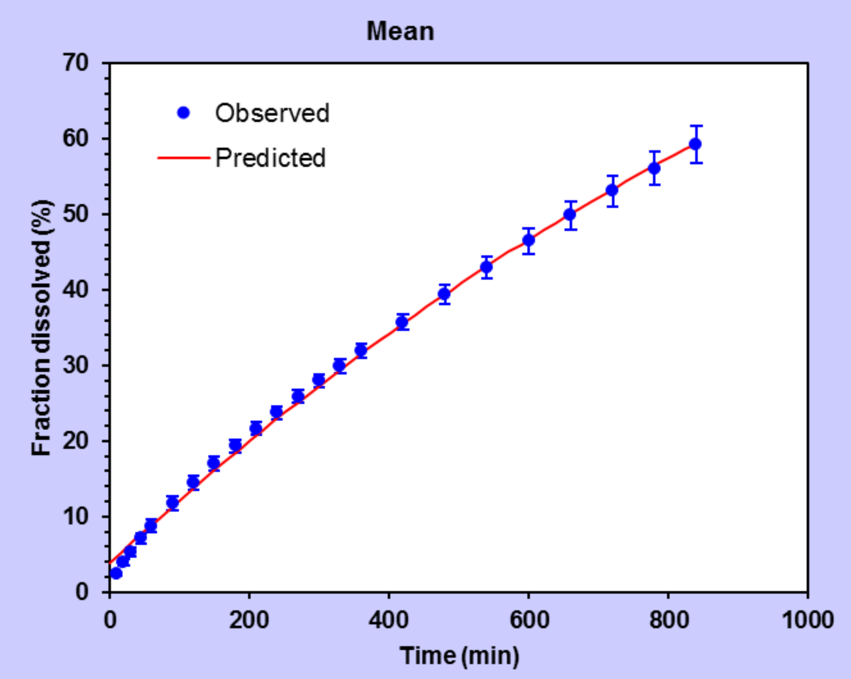

Supplementary materials – Model fitting summary – Avicel® PH-200 (modelling up to app. 60 % of carvedilol released)

Graphical abstract of model fit presented as the fraction % of released carvedilol per tested tablet:

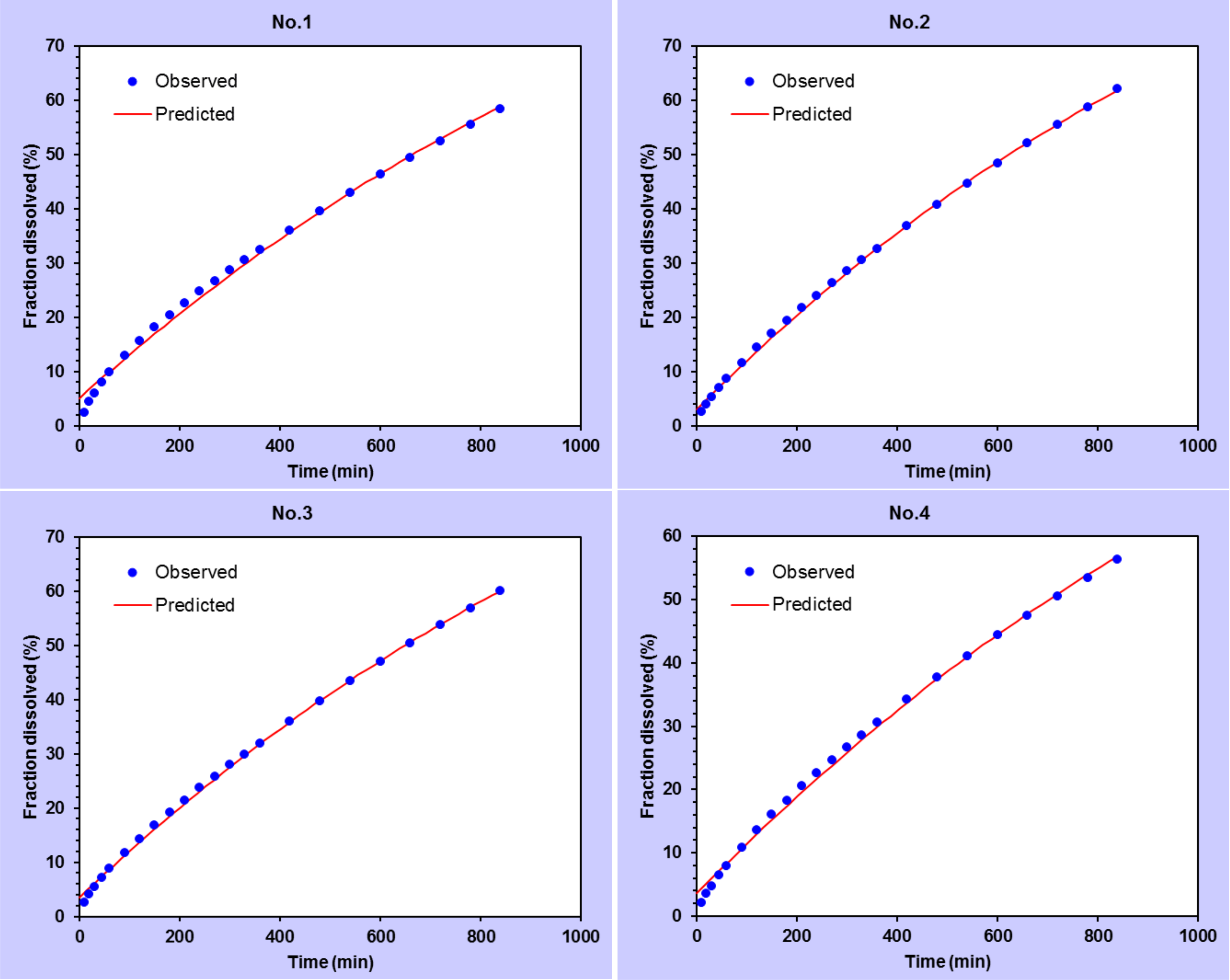

Supplementary materials – Model fitting summary – Avicel® PH-200 (modelling up to app. 60 % of carvedilol released)

Model: **Baker–Lonsdale**

Model equation: $\frac{3}{2}\cdot\left[1-\left(1-\frac{F}{100}\right)^{\frac{2}{3}}\right]-\frac{F}{100}=k_{BL}\cdot t$

Fitted model parameters per tested tablet (N = 4) with statistics – mean, standard deviation (SD), and relative standard deviation expressed in % (RSD%) (output from DDSolver):

Parameter	No.1	No.2	No.3	No.4	Mean	SD	RSD(%)
k_{BL}	0.00005	0.00011	0.00005	0.00004	0.00006	0.00003	49.84724

Number of dissolution data points (N), degrees of freedom (df), and selected goodness of fit criteria – Pearson correlation coefficient (R), coefficient of determination (R^2), adjusted coefficient of determination ($R^2_{adjusted}$), and residual sum of squares (RSS) (manual calculation in MS Excel):

Parameter	No.1	No.2	No.3	No.4
N	23	23	23	23
df	22	22	22	22
R	0.992899644	0.984255221	0.988408935	0.990954506
R^2	0.985849703	0.968758341	0.976952224	0.981990833
$R^2_{adjusted}$	0.985849703	0.968758341	0.976952224	0.981990833
RSS	988.0629829	1811.03271	1029.510043	948.553977

Graphical abstract of model fit presented as mean ± 1 SD of the fraction % of released carvedilol:

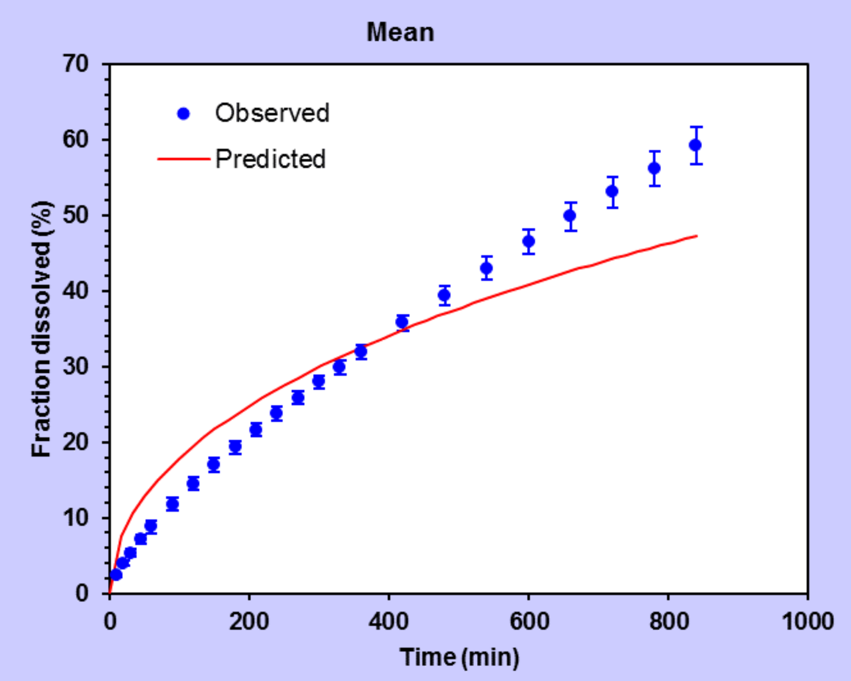

Supplementary materials – Model fitting summary – Avicel® PH-200 (modelling up to app. 60 % of carvedilol released)

Graphical abstract of model fit presented as the fraction % of released carvedilol per tested tablet:

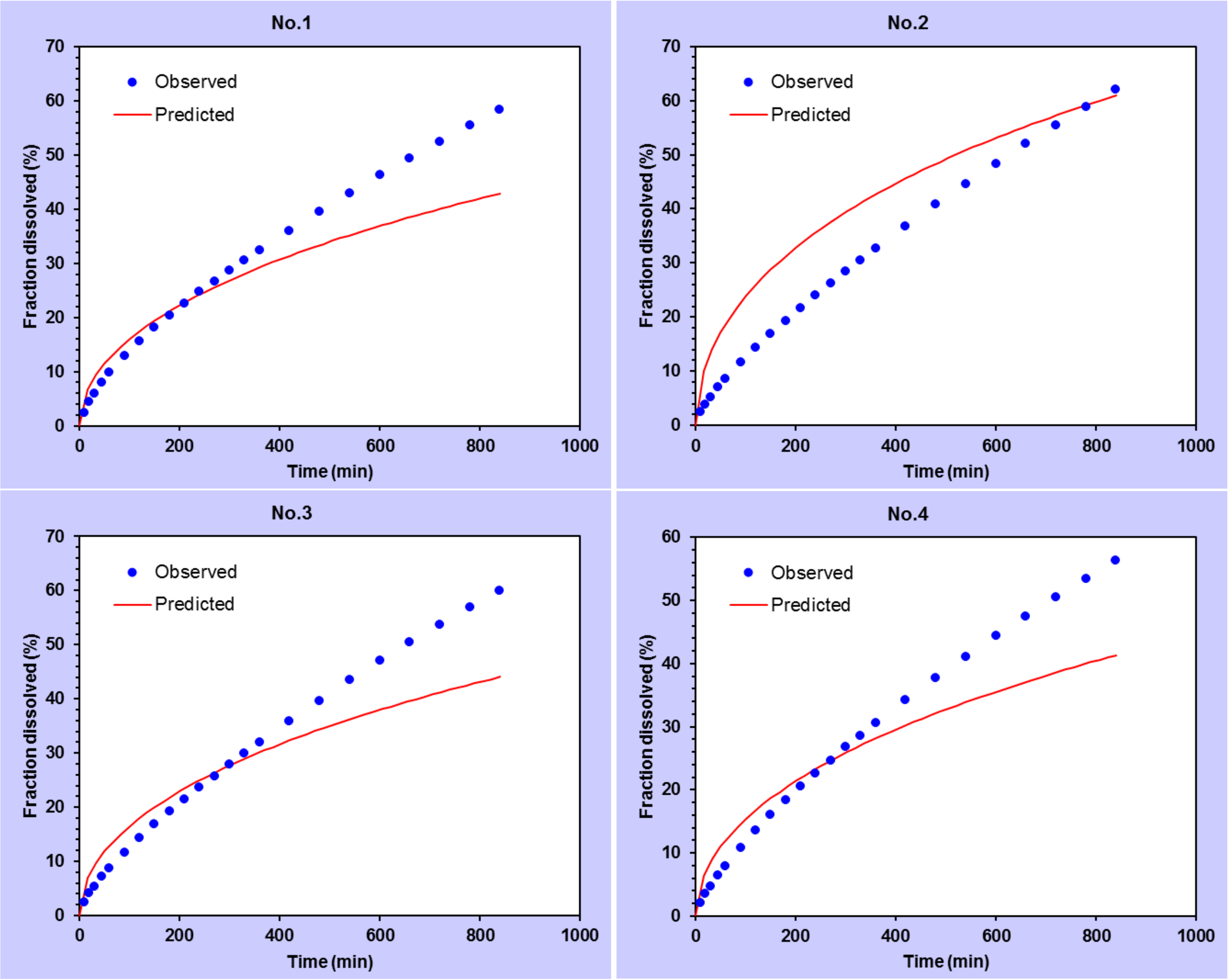

Supplementary materials – Model fitting summary – Avicel® PH-200 (modelling up to app. 60 % of carvedilol released)

Model: **Baker–Lonsdale with T_{lag}**

Model equation: $\frac{3}{2}\cdot\left[1-\left(1-\frac{F}{100}\right)^{\frac{2}{3}}\right]-\frac{F}{100}=k_{BL}\cdot\left(t-T_{lag}\right)$

Fitted model parameters per tested tablet (N = 4) with statistics – mean, standard deviation (SD), and relative standard deviation expressed in % (RSD%) (output from DDSolver):

Parameter	No.1	No.2	No.3	No.4	Mean	SD	RSD(%)
k_{BL}	0.0001	0.0001	0.0001	0.0001	0.0001	0.0000	9.6417
T_{lag}	76.2794	90.4448	87.0319	83.5502	84.3266	6.0584	7.1844

Number of dissolution data points (N), degrees of freedom (df), and selected goodness of fit criteria – Pearson correlation coefficient (R), coefficient of determination (R^2), adjusted coefficient of determination ($R^2_{adjusted}$), and residual sum of squares (RSS) (manual calculation in MS Excel):

Parameter	No.1	No.2	No.3	No.4
N	23	23	23	23
df	21	21	21	21
R	0.981623878	0.975048027	0.978408936	0.982362791
R^2	0.963585439	0.950718654	0.957284047	0.965036653
$R^2_{adjusted}$	0.961851412	0.948371923	0.955249954	0.963371731
RSS	353.8504432	518.0396389	415.4303284	298.1103047

Graphical abstract of model fit presented as mean ± 1 SD of the fraction % of released carvedilol:

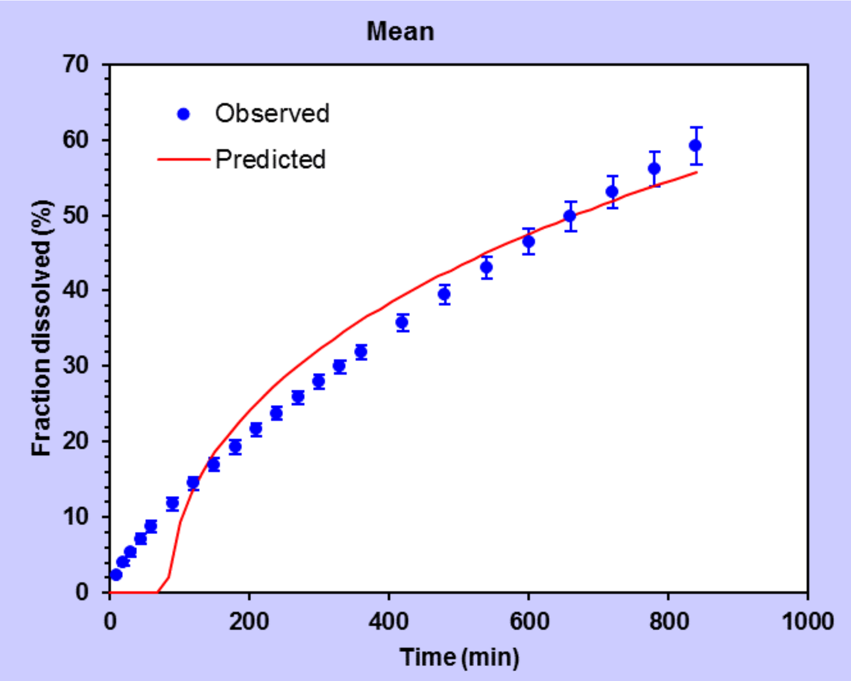

Supplementary materials – Model fitting summary – Avicel® PH-200 (modelling up to app. 60 % of carvedilol released)

Graphical abstract of model fit presented as the fraction % of released carvedilol per tested tablet:

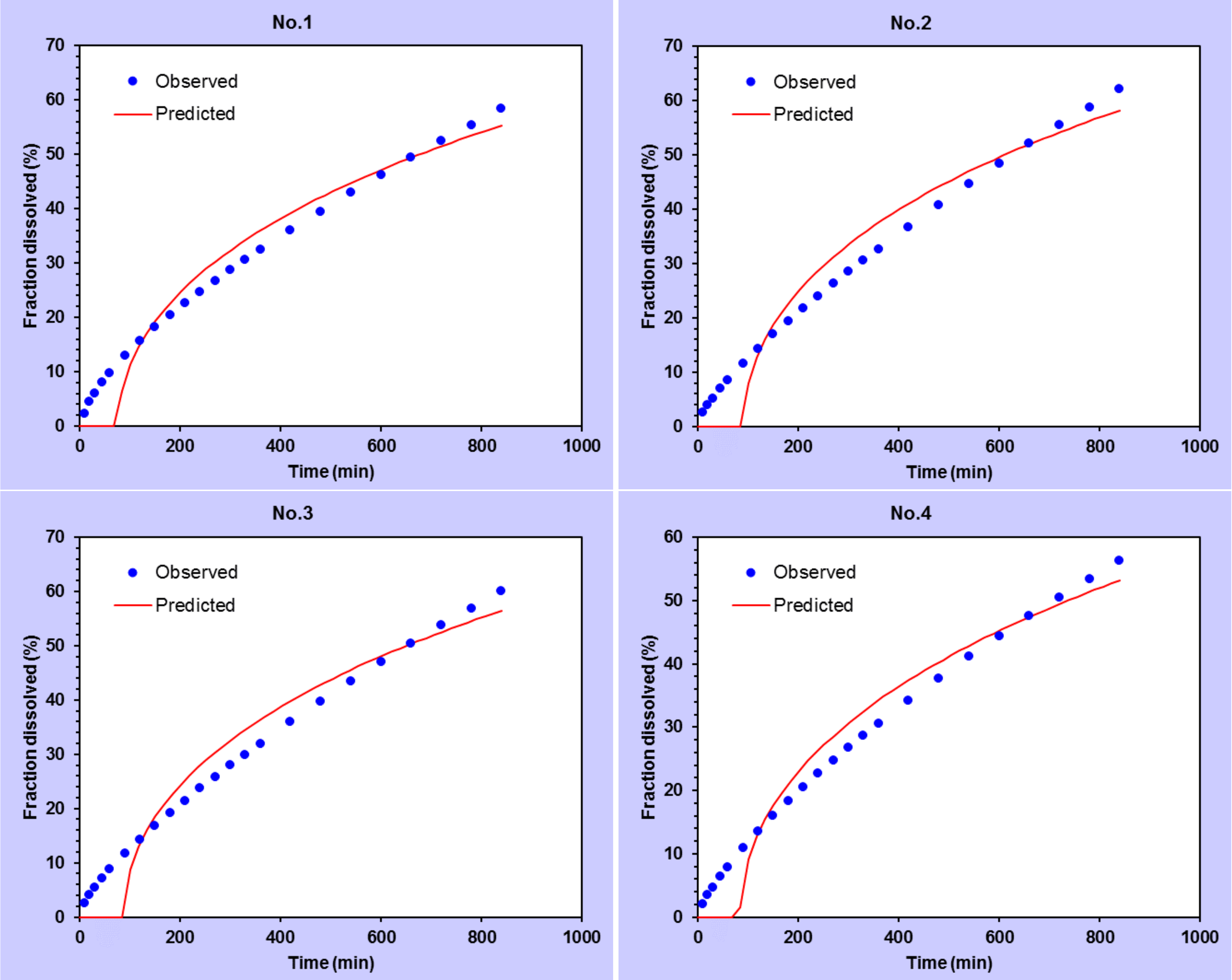

Supplementary materials – Model fitting summary – Avicel® PH-200 (modelling up to app. 60 % of carvedilol released)

Model: **Makoid–Banakar**

Model equation: $F = k_{MB} \cdot t^n \cdot e^{-k \cdot t}$

Fitted model parameters per tested tablet (N = 4) with statistics – mean, standard deviation (SD), and relative standard deviation expressed in % (RSD%) (output from DDSolver):

Parameter	No.1	No.2	No.3	No.4	Mean	SD	RSD(%)
k_{MB}	0.47827	0.46460	0.49738	0.36286	0.45078	0.06013	13.33982
n	0.72996	0.71413	0.70045	0.75647	0.72525	0.02405	3.31664
k	0.00016	-0.00012	-0.00010	0.00006	0.00000	0.00013	-7562.63933

Number of dissolution data points (N), degrees of freedom (df), and selected goodness of fit criteria – Pearson correlation coefficient (R), coefficient of determination (R^2), adjusted coefficient of determination ($R^2_{adjusted}$), and residual sum of squares (RSS) (manual calculation in MS Excel):

Parameter	No.1	No.2	No.3	No.4
N	23	23	23	23
df	20	20	20	20
R	0.999467185	0.99989946	0.999940787	0.999993081
R^2	0.998934654	0.999798929	0.999881577	0.999986162
$R^2_{adjusted}$	0.998828119	0.999778822	0.999869735	0.999984778
RSS	7.165314439	1.617072032	0.851504505	0.092355861

Graphical abstract of model fit presented as mean ± 1 SD of the fraction % of released carvedilol:

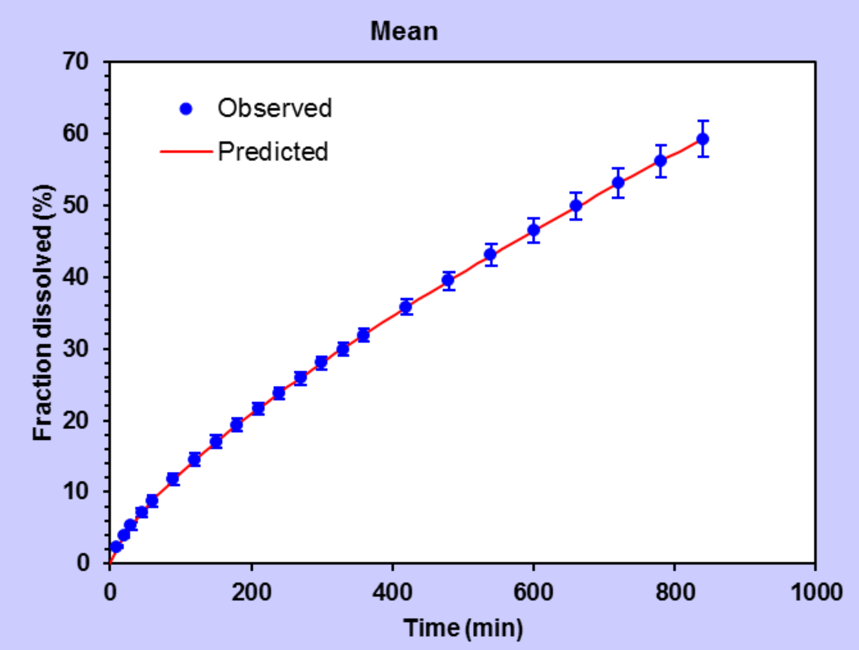

Supplementary materials – Model fitting summary – Avicel® PH-200 (modelling up to app. 60 % of carvedilol released)

Graphical abstract of model fit presented as the fraction % of released carvedilol per tested tablet:

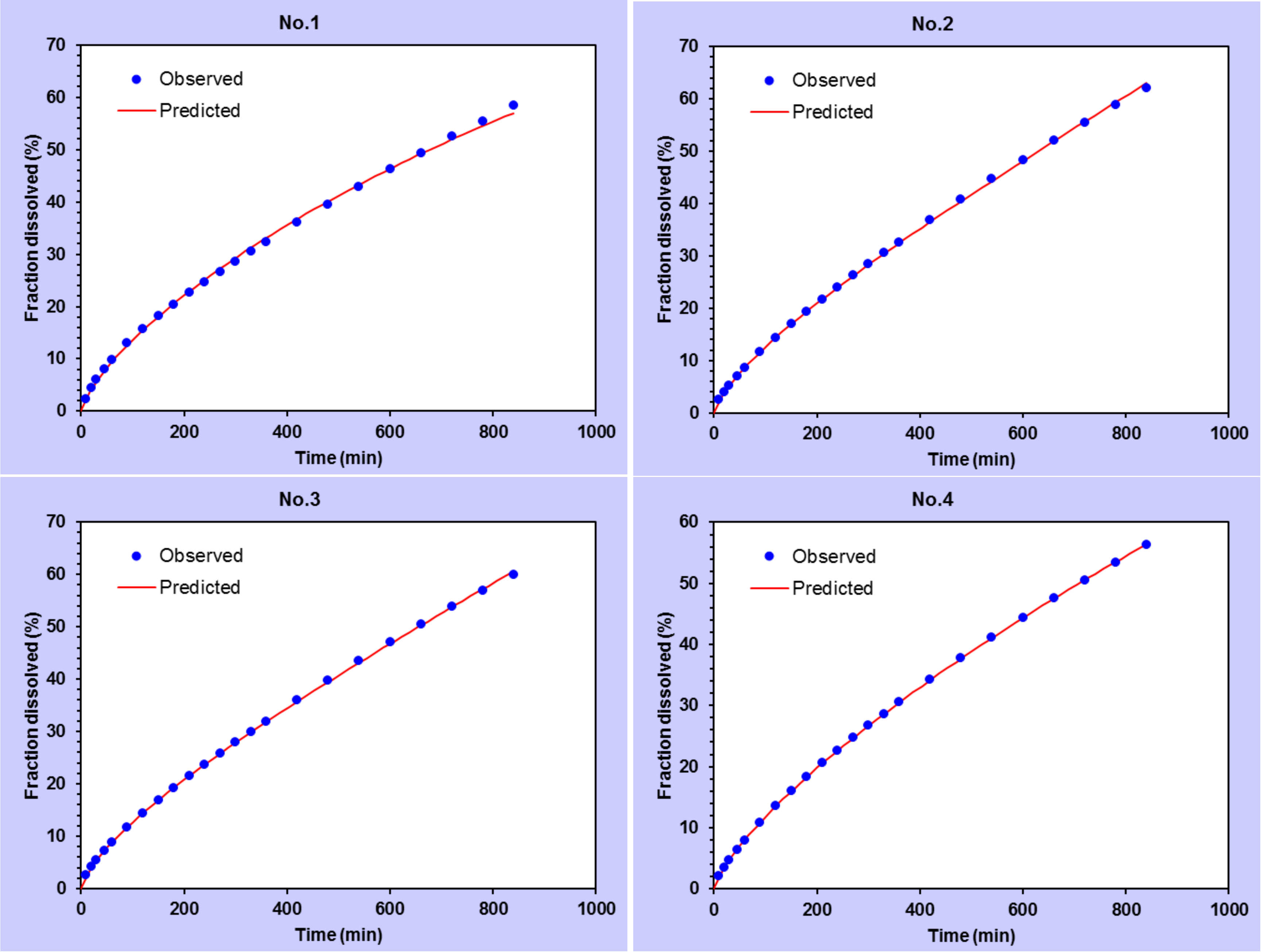

Supplementary materials – Model fitting summary – Avicel® PH-200 (modelling up to app. 60 % of carvedilol released)

Model: **Makoid–Banakar with T_{lag}**

Model equation: $F = k_{MB} \cdot (t - T_{lag})^n \cdot e^{-k \cdot (t - T_{lag})}$

Fitted model parameters per tested tablet (N = 4) with statistics – mean, standard deviation (SD), and relative standard deviation expressed in % (RSD%) (output from DDSolver):

Parameter	No.1	No.2	No.3	No.4	Mean	SD	RSD(%)
k_{MB}	0.74158	0.72624	0.81082	0.60237	0.72025	0.08678	12.04788
n	0.63900	0.62081	0.60357	0.65207	0.62886	0.02118	3.36838
k	-0.00008	-0.00037	-0.00030	-0.00019	-0.00024	0.00013	-54.32654
T_{lag}	4.00000	4.00000	5.16241	5.08937	4.56294	0.65072	14.26088

Number of dissolution data points (N), degrees of freedom (df), and selected goodness of fit criteria – Pearson correlation coefficient (R), coefficient of determination (R^2), adjusted coefficient of determination ($R^2_{adjusted}$), and residual sum of squares (RSS) (manual calculation in MS Excel):

Parameter	No.1	No.2	No.3	No.4
N	23	23	23	23
df	19	19	19	19
R	0.999984674	0.999086802	0.999613363	0.999610257
R^2	0.999969348	0.998174438	0.999226876	0.999220667
$R^2_{adjusted}$	0.999964508	0.997886192	0.999104804	0.999097614
RSS	0.20661661	15.3967182	8.287820077	7.462251508

Graphical abstract of model fit presented as mean ± 1 SD of the fraction % of released carvedilol:

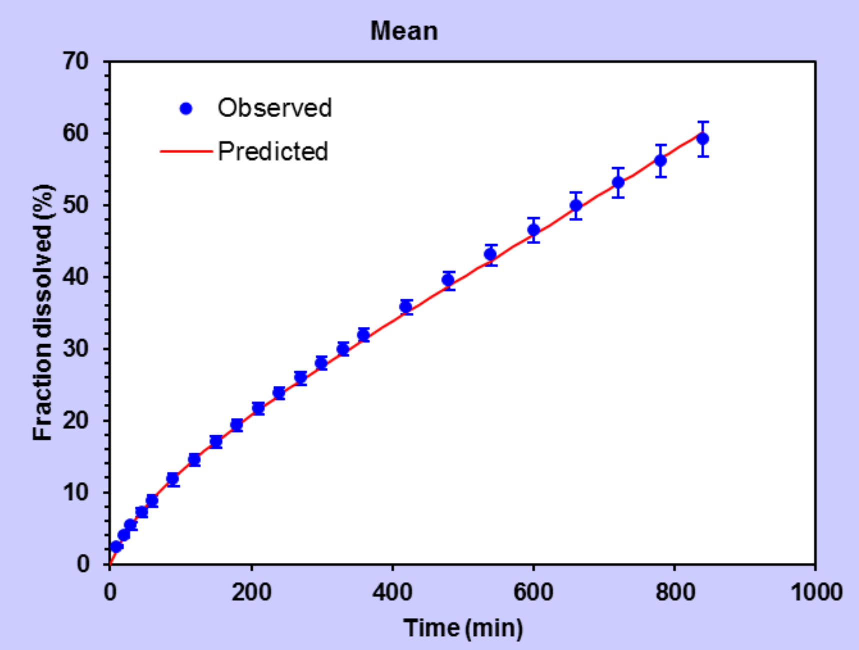

Supplementary materials – Model fitting summary – Avicel® PH-200 (modelling up to app. 60 % of carvedilol released)

Graphical abstract of model fit presented as the fraction % of released carvedilol per tested tablet:

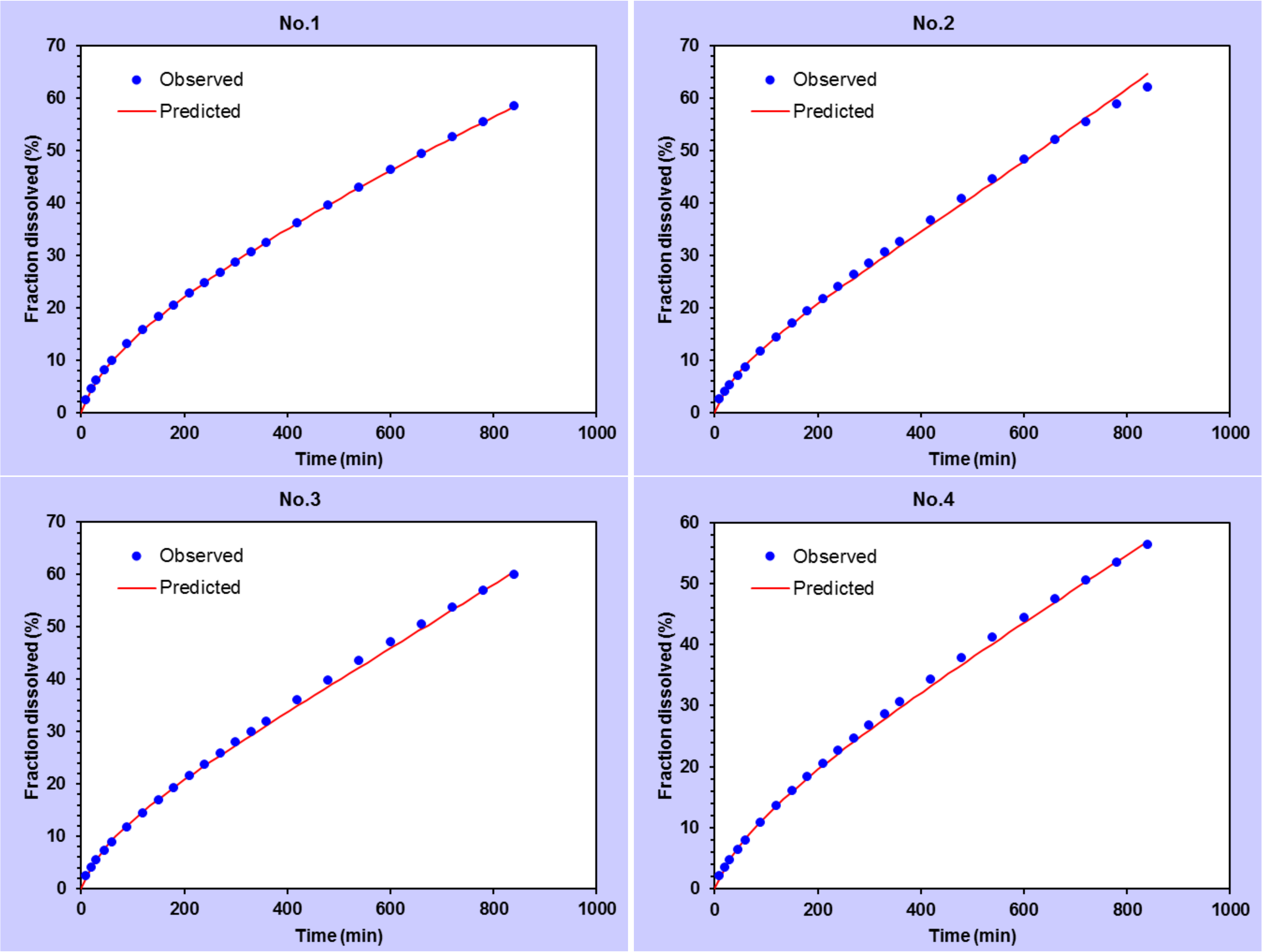

Supplementary materials – Model fitting summary – Avicel® PH-200 (modelling up to app. 60 % of carvedilol released)

Model: **Peppas–Sahlin_1**

Model equation: $F = k_1 \cdot t^m + k_2 \cdot t^{2m}$

Fitted model parameters per tested tablet (N = 4) with statistics – mean, standard deviation (SD), and relative standard deviation expressed in % (RSD%) (output from DDSolver):

Parameter	No.1	No.2	No.3	No.4	Mean	SD	RSD(%)
k_1	1.055	0.705	0.773	0.753	0.822	0.158	19.270
k_2	0.086	0.112	0.104	0.097	0.100	0.011	10.991
m	0.450	0.450	0.450	0.450	0.450	0.000	0.000

Number of dissolution data points (N), degrees of freedom (df), and selected goodness of fit criteria – Pearson correlation coefficient (R), coefficient of determination (R^2), adjusted coefficient of determination ($R^2_{adjusted}$), and residual sum of squares (RSS) (manual calculation in MS Excel):

Parameter	No.1	No.2	No.3	No.4
N	23	23	23	23
df	20	20	20	20
R	0.999699592	0.999880278	0.999884168	0.999565243
R^2	0.999399275	0.999760571	0.999768349	0.999130675
$R^2_{adjusted}$	0.999339202	0.999736628	0.999745184	0.999043742
RSS	4.623542977	2.040688666	1.838105965	6.281465386

Graphical abstract of model fit presented as mean ± 1 SD of the fraction % of released carvedilol:

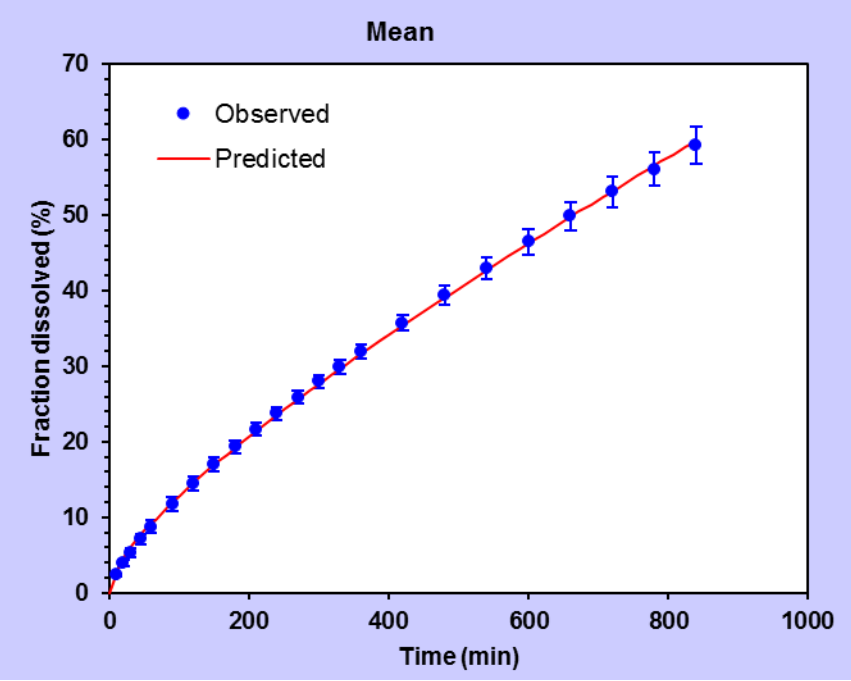

Supplementary materials – Model fitting summary – Avicel® PH-200 (modelling up to app. 60 % of carvedilol released)

Graphical abstract of model fit presented as the fraction % of released carvedilol per tested tablet:

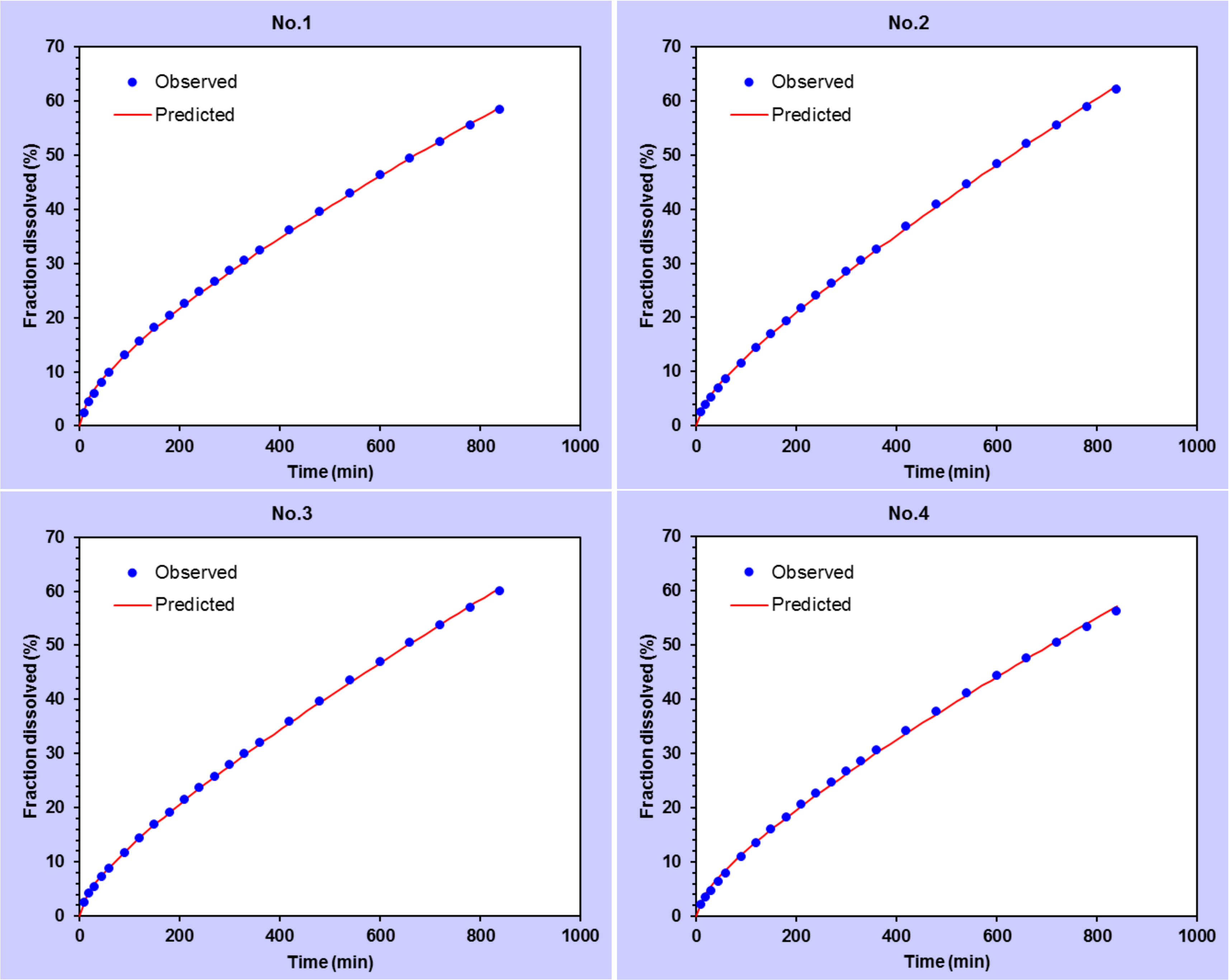

Supplementary materials – Model fitting summary – Avicel® PH-200 (modelling up to app. 60 % of carvedilol released)

Model: **Peppas–Sahlin_1 with T_{lag}**

Model equation: $F = k_1 \cdot (t - T_{lag})^m + k_2 \cdot (t - T_{lag})^{2m}$

Fitted model parameters per tested tablet (N = 4) with statistics – mean, standard deviation (SD), and relative standard deviation expressed in % (RSD%) (output from DDSolver):

Parameter	No.1	No.2	No.3	No.4	Mean	SD	RSD(%)
k_1	1.132	0.778	0.845	0.822	0.895	0.161	17.954
k_2	0.083	0.109	0.101	0.094	0.097	0.011	11.497
m	0.450	0.450	0.450	0.450	0.450	0.000	0.000
T_{lag}	6.000	4.000	4.000	6.000	5.000	1.155	23.094

Number of dissolution data points (N), degrees of freedom (df), and selected goodness of fit criteria – Pearson correlation coefficient (R), coefficient of determination (R^2), adjusted coefficient of determination ($R^2_{adjusted}$), and residual sum of squares (RSS) (manual calculation in MS Excel):

Parameter	No.1	No.2	No.3	No.4
N	23	23	23	23
df	19	19	19	19
R	0.999943491	0.999937799	0.999946257	0.999757041
R^2	0.999886985	0.999875602	0.999892518	0.999514142
$R^2_{adjusted}$	0.999869141	0.99985596	0.999875547	0.999437427
RSS	1.061293121	0.978073355	0.780643032	3.352748134

Graphical abstract of model fit presented as mean ± 1 SD of the fraction % of released carvedilol:

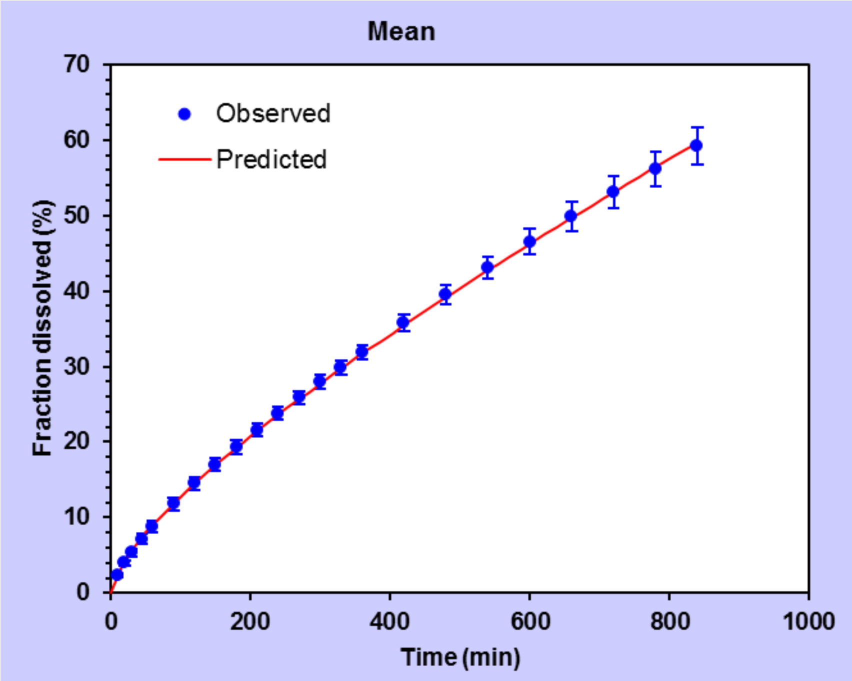

Supplementary materials – Model fitting summary – Avicel® PH-200 (modelling up to app. 60 % of carvedilol released)

Graphical abstract of model fit presented as the fraction % of released carvedilol per tested tablet:

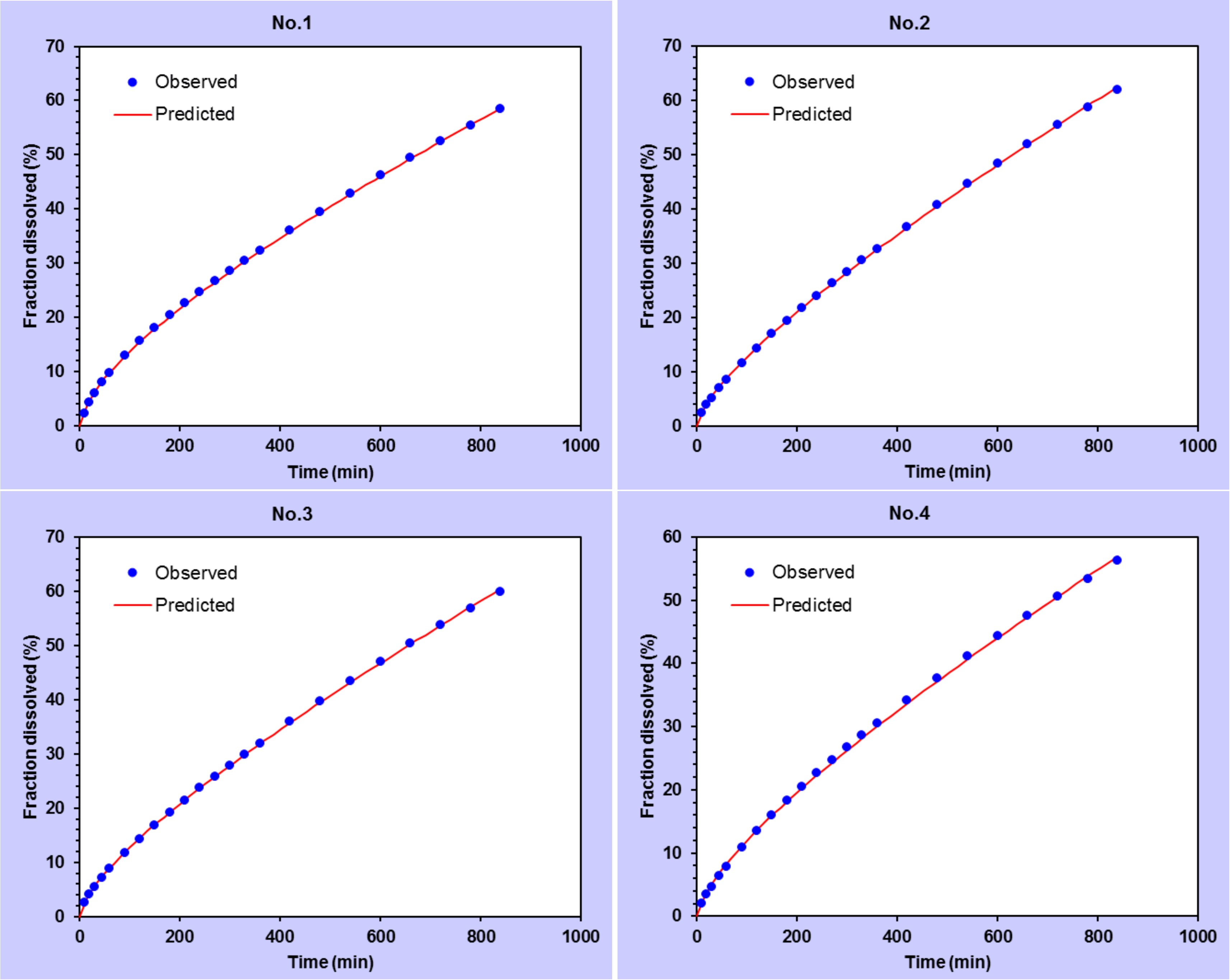

Supplementary materials – Model fitting summary – Avicel® PH-200 (modelling up to app. 60 % of carvedilol released)

Model: **Peppas–Sahlin_2**

Model equation: $F = k_1 \cdot t^{0.5} + k_2 \cdot t$

Fitted model parameters per tested tablet (N = 4) with statistics – mean, standard deviation (SD), and relative standard deviation expressed in % (RSD%) (output from DDSolver):

Parameter	No.1	No.2	No.3	No.4	Mean	SD	RSD(%)
k_1	1.055	0.816	0.855	0.828	0.889	0.112	12.656
k_2	0.034	0.047	0.043	0.040	0.041	0.006	13.531

Number of dissolution data points (N), degrees of freedom (df), and selected goodness of fit criteria – Pearson correlation coefficient (R), coefficient of determination (R^2), adjusted coefficient of determination ($R^2_{adjusted}$), and residual sum of squares (RSS) (manual calculation in MS Excel):

Parameter	No.1	No.2	No.3	No.4
N	23	23	23	23
df	21	21	21	21
R	0.999690531	0.999774529	0.999800498	0.999432238
R^2	0.999381158	0.999549109	0.999601037	0.998864798
$R^2_{adjusted}$	0.99935169	0.999527638	0.999582038	0.998810741
RSS	5.067417244	4.031172306	3.320134466	8.612531046

Graphical abstract of model fit presented as mean ± 1 SD of the fraction % of released carvedilol:

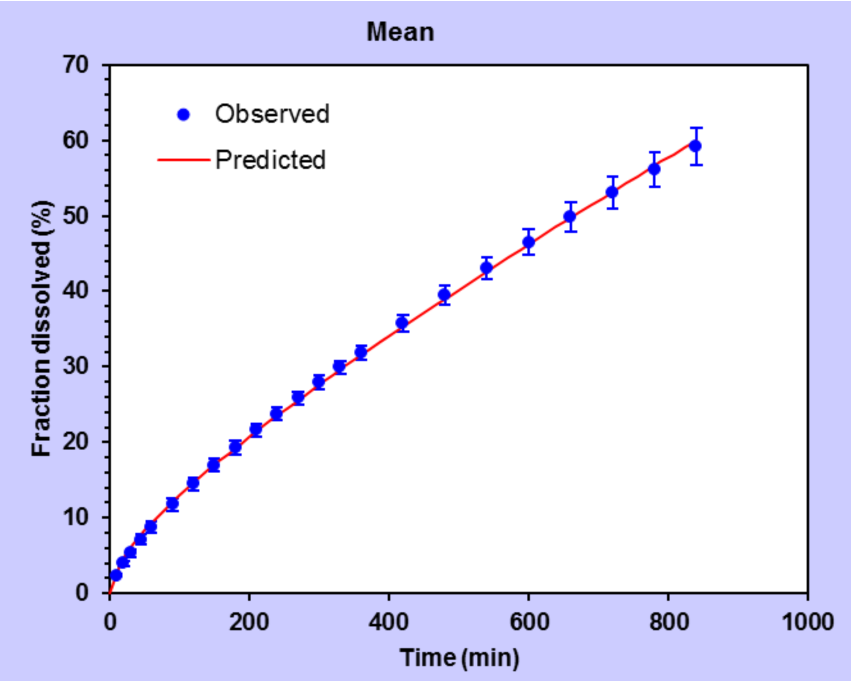

Supplementary materials – Model fitting summary – Avicel® PH-200 (modelling up to app. 60 % of carvedilol released)

Graphical abstract of model fit presented as the fraction % of released carvedilol per tested tablet:

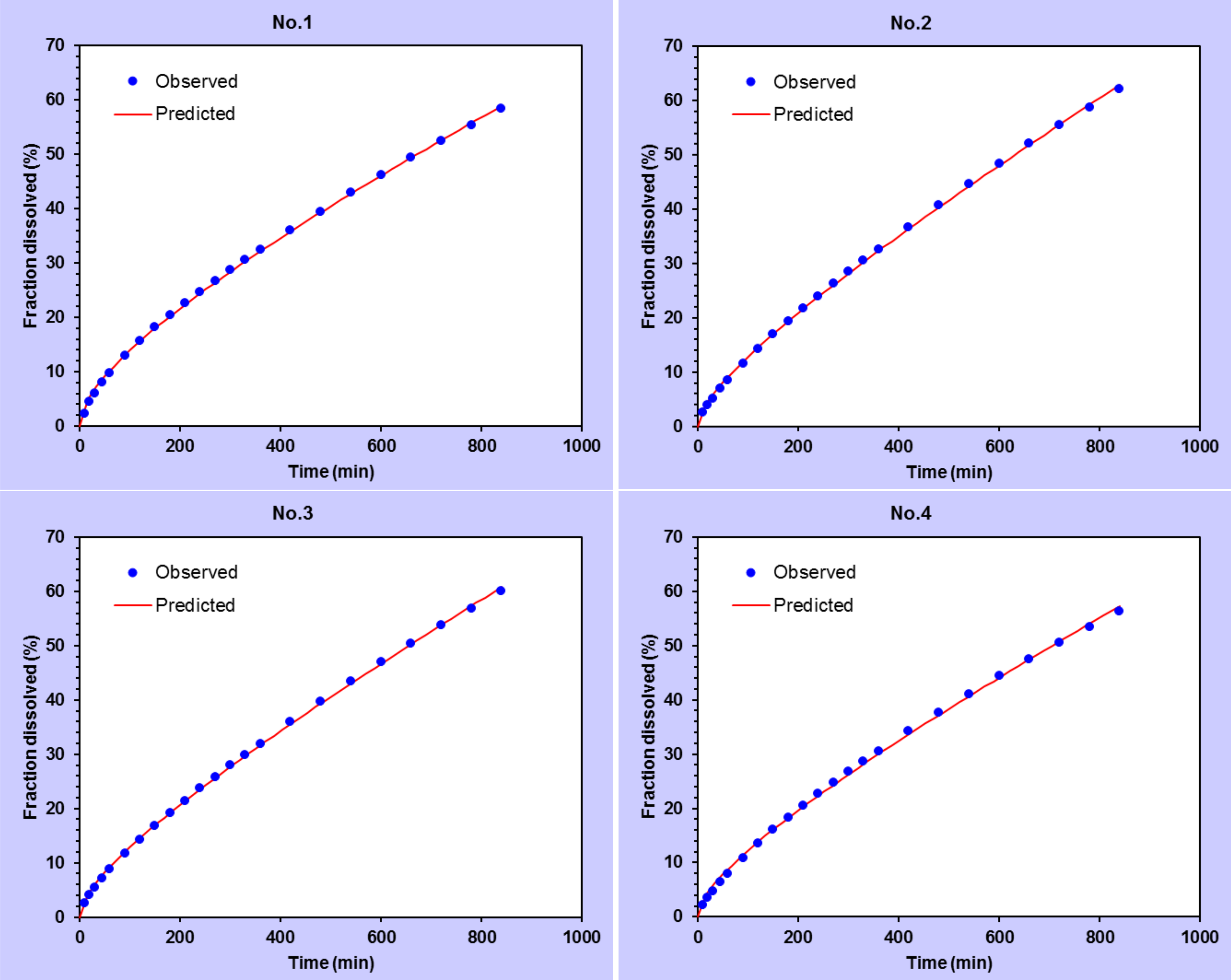

Supplementary materials – Model fitting summary – Avicel® PH-200 (modelling up to app. 60 % of carvedilol released)

Model: **Peppas–Sahlin_2 with T_{lag}**

Model equation: $F = k_1 \cdot (t - T_{lag})^{0.5} + k_2 \cdot (t - T_{lag})$

Fitted model parameters per tested tablet (N = 4) with statistics – mean, standard deviation (SD), and relative standard deviation expressed in % (RSD%) (output from DDSolver):

Parameter	No.1	No.2	No.3	No.4	Mean	SD	RSD(%)
k_1	1.109	0.867	0.906	0.877	0.940	0.114	12.118
k_2	0.032	0.045	0.041	0.038	0.039	0.006	14.265
T_{lag}	6.000	4.000	4.000	6.000	5.000	1.155	23.094

Number of dissolution data points (N), degrees of freedom (df), and selected goodness of fit criteria – Pearson correlation coefficient (R), coefficient of determination (R^2), adjusted coefficient of determination ($R^2_{adjusted}$), and residual sum of squares (RSS) (manual calculation in MS Excel):

Parameter	No.1	No.2	No.3	No.4
N	23	23	23	23
df	20	20	20	20
R	0.999941949	0.99987385	0.999898052	0.999667529
R^2	0.999883902	0.999747717	0.999796115	0.999335169
$R^2_{adjusted}$	0.999872292	0.999722488	0.999775726	0.999268686
RSS	1.056720241	2.087003663	1.548675543	4.532684555

Graphical abstract of model fit presented as mean ± 1 SD of the fraction % of released carvedilol:

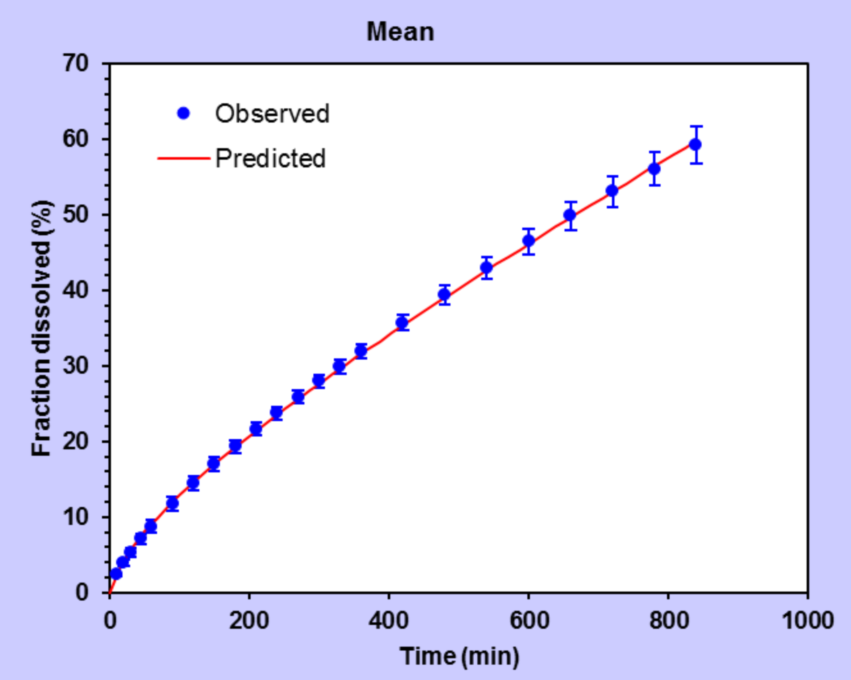

Supplementary materials – Model fitting summary – Avicel® PH-200 (modelling up to app. 60 % of carvedilol released)

Graphical abstract of model fit presented as the fraction % of released carvedilol per tested tablet:

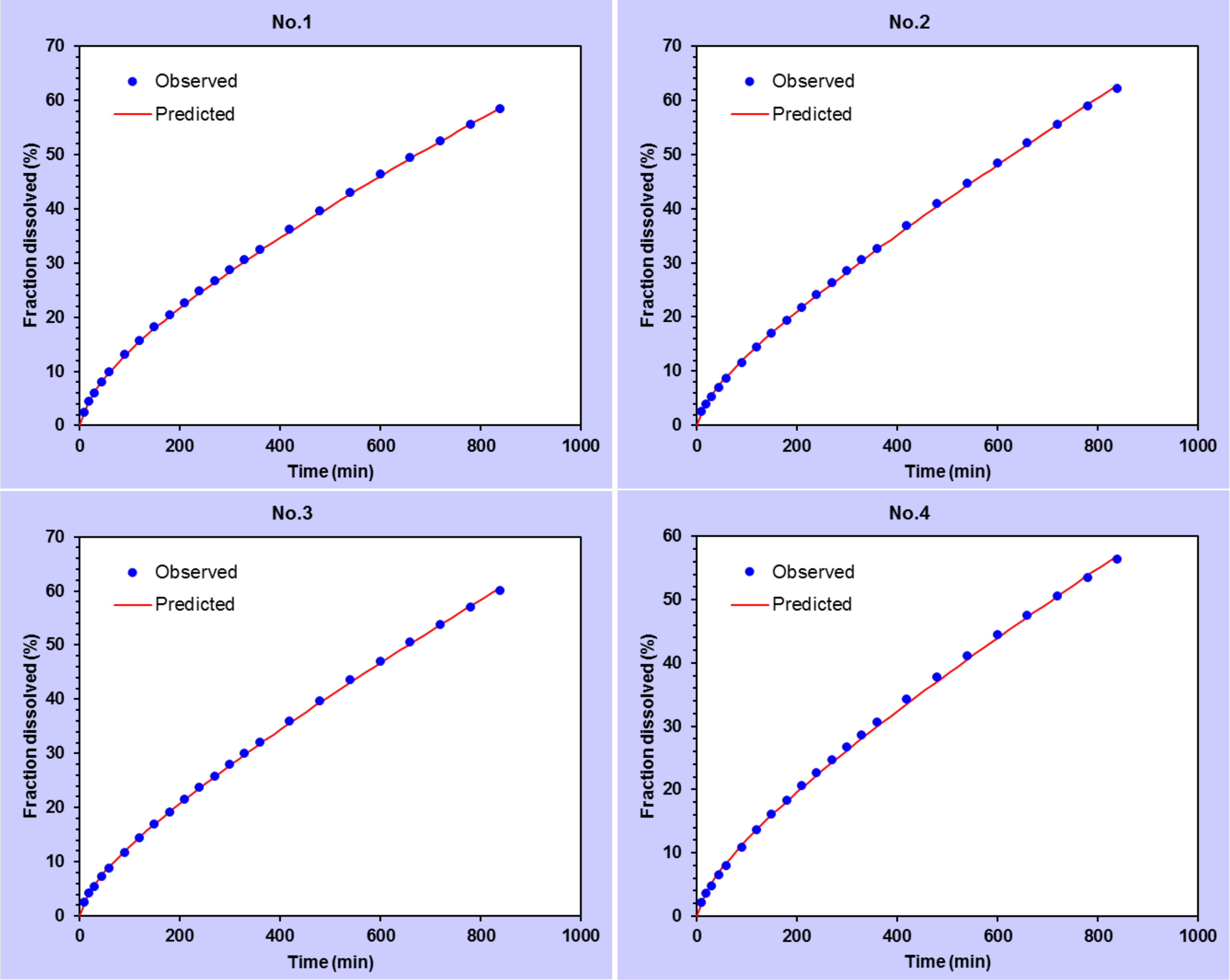

Supplementary materials – Model fitting summary – Avicel® PH-200 (modelling up to app. 60 % of carvedilol released)

Model: **Quadratic**

Model equation: $F = 100 \cdot (k_1 \cdot t^2 + k_2 \cdot t)$

Fitted model parameters per tested tablet (N = 4) with statistics – mean, standard deviation (SD), and relative standard deviation expressed in % (RSD%) (output from DDSolver):

Parameter	No.1	No.2	No.3	No.4	Mean	SD	RSD(%)
k_1	-0.0000006	-0.0000004	-0.0000005	-0.0000005	-0.0000005	0.0000001	-11.2938036
k_2	0.0011303	0.0010860	0.0010747	0.0010302	0.0010803	0.0000411	3.8074551

Number of dissolution data points (N), degrees of freedom (df), and selected goodness of fit criteria – Pearson correlation coefficient (R), coefficient of determination (R^2), adjusted coefficient of determination ($R^2_{adjusted}$), and residual sum of squares (RSS) (manual calculation in MS Excel):

Parameter	No.1	No.2	No.3	No.4
N	23	23	23	23
df	21	21	21	21
R	0.996874399	0.998758761	0.99844561	0.998678741
R^2	0.993758567	0.997519063	0.996893636	0.997359228
$R^2_{adjusted}$	0.993461356	0.997400923	0.996745714	0.997233477
RSS	87.08136185	42.84439636	50.62209846	35.60693331

Graphical abstract of model fit presented as mean ± 1 SD of the fraction % of released carvedilol:

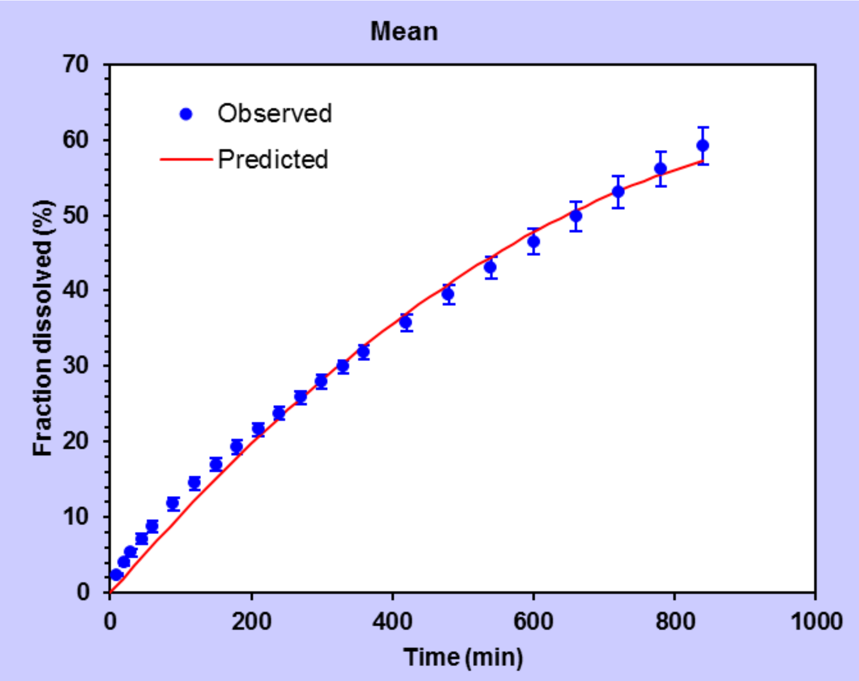

Supplementary materials – Model fitting summary – Avicel® PH-200 (modelling up to app. 60 % of carvedilol released)

Graphical abstract of model fit presented as the fraction % of released carvedilol per tested tablet:

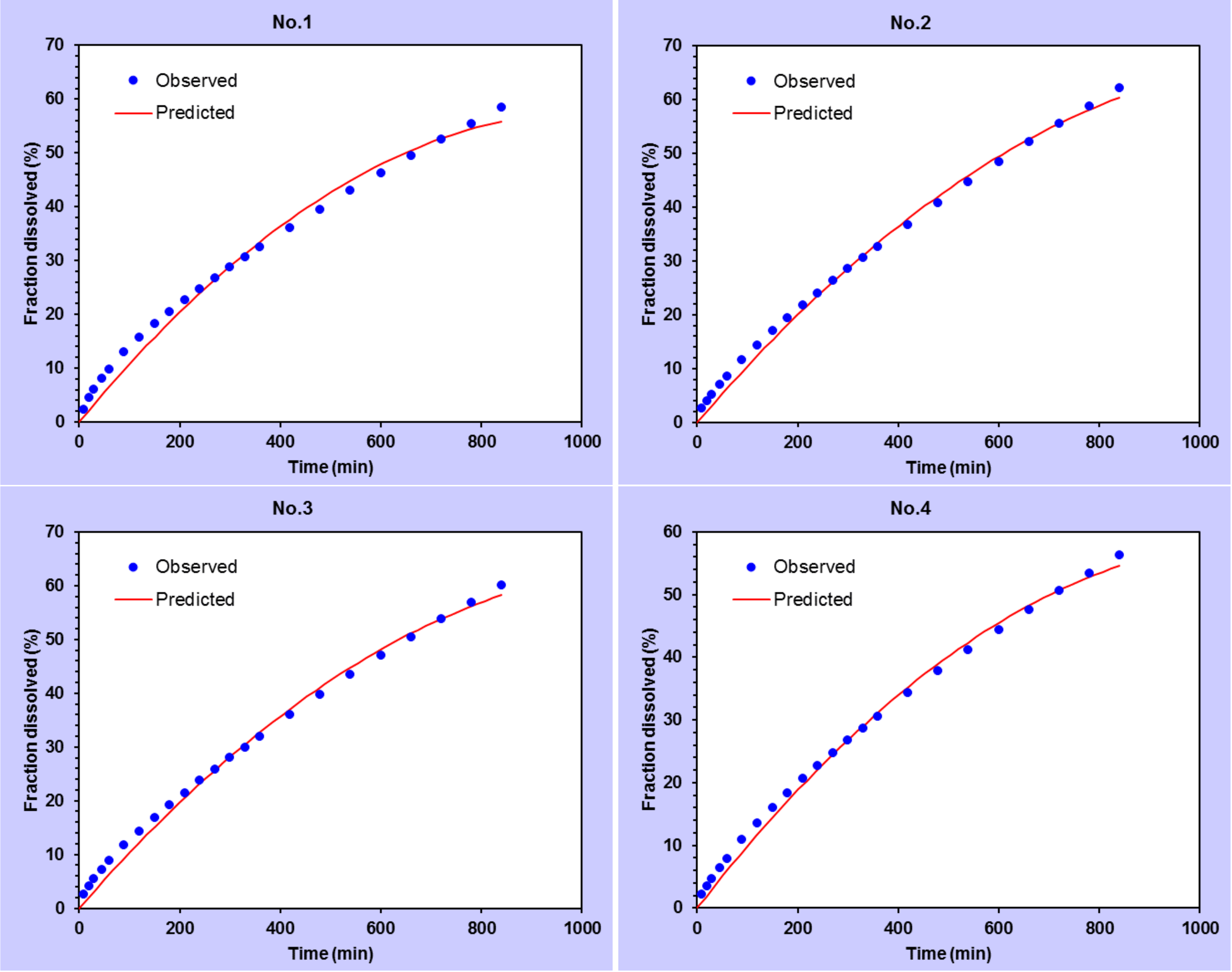

Supplementary materials – Model fitting summary – Avicel® PH-200 (modelling up to app. 60 % of carvedilol released)

Model: **Quadratic with T_{lag}**

Model equation: $F = 100 \cdot \left[k_1 \cdot \left(t - T_{lag}\right)^2 + k_2 \cdot \left(t - T_{lag}\right)\right]$

Fitted model parameters per tested tablet (N = 4) with statistics – mean, standard deviation (SD), and relative standard deviation expressed in % (RSD%) (output from DDSolver):

Parameter	No.1	No.2	No.3	No.4	Mean	SD	RSD(%)
k_1	-0.0000006	-0.0000005	-0.0000005	-0.0000005	-0.0000005	0.0000001	-10.8662868
k_2	0.0011474	0.0011037	0.0010920	0.0010468	0.0010975	0.0000414	3.7689689
T_{lag}	4.0000000	4.0000000	4.0000000	4.0000000	4.0000000	0.0000000	0.0000000

Number of dissolution data points (N), degrees of freedom (df), and selected goodness of fit criteria – Pearson correlation coefficient (R), coefficient of determination (R^2), adjusted coefficient of determination ($R^2_{adjusted}$), and residual sum of squares (RSS) (manual calculation in MS Excel):

Parameter	No.1	No.2	No.3	No.4
N	23	23	23	23
df	20	20	20	20
R	0.996554679	0.998522287	0.998184152	0.9984479
R^2	0.993121229	0.997046758	0.996371601	0.99689821
$R^2_{adjusted}$	0.992433352	0.996751434	0.996008761	0.996588031
RSS	104.6597938	55.50383397	64.1712809	46.22368587

Graphical abstract of model fit presented as mean ± 1 SD of the fraction % of released carvedilol:

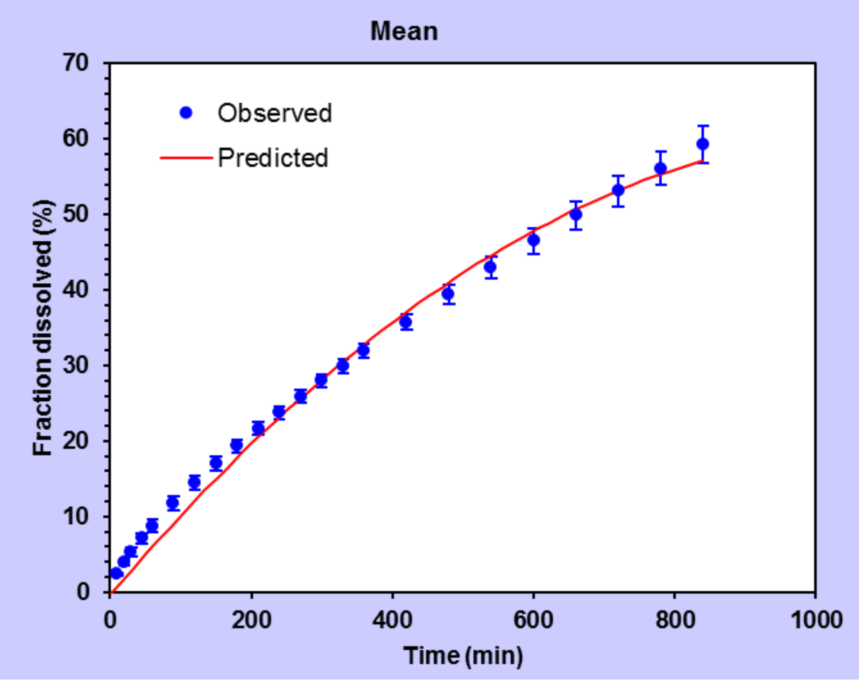

Supplementary materials – Model fitting summary – Avicel® PH-200 (modelling up to app. 60 % of carvedilol released)

Graphical abstract of model fit presented as the fraction % of released carvedilol per tested tablet:

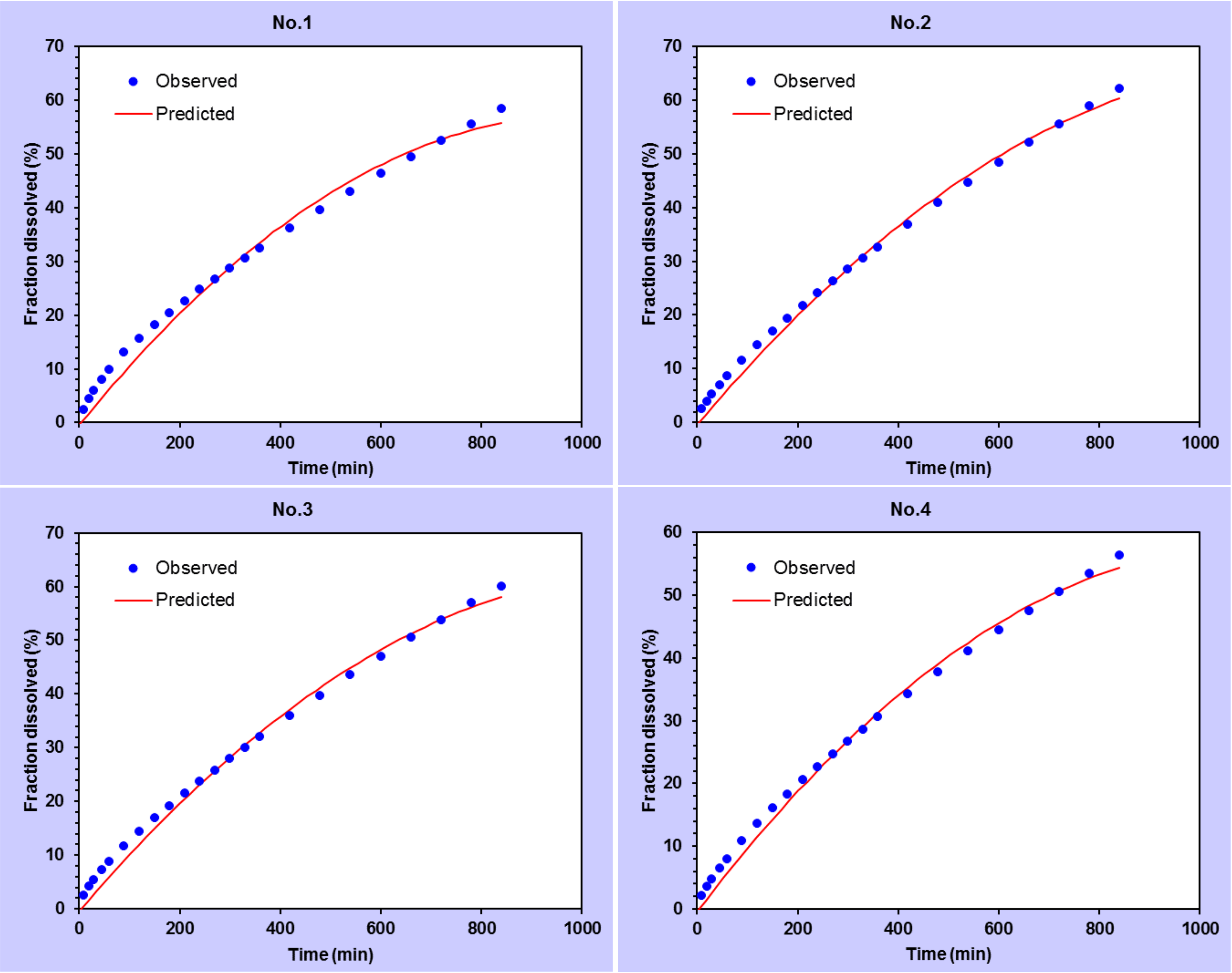

Supplementary materials – Model fitting summary – Avicel® PH-200 (modelling up to app. 60 % of carvedilol released)

Model: **Weibull_1**

Model equation: $F = 100 \cdot \left[1 - e^{-\frac{(t-T_i)^{\beta}}{\alpha}}\right]$

Fitted model parameters per tested tablet (N = 4) with statistics – mean, standard deviation (SD), and relative standard deviation expressed in % (RSD%) (output from DDSolver):

Parameter	No.1	No.2	No.3	No.4	Mean	SD	RSD(%)
α	197.029	222.171	203.971	269.768	223.235	32.784	14.686
β	0.756	0.769	0.750	0.795	0.768	0.020	2.628
T_i	4.733	6.000	6.000	4.733	5.366	0.732	13.635

Number of dissolution data points (N), degrees of freedom (df), and selected goodness of fit criteria – Pearson correlation coefficient (R), coefficient of determination (R^2), adjusted coefficient of determination ($R^2_{adjusted}$), and residual sum of squares (RSS) (manual calculation in MS Excel):

Parameter	No.1	No.2	No.3	No.4
N	23	23	23	23
df	20	20	20	20
R	0.996506883	0.993972123	0.994106399	0.997333622
R^2	0.993025968	0.987980582	0.988247532	0.994674354
$R^2_{adjusted}$	0.992328565	0.98677864	0.987072285	0.99414179
RSS	63.429987	162.2224547	142.6152648	53.15935949

Graphical abstract of model fit presented as mean ± 1 SD of the fraction % of released carvedilol:

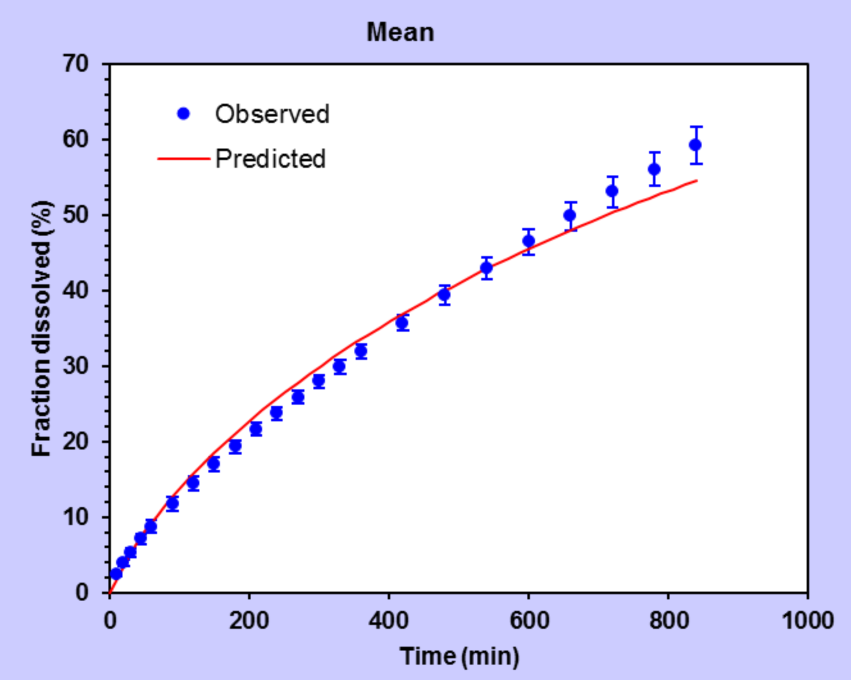

Supplementary materials – Model fitting summary – Avicel® PH-200 (modelling up to app. 60 % of carvedilol released)

Graphical abstract of model fit presented as the fraction % of released carvedilol per tested tablet:

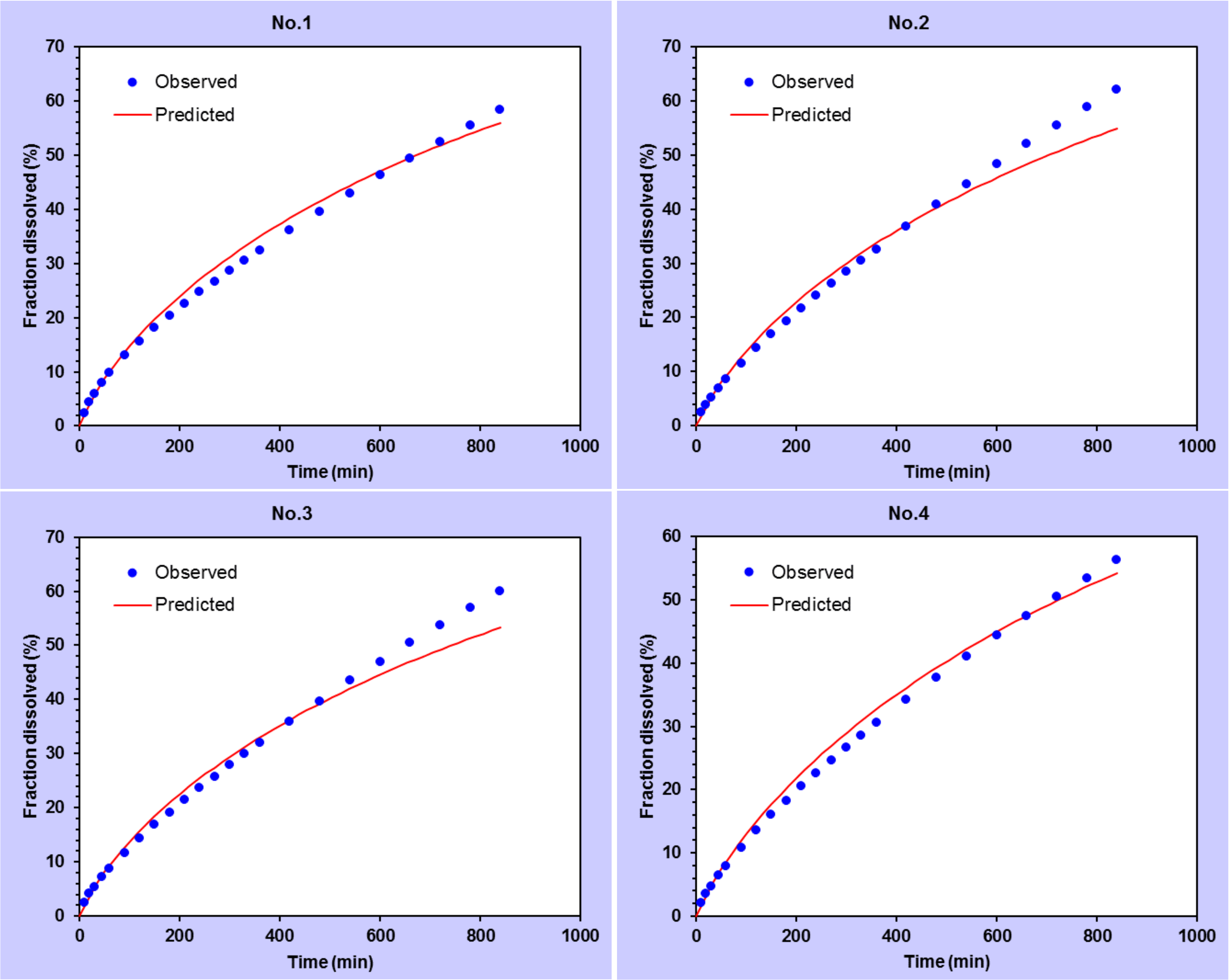

Supplementary materials – Model fitting summary – Avicel® PH-200 (modelling up to app. 60 % of carvedilol released)

Model: **Weibull_2**

Model equation: $F = 100 \cdot \left(1 - e^{-\frac{t^{\beta}}{\alpha}}\right)$

Fitted model parameters per tested tablet (N = 4) with statistics – mean, standard deviation (SD), and relative standard deviation expressed in % (RSD%) (output from DDSolver):

Parameter	No.1	No.2	No.3	No.4	Mean	SD	RSD(%)
α	290.475	319.897	290.438	407.780	327.147	55.518	16.970
β	0.816	0.830	0.809	0.860	0.829	0.023	2.727

Number of dissolution data points (N), degrees of freedom (df), and selected goodness of fit criteria – Pearson correlation coefficient (R), coefficient of determination (R^2), adjusted coefficient of determination ($R^2_{adjusted}$), and residual sum of squares (RSS) (manual calculation in MS Excel):

Parameter	No.1	No.2	No.3	No.4
N	23	23	23	23
df	21	21	21	21
R	0.998212561	0.99649391	0.996619823	0.998939351
R^2	0.996428316	0.993000112	0.993251072	0.997879827
$R^2_{adjusted}$	0.996258236	0.992666784	0.992929694	0.997778866
RSS	28.31395827	87.07276922	75.62322694	20.6982618

Graphical abstract of model fit presented as mean ± 1 SD of the fraction % of released carvedilol:

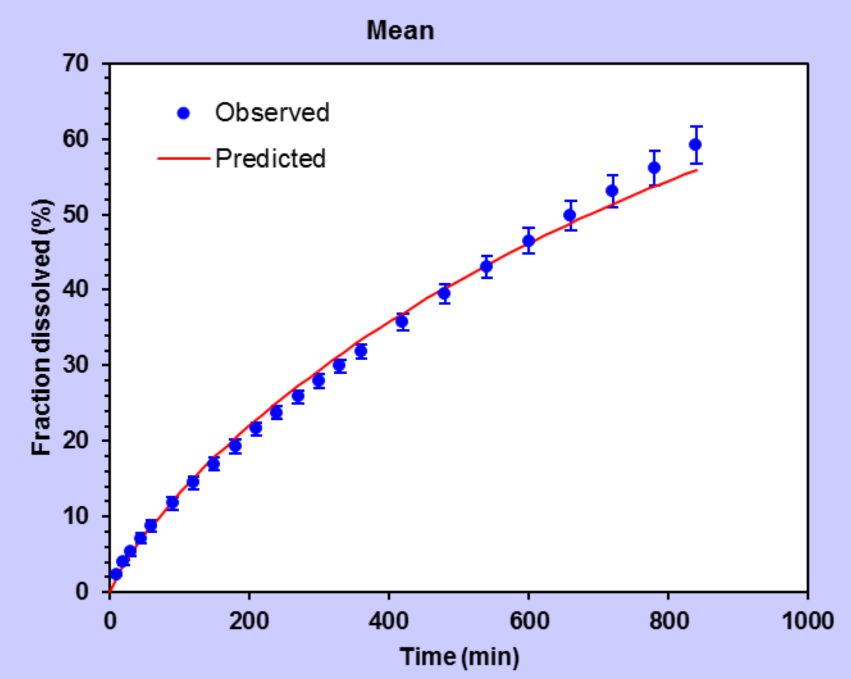

Supplementary materials – Model fitting summary – Avicel® PH-200 (modelling up to app. 60 % of carvedilol released)

Graphical abstract of model fit presented as the fraction % of released carvedilol per tested tablet:

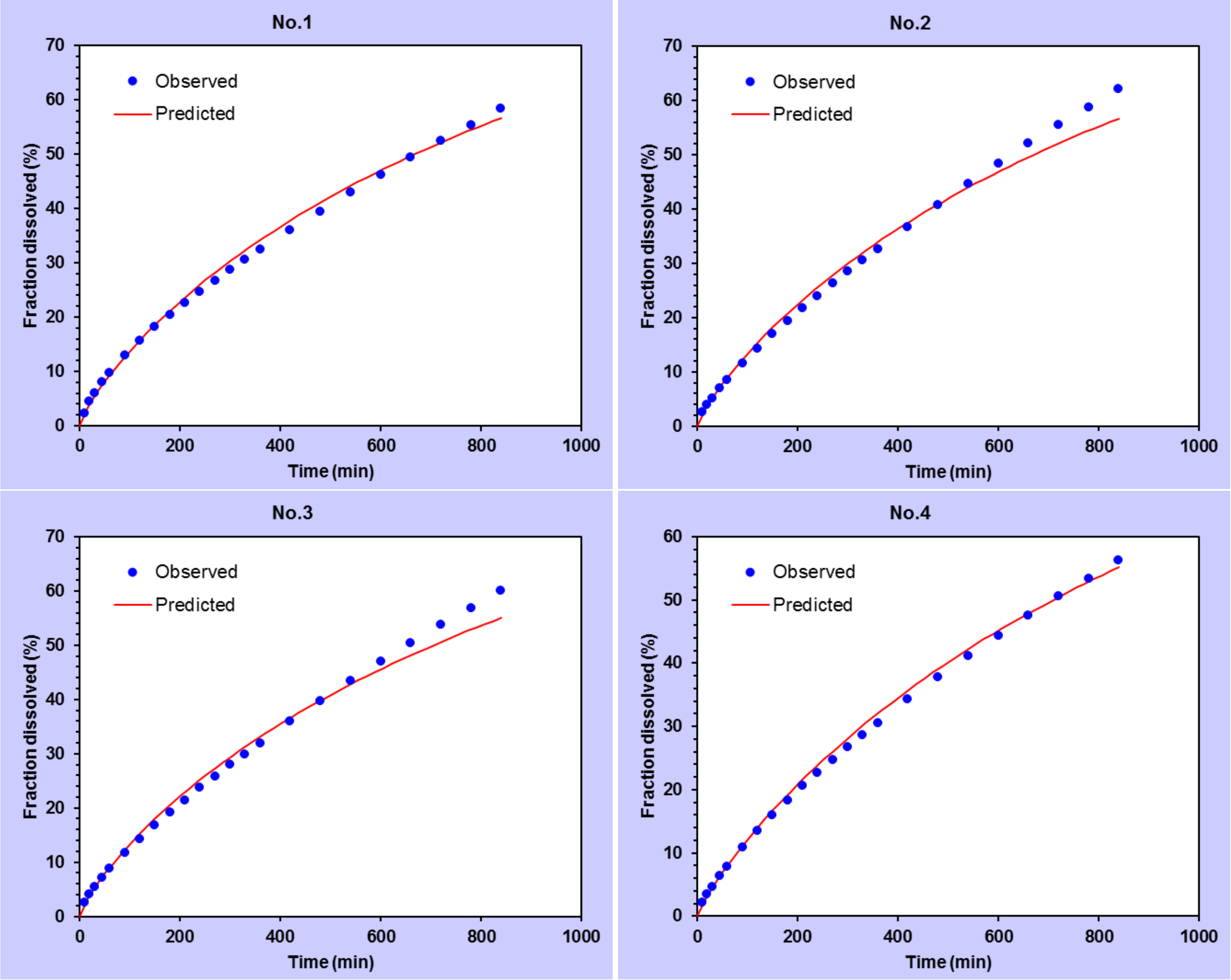

Supplementary materials – Model fitting summary – Avicel® PH-200 (modelling up to app. 60 % of carvedilol released)

Model: **Weibull_3**

Model equation: $F = F_{max} \cdot \left(1 - e^{-\frac{t^{\beta}}{\alpha}}\right)$

Fitted model parameters per tested tablet (N = 4) with statistics – mean, standard deviation (SD), and relative standard deviation expressed in % (RSD%) (output from DDSolver):

Parameter	No.1	No.2	No.3	No.4	Mean	SD	RSD(%)
α	237.583	314.002	280.149	328.154	289.972	40.317	13.904
β	0.905	0.939	0.923	0.952	0.930	0.020	2.188
F_{max}	61.354	65.189	63.003	59.117	62.166	2.569	4.132

Number of dissolution data points (N), degrees of freedom (df), and selected goodness of fit criteria – Pearson correlation coefficient (R), coefficient of determination (R^2), adjusted coefficient of determination ($R^2_{adjusted}$), and residual sum of squares (RSS) (manual calculation in MS Excel):

Parameter	No.1	No.2	No.3	No.4
N	23	23	23	23
df	20	20	20	20
R	0.988915487	0.987678451	0.987326602	0.990269727
R^2	0.977953841	0.975508723	0.974813819	0.980634132
$R^2_{adjusted}$	0.975749225	0.973059595	0.972295201	0.978697545
RSS	160.4181901	226.9396093	212.9906439	146.0669586

Graphical abstract of model fit presented as mean ± 1 SD of the fraction % of released carvedilol:

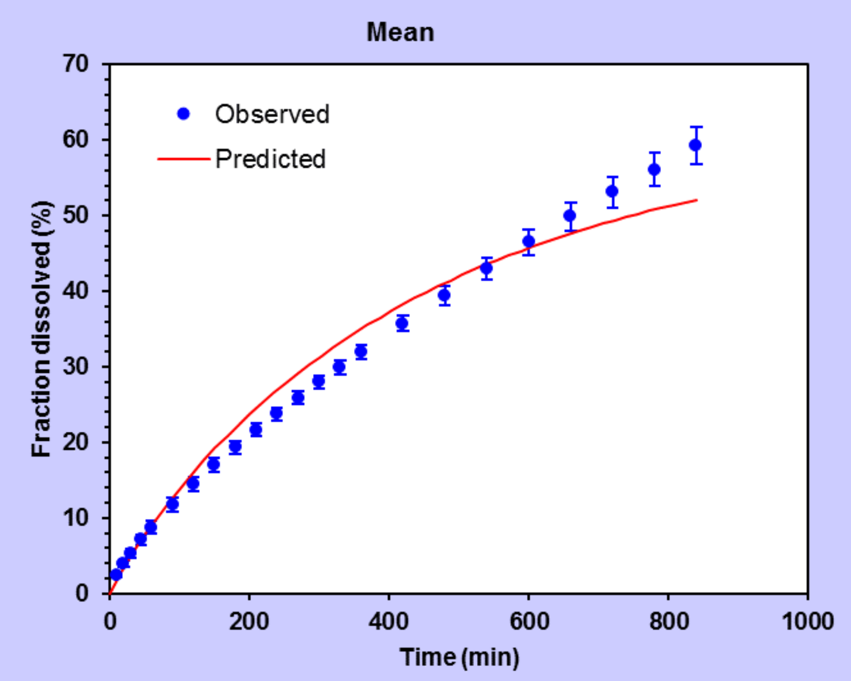

Supplementary materials – Model fitting summary – Avicel® PH-200 (modelling up to app. 60 % of carvedilol released)

Graphical abstract of model fit presented as the fraction % of released carvedilol per tested tablet:

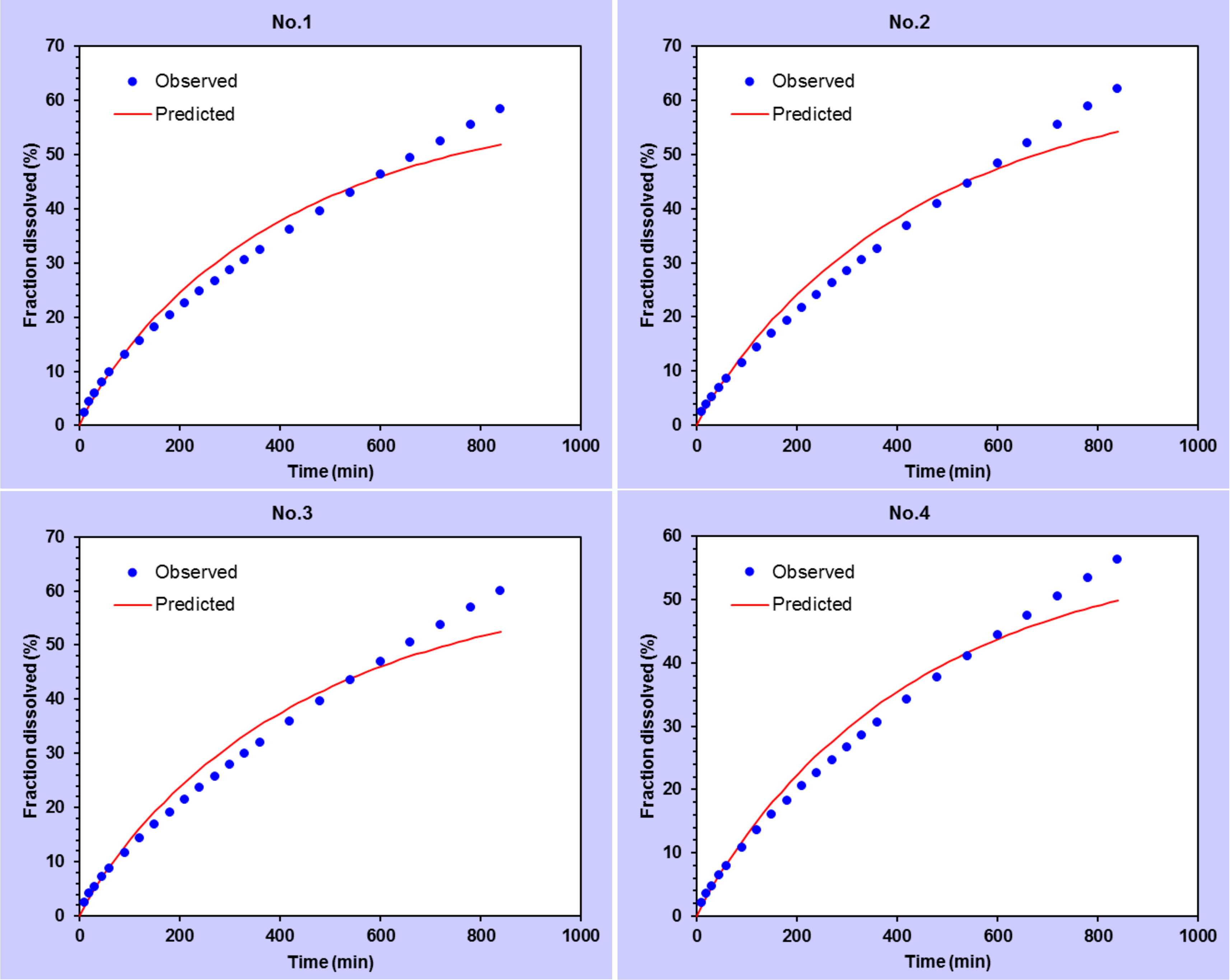

Supplementary materials – Model fitting summary – Avicel® PH-200 (modelling up to app. 60 % of carvedilol released)

Model: **Weibull_4**

Model equation: $F = F_{max} \cdot \left[1 - e^{-\frac{(t-T_i)^\beta}{\alpha}}\right]$

Fitted model parameters per tested tablet (N = 4) with statistics – mean, standard deviation (SD), and relative standard deviation expressed in % (RSD%) (output from DDSolver):

Parameter	No.1	No.2	No.3	No.4	Mean	SD	RSD(%)
α	159.615	205.508	184.928	214.441	191.123	24.371	12.751
β	0.839	0.868	0.854	0.881	0.861	0.018	2.122
T_i	6.000	6.000	6.000	6.000	6.000	0.000	0.000
F_{max}	61.354	65.189	63.003	59.117	62.166	2.569	4.132

Number of dissolution data points (N), degrees of freedom (df), and selected goodness of fit criteria – Pearson correlation coefficient (R), coefficient of determination (R^2), adjusted coefficient of determination ($R^2_{adjusted}$), and residual sum of squares (RSS) (manual calculation in MS Excel):

Parameter	No.1	No.2	No.3	No.4
N	23	23	23	23
df	19	19	19	19
R	0.986040564	0.984341868	0.983917015	0.987428664
R^2	0.972275994	0.968928913	0.968092693	0.975015366
$R^2_{adjusted}$	0.967898519	0.964022952	0.963054697	0.971070424
RSS	203.8858843	295.1046213	275.0566289	193.484665

Graphical abstract of model fit presented as mean ± 1 SD of the fraction % of released carvedilol:

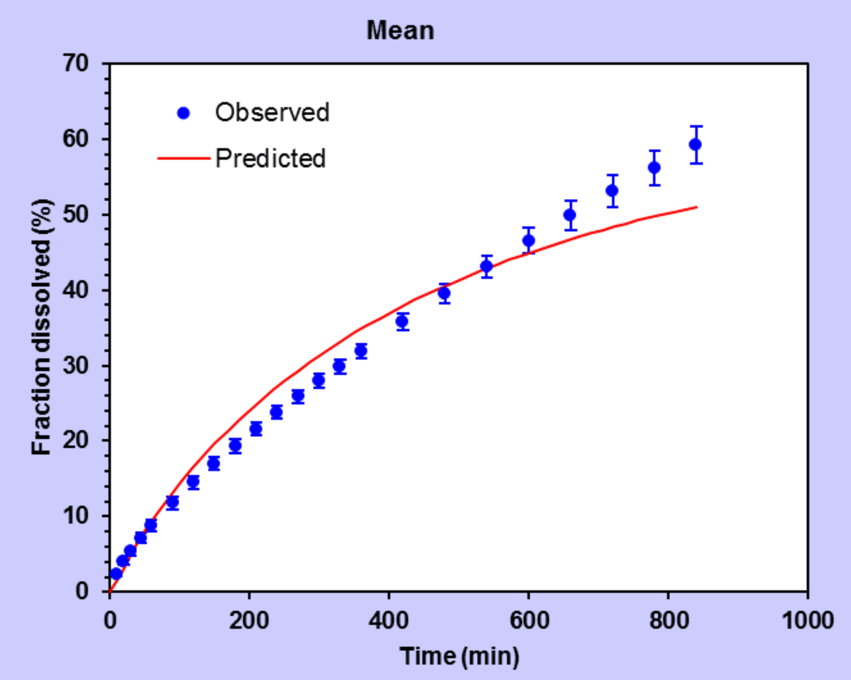

Supplementary materials – Model fitting summary – Avicel® PH-200 (modelling up to app. 60 % of carvedilol released)

Graphical abstract of model fit presented as the fraction % of released carvedilol per tested tablet:

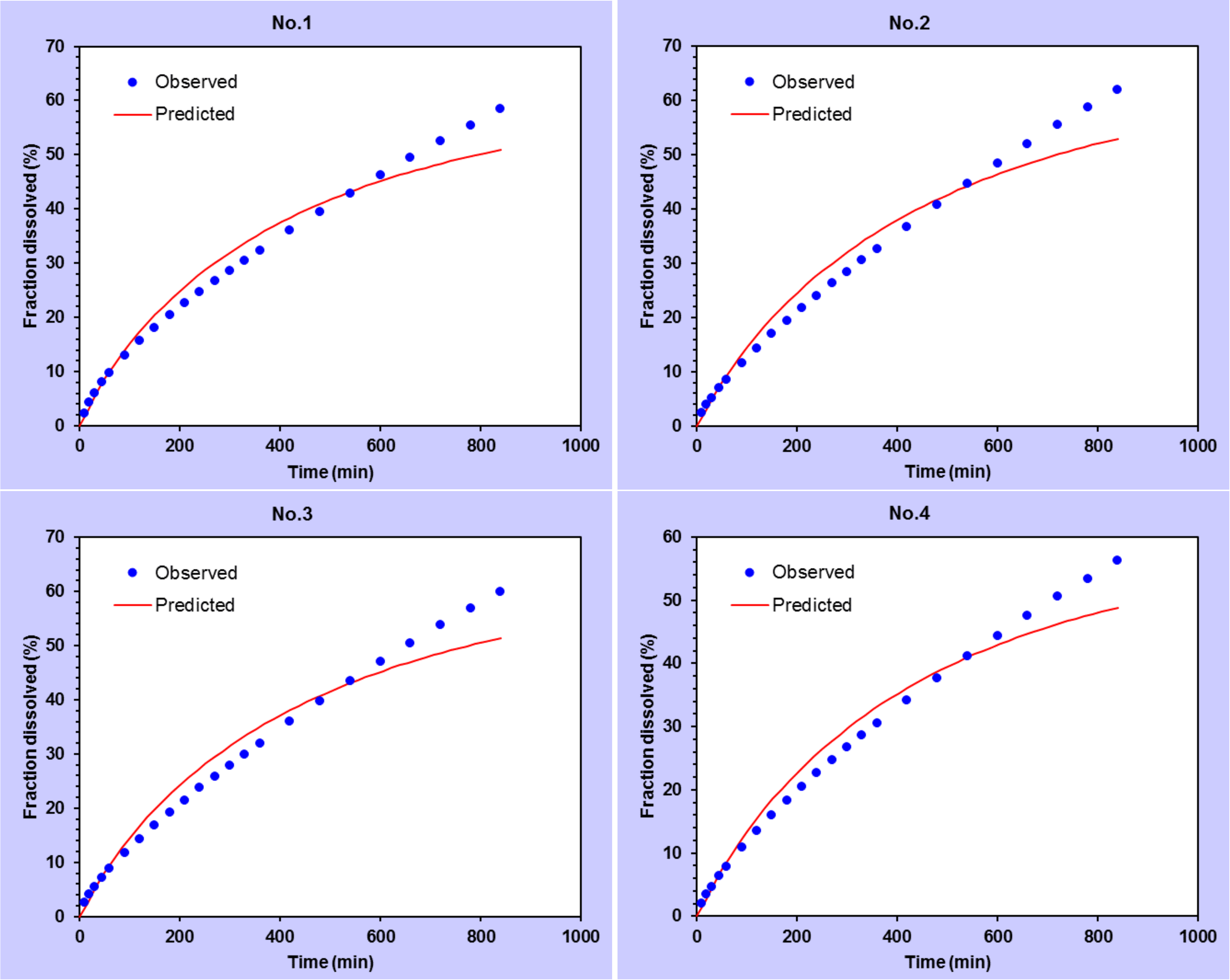

Supplementary materials – Model fitting summary – Avicel® PH-200 (modelling up to app. 60 % of carvedilol released)

Model: **Logistic_1**

Model equation: $F = 100 \cdot \frac{e^{\alpha+\beta\cdot\log(t)}}{1+e^{\alpha+\beta\cdot\log(t)}}$

Fitted model parameters per tested tablet (N = 4) with statistics – mean, standard deviation (SD), and relative standard deviation expressed in % (RSD%) (output from DDSolver):

Parameter	No.1	No.2	No.3	No.4	Mean	SD	RSD(%)
α	-6.333	-6.138	-6.516	-6.180	-6.292	0.171	-2.723
β	2.197	2.158	2.262	2.124	2.185	0.059	2.723

Number of dissolution data points (N), degrees of freedom (df), and selected goodness of fit criteria – Pearson correlation coefficient (R), coefficient of determination (R^2), adjusted coefficient of determination ($R^2_{adjusted}$), and residual sum of squares (RSS) (manual calculation in MS Excel):

Parameter	No.1	No.2	No.3	No.4
N	23	23	23	23
df	21	21	21	21
R	0.995823844	0.990484386	0.994300478	0.994750383
R^2	0.991665129	0.981059318	0.988633441	0.989528324
$R^2_{adjusted}$	0.99126823	0.980157381	0.988092176	0.989029673
RSS	105.105808	199.855356	159.312069	94.14985973

Graphical abstract of model fit presented as mean ± 1 SD of the fraction % of released carvedilol:

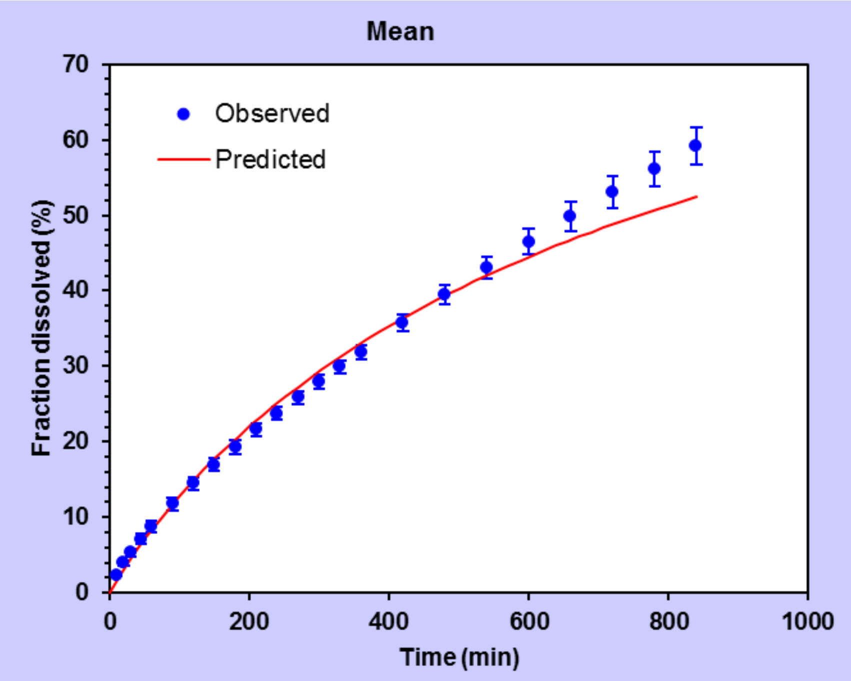

Supplementary materials – Model fitting summary – Avicel® PH-200 (modelling up to app. 60 % of carvedilol released)

Graphical abstract of model fit presented as the fraction % of released carvedilol per tested tablet:

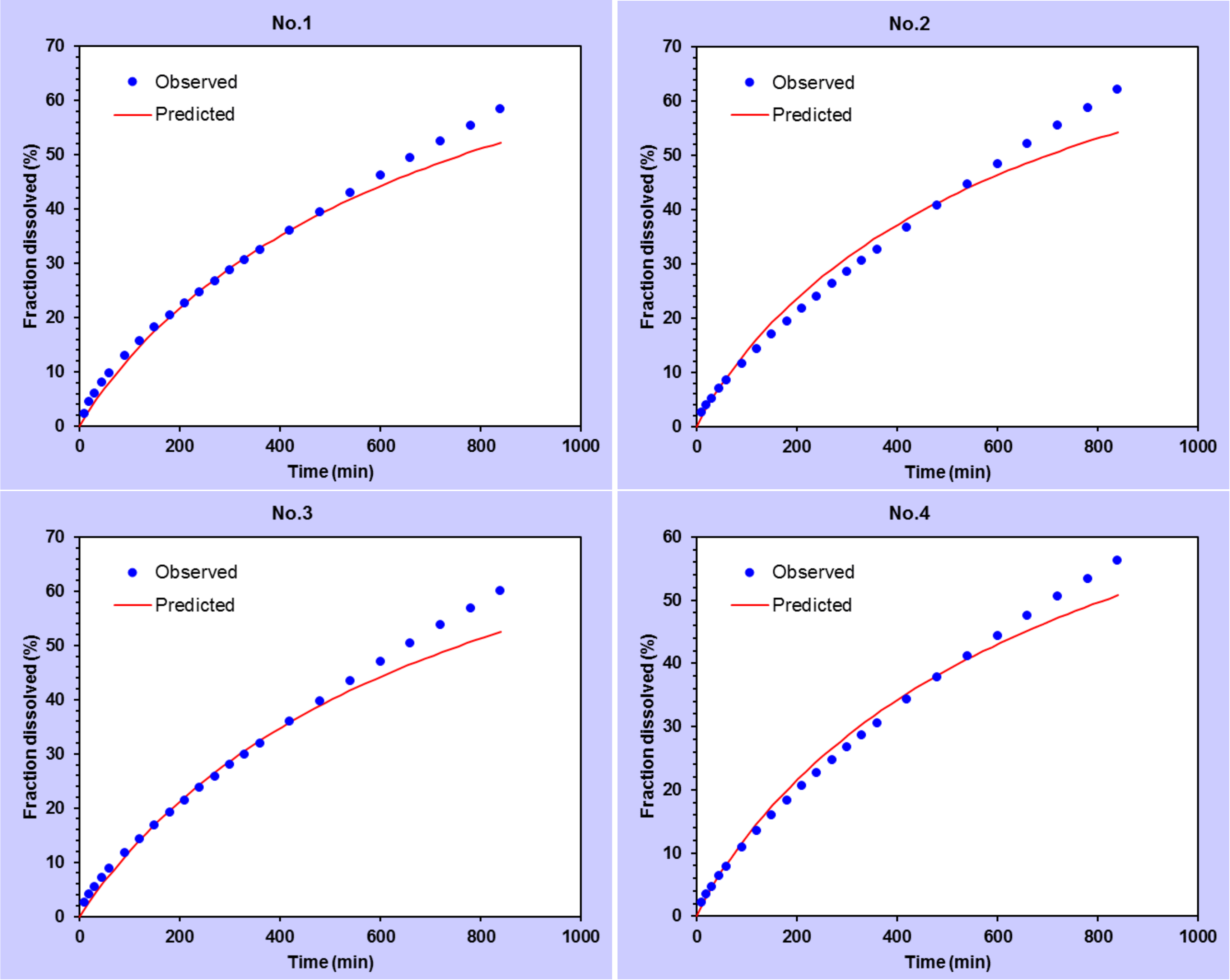

Supplementary materials – Model fitting summary – Avicel® PH-200 (modelling up to app. 60 % of carvedilol released)

Model: **Logistic_2**

Model equation: $F = F_{max} \cdot \frac{e^{\alpha+\beta\cdot\log(t)}}{1+e^{\alpha+\beta\cdot\log(t)}}$

Fitted model parameters per tested tablet (N = 4) with statistics – mean, standard deviation (SD), and relative standard deviation expressed in % (RSD%) (output from DDSolver):

Parameter	No.1	No.2	No.3	No.4	Mean	SD	RSD(%)
α	-7.517	-7.176	-7.709	-7.238	-7.410	0.249	-3.354
β	2.928	2.703	2.965	2.742	2.834	0.131	4.633
F_{max}	67.181	77.703	68.987	70.465	71.084	4.613	6.489

Number of dissolution data points (N), degrees of freedom (df), and selected goodness of fit criteria – Pearson correlation coefficient (R), coefficient of determination (R^2), adjusted coefficient of determination ($R^2_{adjusted}$), and residual sum of squares (RSS) (manual calculation in MS Excel):

Parameter	No.1	No.2	No.3	No.4
N	23	23	23	23
df	20	20	20	20
R	0.989960589	0.989788301	0.98958861	0.992160145
R^2	0.980021968	0.97968088	0.979285618	0.984381753
$R^2_{adjusted}$	0.978024165	0.977648968	0.977214179	0.982819928
RSS	254.5490288	260.3150807	319.1308786	175.9864659

Graphical abstract of model fit presented as mean ± 1 SD of the fraction % of released carvedilol:

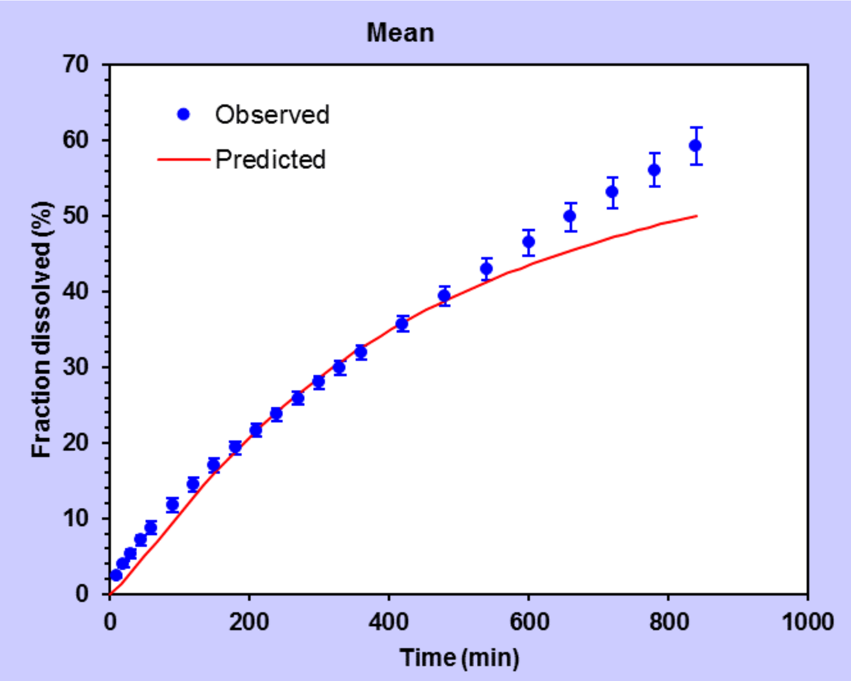

Supplementary materials – Model fitting summary – Avicel® PH-200 (modelling up to app. 60 % of carvedilol released)

Graphical abstract of model fit presented as the fraction % of released carvedilol per tested tablet:

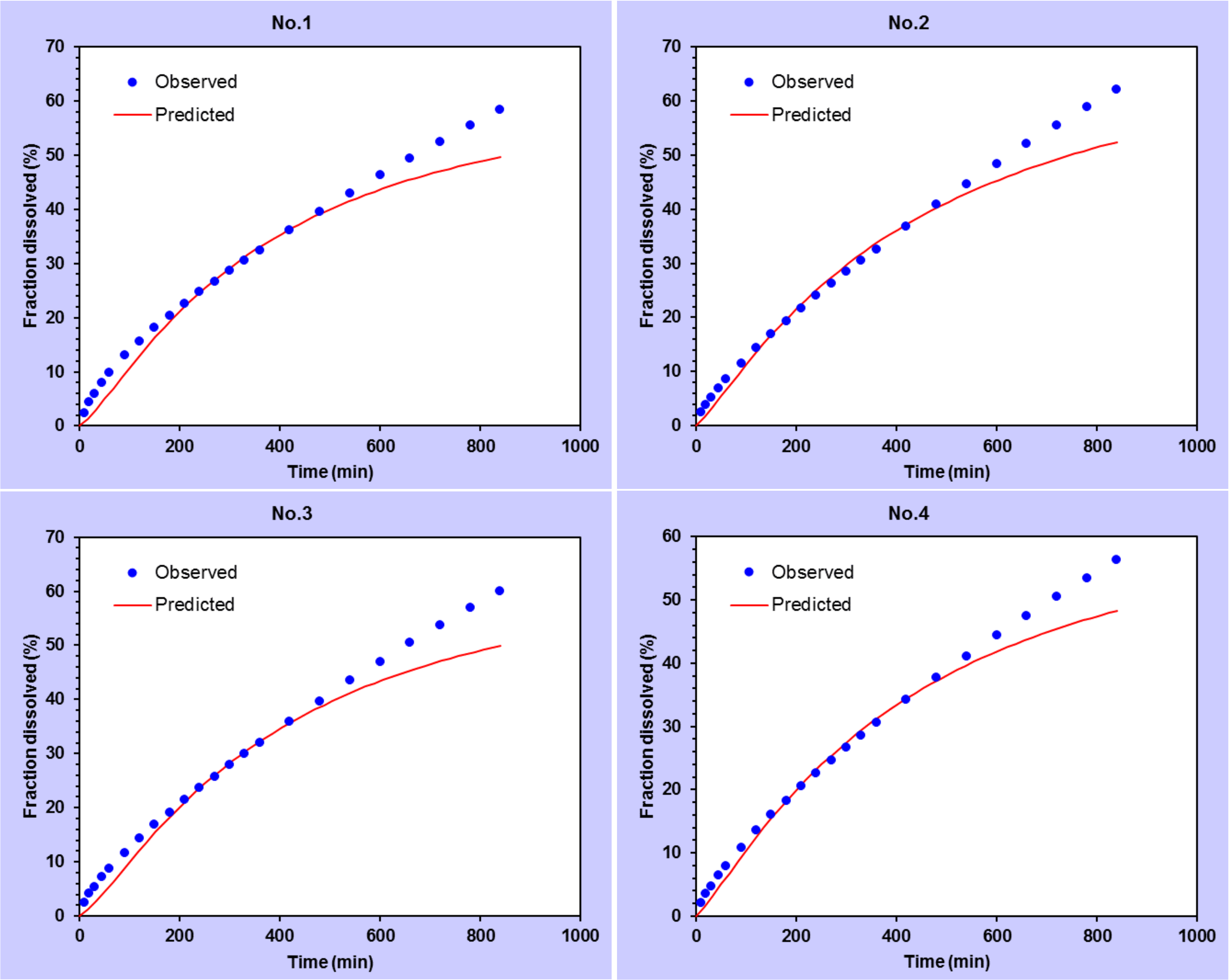

Supplementary materials – Model fitting summary – Avicel® PH-200 (modelling up to app. 60 % of carvedilol released)

Model: **Logistic_3**

Model equation: $F = F_{max} \cdot \frac{1}{1+e^{-k\cdot(t-\gamma)}}$

Fitted model parameters per tested tablet (N = 4) with statistics – mean, standard deviation (SD), and relative standard deviation expressed in % (RSD%) (output from DDSolver):

Parameter	No.1	No.2	No.3	No.4	Mean	SD	RSD(%)
k	0.005	0.006	0.006	0.006	0.006	0.000	7.279
γ	311.248	383.418	378.141	377.370	362.544	34.303	9.462
F_{max}	59.934	65.189	63.003	59.117	61.811	2.806	4.539

Number of dissolution data points (N), degrees of freedom (df), and selected goodness of fit criteria – Pearson correlation coefficient (R), coefficient of determination (R^2), adjusted coefficient of determination ($R^2_{adjusted}$), and residual sum of squares (RSS) (manual calculation in MS Excel):

Parameter	No.1	No.2	No.3	No.4
N	23	23	23	23
df	20	20	20	20
R	0.991959032	0.989256476	0.989325158	0.987300682
R^2	0.983982721	0.978628376	0.978764268	0.974762637
$R^2_{adjusted}$	0.982380993	0.976491213	0.976640695	0.9722389
RSS	207.039246	199.1826617	181.4835769	195.4555949

Graphical abstract of model fit presented as mean ± 1 SD of the fraction % of released carvedilol:

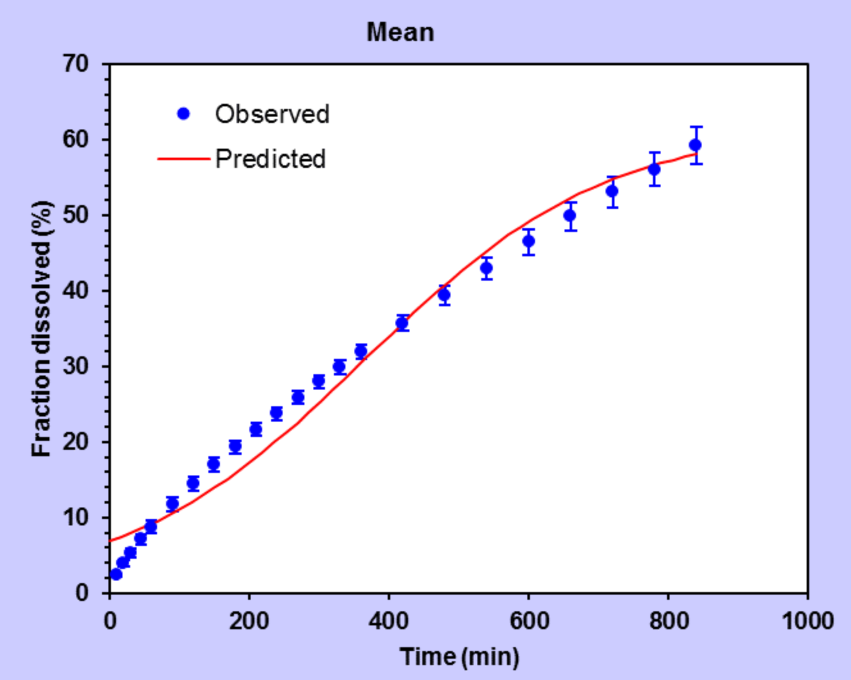

Supplementary materials – Model fitting summary – Avicel® PH-200 (modelling up to app. 60 % of carvedilol released)

Graphical abstract of model fit presented as the fraction % of released carvedilol per tested tablet:

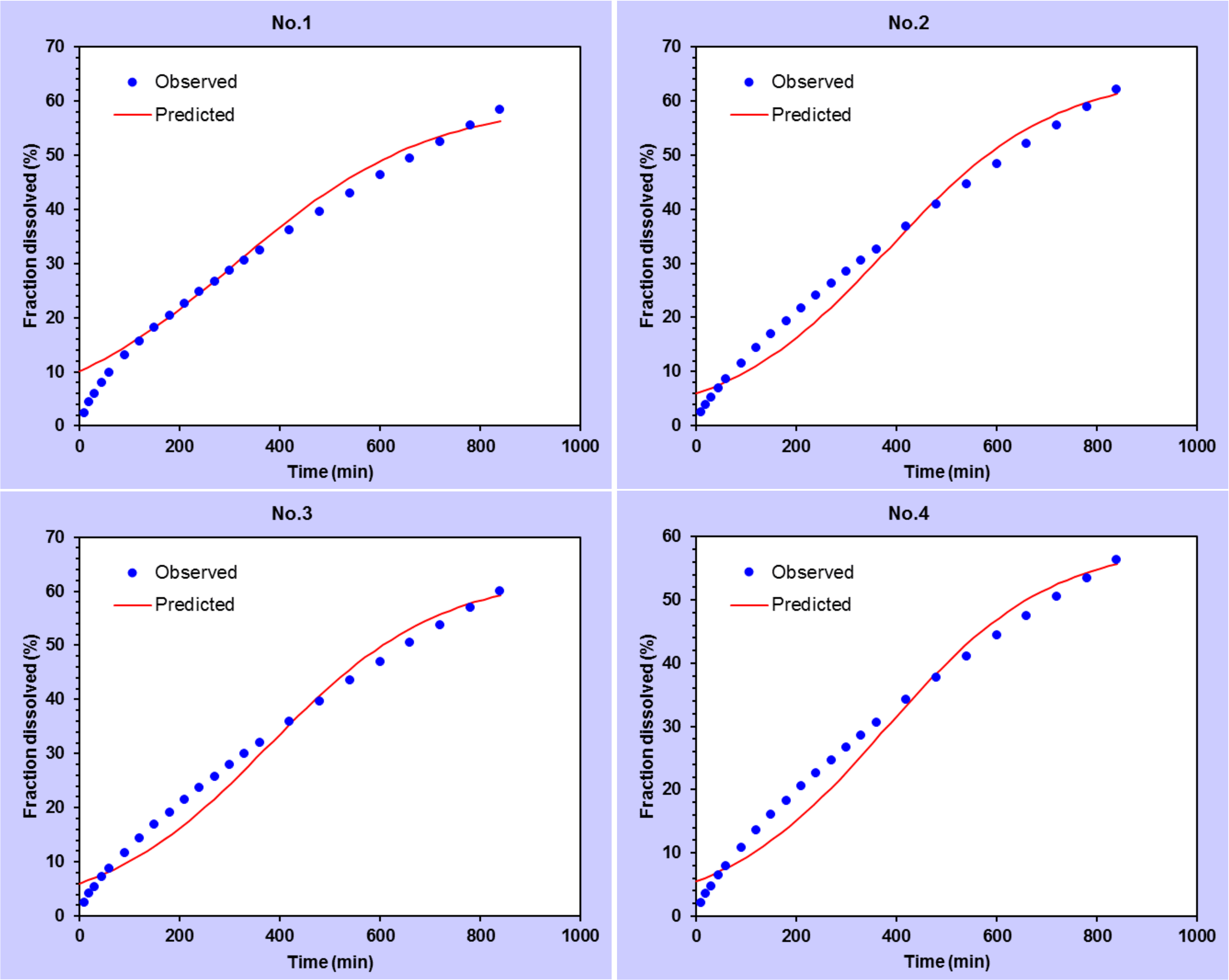

Supplementary materials – Model fitting summary – Avicel® PH-200 (modelling up to app. 60 % of carvedilol released)

Model: **Gompertz_1**

Model equation: $F = 100 \cdot e^{-\alpha \cdot e^{-\beta \cdot \log(t)}}$

Fitted model parameters per tested tablet (N = 4) with statistics – mean, standard deviation (SD), and relative standard deviation expressed in % (RSD%) (output from DDSolver):

Parameter	No.1	No.2	No.3	No.4	Mean	SD	RSD(%)
α	12.881	14.981	13.995	13.938	13.949	0.858	6.149
β	0.989	1.058	1.020	0.996	1.016	0.031	3.051

Number of dissolution data points (N), degrees of freedom (df), and selected goodness of fit criteria – Pearson correlation coefficient (R), coefficient of determination (R^2), adjusted coefficient of determination ($R^2_{adjusted}$), and residual sum of squares (RSS) (manual calculation in MS Excel):

Parameter	No.1	No.2	No.3	No.4
N	23	23	23	23
df	21	21	21	21
R	0.973927507	0.967062503	0.968403329	0.975042373
R^2	0.94853479	0.935209884	0.937805007	0.950707629
$R^2_{adjusted}$	0.946084065	0.93212464	0.93484334	0.948360373
RSS	385.3556967	585.0971962	518.2871754	379.1272177

Graphical abstract of model fit presented as mean ± 1 SD of the fraction % of released carvedilol:

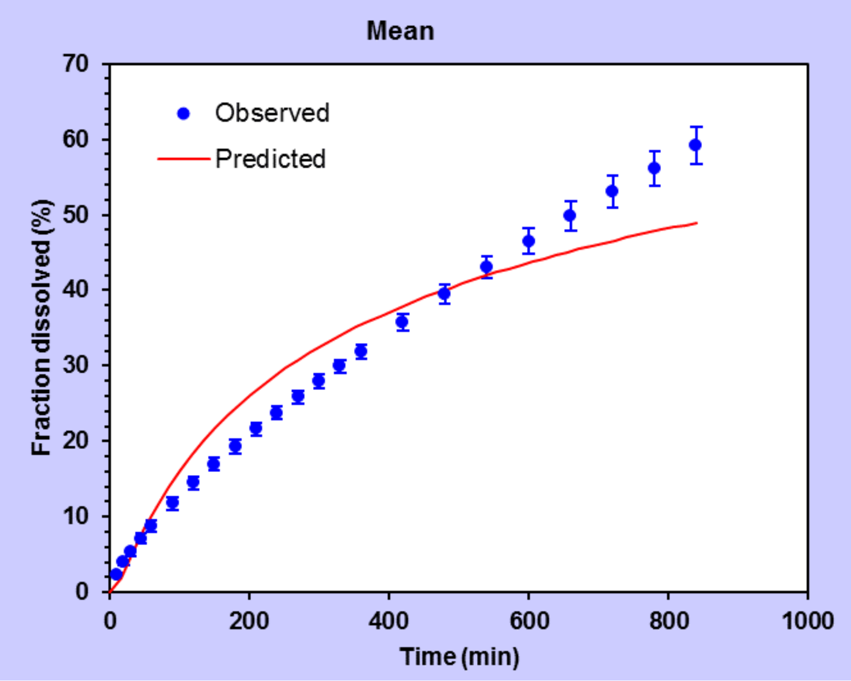

Supplementary materials – Model fitting summary – Avicel® PH-200 (modelling up to app. 60 % of carvedilol released)

Graphical abstract of model fit presented as the fraction % of released carvedilol per tested tablet:

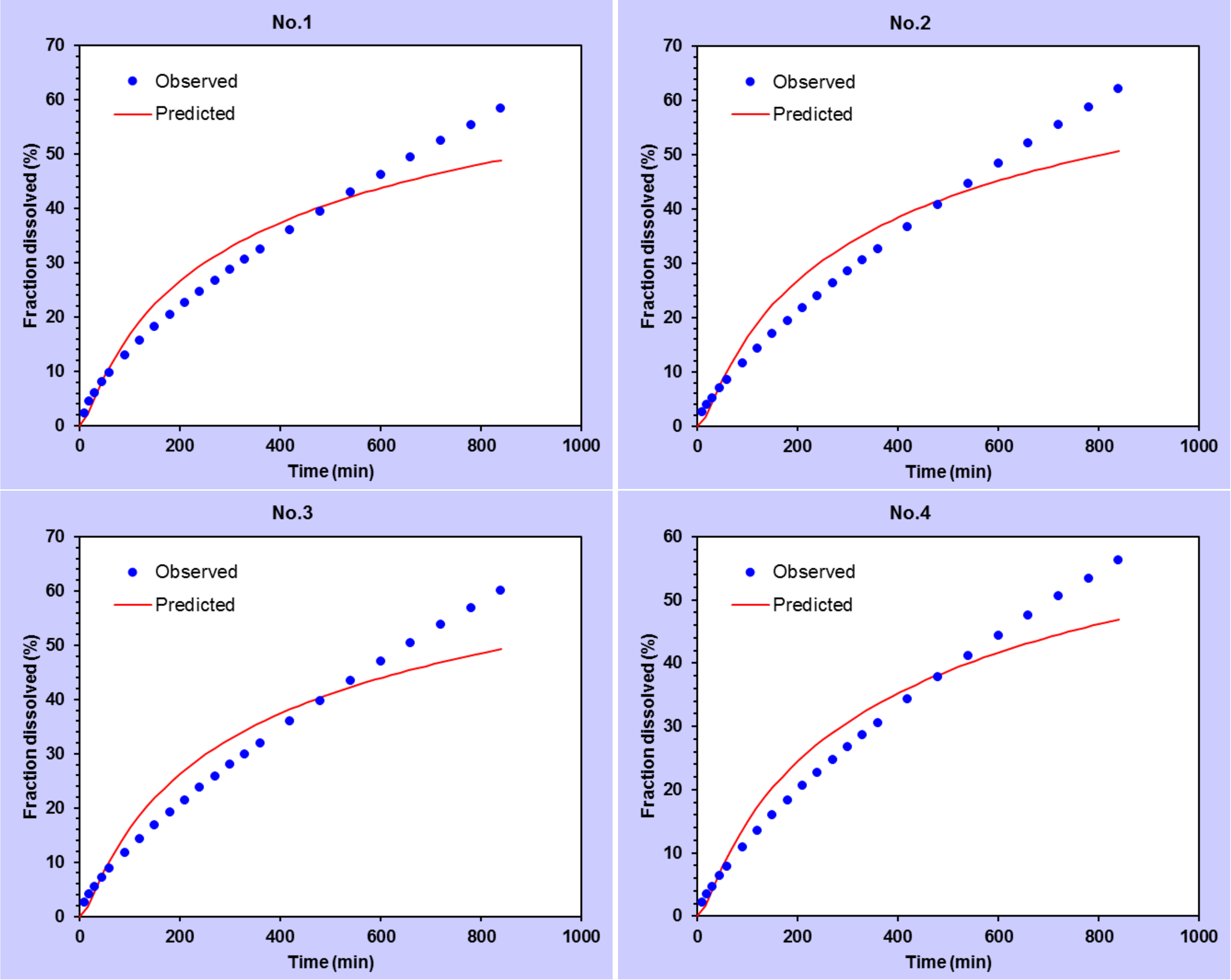

Supplementary materials – Model fitting summary – Avicel® PH-200 (modelling up to app. 60 % of carvedilol released)

Model: **Gompertz_2**

Model equation: $F = F_{max} \cdot e^{-\alpha \cdot e^{-\beta \cdot \log(t)}}$

Fitted model parameters per tested tablet (N = 4) with statistics – mean, standard deviation (SD), and relative standard deviation expressed in % (RSD%) (output from DDSolver):

Parameter	No.1	No.2	No.3	No.4	Mean	SD	RSD(%)
α	58.011	63.842	61.726	53.644	59.306	4.478	7.551
β	1.757	1.768	1.761	1.663	1.737	0.050	2.863
F_{max}	61.354	65.189	63.003	67.122	64.167	2.519	3.926

Number of dissolution data points (N), degrees of freedom (df), and selected goodness of fit criteria – Pearson correlation coefficient (R), coefficient of determination (R^2), adjusted coefficient of determination ($R^2_{adjusted}$), and residual sum of squares (RSS) (manual calculation in MS Excel):

Parameter	No.1	No.2	No.3	No.4
N	23	23	23	23
df	20	20	20	20
R	0.971554912	0.969235548	0.968894906	0.977467593
R^2	0.943918947	0.939417547	0.938757339	0.955442896
$R^2_{adjusted}$	0.938310842	0.933359302	0.932633073	0.950987186
RSS	775.1556242	938.9562371	869.2056763	473.3778808

Graphical abstract of model fit presented as mean ± 1 SD of the fraction % of released carvedilol:

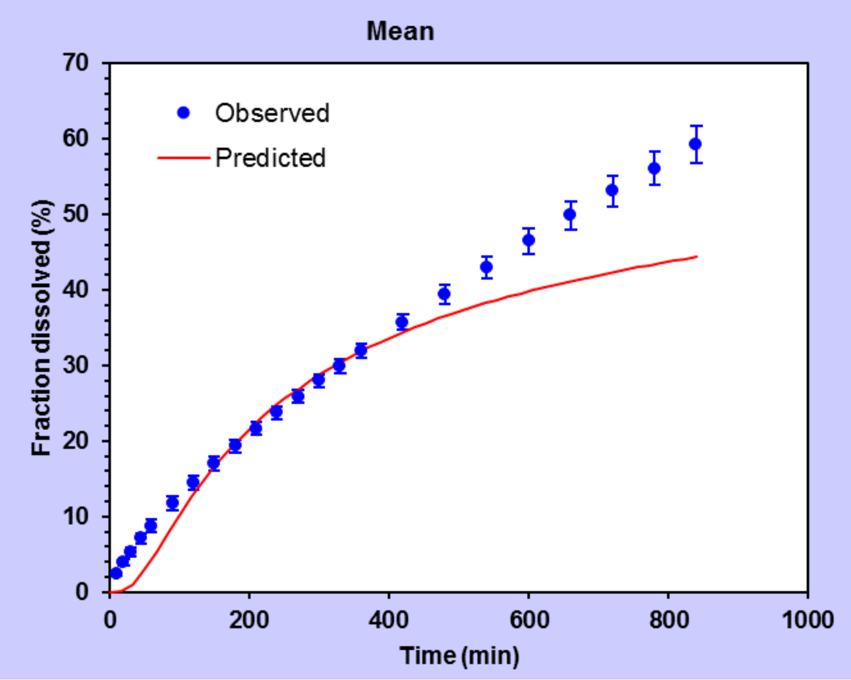

Supplementary materials – Model fitting summary – Avicel® PH-200 (modelling up to app. 60 % of carvedilol released)

Graphical abstract of model fit presented as the fraction % of released carvedilol per tested tablet:

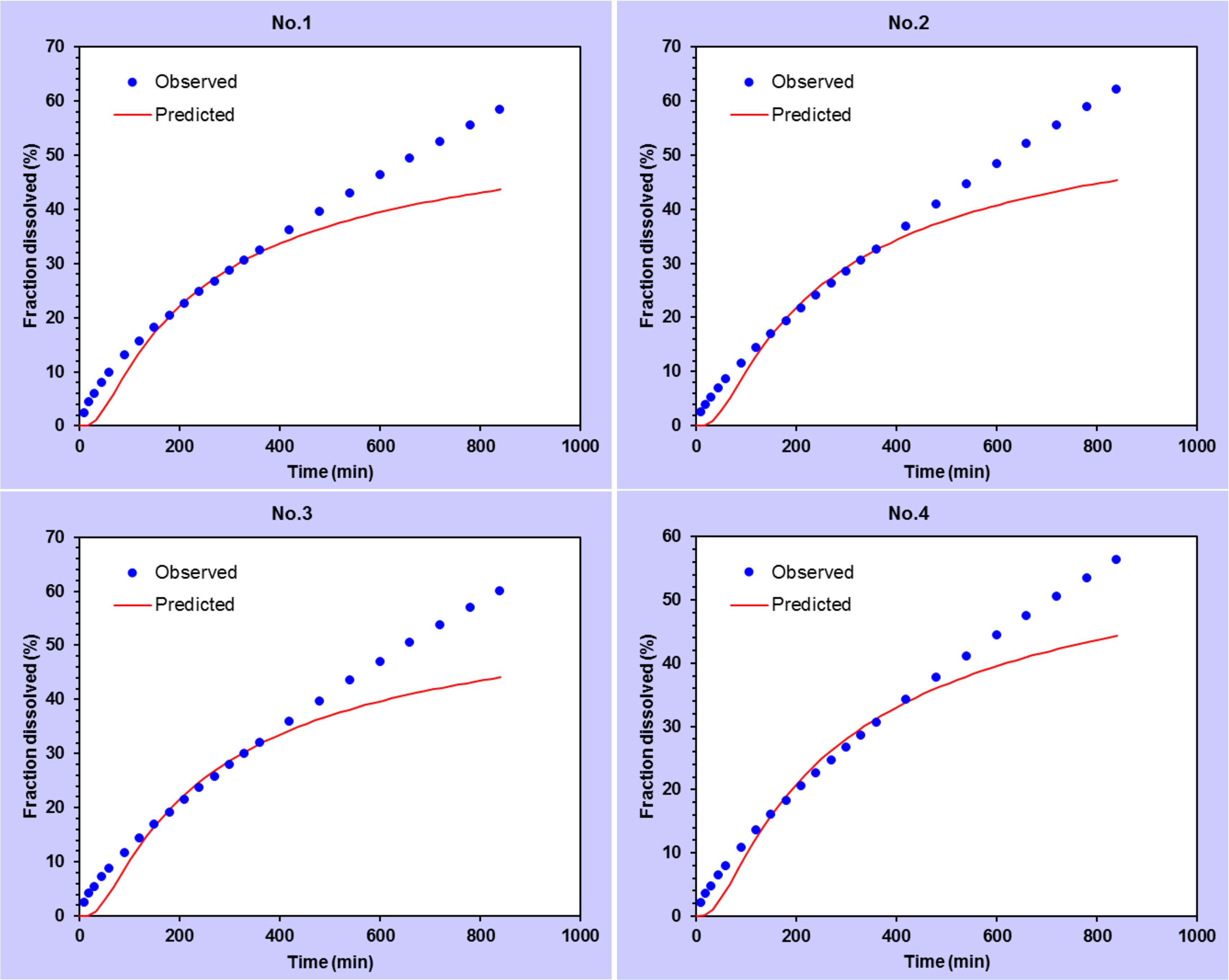

Supplementary materials – Model fitting summary – Avicel® PH-200 (modelling up to app. 60 % of carvedilol released)

Model: **Gompertz_3**

Model equation: $F = F_{max} \cdot e^{-e^{-k \cdot (t - \gamma)}}$

Fitted model parameters per tested tablet (N = 4) with statistics – mean, standard deviation (SD), and relative standard deviation expressed in % (RSD%) (output from DDSolver):

Parameter	No.1	No.2	No.3	No.4	Mean	SD	RSD(%)
k	0.004	0.004	0.004	0.004	0.004	0.000	1.038
γ	233.910	252.514	247.525	246.750	245.175	7.932	3.235
F_{max}	61.354	65.189	63.003	59.117	62.166	2.569	4.132

Number of dissolution data points (N), degrees of freedom (df), and selected goodness of fit criteria – Pearson correlation coefficient (R), coefficient of determination (R^2), adjusted coefficient of determination ($R^2_{adjusted}$), and residual sum of squares (RSS) (manual calculation in MS Excel):

Parameter	No.1	No.2	No.3	No.4
N	23	23	23	23
df	20	20	20	20
R	0.993640276	0.994974547	0.994949207	0.994929248
R^2	0.987320998	0.98997435	0.989923924	0.989884208
$R^2_{adjusted}$	0.986053098	0.988971785	0.988916316	0.988872628
RSS	99.28676704	96.90948794	89.7427859	78.52422239

Graphical abstract of model fit presented as mean ± 1 SD of the fraction % of released carvedilol:

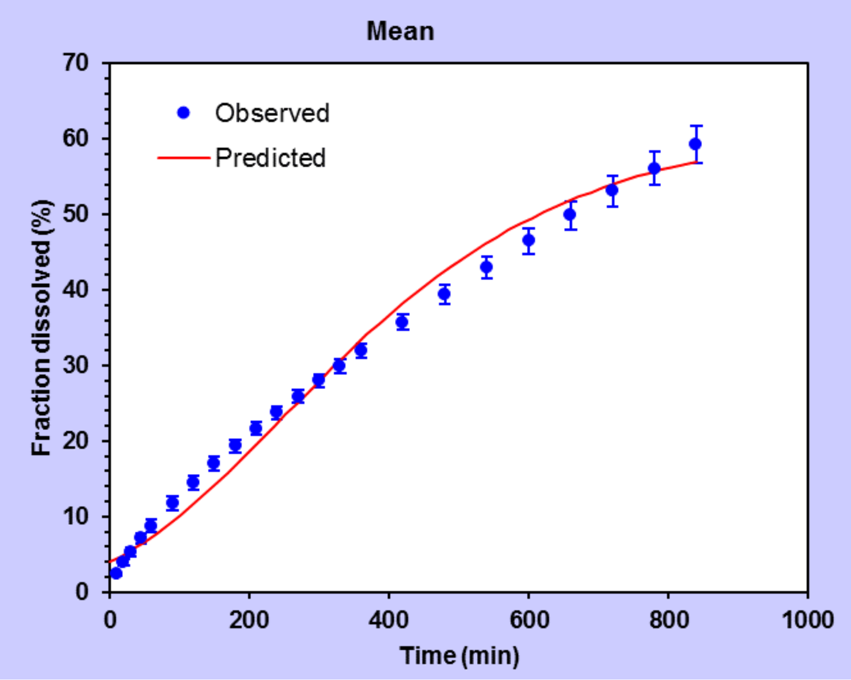

Supplementary materials – Model fitting summary – Avicel® PH-200 (modelling up to app. 60 % of carvedilol released)

Graphical abstract of model fit presented as the fraction % of released carvedilol per tested tablet:

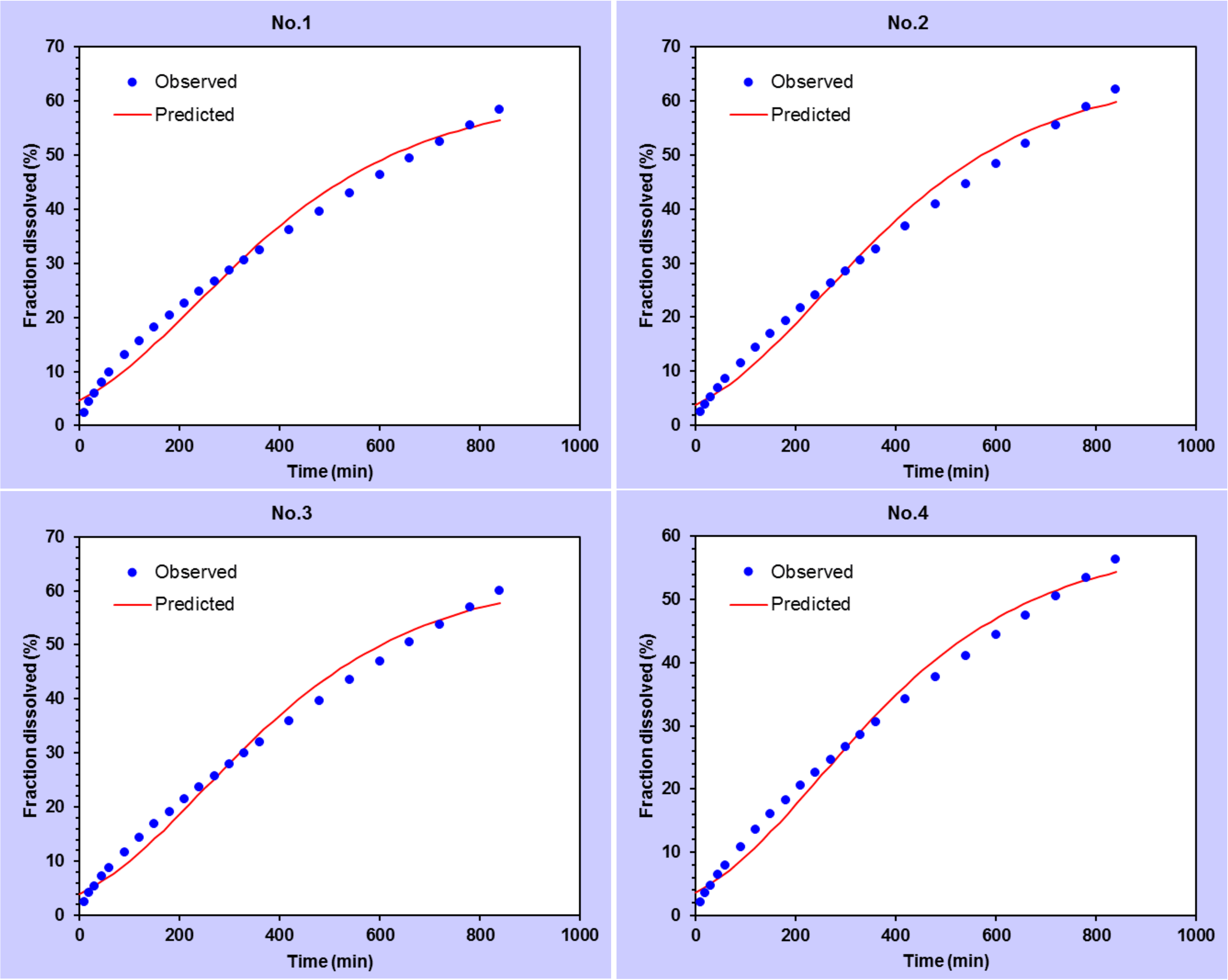

Supplementary materials – Model fitting summary – Avicel® PH-200 (modelling up to app. 60 % of carvedilol released)

Model: **Gompertz_4**

Model equation: $F = F_{max} \cdot e^{-\beta \cdot e^{-k \cdot t}}$

Fitted model parameters per tested tablet (N = 4) with statistics – mean, standard deviation (SD), and relative standard deviation expressed in % (RSD%) (output from DDSolver):

Parameter	No.1	No.2	No.3	No.4	Mean	SD	RSD(%)
k	0.004	0.004	0.004	0.004	0.004	0.000	1.038
β	2.590	2.848	2.779	2.798	2.754	0.113	4.099
F_{max}	61.354	65.189	63.003	59.117	62.166	2.569	4.132

Number of dissolution data points (N), degrees of freedom (df), and selected goodness of fit criteria – Pearson correlation coefficient (R), coefficient of determination (R^2), adjusted coefficient of determination ($R^2_{adjusted}$), and residual sum of squares (RSS) (manual calculation in MS Excel):

Parameter	No.1	No.2	No.3	No.4
N	23	23	23	23
df	20	20	20	20
R	0.993640276	0.994974547	0.994949207	0.994929248
R^2	0.987320998	0.98997435	0.989923924	0.989884208
$R^2_{adjusted}$	0.986053098	0.988971785	0.988916316	0.988872628
RSS	99.28676704	96.90948794	89.7427859	78.52422239

Graphical abstract of model fit presented as mean ± 1 SD of the fraction % of released carvedilol:

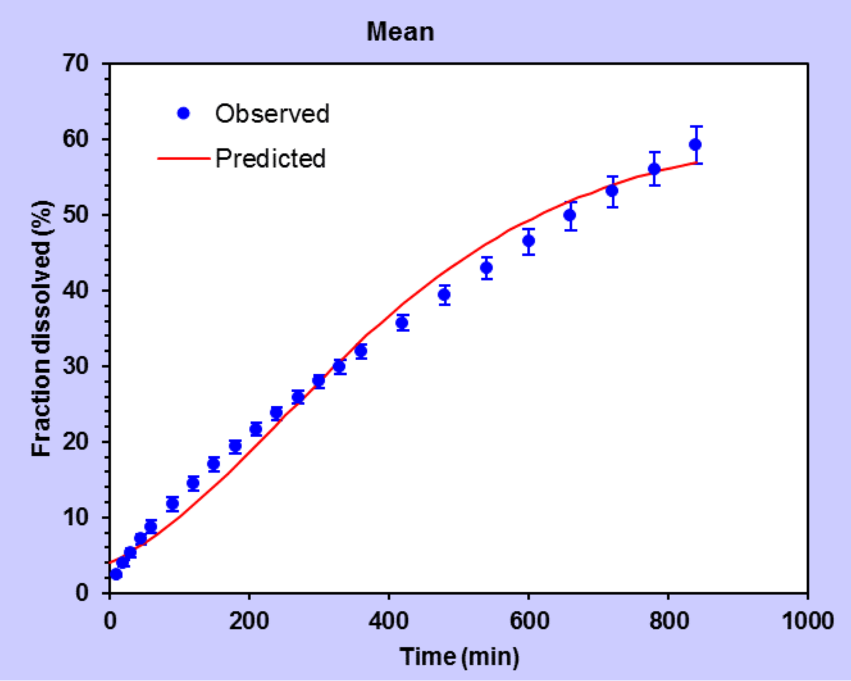

Supplementary materials – Model fitting summary – Avicel® PH-200 (modelling up to app. 60 % of carvedilol released)

Graphical abstract of model fit presented as the fraction % of released carvedilol per tested tablet:

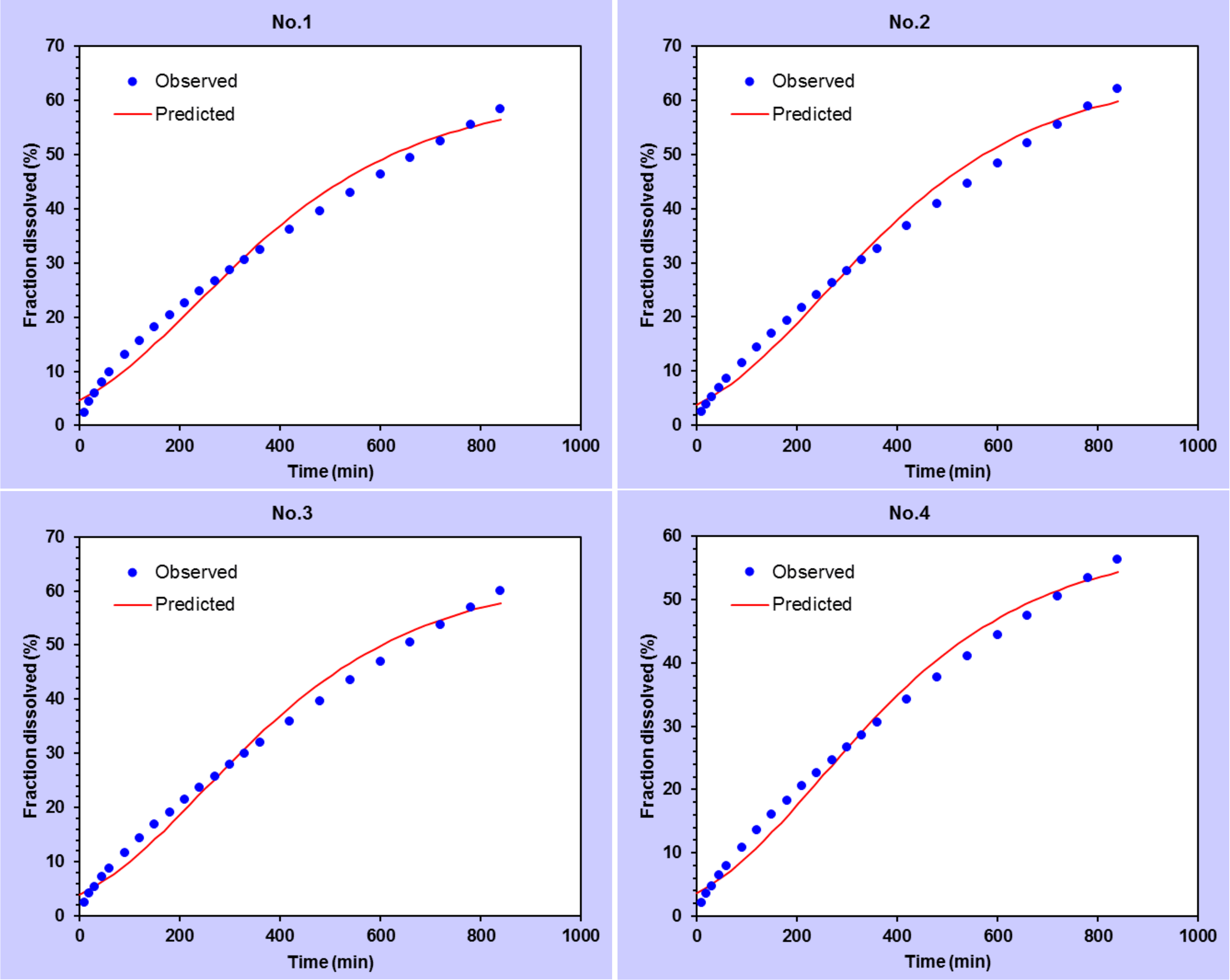

Supplementary materials – Model fitting summary – Avicel® PH-200 (modelling up to app. 60 % of carvedilol released)

Model: **Probit_1**

Model equation: $F = 100 \cdot \phi[\alpha + \beta \cdot \log(t)]$

Fitted model parameters per tested tablet (N = 4) with statistics – mean, standard deviation (SD), and relative standard deviation expressed in % (RSD%) (output from DDSolver):

Parameter	No.1	No.2	No.3	No.4	Mean	SD	RSD(%)
α	-3.265	-3.424	-3.358	-3.408	-3.364	0.072	-2.134
β	1.124	1.193	1.157	1.155	1.157	0.028	2.432

Number of dissolution data points (N), degrees of freedom (df), and selected goodness of fit criteria – Pearson correlation coefficient (R), coefficient of determination (R^2), adjusted coefficient of determination ($R^2_{adjusted}$), and residual sum of squares (RSS) (manual calculation in MS Excel):

Parameter	No.1	No.2	No.3	No.4
N	23	23	23	23
df	21	21	21	21
R	0.987507904	0.983270043	0.983923544	0.988602712
R^2	0.97517186	0.966819978	0.968105541	0.977335322
$R^2_{adjusted}$	0.973989567	0.965239977	0.966586757	0.976256051
RSS	201.9478646	337.741473	298.5357553	197.9773335

Graphical abstract of model fit presented as mean ± 1 SD of the fraction % of released carvedilol:

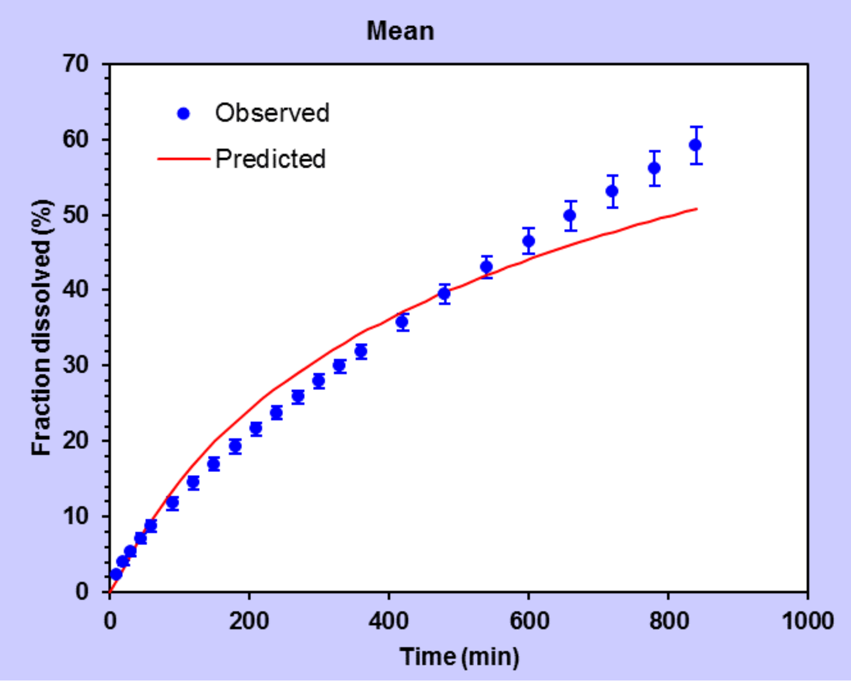

Supplementary materials – Model fitting summary – Avicel® PH-200 (modelling up to app. 60 % of carvedilol released)

Graphical abstract of model fit presented as the fraction % of released carvedilol per tested tablet:

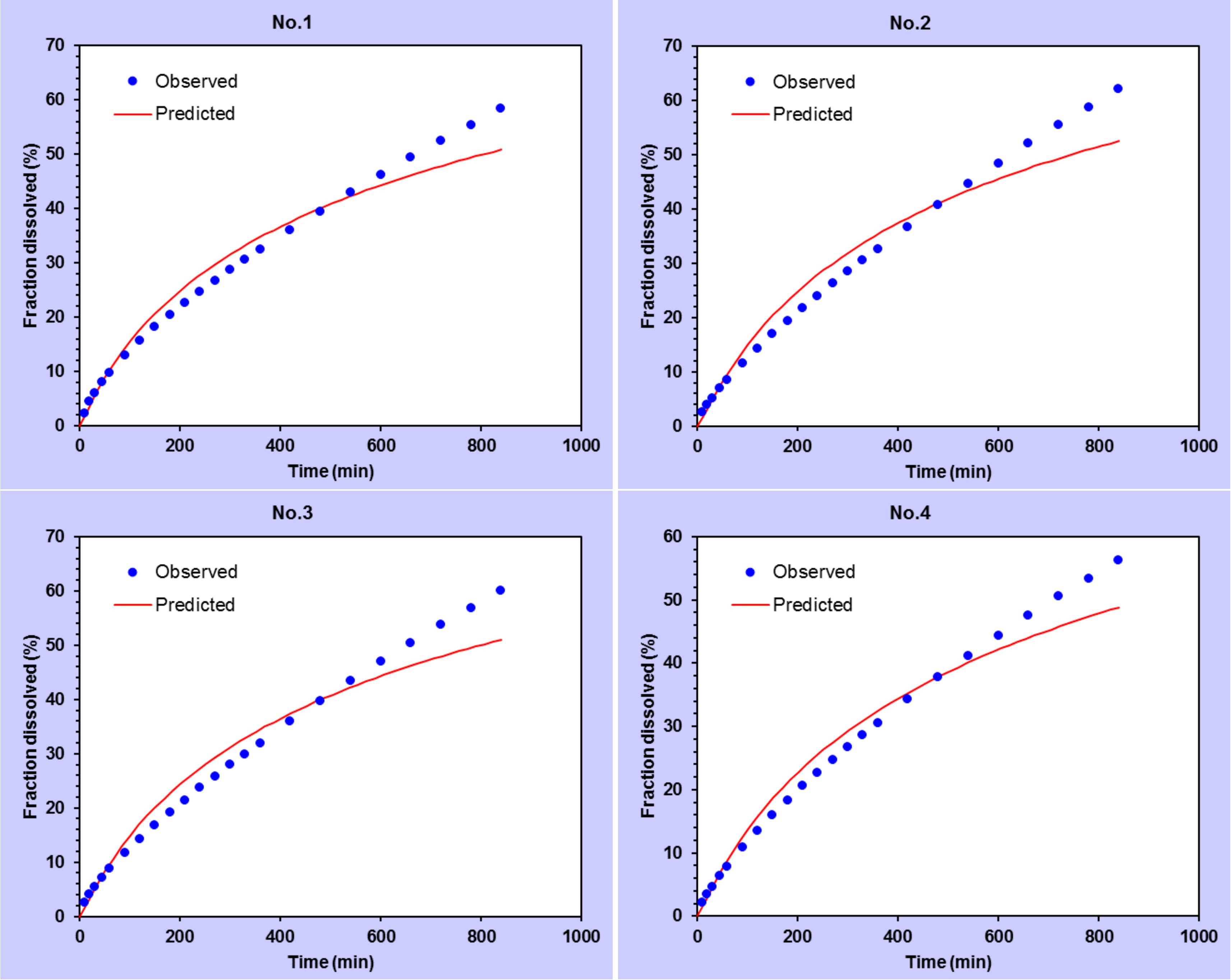

Supplementary materials – Model fitting summary – Avicel® PH-200 (modelling up to app. 60 % of carvedilol released)

Model: **Probit_2**

Model equation: $F = F_{max} \cdot \phi[\alpha + \beta \cdot \log(t)]$

Fitted model parameters per tested tablet (N = 4) with statistics – mean, standard deviation (SD), and relative standard deviation expressed in % (RSD%) (output from DDSolver):

Parameter	No.1	No.2	No.3	No.4	Mean	SD	RSD(%)
α	-4.339	-4.125	-4.442	-4.155	-4.265	0.151	-3.547
β	1.689	1.553	1.708	1.574	1.631	0.079	4.842
F_{max}	67.181	77.703	68.987	70.465	71.084	4.613	6.489

Number of dissolution data points (N), degrees of freedom (df), and selected goodness of fit criteria – Pearson correlation coefficient (R), coefficient of determination (R^2), adjusted coefficient of determination ($R^2_{adjusted}$), and residual sum of squares (RSS) (manual calculation in MS Excel):

Parameter	No.1	No.2	No.3	No.4
N	23	23	23	23
df	20	20	20	20
R	0.986894943	0.984795608	0.985652243	0.987888439
R^2	0.973961629	0.96982239	0.971510345	0.975923567
$R^2_{adjusted}$	0.971357791	0.966804629	0.96866138	0.973515924
RSS	309.2082619	341.5440775	393.9613901	233.6578509

Graphical abstract of model fit presented as mean ± 1 SD of the fraction % of released carvedilol:

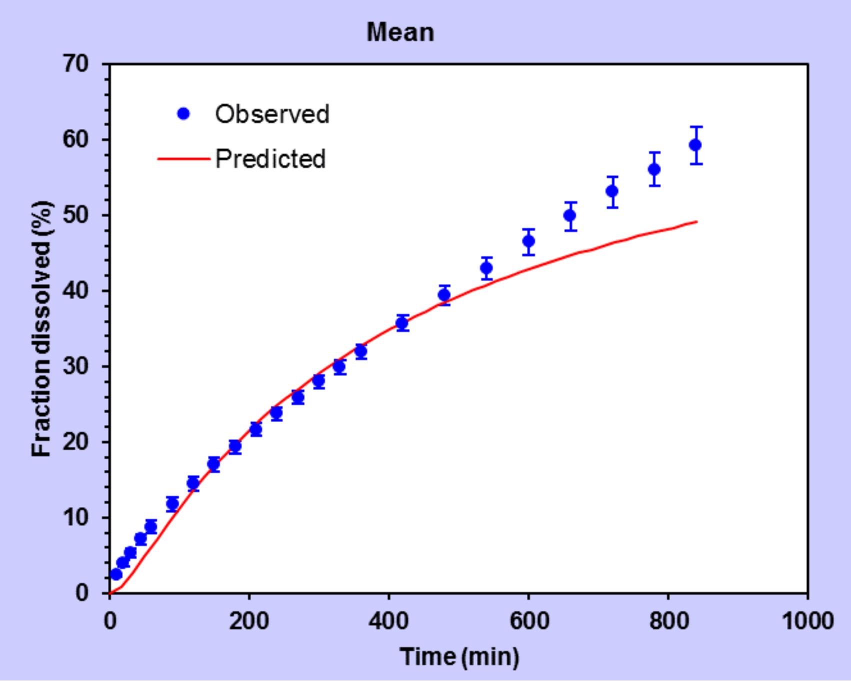

Supplementary materials – Model fitting summary – Avicel® PH-200 (modelling up to app. 60 % of carvedilol released)

Graphical abstract of model fit presented as the fraction % of released carvedilol per tested tablet:

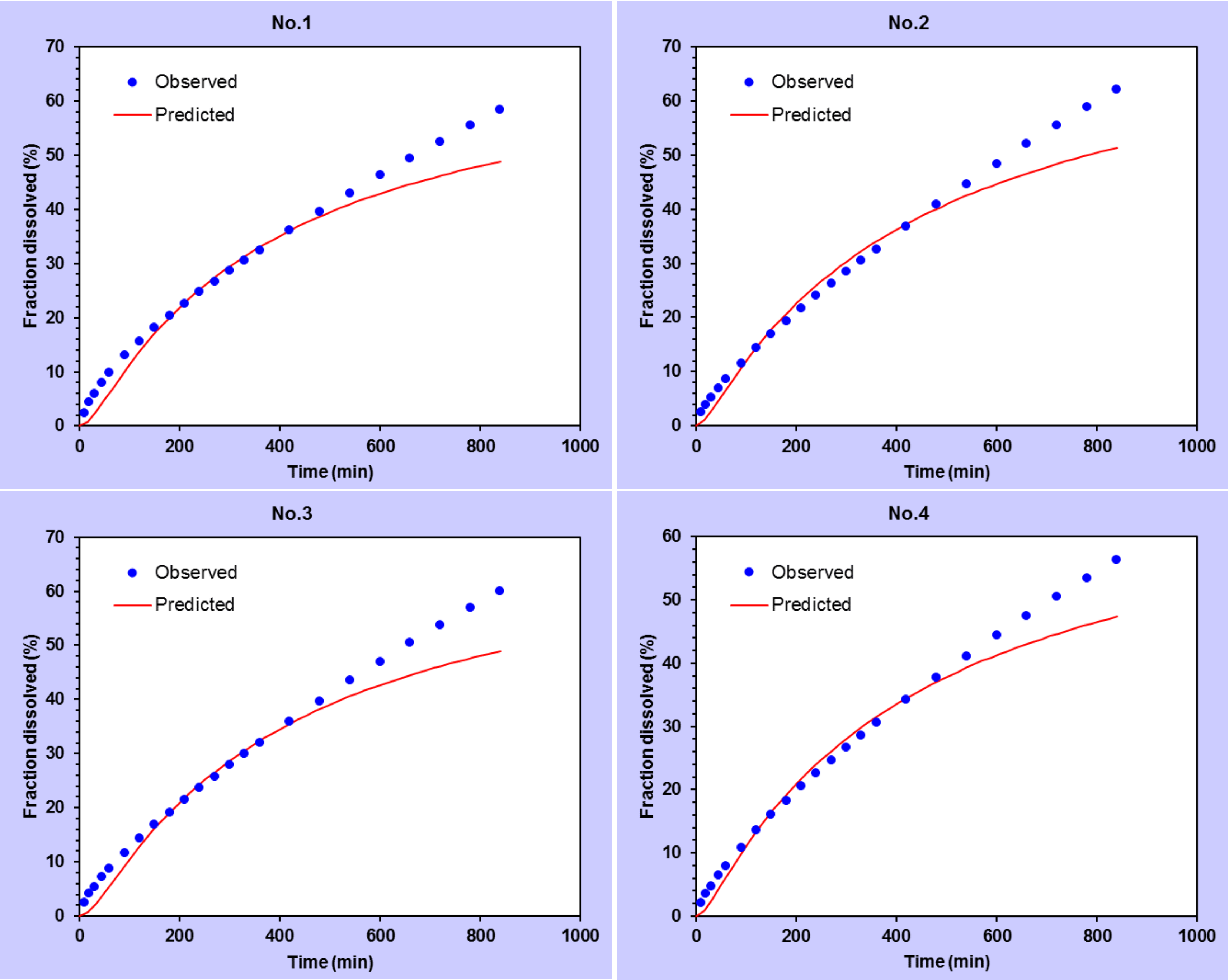

Supplement: Supplementary file 1 [file pharmaceutics-16-00498-s001.zip › Supplementary materials_Model fitting summary_Avicel® PH-200.pdf]
