# Supplementary material for: Comparative Fitting of Mathematical Models to Carvedilol Release Profiles Obtained from Hypromellose Matrix Tablets
Source: Pharmaceutics. 2024 Apr 4;16(4):498. doi: 10.3390/pharmaceutics16040498 (PMC11053526; doi:10.3390/pharmaceutics16040498)

Supplementary materials – Model fitting summary – C*Pharm Mannidex 16700 (modelling the entire relevant dissolution data)

Model: **Zero–order**

Model equation: $F = k_0 \cdot t$

Fitted model parameters per tested tablet (N = 4) with statistics – mean, standard deviation (SD), and relative standard deviation expressed in % (RSD%) (output from DDSolver):

Parameter	No.1	No.2	No.3	No.4	Mean	SD	RSD(%)
k_0	0.295	0.297	0.323	0.356	0.318	0.028	8.924

Number of dissolution data points (N), degrees of freedom (df), and selected goodness of fit criteria – Pearson correlation coefficient (R), coefficient of determination (R^2), adjusted coefficient of determination ($R^2_{adjusted}$), and residual sum of squares (RSS) (manual calculation in MS Excel):

Parameter	No.1	No.2	No.3	No.4
N	16	16	16	16
df	15	15	15	15
R	0.975720399	0.978624062	0.943825474	0.826485003
R^2	0.952030298	0.957705055	0.890806525	0.683077461
$R^2_{adjusted}$	0.952030298	0.957705055	0.890806525	0.683077461
RSS	2028.340002	2444.146761	4323.561847	14221.6603

Graphical abstract of model fit presented as mean ± 1 SD of the fraction % of released carvedilol:

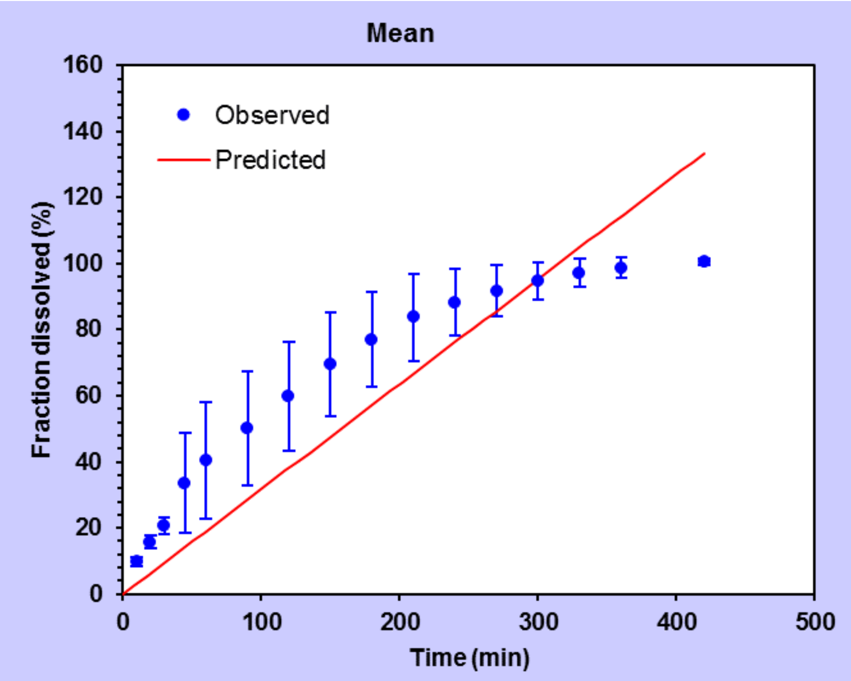

Supplementary materials – Model fitting summary – C*Pharm Mannidex 16700 (modelling the entire relevant dissolution data)

Graphical abstract of model fit presented as the fraction % of released carvedilol per tested tablet:

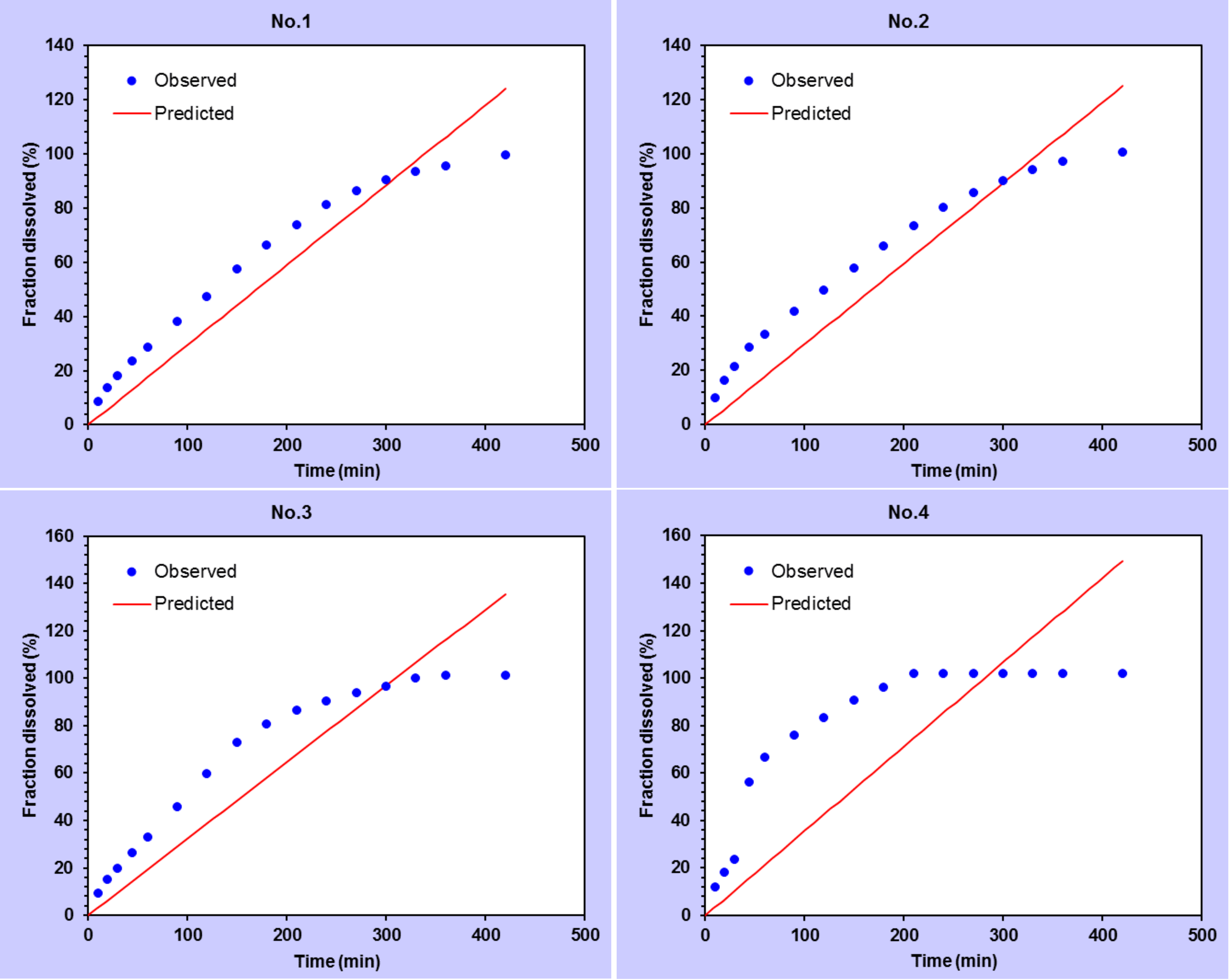

Supplementary materials – Model fitting summary – C*Pharm Mannidex 16700 (modelling the entire relevant dissolution data)

Model: **Zero–order with T_{lag}**

Model equation: $F = k_0 \cdot (t - T_{lag})$

Fitted model parameters per tested tablet (N = 4) with statistics – mean, standard deviation (SD), and relative standard deviation expressed in % (RSD%) (output from DDSolver):

Parameter	No.1	No.2	No.3	No.4	Mean	SD	RSD(%)
k_0	0.238	0.230	0.245	0.204	0.229	0.018	7.832
T_{lag}	-64.168	-79.740	-85.252	-200.351	-107.378	62.622	-58.319

Number of dissolution data points (N), degrees of freedom (df), and selected goodness of fit criteria – Pearson correlation coefficient (R), coefficient of determination (R^2), adjusted coefficient of determination ($R^2_{adjusted}$), and residual sum of squares (RSS) (manual calculation in MS Excel):

Parameter	No.1	No.2	No.3	No.4
N	16	16	16	16
df	14	14	14	14
R	0.975720399	0.978624062	0.943825474	0.826485003
R^2	0.952030298	0.957705055	0.890806525	0.683077461
$R^2_{adjusted}$	0.948603891	0.954683987	0.883006992	0.660440137
RSS	748.0389392	608.6772012	1928.568275	5057.404941

Graphical abstract of model fit presented as mean ± 1 SD of the fraction % of released carvedilol:

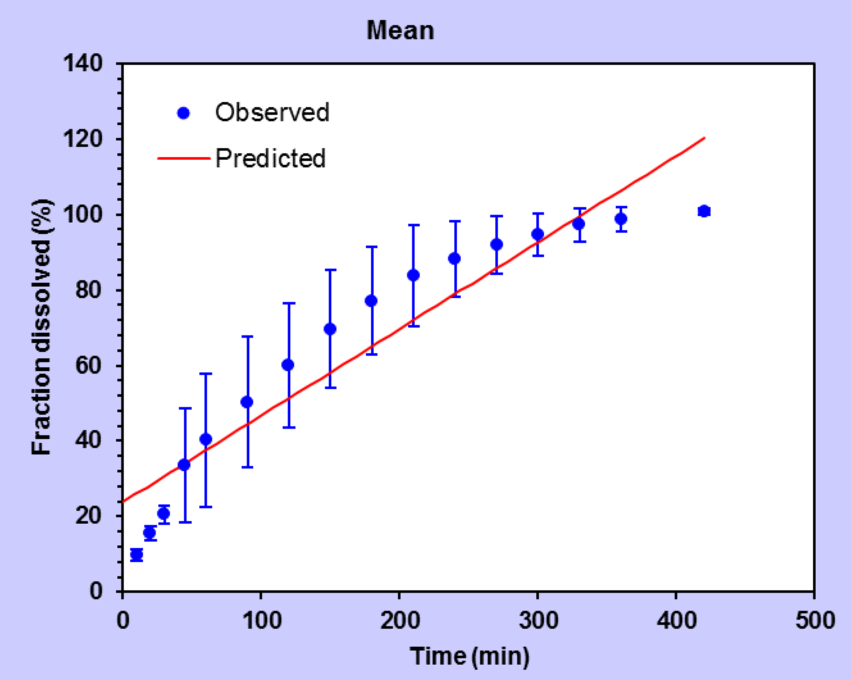

Supplementary materials – Model fitting summary – C*Pharm Mannidex 16700 (modelling the entire relevant dissolution data)

Graphical abstract of model fit presented as the fraction % of released carvedilol per tested tablet:

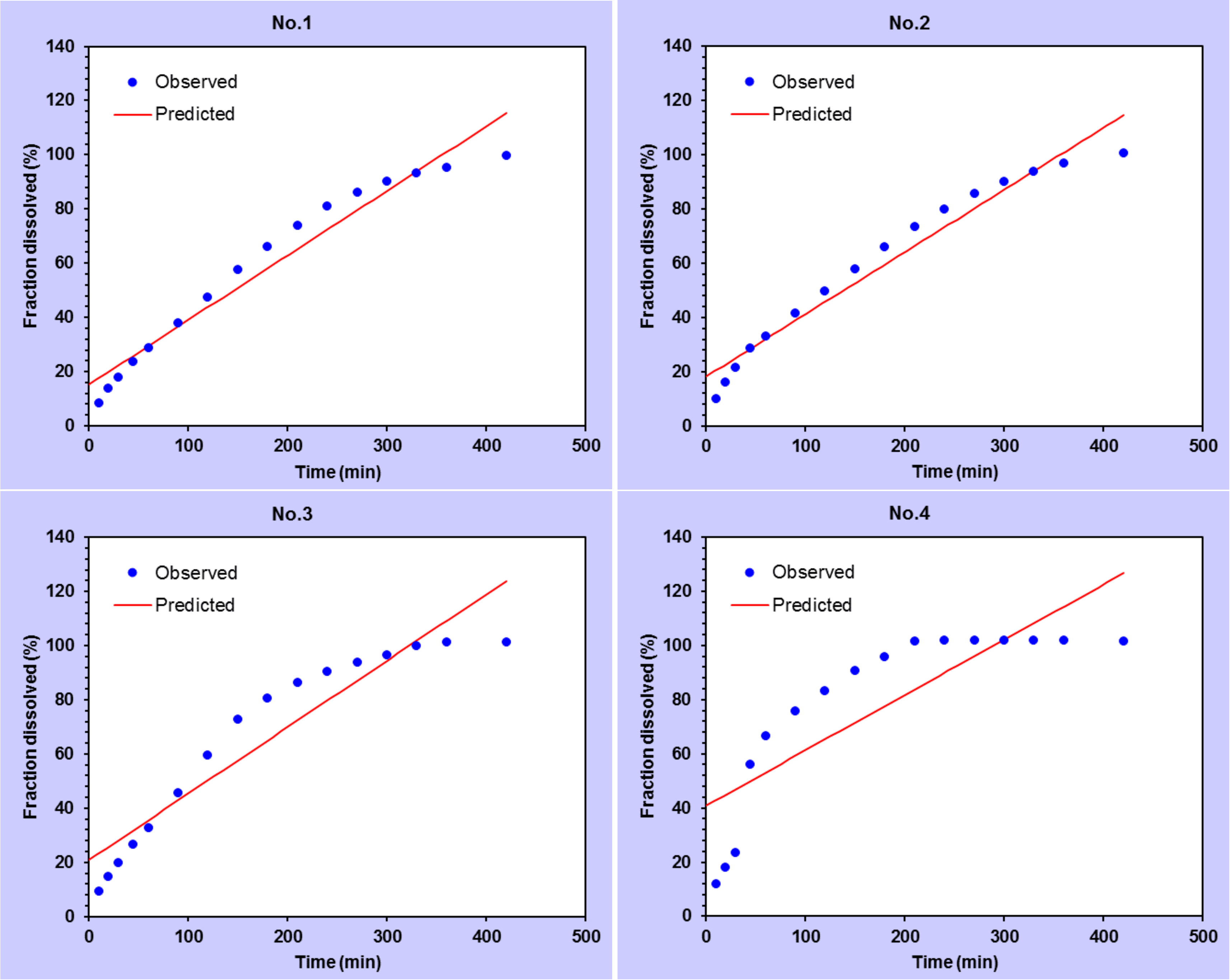

Supplementary materials – Model fitting summary – C*Pharm Mannidex 16700 (modelling the entire relevant dissolution data)

Model: **Zero–order with F_0**

Model equation: $F = F_0 + k_0 \cdot t$

Fitted model parameters per tested tablet (N = 4) with statistics – mean, standard deviation (SD), and relative standard deviation expressed in % (RSD%) (output from DDSolver):

Parameter	No.1	No.2	No.3	No.4	Mean	SD	RSD(%)
k_0	0.238	0.230	0.245	0.204	0.229	0.018	7.832
F_0	15.286	18.303	20.907	40.897	23.849	11.596	48.622

Number of dissolution data points (N), degrees of freedom (df), and selected goodness of fit criteria – Pearson correlation coefficient (R), coefficient of determination (R^2), adjusted coefficient of determination ($R^2_{adjusted}$), and residual sum of squares (RSS) (manual calculation in MS Excel):

Parameter	No.1	No.2	No.3	No.4
N	16	16	16	16
df	14	14	14	14
R	0.975720399	0.978624062	0.943825474	0.826485003
R^2	0.952030298	0.957705055	0.890806525	0.683077461
$R^2_{adjusted}$	0.948603891	0.954683987	0.883006992	0.660440137
RSS	748.0389392	608.6772012	1928.568275	5057.404941

Graphical abstract of model fit presented as mean ± 1 SD of the fraction % of released carvedilol:

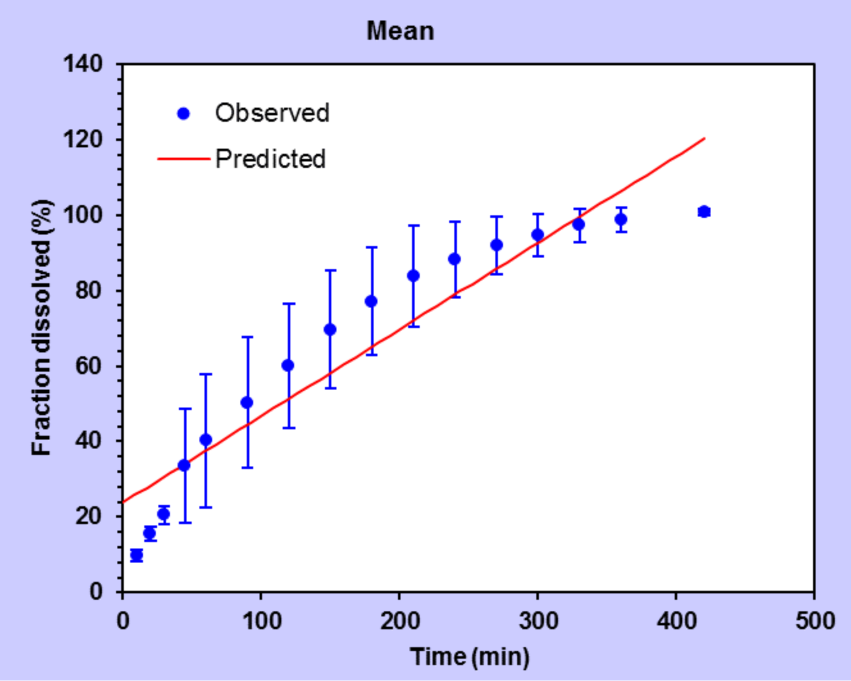

Supplementary materials – Model fitting summary – C*Pharm Mannidex 16700 (modelling the entire relevant dissolution data)

Graphical abstract of model fit presented as the fraction % of released carvedilol per tested tablet:

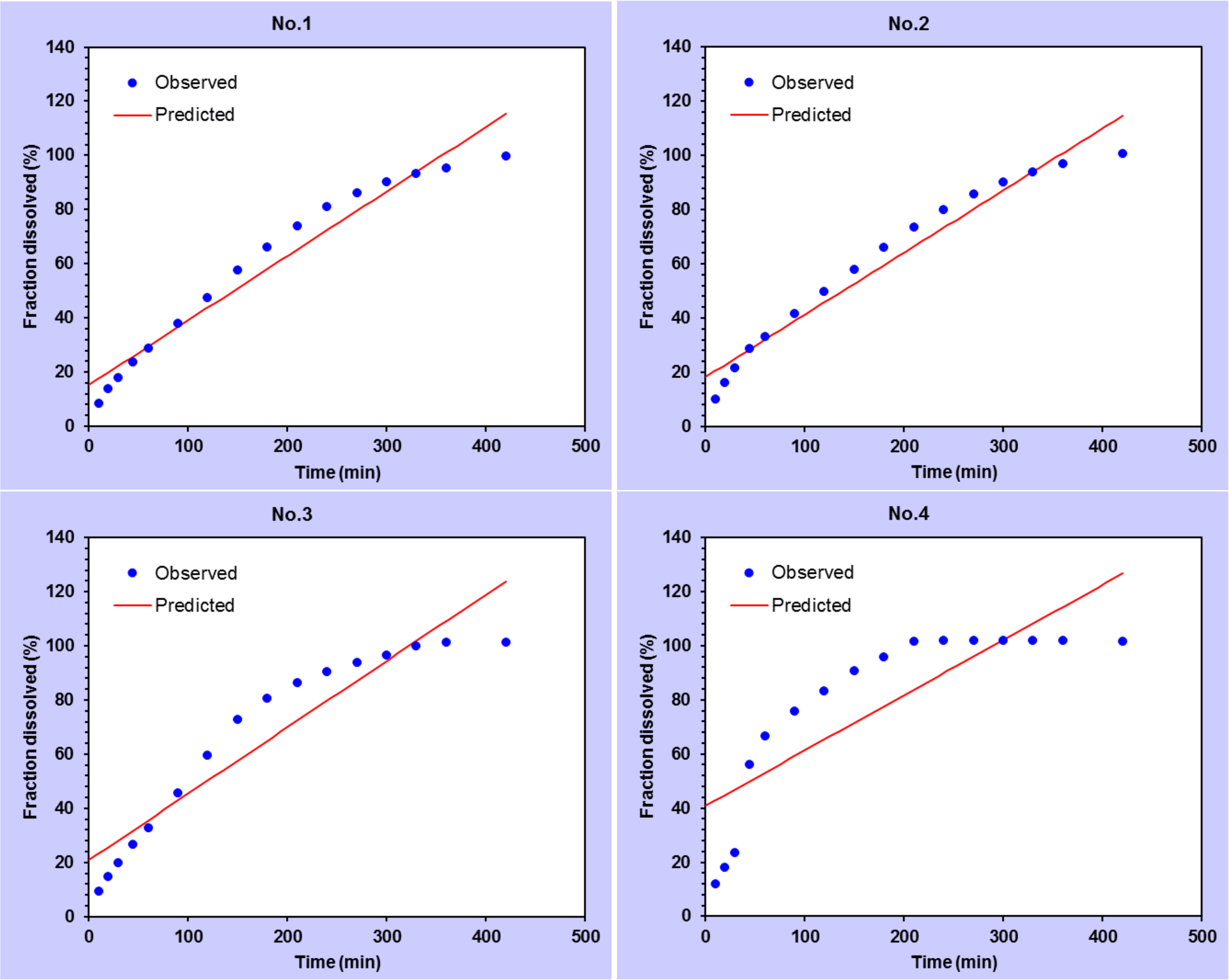

Supplementary materials – Model fitting summary – C*Pharm Mannidex 16700 (modelling the entire relevant dissolution data)

Model: **First–order**

Model equation: $F = 100 \cdot (1 - e^{-k_1 \cdot t})$

Fitted model parameters per tested tablet (N = 4) with statistics – mean, standard deviation (SD), and relative standard deviation expressed in % (RSD%) (output from DDSolver):

Parameter	No.1	No.2	No.3	No.4	Mean	SD	RSD(%)
k_1	0.007	0.006	0.014	0.016	0.011	0.005	49.757

Number of dissolution data points (N), degrees of freedom (df), and selected goodness of fit criteria – Pearson correlation coefficient (R), coefficient of determination (R^2), adjusted coefficient of determination ($R^2_{adjusted}$), and residual sum of squares (RSS) (manual calculation in MS Excel):

Parameter	No.1	No.2	No.3	No.4
N	16	16	16	16
df	15	15	15	15
R	0.992983855	0.995368123	0.967559603	0.991736433
R^2	0.986016937	0.990757701	0.936171585	0.983541153
$R^2_{adjusted}$	0.986016937	0.990757701	0.936171585	0.983541153
RSS	262.3889958	492.9259591	2969.263299	447.0642974

Graphical abstract of model fit presented as mean ± 1 SD of the fraction % of released carvedilol:

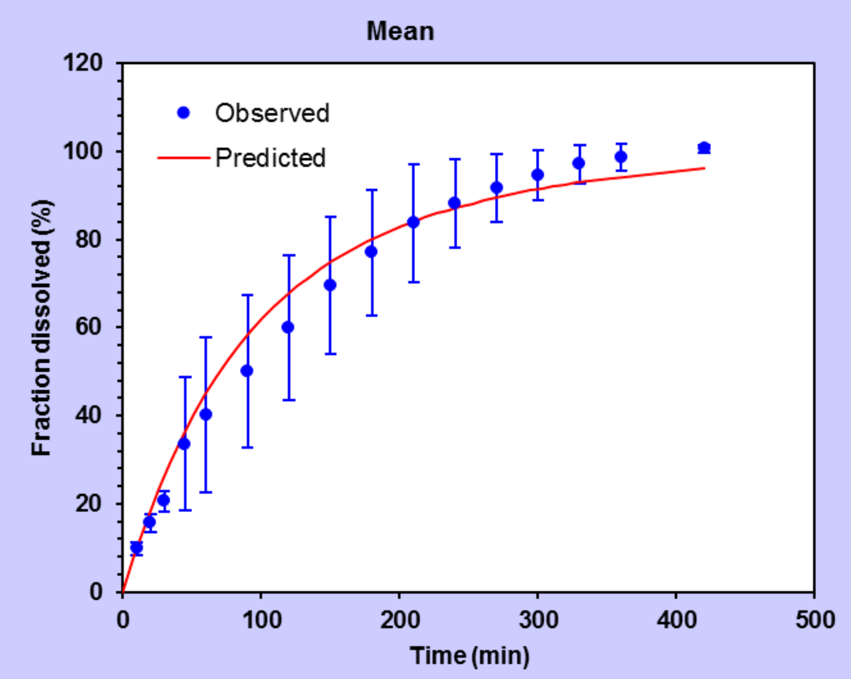

Supplementary materials – Model fitting summary – C*Pharm Mannidex 16700 (modelling the entire relevant dissolution data)

Graphical abstract of model fit presented as the fraction % of released carvedilol per tested tablet:

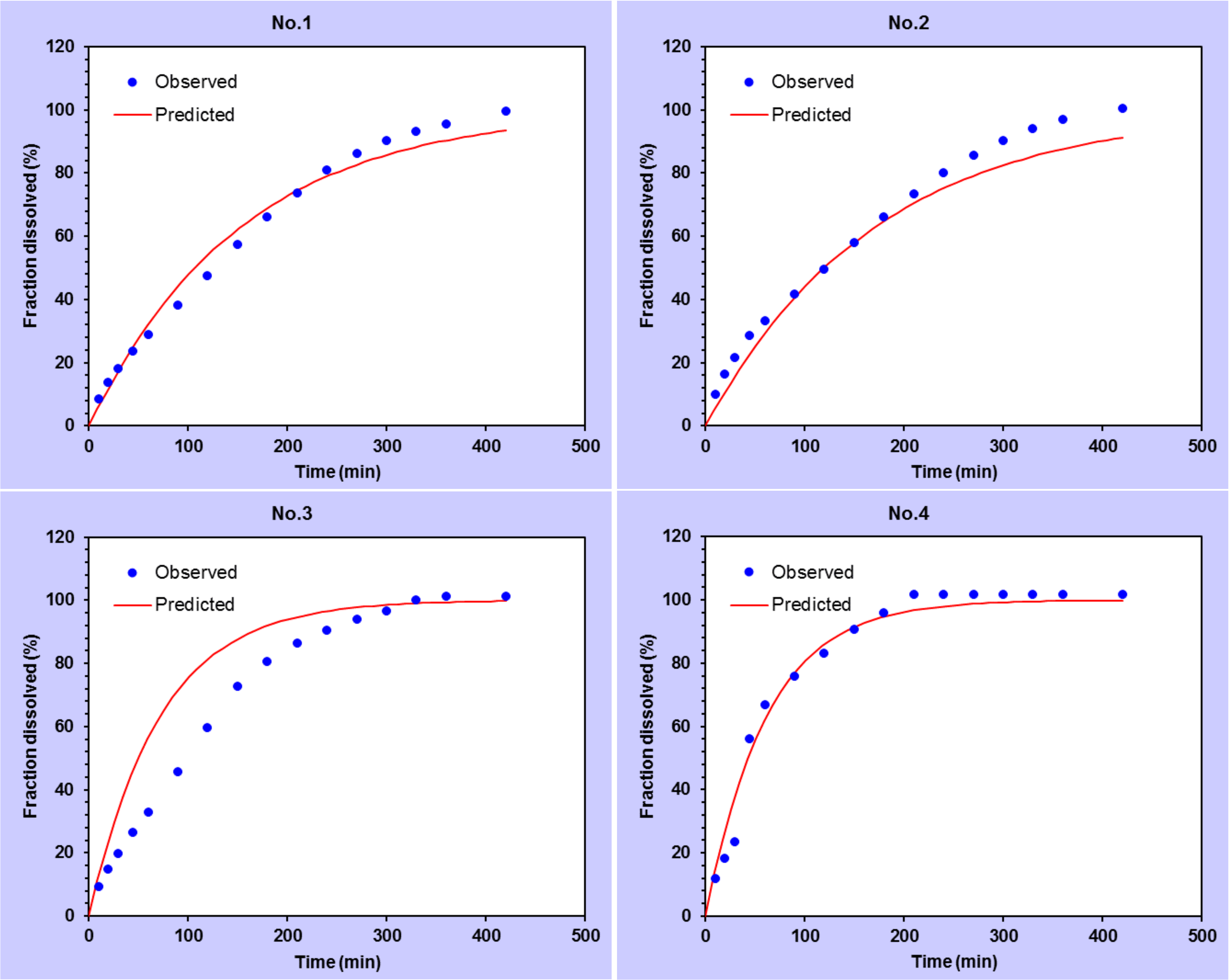

Supplementary materials – Model fitting summary – C*Pharm Mannidex 16700 (modelling the entire relevant dissolution data)

Model: **First–order with T_{lag}**

Model equation: $F = 100 \cdot \left[1 - e^{-k_1 \cdot (t - T_{lag})}\right]$

Fitted model parameters per tested tablet (N = 4) with statistics – mean, standard deviation (SD), and relative standard deviation expressed in % (RSD%) (output from DDSolver):

Parameter	No.1	No.2	No.3	No.4	Mean	SD	RSD(%)
k_1	/	/	/	/	/	/	/
T_{lag}	/	/	/	/	/	/	/

Number of dissolution data points (N), degrees of freedom (df), and selected goodness of fit criteria – Pearson correlation coefficient (R), coefficient of determination (R^2), adjusted coefficient of determination ($R^2_{adjusted}$), and residual sum of squares (RSS) (manual calculation in MS Excel):

Parameter	No.1	No.2	No.3	No.4
N	/	/	/	/
df	/	/	/	/
R	/	/	/	/
R^2	/	/	/	/
$R^2_{adjusted}$	/	/	/	/
RSS	/	/	/	/

Graphical abstract of model fit presented as mean ± 1 SD of the fraction % of released carvedilol: /

Graphical abstract of model fit presented as the fraction % of released carvedilol per tested tablet: /

Note: the model could not be fitted

Supplementary materials – Model fitting summary – C*Pharm Mannidex 16700 (modelling the entire relevant dissolution data)

Model: **First–order with F_{max}**

Model equation: $F = F_{max} \cdot (1 - e^{-k_1 \cdot t})$

Fitted model parameters per tested tablet (N = 4) with statistics – mean, standard deviation (SD), and relative standard deviation expressed in % (RSD%) (output from DDSolver):

Parameter	No.1	No.2	No.3	No.4	Mean	SD	RSD(%)
k_1	0.007	0.006	0.008	0.013	0.008	0.003	36.091
F_{max}	104.499	105.467	106.197	106.848	105.753	1.008	0.954

Number of dissolution data points (N), degrees of freedom (df), and selected goodness of fit criteria – Pearson correlation coefficient (R), coefficient of determination (R^2), adjusted coefficient of determination ($R^2_{adjusted}$), and residual sum of squares (RSS) (manual calculation in MS Excel):

Parameter	No.1	No.2	No.3	No.4
N	16	16	16	16
df	14	14	14	14
R	0.992683437	0.993339538	0.996182659	0.987969382
R^2	0.985420406	0.986723438	0.99237989	0.9760835
$R^2_{adjusted}$	0.984379007	0.985775112	0.991835597	0.974375178
RSS	346.9975696	215.1896582	226.2963361	395.9913079

Graphical abstract of model fit presented as mean ± 1 SD of the fraction % of released carvedilol:

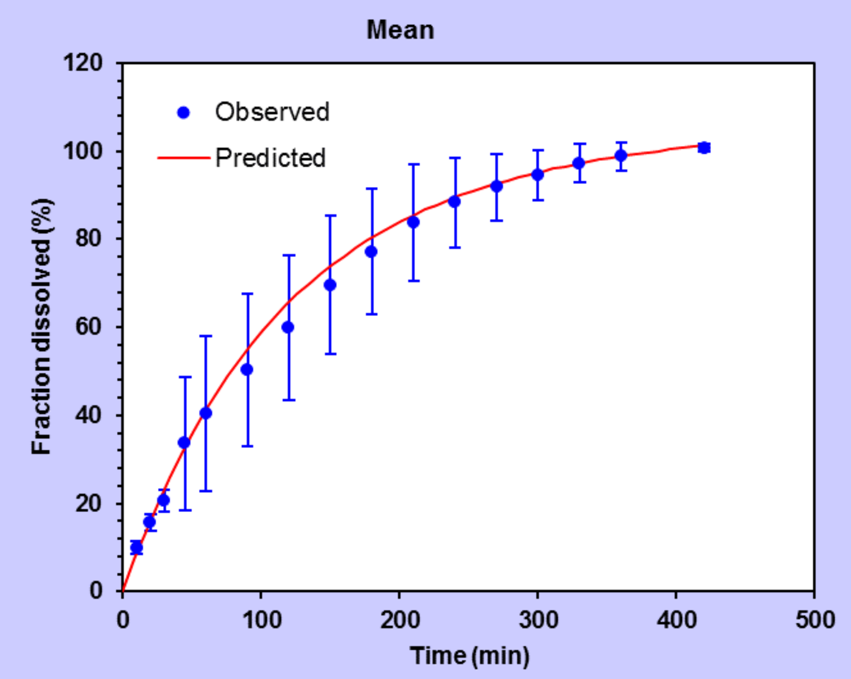

Supplementary materials – Model fitting summary – C*Pharm Mannidex 16700 (modelling the entire relevant dissolution data)

Graphical abstract of model fit presented as the fraction % of released carvedilol per tested tablet:

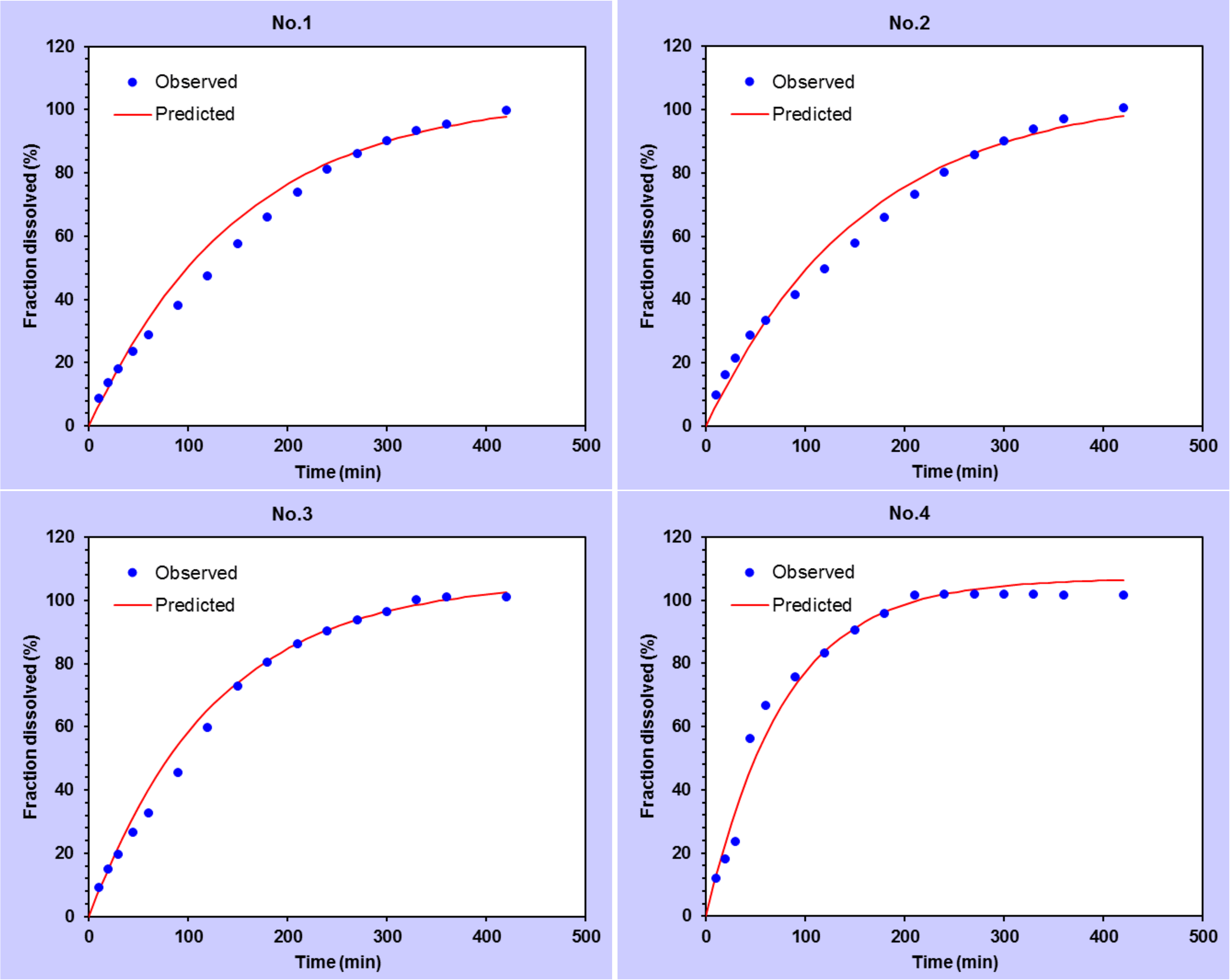

Supplementary materials – Model fitting summary – C*Pharm Mannidex 16700 (modelling the entire relevant dissolution data)

Model: **First–order with T_{lag} and F_{max}**

Model equation: $F = F_{max} \cdot \left[1 - e^{-k_1 \cdot (t - T_{lag})}\right]$

Fitted model parameters per tested tablet (N = 4) with statistics – mean, standard deviation (SD), and relative standard deviation expressed in % (RSD%) (output from DDSolver):

Parameter	No.1	No.2	No.3	No.4	Mean	SD	RSD(%)
k_1	0.007	0.007	0.008	0.008	0.008	0.001	9.558
T_{lag}	16.965	12.644	6.990	-52.998	-4.100	32.854	-801.351
F_{max}	104.499	105.467	106.197	106.848	105.753	1.008	0.954

Number of dissolution data points (N), degrees of freedom (df), and selected goodness of fit criteria – Pearson correlation coefficient (R), coefficient of determination (R^2), adjusted coefficient of determination ($R^2_{adjusted}$), and residual sum of squares (RSS) (manual calculation in MS Excel):

Parameter	No.1	No.2	No.3	No.4
N	16	16	16	16
df	13	13	13	13
R	0.990551663	0.990826684	0.995952508	0.965633301
R^2	0.981192597	0.981737517	0.991921398	0.932447671
$R^2_{adjusted}$	0.97829915	0.978927905	0.990678536	0.922055005
RSS	561.3837456	574.3850964	150.6292614	2908.079718

Graphical abstract of model fit presented as mean ± 1 SD of the fraction % of released carvedilol:

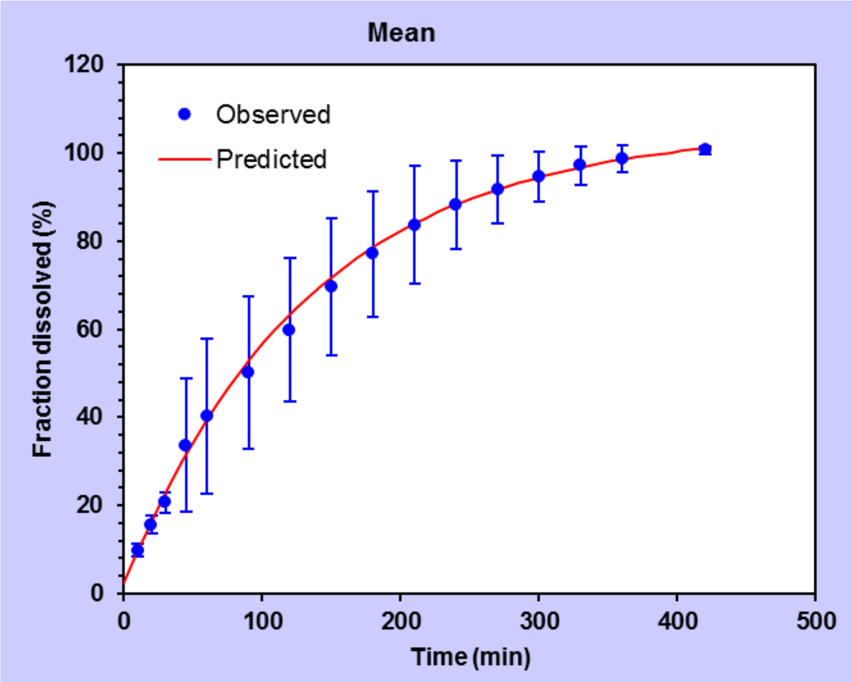

Supplementary materials – Model fitting summary – C*Pharm Mannidex 16700 (modelling the entire relevant dissolution data)

Graphical abstract of model fit presented as the fraction % of released carvedilol per tested tablet:

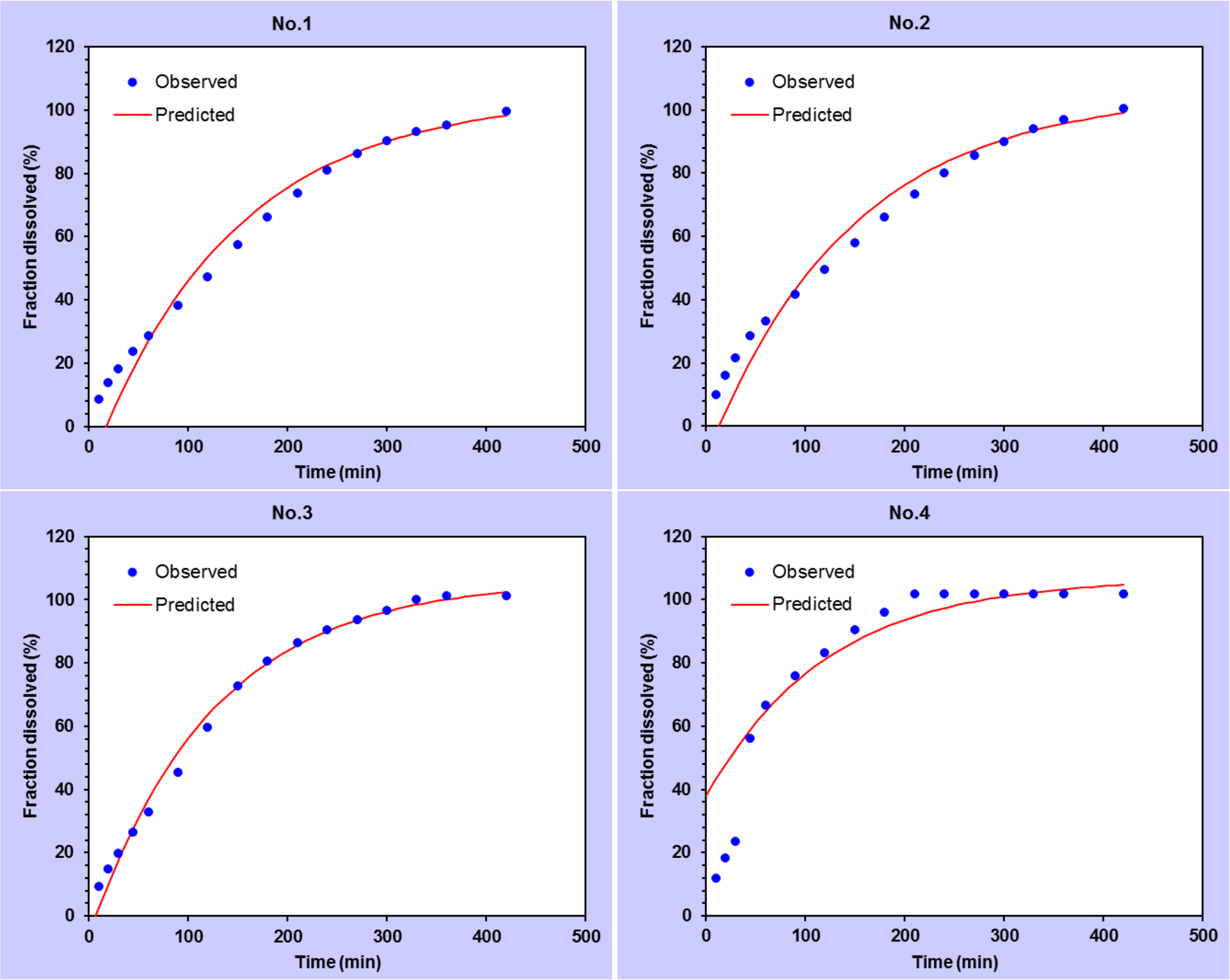

Supplementary materials – Model fitting summary – C*Pharm Mannidex 16700 (modelling the entire relevant dissolution data)

Model: **Higuchi**

Model equation: $F = k_H \cdot t^{0.5}$

Fitted model parameters per tested tablet (N = 4) with statistics – mean, standard deviation (SD), and relative standard deviation expressed in % (RSD%) (output from DDSolver):

Parameter	No.1	No.2	No.3	No.4	Mean	SD	RSD(%)
k_H	4.893	4.960	5.415	6.174	5.361	0.590	10.998

Number of dissolution data points (N), degrees of freedom (df), and selected goodness of fit criteria – Pearson correlation coefficient (R), coefficient of determination (R^2), adjusted coefficient of determination ($R^2_{adjusted}$), and residual sum of squares (RSS) (manual calculation in MS Excel):

Parameter	No.1	No.2	No.3	No.4
N	16	16	16	16
df	15	15	15	15
R	0.995361237	0.997713804	0.984704515	0.914723133
R^2	0.990743991	0.995432835	0.969642981	0.83671841
$R^2_{adjusted}$	0.990743991	0.995432835	0.969642981	0.83671841
RSS	604.0721835	286.2256993	806.0235487	2930.041818

Graphical abstract of model fit presented as mean ± 1 SD of the fraction % of released carvedilol:

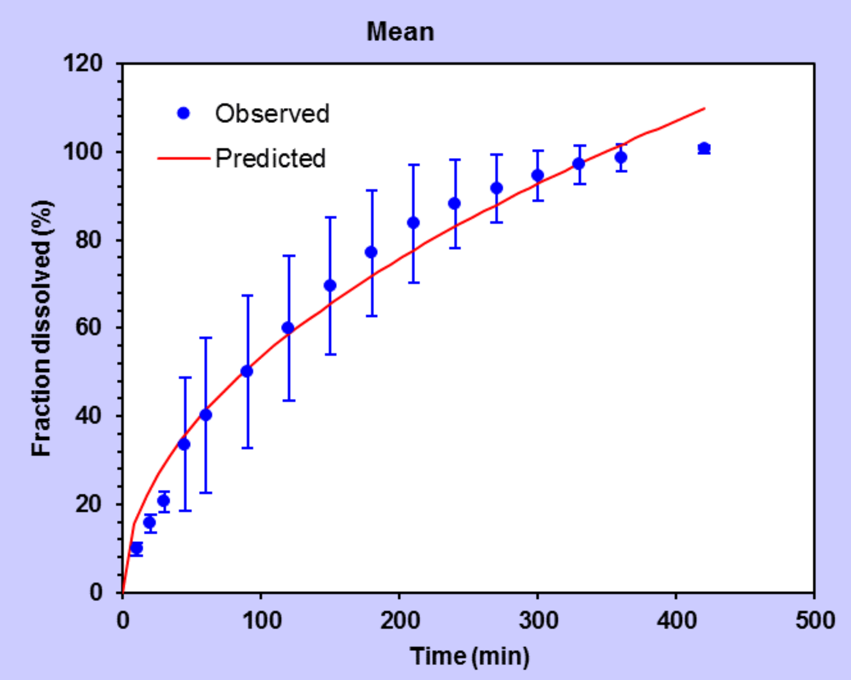

Supplementary materials – Model fitting summary – C*Pharm Mannidex 16700 (modelling the entire relevant dissolution data)

Graphical abstract of model fit presented as the fraction % of released carvedilol per tested tablet:

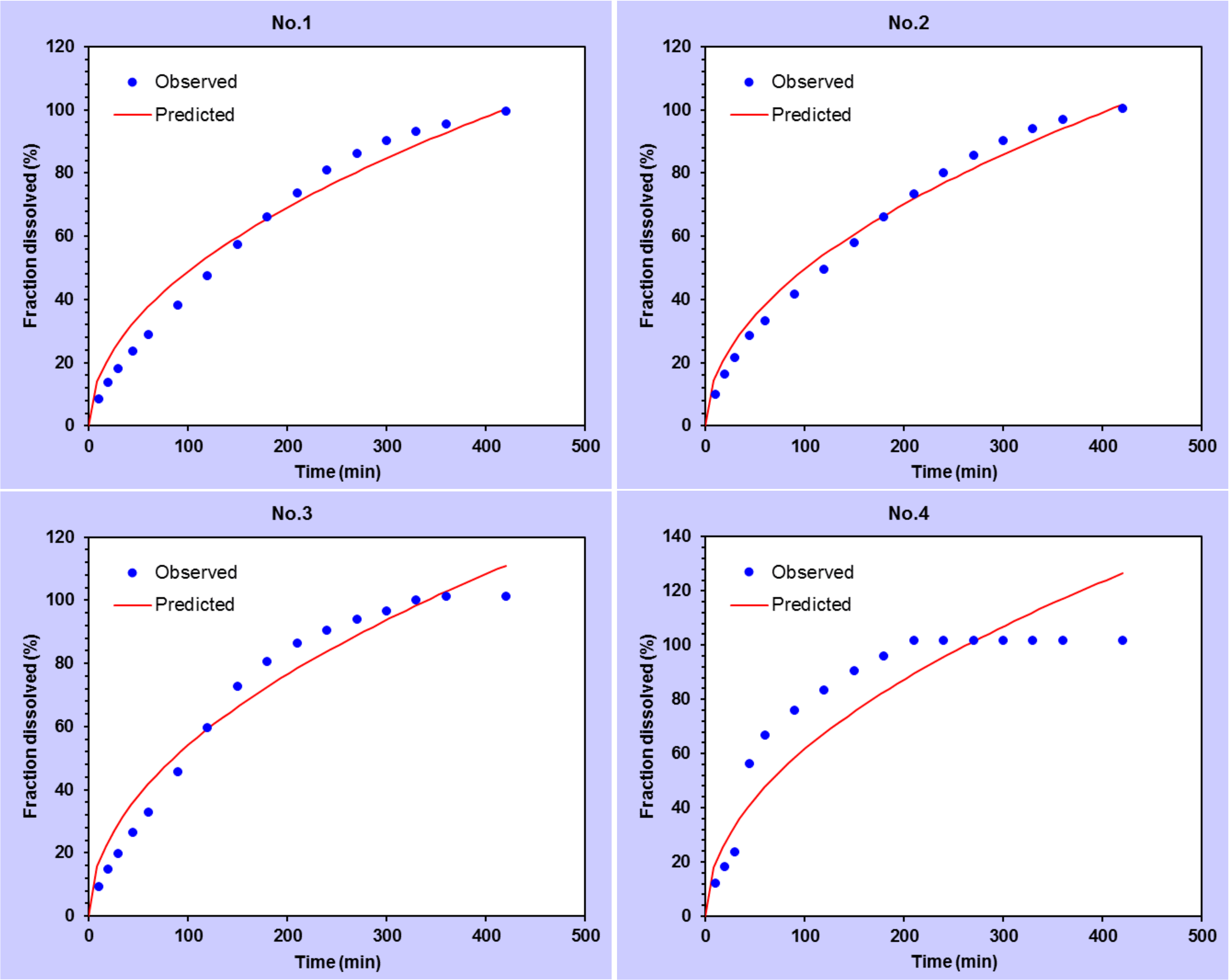

Supplementary materials – Model fitting summary – C*Pharm Mannidex 16700 (modelling the entire relevant dissolution data)

Model: **Higuchi with T_{lag}**

Model equation: $F = k_H \cdot \left(t - T_{lag}\right)^{0.5}$

Fitted model parameters per tested tablet (N = 4) with statistics – mean, standard deviation (SD), and relative standard deviation expressed in % (RSD%) (output from DDSolver):

Parameter	No.1	No.2	No.3	No.4	Mean	SD	RSD(%)
k_H	5.202	5.185	5.446	5.177	5.253	0.130	2.466
T_{lag}	18.994	14.350	0.477	-81.585	-11.941	47.091	-394.366

Number of dissolution data points (N), degrees of freedom (df), and selected goodness of fit criteria – Pearson correlation coefficient (R), coefficient of determination (R^2), adjusted coefficient of determination ($R^2_{adjusted}$), and residual sum of squares (RSS) (manual calculation in MS Excel):

Parameter	No.1	No.2	No.3	No.4
N	16	16	16	16
df	14	14	14	14
R	0.991038475	0.994472585	0.984783069	0.878326434
R^2	0.982157258	0.988975723	0.969797694	0.771457325
$R^2_{adjusted}$	0.980882777	0.988188274	0.967640386	0.755132849
RSS	299.5526132	185.5138961	787.7672017	4565.336567

Graphical abstract of model fit presented as mean ± 1 SD of the fraction % of released carvedilol:

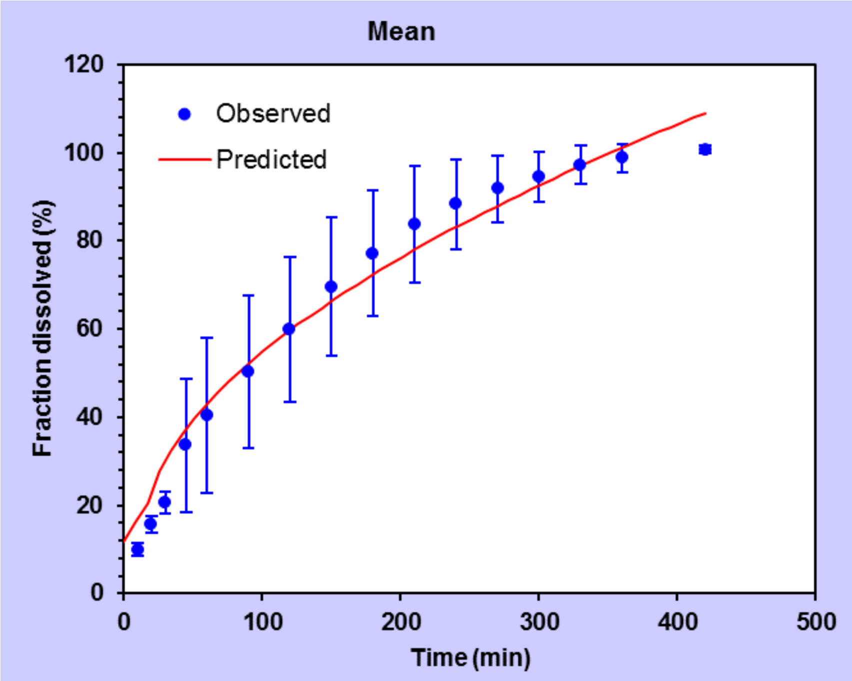

Supplementary materials – Model fitting summary – C*Pharm Mannidex 16700 (modelling the entire relevant dissolution data)

Graphical abstract of model fit presented as the fraction % of released carvedilol per tested tablet:

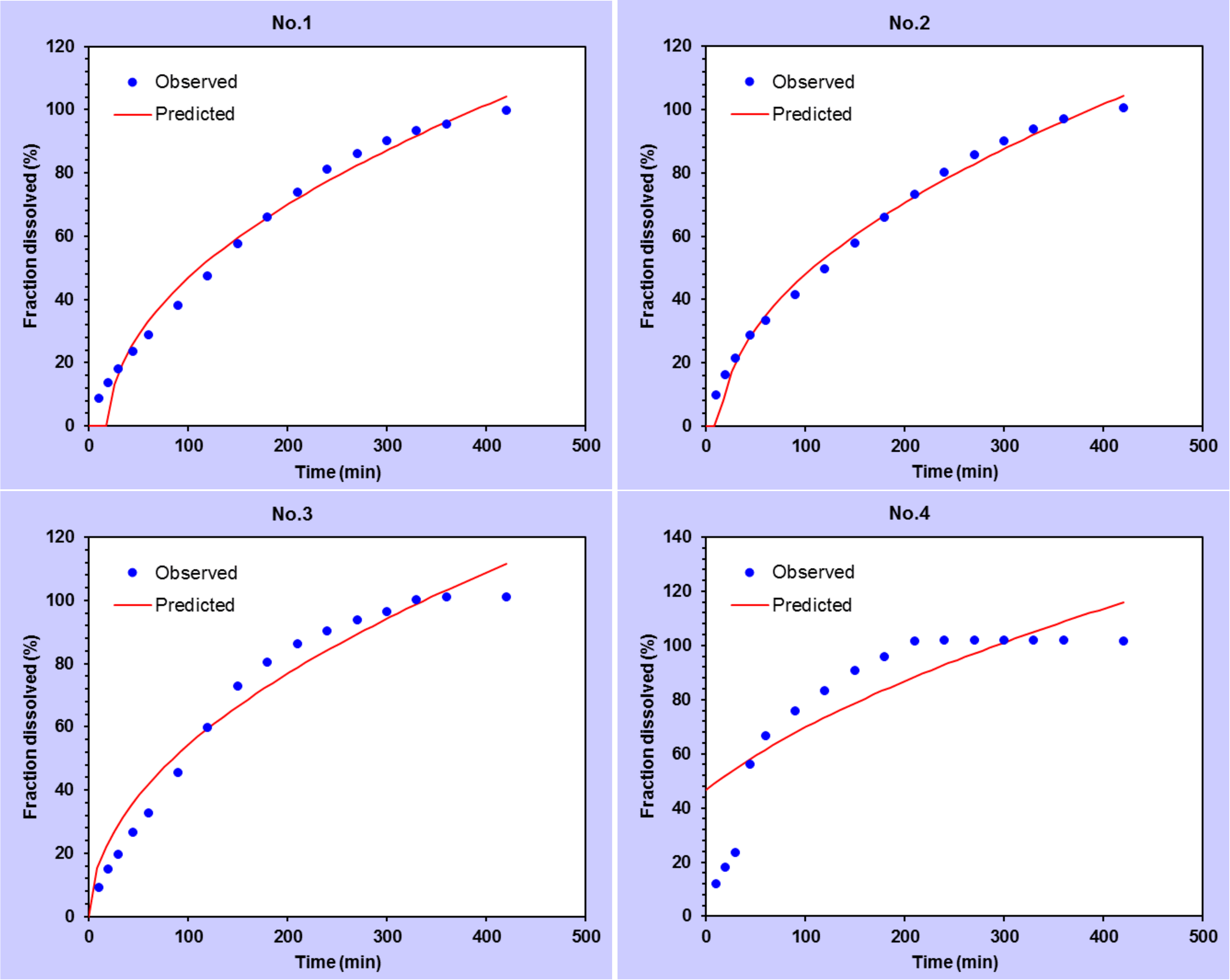

Supplementary materials – Model fitting summary – C*Pharm Mannidex 16700 (modelling the entire relevant dissolution data)

Model: **Higuchi with F_0**

Model equation: $F = F_0 + k_H \cdot t^{0.5}$

Fitted model parameters per tested tablet (N = 4) with statistics – mean, standard deviation (SD), and relative standard deviation expressed in % (RSD%) (output from DDSolver):

Parameter	No.1	No.2	No.3	No.4	Mean	SD	RSD(%)
k_H	5.811	5.596	6.119	5.403	5.732	0.307	5.356
F_0	-13.344	-9.241	-10.224	11.210	-5.400	11.210	-207.601

Number of dissolution data points (N), degrees of freedom (df), and selected goodness of fit criteria – Pearson correlation coefficient (R), coefficient of determination (R^2), adjusted coefficient of determination ($R^2_{adjusted}$), and residual sum of squares (RSS) (manual calculation in MS Excel):

Parameter	No.1	No.2	No.3	No.4
N	16	16	16	16
df	14	14	14	14
R	0.995361237	0.997713804	0.984704515	0.914723133
R^2	0.990743991	0.995432835	0.969642981	0.83671841
$R^2_{adjusted}$	0.990082848	0.995106609	0.967474622	0.82505544
RSS	144.3380869	65.72722144	536.1637607	2605.624457

Graphical abstract of model fit presented as mean ± 1 SD of the fraction % of released carvedilol:

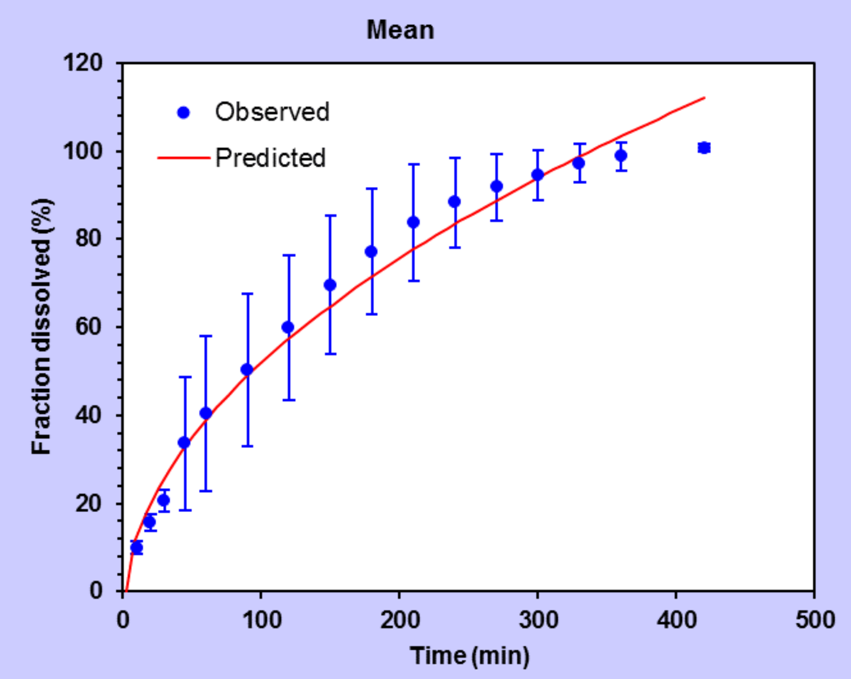

Supplementary materials – Model fitting summary – C*Pharm Mannidex 16700 (modelling the entire relevant dissolution data)

Graphical abstract of model fit presented as the fraction % of released carvedilol per tested tablet:

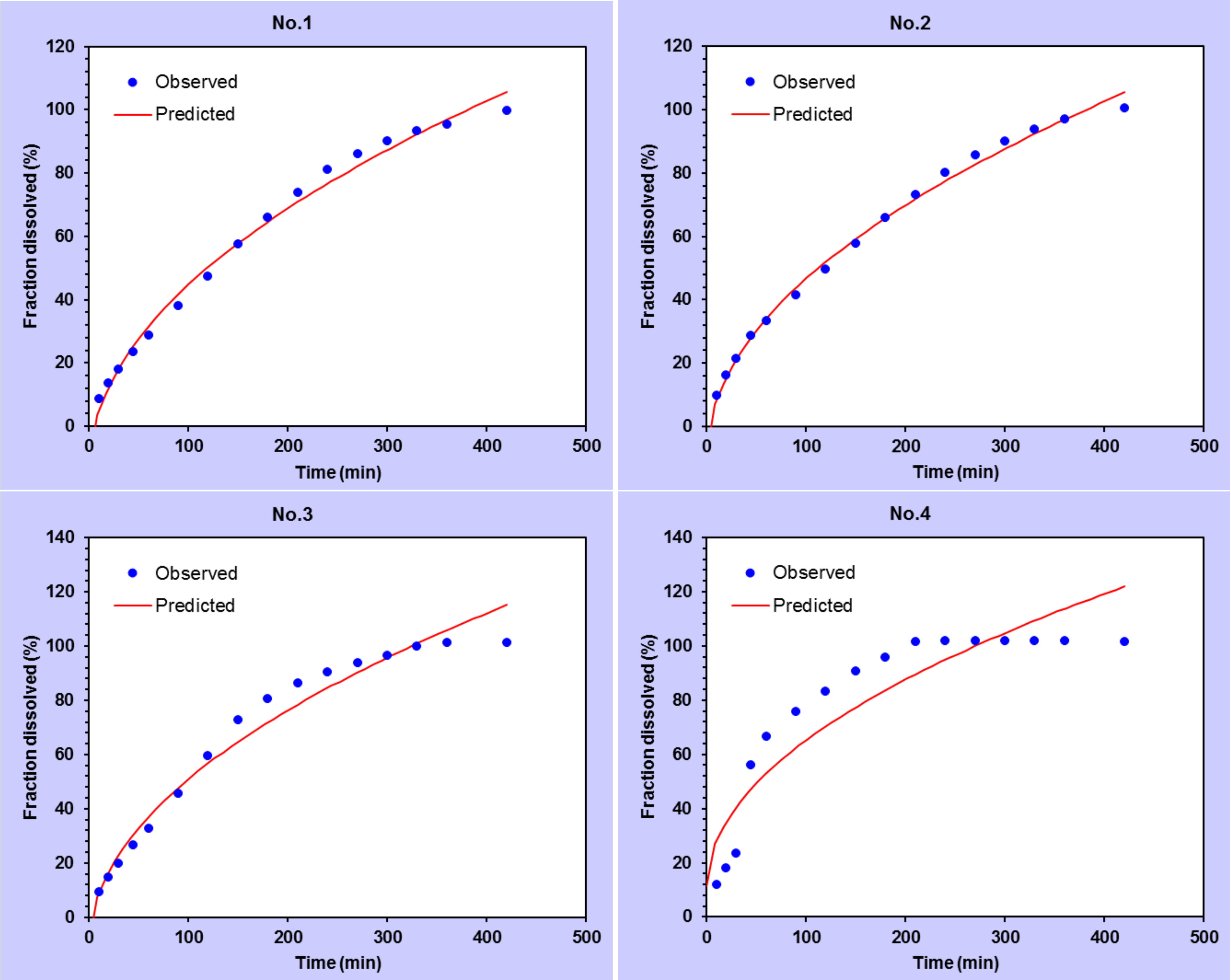

Supplementary materials – Model fitting summary – C*Pharm Mannidex 16700 (modelling the entire relevant dissolution data)

Model: **Korsmeyer–Peppas**

Model equation: $F = k_{KP} \cdot t^n$

Fitted model parameters per tested tablet (N = 4) with statistics – mean, standard deviation (SD), and relative standard deviation expressed in % (RSD%) (output from DDSolver):

Parameter	No.1	No.2	No.3	No.4	Mean	SD	RSD(%)
k_{KP}	1.740	2.392	1.779	2.072	1.996	0.303	15.181
n	0.690	0.637	0.716	0.776	0.705	0.058	8.236

Number of dissolution data points (N), degrees of freedom (df), and selected goodness of fit criteria – Pearson correlation coefficient (R), coefficient of determination (R^2), adjusted coefficient of determination ($R^2_{adjusted}$), and residual sum of squares (RSS) (manual calculation in MS Excel):

Parameter	No.1	No.2	No.3	No.4
N	16	16	16	16
df	14	14	14	14
R	0.992341399	0.996118567	0.971149622	0.86719549
R^2	0.984741452	0.992252199	0.943131589	0.752028018
$R^2_{adjusted}$	0.983651555	0.991698784	0.93906956	0.734315734
RSS	263.1608775	167.3288077	1921.778728	44649.95902

Graphical abstract of model fit presented as mean ± 1 SD of the fraction % of released carvedilol:

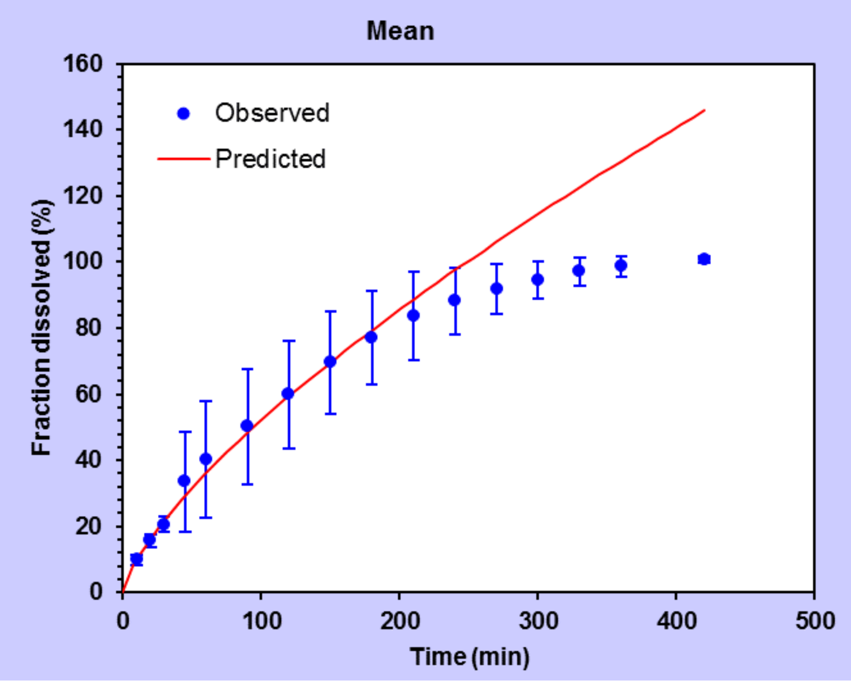

Supplementary materials – Model fitting summary – C*Pharm Mannidex 16700 (modelling the entire relevant dissolution data)

Graphical abstract of model fit presented as the fraction % of released carvedilol per tested tablet:

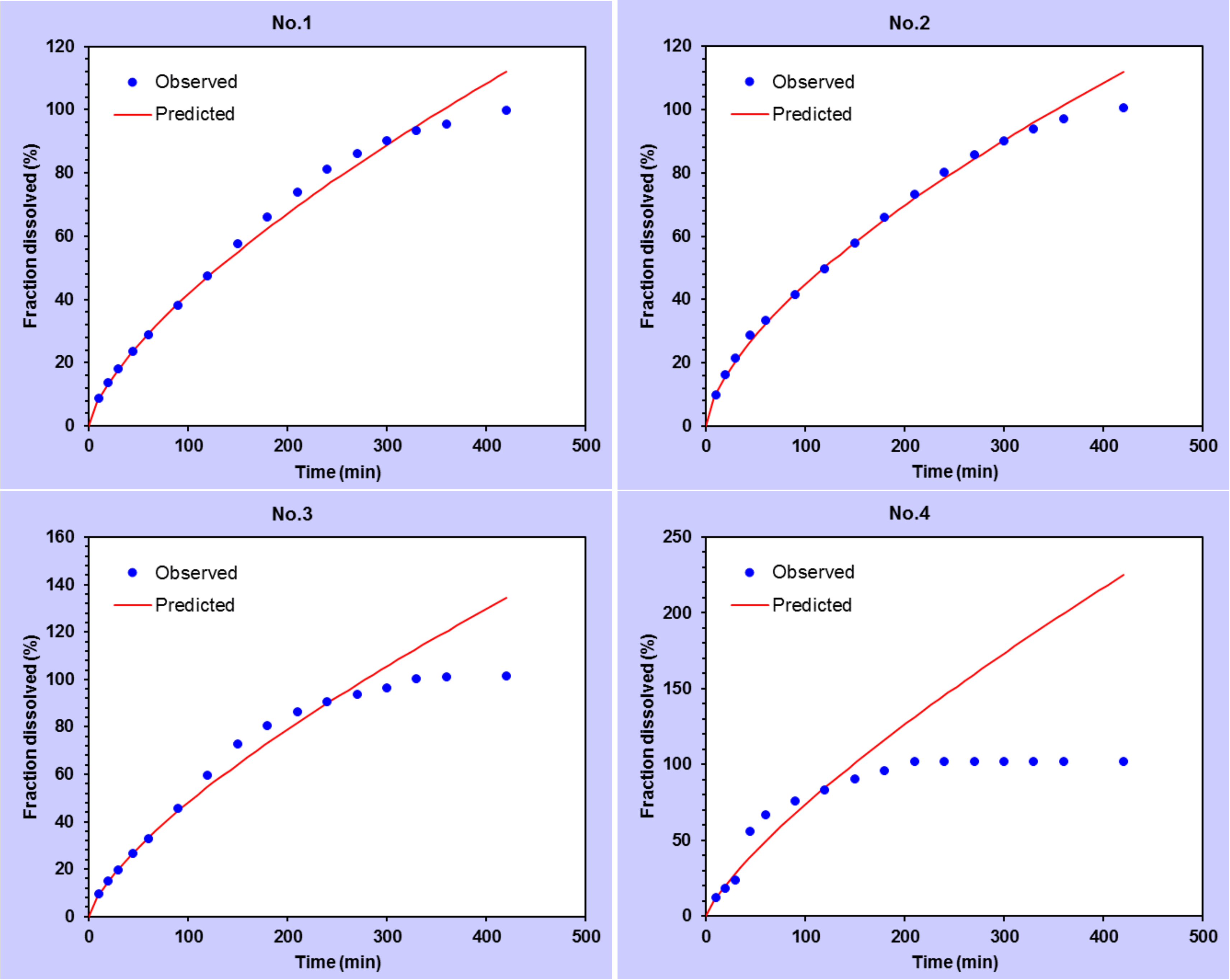

Supplementary materials – Model fitting summary – C*Pharm Mannidex 16700 (modelling the entire relevant dissolution data)

Model: **Korsmeyer–Peppas with T_{lag}**

Model equation: $F = k_{KP} \cdot (t - T_{lag})^n$

Fitted model parameters per tested tablet (N = 4) with statistics – mean, standard deviation (SD), and relative standard deviation expressed in % (RSD%) (output from DDSolver):

Parameter	No.1	No.2	No.3	No.4	Mean	SD	RSD(%)
k_{KP}	2.467	3.368	2.799	5.430	3.516	1.329	37.810
n	0.626	0.573	0.625	0.534	0.589	0.045	7.605
T_{lag}	4.000	4.000	4.000	4.000	4.000	0.000	0.000

Number of dissolution data points (N), degrees of freedom (df), and selected goodness of fit criteria – Pearson correlation coefficient (R), coefficient of determination (R^2), adjusted coefficient of determination ($R^2_{adjusted}$), and residual sum of squares (RSS) (manual calculation in MS Excel):

Parameter	No.1	No.2	No.3	No.4
N	16	16	16	16
df	13	13	13	13
R	0.994252334	0.997405521	0.978779154	0.913525959
R^2	0.988537703	0.994817774	0.958008633	0.834529678
$R^2_{adjusted}$	0.986774272	0.994020508	0.951548423	0.809072705
RSS	179.6804013	74.71109036	824.8466328	3776.518316

Graphical abstract of model fit presented as mean ± 1 SD of the fraction % of released carvedilol:

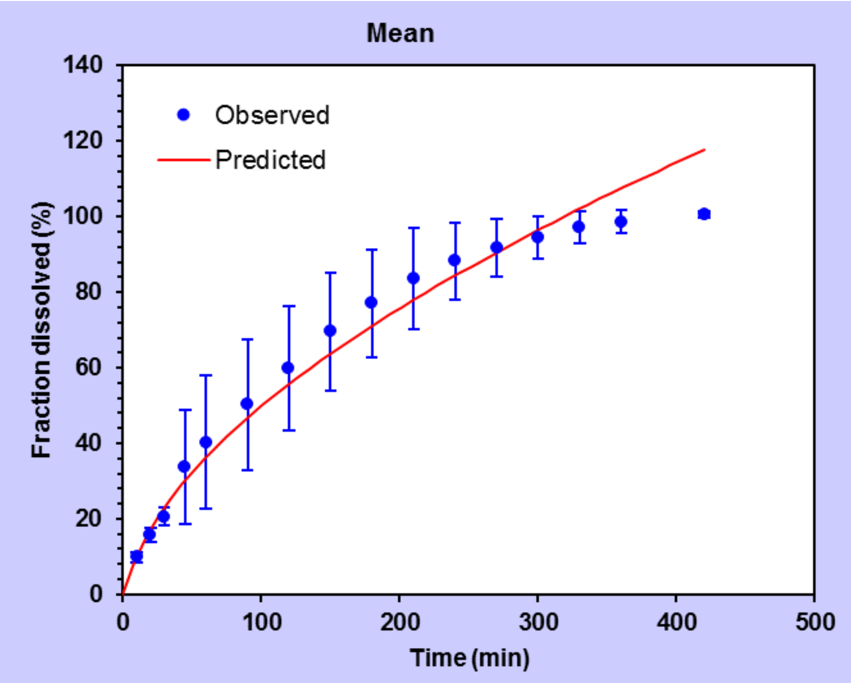

Supplementary materials – Model fitting summary – C*Pharm Mannidex 16700 (modelling the entire relevant dissolution data)

Graphical abstract of model fit presented as the fraction % of released carvedilol per tested tablet:

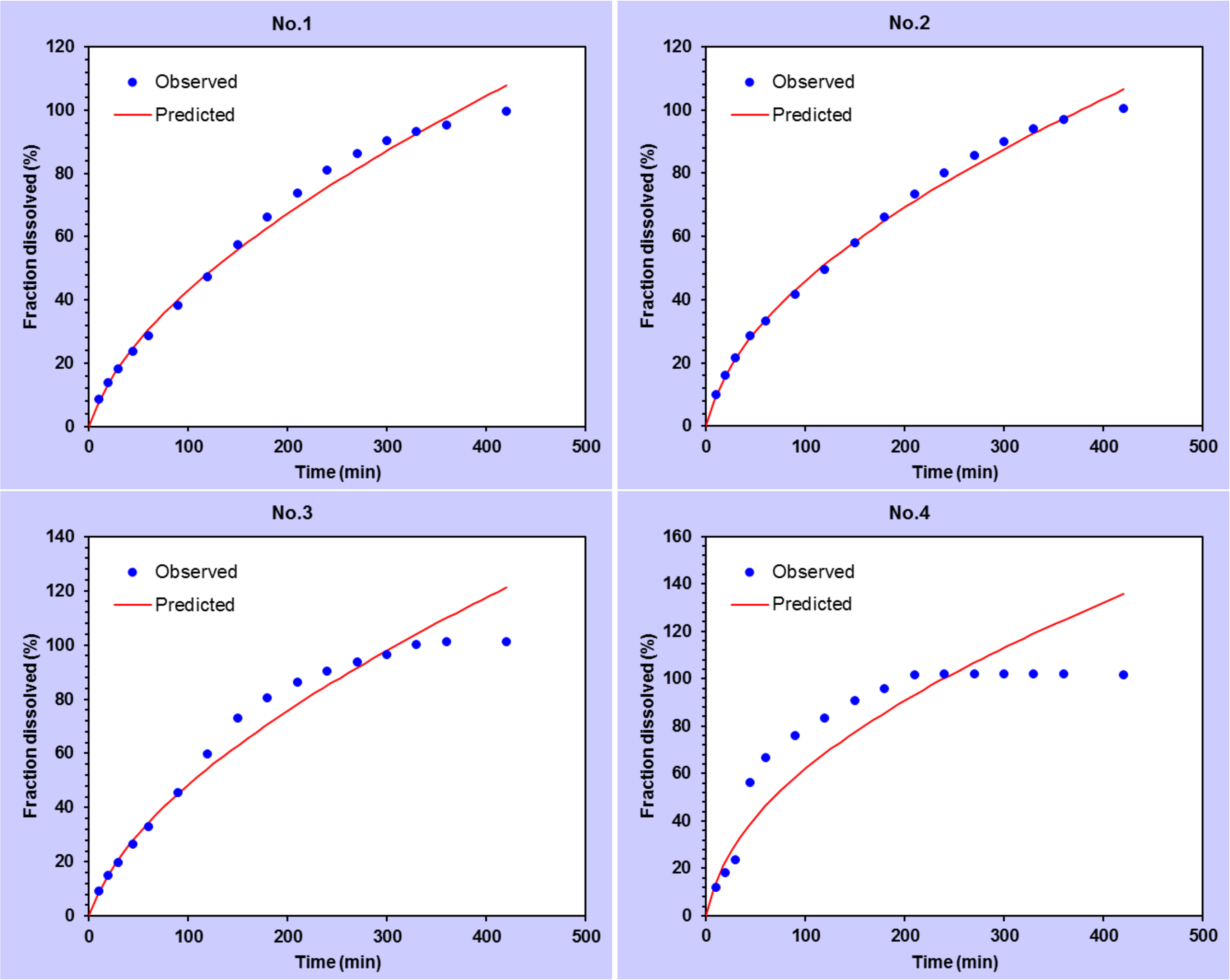

Supplementary materials – Model fitting summary – C*Pharm Mannidex 16700 (modelling the entire relevant dissolution data)

Model: **Korsmeyer–Peppas with F_0**

Model equation: $F = F_0 + k_{KP} \cdot t^n$

Fitted model parameters per tested tablet (N = 4) with statistics – mean, standard deviation (SD), and relative standard deviation expressed in % (RSD%) (output from DDSolver):

Parameter	No.1	No.2	No.3	No.4	Mean	SD	RSD(%)
k_{KP}	0.946	1.490	1.089	2.347	1.468	0.629	42.870
n	0.794	0.699	0.790	0.678	0.740	0.060	8.131
F_0	3.399	4.794	3.679	4.759	4.158	0.723	17.399

Number of dissolution data points (N), degrees of freedom (df), and selected goodness of fit criteria – Pearson correlation coefficient (R), coefficient of determination (R^2), adjusted coefficient of determination ($R^2_{adjusted}$), and residual sum of squares (RSS) (manual calculation in MS Excel):

Parameter	No.1	No.2	No.3	No.4
N	16	16	16	16
df	13	13	13	13
R	0.988143721	0.994354061	0.964908669	0.884655699
R^2	0.976428013	0.98874	0.931048739	0.782615707
$R^2_{adjusted}$	0.972801553	0.987007692	0.920440852	0.749171969
RSS	511.5719148	258.9037631	1789.746285	5969.946127

Graphical abstract of model fit presented as mean ± 1 SD of the fraction % of released carvedilol:

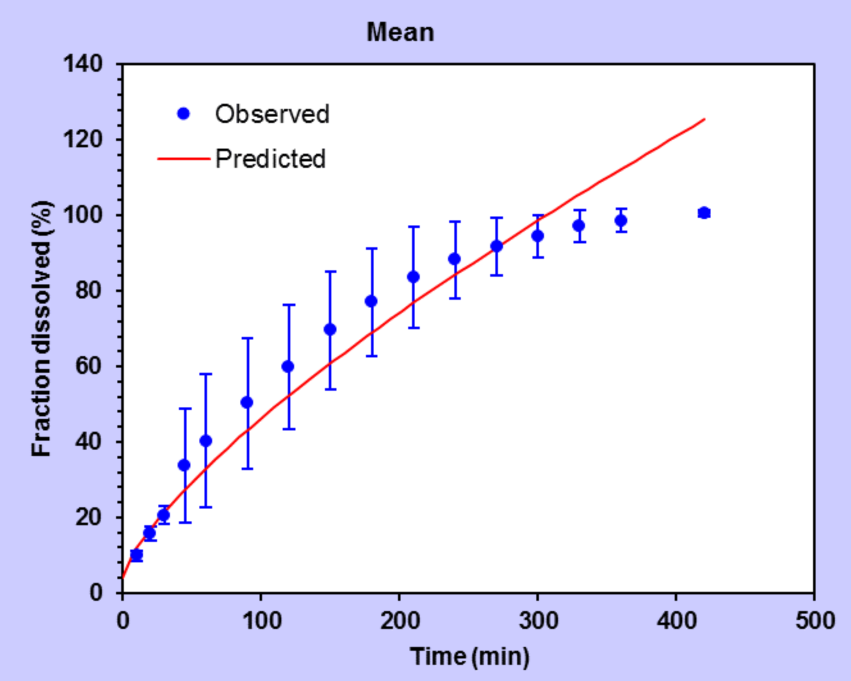

Supplementary materials – Model fitting summary – C*Pharm Mannidex 16700 (modelling the entire relevant dissolution data)

Graphical abstract of model fit presented as the fraction % of released carvedilol per tested tablet:

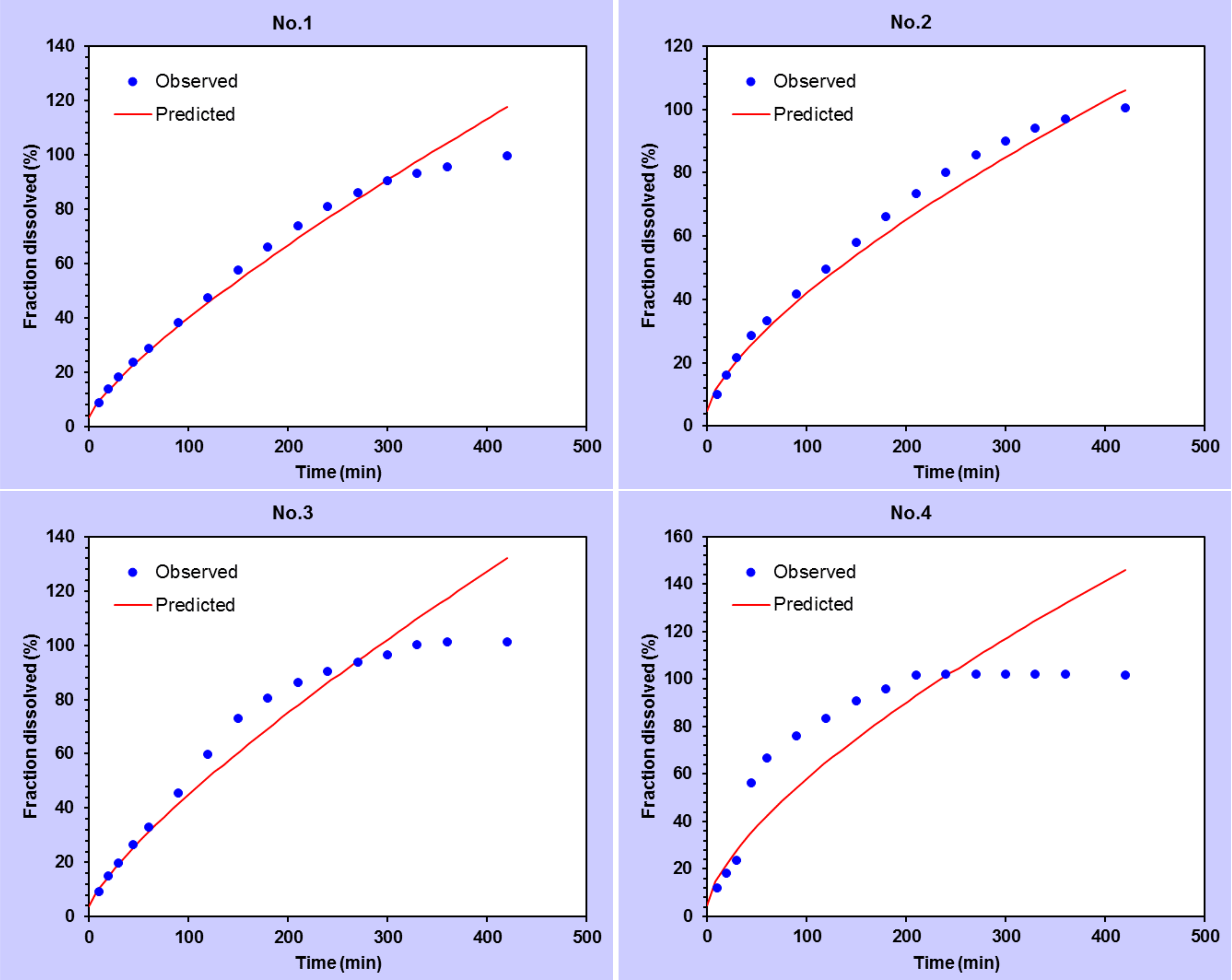

Supplementary materials – Model fitting summary – C*Pharm Mannidex 16700 (modelling the entire relevant dissolution data)

Model: **Hixson–Crowell**

Model equation: $F = 100 \cdot [1 - (1 - k_{HC} \cdot t)^3]$

Fitted model parameters per tested tablet (N = 4) with statistics – mean, standard deviation (SD), and relative standard deviation expressed in % (RSD%) (output from DDSolver):

Parameter	No.1	No.2	No.3	No.4	Mean	SD	RSD(%)
k_{HC}	0.002	0.002	0.002	0.004	0.002	0.001	40.125

Number of dissolution data points (N), degrees of freedom (df), and selected goodness of fit criteria – Pearson correlation coefficient (R), coefficient of determination (R^2), adjusted coefficient of determination ($R^2_{adjusted}$), and residual sum of squares (RSS) (manual calculation in MS Excel):

Parameter	No.1	No.2	No.3	No.4
N	16	16	16	16
df	15	15	15	15
R	0.99755768	0.996767328	0.998935504	0.97689555
R^2	0.995121325	0.993545106	0.997872141	0.954324916
$R^2_{adjusted}$	0.995121325	0.993545106	0.997872141	0.954324916
RSS	98.66793134	185.9854514	107.9244445	803.952613

Graphical abstract of model fit presented as mean ± 1 SD of the fraction % of released carvedilol:

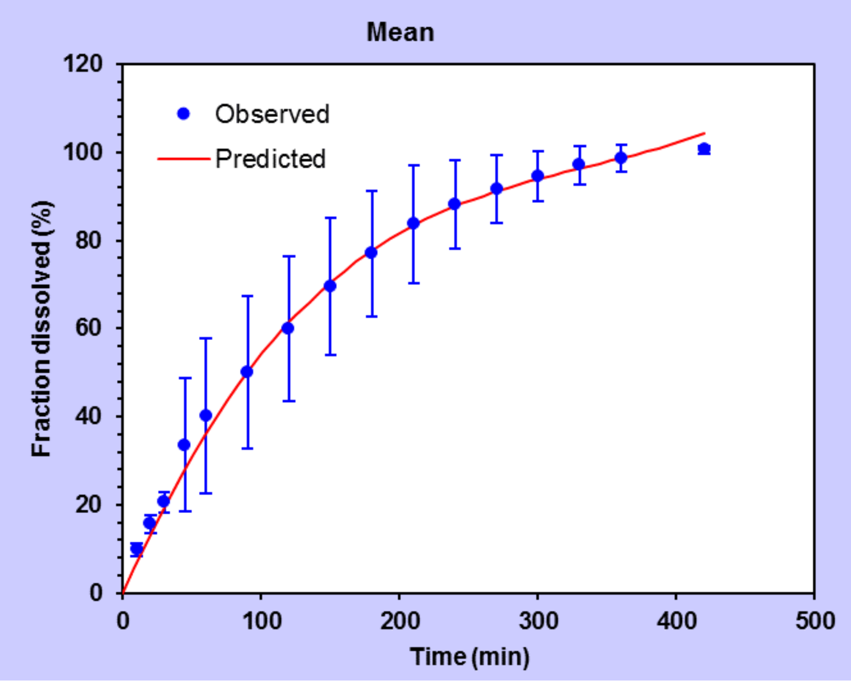

Supplementary materials – Model fitting summary – C*Pharm Mannidex 16700 (modelling the entire relevant dissolution data)

Graphical abstract of model fit presented as the fraction % of released carvedilol per tested tablet:

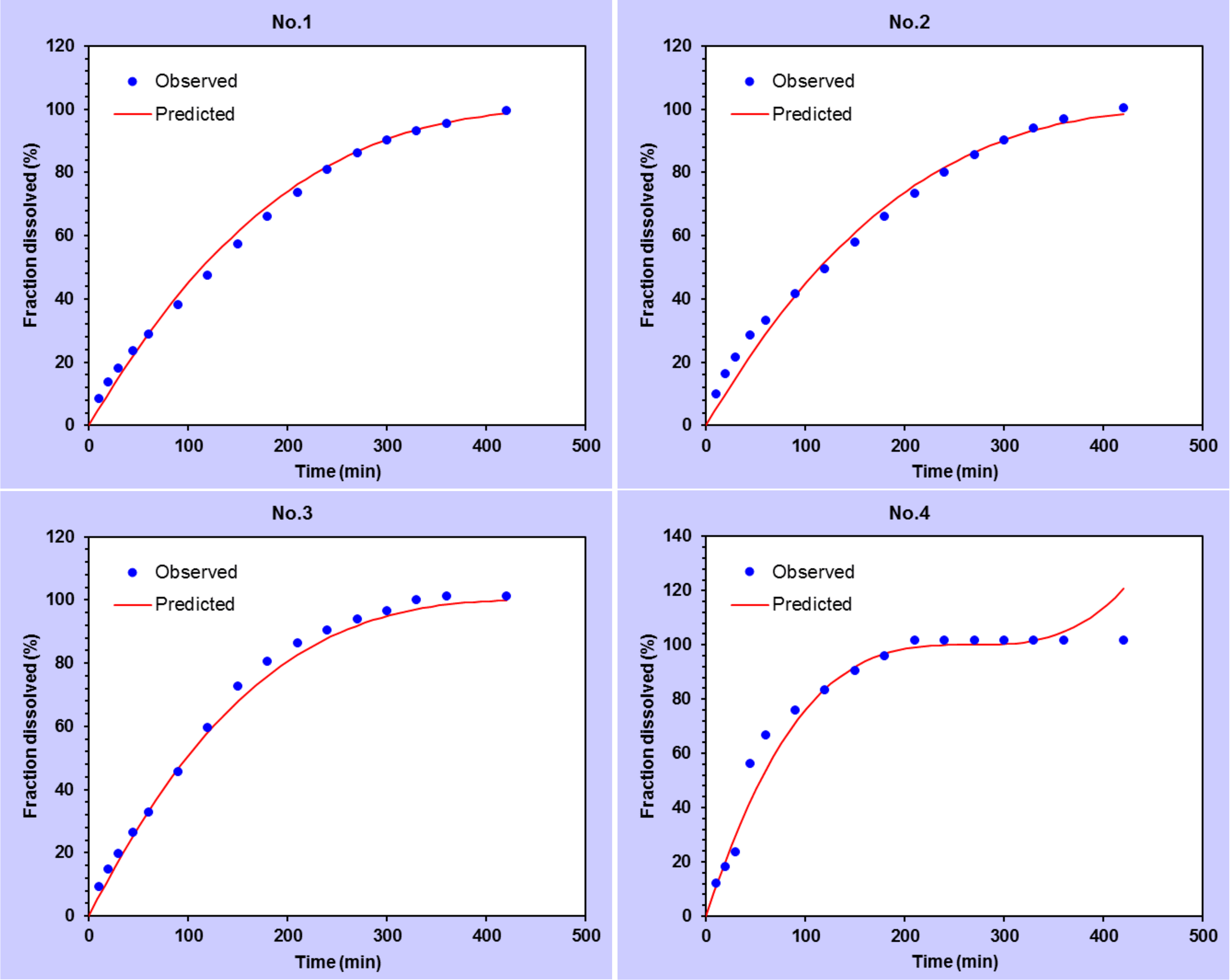

Supplementary materials – Model fitting summary – C*Pharm Mannidex 16700 (modelling the entire relevant dissolution data)

Model: **Hixson–Crowell with T_{lag}**

Model equation: $F = 100 \cdot \left\{1 - \left[1 - k_{HC} \cdot \left(t - T_{lag}\right)\right]^3\right\}$

Fitted model parameters per tested tablet (N = 4) with statistics – mean, standard deviation (SD), and relative standard deviation expressed in % (RSD%) (output from DDSolver):

Parameter	No.1	No.2	No.3	No.4	Mean	SD	RSD(%)
k_{HC}	0.002	0.002	0.002	0.004	0.002	0.001	34.416
T_{lag}	4.993	-7.888	8.374	-7.661	-0.545	8.461	-1551.735

Number of dissolution data points (N), degrees of freedom (df), and selected goodness of fit criteria – Pearson correlation coefficient (R), coefficient of determination (R^2), adjusted coefficient of determination ($R^2_{adjusted}$), and residual sum of squares (RSS) (manual calculation in MS Excel):

Parameter	No.1	No.2	No.3	No.4
N	16	16	16	16
df	14	14	14	14
R	0.99735815	0.996934383	0.997165526	0.980347347
R^2	0.994723279	0.993878165	0.994339085	0.961080921
$R^2_{adjusted}$	0.99434637	0.993440891	0.993934734	0.958300987
RSS	143.6594494	102.0946517	168.5411491	638.8271993

Graphical abstract of model fit presented as mean ± 1 SD of the fraction % of released carvedilol:

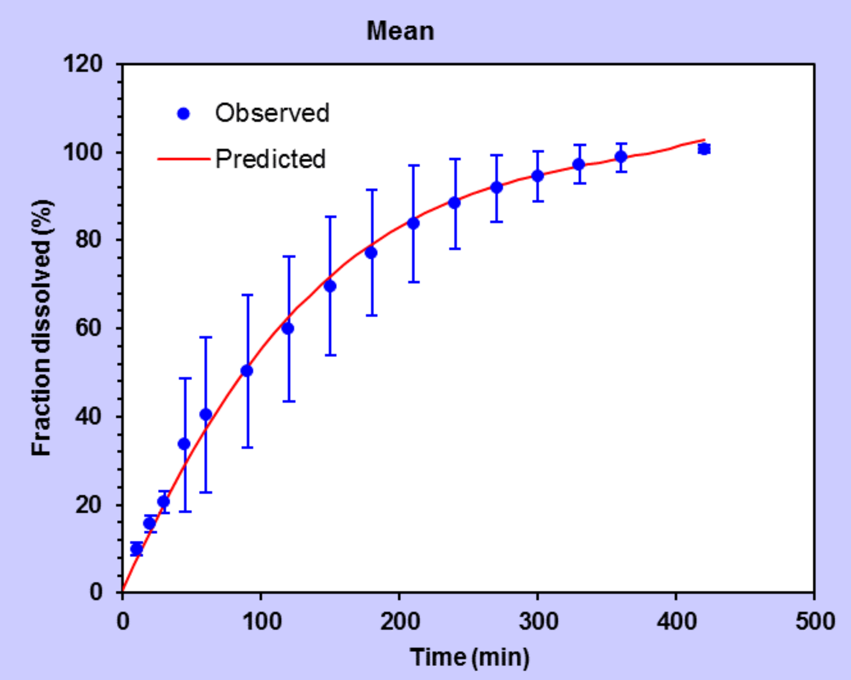

Supplementary materials – Model fitting summary – C*Pharm Mannidex 16700 (modelling the entire relevant dissolution data)

Graphical abstract of model fit presented as the fraction % of released carvedilol per tested tablet:

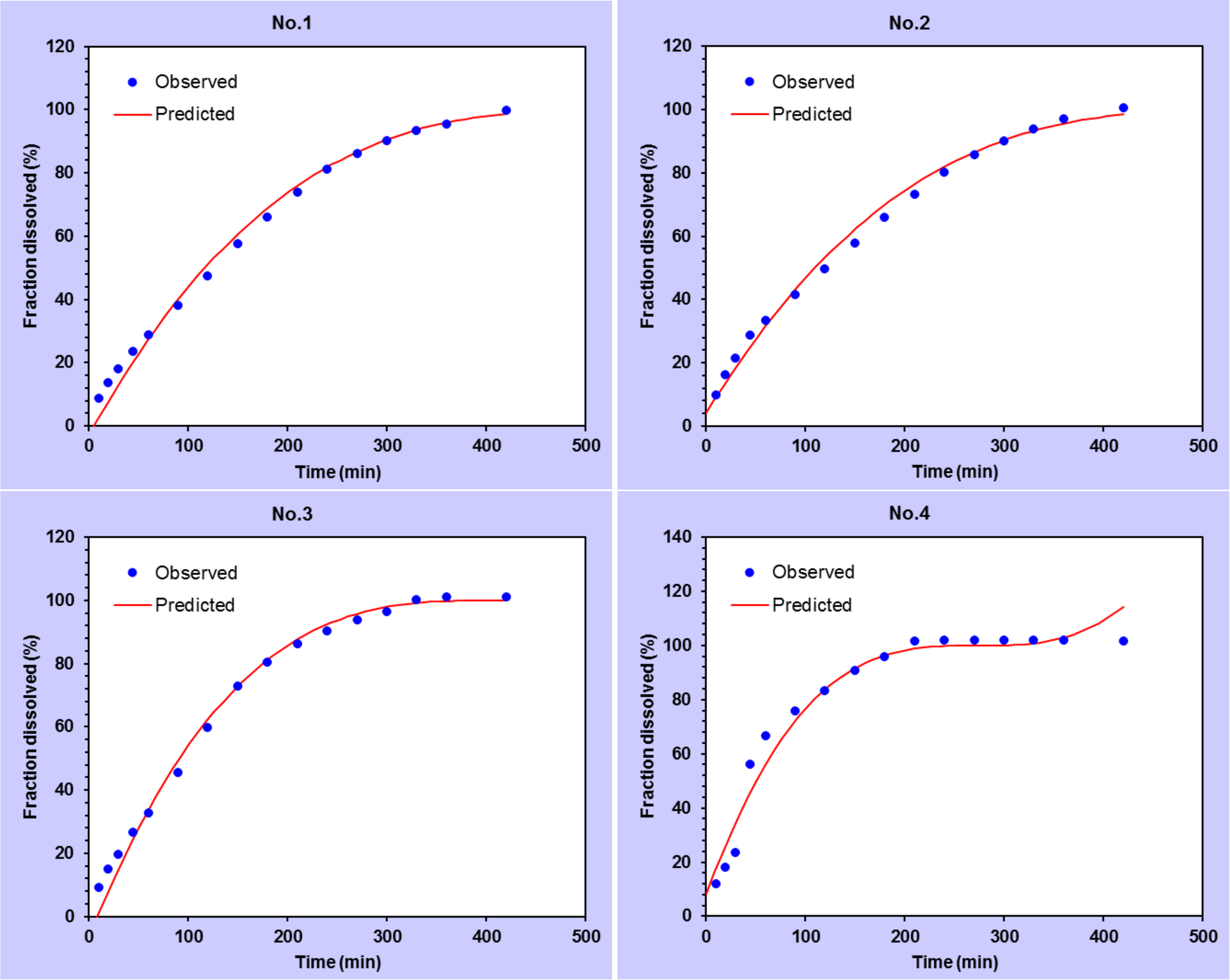

Supplementary materials – Model fitting summary – C*Pharm Mannidex 16700 (modelling the entire relevant dissolution data)

Model: **Hopfenberg**

Model equation: $F = 100 \cdot [1 - (1 - k_{HB} \cdot t)^n]$

Fitted model parameters per tested tablet (N = 4) with statistics – mean, standard deviation (SD), and relative standard deviation expressed in % (RSD%) (output from DDSolver):

Parameter	No.1	No.2	No.3	No.4	Mean	SD	RSD(%)
k_{HB}	0.002	0.001	0.003	0.004	0.002	0.001	44.593
n	3.000	4.125	2.000	3.000	3.031	0.868	28.644

Number of dissolution data points (N), degrees of freedom (df), and selected goodness of fit criteria – Pearson correlation coefficient (R), coefficient of determination (R^2), adjusted coefficient of determination ($R^2_{adjusted}$), and residual sum of squares (RSS) (manual calculation in MS Excel):

Parameter	No.1	No.2	No.3	No.4
N	16	16	16	16
df	14	14	14	14
R	0.99755768	0.996287212	0.999003113	0.988107236
R^2	0.995121325	0.99258821	0.99800722	0.976355909
$R^2_{adjusted}$	0.994772849	0.992058796	0.997864879	0.974667045
RSS	98.66793134	183.996215	83.4524238	436.8365716

Graphical abstract of model fit presented as mean ± 1 SD of the fraction % of released carvedilol:

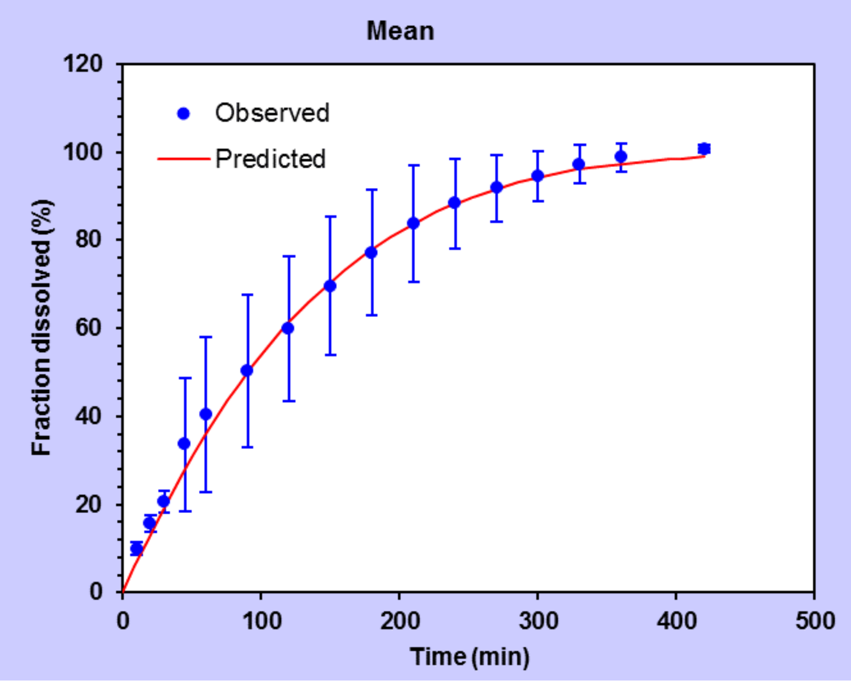

Supplementary materials – Model fitting summary – C*Pharm Mannidex 16700 (modelling the entire relevant dissolution data)

Graphical abstract of model fit presented as the fraction % of released carvedilol per tested tablet:

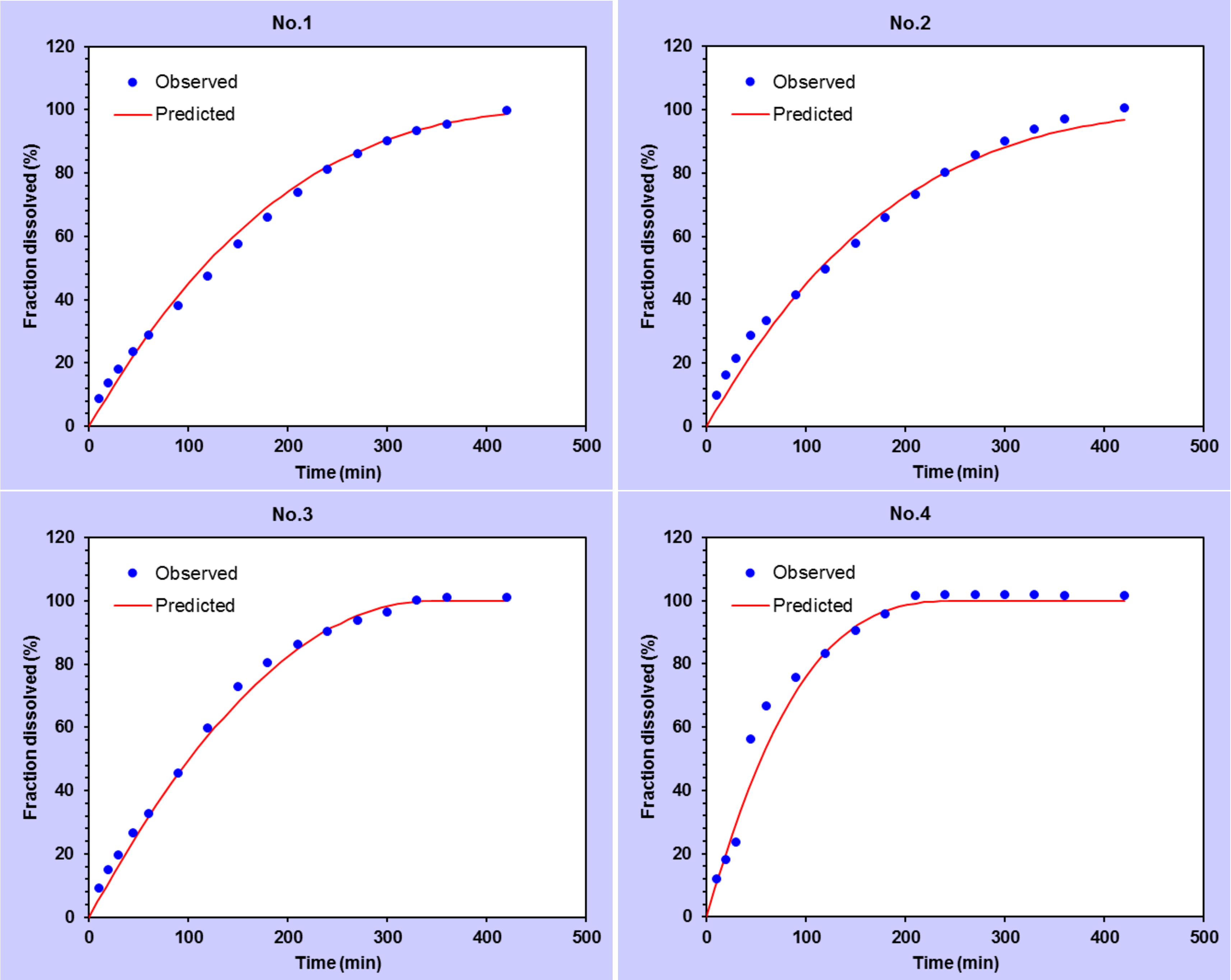

Supplementary materials – Model fitting summary – C*Pharm Mannidex 16700 (modelling the entire relevant dissolution data)

Model: **Hopfenberg with T_{lag}**

Model equation: $F = 100 \cdot \{1 - [1 - k_{HB} \cdot (t - T_{lag})]^n\}$

Fitted model parameters per tested tablet (N = 4) with statistics – mean, standard deviation (SD), and relative standard deviation expressed in % (RSD%) (output from DDSolver):

Parameter	No.1	No.2	No.3	No.4	Mean	SD	RSD(%)
k_{HB}	0.002	0.002	0.003	0.004	0.003	0.001	25.128
n	2.000	2.000	2.000	3.000	2.250	0.500	22.222
T_{lag}	-12.090	-19.238	-7.792	-7.661	-11.695	5.433	-46.456

Number of dissolution data points (N), degrees of freedom (df), and selected goodness of fit criteria – Pearson correlation coefficient (R), coefficient of determination (R^2), adjusted coefficient of determination ($R^2_{adjusted}$), and residual sum of squares (RSS) (manual calculation in MS Excel):

Parameter	No.1	No.2	No.3	No.4
N	16	16	16	16
df	13	13	13	13
R	0.999634723	0.999068389	0.998955995	0.987300167
R^2	0.999269579	0.998137646	0.997913081	0.97476162
$R^2_{adjusted}$	0.999157207	0.99785113	0.997592016	0.970878792
RSS	12.00101602	27.14144839	40.16938763	482.5452714

Graphical abstract of model fit presented as mean ± 1 SD of the fraction % of released carvedilol:

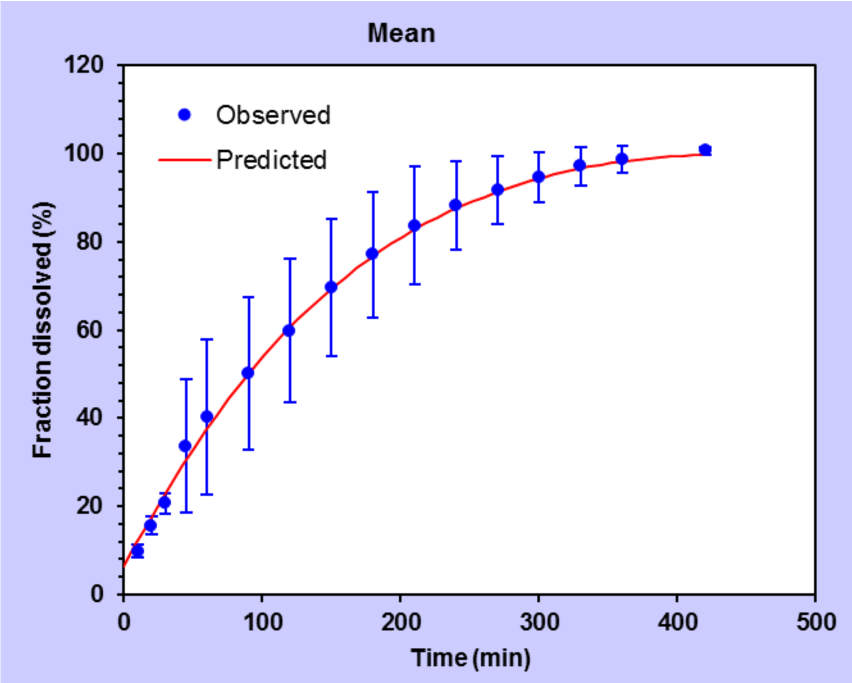

Supplementary materials – Model fitting summary – C*Pharm Mannidex 16700 (modelling the entire relevant dissolution data)

Graphical abstract of model fit presented as the fraction % of released carvedilol per tested tablet:

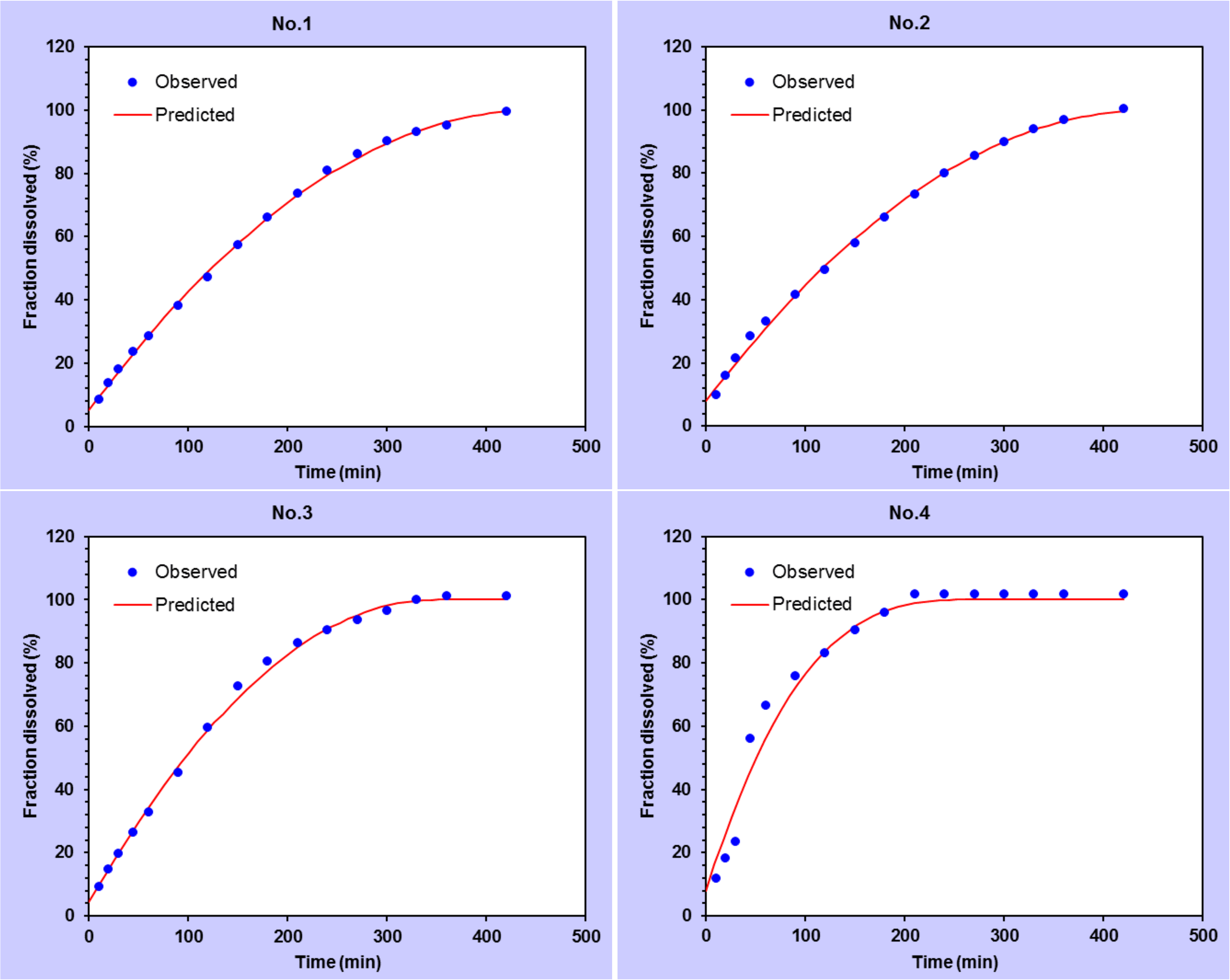

Supplementary materials – Model fitting summary – C*Pharm Mannidex 16700 (modelling the entire relevant dissolution data)

Model: **Baker–Lonsdale**

Model equation: $\frac{3}{2}\cdot\left[1-\left(1-\frac{F}{100}\right)^{\frac{2}{3}}\right]-\frac{F}{100}=k_{BL}\cdot t$

Fitted model parameters per tested tablet (N = 4) with statistics – mean, standard deviation (SD), and relative standard deviation expressed in % (RSD%) (output from DDSolver):

Parameter	No.1	No.2	No.3	No.4	Mean	SD	RSD(%)
k_{BL}	/	/	/	/	/	/	/

Number of dissolution data points (N), degrees of freedom (df), and selected goodness of fit criteria – Pearson correlation coefficient (R), coefficient of determination (R^2), adjusted coefficient of determination ($R^2_{adjusted}$), and residual sum of squares (RSS) (manual calculation in MS Excel):

Parameter	No.1	No.2	No.3	No.4
N	/	/	/	/
df	/	/	/	/
R	/	/	/	/
R^2	/	/	/	/
$R^2_{adjusted}$	/	/	/	/
RSS	/	/	/	/

Graphical abstract of model fit presented as mean ± 1 SD of the fraction % of released carvedilol: /

Graphical abstract of model fit presented as the fraction % of released carvedilol per tested tablet: /

Note: the model could not be fitted

Supplementary materials – Model fitting summary – C*Pharm Mannidex 16700 (modelling the entire relevant dissolution data)

Model: **Baker–Lonsdale with T_{lag}**

Model equation: $\frac{3}{2}\cdot\left[1-\left(1-\frac{F}{100}\right)^{\frac{2}{3}}\right]-\frac{F}{100}=k_{BL}\cdot\left(t-T_{lag}\right)$

Fitted model parameters per tested tablet (N = 4) with statistics – mean, standard deviation (SD), and relative standard deviation expressed in % (RSD%) (output from DDSolver):

Parameter	No.1	No.2	No.3	No.4	Mean	SD	RSD(%)
k_{BL}	/	/	/	/	/	/	/
T_{lag}	/	/	/	/	/	/	/

Number of dissolution data points (N), degrees of freedom (df), and selected goodness of fit criteria – Pearson correlation coefficient (R), coefficient of determination (R^2), adjusted coefficient of determination ($R^2_{adjusted}$), and residual sum of squares (RSS) (manual calculation in MS Excel):

Parameter	No.1	No.2	No.3	No.4
N	/	/	/	/
df	/	/	/	/
R	/	/	/	/
R^2	/	/	/	/
$R^2_{adjusted}$	/	/	/	/
RSS	/	/	/	/

Graphical abstract of model fit presented as mean ± 1 SD of the fraction % of released carvedilol:

Graphical abstract of model fit presented as the fraction % of released carvedilol per tested tablet:

Note: the model could not be fitted

Supplementary materials – Model fitting summary – C*Pharm Mannidex 16700 (modelling the entire relevant dissolution data)

Model: **Makoid–Banakar**

Model equation: $F = k_{MB} \cdot t^n \cdot e^{-k \cdot t}$

Fitted model parameters per tested tablet (N = 4) with statistics – mean, standard deviation (SD), and relative standard deviation expressed in % (RSD%) (output from DDSolver):

Parameter	No.1	No.2	No.3	No.4	Mean	SD	RSD(%)
k_{MB}	1.479	2.074	1.291	1.155	1.500	0.405	26.998
n	0.742	0.685	0.848	0.996	0.818	0.137	16.737
k	0.000	0.001	0.002	0.004	0.002	0.002	95.196

Number of dissolution data points (N), degrees of freedom (df), and selected goodness of fit criteria – Pearson correlation coefficient (R), coefficient of determination (R^2), adjusted coefficient of determination ($R^2_{adjusted}$), and residual sum of squares (RSS) (manual calculation in MS Excel):

Parameter	No.1	No.2	No.3	No.4
N	16	16	16	16
df	13	13	13	13
R	0.99641379	0.998609937	0.997791621	0.981984222
R^2	0.99284044	0.997221806	0.995588118	0.964293013
$R^2_{adjusted}$	0.991738969	0.996794391	0.994909367	0.95879963
RSS	111.9273101	40.01087757	142.7920426	614.8047082

Graphical abstract of model fit presented as mean ± 1 SD of the fraction % of released carvedilol:

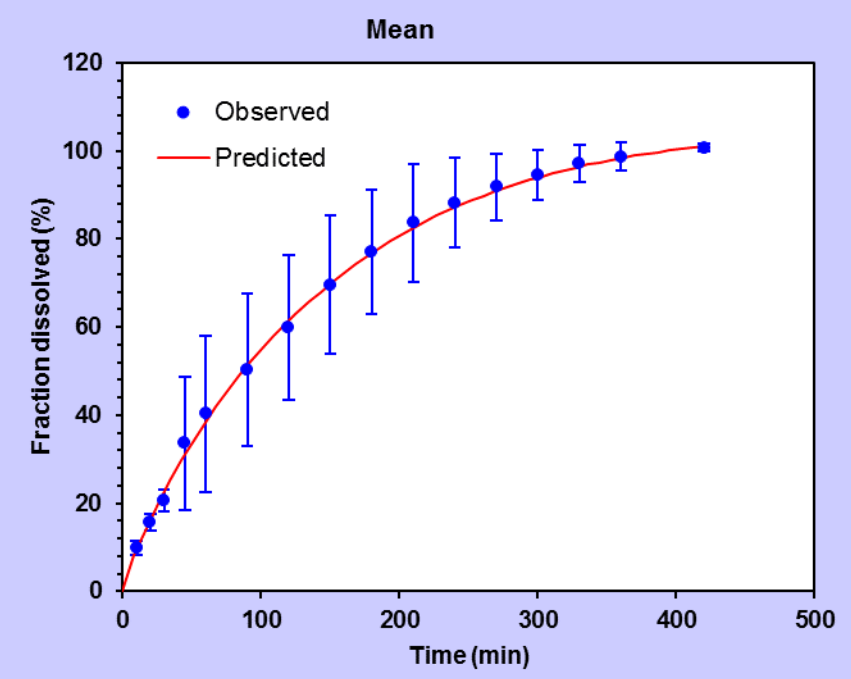

Supplementary materials – Model fitting summary – C*Pharm Mannidex 16700 (modelling the entire relevant dissolution data)

Graphical abstract of model fit presented as the fraction % of released carvedilol per tested tablet:

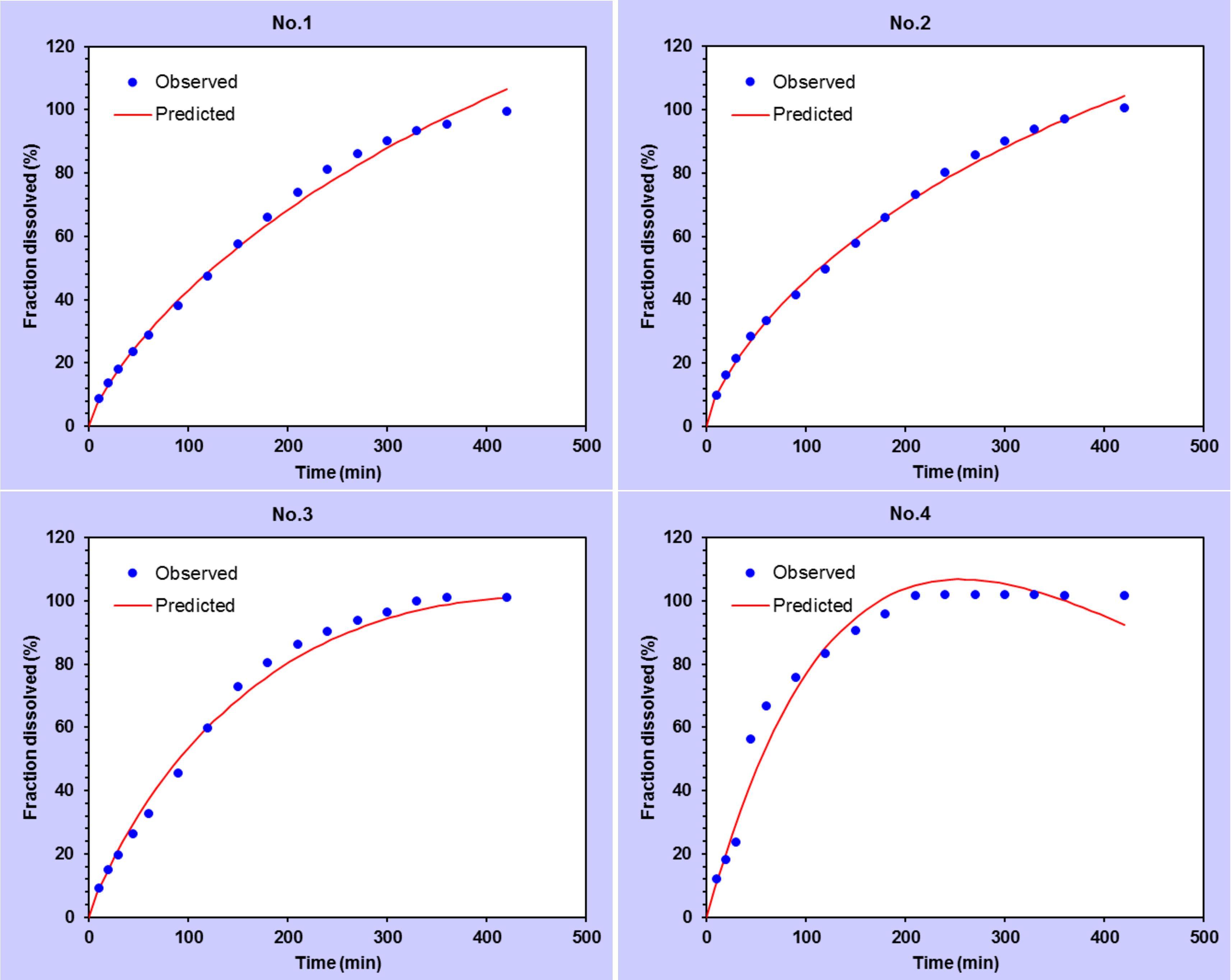

Supplementary materials – Model fitting summary – C*Pharm Mannidex 16700 (modelling the entire relevant dissolution data)

Model: **Makoid–Banakar with T_{lag}**

Model equation: $F = k_{MB} \cdot (t - T_{lag})^n \cdot e^{-k\cdot(t-T_{lag})}$

Fitted model parameters per tested tablet (N = 4) with statistics – mean, standard deviation (SD), and relative standard deviation expressed in % (RSD%) (output from DDSolver):

Parameter	No.1	No.2	No.3	No.4	Mean	SD	RSD(%)
k_{MB}	2.579	3.419	2.290	2.377	2.666	0.516	19.358
n	0.611	0.567	0.695	0.824	0.674	0.113	16.729
k	0.000	0.000	0.001	0.003	0.001	0.001	167.226
T_{lag}	4.000	4.000	4.000	4.000	4.000	0.000	0.000

Number of dissolution data points (N), degrees of freedom (df), and selected goodness of fit criteria – Pearson correlation coefficient (R), coefficient of determination (R^2), adjusted coefficient of determination ($R^2_{adjusted}$), and residual sum of squares (RSS) (manual calculation in MS Excel):

Parameter	No.1	No.2	No.3	No.4
N	16	16	16	16
df	12	12	12	12
R	0.992807113	0.997148461	0.990874822	0.986501796
R^2	0.985665963	0.994305053	0.981832912	0.973185793
$R^2_{adjusted}$	0.982082454	0.992881316	0.97729114	0.966482241
RSS	226.1880634	82.15334426	322.7721795	440.2144737

Graphical abstract of model fit presented as mean ± 1 SD of the fraction % of released carvedilol:

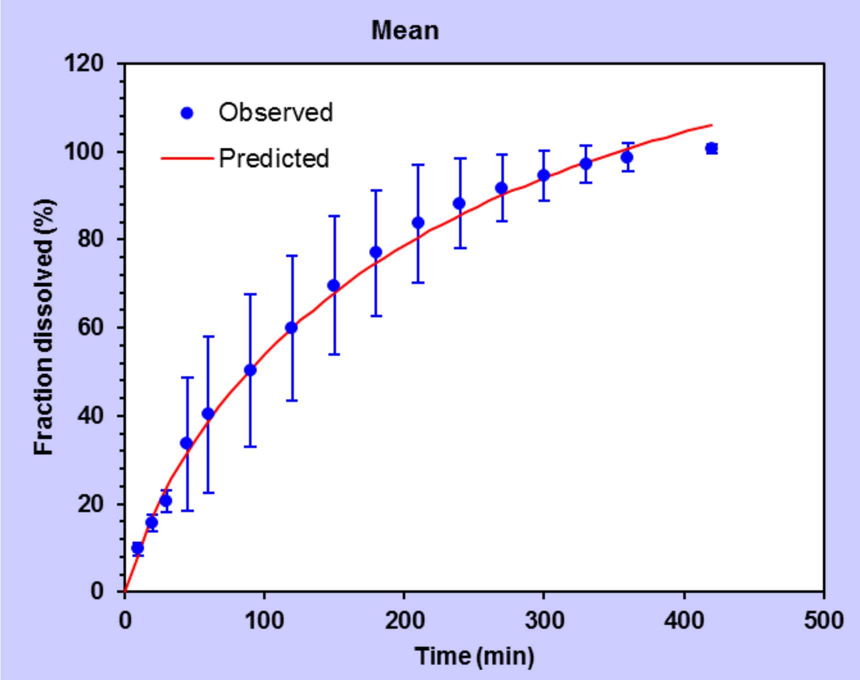

Supplementary materials – Model fitting summary – C*Pharm Mannidex 16700 (modelling the entire relevant dissolution data)

Graphical abstract of model fit presented as the fraction % of released carvedilol per tested tablet:

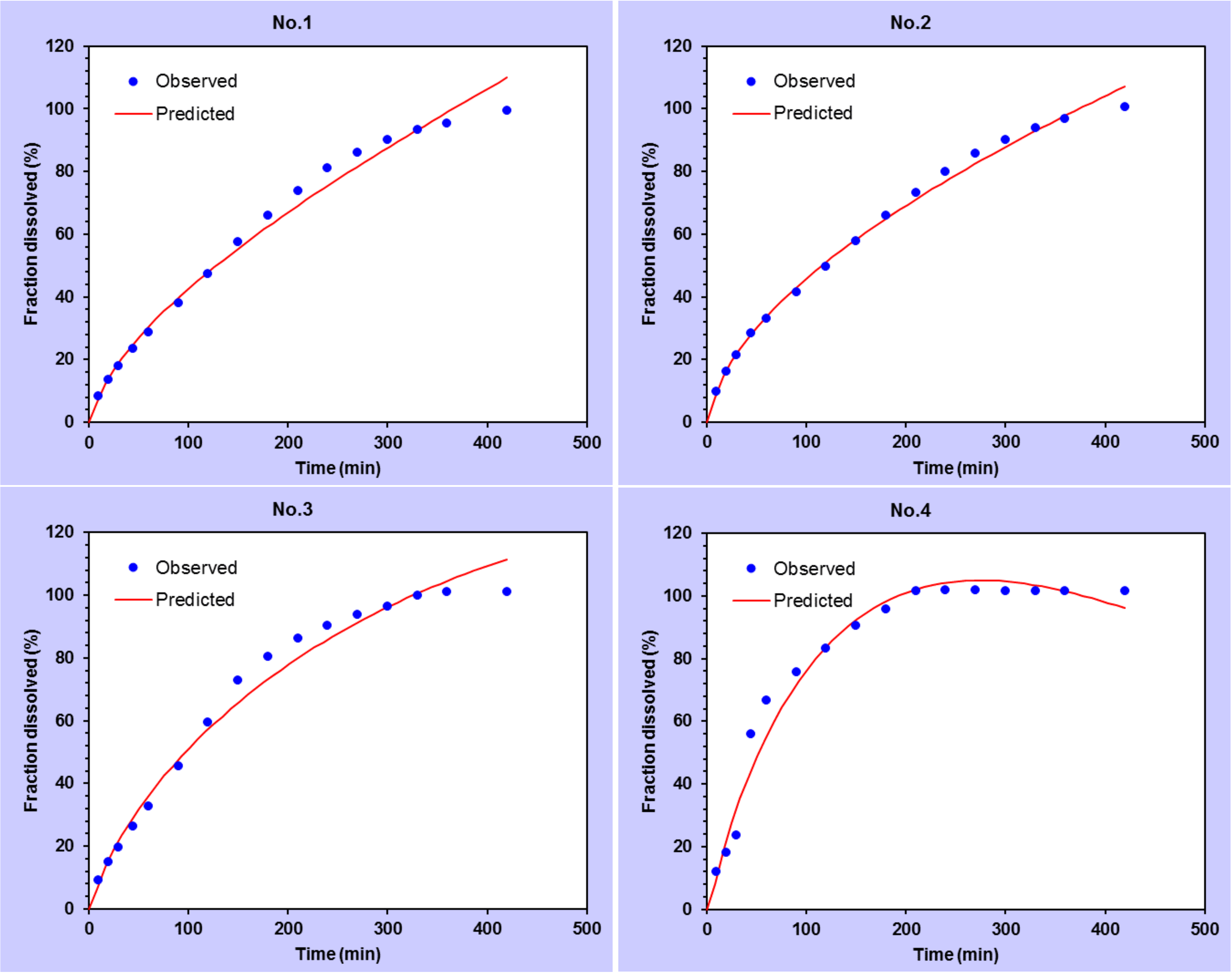

Supplementary materials – Model fitting summary – C*Pharm Mannidex 16700 (modelling the entire relevant dissolution data)

Model: **Peppas–Sahlin_1**

Model equation: $F = k_1 \cdot t^m + k_2 \cdot t^{2m}$

Fitted model parameters per tested tablet (N = 4) with statistics – mean, standard deviation (SD), and relative standard deviation expressed in % (RSD%) (output from DDSolver):

Parameter	No.1	No.2	No.3	No.4	Mean	SD	RSD(%)
k_1	3.477	4.192	5.249	10.991	5.977	3.421	57.232
k_2	0.246	0.194	0.155	-0.241	0.089	0.223	251.315
m	0.450	0.450	0.450	0.450	0.450	0.000	0.000

Number of dissolution data points (N), degrees of freedom (df), and selected goodness of fit criteria – Pearson correlation coefficient (R), coefficient of determination (R^2), adjusted coefficient of determination ($R^2_{adjusted}$), and residual sum of squares (RSS) (manual calculation in MS Excel):

Parameter	No.1	No.2	No.3	No.4
N	16	16	16	16
df	13	13	13	13
R	0.991722733	0.995638076	0.97884307	0.957719772
R^2	0.98351398	0.991295178	0.958133755	0.917227161
$R^2_{adjusted}$	0.980977669	0.989955975	0.951692795	0.904492878
RSS	272.9261865	134.5318229	816.9399549	1723.735696

Graphical abstract of model fit presented as mean ± 1 SD of the fraction % of released carvedilol:

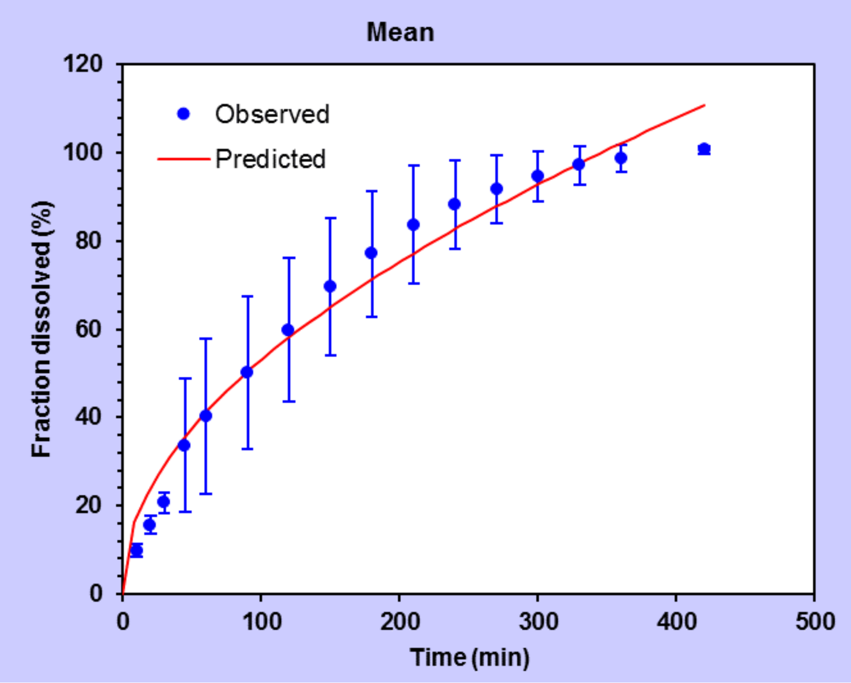

Supplementary materials – Model fitting summary – C*Pharm Mannidex 16700 (modelling the entire relevant dissolution data)

Graphical abstract of model fit presented as the fraction % of released carvedilol per tested tablet:

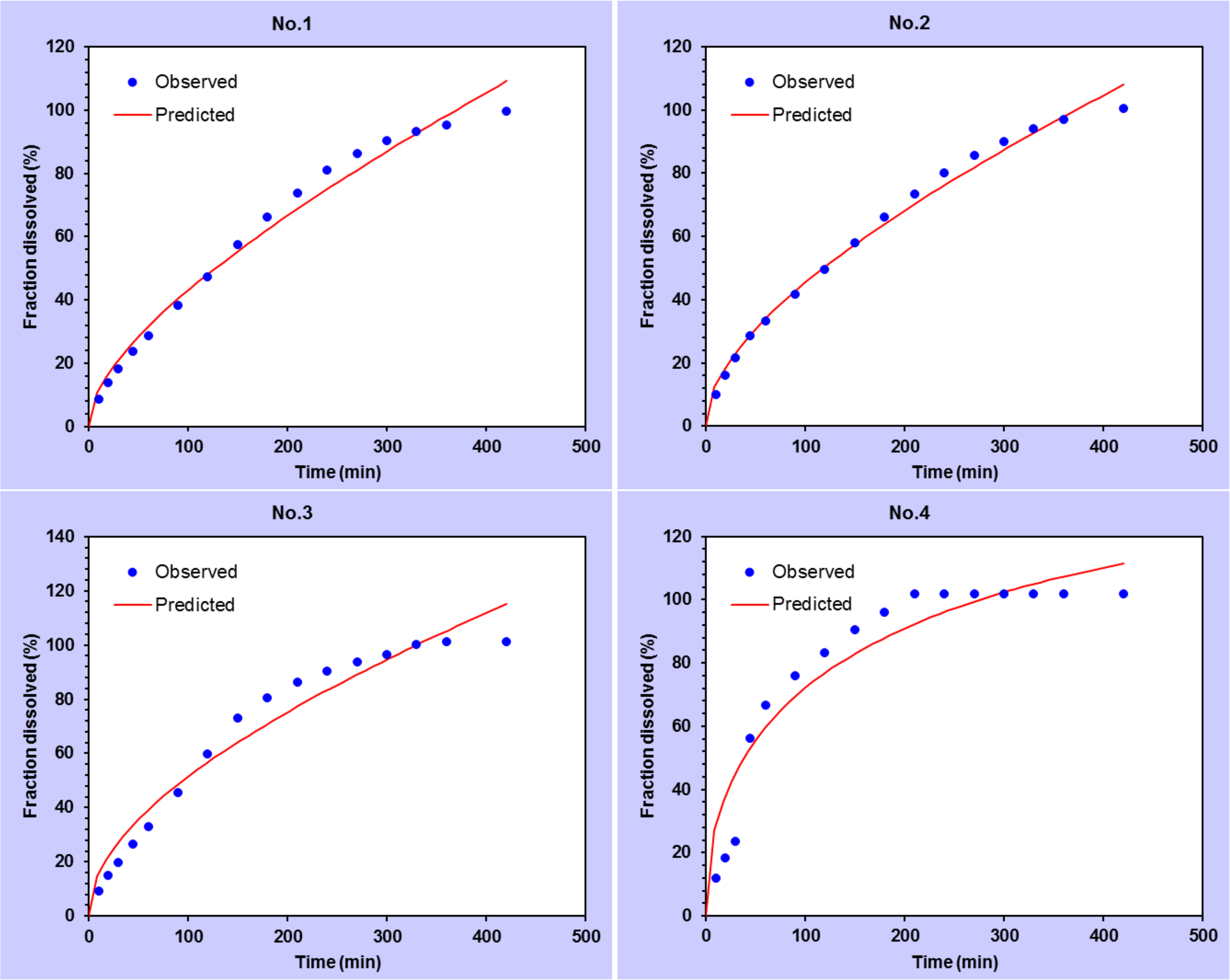

Supplementary materials – Model fitting summary – C*Pharm Mannidex 16700 (modelling the entire relevant dissolution data)

Model: **Peppas–Sahlin_1 with T_{lag}**

Model equation: $F = k_1 \cdot (t - T_{lag})^m + k_2 \cdot (t - T_{lag})^{2m}$

Fitted model parameters per tested tablet (N = 4) with statistics – mean, standard deviation (SD), and relative standard deviation expressed in % (RSD%) (output from DDSolver):

Parameter	No.1	No.2	No.3	No.4	Mean	SD	RSD(%)
k_1	3.888	4.612	5.758	11.711	6.492	3.563	54.881
k_2	0.219	0.166	0.120	-0.294	0.053	0.235	445.235
m	0.450	0.450	0.450	0.450	0.450	0.000	0.000
T_{lag}	6.000	6.000	6.000	6.000	6.000	0.000	0.000

Number of dissolution data points (N), degrees of freedom (df), and selected goodness of fit criteria – Pearson correlation coefficient (R), coefficient of determination (R^2), adjusted coefficient of determination ($R^2_{adjusted}$), and residual sum of squares (RSS) (manual calculation in MS Excel):

Parameter	No.1	No.2	No.3	No.4
N	16	16	16	16
df	12	12	12	12
R	0.992924921	0.9966526	0.982118009	0.969248885
R^2	0.985899898	0.993316405	0.964555784	0.939443401
$R^2_{adjusted}$	0.982374873	0.991645506	0.95569473	0.924304251
RSS	224.0051498	98.79949344	661.4319652	1197.435241

Graphical abstract of model fit presented as mean ± 1 SD of the fraction % of released carvedilol:

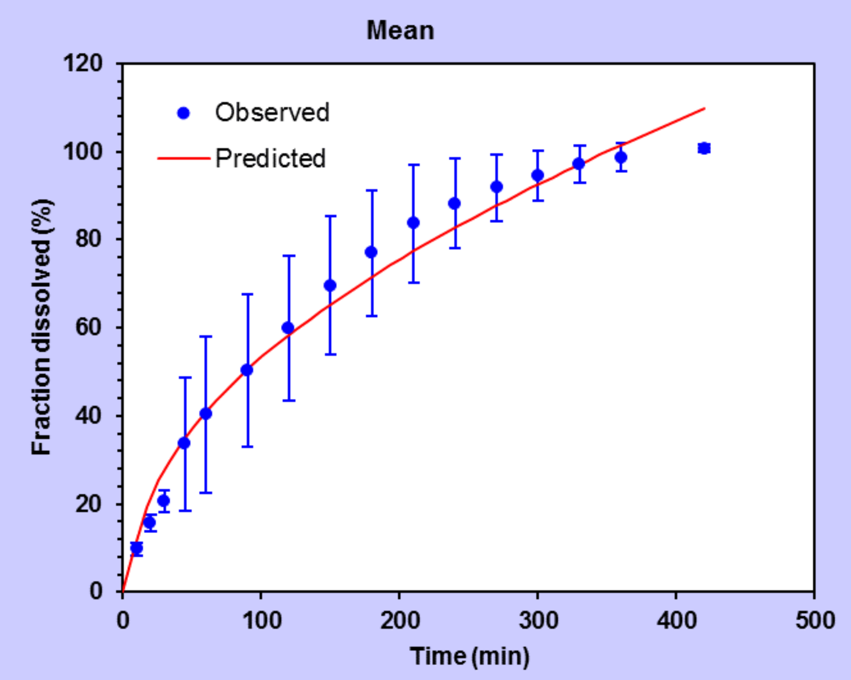

Supplementary materials – Model fitting summary – C*Pharm Mannidex 16700 (modelling the entire relevant dissolution data)

Graphical abstract of model fit presented as the fraction % of released carvedilol per tested tablet:

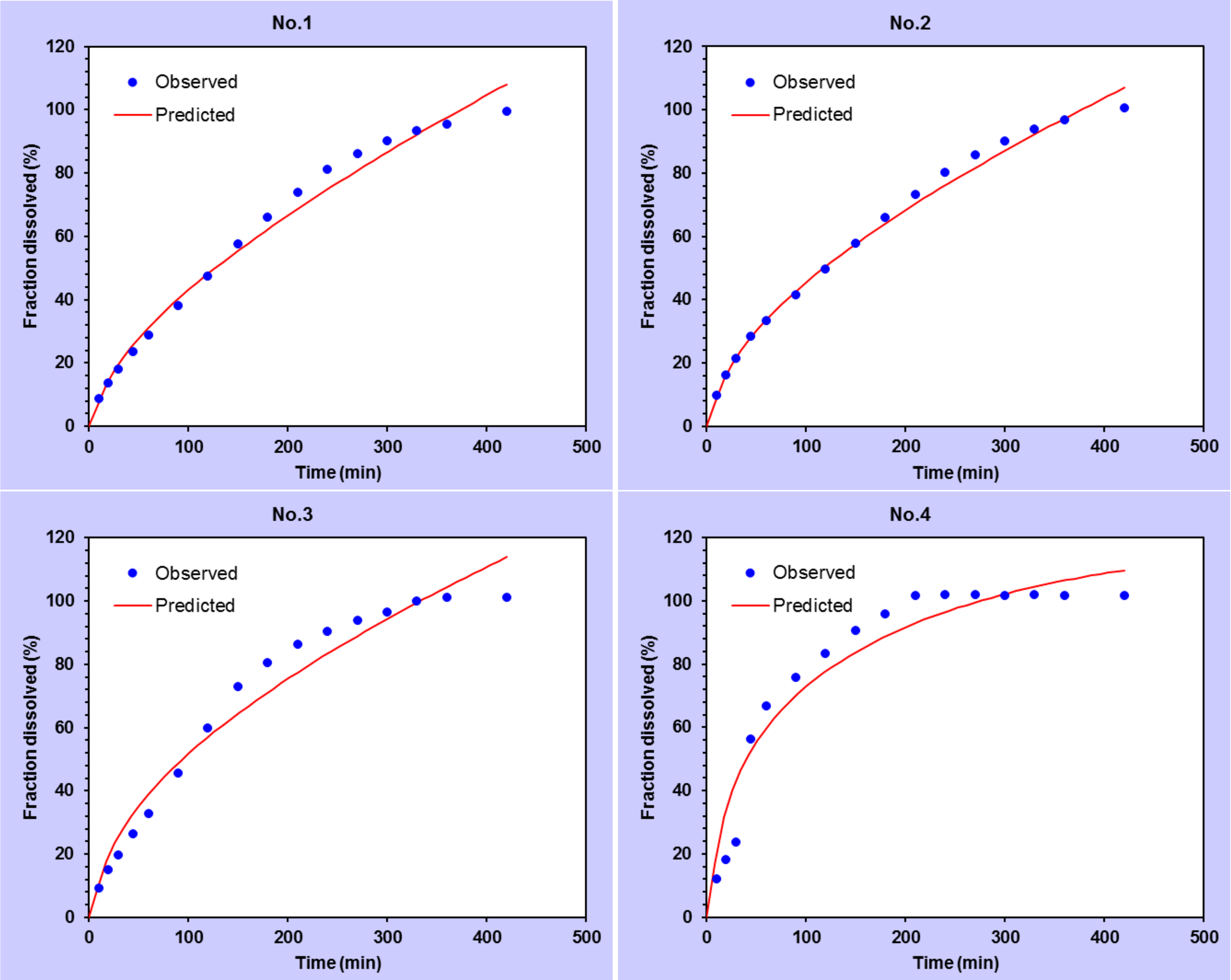

Supplementary materials – Model fitting summary – C*Pharm Mannidex 16700 (modelling the entire relevant dissolution data)

Model: **Peppas–Sahlin_2**

Model equation: $F = k_1 \cdot t^{0.5} + k_2 \cdot t$

Fitted model parameters per tested tablet (N = 4) with statistics – mean, standard deviation (SD), and relative standard deviation expressed in % (RSD%) (output from DDSolver):

Parameter	No.1	No.2	No.3	No.4	Mean	SD	RSD(%)
k_1	3.436	3.947	4.891	9.273	5.387	2.660	49.380
k_2	0.091	0.063	0.033	-0.194	-0.002	0.130	-7946.025

Number of dissolution data points (N), degrees of freedom (df), and selected goodness of fit criteria – Pearson correlation coefficient (R), coefficient of determination (R^2), adjusted coefficient of determination ($R^2_{adjusted}$), and residual sum of squares (RSS) (manual calculation in MS Excel):

Parameter	No.1	No.2	No.3	No.4
N	16	16	16	16
df	14	14	14	14
R	0.992031866	0.996055719	0.981181173	0.968378651
R^2	0.984127223	0.992126995	0.962716495	0.937757213
$R^2_{adjusted}$	0.982993453	0.991564637	0.960053388	0.933311299
RSS	270.5306984	124.9631054	762.8215448	1420.737144

Graphical abstract of model fit presented as mean ± 1 SD of the fraction % of released carvedilol:

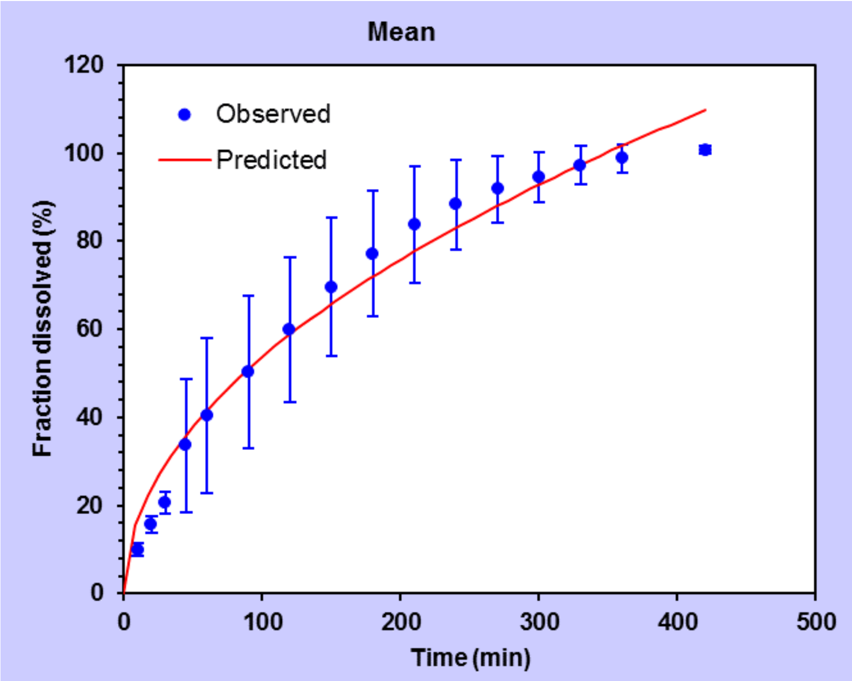

Supplementary materials – Model fitting summary – C*Pharm Mannidex 16700 (modelling the entire relevant dissolution data)

Graphical abstract of model fit presented as the fraction % of released carvedilol per tested tablet:

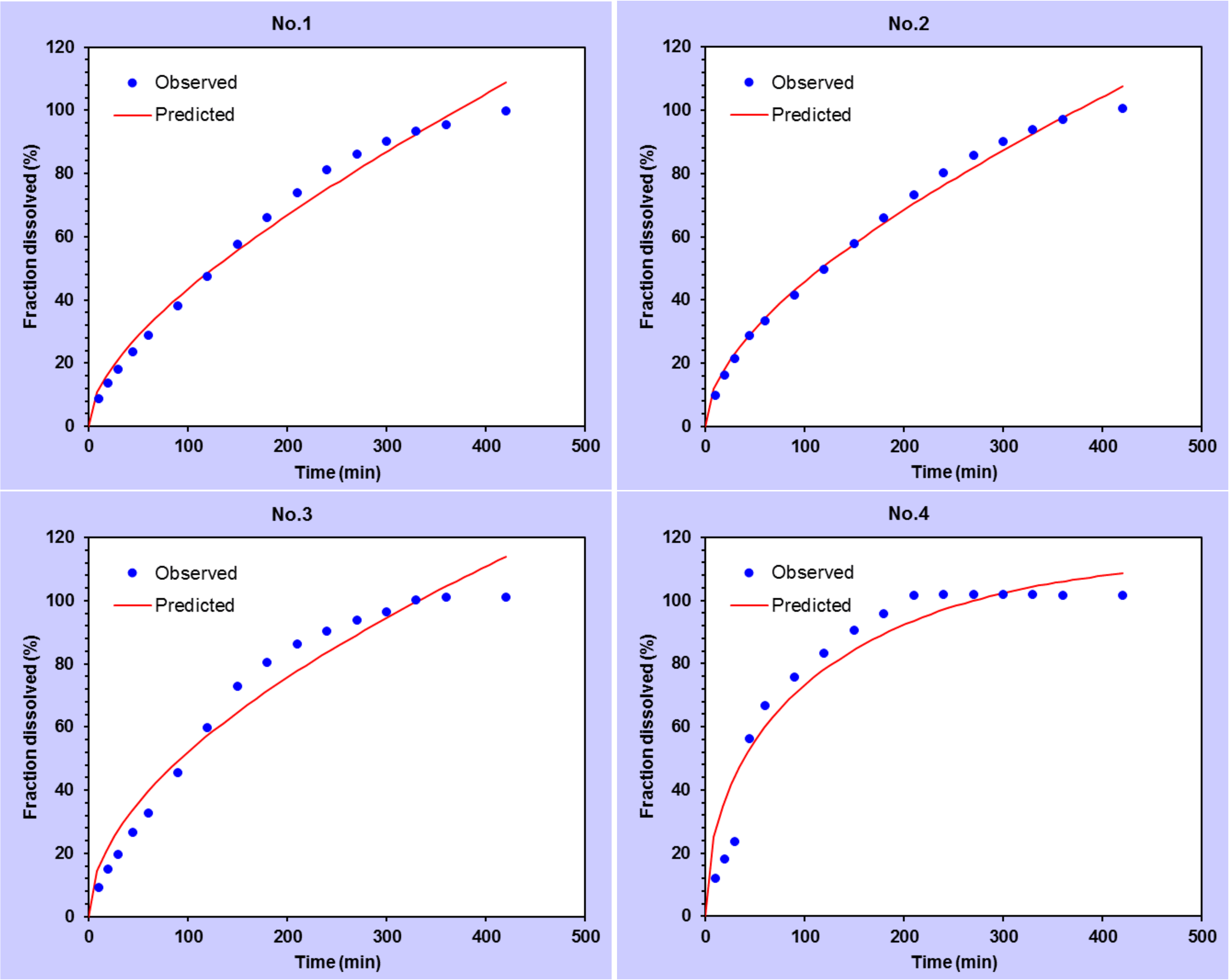

Supplementary materials – Model fitting summary – C*Pharm Mannidex 16700 (modelling the entire relevant dissolution data)

Model: **Peppas–Sahlin_2 with T_{lag}**

Model equation: $F = k_1 \cdot (t - T_{lag})^{0.5} + k_2 \cdot (t - T_{lag})$

Fitted model parameters per tested tablet (N = 4) with statistics – mean, standard deviation (SD), and relative standard deviation expressed in % (RSD%) (output from DDSolver):

Parameter	No.1	No.2	No.3	No.4	Mean	SD	RSD(%)
k_1	3.742	4.257	5.272	9.806	5.770	2.765	47.930
k_2	0.076	0.048	0.013	-0.224	-0.022	0.137	-634.734
T_{lag}	6.000	6.000	6.000	6.000	6.000	0.000	0.000

Number of dissolution data points (N), degrees of freedom (df), and selected goodness of fit criteria – Pearson correlation coefficient (R), coefficient of determination (R^2), adjusted coefficient of determination ($R^2_{adjusted}$), and residual sum of squares (RSS) (manual calculation in MS Excel):

Parameter	No.1	No.2	No.3	No.4
N	16	16	16	16
df	13	13	13	13
R	0.993244017	0.996942721	0.984328289	0.977364563
R^2	0.986533677	0.993894789	0.96890218	0.955241489
$R^2_{adjusted}$	0.984461935	0.992955526	0.964117901	0.948355565
RSS	215.549456	90.44031186	595.4743992	919.6214654

Graphical abstract of model fit presented as mean ± 1 SD of the fraction % of released carvedilol:

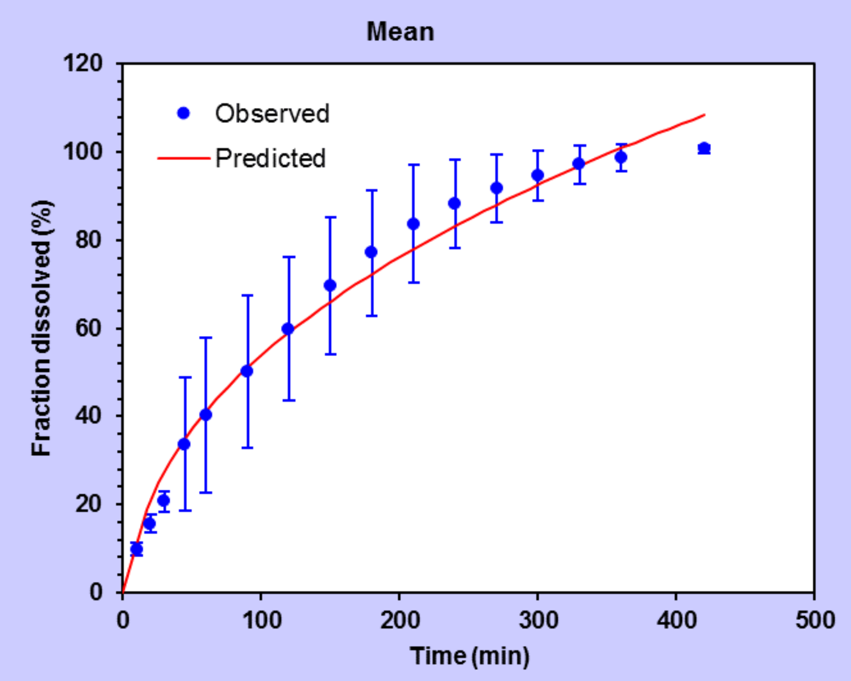

Supplementary materials – Model fitting summary – C*Pharm Mannidex 16700 (modelling the entire relevant dissolution data)

Graphical abstract of model fit presented as the fraction % of released carvedilol per tested tablet:

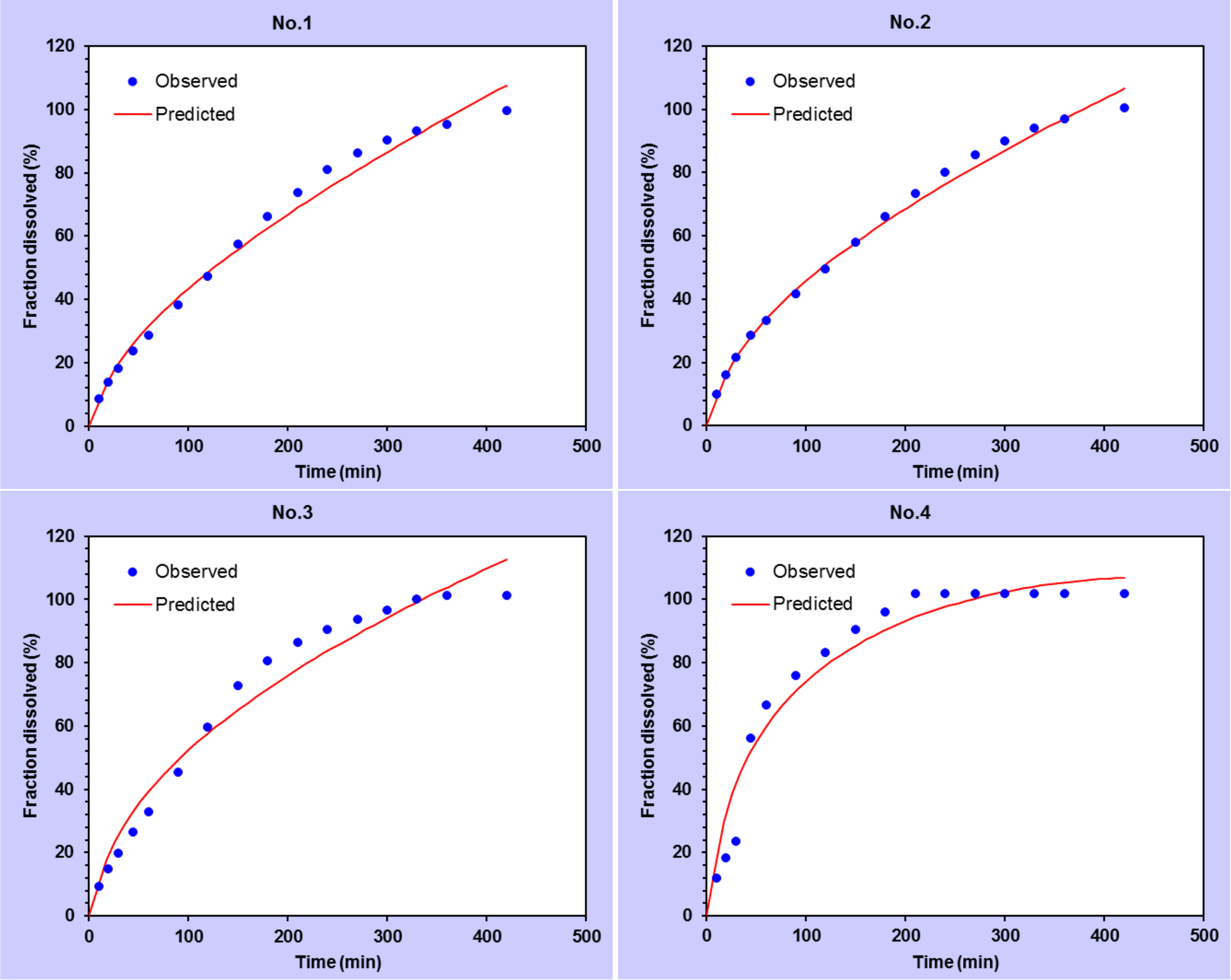

Supplementary materials – Model fitting summary – C*Pharm Mannidex 16700 (modelling the entire relevant dissolution data)

Model: **Quadratic**

Model equation: $F = 100 \cdot (k_1 \cdot t^2 + k_2 \cdot t)$

Fitted model parameters per tested tablet (N = 4) with statistics – mean, standard deviation (SD), and relative standard deviation expressed in % (RSD%) (output from DDSolver):

Parameter	No.1	No.2	No.3	No.4	Mean	SD	RSD(%)
k_1	-0.00001	-0.00001	-0.00001	-0.00001	-0.00001	0.00000	-45.99488
k_2	0.00475	0.00488	0.00588	0.00806	0.00589	0.00153	25.95427

Number of dissolution data points (N), degrees of freedom (df), and selected goodness of fit criteria – Pearson correlation coefficient (R), coefficient of determination (R^2), adjusted coefficient of determination ($R^2_{adjusted}$), and residual sum of squares (RSS) (manual calculation in MS Excel):

Parameter	No.1	No.2	No.3	No.4
N	16	16	16	16
df	14	14	14	14
R	0.999110842	0.997177704	0.99831421	0.959735169
R^2	0.998222475	0.994363373	0.996631263	0.921091595
$R^2_{adjusted}$	0.998095509	0.993960757	0.996390639	0.91545528
RSS	76.22280115	257.6250716	97.89021085	2043.992663

Graphical abstract of model fit presented as mean ± 1 SD of the fraction % of released carvedilol:

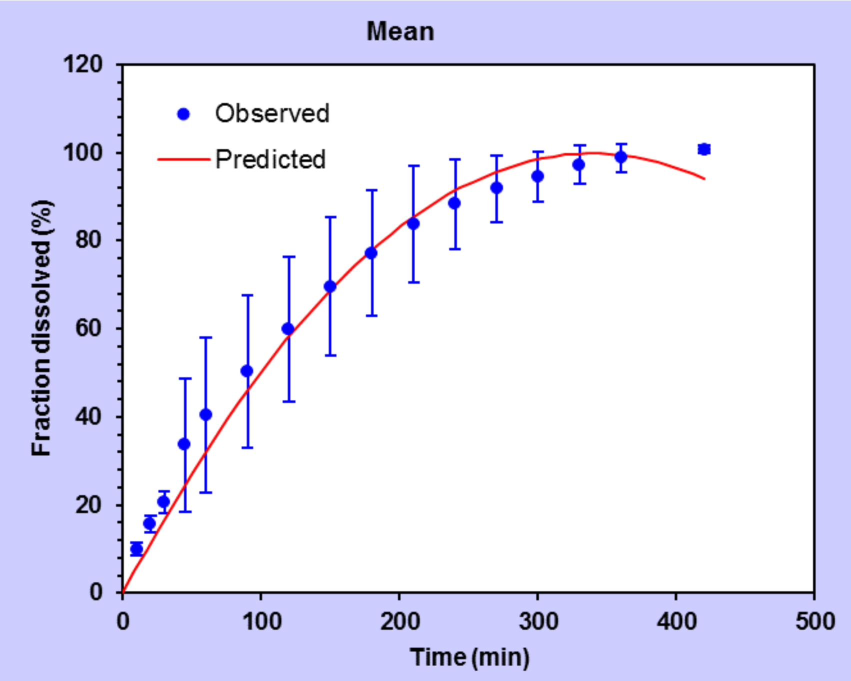

Supplementary materials – Model fitting summary – C*Pharm Mannidex 16700 (modelling the entire relevant dissolution data)

Graphical abstract of model fit presented as the fraction % of released carvedilol per tested tablet:

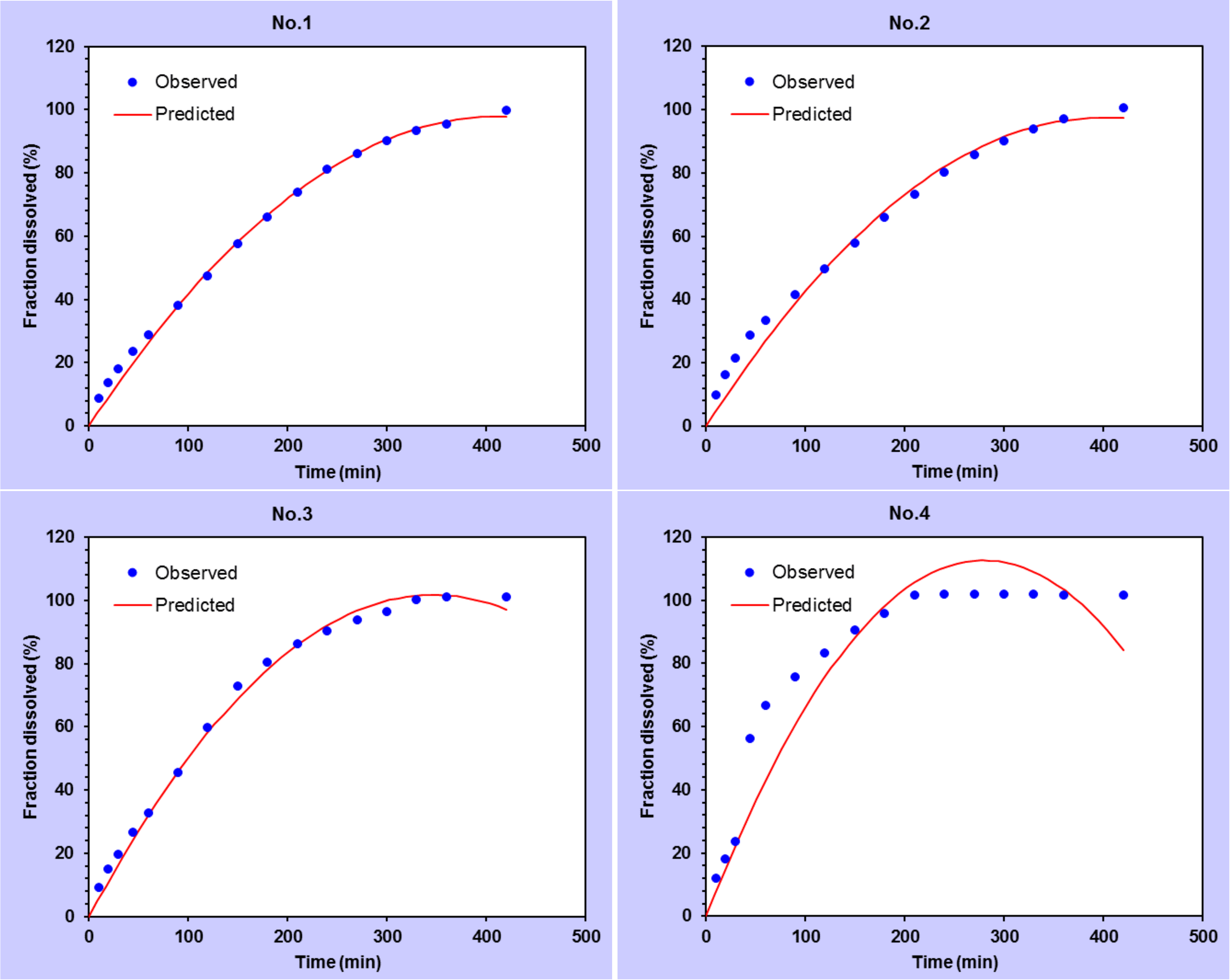

Supplementary materials – Model fitting summary – C*Pharm Mannidex 16700 (modelling the entire relevant dissolution data)

Model: **Quadratic with T_{lag}**

Model equation: $F = 100 \cdot \left[k_1 \cdot \left(t - T_{lag}\right)^2 + k_2 \cdot \left(t - T_{lag}\right)\right]$

Fitted model parameters per tested tablet (N = 4) with statistics – mean, standard deviation (SD), and relative standard deviation expressed in % (RSD%) (output from DDSolver):

Parameter	No.1	No.2	No.3	No.4	Mean	SD	RSD(%)
k_1	-0.00001	-0.00001	-0.00001	-0.00001	-0.00001	0.00000	-44.85213
k_2	0.00489	0.00501	0.00604	0.00823	0.00604	0.00155	25.62586
T_{lag}	4.00000	4.00000	4.00000	4.00000	4.00000	0.00000	0.00000

Number of dissolution data points (N), degrees of freedom (df), and selected goodness of fit criteria – Pearson correlation coefficient (R), coefficient of determination (R^2), adjusted coefficient of determination ($R^2_{adjusted}$), and residual sum of squares (RSS) (manual calculation in MS Excel):

Parameter	No.1	No.2	No.3	No.4
N	16	16	16	16
df	13	13	13	13
R	0.998699431	0.996531975	0.998020754	0.959564194
R^2	0.997400553	0.993075976	0.996045426	0.920763442
$R^2_{adjusted}$	0.997000638	0.992010742	0.99543703	0.908573203
RSS	142.5785145	376.3342949	173.0652866	2407.387021

Graphical abstract of model fit presented as mean ± 1 SD of the fraction % of released carvedilol:

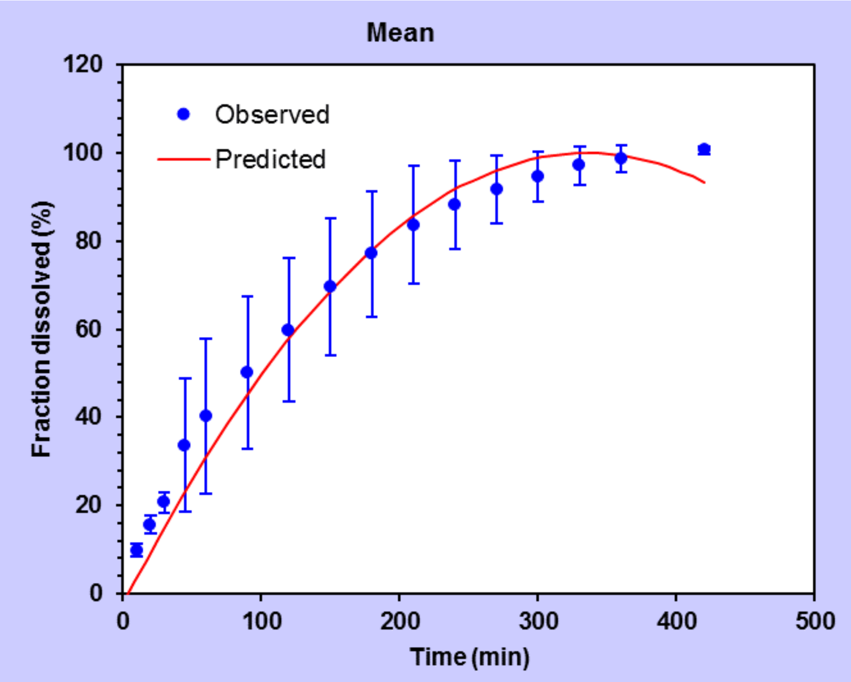

Supplementary materials – Model fitting summary – C*Pharm Mannidex 16700 (modelling the entire relevant dissolution data)

Graphical abstract of model fit presented as the fraction % of released carvedilol per tested tablet:

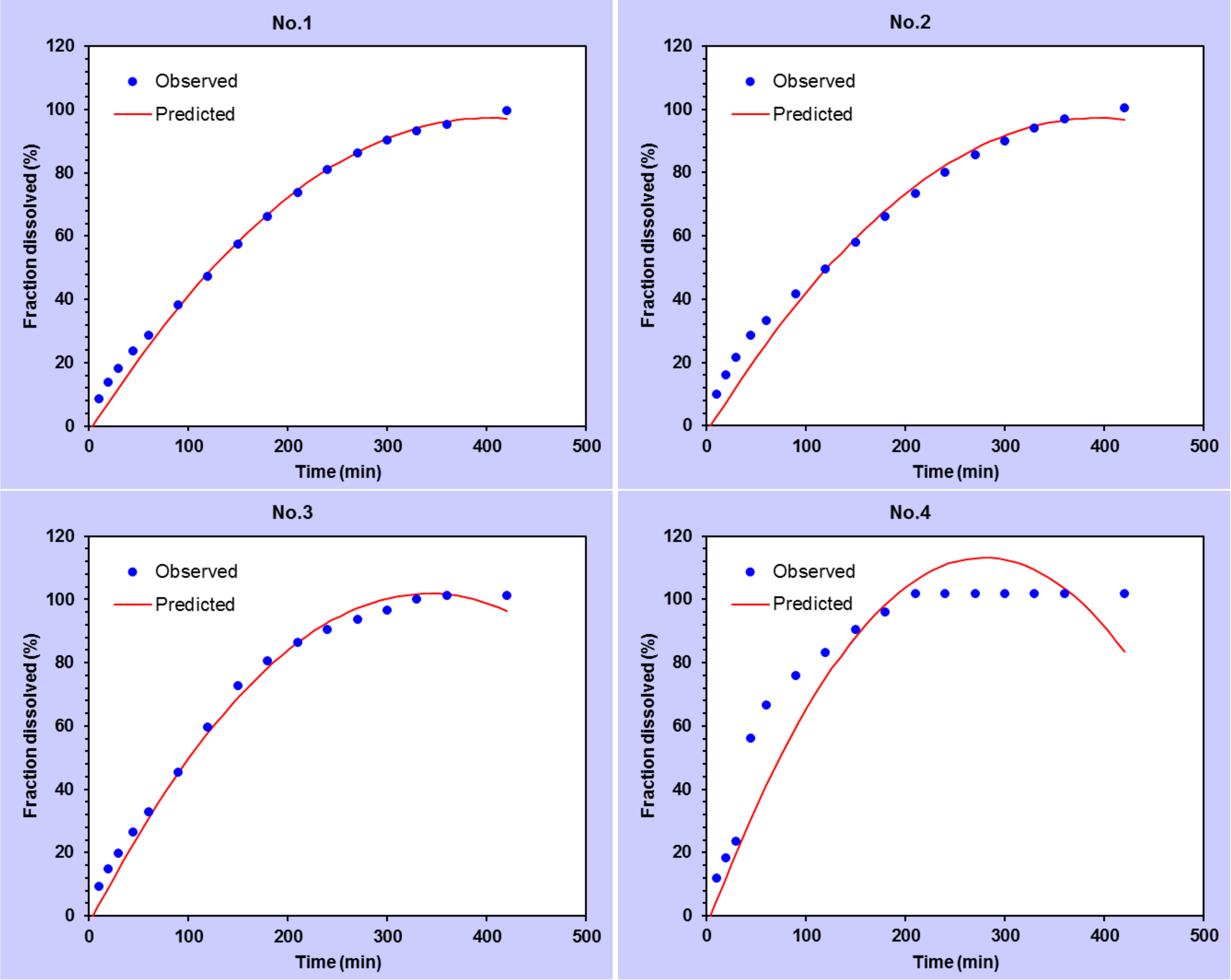

Supplementary materials – Model fitting summary – C*Pharm Mannidex 16700 (modelling the entire relevant dissolution data)

Model: **Weibull_1**

Model equation: $F = 100 \cdot \left[1 - e^{-\frac{(t-T_i)^\beta}{\alpha}}\right]$

Fitted model parameters per tested tablet (N = 4) with statistics – mean, standard deviation (SD), and relative standard deviation expressed in % (RSD%) (output from DDSolver):

Parameter	No.1	No.2	No.3	No.4	Mean	SD	RSD(%)
α	129.067	62.574	115.567	64.161	92.842	34.484	37.142
β	0.952	0.845	1.032	1.010	0.960	0.084	8.708
T_i	4.431	6.000	6.000	4.000	5.108	1.045	20.465

Number of dissolution data points (N), degrees of freedom (df), and selected goodness of fit criteria – Pearson correlation coefficient (R), coefficient of determination (R^2), adjusted coefficient of determination ($R^2_{adjusted}$), and residual sum of squares (RSS) (manual calculation in MS Excel):

Parameter	No.1	No.2	No.3	No.4
N	16	16	16	16
df	13	13	13	13
R	0.993809146	0.984840477	0.989805811	0.991765471
R^2	0.987656619	0.969910766	0.979715544	0.983598749
$R^2_{adjusted}$	0.985757637	0.965281653	0.976594858	0.981075479
RSS	410.9440359	451.4228774	400.3896267	318.1890851

Graphical abstract of model fit presented as mean ± 1 SD of the fraction % of released carvedilol:

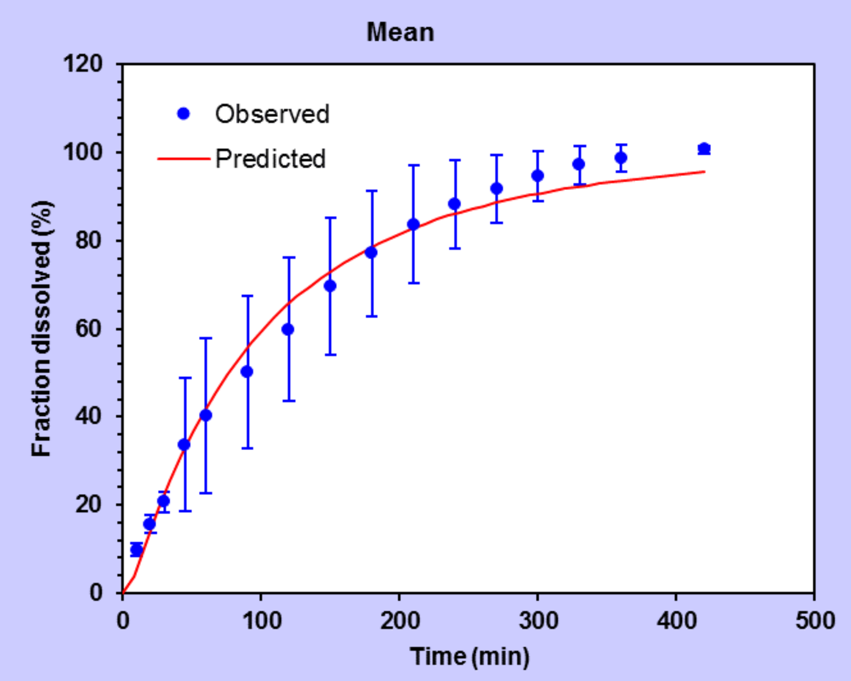

Supplementary materials – Model fitting summary – C*Pharm Mannidex 16700 (modelling the entire relevant dissolution data)

Graphical abstract of model fit presented as the fraction % of released carvedilol per tested tablet:

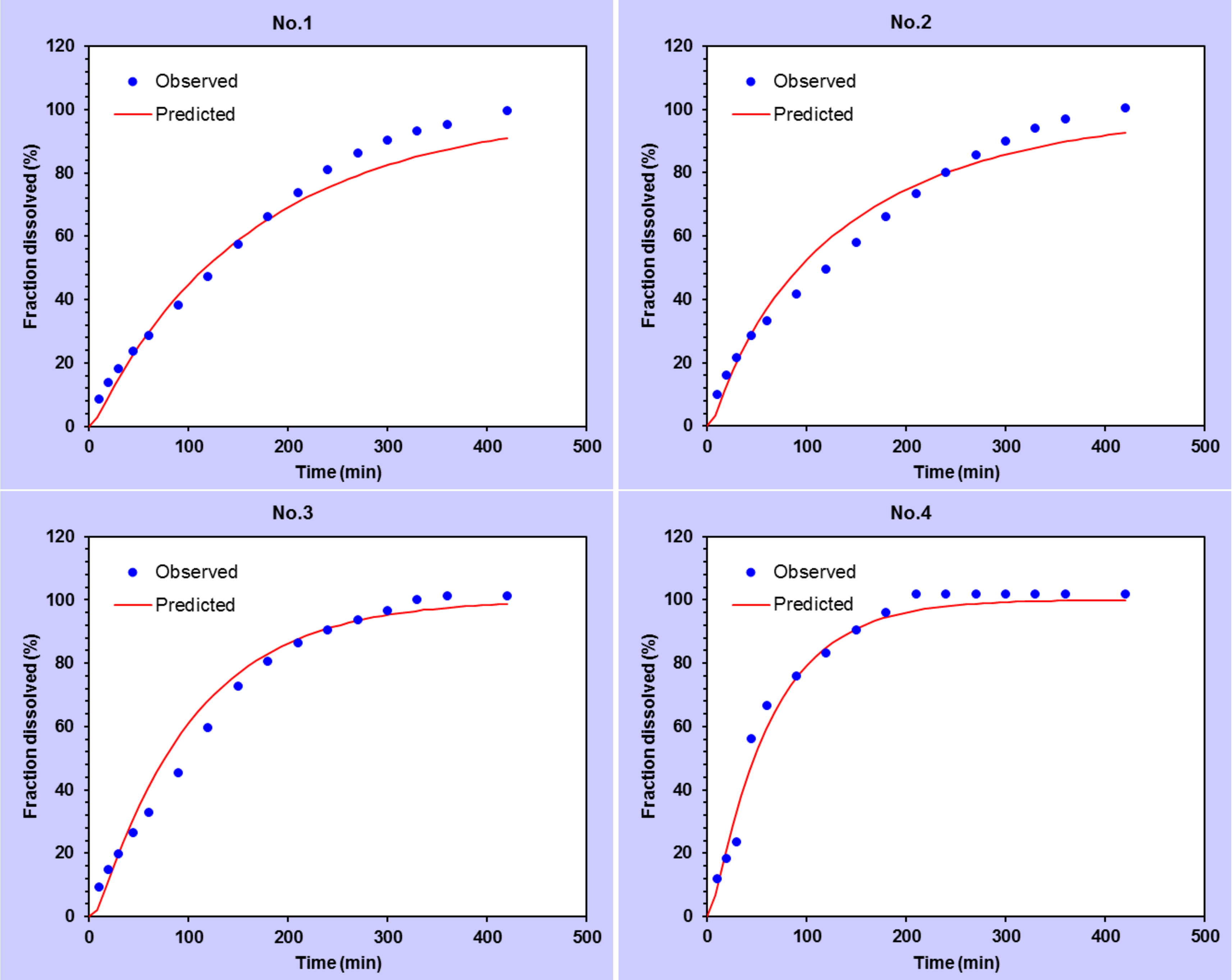

Supplementary materials – Model fitting summary – C*Pharm Mannidex 16700 (modelling the entire relevant dissolution data)

Model: **Weibull_2**

Model equation: $F = 100 \cdot \left(1 - e^{-\frac{t^{\beta}}{\alpha}}\right)$

Fitted model parameters per tested tablet (N = 4) with statistics – mean, standard deviation (SD), and relative standard deviation expressed in % (RSD%) (output from DDSolver):

Parameter	No.1	No.2	No.3	No.4	Mean	SD	RSD(%)
α	232.099	104.455	223.131	135.222	173.727	63.585	36.601
β	1.061	0.940	1.156	1.164	1.080	0.104	9.661

Number of dissolution data points (N), degrees of freedom (df), and selected goodness of fit criteria – Pearson correlation coefficient (R), coefficient of determination (R^2), adjusted coefficient of determination ($R^2_{adjusted}$), and residual sum of squares (RSS) (manual calculation in MS Excel):

Parameter	No.1	No.2	No.3	No.4
N	16	16	16	16
df	14	14	14	14
R	0.996251952	0.988972316	0.992986111	0.991371189
R^2	0.992517953	0.978066241	0.986021417	0.982816834
$R^2_{adjusted}$	0.991983521	0.976499544	0.985022947	0.981589465
RSS	269.953813	341.1327936	311.2030625	312.3972991

Graphical abstract of model fit presented as mean ± 1 SD of the fraction % of released carvedilol:

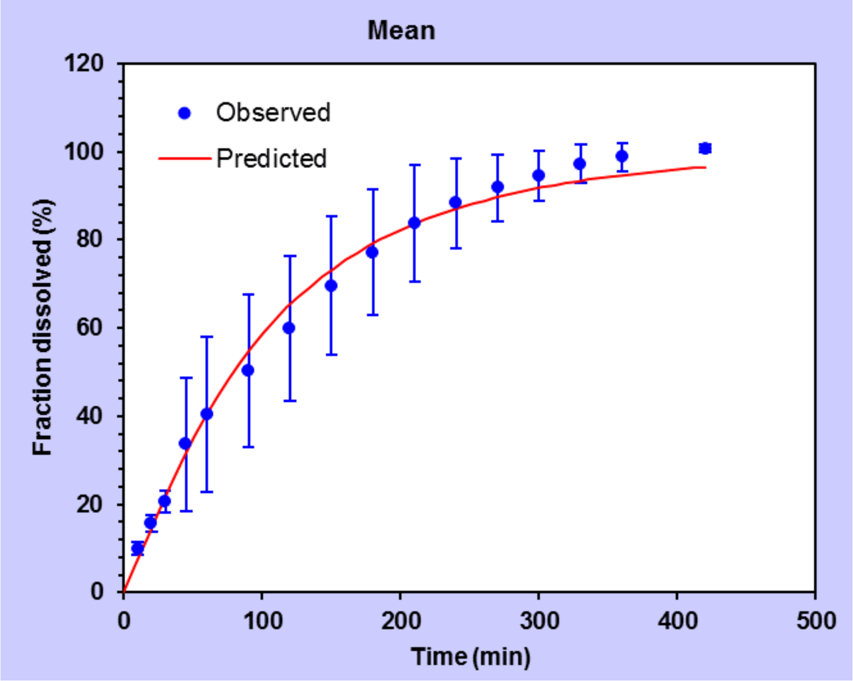

Supplementary materials – Model fitting summary – C*Pharm Mannidex 16700 (modelling the entire relevant dissolution data)

Graphical abstract of model fit presented as the fraction % of released carvedilol per tested tablet:

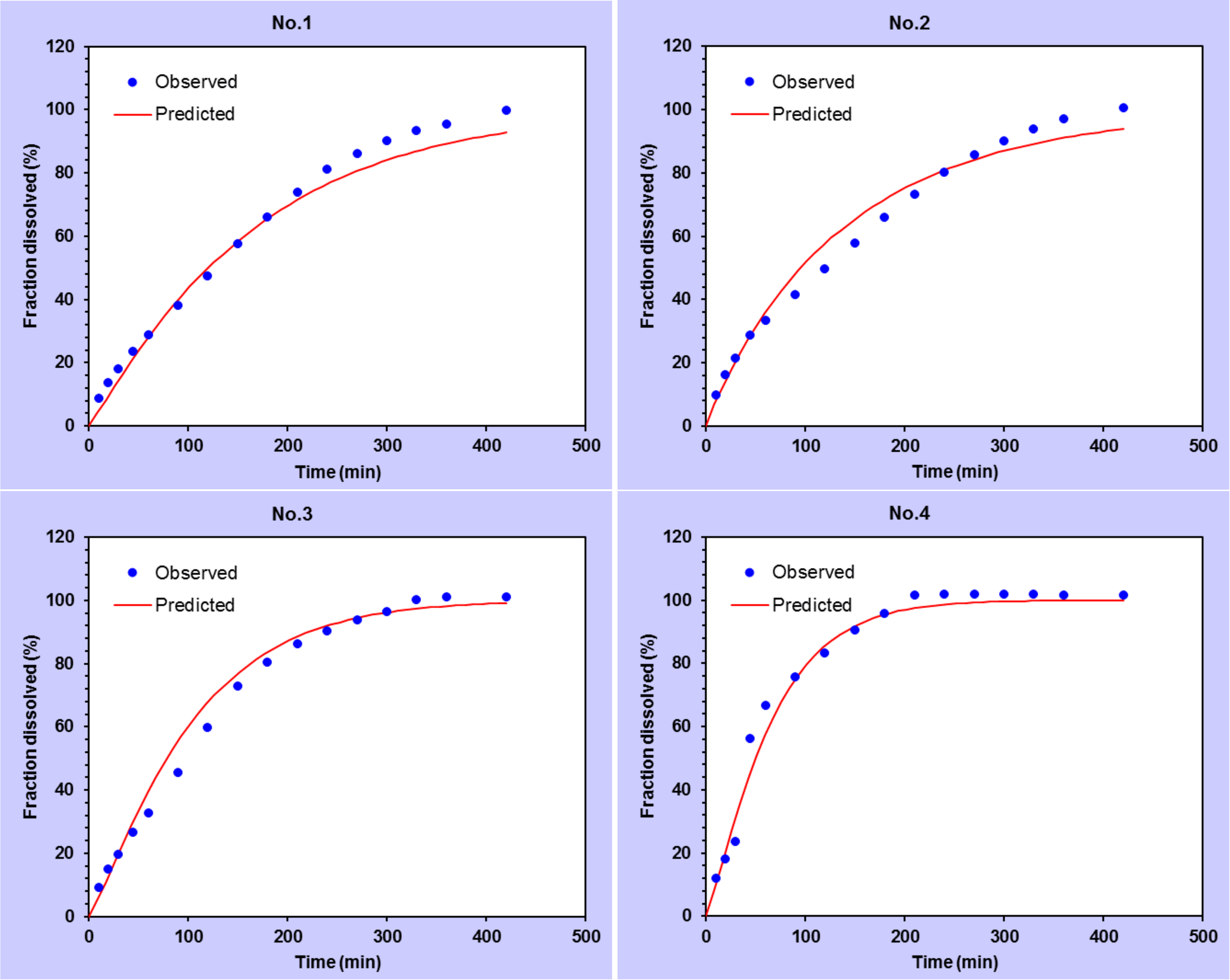

Supplementary materials – Model fitting summary – C*Pharm Mannidex 16700 (modelling the entire relevant dissolution data)

Model: **Weibull_3**

Model equation: $F = F_{max} \cdot \left(1 - e^{-\frac{t^{\beta}}{\alpha}}\right)$

Fitted model parameters per tested tablet (N = 4) with statistics – mean, standard deviation (SD), and relative standard deviation expressed in % (RSD%) (output from DDSolver):

Parameter	No.1	No.2	No.3	No.4	Mean	SD	RSD(%)
α	189.487	114.914	148.923	69.957	130.820	50.750	38.794
β	0.978	0.895	1.023	0.951	0.962	0.054	5.575
F_{max}	112.809	119.761	106.197	106.848	111.404	6.316	5.670

Number of dissolution data points (N), degrees of freedom (df), and selected goodness of fit criteria – Pearson correlation coefficient (R), coefficient of determination (R^2), adjusted coefficient of determination ($R^2_{adjusted}$), and residual sum of squares (RSS) (manual calculation in MS Excel):

Parameter	No.1	No.2	No.3	No.4
N	16	16	16	16
df	13	13	13	13
R	0.998094837	0.996544892	0.997287523	0.983480429
R^2	0.996193304	0.993101722	0.994582404	0.967233754
$R^2_{adjusted}$	0.995607658	0.992040449	0.993748928	0.962192793
RSS	162.3090906	181.1169981	113.9455773	562.4207504

Graphical abstract of model fit presented as mean ± 1 SD of the fraction % of released carvedilol:

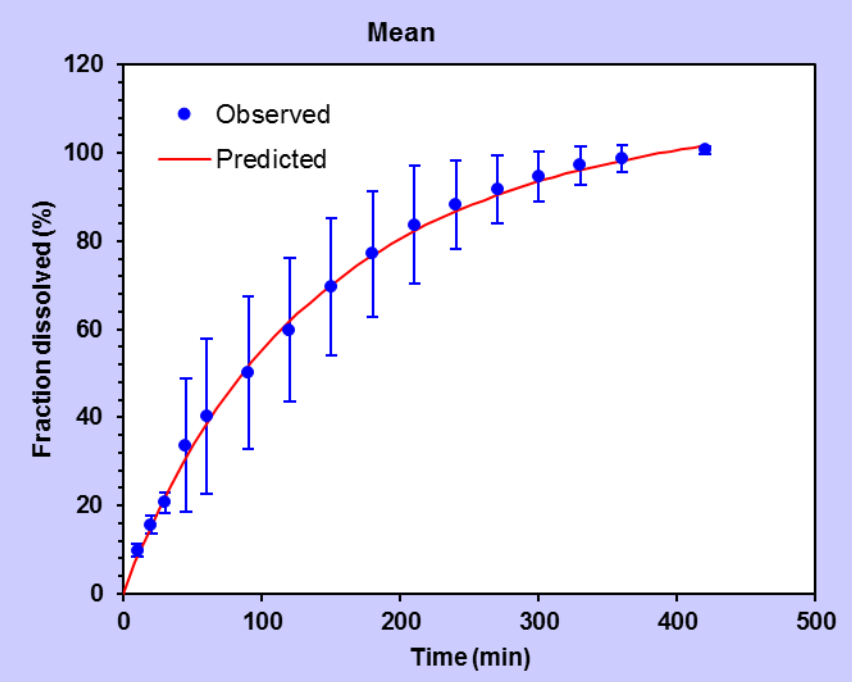

Supplementary materials – Model fitting summary – C*Pharm Mannidex 16700 (modelling the entire relevant dissolution data)

Graphical abstract of model fit presented as the fraction % of released carvedilol per tested tablet:

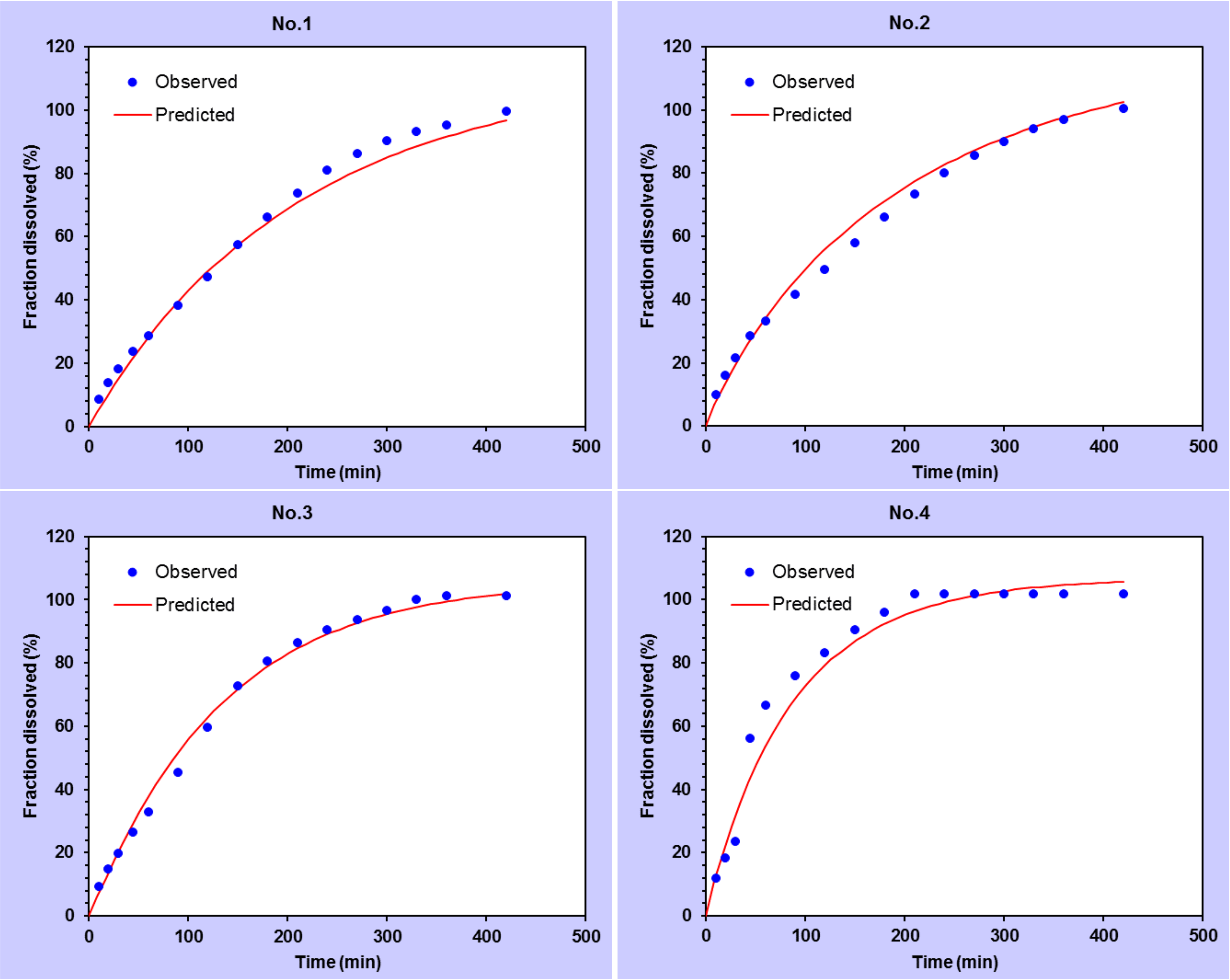

Supplementary materials – Model fitting summary – C*Pharm Mannidex 16700 (modelling the entire relevant dissolution data)

Model: **Weibull_4**

Model equation: $F = F_{max} \cdot \left[1 - e^{-\frac{(t-T_i)^\beta}{\alpha}}\right]$

Fitted model parameters per tested tablet (N = 4) with statistics – mean, standard deviation (SD), and relative standard deviation expressed in % (RSD%) (output from DDSolver):

Parameter	No.1	No.2	No.3	No.4	Mean	SD	RSD(%)
α	87.060	66.418	86.935	43.959	71.093	20.527	28.873
β	0.883	0.797	0.925	0.868	0.868	0.053	6.121
T_i	6.000	4.818	6.000	4.000	5.205	0.977	18.778
F_{max}	104.499	110.540	106.197	106.848	107.021	2.546	2.379

Number of dissolution data points (N), degrees of freedom (df), and selected goodness of fit criteria – Pearson correlation coefficient (R), coefficient of determination (R^2), adjusted coefficient of determination ($R^2_{adjusted}$), and residual sum of squares (RSS) (manual calculation in MS Excel):

Parameter	No.1	No.2	No.3	No.4
N	16	16	16	16
df	12	12	12	12
R	0.990626552	0.993410159	0.994771367	0.986441786
R^2	0.981340966	0.986863744	0.989570072	0.973067398
$R^2_{adjusted}$	0.976676207	0.98357968	0.98696259	0.966334247
RSS	317.7854792	329.8346357	198.7288807	452.0467109

Graphical abstract of model fit presented as mean ± 1 SD of the fraction % of released carvedilol:

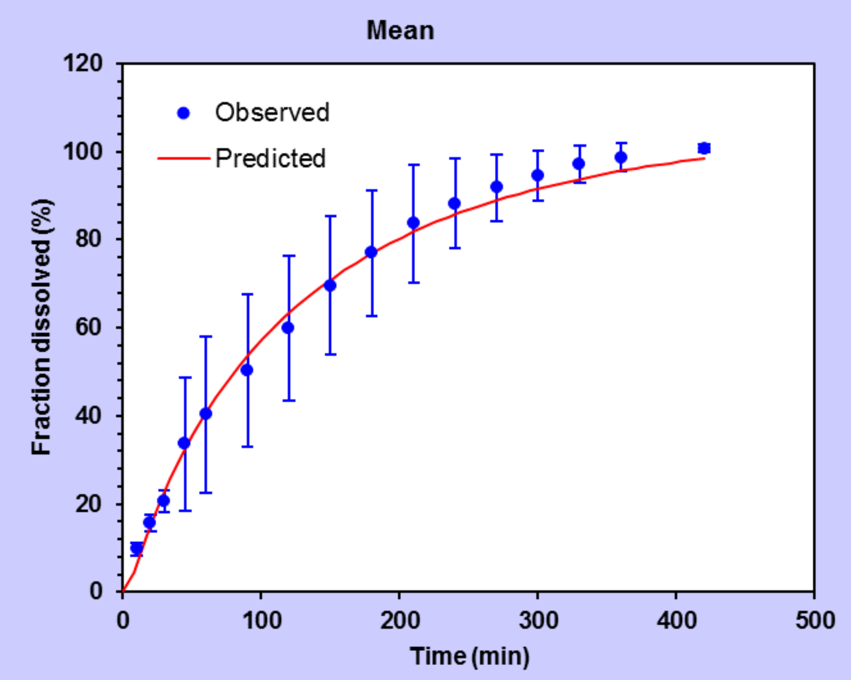

Supplementary materials – Model fitting summary – C*Pharm Mannidex 16700 (modelling the entire relevant dissolution data)

Graphical abstract of model fit presented as the fraction % of released carvedilol per tested tablet:

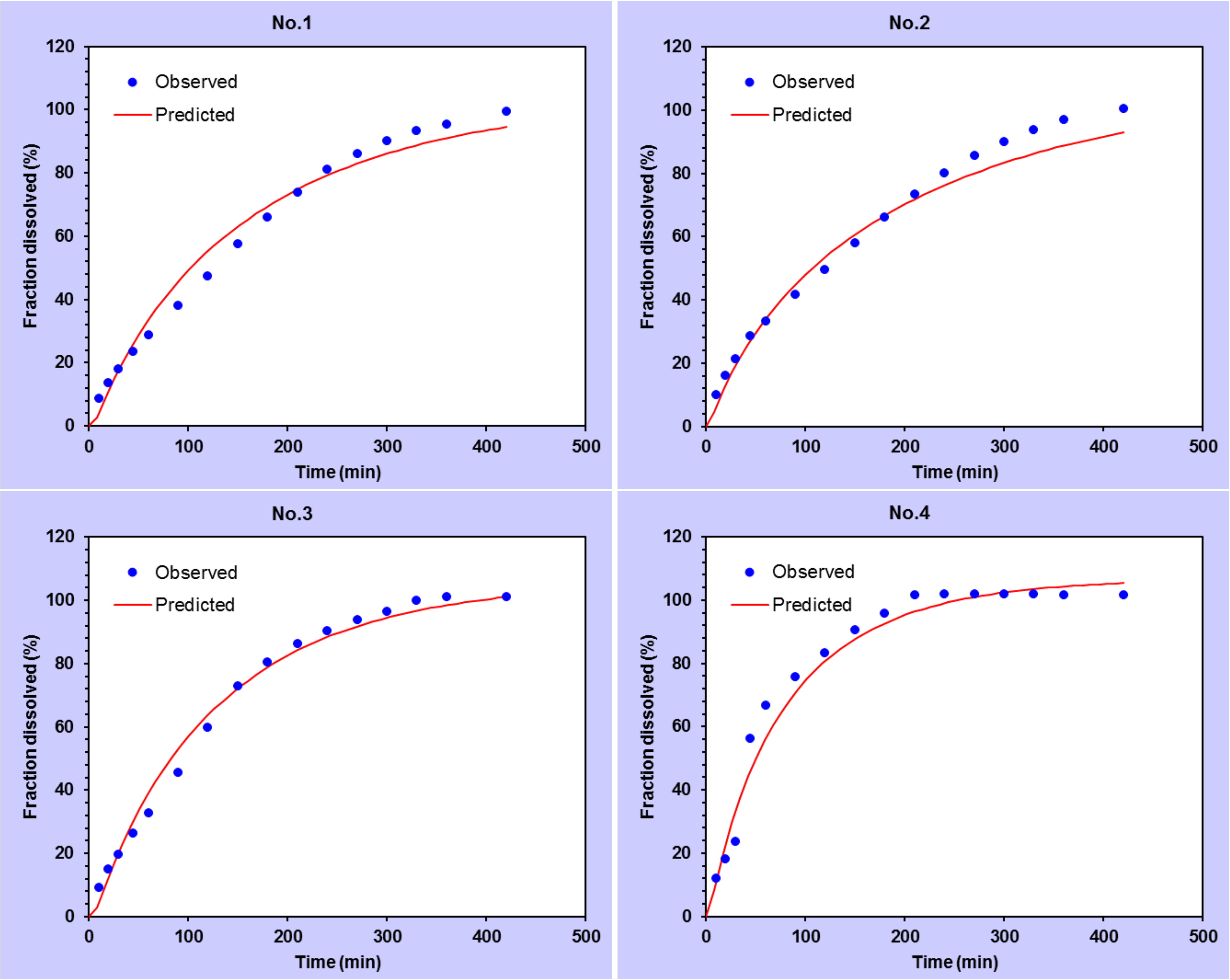

Supplementary materials – Model fitting summary – C*Pharm Mannidex 16700 (modelling the entire relevant dissolution data)

Model: **Logistic_1**

Model equation: $F = 100 \cdot \frac{e^{\alpha+\beta\cdot\log(t)}}{1+e^{\alpha+\beta\cdot\log(t)}}$

Fitted model parameters per tested tablet (N = 4) with statistics – mean, standard deviation (SD), and relative standard deviation expressed in % (RSD%) (output from DDSolver):

Parameter	No.1	No.2	No.3	No.4	Mean	SD	RSD(%)
α	-9.067	-6.142	-10.774	-6.595	-8.145	2.174	-26.688
β	4.300	3.243	5.470	4.065	4.269	0.919	21.536

Number of dissolution data points (N), degrees of freedom (df), and selected goodness of fit criteria – Pearson correlation coefficient (R), coefficient of determination (R^2), adjusted coefficient of determination ($R^2_{adjusted}$), and residual sum of squares (RSS) (manual calculation in MS Excel):

Parameter	No.1	No.2	No.3	No.4
N	16	16	16	16
df	14	14	14	14
R	0.993757988	0.966980715	0.992506798	0.990713207
R^2	0.987554939	0.935051703	0.985069744	0.981512658
$R^2_{adjusted}$	0.986666006	0.930412539	0.984003297	0.980192134
RSS	865.3043026	1028.254055	689.8493171	404.0327169

Graphical abstract of model fit presented as mean ± 1 SD of the fraction % of released carvedilol:

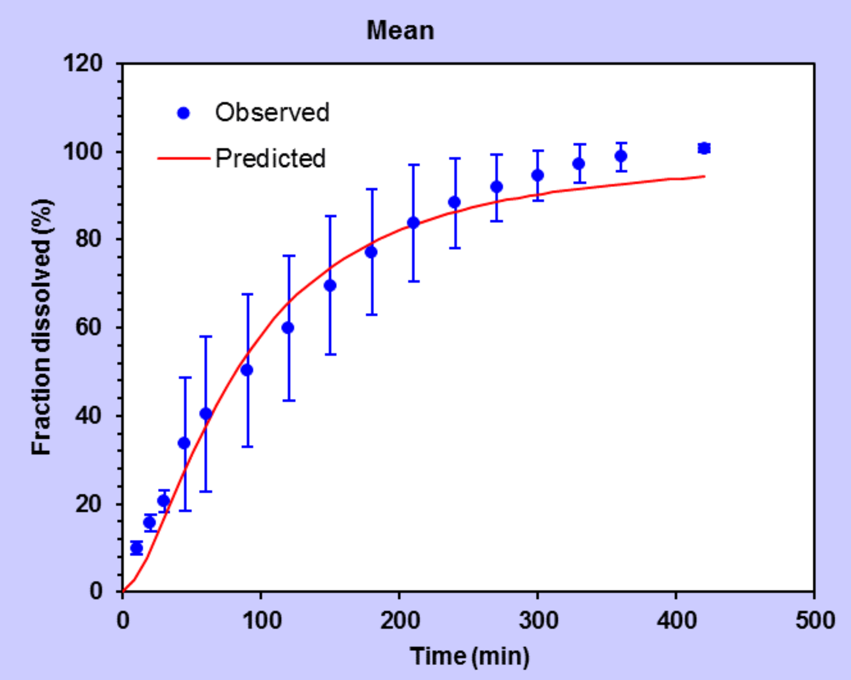

Supplementary materials – Model fitting summary – C*Pharm Mannidex 16700 (modelling the entire relevant dissolution data)

Graphical abstract of model fit presented as the fraction % of released carvedilol per tested tablet:

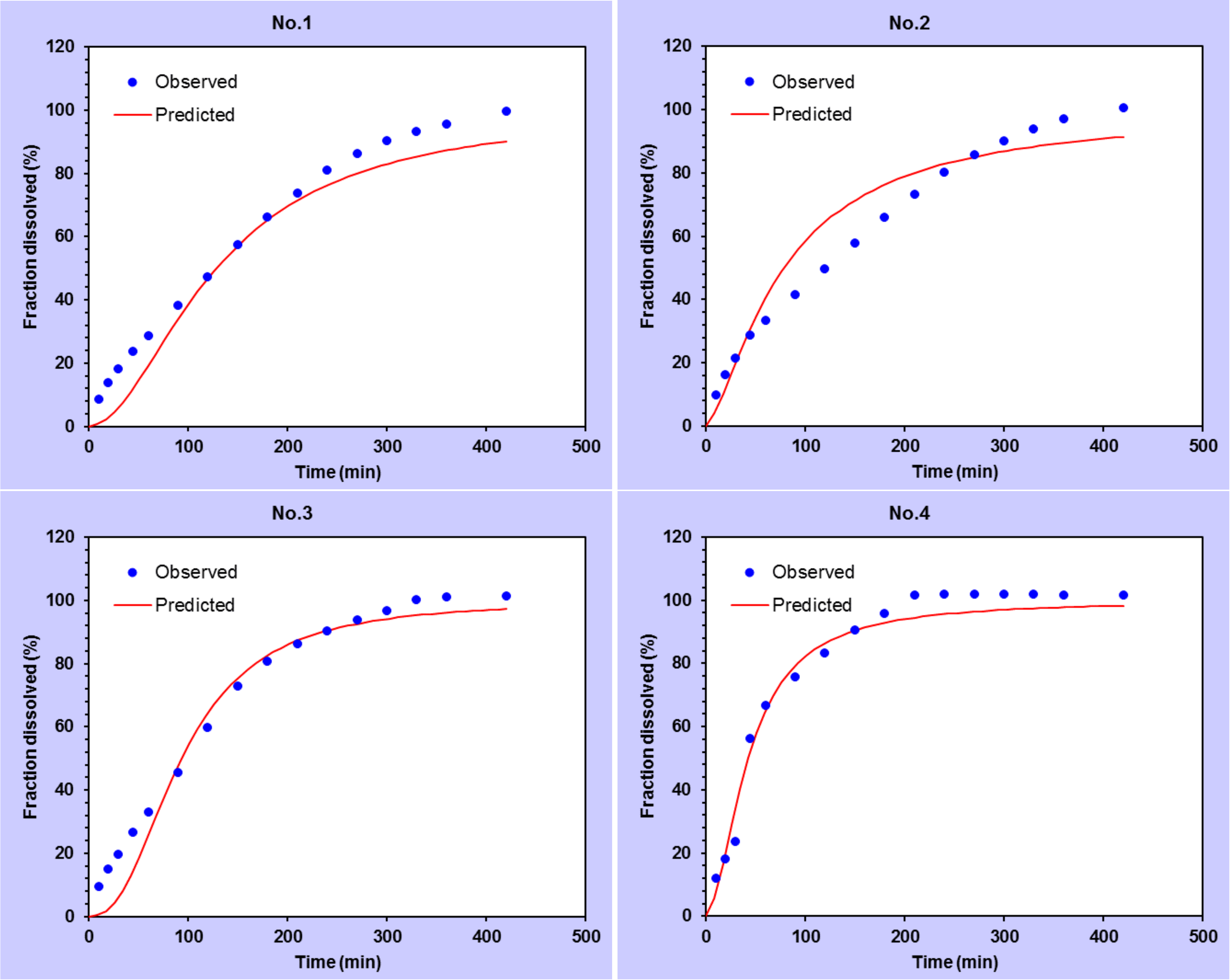

Supplementary materials – Model fitting summary – C*Pharm Mannidex 16700 (modelling the entire relevant dissolution data)

Model: **Logistic_2**

Model equation: $F = F_{max} \cdot \frac{e^{\alpha+\beta\cdot\log(t)}}{1+e^{\alpha+\beta\cdot\log(t)}}$

Fitted model parameters per tested tablet (N = 4) with statistics – mean, standard deviation (SD), and relative standard deviation expressed in % (RSD%) (output from DDSolver):

Parameter	No.1	No.2	No.3	No.4	Mean	SD	RSD(%)
α	-6.367	-6.754	-6.683	-6.076	-6.470	0.312	-4.816
β	3.235	3.317	3.561	3.631	3.436	0.190	5.534
F_{max}	104.499	100.418	106.197	106.848	104.491	2.890	2.766

Number of dissolution data points (N), degrees of freedom (df), and selected goodness of fit criteria – Pearson correlation coefficient (R), coefficient of determination (R^2), adjusted coefficient of determination ($R^2_{adjusted}$), and residual sum of squares (RSS) (manual calculation in MS Excel):

Parameter	No.1	No.2	No.3	No.4
N	16	16	16	16
df	13	13	13	13
R	0.977524952	0.984616295	0.984344559	0.992589674
R^2	0.955555031	0.969469248	0.968934212	0.985234261
$R^2_{adjusted}$	0.948717344	0.96477221	0.96415486	0.982962608
RSS	808.7599986	788.6835383	654.6127202	246.8750622

Graphical abstract of model fit presented as mean ± 1 SD of the fraction % of released carvedilol:

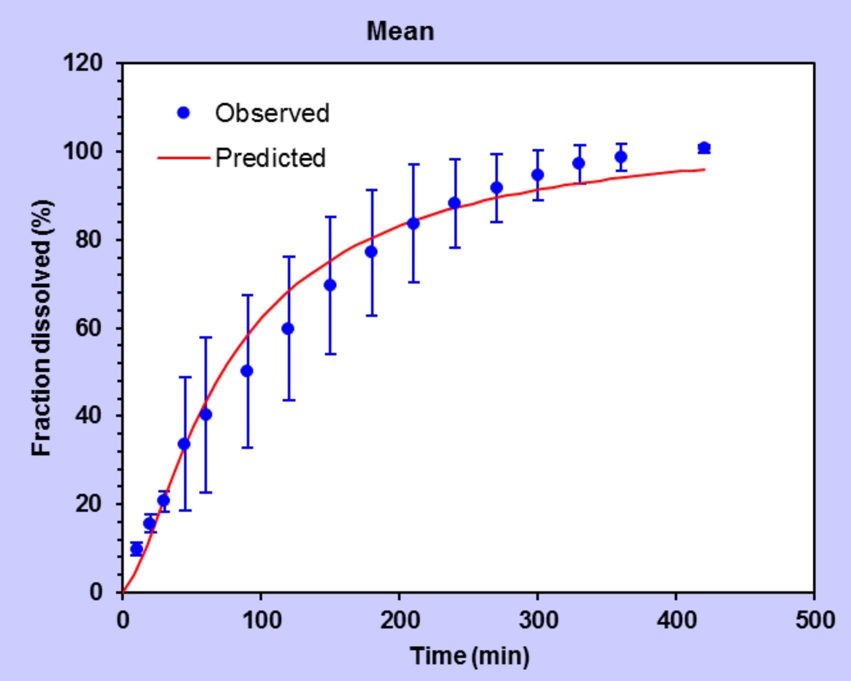

Supplementary materials – Model fitting summary – C*Pharm Mannidex 16700 (modelling the entire relevant dissolution data)

Graphical abstract of model fit presented as the fraction % of released carvedilol per tested tablet:

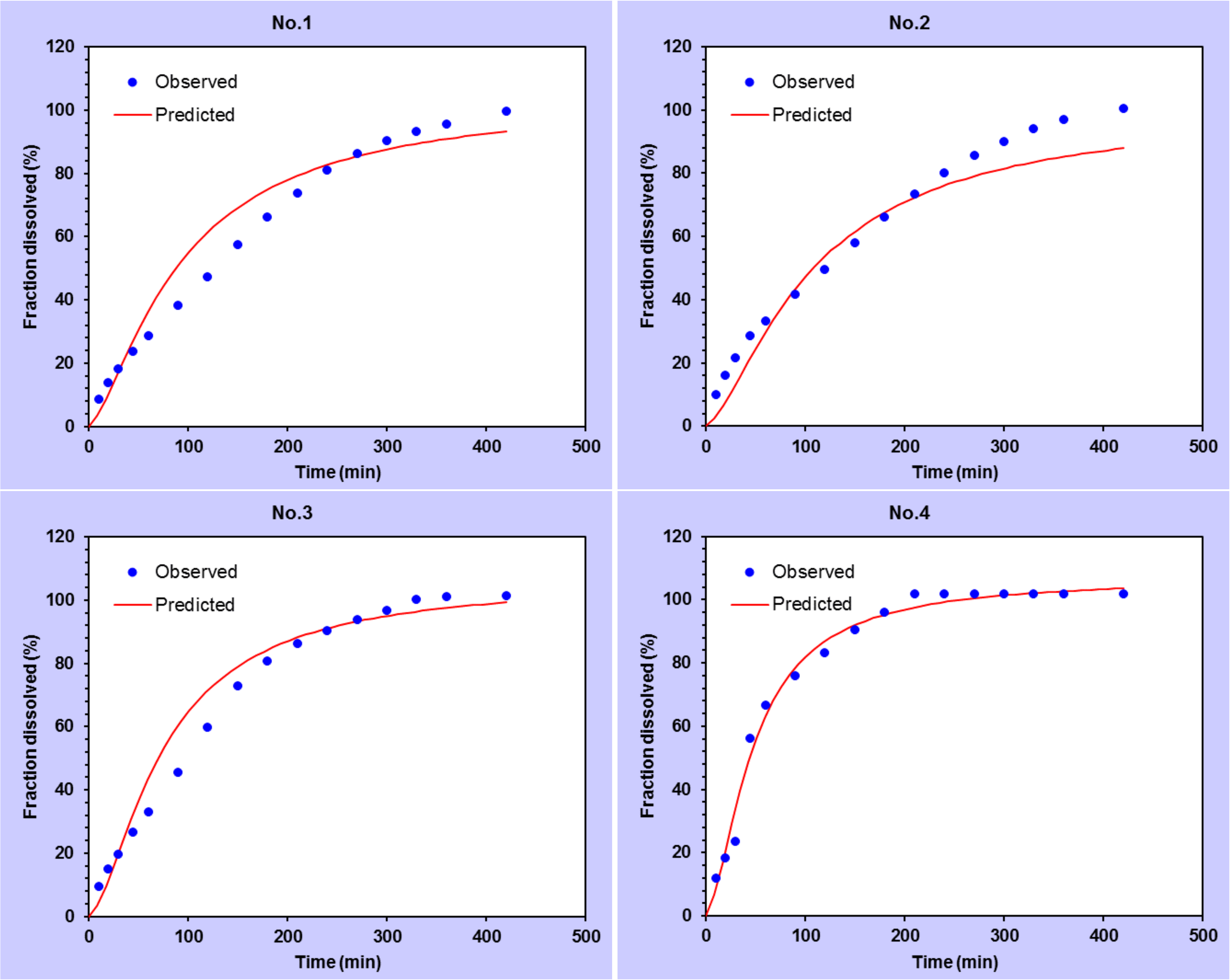

Supplementary materials – Model fitting summary – C*Pharm Mannidex 16700 (modelling the entire relevant dissolution data)

Model: **Logistic_3**

Model equation: $F = F_{max} \cdot \frac{1}{1+e^{-k\cdot(t-\gamma)}}$

Fitted model parameters per tested tablet (N = 4) with statistics – mean, standard deviation (SD), and relative standard deviation expressed in % (RSD%) (output from DDSolver):

Parameter	No.1	No.2	No.3	No.4	Mean	SD	RSD(%)
k	0.015	0.012	0.013	0.018	0.014	0.003	19.332
γ	138.259	131.150	127.261	60.965	114.409	35.919	31.395
F_{max}	104.425	102.023	106.197	106.848	104.873	2.159	2.058

Number of dissolution data points (N), degrees of freedom (df), and selected goodness of fit criteria – Pearson correlation coefficient (R), coefficient of determination (R^2), adjusted coefficient of determination ($R^2_{adjusted}$), and residual sum of squares (RSS) (manual calculation in MS Excel):

Parameter	No.1	No.2	No.3	No.4
N	16	16	16	16
df	13	13	13	13
R	0.996795711	0.995601562	0.98672658	0.962340691
R^2	0.993601689	0.99122247	0.973629344	0.926099606
$R^2_{adjusted}$	0.992617334	0.98987208	0.96957232	0.914730314
RSS	218.9192594	169.2272304	545.923684	1310.97974

Graphical abstract of model fit presented as mean ± 1 SD of the fraction % of released carvedilol:

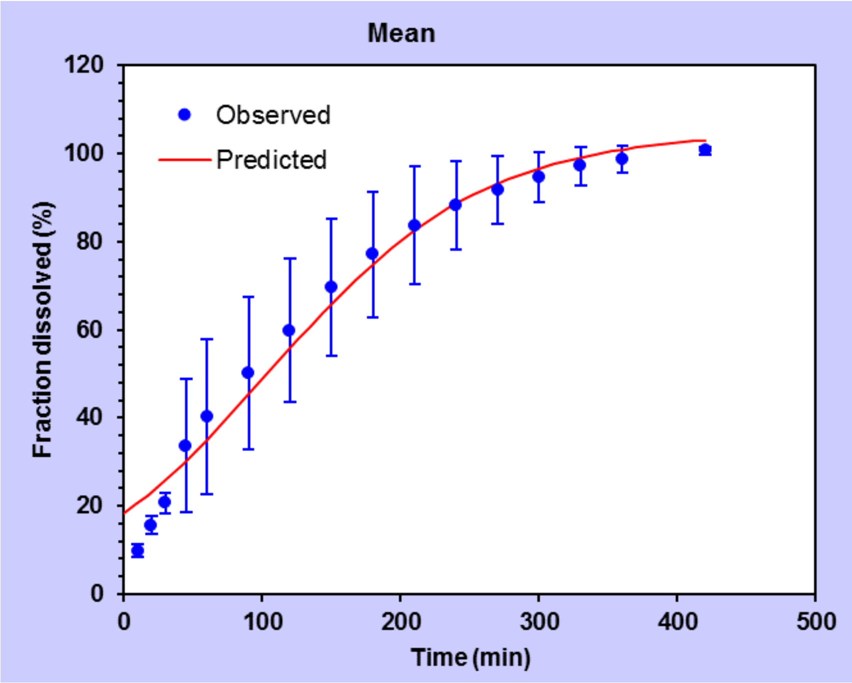

Supplementary materials – Model fitting summary – C*Pharm Mannidex 16700 (modelling the entire relevant dissolution data)

Graphical abstract of model fit presented as the fraction % of released carvedilol per tested tablet:

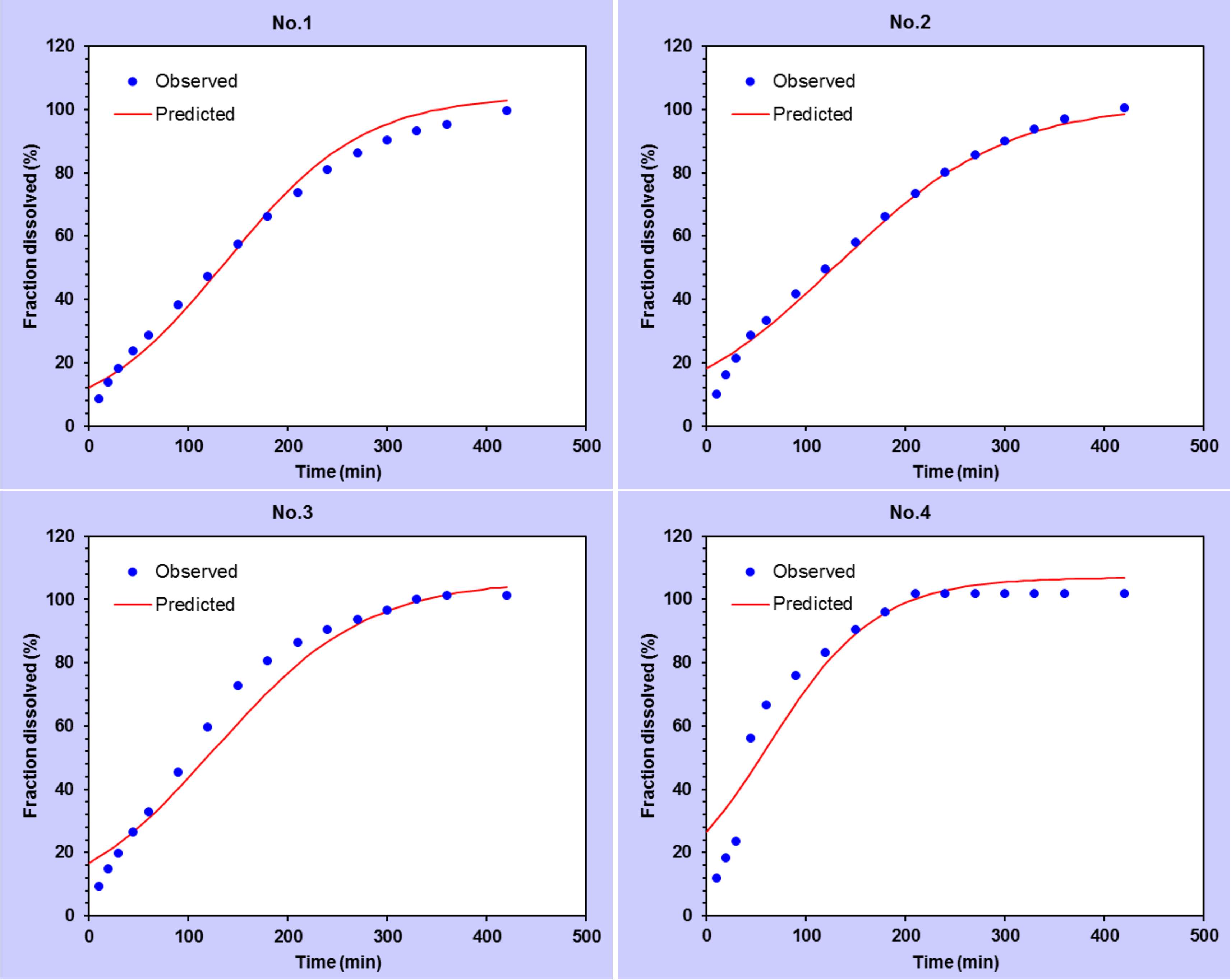

Supplementary materials – Model fitting summary – C*Pharm Mannidex 16700 (modelling the entire relevant dissolution data)

Model: **Gompertz_1**

Model equation: $F = 100 \cdot e^{-\alpha \cdot e^{-\beta \cdot \log(t)}}$

Fitted model parameters per tested tablet (N = 4) with statistics – mean, standard deviation (SD), and relative standard deviation expressed in % (RSD%) (output from DDSolver):

Parameter	No.1	No.2	No.3	No.4	Mean	SD	RSD(%)
α	200.263	80.555	801.689	77.939	290.111	345.792	119.193
β	2.890	2.387	3.898	3.025	3.050	0.629	20.610

Number of dissolution data points (N), degrees of freedom (df), and selected goodness of fit criteria – Pearson correlation coefficient (R), coefficient of determination (R^2), adjusted coefficient of determination ($R^2_{adjusted}$), and residual sum of squares (RSS) (manual calculation in MS Excel):

Parameter	No.1	No.2	No.3	No.4
N	16	16	16	16
df	14	14	14	14
R	0.966727668	0.969752216	0.959539565	0.979318208
R^2	0.934562385	0.94041936	0.920716178	0.959064152
$R^2_{adjusted}$	0.92988827	0.9361636	0.915053047	0.956140163
RSS	1146.637127	1297.488175	1728.640367	761.0009569

Graphical abstract of model fit presented as mean ± 1 SD of the fraction % of released carvedilol:

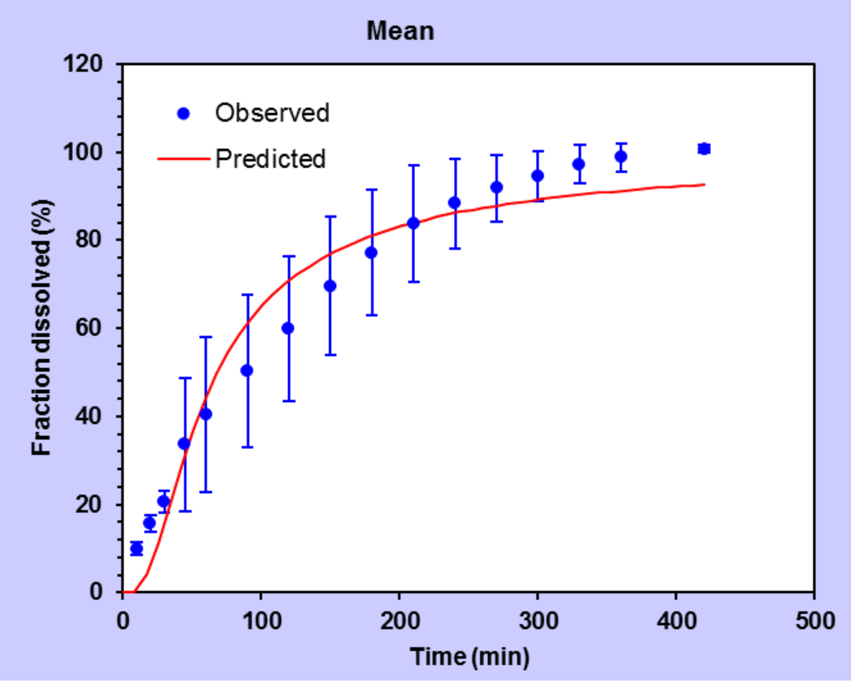

Supplementary materials – Model fitting summary – C*Pharm Mannidex 16700 (modelling the entire relevant dissolution data)

Graphical abstract of model fit presented as the fraction % of released carvedilol per tested tablet:

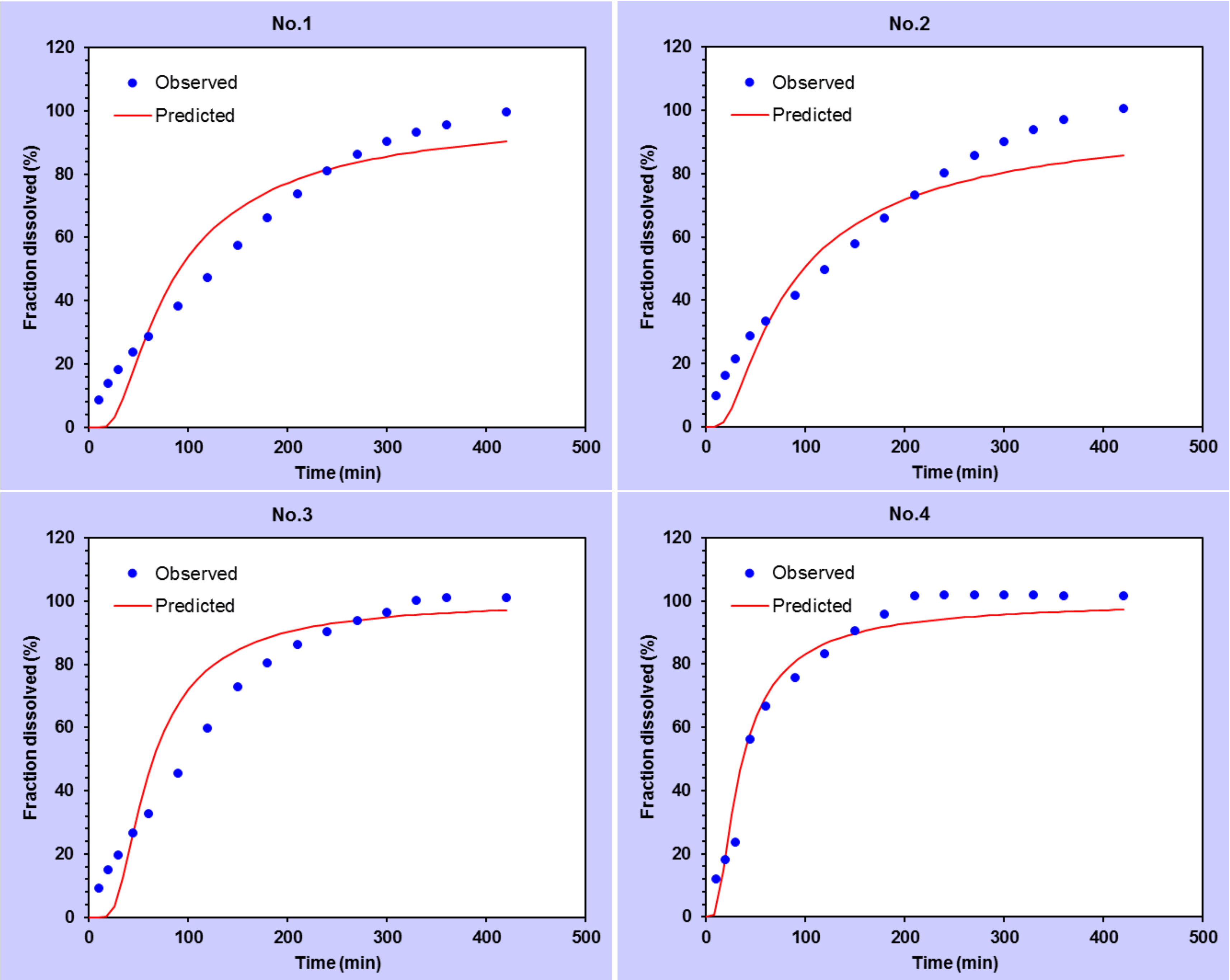

Supplementary materials – Model fitting summary – C*Pharm Mannidex 16700 (modelling the entire relevant dissolution data)

Model: **Gompertz_2**

Model equation: $F = F_{max} \cdot e^{-\alpha \cdot e^{-\beta \cdot \log(t)}}$

Fitted model parameters per tested tablet (N = 4) with statistics – mean, standard deviation (SD), and relative standard deviation expressed in % (RSD%) (output from DDSolver):

Parameter	No.1	No.2	No.3	No.4	Mean	SD	RSD(%)
α	79.603	64.073	118.611	67.873	82.540	24.939	30.214
β	2.296	2.202	2.636	2.862	2.499	0.305	12.208
F_{max}	104.499	105.467	106.197	106.848	105.753	1.008	0.954

Number of dissolution data points (N), degrees of freedom (df), and selected goodness of fit criteria – Pearson correlation coefficient (R), coefficient of determination (R^2), adjusted coefficient of determination ($R^2_{adjusted}$), and residual sum of squares (RSS) (manual calculation in MS Excel):

Parameter	No.1	No.2	No.3	No.4
N	16	16	16	16
df	13	13	13	13
R	0.979568461	0.976725474	0.985980325	0.985155741
R^2	0.959554371	0.953992651	0.972157201	0.970531833
$R^2_{adjusted}$	0.953331966	0.946914598	0.967873693	0.965998269
RSS	1111.221063	1258.677344	802.9390033	510.9552977

Graphical abstract of model fit presented as mean ± 1 SD of the fraction % of released carvedilol:

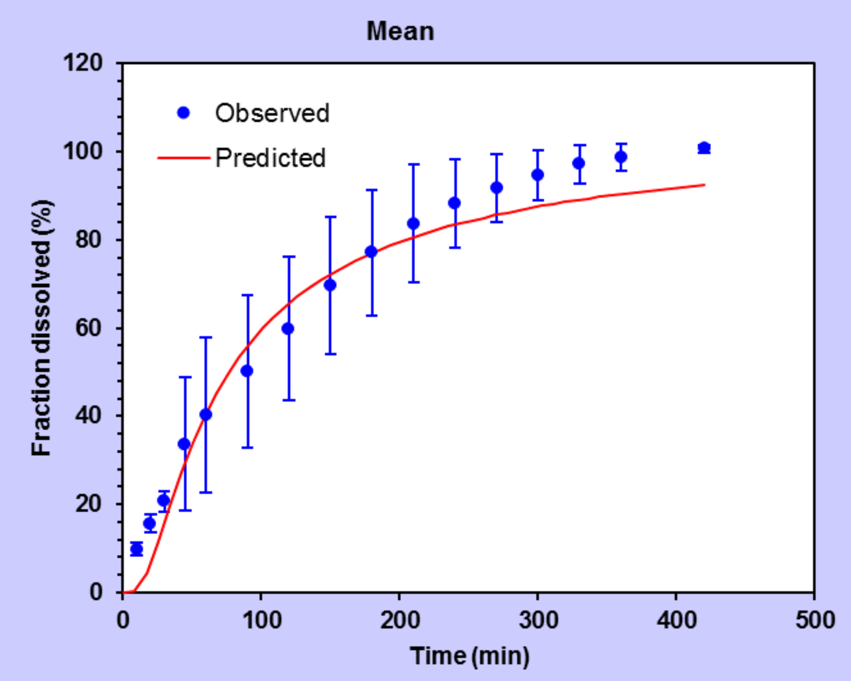

Supplementary materials – Model fitting summary – C*Pharm Mannidex 16700 (modelling the entire relevant dissolution data)

Graphical abstract of model fit presented as the fraction % of released carvedilol per tested tablet:

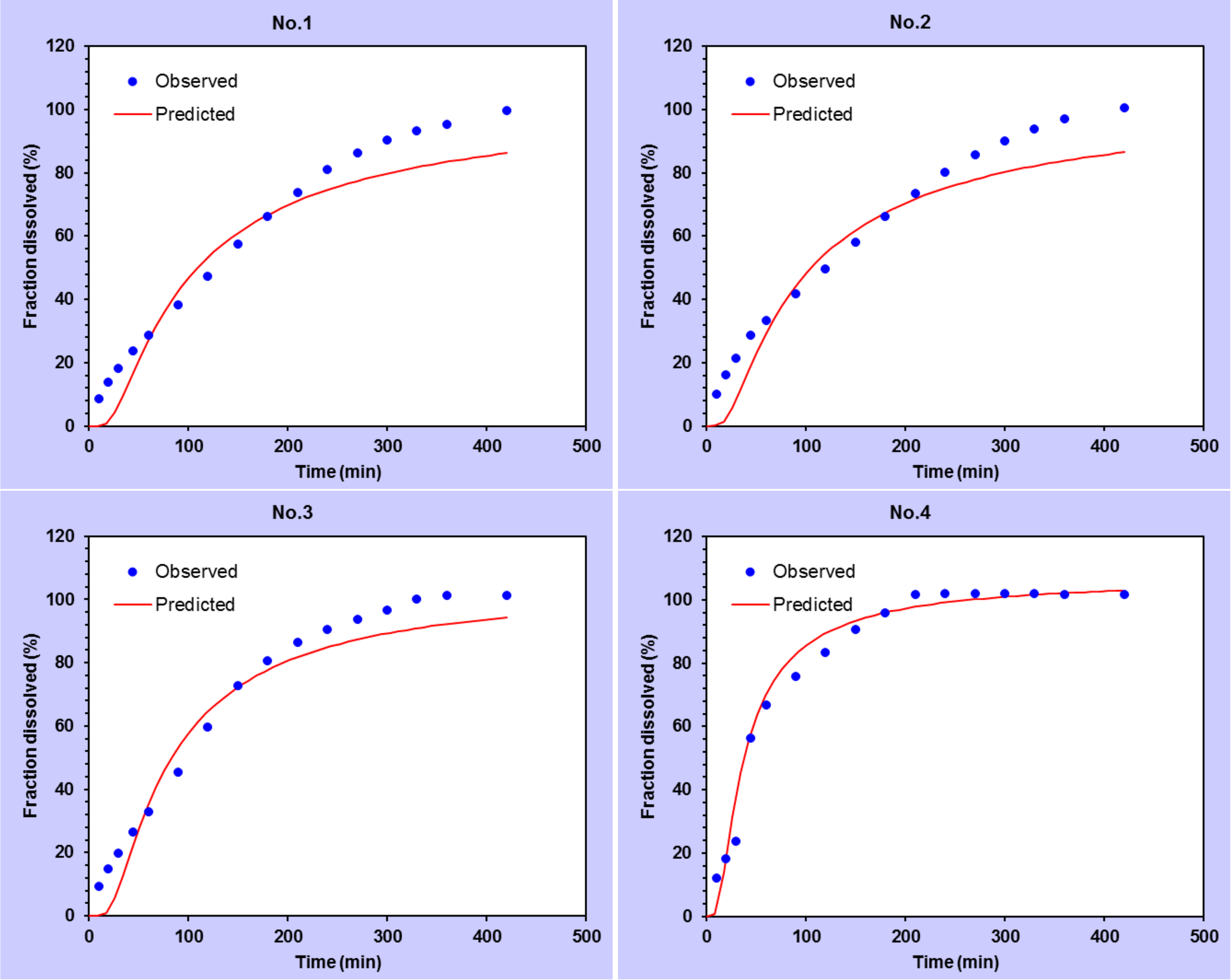

Supplementary materials – Model fitting summary – C*Pharm Mannidex 16700 (modelling the entire relevant dissolution data)

Model: **Gompertz_3**

Model equation: $F = F_{max} \cdot e^{-e^{-k \cdot (t-\gamma)}}$

Fitted model parameters per tested tablet (N = 4) with statistics – mean, standard deviation (SD), and relative standard deviation expressed in % (RSD%) (output from DDSolver):

Parameter	No.1	No.2	No.3	No.4	Mean	SD	RSD(%)
k	0.009	0.009	0.013	0.010	0.010	0.002	20.037
γ	94.608	88.965	77.824	11.387	68.196	38.509	56.469
F_{max}	104.499	105.467	105.644	106.848	105.615	0.964	0.913

Number of dissolution data points (N), degrees of freedom (df), and selected goodness of fit criteria – Pearson correlation coefficient (R), coefficient of determination (R^2), adjusted coefficient of determination ($R^2_{adjusted}$), and residual sum of squares (RSS) (manual calculation in MS Excel):

Parameter	No.1	No.2	No.3	No.4
N	16	16	16	16
df	13	13	13	13
R	0.999245159	0.997555511	0.999325274	0.95056608
R^2	0.998490889	0.995116998	0.998651003	0.903575872
$R^2_{adjusted}$	0.998258718	0.994365766	0.998443465	0.888741391
RSS	23.95664776	75.58294358	130.2983046	2308.850459

Graphical abstract of model fit presented as mean ± 1 SD of the fraction % of released carvedilol:

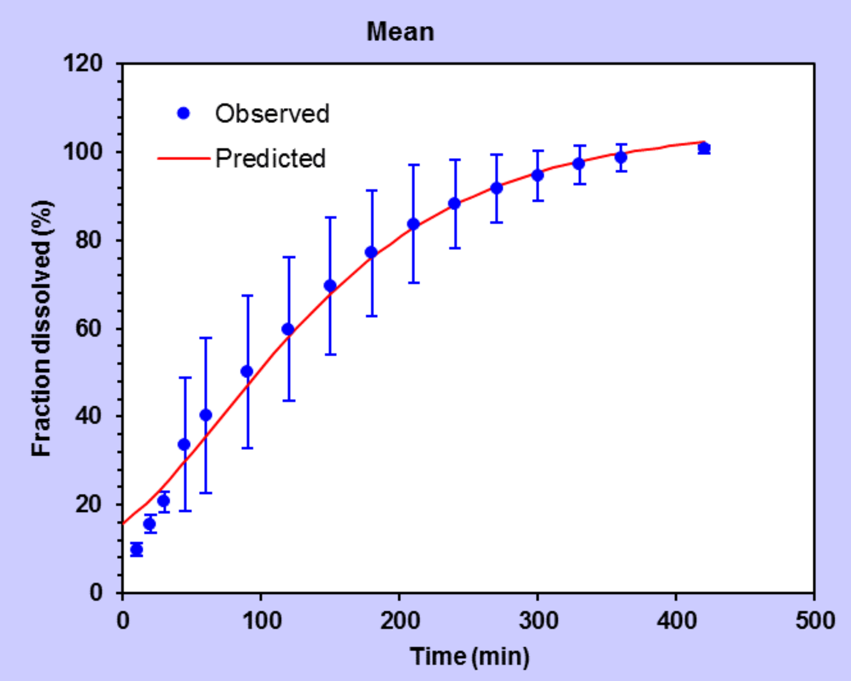

Supplementary materials – Model fitting summary – C*Pharm Mannidex 16700 (modelling the entire relevant dissolution data)

Graphical abstract of model fit presented as the fraction % of released carvedilol per tested tablet:

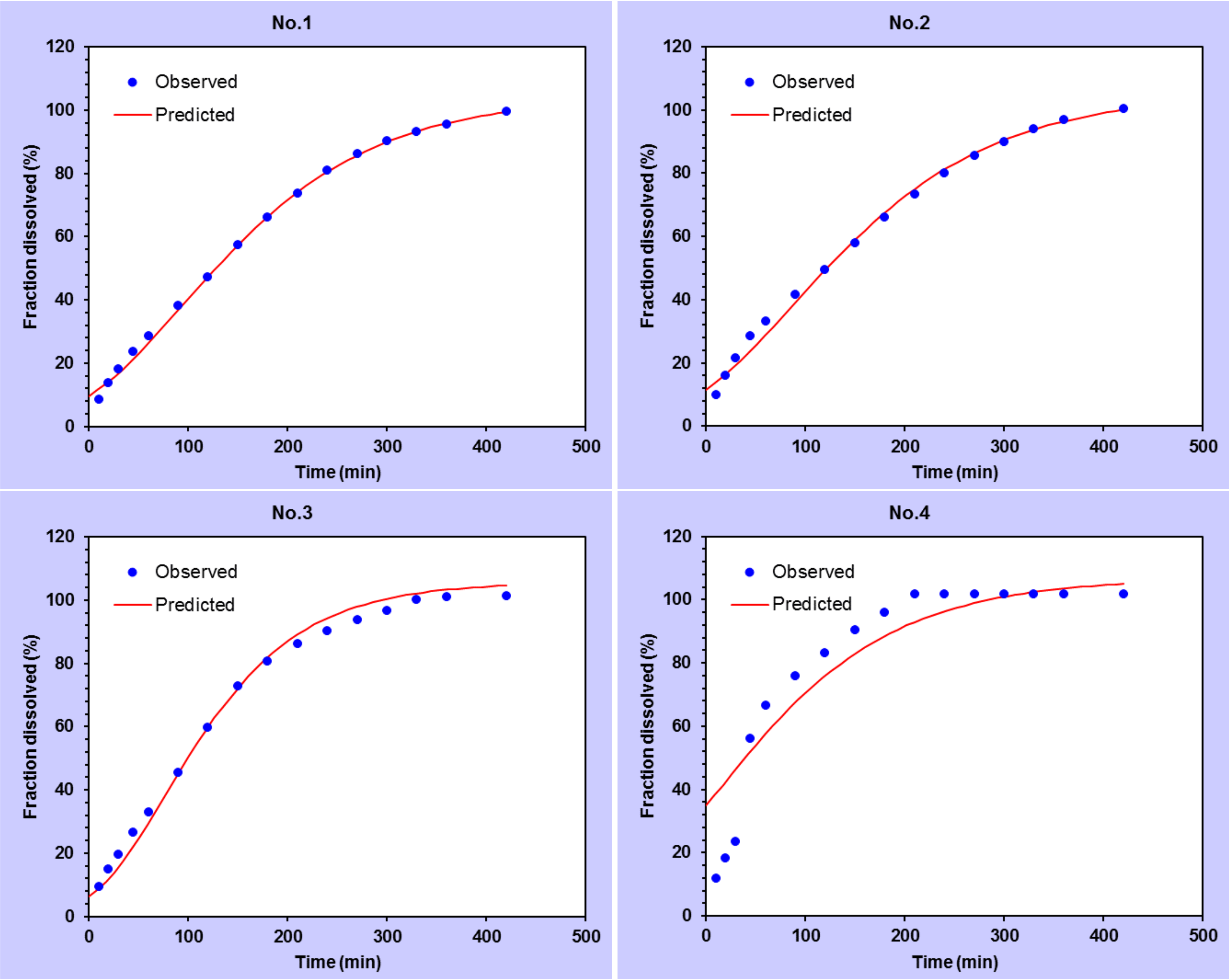

Supplementary materials – Model fitting summary – C*Pharm Mannidex 16700 (modelling the entire relevant dissolution data)

Model: **Gompertz_4**

Model equation: $F = F_{max} \cdot e^{-\beta \cdot e^{-k \cdot t}}$

Fitted model parameters per tested tablet (N = 4) with statistics – mean, standard deviation (SD), and relative standard deviation expressed in % (RSD%) (output from DDSolver):

Parameter	No.1	No.2	No.3	No.4	Mean	SD	RSD(%)
k	0.009	0.009	0.010	0.010	0.010	0.001	6.492
β	2.395	2.207	2.128	1.078	1.952	0.593	30.397
F_{max}	104.499	105.467	106.197	106.848	105.753	1.008	0.954

Number of dissolution data points (N), degrees of freedom (df), and selected goodness of fit criteria – Pearson correlation coefficient (R), coefficient of determination (R^2), adjusted coefficient of determination ($R^2_{adjusted}$), and residual sum of squares (RSS) (manual calculation in MS Excel):

Parameter	No.1	No.2	No.3	No.4
N	16	16	16	16
df	13	13	13	13
R	0.999245159	0.997555511	0.997213574	0.951714722
R^2	0.998490889	0.995116998	0.994434913	0.905760912
$R^2_{adjusted}$	0.998258718	0.994365766	0.993578746	0.89126259
RSS	23.95664776	75.58294358	142.3109704	2440.174444

Graphical abstract of model fit presented as mean ± 1 SD of the fraction % of released carvedilol:

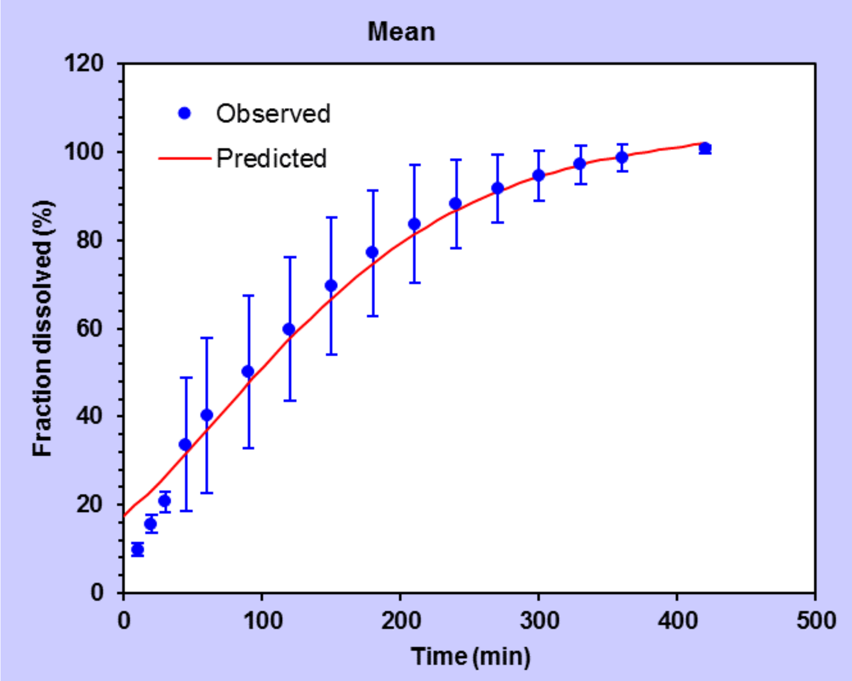

Supplementary materials – Model fitting summary – C*Pharm Mannidex 16700 (modelling the entire relevant dissolution data)

Graphical abstract of model fit presented as the fraction % of released carvedilol per tested tablet:

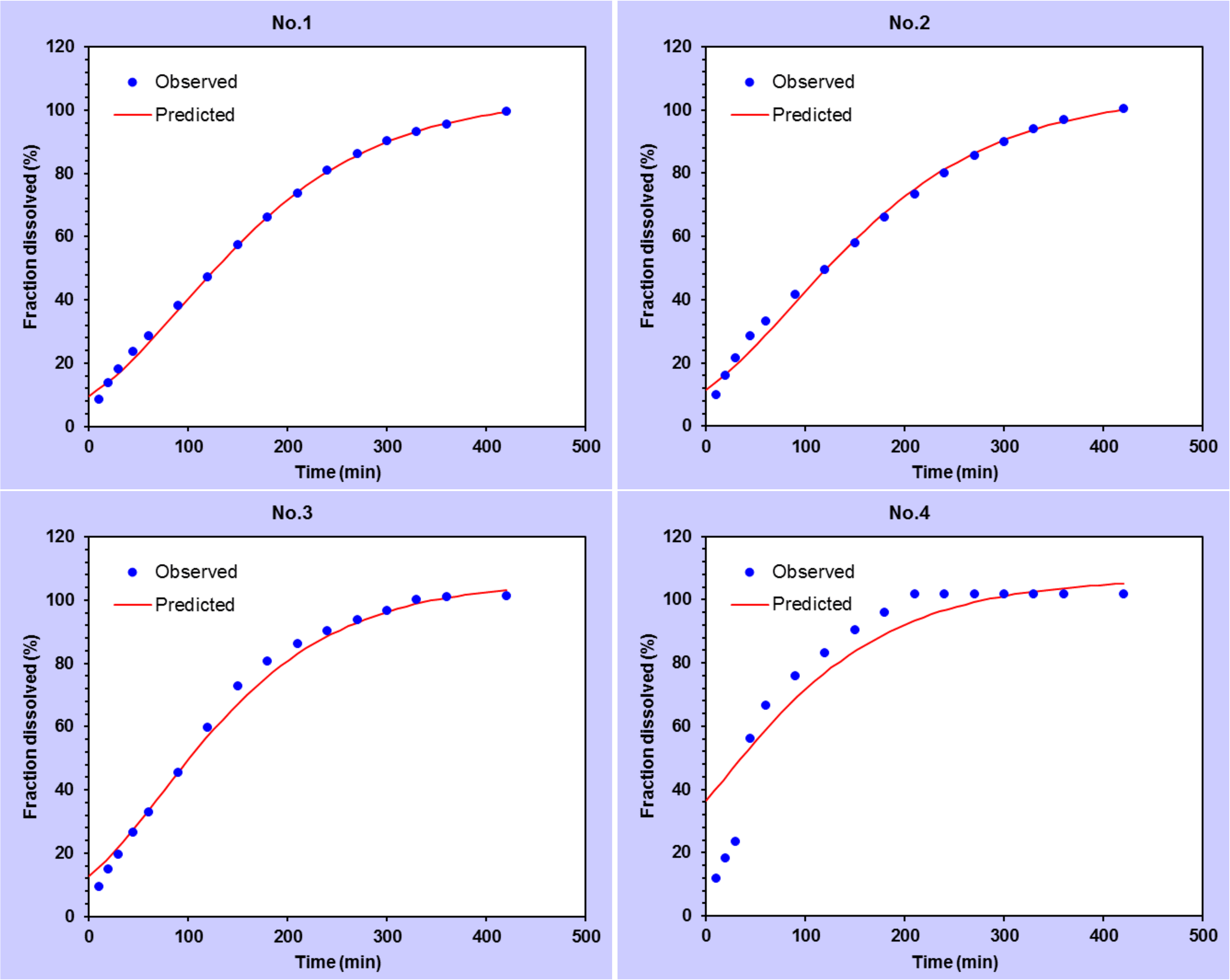

Supplementary materials – Model fitting summary – C*Pharm Mannidex 16700 (modelling the entire relevant dissolution data)

Model: **Probit_1**

Model equation: $F = 100 \cdot \phi[\alpha + \beta \cdot \log(t)]$

Fitted model parameters per tested tablet (N = 4) with statistics – mean, standard deviation (SD), and relative standard deviation expressed in % (RSD%) (output from DDSolver):

Parameter	No.1	No.2	No.3	No.4	Mean	SD	RSD(%)
α	-5.143	-3.594	-5.719	-3.853	-4.577	1.019	-22.266
β	2.423	1.891	2.862	2.369	2.386	0.397	16.648

Number of dissolution data points (N), degrees of freedom (df), and selected goodness of fit criteria – Pearson correlation coefficient (R), coefficient of determination (R^2), adjusted coefficient of determination ($R^2_{adjusted}$), and residual sum of squares (RSS) (manual calculation in MS Excel):

Parameter	No.1	No.2	No.3	No.4
N	16	16	16	16
df	14	14	14	14
R	0.994441941	0.970080587	0.996474152	0.990901527
R^2	0.988914773	0.941056346	0.992960736	0.981885837
$R^2_{adjusted}$	0.988122971	0.936846085	0.992457932	0.980591968
RSS	1137.682495	906.5139568	756.447094	376.7378321

Graphical abstract of model fit presented as mean ± 1 SD of the fraction % of released carvedilol:

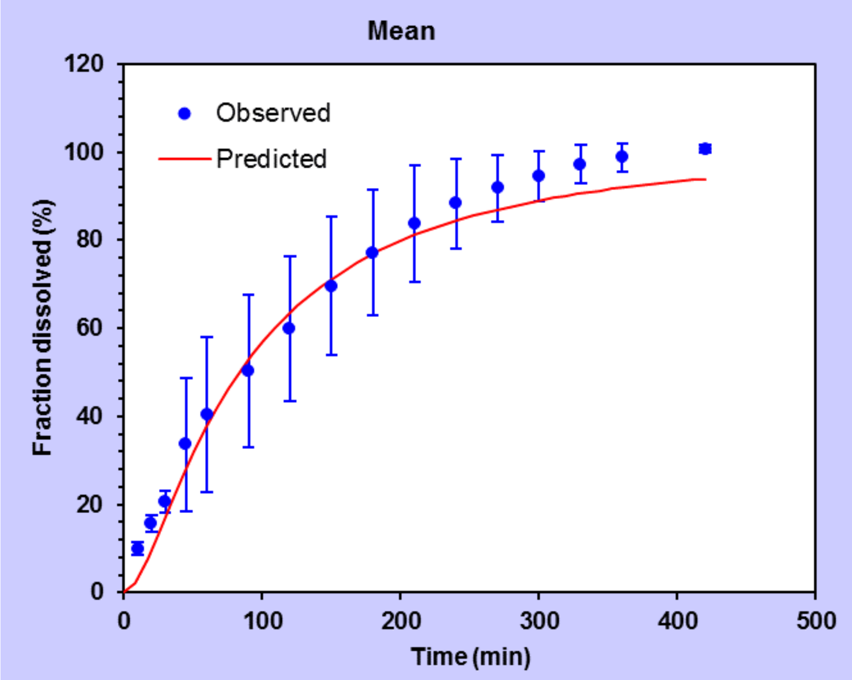

Supplementary materials – Model fitting summary – C*Pharm Mannidex 16700 (modelling the entire relevant dissolution data)

Graphical abstract of model fit presented as the fraction % of released carvedilol per tested tablet:

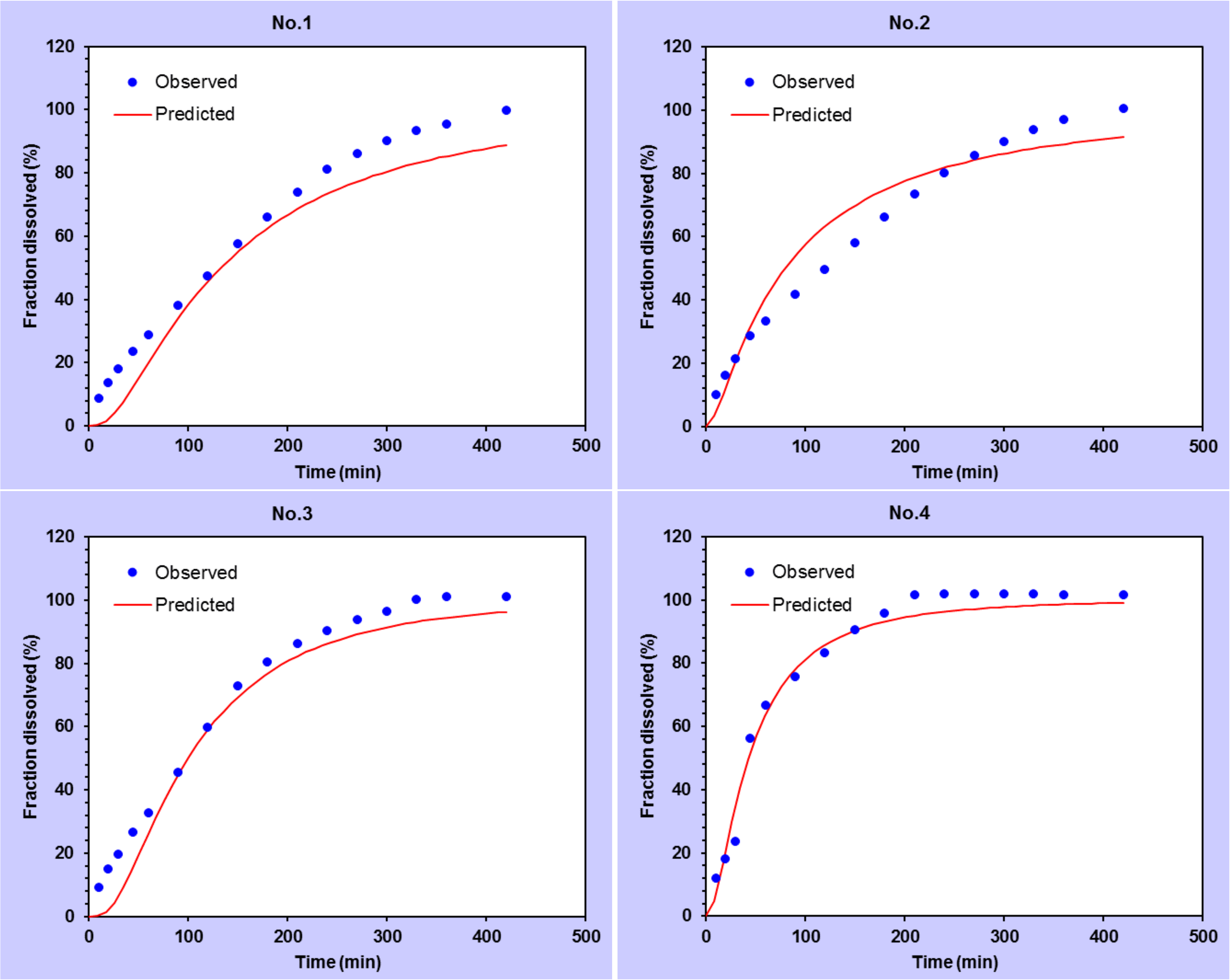

Supplementary materials – Model fitting summary – C*Pharm Mannidex 16700 (modelling the entire relevant dissolution data)

Model: **Probit_2**

Model equation: $F = F_{max} \cdot \phi[\alpha + \beta \cdot \log(t)]$

Fitted model parameters per tested tablet (N = 4) with statistics – mean, standard deviation (SD), and relative standard deviation expressed in % (RSD%) (output from DDSolver):

Parameter	No.1	No.2	No.3	No.4	Mean	SD	RSD(%)
α	-4.314	-3.495	-4.095	-3.473	-3.845	0.426	-11.071
β	2.015	1.794	1.924	2.069	1.950	0.120	6.167
F_{max}	113.784	105.467	128.714	106.848	113.703	10.648	9.365

Number of dissolution data points (N), degrees of freedom (df), and selected goodness of fit criteria – Pearson correlation coefficient (R), coefficient of determination (R^2), adjusted coefficient of determination ($R^2_{adjusted}$), and residual sum of squares (RSS) (manual calculation in MS Excel):

Parameter	No.1	No.2	No.3	No.4
N	16	16	16	16
df	13	13	13	13
R	0.993522188	0.975826499	0.997327622	0.991403245
R^2	0.987086337	0.952237357	0.994662386	0.982880394
$R^2_{adjusted}$	0.98509962	0.944889258	0.993841214	0.980246609
RSS	354.4905747	760.6790573	239.883179	286.3493042

Graphical abstract of model fit presented as mean ± 1 SD of the fraction % of released carvedilol:

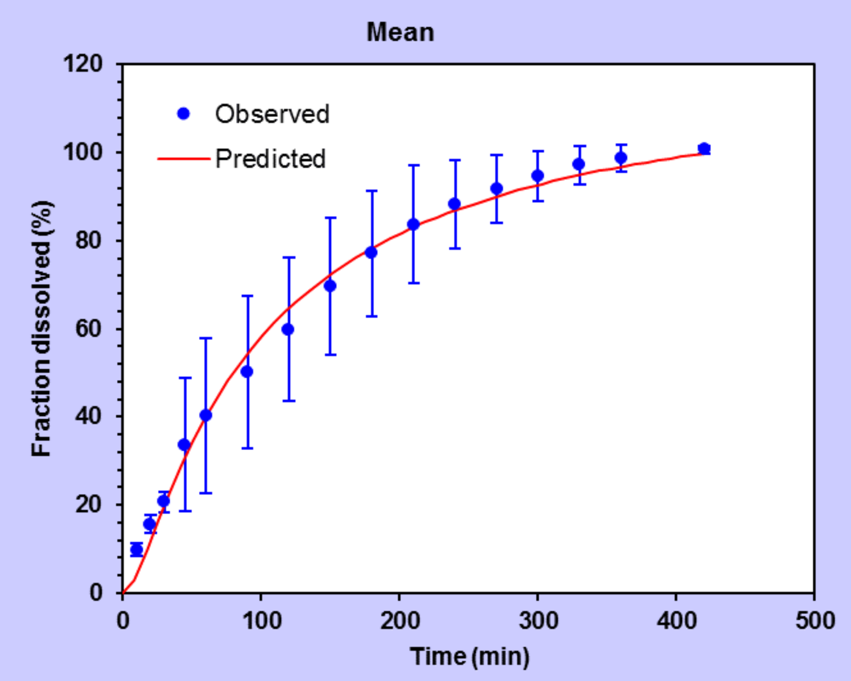

Supplementary materials – Model fitting summary – C*Pharm Mannidex 16700 (modelling the entire relevant dissolution data)

Graphical abstract of model fit presented as the fraction % of released carvedilol per tested tablet:

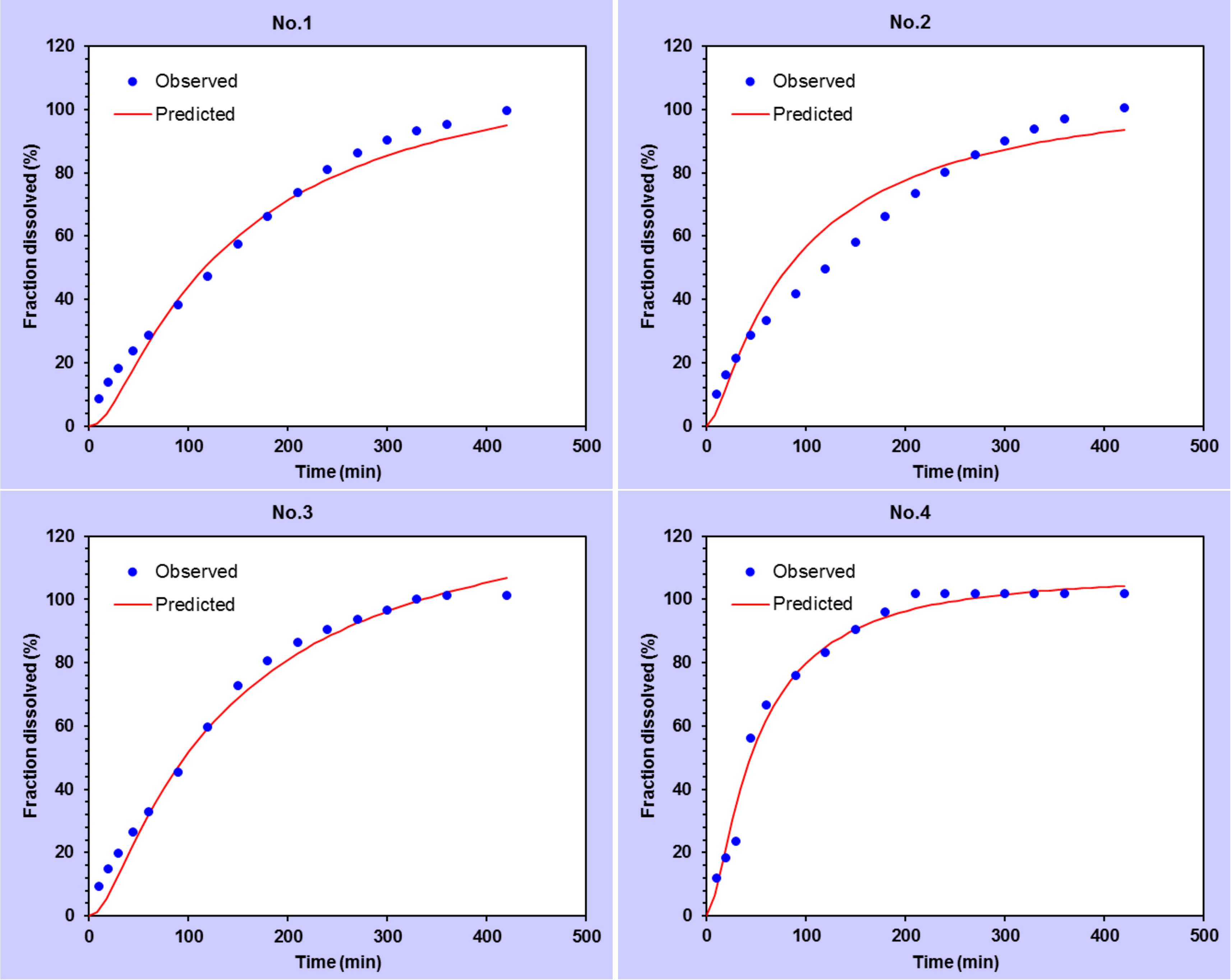

Supplementary materials – Model fitting summary – C*Pharm Mannidex 16700 (modelling up to app. 60 % of carvedilol released)

Model: **Zero–order**

Model equation: $F = k_0 \cdot t$

Fitted model parameters per tested tablet (N = 4) with statistics – mean, standard deviation (SD), and relative standard deviation expressed in % (RSD%) (output from DDSolver):

Parameter	No.1	No.2	No.3	No.4	Mean	SD	RSD(%)
k_0	0.433	0.475	0.521	0.830	0.564	0.181	31.995

Number of dissolution data points (N), degrees of freedom (df), and selected goodness of fit criteria – Pearson correlation coefficient (R), coefficient of determination (R^2), adjusted coefficient of determination ($R^2_{adjusted}$), and residual sum of squares (RSS) (manual calculation in MS Excel):

Parameter	No.1	No.2	No.3	No.4
N	7	7	7	7
df	6	6	6	6
R	0.996666001	0.985812477	0.999519153	0.933989999
R^2	0.993343118	0.971826239	0.999038537	0.872337318
$R^2_{adjusted}$	0.993343118	0.971826239	0.999038537	0.872337318
RSS	113.5106549	249.8308387	72.33885877	920.1172358

Graphical abstract of model fit presented as mean ± 1 SD of the fraction % of released carvedilol:

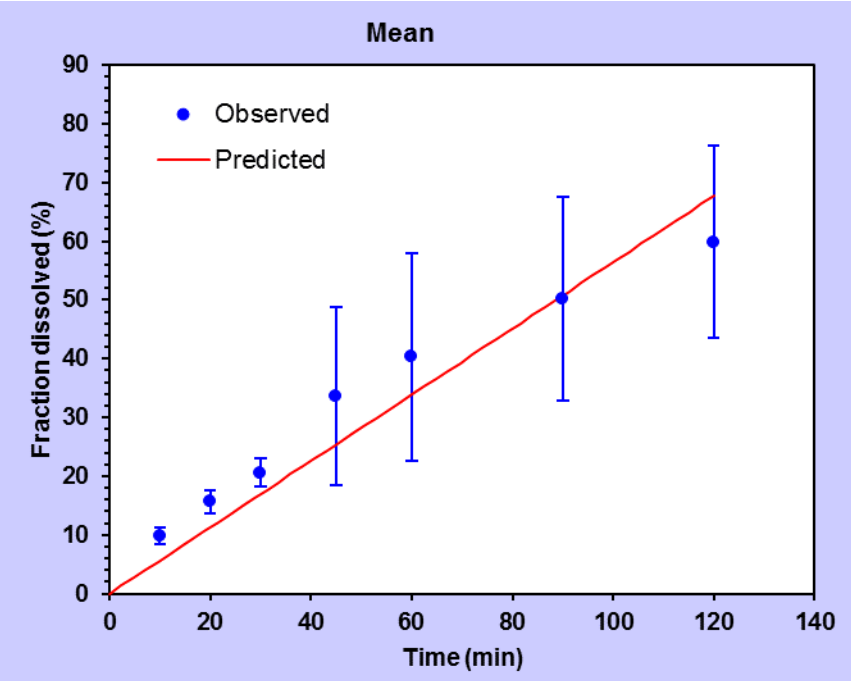

Supplementary materials – Model fitting summary – C*Pharm Mannidex 16700 (modelling up to app. 60 % of carvedilol released)

Graphical abstract of model fit presented as the fraction % of released carvedilol per tested tablet:

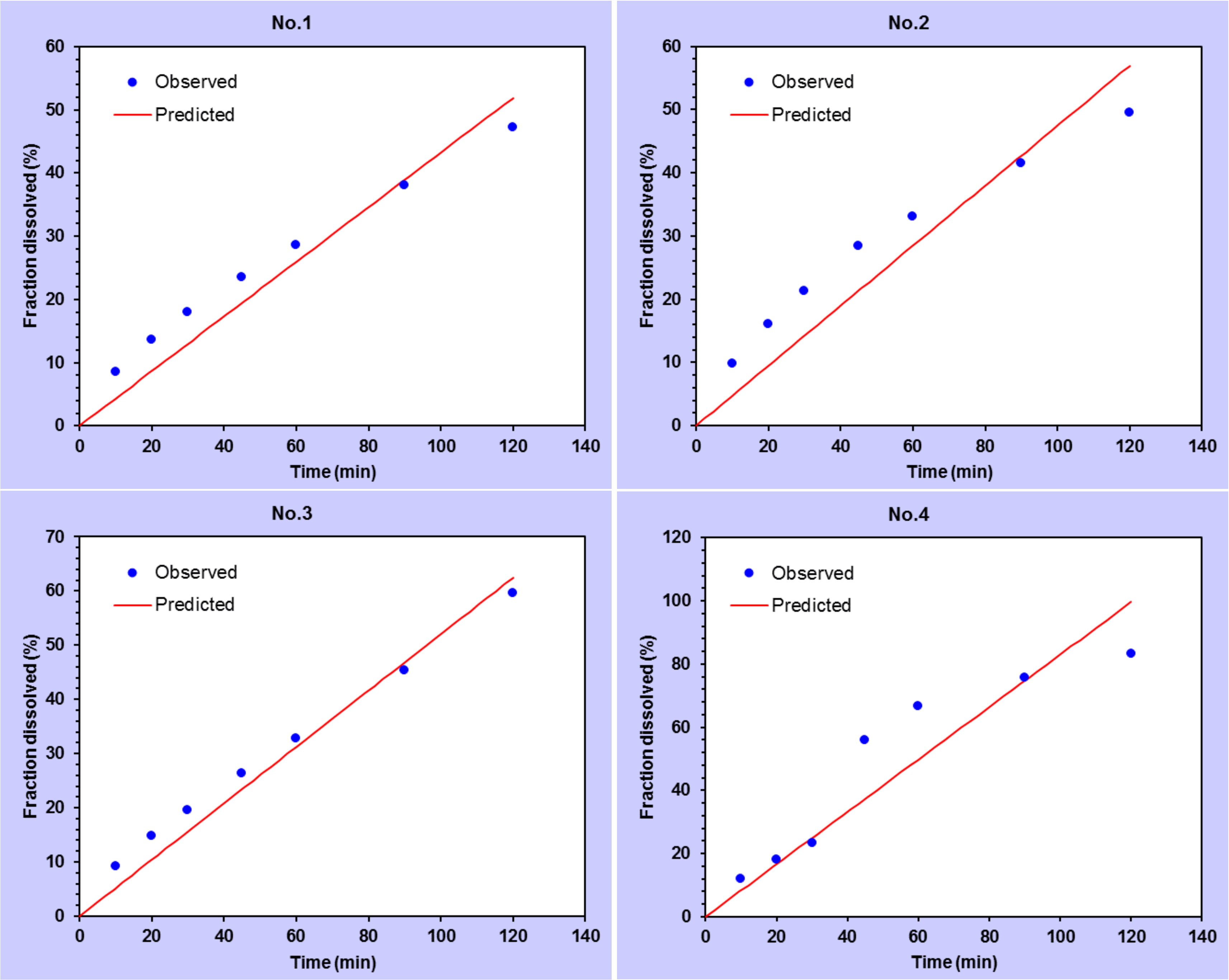

Supplementary materials – Model fitting summary – C*Pharm Mannidex 16700 (modelling up to app. 60 % of carvedilol released)

Model: **Zero–order with T_{lag}**

Model equation: $F = k_0 \cdot (t - T_{lag})$

Fitted model parameters per tested tablet (N = 4) with statistics – mean, standard deviation (SD), and relative standard deviation expressed in % (RSD%) (output from DDSolver):

Parameter	No.1	No.2	No.3	No.4	Mean	SD	RSD(%)
k_0	0.345	0.350	0.449	0.694	0.460	0.164	35.625
T_{lag}	-19.943	-28.123	-12.497	-15.351	-18.978	6.825	-35.960

Number of dissolution data points (N), degrees of freedom (df), and selected goodness of fit criteria – Pearson correlation coefficient (R), coefficient of determination (R^2), adjusted coefficient of determination ($R^2_{adjusted}$), and residual sum of squares (RSS) (manual calculation in MS Excel):

Parameter	No.1	No.2	No.3	No.4
N	7	7	7	7
df	5	5	5	5
R	0.996666001	0.985812477	0.999519153	0.933989999
R^2	0.993343118	0.971826239	0.999038537	0.872337318
$R^2_{adjusted}$	0.992011741	0.966191487	0.998846245	0.846804782
RSS	7.532039867	33.45328274	1.832649172	665.9089668

Graphical abstract of model fit presented as mean ± 1 SD of the fraction % of released carvedilol:

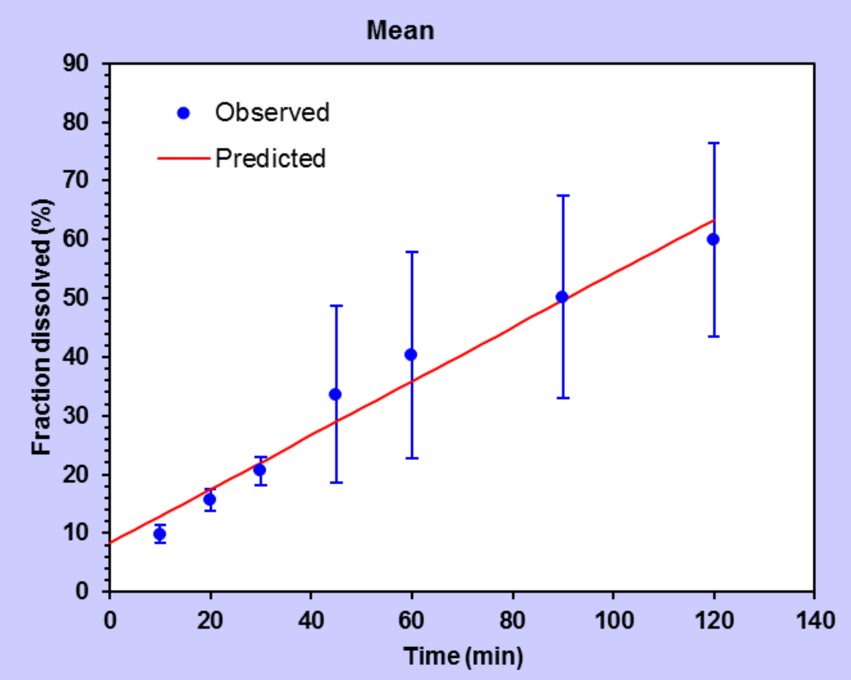

Supplementary materials – Model fitting summary – C*Pharm Mannidex 16700 (modelling up to app. 60 % of carvedilol released)

Graphical abstract of model fit presented as the fraction % of released carvedilol per tested tablet:

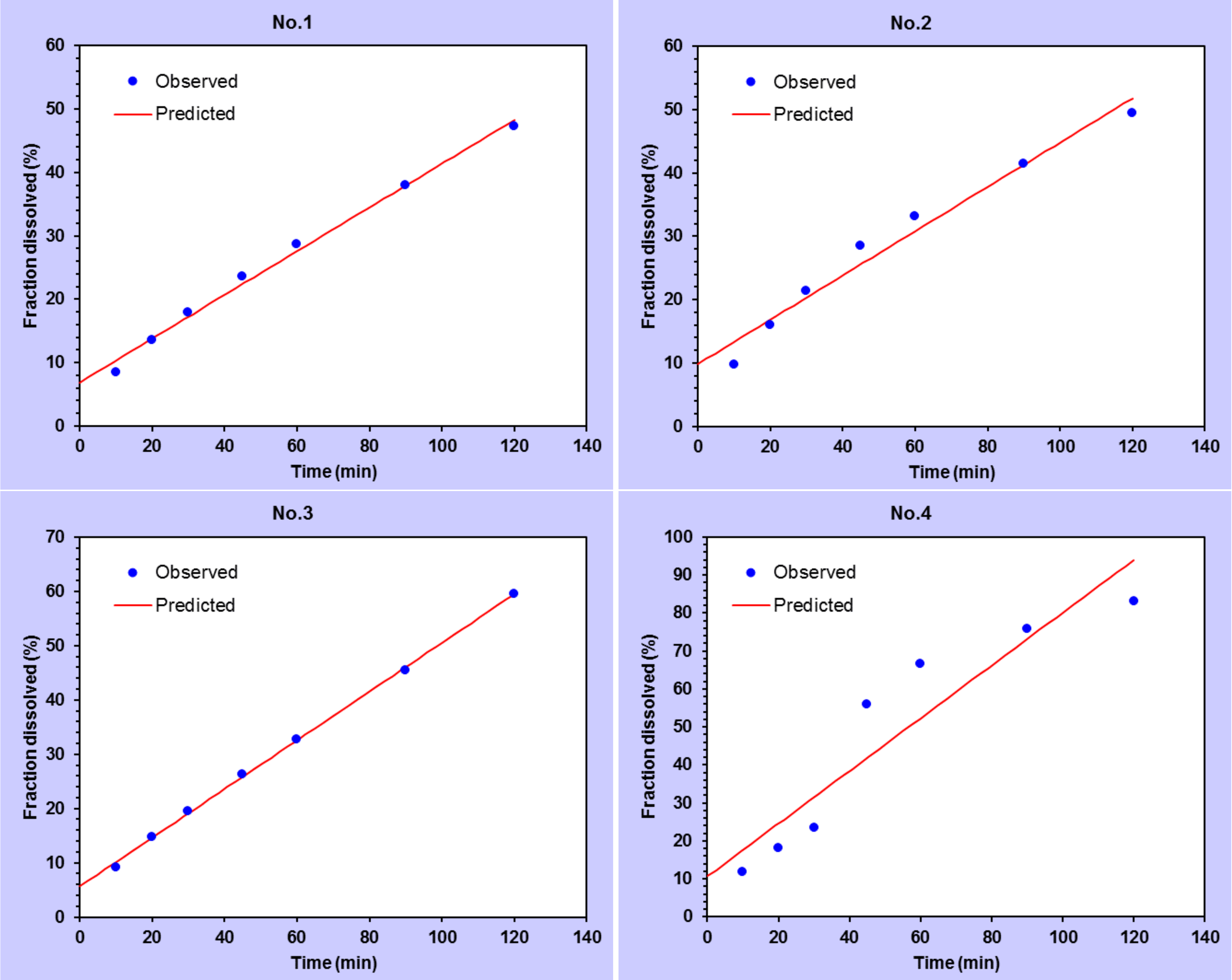

Supplementary materials – Model fitting summary – C*Pharm Mannidex 16700 (modelling up to app. 60 % of carvedilol released)

Model: **Zero–order with F_0**

Model equation: $F = F_0 + k_0 \cdot t$

Fitted model parameters per tested tablet (N = 4) with statistics – mean, standard deviation (SD), and relative standard deviation expressed in % (RSD%) (output from DDSolver):

Parameter	No.1	No.2	No.3	No.4	Mean	SD	RSD(%)
k_0	0.345	0.350	0.449	0.694	0.460	0.164	35.625
F_0	6.883	9.835	5.614	10.660	8.248	2.390	28.978

Number of dissolution data points (N), degrees of freedom (df), and selected goodness of fit criteria – Pearson correlation coefficient (R), coefficient of determination (R^2), adjusted coefficient of determination ($R^2_{adjusted}$), and residual sum of squares (RSS) (manual calculation in MS Excel):

Parameter	No.1	No.2	No.3	No.4
N	7	7	7	7
df	5	5	5	5
R	0.996666001	0.985812477	0.999519153	0.933989999
R^2	0.993343118	0.971826239	0.999038537	0.872337318
$R^2_{adjusted}$	0.992011741	0.966191487	0.998846245	0.846804782
RSS	7.532039867	33.45328274	1.832649172	665.9089668

Graphical abstract of model fit presented as mean ± 1 SD of the fraction % of released carvedilol:

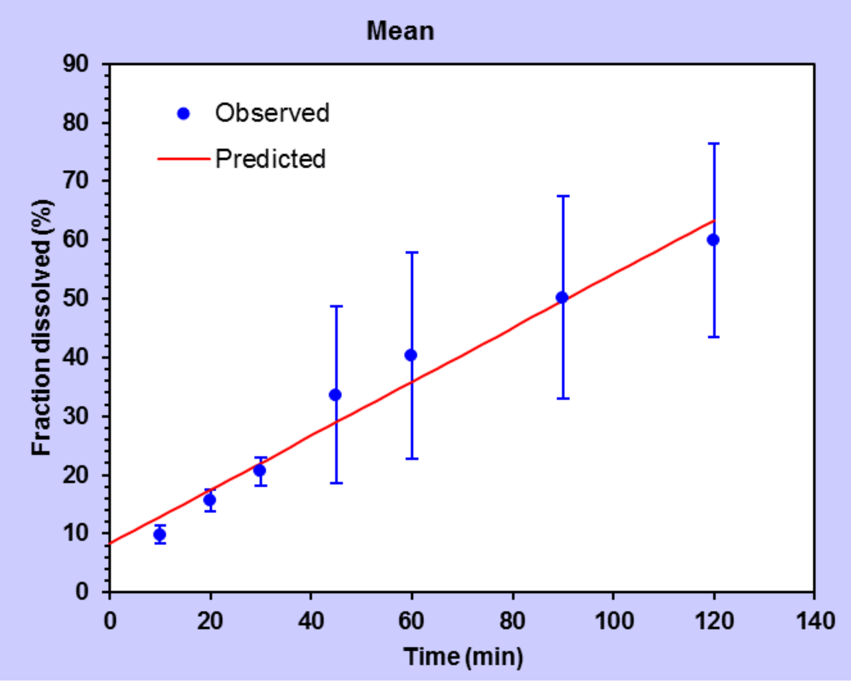

Supplementary materials – Model fitting summary – C*Pharm Mannidex 16700 (modelling up to app. 60 % of carvedilol released)

Graphical abstract of model fit presented as the fraction % of released carvedilol per tested tablet:

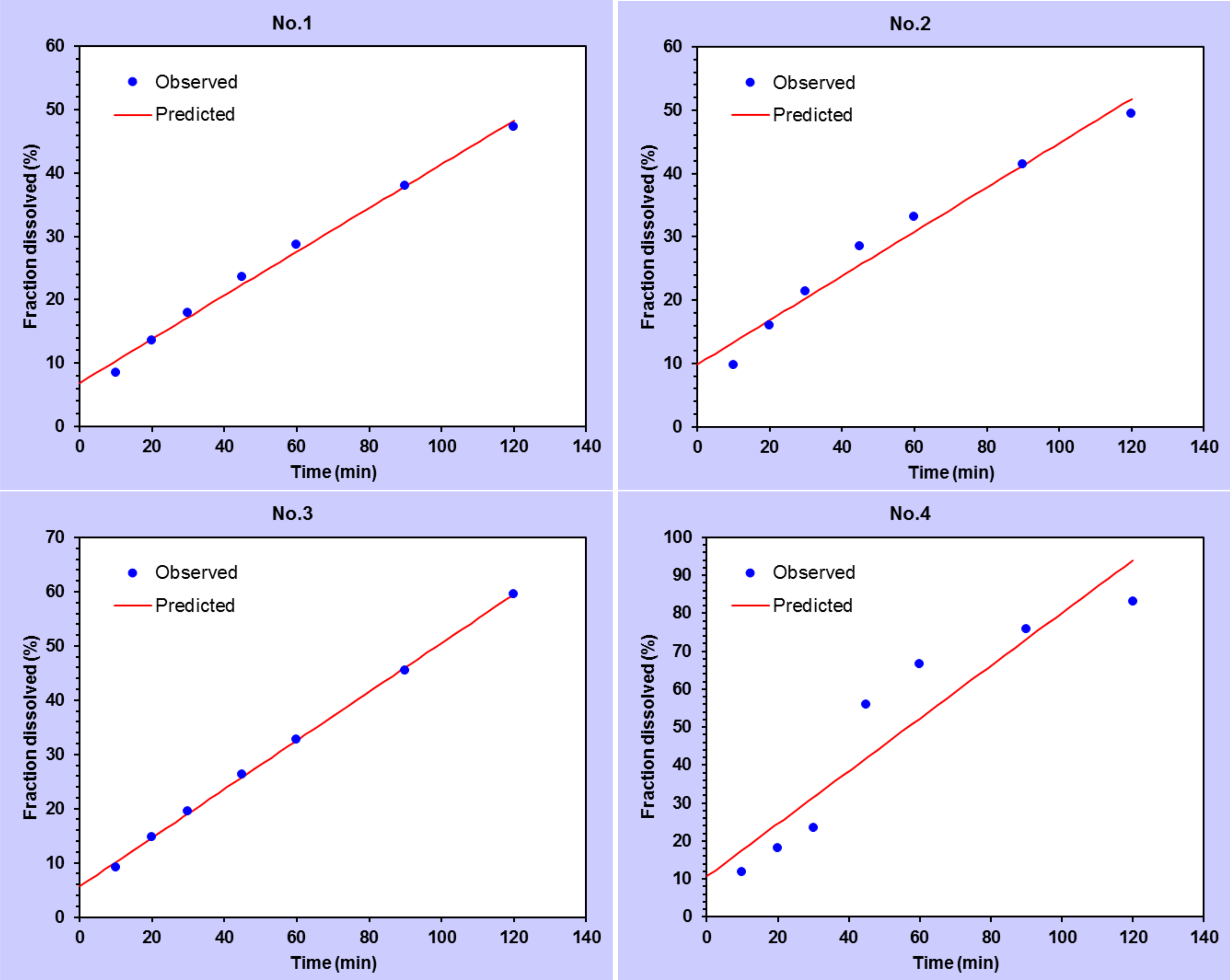

Supplementary materials – Model fitting summary – C*Pharm Mannidex 16700 (modelling up to app. 60 % of carvedilol released)

Model: **First–order**

Model equation: $F = 100 \cdot (1 - e^{-k_1 \cdot t})$

Fitted model parameters per tested tablet (N = 4) with statistics – mean, standard deviation (SD), and relative standard deviation expressed in % (RSD%) (output from DDSolver):

Parameter	No.1	No.2	No.3	No.4	Mean	SD	RSD(%)
k_1	0.005	0.006	0.007	0.015	0.009	0.005	54.426

Number of dissolution data points (N), degrees of freedom (df), and selected goodness of fit criteria – Pearson correlation coefficient (R), coefficient of determination (R^2), adjusted coefficient of determination ($R^2_{adjusted}$), and residual sum of squares (RSS) (manual calculation in MS Excel):

Parameter	No.1	No.2	No.3	No.4
N	7	7	7	7
df	6	6	6	6
R	0.999542431	0.996627231	0.995000789	0.975674112
R^2	0.999085072	0.993265837	0.99002657	0.951939973
$R^2_{adjusted}$	0.999085072	0.993265837	0.99002657	0.951939973
RSS	33.23261076	88.21298328	21.51754541	339.4798798

Graphical abstract of model fit presented as mean ± 1 SD of the fraction % of released carvedilol:

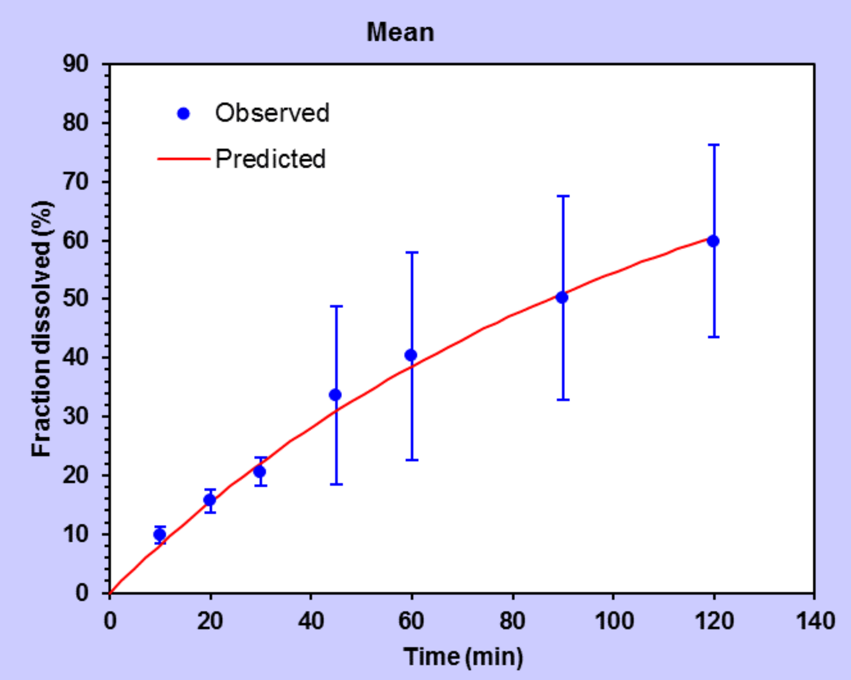

Supplementary materials – Model fitting summary – C*Pharm Mannidex 16700 (modelling up to app. 60 % of carvedilol released)

Graphical abstract of model fit presented as the fraction % of released carvedilol per tested tablet:

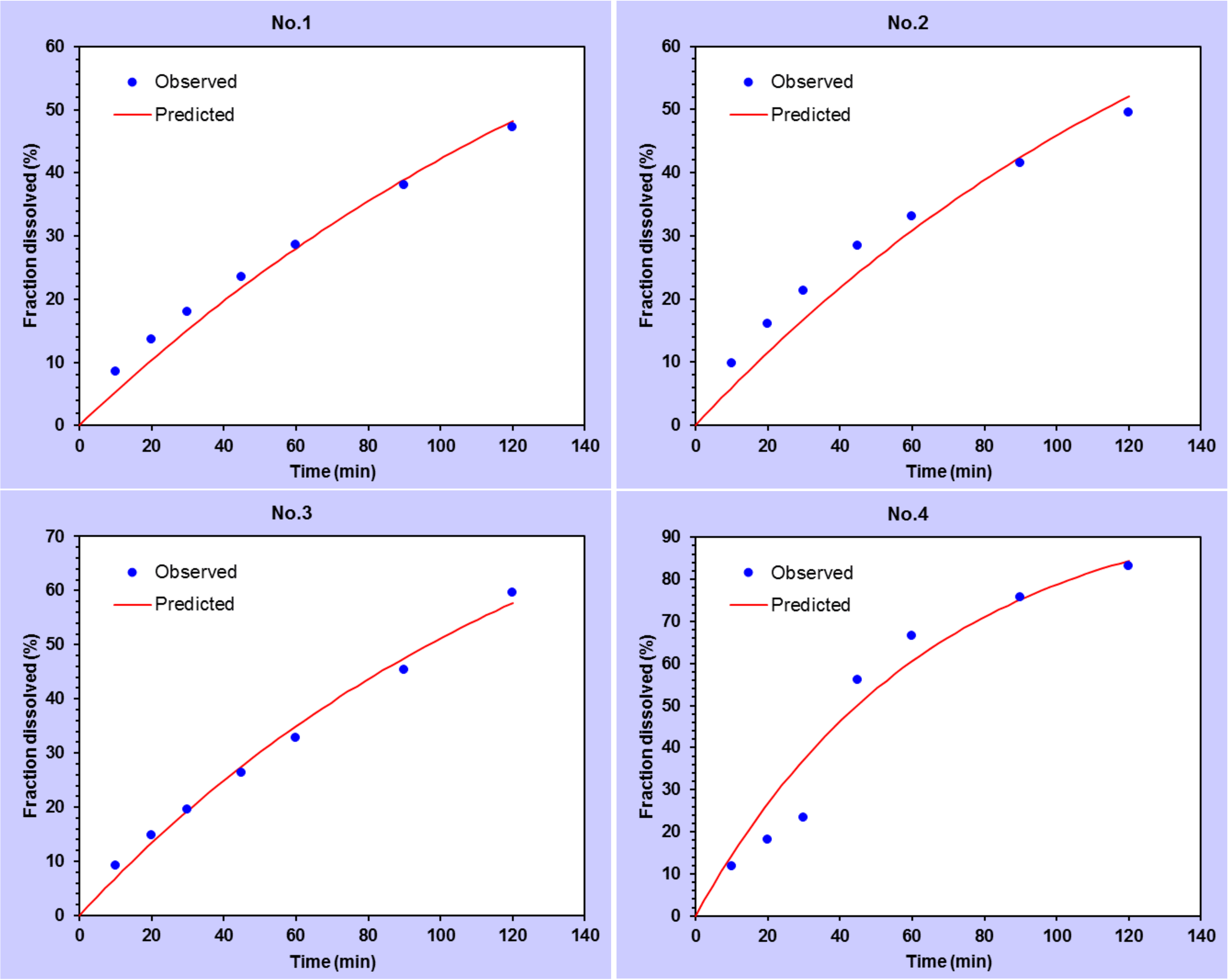

Supplementary materials – Model fitting summary – C*Pharm Mannidex 16700 (modelling up to app. 60 % of carvedilol released)

Model: **First–order with T_{lag}**

Model equation: $F = 100 \cdot \left[1 - e^{-k_1 \cdot (t - T_{lag})}\right]$

Fitted model parameters per tested tablet (N = 4) with statistics – mean, standard deviation (SD), and relative standard deviation expressed in % (RSD%) (output from DDSolver):

Parameter	No.1	No.2	No.3	No.4	Mean	SD	RSD(%)
k_1	0.005	0.005	0.007	0.016	0.008	0.005	63.162
T_{lag}	-9.185	-15.133	-0.257	4.110	-5.116	8.672	-169.509

Number of dissolution data points (N), degrees of freedom (df), and selected goodness of fit criteria – Pearson correlation coefficient (R), coefficient of determination (R^2), adjusted coefficient of determination ($R^2_{adjusted}$), and residual sum of squares (RSS) (manual calculation in MS Excel):

Parameter	No.1	No.2	No.3	No.4
N	7	7	7	7
df	5	5	5	5
R	0.999569021	0.995466986	0.995023137	0.976035706
R^2	0.999138228	0.990954519	0.990071044	0.9526457
$R^2_{adjusted}$	0.998965874	0.989145423	0.988085252	0.94317484
RSS	0.975369606	11.16094691	20.98258211	258.1494981

Graphical abstract of model fit presented as mean ± 1 SD of the fraction % of released carvedilol:

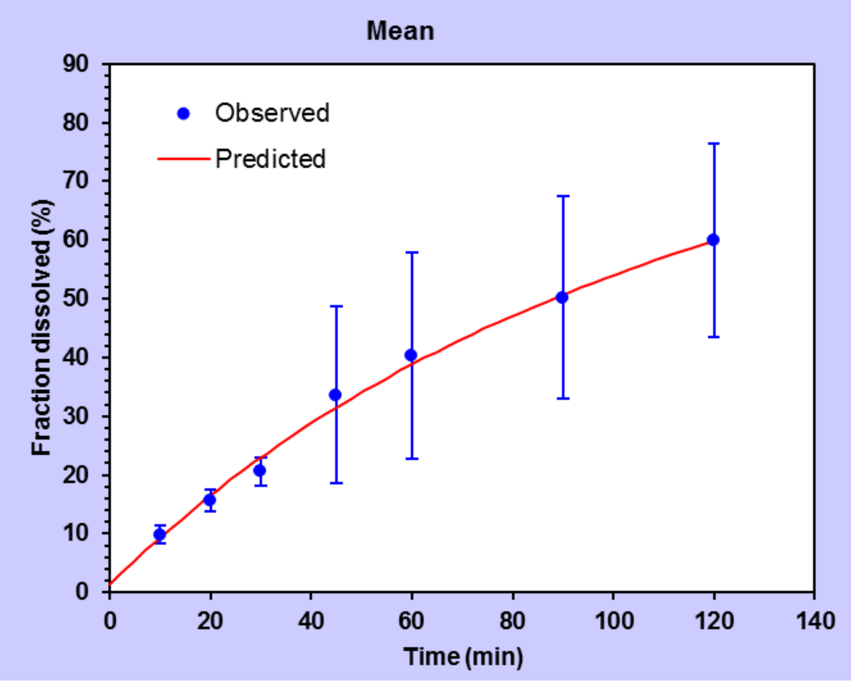

Supplementary materials – Model fitting summary – C*Pharm Mannidex 16700 (modelling up to app. 60 % of carvedilol released)

Graphical abstract of model fit presented as the fraction % of released carvedilol per tested tablet:

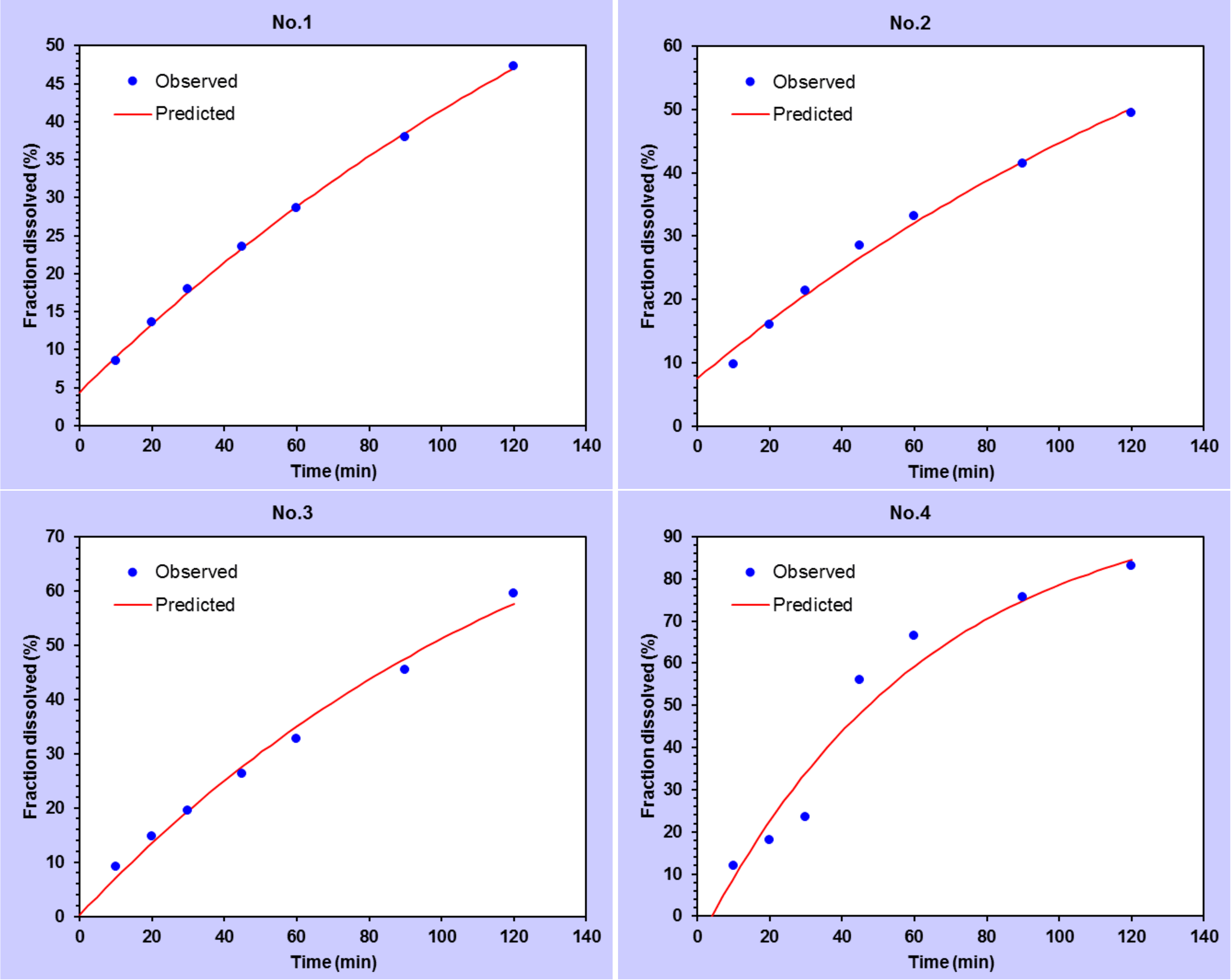

Supplementary materials – Model fitting summary – C*Pharm Mannidex 16700 (modelling up to app. 60 % of carvedilol released)

Model: **First–order with F_{max}**

Model equation: $F = F_{max} \cdot (1 - e^{-k_1 \cdot t})$

Fitted model parameters per tested tablet (N = 4) with statistics – mean, standard deviation (SD), and relative standard deviation expressed in % (RSD%) (output from DDSolver):

Parameter	No.1	No.2	No.3	No.4	Mean	SD	RSD(%)
k_1	0.021	0.021	0.019	0.024	0.021	0.002	8.260
F_{max}	40.326	51.984	62.569	87.327	60.551	20.029	33.078

Number of dissolution data points (N), degrees of freedom (df), and selected goodness of fit criteria – Pearson correlation coefficient (R), coefficient of determination (R^2), adjusted coefficient of determination ($R^2_{adjusted}$), and residual sum of squares (RSS) (manual calculation in MS Excel):

Parameter	No.1	No.2	No.3	No.4
N	7	7	7	7
df	5	5	5	5
R	0.974179148	0.988867552	0.964046013	0.97431011
R^2	0.949025012	0.977859035	0.929384715	0.94928019
$R^2_{adjusted}$	0.938830015	0.973430842	0.915261658	0.939136228
RSS	121.7782834	58.01917688	341.0538728	691.6542113

Graphical abstract of model fit presented as mean ± 1 SD of the fraction % of released carvedilol:

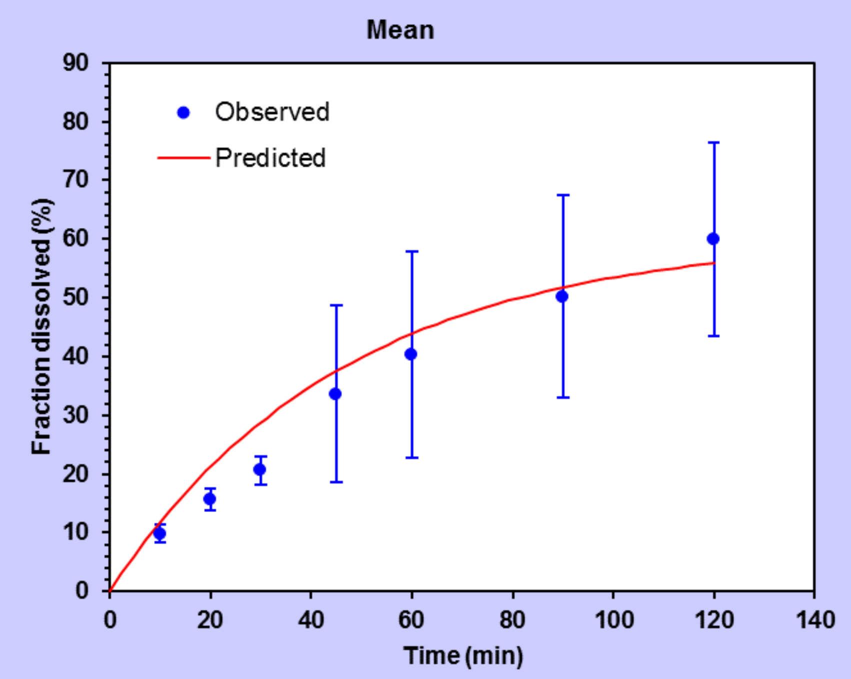

Supplementary materials – Model fitting summary – C*Pharm Mannidex 16700 (modelling up to app. 60 % of carvedilol released)

Graphical abstract of model fit presented as the fraction % of released carvedilol per tested tablet:

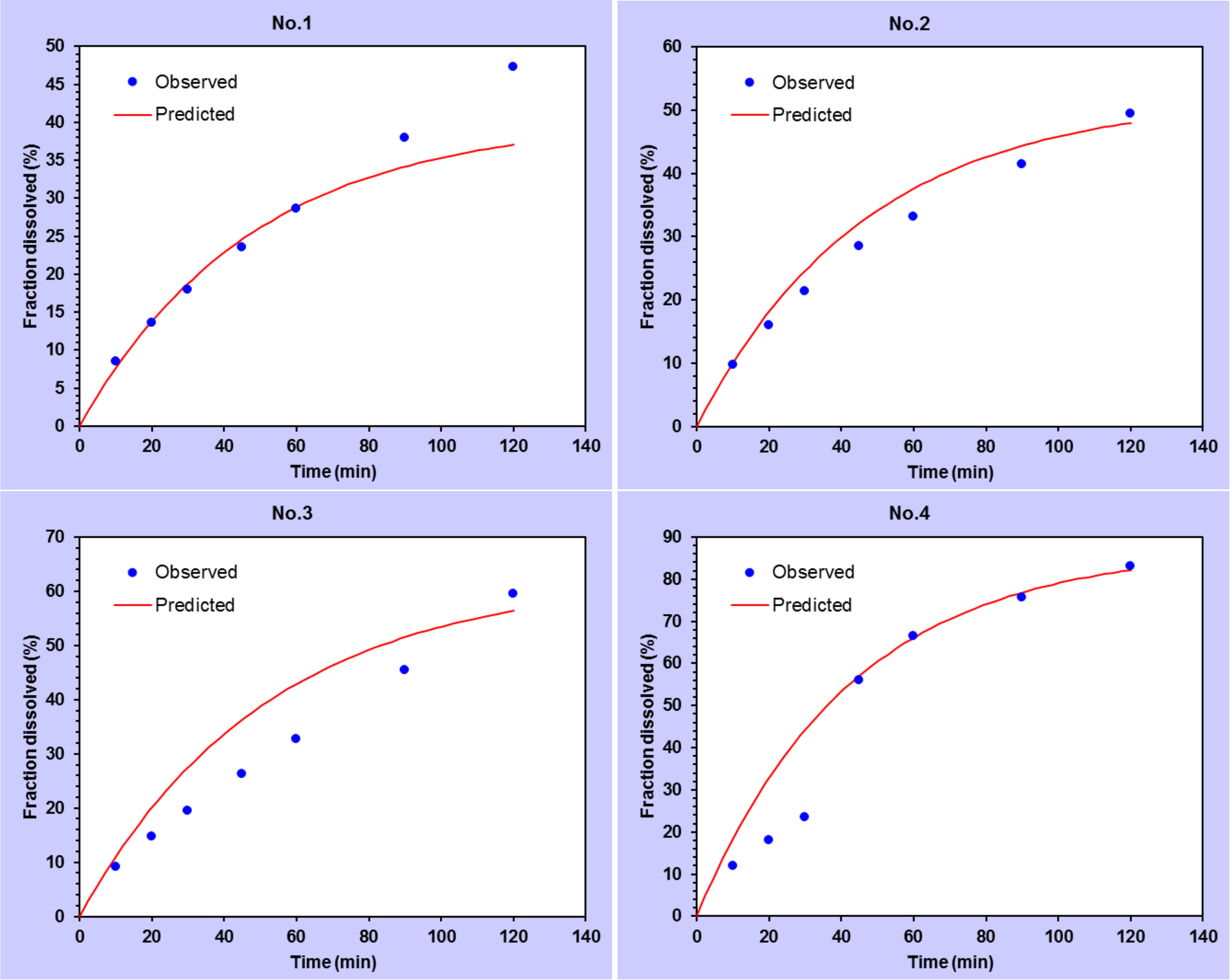

Supplementary materials – Model fitting summary – C*Pharm Mannidex 16700 (modelling up to app. 60 % of carvedilol released)

Model: **First–order with T_{lag} and F_{max}**

Model equation: $F = F_{max} \cdot \left[1 - e^{-k_1 \cdot (t - T_{lag})}\right]$

Fitted model parameters per tested tablet (N = 4) with statistics – mean, standard deviation (SD), and relative standard deviation expressed in % (RSD%) (output from DDSolver):

Parameter	No.1	No.2	No.3	No.4	Mean	SD	RSD(%)
k_1	0.024	0.024	0.024	0.027	0.025	0.002	6.266
T_{lag}	11.987	8.507	14.971	10.064	11.382	2.784	24.458
F_{max}	49.632	51.984	62.569	87.327	62.878	17.243	27.423

Number of dissolution data points (N), degrees of freedom (df), and selected goodness of fit criteria – Pearson correlation coefficient (R), coefficient of determination (R^2), adjusted coefficient of determination ($R^2_{adjusted}$), and residual sum of squares (RSS) (manual calculation in MS Excel):

Parameter	No.1	No.2	No.3	No.4
N	7	7	7	7
df	4	4	4	4
R	0.965065696	0.983724347	0.947388921	0.969977862
R^2	0.931351797	0.967713592	0.897545768	0.940857053
$R^2_{adjusted}$	0.897027696	0.951570387	0.846318652	0.91128558
RSS	200.9353602	104.3015433	501.7184647	330.17623

Graphical abstract of model fit presented as mean ± 1 SD of the fraction % of released carvedilol:

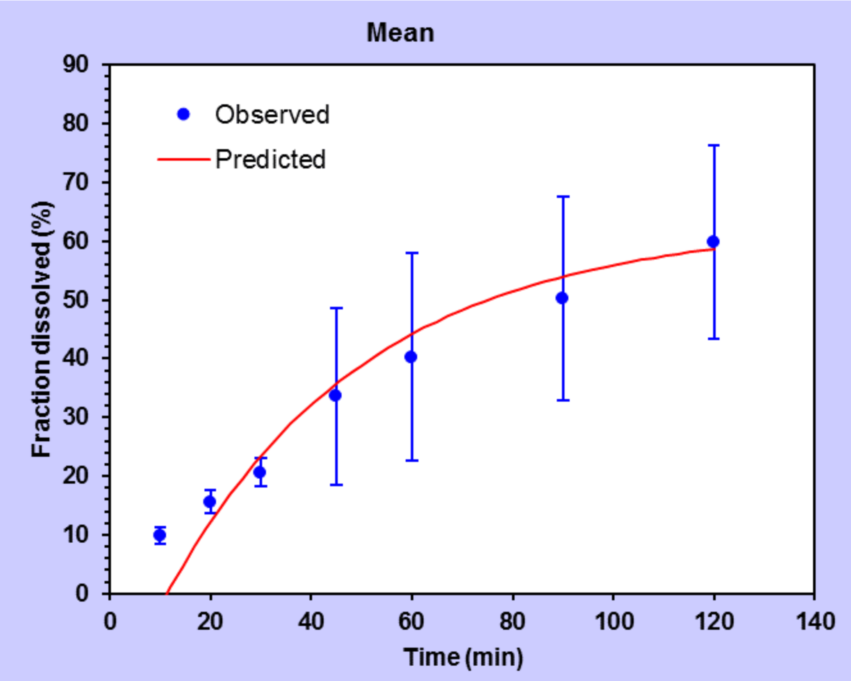

Supplementary materials – Model fitting summary – C*Pharm Mannidex 16700 (modelling up to app. 60 % of carvedilol released)

Graphical abstract of model fit presented as the fraction % of released carvedilol per tested tablet:

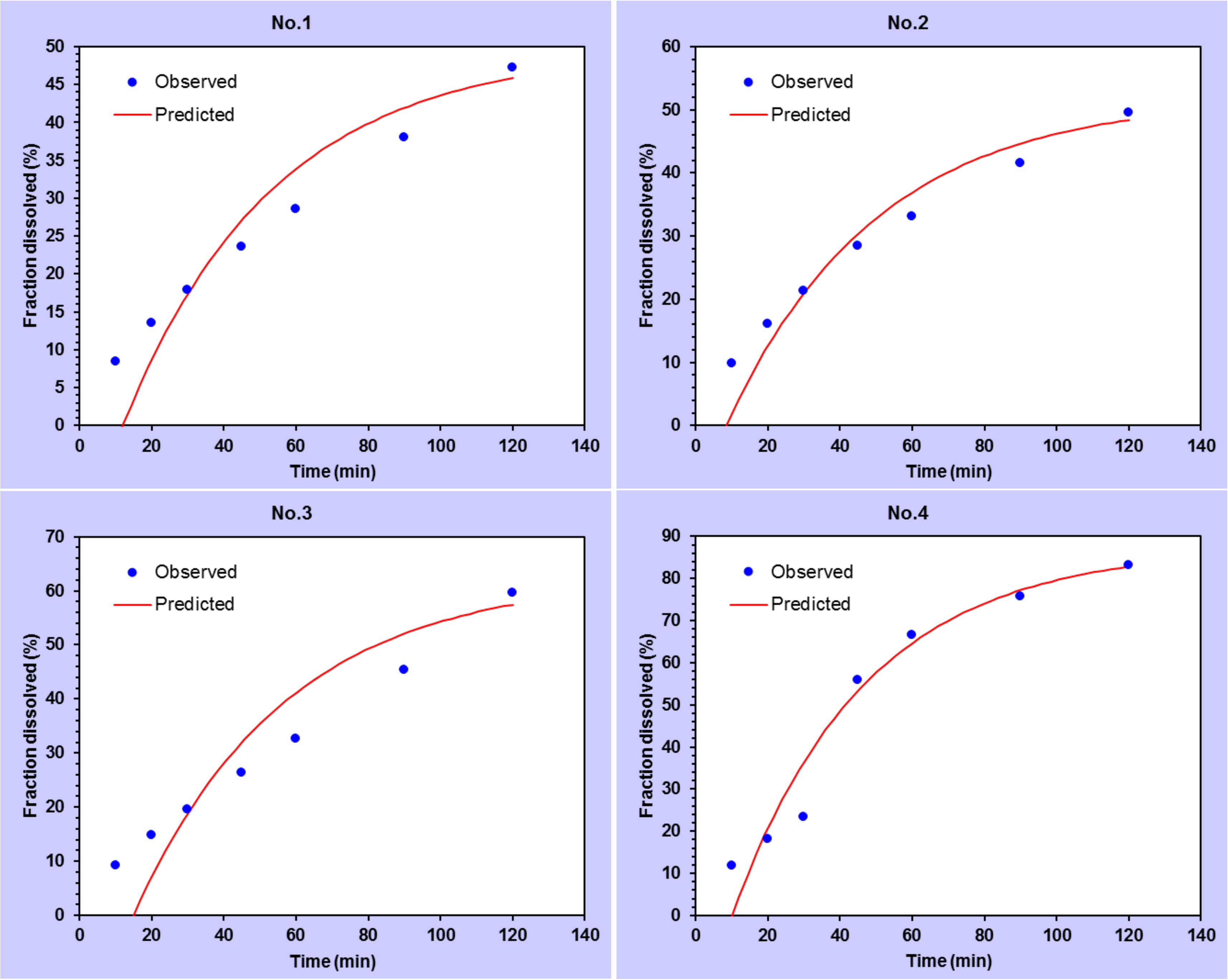

Supplementary materials – Model fitting summary – C*Pharm Mannidex 16700 (modelling up to app. 60 % of carvedilol released)

Model: **Higuchi**

Model equation: $F = k_H \cdot t^{0.5}$

Fitted model parameters per tested tablet (N = 4) with statistics – mean, standard deviation (SD), and relative standard deviation expressed in % (RSD%) (output from DDSolver):

Parameter	No.1	No.2	No.3	No.4	Mean	SD	RSD(%)
k_H	3.853	4.279	4.579	7.383	5.023	1.601	31.871

Number of dissolution data points (N), degrees of freedom (df), and selected goodness of fit criteria – Pearson correlation coefficient (R), coefficient of determination (R^2), adjusted coefficient of determination ($R^2_{adjusted}$), and residual sum of squares (RSS) (manual calculation in MS Excel):

Parameter	No.1	No.2	No.3	No.4
N	7	7	7	7
df	6	6	6	6
R	0.996720072	0.999676899	0.989786405	0.963949503
R^2	0.993450902	0.999353902	0.979677128	0.929198644
$R^2_{adjusted}$	0.993450902	0.999353902	0.979677128	0.929198644
RSS	70.85999633	35.26157963	209.2890357	810.2578624

Graphical abstract of model fit presented as mean ± 1 SD of the fraction % of released carvedilol:

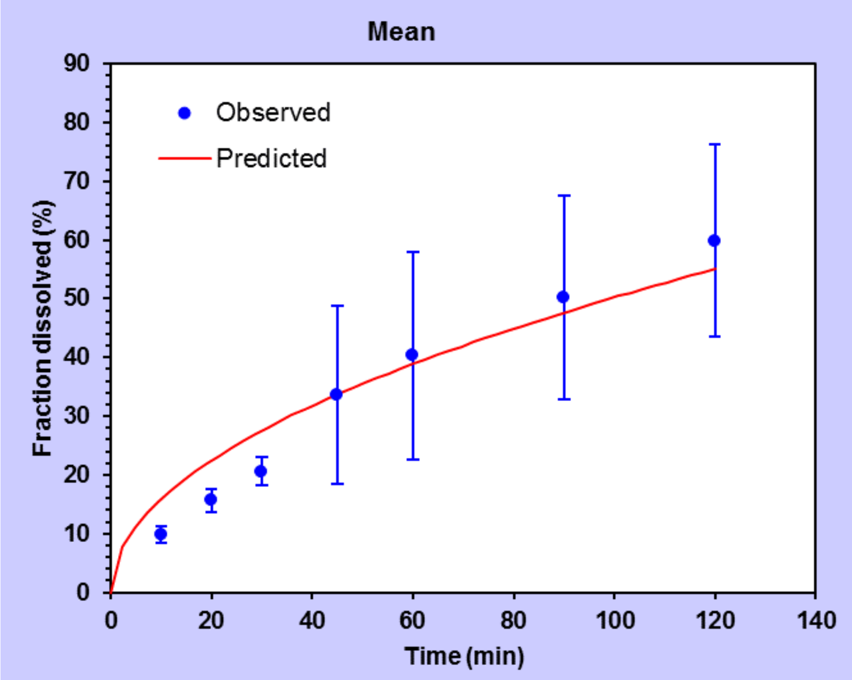

Supplementary materials – Model fitting summary – C*Pharm Mannidex 16700 (modelling up to app. 60 % of carvedilol released)

Graphical abstract of model fit presented as the fraction % of released carvedilol per tested tablet:

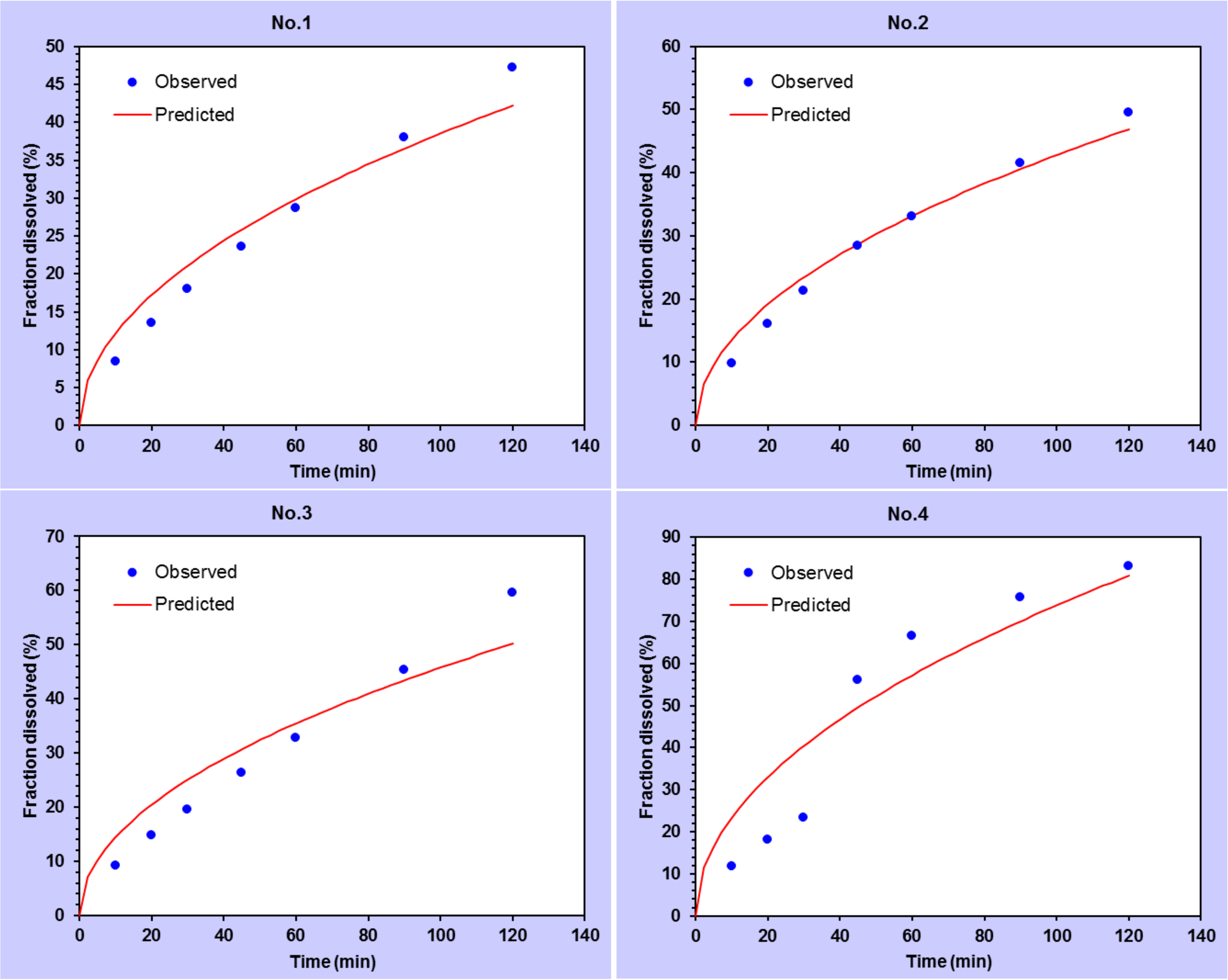

Supplementary materials – Model fitting summary – C*Pharm Mannidex 16700 (modelling up to app. 60 % of carvedilol released)

Model: **Higuchi with T_{lag}**

Model equation: $F = k_H \cdot \left(t - T_{lag}\right)^{0.5}$

Fitted model parameters per tested tablet (N = 4) with statistics – mean, standard deviation (SD), and relative standard deviation expressed in % (RSD%) (output from DDSolver):

Parameter	No.1	No.2	No.3	No.4	Mean	SD	RSD(%)
k_H	4.423	4.484	5.562	8.235	5.676	1.784	31.437
T_{lag}	12.410	5.312	16.299	8.806	10.707	4.722	44.102

Number of dissolution data points (N), degrees of freedom (df), and selected goodness of fit criteria – Pearson correlation coefficient (R), coefficient of determination (R^2), adjusted coefficient of determination ($R^2_{adjusted}$), and residual sum of squares (RSS) (manual calculation in MS Excel):

Parameter	No.1	No.2	No.3	No.4
N	7	7	7	7
df	5	5	5	5
R	0.979472976	0.999098004	0.974696576	0.960907825
R^2	0.95936731	0.998196822	0.950033415	0.923343848
$R^2_{adjusted}$	0.951240772	0.997836186	0.940040098	0.908012618
RSS	83.57792306	4.334890579	144.1090688	423.2030289

Graphical abstract of model fit presented as mean ± 1 SD of the fraction % of released carvedilol:

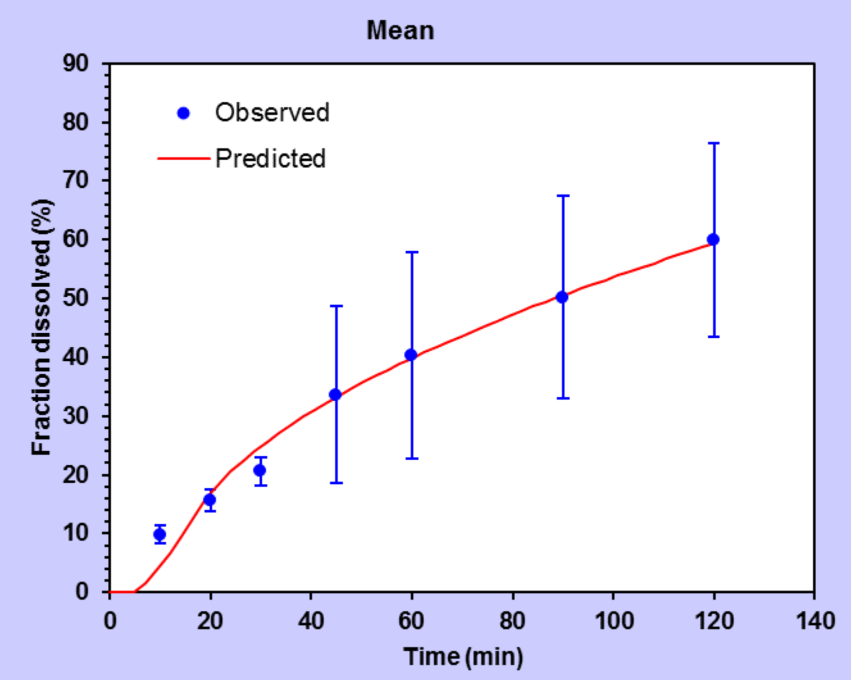

Supplementary materials – Model fitting summary – C*Pharm Mannidex 16700 (modelling up to app. 60 % of carvedilol released)

Graphical abstract of model fit presented as the fraction % of released carvedilol per tested tablet:

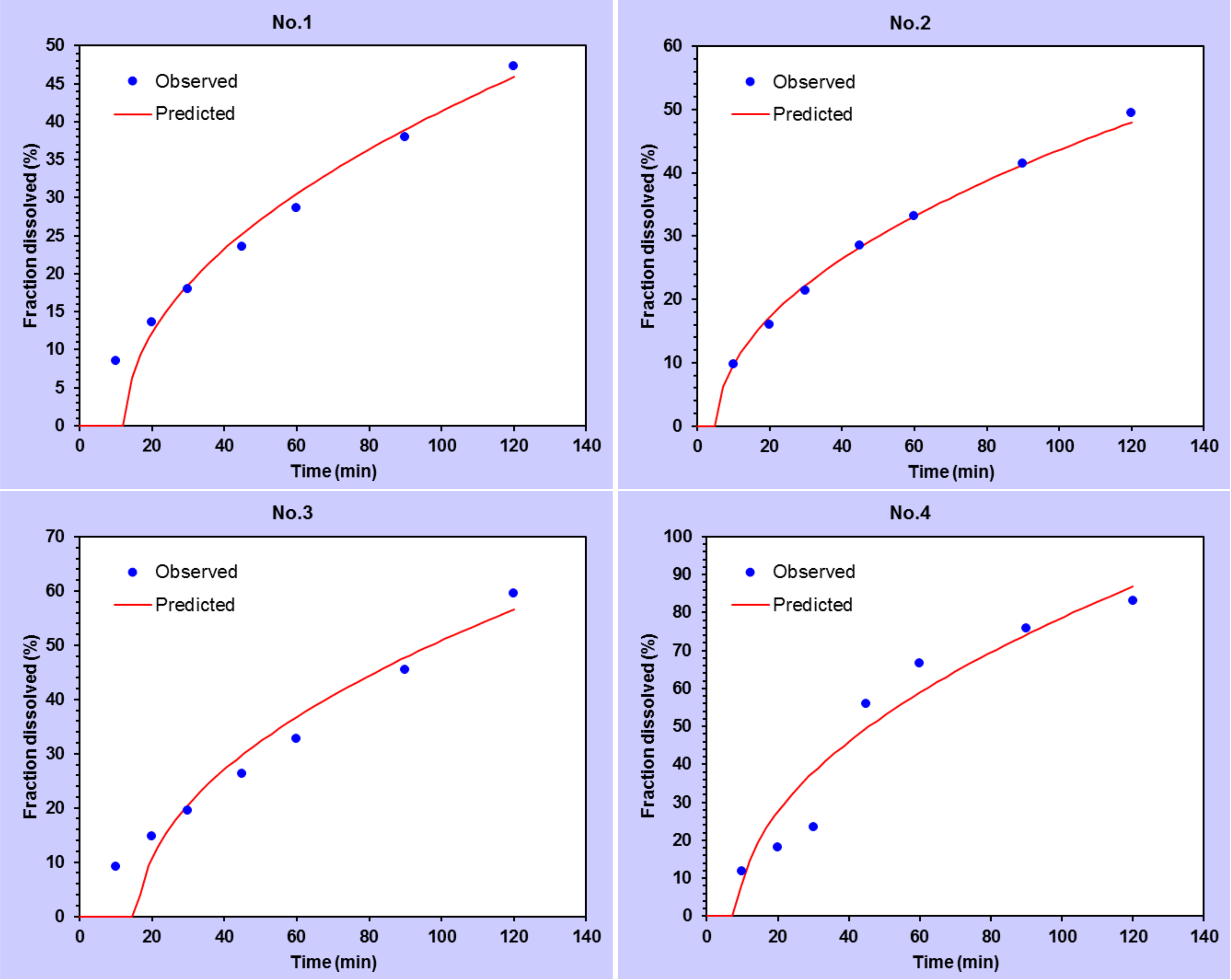

Supplementary materials – Model fitting summary – C*Pharm Mannidex 16700 (modelling up to app. 60 % of carvedilol released)

Model: **Higuchi with F_0**

Model equation: $F = F_0 + k_H \cdot t^{0.5}$

Fitted model parameters per tested tablet (N = 4) with statistics – mean, standard deviation (SD), and relative standard deviation expressed in % (RSD%) (output from DDSolver):

Parameter	No.1	No.2	No.3	No.4	Mean	SD	RSD(%)
k_H	4.956	5.092	6.388	10.292	6.682	2.491	37.285
F_0	-8.619	-6.355	-14.130	-22.720	-12.956	7.283	-56.211

Number of dissolution data points (N), degrees of freedom (df), and selected goodness of fit criteria – Pearson correlation coefficient (R), coefficient of determination (R^2), adjusted coefficient of determination ($R^2_{adjusted}$), and residual sum of squares (RSS) (manual calculation in MS Excel):

Parameter	No.1	No.2	No.3	No.4
N	7	7	7	7
df	5	5	5	5
R	0.996720072	0.999676899	0.989786405	0.963949503
R^2	0.993450902	0.999353902	0.979677128	0.929198644
$R^2_{adjusted}$	0.992141083	0.999224683	0.975612553	0.915038373
RSS	7.410085057	0.767170826	38.7375275	369.3111972

Graphical abstract of model fit presented as mean ± 1 SD of the fraction % of released carvedilol:

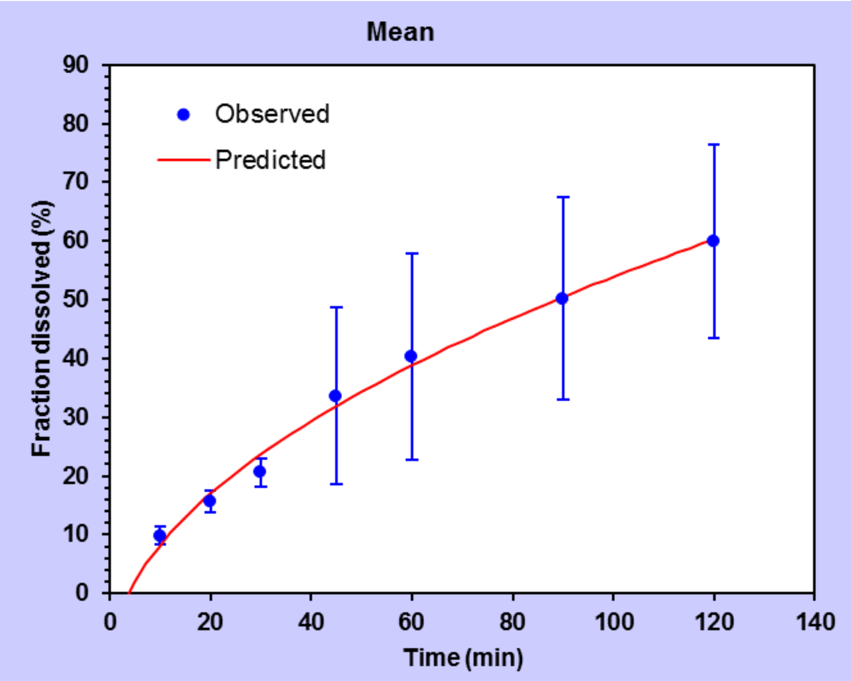

Supplementary materials – Model fitting summary – C*Pharm Mannidex 16700 (modelling up to app. 60 % of carvedilol released)

Graphical abstract of model fit presented as the fraction % of released carvedilol per tested tablet:

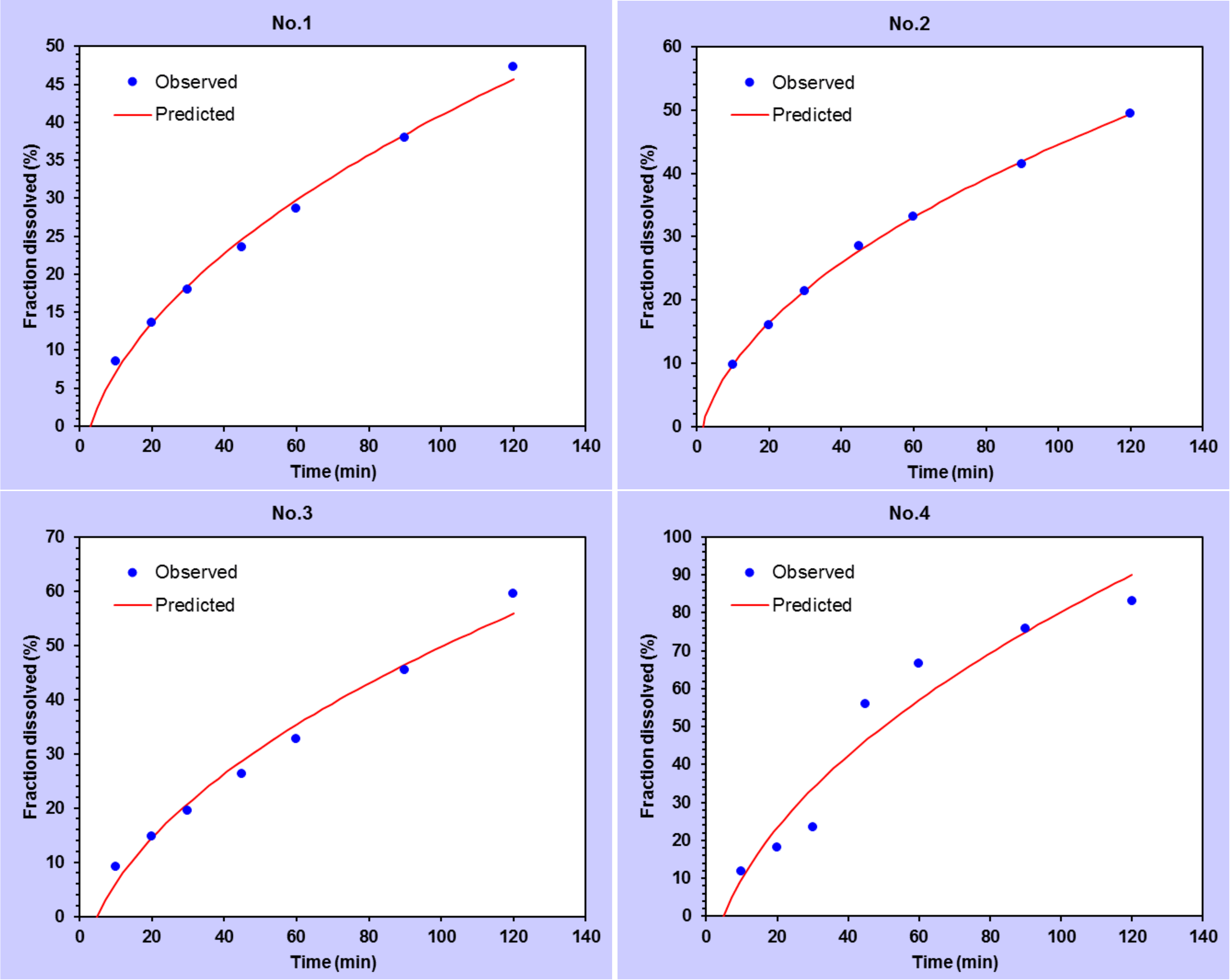

Supplementary materials – Model fitting summary – C*Pharm Mannidex 16700 (modelling up to app. 60 % of carvedilol released)

Model: **Korsmeyer–Peppas**

Model equation: $F = k_{KP} \cdot t^n$

Fitted model parameters per tested tablet (N = 4) with statistics – mean, standard deviation (SD), and relative standard deviation expressed in % (RSD%) (output from DDSolver):

Parameter	No.1	No.2	No.3	No.4	Mean	SD	RSD(%)
k_{KP}	1.738	2.649	1.593	1.509	1.872	0.526	28.111
n	0.687	0.610	0.745	0.874	0.729	0.111	15.240

Number of dissolution data points (N), degrees of freedom (df), and selected goodness of fit criteria – Pearson correlation coefficient (R), coefficient of determination (R^2), adjusted coefficient of determination ($R^2_{adjusted}$), and residual sum of squares (RSS) (manual calculation in MS Excel):

Parameter	No.1	No.2	No.3	No.4
N	7	7	7	7
df	5	5	5	5
R	0.999767897	0.998749238	0.997926409	0.943833424
R^2	0.999535848	0.99750004	0.995857117	0.890821532
$R^2_{adjusted}$	0.999443017	0.997000047	0.99502854	0.868985838
RSS	0.63572561	4.212966127	11.55815585	649.6928482

Graphical abstract of model fit presented as mean ± 1 SD of the fraction % of released carvedilol:

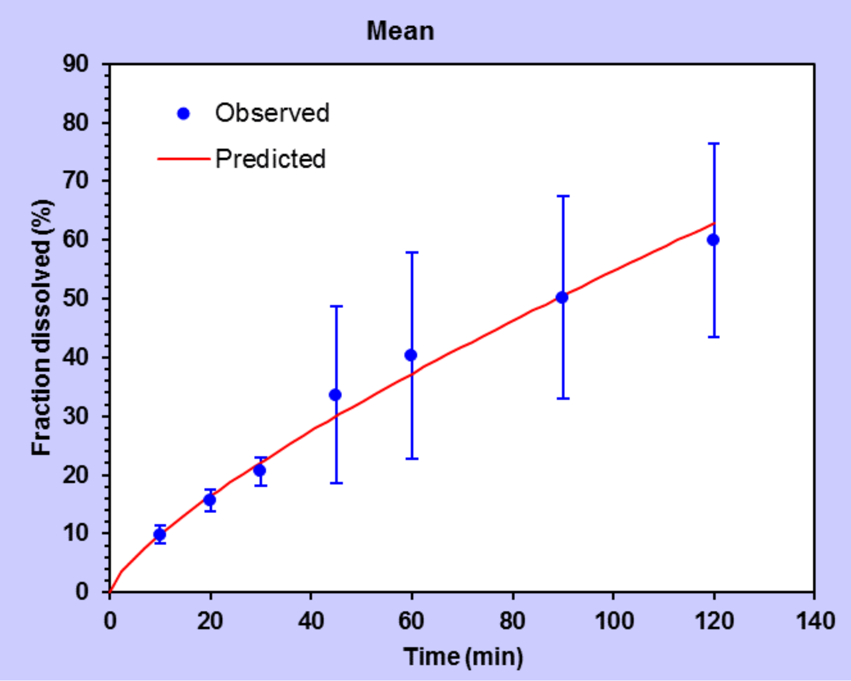

Supplementary materials – Model fitting summary – C*Pharm Mannidex 16700 (modelling up to app. 60 % of carvedilol released)

Graphical abstract of model fit presented as the fraction % of released carvedilol per tested tablet:

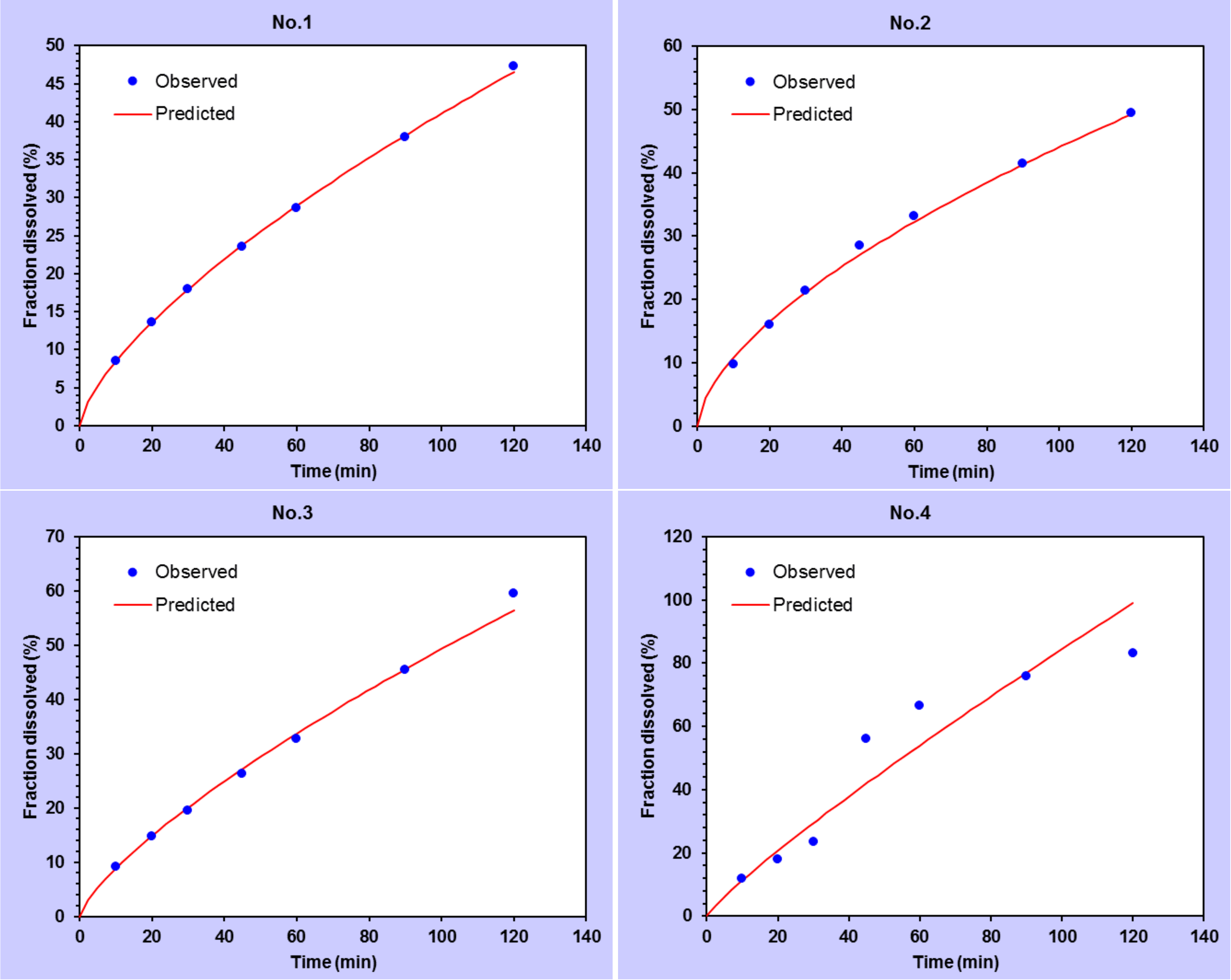

Supplementary materials – Model fitting summary – C*Pharm Mannidex 16700 (modelling up to app. 60 % of carvedilol released)

Model: **Korsmeyer–Peppas with T_{lag}**

Model equation: $F = k_{KP} \cdot (t - T_{lag})^n$

Fitted model parameters per tested tablet (N = 4) with statistics – mean, standard deviation (SD), and relative standard deviation expressed in % (RSD%) (output from DDSolver):

Parameter	No.1	No.2	No.3	No.4	Mean	SD	RSD(%)
k_{KP}	2.823	3.590	2.756	2.794	2.991	0.401	13.394
n	0.581	0.551	0.641	0.739	0.628	0.083	13.213
T_{lag}	4.000	4.000	4.927	4.000	4.232	0.464	10.954

Number of dissolution data points (N), degrees of freedom (df), and selected goodness of fit criteria – Pearson correlation coefficient (R), coefficient of determination (R^2), adjusted coefficient of determination ($R^2_{adjusted}$), and residual sum of squares (RSS) (manual calculation in MS Excel):

Parameter	No.1	No.2	No.3	No.4
N	7	7	7	7
df	4	4	4	4
R	0.99740636	0.999635827	0.993380451	0.95423247
R^2	0.994819447	0.999271787	0.98680472	0.910559607
$R^2_{adjusted}$	0.992229171	0.99890768	0.980207079	0.86583941
RSS	9.336982347	0.877385079	35.03919808	483.7583057

Graphical abstract of model fit presented as mean ± 1 SD of the fraction % of released carvedilol:

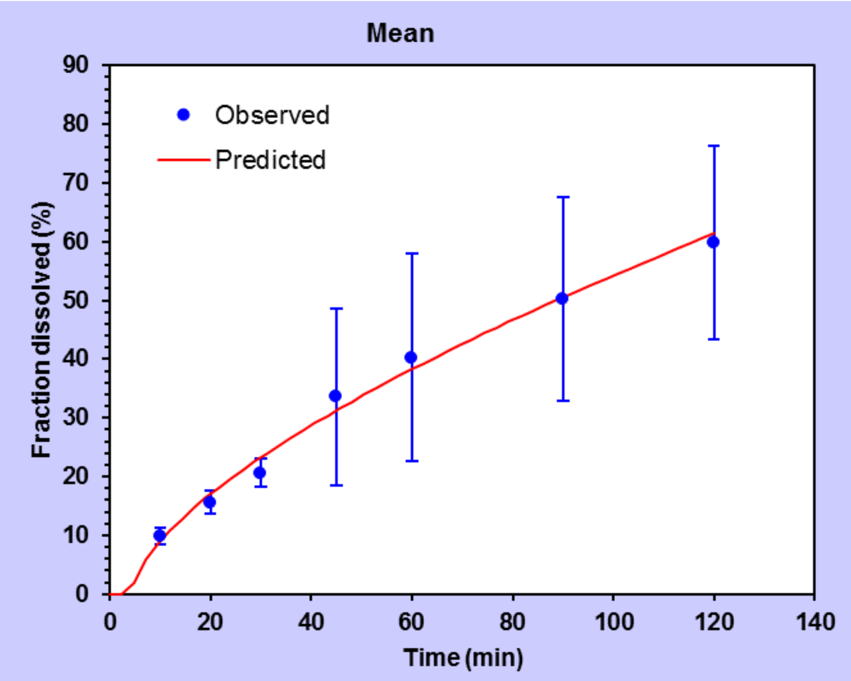

Supplementary materials – Model fitting summary – C*Pharm Mannidex 16700 (modelling up to app. 60 % of carvedilol released)

Graphical abstract of model fit presented as the fraction % of released carvedilol per tested tablet:

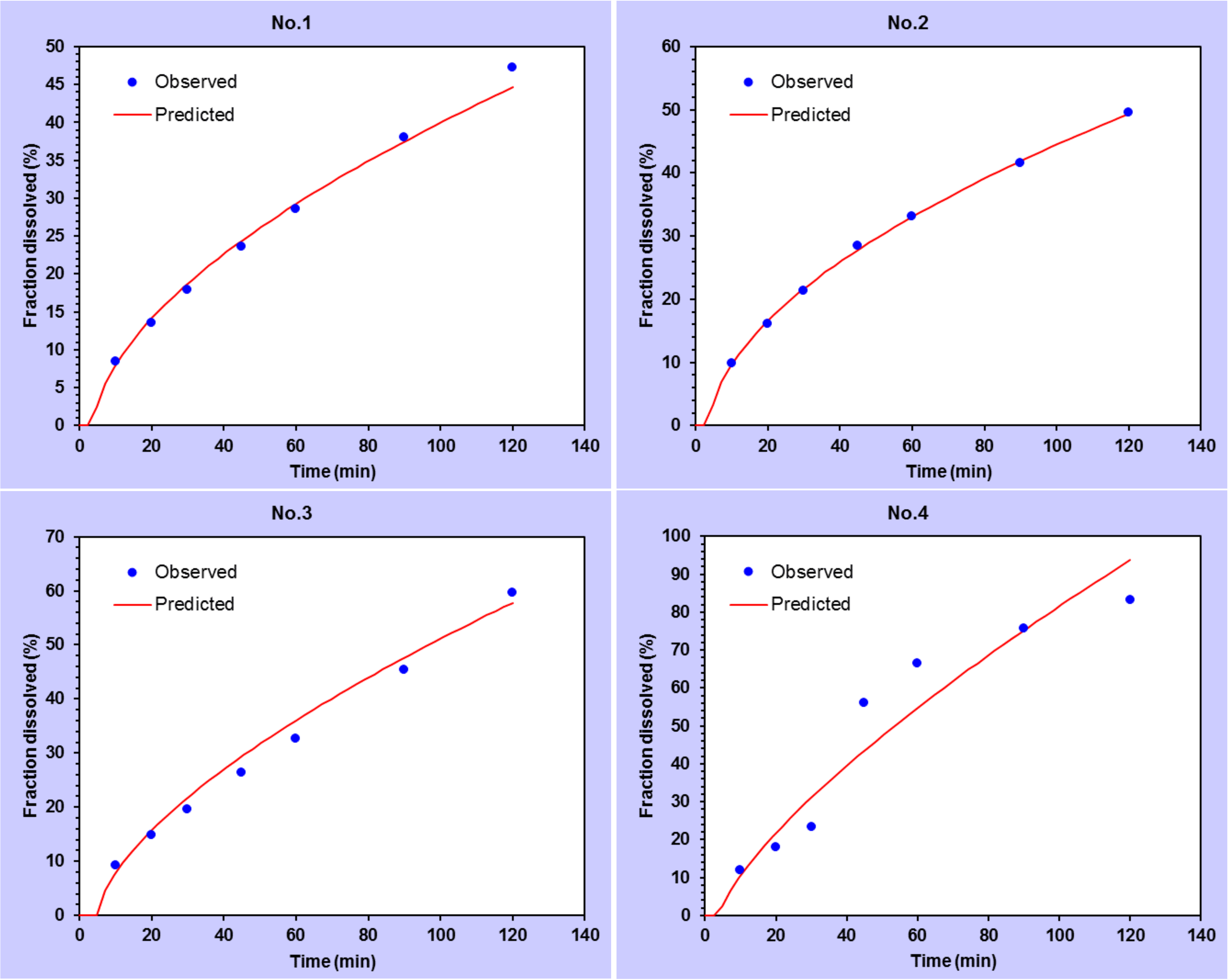

Supplementary materials – Model fitting summary – C*Pharm Mannidex 16700 (modelling up to app. 60 % of carvedilol released)

Model: **Korsmeyer–Peppas with F_0**

Model equation: $F = F_0 + k_{KP} \cdot t^n$

Fitted model parameters per tested tablet (N = 4) with statistics – mean, standard deviation (SD), and relative standard deviation expressed in % (RSD%) (output from DDSolver):

Parameter	No.1	No.2	No.3	No.4	Mean	SD	RSD(%)
k_{KP}	0.762	1.021	0.690	0.629	0.776	0.172	22.233
n	0.853	0.812	0.917	1.059	0.910	0.108	11.897
F_0	3.399	3.919	3.679	4.759	3.939	0.586	14.884

Number of dissolution data points (N), degrees of freedom (df), and selected goodness of fit criteria – Pearson correlation coefficient (R), coefficient of determination (R^2), adjusted coefficient of determination ($R^2_{adjusted}$), and residual sum of squares (RSS) (manual calculation in MS Excel):

Parameter	No.1	No.2	No.3	No.4
N	7	7	7	7
df	4	4	4	4
R	0.999238234	0.993705879	0.999663722	0.929005758
R^2	0.998477048	0.987451374	0.999327557	0.863051698
$R^2_{adjusted}$	0.997715573	0.981177062	0.998991336	0.794577546
RSS	3.905962256	28.0868186	1.359182204	943.8989935

Graphical abstract of model fit presented as mean ± 1 SD of the fraction % of released carvedilol:

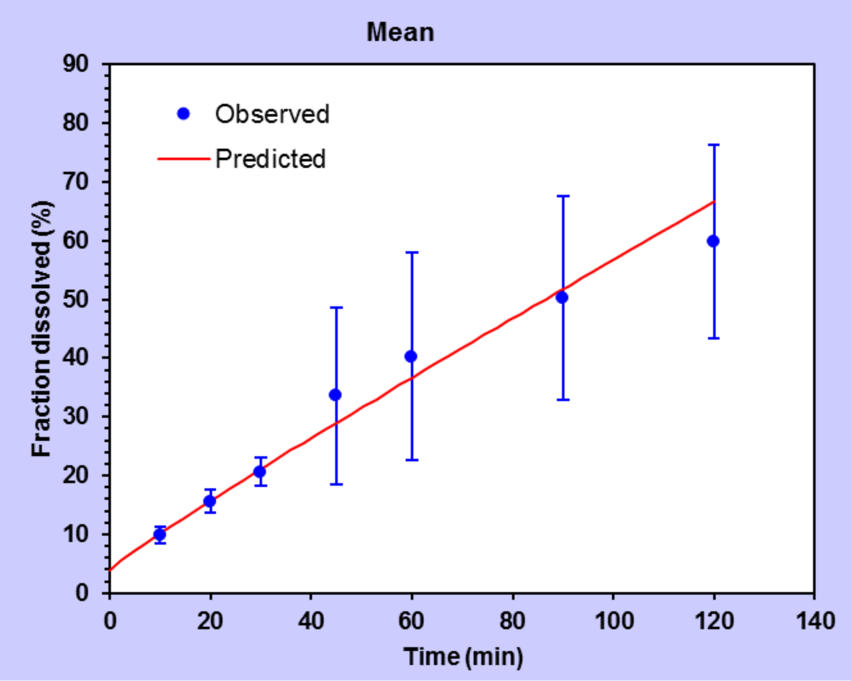

Supplementary materials – Model fitting summary – C*Pharm Mannidex 16700 (modelling up to app. 60 % of carvedilol released)

Graphical abstract of model fit presented as the fraction % of released carvedilol per tested tablet:

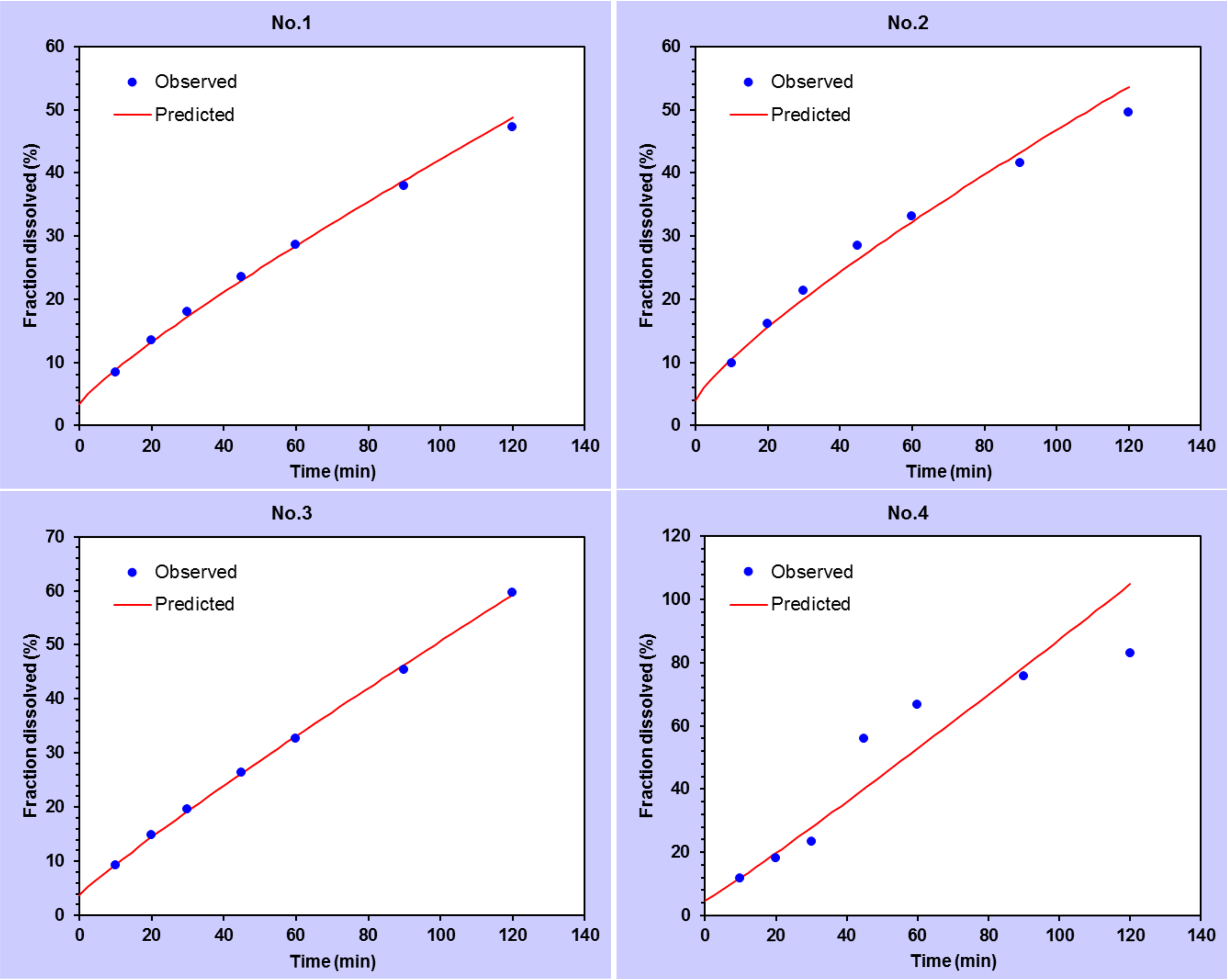

Supplementary materials – Model fitting summary – C*Pharm Mannidex 16700 (modelling up to app. 60 % of carvedilol released)

Model: **Hixson–Crowell**

Model equation: $F = 100 \cdot [1 - (1 - k_{HC} \cdot t)^3]$

Fitted model parameters per tested tablet (N = 4) with statistics – mean, standard deviation (SD), and relative standard deviation expressed in % (RSD%) (output from DDSolver):

Parameter	No.1	No.2	No.3	No.4	Mean	SD	RSD(%)
k_{HC}	0.002	0.002	0.002	0.004	0.002	0.001	45.625

Number of dissolution data points (N), degrees of freedom (df), and selected goodness of fit criteria – Pearson correlation coefficient (R), coefficient of determination (R^2), adjusted coefficient of determination ($R^2_{adjusted}$), and residual sum of squares (RSS) (manual calculation in MS Excel):

Parameter	No.1	No.2	No.3	No.4
N	7	7	7	7
df	6	6	6	6
R	0.999364579	0.994143311	0.997615912	0.970554848
R^2	0.998729562	0.988320922	0.995237509	0.941976713
$R^2_{adjusted}$	0.998729562	0.988320922	0.995237509	0.941976713
RSS	52.54561869	129.6567012	22.36083216	312.8676063

Graphical abstract of model fit presented as mean ± 1 SD of the fraction % of released carvedilol:

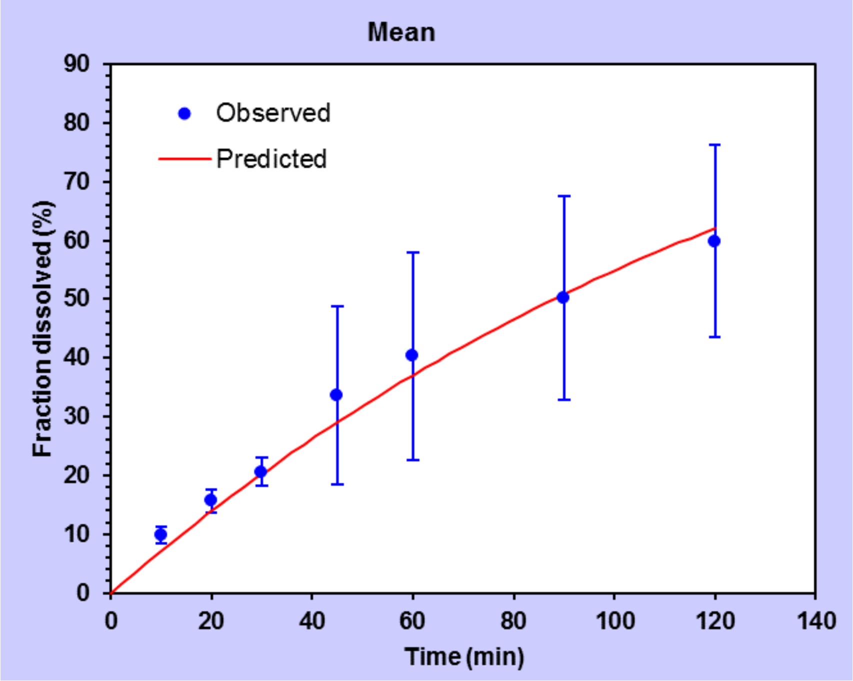

Supplementary materials – Model fitting summary – C*Pharm Mannidex 16700 (modelling up to app. 60 % of carvedilol released)

Graphical abstract of model fit presented as the fraction % of released carvedilol per tested tablet:

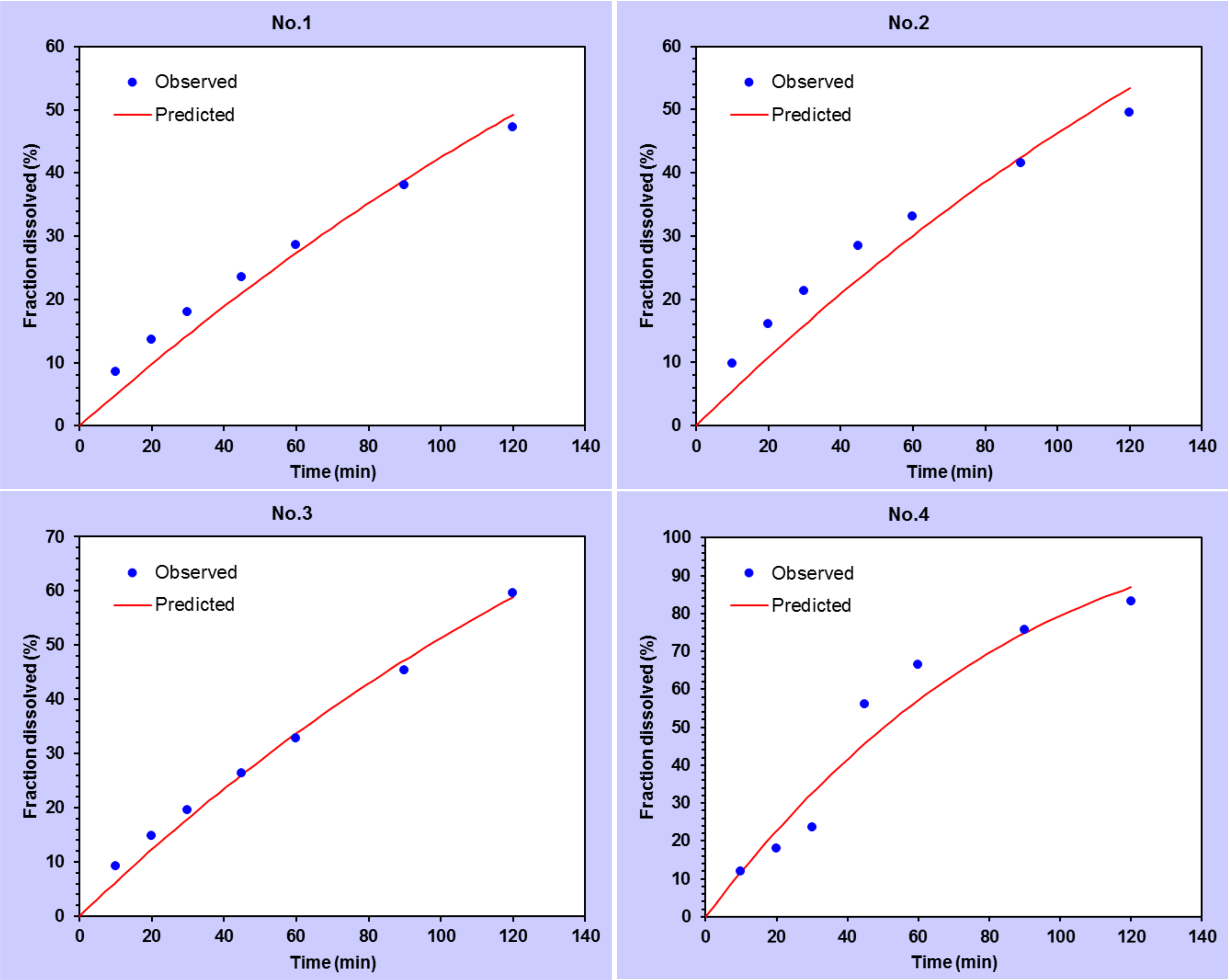

Supplementary materials – Model fitting summary – C*Pharm Mannidex 16700 (modelling up to app. 60 % of carvedilol released)

Model: **Hixson–Crowell with T_{lag}**

Model equation: $F = 100 \cdot \left\{1 - \left[1 - k_{HC} \cdot \left(t - T_{lag}\right)\right]^3\right\}$

Fitted model parameters per tested tablet (N = 4) with statistics – mean, standard deviation (SD), and relative standard deviation expressed in % (RSD%) (output from DDSolver):

Parameter	No.1	No.2	No.3	No.4	Mean	SD	RSD(%)
k_{HC}	0.001	0.002	0.002	0.004	0.002	0.001	52.702
T_{lag}	-12.556	-19.176	-3.978	-2.693	-9.601	7.741	-80.627

Number of dissolution data points (N), degrees of freedom (df), and selected goodness of fit criteria – Pearson correlation coefficient (R), coefficient of determination (R^2), adjusted coefficient of determination ($R^2_{adjusted}$), and residual sum of squares (RSS) (manual calculation in MS Excel):

Parameter	No.1	No.2	No.3	No.4
N	7	7	7	7
df	5	5	5	5
R	0.999199082	0.992883205	0.997829688	0.969894686
R^2	0.998398806	0.985817058	0.995664086	0.940695702
$R^2_{adjusted}$	0.998078567	0.98298047	0.994796903	0.928834843
RSS	1.825754314	17.17417479	8.637396095	342.1364085

Graphical abstract of model fit presented as mean ± 1 SD of the fraction % of released carvedilol:

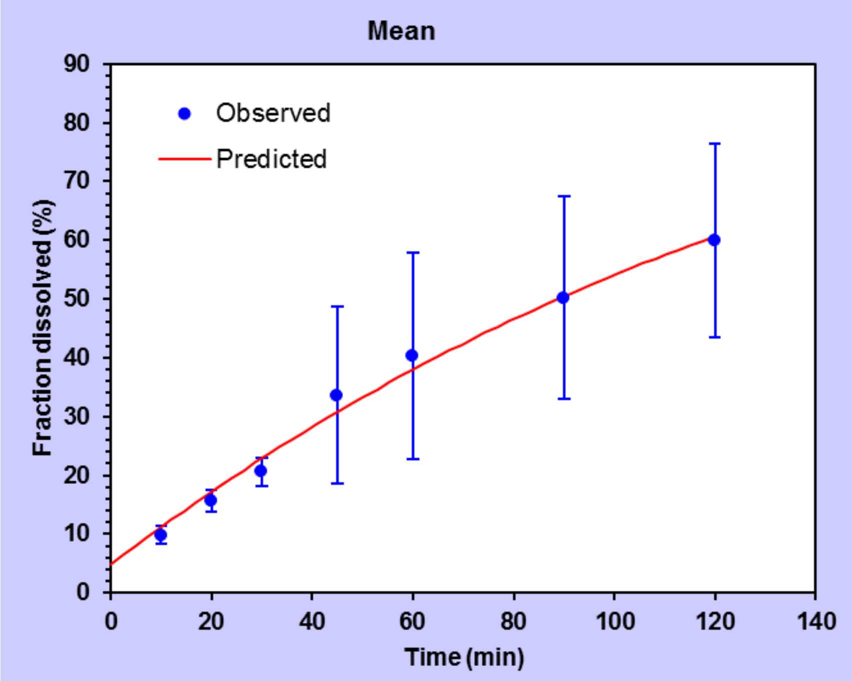

Supplementary materials – Model fitting summary – C*Pharm Mannidex 16700 (modelling up to app. 60 % of carvedilol released)

Graphical abstract of model fit presented as the fraction % of released carvedilol per tested tablet:

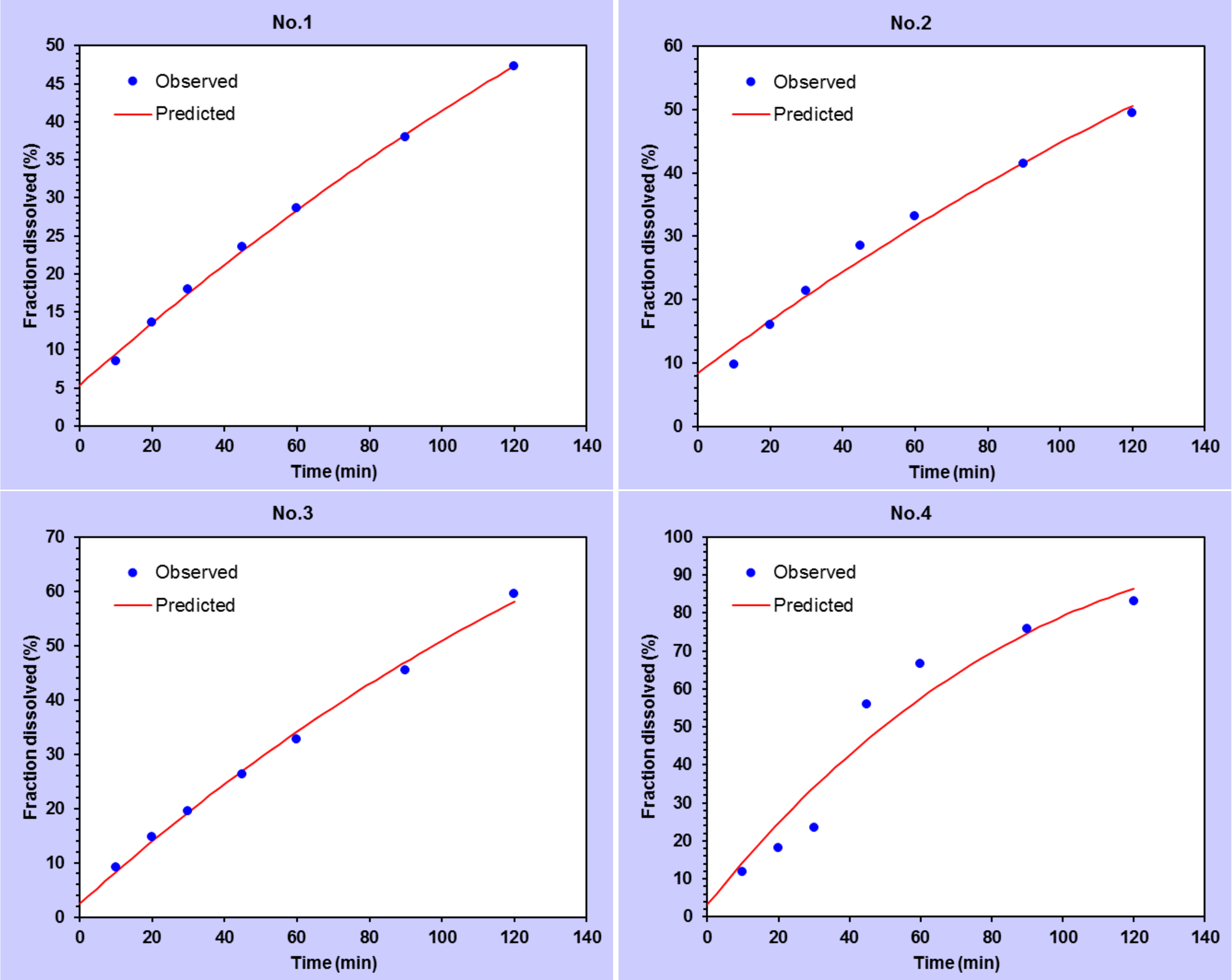

Supplementary materials – Model fitting summary – C*Pharm Mannidex 16700 (modelling up to app. 60 % of carvedilol released)

Model: **Hopfenberg**

Model equation: $F = 100 \cdot [1 - (1 - k_{HB} \cdot t)^n]$

Fitted model parameters per tested tablet (N = 4) with statistics – mean, standard deviation (SD), and relative standard deviation expressed in % (RSD%) (output from DDSolver):

Parameter	No.1	No.2	No.3	No.4	Mean	SD	RSD(%)
k_{HB}	0.002	0.002	0.002	0.004	0.002	0.001	45.625
n	3.000	3.000	3.000	3.000	3.000	0.000	0.000

Number of dissolution data points (N), degrees of freedom (df), and selected goodness of fit criteria – Pearson correlation coefficient (R), coefficient of determination (R^2), adjusted coefficient of determination ($R^2_{adjusted}$), and residual sum of squares (RSS) (manual calculation in MS Excel):

Parameter	No.1	No.2	No.3	No.4
N	7	7	7	7
df	5	5	5	5
R	0.999364579	0.994143311	0.997615912	0.970554848
R^2	0.998729562	0.988320922	0.995237509	0.941976713
$R^2_{adjusted}$	0.998475474	0.985985106	0.99428501	0.930372055
RSS	52.54561869	129.6567012	22.36083216	312.8676063

Graphical abstract of model fit presented as mean ± 1 SD of the fraction % of released carvedilol:

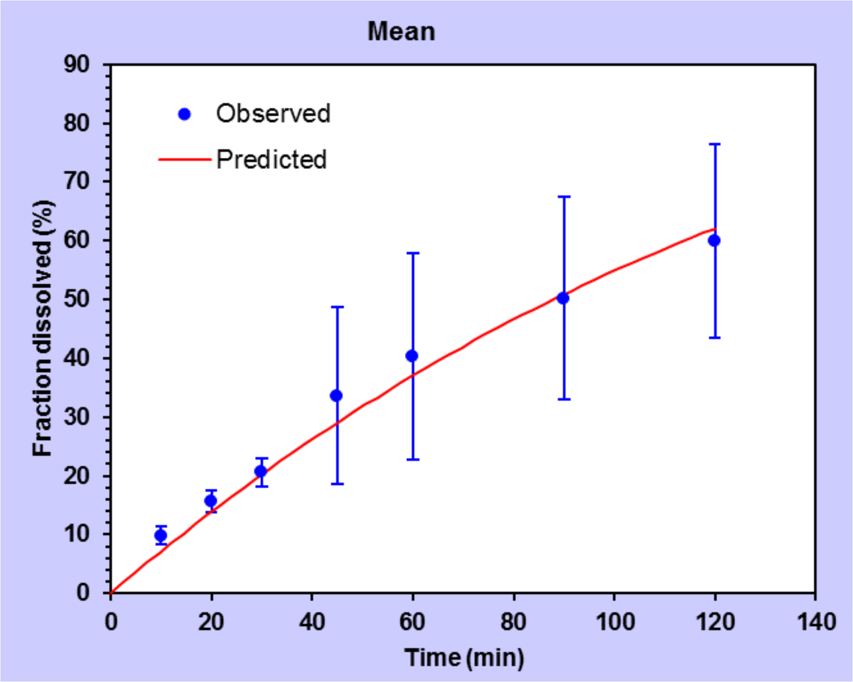

Supplementary materials – Model fitting summary – C*Pharm Mannidex 16700 (modelling up to app. 60 % of carvedilol released)

Graphical abstract of model fit presented as the fraction % of released carvedilol per tested tablet:

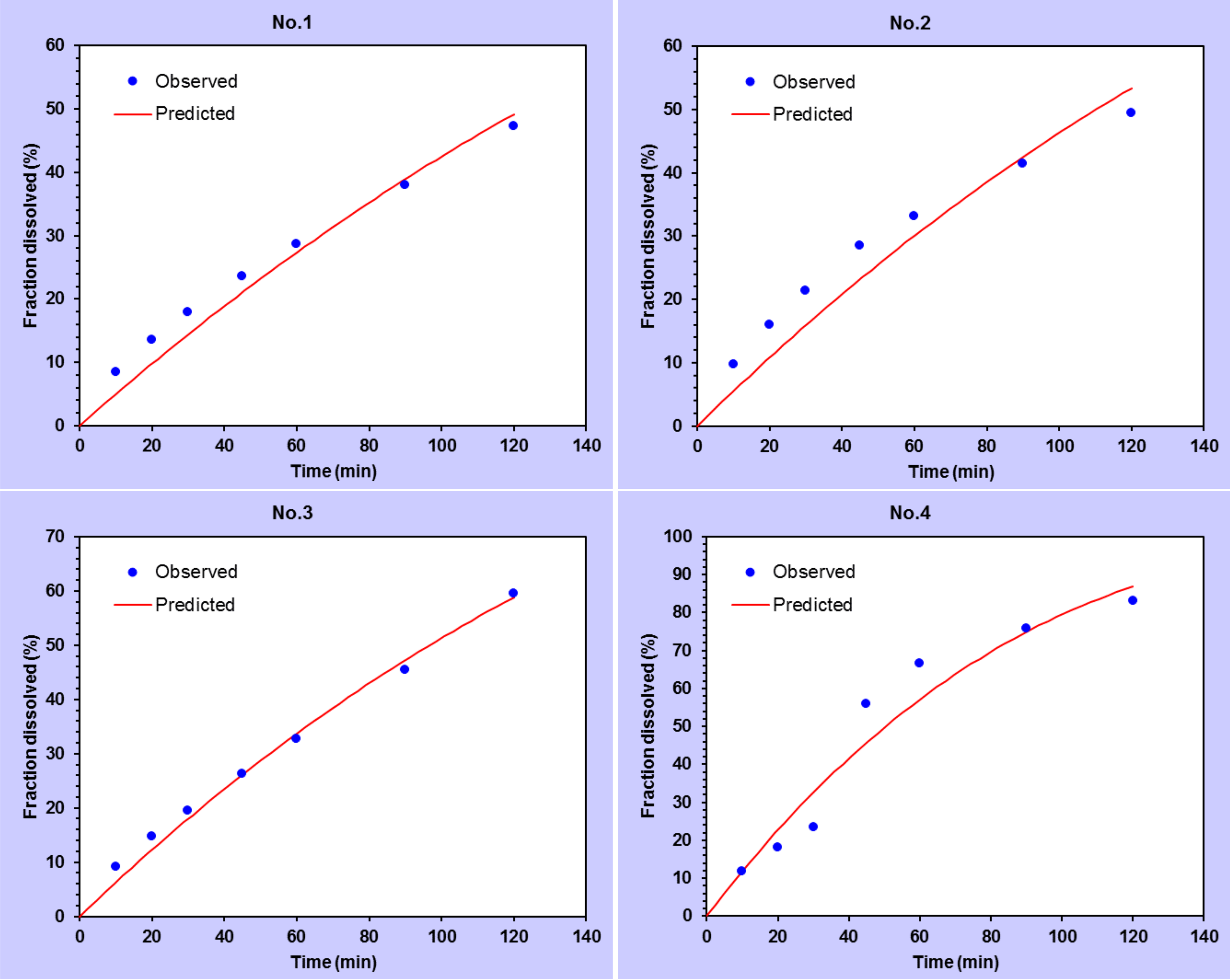

Supplementary materials – Model fitting summary – C*Pharm Mannidex 16700 (modelling up to app. 60 % of carvedilol released)

Model: **Hopfenberg with T_{lag}**

Model equation: $F = 100 \cdot \{1 - [1 - k_{HB} \cdot (t - T_{lag})]^n\}$

Fitted model parameters per tested tablet (N = 4) with statistics – mean, standard deviation (SD), and relative standard deviation expressed in % (RSD%) (output from DDSolver):

Parameter	No.1	No.2	No.3	No.4	Mean	SD	RSD(%)
k_{HB}	0.001	0.002	0.004	0.004	0.003	0.002	58.769
n	3.477	3.000	1.000	3.000	2.619	1.103	42.100
T_{lag}	-11.133	-19.176	-12.497	-2.693	-11.375	6.771	-59.529

Number of dissolution data points (N), degrees of freedom (df), and selected goodness of fit criteria – Pearson correlation coefficient (R), coefficient of determination (R^2), adjusted coefficient of determination ($R^2_{adjusted}$), and residual sum of squares (RSS) (manual calculation in MS Excel):

Parameter	No.1	No.2	No.3	No.4
N	7	7	7	7
df	4	4	4	4
R	0.99929471	0.992883205	0.999519153	0.969894686
R^2	0.998589918	0.985817058	0.999038537	0.940695702
$R^2_{adjusted}$	0.997884877	0.978725587	0.998557806	0.911043553
RSS	1.811501178	17.17417479	1.832649172	342.1364085

Graphical abstract of model fit presented as mean ± 1 SD of the fraction % of released carvedilol:

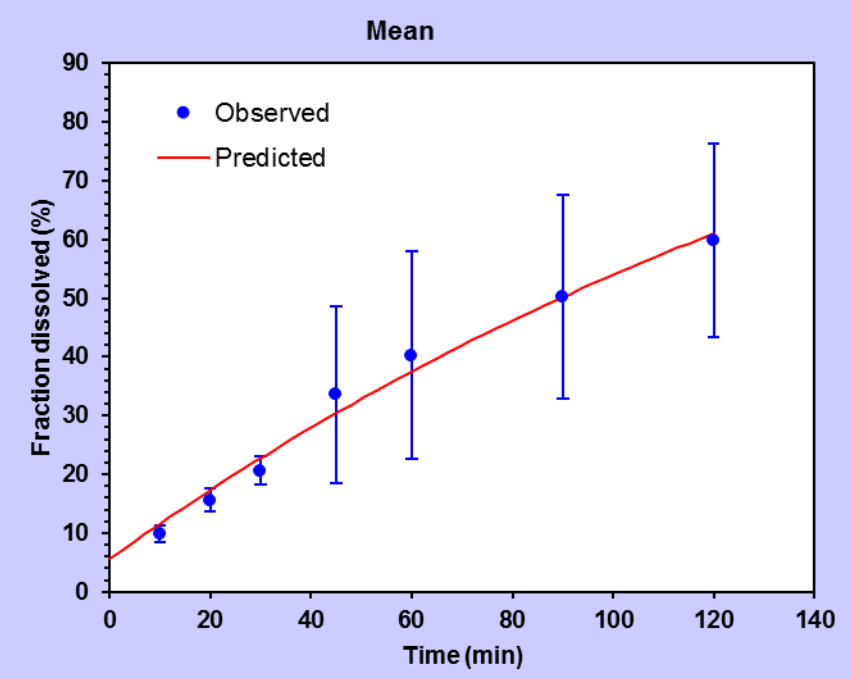

Supplementary materials – Model fitting summary – C*Pharm Mannidex 16700 (modelling up to app. 60 % of carvedilol released)

Graphical abstract of model fit presented as the fraction % of released carvedilol per tested tablet:

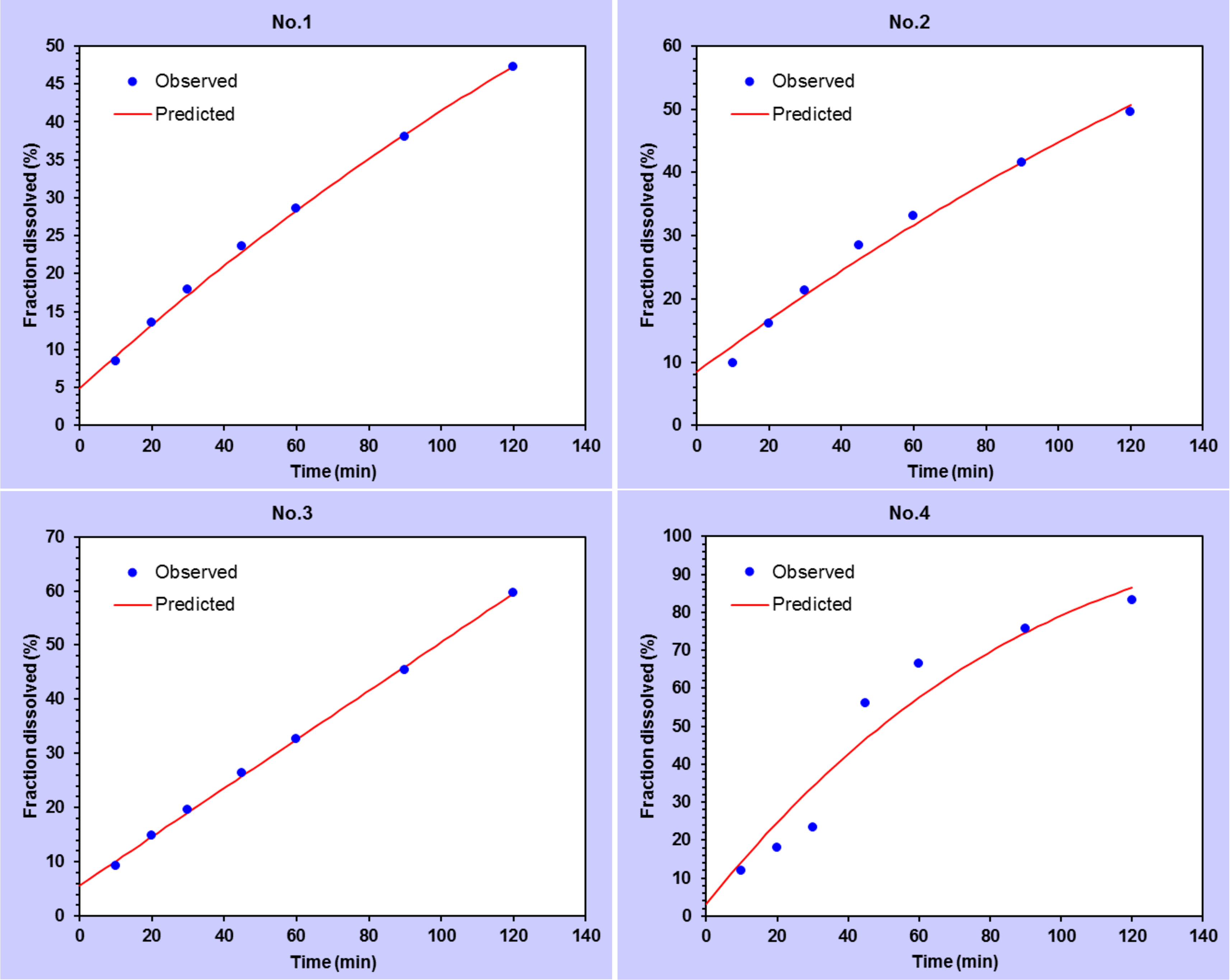

Supplementary materials – Model fitting summary – C*Pharm Mannidex 16700 (modelling up to app. 60 % of carvedilol released)

Model: **Baker–Lonsdale**

Model equation: $\frac{3}{2}\cdot\left[1-\left(1-\frac{F}{100}\right)^{\frac{2}{3}}\right]-\frac{F}{100}=k_{BL}\cdot t$

Fitted model parameters per tested tablet (N = 4) with statistics – mean, standard deviation (SD), and relative standard deviation expressed in % (RSD%) (output from DDSolver):

Parameter	No.1	No.2	No.3	No.4	Mean	SD	RSD(%)
k_{BL}	0.0002	0.0004	0.0007	0.0021	0.0008	0.0008	100.3762

Number of dissolution data points (N), degrees of freedom (df), and selected goodness of fit criteria – Pearson correlation coefficient (R), coefficient of determination (R^2), adjusted coefficient of determination ($R^2_{adjusted}$), and residual sum of squares (RSS) (manual calculation in MS Excel):

Parameter	No.1	No.2	No.3	No.4
N	7	7	7	7
df	6	6	6	6
R	0.994804937	0.999420342	0.982417646	0.970181953
R^2	0.989636863	0.998841021	0.965144431	0.941253022
$R^2_{adjusted}$	0.989636863	0.998841021	0.965144431	0.941253022
RSS	201.4417195	64.56537143	872.467211	1999.086441

Graphical abstract of model fit presented as mean ± 1 SD of the fraction % of released carvedilol:

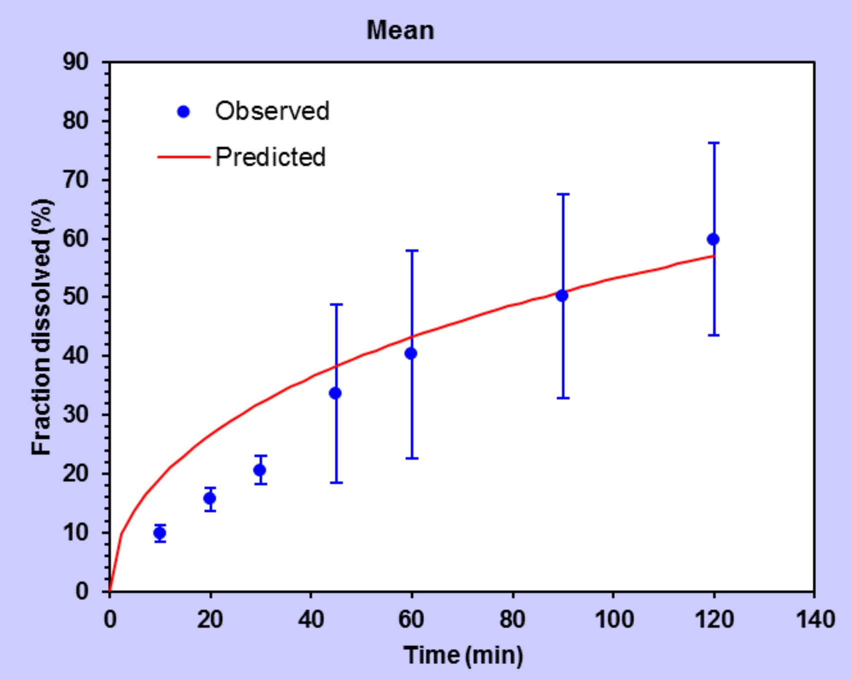

Supplementary materials – Model fitting summary – C*Pharm Mannidex 16700 (modelling up to app. 60 % of carvedilol released)

Graphical abstract of model fit presented as the fraction % of released carvedilol per tested tablet:

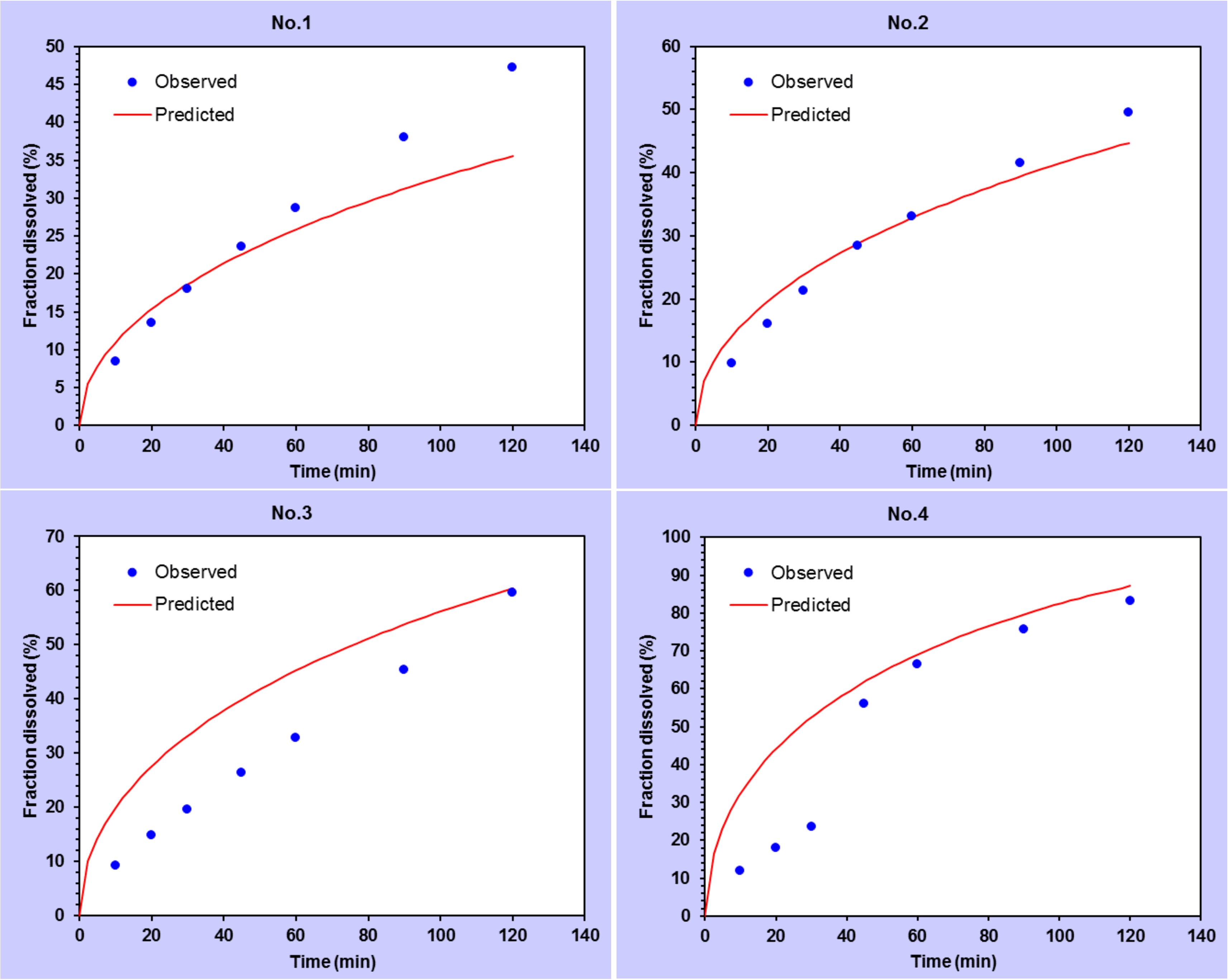

Supplementary materials – Model fitting summary – C*Pharm Mannidex 16700 (modelling up to app. 60 % of carvedilol released)

Model: **Baker–Lonsdale with T_{lag}**

Model equation: $\frac{3}{2}\cdot\left[1-\left(1-\frac{F}{100}\right)^{\frac{2}{3}}\right]-\frac{F}{100}=k_{BL}\cdot\left(t-T_{lag}\right)$

Fitted model parameters per tested tablet (N = 4) with statistics – mean, standard deviation (SD), and relative standard deviation expressed in % (RSD%) (output from DDSolver):

Parameter	No.1	No.2	No.3	No.4	Mean	SD	RSD(%)
k_{BL}	0.0004	0.0005	0.0007	0.0021	0.0009	0.0008	83.5565
T_{lag}	14.9350	10.7047	19.1812	13.6670	14.6220	3.5186	24.0637

Number of dissolution data points (N), degrees of freedom (df), and selected goodness of fit criteria – Pearson correlation coefficient (R), coefficient of determination (R^2), adjusted coefficient of determination ($R^2_{adjusted}$), and residual sum of squares (RSS) (manual calculation in MS Excel):

Parameter	No.1	No.2	No.3	No.4
N	7	7	7	7
df	5	5	5	5
R	0.976681486	0.980648747	0.963085262	0.950848414
R^2	0.953906725	0.961671965	0.927533222	0.904112706
$R^2_{adjusted}$	0.944688069	0.954006358	0.913039867	0.884935247
RSS	95.7561902	99.5928471	236.551769	514.915553

Graphical abstract of model fit presented as mean ± 1 SD of the fraction % of released carvedilol:

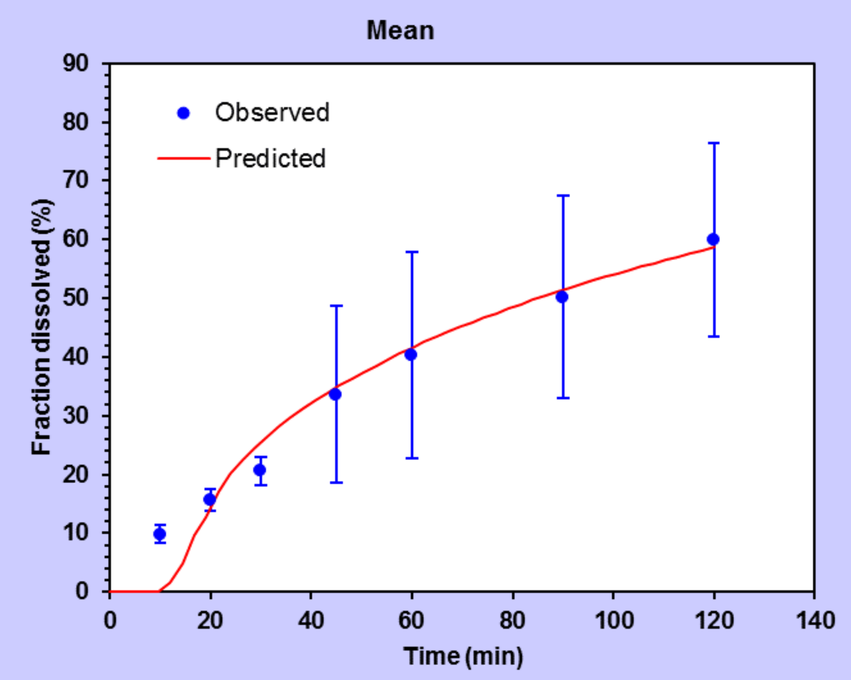

Supplementary materials – Model fitting summary – C*Pharm Mannidex 16700 (modelling up to app. 60 % of carvedilol released)

Graphical abstract of model fit presented as the fraction % of released carvedilol per tested tablet:

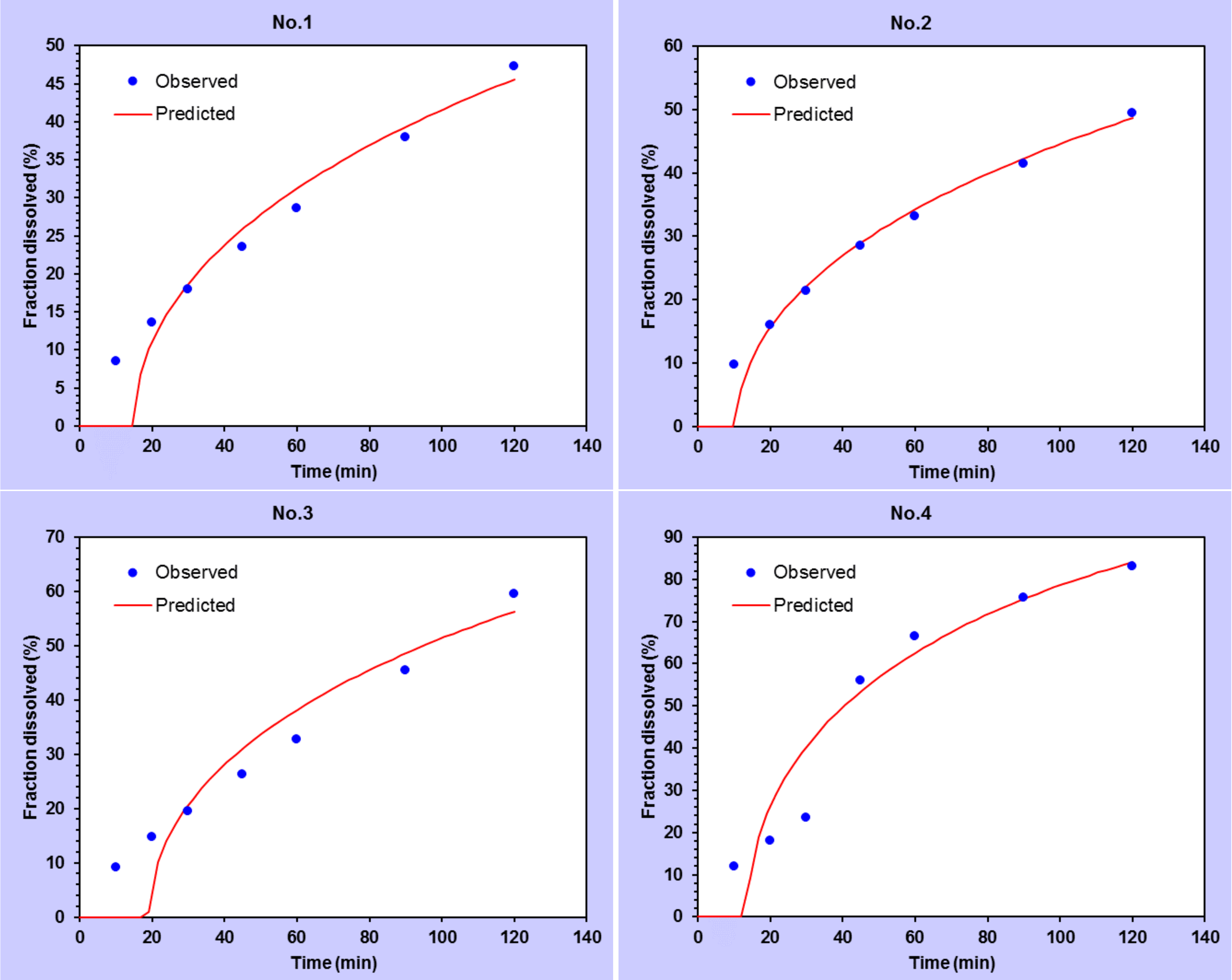

Supplementary materials – Model fitting summary – C*Pharm Mannidex 16700 (modelling up to app. 60 % of carvedilol released)

Model: **Makoid–Banakar**

Model equation: $F = k_{MB} \cdot t^{n} \cdot e^{-k \cdot t}$

Fitted model parameters per tested tablet (N = 4) with statistics – mean, standard deviation (SD), and relative standard deviation expressed in % (RSD%) (output from DDSolver):

Parameter	No.1	No.2	No.3	No.4	Mean	SD	RSD(%)
k_{MB}	1.836	1.787	2.064	0.862	1.637	0.531	32.416
n	0.664	0.750	0.640	1.102	0.789	0.214	27.126
k	-0.001	0.002	-0.002	0.005	0.001	0.003	290.488

Number of dissolution data points (N), degrees of freedom (df), and selected goodness of fit criteria – Pearson correlation coefficient (R), coefficient of determination (R^2), adjusted coefficient of determination ($R^2_{adjusted}$), and residual sum of squares (RSS) (manual calculation in MS Excel):

Parameter	No.1	No.2	No.3	No.4
N	7	7	7	7
df	4	4	4	4
R	0.99994181	0.999454655	0.999936166	0.969496051
R^2	0.999883623	0.998909608	0.999872336	0.939922593
$R^2_{adjusted}$	0.999825435	0.998364411	0.999808504	0.90988389
RSS	0.132365093	1.294796535	0.246695558	317.2795058

Graphical abstract of model fit presented as mean ± 1 SD of the fraction % of released carvedilol:

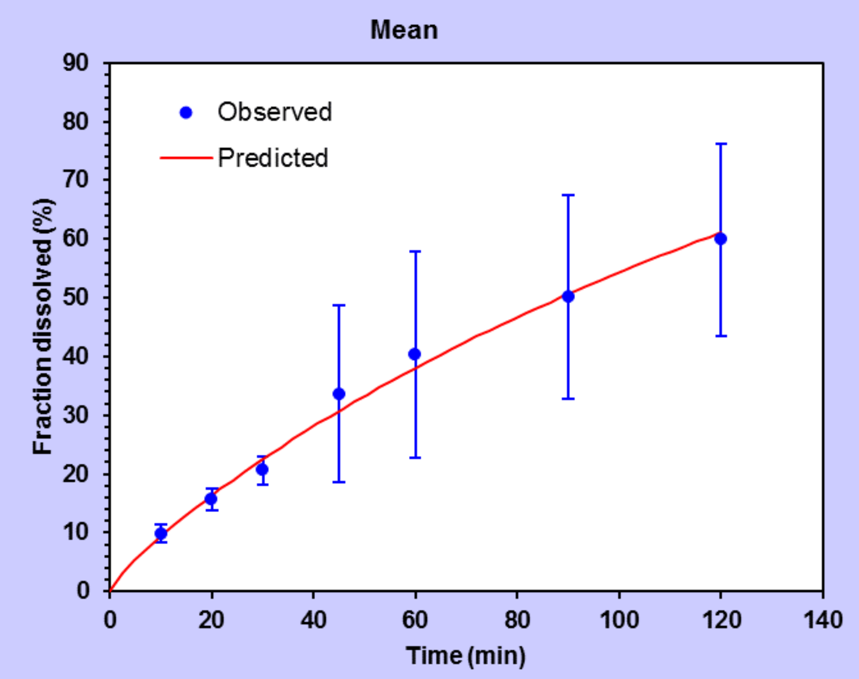

Supplementary materials – Model fitting summary – C*Pharm Mannidex 16700 (modelling up to app. 60 % of carvedilol released)

Graphical abstract of model fit presented as the fraction % of released carvedilol per tested tablet:

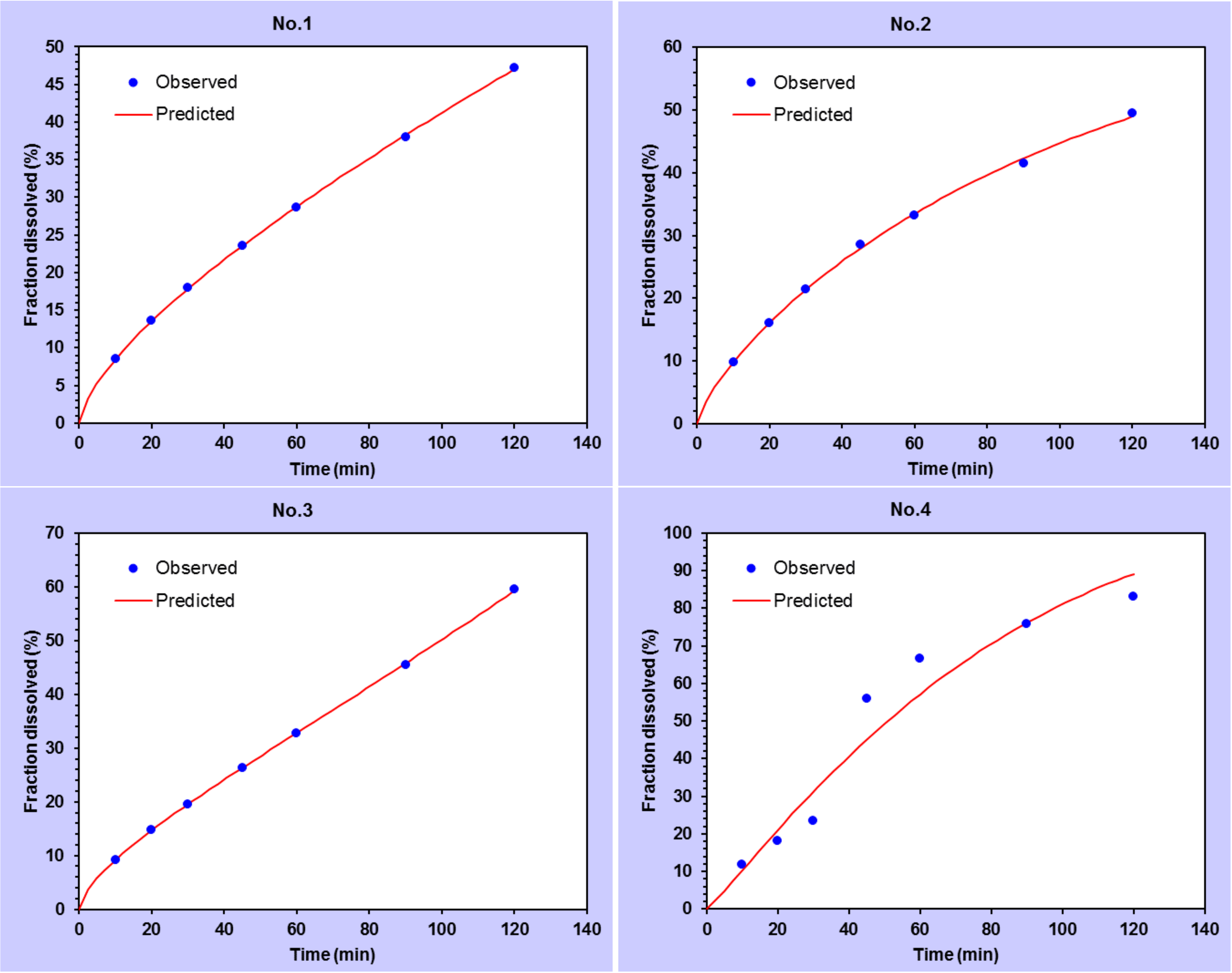

Supplementary materials – Model fitting summary – C*Pharm Mannidex 16700 (modelling up to app. 60 % of carvedilol released)

Model: **Makoid–Banakar with T_{lag}**

Model equation: $F = k_{MB} \cdot (t - T_{lag})^n \cdot e^{-k \cdot (t - T_{lag})}$

Fitted model parameters per tested tablet (N = 4) with statistics – mean, standard deviation (SD), and relative standard deviation expressed in % (RSD%) (output from DDSolver):

Parameter	No.1	No.2	No.3	No.4	Mean	SD	RSD(%)
k_{MB}	3.487	3.645	3.858	2.597	3.397	0.554	16.315
n	0.482	0.544	0.464	0.773	0.566	0.142	25.140
k	-0.003	0.000	-0.005	0.001	-0.002	0.003	-151.724
T_{lag}	4.000	4.000	4.000	4.000	4.000	0.000	0.000

Number of dissolution data points (N), degrees of freedom (df), and selected goodness of fit criteria – Pearson correlation coefficient (R), coefficient of determination (R^2), adjusted coefficient of determination ($R^2_{adjusted}$), and residual sum of squares (RSS) (manual calculation in MS Excel):

Parameter	No.1	No.2	No.3	No.4
N	7	7	7	7
df	3	3	3	3
R	0.999864652	0.999595365	0.99989698	0.959292102
R^2	0.999729323	0.999190894	0.99979397	0.920241338
$R^2_{adjusted}$	0.999458646	0.998381788	0.999587941	0.840482675
RSS	0.313114396	0.962312406	0.411771967	424.0689015

Graphical abstract of model fit presented as mean ± 1 SD of the fraction % of released carvedilol:

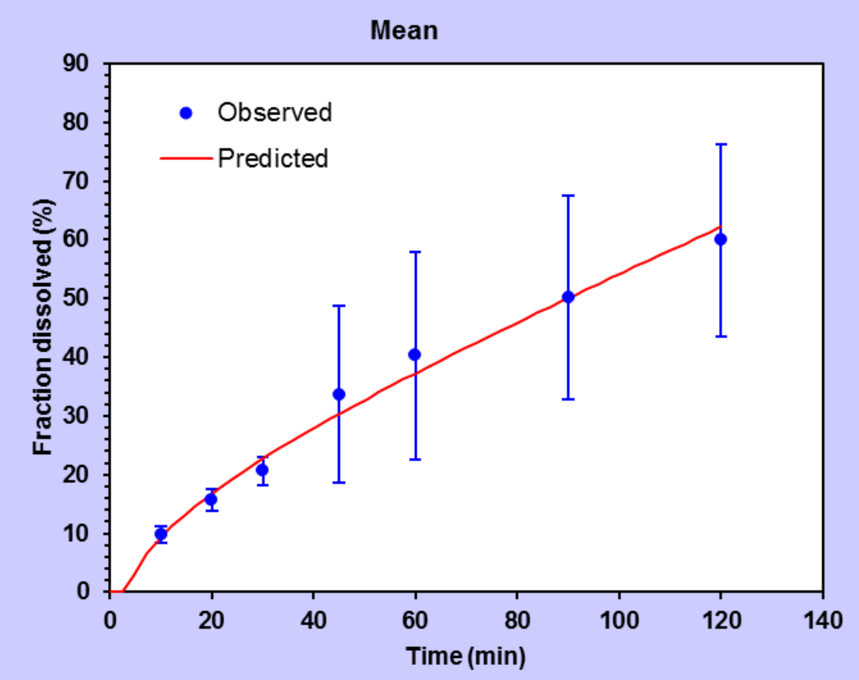

Supplementary materials – Model fitting summary – C*Pharm Mannidex 16700 (modelling up to app. 60 % of carvedilol released)

Graphical abstract of model fit presented as the fraction % of released carvedilol per tested tablet:

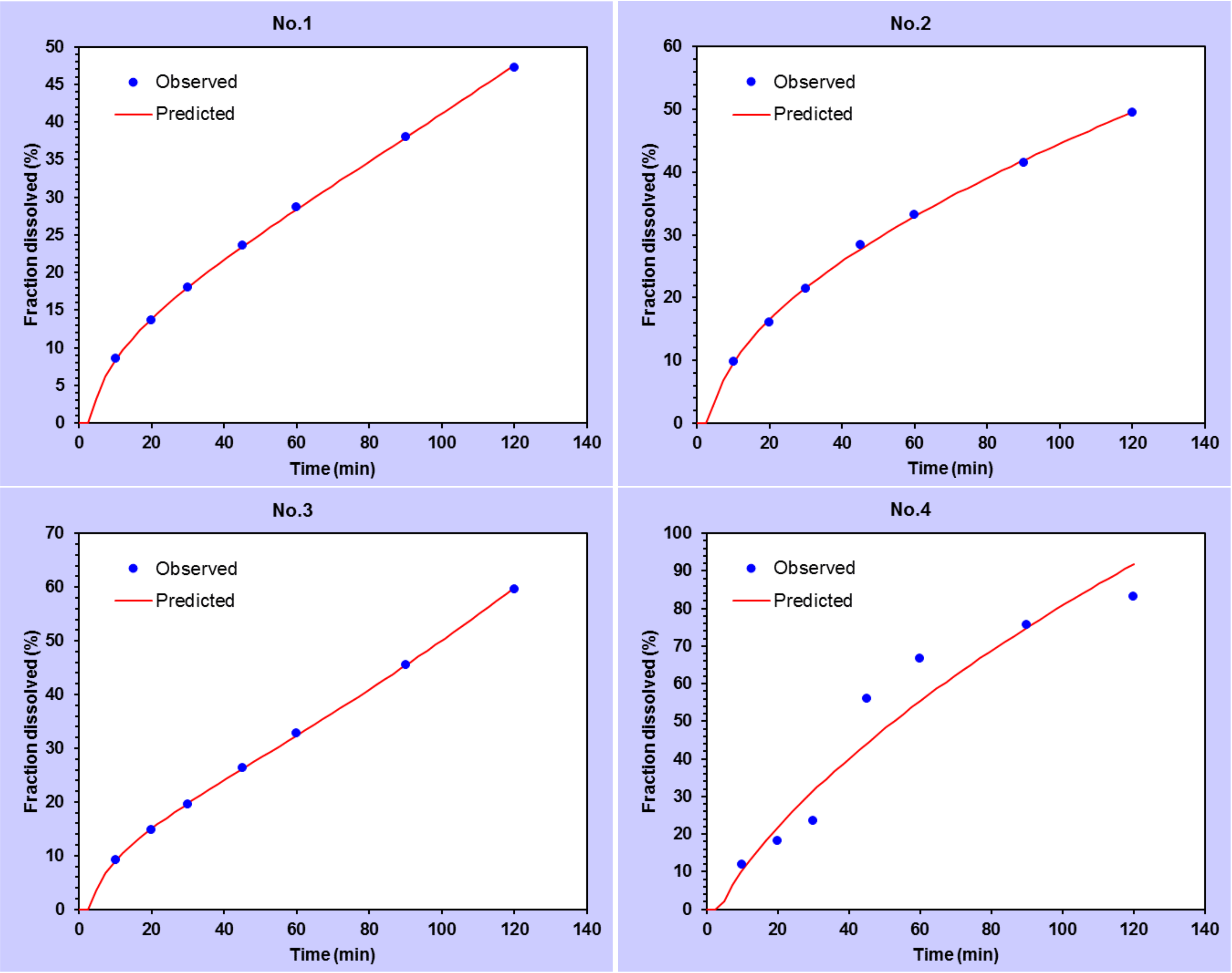

Supplementary materials – Model fitting summary – C*Pharm Mannidex 16700 (modelling up to app. 60 % of carvedilol released)

Model: **Peppas–Sahlin_1**

Model equation: $F = k_1 \cdot t^m + k_2 \cdot t^{2m}$

Fitted model parameters per tested tablet (N = 4) with statistics – mean, standard deviation (SD), and relative standard deviation expressed in % (RSD%) (output from DDSolver):

Parameter	No.1	No.2	No.3	No.4	Mean	SD	RSD(%)
k_1	1.972	3.181	1.221	3.389	2.441	1.025	42.010
k_2	0.406	0.306	0.647	0.835	0.548	0.238	43.454
m	0.450	0.450	0.450	0.450	0.450	0.000	0.000

Number of dissolution data points (N), degrees of freedom (df), and selected goodness of fit criteria – Pearson correlation coefficient (R), coefficient of determination (R^2), adjusted coefficient of determination ($R^2_{adjusted}$), and residual sum of squares (RSS) (manual calculation in MS Excel):

Parameter	No.1	No.2	No.3	No.4
N	7	7	7	7
df	4	4	4	4
R	0.999912038	0.997476623	0.999220061	0.949982017
R^2	0.999824084	0.994959613	0.998440731	0.902465832
$R^2_{adjusted}$	0.999736126	0.99243942	0.997661097	0.853698748
RSS	0.202859963	6.362441975	3.053973192	519.5792725

Graphical abstract of model fit presented as mean ± 1 SD of the fraction % of released carvedilol:

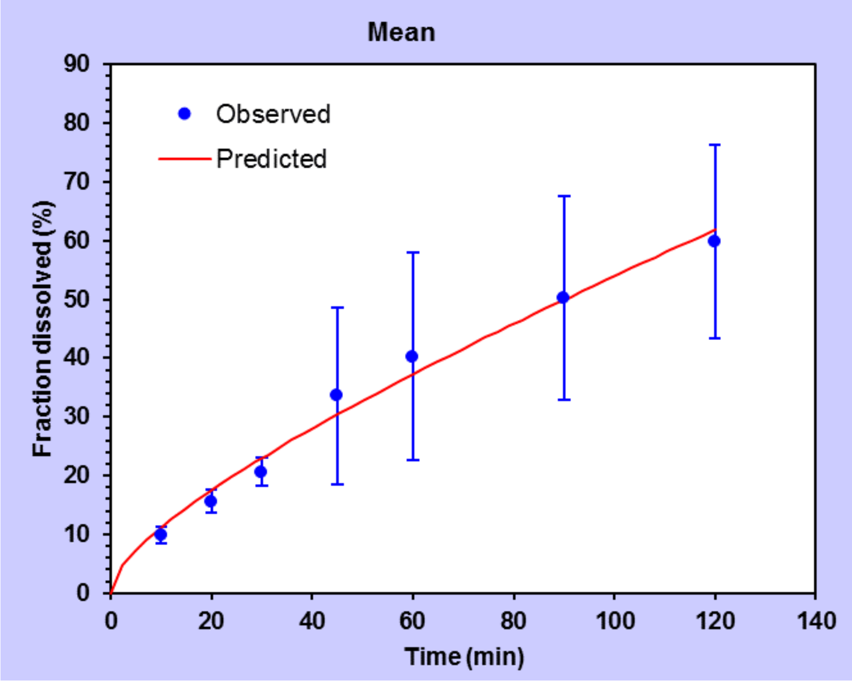

Supplementary materials – Model fitting summary – C*Pharm Mannidex 16700 (modelling up to app. 60 % of carvedilol released)

Graphical abstract of model fit presented as the fraction % of released carvedilol per tested tablet:

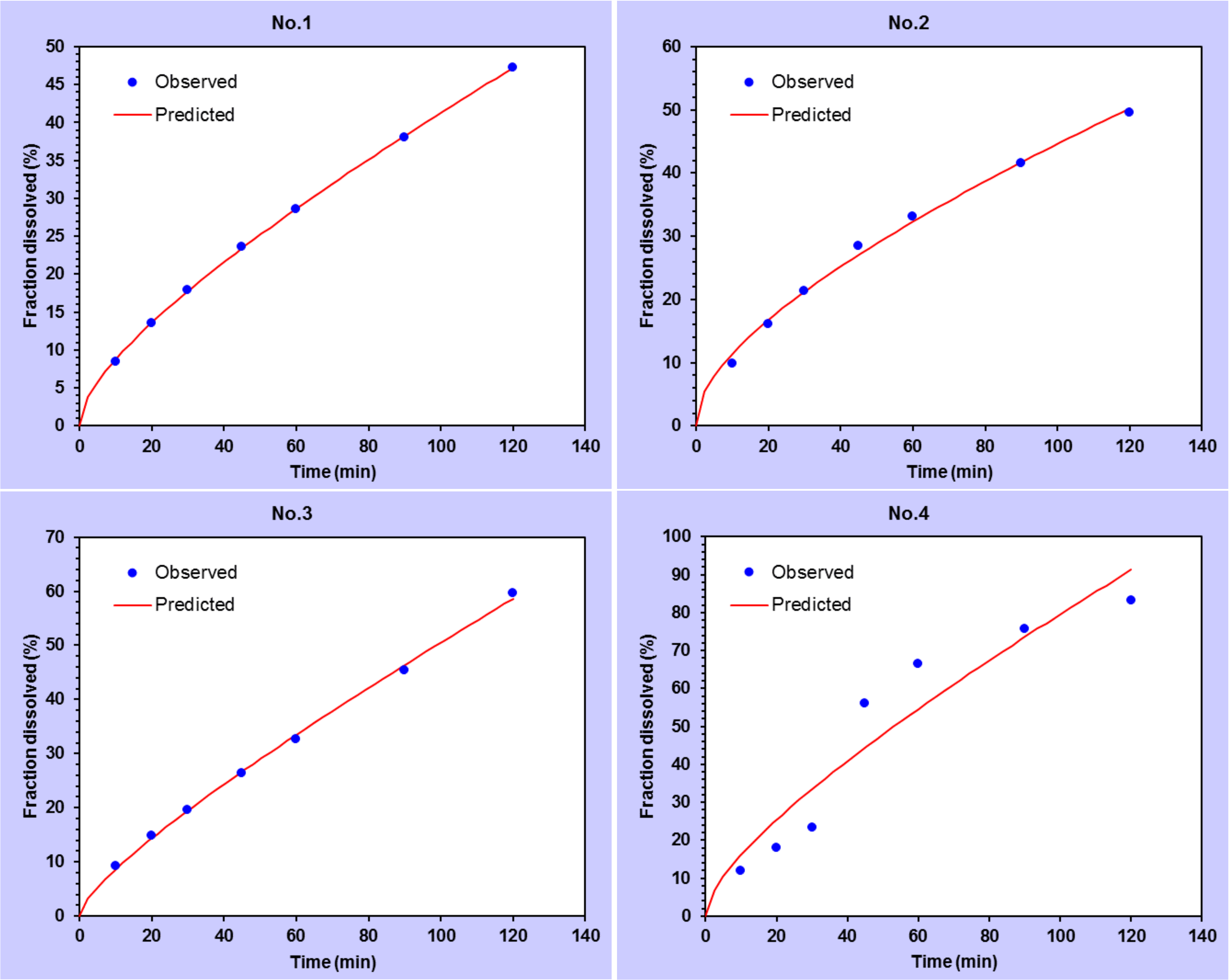

Supplementary materials – Model fitting summary – C*Pharm Mannidex 16700 (modelling up to app. 60 % of carvedilol released)

Model: **Peppas–Sahlin_1 with T_{lag}**

Model equation: $F = k_1 \cdot (t - T_{lag})^m + k_2 \cdot (t - T_{lag})^{2m}$

Fitted model parameters per tested tablet (N = 4) with statistics – mean, standard deviation (SD), and relative standard deviation expressed in % (RSD%) (output from DDSolver):

Parameter	No.1	No.2	No.3	No.4	Mean	SD	RSD(%)
k_1	2.741	4.067	2.108	4.989	3.476	1.297	37.317
k_2	0.327	0.210	0.559	0.667	0.441	0.209	47.443
m	0.450	0.450	0.450	0.450	0.450	0.000	0.000
T_{lag}	4.000	4.000	4.000	6.000	4.500	1.000	22.222

Number of dissolution data points (N), degrees of freedom (df), and selected goodness of fit criteria – Pearson correlation coefficient (R), coefficient of determination (R^2), adjusted coefficient of determination ($R^2_{adjusted}$), and residual sum of squares (RSS) (manual calculation in MS Excel):

Parameter	No.1	No.2	No.3	No.4
N	7	7	7	7
df	3	3	3	3
R	0.999599583	0.999350653	0.998117359	0.956057808
R^2	0.999199326	0.998701727	0.996238263	0.914046533
$R^2_{adjusted}$	0.998398652	0.997403454	0.992476525	0.828093066
RSS	0.993824577	1.6191543	7.693024646	459.0898416

Graphical abstract of model fit presented as mean ± 1 SD of the fraction % of released carvedilol:

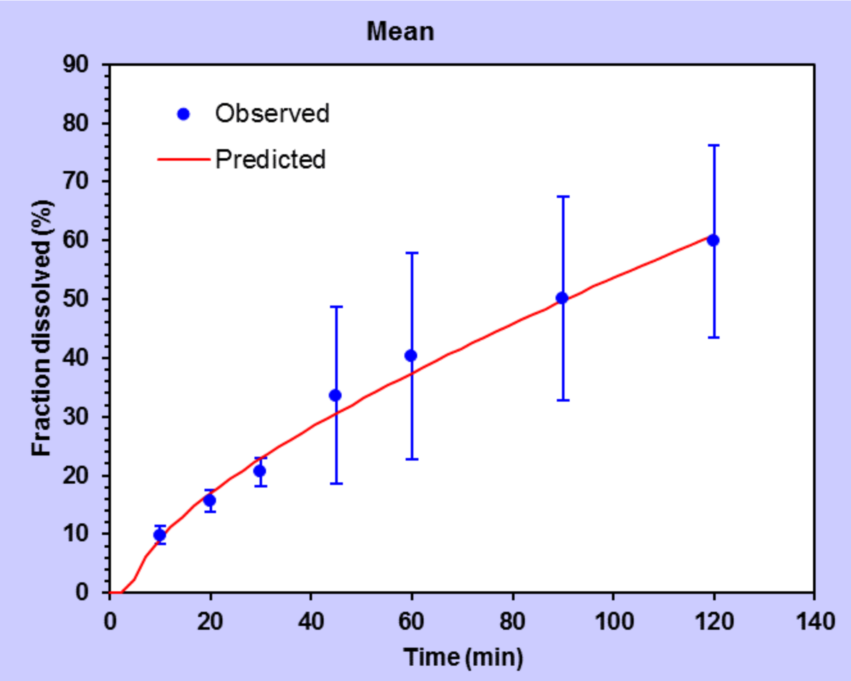

Supplementary materials – Model fitting summary – C*Pharm Mannidex 16700 (modelling up to app. 60 % of carvedilol released)

Graphical abstract of model fit presented as the fraction % of released carvedilol per tested tablet:

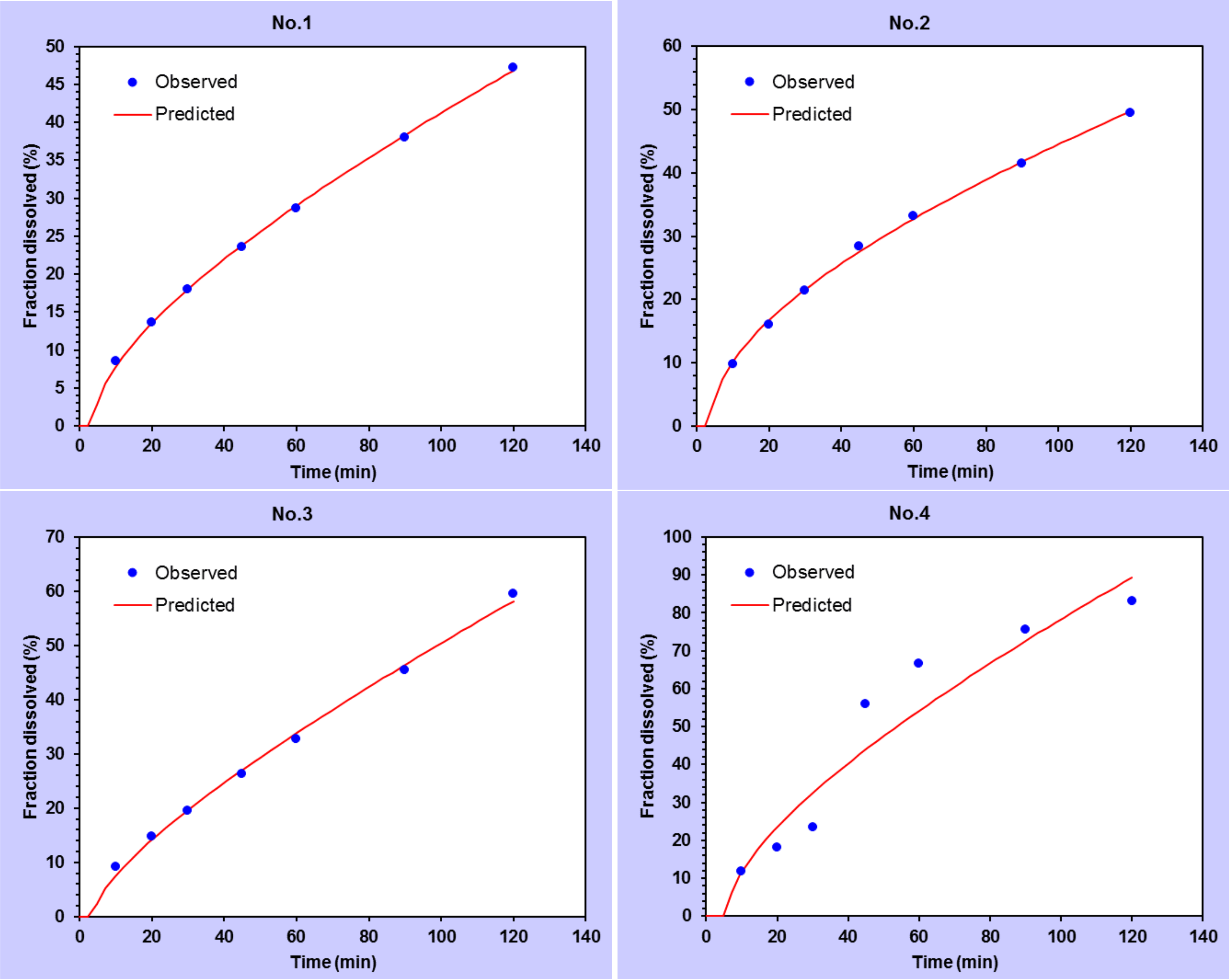

Supplementary materials – Model fitting summary – C*Pharm Mannidex 16700 (modelling up to app. 60 % of carvedilol released)

Model: **Peppas–Sahlin_2**

Model equation: $F = k_1 \cdot t^{0.5} + k_2 \cdot t$

Fitted model parameters per tested tablet (N = 4) with statistics – mean, standard deviation (SD), and relative standard deviation expressed in % (RSD%) (output from DDSolver):

Parameter	No.1	No.2	No.3	No.4	Mean	SD	RSD(%)
k_1	2.184	3.204	1.720	4.056	2.791	1.046	37.492
k_2	0.194	0.125	0.333	0.387	0.260	0.121	46.624

Number of dissolution data points (N), degrees of freedom (df), and selected goodness of fit criteria – Pearson correlation coefficient (R), coefficient of determination (R^2), adjusted coefficient of determination ($R^2_{adjusted}$), and residual sum of squares (RSS) (manual calculation in MS Excel):

Parameter	No.1	No.2	No.3	No.4
N	7	7	7	7
df	5	5	5	5
R	0.999898116	0.997687826	0.999476804	0.949637543
R^2	0.999796243	0.995380999	0.998953881	0.901811463
$R^2_{adjusted}$	0.999755491	0.994457199	0.998744658	0.882173756
RSS	0.240750723	5.991848118	2.061924685	529.5261627

Graphical abstract of model fit presented as mean ± 1 SD of the fraction % of released carvedilol:

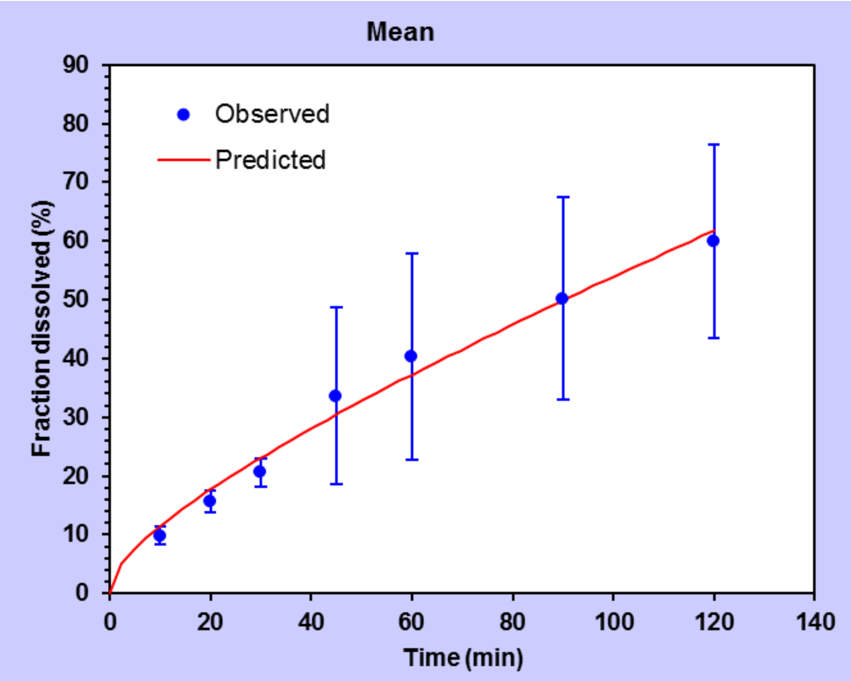

Supplementary materials – Model fitting summary – C*Pharm Mannidex 16700 (modelling up to app. 60 % of carvedilol released)

Graphical abstract of model fit presented as the fraction % of released carvedilol per tested tablet:

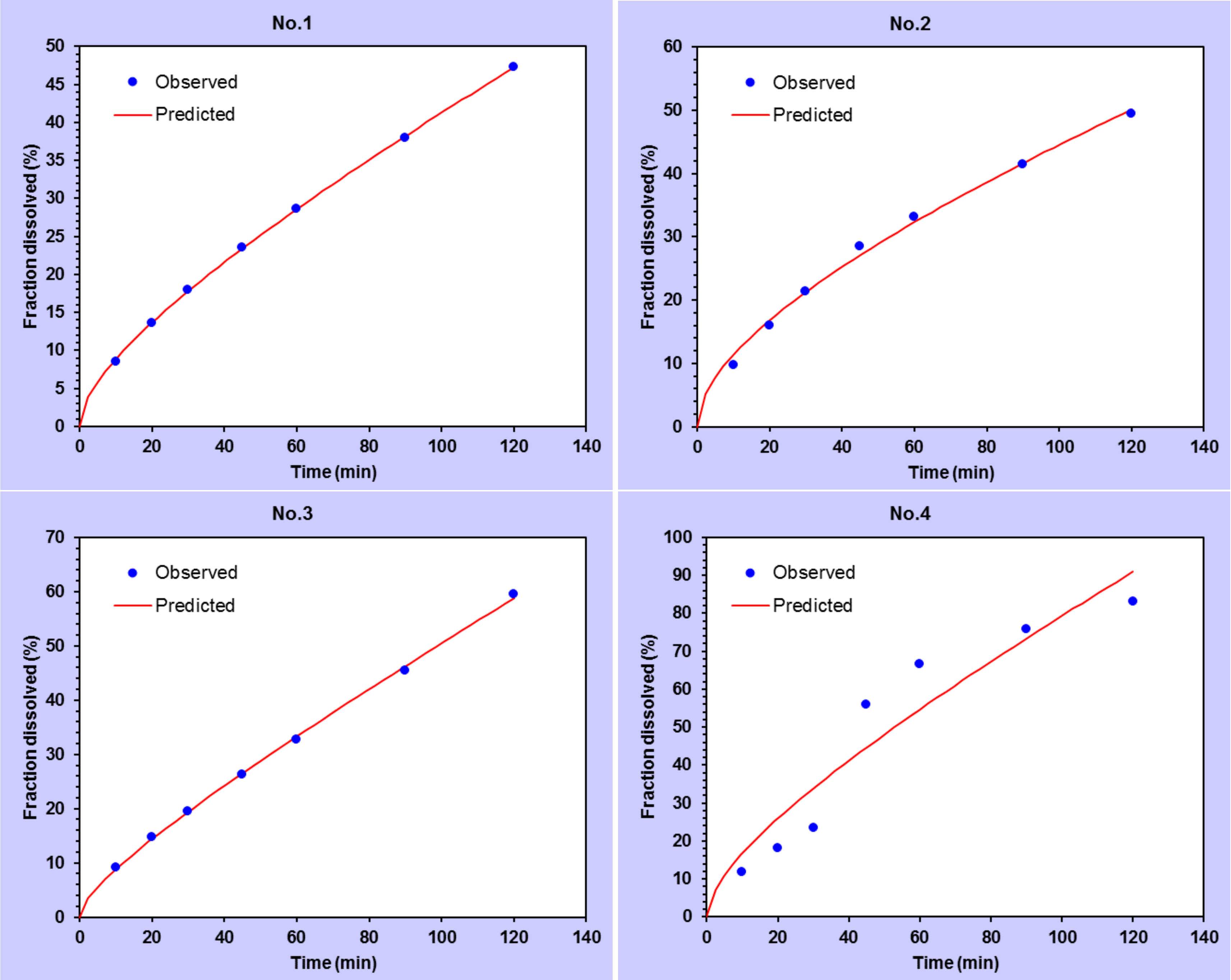

Supplementary materials – Model fitting summary – C*Pharm Mannidex 16700 (modelling up to app. 60 % of carvedilol released)

Model: **Peppas–Sahlin_2 with T_{lag}**

Model equation: $F = k_1 \cdot (t - T_{lag})^{0.5} + k_2 \cdot (t - T_{lag})$

Fitted model parameters per tested tablet (N = 4) with statistics – mean, standard deviation (SD), and relative standard deviation expressed in % (RSD%) (output from DDSolver):

Parameter	No.1	No.2	No.3	No.4	Mean	SD	RSD(%)
k_1	2.791	3.912	2.414	5.347	3.616	1.318	36.437
k_2	0.145	0.064	0.279	0.279	0.192	0.106	55.296
T_{lag}	4.000	4.000	4.000	6.000	4.500	1.000	22.222

Number of dissolution data points (N), degrees of freedom (df), and selected goodness of fit criteria – Pearson correlation coefficient (R), coefficient of determination (R^2), adjusted coefficient of determination ($R^2_{adjusted}$), and residual sum of squares (RSS) (manual calculation in MS Excel):

Parameter	No.1	No.2	No.3	No.4
N	7	7	7	7
df	4	4	4	4
R	0.999584369	0.999508477	0.99844542	0.956641385
R^2	0.999168911	0.999017195	0.996893257	0.91516274
$R^2_{adjusted}$	0.998753367	0.998525792	0.995339886	0.87274411
RSS	1.075646226	1.215679462	6.553196393	454.3061865

Graphical abstract of model fit presented as mean ± 1 SD of the fraction % of released carvedilol:

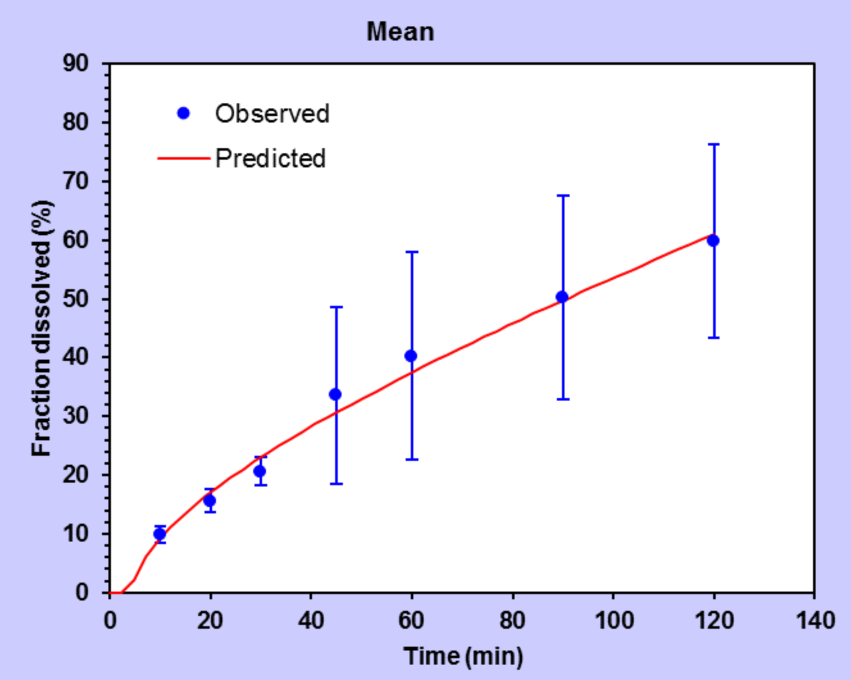

Supplementary materials – Model fitting summary – C*Pharm Mannidex 16700 (modelling up to app. 60 % of carvedilol released)

Graphical abstract of model fit presented as the fraction % of released carvedilol per tested tablet:

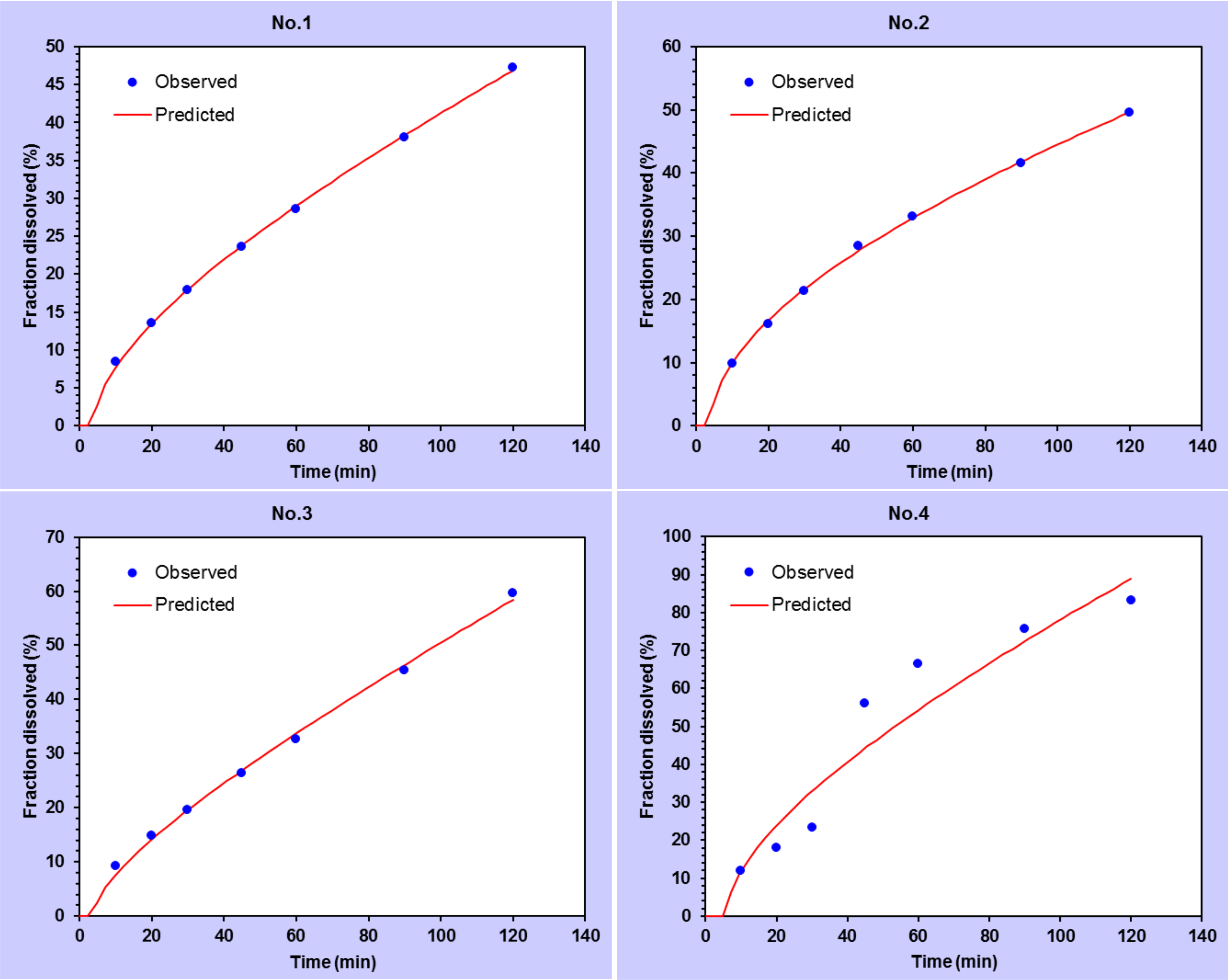

Supplementary materials – Model fitting summary – C*Pharm Mannidex 16700 (modelling up to app. 60 % of carvedilol released)

Model: **Quadratic**

Model equation: $F = 100 \cdot (k_1 \cdot t^2 + k_2 \cdot t)$

Fitted model parameters per tested tablet (N = 4) with statistics – mean, standard deviation (SD), and relative standard deviation expressed in % (RSD%) (output from DDSolver):

Parameter	No.1	No.2	No.3	No.4	Mean	SD	RSD(%)
k_1	-0.00002	-0.00003	-0.00001	-0.00005	-0.00003	0.00002	-57.28477
k_2	0.00616	0.00757	0.00653	0.01313	0.00835	0.00324	38.85466

Number of dissolution data points (N), degrees of freedom (df), and selected goodness of fit criteria – Pearson correlation coefficient (R), coefficient of determination (R^2), adjusted coefficient of determination ($R^2_{adjusted}$), and residual sum of squares (RSS) (manual calculation in MS Excel):

Parameter	No.1	No.2	No.3	No.4
N	7	7	7	7
df	5	5	5	5
R	0.995603417	0.99470855	0.996477192	0.978085317
R^2	0.991226164	0.989445099	0.992966793	0.956650887
$R^2_{adjusted}$	0.989471397	0.987334119	0.991560152	0.947981064
RSS	18.50959959	23.86573558	22.55367698	259.6270571

Graphical abstract of model fit presented as mean ± 1 SD of the fraction % of released carvedilol:

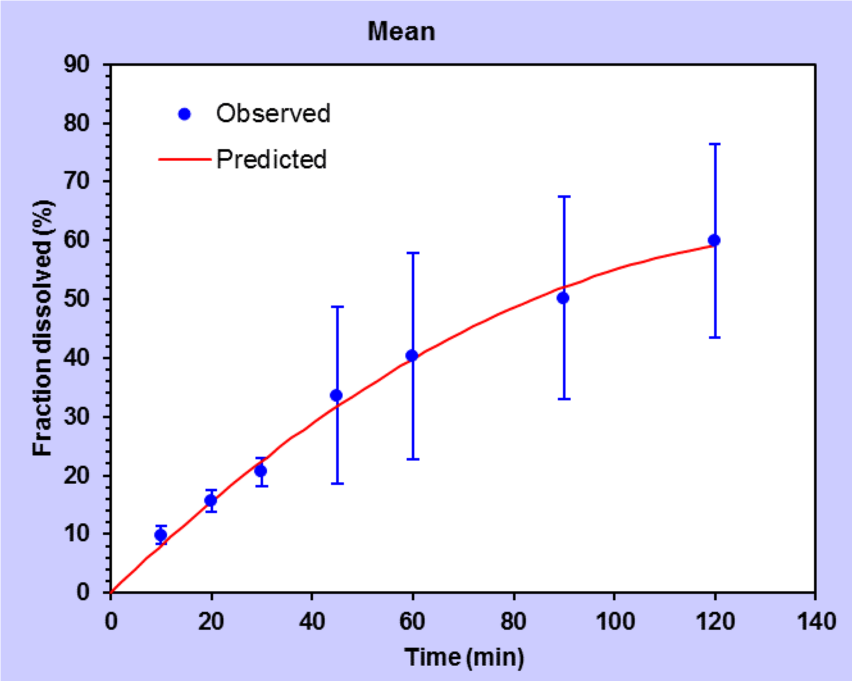

Supplementary materials – Model fitting summary – C*Pharm Mannidex 16700 (modelling up to app. 60 % of carvedilol released)

Graphical abstract of model fit presented as the fraction % of released carvedilol per tested tablet:

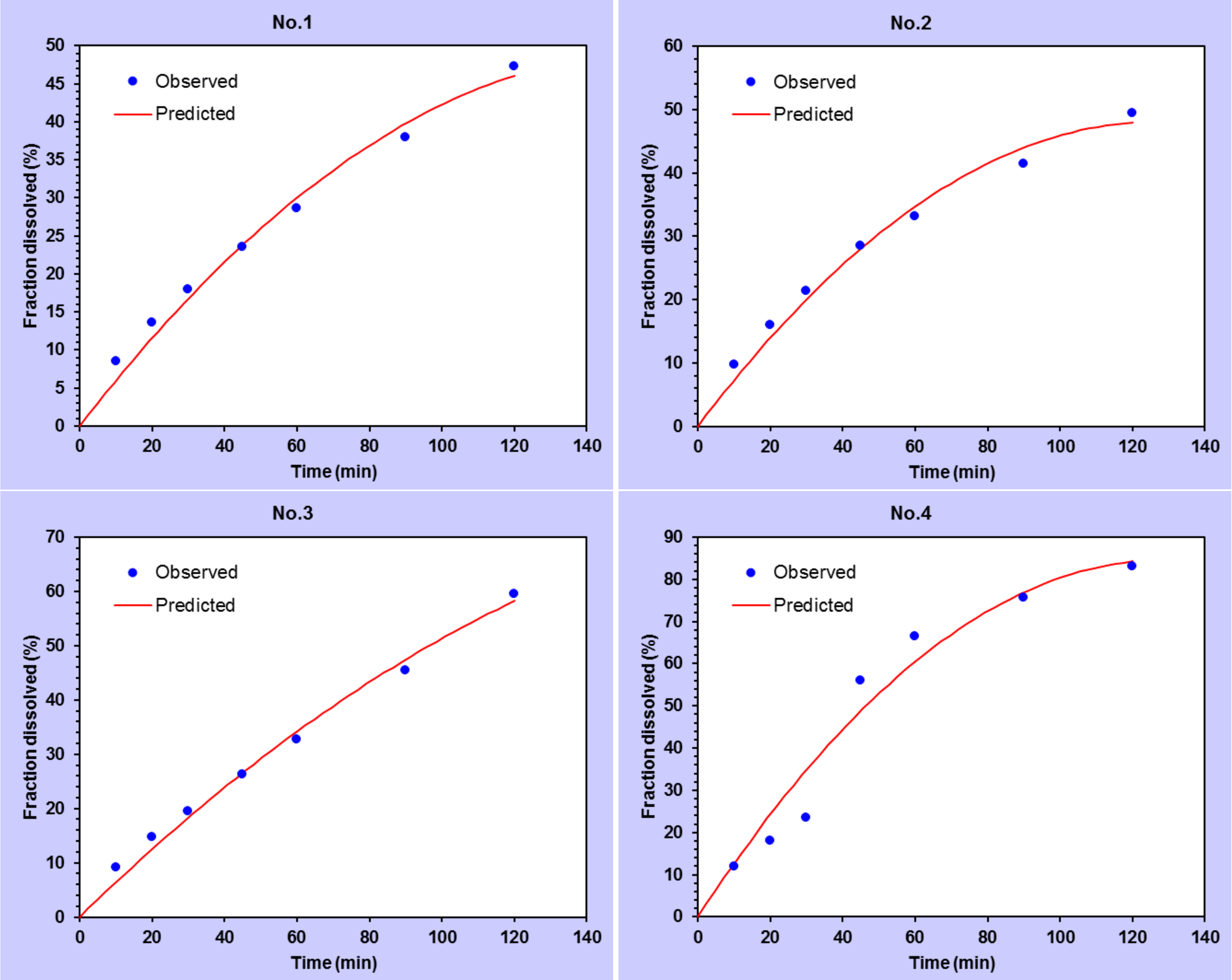

Supplementary materials – Model fitting summary – C*Pharm Mannidex 16700 (modelling up to app. 60 % of carvedilol released)

Model: **Quadratic with T_{lag}**

Model equation: $F = 100 \cdot \left[k_1 \cdot (t - T_{lag})^2 + k_2 \cdot (t - T_{lag})\right]$

Fitted model parameters per tested tablet (N = 4) with statistics – mean, standard deviation (SD), and relative standard deviation expressed in % (RSD%) (output from DDSolver):

Parameter	No.1	No.2	No.3	No.4	Mean	SD	RSD(%)
k_1	-0.00002	-0.00004	-0.00002	-0.00007	-0.00004	0.00002	-55.54956
k_2	0.00683	0.00836	0.00730	0.01476	0.00931	0.00369	39.57831
T_{lag}	4.00000	4.00000	4.00000	4.00000	4.00000	0.00000	0.00000

Number of dissolution data points (N), degrees of freedom (df), and selected goodness of fit criteria – Pearson correlation coefficient (R), coefficient of determination (R^2), adjusted coefficient of determination ($R^2_{adjusted}$), and residual sum of squares (RSS) (manual calculation in MS Excel):

Parameter	No.1	No.2	No.3	No.4
N	7	7	7	7
df	4	4	4	4
R	0.991566089	0.990406676	0.993179485	0.980465156
R^2	0.983203309	0.980905385	0.986405489	0.961311923
$R^2_{adjusted}$	0.974804964	0.971358077	0.979608234	0.941967884
RSS	45.63606939	60.77349959	54.15372444	205.5051212

Graphical abstract of model fit presented as mean ± 1 SD of the fraction % of released carvedilol:

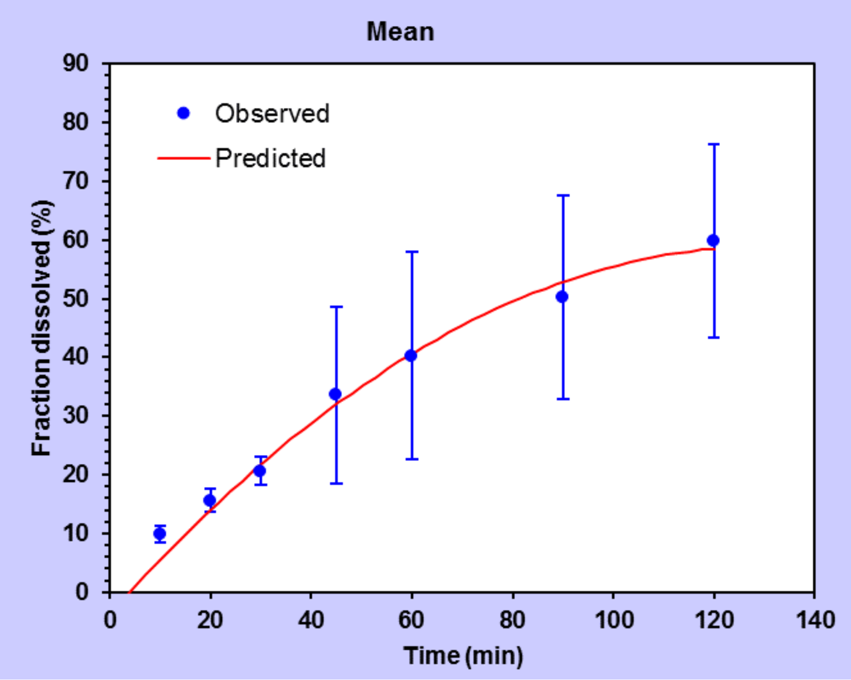

Supplementary materials – Model fitting summary – C*Pharm Mannidex 16700 (modelling up to app. 60 % of carvedilol released)

Graphical abstract of model fit presented as the fraction % of released carvedilol per tested tablet:

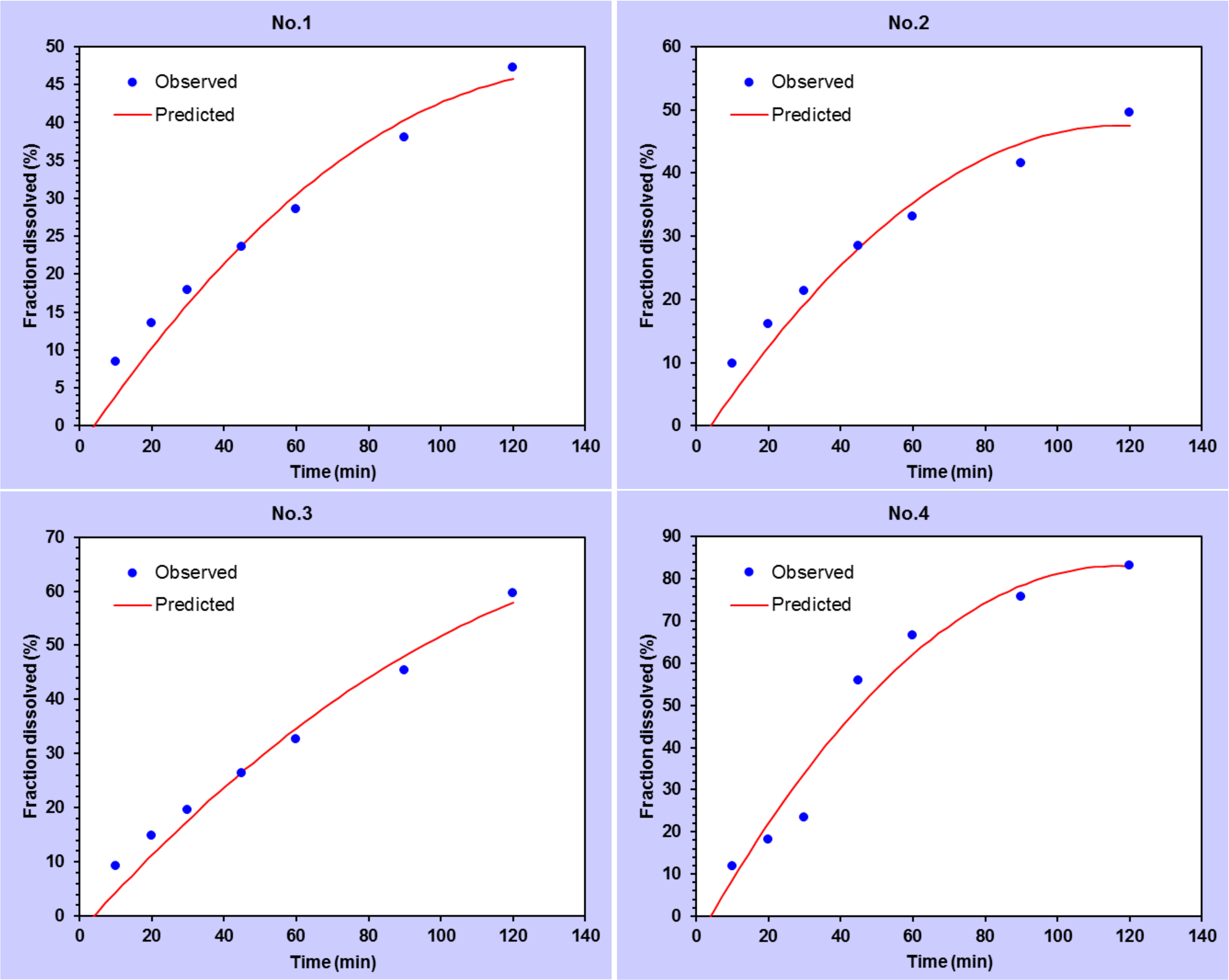

Supplementary materials – Model fitting summary – C*Pharm Mannidex 16700 (modelling up to app. 60 % of carvedilol released)

Model: **Weibull_1**

Model equation: $F = 100 \cdot \left[1 - e^{-\frac{(t-T_i)^{\beta}}{\alpha}}\right]$

Fitted model parameters per tested tablet (N = 4) with statistics – mean, standard deviation (SD), and relative standard deviation expressed in % (RSD%) (output from DDSolver):

Parameter	No.1	No.2	No.3	No.4	Mean	SD	RSD(%)
α	40.916	32.200	50.921	60.575	46.153	12.286	26.620
β	0.664	0.640	0.792	0.991	0.772	0.161	20.834
T_i	4.000	4.000	4.653	4.000	4.163	0.326	7.840

Number of dissolution data points (N), degrees of freedom (df), and selected goodness of fit criteria – Pearson correlation coefficient (R), coefficient of determination (R^2), adjusted coefficient of determination ($R^2_{adjusted}$), and residual sum of squares (RSS) (manual calculation in MS Excel):

Parameter	No.1	No.2	No.3	No.4
N	7	7	7	7
df	4	4	4	4
R	0.993721464	0.9986997	0.985996439	0.975858666
R^2	0.987482349	0.99740109	0.972188977	0.952300135
$R^2_{adjusted}$	0.981223523	0.996101635	0.958283465	0.928450203
RSS	19.26162294	3.967980197	71.24722843	264.4166646

Graphical abstract of model fit presented as mean ± 1 SD of the fraction % of released carvedilol:

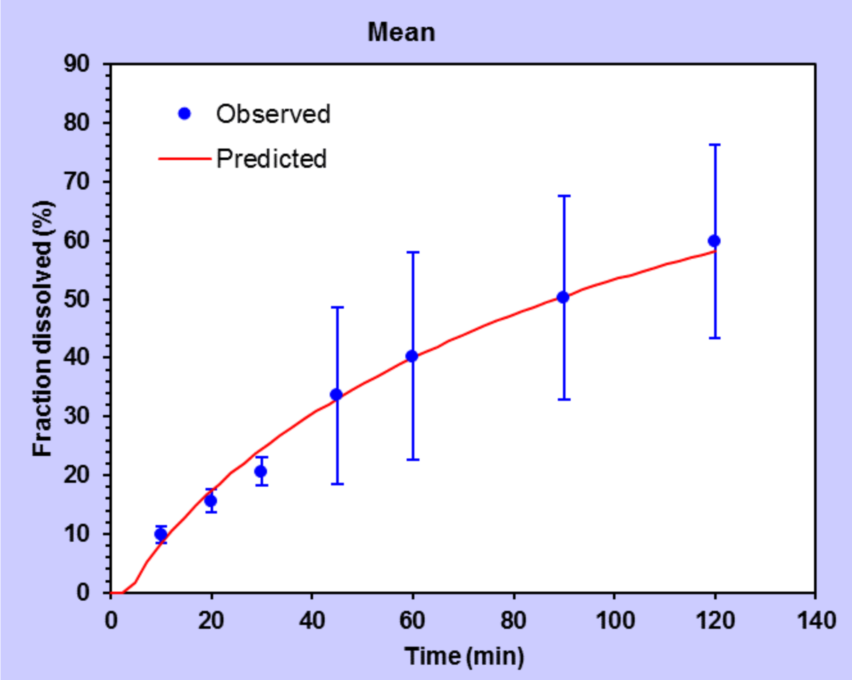

Supplementary materials – Model fitting summary – C*Pharm Mannidex 16700 (modelling up to app. 60 % of carvedilol released)

Graphical abstract of model fit presented as the fraction % of released carvedilol per tested tablet:

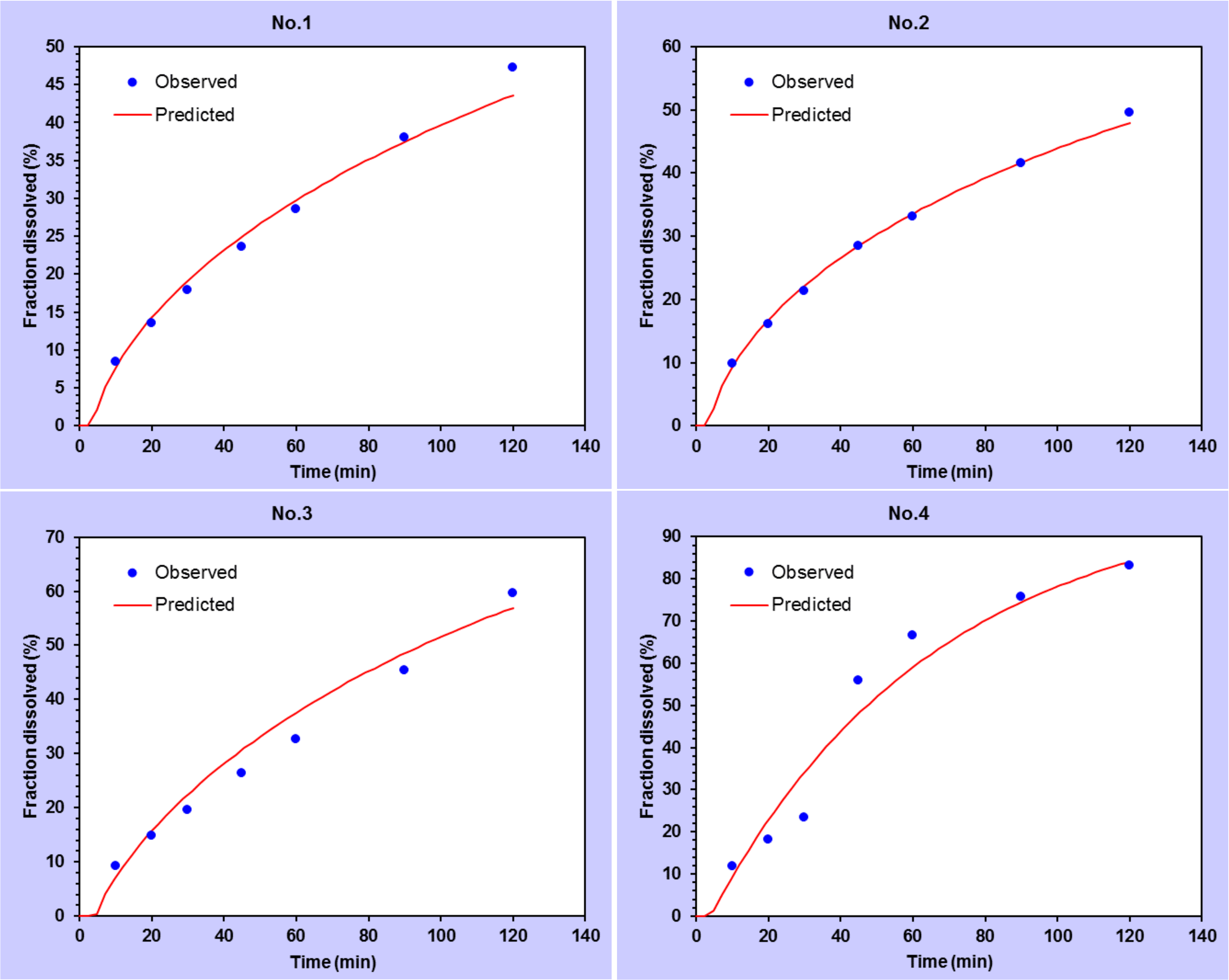

Supplementary materials – Model fitting summary – C*Pharm Mannidex 16700 (modelling up to app. 60 % of carvedilol released)

Model: **Weibull_2**

Model equation: $F = 100 \cdot \left(1 - e^{-\frac{t^{\beta}}{\alpha}}\right)$

Fitted model parameters per tested tablet (N = 4) with statistics – mean, standard deviation (SD), and relative standard deviation expressed in % (RSD%) (output from DDSolver):

Parameter	No.1	No.2	No.3	No.4	Mean	SD	RSD(%)
α	81.182	54.785	87.111	141.339	91.104	36.318	39.864
β	0.821	0.756	0.883	1.178	0.910	0.186	20.451

Number of dissolution data points (N), degrees of freedom (df), and selected goodness of fit criteria – Pearson correlation coefficient (R), coefficient of determination (R^2), adjusted coefficient of determination ($R^2_{adjusted}$), and residual sum of squares (RSS) (manual calculation in MS Excel):

Parameter	No.1	No.2	No.3	No.4
N	7	7	7	7
df	5	5	5	5
R	0.998402786	0.999574083	0.992708983	0.975619734
R^2	0.996808123	0.999148347	0.985471124	0.951833865
$R^2_{adjusted}$	0.996169748	0.998978017	0.982565349	0.942200638
RSS	4.305546505	1.069201257	34.78488027	256.6832843

Graphical abstract of model fit presented as mean ± 1 SD of the fraction % of released carvedilol:

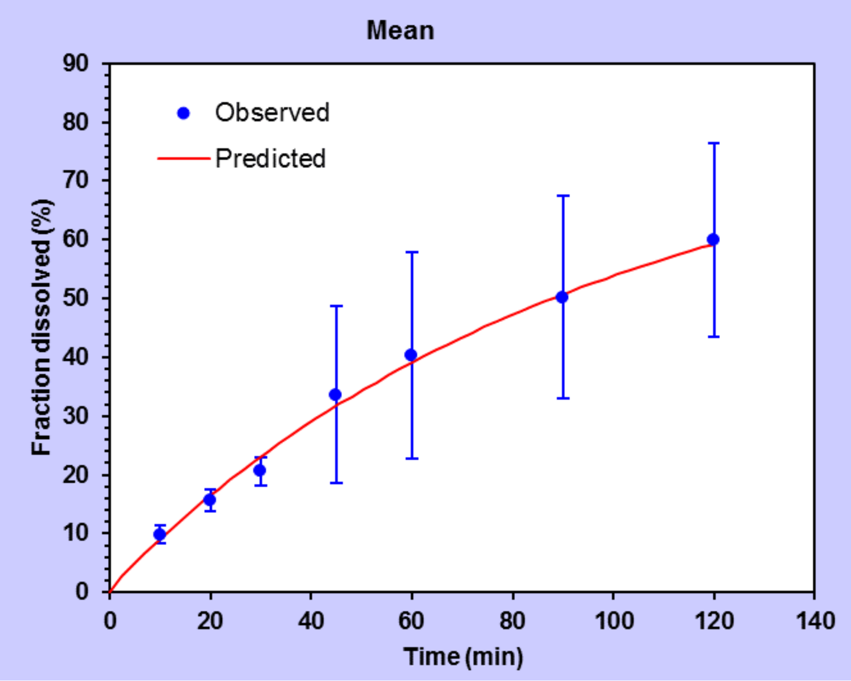

Supplementary materials – Model fitting summary – C*Pharm Mannidex 16700 (modelling up to app. 60 % of carvedilol released)

Graphical abstract of model fit presented as the fraction % of released carvedilol per tested tablet:

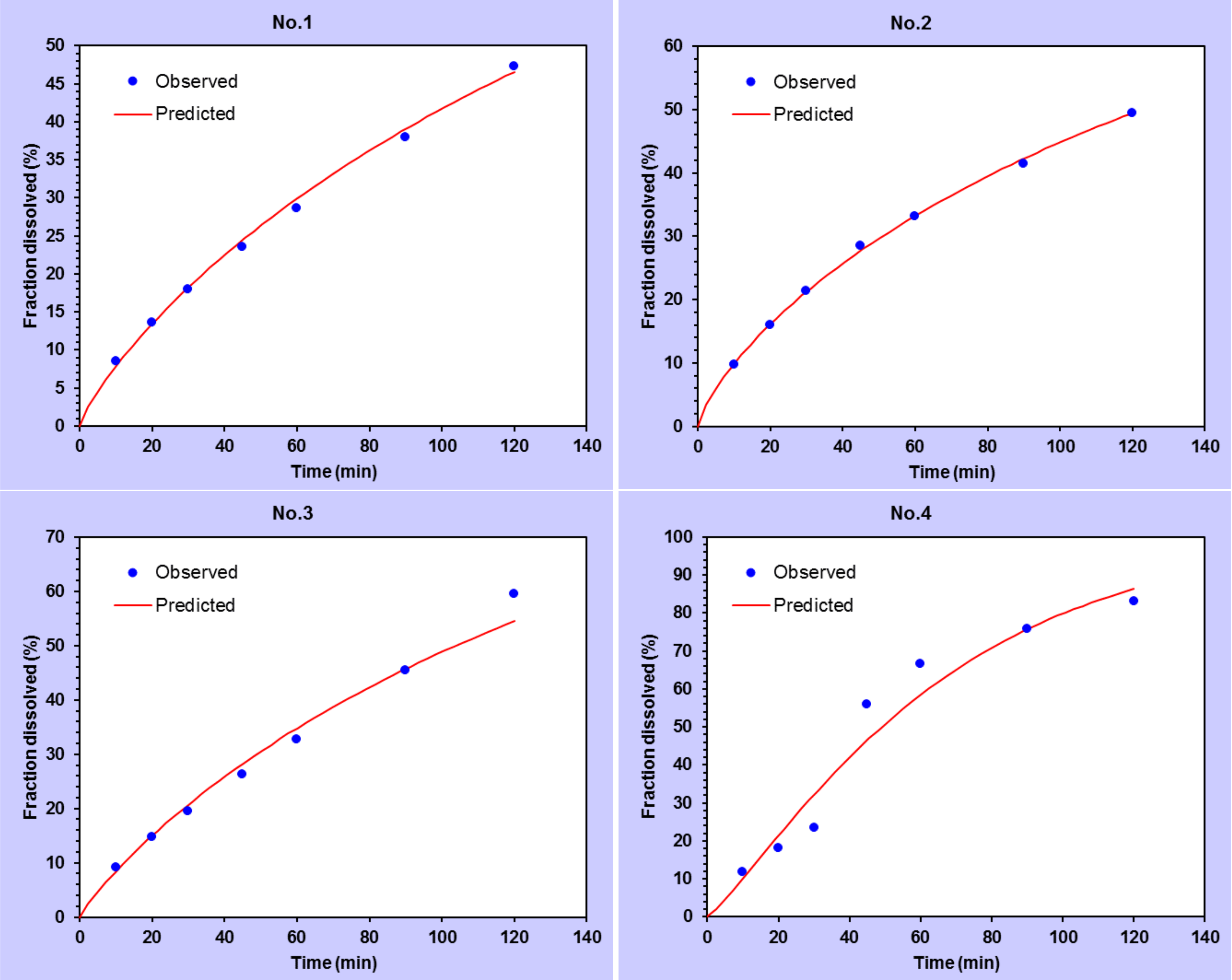

Supplementary materials – Model fitting summary – C*Pharm Mannidex 16700 (modelling up to app. 60 % of carvedilol released)

Model: **Weibull_3**

Model equation: $F = F_{max} \cdot \left(1 - e^{-\frac{t^{\beta}}{\alpha}}\right)$

Fitted model parameters per tested tablet (N = 4) with statistics – mean, standard deviation (SD), and relative standard deviation expressed in % (RSD%) (output from DDSolver):

Parameter	No.1	No.2	No.3	No.4	Mean	SD	RSD(%)
α	72.937	66.557	132.118	183.947	113.890	55.250	48.512
β	1.055	1.029	1.108	1.320	1.128	0.132	11.701
F_{max}	49.632	55.795	66.226	87.327	64.745	16.539	25.545

Number of dissolution data points (N), degrees of freedom (df), and selected goodness of fit criteria – Pearson correlation coefficient (R), coefficient of determination (R^2), adjusted coefficient of determination ($R^2_{adjusted}$), and residual sum of squares (RSS) (manual calculation in MS Excel):

Parameter	No.1	No.2	No.3	No.4
N	7	7	7	7
df	4	4	4	4
R	0.984832338	0.995739385	0.988301253	0.981983709
R^2	0.969894735	0.991496923	0.976739367	0.964292005
$R^2_{adjusted}$	0.954842102	0.987245384	0.965109051	0.946438008
RSS	36.74109324	15.69428902	78.21701114	195.86113

Graphical abstract of model fit presented as mean ± 1 SD of the fraction % of released carvedilol:

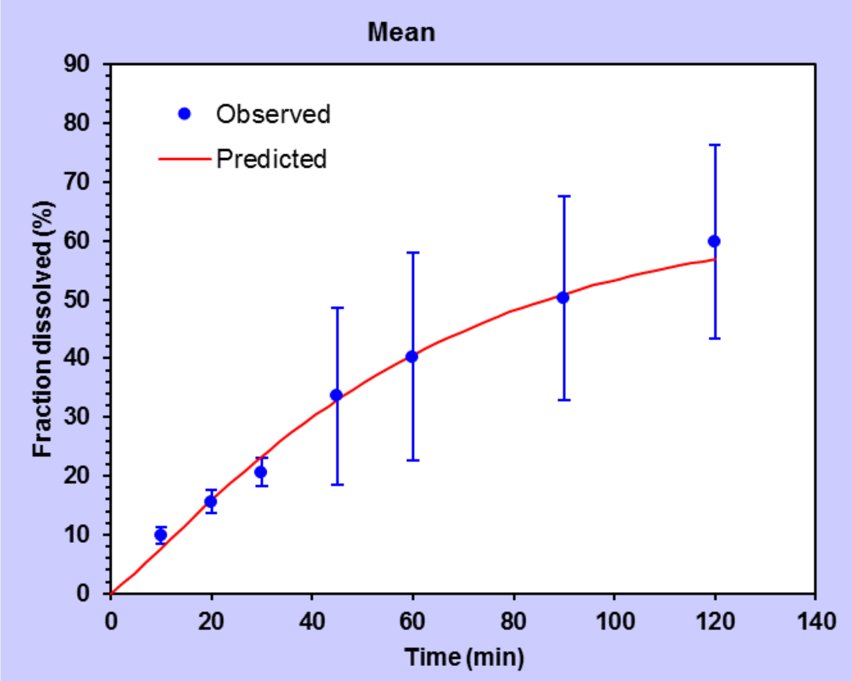

Supplementary materials – Model fitting summary – C*Pharm Mannidex 16700 (modelling up to app. 60 % of carvedilol released)

Graphical abstract of model fit presented as the fraction % of released carvedilol per tested tablet:

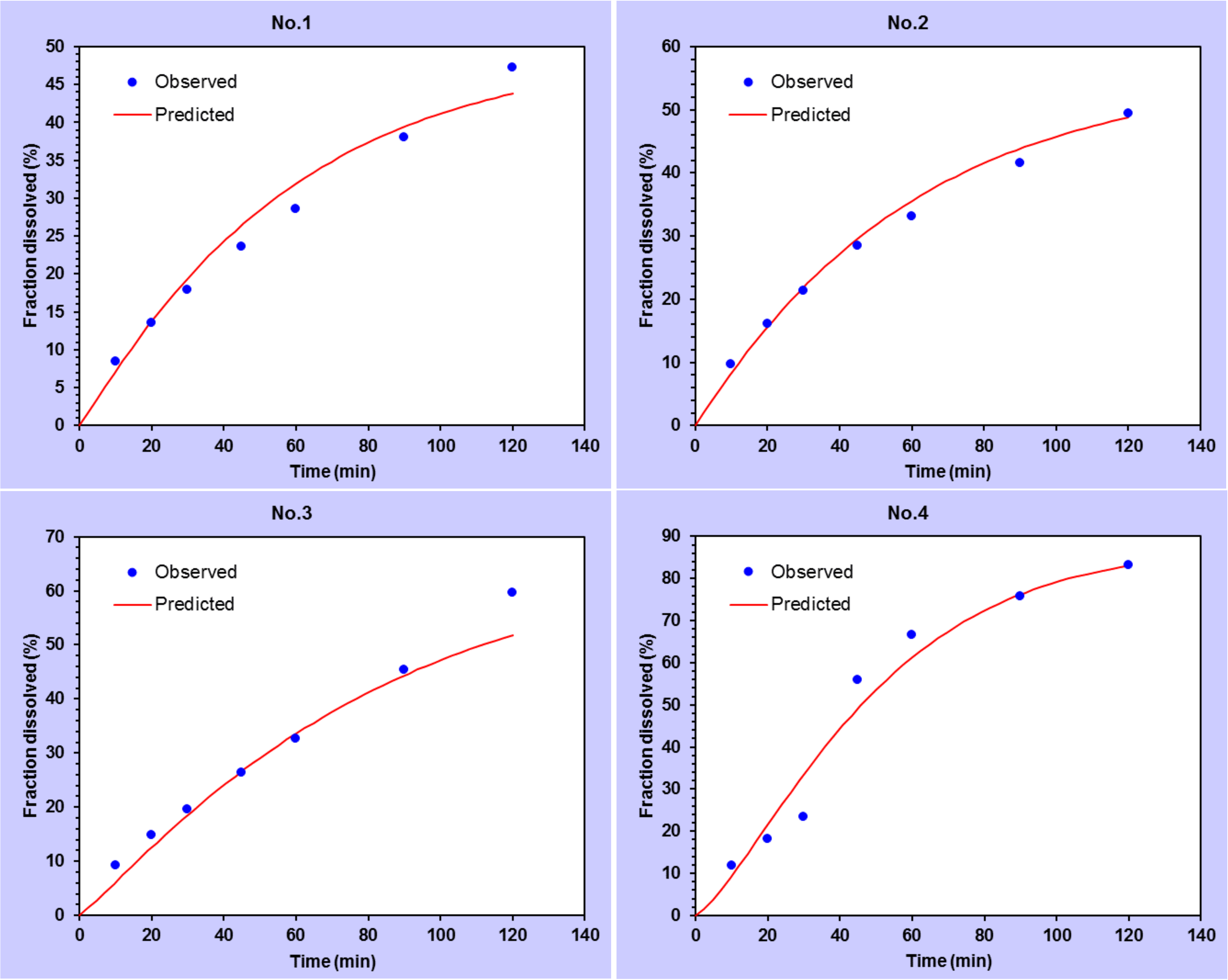

Supplementary materials – Model fitting summary – C*Pharm Mannidex 16700 (modelling up to app. 60 % of carvedilol released)

Model: **Weibull_4**

Model equation: $F = F_{max} \cdot \left[1 - e^{-\frac{(t-T_i)^{\beta}}{\alpha}}\right]$

Fitted model parameters per tested tablet (N = 4) with statistics – mean, standard deviation (SD), and relative standard deviation expressed in % (RSD%) (output from DDSolver):

Parameter	No.1	No.2	No.3	No.4	Mean	SD	RSD(%)
α	33.522	27.360	44.548	70.341	43.943	18.981	43.195
β	0.883	0.862	0.925	1.108	0.944	0.112	11.862
T_i	6.000	4.000	6.000	6.000	5.500	1.000	18.182
F_{max}	49.632	51.984	62.569	87.327	62.878	17.243	27.423

Number of dissolution data points (N), degrees of freedom (df), and selected goodness of fit criteria – Pearson correlation coefficient (R), coefficient of determination (R^2), adjusted coefficient of determination ($R^2_{adjusted}$), and residual sum of squares (RSS) (manual calculation in MS Excel):

Parameter	No.1	No.2	No.3	No.4
N	7	7	7	7
df	3	3	3	3
R	0.976101711	0.987158263	0.969602116	0.979214415
R^2	0.95277455	0.974481436	0.940128263	0.95886087
$R^2_{adjusted}$	0.9055491	0.948962871	0.880256526	0.91772174
RSS	54.05701416	32.96961052	114.3573839	233.1988316

Graphical abstract of model fit presented as mean ± 1 SD of the fraction % of released carvedilol:

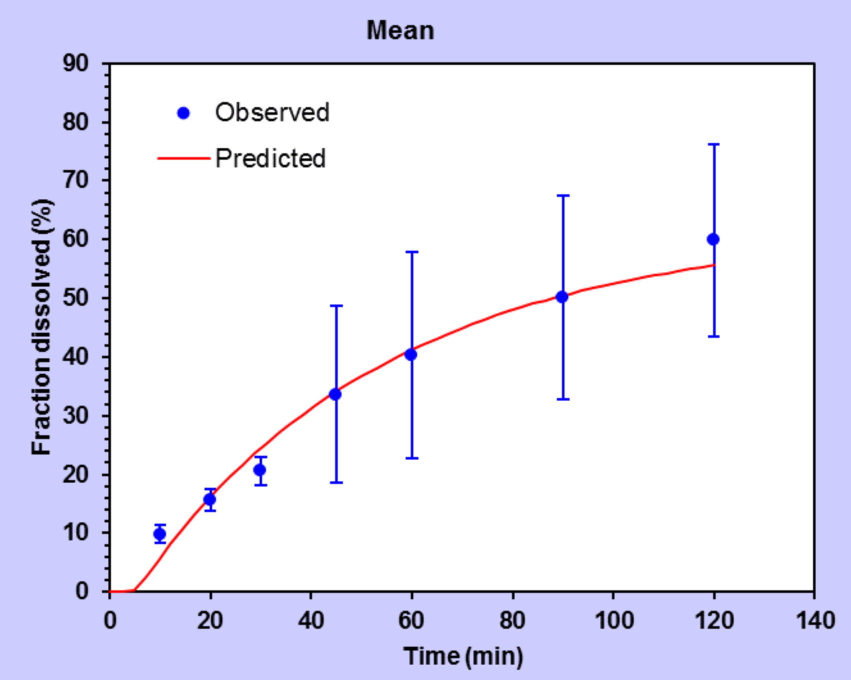

Supplementary materials – Model fitting summary – C*Pharm Mannidex 16700 (modelling up to app. 60 % of carvedilol released)

Graphical abstract of model fit presented as the fraction % of released carvedilol per tested tablet:

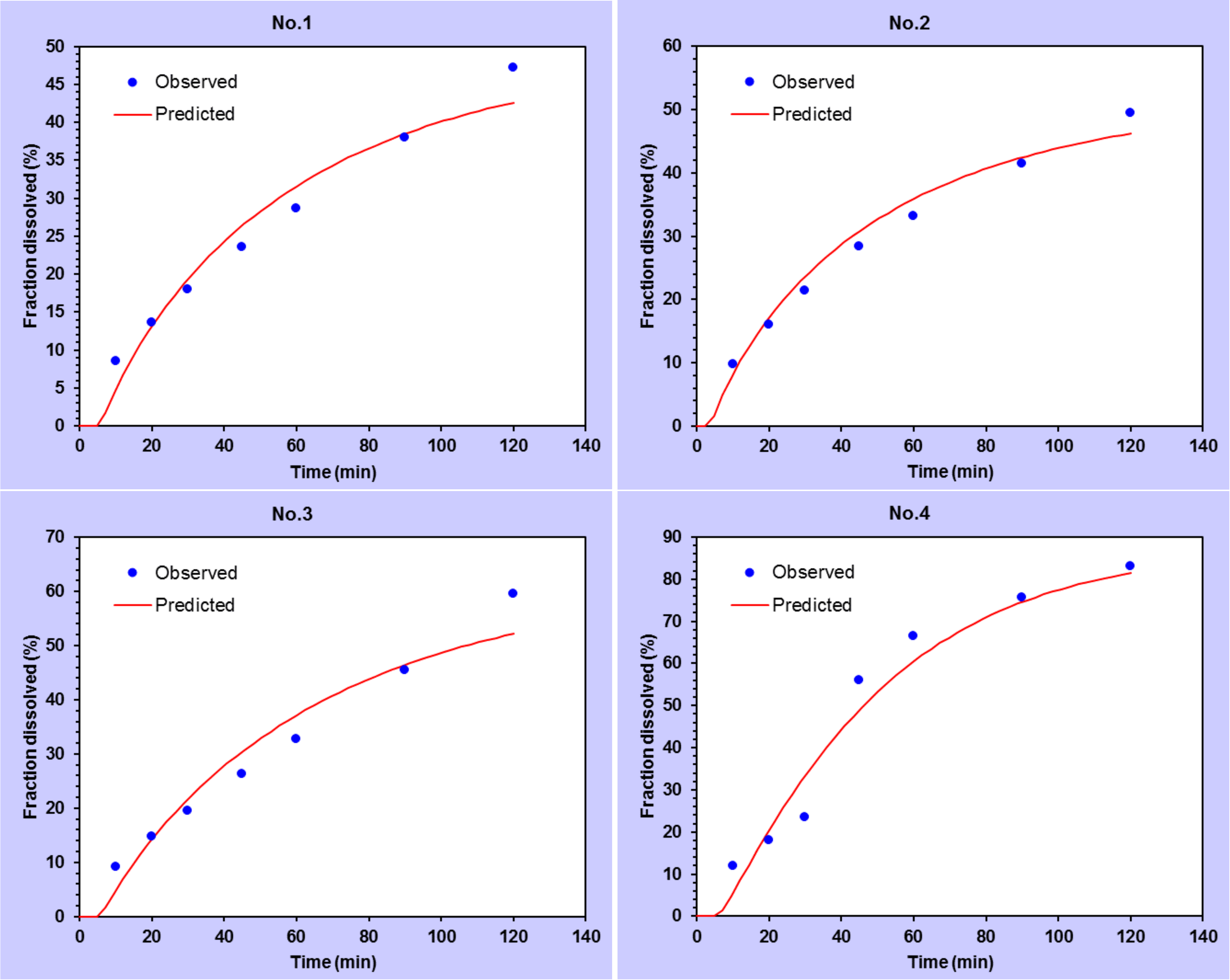

Supplementary materials – Model fitting summary – C*Pharm Mannidex 16700 (modelling up to app. 60 % of carvedilol released)

Model: **Logistic_1**

Model equation: $F = 100 \cdot \frac{e^{\alpha+\beta \cdot \log(t)}}{1+e^{\alpha+\beta \cdot \log(t)}}$

Fitted model parameters per tested tablet (N = 4) with statistics – mean, standard deviation (SD), and relative standard deviation expressed in % (RSD%) (output from DDSolver):

Parameter	No.1	No.2	No.3	No.4	Mean	SD	RSD(%)
α	-4.527	-4.267	-4.859	-5.994	-4.912	0.761	-15.498
β	2.069	2.020	2.403	3.641	2.533	0.758	29.915

Number of dissolution data points (N), degrees of freedom (df), and selected goodness of fit criteria – Pearson correlation coefficient (R), coefficient of determination (R^2), adjusted coefficient of determination ($R^2_{adjusted}$), and residual sum of squares (RSS) (manual calculation in MS Excel):

Parameter	No.1	No.2	No.3	No.4
N	7	7	7	7
df	5	5	5	5
R	0.995260885	0.999127035	0.9862821	0.980444738
R^2	0.990544228	0.998254833	0.97275238	0.961271884
$R^2_{adjusted}$	0.988653074	0.9979058	0.967302856	0.953526261
RSS	12.56747283	2.206234313	59.48495707	213.8385505

Graphical abstract of model fit presented as mean ± 1 SD of the fraction % of released carvedilol:

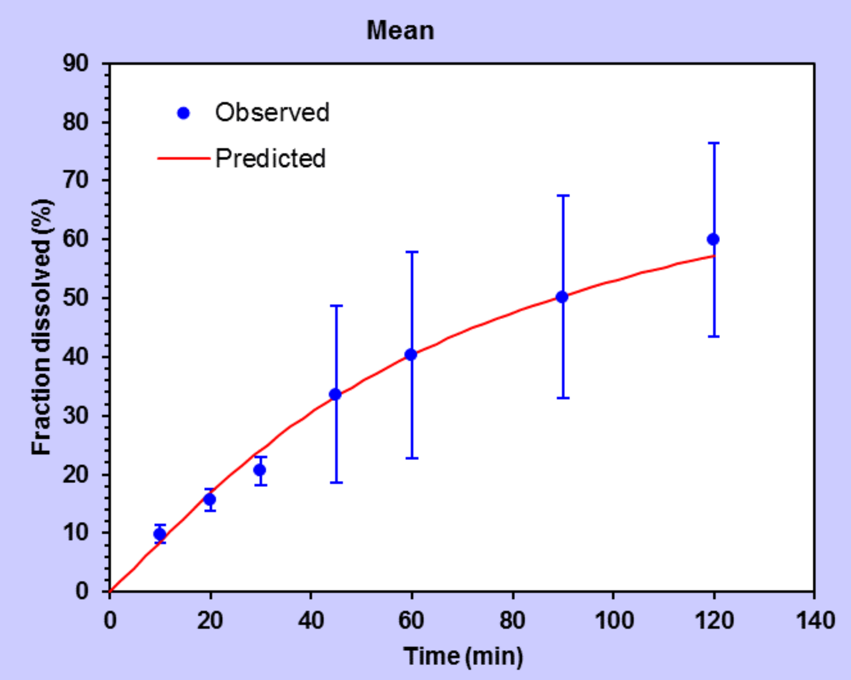

Supplementary materials – Model fitting summary – C*Pharm Mannidex 16700 (modelling up to app. 60 % of carvedilol released)

Graphical abstract of model fit presented as the fraction % of released carvedilol per tested tablet:

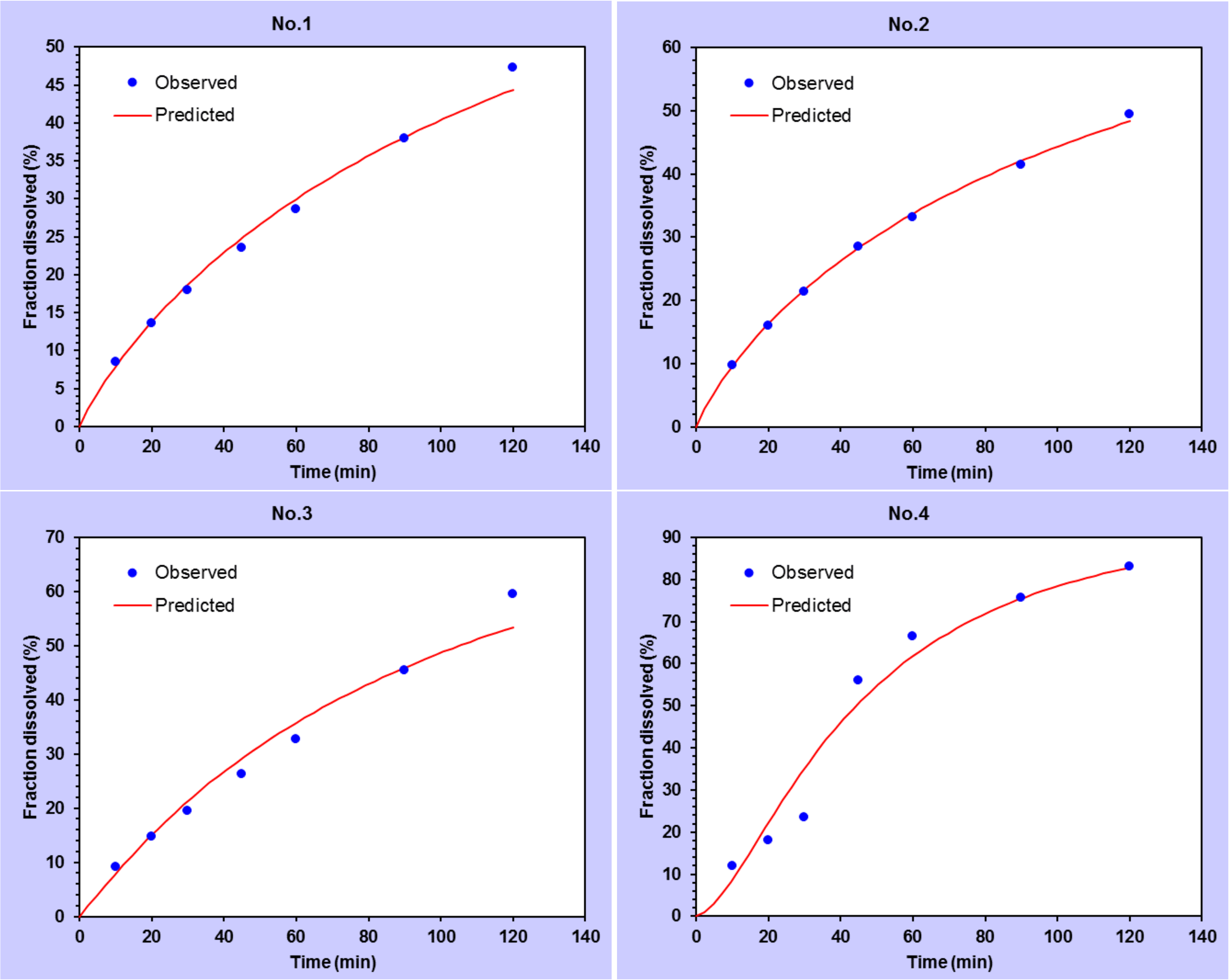

Supplementary materials – Model fitting summary – C*Pharm Mannidex 16700 (modelling up to app. 60 % of carvedilol released)

Model: **Logistic_2**

Model equation: $F = F_{max} \cdot \frac{e^{\alpha+\beta \cdot \log(t)}}{1+e^{\alpha+\beta \cdot \log(t)}}$

Fitted model parameters per tested tablet (N = 4) with statistics – mean, standard deviation (SD), and relative standard deviation expressed in % (RSD%) (output from DDSolver):

Parameter	No.1	No.2	No.3	No.4	Mean	SD	RSD(%)
α	-5.847	-5.622	-6.845	-8.191	-6.626	1.171	-17.671
β	3.754	3.724	3.704	4.925	4.027	0.599	14.884
F_{max}	49.632	51.984	73.323	95.086	67.506	21.252	31.481

Number of dissolution data points (N), degrees of freedom (df), and selected goodness of fit criteria – Pearson correlation coefficient (R), coefficient of determination (R^2), adjusted coefficient of determination ($R^2_{adjusted}$), and residual sum of squares (RSS) (manual calculation in MS Excel):

Parameter	No.1	No.2	No.3	No.4
N	7	7	7	7
df	4	4	4	4
R	0.961416162	0.974803332	0.988474164	0.986840931
R^2	0.924321036	0.950241537	0.977081174	0.973855024
$R^2_{adjusted}$	0.886481554	0.925362305	0.96562176	0.960782536
RSS	105.8919704	74.2573643	174.5257335	234.1992512

Graphical abstract of model fit presented as mean ± 1 SD of the fraction % of released carvedilol:

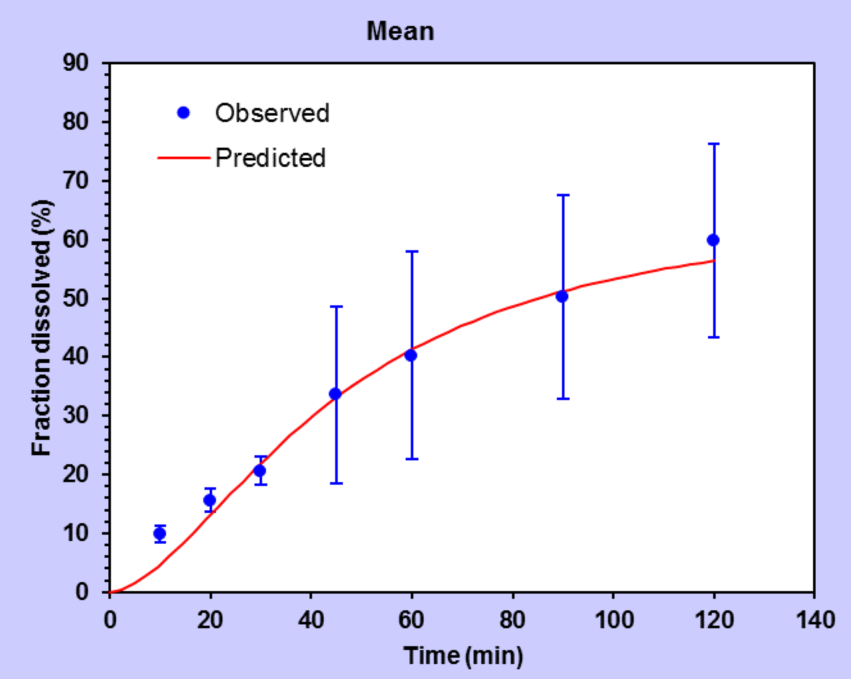

Supplementary materials – Model fitting summary – C*Pharm Mannidex 16700 (modelling up to app. 60 % of carvedilol released)

Graphical abstract of model fit presented as the fraction % of released carvedilol per tested tablet:

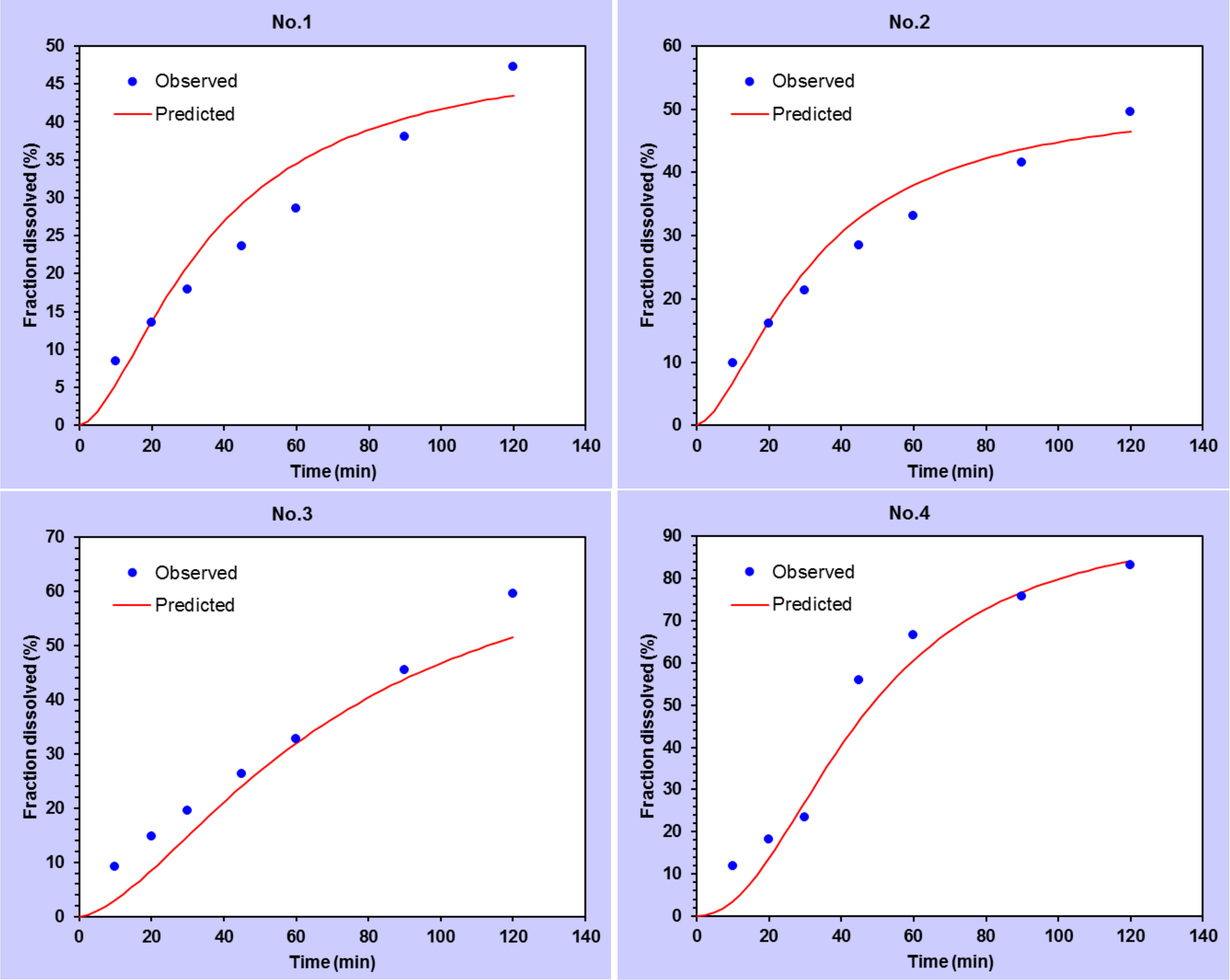

Supplementary materials – Model fitting summary – C*Pharm Mannidex 16700 (modelling up to app. 60 % of carvedilol released)

Model: **Logistic_3**

Model equation: $F = F_{max} \cdot \frac{1}{1+e^{-k\cdot(t-\gamma)}}$

Fitted model parameters per tested tablet (N = 4) with statistics – mean, standard deviation (SD), and relative standard deviation expressed in % (RSD%) (output from DDSolver):

Parameter	No.1	No.2	No.3	No.4	Mean	SD	RSD(%)
k	0.038	0.037	0.026	0.067	0.042	0.017	41.279
γ	48.786	43.888	65.903	45.738	51.079	10.087	19.747
F_{max}	49.632	51.984	69.811	87.327	64.688	17.577	27.172

Number of dissolution data points (N), degrees of freedom (df), and selected goodness of fit criteria – Pearson correlation coefficient (R), coefficient of determination (R^2), adjusted coefficient of determination ($R^2_{adjusted}$), and residual sum of squares (RSS) (manual calculation in MS Excel):

Parameter	No.1	No.2	No.3	No.4
N	7	7	7	7
df	4	4	4	4
R	0.993838059	0.992905155	0.997171263	0.982611305
R^2	0.987714088	0.985860647	0.994350528	0.965524978
$R^2_{adjusted}$	0.981571132	0.97879097	0.991525793	0.948287466
RSS	16.96662253	17.93336751	31.50461713	302.7061301

Graphical abstract of model fit presented as mean ± 1 SD of the fraction % of released carvedilol:

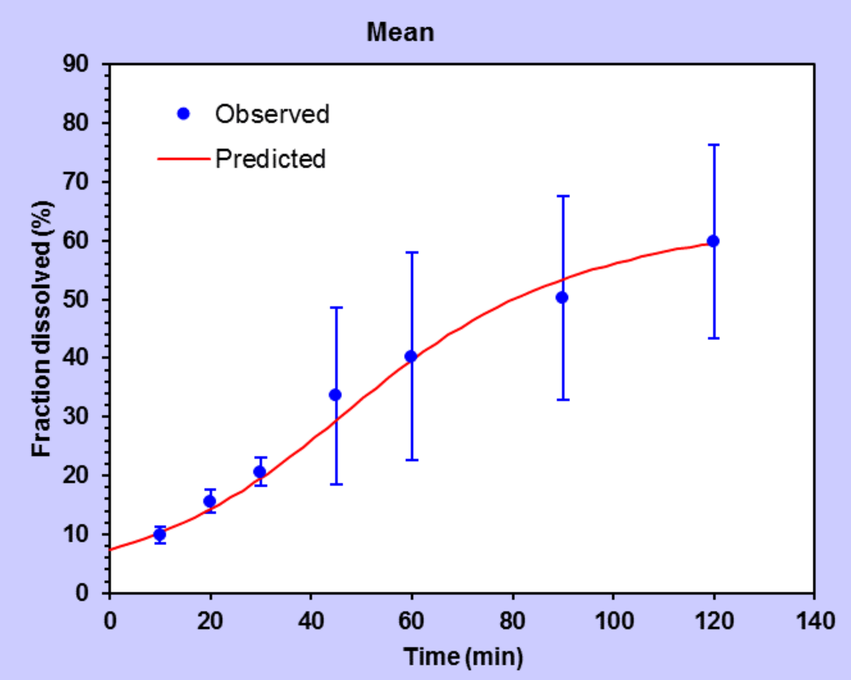

Supplementary materials – Model fitting summary – C*Pharm Mannidex 16700 (modelling up to app. 60 % of carvedilol released)

Graphical abstract of model fit presented as the fraction % of released carvedilol per tested tablet:

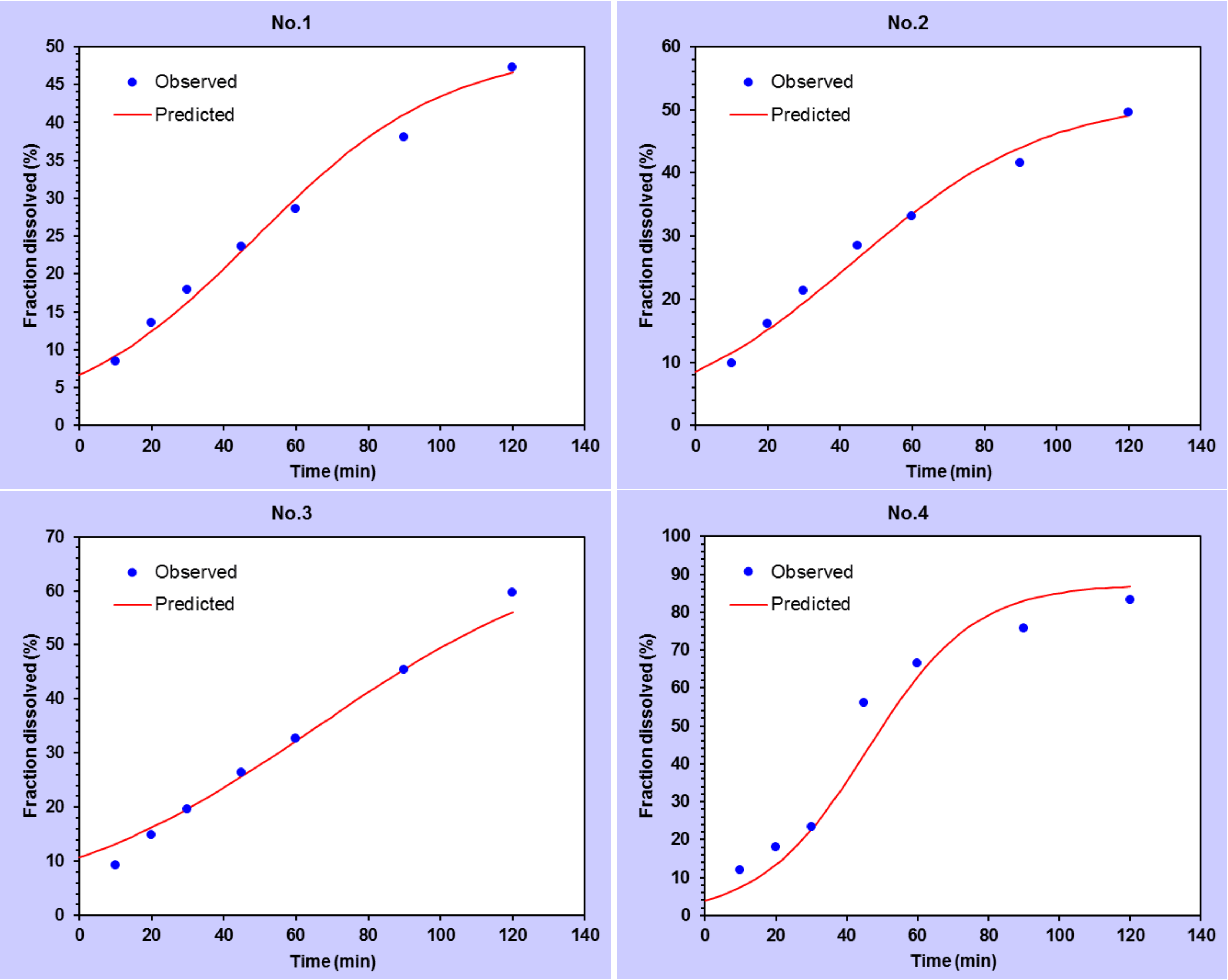

Supplementary materials – Model fitting summary – C*Pharm Mannidex 16700 (modelling up to app. 60 % of carvedilol released)

Model: **Gompertz_1**

Model equation: $F = 100 \cdot e^{-\alpha \cdot e^{-\beta \cdot \log(t)}}$

Fitted model parameters per tested tablet (N = 4) with statistics – mean, standard deviation (SD), and relative standard deviation expressed in % (RSD%) (output from DDSolver):

Parameter	No.1	No.2	No.3	No.4	Mean	SD	RSD(%)
α	7.952	7.421	10.775	34.765	15.228	13.108	86.075
β	1.077	1.095	1.346	2.455	1.493	0.653	43.707

Number of dissolution data points (N), degrees of freedom (df), and selected goodness of fit criteria – Pearson correlation coefficient (R), coefficient of determination (R^2), adjusted coefficient of determination ($R^2_{adjusted}$), and residual sum of squares (RSS) (manual calculation in MS Excel):

Parameter	No.1	No.2	No.3	No.4
N	7	7	7	7
df	5	5	5	5
R	0.983782897	0.993248538	0.967897416	0.966850987
R^2	0.967828789	0.986542659	0.936825408	0.934800832
$R^2_{adjusted}$	0.961394547	0.983851191	0.92419049	0.921760998
RSS	38.48524574	16.65330216	125.3934608	354.7382673

Graphical abstract of model fit presented as mean ± 1 SD of the fraction % of released carvedilol:

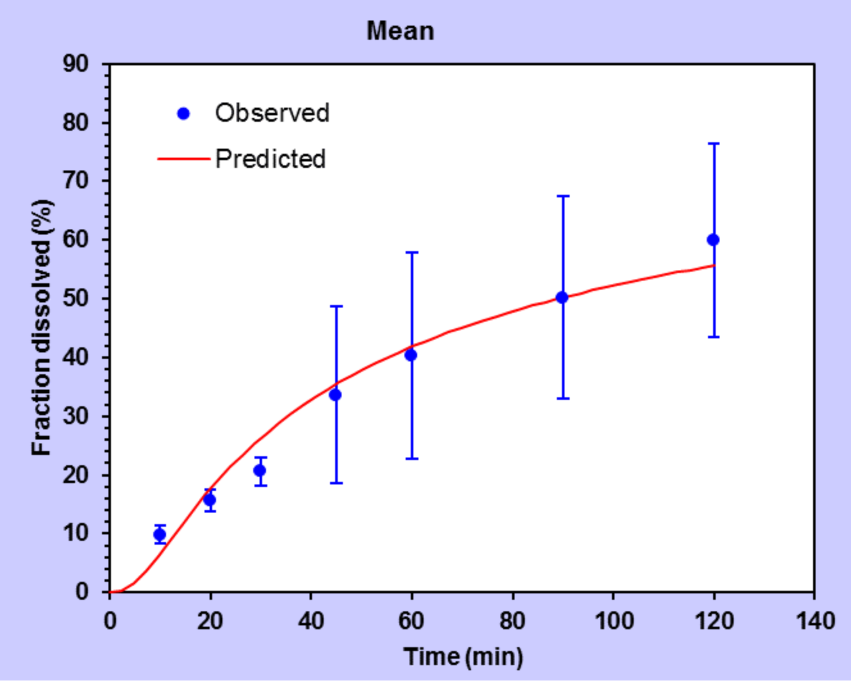

Supplementary materials – Model fitting summary – C*Pharm Mannidex 16700 (modelling up to app. 60 % of carvedilol released)

Graphical abstract of model fit presented as the fraction % of released carvedilol per tested tablet:

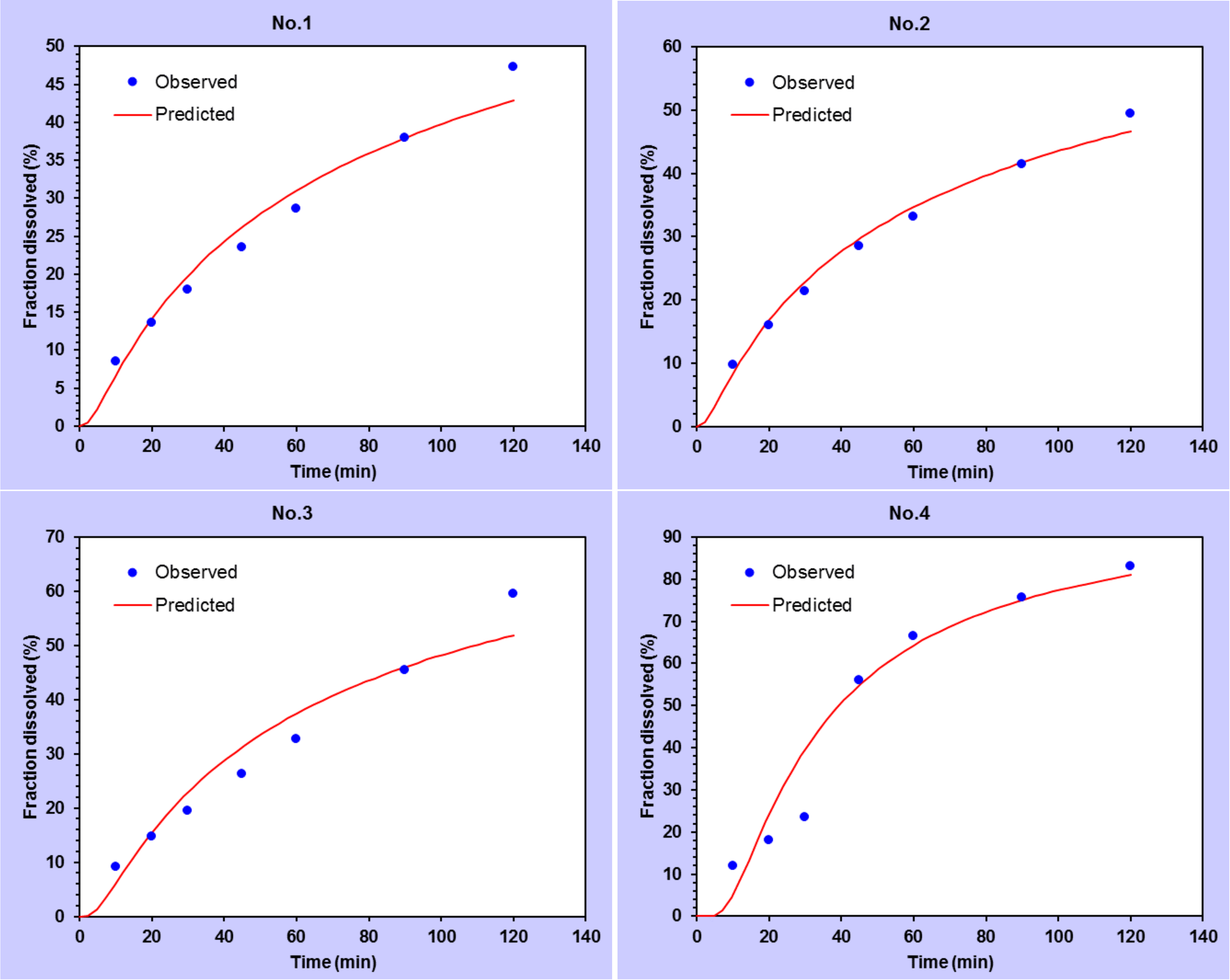

Supplementary materials – Model fitting summary – C*Pharm Mannidex 16700 (modelling up to app. 60 % of carvedilol released)

Model: **Gompertz_2**

Model equation: $F = F_{max} \cdot e^{-\alpha \cdot e^{-\beta \cdot \log(t)}}$

Fitted model parameters per tested tablet (N = 4) with statistics – mean, standard deviation (SD), and relative standard deviation expressed in % (RSD%) (output from DDSolver):

Parameter	No.1	No.2	No.3	No.4	Mean	SD	RSD(%)
α	77.508	47.628	87.402	143.802	89.085	40.206	45.132
β	2.847	2.874	2.849	3.192	2.941	0.168	5.713
F_{max}	49.632	51.984	62.569	97.737	65.480	22.229	33.947

Number of dissolution data points (N), degrees of freedom (df), and selected goodness of fit criteria – Pearson correlation coefficient (R), coefficient of determination (R^2), adjusted coefficient of determination ($R^2_{adjusted}$), and residual sum of squares (RSS) (manual calculation in MS Excel):

Parameter	No.1	No.2	No.3	No.4
N	7	7	7	7
df	4	4	4	4
R	0.966079839	0.9524491	0.957754405	0.986967487
R^2	0.933310256	0.907159289	0.917293501	0.974104821
$R^2_{adjusted}$	0.899965384	0.860738933	0.875940252	0.961157232
RSS	161.4816723	149.8837187	256.798792	350.0353682

Graphical abstract of model fit presented as mean ± 1 SD of the fraction % of released carvedilol:

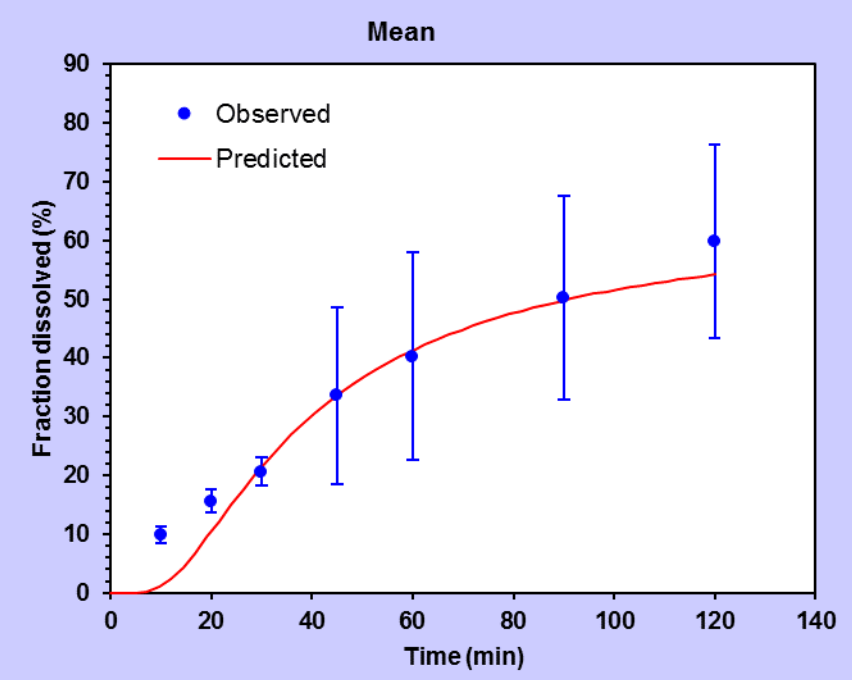

Supplementary materials – Model fitting summary – C*Pharm Mannidex 16700 (modelling up to app. 60 % of carvedilol released)

Graphical abstract of model fit presented as the fraction % of released carvedilol per tested tablet:

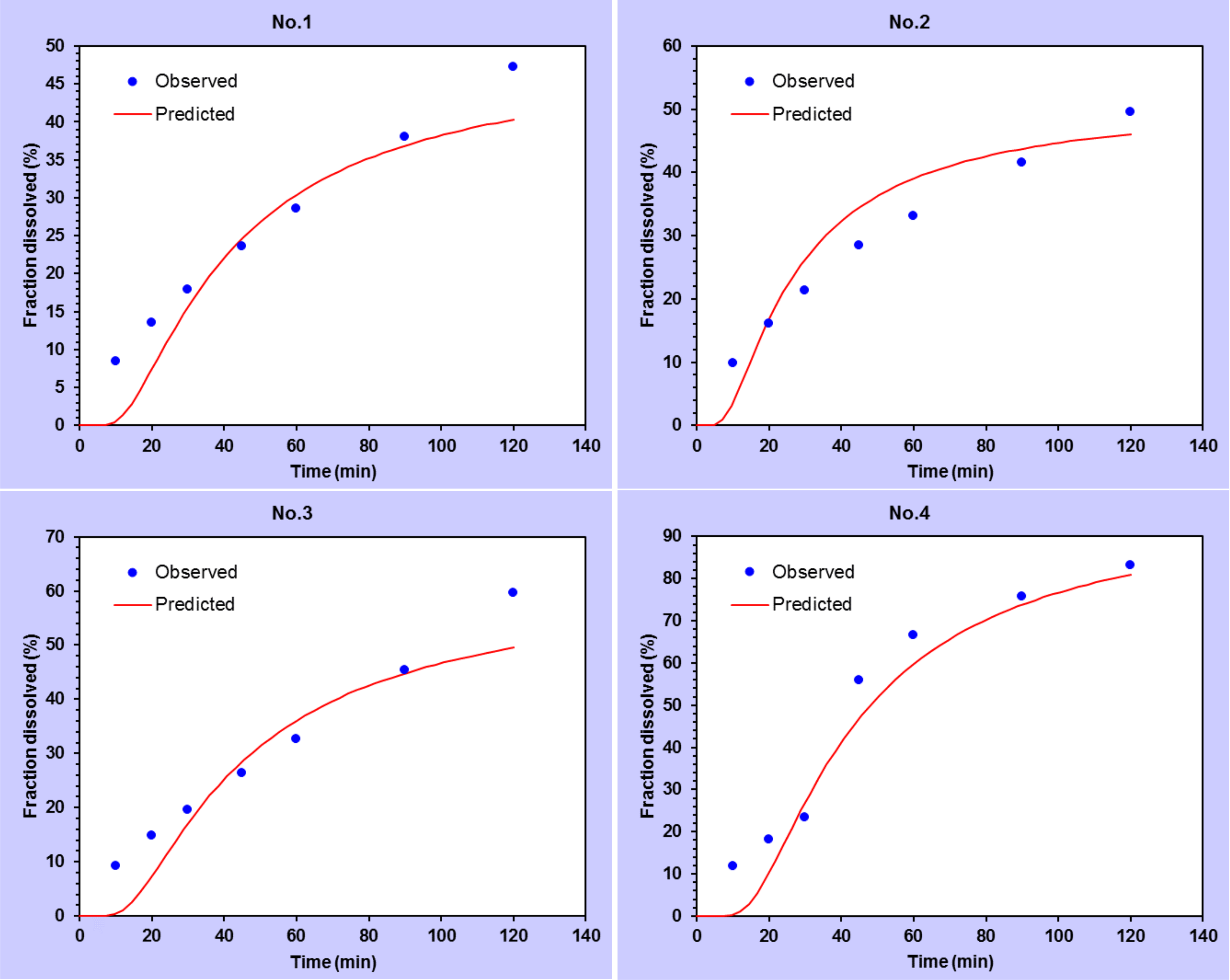

Supplementary materials – Model fitting summary – C*Pharm Mannidex 16700 (modelling up to app. 60 % of carvedilol released)

Model: **Gompertz_3**

Model equation: $F = F_{max} \cdot e^{-e^{-k\cdot(t-\gamma)}}$

Fitted model parameters per tested tablet (N = 4) with statistics – mean, standard deviation (SD), and relative standard deviation expressed in % (RSD%) (output from DDSolver):

Parameter	No.1	No.2	No.3	No.4	Mean	SD	RSD(%)
k	0.021	0.030	0.031	0.034	0.029	0.006	20.426
γ	35.937	28.262	36.826	30.028	32.763	4.255	12.988
F_{max}	51.441	51.984	62.569	87.327	63.330	16.798	26.524

Number of dissolution data points (N), degrees of freedom (df), and selected goodness of fit criteria – Pearson correlation coefficient (R), coefficient of determination (R^2), adjusted coefficient of determination ($R^2_{adjusted}$), and residual sum of squares (RSS) (manual calculation in MS Excel):

Parameter	No.1	No.2	No.3	No.4
N	7	7	7	7
df	4	4	4	4
R	0.997450778	0.995010042	0.98389286	0.984000889
R^2	0.994908055	0.990044984	0.968045159	0.968257749
$R^2_{adjusted}$	0.992362082	0.985067476	0.952067739	0.952386624
RSS	23.8301964	17.22044341	91.50079008	179.3405689

Graphical abstract of model fit presented as mean ± 1 SD of the fraction % of released carvedilol:

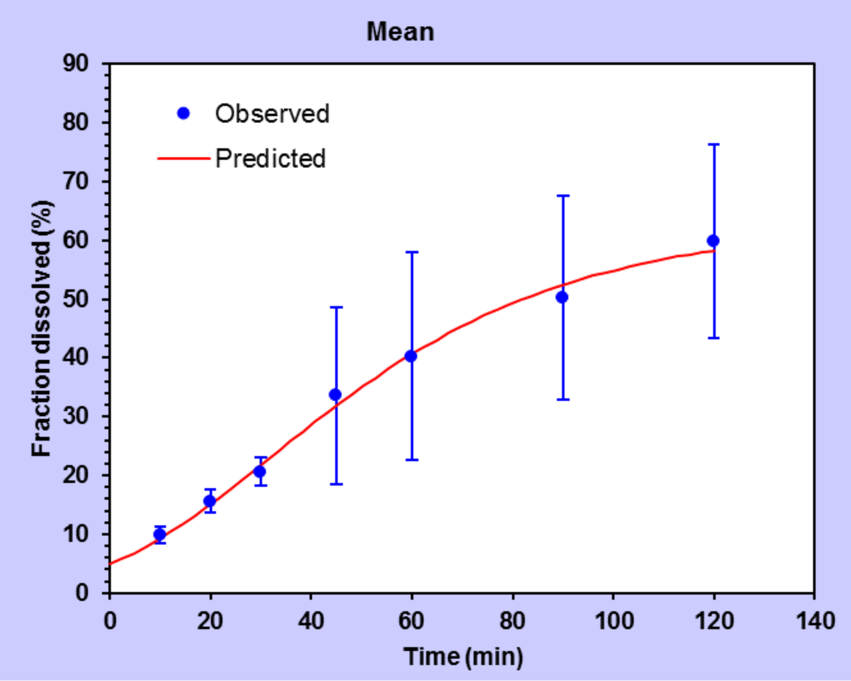

Supplementary materials – Model fitting summary – C*Pharm Mannidex 16700 (modelling up to app. 60 % of carvedilol released)

Graphical abstract of model fit presented as the fraction % of released carvedilol per tested tablet:

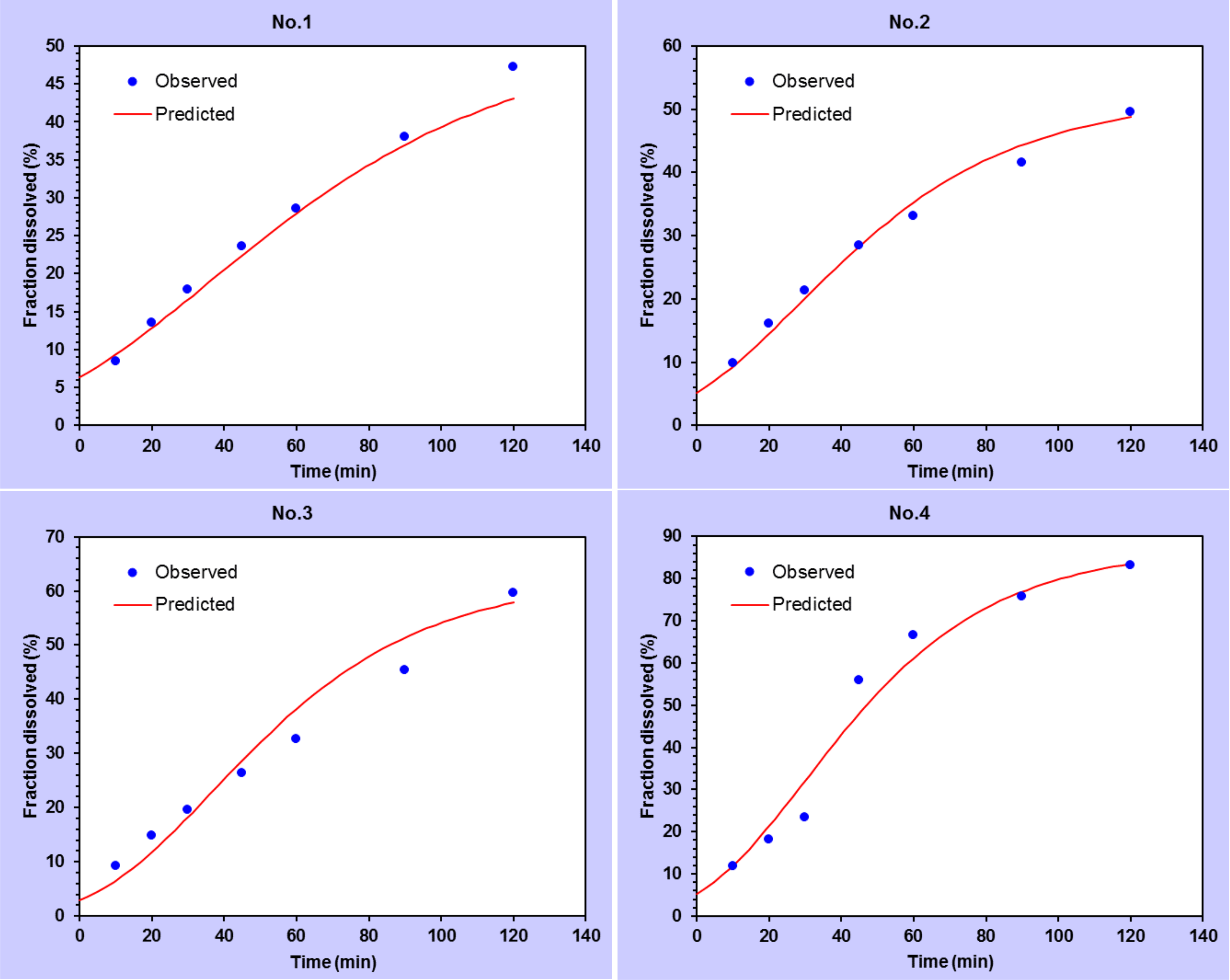

Supplementary materials – Model fitting summary – C*Pharm Mannidex 16700 (modelling up to app. 60 % of carvedilol released)

Model: **Gompertz_4**

Model equation: $F = F_{max} \cdot e^{-\beta \cdot e^{-k \cdot t}}$

Fitted model parameters per tested tablet (N = 4) with statistics – mean, standard deviation (SD), and relative standard deviation expressed in % (RSD%) (output from DDSolver):

Parameter	No.1	No.2	No.3	No.4	Mean	SD	RSD(%)
k	0.030	0.030	0.031	0.034	0.031	0.002	6.973
β	2.675	2.318	3.076	2.811	2.720	0.315	11.588
F_{max}	49.632	51.984	62.569	87.327	62.878	17.243	27.423

Number of dissolution data points (N), degrees of freedom (df), and selected goodness of fit criteria – Pearson correlation coefficient (R), coefficient of determination (R^2), adjusted coefficient of determination ($R^2_{adjusted}$), and residual sum of squares (RSS) (manual calculation in MS Excel):

Parameter	No.1	No.2	No.3	No.4
N	7	7	7	7
df	4	4	4	4
R	0.989924522	0.995010042	0.98389286	0.984000889
R^2	0.979950559	0.990044984	0.968045159	0.968257749
$R^2_{adjusted}$	0.969925838	0.985067476	0.952067739	0.952386624
RSS	34.84419197	17.22044341	91.50079008	179.3405689

Graphical abstract of model fit presented as mean ± 1 SD of the fraction % of released carvedilol:

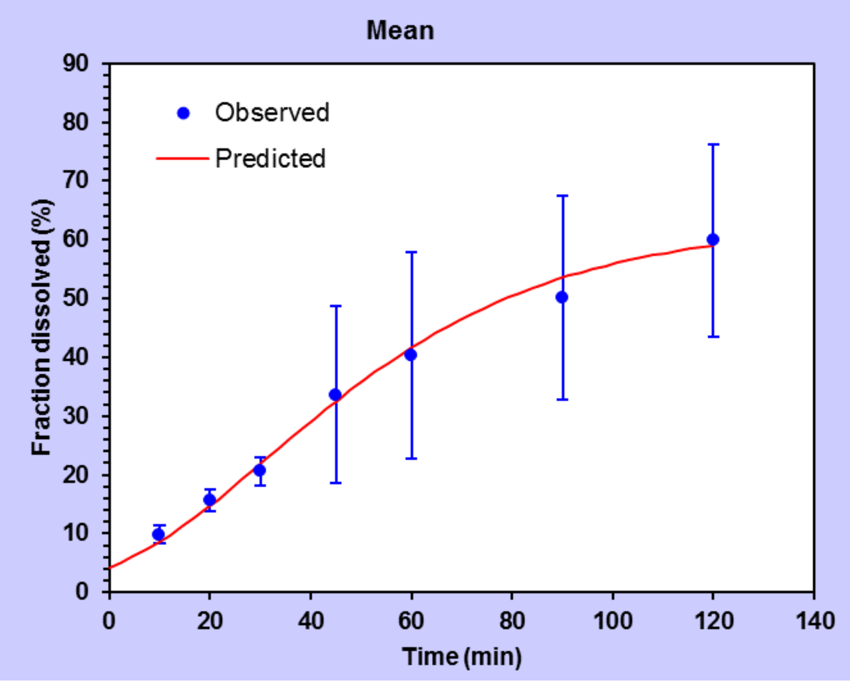

Supplementary materials – Model fitting summary – C*Pharm Mannidex 16700 (modelling up to app. 60 % of carvedilol released)

Graphical abstract of model fit presented as the fraction % of released carvedilol per tested tablet:

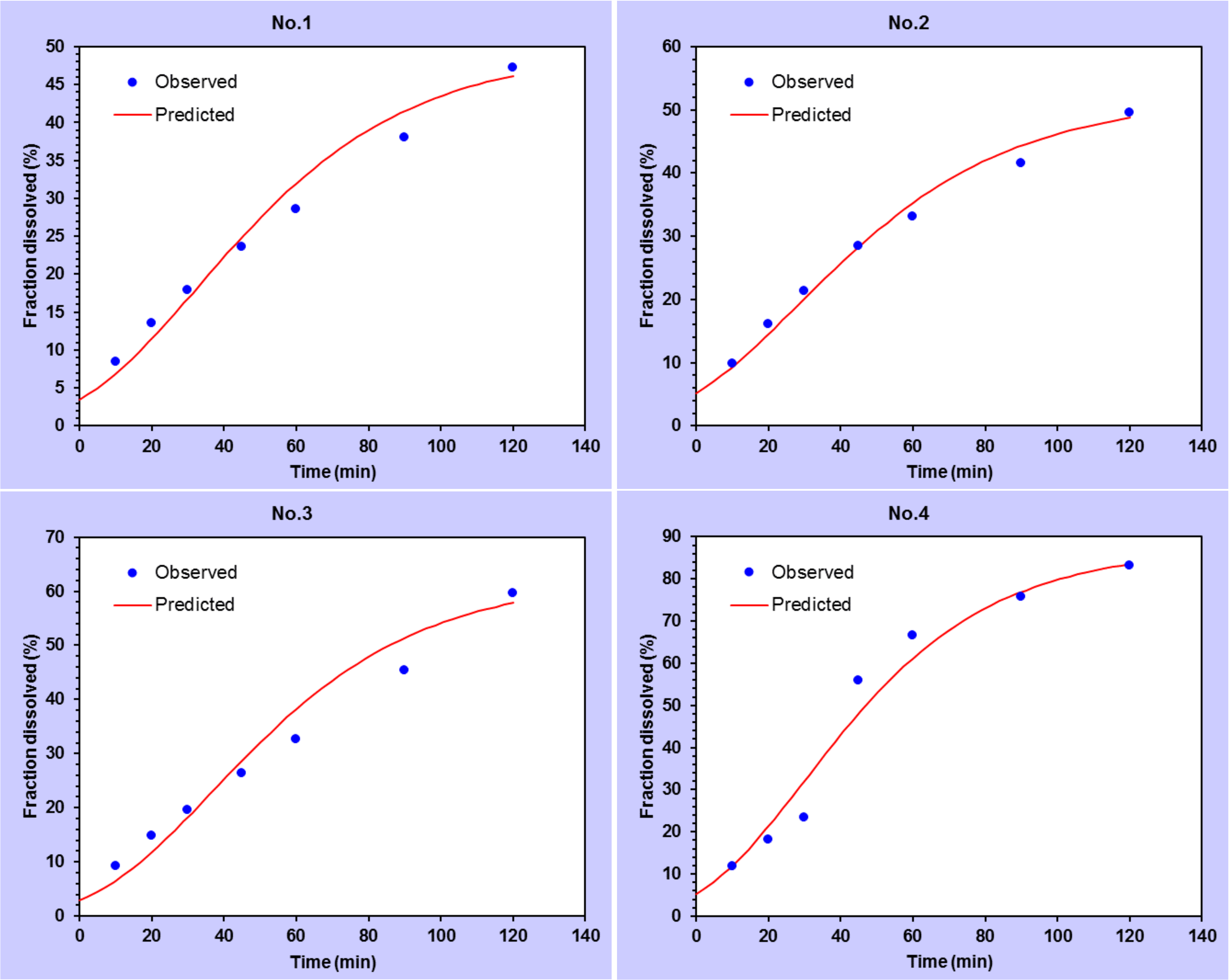

Supplementary materials – Model fitting summary – C*Pharm Mannidex 16700 (modelling up to app. 60 % of carvedilol released)

Model: **Probit_1**

Model equation: $F = 100 \cdot \phi[\alpha + \beta \cdot \log(t)]$

Fitted model parameters per tested tablet (N = 4) with statistics – mean, standard deviation (SD), and relative standard deviation expressed in % (RSD%) (output from DDSolver):

Parameter	No.1	No.2	No.3	No.4	Mean	SD	RSD(%)
α	-2.829	-2.508	-2.860	-3.591	-2.947	0.458	-15.527
β	1.313	1.179	1.409	2.184	1.521	0.452	29.720

Number of dissolution data points (N), degrees of freedom (df), and selected goodness of fit criteria – Pearson correlation coefficient (R), coefficient of determination (R^2), adjusted coefficient of determination ($R^2_{adjusted}$), and residual sum of squares (RSS) (manual calculation in MS Excel):

Parameter	No.1	No.2	No.3	No.4
N	7	7	7	7
df	5	5	5	5
R	0.993321452	0.997981009	0.981456668	0.977674519
R^2	0.986687508	0.995966095	0.963257191	0.955847465
$R^2_{adjusted}$	0.984025009	0.995159314	0.955908629	0.947016958
RSS	18.20388021	5.26100684	78.07715294	241.3915483

Graphical abstract of model fit presented as mean ± 1 SD of the fraction % of released carvedilol:

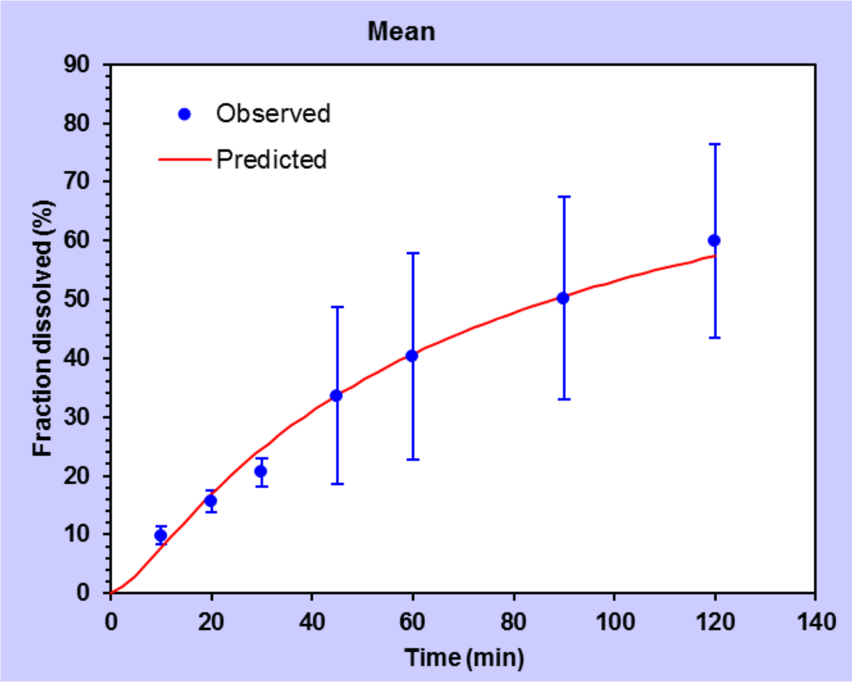

Supplementary materials – Model fitting summary – C*Pharm Mannidex 16700 (modelling up to app. 60 % of carvedilol released)

Graphical abstract of model fit presented as the fraction % of released carvedilol per tested tablet:

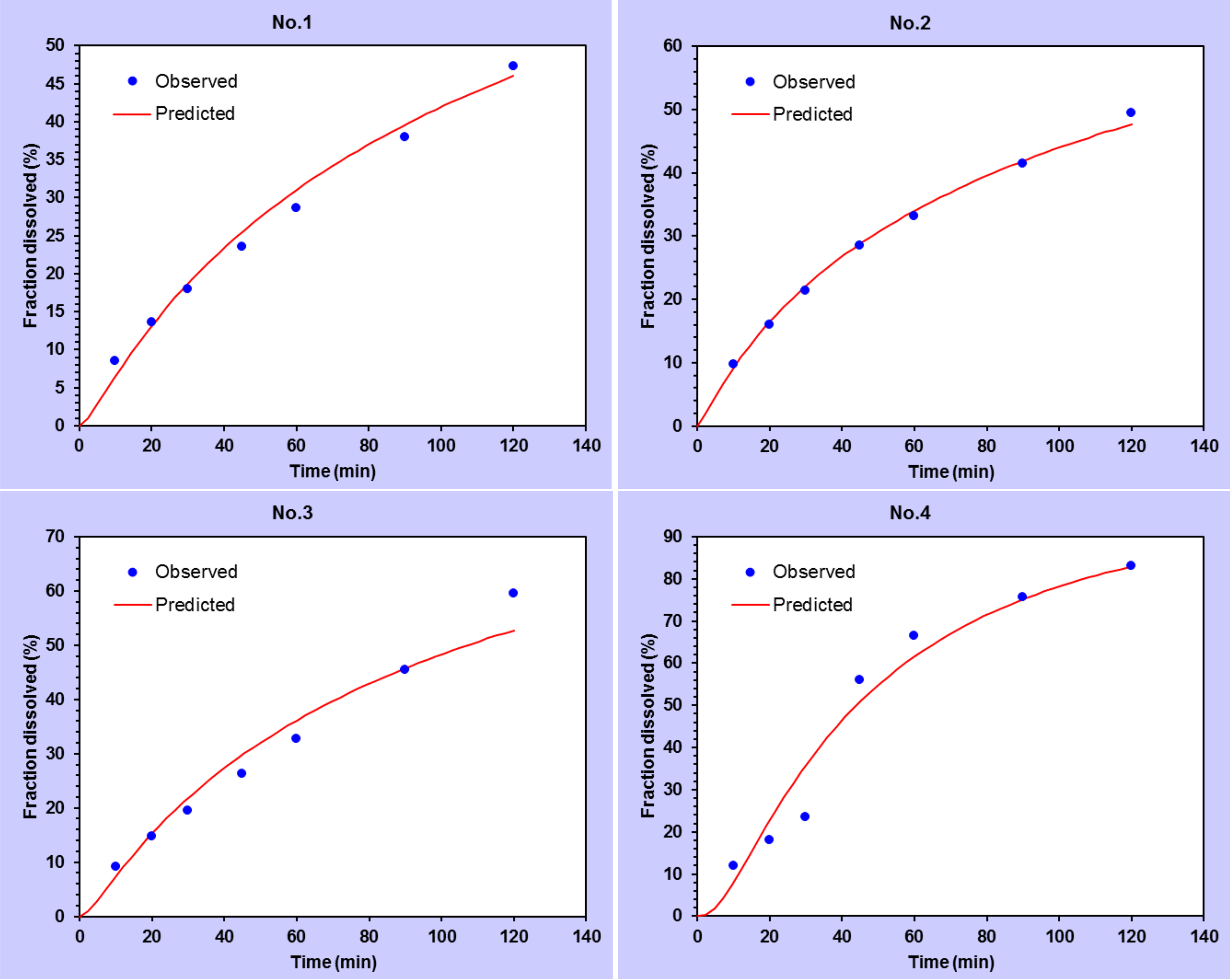

Supplementary materials – Model fitting summary – C*Pharm Mannidex 16700 (modelling up to app. 60 % of carvedilol released)

Model: **Probit_2**

Model equation: $F = F_{max} \cdot \phi[\alpha + \beta \cdot \log(t)]$

Fitted model parameters per tested tablet (N = 4) with statistics – mean, standard deviation (SD), and relative standard deviation expressed in % (RSD%) (output from DDSolver):

Parameter	No.1	No.2	No.3	No.4	Mean	SD	RSD(%)
α	-3.441	-3.309	-3.624	-4.794	-3.792	0.680	-17.943
β	2.198	2.182	2.245	2.910	2.384	0.352	14.759
F_{max}	49.632	51.984	62.569	94.318	64.625	20.579	31.843

Number of dissolution data points (N), degrees of freedom (df), and selected goodness of fit criteria – Pearson correlation coefficient (R), coefficient of determination (R^2), adjusted coefficient of determination ($R^2_{adjusted}$), and residual sum of squares (RSS) (manual calculation in MS Excel):

Parameter	No.1	No.2	No.3	No.4
N	7	7	7	7
df	4	4	4	4
R	0.964508792	0.977931356	0.955319609	0.984802885
R^2	0.930277209	0.956349737	0.912635555	0.969836723
$R^2_{adjusted}$	0.895415814	0.934524606	0.868953333	0.954755084
RSS	90.43560799	60.3253055	189.569504	226.9701837

Graphical abstract of model fit presented as mean ± 1 SD of the fraction % of released carvedilol:

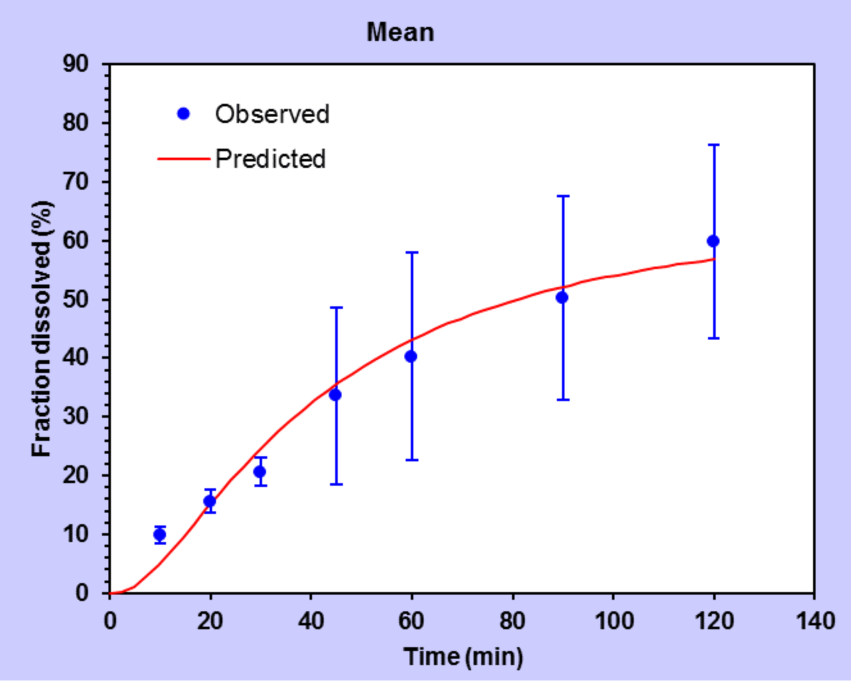

Supplementary materials – Model fitting summary – C*Pharm Mannidex 16700 (modelling up to app. 60 % of carvedilol released)

Graphical abstract of model fit presented as the fraction % of released carvedilol per tested tablet:

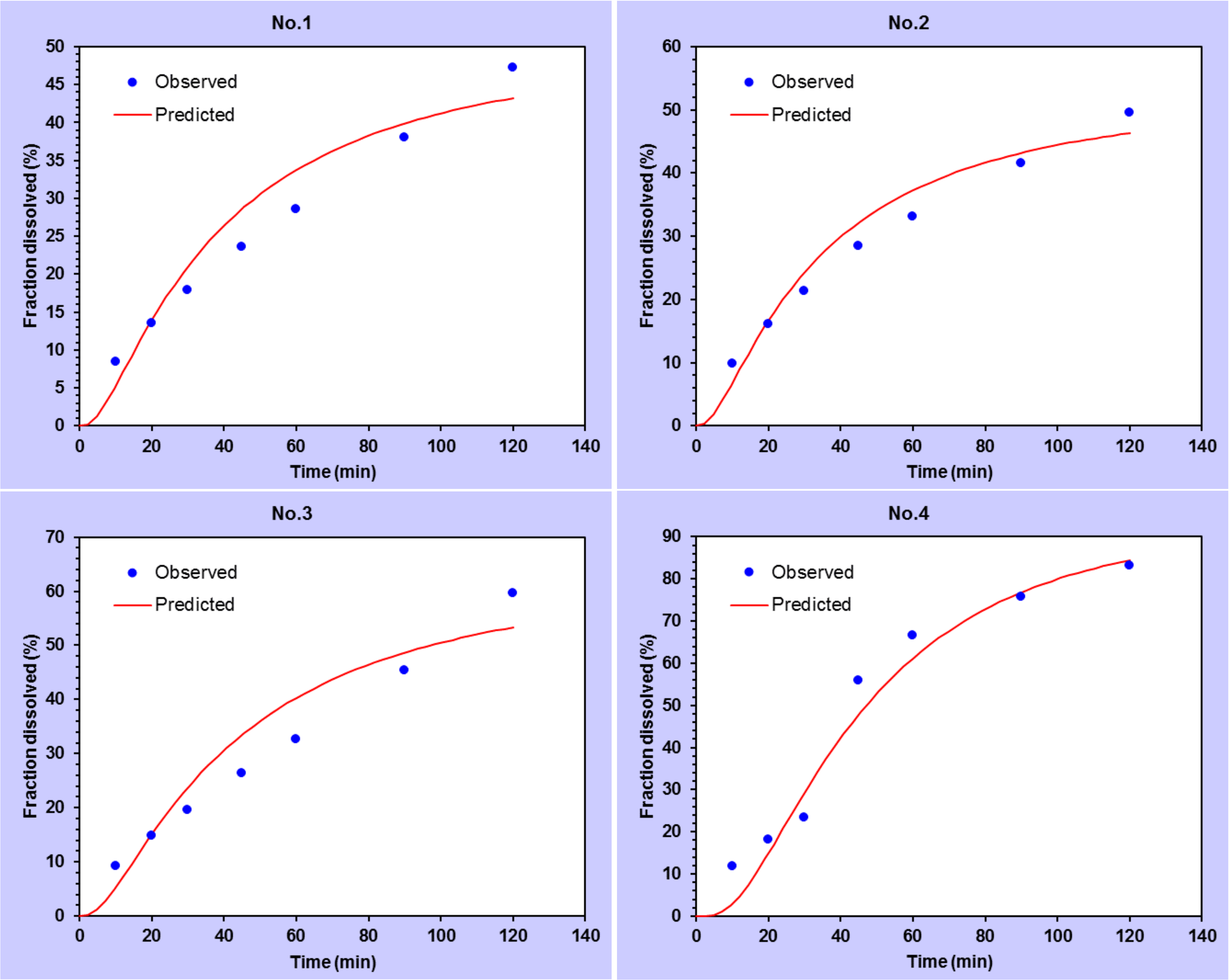

Supplement: Supplementary file 1 [file pharmaceutics-16-00498-s001.zip › Supplementary materials_Model fitting summary_CPharmMannidex 16700.pdf]
