# Supplementary material for: Comparative Fitting of Mathematical Models to Carvedilol Release Profiles Obtained from Hypromellose Matrix Tablets
Source: Pharmaceutics. 2024 Apr 4;16(4):498. doi: 10.3390/pharmaceutics16040498 (PMC11053526; doi:10.3390/pharmaceutics16040498)

Supplementary materials – Model fitting summary – Di-Cafos® A12 (modelling the entire relevant dissolution data)

Model: **Zero–order**

Model equation: $F = k_0 \cdot t$

Fitted model parameters per tested tablet (N = 4) with statistics – mean, standard deviation (SD), and relative standard deviation expressed in % (RSD%) (output from DDSolver):

Parameter	No.1	No.2	No.3	No.4	Mean	SD	RSD(%)
k_0	0.079	0.069	0.074	0.076	0.074	0.004	5.935

Number of dissolution data points (N), degrees of freedom (df), and selected goodness of fit criteria – Pearson correlation coefficient (R), coefficient of determination (R^2), adjusted coefficient of determination ($R^2_{adjusted}$), and residual sum of squares (RSS) (manual calculation in MS Excel):

Parameter	No.1	No.2	No.3	No.4
N	33	33	33	33
df	32	32	32	32
R	0.986669513	0.990299515	0.990474283	0.990646426
R^2	0.973516728	0.98069313	0.981039306	0.98138034
$R^2_{adjusted}$	0.973516728	0.98069313	0.981039306	0.98138034
RSS	2571.566904	1813.404513	1956.742601	2096.480183

Graphical abstract of model fit presented as mean ± 1 SD of the fraction % of released carvedilol:

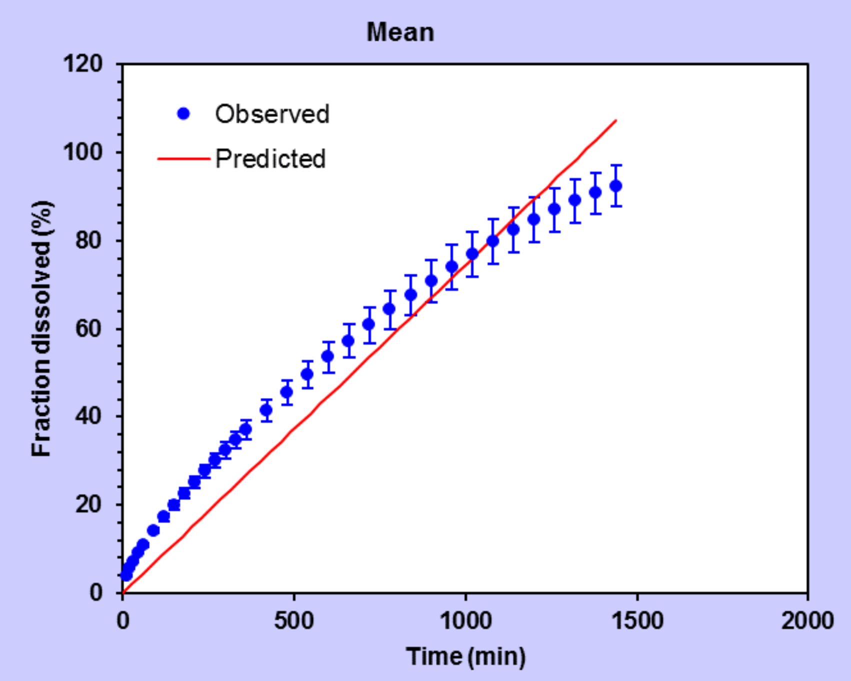

Supplementary materials – Model fitting summary – Di-Cafos® A12 (modelling the entire relevant dissolution data)

Graphical abstract of model fit presented as the fraction % of released carvedilol per tested tablet:

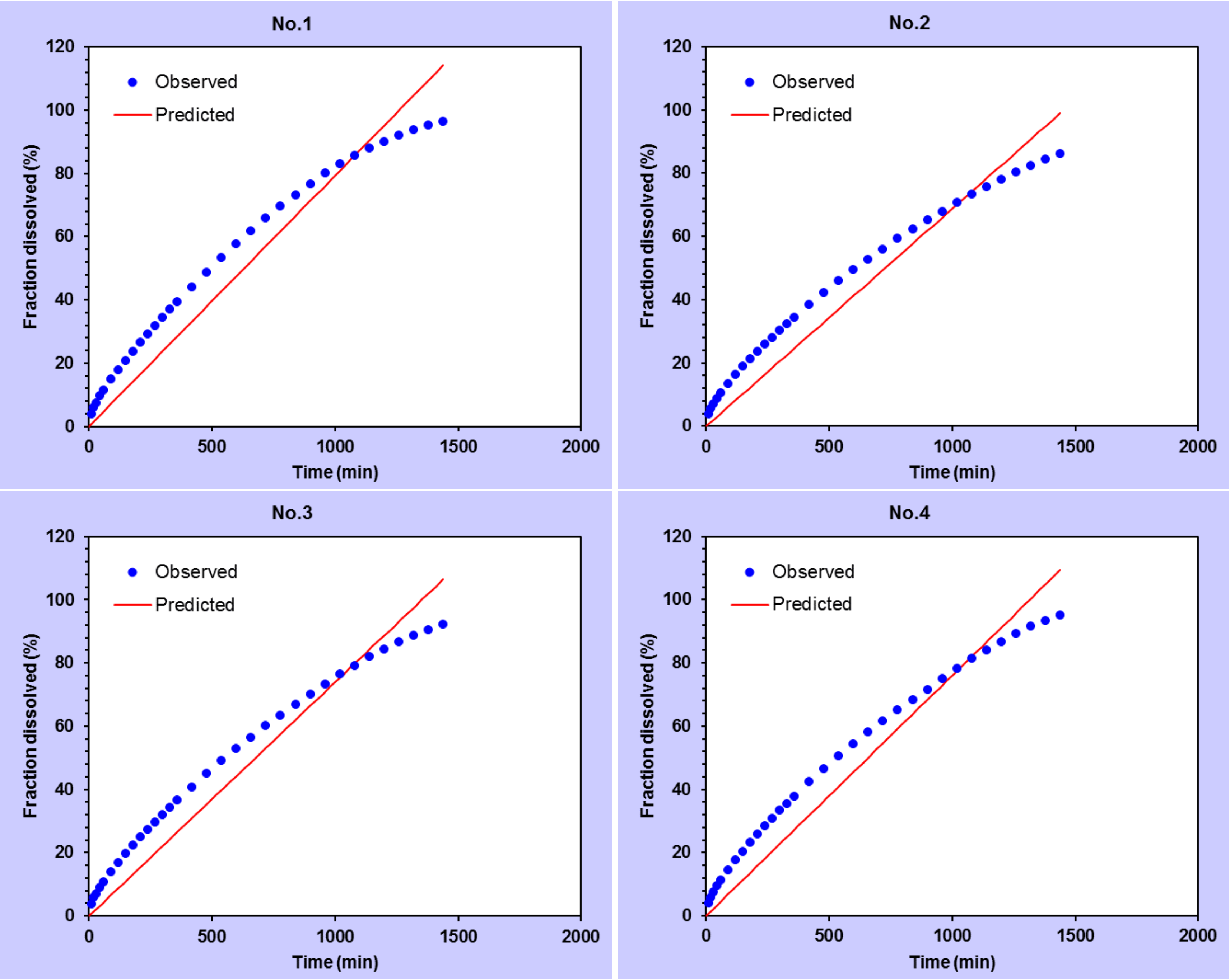

Supplementary materials – Model fitting summary – Di-Cafos® A12 (modelling the entire relevant dissolution data)

Model: **Zero–order with T_{lag}**

Model equation: $F = k_0 \cdot (t - T_{lag})$

Fitted model parameters per tested tablet (N = 4) with statistics – mean, standard deviation (SD), and relative standard deviation expressed in % (RSD%) (output from DDSolver):

Parameter	No.1	No.2	No.3	No.4	Mean	SD	RSD(%)
k_0	0.067	0.058	0.062	0.064	0.063	0.004	6.099
T_{lag}	-177.671	-181.976	-172.318	-175.020	-176.746	4.115	-2.328

Number of dissolution data points (N), degrees of freedom (df), and selected goodness of fit criteria – Pearson correlation coefficient (R), coefficient of determination (R^2), adjusted coefficient of determination ($R^2_{adjusted}$), and residual sum of squares (RSS) (manual calculation in MS Excel):

Parameter	No.1	No.2	No.3	No.4
N	33	33	33	33
df	31	31	31	31
R	0.986669513	0.990299515	0.990474283	0.990646426
R^2	0.973516728	0.98069313	0.981039306	0.98138034
$R^2_{adjusted}$	0.972662428	0.980070328	0.98042767	0.980779706
RSS	819.1560766	442.1790212	510.5794585	527.2213877

Graphical abstract of model fit presented as mean ± 1 SD of the fraction % of released carvedilol:

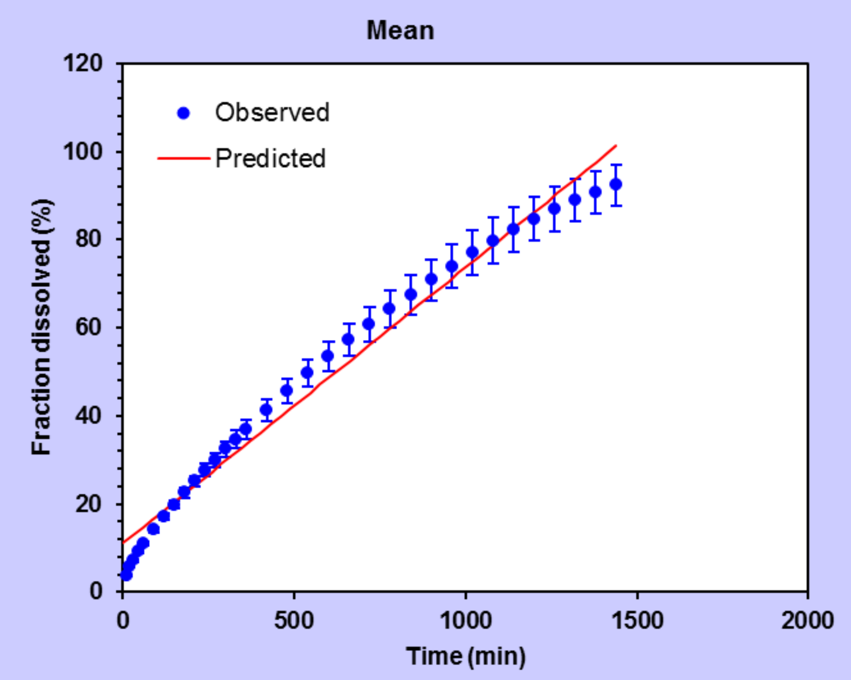

Supplementary materials – Model fitting summary – Di-Cafos® A12 (modelling the entire relevant dissolution data)

Graphical abstract of model fit presented as the fraction % of released carvedilol per tested tablet:

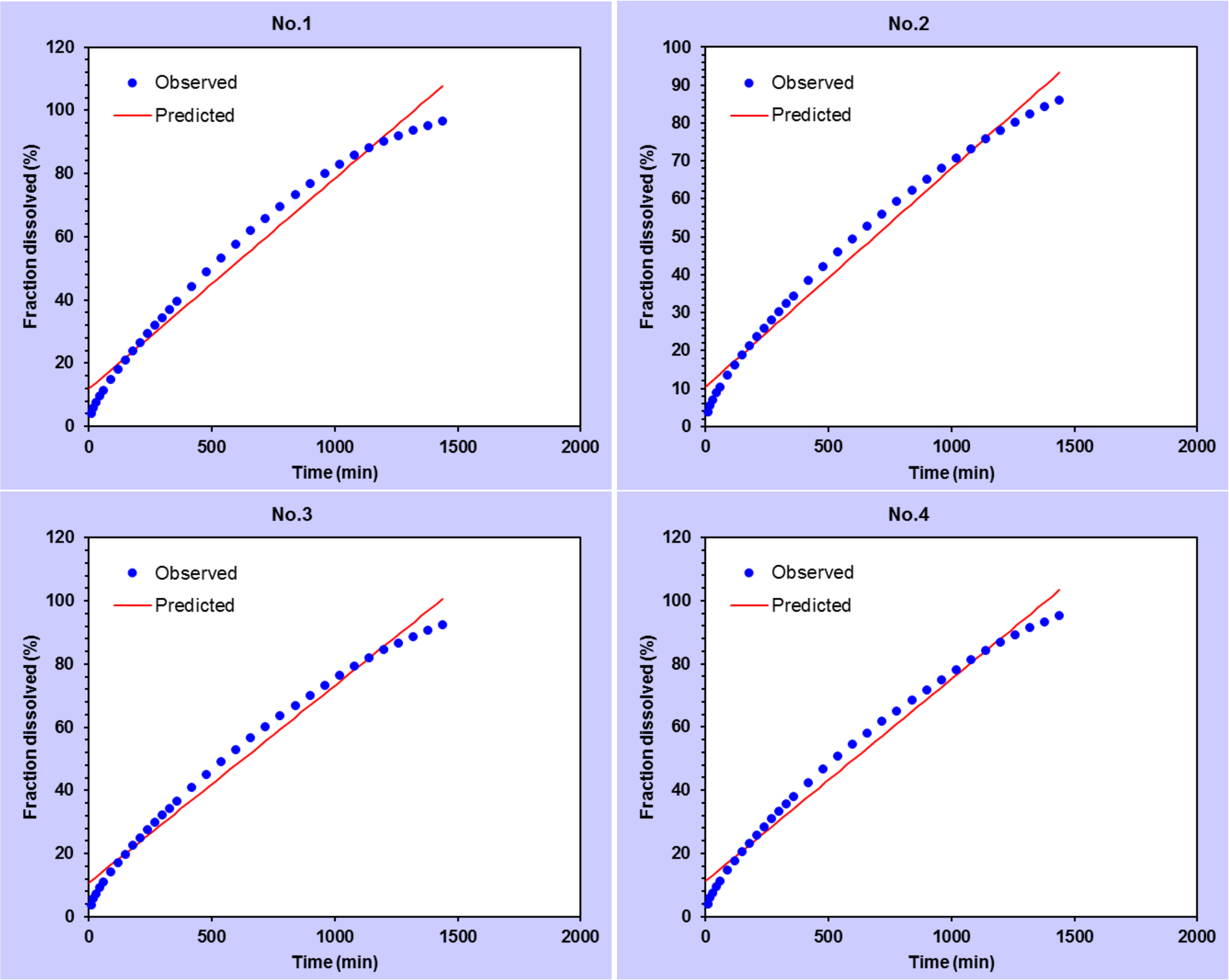

Supplementary materials – Model fitting summary – Di-Cafos® A12 (modelling the entire relevant dissolution data)

Model: **Zero–order with F_0**

Model equation: $F = F_0 + k_0 \cdot t$

Fitted model parameters per tested tablet (N = 4) with statistics – mean, standard deviation (SD), and relative standard deviation expressed in % (RSD%) (output from DDSolver):

Parameter	No.1	No.2	No.3	No.4	Mean	SD	RSD(%)
k_0	0.067	0.058	0.062	0.064	0.063	0.004	6.099
F_0	11.839	10.473	10.755	11.204	11.068	0.596	5.384

Number of dissolution data points (N), degrees of freedom (df), and selected goodness of fit criteria – Pearson correlation coefficient (R), coefficient of determination (R^2), adjusted coefficient of determination ($R^2_{adjusted}$), and residual sum of squares (RSS) (manual calculation in MS Excel):

Parameter	No.1	No.2	No.3	No.4
N	33	33	33	33
df	31	31	31	31
R	0.986669513	0.990299515	0.990474283	0.990646426
R^2	0.973516728	0.98069313	0.981039306	0.98138034
$R^2_{adjusted}$	0.972662428	0.980070328	0.98042767	0.980779706
RSS	819.1560766	442.1790212	510.5794585	527.2213877

Graphical abstract of model fit presented as mean ± 1 SD of the fraction % of released carvedilol:

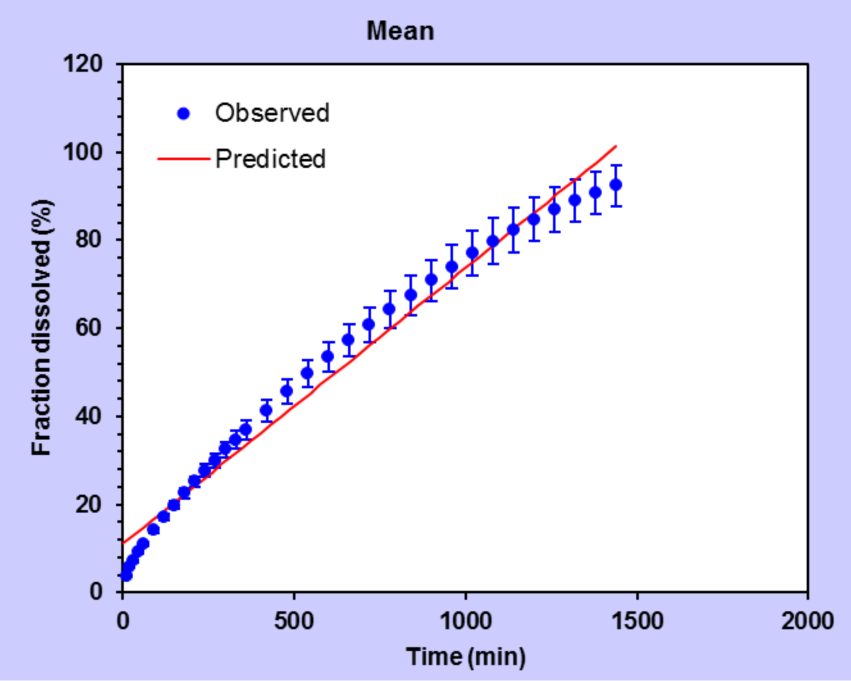

Supplementary materials – Model fitting summary – Di-Cafos® A12 (modelling the entire relevant dissolution data)

Graphical abstract of model fit presented as the fraction % of released carvedilol per tested tablet:

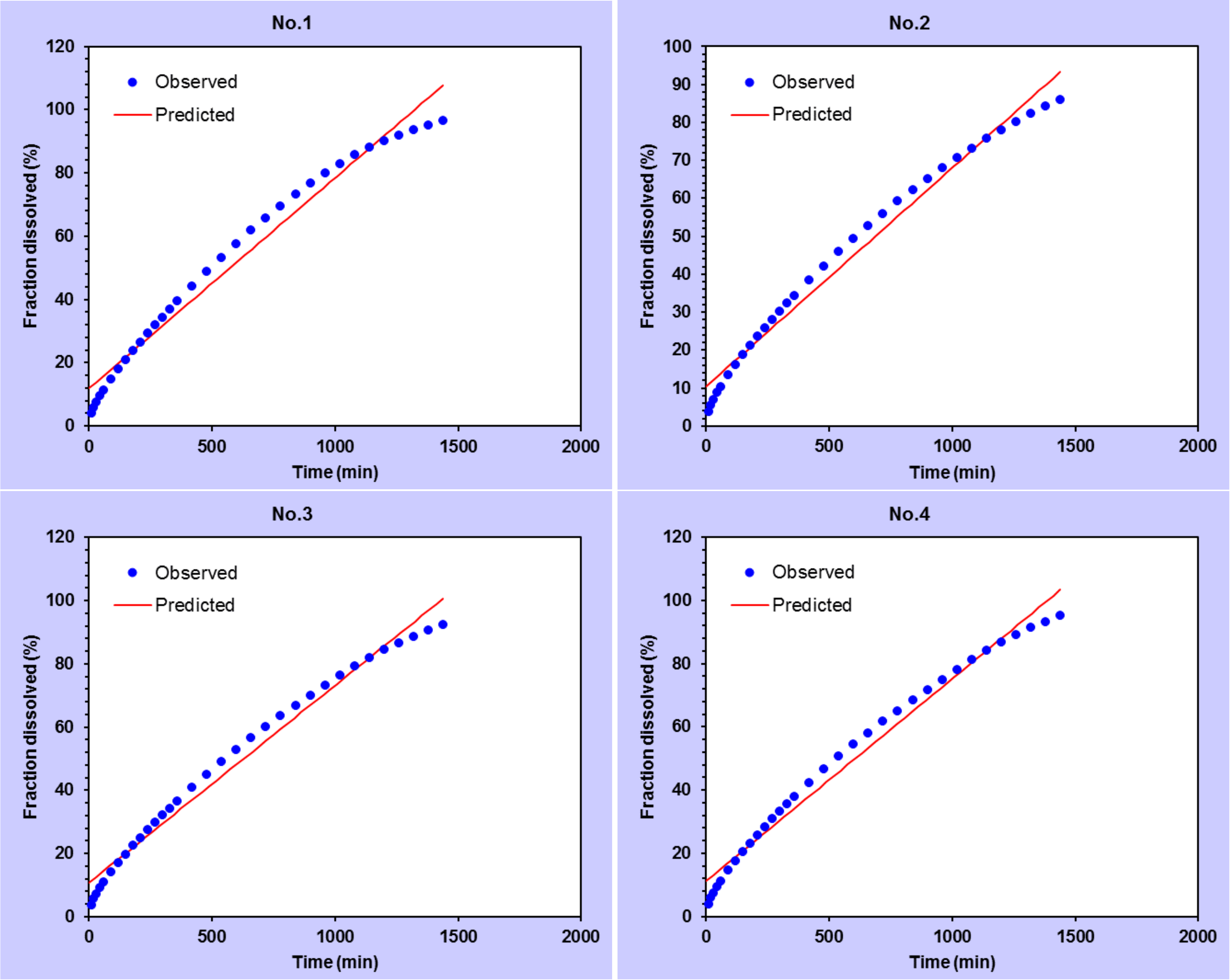

Supplementary materials – Model fitting summary – Di-Cafos® A12 (modelling the entire relevant dissolution data)

Model: **First–order**

Model equation: $F = 100 \cdot (1 - e^{-k_1 \cdot t})$

Fitted model parameters per tested tablet (N = 4) with statistics – mean, standard deviation (SD), and relative standard deviation expressed in % (RSD%) (output from DDSolver):

Parameter	No.1	No.2	No.3	No.4	Mean	SD	RSD(%)
k_1	0.001	0.001	0.001	0.001	0.001	0.000	6.461

Number of dissolution data points (N), degrees of freedom (df), and selected goodness of fit criteria – Pearson correlation coefficient (R), coefficient of determination (R^2), adjusted coefficient of determination ($R^2_{adjusted}$), and residual sum of squares (RSS) (manual calculation in MS Excel):

Parameter	No.1	No.2	No.3	No.4
N	33	33	33	33
df	32	32	32	32
R	0.996636208	0.996939495	0.995879118	0.993825382
R^2	0.993283731	0.993888356	0.991775218	0.987688891
$R^2_{adjusted}$	0.993283731	0.993888356	0.991775218	0.987688891
RSS	764.9530781	178.0224546	307.3387344	362.1408908

Graphical abstract of model fit presented as mean ± 1 SD of the fraction % of released carvedilol:

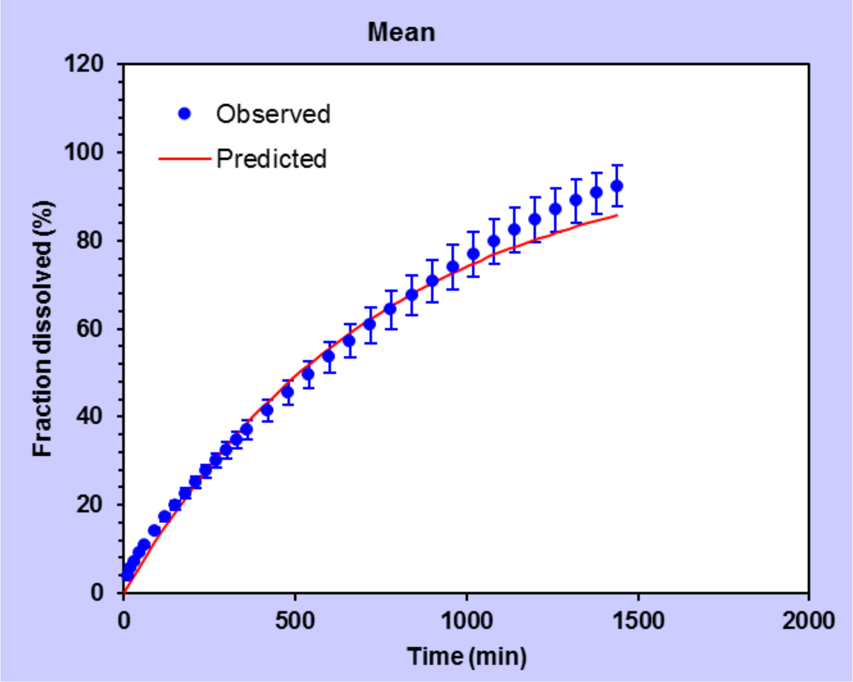

Supplementary materials – Model fitting summary – Di-Cafos® A12 (modelling the entire relevant dissolution data)

Graphical abstract of model fit presented as the fraction % of released carvedilol per tested tablet:

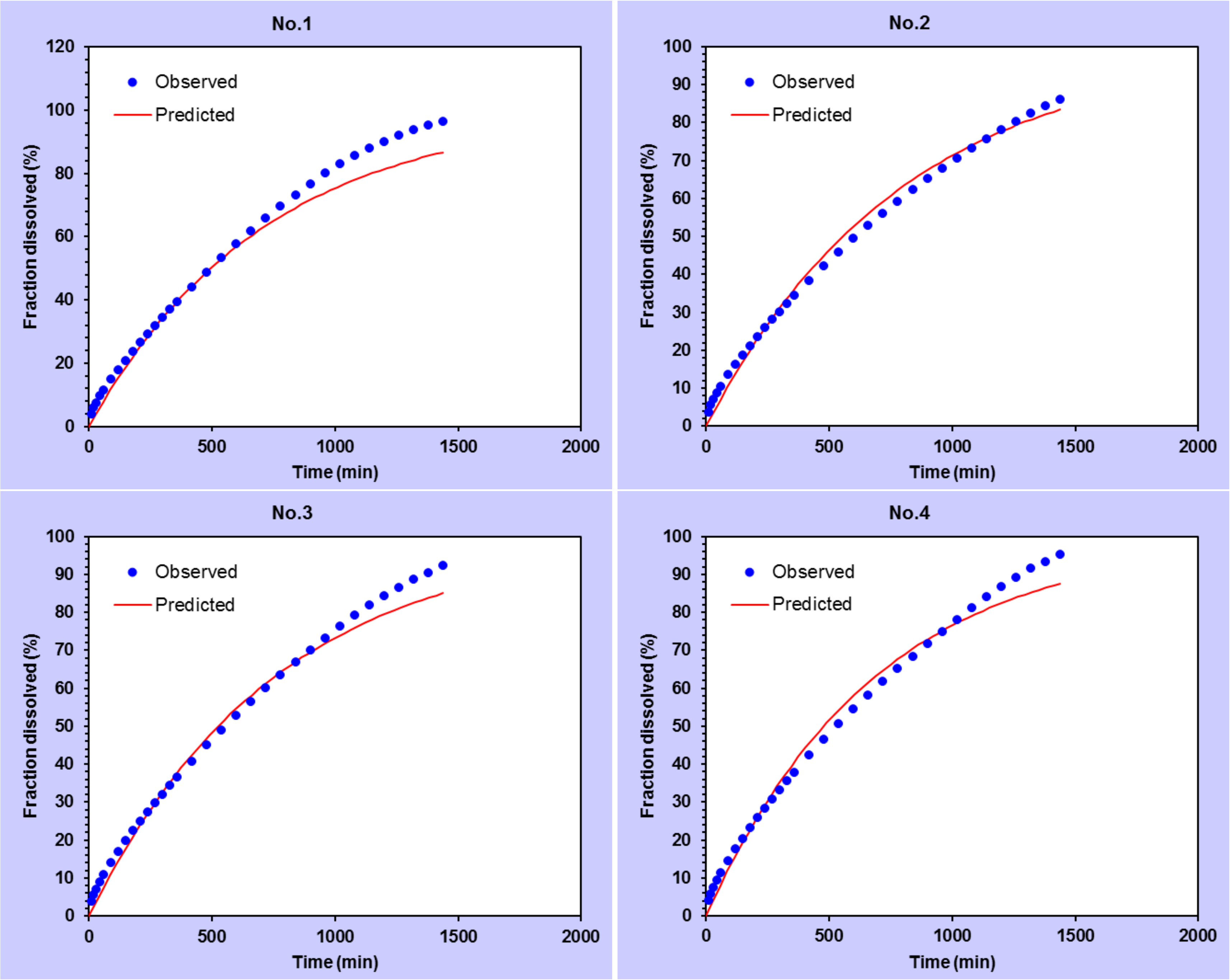

Supplementary materials – Model fitting summary – Di-Cafos® A12 (modelling the entire relevant dissolution data)

Model: **First–order with T_{lag}**

Model equation: $F = 100 \cdot \left[1 - e^{-k_1 \cdot (t - T_{lag})}\right]$

Fitted model parameters per tested tablet (N = 4) with statistics – mean, standard deviation (SD), and relative standard deviation expressed in % (RSD%) (output from DDSolver):

Parameter	No.1	No.2	No.3	No.4	Mean	SD	RSD(%)
k_1	0.002	0.001	0.002	0.002	0.002	0.000	19.389
T_{lag}	75.484	10.634	47.259	64.824	49.550	28.435	57.386

Number of dissolution data points (N), degrees of freedom (df), and selected goodness of fit criteria – Pearson correlation coefficient (R), coefficient of determination (R^2), adjusted coefficient of determination ($R^2_{adjusted}$), and residual sum of squares (RSS) (manual calculation in MS Excel):

Parameter	No.1	No.2	No.3	No.4
N	33	33	33	33
df	31	31	31	31
R	0.984868084	0.996765556	0.991387352	0.987305932
R^2	0.969965142	0.993541574	0.982848881	0.974773002
$R^2_{adjusted}$	0.968996276	0.993333238	0.982295619	0.973959228
RSS	2201.783207	228.739076	874.6812216	1603.96885

Graphical abstract of model fit presented as mean ± 1 SD of the fraction % of released carvedilol:

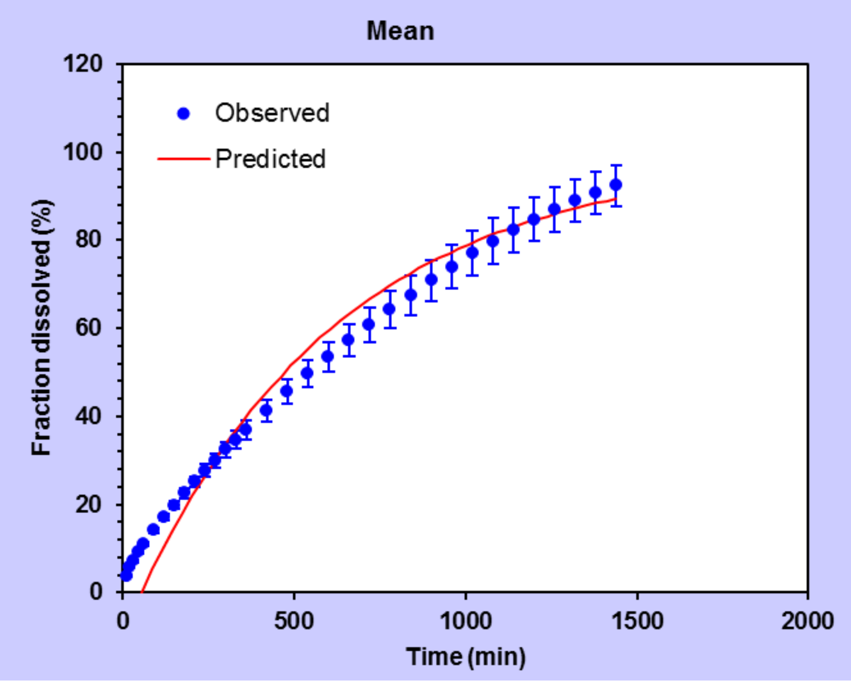

Supplementary materials – Model fitting summary – Di-Cafos® A12 (modelling the entire relevant dissolution data)

Graphical abstract of model fit presented as the fraction % of released carvedilol per tested tablet:

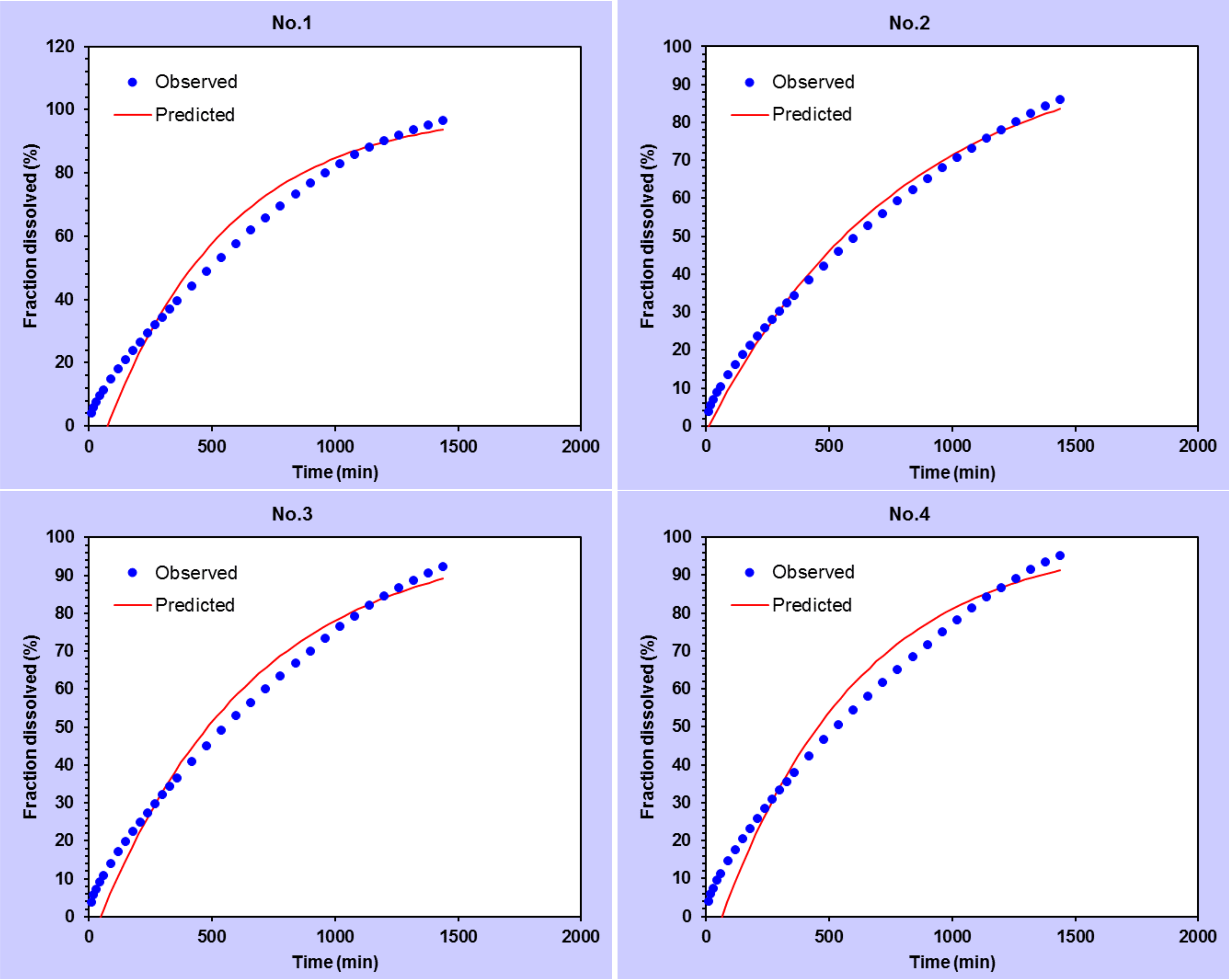

Supplementary materials – Model fitting summary – Di-Cafos® A12 (modelling the entire relevant dissolution data)

Model: **First–order with F_{max}**

Model equation: $F = F_{max} \cdot (1 - e^{-k_1 \cdot t})$

Fitted model parameters per tested tablet (N = 4) with statistics – mean, standard deviation (SD), and relative standard deviation expressed in % (RSD%) (output from DDSolver):

Parameter	No.1	No.2	No.3	No.4	Mean	SD	RSD(%)
k_1	0.002	0.002	0.002	0.002	0.002	0.000	0.652
F_{max}	93.290	90.347	96.822	99.832	95.072	4.132	4.346

Number of dissolution data points (N), degrees of freedom (df), and selected goodness of fit criteria – Pearson correlation coefficient (R), coefficient of determination (R^2), adjusted coefficient of determination ($R^2_{adjusted}$), and residual sum of squares (RSS) (manual calculation in MS Excel):

Parameter	No.1	No.2	No.3	No.4
N	33	33	33	33
df	31	31	31	31
R	0.992806832	0.990356837	0.989614896	0.98976166
R^2	0.985665406	0.980806665	0.979337642	0.979628143
$R^2_{adjusted}$	0.985202999	0.980187525	0.978671115	0.978970986
RSS	912.9737737	657.9875476	860.8412971	887.9018916

Graphical abstract of model fit presented as mean ± 1 SD of the fraction % of released carvedilol:

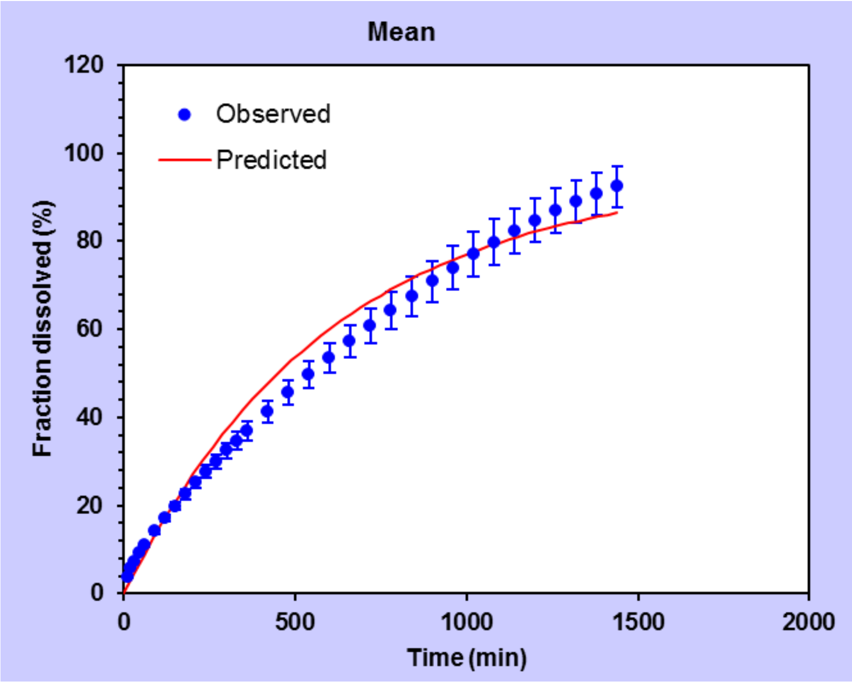

Supplementary materials – Model fitting summary – Di-Cafos® A12 (modelling the entire relevant dissolution data)

Graphical abstract of model fit presented as the fraction % of released carvedilol per tested tablet:

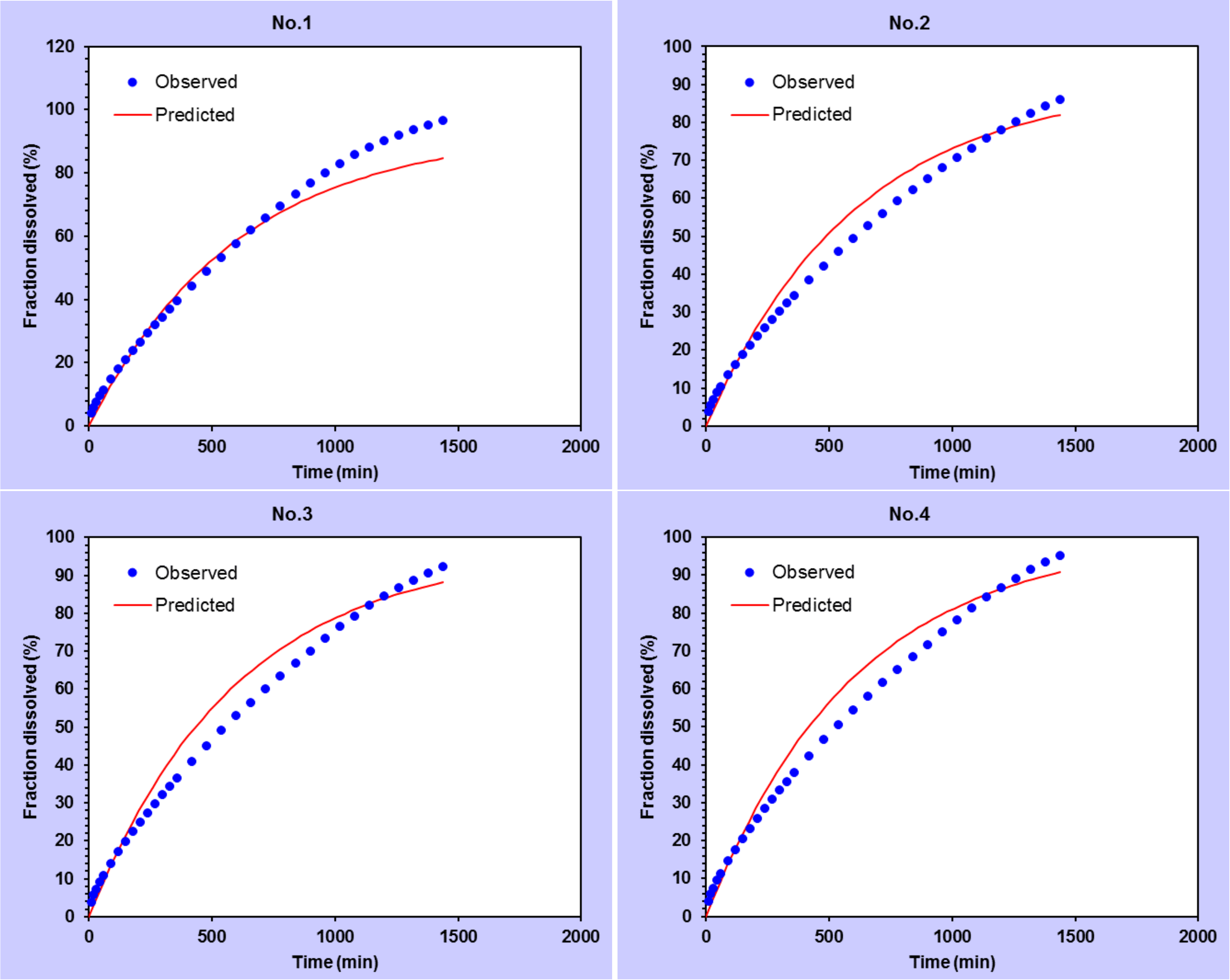

Supplementary materials – Model fitting summary – Di-Cafos® A12 (modelling the entire relevant dissolution data)

Model: **First–order with T_{lag} and F_{max}**

Model equation: $F = F_{max} \cdot \left[1 - e^{-k_1 \cdot (t - T_{lag})}\right]$

Fitted model parameters per tested tablet (N = 4) with statistics – mean, standard deviation (SD), and relative standard deviation expressed in % (RSD%) (output from DDSolver):

Parameter	No.1	No.2	No.3	No.4	Mean	SD	RSD(%)
k_1	0.002	0.002	0.002	0.002	0.002	0.000	3.460
T_{lag}	65.934	61.549	67.162	66.031	65.169	2.477	3.800
F_{max}	101.196	90.347	96.822	99.832	97.049	4.827	4.974

Number of dissolution data points (N), degrees of freedom (df), and selected goodness of fit criteria – Pearson correlation coefficient (R), coefficient of determination (R^2), adjusted coefficient of determination ($R^2_{adjusted}$), and residual sum of squares (RSS) (manual calculation in MS Excel):

Parameter	No.1	No.2	No.3	No.4
N	33	33	33	33
df	30	30	30	30
R	0.987592466	0.98788294	0.986749884	0.987001613
R^2	0.975338879	0.975912703	0.973675334	0.974172185
$R^2_{adjusted}$	0.973694804	0.974306883	0.971920356	0.972450331
RSS	1649.547795	1243.626593	1586.343988	1659.970242

Graphical abstract of model fit presented as mean ± 1 SD of the fraction % of released carvedilol:

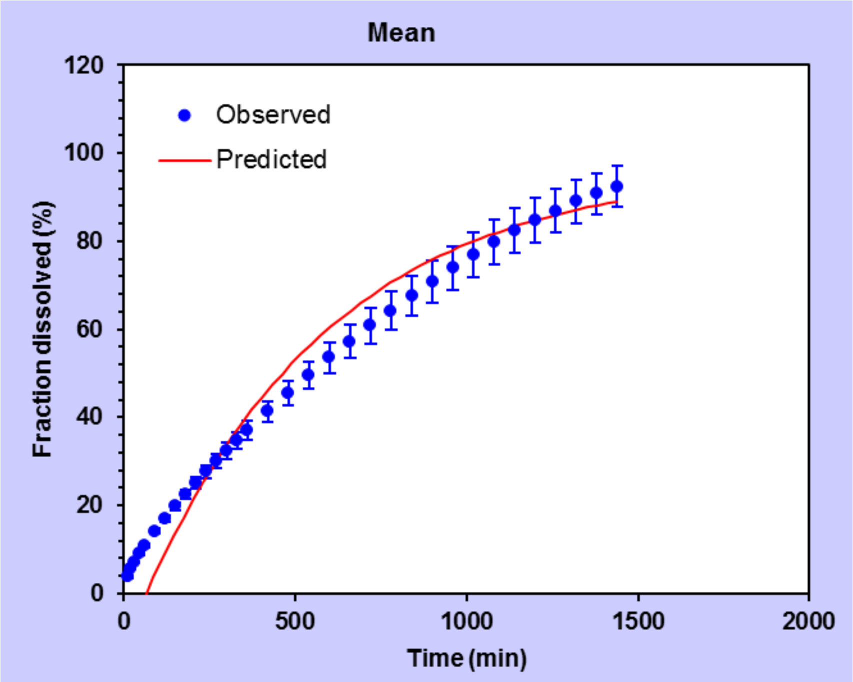

Supplementary materials – Model fitting summary – Di-Cafos® A12 (modelling the entire relevant dissolution data)

Graphical abstract of model fit presented as the fraction % of released carvedilol per tested tablet:

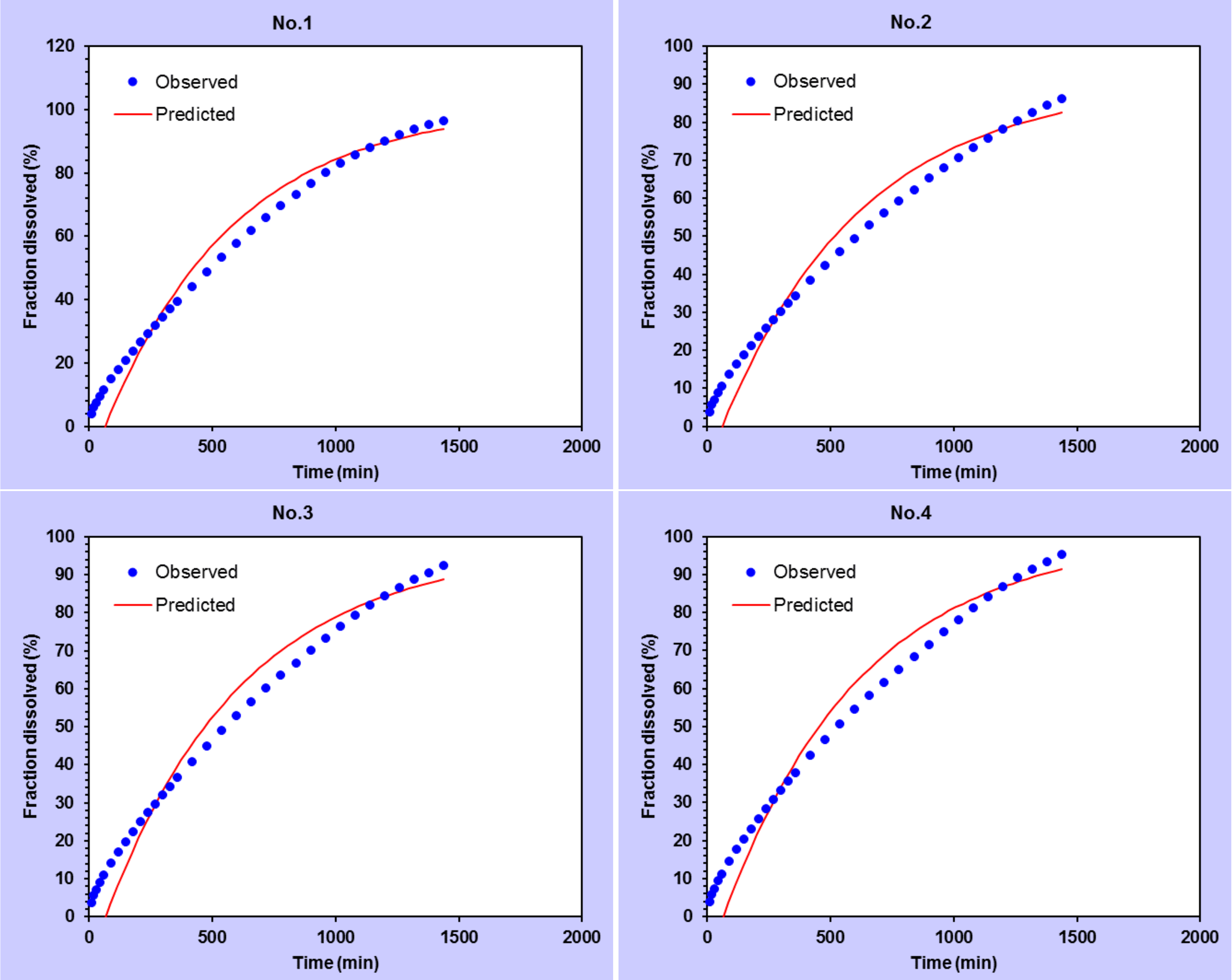

Supplementary materials – Model fitting summary – Di-Cafos® A12 (modelling the entire relevant dissolution data)

Model: **Higuchi**

Model equation: $F = k_H \cdot t^{0.5}$

Fitted model parameters per tested tablet (N = 4) with statistics – mean, standard deviation (SD), and relative standard deviation expressed in % (RSD%) (output from DDSolver):

Parameter	No.1	No.2	No.3	No.4	Mean	SD	RSD(%)
k_H	2.439	2.114	2.270	2.335	2.289	0.136	5.943

Number of dissolution data points (N), degrees of freedom (df), and selected goodness of fit criteria – Pearson correlation coefficient (R), coefficient of determination (R^2), adjusted coefficient of determination ($R^2_{adjusted}$), and residual sum of squares (RSS) (manual calculation in MS Excel):

Parameter	No.1	No.2	No.3	No.4
N	33	33	33	33
df	32	32	32	32
R	0.996047183	0.996255145	0.995896862	0.996068951
R^2	0.99210999	0.992524315	0.991810559	0.992153354
$R^2_{adjusted}$	0.99210999	0.992524315	0.991810559	0.992153354
RSS	1195.628878	824.0031465	1069.826974	1089.774422

Graphical abstract of model fit presented as mean ± 1 SD of the fraction % of released carvedilol:

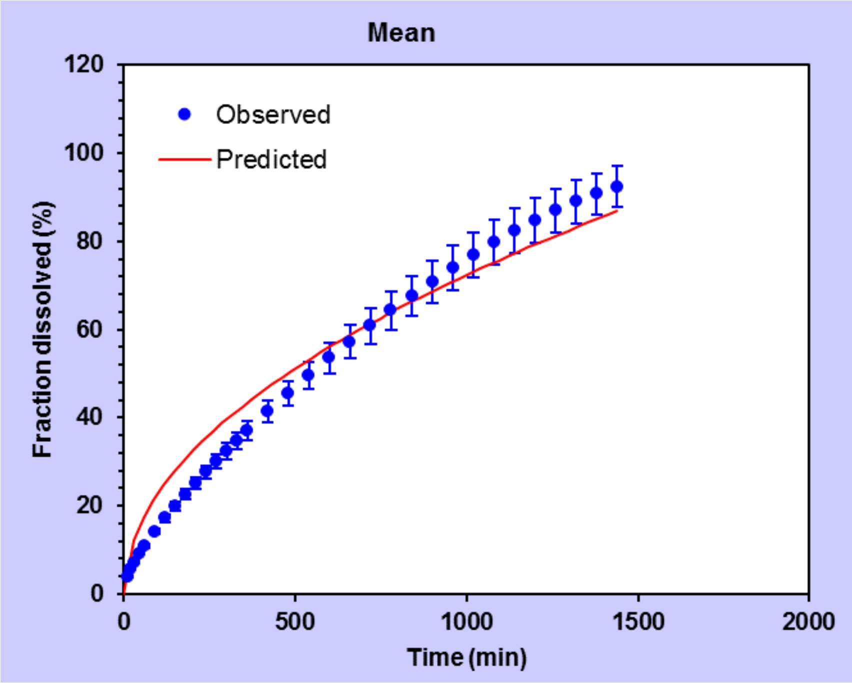

Supplementary materials – Model fitting summary – Di-Cafos® A12 (modelling the entire relevant dissolution data)

Graphical abstract of model fit presented as the fraction % of released carvedilol per tested tablet:

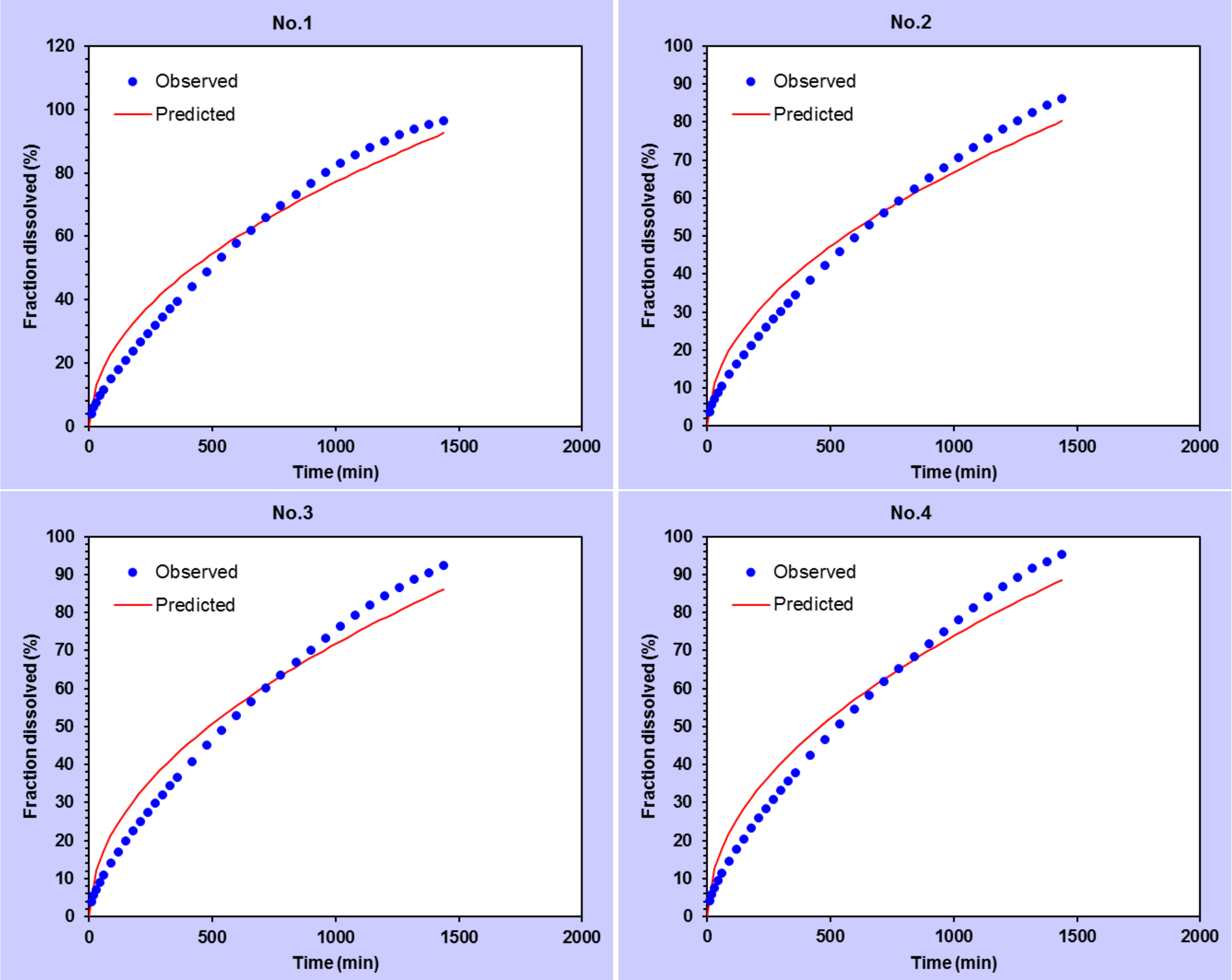

Supplementary materials – Model fitting summary – Di-Cafos® A12 (modelling the entire relevant dissolution data)

Model: **Higuchi with T_{lag}**

Model equation: $F = k_H \cdot (t - T_{lag})^{0.5}$

Fitted model parameters per tested tablet (N = 4) with statistics – mean, standard deviation (SD), and relative standard deviation expressed in % (RSD%) (output from DDSolver):

Parameter	No.1	No.2	No.3	No.4	Mean	SD	RSD(%)
k_H	2.653	2.309	2.490	2.558	2.502	0.145	5.808
T_{lag}	84.519	89.040	92.656	92.079	89.574	3.725	4.158

Number of dissolution data points (N), degrees of freedom (df), and selected goodness of fit criteria – Pearson correlation coefficient (R), coefficient of determination (R^2), adjusted coefficient of determination ($R^2_{adjusted}$), and residual sum of squares (RSS) (manual calculation in MS Excel):

Parameter	No.1	No.2	No.3	No.4
N	33	33	33	33
df	31	31	31	31
R	0.991982069	0.99113716	0.990405052	0.990529099
R^2	0.984028426	0.982352869	0.980902167	0.981147896
$R^2_{adjusted}$	0.983513214	0.981783607	0.980286108	0.980539764
RSS	592.0585668	538.5723749	667.7163986	705.5027496

Graphical abstract of model fit presented as mean ± 1 SD of the fraction % of released carvedilol:

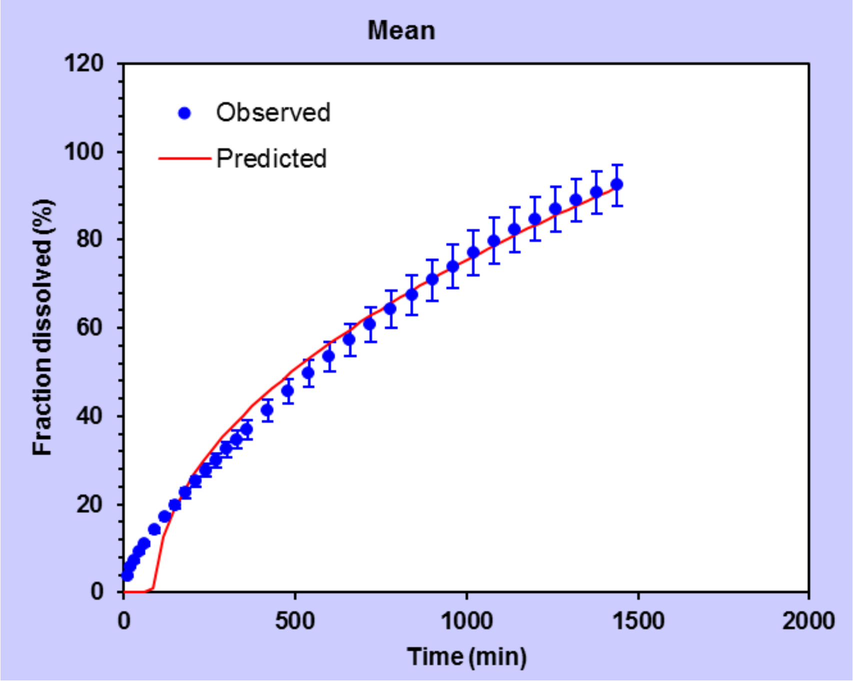

Supplementary materials – Model fitting summary – Di-Cafos® A12 (modelling the entire relevant dissolution data)

Graphical abstract of model fit presented as the fraction % of released carvedilol per tested tablet:

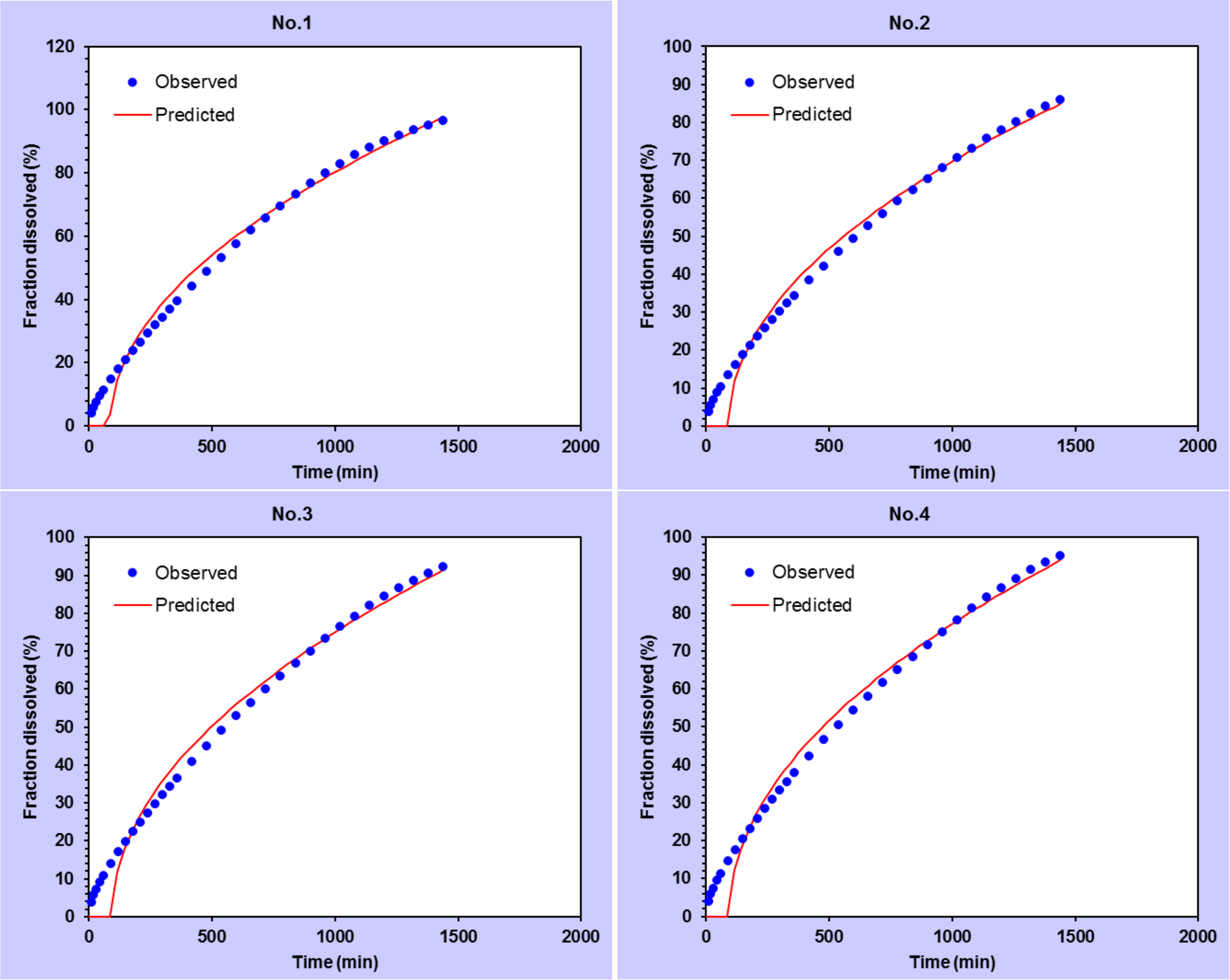

Supplementary materials – Model fitting summary – Di-Cafos® A12 (modelling the entire relevant dissolution data)

Model: **Higuchi with F_0**

Model equation: $F = F_0 + k_H \cdot t^{0.5}$

Fitted model parameters per tested tablet (N = 4) with statistics – mean, standard deviation (SD), and relative standard deviation expressed in % (RSD%) (output from DDSolver):

Parameter	No.1	No.2	No.3	No.4	Mean	SD	RSD(%)
k_H	2.898	2.494	2.704	2.773	2.717	0.169	6.220
F_0	-12.296	-10.184	-11.616	-11.741	-11.459	0.900	-7.854

Number of dissolution data points (N), degrees of freedom (df), and selected goodness of fit criteria – Pearson correlation coefficient (R), coefficient of determination (R^2), adjusted coefficient of determination ($R^2_{adjusted}$), and residual sum of squares (RSS) (manual calculation in MS Excel):

Parameter	No.1	No.2	No.3	No.4
N	33	33	33	33
df	31	31	31	31
R	0.996047183	0.996255145	0.995896862	0.996068951
R^2	0.99210999	0.992524315	0.991810559	0.992153354
$R^2_{adjusted}$	0.991855474	0.992283164	0.991546383	0.991900237
RSS	244.0464868	171.2132122	220.5278095	222.1801855

Graphical abstract of model fit presented as mean ± 1 SD of the fraction % of released carvedilol:

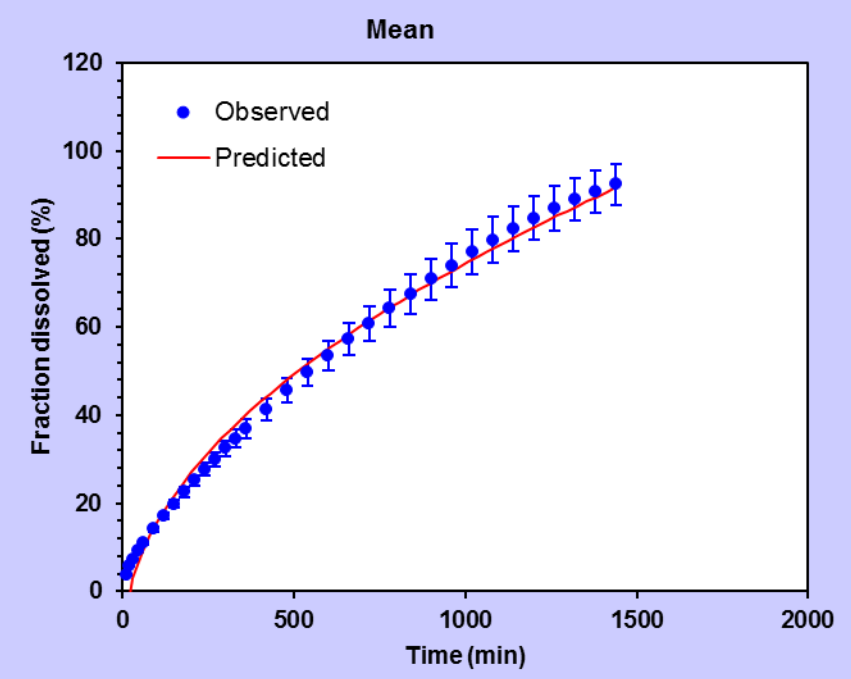

Supplementary materials – Model fitting summary – Di-Cafos® A12 (modelling the entire relevant dissolution data)

Graphical abstract of model fit presented as the fraction % of released carvedilol per tested tablet:

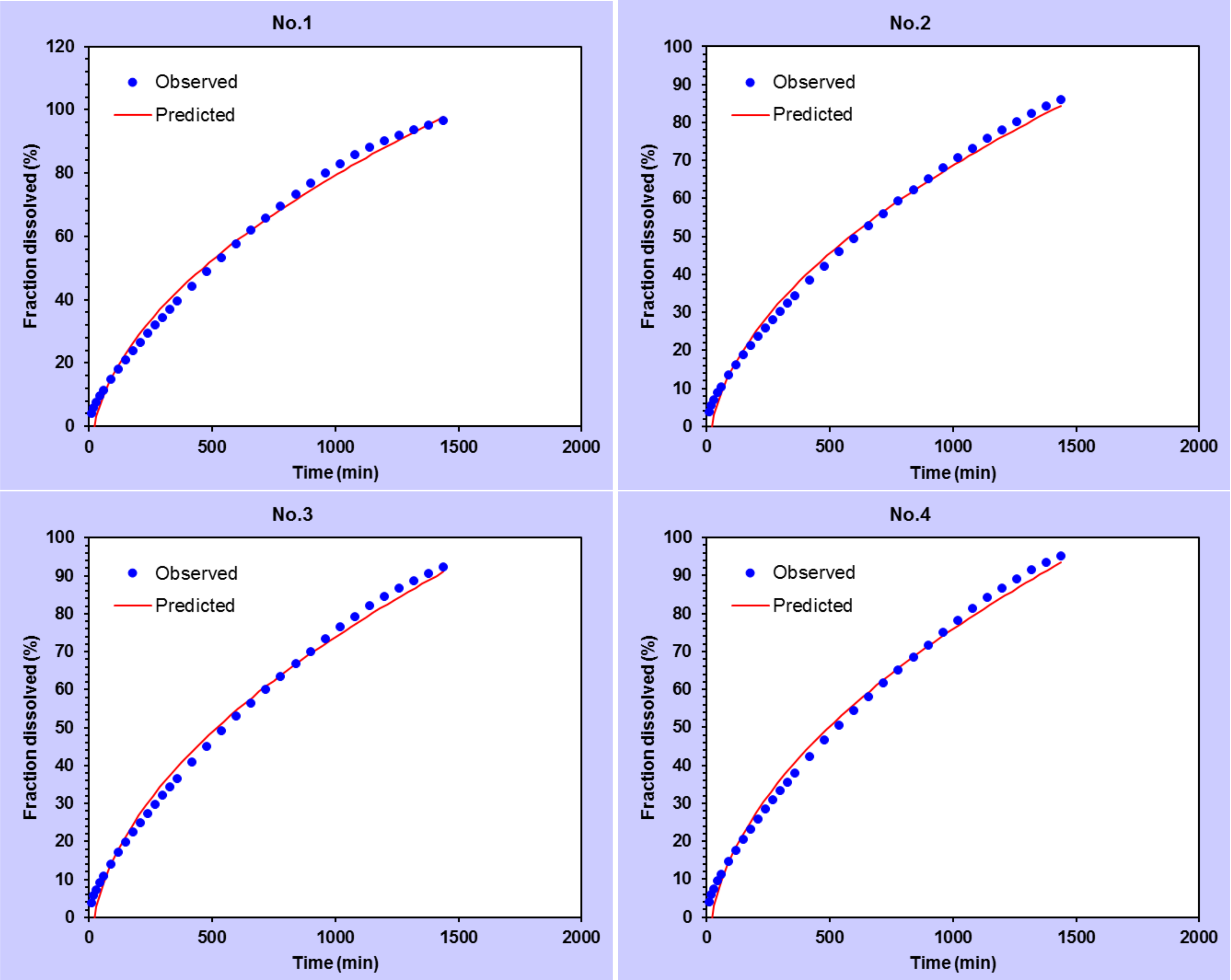

Supplementary materials – Model fitting summary – Di-Cafos® A12 (modelling the entire relevant dissolution data)

Model: **Korsmeyer–Peppas**

Model equation: $F = k_{KP} \cdot t^n$

Fitted model parameters per tested tablet (N = 4) with statistics – mean, standard deviation (SD), and relative standard deviation expressed in % (RSD%) (output from DDSolver):

Parameter	No.1	No.2	No.3	No.4	Mean	SD	RSD(%)
k_{KP}	0.737	0.735	0.713	0.754	0.735	0.017	2.268
n	0.677	0.656	0.671	0.667	0.668	0.009	1.347

Number of dissolution data points (N), degrees of freedom (df), and selected goodness of fit criteria – Pearson correlation coefficient (R), coefficient of determination (R^2), adjusted coefficient of determination ($R^2_{adjusted}$), and residual sum of squares (RSS) (manual calculation in MS Excel):

Parameter	No.1	No.2	No.3	No.4
N	33	33	33	33
df	31	31	31	31
R	0.998304116	0.999663602	0.999545229	0.99968372
R^2	0.996611107	0.999327317	0.999090665	0.99936754
$R^2_{adjusted}$	0.996501788	0.999305618	0.999061332	0.999347138
RSS	106.9136329	27.48077202	34.44612629	28.98574576

Graphical abstract of model fit presented as mean ± 1 SD of the fraction % of released carvedilol:

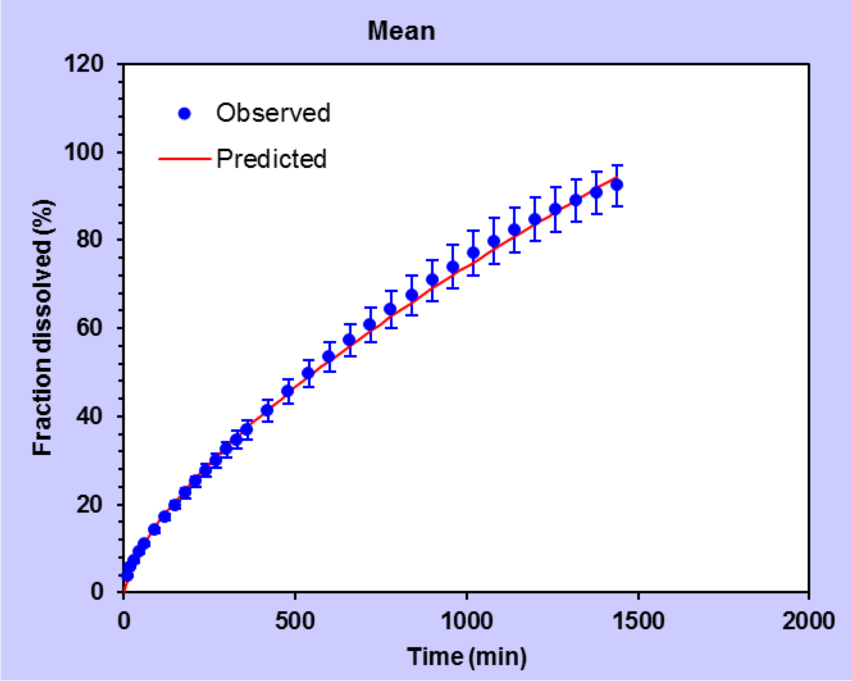

Supplementary materials – Model fitting summary – Di-Cafos® A12 (modelling the entire relevant dissolution data)

Graphical abstract of model fit presented as the fraction % of released carvedilol per tested tablet:

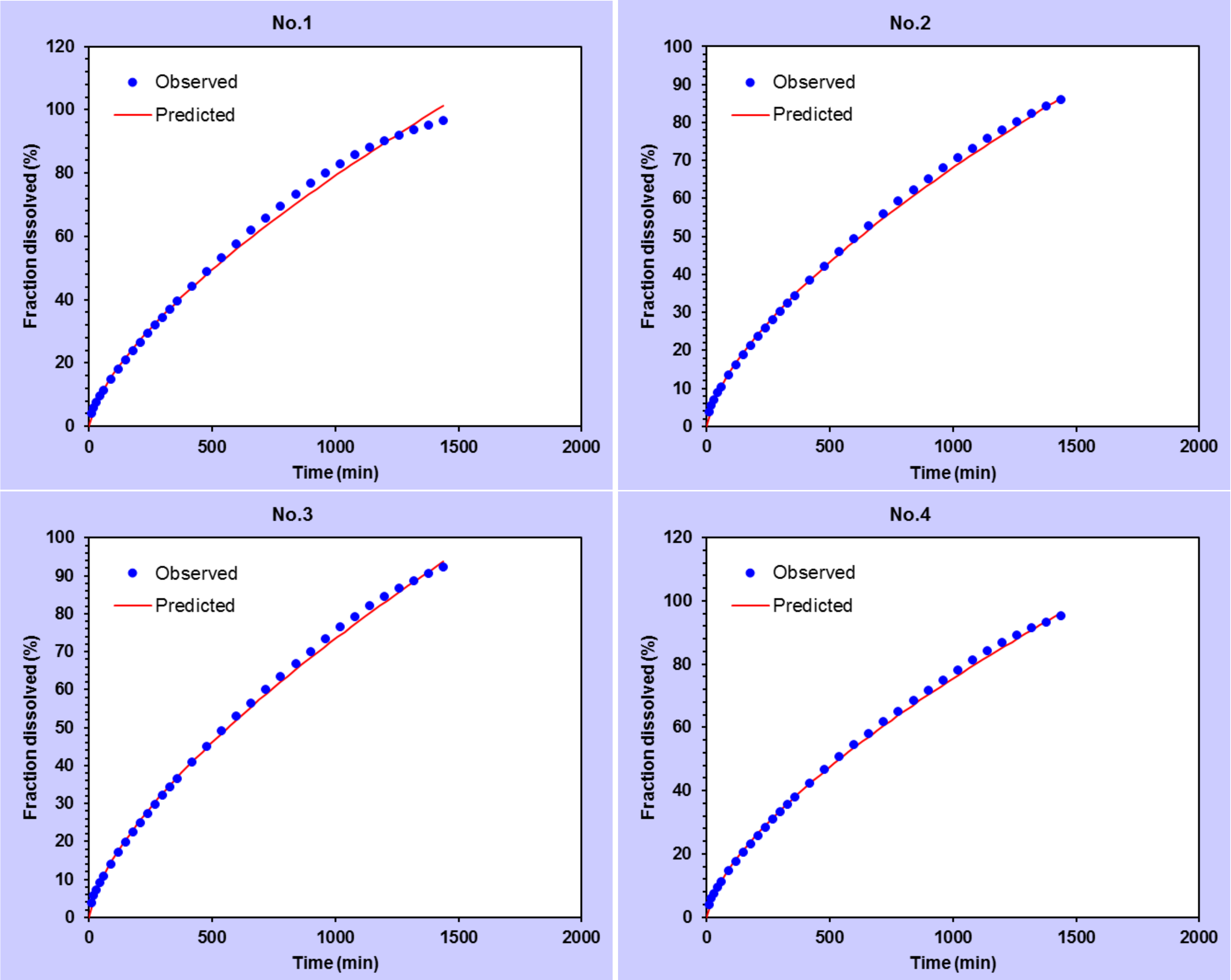

Supplementary materials – Model fitting summary – Di-Cafos® A12 (modelling the entire relevant dissolution data)

Model: **Korsmeyer–Peppas with T_{lag}**

Model equation: $F = k_{KP} \cdot (t - T_{lag})^n$

Fitted model parameters per tested tablet (N = 4) with statistics – mean, standard deviation (SD), and relative standard deviation expressed in % (RSD%) (output from DDSolver):

Parameter	No.1	No.2	No.3	No.4	Mean	SD	RSD(%)
k_{KP}	0.943	1.023	0.999	1.054	1.005	0.047	4.697
n	0.639	0.612	0.626	0.622	0.625	0.011	1.825
T_{lag}	4.000	4.831	4.831	4.831	4.623	0.415	8.987

Number of dissolution data points (N), degrees of freedom (df), and selected goodness of fit criteria – Pearson correlation coefficient (R), coefficient of determination (R^2), adjusted coefficient of determination ($R^2_{adjusted}$), and residual sum of squares (RSS) (manual calculation in MS Excel):

Parameter	No.1	No.2	No.3	No.4
N	33	33	33	33
df	30	30	30	30
R	0.998421715	0.999130084	0.999131768	0.999228759
R^2	0.996845921	0.998260926	0.99826429	0.998458113
$R^2_{adjusted}$	0.996635649	0.998144987	0.998148576	0.998355321
RSS	159.2193293	107.9123046	118.3994434	121.5844086

Graphical abstract of model fit presented as mean ± 1 SD of the fraction % of released carvedilol:

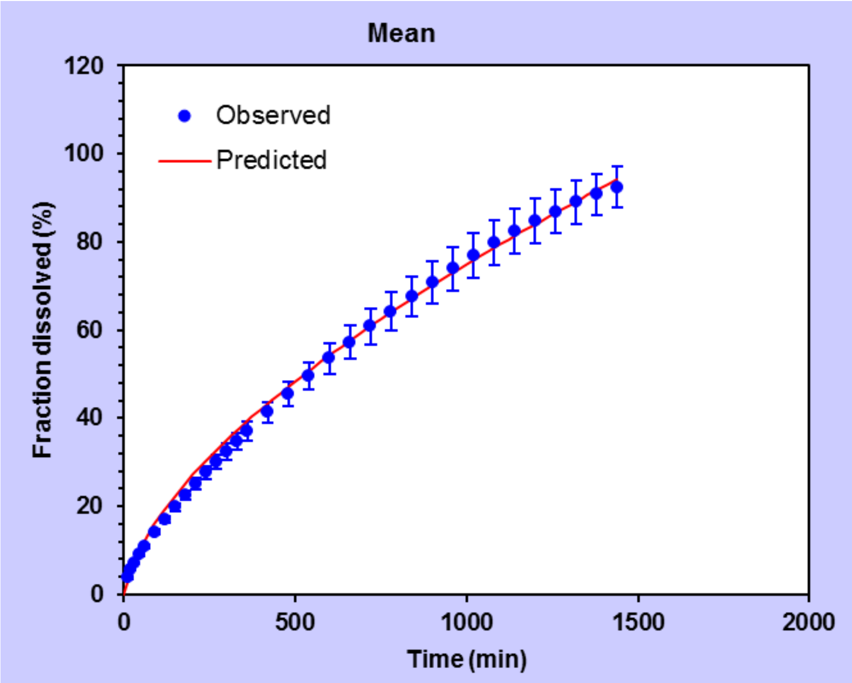

Supplementary materials – Model fitting summary – Di-Cafos® A12 (modelling the entire relevant dissolution data)

Graphical abstract of model fit presented as the fraction % of released carvedilol per tested tablet:

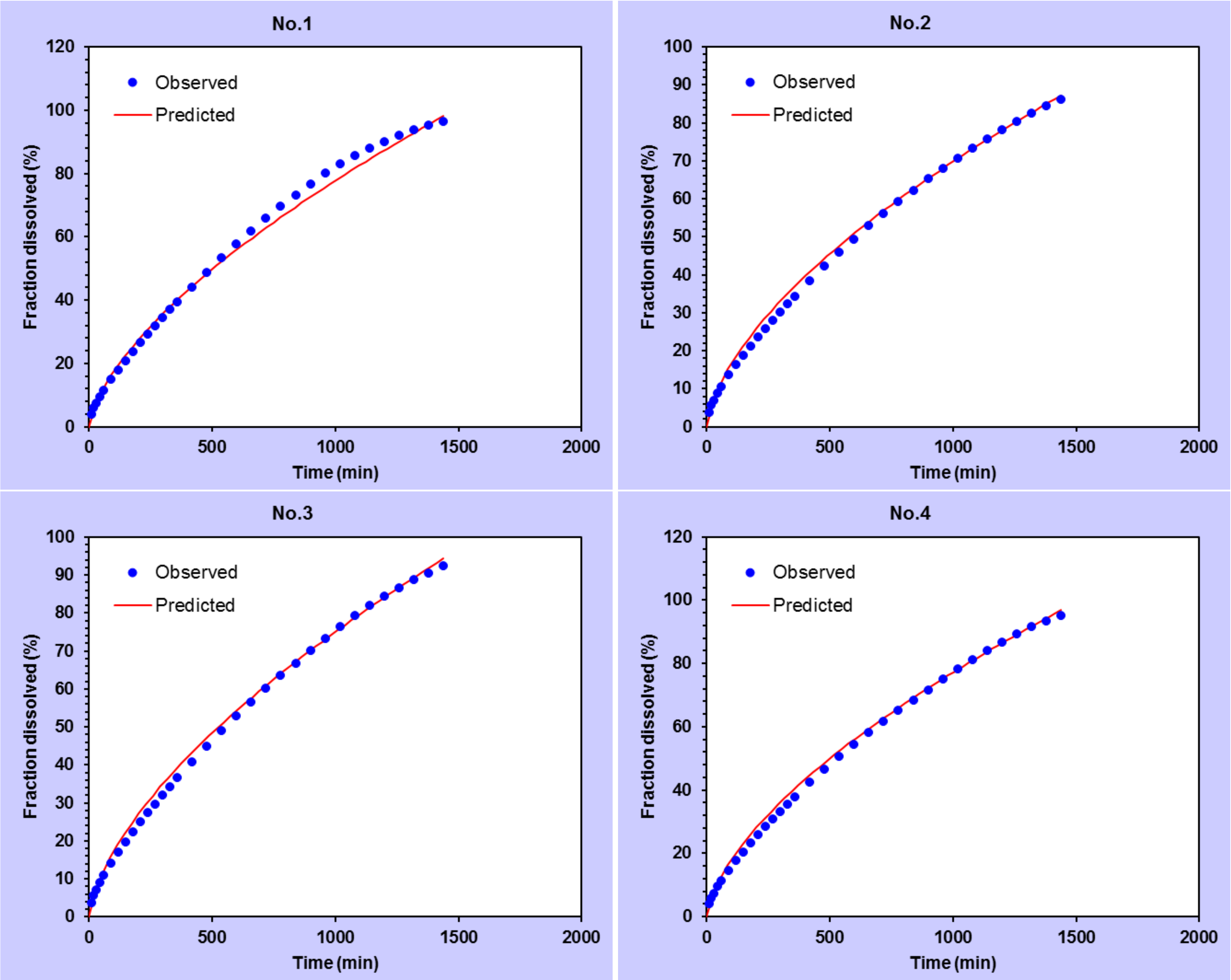

Supplementary materials – Model fitting summary – Di-Cafos® A12 (modelling the entire relevant dissolution data)

Model: **Korsmeyer–Peppas with F_0**

Model equation: $F = F_0 + k_{KP} \cdot t^n$

Fitted model parameters per tested tablet (N = 4) with statistics – mean, standard deviation (SD), and relative standard deviation expressed in % (RSD%) (output from DDSolver):

Parameter	No.1	No.2	No.3	No.4	Mean	SD	RSD(%)
k_{KP}	0.456	0.449	0.478	0.503	0.472	0.024	5.164
n	0.747	0.723	0.724	0.721	0.729	0.012	1.682
F_0	1.560	1.859	1.807	1.904	1.782	0.154	8.625

Number of dissolution data points (N), degrees of freedom (df), and selected goodness of fit criteria – Pearson correlation coefficient (R), coefficient of determination (R^2), adjusted coefficient of determination ($R^2_{adjusted}$), and residual sum of squares (RSS) (manual calculation in MS Excel):

Parameter	No.1	No.2	No.3	No.4
N	33	33	33	33
df	30	30	30	30
R	0.997180803	0.99934507	0.999266428	0.999439901
R^2	0.994369554	0.998690569	0.998533395	0.998880115
$R^2_{adjusted}$	0.993994191	0.998603274	0.998435621	0.998805456
RSS	231.3924264	41.27231201	67.16267031	59.3031685

Graphical abstract of model fit presented as mean ± 1 SD of the fraction % of released carvedilol:

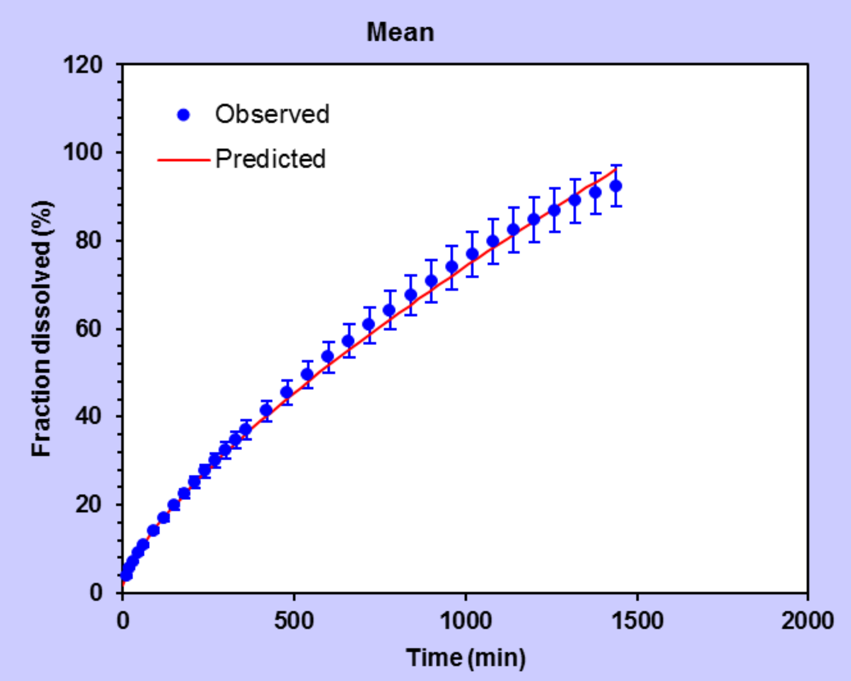

Supplementary materials – Model fitting summary – Di-Cafos® A12 (modelling the entire relevant dissolution data)

Graphical abstract of model fit presented as the fraction % of released carvedilol per tested tablet:

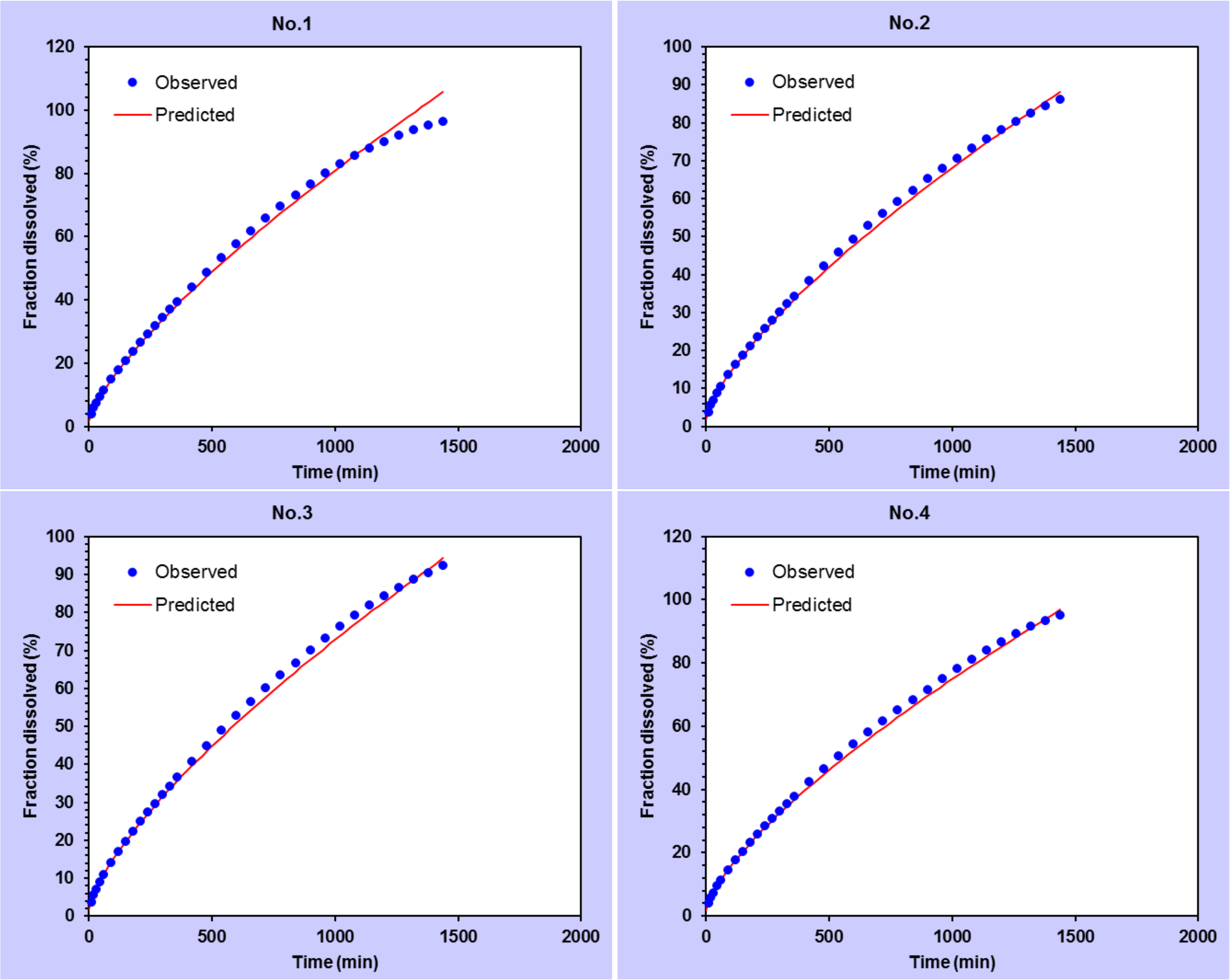

Supplementary materials – Model fitting summary – Di-Cafos® A12 (modelling the entire relevant dissolution data)

Model: **Hixson–Crowell**

Model equation: $F = 100 \cdot [1 - (1 - k_{HC} \cdot t)^3]$

Fitted model parameters per tested tablet (N = 4) with statistics – mean, standard deviation (SD), and relative standard deviation expressed in % (RSD%) (output from DDSolver):

Parameter	No.1	No.2	No.3	No.4	Mean	SD	RSD(%)
k_{HC}	0.0004	0.0003	0.0004	0.0004	0.0004	0.0000	11.6976

Number of dissolution data points (N), degrees of freedom (df), and selected goodness of fit criteria – Pearson correlation coefficient (R), coefficient of determination (R^2), adjusted coefficient of determination ($R^2_{adjusted}$), and residual sum of squares (RSS) (manual calculation in MS Excel):

Parameter	No.1	No.2	No.3	No.4
N	33	33	33	33
df	32	32	32	32
R	0.998486935	0.999607975	0.998882847	0.99805373
R^2	0.996976159	0.999216104	0.997766942	0.996111248
$R^2_{adjusted}$	0.996976159	0.999216104	0.997766942	0.996111248
RSS	143.7188015	226.9880106	161.4786253	200.827795

Graphical abstract of model fit presented as mean ± 1 SD of the fraction % of released carvedilol:

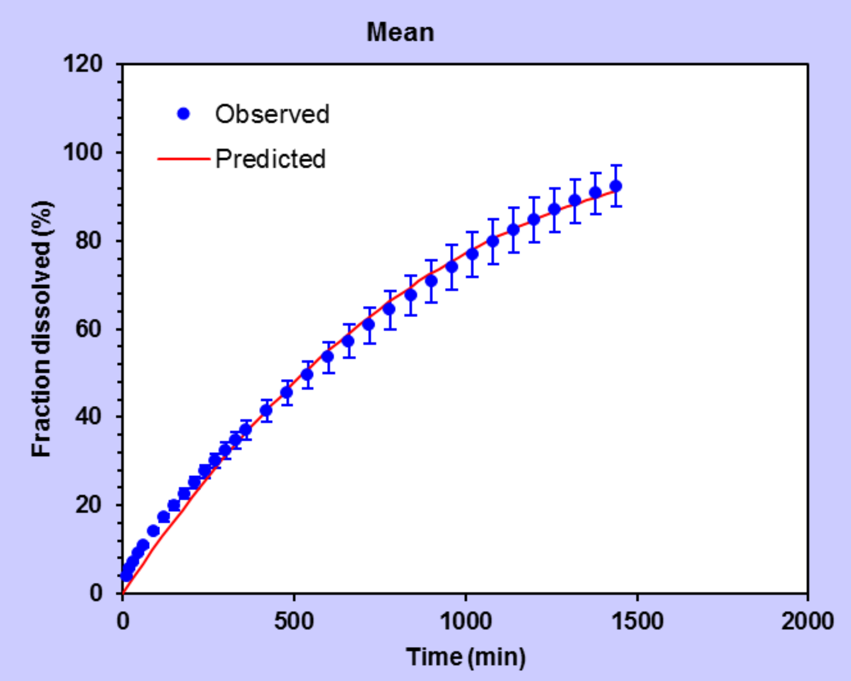

Supplementary materials – Model fitting summary – Di-Cafos® A12 (modelling the entire relevant dissolution data)

Graphical abstract of model fit presented as the fraction % of released carvedilol per tested tablet:

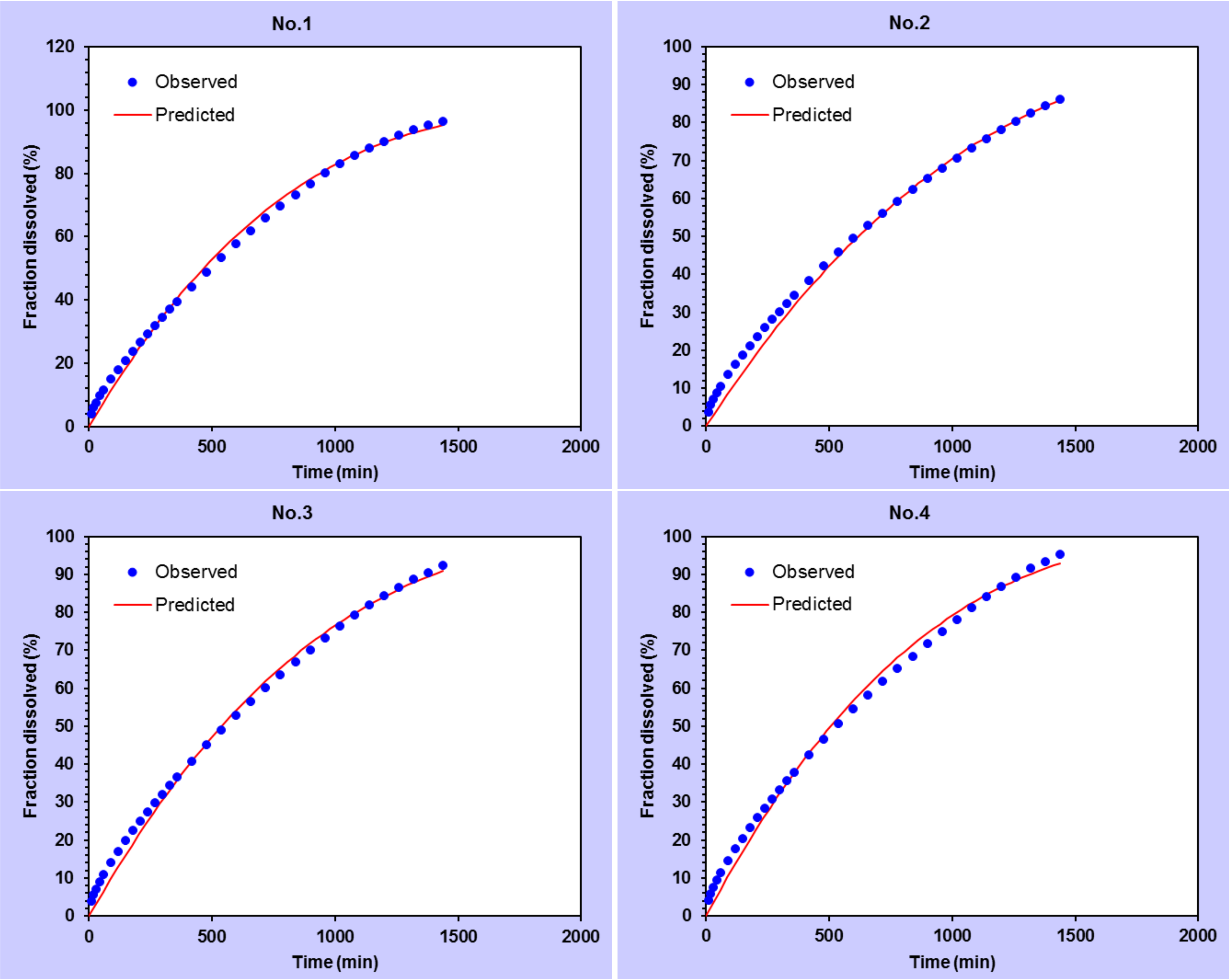

Supplementary materials – Model fitting summary – Di-Cafos® A12 (modelling the entire relevant dissolution data)

Model: **Hixson–Crowell with T_{lag}**

Model equation: $F = 100 \cdot \left\{1 - \left[1 - k_{HC} \cdot \left(t - T_{lag}\right)\right]^3\right\}$

Fitted model parameters per tested tablet (N = 4) with statistics – mean, standard deviation (SD), and relative standard deviation expressed in % (RSD%) (output from DDSolver):

Parameter	No.1	No.2	No.3	No.4	Mean	SD	RSD(%)
k_{HC}	0.0004	0.0003	0.0004	0.0004	0.0004	0.0001	13.6618
T_{lag}	-2.2151	-48.0553	-19.4938	-12.6211	-20.5963	19.6357	-95.3360

Number of dissolution data points (N), degrees of freedom (df), and selected goodness of fit criteria – Pearson correlation coefficient (R), coefficient of determination (R^2), adjusted coefficient of determination ($R^2_{adjusted}$), and residual sum of squares (RSS) (manual calculation in MS Excel):

Parameter	No.1	No.2	No.3	No.4
N	33	33	33	33
df	31	31	31	31
R	0.998495252	0.999667362	0.99899793	0.998105033
R^2	0.996992768	0.999334834	0.997996864	0.996213657
$R^2_{adjusted}$	0.996895761	0.999313377	0.997932246	0.996091517
RSS	134.6511151	15.81171007	70.72793381	147.7926276

Graphical abstract of model fit presented as mean ± 1 SD of the fraction % of released carvedilol:

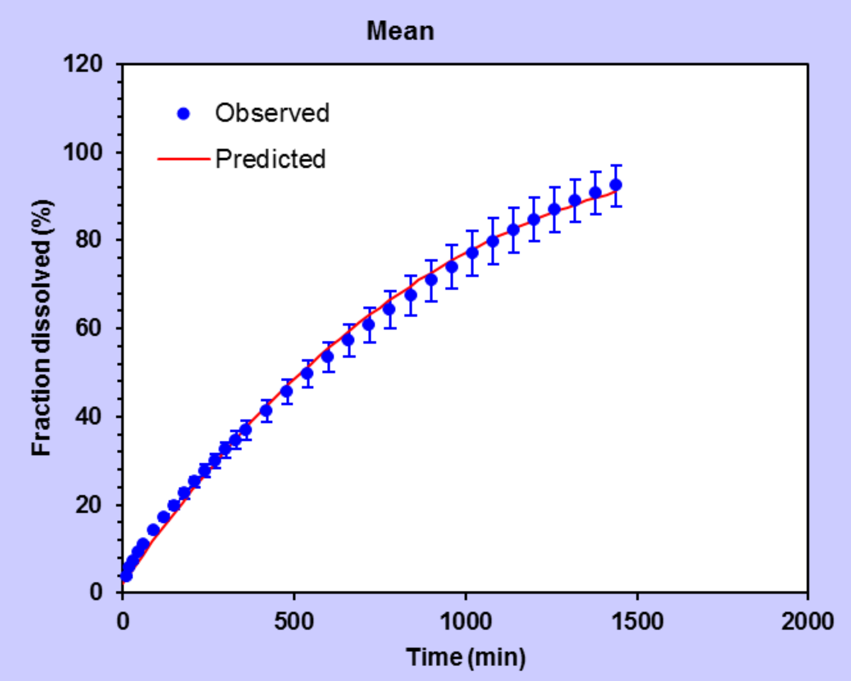

Supplementary materials – Model fitting summary – Di-Cafos® A12 (modelling the entire relevant dissolution data)

Graphical abstract of model fit presented as the fraction % of released carvedilol per tested tablet:

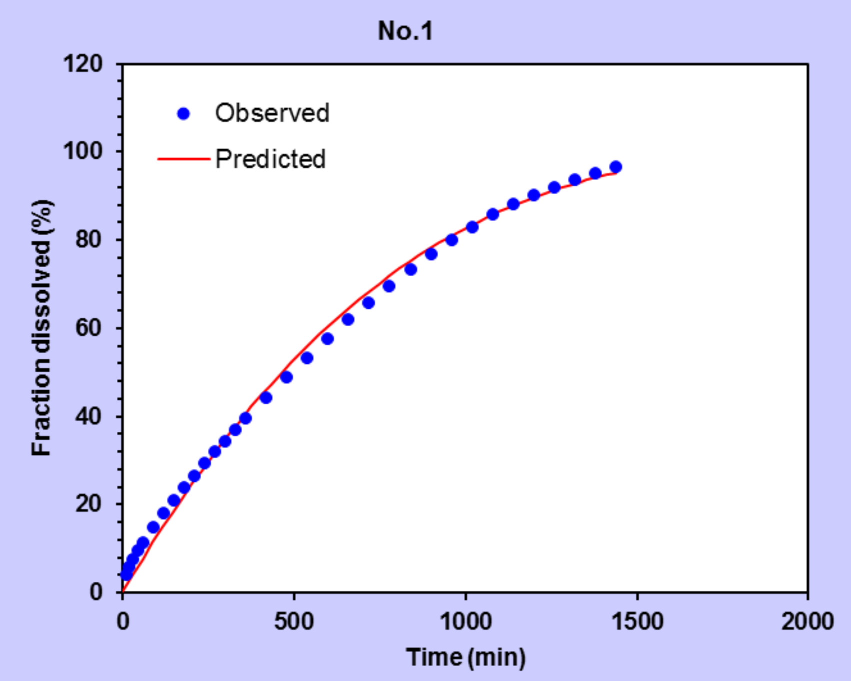

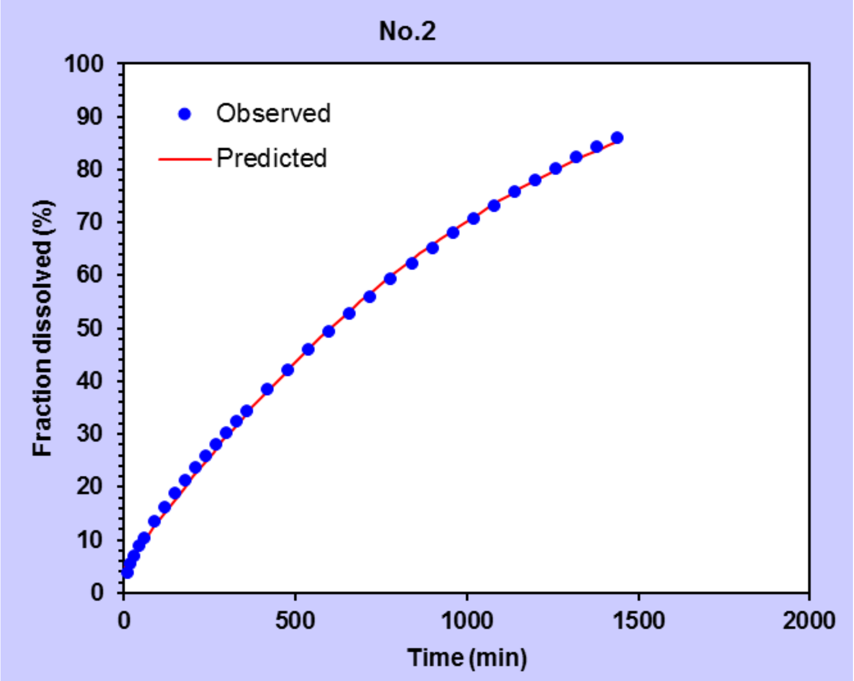

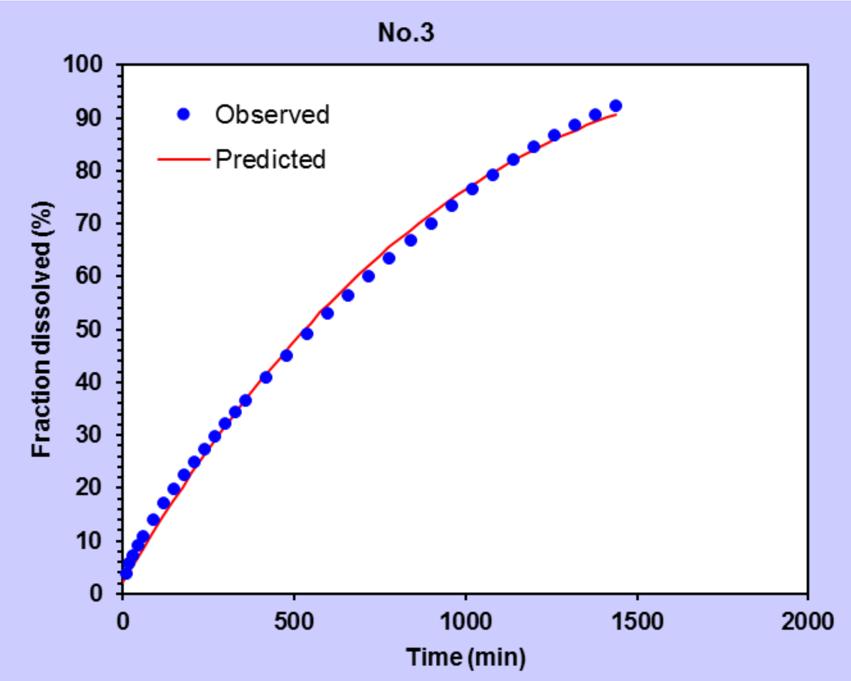

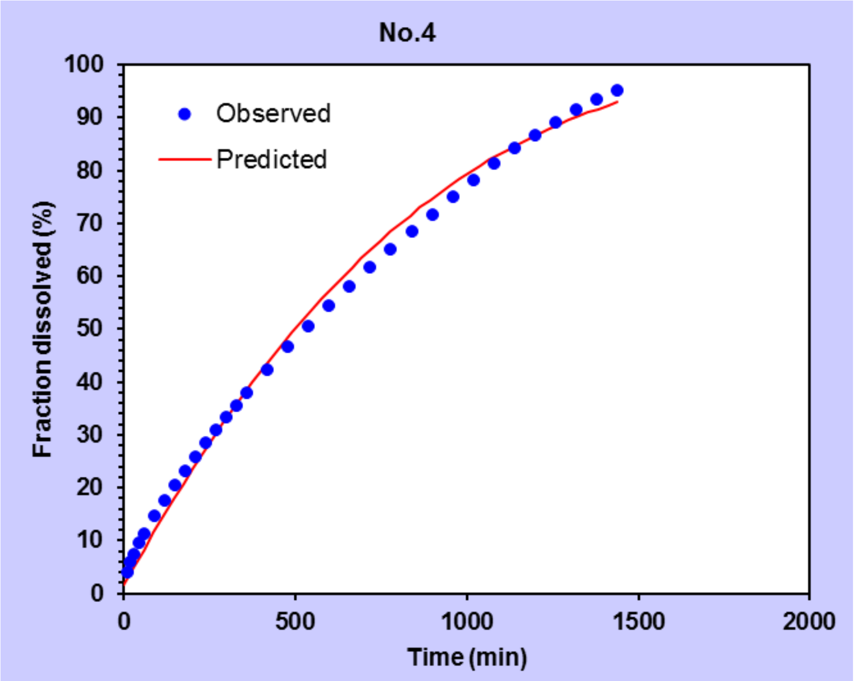

Supplementary materials – Model fitting summary – Di-Cafos® A12 (modelling the entire relevant dissolution data)

Model: **Hopfenberg**

Model equation: $F = 100 \cdot [1 - (1 - k_{HB} \cdot t)^n]$

Fitted model parameters per tested tablet (N = 4) with statistics – mean, standard deviation (SD), and relative standard deviation expressed in % (RSD%) (output from DDSolver):

Parameter	No.1	No.2	No.3	No.4	Mean	SD	RSD(%)
k_{HB}	0.0004	0.0003	0.0004	0.0004	0.0004	0.0001	22.6588
n	3.0000	4.1250	3.0000	3.0000	3.2813	0.5625	17.1429

Number of dissolution data points (N), degrees of freedom (df), and selected goodness of fit criteria – Pearson correlation coefficient (R), coefficient of determination (R^2), adjusted coefficient of determination ($R^2_{adjusted}$), and residual sum of squares (RSS) (manual calculation in MS Excel):

Parameter	No.1	No.2	No.3	No.4
N	33	33	33	33
df	31	31	31	31
R	0.998486935	0.999421932	0.998882847	0.99805373
R^2	0.996976159	0.998844197	0.997766942	0.996111248
$R^2_{adjusted}$	0.996878615	0.998806913	0.997694908	0.995985804
RSS	143.7188015	204.7726519	161.4786253	200.827795

Graphical abstract of model fit presented as mean ± 1 SD of the fraction % of released carvedilol:

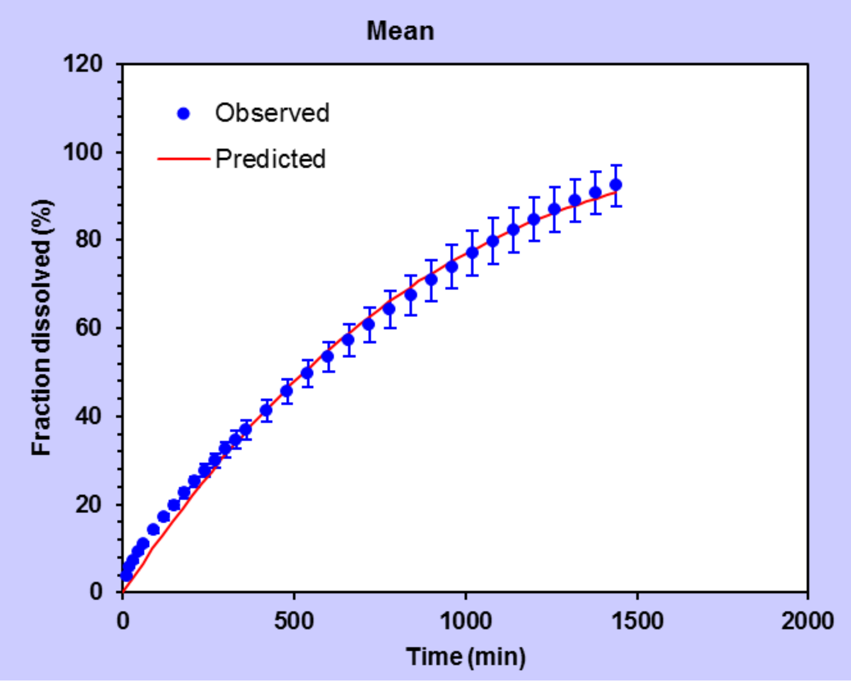

Supplementary materials – Model fitting summary – Di-Cafos® A12 (modelling the entire relevant dissolution data)

Graphical abstract of model fit presented as the fraction % of released carvedilol per tested tablet:

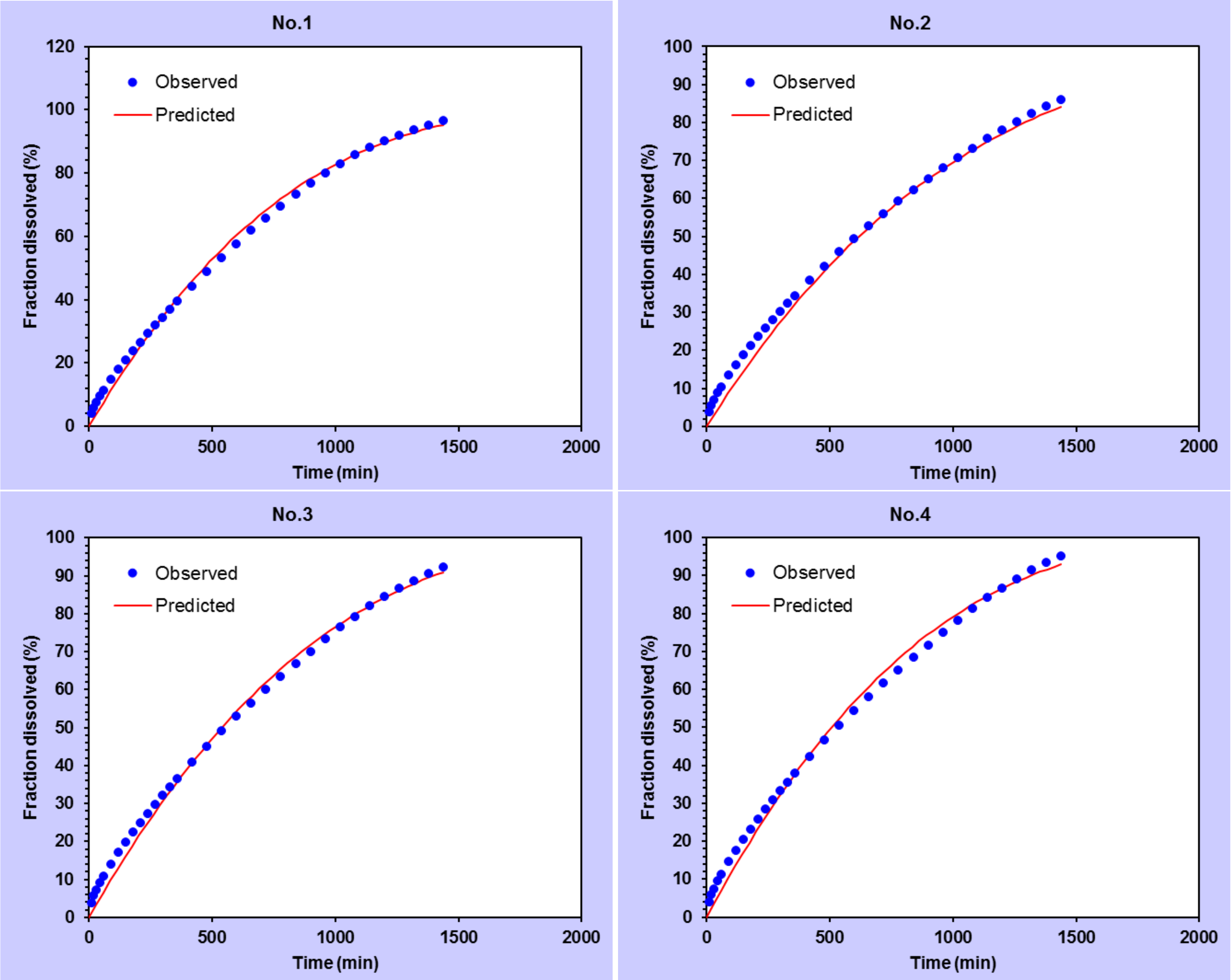

Supplementary materials – Model fitting summary – Di-Cafos® A12 (modelling the entire relevant dissolution data)

Model: **Hopfenberg with T_{lag}**

Model equation: $F = 100 \cdot \{1 - [1 - k_{HB} \cdot (t - T_{lag})]^n\}$

Fitted model parameters per tested tablet (N = 4) with statistics – mean, standard deviation (SD), and relative standard deviation expressed in % (RSD%) (output from DDSolver):

Parameter	No.1	No.2	No.3	No.4	Mean	SD	RSD(%)
k_{HB}	0.0005	0.0003	0.0005	0.0005	0.0005	0.0001	21.8444
n	2.0000	3.0000	2.0000	2.0000	2.2500	0.5000	22.2222
T_{lag}	-43.1807	-48.0553	-55.5091	-47.8795	-48.6562	5.0960	-10.4735

Number of dissolution data points (N), degrees of freedom (df), and selected goodness of fit criteria – Pearson correlation coefficient (R), coefficient of determination (R^2), adjusted coefficient of determination ($R^2_{adjusted}$), and residual sum of squares (RSS) (manual calculation in MS Excel):

Parameter	No.1	No.2	No.3	No.4
N	33	33	33	33
df	30	30	30	30
R	0.999840925	0.999667362	0.999639892	0.999470221
R^2	0.999681875	0.999334834	0.999279913	0.998940722
$R^2_{adjusted}$	0.999660667	0.999290489	0.999231907	0.998870103
RSS	9.840070131	15.81171007	19.39224088	31.98143785

Graphical abstract of model fit presented as mean ± 1 SD of the fraction % of released carvedilol:

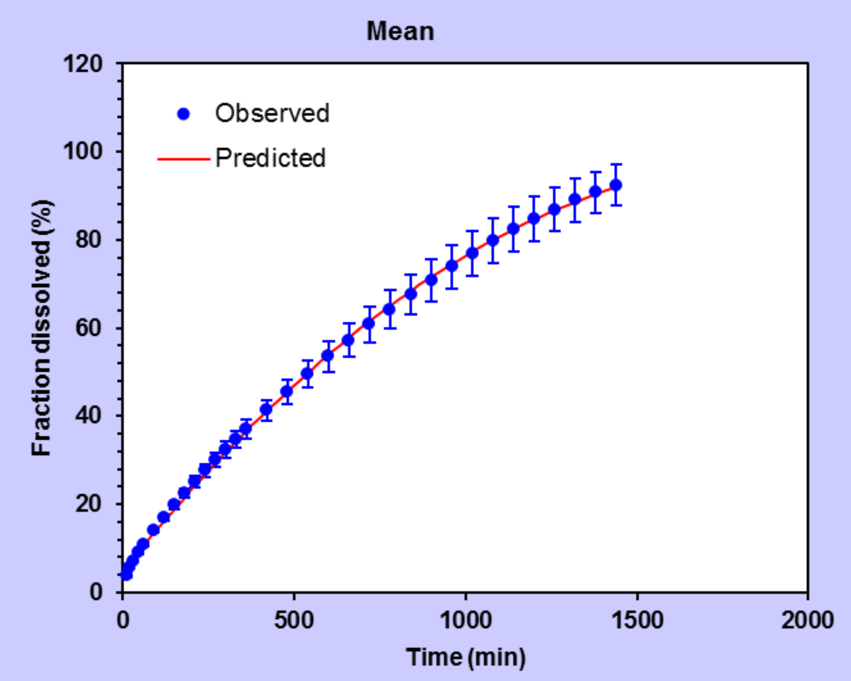

Supplementary materials – Model fitting summary – Di-Cafos® A12 (modelling the entire relevant dissolution data)

Graphical abstract of model fit presented as the fraction % of released carvedilol per tested tablet:

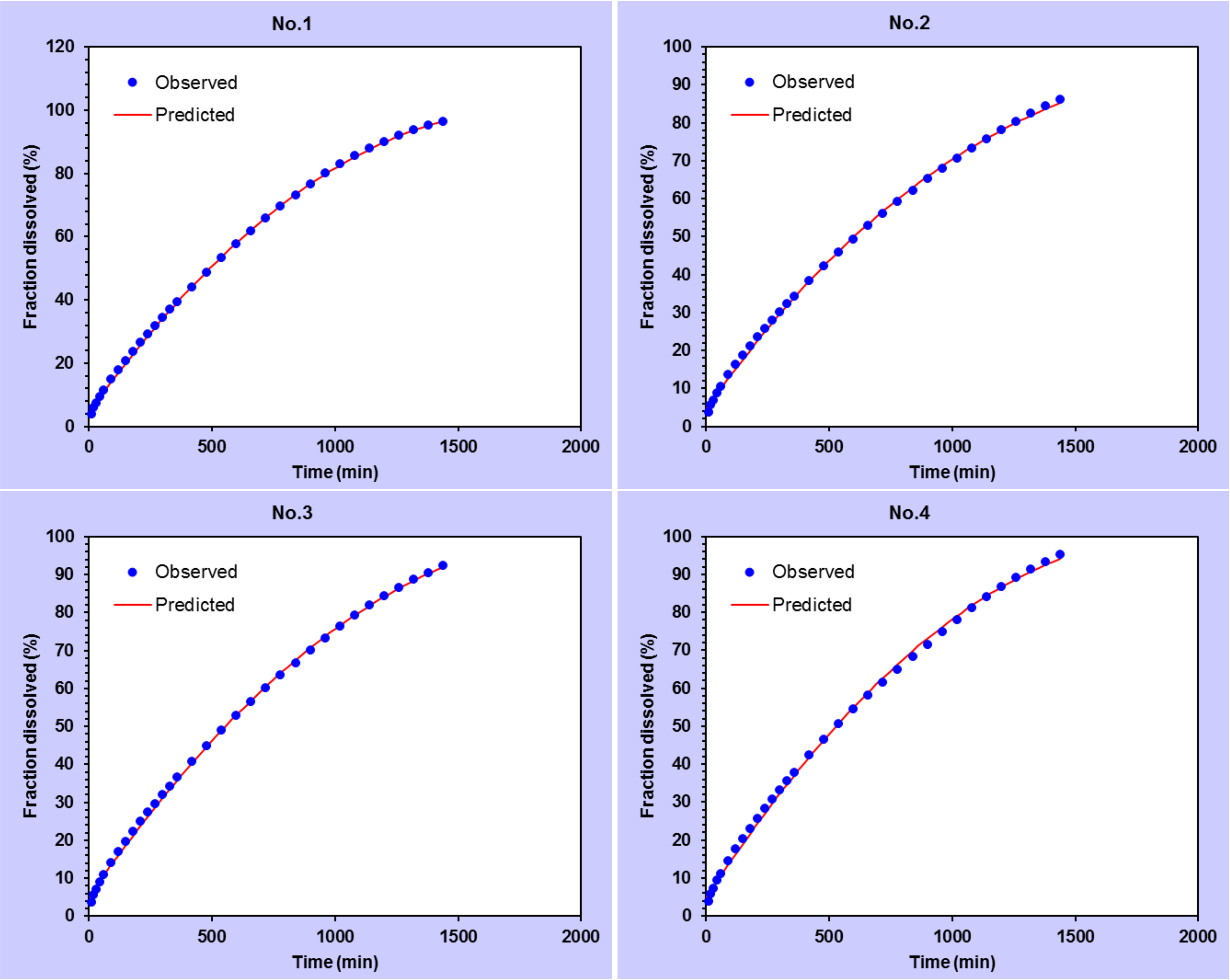

Supplementary materials – Model fitting summary – Di-Cafos® A12 (modelling the entire relevant dissolution data)

Model: **Baker–Lonsdale**

Model equation: $\frac{3}{2} \cdot \left[1 - \left(1 - \frac{F}{100}\right)^{\frac{2}{3}}\right] - \frac{F}{100} = k_{BL} \cdot t$

Fitted model parameters per tested tablet (N = 4) with statistics – mean, standard deviation (SD), and relative standard deviation expressed in % (RSD%) (output from DDSolver):

Parameter	No.1	No.2	No.3	No.4	Mean	SD	RSD(%)
k_{BL}	0.0001	0.0002	0.0002	0.0002	0.0002	0.0000	24.5729

Number of dissolution data points (N), degrees of freedom (df), and selected goodness of fit criteria – Pearson correlation coefficient (R), coefficient of determination (R^2), adjusted coefficient of determination ($R^2_{adjusted}$), and residual sum of squares (RSS) (manual calculation in MS Excel):

Parameter	No.1	No.2	No.3	No.4
N	33	33	33	33
df	32	32	32	32
R	0.987621465	0.983970828	0.979449386	0.977371968
R^2	0.975396158	0.96819859	0.9593211	0.955255964
$R^2_{adjusted}$	0.975396158	0.96819859	0.9593211	0.955255964
RSS	3437.713227	4468.491477	6389.846712	7245.046856

Graphical abstract of model fit presented as mean ± 1 SD of the fraction % of released carvedilol:

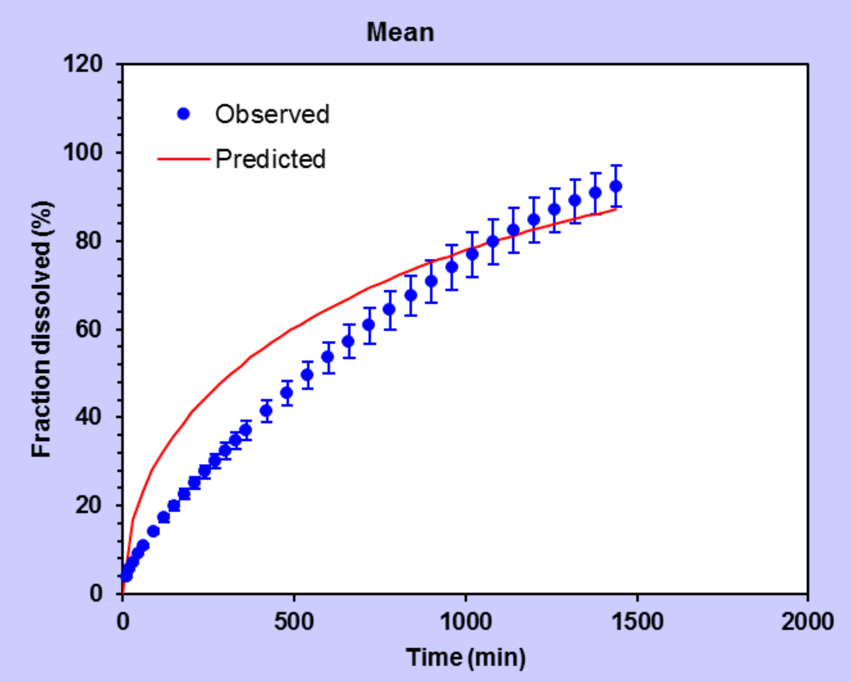

Supplementary materials – Model fitting summary – Di-Cafos® A12 (modelling the entire relevant dissolution data)

Graphical abstract of model fit presented as the fraction % of released carvedilol per tested tablet:

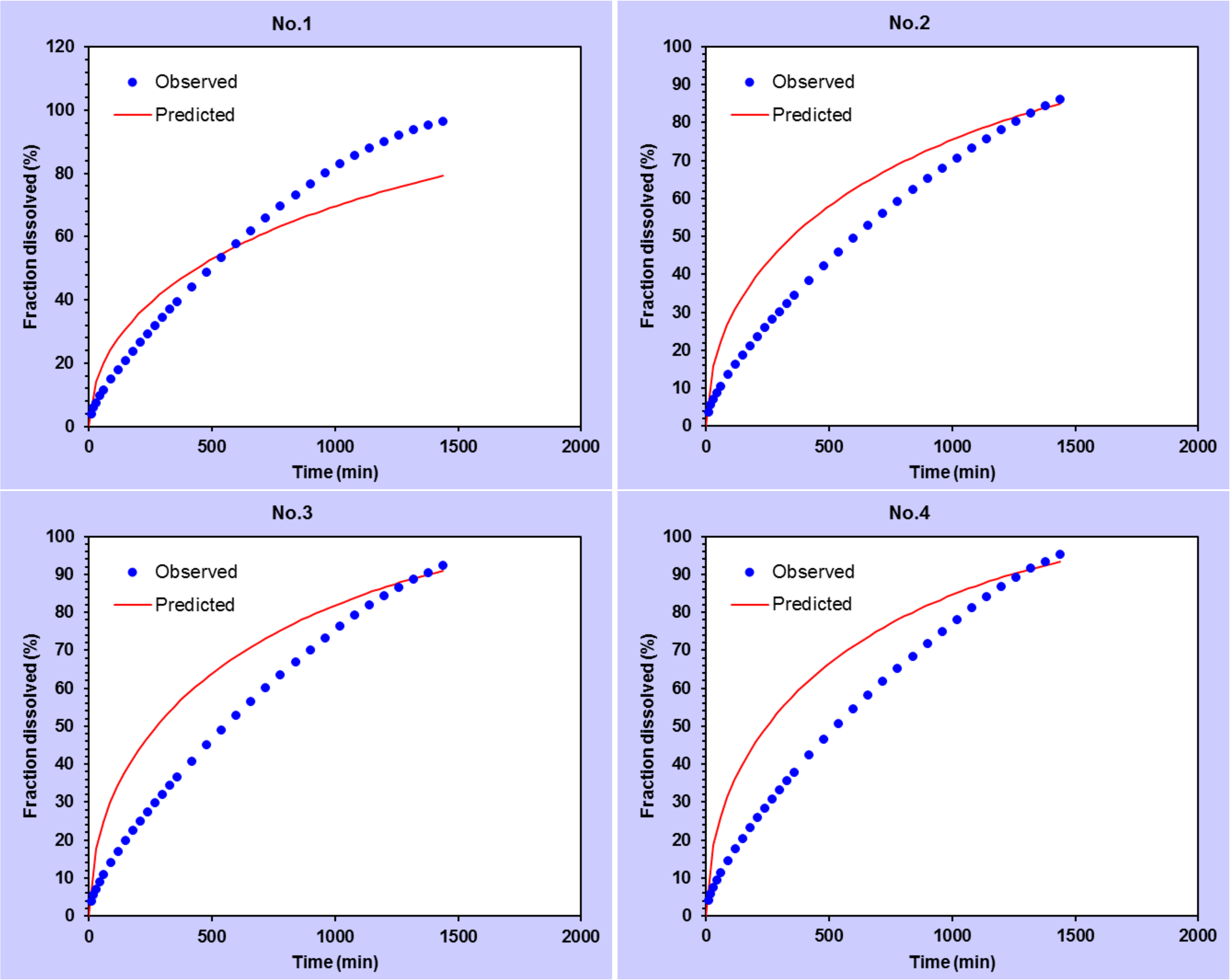

Supplementary materials – Model fitting summary – Di-Cafos® A12 (modelling the entire relevant dissolution data)

Model: **Baker–Lonsdale with T_{lag}**

Model equation: $\frac{3}{2}\cdot\left[1-\left(1-\frac{F}{100}\right)^{\frac{2}{3}}\right]-\frac{F}{100}=k_{BL}\cdot(t-T_{lag})$

Fitted model parameters per tested tablet (N = 4) with statistics – mean, standard deviation (SD), and relative standard deviation expressed in % (RSD%) (output from DDSolver):

Parameter	No.1	No.2	No.3	No.4	Mean	SD	RSD(%)
k_{BL}	0.0003	0.0002	0.0002	0.0002	0.0002	0.0000	19.0417
T_{lag}	154.4049	141.4717	153.0607	158.3744	151.8279	7.2633	4.7839

Number of dissolution data points (N), degrees of freedom (df), and selected goodness of fit criteria – Pearson correlation coefficient (R), coefficient of determination (R^2), adjusted coefficient of determination ($R^2_{adjusted}$), and residual sum of squares (RSS) (manual calculation in MS Excel):

Parameter	No.1	No.2	No.3	No.4
N	33	33	33	33
df	31	31	31	31
R	0.975553948	0.980096473	0.975651503	0.97509493
R^2	0.951705506	0.960589097	0.951895855	0.950810122
$R^2_{adjusted}$	0.950147619	0.959317777	0.950344108	0.949223352
RSS	2190.276049	1309.990768	1913.127653	2126.391292

Graphical abstract of model fit presented as mean ± 1 SD of the fraction % of released carvedilol:

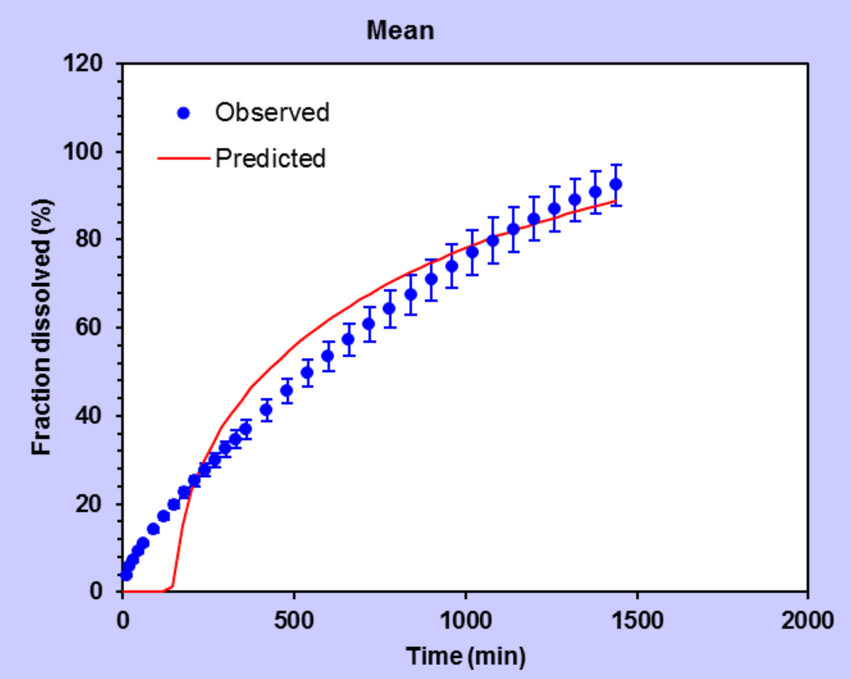

Supplementary materials – Model fitting summary – Di-Cafos® A12 (modelling the entire relevant dissolution data)

Graphical abstract of model fit presented as the fraction % of released carvedilol per tested tablet:

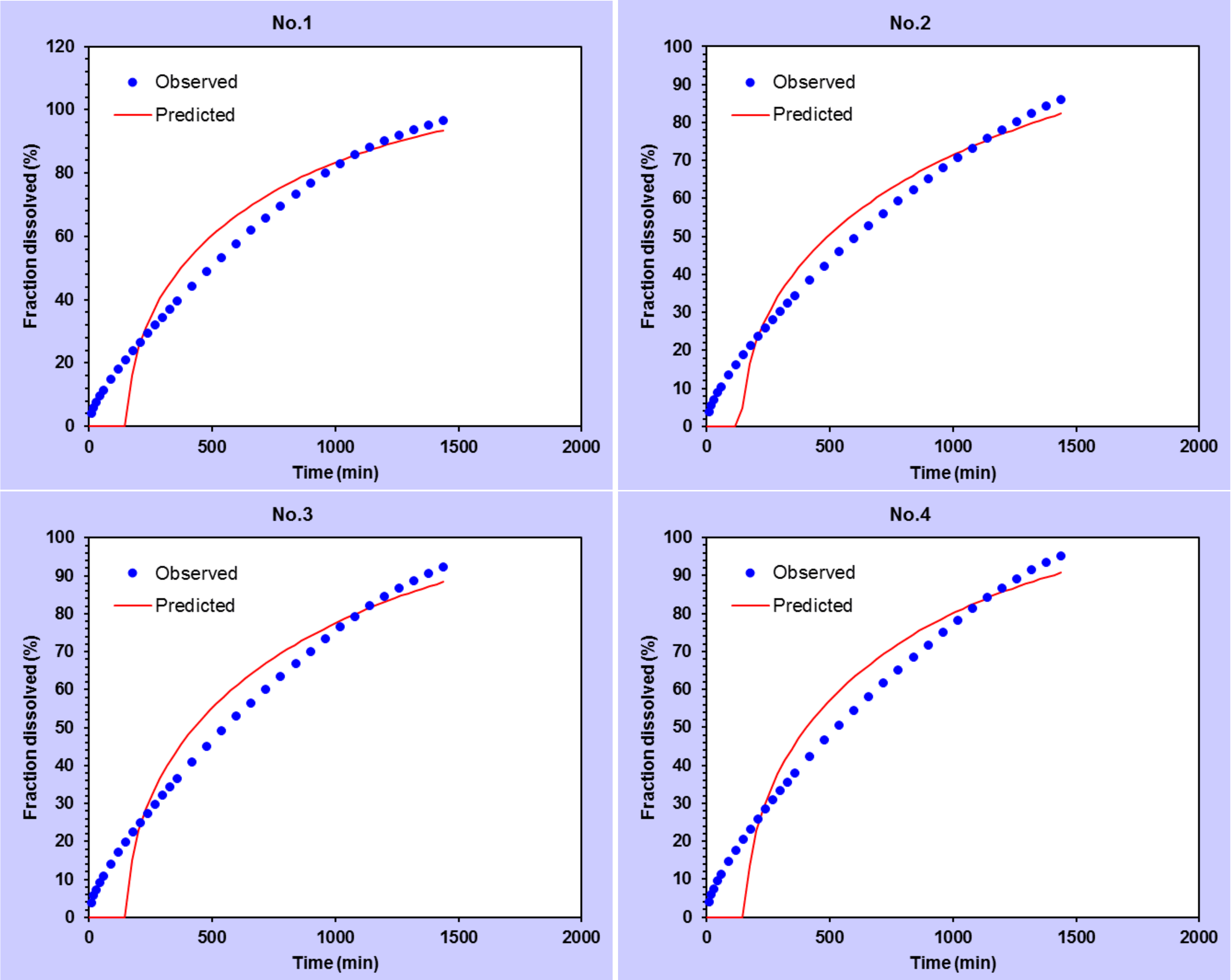

Supplementary materials – Model fitting summary – Di-Cafos® A12 (modelling the entire relevant dissolution data)

Model: **Makoid–Banakar**

Model equation: $F = k_{MB} \cdot t^n \cdot e^{-k \cdot t}$

Fitted model parameters per tested tablet (N = 4) with statistics – mean, standard deviation (SD), and relative standard deviation expressed in % (RSD%) (output from DDSolver):

Parameter	No.1	No.2	No.3	No.4	Mean	SD	RSD(%)
k_{MB}	0.778339	0.817358	0.783008	0.828884	0.801897	0.025027	3.121003
n	0.663011	0.628382	0.646890	0.642444	0.645182	0.014266	2.211125
k	-0.000047	-0.000090	-0.000079	-0.000080	-0.000074	0.000019	-25.570739

Number of dissolution data points (N), degrees of freedom (df), and selected goodness of fit criteria – Pearson correlation coefficient (R), coefficient of determination (R^2), adjusted coefficient of determination ($R^2_{adjusted}$), and residual sum of squares (RSS) (manual calculation in MS Excel):

Parameter	No.1	No.2	No.3	No.4
N	33	33	33	33
df	30	30	30	30
R	0.99755799	0.999015514	0.99889871	0.999110152
R^2	0.995121943	0.998031996	0.997798633	0.998221095
$R^2_{adjusted}$	0.994796739	0.997900796	0.997651876	0.998102501
RSS	153.8687979	46.32584492	60.8108314	51.86296001

Graphical abstract of model fit presented as mean ± 1 SD of the fraction % of released carvedilol:

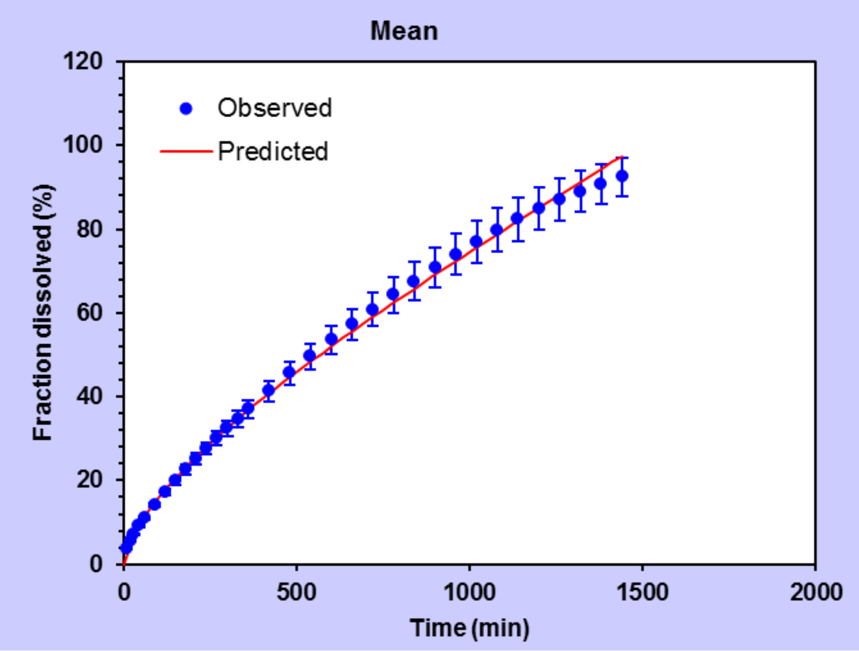

Supplementary materials – Model fitting summary – Di-Cafos® A12 (modelling the entire relevant dissolution data)

Graphical abstract of model fit presented as the fraction % of released carvedilol per tested tablet:

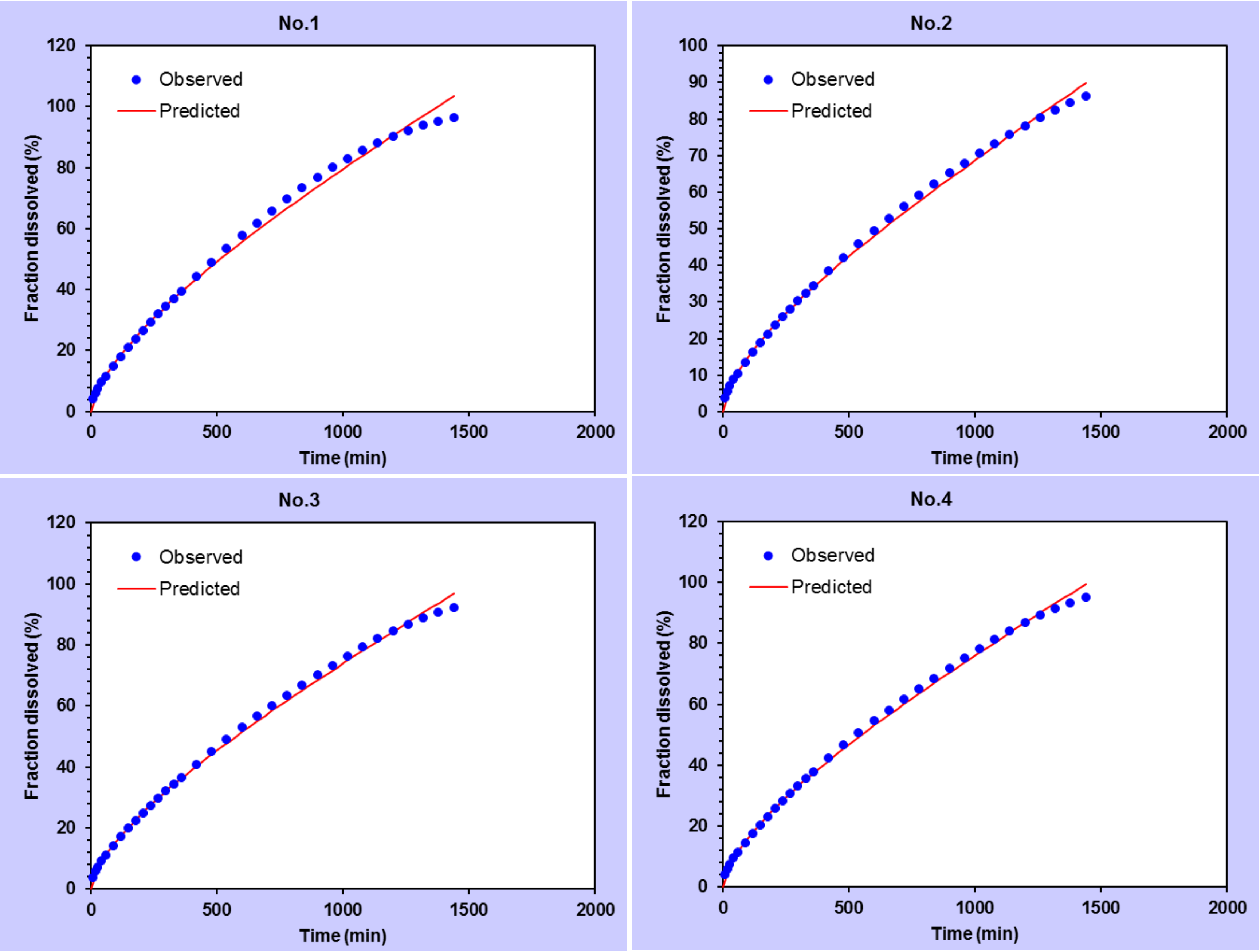

Supplementary materials – Model fitting summary – Di-Cafos® A12 (modelling the entire relevant dissolution data)

Model: **Makoid–Banakar with T_{lag}**

Model equation: $F = k_{MB} \cdot (t - T_{lag})^n \cdot e^{-k \cdot (t - T_{lag})}$

Fitted model parameters per tested tablet (N = 4) with statistics – mean, standard deviation (SD), and relative standard deviation expressed in % (RSD%) (output from DDSolver):

Parameter	No.1	No.2	No.3	No.4	Mean	SD	RSD(%)
k_{MB}	1.12653	1.15838	1.12096	1.18414	1.14750	0.02947	2.56812
n	0.59132	0.56088	0.57743	0.57337	0.57575	0.01254	2.17842
k	-0.00017	-0.00021	-0.00020	-0.00020	-0.00019	0.00002	-8.20371
T_{lag}	4.00000	4.00000	4.00000	4.00000	4.00000	0.00000	0.00000

Number of dissolution data points (N), degrees of freedom (df), and selected goodness of fit criteria – Pearson correlation coefficient (R), coefficient of determination (R^2), adjusted coefficient of determination ($R^2_{adjusted}$), and residual sum of squares (RSS) (manual calculation in MS Excel):

Parameter	No.1	No.2	No.3	No.4
N	33	33	33	33
df	29	29	29	29
R	0.995482528	0.997628158	0.997447816	0.997755801
R^2	0.990985464	0.995261941	0.994902145	0.995516639
$R^2_{adjusted}$	0.990052926	0.994771797	0.994374781	0.995052843
RSS	290.6323549	114.3817419	144.5978124	134.2499208

Graphical abstract of model fit presented as mean ± 1 SD of the fraction % of released carvedilol:

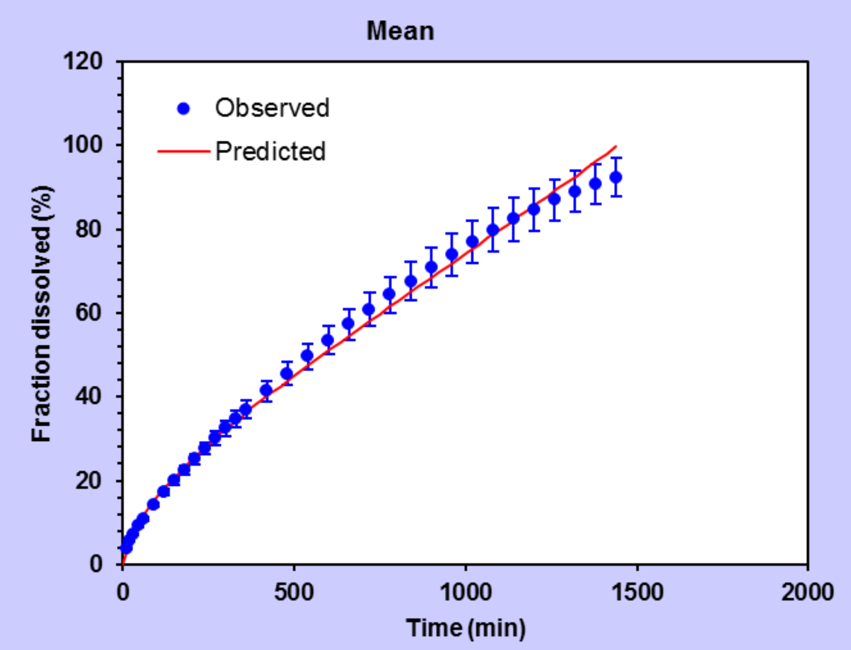

Supplementary materials – Model fitting summary – Di-Cafos® A12 (modelling the entire relevant dissolution data)

Graphical abstract of model fit presented as the fraction % of released carvedilol per tested tablet:

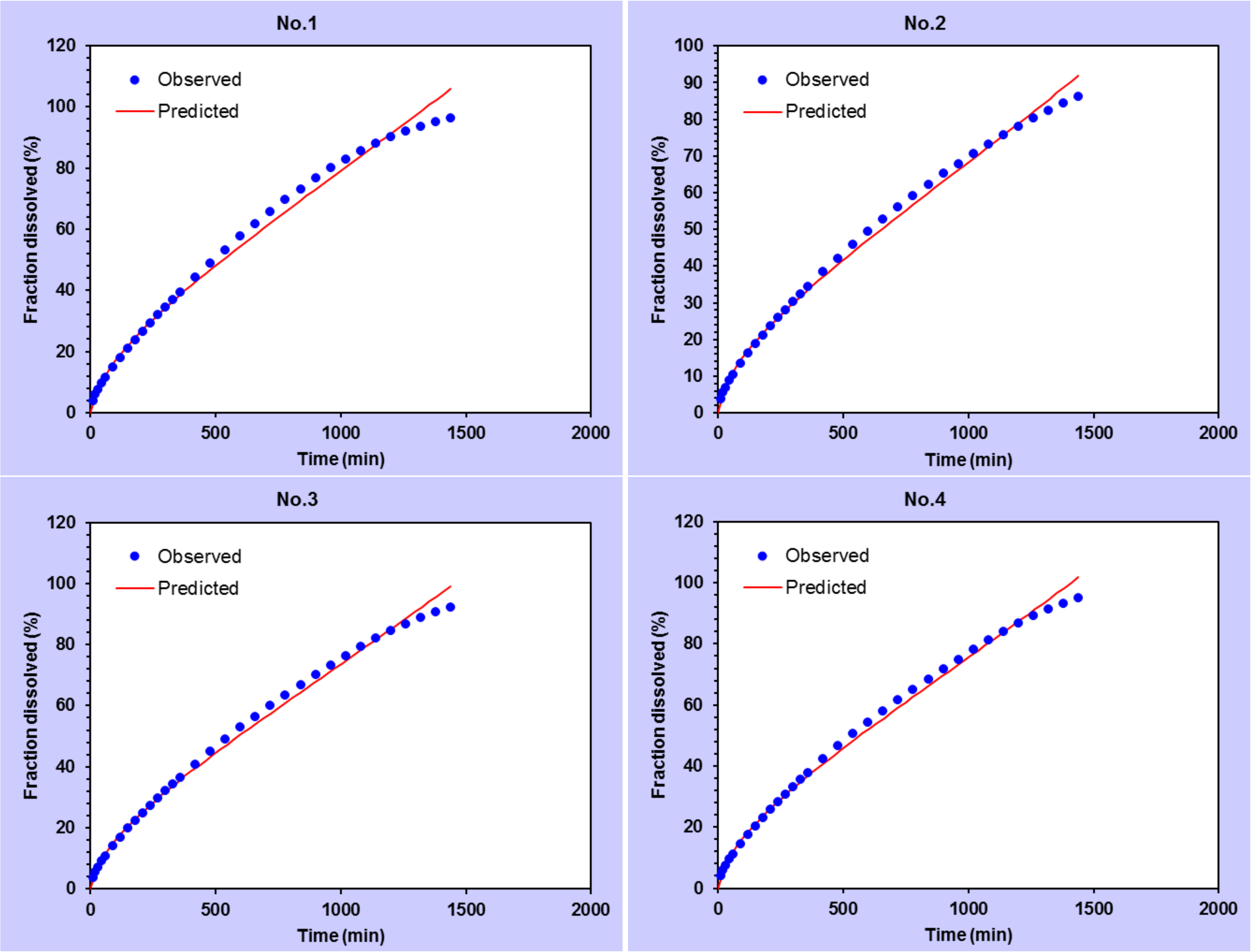

Supplementary materials – Model fitting summary – Di-Cafos® A12 (modelling the entire relevant dissolution data)

Model: **Peppas–Sahlin_1**

Model equation: $F = k_1 \cdot t^m + k_2 \cdot t^{2m}$

Fitted model parameters per tested tablet (N = 4) with statistics – mean, standard deviation (SD), and relative standard deviation expressed in % (RSD%) (output from DDSolver):

Parameter	No.1	No.2	No.3	No.4	Mean	SD	RSD(%)
k_1	1.549	1.340	1.363	1.423	1.419	0.094	6.593
k_2	0.089	0.077	0.086	0.088	0.085	0.005	6.330
m	0.450	0.450	0.450	0.450	0.450	0.000	0.000

Number of dissolution data points (N), degrees of freedom (df), and selected goodness of fit criteria – Pearson correlation coefficient (R), coefficient of determination (R^2), adjusted coefficient of determination ($R^2_{adjusted}$), and residual sum of squares (RSS) (manual calculation in MS Excel):

Parameter	No.1	No.2	No.3	No.4
N	33	33	33	33
df	30	30	30	30
R	0.997349227	0.999180731	0.998983561	0.999177606
R^2	0.994705481	0.998362133	0.997968155	0.998355888
$R^2_{adjusted}$	0.994352513	0.998252942	0.997832699	0.99824628
RSS	173.2204134	40.18247866	58.30555639	50.0544335

Graphical abstract of model fit presented as mean ± 1 SD of the fraction % of released carvedilol:

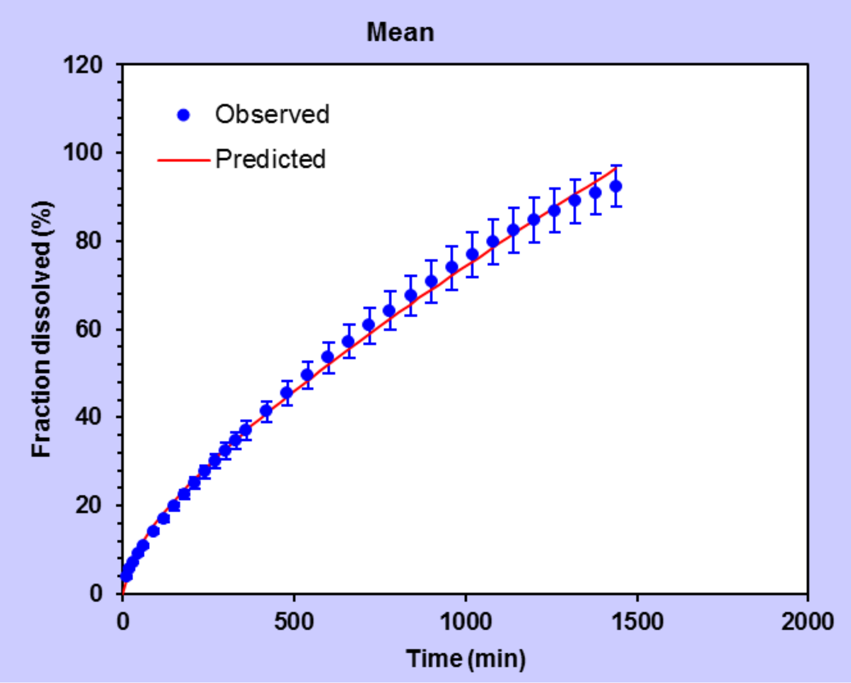

Supplementary materials – Model fitting summary – Di-Cafos® A12 (modelling the entire relevant dissolution data)

Graphical abstract of model fit presented as the fraction % of released carvedilol per tested tablet:

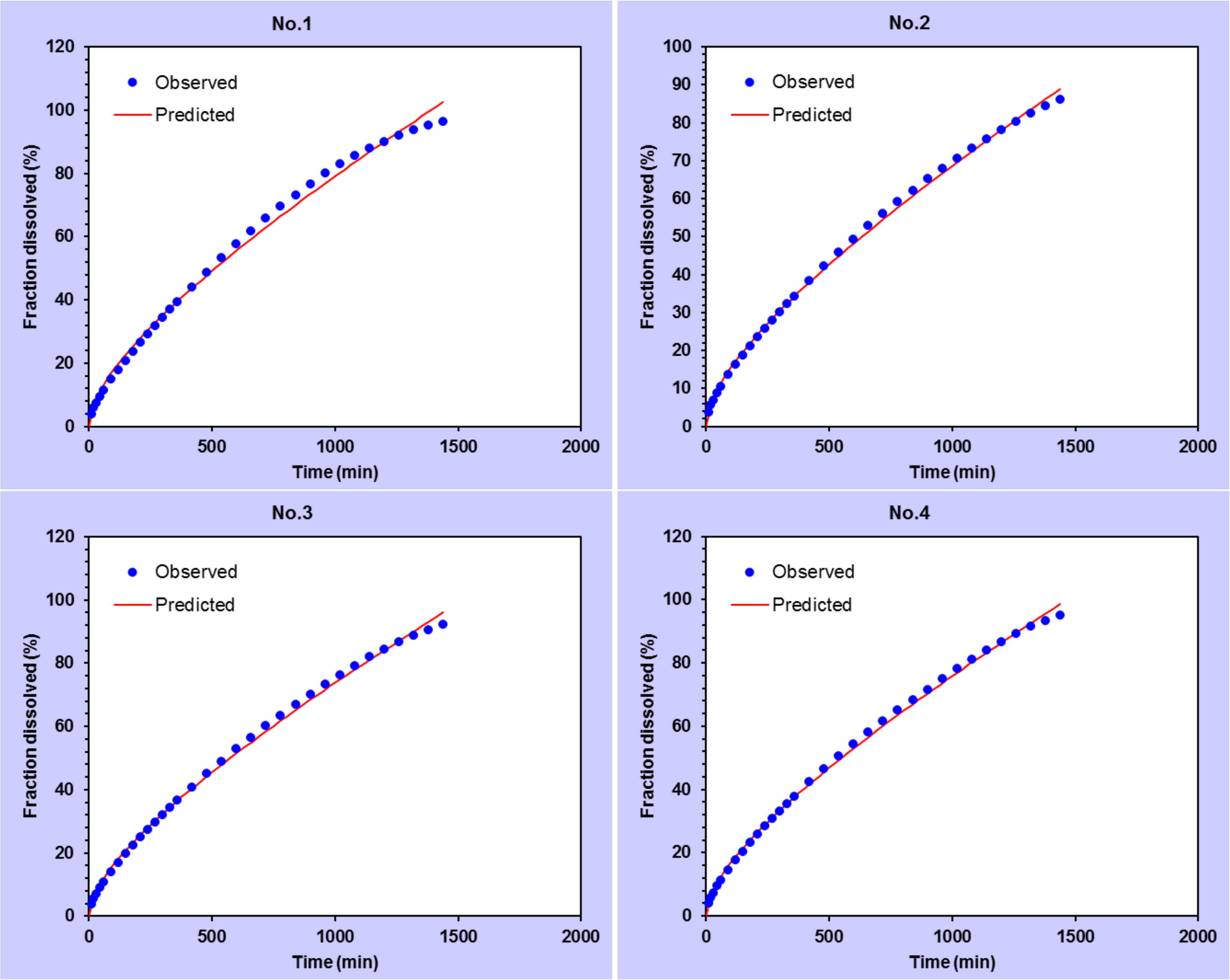

Supplementary materials – Model fitting summary – Di-Cafos® A12 (modelling the entire relevant dissolution data)

Model: **Peppas–Sahlin_1 with T_{lag}**

Model equation: $F = k_1 \cdot (t - T_{lag})^m + k_2 \cdot (t - T_{lag})^{2m}$

Fitted model parameters per tested tablet (N = 4) with statistics – mean, standard deviation (SD), and relative standard deviation expressed in % (RSD%) (output from DDSolver):

Parameter	No.1	No.2	No.3	No.4	Mean	SD	RSD(%)
k_1	1.614	1.395	1.422	1.483	1.478	0.097	6.592
k_2	0.086	0.075	0.084	0.086	0.083	0.005	6.338
m	0.450	0.450	0.450	0.450	0.450	0.000	0.000
T_{lag}	6.000	6.000	6.000	6.000	6.000	0.000	0.000

Number of dissolution data points (N), degrees of freedom (df), and selected goodness of fit criteria – Pearson correlation coefficient (R), coefficient of determination (R^2), adjusted coefficient of determination ($R^2_{adjusted}$), and residual sum of squares (RSS) (manual calculation in MS Excel):

Parameter	No.1	No.2	No.3	No.4
N	33	33	33	33
df	29	29	29	29
R	0.99751803	0.999267762	0.999083654	0.999273673
R^2	0.995042219	0.998536061	0.998168149	0.998547873
$R^2_{adjusted}$	0.994529345	0.998384619	0.997978647	0.998397653
RSS	156.4057643	33.94969716	49.98721958	41.71880903

Graphical abstract of model fit presented as mean ± 1 SD of the fraction % of released carvedilol:

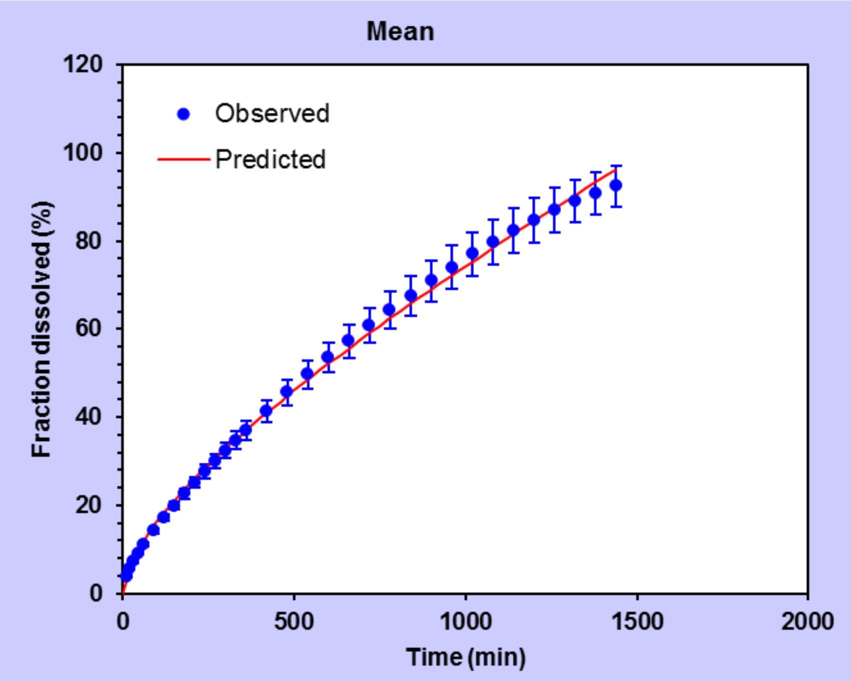

Supplementary materials – Model fitting summary – Di-Cafos® A12 (modelling the entire relevant dissolution data)

Graphical abstract of model fit presented as the fraction % of released carvedilol per tested tablet:

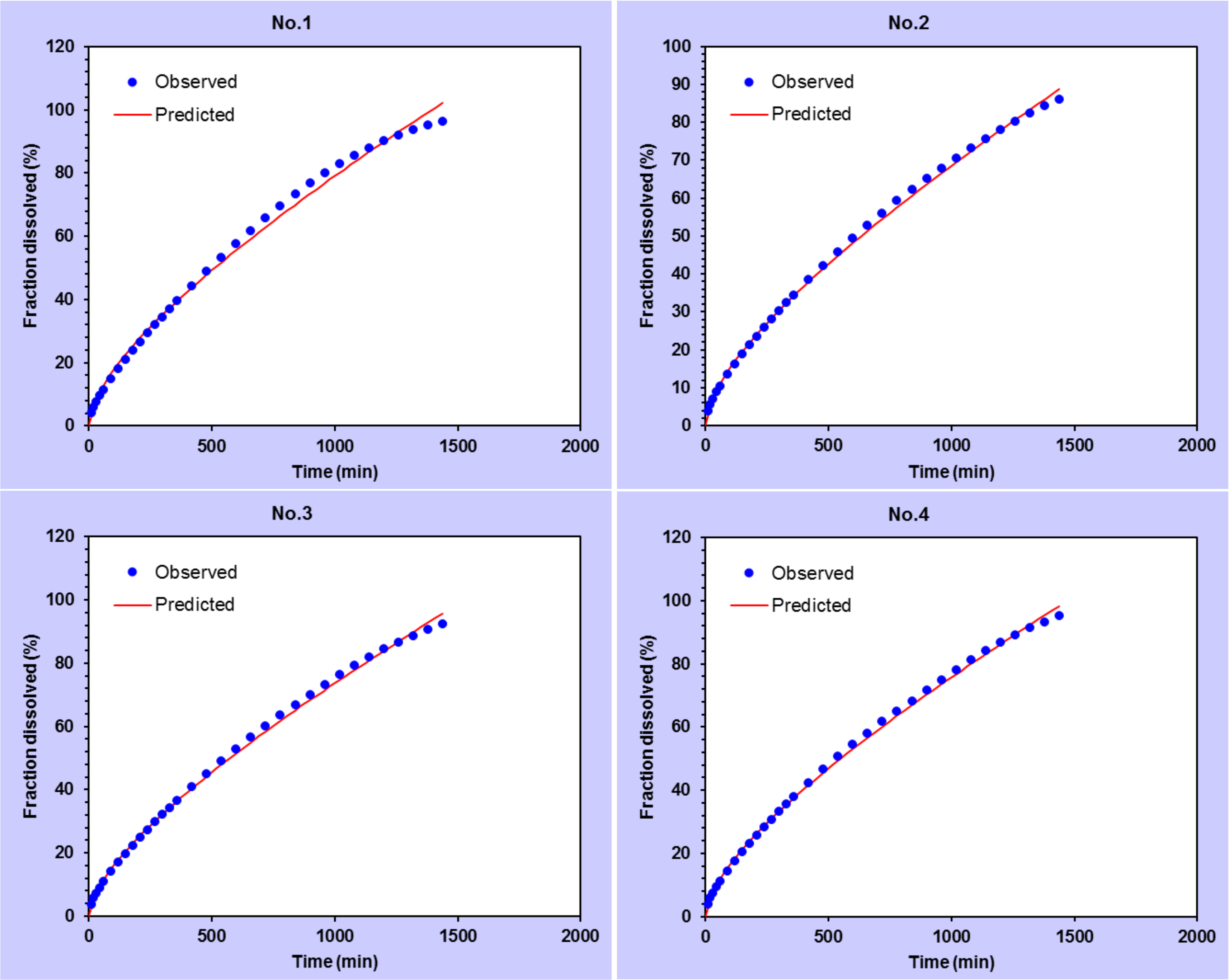

Supplementary materials – Model fitting summary – Di-Cafos® A12 (modelling the entire relevant dissolution data)

Model: **Peppas–Sahlin_2**

Model equation: $F = k_1 \cdot t^{0.5} + k_2 \cdot t$

Fitted model parameters per tested tablet (N = 4) with statistics – mean, standard deviation (SD), and relative standard deviation expressed in % (RSD%) (output from DDSolver):

Parameter	No.1	No.2	No.3	No.4	Mean	SD	RSD(%)
k_1	1.494	1.285	1.329	1.380	1.372	0.090	6.556
k_2	0.032	0.028	0.032	0.032	0.031	0.002	6.457

Number of dissolution data points (N), degrees of freedom (df), and selected goodness of fit criteria – Pearson correlation coefficient (R), coefficient of determination (R^2), adjusted coefficient of determination ($R^2_{adjusted}$), and residual sum of squares (RSS) (manual calculation in MS Excel):

Parameter	No.1	No.2	No.3	No.4
N	33	33	33	33
df	31	31	31	31
R	0.997308976	0.999141628	0.998918685	0.999125107
R^2	0.994625194	0.998283993	0.99783854	0.99825098
$R^2_{adjusted}$	0.994451813	0.998228638	0.997768815	0.99819456
RSS	180.2229921	43.24020222	63.74557467	54.83531726

Graphical abstract of model fit presented as mean ± 1 SD of the fraction % of released carvedilol:

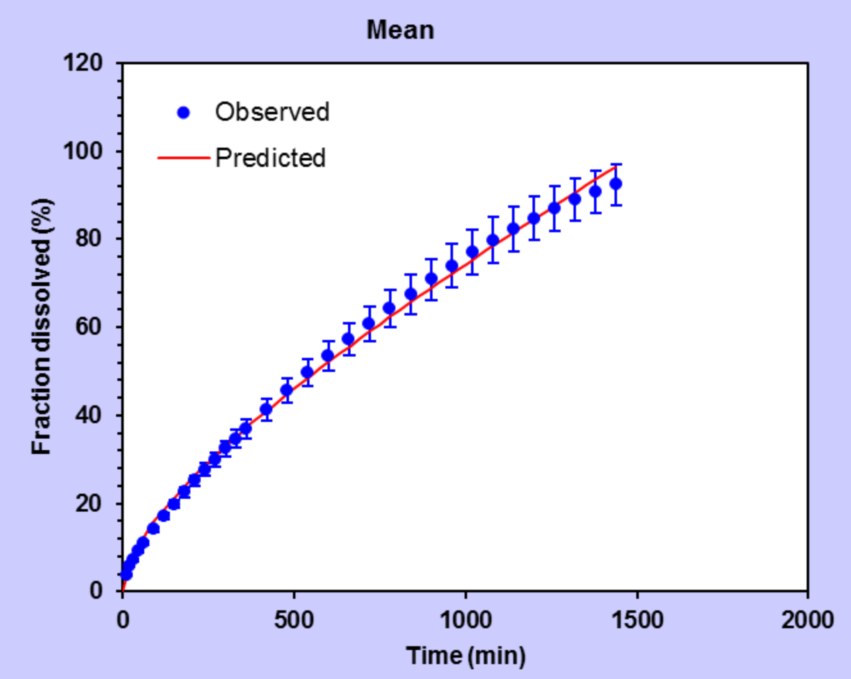

Supplementary materials – Model fitting summary – Di-Cafos® A12 (modelling the entire relevant dissolution data)

Graphical abstract of model fit presented as the fraction % of released carvedilol per tested tablet:

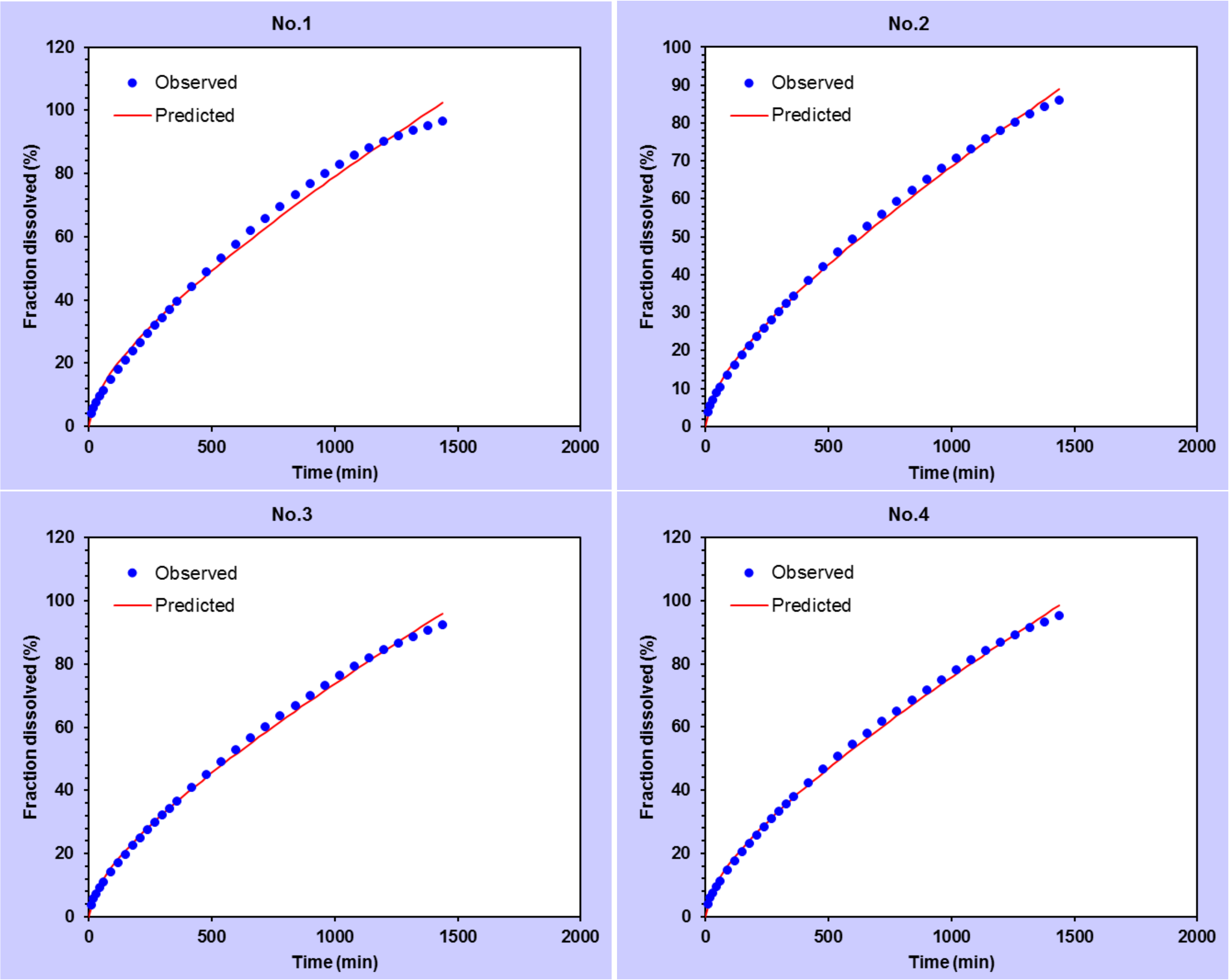

Supplementary materials – Model fitting summary – Di-Cafos® A12 (modelling the entire relevant dissolution data)

Model: **Peppas–Sahlin_2 with T_{lag}**

Model equation: $F = k_1 \cdot (t - T_{lag})^{0.5} + k_2 \cdot (t - T_{lag})$

Fitted model parameters per tested tablet (N = 4) with statistics – mean, standard deviation (SD), and relative standard deviation expressed in % (RSD%) (output from DDSolver):

Parameter	No.1	No.2	No.3	No.4	Mean	SD	RSD(%)
k_1	1.538	1.322	1.369	1.421	1.413	0.093	6.566
k_2	0.031	0.027	0.031	0.031	0.030	0.002	6.493
T_{lag}	6.000	6.000	6.000	6.000	6.000	0.000	0.000

Number of dissolution data points (N), degrees of freedom (df), and selected goodness of fit criteria – Pearson correlation coefficient (R), coefficient of determination (R^2), adjusted coefficient of determination ($R^2_{adjusted}$), and residual sum of squares (RSS) (manual calculation in MS Excel):

Parameter	No.1	No.2	No.3	No.4
N	33	33	33	33
df	30	30	30	30
R	0.99747448	0.999223281	0.999019265	0.999220037
R^2	0.994955338	0.998447165	0.998039493	0.998440683
$R^2_{adjusted}$	0.994619027	0.998343643	0.997908792	0.998336728
RSS	161.0578726	36.19852249	53.93466167	45.13976531

Graphical abstract of model fit presented as mean ± 1 SD of the fraction % of released carvedilol:

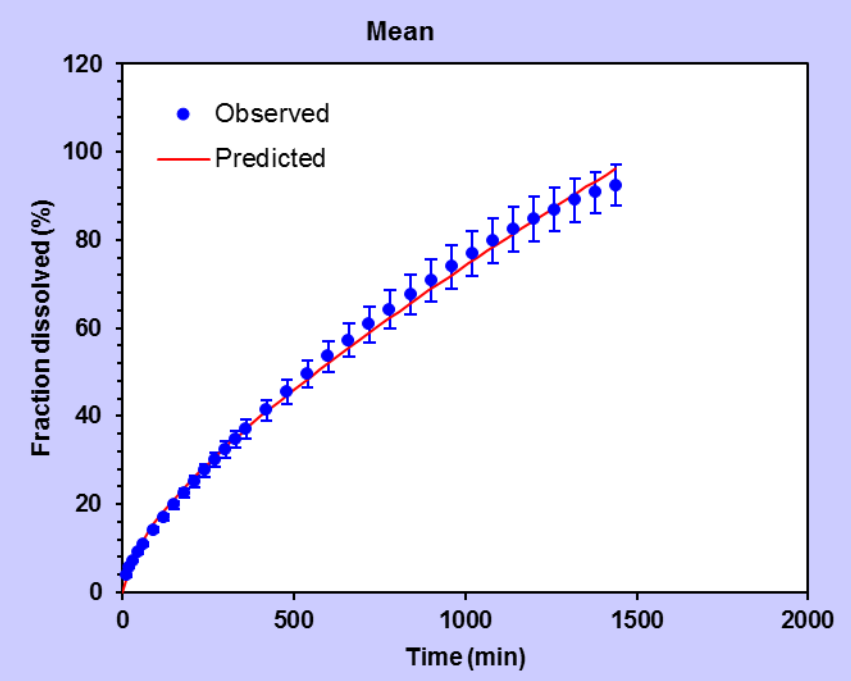

Supplementary materials – Model fitting summary – Di-Cafos® A12 (modelling the entire relevant dissolution data)

Graphical abstract of model fit presented as the fraction % of released carvedilol per tested tablet:

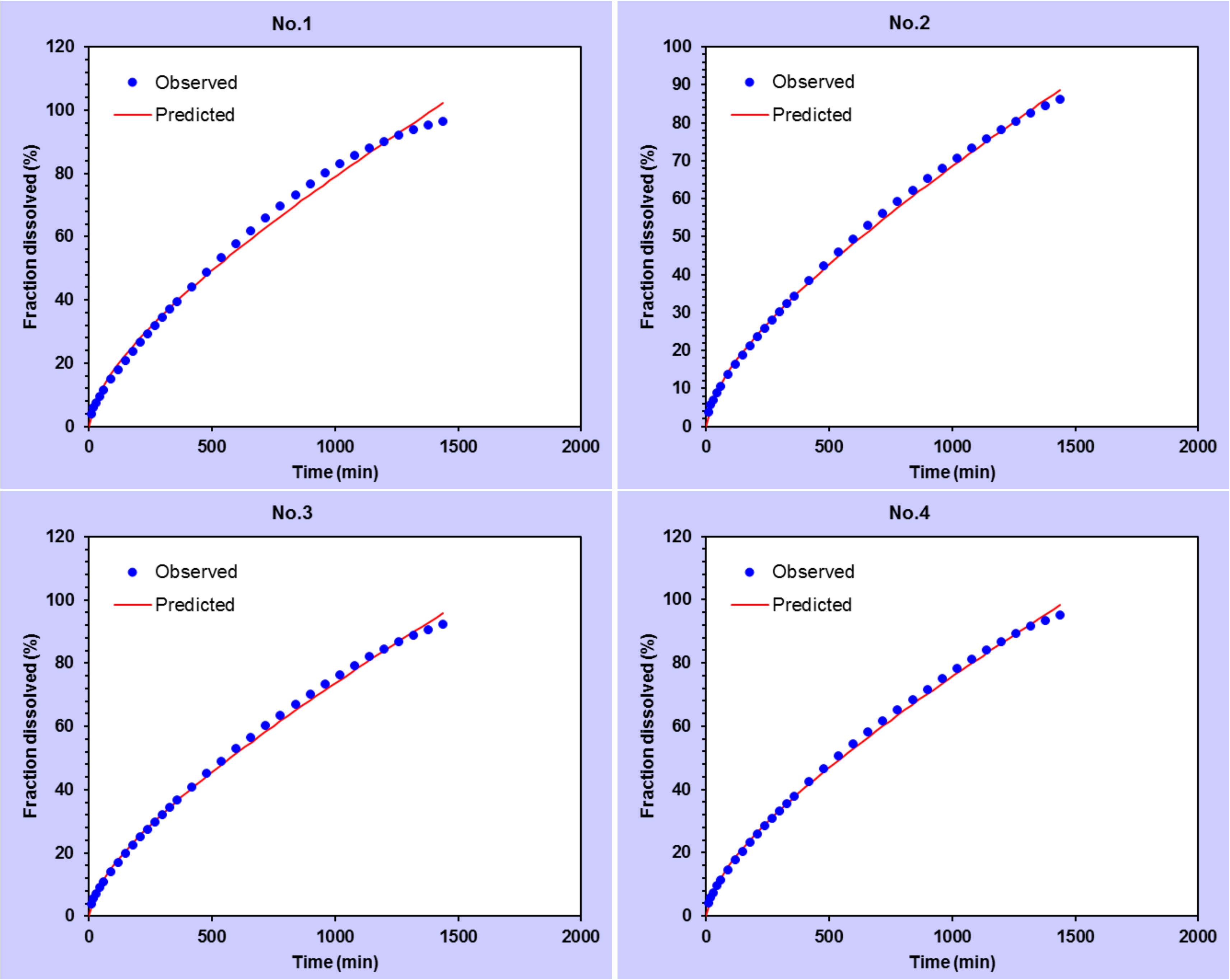

Supplementary materials – Model fitting summary – Di-Cafos® A12 (modelling the entire relevant dissolution data)

Model: **Quadratic**

Model equation: $F = 100 \cdot (k_1 \cdot t^2 + k_2 \cdot t)$

Fitted model parameters per tested tablet (N = 4) with statistics – mean, standard deviation (SD), and relative standard deviation expressed in % (RSD%) (output from DDSolver):

Parameter	No.1	No.2	No.3	No.4	Mean	SD	RSD(%)
k_1	-0.0000004	-0.0000003	-0.0000003	-0.0000003	-0.0000003	0.0000000	-9.0714237
k_2	0.0012152	0.0010312	0.0010991	0.0011303	0.0011190	0.0000763	6.8221639

Number of dissolution data points (N), degrees of freedom (df), and selected goodness of fit criteria – Pearson correlation coefficient (R), coefficient of determination (R^2), adjusted coefficient of determination ($R^2_{adjusted}$), and residual sum of squares (RSS) (manual calculation in MS Excel):

Parameter	No.1	No.2	No.3	No.4
N	33	33	33	33
df	31	31	31	31
R	0.999133831	0.998280517	0.998598849	0.99838501
R^2	0.998268413	0.996563991	0.99719966	0.996772629
$R^2_{adjusted}$	0.998212555	0.996453153	0.997109327	0.99666852
RSS	163.0555495	217.2774395	206.618661	243.3749593

Graphical abstract of model fit presented as mean ± 1 SD of the fraction % of released carvedilol:

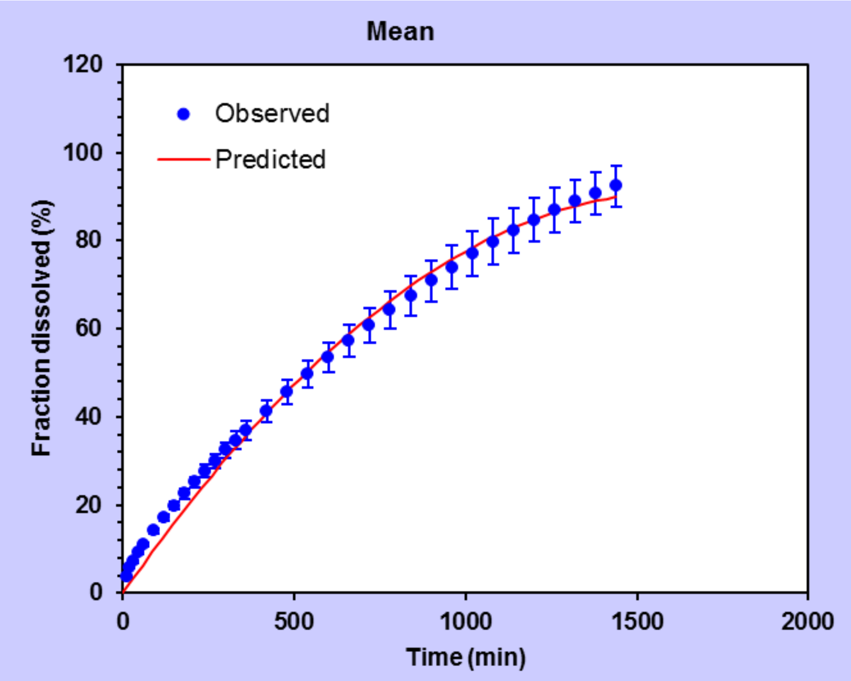

Supplementary materials – Model fitting summary – Di-Cafos® A12 (modelling the entire relevant dissolution data)

Graphical abstract of model fit presented as the fraction % of released carvedilol per tested tablet:

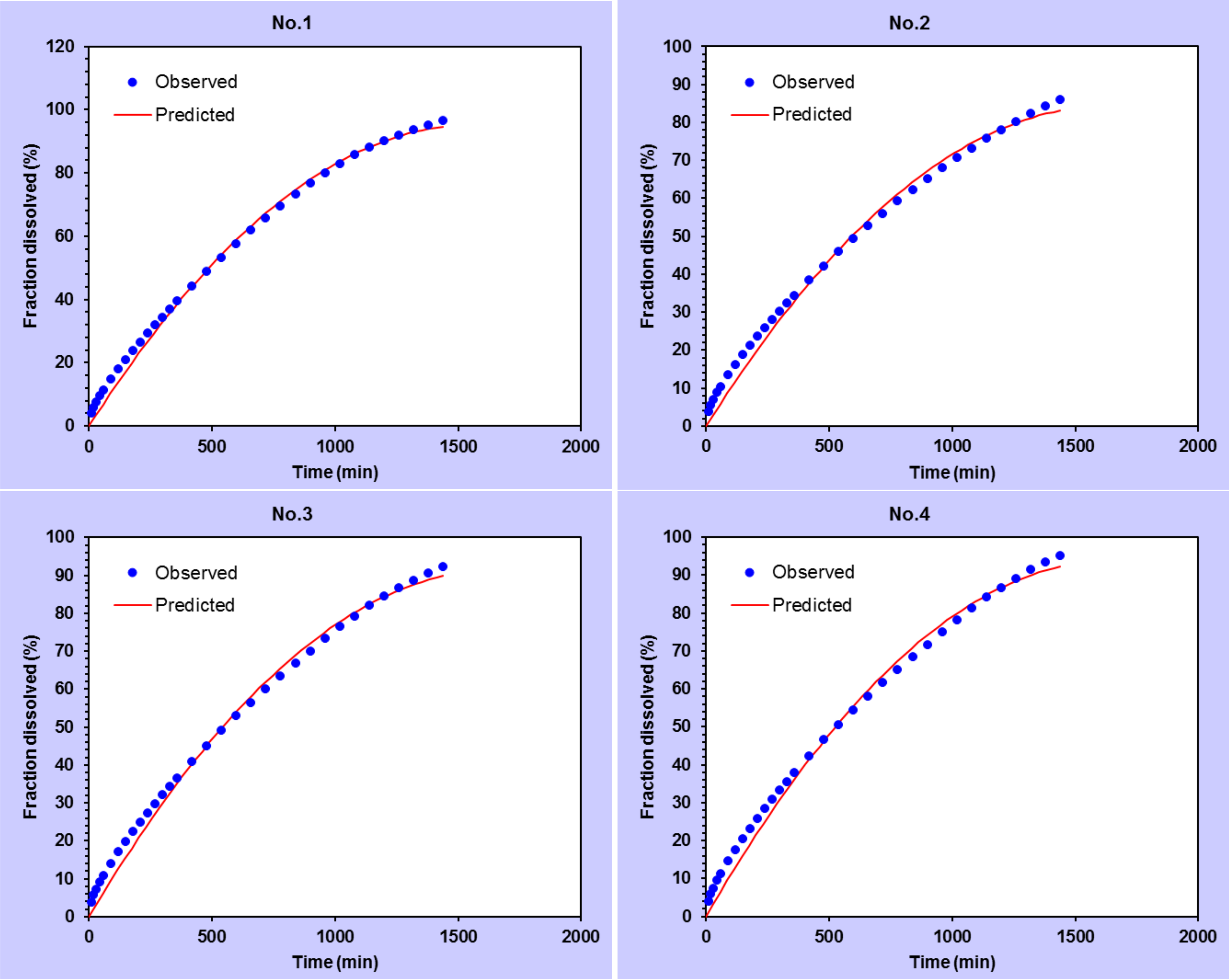

Supplementary materials – Model fitting summary – Di-Cafos® A12 (modelling the entire relevant dissolution data)

Model: **Quadratic with T_{lag}**

Model equation: $F = 100 \cdot \left[k_1 \cdot \left(t - T_{lag}\right)^2 + k_2 \cdot \left(t - T_{lag}\right)\right]$

Fitted model parameters per tested tablet (N = 4) with statistics – mean, standard deviation (SD), and relative standard deviation expressed in % (RSD%) (output from DDSolver):

Parameter	No.1	No.2	No.3	No.4	Mean	SD	RSD(%)
k_1	-0.0000004	-0.0000003	-0.0000003	-0.0000003	-0.0000004	0.0000000	-9.0549231
k_2	0.0012261	0.0010403	0.0011089	0.0011403	0.0011289	0.0000771	6.8291508
T_{lag}	4.0000000	4.0000000	4.0000000	4.0000000	4.0000000	0.0000000	0.0000000

Number of dissolution data points (N), degrees of freedom (df), and selected goodness of fit criteria – Pearson correlation coefficient (R), coefficient of determination (R^2), adjusted coefficient of determination ($R^2_{adjusted}$), and residual sum of squares (RSS) (manual calculation in MS Excel):

Parameter	No.1	No.2	No.3	No.4
N	33	33	33	33
df	30	30	30	30
R	0.99900863	0.998124065	0.998454379	0.998235016
R^2	0.998018242	0.99625165	0.996911148	0.996473147
$R^2_{adjusted}$	0.997886125	0.99600176	0.996705224	0.996238024
RSS	196.8765367	249.3907793	240.1383714	280.404017

Graphical abstract of model fit presented as mean ± 1 SD of the fraction % of released carvedilol:

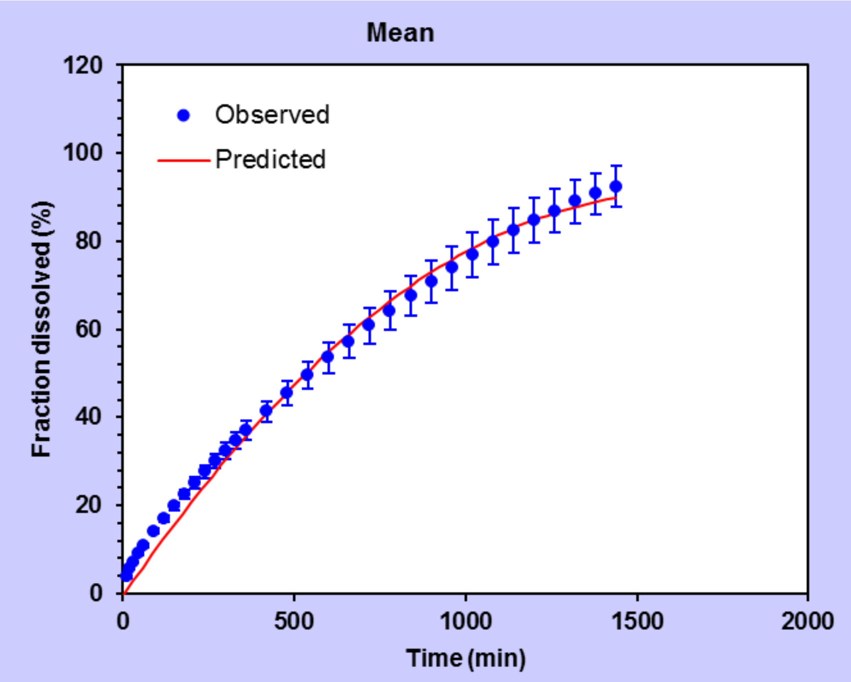

Supplementary materials – Model fitting summary – Di-Cafos® A12 (modelling the entire relevant dissolution data)

Graphical abstract of model fit presented as the fraction % of released carvedilol per tested tablet:

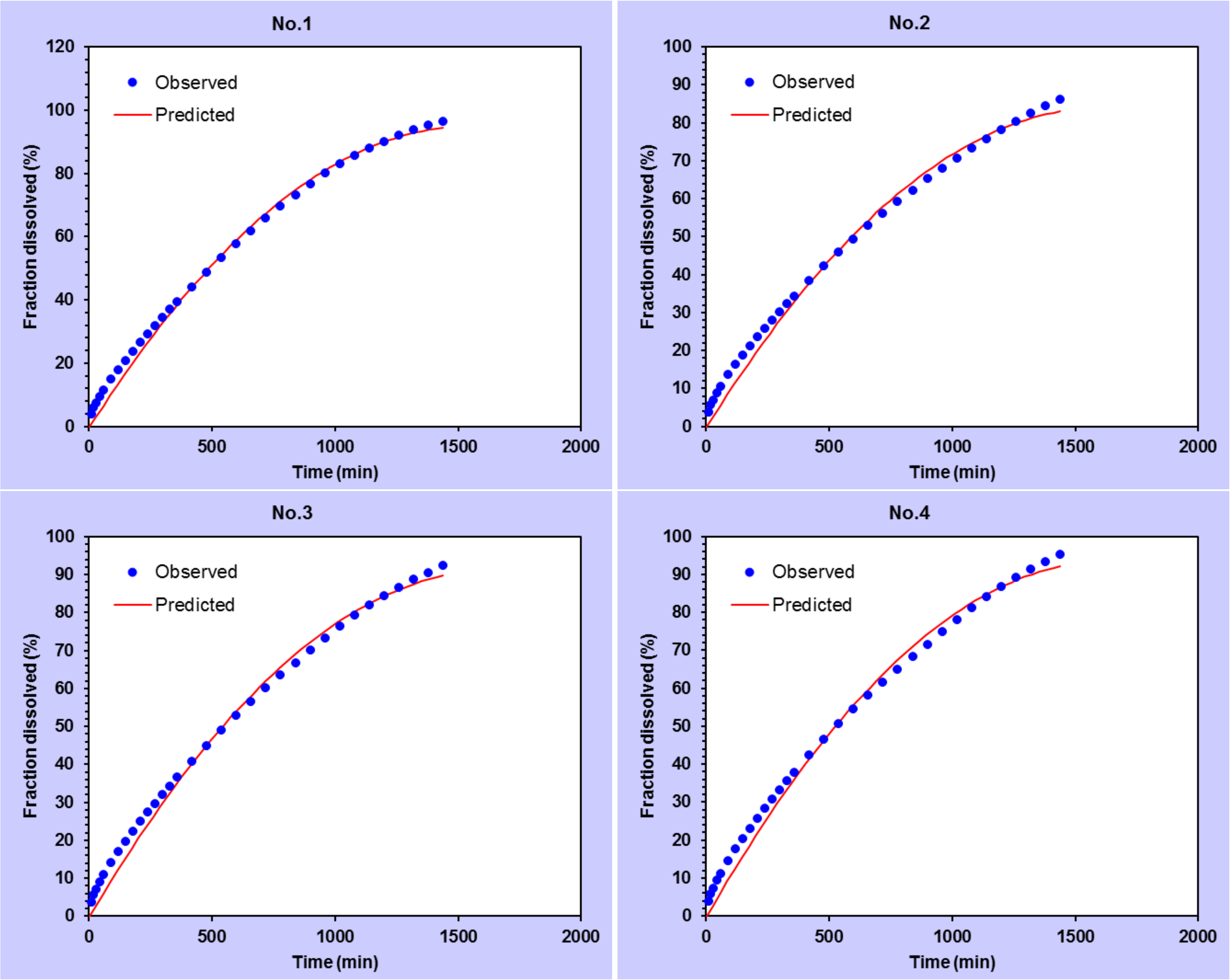

Supplementary materials – Model fitting summary – Di-Cafos® A12 (modelling the entire relevant dissolution data)

Model: **Weibull_1**

Model equation: $F = 100 \cdot \left[1 - e^{-\frac{(t-T_i)^{\beta}}{\alpha}}\right]$

Fitted model parameters per tested tablet (N = 4) with statistics – mean, standard deviation (SD), and relative standard deviation expressed in % (RSD%) (output from DDSolver):

Parameter	No.1	No.2	No.3	No.4	Mean	SD	RSD(%)
α	235.615	183.880	211.157	211.724	210.594	21.145	10.040
β	0.853	0.769	0.811	0.821	0.814	0.035	4.268
T_i	6.000	6.000	6.000	6.000	6.000	0.000	0.000

Number of dissolution data points (N), degrees of freedom (df), and selected goodness of fit criteria – Pearson correlation coefficient (R), coefficient of determination (R^2), adjusted coefficient of determination ($R^2_{adjusted}$), and residual sum of squares (RSS) (manual calculation in MS Excel):

Parameter	No.1	No.2	No.3	No.4
N	33	33	33	33
df	30	30	30	30
R	0.987752384	0.990686878	0.988607628	0.987091972
R^2	0.975654772	0.98146049	0.977345042	0.974350561
$R^2_{adjusted}$	0.974031757	0.980224522	0.975834711	0.972640599
RSS	978.5649162	623.6811283	827.9347882	940.5430899

Graphical abstract of model fit presented as mean ± 1 SD of the fraction % of released carvedilol:

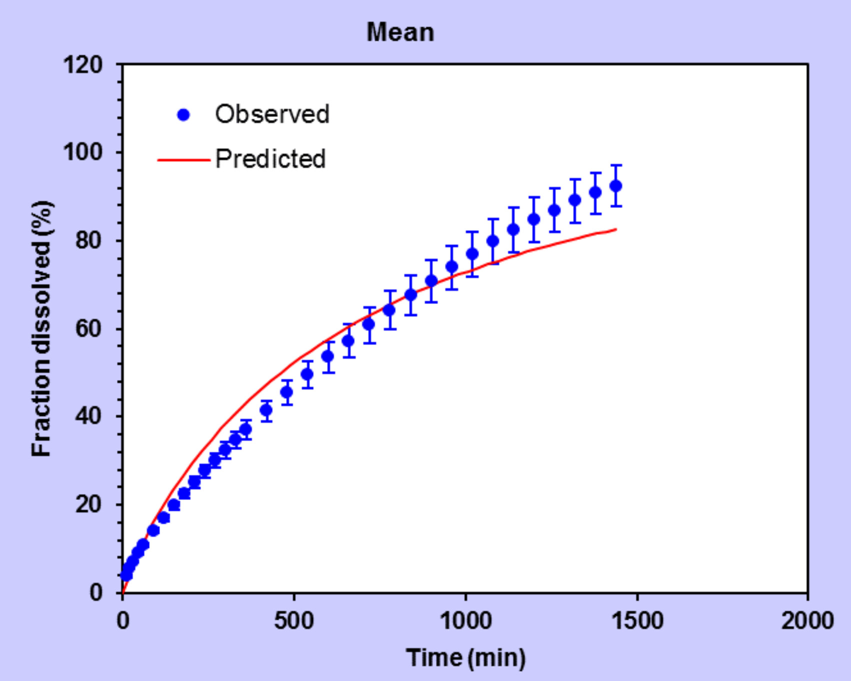

Supplementary materials – Model fitting summary – Di-Cafos® A12 (modelling the entire relevant dissolution data)

Graphical abstract of model fit presented as the fraction % of released carvedilol per tested tablet:

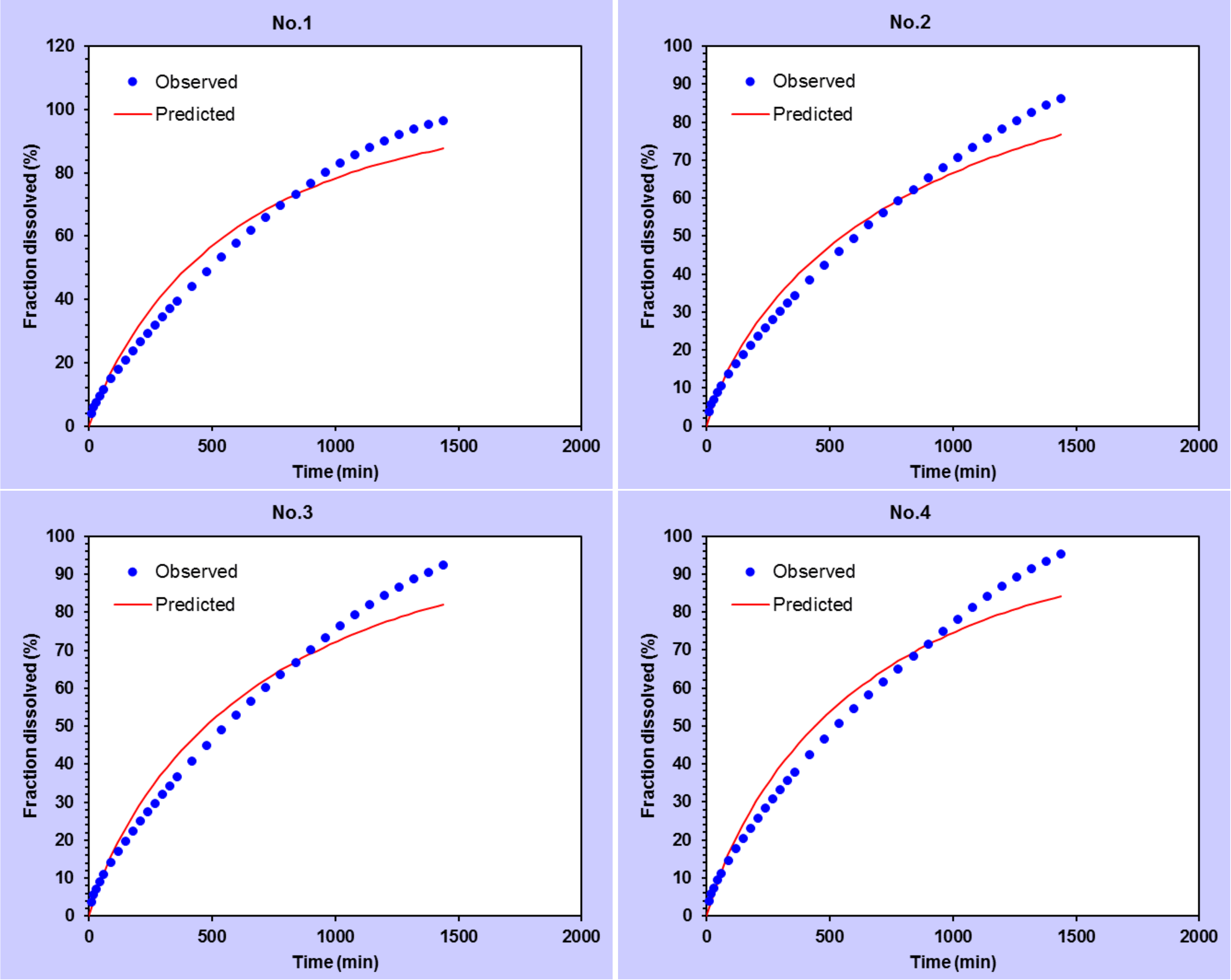

Supplementary materials – Model fitting summary – Di-Cafos® A12 (modelling the entire relevant dissolution data)

Model: **Weibull_2**

Model equation: $F = 100 \cdot \left(1 - e^{-\frac{t^{\beta}}{\alpha}}\right)$

Fitted model parameters per tested tablet (N = 4) with statistics – mean, standard deviation (SD), and relative standard deviation expressed in % (RSD%) (output from DDSolver):

Parameter	No.1	No.2	No.3	No.4	Mean	SD	RSD(%)
α	335.041	251.229	294.171	296.736	294.294	34.259	11.641
β	0.908	0.817	0.863	0.874	0.865	0.037	4.314

Number of dissolution data points (N), degrees of freedom (df), and selected goodness of fit criteria – Pearson correlation coefficient (R), coefficient of determination (R^2), adjusted coefficient of determination ($R^2_{adjusted}$), and residual sum of squares (RSS) (manual calculation in MS Excel):

Parameter	No.1	No.2	No.3	No.4
N	33	33	33	33
df	31	31	31	31
R	0.990265705	0.993102718	0.991088079	0.989628251
R^2	0.980626166	0.986253008	0.982255581	0.979364075
$R^2_{adjusted}$	0.980001204	0.985809556	0.98168318	0.9786984
RSS	768.571998	446.72381	629.4745886	739.1477174

Graphical abstract of model fit presented as mean ± 1 SD of the fraction % of released carvedilol:

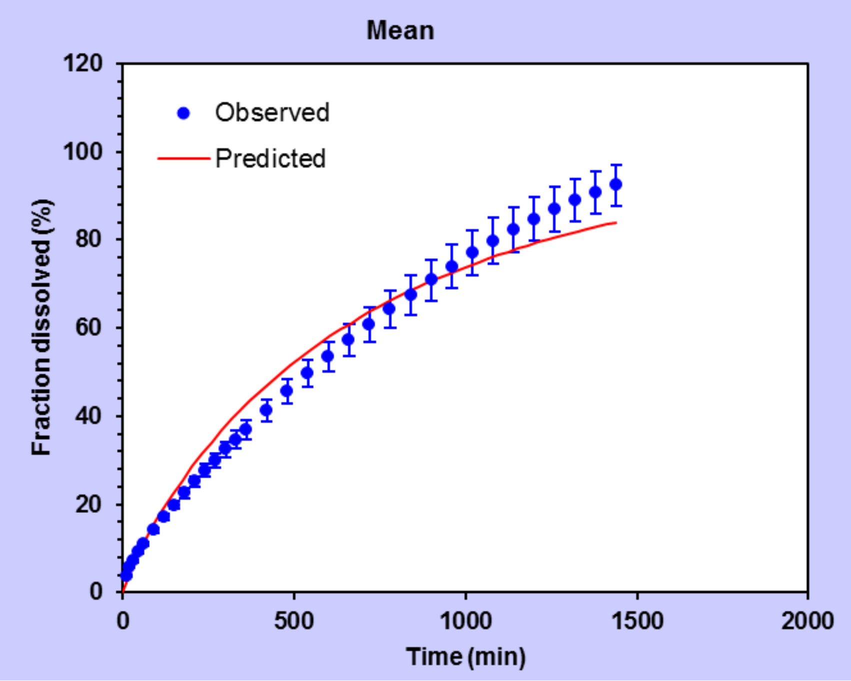

Supplementary materials – Model fitting summary – Di-Cafos® A12 (modelling the entire relevant dissolution data)

Graphical abstract of model fit presented as the fraction % of released carvedilol per tested tablet:

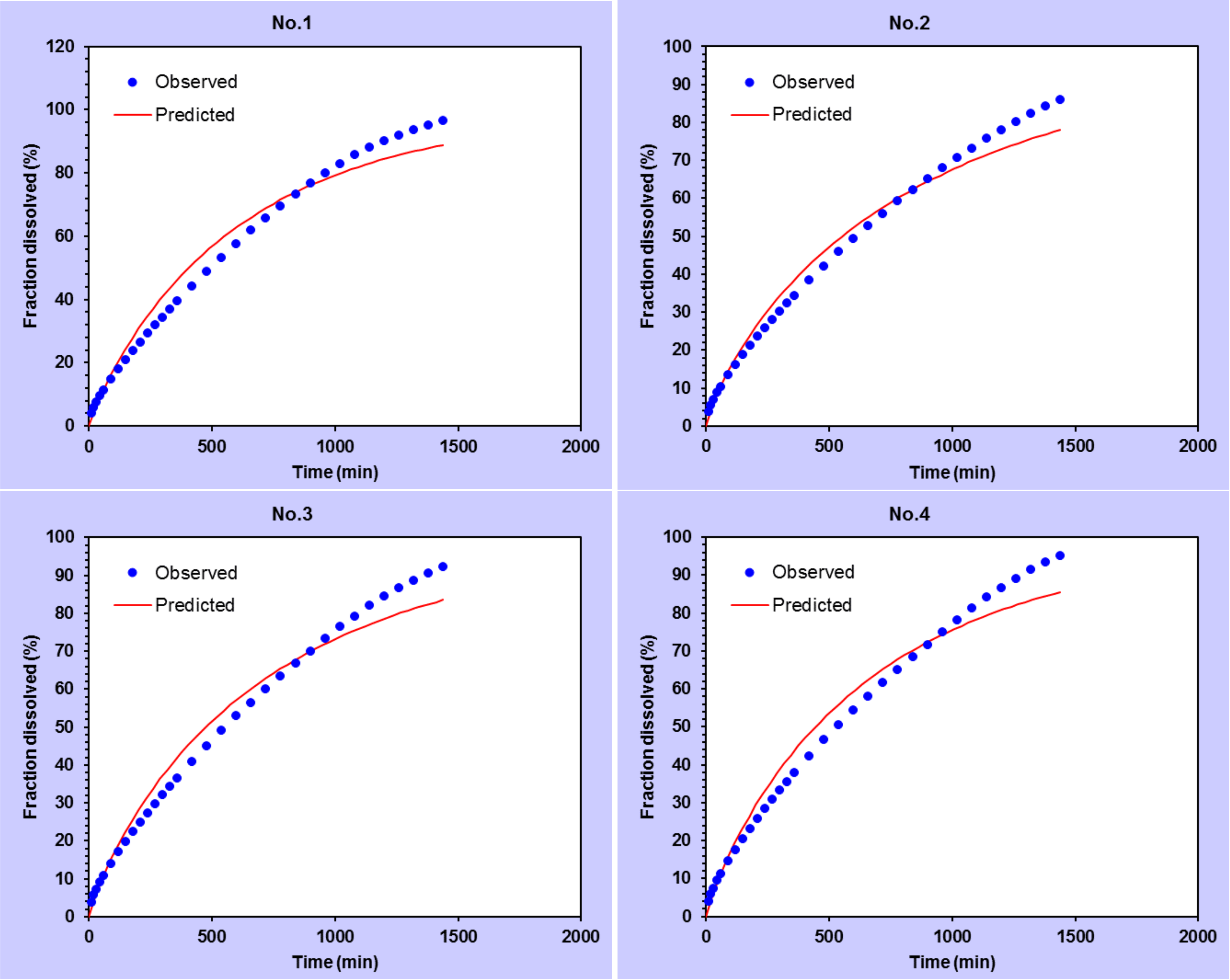

Supplementary materials – Model fitting summary – Di-Cafos® A12 (modelling the entire relevant dissolution data)

Model: **Weibull_3**

Model equation: $F = F_{max} \cdot \left(1 - e^{-\frac{t^{\beta}}{\alpha}}\right)$

Fitted model parameters per tested tablet (N = 4) with statistics – mean, standard deviation (SD), and relative standard deviation expressed in % (RSD%) (output from DDSolver):

Parameter	No.1	No.2	No.3	No.4	Mean	SD	RSD(%)
α	366.293	273.976	307.323	297.566	311.289	39.250	12.609
β	0.868	0.862	0.880	0.875	0.871	0.008	0.906
F_{max}	113.581	90.347	96.822	99.832	100.145	9.793	9.778

Number of dissolution data points (N), degrees of freedom (df), and selected goodness of fit criteria – Pearson correlation coefficient (R), coefficient of determination (R^2), adjusted coefficient of determination ($R^2_{adjusted}$), and residual sum of squares (RSS) (manual calculation in MS Excel):

Parameter	No.1	No.2	No.3	No.4
N	33	33	33	33
df	30	30	30	30
R	0.996678275	0.989434357	0.989611801	0.989541539
R^2	0.993367583	0.978980346	0.979331516	0.979192458
$R^2_{adjusted}$	0.992925422	0.977579036	0.977953617	0.977805288
RSS	586.2814622	609.1788994	706.0923322	743.9162527

Graphical abstract of model fit presented as mean ± 1 SD of the fraction % of released carvedilol:

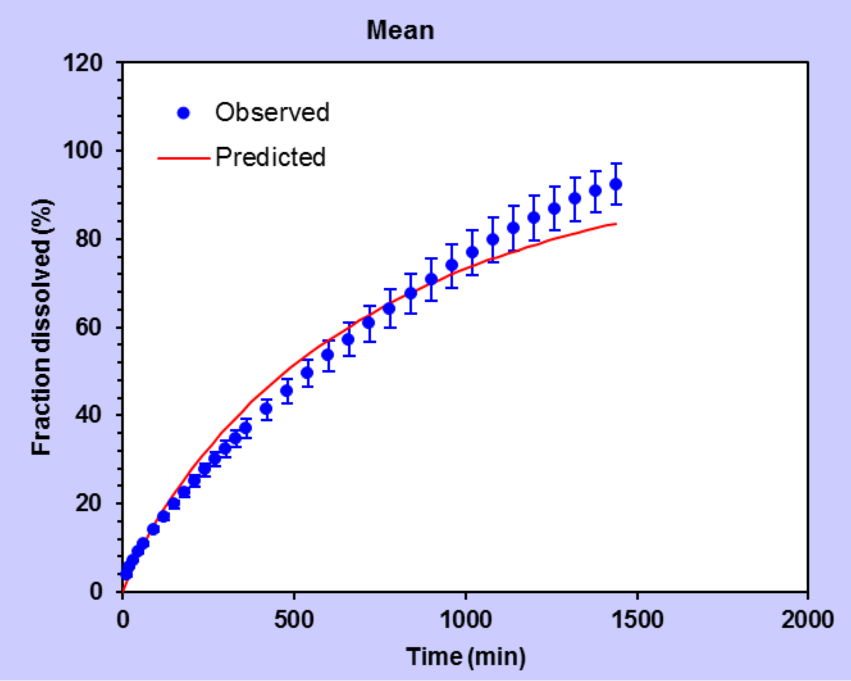

Supplementary materials – Model fitting summary – Di-Cafos® A12 (modelling the entire relevant dissolution data)

Graphical abstract of model fit presented as the fraction % of released carvedilol per tested tablet:

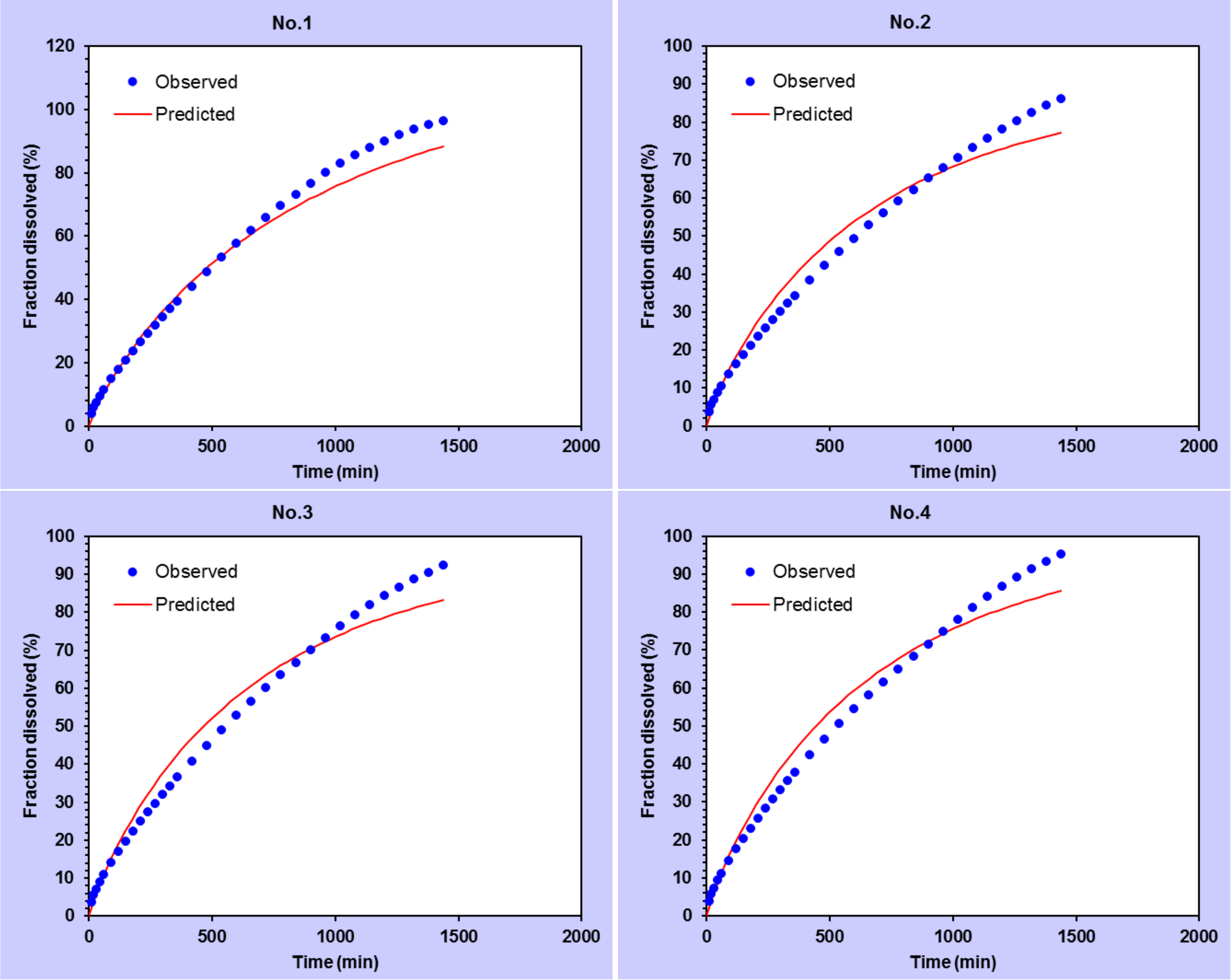

Supplementary materials – Model fitting summary – Di-Cafos® A12 (modelling the entire relevant dissolution data)

Model: **Weibull_4**

Model equation: $F = F_{max} \cdot \left[1 - e^{-\frac{(t-T_i)^{\beta}}{\alpha}}\right]$

Fitted model parameters per tested tablet (N = 4) with statistics – mean, standard deviation (SD), and relative standard deviation expressed in % (RSD%) (output from DDSolver):

Parameter	No.1	No.2	No.3	No.4	Mean	SD	RSD(%)
α	230.489	196.284	218.710	212.208	214.423	14.264	6.652
β	0.845	0.811	0.828	0.822	0.826	0.014	1.724
T_i	6.000	6.000	6.000	6.000	6.000	0.000	0.000
F_{max}	101.196	90.347	96.822	99.832	97.049	4.827	4.974

Number of dissolution data points (N), degrees of freedom (df), and selected goodness of fit criteria – Pearson correlation coefficient (R), coefficient of determination (R^2), adjusted coefficient of determination ($R^2_{adjusted}$), and residual sum of squares (RSS) (manual calculation in MS Excel):

Parameter	No.1	No.2	No.3	No.4
N	33	33	33	33
df	29	29	29	29
R	0.988461214	0.986806146	0.987068607	0.987002354
R^2	0.977055572	0.973786371	0.974304436	0.974173646
$R^2_{adjusted}$	0.97468201	0.971074616	0.971646274	0.971501954
RSS	939.0639589	775.3782282	897.8984966	944.8360554

Graphical abstract of model fit presented as mean ± 1 SD of the fraction % of released carvedilol:

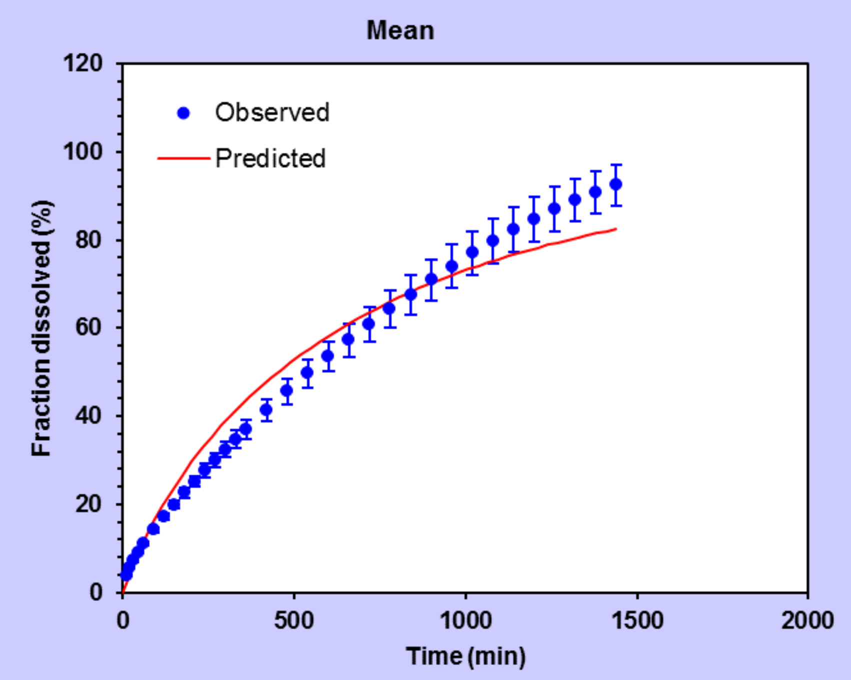

Supplementary materials – Model fitting summary – Di-Cafos® A12 (modelling the entire relevant dissolution data)

Graphical abstract of model fit presented as the fraction % of released carvedilol per tested tablet:

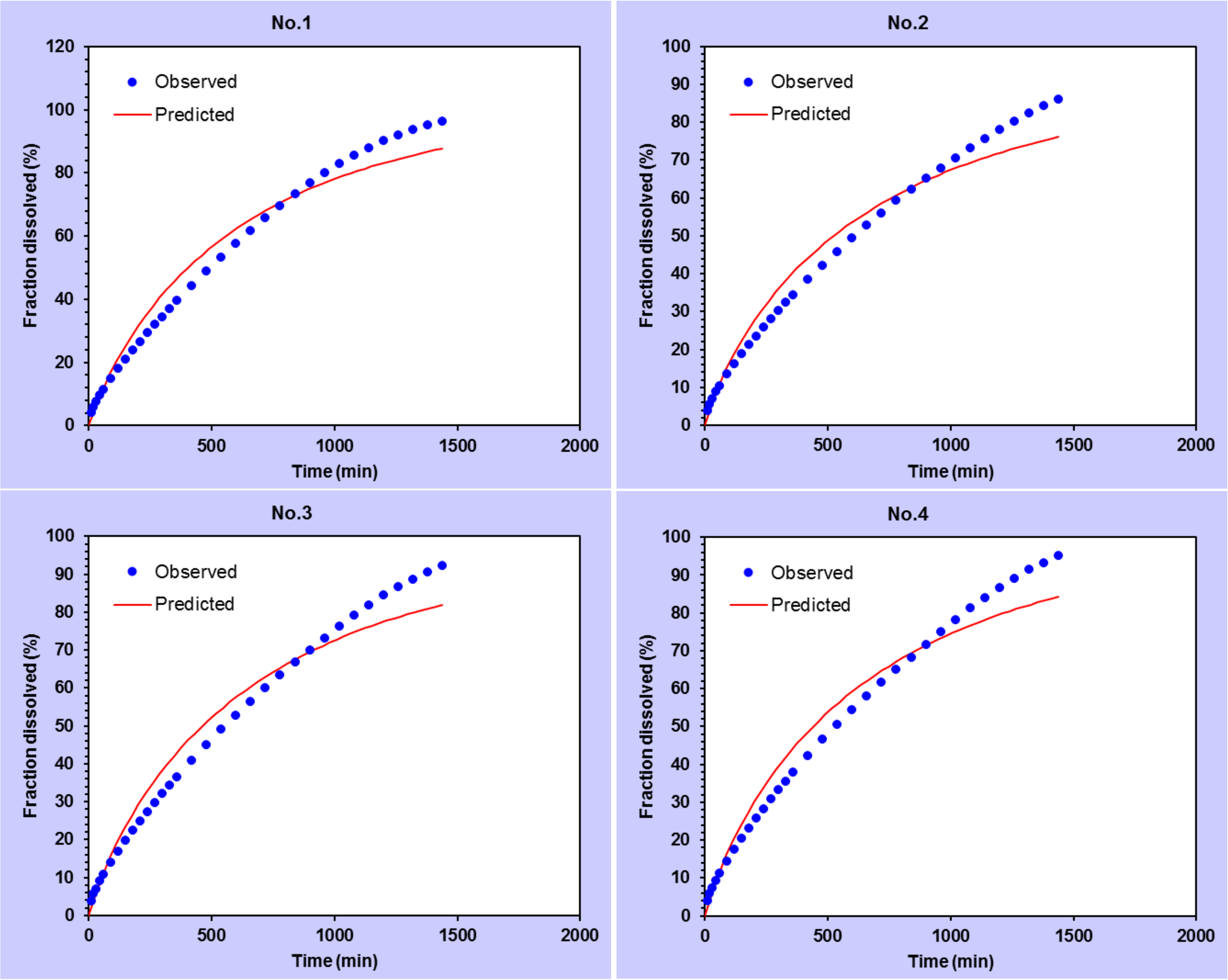

Supplementary materials – Model fitting summary – Di-Cafos® A12 (modelling the entire relevant dissolution data)

Model: **Logistic_1**

Model equation: $F = 100 \cdot \frac{e^{\alpha+\beta \cdot \log(t)}}{1+e^{\alpha+\beta \cdot \log(t)}}$

Fitted model parameters per tested tablet (N = 4) with statistics – mean, standard deviation (SD), and relative standard deviation expressed in % (RSD%) (output from DDSolver):

Parameter	No.1	No.2	No.3	No.4	Mean	SD	RSD(%)
α	-7.198	-7.285	-7.760	-6.879	-7.280	0.364	-5.002
β	2.872	2.611	2.828	2.688	2.750	0.121	4.408

Number of dissolution data points (N), degrees of freedom (df), and selected goodness of fit criteria – Pearson correlation coefficient (R), coefficient of determination (R^2), adjusted coefficient of determination ($R^2_{adjusted}$), and residual sum of squares (RSS) (manual calculation in MS Excel):

Parameter	No.1	No.2	No.3	No.4
N	33	33	33	33
df	31	31	31	31
R	0.968337076	0.991110056	0.989946723	0.970250504
R^2	0.937676693	0.982299143	0.979994515	0.941386041
$R^2_{adjusted}$	0.935666264	0.981728148	0.979349176	0.939495268
RSS	2411.014588	1069.588638	1555.439576	2009.573797

Graphical abstract of model fit presented as mean ± 1 SD of the fraction % of released carvedilol:

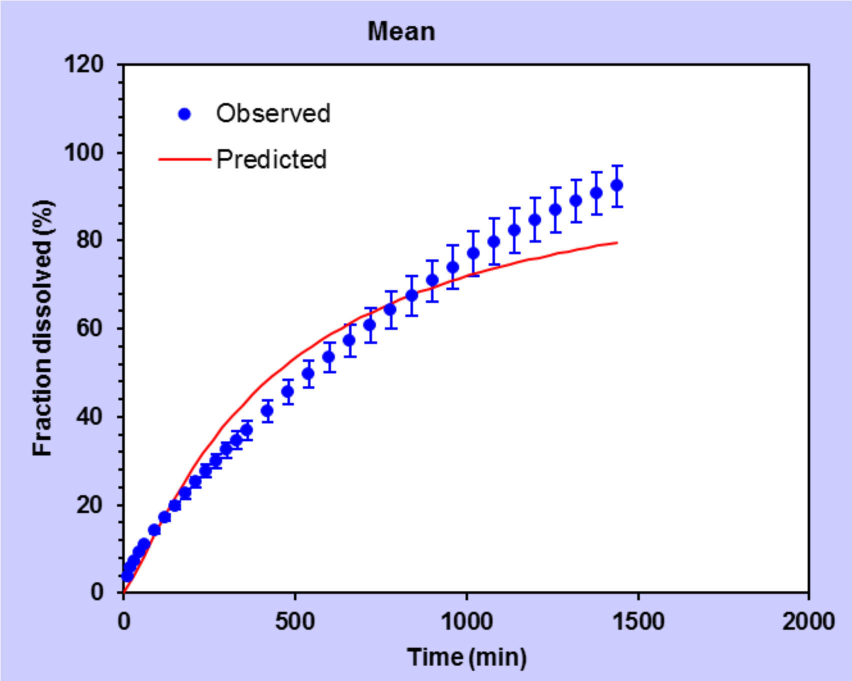

Supplementary materials – Model fitting summary – Di-Cafos® A12 (modelling the entire relevant dissolution data)

Graphical abstract of model fit presented as the fraction % of released carvedilol per tested tablet:

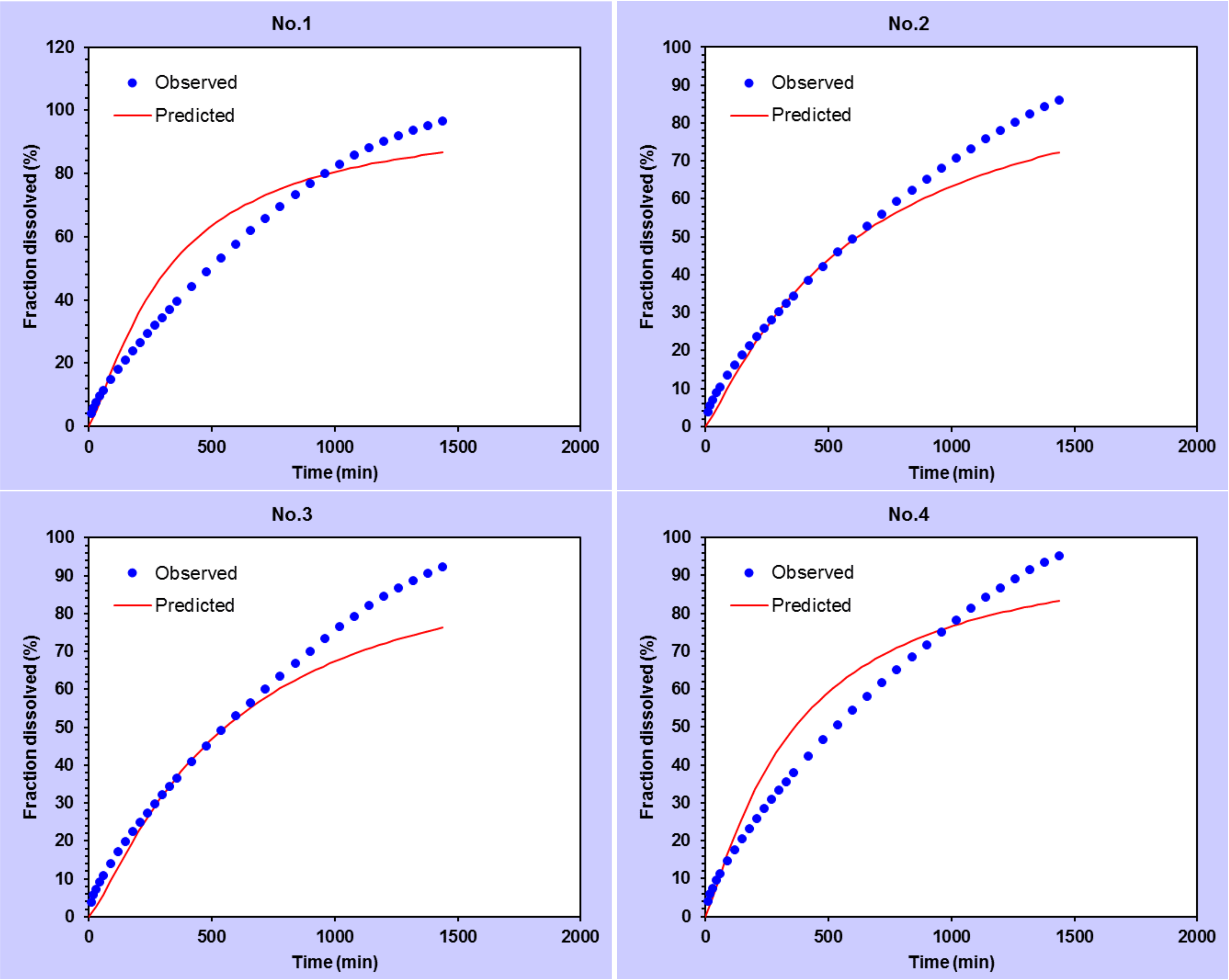

Supplementary materials – Model fitting summary – Di-Cafos® A12 (modelling the entire relevant dissolution data)

Model: **Logistic_2**

Model equation: $F = F_{max} \cdot \frac{e^{\alpha+\beta \cdot \log(t)}}{1+e^{\alpha+\beta \cdot \log(t)}}$

Fitted model parameters per tested tablet (N = 4) with statistics – mean, standard deviation (SD), and relative standard deviation expressed in % (RSD%) (output from DDSolver):

Parameter	No.1	No.2	No.3	No.4	Mean	SD	RSD(%)
α	-8.185	-7.849	-8.013	-7.962	-8.002	0.140	-1.747
β	2.985	2.830	2.888	2.867	2.893	0.066	2.293
F_{max}	109.775	98.006	105.030	108.295	105.277	5.237	4.974

Number of dissolution data points (N), degrees of freedom (df), and selected goodness of fit criteria – Pearson correlation coefficient (R), coefficient of determination (R^2), adjusted coefficient of determination ($R^2_{adjusted}$), and residual sum of squares (RSS) (manual calculation in MS Excel):

Parameter	No.1	No.2	No.3	No.4
N	33	33	33	33
df	30	30	30	30
R	0.993656966	0.992187375	0.99243062	0.992304298
R^2	0.987354165	0.984435786	0.984918535	0.984667821
$R^2_{adjusted}$	0.986511109	0.983398172	0.983913104	0.983645675
RSS	1035.887241	973.6020354	1134.082853	1208.273465

Graphical abstract of model fit presented as mean ± 1 SD of the fraction % of released carvedilol:

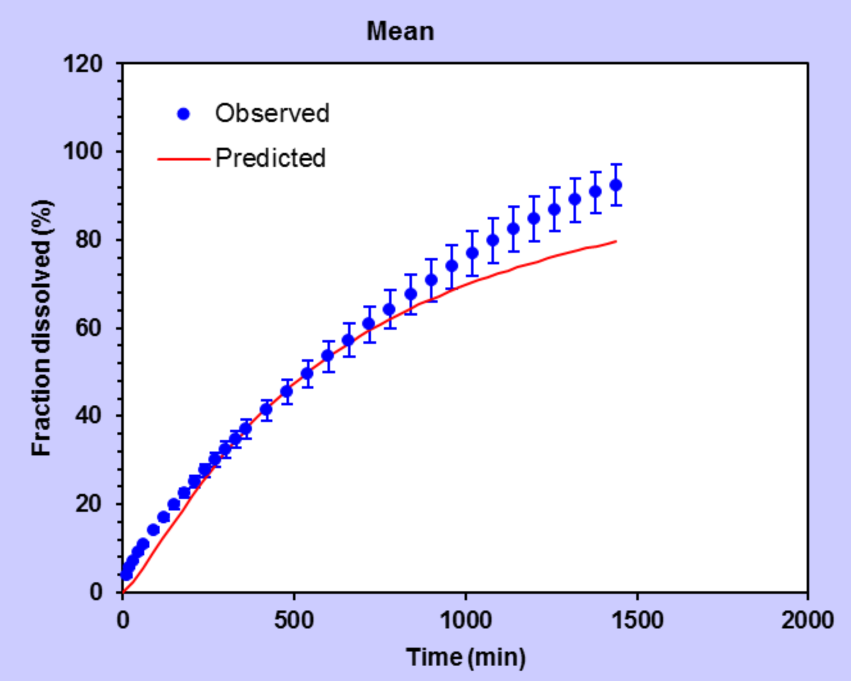

Supplementary materials – Model fitting summary – Di-Cafos® A12 (modelling the entire relevant dissolution data)

Graphical abstract of model fit presented as the fraction % of released carvedilol per tested tablet:

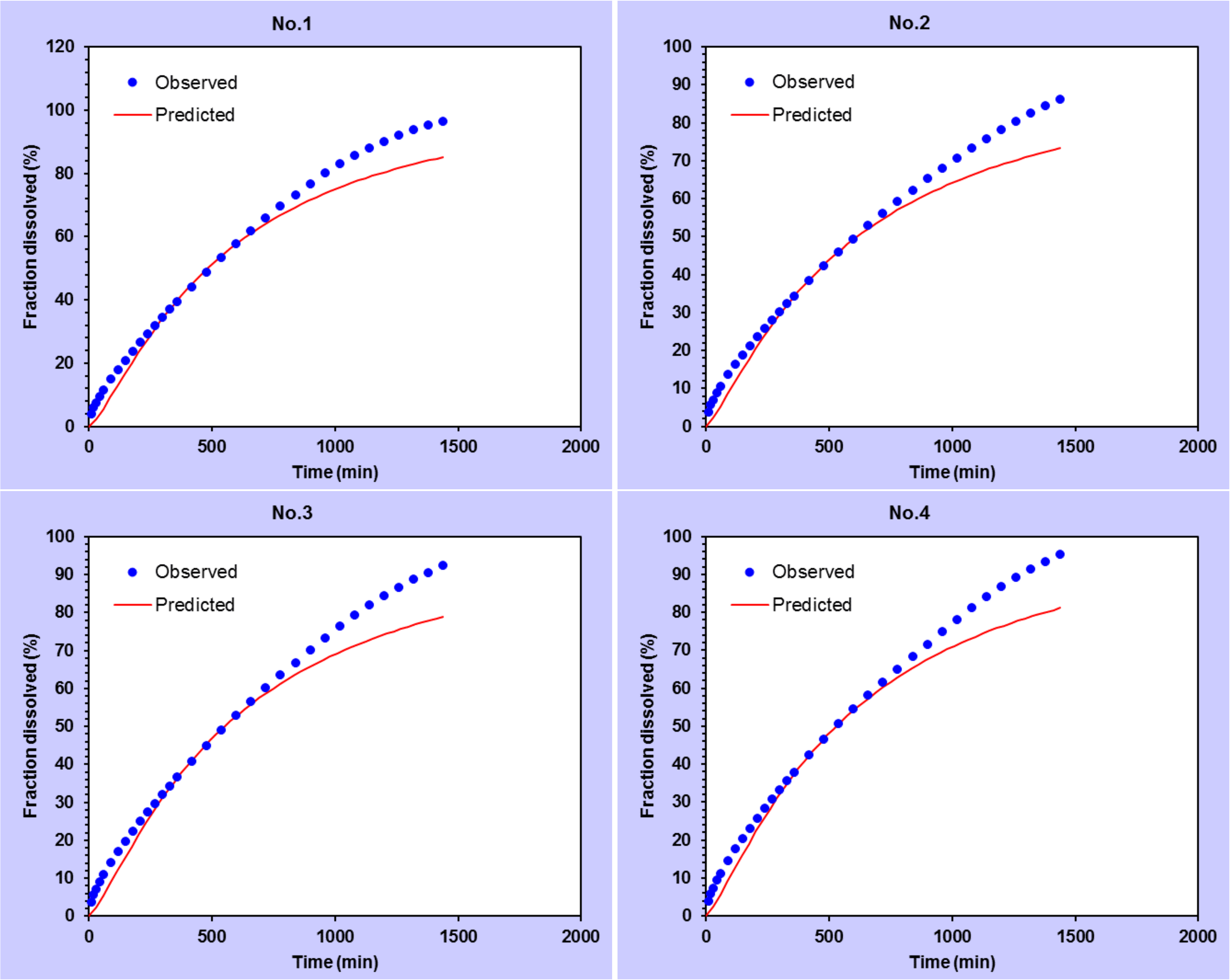

Supplementary materials – Model fitting summary – Di-Cafos® A12 (modelling the entire relevant dissolution data)

Model: **Logistic_3**

Model equation: $F = F_{max} \cdot \frac{1}{1+e^{-k\cdot(t-\gamma)}}$

Fitted model parameters per tested tablet (N = 4) with statistics – mean, standard deviation (SD), and relative standard deviation expressed in % (RSD%) (output from DDSolver):

Parameter	No.1	No.2	No.3	No.4	Mean	SD	RSD(%)
k	0.003	0.003	0.003	0.003	0.003	0.000	4.776
γ	495.776	603.746	515.677	516.886	533.021	48.133	9.030
F_{max}	98.853	90.347	94.580	97.521	95.325	3.769	3.953

Number of dissolution data points (N), degrees of freedom (df), and selected goodness of fit criteria – Pearson correlation coefficient (R), coefficient of determination (R^2), adjusted coefficient of determination ($R^2_{adjusted}$), and residual sum of squares (RSS) (manual calculation in MS Excel):

Parameter	No.1	No.2	No.3	No.4
N	33	33	33	33
df	30	30	30	30
R	0.995473432	0.989697017	0.994781736	0.994342352
R^2	0.990967355	0.979500186	0.989590702	0.988716713
$R^2_{adjusted}$	0.990365178	0.978133532	0.988896749	0.987964494
RSS	673.3226681	553.0495403	630.1975485	686.70458

Graphical abstract of model fit presented as mean ± 1 SD of the fraction % of released carvedilol:

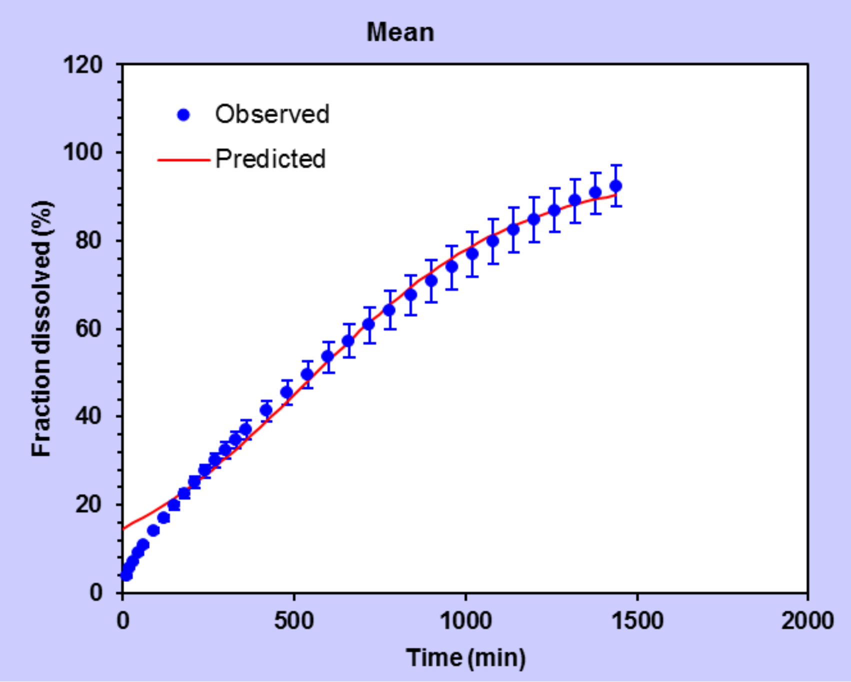

Supplementary materials – Model fitting summary – Di-Cafos® A12 (modelling the entire relevant dissolution data)

Graphical abstract of model fit presented as the fraction % of released carvedilol per tested tablet:

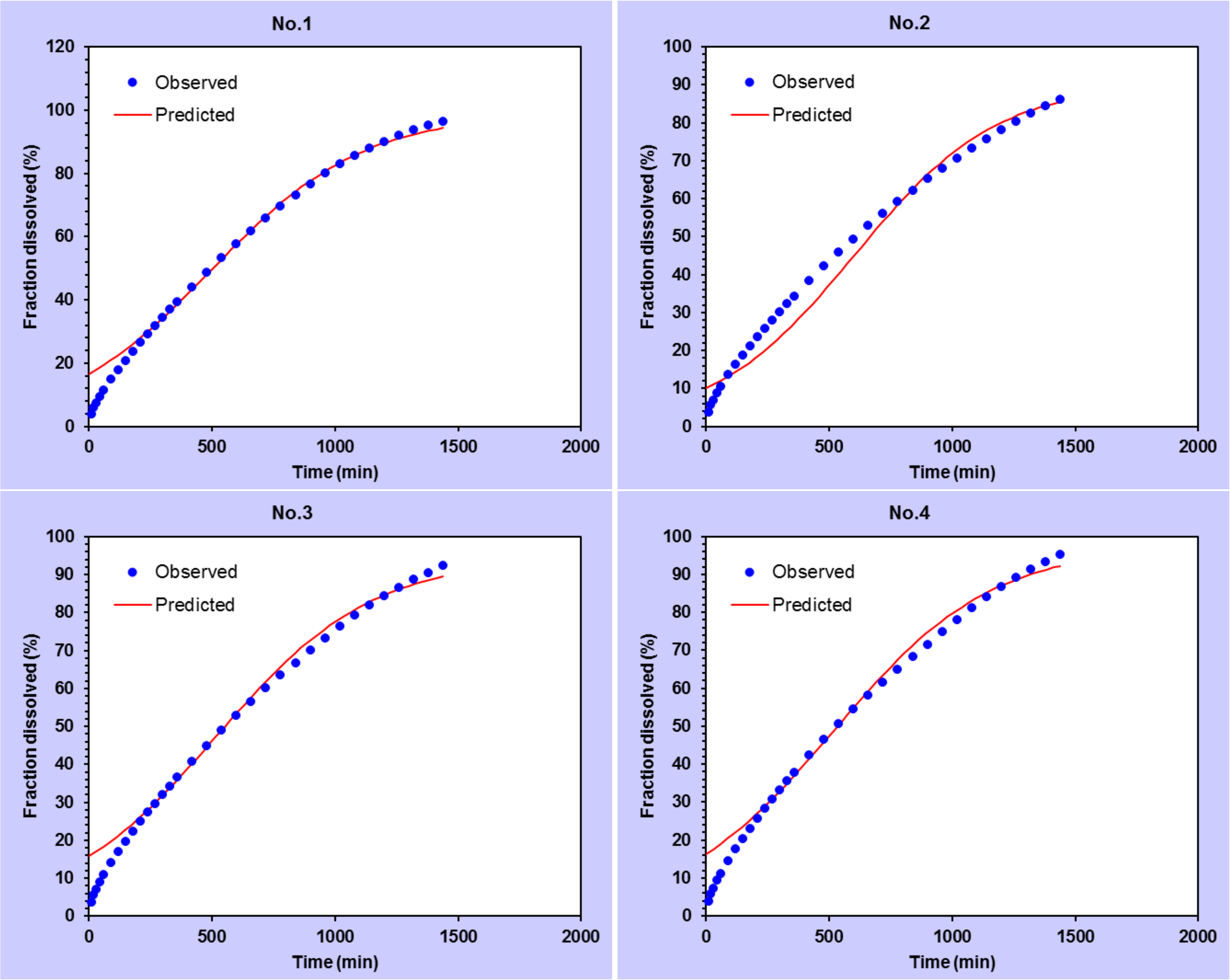

Supplementary materials – Model fitting summary – Di-Cafos® A12 (modelling the entire relevant dissolution data)

Model: **Gompertz_1**

Model equation: $F = 100 \cdot e^{-\alpha \cdot e^{-\beta \cdot \log(t)}}$

Fitted model parameters per tested tablet (N = 4) with statistics – mean, standard deviation (SD), and relative standard deviation expressed in % (RSD%) (output from DDSolver):

Parameter	No.1	No.2	No.3	No.4	Mean	SD	RSD(%)
α	104.117	28.741	63.214	76.287	68.090	31.290	45.954
β	1.921	1.429	1.640	1.748	1.684	0.206	12.223

Number of dissolution data points (N), degrees of freedom (df), and selected goodness of fit criteria – Pearson correlation coefficient (R), coefficient of determination (R^2), adjusted coefficient of determination ($R^2_{adjusted}$), and residual sum of squares (RSS) (manual calculation in MS Excel):

Parameter	No.1	No.2	No.3	No.4
N	33	33	33	33
df	31	31	31	31
R	0.969652656	0.951476633	0.975820715	0.972014609
R^2	0.940226272	0.905307782	0.952226069	0.944812399
$R^2_{adjusted}$	0.938298088	0.902253195	0.950684974	0.943032154
RSS	2533.49084	2503.654689	3252.160432	2855.398403

Graphical abstract of model fit presented as mean ± 1 SD of the fraction % of released carvedilol:

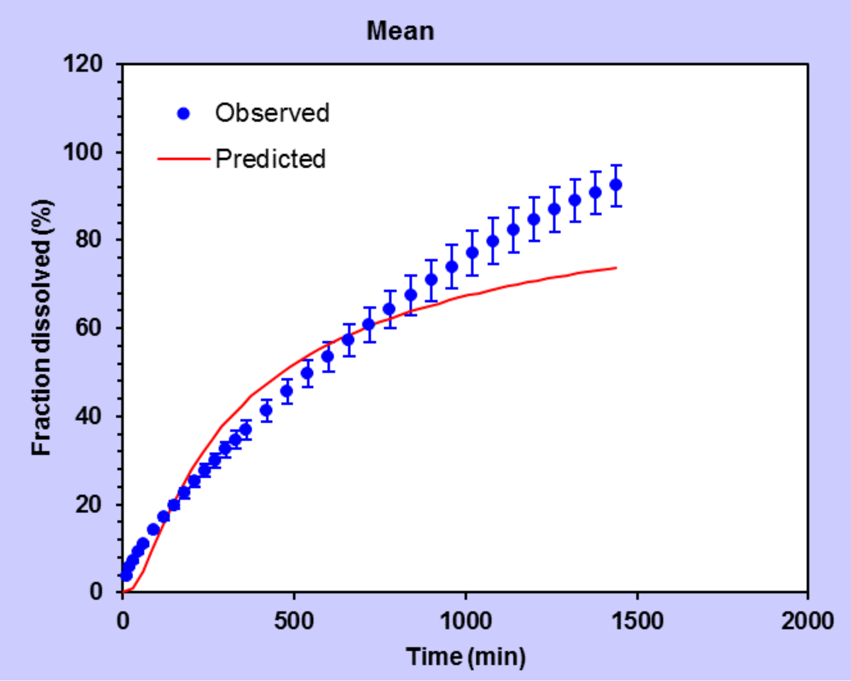

Supplementary materials – Model fitting summary – Di-Cafos® A12 (modelling the entire relevant dissolution data)

Graphical abstract of model fit presented as the fraction % of released carvedilol per tested tablet:

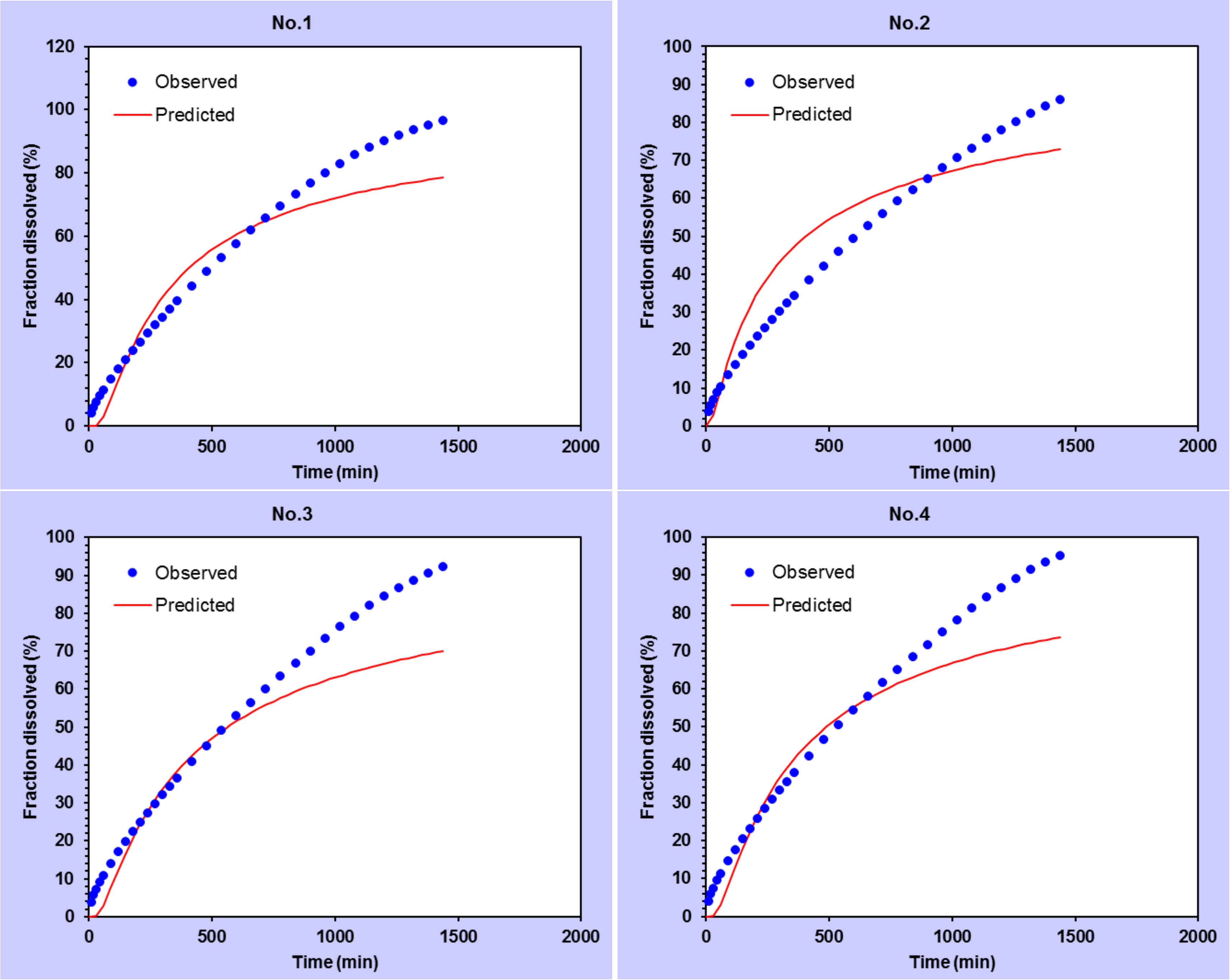

Supplementary materials – Model fitting summary – Di-Cafos® A12 (modelling the entire relevant dissolution data)

Model: **Gompertz_2**

Model equation: $F = F_{max} \cdot e^{-\alpha \cdot e^{-\beta \cdot \log(t)}}$

Fitted model parameters per tested tablet (N = 4) with statistics – mean, standard deviation (SD), and relative standard deviation expressed in % (RSD%) (output from DDSolver):

Parameter	No.1	No.2	No.3	No.4	Mean	SD	RSD(%)
α	91.966	58.600	63.139	61.406	68.778	15.571	22.640
β	1.853	1.548	1.576	1.565	1.636	0.145	8.894
F_{max}	101.196	110.912	118.861	122.556	113.381	9.465	8.348

Number of dissolution data points (N), degrees of freedom (df), and selected goodness of fit criteria – Pearson correlation coefficient (R), coefficient of determination (R^2), adjusted coefficient of determination ($R^2_{adjusted}$), and residual sum of squares (RSS) (manual calculation in MS Excel):

Parameter	No.1	No.2	No.3	No.4
N	33	33	33	33
df	30	30	30	30
R	0.97196946	0.984190228	0.984341351	0.984307612
R^2	0.944724631	0.968630404	0.968927895	0.968861475
$R^2_{adjusted}$	0.941039607	0.966539098	0.966856421	0.966785573
RSS	2701.076687	1421.703073	1629.900704	1739.423882

Graphical abstract of model fit presented as mean ± 1 SD of the fraction % of released carvedilol:

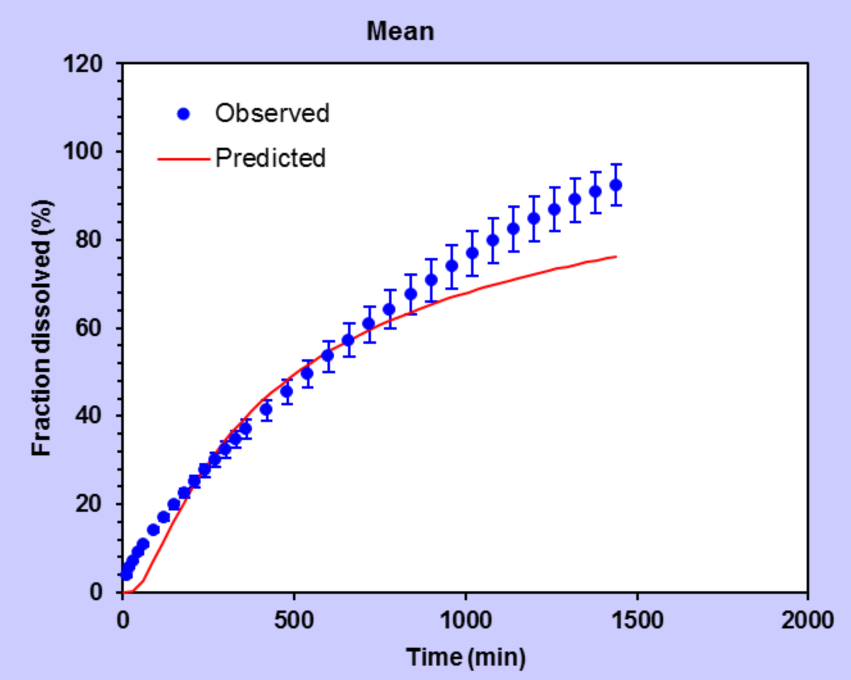

Supplementary materials – Model fitting summary – Di-Cafos® A12 (modelling the entire relevant dissolution data)

Graphical abstract of model fit presented as the fraction % of released carvedilol per tested tablet:

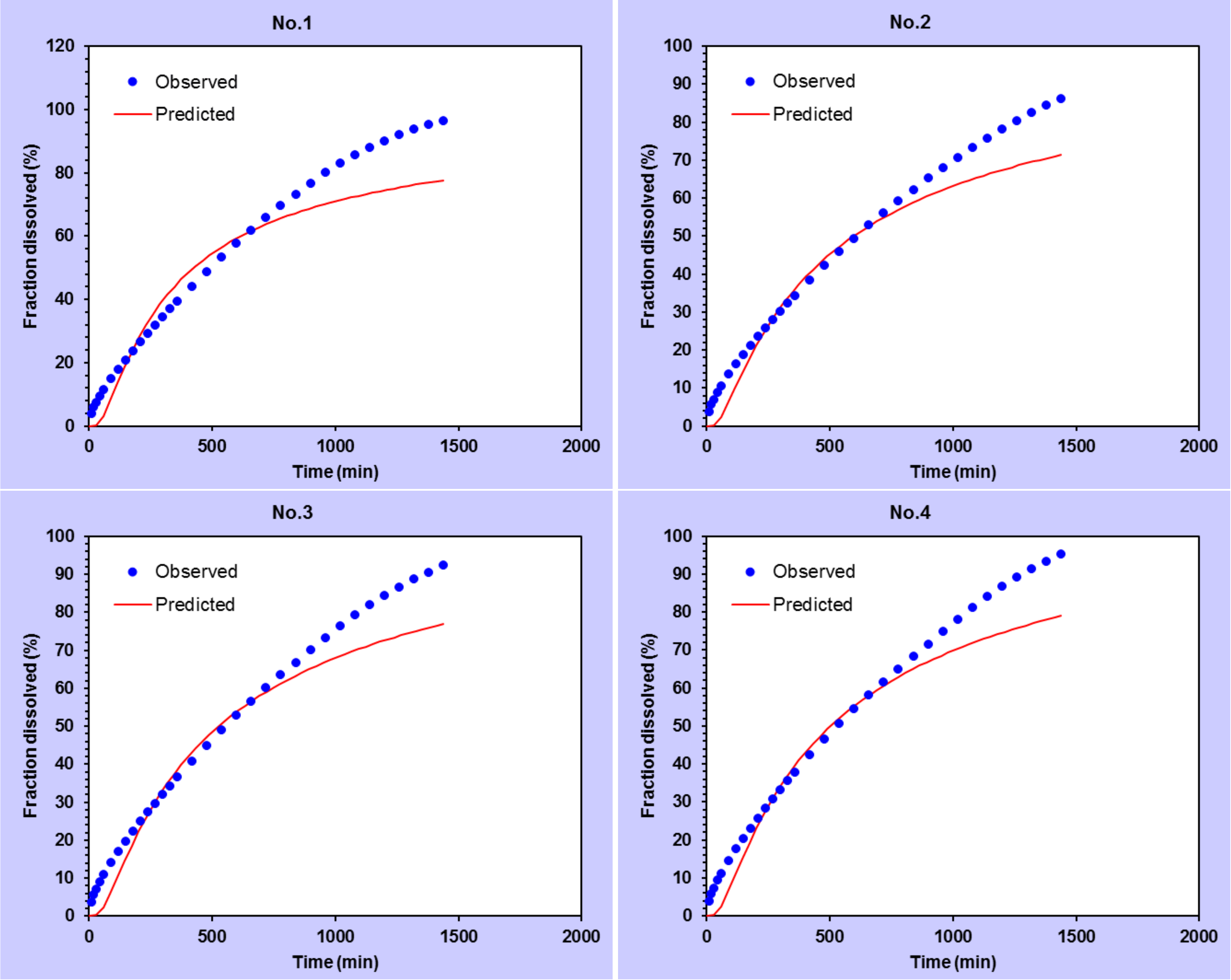

Supplementary materials – Model fitting summary – Di-Cafos® A12 (modelling the entire relevant dissolution data)

Model: **Gompertz_3**

Model equation: $F = F_{max} \cdot e^{-e^{-k\cdot(t-\gamma)}}$

Fitted model parameters per tested tablet (N = 4) with statistics – mean, standard deviation (SD), and relative standard deviation expressed in % (RSD%) (output from DDSolver):

Parameter	No.1	No.2	No.3	No.4	Mean	SD	RSD(%)
k	0.003	0.002	0.002	0.002	0.003	0.000	2.766
γ	365.202	380.795	382.795	383.496	378.072	8.656	2.289
F_{max}	101.196	90.347	96.822	99.832	97.049	4.827	4.974

Number of dissolution data points (N), degrees of freedom (df), and selected goodness of fit criteria – Pearson correlation coefficient (R), coefficient of determination (R^2), adjusted coefficient of determination ($R^2_{adjusted}$), and residual sum of squares (RSS) (manual calculation in MS Excel):

Parameter	No.1	No.2	No.3	No.4
N	33	33	33	33
df	30	30	30	30
R	0.997455185	0.996200838	0.996309455	0.995947803
R^2	0.994916846	0.992416109	0.99263253	0.991912026
$R^2_{adjusted}$	0.994577969	0.991910517	0.992141366	0.991372827
RSS	179.6759639	204.4966425	234.6641974	271.1820649

Graphical abstract of model fit presented as mean ± 1 SD of the fraction % of released carvedilol:

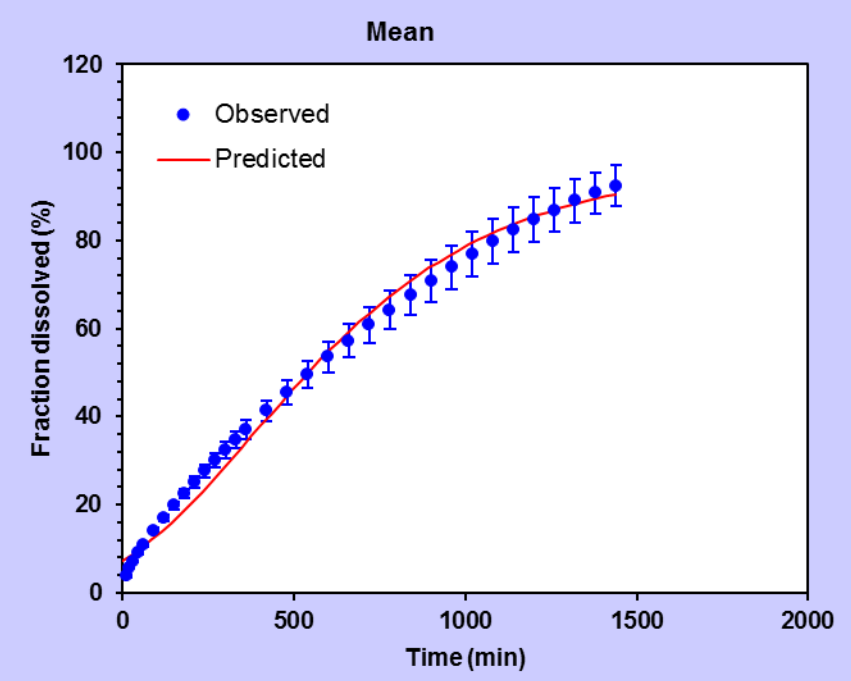

Supplementary materials – Model fitting summary – Di-Cafos® A12 (modelling the entire relevant dissolution data)

Graphical abstract of model fit presented as the fraction % of released carvedilol per tested tablet:

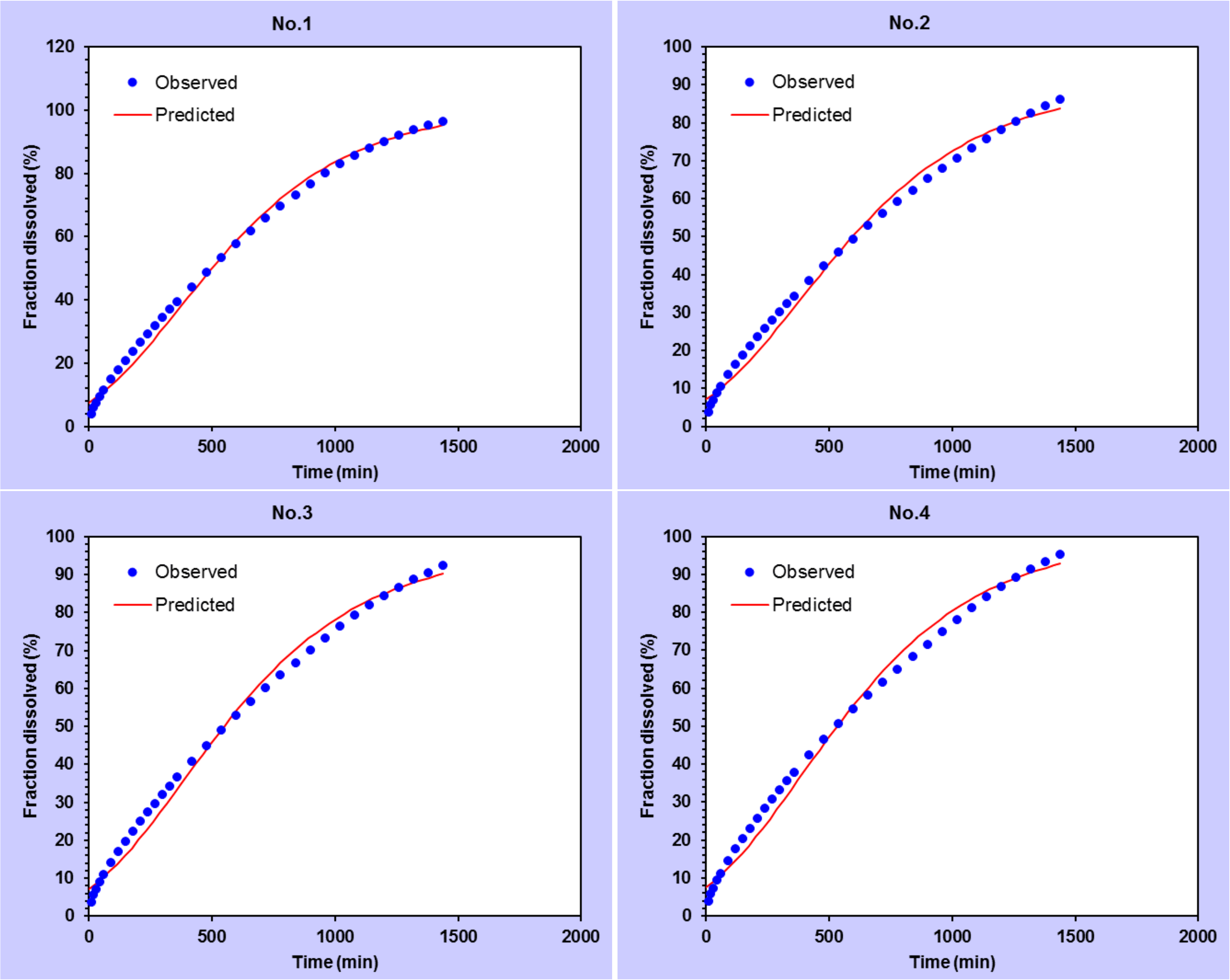

Supplementary materials – Model fitting summary – Di-Cafos® A12 (modelling the entire relevant dissolution data)

Model: **Gompertz_4**

Model equation: $F = F_{max} \cdot e^{-\beta \cdot e^{-k \cdot t}}$

Fitted model parameters per tested tablet (N = 4) with statistics – mean, standard deviation (SD), and relative standard deviation expressed in % (RSD%) (output from DDSolver):

Parameter	No.1	No.2	No.3	No.4	Mean	SD	RSD(%)
k	0.003	0.002	0.002	0.002	0.003	0.000	2.766
β	2.594	2.543	2.602	2.588	2.582	0.026	1.015
F_{max}	101.196	90.347	96.822	99.832	97.049	4.827	4.974

Number of dissolution data points (N), degrees of freedom (df), and selected goodness of fit criteria – Pearson correlation coefficient (R), coefficient of determination (R^2), adjusted coefficient of determination ($R^2_{adjusted}$), and residual sum of squares (RSS) (manual calculation in MS Excel):

Parameter	No.1	No.2	No.3	No.4
N	33	33	33	33
df	30	30	30	30
R	0.997455185	0.996200838	0.996309455	0.995947803
R^2	0.994916846	0.992416109	0.99263253	0.991912026
$R^2_{adjusted}$	0.994577969	0.991910517	0.992141366	0.991372827
RSS	179.6759639	204.4966425	234.6641974	271.1820649

Graphical abstract of model fit presented as mean ± 1 SD of the fraction % of released carvedilol:

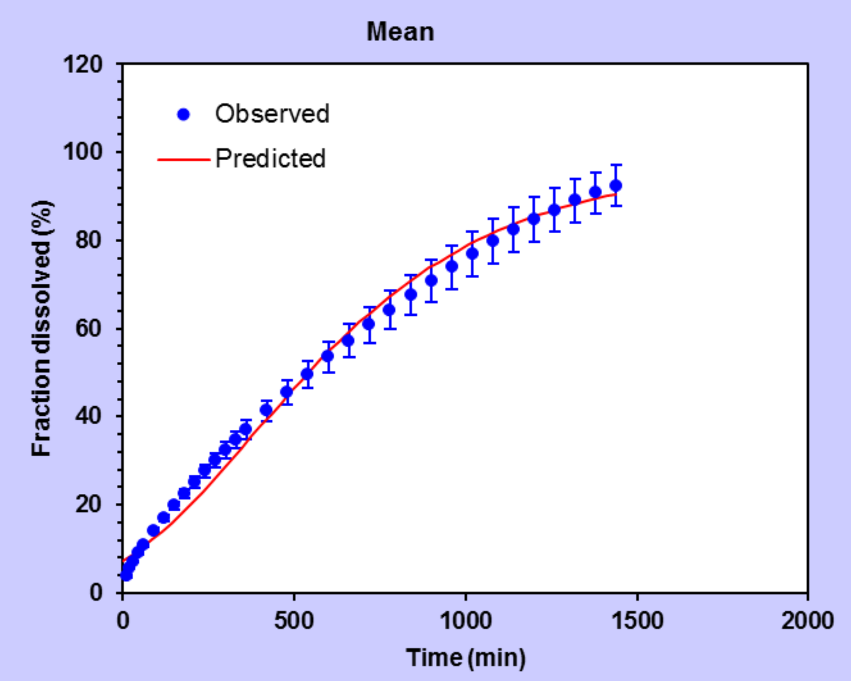

Supplementary materials – Model fitting summary – Di-Cafos® A12 (modelling the entire relevant dissolution data)

Graphical abstract of model fit presented as the fraction % of released carvedilol per tested tablet:

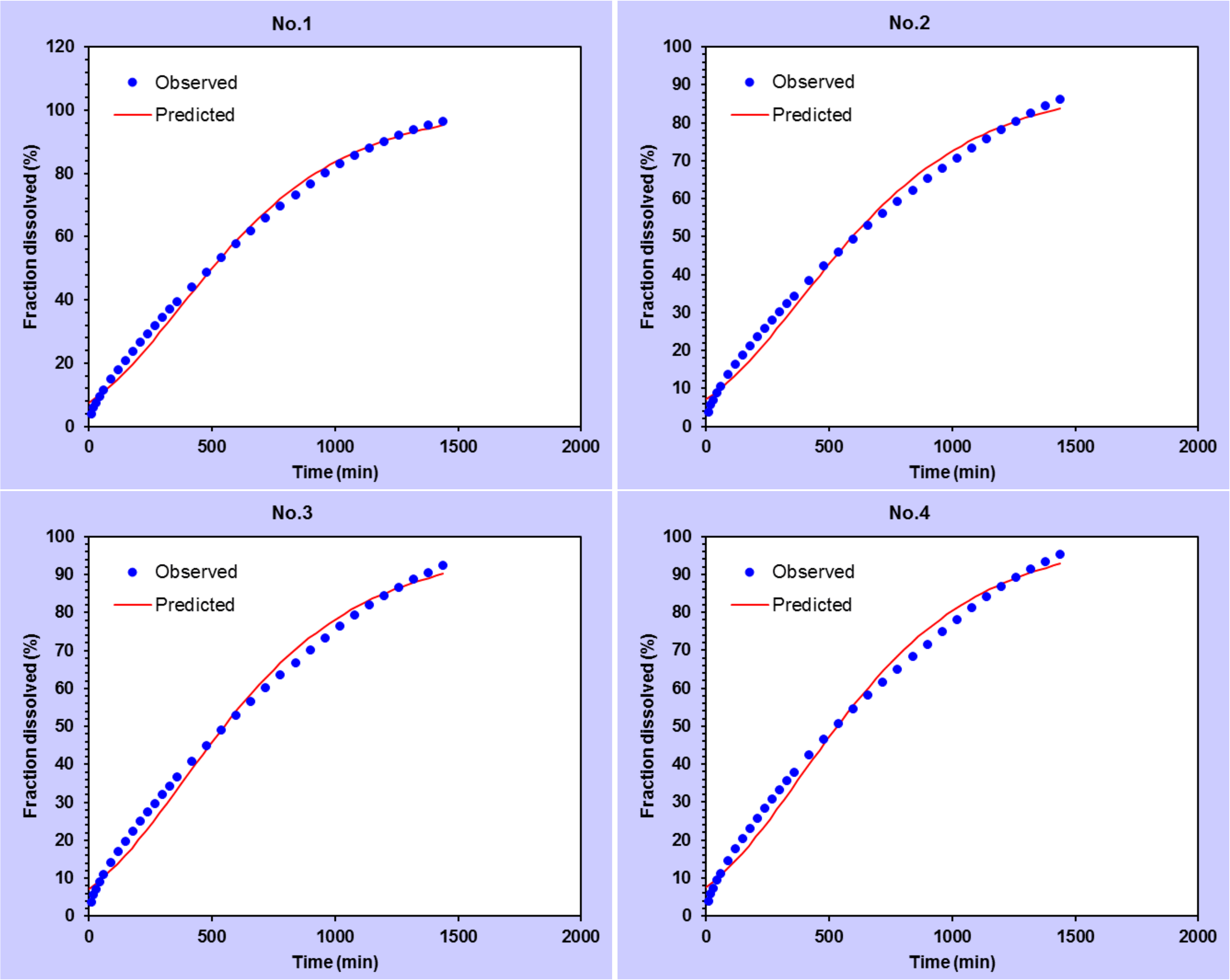

Supplementary materials – Model fitting summary – Di-Cafos® A12 (modelling the entire relevant dissolution data)

Model: **Probit_1**

Model equation: $F = 100 \cdot \phi[\alpha + \beta \cdot \log(t)]$

Fitted model parameters per tested tablet (N = 4) with statistics – mean, standard deviation (SD), and relative standard deviation expressed in % (RSD%) (output from DDSolver):

Parameter	No.1	No.2	No.3	No.4	Mean	SD	RSD(%)
α	-4.143	-4.224	-4.492	-3.975	-4.209	0.215	-5.118
β	1.652	1.517	1.639	1.553	1.590	0.066	4.130

Number of dissolution data points (N), degrees of freedom (df), and selected goodness of fit criteria – Pearson correlation coefficient (R), coefficient of determination (R^2), adjusted coefficient of determination ($R^2_{adjusted}$), and residual sum of squares (RSS) (manual calculation in MS Excel):

Parameter	No.1	No.2	No.3	No.4
N	33	33	33	33
df	31	31	31	31
R	0.966816468	0.987175028	0.986536514	0.967503466
R^2	0.934734083	0.974514537	0.973254293	0.936062956
$R^2_{adjusted}$	0.932628731	0.973692425	0.972391529	0.934000471
RSS	2447.519192	1263.735127	1823.279367	2150.661146

Graphical abstract of model fit presented as mean ± 1 SD of the fraction % of released carvedilol:

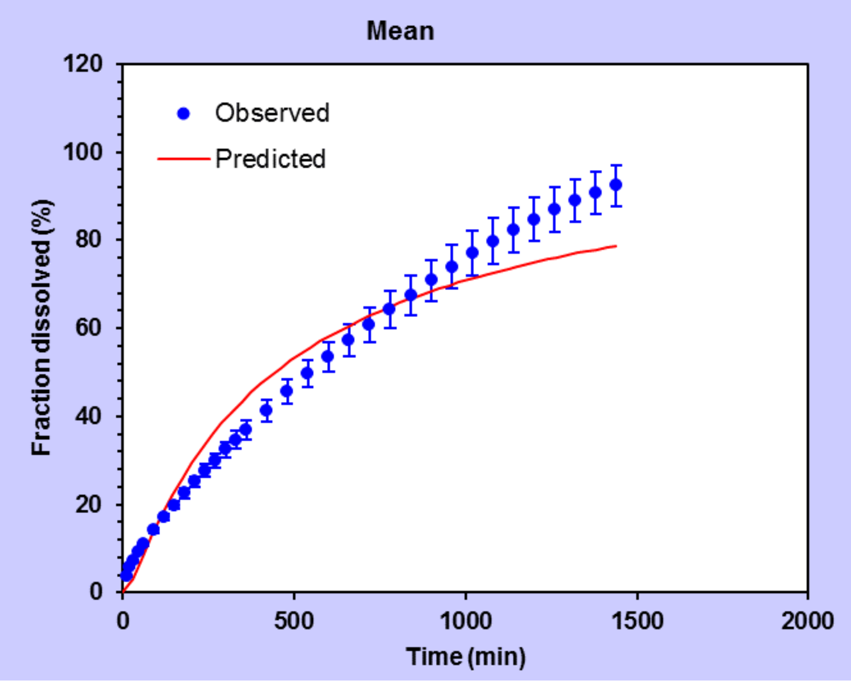

Supplementary materials – Model fitting summary – Di-Cafos® A12 (modelling the entire relevant dissolution data)

Graphical abstract of model fit presented as the fraction % of released carvedilol per tested tablet:

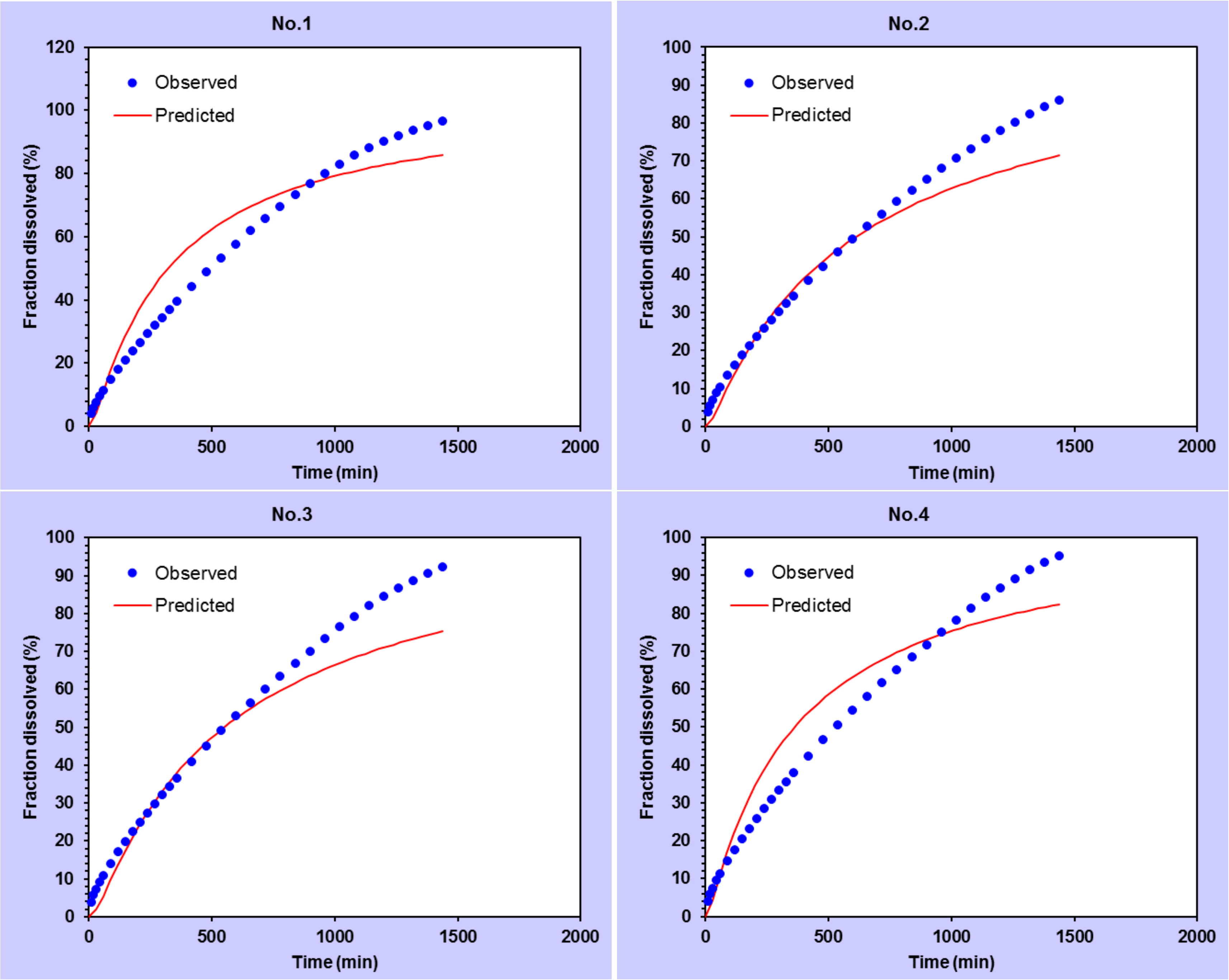

Supplementary materials – Model fitting summary – Di-Cafos® A12 (modelling the entire relevant dissolution data)

Model: **Probit_2**

Model equation: $F = F_{max} \cdot \phi[\alpha + \beta \cdot \log(t)]$

Fitted model parameters per tested tablet (N = 4) with statistics – mean, standard deviation (SD), and relative standard deviation expressed in % (RSD%) (output from DDSolver):

Parameter	No.1	No.2	No.3	No.4	Mean	SD	RSD(%)
α	-4.722	-4.544	-4.627	-4.600	-4.623	0.075	-1.613
β	1.722	1.638	1.668	1.657	1.671	0.036	2.165
F_{max}	109.775	98.006	105.030	108.295	105.277	5.237	4.974

Number of dissolution data points (N), degrees of freedom (df), and selected goodness of fit criteria – Pearson correlation coefficient (R), coefficient of determination (R^2), adjusted coefficient of determination ($R^2_{adjusted}$), and residual sum of squares (RSS) (manual calculation in MS Excel):

Parameter	No.1	No.2	No.3	No.4
N	33	33	33	33
df	30	30	30	30
R	0.990911109	0.989150755	0.989404678	0.989316364
R^2	0.981904826	0.978419216	0.978921616	0.978746867
$R^2_{adjusted}$	0.980698481	0.976980497	0.97751639	0.977329992
RSS	1336.886941	1199.18948	1402.252028	1486.961489

Graphical abstract of model fit presented as mean ± 1 SD of the fraction % of released carvedilol:

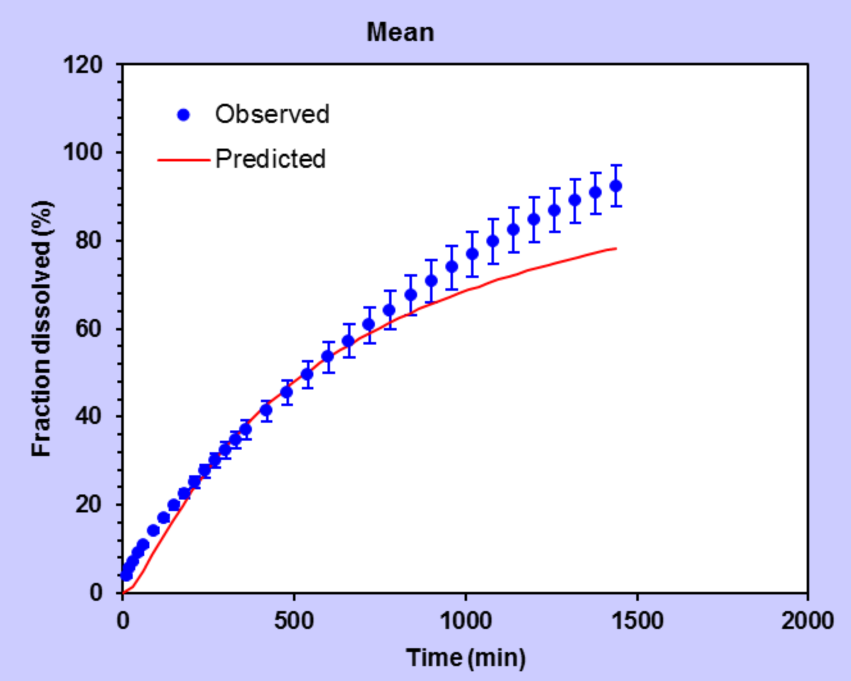

Supplementary materials – Model fitting summary – Di-Cafos® A12 (modelling the entire relevant dissolution data)

Graphical abstract of model fit presented as the fraction % of released carvedilol per tested tablet:

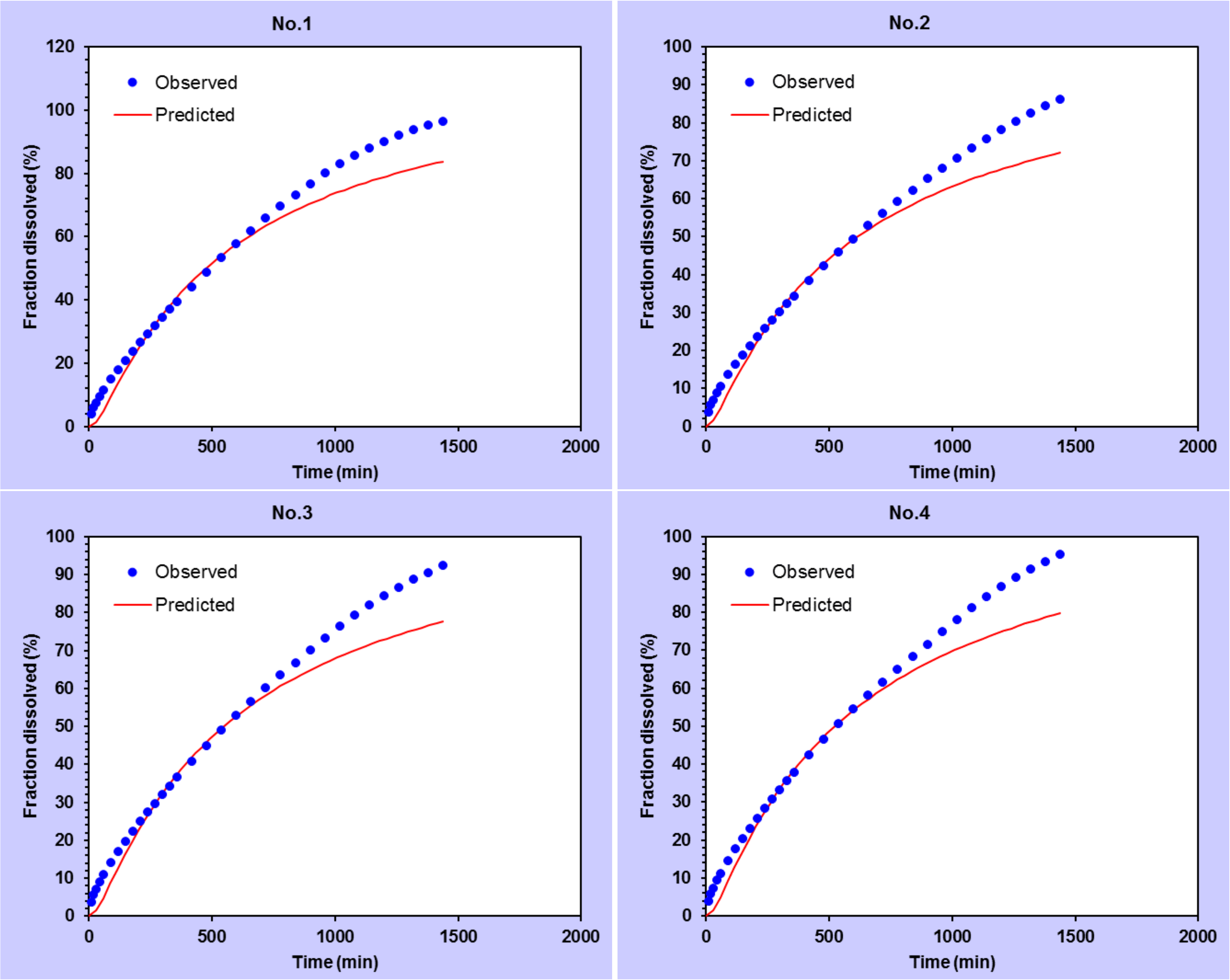

Supplementary materials – Model fitting summary – Di-Cafos® A12 (modelling up to app. 60 % of carvedilol released)

Model: **Zero–order**

Model equation: $F = k_0 \cdot t$

Fitted model parameters per tested tablet (N = 4) with statistics – mean, standard deviation (SD), and relative standard deviation expressed in % (RSD%) (output from DDSolver):

Parameter	No.1	No.2	No.3	No.4	Mean	SD	RSD(%)
k_0	0.101	0.088	0.093	0.096	0.095	0.006	6.070

Number of dissolution data points (N), degrees of freedom (df), and selected goodness of fit criteria – Pearson correlation coefficient (R), coefficient of determination (R^2), adjusted coefficient of determination ($R^2_{adjusted}$), and residual sum of squares (RSS) (manual calculation in MS Excel):

Parameter	No.1	No.2	No.3	No.4
N	21	21	21	21
df	20	20	20	20
R	0.995654453	0.994123283	0.994546734	0.994072946
R^2	0.99132779	0.988281102	0.989123207	0.988181021
$R^2_{adjusted}$	0.99132779	0.988281102	0.989123207	0.988181021
RSS	420.796285	407.2987379	416.6813538	463.2523335

Graphical abstract of model fit presented as mean ± 1 SD of the fraction % of released carvedilol:

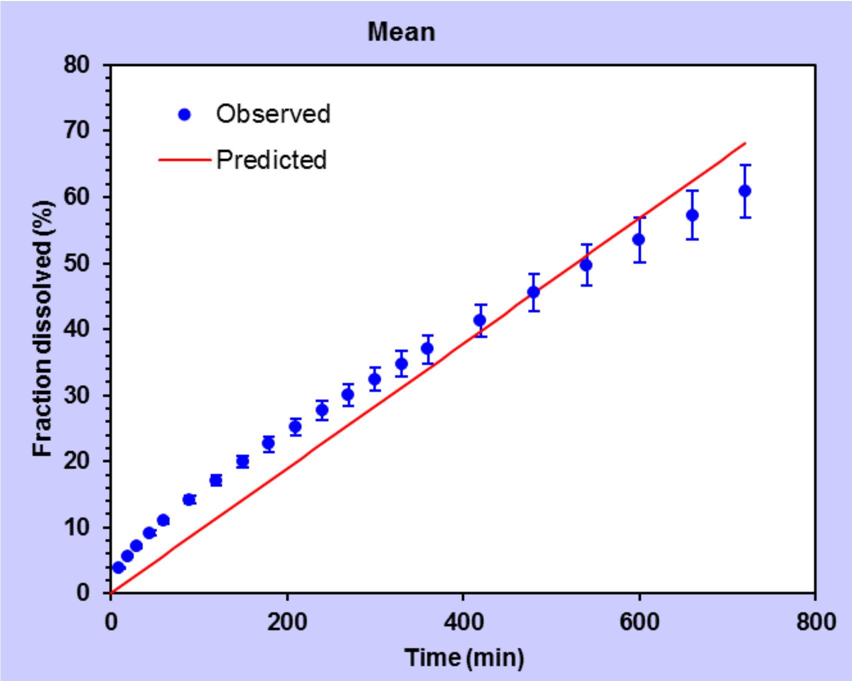

Supplementary materials – Model fitting summary – Di-Cafos® A12 (modelling up to app. 60 % of carvedilol released)

Graphical abstract of model fit presented as the fraction % of released carvedilol per tested tablet:

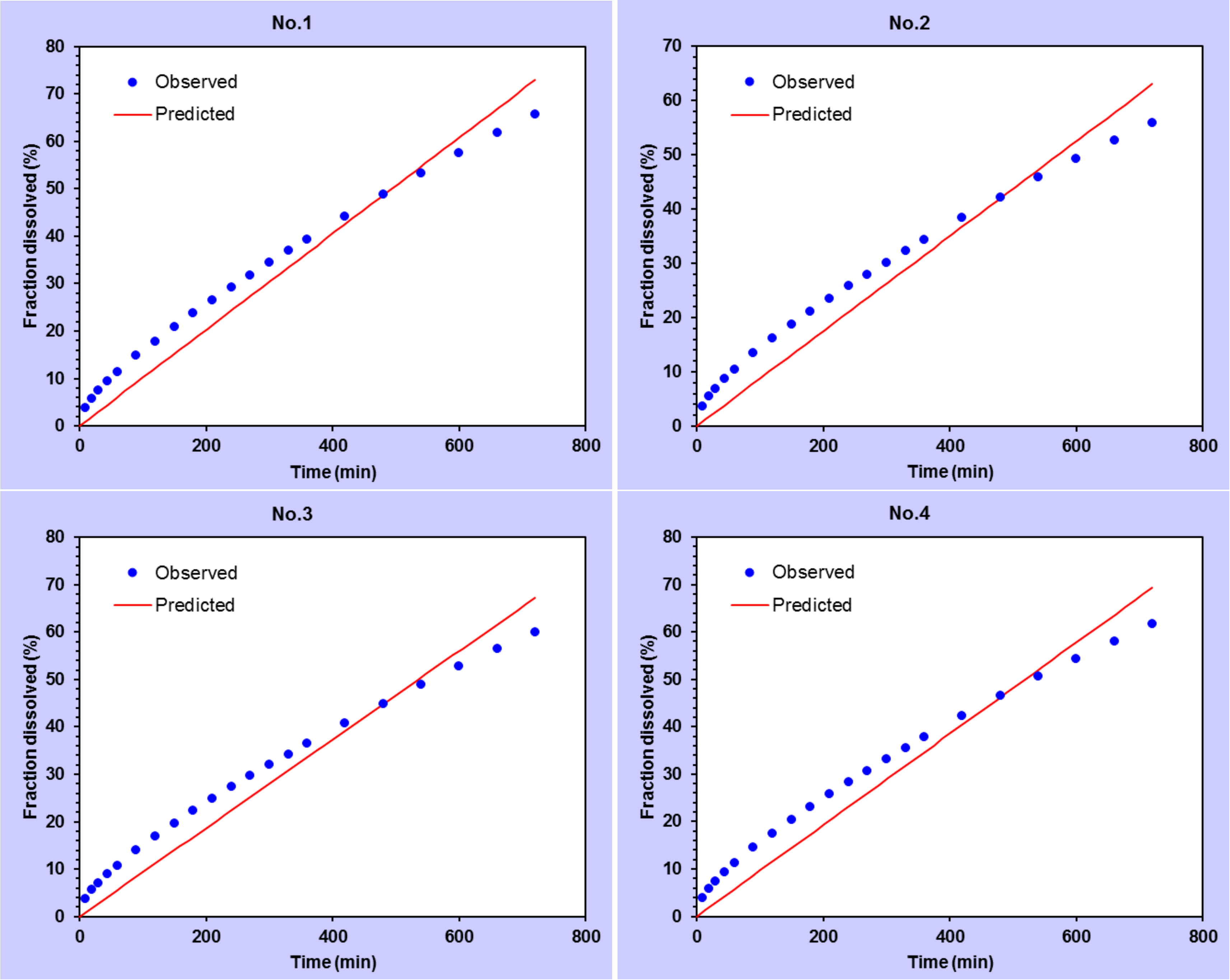

Supplementary materials – Model fitting summary – Di-Cafos® A12 (modelling up to app. 60 % of carvedilol released)

Model: **Zero–order with T_{lag}**

Model equation: $F = k_0 \cdot (t - T_{lag})$

Fitted model parameters per tested tablet (N = 4) with statistics – mean, standard deviation (SD), and relative standard deviation expressed in % (RSD%) (output from DDSolver):

Parameter	No.1	No.2	No.3	No.4	Mean	SD	RSD(%)
k_0	0.086	0.073	0.078	0.081	0.080	0.006	7.028
T_{lag}	-77.947	-90.967	-84.898	-86.821	-85.158	5.434	-6.381

Number of dissolution data points (N), degrees of freedom (df), and selected goodness of fit criteria – Pearson correlation coefficient (R), coefficient of determination (R^2), adjusted coefficient of determination ($R^2_{adjusted}$), and residual sum of squares (RSS) (manual calculation in MS Excel):

Parameter	No.1	No.2	No.3	No.4
N	21	21	21	21
df	19	19	19	19
R	0.995654453	0.994123283	0.994546734	0.994072946
R^2	0.99132779	0.988281102	0.989123207	0.988181021
$R^2_{adjusted}$	0.990871358	0.987664318	0.988550744	0.98755897
RSS	63.66263536	61.37075285	66.19997978	76.06093759

Graphical abstract of model fit presented as mean ± 1 SD of the fraction % of released carvedilol:

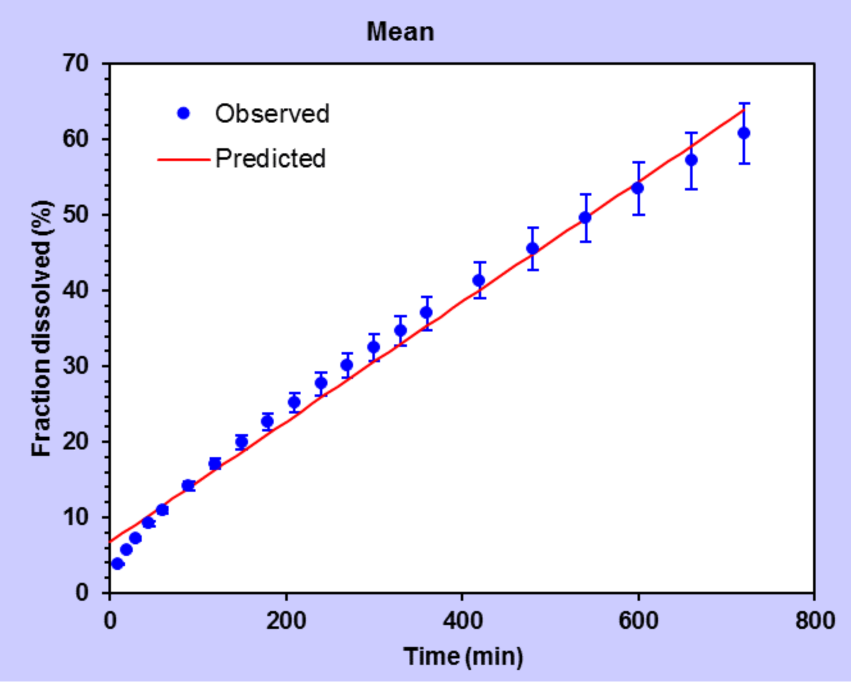

Supplementary materials – Model fitting summary – Di-Cafos® A12 (modelling up to app. 60 % of carvedilol released)

Graphical abstract of model fit presented as the fraction % of released carvedilol per tested tablet:

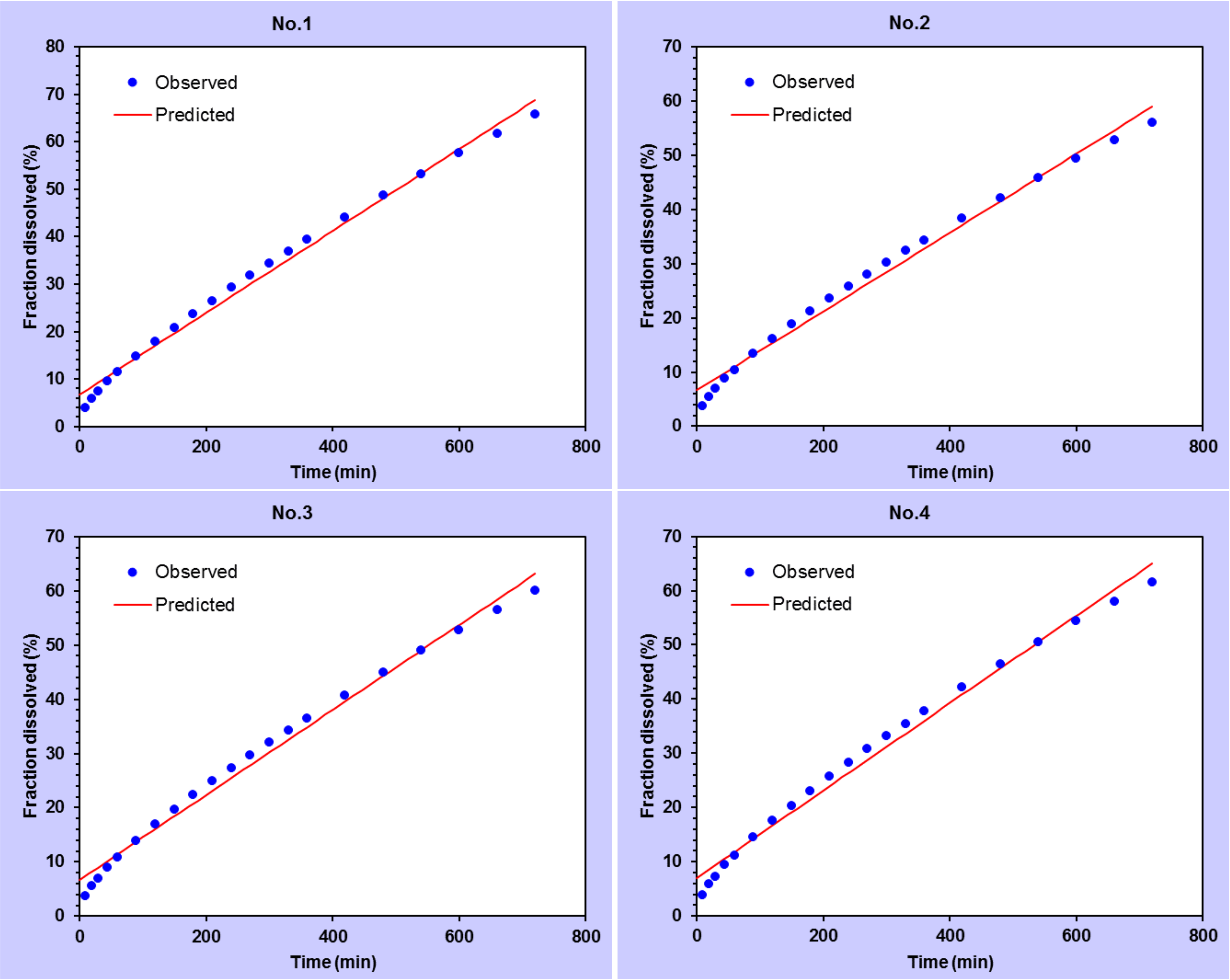

Supplementary materials – Model fitting summary – Di-Cafos® A12 (modelling up to app. 60 % of carvedilol released)

Model: **Zero–order with F_0**

Model equation: $F = F_0 + k_0 \cdot t$

Fitted model parameters per tested tablet (N = 4) with statistics – mean, standard deviation (SD), and relative standard deviation expressed in % (RSD%) (output from DDSolver):

Parameter	No.1	No.2	No.3	No.4	Mean	SD	RSD(%)
k_0	0.086	0.073	0.078	0.081	0.080	0.006	7.028
F_0	6.722	6.615	6.659	6.999	6.749	0.172	2.554

Number of dissolution data points (N), degrees of freedom (df), and selected goodness of fit criteria – Pearson correlation coefficient (R), coefficient of determination (R^2), adjusted coefficient of determination ($R^2_{adjusted}$), and residual sum of squares (RSS) (manual calculation in MS Excel):

Parameter	No.1	No.2	No.3	No.4
N	21	21	21	21
df	19	19	19	19
R	0.995654453	0.994123283	0.994546734	0.994072946
R^2	0.99132779	0.988281102	0.989123207	0.988181021
$R^2_{adjusted}$	0.990871358	0.987664318	0.988550744	0.98755897
RSS	63.66263536	61.37075285	66.19997978	76.06093759

Graphical abstract of model fit presented as mean ± 1 SD of the fraction % of released carvedilol:

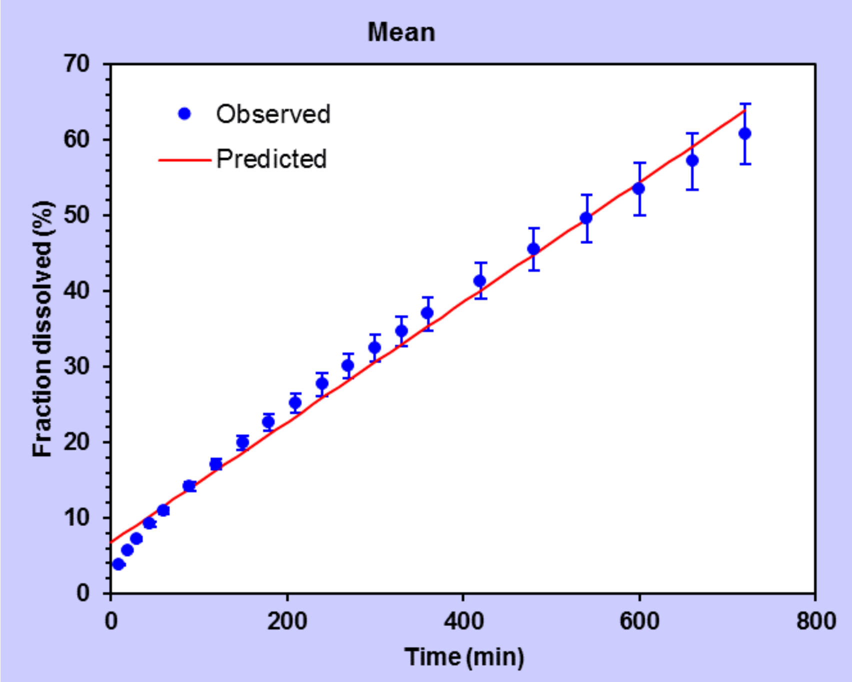

Supplementary materials – Model fitting summary – Di-Cafos® A12 (modelling up to app. 60 % of carvedilol released)

Graphical abstract of model fit presented as the fraction % of released carvedilol per tested tablet:

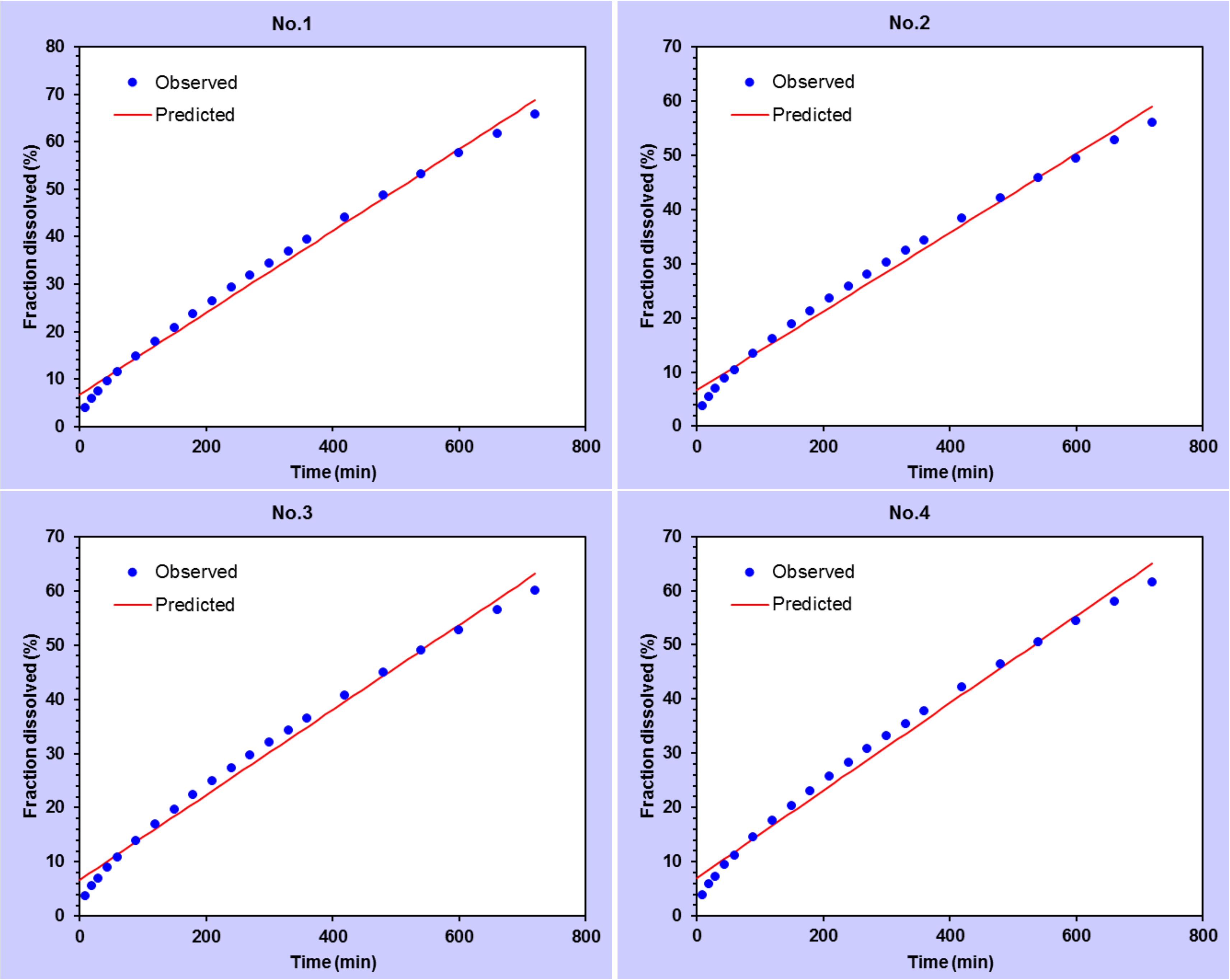

Supplementary materials – Model fitting summary – Di-Cafos® A12 (modelling up to app. 60 % of carvedilol released)

Model: **First–order**

Model equation: $F = 100 \cdot (1 - e^{-k_1 \cdot t})$

Fitted model parameters per tested tablet (N = 4) with statistics – mean, standard deviation (SD), and relative standard deviation expressed in % (RSD%) (output from DDSolver):

Parameter	No.1	No.2	No.3	No.4	Mean	SD	RSD(%)
k_1	0.001	0.001	0.001	0.001	0.001	0.000	8.929

Number of dissolution data points (N), degrees of freedom (df), and selected goodness of fit criteria – Pearson correlation coefficient (R), coefficient of determination (R^2), adjusted coefficient of determination ($R^2_{adjusted}$), and residual sum of squares (RSS) (manual calculation in MS Excel):

Parameter	No.1	No.2	No.3	No.4
N	21	21	21	21
df	20	20	20	20
R	0.998571581	0.999555165	0.999396773	0.999460667
R^2	0.997145203	0.999110527	0.99879391	0.998921625
$R^2_{adjusted}$	0.997145203	0.999110527	0.99879391	0.998921625
RSS	66.48426904	102.3246467	81.66992714	84.59863281

Graphical abstract of model fit presented as mean ± 1 SD of the fraction % of released carvedilol:

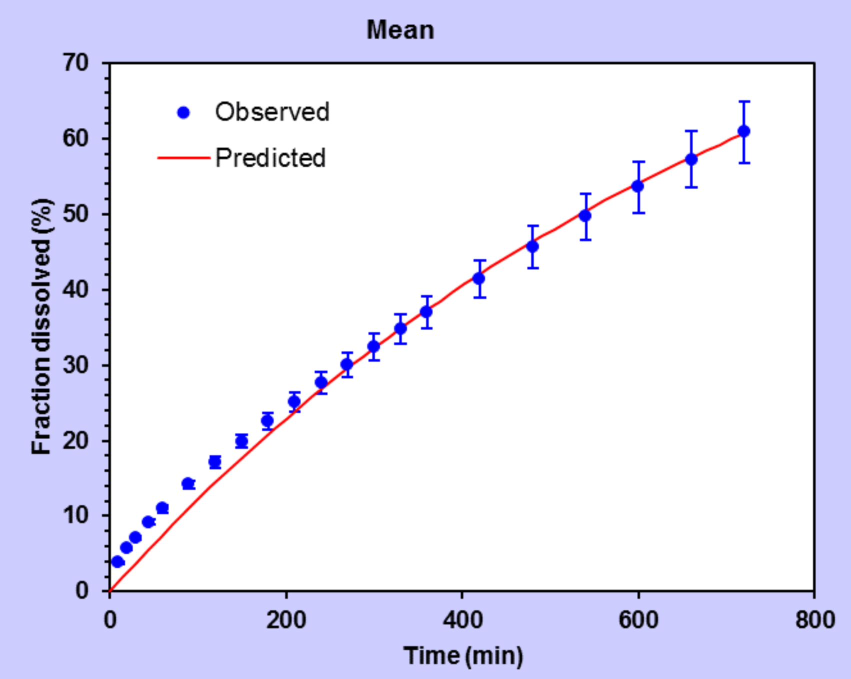

Supplementary materials – Model fitting summary – Di-Cafos® A12 (modelling up to app. 60 % of carvedilol released)

Graphical abstract of model fit presented as the fraction % of released carvedilol per tested tablet:

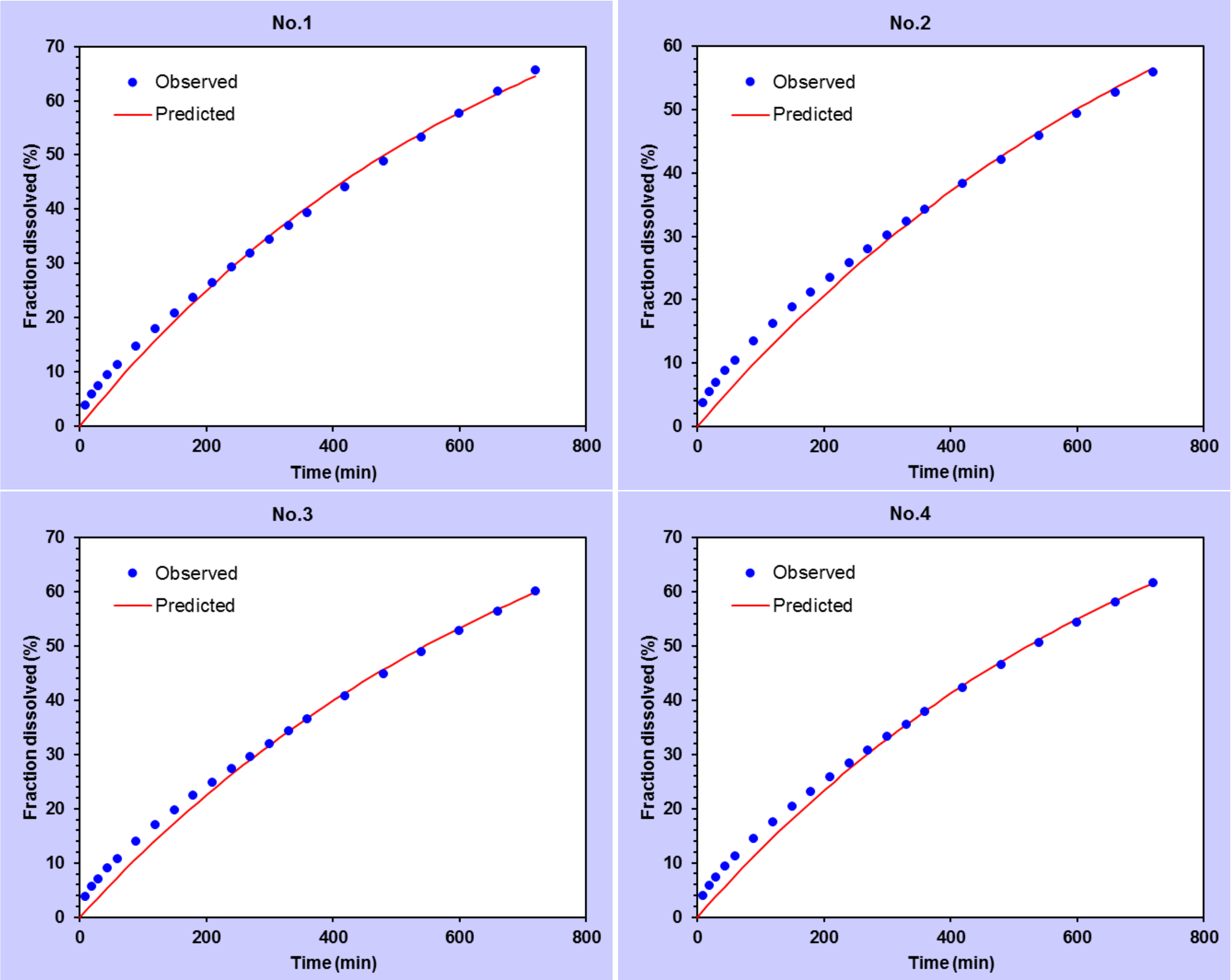

Supplementary materials – Model fitting summary – Di-Cafos® A12 (modelling up to app. 60 % of carvedilol released)

Model: **First–order with T_{lag}**

Model equation: $F = 100 \cdot \left[1 - e^{-k_1 \cdot (t - T_{lag})}\right]$

Fitted model parameters per tested tablet (N = 4) with statistics – mean, standard deviation (SD), and relative standard deviation expressed in % (RSD%) (output from DDSolver):

Parameter	No.1	No.2	No.3	No.4	Mean	SD	RSD(%)
k_1	0.001	0.001	0.001	0.001	0.001	0.000	10.795
T_{lag}	-16.168	-37.990	-28.588	-28.039	-27.696	8.940	-32.279

Number of dissolution data points (N), degrees of freedom (df), and selected goodness of fit criteria – Pearson correlation coefficient (R), coefficient of determination (R^2), adjusted coefficient of determination ($R^2_{adjusted}$), and residual sum of squares (RSS) (manual calculation in MS Excel):

Parameter	No.1	No.2	No.3	No.4
N	21	21	21	21
df	19	19	19	19
R	0.998779972	0.999574978	0.999519443	0.999587594
R^2	0.997561432	0.999150137	0.999039118	0.999175358
$R^2_{adjusted}$	0.997433086	0.999105407	0.998988545	0.999131955
RSS	20.65488314	4.452152069	6.087092591	5.572081931

Graphical abstract of model fit presented as mean ± 1 SD of the fraction % of released carvedilol:

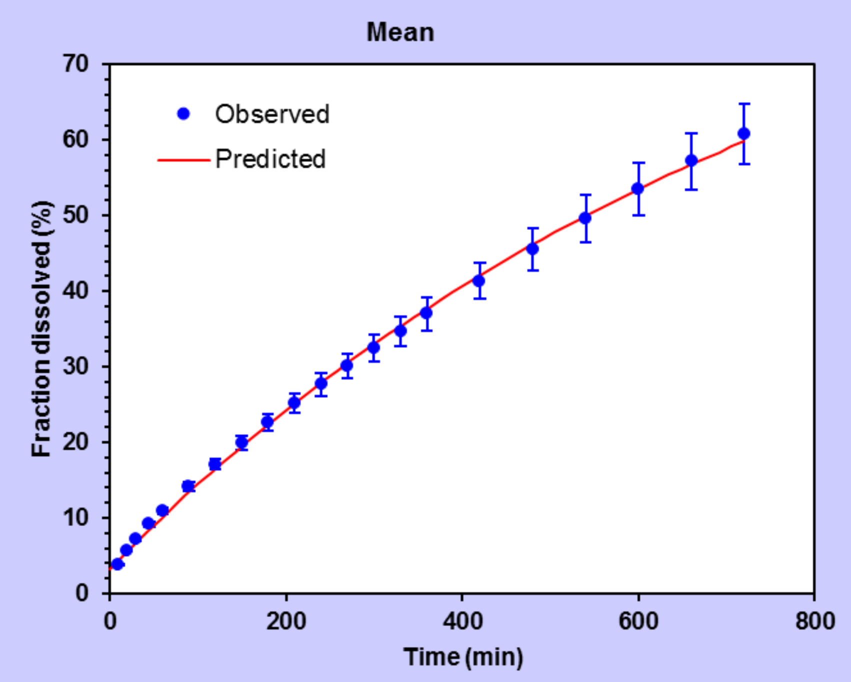

Supplementary materials – Model fitting summary – Di-Cafos® A12 (modelling up to app. 60 % of carvedilol released)

Graphical abstract of model fit presented as the fraction % of released carvedilol per tested tablet:

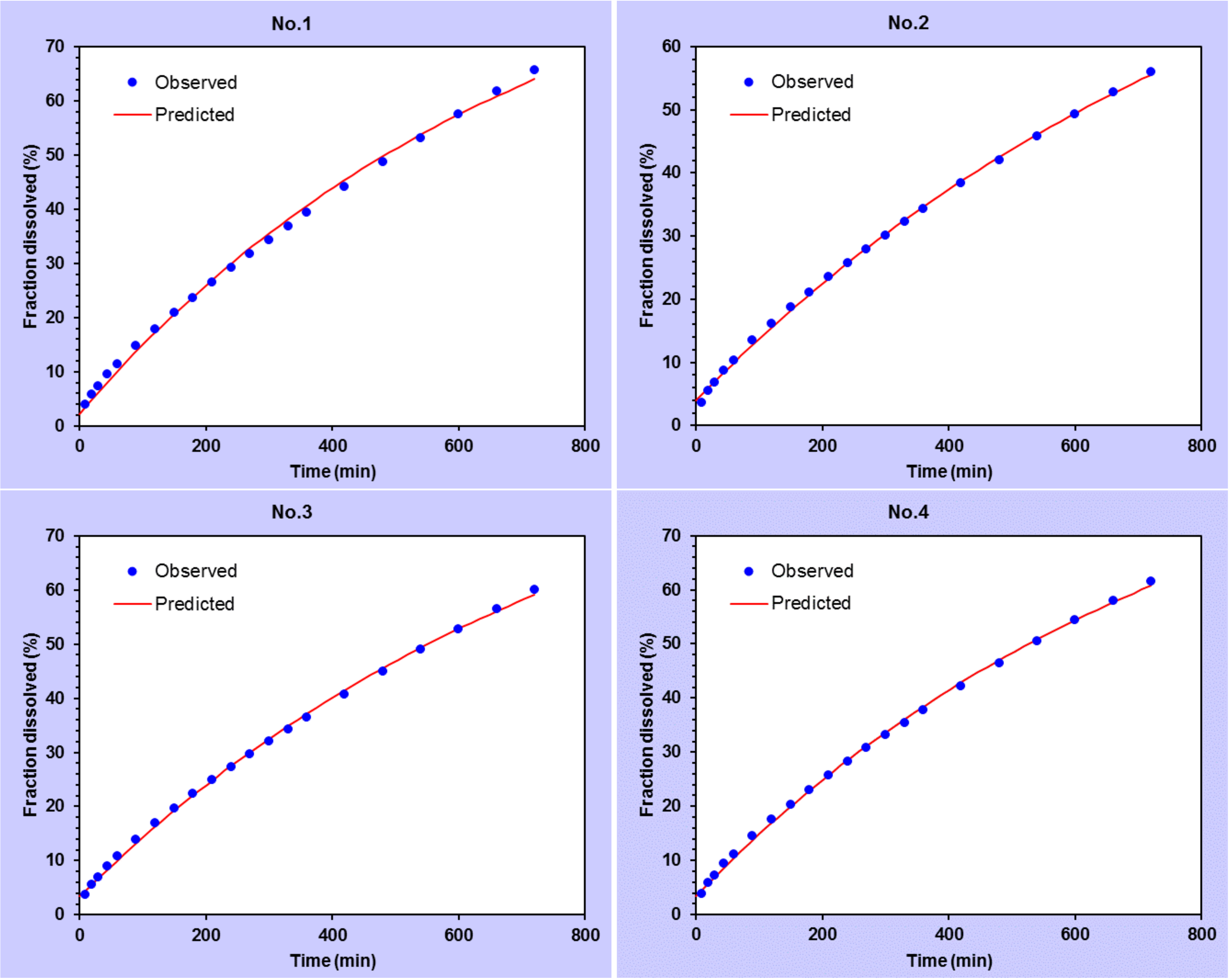

Supplementary materials – Model fitting summary – Di-Cafos® A12 (modelling up to app. 60 % of carvedilol released)

Model: **First–order with F_{max}**

Model equation: $F = F_{max} \cdot (1 - e^{-k_1 \cdot t})$

Fitted model parameters per tested tablet (N = 4) with statistics – mean, standard deviation (SD), and relative standard deviation expressed in % (RSD%) (output from DDSolver):

Parameter	No.1	No.2	No.3	No.4	Mean	SD	RSD(%)
k_1	0.003	0.003	0.003	0.003	0.003	0.000	0.879
F_{max}	68.971	58.770	63.037	64.680	63.865	4.218	6.605

Number of dissolution data points (N), degrees of freedom (df), and selected goodness of fit criteria – Pearson correlation coefficient (R), coefficient of determination (R^2), adjusted coefficient of determination ($R^2_{adjusted}$), and residual sum of squares (RSS) (manual calculation in MS Excel):

Parameter	No.1	No.2	No.3	No.4
N	21	21	21	21
df	19	19	19	19
R	0.982356808	0.984171015	0.983894784	0.984412226
R^2	0.965024897	0.968592587	0.968048946	0.969067431
$R^2_{adjusted}$	0.963184103	0.966939565	0.966367312	0.967439402
RSS	428.8309885	261.4609065	317.0532264	322.3293208

Graphical abstract of model fit presented as mean ± 1 SD of the fraction % of released carvedilol:

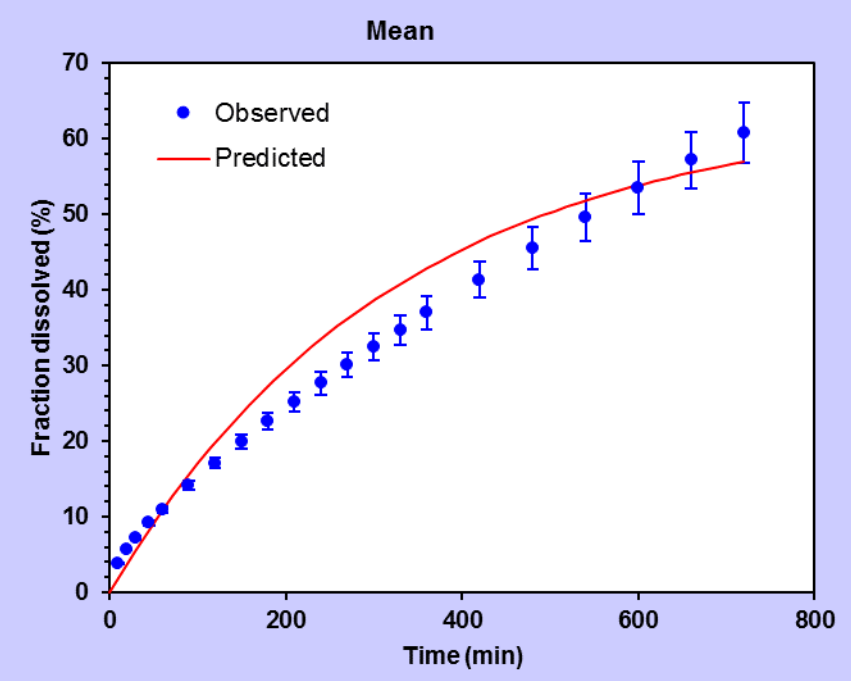

Supplementary materials – Model fitting summary – Di-Cafos® A12 (modelling up to app. 60 % of carvedilol released)

Graphical abstract of model fit presented as the fraction % of released carvedilol per tested tablet:

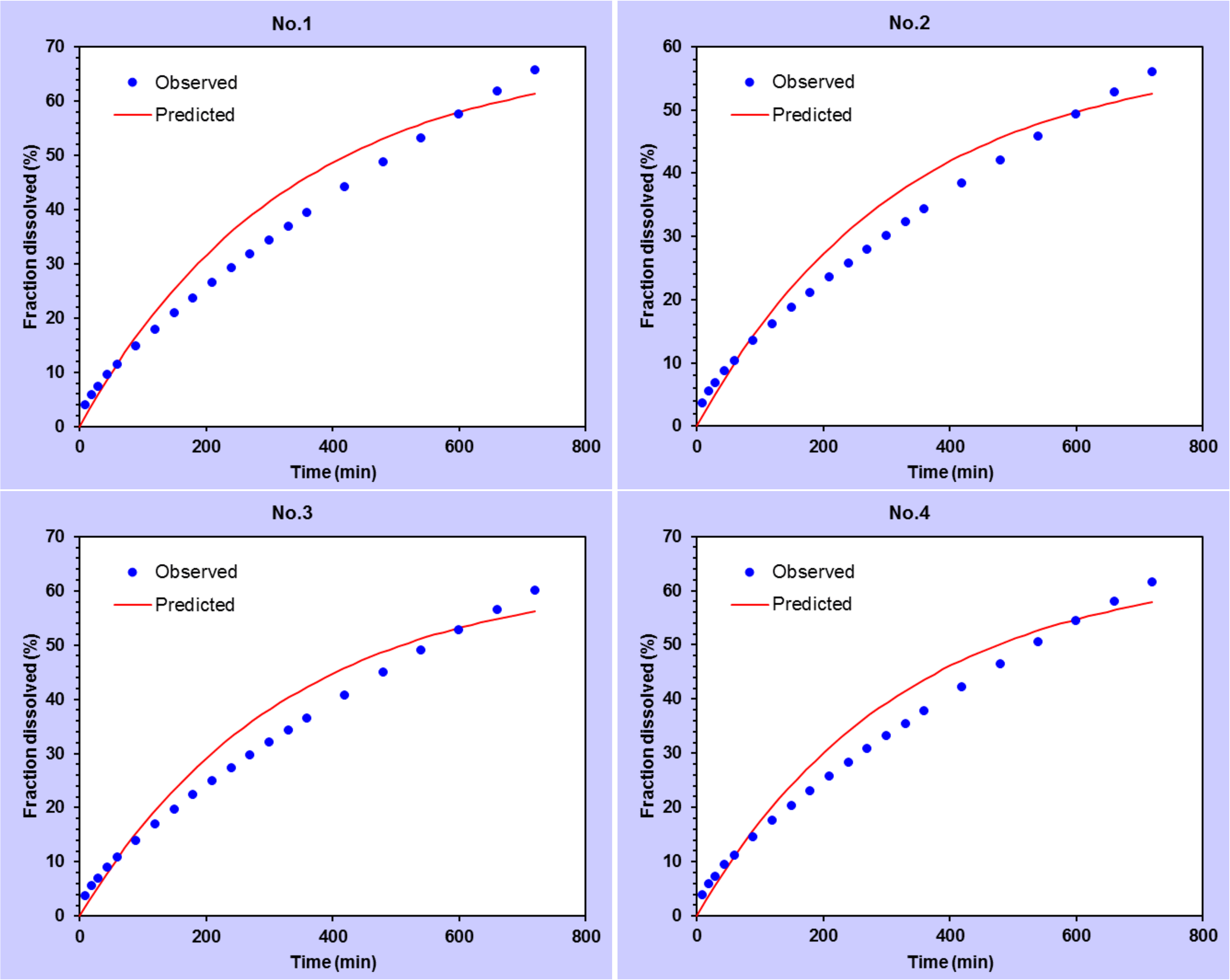

Supplementary materials – Model fitting summary – Di-Cafos® A12 (modelling up to app. 60 % of carvedilol released)

Model: **First–order with T_{lag} and F_{max}**

Model equation: $F = F_{max} \cdot \left[1 - e^{-k_1 \cdot (t - T_{lag})}\right]$

Fitted model parameters per tested tablet (N = 4) with statistics – mean, standard deviation (SD), and relative standard deviation expressed in % (RSD%) (output from DDSolver):

Parameter	No.1	No.2	No.3	No.4	Mean	SD	RSD(%)
k_1	0.003	0.003	0.003	0.003	0.003	0.000	0.352
T_{lag}	43.237	37.524	39.908	39.018	39.922	2.419	6.060
F_{max}	68.971	58.770	63.037	64.680	63.865	4.218	6.605

Number of dissolution data points (N), degrees of freedom (df), and selected goodness of fit criteria – Pearson correlation coefficient (R), coefficient of determination (R^2), adjusted coefficient of determination ($R^2_{adjusted}$), and residual sum of squares (RSS) (manual calculation in MS Excel):

Parameter	No.1	No.2	No.3	No.4
N	21	21	21	21
df	18	18	18	18
R	0.977289533	0.980048619	0.979455339	0.980106791
R^2	0.955094831	0.960495296	0.959332761	0.960609323
$R^2_{adjusted}$	0.950105368	0.956105884	0.954814179	0.956232581
RSS	795.9962544	504.7689099	602.5448194	616.7118924

Graphical abstract of model fit presented as mean ± 1 SD of the fraction % of released carvedilol:

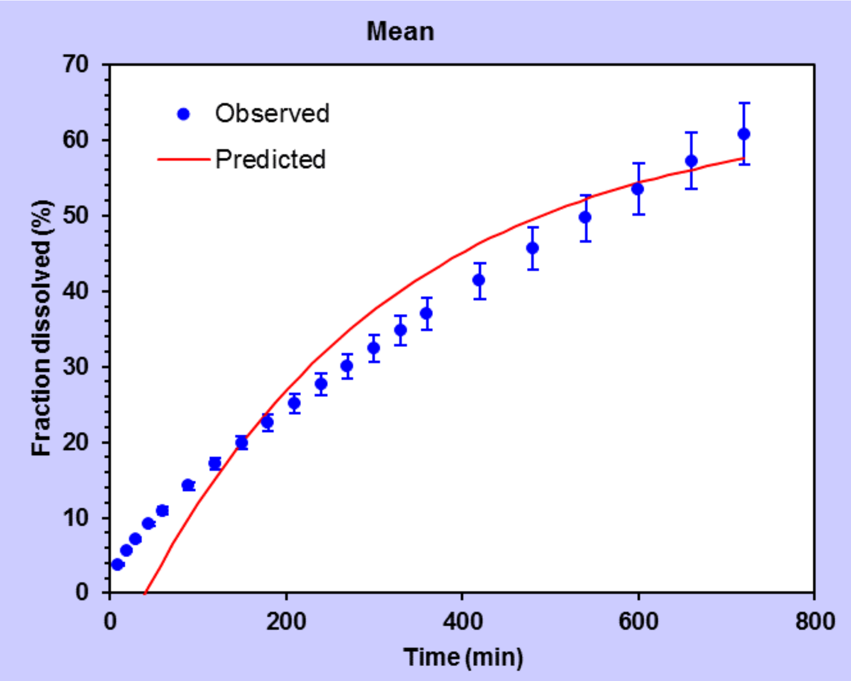

Supplementary materials – Model fitting summary – Di-Cafos® A12 (modelling up to app. 60 % of carvedilol released)

Graphical abstract of model fit presented as the fraction % of released carvedilol per tested tablet:

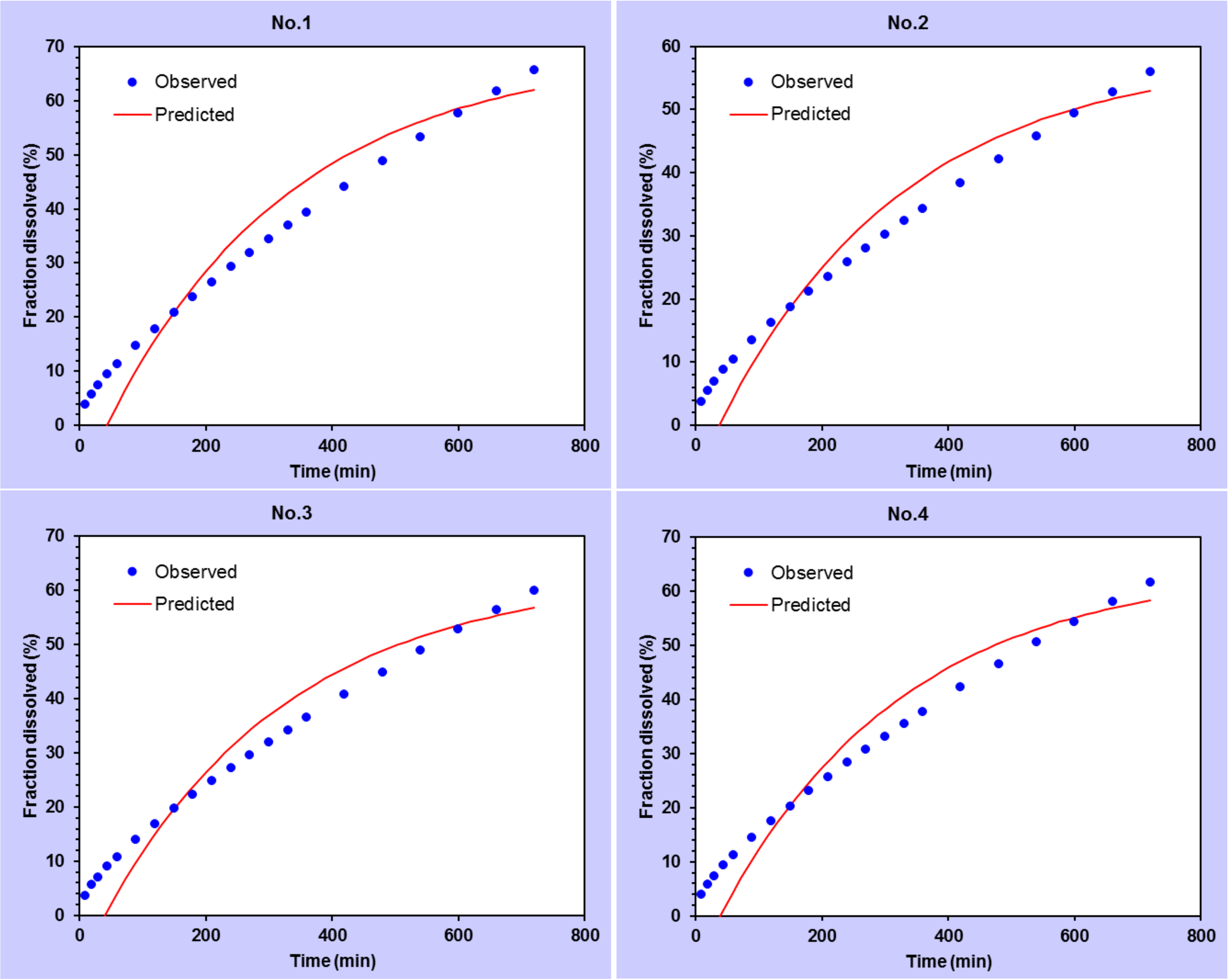

Supplementary materials – Model fitting summary – Di-Cafos® A12 (modelling up to app. 60 % of carvedilol released)

Model: **Higuchi**

Model equation: $F = k_H \cdot t^{0.5}$

Fitted model parameters per tested tablet (N = 4) with statistics – mean, standard deviation (SD), and relative standard deviation expressed in % (RSD%) (output from DDSolver):

Parameter	No.1	No.2	No.3	No.4	Mean	SD	RSD(%)
k_H	2.139	1.856	1.976	2.040	2.003	0.118	5.907

Number of dissolution data points (N), degrees of freedom (df), and selected goodness of fit criteria – Pearson correlation coefficient (R), coefficient of determination (R^2), adjusted coefficient of determination ($R^2_{adjusted}$), and residual sum of squares (RSS) (manual calculation in MS Excel):

Parameter	No.1	No.2	No.3	No.4
N	21	21	21	21
df	20	20	20	20
R	0.992079324	0.993872266	0.993429341	0.993831926
R^2	0.984221386	0.987782082	0.986901855	0.987701898
$R^2_{adjusted}$	0.984221386	0.987782082	0.986901855	0.987701898
RSS	424.2558579	238.037904	307.4300283	312.4018467

Graphical abstract of model fit presented as mean ± 1 SD of the fraction % of released carvedilol:

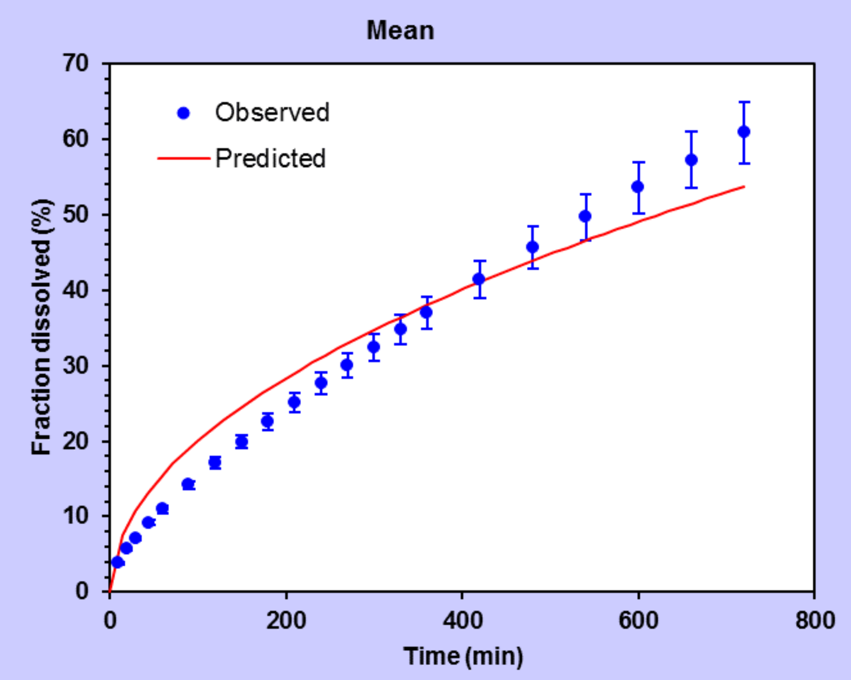

Supplementary materials – Model fitting summary – Di-Cafos® A12 (modelling up to app. 60 % of carvedilol released)

Graphical abstract of model fit presented as the fraction % of released carvedilol per tested tablet:

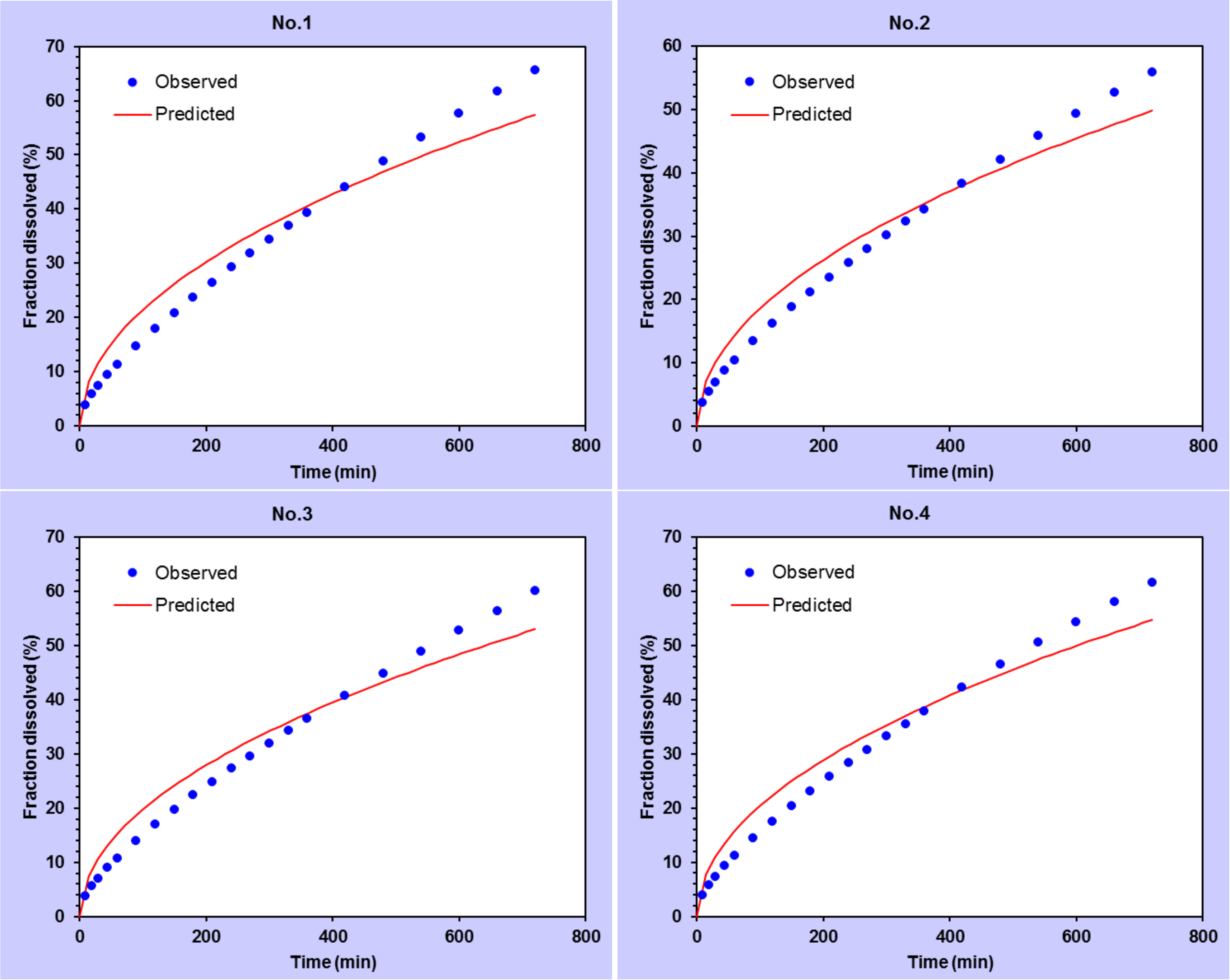

Supplementary materials – Model fitting summary – Di-Cafos® A12 (modelling up to app. 60 % of carvedilol released)

Model: **Higuchi with T_{lag}**

Model equation: $F = k_H \cdot (t - T_{lag})^{0.5}$

Fitted model parameters per tested tablet (N = 4) with statistics – mean, standard deviation (SD), and relative standard deviation expressed in % (RSD%) (output from DDSolver):

Parameter	No.1	No.2	No.3	No.4	Mean	SD	RSD(%)
k_H	2.433	2.078	2.226	2.290	2.257	0.147	6.531
T_{lag}	59.711	53.396	55.992	54.710	55.952	2.721	4.863

Number of dissolution data points (N), degrees of freedom (df), and selected goodness of fit criteria – Pearson correlation coefficient (R), coefficient of determination (R^2), adjusted coefficient of determination ($R^2_{adjusted}$), and residual sum of squares (RSS) (manual calculation in MS Excel):

Parameter	No.1	No.2	No.3	No.4
N	21	21	21	21
df	19	19	19	19
R	0.981877855	0.984861106	0.984649937	0.985112518
R^2	0.964084122	0.969951399	0.969535498	0.970446673
$R^2_{adjusted}$	0.962193813	0.968369893	0.967932104	0.968891235
RSS	397.198811	249.566867	284.8428251	293.5453384

Graphical abstract of model fit presented as mean ± 1 SD of the fraction % of released carvedilol:

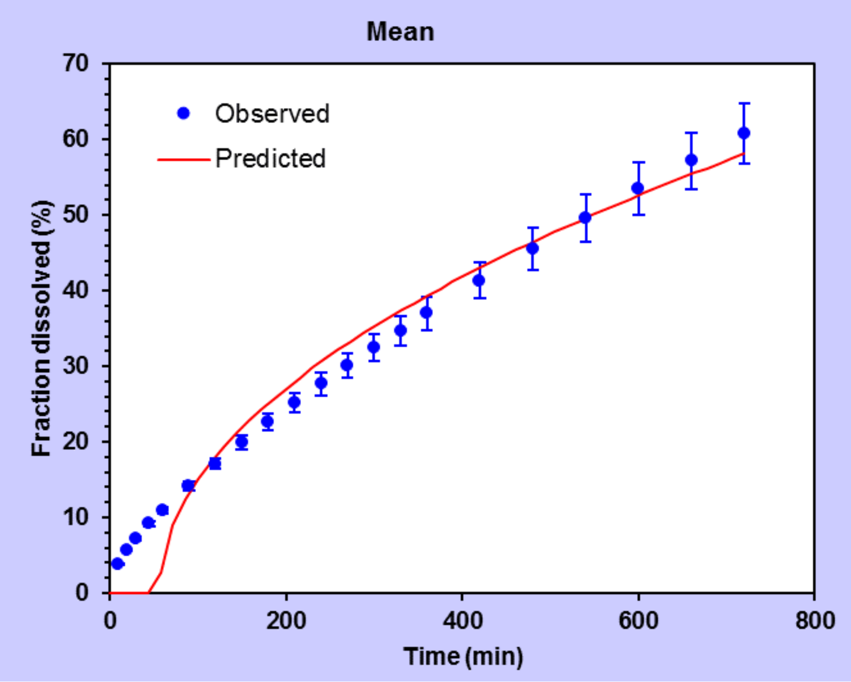

Supplementary materials – Model fitting summary – Di-Cafos® A12 (modelling up to app. 60 % of carvedilol released)

Graphical abstract of model fit presented as the fraction % of released carvedilol per tested tablet:

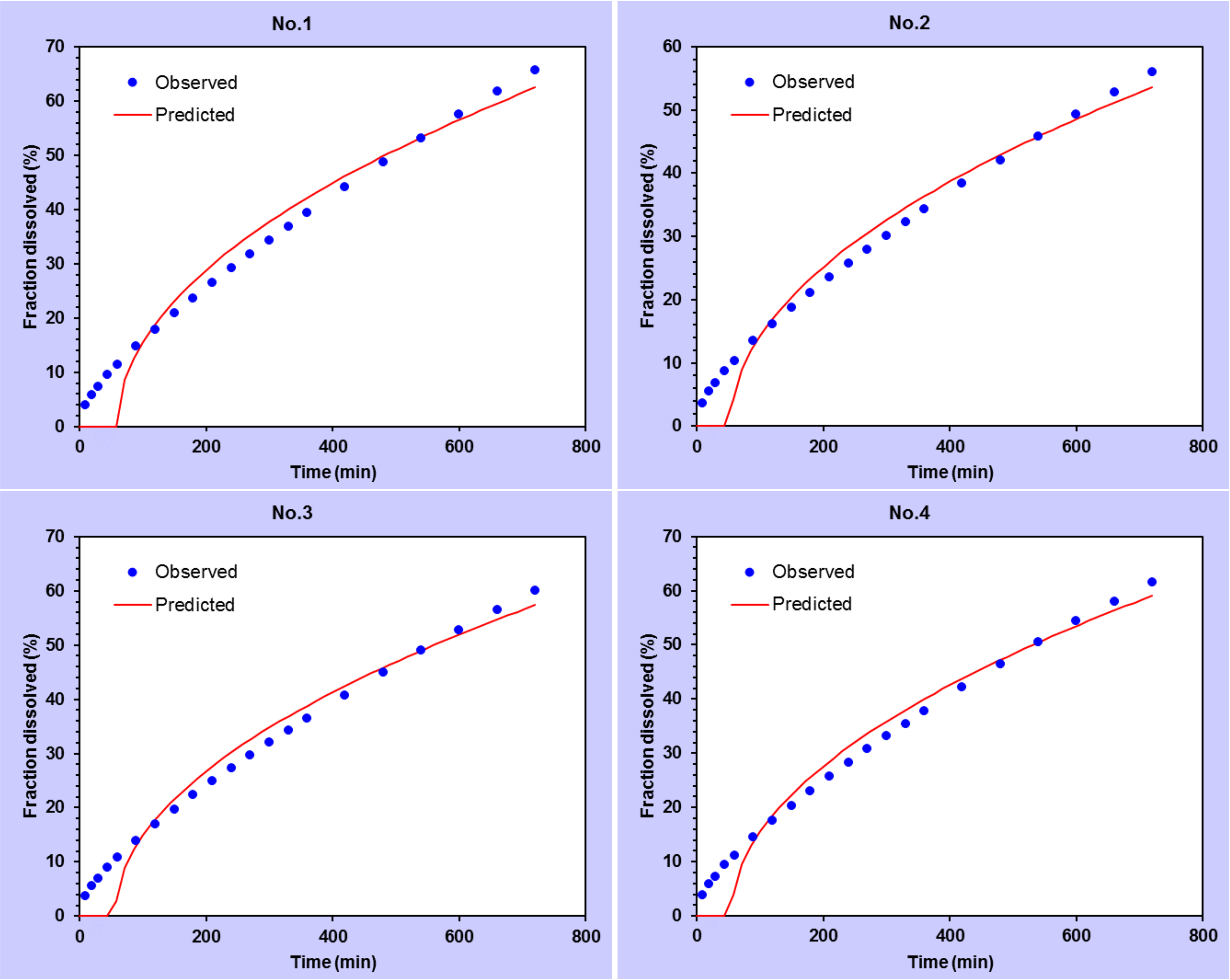

Supplementary materials – Model fitting summary – Di-Cafos® A12 (modelling up to app. 60 % of carvedilol released)

Model: **Higuchi with F_0**

Model equation: $F = F_0 + k_H \cdot t^{0.5}$

Fitted model parameters per tested tablet (N = 4) with statistics – mean, standard deviation (SD), and relative standard deviation expressed in % (RSD%) (output from DDSolver):

Parameter	No.1	No.2	No.3	No.4	Mean	SD	RSD(%)
k_H	2.631	2.226	2.399	2.468	2.431	0.168	6.897
F_0	-9.062	-6.807	-7.786	-7.881	-7.884	0.923	-11.707

Number of dissolution data points (N), degrees of freedom (df), and selected goodness of fit criteria – Pearson correlation coefficient (R), coefficient of determination (R^2), adjusted coefficient of determination ($R^2_{adjusted}$), and residual sum of squares (RSS) (manual calculation in MS Excel):

Parameter	No.1	No.2	No.3	No.4
N	21	21	21	21
df	19	19	19	19
R	0.992079324	0.993872266	0.993429341	0.993831926
R^2	0.984221386	0.987782082	0.986901855	0.987701898
$R^2_{adjusted}$	0.983390932	0.987139033	0.986212479	0.987054629
RSS	115.8306973	63.98407403	79.71990761	79.14433258

Graphical abstract of model fit presented as mean ± 1 SD of the fraction % of released carvedilol:

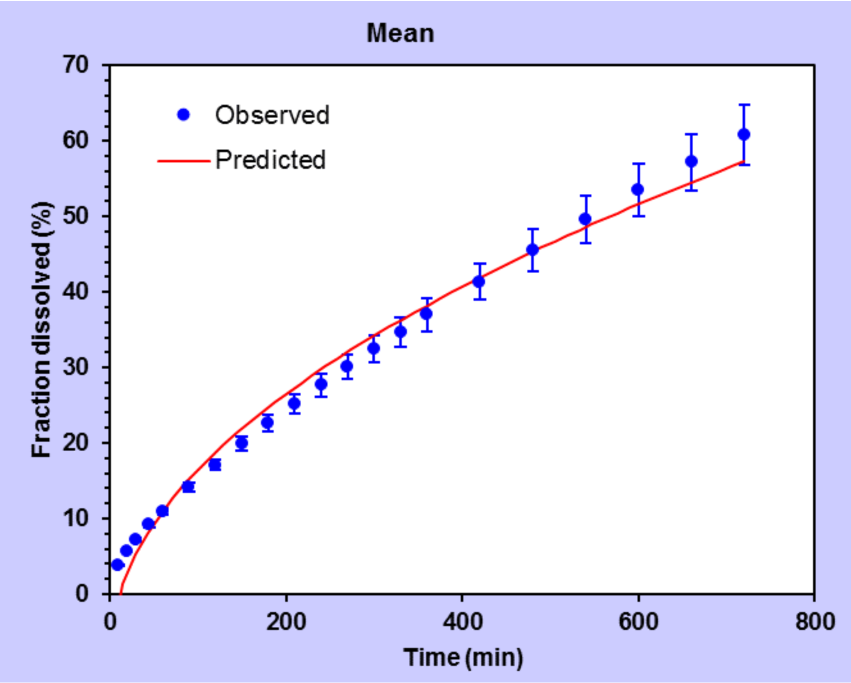

Supplementary materials – Model fitting summary – Di-Cafos® A12 (modelling up to app. 60 % of carvedilol released)

Graphical abstract of model fit presented as the fraction % of released carvedilol per tested tablet:

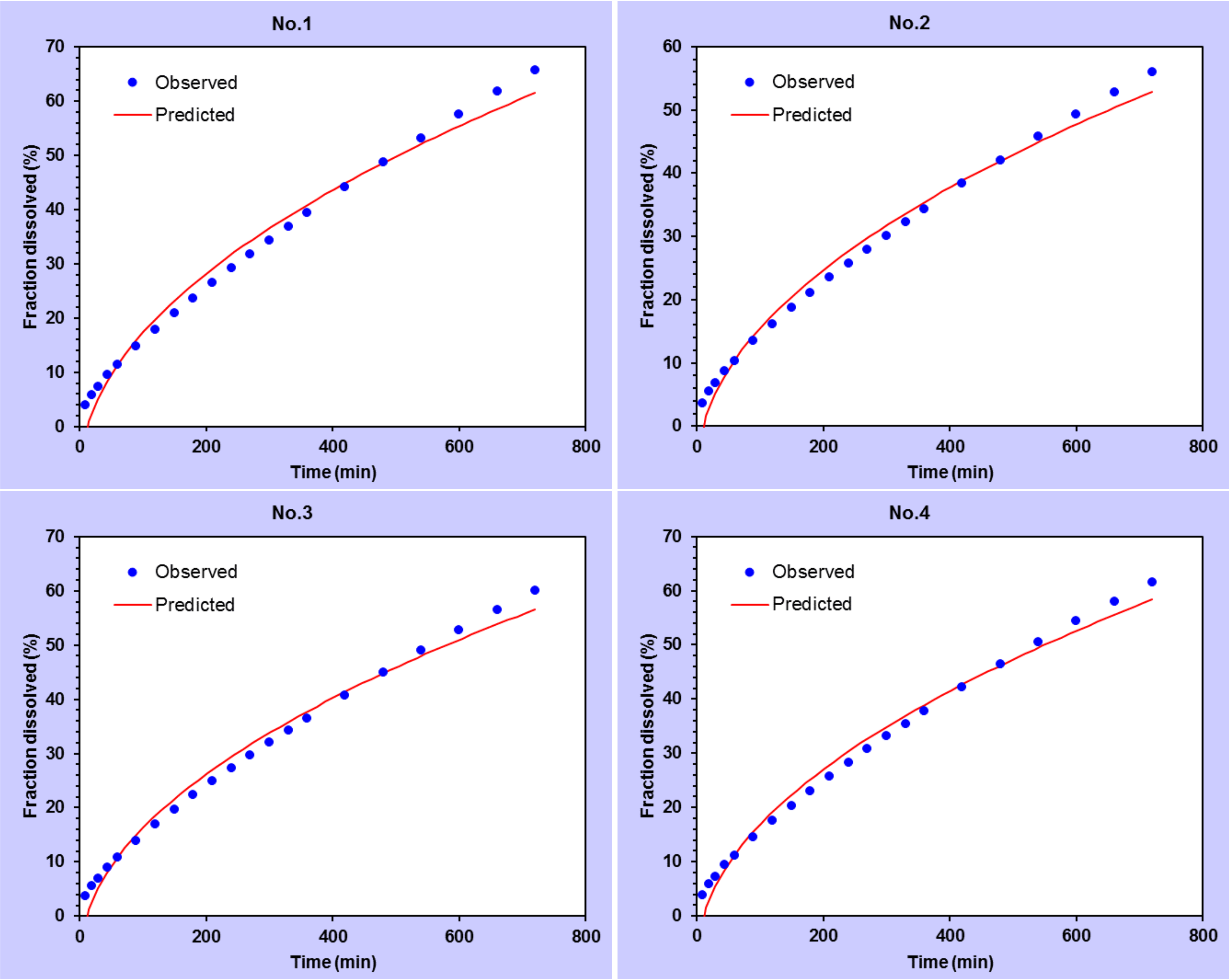

Supplementary materials – Model fitting summary – Di-Cafos® A12 (modelling up to app. 60 % of carvedilol released)

Model: **Korsmeyer–Peppas**

Model equation: $F = k_{KP} \cdot t^n$

Fitted model parameters per tested tablet (N = 4) with statistics – mean, standard deviation (SD), and relative standard deviation expressed in % (RSD%) (output from DDSolver):

Parameter	No.1	No.2	No.3	No.4	Mean	SD	RSD(%)
k_{KP}	0.752	0.771	0.744	0.785	0.763	0.018	2.424
n	0.672	0.645	0.661	0.657	0.659	0.011	1.703

Number of dissolution data points (N), degrees of freedom (df), and selected goodness of fit criteria – Pearson correlation coefficient (R), coefficient of determination (R^2), adjusted coefficient of determination ($R^2_{adjusted}$), and residual sum of squares (RSS) (manual calculation in MS Excel):

Parameter	No.1	No.2	No.3	No.4
N	21	21	21	21
df	19	19	19	19
R	0.999033158	0.999148656	0.9992938	0.999359108
R^2	0.998067251	0.998298037	0.998588099	0.998718626
$R^2_{adjusted}$	0.997965527	0.99820846	0.998513789	0.998651185
RSS	31.55199584	18.94594021	19.00222427	18.96566498

Graphical abstract of model fit presented as mean ± 1 SD of the fraction % of released carvedilol:

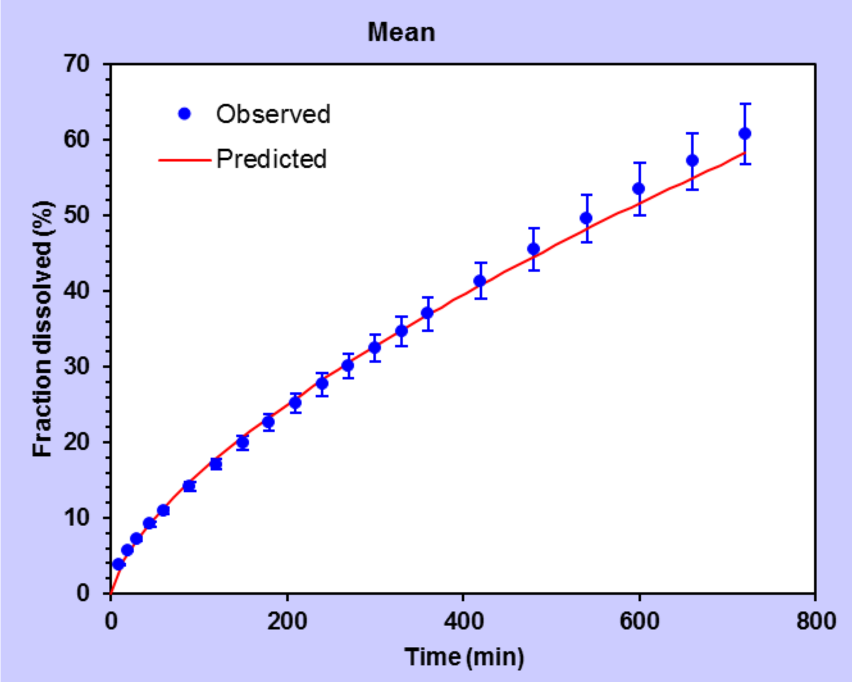

Supplementary materials – Model fitting summary – Di-Cafos® A12 (modelling up to app. 60 % of carvedilol released)

Graphical abstract of model fit presented as the fraction % of released carvedilol per tested tablet:

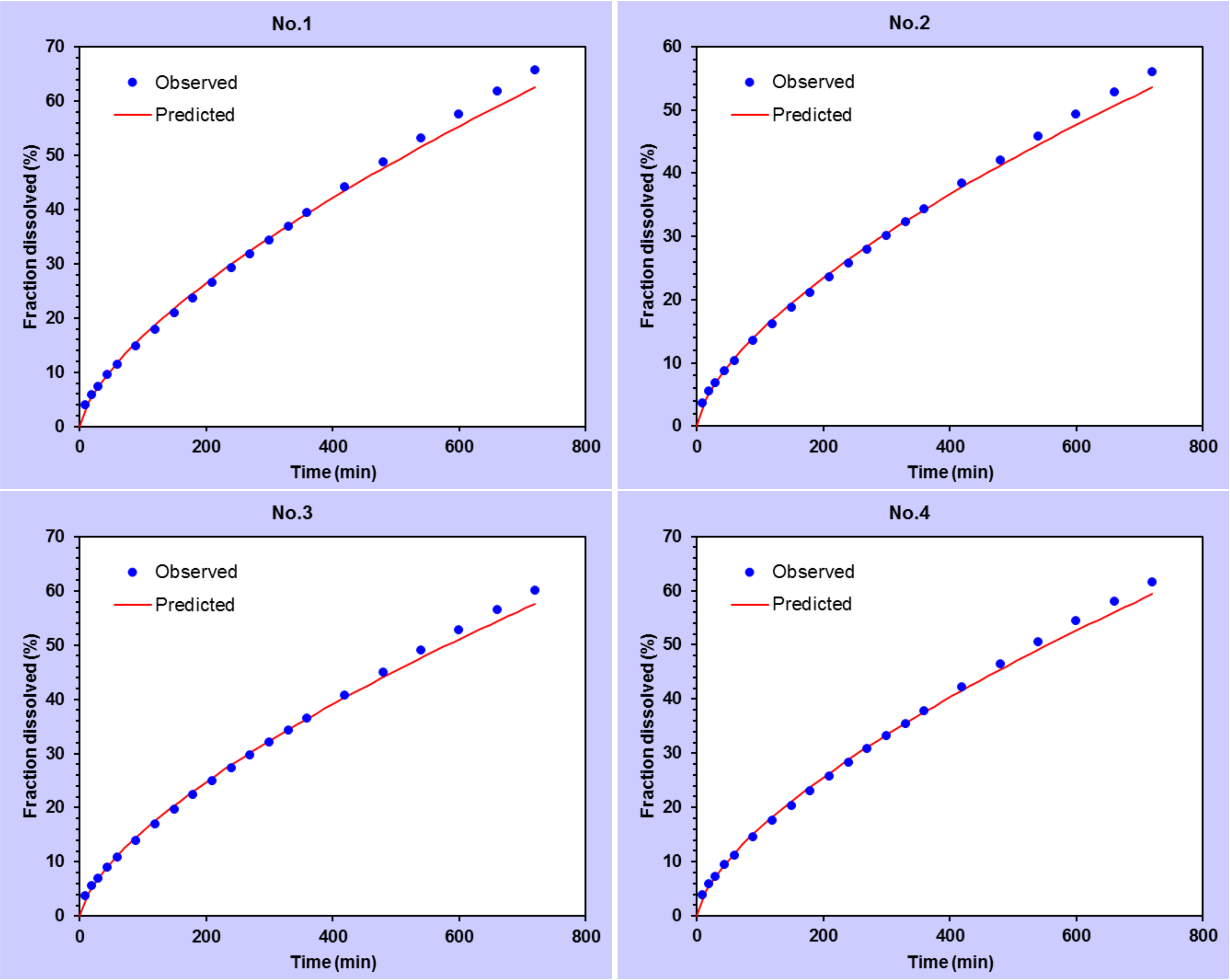

Supplementary materials – Model fitting summary – Di-Cafos® A12 (modelling up to app. 60 % of carvedilol released)

Model: **Korsmeyer–Peppas with T_{lag}**

Model equation: $F = k_{KP} \cdot (t - T_{lag})^n$

Fitted model parameters per tested tablet (N = 4) with statistics – mean, standard deviation (SD), and relative standard deviation expressed in % (RSD%) (output from DDSolver):

Parameter	No.1	No.2	No.3	No.4	Mean	SD	RSD(%)
k_{KP}	1.021	1.033	1.004	1.058	1.029	0.023	2.211
n	0.620	0.595	0.610	0.607	0.608	0.010	1.697
T_{lag}	4.000	4.000	4.000	4.000	4.000	0.000	0.000

Number of dissolution data points (N), degrees of freedom (df), and selected goodness of fit criteria – Pearson correlation coefficient (R), coefficient of determination (R^2), adjusted coefficient of determination ($R^2_{adjusted}$), and residual sum of squares (RSS) (manual calculation in MS Excel):

Parameter	No.1	No.2	No.3	No.4
N	21	21	21	21
df	18	18	18	18
R	0.997215307	0.997410249	0.997669078	0.997785772
R^2	0.994438368	0.994827204	0.99534359	0.995576448
$R^2_{adjusted}$	0.993820409	0.994252449	0.994826211	0.995084942
RSS	99.14676896	63.13864328	68.74308817	70.64127883

Graphical abstract of model fit presented as mean ± 1 SD of the fraction % of released carvedilol:

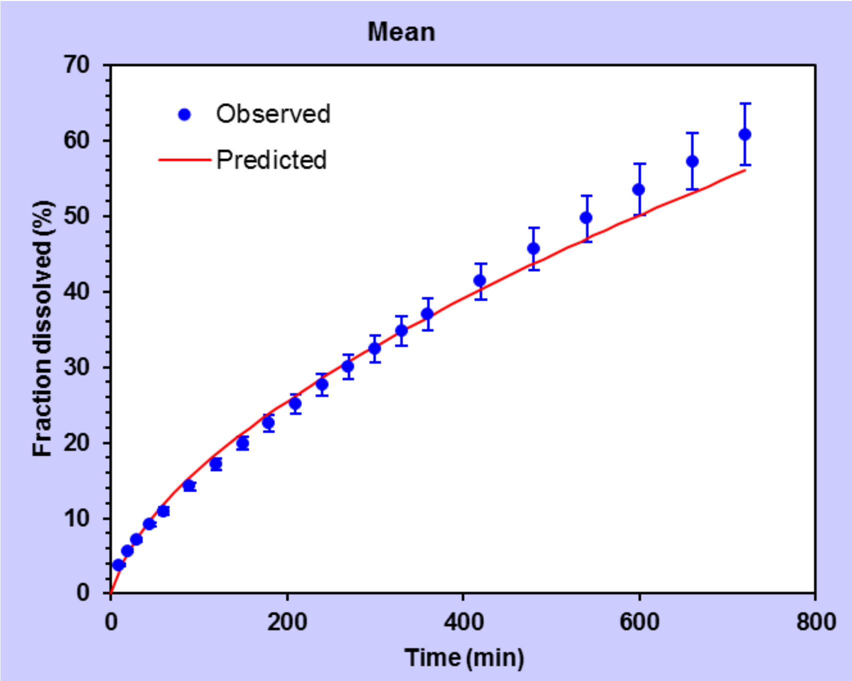

Supplementary materials – Model fitting summary – Di-Cafos® A12 (modelling up to app. 60 % of carvedilol released)

Graphical abstract of model fit presented as the fraction % of released carvedilol per tested tablet:

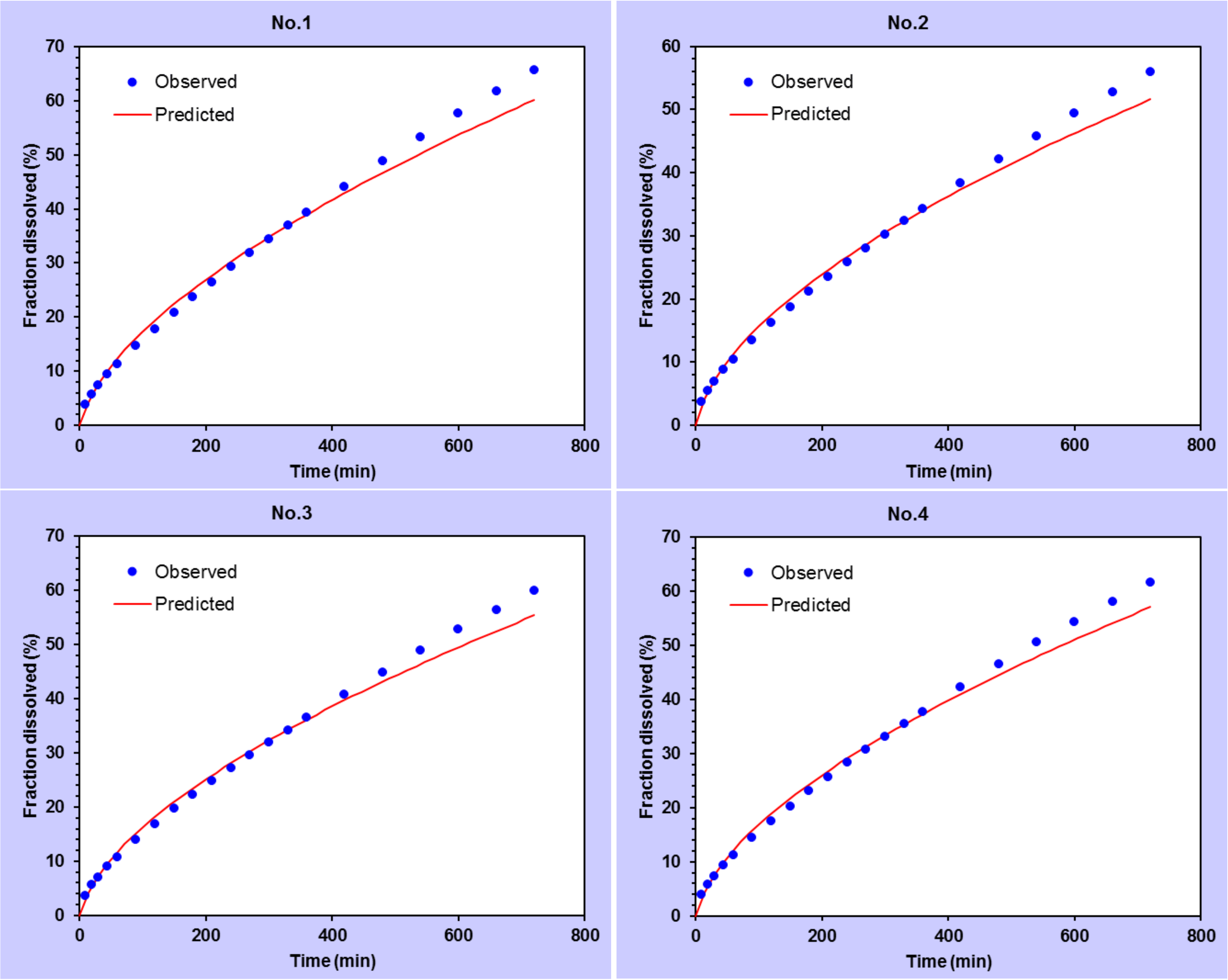

Supplementary materials – Model fitting summary – Di-Cafos® A12 (modelling up to app. 60 % of carvedilol released)

Model: **Korsmeyer–Peppas with F_0**

Model equation: $F = F_0 + k_{KP} \cdot t^n$

Fitted model parameters per tested tablet (N = 4) with statistics – mean, standard deviation (SD), and relative standard deviation expressed in % (RSD%) (output from DDSolver):

Parameter	No.1	No.2	No.3	No.4	Mean	SD	RSD(%)
k_{KP}	0.424	0.433	0.415	0.436	0.427	0.010	2.229
n	0.763	0.736	0.751	0.748	0.750	0.011	1.504
F_0	1.560	1.480	1.798	1.895	1.683	0.195	11.612

Number of dissolution data points (N), degrees of freedom (df), and selected goodness of fit criteria – Pearson correlation coefficient (R), coefficient of determination (R^2), adjusted coefficient of determination ($R^2_{adjusted}$), and residual sum of squares (RSS) (manual calculation in MS Excel):

Parameter	No.1	No.2	No.3	No.4
N	21	21	21	21
df	18	18	18	18
R	0.999979925	0.999984433	0.999984623	0.999957452
R^2	0.999959851	0.999968866	0.999969246	0.999914906
$R^2_{adjusted}$	0.99995539	0.999965407	0.999965829	0.999905451
RSS	0.30707142	0.297513086	0.550280809	0.861186791

Graphical abstract of model fit presented as mean ± 1 SD of the fraction % of released carvedilol:

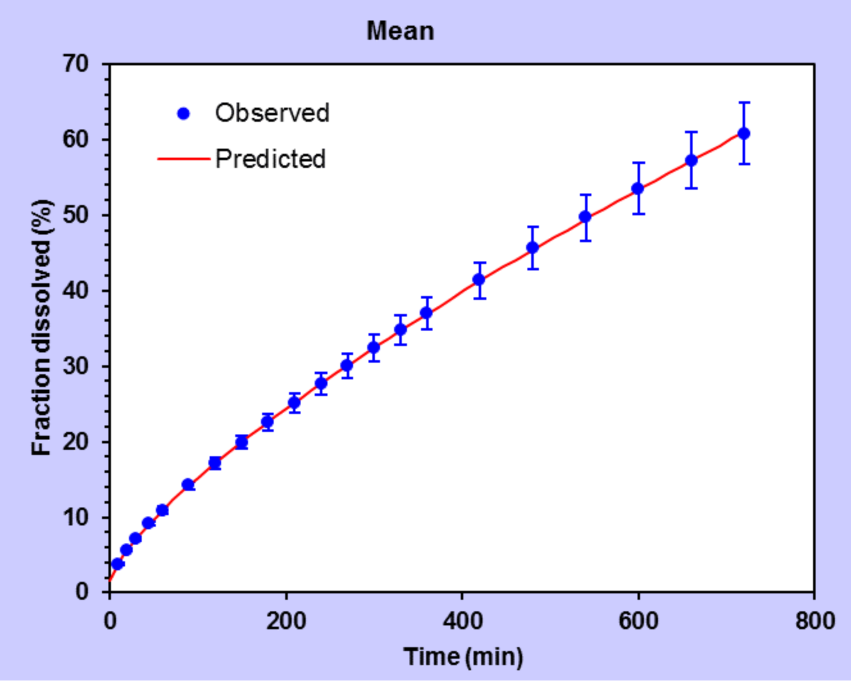

Supplementary materials – Model fitting summary – Di-Cafos® A12 (modelling up to app. 60 % of carvedilol released)

Graphical abstract of model fit presented as the fraction % of released carvedilol per tested tablet:

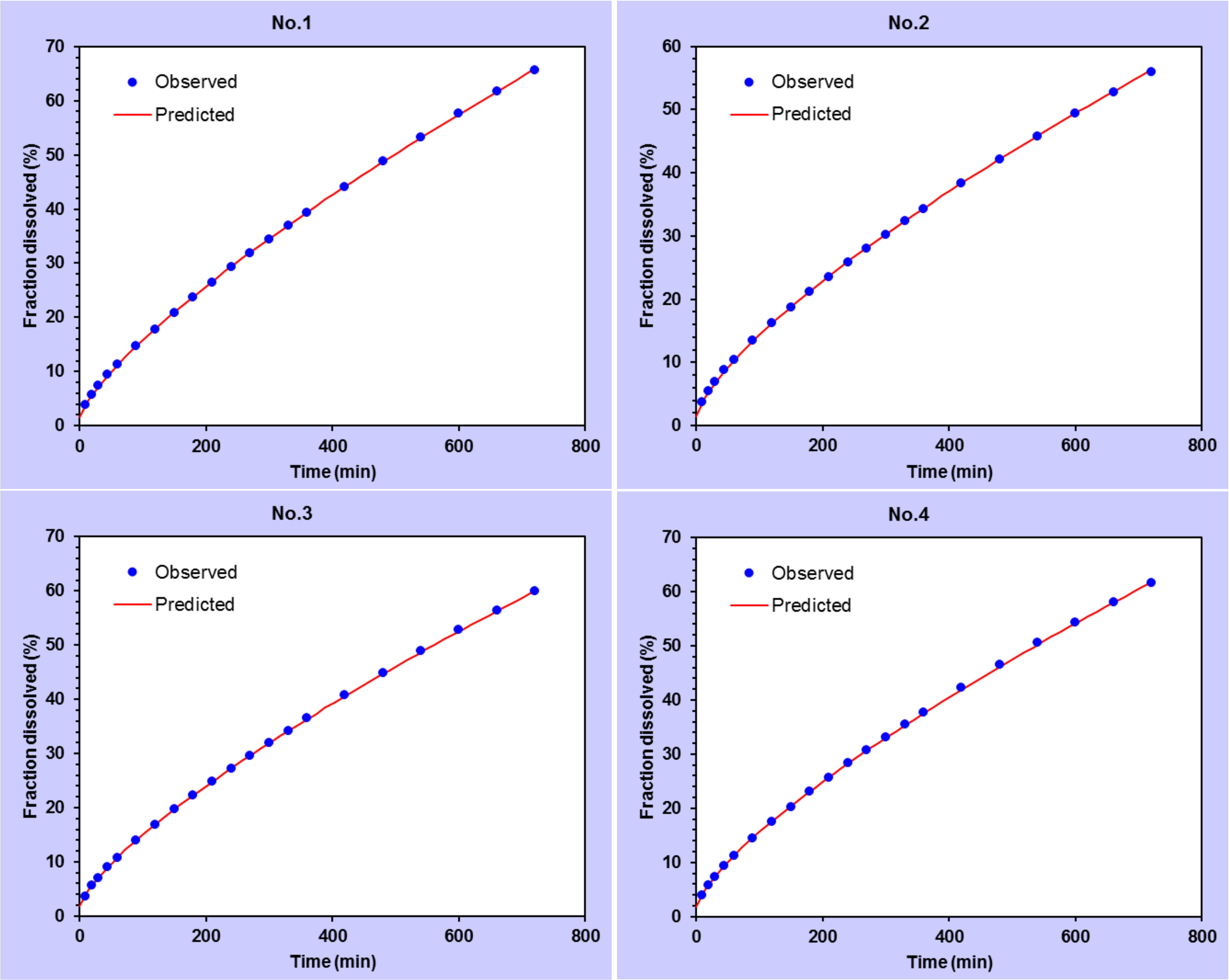

Supplementary materials – Model fitting summary – Di-Cafos® A12 (modelling up to app. 60 % of carvedilol released)

Model: **Hixson–Crowell**

Model equation: $F = 100 \cdot [1 - (1 - k_{HC} \cdot t)^3]$

Fitted model parameters per tested tablet (N = 4) with statistics – mean, standard deviation (SD), and relative standard deviation expressed in % (RSD%) (output from DDSolver):

Parameter	No.1	No.2	No.3	No.4	Mean	SD	RSD(%)
k_{HC}	0.0004	0.0004	0.0004	0.0004	0.0004	0.0000	7.8564

Number of dissolution data points (N), degrees of freedom (df), and selected goodness of fit criteria – Pearson correlation coefficient (R), coefficient of determination (R^2), adjusted coefficient of determination ($R^2_{adjusted}$), and residual sum of squares (RSS) (manual calculation in MS Excel):

Parameter	No.1	No.2	No.3	No.4
N	21	21	21	21
df	20	20	20	20
R	0.999619391	0.999147831	0.999423797	0.999472923
R^2	0.999238926	0.998296388	0.998847925	0.998946123
$R^2_{adjusted}$	0.999238926	0.998296388	0.998847925	0.998946123
RSS	115.7520085	167.6301571	145.4541816	155.374558

Graphical abstract of model fit presented as mean ± 1 SD of the fraction % of released carvedilol:

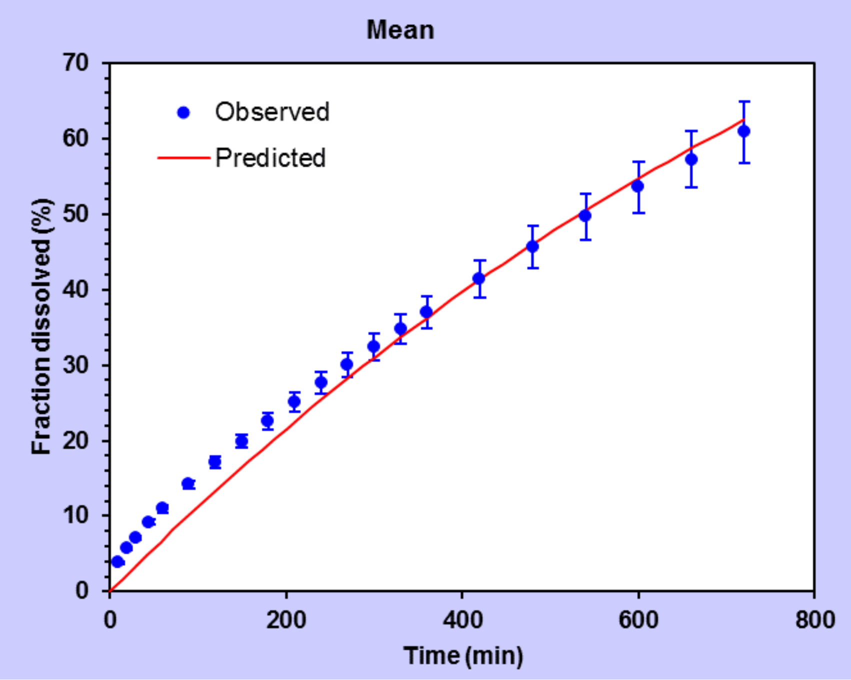

Supplementary materials – Model fitting summary – Di-Cafos® A12 (modelling up to app. 60 % of carvedilol released)

Graphical abstract of model fit presented as the fraction % of released carvedilol per tested tablet:

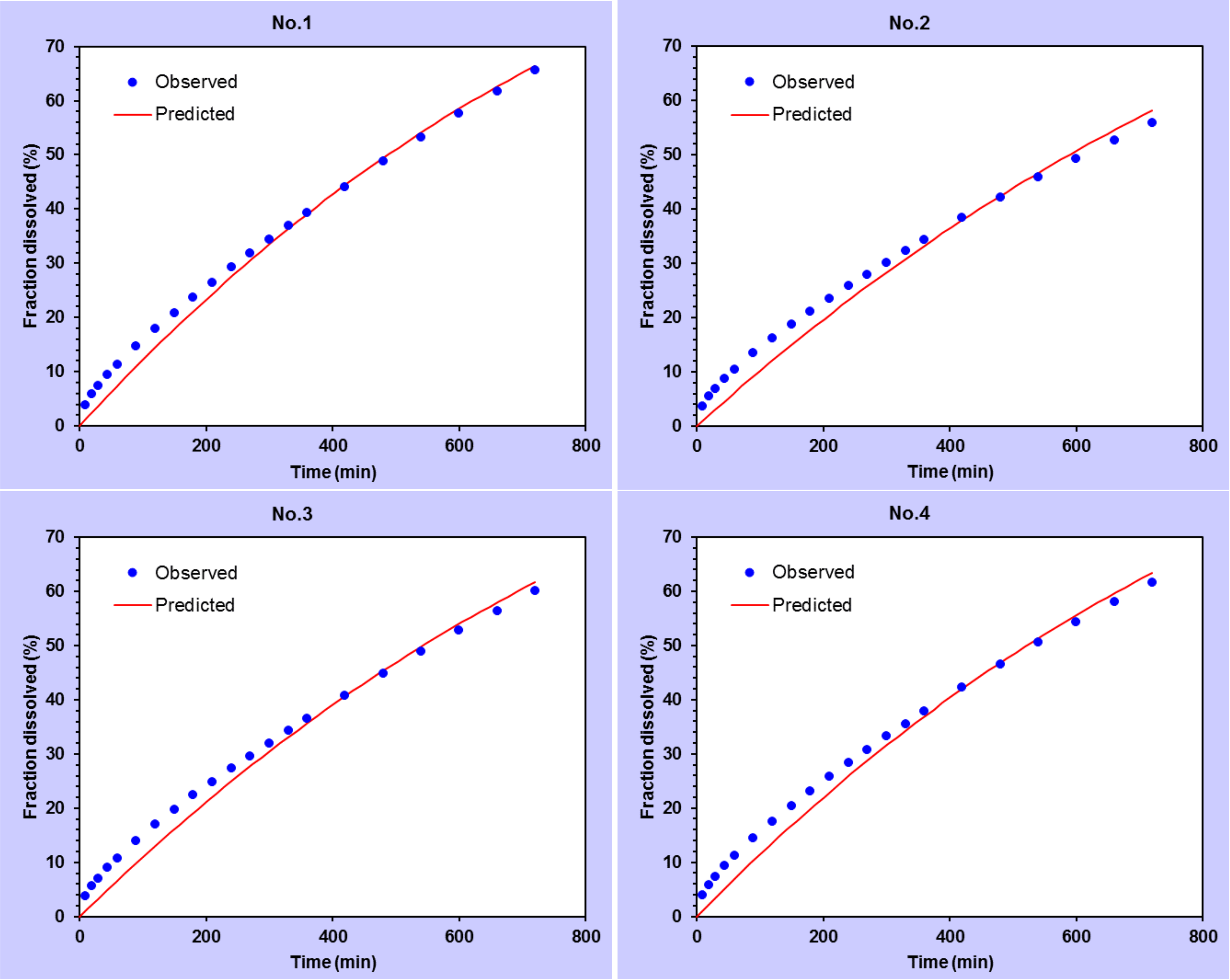

Supplementary materials – Model fitting summary – Di-Cafos® A12 (modelling up to app. 60 % of carvedilol released)

Model: **Hixson–Crowell with T_{lag}**

Model equation: $F = 100 \cdot \left\{1 - \left[1 - k_{HC} \cdot \left(t - T_{lag}\right)\right]^3\right\}$

Fitted model parameters per tested tablet (N = 4) with statistics – mean, standard deviation (SD), and relative standard deviation expressed in % (RSD%) (output from DDSolver):

Parameter	No.1	No.2	No.3	No.4	Mean	SD	RSD(%)
k_{HC}	0.0004	0.0003	0.0003	0.0004	0.0004	0.0000	9.4252
T_{lag}	-35.5633	-54.7501	-46.3510	-46.5382	-45.8006	7.8685	-17.1800

Number of dissolution data points (N), degrees of freedom (df), and selected goodness of fit criteria – Pearson correlation coefficient (R), coefficient of determination (R^2), adjusted coefficient of determination ($R^2_{adjusted}$), and residual sum of squares (RSS) (manual calculation in MS Excel):

Parameter	No.1	No.2	No.3	No.4
N	21	21	21	21
df	19	19	19	19
R	0.999640361	0.99890383	0.99928092	0.999310848
R^2	0.999280851	0.997808863	0.998562357	0.99862217
$R^2_{adjusted}$	0.999243001	0.99769354	0.998486692	0.998549653
RSS	5.282672223	11.72112755	8.892569774	9.065727424

Graphical abstract of model fit presented as mean ± 1 SD of the fraction % of released carvedilol:

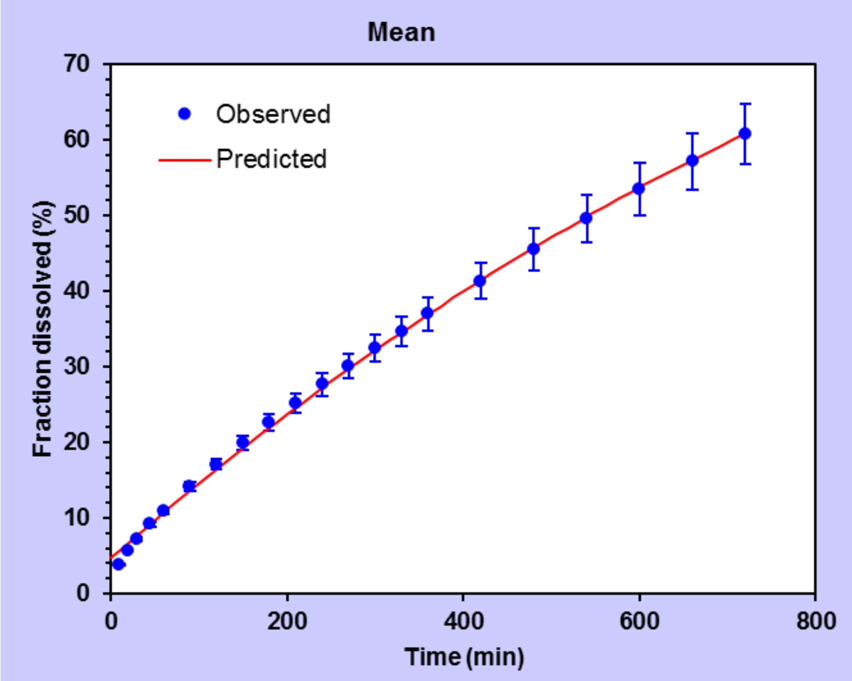

Supplementary materials – Model fitting summary – Di-Cafos® A12 (modelling up to app. 60 % of carvedilol released)

Graphical abstract of model fit presented as the fraction % of released carvedilol per tested tablet:

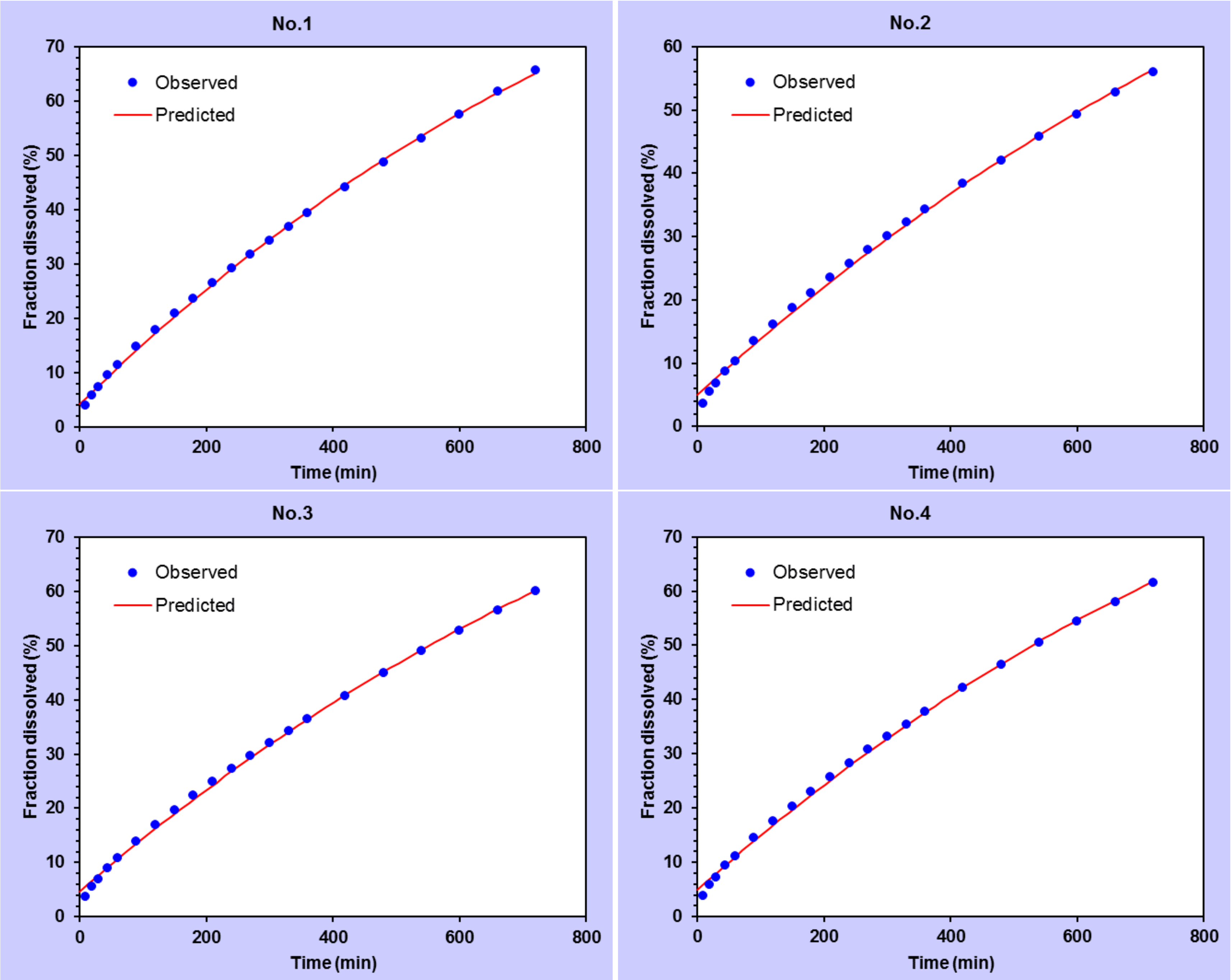

Supplementary materials – Model fitting summary – Di-Cafos® A12 (modelling up to app. 60 % of carvedilol released)

Model: **Hopfenberg**

Model equation: $F = 100 \cdot [1 - (1 - k_{HB} \cdot t)^n]$

Fitted model parameters per tested tablet (N = 4) with statistics – mean, standard deviation (SD), and relative standard deviation expressed in % (RSD%) (output from DDSolver):

Parameter	No.1	No.2	No.3	No.4	Mean	SD	RSD(%)
k_{HB}	0.0004	0.0004	0.0004	0.0004	0.0004	0.0000	7.8564
n	3.0000	3.0000	3.0000	3.0000	3.0000	0.0000	0.0000

Number of dissolution data points (N), degrees of freedom (df), and selected goodness of fit criteria – Pearson correlation coefficient (R), coefficient of determination (R^2), adjusted coefficient of determination ($R^2_{adjusted}$), and residual sum of squares (RSS) (manual calculation in MS Excel):

Parameter	No.1	No.2	No.3	No.4
N	21	21	21	21
df	19	19	19	19
R	0.999619391	0.999147831	0.999423797	0.999472923
R^2	0.999238926	0.998296388	0.998847925	0.998946123
$R^2_{adjusted}$	0.999198869	0.998206725	0.99878729	0.998890656
RSS	115.7520085	167.6301571	145.4541816	155.374558

Graphical abstract of model fit presented as mean ± 1 SD of the fraction % of released carvedilol:

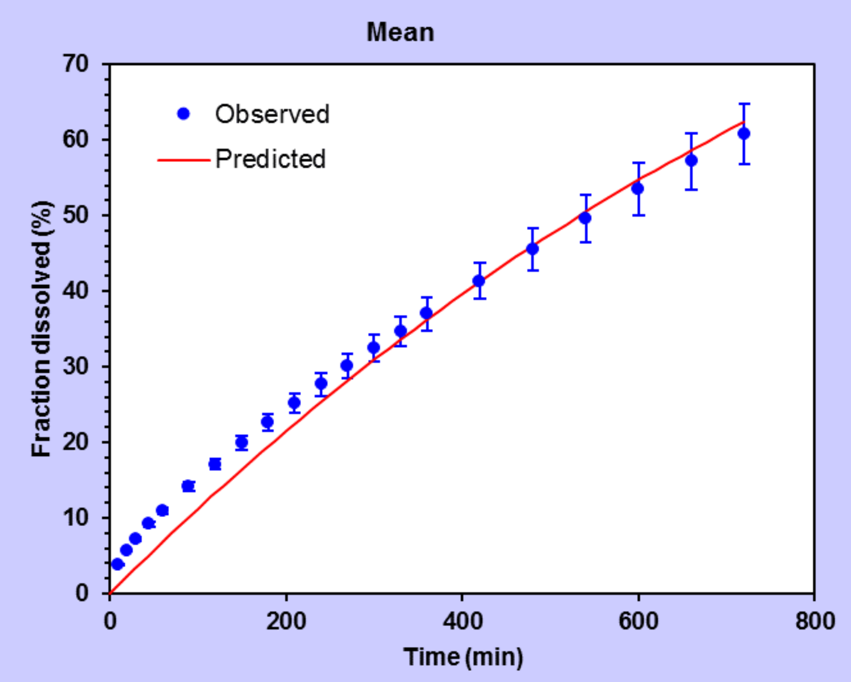

Supplementary materials – Model fitting summary – Di-Cafos® A12 (modelling up to app. 60 % of carvedilol released)

Graphical abstract of model fit presented as the fraction % of released carvedilol per tested tablet:

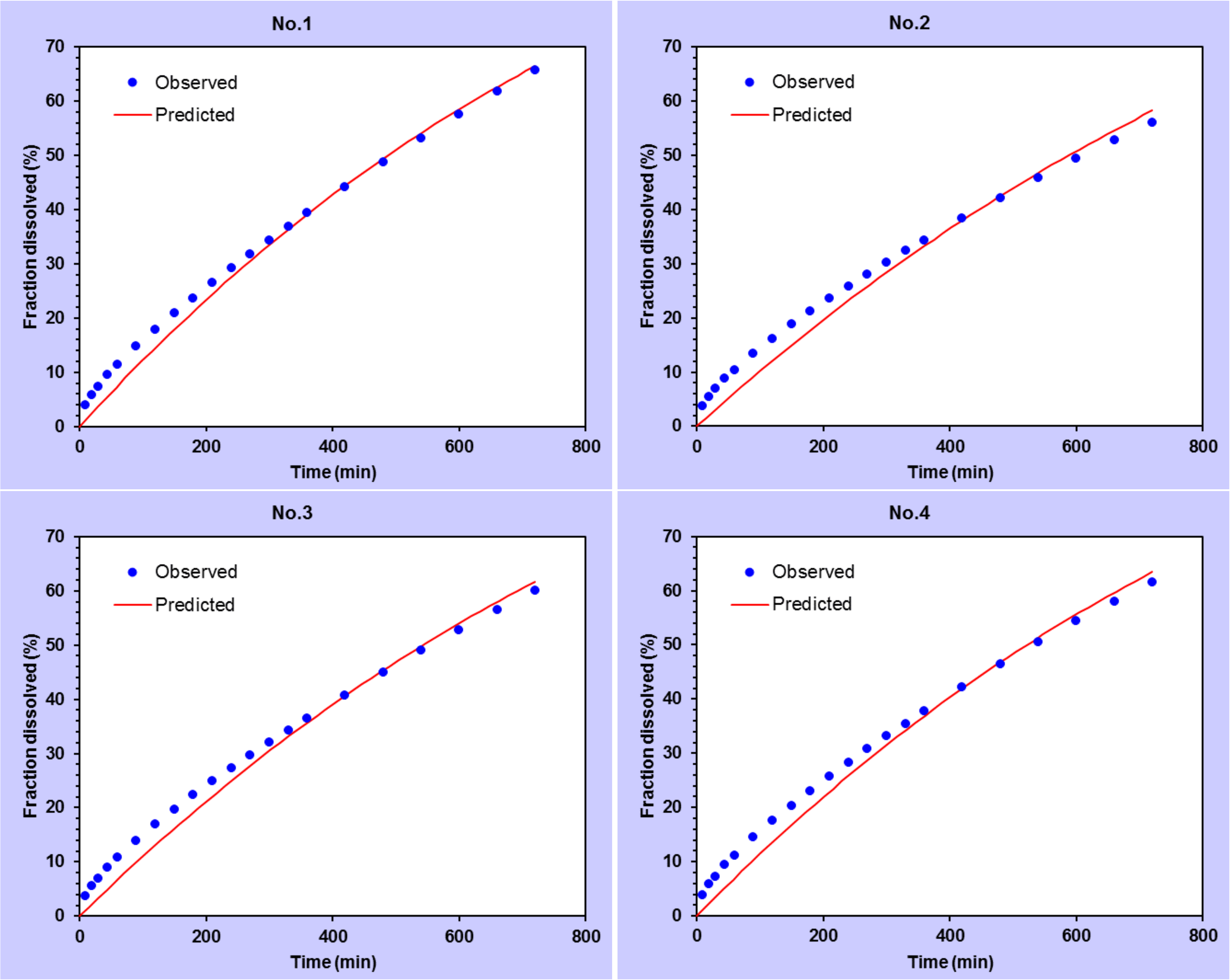

Supplementary materials – Model fitting summary – Di-Cafos® A12 (modelling up to app. 60 % of carvedilol released)

Model: **Hopfenberg with T_{lag}**

Model equation: $F = 100 \cdot \{1 - [1 - k_{HB} \cdot (t - T_{lag})]^n\}$

Fitted model parameters per tested tablet (N = 4) with statistics – mean, standard deviation (SD), and relative standard deviation expressed in % (RSD%) (output from DDSolver):

Parameter	No.1	No.2	No.3	No.4	Mean	SD	RSD(%)
k_{HB}	0.0004	0.0003	0.0003	0.0003	0.0003	0.0000	12.1104
n	3.0000	3.0000	3.0000	3.7120	3.1780	0.3560	11.2027
T_{lag}	-35.5633	-54.7501	-46.3510	-36.9632	-43.4069	8.9513	-20.6219

Number of dissolution data points (N), degrees of freedom (df), and selected goodness of fit criteria – Pearson correlation coefficient (R), coefficient of determination (R^2), adjusted coefficient of determination ($R^2_{adjusted}$), and residual sum of squares (RSS) (manual calculation in MS Excel):

Parameter	No.1	No.2	No.3	No.4
N	21	21	21	21
df	18	18	18	18
R	0.999640361	0.99890383	0.99928092	0.9994968
R^2	0.999280851	0.997808863	0.998562357	0.998993852
$R^2_{adjusted}$	0.999200945	0.997565403	0.998402619	0.998882058
RSS	5.282672223	11.72112755	8.892569774	8.238718463

Graphical abstract of model fit presented as mean ± 1 SD of the fraction % of released carvedilol:

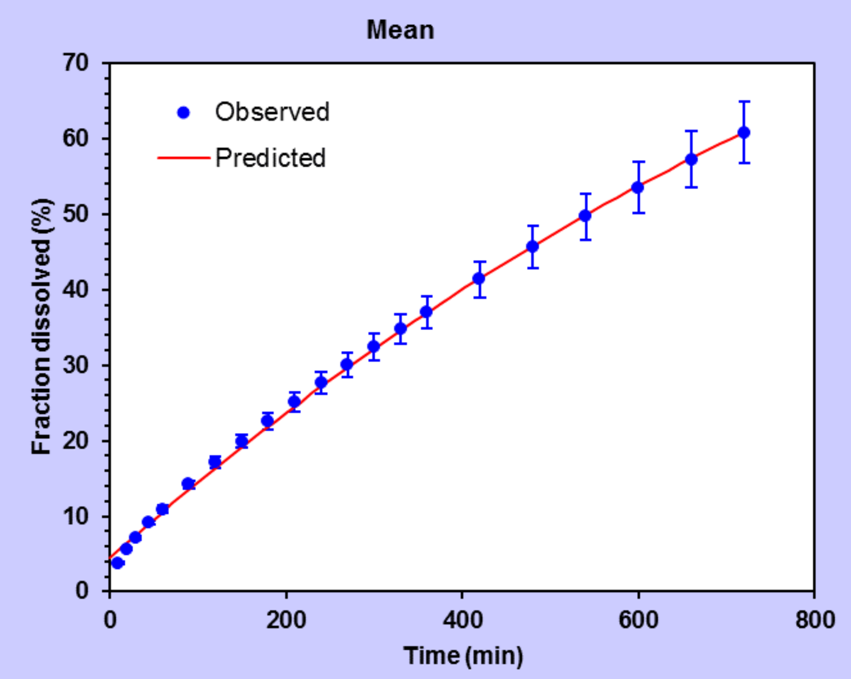

Supplementary materials – Model fitting summary – Di-Cafos® A12 (modelling up to app. 60 % of carvedilol released)

Graphical abstract of model fit presented as the fraction % of released carvedilol per tested tablet:

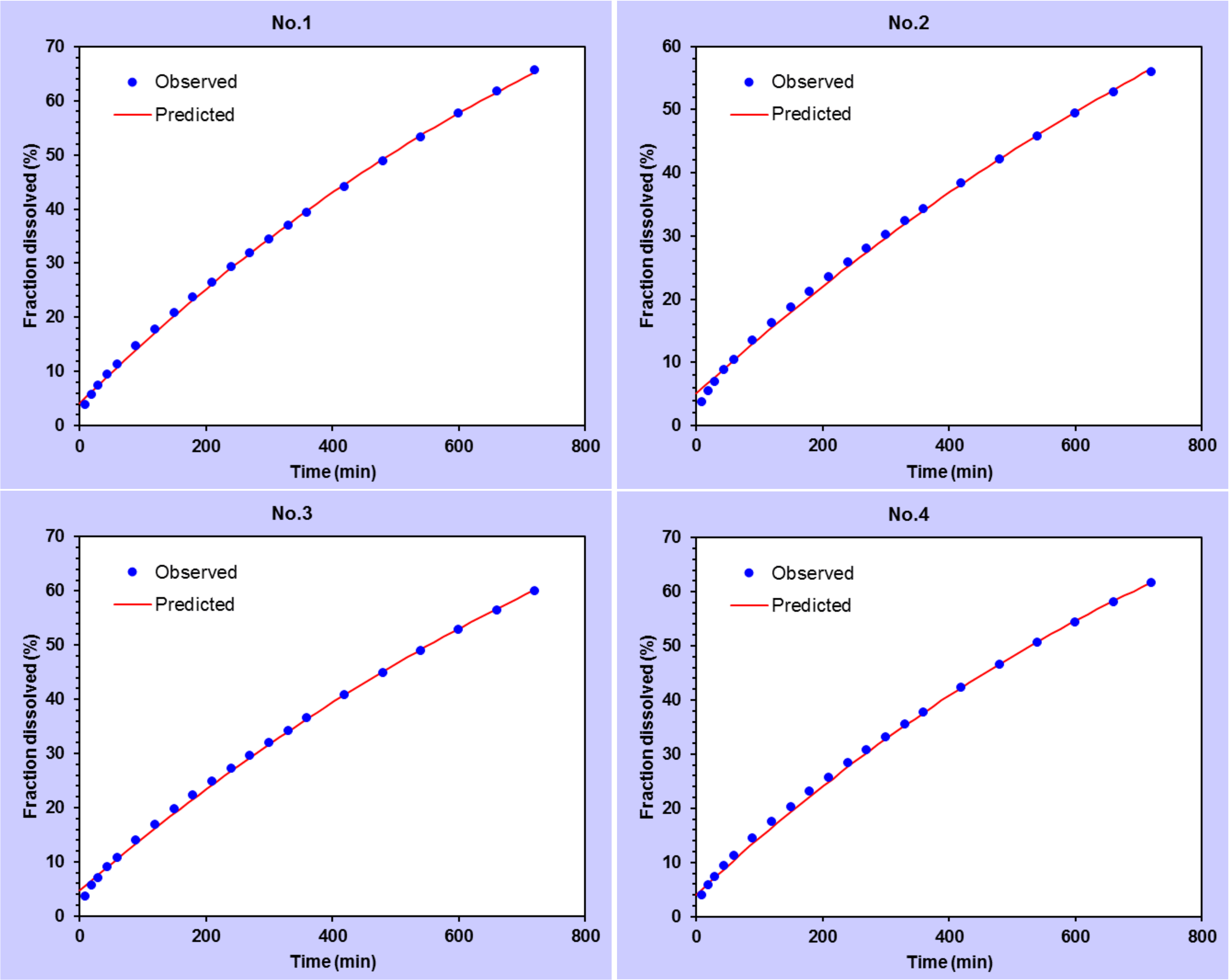

Supplementary materials – Model fitting summary – Di-Cafos® A12 (modelling up to app. 60 % of carvedilol released)

Model: **Baker–Lonsdale**

Model equation: $\frac{3}{2}\cdot\left[1-\left(1-\frac{F}{100}\right)^{\frac{2}{3}}\right]-\frac{F}{100}=k_{BL}\cdot t$

Fitted model parameters per tested tablet (N = 4) with statistics – mean, standard deviation (SD), and relative standard deviation expressed in % (RSD%) (output from DDSolver):

Parameter	No.1	No.2	No.3	No.4	Mean	SD	RSD(%)
k_{BL}	0.00007	0.00005	0.00006	0.00006	0.00006	0.00001	15.84556

Number of dissolution data points (N), degrees of freedom (df), and selected goodness of fit criteria – Pearson correlation coefficient (R), coefficient of determination (R^2), adjusted coefficient of determination ($R^2_{adjusted}$), and residual sum of squares (RSS) (manual calculation in MS Excel):

Parameter	No.1	No.2	No.3	No.4
N	21	21	21	21
df	20	20	20	20
R	0.986861391	0.990256727	0.989274442	0.989614775
R^2	0.973895405	0.980608386	0.978663921	0.979337403
$R^2_{adjusted}$	0.973895405	0.980608386	0.978663921	0.979337403
RSS	1030.041562	808.0850858	897.1550231	945.0997834

Graphical abstract of model fit presented as mean ± 1 SD of the fraction % of released carvedilol:

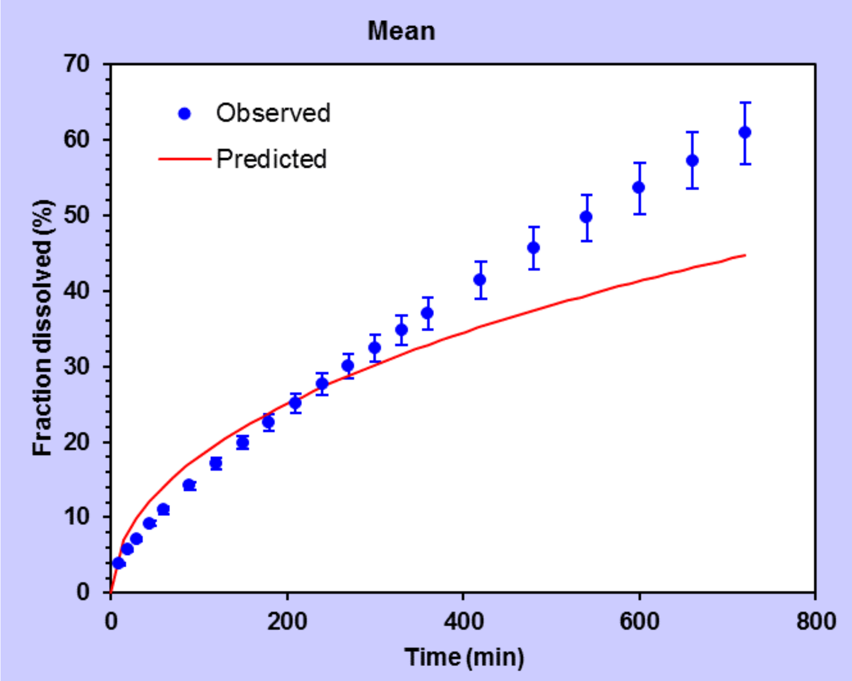

Supplementary materials – Model fitting summary – Di-Cafos® A12 (modelling up to app. 60 % of carvedilol released)

Graphical abstract of model fit presented as the fraction % of released carvedilol per tested tablet:

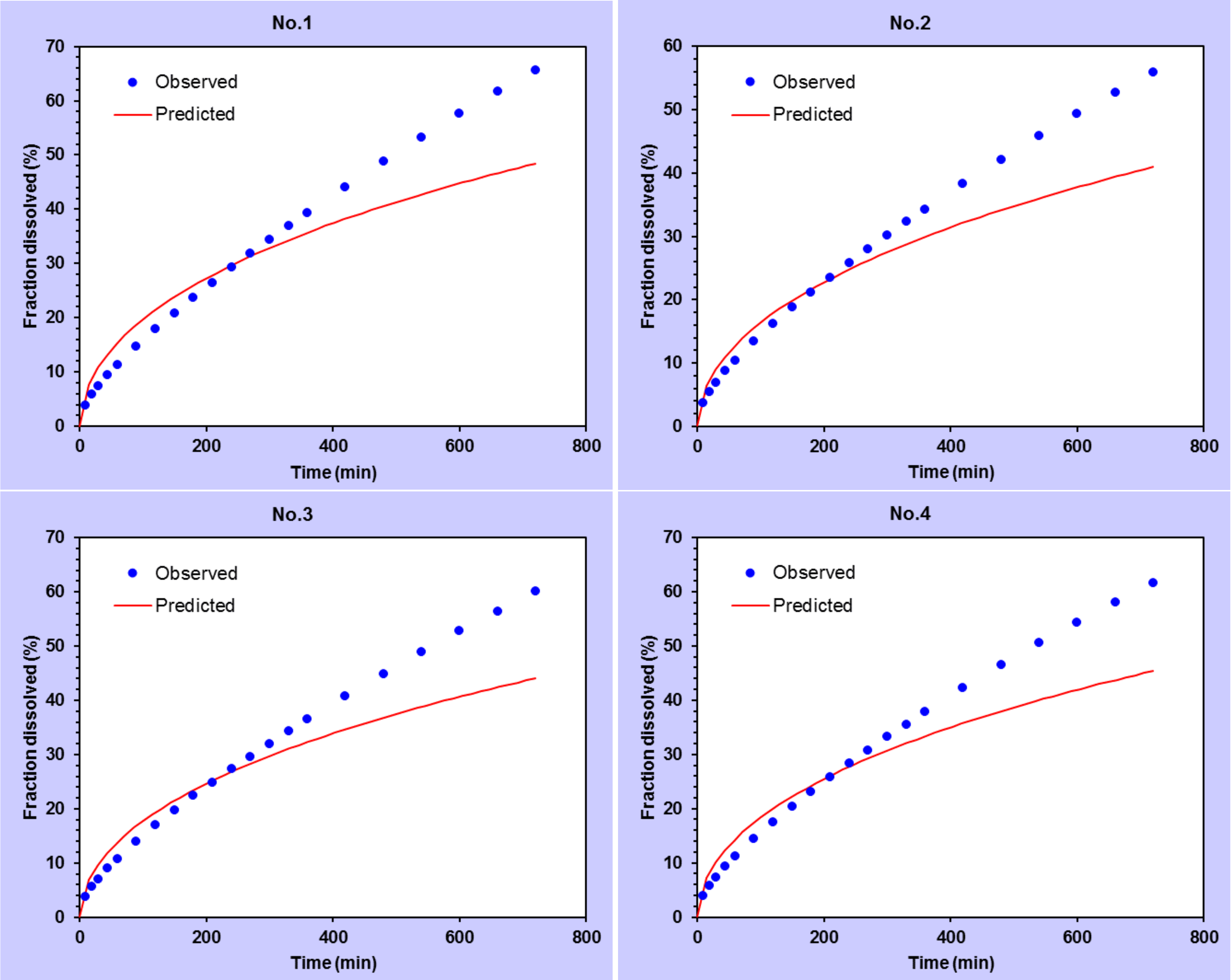

Supplementary materials – Model fitting summary – Di-Cafos® A12 (modelling up to app. 60 % of carvedilol released)

Model: **Baker–Lonsdale with T_{lag}**

Model equation: $\frac{3}{2}\cdot\left[1-\left(1-\frac{F}{100}\right)^{\frac{2}{3}}\right]-\frac{F}{100}=k_{BL}\cdot\left(t-T_{lag}\right)$

Fitted model parameters per tested tablet (N = 4) with statistics – mean, standard deviation (SD), and relative standard deviation expressed in % (RSD%) (output from DDSolver):

Parameter	No.1	No.2	No.3	No.4	Mean	SD	RSD(%)
k_{BL}	0.00014	0.00010	0.00011	0.00012	0.00012	0.00002	15.84556
T_{lag}	76.25954	67.35902	70.98820	70.37518	71.24548	3.69995	5.19325

Number of dissolution data points (N), degrees of freedom (df), and selected goodness of fit criteria – Pearson correlation coefficient (R), coefficient of determination (R^2), adjusted coefficient of determination ($R^2_{adjusted}$), and residual sum of squares (RSS) (manual calculation in MS Excel):

Parameter	No.1	No.2	No.3	No.4
N	21	21	21	21
df	19	19	19	19
R	0.974985389	0.977081048	0.976833284	0.97694823
R^2	0.950596509	0.954687375	0.954203264	0.954427845
$R^2_{adjusted}$	0.947996325	0.9523025	0.95179291	0.95202931
RSS	544.6584262	385.900757	435.4972407	462.4846877

Graphical abstract of model fit presented as mean ± 1 SD of the fraction % of released carvedilol:

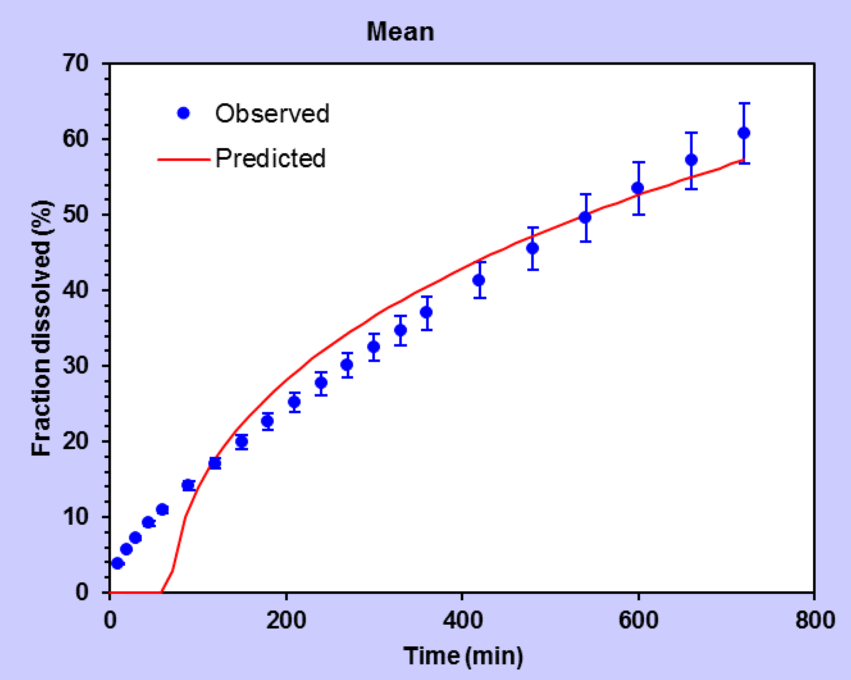

Supplementary materials – Model fitting summary – Di-Cafos® A12 (modelling up to app. 60 % of carvedilol released)

Graphical abstract of model fit presented as the fraction % of released carvedilol per tested tablet:

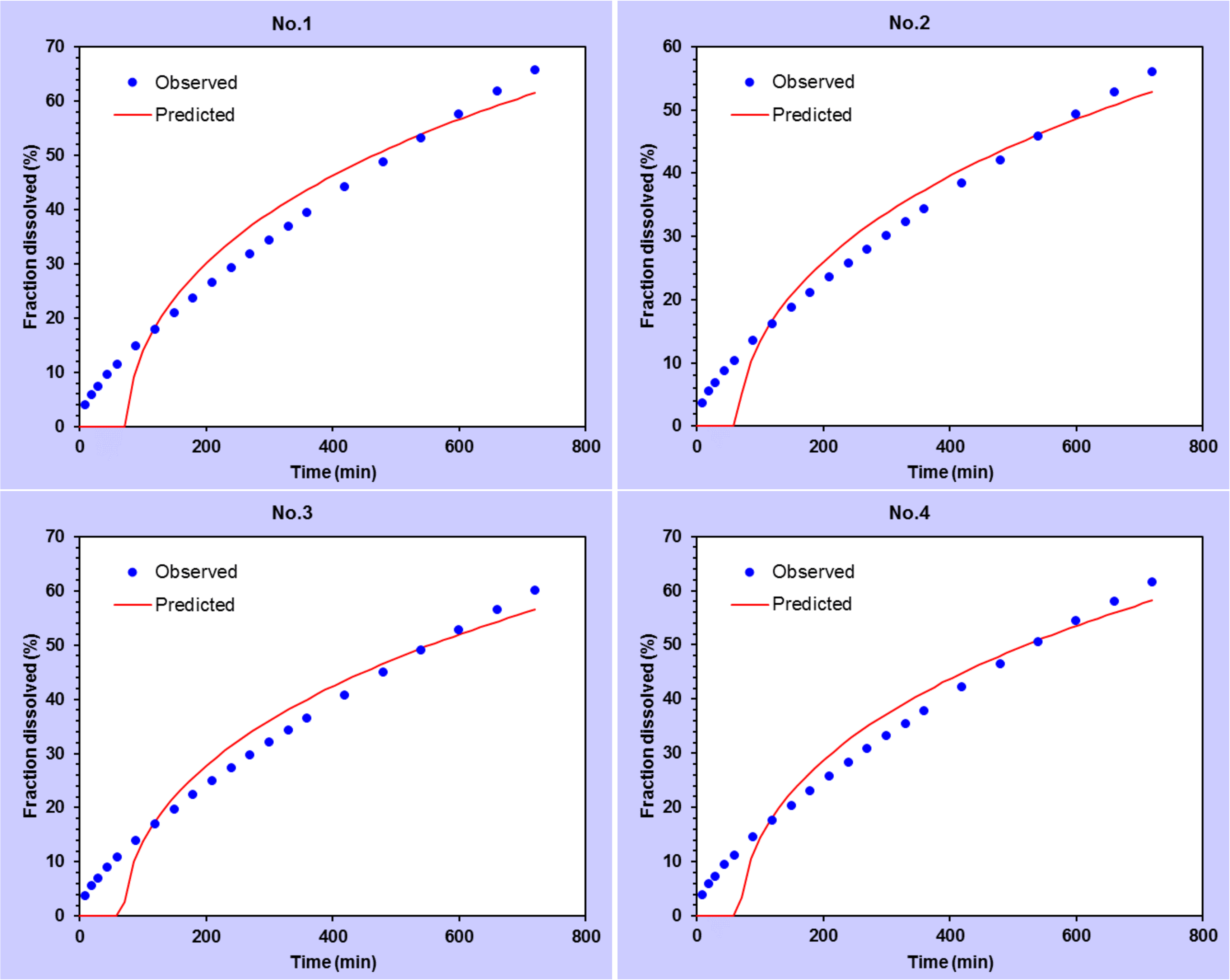

Supplementary materials – Model fitting summary – Di-Cafos® A12 (modelling up to app. 60 % of carvedilol released)

Model: **Makoid–Banakar**

Model equation: $F = k_{MB} \cdot t^{n} \cdot e^{-k \cdot t}$

Fitted model parameters per tested tablet (N = 4) with statistics – mean, standard deviation (SD), and relative standard deviation expressed in % (RSD%) (output from DDSolver):

Parameter	No.1	No.2	No.3	No.4	Mean	SD	RSD(%)
k_{MB}	0.91073	0.91439	0.87485	0.92334	0.90583	0.02132	2.35366
n	0.61598	0.59462	0.61358	0.60991	0.60852	0.00960	1.57692
k	-0.00035	-0.00031	-0.00029	-0.00029	-0.00031	0.00002	-7.92759

Number of dissolution data points (N), degrees of freedom (df), and selected goodness of fit criteria – Pearson correlation coefficient (R), coefficient of determination (R^2), adjusted coefficient of determination ($R^2_{adjusted}$), and residual sum of squares (RSS) (manual calculation in MS Excel):

Parameter	No.1	No.2	No.3	No.4
N	21	21	21	21
df	18	18	18	18
R	0.999661758	0.999713731	0.999742227	0.999613085
R^2	0.99932363	0.999427543	0.999484521	0.99922632
$R^2_{adjusted}$	0.999248478	0.999363937	0.999427246	0.999140355
RSS	5.175432083	3.097289528	3.257105337	5.154318928

Graphical abstract of model fit presented as mean ± 1 SD of the fraction % of released carvedilol:

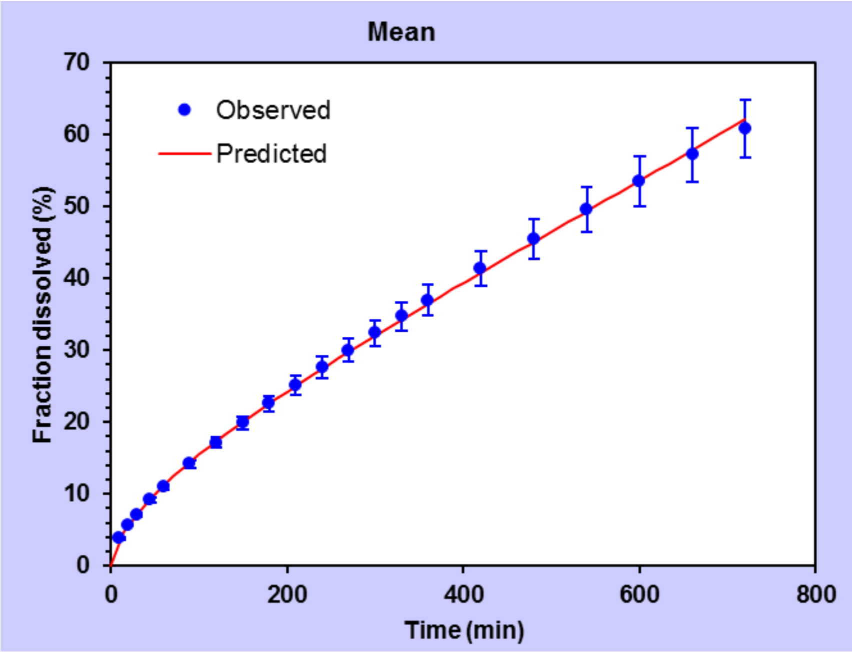

Supplementary materials – Model fitting summary – Di-Cafos® A12 (modelling up to app. 60 % of carvedilol released)

Graphical abstract of model fit presented as the fraction % of released carvedilol per tested tablet:

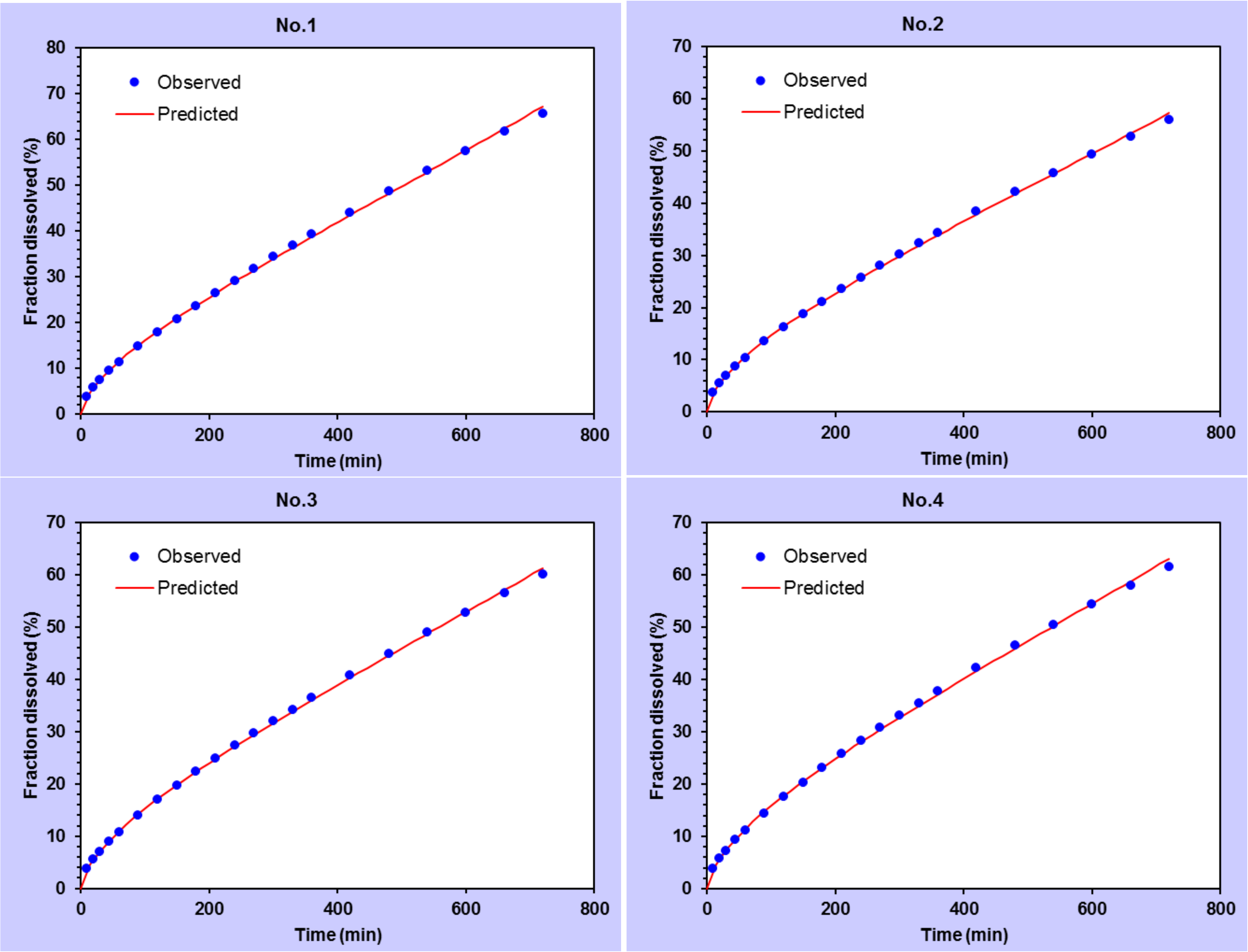

Supplementary materials – Model fitting summary – Di-Cafos® A12 (modelling up to app. 60 % of carvedilol released)

Model: **Makoid–Banakar with T_{lag}**

Model equation: $F = k_{MB} \cdot (t - T_{lag})^n \cdot e^{-k\cdot(t-T_{lag})}$

Fitted model parameters per tested tablet (N = 4) with statistics – mean, standard deviation (SD), and relative standard deviation expressed in % (RSD%) (output from DDSolver):

Parameter	No.1	No.2	No.3	No.4	Mean	SD	RSD(%)
k_{MB}	1.36102	1.34667	1.30437	1.37455	1.34665	0.03040	2.25733
n	0.53018	0.51197	0.52827	0.52488	0.52383	0.00821	1.56638
k	-0.00061	-0.00057	-0.00056	-0.00056	-0.00058	0.00003	-4.54573
T_{lag}	4.00000	4.00000	4.00000	4.00000	4.00000	0.00000	0.00000

Number of dissolution data points (N), degrees of freedom (df), and selected goodness of fit criteria – Pearson correlation coefficient (R), coefficient of determination (R^2), adjusted coefficient of determination ($R^2_{adjusted}$), and residual sum of squares (RSS) (manual calculation in MS Excel):

Parameter	No.1	No.2	No.3	No.4
N	21	21	21	21
df	17	17	17	17
R	0.998597635	0.998716811	0.998744981	0.998480543
R^2	0.997197236	0.997435268	0.997491536	0.996963395
$R^2_{adjusted}$	0.996702631	0.996982668	0.997048866	0.996427523
RSS	22.31253513	14.37020776	16.43554862	20.94902925

Graphical abstract of model fit presented as mean ± 1 SD of the fraction % of released carvedilol:

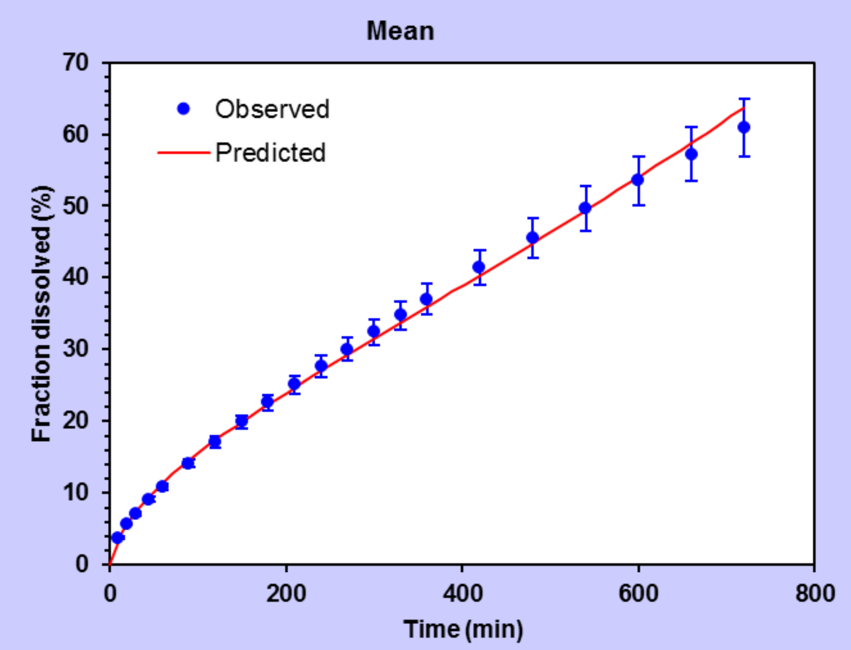

Supplementary materials – Model fitting summary – Di-Cafos® A12 (modelling up to app. 60 % of carvedilol released)

Graphical abstract of model fit presented as the fraction % of released carvedilol per tested tablet:

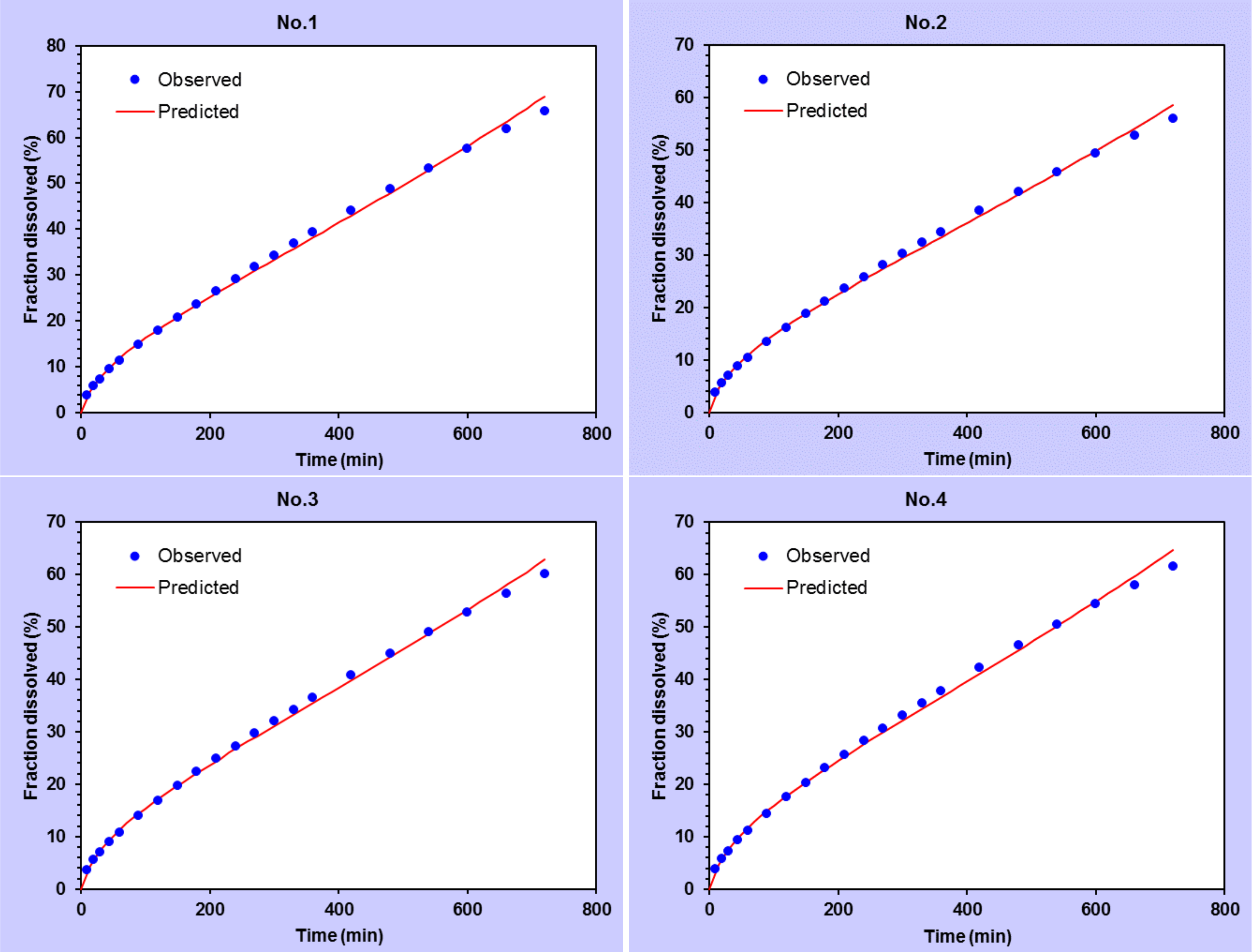

Supplementary materials – Model fitting summary – Di-Cafos® A12 (modelling up to app. 60 % of carvedilol released)

Model: **Peppas–Sahlin_1**

Model equation: $F = k_1 \cdot t^m + k_2 \cdot t^{2m}$

Fitted model parameters per tested tablet (N = 4) with statistics – mean, standard deviation (SD), and relative standard deviation expressed in % (RSD%) (output from DDSolver):

Parameter	No.1	No.2	No.3	No.4	Mean	SD	RSD(%)
k_1	1.014	1.057	1.044	1.109	1.056	0.039	3.737
k_2	0.125	0.096	0.108	0.109	0.110	0.012	10.577
m	0.450	0.450	0.450	0.450	0.450	0.000	0.000

Number of dissolution data points (N), degrees of freedom (df), and selected goodness of fit criteria – Pearson correlation coefficient (R), coefficient of determination (R^2), adjusted coefficient of determination ($R^2_{adjusted}$), and residual sum of squares (RSS) (manual calculation in MS Excel):

Parameter	No.1	No.2	No.3	No.4
N	21	21	21	21
df	18	18	18	18
R	0.999977121	0.999968934	0.999967614	0.999917338
R^2	0.999954242	0.999937868	0.99993523	0.999834683
$R^2_{adjusted}$	0.999949158	0.999930965	0.999928033	0.999816314
RSS	0.335908712	0.331508543	0.41224637	1.10332627

Graphical abstract of model fit presented as mean ± 1 SD of the fraction % of released carvedilol:

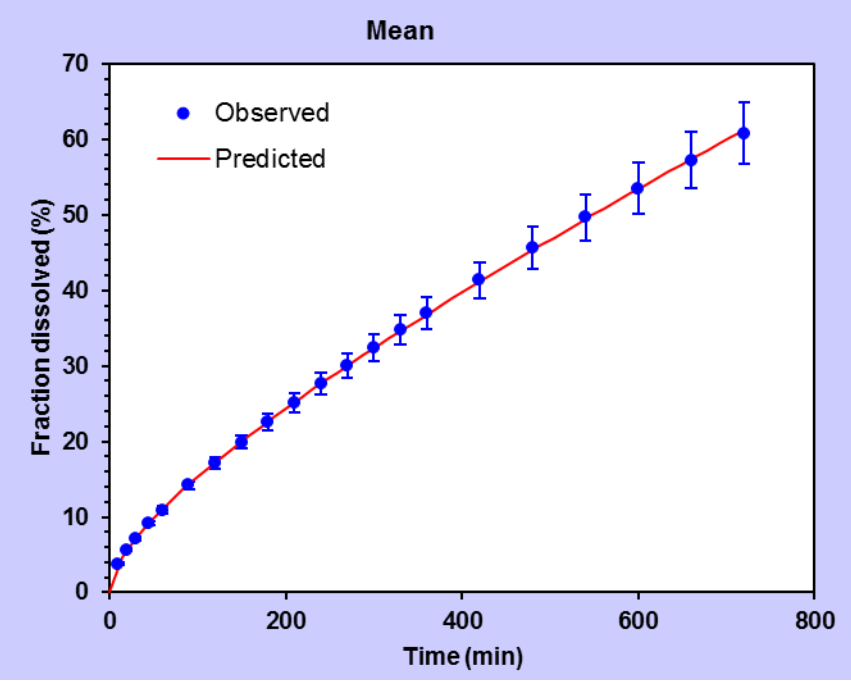

Supplementary materials – Model fitting summary – Di-Cafos® A12 (modelling up to app. 60 % of carvedilol released)

Graphical abstract of model fit presented as the fraction % of released carvedilol per tested tablet:

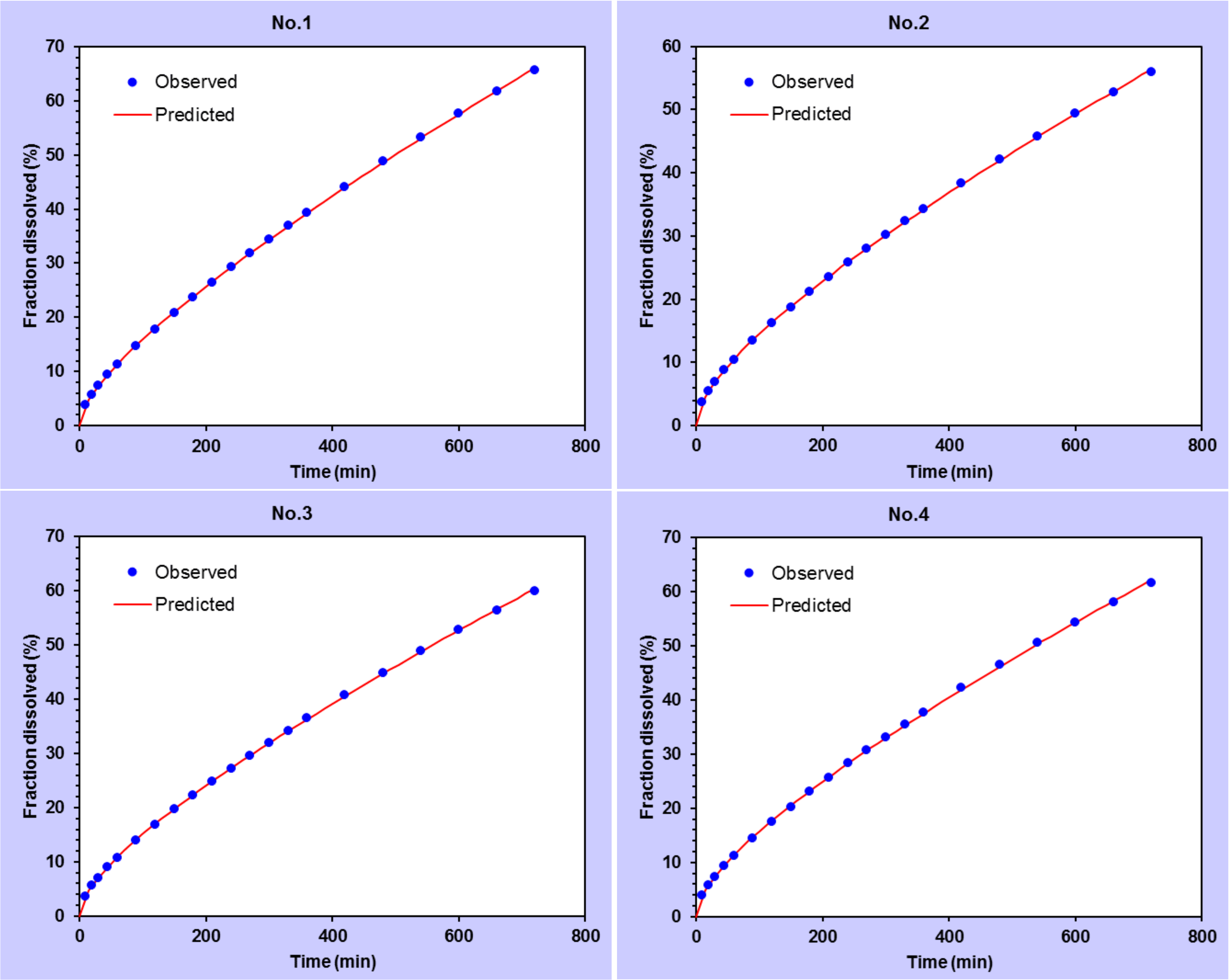

Supplementary materials – Model fitting summary – Di-Cafos® A12 (modelling up to app. 60 % of carvedilol released)

Model: **Peppas–Sahlin_1 with T_{lag}**

Model equation: $F = k_1 \cdot (t - T_{lag})^m + k_2 \cdot (t - T_{lag})^{2m}$

Fitted model parameters per tested tablet (N = 4) with statistics – mean, standard deviation (SD), and relative standard deviation expressed in % (RSD%) (output from DDSolver):

Parameter	No.1	No.2	No.3	No.4	Mean	SD	RSD(%)
k_1	1.111	1.143	1.135	1.203	1.148	0.039	3.414
k_2	0.120	0.092	0.103	0.105	0.105	0.011	10.861
m	0.450	0.450	0.450	0.450	0.450	0.000	0.000
T_{lag}	4.000	4.000	4.000	4.000	4.000	0.000	0.000

Number of dissolution data points (N), degrees of freedom (df), and selected goodness of fit criteria – Pearson correlation coefficient (R), coefficient of determination (R^2), adjusted coefficient of determination ($R^2_{adjusted}$), and residual sum of squares (RSS) (manual calculation in MS Excel):

Parameter	No.1	No.2	No.3	No.4
N	21	21	21	21
df	17	17	17	17
R	0.999912223	0.999915119	0.999935745	0.999896143
R^2	0.999824454	0.999830245	0.999871495	0.999792296
$R^2_{adjusted}$	0.999793475	0.999800288	0.999848818	0.999755642
RSS	1.463019502	0.991015289	0.852989698	1.384766206

Graphical abstract of model fit presented as mean ± 1 SD of the fraction % of released carvedilol:

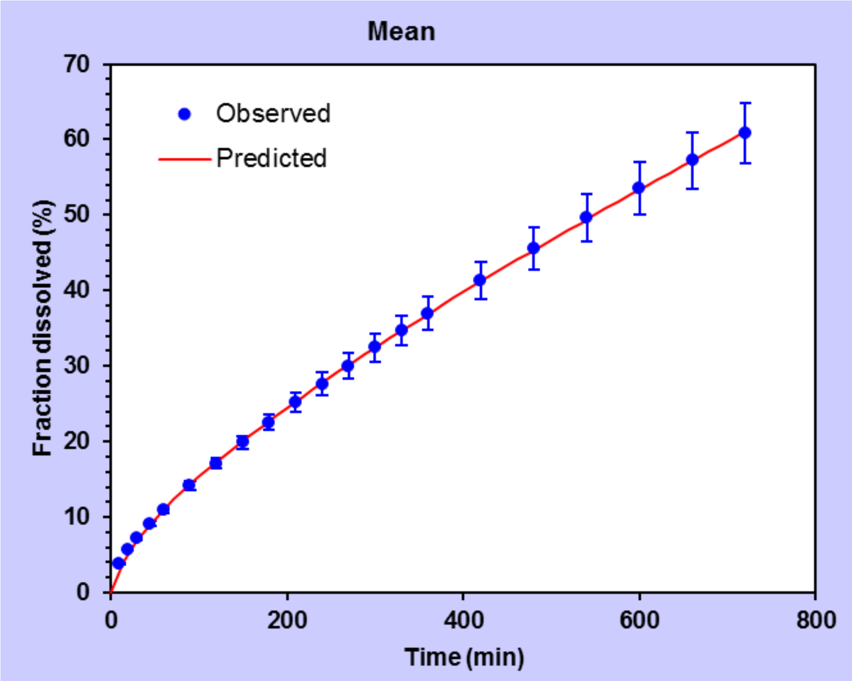

Supplementary materials – Model fitting summary – Di-Cafos® A12 (modelling up to app. 60 % of carvedilol released)

Graphical abstract of model fit presented as the fraction % of released carvedilol per tested tablet:

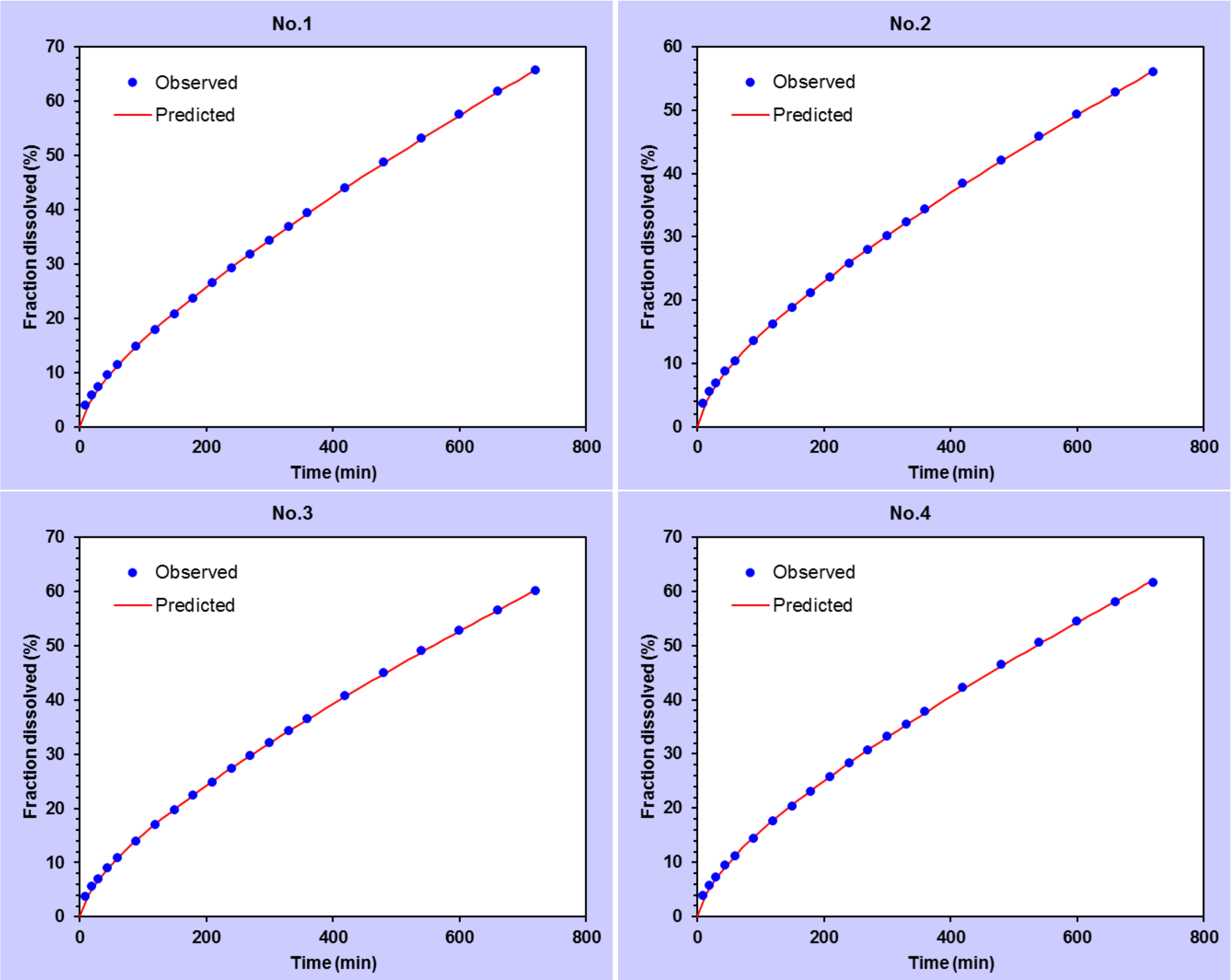

Supplementary materials – Model fitting summary – Di-Cafos® A12 (modelling up to app. 60 % of carvedilol released)

Model: **Peppas–Sahlin_2**

Model equation: $F = k_1 \cdot t^{0.5} + k_2 \cdot t$

Fitted model parameters per tested tablet (N = 4) with statistics – mean, standard deviation (SD), and relative standard deviation expressed in % (RSD%) (output from DDSolver):

Parameter	No.1	No.2	No.3	No.4	Mean	SD	RSD(%)
k_1	1.084	1.067	1.079	1.136	1.091	0.031	2.822
k_2	0.052	0.039	0.044	0.044	0.045	0.005	11.953

Number of dissolution data points (N), degrees of freedom (df), and selected goodness of fit criteria – Pearson correlation coefficient (R), coefficient of determination (R^2), adjusted coefficient of determination ($R^2_{adjusted}$), and residual sum of squares (RSS) (manual calculation in MS Excel):

Parameter	No.1	No.2	No.3	No.4
N	21	21	21	21
df	19	19	19	19
R	0.999952331	0.999948068	0.999937225	0.99987704
R^2	0.999904664	0.999896139	0.999874454	0.999754096
$R^2_{adjusted}$	0.999899646	0.999890673	0.999867846	0.999741154
RSS	0.727422815	0.570530807	0.834571952	1.69559774

Graphical abstract of model fit presented as mean ± 1 SD of the fraction % of released carvedilol:

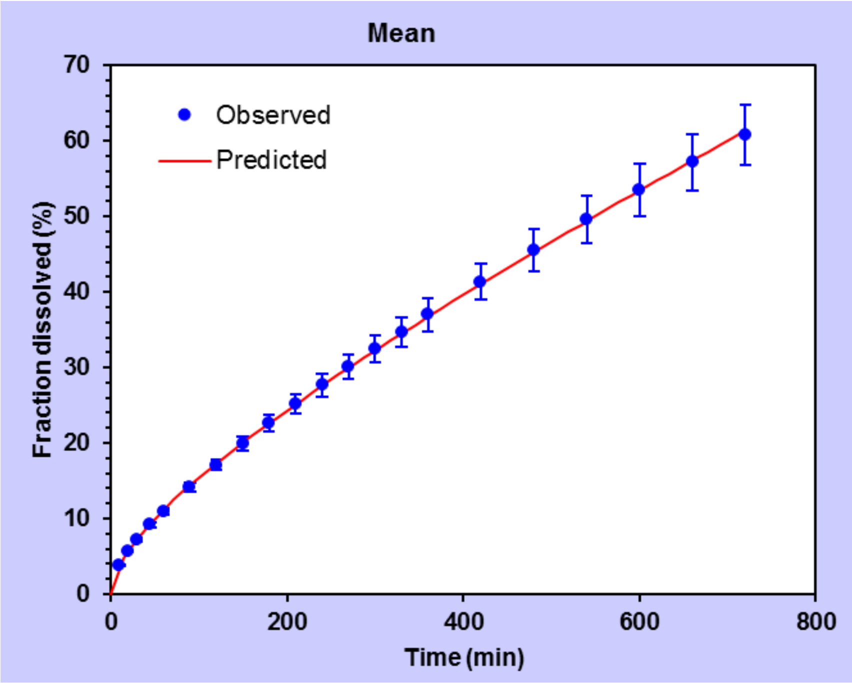

Supplementary materials – Model fitting summary – Di-Cafos® A12 (modelling up to app. 60 % of carvedilol released)

Graphical abstract of model fit presented as the fraction % of released carvedilol per tested tablet:

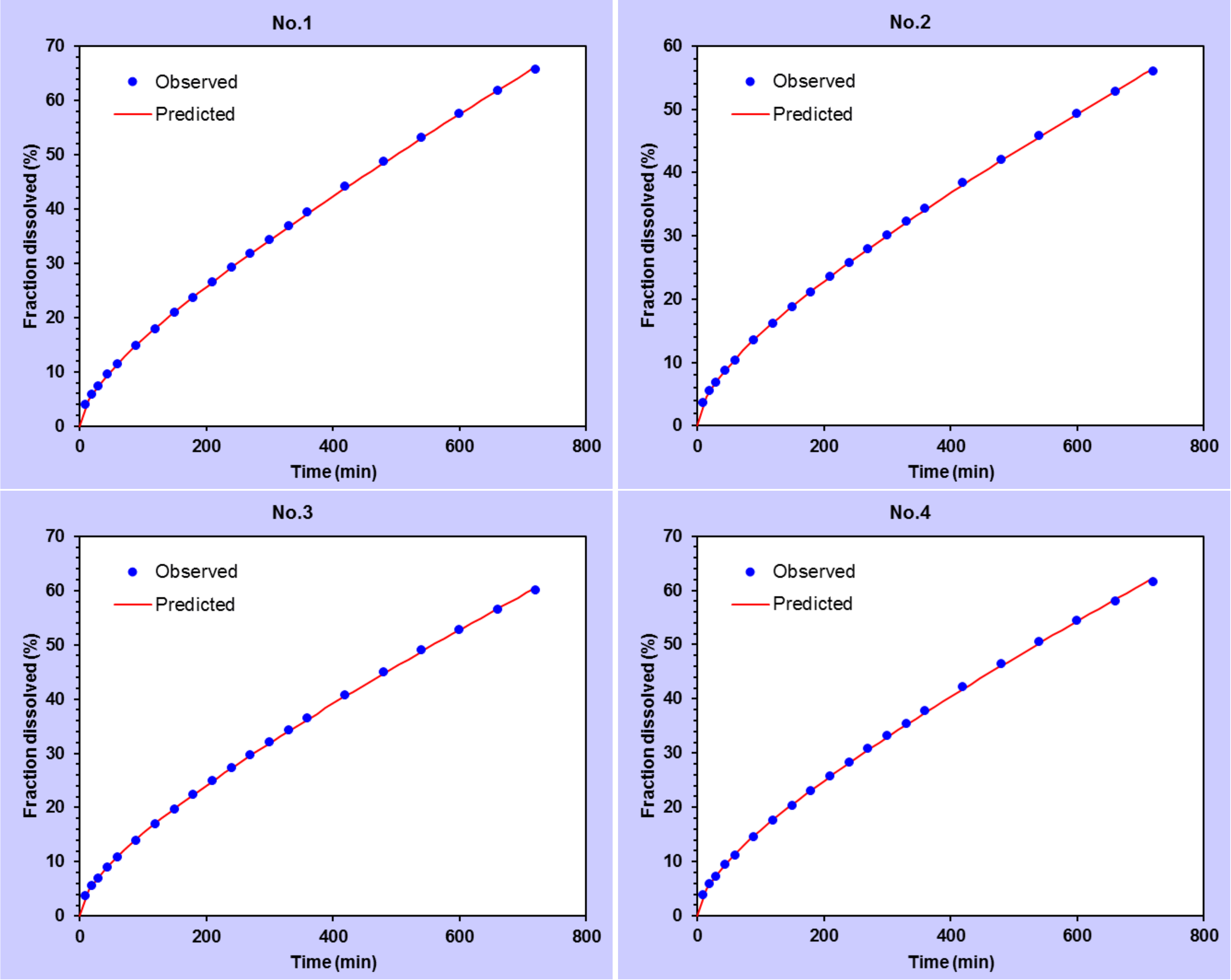

Supplementary materials – Model fitting summary – Di-Cafos® A12 (modelling up to app. 60 % of carvedilol released)

Model: **Peppas–Sahlin_2 with T_{lag}**

Model equation: $F = k_1 \cdot (t - T_{lag})^{0.5} + k_2 \cdot (t - T_{lag})$

Fitted model parameters per tested tablet (N = 4) with statistics – mean, standard deviation (SD), and relative standard deviation expressed in % (RSD%) (output from DDSolver):

Parameter	No.1	No.2	No.3	No.4	Mean	SD	RSD(%)
k_1	1.153	1.127	1.143	1.203	1.156	0.033	2.831
k_2	0.049	0.036	0.042	0.042	0.042	0.005	12.387
T_{lag}	4.000	4.000	4.000	4.000	4.000	0.000	0.000

Number of dissolution data points (N), degrees of freedom (df), and selected goodness of fit criteria – Pearson correlation coefficient (R), coefficient of determination (R^2), adjusted coefficient of determination ($R^2_{adjusted}$), and residual sum of squares (RSS) (manual calculation in MS Excel):

Parameter	No.1	No.2	No.3	No.4
N	21	21	21	21
df	18	18	18	18
R	0.99991453	0.999902904	0.999923492	0.999871638
R^2	0.999829067	0.999805818	0.99984699	0.999743292
$R^2_{adjusted}$	0.999810074	0.999784242	0.999829989	0.999714768
RSS	1.373900883	1.123070155	0.982792184	1.683861161

Graphical abstract of model fit presented as mean ± 1 SD of the fraction % of released carvedilol:

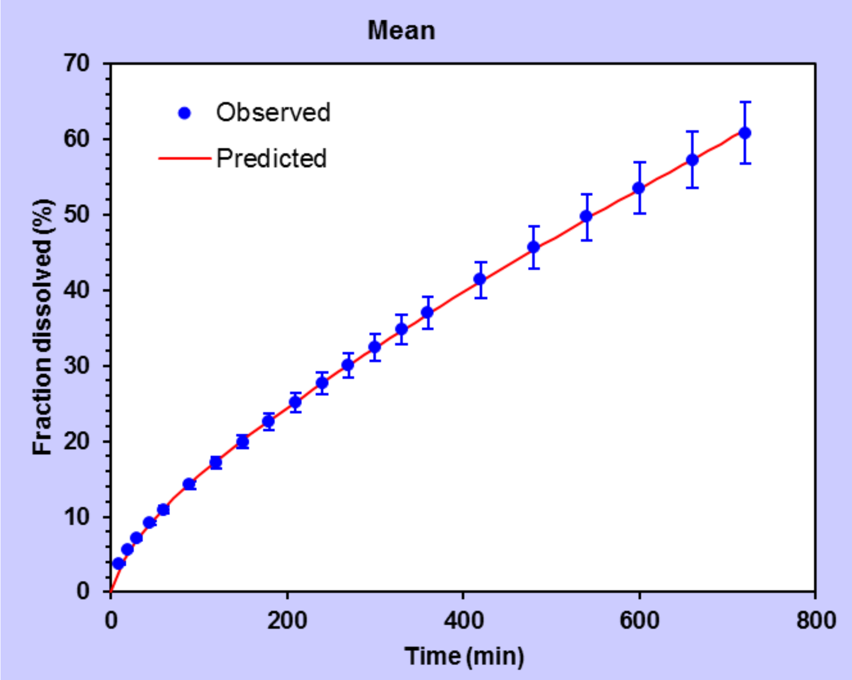

Supplementary materials – Model fitting summary – Di-Cafos® A12 (modelling up to app. 60 % of carvedilol released)

Graphical abstract of model fit presented as the fraction % of released carvedilol per tested tablet:

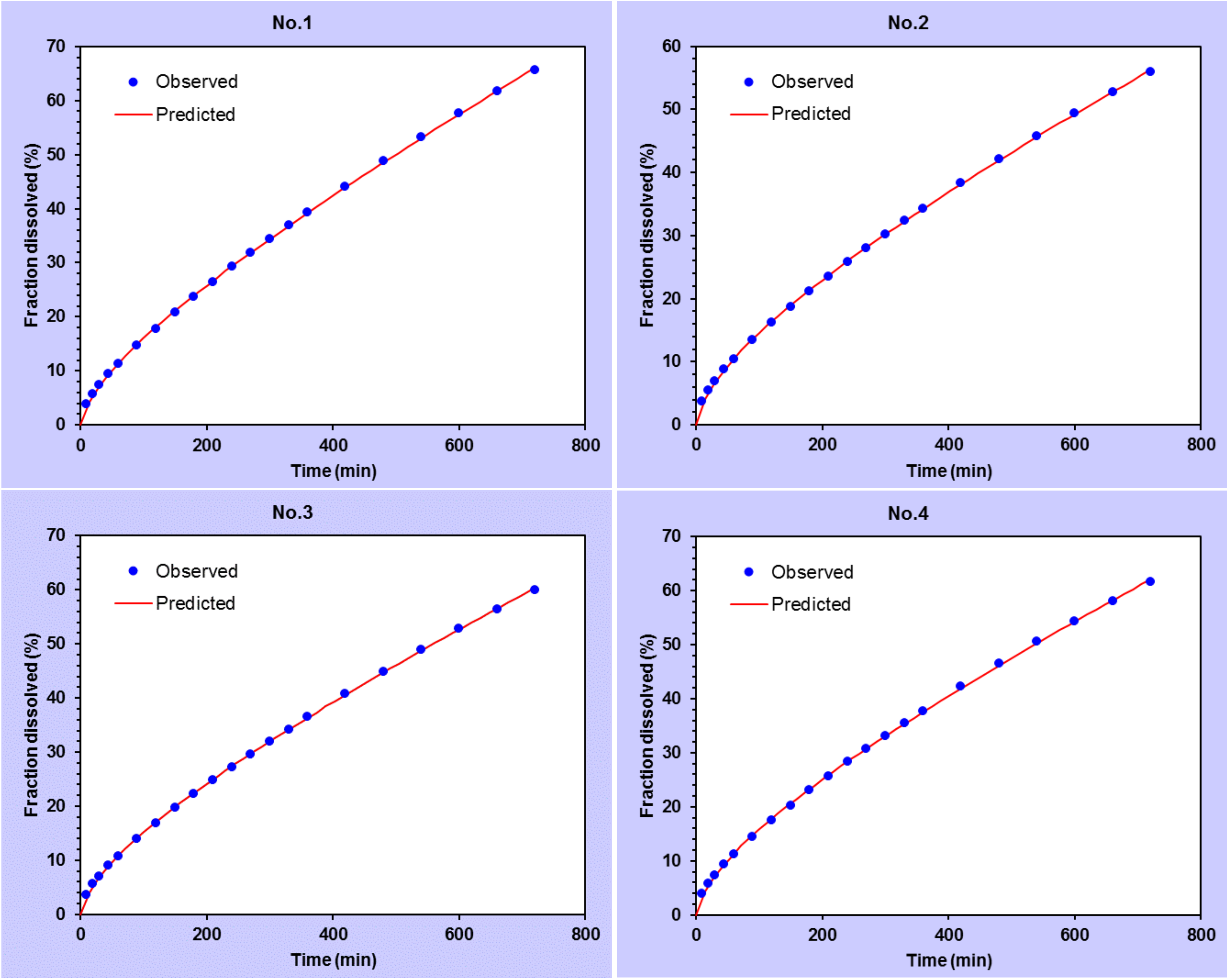

Supplementary materials – Model fitting summary – Di-Cafos® A12 (modelling up to app. 60 % of carvedilol released)

Model: **Quadratic**

Model equation: $F = 100 \cdot (k_1 \cdot t^2 + k_2 \cdot t)$

Fitted model parameters per tested tablet (N = 4) with statistics – mean, standard deviation (SD), and relative standard deviation expressed in % (RSD%) (output from DDSolver):

Parameter	No.1	No.2	No.3	No.4	Mean	SD	RSD(%)
k_1	-0.000001	-0.000001	-0.000001	-0.000001	-0.000001	0.000000	-3.370520
k_2	0.001381	0.001237	0.001301	0.001353	0.001318	0.000063	4.804215

Number of dissolution data points (N), degrees of freedom (df), and selected goodness of fit criteria – Pearson correlation coefficient (R), coefficient of determination (R^2), adjusted coefficient of determination ($R^2_{adjusted}$), and residual sum of squares (RSS) (manual calculation in MS Excel):

Parameter	No.1	No.2	No.3	No.4
N	21	21	21	21
df	19	19	19	19
R	0.997796873	0.997090333	0.997474577	0.997560636
R^2	0.9955986	0.994189132	0.994955532	0.995127222
$R^2_{adjusted}$	0.995366947	0.993883297	0.994690034	0.99487076
RSS	83.59298743	81.74668035	80.23655847	84.34461488

Graphical abstract of model fit presented as mean ± 1 SD of the fraction % of released carvedilol:

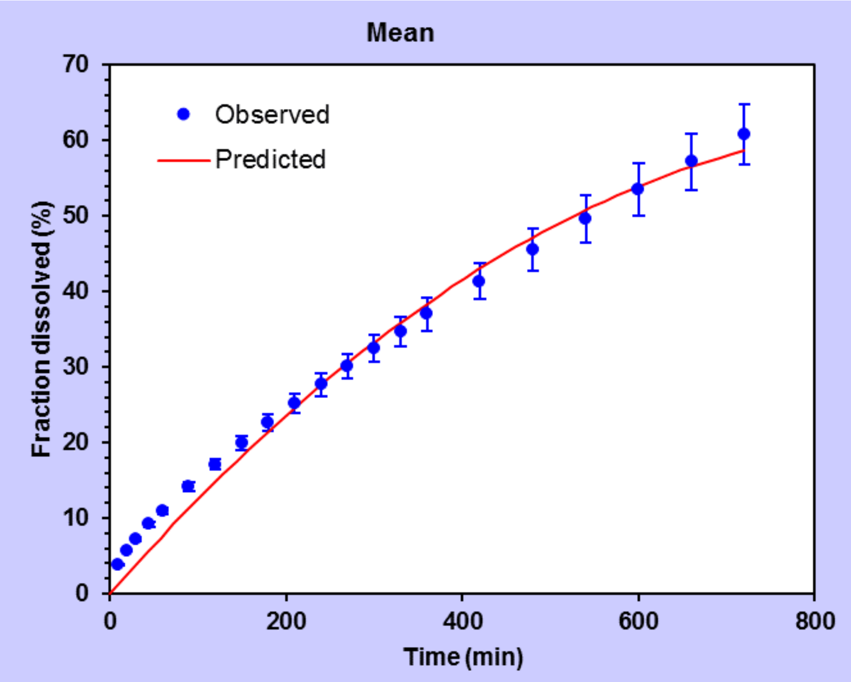

Supplementary materials – Model fitting summary – Di-Cafos® A12 (modelling up to app. 60 % of carvedilol released)

Graphical abstract of model fit presented as the fraction % of released carvedilol per tested tablet:

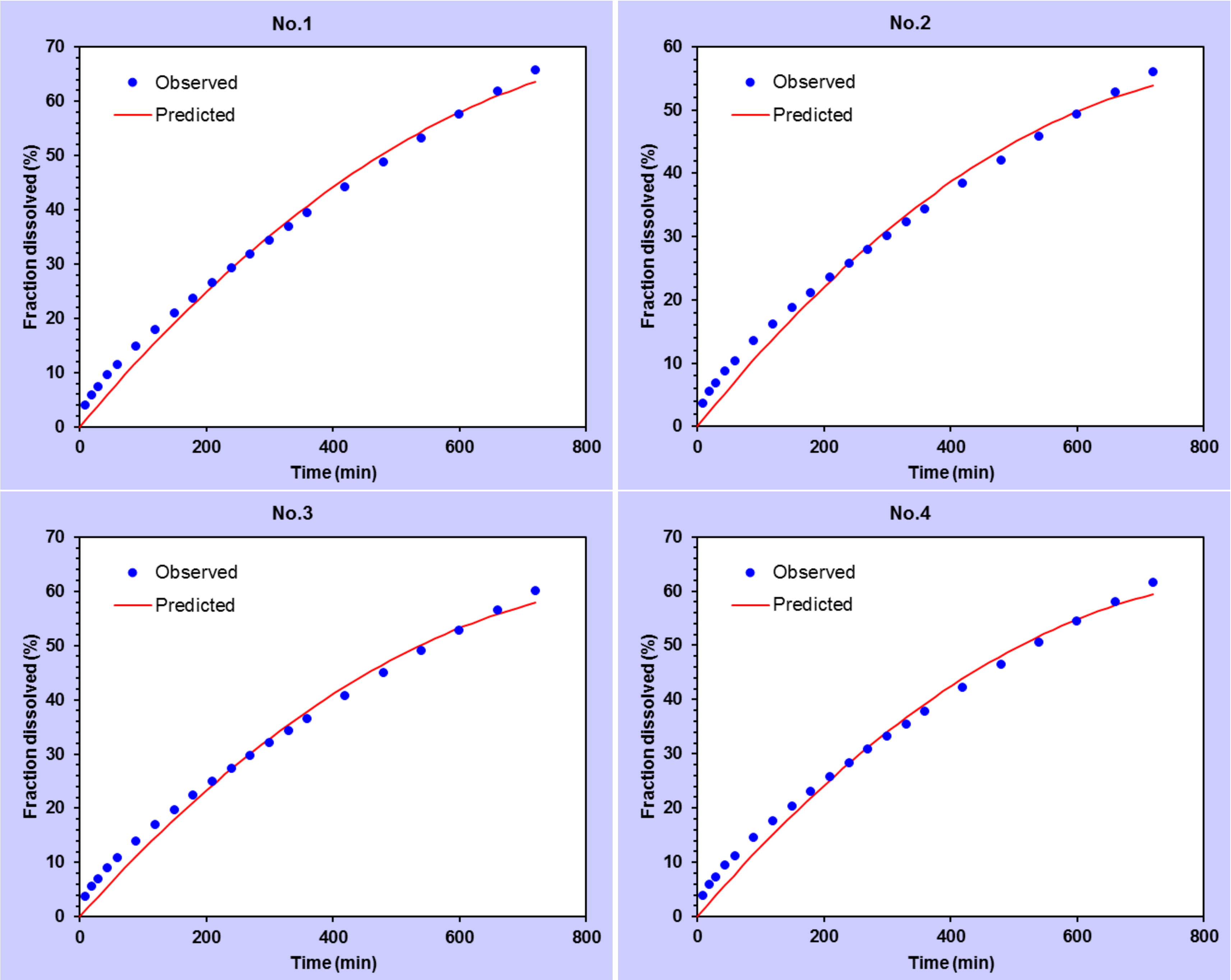

Supplementary materials – Model fitting summary – Di-Cafos® A12 (modelling up to app. 60 % of carvedilol released)

Model: **Quadratic with T_{lag}**

Model equation: $F = 100 \cdot \left[k_1 \cdot \left(t - T_{lag}\right)^2 + k_2 \cdot \left(t - T_{lag}\right)\right]$

Fitted model parameters per tested tablet (N = 4) with statistics – mean, standard deviation (SD), and relative standard deviation expressed in % (RSD%) (output from DDSolver):

Parameter	No.1	No.2	No.3	No.4	Mean	SD	RSD(%)
k_1	-0.000001	-0.000001	-0.000001	-0.000001	-0.000001	0.000000	-3.357162
k_2	0.001406	0.001258	0.001324	0.001377	0.001341	0.000065	4.826335
T_{lag}	4.000000	4.000000	4.000000	4.000000	4.000000	0.000000	0.000000

Number of dissolution data points (N), degrees of freedom (df), and selected goodness of fit criteria – Pearson correlation coefficient (R), coefficient of determination (R^2), adjusted coefficient of determination ($R^2_{adjusted}$), and residual sum of squares (RSS) (manual calculation in MS Excel):

Parameter	No.1	No.2	No.3	No.4
N	21	21	21	21
df	18	18	18	18
R	0.997423913	0.996673362	0.997082638	0.997168203
R^2	0.994854462	0.993357791	0.994173787	0.994344425
$R^2_{adjusted}$	0.994282735	0.992619768	0.99352643	0.993716028
RSS	105.5555625	101.0517384	100.3646763	105.9386568

Graphical abstract of model fit presented as mean ± 1 SD of the fraction % of released carvedilol:

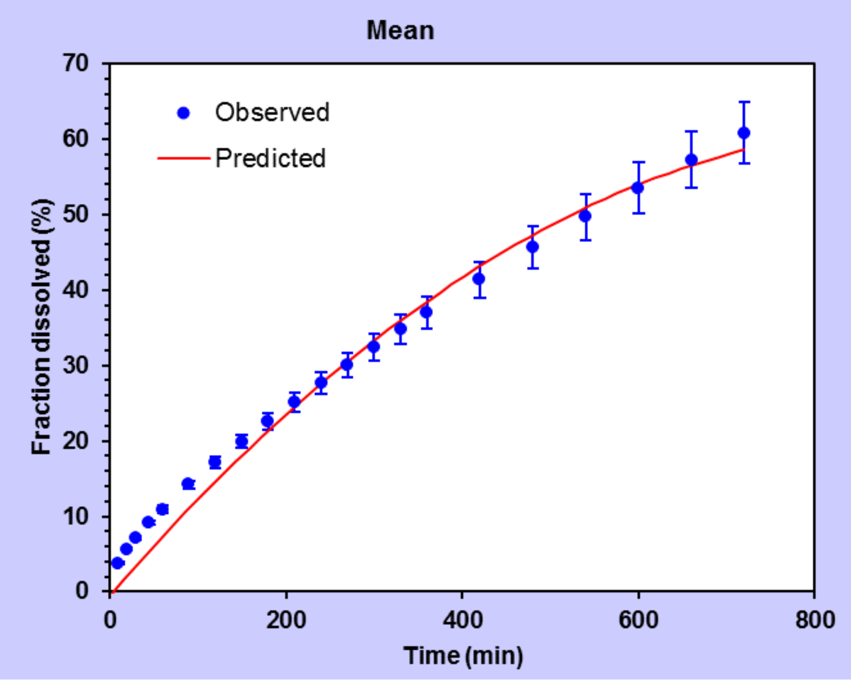

Supplementary materials – Model fitting summary – Di-Cafos® A12 (modelling up to app. 60 % of carvedilol released)

Graphical abstract of model fit presented as the fraction % of released carvedilol per tested tablet:

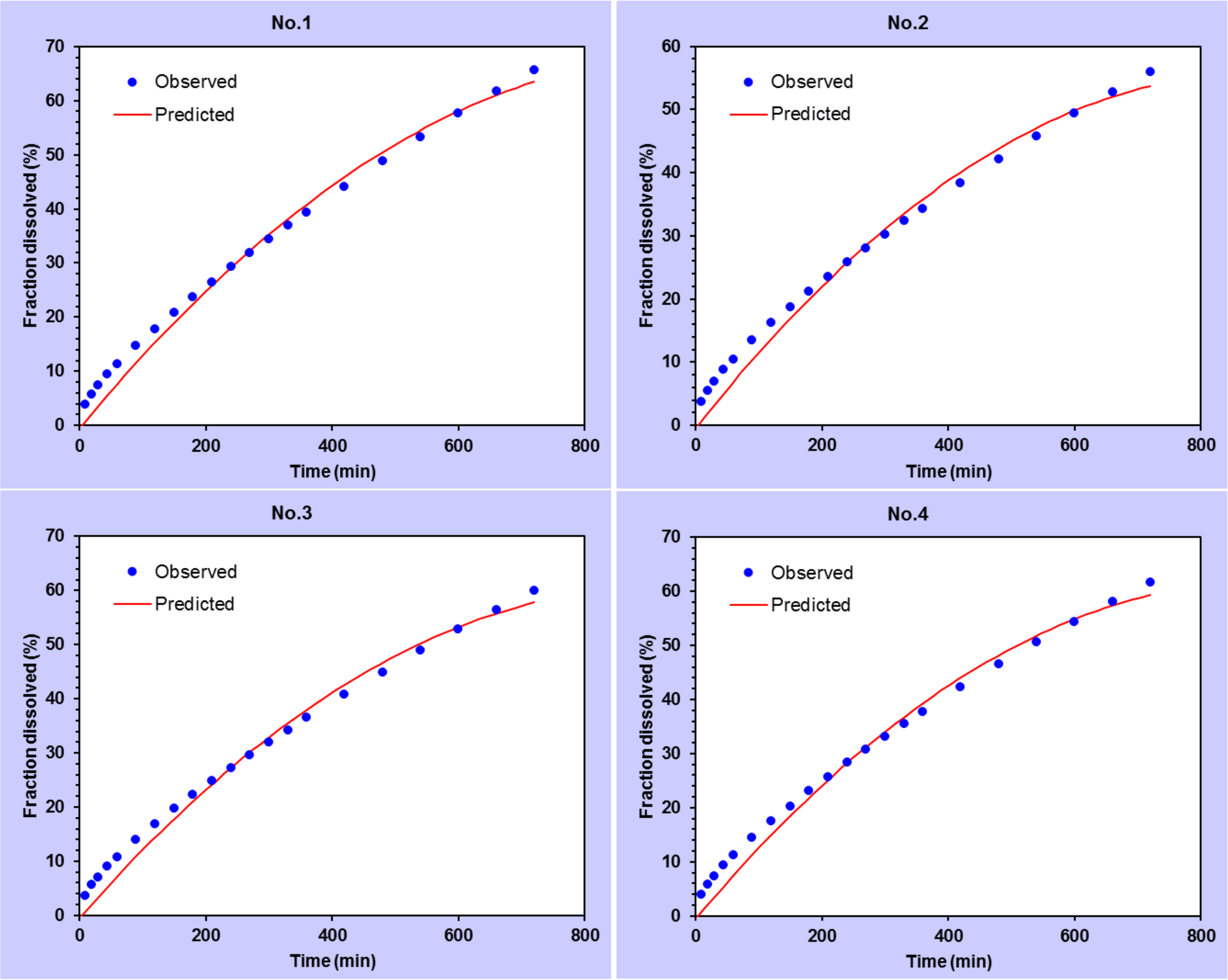

Supplementary materials – Model fitting summary – Di-Cafos® A12 (modelling up to app. 60 % of carvedilol released)

Model: **Weibull_1**

Model equation: $F = 100 \cdot \left[1 - e^{-\frac{(t-T_i)^\beta}{\alpha}}\right]$

Fitted model parameters per tested tablet (N = 4) with statistics – mean, standard deviation (SD), and relative standard deviation expressed in % (RSD%) (output from DDSolver):

Parameter	No.1	No.2	No.3	No.4	Mean	SD	RSD(%)
α	128.989	119.862	126.481	121.225	124.139	4.312	3.474
β	0.712	0.669	0.692	0.692	0.691	0.018	2.570
T_i	6.000	6.000	6.000	6.000	6.000	0.000	0.000

Number of dissolution data points (N), degrees of freedom (df), and selected goodness of fit criteria – Pearson correlation coefficient (R), coefficient of determination (R^2), adjusted coefficient of determination ($R^2_{adjusted}$), and residual sum of squares (RSS) (manual calculation in MS Excel):

Parameter	No.1	No.2	No.3	No.4
N	21	21	21	21
df	18	18	18	18
R	0.989509203	0.991644484	0.991387916	0.991363404
R^2	0.979128462	0.983358783	0.982849999	0.982801398
$R^2_{adjusted}$	0.976809402	0.981509759	0.980944444	0.980890443
RSS	235.0366616	137.4632168	163.9181191	173.2074482

Graphical abstract of model fit presented as mean ± 1 SD of the fraction % of released carvedilol:

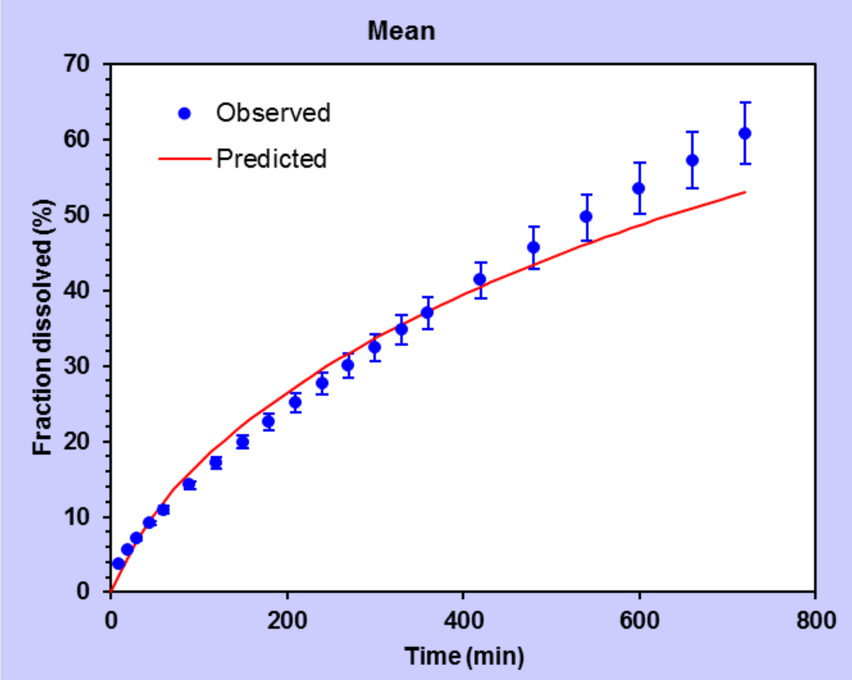

Supplementary materials – Model fitting summary – Di-Cafos® A12 (modelling up to app. 60 % of carvedilol released)

Graphical abstract of model fit presented as the fraction % of released carvedilol per tested tablet:

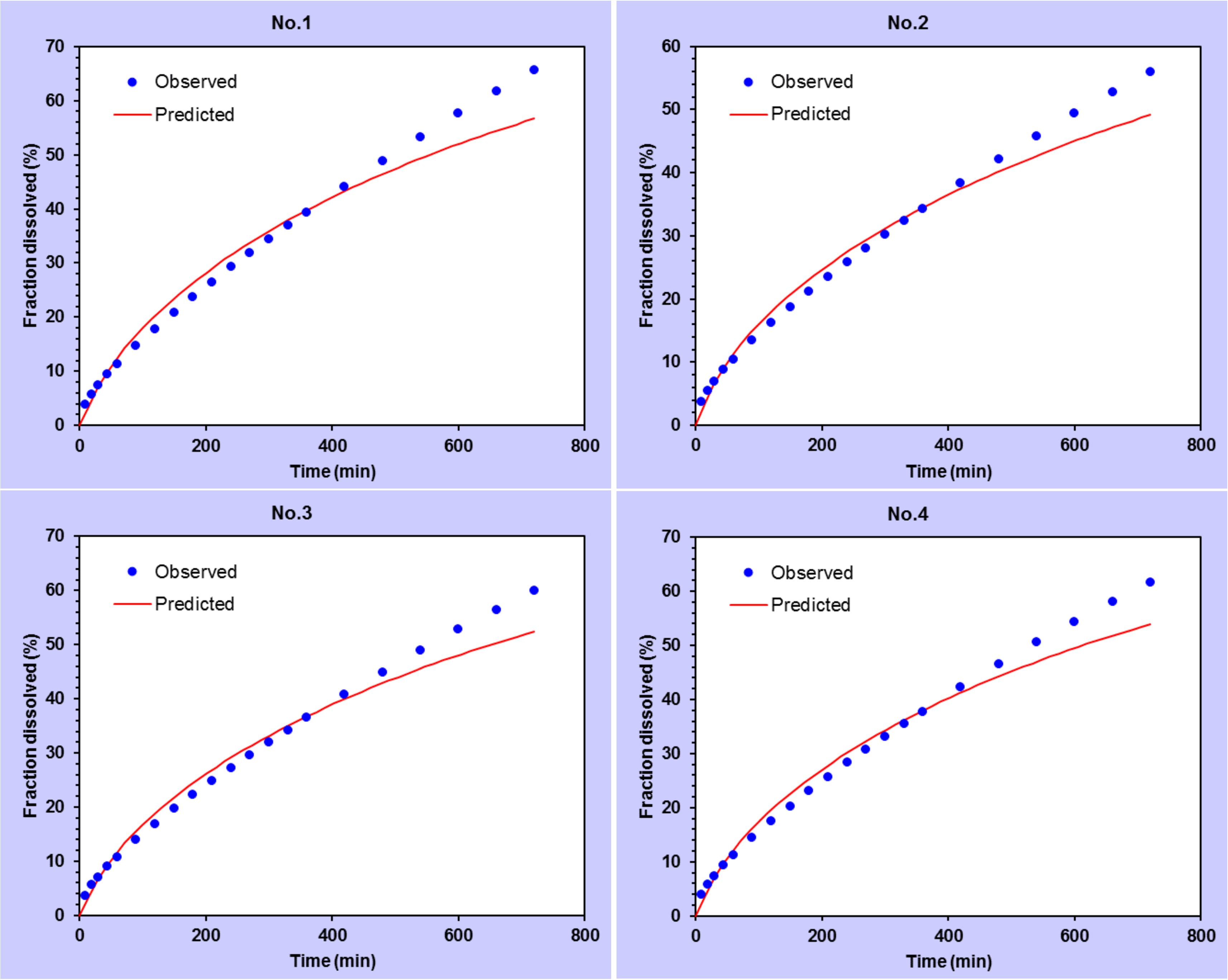

Supplementary materials – Model fitting summary – Di-Cafos® A12 (modelling up to app. 60 % of carvedilol released)

Model: **Weibull_2**

Model equation: $F = 100 \cdot \left(1 - e^{-\frac{t^\beta}{\alpha}}\right)$

Fitted model parameters per tested tablet (N = 4) with statistics – mean, standard deviation (SD), and relative standard deviation expressed in % (RSD%) (output from DDSolver):

Parameter	No.1	No.2	No.3	No.4	Mean	SD	RSD(%)
α	184.870	167.675	202.571	194.287	187.351	14.979	7.995
β	0.774	0.726	0.784	0.784	0.767	0.027	3.583

Number of dissolution data points (N), degrees of freedom (df), and selected goodness of fit criteria – Pearson correlation coefficient (R), coefficient of determination (R^2), adjusted coefficient of determination ($R^2_{adjusted}$), and residual sum of squares (RSS) (manual calculation in MS Excel):

Parameter	No.1	No.2	No.3	No.4
N	21	21	21	21
df	19	19	19	19
R	0.993341096	0.995195636	0.995277231	0.995261029
R^2	0.986726533	0.990414354	0.990576767	0.990544517
$R^2_{adjusted}$	0.98602793	0.989909846	0.990080808	0.99004686
RSS	145.4999803	77.63507884	88.73448848	93.88135435

Graphical abstract of model fit presented as mean ± 1 SD of the fraction % of released carvedilol:

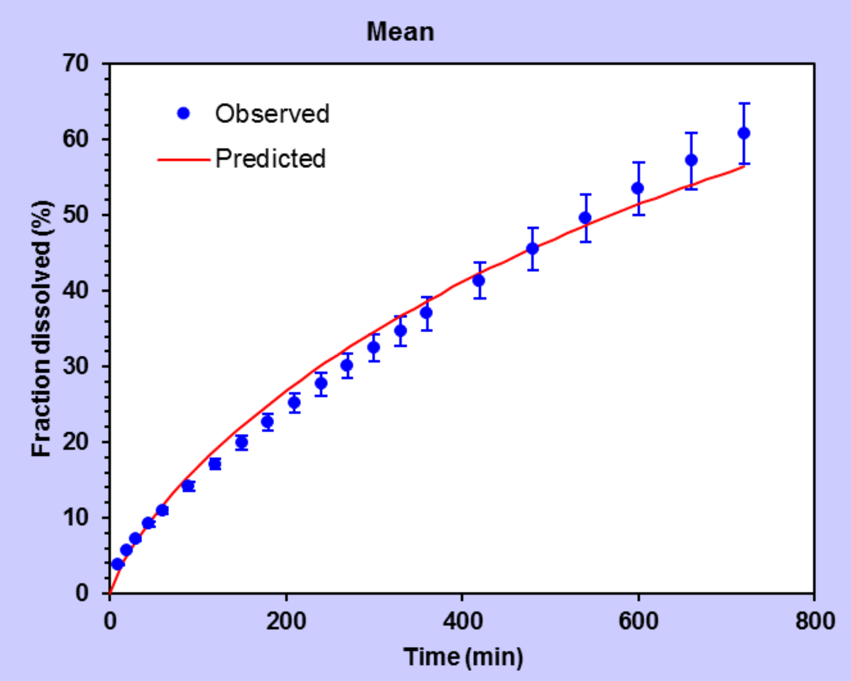

Supplementary materials – Model fitting summary – Di-Cafos® A12 (modelling up to app. 60 % of carvedilol released)

Graphical abstract of model fit presented as the fraction % of released carvedilol per tested tablet:

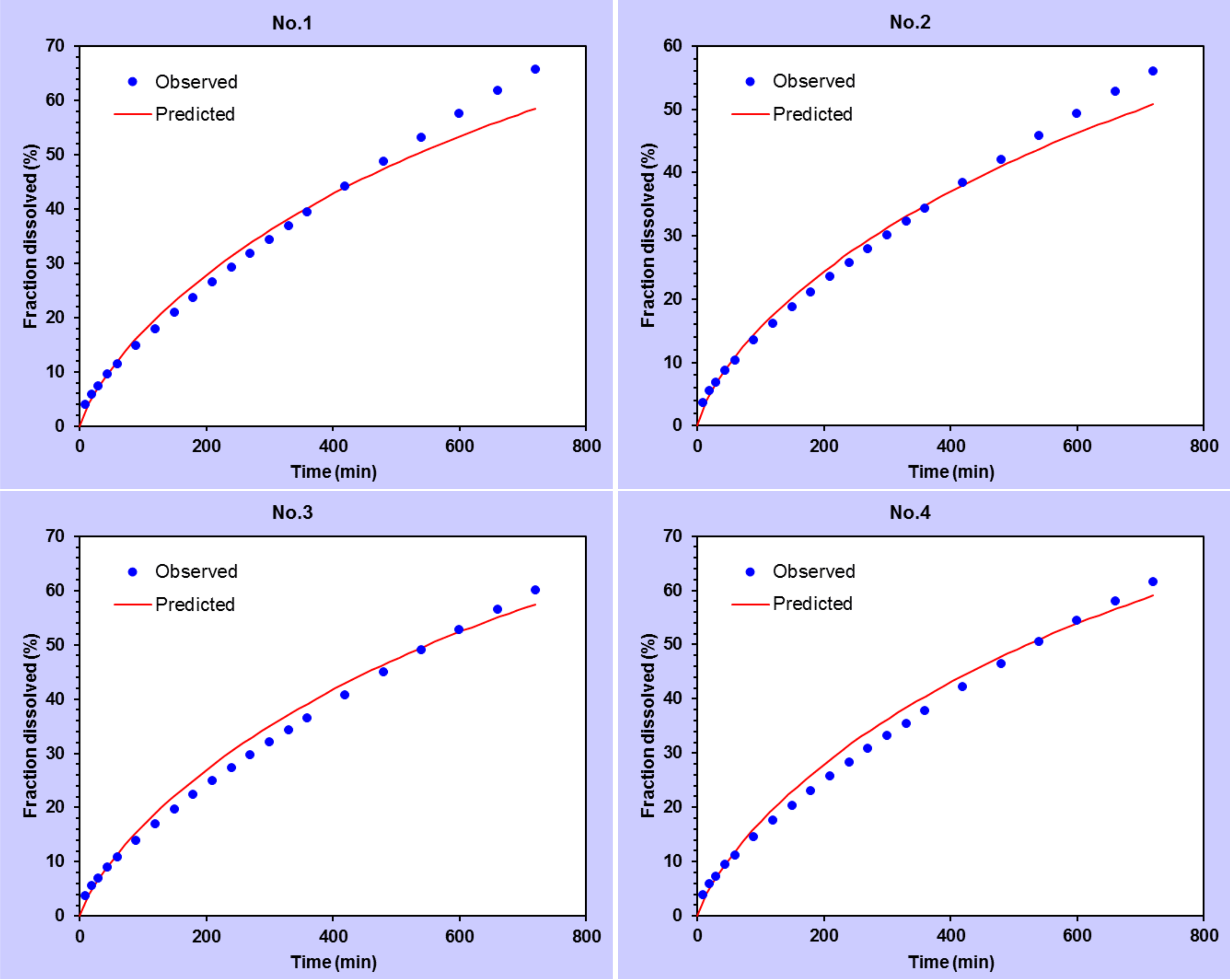

Supplementary materials – Model fitting summary – Di-Cafos® A12 (modelling up to app. 60 % of carvedilol released)

Model: **Weibull_3**

Model equation: $F = F_{max} \cdot \left(1 - e^{-\frac{t^{\beta}}{\alpha}}\right)$

Fitted model parameters per tested tablet (N = 4) with statistics – mean, standard deviation (SD), and relative standard deviation expressed in % (RSD%) (output from DDSolver):

Parameter	No.1	No.2	No.3	No.4	Mean	SD	RSD(%)
α	185.117	153.777	171.134	167.076	169.276	12.903	7.622
β	0.877	0.851	0.867	0.865	0.865	0.011	1.217
F_{max}	68.971	58.770	63.037	64.680	63.865	4.218	6.605

Number of dissolution data points (N), degrees of freedom (df), and selected goodness of fit criteria – Pearson correlation coefficient (R), coefficient of determination (R^2), adjusted coefficient of determination ($R^2_{adjusted}$), and residual sum of squares (RSS) (manual calculation in MS Excel):

Parameter	No.1	No.2	No.3	No.4
N	21	21	21	21
df	18	18	18	18
R	0.983844148	0.984105068	0.984625232	0.984941036
R^2	0.967949307	0.968462784	0.969486847	0.970108844
$R^2_{adjusted}$	0.964388119	0.964958649	0.966096496	0.966787604
RSS	276.8969551	191.7495386	216.8569411	225.2544537

Graphical abstract of model fit presented as mean ± 1 SD of the fraction % of released carvedilol:

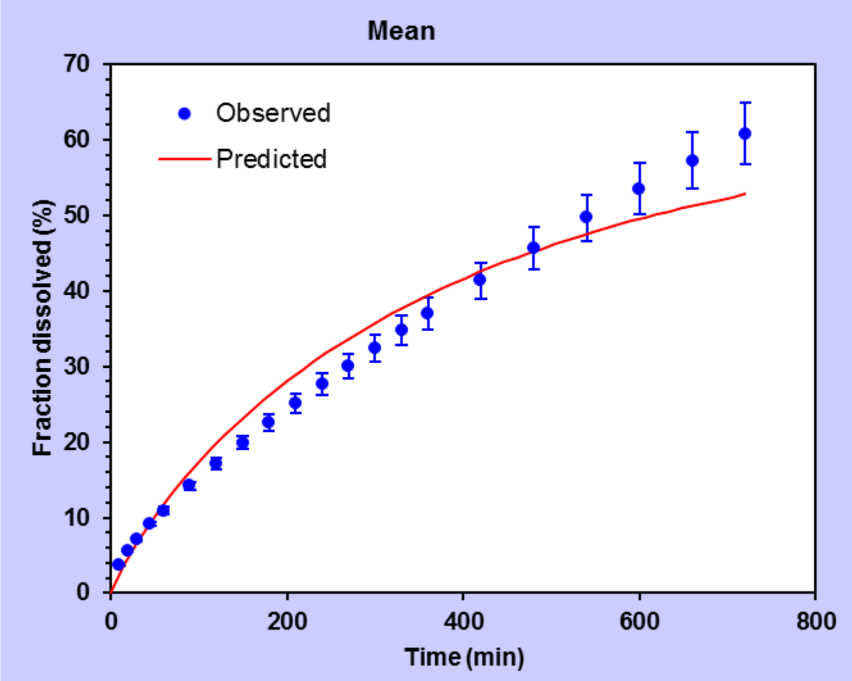

Supplementary materials – Model fitting summary – Di-Cafos® A12 (modelling up to app. 60 % of carvedilol released)

Graphical abstract of model fit presented as the fraction % of released carvedilol per tested tablet:

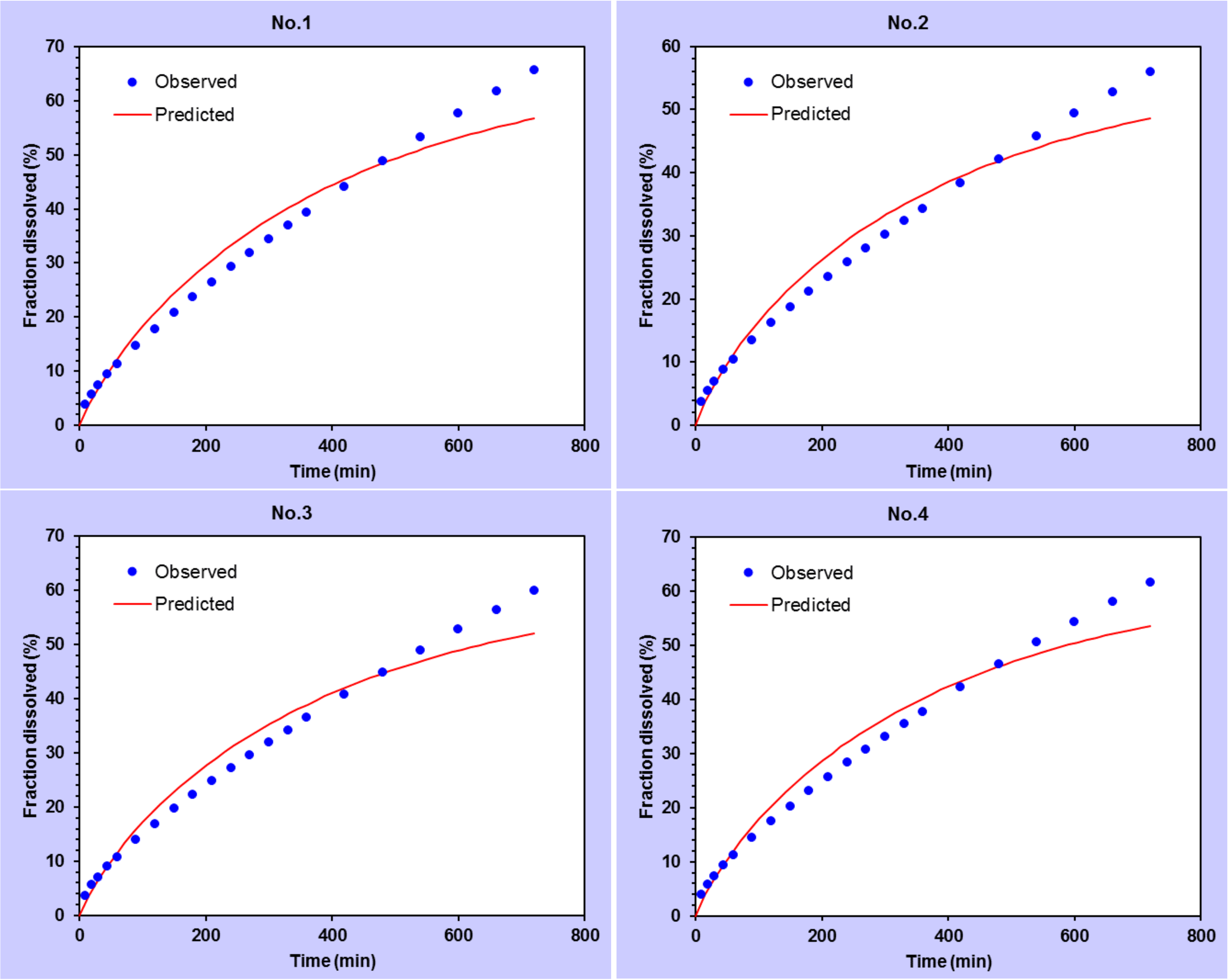

Supplementary materials – Model fitting summary – Di-Cafos® A12 (modelling up to app. 60 % of carvedilol released)

Model: **Weibull_4**

Model equation: $F = F_{max} \cdot \left[1 - e^{-\frac{(t-T_i)^\beta}{\alpha}}\right]$

Fitted model parameters per tested tablet (N = 4) with statistics – mean, standard deviation (SD), and relative standard deviation expressed in % (RSD%) (output from DDSolver):

Parameter	No.1	No.2	No.3	No.4	Mean	SD	RSD(%)
α	121.886	102.510	113.296	110.674	112.091	7.983	7.122
β	0.805	0.782	0.796	0.794	0.794	0.010	1.217
T_i	6.000	6.000	6.000	6.000	6.000	0.000	0.000
F_{max}	68.971	58.770	63.037	64.680	63.865	4.218	6.605

Number of dissolution data points (N), degrees of freedom (df), and selected goodness of fit criteria – Pearson correlation coefficient (R), coefficient of determination (R^2), adjusted coefficient of determination ($R^2_{adjusted}$), and residual sum of squares (RSS) (manual calculation in MS Excel):

Parameter	No.1	No.2	No.3	No.4
N	21	21	21	21
df	17	17	17	17
R	0.979361428	0.97954893	0.980230483	0.98052031
R^2	0.959148807	0.959516107	0.960851801	0.961420078
$R^2_{adjusted}$	0.951939773	0.952371891	0.953943295	0.954611856
RSS	353.8999094	245.12926	278.1349656	290.0849071

Graphical abstract of model fit presented as mean ± 1 SD of the fraction % of released carvedilol:

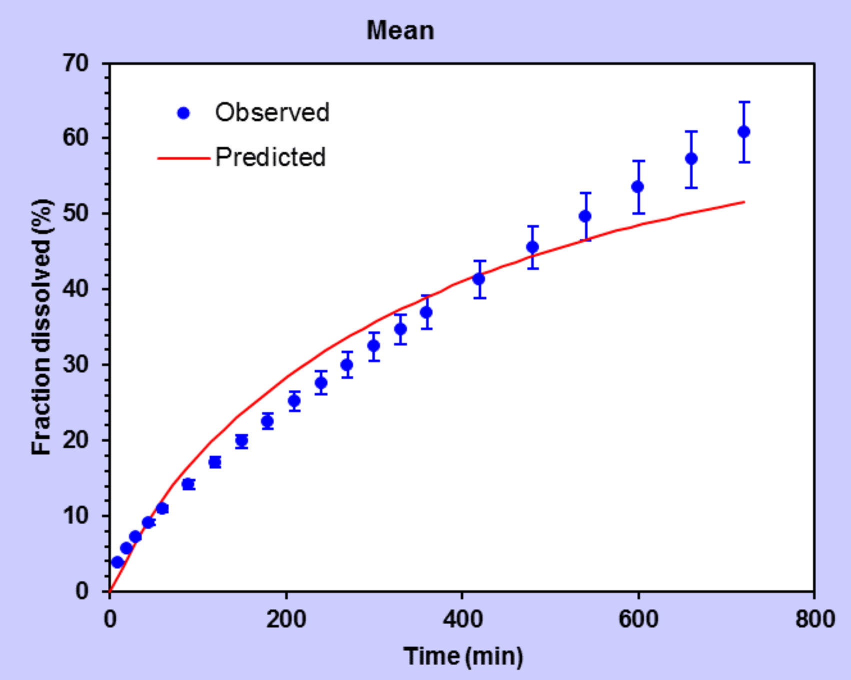

Supplementary materials – Model fitting summary – Di-Cafos® A12 (modelling up to app. 60 % of carvedilol released)

Graphical abstract of model fit presented as the fraction % of released carvedilol per tested tablet:

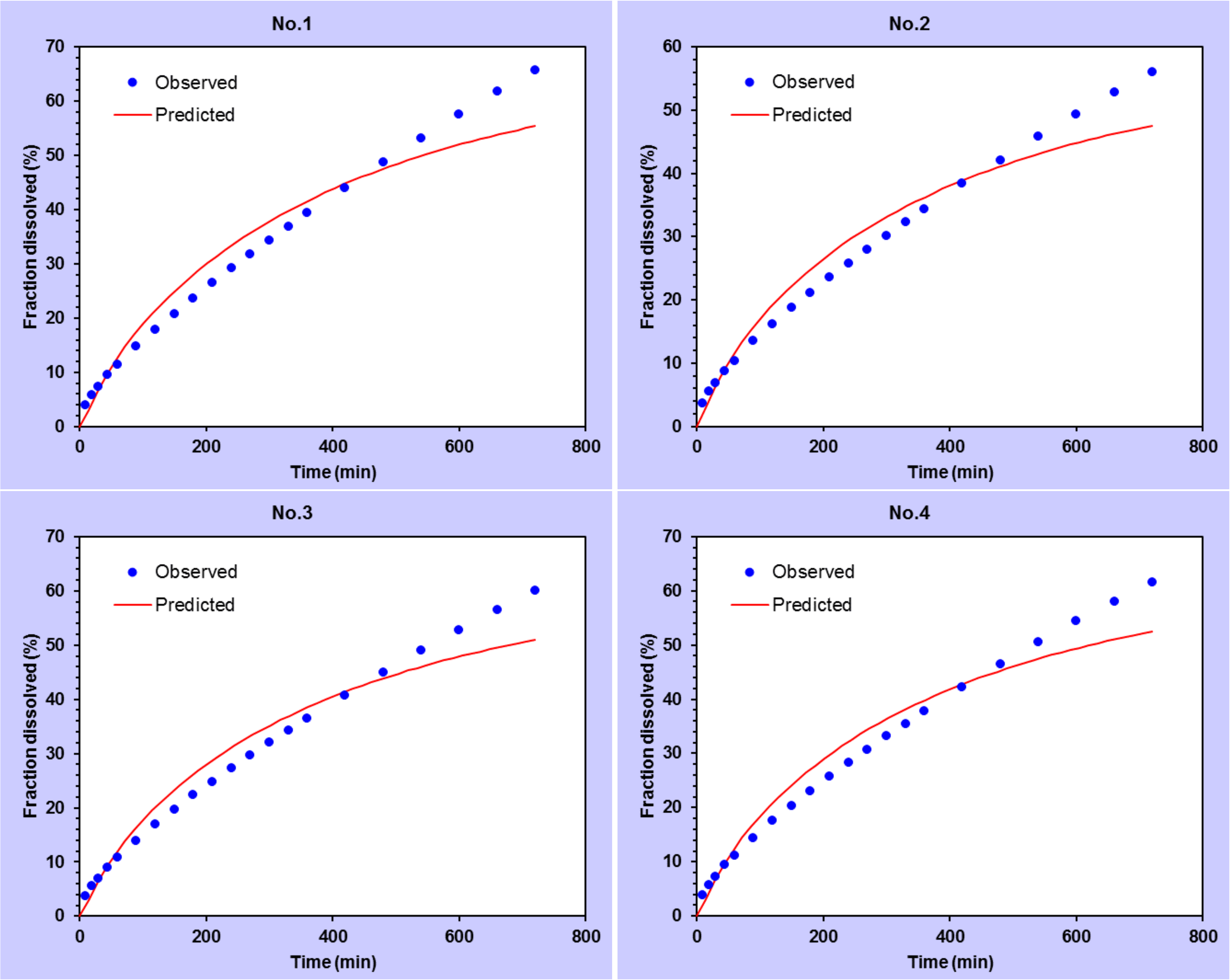

Supplementary materials – Model fitting summary – Di-Cafos® A12 (modelling up to app. 60 % of carvedilol released)

Model: **Logistic_1**

Model equation: $F = 100 \cdot \frac{e^{\alpha+\beta \cdot \log(t)}}{1+e^{\alpha+\beta \cdot \log(t)}}$

Fitted model parameters per tested tablet (N = 4) with statistics – mean, standard deviation (SD), and relative standard deviation expressed in % (RSD%) (output from DDSolver):

Parameter	No.1	No.2	No.3	No.4	Mean	SD	RSD(%)
α	-5.611	-5.417	-5.521	-5.496	-5.511	0.080	-1.455
β	2.054	1.883	1.964	1.976	1.969	0.070	3.548

Number of dissolution data points (N), degrees of freedom (df), and selected goodness of fit criteria – Pearson correlation coefficient (R), coefficient of determination (R^2), adjusted coefficient of determination ($R^2_{adjusted}$), and residual sum of squares (RSS) (manual calculation in MS Excel):

Parameter	No.1	No.2	No.3	No.4
N	21	21	21	21
df	19	19	19	19
R	0.985487892	0.989662936	0.988632838	0.988394101
R^2	0.971186385	0.979432726	0.977394889	0.976922899
$R^2_{adjusted}$	0.969669879	0.978350238	0.976205146	0.975708315
RSS	270.5851555	144.5237356	181.3115672	194.5816137

Graphical abstract of model fit presented as mean ± 1 SD of the fraction % of released carvedilol:

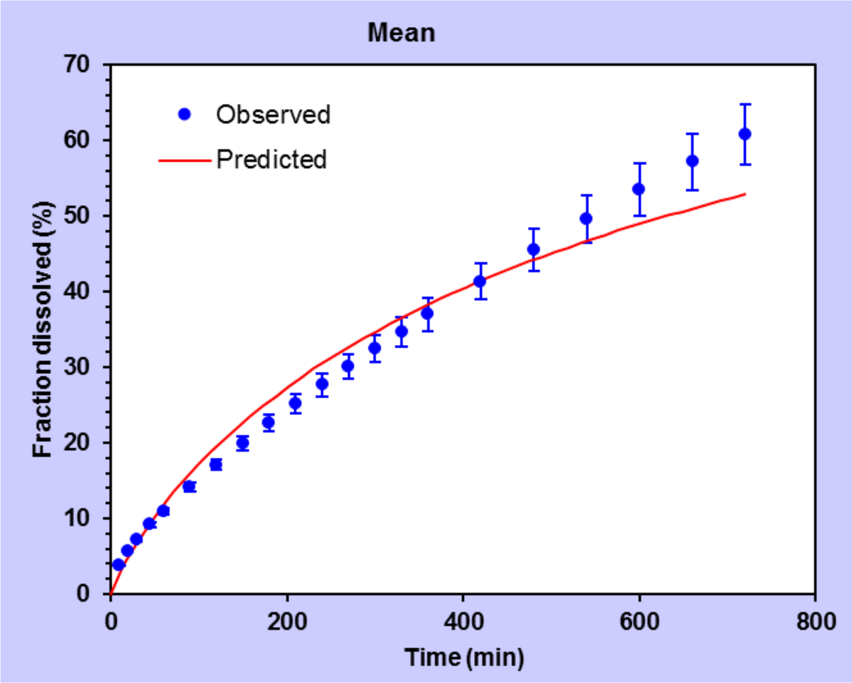

Supplementary materials – Model fitting summary – Di-Cafos® A12 (modelling up to app. 60 % of carvedilol released)

Graphical abstract of model fit presented as the fraction % of released carvedilol per tested tablet:

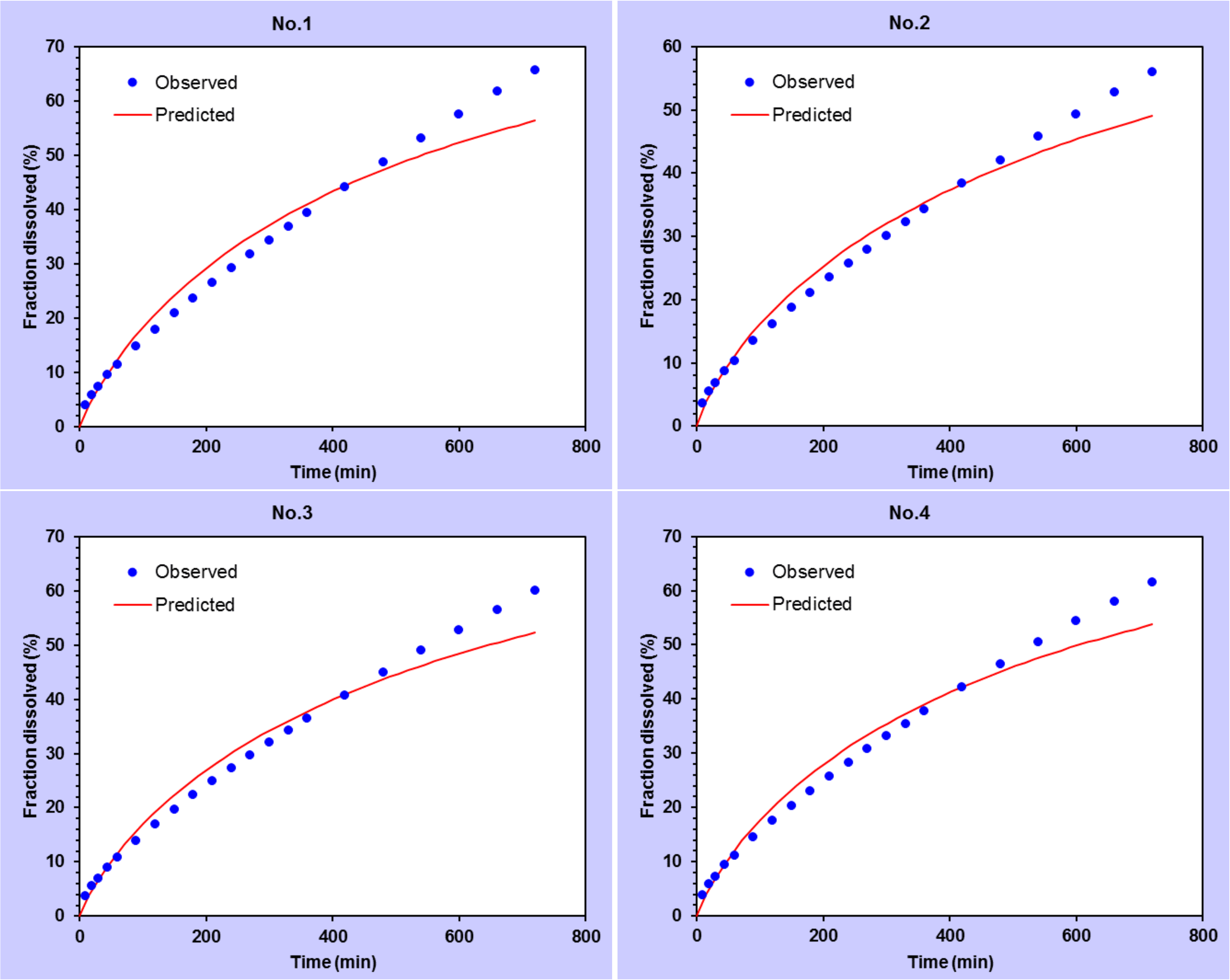

Supplementary materials – Model fitting summary – Di-Cafos® A12 (modelling up to app. 60 % of carvedilol released)

Model: **Logistic_2**

Model equation: $F = F_{max} \cdot \frac{e^{\alpha+\beta \cdot \log(t)}}{1+e^{\alpha+\beta \cdot \log(t)}}$

Fitted model parameters per tested tablet (N = 4) with statistics – mean, standard deviation (SD), and relative standard deviation expressed in % (RSD%) (output from DDSolver):

Parameter	No.1	No.2	No.3	No.4	Mean	SD	RSD(%)
α	-7.202	-6.994	-7.115	-7.096	-7.102	0.086	-1.205
β	2.857	2.803	2.838	2.838	2.834	0.023	0.801
F_{max}	75.521	64.351	69.024	70.823	69.930	4.619	6.605

Number of dissolution data points (N), degrees of freedom (df), and selected goodness of fit criteria – Pearson correlation coefficient (R), coefficient of determination (R^2), adjusted coefficient of determination ($R^2_{adjusted}$), and residual sum of squares (RSS) (manual calculation in MS Excel):

Parameter	No.1	No.2	No.3	No.4
N	21	21	21	21
df	18	18	18	18
R	0.985770593	0.985408817	0.986182098	0.986435529
R^2	0.971743662	0.971030536	0.972555131	0.973055054
$R^2_{adjusted}$	0.968604069	0.967811707	0.969505701	0.970061171
RSS	339.7121617	223.5283564	263.9467169	269.3405991

Graphical abstract of model fit presented as mean ± 1 SD of the fraction % of released carvedilol:

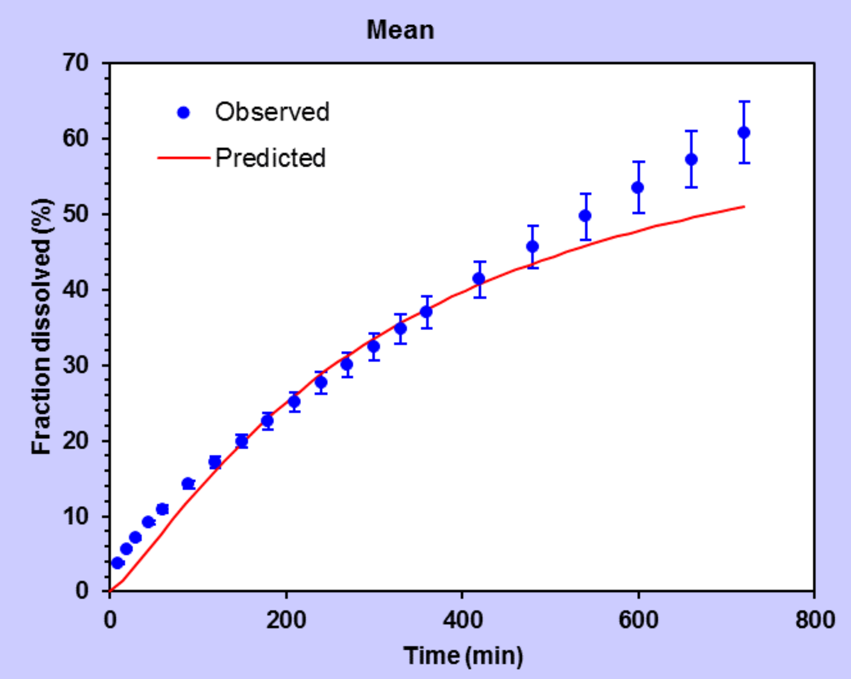

Supplementary materials – Model fitting summary – Di-Cafos® A12 (modelling up to app. 60 % of carvedilol released)

Graphical abstract of model fit presented as the fraction % of released carvedilol per tested tablet:

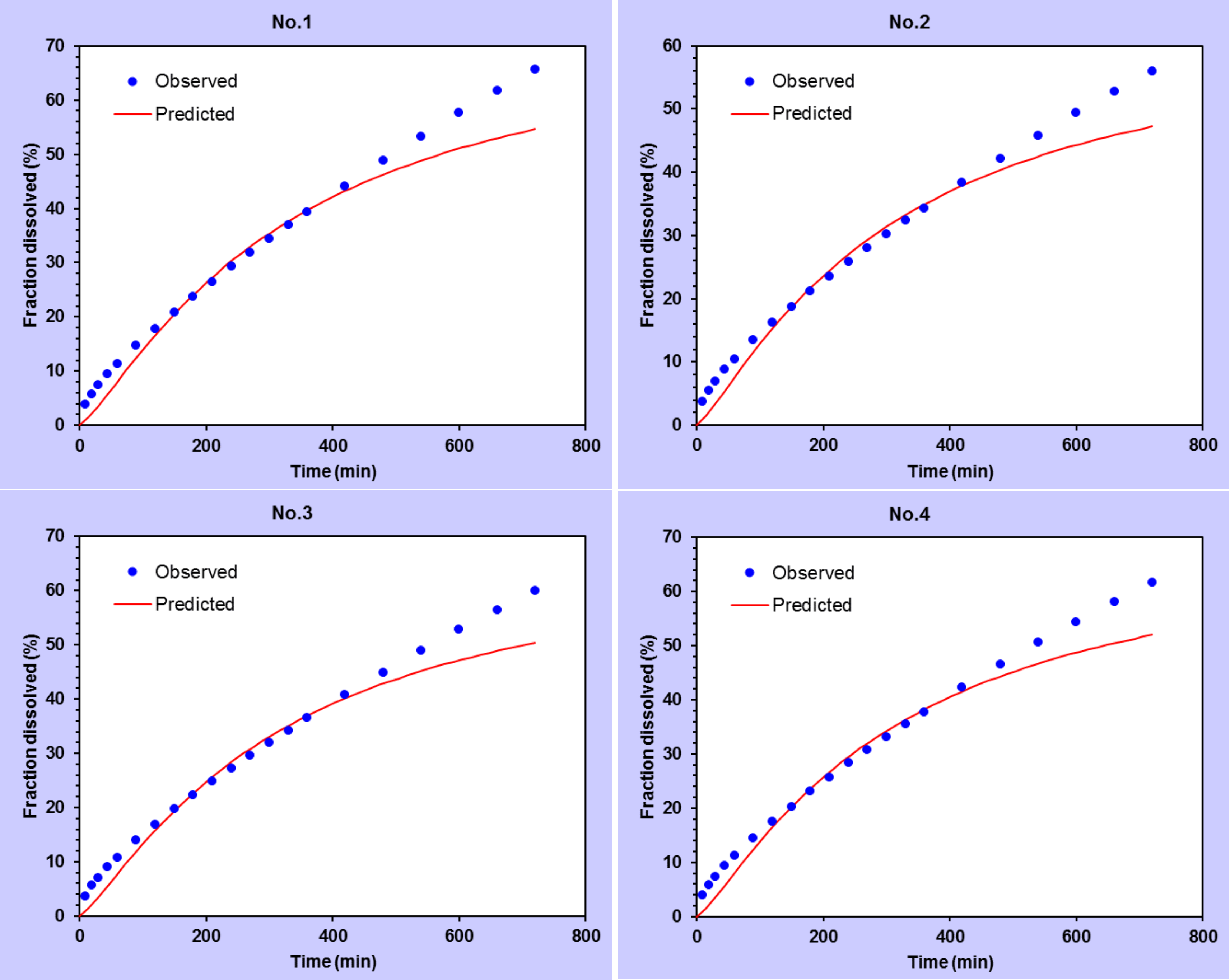

Supplementary materials – Model fitting summary – Di-Cafos® A12 (modelling up to app. 60 % of carvedilol released)

Model: **Logistic_3**

Model equation: $F = F_{max} \cdot \frac{1}{1+e^{-k\cdot(t-\gamma)}}$

Fitted model parameters per tested tablet (N = 4) with statistics – mean, standard deviation (SD), and relative standard deviation expressed in % (RSD%) (output from DDSolver):

Parameter	No.1	No.2	No.3	No.4	Mean	SD	RSD(%)
k	0.007	0.007	0.007	0.007	0.007	0.000	0.767
γ	318.267	308.835	313.283	310.634	312.755	4.104	1.312
F_{max}	68.971	58.770	63.037	64.680	63.865	4.218	6.605

Number of dissolution data points (N), degrees of freedom (df), and selected goodness of fit criteria – Pearson correlation coefficient (R), coefficient of determination (R^2), adjusted coefficient of determination ($R^2_{adjusted}$), and residual sum of squares (RSS) (manual calculation in MS Excel):

Parameter	No.1	No.2	No.3	No.4
N	21	21	21	21
df	18	18	18	18
R	0.991405991	0.991120717	0.990898701	0.991159301
R^2	0.982885839	0.982320276	0.981880235	0.982396759
$R^2_{adjusted}$	0.980984265	0.980355862	0.979866928	0.980440844
RSS	145.2456869	105.2654164	126.4676753	129.4089751

Graphical abstract of model fit presented as mean ± 1 SD of the fraction % of released carvedilol:

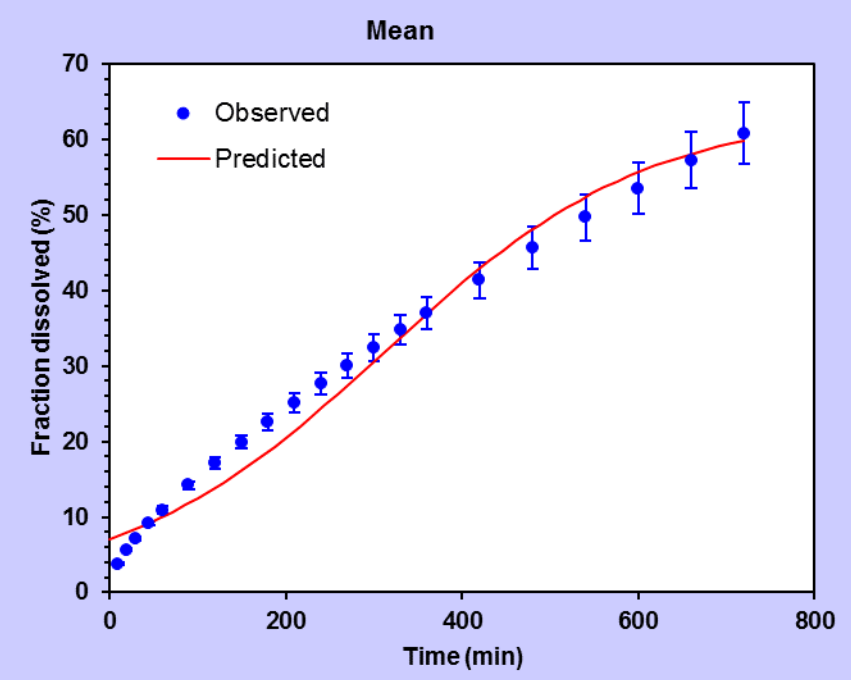

Supplementary materials – Model fitting summary – Di-Cafos® A12 (modelling up to app. 60 % of carvedilol released)

Graphical abstract of model fit presented as the fraction % of released carvedilol per tested tablet:

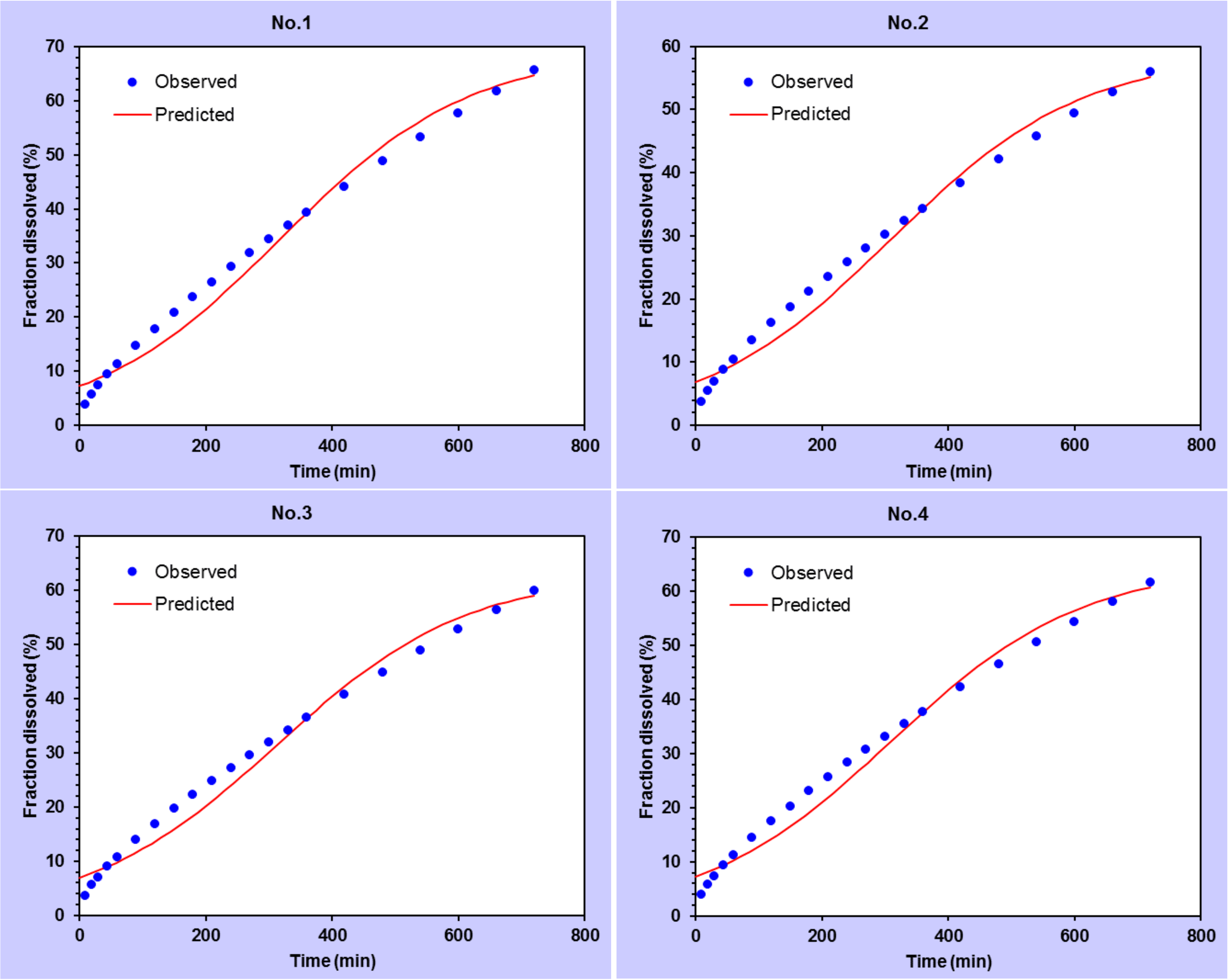

Supplementary materials – Model fitting summary – Di-Cafos® A12 (modelling up to app. 60 % of carvedilol released)

Model: **Gompertz_1**

Model equation: $F = 100 \cdot e^{-\alpha \cdot e^{-\beta \cdot \log(t)}}$

Fitted model parameters per tested tablet (N = 4) with statistics – mean, standard deviation (SD), and relative standard deviation expressed in % (RSD%) (output from DDSolver):

Parameter	No.1	No.2	No.3	No.4	Mean	SD	RSD(%)
α	13.340	10.912	11.927	12.138	12.079	0.996	8.249
β	1.070	0.928	0.989	1.010	1.000	0.059	5.878

Number of dissolution data points (N), degrees of freedom (df), and selected goodness of fit criteria – Pearson correlation coefficient (R), coefficient of determination (R^2), adjusted coefficient of determination ($R^2_{adjusted}$), and residual sum of squares (RSS) (manual calculation in MS Excel):

Parameter	No.1	No.2	No.3	No.4
N	21	21	21	21
df	19	19	19	19
R	0.9612153	0.968748153	0.966594676	0.966189757
R^2	0.923934854	0.938472985	0.934305268	0.933522646
$R^2_{adjusted}$	0.919931425	0.935234721	0.930847651	0.930023838
RSS	624.2543689	367.4676904	452.9381216	482.2648865

Graphical abstract of model fit presented as mean ± 1 SD of the fraction % of released carvedilol:

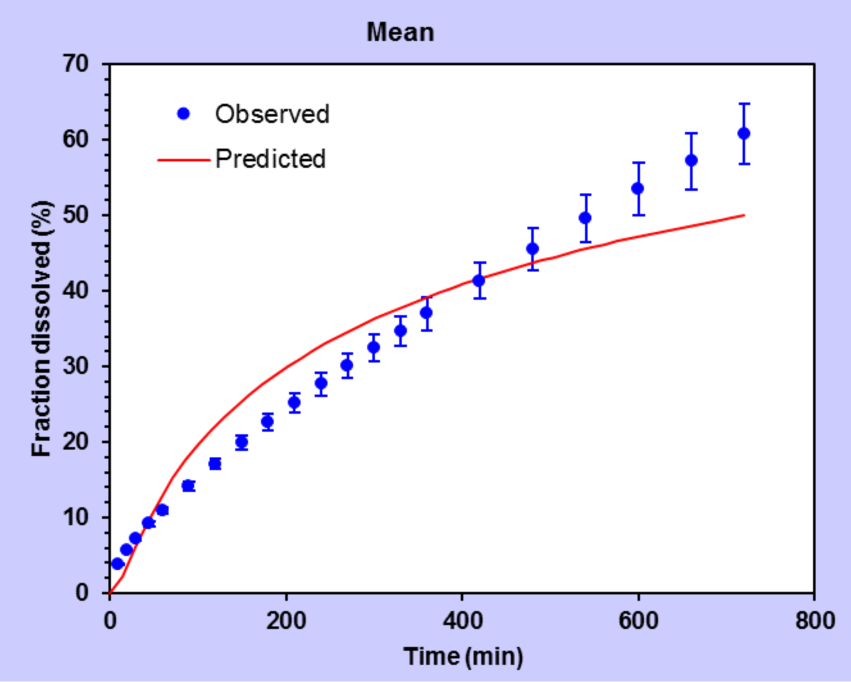

Supplementary materials – Model fitting summary – Di-Cafos® A12 (modelling up to app. 60 % of carvedilol released)

Graphical abstract of model fit presented as the fraction % of released carvedilol per tested tablet:

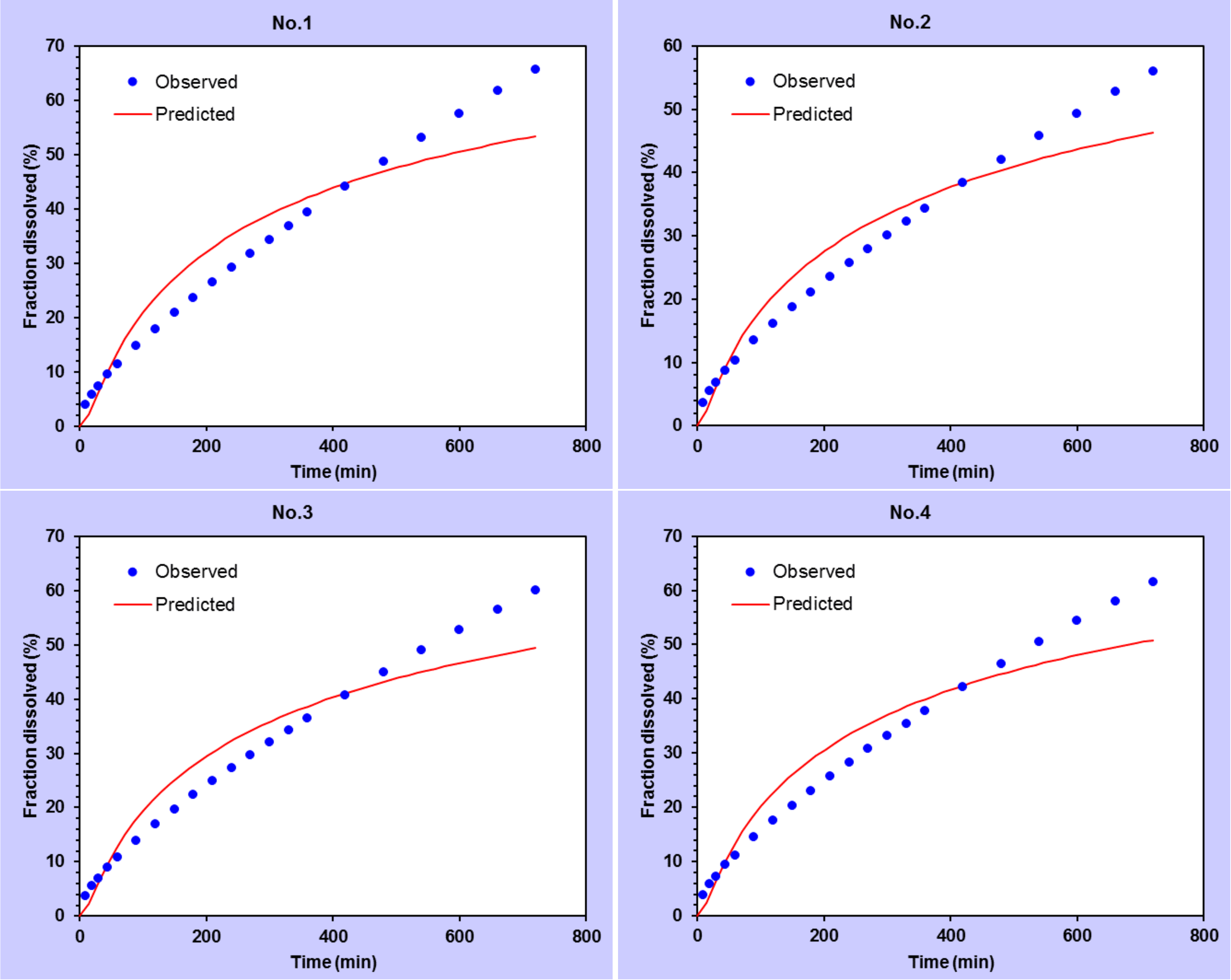

Supplementary materials – Model fitting summary – Di-Cafos® A12 (modelling up to app. 60 % of carvedilol released)

Model: **Gompertz_2**

Model equation: $F = F_{max} \cdot e^{-\alpha \cdot e^{-\beta \cdot \log(t)}}$

Fitted model parameters per tested tablet (N = 4) with statistics – mean, standard deviation (SD), and relative standard deviation expressed in % (RSD%) (output from DDSolver):

Parameter	No.1	No.2	No.3	No.4	Mean	SD	RSD(%)
α	52.145	39.162	40.482	40.650	43.110	6.060	14.057
β	1.740	1.544	1.550	1.557	1.598	0.095	5.937
F_{max}	68.971	72.147	77.386	79.403	74.477	4.777	6.415

Number of dissolution data points (N), degrees of freedom (df), and selected goodness of fit criteria – Pearson correlation coefficient (R), coefficient of determination (R^2), adjusted coefficient of determination ($R^2_{adjusted}$), and residual sum of squares (RSS) (manual calculation in MS Excel):

Parameter	No.1	No.2	No.3	No.4
N	21	21	21	21
df	18	18	18	18
R	0.965629125	0.976820651	0.977403978	0.977819567
R^2	0.932439607	0.954178584	0.955318537	0.956131105
$R^2_{adjusted}$	0.924932897	0.949087315	0.95035393	0.951256784
RSS	978.6500479	434.7026782	511.7859425	526.7053559

Graphical abstract of model fit presented as mean ± 1 SD of the fraction % of released carvedilol:

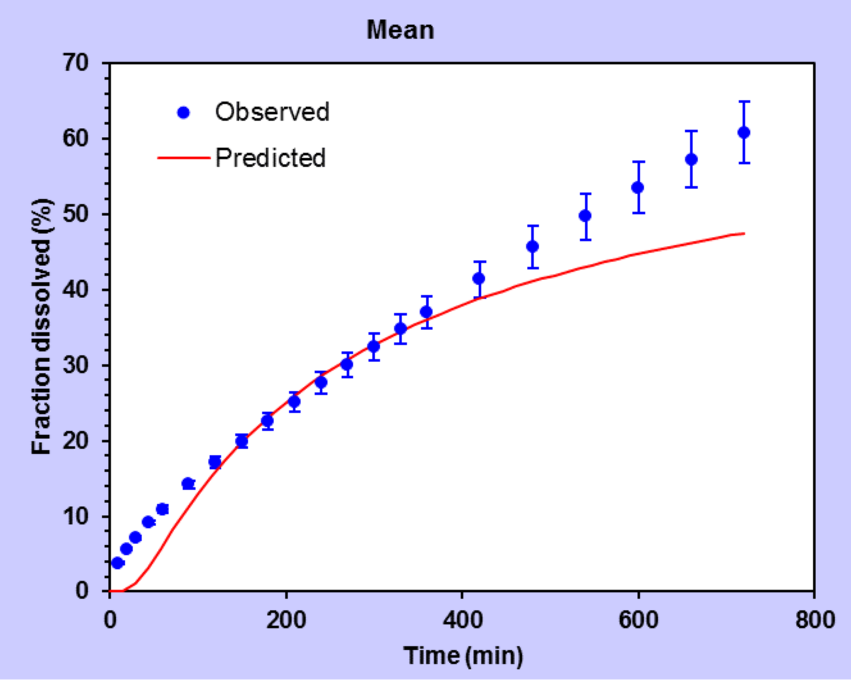

Supplementary materials – Model fitting summary – Di-Cafos® A12 (modelling up to app. 60 % of carvedilol released)

Graphical abstract of model fit presented as the fraction % of released carvedilol per tested tablet:

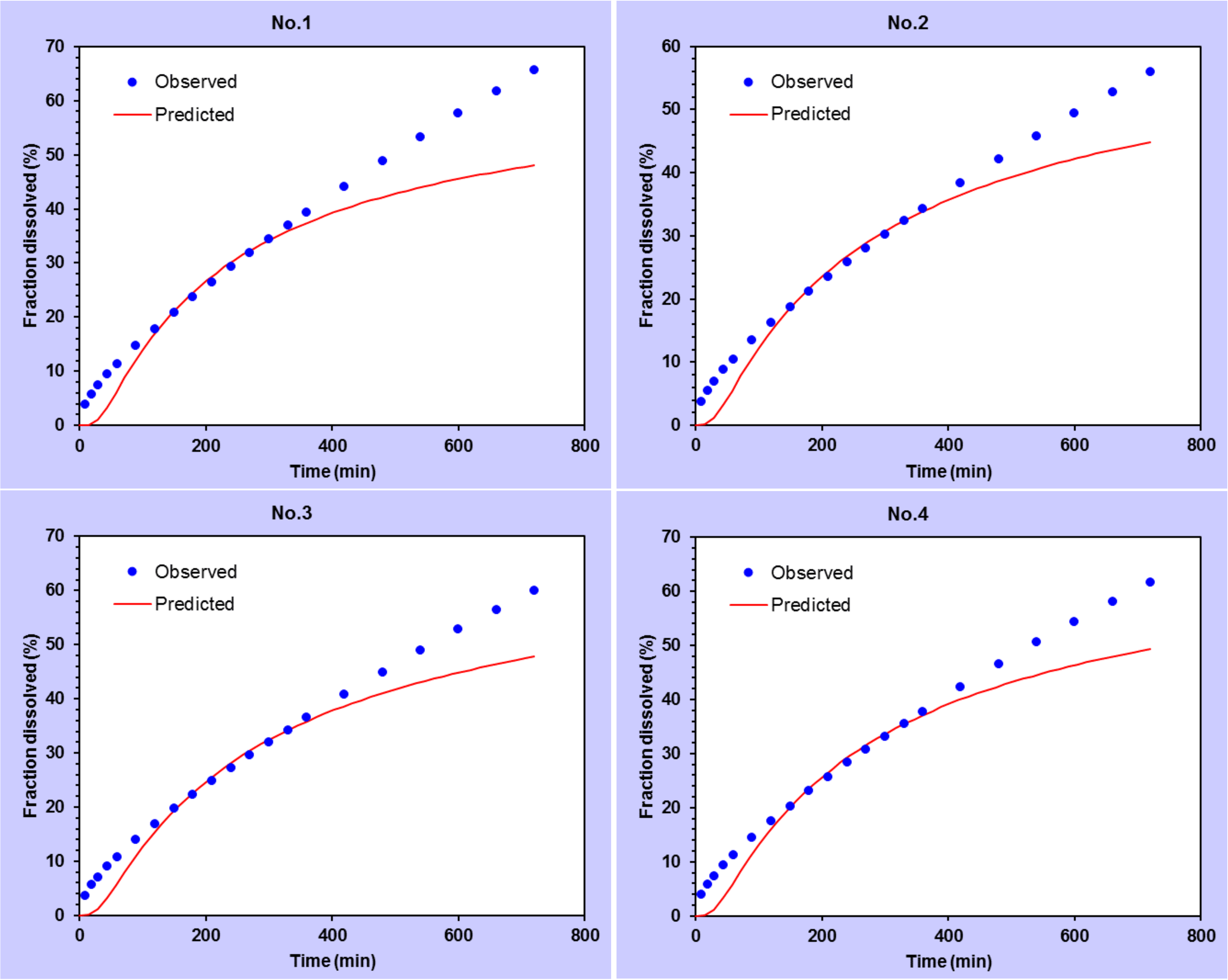

Supplementary materials – Model fitting summary – Di-Cafos® A12 (modelling up to app. 60 % of carvedilol released)

Model: **Gompertz_3**

Model equation: $F = F_{max} \cdot e^{-e^{-k\cdot(t-\gamma)}}$

Fitted model parameters per tested tablet (N = 4) with statistics – mean, standard deviation (SD), and relative standard deviation expressed in % (RSD%) (output from DDSolver):

Parameter	No.1	No.2	No.3	No.4	Mean	SD	RSD(%)
k	0.004	0.005	0.005	0.005	0.005	0.000	5.006
γ	232.776	198.834	202.927	200.695	208.808	16.066	7.694
F_{max}	74.058	58.770	63.037	64.680	65.136	6.448	9.900

Number of dissolution data points (N), degrees of freedom (df), and selected goodness of fit criteria – Pearson correlation coefficient (R), coefficient of determination (R^2), adjusted coefficient of determination ($R^2_{adjusted}$), and residual sum of squares (RSS) (manual calculation in MS Excel):

Parameter	No.1	No.2	No.3	No.4
N	21	21	21	21
df	18	18	18	18
R	0.996059337	0.995056611	0.994997554	0.995386912
R^2	0.992134203	0.99013766	0.990020132	0.990795104
$R^2_{adjusted}$	0.991260226	0.989041844	0.988911258	0.989772338
RSS	97.62858846	66.89462565	78.60918799	76.5535518

Graphical abstract of model fit presented as mean ± 1 SD of the fraction % of released carvedilol:

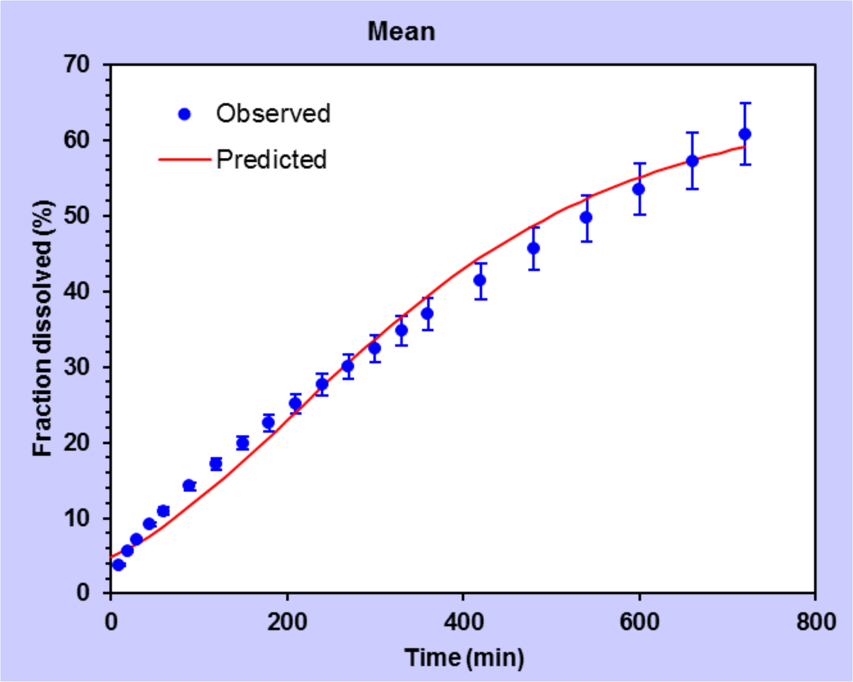

Supplementary materials – Model fitting summary – Di-Cafos® A12 (modelling up to app. 60 % of carvedilol released)

Graphical abstract of model fit presented as the fraction % of released carvedilol per tested tablet:

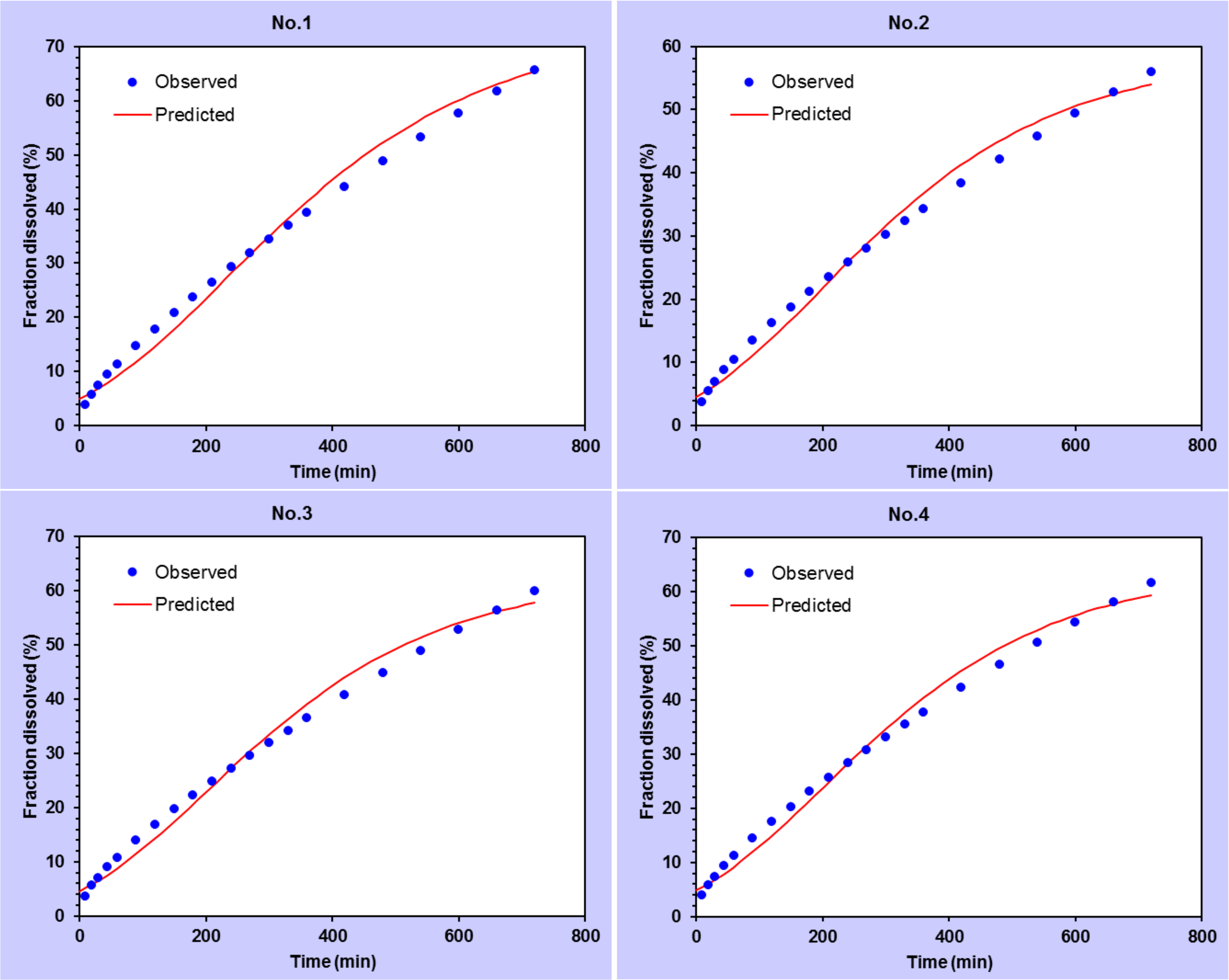

Supplementary materials – Model fitting summary – Di-Cafos® A12 (modelling up to app. 60 % of carvedilol released)

Model: **Gompertz_4**

Model equation: $F = F_{max} \cdot e^{-\beta \cdot e^{-k \cdot t}}$

Fitted model parameters per tested tablet (N = 4) with statistics – mean, standard deviation (SD), and relative standard deviation expressed in % (RSD%) (output from DDSolver):

Parameter	No.1	No.2	No.3	No.4	Mean	SD	RSD(%)
k	0.005	0.005	0.005	0.005	0.005	0.000	0.320
β	2.678	2.552	2.610	2.591	2.608	0.053	2.016
F_{max}	68.971	58.770	63.037	64.680	63.865	4.218	6.605

Number of dissolution data points (N), degrees of freedom (df), and selected goodness of fit criteria – Pearson correlation coefficient (R), coefficient of determination (R^2), adjusted coefficient of determination ($R^2_{adjusted}$), and residual sum of squares (RSS) (manual calculation in MS Excel):

Parameter	No.1	No.2	No.3	No.4
N	21	21	21	21
df	18	18	18	18
R	0.994923576	0.995056611	0.994997554	0.995386912
R^2	0.989872922	0.99013766	0.990020132	0.990795104
$R^2_{adjusted}$	0.988747691	0.989041844	0.988911258	0.989772338
RSS	97.71661889	66.89462565	78.60918799	76.5535518

Graphical abstract of model fit presented as mean ± 1 SD of the fraction % of released carvedilol:

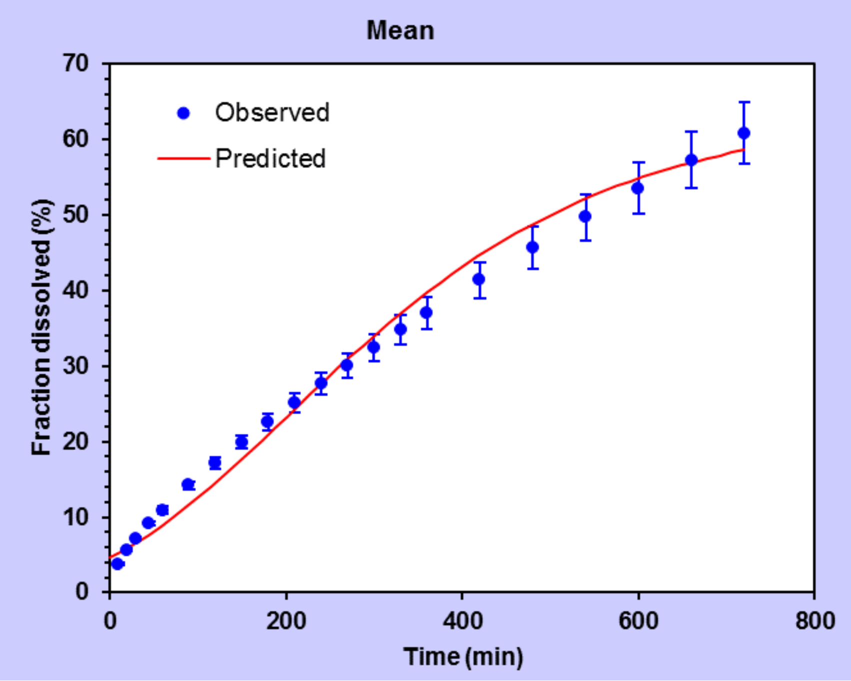

Supplementary materials – Model fitting summary – Di-Cafos® A12 (modelling up to app. 60 % of carvedilol released)

Graphical abstract of model fit presented as the fraction % of released carvedilol per tested tablet:

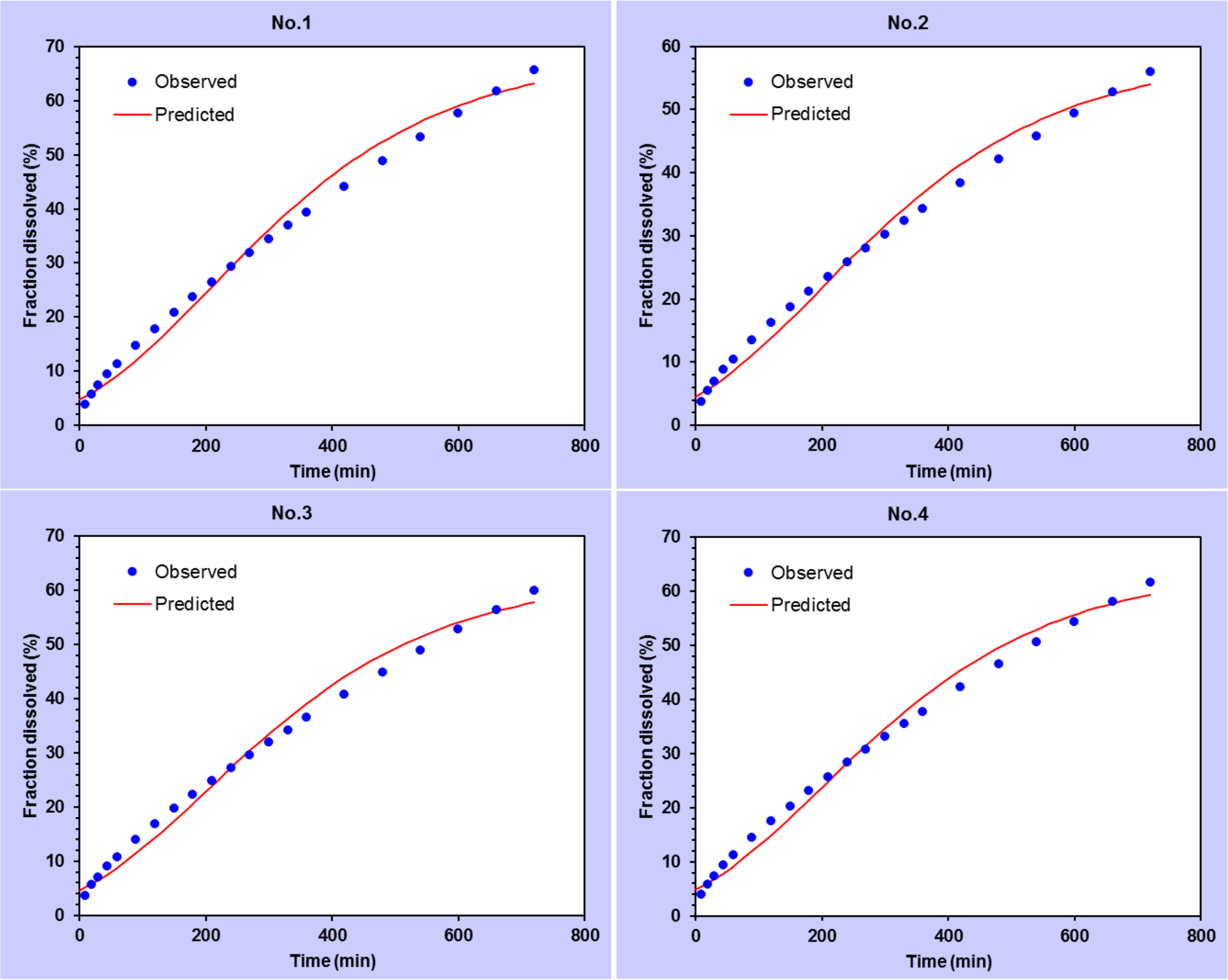

Supplementary materials – Model fitting summary – Di-Cafos® A12 (modelling up to app. 60 % of carvedilol released)

Model: **Probit_1**

Model equation: $F = 100 \cdot \phi[\alpha + \beta \cdot \log(t)]$

Fitted model parameters per tested tablet (N = 4) with statistics – mean, standard deviation (SD), and relative standard deviation expressed in % (RSD%) (output from DDSolver):

Parameter	No.1	No.2	No.3	No.4	Mean	SD	RSD(%)
α	-3.200	-3.381	-3.128	-3.126	-3.209	0.120	-3.734
β	1.164	1.207	1.103	1.115	1.147	0.048	4.156

Number of dissolution data points (N), degrees of freedom (df), and selected goodness of fit criteria – Pearson correlation coefficient (R), coefficient of determination (R^2), adjusted coefficient of determination ($R^2_{adjusted}$), and residual sum of squares (RSS) (manual calculation in MS Excel):

Parameter	No.1	No.2	No.3	No.4
N	21	21	21	21
df	19	19	19	19
R	0.978369359	0.985840921	0.981796056	0.981680094
R^2	0.957206603	0.971882321	0.963923495	0.963695807
$R^2_{adjusted}$	0.954954319	0.970402443	0.962024732	0.961785061
RSS	387.7918914	208.6749855	276.6121808	292.7823727

Graphical abstract of model fit presented as mean ± 1 SD of the fraction % of released carvedilol:

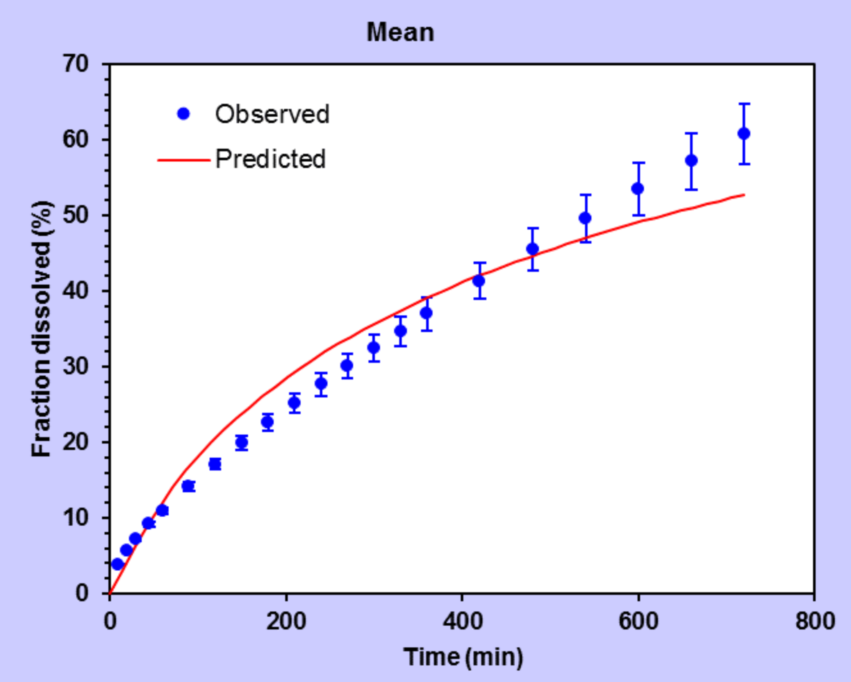

Supplementary materials – Model fitting summary – Di-Cafos® A12 (modelling up to app. 60 % of carvedilol released)

Graphical abstract of model fit presented as the fraction % of released carvedilol per tested tablet:

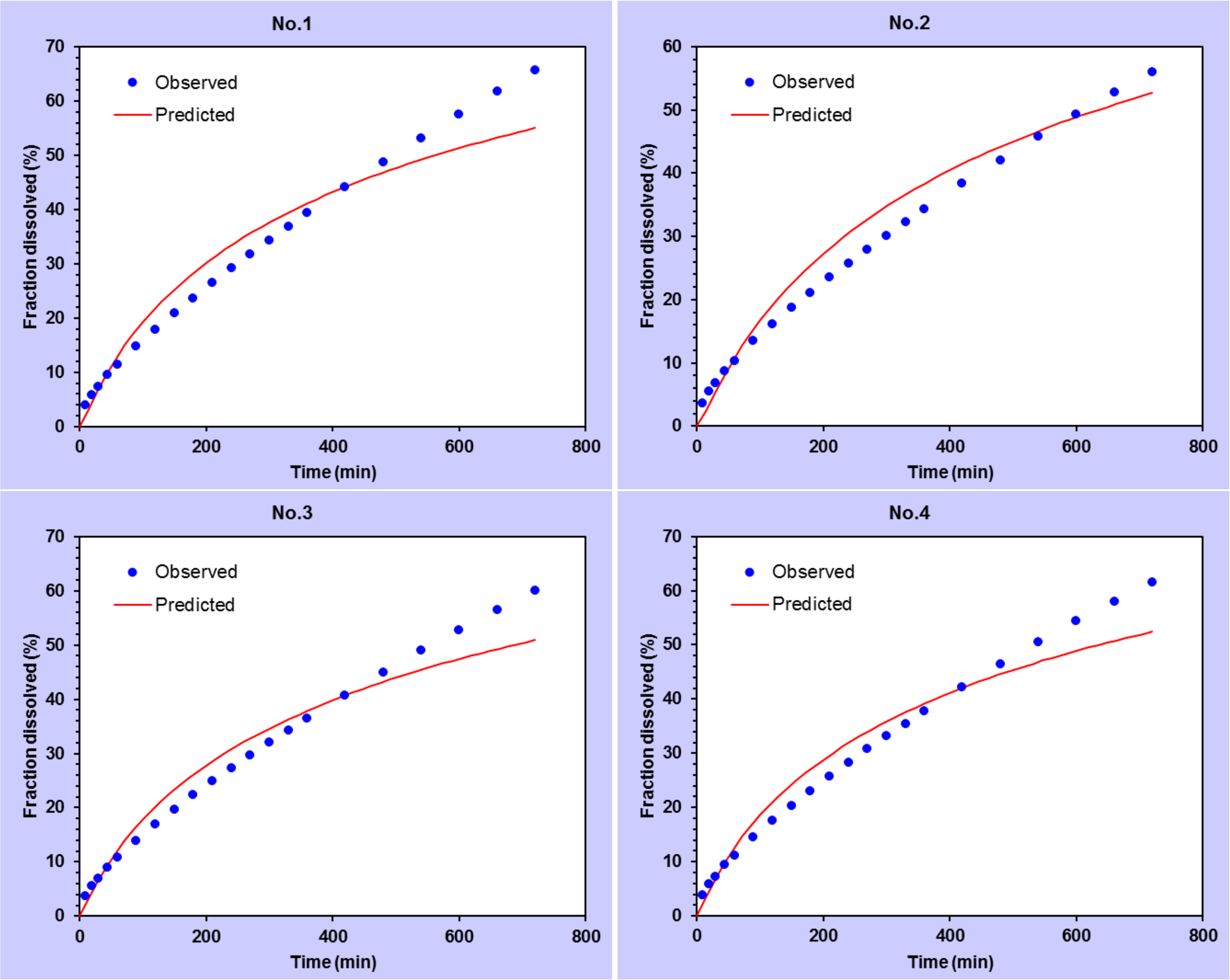

Supplementary materials – Model fitting summary – Di-Cafos® A12 (modelling up to app. 60 % of carvedilol released)

Model: **Probit_2**

Model equation: $F = F_{max} \cdot \phi[\alpha + \beta \cdot \log(t)]$

Fitted model parameters per tested tablet (N = 4) with statistics – mean, standard deviation (SD), and relative standard deviation expressed in % (RSD%) (output from DDSolver):

Parameter	No.1	No.2	No.3	No.4	Mean	SD	RSD(%)
α	-4.191	-4.086	-4.147	-4.140	-4.141	0.043	-1.037
β	1.660	1.635	1.652	1.654	1.650	0.011	0.646
F_{max}	75.521	64.351	69.024	70.823	69.930	4.619	6.605

Number of dissolution data points (N), degrees of freedom (df), and selected goodness of fit criteria – Pearson correlation coefficient (R), coefficient of determination (R^2), adjusted coefficient of determination ($R^2_{adjusted}$), and residual sum of squares (RSS) (manual calculation in MS Excel):

Parameter	No.1	No.2	No.3	No.4
N	21	21	21	21
df	18	18	18	18
R	0.981732296	0.981792036	0.982459202	0.982787511
R^2	0.9637983	0.963915603	0.965226084	0.965871291
$R^2_{adjusted}$	0.959775889	0.959906225	0.961362315	0.962079213
RSS	422.7052235	278.41315	328.6982908	336.8868137

Graphical abstract of model fit presented as mean ± 1 SD of the fraction % of released carvedilol:

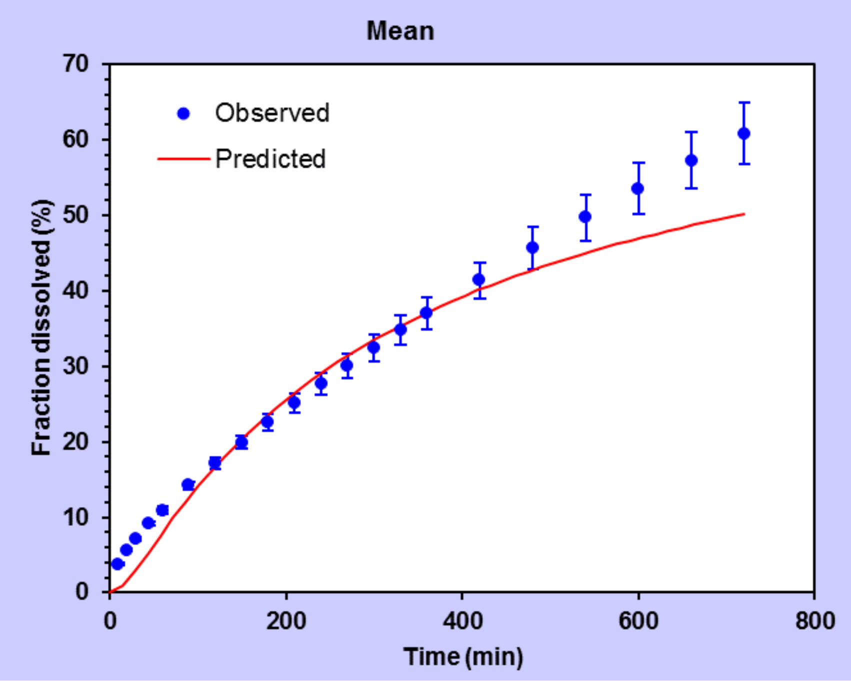

Supplementary materials – Model fitting summary – Di-Cafos® A12 (modelling up to app. 60 % of carvedilol released)

Graphical abstract of model fit presented as the fraction % of released carvedilol per tested tablet:

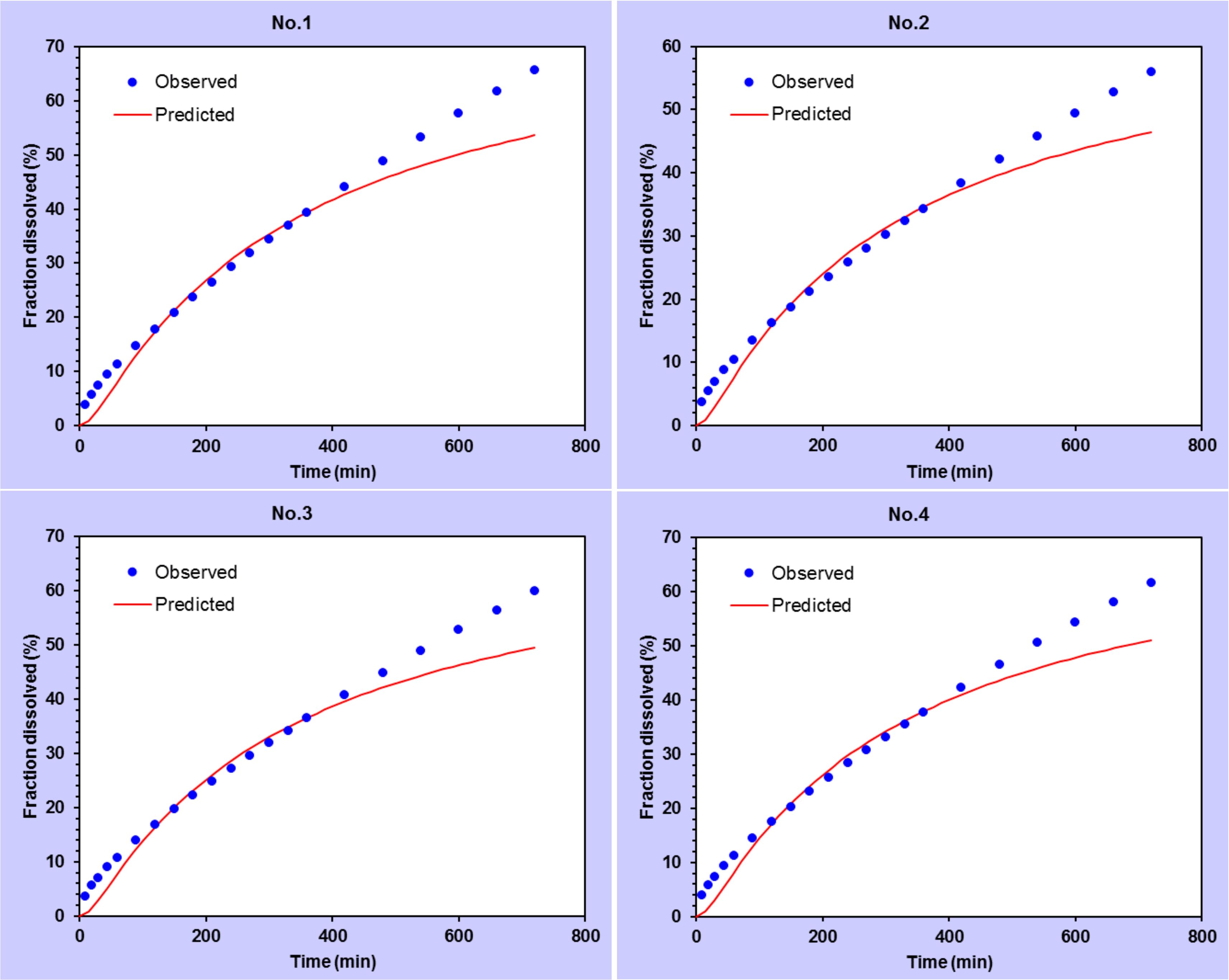

Supplement: Supplementary file 1 [file pharmaceutics-16-00498-s001.zip › Supplementary materials_Model fitting summary_Di-Cafos® A12.pdf]
