# Supplementary material for: Comparative Fitting of Mathematical Models to Carvedilol Release Profiles Obtained from Hypromellose Matrix Tablets
Source: Pharmaceutics. 2024 Apr 4;16(4):498. doi: 10.3390/pharmaceutics16040498 (PMC11053526; doi:10.3390/pharmaceutics16040498)

Supplementary materials – Model fitting summary – Emcompress® Anhydrous (modelling the entire relevant dissolution data)

Model: **Zero–order**

Model equation: $F = k_0 \cdot t$

Fitted model parameters per tested tablet (N = 4) with statistics – mean, standard deviation (SD), and relative standard deviation expressed in % (RSD%) (output from DDSolver):

Parameter	No.1	No.2	No.3	No.4	Mean	SD	RSD(%)
k_0	0.065	0.079	0.067	0.071	0.071	0.006	8.623

Number of dissolution data points (N), degrees of freedom (df), and selected goodness of fit criteria – Pearson correlation coefficient (R), coefficient of determination (R^2), adjusted coefficient of determination ($R^2_{adjusted}$), and residual sum of squares (RSS) (manual calculation in MS Excel):

Parameter	No.1	No.2	No.3	No.4
N	33	33	33	33
df	32	32	32	32
R	0.985213317	0.98388116	0.984370006	0.985031137
R^2	0.97064528	0.968022137	0.968984309	0.970286341
$R^2_{adjusted}$	0.97064528	0.968022137	0.968984309	0.970286341
RSS	3366.417306	4429.442459	3558.185124	3574.059795

Graphical abstract of model fit presented as mean ± 1 SD of the fraction % of released carvedilol:

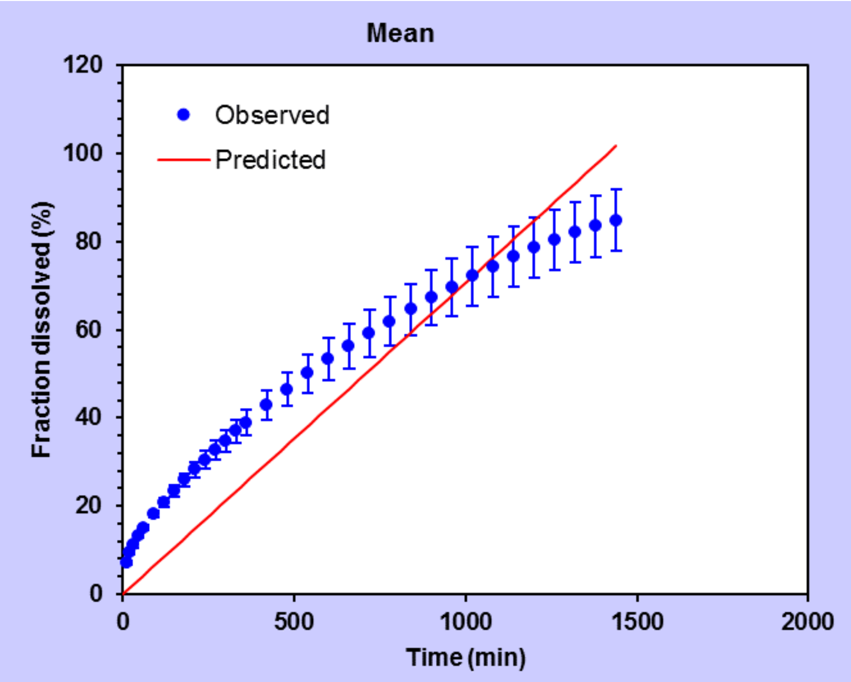

Supplementary materials – Model fitting summary – Emcompress® Anhydrous (modelling the entire relevant dissolution data)

Graphical abstract of model fit presented as the fraction % of released carvedilol per tested tablet:

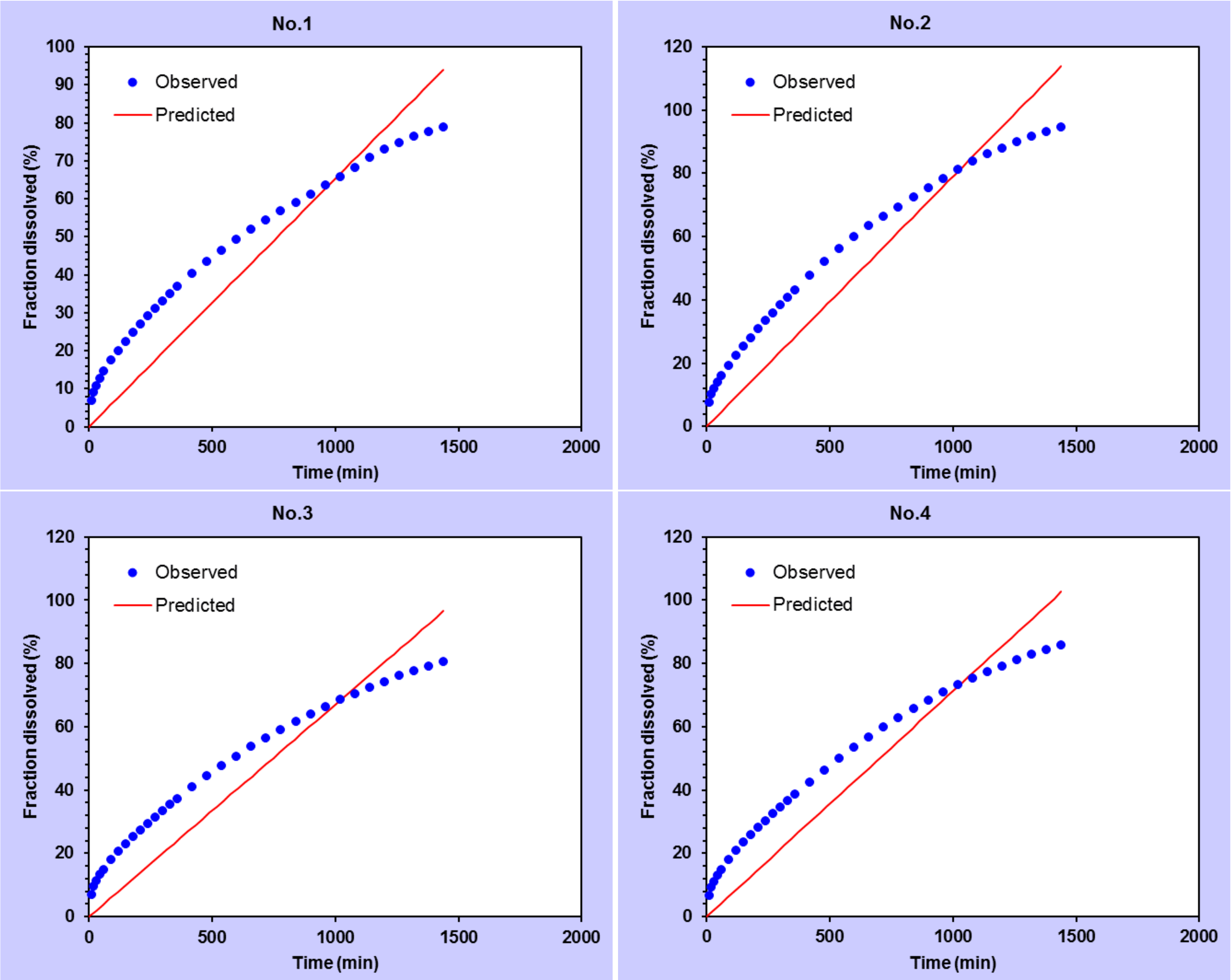

Supplementary materials – Model fitting summary – Emcompress® Anhydrous (modelling the entire relevant dissolution data)

Model: **Zero–order with T_{lag}**

Model equation: $F = k_0 \cdot (t - T_{lag})$

Fitted model parameters per tested tablet (N = 4) with statistics – mean, standard deviation (SD), and relative standard deviation expressed in % (RSD%) (output from DDSolver):

Parameter	No.1	No.2	No.3	No.4	Mean	SD	RSD(%)
k_0	0.049	0.061	0.050	0.055	0.054	0.005	9.820
T_{lag}	-308.551	-278.889	-307.309	-280.400	-293.787	16.350	-5.565

Number of dissolution data points (N), degrees of freedom (df), and selected goodness of fit criteria – Pearson correlation coefficient (R), coefficient of determination (R^2), adjusted coefficient of determination ($R^2_{adjusted}$), and residual sum of squares (RSS) (manual calculation in MS Excel):

Parameter	No.1	No.2	No.3	No.4
N	33	33	33	33
df	31	31	31	31
R	0.985213317	0.98388116	0.984370006	0.985031137
R^2	0.97064528	0.968022137	0.968984309	0.970286341
$R^2_{adjusted}$	0.969698354	0.966990593	0.967983802	0.969327836
RSS	494.798138	829.3732667	552.5675462	623.3879832

Graphical abstract of model fit presented as mean ± 1 SD of the fraction % of released carvedilol:

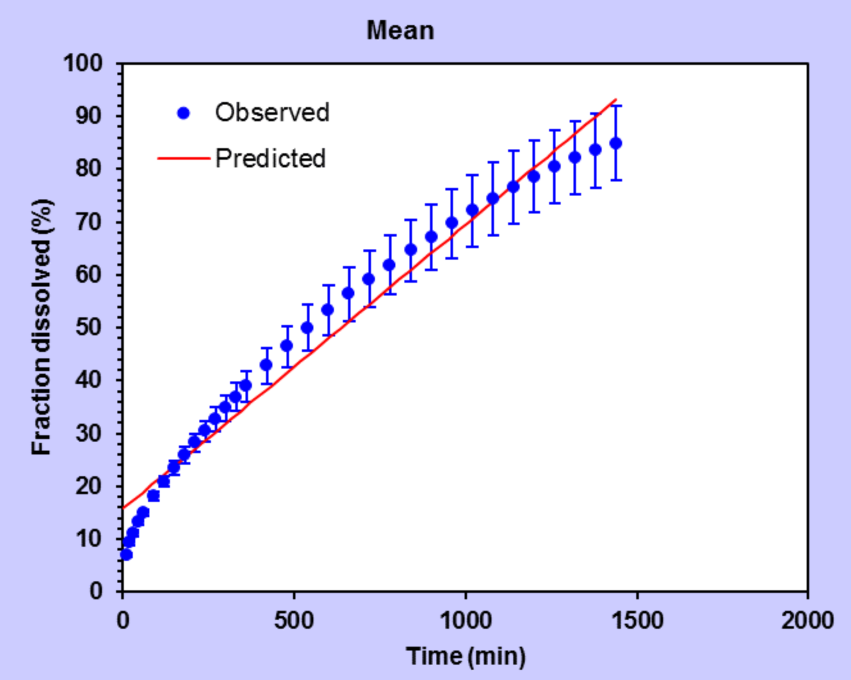

Supplementary materials – Model fitting summary – Emcompress® Anhydrous (modelling the entire relevant dissolution data)

Graphical abstract of model fit presented as the fraction % of released carvedilol per tested tablet:

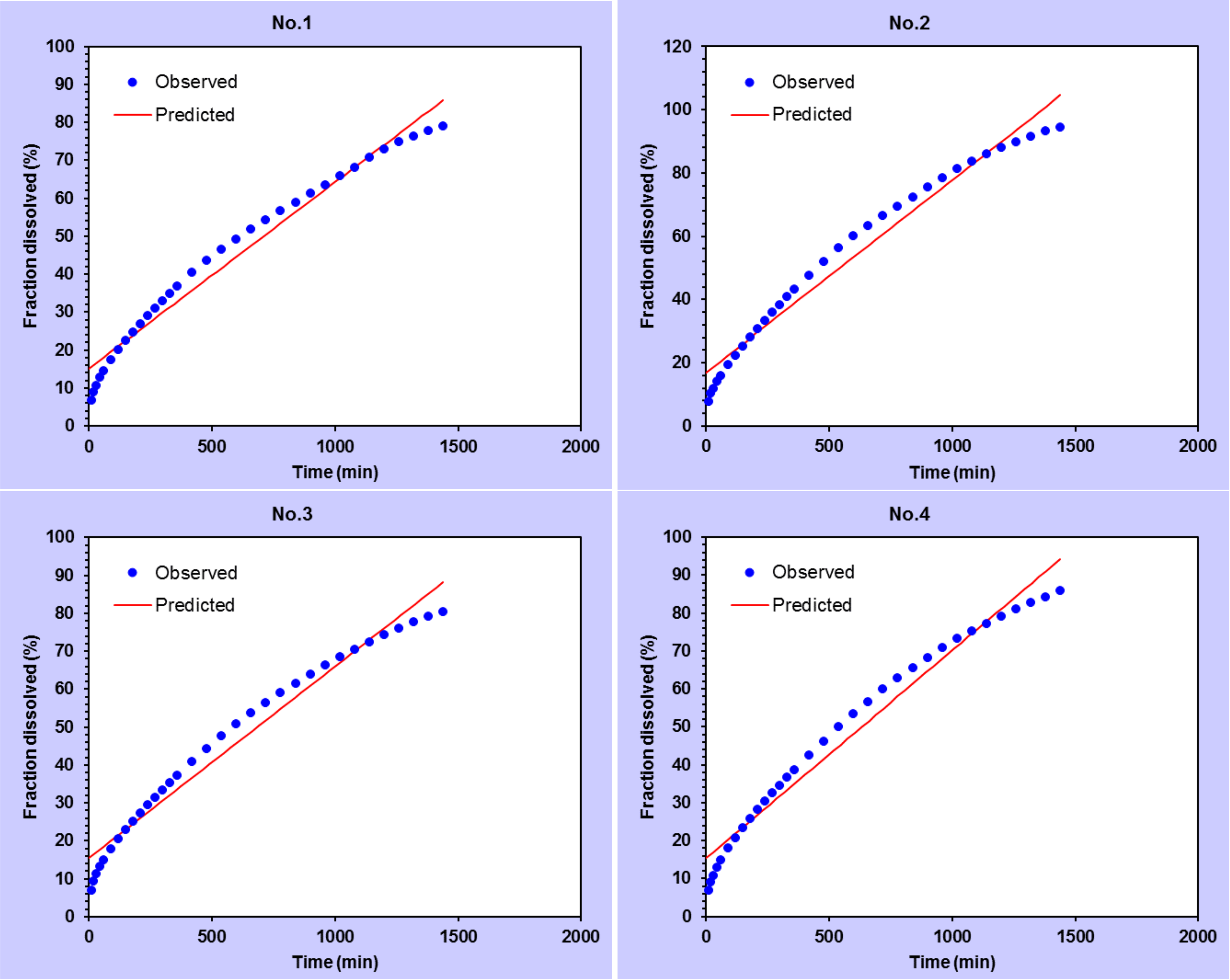

Supplementary materials – Model fitting summary – Emcompress® Anhydrous (modelling the entire relevant dissolution data)

Model: **Zero–order with F_0**

Model equation: $F = F_0 + k_0 \cdot t$

Fitted model parameters per tested tablet (N = 4) with statistics – mean, standard deviation (SD), and relative standard deviation expressed in % (RSD%) (output from DDSolver):

Parameter	No.1	No.2	No.3	No.4	Mean	SD	RSD(%)
k_0	0.049	0.061	0.050	0.055	0.054	0.005	9.820
F_0	15.156	16.969	15.505	15.363	15.748	0.827	5.249

Number of dissolution data points (N), degrees of freedom (df), and selected goodness of fit criteria – Pearson correlation coefficient (R), coefficient of determination (R^2), adjusted coefficient of determination ($R^2_{adjusted}$), and residual sum of squares (RSS) (manual calculation in MS Excel):

Parameter	No.1	No.2	No.3	No.4
N	33	33	33	33
df	31	31	31	31
R	0.985213317	0.98388116	0.984370006	0.985031137
R^2	0.97064528	0.968022137	0.968984309	0.970286341
$R^2_{adjusted}$	0.969698354	0.966990593	0.967983802	0.969327836
RSS	494.798138	829.3732667	552.5675462	623.3879832

Graphical abstract of model fit presented as mean ± 1 SD of the fraction % of released carvedilol:

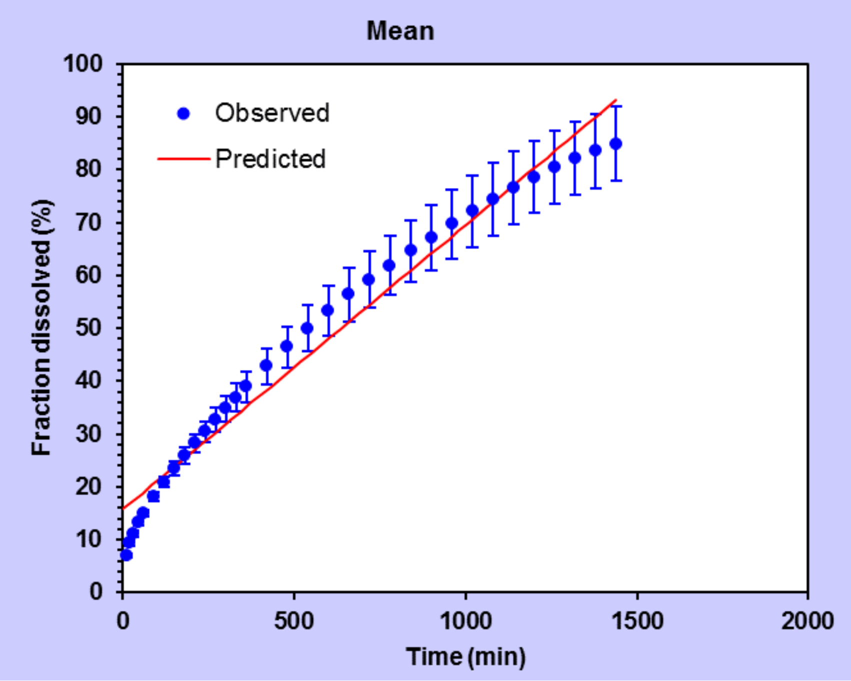

Supplementary materials – Model fitting summary – Emcompress® Anhydrous (modelling the entire relevant dissolution data)

Graphical abstract of model fit presented as the fraction % of released carvedilol per tested tablet:

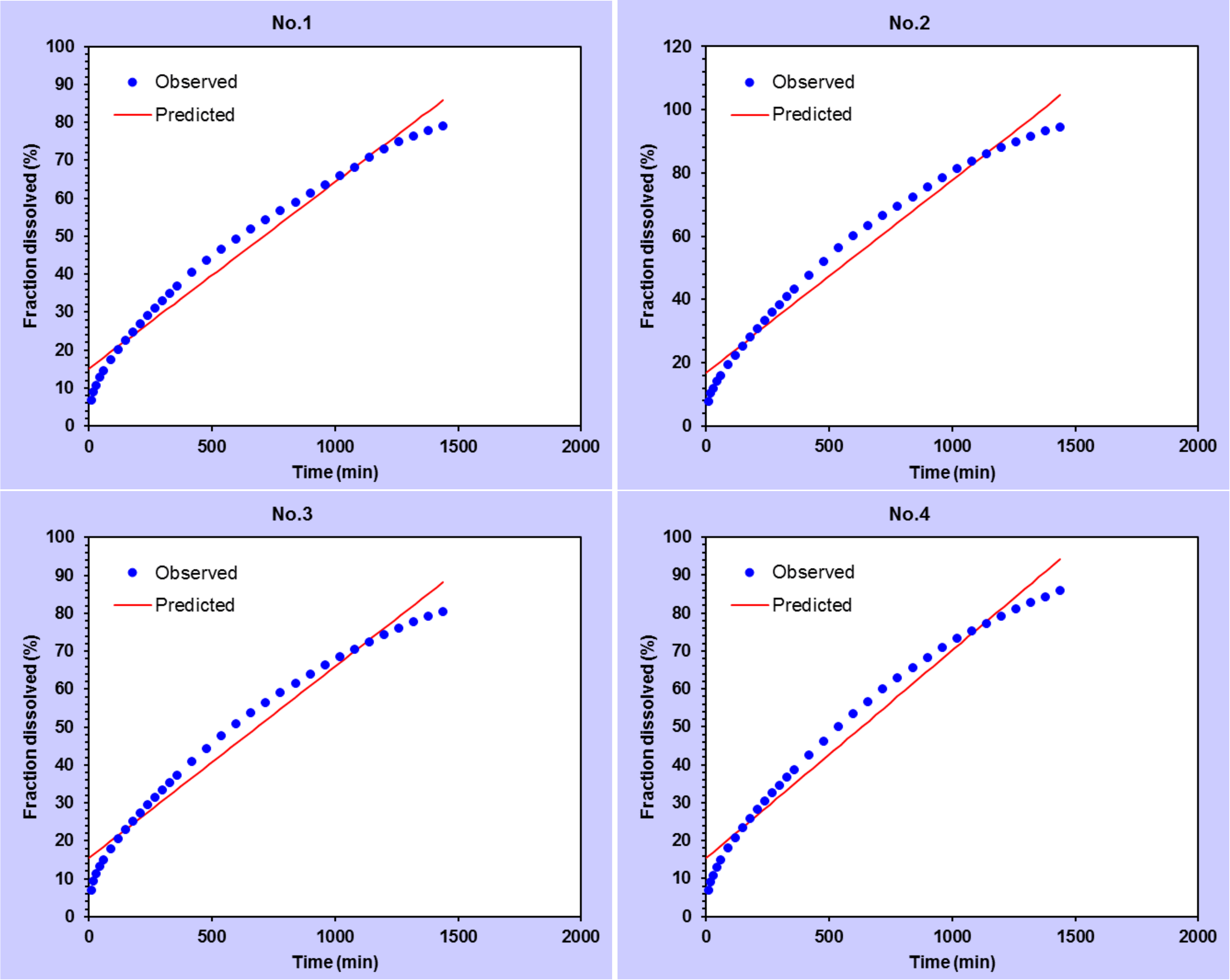

Supplementary materials – Model fitting summary – Emcompress® Anhydrous (modelling the entire relevant dissolution data)

Model: **First–order**

Model equation: $F = 100 \cdot (1 - e^{-k_1 \cdot t})$

Fitted model parameters per tested tablet (N = 4) with statistics – mean, standard deviation (SD), and relative standard deviation expressed in % (RSD%) (output from DDSolver):

Parameter	No.1	No.2	No.3	No.4	Mean	SD	RSD(%)
k_1	0.001	0.002	0.001	0.001	0.001	0.000	22.281

Number of dissolution data points (N), degrees of freedom (df), and selected goodness of fit criteria – Pearson correlation coefficient (R), coefficient of determination (R^2), adjusted coefficient of determination ($R^2_{adjusted}$), and residual sum of squares (RSS) (manual calculation in MS Excel):

Parameter	No.1	No.2	No.3	No.4
N	33	33	33	33
df	32	32	32	32
R	0.998798283	0.993697173	0.999289447	0.998272581
R^2	0.997598009	0.987434072	0.9985794	0.996548146
$R^2_{adjusted}$	0.997598009	0.987434072	0.9985794	0.996548146
RSS	692.7517805	505.1275345	636.1112528	412.5325084

Graphical abstract of model fit presented as mean ± 1 SD of the fraction % of released carvedilol:

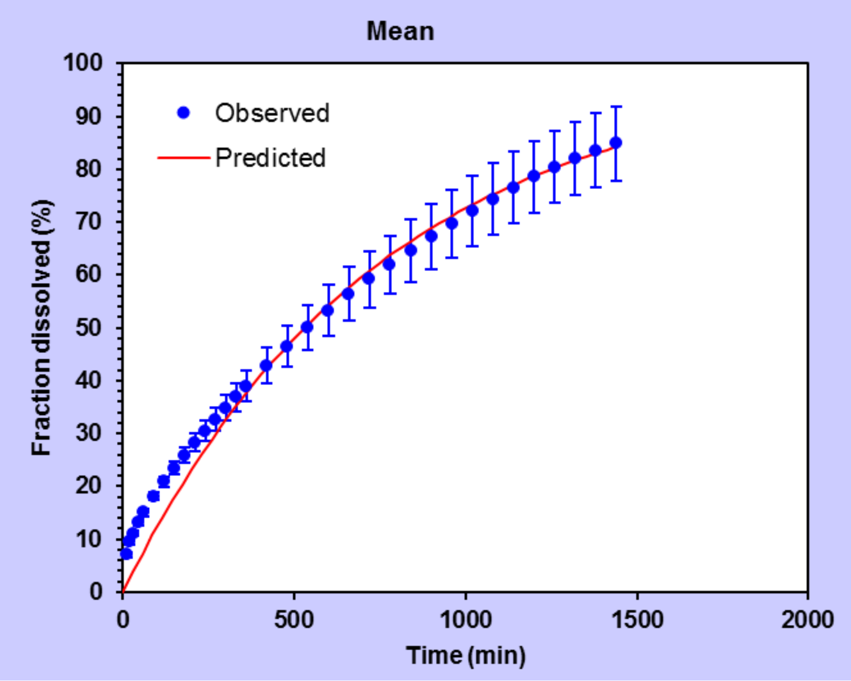

Supplementary materials – Model fitting summary – Emcompress® Anhydrous (modelling the entire relevant dissolution data)

Graphical abstract of model fit presented as the fraction % of released carvedilol per tested tablet:

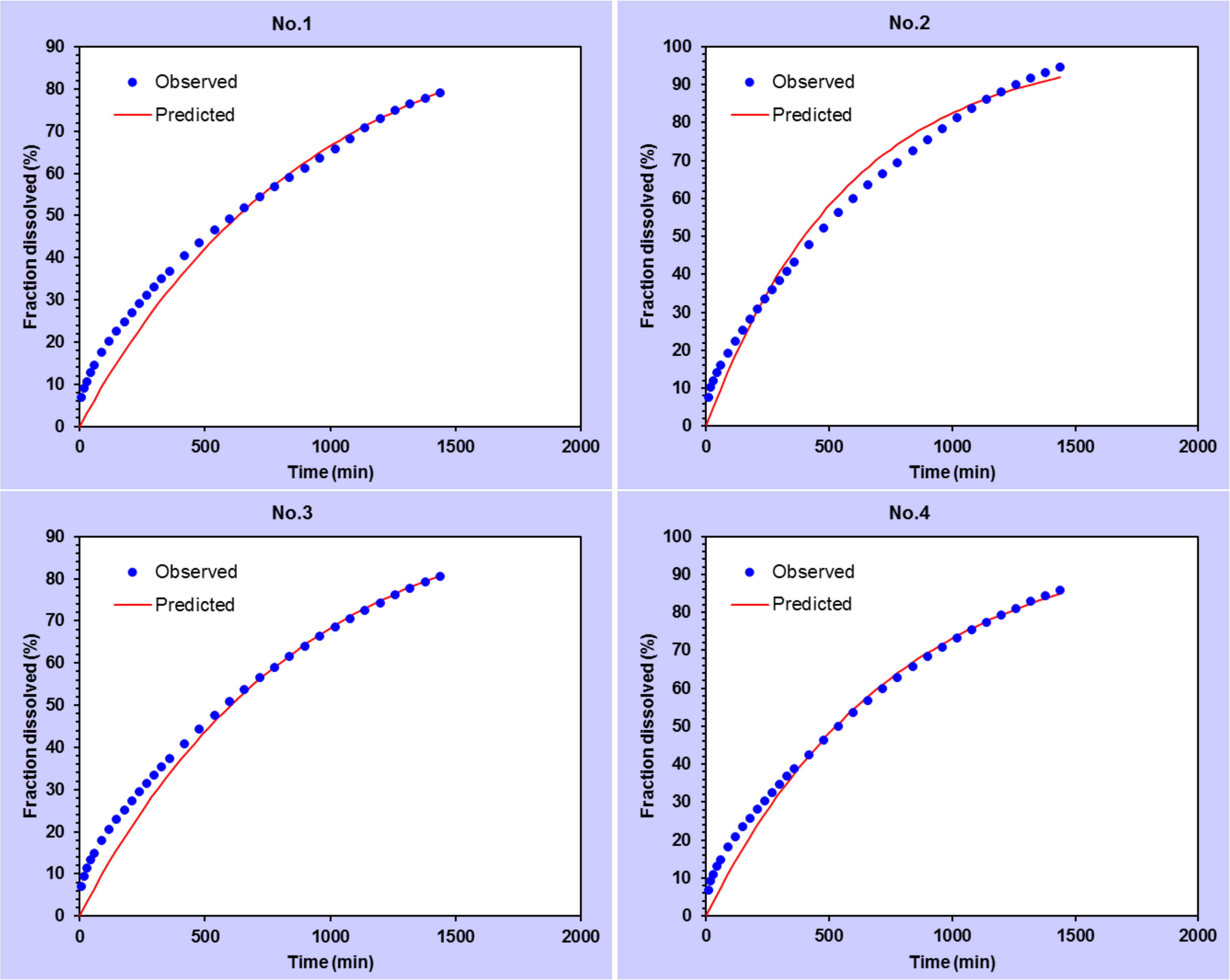

Supplementary materials – Model fitting summary – Emcompress® Anhydrous (modelling the entire relevant dissolution data)

Model: **First–order with T_{lag}**

Model equation: $F = 100 \cdot \left[1 - e^{-k_1 \cdot (t - T_{lag})}\right]$

Fitted model parameters per tested tablet (N = 4) with statistics – mean, standard deviation (SD), and relative standard deviation expressed in % (RSD%) (output from DDSolver):

Parameter	No.1	No.2	No.3	No.4	Mean	SD	RSD(%)
k_1	0.001	0.002	0.001	0.001	0.001	0.000	27.895
T_{lag}	-87.889	18.086	-81.214	-40.821	-47.959	48.693	-101.531

Number of dissolution data points (N), degrees of freedom (df), and selected goodness of fit criteria – Pearson correlation coefficient (R), coefficient of determination (R^2), adjusted coefficient of determination ($R^2_{adjusted}$), and residual sum of squares (RSS) (manual calculation in MS Excel):

Parameter	No.1	No.2	No.3	No.4
N	33	33	33	33
df	31	31	31	31
R	0.998964837	0.99313283	0.999500788	0.998687504
R^2	0.997930745	0.986312819	0.999001826	0.997376731
$R^2_{adjusted}$	0.997863995	0.985871297	0.998969627	0.997292109
RSS	36.07721452	766.9718403	18.73254144	77.97472592

Graphical abstract of model fit presented as mean ± 1 SD of the fraction % of released carvedilol:

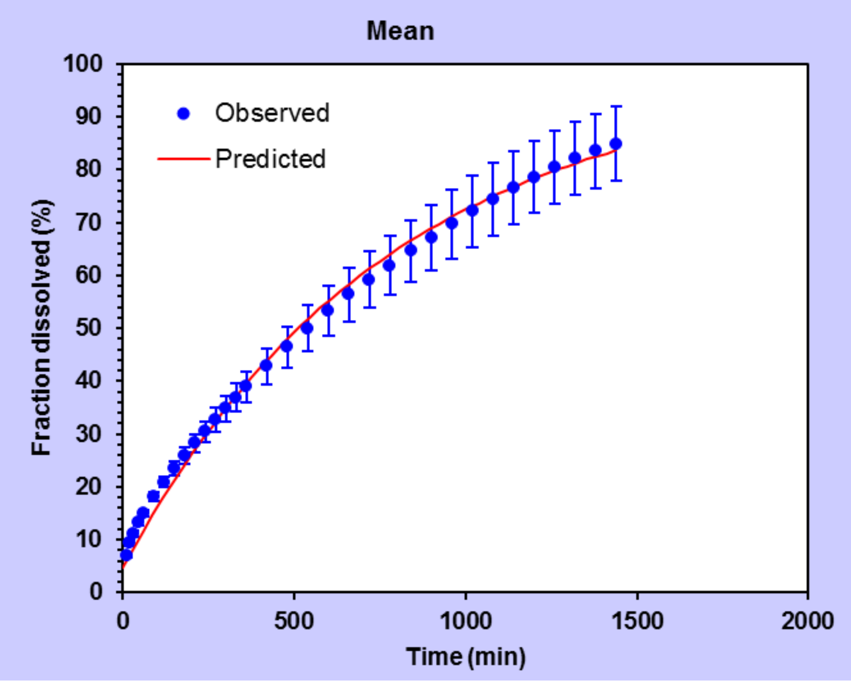

Supplementary materials – Model fitting summary – Emcompress® Anhydrous (modelling the entire relevant dissolution data)

Graphical abstract of model fit presented as the fraction % of released carvedilol per tested tablet:

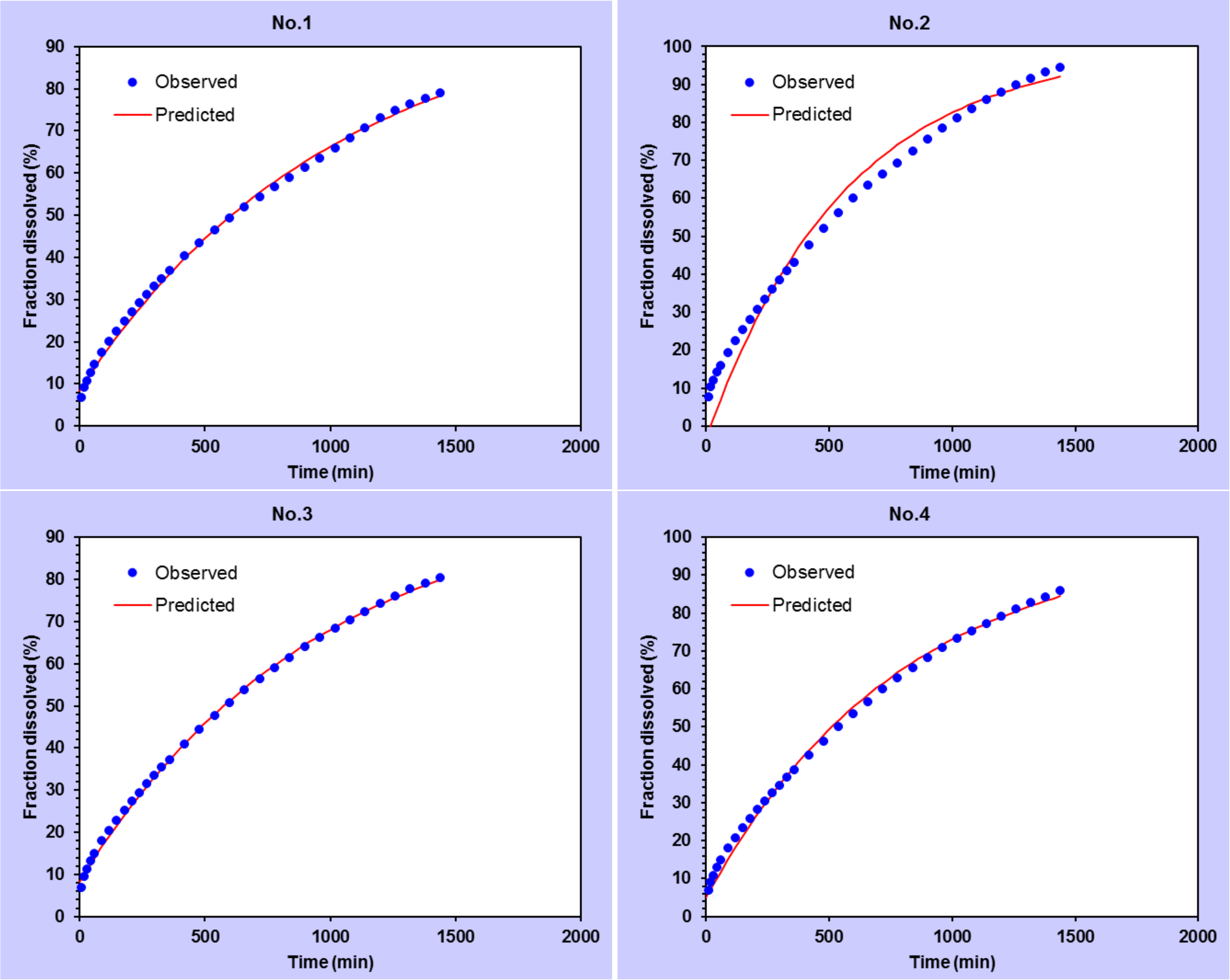

Supplementary materials – Model fitting summary – Emcompress® Anhydrous (modelling the entire relevant dissolution data)

Model: **First–order with F_{max}**

Model equation: $F = F_{max} \cdot (1 - e^{-k_1 \cdot t})$

Fitted model parameters per tested tablet (N = 4) with statistics – mean, standard deviation (SD), and relative standard deviation expressed in % (RSD%) (output from DDSolver):

Parameter	No.1	No.2	No.3	No.4	Mean	SD	RSD(%)
k_1	0.002	0.002	0.002	0.002	0.002	0.000	1.231
F_{max}	82.875	99.129	84.448	90.031	89.121	7.345	8.241

Number of dissolution data points (N), degrees of freedom (df), and selected goodness of fit criteria – Pearson correlation coefficient (R), coefficient of determination (R^2), adjusted coefficient of determination ($R^2_{adjusted}$), and residual sum of squares (RSS) (manual calculation in MS Excel):

Parameter	No.1	No.2	No.3	No.4
N	33	33	33	33
df	31	31	31	31
R	0.992091201	0.992752127	0.992755569	0.992382702
R^2	0.984244951	0.985556786	0.98556362	0.984823428
$R^2_{adjusted}$	0.983736724	0.985090876	0.985097931	0.984333861
RSS	488.5286409	559.7780417	462.9359686	485.5758733

Graphical abstract of model fit presented as mean ± 1 SD of the fraction % of released carvedilol:

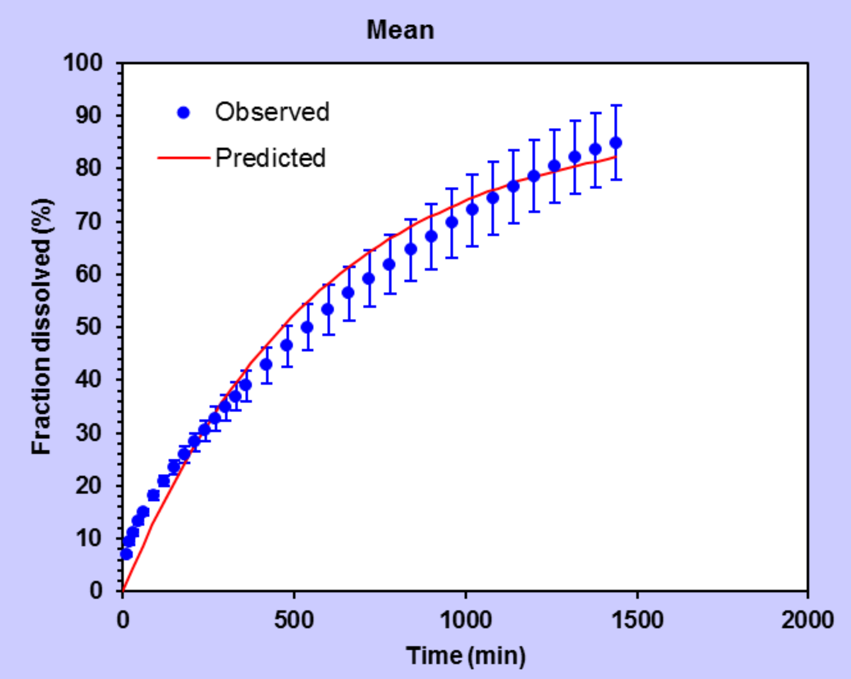

Supplementary materials – Model fitting summary – Emcompress® Anhydrous (modelling the entire relevant dissolution data)

Graphical abstract of model fit presented as the fraction % of released carvedilol per tested tablet:

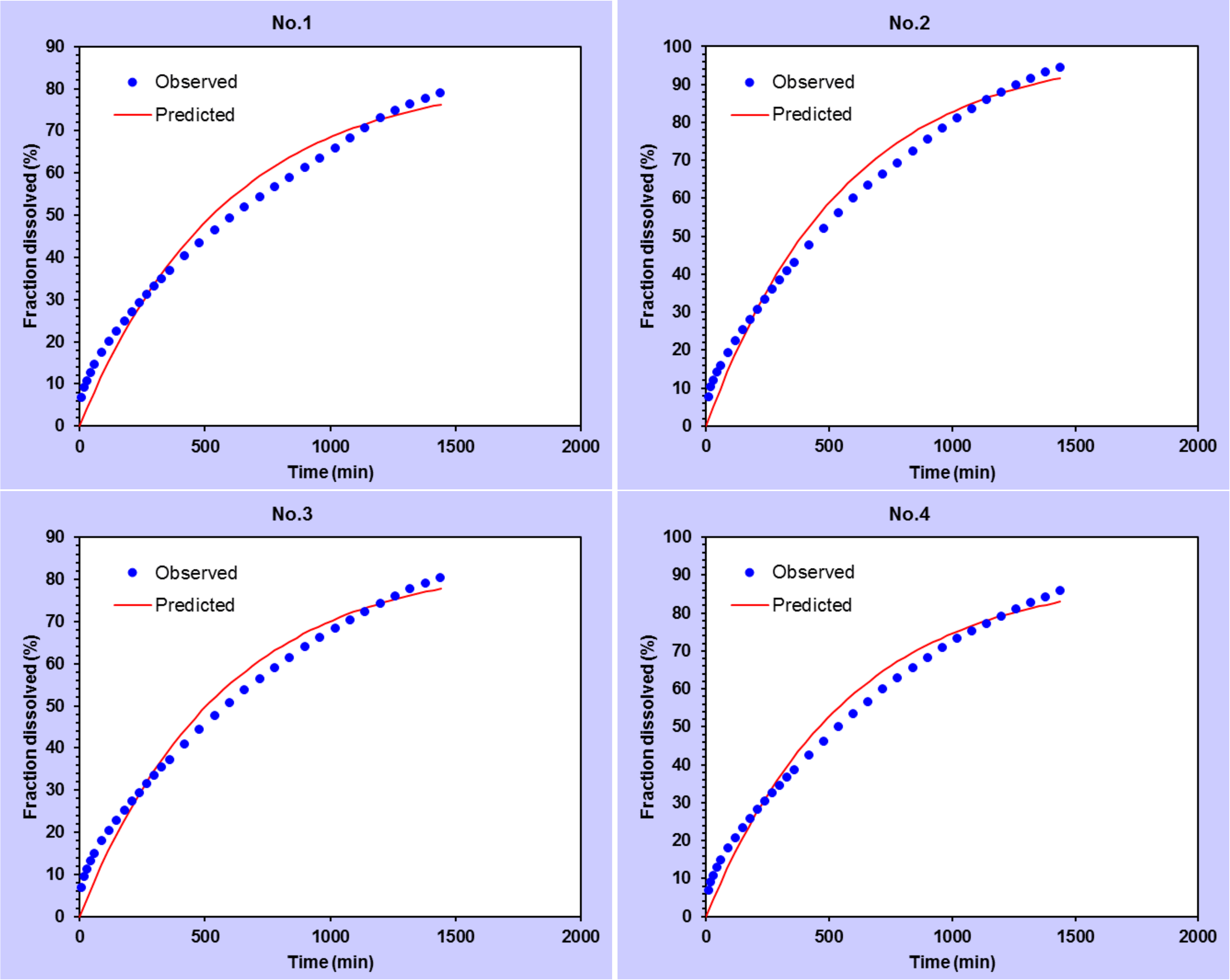

Supplementary materials – Model fitting summary – Emcompress® Anhydrous (modelling the entire relevant dissolution data)

Model: **First–order with T_{lag} and F_{max}**

Model equation: $F = F_{max} \cdot \left[1 - e^{-k_1 \cdot (t - T_{lag})}\right]$

Fitted model parameters per tested tablet (N = 4) with statistics – mean, standard deviation (SD), and relative standard deviation expressed in % (RSD%) (output from DDSolver):

Parameter	No.1	No.2	No.3	No.4	Mean	SD	RSD(%)
k_1	0.002	0.002	0.002	0.002	0.002	0.000	1.780
T_{lag}	14.201	25.549	12.686	22.725	18.790	6.311	33.587
F_{max}	82.875	99.129	84.448	90.031	89.121	7.345	8.241

Number of dissolution data points (N), degrees of freedom (df), and selected goodness of fit criteria – Pearson correlation coefficient (R), coefficient of determination (R^2), adjusted coefficient of determination ($R^2_{adjusted}$), and residual sum of squares (RSS) (manual calculation in MS Excel):

Parameter	No.1	No.2	No.3	No.4
N	33	33	33	33
df	30	30	30	30
R	0.991636059	0.991864369	0.992343325	0.991599685
R^2	0.983342073	0.983794927	0.984745275	0.983269936
$R^2_{adjusted}$	0.982231545	0.982714589	0.983728293	0.982154598
RSS	664.7128325	961.3605641	619.8373616	780.4354421

Graphical abstract of model fit presented as mean ± 1 SD of the fraction % of released carvedilol:

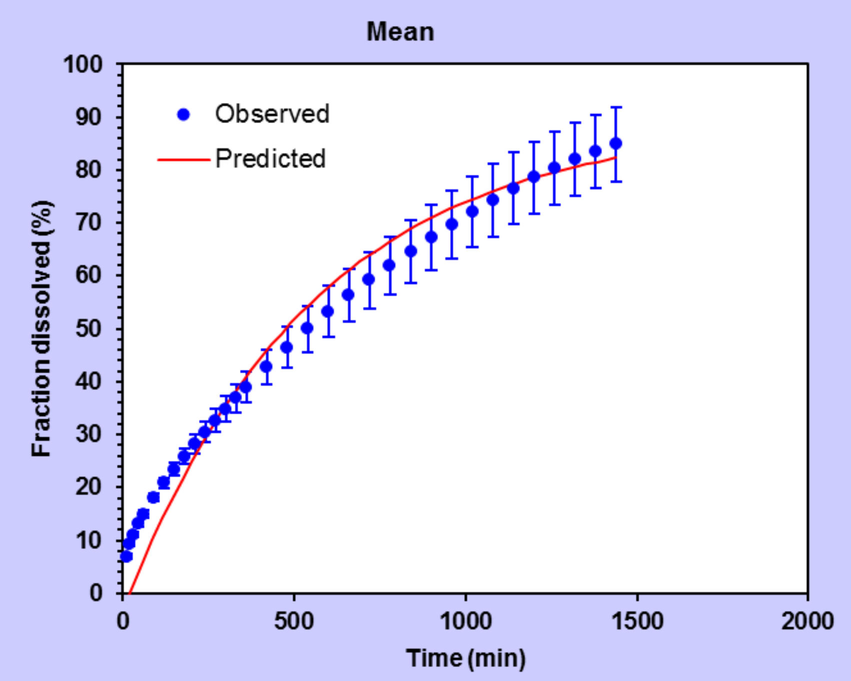

Supplementary materials – Model fitting summary – Emcompress® Anhydrous (modelling the entire relevant dissolution data)

Graphical abstract of model fit presented as the fraction % of released carvedilol per tested tablet:

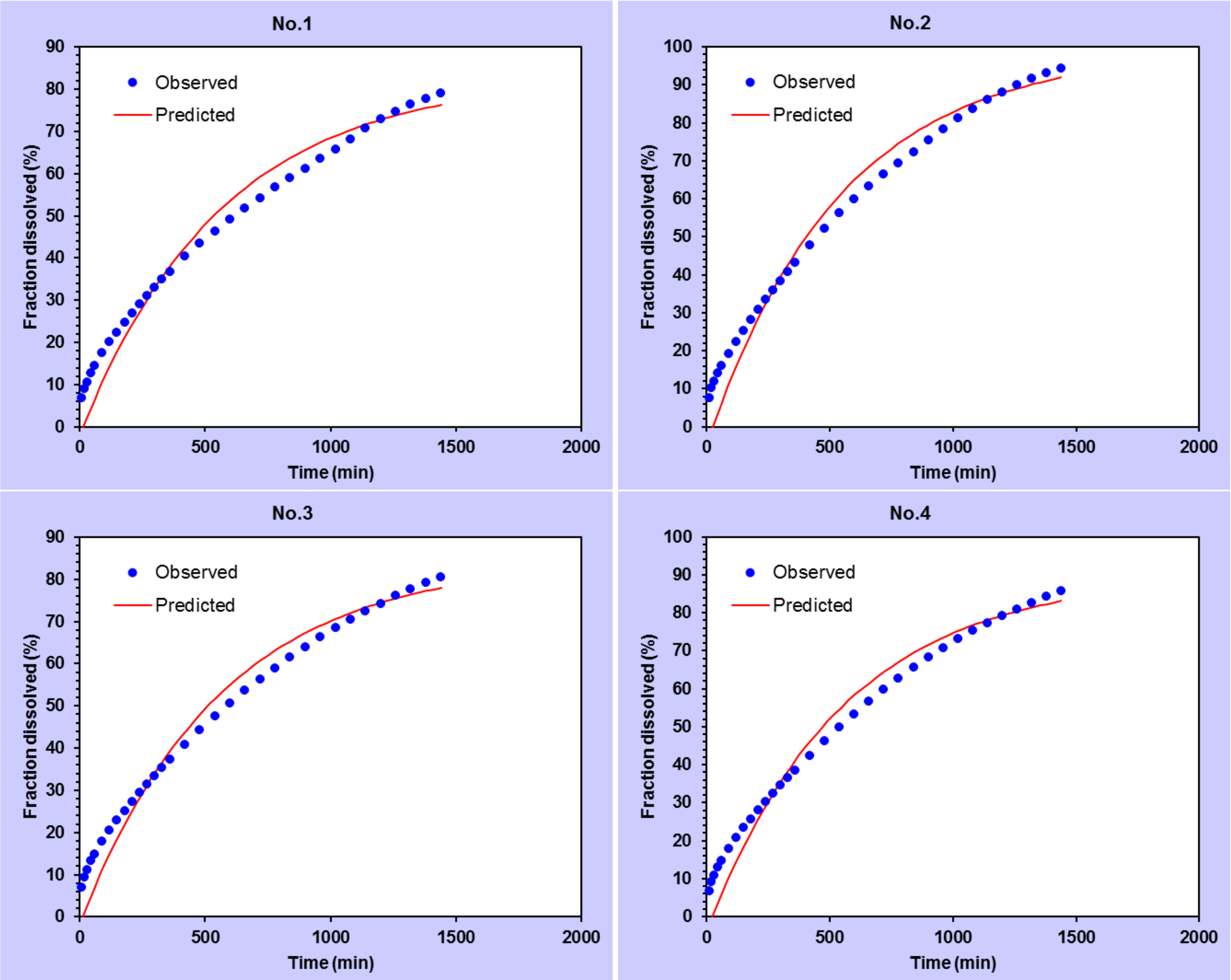

Supplementary materials – Model fitting summary – Emcompress® Anhydrous (modelling the entire relevant dissolution data)

Model: **Higuchi**

Model equation: $F = k_H \cdot t^{0.5}$

Fitted model parameters per tested tablet (N = 4) with statistics – mean, standard deviation (SD), and relative standard deviation expressed in % (RSD%) (output from DDSolver):

Parameter	No.1	No.2	No.3	No.4	Mean	SD	RSD(%)
k_H	2.040	2.460	2.093	2.217	2.202	0.187	8.486

Number of dissolution data points (N), degrees of freedom (df), and selected goodness of fit criteria – Pearson correlation coefficient (R), coefficient of determination (R^2), adjusted coefficient of determination ($R^2_{adjusted}$), and residual sum of squares (RSS) (manual calculation in MS Excel):

Parameter	No.1	No.2	No.3	No.4
N	33	33	33	33
df	32	32	32	32
R	0.998776605	0.997958274	0.998473973	0.997978919
R^2	0.997554706	0.995920716	0.996950275	0.995961922
$R^2_{adjusted}$	0.997554706	0.995920716	0.996950275	0.995961922
RSS	91.63802544	285.3906816	110.844431	221.8427796

Graphical abstract of model fit presented as mean ± 1 SD of the fraction % of released carvedilol:

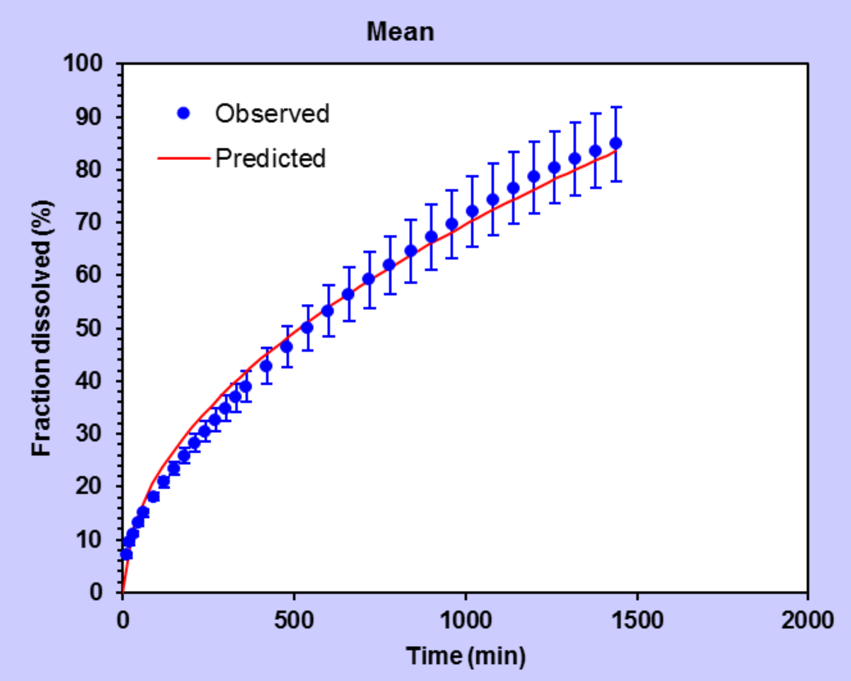

Supplementary materials – Model fitting summary – Emcompress® Anhydrous (modelling the entire relevant dissolution data)

Graphical abstract of model fit presented as the fraction % of released carvedilol per tested tablet:

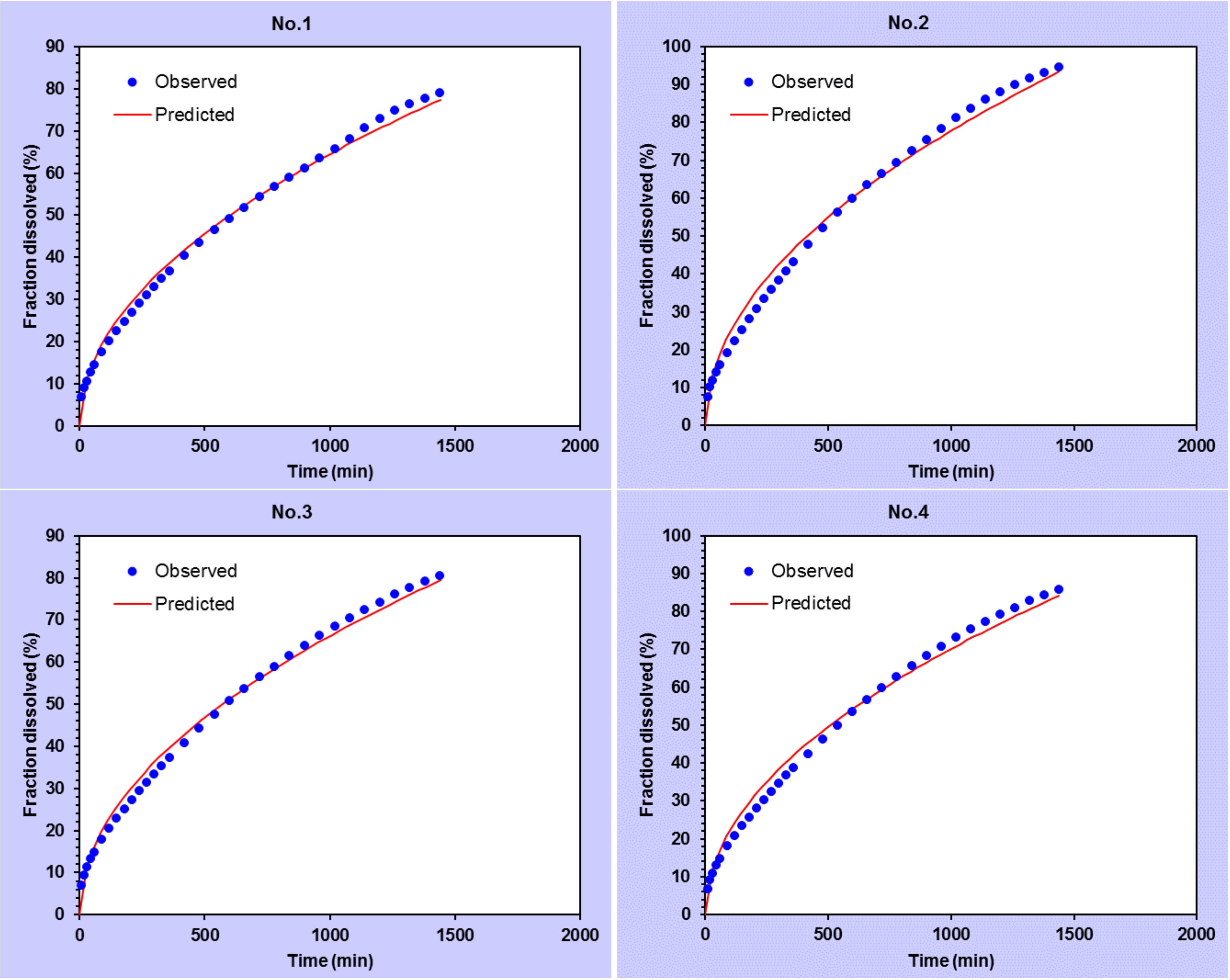

Supplementary materials – Model fitting summary – Emcompress® Anhydrous (modelling the entire relevant dissolution data)

Model: **Higuchi with T_{lag}**

Model equation: $F = k_H \cdot (t - T_{lag})^{0.5}$

Fitted model parameters per tested tablet (N = 4) with statistics – mean, standard deviation (SD), and relative standard deviation expressed in % (RSD%) (output from DDSolver):

Parameter	No.1	No.2	No.3	No.4	Mean	SD	RSD(%)
k_H	2.107	2.563	2.160	2.313	2.286	0.204	8.929
T_{lag}	36.115	44.470	34.830	45.757	40.293	5.616	13.937

Number of dissolution data points (N), degrees of freedom (df), and selected goodness of fit criteria – Pearson correlation coefficient (R), coefficient of determination (R^2), adjusted coefficient of determination ($R^2_{adjusted}$), and residual sum of squares (RSS) (manual calculation in MS Excel):

Parameter	No.1	No.2	No.3	No.4
N	33	33	33	33
df	31	31	31	31
R	0.994429977	0.992880205	0.993803902	0.992233367
R^2	0.98889098	0.985811102	0.987646195	0.984527055
$R^2_{adjusted}$	0.988532624	0.985353396	0.987247685	0.984027928
RSS	320.5419397	546.6726509	346.437027	504.6173007

Graphical abstract of model fit presented as mean ± 1 SD of the fraction % of released carvedilol:

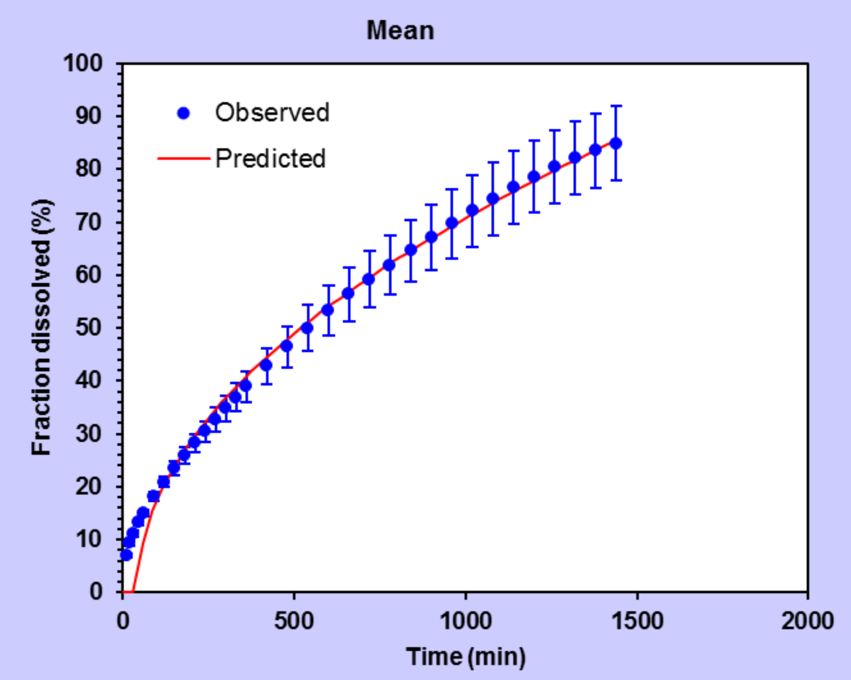

Supplementary materials – Model fitting summary – Emcompress® Anhydrous (modelling the entire relevant dissolution data)

Graphical abstract of model fit presented as the fraction % of released carvedilol per tested tablet:

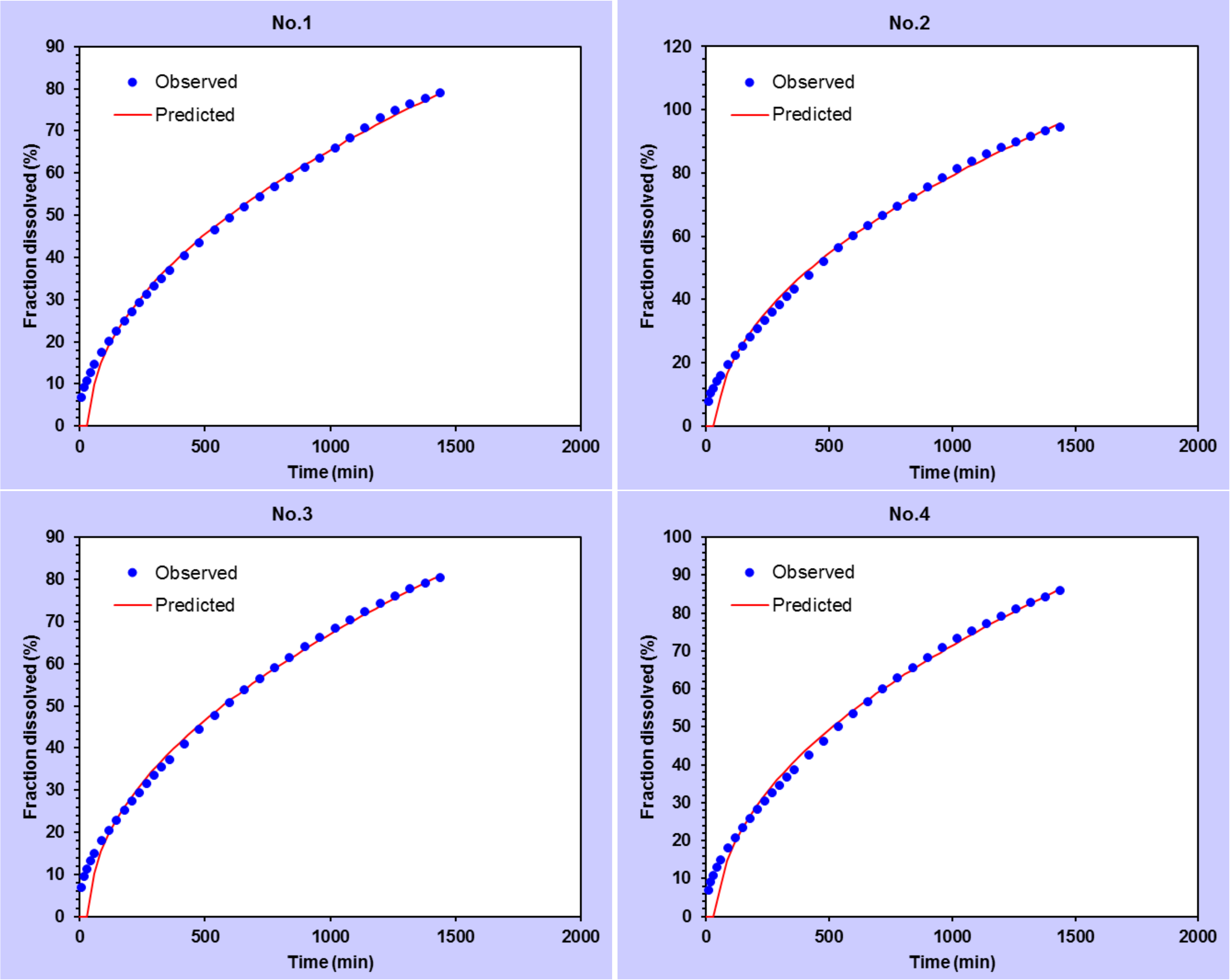

Supplementary materials – Model fitting summary – Emcompress® Anhydrous (modelling the entire relevant dissolution data)

Model: **Higuchi with F_0**

Model equation: $F = F_0 + k_H \cdot t^{0.5}$

Fitted model parameters per tested tablet (N = 4) with statistics – mean, standard deviation (SD), and relative standard deviation expressed in % (RSD%) (output from DDSolver):

Parameter	No.1	No.2	No.3	No.4	Mean	SD	RSD(%)
k_H	2.145	2.659	2.205	2.392	2.350	0.231	9.830
F_0	-2.830	-5.342	-2.996	-4.668	-3.959	1.240	-31.326

Number of dissolution data points (N), degrees of freedom (df), and selected goodness of fit criteria – Pearson correlation coefficient (R), coefficient of determination (R^2), adjusted coefficient of determination ($R^2_{adjusted}$), and residual sum of squares (RSS) (manual calculation in MS Excel):

Parameter	No.1	No.2	No.3	No.4
N	33	33	33	33
df	31	31	31	31
R	0.998776605	0.997958274	0.998473973	0.997978919
R^2	0.997554706	0.995920716	0.996950275	0.995961922
$R^2_{adjusted}$	0.997475825	0.995789126	0.996851897	0.995831661
RSS	41.21745997	105.7997311	54.3331063	84.71825701

Graphical abstract of model fit presented as mean ± 1 SD of the fraction % of released carvedilol:

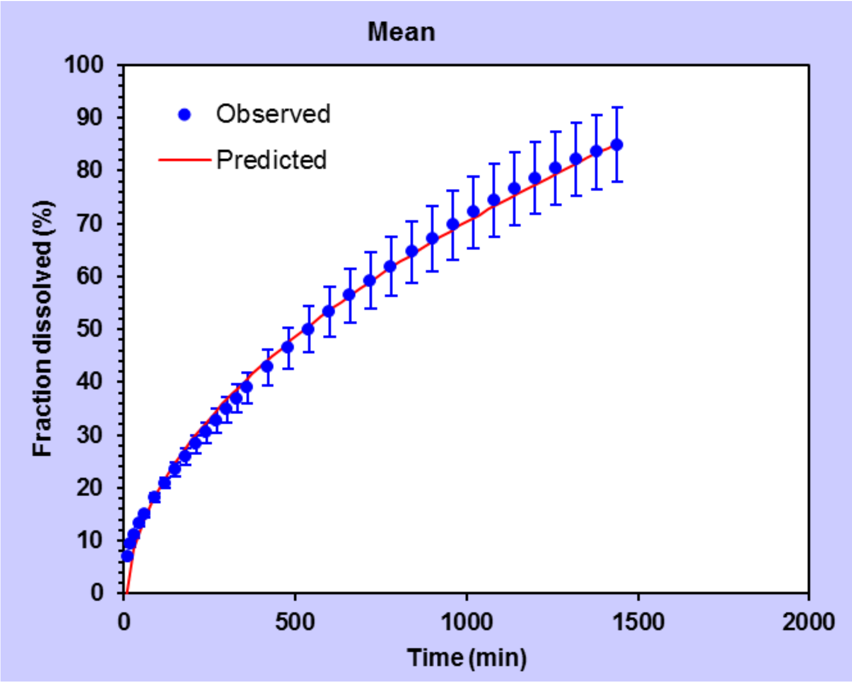

Supplementary materials – Model fitting summary – Emcompress® Anhydrous (modelling the entire relevant dissolution data)

Graphical abstract of model fit presented as the fraction % of released carvedilol per tested tablet:

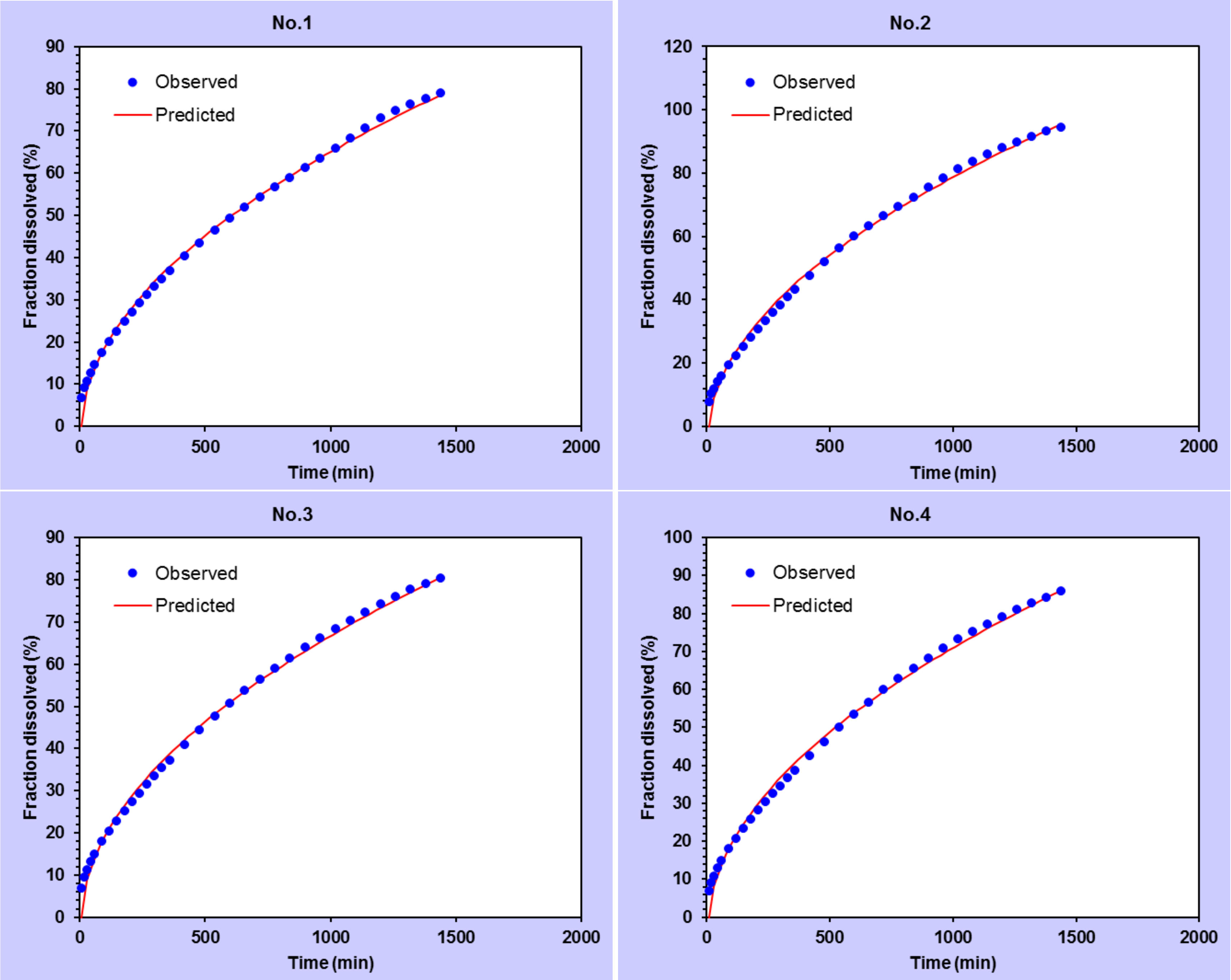

Supplementary materials – Model fitting summary – Emcompress® Anhydrous (modelling the entire relevant dissolution data)

Model: **Korsmeyer–Peppas**

Model equation: $F = k_{KP} \cdot t^n$

Fitted model parameters per tested tablet (N = 4) with statistics – mean, standard deviation (SD), and relative standard deviation expressed in % (RSD%) (output from DDSolver):

Parameter	No.1	No.2	No.3	No.4	Mean	SD	RSD(%)
k_{KP}	1.791	1.825	1.844	1.671	1.783	0.078	4.373
n	0.517	0.542	0.517	0.540	0.529	0.014	2.635

Number of dissolution data points (N), degrees of freedom (df), and selected goodness of fit criteria – Pearson correlation coefficient (R), coefficient of determination (R^2), adjusted coefficient of determination ($R^2_{adjusted}$), and residual sum of squares (RSS) (manual calculation in MS Excel):

Parameter	No.1	No.2	No.3	No.4
N	33	33	33	33
df	31	31	31	31
R	0.9991528	0.998767547	0.998834676	0.9988383
R^2	0.998306317	0.997536614	0.997670711	0.997677949
$R^2_{adjusted}$	0.998251682	0.99745715	0.997595572	0.997603044
RSS	62.59950717	123.1920804	83.67444506	94.82910251

Graphical abstract of model fit presented as mean ± 1 SD of the fraction % of released carvedilol:

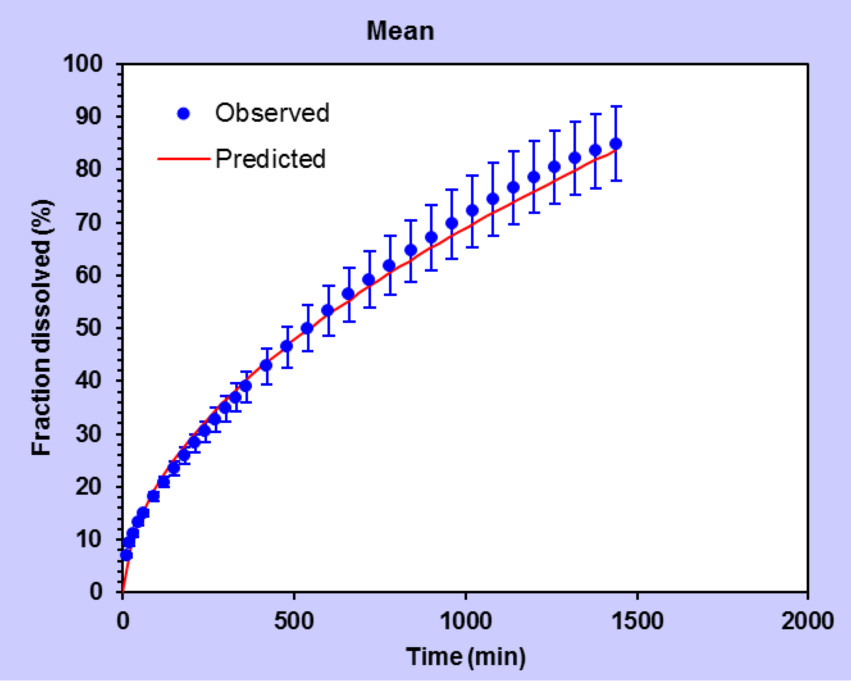

Supplementary materials – Model fitting summary – Emcompress® Anhydrous (modelling the entire relevant dissolution data)

Graphical abstract of model fit presented as the fraction % of released carvedilol per tested tablet:

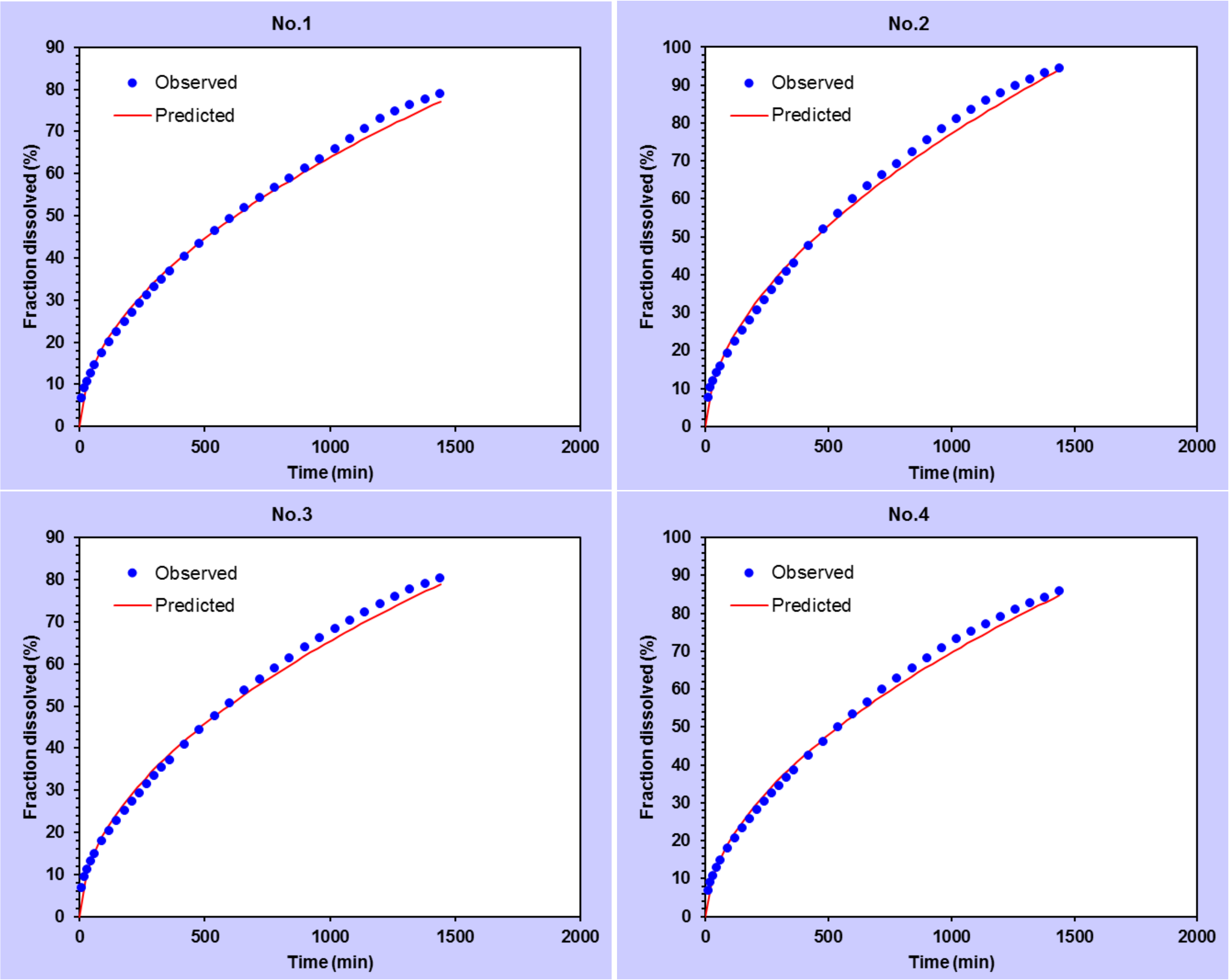

Supplementary materials – Model fitting summary – Emcompress® Anhydrous (modelling the entire relevant dissolution data)

Model: **Korsmeyer–Peppas with T_{lag}**

Model equation: $F = k_{KP} \cdot (t - T_{lag})^n$

Fitted model parameters per tested tablet (N = 4) with statistics – mean, standard deviation (SD), and relative standard deviation expressed in % (RSD%) (output from DDSolver):

Parameter	No.1	No.2	No.3	No.4	Mean	SD	RSD(%)
k_{KP}	2.170	2.235	2.234	2.041	2.170	0.091	4.196
n	0.488	0.511	0.487	0.509	0.499	0.013	2.617
T_{lag}	4.000	4.000	4.000	4.000	4.000	0.000	0.000

Number of dissolution data points (N), degrees of freedom (df), and selected goodness of fit criteria – Pearson correlation coefficient (R), coefficient of determination (R^2), adjusted coefficient of determination ($R^2_{adjusted}$), and residual sum of squares (RSS) (manual calculation in MS Excel):

Parameter	No.1	No.2	No.3	No.4
N	33	33	33	33
df	30	30	30	30
R	0.997997624	0.997810623	0.997654351	0.997802735
R^2	0.995999257	0.995626039	0.995314204	0.995610298
$R^2_{adjusted}$	0.995732541	0.995334441	0.995001818	0.995317651
RSS	161.1268656	276.0010794	192.2151235	218.2540214

Graphical abstract of model fit presented as mean ± 1 SD of the fraction % of released carvedilol:

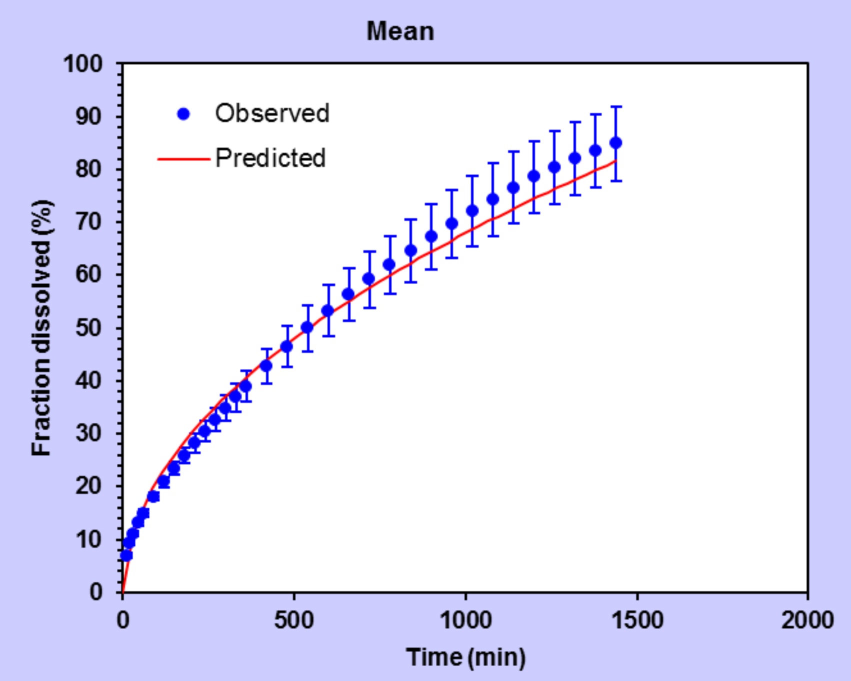

Supplementary materials – Model fitting summary – Emcompress® Anhydrous (modelling the entire relevant dissolution data)

Graphical abstract of model fit presented as the fraction % of released carvedilol per tested tablet:

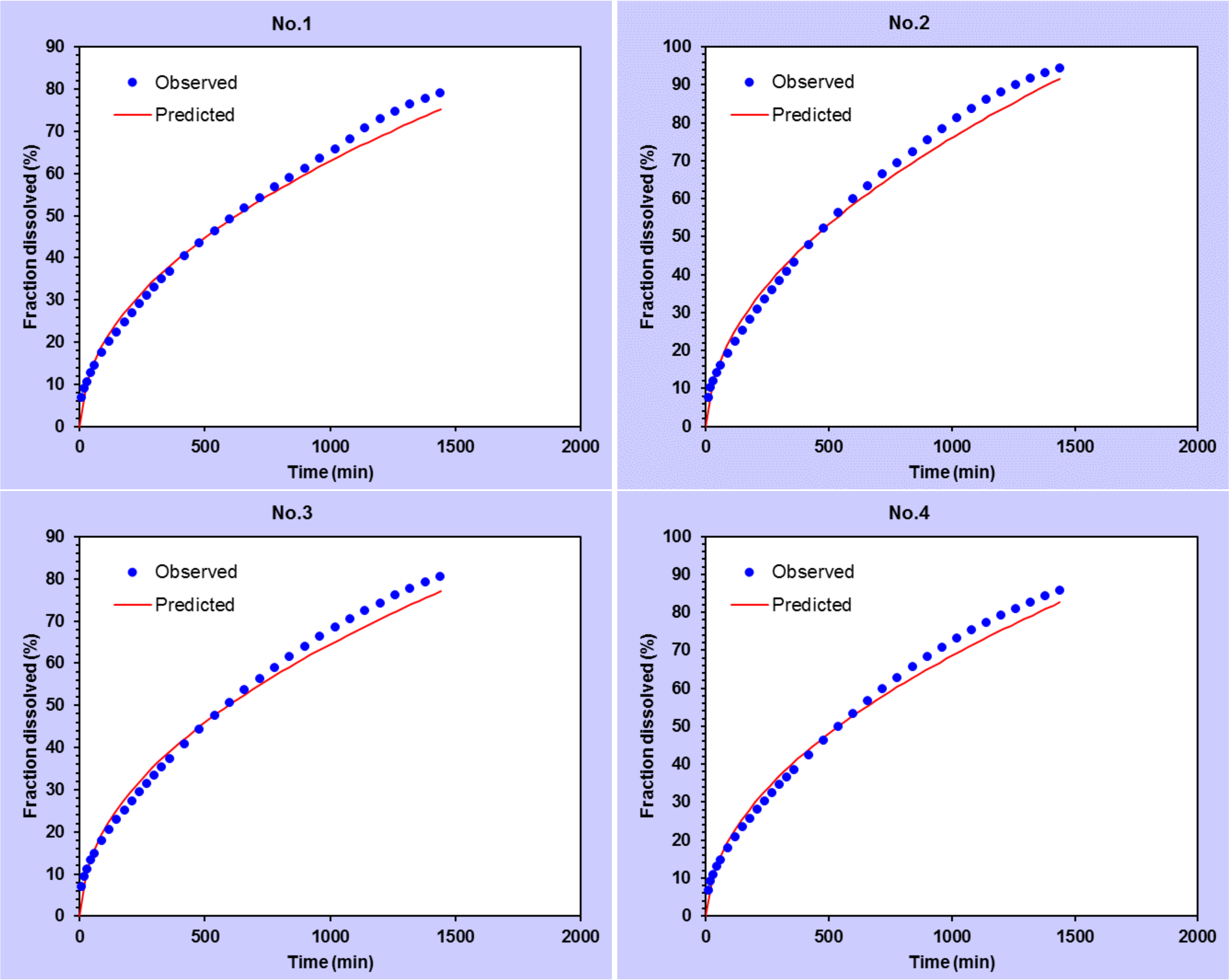

Supplementary materials – Model fitting summary – Emcompress® Anhydrous (modelling the entire relevant dissolution data)

Model: **Korsmeyer–Peppas with F_0**

Model equation: $F = F_0 + k_{KP} \cdot t^n$

Fitted model parameters per tested tablet (N = 4) with statistics – mean, standard deviation (SD), and relative standard deviation expressed in % (RSD%) (output from DDSolver):

Parameter	No.1	No.2	No.3	No.4	Mean	SD	RSD(%)
k_{KP}	1.034	1.051	1.075	0.975	1.034	0.043	4.126
n	0.593	0.619	0.591	0.615	0.605	0.014	2.383
F_0	2.719	3.039	2.759	2.679	2.799	0.163	5.829

Number of dissolution data points (N), degrees of freedom (df), and selected goodness of fit criteria – Pearson correlation coefficient (R), coefficient of determination (R^2), adjusted coefficient of determination ($R^2_{adjusted}$), and residual sum of squares (RSS) (manual calculation in MS Excel):

Parameter	No.1	No.2	No.3	No.4
N	33	33	33	33
df	30	30	30	30
R	0.999844101	0.999002924	0.999515745	0.999285057
R^2	0.999688226	0.998006842	0.999031724	0.998570626
$R^2_{adjusted}$	0.999667441	0.997873965	0.998967172	0.998475335
RSS	5.591420643	52.98016104	18.83631774	30.72815912

Graphical abstract of model fit presented as mean ± 1 SD of the fraction % of released carvedilol:

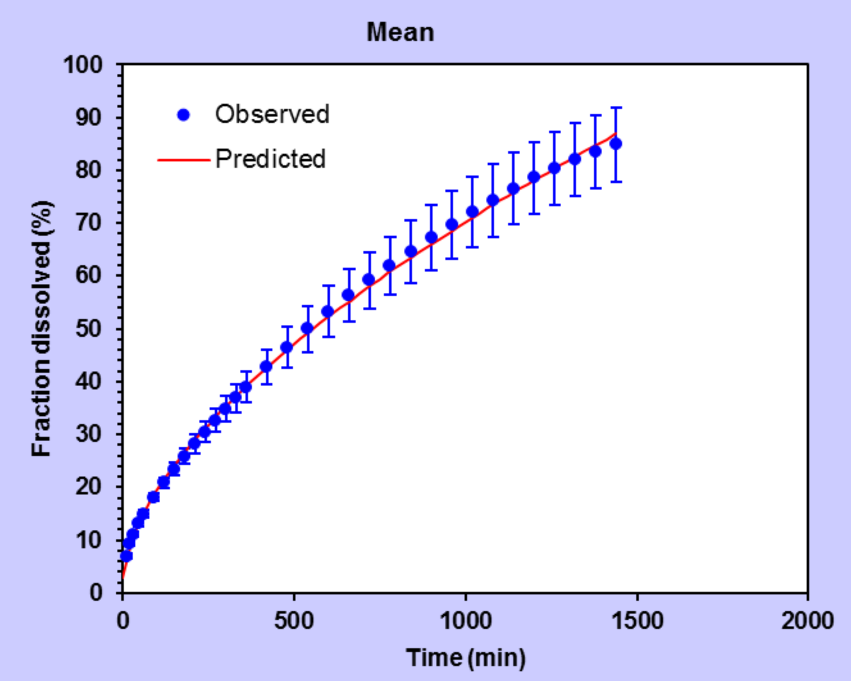

Supplementary materials – Model fitting summary – Emcompress® Anhydrous (modelling the entire relevant dissolution data)

Graphical abstract of model fit presented as the fraction % of released carvedilol per tested tablet:

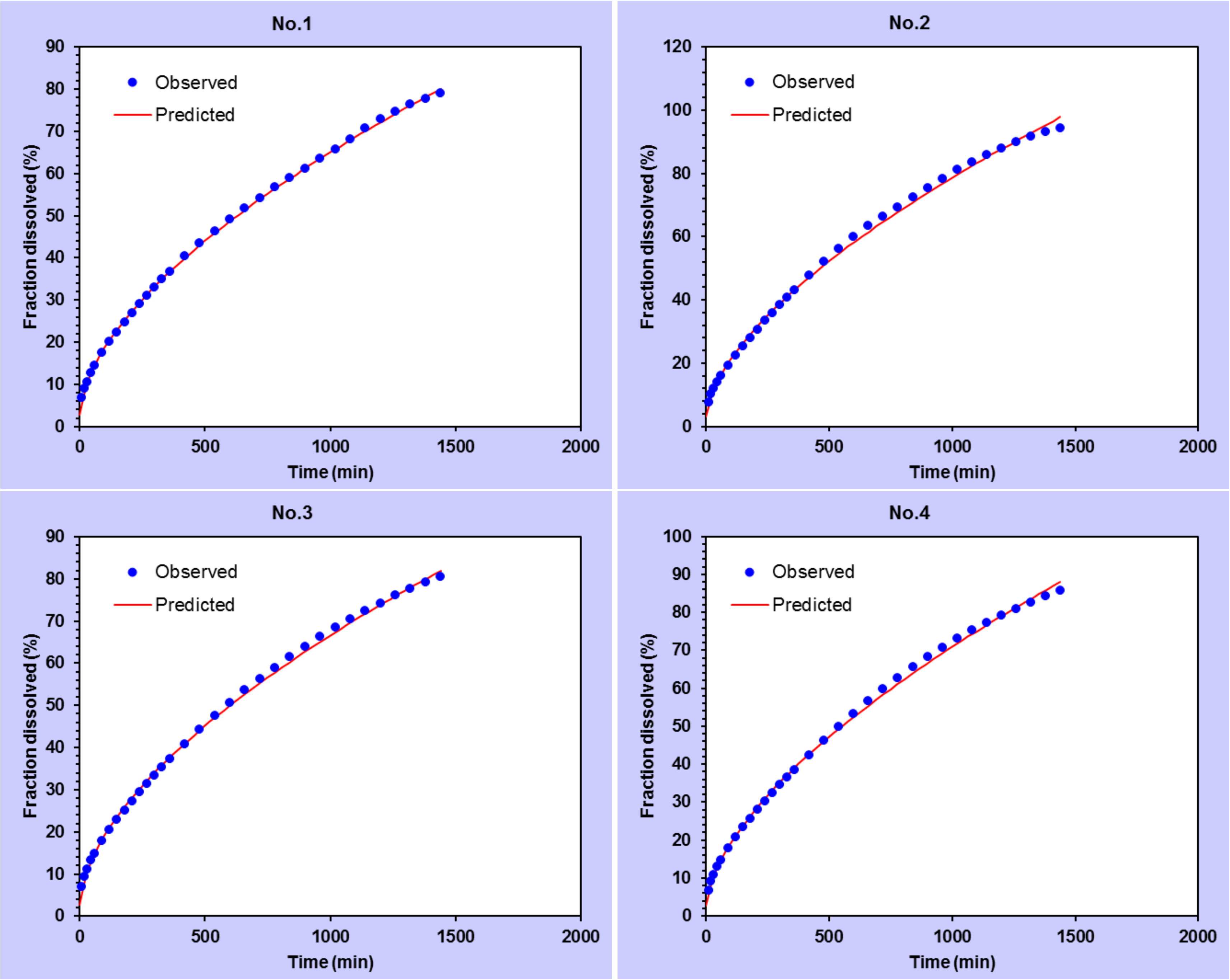

Supplementary materials – Model fitting summary – Emcompress® Anhydrous (modelling the entire relevant dissolution data)

Model: **Hixson–Crowell**

Model equation: $F = 100 \cdot [1 - (1 - k_{HC} \cdot t)^3]$

Fitted model parameters per tested tablet (N = 4) with statistics – mean, standard deviation (SD), and relative standard deviation expressed in % (RSD%) (output from DDSolver):

Parameter	No.1	No.2	No.3	No.4	Mean	SD	RSD(%)
k_{HC}	0.0003	0.0004	0.0003	0.0003	0.0003	0.0001	16.1760

Number of dissolution data points (N), degrees of freedom (df), and selected goodness of fit criteria – Pearson correlation coefficient (R), coefficient of determination (R^2), adjusted coefficient of determination ($R^2_{adjusted}$), and residual sum of squares (RSS) (manual calculation in MS Excel):

Parameter	No.1	No.2	No.3	No.4
N	33	33	33	33
df	32	32	32	32
R	0.998055239	0.999334088	0.998704366	0.999417127
R^2	0.99611426	0.99866862	0.99741041	0.998834594
$R^2_{adjusted}$	0.99611426	0.99866862	0.99741041	0.998834594
RSS	1250.353416	702.181495	1218.519594	912.7747578

Graphical abstract of model fit presented as mean ± 1 SD of the fraction % of released carvedilol:

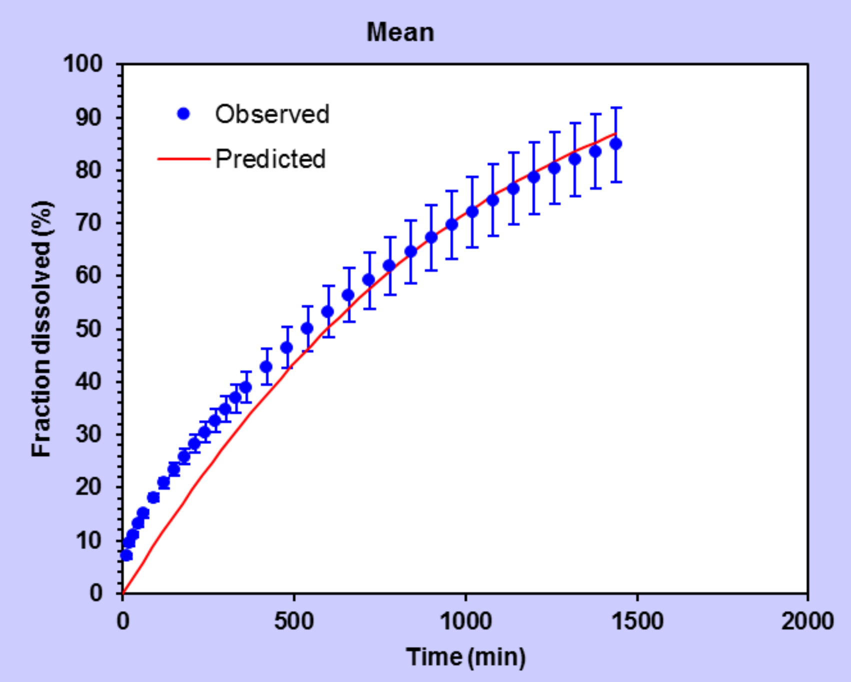

Supplementary materials – Model fitting summary – Emcompress® Anhydrous (modelling the entire relevant dissolution data)

Graphical abstract of model fit presented as the fraction % of released carvedilol per tested tablet:

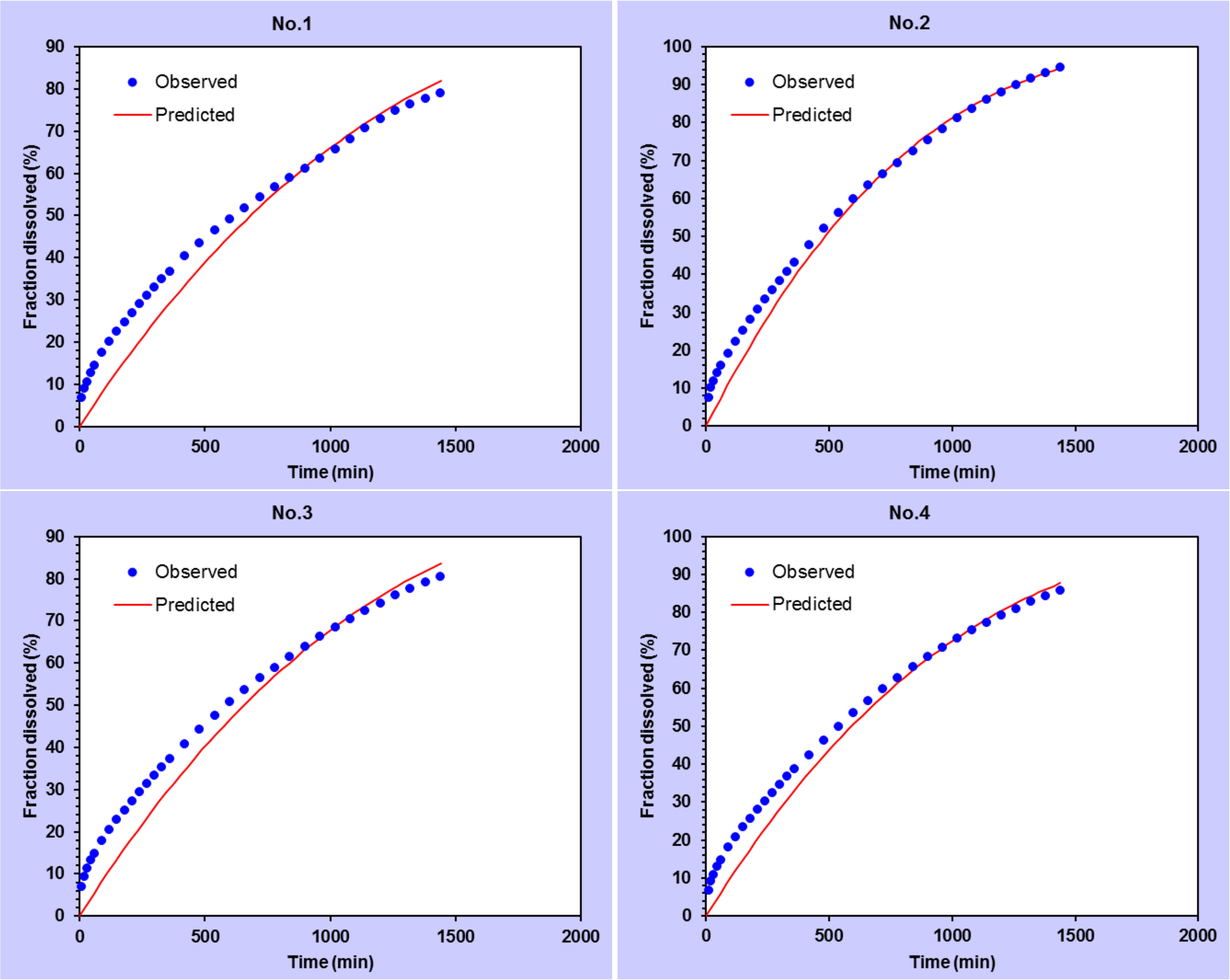

Supplementary materials – Model fitting summary – Emcompress® Anhydrous (modelling the entire relevant dissolution data)

Model: **Hixson–Crowell with T_{lag}**

Model equation: $F = 100 \cdot \left\{1 - \left[1 - k_{HC} \cdot \left(t - T_{lag}\right)\right]^3\right\}$

Fitted model parameters per tested tablet (N = 4) with statistics – mean, standard deviation (SD), and relative standard deviation expressed in % (RSD%) (output from DDSolver):

Parameter	No.1	No.2	No.3	No.4	Mean	SD	RSD(%)
k_{HC}	0.0003	0.0004	0.0003	0.0003	0.0003	0.0001	20.2000
T_{lag}	-154.2297	-69.5938	-148.8609	-112.1306	-121.2037	39.1643	-32.3128

Number of dissolution data points (N), degrees of freedom (df), and selected goodness of fit criteria – Pearson correlation coefficient (R), coefficient of determination (R^2), adjusted coefficient of determination ($R^2_{adjusted}$), and residual sum of squares (RSS) (manual calculation in MS Excel):

Parameter	No.1	No.2	No.3	No.4
N	33	33	33	33
df	31	31	31	31
R	0.997399579	0.999546462	0.998004095	0.999141695
R^2	0.994805921	0.999093129	0.996012173	0.998284126
$R^2_{adjusted}$	0.99463837	0.999063875	0.995883533	0.998228775
RSS	91.36371748	28.16911464	76.77033457	38.40714247

Graphical abstract of model fit presented as mean ± 1 SD of the fraction % of released carvedilol:

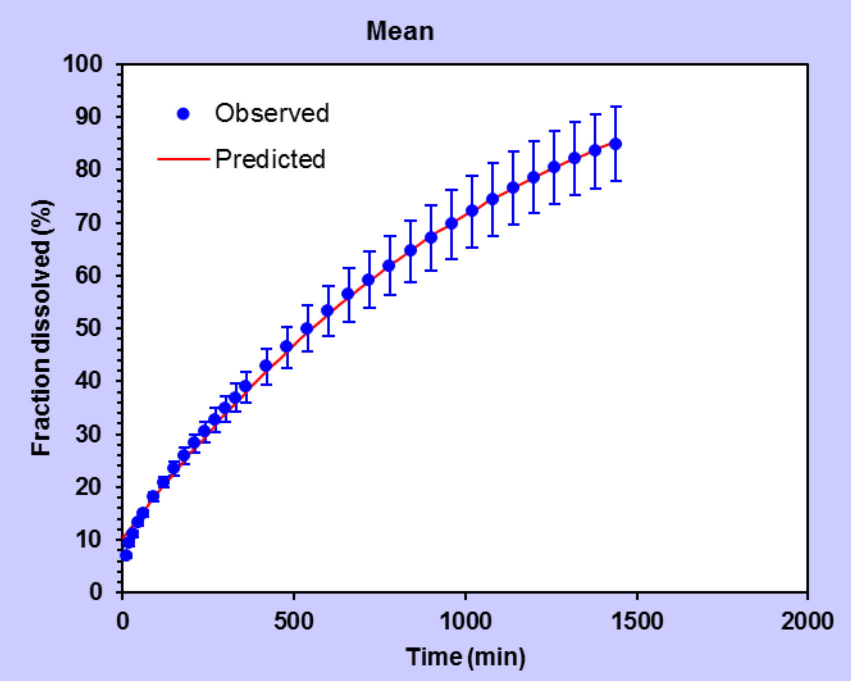

Supplementary materials – Model fitting summary – Emcompress® Anhydrous (modelling the entire relevant dissolution data)

Graphical abstract of model fit presented as the fraction % of released carvedilol per tested tablet:

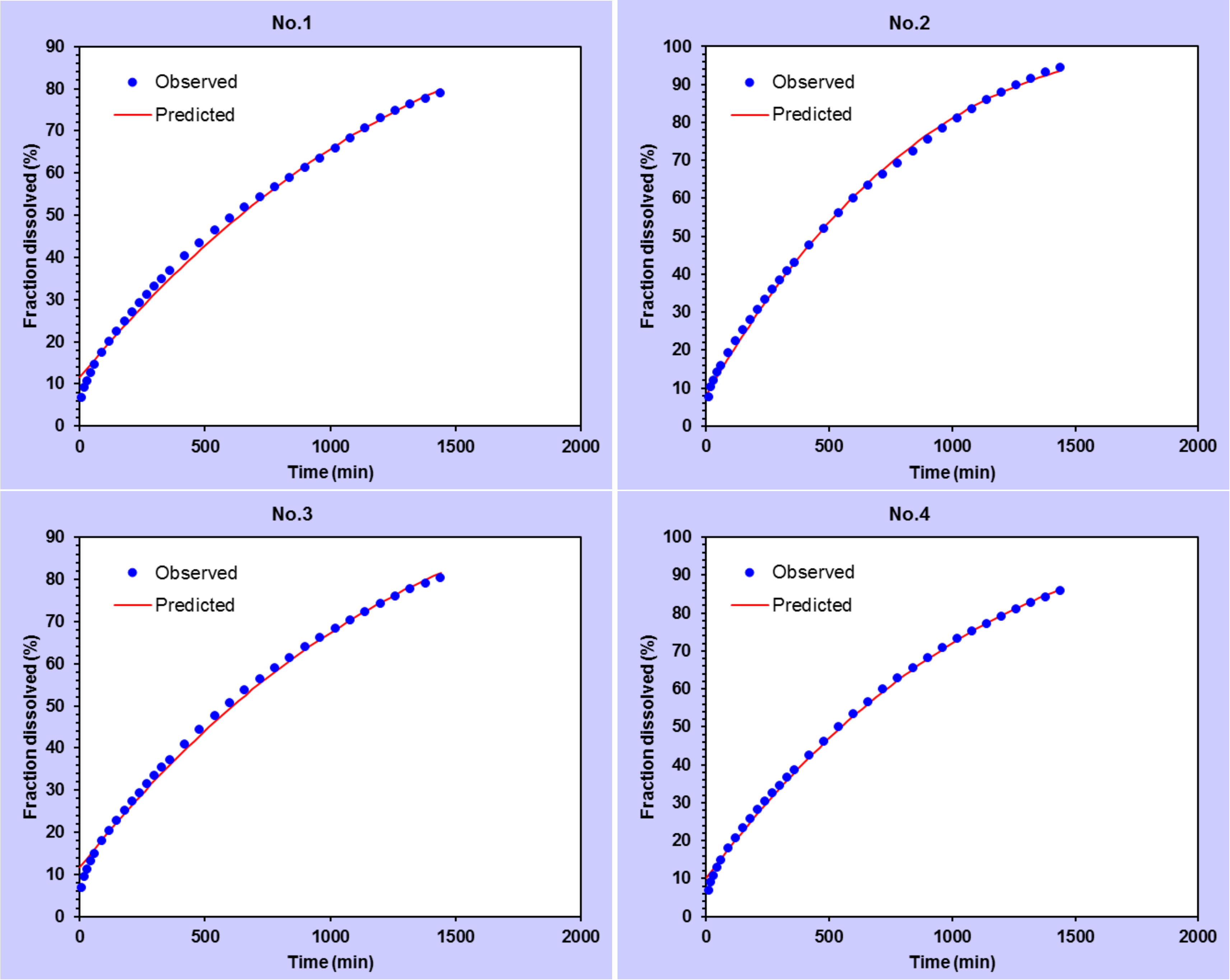

Supplementary materials – Model fitting summary – Emcompress® Anhydrous (modelling the entire relevant dissolution data)

Model: **Hopfenberg**

Model equation: $F = 100 \cdot [1 - (1 - k_{HB} \cdot t)^n]$

Fitted model parameters per tested tablet (N = 4) with statistics – mean, standard deviation (SD), and relative standard deviation expressed in % (RSD%) (output from DDSolver):

Parameter	No.1	No.2	No.3	No.4	Mean	SD	RSD(%)
k_{HB}	0.0003	0.0004	0.0003	0.0003	0.0003	0.0001	16.1760
n	3.0000	3.0000	3.0000	3.0000	3.0000	0.0000	0.0000

Number of dissolution data points (N), degrees of freedom (df), and selected goodness of fit criteria – Pearson correlation coefficient (R), coefficient of determination (R^2), adjusted coefficient of determination ($R^2_{adjusted}$), and residual sum of squares (RSS) (manual calculation in MS Excel):

Parameter	No.1	No.2	No.3	No.4
N	33	33	33	33
df	31	31	31	31
R	0.998055239	0.999334088	0.998704366	0.999417127
R^2	0.99611426	0.99866862	0.99741041	0.998834594
$R^2_{adjusted}$	0.995988914	0.998625672	0.997326875	0.998797
RSS	1250.353416	702.181495	1218.519594	912.7747578

Graphical abstract of model fit presented as mean ± 1 SD of the fraction % of released carvedilol:

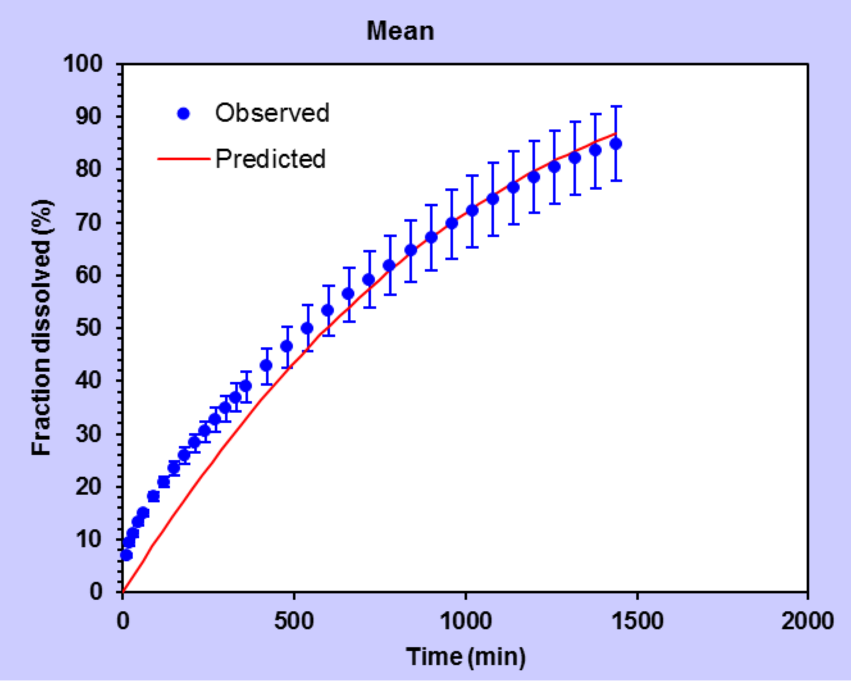

Supplementary materials – Model fitting summary – Emcompress® Anhydrous (modelling the entire relevant dissolution data)

Graphical abstract of model fit presented as the fraction % of released carvedilol per tested tablet:

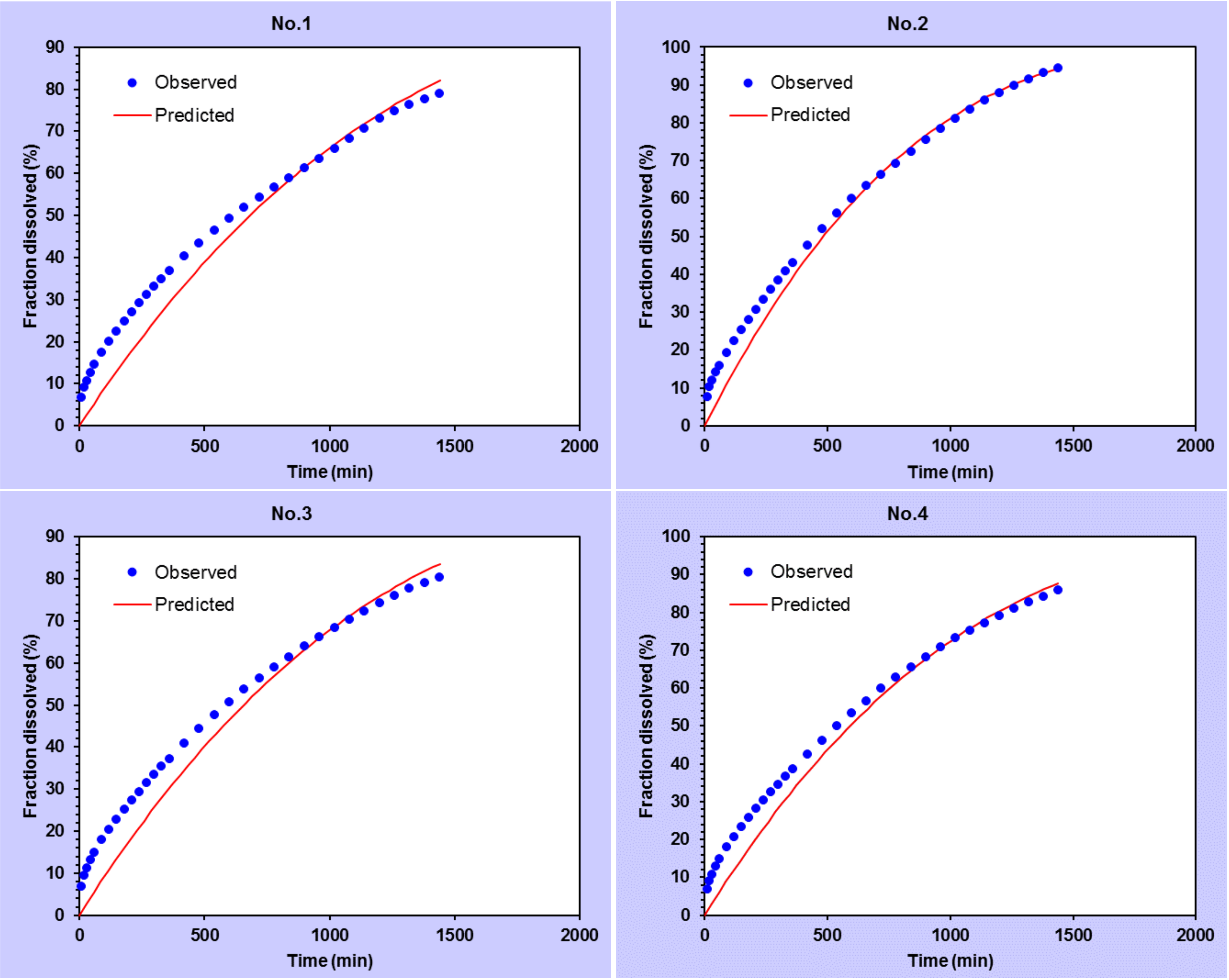

Supplementary materials – Model fitting summary – Emcompress® Anhydrous (modelling the entire relevant dissolution data)

Model: **Hopfenberg with T_{lag}**

Model equation: $F = 100 \cdot \{1 - [1 - k_{HB} \cdot (t - T_{lag})]^n\}$

Fitted model parameters per tested tablet (N = 4) with statistics – mean, standard deviation (SD), and relative standard deviation expressed in % (RSD%) (output from DDSolver):

Parameter	No.1	No.2	No.3	No.4	Mean	SD	RSD(%)
k_{HB}	0.0003	0.0004	0.0003	0.0003	0.0003	0.0001	20.2000
n	3.0000	3.0000	3.0000	3.0000	3.0000	0.0000	0.0000
T_{lag}	-154.2297	-69.5938	-148.8609	-112.1306	-121.2037	39.1643	-32.3128

Number of dissolution data points (N), degrees of freedom (df), and selected goodness of fit criteria – Pearson correlation coefficient (R), coefficient of determination (R^2), adjusted coefficient of determination ($R^2_{adjusted}$), and residual sum of squares (RSS) (manual calculation in MS Excel):

Parameter	No.1	No.2	No.3	No.4
N	33	33	33	33
df	30	30	30	30
R	0.997399579	0.999546462	0.998004095	0.999141695
R^2	0.994805921	0.999093129	0.996012173	0.998284126
$R^2_{adjusted}$	0.994459649	0.999032671	0.995746318	0.998169735
RSS	91.36371748	28.16911464	76.77033457	38.40714247

Graphical abstract of model fit presented as mean ± 1 SD of the fraction % of released carvedilol:

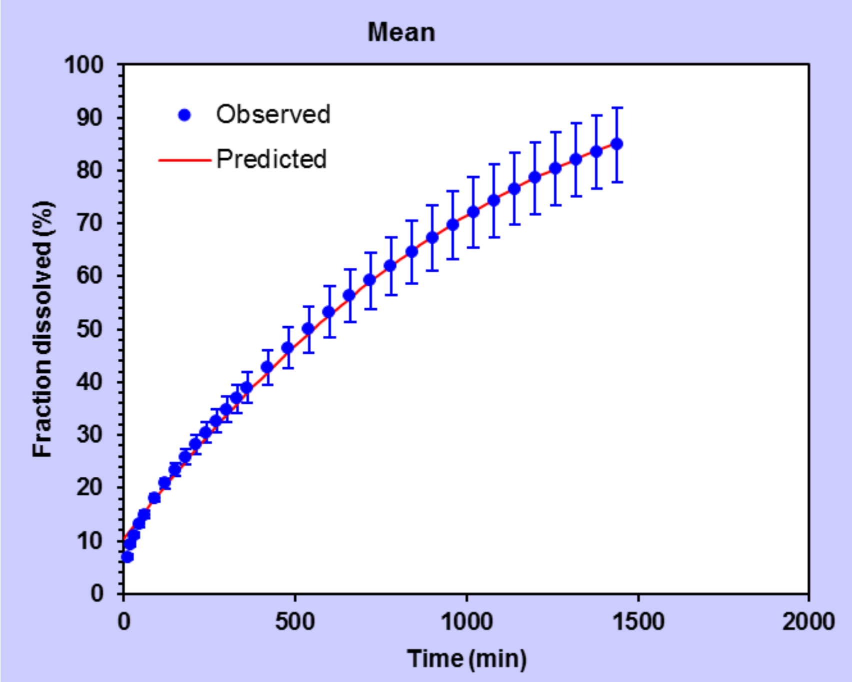

Supplementary materials – Model fitting summary – Emcompress® Anhydrous (modelling the entire relevant dissolution data)

Graphical abstract of model fit presented as the fraction % of released carvedilol per tested tablet:

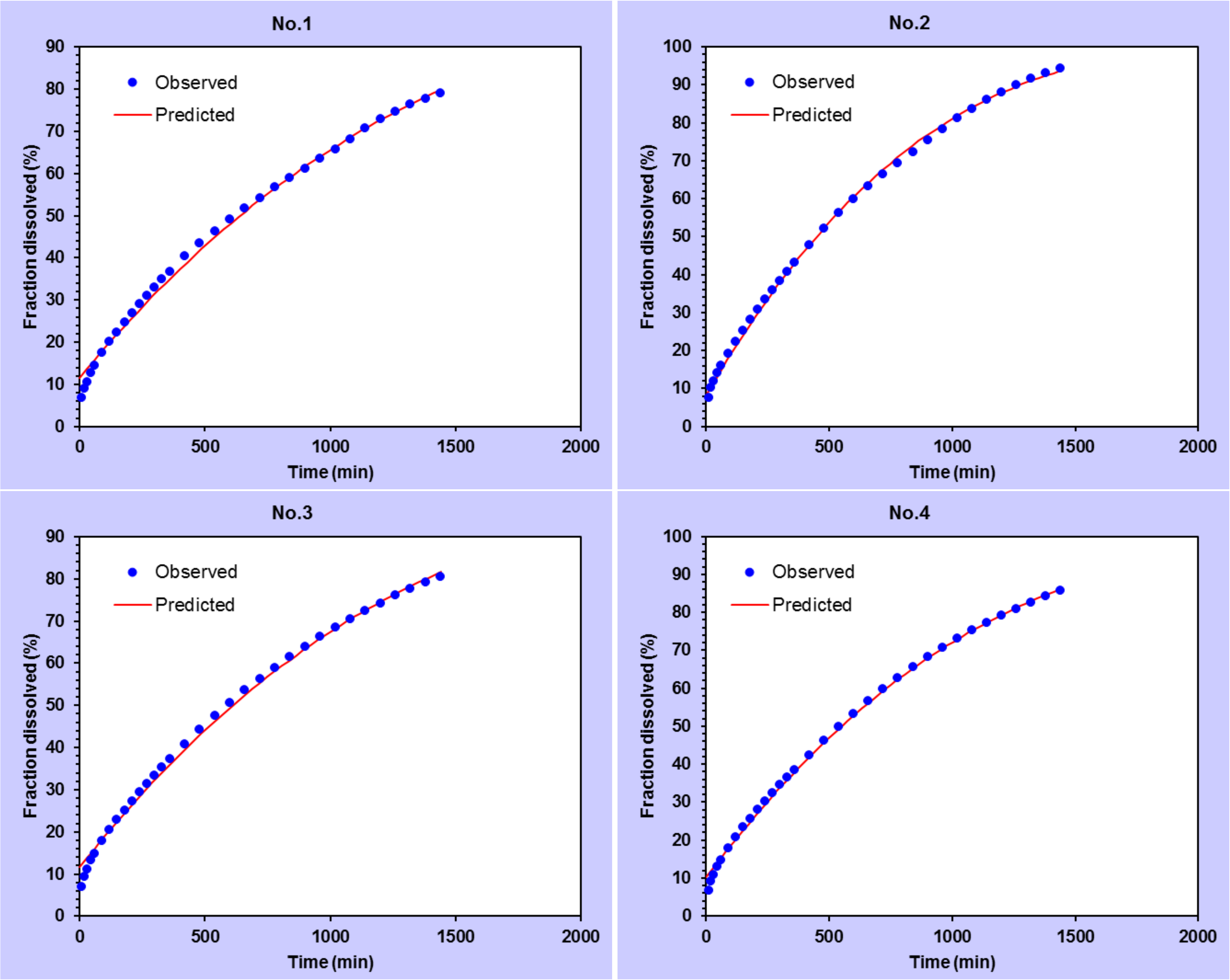

Supplementary materials – Model fitting summary – Emcompress® Anhydrous (modelling the entire relevant dissolution data)

Model: **Baker–Lonsdale**

Model equation: $\frac{3}{2}\cdot\left[1-\left(1-\frac{F}{100}\right)^{\frac{2}{3}}\right]-\frac{F}{100}=k_{BL}\cdot t$

Fitted model parameters per tested tablet (N = 4) with statistics – mean, standard deviation (SD), and relative standard deviation expressed in % (RSD%) (output from DDSolver):

Parameter	No.1	No.2	No.3	No.4	Mean	SD	RSD(%)
k_{BL}	0.0001	0.0002	0.0001	0.0001	0.0001	0.0001	46.6934

Number of dissolution data points (N), degrees of freedom (df), and selected goodness of fit criteria – Pearson correlation coefficient (R), coefficient of determination (R^2), adjusted coefficient of determination ($R^2_{adjusted}$), and residual sum of squares (RSS) (manual calculation in MS Excel):

Parameter	No.1	No.2	No.3	No.4
N	33	33	33	33
df	32	32	32	32
R	0.993742444	0.984049099	0.993284732	0.991187105
R^2	0.987524046	0.96835263	0.986614558	0.982451876
$R^2_{adjusted}$	0.987524046	0.96835263	0.986614558	0.982451876
RSS	560.0110788	3991.604732	625.749438	973.0569323

Graphical abstract of model fit presented as mean ± 1 SD of the fraction % of released carvedilol:

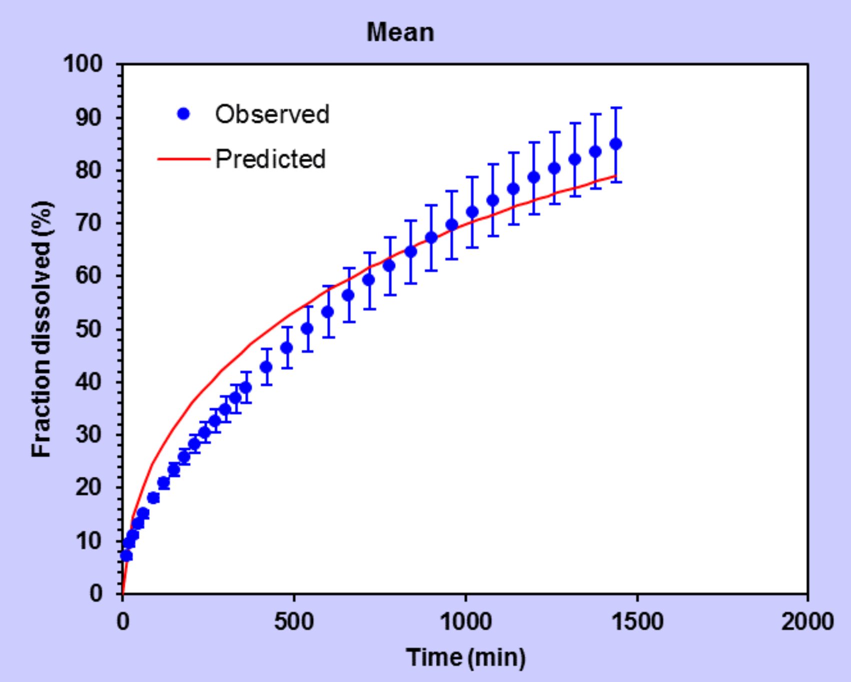

Supplementary materials – Model fitting summary – Emcompress® Anhydrous (modelling the entire relevant dissolution data)

Graphical abstract of model fit presented as the fraction % of released carvedilol per tested tablet:

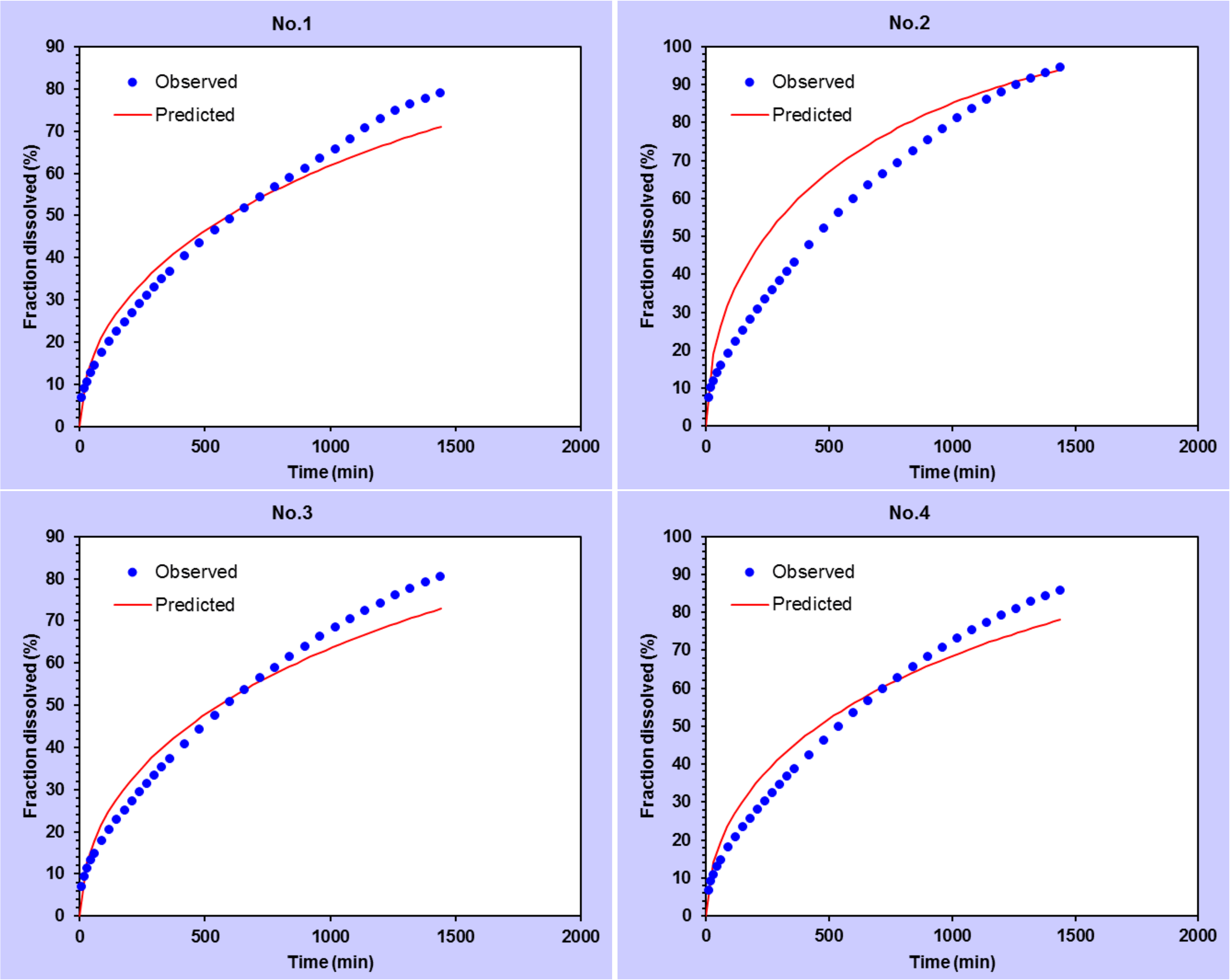

Supplementary materials – Model fitting summary – Emcompress® Anhydrous (modelling the entire relevant dissolution data)

Model: **Baker–Lonsdale with T_{lag}**

Model equation: $\frac{3}{2} \cdot \left[1 - \left(1 - \frac{F}{100}\right)^{\frac{2}{3}}\right] - \frac{F}{100} = k_{BL} \cdot (t - T_{lag})$

Fitted model parameters per tested tablet (N = 4) with statistics – mean, standard deviation (SD), and relative standard deviation expressed in % (RSD%) (output from DDSolver):

Parameter	No.1	No.2	No.3	No.4	Mean	SD	RSD(%)
k_{BL}	0.0001	0.0002	0.0001	0.0002	0.0002	0.0000	29.3981
T_{lag}	93.9298	124.4283	93.6714	108.8881	105.2294	14.6430	13.9153

Number of dissolution data points (N), degrees of freedom (df), and selected goodness of fit criteria – Pearson correlation coefficient (R), coefficient of determination (R^2), adjusted coefficient of determination ($R^2_{adjusted}$), and residual sum of squares (RSS) (manual calculation in MS Excel):

Parameter	No.1	No.2	No.3	No.4
N	33	33	33	33
df	31	31	31	31
R	0.983228353	0.978291203	0.982416277	0.981781593
R^2	0.966737993	0.957053678	0.965141741	0.963895096
$R^2_{adjusted}$	0.965665026	0.955668313	0.964017281	0.962730422
RSS	1115.471557	2103.086992	1180.018422	1345.695098

Graphical abstract of model fit presented as mean ± 1 SD of the fraction % of released carvedilol:

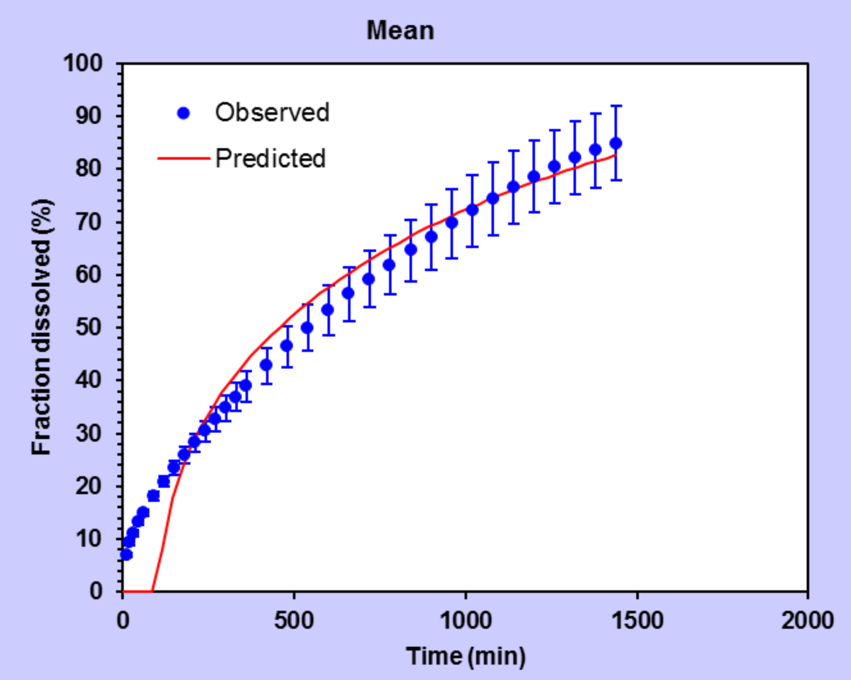

Supplementary materials – Model fitting summary – Emcompress® Anhydrous (modelling the entire relevant dissolution data)

Graphical abstract of model fit presented as the fraction % of released carvedilol per tested tablet:

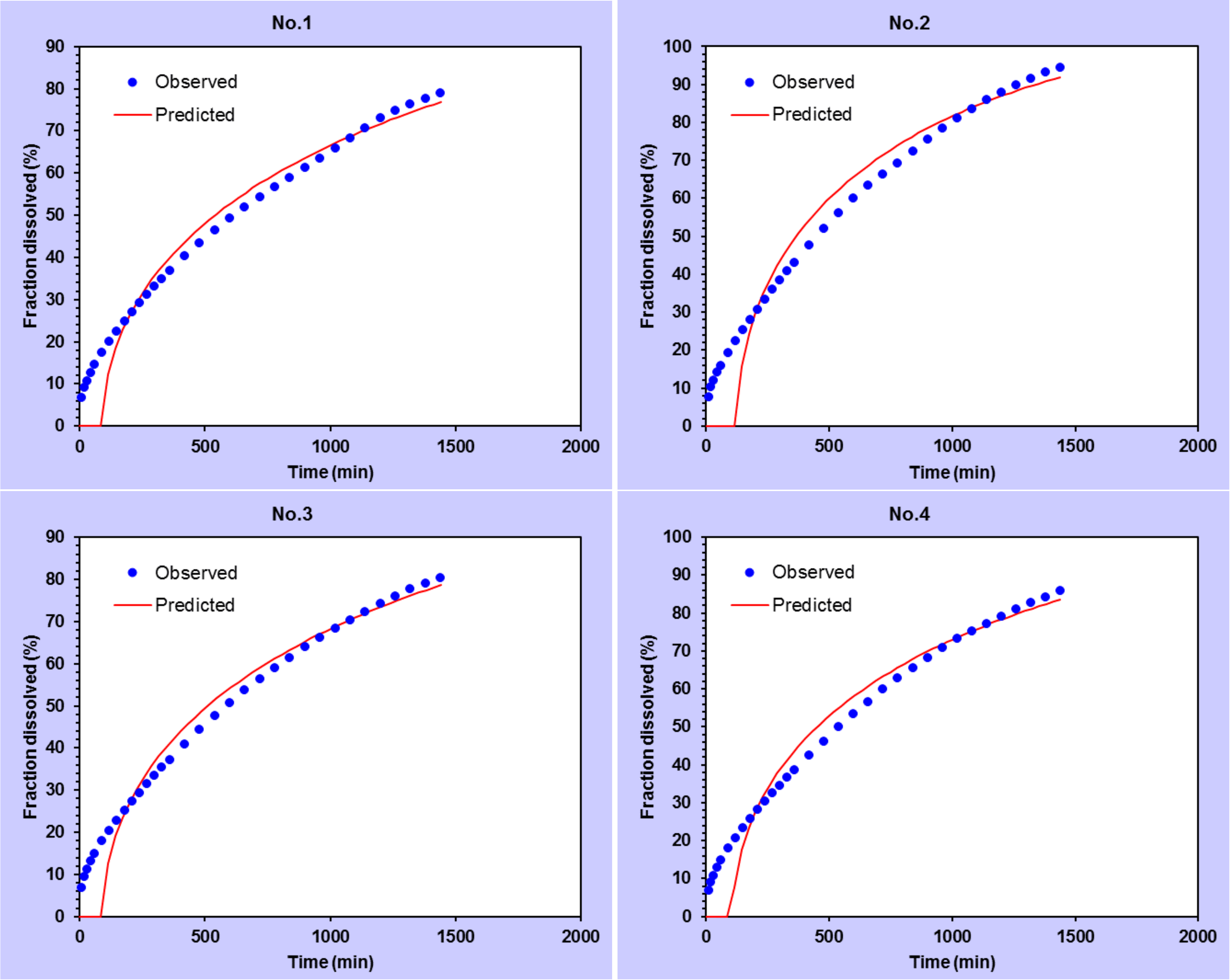

Supplementary materials – Model fitting summary – Emcompress® Anhydrous (modelling the entire relevant dissolution data)

Model: **Makoid–Banakar**

Model equation: $F = k_{MB} \cdot t^n \cdot e^{-k \cdot t}$

Fitted model parameters per tested tablet (N = 4) with statistics – mean, standard deviation (SD), and relative standard deviation expressed in % (RSD%) (output from DDSolver):

Parameter	No.1	No.2	No.3	No.4	Mean	SD	RSD(%)
k_{MB}	2.15150	2.24093	2.25250	2.03229	2.16931	0.10188	4.69665
n	0.47027	0.48939	0.46529	0.48962	0.47864	0.01270	2.65427
k	-0.00016	-0.00017	-0.00017	-0.00017	-0.00017	0.00001	-4.82621

Number of dissolution data points (N), degrees of freedom (df), and selected goodness of fit criteria – Pearson correlation coefficient (R), coefficient of determination (R^2), adjusted coefficient of determination ($R^2_{adjusted}$), and residual sum of squares (RSS) (manual calculation in MS Excel):

Parameter	No.1	No.2	No.3	No.4
N	33	33	33	33
df	30	30	30	30
R	0.999046715	0.996930562	0.998065316	0.99769909
R^2	0.998094339	0.993870545	0.996134376	0.995403475
$R^2_{adjusted}$	0.997967295	0.993461914	0.995876668	0.99509704
RSS	32.67705334	162.3362588	69.86728908	97.98710062

Graphical abstract of model fit presented as mean ± 1 SD of the fraction % of released carvedilol:

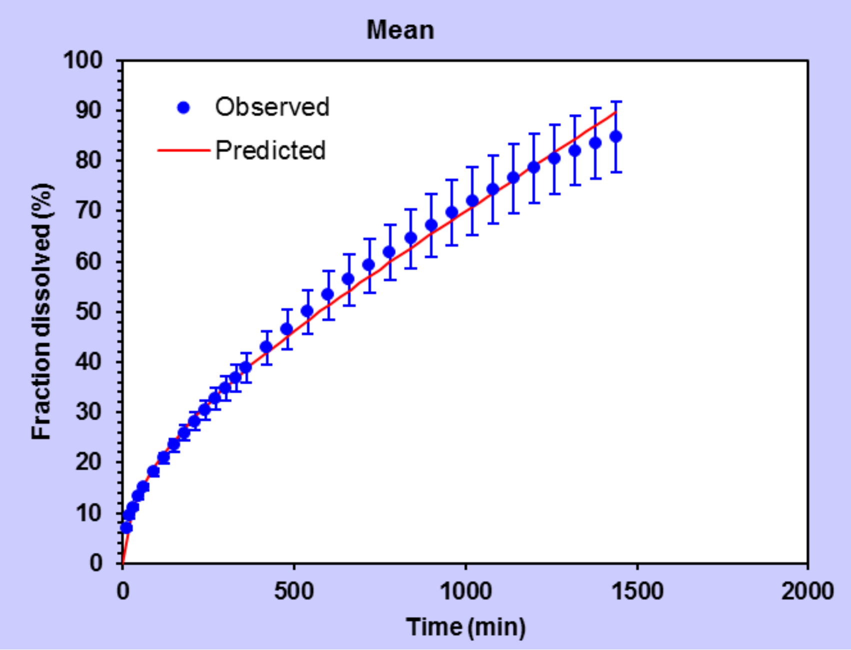

Supplementary materials – Model fitting summary – Emcompress® Anhydrous (modelling the entire relevant dissolution data)

Graphical abstract of model fit presented as the fraction % of released carvedilol per tested tablet:

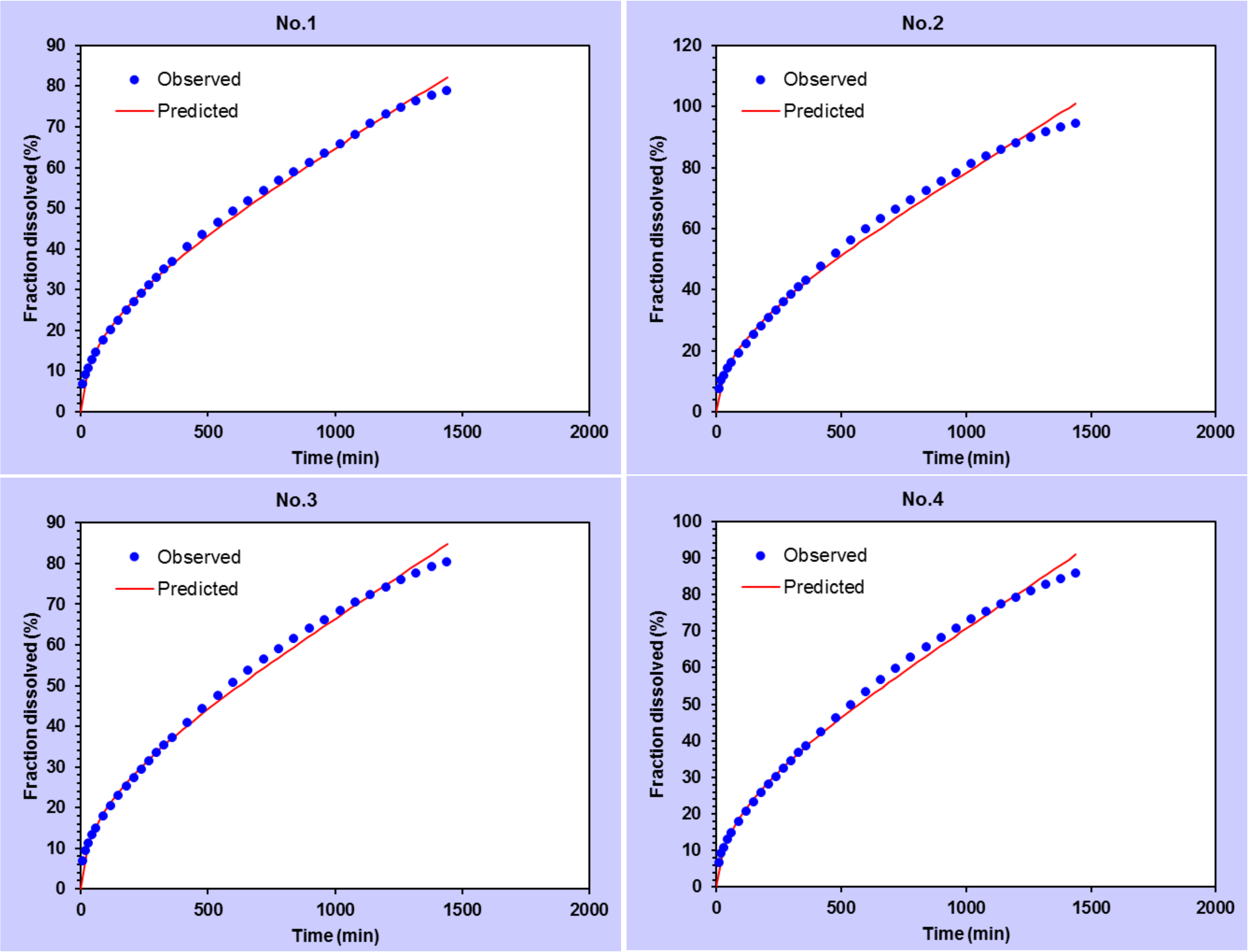

Supplementary materials – Model fitting summary – Emcompress® Anhydrous (modelling the entire relevant dissolution data)

Model: **Makoid–Banakar with T_{lag}**

Model equation: $F = k_{MB} \cdot (t - T_{lag})^n \cdot e^{-k \cdot (t - T_{lag})}$

Fitted model parameters per tested tablet (N = 4) with statistics – mean, standard deviation (SD), and relative standard deviation expressed in % (RSD%) (output from DDSolver):

Parameter	No.1	No.2	No.3	No.4	Mean	SD	RSD(%)
k_{MB}	2.79953	2.95800	2.92049	2.67375	2.83794	0.12866	4.53373
n	0.41929	0.43550	0.41505	0.43650	0.42659	0.01101	2.58180
k	-0.00024	-0.00027	-0.00026	-0.00026	-0.00026	0.00001	-3.95009
T_{lag}	4.00000	4.00000	4.00000	4.00000	4.00000	0.00000	0.00000

Number of dissolution data points (N), degrees of freedom (df), and selected goodness of fit criteria – Pearson correlation coefficient (R), coefficient of determination (R^2), adjusted coefficient of determination ($R^2_{adjusted}$), and residual sum of squares (RSS) (manual calculation in MS Excel):

Parameter	No.1	No.2	No.3	No.4
N	33	33	33	33
df	29	29	29	29
R	0.99789239	0.994963143	0.996584864	0.996057123
R^2	0.995789222	0.989951656	0.993181392	0.992129792
$R^2_{adjusted}$	0.995353624	0.988912172	0.992476018	0.991315632
RSS	73.26519314	270.0708596	124.8289026	170.1938533

Graphical abstract of model fit presented as mean ± 1 SD of the fraction % of released carvedilol:

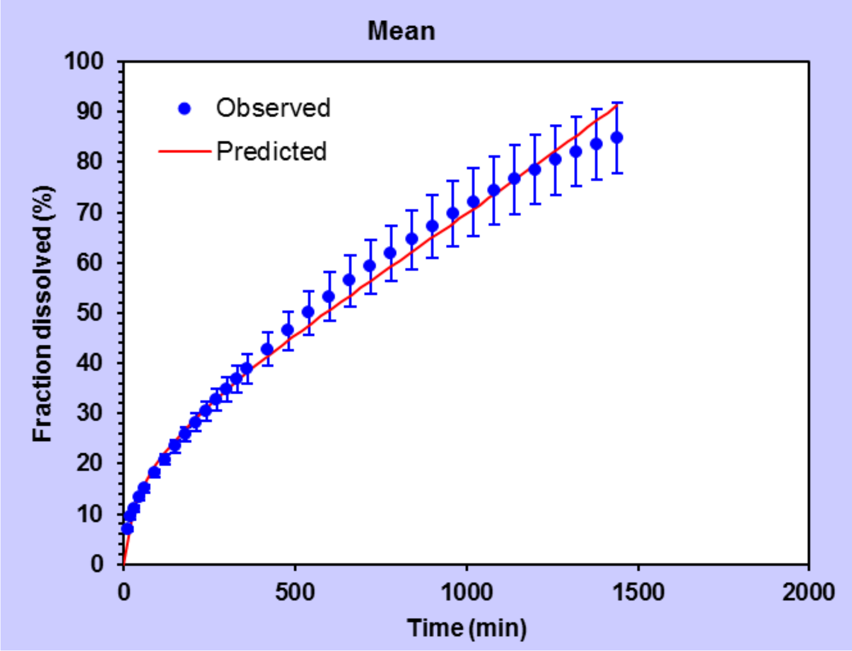

Supplementary materials – Model fitting summary – Emcompress® Anhydrous (modelling the entire relevant dissolution data)

Graphical abstract of model fit presented as the fraction % of released carvedilol per tested tablet:

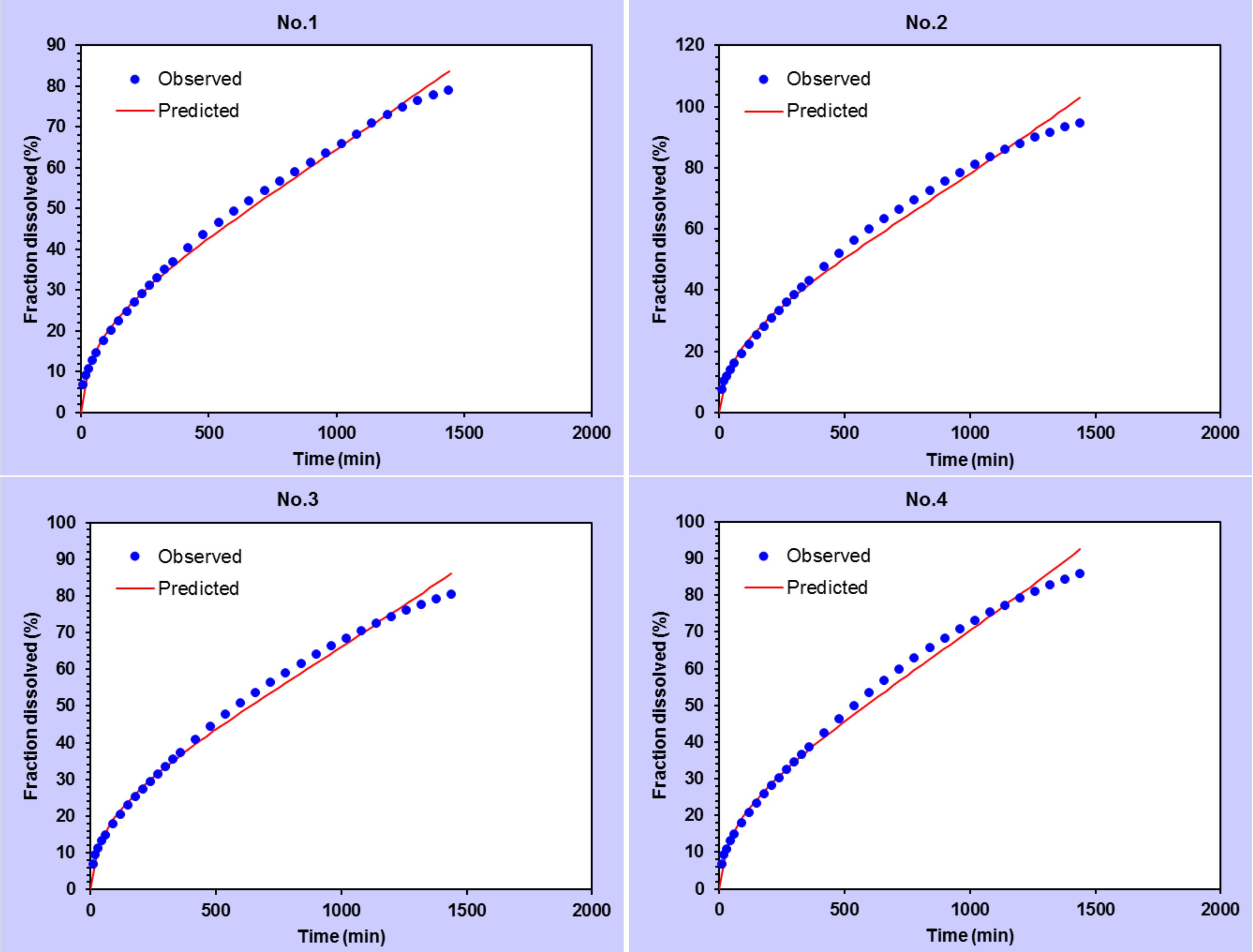

Supplementary materials – Model fitting summary – Emcompress® Anhydrous (modelling the entire relevant dissolution data)

Model: **Peppas–Sahlin_1**

Model equation: $F = k_1 \cdot t^m + k_2 \cdot t^{2m}$

Fitted model parameters per tested tablet (N = 4) with statistics – mean, standard deviation (SD), and relative standard deviation expressed in % (RSD%) (output from DDSolver):

Parameter	No.1	No.2	No.3	No.4	Mean	SD	RSD(%)
k_1	2.074	2.321	2.125	2.091	2.153	0.114	5.296
k_2	0.037	0.053	0.038	0.048	0.044	0.008	17.777
m	0.450	0.450	0.450	0.450	0.450	0.000	0.000

Number of dissolution data points (N), degrees of freedom (df), and selected goodness of fit criteria – Pearson correlation coefficient (R), coefficient of determination (R^2), adjusted coefficient of determination ($R^2_{adjusted}$), and residual sum of squares (RSS) (manual calculation in MS Excel):

Parameter	No.1	No.2	No.3	No.4
N	33	33	33	33
df	30	30	30	30
R	0.999734137	0.998669849	0.999298958	0.999006693
R^2	0.999468346	0.997341466	0.998598406	0.998014373
$R^2_{adjusted}$	0.999432902	0.997164231	0.998504967	0.997881998
RSS	8.968142294	70.34241593	25.02243021	42.16096997

Graphical abstract of model fit presented as mean ± 1 SD of the fraction % of released carvedilol:

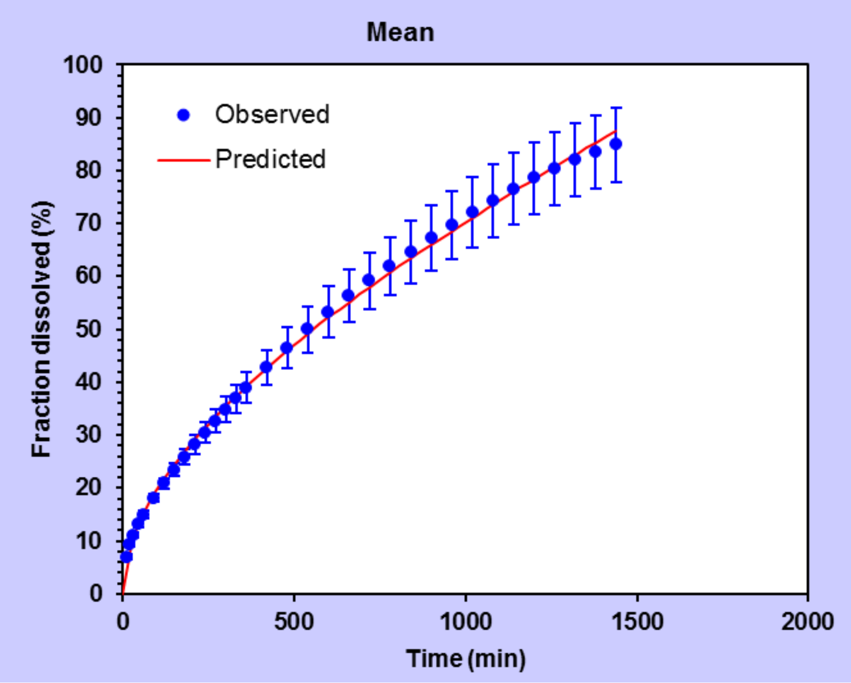

Supplementary materials – Model fitting summary – Emcompress® Anhydrous (modelling the entire relevant dissolution data)

Graphical abstract of model fit presented as the fraction % of released carvedilol per tested tablet:

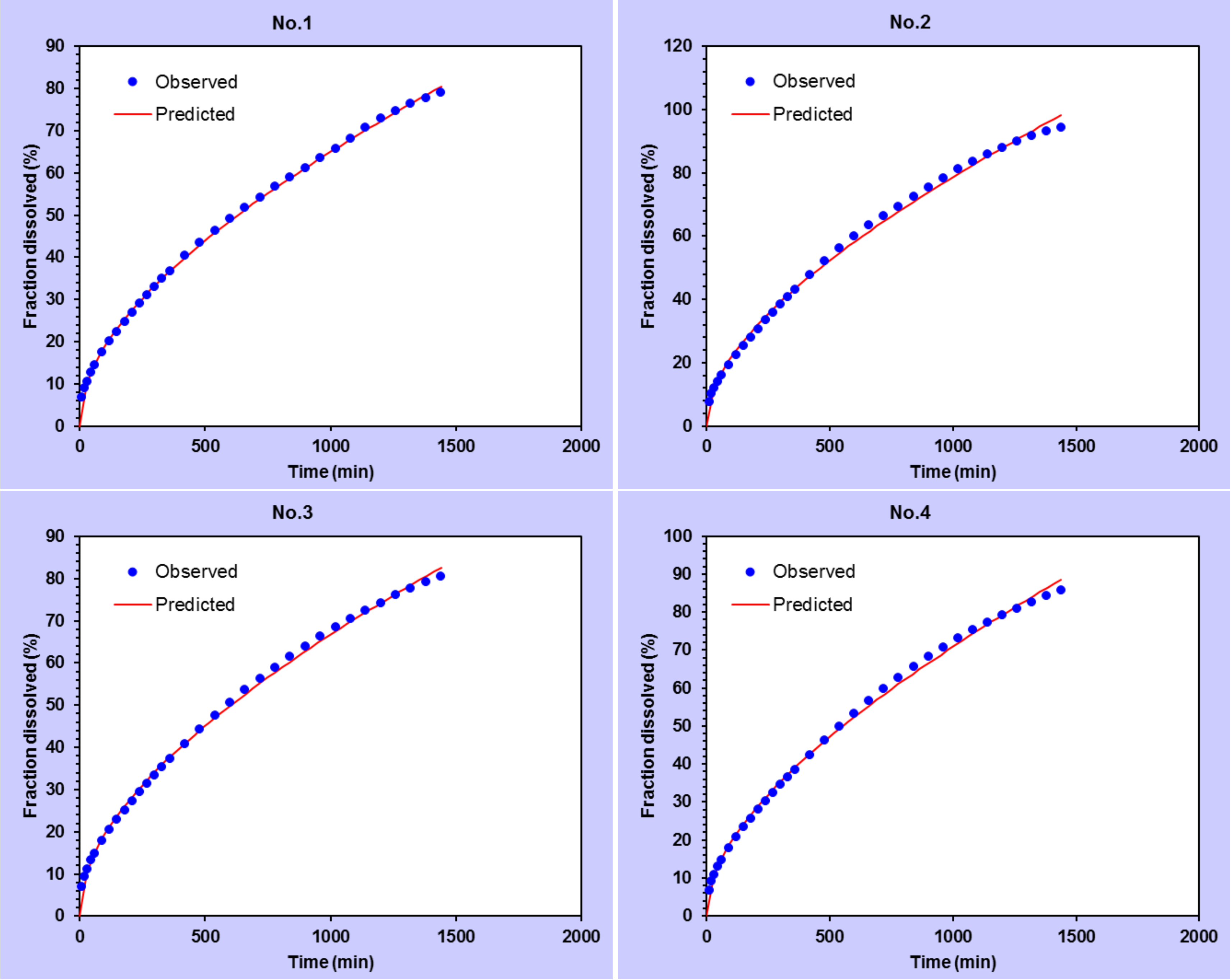

Supplementary materials – Model fitting summary – Emcompress® Anhydrous (modelling the entire relevant dissolution data)

Model: **Peppas–Sahlin_1 with T_{lag}**

Model equation: $F = k_1 \cdot (t - T_{lag})^m + k_2 \cdot (t - T_{lag})^{2m}$

Fitted model parameters per tested tablet (N = 4) with statistics – mean, standard deviation (SD), and relative standard deviation expressed in % (RSD%) (output from DDSolver):

Parameter	No.1	No.2	No.3	No.4	Mean	SD	RSD(%)
k_1	2.129	2.386	2.181	2.150	2.211	0.119	5.361
k_2	0.035	0.051	0.036	0.046	0.042	0.008	18.329
m	0.450	0.450	0.450	0.450	0.450	0.000	0.000
T_{lag}	4.000	4.000	4.000	4.000	4.000	0.000	0.000

Number of dissolution data points (N), degrees of freedom (df), and selected goodness of fit criteria – Pearson correlation coefficient (R), coefficient of determination (R^2), adjusted coefficient of determination ($R^2_{adjusted}$), and residual sum of squares (RSS) (manual calculation in MS Excel):

Parameter	No.1	No.2	No.3	No.4
N	33	33	33	33
df	29	29	29	29
R	0.999631421	0.998637554	0.999192207	0.998959223
R^2	0.999262978	0.997276965	0.998385066	0.997919529
$R^2_{adjusted}$	0.999186734	0.996995272	0.998218004	0.997704307
RSS	12.8934245	70.74008061	29.08524523	43.65072843

Graphical abstract of model fit presented as mean ± 1 SD of the fraction % of released carvedilol:

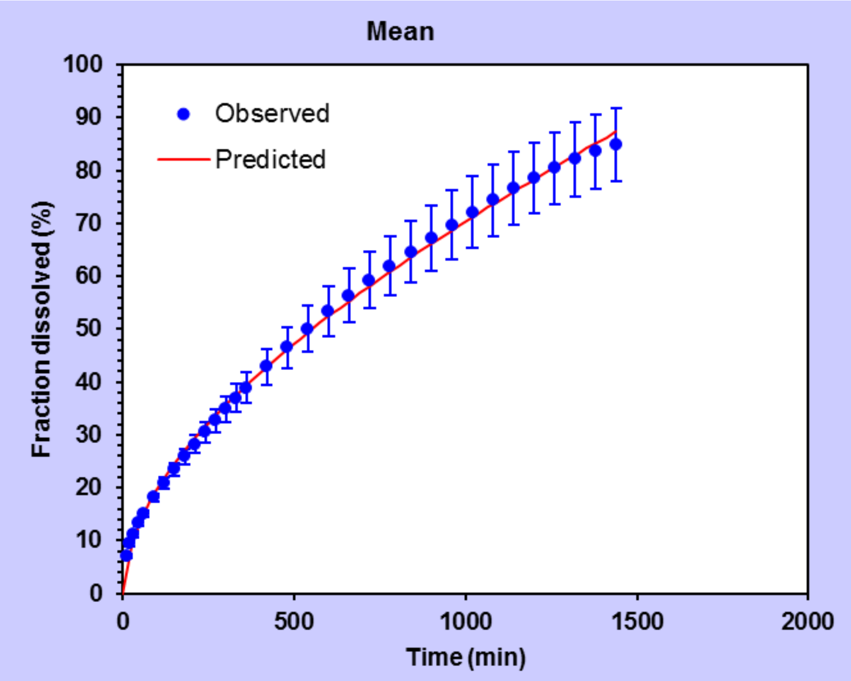

Supplementary materials – Model fitting summary – Emcompress® Anhydrous (modelling the entire relevant dissolution data)

Graphical abstract of model fit presented as the fraction % of released carvedilol per tested tablet:

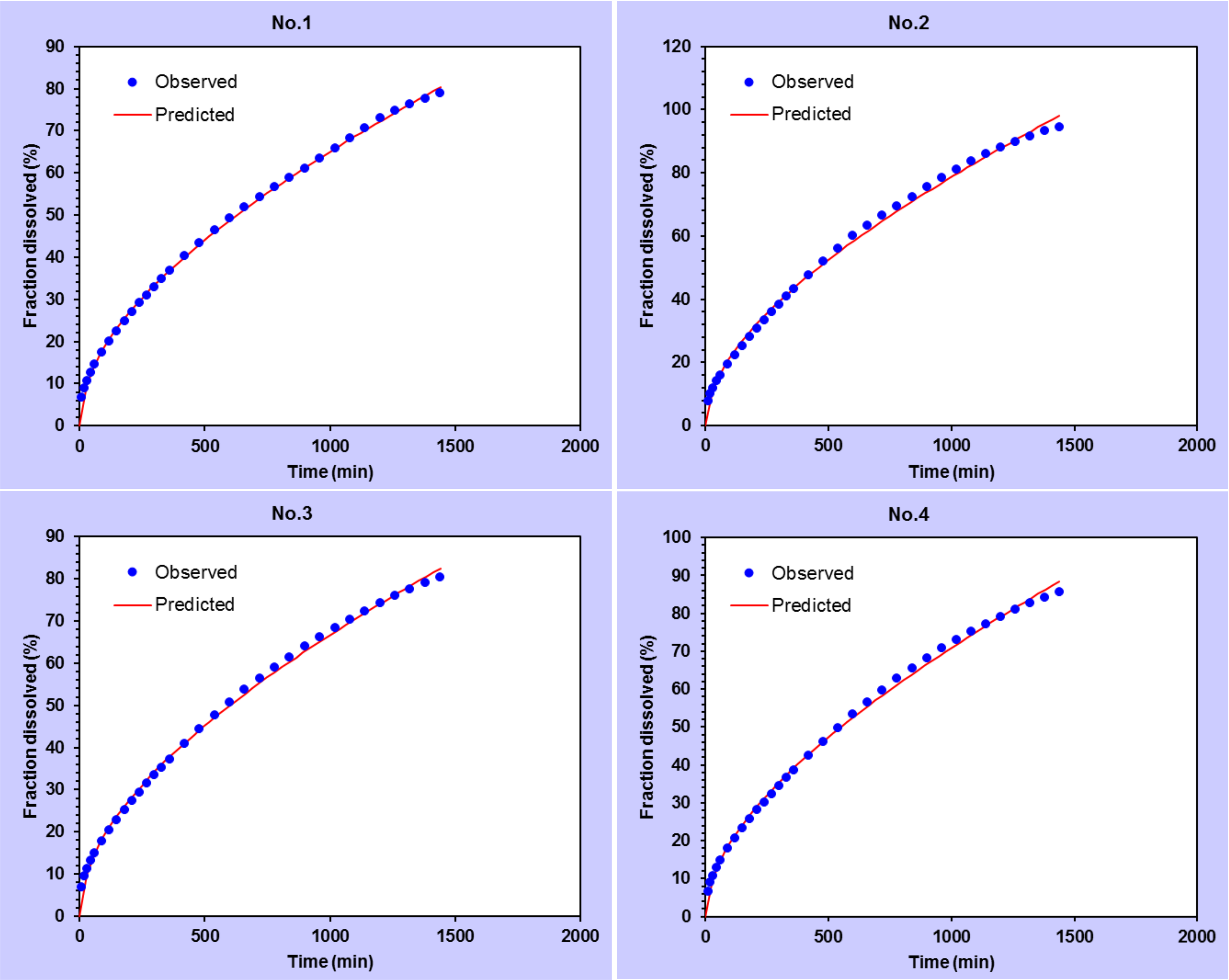

Supplementary materials – Model fitting summary – Emcompress® Anhydrous (modelling the entire relevant dissolution data)

Model: **Peppas–Sahlin_2**

Model equation: $F = k_1 \cdot t^{0.5} + k_2 \cdot t$

Fitted model parameters per tested tablet (N = 4) with statistics – mean, standard deviation (SD), and relative standard deviation expressed in % (RSD%) (output from DDSolver):

Parameter	No.1	No.2	No.3	No.4	Mean	SD	RSD(%)
k_1	1.770	2.018	1.816	1.816	1.855	0.111	5.978
k_2	0.009	0.015	0.009	0.014	0.012	0.003	25.029

Number of dissolution data points (N), degrees of freedom (df), and selected goodness of fit criteria – Pearson correlation coefficient (R), coefficient of determination (R^2), adjusted coefficient of determination ($R^2_{adjusted}$), and residual sum of squares (RSS) (manual calculation in MS Excel):

Parameter	No.1	No.2	No.3	No.4
N	33	33	33	33
df	31	31	31	31
R	0.999742542	0.998784369	0.999340661	0.999076523
R^2	0.999485149	0.997570216	0.998681757	0.998153899
$R^2_{adjusted}$	0.999468541	0.997491836	0.998639233	0.998094347
RSS	8.872599979	63.92658878	23.54302632	38.91511342

Graphical abstract of model fit presented as mean ± 1 SD of the fraction % of released carvedilol:

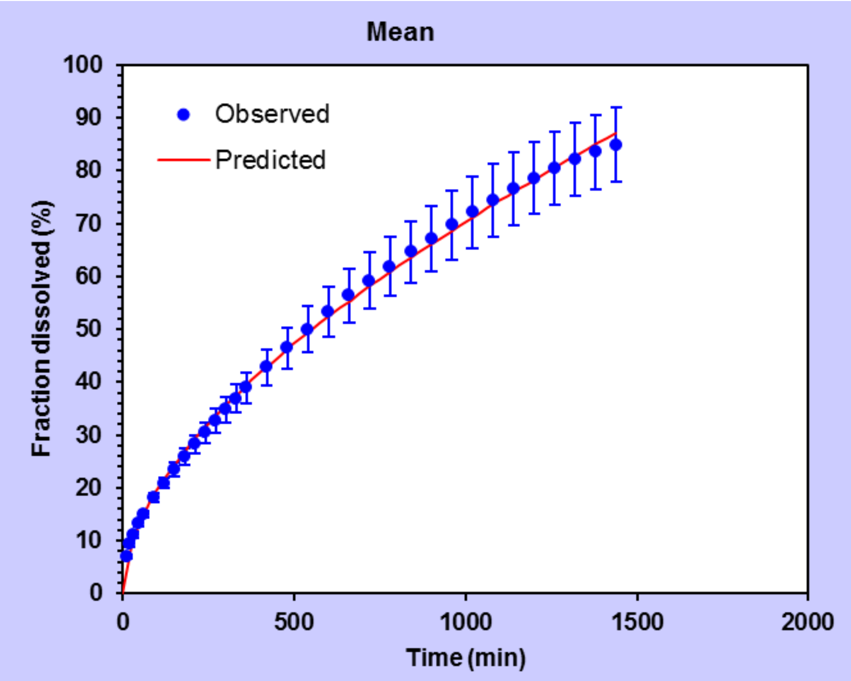

Supplementary materials – Model fitting summary – Emcompress® Anhydrous (modelling the entire relevant dissolution data)

Graphical abstract of model fit presented as the fraction % of released carvedilol per tested tablet:

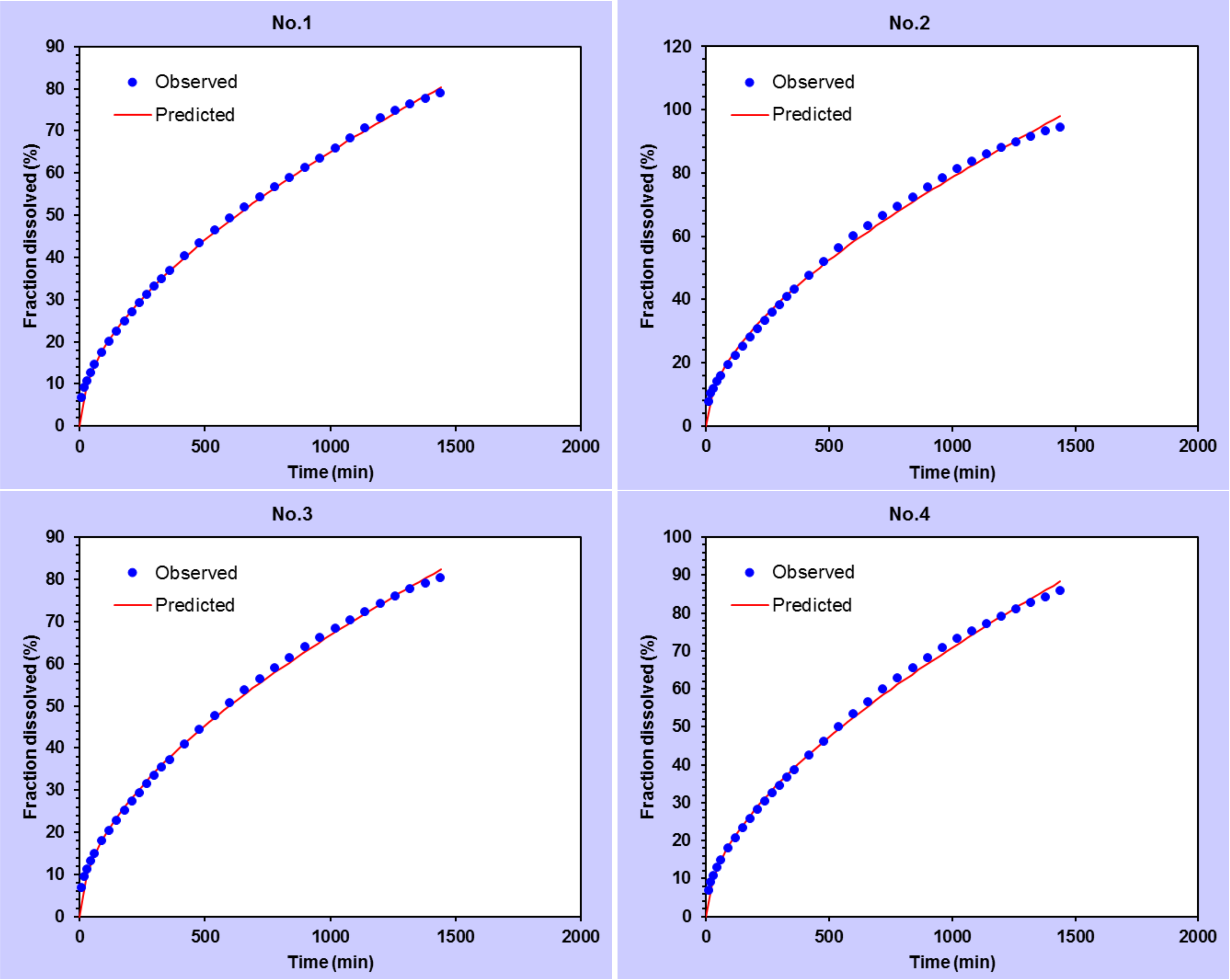

Supplementary materials – Model fitting summary – Emcompress® Anhydrous (modelling the entire relevant dissolution data)

Model: **Peppas–Sahlin_2 with T_{lag}**

Model equation: $F = k_1 \cdot (t - T_{lag})^{0.5} + k_2 \cdot (t - T_{lag})$

Fitted model parameters per tested tablet (N = 4) with statistics – mean, standard deviation (SD), and relative standard deviation expressed in % (RSD%) (output from DDSolver):

Parameter	No.1	No.2	No.3	No.4	Mean	SD	RSD(%)
k_1	1.806	2.062	1.853	1.856	1.894	0.114	6.030
k_2	0.008	0.014	0.008	0.012	0.011	0.003	26.749
T_{lag}	4.000	4.000	4.000	4.000	4.000	0.000	0.000

Number of dissolution data points (N), degrees of freedom (df), and selected goodness of fit criteria – Pearson correlation coefficient (R), coefficient of determination (R^2), adjusted coefficient of determination ($R^2_{adjusted}$), and residual sum of squares (RSS) (manual calculation in MS Excel):

Parameter	No.1	No.2	No.3	No.4
N	33	33	33	33
df	30	30	30	30
R	0.999575937	0.998701932	0.999170682	0.998978919
R^2	0.999152055	0.997405549	0.998342051	0.99795888
$R^2_{adjusted}$	0.999095525	0.997232586	0.998231521	0.997822806
RSS	16.12648718	67.29209596	30.92478386	43.05543668

Graphical abstract of model fit presented as mean ± 1 SD of the fraction % of released carvedilol:

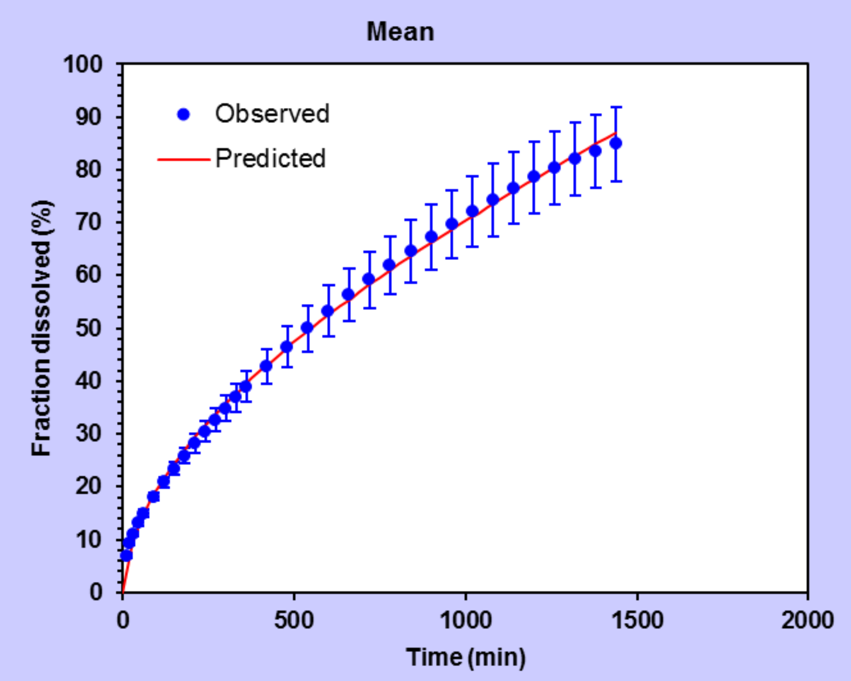

Supplementary materials – Model fitting summary – Emcompress® Anhydrous (modelling the entire relevant dissolution data)

Graphical abstract of model fit presented as the fraction % of released carvedilol per tested tablet:

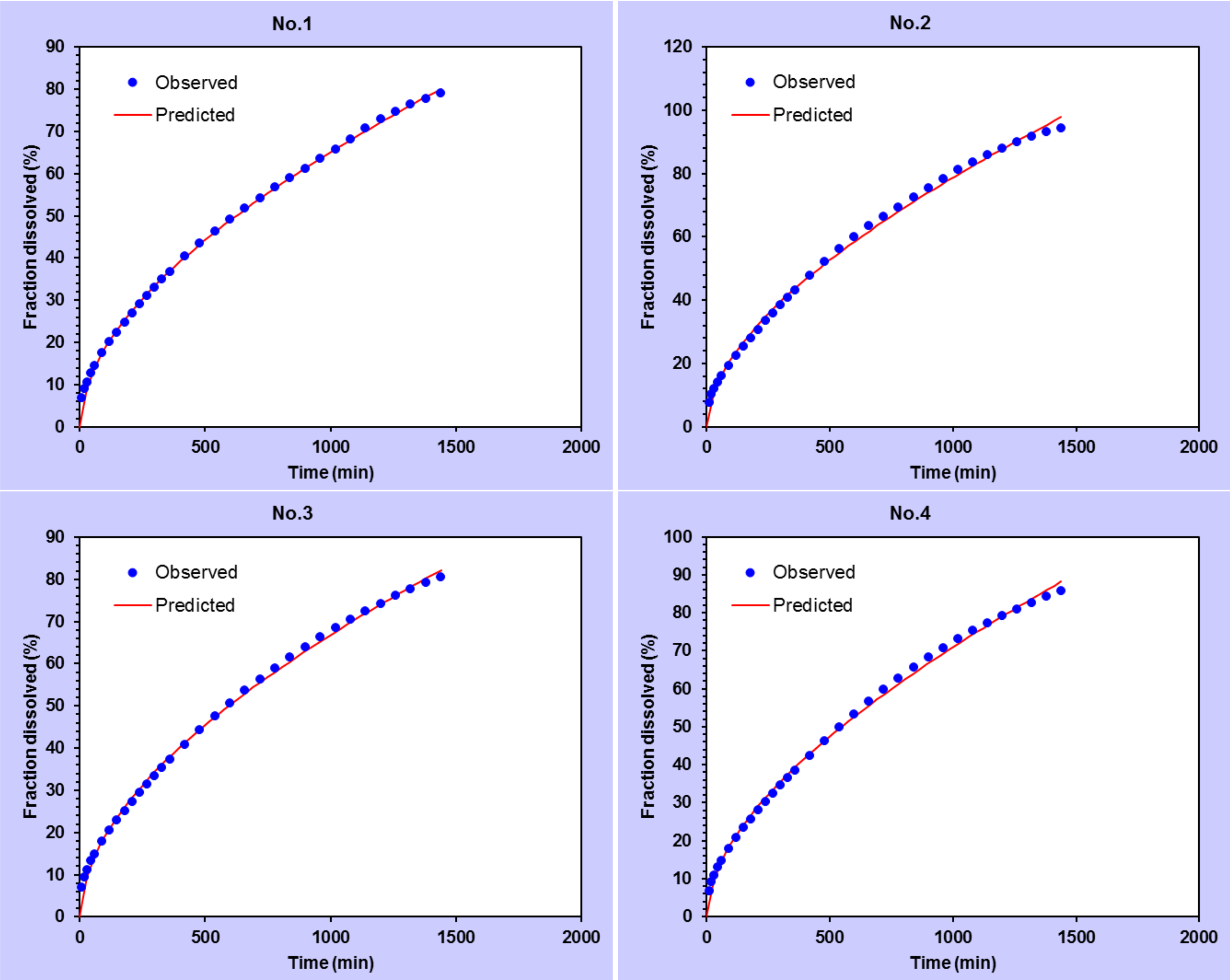

Supplementary materials – Model fitting summary – Emcompress® Anhydrous (modelling the entire relevant dissolution data)

Model: **Quadratic**

Model equation: $F = 100 \cdot (k_1 \cdot t^2 + k_2 \cdot t)$

Fitted model parameters per tested tablet (N = 4) with statistics – mean, standard deviation (SD), and relative standard deviation expressed in % (RSD%) (output from DDSolver):

Parameter	No.1	No.2	No.3	No.4	Mean	SD	RSD(%)
k_1	-0.0000004	-0.0000005	-0.0000004	-0.0000004	-0.0000004	0.0000000	-7.7091021
k_2	0.0010932	0.0013146	0.0011297	0.0011792	0.0011792	0.0000969	8.2188514

Number of dissolution data points (N), degrees of freedom (df), and selected goodness of fit criteria – Pearson correlation coefficient (R), coefficient of determination (R^2), adjusted coefficient of determination ($R^2_{adjusted}$), and residual sum of squares (RSS) (manual calculation in MS Excel):

Parameter	No.1	No.2	No.3	No.4
N	33	33	33	33
df	31	31	31	31
R	0.993178241	0.996197512	0.99448612	0.99575524
R^2	0.986403019	0.992409482	0.989002642	0.991528497
$R^2_{adjusted}$	0.985964406	0.992164627	0.988647889	0.991255223
RSS	752.2955667	710.8970629	707.3098698	627.2306643

Graphical abstract of model fit presented as mean ± 1 SD of the fraction % of released carvedilol:

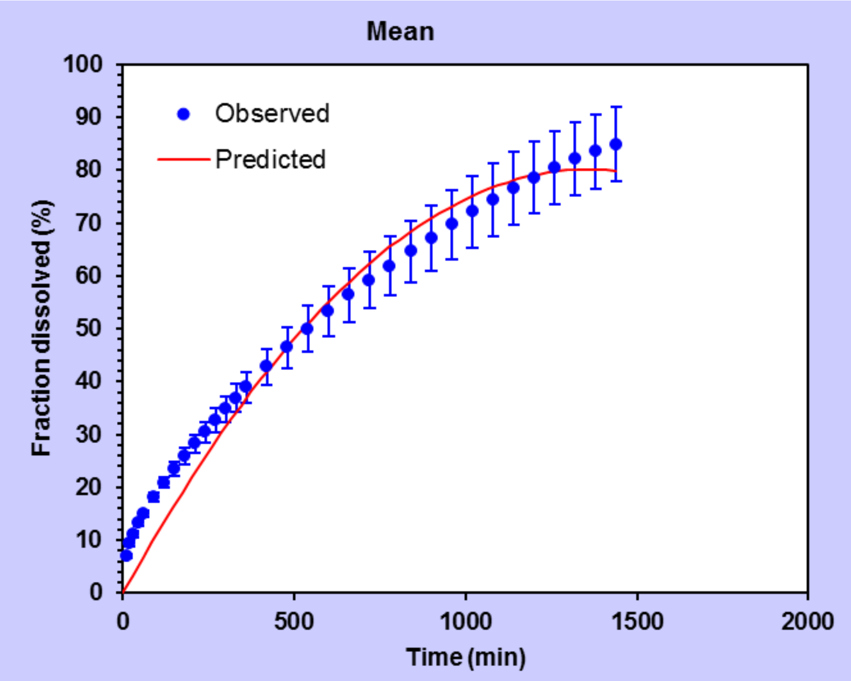

Supplementary materials – Model fitting summary – Emcompress® Anhydrous (modelling the entire relevant dissolution data)

Graphical abstract of model fit presented as the fraction % of released carvedilol per tested tablet:

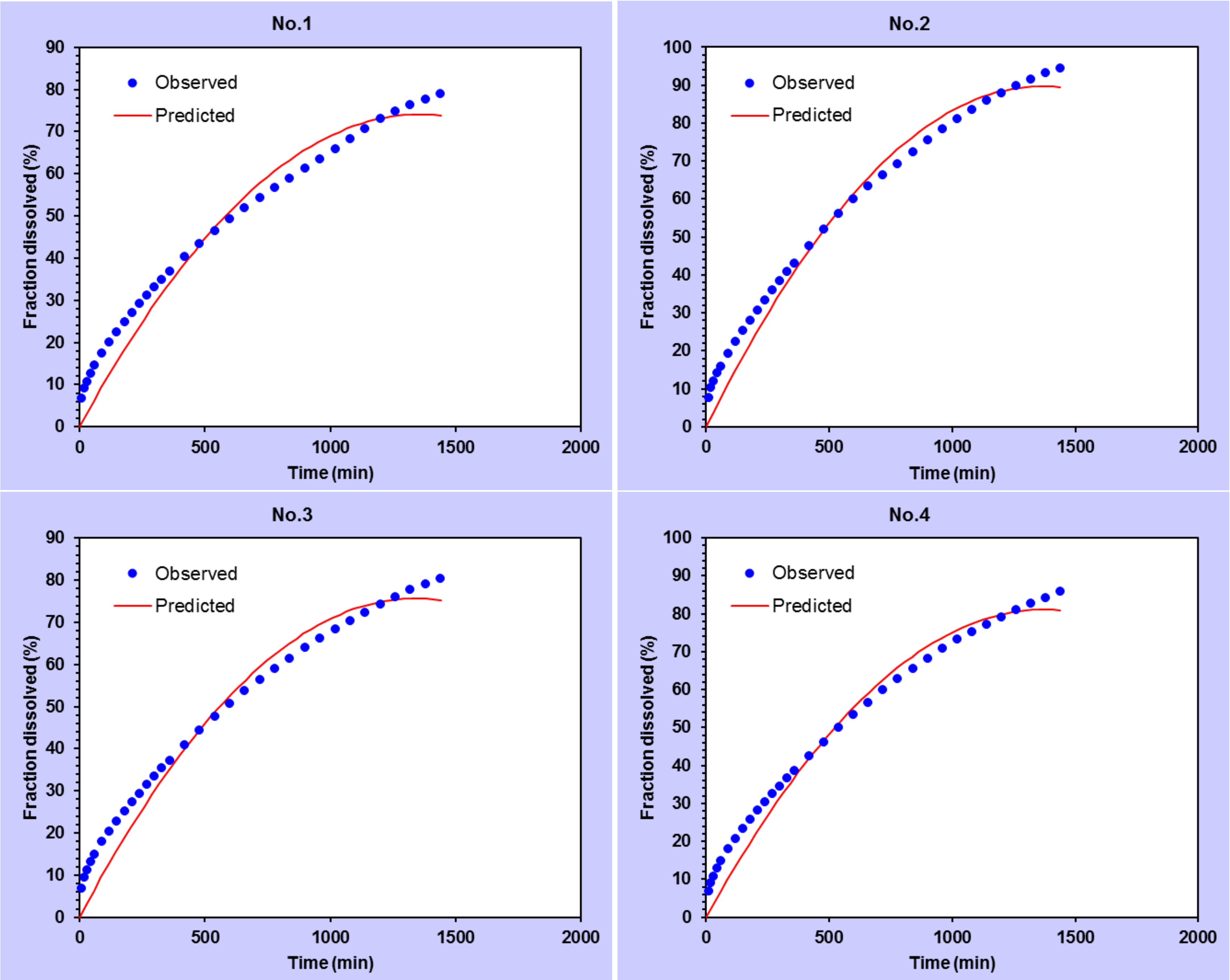

Supplementary materials – Model fitting summary – Emcompress® Anhydrous (modelling the entire relevant dissolution data)

Model: **Quadratic with T_{lag}**

Model equation: $F = 100 \cdot \left[k_1 \cdot (t - T_{lag})^2 + k_2 \cdot (t - T_{lag})\right]$

Fitted model parameters per tested tablet (N = 4) with statistics – mean, standard deviation (SD), and relative standard deviation expressed in % (RSD%) (output from DDSolver):

Parameter	No.1	No.2	No.3	No.4	Mean	SD	RSD(%)
k_1	-0.0000004	-0.0000005	-0.0000004	-0.0000004	-0.0000004	0.0000000	-7.7499241
k_2	0.0011012	0.0013247	0.0011381	0.0011883	0.0011881	0.0000978	8.2358290
T_{lag}	4.0000000	4.0000000	4.0000000	4.0000000	4.0000000	0.0000000	0.0000000

Number of dissolution data points (N), degrees of freedom (df), and selected goodness of fit criteria – Pearson correlation coefficient (R), coefficient of determination (R^2), adjusted coefficient of determination ($R^2_{adjusted}$), and residual sum of squares (RSS) (manual calculation in MS Excel):

Parameter	No.1	No.2	No.3	No.4
N	33	33	33	33
df	30	30	30	30
R	0.992931343	0.99597865	0.994246049	0.995529925
R^2	0.985912652	0.991973471	0.988525205	0.991079831
$R^2_{adjusted}$	0.984973495	0.991438369	0.987760219	0.990485154
RSS	814.9024093	786.0308798	771.1923882	690.2954668

Graphical abstract of model fit presented as mean ± 1 SD of the fraction % of released carvedilol:

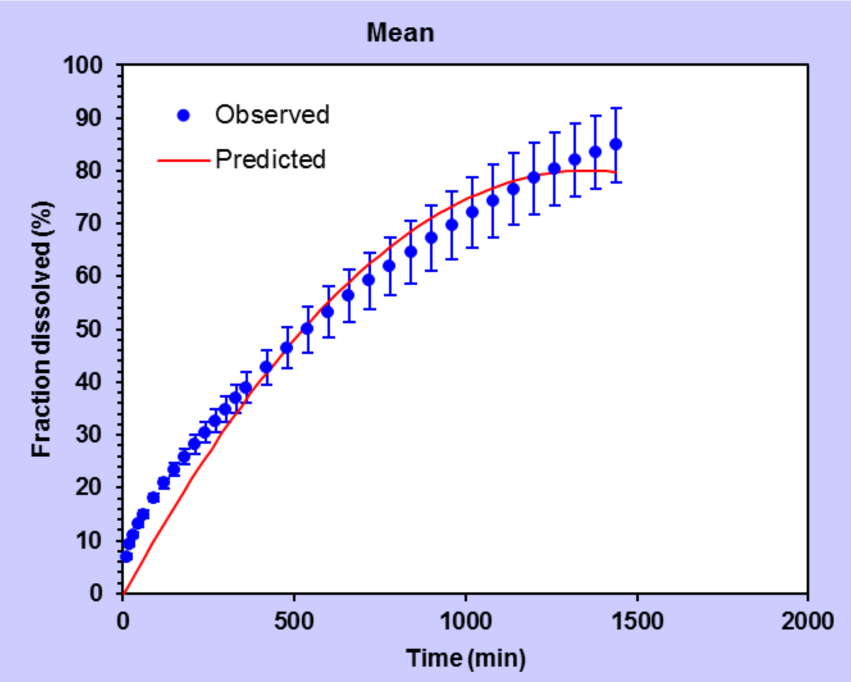

Supplementary materials – Model fitting summary – Emcompress® Anhydrous (modelling the entire relevant dissolution data)

Graphical abstract of model fit presented as the fraction % of released carvedilol per tested tablet:

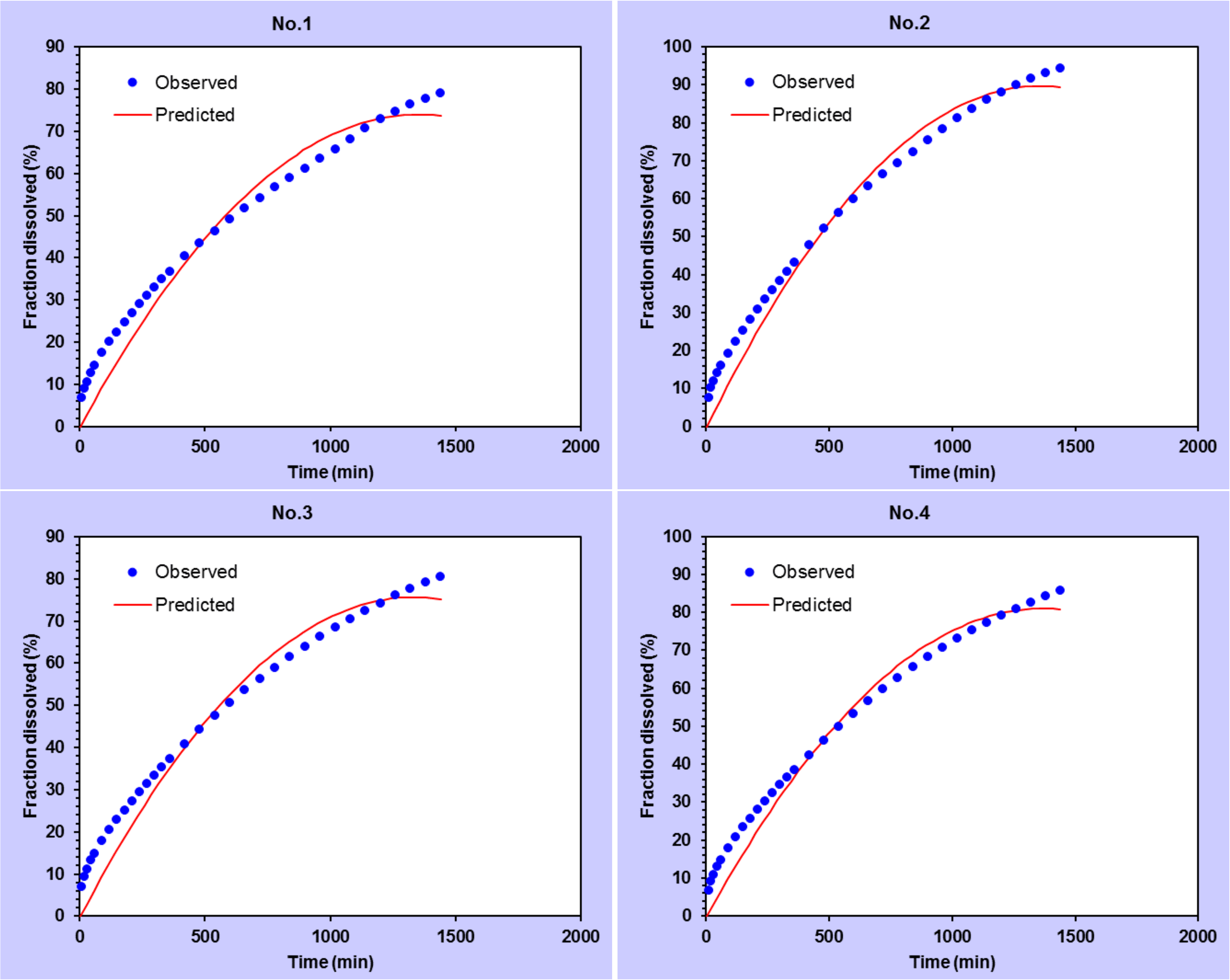

Supplementary materials – Model fitting summary – Emcompress® Anhydrous (modelling the entire relevant dissolution data)

Model: **Weibull_1**

Model equation: $F = 100 \cdot \left[1 - e^{-\frac{(t-T_i)^{\beta}}{\alpha}}\right]$

Fitted model parameters per tested tablet (N = 4) with statistics – mean, standard deviation (SD), and relative standard deviation expressed in % (RSD%) (output from DDSolver):

Parameter	No.1	No.2	No.3	No.4	Mean	SD	RSD(%)
α	69.747	89.194	69.290	82.132	77.591	9.758	12.577
β	0.613	0.705	0.618	0.660	0.649	0.043	6.634
T_i	6.000	6.000	6.000	6.000	6.000	0.000	0.000

Number of dissolution data points (N), degrees of freedom (df), and selected goodness of fit criteria – Pearson correlation coefficient (R), coefficient of determination (R^2), adjusted coefficient of determination ($R^2_{adjusted}$), and residual sum of squares (RSS) (manual calculation in MS Excel):

Parameter	No.1	No.2	No.3	No.4
N	33	33	33	33
df	30	30	30	30
R	0.988460086	0.983075744	0.987822666	0.986744004
R^2	0.977053342	0.966437918	0.97579362	0.97366373
$R^2_{adjusted}$	0.975523564	0.964200445	0.974179862	0.971907979
RSS	519.5199173	1068.008767	579.4791182	729.6989013

Graphical abstract of model fit presented as mean ± 1 SD of the fraction % of released carvedilol:

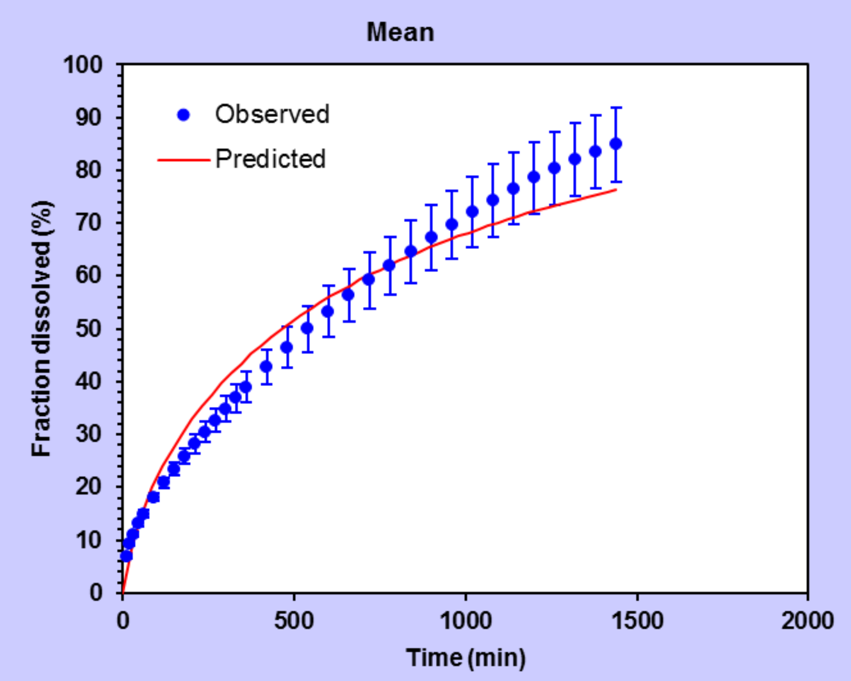

Supplementary materials – Model fitting summary – Emcompress® Anhydrous (modelling the entire relevant dissolution data)

Graphical abstract of model fit presented as the fraction % of released carvedilol per tested tablet:

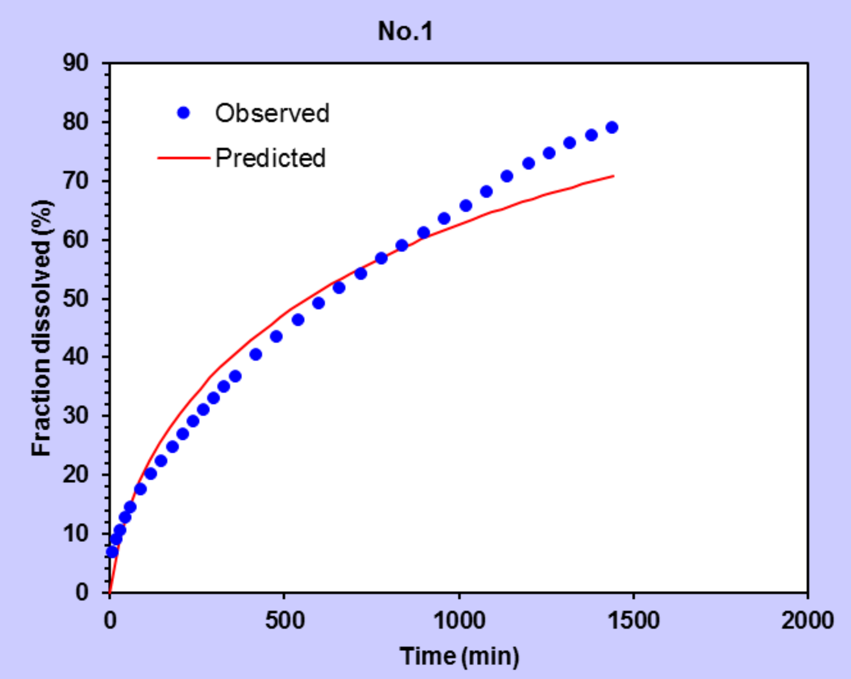

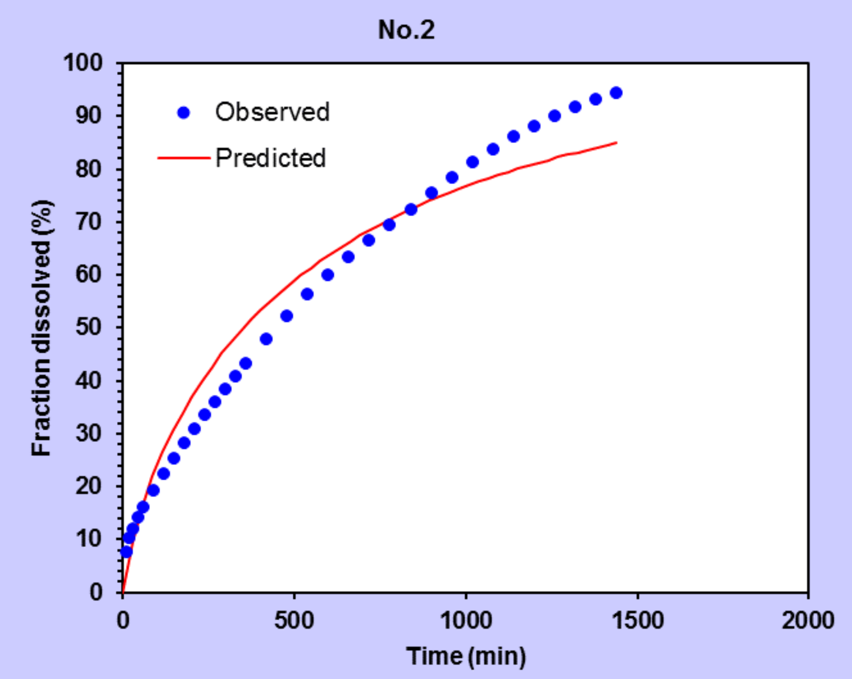

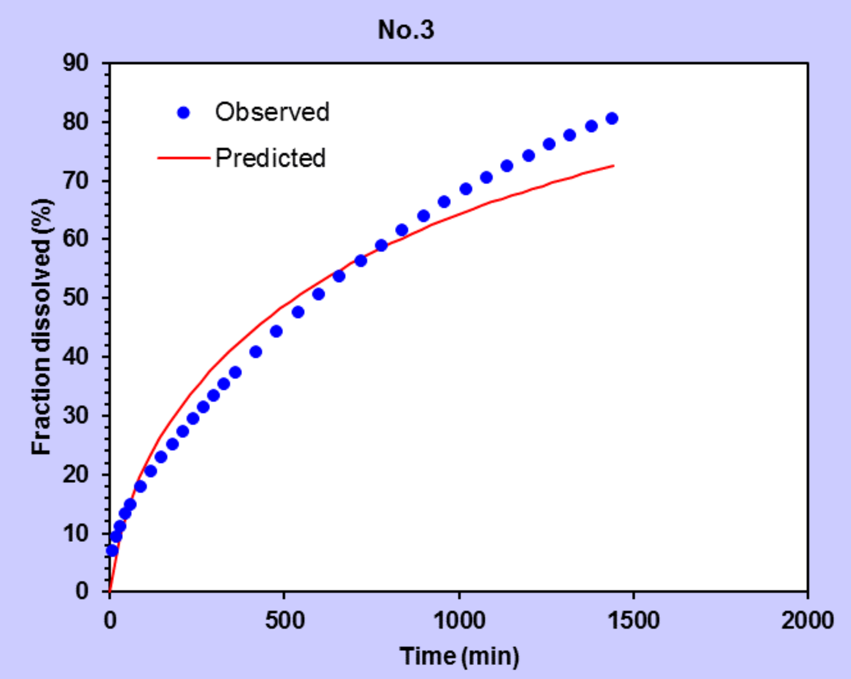

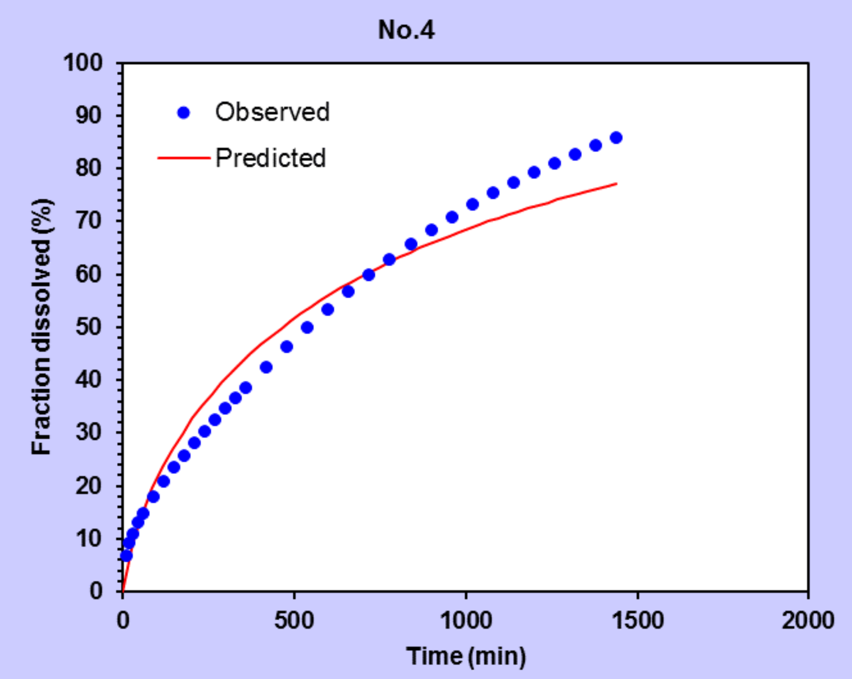

Supplementary materials – Model fitting summary – Emcompress® Anhydrous (modelling the entire relevant dissolution data)

Model: **Weibull_2**

Model equation: $F = 100 \cdot \left(1 - e^{-\frac{t^{\beta}}{\alpha}}\right)$

Fitted model parameters per tested tablet (N = 4) with statistics – mean, standard deviation (SD), and relative standard deviation expressed in % (RSD%) (output from DDSolver):

Parameter	No.1	No.2	No.3	No.4	Mean	SD	RSD(%)
α	89.664	120.088	89.330	107.879	101.740	14.991	14.734
β	0.652	0.752	0.658	0.702	0.691	0.046	6.720

Number of dissolution data points (N), degrees of freedom (df), and selected goodness of fit criteria – Pearson correlation coefficient (R), coefficient of determination (R^2), adjusted coefficient of determination ($R^2_{adjusted}$), and residual sum of squares (RSS) (manual calculation in MS Excel):

Parameter	No.1	No.2	No.3	No.4
N	33	33	33	33
df	31	31	31	31
R	0.991762562	0.986804976	0.991303031	0.990193985
R^2	0.98359298	0.97378406	0.9826817	0.980484129
$R^2_{adjusted}$	0.983063721	0.972938385	0.982123045	0.979854585
RSS	372.147587	841.4880166	418.1808784	542.4005717

Graphical abstract of model fit presented as mean ± 1 SD of the fraction % of released carvedilol:

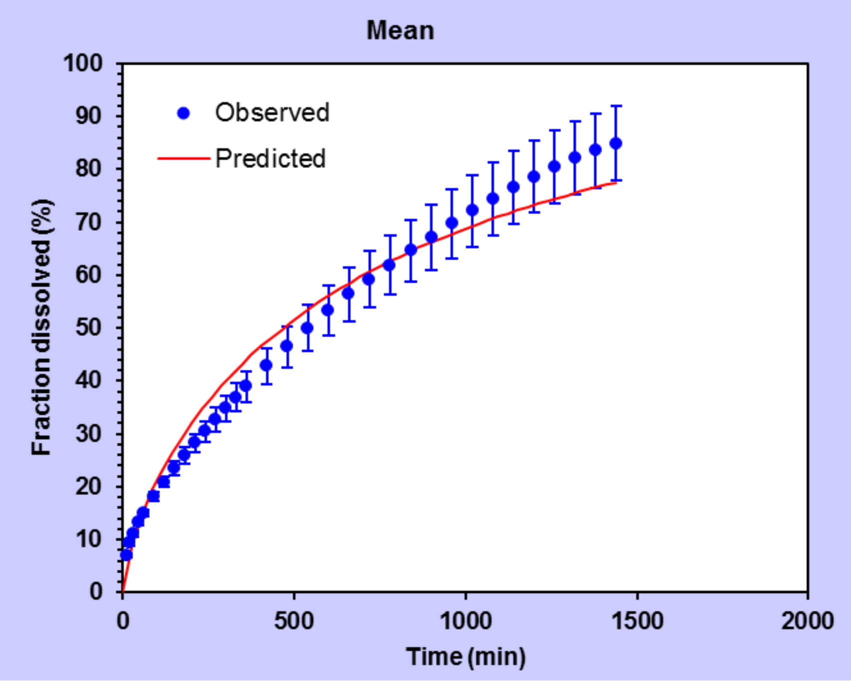

Supplementary materials – Model fitting summary – Emcompress® Anhydrous (modelling the entire relevant dissolution data)

Graphical abstract of model fit presented as the fraction % of released carvedilol per tested tablet:

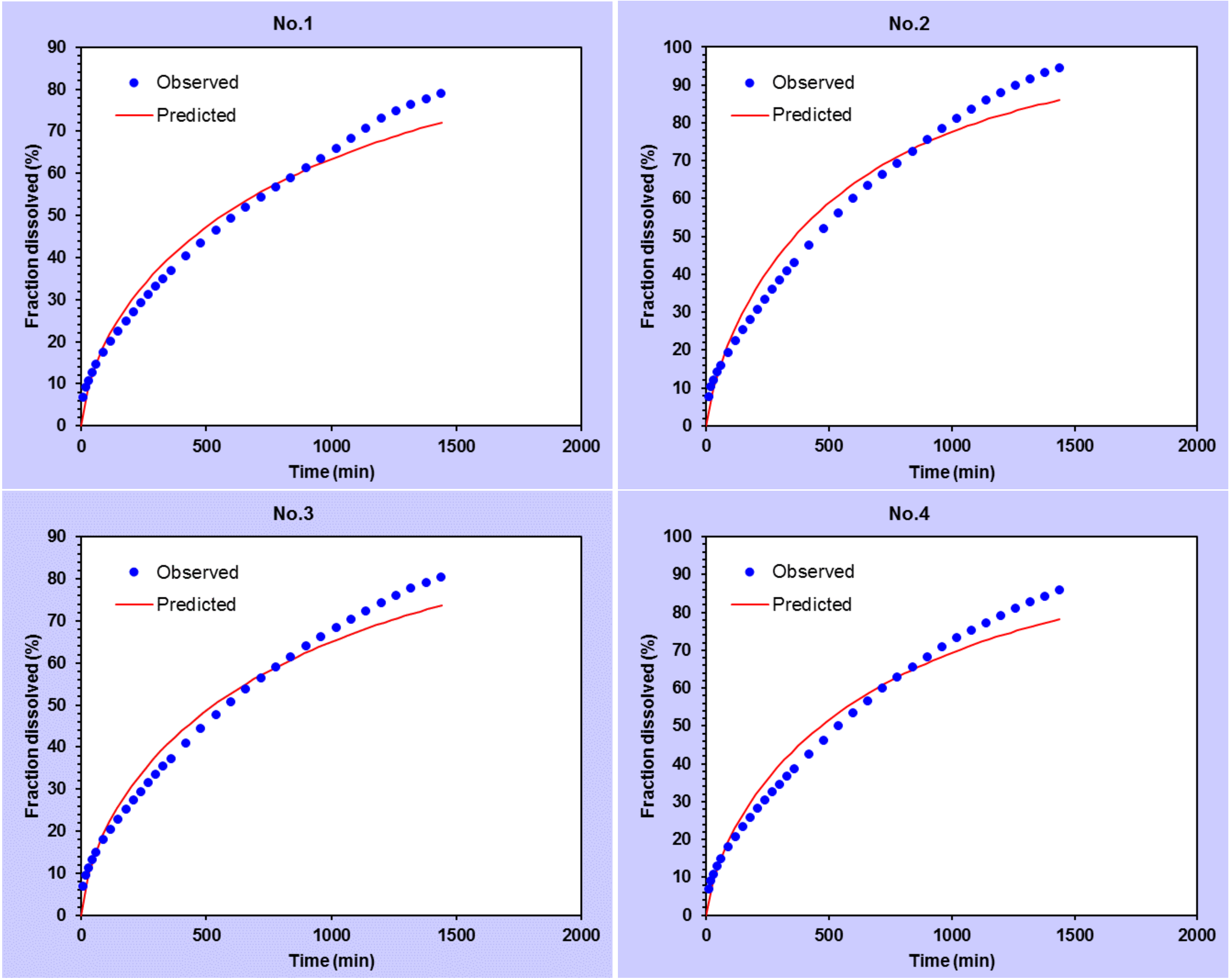

Supplementary materials – Model fitting summary – Emcompress® Anhydrous (modelling the entire relevant dissolution data)

Model: **Weibull_3**

Model equation: $F = F_{max} \cdot \left(1 - e^{-\frac{t^{\beta}}{\alpha}}\right)$

Fitted model parameters per tested tablet (N = 4) with statistics – mean, standard deviation (SD), and relative standard deviation expressed in % (RSD%) (output from DDSolver):

Parameter	No.1	No.2	No.3	No.4	Mean	SD	RSD(%)
α	111.727	136.932	111.760	133.405	123.456	13.601	11.017
β	0.704	0.737	0.706	0.731	0.720	0.017	2.309
F_{max}	98.573	117.905	100.443	107.083	106.001	8.736	8.241

Number of dissolution data points (N), degrees of freedom (df), and selected goodness of fit criteria – Pearson correlation coefficient (R), coefficient of determination (R^2), adjusted coefficient of determination ($R^2_{adjusted}$), and residual sum of squares (RSS) (manual calculation in MS Excel):

Parameter	No.1	No.2	No.3	No.4
N	33	33	33	33
df	30	30	30	30
R	0.991368886	0.99264121	0.991399377	0.992056276
R^2	0.982812268	0.985336572	0.982872724	0.984175656
$R^2_{adjusted}$	0.981666419	0.98435901	0.981730906	0.983120699
RSS	500.9617807	669.1347719	532.6810274	556.6313337

Graphical abstract of model fit presented as mean ± 1 SD of the fraction % of released carvedilol:

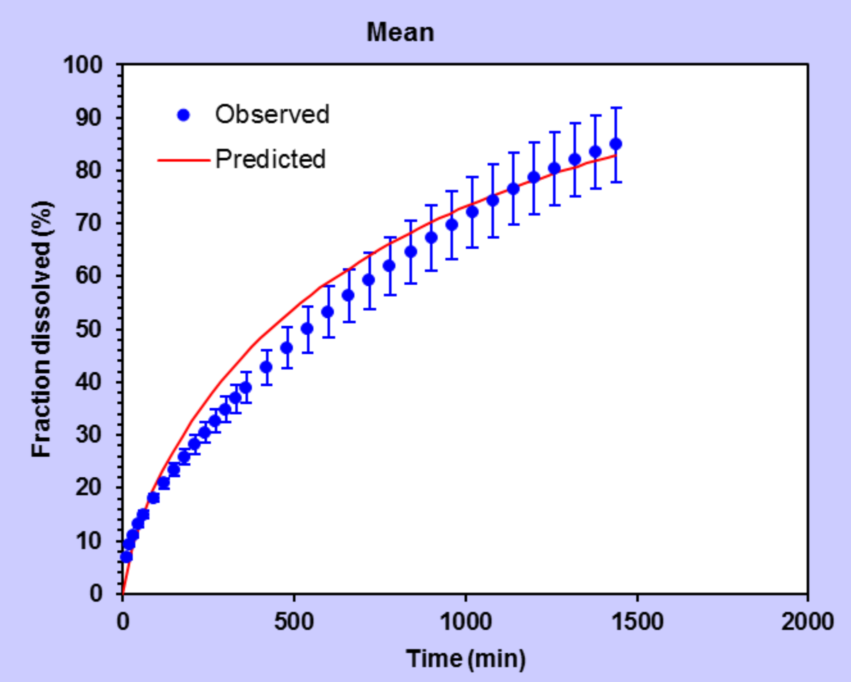

Supplementary materials – Model fitting summary – Emcompress® Anhydrous (modelling the entire relevant dissolution data)

Graphical abstract of model fit presented as the fraction % of released carvedilol per tested tablet:

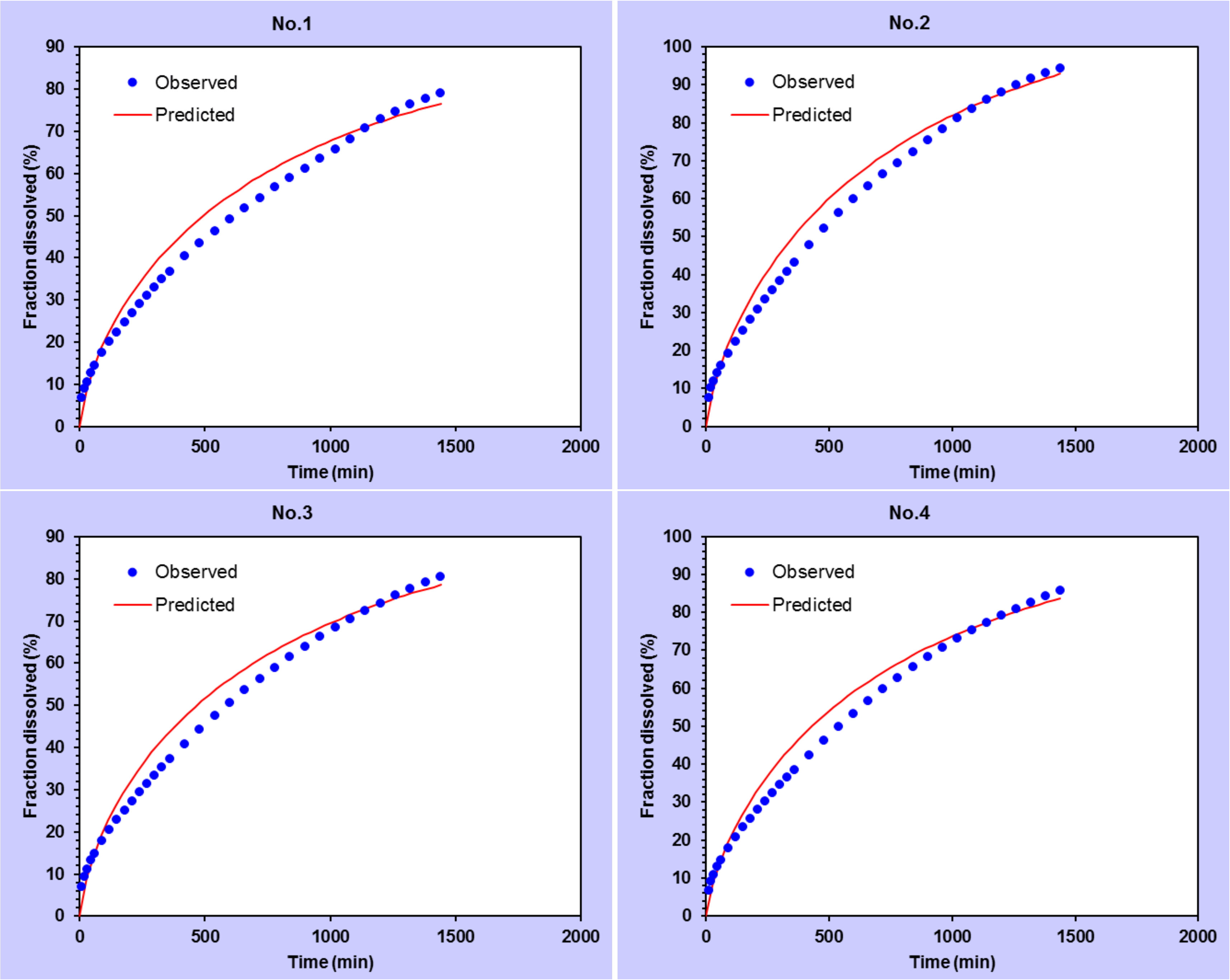

Supplementary materials – Model fitting summary – Emcompress® Anhydrous (modelling the entire relevant dissolution data)

Model: **Weibull_4**

Model equation: $F = F_{max} \cdot \left[1 - e^{-\frac{(t-T_i)^\beta}{\alpha}}\right]$

Fitted model parameters per tested tablet (N = 4) with statistics – mean, standard deviation (SD), and relative standard deviation expressed in % (RSD%) (output from DDSolver):

Parameter	No.1	No.2	No.3	No.4	Mean	SD	RSD(%)
α	74.838	90.351	74.780	88.403	82.093	8.448	10.291
β	0.680	0.711	0.682	0.705	0.694	0.016	2.294
T_i	6.000	6.000	6.000	6.000	6.000	0.000	0.000
F_{max}	82.875	99.129	84.448	90.031	89.121	7.345	8.241

Number of dissolution data points (N), degrees of freedom (df), and selected goodness of fit criteria – Pearson correlation coefficient (R), coefficient of determination (R^2), adjusted coefficient of determination ($R^2_{adjusted}$), and residual sum of squares (RSS) (manual calculation in MS Excel):

Parameter	No.1	No.2	No.3	No.4
N	33	33	33	33
df	29	29	29	29
R	0.981154367	0.982523676	0.980901553	0.981899705
R^2	0.962663892	0.965352774	0.962167856	0.964127031
$R^2_{adjusted}$	0.958801536	0.961768578	0.958254186	0.960416034
RSS	734.6030251	1093.207789	793.4202864	904.1742731

Graphical abstract of model fit presented as mean ± 1 SD of the fraction % of released carvedilol:

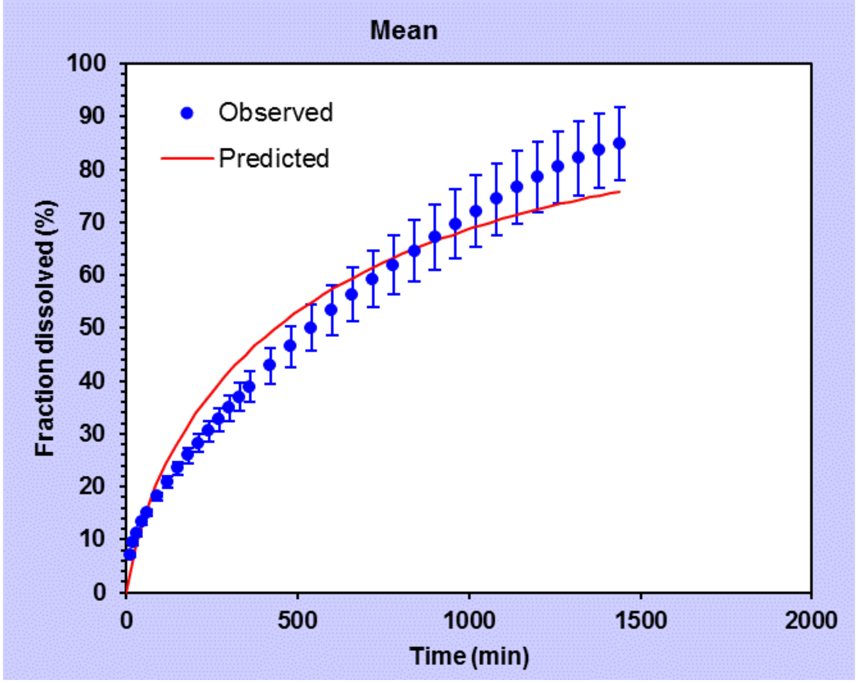

Supplementary materials – Model fitting summary – Emcompress® Anhydrous (modelling the entire relevant dissolution data)

Graphical abstract of model fit presented as the fraction % of released carvedilol per tested tablet:

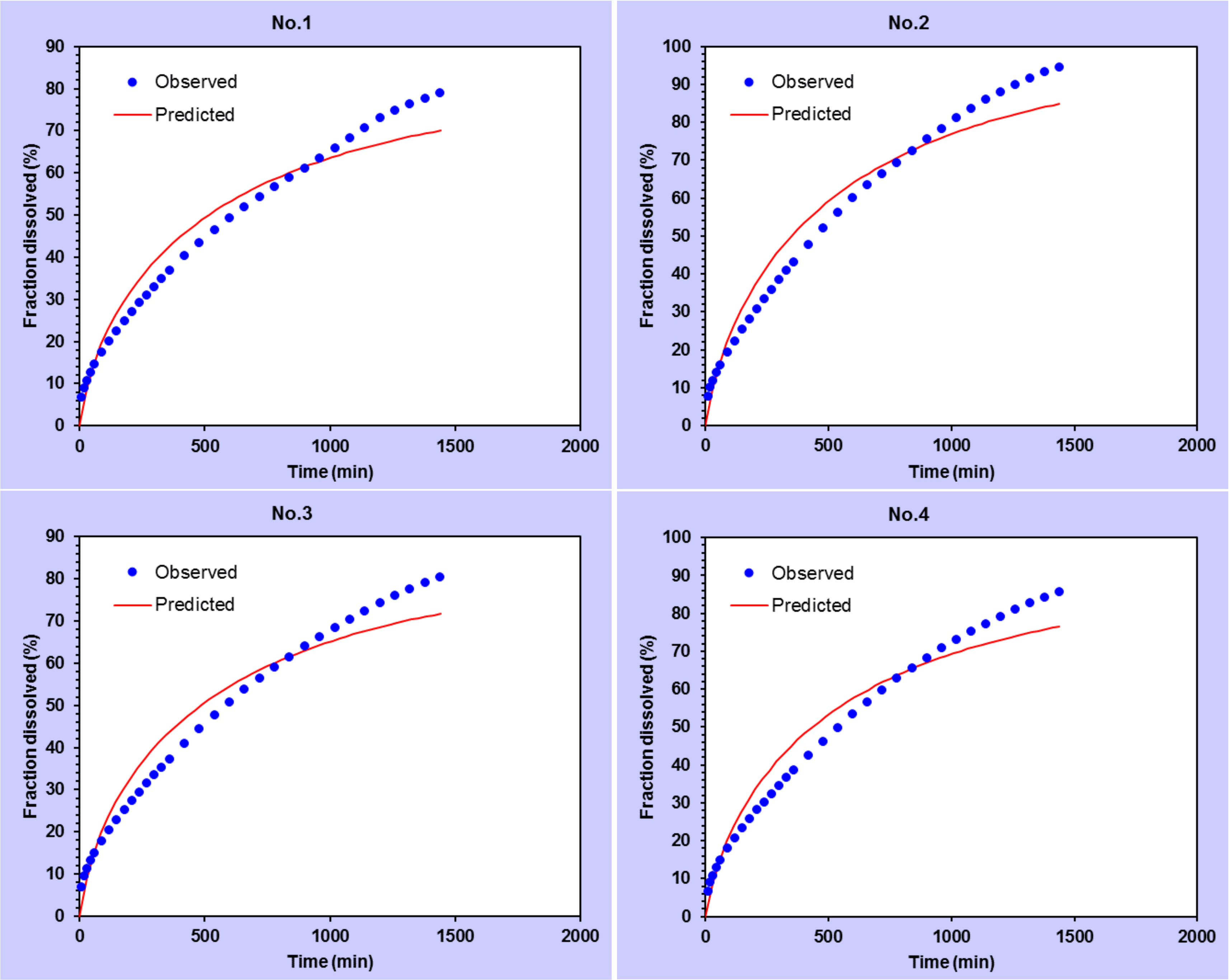

Supplementary materials – Model fitting summary – Emcompress® Anhydrous (modelling the entire relevant dissolution data)

Model: **Logistic_1**

Model equation: $F = 100 \cdot \frac{e^{\alpha+\beta\cdot\log(t)}}{1+e^{\alpha+\beta\cdot\log(t)}}$

Fitted model parameters per tested tablet (N = 4) with statistics – mean, standard deviation (SD), and relative standard deviation expressed in % (RSD%) (output from DDSolver):

Parameter	No.1	No.2	No.3	No.4	Mean	SD	RSD(%)
α	-5.113	-5.970	-5.150	-6.298	-5.633	0.594	-10.547
β	1.888	2.424	1.925	2.326	2.141	0.274	12.792

Number of dissolution data points (N), degrees of freedom (df), and selected goodness of fit criteria – Pearson correlation coefficient (R), coefficient of determination (R^2), adjusted coefficient of determination ($R^2_{adjusted}$), and residual sum of squares (RSS) (manual calculation in MS Excel):

Parameter	No.1	No.2	No.3	No.4
N	33	33	33	33
df	31	31	31	31
R	0.980669561	0.965588977	0.979344064	0.986845408
R^2	0.961712788	0.932362072	0.959114797	0.973863858
$R^2_{adjusted}$	0.960477717	0.930180203	0.957795919	0.973020757
RSS	756.3722057	2068.917304	852.9036708	936.37998

Graphical abstract of model fit presented as mean ± 1 SD of the fraction % of released carvedilol:

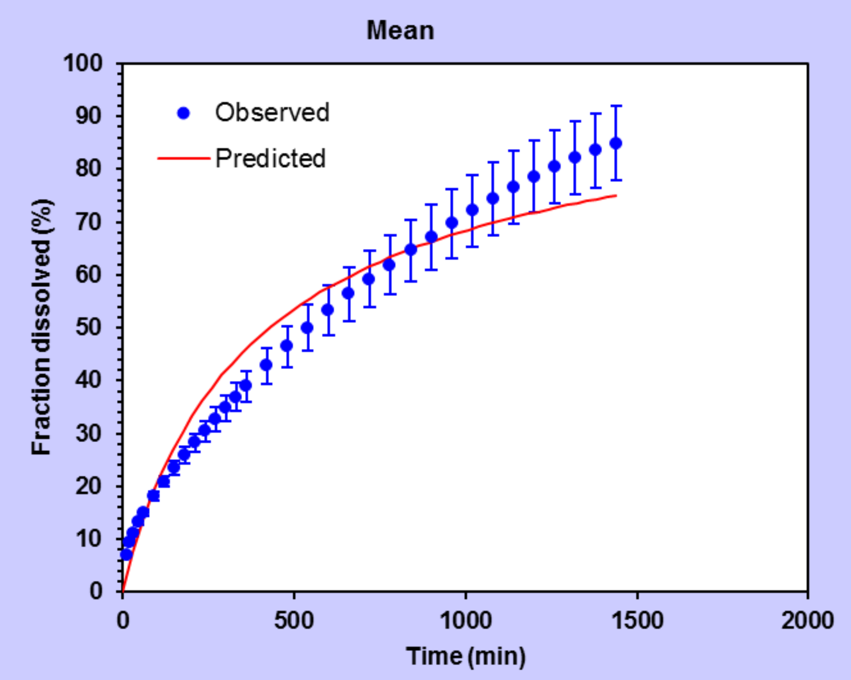

Supplementary materials – Model fitting summary – Emcompress® Anhydrous (modelling the entire relevant dissolution data)

Graphical abstract of model fit presented as the fraction % of released carvedilol per tested tablet:

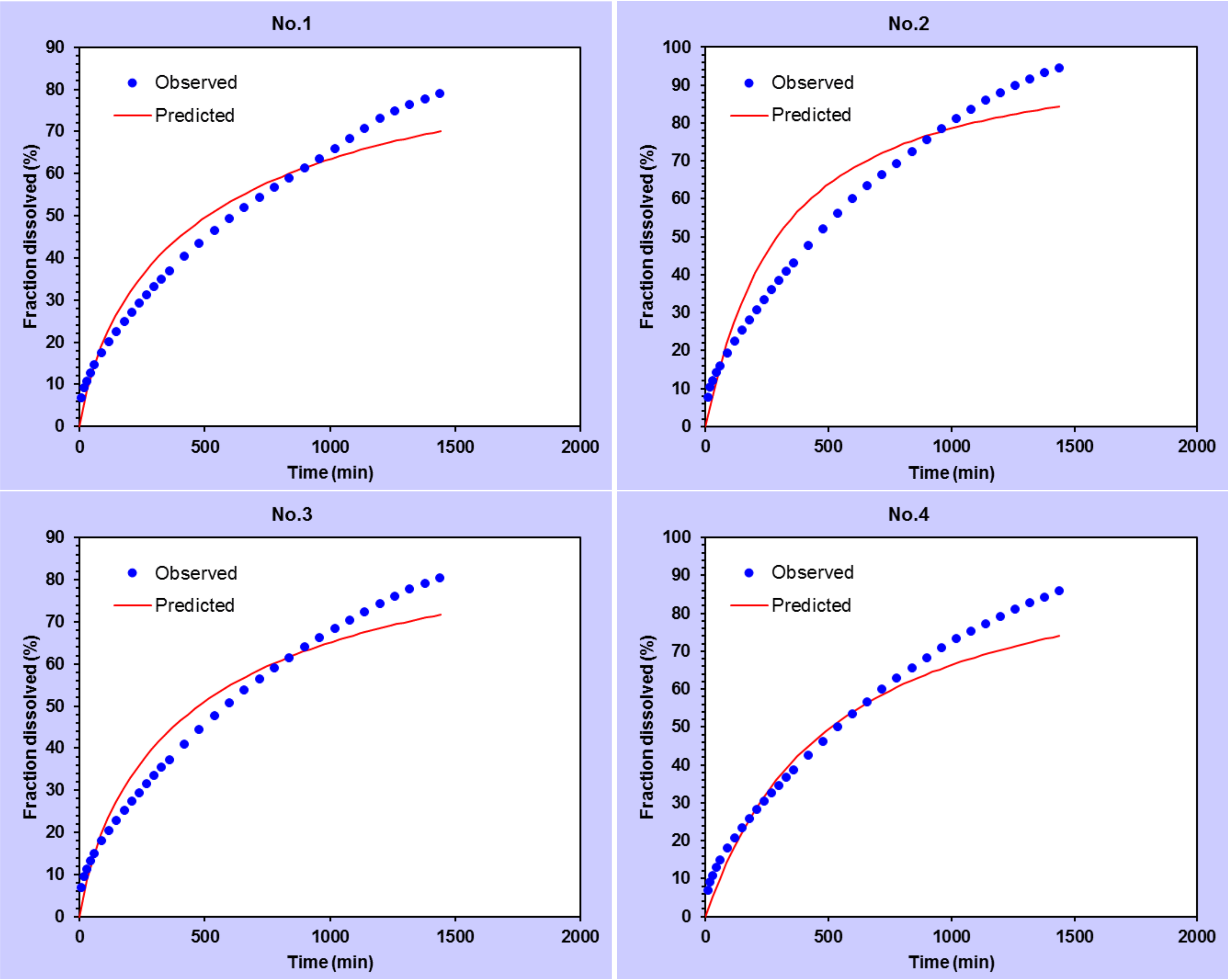

Supplementary materials – Model fitting summary – Emcompress® Anhydrous (modelling the entire relevant dissolution data)

Model: **Logistic_2**

Model equation: $F = F_{max} \cdot \frac{e^{\alpha+\beta\cdot\log(t)}}{1+e^{\alpha+\beta\cdot\log(t)}}$

Fitted model parameters per tested tablet (N = 4) with statistics – mean, standard deviation (SD), and relative standard deviation expressed in % (RSD%) (output from DDSolver):

Parameter	No.1	No.2	No.3	No.4	Mean	SD	RSD(%)
α	-5.770	-6.040	-5.787	-5.979	-5.894	0.136	-2.301
β	2.357	2.466	2.371	2.432	2.406	0.051	2.131
F_{max}	82.875	99.129	84.448	90.031	89.121	7.345	8.241

Number of dissolution data points (N), degrees of freedom (df), and selected goodness of fit criteria – Pearson correlation coefficient (R), coefficient of determination (R^2), adjusted coefficient of determination ($R^2_{adjusted}$), and residual sum of squares (RSS) (manual calculation in MS Excel):

Parameter	No.1	No.2	No.3	No.4
N	33	33	33	33
df	30	30	30	30
R	0.963388348	0.964074874	0.962563585	0.963525269
R^2	0.92811711	0.929440363	0.926528656	0.928380944
$R^2_{adjusted}$	0.923324917	0.924736388	0.921630566	0.92360634
RSS	1417.709325	2175.406467	1528.972331	1768.558564

Graphical abstract of model fit presented as mean ± 1 SD of the fraction % of released carvedilol:

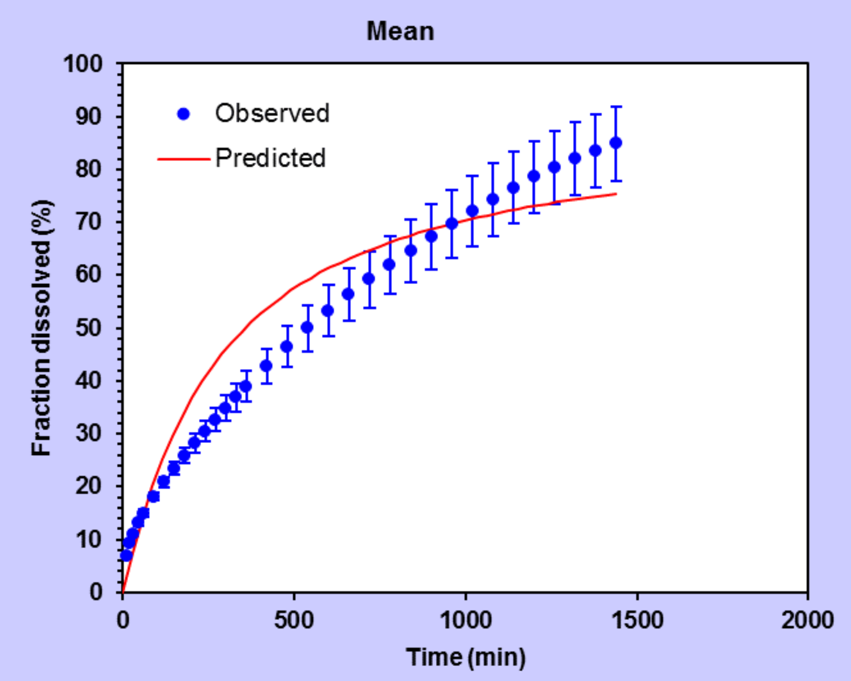

Supplementary materials – Model fitting summary – Emcompress® Anhydrous (modelling the entire relevant dissolution data)

Graphical abstract of model fit presented as the fraction % of released carvedilol per tested tablet:

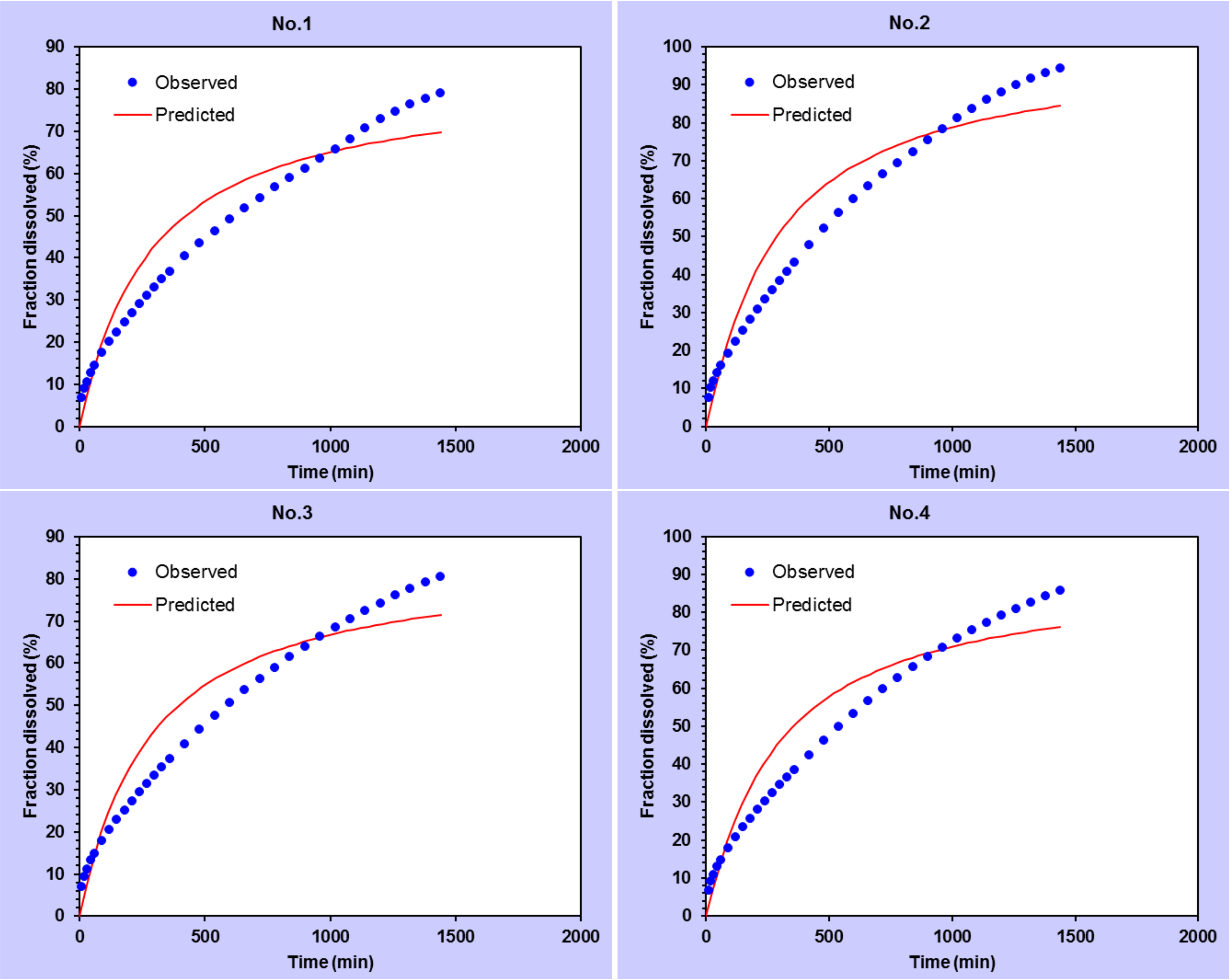

Supplementary materials – Model fitting summary – Emcompress® Anhydrous (modelling the entire relevant dissolution data)

Model: **Logistic_3**

Model equation: $F = F_{max} \cdot \frac{1}{1+e^{-k\cdot(t-\gamma)}}$

Fitted model parameters per tested tablet (N = 4) with statistics – mean, standard deviation (SD), and relative standard deviation expressed in % (RSD%) (output from DDSolver):

Parameter	No.1	No.2	No.3	No.4	Mean	SD	RSD(%)
k	0.003	0.003	0.003	0.003	0.003	0.000	2.068
γ	523.069	523.901	517.229	530.401	523.650	5.391	1.030
F_{max}	82.875	99.129	84.448	90.031	89.121	7.345	8.241

Number of dissolution data points (N), degrees of freedom (df), and selected goodness of fit criteria – Pearson correlation coefficient (R), coefficient of determination (R^2), adjusted coefficient of determination ($R^2_{adjusted}$), and residual sum of squares (RSS) (manual calculation in MS Excel):

Parameter	No.1	No.2	No.3	No.4
N	33	33	33	33
df	30	30	30	30
R	0.989097991	0.991026088	0.990852218	0.991239143
R^2	0.978314836	0.982132708	0.981788118	0.982555039
$R^2_{adjusted}$	0.976869159	0.980941555	0.980573992	0.981392042
RSS	394.8278246	505.0927056	350.1414326	400.0778555

Graphical abstract of model fit presented as mean ± 1 SD of the fraction % of released carvedilol:

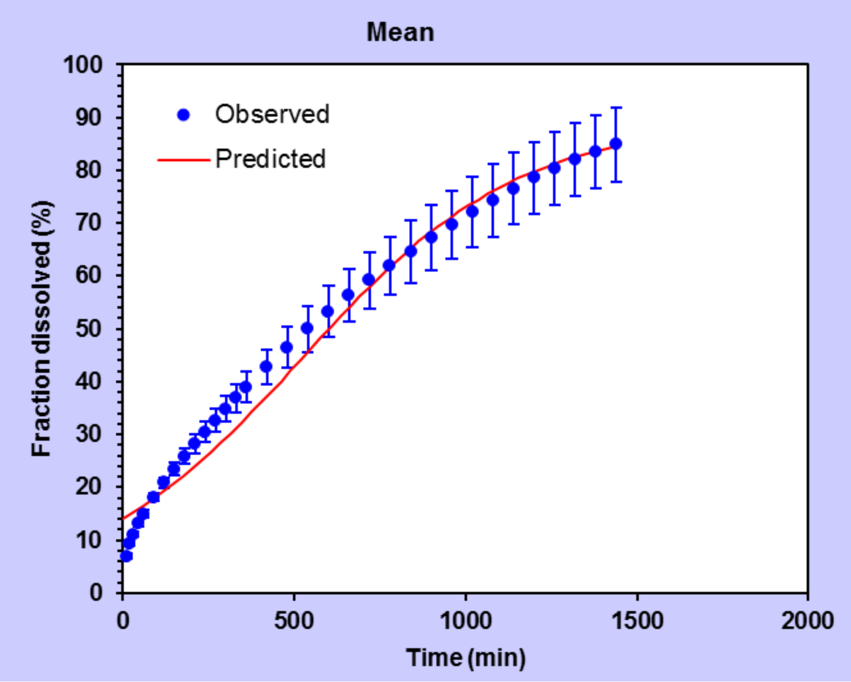

Supplementary materials – Model fitting summary – Emcompress® Anhydrous (modelling the entire relevant dissolution data)

Graphical abstract of model fit presented as the fraction % of released carvedilol per tested tablet:

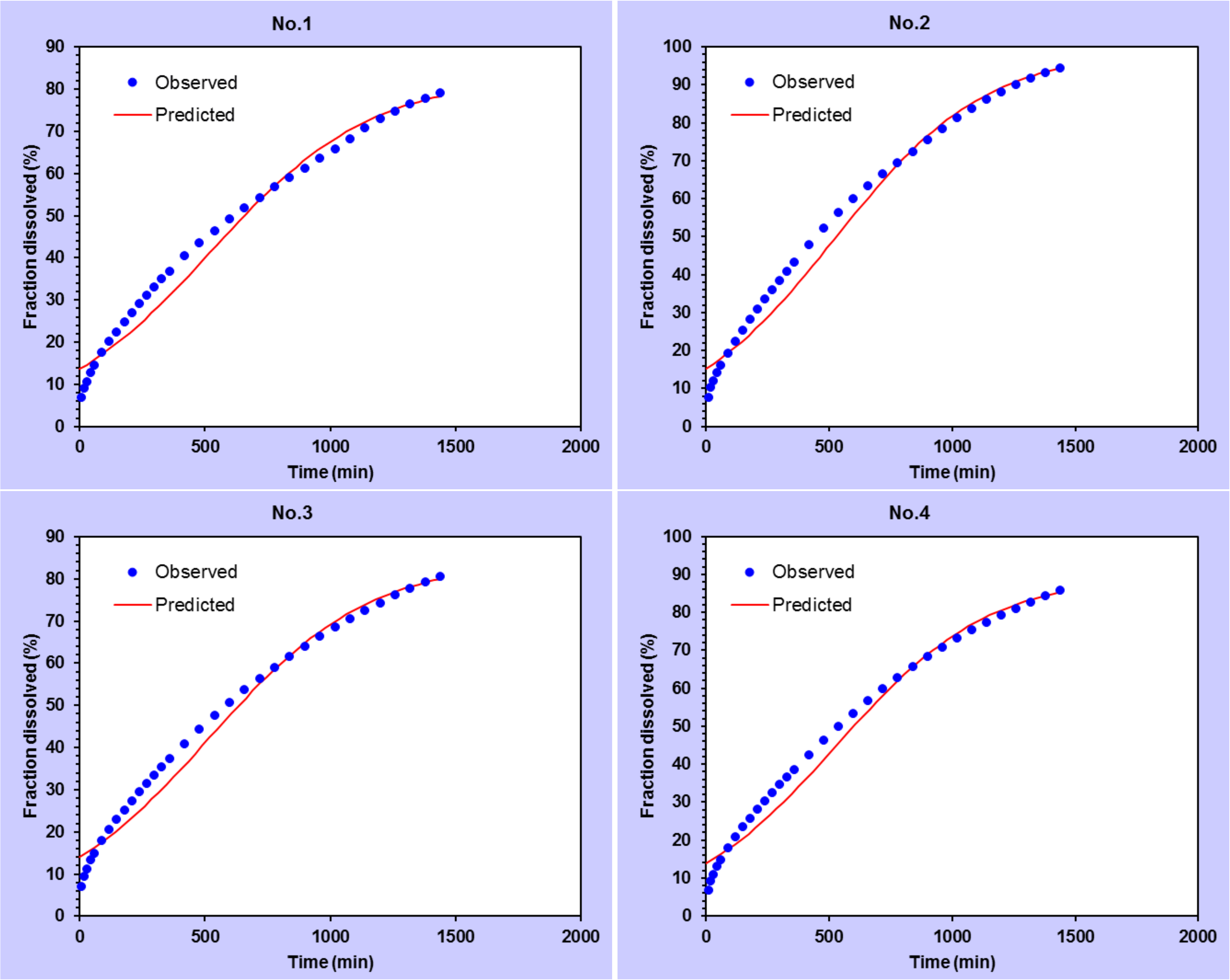

Supplementary materials – Model fitting summary – Emcompress® Anhydrous (modelling the entire relevant dissolution data)

Model: **Gompertz_1**

Model equation: $F = 100 \cdot e^{-\alpha \cdot e^{-\beta \cdot \log(t)}}$

Fitted model parameters per tested tablet (N = 4) with statistics – mean, standard deviation (SD), and relative standard deviation expressed in % (RSD%) (output from DDSolver):

Parameter	No.1	No.2	No.3	No.4	Mean	SD	RSD(%)
α	19.815	56.307	16.228	21.412	28.440	18.704	65.764
β	1.204	1.683	1.208	1.357	1.363	0.225	16.521

Number of dissolution data points (N), degrees of freedom (df), and selected goodness of fit criteria – Pearson correlation coefficient (R), coefficient of determination (R^2), adjusted coefficient of determination ($R^2_{adjusted}$), and residual sum of squares (RSS) (manual calculation in MS Excel):

Parameter	No.1	No.2	No.3	No.4
N	33	33	33	33
df	31	31	31	31
R	0.970423717	0.970017003	0.953894315	0.948026888
R^2	0.941722191	0.940932986	0.909914364	0.898754981
$R^2_{adjusted}$	0.939842262	0.939027598	0.907008376	0.895489012
RSS	1465.865988	2773.994949	1743.909942	2347.750307

Graphical abstract of model fit presented as mean ± 1 SD of the fraction % of released carvedilol:

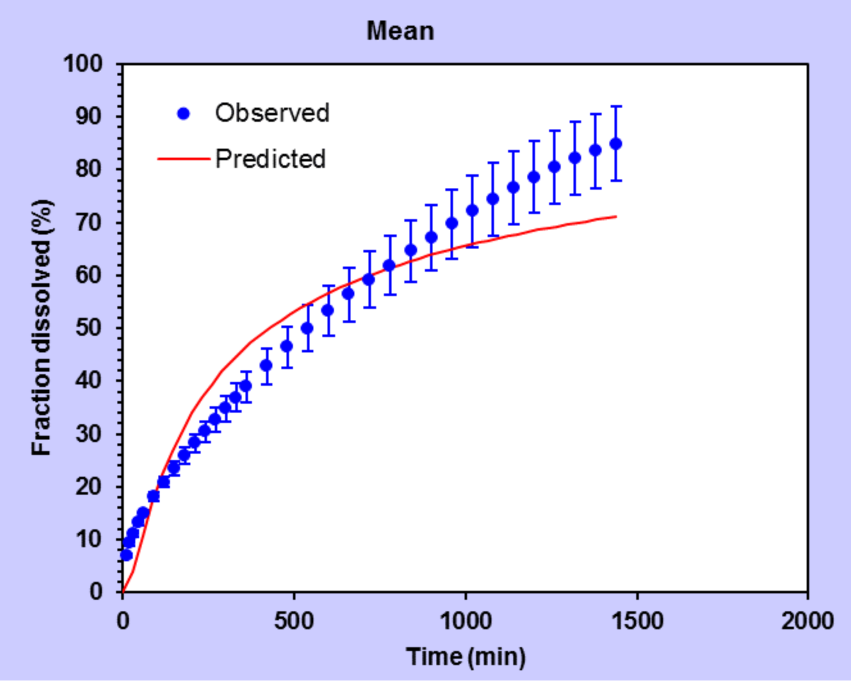

Supplementary materials – Model fitting summary – Emcompress® Anhydrous (modelling the entire relevant dissolution data)

Graphical abstract of model fit presented as the fraction % of released carvedilol per tested tablet:

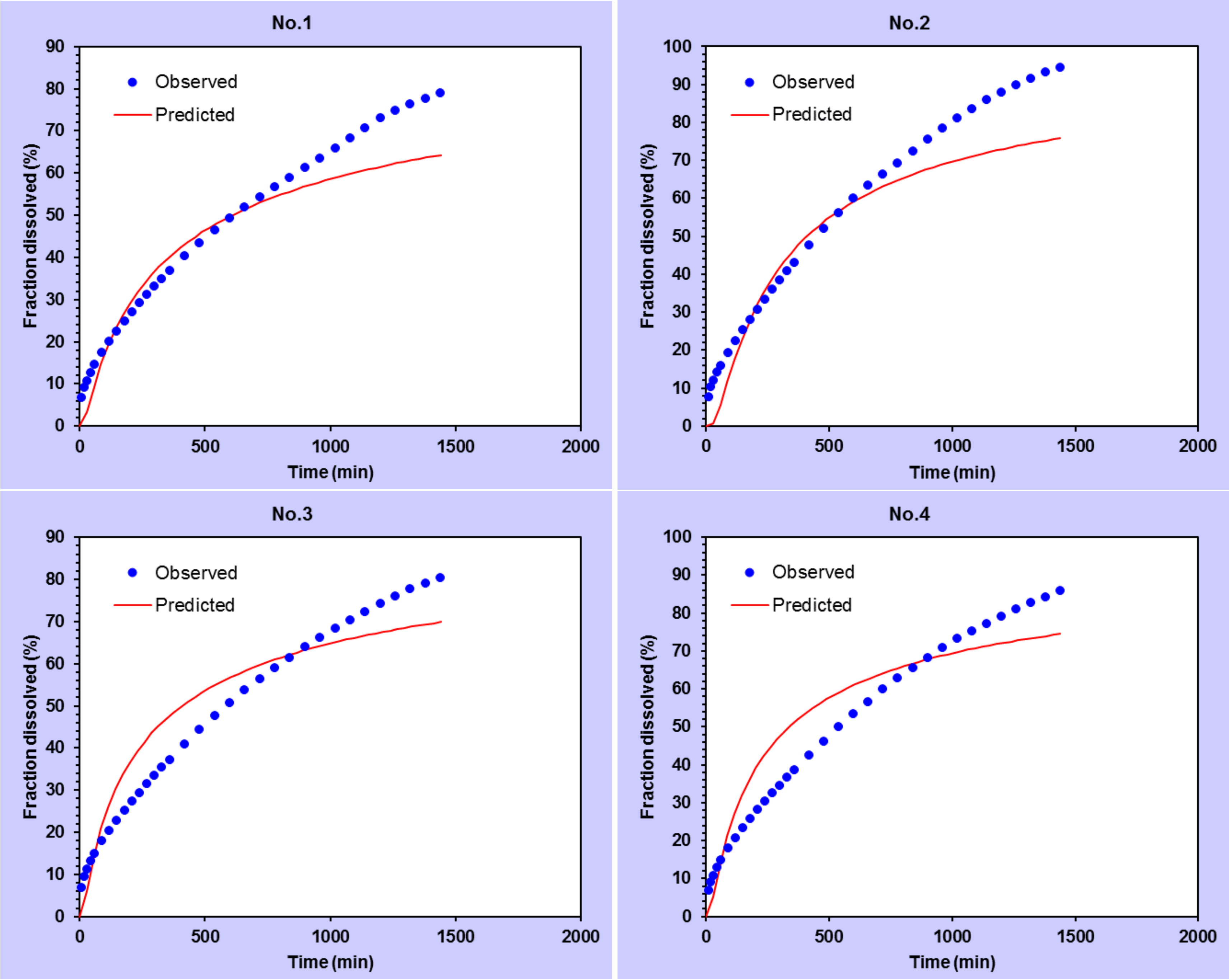

Supplementary materials – Model fitting summary – Emcompress® Anhydrous (modelling the entire relevant dissolution data)

Model: **Gompertz_2**

Model equation: $F = F_{max} \cdot e^{-\alpha \cdot e^{-\beta \cdot \log(t)}}$

Fitted model parameters per tested tablet (N = 4) with statistics – mean, standard deviation (SD), and relative standard deviation expressed in % (RSD%) (output from DDSolver):

Parameter	No.1	No.2	No.3	No.4	Mean	SD	RSD(%)
α	42.158	49.869	43.250	47.059	45.584	3.546	7.779
β	1.521	1.588	1.536	1.559	1.551	0.029	1.890
F_{max}	92.755	110.947	94.515	100.763	99.745	8.220	8.241

Number of dissolution data points (N), degrees of freedom (df), and selected goodness of fit criteria – Pearson correlation coefficient (R), coefficient of determination (R^2), adjusted coefficient of determination ($R^2_{adjusted}$), and residual sum of squares (RSS) (manual calculation in MS Excel):

Parameter	No.1	No.2	No.3	No.4
N	33	33	33	33
df	30	30	30	30
R	0.974813035	0.976489277	0.974475096	0.975367264
R^2	0.950260452	0.953531307	0.949601714	0.9513413
$R^2_{adjusted}$	0.946944482	0.950433395	0.946241828	0.948097387
RSS	1392.584552	1925.690919	1452.137025	1671.075933

Graphical abstract of model fit presented as mean ± 1 SD of the fraction % of released carvedilol:

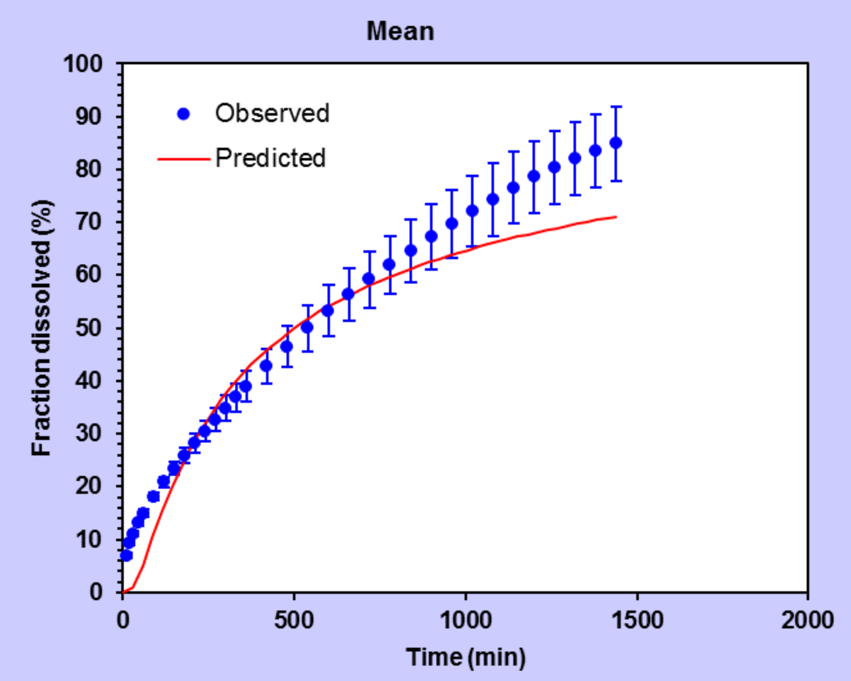

Supplementary materials – Model fitting summary – Emcompress® Anhydrous (modelling the entire relevant dissolution data)

Graphical abstract of model fit presented as the fraction % of released carvedilol per tested tablet:

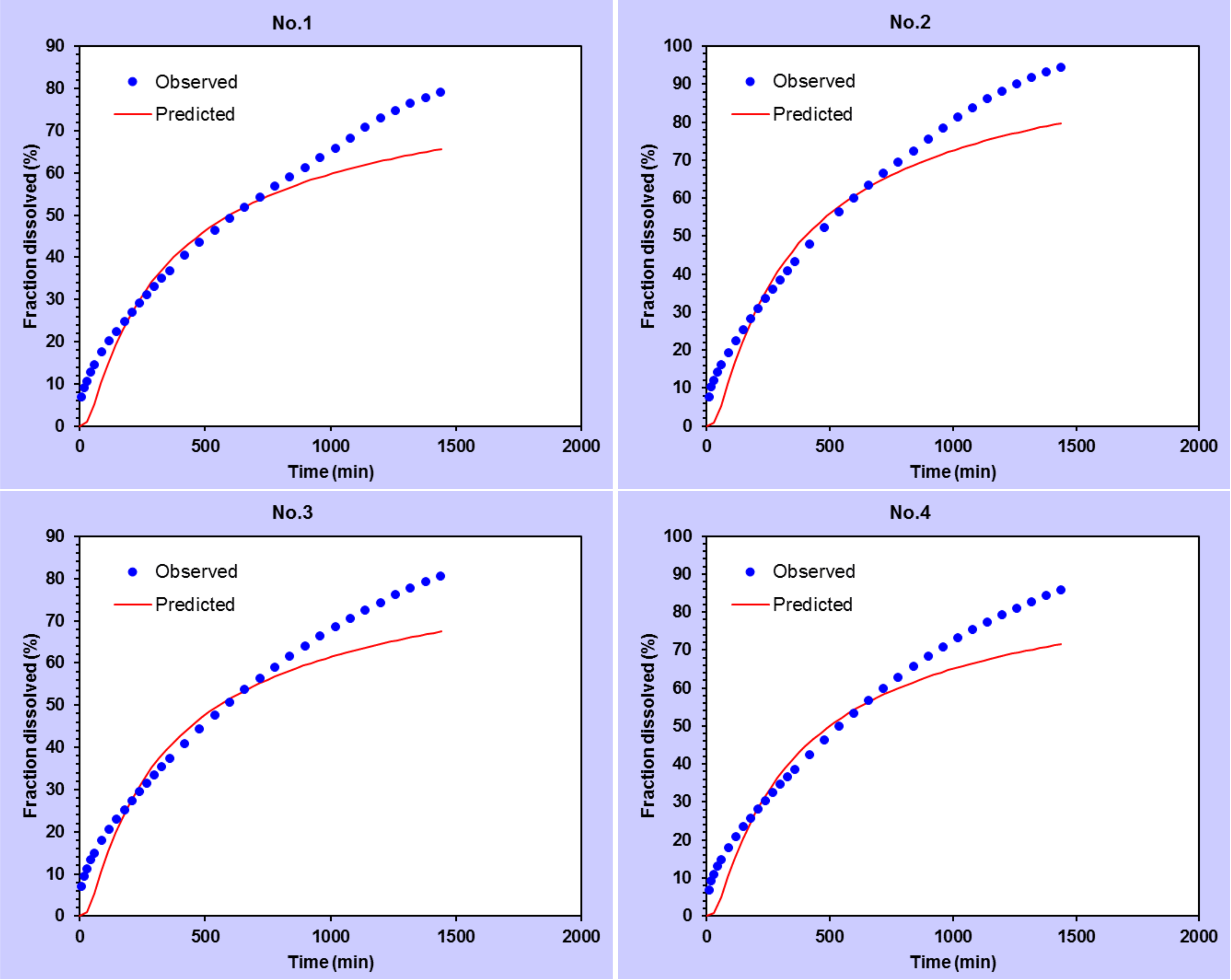

Supplementary materials – Model fitting summary – Emcompress® Anhydrous (modelling the entire relevant dissolution data)

Model: **Gompertz_3**

Model equation: $F = F_{max} \cdot e^{-e^{-k \cdot (t-\gamma)}}$

Fitted model parameters per tested tablet (N = 4) with statistics – mean, standard deviation (SD), and relative standard deviation expressed in % (RSD%) (output from DDSolver):

Parameter	No.1	No.2	No.3	No.4	Mean	SD	RSD(%)
k	0.002	0.002	0.002	0.002	0.002	0.000	1.863
γ	307.741	312.308	303.343	315.911	309.826	5.464	1.764
F_{max}	82.875	99.129	84.448	90.031	89.121	7.345	8.241

Number of dissolution data points (N), degrees of freedom (df), and selected goodness of fit criteria – Pearson correlation coefficient (R), coefficient of determination (R^2), adjusted coefficient of determination ($R^2_{adjusted}$), and residual sum of squares (RSS) (manual calculation in MS Excel):

Parameter	No.1	No.2	No.3	No.4
N	33	33	33	33
df	30	30	30	30
R	0.995028163	0.997209969	0.996641993	0.996998385
R^2	0.990081045	0.994427723	0.993295262	0.994005779
$R^2_{adjusted}$	0.989419781	0.994056238	0.99284828	0.993606164
RSS	188.7495552	160.2500744	132.634877	139.8971917

Graphical abstract of model fit presented as mean ± 1 SD of the fraction % of released carvedilol:

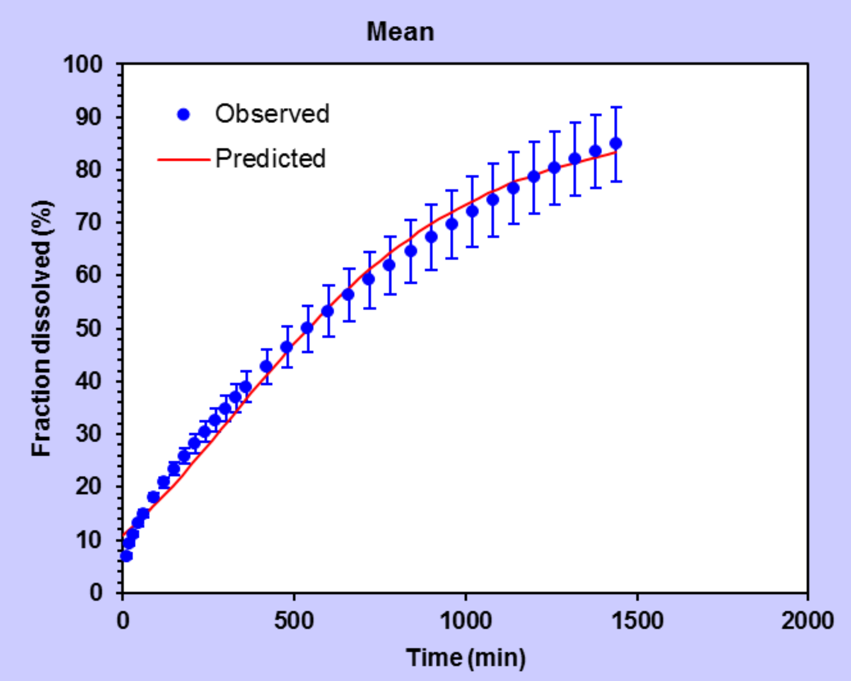

Supplementary materials – Model fitting summary – Emcompress® Anhydrous (modelling the entire relevant dissolution data)

Graphical abstract of model fit presented as the fraction % of released carvedilol per tested tablet:

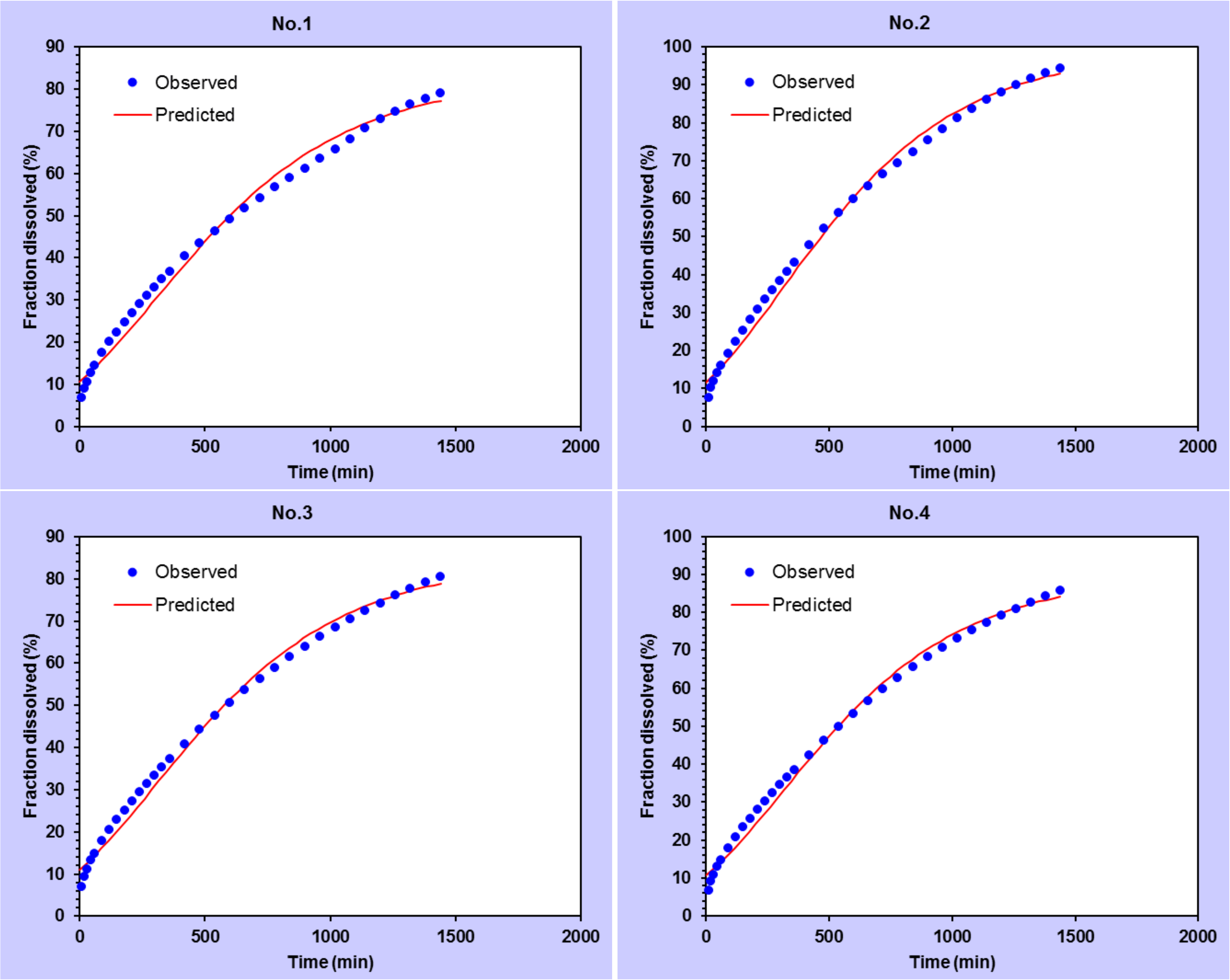

Supplementary materials – Model fitting summary – Emcompress® Anhydrous (modelling the entire relevant dissolution data)

Model: **Gompertz_4**

Model equation: $F = F_{max} \cdot e^{-\beta \cdot e^{-k \cdot t}}$

Fitted model parameters per tested tablet (N = 4) with statistics – mean, standard deviation (SD), and relative standard deviation expressed in % (RSD%) (output from DDSolver):

Parameter	No.1	No.2	No.3	No.4	Mean	SD	RSD(%)
k	0.002	0.002	0.002	0.002	0.002	0.000	1.863
β	2.056	2.145	2.047	2.132	2.095	0.051	2.415
F_{max}	82.875	99.129	84.448	90.031	89.121	7.345	8.241

Number of dissolution data points (N), degrees of freedom (df), and selected goodness of fit criteria – Pearson correlation coefficient (R), coefficient of determination (R^2), adjusted coefficient of determination ($R^2_{adjusted}$), and residual sum of squares (RSS) (manual calculation in MS Excel):

Parameter	No.1	No.2	No.3	No.4
N	33	33	33	33
df	30	30	30	30
R	0.995028163	0.997209969	0.996641993	0.996998385
R^2	0.990081045	0.994427723	0.993295262	0.994005779
$R^2_{adjusted}$	0.989419781	0.994056238	0.99284828	0.993606164
RSS	188.7495552	160.2500744	132.634877	139.8971917

Graphical abstract of model fit presented as mean ± 1 SD of the fraction % of released carvedilol:

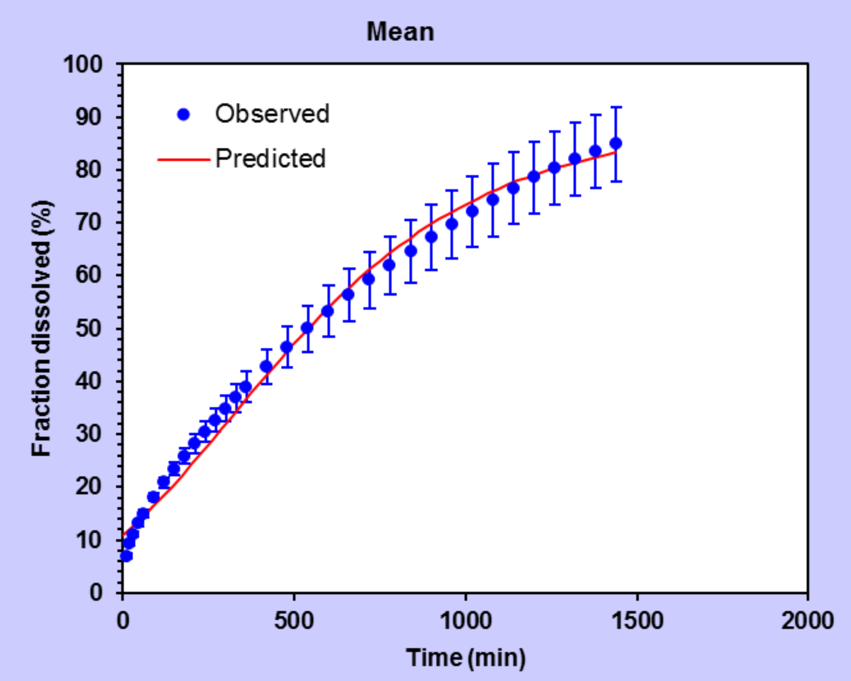

Supplementary materials – Model fitting summary – Emcompress® Anhydrous (modelling the entire relevant dissolution data)

Graphical abstract of model fit presented as the fraction % of released carvedilol per tested tablet:

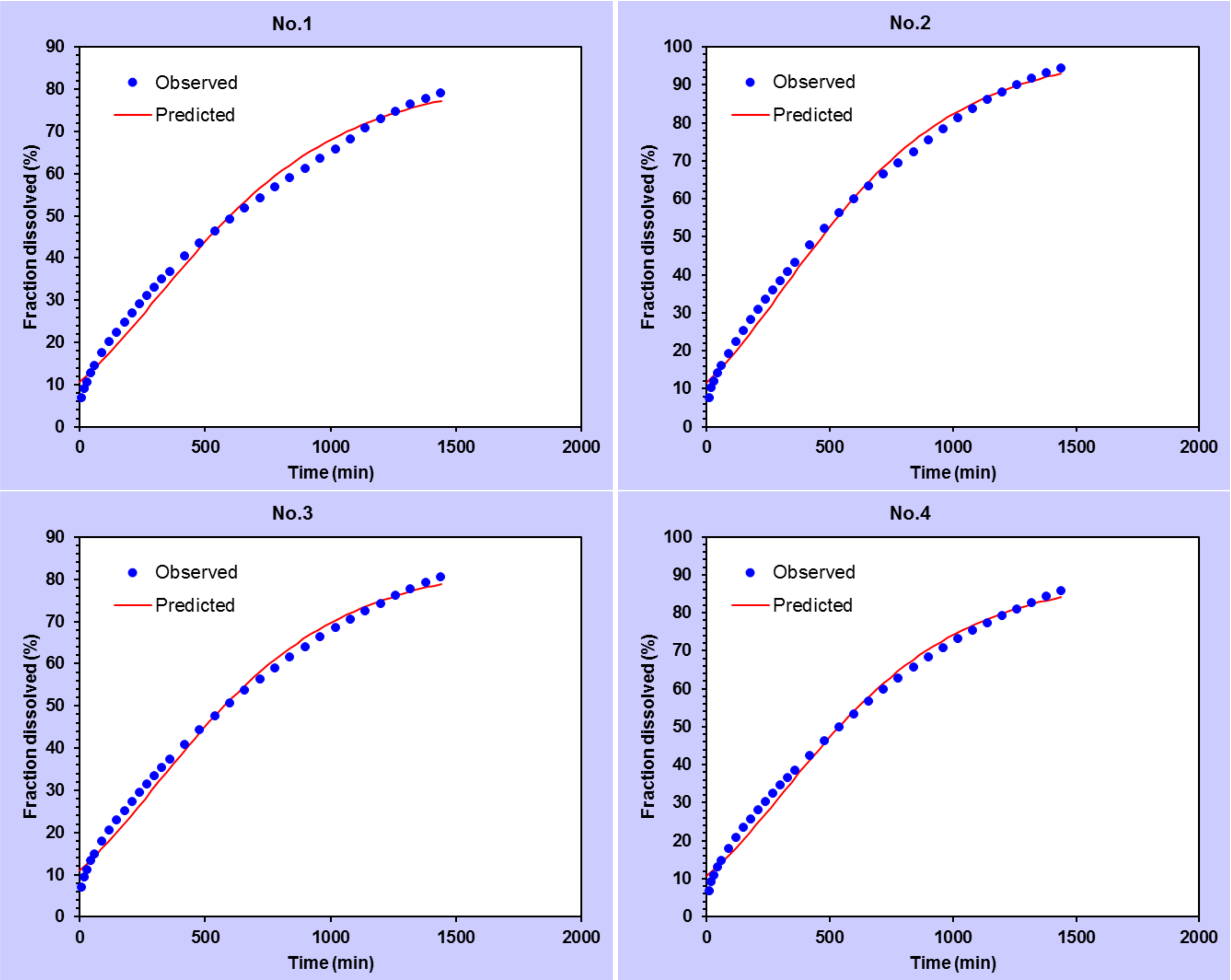

Supplementary materials – Model fitting summary – Emcompress® Anhydrous (modelling the entire relevant dissolution data)

Model: **Probit_1**

Model equation: $F = 100 \cdot \phi[\alpha + \beta \cdot \log(t)]$

Fitted model parameters per tested tablet (N = 4) with statistics – mean, standard deviation (SD), and relative standard deviation expressed in % (RSD%) (output from DDSolver):

Parameter	No.1	No.2	No.3	No.4	Mean	SD	RSD(%)
α	-3.032	-3.519	-3.059	-3.733	-3.336	0.347	-10.390
β	1.121	1.427	1.145	1.381	1.268	0.158	12.448

Number of dissolution data points (N), degrees of freedom (df), and selected goodness of fit criteria – Pearson correlation coefficient (R), coefficient of determination (R^2), adjusted coefficient of determination ($R^2_{adjusted}$), and residual sum of squares (RSS) (manual calculation in MS Excel):

Parameter	No.1	No.2	No.3	No.4
N	33	33	33	33
df	31	31	31	31
R	0.97622383	0.964293415	0.974939626	0.983277846
R^2	0.953012966	0.92986179	0.950507275	0.966835323
$R^2_{adjusted}$	0.951497255	0.927599267	0.948910736	0.965765495
RSS	912.760637	2080.088791	1013.878669	1103.810835

Graphical abstract of model fit presented as mean ± 1 SD of the fraction % of released carvedilol:

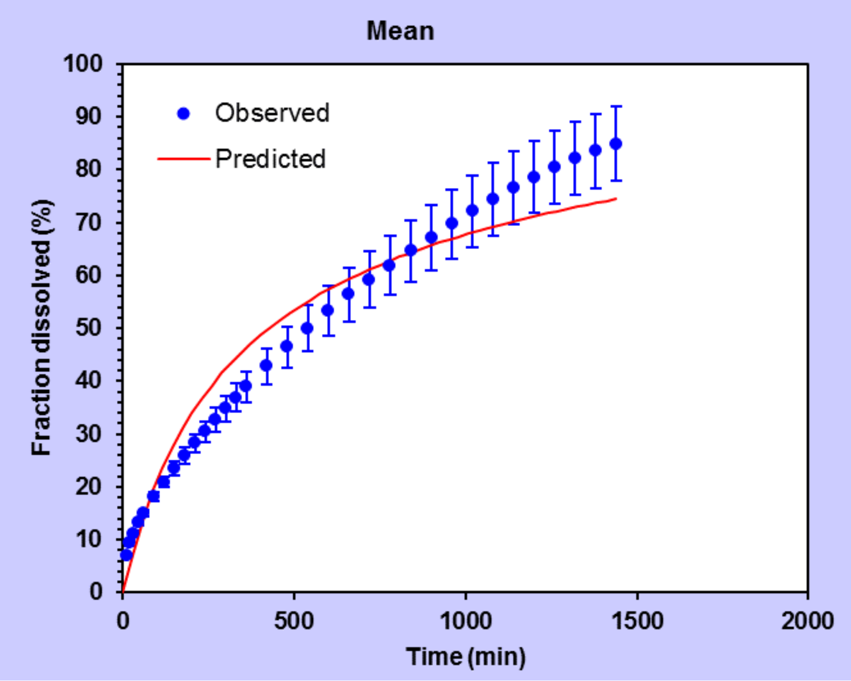

Supplementary materials – Model fitting summary – Emcompress® Anhydrous (modelling the entire relevant dissolution data)

Graphical abstract of model fit presented as the fraction % of released carvedilol per tested tablet:

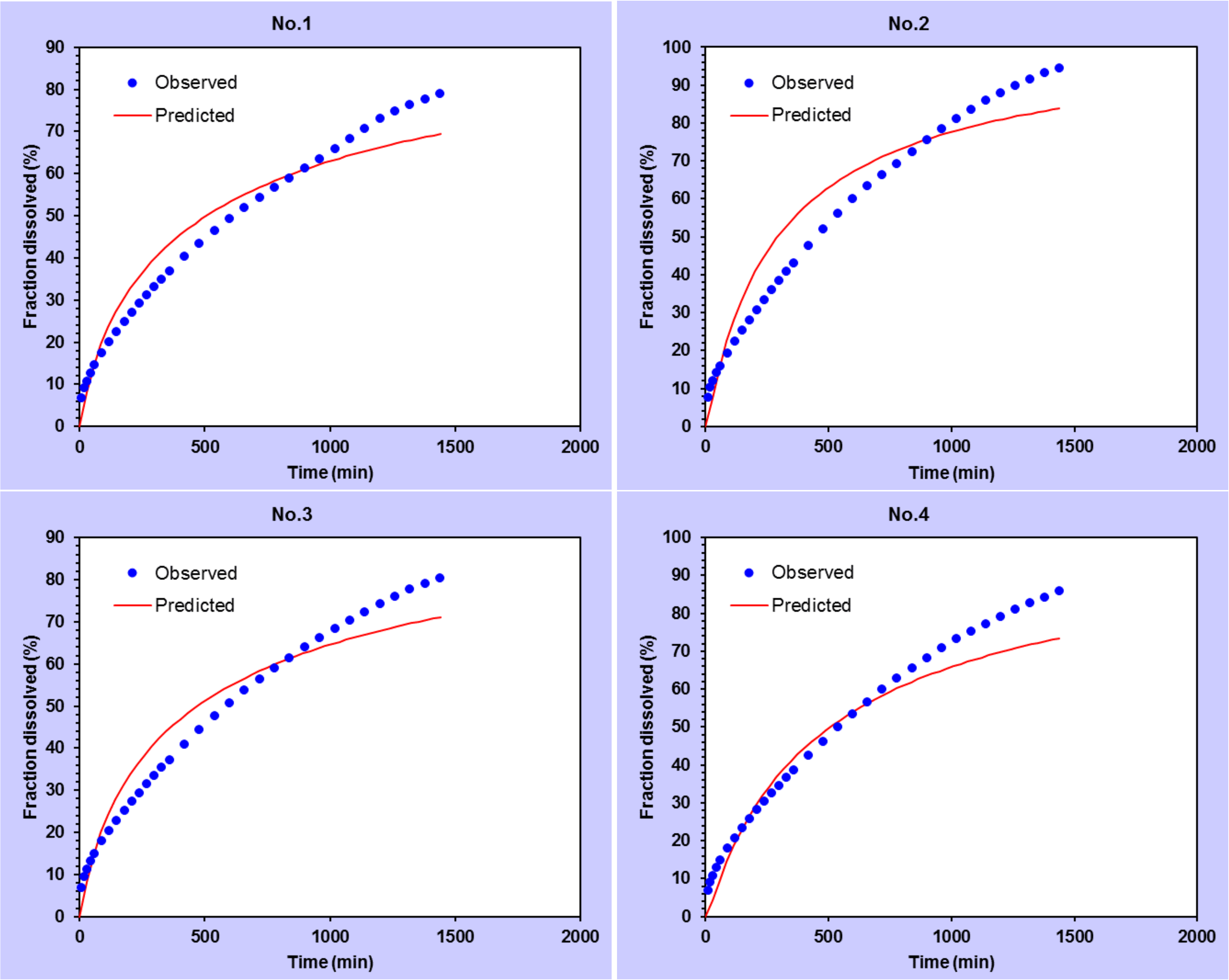

Supplementary materials – Model fitting summary – Emcompress® Anhydrous (modelling the entire relevant dissolution data)

Model: **Probit_2**

Model equation: $F = F_{max} \cdot \phi[\alpha + \beta \cdot \log(t)]$

Fitted model parameters per tested tablet (N = 4) with statistics – mean, standard deviation (SD), and relative standard deviation expressed in % (RSD%) (output from DDSolver):

Parameter	No.1	No.2	No.3	No.4	Mean	SD	RSD(%)
α	-3.404	-3.553	-3.416	-3.520	-3.473	0.074	-2.144
β	1.388	1.448	1.397	1.430	1.416	0.028	1.984
F_{max}	82.875	99.129	84.448	90.031	89.121	7.345	8.241

Number of dissolution data points (N), degrees of freedom (df), and selected goodness of fit criteria – Pearson correlation coefficient (R), coefficient of determination (R^2), adjusted coefficient of determination ($R^2_{adjusted}$), and residual sum of squares (RSS) (manual calculation in MS Excel):

Parameter	No.1	No.2	No.3	No.4
N	33	33	33	33
df	30	30	30	30
R	0.962681605	0.963203087	0.961879295	0.96254267
R^2	0.926755873	0.927760187	0.925211778	0.926488391
$R^2_{adjusted}$	0.921872931	0.922944199	0.920225896	0.921587617
RSS	1393.045627	2149.952773	1503.02558	1756.439227

Graphical abstract of model fit presented as mean ± 1 SD of the fraction % of released carvedilol:

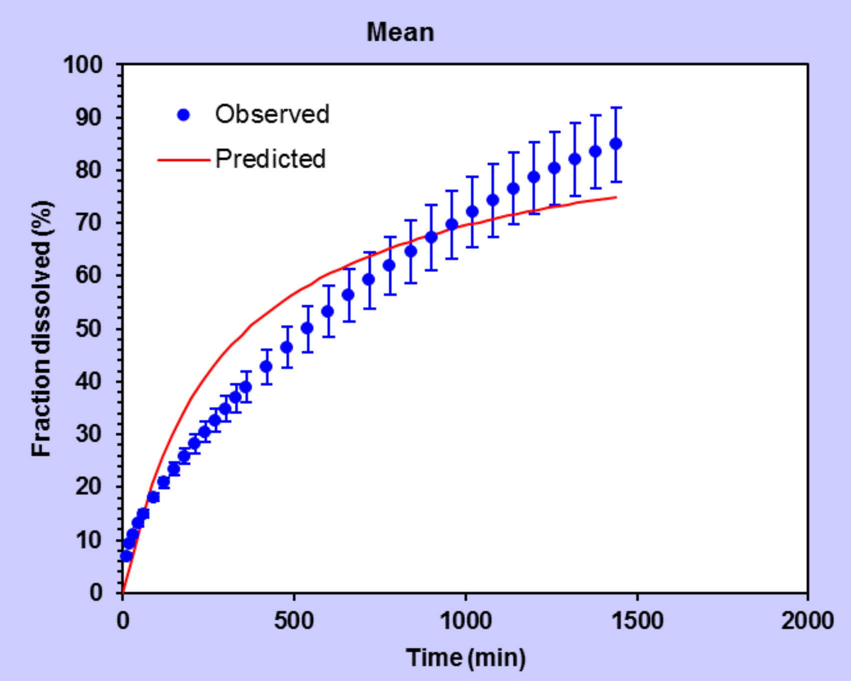

Supplementary materials – Model fitting summary – Emcompress® Anhydrous (modelling the entire relevant dissolution data)

Graphical abstract of model fit presented as the fraction % of released carvedilol per tested tablet:

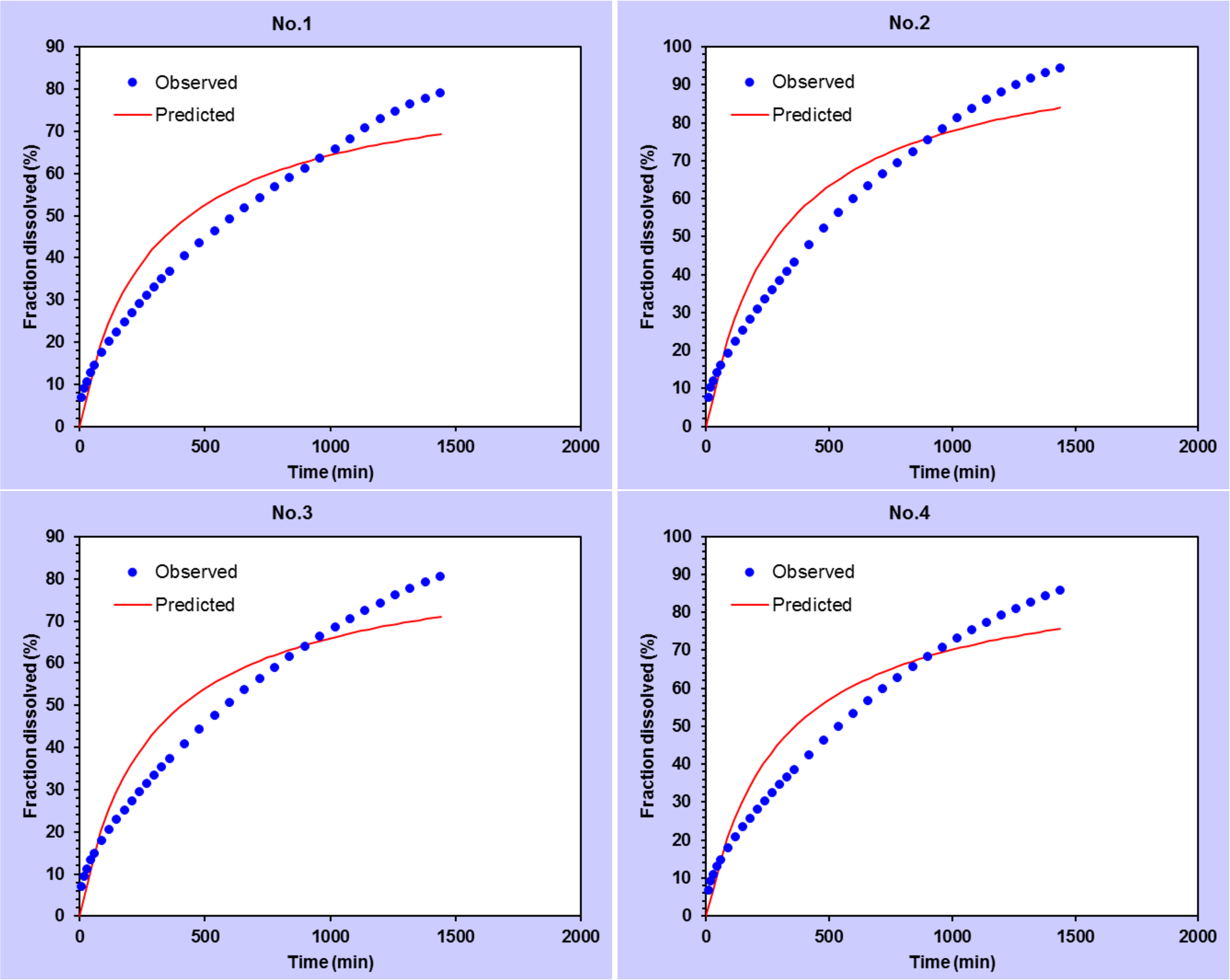

Supplementary materials – Model fitting summary – Emcompress® Anhydrous (modelling up to app. 60 % of carvedilol released)

Model: **Zero–order**

Model equation: $F = k_0 \cdot t$

Fitted model parameters per tested tablet (N = 4) with statistics – mean, standard deviation (SD), and relative standard deviation expressed in % (RSD%) (output from DDSolver):

Parameter	No.1	No.2	No.3	No.4	Mean	SD	RSD(%)
k_0	0.090	0.108	0.093	0.097	0.097	0.008	8.095

Number of dissolution data points (N), degrees of freedom (df), and selected goodness of fit criteria – Pearson correlation coefficient (R), coefficient of determination (R^2), adjusted coefficient of determination ($R^2_{adjusted}$), and residual sum of squares (RSS) (manual calculation in MS Excel):

Parameter	No.1	No.2	No.3	No.4
N	21	21	21	21
df	20	20	20	20
R	0.987380349	0.992631635	0.990333268	0.991660125
R^2	0.974919954	0.985317562	0.980759982	0.983389803
$R^2_{adjusted}$	0.974919954	0.985317562	0.980759982	0.983389803
RSS	1081.670402	1161.383804	1077.11899	1012.364174

Graphical abstract of model fit presented as mean ± 1 SD of the fraction % of released carvedilol:

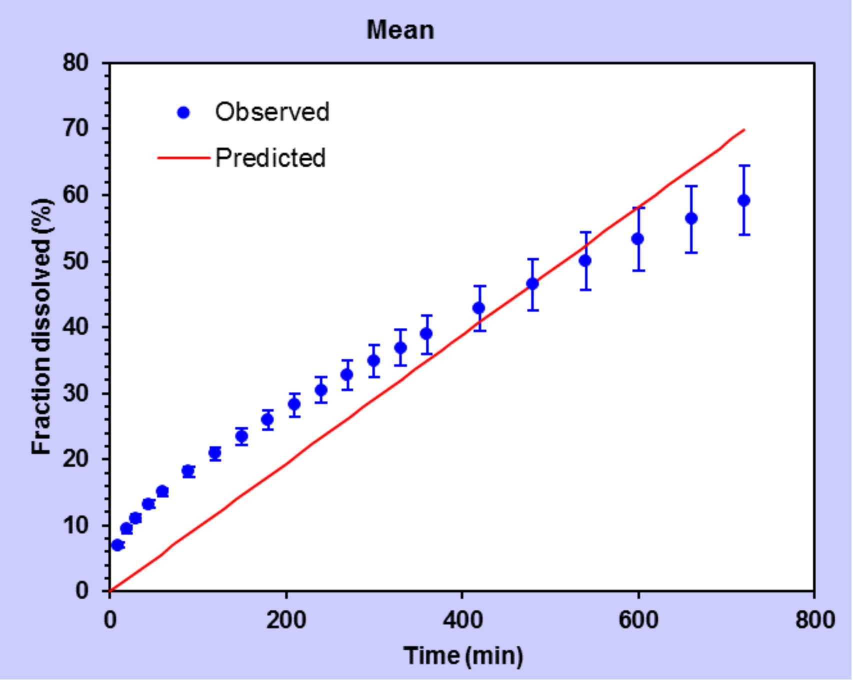

Supplementary materials – Model fitting summary – Emcompress® Anhydrous (modelling up to app. 60 % of carvedilol released)

Graphical abstract of model fit presented as the fraction % of released carvedilol per tested tablet:

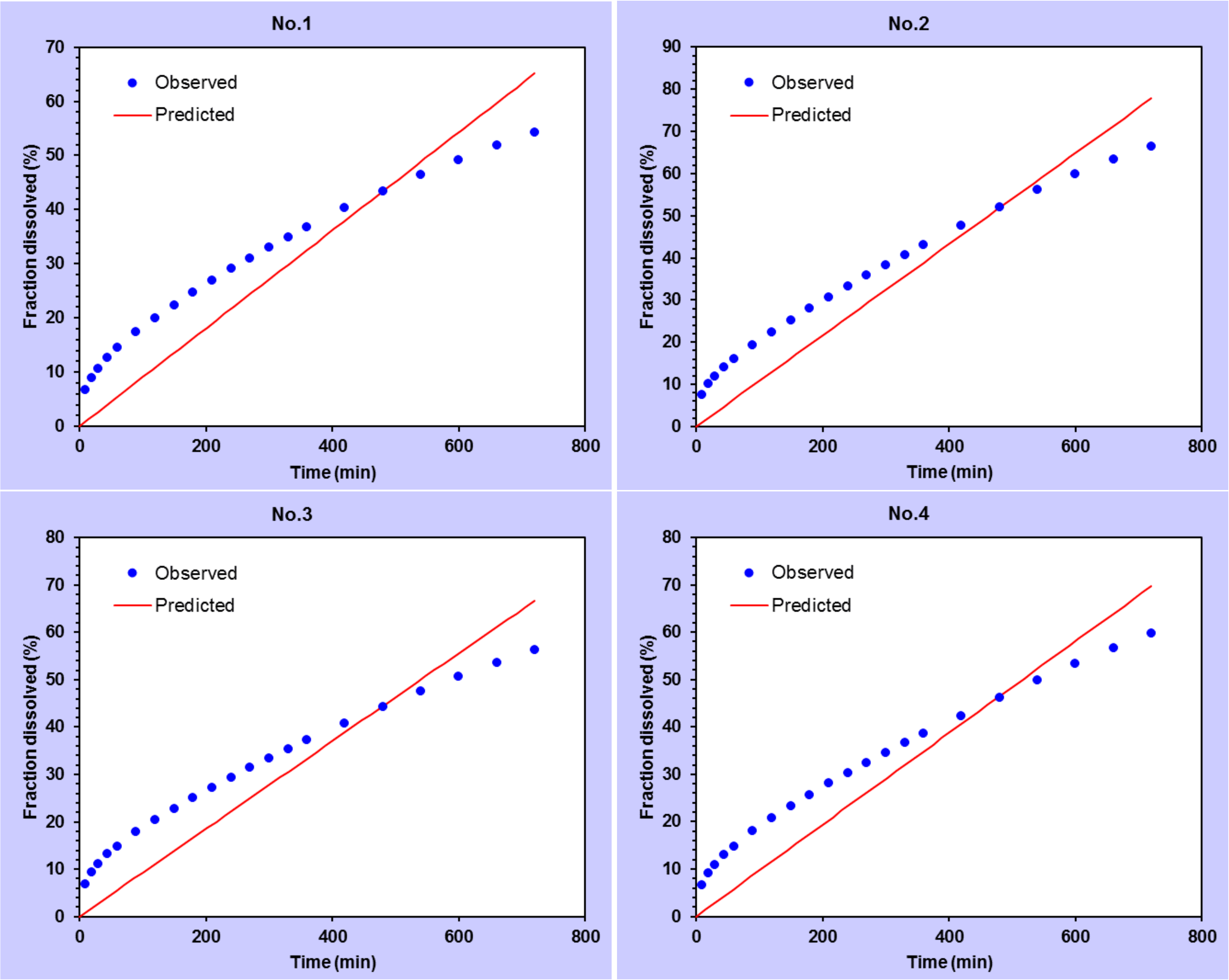

Supplementary materials – Model fitting summary – Emcompress® Anhydrous (modelling up to app. 60 % of carvedilol released)

Model: **Zero–order with T_{lag}**

Model equation: $F = k_0 \cdot (t - T_{lag})$

Fitted model parameters per tested tablet (N = 4) with statistics – mean, standard deviation (SD), and relative standard deviation expressed in % (RSD%) (output from DDSolver):

Parameter	No.1	No.2	No.3	No.4	Mean	SD	RSD(%)
k_0	0.066	0.082	0.068	0.073	0.072	0.007	10.258
T_{lag}	-169.355	-141.323	-165.660	-149.071	-156.352	13.349	-8.538

Number of dissolution data points (N), degrees of freedom (df), and selected goodness of fit criteria – Pearson correlation coefficient (R), coefficient of determination (R^2), adjusted coefficient of determination ($R^2_{adjusted}$), and residual sum of squares (RSS) (manual calculation in MS Excel):

Parameter	No.1	No.2	No.3	No.4
N	21	21	21	21
df	19	19	19	19
R	0.987380349	0.992631635	0.990333268	0.991660125
R^2	0.974919954	0.985317562	0.980759982	0.983389803
$R^2_{adjusted}$	0.973599951	0.984544802	0.97974735	0.982515583
RSS	108.1102811	98.20665498	87.57550384	87.07284242

Graphical abstract of model fit presented as mean ± 1 SD of the fraction % of released carvedilol:

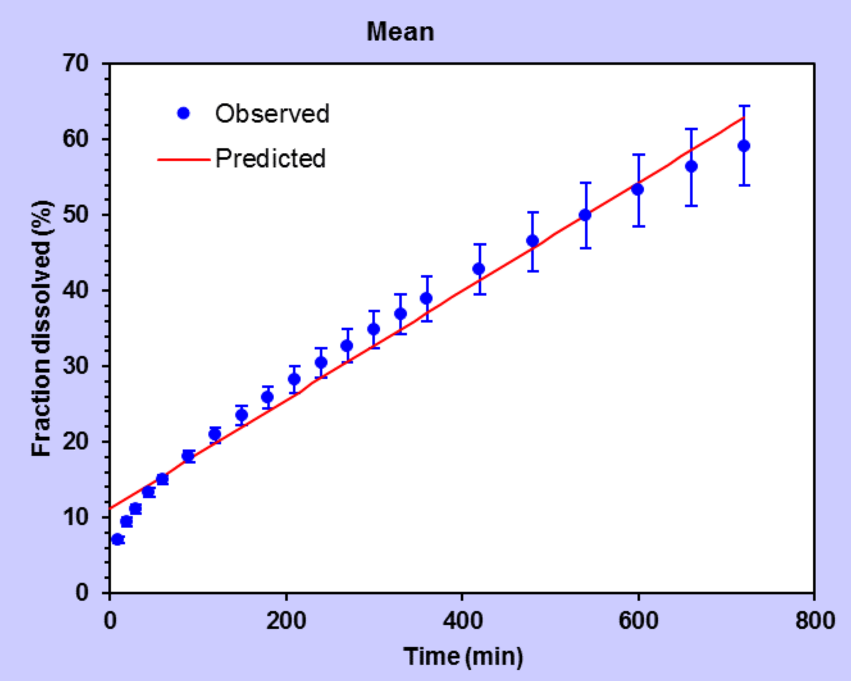

Supplementary materials – Model fitting summary – Emcompress® Anhydrous (modelling up to app. 60 % of carvedilol released)

Graphical abstract of model fit presented as the fraction % of released carvedilol per tested tablet:

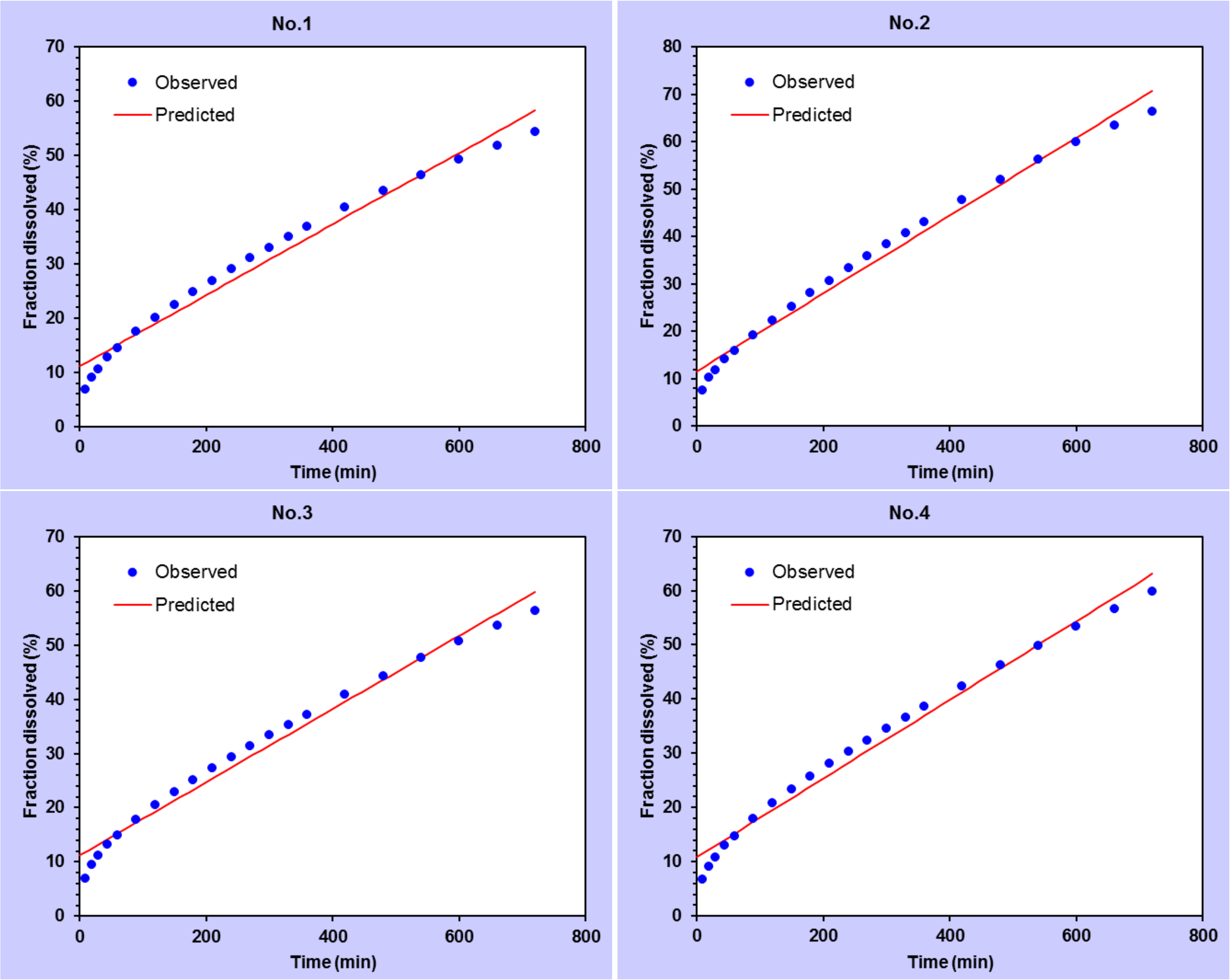

Supplementary materials – Model fitting summary – Emcompress® Anhydrous (modelling up to app. 60 % of carvedilol released)

Model: **Zero–order with F_0**

Model equation: $F = F_0 + k_0 \cdot t$

Fitted model parameters per tested tablet (N = 4) with statistics – mean, standard deviation (SD), and relative standard deviation expressed in % (RSD%) (output from DDSolver):

Parameter	No.1	No.2	No.3	No.4	Mean	SD	RSD(%)
k_0	0.066	0.082	0.068	0.073	0.072	0.007	10.258
F_0	11.098	11.598	11.189	10.819	11.176	0.322	2.882

Number of dissolution data points (N), degrees of freedom (df), and selected goodness of fit criteria – Pearson correlation coefficient (R), coefficient of determination (R^2), adjusted coefficient of determination ($R^2_{adjusted}$), and residual sum of squares (RSS) (manual calculation in MS Excel):

Parameter	No.1	No.2	No.3	No.4
N	21	21	21	21
df	19	19	19	19
R	0.987380349	0.992631635	0.990333268	0.991660125
R^2	0.974919954	0.985317562	0.980759982	0.983389803
$R^2_{adjusted}$	0.973599951	0.984544802	0.97974735	0.982515583
RSS	108.1102811	98.20665498	87.57550384	87.07284242

Graphical abstract of model fit presented as mean ± 1 SD of the fraction % of released carvedilol:

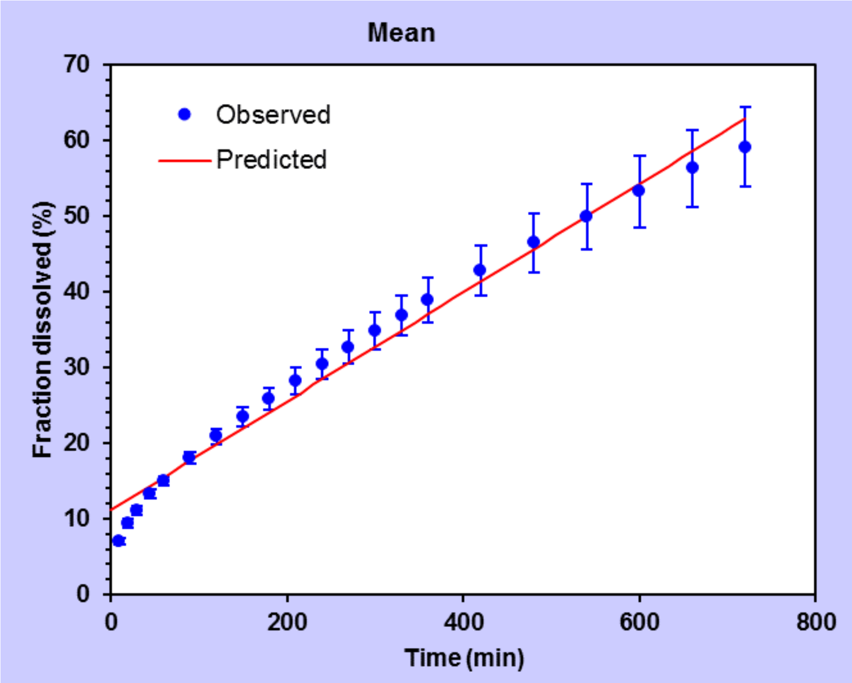

Supplementary materials – Model fitting summary – Emcompress® Anhydrous (modelling up to app. 60 % of carvedilol released)

Graphical abstract of model fit presented as the fraction % of released carvedilol per tested tablet:

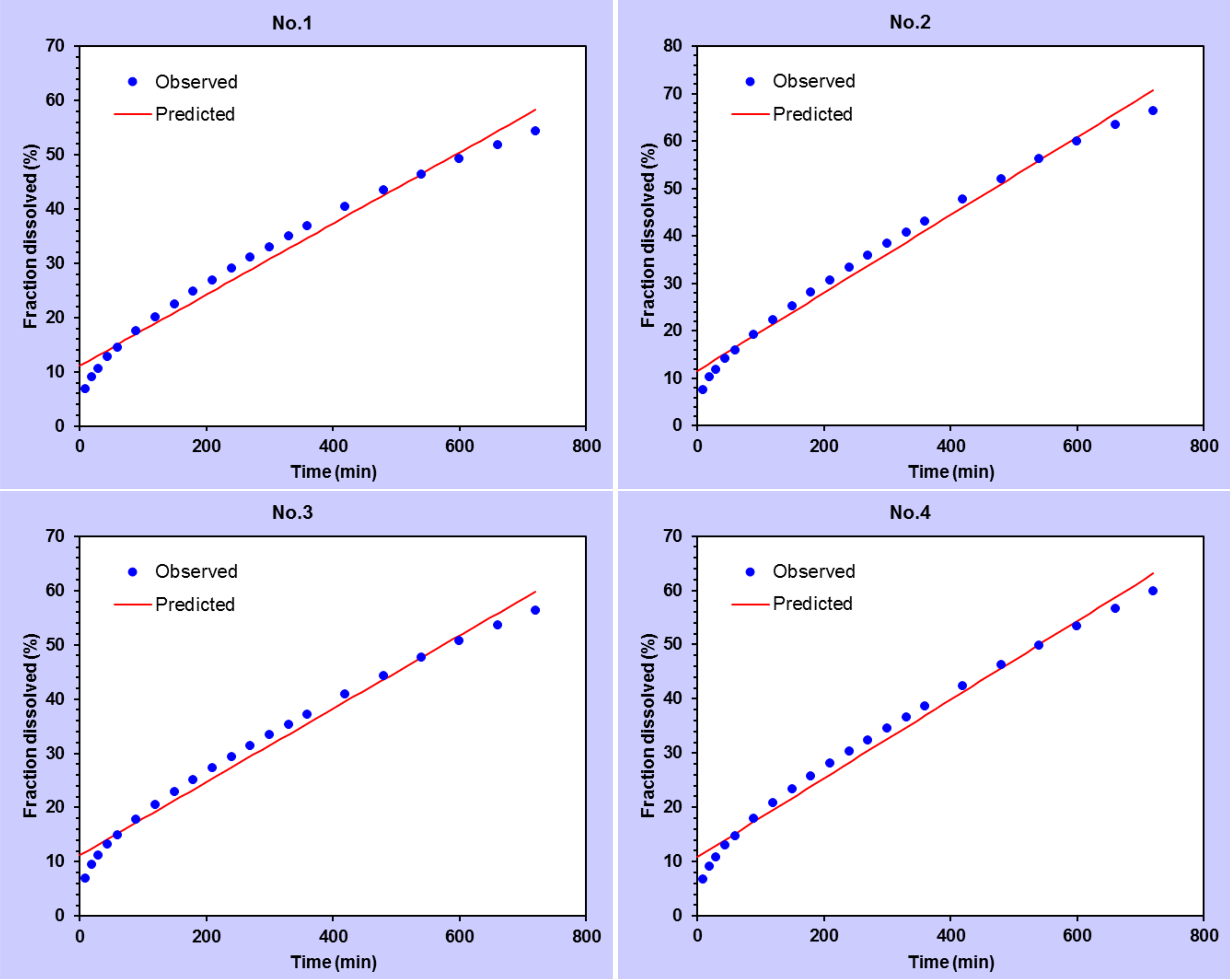

Supplementary materials – Model fitting summary – Emcompress® Anhydrous (modelling up to app. 60 % of carvedilol released)

Model: **First–order**

Model equation: $F = 100 \cdot (1 - e^{-k_1 \cdot t})$

Fitted model parameters per tested tablet (N = 4) with statistics – mean, standard deviation (SD), and relative standard deviation expressed in % (RSD%) (output from DDSolver):

Parameter	No.1	No.2	No.3	No.4	Mean	SD	RSD(%)
k_1	0.001	0.002	0.001	0.001	0.001	0.000	12.255

Number of dissolution data points (N), degrees of freedom (df), and selected goodness of fit criteria – Pearson correlation coefficient (R), coefficient of determination (R^2), adjusted coefficient of determination ($R^2_{adjusted}$), and residual sum of squares (RSS) (manual calculation in MS Excel):

Parameter	No.1	No.2	No.3	No.4
N	21	21	21	21
df	20	20	20	20
R	0.998409152	0.999055842	0.99879842	0.998949619
R^2	0.996820835	0.998112576	0.997598284	0.997900341
$R^2_{adjusted}$	0.996820835	0.998112576	0.997598284	0.997900341
RSS	493.1964798	359.9435486	482.2302296	395.7330085

Graphical abstract of model fit presented as mean ± 1 SD of the fraction % of released carvedilol:

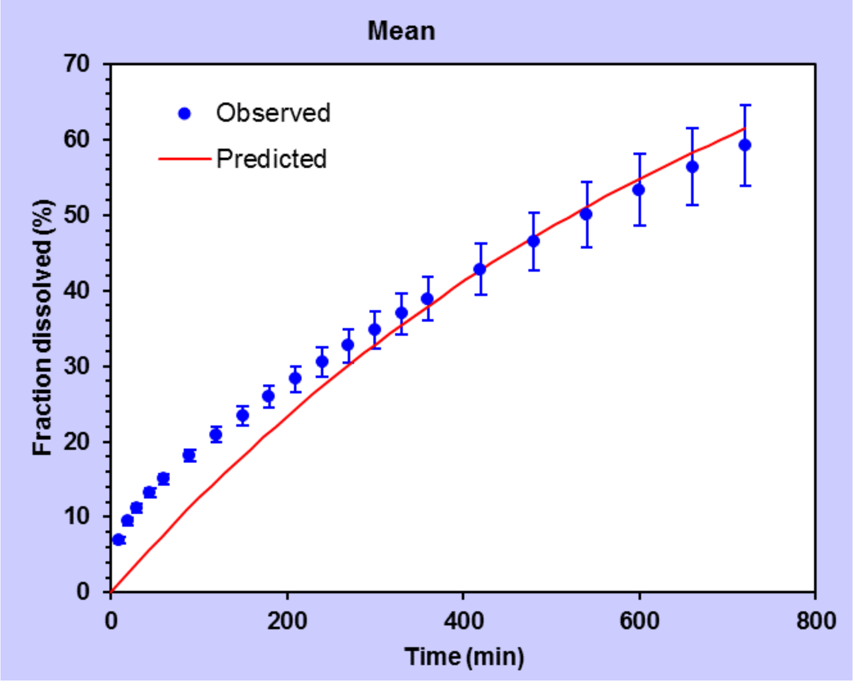

Supplementary materials – Model fitting summary – Emcompress® Anhydrous (modelling up to app. 60 % of carvedilol released)

Graphical abstract of model fit presented as the fraction % of released carvedilol per tested tablet:

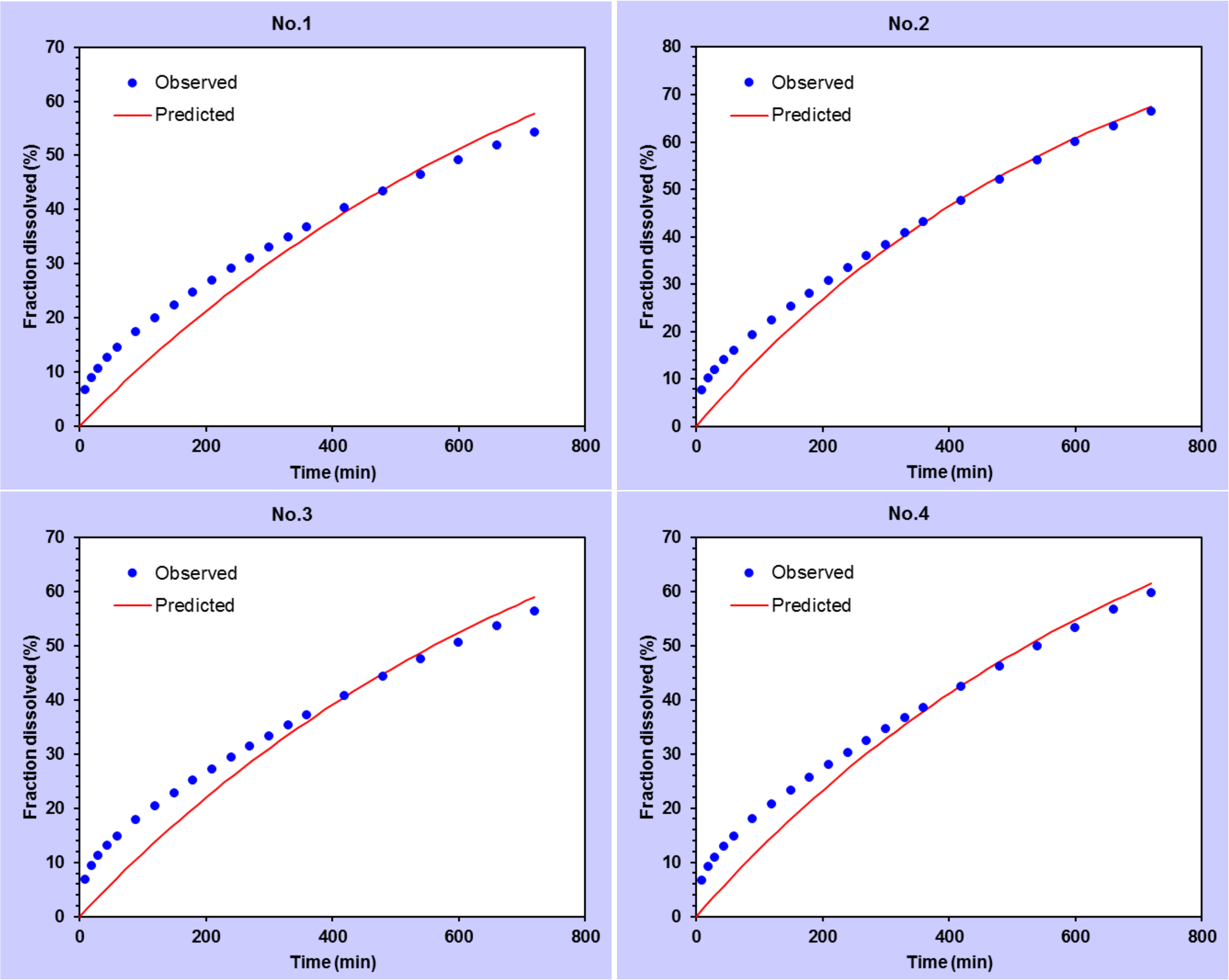

Supplementary materials – Model fitting summary – Emcompress® Anhydrous (modelling up to app. 60 % of carvedilol released)

Model: **First–order with T_{lag}**

Model equation: $F = 100 \cdot \left[1 - e^{-k_1 \cdot (t - T_{lag})}\right]$

Fitted model parameters per tested tablet (N = 4) with statistics – mean, standard deviation (SD), and relative standard deviation expressed in % (RSD%) (output from DDSolver):

Parameter	No.1	No.2	No.3	No.4	Mean	SD	RSD(%)
k_1	0.001	0.001	0.001	0.001	0.001	0.000	15.956
T_{lag}	-98.307	-56.784	-91.523	-74.112	-80.182	18.632	-23.237

Number of dissolution data points (N), degrees of freedom (df), and selected goodness of fit criteria – Pearson correlation coefficient (R), coefficient of determination (R^2), adjusted coefficient of determination ($R^2_{adjusted}$), and residual sum of squares (RSS) (manual calculation in MS Excel):

Parameter	No.1	No.2	No.3	No.4
N	21	21	21	21
df	19	19	19	19
R	0.997398338	0.999427546	0.998346641	0.99887358
R^2	0.994803445	0.998855419	0.996696016	0.997748428
$R^2_{adjusted}$	0.994529942	0.998795178	0.996522122	0.997629925
RSS	23.53040525	8.009822493	15.37077408	11.83464486

Graphical abstract of model fit presented as mean ± 1 SD of the fraction % of released carvedilol:

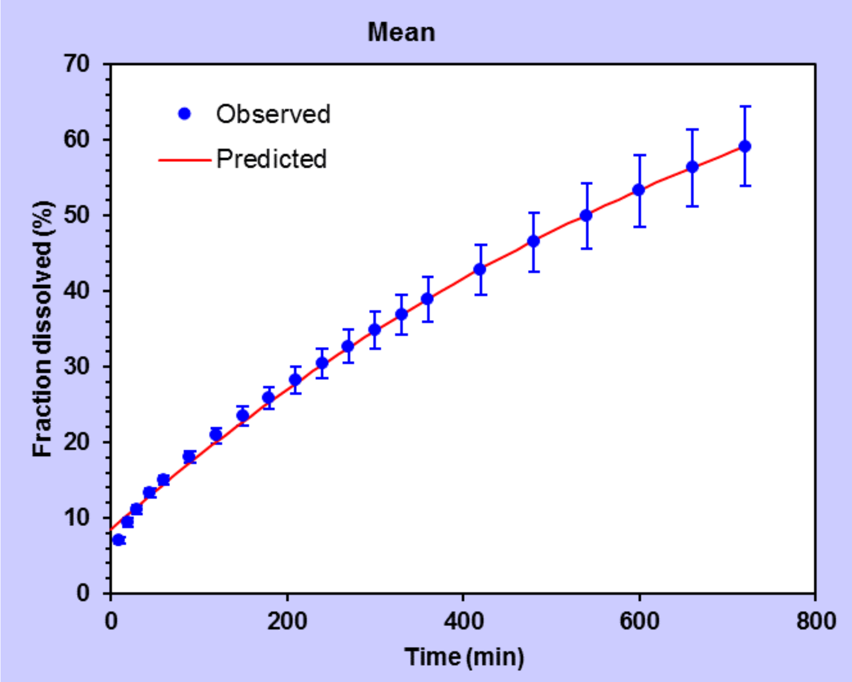

Supplementary materials – Model fitting summary – Emcompress® Anhydrous (modelling up to app. 60 % of carvedilol released)

Graphical abstract of model fit presented as the fraction % of released carvedilol per tested tablet:

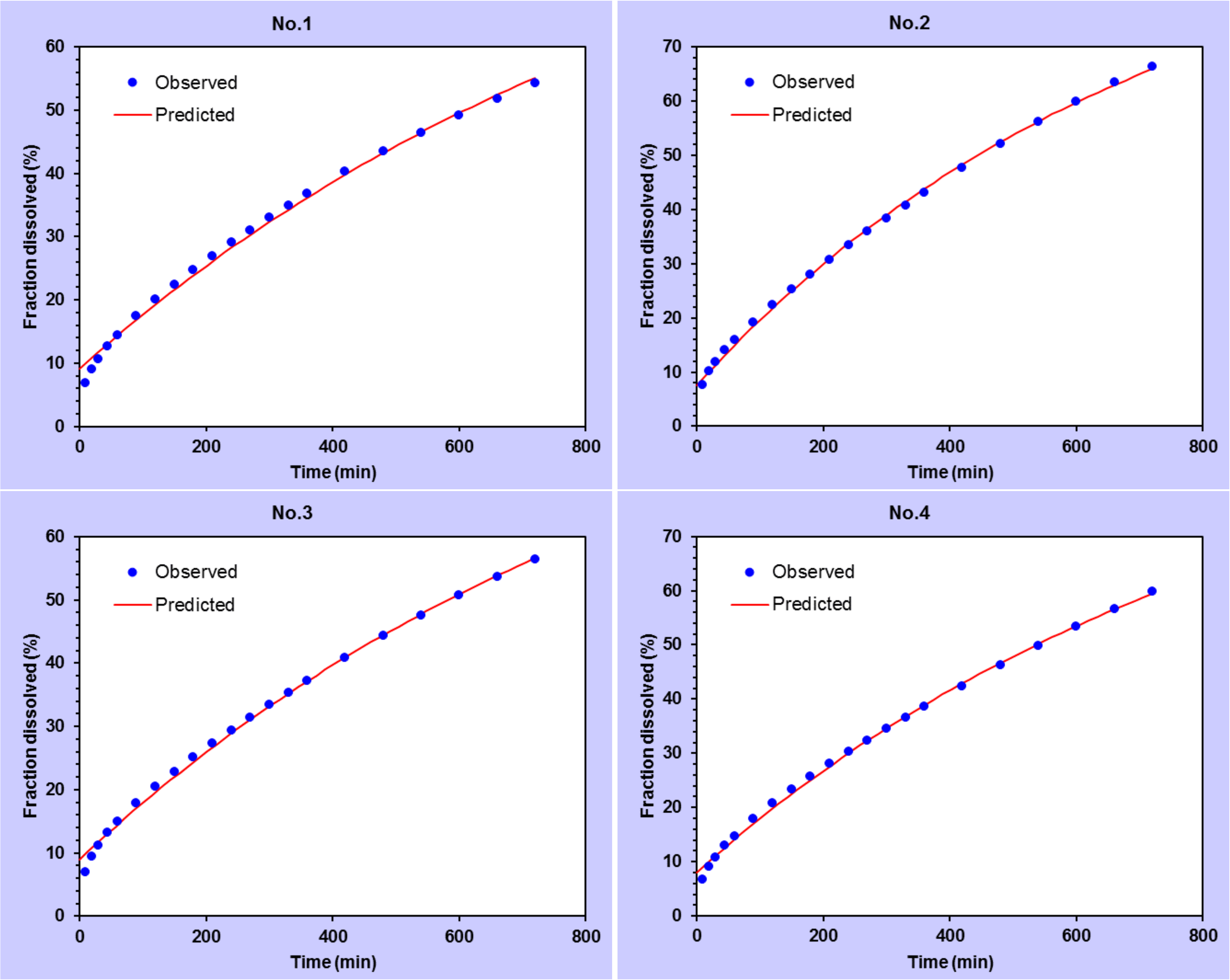

Supplementary materials – Model fitting summary – Emcompress® Anhydrous (modelling up to app. 60 % of carvedilol released)

Model: **First–order with F_{max}**

Model equation: $F = F_{max} \cdot (1 - e^{-k_1 \cdot t})$

Fitted model parameters per tested tablet (N = 4) with statistics – mean, standard deviation (SD), and relative standard deviation expressed in % (RSD%) (output from DDSolver):

Parameter	No.1	No.2	No.3	No.4	Mean	SD	RSD(%)
k_1	0.003	0.003	0.003	0.003	0.003	0.000	1.905
F_{max}	56.980	69.711	59.162	62.776	62.157	5.574	8.968

Number of dissolution data points (N), degrees of freedom (df), and selected goodness of fit criteria – Pearson correlation coefficient (R), coefficient of determination (R^2), adjusted coefficient of determination ($R^2_{adjusted}$), and residual sum of squares (RSS) (manual calculation in MS Excel):

Parameter	No.1	No.2	No.3	No.4
N	21	21	21	21
df	19	19	19	19
R	0.988292547	0.983554442	0.985444188	0.984940138
R^2	0.976722159	0.96737934	0.971100248	0.970107075
$R^2_{adjusted}$	0.975497009	0.965662464	0.969579208	0.968533763
RSS	204.0907621	346.4215383	252.9700432	268.9024031

Graphical abstract of model fit presented as mean ± 1 SD of the fraction % of released carvedilol:

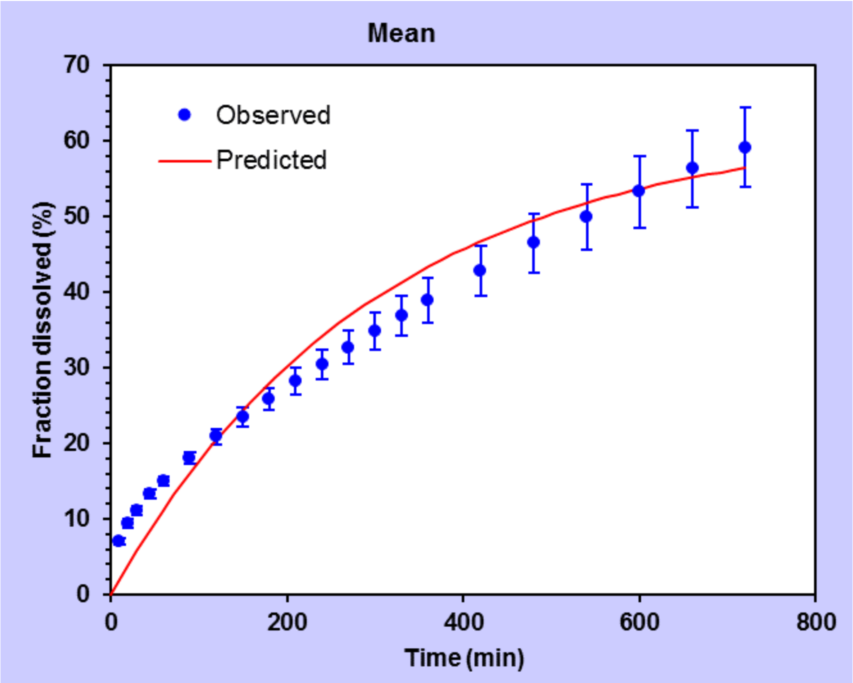

Supplementary materials – Model fitting summary – Emcompress® Anhydrous (modelling up to app. 60 % of carvedilol released)

Graphical abstract of model fit presented as the fraction % of released carvedilol per tested tablet:

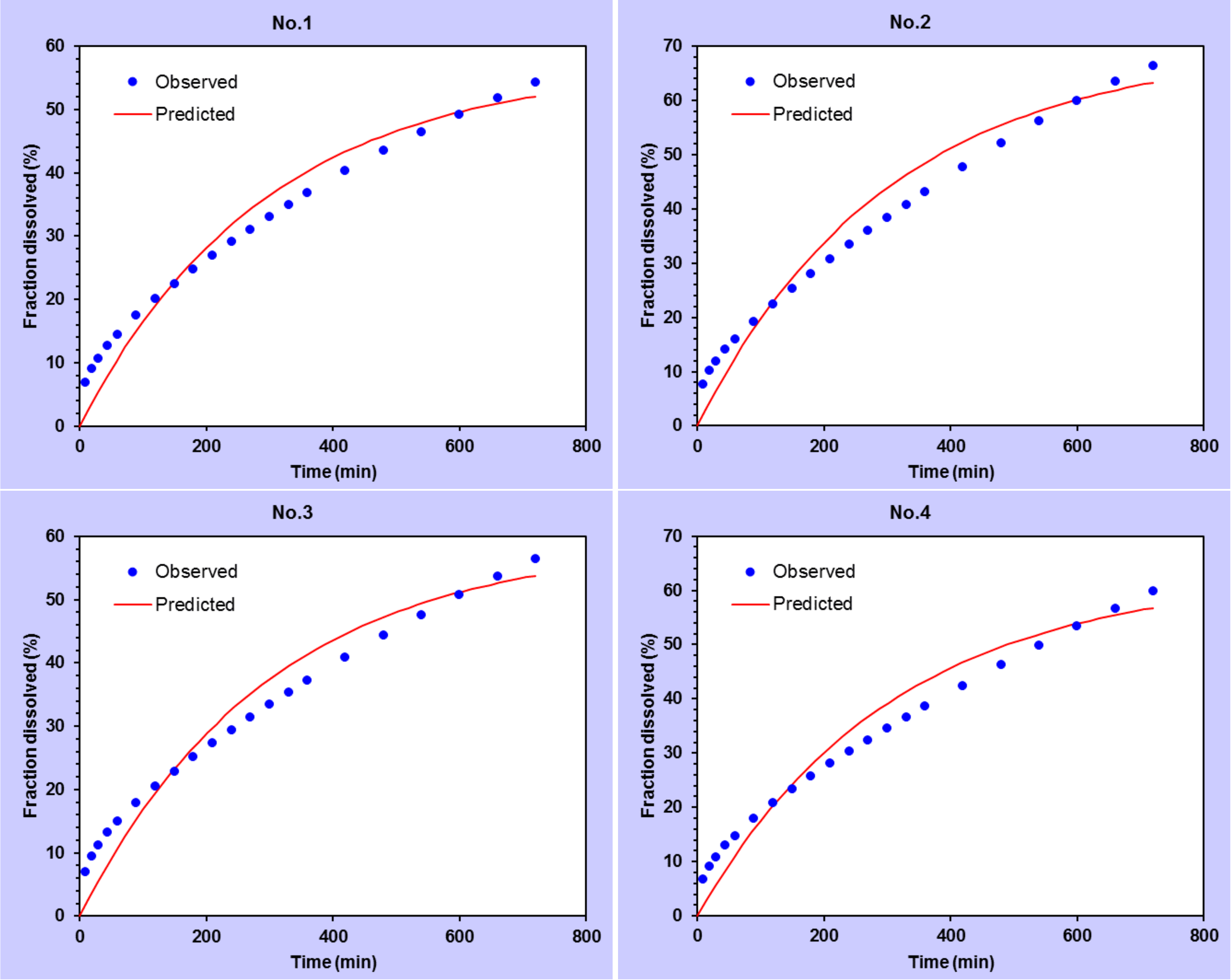

Supplementary materials – Model fitting summary – Emcompress® Anhydrous (modelling up to app. 60 % of carvedilol released)

Model: **First–order with T_{lag} and F_{max}**

Model equation: $F = F_{max} \cdot \left[1 - e^{-k_1 \cdot (t - T_{lag})}\right]$

Fitted model parameters per tested tablet (N = 4) with statistics – mean, standard deviation (SD), and relative standard deviation expressed in % (RSD%) (output from DDSolver):

Parameter	No.1	No.2	No.3	No.4	Mean	SD	RSD(%)
k_1	0.003	0.003	0.003	0.003	0.003	0.000	1.339
T_{lag}	6.304	19.051	8.619	14.645	12.155	5.787	47.614
F_{max}	56.980	69.711	59.162	62.776	62.157	5.574	8.968

Number of dissolution data points (N), degrees of freedom (df), and selected goodness of fit criteria – Pearson correlation coefficient (R), coefficient of determination (R^2), adjusted coefficient of determination ($R^2_{adjusted}$), and residual sum of squares (RSS) (manual calculation in MS Excel):

Parameter	No.1	No.2	No.3	No.4
N	21	21	21	21
df	18	18	18	18
R	0.987738953	0.981471656	0.984615625	0.983496741
R^2	0.97562824	0.963286612	0.96946793	0.96726584
$R^2_{adjusted}$	0.972920266	0.959207347	0.966075478	0.963628712
RSS	257.6065132	586.2344489	334.7904957	415.6363645

Graphical abstract of model fit presented as mean ± 1 SD of the fraction % of released carvedilol:

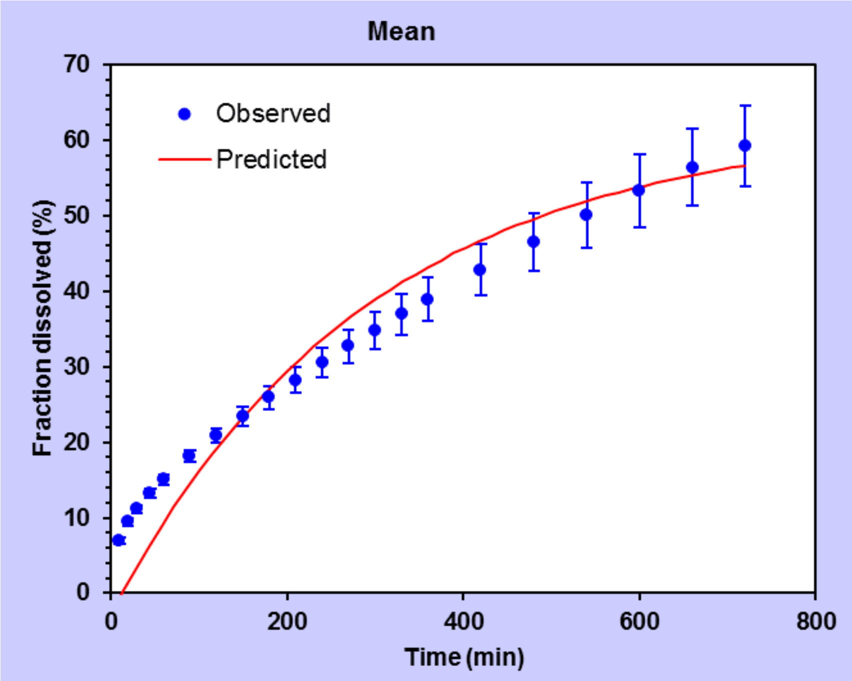

Supplementary materials – Model fitting summary – Emcompress® Anhydrous (modelling up to app. 60 % of carvedilol released)

Graphical abstract of model fit presented as the fraction % of released carvedilol per tested tablet:

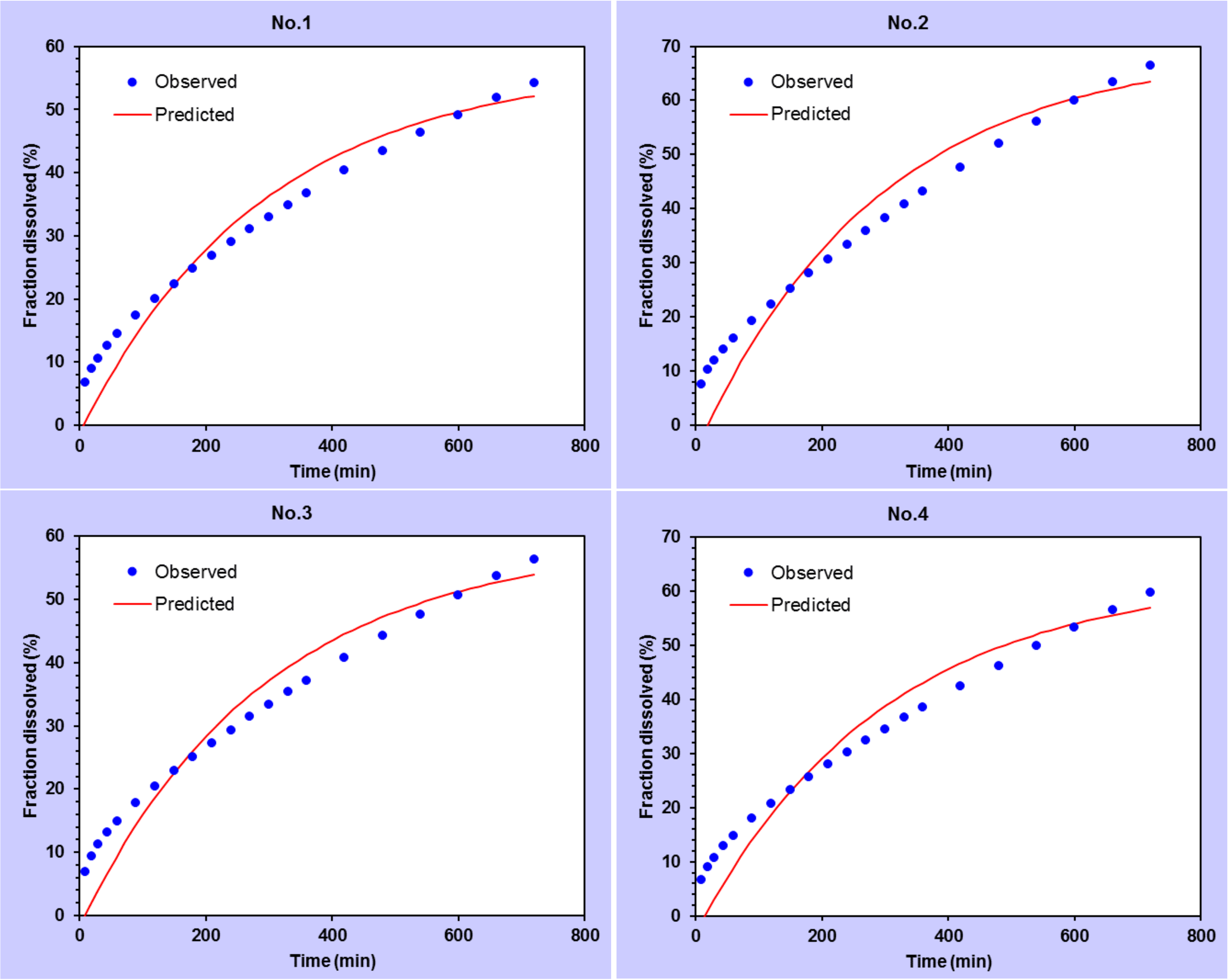

Supplementary materials – Model fitting summary – Emcompress® Anhydrous (modelling up to app. 60 % of carvedilol released)

Model: **Higuchi**

Model equation: $F = k_H \cdot t^{0.5}$

Fitted model parameters per tested tablet (N = 4) with statistics – mean, standard deviation (SD), and relative standard deviation expressed in % (RSD%) (output from DDSolver):

Parameter	No.1	No.2	No.3	No.4	Mean	SD	RSD(%)
k_H	1.956	2.321	2.001	2.084	2.090	0.162	7.770

Number of dissolution data points (N), degrees of freedom (df), and selected goodness of fit criteria – Pearson correlation coefficient (R), coefficient of determination (R^2), adjusted coefficient of determination ($R^2_{adjusted}$), and residual sum of squares (RSS) (manual calculation in MS Excel):

Parameter	No.1	No.2	No.3	No.4
N	21	21	21	21
df	20	20	20	20
R	0.998050146	0.994940933	0.996728927	0.995972521
R^2	0.996104095	0.989907459	0.993468554	0.991961262
$R^2_{adjusted}$	0.996104095	0.989907459	0.993468554	0.991961262
RSS	23.46357585	117.4710031	38.03638008	70.1599552

Graphical abstract of model fit presented as mean ± 1 SD of the fraction % of released carvedilol:

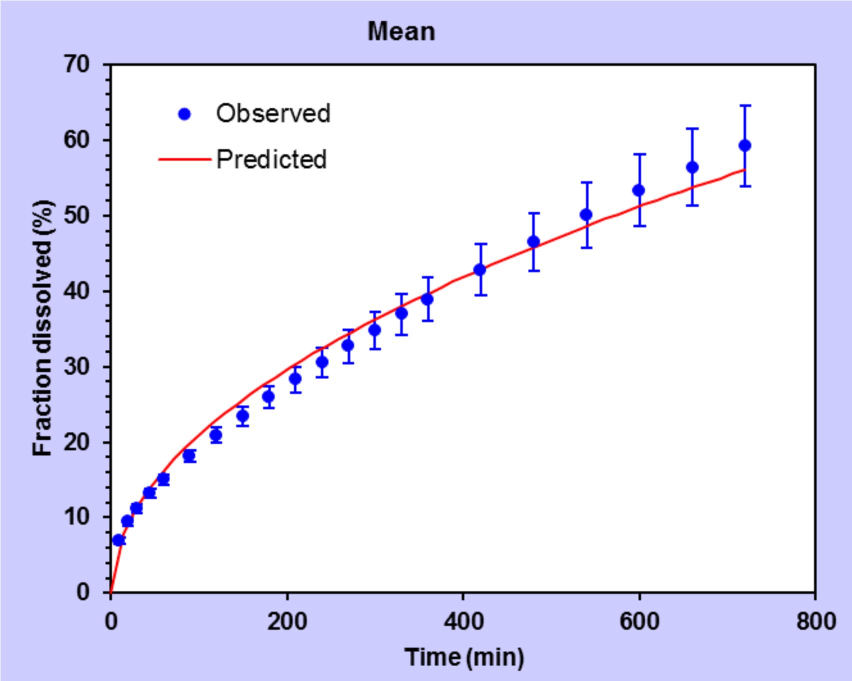

Supplementary materials – Model fitting summary – Emcompress® Anhydrous (modelling up to app. 60 % of carvedilol released)

Graphical abstract of model fit presented as the fraction % of released carvedilol per tested tablet:

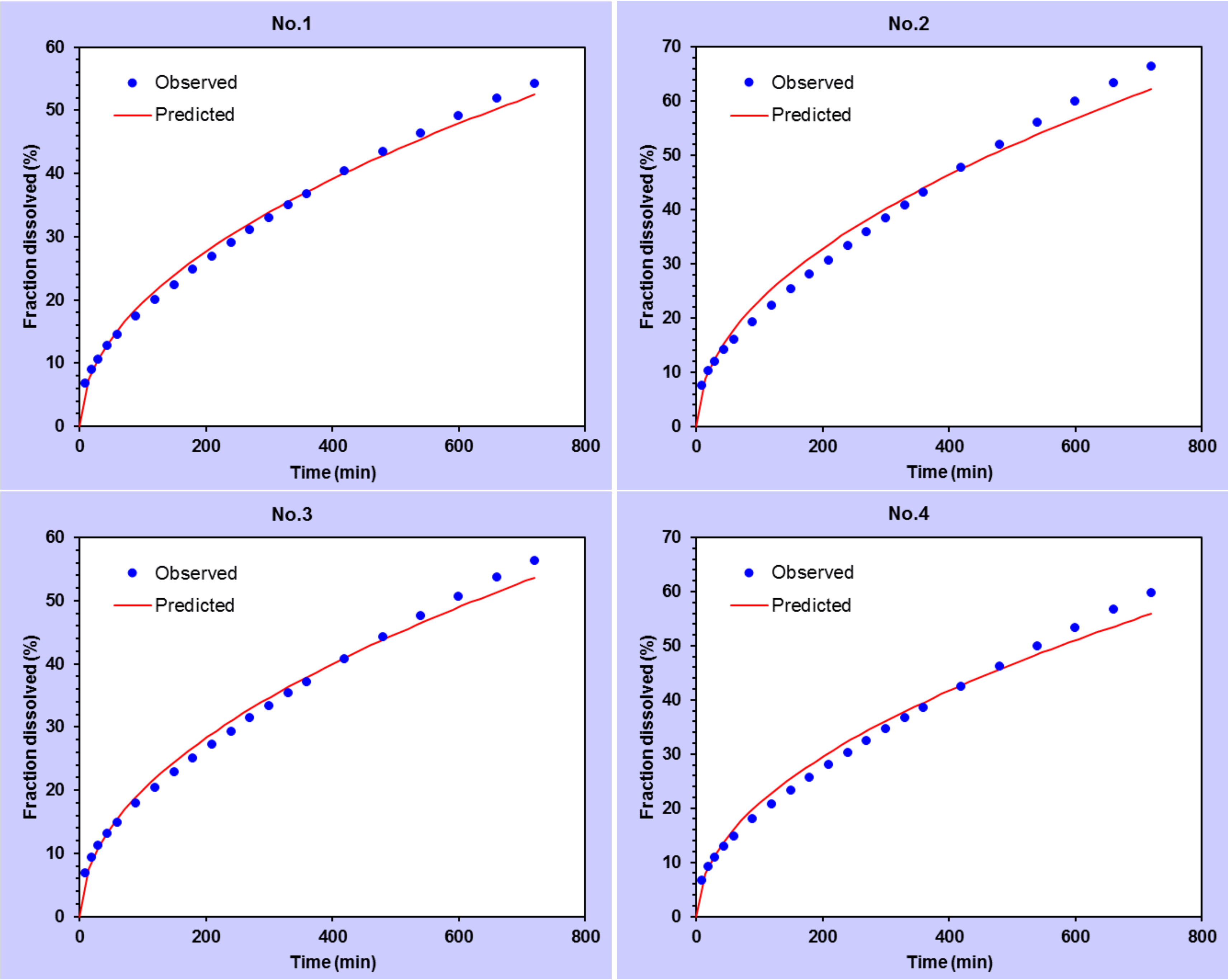

Supplementary materials – Model fitting summary – Emcompress® Anhydrous (modelling up to app. 60 % of carvedilol released)

Model: **Higuchi with T_{lag}**

Model equation: $F = k_H \cdot (t - T_{lag})^{0.5}$

Fitted model parameters per tested tablet (N = 4) with statistics – mean, standard deviation (SD), and relative standard deviation expressed in % (RSD%) (output from DDSolver):

Parameter	No.1	No.2	No.3	No.4	Mean	SD	RSD(%)
k_H	2.023	2.478	2.087	2.208	2.199	0.201	9.143
T_{lag}	17.938	33.263	22.070	29.733	25.751	6.997	27.173

Number of dissolution data points (N), degrees of freedom (df), and selected goodness of fit criteria – Pearson correlation coefficient (R), coefficient of determination (R^2), adjusted coefficient of determination ($R^2_{adjusted}$), and residual sum of squares (RSS) (manual calculation in MS Excel):

Parameter	No.1	No.2	No.3	No.4
N	21	21	21	21
df	19	19	19	19
R	0.993050663	0.984830691	0.989050738	0.986853763
R^2	0.986149619	0.969891489	0.978221361	0.97388035
$R^2_{adjusted}$	0.985420652	0.968306831	0.977075117	0.972505631
RSS	110.9593582	383.6546831	195.462621	274.2287242

Graphical abstract of model fit presented as mean ± 1 SD of the fraction % of released carvedilol:

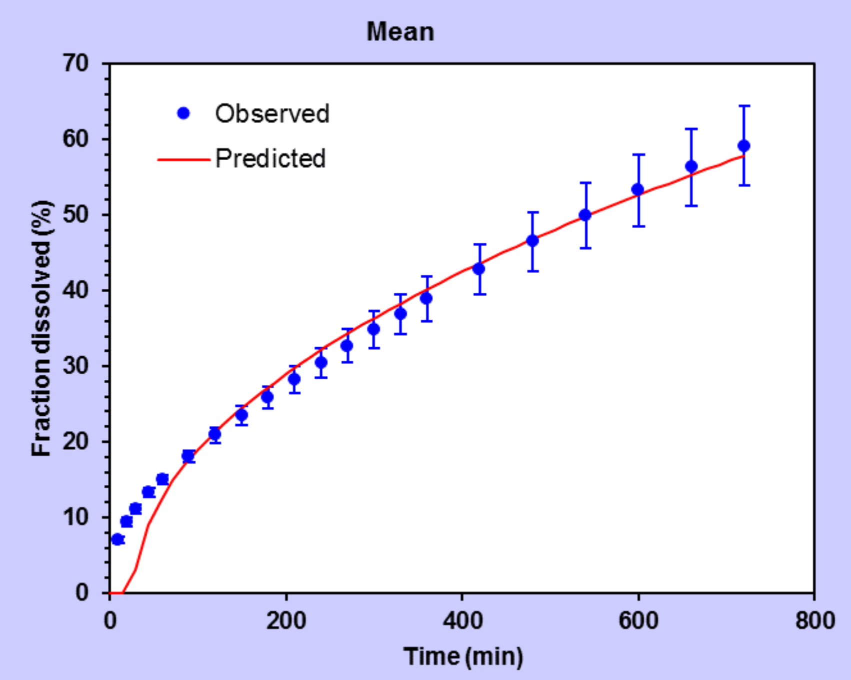

Supplementary materials – Model fitting summary – Emcompress® Anhydrous (modelling up to app. 60 % of carvedilol released)

Graphical abstract of model fit presented as the fraction % of released carvedilol per tested tablet:

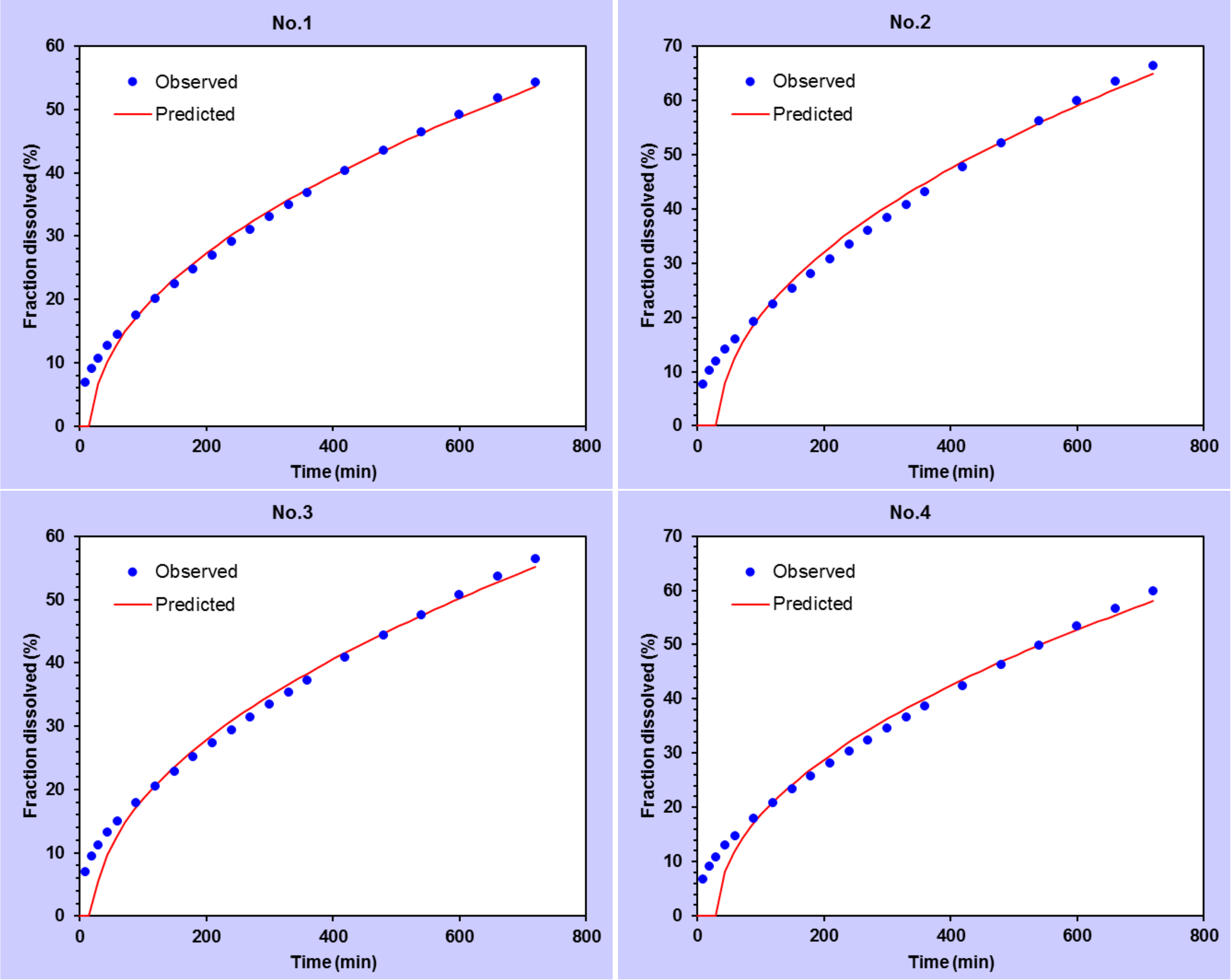

Supplementary materials – Model fitting summary – Emcompress® Anhydrous (modelling up to app. 60 % of carvedilol released)

Model: **Higuchi with F_0**

Model equation: $F = F_0 + k_H \cdot t^{0.5}$

Fitted model parameters per tested tablet (N = 4) with statistics – mean, standard deviation (SD), and relative standard deviation expressed in % (RSD%) (output from DDSolver):

Parameter	No.1	No.2	No.3	No.4	Mean	SD	RSD(%)
k_H	2.028	2.519	2.082	2.232	2.215	0.220	9.932
F_0	-1.333	-3.647	-1.487	-2.731	-2.300	1.095	-47.622

Number of dissolution data points (N), degrees of freedom (df), and selected goodness of fit criteria – Pearson correlation coefficient (R), coefficient of determination (R^2), adjusted coefficient of determination ($R^2_{adjusted}$), and residual sum of squares (RSS) (manual calculation in MS Excel):

Parameter	No.1	No.2	No.3	No.4
N	21	21	21	21
df	19	19	19	19
R	0.998050146	0.994940933	0.996728927	0.995972521
R^2	0.996104095	0.989907459	0.993468554	0.991961262
$R^2_{adjusted}$	0.995899047	0.989376273	0.993124794	0.99153817
RSS	16.79372521	67.50613621	29.72942337	42.14012748

Graphical abstract of model fit presented as mean ± 1 SD of the fraction % of released carvedilol:

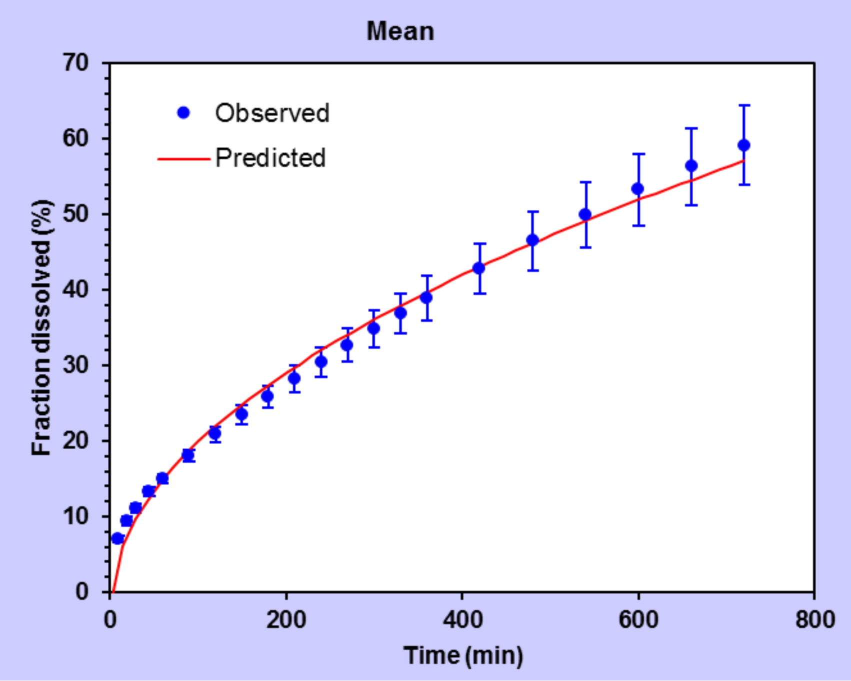

Supplementary materials – Model fitting summary – Emcompress® Anhydrous (modelling up to app. 60 % of carvedilol released)

Graphical abstract of model fit presented as the fraction % of released carvedilol per tested tablet:

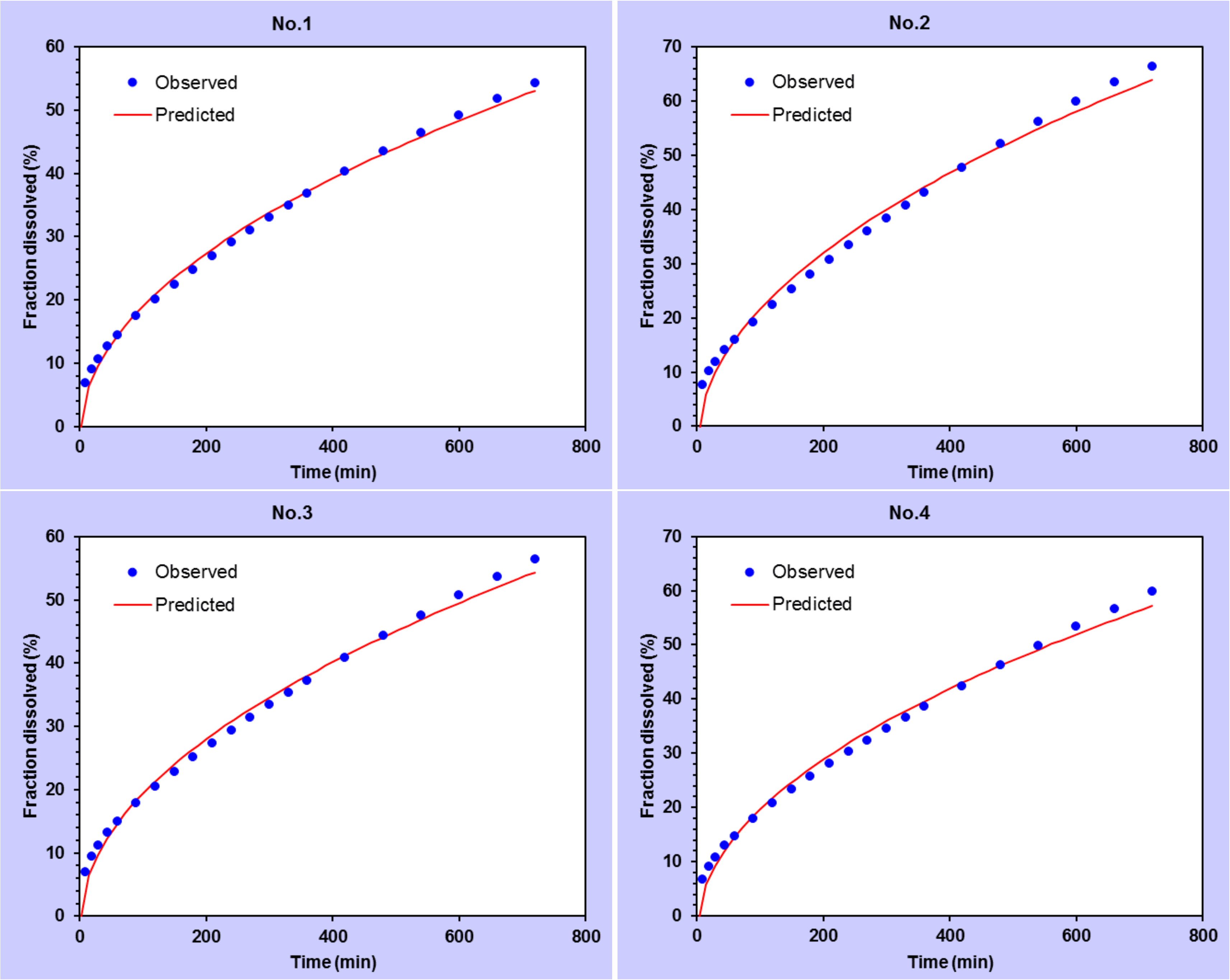

Supplementary materials – Model fitting summary – Emcompress® Anhydrous (modelling up to app. 60 % of carvedilol released)

Model: **Korsmeyer–Peppas**

Model equation: $F = k_{KP} \cdot t^n$

Fitted model parameters per tested tablet (N = 4) with statistics – mean, standard deviation (SD), and relative standard deviation expressed in % (RSD%) (output from DDSolver):

Parameter	No.1	No.2	No.3	No.4	Mean	SD	RSD(%)
k_{KP}	1.949	1.997	2.030	1.838	1.954	0.084	4.303
n	0.498	0.521	0.495	0.518	0.508	0.014	2.676

Number of dissolution data points (N), degrees of freedom (df), and selected goodness of fit criteria – Pearson correlation coefficient (R), coefficient of determination (R^2), adjusted coefficient of determination ($R^2_{adjusted}$), and residual sum of squares (RSS) (manual calculation in MS Excel):

Parameter	No.1	No.2	No.3	No.4
N	21	21	21	21
df	19	19	19	19
R	0.997995201	0.995940453	0.996516814	0.996722792
R^2	0.995994421	0.991897385	0.993045761	0.993456325
$R^2_{adjusted}$	0.995783601	0.991470932	0.992679748	0.993111921
RSS	29.27728796	94.21130565	49.46142619	55.79767427

Graphical abstract of model fit presented as mean ± 1 SD of the fraction % of released carvedilol:

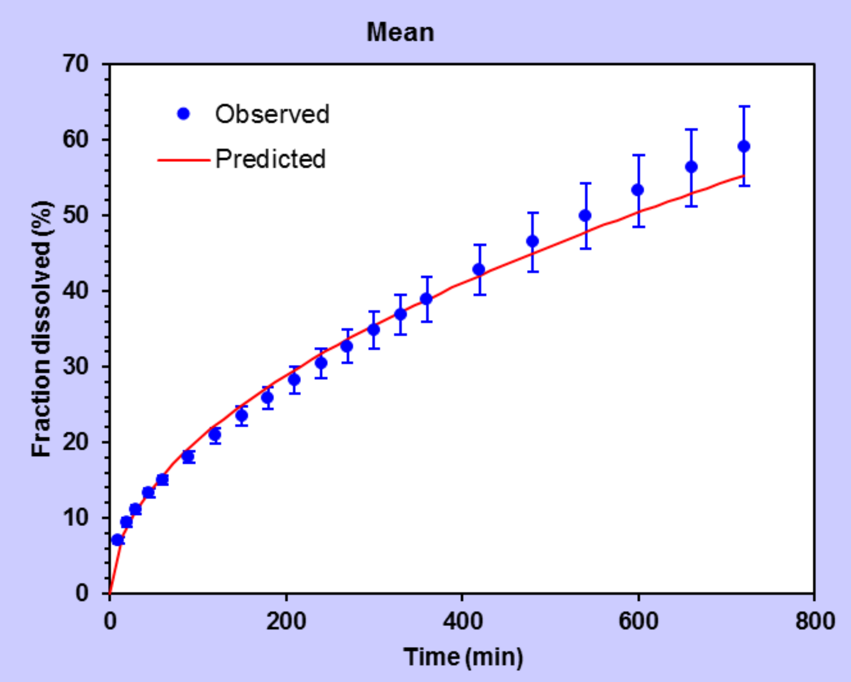

Supplementary materials – Model fitting summary – Emcompress® Anhydrous (modelling up to app. 60 % of carvedilol released)

Graphical abstract of model fit presented as the fraction % of released carvedilol per tested tablet:

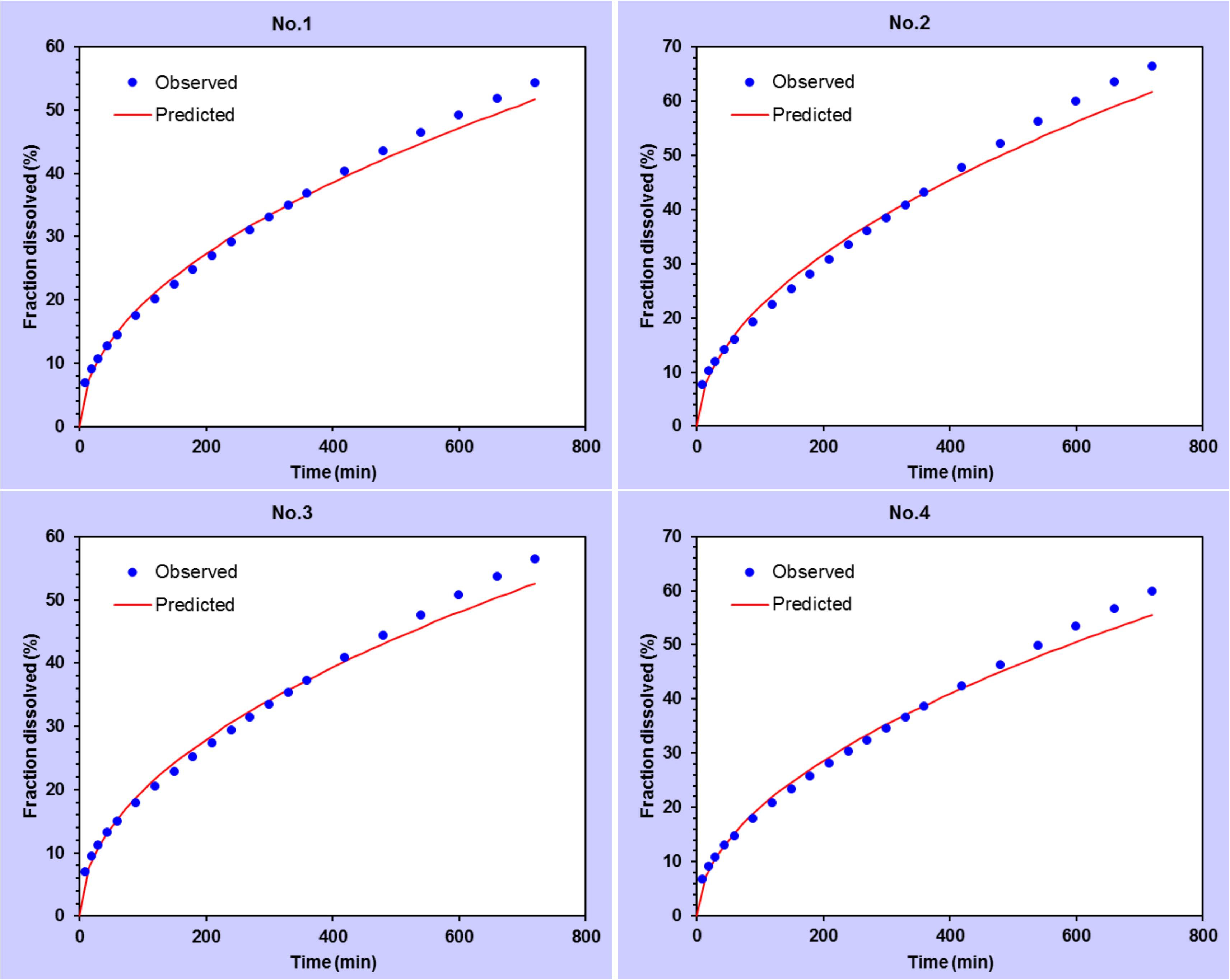

Supplementary materials – Model fitting summary – Emcompress® Anhydrous (modelling up to app. 60 % of carvedilol released)

Model: **Korsmeyer–Peppas with T_{lag}**

Model equation: $F = k_{KP} \cdot (t - T_{lag})^n$

Fitted model parameters per tested tablet (N = 4) with statistics – mean, standard deviation (SD), and relative standard deviation expressed in % (RSD%) (output from DDSolver):

Parameter	No.1	No.2	No.3	No.4	Mean	SD	RSD(%)
k_{KP}	2.449	2.544	2.547	2.332	2.468	0.102	4.119
n	0.459	0.480	0.456	0.478	0.468	0.012	2.636
T_{lag}	4.000	4.000	4.000	4.000	4.000	0.000	0.000

Number of dissolution data points (N), degrees of freedom (df), and selected goodness of fit criteria – Pearson correlation coefficient (R), coefficient of determination (R^2), adjusted coefficient of determination ($R^2_{adjusted}$), and residual sum of squares (RSS) (manual calculation in MS Excel):

Parameter	No.1	No.2	No.3	No.4
N	21	21	21	21
df	18	18	18	18
R	0.995483971	0.992604939	0.993492897	0.993767539
R^2	0.990988336	0.985264565	0.987028135	0.987573921
$R^2_{adjusted}$	0.98998704	0.983627295	0.985586817	0.986193246
RSS	69.704093	179.0374465	98.52603889	113.4532892

Graphical abstract of model fit presented as mean ± 1 SD of the fraction % of released carvedilol:

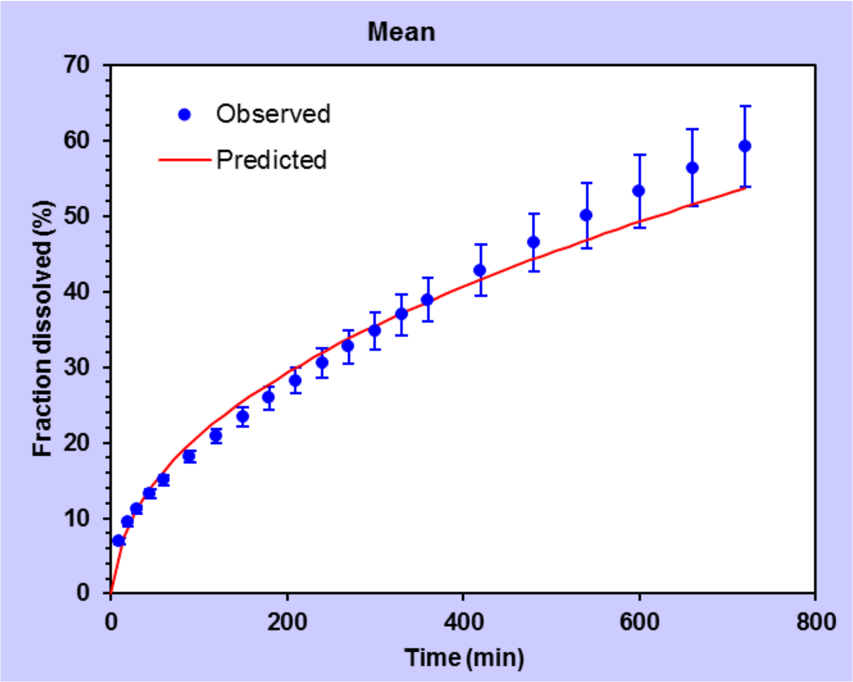

Supplementary materials – Model fitting summary – Emcompress® Anhydrous (modelling up to app. 60 % of carvedilol released)

Graphical abstract of model fit presented as the fraction % of released carvedilol per tested tablet:

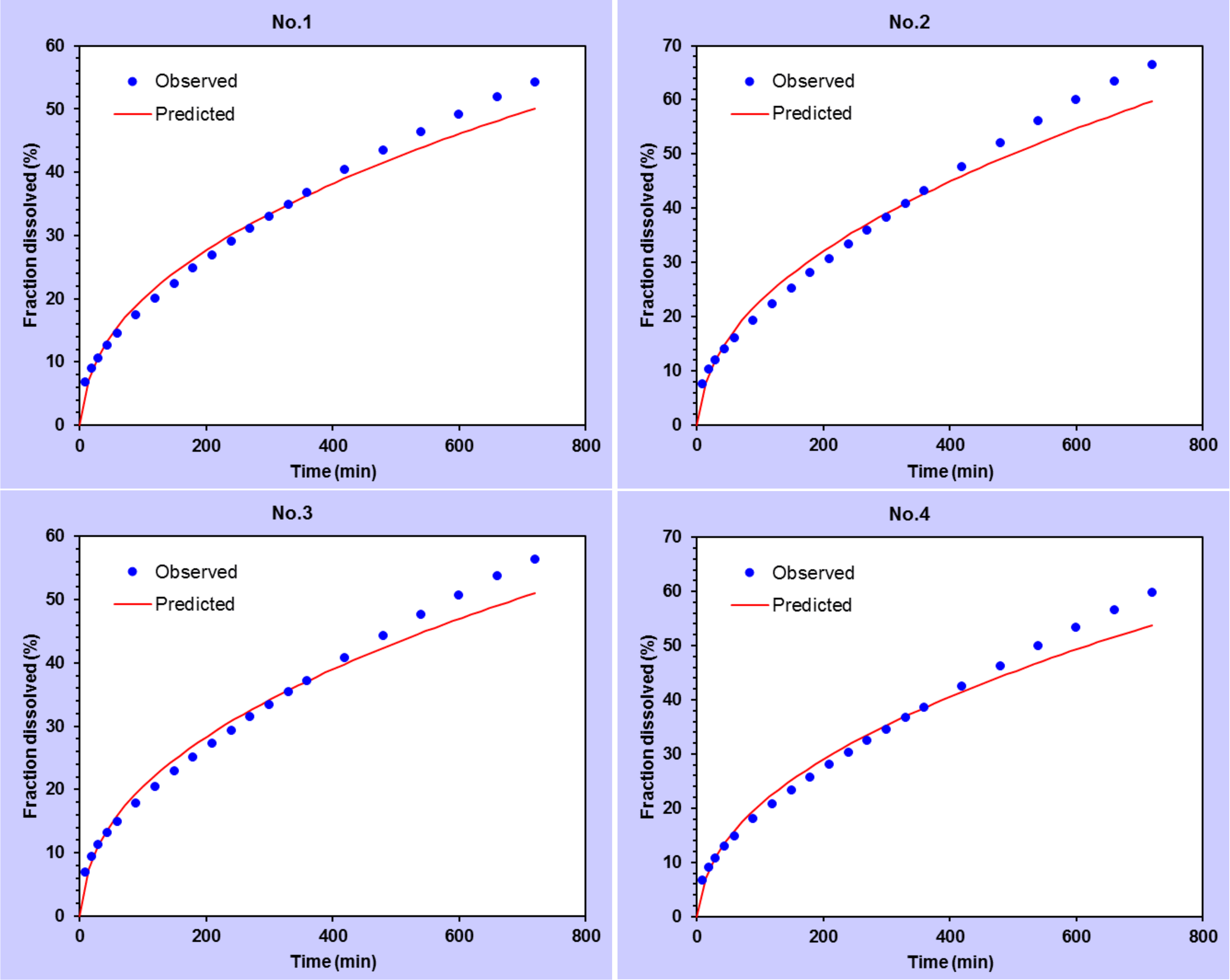

Supplementary materials – Model fitting summary – Emcompress® Anhydrous (modelling up to app. 60 % of carvedilol released)

Model: **Korsmeyer–Peppas with F_0**

Model equation: $F = F_0 + k_{KP} \cdot t^n$

Fitted model parameters per tested tablet (N = 4) with statistics – mean, standard deviation (SD), and relative standard deviation expressed in % (RSD%) (output from DDSolver):

Parameter	No.1	No.2	No.3	No.4	Mean	SD	RSD(%)
k_{KP}	1.081	1.062	1.096	0.990	1.057	0.047	4.437
n	0.589	0.616	0.587	0.611	0.601	0.015	2.518
F_0	2.293	3.039	2.759	2.679	2.693	0.308	11.430

Number of dissolution data points (N), degrees of freedom (df), and selected goodness of fit criteria – Pearson correlation coefficient (R), coefficient of determination (R^2), adjusted coefficient of determination ($R^2_{adjusted}$), and residual sum of squares (RSS) (manual calculation in MS Excel):

Parameter	No.1	No.2	No.3	No.4
N	21	21	21	21
df	18	18	18	18
R	0.999763235	0.998950266	0.999170568	0.999286332
R^2	0.999526526	0.997901635	0.998341823	0.998573173
$R^2_{adjusted}$	0.999473917	0.997668483	0.998157581	0.998414637
RSS	2.789091858	26.02239005	10.89216371	11.22725493

Graphical abstract of model fit presented as mean ± 1 SD of the fraction % of released carvedilol:

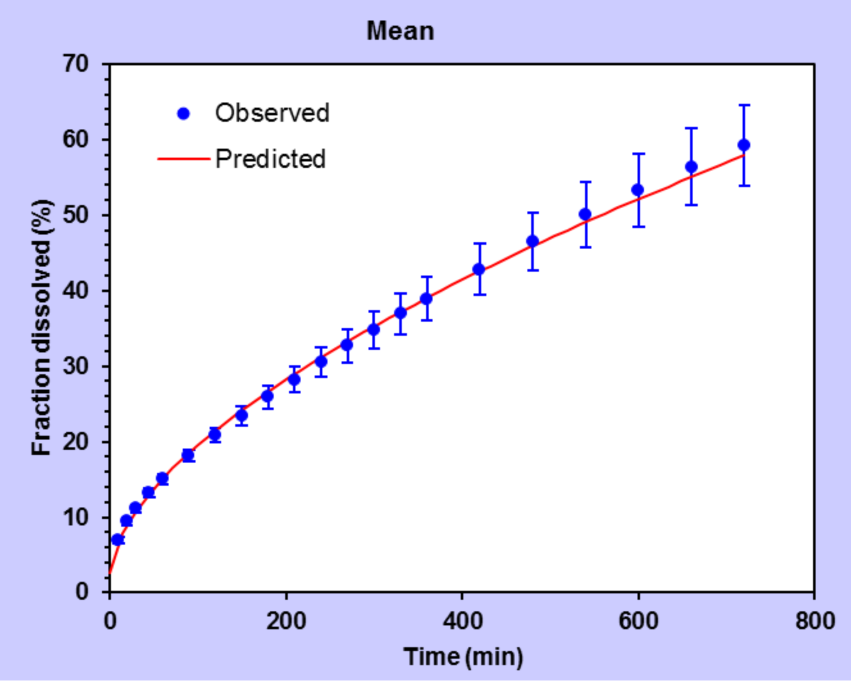

Supplementary materials – Model fitting summary – Emcompress® Anhydrous (modelling up to app. 60 % of carvedilol released)

Graphical abstract of model fit presented as the fraction % of released carvedilol per tested tablet:

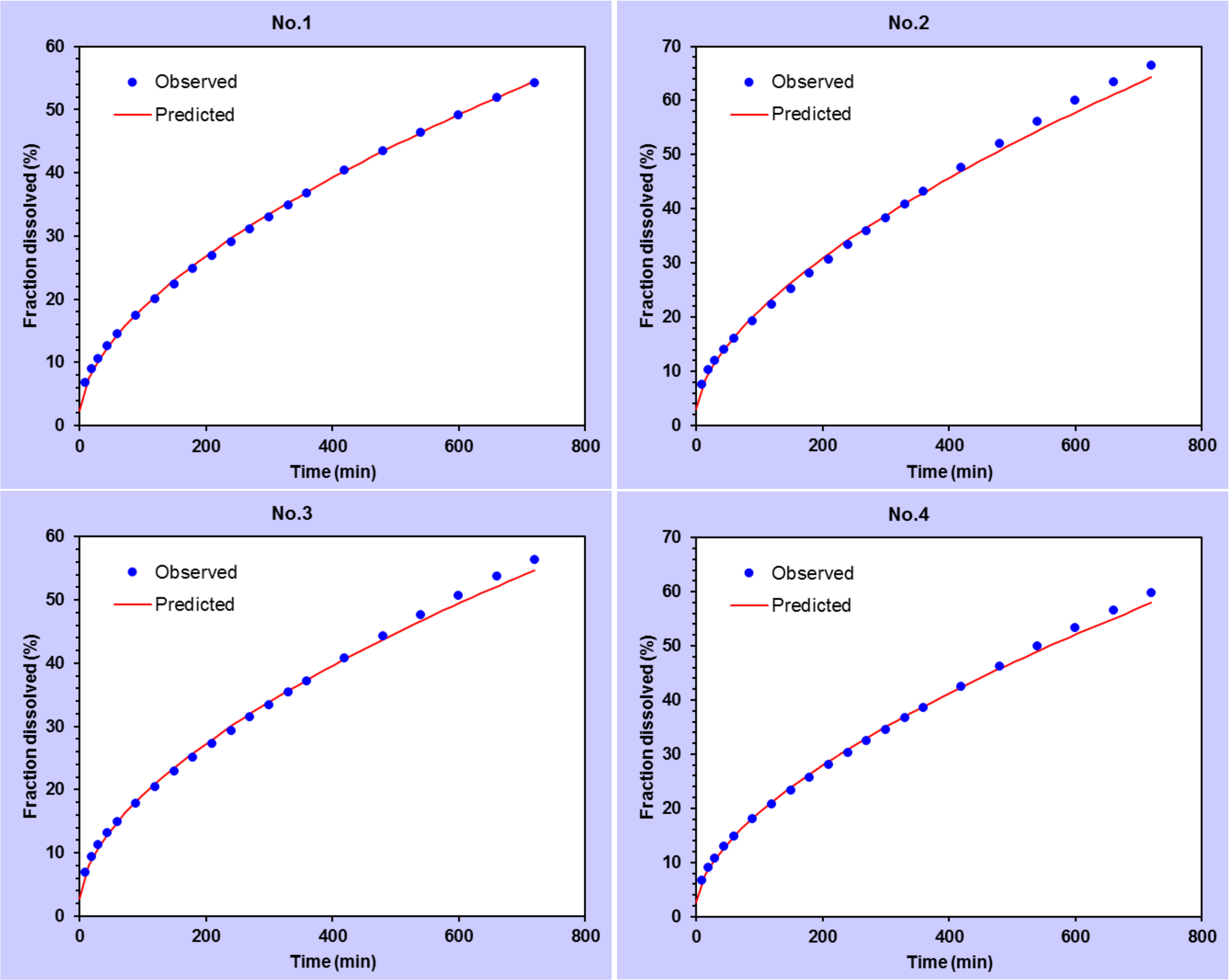

Supplementary materials – Model fitting summary – Emcompress® Anhydrous (modelling up to app. 60 % of carvedilol released)

Model: **Hixson–Crowell**

Model equation: $F = 100 \cdot [1 - (1 - k_{HC} \cdot t)^3]$

Fitted model parameters per tested tablet (N = 4) with statistics – mean, standard deviation (SD), and relative standard deviation expressed in % (RSD%) (output from DDSolver):

Parameter	No.1	No.2	No.3	No.4	Mean	SD	RSD(%)
k_{HC}	0.0004	0.0005	0.0004	0.0004	0.0004	0.0000	10.6859

Number of dissolution data points (N), degrees of freedom (df), and selected goodness of fit criteria – Pearson correlation coefficient (R), coefficient of determination (R^2), adjusted coefficient of determination ($R^2_{adjusted}$), and residual sum of squares (RSS) (manual calculation in MS Excel):

Parameter	No.1	No.2	No.3	No.4
N	21	21	21	21
df	20	20	20	20
R	0.996349275	0.999387604	0.997607164	0.998302409
R^2	0.992711878	0.998775584	0.995220053	0.996607701
$R^2_{adjusted}$	0.992711878	0.998775584	0.995220053	0.996607701
RSS	643.3822754	533.5594434	631.5851845	543.7481361

Graphical abstract of model fit presented as mean ± 1 SD of the fraction % of released carvedilol:

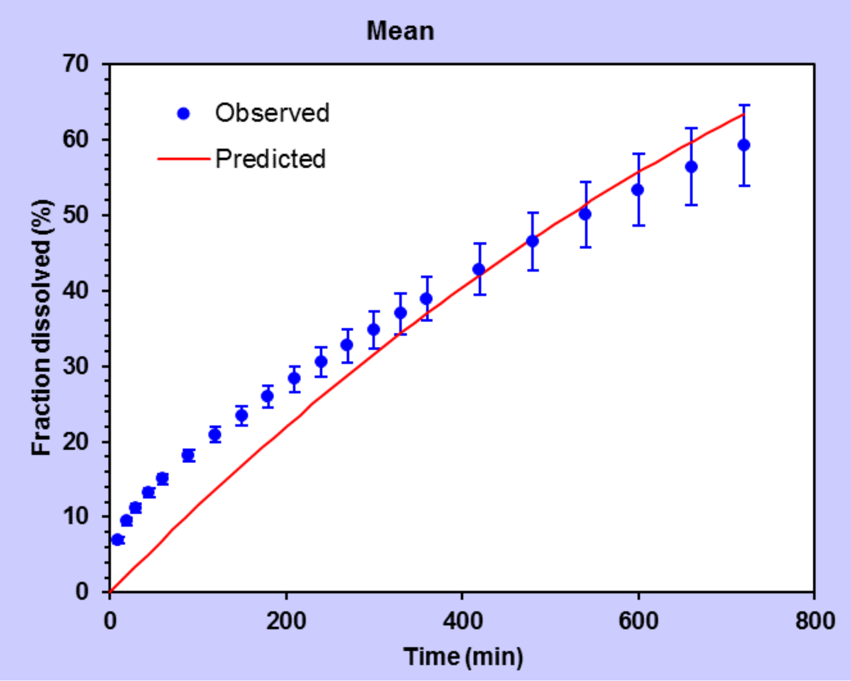

Supplementary materials – Model fitting summary – Emcompress® Anhydrous (modelling up to app. 60 % of carvedilol released)

Graphical abstract of model fit presented as the fraction % of released carvedilol per tested tablet:

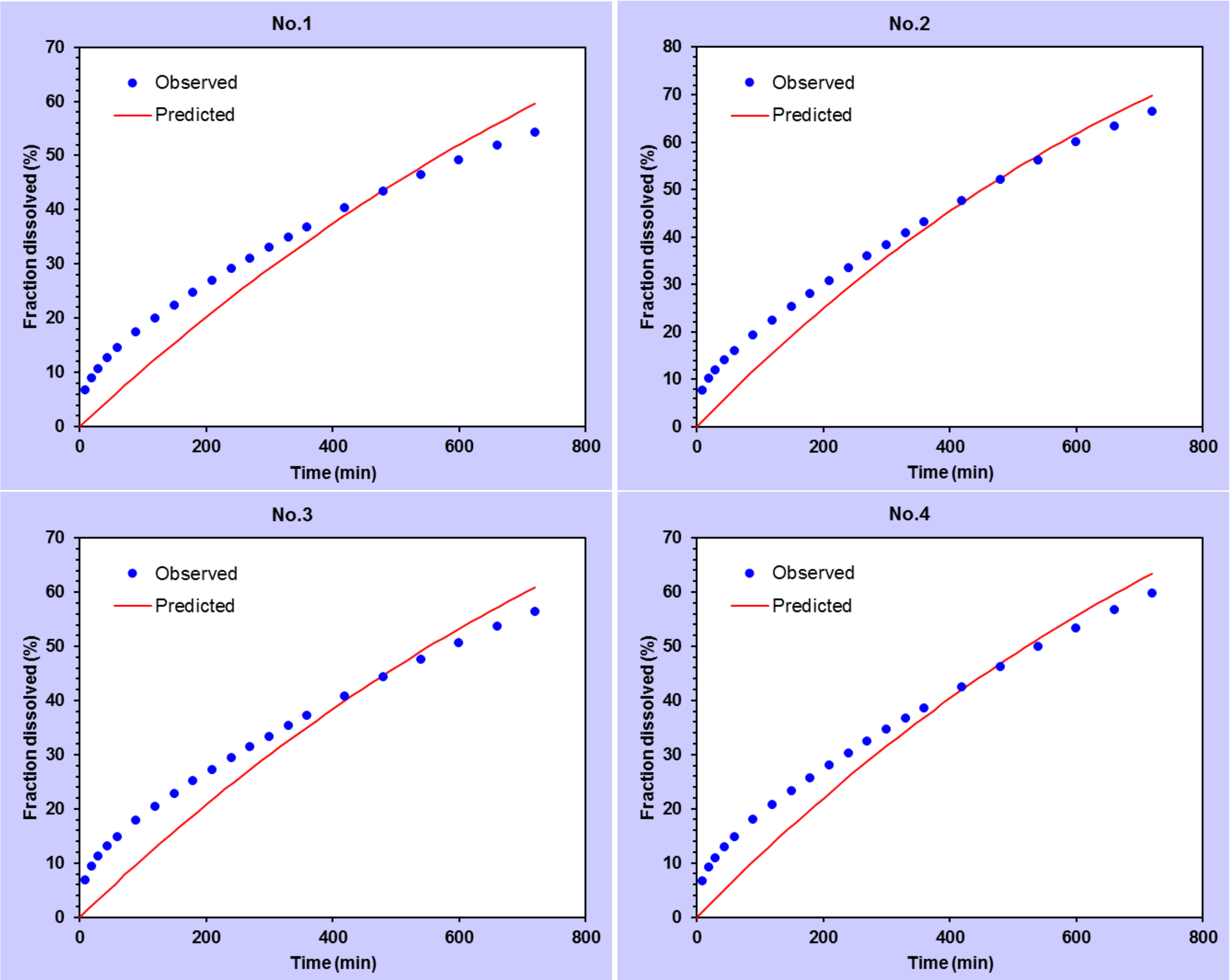

Supplementary materials – Model fitting summary – Emcompress® Anhydrous (modelling up to app. 60 % of carvedilol released)

Model: **Hixson–Crowell with T_{lag}**

Model equation: $F = 100 \cdot \left\{1 - \left[1 - k_{HC} \cdot \left(t - T_{lag}\right)\right]^3\right\}$

Fitted model parameters per tested tablet (N = 4) with statistics – mean, standard deviation (SD), and relative standard deviation expressed in % (RSD%) (output from DDSolver):

Parameter	No.1	No.2	No.3	No.4	Mean	SD	RSD(%)
k_{HC}	0.0003	0.0004	0.0003	0.0003	0.0003	0.0000	13.8828
T_{lag}	-120.4986	-82.8068	-114.6189	-97.4299	-103.8386	17.0995	-16.4674

Number of dissolution data points (N), degrees of freedom (df), and selected goodness of fit criteria – Pearson correlation coefficient (R), coefficient of determination (R^2), adjusted coefficient of determination ($R^2_{adjusted}$), and residual sum of squares (RSS) (manual calculation in MS Excel):

Parameter	No.1	No.2	No.3	No.4
N	21	21	21	21
df	19	19	19	19
R	0.995042791	0.999082349	0.996725934	0.997722425
R^2	0.990110157	0.99816554	0.993462588	0.995450037
$R^2_{adjusted}$	0.989589639	0.998068989	0.993118513	0.995210565
RSS	43.84716575	12.60938756	30.44808648	24.34762265

Graphical abstract of model fit presented as mean ± 1 SD of the fraction % of released carvedilol:

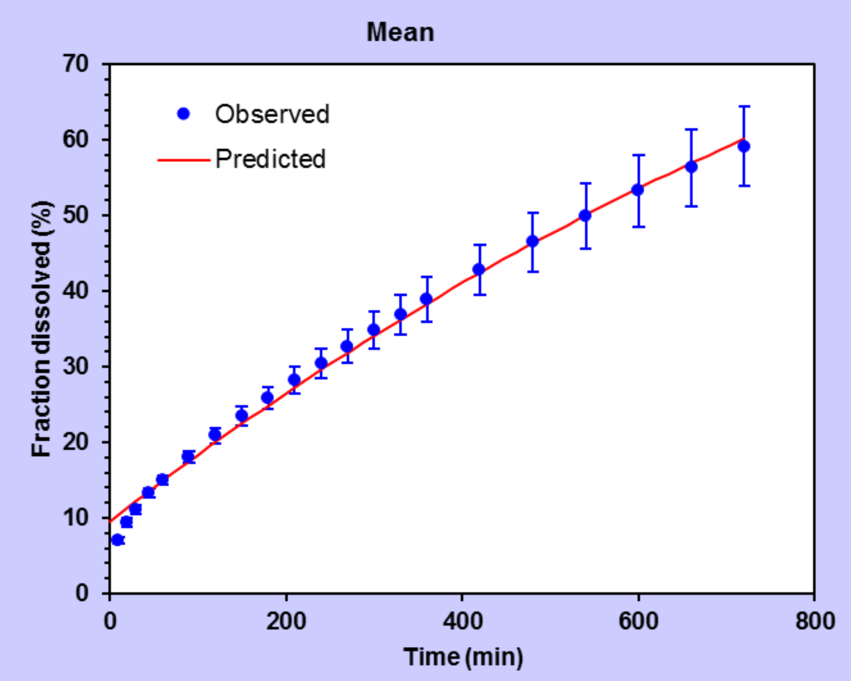

Supplementary materials – Model fitting summary – Emcompress® Anhydrous (modelling up to app. 60 % of carvedilol released)

Graphical abstract of model fit presented as the fraction % of released carvedilol per tested tablet:

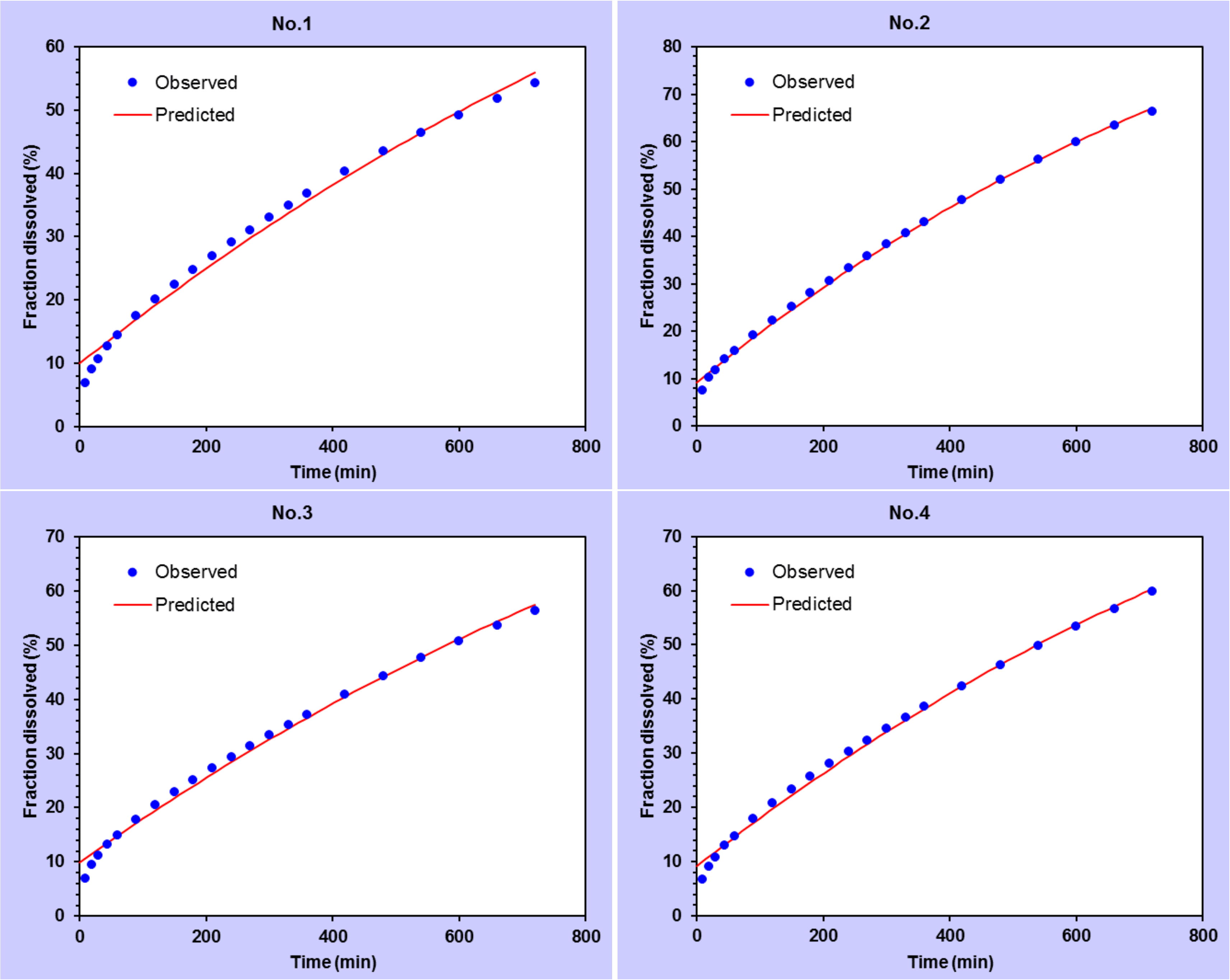

Supplementary materials – Model fitting summary – Emcompress® Anhydrous (modelling up to app. 60 % of carvedilol released)

Model: **Hopfenberg**

Model equation: $F = 100 \cdot [1 - (1 - k_{HB} \cdot t)^n]$

Fitted model parameters per tested tablet (N = 4) with statistics – mean, standard deviation (SD), and relative standard deviation expressed in % (RSD%) (output from DDSolver):

Parameter	No.1	No.2	No.3	No.4	Mean	SD	RSD(%)
k_{HB}	0.0004	0.0003	0.0004	0.0004	0.0004	0.0000	6.0016
n	3.0000	4.1250	3.0000	3.0000	3.2813	0.5625	17.1429

Number of dissolution data points (N), degrees of freedom (df), and selected goodness of fit criteria – Pearson correlation coefficient (R), coefficient of determination (R^2), adjusted coefficient of determination ($R^2_{adjusted}$), and residual sum of squares (RSS) (manual calculation in MS Excel):

Parameter	No.1	No.2	No.3	No.4
N	21	21	21	21
df	19	19	19	19
R	0.996349275	0.999491861	0.997607164	0.998302409
R^2	0.992711878	0.998983981	0.995220053	0.996607701
$R^2_{adjusted}$	0.992328293	0.998930506	0.994968477	0.996429159
RSS	643.3822754	484.7403238	631.5851845	543.7481361

Graphical abstract of model fit presented as mean ± 1 SD of the fraction % of released carvedilol:

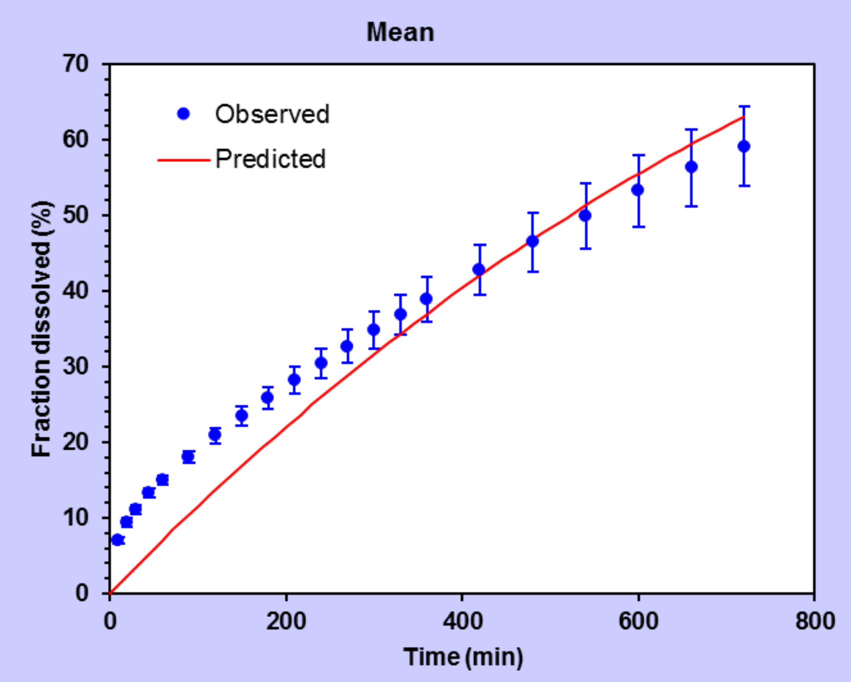

Supplementary materials – Model fitting summary – Emcompress® Anhydrous (modelling up to app. 60 % of carvedilol released)

Graphical abstract of model fit presented as the fraction % of released carvedilol per tested tablet:

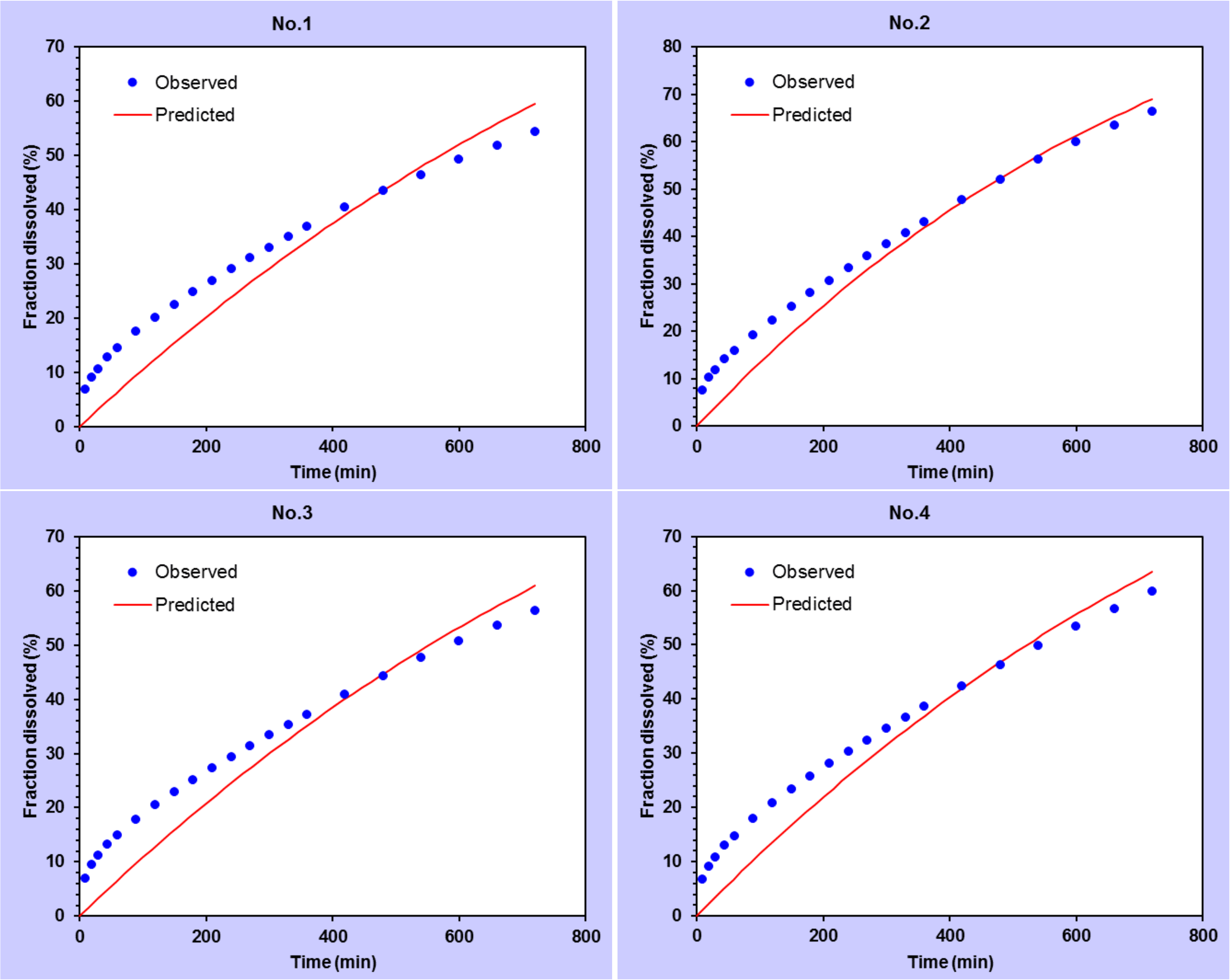

Supplementary materials – Model fitting summary – Emcompress® Anhydrous (modelling up to app. 60 % of carvedilol released)

Model: **Hopfenberg with T_{lag}**

Model equation: $F = 100 \cdot \{1 - [1 - k_{HB} \cdot (t - T_{lag})]^n\}$

Fitted model parameters per tested tablet (N = 4) with statistics – mean, standard deviation (SD), and relative standard deviation expressed in % (RSD%) (output from DDSolver):

Parameter	No.1	No.2	No.3	No.4	Mean	SD	RSD(%)
k_{HB}	0.0003	0.0004	0.0003	0.0003	0.0003	0.0000	15.7767
n	3.0000	3.0000	3.0000	3.6265	3.1566	0.3133	9.9242
T_{lag}	-120.4986	-82.8068	-114.6189	-83.4970	-100.3553	20.0113	-19.9404

Number of dissolution data points (N), degrees of freedom (df), and selected goodness of fit criteria – Pearson correlation coefficient (R), coefficient of determination (R^2), adjusted coefficient of determination ($R^2_{adjusted}$), and residual sum of squares (RSS) (manual calculation in MS Excel):

Parameter	No.1	No.2	No.3	No.4
N	21	21	21	21
df	18	18	18	18
R	0.995042791	0.999082349	0.996725934	0.998072335
R^2	0.990110157	0.99816554	0.993462588	0.996148386
$R^2_{adjusted}$	0.989011285	0.997961711	0.992736208	0.995720429
RSS	43.84716575	12.60938756	30.44808648	21.99952924

Graphical abstract of model fit presented as mean ± 1 SD of the fraction % of released carvedilol:

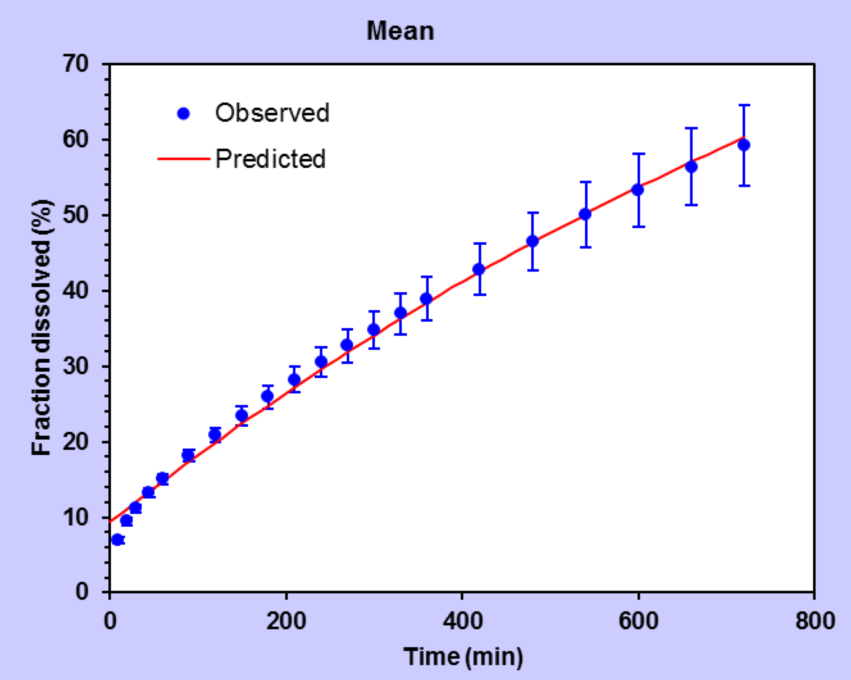

Supplementary materials – Model fitting summary – Emcompress® Anhydrous (modelling up to app. 60 % of carvedilol released)

Graphical abstract of model fit presented as the fraction % of released carvedilol per tested tablet:

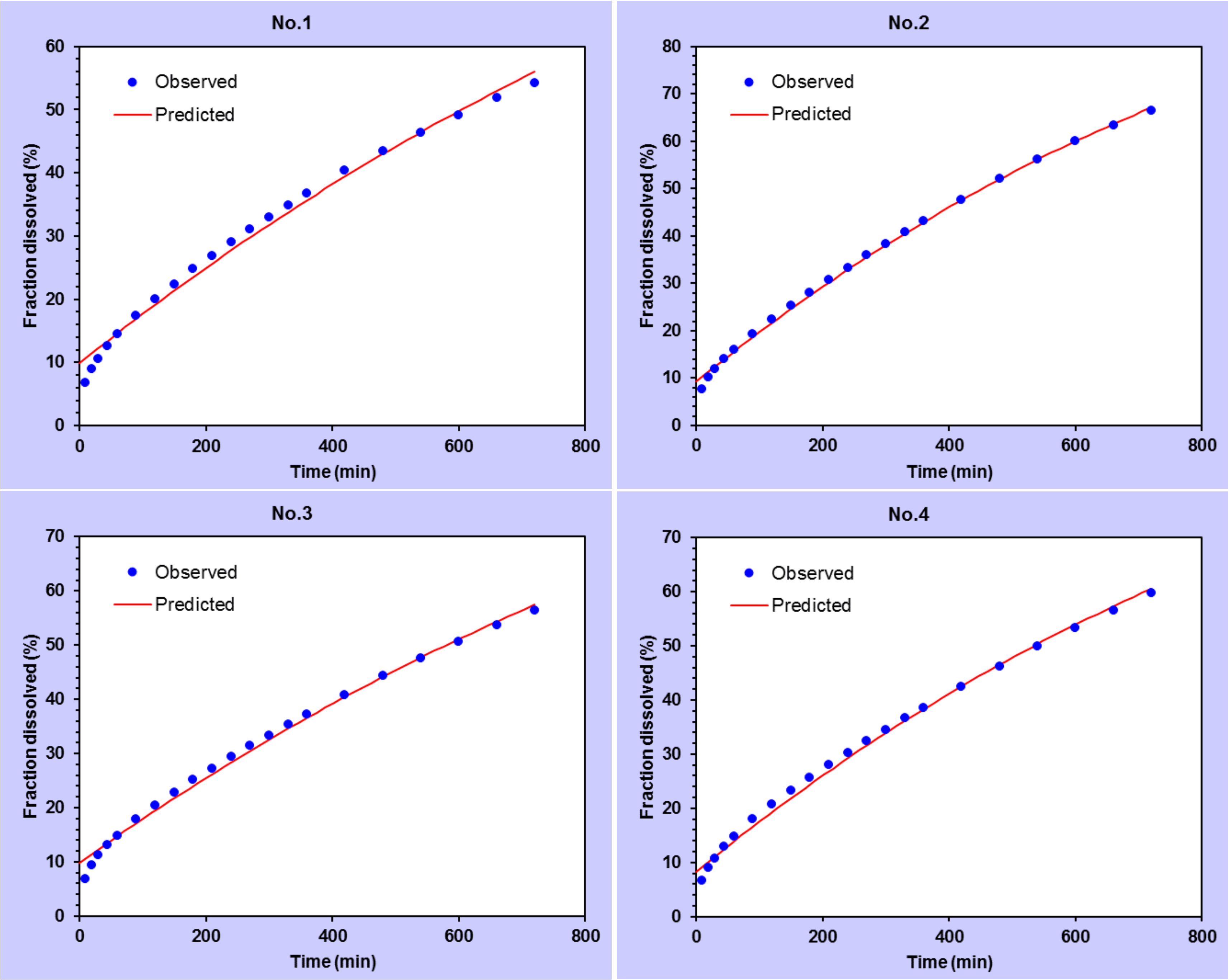

Supplementary materials – Model fitting summary – Emcompress® Anhydrous (modelling up to app. 60 % of carvedilol released)

Model: **Baker–Lonsdale**

Model equation: $\frac{3}{2} \cdot \left[1-\left(1-\frac{F}{100}\right)^{\frac{2}{3}}\right]-\frac{F}{100}=k_{BL} \cdot t$

Fitted model parameters per tested tablet (N = 4) with statistics – mean, standard deviation (SD), and relative standard deviation expressed in % (RSD%) (output from DDSolver):

Parameter	No.1	No.2	No.3	No.4	Mean	SD	RSD(%)
k_{BL}	0.00007	0.00011	0.00007	0.00008	0.00009	0.00002	22.98649

Number of dissolution data points (N), degrees of freedom (df), and selected goodness of fit criteria – Pearson correlation coefficient (R), coefficient of determination (R^2), adjusted coefficient of determination ($R^2_{adjusted}$), and residual sum of squares (RSS) (manual calculation in MS Excel):

Parameter	No.1	No.2	No.3	No.4
N	21	21	21	21
df	20	20	20	20
R	0.99526177	0.989012904	0.993048626	0.991529428
R^2	0.99054599	0.978146525	0.986145574	0.983130606
$R^2_{adjusted}$	0.99054599	0.978146525	0.986145574	0.983130606
RSS	146.7512266	289.844131	152.4422298	188.6769245

Graphical abstract of model fit presented as mean ± 1 SD of the fraction % of released carvedilol:

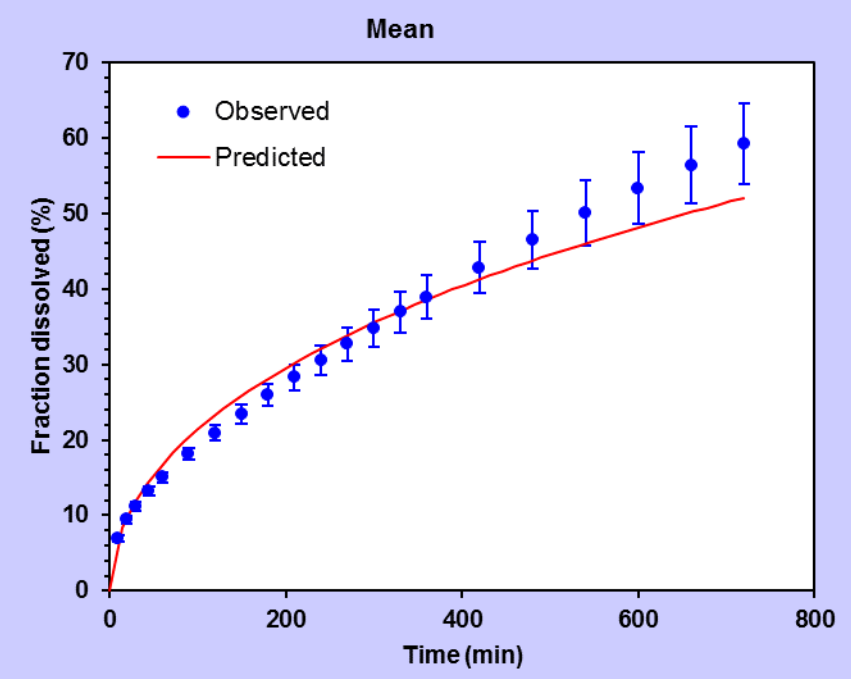

Supplementary materials – Model fitting summary – Emcompress® Anhydrous (modelling up to app. 60 % of carvedilol released)

Graphical abstract of model fit presented as the fraction % of released carvedilol per tested tablet:

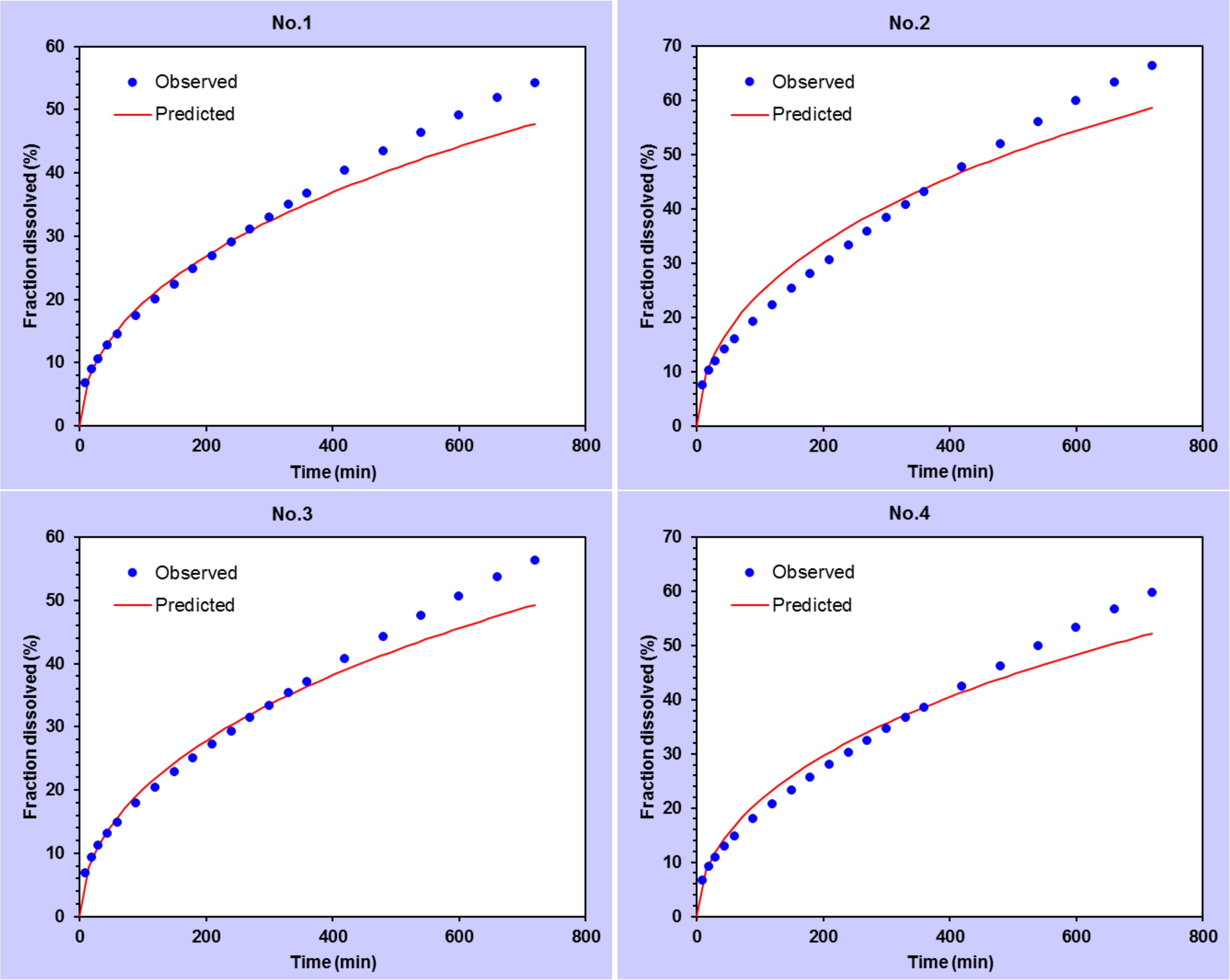

Supplementary materials – Model fitting summary – Emcompress® Anhydrous (modelling up to app. 60 % of carvedilol released)

Model: **Baker–Lonsdale with T_{lag}**

Model equation: $\frac{3}{2} \cdot \left[1 - \left(1 - \frac{F}{100}\right)^{\frac{2}{3}}\right] - \frac{F}{100} = k_{BL} \cdot (t - T_{lag})$

Fitted model parameters per tested tablet (N = 4) with statistics – mean, standard deviation (SD), and relative standard deviation expressed in % (RSD%) (output from DDSolver):

Parameter	No.1	No.2	No.3	No.4	Mean	SD	RSD(%)
k_{BL}	0.00009	0.00015	0.00010	0.00011	0.00011	0.00003	22.98649
T_{lag}	35.86758	54.82483	40.74440	48.84372	45.07013	8.42206	18.68656

Number of dissolution data points (N), degrees of freedom (df), and selected goodness of fit criteria – Pearson correlation coefficient (R), coefficient of determination (R^2), adjusted coefficient of determination ($R^2_{adjusted}$), and residual sum of squares (RSS) (manual calculation in MS Excel):

Parameter	No.1	No.2	No.3	No.4
N	21	21	21	21
df	19	19	19	19
R	0.983776322	0.974770251	0.980506386	0.976985413
R^2	0.967815851	0.950177043	0.961392773	0.954500498
$R^2_{adjusted}$	0.966121948	0.947554782	0.959360813	0.952105788
RSS	310.9514201	699.3249166	395.6795798	524.1565396

Graphical abstract of model fit presented as mean ± 1 SD of the fraction % of released carvedilol:

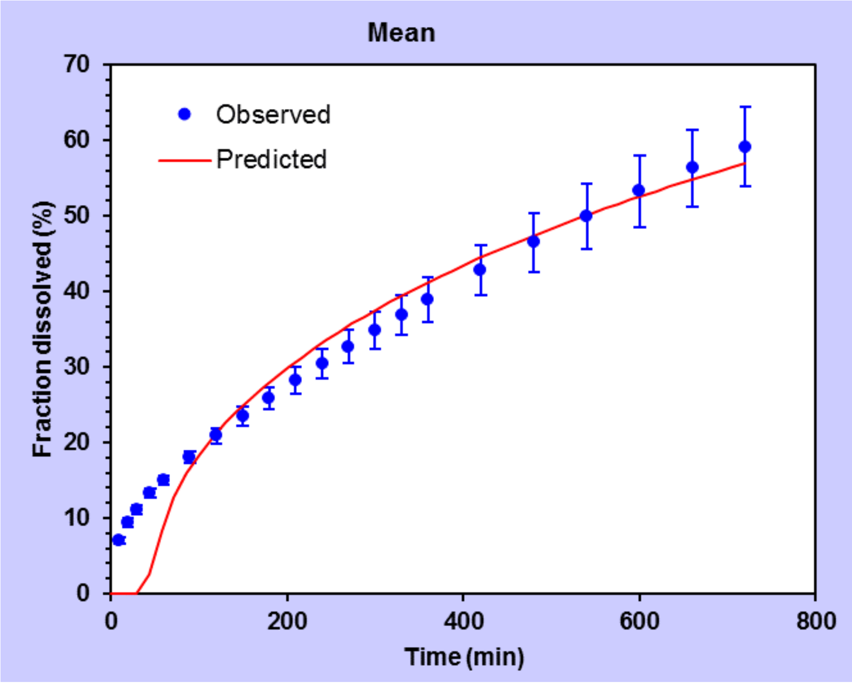

Supplementary materials – Model fitting summary – Emcompress® Anhydrous (modelling up to app. 60 % of carvedilol released)

Graphical abstract of model fit presented as the fraction % of released carvedilol per tested tablet:

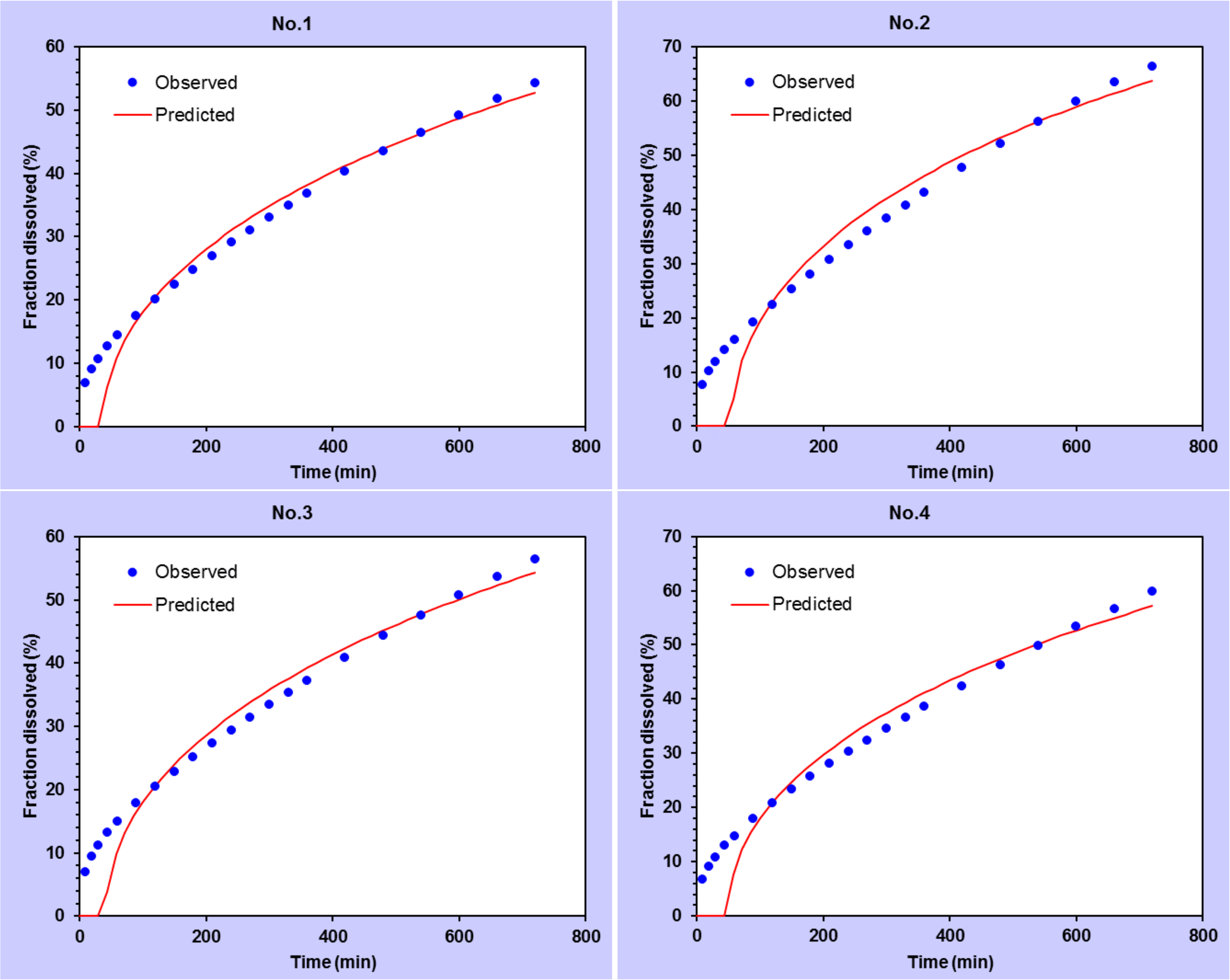

Supplementary materials – Model fitting summary – Emcompress® Anhydrous (modelling up to app. 60 % of carvedilol released)

Model: **Makoid–Banakar**

Model equation: $F = k_{MB} \cdot t^n \cdot e^{-k \cdot t}$

Fitted model parameters per tested tablet (N = 4) with statistics – mean, standard deviation (SD), and relative standard deviation expressed in % (RSD%) (output from DDSolver):

Parameter	No.1	No.2	No.3	No.4	Mean	SD	RSD(%)
k_{MB}	2.3945	2.4871	2.5834	2.3536	2.4547	0.1024	4.1727
n	0.4378	0.4561	0.4241	0.4456	0.4409	0.0135	3.0568
k	-0.0004	-0.0005	-0.0004	-0.0005	-0.0004	0.0000	-8.6110

Number of dissolution data points (N), degrees of freedom (df), and selected goodness of fit criteria – Pearson correlation coefficient (R), coefficient of determination (R^2), adjusted coefficient of determination ($R^2_{adjusted}$), and residual sum of squares (RSS) (manual calculation in MS Excel):

Parameter	No.1	No.2	No.3	No.4
N	21	21	21	21
df	18	18	18	18
R	0.999257168	0.999204076	0.999600397	0.99970198
R^2	0.998514887	0.998408786	0.999200955	0.999404048
$R^2_{adjusted}$	0.998349874	0.998231984	0.999112172	0.999337831
RSS	6.487130552	16.02063584	3.674744288	3.181931689

Graphical abstract of model fit presented as mean ± 1 SD of the fraction % of released carvedilol:

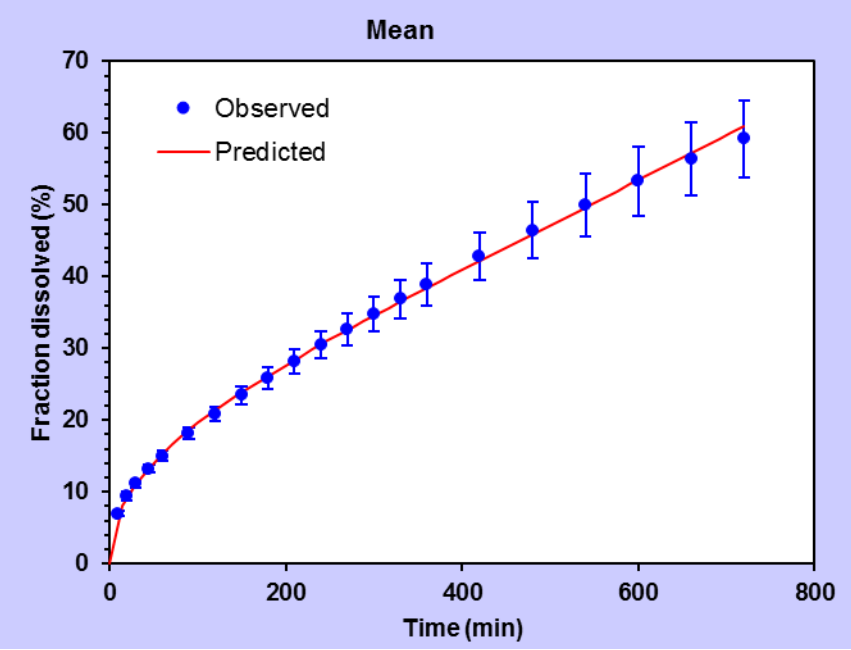

Supplementary materials – Model fitting summary – Emcompress® Anhydrous (modelling up to app. 60 % of carvedilol released)

Graphical abstract of model fit presented as the fraction % of released carvedilol per tested tablet:

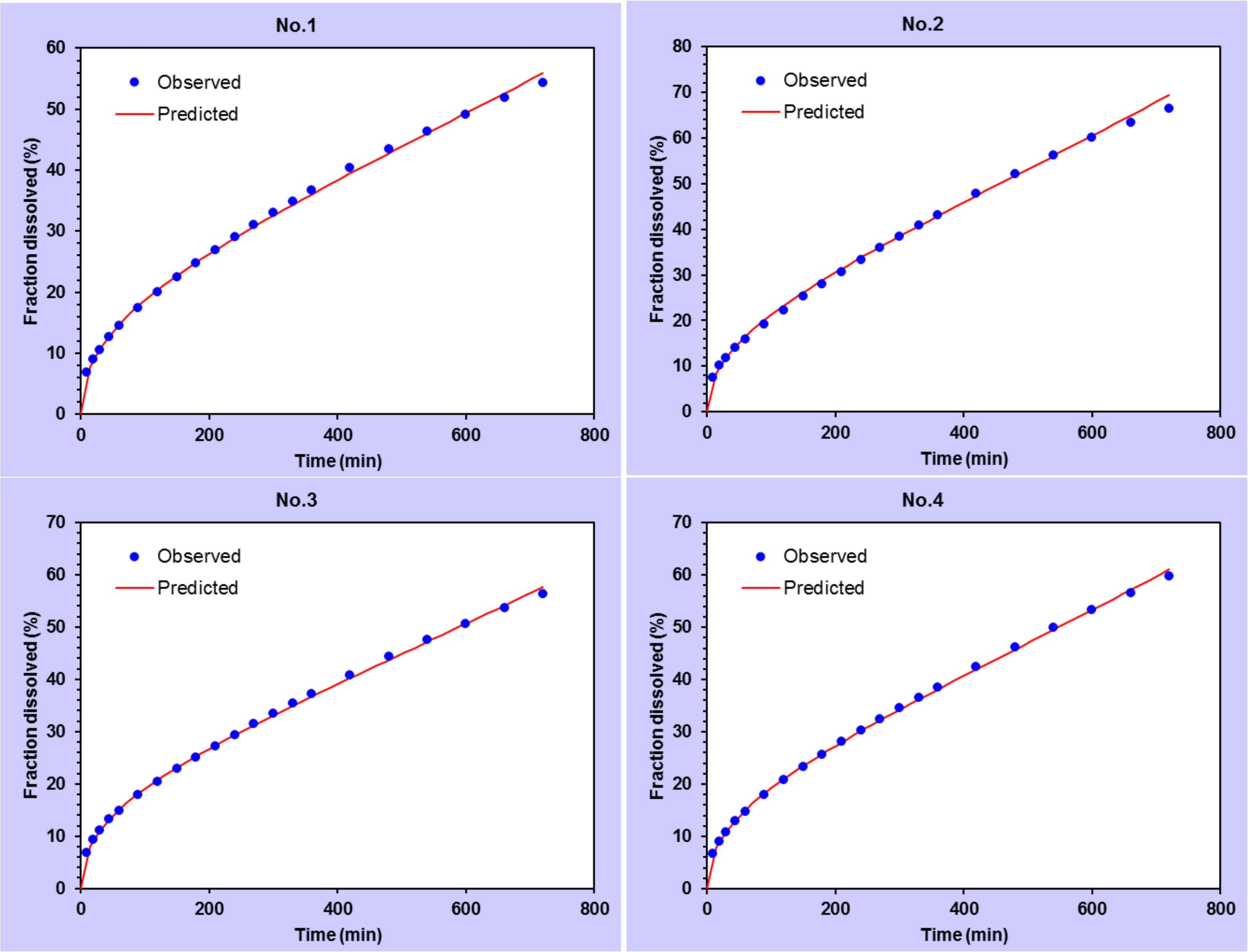

Supplementary materials – Model fitting summary – Emcompress® Anhydrous (modelling up to app. 60 % of carvedilol released)

Model: **Makoid–Banakar with T_{lag}**

Model equation: $F = k_{MB} \cdot (t - T_{lag})^n \cdot e^{-k \cdot (t - T_{lag})}$

Fitted model parameters per tested tablet (N = 4) with statistics – mean, standard deviation (SD), and relative standard deviation expressed in % (RSD%) (output from DDSolver):

Parameter	No.1	No.2	No.3	No.4	Mean	SD	RSD(%)
k_{MB}	3.1924	3.6093	3.4018	3.1474	3.3377	0.2123	6.3606
n	0.3764	0.3708	0.3656	0.3837	0.3741	0.0078	2.0790
k	-0.0006	-0.0007	-0.0006	-0.0006	-0.0006	0.0001	-11.8440
T_{lag}	4.0000	4.0000	4.0000	4.0000	4.0000	0.0000	0.0000

Number of dissolution data points (N), degrees of freedom (df), and selected goodness of fit criteria – Pearson correlation coefficient (R), coefficient of determination (R^2), adjusted coefficient of determination ($R^2_{adjusted}$), and residual sum of squares (RSS) (manual calculation in MS Excel):

Parameter	No.1	No.2	No.3	No.4
N	21	21	21	21
df	17	17	17	17
R	0.99810742	0.997406625	0.998836199	0.998916751
R^2	0.996218421	0.994819976	0.997673753	0.997834675
$R^2_{adjusted}$	0.995551084	0.993905855	0.997263239	0.997452559
RSS	16.78599968	36.26766686	10.90815662	11.82861229

Graphical abstract of model fit presented as mean ± 1 SD of the fraction % of released carvedilol:

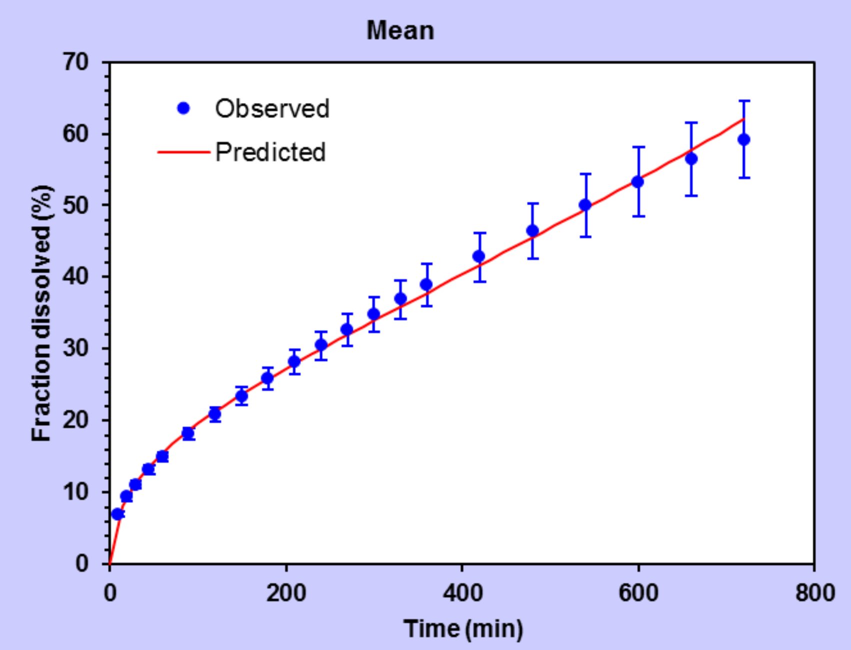

Supplementary materials – Model fitting summary – Emcompress® Anhydrous (modelling up to app. 60 % of carvedilol released)

Graphical abstract of model fit presented as the fraction % of released carvedilol per tested tablet:

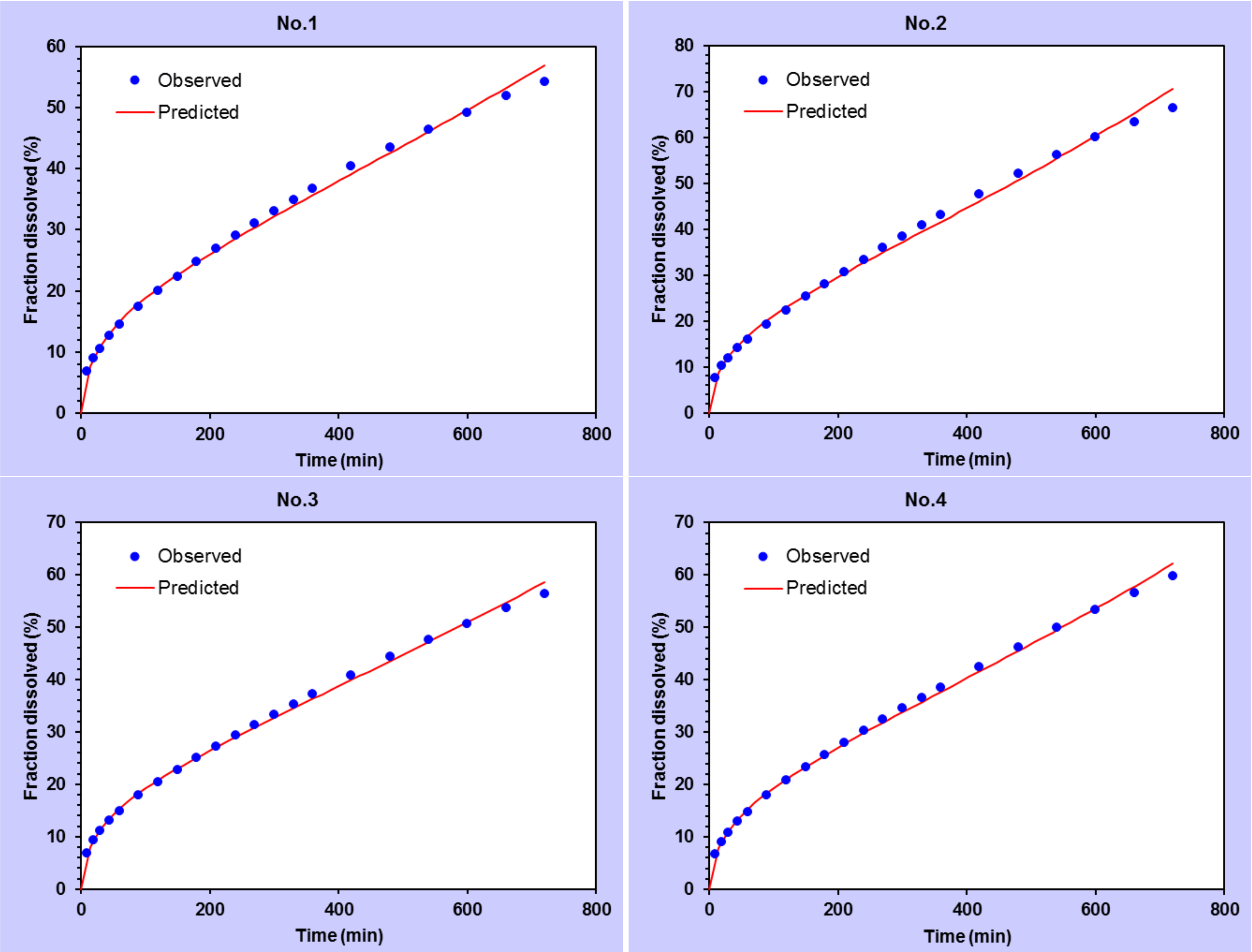

Supplementary materials – Model fitting summary – Emcompress® Anhydrous (modelling up to app. 60 % of carvedilol released)

Model: **Peppas–Sahlin_1**

Model equation: $F = k_1 \cdot t^m + k_2 \cdot t^{2m}$

Fitted model parameters per tested tablet (N = 4) with statistics – mean, standard deviation (SD), and relative standard deviation expressed in % (RSD%) (output from DDSolver):

Parameter	No.1	No.2	No.3	No.4	Mean	SD	RSD(%)
k_1	1.984	1.984	1.969	1.876	1.953	0.052	2.658
k_2	0.043	0.076	0.048	0.062	0.058	0.015	25.645
m	0.450	0.450	0.450	0.450	0.450	0.000	0.000

Number of dissolution data points (N), degrees of freedom (df), and selected goodness of fit criteria – Pearson correlation coefficient (R), coefficient of determination (R^2), adjusted coefficient of determination ($R^2_{adjusted}$), and residual sum of squares (RSS) (manual calculation in MS Excel):

Parameter	No.1	No.2	No.3	No.4
N	21	21	21	21
df	18	18	18	18
R	0.999703282	0.99940622	0.999423487	0.999618126
R^2	0.999406653	0.998812793	0.998847307	0.999236397
$R^2_{adjusted}$	0.999340725	0.998680882	0.99871923	0.999151552
RSS	2.901471483	9.144719274	6.33612528	4.82977928

Graphical abstract of model fit presented as mean ± 1 SD of the fraction % of released carvedilol:

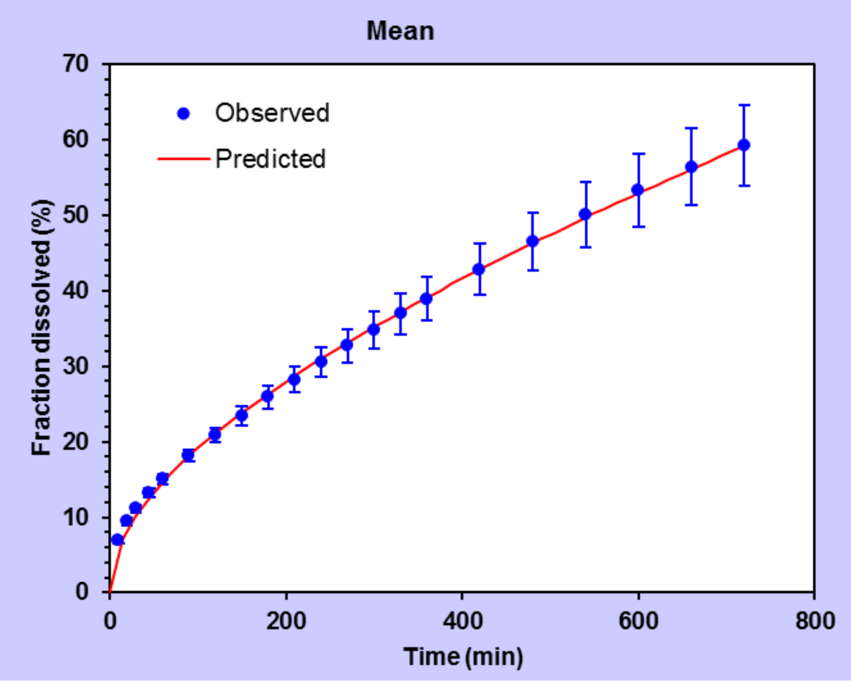

Supplementary materials – Model fitting summary – Emcompress® Anhydrous (modelling up to app. 60 % of carvedilol released)

Graphical abstract of model fit presented as the fraction % of released carvedilol per tested tablet:

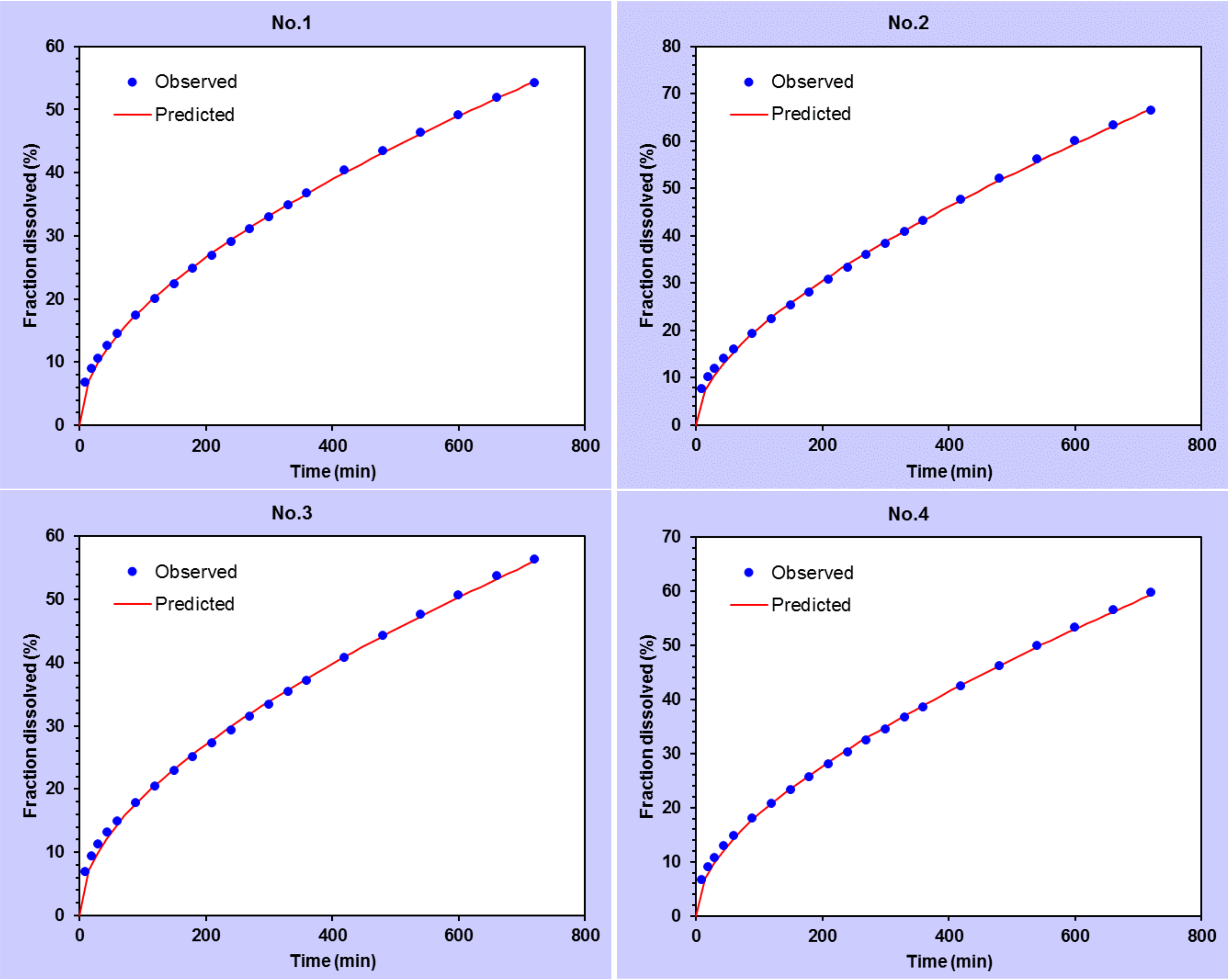

Supplementary materials – Model fitting summary – Emcompress® Anhydrous (modelling up to app. 60 % of carvedilol released)

Model: **Peppas–Sahlin_1 with T_{lag}**

Model equation: $F = k_1 \cdot (t - T_{lag})^m + k_2 \cdot (t - T_{lag})^{2m}$

Fitted model parameters per tested tablet (N = 4) with statistics – mean, standard deviation (SD), and relative standard deviation expressed in % (RSD%) (output from DDSolver):

Parameter	No.1	No.2	No.3	No.4	Mean	SD	RSD(%)
k_1	2.079	2.091	2.064	1.974	2.052	0.053	2.585
k_2	0.038	0.071	0.043	0.057	0.052	0.015	27.752
m	0.450	0.450	0.450	0.450	0.450	0.000	0.000
T_{lag}	4.000	4.000	4.000	4.000	4.000	0.000	0.000

Number of dissolution data points (N), degrees of freedom (df), and selected goodness of fit criteria – Pearson correlation coefficient (R), coefficient of determination (R^2), adjusted coefficient of determination ($R^2_{adjusted}$), and residual sum of squares (RSS) (manual calculation in MS Excel):

Parameter	No.1	No.2	No.3	No.4
N	21	21	21	21
df	17	17	17	17
R	0.999193755	0.998841553	0.998781268	0.999100915
R^2	0.998388159	0.997684448	0.997564022	0.998202638
$R^2_{adjusted}$	0.998103717	0.997275821	0.997134143	0.997885456
RSS	8.632608128	18.9772454	14.2519745	12.01668887

Graphical abstract of model fit presented as mean ± 1 SD of the fraction % of released carvedilol:

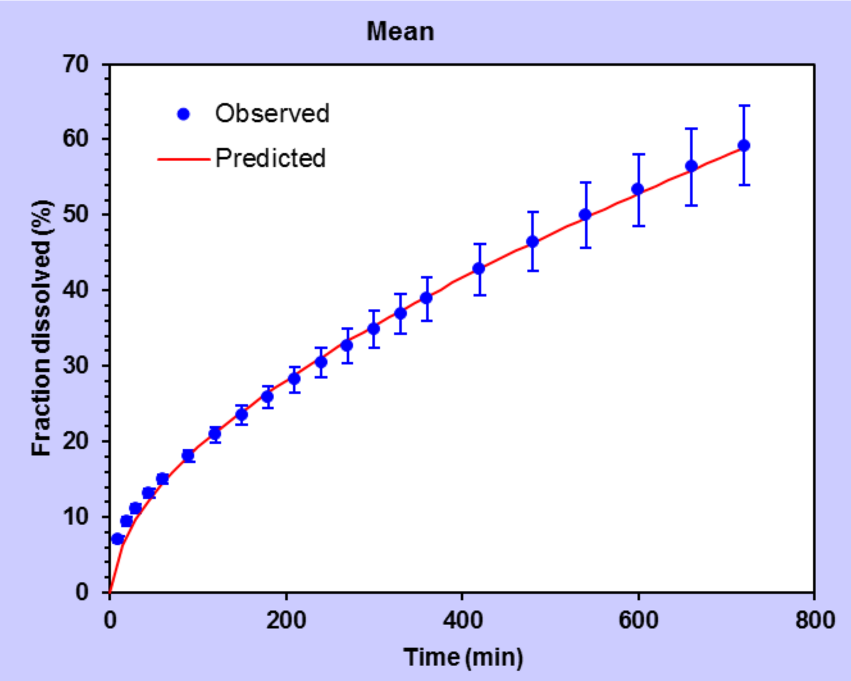

Supplementary materials – Model fitting summary – Emcompress® Anhydrous (modelling up to app. 60 % of carvedilol released)

Graphical abstract of model fit presented as the fraction % of released carvedilol per tested tablet:

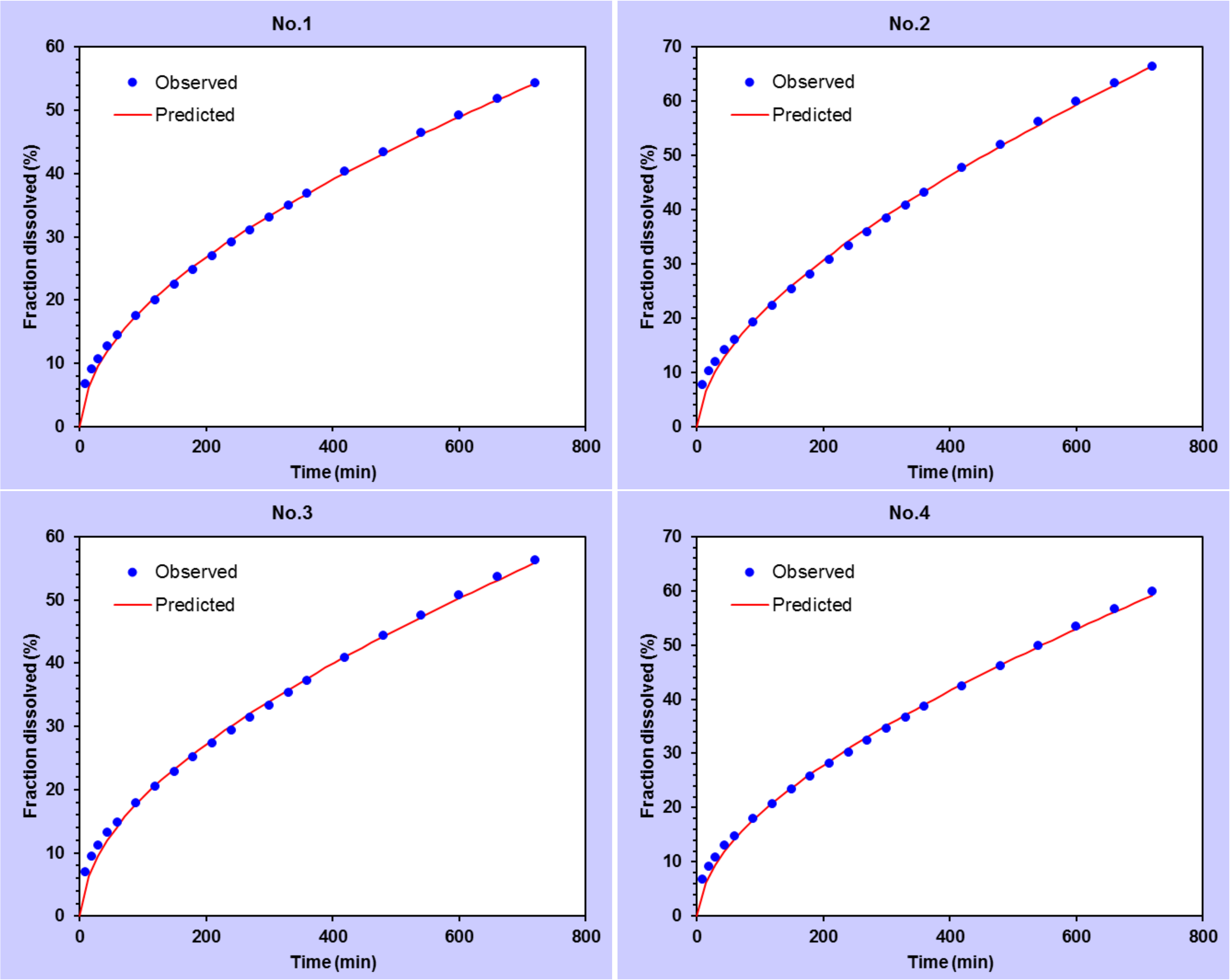

Supplementary materials – Model fitting summary – Emcompress® Anhydrous (modelling up to app. 60 % of carvedilol released)

Model: **Peppas–Sahlin_2**

Model equation: $F = k_1 \cdot t^{0.5} + k_2 \cdot t$

Fitted model parameters per tested tablet (N = 4) with statistics – mean, standard deviation (SD), and relative standard deviation expressed in % (RSD%) (output from DDSolver):

Parameter	No.1	No.2	No.3	No.4	Mean	SD	RSD(%)
k_1	1.735	1.793	1.728	1.677	1.733	0.048	2.752
k_2	0.011	0.026	0.013	0.020	0.017	0.007	38.691

Number of dissolution data points (N), degrees of freedom (df), and selected goodness of fit criteria – Pearson correlation coefficient (R), coefficient of determination (R^2), adjusted coefficient of determination ($R^2_{adjusted}$), and residual sum of squares (RSS) (manual calculation in MS Excel):

Parameter	No.1	No.2	No.3	No.4
N	21	21	21	21
df	19	19	19	19
R	0.999540132	0.999288537	0.999185347	0.99947772
R^2	0.999080475	0.99857758	0.998371357	0.998955713
$R^2_{adjusted}$	0.999032079	0.998502716	0.998285639	0.998900751
RSS	4.95247751	11.63179698	9.699642921	7.129080304

Graphical abstract of model fit presented as mean ± 1 SD of the fraction % of released carvedilol:

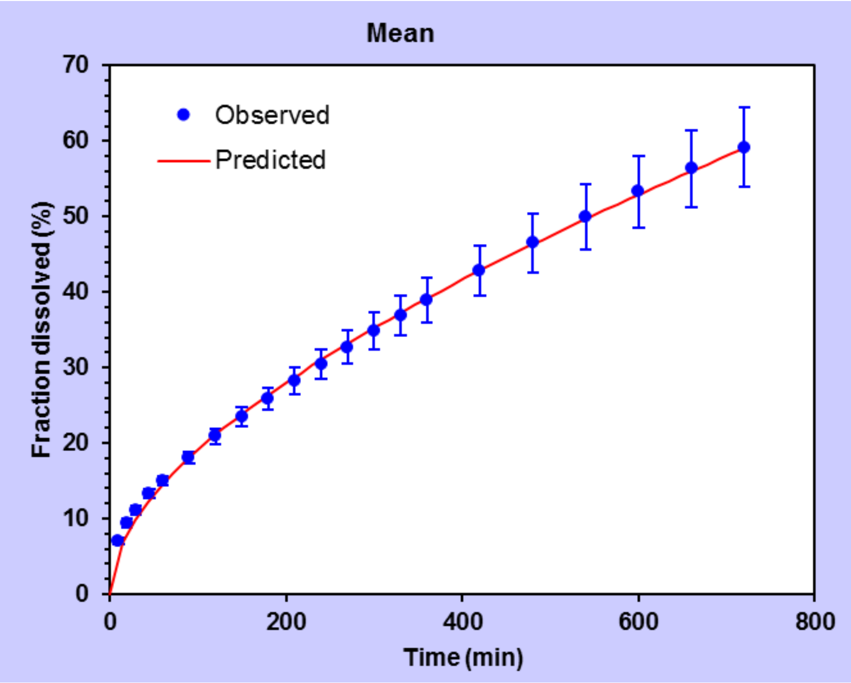

Supplementary materials – Model fitting summary – Emcompress® Anhydrous (modelling up to app. 60 % of carvedilol released)

Graphical abstract of model fit presented as the fraction % of released carvedilol per tested tablet:

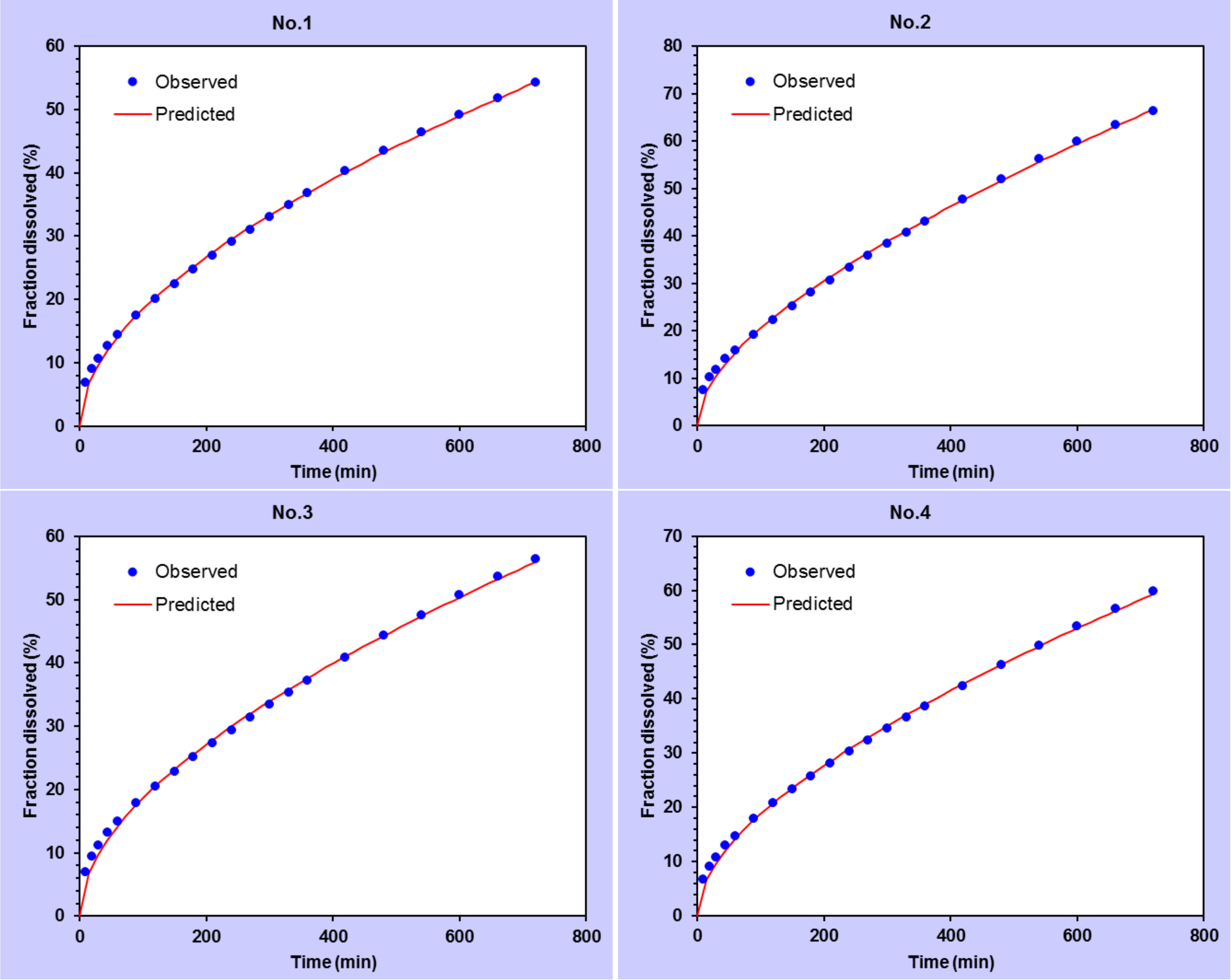

Supplementary materials – Model fitting summary – Emcompress® Anhydrous (modelling up to app. 60 % of carvedilol released)

Model: **Peppas–Sahlin_2 with T_{lag}**

Model equation: $F = k_1 \cdot (t - T_{lag})^{0.5} + k_2 \cdot (t - T_{lag})$

Fitted model parameters per tested tablet (N = 4) with statistics – mean, standard deviation (SD), and relative standard deviation expressed in % (RSD%) (output from DDSolver):

Parameter	No.1	No.2	No.3	No.4	Mean	SD	RSD(%)
k_1	1.801	1.868	1.794	1.745	1.802	0.051	2.804
k_2	0.008	0.023	0.011	0.017	0.015	0.007	44.632
T_{lag}	4.000	4.000	4.000	4.000	4.000	0.000	0.000

Number of dissolution data points (N), degrees of freedom (df), and selected goodness of fit criteria – Pearson correlation coefficient (R), coefficient of determination (R^2), adjusted coefficient of determination ($R^2_{adjusted}$), and residual sum of squares (RSS) (manual calculation in MS Excel):

Parameter	No.1	No.2	No.3	No.4
N	21	21	21	21
df	18	18	18	18
R	0.998898151	0.998655676	0.998428335	0.998873612
R^2	0.997797515	0.997313159	0.996859139	0.997748492
$R^2_{adjusted}$	0.997552795	0.997014621	0.996510155	0.997498325
RSS	12.95093037	23.63528136	20.04915256	16.35677758

Graphical abstract of model fit presented as mean ± 1 SD of the fraction % of released carvedilol:

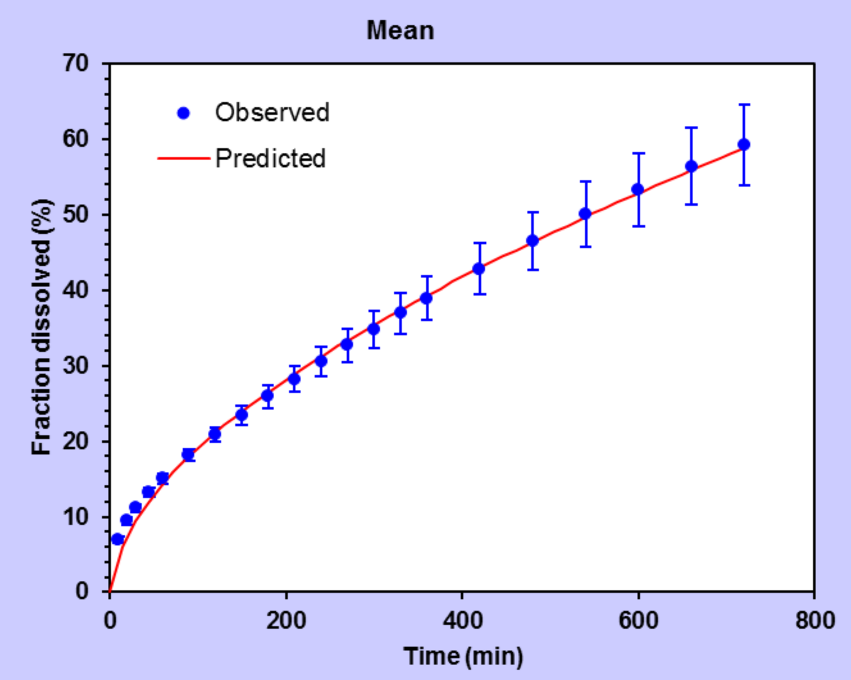

Supplementary materials – Model fitting summary – Emcompress® Anhydrous (modelling up to app. 60 % of carvedilol released)

Graphical abstract of model fit presented as the fraction % of released carvedilol per tested tablet:

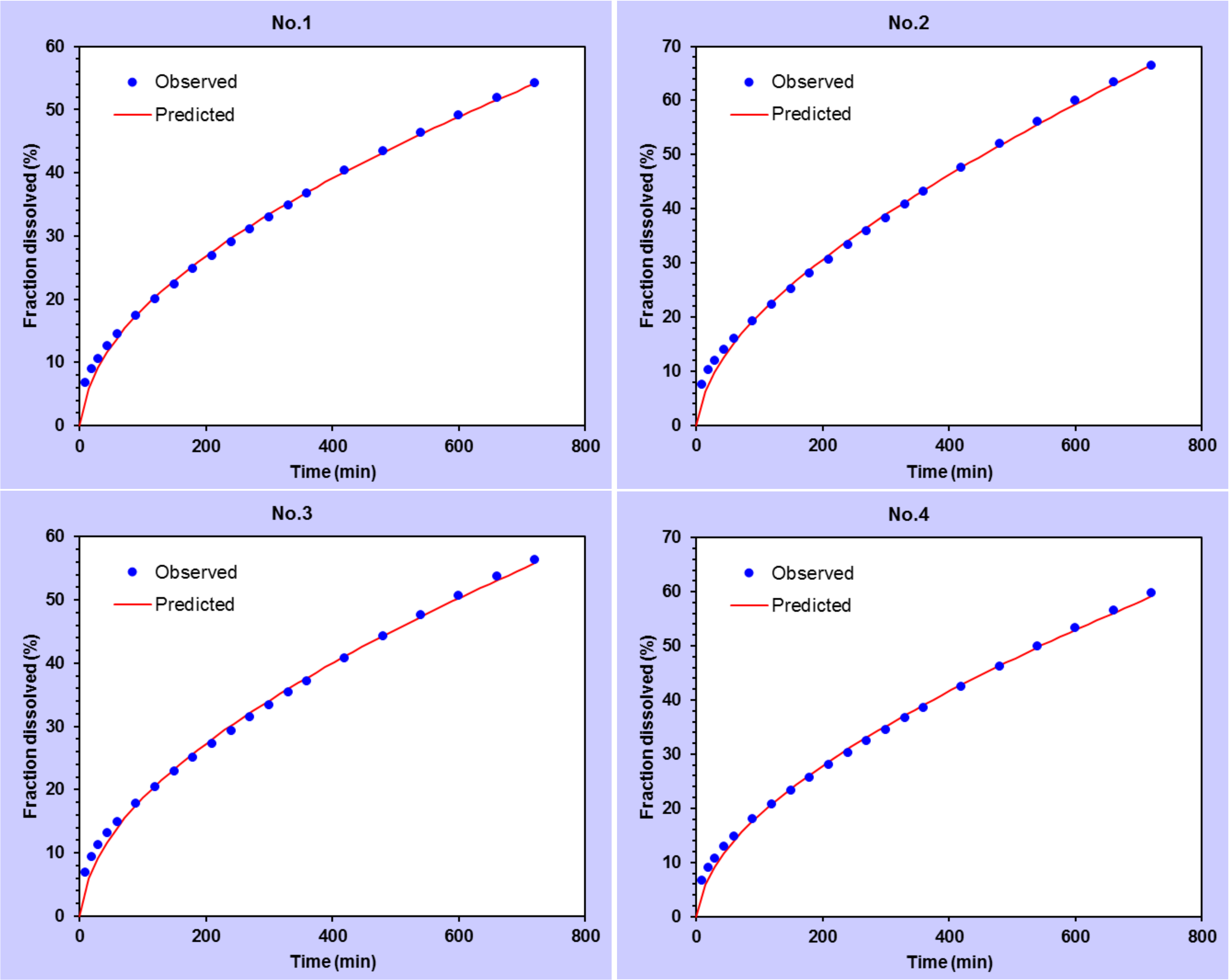

Supplementary materials – Model fitting summary – Emcompress® Anhydrous (modelling up to app. 60 % of carvedilol released)

Model: **Quadratic**

Model equation: $F = 100 \cdot (k_1 \cdot t^2 + k_2 \cdot t)$

Fitted model parameters per tested tablet (N = 4) with statistics – mean, standard deviation (SD), and relative standard deviation expressed in % (RSD%) (output from DDSolver):

Parameter	No.1	No.2	No.3	No.4	Mean	SD	RSD(%)
k_1	-0.000001	-0.000001	-0.000001	-0.000001	-0.000001	0.000000	-3.377289
k_2	0.001472	0.001662	0.001479	0.001506	0.001530	0.000089	5.840234

Number of dissolution data points (N), degrees of freedom (df), and selected goodness of fit criteria – Pearson correlation coefficient (R), coefficient of determination (R^2), adjusted coefficient of determination ($R^2_{adjusted}$), and residual sum of squares (RSS) (manual calculation in MS Excel):

Parameter	No.1	No.2	No.3	No.4
N	21	21	21	21
df	19	19	19	19
R	0.991187956	0.992949094	0.990036255	0.991450306
R^2	0.982453563	0.985947904	0.980171786	0.982973709
$R^2_{adjusted}$	0.981530067	0.98520832	0.979128196	0.982077588
RSS	277.8246763	319.1555976	315.450759	292.1865222

Graphical abstract of model fit presented as mean ± 1 SD of the fraction % of released carvedilol:

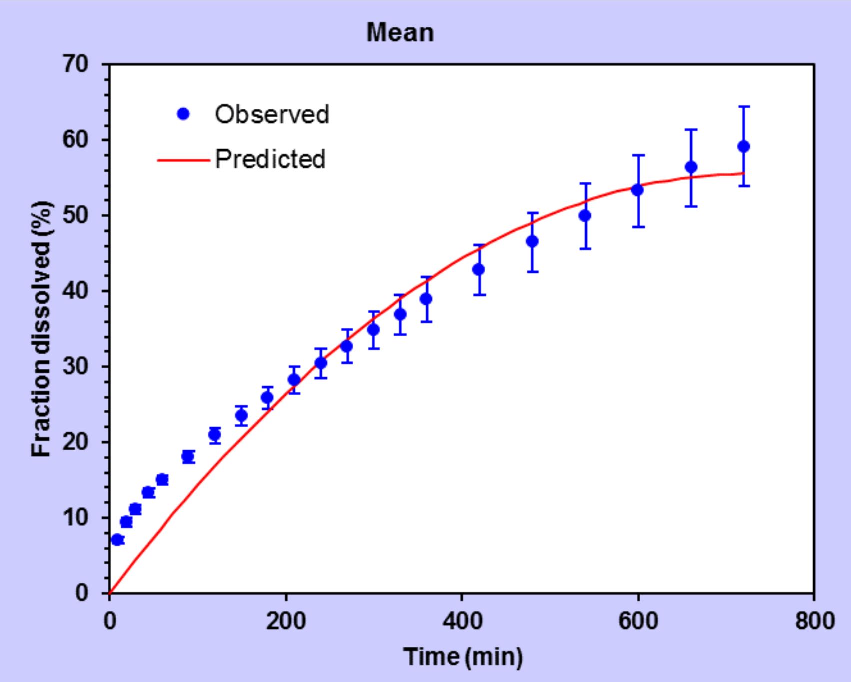

Supplementary materials – Model fitting summary – Emcompress® Anhydrous (modelling up to app. 60 % of carvedilol released)

Graphical abstract of model fit presented as the fraction % of released carvedilol per tested tablet:

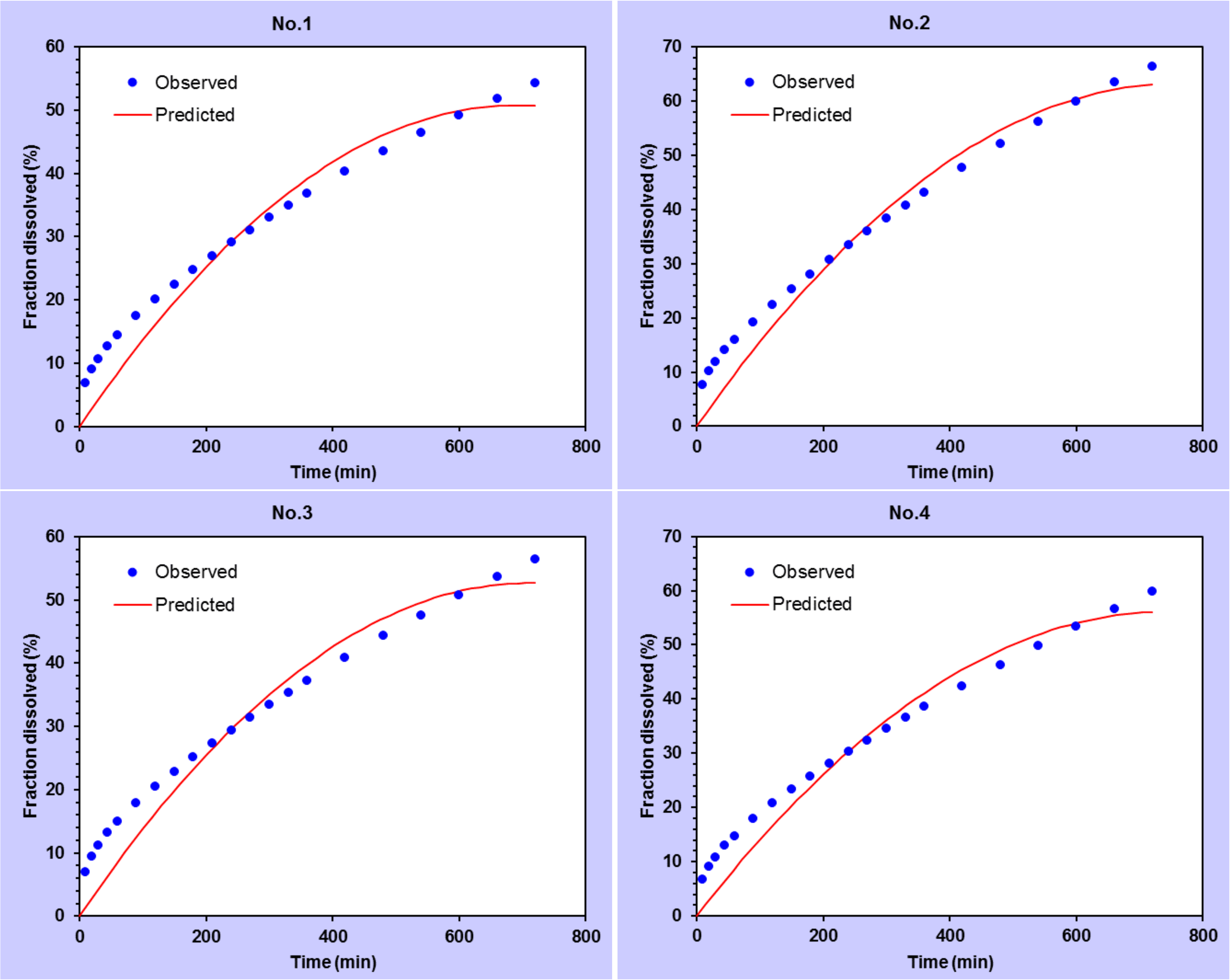

Supplementary materials – Model fitting summary – Emcompress® Anhydrous (modelling up to app. 60 % of carvedilol released)

Model: **Quadratic with T_{lag}**

Model equation: $F = 100 \cdot \left[k_1 \cdot (t - T_{lag})^2 + k_2 \cdot (t - T_{lag})\right]$

Fitted model parameters per tested tablet (N = 4) with statistics – mean, standard deviation (SD), and relative standard deviation expressed in % (RSD%) (output from DDSolver):

Parameter	No.1	No.2	No.3	No.4	Mean	SD	RSD(%)
k_1	-0.000001	-0.000001	-0.000001	-0.000001	-0.000001	0.000000	-3.417128
k_2	0.001493	0.001687	0.001500	0.001528	0.001552	0.000091	5.874416
T_{lag}	4.000000	4.000000	4.000000	4.000000	4.000000	0.000000	0.000000

Number of dissolution data points (N), degrees of freedom (df), and selected goodness of fit criteria – Pearson correlation coefficient (R), coefficient of determination (R^2), adjusted coefficient of determination ($R^2_{adjusted}$), and residual sum of squares (RSS) (manual calculation in MS Excel):

Parameter	No.1	No.2	No.3	No.4
N	21	21	21	21
df	18	18	18	18
R	0.990590651	0.992363351	0.989416987	0.990849306
R^2	0.981269838	0.98478502	0.978945973	0.981782348
$R^2_{adjusted}$	0.979188709	0.983094467	0.976606637	0.979758164
RSS	320.0454404	370.6987202	360.4718613	336.2567786

Graphical abstract of model fit presented as mean ± 1 SD of the fraction % of released carvedilol:

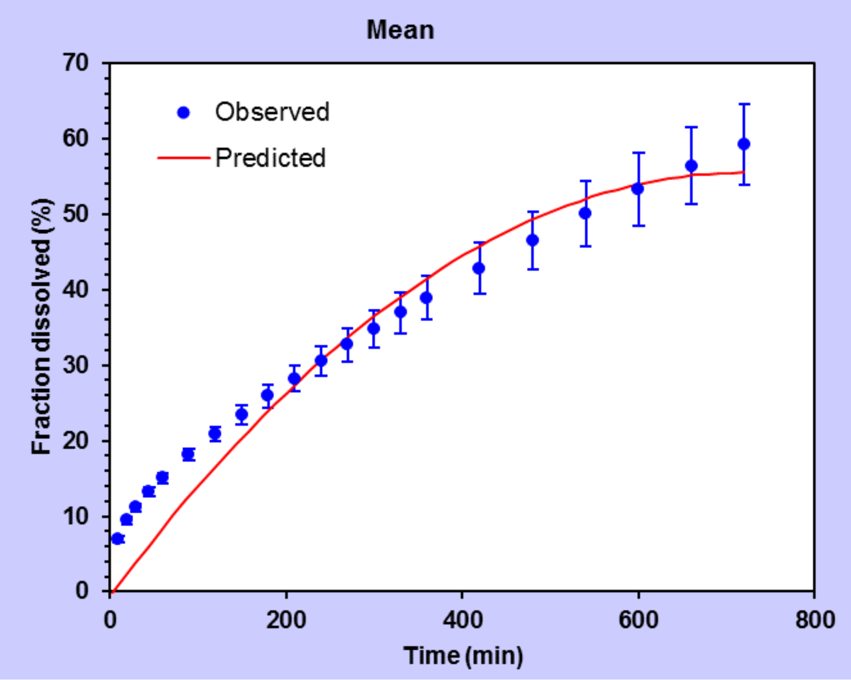

Supplementary materials – Model fitting summary – Emcompress® Anhydrous (modelling up to app. 60 % of carvedilol released)

Graphical abstract of model fit presented as the fraction % of released carvedilol per tested tablet:

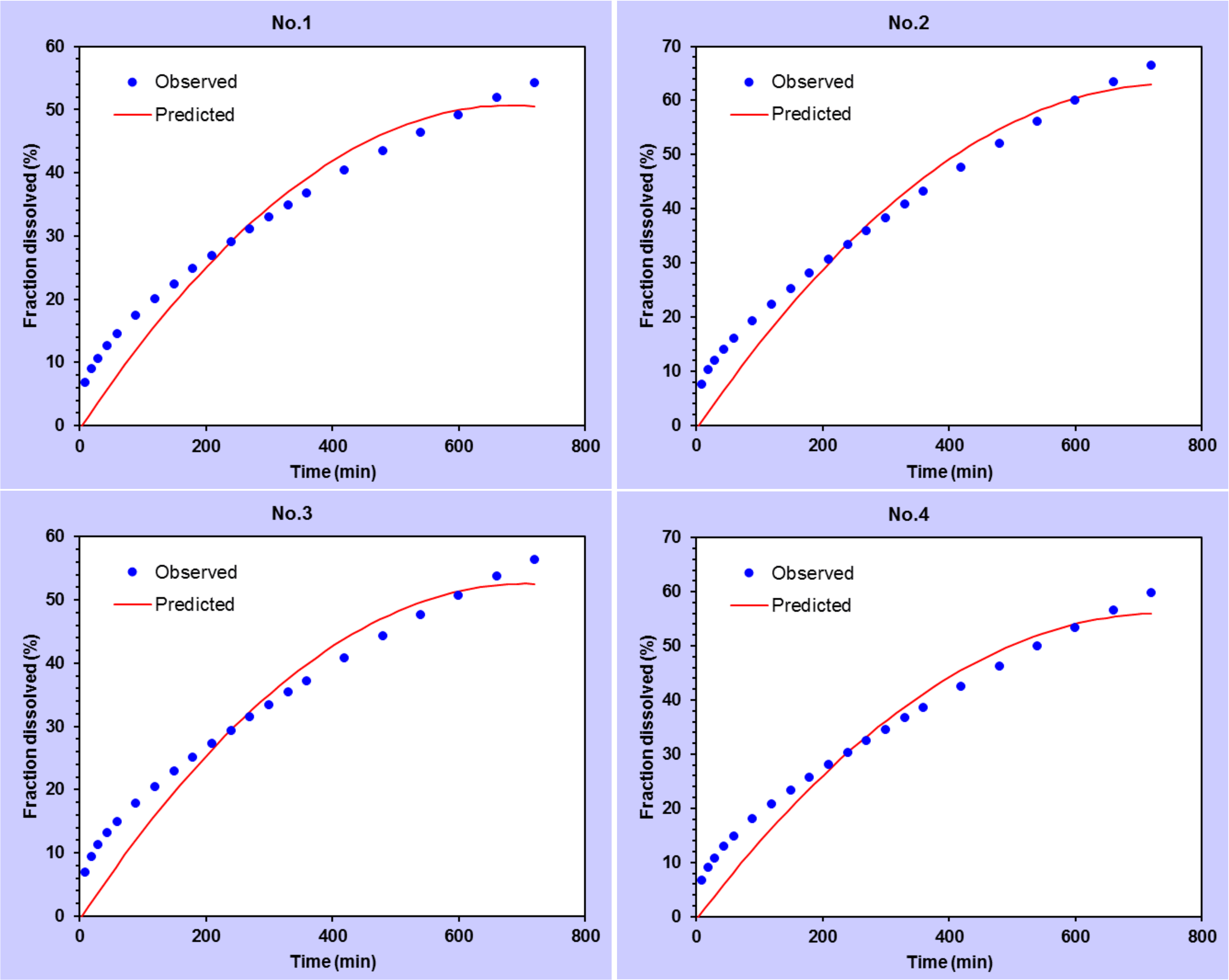

Supplementary materials – Model fitting summary – Emcompress® Anhydrous (modelling up to app. 60 % of carvedilol released)

Model: **Weibull_1**

Model equation: $F = 100 \cdot \left[1 - e^{-\frac{(t-T_i)^{\beta}}{\alpha}}\right]$

Fitted model parameters per tested tablet (N = 4) with statistics – mean, standard deviation (SD), and relative standard deviation expressed in % (RSD%) (output from DDSolver):

Parameter	No.1	No.2	No.3	No.4	Mean	SD	RSD(%)
α	48.701	50.552	47.165	52.738	49.789	2.405	4.830
β	0.529	0.573	0.528	0.556	0.546	0.022	4.005
T_i	4.000	4.000	4.000	4.000	4.000	0.000	0.000

Number of dissolution data points (N), degrees of freedom (df), and selected goodness of fit criteria – Pearson correlation coefficient (R), coefficient of determination (R^2), adjusted coefficient of determination ($R^2_{adjusted}$), and residual sum of squares (RSS) (manual calculation in MS Excel):

Parameter	No.1	No.2	No.3	No.4
N	21	21	21	21
df	18	18	18	18
R	0.989957432	0.983321843	0.986745961	0.98637485
R^2	0.980015716	0.966921847	0.973667591	0.972935344
$R^2_{adjusted}$	0.977795241	0.963246497	0.970741768	0.96992816
RSS	124.8758063	307.4365669	165.7657403	198.6727013

Graphical abstract of model fit presented as mean ± 1 SD of the fraction % of released carvedilol:

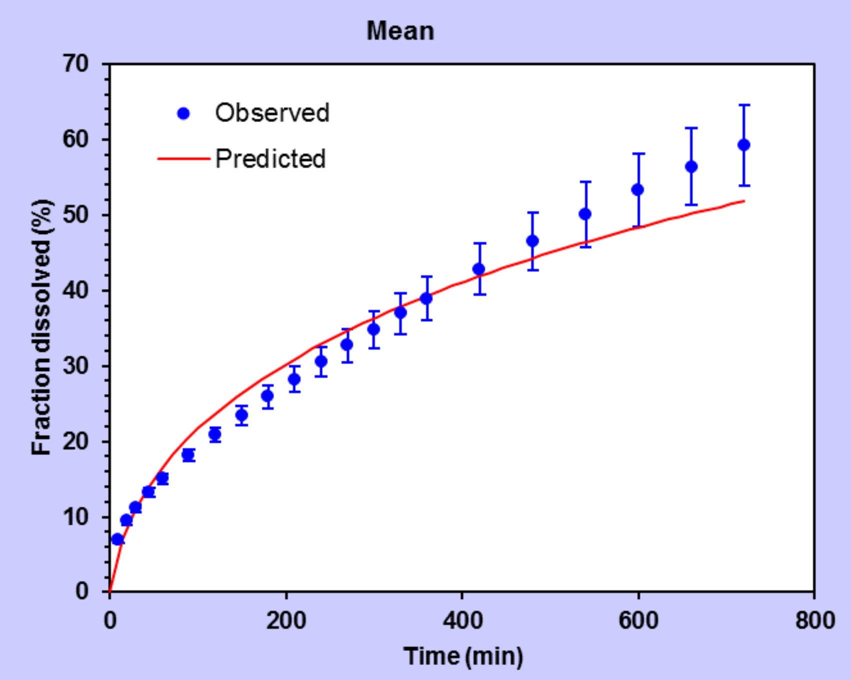

Supplementary materials – Model fitting summary – Emcompress® Anhydrous (modelling up to app. 60 % of carvedilol released)

Graphical abstract of model fit presented as the fraction % of released carvedilol per tested tablet:

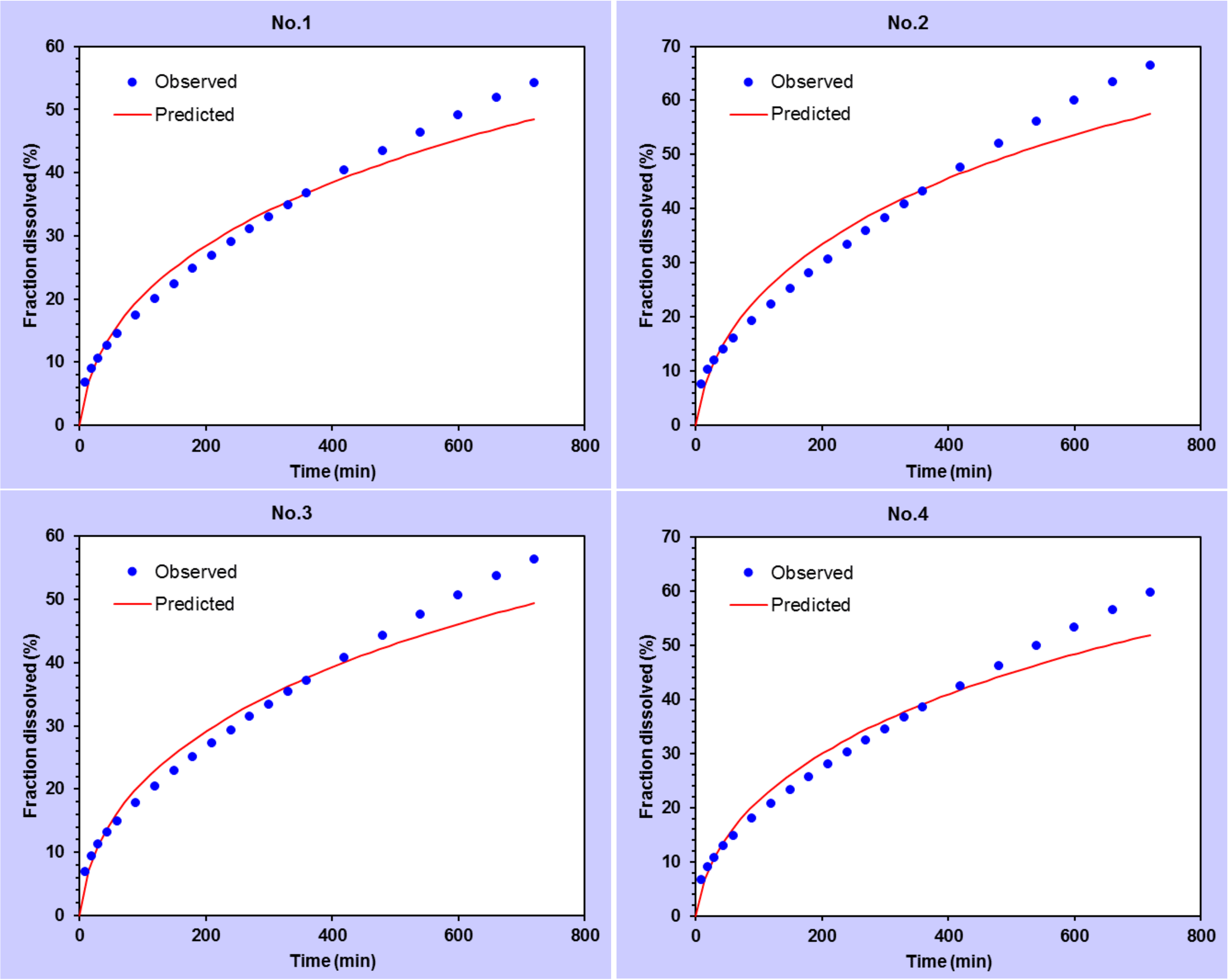

Supplementary materials – Model fitting summary – Emcompress® Anhydrous (modelling up to app. 60 % of carvedilol released)

Model: **Weibull_2**

Model equation: $F = 100 \cdot \left(1 - e^{-\frac{t^{\beta}}{\alpha}}\right)$

Fitted model parameters per tested tablet (N = 4) with statistics – mean, standard deviation (SD), and relative standard deviation expressed in % (RSD%) (output from DDSolver):

Parameter	No.1	No.2	No.3	No.4	Mean	SD	RSD(%)
α	63.704	67.970	61.666	69.996	65.834	3.821	5.804
β	0.575	0.624	0.574	0.604	0.594	0.024	4.075

Number of dissolution data points (N), degrees of freedom (df), and selected goodness of fit criteria – Pearson correlation coefficient (R), coefficient of determination (R^2), adjusted coefficient of determination ($R^2_{adjusted}$), and residual sum of squares (RSS) (manual calculation in MS Excel):

Parameter	No.1	No.2	No.3	No.4
N	21	21	21	21
df	19	19	19	19
R	0.993707743	0.988092355	0.990945364	0.990573867
R^2	0.987455079	0.976326502	0.981972714	0.981236586
$R^2_{adjusted}$	0.98679482	0.975080529	0.98102391	0.980249038
RSS	74.71361592	211.5219226	108.2541626	131.0256479

Graphical abstract of model fit presented as mean ± 1 SD of the fraction % of released carvedilol:

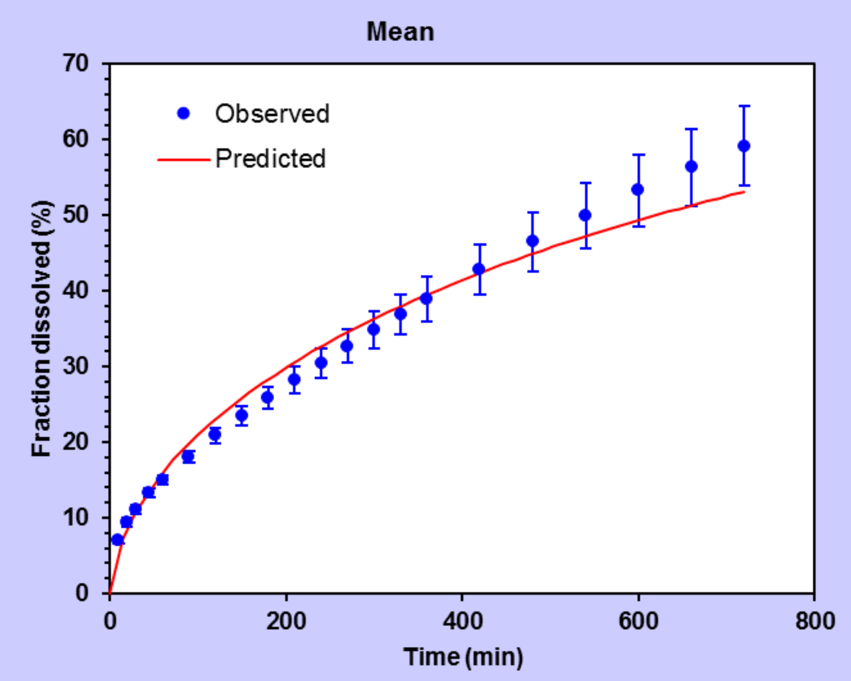

Supplementary materials – Model fitting summary – Emcompress® Anhydrous (modelling up to app. 60 % of carvedilol released)

Graphical abstract of model fit presented as the fraction % of released carvedilol per tested tablet:

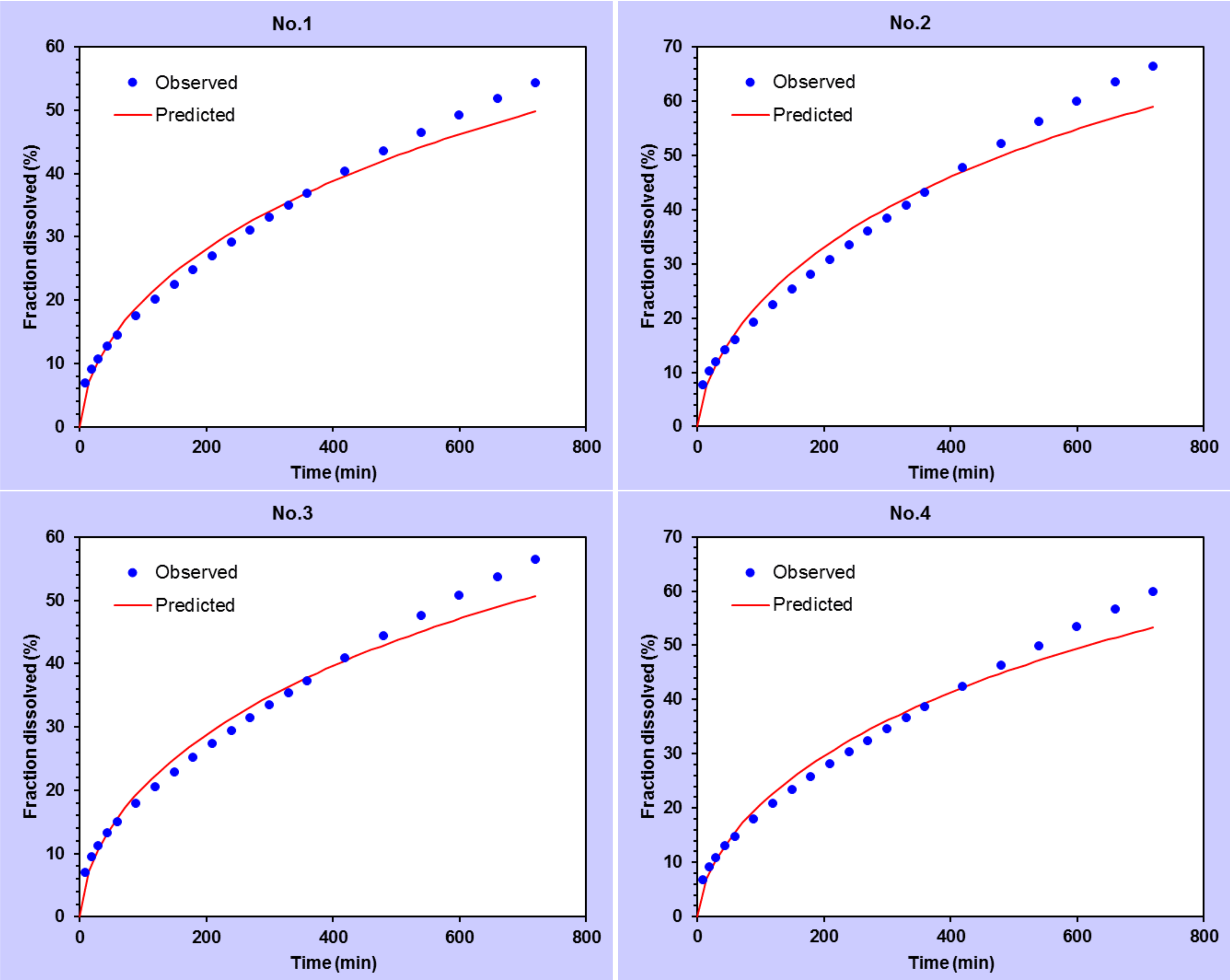

Supplementary materials – Model fitting summary – Emcompress® Anhydrous (modelling up to app. 60 % of carvedilol released)

Model: **Weibull_3**

Model equation: $F = F_{max} \cdot \left(1 - e^{-\frac{t^{\beta}}{\alpha}}\right)$

Fitted model parameters per tested tablet (N = 4) with statistics – mean, standard deviation (SD), and relative standard deviation expressed in % (RSD%) (output from DDSolver):

Parameter	No.1	No.2	No.3	No.4	Mean	SD	RSD(%)
α	57.312	68.831	56.263	66.070	62.119	6.273	10.099
β	0.709	0.730	0.701	0.722	0.715	0.013	1.818
F_{max}	56.980	69.711	59.162	62.776	62.157	5.574	8.968

Number of dissolution data points (N), degrees of freedom (df), and selected goodness of fit criteria – Pearson correlation coefficient (R), coefficient of determination (R^2), adjusted coefficient of determination ($R^2_{adjusted}$), and residual sum of squares (RSS) (manual calculation in MS Excel):

Parameter	No.1	No.2	No.3	No.4
N	21	21	21	21
df	18	18	18	18
R	0.980675638	0.976532476	0.977375609	0.978087774
R^2	0.961724706	0.953615677	0.95526308	0.956655693
$R^2_{adjusted}$	0.957471896	0.948461863	0.950292311	0.951839659
RSS	181.9699863	347.0407483	222.7584182	251.1511674

Graphical abstract of model fit presented as mean ± 1 SD of the fraction % of released carvedilol:

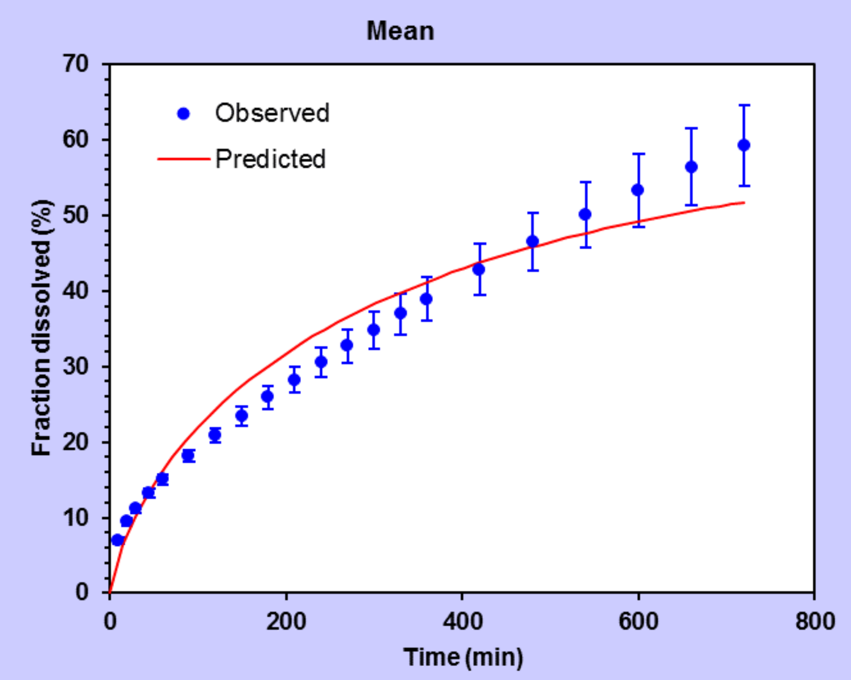

Supplementary materials – Model fitting summary – Emcompress® Anhydrous (modelling up to app. 60 % of carvedilol released)

Graphical abstract of model fit presented as the fraction % of released carvedilol per tested tablet:

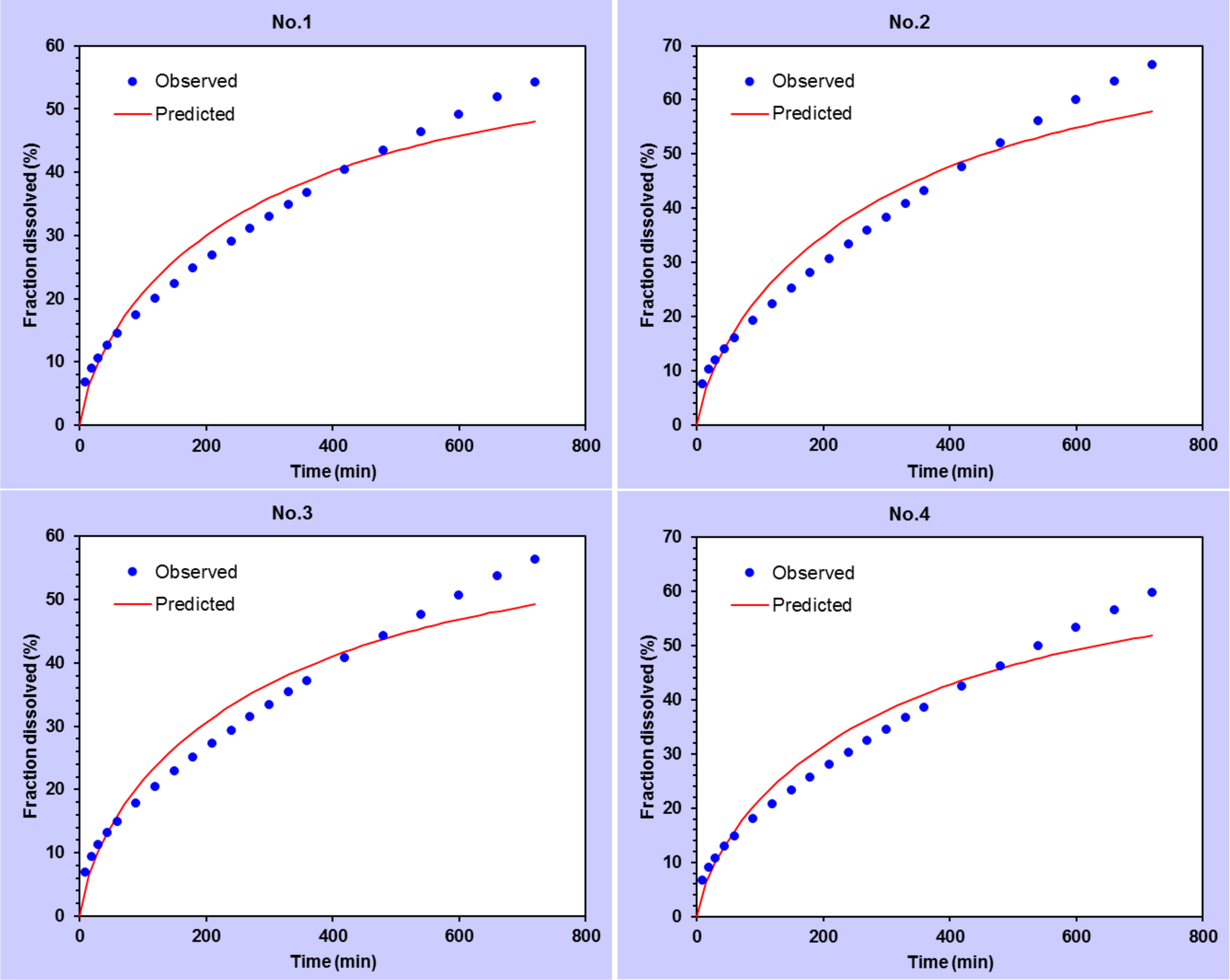

Supplementary materials – Model fitting summary – Emcompress® Anhydrous (modelling up to app. 60 % of carvedilol released)

Model: **Weibull_4**

Model equation: $F = F_{max} \cdot \left[1 - e^{-\frac{(t-T_i)^{\beta}}{\alpha}}\right]$

Fitted model parameters per tested tablet (N = 4) with statistics – mean, standard deviation (SD), and relative standard deviation expressed in % (RSD%) (output from DDSolver):

Parameter	No.1	No.2	No.3	No.4	Mean	SD	RSD(%)
α	40.678	48.230	40.107	46.616	43.908	4.119	9.381
β	0.650	0.669	0.643	0.662	0.656	0.012	1.785
T_i	6.000	6.000	6.000	6.000	6.000	0.000	0.000
F_{max}	56.980	69.711	59.162	62.776	62.157	5.574	8.968

Number of dissolution data points (N), degrees of freedom (df), and selected goodness of fit criteria – Pearson correlation coefficient (R), coefficient of determination (R^2), adjusted coefficient of determination ($R^2_{adjusted}$), and residual sum of squares (RSS) (manual calculation in MS Excel):

Parameter	No.1	No.2	No.3	No.4
N	21	21	21	21
df	17	17	17	17
R	0.974441174	0.969870475	0.970875164	0.971878137
R^2	0.949535602	0.940648738	0.942598584	0.944547113
$R^2_{adjusted}$	0.94063012	0.930174985	0.932468923	0.93476131
RSS	232.3204674	433.0639948	277.9650657	313.6016385

Graphical abstract of model fit presented as mean ± 1 SD of the fraction % of released carvedilol:

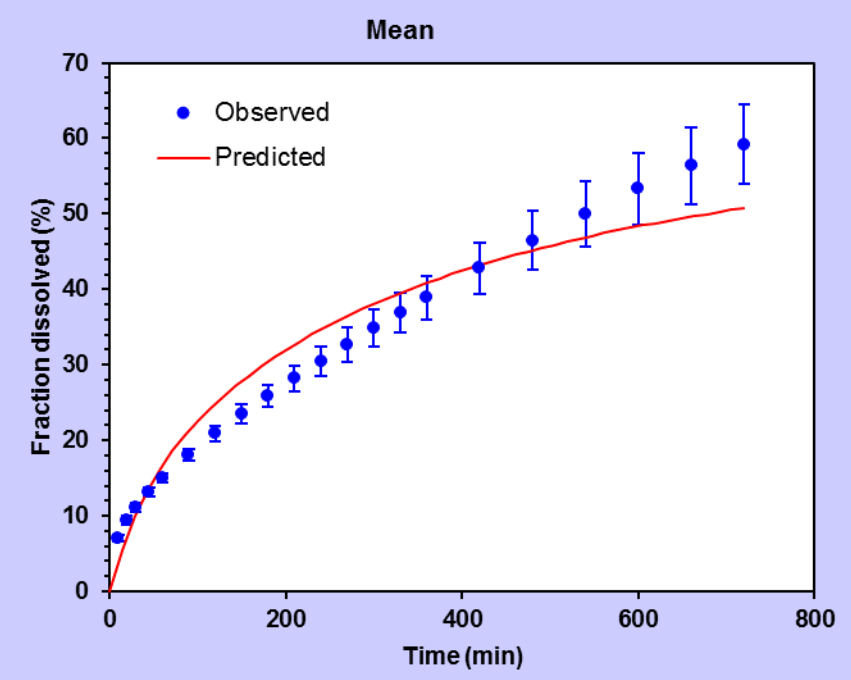

Supplementary materials – Model fitting summary – Emcompress® Anhydrous (modelling up to app. 60 % of carvedilol released)

Graphical abstract of model fit presented as the fraction % of released carvedilol per tested tablet:

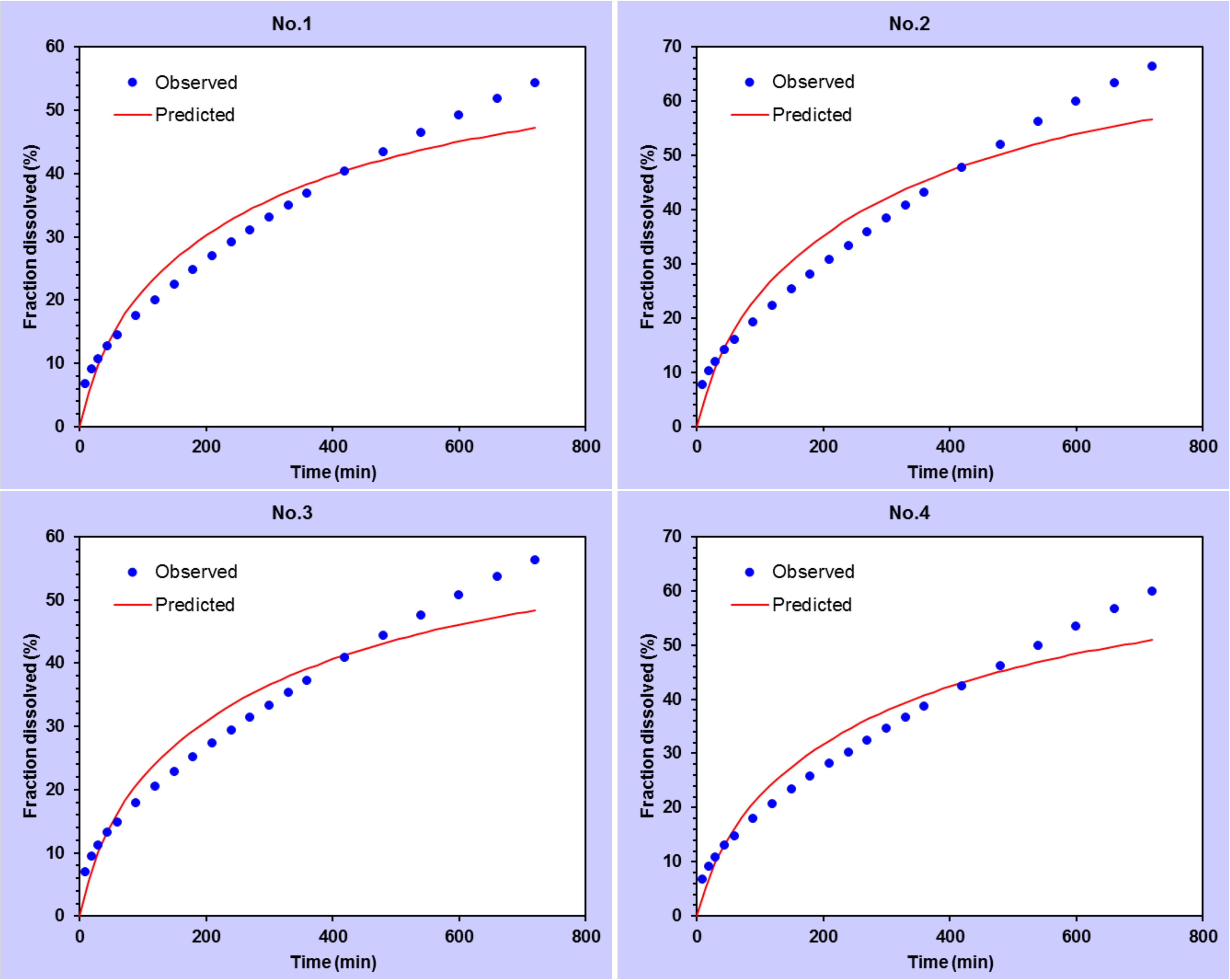

Supplementary materials – Model fitting summary – Emcompress® Anhydrous (modelling up to app. 60 % of carvedilol released)

Model: **Logistic_1**

Model equation: $F = 100 \cdot \frac{e^{\alpha+\beta \cdot \log(t)}}{1+e^{\alpha+\beta \cdot \log(t)}}$

Fitted model parameters per tested tablet (N = 4) with statistics – mean, standard deviation (SD), and relative standard deviation expressed in % (RSD%) (output from DDSolver):

Parameter	No.1	No.2	No.3	No.4	Mean	SD	RSD(%)
α	-4.407	-4.592	-4.386	-4.547	-4.483	0.102	-2.273
β	1.523	1.713	1.529	1.619	1.596	0.090	5.610

Number of dissolution data points (N), degrees of freedom (df), and selected goodness of fit criteria – Pearson correlation coefficient (R), coefficient of determination (R^2), adjusted coefficient of determination ($R^2_{adjusted}$), and residual sum of squares (RSS) (manual calculation in MS Excel):

Parameter	No.1	No.2	No.3	No.4
N	21	21	21	21
df	19	19	19	19
R	0.988291079	0.978874529	0.984478737	0.983398191
R^2	0.976719256	0.958195344	0.969198384	0.967072002
$R^2_{adjusted}$	0.975493954	0.955995099	0.967577246	0.96533895
RSS	123.9691558	329.6811259	167.9388288	206.9473895

Graphical abstract of model fit presented as mean ± 1 SD of the fraction % of released carvedilol:

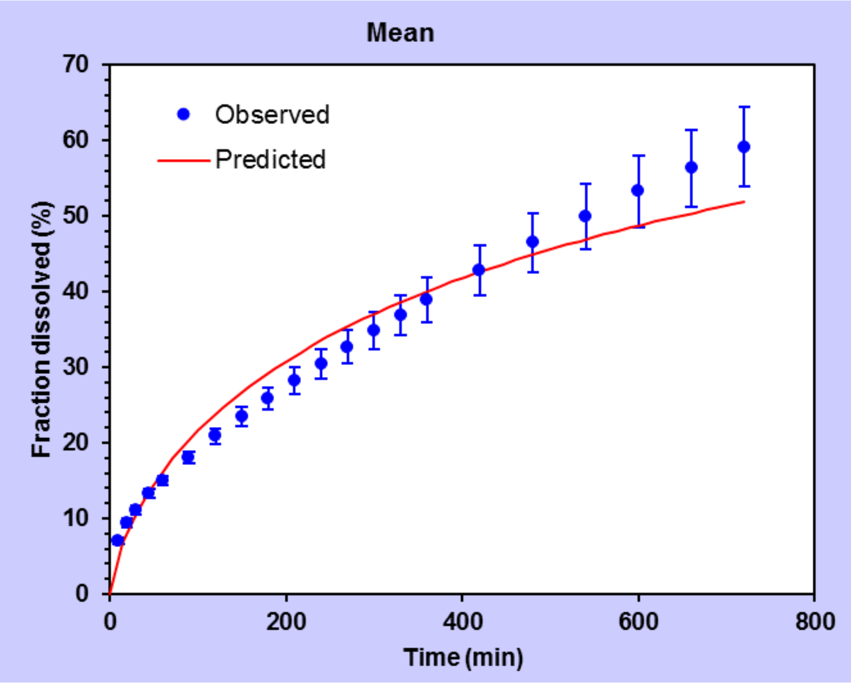

Supplementary materials – Model fitting summary – Emcompress® Anhydrous (modelling up to app. 60 % of carvedilol released)

Graphical abstract of model fit presented as the fraction % of released carvedilol per tested tablet:

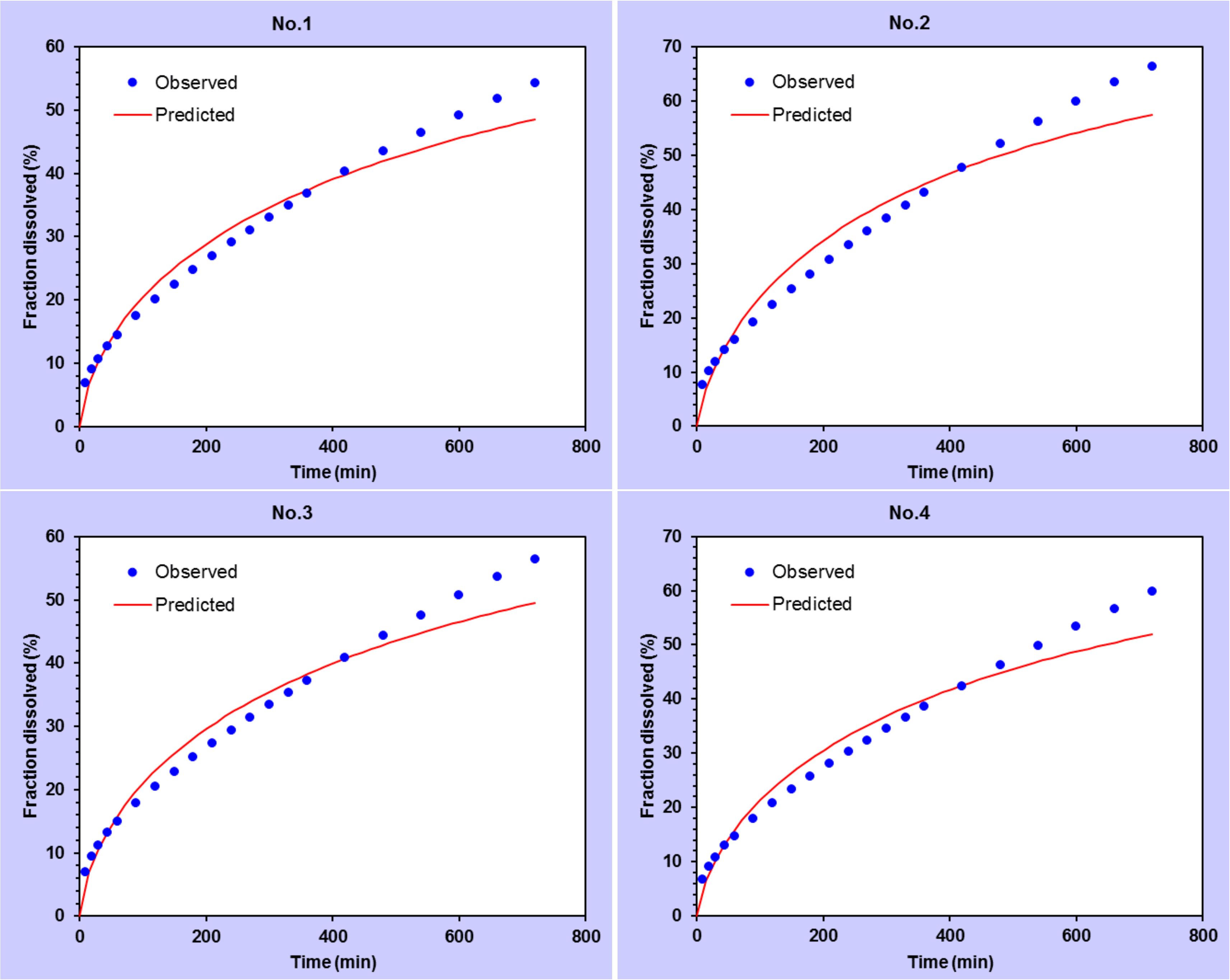

Supplementary materials – Model fitting summary – Emcompress® Anhydrous (modelling up to app. 60 % of carvedilol released)

Model: **Logistic_2**

Model equation: $F = F_{max} \cdot \frac{e^{\alpha+\beta\cdot\log(t)}}{1+e^{\alpha+\beta\cdot\log(t)}}$

Fitted model parameters per tested tablet (N = 4) with statistics – mean, standard deviation (SD), and relative standard deviation expressed in % (RSD%) (output from DDSolver):

Parameter	No.1	No.2	No.3	No.4	Mean	SD	RSD(%)
α	-5.087	-5.269	-5.043	-5.196	-5.149	0.103	-1.996
β	2.335	2.371	2.298	2.336	2.335	0.030	1.280
F_{max}	56.980	69.711	59.162	62.776	62.157	5.574	8.968

Number of dissolution data points (N), degrees of freedom (df), and selected goodness of fit criteria – Pearson correlation coefficient (R), coefficient of determination (R^2), adjusted coefficient of determination ($R^2_{adjusted}$), and residual sum of squares (RSS) (manual calculation in MS Excel):

Parameter	No.1	No.2	No.3	No.4
N	21	21	21	21
df	18	18	18	18
R	0.958545736	0.952906278	0.954572516	0.95578128
R^2	0.918809928	0.908030375	0.911208689	0.913517855
$R^2_{adjusted}$	0.909788808	0.897811528	0.901342988	0.903908728
RSS	395.3316401	694.6877571	454.1870897	510.3604715

Graphical abstract of model fit presented as mean ± 1 SD of the fraction % of released carvedilol:

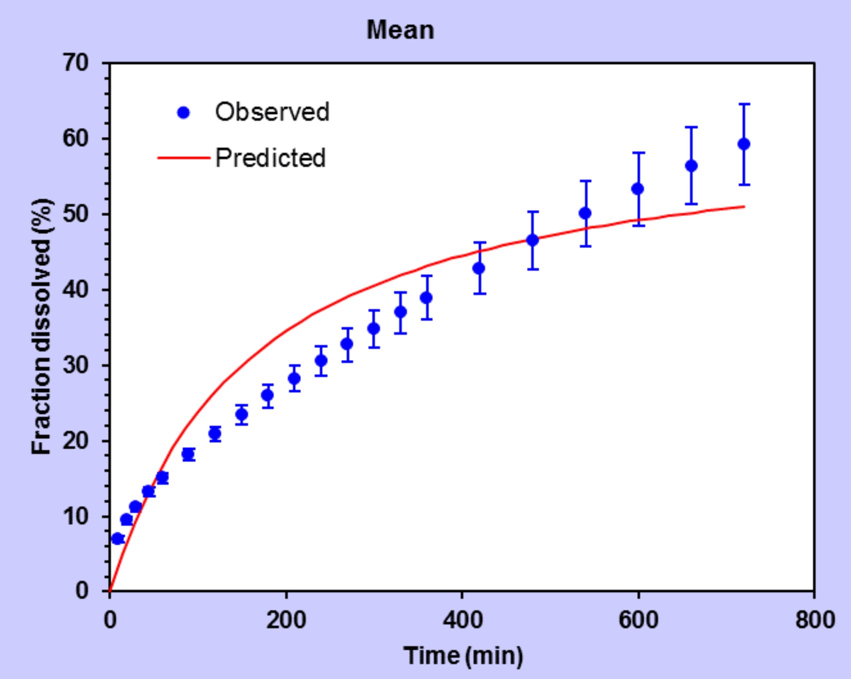

Supplementary materials – Model fitting summary – Emcompress® Anhydrous (modelling up to app. 60 % of carvedilol released)

Graphical abstract of model fit presented as the fraction % of released carvedilol per tested tablet:

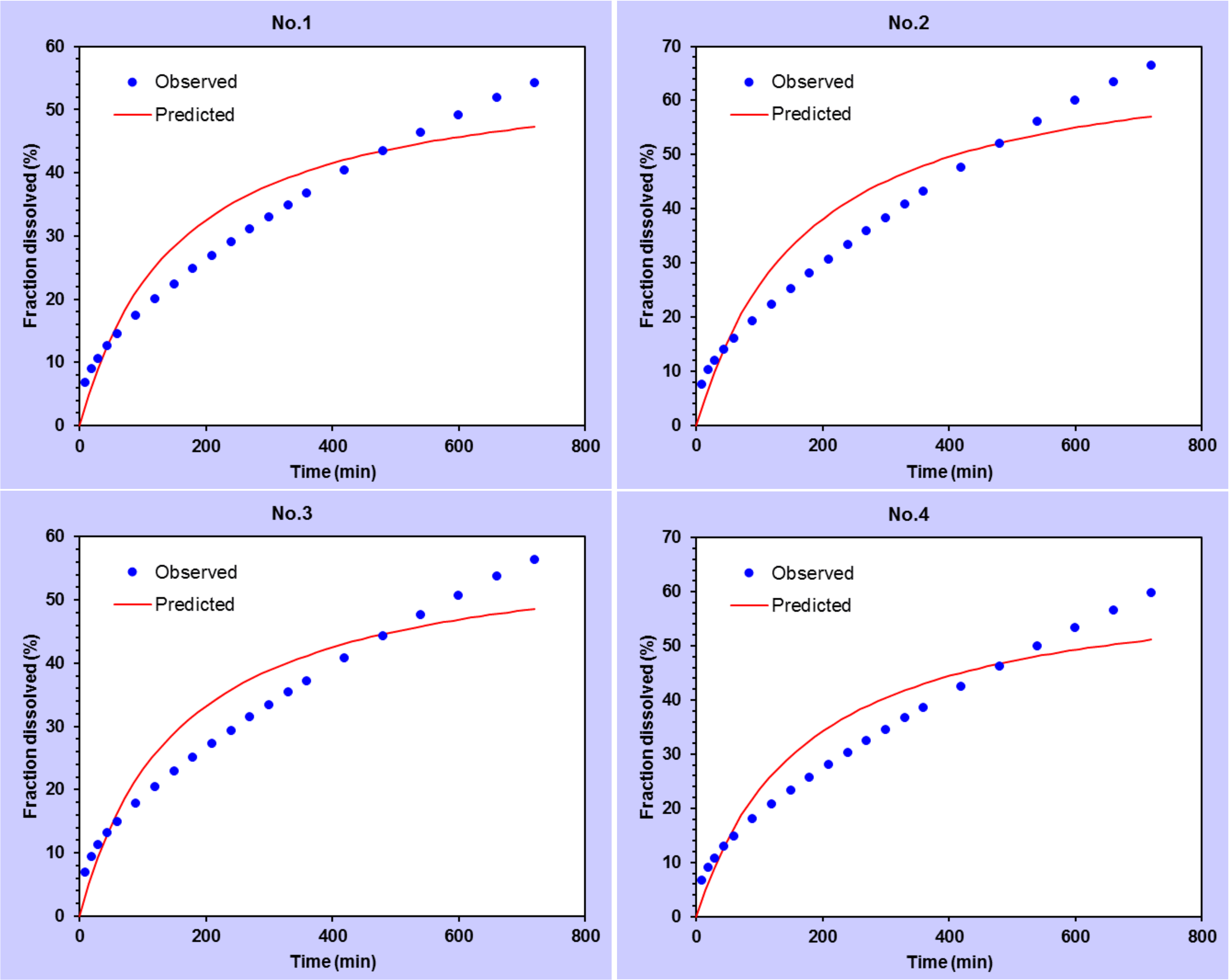

Supplementary materials – Model fitting summary – Emcompress® Anhydrous (modelling up to app. 60 % of carvedilol released)

Model: **Logistic_3**

Model equation: $F = F_{max} \cdot \frac{1}{1+e^{-k\cdot(t-\gamma)}}$

Fitted model parameters per tested tablet (N = 4) with statistics – mean, standard deviation (SD), and relative standard deviation expressed in % (RSD%) (output from DDSolver):

Parameter	No.1	No.2	No.3	No.4	Mean	SD	RSD(%)
k	0.006	0.006	0.006	0.006	0.006	0.000	1.521
γ	257.365	274.446	263.626	275.221	267.664	8.669	3.239
F_{max}	56.980	69.711	59.162	62.776	62.157	5.574	8.968

Number of dissolution data points (N), degrees of freedom (df), and selected goodness of fit criteria – Pearson correlation coefficient (R), coefficient of determination (R^2), adjusted coefficient of determination ($R^2_{adjusted}$), and residual sum of squares (RSS) (manual calculation in MS Excel):

Parameter	No.1	No.2	No.3	No.4
N	21	21	21	21
df	18	18	18	18
R	0.99240036	0.994263648	0.992596754	0.992145055
R^2	0.984858475	0.988560202	0.985248317	0.984351811
$R^2_{adjusted}$	0.983176084	0.987289114	0.983609241	0.982613123
RSS	68.88043725	82.454253	71.68435597	88.56677941

Graphical abstract of model fit presented as mean ± 1 SD of the fraction % of released carvedilol:

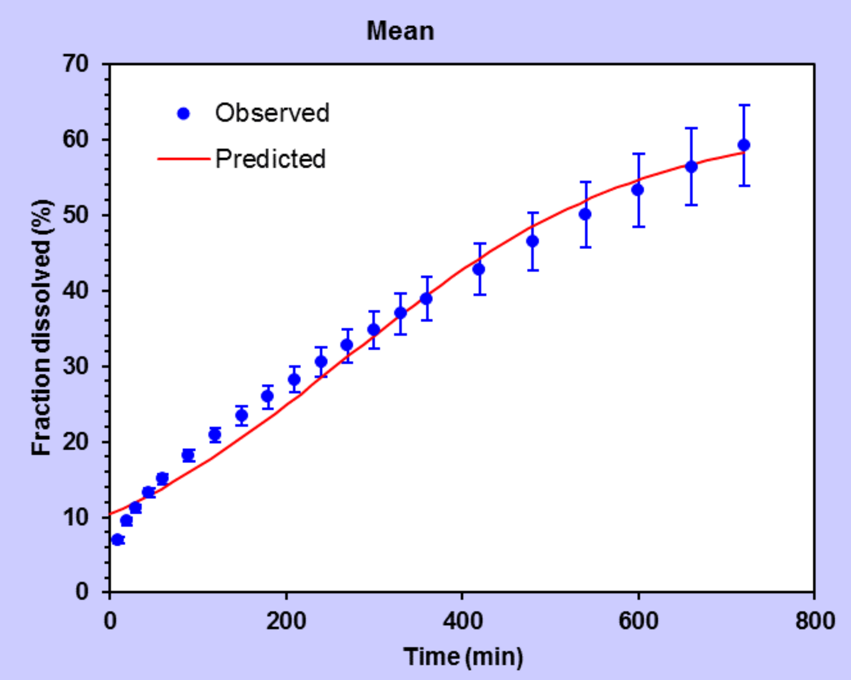

Supplementary materials – Model fitting summary – Emcompress® Anhydrous (modelling up to app. 60 % of carvedilol released)

Graphical abstract of model fit presented as the fraction % of released carvedilol per tested tablet:

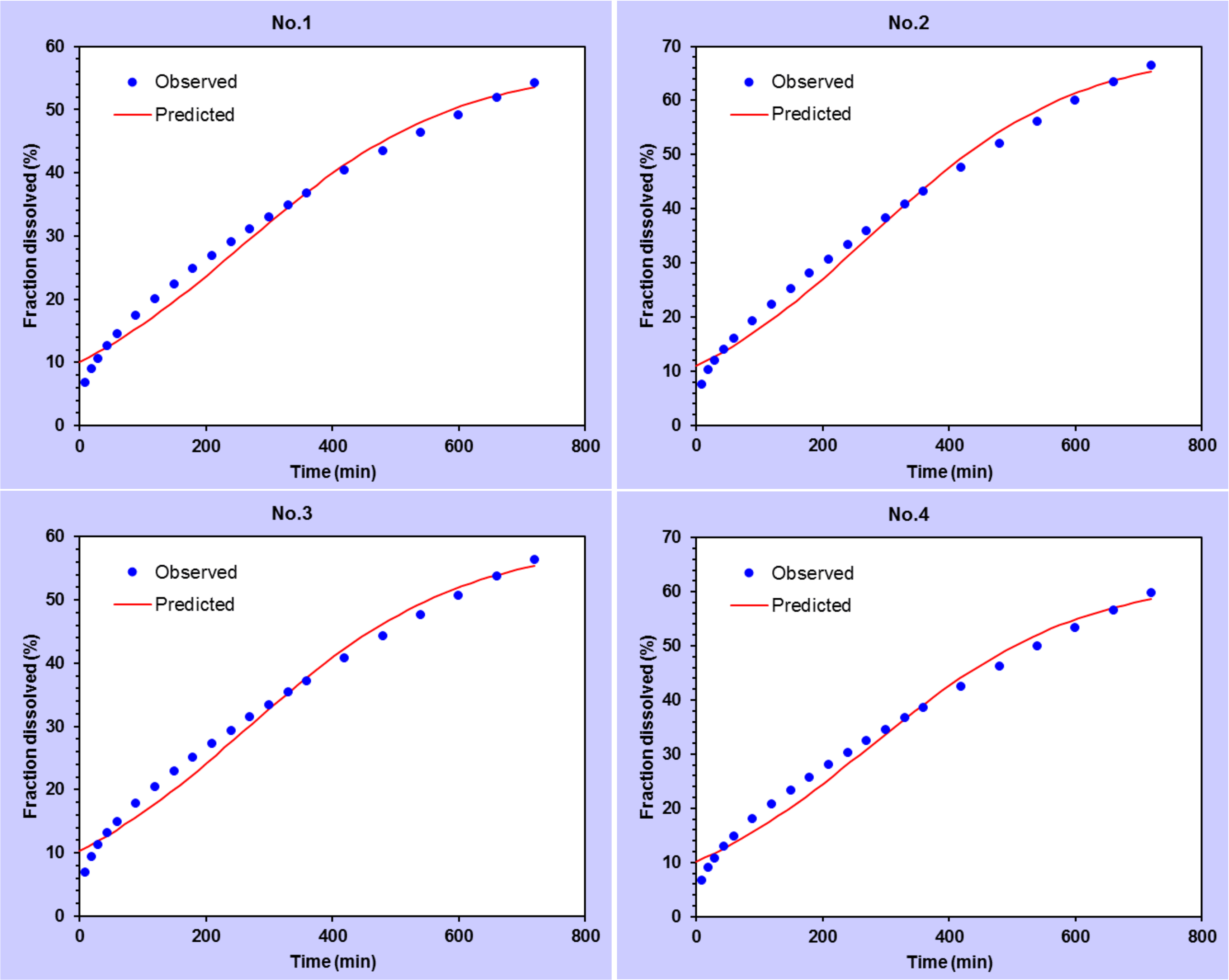

Supplementary materials – Model fitting summary – Emcompress® Anhydrous (modelling up to app. 60 % of carvedilol released)

Model: **Gompertz_1**

Model equation: $F = 100 \cdot e^{-\alpha \cdot e^{-\beta \cdot \log(t)}}$

Fitted model parameters per tested tablet (N = 4) with statistics – mean, standard deviation (SD), and relative standard deviation expressed in % (RSD%) (output from DDSolver):

Parameter	No.1	No.2	No.3	No.4	Mean	SD	RSD(%)
α	7.574	9.496	7.650	8.452	8.293	0.895	10.791
β	0.805	0.975	0.818	0.876	0.868	0.077	8.907

Number of dissolution data points (N), degrees of freedom (df), and selected goodness of fit criteria – Pearson correlation coefficient (R), coefficient of determination (R^2), adjusted coefficient of determination ($R^2_{adjusted}$), and residual sum of squares (RSS) (manual calculation in MS Excel):

Parameter	No.1	No.2	No.3	No.4
N	21	21	21	21
df	19	19	19	19
R	0.970505755	0.955677935	0.965106882	0.96280912
R^2	0.941881421	0.913320315	0.931431293	0.927001401
$R^2_{adjusted}$	0.938822549	0.908758226	0.927822414	0.92315937
RSS	270.0676324	613.194037	332.77619	408.7399135

Graphical abstract of model fit presented as mean ± 1 SD of the fraction % of released carvedilol:

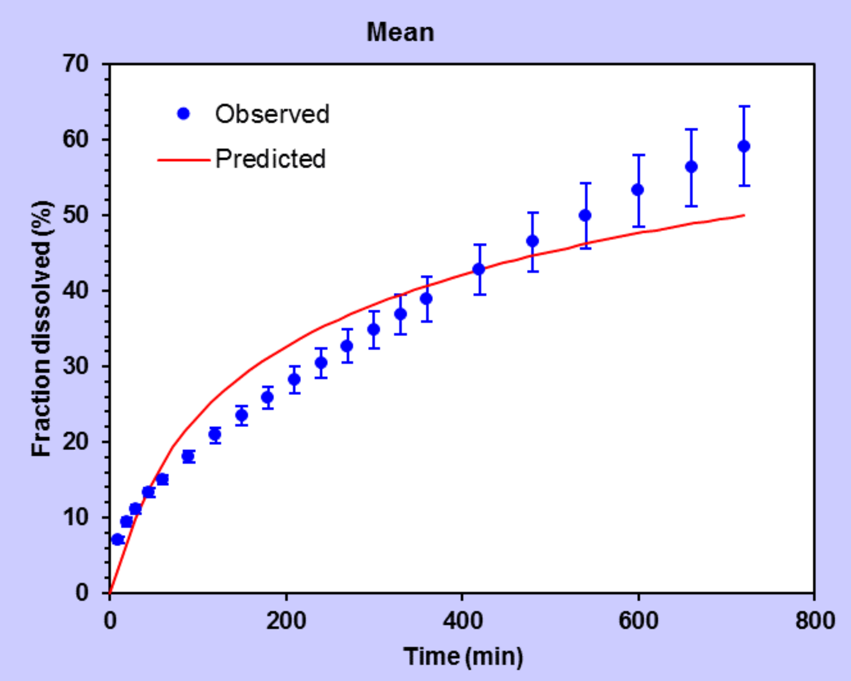

Supplementary materials – Model fitting summary – Emcompress® Anhydrous (modelling up to app. 60 % of carvedilol released)

Graphical abstract of model fit presented as the fraction % of released carvedilol per tested tablet:

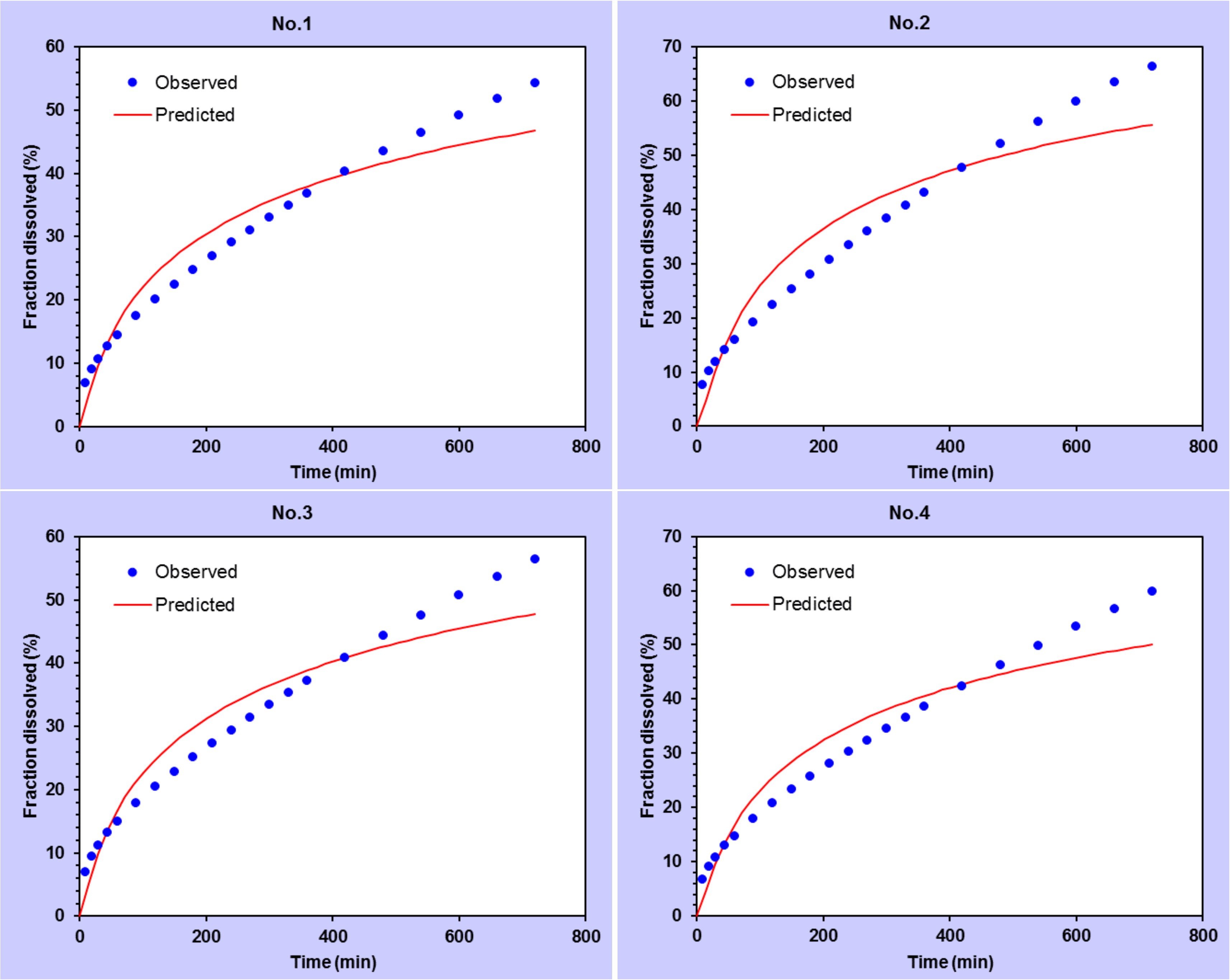

Supplementary materials – Model fitting summary – Emcompress® Anhydrous (modelling up to app. 60 % of carvedilol released)

Model: **Gompertz_2**

Model equation: $F = F_{max} \cdot e^{-\alpha \cdot e^{-\beta \cdot \log(t)}}$

Fitted model parameters per tested tablet (N = 4) with statistics – mean, standard deviation (SD), and relative standard deviation expressed in % (RSD%) (output from DDSolver):

Parameter	No.1	No.2	No.3	No.4	Mean	SD	RSD(%)
α	34.831	37.283	33.349	35.029	35.123	1.623	4.621
β	1.666	1.666	1.633	1.635	1.650	0.019	1.128
F_{max}	56.980	69.711	59.162	62.776	62.157	5.574	8.968

Number of dissolution data points (N), degrees of freedom (df), and selected goodness of fit criteria – Pearson correlation coefficient (R), coefficient of determination (R^2), adjusted coefficient of determination ($R^2_{adjusted}$), and residual sum of squares (RSS) (manual calculation in MS Excel):

Parameter	No.1	No.2	No.3	No.4
N	21	21	21	21
df	18	18	18	18
R	0.963539336	0.958131881	0.959546315	0.960597553
R^2	0.928408052	0.918016702	0.920729131	0.922747659
$R^2_{adjusted}$	0.920453391	0.908907447	0.911921257	0.914164066
RSS	663.7067993	1042.584817	754.590804	843.5712093

Graphical abstract of model fit presented as mean ± 1 SD of the fraction % of released carvedilol:

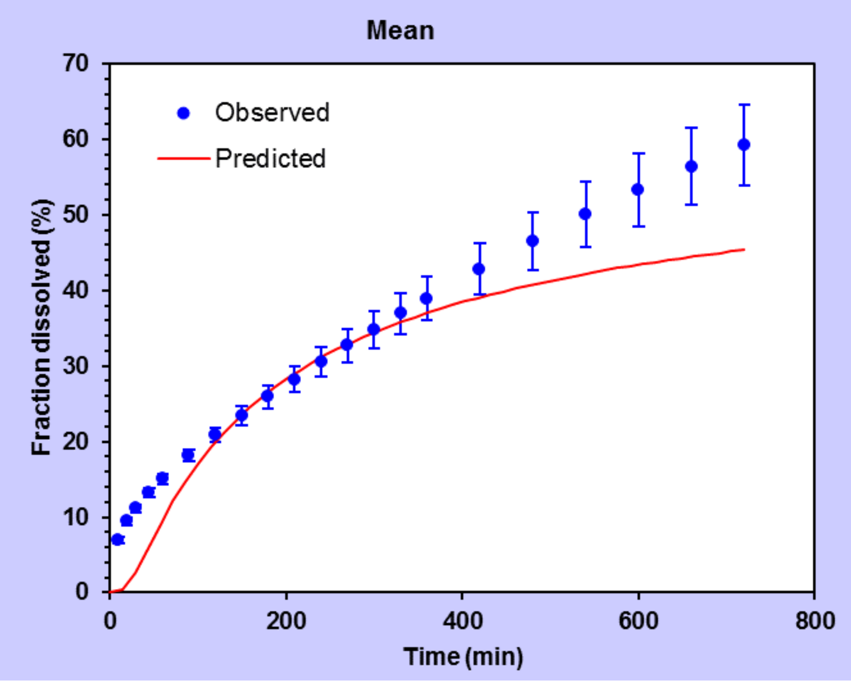

Supplementary materials – Model fitting summary – Emcompress® Anhydrous (modelling up to app. 60 % of carvedilol released)

Graphical abstract of model fit presented as the fraction % of released carvedilol per tested tablet:

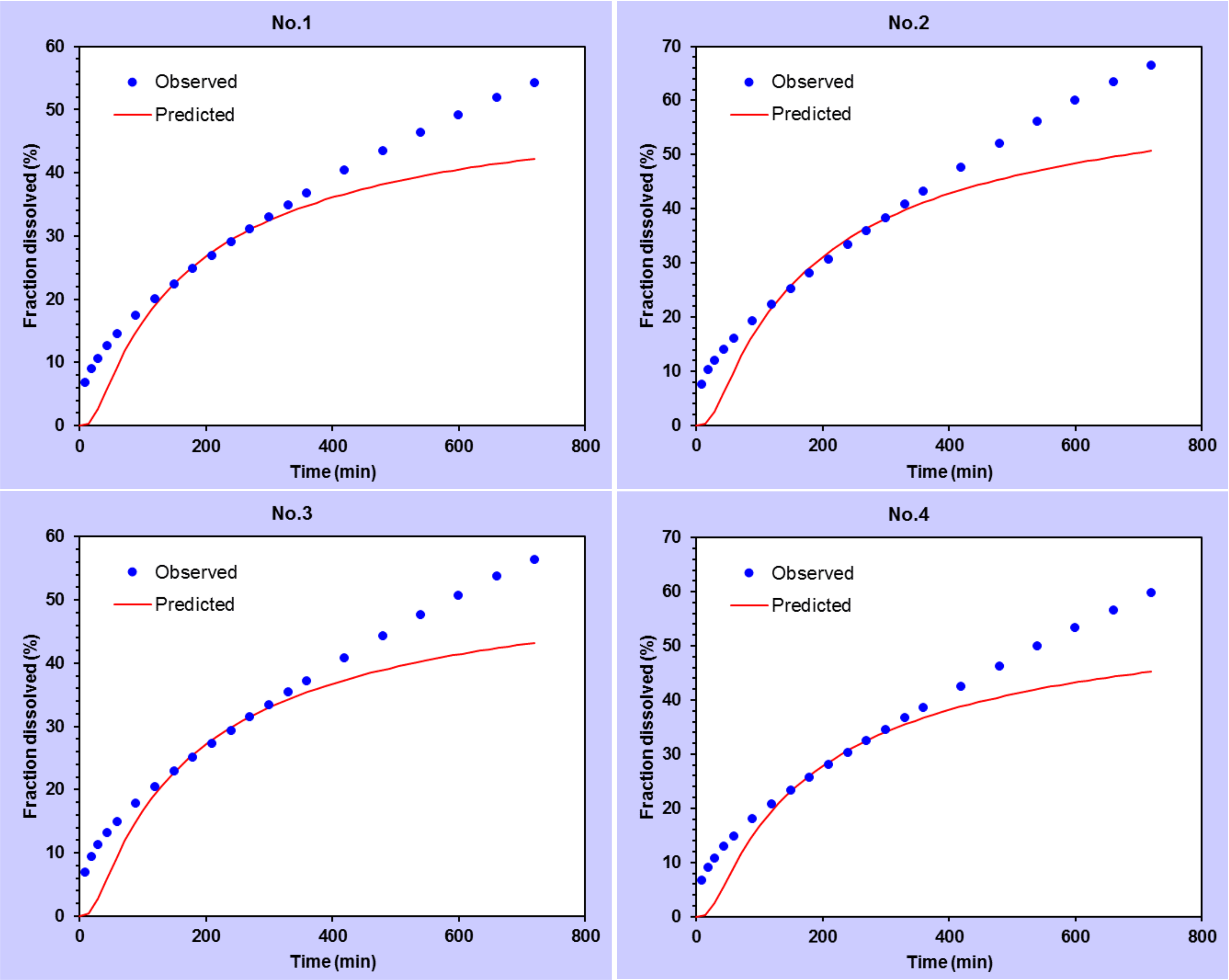

Supplementary materials – Model fitting summary – Emcompress® Anhydrous (modelling up to app. 60 % of carvedilol released)

Model: **Gompertz_3**

Model equation: $F = F_{max} \cdot e^{-e^{-k\cdot(t-\gamma)}}$

Fitted model parameters per tested tablet (N = 4) with statistics – mean, standard deviation (SD), and relative standard deviation expressed in % (RSD%) (output from DDSolver):

Parameter	No.1	No.2	No.3	No.4	Mean	SD	RSD(%)
k	0.005	0.005	0.004	0.004	0.005	0.000	1.219
γ	151.532	167.750	156.551	166.760	160.648	7.910	4.924
F_{max}	56.980	69.711	59.162	62.776	62.157	5.574	8.968

Number of dissolution data points (N), degrees of freedom (df), and selected goodness of fit criteria – Pearson correlation coefficient (R), coefficient of determination (R^2), adjusted coefficient of determination ($R^2_{adjusted}$), and residual sum of squares (RSS) (manual calculation in MS Excel):

Parameter	No.1	No.2	No.3	No.4
N	21	21	21	21
df	18	18	18	18
R	0.99598995	0.995672131	0.994871682	0.994598692
R^2	0.99199598	0.991362992	0.989769663	0.989226558
$R^2_{adjusted}$	0.991106645	0.990403325	0.988632959	0.988029509
RSS	42.95900201	77.99968876	60.18496168	73.3769994

Graphical abstract of model fit presented as mean ± 1 SD of the fraction % of released carvedilol:

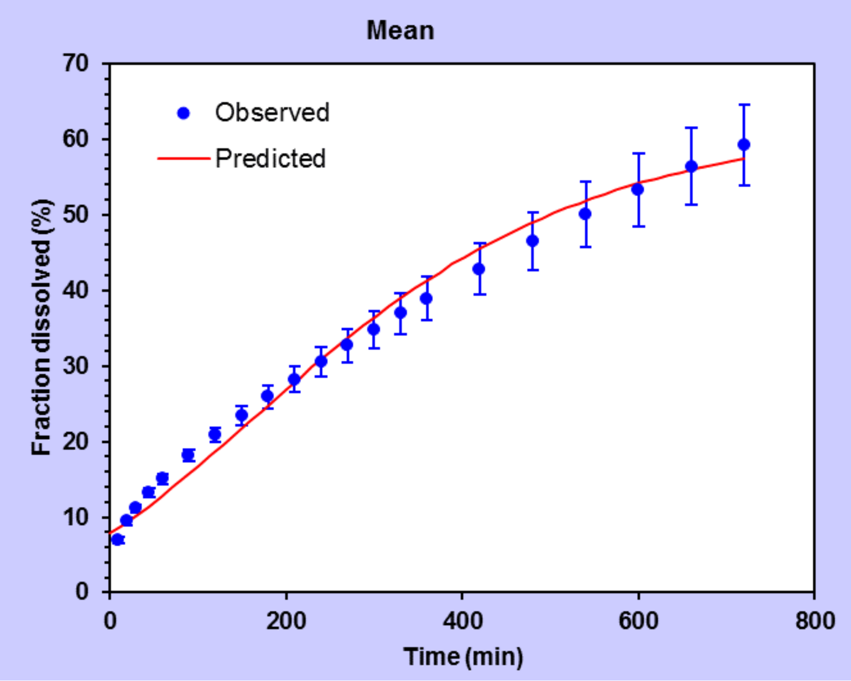

Supplementary materials – Model fitting summary – Emcompress® Anhydrous (modelling up to app. 60 % of carvedilol released)

Graphical abstract of model fit presented as the fraction % of released carvedilol per tested tablet:

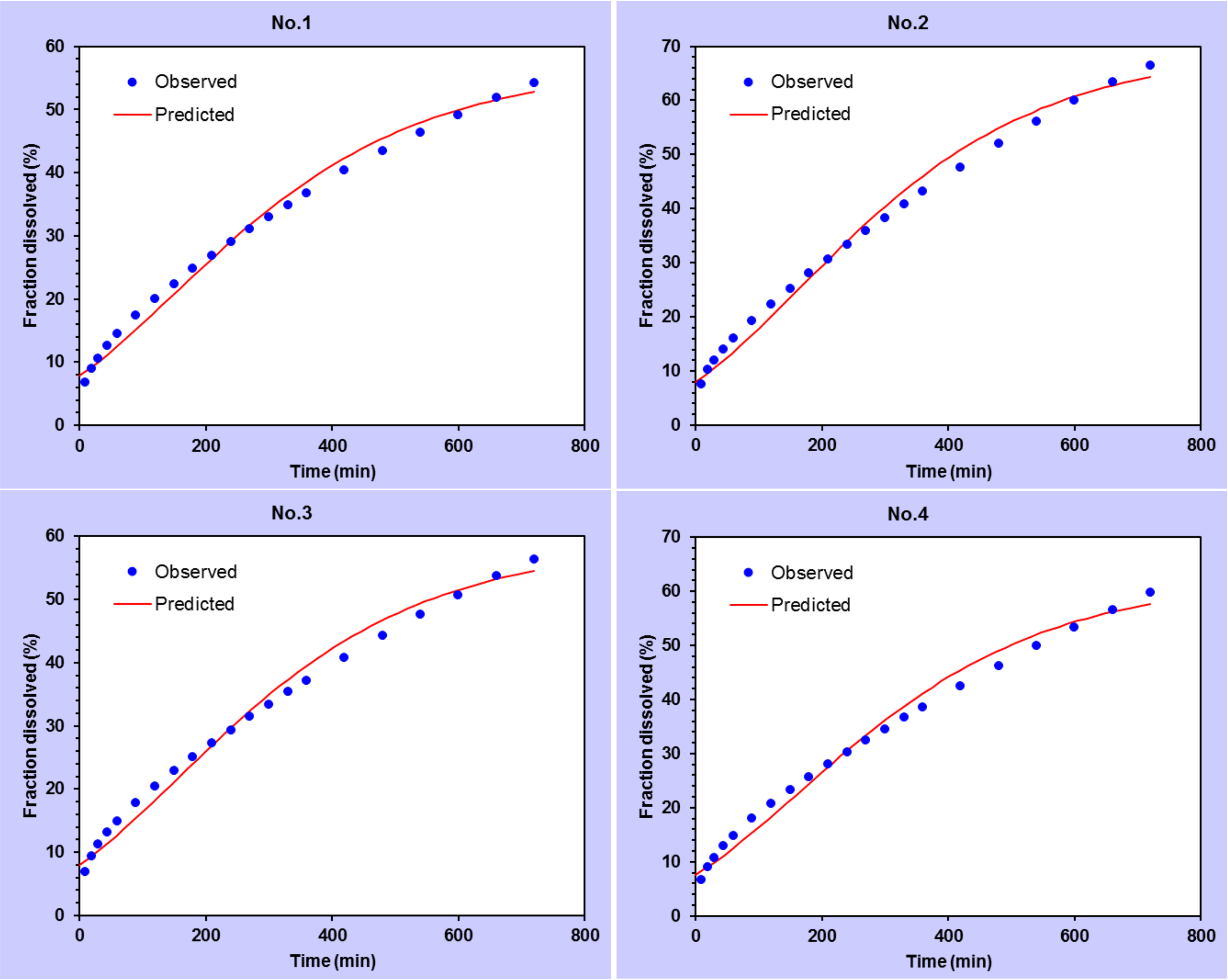

Supplementary materials – Model fitting summary – Emcompress® Anhydrous (modelling up to app. 60 % of carvedilol released)

Model: **Gompertz_4**

Model equation: $F = F_{max} \cdot e^{-\beta \cdot e^{-k \cdot t}}$

Fitted model parameters per tested tablet (N = 4) with statistics – mean, standard deviation (SD), and relative standard deviation expressed in % (RSD%) (output from DDSolver):

Parameter	No.1	No.2	No.3	No.4	Mean	SD	RSD(%)
k	0.005	0.005	0.004	0.004	0.005	0.000	1.219
β	1.985	2.157	2.012	2.108	2.065	0.081	3.912
F_{max}	56.980	69.711	59.162	62.776	62.157	5.574	8.968

Number of dissolution data points (N), degrees of freedom (df), and selected goodness of fit criteria – Pearson correlation coefficient (R), coefficient of determination (R^2), adjusted coefficient of determination ($R^2_{adjusted}$), and residual sum of squares (RSS) (manual calculation in MS Excel):

Parameter	No.1	No.2	No.3	No.4
N	21	21	21	21
df	18	18	18	18
R	0.99598995	0.995672131	0.994871682	0.994598692
R^2	0.99199598	0.991362992	0.989769663	0.989226558
$R^2_{adjusted}$	0.991106645	0.990403325	0.988632959	0.988029509
RSS	42.95900201	77.99968876	60.18496168	73.3769994

Graphical abstract of model fit presented as mean ± 1 SD of the fraction % of released carvedilol:

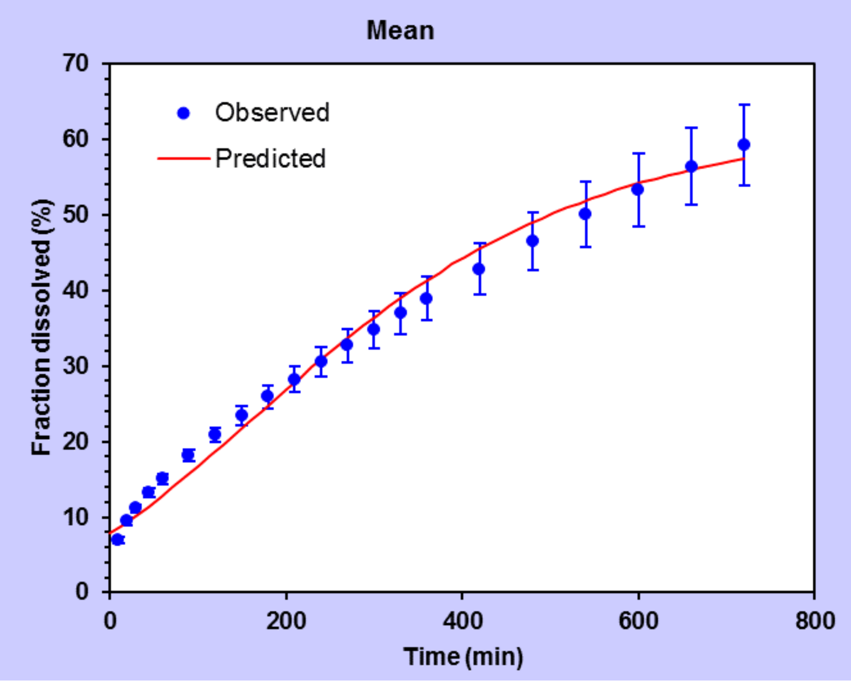

Supplementary materials – Model fitting summary – Emcompress® Anhydrous (modelling up to app. 60 % of carvedilol released)

Graphical abstract of model fit presented as the fraction % of released carvedilol per tested tablet:

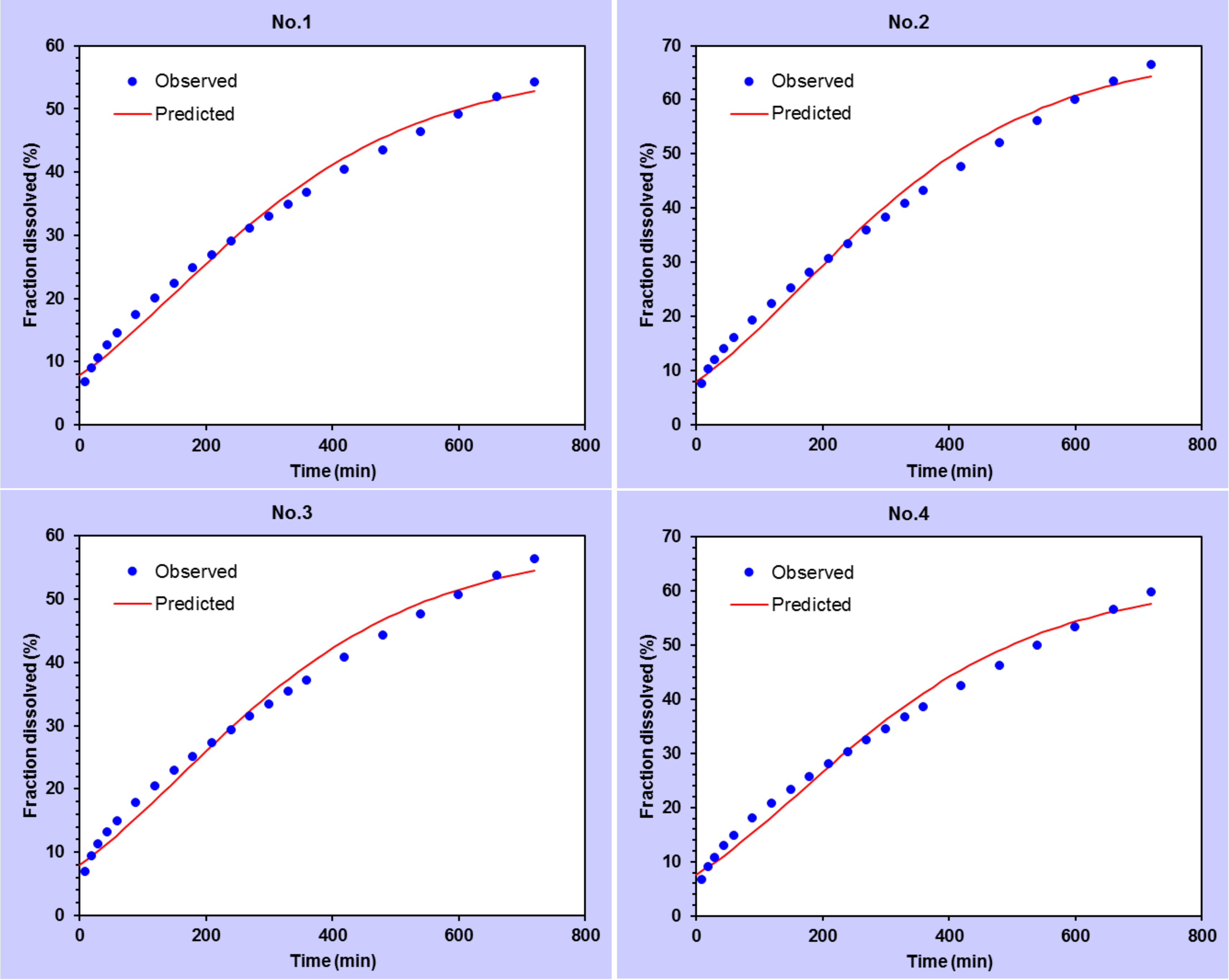

Supplementary materials – Model fitting summary – Emcompress® Anhydrous (modelling up to app. 60 % of carvedilol released)

Model: **Probit_1**

Model equation: $F = 100 \cdot \phi[\alpha + \beta \cdot \log(t)]$

Fitted model parameters per tested tablet (N = 4) with statistics – mean, standard deviation (SD), and relative standard deviation expressed in % (RSD%) (output from DDSolver):

Parameter	No.1	No.2	No.3	No.4	Mean	SD	RSD(%)
α	-2.562	-2.705	-2.827	-2.652	-2.687	0.111	-4.120
β	0.878	1.006	1.016	0.938	0.959	0.065	6.724

Number of dissolution data points (N), degrees of freedom (df), and selected goodness of fit criteria – Pearson correlation coefficient (R), coefficient of determination (R^2), adjusted coefficient of determination ($R^2_{adjusted}$), and residual sum of squares (RSS) (manual calculation in MS Excel):

Parameter	No.1	No.2	No.3	No.4
N	21	21	21	21
df	19	19	19	19
R	0.982890824	0.972783017	0.981461188	0.977390563
R^2	0.966074372	0.946306797	0.963266063	0.955292312
$R^2_{adjusted}$	0.964288813	0.943480839	0.961332698	0.952939276
RSS	171.9221566	409.4973862	218.106898	270.7574313

Graphical abstract of model fit presented as mean ± 1 SD of the fraction % of released carvedilol:

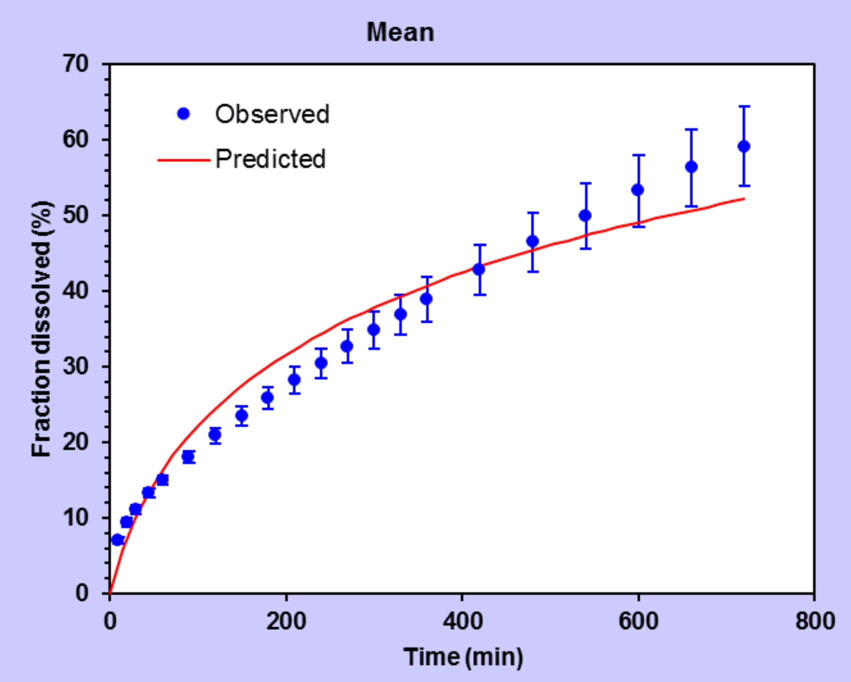

Supplementary materials – Model fitting summary – Emcompress® Anhydrous (modelling up to app. 60 % of carvedilol released)

Graphical abstract of model fit presented as the fraction % of released carvedilol per tested tablet:

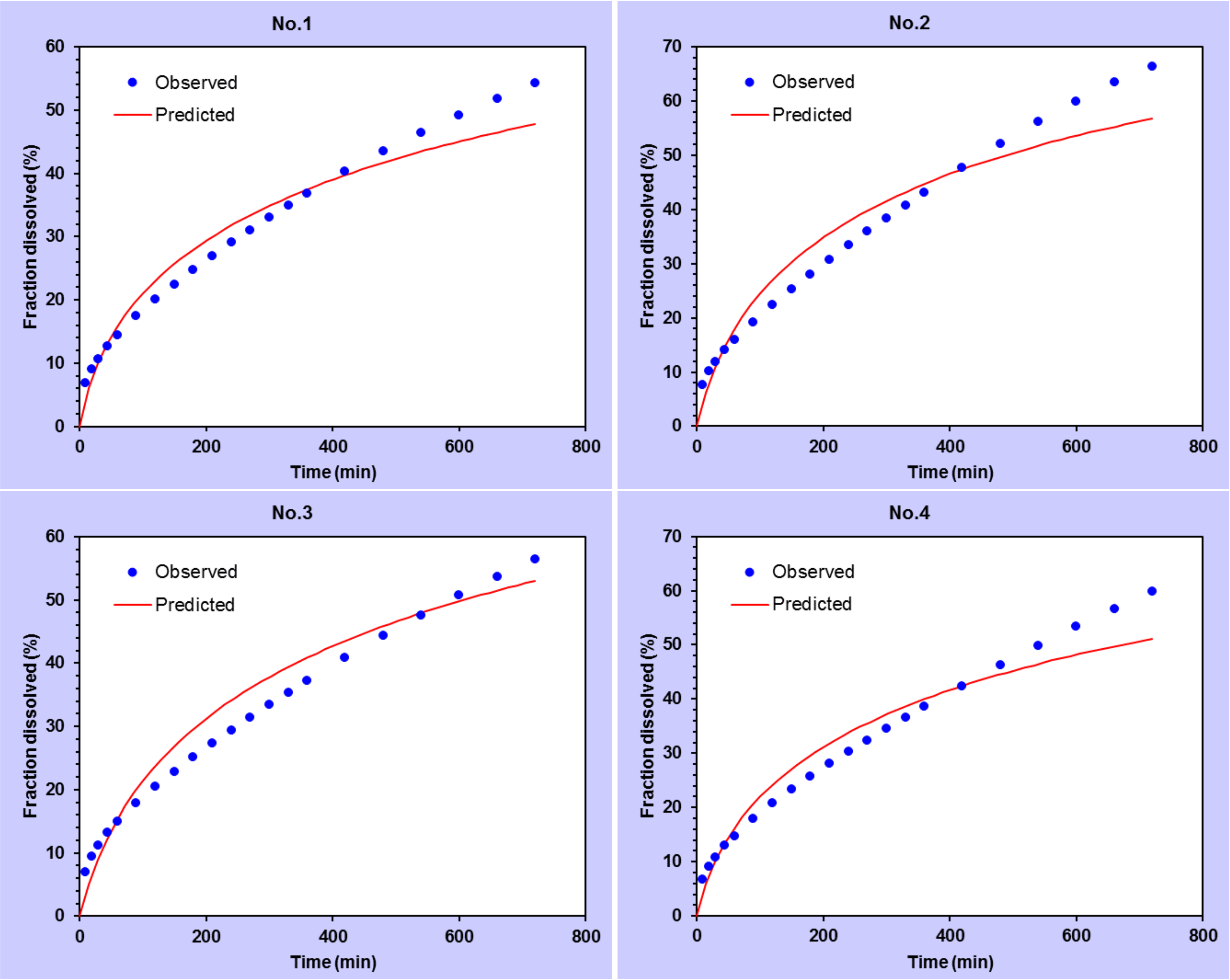

Supplementary materials – Model fitting summary – Emcompress® Anhydrous (modelling up to app. 60 % of carvedilol released)

Model: **Probit_2**

Model equation: $F = F_{max} \cdot \phi[\alpha + \beta \cdot \log(t)]$

Fitted model parameters per tested tablet (N = 4) with statistics – mean, standard deviation (SD), and relative standard deviation expressed in % (RSD%) (output from DDSolver):

Parameter	No.1	No.2	No.3	No.4	Mean	SD	RSD(%)
α	-3.025	-3.127	-3.000	-3.084	-3.059	0.057	-1.872
β	1.385	1.403	1.363	1.383	1.384	0.016	1.182
F_{max}	56.980	69.711	59.162	62.776	62.157	5.574	8.968

Number of dissolution data points (N), degrees of freedom (df), and selected goodness of fit criteria – Pearson correlation coefficient (R), coefficient of determination (R^2), adjusted coefficient of determination ($R^2_{adjusted}$), and residual sum of squares (RSS) (manual calculation in MS Excel):

Parameter	No.1	No.2	No.3	No.4
N	21	21	21	21
df	18	18	18	18
R	0.958571589	0.952145687	0.954423306	0.955080529
R^2	0.918859491	0.906581408	0.910923847	0.912178816
$R^2_{adjusted}$	0.909843879	0.896201565	0.901026496	0.902420907
RSS	379.1975051	678.8139265	437.7290834	498.6574359

Graphical abstract of model fit presented as mean ± 1 SD of the fraction % of released carvedilol:

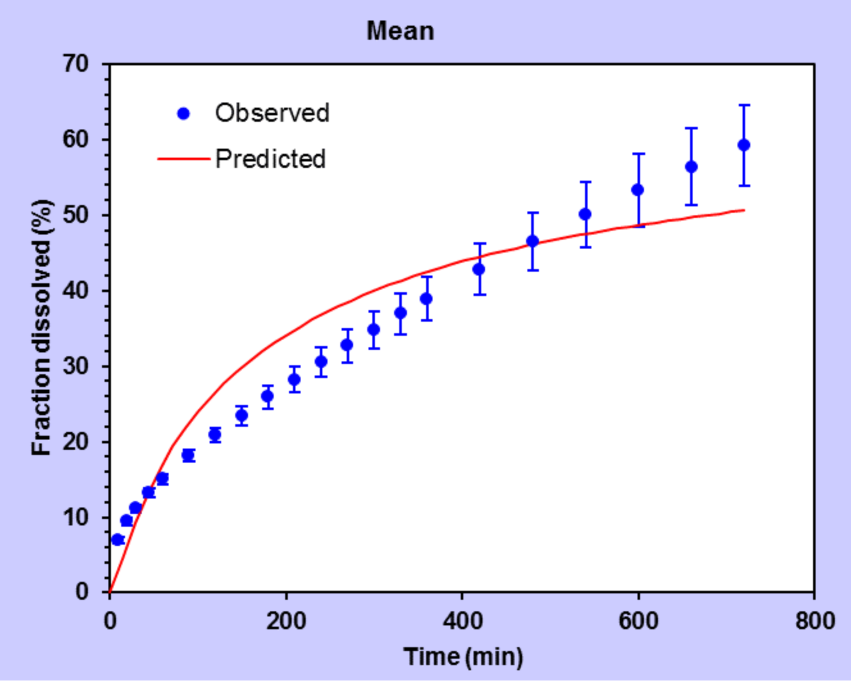

Supplementary materials – Model fitting summary – Emcompress® Anhydrous (modelling up to app. 60 % of carvedilol released)

Graphical abstract of model fit presented as the fraction % of released carvedilol per tested tablet:

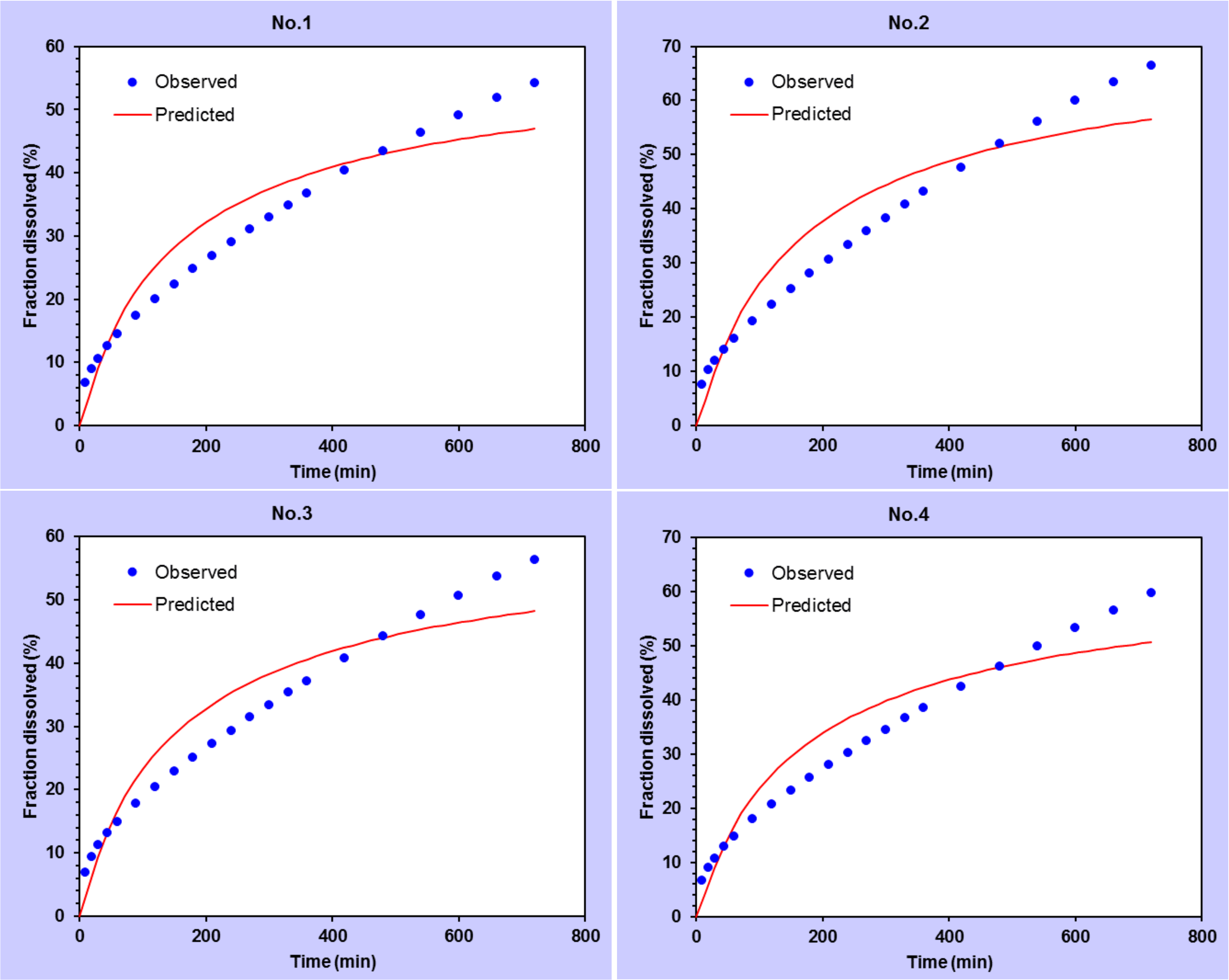

Supplement: Supplementary file 1 [file pharmaceutics-16-00498-s001.zip › Supplementary materials_Model fitting summary_Emcompress® Anhydrous.pdf]
