# Supplementary material for: Comparative Fitting of Mathematical Models to Carvedilol Release Profiles Obtained from Hypromellose Matrix Tablets
Source: Pharmaceutics. 2024 Apr 4;16(4):498. doi: 10.3390/pharmaceutics16040498 (PMC11053526; doi:10.3390/pharmaceutics16040498)

Supplementary materials – Model fitting summary – Ethocel™ Standard 20 Premium (modelling the entire relevant dissolution data)

Model: **Zero–order**

Model equation: $F = k_0 \cdot t$

Fitted model parameters per tested tablet (N = 4) with statistics – mean, standard deviation (SD), and relative standard deviation expressed in % (RSD%) (output from DDSolver):

Parameter	No.1	No.2	No.3	No.4	Mean	SD	RSD(%)
k_0	0.065	0.065	0.069	0.063	0.066	0.002	3.663

Number of dissolution data points (N), degrees of freedom (df), and selected goodness of fit criteria – Pearson correlation coefficient (R), coefficient of determination (R^2), adjusted coefficient of determination ($R^2_{adjusted}$), and residual sum of squares (RSS) (manual calculation in MS Excel):

Parameter	No.1	No.2	No.3	No.4
N	33	33	33	33
df	32	32	32	32
R	0.987306784	0.98645439	0.991484385	0.988513637
R^2	0.974774686	0.973092264	0.983041286	0.977159211
$R^2_{adjusted}$	0.974774686	0.973092264	0.983041286	0.977159211
RSS	1322.068532	1638.641589	1162.542739	1325.124809

Graphical abstract of model fit presented as mean ± 1 SD of the fraction % of released carvedilol:

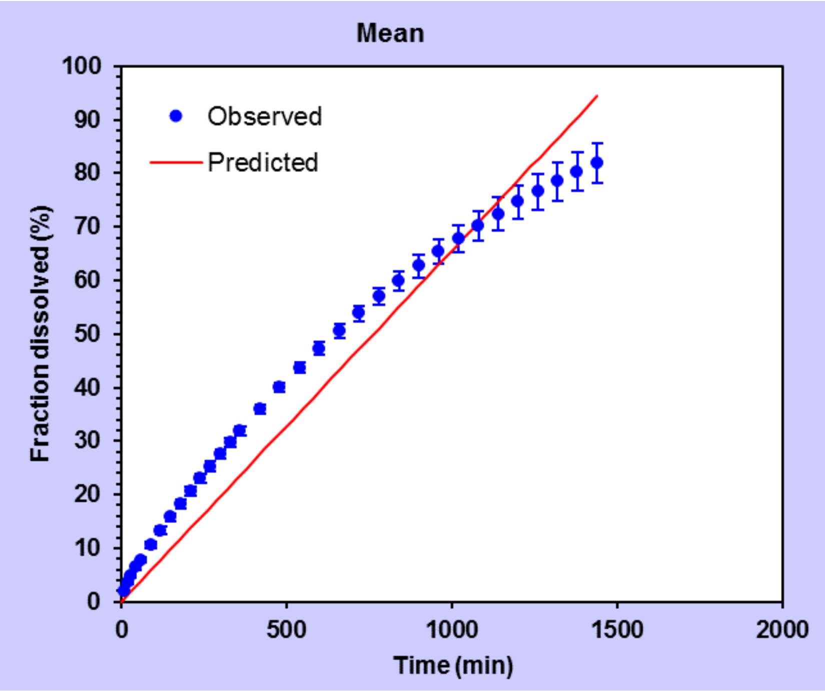

Supplementary materials – Model fitting summary – Ethocel^{TM} Standard 20 Premium (modelling the entire relevant dissolution data)

Graphical abstract of model fit presented as the fraction % of released carvedilol per tested tablet:

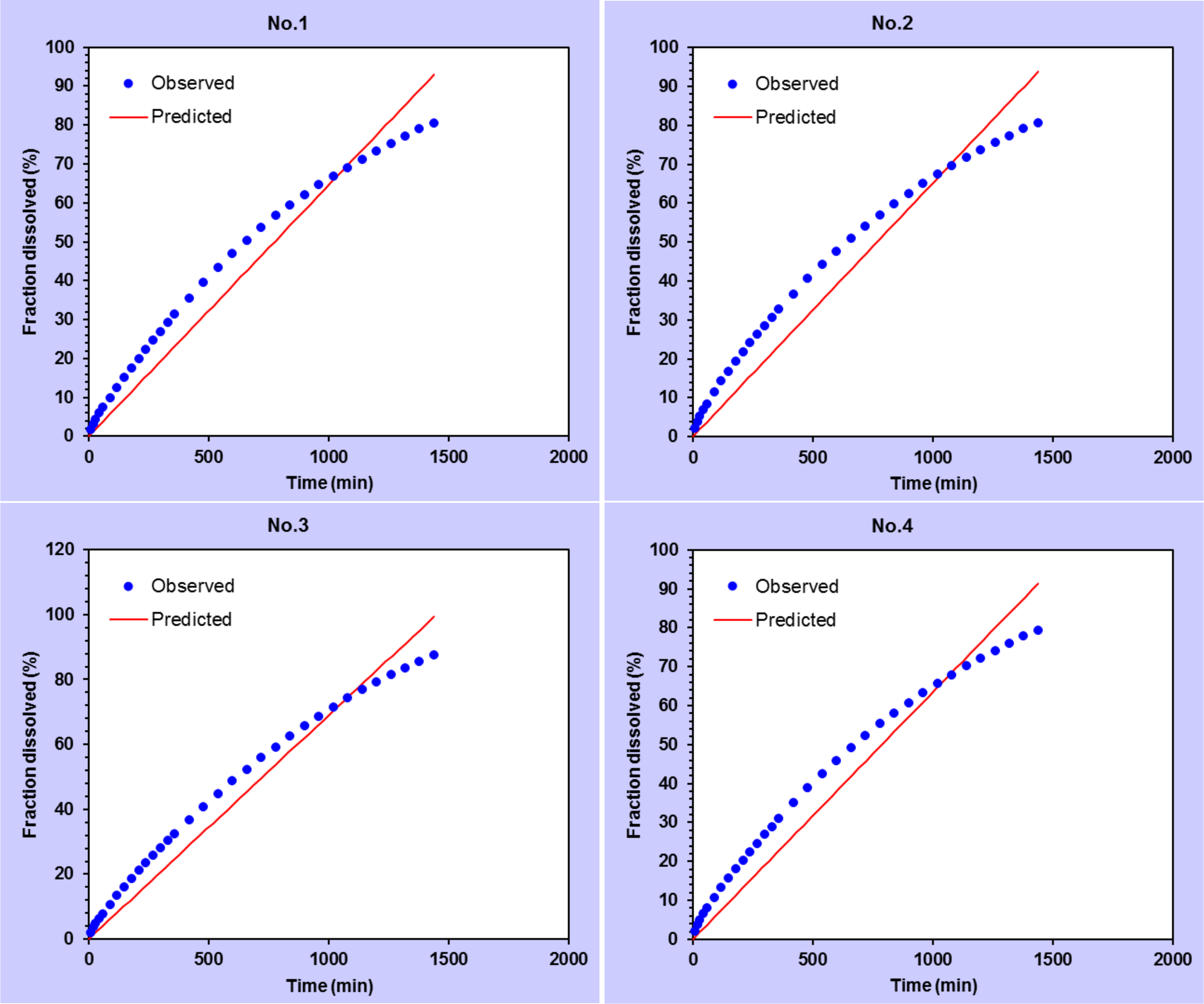

Supplementary materials – Model fitting summary – Ethocel™ Standard 20 Premium (modelling the entire relevant dissolution data)

Model: **Zero–order with T_{lag}**

Model equation: $F = k_0 \cdot (t - T_{lag})$

Fitted model parameters per tested tablet (N = 4) with statistics – mean, standard deviation (SD), and relative standard deviation expressed in % (RSD%) (output from DDSolver):

Parameter	No.1	No.2	No.3	No.4	Mean	SD	RSD(%)
k_0	0.056	0.055	0.061	0.055	0.057	0.003	4.931
T_{lag}	-139.502	-166.940	-125.574	-150.986	-145.751	17.536	-12.032

Number of dissolution data points (N), degrees of freedom (df), and selected goodness of fit criteria – Pearson correlation coefficient (R), coefficient of determination (R^2), adjusted coefficient of determination ($R^2_{adjusted}$), and residual sum of squares (RSS) (manual calculation in MS Excel):

Parameter	No.1	No.2	No.3	No.4
N	33	33	33	33
df	31	31	31	31
R	0.987306784	0.98645439	0.991484385	0.988513637
R^2	0.974774686	0.973092264	0.983041286	0.977159211
$R^2_{adjusted}$	0.973960966	0.972224272	0.98249423	0.976422412
RSS	553.9986981	573.3383327	432.9531736	473.5963054

Graphical abstract of model fit presented as mean ± 1 SD of the fraction % of released carvedilol:

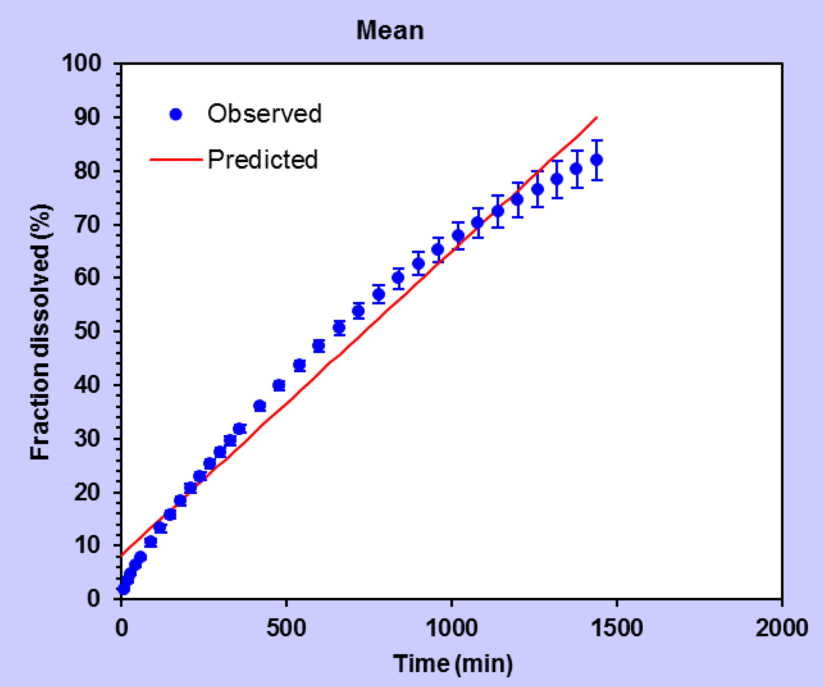

Supplementary materials – Model fitting summary – Ethocel^{TM} Standard 20 Premium (modelling the entire relevant dissolution data)

Graphical abstract of model fit presented as the fraction % of released carvedilol per tested tablet:

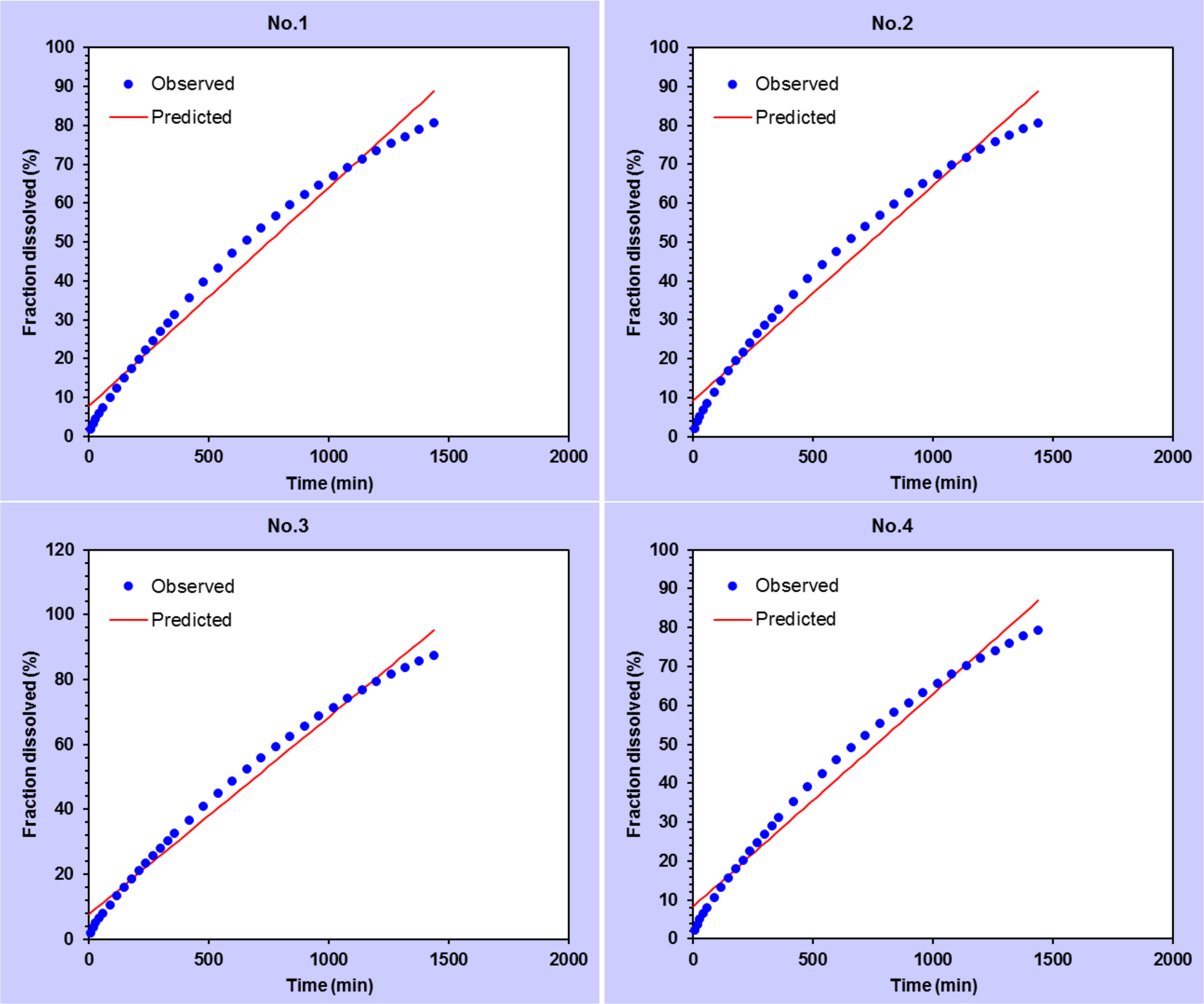

Supplementary materials – Model fitting summary – Ethocel™ Standard 20 Premium (modelling the entire relevant dissolution data)

Model: **Zero–order with F_0**

Model equation: $F = F_0 + k_0 \cdot t$

Fitted model parameters per tested tablet (N = 4) with statistics – mean, standard deviation (SD), and relative standard deviation expressed in % (RSD%) (output from DDSolver):

Parameter	No.1	No.2	No.3	No.4	Mean	SD	RSD(%)
k_0	0.056	0.055	0.061	0.055	0.057	0.003	4.931
F_0	7.838	9.231	7.639	8.253	8.240	0.708	8.594

Number of dissolution data points (N), degrees of freedom (df), and selected goodness of fit criteria – Pearson correlation coefficient (R), coefficient of determination (R^2), adjusted coefficient of determination ($R^2_{adjusted}$), and residual sum of squares (RSS) (manual calculation in MS Excel):

Parameter	No.1	No.2	No.3	No.4
N	33	33	33	33
df	31	31	31	31
R	0.987306784	0.98645439	0.991484385	0.988513637
R^2	0.974774686	0.973092264	0.983041286	0.977159211
$R^2_{adjusted}$	0.973960966	0.972224272	0.98249423	0.976422412
RSS	553.9986981	573.3383327	432.9531736	473.5963054

Graphical abstract of model fit presented as mean ± 1 SD of the fraction % of released carvedilol:

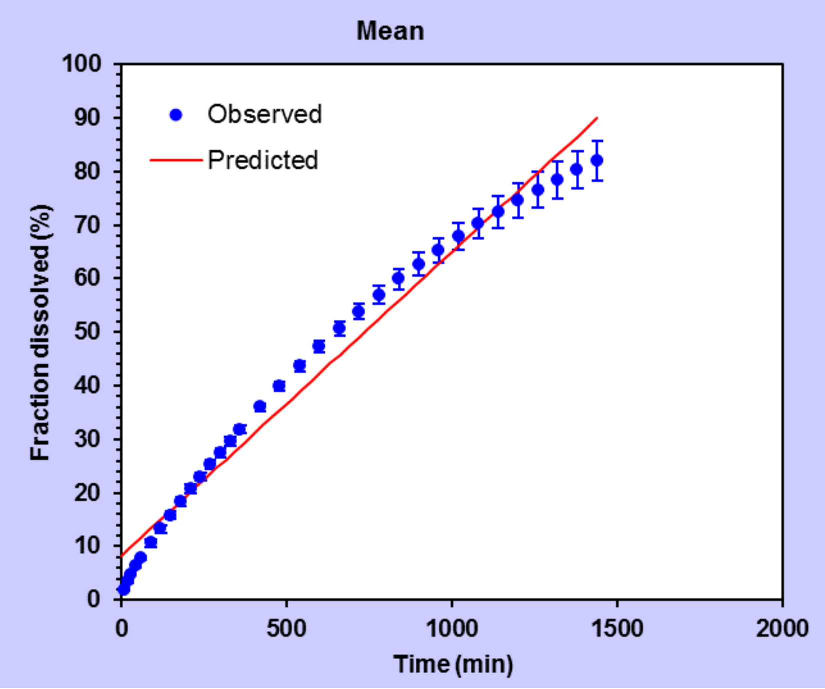

Supplementary materials – Model fitting summary – Ethocel™ Standard 20 Premium (modelling the entire relevant dissolution data)

Graphical abstract of model fit presented as the fraction % of released carvedilol per tested tablet:

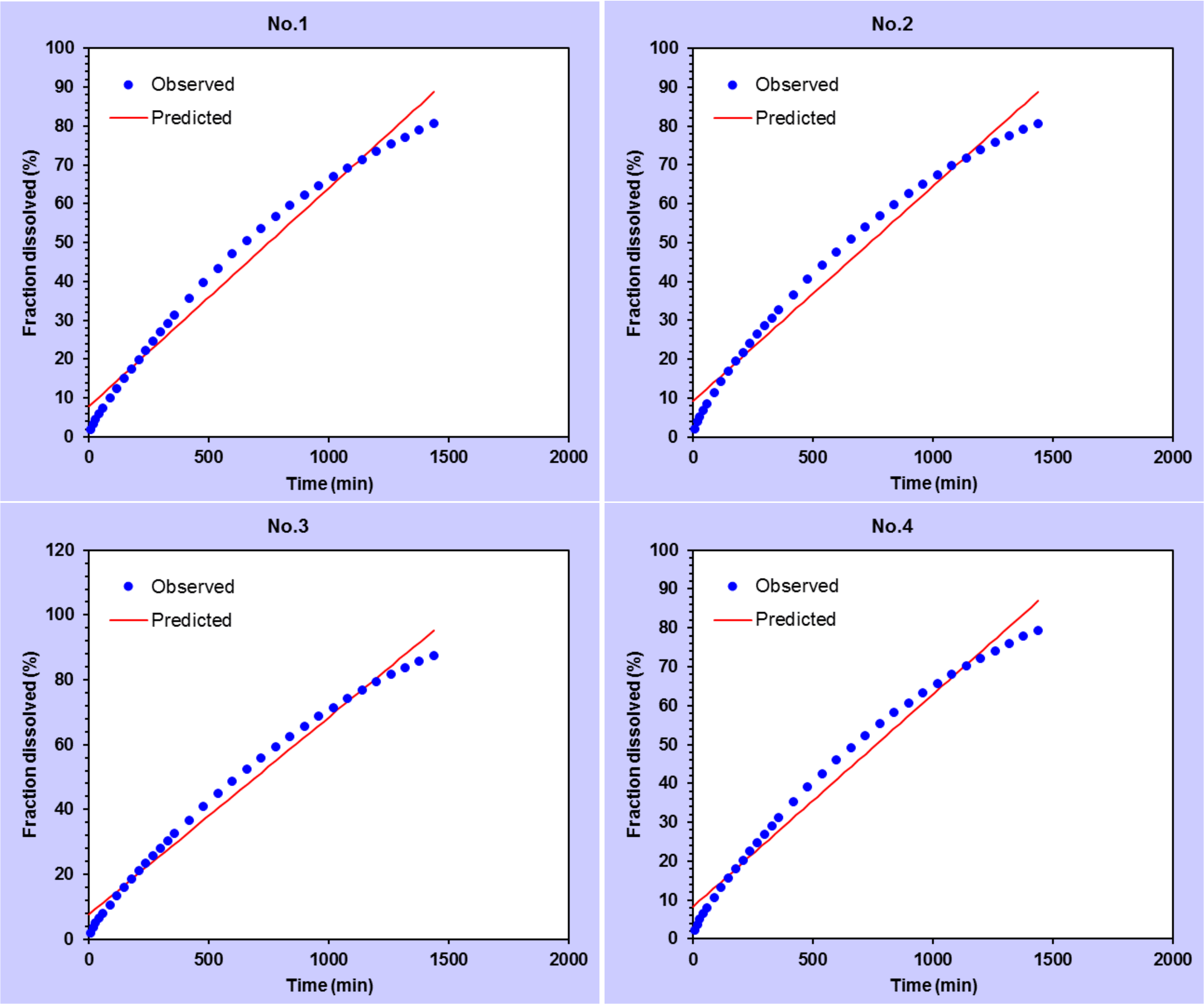

Supplementary materials – Model fitting summary – Ethocel™ Standard 20 Premium (modelling the entire relevant dissolution data)

Model: **First–order**

Model equation: $F = 100 \cdot (1 - e^{-k_1 \cdot t})$

Fitted model parameters per tested tablet (N = 4) with statistics – mean, standard deviation (SD), and relative standard deviation expressed in % (RSD%) (output from DDSolver):

Parameter	No.1	No.2	No.3	No.4	Mean	SD	RSD(%)
k_1	0.001	0.001	0.001	0.001	0.001	0.000	2.437

Number of dissolution data points (N), degrees of freedom (df), and selected goodness of fit criteria – Pearson correlation coefficient (R), coefficient of determination (R^2), adjusted coefficient of determination ($R^2_{adjusted}$), and residual sum of squares (RSS) (manual calculation in MS Excel):

Parameter	No.1	No.2	No.3	No.4
N	33	33	33	33
df	32	32	32	32
R	0.999475983	0.999522261	0.997889383	0.999421336
R^2	0.99895224	0.99904475	0.99578322	0.998843006
$R^2_{adjusted}$	0.99895224	0.99904475	0.99578322	0.998843006
RSS	26.22185896	36.38417747	277.6538063	31.68197007

Graphical abstract of model fit presented as mean ± 1 SD of the fraction % of released carvedilol:

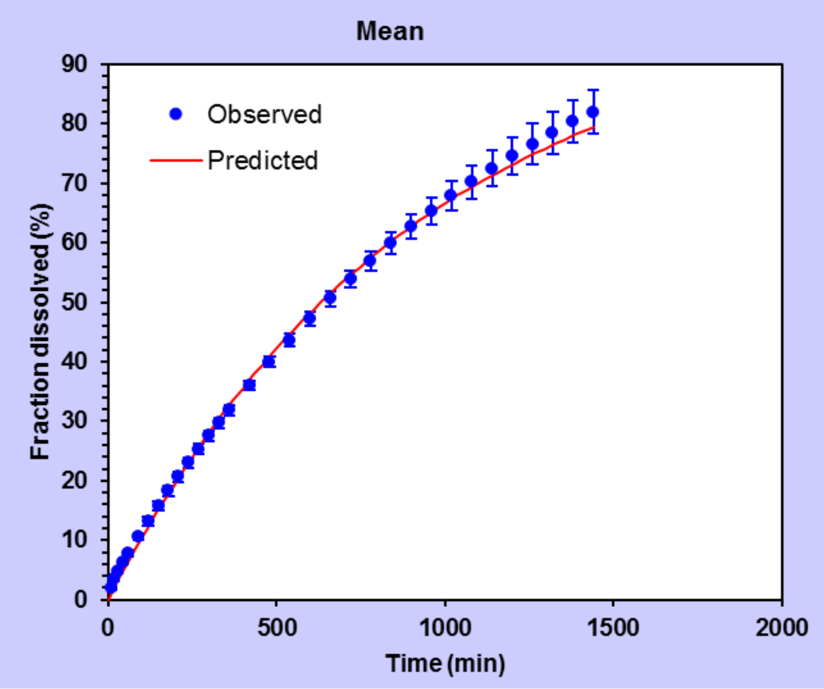

Supplementary materials – Model fitting summary – Ethocel^{TM} Standard 20 Premium (modelling the entire relevant dissolution data)

Graphical abstract of model fit presented as the fraction % of released carvedilol per tested tablet:

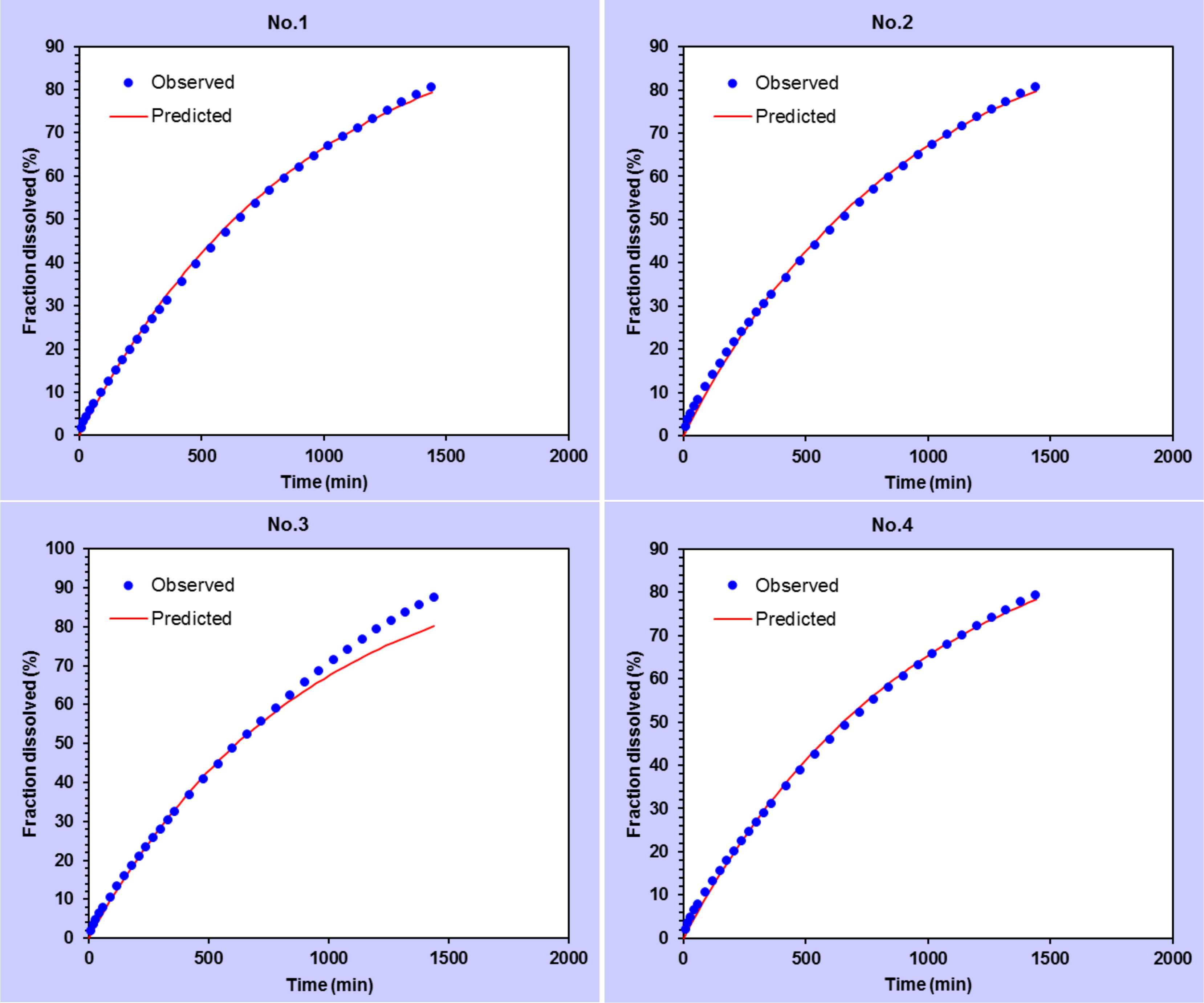

Supplementary materials – Model fitting summary – Ethocel™ Standard 20 Premium (modelling the entire relevant dissolution data)

Model: **First–order with T_{lag}**

Model equation: $F = 100 \cdot \left[1 - e^{-k_1 \cdot (t - T_{lag})}\right]$

Fitted model parameters per tested tablet (N = 4) with statistics – mean, standard deviation (SD), and relative standard deviation expressed in % (RSD%) (output from DDSolver):

Parameter	No.1	No.2	No.3	No.4	Mean	SD	RSD(%)
k_1	0.001	0.001	0.001	0.001	0.001	0.000	11.266
T_{lag}	9.516	-7.536	45.063	0.283	11.831	23.225	196.298

Number of dissolution data points (N), degrees of freedom (df), and selected goodness of fit criteria – Pearson correlation coefficient (R), coefficient of determination (R^2), adjusted coefficient of determination ($R^2_{adjusted}$), and residual sum of squares (RSS) (manual calculation in MS Excel):

Parameter	No.1	No.2	No.3	No.4
N	33	33	33	33
df	31	31	31	31
R	0.999416216	0.999548202	0.994933083	0.999419608
R^2	0.998832772	0.999096608	0.98989184	0.998839553
$R^2_{adjusted}$	0.998795119	0.999067467	0.98956577	0.998802119
RSS	34.33027343	24.03966472	411.8630397	32.15680235

Graphical abstract of model fit presented as mean ± 1 SD of the fraction % of released carvedilol:

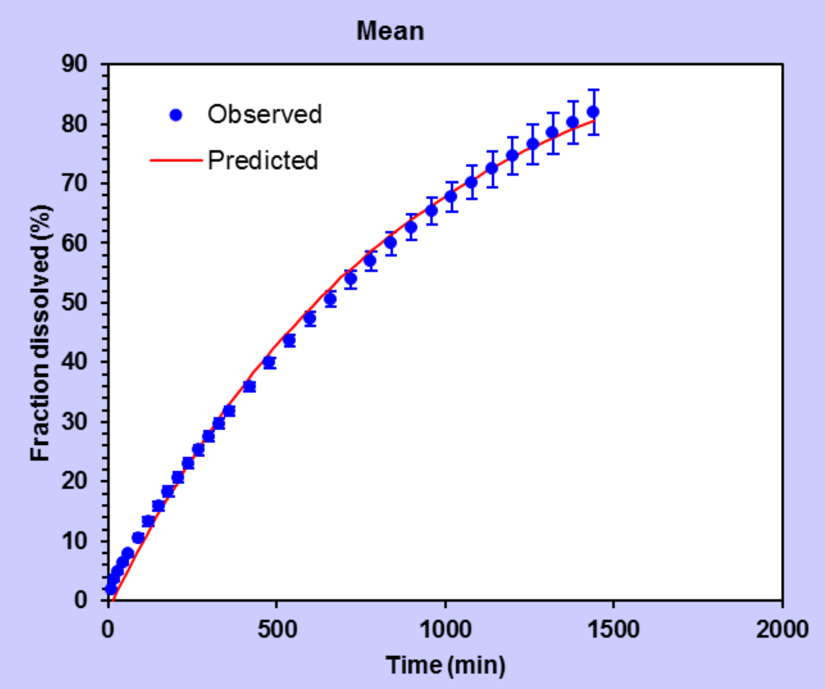

Supplementary materials – Model fitting summary – Ethocel^{TM} Standard 20 Premium (modelling the entire relevant dissolution data)

Graphical abstract of model fit presented as the fraction % of released carvedilol per tested tablet:

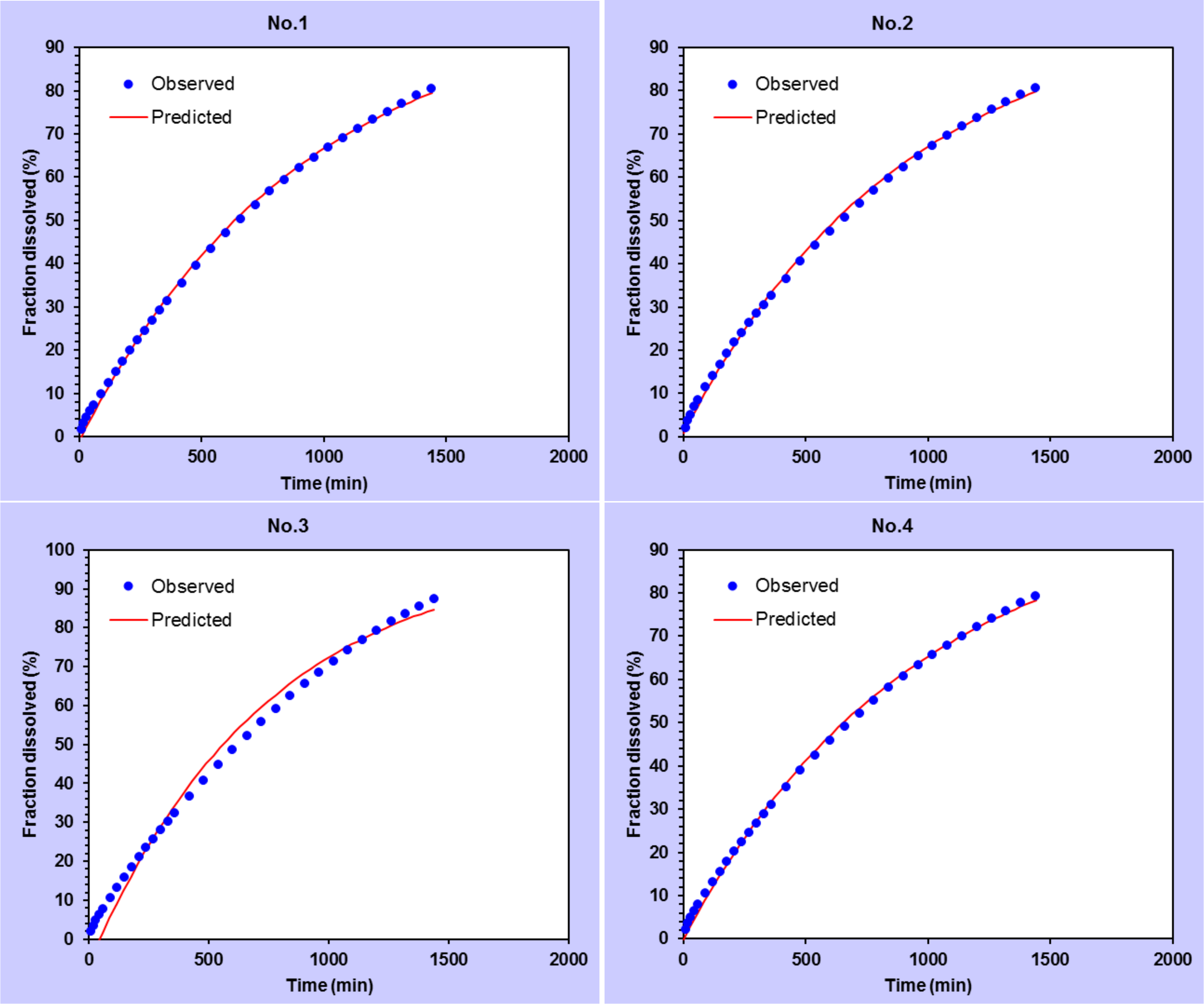

Supplementary materials – Model fitting summary – Ethocel™ Standard 20 Premium (modelling the entire relevant dissolution data)

Model: **First–order with F_{max}**

Model equation: $F = F_{max} \cdot (1 - e^{-k_1 \cdot t})$

Fitted model parameters per tested tablet (N = 4) with statistics – mean, standard deviation (SD), and relative standard deviation expressed in % (RSD%) (output from DDSolver):

Parameter	No.1	No.2	No.3	No.4	Mean	SD	RSD(%)
k_1	0.002	0.002	0.002	0.002	0.002	0.000	1.622
F_{max}	84.520	84.597	91.858	83.263	86.059	3.913	4.547

Number of dissolution data points (N), degrees of freedom (df), and selected goodness of fit criteria – Pearson correlation coefficient (R), coefficient of determination (R^2), adjusted coefficient of determination ($R^2_{adjusted}$), and residual sum of squares (RSS) (manual calculation in MS Excel):

Parameter	No.1	No.2	No.3	No.4
N	33	33	33	33
df	31	31	31	31
R	0.992508183	0.992877646	0.989598194	0.99179032
R^2	0.985072493	0.98580602	0.979304586	0.983648039
$R^2_{adjusted}$	0.984590961	0.985348149	0.978636992	0.983120556
RSS	650.6797405	521.3181103	1007.209069	606.2994403

Graphical abstract of model fit presented as mean ± 1 SD of the fraction % of released carvedilol:

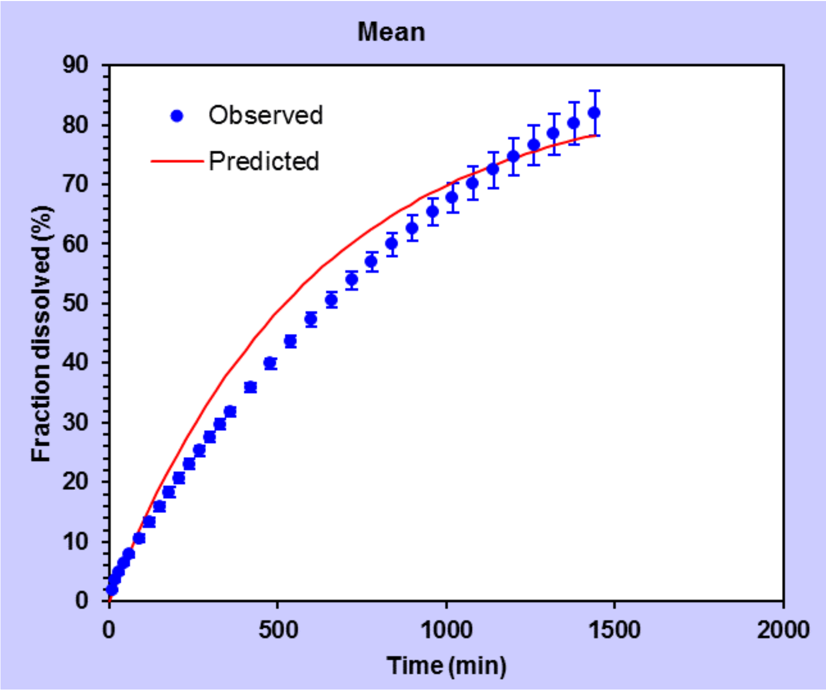

Supplementary materials – Model fitting summary – Ethocel^{TM} Standard 20 Premium (modelling the entire relevant dissolution data)

Graphical abstract of model fit presented as the fraction % of released carvedilol per tested tablet:

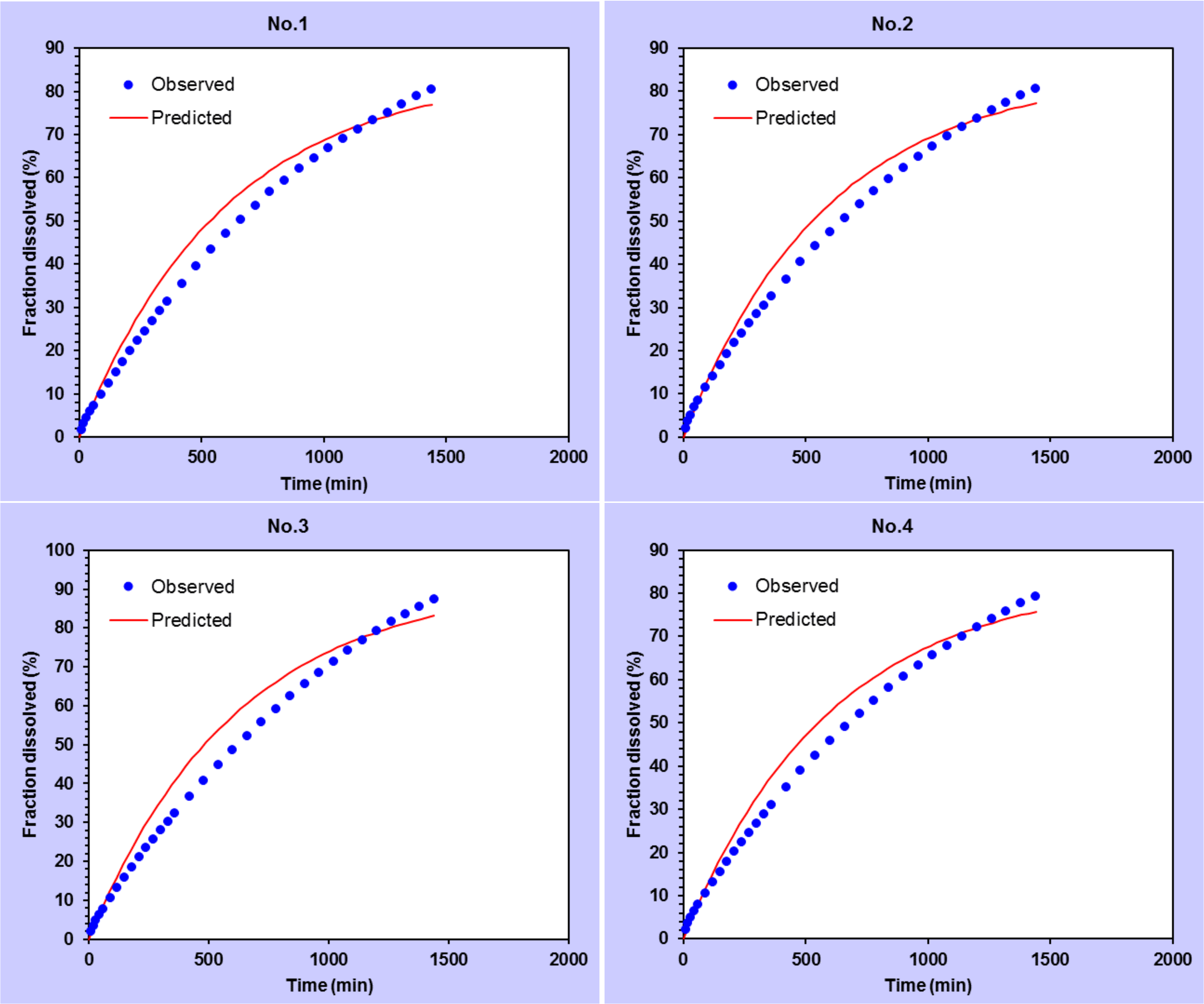

Supplementary materials – Model fitting summary – Ethocel™ Standard 20 Premium (modelling the entire relevant dissolution data)

Model: **First–order with T_{lag} and F_{max}**

Model equation: $F = F_{max} \cdot \left[1 - e^{-k_1 \cdot (t - T_{lag})}\right]$

Fitted model parameters per tested tablet (N = 4) with statistics – mean, standard deviation (SD), and relative standard deviation expressed in % (RSD%) (output from DDSolver):

Parameter	No.1	No.2	No.3	No.4	Mean	SD	RSD(%)
k_1	0.002	0.002	0.002	0.002	0.002	0.000	0.788
T_{lag}	75.321	65.367	84.244	71.995	74.232	7.853	10.579
F_{max}	84.520	84.597	91.858	83.263	86.059	3.913	4.547

Number of dissolution data points (N), degrees of freedom (df), and selected goodness of fit criteria – Pearson correlation coefficient (R), coefficient of determination (R^2), adjusted coefficient of determination ($R^2_{adjusted}$), and residual sum of squares (RSS) (manual calculation in MS Excel):

Parameter	No.1	No.2	No.3	No.4
N	33	33	33	33
df	30	30	30	30
R	0.98964225	0.990510189	0.985904775	0.988980934
R^2	0.979391782	0.981110434	0.972008226	0.978083287
$R^2_{adjusted}$	0.978017901	0.97985113	0.970142108	0.976622173
RSS	1003.522485	921.4526246	1578.924006	1017.769399

Graphical abstract of model fit presented as mean ± 1 SD of the fraction % of released carvedilol:

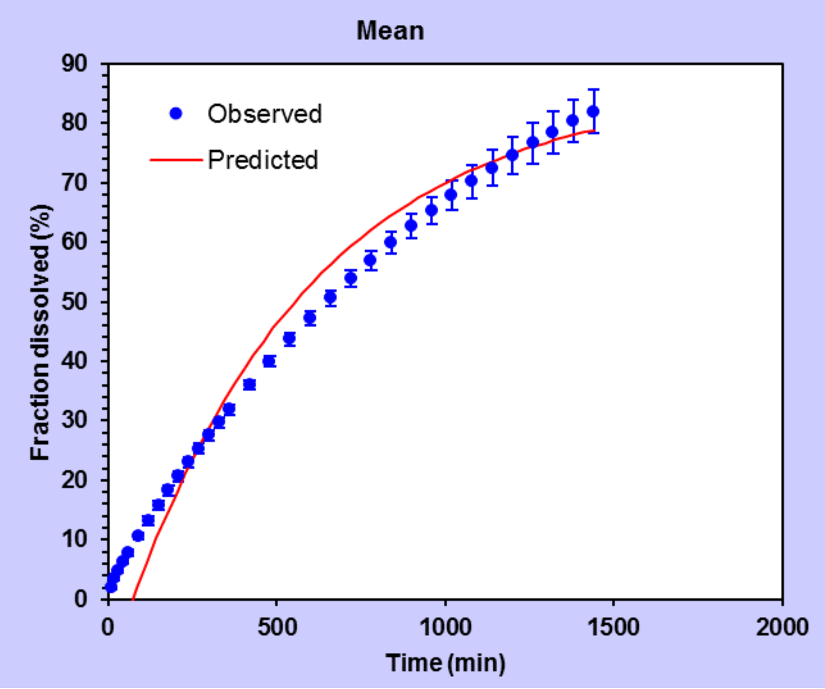

Supplementary materials – Model fitting summary – Ethocel^{TM} Standard 20 Premium (modelling the entire relevant dissolution data)

Graphical abstract of model fit presented as the fraction % of released carvedilol per tested tablet:

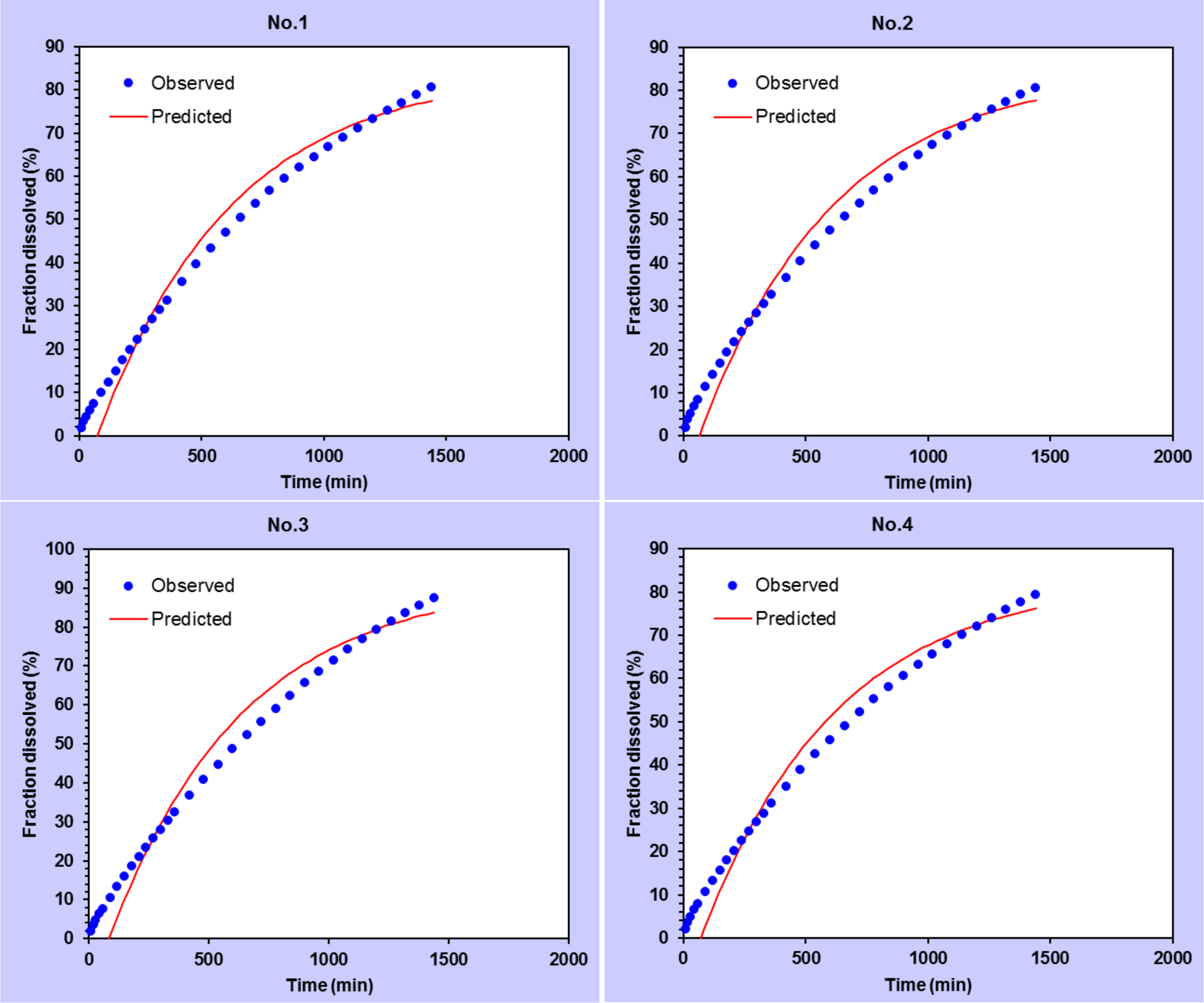

Supplementary materials – Model fitting summary – Ethocel™ Standard 20 Premium (modelling the entire relevant dissolution data)

Model: **Higuchi**

Model equation: $F = k_H \cdot t^{0.5}$

Fitted model parameters per tested tablet (N = 4) with statistics – mean, standard deviation (SD), and relative standard deviation expressed in % (RSD%) (output from DDSolver):

Parameter	No.1	No.2	No.3	No.4	Mean	SD	RSD(%)
k_H	1.976	2.002	2.105	1.946	2.007	0.069	3.453

Number of dissolution data points (N), degrees of freedom (df), and selected goodness of fit criteria – Pearson correlation coefficient (R), coefficient of determination (R^2), adjusted coefficient of determination ($R^2_{adjusted}$), and residual sum of squares (RSS) (manual calculation in MS Excel):

Parameter	No.1	No.2	No.3	No.4
N	33	33	33	33
df	32	32	32	32
R	0.99605536	0.997302108	0.994797691	0.996228878
R^2	0.992126279	0.994611494	0.989622447	0.992471978
$R^2_{adjusted}$	0.992126279	0.994611494	0.989622447	0.992471978
RSS	1152.97278	858.9853425	1506.444052	982.3973912

Graphical abstract of model fit presented as mean ± 1 SD of the fraction % of released carvedilol:

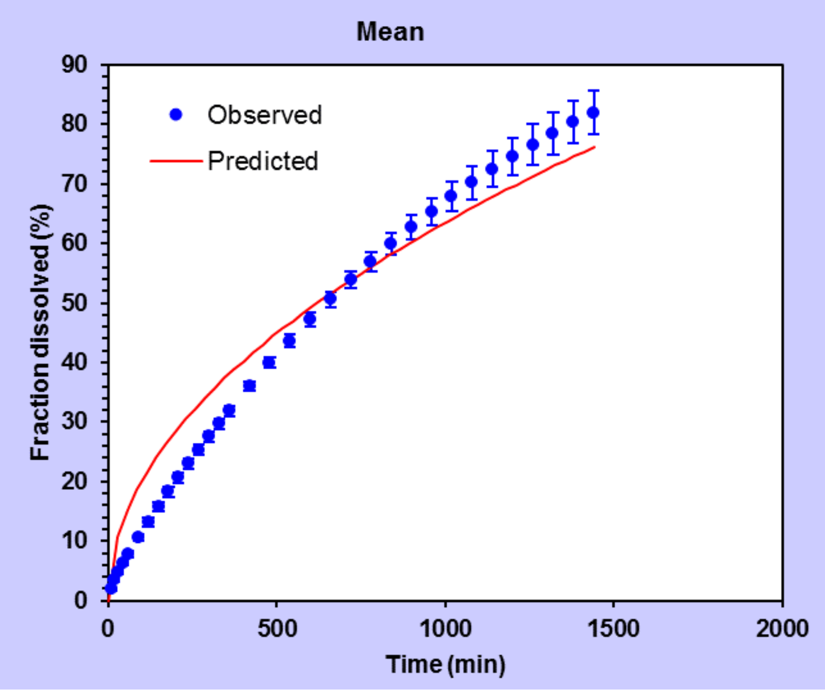

Supplementary materials – Model fitting summary – Ethocel™ Standard 20 Premium (modelling the entire relevant dissolution data)

Graphical abstract of model fit presented as the fraction % of released carvedilol per tested tablet:

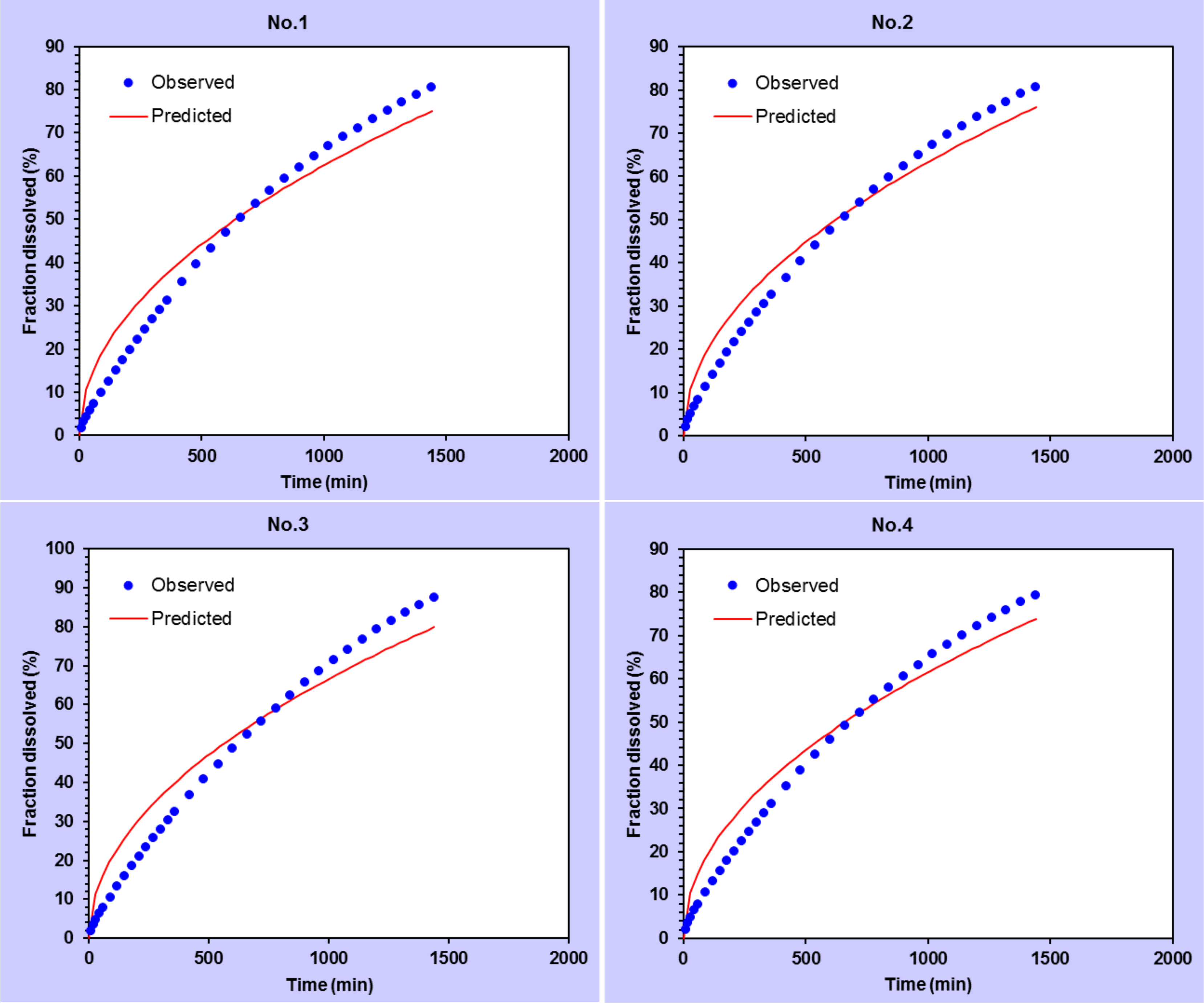

Supplementary materials – Model fitting summary – Ethocel™ Standard 20 Premium (modelling the entire relevant dissolution data)

Model: **Higuchi with T_{lag}**

Model equation: $F = k_H \cdot (t - T_{lag})^{0.5}$

Fitted model parameters per tested tablet (N = 4) with statistics – mean, standard deviation (SD), and relative standard deviation expressed in % (RSD%) (output from DDSolver):

Parameter	No.1	No.2	No.3	No.4	Mean	SD	RSD(%)
k_H	2.183	2.184	2.358	2.144	2.217	0.096	4.318
T_{lag}	97.475	87.191	109.571	95.890	97.532	9.212	9.445

Number of dissolution data points (N), degrees of freedom (df), and selected goodness of fit criteria – Pearson correlation coefficient (R), coefficient of determination (R^2), adjusted coefficient of determination ($R^2_{adjusted}$), and residual sum of squares (RSS) (manual calculation in MS Excel):

Parameter	No.1	No.2	No.3	No.4
N	33	33	33	33
df	31	31	31	31
R	0.992522433	0.993543523	0.991396486	0.991933437
R^2	0.98510078	0.987128733	0.982866992	0.983931943
$R^2_{adjusted}$	0.98462016	0.986713531	0.982314315	0.983413619
RSS	364.295396	324.8220223	496.6801147	390.5616147

Graphical abstract of model fit presented as mean ± 1 SD of the fraction % of released carvedilol:

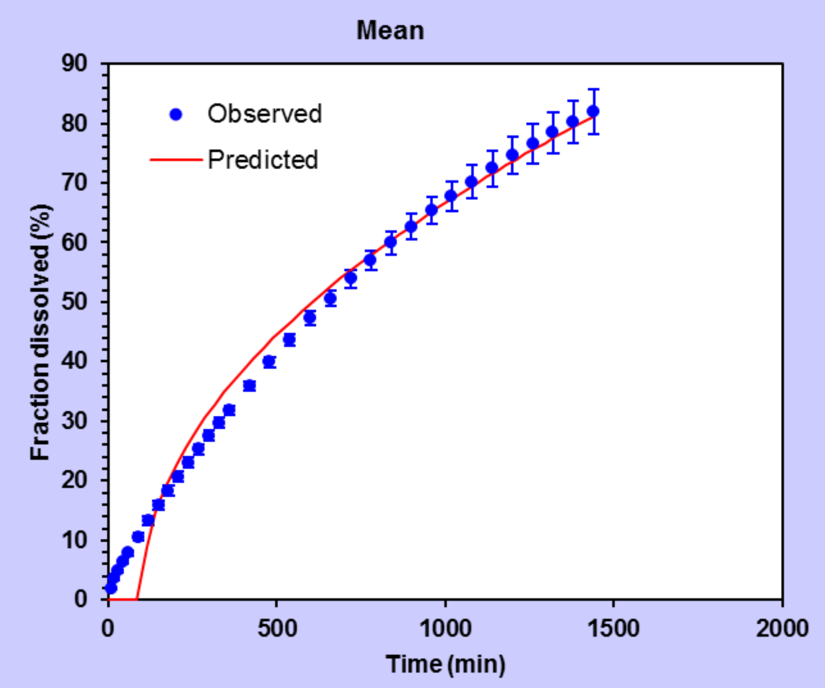

Supplementary materials – Model fitting summary – Ethocel™ Standard 20 Premium (modelling the entire relevant dissolution data)

Graphical abstract of model fit presented as the fraction % of released carvedilol per tested tablet:

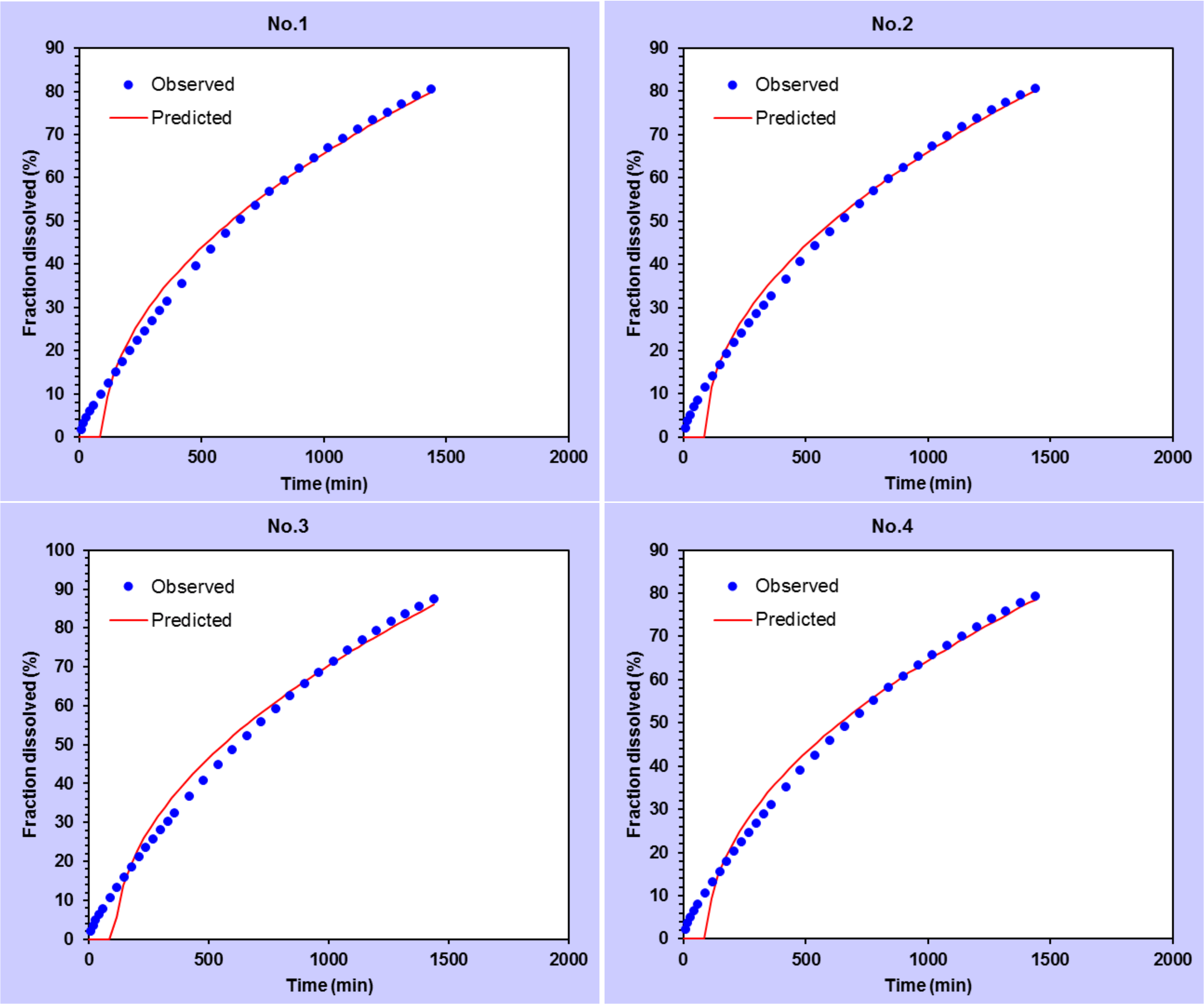

Supplementary materials – Model fitting summary – Ethocel™ Standard 20 Premium (modelling the entire relevant dissolution data)

Model: **Higuchi with F_0**

Model equation: $F = F_0 + k_H \cdot t^{0.5}$

Fitted model parameters per tested tablet (N = 4) with statistics – mean, standard deviation (SD), and relative standard deviation expressed in % (RSD%) (output from DDSolver):

Parameter	No.1	No.2	No.3	No.4	Mean	SD	RSD(%)
k_H	2.442	2.408	2.630	2.373	2.463	0.114	4.643
F_0	-12.478	-10.874	-14.045	-11.458	-12.214	1.389	-11.374

Number of dissolution data points (N), degrees of freedom (df), and selected goodness of fit criteria – Pearson correlation coefficient (R), coefficient of determination (R^2), adjusted coefficient of determination ($R^2_{adjusted}$), and residual sum of squares (RSS) (manual calculation in MS Excel):

Parameter	No.1	No.2	No.3	No.4
N	33	33	33	33
df	31	31	31	31
R	0.99605536	0.997302108	0.994797691	0.996228878
R^2	0.992126279	0.994611494	0.989622447	0.992471978
$R^2_{adjusted}$	0.991872288	0.994437671	0.989287687	0.992229138
RSS	172.9227641	114.815937	264.9372212	156.0910905

Graphical abstract of model fit presented as mean ± 1 SD of the fraction % of released carvedilol:

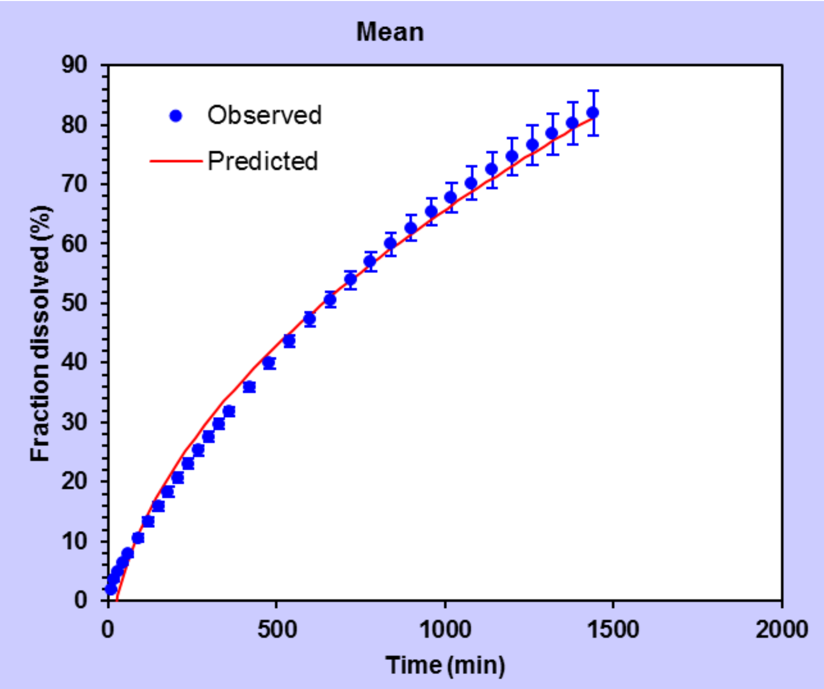

Supplementary materials – Model fitting summary – Ethocel™ Standard 20 Premium (modelling the entire relevant dissolution data)

Graphical abstract of model fit presented as the fraction % of released carvedilol per tested tablet:

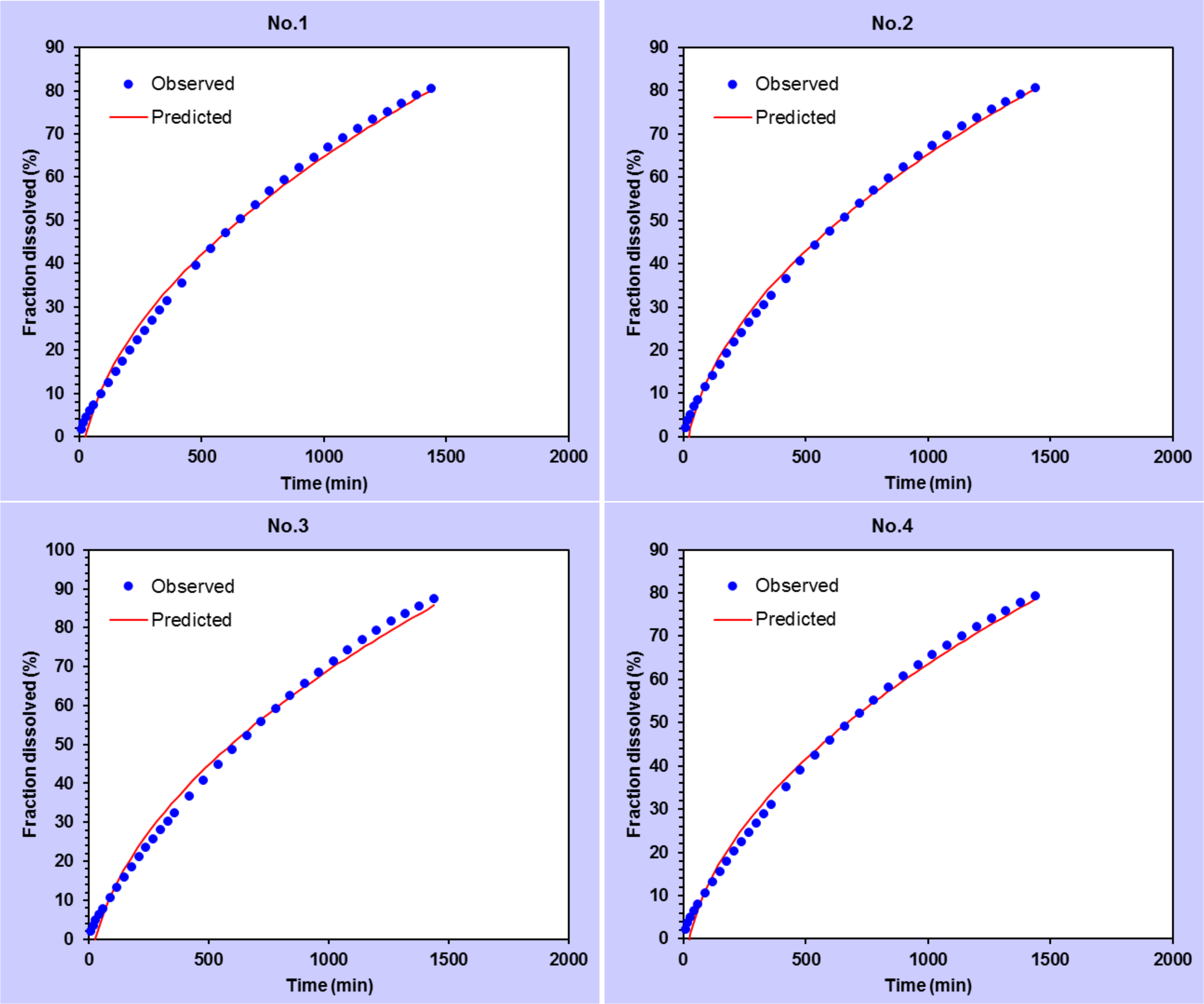

Supplementary materials – Model fitting summary – Ethocel™ Standard 20 Premium (modelling the entire relevant dissolution data)

Model: **Korsmeyer–Peppas**

Model equation: $F = k_{KP} \cdot t^n$

Fitted model parameters per tested tablet (N = 4) with statistics – mean, standard deviation (SD), and relative standard deviation expressed in % (RSD%) (output from DDSolver):

Parameter	No.1	No.2	No.3	No.4	Mean	SD	RSD(%)
k_{KP}	0.307	0.416	0.334	0.390	0.362	0.050	13.878
n	0.778	0.735	0.774	0.739	0.757	0.023	2.976

Number of dissolution data points (N), degrees of freedom (df), and selected goodness of fit criteria – Pearson correlation coefficient (R), coefficient of determination (R^2), adjusted coefficient of determination ($R^2_{adjusted}$), and residual sum of squares (RSS) (manual calculation in MS Excel):

Parameter	No.1	No.2	No.3	No.4
N	33	33	33	33
df	31	31	31	31
R	0.996781034	0.997820713	0.998739203	0.998333248
R^2	0.993572429	0.995646174	0.997479995	0.996669275
$R^2_{adjusted}$	0.993365088	0.995505728	0.997398705	0.996561832
RSS	204.3734076	149.2800747	82.81889382	85.86219878

Graphical abstract of model fit presented as mean ± 1 SD of the fraction % of released carvedilol:

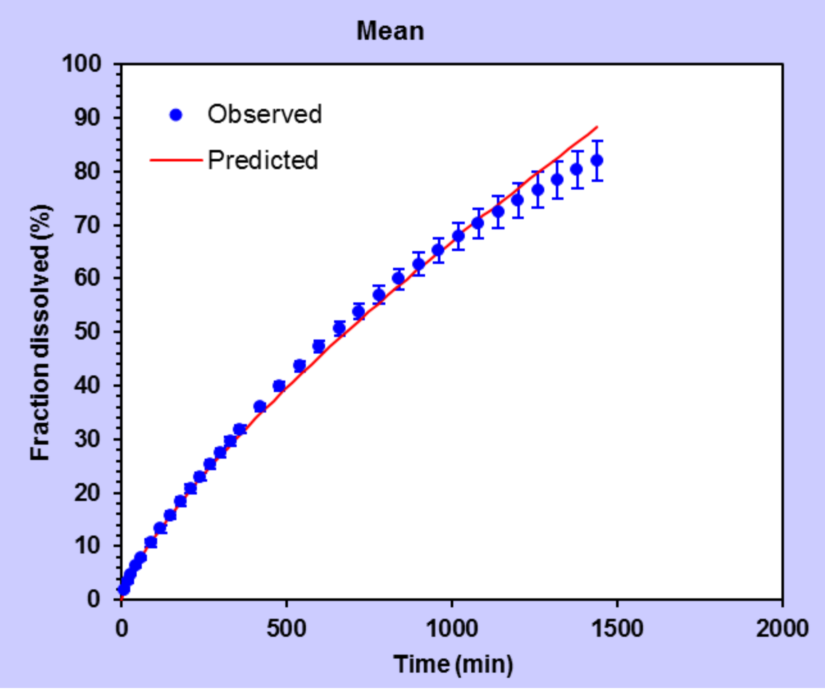

Supplementary materials – Model fitting summary – Ethocel^{TM} Standard 20 Premium (modelling the entire relevant dissolution data)

Graphical abstract of model fit presented as the fraction % of released carvedilol per tested tablet:

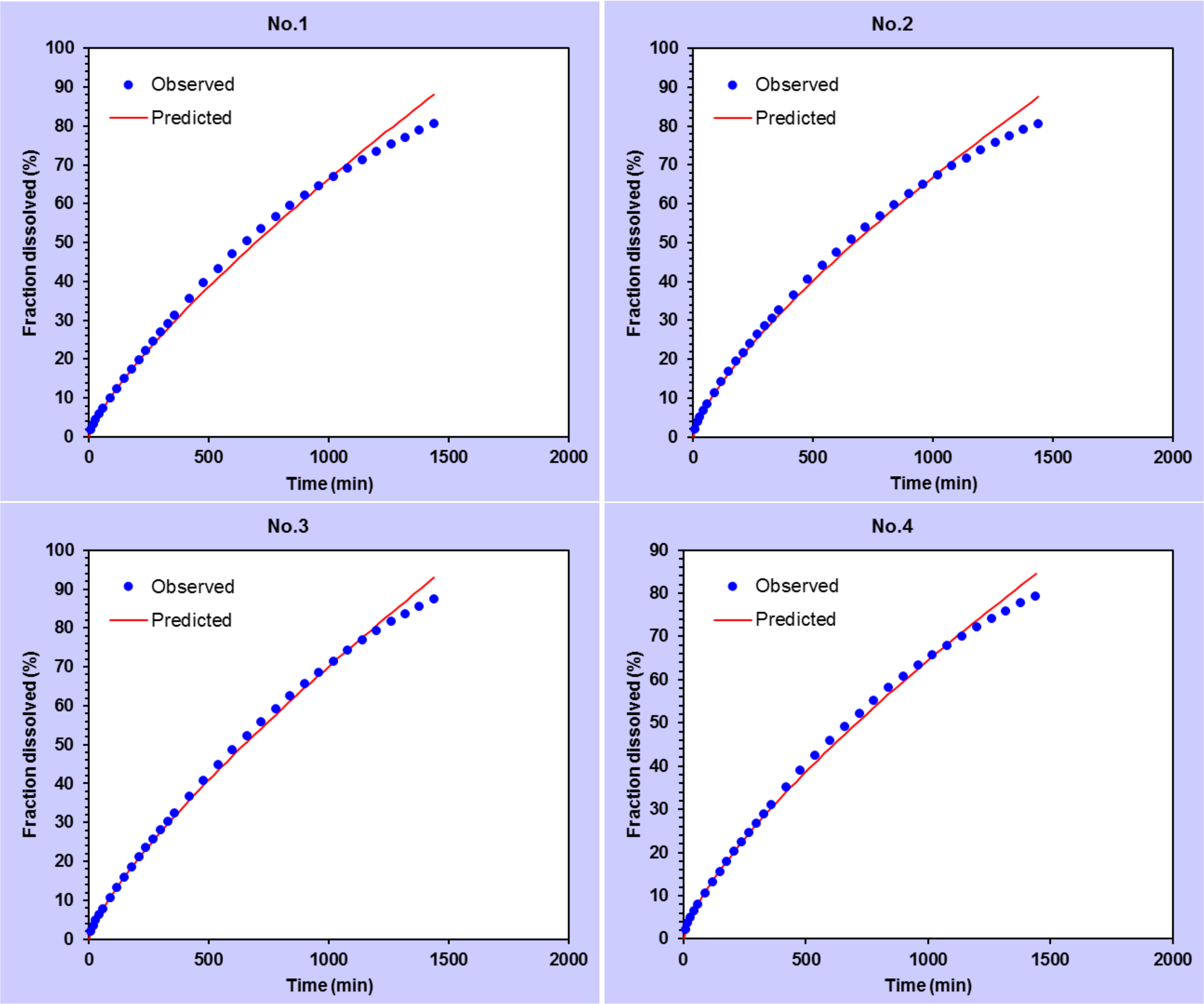

Supplementary materials – Model fitting summary – Ethocel™ Standard 20 Premium (modelling the entire relevant dissolution data)

Model: **Korsmeyer–Peppas with T_{lag}**

Model equation: $F = k_{KP} \cdot (t - T_{lag})^n$

Fitted model parameters per tested tablet (N = 4) with statistics – mean, standard deviation (SD), and relative standard deviation expressed in % (RSD%) (output from DDSolver):

Parameter	No.1	No.2	No.3	No.4	Mean	SD	RSD(%)
k_{KP}	0.404	0.538	0.439	0.508	0.472	0.062	13.040
n	0.736	0.696	0.732	0.699	0.716	0.021	2.965
T_{lag}	4.000	4.000	4.000	4.000	4.000	0.000	0.000

Number of dissolution data points (N), degrees of freedom (df), and selected goodness of fit criteria – Pearson correlation coefficient (R), coefficient of determination (R^2), adjusted coefficient of determination ($R^2_{adjusted}$), and residual sum of squares (RSS) (manual calculation in MS Excel):

Parameter	No.1	No.2	No.3	No.4
N	33	33	33	33
df	30	30	30	30
R	0.997827905	0.99867198	0.999244958	0.998928552
R^2	0.995660527	0.997345724	0.998490486	0.997858252
$R^2_{adjusted}$	0.995371229	0.997168773	0.998389851	0.997715468
RSS	99.64239465	60.46131267	43.9977619	47.58717388

Graphical abstract of model fit presented as mean ± 1 SD of the fraction % of released carvedilol:

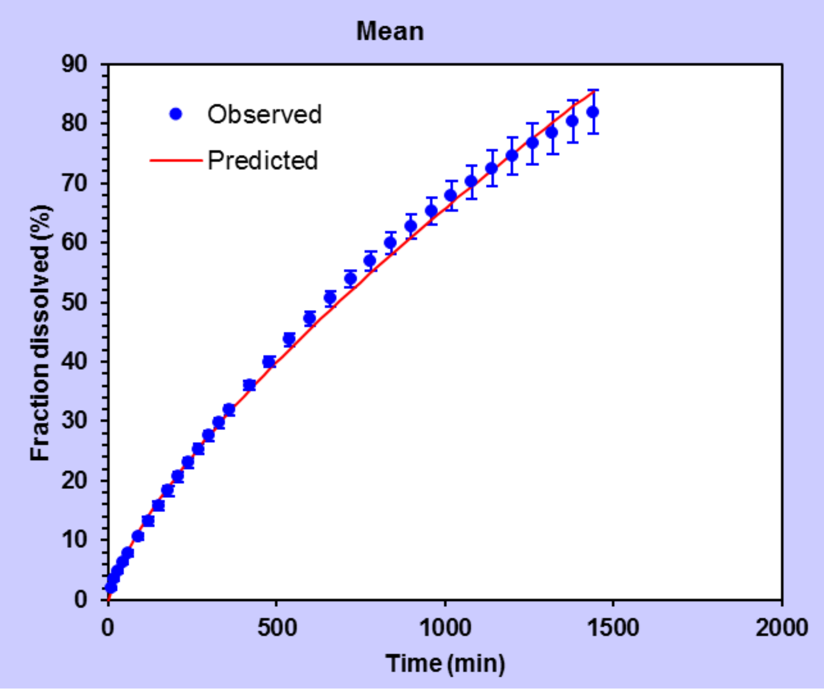

Supplementary materials – Model fitting summary – Ethocel™ Standard 20 Premium (modelling the entire relevant dissolution data)

Graphical abstract of model fit presented as the fraction % of released carvedilol per tested tablet:

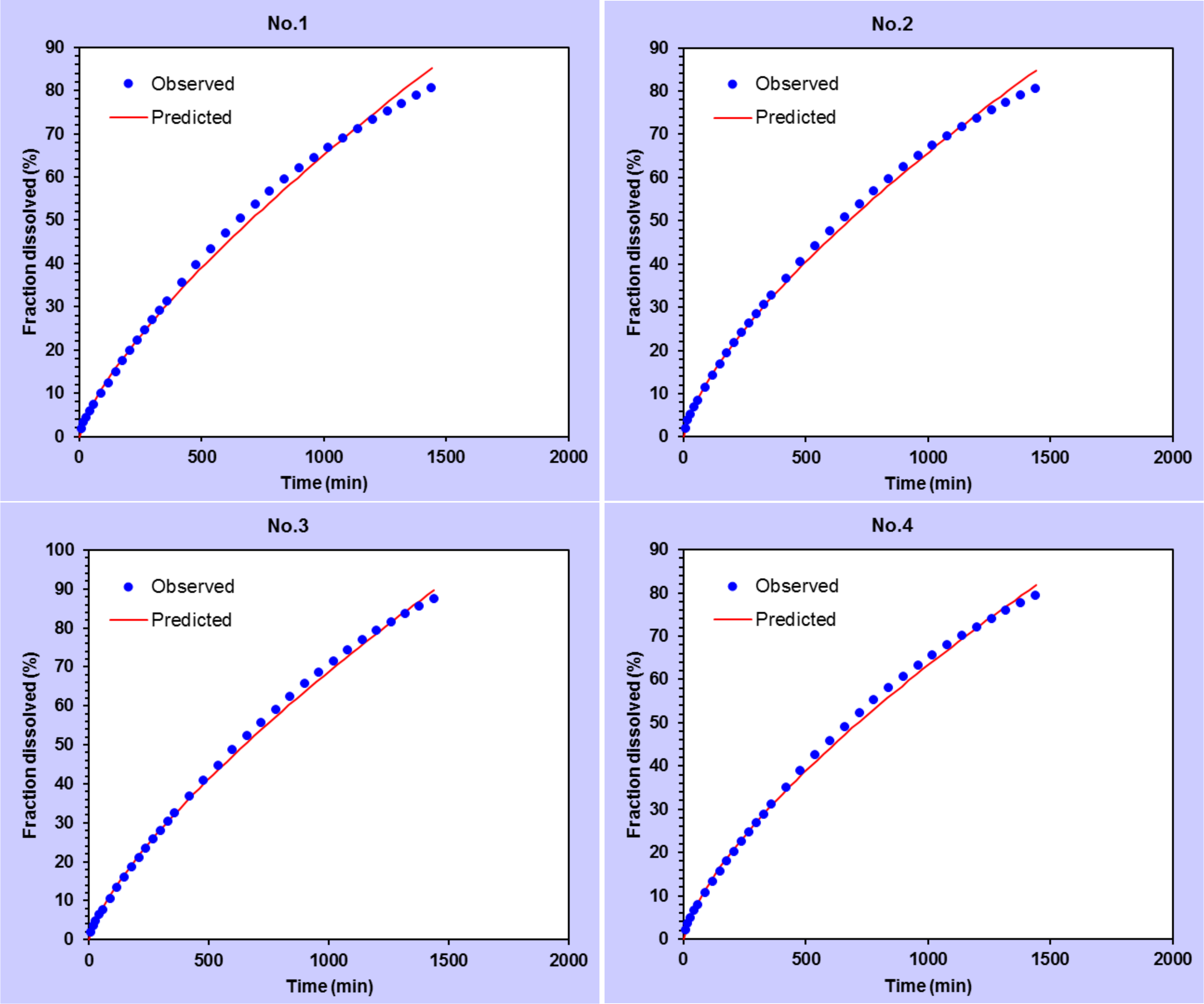

Supplementary materials – Model fitting summary – Ethocel™ Standard 20 Premium (modelling the entire relevant dissolution data)

Model: **Korsmeyer–Peppas with F_0**

Model equation: $F = F_0 + k_{KP} \cdot t^n$

Fitted model parameters per tested tablet (N = 4) with statistics – mean, standard deviation (SD), and relative standard deviation expressed in % (RSD%) (output from DDSolver):

Parameter	No.1	No.2	No.3	No.4	Mean	SD	RSD(%)
k_{KP}	0.206	0.308	0.222	0.256	0.248	0.045	18.171
n	0.838	0.766	0.835	0.802	0.810	0.034	4.162
F_0	0.680	0.973	0.760	0.840	0.813	0.125	15.367

Number of dissolution data points (N), degrees of freedom (df), and selected goodness of fit criteria – Pearson correlation coefficient (R), coefficient of determination (R^2), adjusted coefficient of determination ($R^2_{adjusted}$), and residual sum of squares (RSS) (manual calculation in MS Excel):

Parameter	No.1	No.2	No.3	No.4
N	33	33	33	33
df	30	30	30	30
R	0.994879655	0.997023719	0.997492247	0.996851851
R^2	0.989785527	0.994056296	0.994990783	0.993713612
$R^2_{adjusted}$	0.989104562	0.993660049	0.994656836	0.99329452
RSS	472.0072362	380.6076372	281.76491	263.0839509

Graphical abstract of model fit presented as mean ± 1 SD of the fraction % of released carvedilol:

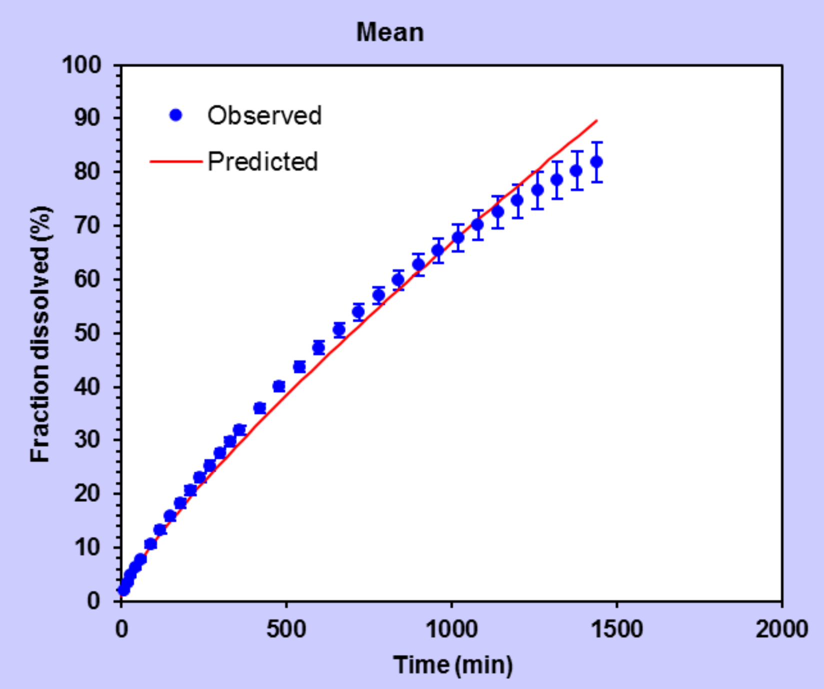

Supplementary materials – Model fitting summary – Ethocel™ Standard 20 Premium (modelling the entire relevant dissolution data)

Graphical abstract of model fit presented as the fraction % of released carvedilol per tested tablet:

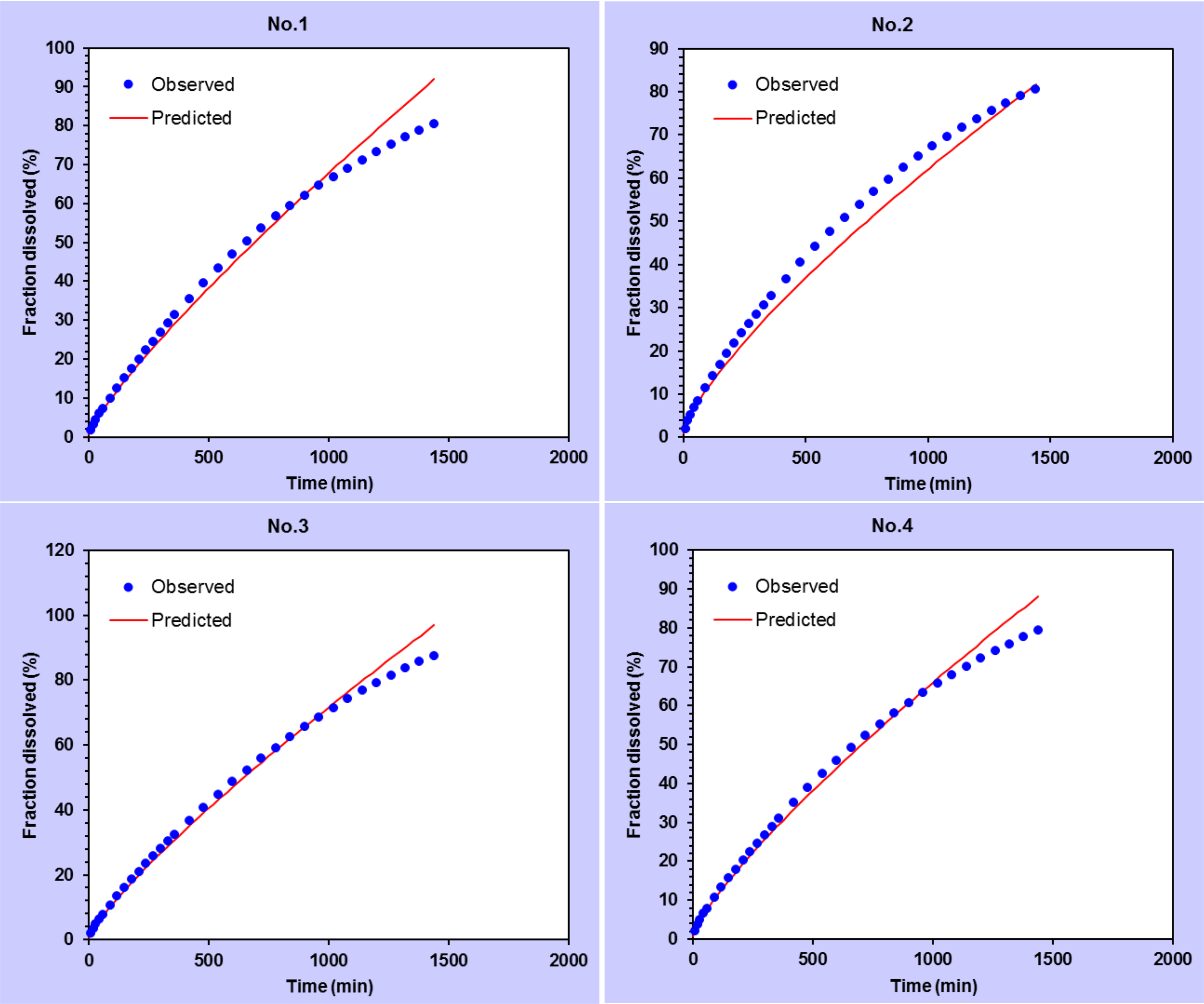

Supplementary materials – Model fitting summary – Ethocel™ Standard 20 Premium (modelling the entire relevant dissolution data)

Model: **Hixson–Crowell**

Model equation: $F = 100 \cdot [1 - (1 - k_{HC} \cdot t)^3]$

Fitted model parameters per tested tablet (N = 4) with statistics – mean, standard deviation (SD), and relative standard deviation expressed in % (RSD%) (output from DDSolver):

Parameter	No.1	No.2	No.3	No.4	Mean	SD	RSD(%)
k_{HC}	0.0003	0.0003	0.0003	0.0003	0.0003	0.0000	6.5497

Number of dissolution data points (N), degrees of freedom (df), and selected goodness of fit criteria – Pearson correlation coefficient (R), coefficient of determination (R^2), adjusted coefficient of determination ($R^2_{adjusted}$), and residual sum of squares (RSS) (manual calculation in MS Excel):

Parameter	No.1	No.2	No.3	No.4
N	33	33	33	33
df	32	32	32	32
R	0.999616839	0.999364846	0.999662612	0.999659174
R^2	0.999233826	0.998730096	0.999325338	0.999318464
$R^2_{adjusted}$	0.999233826	0.998730096	0.999325338	0.999318464
RSS	88.82542102	196.4959251	33.3071438	132.6882622

Graphical abstract of model fit presented as mean ± 1 SD of the fraction % of released carvedilol:

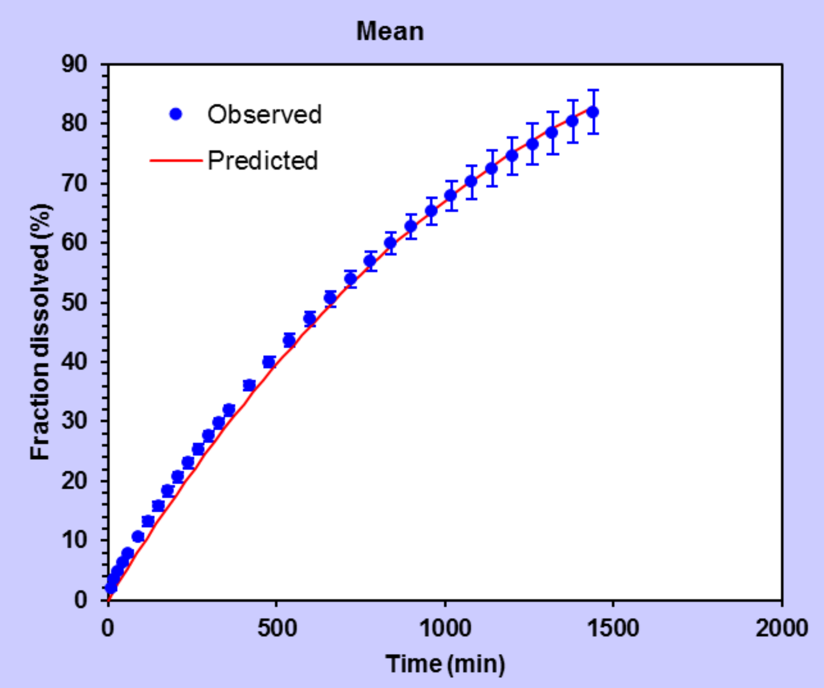

Supplementary materials – Model fitting summary – Ethocel™ Standard 20 Premium (modelling the entire relevant dissolution data)

Graphical abstract of model fit presented as the fraction % of released carvedilol per tested tablet:

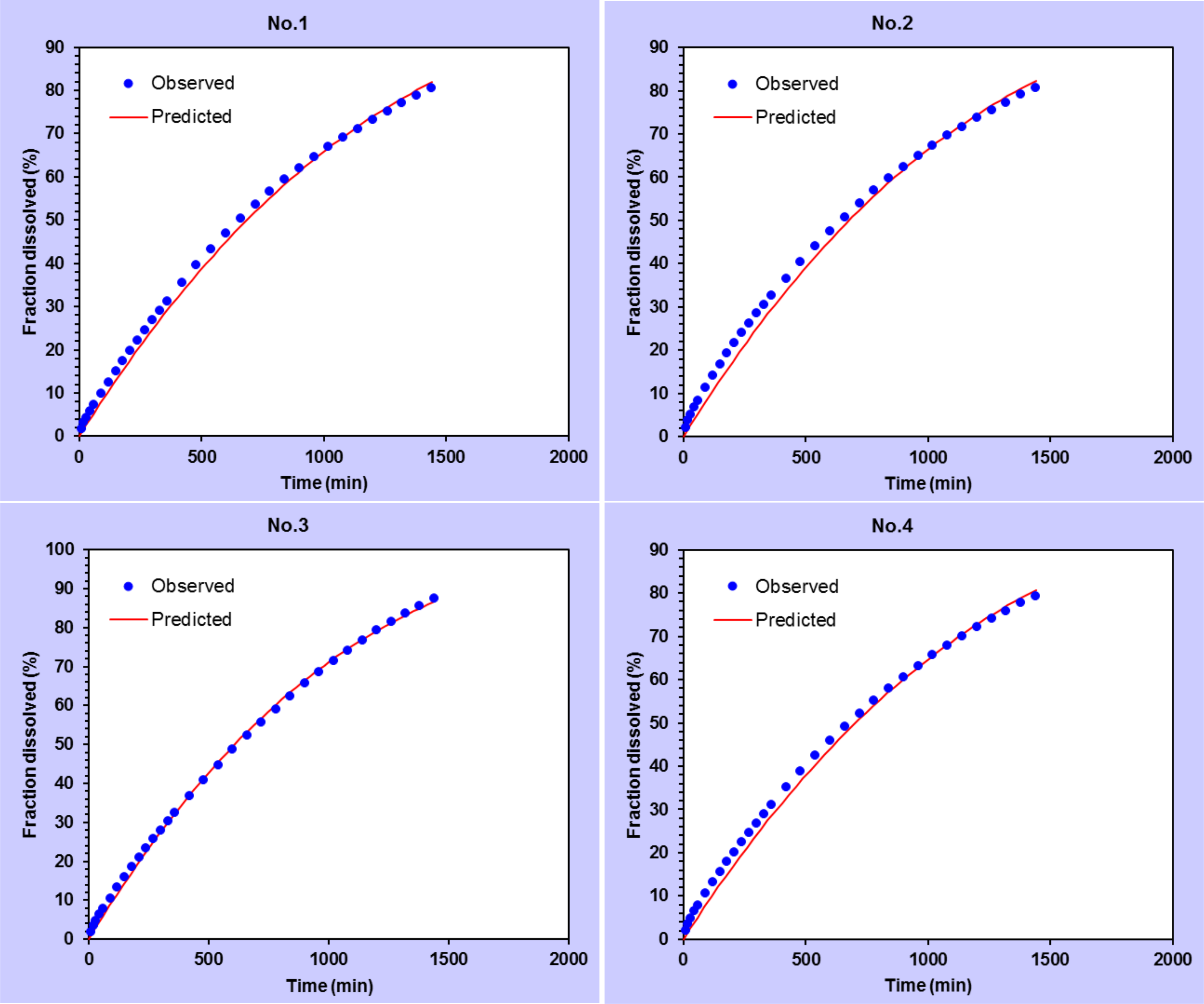

Supplementary materials – Model fitting summary – Ethocel™ Standard 20 Premium (modelling the entire relevant dissolution data)

Model: **Hixson–Crowell with T_{lag}**

Model equation: $F = 100 \cdot \left\{1 - \left[1 - k_{HC} \cdot \left(t - T_{lag}\right)\right]^3\right\}$

Fitted model parameters per tested tablet (N = 4) with statistics – mean, standard deviation (SD), and relative standard deviation expressed in % (RSD%) (output from DDSolver):

Parameter	No.1	No.2	No.3	No.4	Mean	SD	RSD(%)
k_{HC}	0.0003	0.0003	0.0003	0.0003	0.0003	0.0000	8.6360
T_{lag}	-36.6683	-54.8950	-7.6255	-46.4867	-36.4189	20.5901	-56.5368

Number of dissolution data points (N), degrees of freedom (df), and selected goodness of fit criteria – Pearson correlation coefficient (R), coefficient of determination (R^2), adjusted coefficient of determination ($R^2_{adjusted}$), and residual sum of squares (RSS) (manual calculation in MS Excel):

Parameter	No.1	No.2	No.3	No.4
N	33	33	33	33
df	31	31	31	31
R	0.999493632	0.99915667	0.999686099	0.999526146
R^2	0.99898752	0.998314052	0.999372297	0.999052516
$R^2_{adjusted}$	0.998954859	0.998259666	0.999352049	0.999021952
RSS	25.4282268	39.73797869	19.68647844	21.79594433

Graphical abstract of model fit presented as mean ± 1 SD of the fraction % of released carvedilol:

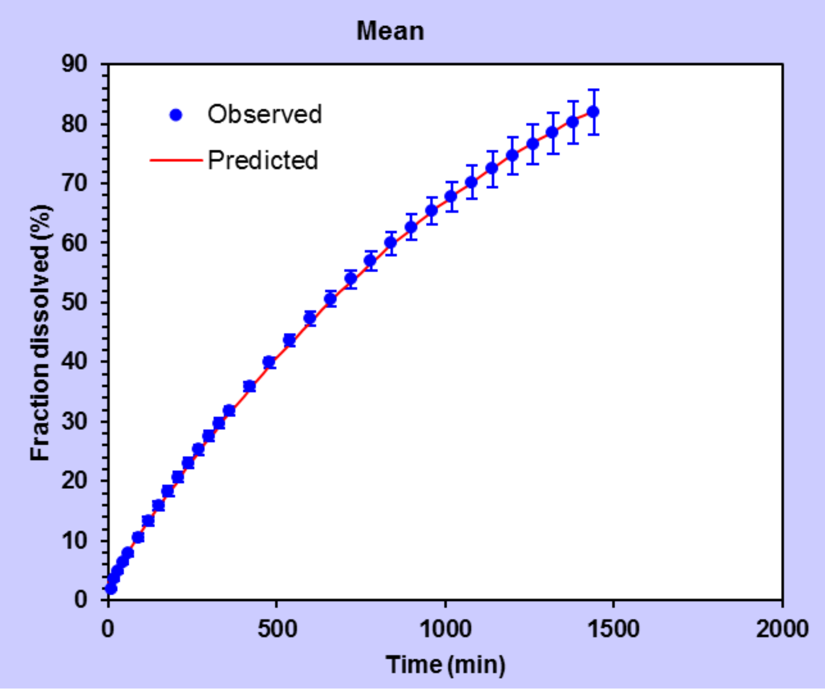

Supplementary materials – Model fitting summary – Ethocel^{TM} Standard 20 Premium (modelling the entire relevant dissolution data)

Graphical abstract of model fit presented as the fraction % of released carvedilol per tested tablet:

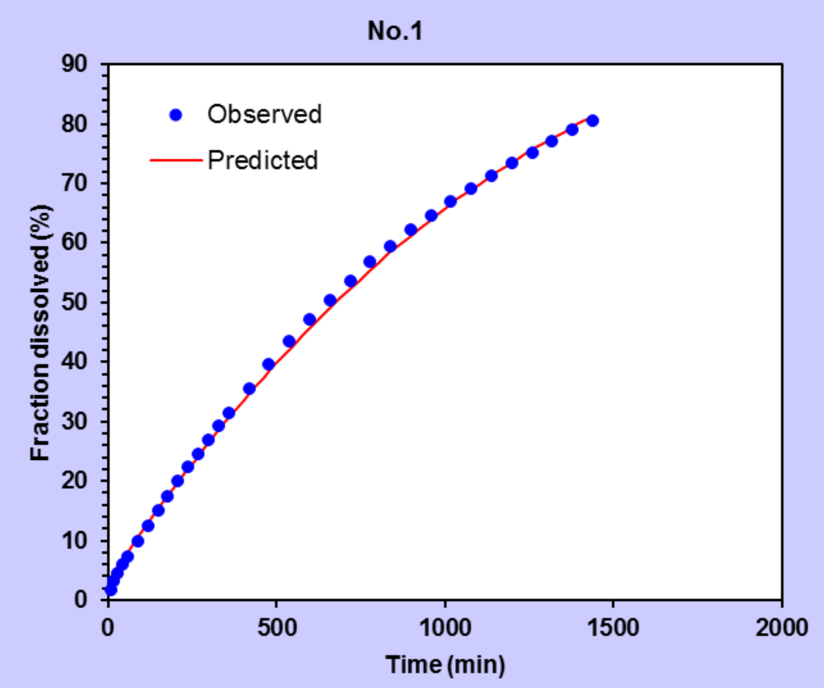

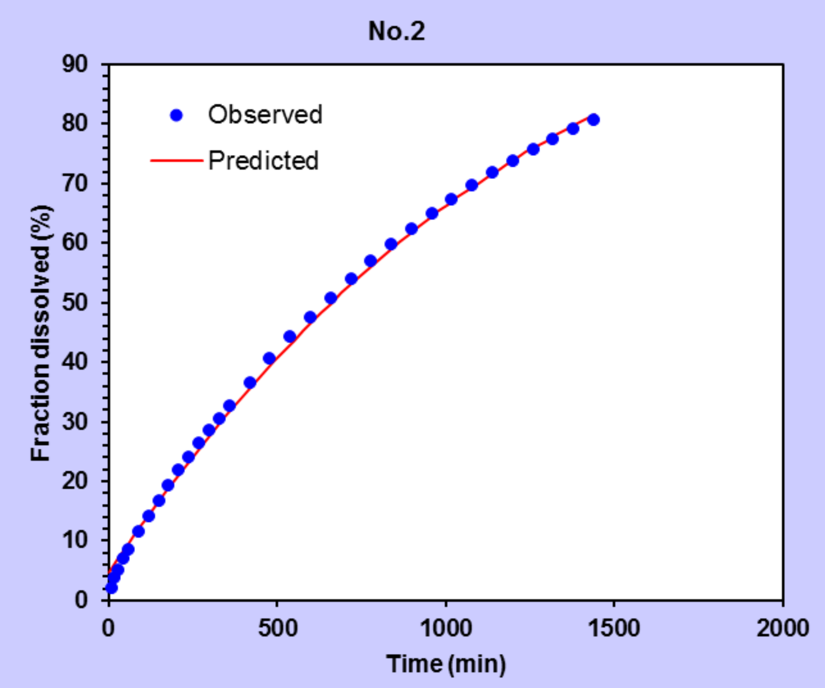

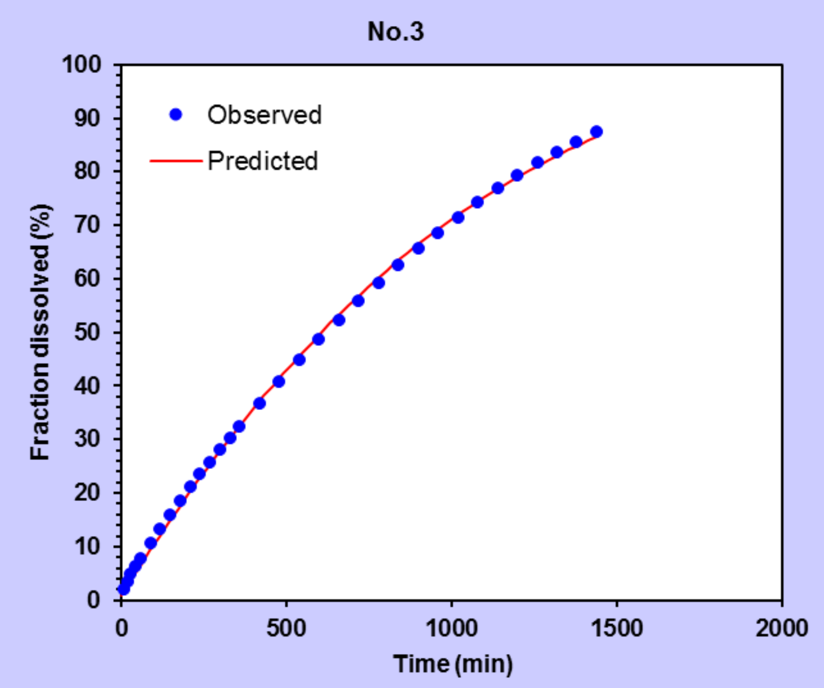

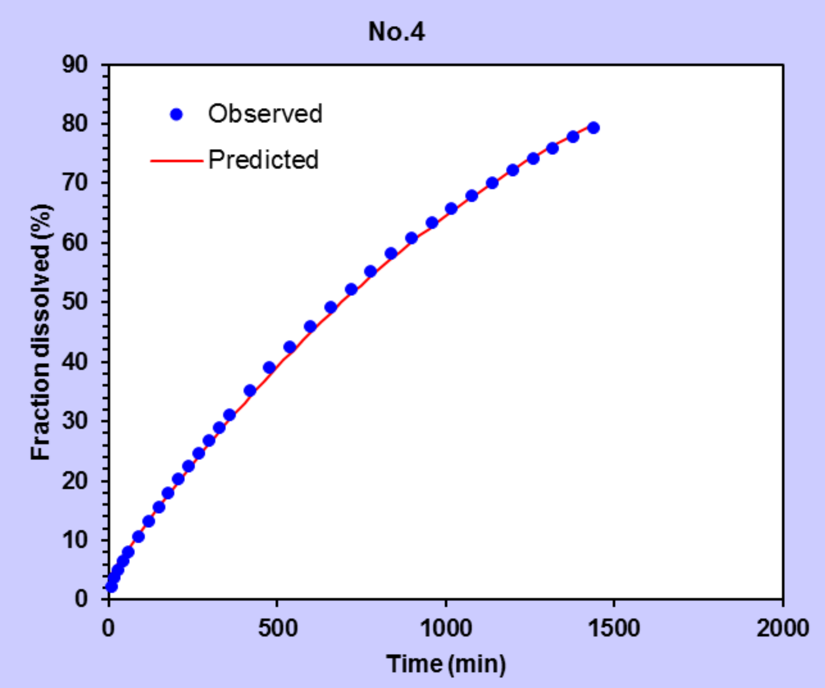

Supplementary materials – Model fitting summary – Ethocel™ Standard 20 Premium (modelling the entire relevant dissolution data)

Model: **Hopfenberg**

Model equation: $F = 100 \cdot [1 - (1 - k_{HB} \cdot t)^n]$

Fitted model parameters per tested tablet (N = 4) with statistics – mean, standard deviation (SD), and relative standard deviation expressed in % (RSD%) (output from DDSolver):

Parameter	No.1	No.2	No.3	No.4	Mean	SD	RSD(%)
k_{HB}	0.0002	0.0002	0.0003	0.0002	0.0003	0.0001	22.6095
n	4.1250	4.1250	3.0000	4.1250	3.8438	0.5625	14.6341

Number of dissolution data points (N), degrees of freedom (df), and selected goodness of fit criteria – Pearson correlation coefficient (R), coefficient of determination (R^2), adjusted coefficient of determination ($R^2_{adjusted}$), and residual sum of squares (RSS) (manual calculation in MS Excel):

Parameter	No.1	No.2	No.3	No.4
N	33	33	33	33
df	31	31	31	31
R	0.999879981	0.999692166	0.999662612	0.999883132
R^2	0.999759976	0.999384427	0.999325338	0.999766278
$R^2_{adjusted}$	0.999752234	0.99936457	0.999303575	0.999758738
RSS	68.43918933	161.3643531	33.3071438	104.9394514

Graphical abstract of model fit presented as mean ± 1 SD of the fraction % of released carvedilol:

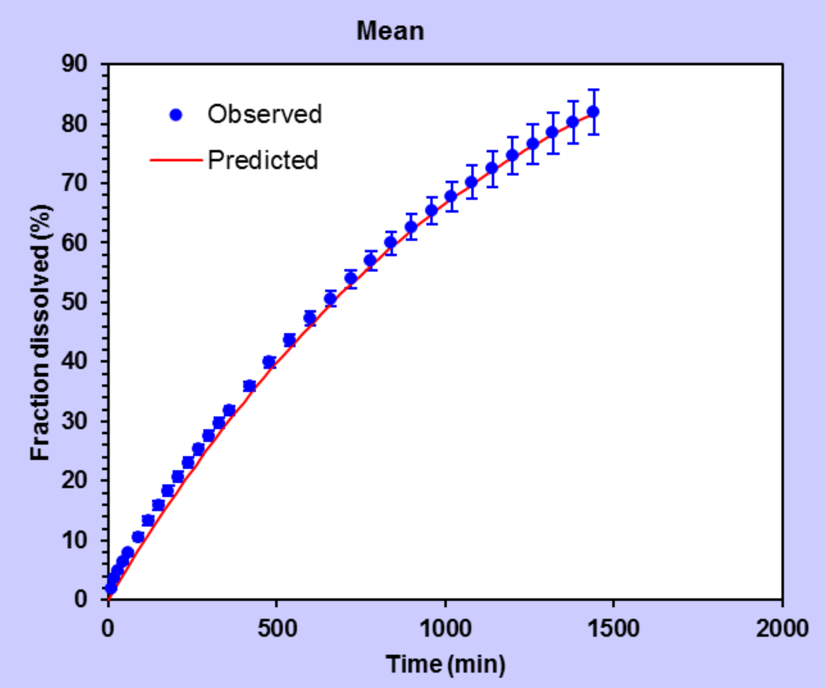

Supplementary materials – Model fitting summary – Ethocel™ Standard 20 Premium (modelling the entire relevant dissolution data)

Graphical abstract of model fit presented as the fraction % of released carvedilol per tested tablet:

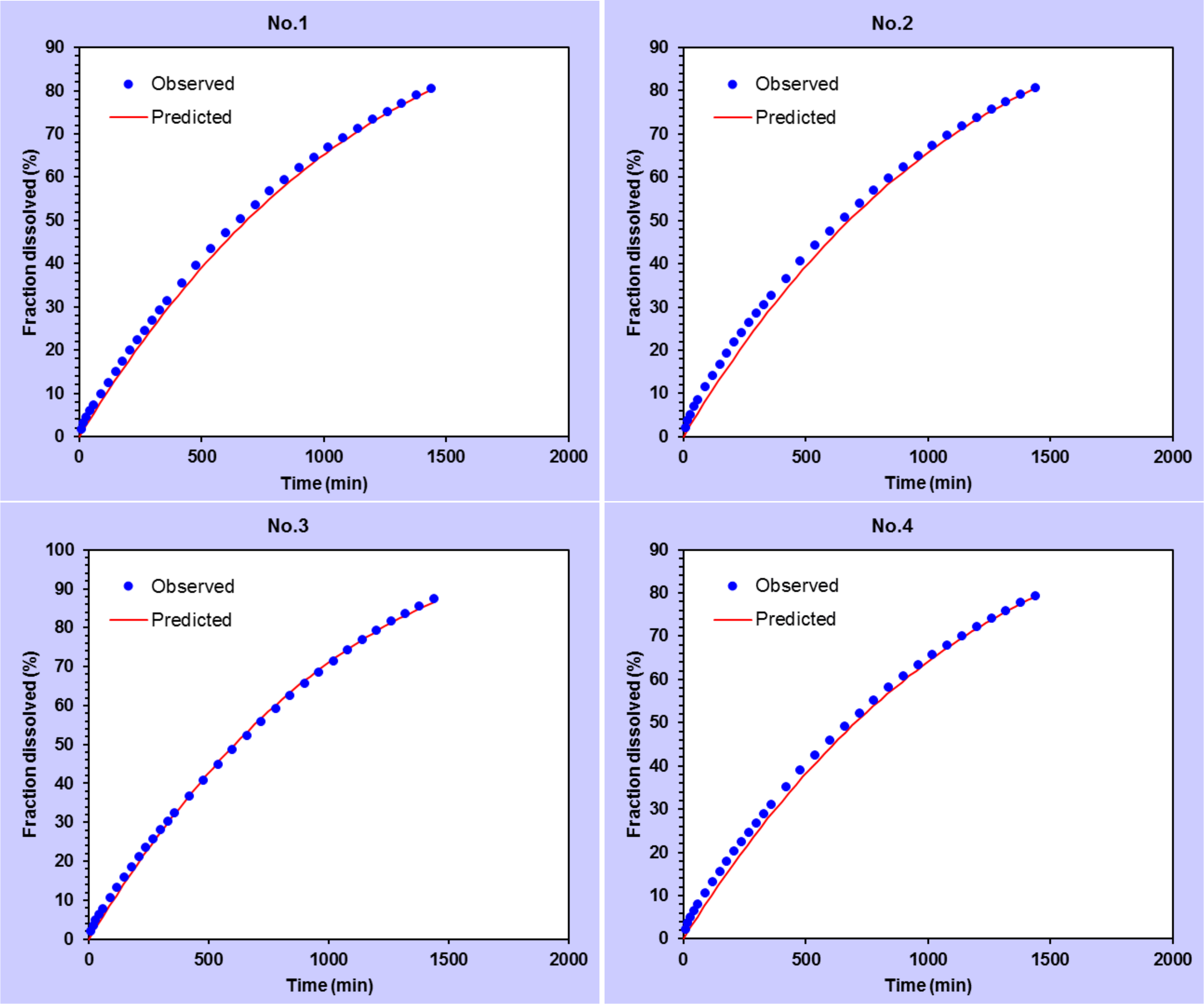

Supplementary materials – Model fitting summary – Ethocel™ Standard 20 Premium (modelling the entire relevant dissolution data)

Model: **Hopfenberg with T_{lag}**

Model equation: $F = 100 \cdot \{1 - [1 - k_{HB} \cdot (t - T_{lag})]^n\}$

Fitted model parameters per tested tablet (N = 4) with statistics – mean, standard deviation (SD), and relative standard deviation expressed in % (RSD%) (output from DDSolver):

Parameter	No.1	No.2	No.3	No.4	Mean	SD	RSD(%)
k_{HB}	0.0003	0.0003	0.0004	0.0003	0.0003	0.0001	27.1283
n	3.0000	3.5469	2.0000	3.0000	2.8867	0.6449	22.3407
T_{lag}	-36.6683	-42.7914	-35.6746	-46.4867	-40.4053	5.1323	-12.7021

Number of dissolution data points (N), degrees of freedom (df), and selected goodness of fit criteria – Pearson correlation coefficient (R), coefficient of determination (R^2), adjusted coefficient of determination ($R^2_{adjusted}$), and residual sum of squares (RSS) (manual calculation in MS Excel):

Parameter	No.1	No.2	No.3	No.4
N	33	33	33	33
df	30	30	30	30
R	0.999493632	0.999429112	0.999788579	0.999526146
R^2	0.99898752	0.998858551	0.999577202	0.999052516
$R^2_{adjusted}$	0.998920021	0.998782454	0.999549016	0.998989351
RSS	25.4282268	34.84600978	11.34760569	21.79594433

Graphical abstract of model fit presented as mean ± 1 SD of the fraction % of released carvedilol:

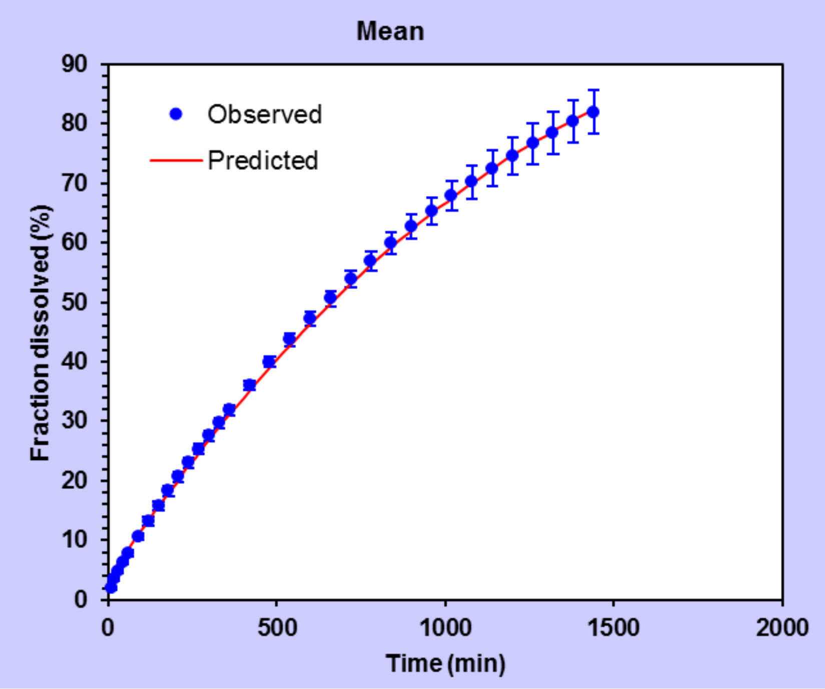

Supplementary materials – Model fitting summary – Ethocel™ Standard 20 Premium (modelling the entire relevant dissolution data)

Graphical abstract of model fit presented as the fraction % of released carvedilol per tested tablet:

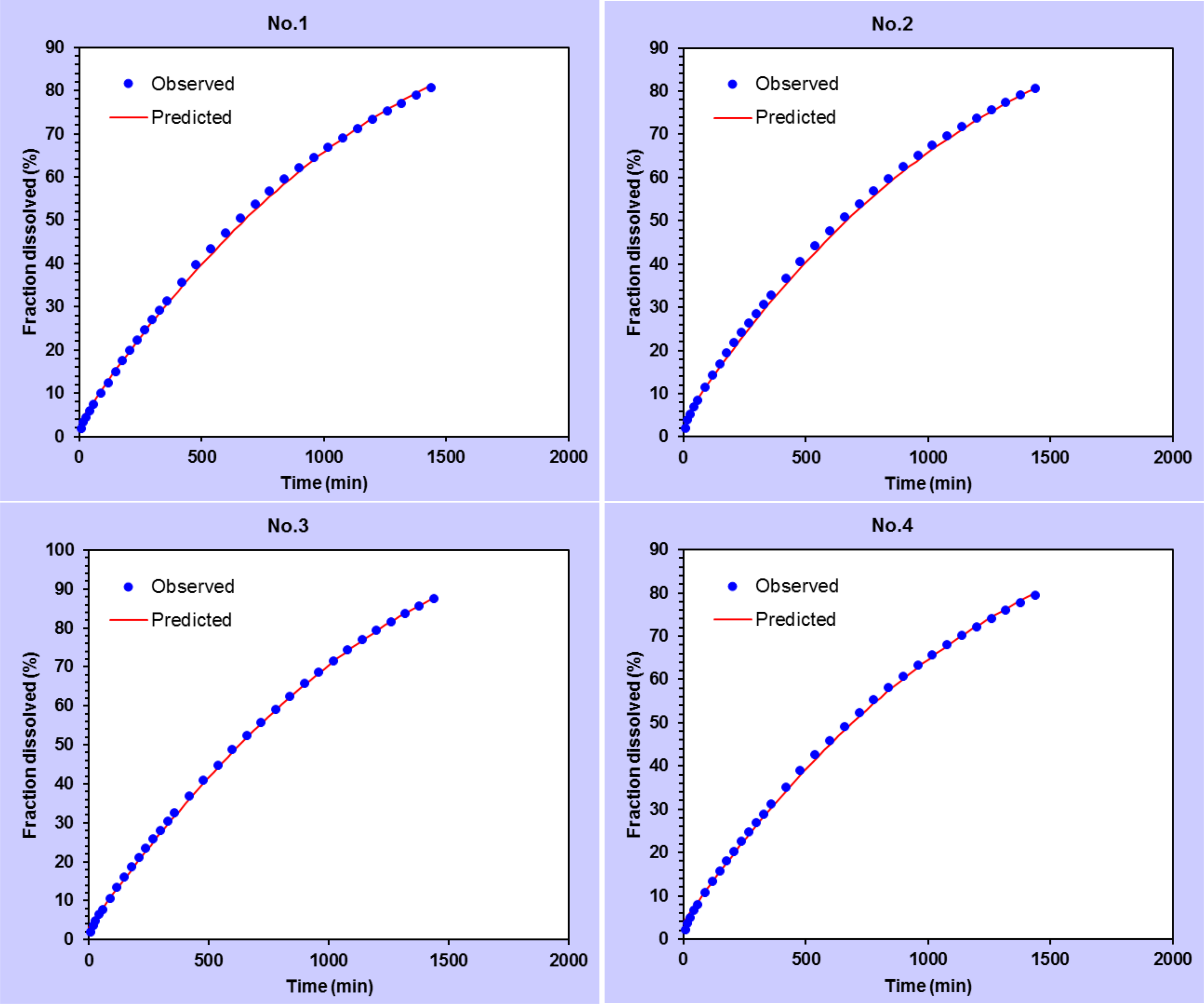

Supplementary materials – Model fitting summary – Ethocel™ Standard 20 Premium (modelling the entire relevant dissolution data)

Model: **Baker–Lonsdale**

Model equation: $\frac{3}{2}\cdot\left[1-\left(1-\frac{F}{100}\right)^{\frac{2}{3}}\right]-\frac{F}{100}=k_{BL}\cdot t$

Fitted model parameters per tested tablet (N = 4) with statistics – mean, standard deviation (SD), and relative standard deviation expressed in % (RSD%) (output from DDSolver):

Parameter	No.1	No.2	No.3	No.4	Mean	SD	RSD(%)
k_{BL}	0.0001	0.0001	0.0002	0.0001	0.0001	0.0000	13.2428

Number of dissolution data points (N), degrees of freedom (df), and selected goodness of fit criteria – Pearson correlation coefficient (R), coefficient of determination (R^2), adjusted coefficient of determination ($R^2_{adjusted}$), and residual sum of squares (RSS) (manual calculation in MS Excel):

Parameter	No.1	No.2	No.3	No.4
N	33	33	33	33
df	32	32	32	32
R	0.986963361	0.988949713	0.980560685	0.987170067
R^2	0.974096675	0.978021534	0.961499257	0.974504741
$R^2_{adjusted}$	0.974096675	0.978021534	0.961499257	0.974504741
RSS	4368.665721	3706.779894	6477.257371	3919.77376

Graphical abstract of model fit presented as mean ± 1 SD of the fraction % of released carvedilol:

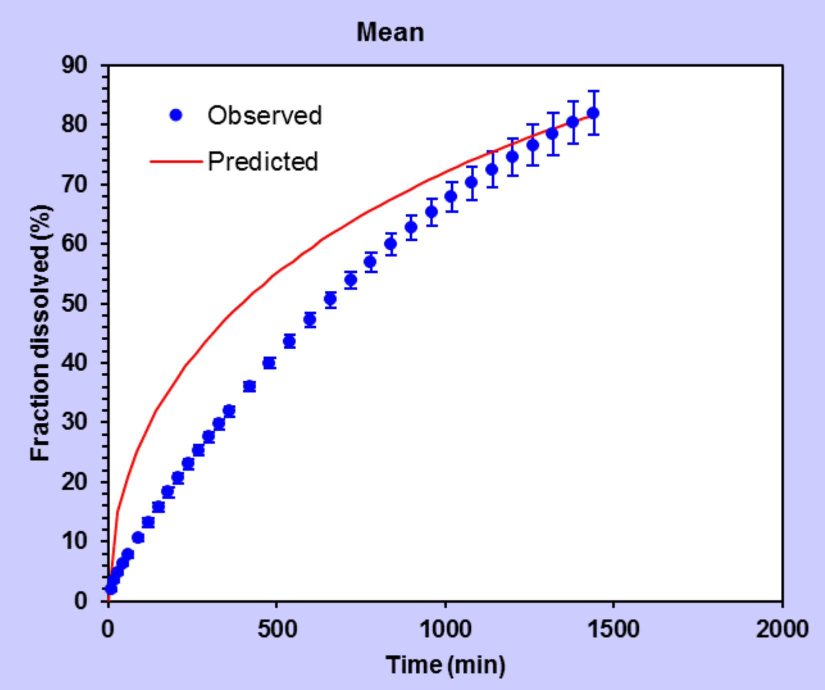

Supplementary materials – Model fitting summary – Ethocel^{TM} Standard 20 Premium (modelling the entire relevant dissolution data)

Graphical abstract of model fit presented as the fraction % of released carvedilol per tested tablet:

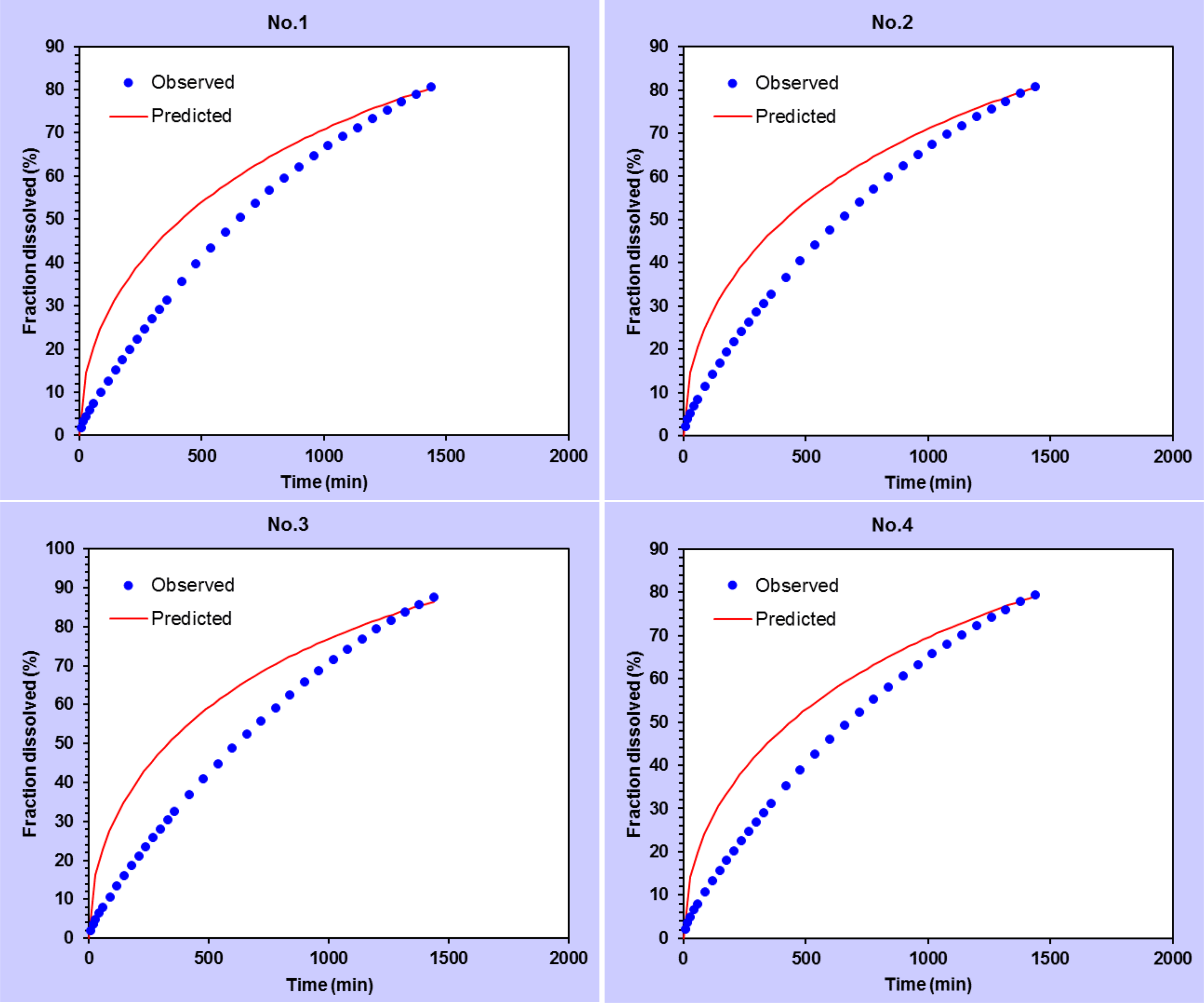

Supplementary materials – Model fitting summary – Ethocel™ Standard 20 Premium (modelling the entire relevant dissolution data)

Model: **Baker–Lonsdale with T_{lag}**

Model equation: $\frac{3}{2} \cdot \left[1 - \left(1 - \frac{F}{100}\right)^{\frac{2}{3}}\right] - \frac{F}{100} = k_{BL} \cdot (t - T_{lag})$

Fitted model parameters per tested tablet (N = 4) with statistics – mean, standard deviation (SD), and relative standard deviation expressed in % (RSD%) (output from DDSolver):

Parameter	No.1	No.2	No.3	No.4	Mean	SD	RSD(%)
k_{BL}	0.0001	0.0001	0.0002	0.0001	0.0001	0.0000	13.2428
T_{lag}	139.4600	132.2723	157.5955	137.8040	141.7830	10.9806	7.7446

Number of dissolution data points (N), degrees of freedom (df), and selected goodness of fit criteria – Pearson correlation coefficient (R), coefficient of determination (R^2), adjusted coefficient of determination ($R^2_{adjusted}$), and residual sum of squares (RSS) (manual calculation in MS Excel):

Parameter	No.1	No.2	No.3	No.4
N	33	33	33	33
df	31	31	31	31
R	0.985162094	0.984655237	0.978844192	0.984110549
R^2	0.970544352	0.969545936	0.958135951	0.968473572
$R^2_{adjusted}$	0.969594169	0.968563547	0.956785498	0.96745659
RSS	795.3721457	850.2329362	1383.396766	834.0696604

Graphical abstract of model fit presented as mean ± 1 SD of the fraction % of released carvedilol:

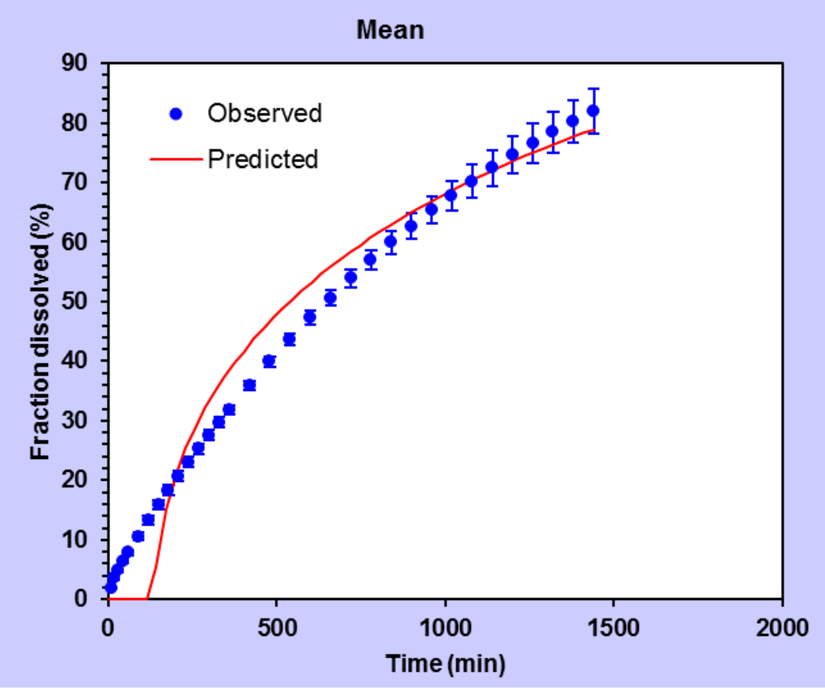

Supplementary materials – Model fitting summary – Ethocel^{TM} Standard 20 Premium (modelling the entire relevant dissolution data)

Graphical abstract of model fit presented as the fraction % of released carvedilol per tested tablet:

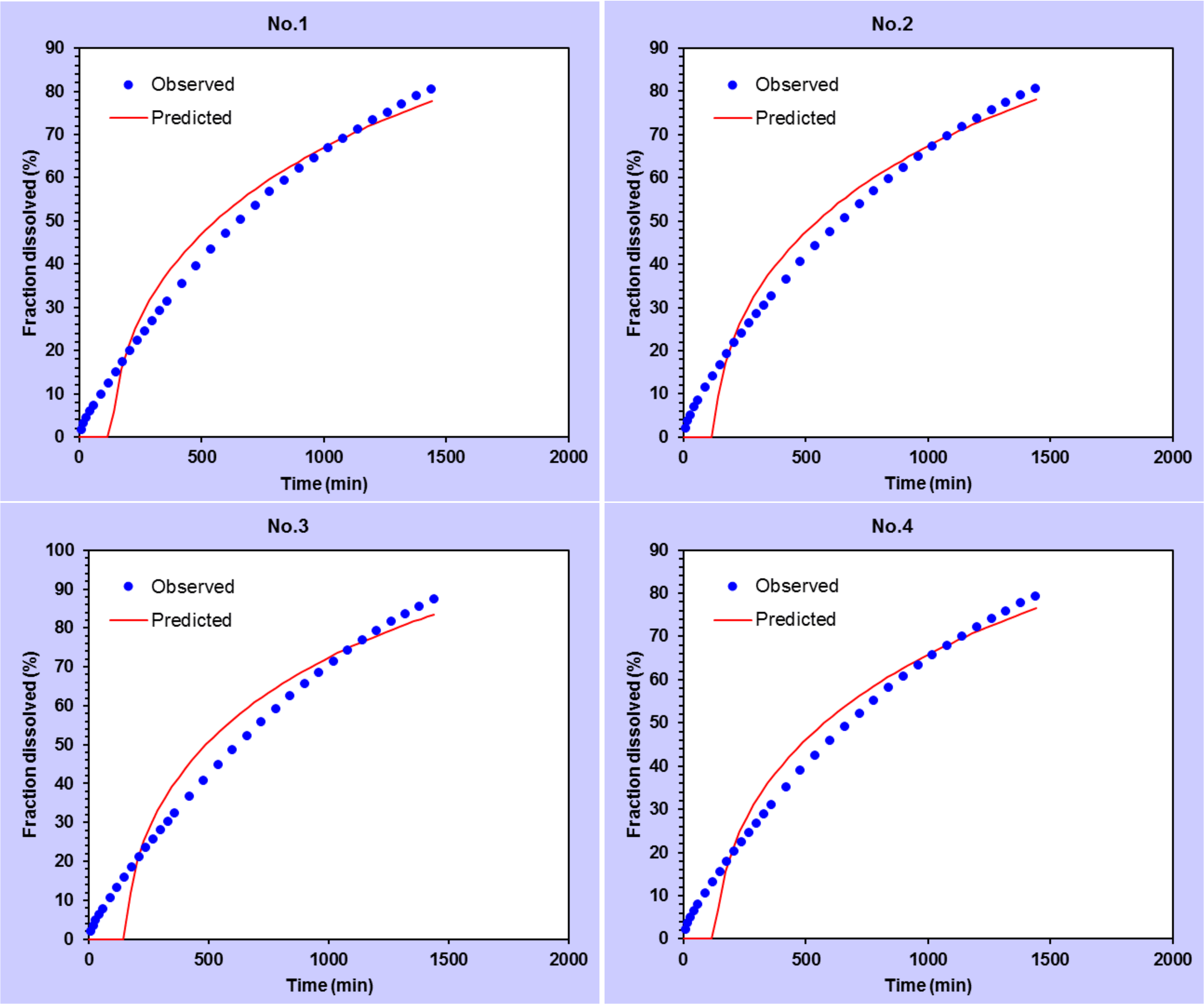

Supplementary materials – Model fitting summary – Ethocel™ Standard 20 Premium (modelling the entire relevant dissolution data)

Model: **Makoid–Banakar**

Model equation: $F = k_{MB} \cdot t^n \cdot e^{-k \cdot t}$

Fitted model parameters per tested tablet (N = 4) with statistics – mean, standard deviation (SD), and relative standard deviation expressed in % (RSD%) (output from DDSolver):

Parameter	No.1	No.2	No.3	No.4	Mean	SD	RSD(%)
k_{MB}	0.28486	0.34679	0.31131	0.36264	0.32640	0.03504	10.73384
n	0.81303	0.78212	0.79515	0.76147	0.78794	0.02173	2.75748
k	0.00017	0.00015	0.00009	0.00010	0.00013	0.00004	31.32838

Number of dissolution data points (N), degrees of freedom (df), and selected goodness of fit criteria – Pearson correlation coefficient (R), coefficient of determination (R^2), adjusted coefficient of determination ($R^2_{adjusted}$), and residual sum of squares (RSS) (manual calculation in MS Excel):

Parameter	No.1	No.2	No.3	No.4
N	33	33	33	33
df	30	30	30	30
R	0.999619164	0.999817927	0.999709673	0.999573107
R^2	0.999238473	0.999635887	0.999419431	0.999146396
$R^2_{adjusted}$	0.999187704	0.999611612	0.999380726	0.999089489
RSS	26.65393005	7.773495717	22.63399686	25.84836358

Graphical abstract of model fit presented as mean ± 1 SD of the fraction % of released carvedilol:

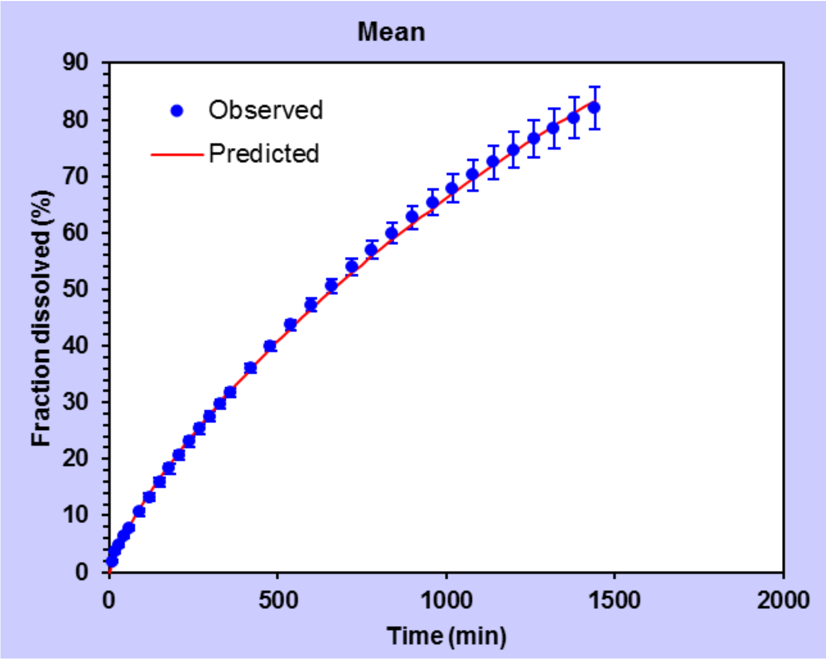

Supplementary materials – Model fitting summary – Ethocel™ Standard 20 Premium (modelling the entire relevant dissolution data)

Graphical abstract of model fit presented as the fraction % of released carvedilol per tested tablet:

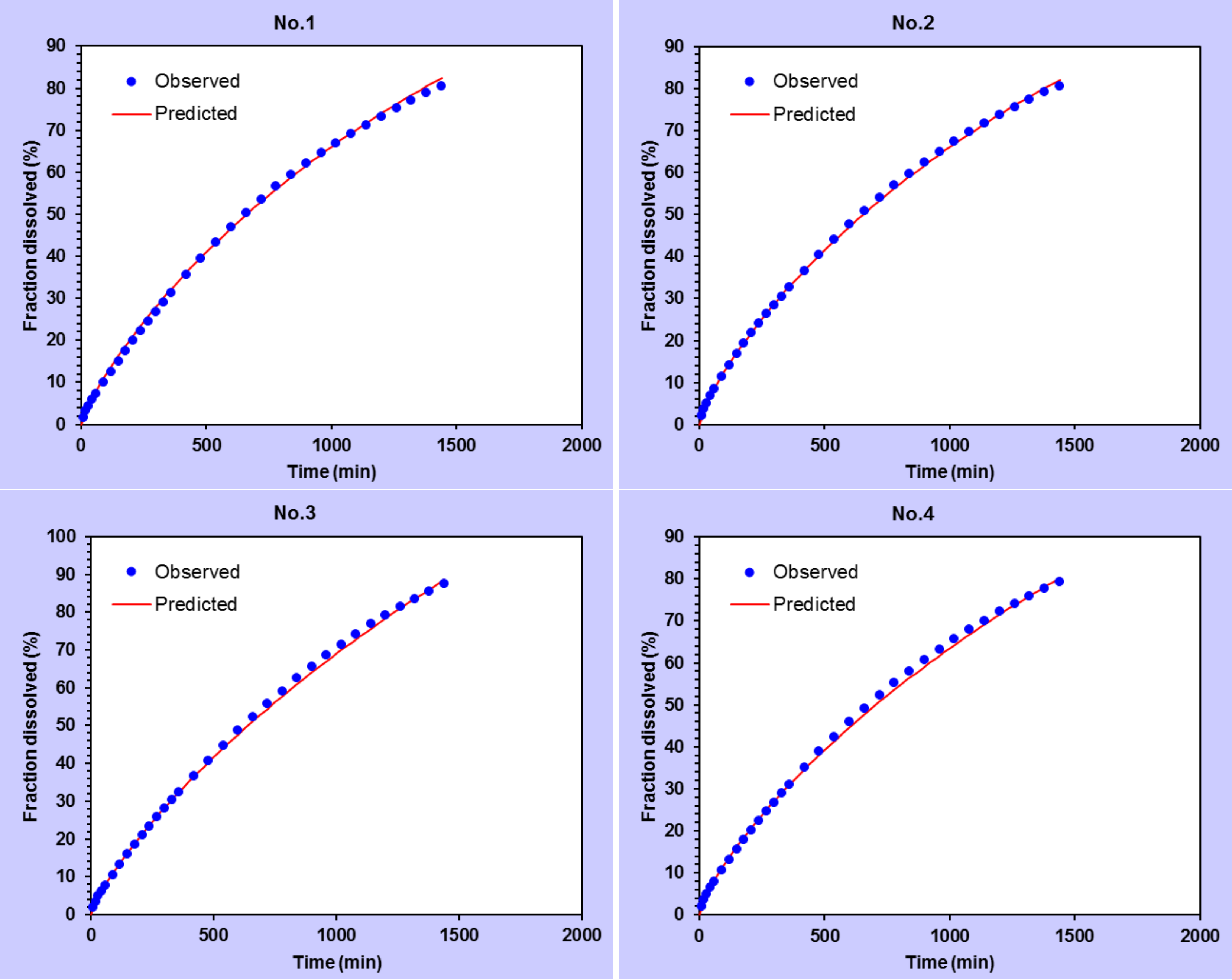

Supplementary materials – Model fitting summary – Ethocel™ Standard 20 Premium (modelling the entire relevant dissolution data)

Model: **Makoid–Banakar with T_{lag}**

Model equation: $F = k_{MB} \cdot (t - T_{lag})^n \cdot e^{-k \cdot (t - T_{lag})}$

Fitted model parameters per tested tablet (N = 4) with statistics – mean, standard deviation (SD), and relative standard deviation expressed in % (RSD%) (output from DDSolver):

Parameter	No.1	No.2	No.3	No.4	Mean	SD	RSD(%)
k_{MB}	0.4038371	0.5288887	0.4744307	0.5435249	0.4876704	0.0633035	12.9807946
n	0.7361852	0.7006504	0.7112533	0.6807293	0.7072046	0.0230952	3.2657041
k	-0.0000004	0.0000245	-0.0000734	-0.0000650	-0.0000286	0.0000481	-168.3769476
T_{lag}	4.0000000	4.0000000	4.0000000	4.0000000	4.0000000	0.0000000	0.0000000

Number of dissolution data points (N), degrees of freedom (df), and selected goodness of fit criteria – Pearson correlation coefficient (R), coefficient of determination (R^2), adjusted coefficient of determination ($R^2_{adjusted}$), and residual sum of squares (RSS) (manual calculation in MS Excel):

Parameter	No.1	No.2	No.3	No.4
N	33	33	33	33
df	29	29	29	29
R	0.997819413	0.999051422	0.998284208	0.997922034
R^2	0.995643581	0.998103744	0.99657136	0.995848386
$R^2_{adjusted}$	0.995192917	0.997907579	0.996216673	0.995418909
RSS	100.1020471	44.36625956	92.6025802	90.12813693

Graphical abstract of model fit presented as mean ± 1 SD of the fraction % of released carvedilol:

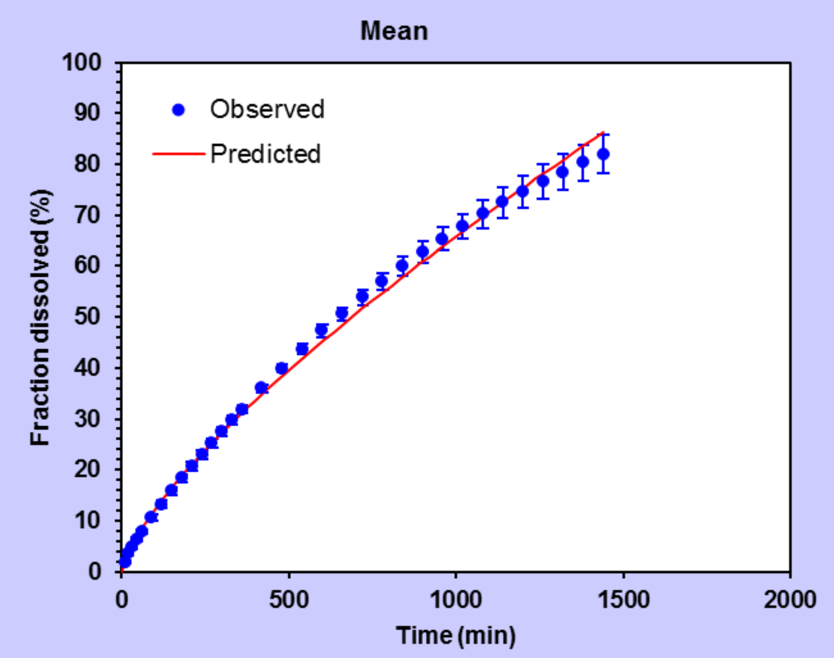

Supplementary materials – Model fitting summary – Ethocel™ Standard 20 Premium (modelling the entire relevant dissolution data)

Graphical abstract of model fit presented as the fraction % of released carvedilol per tested tablet:

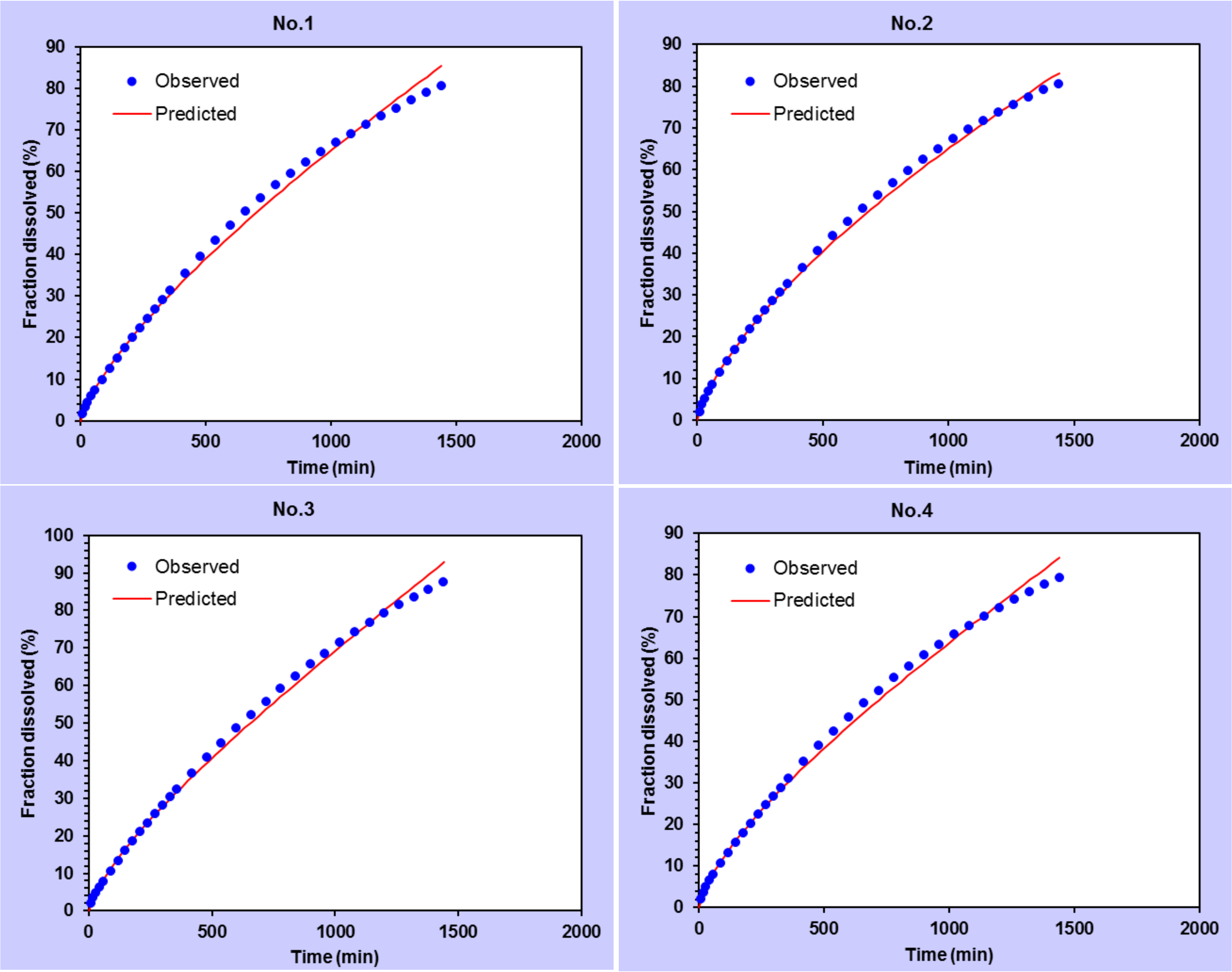

Supplementary materials – Model fitting summary – Ethocel™ Standard 20 Premium (modelling the entire relevant dissolution data)

Model: **Peppas–Sahlin_1**

Model equation: $F = k_1 \cdot t^m + k_2 \cdot t^{2m}$

Fitted model parameters per tested tablet (N = 4) with statistics – mean, standard deviation (SD), and relative standard deviation expressed in % (RSD%) (output from DDSolver):

Parameter	No.1	No.2	No.3	No.4	Mean	SD	RSD(%)
k_1	1.002	1.219	0.911	1.053	1.046	0.129	12.375
k_2	0.084	0.075	0.097	0.079	0.084	0.009	11.154
m	0.450	0.450	0.450	0.450	0.450	0.000	0.000

Number of dissolution data points (N), degrees of freedom (df), and selected goodness of fit criteria – Pearson correlation coefficient (R), coefficient of determination (R^2), adjusted coefficient of determination ($R^2_{adjusted}$), and residual sum of squares (RSS) (manual calculation in MS Excel):

Parameter	No.1	No.2	No.3	No.4
N	33	33	33	33
df	30	30	30	30
R	0.996932199	0.997701488	0.998413442	0.997866568
R^2	0.99387381	0.995408259	0.9968294	0.995737687
$R^2_{adjusted}$	0.993465398	0.995102143	0.996618027	0.995453533
RSS	146.1149066	108.4661958	87.40876013	96.10145313

Graphical abstract of model fit presented as mean ± 1 SD of the fraction % of released carvedilol:

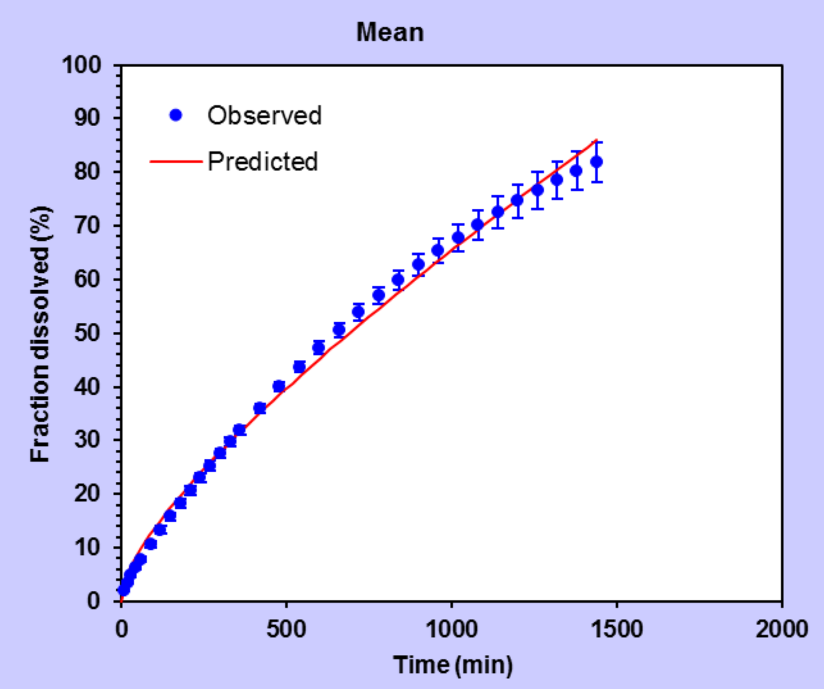

Supplementary materials – Model fitting summary – Ethocel^{TM} Standard 20 Premium (modelling the entire relevant dissolution data)

Graphical abstract of model fit presented as the fraction % of released carvedilol per tested tablet:

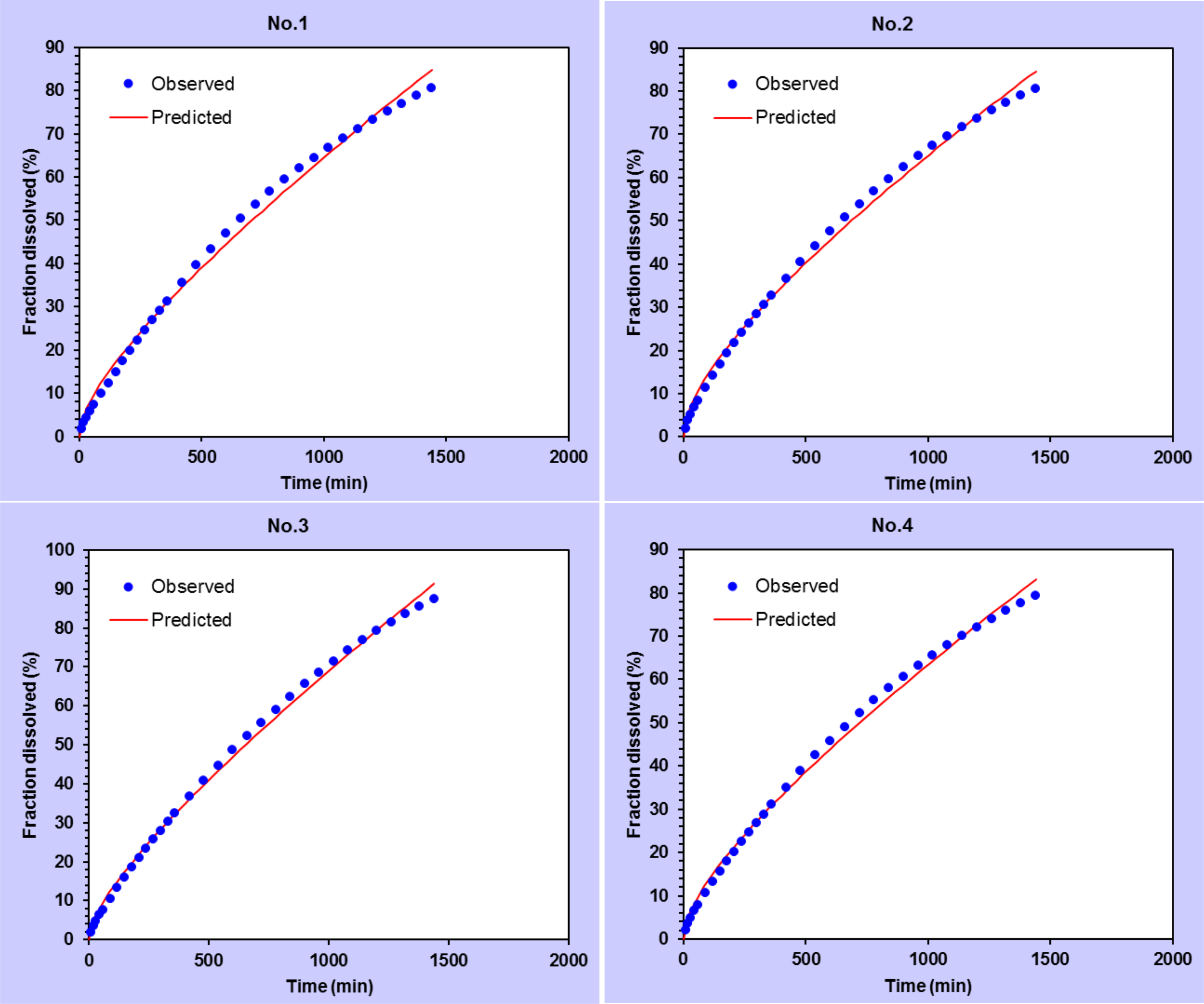

Supplementary materials – Model fitting summary – Ethocel™ Standard 20 Premium (modelling the entire relevant dissolution data)

Model: **Peppas–Sahlin_1 with T_{lag}**

Model equation: $F = k_1 \cdot (t - T_{lag})^m + k_2 \cdot (t - T_{lag})^{2m}$

Fitted model parameters per tested tablet (N = 4) with statistics – mean, standard deviation (SD), and relative standard deviation expressed in % (RSD%) (output from DDSolver):

Parameter	No.1	No.2	No.3	No.4	Mean	SD	RSD(%)
k_1	1.055	1.274	0.964	1.105	1.100	0.130	11.823
k_2	0.082	0.073	0.095	0.078	0.082	0.009	11.447
m	0.450	0.450	0.450	0.450	0.450	0.000	0.000
T_{lag}	6.000	6.000	6.000	6.000	6.000	0.000	0.000

Number of dissolution data points (N), degrees of freedom (df), and selected goodness of fit criteria – Pearson correlation coefficient (R), coefficient of determination (R^2), adjusted coefficient of determination ($R^2_{adjusted}$), and residual sum of squares (RSS) (manual calculation in MS Excel):

Parameter	No.1	No.2	No.3	No.4
N	33	33	33	33
df	29	29	29	29
R	0.997199982	0.997991845	0.998582321	0.998087833
R^2	0.994407804	0.995987723	0.997166652	0.996179322
$R^2_{adjusted}$	0.993829301	0.995572659	0.996873547	0.995784079
RSS	128.838721	90.39430081	74.8971414	82.50370277

Graphical abstract of model fit presented as mean ± 1 SD of the fraction % of released carvedilol:

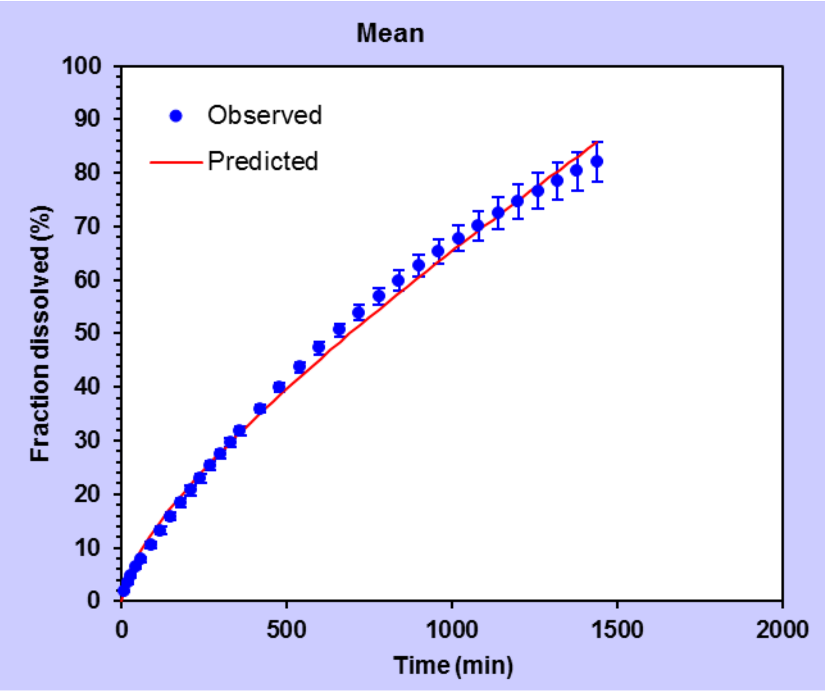

Supplementary materials – Model fitting summary – Ethocel™ Standard 20 Premium (modelling the entire relevant dissolution data)

Graphical abstract of model fit presented as the fraction % of released carvedilol per tested tablet:

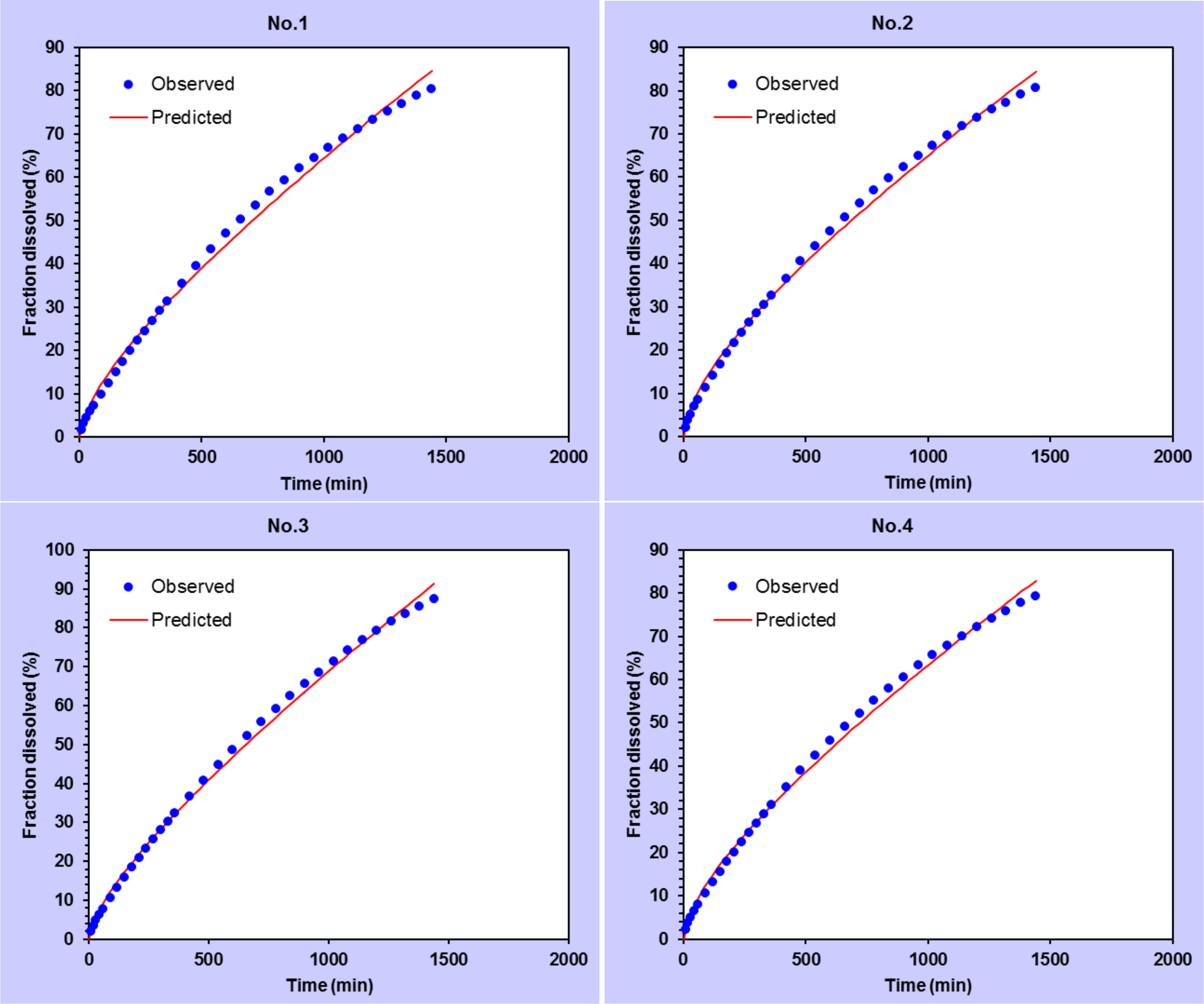

Supplementary materials – Model fitting summary – Ethocel™ Standard 20 Premium (modelling the entire relevant dissolution data)

Model: **Peppas–Sahlin_2**

Model equation: $F = k_1 \cdot t^{0.5} + k_2 \cdot t$

Fitted model parameters per tested tablet (N = 4) with statistics – mean, standard deviation (SD), and relative standard deviation expressed in % (RSD%) (output from DDSolver):

Parameter	No.1	No.2	No.3	No.4	Mean	SD	RSD(%)
k_1	1.041	1.193	0.994	1.067	1.074	0.085	7.905
k_2	0.031	0.027	0.037	0.030	0.031	0.004	13.814

Number of dissolution data points (N), degrees of freedom (df), and selected goodness of fit criteria – Pearson correlation coefficient (R), coefficient of determination (R^2), adjusted coefficient of determination ($R^2_{adjusted}$), and residual sum of squares (RSS) (manual calculation in MS Excel):

Parameter	No.1	No.2	No.3	No.4
N	33	33	33	33
df	31	31	31	31
R	0.996764493	0.997682168	0.998189121	0.997746882
R^2	0.993539454	0.995369709	0.996381522	0.995498841
$R^2_{adjusted}$	0.993331049	0.995220345	0.996264796	0.995353643
RSS	159.9898076	113.9879761	103.5728009	105.3003237

Graphical abstract of model fit presented as mean ± 1 SD of the fraction % of released carvedilol:

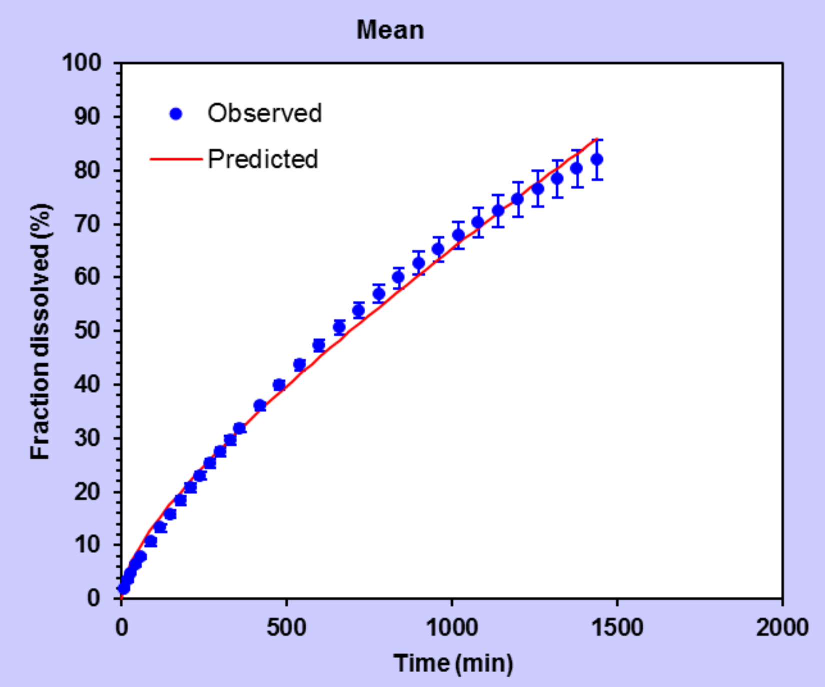

Supplementary materials – Model fitting summary – Ethocel™ Standard 20 Premium (modelling the entire relevant dissolution data)

Graphical abstract of model fit presented as the fraction % of released carvedilol per tested tablet:

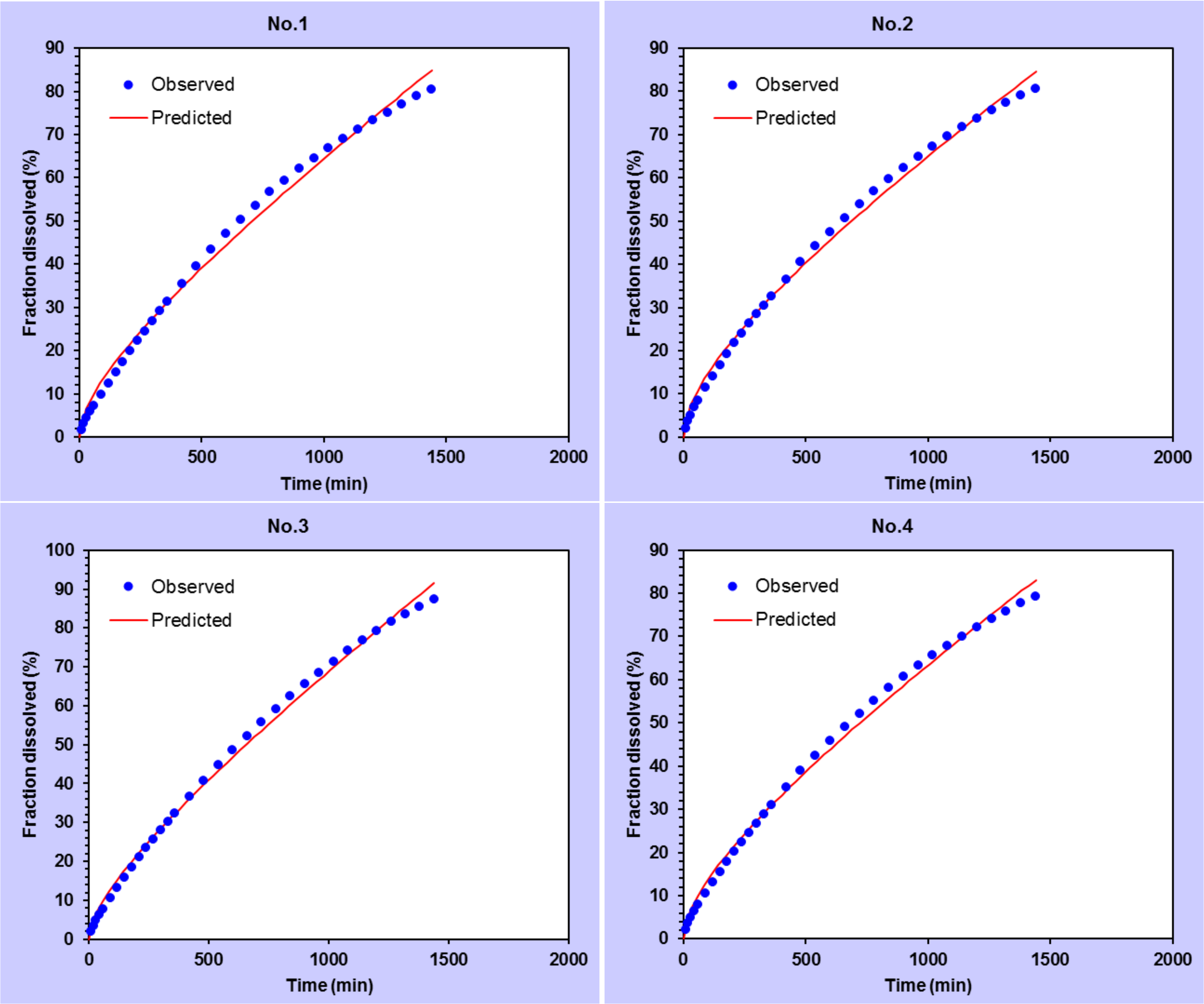

Supplementary materials – Model fitting summary – Ethocel™ Standard 20 Premium (modelling the entire relevant dissolution data)

Model: **Peppas–Sahlin_2 with T_{lag}**

Model equation: $F = k_1 \cdot (t - T_{lag})^{0.5} + k_2 \cdot (t - T_{lag})$

Fitted model parameters per tested tablet (N = 4) with statistics – mean, standard deviation (SD), and relative standard deviation expressed in % (RSD%) (output from DDSolver):

Parameter	No.1	No.2	No.3	No.4	Mean	SD	RSD(%)
k_1	1.078	1.230	1.031	1.102	1.110	0.085	7.671
k_2	0.031	0.026	0.036	0.029	0.030	0.004	14.269
T_{lag}	6.000	6.000	6.000	6.000	6.000	0.000	0.000

Number of dissolution data points (N), degrees of freedom (df), and selected goodness of fit criteria – Pearson correlation coefficient (R), coefficient of determination (R^2), adjusted coefficient of determination ($R^2_{adjusted}$), and residual sum of squares (RSS) (manual calculation in MS Excel):

Parameter	No.1	No.2	No.3	No.4
N	33	33	33	33
df	30	30	30	30
R	0.997055808	0.997977611	0.998386027	0.997983045
R^2	0.994120284	0.995959311	0.996774658	0.995970157
$R^2_{adjusted}$	0.993728303	0.995689932	0.996559635	0.995701501
RSS	139.315108	93.59907548	87.52560423	89.17864924

Graphical abstract of model fit presented as mean ± 1 SD of the fraction % of released carvedilol:

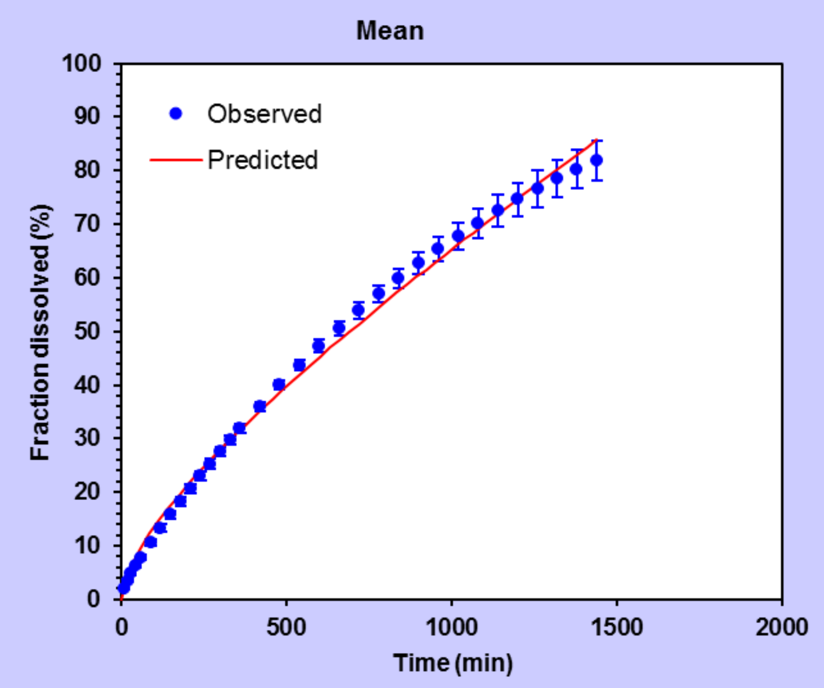

Supplementary materials – Model fitting summary – Ethocel^{TM} Standard 20 Premium (modelling the entire relevant dissolution data)

Graphical abstract of model fit presented as the fraction % of released carvedilol per tested tablet:

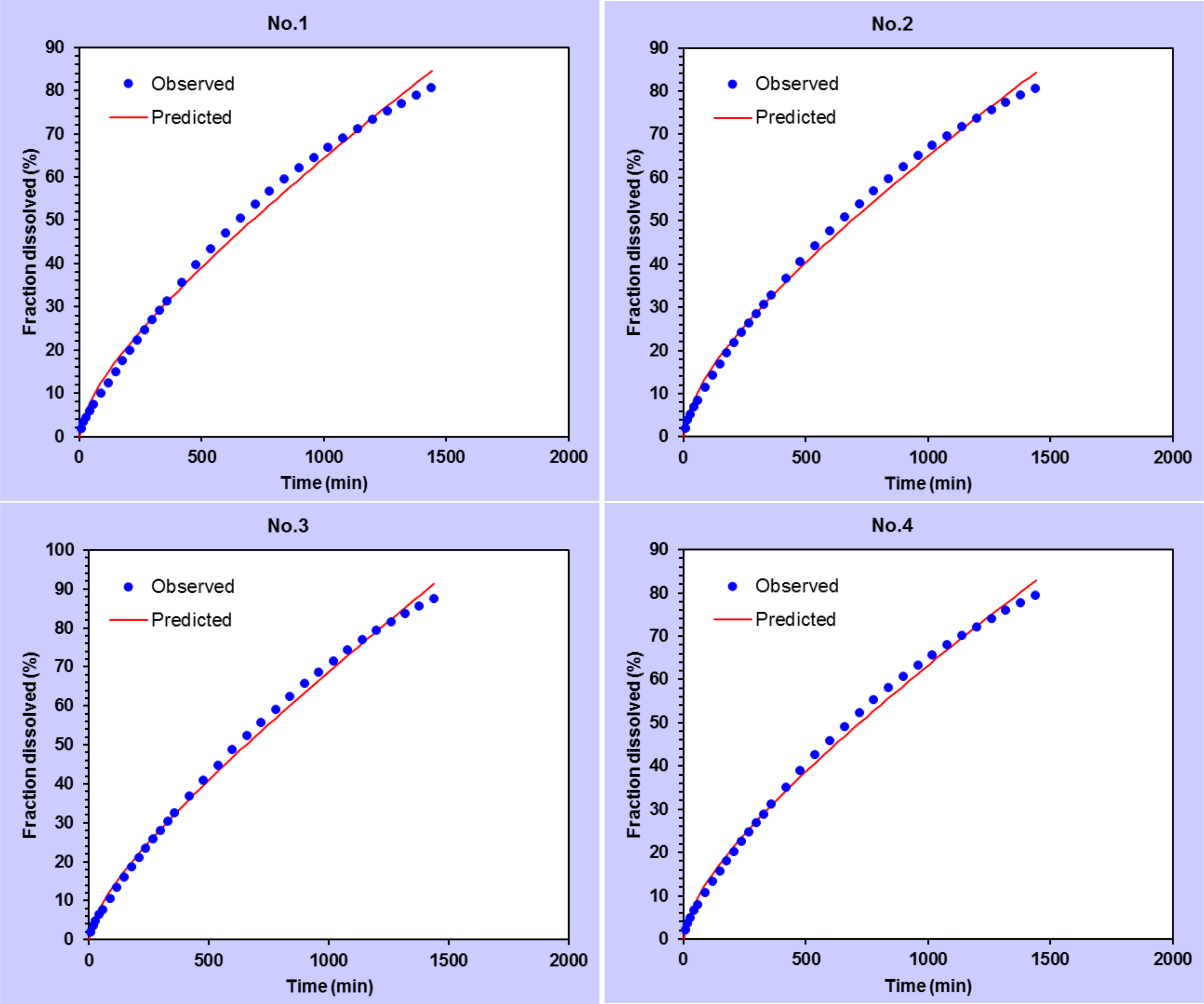

Supplementary materials – Model fitting summary – Ethocel™ Standard 20 Premium (modelling the entire relevant dissolution data)

Model: **Quadratic**

Model equation: $F = 100 \cdot (k_1 \cdot t^2 + k_2 \cdot t)$

Fitted model parameters per tested tablet (N = 4) with statistics – mean, standard deviation (SD), and relative standard deviation expressed in % (RSD%) (output from DDSolver):

Parameter	No.1	No.2	No.3	No.4	Mean	SD	RSD(%)
k_1	-0.0000003	-0.0000003	-0.0000003	-0.0000003	-0.0000003	0.0000000	-6.8077312
k_2	0.0009547	0.0009899	0.0009773	0.0009404	0.0009656	0.0000222	2.3038732

Number of dissolution data points (N), degrees of freedom (df), and selected goodness of fit criteria – Pearson correlation coefficient (R), coefficient of determination (R^2), adjusted coefficient of determination ($R^2_{adjusted}$), and residual sum of squares (RSS) (manual calculation in MS Excel):

Parameter	No.1	No.2	No.3	No.4
N	33	33	33	33
df	31	31	31	31
R	0.999685216	0.999086314	0.999619951	0.999394505
R^2	0.99937053	0.998173463	0.999240045	0.998789377
$R^2_{adjusted}$	0.999350225	0.998114543	0.999215531	0.998750325
RSS	32.33816193	92.88254878	47.65176709	64.09705957

Graphical abstract of model fit presented as mean ± 1 SD of the fraction % of released carvedilol:

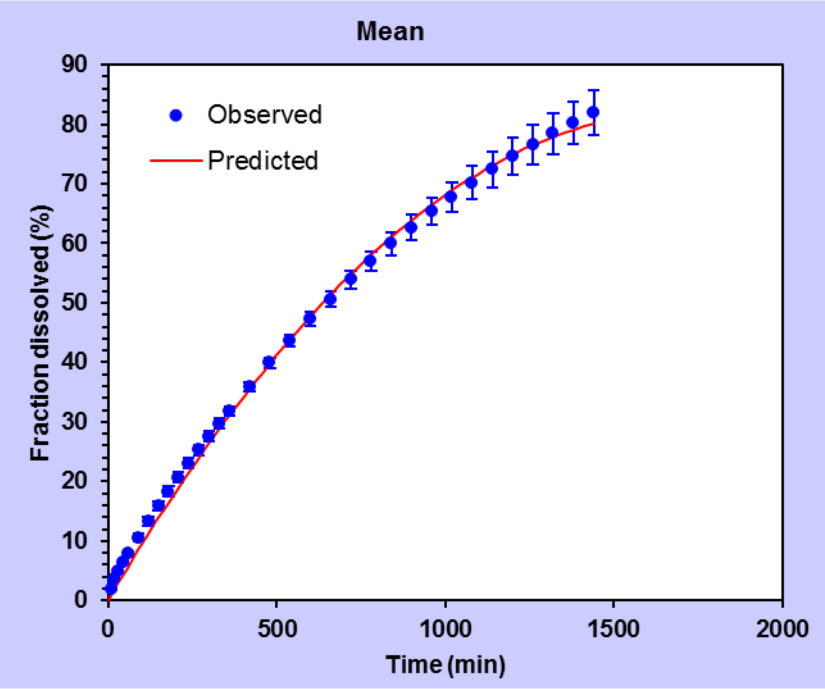

Supplementary materials – Model fitting summary – Ethocel™ Standard 20 Premium (modelling the entire relevant dissolution data)

Graphical abstract of model fit presented as the fraction % of released carvedilol per tested tablet:

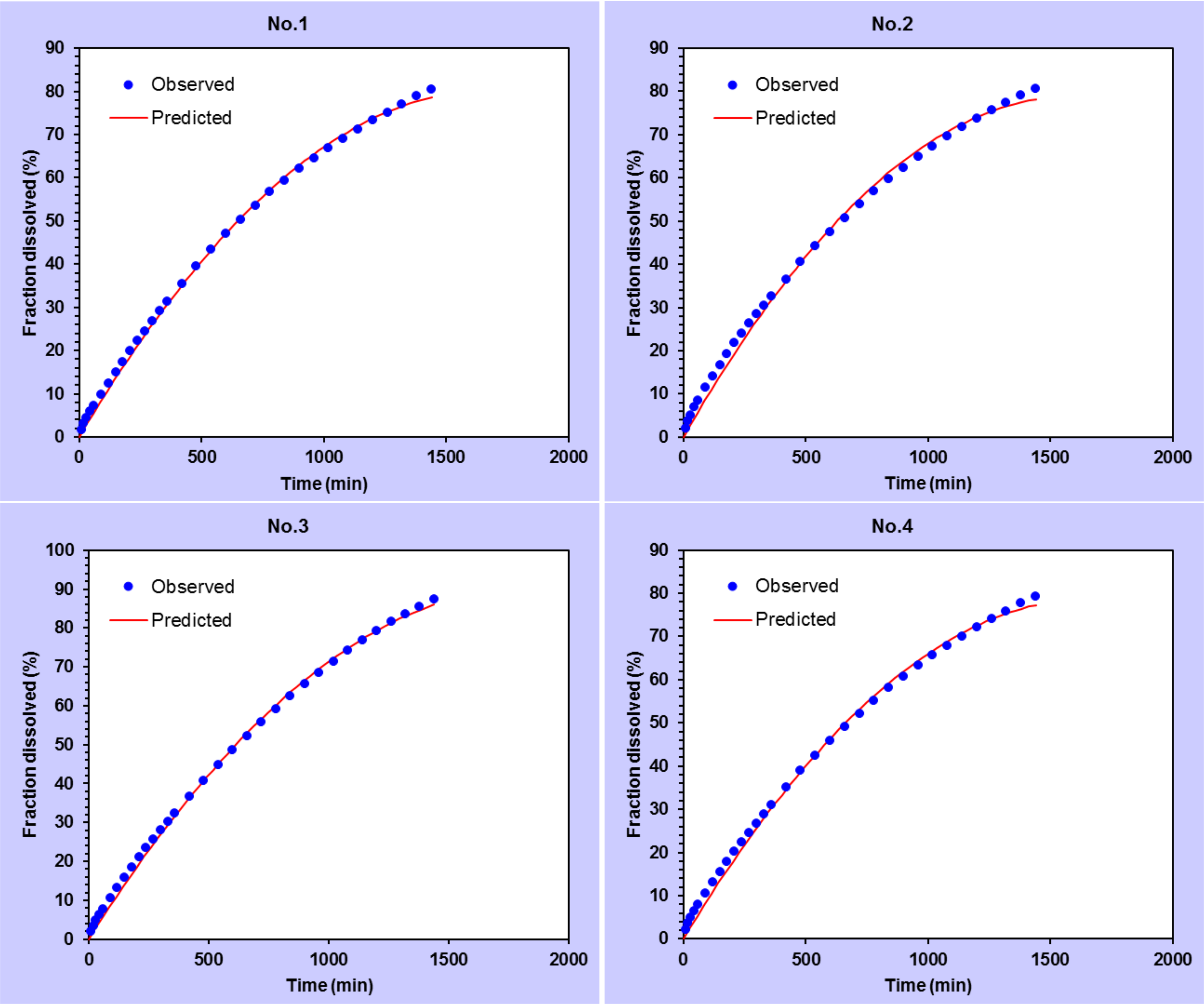

Supplementary materials – Model fitting summary – Ethocel™ Standard 20 Premium (modelling the entire relevant dissolution data)

Model: **Quadratic with T_{lag}**

Model equation: $F = 100 \cdot \left[k_1 \cdot (t - T_{lag})^2 + k_2 \cdot (t - T_{lag})\right]$

Fitted model parameters per tested tablet (N = 4) with statistics – mean, standard deviation (SD), and relative standard deviation expressed in % (RSD%) (output from DDSolver):

Parameter	No.1	No.2	No.3	No.4	Mean	SD	RSD(%)
k_1	-0.0000003	-0.0000003	-0.0000003	-0.0000003	-0.0000003	0.0000000	-6.6499086
k_2	0.0009637	0.0009989	0.0009867	0.0009491	0.0009746	0.0000224	2.2988891
T_{lag}	4.0000000	4.0000000	4.0000000	4.0000000	4.0000000	0.0000000	0.0000000

Number of dissolution data points (N), degrees of freedom (df), and selected goodness of fit criteria – Pearson correlation coefficient (R), coefficient of determination (R^2), adjusted coefficient of determination ($R^2_{adjusted}$), and residual sum of squares (RSS) (manual calculation in MS Excel):

Parameter	No.1	No.2	No.3	No.4
N	33	33	33	33
df	30	30	30	30
R	0.999614861	0.998976486	0.999539898	0.999296111
R^2	0.99922987	0.99795402	0.999080008	0.998592718
$R^2_{adjusted}$	0.999178528	0.997817621	0.999018675	0.998498899
RSS	43.88847966	112.434937	62.22265153	80.12044458

Graphical abstract of model fit presented as mean ± 1 SD of the fraction % of released carvedilol:

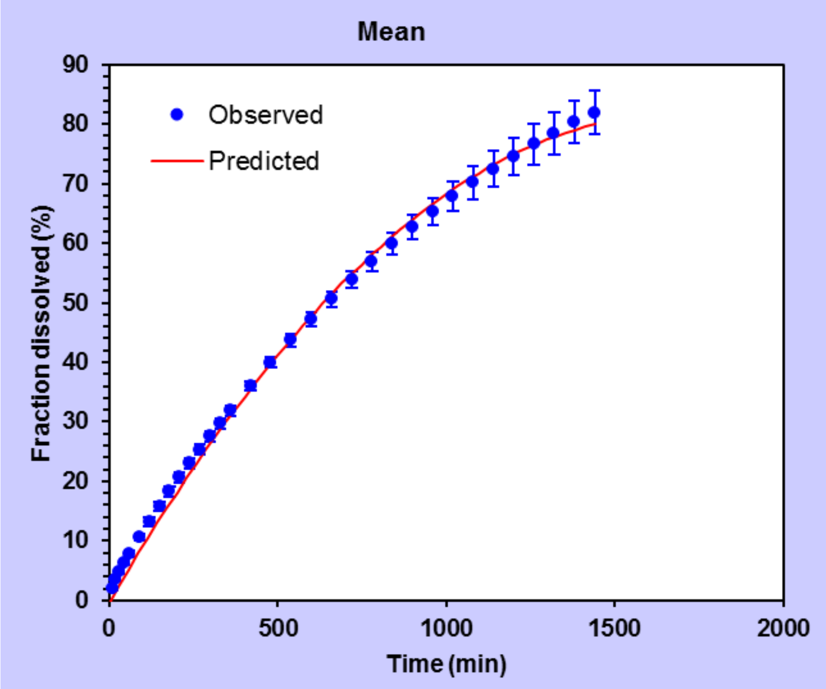

Supplementary materials – Model fitting summary – Ethocel™ Standard 20 Premium (modelling the entire relevant dissolution data)

Graphical abstract of model fit presented as the fraction % of released carvedilol per tested tablet:

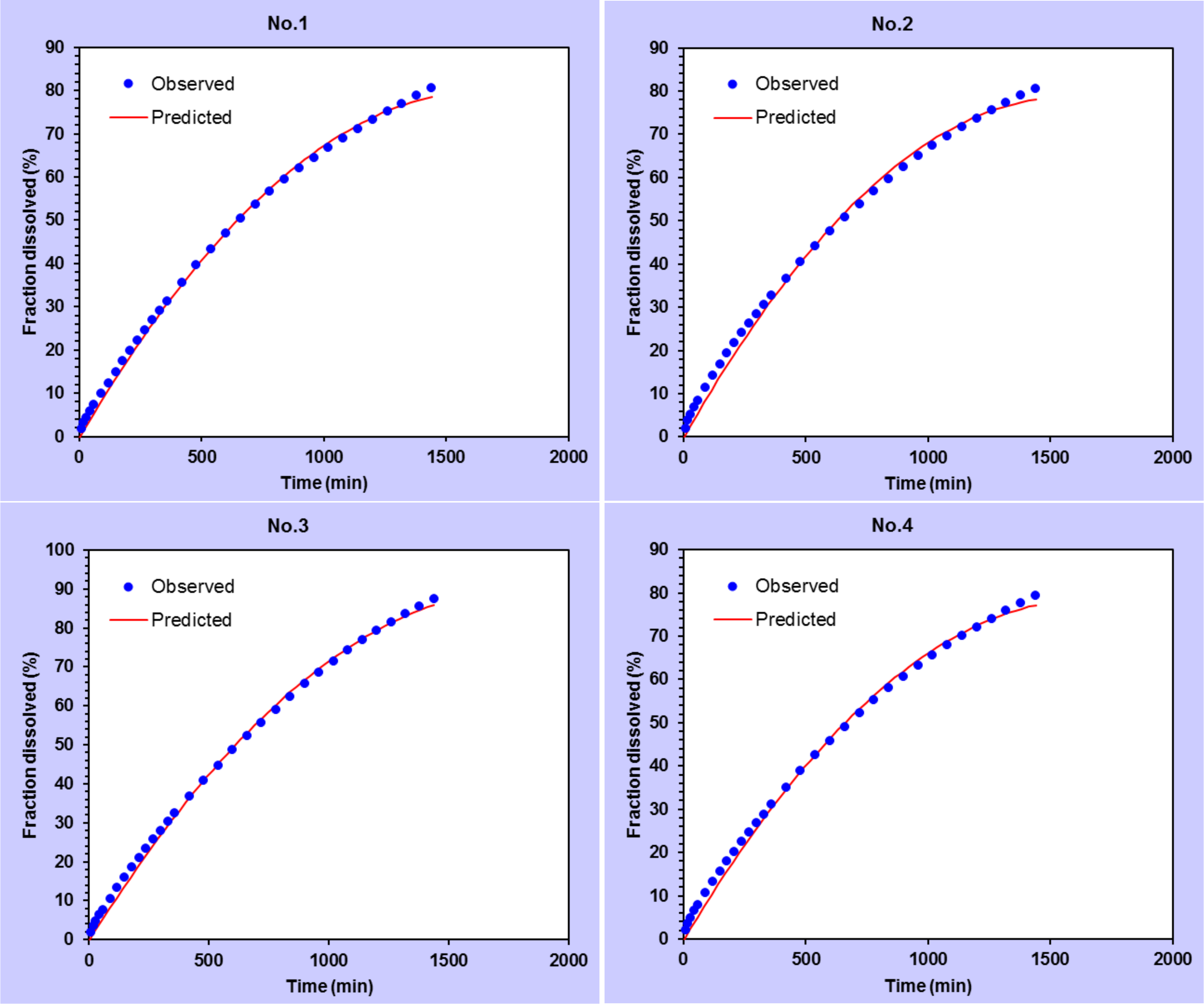

Supplementary materials – Model fitting summary – Ethocel™ Standard 20 Premium (modelling the entire relevant dissolution data)

Model: **Weibull_1**

Model equation: $F = 100 \cdot \left[1 - e^{-\frac{(t-T_i)^\beta}{\alpha}}\right]$

Fitted model parameters per tested tablet (N = 4) with statistics – mean, standard deviation (SD), and relative standard deviation expressed in % (RSD%) (output from DDSolver):

Parameter	No.1	No.2	No.3	No.4	Mean	SD	RSD(%)
α	408.446	304.364	408.651	317.212	359.668	56.685	15.760
β	0.873	0.833	0.889	0.831	0.856	0.029	3.434
T_i	6.000	6.000	6.000	6.000	6.000	0.000	0.000

Number of dissolution data points (N), degrees of freedom (df), and selected goodness of fit criteria – Pearson correlation coefficient (R), coefficient of determination (R^2), adjusted coefficient of determination ($R^2_{adjusted}$), and residual sum of squares (RSS) (manual calculation in MS Excel):

Parameter	No.1	No.2	No.3	No.4
N	33	33	33	33
df	30	30	30	30
R	0.997655028	0.997045351	0.994336836	0.99652451
R^2	0.995315555	0.994099433	0.988705744	0.9930611
$R^2_{adjusted}$	0.995003259	0.993706062	0.987952794	0.992598507
RSS	204.9920602	210.9126236	455.1170909	263.9084864

Graphical abstract of model fit presented as mean ± 1 SD of the fraction % of released carvedilol:

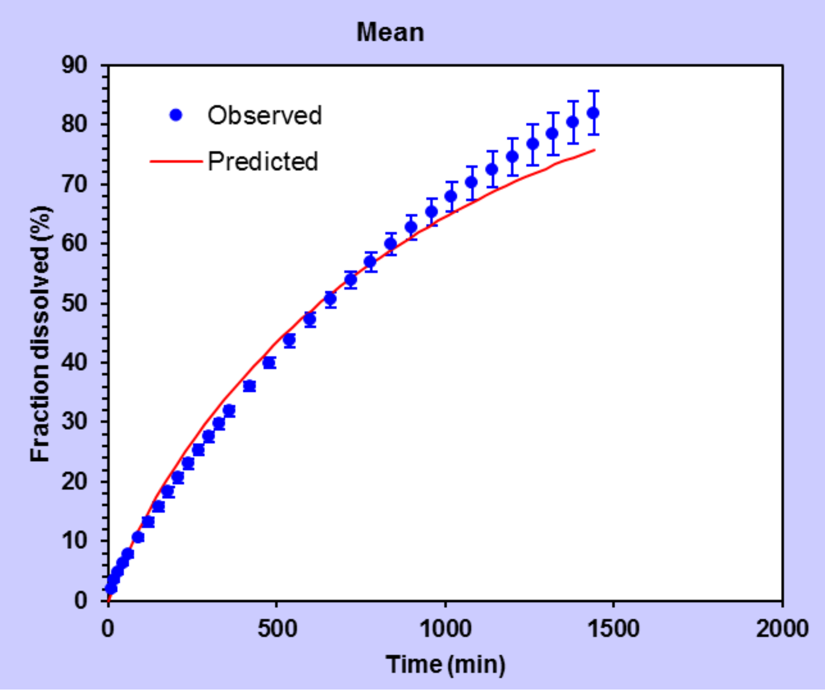

Supplementary materials – Model fitting summary – Ethocel^{TM} Standard 20 Premium (modelling the entire relevant dissolution data)

Graphical abstract of model fit presented as the fraction % of released carvedilol per tested tablet:

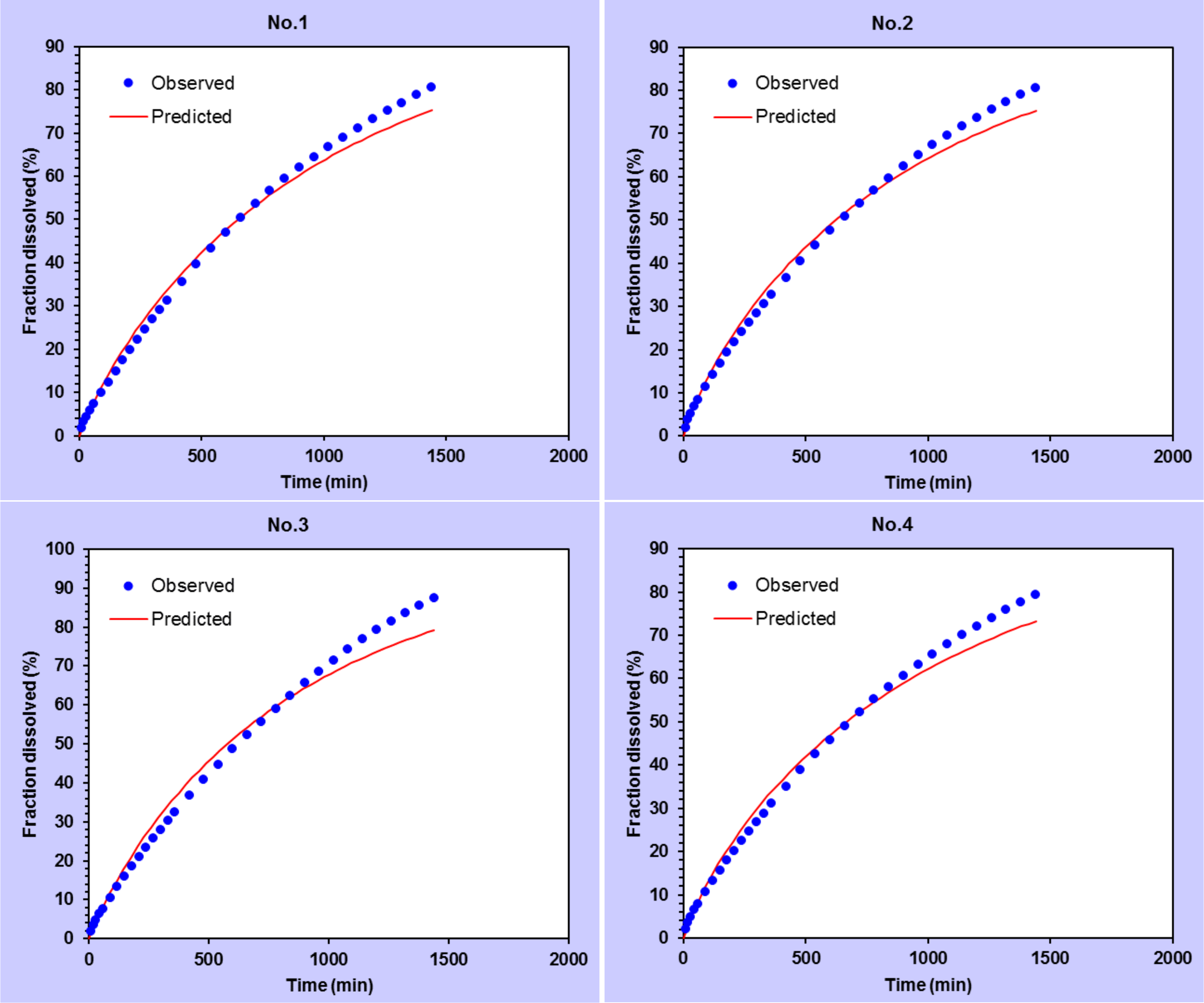

Supplementary materials – Model fitting summary – Ethocel™ Standard 20 Premium (modelling the entire relevant dissolution data)

Model: **Weibull_2**

Model equation: $F = 100 \cdot \left(1 - e^{-\frac{t^{\beta}}{\alpha}}\right)$

Fitted model parameters per tested tablet (N = 4) with statistics – mean, standard deviation (SD), and relative standard deviation expressed in % (RSD%) (output from DDSolver):

Parameter	No.1	No.2	No.3	No.4	Mean	SD	RSD(%)
α	574.290	420.541	688.971	439.792	530.898	125.630	23.664
β	0.926	0.883	0.977	0.881	0.916	0.045	4.942

Number of dissolution data points (N), degrees of freedom (df), and selected goodness of fit criteria – Pearson correlation coefficient (R), coefficient of determination (R^2), adjusted coefficient of determination ($R^2_{adjusted}$), and residual sum of squares (RSS) (manual calculation in MS Excel):

Parameter	No.1	No.2	No.3	No.4
N	33	33	33	33
df	31	31	31	31
R	0.998754314	0.998272609	0.995893587	0.997923659
R^2	0.997510181	0.996548201	0.991804037	0.995851629
$R^2_{adjusted}$	0.997429864	0.996436853	0.991539651	0.995717811
RSS	100.4580927	112.3271744	273.2220796	146.8506757

Graphical abstract of model fit presented as mean ± 1 SD of the fraction % of released carvedilol:

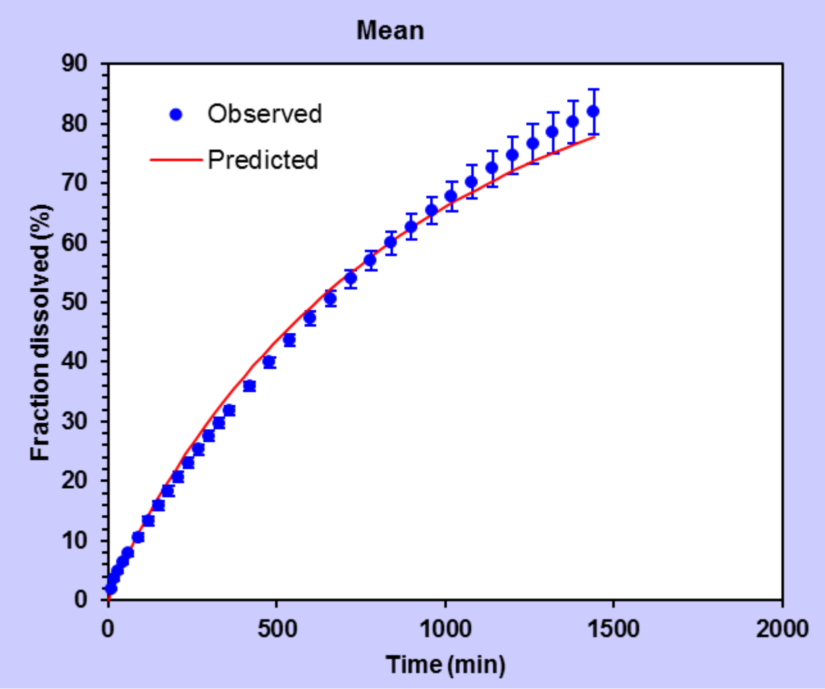

Supplementary materials – Model fitting summary – Ethocel™ Standard 20 Premium (modelling the entire relevant dissolution data)

Graphical abstract of model fit presented as the fraction % of released carvedilol per tested tablet:

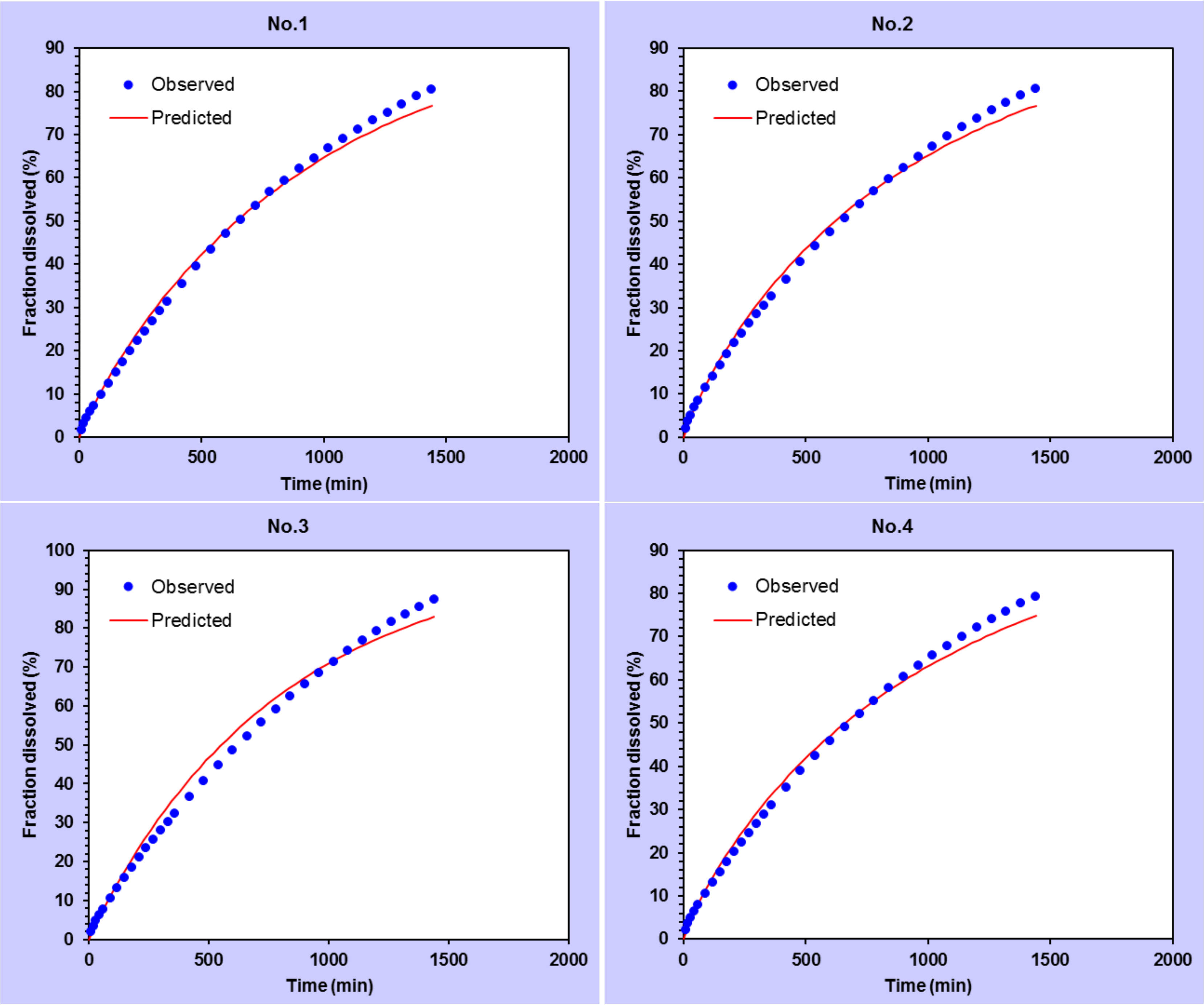

Supplementary materials – Model fitting summary – Ethocel™ Standard 20 Premium (modelling the entire relevant dissolution data)

Model: **Weibull_3**

Model equation: $F = F_{max} \cdot \left(1 - e^{-\frac{t^{\beta}}{\alpha}}\right)$

Fitted model parameters per tested tablet (N = 4) with statistics – mean, standard deviation (SD), and relative standard deviation expressed in % (RSD%) (output from DDSolver):

Parameter	No.1	No.2	No.3	No.4	Mean	SD	RSD(%)
α	641.168	539.439	707.140	489.962	594.427	98.023	16.490
β	0.992	0.945	0.963	0.951	0.963	0.021	2.193
F_{max}	84.520	95.454	108.629	83.263	92.966	11.790	12.682

Number of dissolution data points (N), degrees of freedom (df), and selected goodness of fit criteria – Pearson correlation coefficient (R), coefficient of determination (R^2), adjusted coefficient of determination ($R^2_{adjusted}$), and residual sum of squares (RSS) (manual calculation in MS Excel):

Parameter	No.1	No.2	No.3	No.4
N	33	33	33	33
df	30	30	30	30
R	0.995482392	0.997511018	0.997370706	0.993784584
R^2	0.990985193	0.995028231	0.994748325	0.987607799
$R^2_{adjusted}$	0.990384206	0.994696779	0.994398213	0.986781652
RSS	265.6109671	210.2166674	246.539549	335.5726545

Graphical abstract of model fit presented as mean ± 1 SD of the fraction % of released carvedilol:

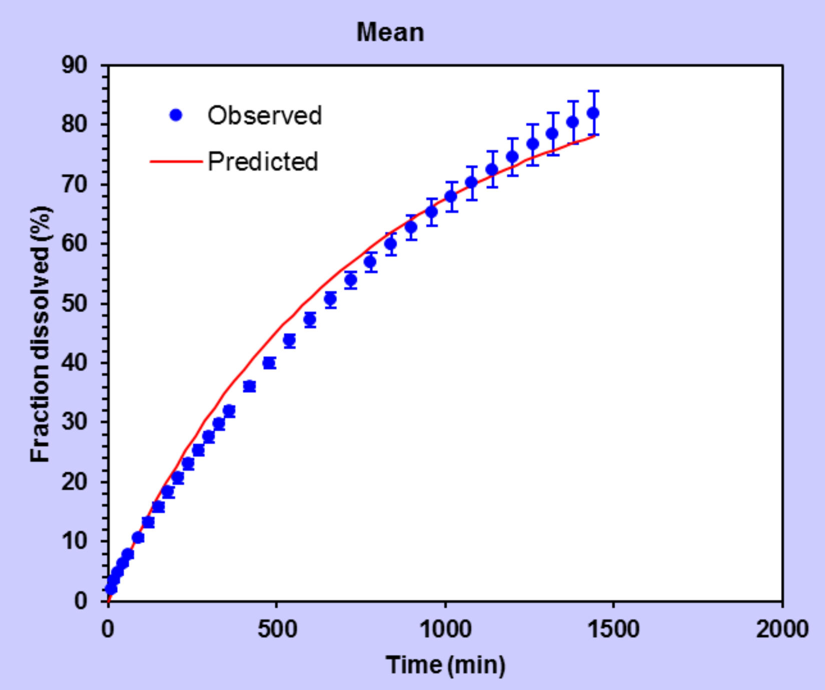

Supplementary materials – Model fitting summary – Ethocel™ Standard 20 Premium (modelling the entire relevant dissolution data)

Graphical abstract of model fit presented as the fraction % of released carvedilol per tested tablet:

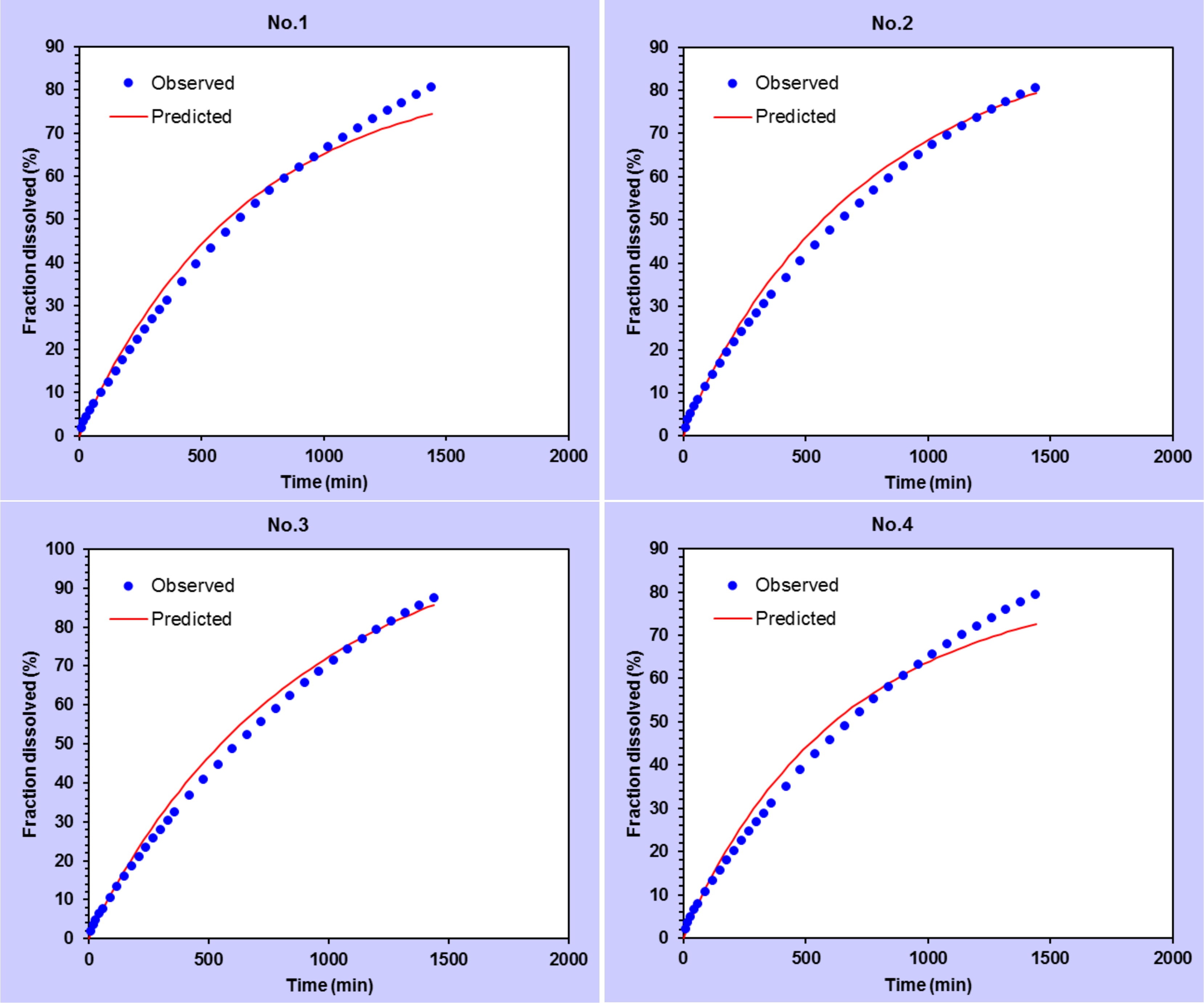

Supplementary materials – Model fitting summary – Ethocel™ Standard 20 Premium (modelling the entire relevant dissolution data)

Model: **Weibull_4**

Model equation: $F = F_{max} \cdot \left[1 - e^{-\frac{(t-T_i)^{\beta}}{\alpha}}\right]$

Fitted model parameters per tested tablet (N = 4) with statistics – mean, standard deviation (SD), and relative standard deviation expressed in % (RSD%) (output from DDSolver):

Parameter	No.1	No.2	No.3	No.4	Mean	SD	RSD(%)
α	441.872	329.810	434.824	341.914	387.105	59.446	15.357
β	0.935	0.895	0.926	0.895	0.913	0.021	2.257
T_i	6.000	6.000	6.000	6.000	6.000	0.000	0.000
F_{max}	84.520	84.597	91.858	83.263	86.059	3.913	4.547

Number of dissolution data points (N), degrees of freedom (df), and selected goodness of fit criteria – Pearson correlation coefficient (R), coefficient of determination (R^2), adjusted coefficient of determination ($R^2_{adjusted}$), and residual sum of squares (RSS) (manual calculation in MS Excel):

Parameter	No.1	No.2	No.3	No.4
N	33	33	33	33
df	29	29	29	29
R	0.993924316	0.992750497	0.991514099	0.991927015
R^2	0.987885545	0.985553549	0.983100208	0.983919202
$R^2_{adjusted}$	0.986632326	0.984059089	0.981351954	0.982255671
RSS	375.5088352	400.3684762	587.685329	453.0295164

Graphical abstract of model fit presented as mean ± 1 SD of the fraction % of released carvedilol:

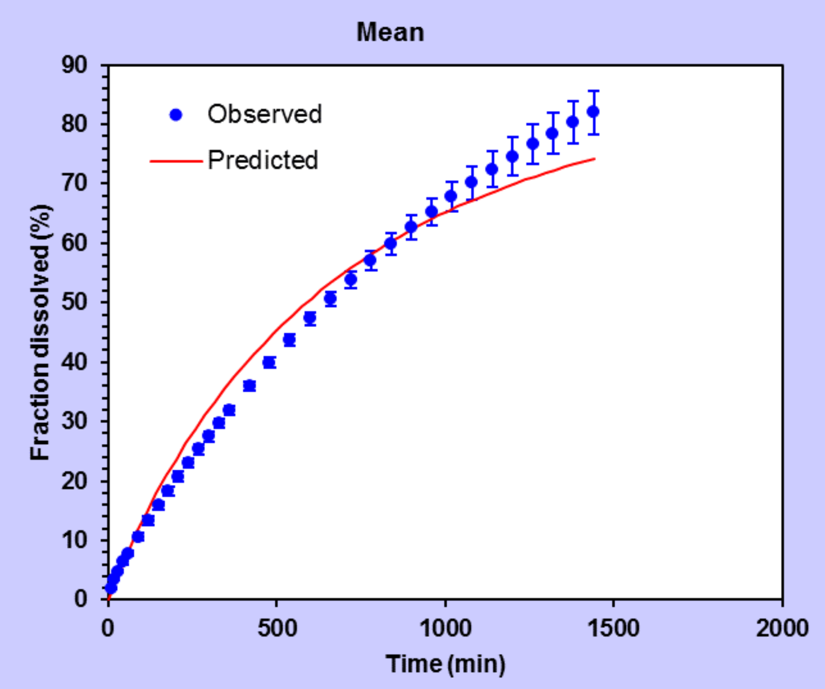

Supplementary materials – Model fitting summary – Ethocel™ Standard 20 Premium (modelling the entire relevant dissolution data)

Graphical abstract of model fit presented as the fraction % of released carvedilol per tested tablet:

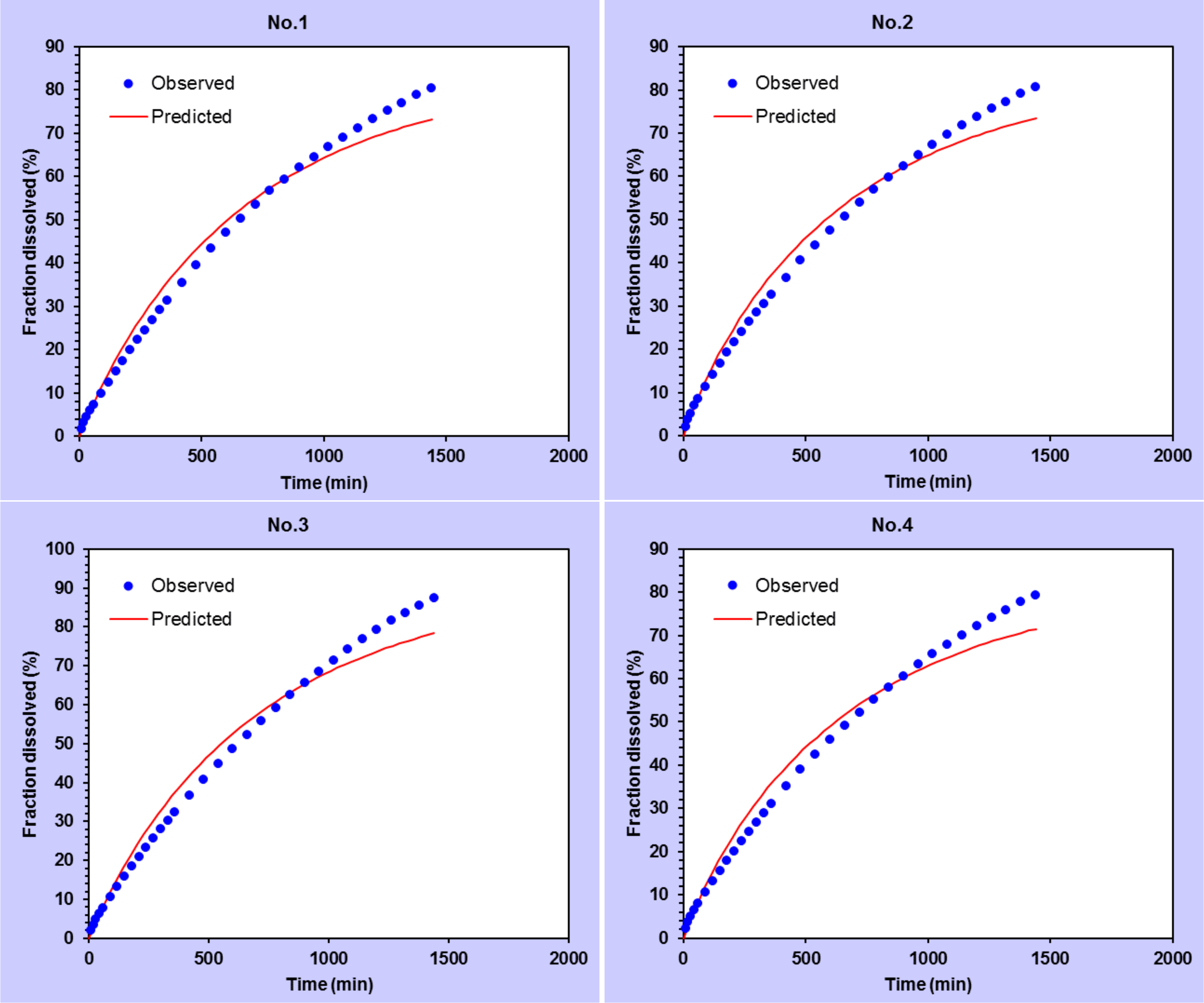

Supplementary materials – Model fitting summary – Ethocel™ Standard 20 Premium (modelling the entire relevant dissolution data)

Model: **Logistic_1**

Model equation: $F = 100 \cdot \frac{e^{\alpha+\beta \cdot \log(t)}}{1+e^{\alpha+\beta \cdot \log(t)}}$

Fitted model parameters per tested tablet (N = 4) with statistics – mean, standard deviation (SD), and relative standard deviation expressed in % (RSD%) (output from DDSolver):

Parameter	No.1	No.2	No.3	No.4	Mean	SD	RSD(%)
α	-7.074	-6.757	-7.248	-6.770	-6.962	0.240	-3.449
β	2.552	2.454	2.680	2.431	2.530	0.113	4.481

Number of dissolution data points (N), degrees of freedom (df), and selected goodness of fit criteria – Pearson correlation coefficient (R), coefficient of determination (R^2), adjusted coefficient of determination ($R^2_{adjusted}$), and residual sum of squares (RSS) (manual calculation in MS Excel):

Parameter	No.1	No.2	No.3	No.4
N	33	33	33	33
df	31	31	31	31
R	0.991631396	0.990410625	0.984123787	0.990076224
R^2	0.983332825	0.980913205	0.968499628	0.980250929
$R^2_{adjusted}$	0.982795174	0.980297502	0.967483487	0.979613863
RSS	492.2081585	516.3674273	996.2145414	540.6493436

Graphical abstract of model fit presented as mean ± 1 SD of the fraction % of released carvedilol:

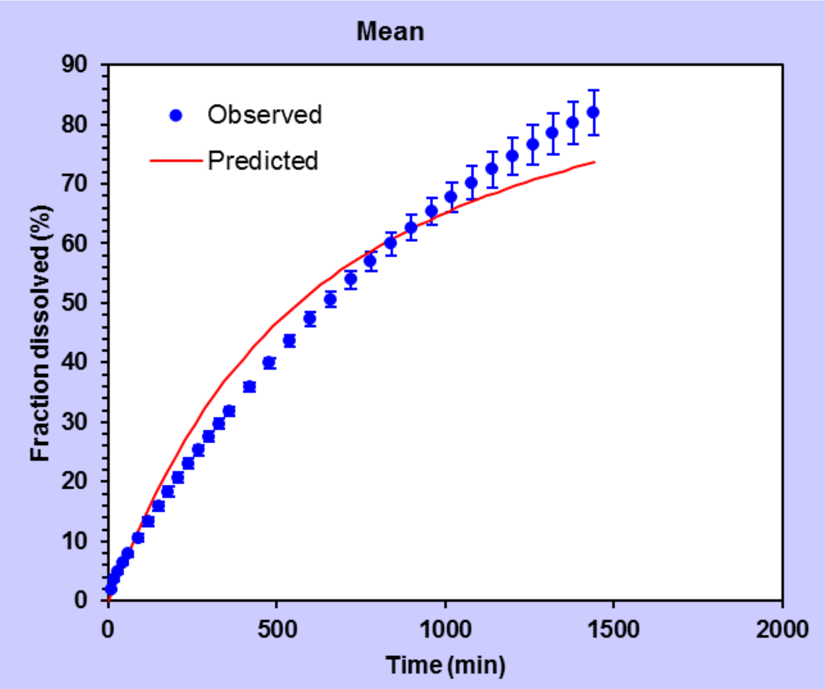

Supplementary materials – Model fitting summary – Ethocel^{TM} Standard 20 Premium (modelling the entire relevant dissolution data)

Graphical abstract of model fit presented as the fraction % of released carvedilol per tested tablet:

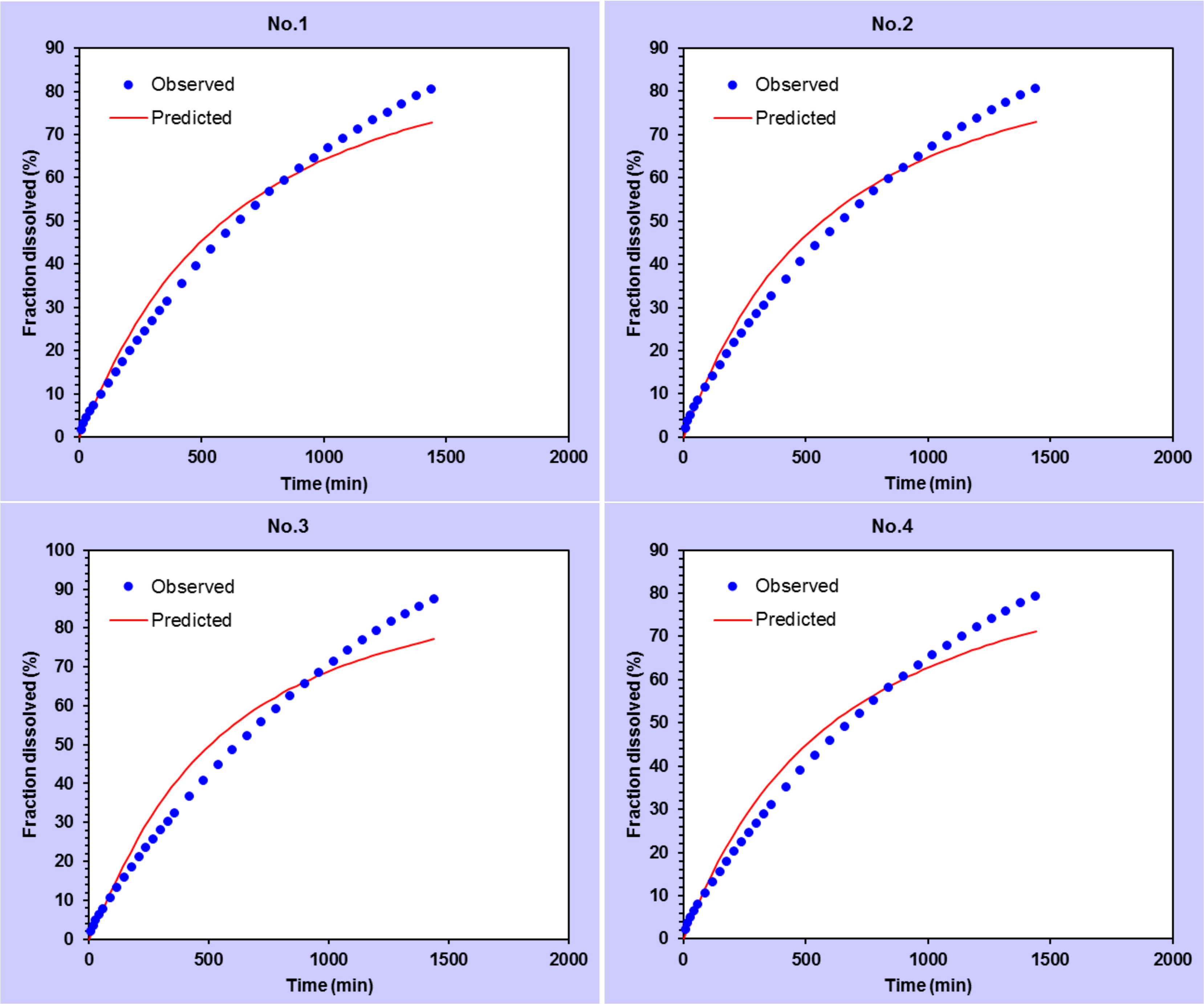

Supplementary materials – Model fitting summary – Ethocel™ Standard 20 Premium (modelling the entire relevant dissolution data)

Model: **Logistic_2**

Model equation: $F = F_{max} \cdot \frac{e^{\alpha+\beta \cdot \log(t)}}{1+e^{\alpha+\beta \cdot \log(t)}}$

Fitted model parameters per tested tablet (N = 4) with statistics – mean, standard deviation (SD), and relative standard deviation expressed in % (RSD%) (output from DDSolver):

Parameter	No.1	No.2	No.3	No.4	Mean	SD	RSD(%)
α	-8.834	-8.535	-8.782	-8.508	-8.665	0.167	-1.932
β	3.148	3.072	3.107	3.041	3.092	0.046	1.497
F_{max}	94.395	91.770	102.590	92.991	95.436	4.888	5.122

Number of dissolution data points (N), degrees of freedom (df), and selected goodness of fit criteria – Pearson correlation coefficient (R), coefficient of determination (R^2), adjusted coefficient of determination ($R^2_{adjusted}$), and residual sum of squares (RSS) (manual calculation in MS Excel):

Parameter	No.1	No.2	No.3	No.4
N	33	33	33	33
df	30	30	30	30
R	0.997795624	0.99649518	0.996101299	0.996310947
R^2	0.995596107	0.993002644	0.992217797	0.992635503
$R^2_{adjusted}$	0.995302515	0.992536154	0.991698984	0.992144536
RSS	645.2669161	843.1395793	907.6195084	590.4477755

Graphical abstract of model fit presented as mean ± 1 SD of the fraction % of released carvedilol:

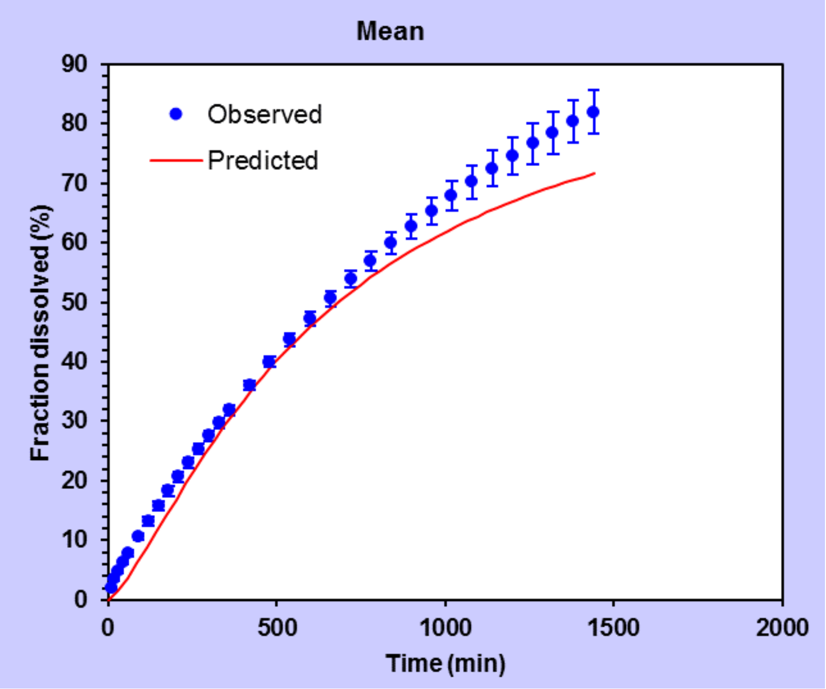

Supplementary materials – Model fitting summary – Ethocel™ Standard 20 Premium (modelling the entire relevant dissolution data)

Graphical abstract of model fit presented as the fraction % of released carvedilol per tested tablet:

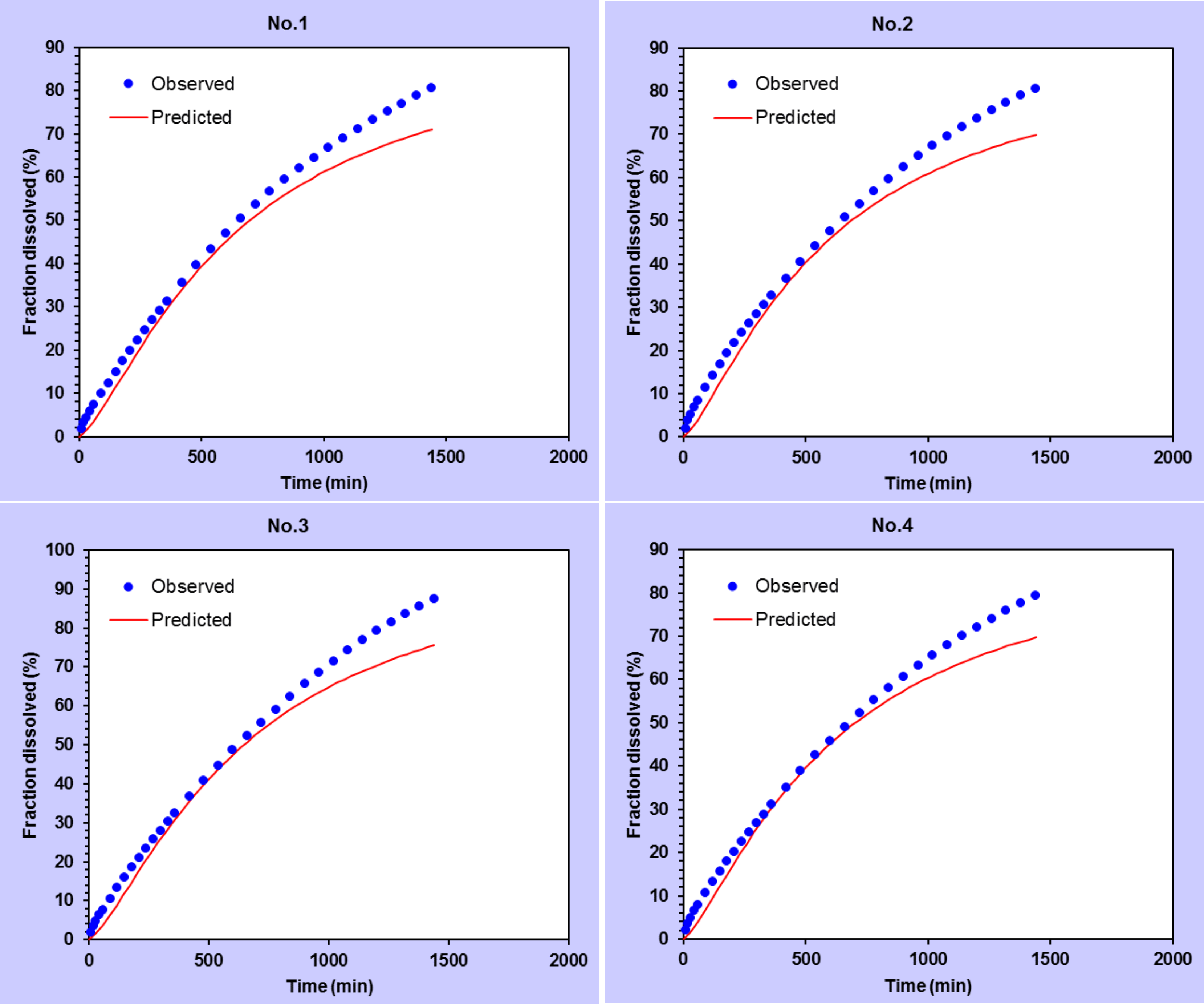

Supplementary materials – Model fitting summary – Ethocel™ Standard 20 Premium (modelling the entire relevant dissolution data)

Model: **Logistic_3**

Model equation: $F = F_{max} \cdot \frac{1}{1+e^{-k\cdot(t-\gamma)}}$

Fitted model parameters per tested tablet (N = 4) with statistics – mean, standard deviation (SD), and relative standard deviation expressed in % (RSD%) (output from DDSolver):

Parameter	No.1	No.2	No.3	No.4	Mean	SD	RSD(%)
k	0.004	0.004	0.003	0.004	0.004	0.000	5.196
γ	627.019	607.862	547.592	620.557	600.758	36.326	6.047
F_{max}	84.520	84.597	89.731	83.263	85.528	2.868	3.354

Number of dissolution data points (N), degrees of freedom (df), and selected goodness of fit criteria – Pearson correlation coefficient (R), coefficient of determination (R^2), adjusted coefficient of determination ($R^2_{adjusted}$), and residual sum of squares (RSS) (manual calculation in MS Excel):

Parameter	No.1	No.2	No.3	No.4
N	33	33	33	33
df	30	30	30	30
R	0.986202795	0.986441611	0.994471721	0.987537741
R^2	0.972595953	0.973067052	0.988974005	0.97523079
$R^2_{adjusted}$	0.970769017	0.971271522	0.988238939	0.973579509
RSS	765.7106588	715.6208693	538.666598	639.078682

Graphical abstract of model fit presented as mean ± 1 SD of the fraction % of released carvedilol:

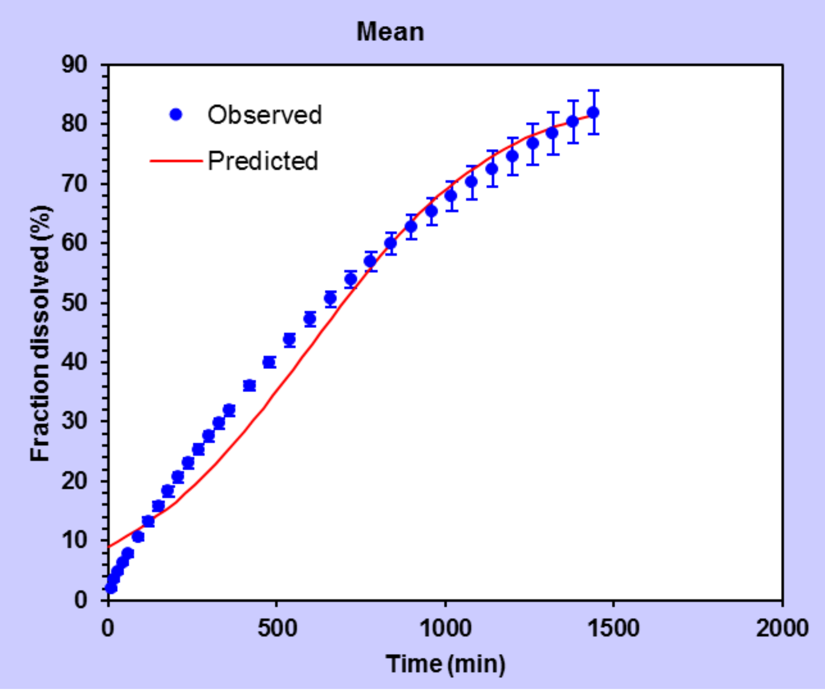

Supplementary materials – Model fitting summary – Ethocel™ Standard 20 Premium (modelling the entire relevant dissolution data)

Graphical abstract of model fit presented as the fraction % of released carvedilol per tested tablet:

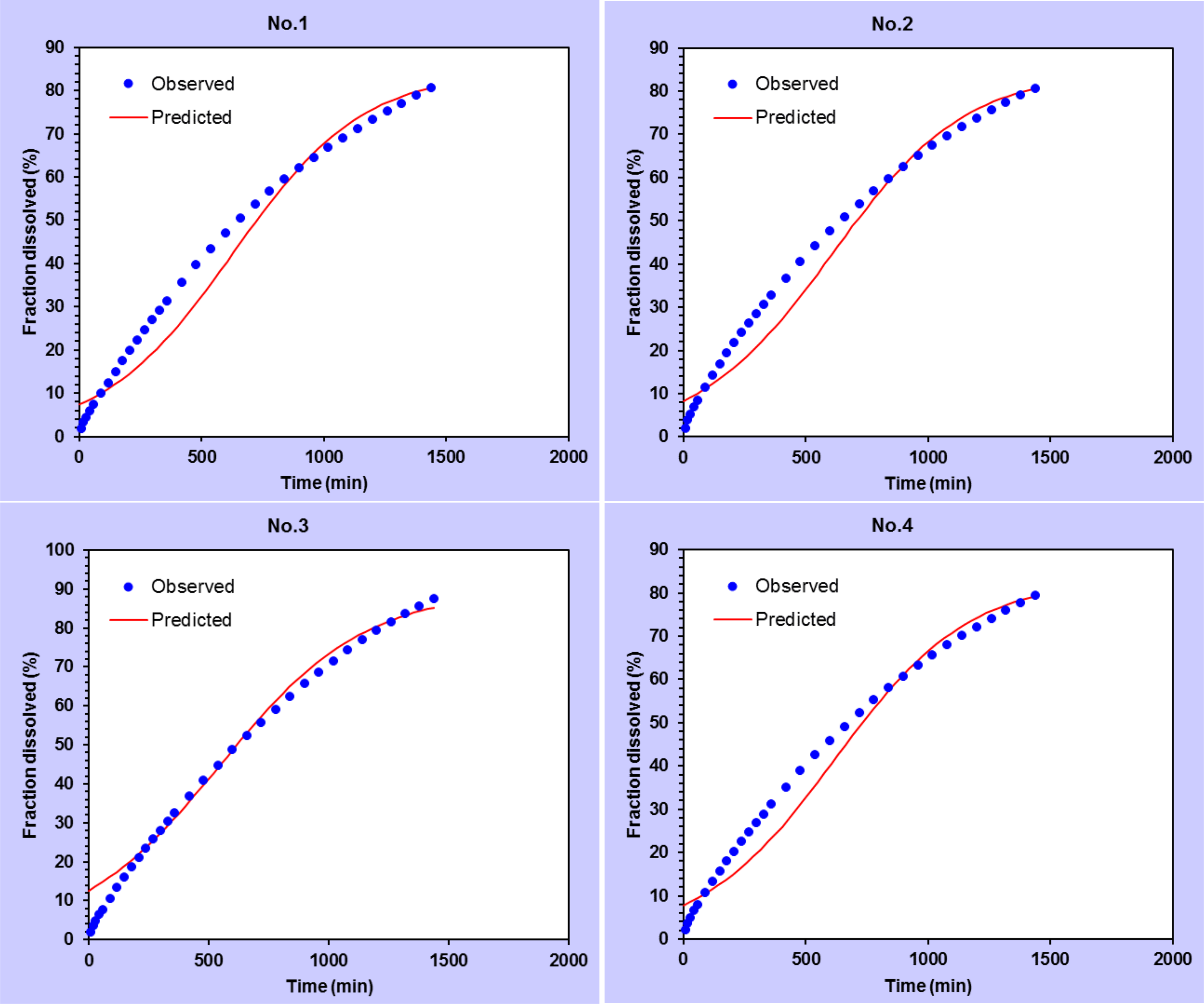

Supplementary materials – Model fitting summary – Ethocel™ Standard 20 Premium (modelling the entire relevant dissolution data)

Model: **Gompertz_1**

Model equation: $F = 100 \cdot e^{-\alpha \cdot e^{-\beta \cdot \log(t)}}$

Fitted model parameters per tested tablet (N = 4) with statistics – mean, standard deviation (SD), and relative standard deviation expressed in % (RSD%) (output from DDSolver):

Parameter	No.1	No.2	No.3	No.4	Mean	SD	RSD(%)
α	31.583	34.850	40.568	27.688	33.672	5.450	16.187
β	1.409	1.469	1.553	1.351	1.445	0.086	5.969

Number of dissolution data points (N), degrees of freedom (df), and selected goodness of fit criteria – Pearson correlation coefficient (R), coefficient of determination (R^2), adjusted coefficient of determination ($R^2_{adjusted}$), and residual sum of squares (RSS) (manual calculation in MS Excel):

Parameter	No.1	No.2	No.3	No.4
N	33	33	33	33
df	31	31	31	31
R	0.966329654	0.966495265	0.954210453	0.964709291
R^2	0.933793001	0.934113097	0.910517589	0.930664017
$R^2_{adjusted}$	0.931657291	0.931987713	0.90763106	0.928427372
RSS	1774.725879	1708.544797	2749.262254	1723.741183

Graphical abstract of model fit presented as mean ± 1 SD of the fraction % of released carvedilol:

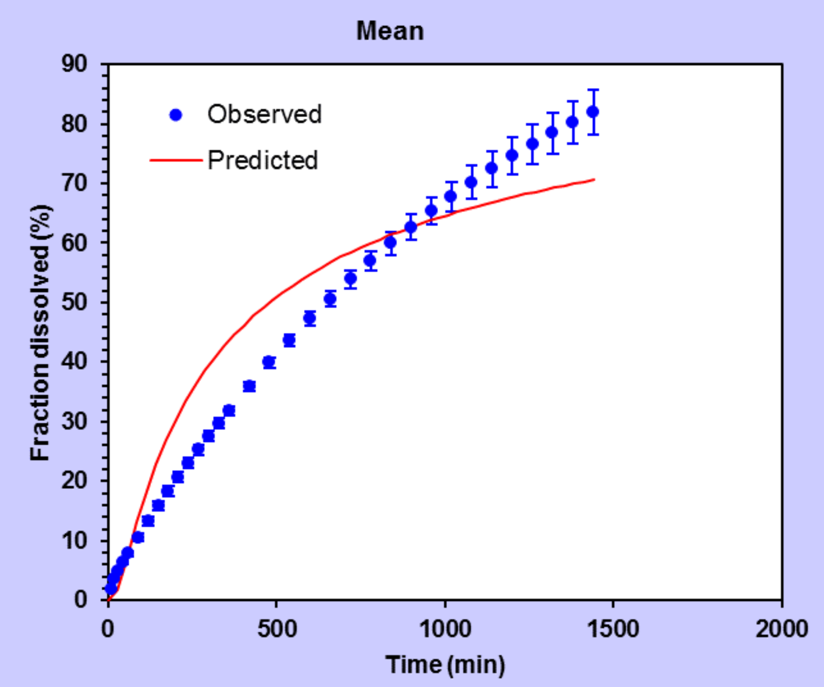

Supplementary materials – Model fitting summary – Ethocel™ Standard 20 Premium (modelling the entire relevant dissolution data)

Graphical abstract of model fit presented as the fraction % of released carvedilol per tested tablet:

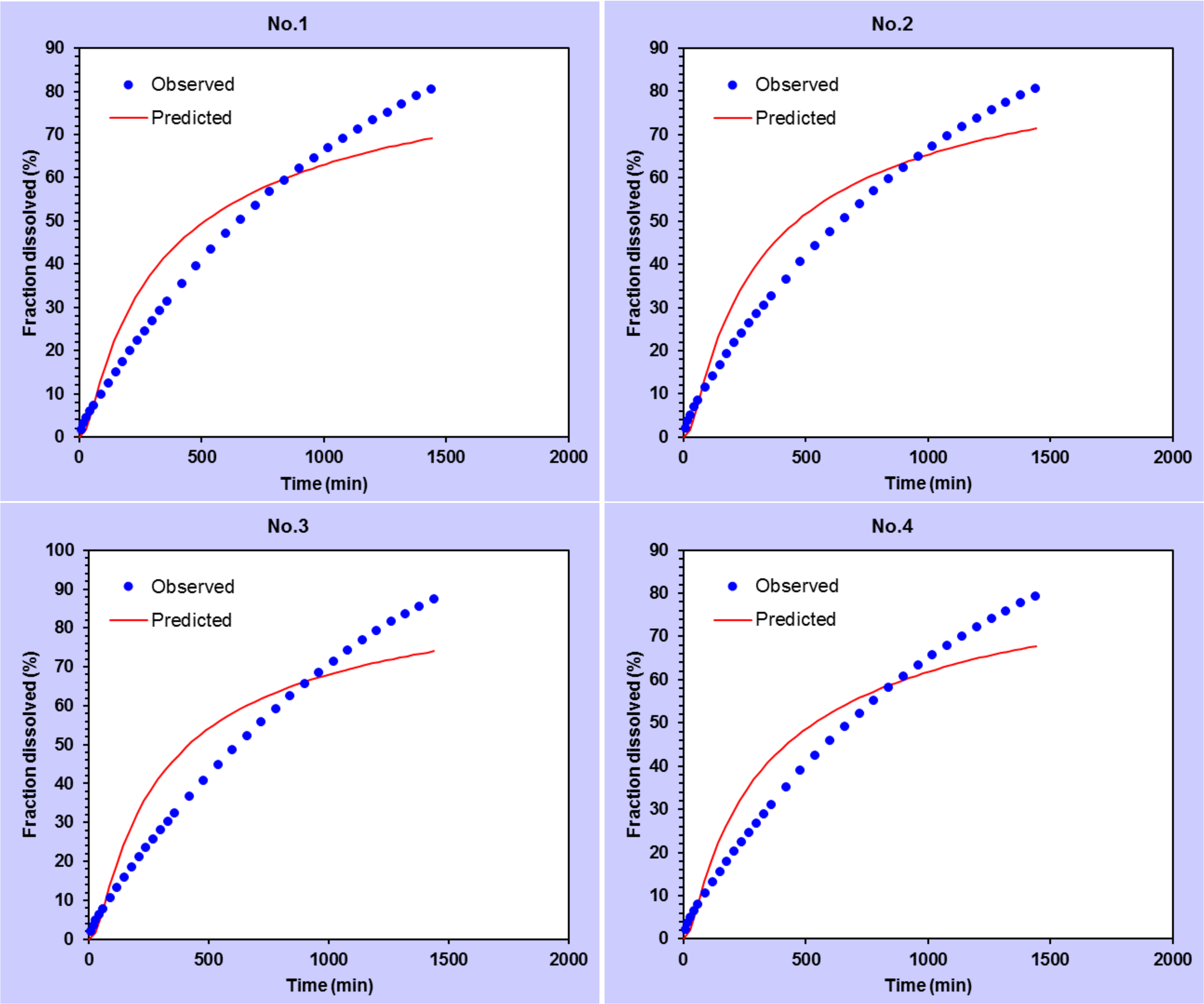

Supplementary materials – Model fitting summary – Ethocel™ Standard 20 Premium (modelling the entire relevant dissolution data)

Model: **Gompertz_2**

Model equation: $F = F_{max} \cdot e^{-\alpha \cdot e^{-\beta \cdot \log(t)}}$

Fitted model parameters per tested tablet (N = 4) with statistics – mean, standard deviation (SD), and relative standard deviation expressed in % (RSD%) (output from DDSolver):

Parameter	No.1	No.2	No.3	No.4	Mean	SD	RSD(%)
α	83.586	74.222	79.849	75.487	78.286	4.277	5.464
β	1.722	1.635	1.691	1.687	1.684	0.036	2.130
F_{max}	95.966	103.854	104.297	94.538	99.663	5.131	5.148

Number of dissolution data points (N), degrees of freedom (df), and selected goodness of fit criteria – Pearson correlation coefficient (R), coefficient of determination (R^2), adjusted coefficient of determination ($R^2_{adjusted}$), and residual sum of squares (RSS) (manual calculation in MS Excel):

Parameter	No.1	No.2	No.3	No.4
N	33	33	33	33
df	30	30	30	30
R	0.985290867	0.989808416	0.981825306	0.982842098
R^2	0.970798092	0.979720701	0.963980931	0.965978589
$R^2_{adjusted}$	0.968851298	0.978368747	0.96157966	0.963710495
RSS	1158.051052	1133.022584	1582.079886	1161.601161

Graphical abstract of model fit presented as mean ± 1 SD of the fraction % of released carvedilol:

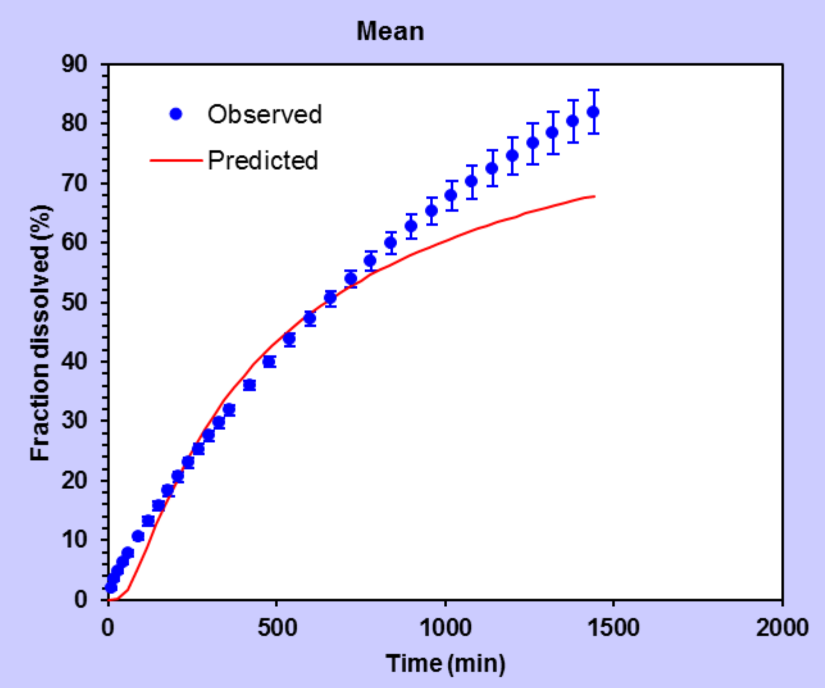

Supplementary materials – Model fitting summary – Ethocel™ Standard 20 Premium (modelling the entire relevant dissolution data)

Graphical abstract of model fit presented as the fraction % of released carvedilol per tested tablet:

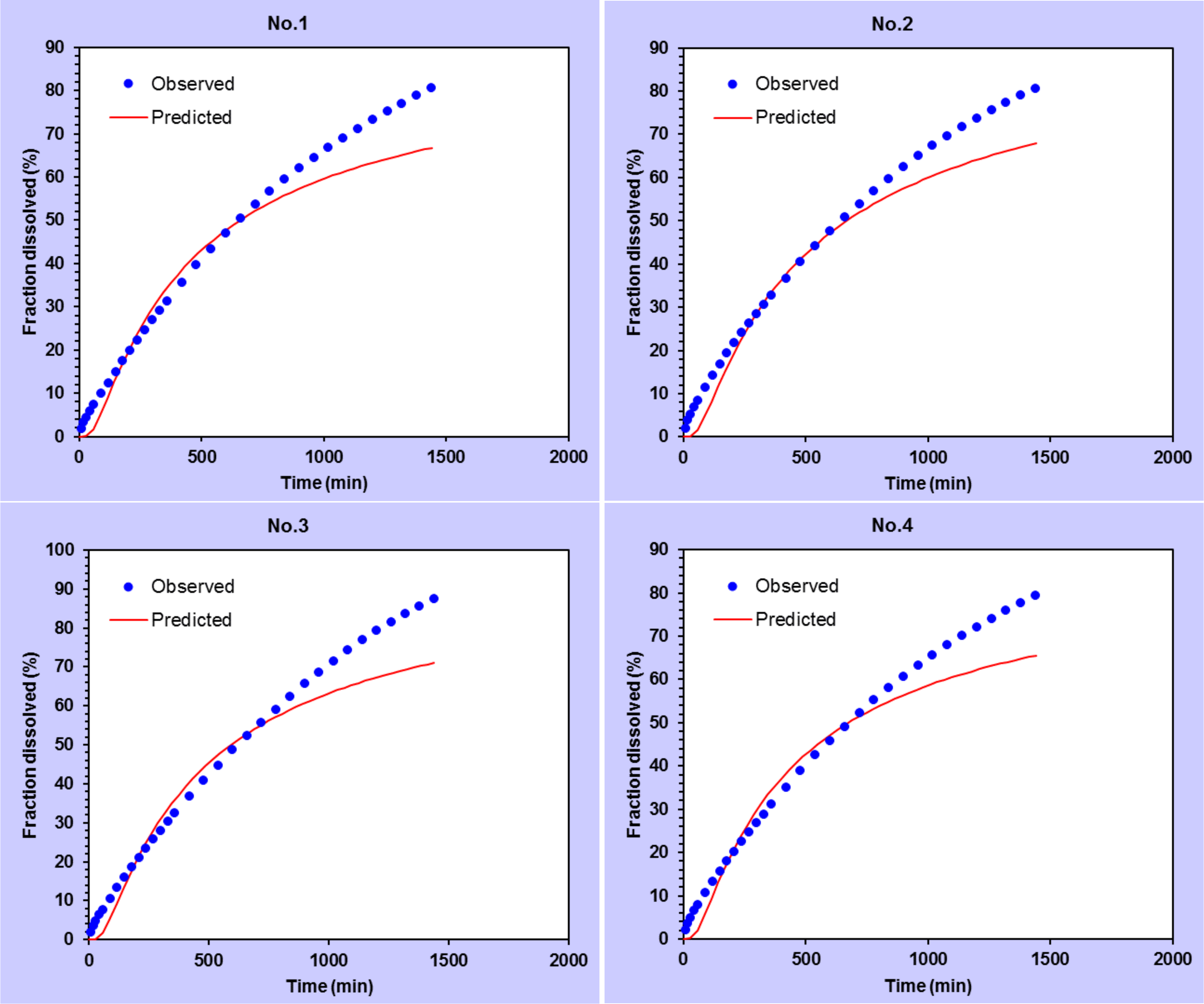

Supplementary materials – Model fitting summary – Ethocel™ Standard 20 Premium (modelling the entire relevant dissolution data)

Model: **Gompertz_3**

Model equation: $F = F_{max} \cdot e^{-e^{-k\cdot(t-\gamma)}}$

Fitted model parameters per tested tablet (N = 4) with statistics – mean, standard deviation (SD), and relative standard deviation expressed in % (RSD%) (output from DDSolver):

Parameter	No.1	No.2	No.3	No.4	Mean	SD	RSD(%)
k	0.003	0.003	0.003	0.003	0.003	0.000	0.650
γ	401.648	384.238	415.570	396.049	399.376	13.008	3.257
F_{max}	84.520	84.597	91.858	83.263	86.059	3.913	4.547

Number of dissolution data points (N), degrees of freedom (df), and selected goodness of fit criteria – Pearson correlation coefficient (R), coefficient of determination (R^2), adjusted coefficient of determination ($R^2_{adjusted}$), and residual sum of squares (RSS) (manual calculation in MS Excel):

Parameter	No.1	No.2	No.3	No.4
N	33	33	33	33
df	30	30	30	30
R	0.99664489	0.995979456	0.99640108	0.996540523
R^2	0.993301037	0.991975077	0.992815113	0.993093015
$R^2_{adjusted}$	0.99285444	0.991440083	0.99233612	0.992632549
RSS	170.9708704	196.3085001	220.777364	166.9115356

Graphical abstract of model fit presented as mean ± 1 SD of the fraction % of released carvedilol:

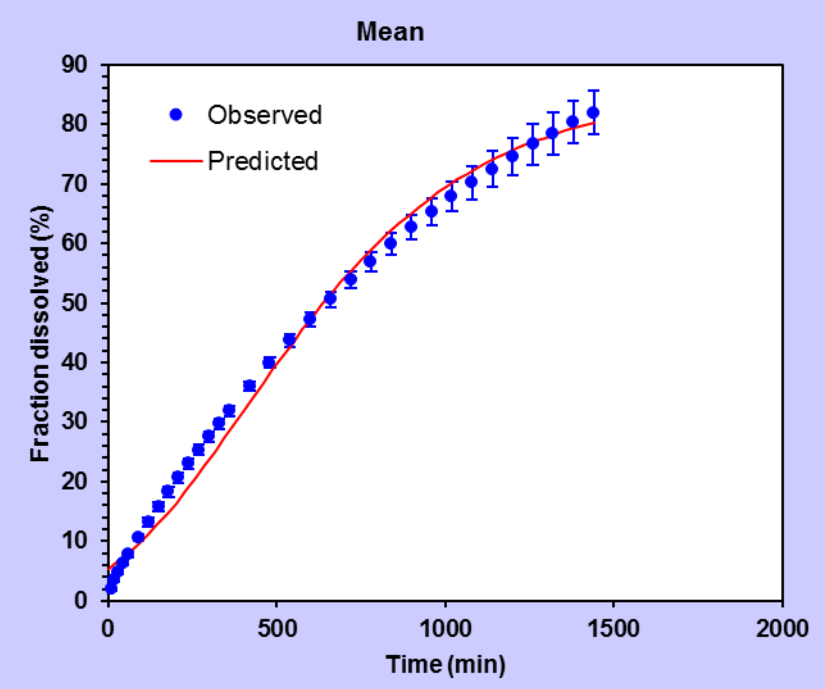

Supplementary materials – Model fitting summary – Ethocel™ Standard 20 Premium (modelling the entire relevant dissolution data)

Graphical abstract of model fit presented as the fraction % of released carvedilol per tested tablet:

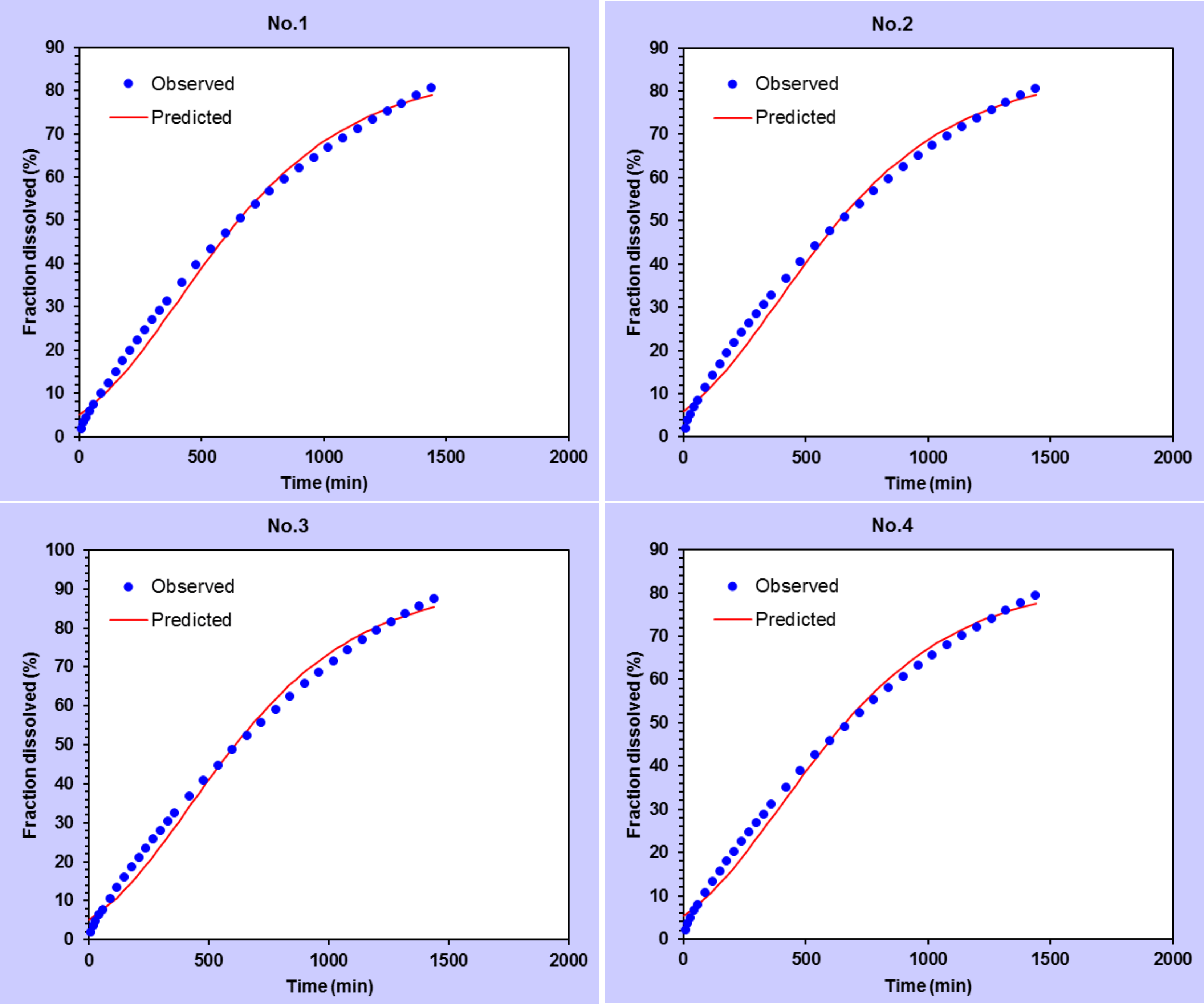

Supplementary materials – Model fitting summary – Ethocel™ Standard 20 Premium (modelling the entire relevant dissolution data)

Model: **Gompertz_4**

Model equation: $F = F_{max} \cdot e^{-\beta \cdot e^{-k \cdot t}}$

Fitted model parameters per tested tablet (N = 4) with statistics – mean, standard deviation (SD), and relative standard deviation expressed in % (RSD%) (output from DDSolver):

Parameter	No.1	No.2	No.3	No.4	Mean	SD	RSD(%)
k	0.003	0.003	0.003	0.003	0.003	0.000	0.650
β	2.816	2.661	2.889	2.733	2.775	0.099	3.566
F_{max}	84.520	84.597	91.858	83.263	86.059	3.913	4.547

Number of dissolution data points (N), degrees of freedom (df), and selected goodness of fit criteria – Pearson correlation coefficient (R), coefficient of determination (R^2), adjusted coefficient of determination ($R^2_{adjusted}$), and residual sum of squares (RSS) (manual calculation in MS Excel):

Parameter	No.1	No.2	No.3	No.4
N	33	33	33	33
df	30	30	30	30
R	0.99664489	0.995979456	0.99640108	0.996540523
R^2	0.993301037	0.991975077	0.992815113	0.993093015
$R^2_{adjusted}$	0.99285444	0.991440083	0.99233612	0.992632549
RSS	170.9708704	196.3085001	220.777364	166.9115356

Graphical abstract of model fit presented as mean ± 1 SD of the fraction % of released carvedilol:

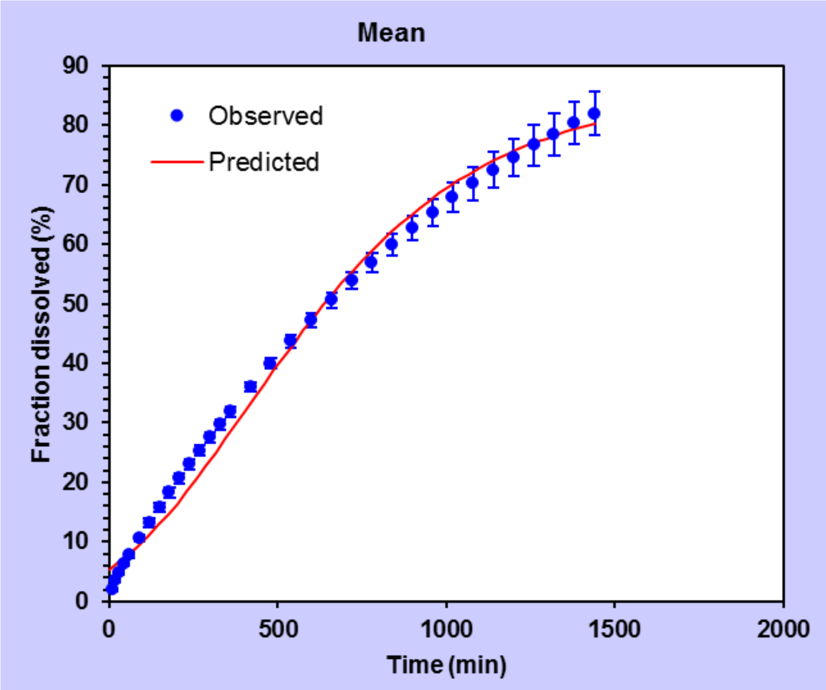

Supplementary materials – Model fitting summary – Ethocel^{TM} Standard 20 Premium (modelling the entire relevant dissolution data)

Graphical abstract of model fit presented as the fraction % of released carvedilol per tested tablet:

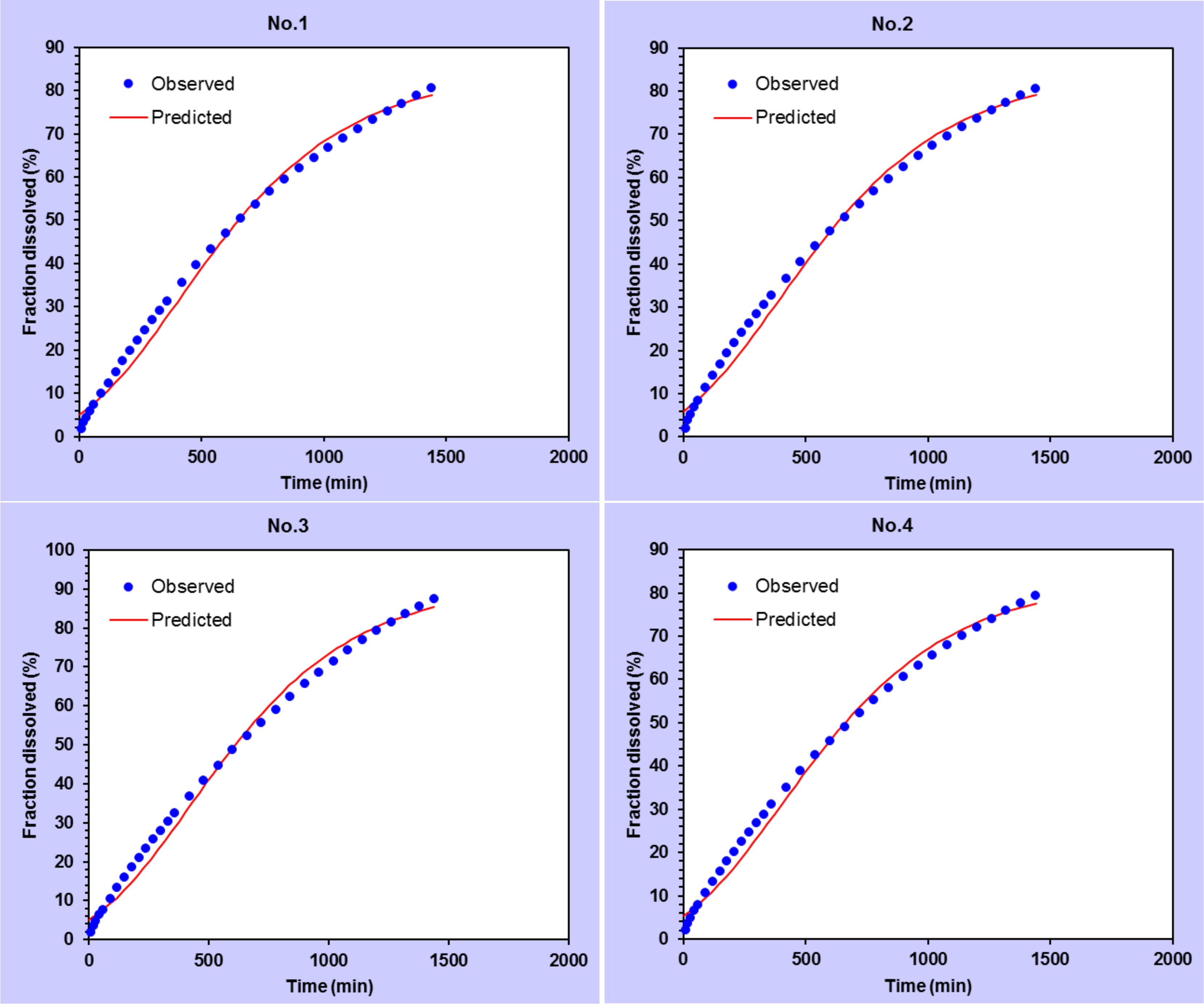

Supplementary materials – Model fitting summary – Ethocel™ Standard 20 Premium (modelling the entire relevant dissolution data)

Model: **Probit_1**

Model equation: $F = 100 \cdot \phi[\alpha + \beta \cdot \log(t)]$

Fitted model parameters per tested tablet (N = 4) with statistics – mean, standard deviation (SD), and relative standard deviation expressed in % (RSD%) (output from DDSolver):

Parameter	No.1	No.2	No.3	No.4	Mean	SD	RSD(%)
α	-4.438	-4.274	-4.721	-3.843	-4.319	0.367	-8.501
β	1.563	1.515	1.684	1.381	1.536	0.125	8.142

Number of dissolution data points (N), degrees of freedom (df), and selected goodness of fit criteria – Pearson correlation coefficient (R), coefficient of determination (R^2), adjusted coefficient of determination ($R^2_{adjusted}$), and residual sum of squares (RSS) (manual calculation in MS Excel):

Parameter	No.1	No.2	No.3	No.4
N	33	33	33	33
df	31	31	31	31
R	0.993553204	0.992522468	0.990689108	0.984531394
R^2	0.987147968	0.98510085	0.981464908	0.969302066
$R^2_{adjusted}$	0.986733387	0.984620233	0.980867002	0.96831181
RSS	810.8388417	757.5289156	1322.849722	841.4728943

Graphical abstract of model fit presented as mean ± 1 SD of the fraction % of released carvedilol:

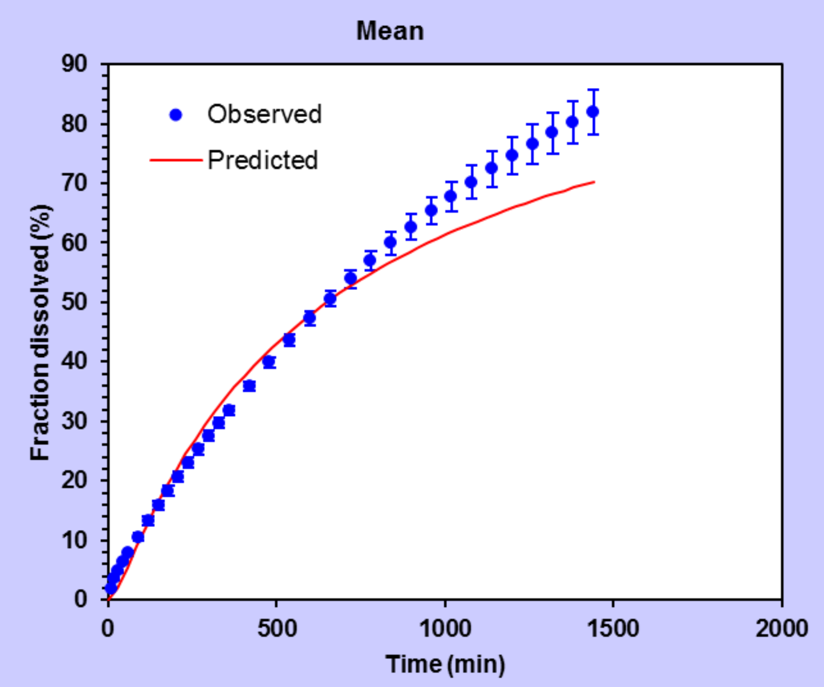

Supplementary materials – Model fitting summary – Ethocel™ Standard 20 Premium (modelling the entire relevant dissolution data)

Graphical abstract of model fit presented as the fraction % of released carvedilol per tested tablet:

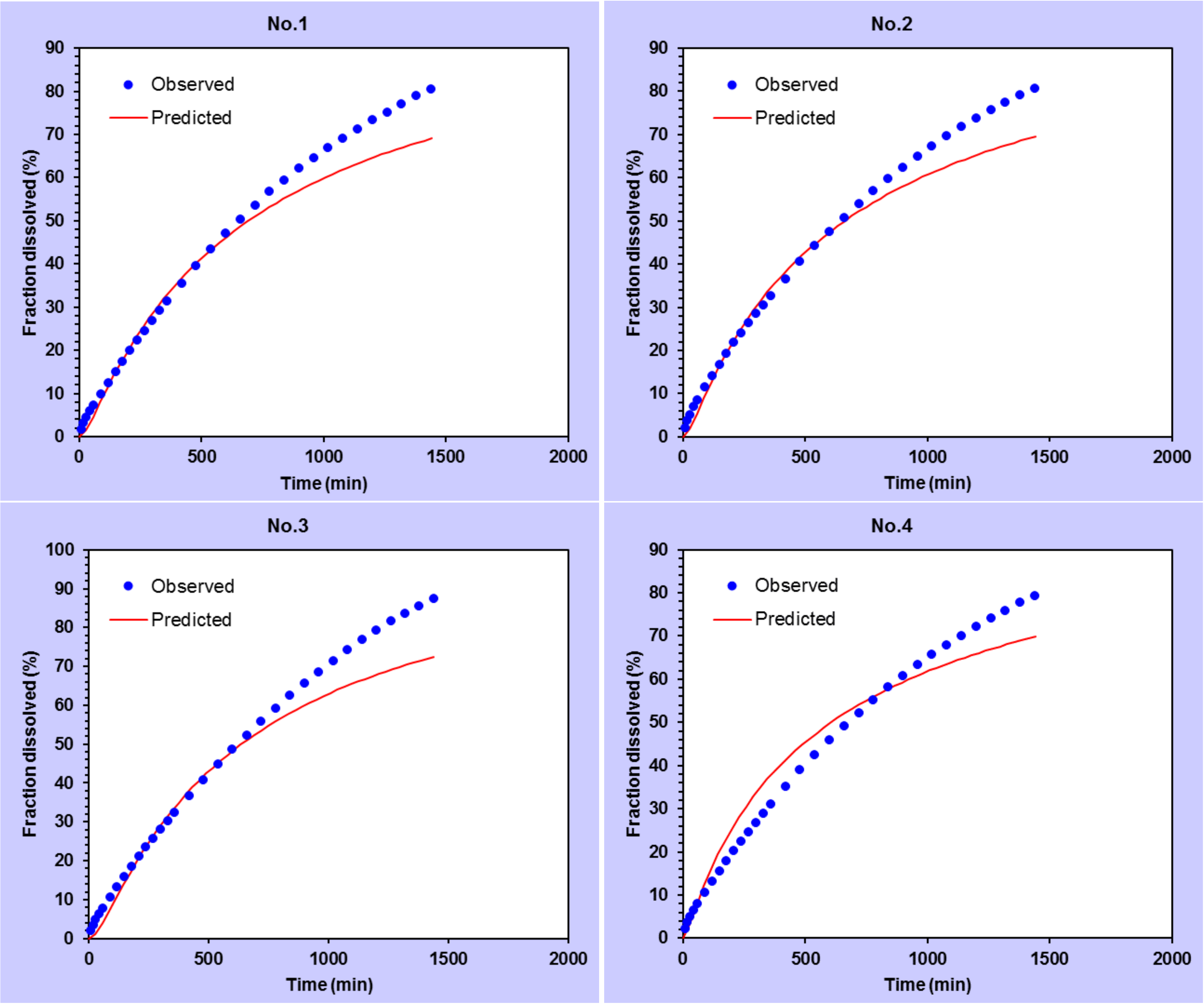

Supplementary materials – Model fitting summary – Ethocel™ Standard 20 Premium (modelling the entire relevant dissolution data)

Model: **Probit_2**

Model equation: $F = F_{max} \cdot \phi[\alpha + \beta \cdot \log(t)]$

Fitted model parameters per tested tablet (N = 4) with statistics – mean, standard deviation (SD), and relative standard deviation expressed in % (RSD%) (output from DDSolver):

Parameter	No.1	No.2	No.3	No.4	Mean	SD	RSD(%)
α	-5.012	-4.873	-4.983	-4.858	-4.931	0.078	-1.574
β	1.789	1.756	1.765	1.738	1.762	0.021	1.203
F_{max}	94.395	91.770	102.590	92.991	95.436	4.888	5.122

Number of dissolution data points (N), degrees of freedom (df), and selected goodness of fit criteria – Pearson correlation coefficient (R), coefficient of determination (R^2), adjusted coefficient of determination ($R^2_{adjusted}$), and residual sum of squares (RSS) (manual calculation in MS Excel):

Parameter	No.1	No.2	No.3	No.4
N	33	33	33	33
df	30	30	30	30
R	0.995524912	0.994291344	0.993235539	0.993687115
R^2	0.99106985	0.988615277	0.986516836	0.987414082
$R^2_{adjusted}$	0.990474507	0.987856296	0.985617959	0.986575021
RSS	771.7639119	988.9355012	1093.027434	736.8790128

Graphical abstract of model fit presented as mean ± 1 SD of the fraction % of released carvedilol:

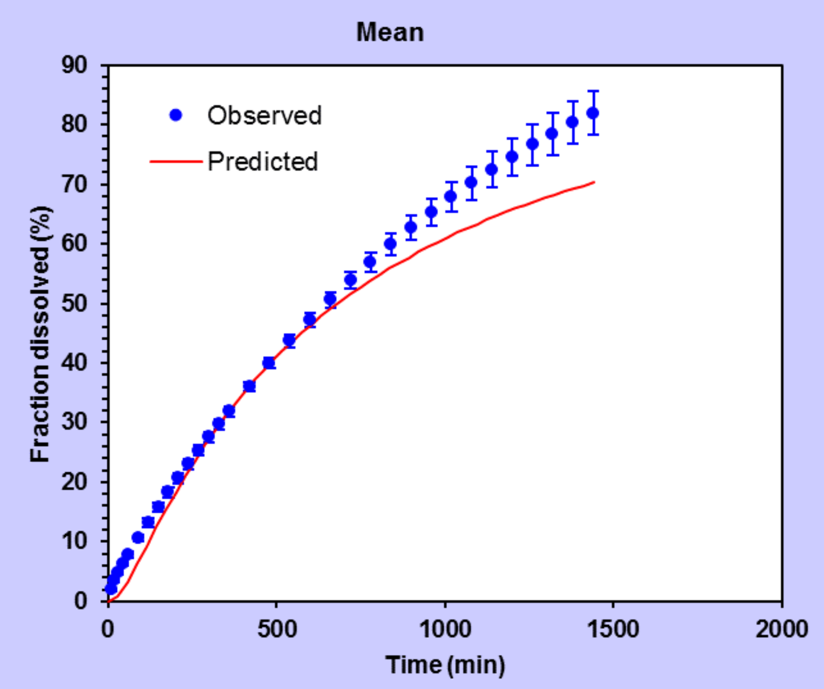

Supplementary materials – Model fitting summary – Ethocel™ Standard 20 Premium (modelling the entire relevant dissolution data)

Graphical abstract of model fit presented as the fraction % of released carvedilol per tested tablet:

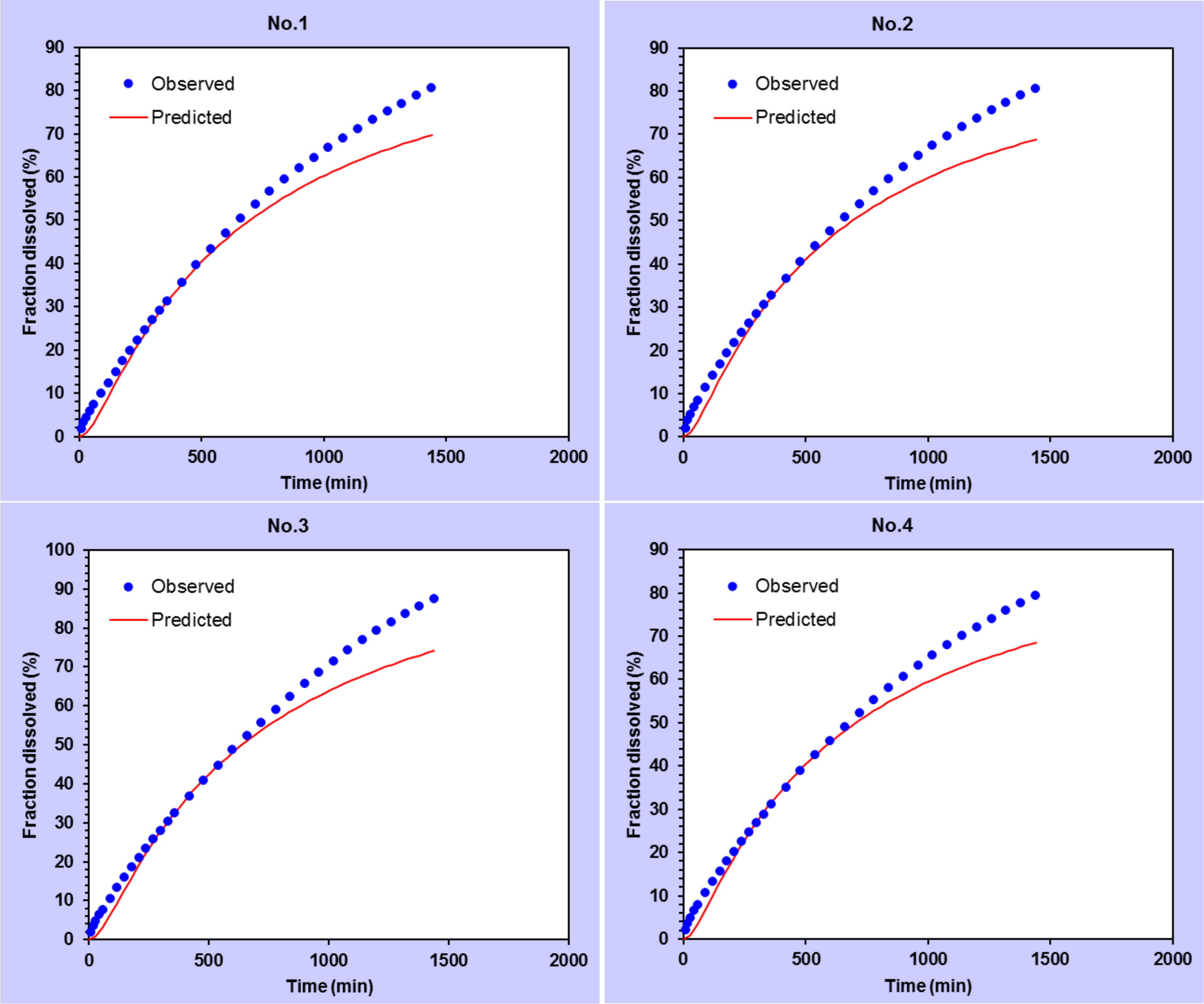

Supplementary materials – Model fitting summary – Ethocel™ Standard 20 Premium (modelling up to app. 60 % of carvedilol released)

Model: **Zero–order**

Model equation: $F = k_0 \cdot t$

Fitted model parameters per tested tablet (N = 4) with statistics – mean, standard deviation (SD), and relative standard deviation expressed in % (RSD%) (output from DDSolver):

Parameter	No.1	No.2	No.3	No.4	Mean	SD	RSD(%)
k_0	0.078	0.080	0.081	0.077	0.079	0.002	2.525

Number of dissolution data points (N), degrees of freedom (df), and selected goodness of fit criteria – Pearson correlation coefficient (R), coefficient of determination (R^2), adjusted coefficient of determination ($R^2_{adjusted}$), and residual sum of squares (RSS) (manual calculation in MS Excel):

Parameter	No.1	No.2	No.3	No.4
N	23	23	23	23
df	22	22	22	22
R	0.995631145	0.993142788	0.996150978	0.995030879
R^2	0.991281376	0.986332598	0.992316772	0.99008645
$R^2_{adjusted}$	0.991281376	0.986332598	0.992316772	0.99008645
RSS	206.2404332	361.6687992	228.261752	268.594635

Graphical abstract of model fit presented as mean ± 1 SD of the fraction % of released carvedilol:

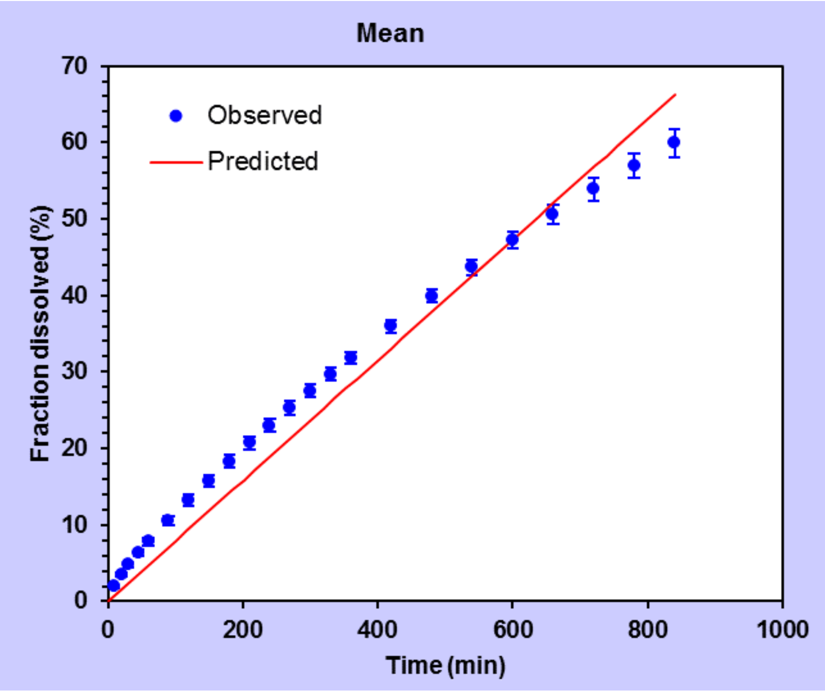

Supplementary materials – Model fitting summary – Ethocel™ Standard 20 Premium (modelling up to app. 60 % of carvedilol released)

Graphical abstract of model fit presented as the fraction % of released carvedilol per tested tablet:

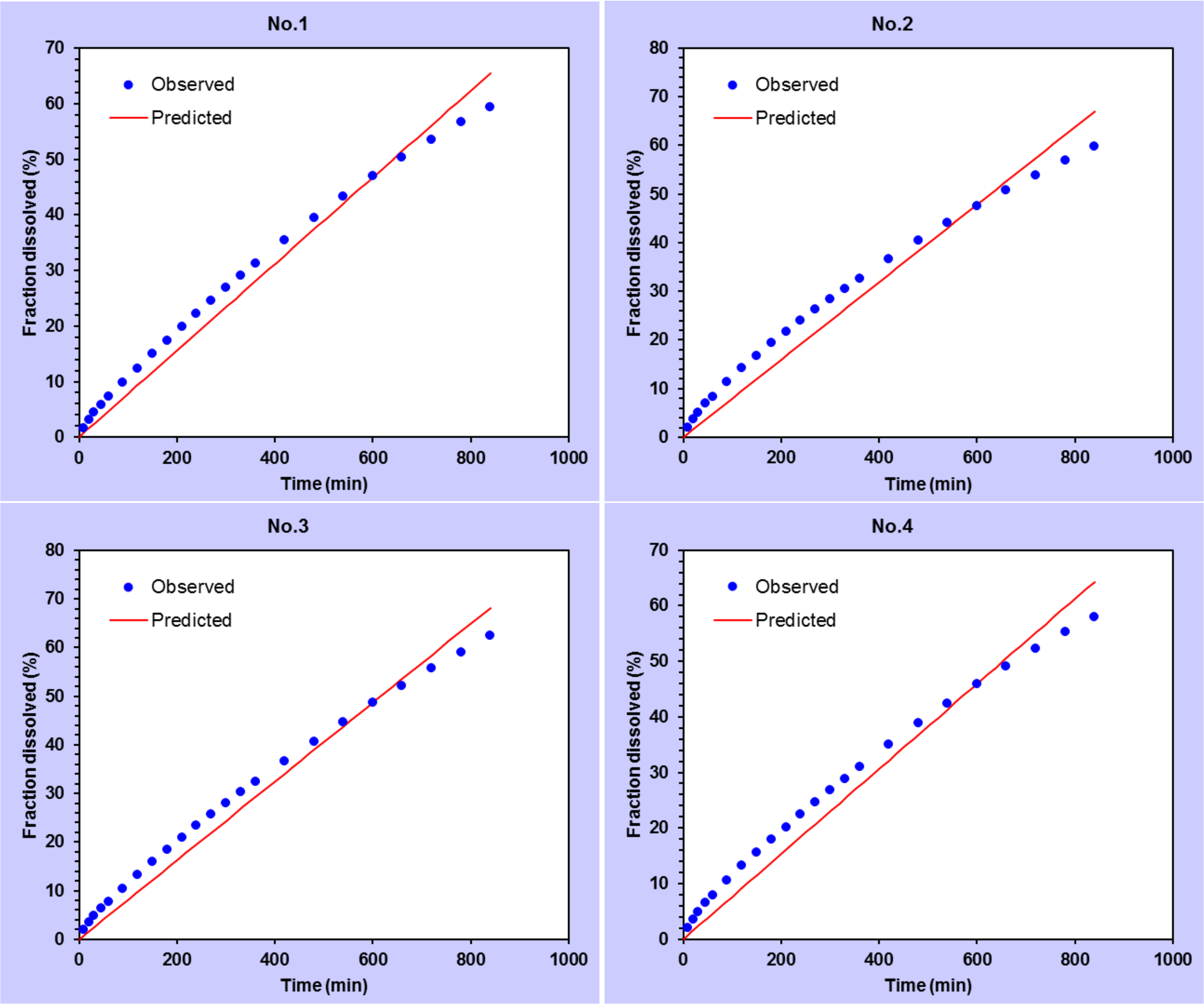

Supplementary materials – Model fitting summary – Ethocel™ Standard 20 Premium (modelling up to app. 60 % of carvedilol released)

Model: **Zero–order with T_{lag}**

Model equation: $F = k_0 \cdot (t - T_{lag})$

Fitted model parameters per tested tablet (N = 4) with statistics – mean, standard deviation (SD), and relative standard deviation expressed in % (RSD%) (output from DDSolver):

Parameter	No.1	No.2	No.3	No.4	Mean	SD	RSD(%)
k_0	0.070	0.069	0.073	0.067	0.070	0.002	3.227
T_{lag}	-56.928	-78.873	-59.711	-70.676	-66.547	10.137	-15.232

Number of dissolution data points (N), degrees of freedom (df), and selected goodness of fit criteria – Pearson correlation coefficient (R), coefficient of determination (R^2), adjusted coefficient of determination ($R^2_{adjusted}$), and residual sum of squares (RSS) (manual calculation in MS Excel):

Parameter	No.1	No.2	No.3	No.4
N	23	23	23	23
df	21	21	21	21
R	0.995631145	0.993142788	0.996150978	0.995030879
R^2	0.991281376	0.986332598	0.992316772	0.99008645
$R^2_{adjusted}$	0.990866204	0.985681769	0.991950904	0.989614376
RSS	65.15726199	99.40789175	61.59242837	68.31736429

Graphical abstract of model fit presented as mean ± 1 SD of the fraction % of released carvedilol:

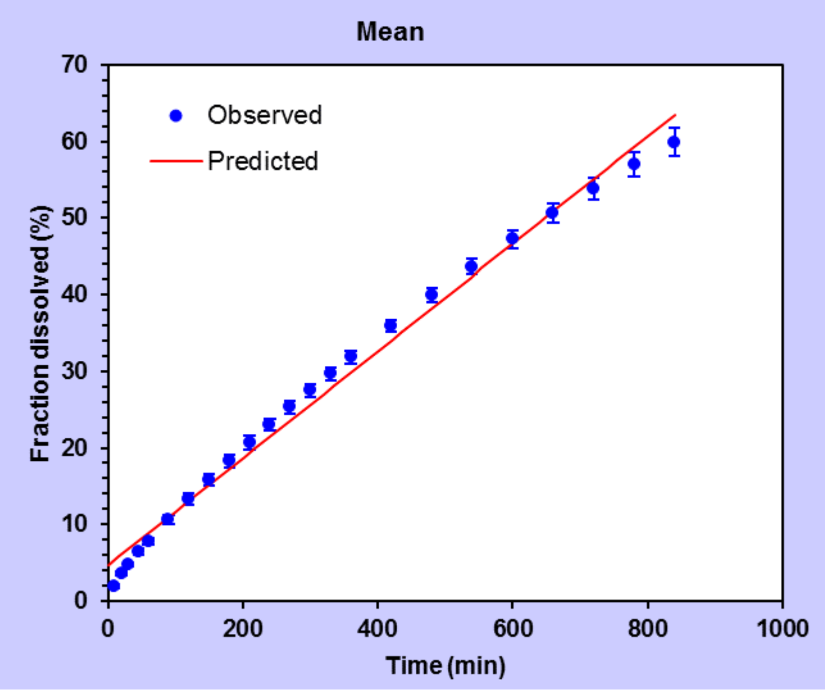

Supplementary materials – Model fitting summary – Ethocel™ Standard 20 Premium (modelling up to app. 60 % of carvedilol released)

Graphical abstract of model fit presented as the fraction % of released carvedilol per tested tablet:

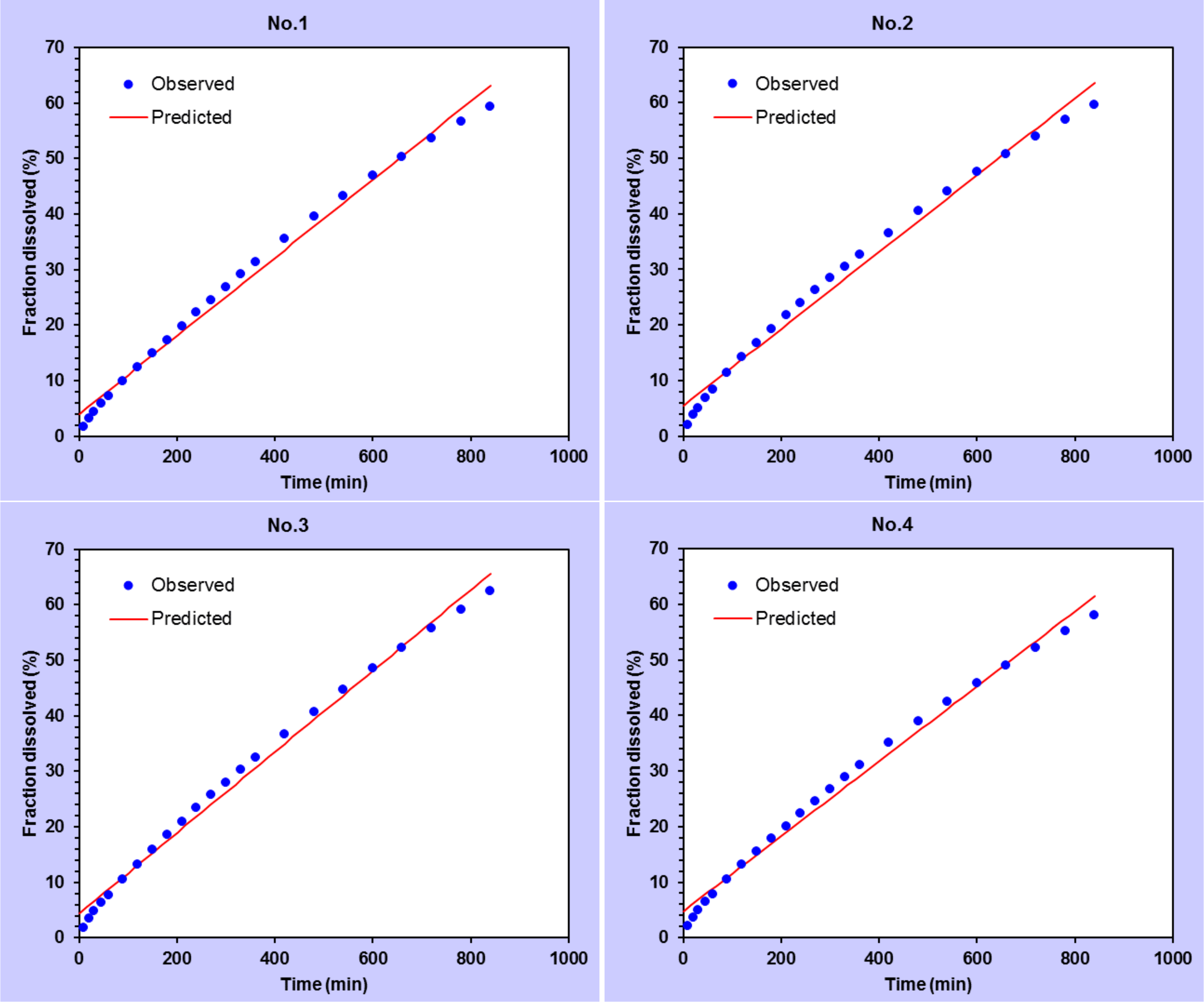

Supplementary materials – Model fitting summary – Ethocel™ Standard 20 Premium (modelling up to app. 60 % of carvedilol released)

Model: **Zero–order with F_0**

Model equation: $F = F_0 + k_0 \cdot t$

Fitted model parameters per tested tablet (N = 4) with statistics – mean, standard deviation (SD), and relative standard deviation expressed in % (RSD%) (output from DDSolver):

Parameter	No.1	No.2	No.3	No.4	Mean	SD	RSD(%)
k_0	0.070	0.069	0.073	0.067	0.070	0.002	3.227
F_0	4.004	5.459	4.352	4.771	4.646	0.626	13.471

Number of dissolution data points (N), degrees of freedom (df), and selected goodness of fit criteria – Pearson correlation coefficient (R), coefficient of determination (R^2), adjusted coefficient of determination ($R^2_{adjusted}$), and residual sum of squares (RSS) (manual calculation in MS Excel):

Parameter	No.1	No.2	No.3	No.4
N	23	23	23	23
df	21	21	21	21
R	0.995631145	0.993142788	0.996150978	0.995030879
R^2	0.991281376	0.986332598	0.992316772	0.99008645
$R^2_{adjusted}$	0.990866204	0.985681769	0.991950904	0.989614376
RSS	65.15726199	99.40789175	61.59242837	68.31736429

Graphical abstract of model fit presented as mean ± 1 SD of the fraction % of released carvedilol:

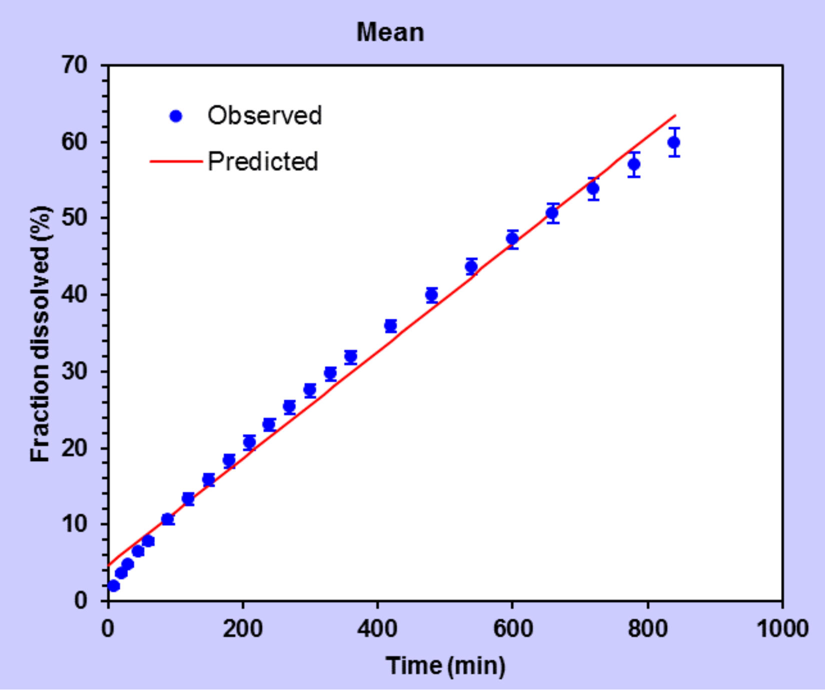

Supplementary materials – Model fitting summary – Ethocel™ Standard 20 Premium (modelling up to app. 60 % of carvedilol released)

Graphical abstract of model fit presented as the fraction % of released carvedilol per tested tablet:

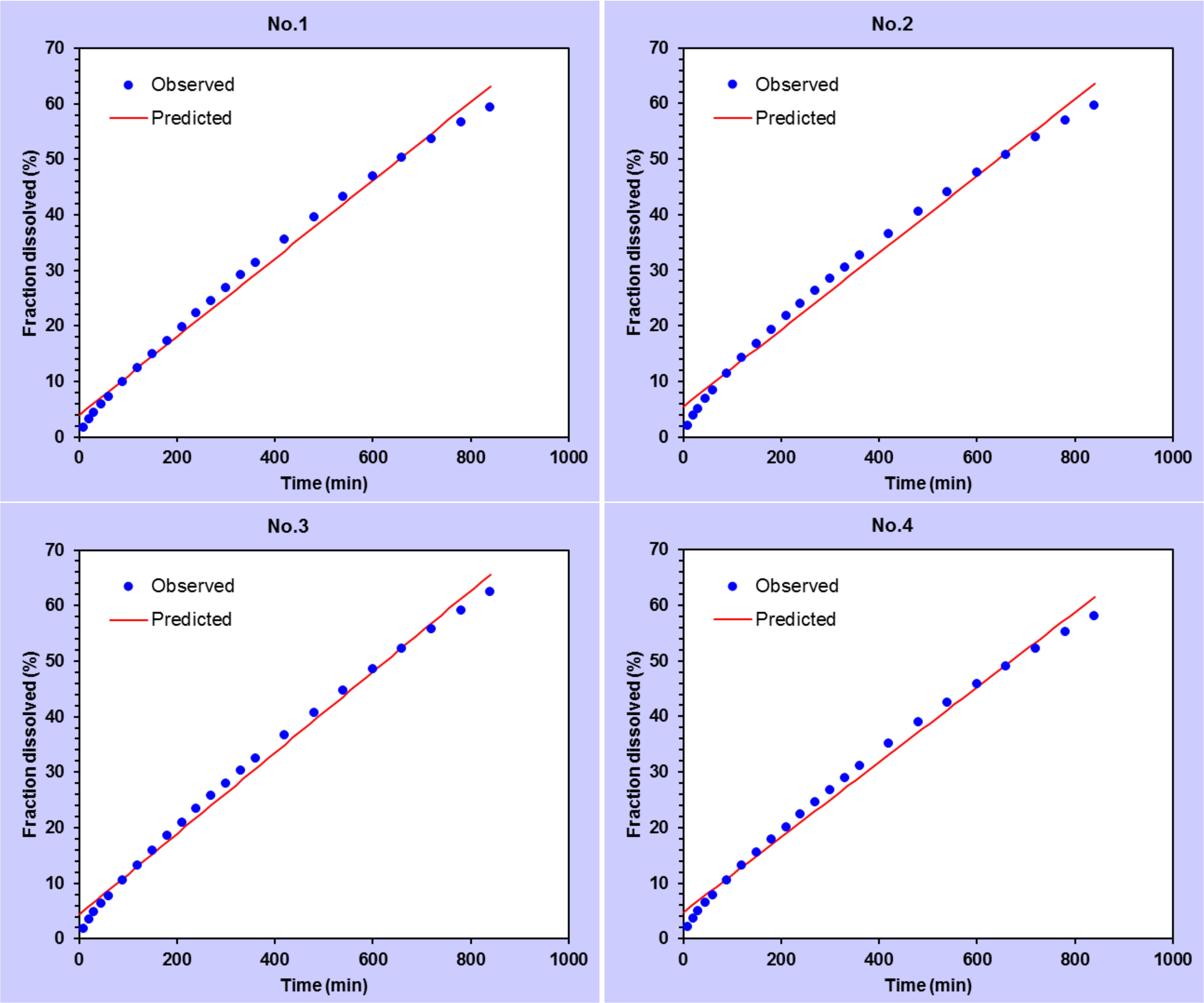

Supplementary materials – Model fitting summary – Ethocel™ Standard 20 Premium (modelling up to app. 60 % of carvedilol released)

Model: **First–order**

Model equation: $F = 100 \cdot (1 - e^{-k_1 \cdot t})$

Fitted model parameters per tested tablet (N = 4) with statistics – mean, standard deviation (SD), and relative standard deviation expressed in % (RSD%) (output from DDSolver):

Parameter	No.1	No.2	No.3	No.4	Mean	SD	RSD(%)
k_1	0.001	0.001	0.001	0.001	0.001	0.000	3.715

Number of dissolution data points (N), degrees of freedom (df), and selected goodness of fit criteria – Pearson correlation coefficient (R), coefficient of determination (R^2), adjusted coefficient of determination ($R^2_{adjusted}$), and residual sum of squares (RSS) (manual calculation in MS Excel):

Parameter	No.1	No.2	No.3	No.4
N	23	23	23	23
df	22	22	22	22
R	0.999652924	0.999780365	0.999225564	0.999770311
R^2	0.999305969	0.999560779	0.998451728	0.999540674
$R^2_{adjusted}$	0.999305969	0.999560779	0.998451728	0.999540674
RSS	8.472484463	34.12348614	16.37521233	23.20631201

Graphical abstract of model fit presented as mean ± 1 SD of the fraction % of released carvedilol:

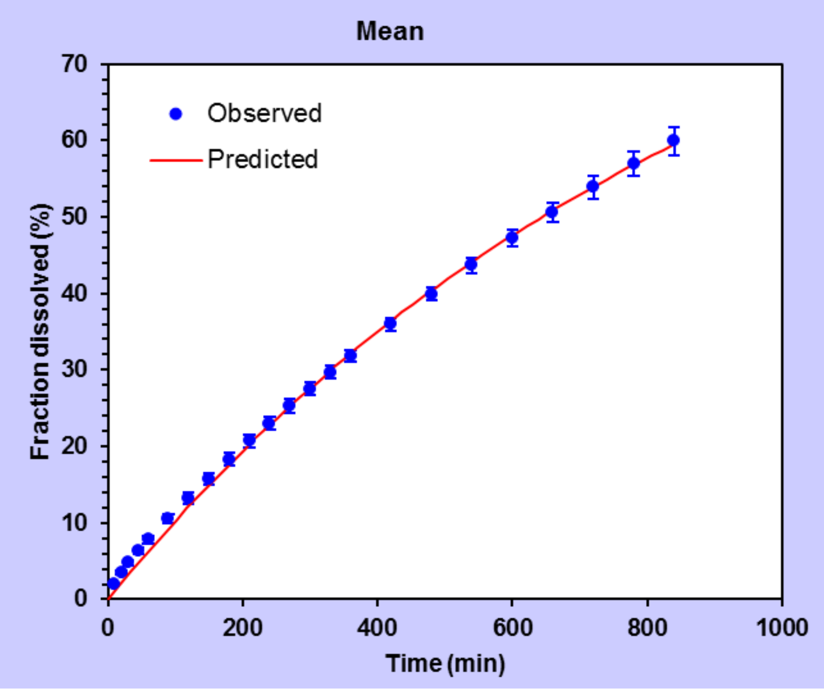

Supplementary materials – Model fitting summary – Ethocel™ Standard 20 Premium (modelling up to app. 60 % of carvedilol released)

Graphical abstract of model fit presented as the fraction % of released carvedilol per tested tablet:

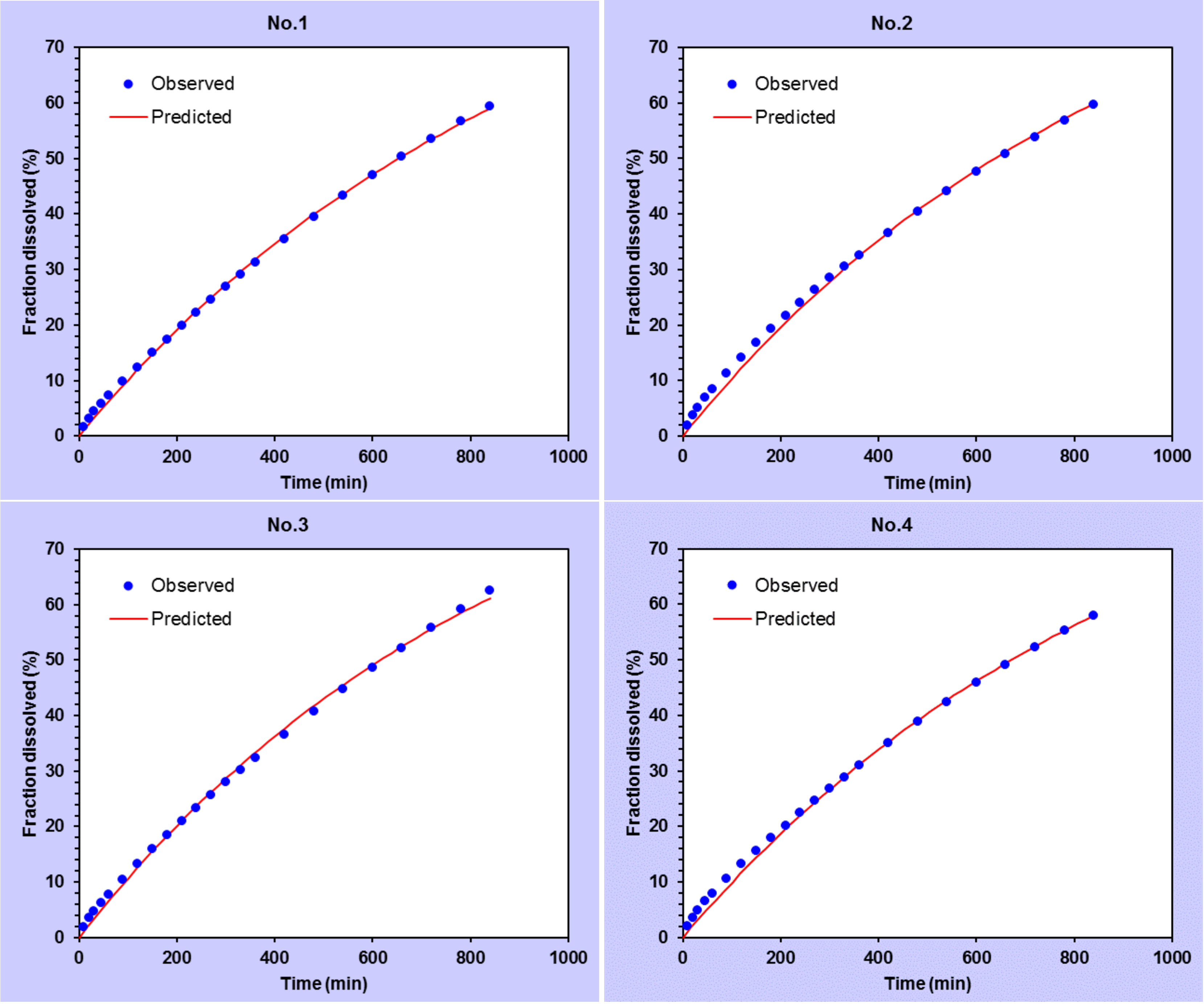

Supplementary materials – Model fitting summary – Ethocel™ Standard 20 Premium (modelling up to app. 60 % of carvedilol released)

Model: **First–order with T_{lag}**

Model equation: $F = 100 \cdot \left[1 - e^{-k_1 \cdot (t - T_{lag})}\right]$

Fitted model parameters per tested tablet (N = 4) with statistics – mean, standard deviation (SD), and relative standard deviation expressed in % (RSD%) (output from DDSolver):

Parameter	No.1	No.2	No.3	No.4	Mean	SD	RSD(%)
k_1	0.001	0.001	0.001	0.001	0.001	0.000	4.663
T_{lag}	-4.410	-20.579	-4.177	-16.198	-11.341	8.332	-73.472

Number of dissolution data points (N), degrees of freedom (df), and selected goodness of fit criteria – Pearson correlation coefficient (R), coefficient of determination (R^2), adjusted coefficient of determination ($R^2_{adjusted}$), and residual sum of squares (RSS) (manual calculation in MS Excel):

Parameter	No.1	No.2	No.3	No.4
N	23	23	23	23
df	21	21	21	21
R	0.999675822	0.999786438	0.999248748	0.999814932
R^2	0.999351749	0.999572922	0.998498061	0.999629898
$R^2_{adjusted}$	0.99932088	0.999552585	0.99842654	0.999612274
RSS	5.349495854	3.109927383	13.55441724	2.681132858

Graphical abstract of model fit presented as mean ± 1 SD of the fraction % of released carvedilol:

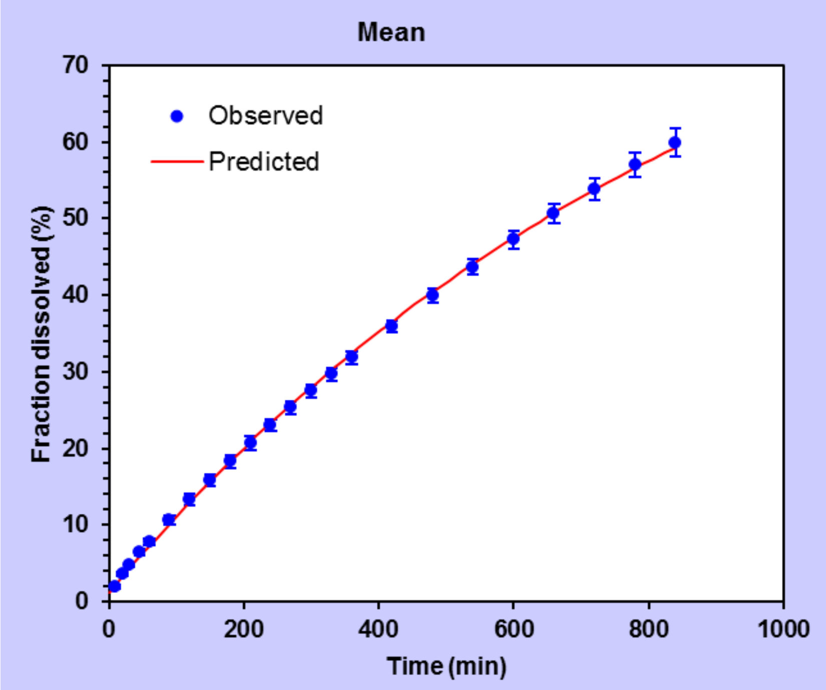

Supplementary materials – Model fitting summary – Ethocel™ Standard 20 Premium (modelling up to app. 60 % of carvedilol released)

Graphical abstract of model fit presented as the fraction % of released carvedilol per tested tablet:

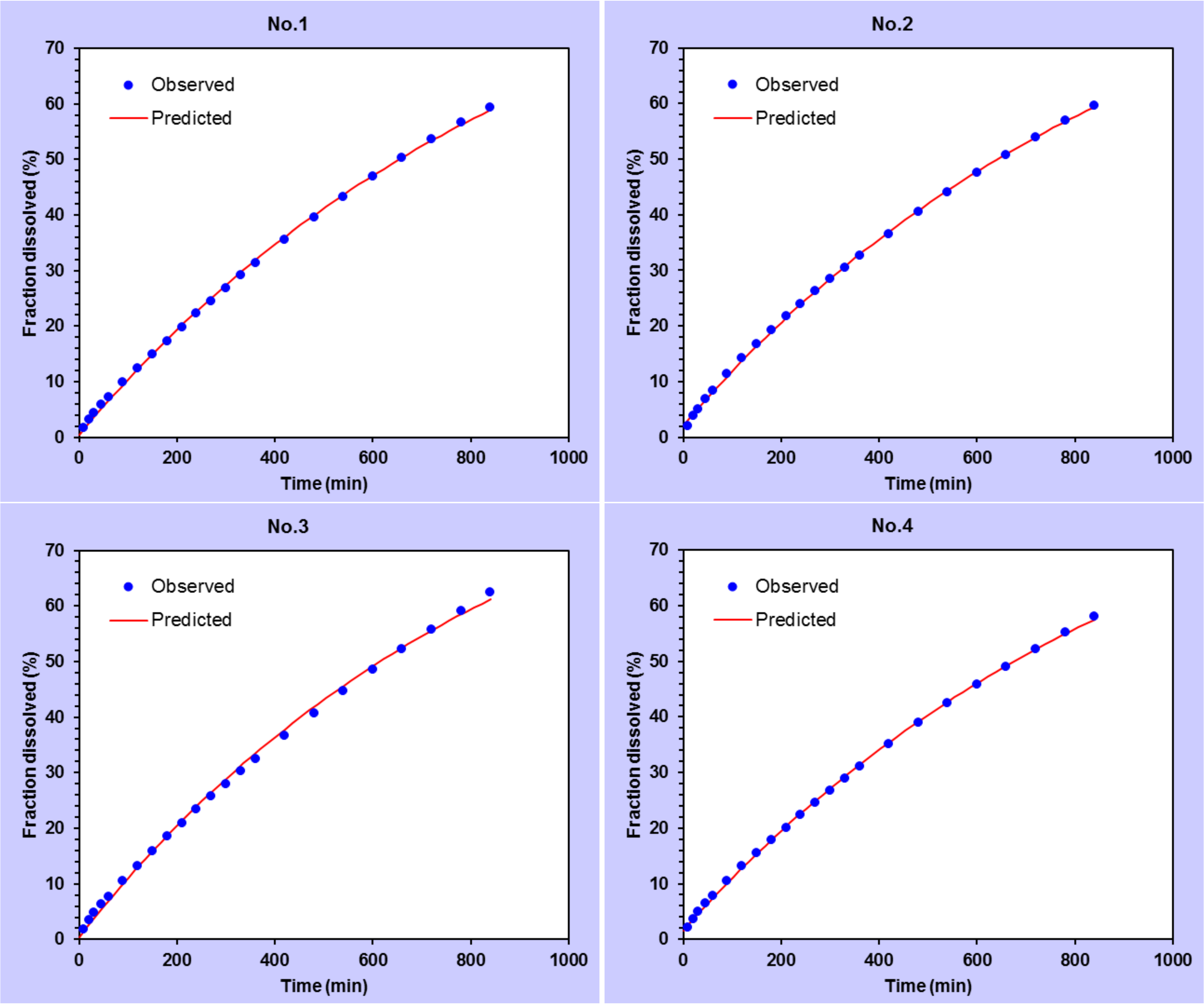

Supplementary materials – Model fitting summary – Ethocel™ Standard 20 Premium (modelling up to app. 60 % of carvedilol released)

Model: **First–order with F_{max}**

Model equation: $F = F_{max} \cdot (1 - e^{-k_1 \cdot t})$

Fitted model parameters per tested tablet (N = 4) with statistics – mean, standard deviation (SD), and relative standard deviation expressed in % (RSD%) (output from DDSolver):

Parameter	No.1	No.2	No.3	No.4	Mean	SD	RSD(%)
k_1	0.003	0.003	0.003	0.003	0.003	0.000	1.526
F_{max}	62.383	62.702	65.584	60.940	62.902	1.946	3.093

Number of dissolution data points (N), degrees of freedom (df), and selected goodness of fit criteria – Pearson correlation coefficient (R), coefficient of determination (R^2), adjusted coefficient of determination ($R^2_{adjusted}$), and residual sum of squares (RSS) (manual calculation in MS Excel):

Parameter	No.1	No.2	No.3	No.4
N	23	23	23	23
df	21	21	21	21
R	0.982974012	0.986524857	0.983116021	0.984081476
R^2	0.966237908	0.973231294	0.96651711	0.968416352
$R^2_{adjusted}$	0.964630189	0.971956594	0.964922687	0.966912369
RSS	517.3916296	363.9304424	534.3211532	409.3380031

Graphical abstract of model fit presented as mean ± 1 SD of the fraction % of released carvedilol:

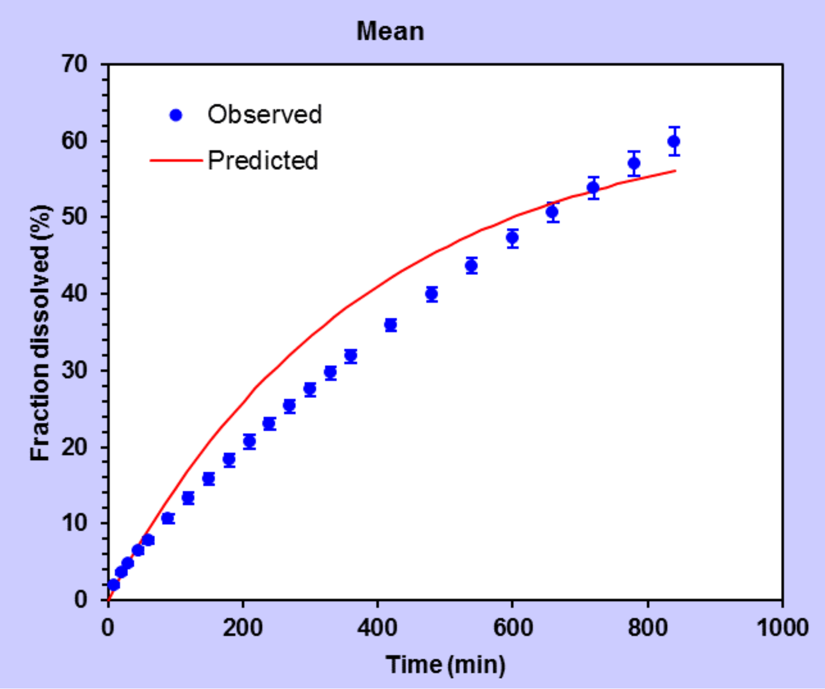

Supplementary materials – Model fitting summary – Ethocel™ Standard 20 Premium (modelling up to app. 60 % of carvedilol released)

Graphical abstract of model fit presented as the fraction % of released carvedilol per tested tablet:

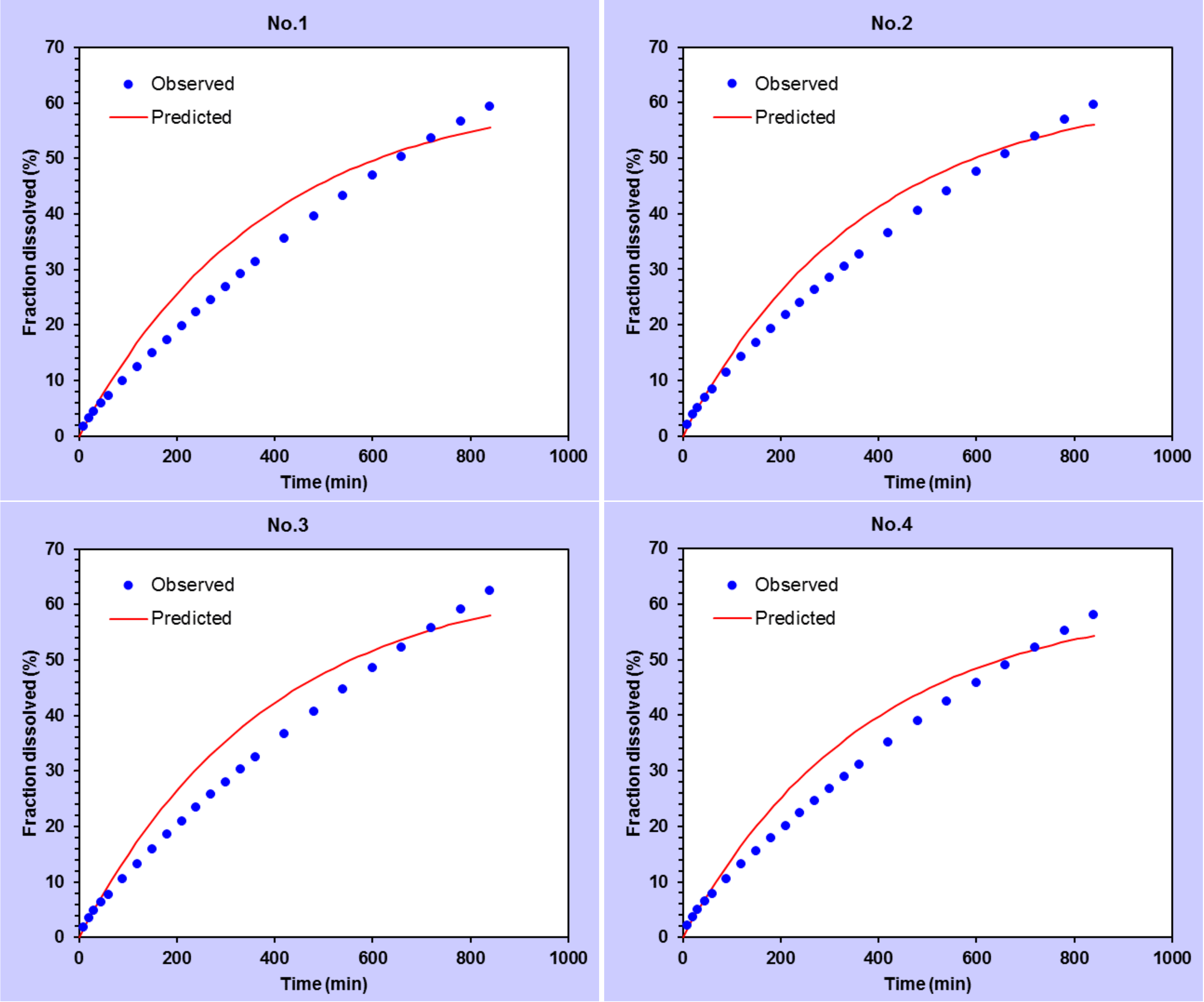

Supplementary materials – Model fitting summary – Ethocel™ Standard 20 Premium (modelling up to app. 60 % of carvedilol released)

Model: **First–order with T_{lag} and F_{max}**

Model equation: $F = F_{max} \cdot \left[1 - e^{-k_1 \cdot (t - T_{lag})}\right]$

Fitted model parameters per tested tablet (N = 4) with statistics – mean, standard deviation (SD), and relative standard deviation expressed in % (RSD%) (output from DDSolver):

Parameter	No.1	No.2	No.3	No.4	Mean	SD	RSD(%)
k_1	0.003	0.003	0.003	0.003	0.003	0.000	1.063
T_{lag}	62.139	51.899	60.548	55.990	57.644	4.632	8.036
F_{max}	62.383	62.702	65.584	60.940	62.902	1.946	3.093

Number of dissolution data points (N), degrees of freedom (df), and selected goodness of fit criteria – Pearson correlation coefficient (R), coefficient of determination (R^2), adjusted coefficient of determination ($R^2_{adjusted}$), and residual sum of squares (RSS) (manual calculation in MS Excel):

Parameter	No.1	No.2	No.3	No.4
N	23	23	23	23
df	20	20	20	20
R	0.976493542	0.981846117	0.977015361	0.978564436
R^2	0.953539638	0.964021797	0.954559016	0.957588356
$R^2_{adjusted}$	0.948893601	0.960423977	0.950014917	0.953347191
RSS	791.305702	615.8055345	836.1253571	671.602195

Graphical abstract of model fit presented as mean ± 1 SD of the fraction % of released carvedilol:

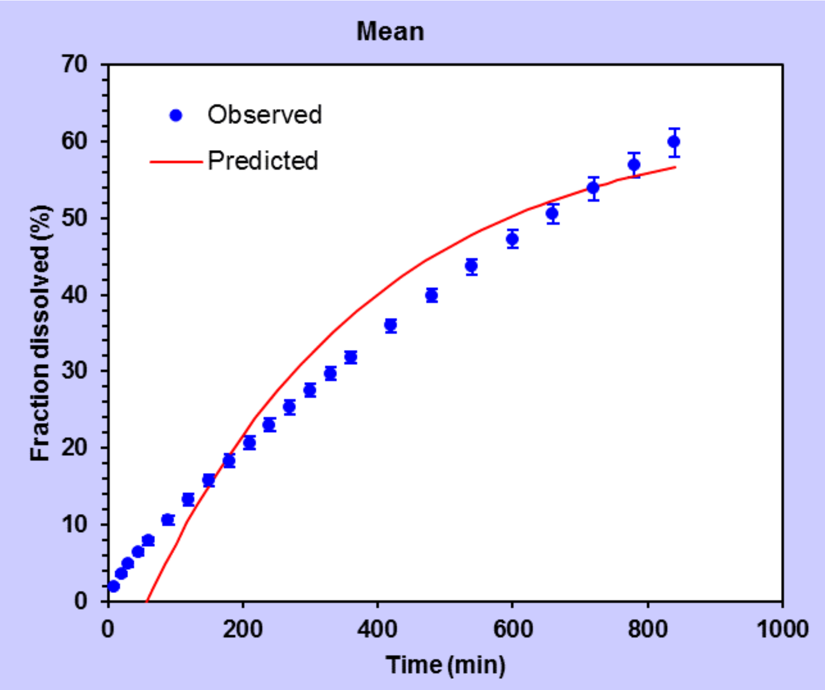

Supplementary materials – Model fitting summary – Ethocel™ Standard 20 Premium (modelling up to app. 60 % of carvedilol released)

Graphical abstract of model fit presented as the fraction % of released carvedilol per tested tablet:

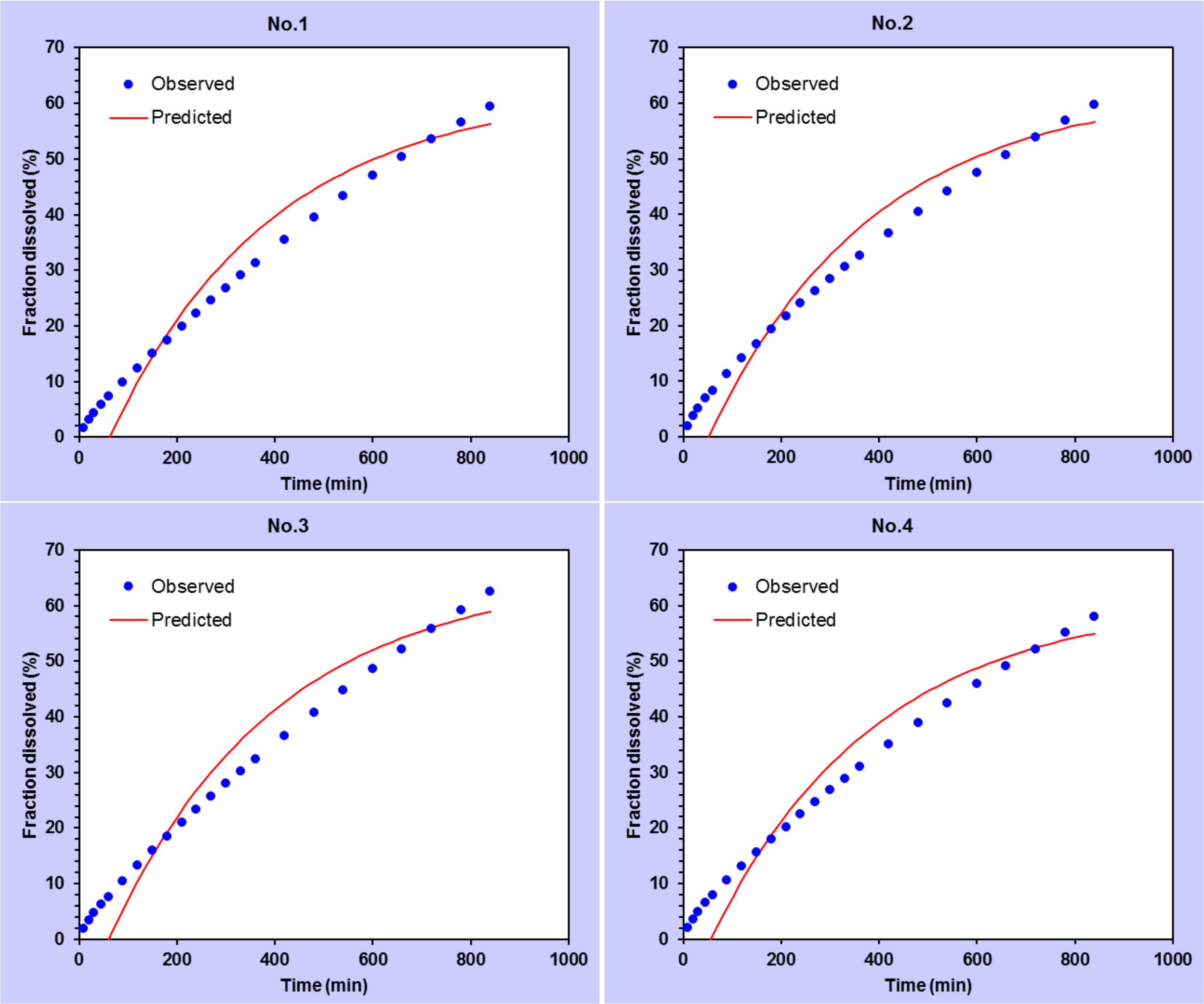

Supplementary materials – Model fitting summary – Ethocel™ Standard 20 Premium (modelling up to app. 60 % of carvedilol released)

Model: **Higuchi**

Model equation: $F = k_H \cdot t^{0.5}$

Fitted model parameters per tested tablet (N = 4) with statistics – mean, standard deviation (SD), and relative standard deviation expressed in % (RSD%) (output from DDSolver):

Parameter	No.1	No.2	No.3	No.4	Mean	SD	RSD(%)
k_H	1.770	1.821	1.844	1.746	1.795	0.045	2.509

Number of dissolution data points (N), degrees of freedom (df), and selected goodness of fit criteria – Pearson correlation coefficient (R), coefficient of determination (R^2), adjusted coefficient of determination ($R^2_{adjusted}$), and residual sum of squares (RSS) (manual calculation in MS Excel):

Parameter	No.1	No.2	No.3	No.4
N	23	23	23	23
df	22	22	22	22
R	0.990953743	0.994365188	0.990884018	0.99220989
R^2	0.98198932	0.988762127	0.981851137	0.984480466
$R^2_{adjusted}$	0.98198932	0.988762127	0.981851137	0.984480466
RSS	632.0618742	453.4455557	658.4091973	492.5320009

Graphical abstract of model fit presented as mean ± 1 SD of the fraction % of released carvedilol:

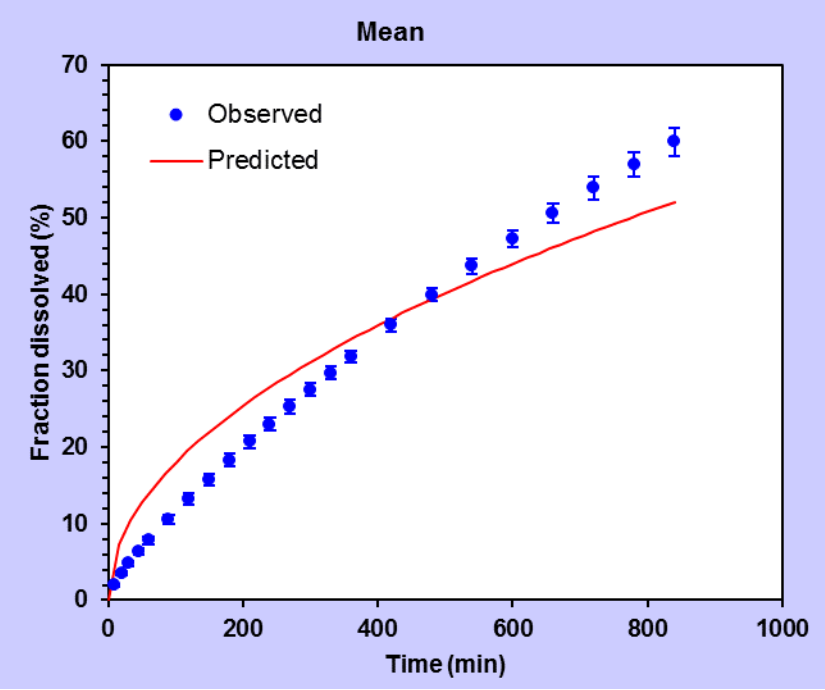

Supplementary materials – Model fitting summary – Ethocel™ Standard 20 Premium (modelling up to app. 60 % of carvedilol released)

Graphical abstract of model fit presented as the fraction % of released carvedilol per tested tablet:

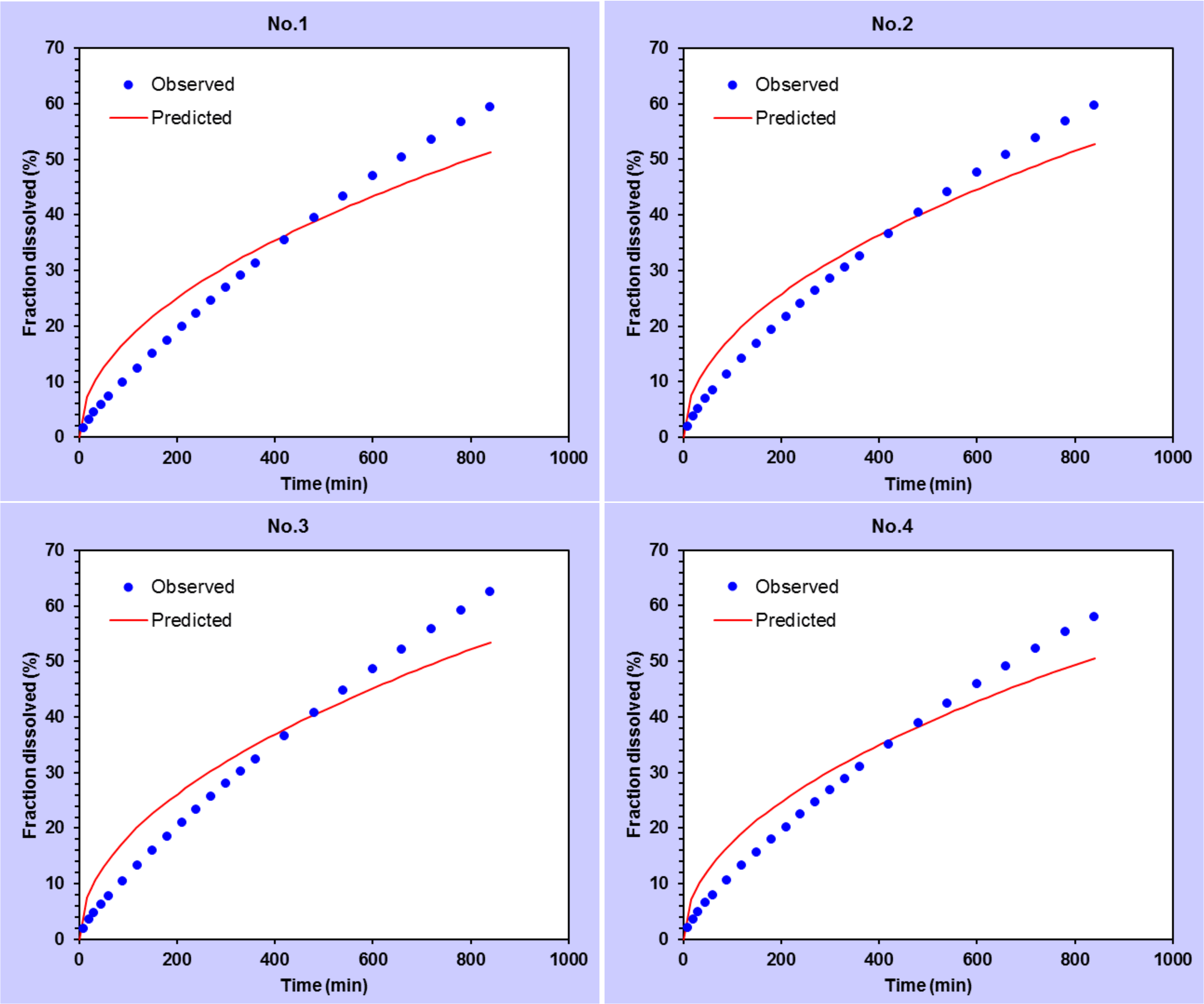

Supplementary materials – Model fitting summary – Ethocel™ Standard 20 Premium (modelling up to app. 60 % of carvedilol released)

Model: **Higuchi with T_{lag}**

Model equation: $F = k_H \cdot (t - T_{lag})^{0.5}$

Fitted model parameters per tested tablet (N = 4) with statistics – mean, standard deviation (SD), and relative standard deviation expressed in % (RSD%) (output from DDSolver):

Parameter	No.1	No.2	No.3	No.4	Mean	SD	RSD(%)
k_H	2.065	2.071	2.150	2.010	2.074	0.058	2.775
T_{lag}	79.428	69.065	79.367	74.180	75.510	4.951	6.557

Number of dissolution data points (N), degrees of freedom (df), and selected goodness of fit criteria – Pearson correlation coefficient (R), coefficient of determination (R^2), adjusted coefficient of determination ($R^2_{adjusted}$), and residual sum of squares (RSS) (manual calculation in MS Excel):

Parameter	No.1	No.2	No.3	No.4
N	23	23	23	23
df	21	21	21	21
R	0.985468582	0.986975549	0.985229038	0.985416282
R^2	0.971148325	0.974120735	0.970676258	0.971045249
$R^2_{adjusted}$	0.969774436	0.972888389	0.969279889	0.969666452
RSS	253.9626118	244.8377797	282.9199278	249.9710657

Graphical abstract of model fit presented as mean ± 1 SD of the fraction % of released carvedilol:

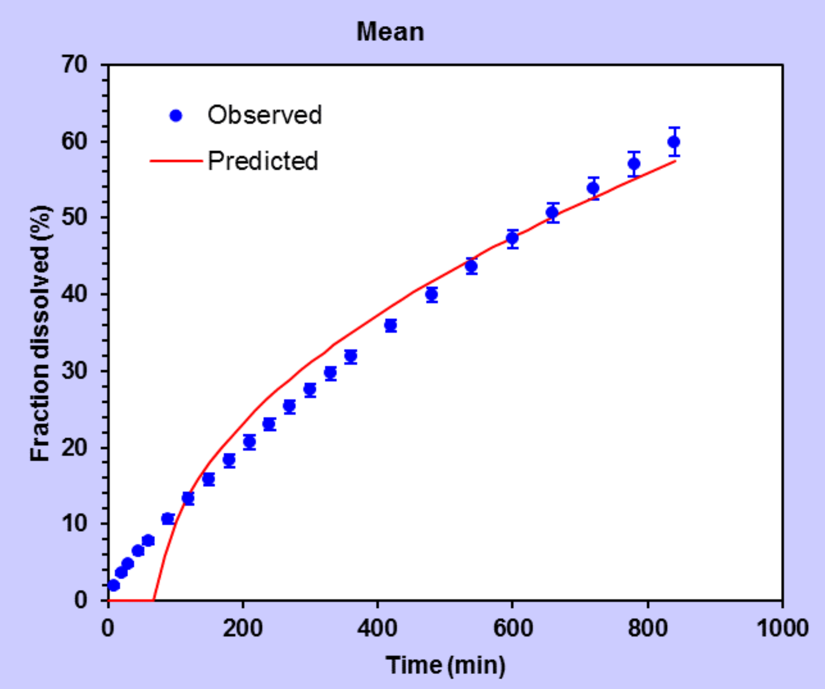

Supplementary materials – Model fitting summary – Ethocel™ Standard 20 Premium (modelling up to app. 60 % of carvedilol released)

Graphical abstract of model fit presented as the fraction % of released carvedilol per tested tablet:

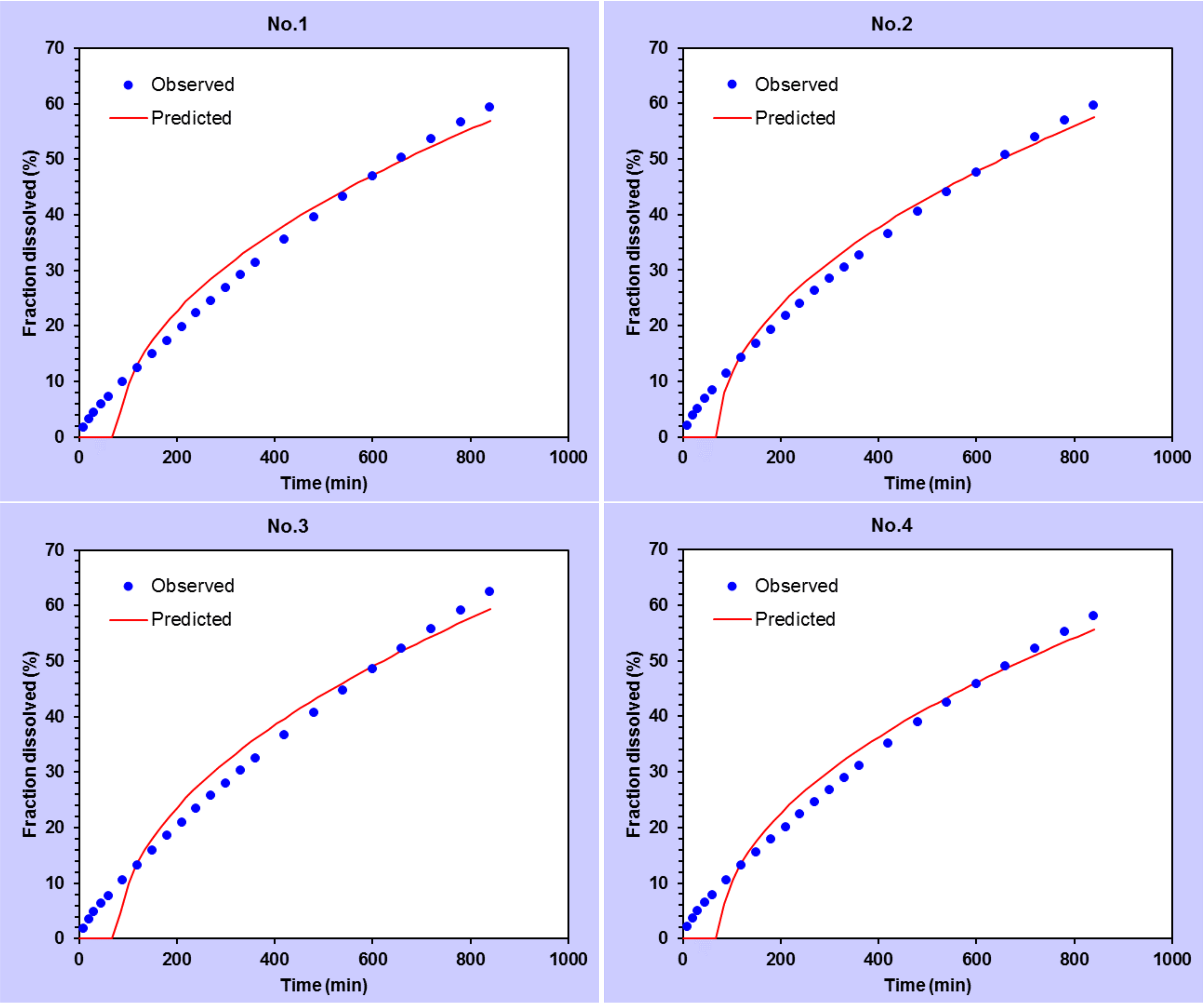

Supplementary materials – Model fitting summary – Ethocel™ Standard 20 Premium (modelling up to app. 60 % of carvedilol released)

Model: **Higuchi with F_0**

Model equation: $F = F_0 + k_H \cdot t^{0.5}$

Fitted model parameters per tested tablet (N = 4) with statistics – mean, standard deviation (SD), and relative standard deviation expressed in % (RSD%) (output from DDSolver):

Parameter	No.1	No.2	No.3	No.4	Mean	SD	RSD(%)
k_H	2.315	2.291	2.397	2.226	2.307	0.071	3.072
F_0	-10.850	-9.379	-11.017	-9.552	-10.200	0.853	-8.366

Number of dissolution data points (N), degrees of freedom (df), and selected goodness of fit criteria – Pearson correlation coefficient (R), coefficient of determination (R^2), adjusted coefficient of determination ($R^2_{adjusted}$), and residual sum of squares (RSS) (manual calculation in MS Excel):

Parameter	No.1	No.2	No.3	No.4
N	23	23	23	23
df	21	21	21	21
R	0.990953743	0.994365188	0.990884018	0.99220989
R^2	0.98198932	0.988762127	0.981851137	0.984480466
$R^2_{adjusted}$	0.981131669	0.98822699	0.980986905	0.983741441
RSS	134.5999805	81.73706231	145.4899548	106.9499486

Graphical abstract of model fit presented as mean ± 1 SD of the fraction % of released carvedilol:

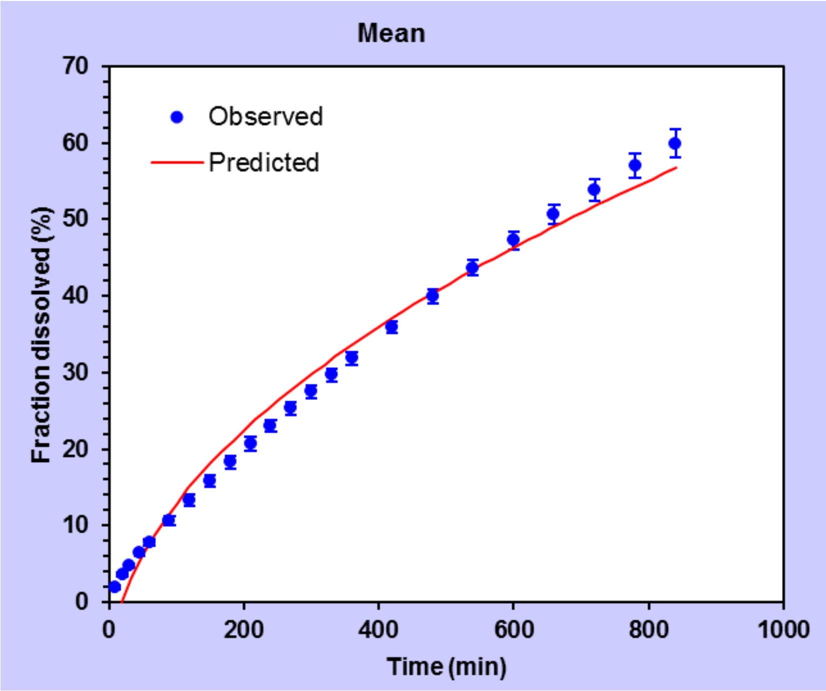

Supplementary materials – Model fitting summary – Ethocel^{TM} Standard 20 Premium (modelling up to app. 60 % of carvedilol released)

Graphical abstract of model fit presented as the fraction % of released carvedilol per tested tablet:

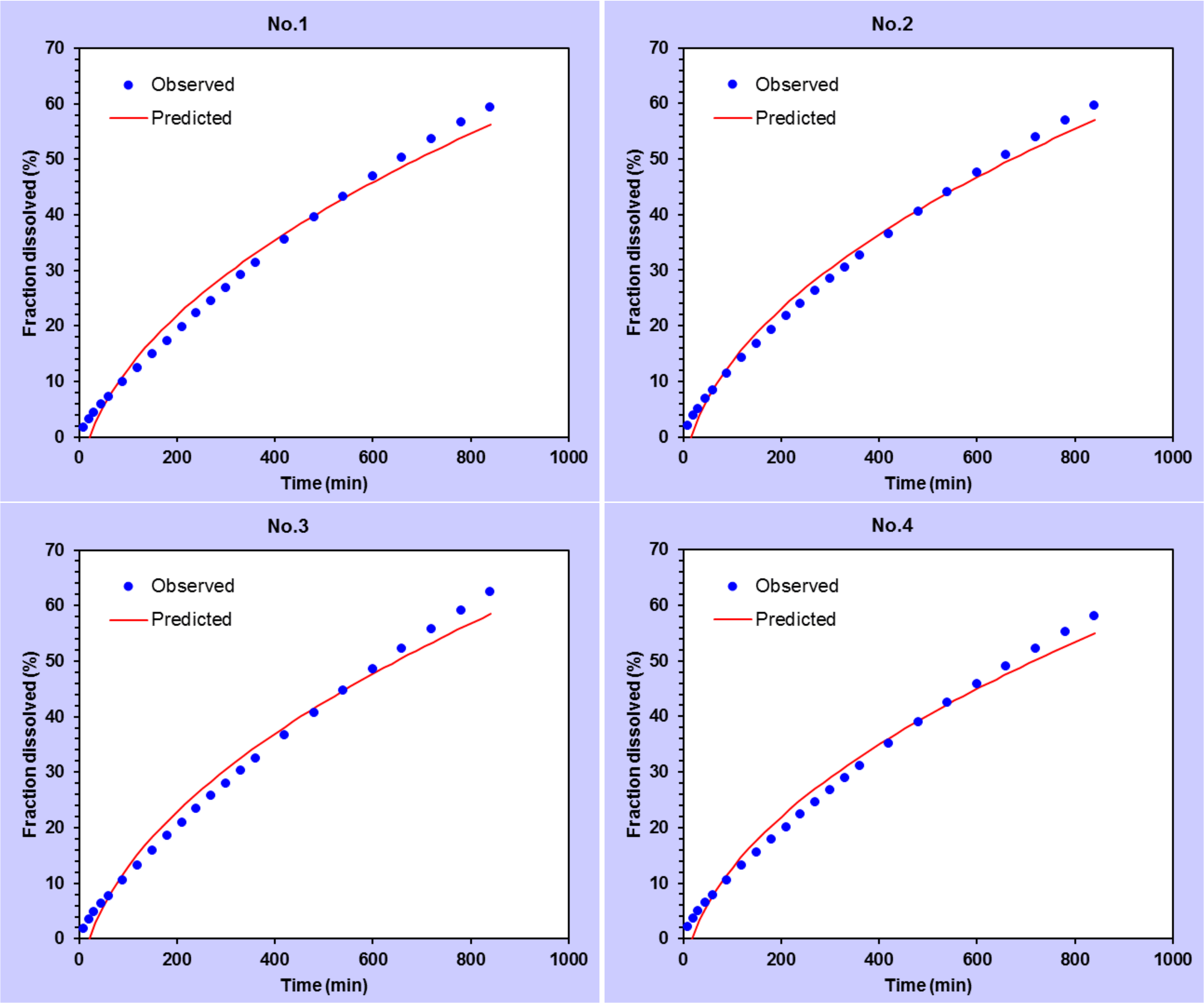

Supplementary materials – Model fitting summary – Ethocel™ Standard 20 Premium (modelling up to app. 60 % of carvedilol released)

Model: **Korsmeyer–Peppas**

Model equation: $F = k_{KP} \cdot t^n$

Fitted model parameters per tested tablet (N = 4) with statistics – mean, standard deviation (SD), and relative standard deviation expressed in % (RSD%) (output from DDSolver):

Parameter	No.1	No.2	No.3	No.4	Mean	SD	RSD(%)
k_{KP}	0.279	0.430	0.318	0.370	0.349	0.065	18.743
n	0.800	0.733	0.785	0.751	0.767	0.030	3.964

Number of dissolution data points (N), degrees of freedom (df), and selected goodness of fit criteria – Pearson correlation coefficient (R), coefficient of determination (R^2), adjusted coefficient of determination ($R^2_{adjusted}$), and residual sum of squares (RSS) (manual calculation in MS Excel):

Parameter	No.1	No.2	No.3	No.4
N	23	23	23	23
df	21	21	21	21
R	0.999681636	0.999890278	0.999931893	0.999848958
R^2	0.999363373	0.999780568	0.999863791	0.99969794
$R^2_{adjusted}$	0.999333057	0.999770118	0.999857305	0.999683556
RSS	4.774746335	3.990999807	1.405790404	2.952785826

Graphical abstract of model fit presented as mean ± 1 SD of the fraction % of released carvedilol:

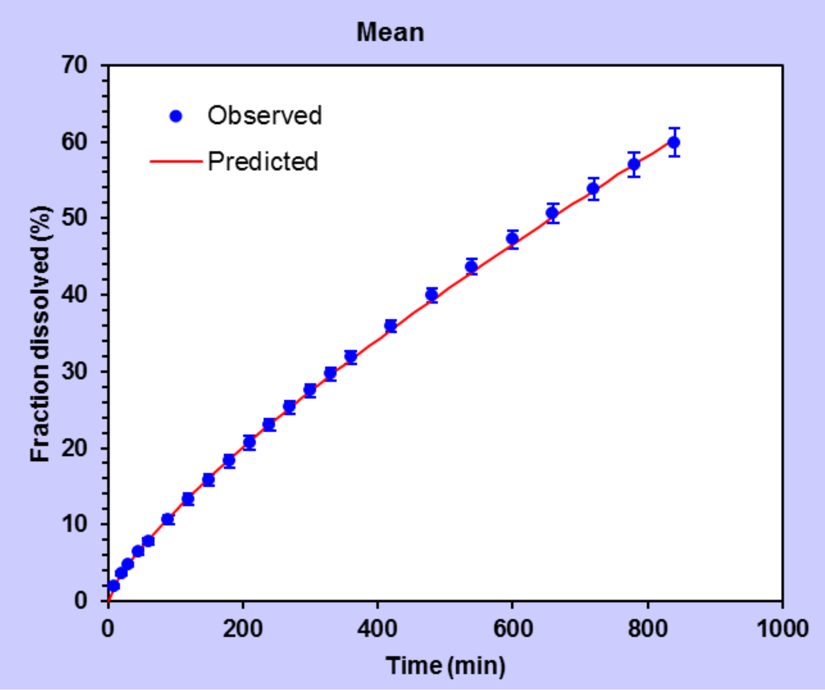

Supplementary materials – Model fitting summary – Ethocel^{TM} Standard 20 Premium (modelling up to app. 60 % of carvedilol released)

Graphical abstract of model fit presented as the fraction % of released carvedilol per tested tablet:

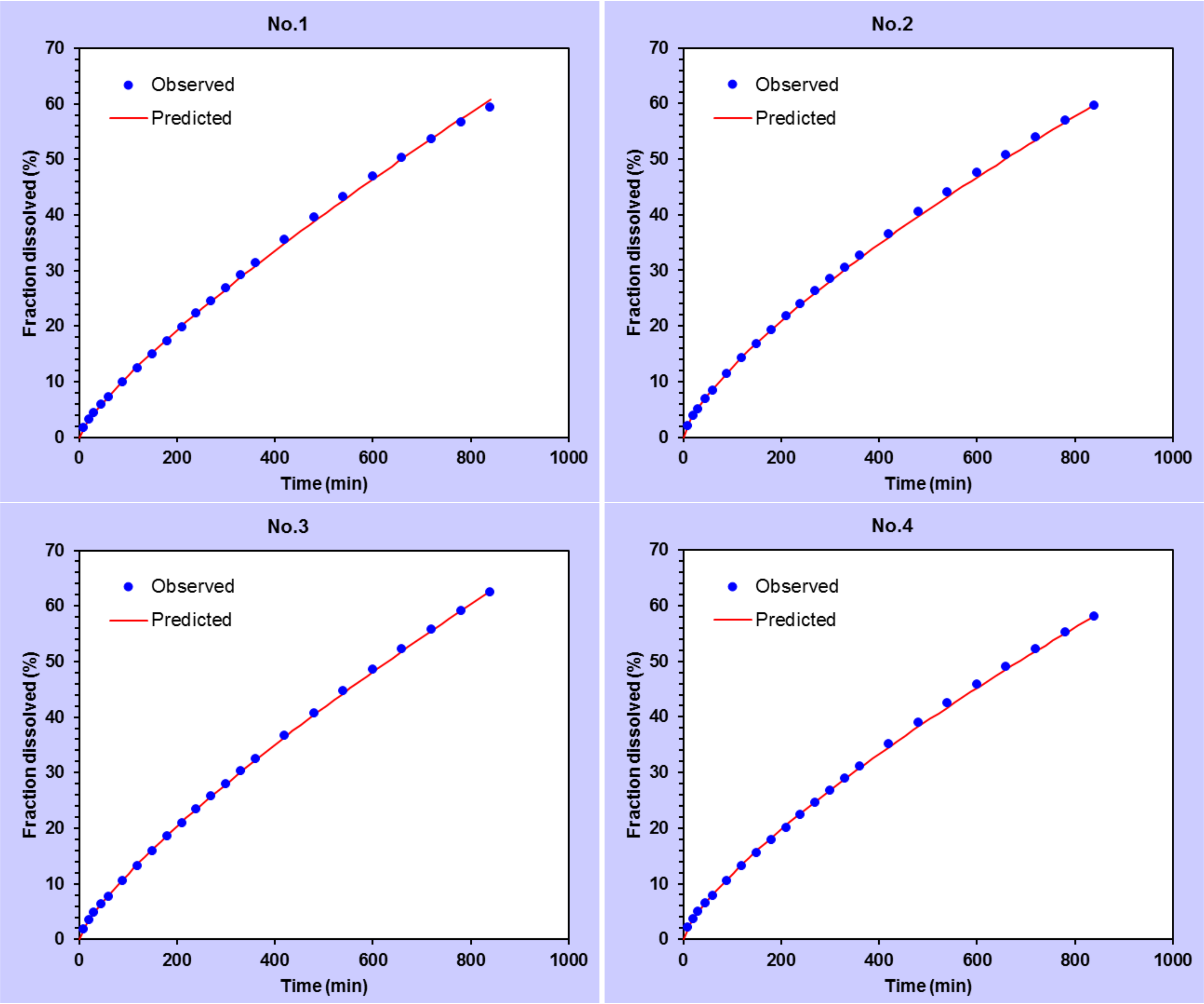

Supplementary materials – Model fitting summary – Ethocel™ Standard 20 Premium (modelling up to app. 60 % of carvedilol released)

Model: **Korsmeyer–Peppas with T_{lag}**

Model equation: $F = k_{KP} \cdot (t - T_{lag})^n$

Fitted model parameters per tested tablet (N = 4) with statistics – mean, standard deviation (SD), and relative standard deviation expressed in % (RSD%) (output from DDSolver):

Parameter	No.1	No.2	No.3	No.4	Mean	SD	RSD(%)
k_{KP}	0.429	0.528	0.487	0.509	0.488	0.043	8.835
n	0.735	0.705	0.721	0.698	0.715	0.016	2.298
T_{lag}	4.831	4.975	4.831	4.000	4.659	0.445	9.546

Number of dissolution data points (N), degrees of freedom (df), and selected goodness of fit criteria – Pearson correlation coefficient (R), coefficient of determination (R^2), adjusted coefficient of determination ($R^2_{adjusted}$), and residual sum of squares (RSS) (manual calculation in MS Excel):

Parameter	No.1	No.2	No.3	No.4
N	23	23	23	23
df	20	20	20	20
R	0.99941555	0.999825598	0.999366401	0.999331031
R^2	0.998831442	0.999651226	0.998733204	0.99866251
$R^2_{adjusted}$	0.998714587	0.999616349	0.998606524	0.998528761
RSS	20.87727426	6.864754287	24.82734028	30.5476171

Graphical abstract of model fit presented as mean ± 1 SD of the fraction % of released carvedilol:

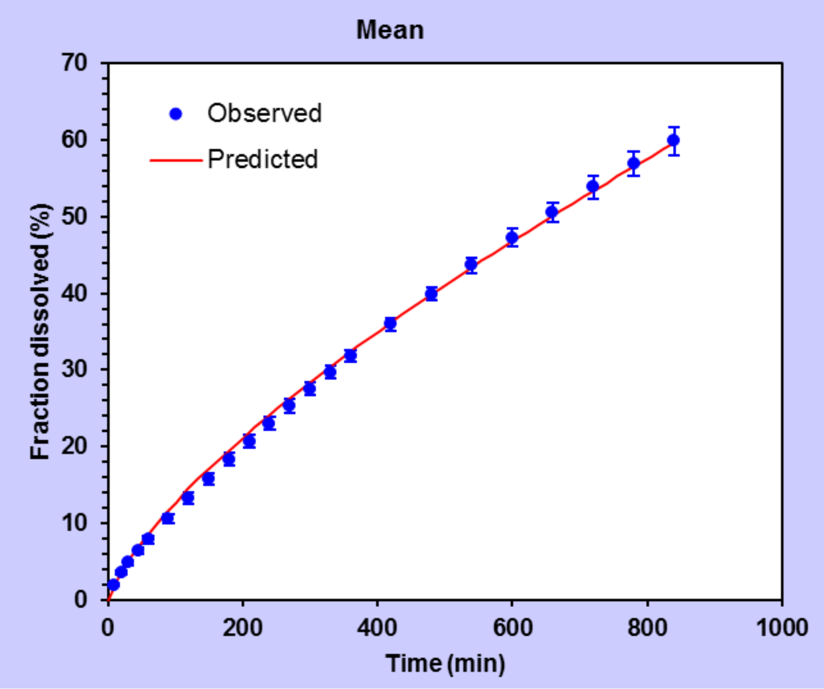

Supplementary materials – Model fitting summary – Ethocel^{TM} Standard 20 Premium (modelling up to app. 60 % of carvedilol released)

Graphical abstract of model fit presented as the fraction % of released carvedilol per tested tablet:

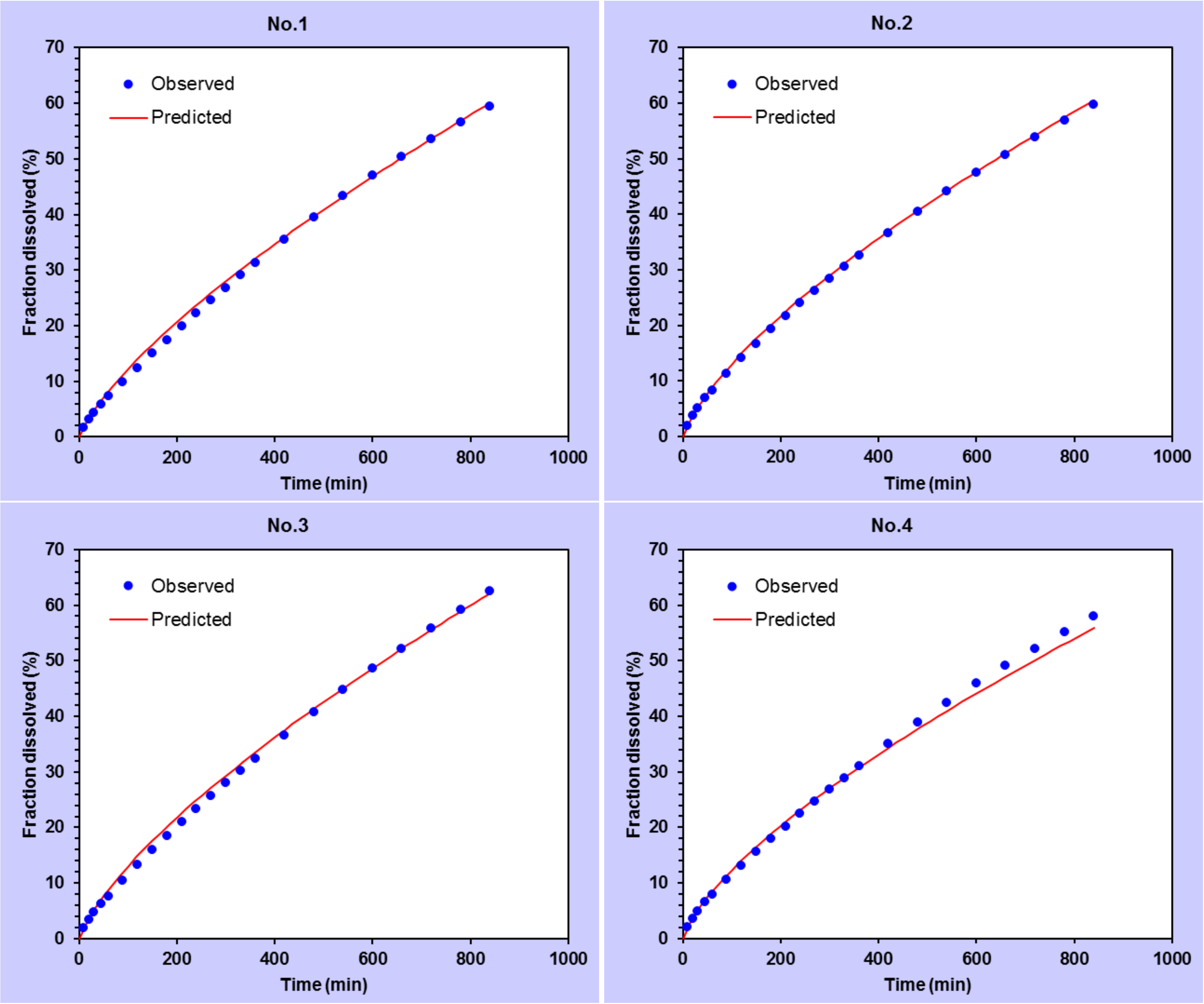

Supplementary materials – Model fitting summary – Ethocel™ Standard 20 Premium (modelling up to app. 60 % of carvedilol released)

Model: **Korsmeyer–Peppas with F_0**

Model equation: $F = F_0 + k_{KP} \cdot t^n$

Fitted model parameters per tested tablet (N = 4) with statistics – mean, standard deviation (SD), and relative standard deviation expressed in % (RSD%) (output from DDSolver):

Parameter	No.1	No.2	No.3	No.4	Mean	SD	RSD(%)
k_{KP}	0.188	0.231	0.213	0.240	0.218	0.023	10.623
n	0.857	0.824	0.843	0.813	0.834	0.019	2.326
F_0	0.830	1.016	0.928	1.030	0.951	0.092	9.700

Number of dissolution data points (N), degrees of freedom (df), and selected goodness of fit criteria – Pearson correlation coefficient (R), coefficient of determination (R^2), adjusted coefficient of determination ($R^2_{adjusted}$), and residual sum of squares (RSS) (manual calculation in MS Excel):

Parameter	No.1	No.2	No.3	No.4
N	23	23	23	23
df	20	20	20	20
R	0.999162495	0.998846132	0.999627848	0.999583201
R^2	0.998325691	0.997693595	0.999255835	0.999166575
$R^2_{adjusted}$	0.99815826	0.997462955	0.999181419	0.999083232
RSS	19.09236479	52.50851048	12.82609031	14.34464585

Graphical abstract of model fit presented as mean ± 1 SD of the fraction % of released carvedilol:

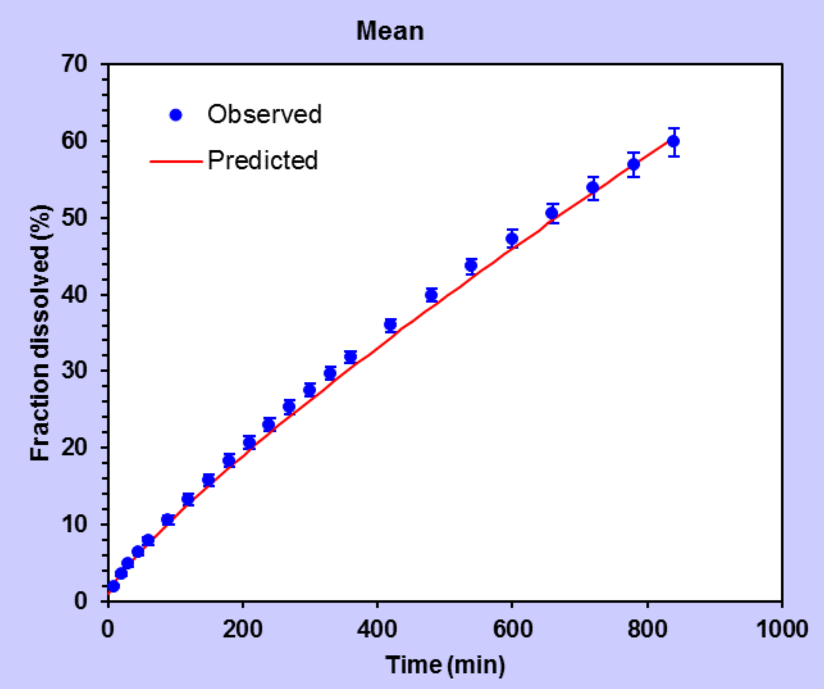

Supplementary materials – Model fitting summary – Ethocel™ Standard 20 Premium (modelling up to app. 60 % of carvedilol released)

Graphical abstract of model fit presented as the fraction % of released carvedilol per tested tablet:

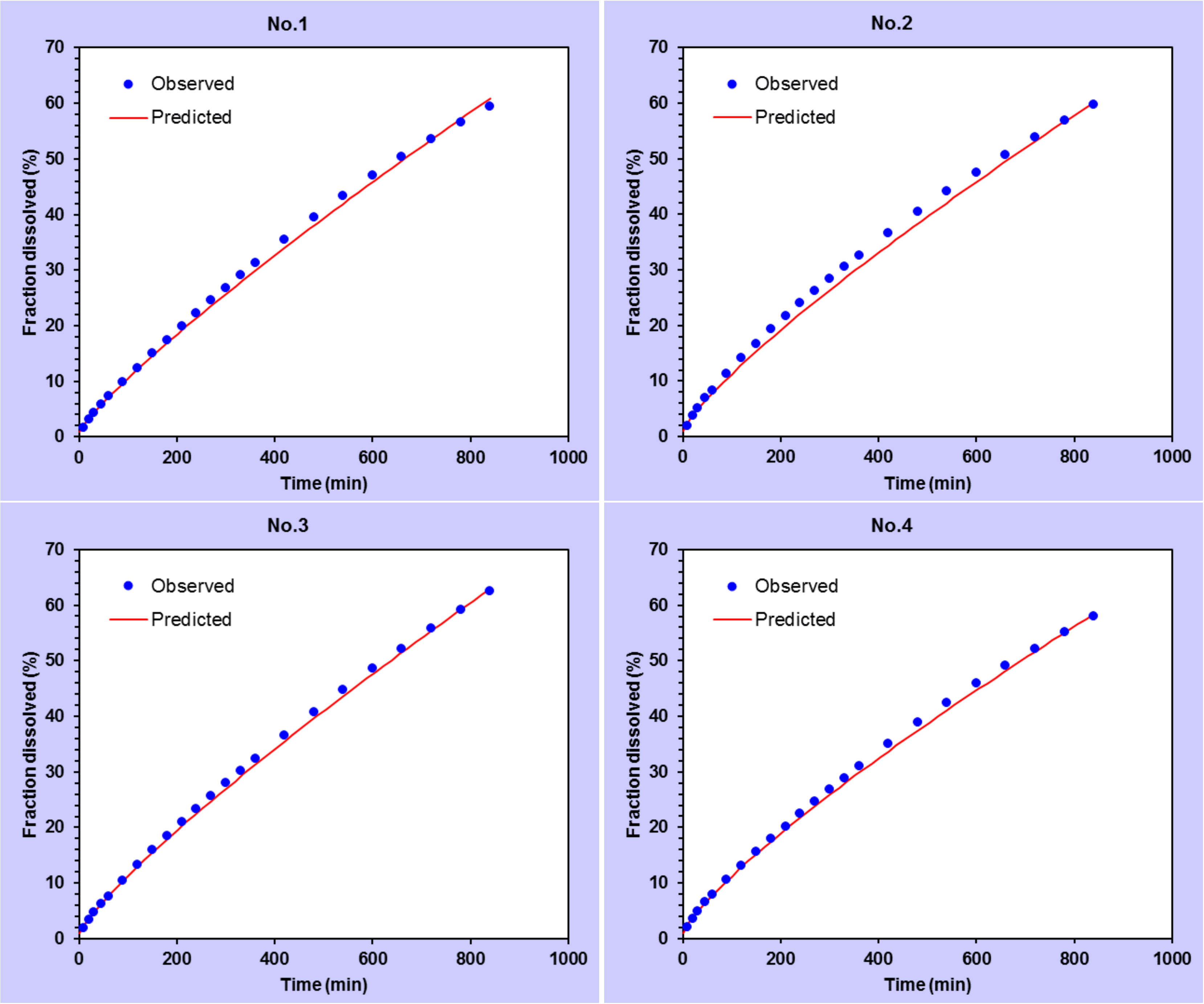

Supplementary materials – Model fitting summary – Ethocel™ Standard 20 Premium (modelling up to app. 60 % of carvedilol released)

Model: **Hixson–Crowell**

Model equation: $F = 100 \cdot [1 - (1 - k_{HC} \cdot t)^3]$

Fitted model parameters per tested tablet (N = 4) with statistics – mean, standard deviation (SD), and relative standard deviation expressed in % (RSD%) (output from DDSolver):

Parameter	No.1	No.2	No.3	No.4	Mean	SD	RSD(%)
k_{HC}	0.0003	0.0003	0.0003	0.0003	0.0003	0.0000	3.2660

Number of dissolution data points (N), degrees of freedom (df), and selected goodness of fit criteria – Pearson correlation coefficient (R), coefficient of determination (R^2), adjusted coefficient of determination ($R^2_{adjusted}$), and residual sum of squares (RSS) (manual calculation in MS Excel):

Parameter	No.1	No.2	No.3	No.4
N	23	23	23	23
df	22	22	22	22
R	0.99987627	0.99919287	0.999877082	0.99965939
R^2	0.999752555	0.998386391	0.999754179	0.999318897
$R^2_{adjusted}$	0.999752555	0.998386391	0.999754179	0.999318897
RSS	26.45789074	91.15356459	30.43782359	61.34916433

Graphical abstract of model fit presented as mean ± 1 SD of the fraction % of released carvedilol:

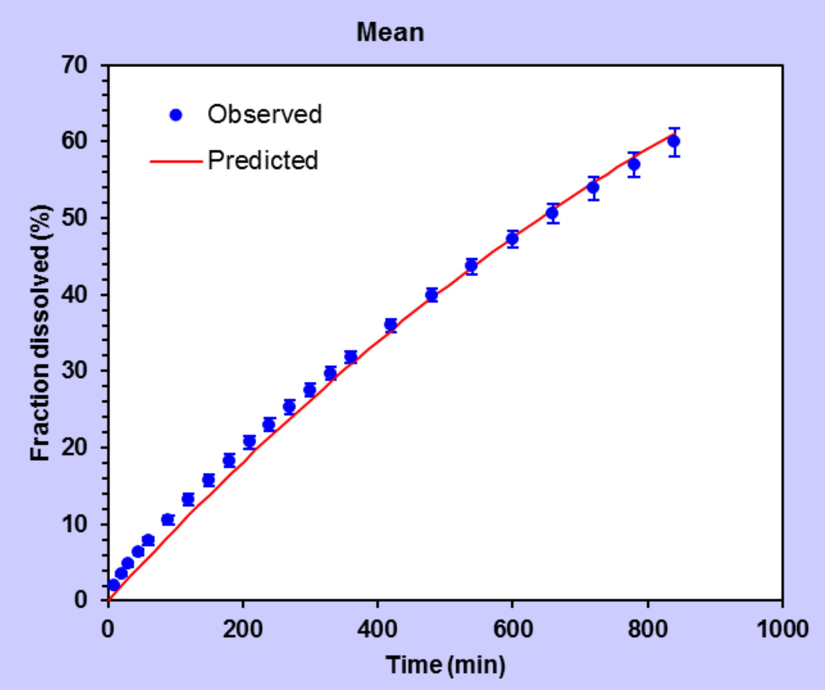

Supplementary materials – Model fitting summary – Ethocel™ Standard 20 Premium (modelling up to app. 60 % of carvedilol released)

Graphical abstract of model fit presented as the fraction % of released carvedilol per tested tablet:

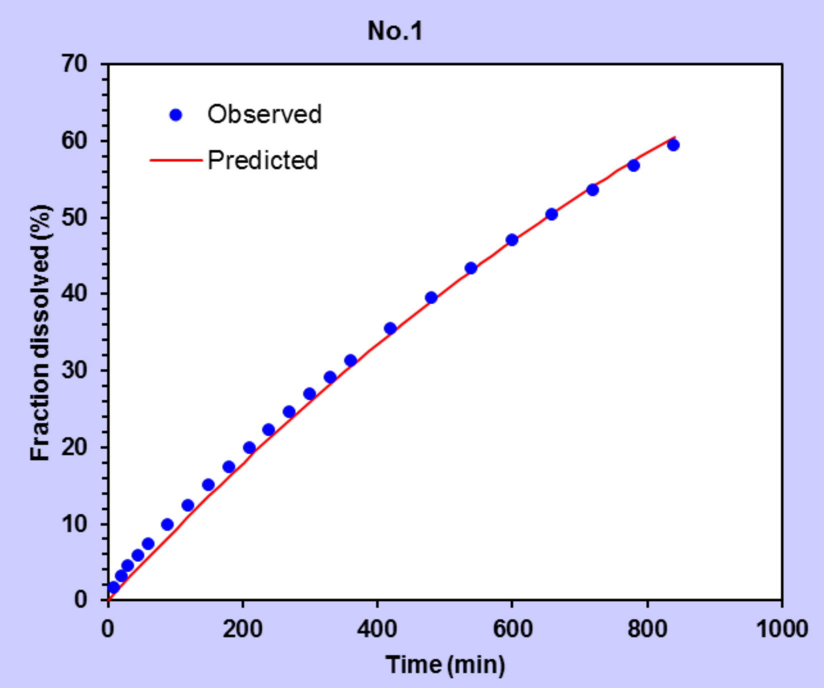

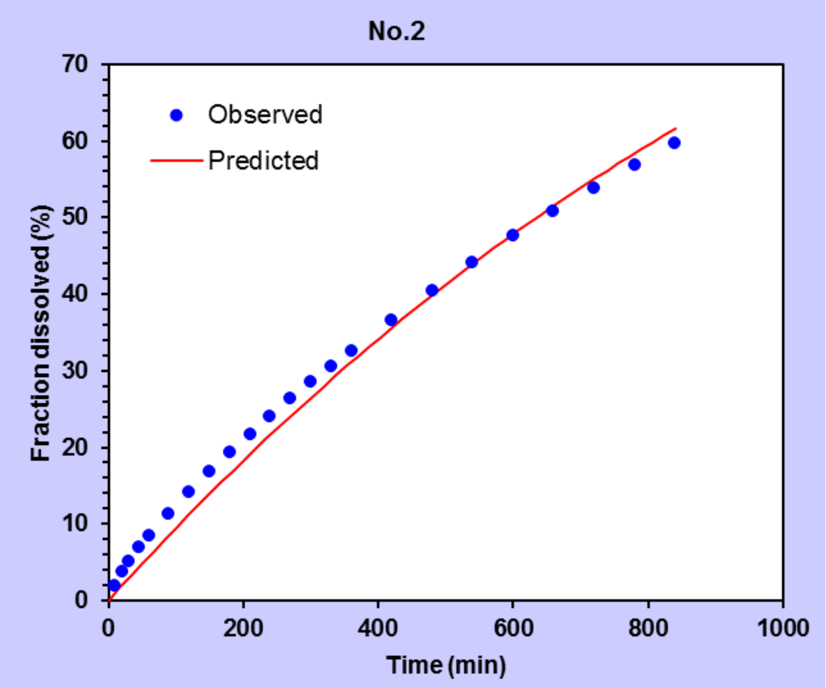

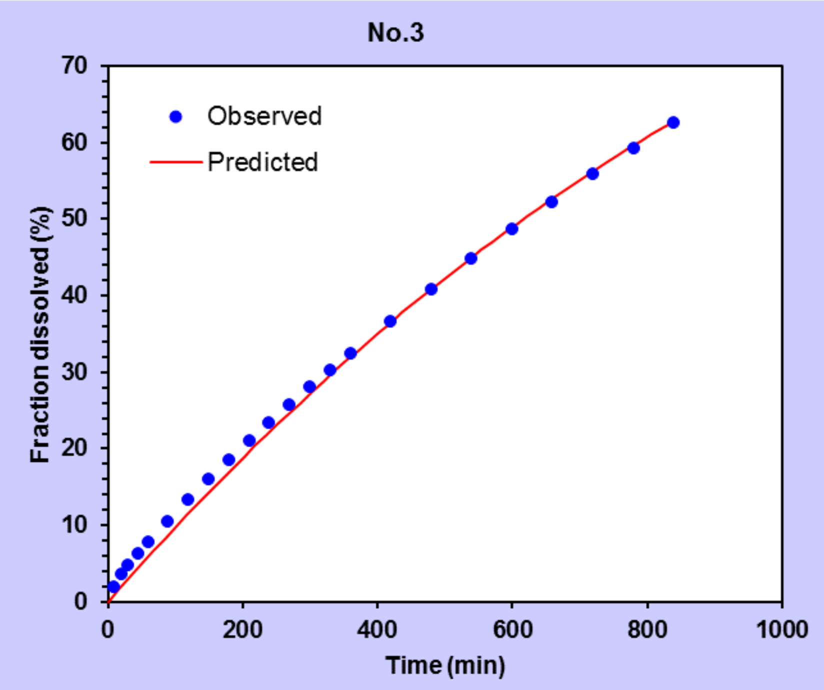

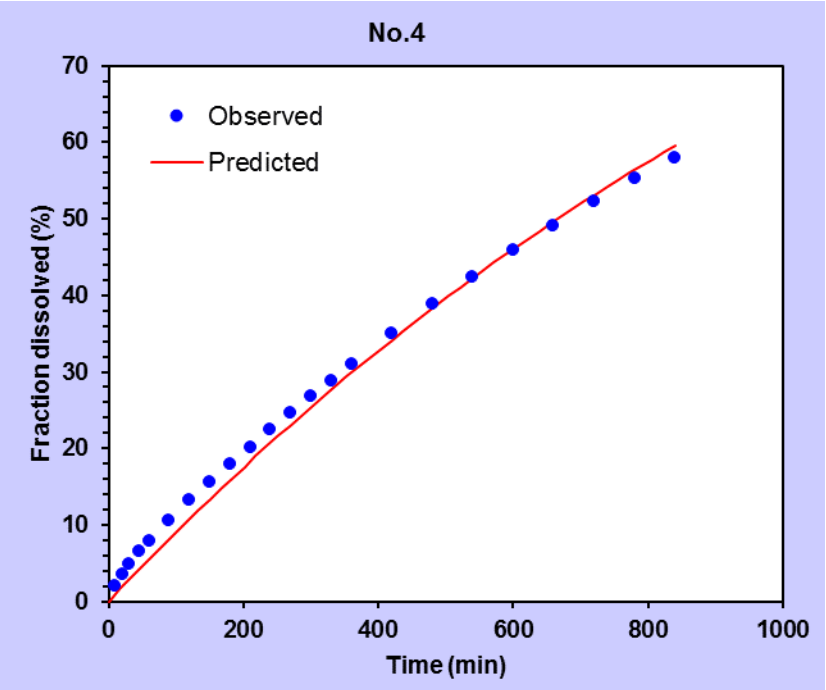

Supplementary materials – Model fitting summary – Ethocel™ Standard 20 Premium (modelling up to app. 60 % of carvedilol released)

Model: **Hixson–Crowell with T_{lag}**

Model equation: $F = 100 \cdot \left\{1 - \left[1 - k_{HC} \cdot (t - T_{lag})\right]^3\right\}$

Fitted model parameters per tested tablet (N = 4) with statistics – mean, standard deviation (SD), and relative standard deviation expressed in % (RSD%) (output from DDSolver):

Parameter	No.1	No.2	No.3	No.4	Mean	SD	RSD(%)
k_{HC}	0.0003	0.0003	0.0003	0.0003	0.0003	0.0000	4.1229
T_{lag}	-21.0964	-39.0425	-20.8271	-33.4850	-28.6128	9.1220	-31.8808

Number of dissolution data points (N), degrees of freedom (df), and selected goodness of fit criteria – Pearson correlation coefficient (R), coefficient of determination (R^2), adjusted coefficient of determination ($R^2_{adjusted}$), and residual sum of squares (RSS) (manual calculation in MS Excel):

Parameter	No.1	No.2	No.3	No.4
N	23	23	23	23
df	21	21	21	21
R	0.999836215	0.999005439	0.999869405	0.9995614
R^2	0.999672457	0.998011867	0.999738827	0.999122993
$R^2_{adjusted}$	0.99965686	0.997917194	0.99972639	0.999081231
RSS	2.542835867	14.97673332	2.097047891	6.235707526

Graphical abstract of model fit presented as mean ± 1 SD of the fraction % of released carvedilol:

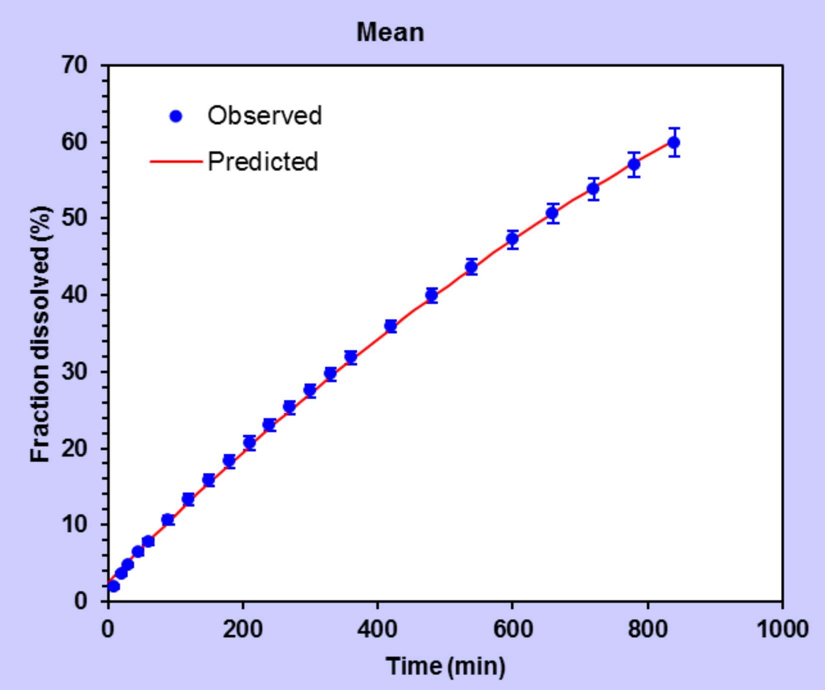

Supplementary materials – Model fitting summary – Ethocel™ Standard 20 Premium (modelling up to app. 60 % of carvedilol released)

Graphical abstract of model fit presented as the fraction % of released carvedilol per tested tablet:

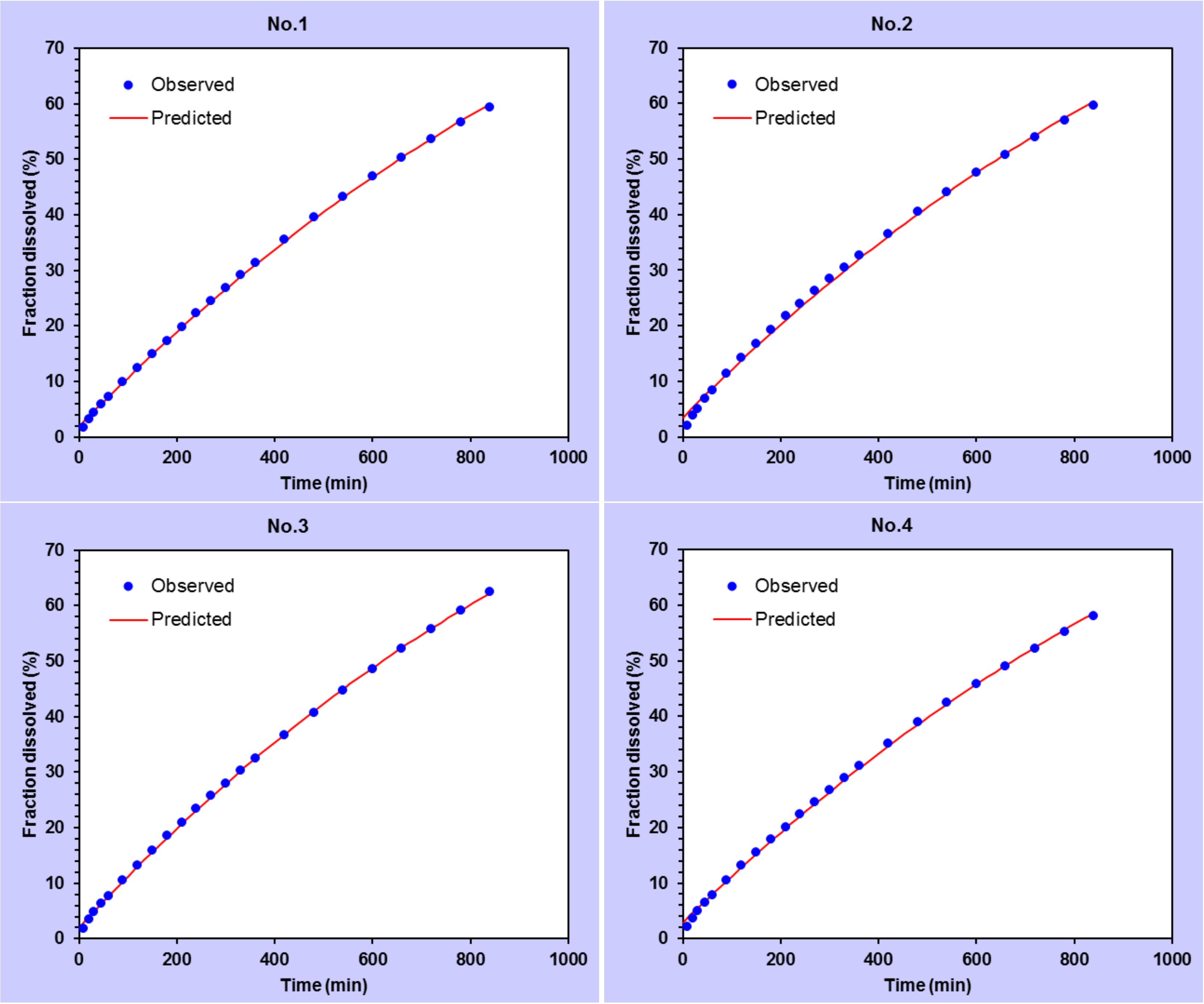

Supplementary materials – Model fitting summary – Ethocel™ Standard 20 Premium (modelling up to app. 60 % of carvedilol released)

Model: **Hopfenberg**

Model equation: $F = 100 \cdot [1 - (1 - k_{HB} \cdot t)^n]$

Fitted model parameters per tested tablet (N = 4) with statistics – mean, standard deviation (SD), and relative standard deviation expressed in % (RSD%) (output from DDSolver):

Parameter	No.1	No.2	No.3	No.4	Mean	SD	RSD(%)
k_{HB}	0.0003	0.0003	0.0003	0.0003	0.0003	0.0000	3.8252
n	3.3340	3.0000	3.3340	3.0000	3.1670	0.1928	6.0886

Number of dissolution data points (N), degrees of freedom (df), and selected goodness of fit criteria – Pearson correlation coefficient (R), coefficient of determination (R^2), adjusted coefficient of determination ($R^2_{adjusted}$), and residual sum of squares (RSS) (manual calculation in MS Excel):

Parameter	No.1	No.2	No.3	No.4
N	23	23	23	23
df	21	21	21	21
R	0.999932224	0.99919287	0.999868159	0.99965939
R^2	0.999864453	0.998386391	0.999736336	0.999318897
$R^2_{adjusted}$	0.999857999	0.998309552	0.99972378	0.999286463
RSS	25.43503251	91.15356459	30.24647671	61.34916433

Graphical abstract of model fit presented as mean ± 1 SD of the fraction % of released carvedilol:

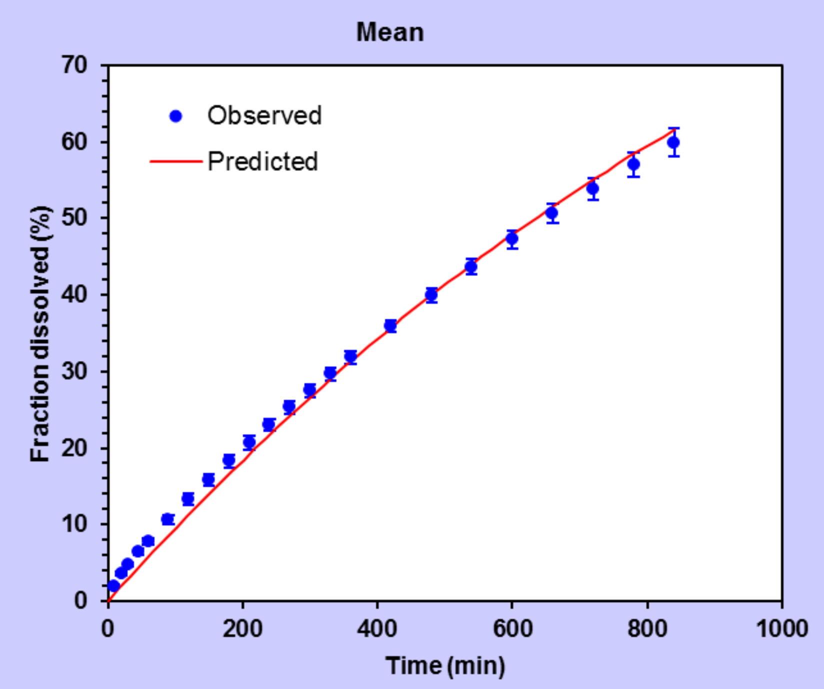

Supplementary materials – Model fitting summary – Ethocel™ Standard 20 Premium (modelling up to app. 60 % of carvedilol released)

Graphical abstract of model fit presented as the fraction % of released carvedilol per tested tablet:

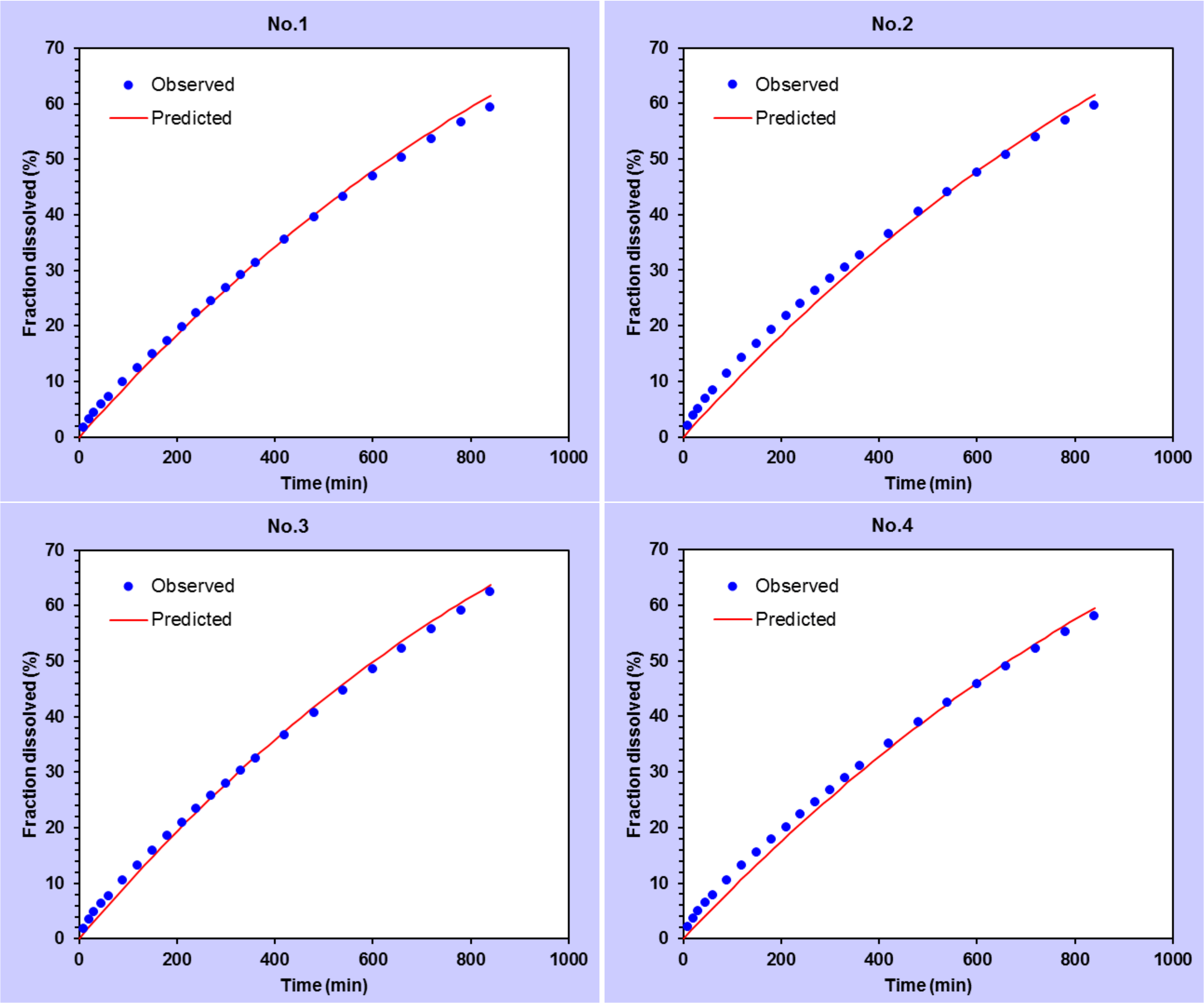

Supplementary materials – Model fitting summary – Ethocel™ Standard 20 Premium (modelling up to app. 60 % of carvedilol released)

Model: **Hopfenberg with T_{lag}**

Model equation: $F = 100 \cdot \{1 - [1 - k_{HB} \cdot (t - T_{lag})]^n\}$

Fitted model parameters per tested tablet (N = 4) with statistics – mean, standard deviation (SD), and relative standard deviation expressed in % (RSD%) (output from DDSolver):

Parameter	No.1	No.2	No.3	No.4	Mean	SD	RSD(%)
k_{HB}	0.0003	0.0003	0.0003	0.0003	0.0003	0.0000	4.1229
n	3.0000	3.0000	3.0000	3.0000	3.0000	0.0000	0.0000
T_{lag}	-21.0964	-39.0425	-20.8271	-33.4850	-28.6128	9.1220	-31.8808

Number of dissolution data points (N), degrees of freedom (df), and selected goodness of fit criteria – Pearson correlation coefficient (R), coefficient of determination (R^2), adjusted coefficient of determination ($R^2_{adjusted}$), and residual sum of squares (RSS) (manual calculation in MS Excel):

Parameter	No.1	No.2	No.3	No.4
N	23	23	23	23
df	20	20	20	20
R	0.999836215	0.999005439	0.999869405	0.9995614
R^2	0.999672457	0.998011867	0.999738827	0.999122993
$R^2_{adjusted}$	0.999639703	0.997813054	0.99971271	0.999035292
RSS	2.542835867	14.97673332	2.097047891	6.235707526

Graphical abstract of model fit presented as mean ± 1 SD of the fraction % of released carvedilol:

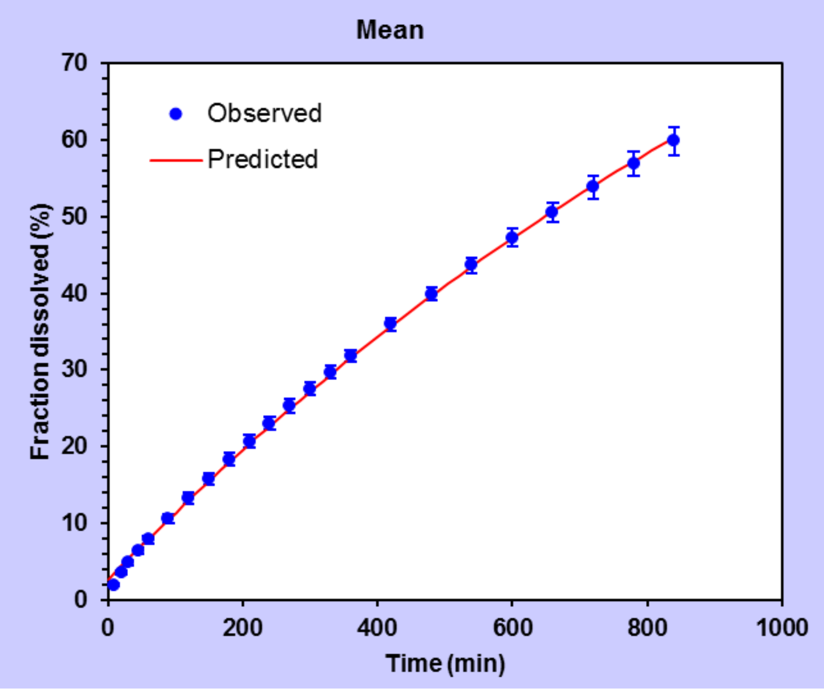

Supplementary materials – Model fitting summary – Ethocel™ Standard 20 Premium (modelling up to app. 60 % of carvedilol released)

Graphical abstract of model fit presented as the fraction % of released carvedilol per tested tablet:

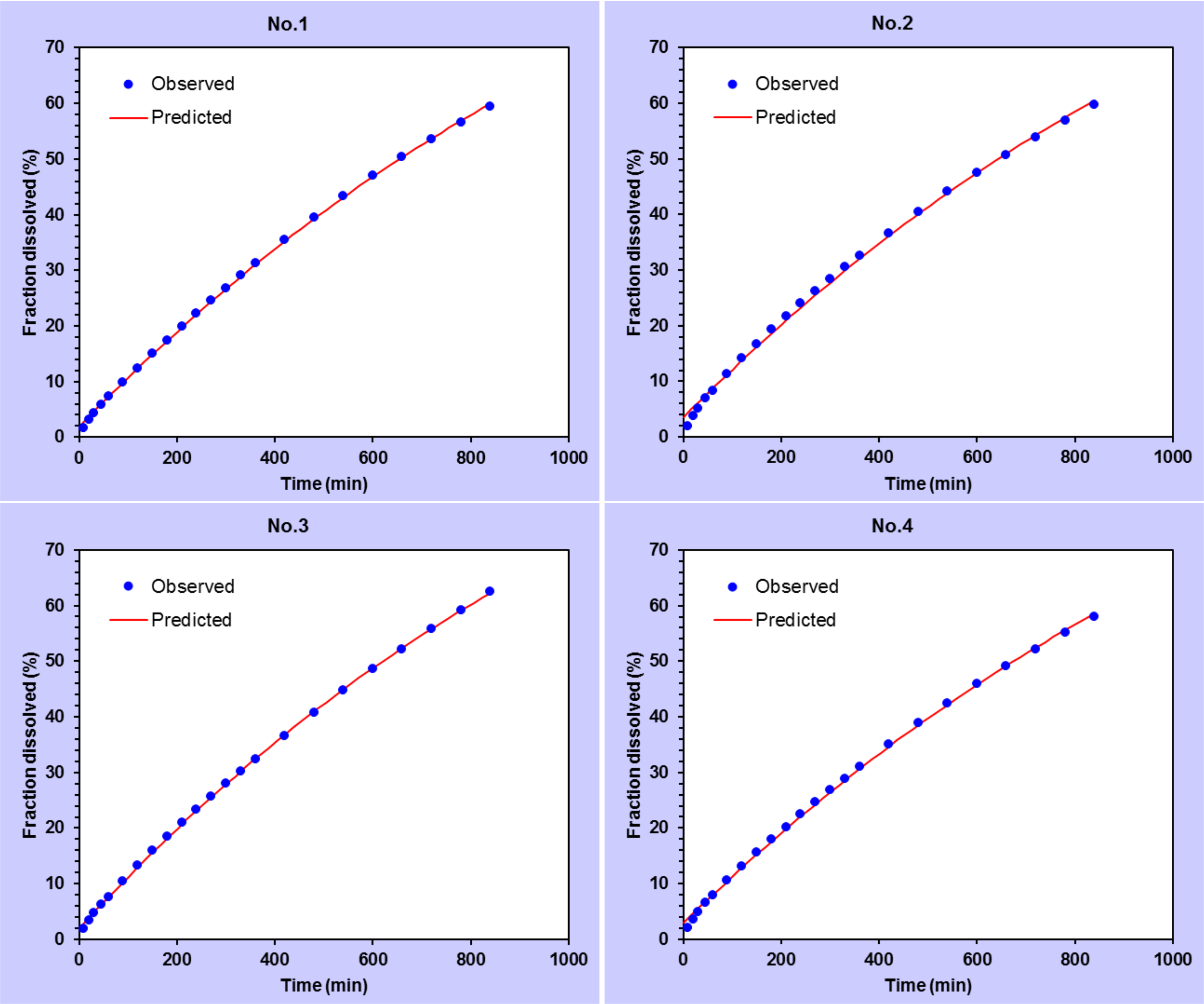

Supplementary materials – Model fitting summary – Ethocel™ Standard 20 Premium (modelling up to app. 60 % of carvedilol released)

Model: **Baker–Lonsdale**

Model equation: $\frac{3}{2}\cdot\left[1-\left(1-\frac{F}{100}\right)^{\frac{2}{3}}\right]-\frac{F}{100}=k_{BL}\cdot t$

Fitted model parameters per tested tablet (N = 4) with statistics – mean, standard deviation (SD), and relative standard deviation expressed in % (RSD%) (output from DDSolver):

Parameter	No.1	No.2	No.3	No.4	Mean	SD	RSD(%)
k_{BL}	0.00010	0.00005	0.00011	0.00005	0.00007	0.00003	42.79662

Number of dissolution data points (N), degrees of freedom (df), and selected goodness of fit criteria – Pearson correlation coefficient (R), coefficient of determination (R^2), adjusted coefficient of determination ($R^2_{adjusted}$), and residual sum of squares (RSS) (manual calculation in MS Excel):

Parameter	No.1	No.2	No.3	No.4
N	23	23	23	23
df	22	22	22	22
R	0.983402222	0.990502017	0.982605751	0.987964756
R^2	0.96707993	0.981094246	0.965514063	0.976074359
$R^2_{adjusted}$	0.96707993	0.981094246	0.965514063	0.976074359
RSS	1933.79248	1068.182706	2117.758743	1020.162784

Graphical abstract of model fit presented as mean ± 1 SD of the fraction % of released carvedilol:

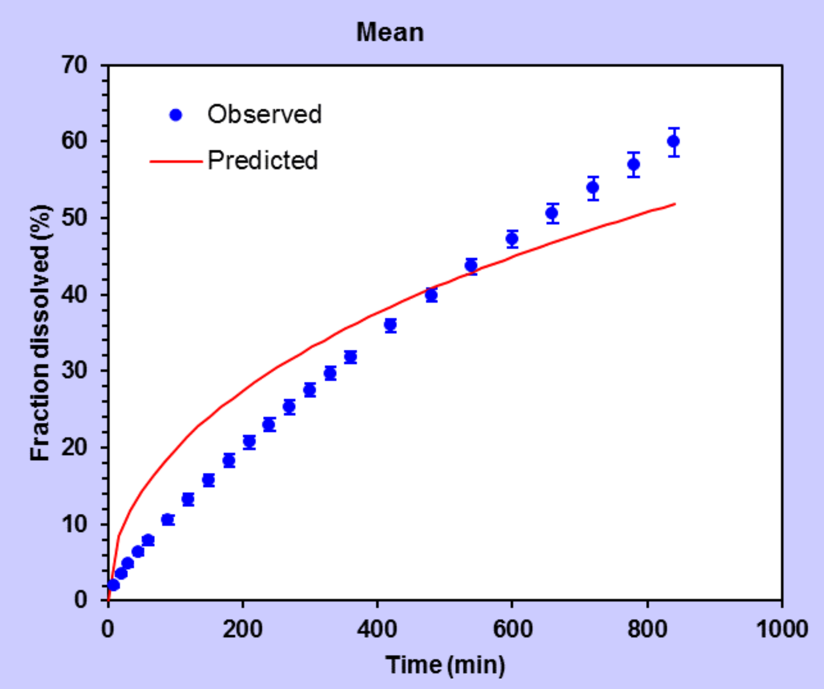

Supplementary materials – Model fitting summary – Ethocel™ Standard 20 Premium (modelling up to app. 60 % of carvedilol released)

Graphical abstract of model fit presented as the fraction % of released carvedilol per tested tablet:

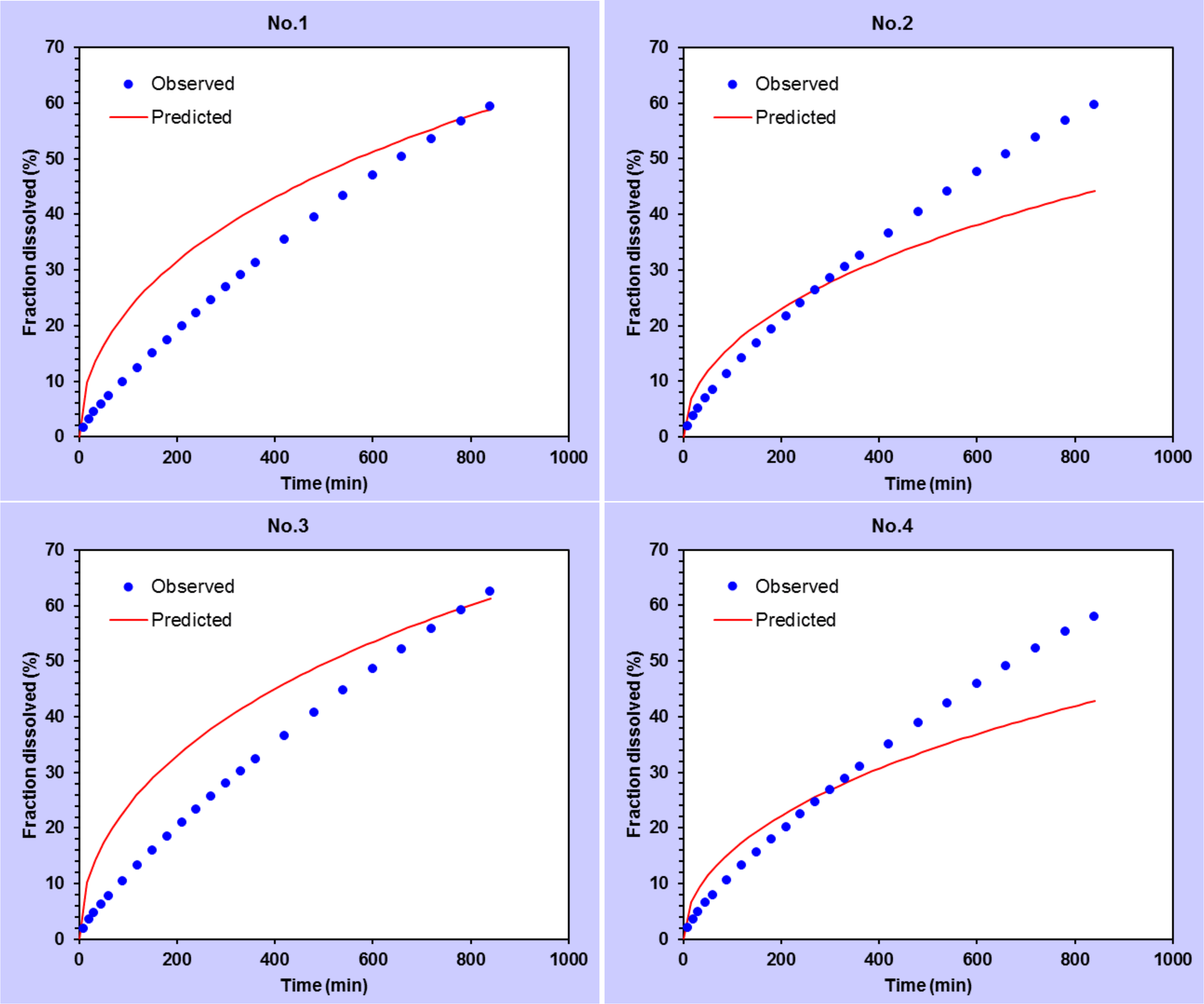

Supplementary materials – Model fitting summary – Ethocel™ Standard 20 Premium (modelling up to app. 60 % of carvedilol released)

Model: **Baker–Lonsdale with T_{lag}**

Model equation: $\frac{3}{2}\cdot\left[1-\left(1-\frac{F}{100}\right)^{\frac{2}{3}}\right]-\frac{F}{100}=k_{BL}\cdot(t-T_{lag})$

Fitted model parameters per tested tablet (N = 4) with statistics – mean, standard deviation (SD), and relative standard deviation expressed in % (RSD%) (output from DDSolver):

Parameter	No.1	No.2	No.3	No.4	Mean	SD	RSD(%)
k_{BL}	0.0001	0.0001	0.0001	0.0001	0.0001	0.0000	6.7923
T_{lag}	93.6644	84.8980	94.8483	88.7664	90.5443	4.5935	5.0732

Number of dissolution data points (N), degrees of freedom (df), and selected goodness of fit criteria – Pearson correlation coefficient (R), coefficient of determination (R^2), adjusted coefficient of determination ($R^2_{adjusted}$), and residual sum of squares (RSS) (manual calculation in MS Excel):

Parameter	No.1	No.2	No.3	No.4
N	23	23	23	23
df	21	21	21	21
R	0.976657705	0.981599469	0.97593111	0.978475454
R^2	0.953860273	0.963537517	0.952441531	0.957414215
$R^2_{adjusted}$	0.951663143	0.961801208	0.950176842	0.95538632
RSS	433.5844617	352.2471133	490.6310597	384.1146193

Graphical abstract of model fit presented as mean ± 1 SD of the fraction % of released carvedilol:

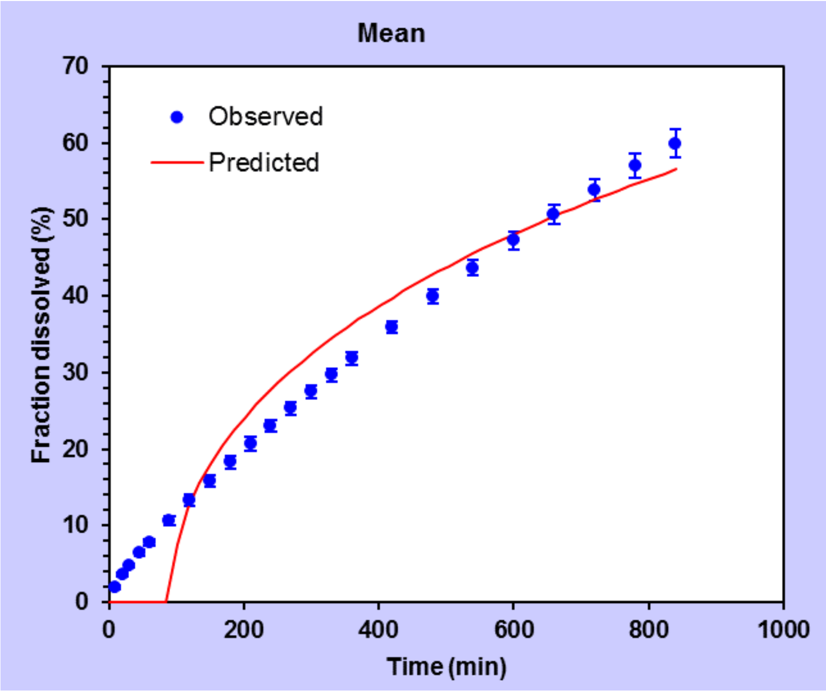

Supplementary materials – Model fitting summary – Ethocel™ Standard 20 Premium (modelling up to app. 60 % of carvedilol released)

Graphical abstract of model fit presented as the fraction % of released carvedilol per tested tablet:

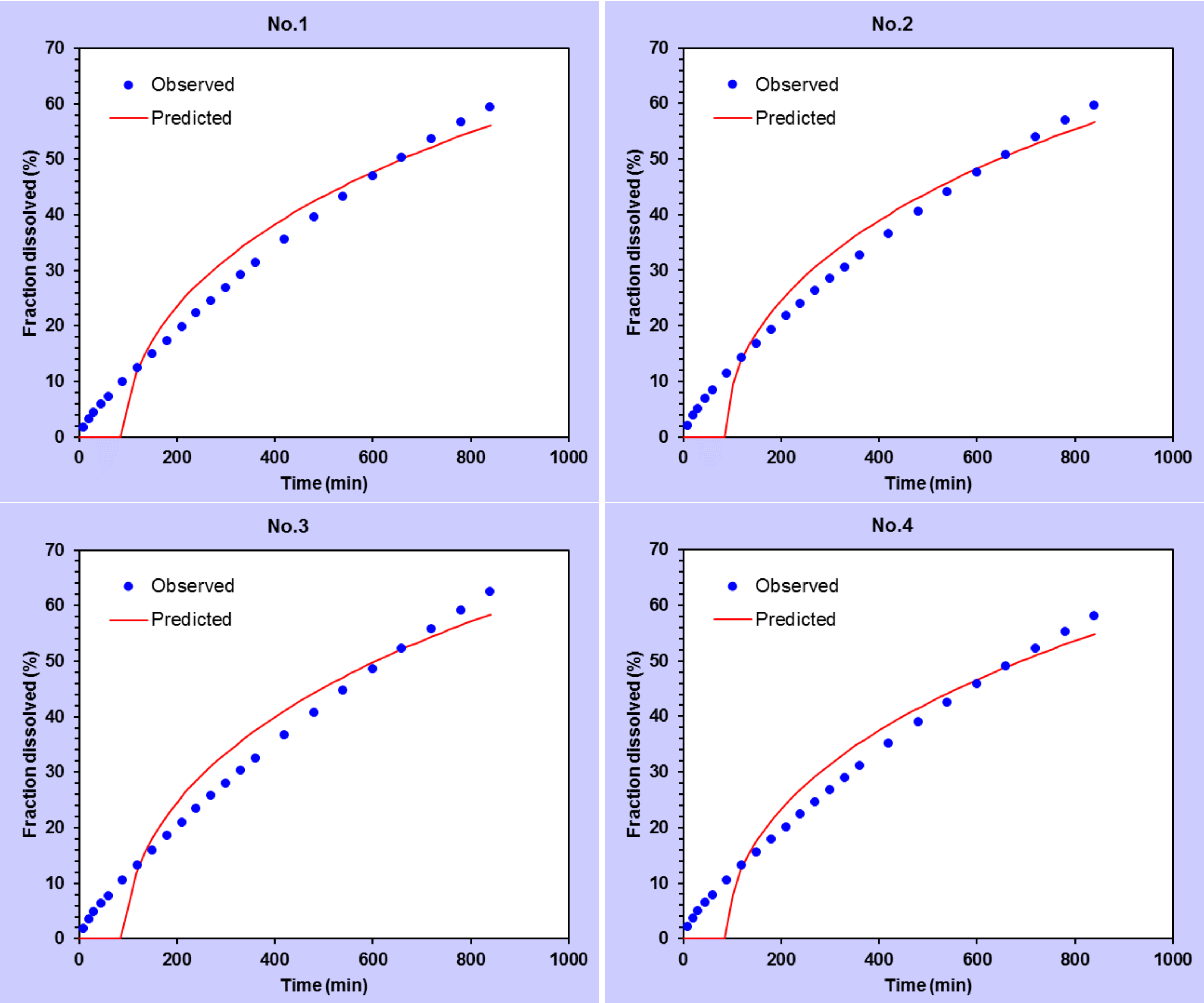

Supplementary materials – Model fitting summary – Ethocel™ Standard 20 Premium (modelling up to app. 60 % of carvedilol released)

Model: **Makoid–Banakar**

Model equation: $F = k_{MB} \cdot t^{n} \cdot e^{-k \cdot t}$

Fitted model parameters per tested tablet (N = 4) with statistics – mean, standard deviation (SD), and relative standard deviation expressed in % (RSD%) (output from DDSolver):

Parameter	No.1	No.2	No.3	No.4	Mean	SD	RSD(%)
k_{MB}	0.28039	0.35777	0.32753	0.38786	0.33839	0.04584	13.54796
n	0.79797	0.77292	0.77638	0.73776	0.77126	0.02493	3.23236
k	-0.00001	0.00010	-0.00004	-0.00007	-0.00001	0.00008	-1111.51342

Number of dissolution data points (N), degrees of freedom (df), and selected goodness of fit criteria – Pearson correlation coefficient (R), coefficient of determination (R^2), adjusted coefficient of determination ($R^2_{adjusted}$), and residual sum of squares (RSS) (manual calculation in MS Excel):

Parameter	No.1	No.2	No.3	No.4
N	23	23	23	23
df	20	20	20	20
R	0.999652414	0.999969949	0.999866884	0.999722835
R^2	0.999304949	0.999939899	0.999733786	0.999445747
$R^2_{adjusted}$	0.999235444	0.999933889	0.999707164	0.999390321
RSS	5.260696985	0.447095232	2.176182854	3.873024913

Graphical abstract of model fit presented as mean ± 1 SD of the fraction % of released carvedilol:

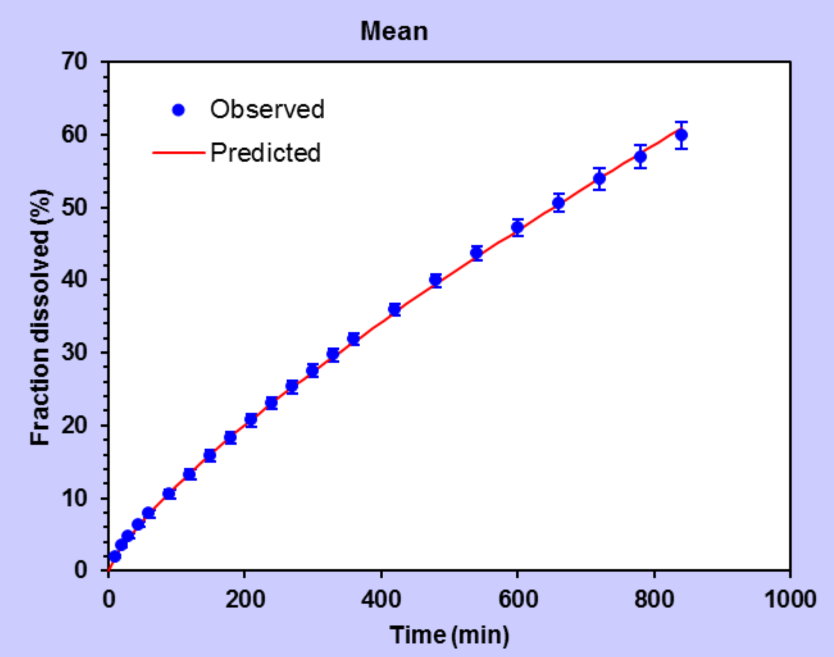

Supplementary materials – Model fitting summary – Ethocel™ Standard 20 Premium (modelling up to app. 60 % of carvedilol released)

Graphical abstract of model fit presented as the fraction % of released carvedilol per tested tablet:

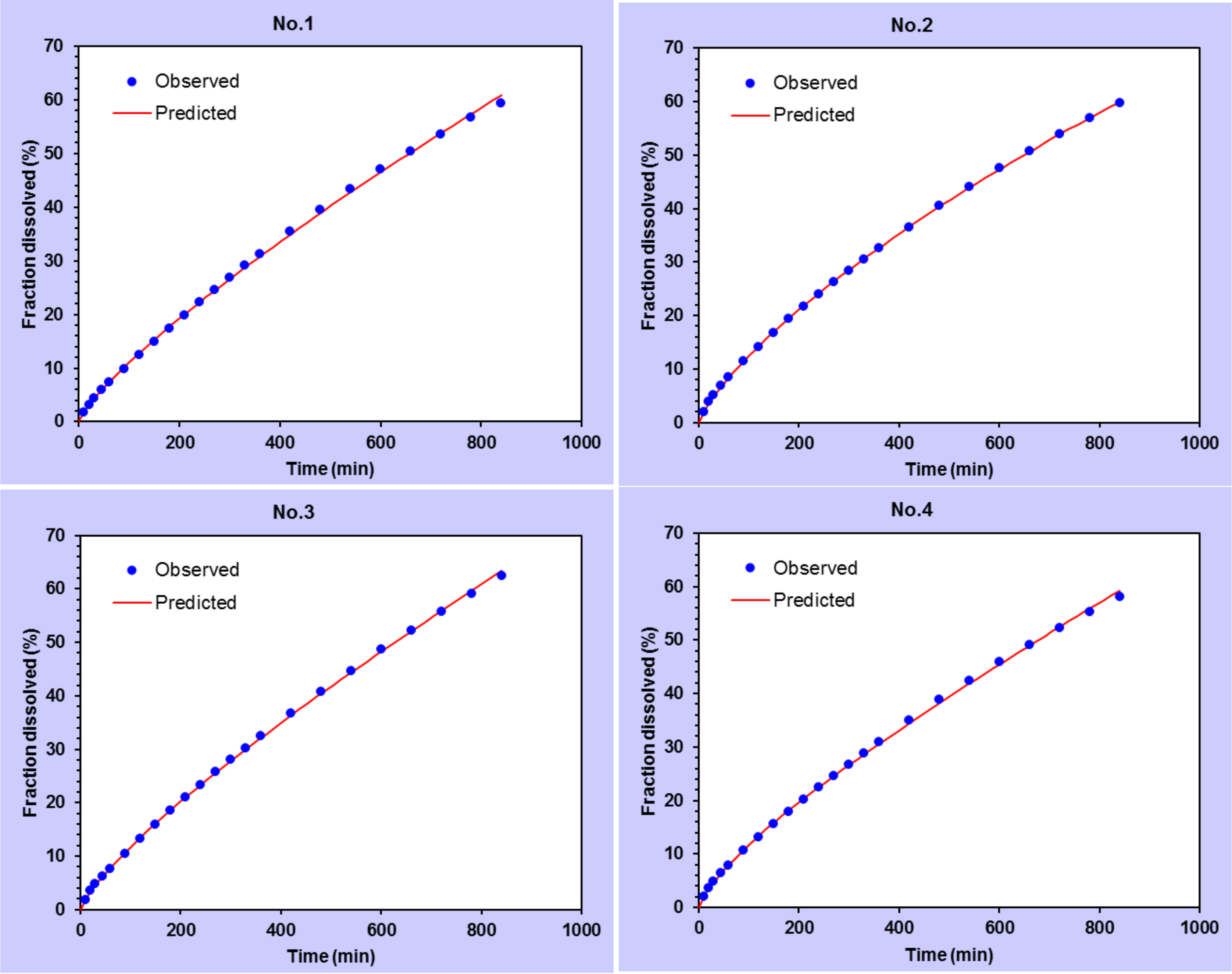

Supplementary materials – Model fitting summary – Ethocel™ Standard 20 Premium (modelling up to app. 60 % of carvedilol released)

Model: **Makoid–Banakar with T_{lag}**

Model equation: $F = k_{MB} \cdot (t - T_{lag})^n \cdot e^{-k \cdot (t - T_{lag})}$

Fitted model parameters per tested tablet (N = 4) with statistics – mean, standard deviation (SD), and relative standard deviation expressed in % (RSD%) (output from DDSolver):

Parameter	No.1	No.2	No.3	No.4	Mean	SD	RSD(%)
k_{MB}	0.45857	0.57404	0.52889	0.61246	0.54349	0.06612	12.16563
n	0.69546	0.67448	0.67653	0.64252	0.67225	0.02195	3.26539
k	-0.00028	-0.00016	-0.00031	-0.00033	-0.00027	0.00007	-27.48824
T_{lag}	4.00000	4.00000	4.00000	4.00000	4.00000	0.00000	0.00000

Number of dissolution data points (N), degrees of freedom (df), and selected goodness of fit criteria – Pearson correlation coefficient (R), coefficient of determination (R^2), adjusted coefficient of determination ($R^2_{adjusted}$), and residual sum of squares (RSS) (manual calculation in MS Excel):

Parameter	No.1	No.2	No.3	No.4
N	23	23	23	23
df	19	19	19	19
R	0.998520476	0.999465958	0.9990004	0.998723719
R^2	0.997043142	0.998932201	0.9980018	0.997449067
$R^2_{adjusted}$	0.99657627	0.998763601	0.997686294	0.997046288
RSS	23.5877483	8.142812406	17.36310625	18.73794153

Graphical abstract of model fit presented as mean ± 1 SD of the fraction % of released carvedilol:

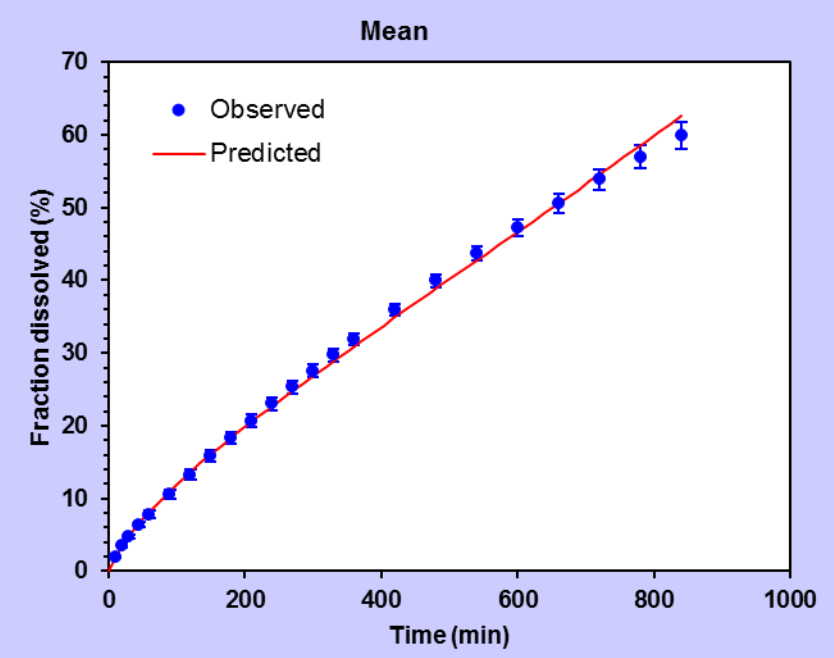

Supplementary materials – Model fitting summary – Ethocel™ Standard 20 Premium (modelling up to app. 60 % of carvedilol released)

Graphical abstract of model fit presented as the fraction % of released carvedilol per tested tablet:

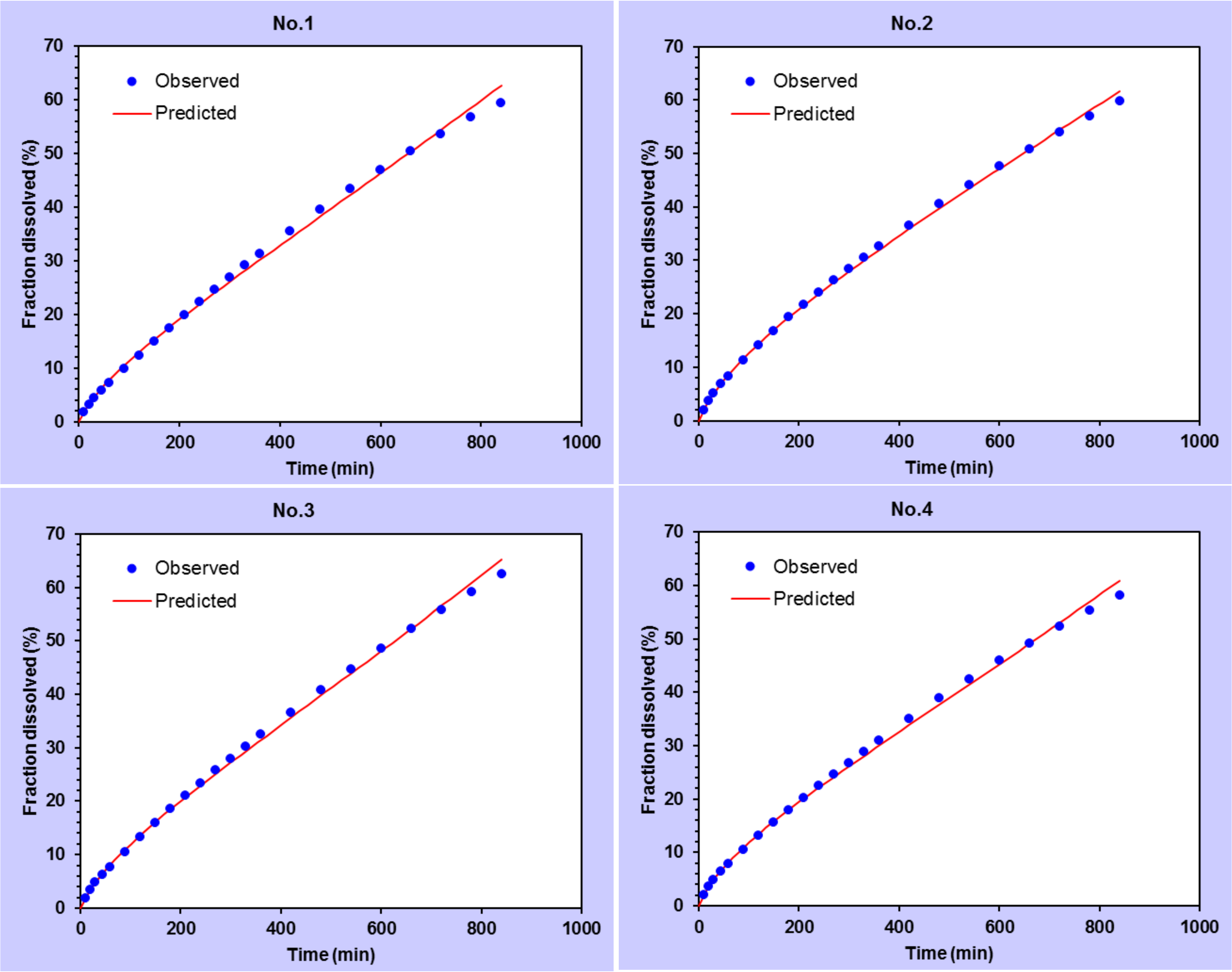

Supplementary materials – Model fitting summary – Ethocel™ Standard 20 Premium (modelling up to app. 60 % of carvedilol released)

Model: **Peppas–Sahlin_1**

Model equation: $F = k_1 \cdot t^m + k_2 \cdot t^{2m}$

Fitted model parameters per tested tablet (N = 4) with statistics – mean, standard deviation (SD), and relative standard deviation expressed in % (RSD%) (output from DDSolver):

Parameter	No.1	No.2	No.3	No.4	Mean	SD	RSD(%)
k_1	0.481	0.786	0.530	0.639	0.609	0.135	22.202
k_2	0.119	0.105	0.122	0.107	0.113	0.009	7.715
m	0.450	0.450	0.450	0.450	0.450	0.000	0.000

Number of dissolution data points (N), degrees of freedom (df), and selected goodness of fit criteria – Pearson correlation coefficient (R), coefficient of determination (R^2), adjusted coefficient of determination ($R^2_{adjusted}$), and residual sum of squares (RSS) (manual calculation in MS Excel):

Parameter	No.1	No.2	No.3	No.4
N	23	23	23	23
df	20	20	20	20
R	0.99941395	0.999434382	0.999745802	0.999616006
R^2	0.998828244	0.998869084	0.999491668	0.99923216
$R^2_{adjusted}$	0.998711069	0.998755993	0.999440835	0.999155376
RSS	9.200043806	9.154541007	4.376538018	5.623866982

Graphical abstract of model fit presented as mean ± 1 SD of the fraction % of released carvedilol:

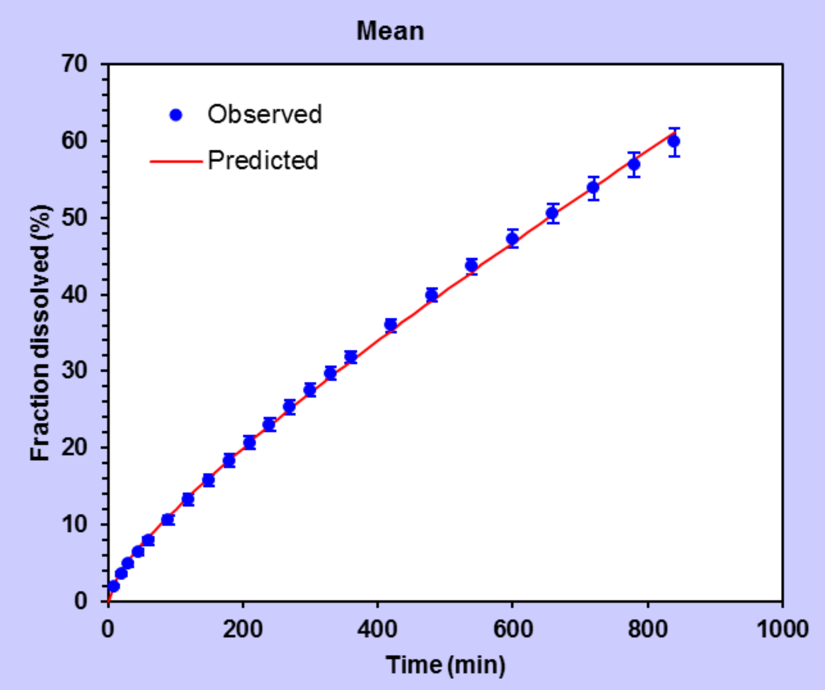

Supplementary materials – Model fitting summary – Ethocel™ Standard 20 Premium (modelling up to app. 60 % of carvedilol released)

Graphical abstract of model fit presented as the fraction % of released carvedilol per tested tablet:

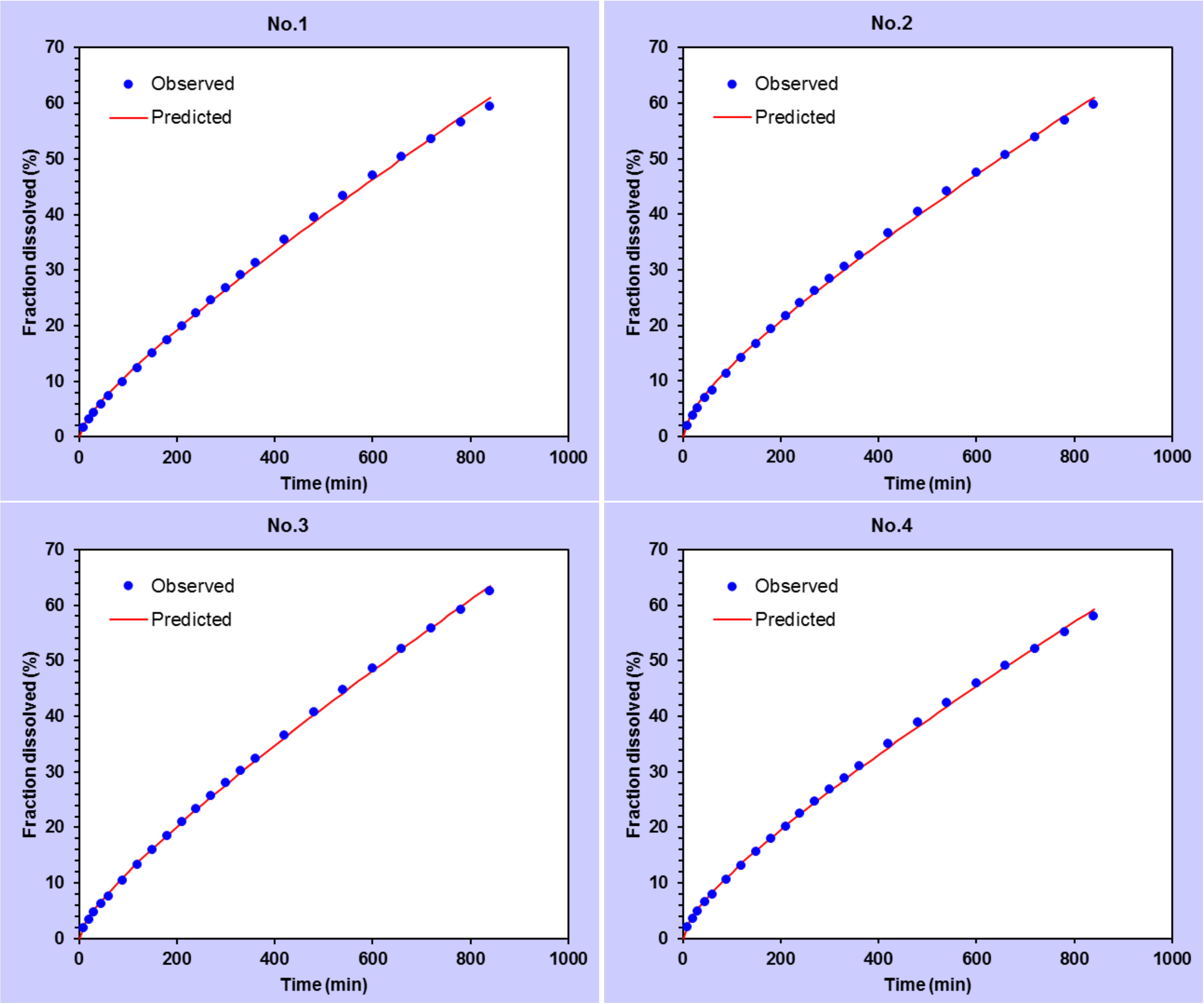

Supplementary materials – Model fitting summary – Ethocel™ Standard 20 Premium (modelling up to app. 60 % of carvedilol released)

Model: **Peppas–Sahlin_1 with T_{lag}**

Model equation: $F = k_1 \cdot (t - T_{lag})^m + k_2 \cdot (t - T_{lag})^{2m}$

Fitted model parameters per tested tablet (N = 4) with statistics – mean, standard deviation (SD), and relative standard deviation expressed in % (RSD%) (output from DDSolver):

Parameter	No.1	No.2	No.3	No.4	Mean	SD	RSD(%)
k_1	0.550	0.861	0.602	0.708	0.680	0.137	20.184
k_2	0.116	0.101	0.119	0.104	0.110	0.009	8.027
m	0.450	0.450	0.450	0.450	0.450	0.000	0.000
T_{lag}	4.000	6.000	4.000	4.000	4.500	1.000	22.222

Number of dissolution data points (N), degrees of freedom (df), and selected goodness of fit criteria – Pearson correlation coefficient (R), coefficient of determination (R^2), adjusted coefficient of determination ($R^2_{adjusted}$), and residual sum of squares (RSS) (manual calculation in MS Excel):

Parameter	No.1	No.2	No.3	No.4
N	23	23	23	23
df	19	19	19	19
R	0.999509537	0.999637829	0.999818908	0.999702713
R^2	0.999019315	0.999275789	0.999637848	0.999405514
$R^2_{adjusted}$	0.99886447	0.99916144	0.999580666	0.999311648
RSS	7.51664512	5.564843519	2.989629536	4.192504412

Graphical abstract of model fit presented as mean ± 1 SD of the fraction % of released carvedilol:

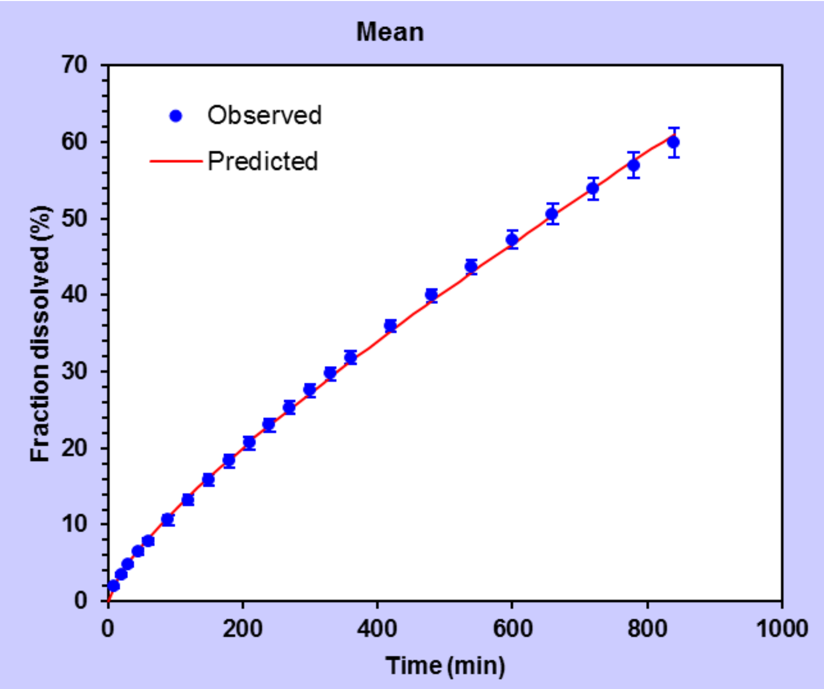

Supplementary materials – Model fitting summary – Ethocel^{TM} Standard 20 Premium (modelling up to app. 60 % of carvedilol released)

Graphical abstract of model fit presented as the fraction % of released carvedilol per tested tablet:

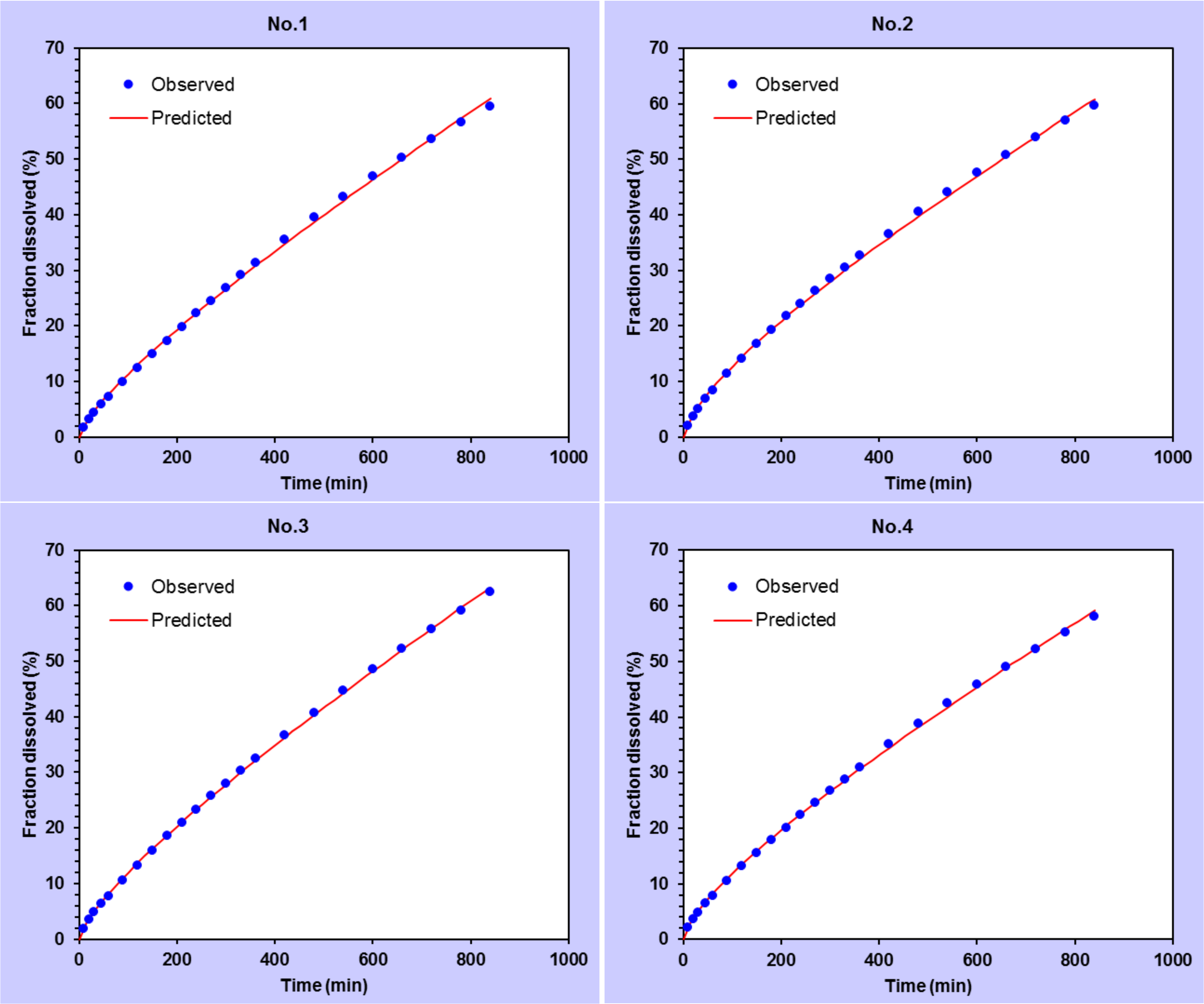

Supplementary materials – Model fitting summary – Ethocel™ Standard 20 Premium (modelling up to app. 60 % of carvedilol released)

Model: **Peppas–Sahlin_2**

Model equation: $F = k_1 \cdot t^{0.5} + k_2 \cdot t$

Fitted model parameters per tested tablet (N = 4) with statistics – mean, standard deviation (SD), and relative standard deviation expressed in % (RSD%) (output from DDSolver):

Parameter	No.1	No.2	No.3	No.4	Mean	SD	RSD(%)
k_1	0.647	0.872	0.692	0.752	0.741	0.097	13.150
k_2	0.051	0.043	0.052	0.045	0.048	0.004	9.335

Number of dissolution data points (N), degrees of freedom (df), and selected goodness of fit criteria – Pearson correlation coefficient (R), coefficient of determination (R^2), adjusted coefficient of determination ($R^2_{adjusted}$), and residual sum of squares (RSS) (manual calculation in MS Excel):

Parameter	No.1	No.2	No.3	No.4
N	23	23	23	23
df	21	21	21	21
R	0.999155046	0.999277482	0.999556816	0.999447378
R^2	0.998310806	0.998555486	0.999113829	0.998895061
$R^2_{adjusted}$	0.998230368	0.9984867	0.99907163	0.998842445
RSS	13.8243143	12.24163494	8.004753062	8.446908835

Graphical abstract of model fit presented as mean ± 1 SD of the fraction % of released carvedilol:

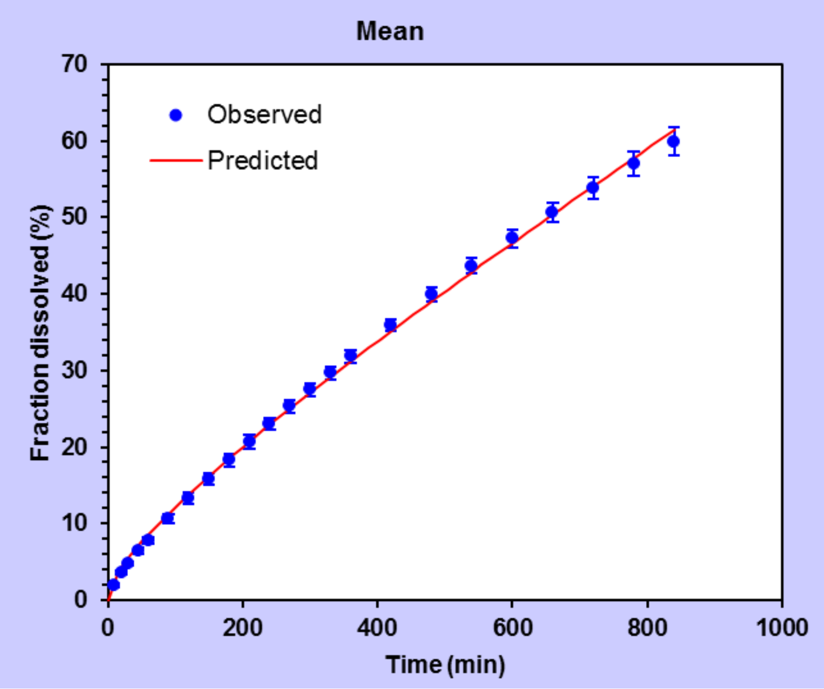

Supplementary materials – Model fitting summary – Ethocel™ Standard 20 Premium (modelling up to app. 60 % of carvedilol released)

Graphical abstract of model fit presented as the fraction % of released carvedilol per tested tablet:

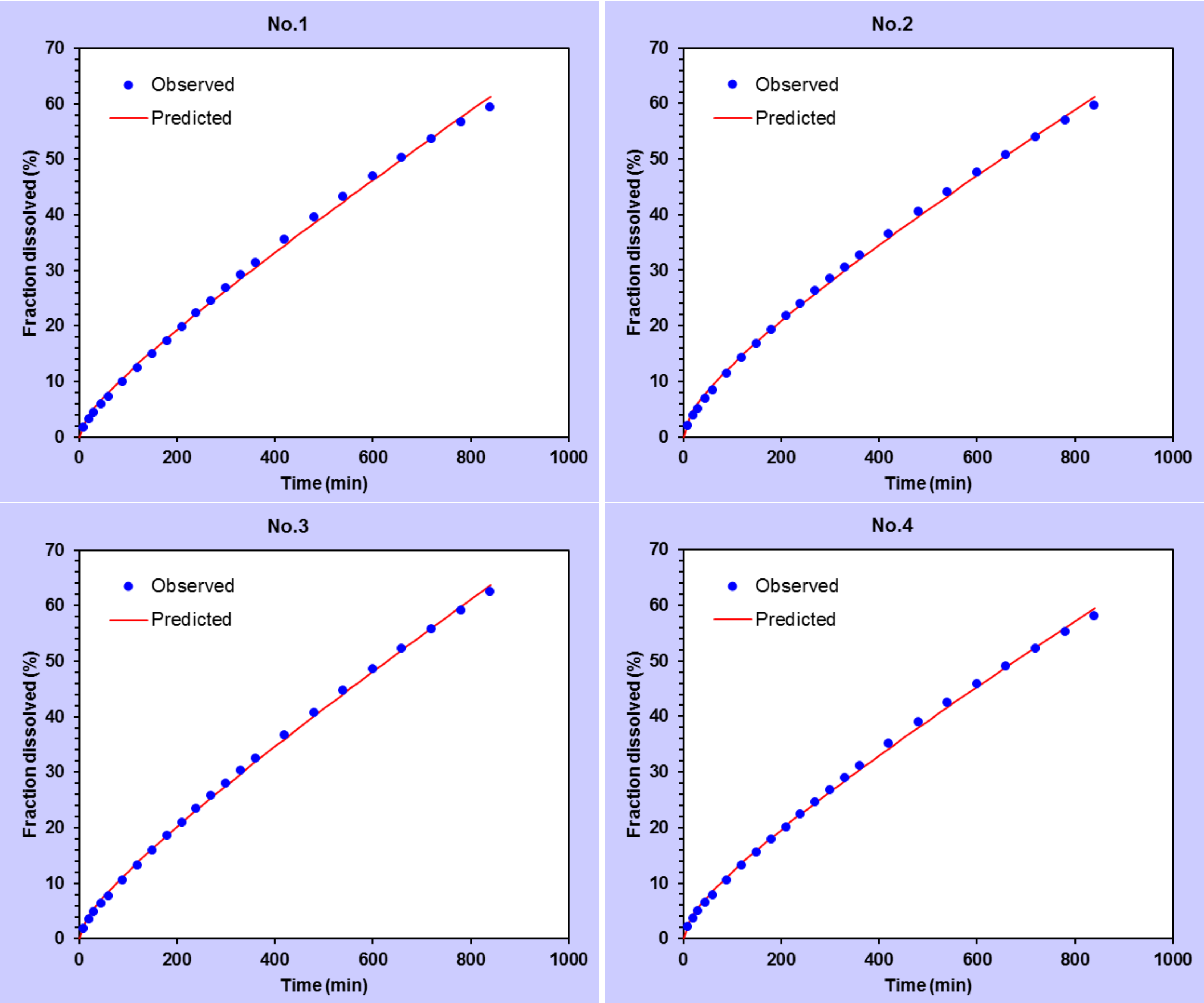

Supplementary materials – Model fitting summary – Ethocel™ Standard 20 Premium (modelling up to app. 60 % of carvedilol released)

Model: **Peppas–Sahlin_2 with T_{lag}**

Model equation: $F = k_1 \cdot (t - T_{lag})^{0.5} + k_2 \cdot (t - T_{lag})$

Fitted model parameters per tested tablet (N = 4) with statistics – mean, standard deviation (SD), and relative standard deviation expressed in % (RSD%) (output from DDSolver):

Parameter	No.1	No.2	No.3	No.4	Mean	SD	RSD(%)
k_1	0.696	0.925	0.743	0.801	0.791	0.099	12.486
k_2	0.049	0.041	0.050	0.043	0.046	0.004	9.771
T_{lag}	6.000	6.000	6.000	6.000	6.000	0.000	0.000

Number of dissolution data points (N), degrees of freedom (df), and selected goodness of fit criteria – Pearson correlation coefficient (R), coefficient of determination (R^2), adjusted coefficient of determination ($R^2_{adjusted}$), and residual sum of squares (RSS) (manual calculation in MS Excel):

Parameter	No.1	No.2	No.3	No.4
N	23	23	23	23
df	20	20	20	20
R	0.999320716	0.999527612	0.999692321	0.999585852
R^2	0.998641893	0.999055447	0.999384737	0.999171875
$R^2_{adjusted}$	0.998506083	0.998960992	0.99932321	0.999089063
RSS	10.48508197	7.250901615	5.254925495	5.991163172

Graphical abstract of model fit presented as mean ± 1 SD of the fraction % of released carvedilol:

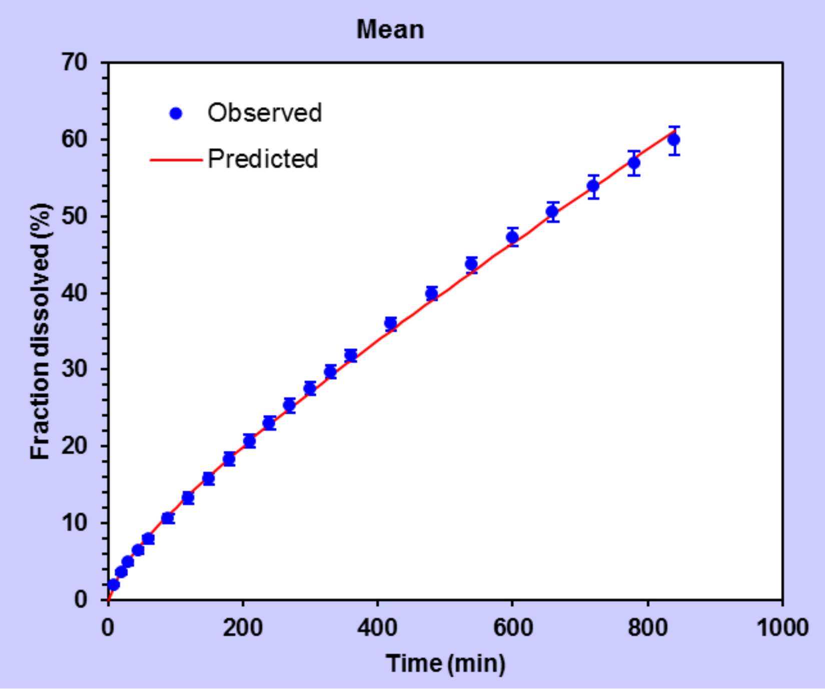

Supplementary materials – Model fitting summary – Ethocel™ Standard 20 Premium (modelling up to app. 60 % of carvedilol released)

Graphical abstract of model fit presented as the fraction % of released carvedilol per tested tablet:

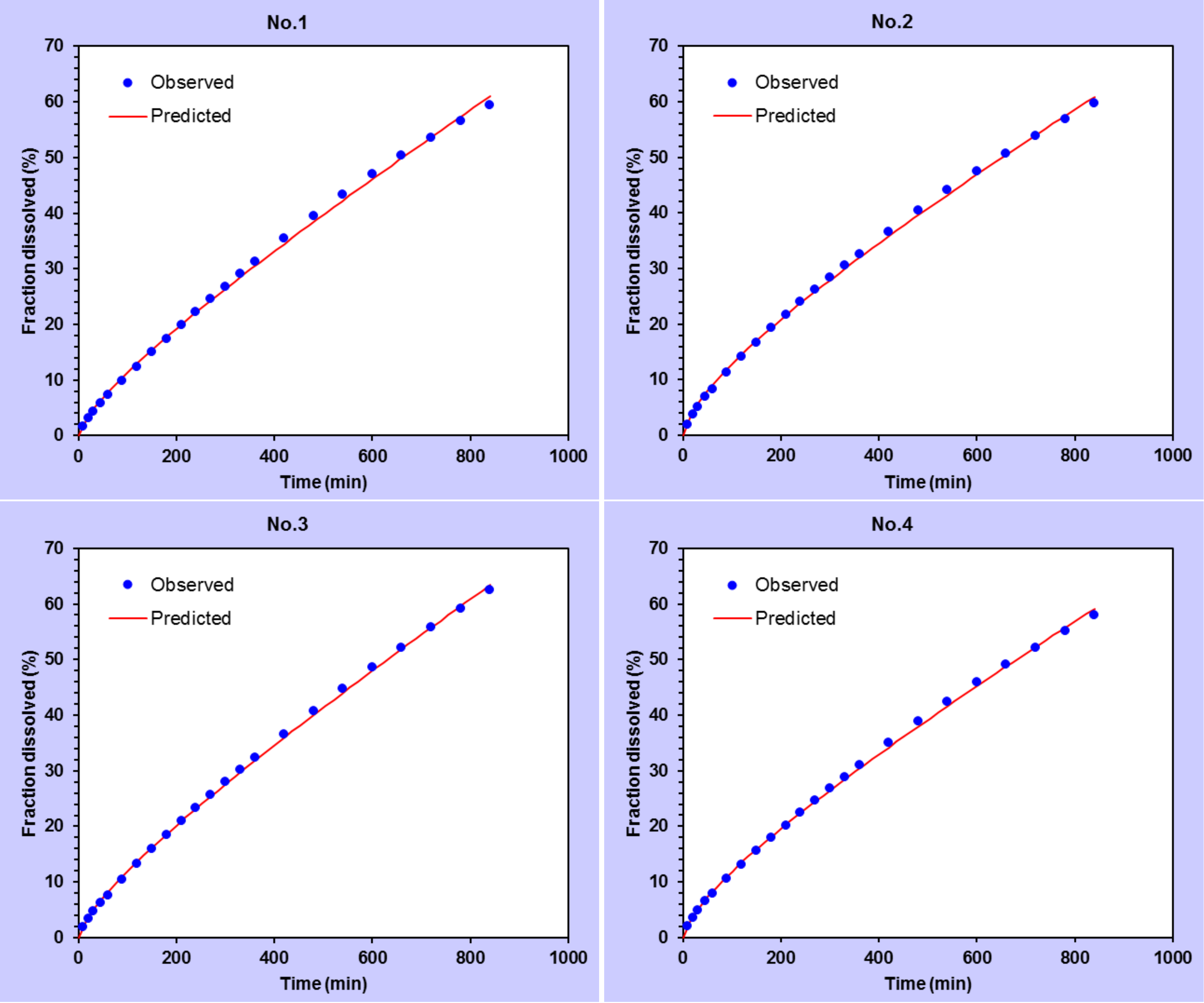

Supplementary materials – Model fitting summary – Ethocel™ Standard 20 Premium (modelling up to app. 60 % of carvedilol released)

Model: **Quadratic**

Model equation: $F = 100 \cdot (k_1 \cdot t^2 + k_2 \cdot t)$

Fitted model parameters per tested tablet (N = 4) with statistics – mean, standard deviation (SD), and relative standard deviation expressed in % (RSD%) (output from DDSolver):

Parameter	No.1	No.2	No.3	No.4	Mean	SD	RSD(%)
k_1	-0.0000004	-0.0000005	-0.0000004	-0.0000004	-0.0000004	0.0000000	-12.0140457
k_2	0.0010128	0.0010989	0.0010526	0.0010261	0.0010476	0.0000380	3.6256687

Number of dissolution data points (N), degrees of freedom (df), and selected goodness of fit criteria – Pearson correlation coefficient (R), coefficient of determination (R^2), adjusted coefficient of determination ($R^2_{adjusted}$), and residual sum of squares (RSS) (manual calculation in MS Excel):

Parameter	No.1	No.2	No.3	No.4
N	23	23	23	23
df	21	21	21	21
R	0.999676716	0.998964081	0.999425576	0.999206863
R^2	0.999353536	0.997929236	0.998851483	0.998414355
$R^2_{adjusted}$	0.999322752	0.997830628	0.998796791	0.998338848
RSS	11.65324847	33.70975953	20.70299941	26.358944

Graphical abstract of model fit presented as mean ± 1 SD of the fraction % of released carvedilol:

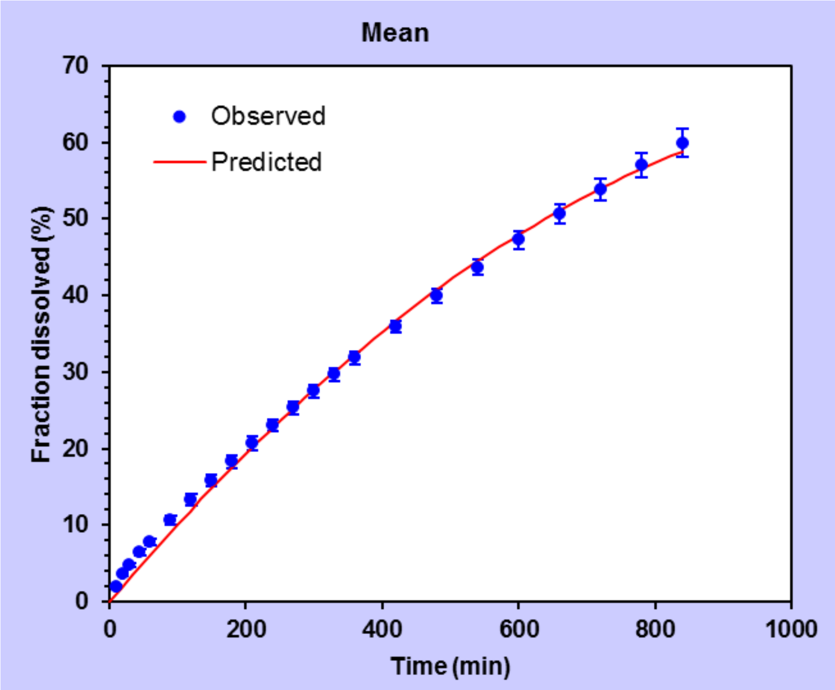

Supplementary materials – Model fitting summary – Ethocel^{TM} Standard 20 Premium (modelling up to app. 60 % of carvedilol released)

Graphical abstract of model fit presented as the fraction % of released carvedilol per tested tablet:

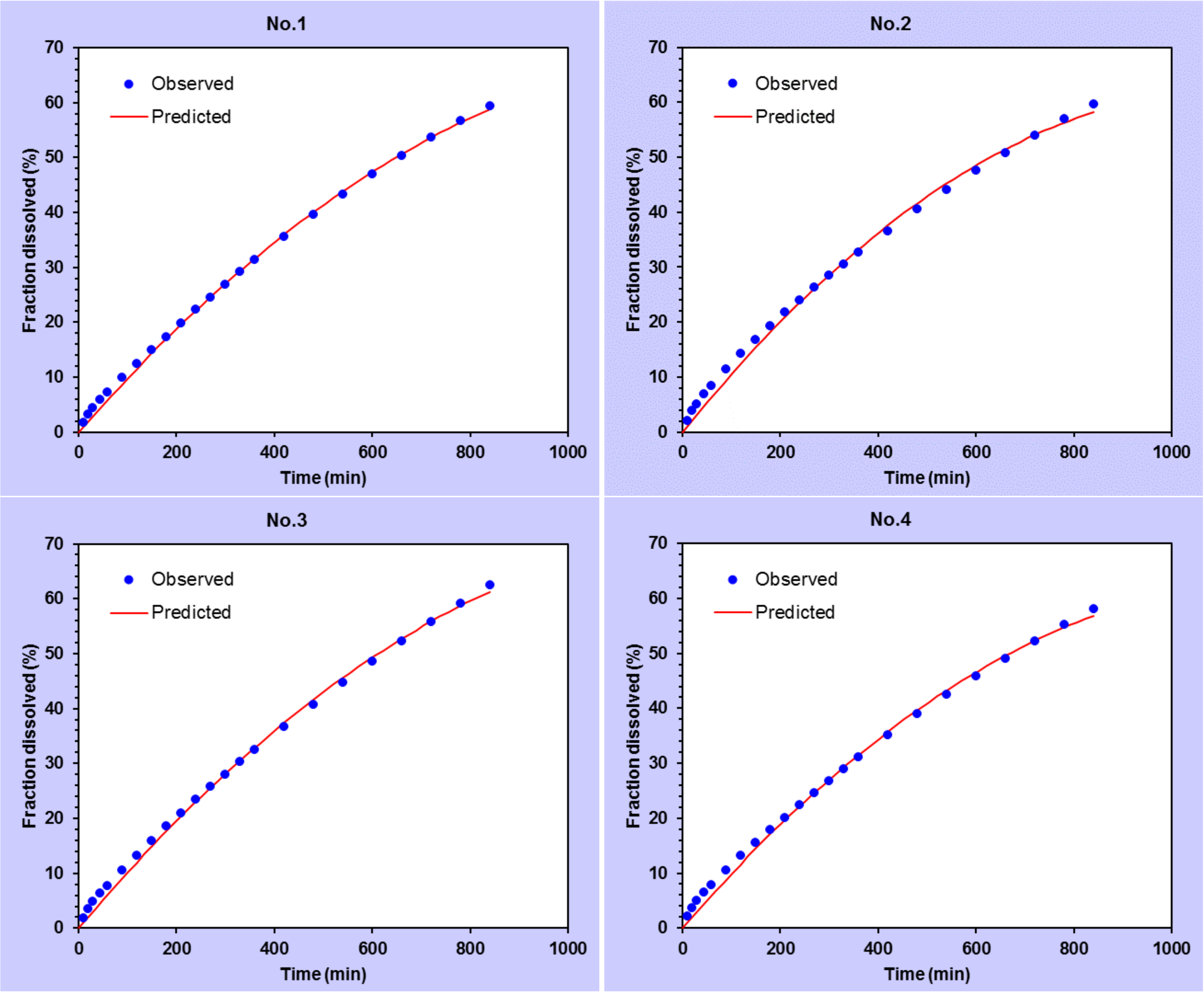

Supplementary materials – Model fitting summary – Ethocel™ Standard 20 Premium (modelling up to app. 60 % of carvedilol released)

Model: **Quadratic with T_{lag}**

Model equation: $F = 100 \cdot \left[k_1 \cdot \left(t - T_{lag}\right)^2 + k_2 \cdot \left(t - T_{lag}\right)\right]$

Fitted model parameters per tested tablet (N = 4) with statistics – mean, standard deviation (SD), and relative standard deviation expressed in % (RSD%) (output from DDSolver):

Parameter	No.1	No.2	No.3	No.4	Mean	SD	RSD(%)
k_1	-0.0000004	-0.0000005	-0.0000004	-0.0000004	-0.0000004	0.0000001	-11.5284863
k_2	0.0010303	0.0011167	0.0010705	0.0010430	0.0010651	0.0000383	3.5923717
T_{lag}	4.0000000	4.0000000	4.0000000	4.0000000	4.0000000	0.0000000	0.0000000

Number of dissolution data points (N), degrees of freedom (df), and selected goodness of fit criteria – Pearson correlation coefficient (R), coefficient of determination (R^2), adjusted coefficient of determination ($R^2_{adjusted}$), and residual sum of squares (RSS) (manual calculation in MS Excel):

Parameter	No.1	No.2	No.3	No.4
N	23	23	23	23
df	20	20	20	20
R	0.9995321	0.998746013	0.99925189	0.998999246
R^2	0.999064419	0.997493598	0.99850434	0.997999494
$R^2_{adjusted}$	0.998970861	0.997242958	0.998354774	0.997799443
RSS	18.38005187	45.04937523	29.56418458	36.10688041

Graphical abstract of model fit presented as mean ± 1 SD of the fraction % of released carvedilol:

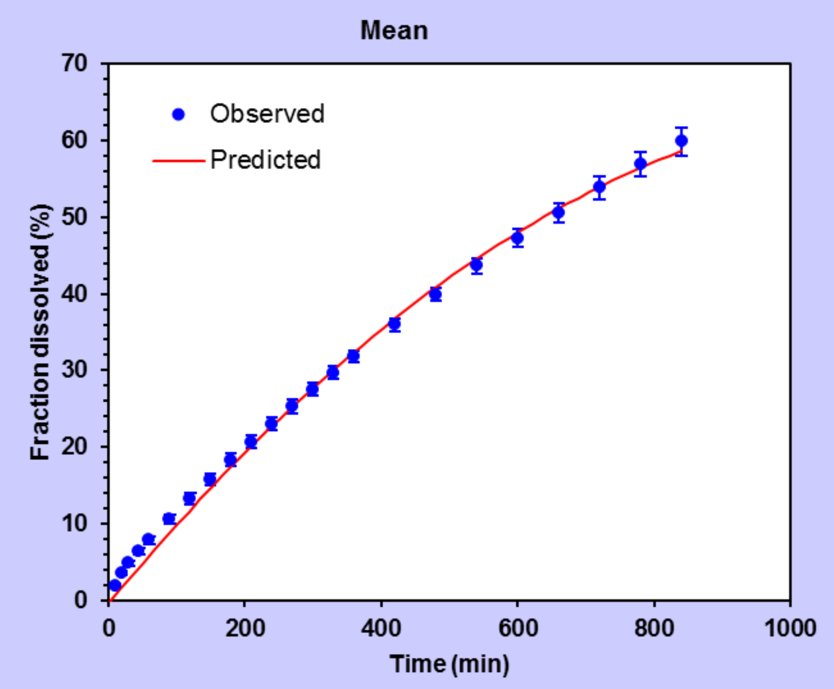

Supplementary materials – Model fitting summary – Ethocel™ Standard 20 Premium (modelling up to app. 60 % of carvedilol released)

Graphical abstract of model fit presented as the fraction % of released carvedilol per tested tablet:

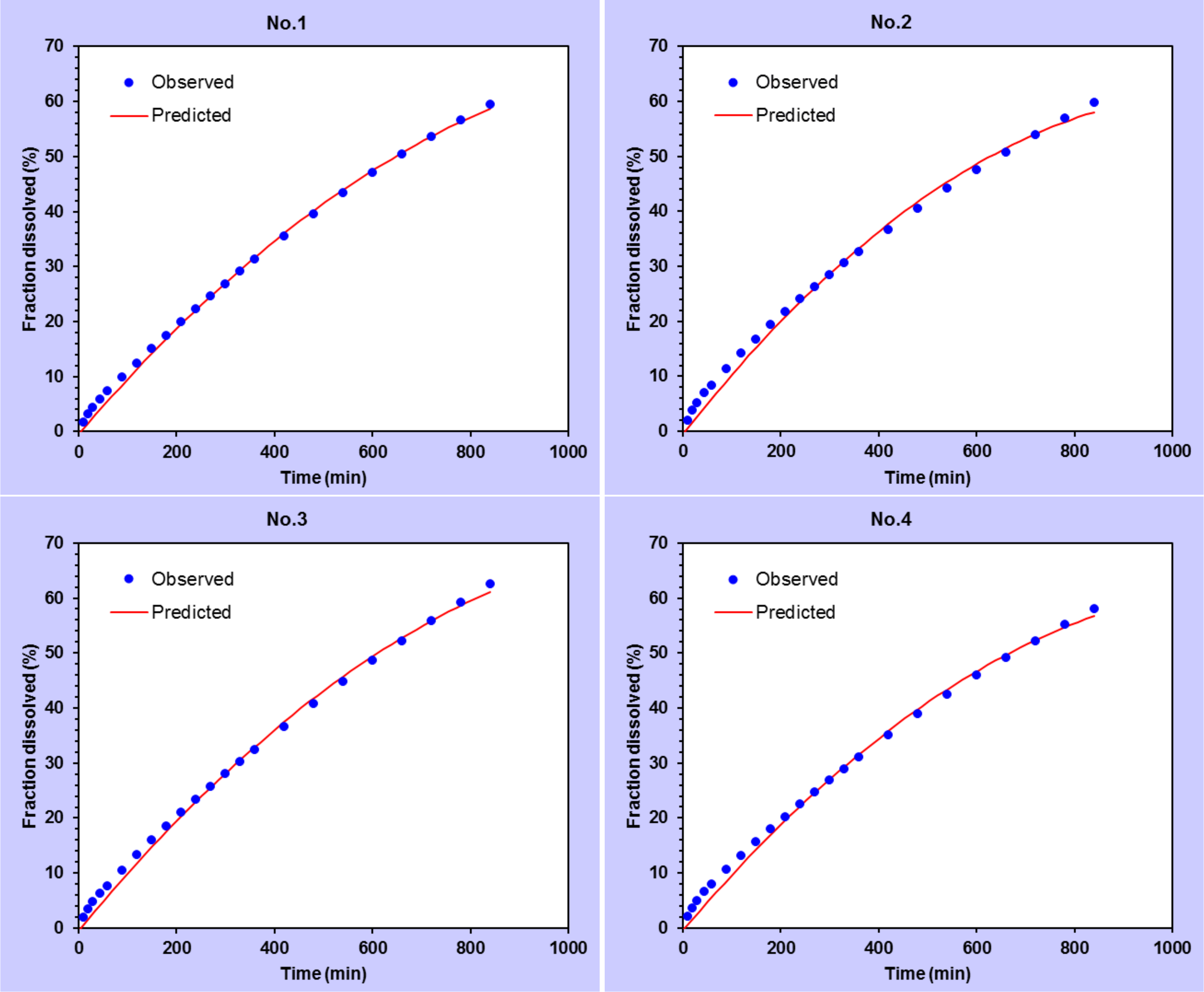

Supplementary materials – Model fitting summary – Ethocel™ Standard 20 Premium (modelling up to app. 60 % of carvedilol released)

Model: **Weibull_1**

Model equation: $F = 100 \cdot \left[1 - e^{-\frac{(t-T_i)^{\beta}}{\alpha}}\right]$

Fitted model parameters per tested tablet (N = 4) with statistics – mean, standard deviation (SD), and relative standard deviation expressed in % (RSD%) (output from DDSolver):

Parameter	No.1	No.2	No.3	No.4	Mean	SD	RSD(%)
α	333.088	272.371	297.526	251.877	288.716	34.980	12.116
β	0.826	0.812	0.816	0.777	0.808	0.021	2.623
T_i	6.000	4.733	6.000	6.000	5.683	0.634	11.150

Number of dissolution data points (N), degrees of freedom (df), and selected goodness of fit criteria – Pearson correlation coefficient (R), coefficient of determination (R^2), adjusted coefficient of determination ($R^2_{adjusted}$), and residual sum of squares (RSS) (manual calculation in MS Excel):

Parameter	No.1	No.2	No.3	No.4
N	23	23	23	23
df	20	20	20	20
R	0.996313452	0.997131141	0.995212879	0.995467042
R^2	0.992640495	0.994270512	0.990448675	0.990954632
$R^2_{adjusted}$	0.991904545	0.993697563	0.989493542	0.990050096
RSS	113.0832334	66.58617872	144.3435505	118.4257792

Graphical abstract of model fit presented as mean ± 1 SD of the fraction % of released carvedilol:

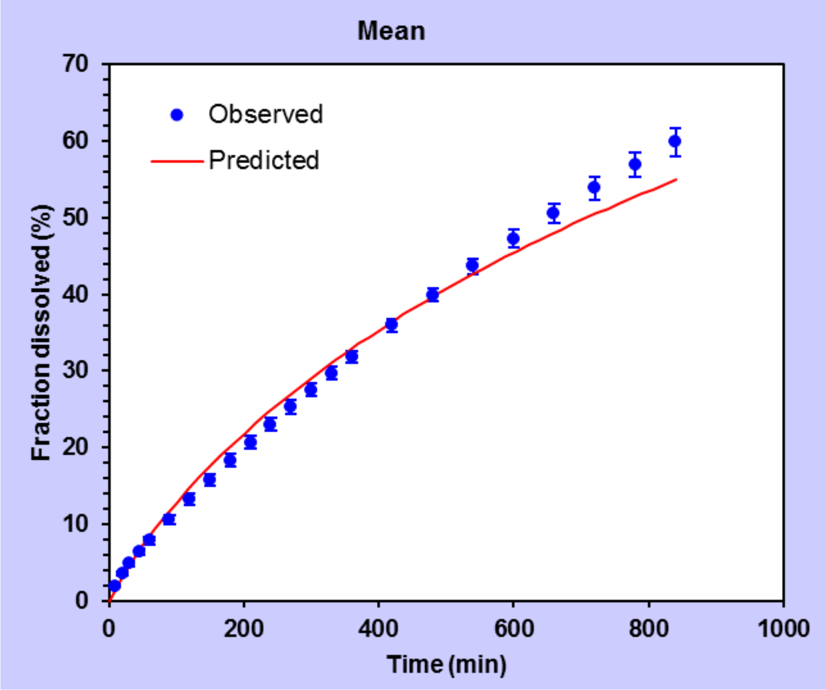

Supplementary materials – Model fitting summary – Ethocel^{TM} Standard 20 Premium (modelling up to app. 60 % of carvedilol released)

Graphical abstract of model fit presented as the fraction % of released carvedilol per tested tablet:

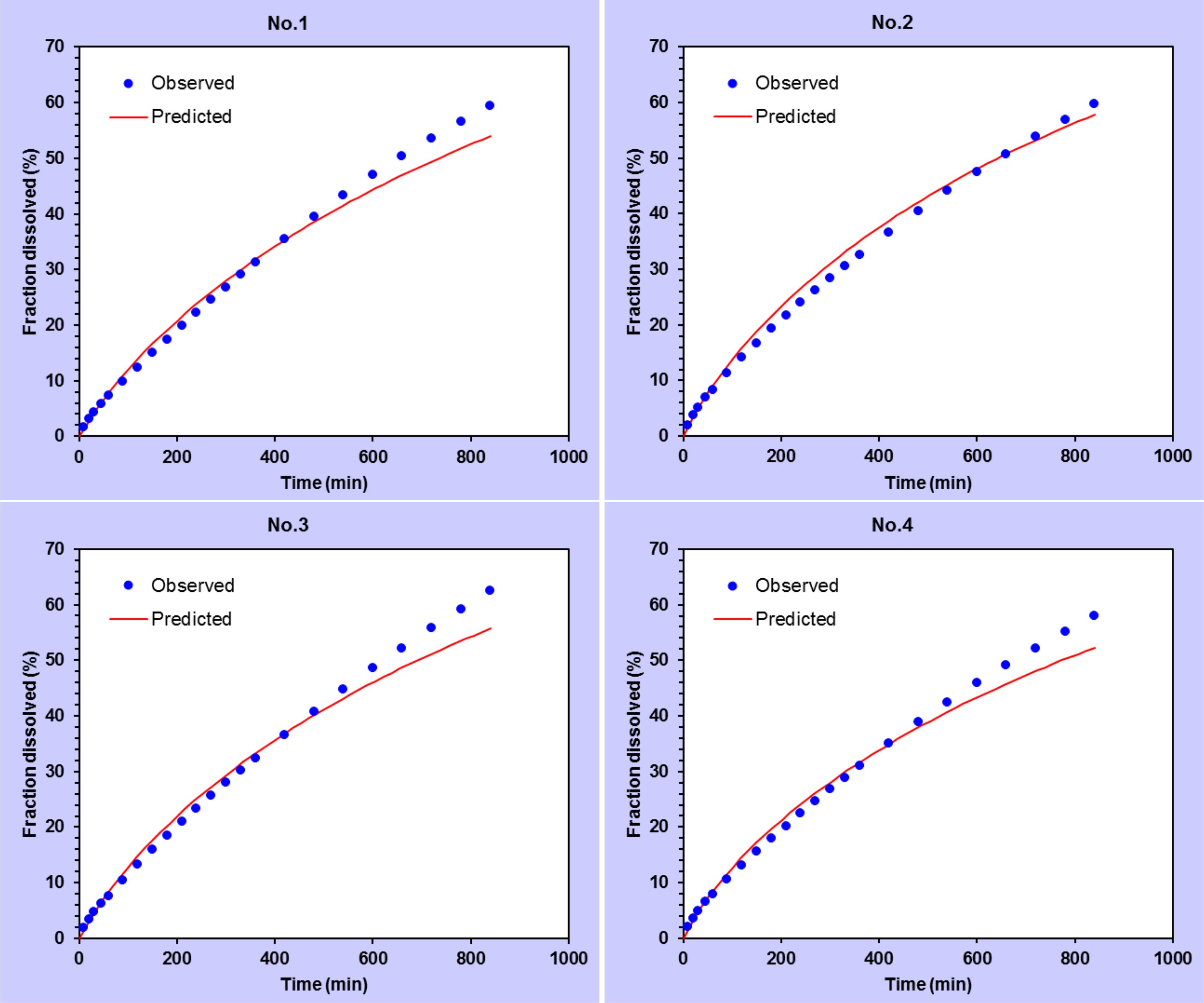

Supplementary materials – Model fitting summary – Ethocel™ Standard 20 Premium (modelling up to app. 60 % of carvedilol released)

Model: **Weibull_2**

Model equation: $F = 100 \cdot \left(1 - e^{-\frac{t^{\beta}}{\alpha}}\right)$

Fitted model parameters per tested tablet (N = 4) with statistics – mean, standard deviation (SD), and relative standard deviation expressed in % (RSD%) (output from DDSolver):

Parameter	No.1	No.2	No.3	No.4	Mean	SD	RSD(%)
α	568.788	413.567	506.506	362.767	462.907	92.329	19.945
β	0.923	0.877	0.912	0.838	0.888	0.039	4.344

Number of dissolution data points (N), degrees of freedom (df), and selected goodness of fit criteria – Pearson correlation coefficient (R), coefficient of determination (R^2), adjusted coefficient of determination ($R^2_{adjusted}$), and residual sum of squares (RSS) (manual calculation in MS Excel):

Parameter	No.1	No.2	No.3	No.4
N	23	23	23	23
df	21	21	21	21
R	0.998544651	0.998825194	0.997727808	0.997673084
R^2	0.997091419	0.997651768	0.99546078	0.995351583
$R^2_{adjusted}$	0.996952915	0.997539947	0.995244626	0.99513023
RSS	36.14073925	27.66050724	50.53840755	56.1991018

Graphical abstract of model fit presented as mean ± 1 SD of the fraction % of released carvedilol:

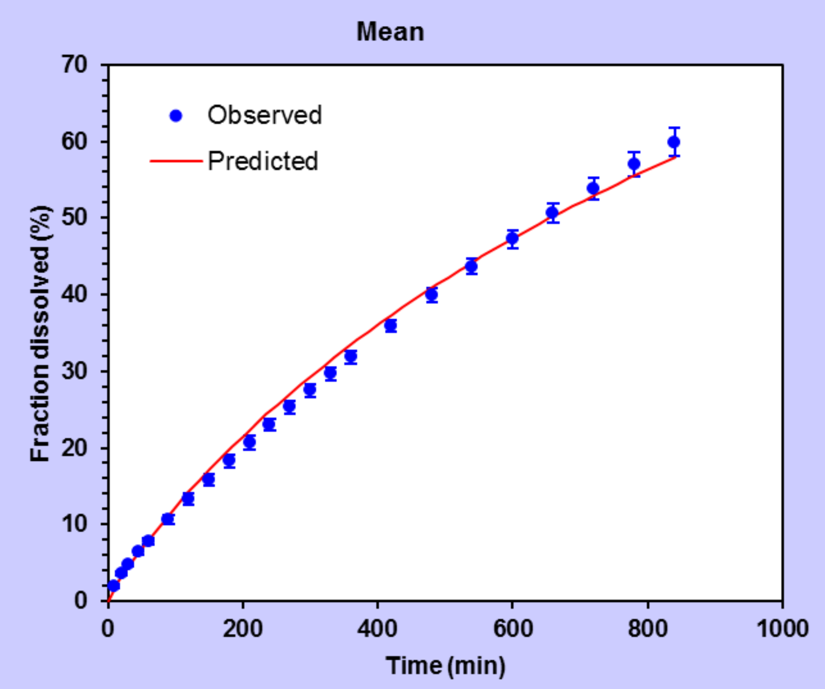

Supplementary materials – Model fitting summary – Ethocel™ Standard 20 Premium (modelling up to app. 60 % of carvedilol released)

Graphical abstract of model fit presented as the fraction % of released carvedilol per tested tablet:

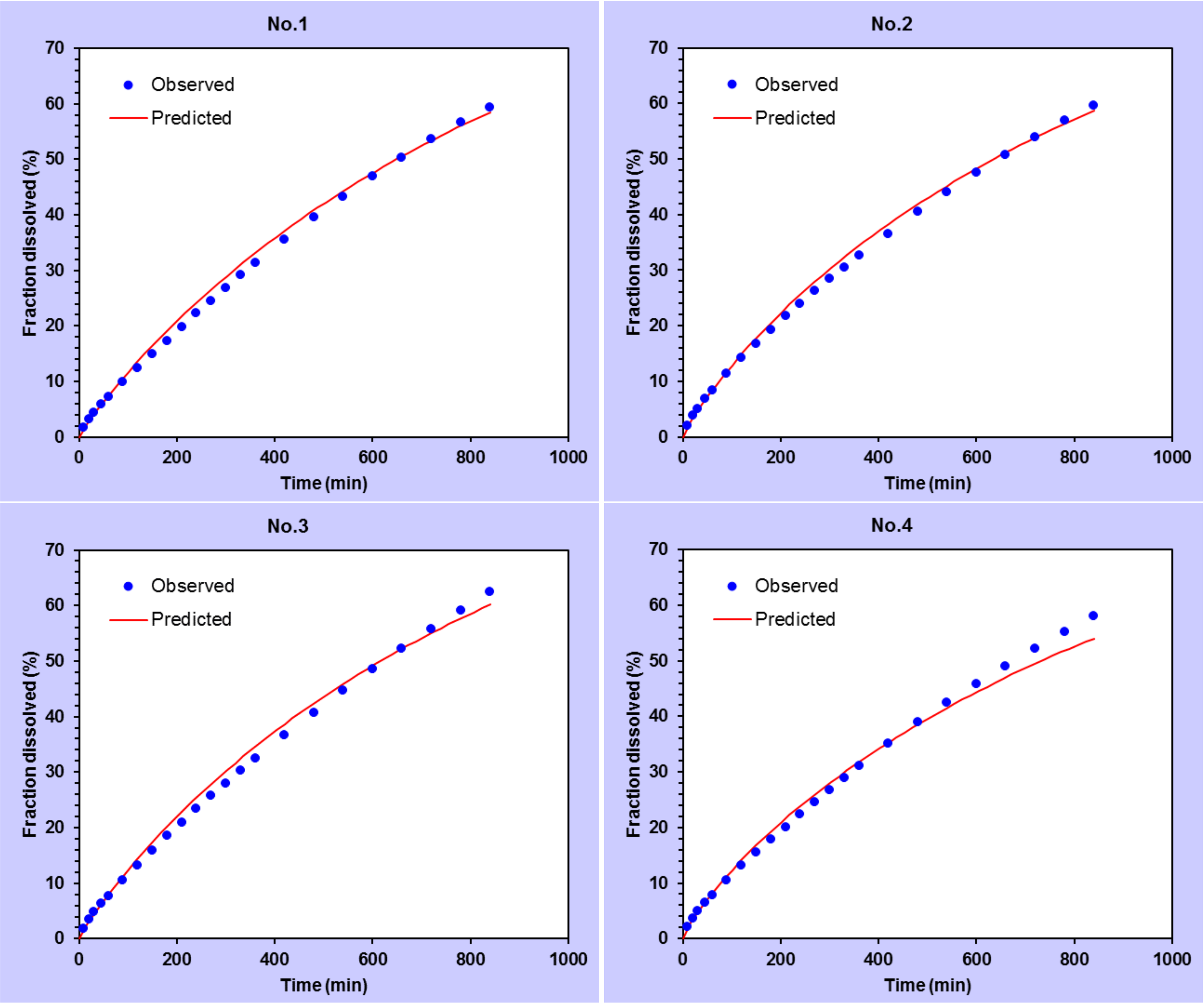

Supplementary materials – Model fitting summary – Ethocel™ Standard 20 Premium (modelling up to app. 60 % of carvedilol released)

Model: **Weibull_3**

Model equation: $F = F_{max} \cdot \left(1 - e^{-\frac{t^{\beta}}{\alpha}}\right)$

Fitted model parameters per tested tablet (N = 4) with statistics – mean, standard deviation (SD), and relative standard deviation expressed in % (RSD%) (output from DDSolver):

Parameter	No.1	No.2	No.3	No.4	Mean	SD	RSD(%)
α	477.207	347.073	430.798	346.612	400.423	64.705	16.159
β	1.008	0.964	0.988	0.958	0.980	0.023	2.366
F_{max}	62.383	62.702	65.584	60.940	62.902	1.946	3.093

Number of dissolution data points (N), degrees of freedom (df), and selected goodness of fit criteria – Pearson correlation coefficient (R), coefficient of determination (R^2), adjusted coefficient of determination ($R^2_{adjusted}$), and residual sum of squares (RSS) (manual calculation in MS Excel):

Parameter	No.1	No.2	No.3	No.4
N	23	23	23	23
df	20	20	20	20
R	0.990114459	0.990504333	0.989281552	0.988763574
R^2	0.980326642	0.981098834	0.978677989	0.977653406
$R^2_{adjusted}$	0.978359307	0.979208717	0.976545788	0.975418747
RSS	183.068547	162.8117133	210.0458802	188.0313878

Graphical abstract of model fit presented as mean ± 1 SD of the fraction % of released carvedilol:

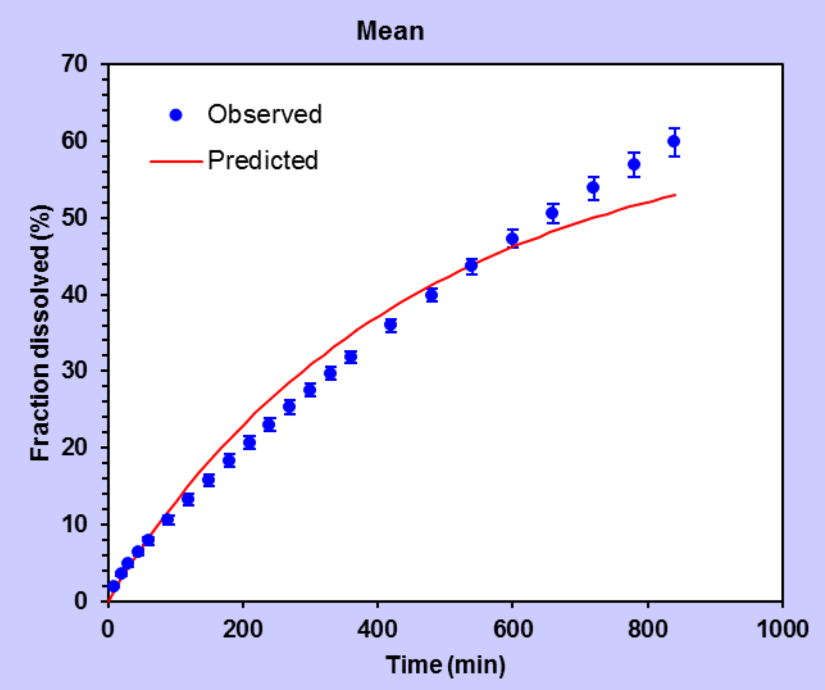

Supplementary materials – Model fitting summary – Ethocel™ Standard 20 Premium (modelling up to app. 60 % of carvedilol released)

Graphical abstract of model fit presented as the fraction % of released carvedilol per tested tablet:

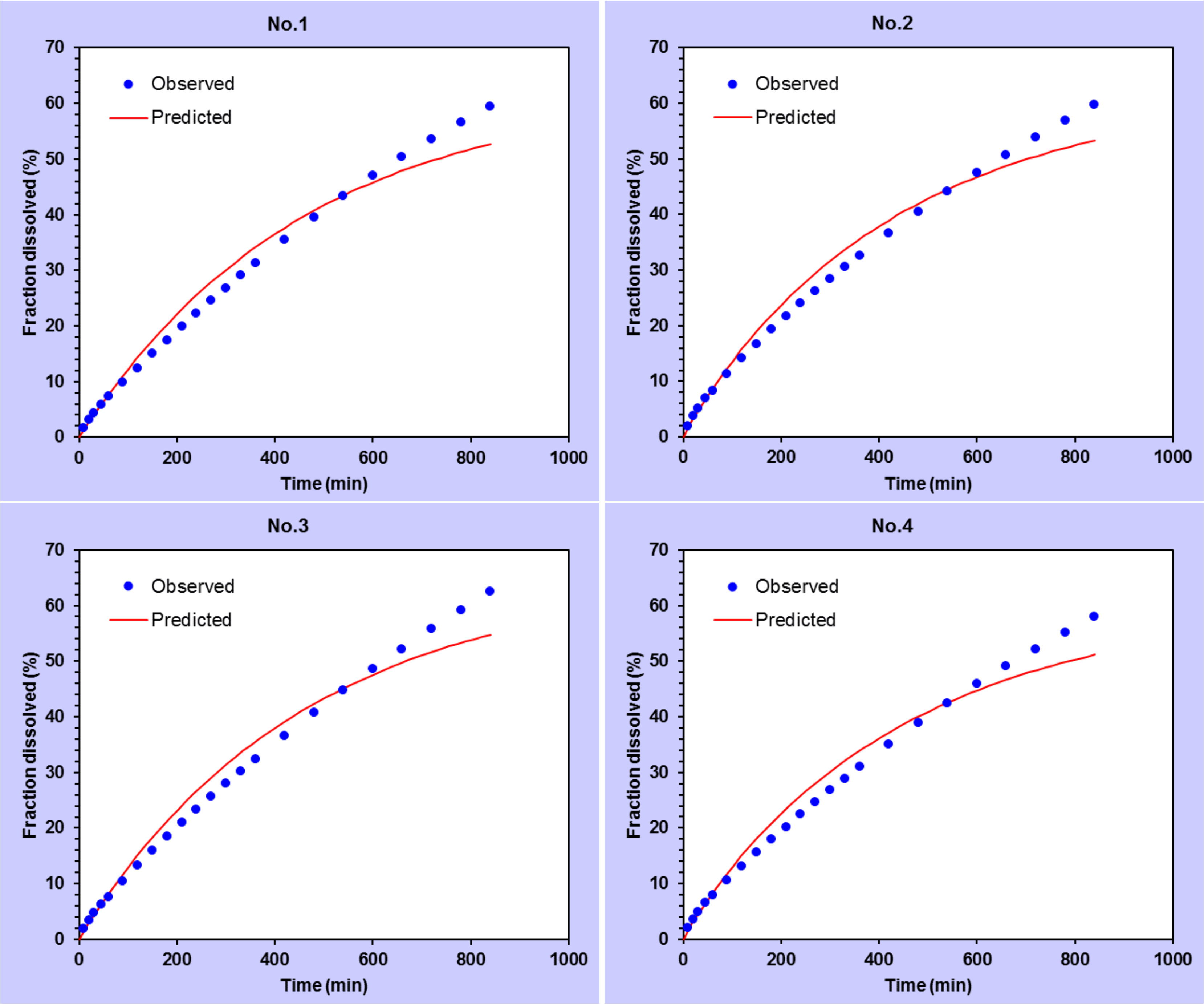

Supplementary materials – Model fitting summary – Ethocel™ Standard 20 Premium (modelling up to app. 60 % of carvedilol released)

Model: **Weibull_4**

Model equation: $F = F_{max} \cdot \left[1 - e^{-\frac{(t-T_i)^{\beta}}{\alpha}}\right]$

Fitted model parameters per tested tablet (N = 4) with statistics – mean, standard deviation (SD), and relative standard deviation expressed in % (RSD%) (output from DDSolver):

Parameter	No.1	No.2	No.3	No.4	Mean	SD	RSD(%)
α	304.283	226.380	276.973	225.468	258.276	38.987	15.095
β	0.933	0.892	0.914	0.886	0.906	0.021	2.367
T_i	6.000	6.000	6.000	6.000	6.000	0.000	0.000
F_{max}	62.383	62.702	65.584	60.940	62.902	1.946	3.093

Number of dissolution data points (N), degrees of freedom (df), and selected goodness of fit criteria – Pearson correlation coefficient (R), coefficient of determination (R^2), adjusted coefficient of determination ($R^2_{adjusted}$), and residual sum of squares (RSS) (manual calculation in MS Excel):

Parameter	No.1	No.2	No.3	No.4
N	23	23	23	23
df	19	19	19	19
R	0.987177717	0.987682141	0.986259936	0.985527178
R^2	0.974519844	0.975516011	0.972708661	0.971263819
$R^2_{adjusted}$	0.970496662	0.971650118	0.968399502	0.966726527
RSS	245.3159853	215.9196121	278.2217466	247.5970441

Graphical abstract of model fit presented as mean ± 1 SD of the fraction % of released carvedilol:

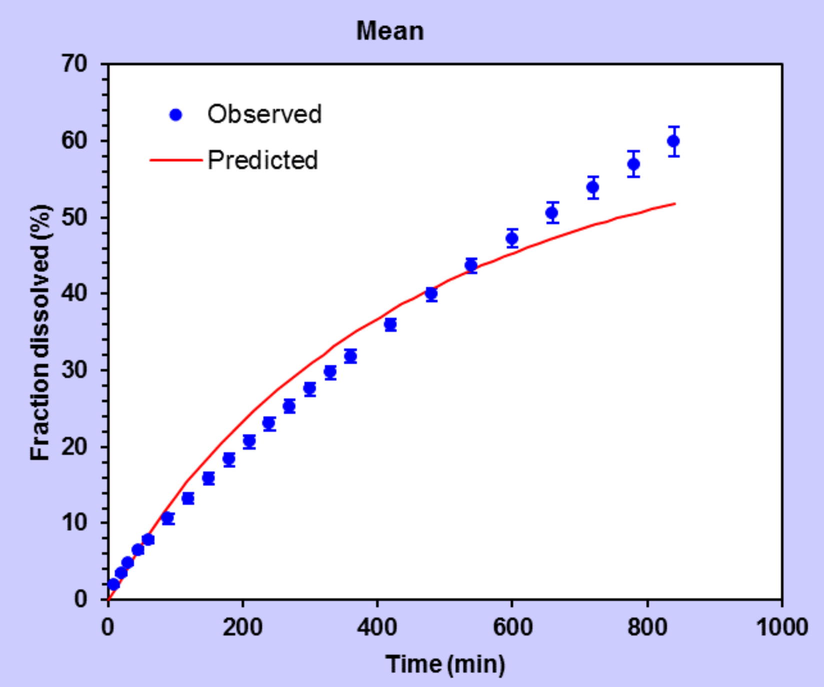

Supplementary materials – Model fitting summary – Ethocel™ Standard 20 Premium (modelling up to app. 60 % of carvedilol released)

Graphical abstract of model fit presented as the fraction % of released carvedilol per tested tablet:

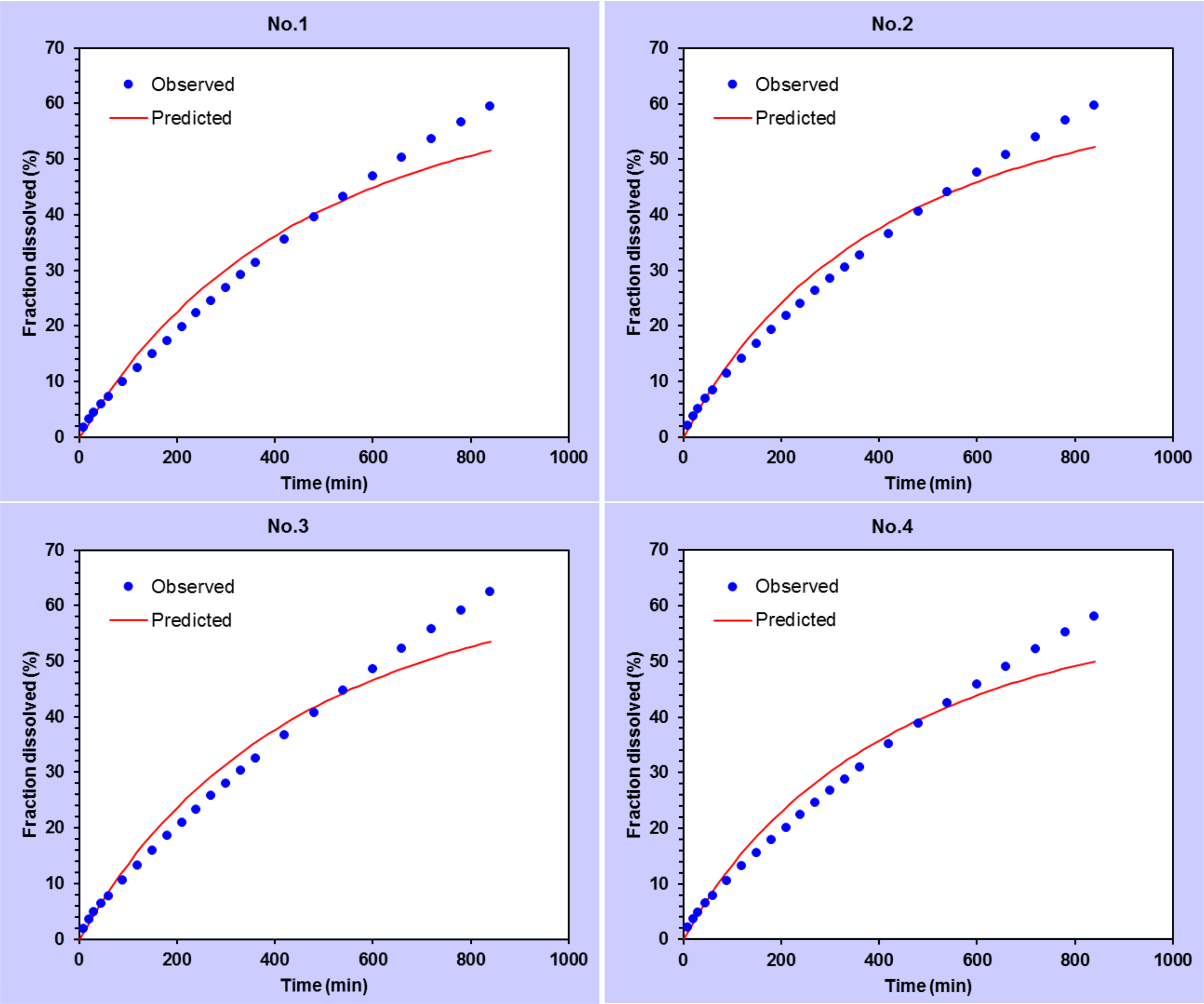

Supplementary materials – Model fitting summary – Ethocel™ Standard 20 Premium (modelling up to app. 60 % of carvedilol released)

Model: **Logistic_1**

Model equation: $F = 100 \cdot \frac{e^{\alpha+\beta\cdot\log(t)}}{1+e^{\alpha+\beta\cdot\log(t)}}$

Fitted model parameters per tested tablet (N = 4) with statistics – mean, standard deviation (SD), and relative standard deviation expressed in % (RSD%) (output from DDSolver):

Parameter	No.1	No.2	No.3	No.4	Mean	SD	RSD(%)
α	-7.093	-6.744	-6.992	-6.745	-6.894	0.177	-2.566
β	2.466	2.357	2.456	2.325	2.401	0.071	2.944

Number of dissolution data points (N), degrees of freedom (df), and selected goodness of fit criteria – Pearson correlation coefficient (R), coefficient of determination (R^2), adjusted coefficient of determination ($R^2_{adjusted}$), and residual sum of squares (RSS) (manual calculation in MS Excel):

Parameter	No.1	No.2	No.3	No.4
N	23	23	23	23
df	21	21	21	21
R	0.996561711	0.996745902	0.995037364	0.995950691
R^2	0.993135244	0.993502393	0.990099355	0.991917779
$R^2_{adjusted}$	0.992808351	0.993192983	0.989627896	0.991532912
RSS	136.6612596	109.6578426	161.9627958	140.5748542

Graphical abstract of model fit presented as mean ± 1 SD of the fraction % of released carvedilol:

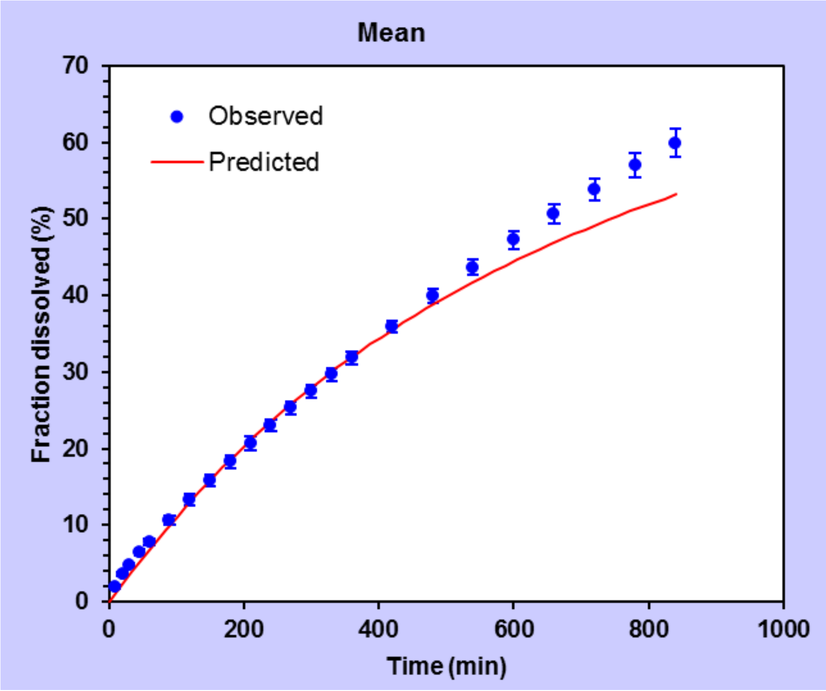

Supplementary materials – Model fitting summary – Ethocel^{TM} Standard 20 Premium (modelling up to app. 60 % of carvedilol released)

Graphical abstract of model fit presented as the fraction % of released carvedilol per tested tablet:

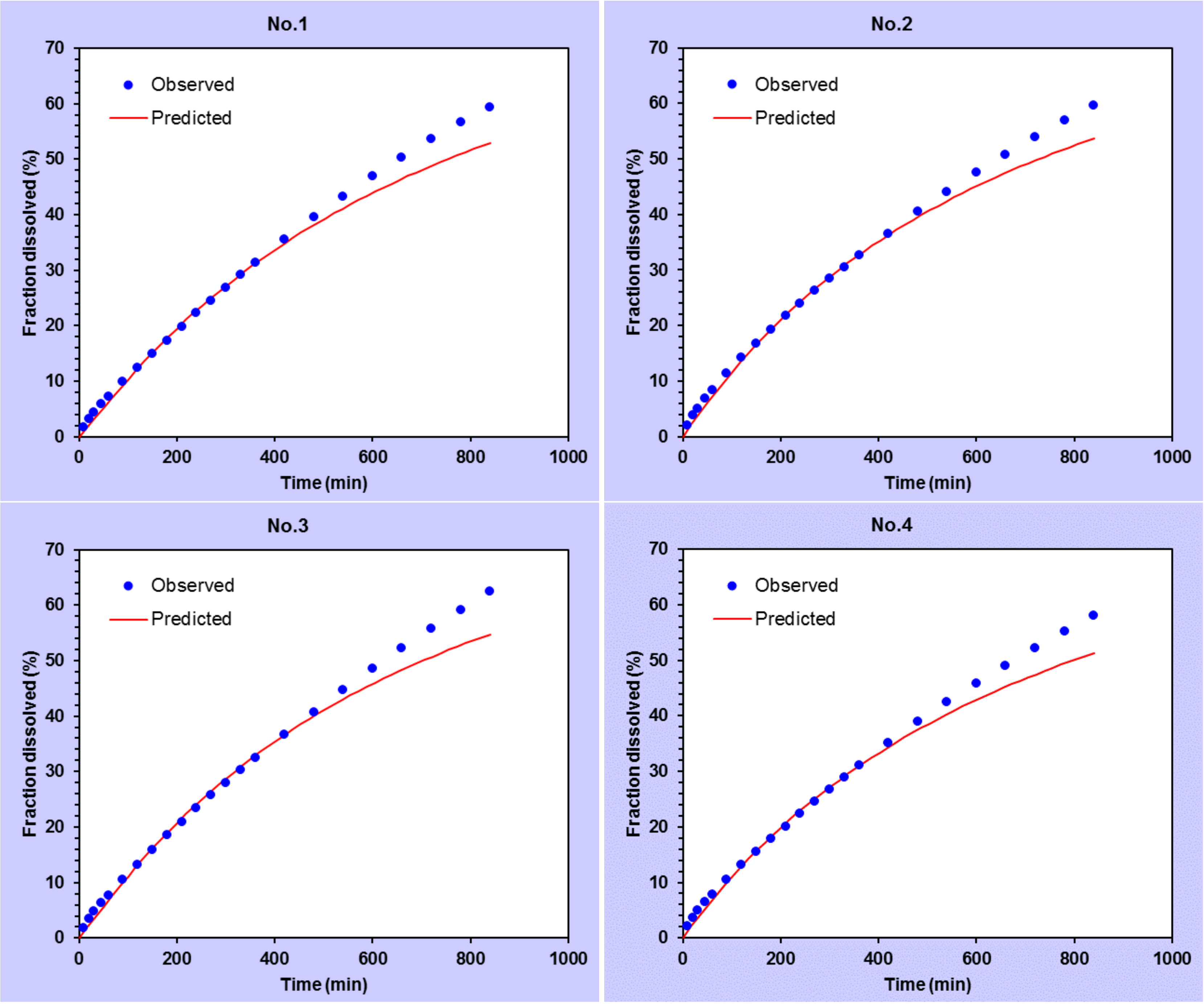

Supplementary materials – Model fitting summary – Ethocel™ Standard 20 Premium (modelling up to app. 60 % of carvedilol released)

Model: **Logistic_2**

Model equation: $F = F_{max} \cdot \frac{e^{\alpha+\beta\cdot\log(t)}}{1+e^{\alpha+\beta\cdot\log(t)}}$

Fitted model parameters per tested tablet (N = 4) with statistics – mean, standard deviation (SD), and relative standard deviation expressed in % (RSD%) (output from DDSolver):

Parameter	No.1	No.2	No.3	No.4	Mean	SD	RSD(%)
α	-8.340	-7.966	-7.519	-7.307	-7.783	0.462	-5.935
β	3.164	3.058	2.812	2.758	2.948	0.194	6.588
F_{max}	69.671	70.027	78.175	72.638	72.628	3.927	5.407

Number of dissolution data points (N), degrees of freedom (df), and selected goodness of fit criteria – Pearson correlation coefficient (R), coefficient of determination (R^2), adjusted coefficient of determination ($R^2_{adjusted}$), and residual sum of squares (RSS) (manual calculation in MS Excel):

Parameter	No.1	No.2	No.3	No.4
N	23	23	23	23
df	20	20	20	20
R	0.993753358	0.993178816	0.99179065	0.991036799
R^2	0.987545737	0.98640416	0.983648693	0.982153936
$R^2_{adjusted}$	0.98630031	0.985044576	0.982013562	0.98036933
RSS	337.371049	273.282405	291.9242645	218.3428939

Graphical abstract of model fit presented as mean ± 1 SD of the fraction % of released carvedilol:

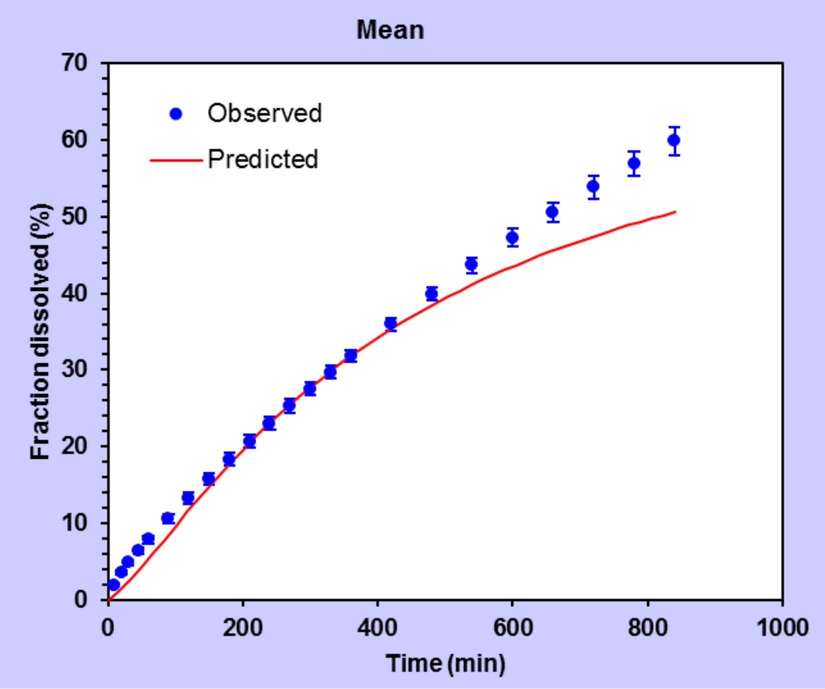

Supplementary materials – Model fitting summary – Ethocel™ Standard 20 Premium (modelling up to app. 60 % of carvedilol released)

Graphical abstract of model fit presented as the fraction % of released carvedilol per tested tablet:

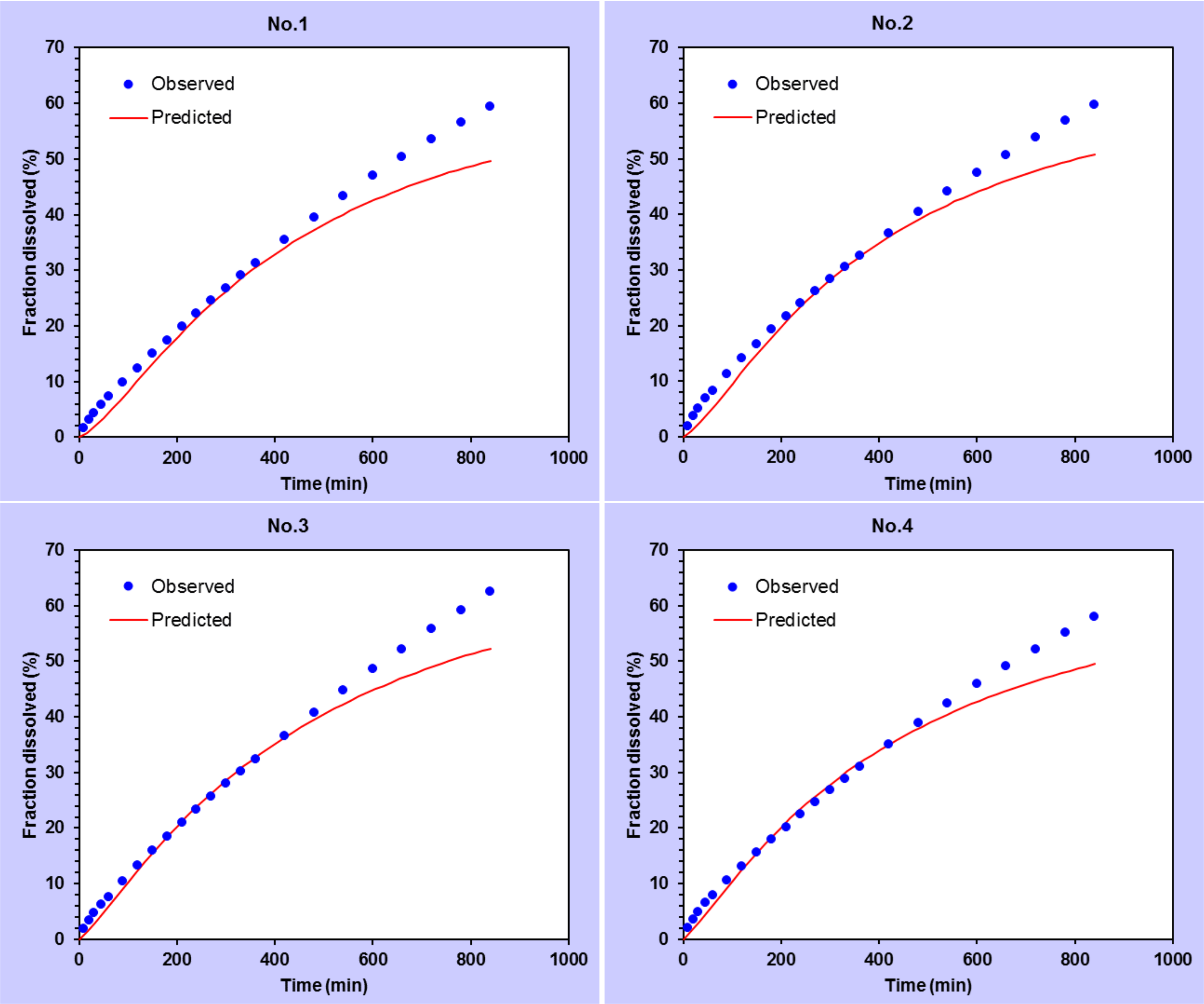

Supplementary materials – Model fitting summary – Ethocel™ Standard 20 Premium (modelling up to app. 60 % of carvedilol released)

Model: **Logistic_3**

Model equation: $F = F_{max} \cdot \frac{1}{1+e^{-k\cdot(t-\gamma)}}$

Fitted model parameters per tested tablet (N = 4) with statistics – mean, standard deviation (SD), and relative standard deviation expressed in % (RSD%) (output from DDSolver):

Parameter	No.1	No.2	No.3	No.4	Mean	SD	RSD(%)
k	0.006	0.006	0.006	0.006	0.006	0.000	1.736
γ	388.705	375.340	391.971	381.099	384.279	7.501	1.952
F_{max}	62.383	62.702	65.584	60.940	62.902	1.946	3.093

Number of dissolution data points (N), degrees of freedom (df), and selected goodness of fit criteria – Pearson correlation coefficient (R), coefficient of determination (R^2), adjusted coefficient of determination ($R^2_{adjusted}$), and residual sum of squares (RSS) (manual calculation in MS Excel):

Parameter	No.1	No.2	No.3	No.4
N	23	23	23	23
df	20	20	20	20
R	0.98985239	0.988053774	0.989165589	0.989933605
R^2	0.979807754	0.97625026	0.978448563	0.979968542
$R^2_{adjusted}$	0.97778853	0.973875286	0.97629342	0.977965396
RSS	190.2968683	212.0345642	215.8611561	169.1491073

Graphical abstract of model fit presented as mean ± 1 SD of the fraction % of released carvedilol:

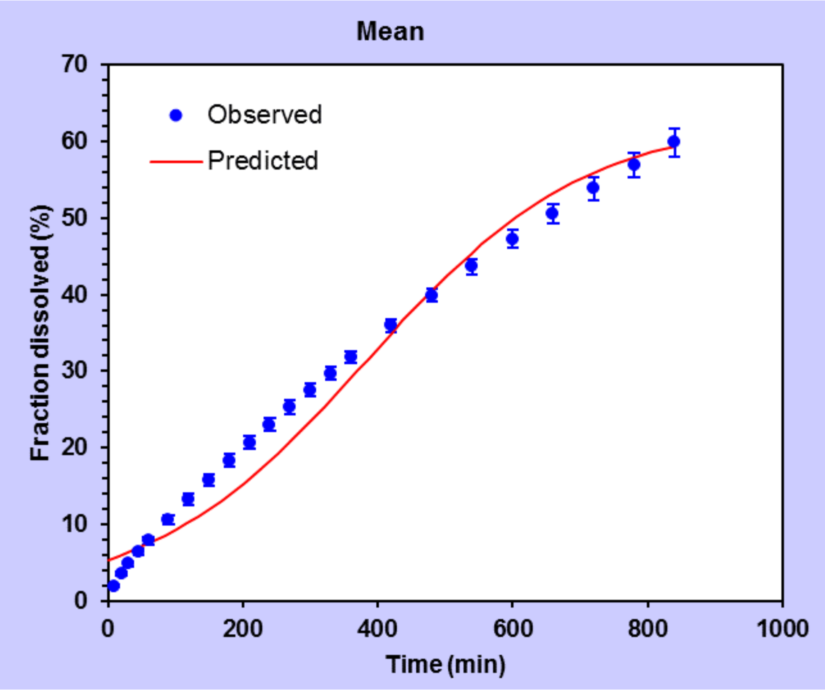

Supplementary materials – Model fitting summary – Ethocel™ Standard 20 Premium (modelling up to app. 60 % of carvedilol released)

Graphical abstract of model fit presented as the fraction % of released carvedilol per tested tablet:

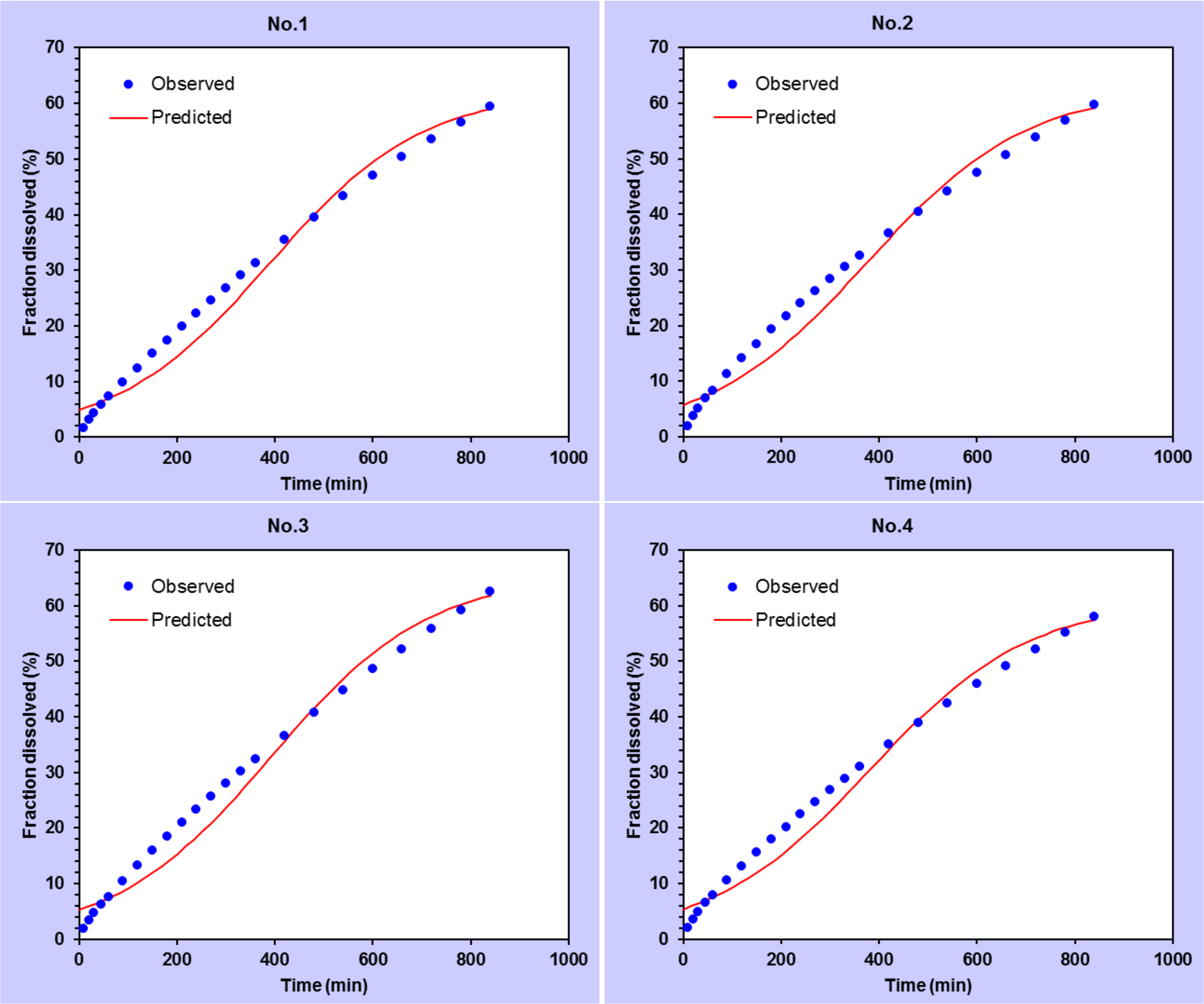

Supplementary materials – Model fitting summary – Ethocel™ Standard 20 Premium (modelling up to app. 60 % of carvedilol released)

Model: **Gompertz_1**

Model equation: $F = 100 \cdot e^{-\alpha \cdot e^{-\beta \cdot \log(t)}}$

Fitted model parameters per tested tablet (N = 4) with statistics – mean, standard deviation (SD), and relative standard deviation expressed in % (RSD%) (output from DDSolver):

Parameter	No.1	No.2	No.3	No.4	Mean	SD	RSD(%)
α	16.415	14.949	16.597	14.590	15.638	1.016	6.498
β	1.073	1.049	1.095	1.022	1.060	0.031	2.946

Number of dissolution data points (N), degrees of freedom (df), and selected goodness of fit criteria – Pearson correlation coefficient (R), coefficient of determination (R^2), adjusted coefficient of determination ($R^2_{adjusted}$), and residual sum of squares (RSS) (manual calculation in MS Excel):

Parameter	No.1	No.2	No.3	No.4
N	23	23	23	23
df	21	21	21	21
R	0.970493745	0.973184466	0.967745912	0.970563624
R^2	0.941858109	0.947088006	0.936532149	0.941993748
$R^2_{adjusted}$	0.939089448	0.944568387	0.933509871	0.939231545
RSS	532.2670352	458.7901912	609.85601	481.7692573

Graphical abstract of model fit presented as mean ± 1 SD of the fraction % of released carvedilol:

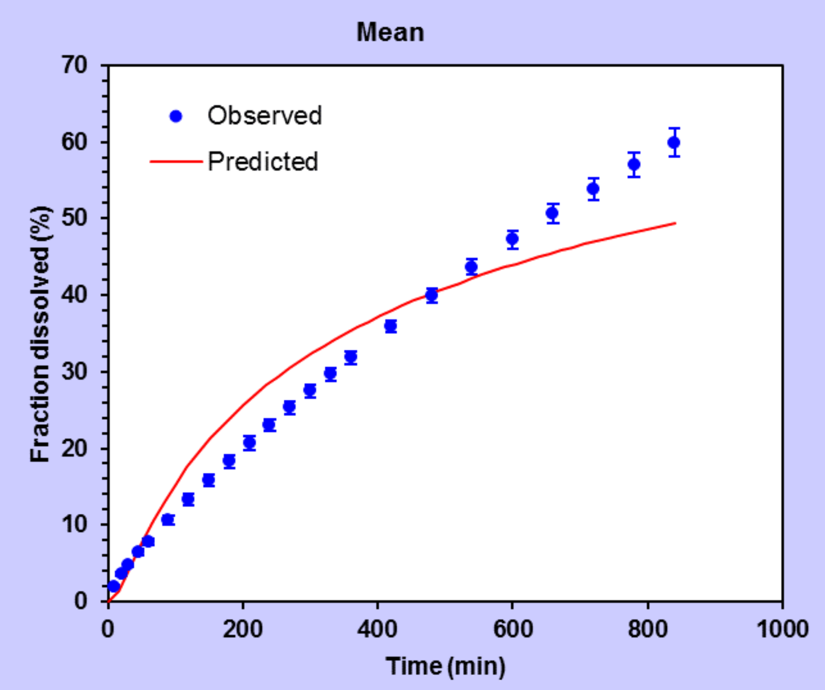

Supplementary materials – Model fitting summary – Ethocel^{TM} Standard 20 Premium (modelling up to app. 60 % of carvedilol released)

Graphical abstract of model fit presented as the fraction % of released carvedilol per tested tablet:

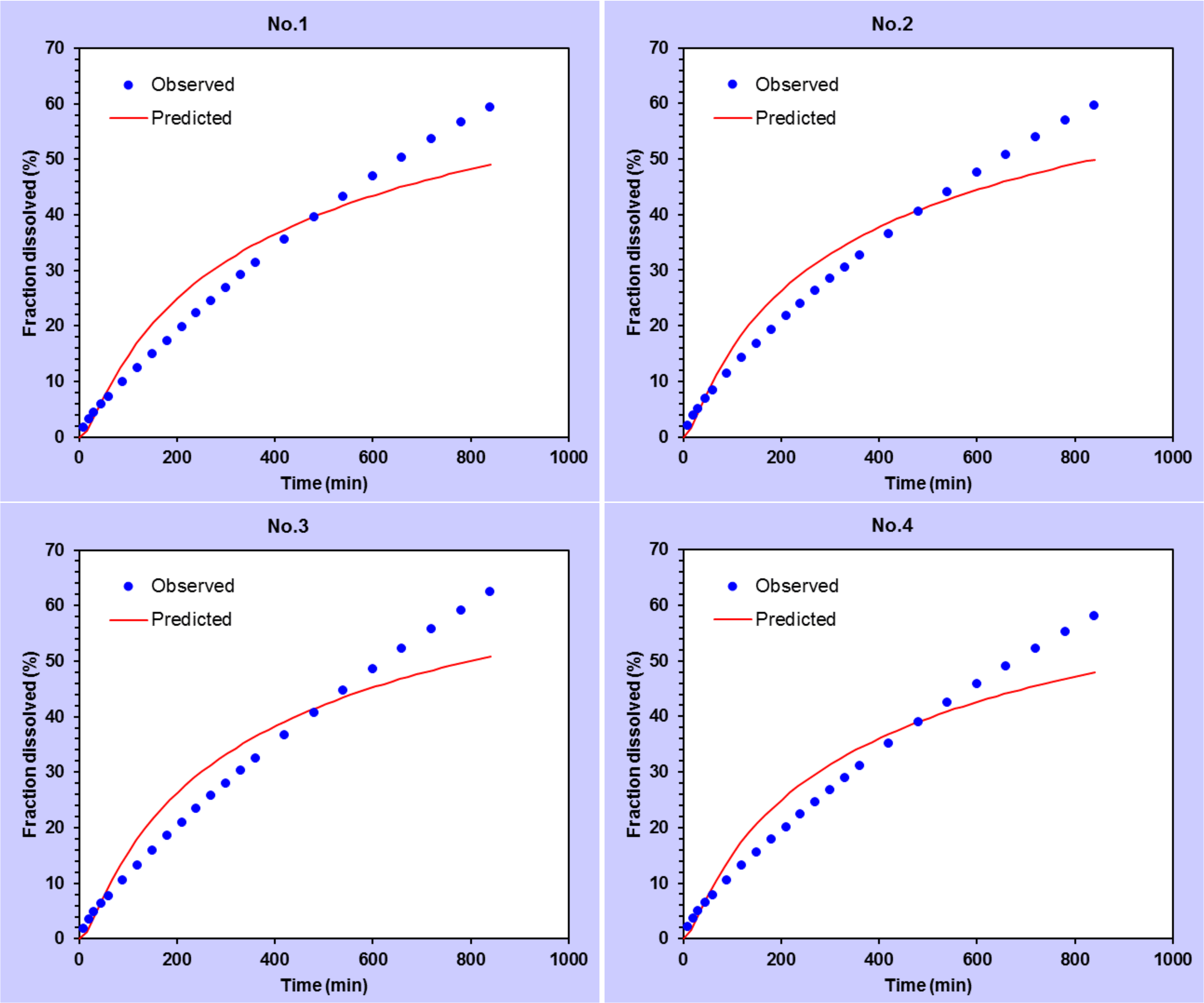

Supplementary materials – Model fitting summary – Ethocel™ Standard 20 Premium (modelling up to app. 60 % of carvedilol released)

Model: **Gompertz_2**

Model equation: $F = F_{max} \cdot e^{-\alpha \cdot e^{-\beta \cdot \log(t)}}$

Fitted model parameters per tested tablet (N = 4) with statistics – mean, standard deviation (SD), and relative standard deviation expressed in % (RSD%) (output from DDSolver):

Parameter	No.1	No.2	No.3	No.4	Mean	SD	RSD(%)
α	62.521	56.013	57.808	55.490	57.958	3.200	5.521
β	1.708	1.684	1.670	1.671	1.683	0.018	1.055
F_{max}	70.831	71.192	74.466	69.192	71.420	2.209	3.093

Number of dissolution data points (N), degrees of freedom (df), and selected goodness of fit criteria – Pearson correlation coefficient (R), coefficient of determination (R^2), adjusted coefficient of determination ($R^2_{adjusted}$), and residual sum of squares (RSS) (manual calculation in MS Excel):

Parameter	No.1	No.2	No.3	No.4
N	23	23	23	23
df	20	20	20	20
R	0.976311391	0.977447468	0.975549899	0.974912607
R^2	0.953183932	0.955403552	0.951697605	0.950454591
$R^2_{adjusted}$	0.948502325	0.950943908	0.946867365	0.94550005
RSS	586.7975128	528.579025	663.1209747	544.1977311

Graphical abstract of model fit presented as mean ± 1 SD of the fraction % of released carvedilol:

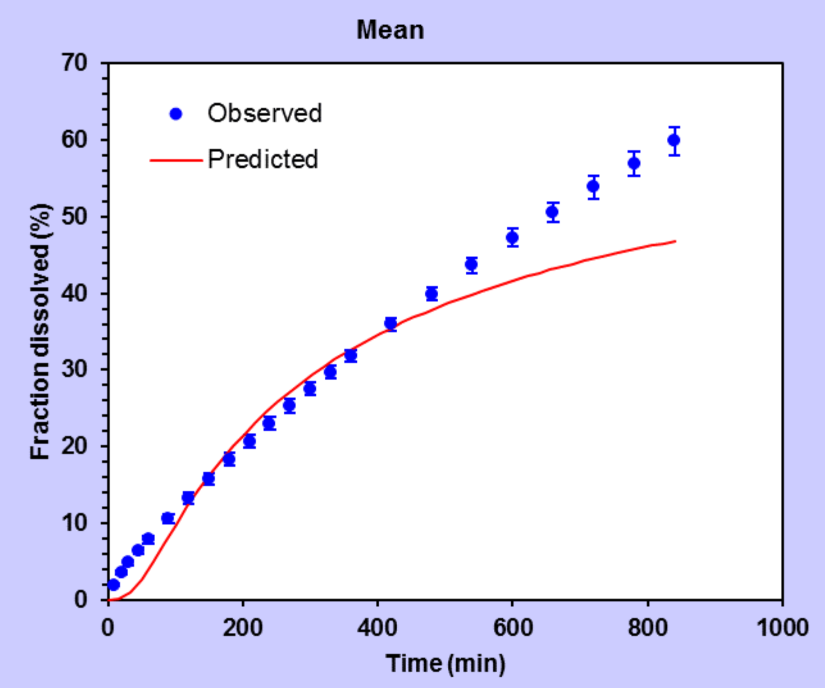

Supplementary materials – Model fitting summary – Ethocel^{TM} Standard 20 Premium (modelling up to app. 60 % of carvedilol released)

Graphical abstract of model fit presented as the fraction % of released carvedilol per tested tablet:

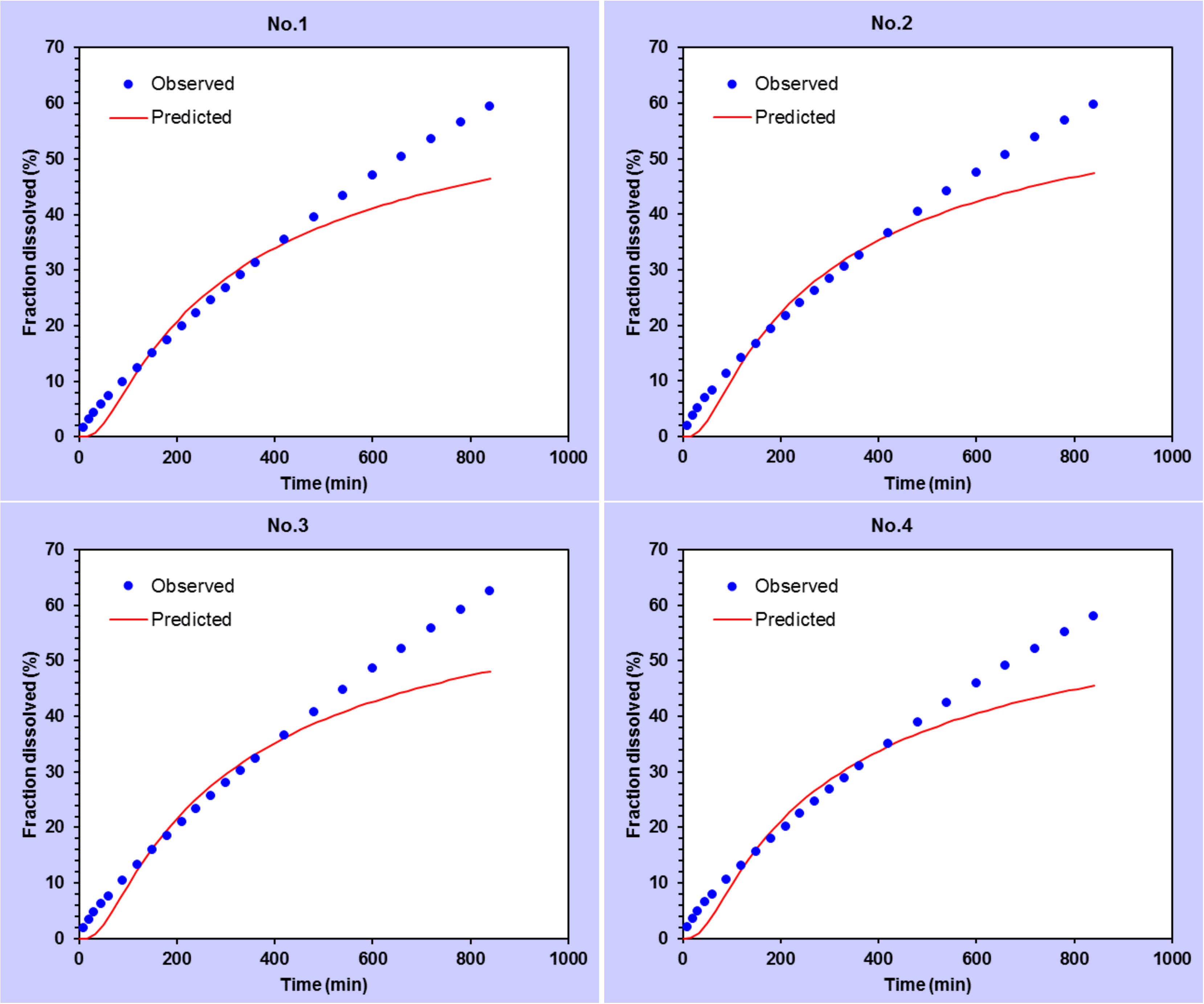

Supplementary materials – Model fitting summary – Ethocel™ Standard 20 Premium (modelling up to app. 60 % of carvedilol released)

Model: **Gompertz_3**

Model equation: $F = F_{max} \cdot e^{-e^{-k\cdot(t-\gamma)}}$

Fitted model parameters per tested tablet (N = 4) with statistics – mean, standard deviation (SD), and relative standard deviation expressed in % (RSD%) (output from DDSolver):

Parameter	No.1	No.2	No.3	No.4	Mean	SD	RSD(%)
k	0.004	0.004	0.003	0.004	0.004	0.001	13.368
γ	258.840	245.467	300.876	251.134	264.079	25.136	9.518
F_{max}	62.383	62.702	70.658	60.940	64.171	4.393	6.845

Number of dissolution data points (N), degrees of freedom (df), and selected goodness of fit criteria – Pearson correlation coefficient (R), coefficient of determination (R^2), adjusted coefficient of determination ($R^2_{adjusted}$), and residual sum of squares (RSS) (manual calculation in MS Excel):

Parameter	No.1	No.2	No.3	No.4
N	23	23	23	23
df	20	20	20	20
R	0.996329168	0.995522296	0.996809612	0.995986462
R^2	0.992671811	0.991064642	0.993629402	0.991989032
$R^2_{adjusted}$	0.991938993	0.990171106	0.992992342	0.991187935
RSS	69.9646821	79.8393754	84.4512487	69.87692844

Graphical abstract of model fit presented as mean ± 1 SD of the fraction % of released carvedilol:

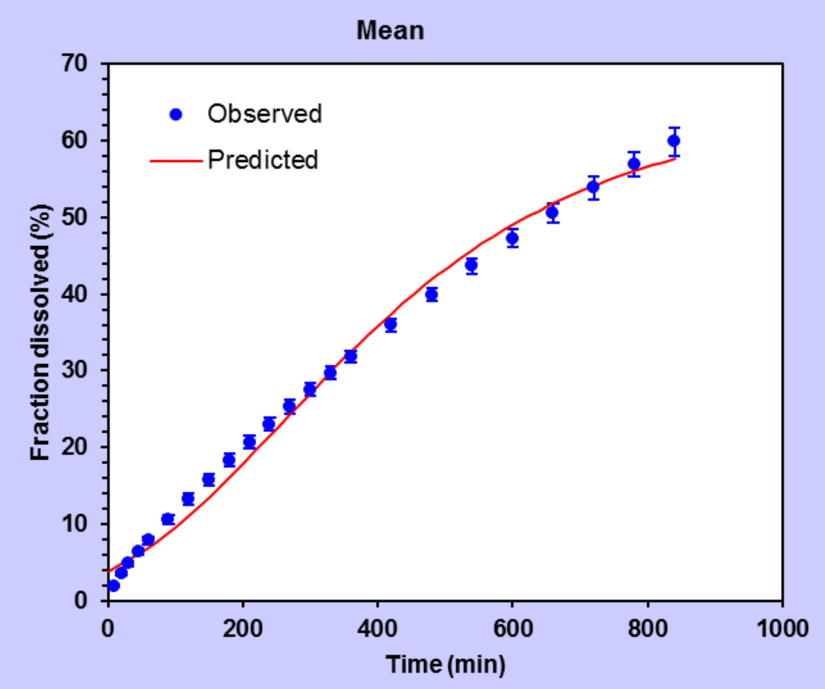

Supplementary materials – Model fitting summary – Ethocel™ Standard 20 Premium (modelling up to app. 60 % of carvedilol released)

Graphical abstract of model fit presented as the fraction % of released carvedilol per tested tablet:

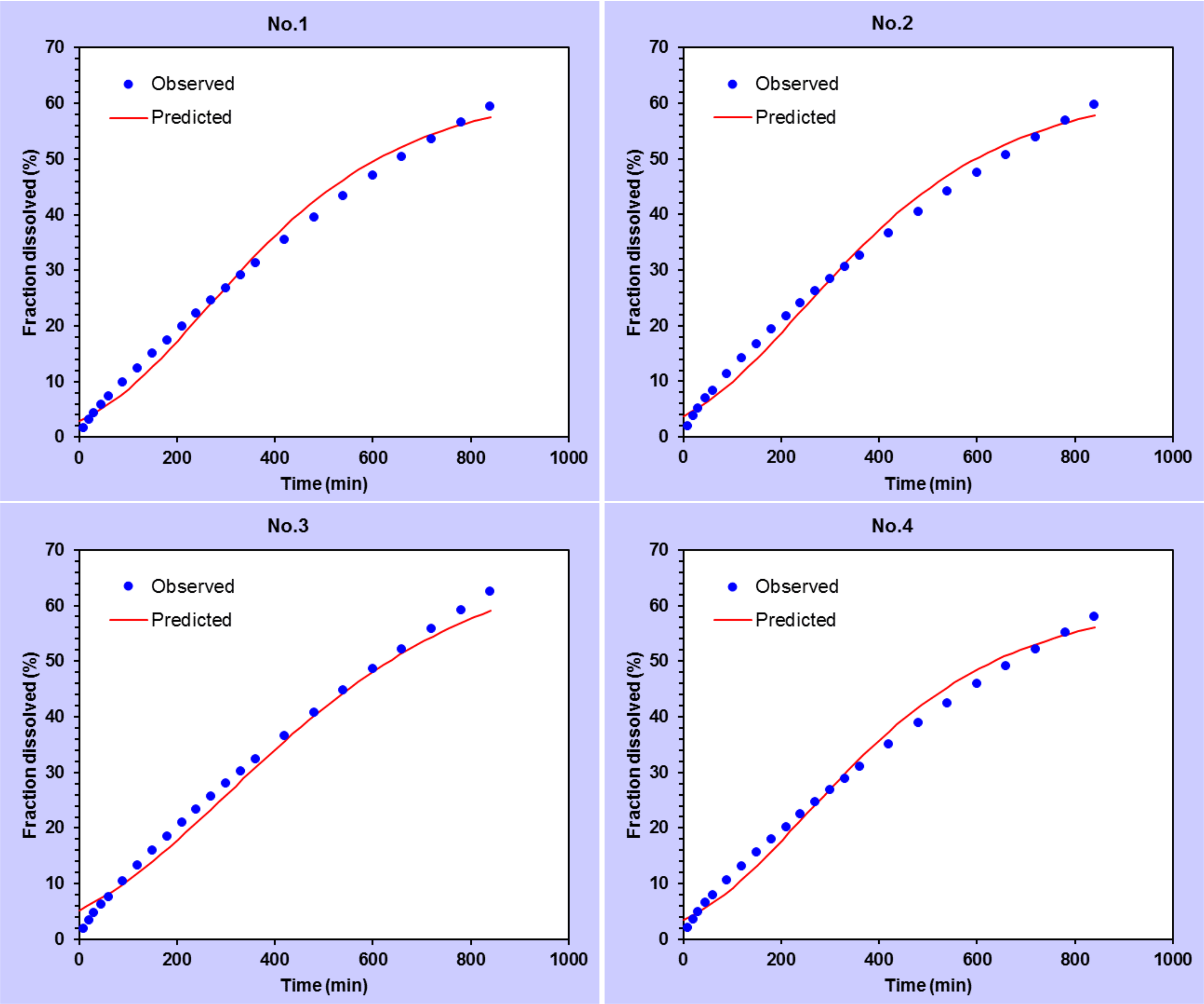

Supplementary materials – Model fitting summary – Ethocel™ Standard 20 Premium (modelling up to app. 60 % of carvedilol released)

Model: **Gompertz_4**

Model equation: $F = F_{max} \cdot e^{-\beta \cdot e^{-k \cdot t}}$

Fitted model parameters per tested tablet (N = 4) with statistics – mean, standard deviation (SD), and relative standard deviation expressed in % (RSD%) (output from DDSolver):

Parameter	No.1	No.2	No.3	No.4	Mean	SD	RSD(%)
k	0.004	0.004	0.004	0.004	0.004	0.000	1.078
β	3.052	2.819	3.005	2.886	2.941	0.107	3.642
F_{max}	62.383	62.702	65.584	60.940	62.902	1.946	3.093

Number of dissolution data points (N), degrees of freedom (df), and selected goodness of fit criteria – Pearson correlation coefficient (R), coefficient of determination (R^2), adjusted coefficient of determination ($R^2_{adjusted}$), and residual sum of squares (RSS) (manual calculation in MS Excel):

Parameter	No.1	No.2	No.3	No.4
N	23	23	23	23
df	20	20	20	20
R	0.996329168	0.995522296	0.995539105	0.995986462
R^2	0.992671811	0.991064642	0.991098109	0.991989032
$R^2_{adjusted}$	0.991938993	0.990171106	0.99020792	0.991187935
RSS	69.9646821	79.8393754	90.78804901	69.87692844

Graphical abstract of model fit presented as mean ± 1 SD of the fraction % of released carvedilol:

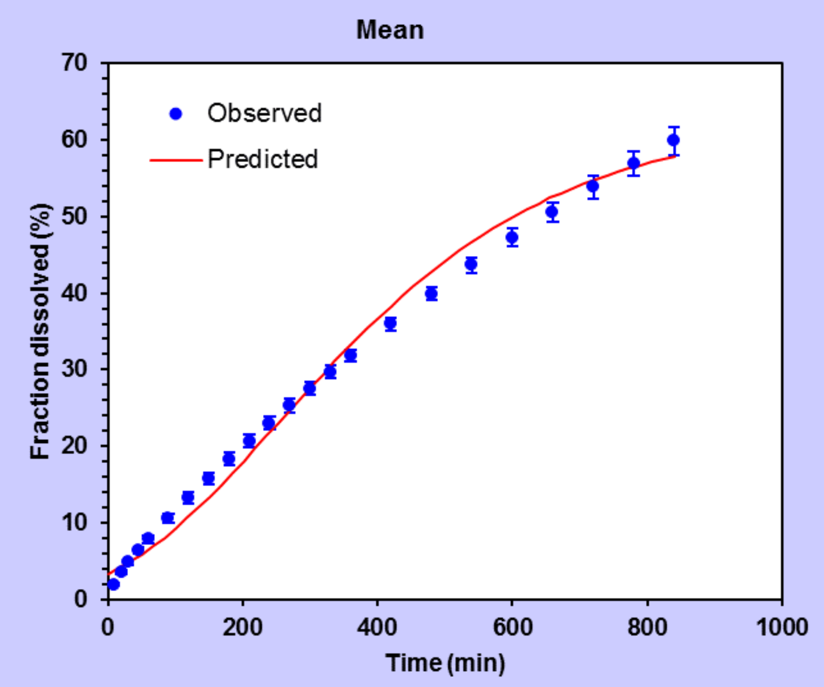

Supplementary materials – Model fitting summary – Ethocel^{TM} Standard 20 Premium (modelling up to app. 60 % of carvedilol released)

Graphical abstract of model fit presented as the fraction % of released carvedilol per tested tablet:

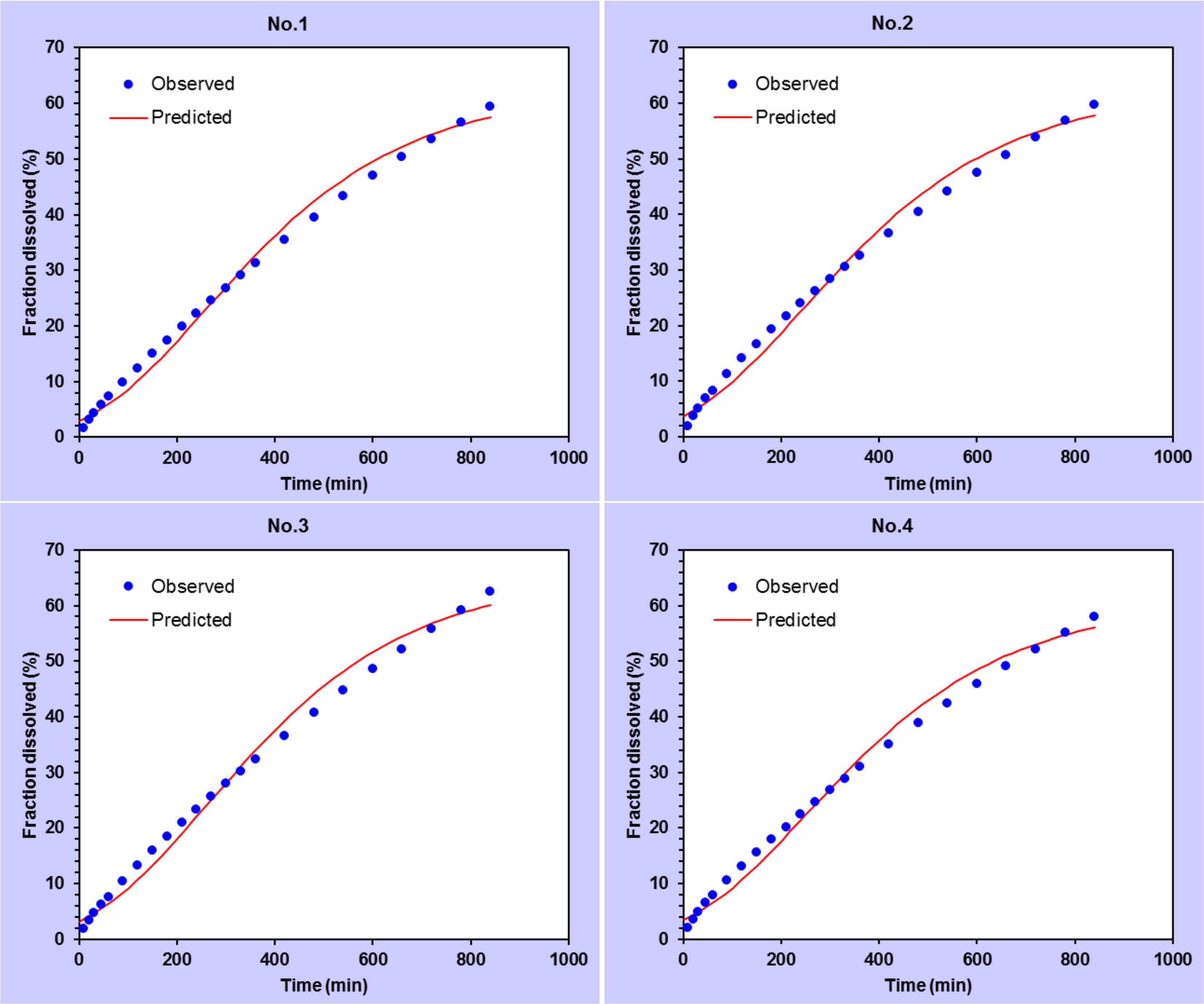

Supplementary materials – Model fitting summary – Ethocel^{TM} Standard 20 Premium (modelling up to app. 60 % of carvedilol released)

Model: **Probit_1**

Model equation: $F = 100 \cdot \phi[\alpha + \beta \cdot \log(t)]$

Fitted model parameters per tested tablet (N = 4) with statistics – mean, standard deviation (SD), and relative standard deviation expressed in % (RSD%) (output from DDSolver):

Parameter	No.1	No.2	No.3	No.4	Mean	SD	RSD(%)
α	-3.596	-3.452	-3.570	-3.444	-3.516	0.079	-2.234
β	1.239	1.197	1.246	1.176	1.215	0.033	2.737

Number of dissolution data points (N), degrees of freedom (df), and selected goodness of fit criteria – Pearson correlation coefficient (R), coefficient of determination (R^2), adjusted coefficient of determination ($R^2_{adjusted}$), and residual sum of squares (RSS) (manual calculation in MS Excel):

Parameter	No.1	No.2	No.3	No.4
N	23	23	23	23
df	21	21	21	21
R	0.986275307	0.987778076	0.984328262	0.985735854
R^2	0.972738982	0.975705528	0.968902128	0.971675175
$R^2_{adjusted}$	0.971440838	0.974548648	0.967421277	0.970326373
RSS	288.626487	237.8502383	340.4456193	269.1078841

Graphical abstract of model fit presented as mean ± 1 SD of the fraction % of released carvedilol:

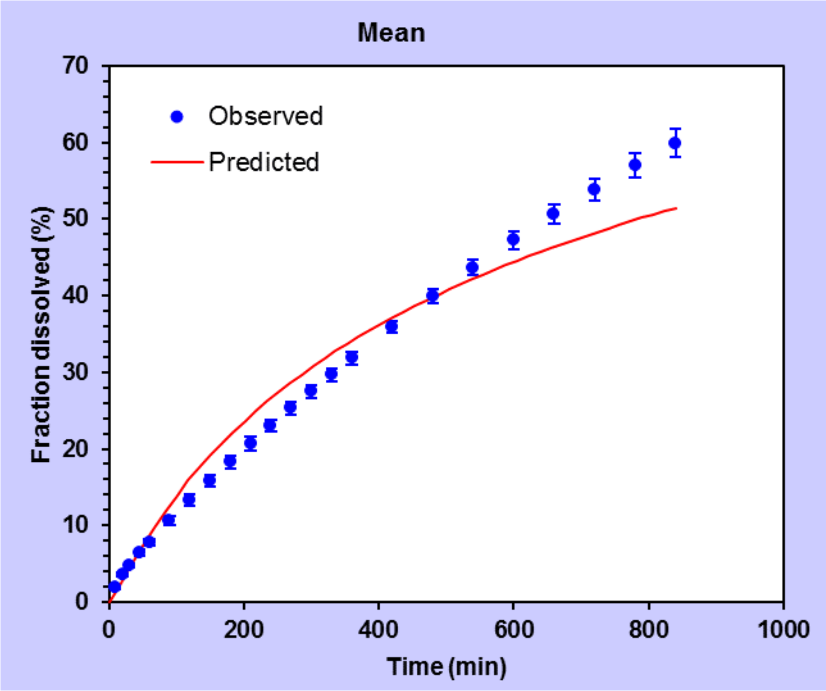

Supplementary materials – Model fitting summary – Ethocel™ Standard 20 Premium (modelling up to app. 60 % of carvedilol released)

Graphical abstract of model fit presented as the fraction % of released carvedilol per tested tablet:

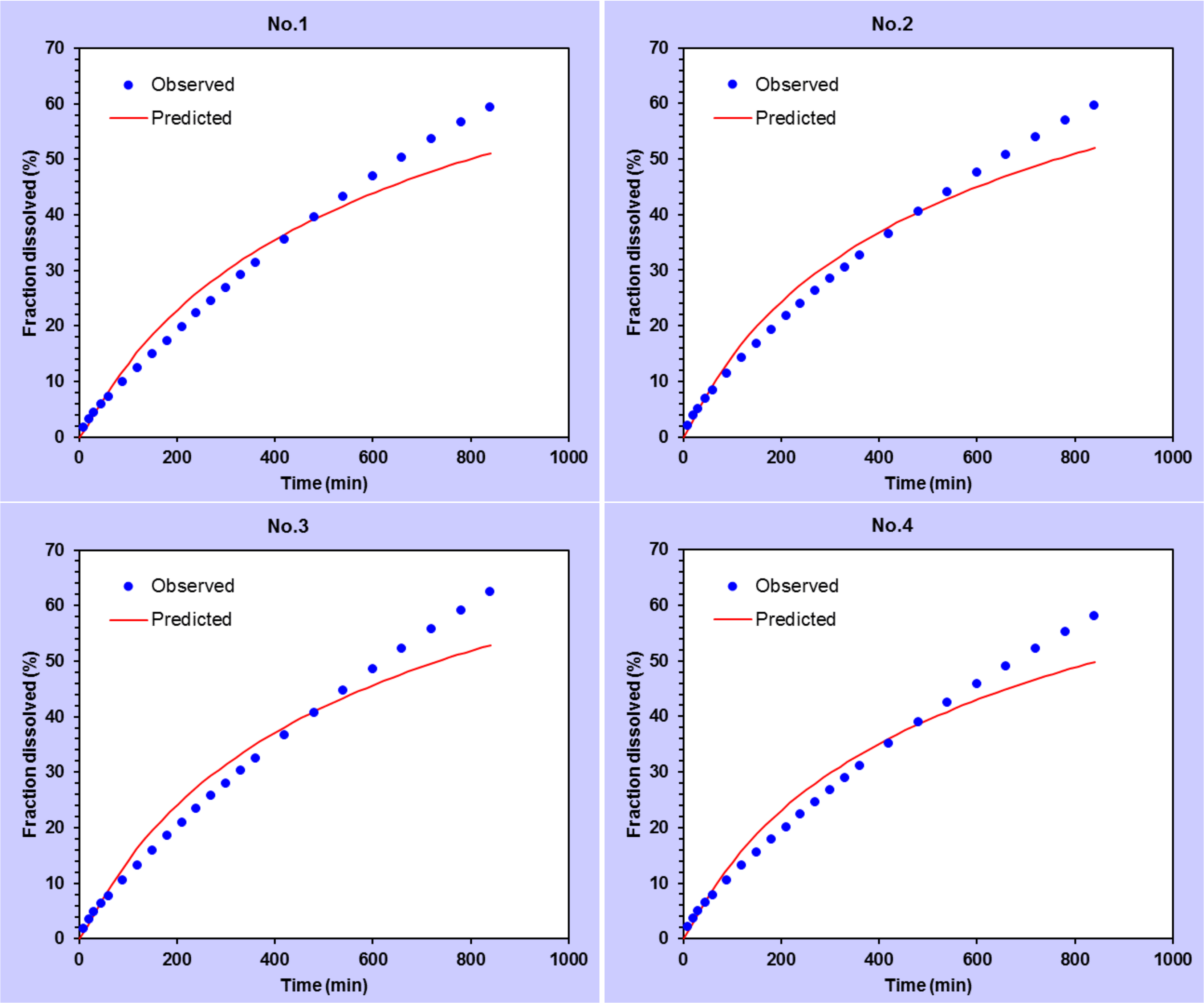

Supplementary materials – Model fitting summary – Ethocel™ Standard 20 Premium (modelling up to app. 60 % of carvedilol released)

Model: **Probit_2**

Model equation: $F = F_{max} \cdot \phi[\alpha + \beta \cdot \log(t)]$

Fitted model parameters per tested tablet (N = 4) with statistics – mean, standard deviation (SD), and relative standard deviation expressed in % (RSD%) (output from DDSolver):

Parameter	No.1	No.2	No.3	No.4	Mean	SD	RSD(%)
α	-4.361	-4.194	-4.684	-4.192	-4.358	0.231	-5.311
β	1.635	1.592	1.780	1.581	1.647	0.092	5.562
F_{max}	74.359	74.738	71.813	72.638	73.387	1.391	1.896

Number of dissolution data points (N), degrees of freedom (df), and selected goodness of fit criteria – Pearson correlation coefficient (R), coefficient of determination (R^2), adjusted coefficient of determination ($R^2_{adjusted}$), and residual sum of squares (RSS) (manual calculation in MS Excel):

Parameter	No.1	No.2	No.3	No.4
N	23	23	23	23
df	20	20	20	20
R	0.987664755	0.98815375	0.988067403	0.986054956
R^2	0.975481668	0.976447835	0.976277193	0.972304377
$R^2_{adjusted}$	0.973029835	0.974092618	0.973904913	0.969534815
RSS	333.2959242	261.4612454	481.5889116	291.5686083

Graphical abstract of model fit presented as mean ± 1 SD of the fraction % of released carvedilol:

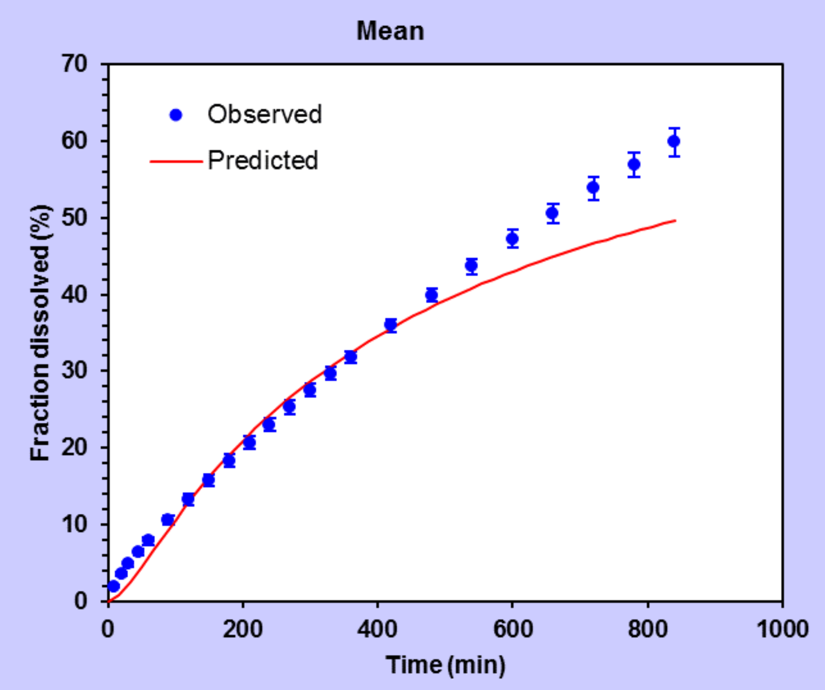

Supplementary materials – Model fitting summary – Ethocel™ Standard 20 Premium (modelling up to app. 60 % of carvedilol released)

Graphical abstract of model fit presented as the fraction % of released carvedilol per tested tablet:

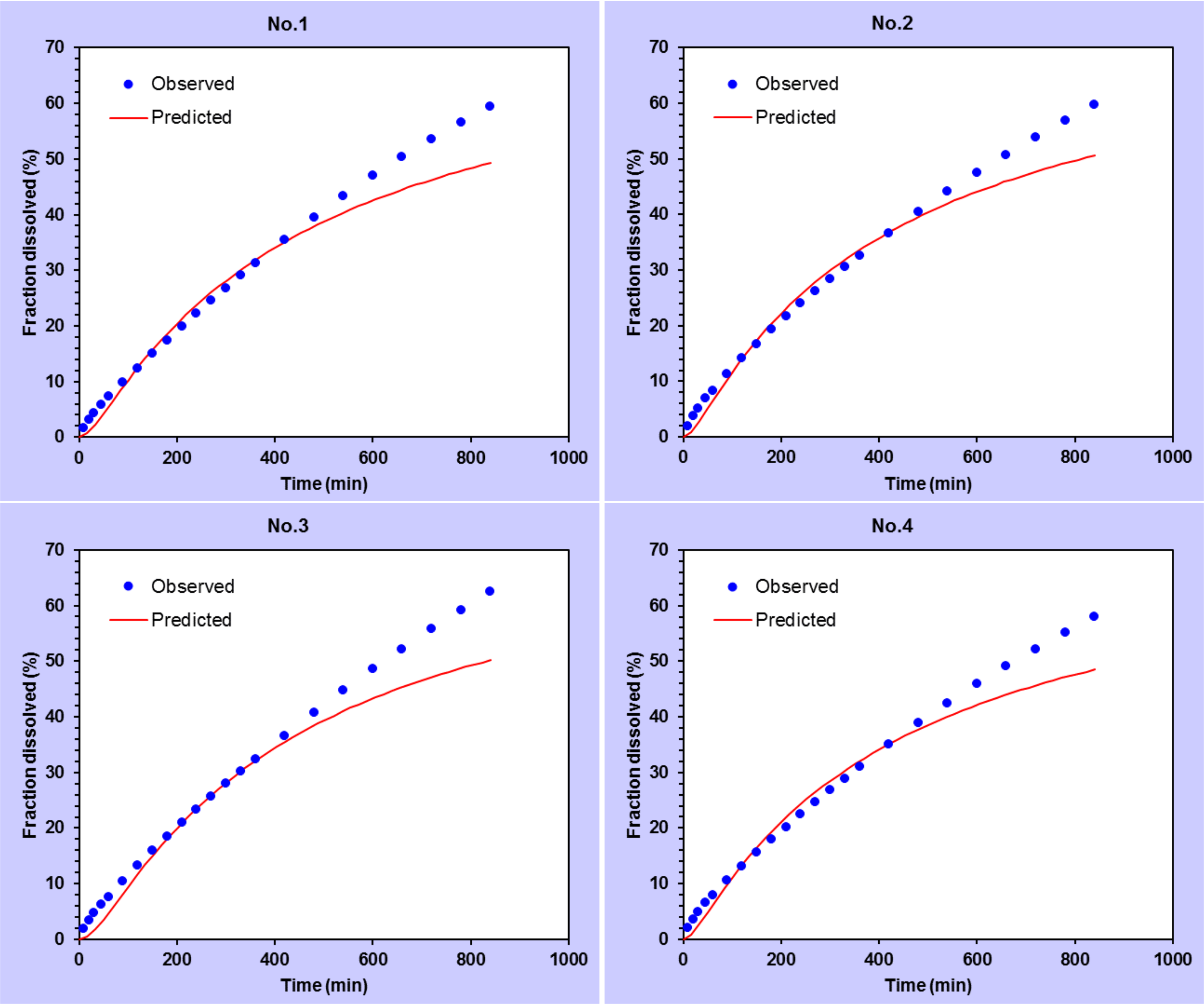

Supplement: Supplementary file 1 [file pharmaceutics-16-00498-s001.zip › Supplementary materials_Model fitting summary_EthocelTM Standard 20 Premium.pdf]
