# Supplementary material for: Comparative Fitting of Mathematical Models to Carvedilol Release Profiles Obtained from Hypromellose Matrix Tablets
Source: Pharmaceutics. 2024 Apr 4;16(4):498. doi: 10.3390/pharmaceutics16040498 (PMC11053526; doi:10.3390/pharmaceutics16040498)

Supplementary materials – Model fitting summary – FlowLac® 100 (modelling the entire relevant dissolution data)

Model: **Zero–order**

Model equation: $F = k_0 \cdot t$

Fitted model parameters per tested tablet (N = 4) with statistics – mean, standard deviation (SD), and relative standard deviation expressed in % (RSD%) (output from DDSolver):

Parameter	No.1	No.2	No.3	No.4	Mean	SD	RSD(%)
k_0	0.133	0.133	0.143	0.136	0.136	0.005	3.543

Number of dissolution data points (N), degrees of freedom (df), and selected goodness of fit criteria – Pearson correlation coefficient (R), coefficient of determination (R^2), adjusted coefficient of determination ($R^2_{adjusted}$), and residual sum of squares (RSS) (manual calculation in MS Excel):

Parameter	No.1	No.2	No.3	No.4
N	23	23	23	23
df	22	22	22	22
R	0.995431174	0.994660468	0.991060273	0.994612142
R^2	0.990883222	0.989349446	0.982200465	0.989253312
$R^2_{adjusted}$	0.990883222	0.989349446	0.982200465	0.989253312
RSS	1545.419385	1811.534588	2978.125745	1692.401154

Graphical abstract of model fit presented as mean ± 1 SD of the fraction % of released carvedilol:

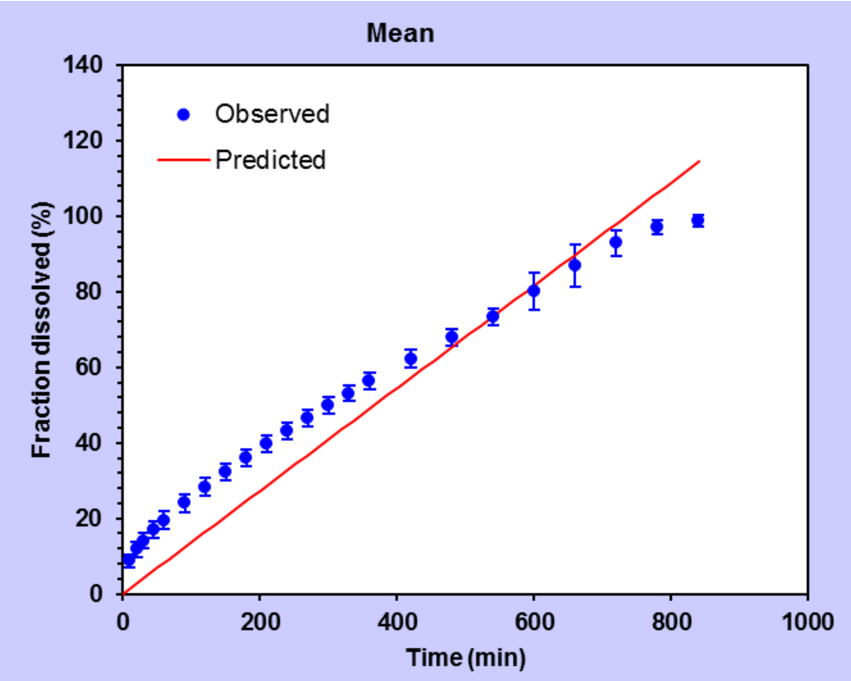

Supplementary materials – Model fitting summary – FlowLac® 100 (modelling the entire relevant dissolution data)

Graphical abstract of model fit presented as the fraction % of released carvedilol per tested tablet:

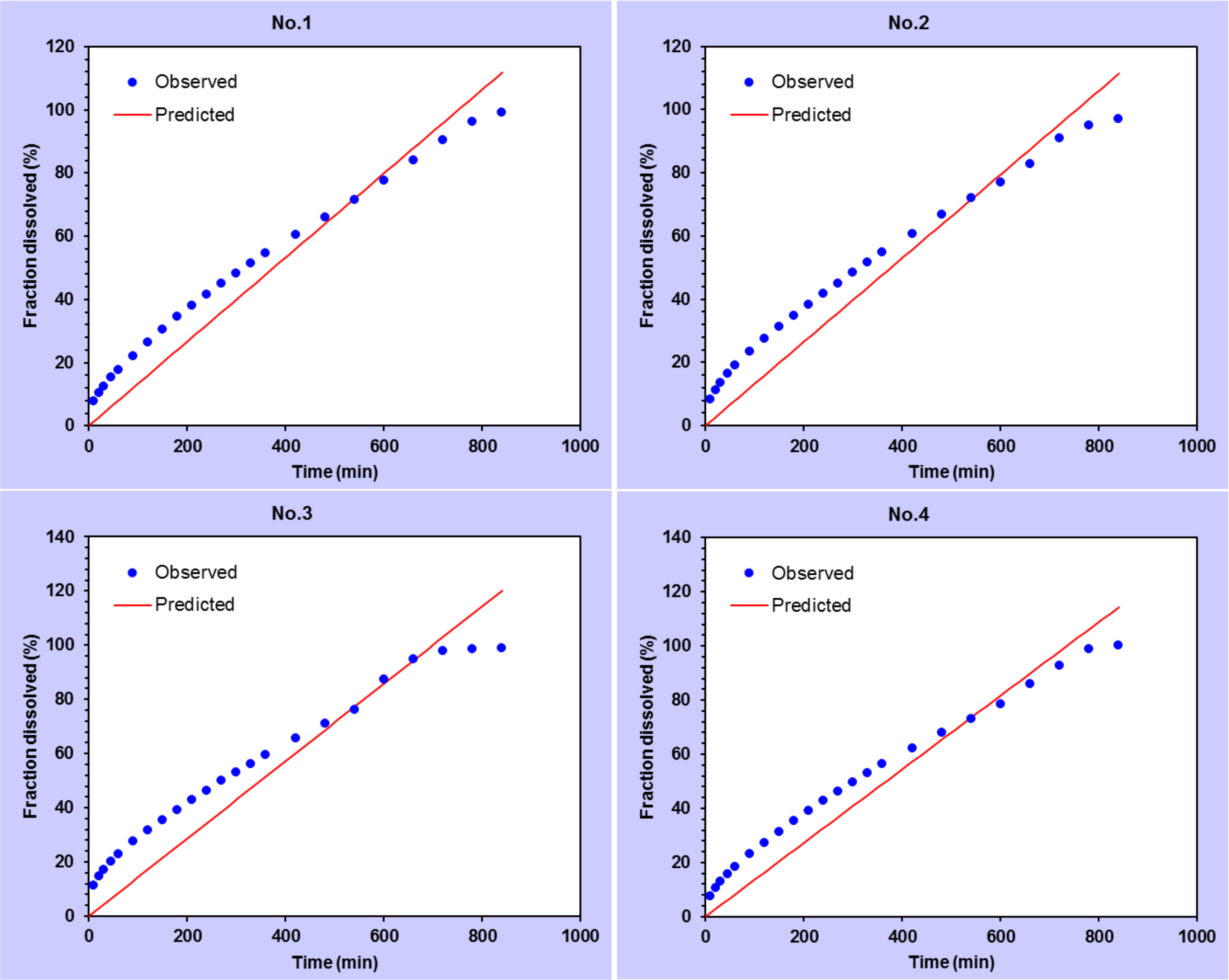

Supplementary materials – Model fitting summary – FlowLac® 100 (modelling the entire relevant dissolution data)

Model: **Zero–order with T_{lag}**

Model equation: $F = k_0 \cdot (t - T_{lag})$

Fitted model parameters per tested tablet (N = 4) with statistics – mean, standard deviation (SD), and relative standard deviation expressed in % (RSD%) (output from DDSolver):

Parameter	No.1	No.2	No.3	No.4	Mean	SD	RSD(%)
k_0	0.109	0.107	0.110	0.111	0.109	0.002	1.822
T_{lag}	-114.581	-127.540	-157.679	-116.845	-129.161	19.834	-15.356

Number of dissolution data points (N), degrees of freedom (df), and selected goodness of fit criteria – Pearson correlation coefficient (R), coefficient of determination (R^2), adjusted coefficient of determination ($R^2_{adjusted}$), and residual sum of squares (RSS) (manual calculation in MS Excel):

Parameter	No.1	No.2	No.3	No.4
N	23	23	23	23
df	21	21	21	21
R	0.995431174	0.994660468	0.991060273	0.994612142
R^2	0.990883222	0.989349446	0.982200465	0.989253312
$R^2_{adjusted}$	0.990449089	0.988842277	0.981352868	0.988741565
RSS	164.6630878	183.3648406	328.6323032	201.828923

Graphical abstract of model fit presented as mean ± 1 SD of the fraction % of released carvedilol:

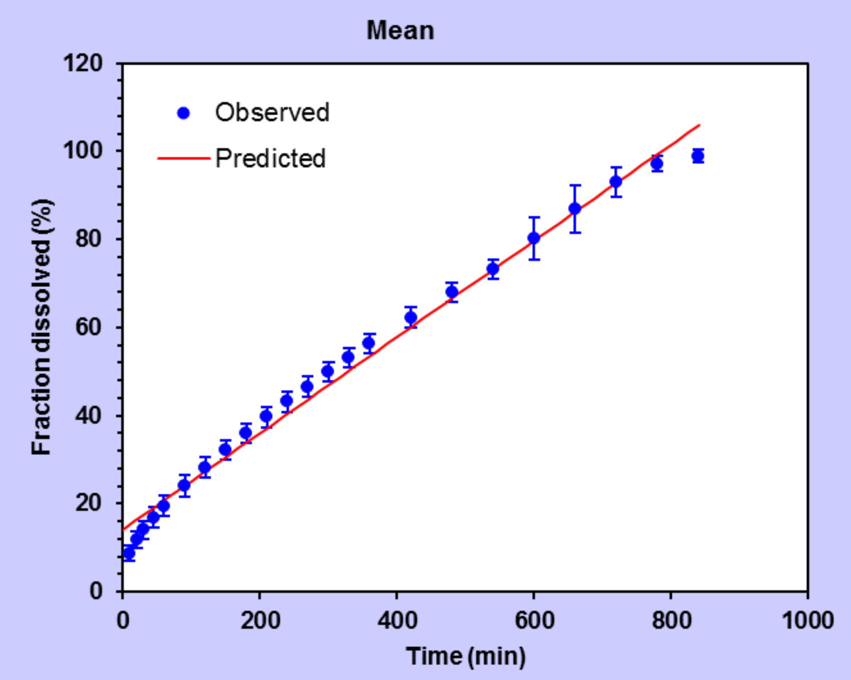

Supplementary materials – Model fitting summary – FlowLac® 100 (modelling the entire relevant dissolution data)

Graphical abstract of model fit presented as the fraction % of released carvedilol per tested tablet:

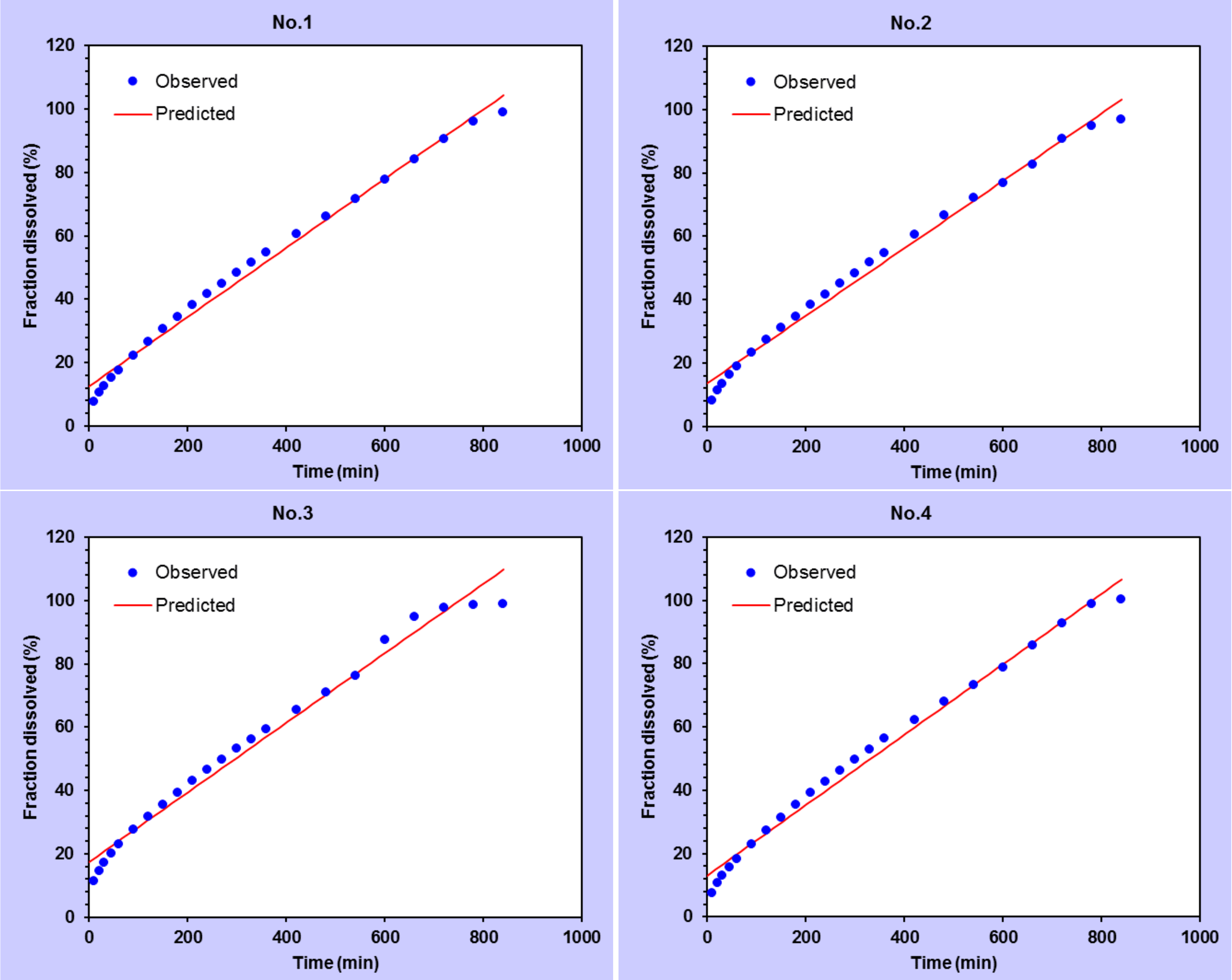

Supplementary materials – Model fitting summary – FlowLac® 100 (modelling the entire relevant dissolution data)

Model: **Zero–order with F_0**

Model equation: $F = F_0 + k_0 \cdot t$

Fitted model parameters per tested tablet (N = 4) with statistics – mean, standard deviation (SD), and relative standard deviation expressed in % (RSD%) (output from DDSolver):

Parameter	No.1	No.2	No.3	No.4	Mean	SD	RSD(%)
k_0	0.109	0.107	0.110	0.111	0.109	0.002	1.822
F_0	12.526	13.602	17.351	13.015	14.124	2.196	15.552

Number of dissolution data points (N), degrees of freedom (df), and selected goodness of fit criteria – Pearson correlation coefficient (R), coefficient of determination (R^2), adjusted coefficient of determination ($R^2_{adjusted}$), and residual sum of squares (RSS) (manual calculation in MS Excel):

Parameter	No.1	No.2	No.3	No.4
N	23	23	23	23
df	21	21	21	21
R	0.995431174	0.994660468	0.991060273	0.994612142
R^2	0.990883222	0.989349446	0.982200465	0.989253312
$R^2_{adjusted}$	0.990449089	0.988842277	0.981352868	0.988741565
RSS	164.6630878	183.3648406	328.6323032	201.828923

Graphical abstract of model fit presented as mean ± 1 SD of the fraction % of released carvedilol:

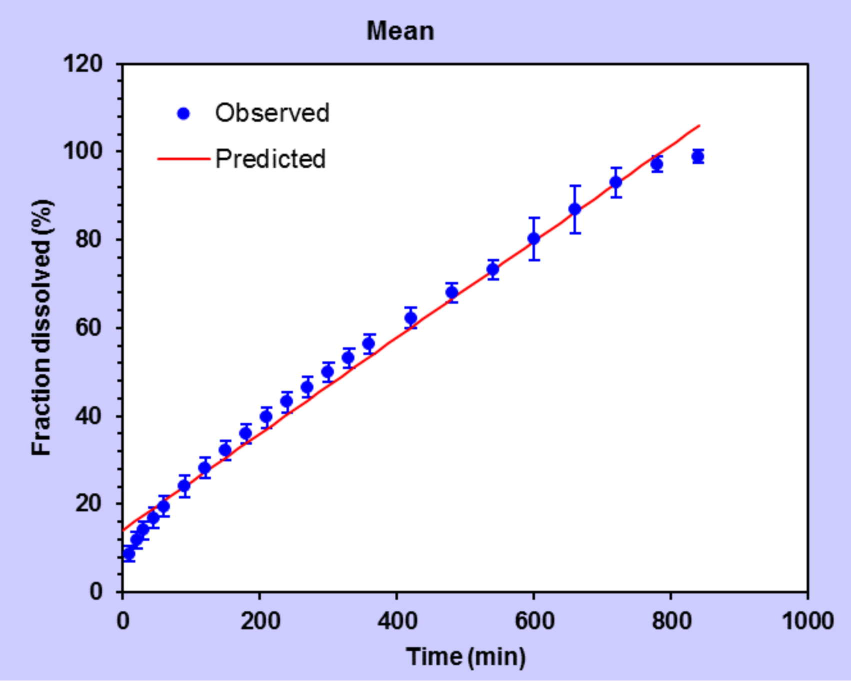

Supplementary materials – Model fitting summary – FlowLac® 100 (modelling the entire relevant dissolution data)

Graphical abstract of model fit presented as the fraction % of released carvedilol per tested tablet:

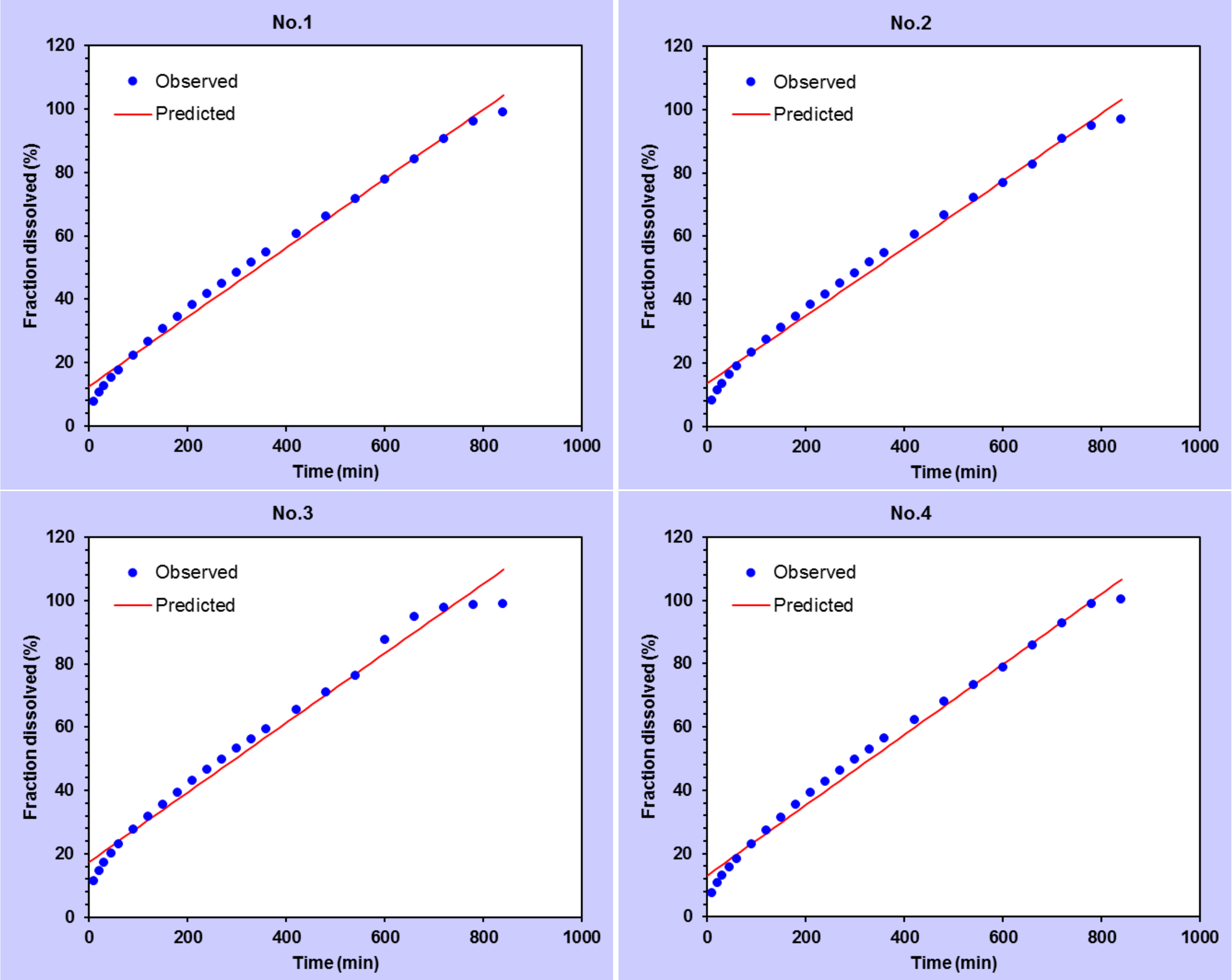

Supplementary materials – Model fitting summary – FlowLac® 100 (modelling the entire relevant dissolution data)

Model: **First–order**

Model equation: $F = 100 \cdot (1 - e^{-k_1 \cdot t})$

Fitted model parameters per tested tablet (N = 4) with statistics – mean, standard deviation (SD), and relative standard deviation expressed in % (RSD%) (output from DDSolver):

Parameter	No.1	No.2	No.3	No.4	Mean	SD	RSD(%)
k_1	0.003	0.002	0.002	0.002	0.002	0.000	8.163

Number of dissolution data points (N), degrees of freedom (df), and selected goodness of fit criteria – Pearson correlation coefficient (R), coefficient of determination (R^2), adjusted coefficient of determination ($R^2_{adjusted}$), and residual sum of squares (RSS) (manual calculation in MS Excel):

Parameter	No.1	No.2	No.3	No.4
N	23	23	23	23
df	22	22	22	22
R	0.984178261	0.988534251	0.990607059	0.985572934
R^2	0.96860685	0.977199966	0.981302346	0.971354007
$R^2_{adjusted}$	0.96860685	0.977199966	0.981302346	0.971354007
RSS	577.7127363	655.1107134	2857.223893	652.6955153

Graphical abstract of model fit presented as mean ± 1 SD of the fraction % of released carvedilol:

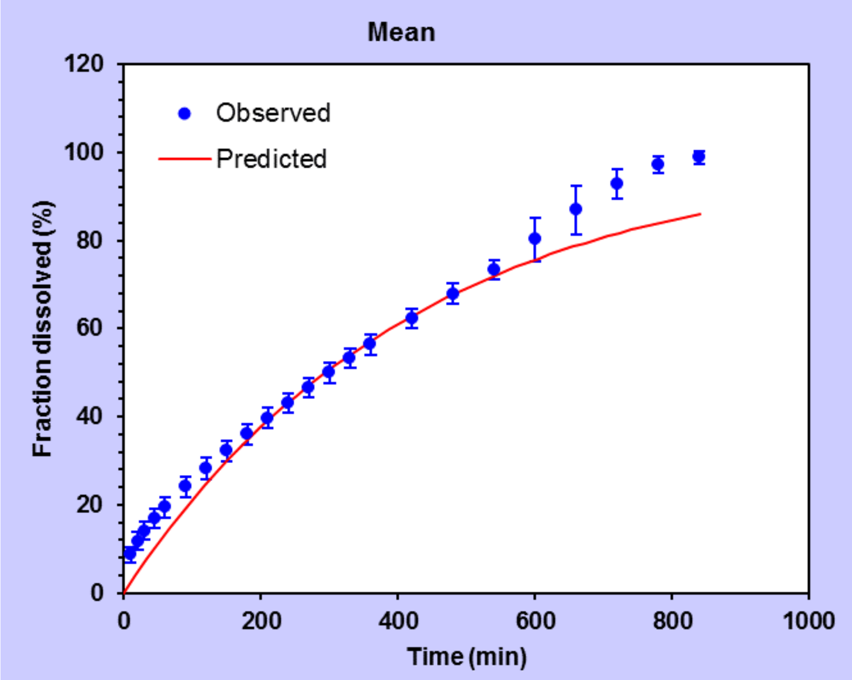

Supplementary materials – Model fitting summary – FlowLac® 100 (modelling the entire relevant dissolution data)

Graphical abstract of model fit presented as the fraction % of released carvedilol per tested tablet:

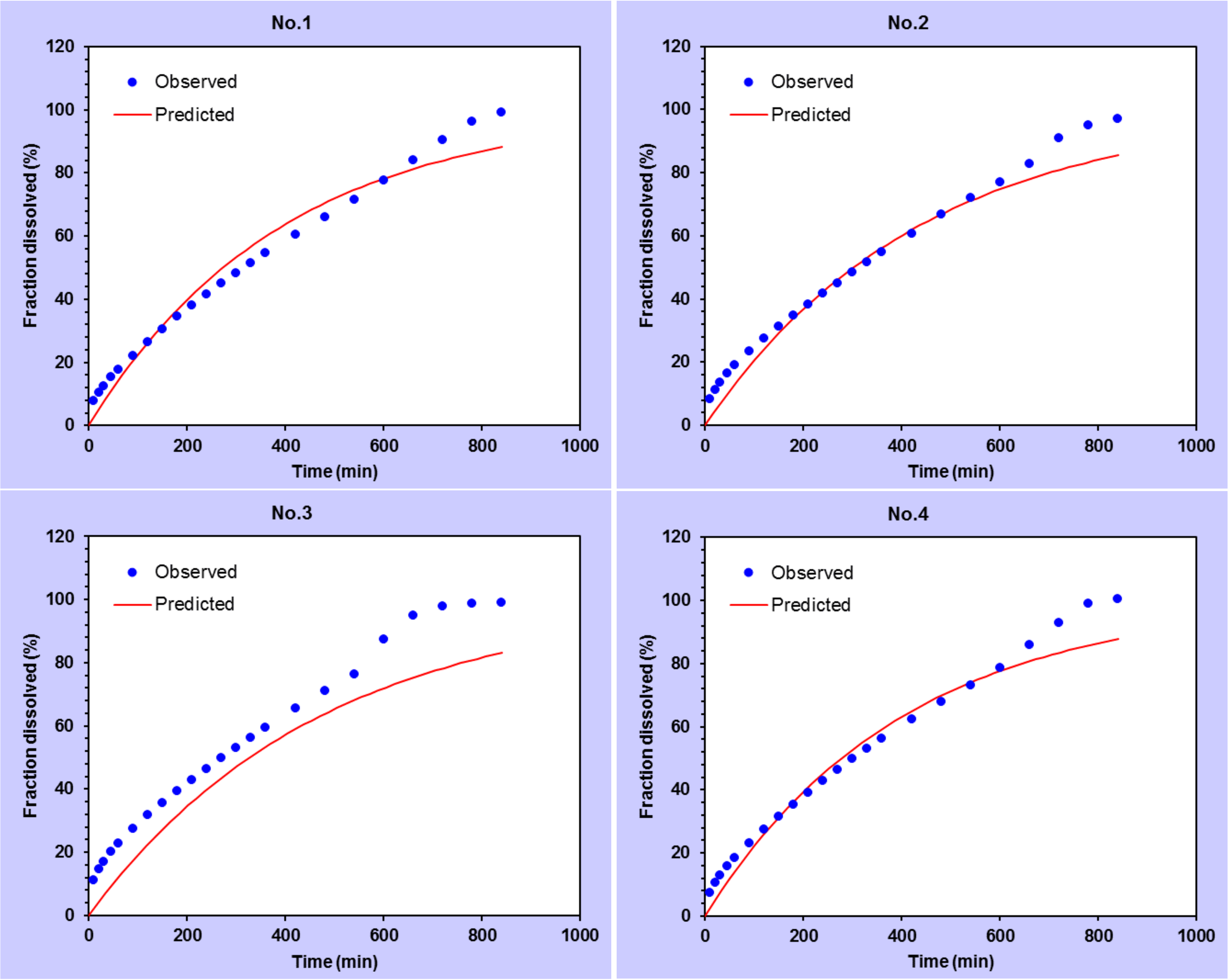

Supplementary materials – Model fitting summary – FlowLac® 100 (modelling the entire relevant dissolution data)

Model: **First–order with T_{lag}**

Model equation: $F = 100 \cdot \left[1 - e^{-k_1 \cdot (t - T_{lag})}\right]$

Fitted model parameters per tested tablet (N = 4) with statistics – mean, standard deviation (SD), and relative standard deviation expressed in % (RSD%) (output from DDSolver):

Parameter	No.1	No.2	No.3	No.4	Mean	SD	RSD(%)
k_1	/	/	/	/	/	/	/
T_{lag}	/	/	/	/	/	/	/

Number of dissolution data points (N), degrees of freedom (df), and selected goodness of fit criteria – Pearson correlation coefficient (R), coefficient of determination (R^2), adjusted coefficient of determination ($R^2_{adjusted}$), and residual sum of squares (RSS) (manual calculation in MS Excel):

Parameter	No.1	No.2	No.3	No.4
N	/	/	/	/
df	/	/	/	/
R	/	/	/	/
R^2	/	/	/	/
$R^2_{adjusted}$	/	/	/	/
RSS	/	/	/	/

Graphical abstract of model fit presented as mean ± 1 SD of the fraction % of released carvedilol: /

Graphical abstract of model fit presented as the fraction % of released carvedilol per tested tablet: /

Note: the model could not be fitted

Supplementary materials – Model fitting summary – FlowLac® 100 (modelling the entire relevant dissolution data)

Model: **First–order with F_{max}**

Model equation: $F = F_{max} \cdot (1 - e^{-k_1 \cdot t})$

Fitted model parameters per tested tablet (N = 4) with statistics – mean, standard deviation (SD), and relative standard deviation expressed in % (RSD%) (output from DDSolver):

Parameter	No.1	No.2	No.3	No.4	Mean	SD	RSD(%)
k_1	0.003	0.003	0.003	0.003	0.003	0.000	9.697
F_{max}	104.053	101.773	103.988	105.401	103.804	1.502	1.447

Number of dissolution data points (N), degrees of freedom (df), and selected goodness of fit criteria – Pearson correlation coefficient (R), coefficient of determination (R^2), adjusted coefficient of determination ($R^2_{adjusted}$), and residual sum of squares (RSS) (manual calculation in MS Excel):

Parameter	No.1	No.2	No.3	No.4
N	23	23	23	23
df	21	21	21	21
R	0.980908238	0.980002422	0.972761525	0.983481492
R^2	0.962180971	0.960404747	0.946264985	0.967235845
$R^2_{adjusted}$	0.960380065	0.958519259	0.943706174	0.965675647
RSS	1047.479337	1029.273089	1448.913545	747.2197318

Graphical abstract of model fit presented as mean ± 1 SD of the fraction % of released carvedilol:

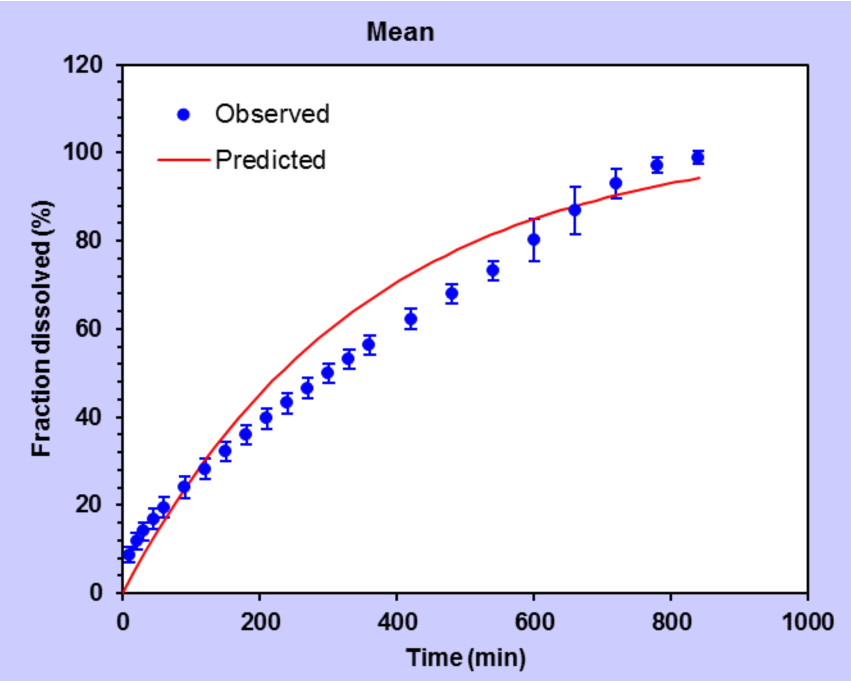

Supplementary materials – Model fitting summary – FlowLac® 100 (modelling the entire relevant dissolution data)

Graphical abstract of model fit presented as the fraction % of released carvedilol per tested tablet:

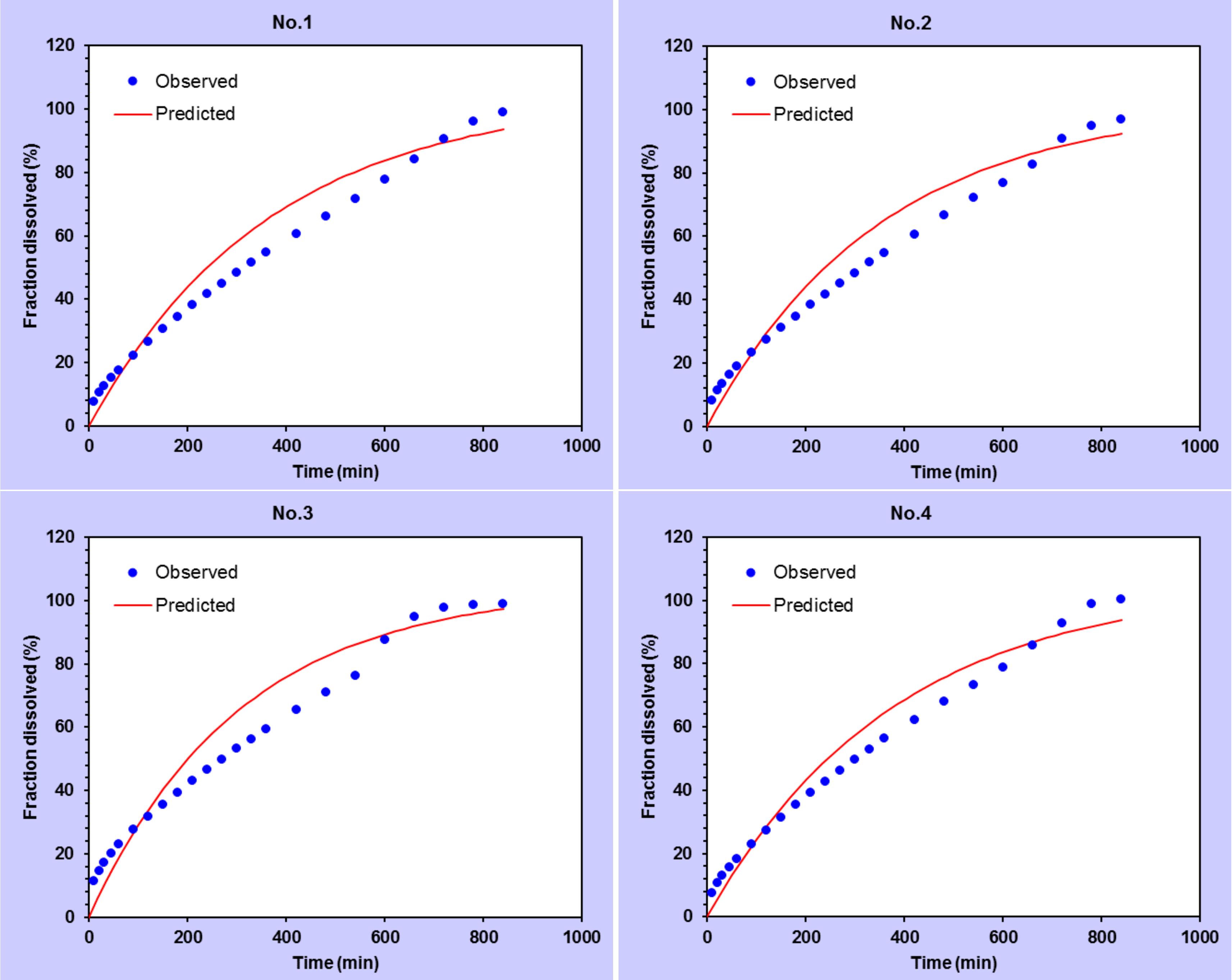

Supplementary materials – Model fitting summary – FlowLac® 100 (modelling the entire relevant dissolution data)

Model: **First–order with T_{lag} and F_{max}**

Model equation: $F = F_{max} \cdot \left[1 - e^{-k_1 \cdot (t - T_{lag})}\right]$

Fitted model parameters per tested tablet (N = 4) with statistics – mean, standard deviation (SD), and relative standard deviation expressed in % (RSD%) (output from DDSolver):

Parameter	No.1	No.2	No.3	No.4	Mean	SD	RSD(%)
k_1	0.003	0.003	0.004	0.003	0.003	0.000	7.634
T_{lag}	42.103	39.261	36.684	43.490	40.384	3.030	7.504
F_{max}	104.053	101.773	103.988	105.401	103.804	1.502	1.447

Number of dissolution data points (N), degrees of freedom (df), and selected goodness of fit criteria – Pearson correlation coefficient (R), coefficient of determination (R^2), adjusted coefficient of determination ($R^2_{adjusted}$), and residual sum of squares (RSS) (manual calculation in MS Excel):

Parameter	No.1	No.2	No.3	No.4
N	23	23	23	23
df	20	20	20	20
R	0.976620581	0.975847036	0.967961655	0.975955441
R^2	0.953787759	0.952277438	0.936949766	0.952489024
$R^2_{adjusted}$	0.949166535	0.947505182	0.930644742	0.947737926
RSS	1987.046198	1976.452775	2751.09914	2196.683866

Graphical abstract of model fit presented as mean ± 1 SD of the fraction % of released carvedilol:

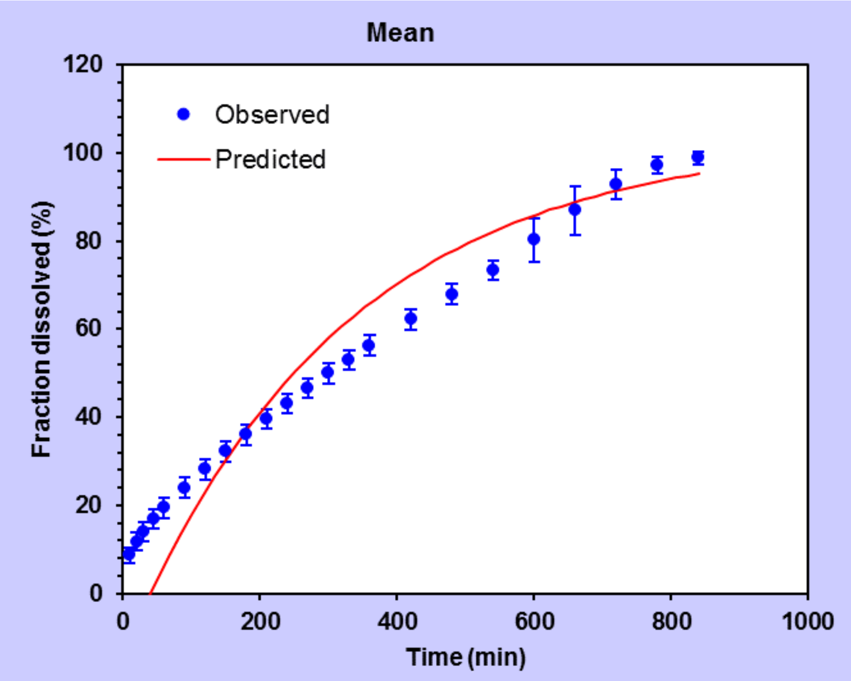

Supplementary materials – Model fitting summary – FlowLac® 100 (modelling the entire relevant dissolution data)

Graphical abstract of model fit presented as the fraction % of released carvedilol per tested tablet:

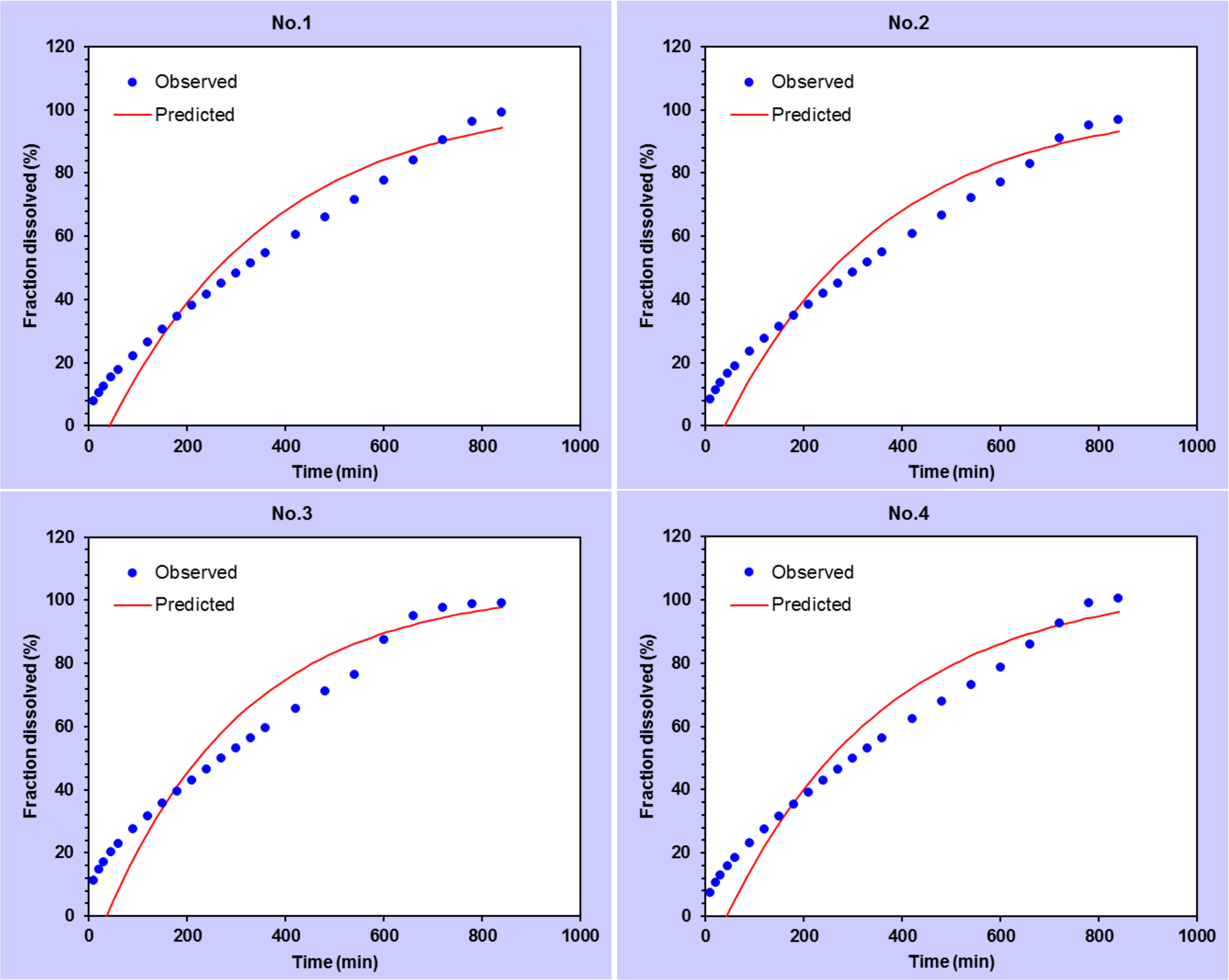

Supplementary materials – Model fitting summary – FlowLac® 100 (modelling the entire relevant dissolution data)

Model: **Higuchi**

Model equation: $F = k_H \cdot t^{0.5}$

Fitted model parameters per tested tablet (N = 4) with statistics – mean, standard deviation (SD), and relative standard deviation expressed in % (RSD%) (output from DDSolver):

Parameter	No.1	No.2	No.3	No.4	Mean	SD	RSD(%)
k_H	3.069	3.064	3.329	3.140	3.150	0.124	3.944

Number of dissolution data points (N), degrees of freedom (df), and selected goodness of fit criteria – Pearson correlation coefficient (R), coefficient of determination (R^2), adjusted coefficient of determination ($R^2_{adjusted}$), and residual sum of squares (RSS) (manual calculation in MS Excel):

Parameter	No.1	No.2	No.3	No.4
N	23	23	23	23
df	22	22	22	22
R	0.991941259	0.992487888	0.991269943	0.992555274
R^2	0.983947462	0.985032208	0.982616101	0.985165973
$R^2_{adjusted}$	0.983947462	0.985032208	0.982616101	0.985165973
RSS	767.5670603	604.9836971	482.3132242	759.3368346

Graphical abstract of model fit presented as mean ± 1 SD of the fraction % of released carvedilol:

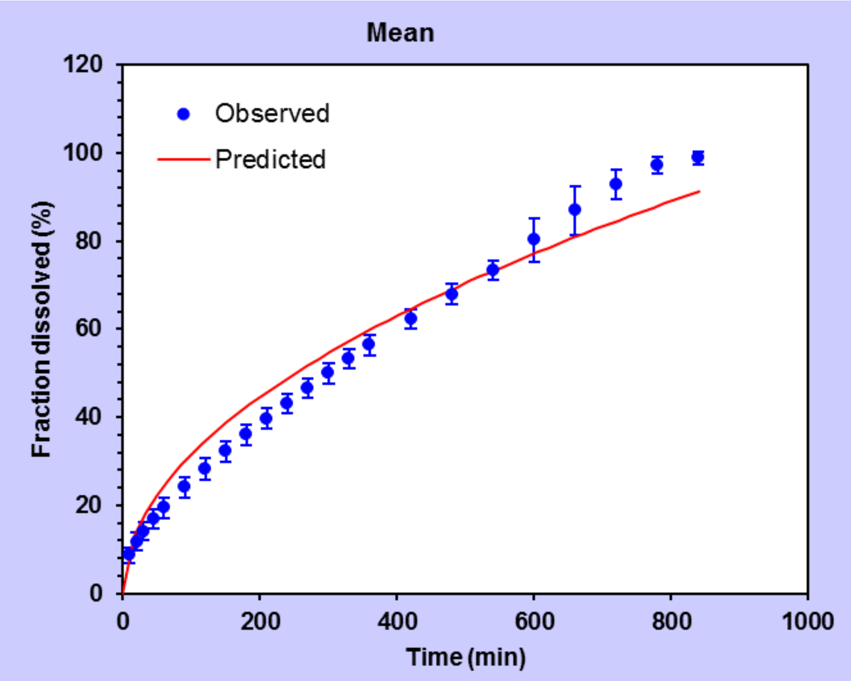

Supplementary materials – Model fitting summary – FlowLac® 100 (modelling the entire relevant dissolution data)

Graphical abstract of model fit presented as the fraction % of released carvedilol per tested tablet:

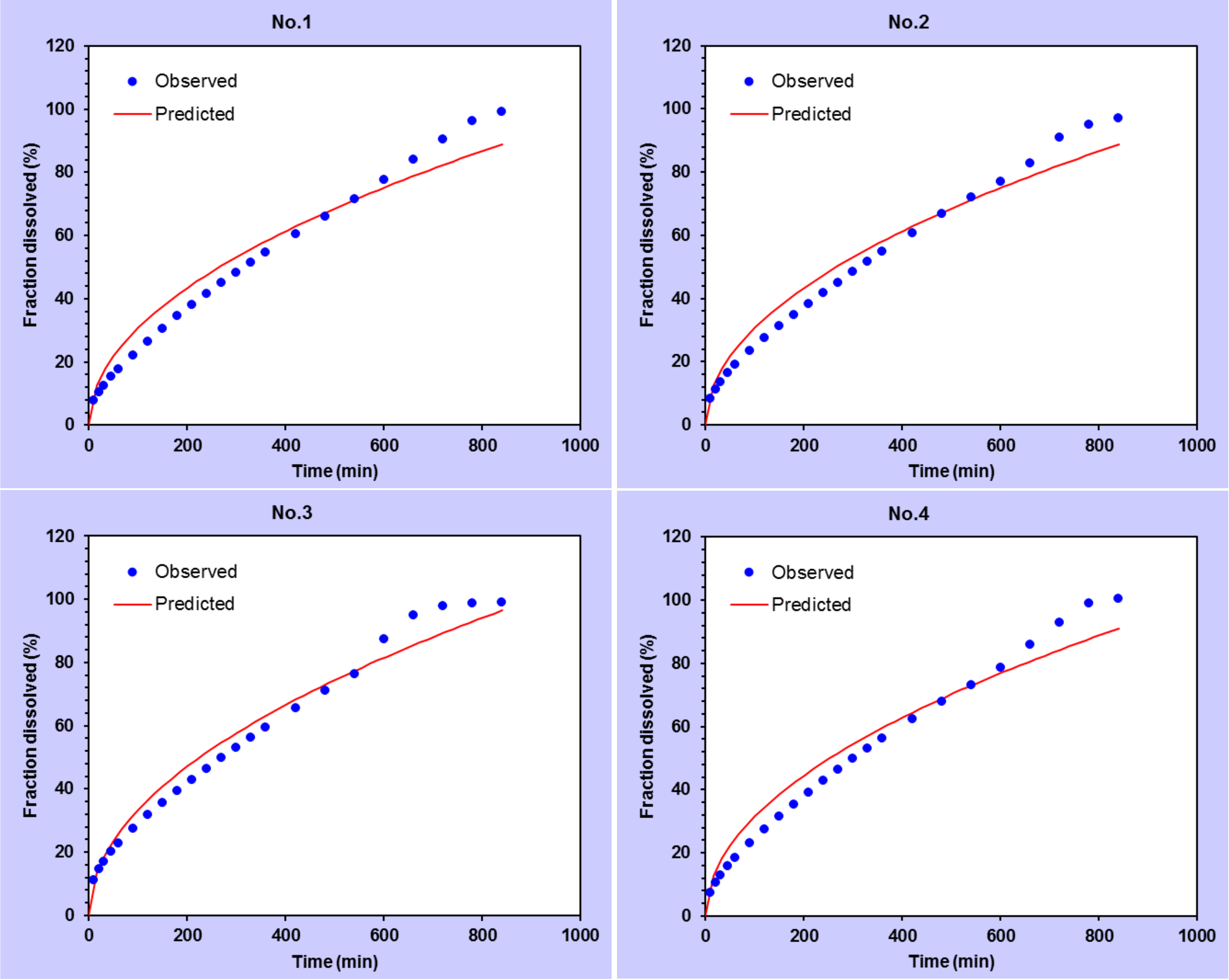

Supplementary materials – Model fitting summary – FlowLac® 100 (modelling the entire relevant dissolution data)

Model: **Higuchi with T_{lag}**

Model equation: $F = k_H \cdot (t - T_{lag})^{0.5}$

Fitted model parameters per tested tablet (N = 4) with statistics – mean, standard deviation (SD), and relative standard deviation expressed in % (RSD%) (output from DDSolver):

Parameter	No.1	No.2	No.3	No.4	Mean	SD	RSD(%)
k_H	3.420	3.376	3.577	3.489	3.465	0.087	2.524
T_{lag}	60.385	54.851	41.629	58.804	53.917	8.517	15.796

Number of dissolution data points (N), degrees of freedom (df), and selected goodness of fit criteria – Pearson correlation coefficient (R), coefficient of determination (R^2), adjusted coefficient of determination ($R^2_{adjusted}$), and residual sum of squares (RSS) (manual calculation in MS Excel):

Parameter	No.1	No.2	No.3	No.4
N	23	23	23	23
df	21	21	21	21
R	0.981350558	0.983022644	0.98204599	0.983072355
R^2	0.963048918	0.966333518	0.964414326	0.966431256
$R^2_{adjusted}$	0.961289342	0.964730352	0.96271977	0.964832744
RSS	1101.769287	972.1337673	1110.997525	1024.995445

Graphical abstract of model fit presented as mean ± 1 SD of the fraction % of released carvedilol:

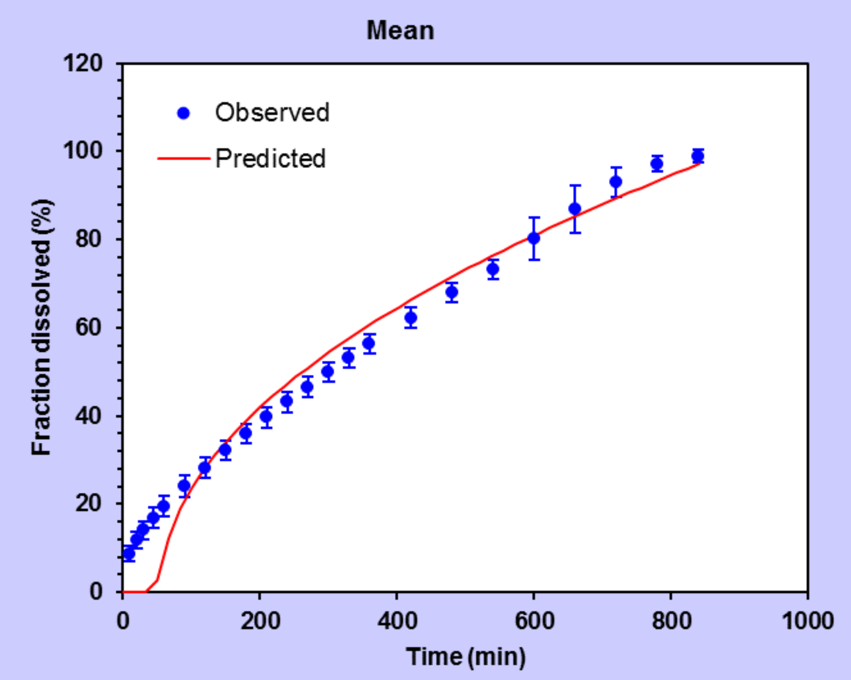

Supplementary materials – Model fitting summary – FlowLac® 100 (modelling the entire relevant dissolution data)

Graphical abstract of model fit presented as the fraction % of released carvedilol per tested tablet:

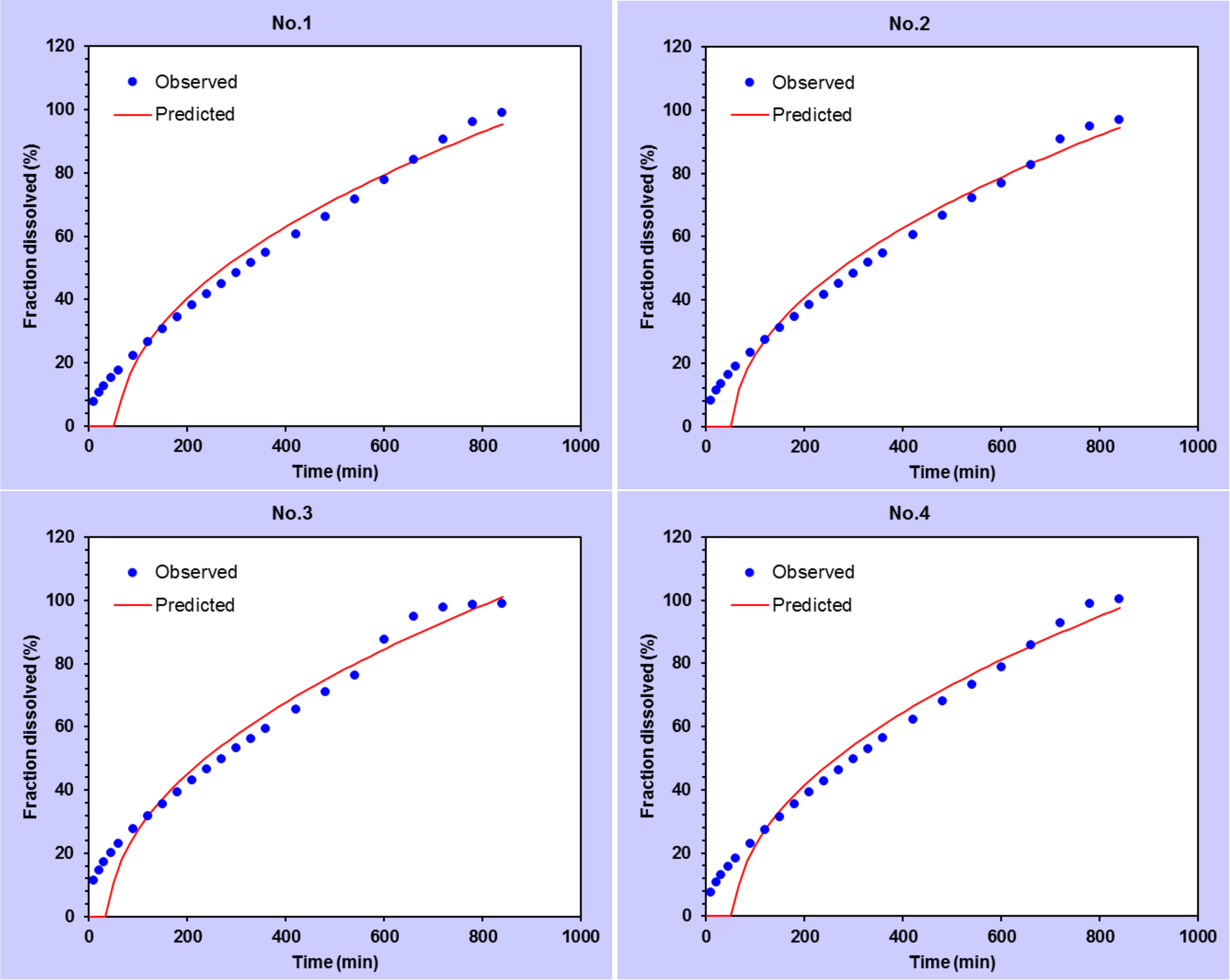

Supplementary materials – Model fitting summary – FlowLac® 100 (modelling the entire relevant dissolution data)

Model: **Higuchi with F_0**

Model equation: $F = F_0 + k_H \cdot t^{0.5}$

Fitted model parameters per tested tablet (N = 4) with statistics – mean, standard deviation (SD), and relative standard deviation expressed in % (RSD%) (output from DDSolver):

Parameter	No.1	No.2	No.3	No.4	Mean	SD	RSD(%)
k_H	3.602	3.519	3.639	3.675	3.609	0.067	1.860
F_0	-10.632	-9.066	-6.179	-10.666	-9.136	2.108	-23.069

Number of dissolution data points (N), degrees of freedom (df), and selected goodness of fit criteria – Pearson correlation coefficient (R), coefficient of determination (R^2), adjusted coefficient of determination ($R^2_{adjusted}$), and residual sum of squares (RSS) (manual calculation in MS Excel):

Parameter	No.1	No.2	No.3	No.4
N	23	23	23	23
df	21	21	21	21
R	0.991941259	0.992487888	0.991269943	0.992555274
R^2	0.983947462	0.985032208	0.982616101	0.985165973
$R^2_{adjusted}$	0.983183055	0.984319456	0.981788296	0.984459591
RSS	289.93362	257.6924097	320.9584295	278.5914848

Graphical abstract of model fit presented as mean ± 1 SD of the fraction % of released carvedilol:

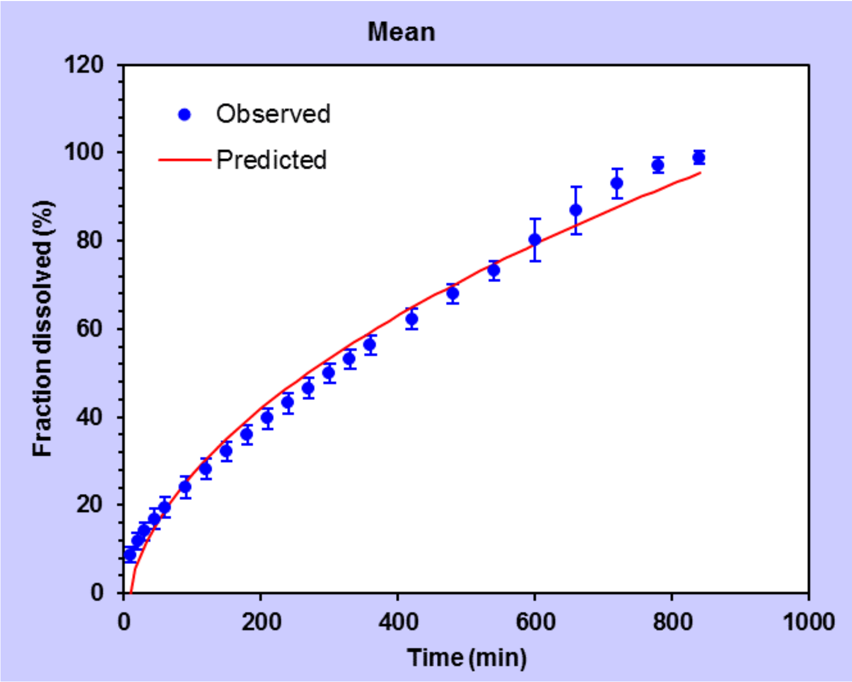

Supplementary materials – Model fitting summary – FlowLac® 100 (modelling the entire relevant dissolution data)

Graphical abstract of model fit presented as the fraction % of released carvedilol per tested tablet:

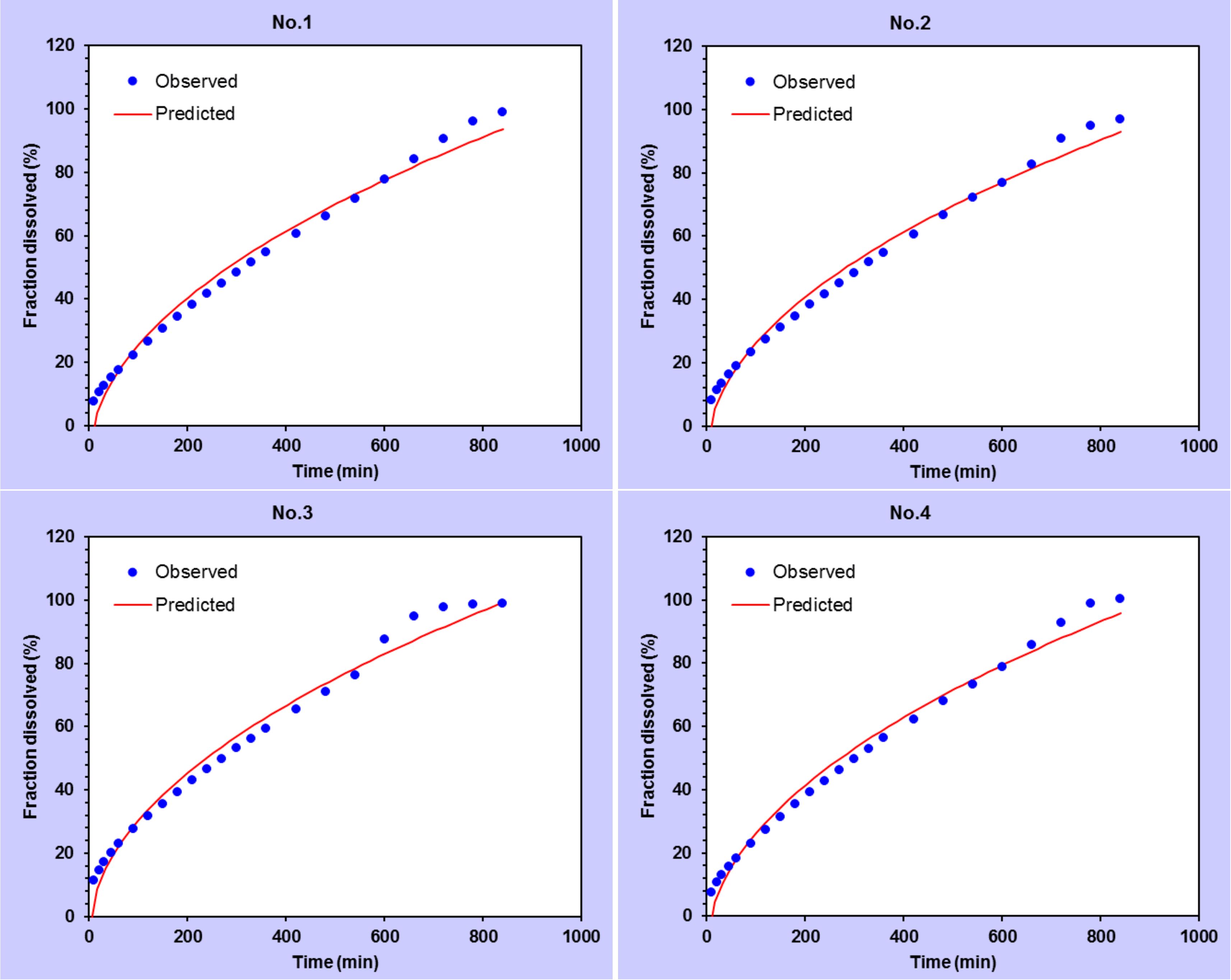

Supplementary materials – Model fitting summary – FlowLac® 100 (modelling the entire relevant dissolution data)

Model: **Korsmeyer–Peppas**

Model equation: $F = k_{KP} \cdot t^n$

Fitted model parameters per tested tablet (N = 4) with statistics – mean, standard deviation (SD), and relative standard deviation expressed in % (RSD%) (output from DDSolver):

Parameter	No.1	No.2	No.3	No.4	Mean	SD	RSD(%)
k_{KP}	1.645	1.896	2.874	1.661	2.019	0.581	28.795
n	0.597	0.574	0.519	0.599	0.573	0.037	6.503

Number of dissolution data points (N), degrees of freedom (df), and selected goodness of fit criteria – Pearson correlation coefficient (R), coefficient of determination (R^2), adjusted coefficient of determination ($R^2_{adjusted}$), and residual sum of squares (RSS) (manual calculation in MS Excel):

Parameter	No.1	No.2	No.3	No.4
N	23	23	23	23
df	21	21	21	21
R	0.996811735	0.996204336	0.992301842	0.997162845
R^2	0.993633635	0.992423078	0.984662946	0.994333739
$R^2_{adjusted}$	0.993330475	0.992062273	0.98393261	0.994063917
RSS	231.6954623	241.5317179	439.3755229	221.0253974

Graphical abstract of model fit presented as mean ± 1 SD of the fraction % of released carvedilol:

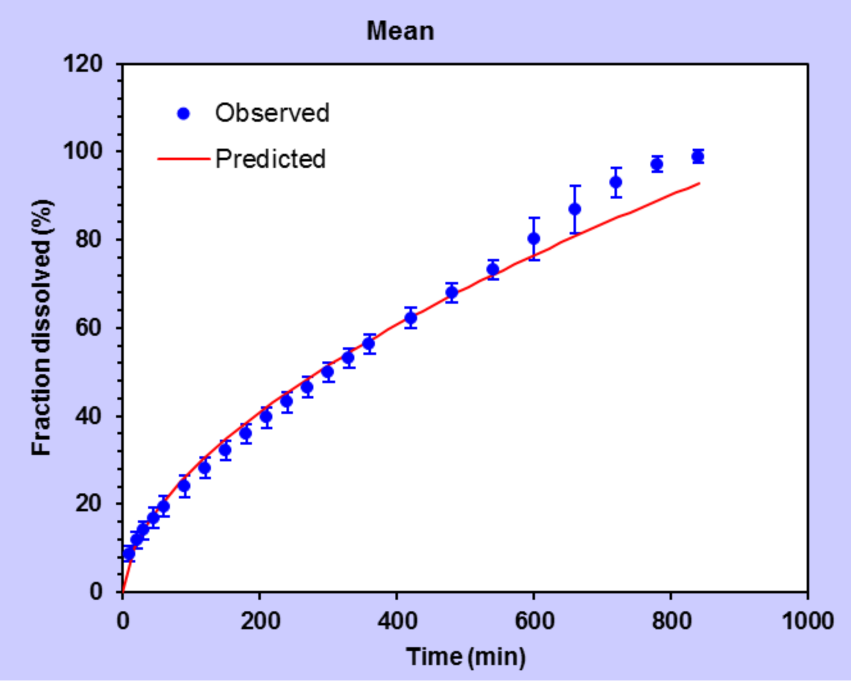

Supplementary materials – Model fitting summary – FlowLac® 100 (modelling the entire relevant dissolution data)

Graphical abstract of model fit presented as the fraction % of released carvedilol per tested tablet:

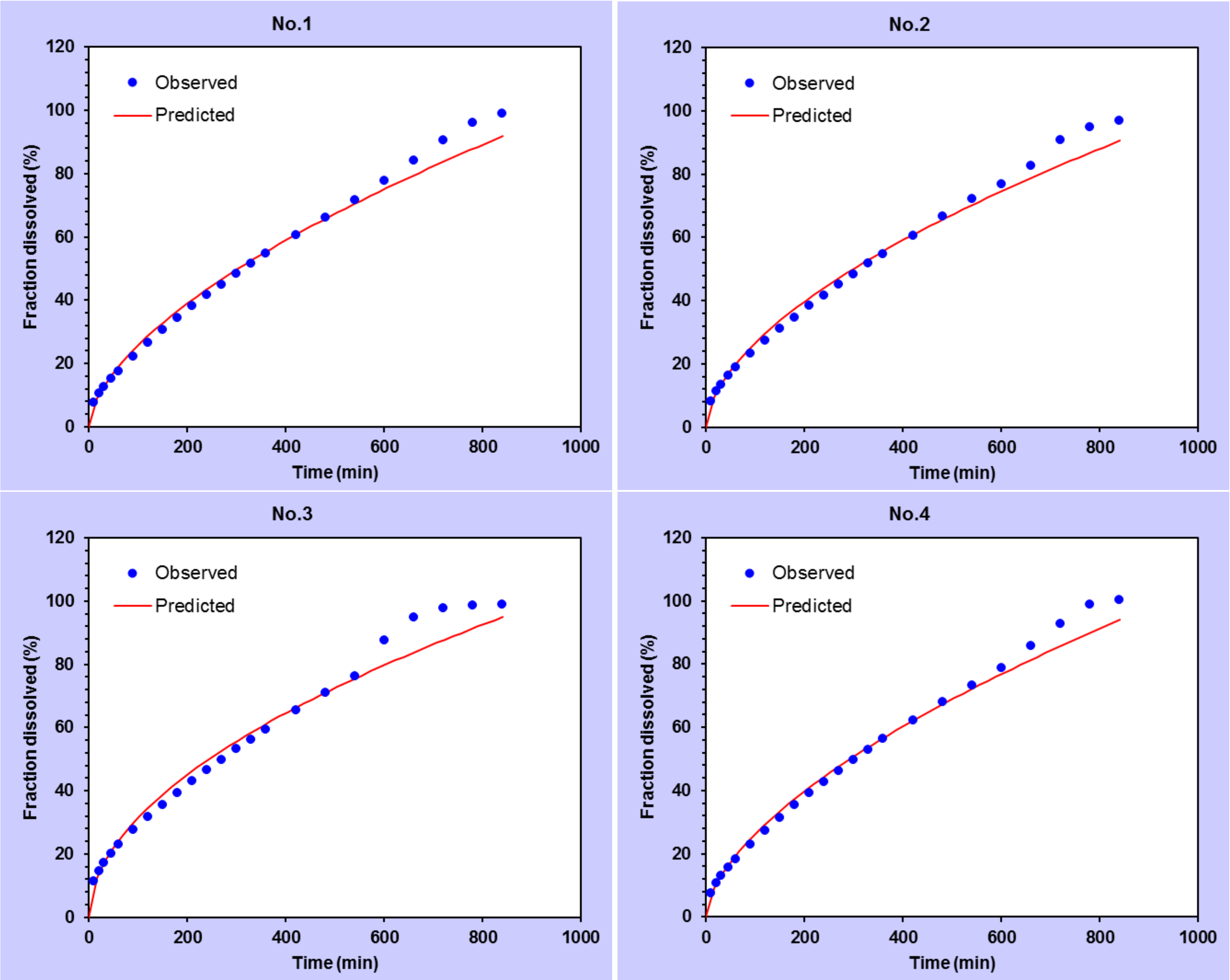

Supplementary materials – Model fitting summary – FlowLac® 100 (modelling the entire relevant dissolution data)

Model: **Korsmeyer–Peppas with T_{lag}**

Model equation: $F = k_{KP} \cdot (t - T_{lag})^n$

Fitted model parameters per tested tablet (N = 4) with statistics – mean, standard deviation (SD), and relative standard deviation expressed in % (RSD%) (output from DDSolver):

Parameter	No.1	No.2	No.3	No.4	Mean	SD	RSD(%)
k_{KP}	2.162	2.445	3.626	2.155	2.597	0.699	26.922
n	0.565	0.532	0.481	0.571	0.537	0.041	7.710
T_{lag}	4.939	4.000	4.000	4.939	4.470	0.542	12.135

Number of dissolution data points (N), degrees of freedom (df), and selected goodness of fit criteria – Pearson correlation coefficient (R), coefficient of determination (R^2), adjusted coefficient of determination ($R^2_{adjusted}$), and residual sum of squares (RSS) (manual calculation in MS Excel):

Parameter	No.1	No.2	No.3	No.4
N	23	23	23	23
df	20	20	20	20
R	0.994552141	0.993351868	0.988834483	0.995279099
R^2	0.989133961	0.986747934	0.977793636	0.990580484
$R^2_{adjusted}$	0.988047357	0.985422728	0.975572999	0.989638533
RSS	390.2880335	455.2881044	692.2933222	396.9474922

Graphical abstract of model fit presented as mean ± 1 SD of the fraction % of released carvedilol:

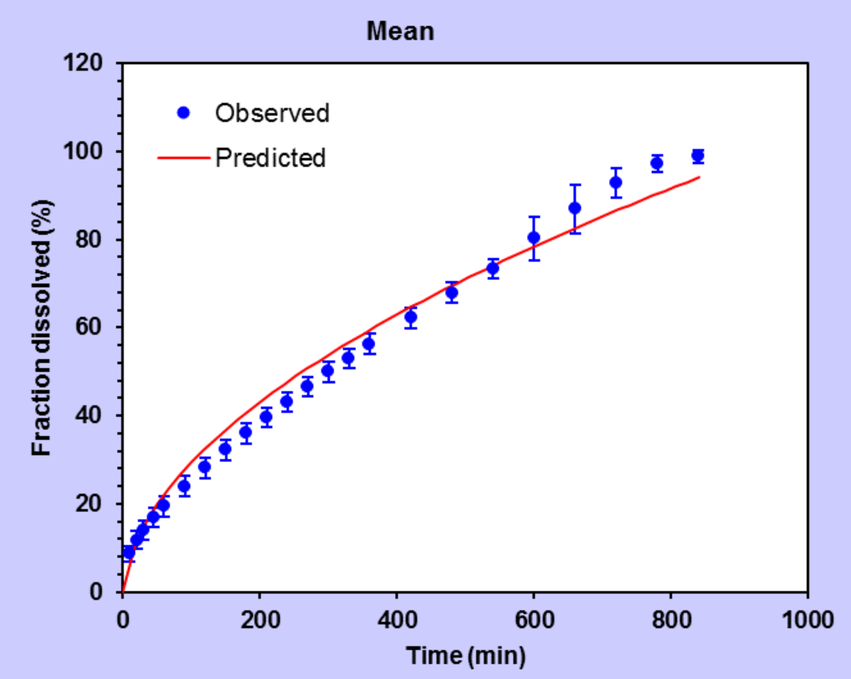

Supplementary materials – Model fitting summary – FlowLac® 100 (modelling the entire relevant dissolution data)

Graphical abstract of model fit presented as the fraction % of released carvedilol per tested tablet:

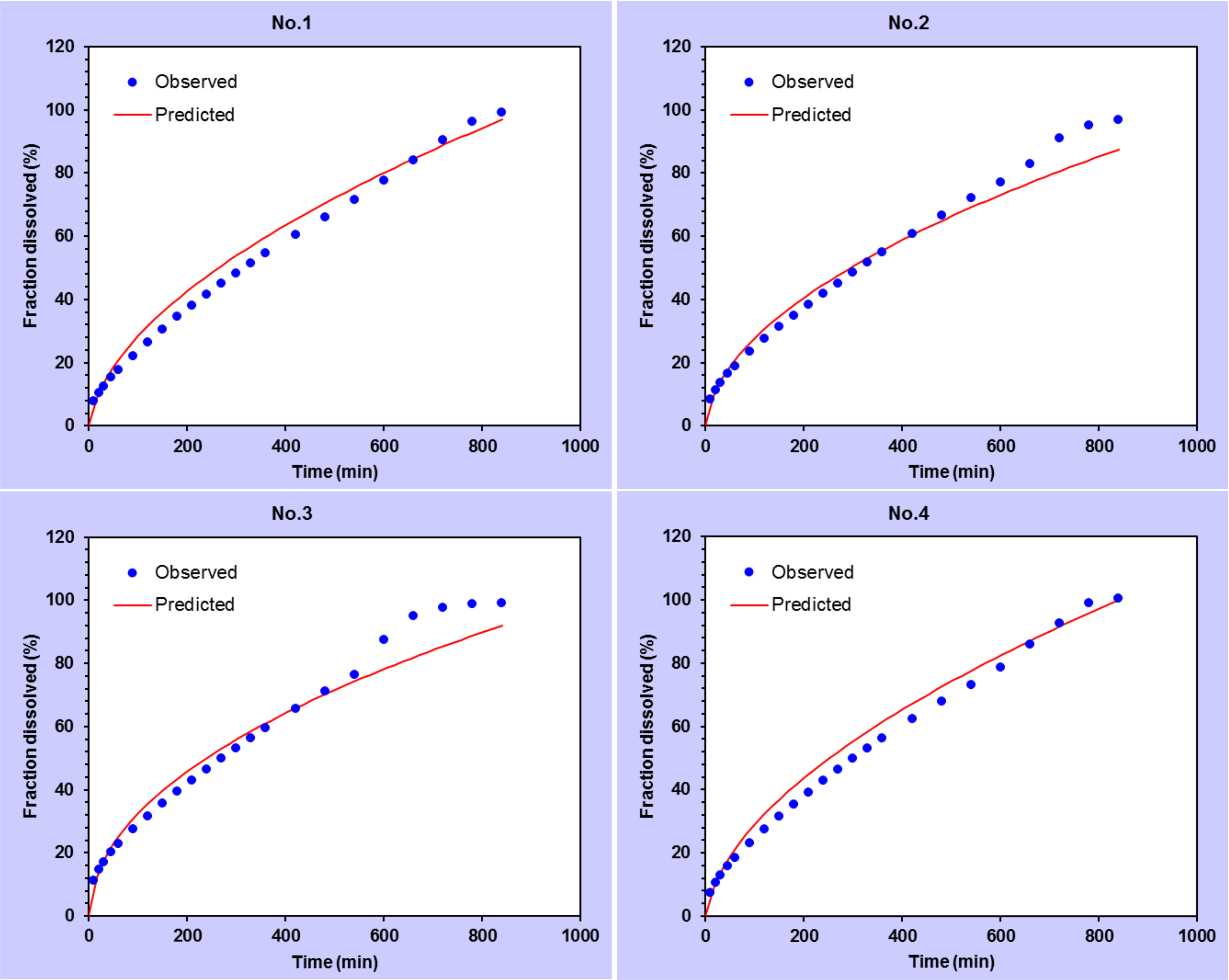

Supplementary materials – Model fitting summary – FlowLac® 100 (modelling the entire relevant dissolution data)

Model: **Korsmeyer–Peppas with F_0**

Model equation: $F = F_0 + k_{KP} \cdot t^n$

Fitted model parameters per tested tablet (N = 4) with statistics – mean, standard deviation (SD), and relative standard deviation expressed in % (RSD%) (output from DDSolver):

Parameter	No.1	No.2	No.3	No.4	Mean	SD	RSD(%)
k_{KP}	0.896	1.038	1.532	0.922	1.097	0.296	27.023
n	0.690	0.665	0.612	0.690	0.664	0.037	5.528
F_0	3.120	3.319	4.519	2.960	3.479	0.709	20.363

Number of dissolution data points (N), degrees of freedom (df), and selected goodness of fit criteria – Pearson correlation coefficient (R), coefficient of determination (R^2), adjusted coefficient of determination ($R^2_{adjusted}$), and residual sum of squares (RSS) (manual calculation in MS Excel):

Parameter	No.1	No.2	No.3	No.4
N	23	23	23	23
df	20	20	20	20
R	0.99921626	0.99883013	0.995805792	0.999218773
R^2	0.998433134	0.997661629	0.991629176	0.998438155
$R^2_{adjusted}$	0.998276447	0.997427792	0.990792093	0.998281971
RSS	55.07203956	68.16830056	217.1200689	41.60340454

Graphical abstract of model fit presented as mean ± 1 SD of the fraction % of released carvedilol:

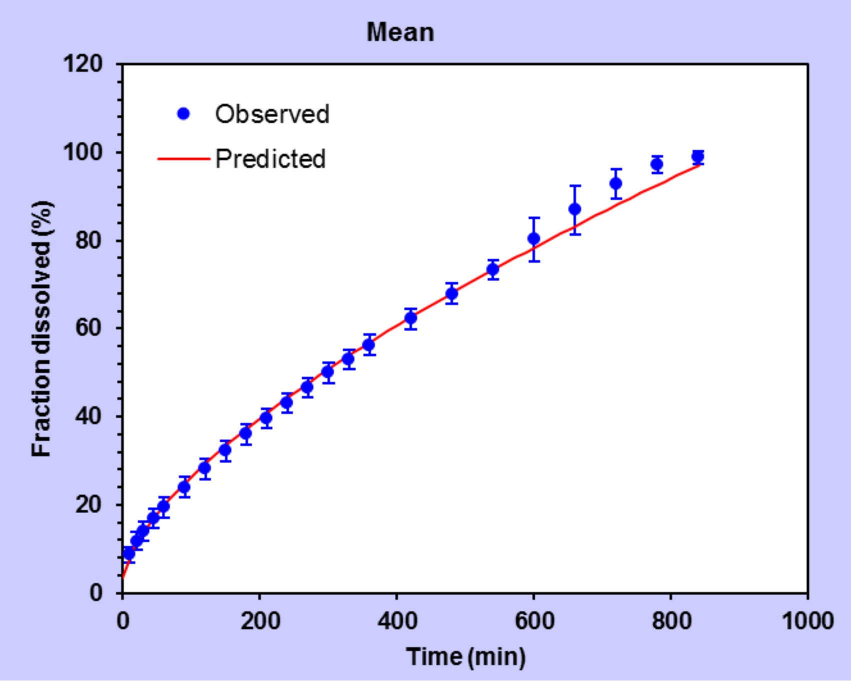

Supplementary materials – Model fitting summary – FlowLac® 100 (modelling the entire relevant dissolution data)

Graphical abstract of model fit presented as the fraction % of released carvedilol per tested tablet:

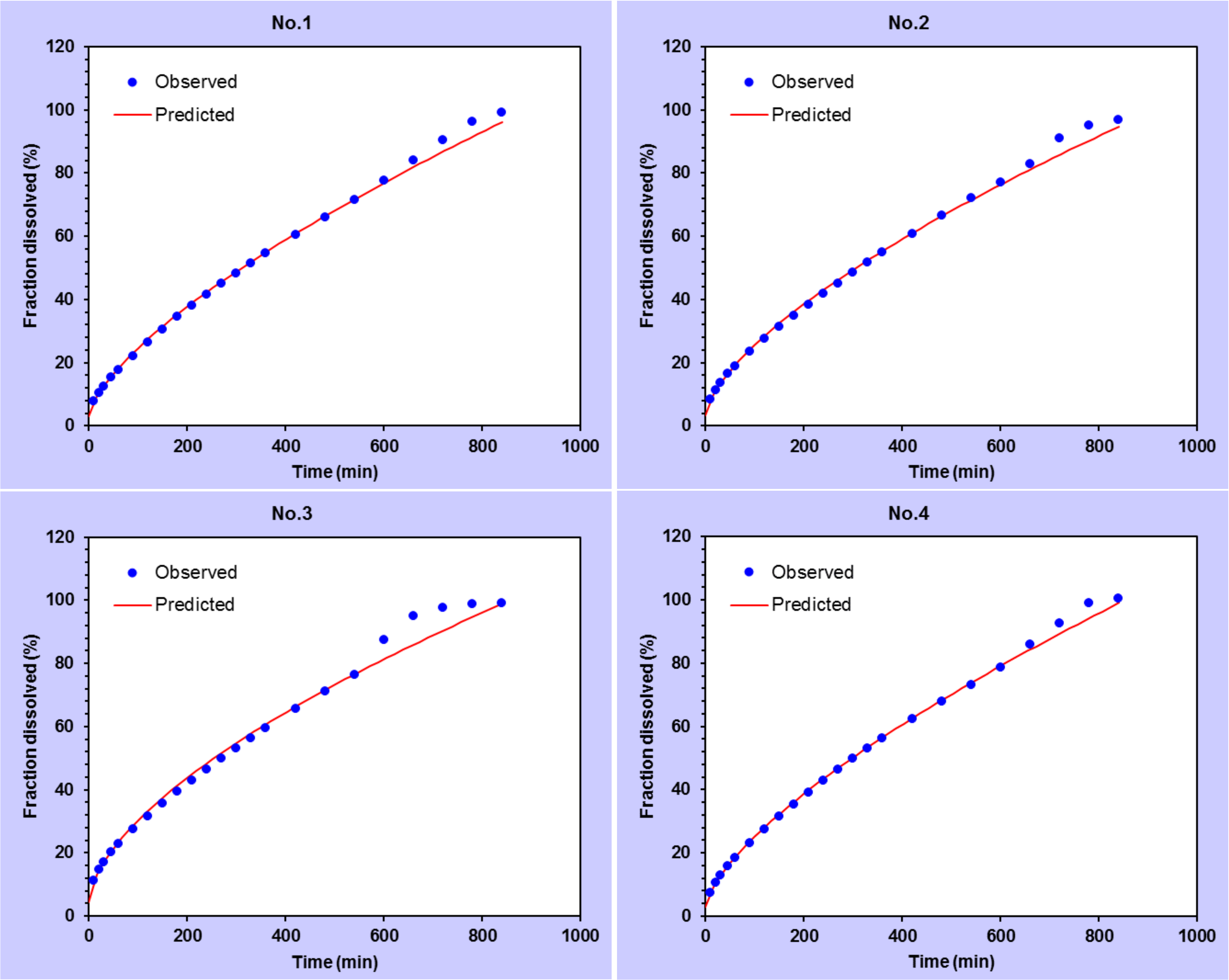

Supplementary materials – Model fitting summary – FlowLac® 100 (modelling the entire relevant dissolution data)

Model: **Hixson–Crowell**

Model equation: $F = 100 \cdot [1 - (1 - k_{HC} \cdot t)^3]$

Fitted model parameters per tested tablet (N = 4) with statistics – mean, standard deviation (SD), and relative standard deviation expressed in % (RSD%) (output from DDSolver):

Parameter	No.1	No.2	No.3	No.4	Mean	SD	RSD(%)
k_{HC}	0.001	0.001	0.001	0.001	0.001	0.000	6.904

Number of dissolution data points (N), degrees of freedom (df), and selected goodness of fit criteria – Pearson correlation coefficient (R), coefficient of determination (R^2), adjusted coefficient of determination ($R^2_{adjusted}$), and residual sum of squares (RSS) (manual calculation in MS Excel):

Parameter	No.1	No.2	No.3	No.4
N	23	23	23	23
df	22	22	22	22
R	0.994036803	0.991979769	0.990171291	0.989890903
R^2	0.988109166	0.984023863	0.980439186	0.979884001
$R^2_{adjusted}$	0.988109166	0.984023863	0.980439186	0.979884001
RSS	442.3538741	489.8551821	824.3868243	517.6914668

Graphical abstract of model fit presented as mean ± 1 SD of the fraction % of released carvedilol:

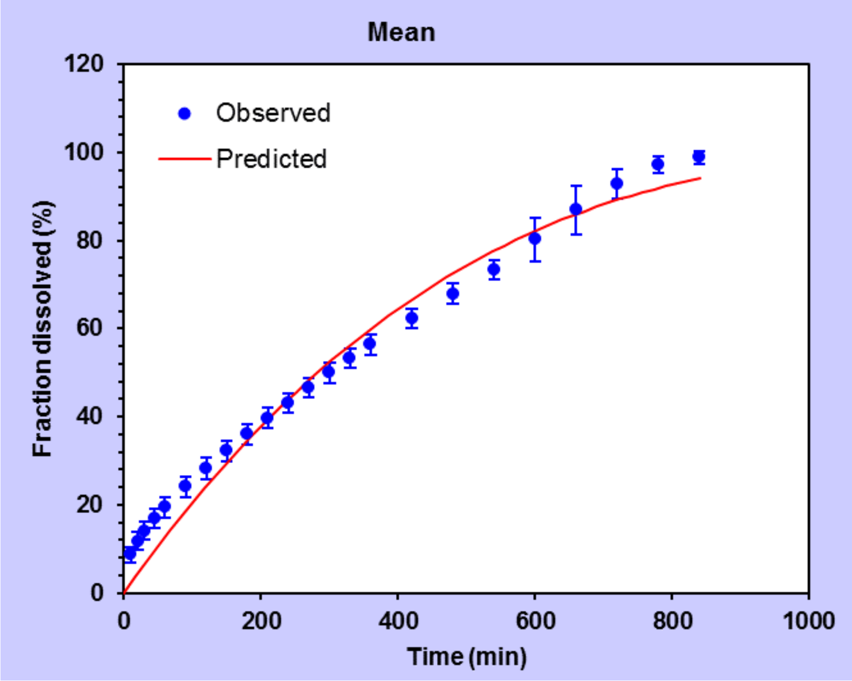

Supplementary materials – Model fitting summary – FlowLac® 100 (modelling the entire relevant dissolution data)

Graphical abstract of model fit presented as the fraction % of released carvedilol per tested tablet:

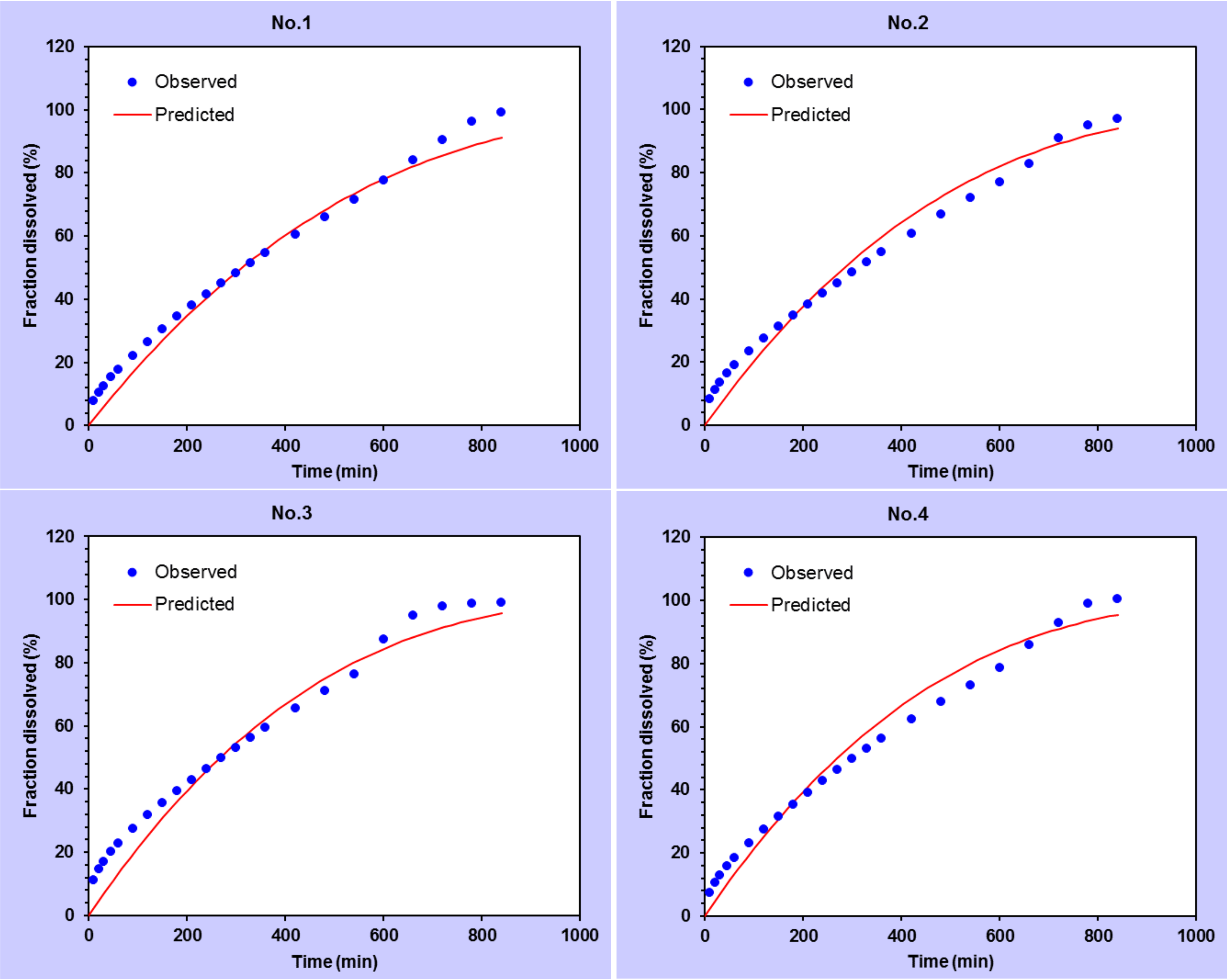

Supplementary materials – Model fitting summary – FlowLac® 100 (modelling the entire relevant dissolution data)

Model: **Hixson–Crowell with T_{lag}**

Model equation: $F = 100 \cdot \left\{1 - \left[1 - k_{HC} \cdot \left(t - T_{lag}\right)\right]^3\right\}$

Fitted model parameters per tested tablet (N = 4) with statistics – mean, standard deviation (SD), and relative standard deviation expressed in % (RSD%) (output from DDSolver):

Parameter	No.1	No.2	No.3	No.4	Mean	SD	RSD(%)
k_{HC}	0.001	0.001	0.001	0.001	0.001	0.000	8.872
T_{lag}	12.333	-6.090	4.373	5.350	3.992	7.598	190.356

Number of dissolution data points (N), degrees of freedom (df), and selected goodness of fit criteria – Pearson correlation coefficient (R), coefficient of determination (R^2), adjusted coefficient of determination ($R^2_{adjusted}$), and residual sum of squares (RSS) (manual calculation in MS Excel):

Parameter	No.1	No.2	No.3	No.4
N	23	23	23	23
df	21	21	21	21
R	0.98940834	0.992083812	0.983922865	0.989621898
R^2	0.978928864	0.98423029	0.968104205	0.979351502
$R^2_{adjusted}$	0.977925477	0.983479352	0.966585357	0.97836824
RSS	732.1340225	427.4161489	1059.009072	584.9607748

Graphical abstract of model fit presented as mean ± 1 SD of the fraction % of released carvedilol:

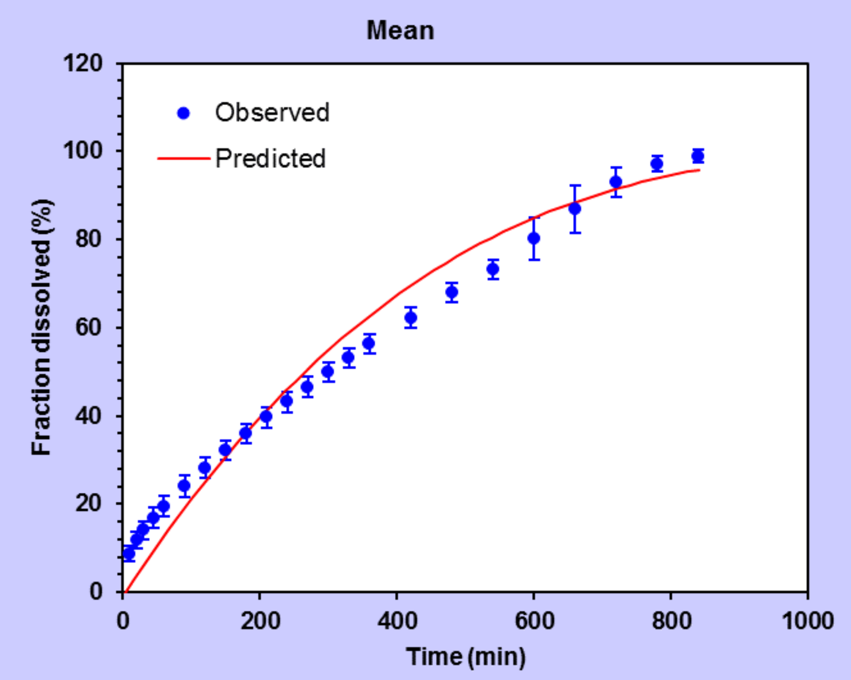

Supplementary materials – Model fitting summary – FlowLac® 100 (modelling the entire relevant dissolution data)

Graphical abstract of model fit presented as the fraction % of released carvedilol per tested tablet:

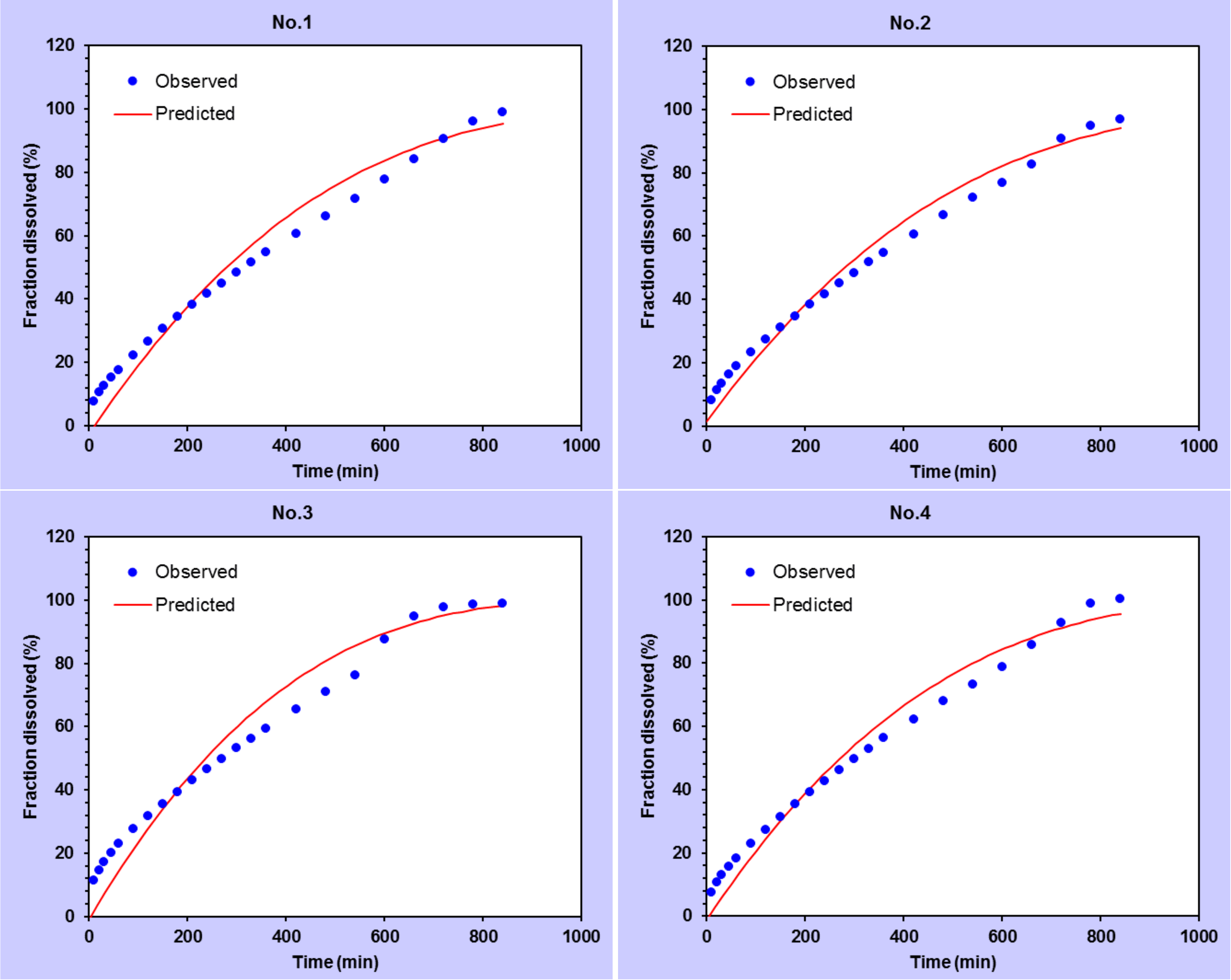

Supplementary materials – Model fitting summary – FlowLac® 100 (modelling the entire relevant dissolution data)

Model: **Hopfenberg**

Model equation: $F = 100 \cdot [1 - (1 - k_{HB} \cdot t)^n]$

Fitted model parameters per tested tablet (N = 4) with statistics – mean, standard deviation (SD), and relative standard deviation expressed in % (RSD%) (output from DDSolver):

Parameter	No.1	No.2	No.3	No.4	Mean	SD	RSD(%)
k_{HB}	0.001	0.001	0.001	0.001	0.001	0.000	25.615
n	2.750	4.031	2.750	2.000	2.883	0.843	29.253

Number of dissolution data points (N), degrees of freedom (df), and selected goodness of fit criteria – Pearson correlation coefficient (R), coefficient of determination (R^2), adjusted coefficient of determination ($R^2_{adjusted}$), and residual sum of squares (RSS) (manual calculation in MS Excel):

Parameter	No.1	No.2	No.3	No.4
N	23	23	23	23
df	21	21	21	21
R	0.994075041	0.992575009	0.991058154	0.994890431
R^2	0.988185187	0.985205148	0.982196265	0.98980697
$R^2_{adjusted}$	0.987622576	0.984500631	0.981348468	0.989321588
RSS	401.3120619	467.655458	840.1798883	439.5057067

Graphical abstract of model fit presented as mean ± 1 SD of the fraction % of released carvedilol:

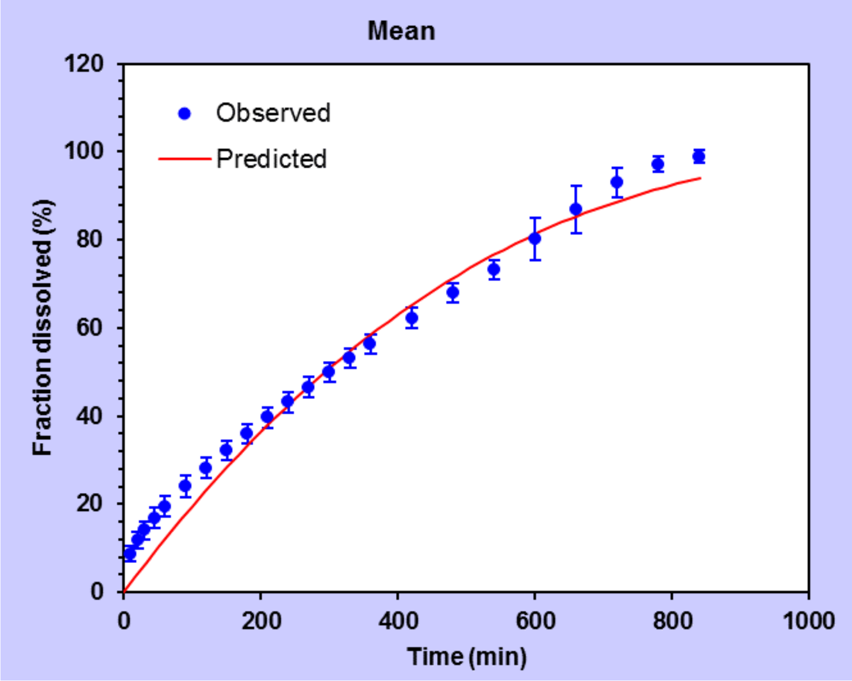

Supplementary materials – Model fitting summary – FlowLac® 100 (modelling the entire relevant dissolution data)

Graphical abstract of model fit presented as the fraction % of released carvedilol per tested tablet:

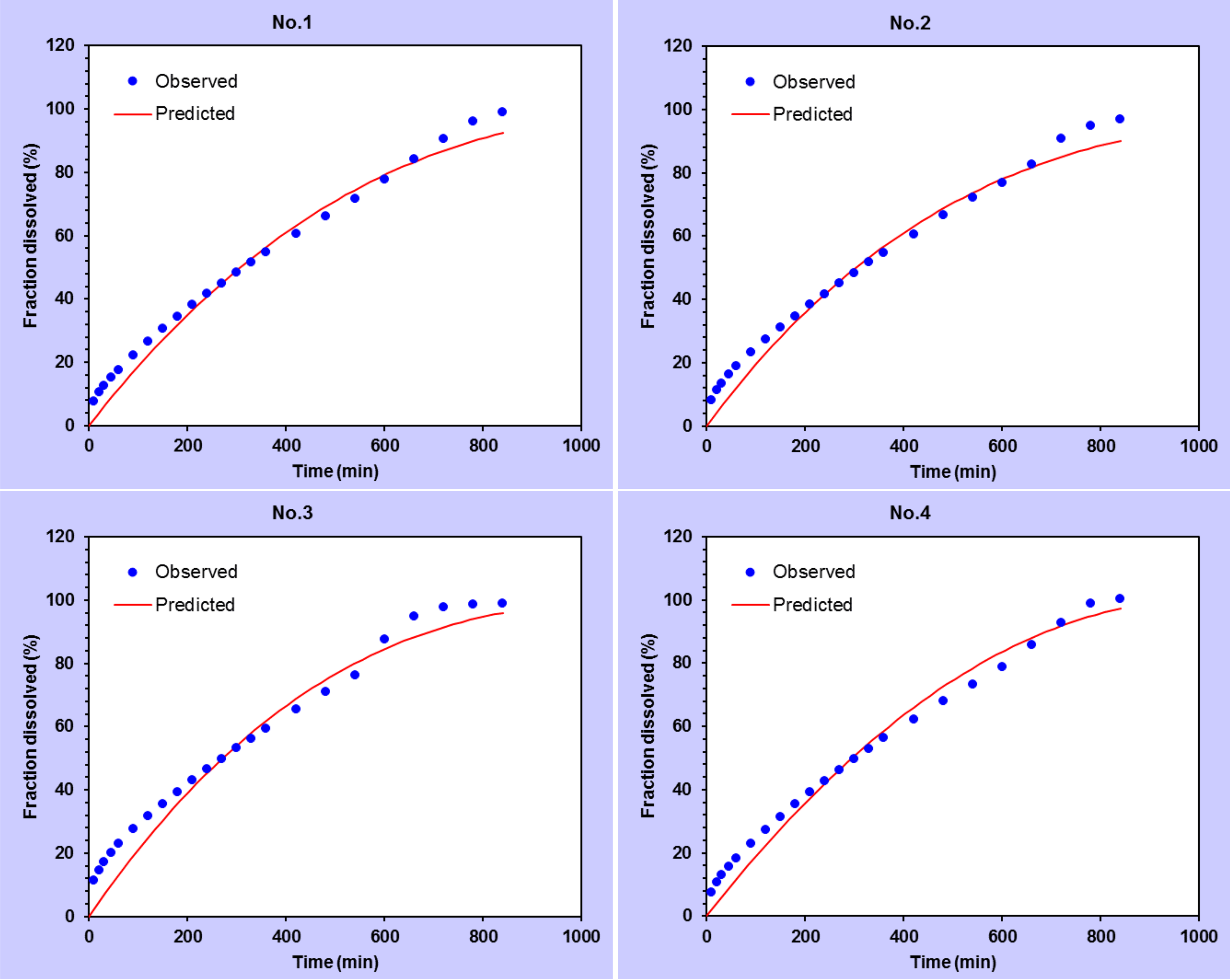

Supplementary materials – Model fitting summary – FlowLac® 100 (modelling the entire relevant dissolution data)

Model: **Hopfenberg with T_{lag}**

Model equation: $F = 100 \cdot \{1 - [1 - k_{HB} \cdot (t - T_{lag})]^n\}$

Fitted model parameters per tested tablet (N = 4) with statistics – mean, standard deviation (SD), and relative standard deviation expressed in % (RSD%) (output from DDSolver):

Parameter	No.1	No.2	No.3	No.4	Mean	SD	RSD(%)
k_{HB}	0.001	0.001	0.001	0.001	0.001	0.000	12.640
n	1.000	2.000	1.185	1.000	1.296	0.477	36.812
T_{lag}	-114.581	-33.208	-105.119	-109.288	-90.549	38.423	-42.433

Number of dissolution data points (N), degrees of freedom (df), and selected goodness of fit criteria – Pearson correlation coefficient (R), coefficient of determination (R^2), adjusted coefficient of determination ($R^2_{adjusted}$), and residual sum of squares (RSS) (manual calculation in MS Excel):

Parameter	No.1	No.2	No.3	No.4
N	23	23	23	23
df	20	20	20	20
R	0.996299575	0.996850337	0.997888709	0.99609183
R^2	0.992612843	0.993710594	0.995781876	0.992198934
$R^2_{adjusted}$	0.991874128	0.993081654	0.995360063	0.991418828
RSS	137.8451706	140.4232467	147.5453578	147.1940189

Graphical abstract of model fit presented as mean ± 1 SD of the fraction % of released carvedilol:

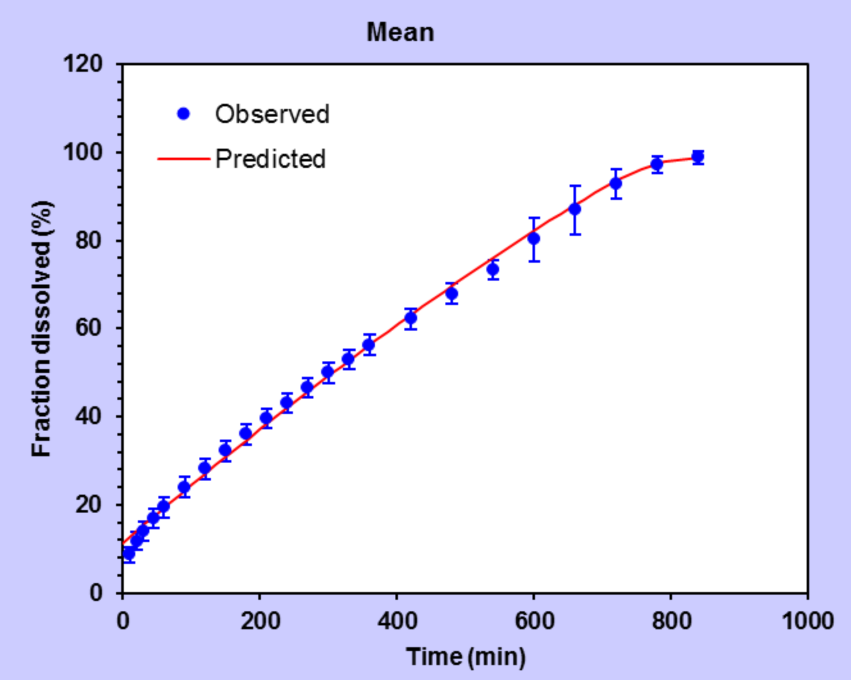

Supplementary materials – Model fitting summary – FlowLac® 100 (modelling the entire relevant dissolution data)

Graphical abstract of model fit presented as the fraction % of released carvedilol per tested tablet:

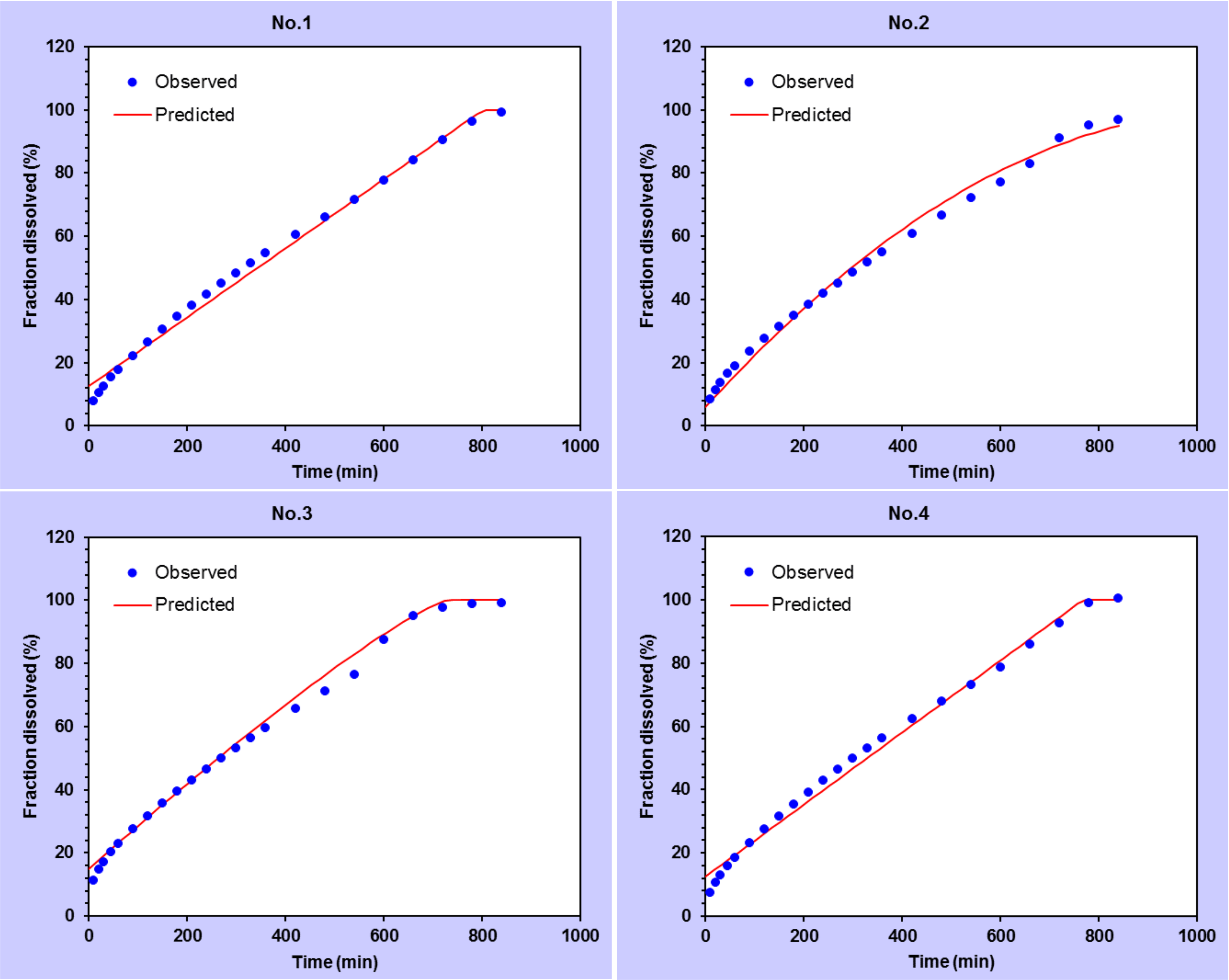

Supplementary materials – Model fitting summary – FlowLac® 100 (modelling the entire relevant dissolution data)

Model: **Baker–Lonsdale**

Model equation: $\frac{3}{2}\cdot\left[1-\left(1-\frac{F}{100}\right)^{\frac{2}{3}}\right]-\frac{F}{100}=k_{BL}\cdot t$

Fitted model parameters per tested tablet (N = 4) with statistics – mean, standard deviation (SD), and relative standard deviation expressed in % (RSD%) (output from DDSolver):

Parameter	No.1	No.2	No.3	No.4	Mean	SD	RSD(%)
k_{BL}	0.0002	0.0002	0.0003	0.0002	0.0002	0.0000	11.8711

Number of dissolution data points (N), degrees of freedom (df), and selected goodness of fit criteria – Pearson correlation coefficient (R), coefficient of determination (R^2), adjusted coefficient of determination ($R^2_{adjusted}$), and residual sum of squares (RSS) (manual calculation in MS Excel):

Parameter	No.1	No.2	No.3	No.4
N	23	23	23	23
df	22	22	22	22
R	0.977662211	0.979583508	0.975857596	0.979066371
R^2	0.9558234	0.959583849	0.952298048	0.95857096
$R^2_{adjusted}$	0.9558234	0.959583849	0.952298048	0.95857096
RSS	1919.002375	1724.070627	1679.170735	2082.460802

Graphical abstract of model fit presented as mean ± 1 SD of the fraction % of released carvedilol:

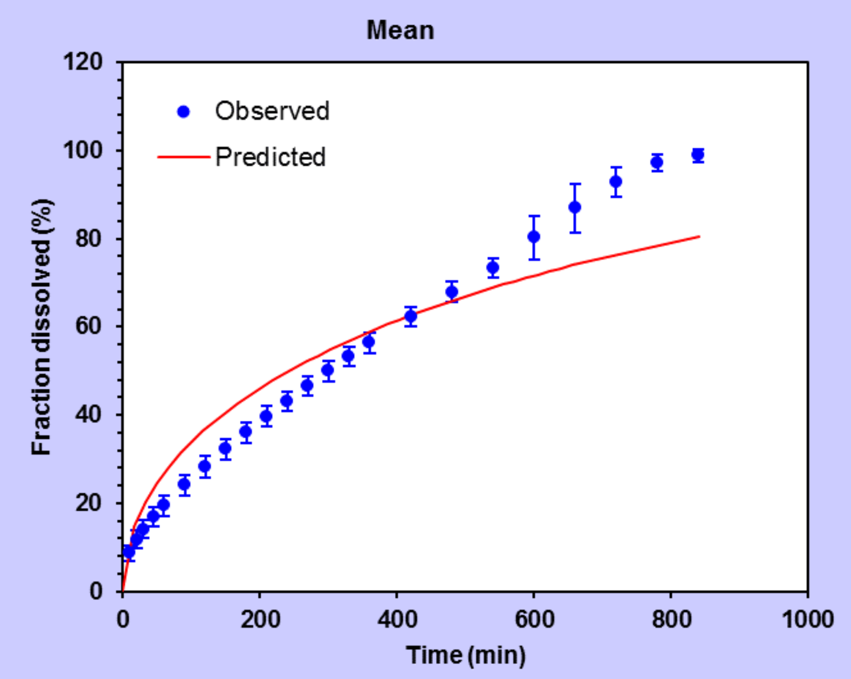

Supplementary materials – Model fitting summary – FlowLac® 100 (modelling the entire relevant dissolution data)

Graphical abstract of model fit presented as the fraction % of released carvedilol per tested tablet:

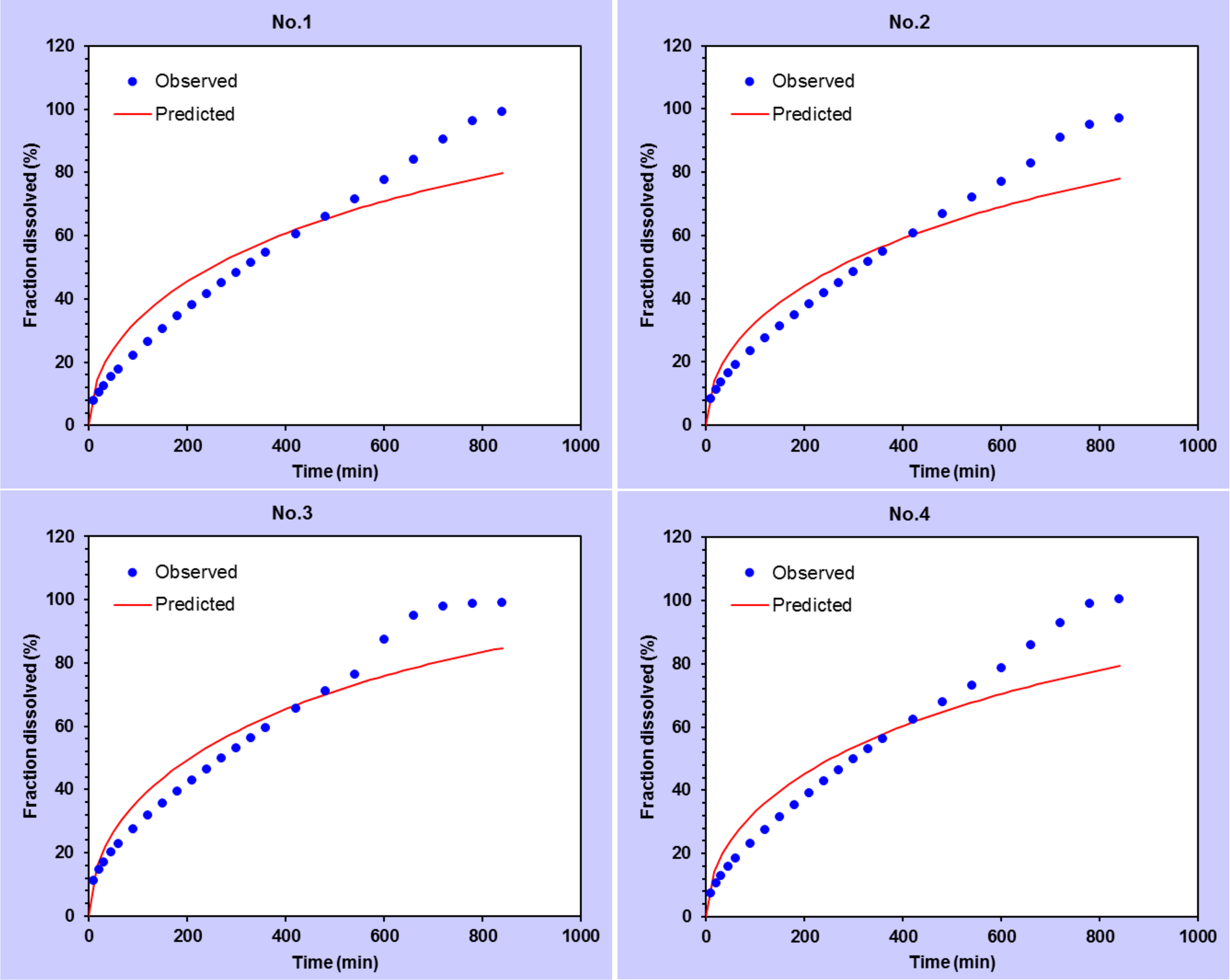

Supplementary materials – Model fitting summary – FlowLac® 100 (modelling the entire relevant dissolution data)

Model: **Baker–Lonsdale with T_{lag}**

Model equation: $\frac{3}{2}\cdot\left[1-\left(1-\frac{F}{100}\right)^{\frac{2}{3}}\right]-\frac{F}{100}=k_{BL}\cdot(t-T_{lag})$

Fitted model parameters per tested tablet (N = 4) with statistics – mean, standard deviation (SD), and relative standard deviation expressed in % (RSD%) (output from DDSolver):

Parameter	No.1	No.2	No.3	No.4	Mean	SD	RSD(%)
k_{BL}	0.000	0.000	0.001	0.000	0.000	0.000	11.871
T_{lag}	109.001	101.791	100.153	100.719	102.916	4.113	3.996

Number of dissolution data points (N), degrees of freedom (df), and selected goodness of fit criteria – Pearson correlation coefficient (R), coefficient of determination (R^2), adjusted coefficient of determination ($R^2_{adjusted}$), and residual sum of squares (RSS) (manual calculation in MS Excel):

Parameter	No.1	No.2	No.3	No.4
N	23	23	23	23
df	21	21	21	21
R	0.96185056	0.961009889	0.952453065	0.959824345
R^2	0.925156499	0.923540006	0.90716684	0.921262773
$R^2_{adjusted}$	0.921592523	0.919899054	0.902746214	0.917513382
RSS	2561.41802	2506.062131	3593.892856	2472.213323

Graphical abstract of model fit presented as mean ± 1 SD of the fraction % of released carvedilol:

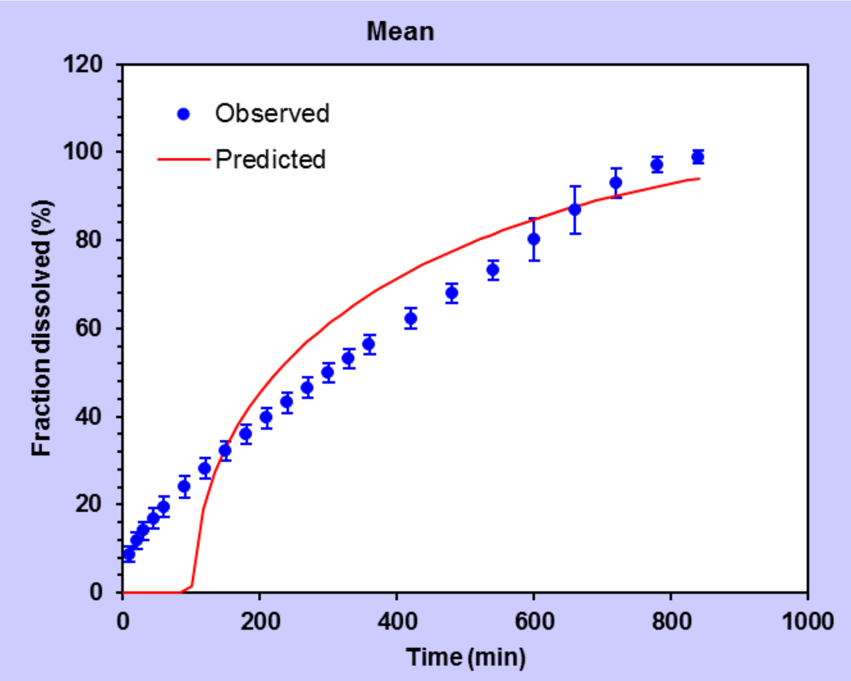

Supplementary materials – Model fitting summary – FlowLac® 100 (modelling the entire relevant dissolution data)

Graphical abstract of model fit presented as the fraction % of released carvedilol per tested tablet:

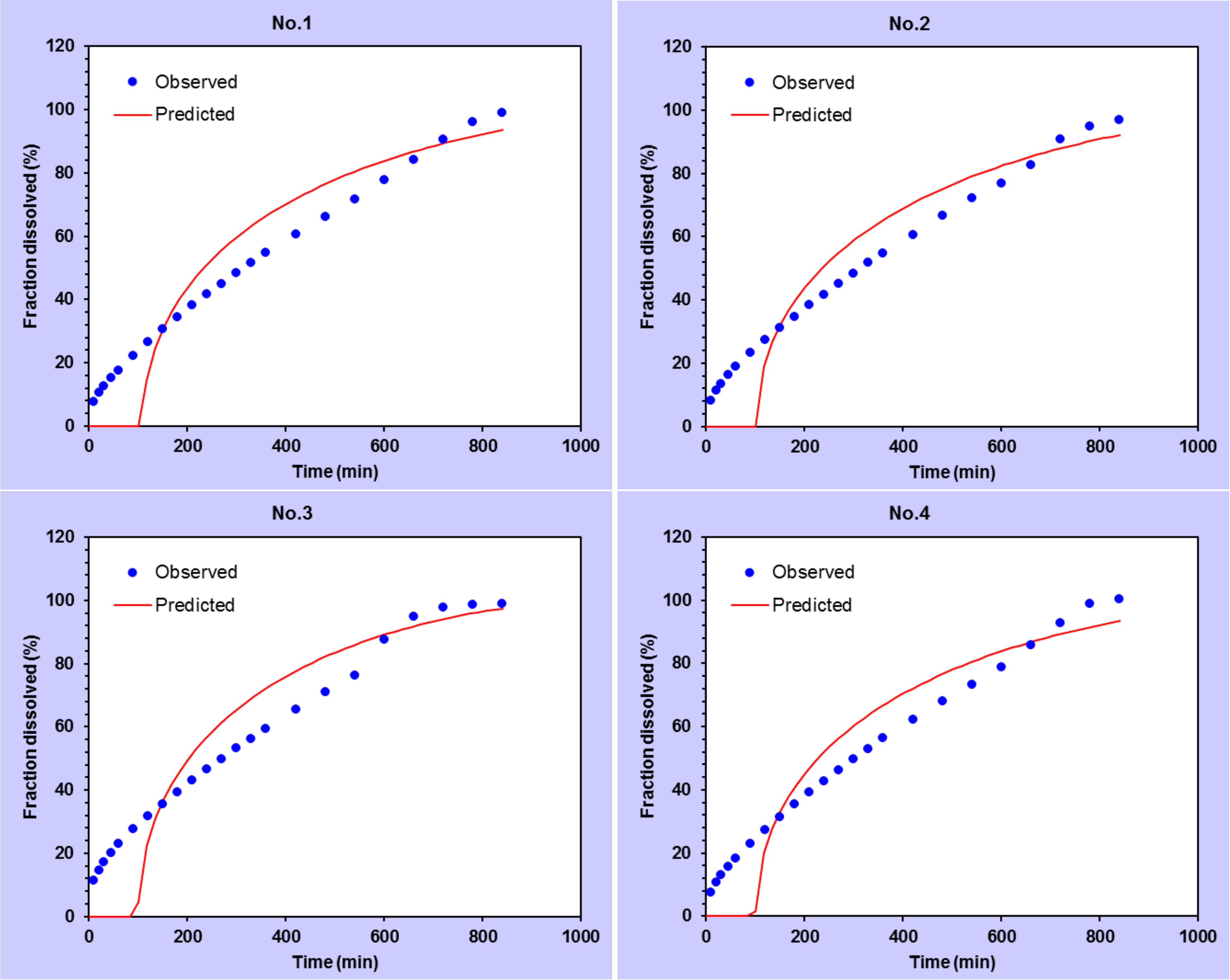

Supplementary materials – Model fitting summary – FlowLac® 100 (modelling the entire relevant dissolution data)

Model: **Makoid–Banakar**

Model equation: $F = k_{MB} \cdot t^{n} \cdot e^{-k \cdot t}$

Fitted model parameters per tested tablet (N = 4) with statistics – mean, standard deviation (SD), and relative standard deviation expressed in % (RSD%) (output from DDSolver):

Parameter	No.1	No.2	No.3	No.4	Mean	SD	RSD(%)
k_{MB}	2.2572	2.5899	4.1122	2.1373	2.7742	0.9124	32.8875
n	0.5075	0.4855	0.4176	0.5272	0.4844	0.0477	9.8437
k	-0.0005	-0.0005	-0.0006	-0.0004	-0.0005	0.0001	-12.3472

Number of dissolution data points (N), degrees of freedom (df), and selected goodness of fit criteria – Pearson correlation coefficient (R), coefficient of determination (R^2), adjusted coefficient of determination ($R^2_{adjusted}$), and residual sum of squares (RSS) (manual calculation in MS Excel):

Parameter	No.1	No.2	No.3	No.4
N	23	23	23	23
df	20	20	20	20
R	0.999161411	0.998954592	0.995342326	0.999286847
R^2	0.998323525	0.997910276	0.990706345	0.998574203
$R^2_{adjusted}$	0.998155878	0.997701304	0.98977698	0.998431623
RSS	31.78450464	36.87556311	173.2087285	27.33889739

Graphical abstract of model fit presented as mean ± 1 SD of the fraction % of released carvedilol:

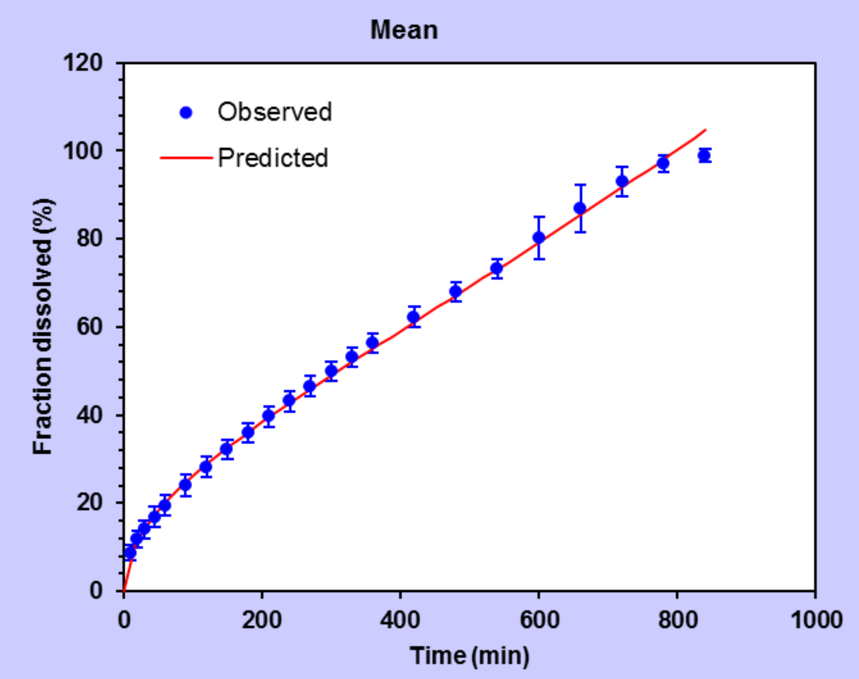

Supplementary materials – Model fitting summary – FlowLac® 100 (modelling the entire relevant dissolution data)

Graphical abstract of model fit presented as the fraction % of released carvedilol per tested tablet:

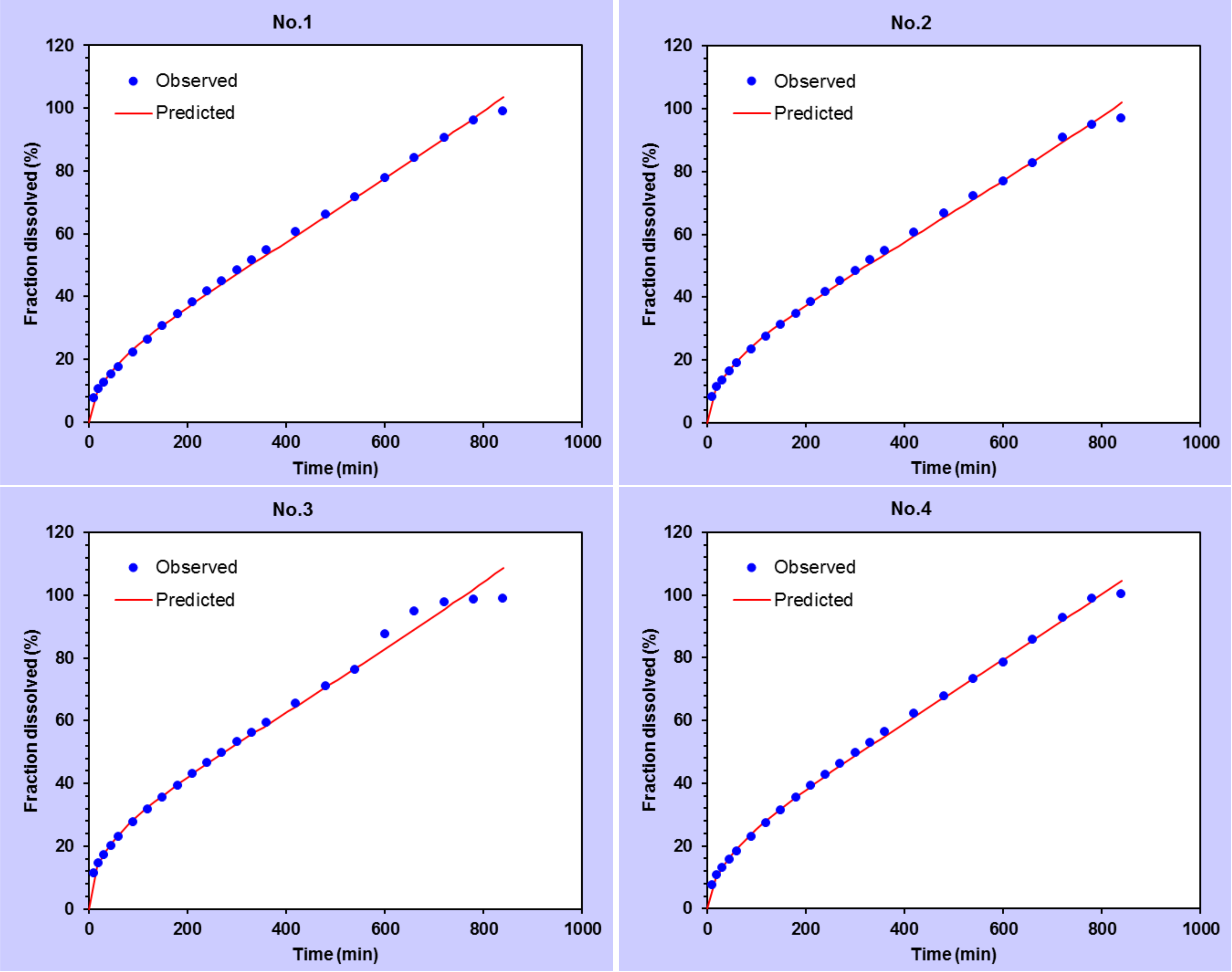

Supplementary materials – Model fitting summary – FlowLac® 100 (modelling the entire relevant dissolution data)

Model: **Makoid–Banakar with T_{lag}**

Model equation: $F = k_{MB} \cdot (t - T_{lag})^n \cdot e^{-k \cdot (t - T_{lag})}$

Fitted model parameters per tested tablet (N = 4) with statistics – mean, standard deviation (SD), and relative standard deviation expressed in % (RSD%) (output from DDSolver):

Parameter	No.1	No.2	No.3	No.4	Mean	SD	RSD(%)
k_{MB}	3.1244	3.5255	5.3717	2.9796	3.7503	1.1053	29.4728
n	0.4395	0.4213	0.3620	0.4580	0.4202	0.0416	9.8992
k	-0.0007	-0.0007	-0.0007	-0.0006	-0.0007	0.0000	-7.0358
T_{lag}	4.0000	4.0000	4.0000	4.0000	4.0000	0.0000	0.0000

Number of dissolution data points (N), degrees of freedom (df), and selected goodness of fit criteria – Pearson correlation coefficient (R), coefficient of determination (R^2), adjusted coefficient of determination ($R^2_{adjusted}$), and residual sum of squares (RSS) (manual calculation in MS Excel):

Parameter	No.1	No.2	No.3	No.4
N	23	23	23	23
df	19	19	19	19
R	0.998015011	0.997893811	0.993901994	0.99838717
R^2	0.996033962	0.995792057	0.987841173	0.996776941
$R^2_{adjusted}$	0.995407745	0.995127645	0.985921358	0.996268037
RSS	77.69350256	76.52233702	229.8363583	64.02805082

Graphical abstract of model fit presented as mean ± 1 SD of the fraction % of released carvedilol:

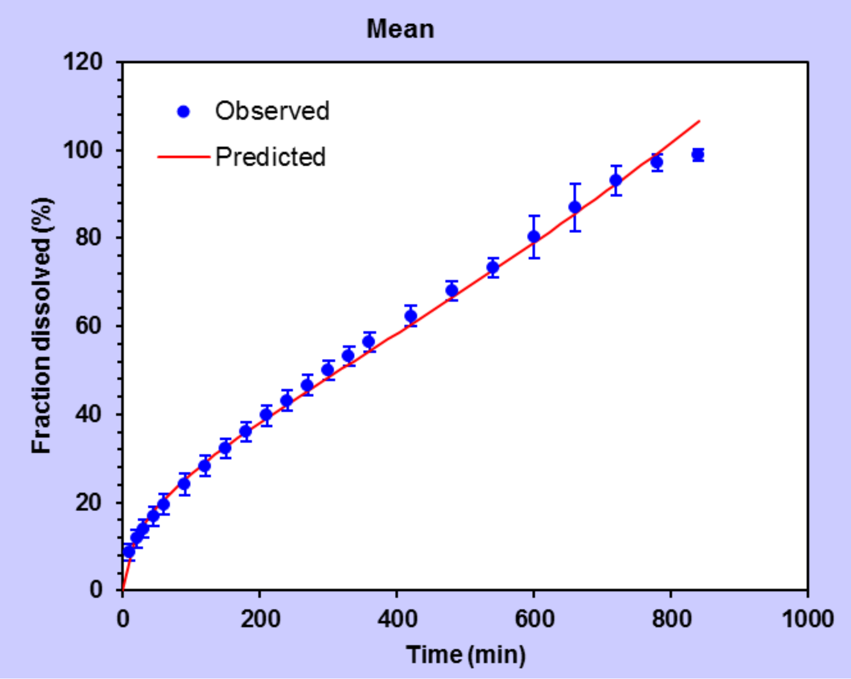

Supplementary materials – Model fitting summary – FlowLac® 100 (modelling the entire relevant dissolution data)

Graphical abstract of model fit presented as the fraction % of released carvedilol per tested tablet:

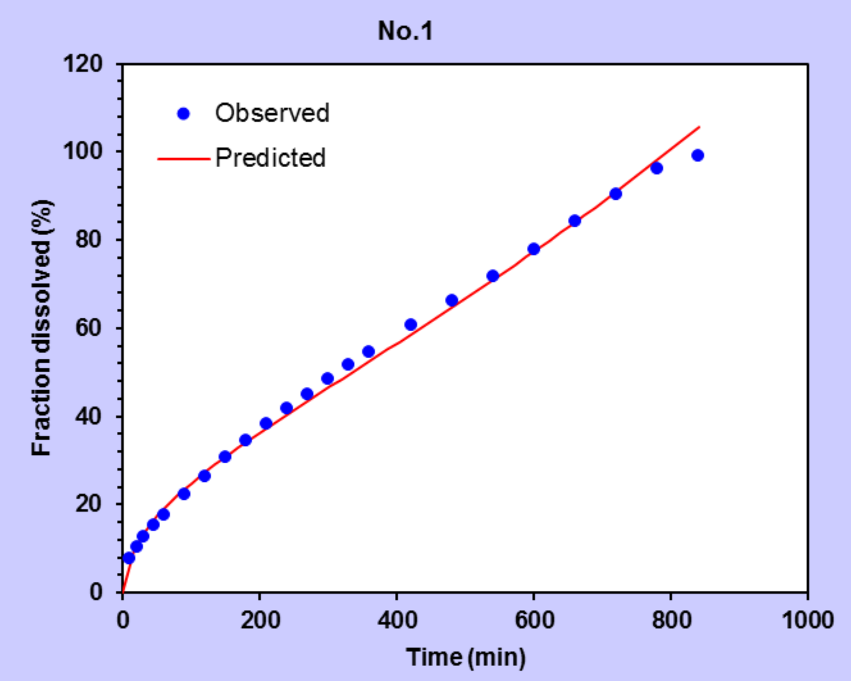

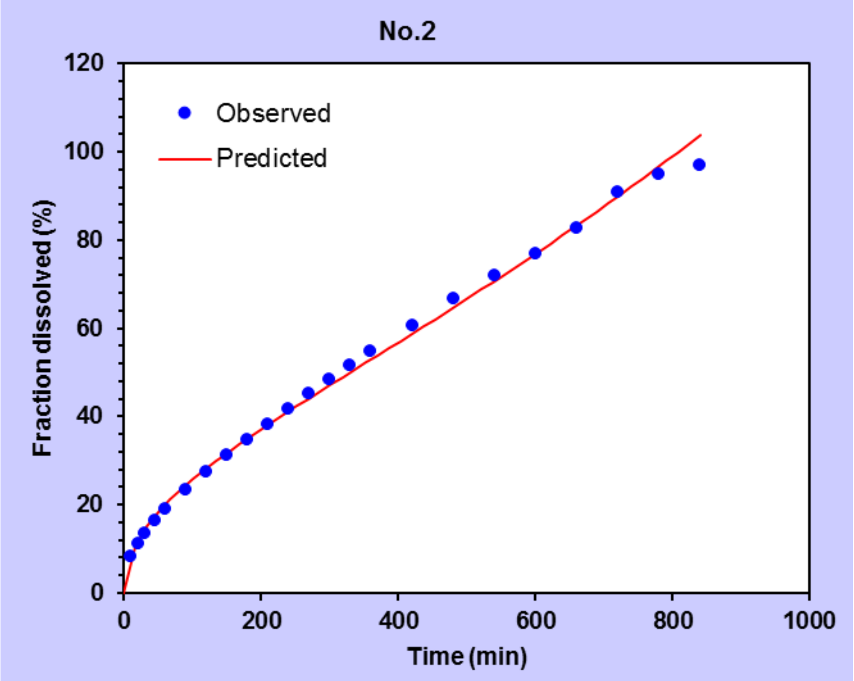

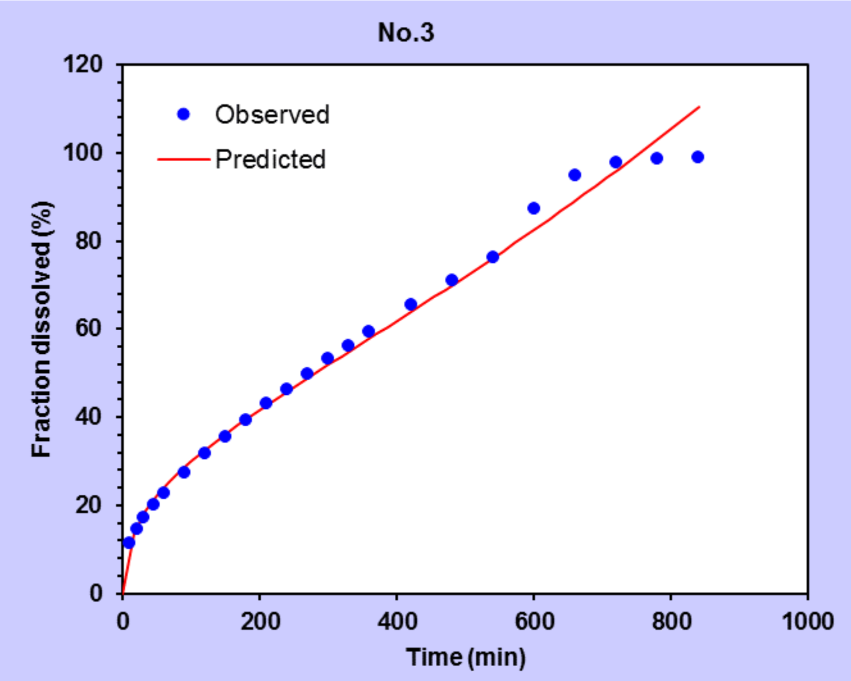

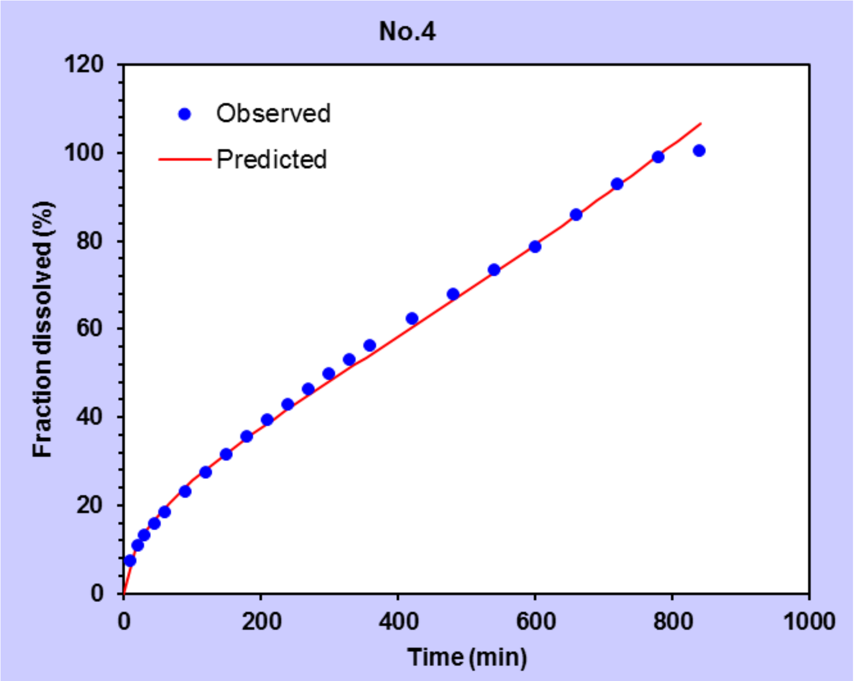

Supplementary materials – Model fitting summary – FlowLac® 100 (modelling the entire relevant dissolution data)

Model: **Peppas–Sahlin_1**

Model equation: $F = k_1 \cdot t^m + k_2 \cdot t^{2m}$

Fitted model parameters per tested tablet (N = 4) with statistics – mean, standard deviation (SD), and relative standard deviation expressed in % (RSD%) (output from DDSolver):

Parameter	No.1	No.2	No.3	No.4	Mean	SD	RSD(%)
k_1	1.865	2.077	2.748	1.963	2.163	0.399	18.462
k_2	0.143	0.129	0.110	0.143	0.131	0.016	11.839
m	0.450	0.450	0.450	0.450	0.450	0.000	0.000

Number of dissolution data points (N), degrees of freedom (df), and selected goodness of fit criteria – Pearson correlation coefficient (R), coefficient of determination (R^2), adjusted coefficient of determination ($R^2_{adjusted}$), and residual sum of squares (RSS) (manual calculation in MS Excel):

Parameter	No.1	No.2	No.3	No.4
N	23	23	23	23
df	20	20	20	20
R	0.99968564	0.999422903	0.996650776	0.999556536
R^2	0.999371379	0.998846138	0.99331277	0.999113268
$R^2_{adjusted}$	0.999308517	0.998730752	0.992644047	0.999024595
RSS	12.3047949	21.32969093	127.9120046	17.21437286

Graphical abstract of model fit presented as mean ± 1 SD of the fraction % of released carvedilol:

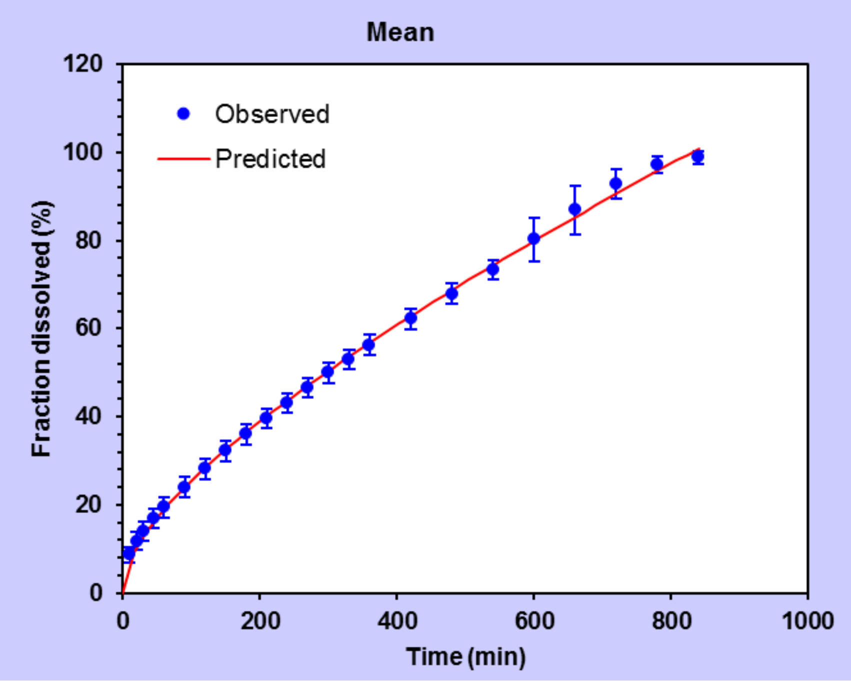

Supplementary materials – Model fitting summary – FlowLac® 100 (modelling the entire relevant dissolution data)

Graphical abstract of model fit presented as the fraction % of released carvedilol per tested tablet:

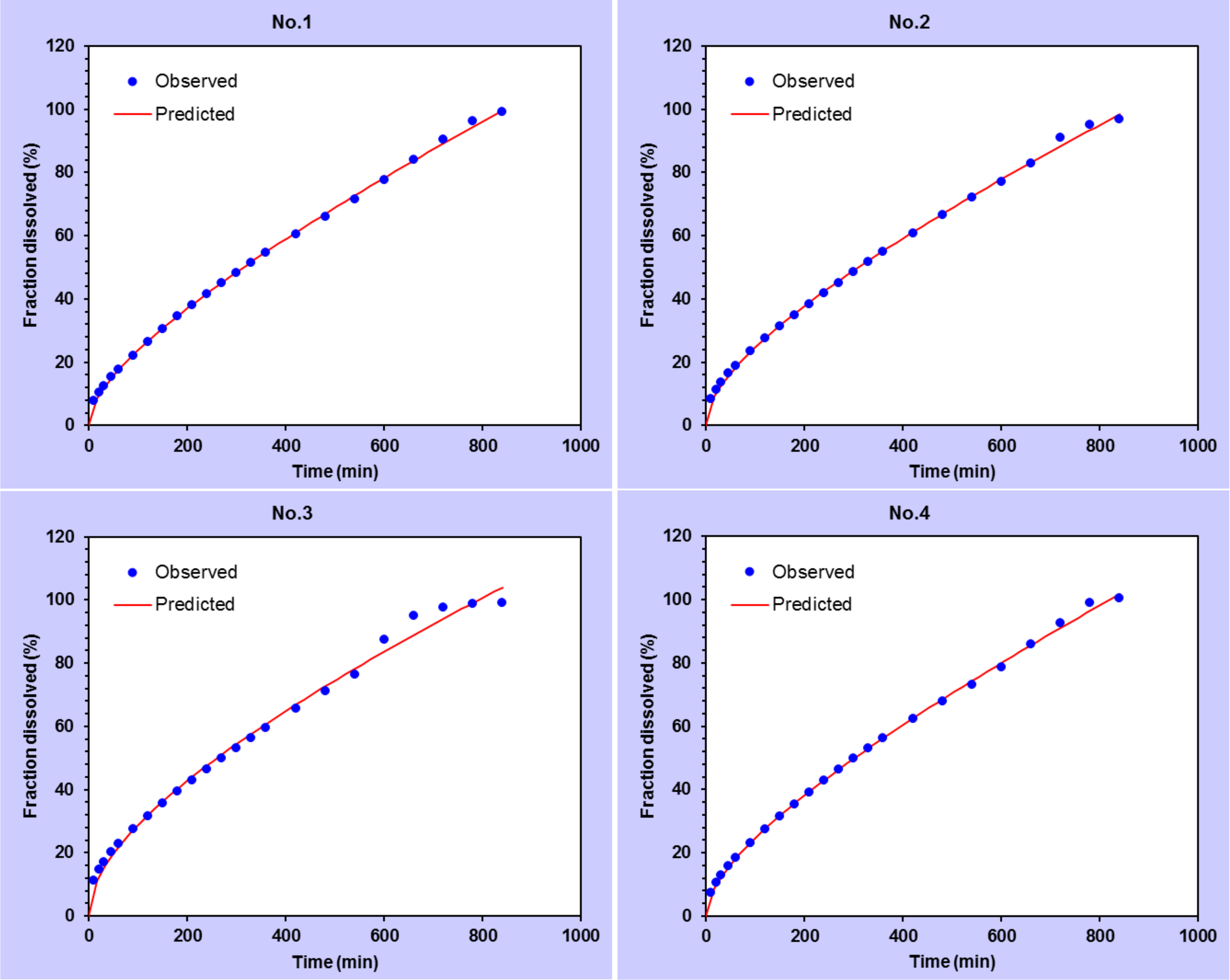

Supplementary materials – Model fitting summary – FlowLac® 100 (modelling the entire relevant dissolution data)

Model: **Peppas–Sahlin_1 with T_{lag}**

Model equation: $F = k_1 \cdot (t - T_{lag})^m + k_2 \cdot (t - T_{lag})^{2m}$

Fitted model parameters per tested tablet (N = 4) with statistics – mean, standard deviation (SD), and relative standard deviation expressed in % (RSD%) (output from DDSolver):

Parameter	No.1	No.2	No.3	No.4	Mean	SD	RSD(%)
k_1	1.984	2.197	2.879	2.087	2.287	0.404	17.676
k_2	0.137	0.124	0.104	0.137	0.125	0.016	12.597
m	0.450	0.450	0.450	0.450	0.450	0.000	0.000
T_{lag}	4.000	4.000	4.000	4.000	4.000	0.000	0.000

Number of dissolution data points (N), degrees of freedom (df), and selected goodness of fit criteria – Pearson correlation coefficient (R), coefficient of determination (R^2), adjusted coefficient of determination ($R^2_{adjusted}$), and residual sum of squares (RSS) (manual calculation in MS Excel):

Parameter	No.1	No.2	No.3	No.4
N	23	23	23	23
df	19	19	19	19
R	0.999446566	0.999125428	0.996120968	0.999364865
R^2	0.998893438	0.99825162	0.992256982	0.998730133
$R^2_{adjusted}$	0.998718718	0.99797556	0.9910344	0.998529627
RSS	23.10129781	34.37150279	153.4763454	26.23417409

Graphical abstract of model fit presented as mean ± 1 SD of the fraction % of released carvedilol:

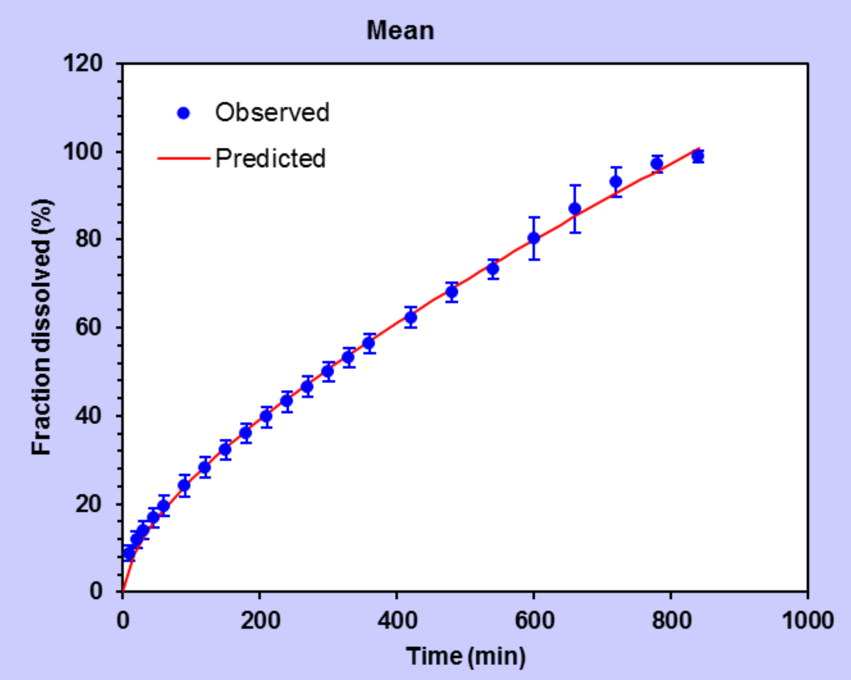

Supplementary materials – Model fitting summary – FlowLac® 100 (modelling the entire relevant dissolution data)

Graphical abstract of model fit presented as the fraction % of released carvedilol per tested tablet:

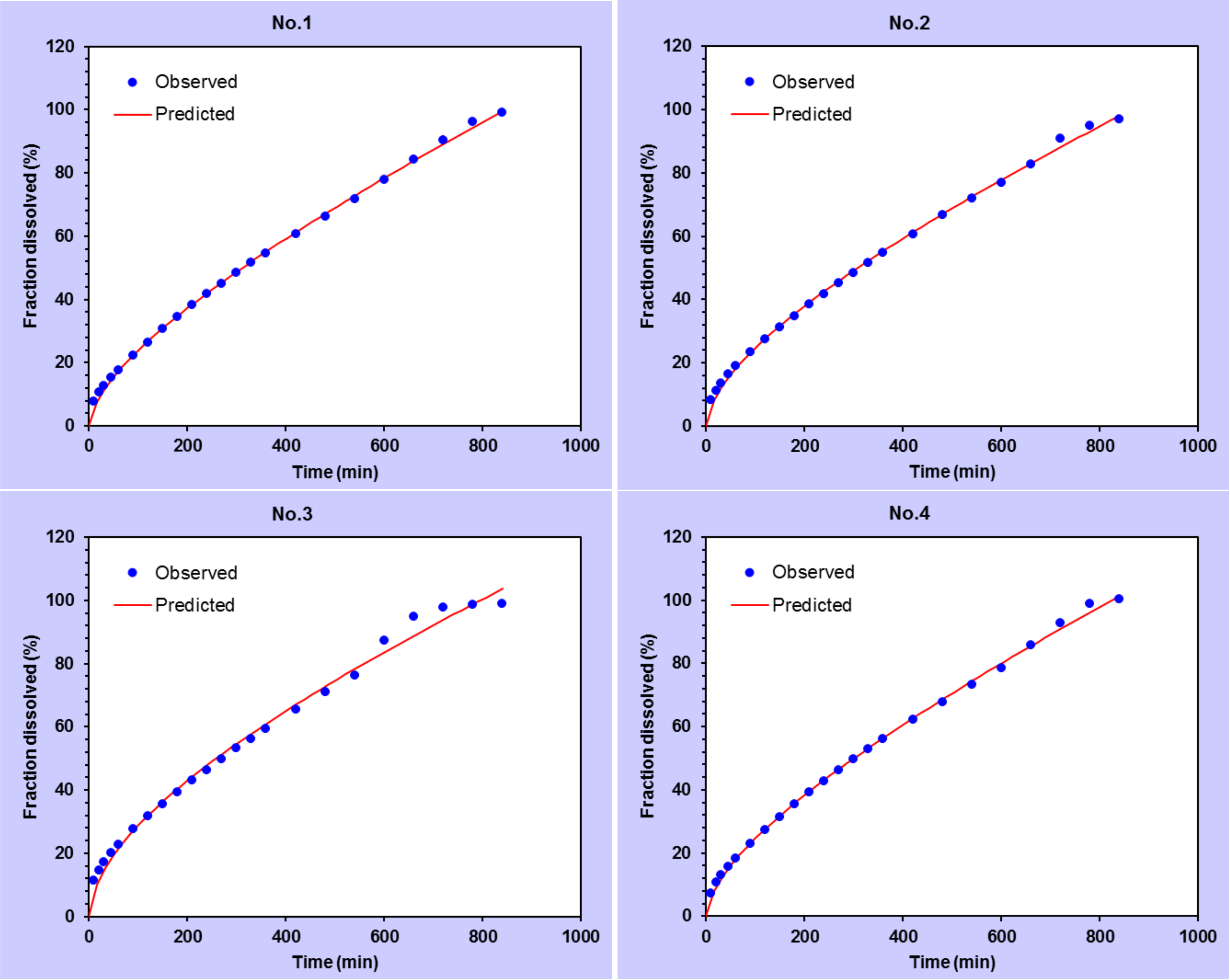

Supplementary materials – Model fitting summary – FlowLac® 100 (modelling the entire relevant dissolution data)

Model: **Peppas–Sahlin_2**

Model equation: $F = k_1 \cdot t^{0.5} + k_2 \cdot t$

Fitted model parameters per tested tablet (N = 4) with statistics – mean, standard deviation (SD), and relative standard deviation expressed in % (RSD%) (output from DDSolver):

Parameter	No.1	No.2	No.3	No.4	Mean	SD	RSD(%)
k_1	1.827	1.973	2.487	1.909	2.049	0.298	14.552
k_2	0.056	0.049	0.038	0.055	0.050	0.008	16.885

Number of dissolution data points (N), degrees of freedom (df), and selected goodness of fit criteria – Pearson correlation coefficient (R), coefficient of determination (R^2), adjusted coefficient of determination ($R^2_{adjusted}$), and residual sum of squares (RSS) (manual calculation in MS Excel):

Parameter	No.1	No.2	No.3	No.4
N	23	23	23	23
df	21	21	21	21
R	0.999706979	0.999420791	0.9965391	0.999569481
R^2	0.999414044	0.998841918	0.993090178	0.999139147
$R^2_{adjusted}$	0.999386142	0.998786772	0.992761139	0.999098153
RSS	11.69181562	21.96144716	134.6419566	16.84544033

Graphical abstract of model fit presented as mean ± 1 SD of the fraction % of released carvedilol:

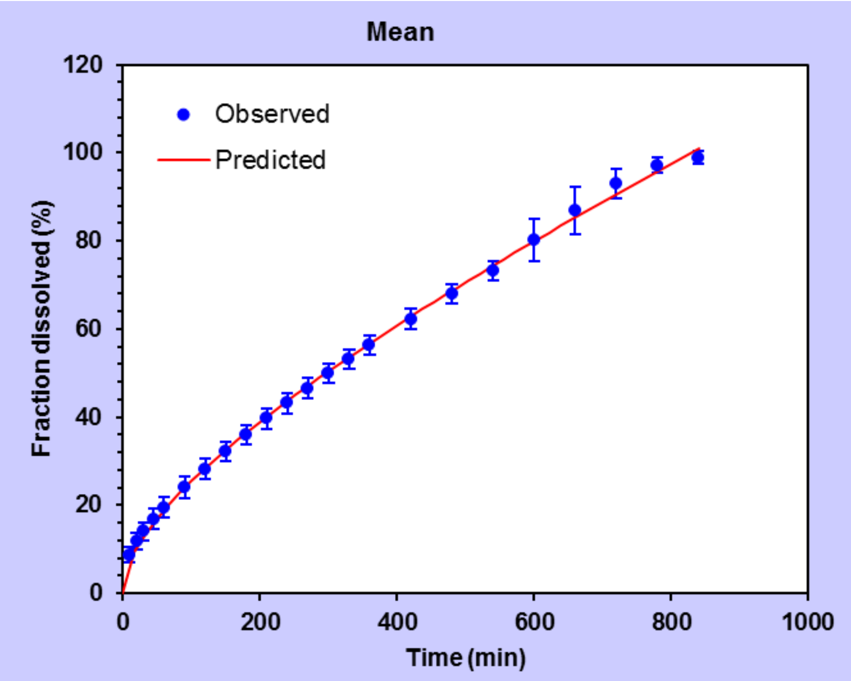

Supplementary materials – Model fitting summary – FlowLac® 100 (modelling the entire relevant dissolution data)

Graphical abstract of model fit presented as the fraction % of released carvedilol per tested tablet:

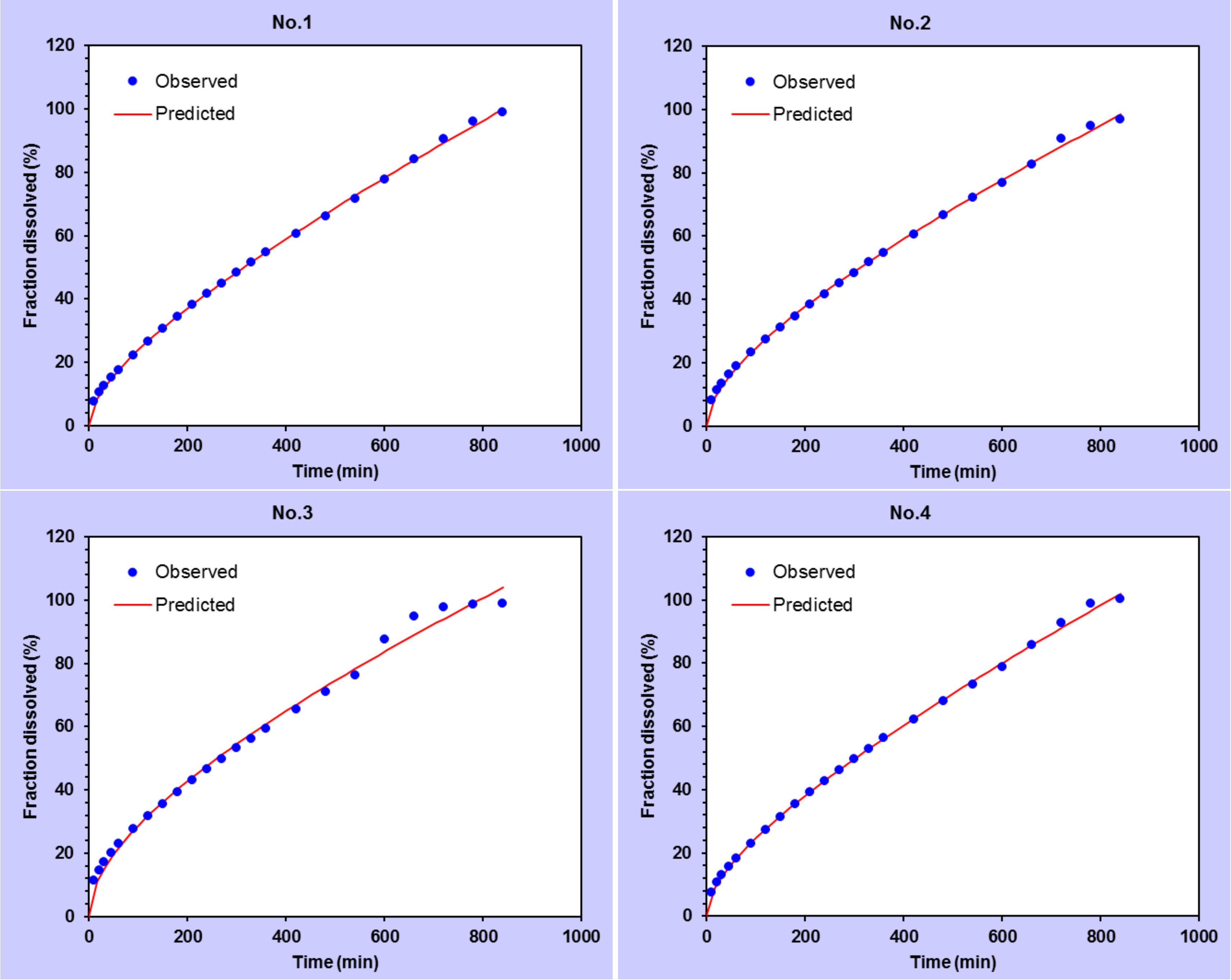

Supplementary materials – Model fitting summary – FlowLac® 100 (modelling the entire relevant dissolution data)

Model: **Peppas–Sahlin_2 with T_{lag}**

Model equation: $F = k_1 \cdot (t - T_{lag})^{0.5} + k_2 \cdot (t - T_{lag})$

Fitted model parameters per tested tablet (N = 4) with statistics – mean, standard deviation (SD), and relative standard deviation expressed in % (RSD%) (output from DDSolver):

Parameter	No.1	No.2	No.3	No.4	Mean	SD	RSD(%)
k_1	1.910	2.056	2.577	1.995	2.135	0.301	14.105
k_2	0.053	0.046	0.035	0.053	0.047	0.008	18.174
T_{lag}	4.000	4.000	4.000	4.000	4.000	0.000	0.000

Number of dissolution data points (N), degrees of freedom (df), and selected goodness of fit criteria – Pearson correlation coefficient (R), coefficient of determination (R^2), adjusted coefficient of determination ($R^2_{adjusted}$), and residual sum of squares (RSS) (manual calculation in MS Excel):

Parameter	No.1	No.2	No.3	No.4
N	23	23	23	23
df	20	20	20	20
R	0.999464239	0.999106118	0.995962783	0.999371149
R^2	0.998928766	0.998213034	0.991941865	0.998742694
$R^2_{adjusted}$	0.998821642	0.998034338	0.991136051	0.998616963
RSS	23.32826494	36.74031683	164.7749476	26.78167365

Graphical abstract of model fit presented as mean ± 1 SD of the fraction % of released carvedilol:

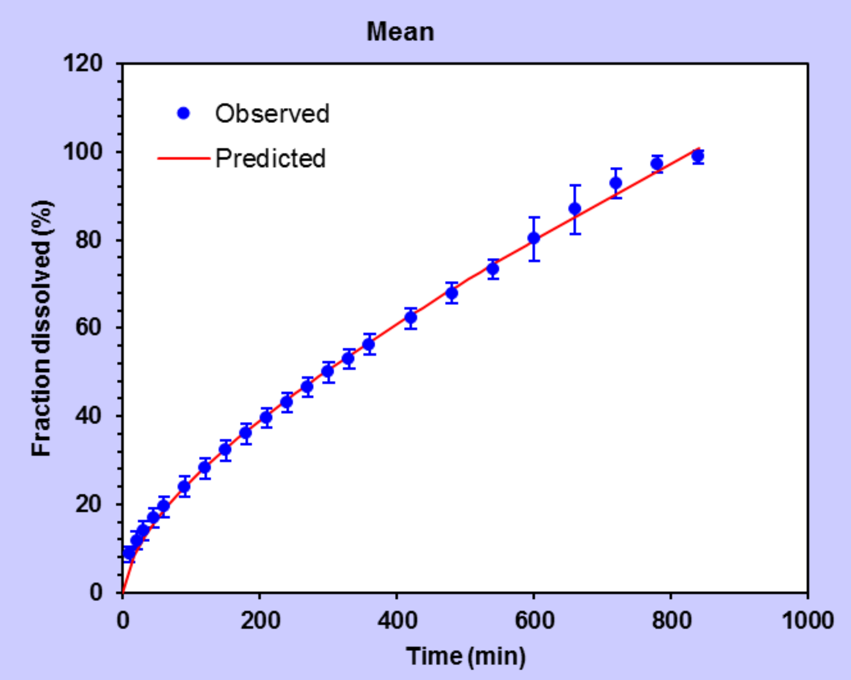

Supplementary materials – Model fitting summary – FlowLac® 100 (modelling the entire relevant dissolution data)

Graphical abstract of model fit presented as the fraction % of released carvedilol per tested tablet:

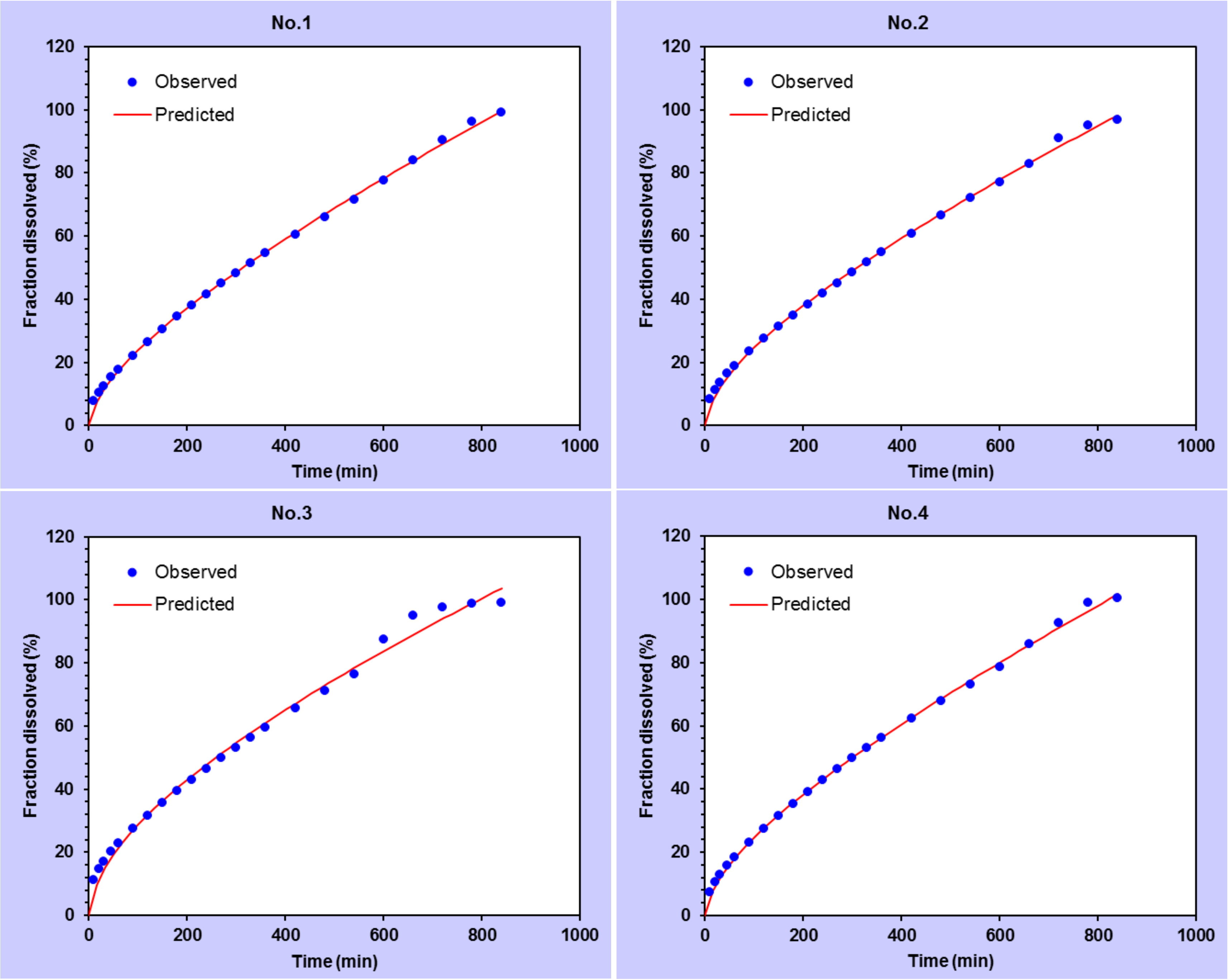

Supplementary materials – Model fitting summary – FlowLac® 100 (modelling the entire relevant dissolution data)

Model: **Quadratic**

Model equation: $F = 100 \cdot (k_1 \cdot t^2 + k_2 \cdot t)$

Fitted model parameters per tested tablet (N = 4) with statistics – mean, standard deviation (SD), and relative standard deviation expressed in % (RSD%) (output from DDSolver):

Parameter	No.1	No.2	No.3	No.4	Mean	SD	RSD(%)
k_1	-0.000001	-0.000001	-0.000001	-0.000001	-0.000001	0.000000	-14.728275
k_2	0.001896	0.001934	0.002202	0.001954	0.001997	0.000139	6.978453

Number of dissolution data points (N), degrees of freedom (df), and selected goodness of fit criteria – Pearson correlation coefficient (R), coefficient of determination (R^2), adjusted coefficient of determination ($R^2_{adjusted}$), and residual sum of squares (RSS) (manual calculation in MS Excel):

Parameter	No.1	No.2	No.3	No.4
N	23	23	23	23
df	21	21	21	21
R	0.995763895	0.994849518	0.991543262	0.995464838
R^2	0.991545735	0.989725563	0.983158041	0.990950244
$R^2_{adjusted}$	0.991143151	0.989236304	0.982356043	0.990519303
RSS	406.6344852	489.2850933	848.9876062	436.1756583

Graphical abstract of model fit presented as mean ± 1 SD of the fraction % of released carvedilol:

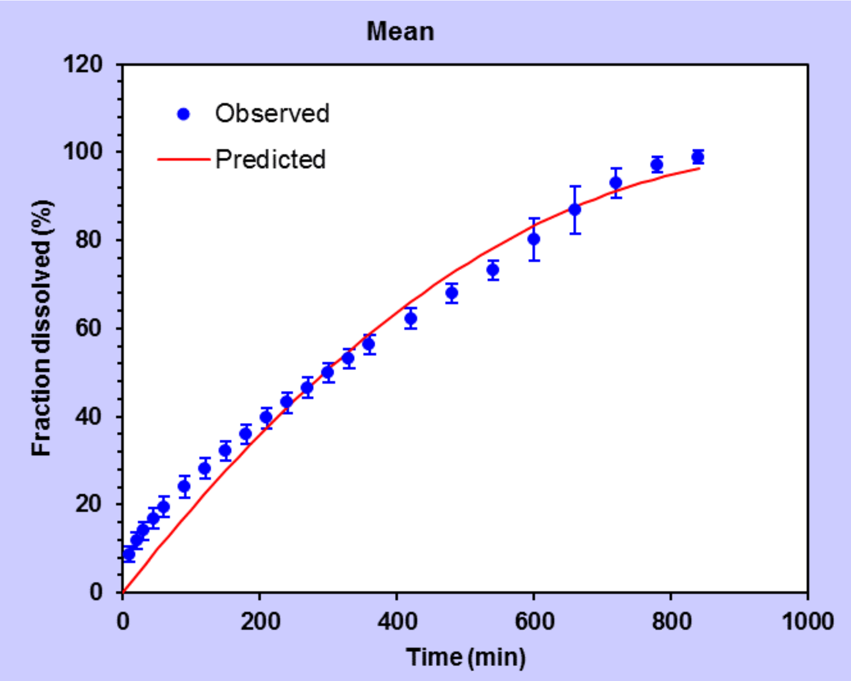

Supplementary materials – Model fitting summary – FlowLac® 100 (modelling the entire relevant dissolution data)

Graphical abstract of model fit presented as the fraction % of released carvedilol per tested tablet:

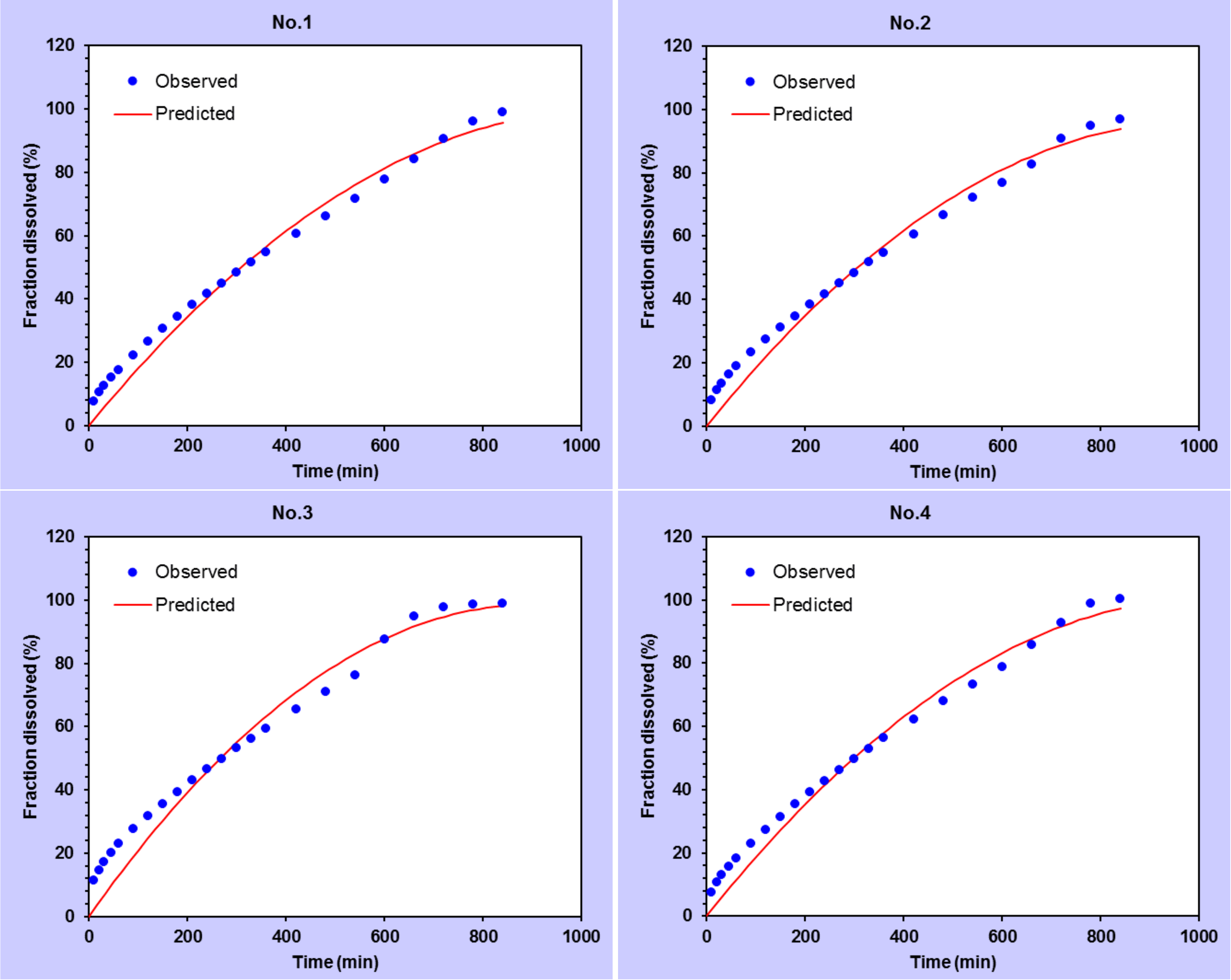

Supplementary materials – Model fitting summary – FlowLac® 100 (modelling the entire relevant dissolution data)

Model: **Quadratic with T_{lag}**

Model equation: $F = 100 \cdot \left[k_1 \cdot \left(t - T_{lag}\right)^2 + k_2 \cdot \left(t - T_{lag}\right)\right]$

Fitted model parameters per tested tablet (N = 4) with statistics – mean, standard deviation (SD), and relative standard deviation expressed in % (RSD%) (output from DDSolver):

Parameter	No.1	No.2	No.3	No.4	Mean	SD	RSD(%)
k_1	-0.000001	-0.000001	-0.000001	-0.000001	-0.000001	0.000000	-14.389075
k_2	0.001923	0.001961	0.002231	0.001982	0.002024	0.000140	6.919501
T_{lag}	4.000000	4.000000	4.000000	4.000000	4.000000	0.000000	0.000000

Number of dissolution data points (N), degrees of freedom (df), and selected goodness of fit criteria – Pearson correlation coefficient (R), coefficient of determination (R^2), adjusted coefficient of determination ($R^2_{adjusted}$), and residual sum of squares (RSS) (manual calculation in MS Excel):

Parameter	No.1	No.2	No.3	No.4
N	23	23	23	23
df	20	20	20	20
R	0.995353863	0.994407529	0.991048669	0.995054779
R^2	0.990729312	0.988846333	0.982177465	0.990134013
$R^2_{adjusted}$	0.989802243	0.987730966	0.980395211	0.989147414
RSS	474.8088891	565.467421	960.0553065	507.9415175

Graphical abstract of model fit presented as mean ± 1 SD of the fraction % of released carvedilol:

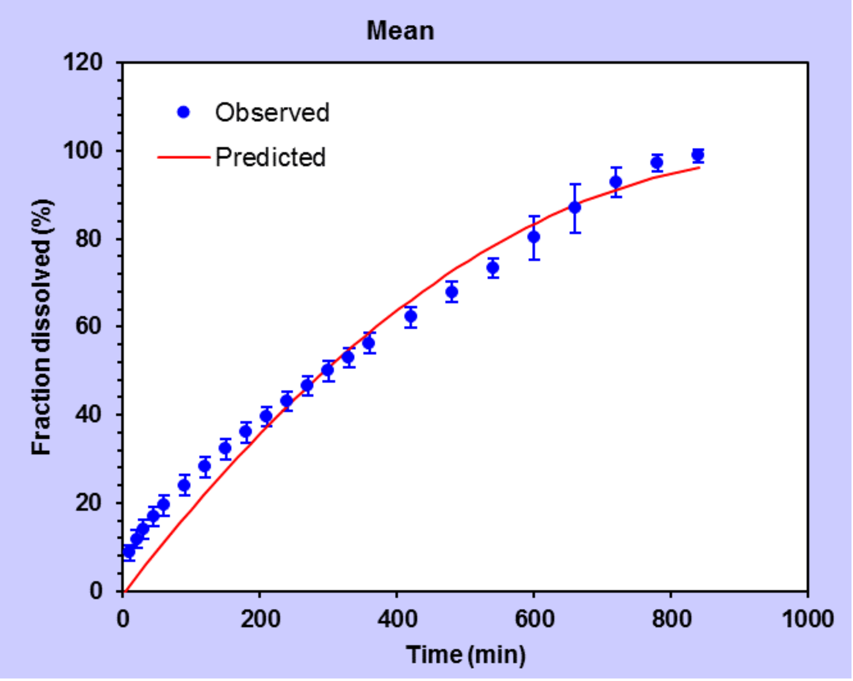

Supplementary materials – Model fitting summary – FlowLac® 100 (modelling the entire relevant dissolution data)

Graphical abstract of model fit presented as the fraction % of released carvedilol per tested tablet:

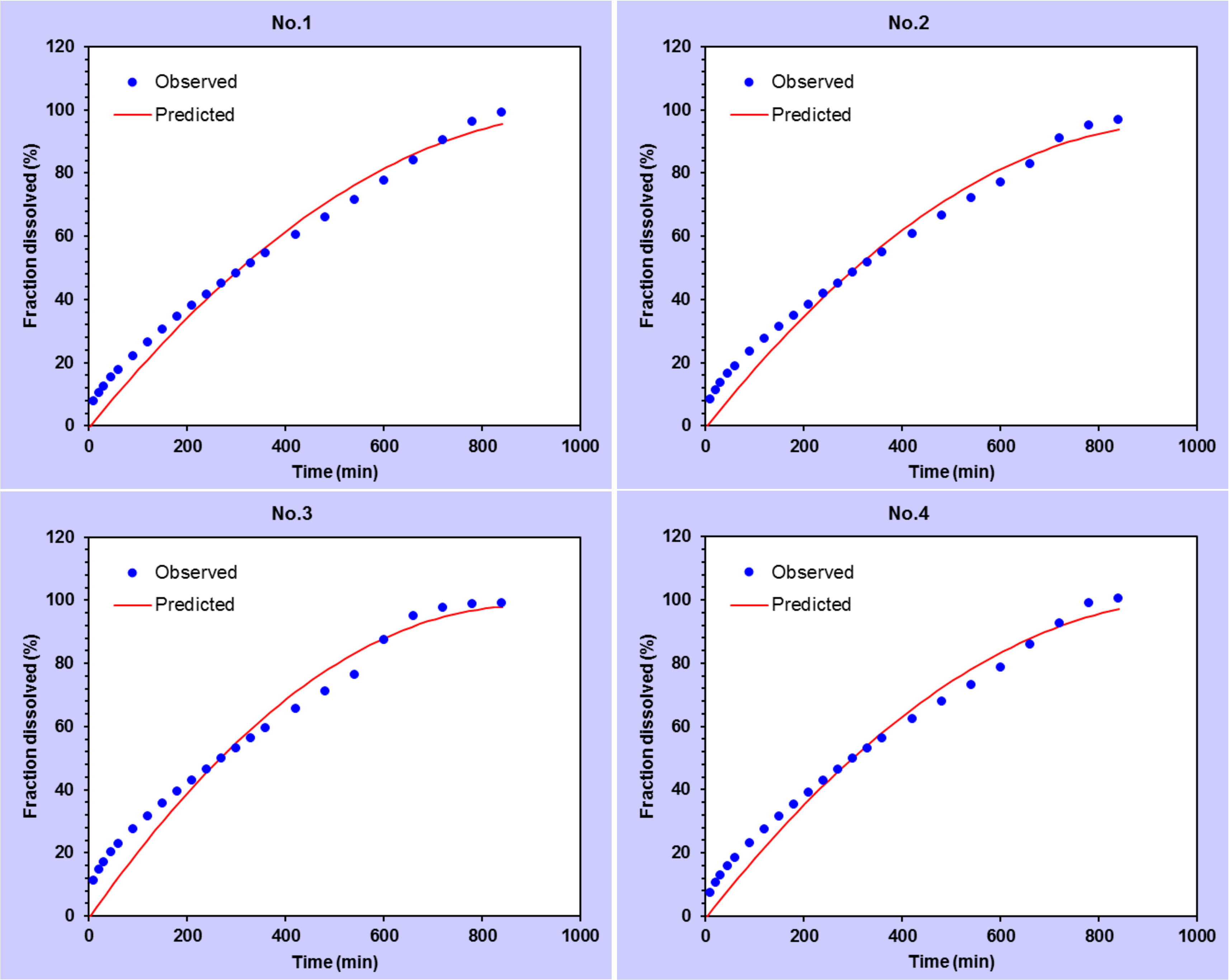

Supplementary materials – Model fitting summary – FlowLac® 100 (modelling the entire relevant dissolution data)

Model: **Weibull_1**

Model equation: $F = 100 \cdot \left[1 - e^{-\frac{(t-T_i)^\beta}{\alpha}}\right]$

Fitted model parameters per tested tablet (N = 4) with statistics – mean, standard deviation (SD), and relative standard deviation expressed in % (RSD%) (output from DDSolver):

Parameter	No.1	No.2	No.3	No.4	Mean	SD	RSD(%)
α	96.385	79.058	64.672	88.617	82.183	13.656	16.617
β	0.771	0.734	0.739	0.756	0.750	0.017	2.257
T_i	6.000	6.000	6.000	6.000	6.000	0.000	0.000

Number of dissolution data points (N), degrees of freedom (df), and selected goodness of fit criteria – Pearson correlation coefficient (R), coefficient of determination (R^2), adjusted coefficient of determination ($R^2_{adjusted}$), and residual sum of squares (RSS) (manual calculation in MS Excel):

Parameter	No.1	No.2	No.3	No.4
N	23	23	23	23
df	20	20	20	20
R	0.970387096	0.970090951	0.957010683	0.970868667
R^2	0.941651115	0.941076452	0.915869448	0.942585968
$R^2_{adjusted}$	0.935816227	0.935184097	0.907456392	0.936844565
RSS	1189.181632	1138.67397	1731.26727	1254.064471

Graphical abstract of model fit presented as mean ± 1 SD of the fraction % of released carvedilol:

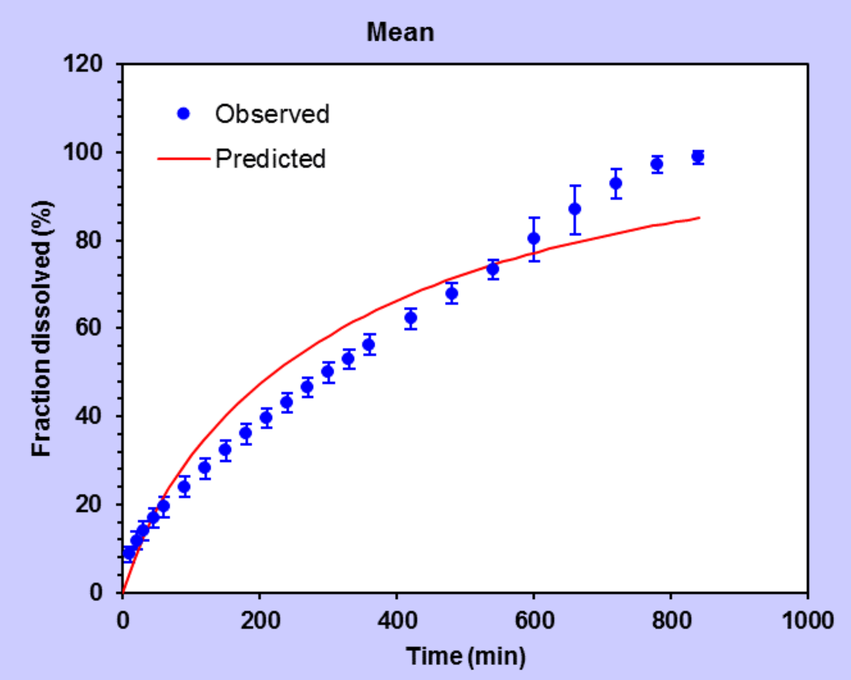

Supplementary materials – Model fitting summary – FlowLac® 100 (modelling the entire relevant dissolution data)

Graphical abstract of model fit presented as the fraction % of released carvedilol per tested tablet:

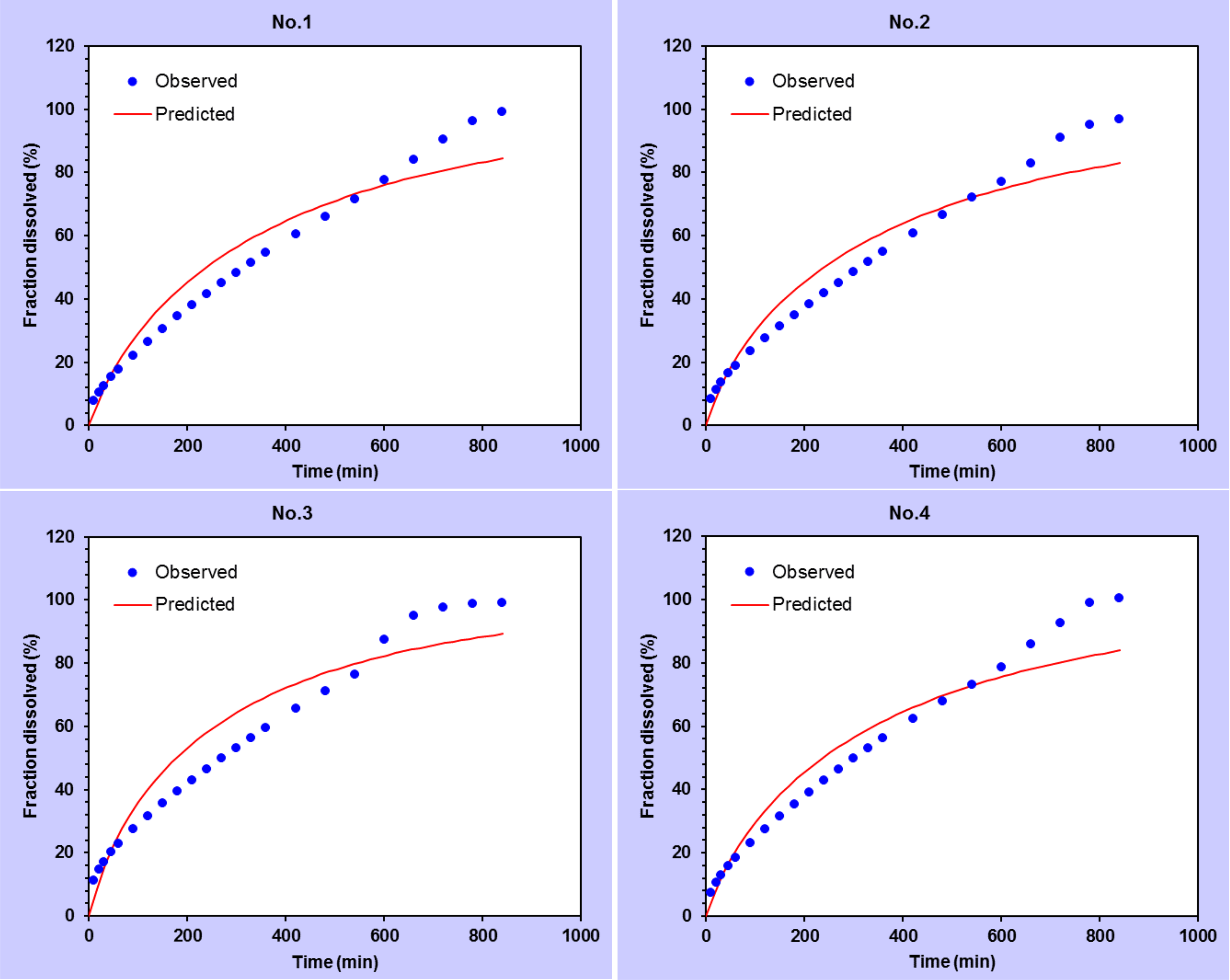

Supplementary materials – Model fitting summary – FlowLac® 100 (modelling the entire relevant dissolution data)

Model: **Weibull_2**

Model equation: $F = 100 \cdot \left(1 - e^{-\frac{t^{\beta}}{\alpha}}\right)$

Fitted model parameters per tested tablet (N = 4) with statistics – mean, standard deviation (SD), and relative standard deviation expressed in % (RSD%) (output from DDSolver):

Parameter	No.1	No.2	No.3	No.4	Mean	SD	RSD(%)
α	142.934	114.766	95.083	130.885	120.917	20.731	17.145
β	0.838	0.797	0.804	0.823	0.815	0.018	2.253

Number of dissolution data points (N), degrees of freedom (df), and selected goodness of fit criteria – Pearson correlation coefficient (R), coefficient of determination (R^2), adjusted coefficient of determination ($R^2_{adjusted}$), and residual sum of squares (RSS) (manual calculation in MS Excel):

Parameter	No.1	No.2	No.3	No.4
N	23	23	23	23
df	21	21	21	21
R	0.975564248	0.975611045	0.963353481	0.975954659
R^2	0.951725603	0.95181691	0.92804993	0.952487497
$R^2_{adjusted}$	0.949426822	0.949522478	0.924623736	0.950224997
RSS	1004.089437	945.0502889	1533.287598	1034.586559

Graphical abstract of model fit presented as mean ± 1 SD of the fraction % of released carvedilol:

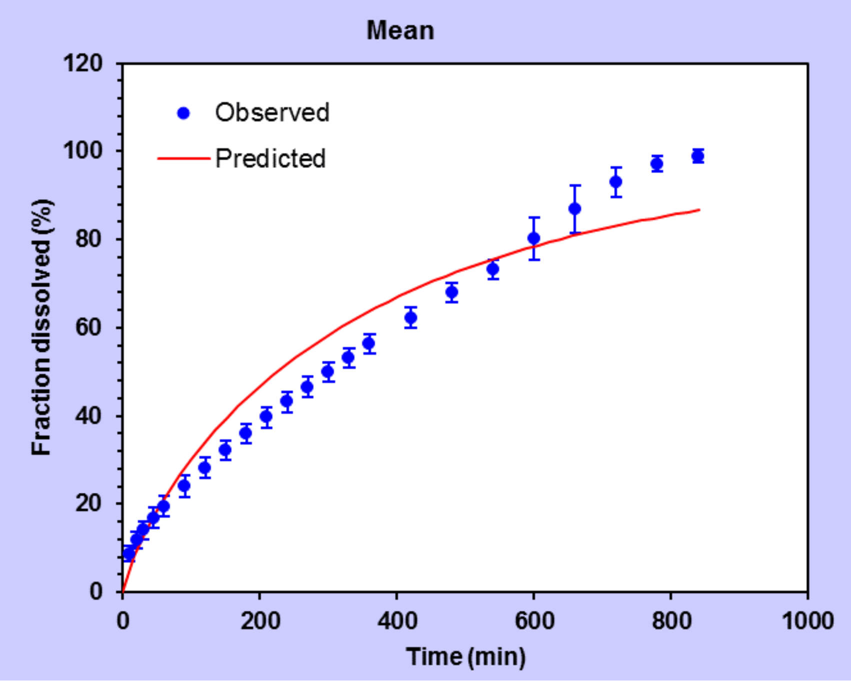

Supplementary materials – Model fitting summary – FlowLac® 100 (modelling the entire relevant dissolution data)

Graphical abstract of model fit presented as the fraction % of released carvedilol per tested tablet:

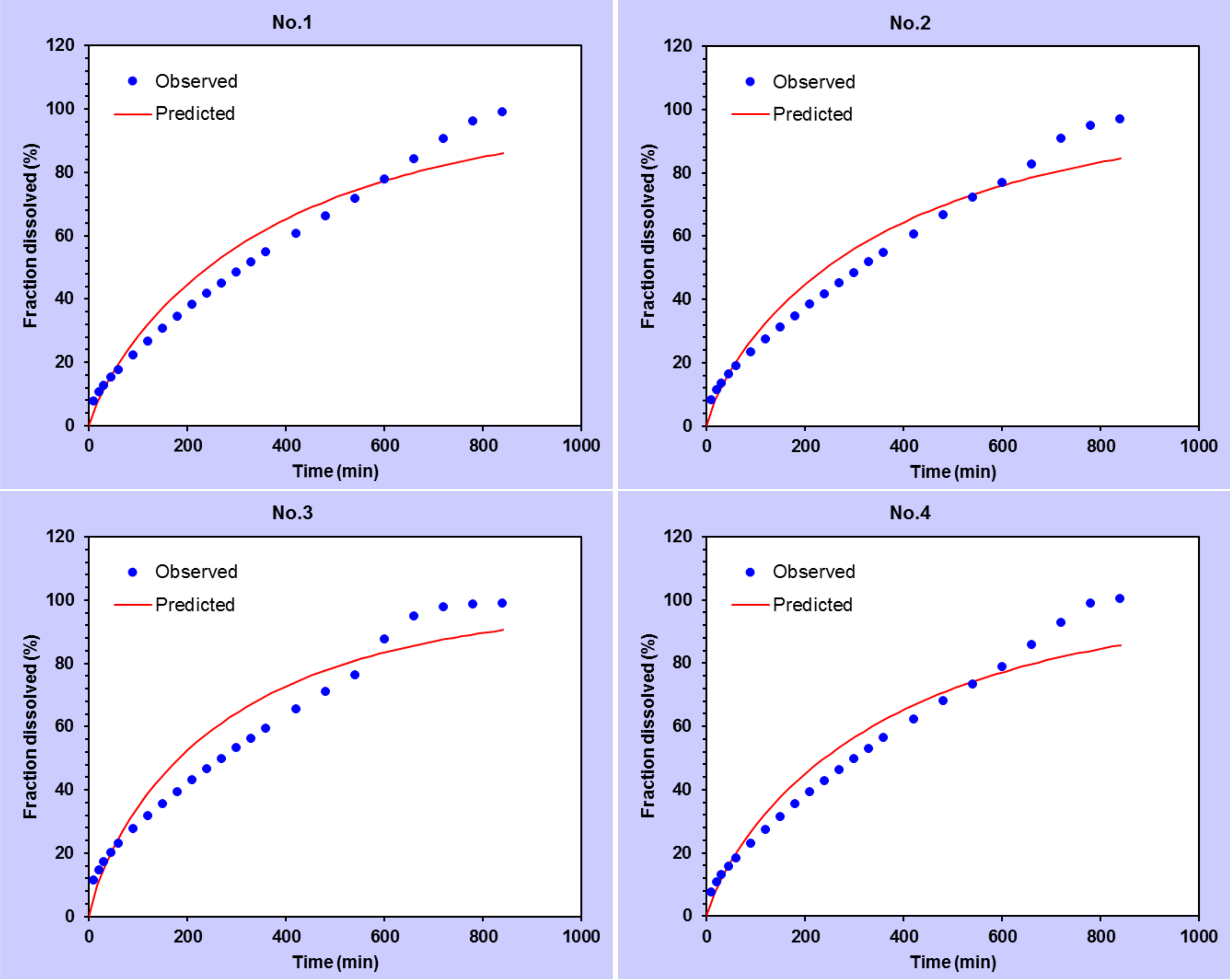

Supplementary materials – Model fitting summary – FlowLac® 100 (modelling the entire relevant dissolution data)

Model: **Weibull_3**

Model equation: $F = F_{max} \cdot \left(1 - e^{-\frac{t^{\beta}}{\alpha}}\right)$

Fitted model parameters per tested tablet (N = 4) with statistics – mean, standard deviation (SD), and relative standard deviation expressed in % (RSD%) (output from DDSolver):

Parameter	No.1	No.2	No.3	No.4	Mean	SD	RSD(%)
α	168.019	122.884	80.378	173.581	136.215	43.601	32.009
β	0.814	0.738	0.752	0.824	0.782	0.043	5.510
F_{max}	108.670	119.462	103.988	110.077	110.549	6.487	5.868

Number of dissolution data points (N), degrees of freedom (df), and selected goodness of fit criteria – Pearson correlation coefficient (R), coefficient of determination (R^2), adjusted coefficient of determination ($R^2_{adjusted}$), and residual sum of squares (RSS) (manual calculation in MS Excel):

Parameter	No.1	No.2	No.3	No.4
N	23	23	23	23
df	20	20	20	20
R	0.98604186	0.986907882	0.968850977	0.986501273
R^2	0.972278549	0.973987168	0.938672215	0.973184762
$R^2_{adjusted}$	0.969506404	0.971385885	0.932539437	0.970503239
RSS	875.2205559	799.0878606	1260.157681	836.5159391

Graphical abstract of model fit presented as mean ± 1 SD of the fraction % of released carvedilol:

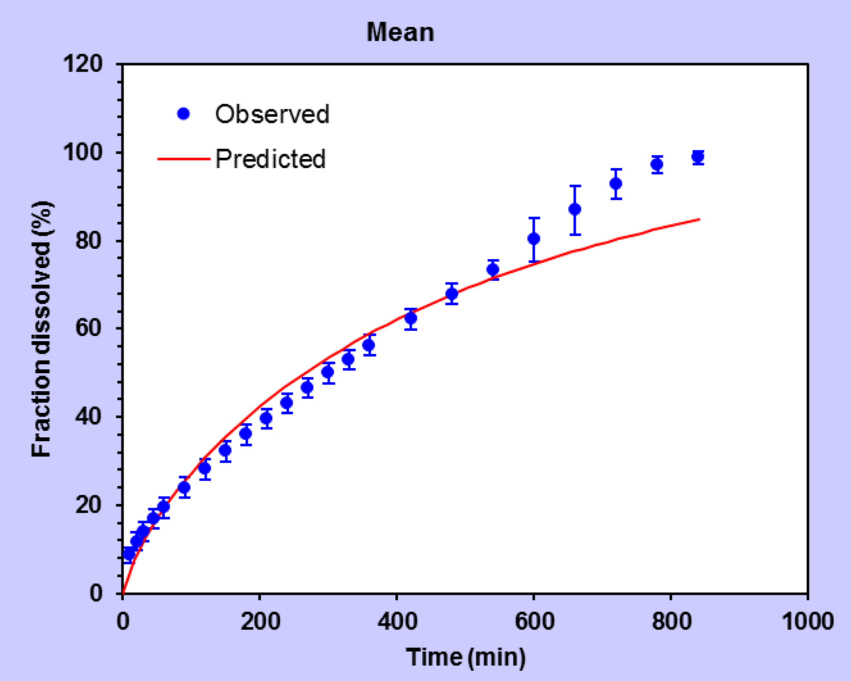

Supplementary materials – Model fitting summary – FlowLac® 100 (modelling the entire relevant dissolution data)

Graphical abstract of model fit presented as the fraction % of released carvedilol per tested tablet:

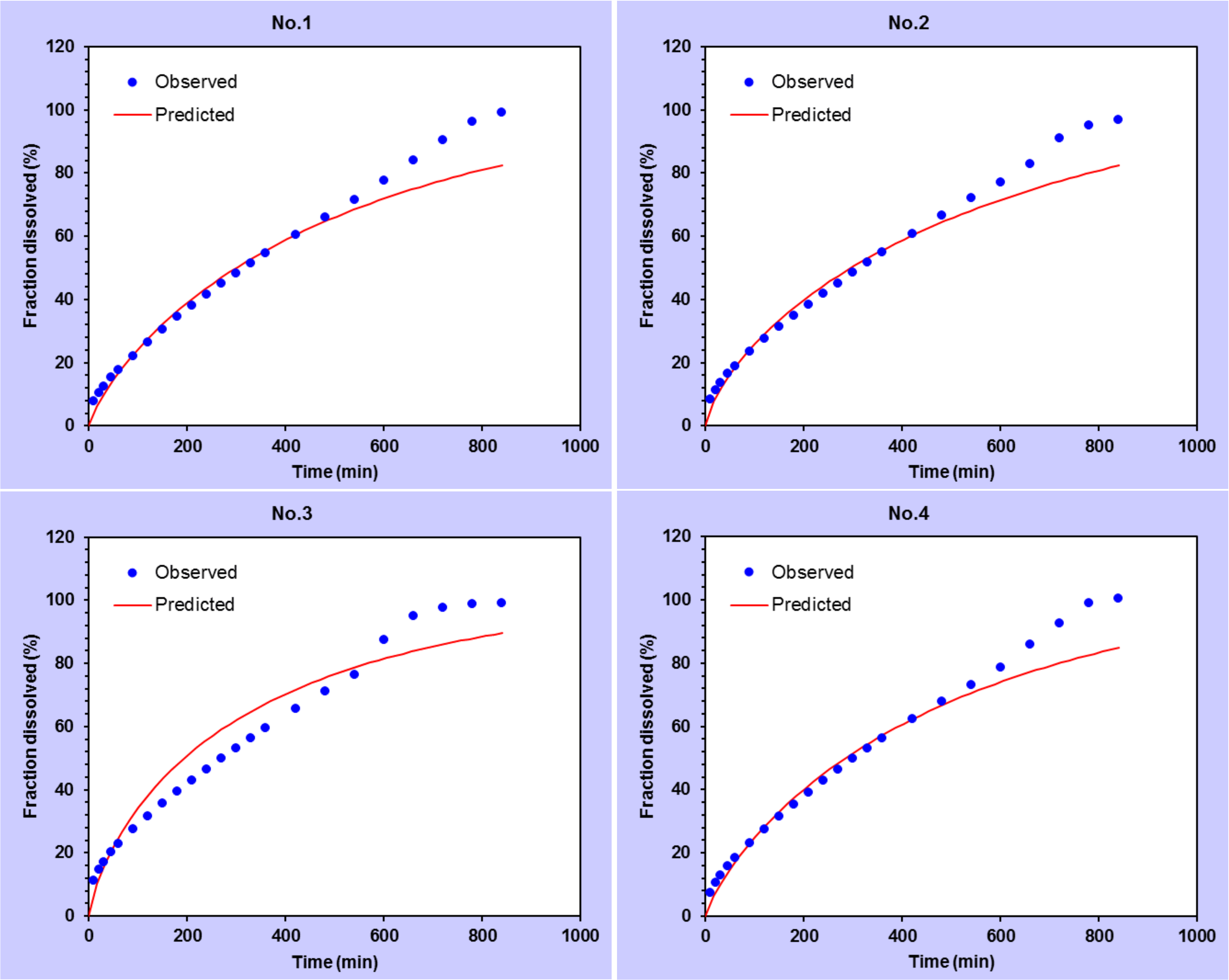

Supplementary materials – Model fitting summary – FlowLac® 100 (modelling the entire relevant dissolution data)

Model: **Weibull_4**

Model equation: $F = F_{max} \cdot \left[1 - e^{-\frac{(t-T_i)^{\beta}}{\alpha}}\right]$

Fitted model parameters per tested tablet (N = 4) with statistics – mean, standard deviation (SD), and relative standard deviation expressed in % (RSD%) (output from DDSolver):

Parameter	No.1	No.2	No.3	No.4	Mean	SD	RSD(%)
α	88.701	76.909	56.303	91.512	78.356	16.005	20.426
β	0.739	0.722	0.692	0.748	0.725	0.025	3.390
T_i	6.000	6.000	6.000	6.000	6.000	0.000	0.000
F_{max}	104.053	101.773	103.988	105.401	103.804	1.502	1.447

Number of dissolution data points (N), degrees of freedom (df), and selected goodness of fit criteria – Pearson correlation coefficient (R), coefficient of determination (R^2), adjusted coefficient of determination ($R^2_{adjusted}$), and residual sum of squares (RSS) (manual calculation in MS Excel):

Parameter	No.1	No.2	No.3	No.4
N	23	23	23	23
df	19	19	19	19
R	0.973464515	0.971405788	0.96228693	0.974231389
R^2	0.947633161	0.943629204	0.925996135	0.949126799
$R^2_{adjusted}$	0.939364713	0.934728552	0.914311314	0.941094188
RSS	1101.828562	1103.211066	1489.376848	1096.384656

Graphical abstract of model fit presented as mean ± 1 SD of the fraction % of released carvedilol:

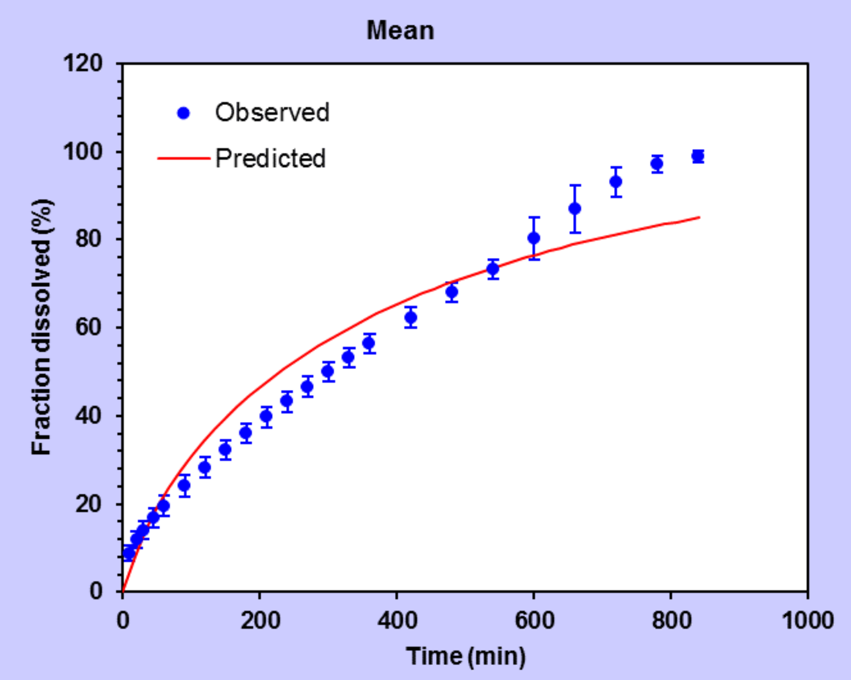

Supplementary materials – Model fitting summary – FlowLac® 100 (modelling the entire relevant dissolution data)

Graphical abstract of model fit presented as the fraction % of released carvedilol per tested tablet:

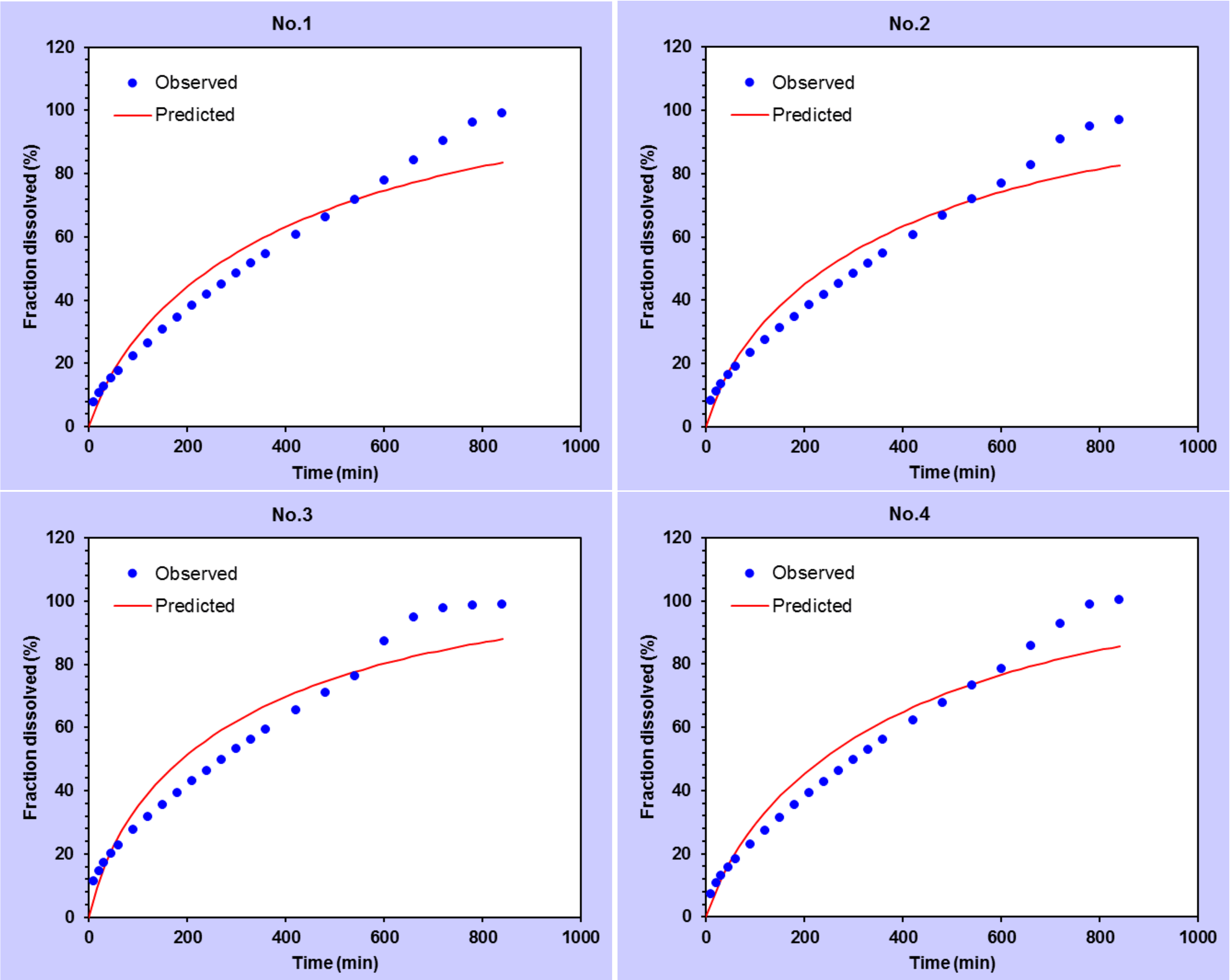

Supplementary materials – Model fitting summary – FlowLac® 100 (modelling the entire relevant dissolution data)

Model: **Logistic_1**

Model equation: $F = 100 \cdot \frac{e^{\alpha+\beta \cdot \log(t)}}{1+e^{\alpha+\beta \cdot \log(t)}}$

Fitted model parameters per tested tablet (N = 4) with statistics – mean, standard deviation (SD), and relative standard deviation expressed in % (RSD%) (output from DDSolver):

Parameter	No.1	No.2	No.3	No.4	Mean	SD	RSD(%)
α	-8.019	-5.972	-8.045	-6.154	-7.048	1.139	-16.161
β	3.168	2.604	3.373	2.694	2.960	0.370	12.510

Number of dissolution data points (N), degrees of freedom (df), and selected goodness of fit criteria – Pearson correlation coefficient (R), coefficient of determination (R^2), adjusted coefficient of determination ($R^2_{adjusted}$), and residual sum of squares (RSS) (manual calculation in MS Excel):

Parameter	No.1	No.2	No.3	No.4
N	23	23	23	23
df	21	21	21	21
R	0.984168935	0.948794245	0.967945134	0.948104235
R^2	0.968588493	0.90021052	0.936917782	0.89890164
$R^2_{adjusted}$	0.967092707	0.89545864	0.933913867	0.894087433
RSS	2095.754445	2061.741182	1755.06291	2187.744551

Graphical abstract of model fit presented as mean ± 1 SD of the fraction % of released carvedilol:

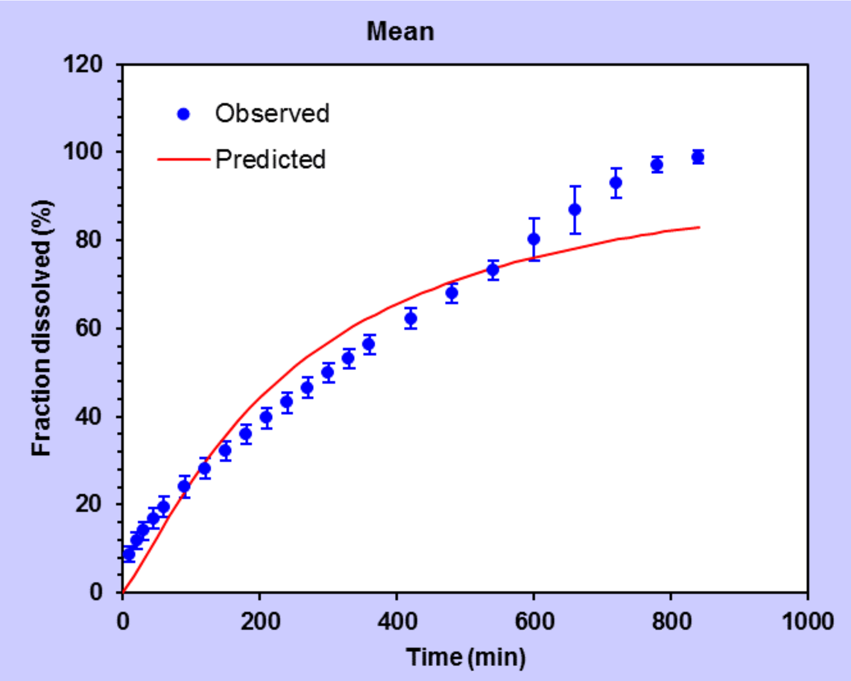

Supplementary materials – Model fitting summary – FlowLac® 100 (modelling the entire relevant dissolution data)

Graphical abstract of model fit presented as the fraction % of released carvedilol per tested tablet:

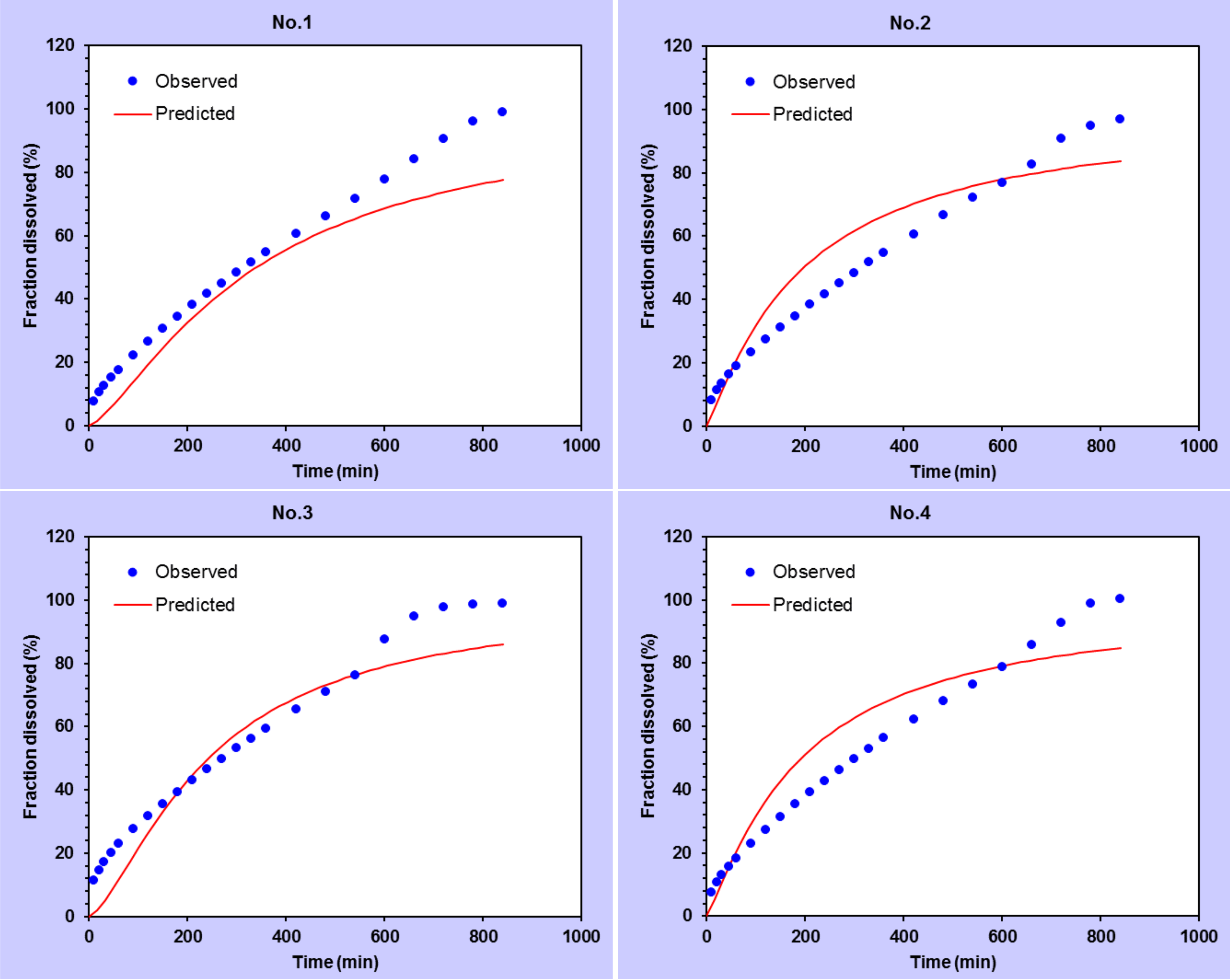

Supplementary materials – Model fitting summary – FlowLac® 100 (modelling the entire relevant dissolution data)

Model: **Logistic_2**

Model equation: $F = F_{max} \cdot \frac{e^{\alpha+\beta\cdot\log(t)}}{1+e^{\alpha+\beta\cdot\log(t)}}$

Fitted model parameters per tested tablet (N = 4) with statistics – mean, standard deviation (SD), and relative standard deviation expressed in % (RSD%) (output from DDSolver):

Parameter	No.1	No.2	No.3	No.4	Mean	SD	RSD(%)
α	-5.925	-5.810	-6.210	-5.995	-5.985	0.168	-2.811
β	2.518	2.502	2.349	2.562	2.483	0.093	3.734
Fmax	104.053	101.773	130.961	105.401	110.547	13.691	12.385

Number of dissolution data points (N), degrees of freedom (df), and selected goodness of fit criteria – Pearson correlation coefficient (R), coefficient of determination (R^2), adjusted coefficient of determination ($R^2_{adjusted}$), and residual sum of squares (RSS) (manual calculation in MS Excel):

Parameter	No.1	No.2	No.3	No.4
N	23	23	23	23
df	20	20	20	20
R	0.955622759	0.952481386	0.983498504	0.955723722
R^2	0.913214858	0.90722079	0.967269307	0.913407834
$R^2_{adjusted}$	0.904536344	0.897942869	0.963996237	0.904748617
RSS	1810.300788	1847.100393	1503.55401	1893.380106

Graphical abstract of model fit presented as mean ± 1 SD of the fraction % of released carvedilol:

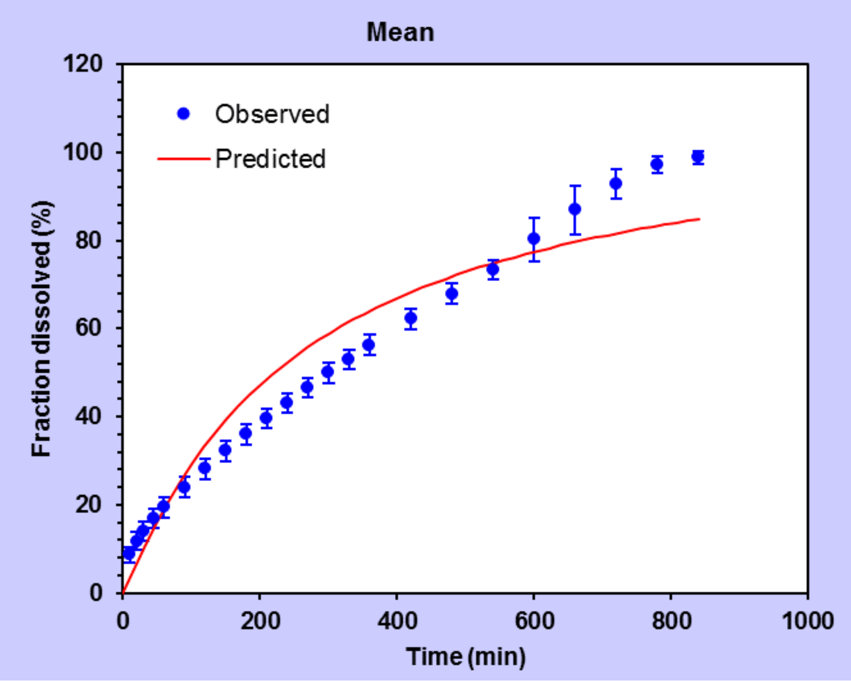

Supplementary materials – Model fitting summary – FlowLac® 100 (modelling the entire relevant dissolution data)

Graphical abstract of model fit presented as the fraction % of released carvedilol per tested tablet:

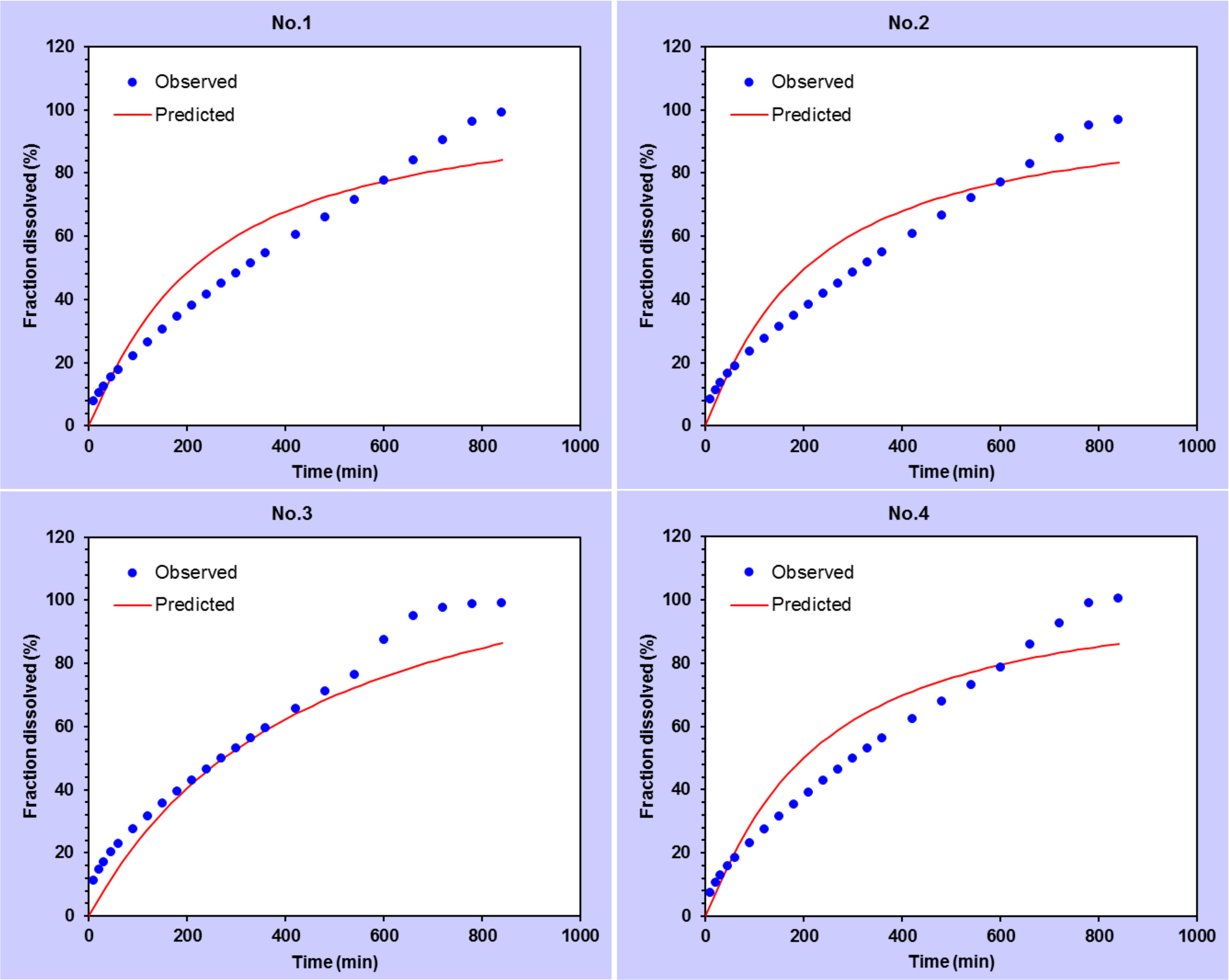

Supplementary materials – Model fitting summary – FlowLac® 100 (modelling the entire relevant dissolution data)

Model: **Logistic_3**

Model equation: $F = F_{max} \cdot \frac{1}{1+e^{-k\cdot(t-\gamma)}}$

Fitted model parameters per tested tablet (N = 4) with statistics – mean, standard deviation (SD), and relative standard deviation expressed in % (RSD%) (output from DDSolver):

Parameter	No.1	No.2	No.3	No.4	Mean	SD	RSD(%)
k	0.006	0.006	0.006	0.006	0.006	0.000	1.882
γ	352.624	338.768	296.843	346.678	333.728	25.237	7.562
F_{max}	104.053	101.773	103.988	105.401	103.804	1.502	1.447

Number of dissolution data points (N), degrees of freedom (df), and selected goodness of fit criteria – Pearson correlation coefficient (R), coefficient of determination (R^2), adjusted coefficient of determination ($R^2_{adjusted}$), and residual sum of squares (RSS) (manual calculation in MS Excel):

Parameter	No.1	No.2	No.3	No.4
N	23	23	23	23
df	20	20	20	20
R	0.99155308	0.992358074	0.993354692	0.990895125
R^2	0.98317751	0.984774548	0.986753544	0.981873148
$R^2_{adjusted}$	0.981495261	0.983252002	0.985428898	0.980060463
RSS	341.5655001	292.3052473	261.6477254	384.1228138

Graphical abstract of model fit presented as mean ± 1 SD of the fraction % of released carvedilol:

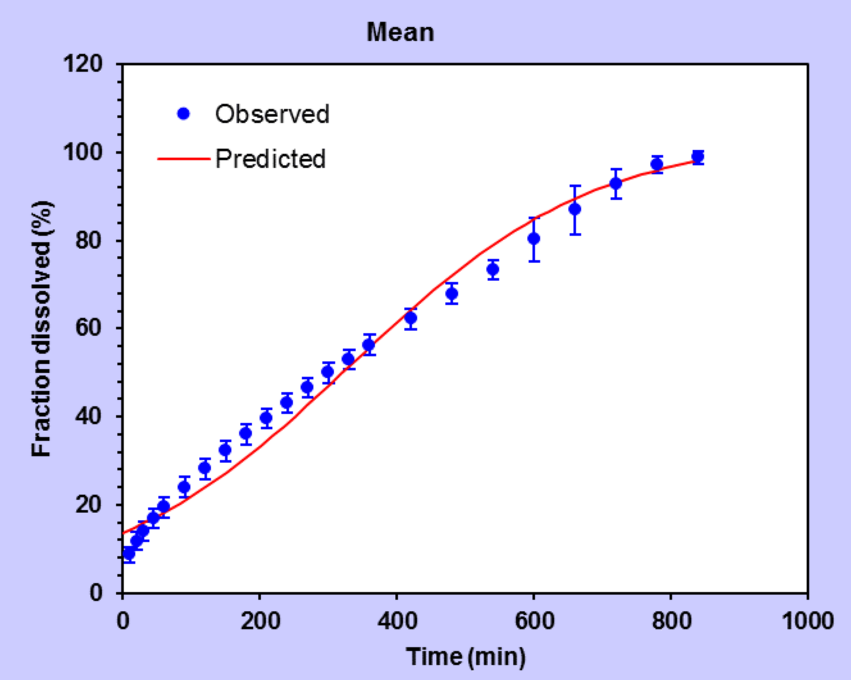

Supplementary materials – Model fitting summary – FlowLac® 100 (modelling the entire relevant dissolution data)

Graphical abstract of model fit presented as the fraction % of released carvedilol per tested tablet:

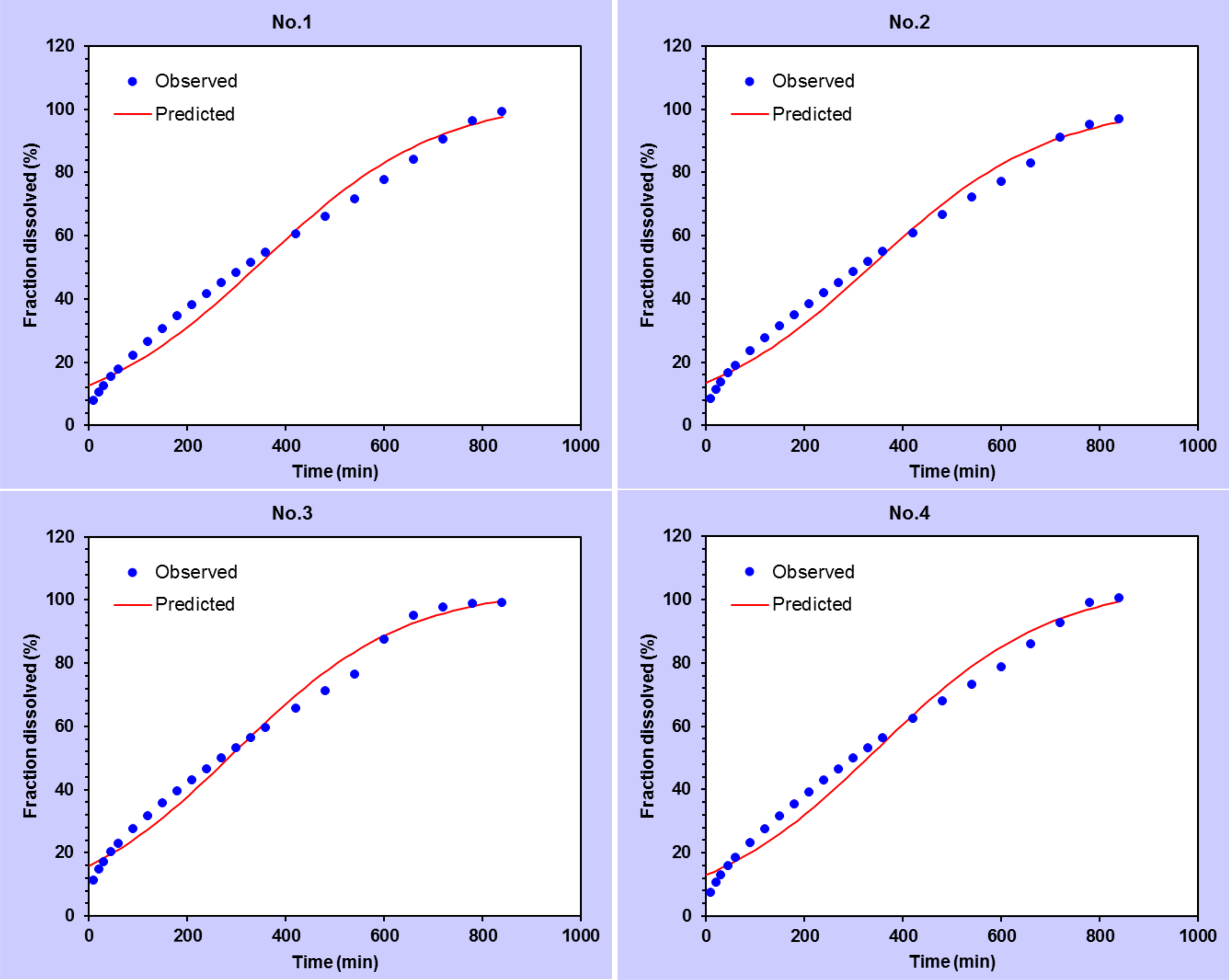

Supplementary materials – Model fitting summary – FlowLac® 100 (modelling the entire relevant dissolution data)

Model: **Gompertz_1**

Model equation: $F = 100 \cdot e^{-\alpha \cdot e^{-\beta \cdot \log(t)}}$

Fitted model parameters per tested tablet (N = 4) with statistics – mean, standard deviation (SD), and relative standard deviation expressed in % (RSD%) (output from DDSolver):

Parameter	No.1	No.2	No.3	No.4	Mean	SD	RSD(%)
α	81.723	57.574	121.309	63.004	80.902	28.856	35.668
β	1.995	1.818	2.302	1.868	1.996	0.217	10.896

Number of dissolution data points (N), degrees of freedom (df), and selected goodness of fit criteria – Pearson correlation coefficient (R), coefficient of determination (R^2), adjusted coefficient of determination ($R^2_{adjusted}$), and residual sum of squares (RSS) (manual calculation in MS Excel):

Parameter	No.1	No.2	No.3	No.4
N	23	23	23	23
df	21	21	21	21
R	0.949492122	0.953124548	0.926297912	0.95213685
R^2	0.90153529	0.908446403	0.858027821	0.906564582
$R^2_{adjusted}$	0.896846494	0.904086708	0.851267241	0.902115276
RSS	1947.164008	2118.617279	2947.162965	2364.090716

Graphical abstract of model fit presented as mean ± 1 SD of the fraction % of released carvedilol:

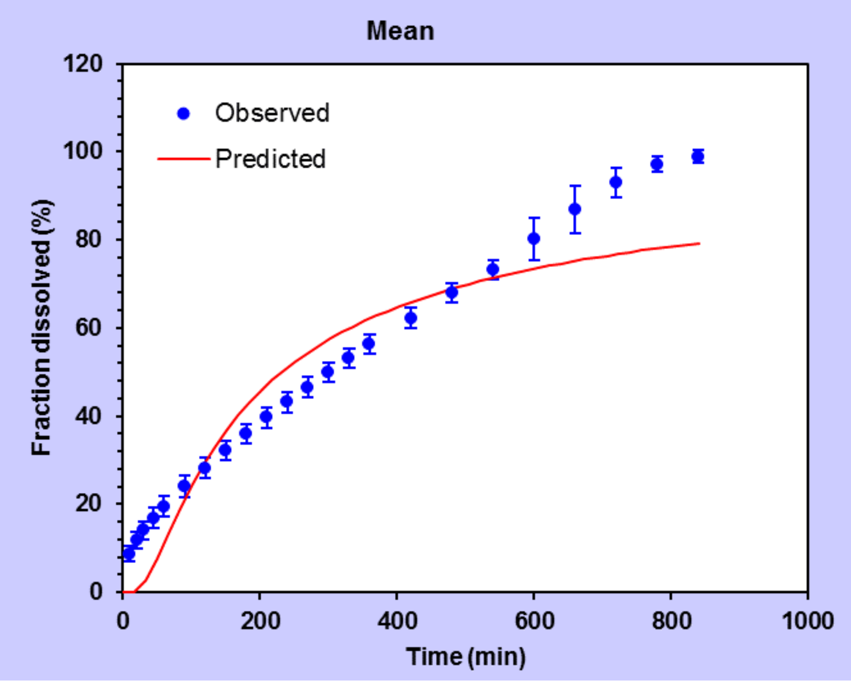

Supplementary materials – Model fitting summary – FlowLac® 100 (modelling the entire relevant dissolution data)

Graphical abstract of model fit presented as the fraction % of released carvedilol per tested tablet:

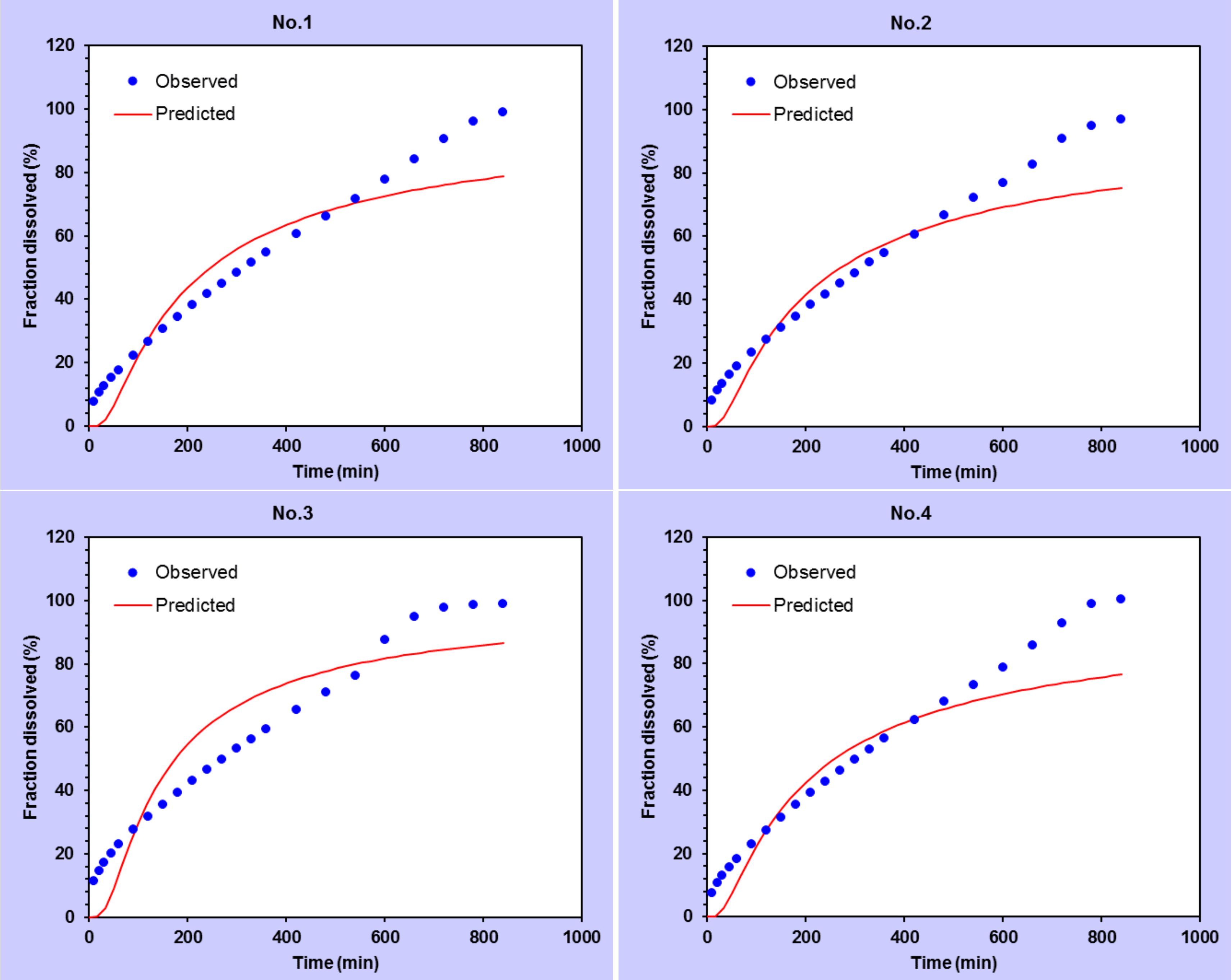

Supplementary materials – Model fitting summary – FlowLac® 100 (modelling the entire relevant dissolution data)

Model: **Gompertz_2**

Model equation: $F = F_{max} \cdot e^{-\alpha \cdot e^{-\beta \cdot \log(t)}}$

Fitted model parameters per tested tablet (N = 4) with statistics – mean, standard deviation (SD), and relative standard deviation expressed in % (RSD%) (output from DDSolver):

Parameter	No.1	No.2	No.3	No.4	Mean	SD	RSD(%)
α	37.161	39.587	54.700	51.449	45.724	8.647	18.912
β	1.583	1.577	1.845	1.730	1.684	0.129	7.660
F_{max}	131.503	113.906	103.988	105.401	113.699	12.652	11.127

Number of dissolution data points (N), degrees of freedom (df), and selected goodness of fit criteria – Pearson correlation coefficient (R), coefficient of determination (R^2), adjusted coefficient of determination ($R^2_{adjusted}$), and residual sum of squares (RSS) (manual calculation in MS Excel):

Parameter	No.1	No.2	No.3	No.4
N	23	23	23	23
df	20	20	20	20
R	0.956920477	0.963952644	0.944192113	0.959560401
R^2	0.915696799	0.9292047	0.891498745	0.920756164
$R^2_{adjusted}$	0.907266479	0.92212517	0.88064862	0.91283178
RSS	2246.957133	1870.604628	2545.825697	2447.867853

Graphical abstract of model fit presented as mean ± 1 SD of the fraction % of released carvedilol:

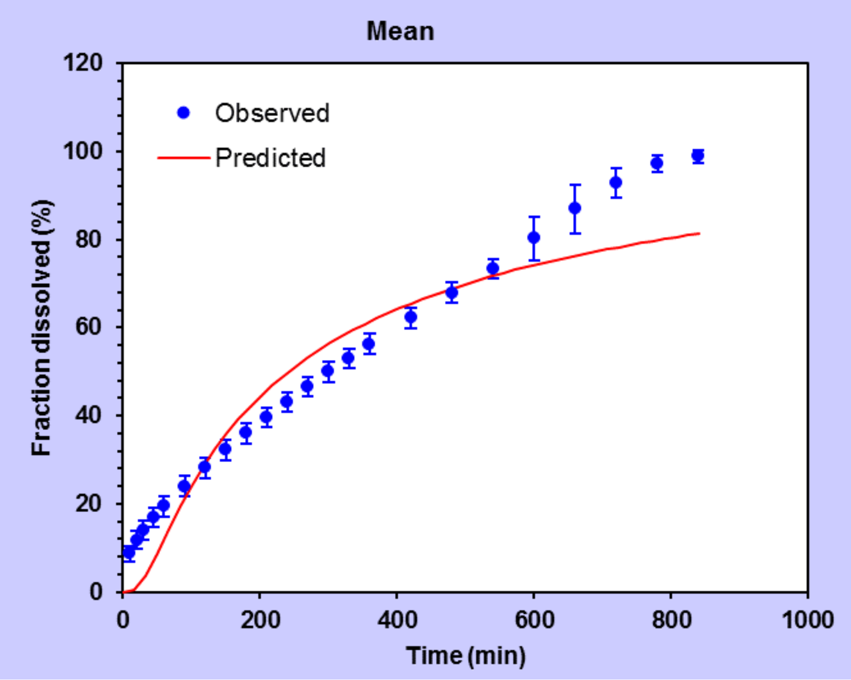

Supplementary materials – Model fitting summary – FlowLac® 100 (modelling the entire relevant dissolution data)

Graphical abstract of model fit presented as the fraction % of released carvedilol per tested tablet:

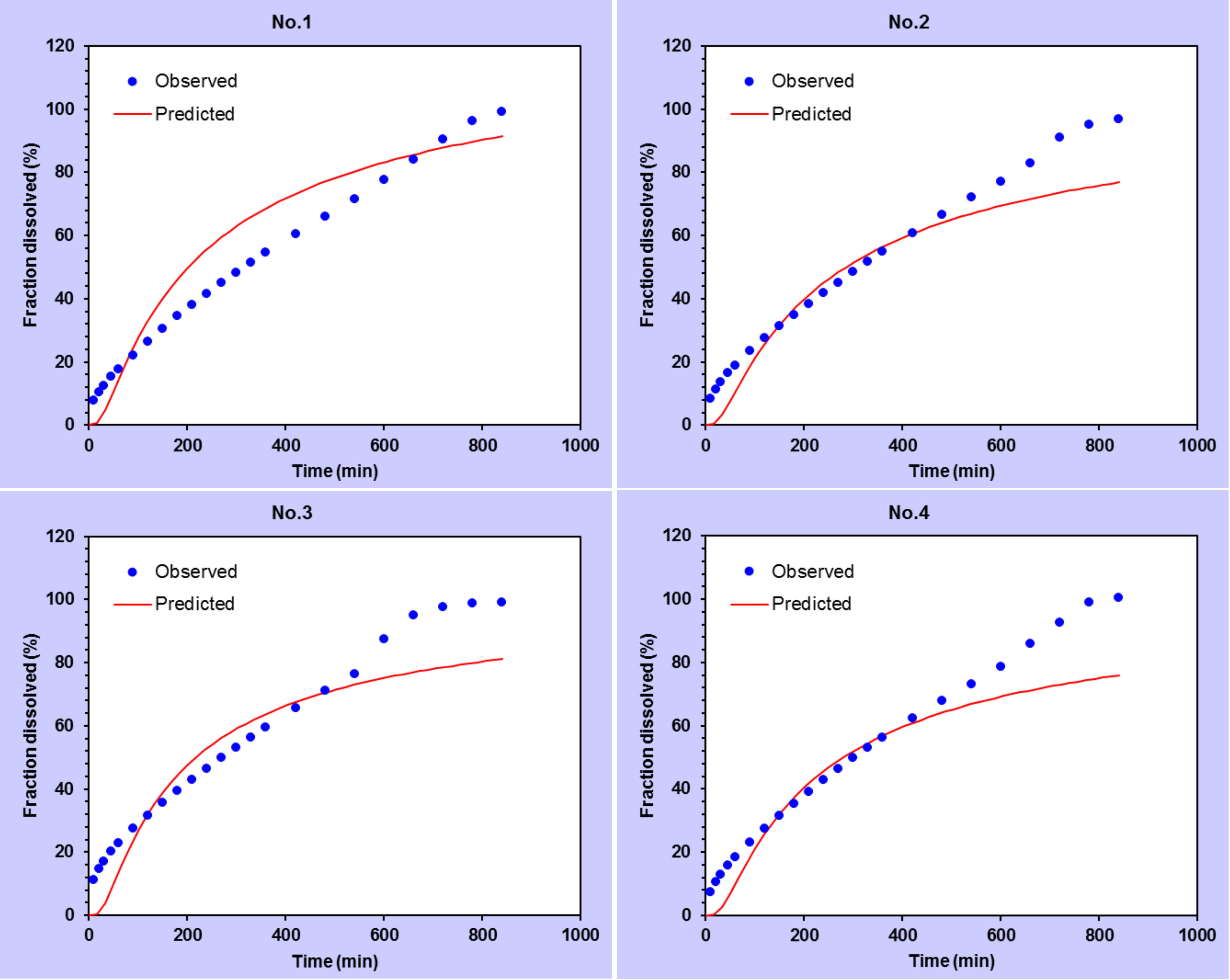

Supplementary materials – Model fitting summary – FlowLac® 100 (modelling the entire relevant dissolution data)

Model: **Gompertz_3**

Model equation: $F = F_{max} \cdot e^{-e^{-k\cdot(t-\gamma)}}$

Fitted model parameters per tested tablet (N = 4) with statistics – mean, standard deviation (SD), and relative standard deviation expressed in % (RSD%) (output from DDSolver):

Parameter	No.1	No.2	No.3	No.4	Mean	SD	RSD(%)
k	0.004	0.004	0.004	0.004	0.004	0.000	4.669
γ	225.117	214.084	183.985	221.130	211.079	18.630	8.826
F_{max}	104.053	101.773	103.988	105.401	103.804	1.502	1.447

Number of dissolution data points (N), degrees of freedom (df), and selected goodness of fit criteria – Pearson correlation coefficient (R), coefficient of determination (R^2), adjusted coefficient of determination ($R^2_{adjusted}$), and residual sum of squares (RSS) (manual calculation in MS Excel):

Parameter	No.1	No.2	No.3	No.4
N	23	23	23	23
df	20	20	20	20
R	0.993879139	0.993741565	0.990785591	0.993255202
R^2	0.987795742	0.987522299	0.981656088	0.986555897
$R^2_{adjusted}$	0.986575316	0.986274529	0.979821697	0.985211487
RSS	290.2724472	286.7045205	440.2908769	335.1789234

Graphical abstract of model fit presented as mean ± 1 SD of the fraction % of released carvedilol:

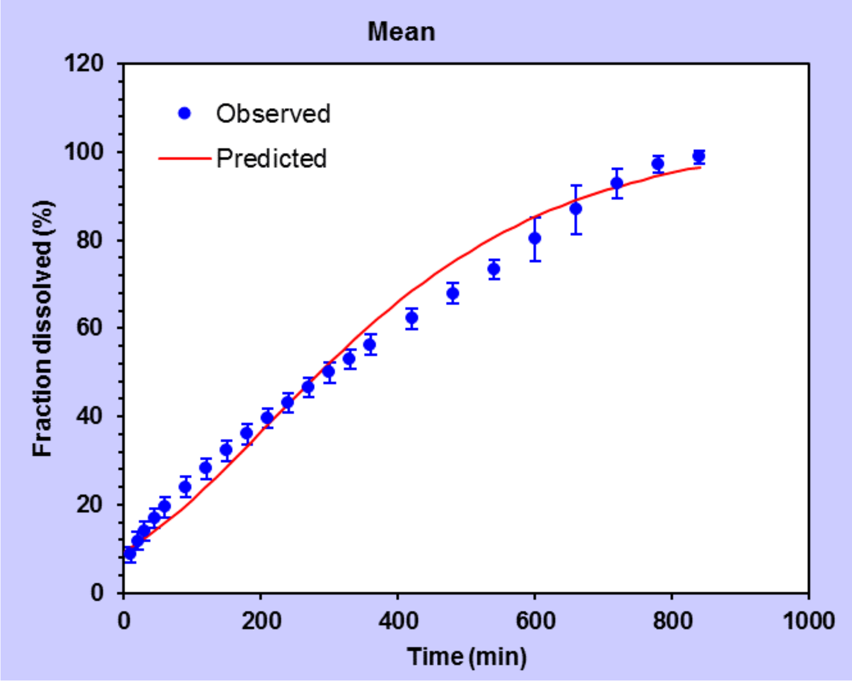

Supplementary materials – Model fitting summary – FlowLac® 100 (modelling the entire relevant dissolution data)

Graphical abstract of model fit presented as the fraction % of released carvedilol per tested tablet:

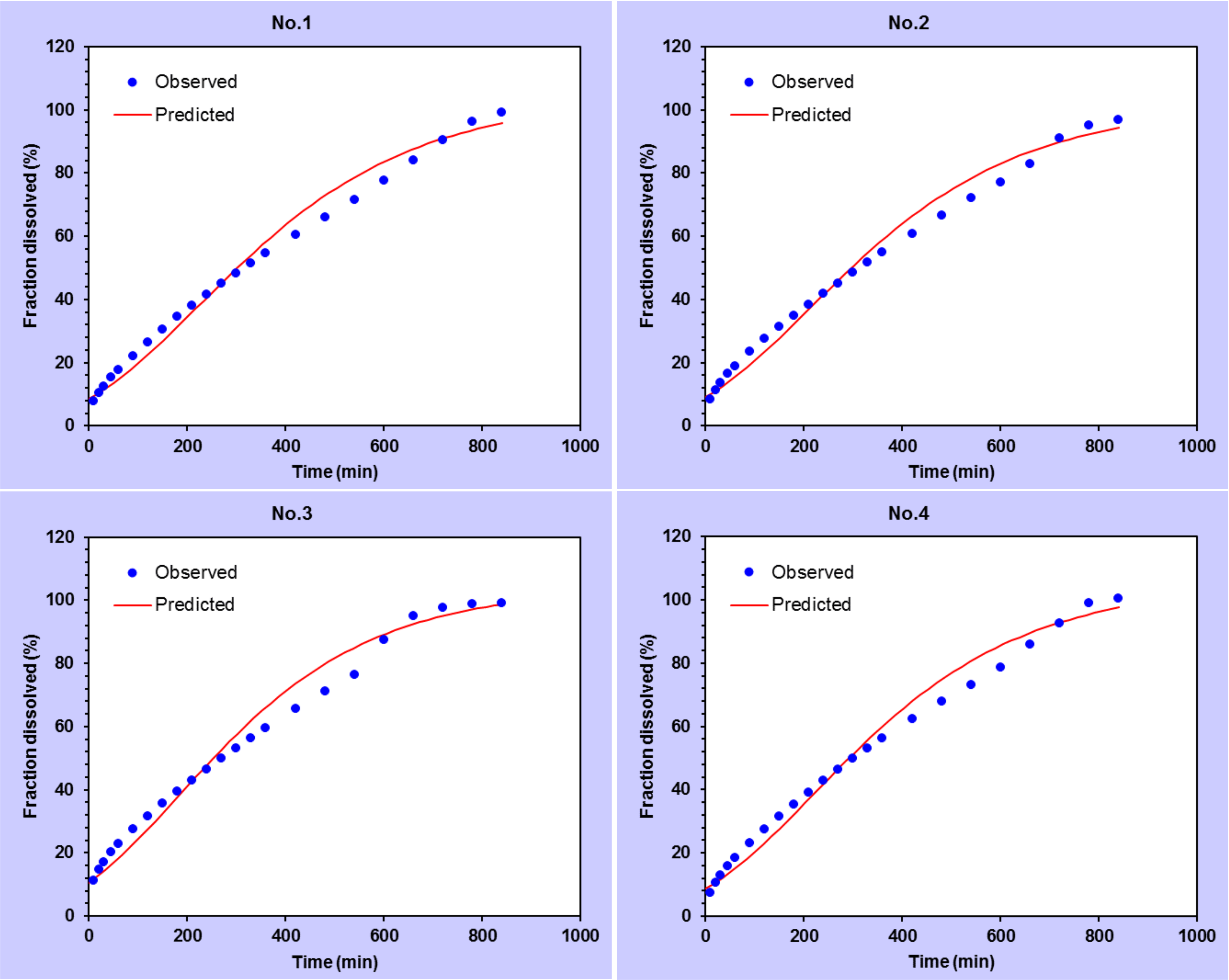

Supplementary materials – Model fitting summary – FlowLac® 100 (modelling the entire relevant dissolution data)

Model: **Gompertz_4**

Model equation: $F = F_{max} \cdot e^{-\beta \cdot e^{-k \cdot t}}$

Fitted model parameters per tested tablet (N = 4) with statistics – mean, standard deviation (SD), and relative standard deviation expressed in % (RSD%) (output from DDSolver):

Parameter	No.1	No.2	No.3	No.4	Mean	SD	RSD(%)
k	0.004	0.004	0.004	0.004	0.004	0.000	4.669
β	2.490	2.414	2.284	2.500	2.422	0.100	4.116
F_{max}	104.053	101.773	103.988	105.401	103.804	1.502	1.447

Number of dissolution data points (N), degrees of freedom (df), and selected goodness of fit criteria – Pearson correlation coefficient (R), coefficient of determination (R^2), adjusted coefficient of determination ($R^2_{adjusted}$), and residual sum of squares (RSS) (manual calculation in MS Excel):

Parameter	No.1	No.2	No.3	No.4
N	23	23	23	23
df	20	20	20	20
R	0.993879139	0.993741565	0.990785591	0.993255202
R^2	0.987795742	0.987522299	0.981656088	0.986555897
$R^2_{adjusted}$	0.986575316	0.986274529	0.979821697	0.985211487
RSS	290.2724472	286.7045205	440.2908769	335.1789234

Graphical abstract of model fit presented as mean ± 1 SD of the fraction % of released carvedilol:

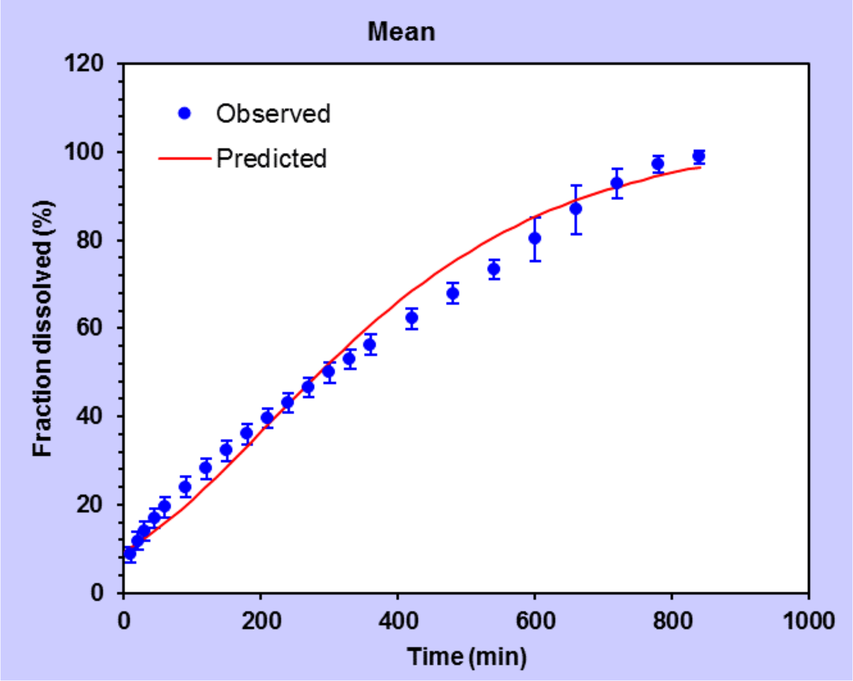

Supplementary materials – Model fitting summary – FlowLac® 100 (modelling the entire relevant dissolution data)

Graphical abstract of model fit presented as the fraction % of released carvedilol per tested tablet:

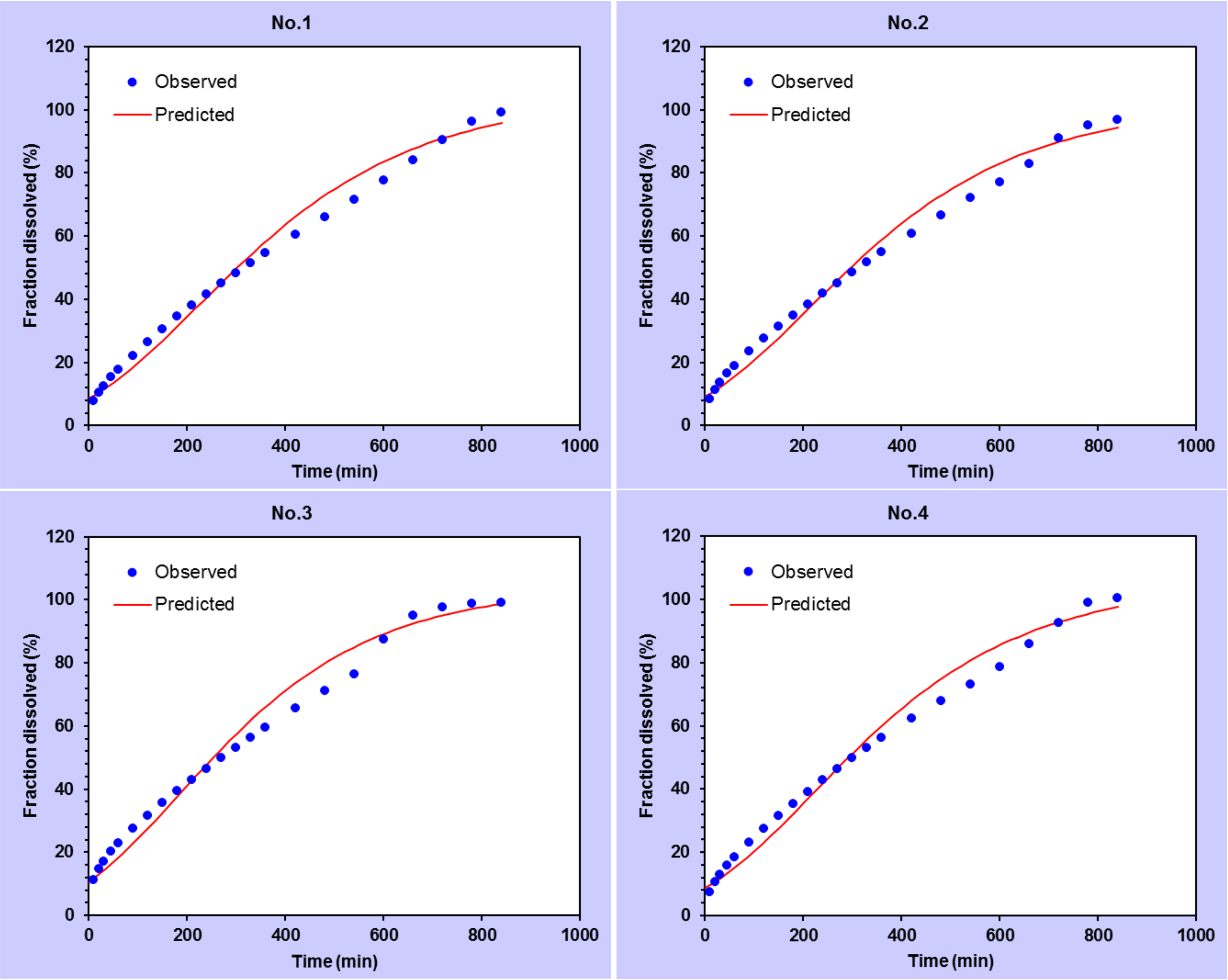

Supplementary materials – Model fitting summary – FlowLac® 100 (modelling the entire relevant dissolution data)

Model: **Probit_1**

Model equation: $F = 100 \cdot \phi[\alpha + \beta \cdot \log(t)]$

Fitted model parameters per tested tablet (N = 4) with statistics – mean, standard deviation (SD), and relative standard deviation expressed in % (RSD%) (output from DDSolver):

Parameter	No.1	No.2	No.3	No.4	Mean	SD	RSD(%)
α	-3.693	-3.491	-4.575	-3.560	-3.830	0.504	-13.148
β	1.610	1.516	1.899	1.550	1.644	0.174	10.612

Number of dissolution data points (N), degrees of freedom (df), and selected goodness of fit criteria – Pearson correlation coefficient (R), coefficient of determination (R^2), adjusted coefficient of determination ($R^2_{adjusted}$), and residual sum of squares (RSS) (manual calculation in MS Excel):

Parameter	No.1	No.2	No.3	No.4
N	23	23	23	23
df	21	21	21	21
R	0.947105786	0.948490247	0.969249147	0.948586174
R^2	0.89700937	0.899633749	0.939443909	0.899815729
$R^2_{adjusted}$	0.892105054	0.894854404	0.936560286	0.895045049
RSS	2222.899379	1955.759125	2111.098278	2065.513864

Graphical abstract of model fit presented as mean ± 1 SD of the fraction % of released carvedilol:

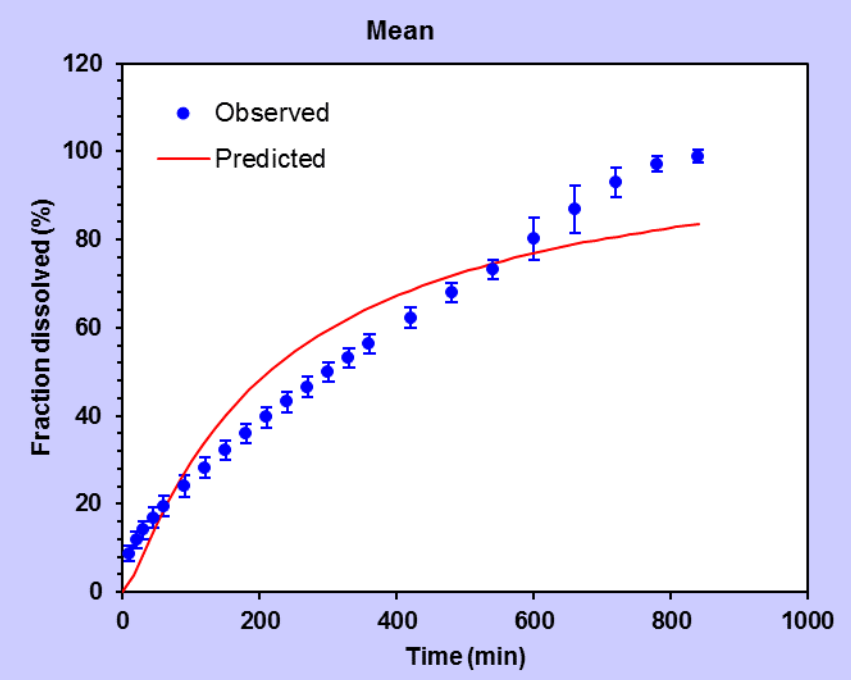

Supplementary materials – Model fitting summary – FlowLac® 100 (modelling the entire relevant dissolution data)

Graphical abstract of model fit presented as the fraction % of released carvedilol per tested tablet:

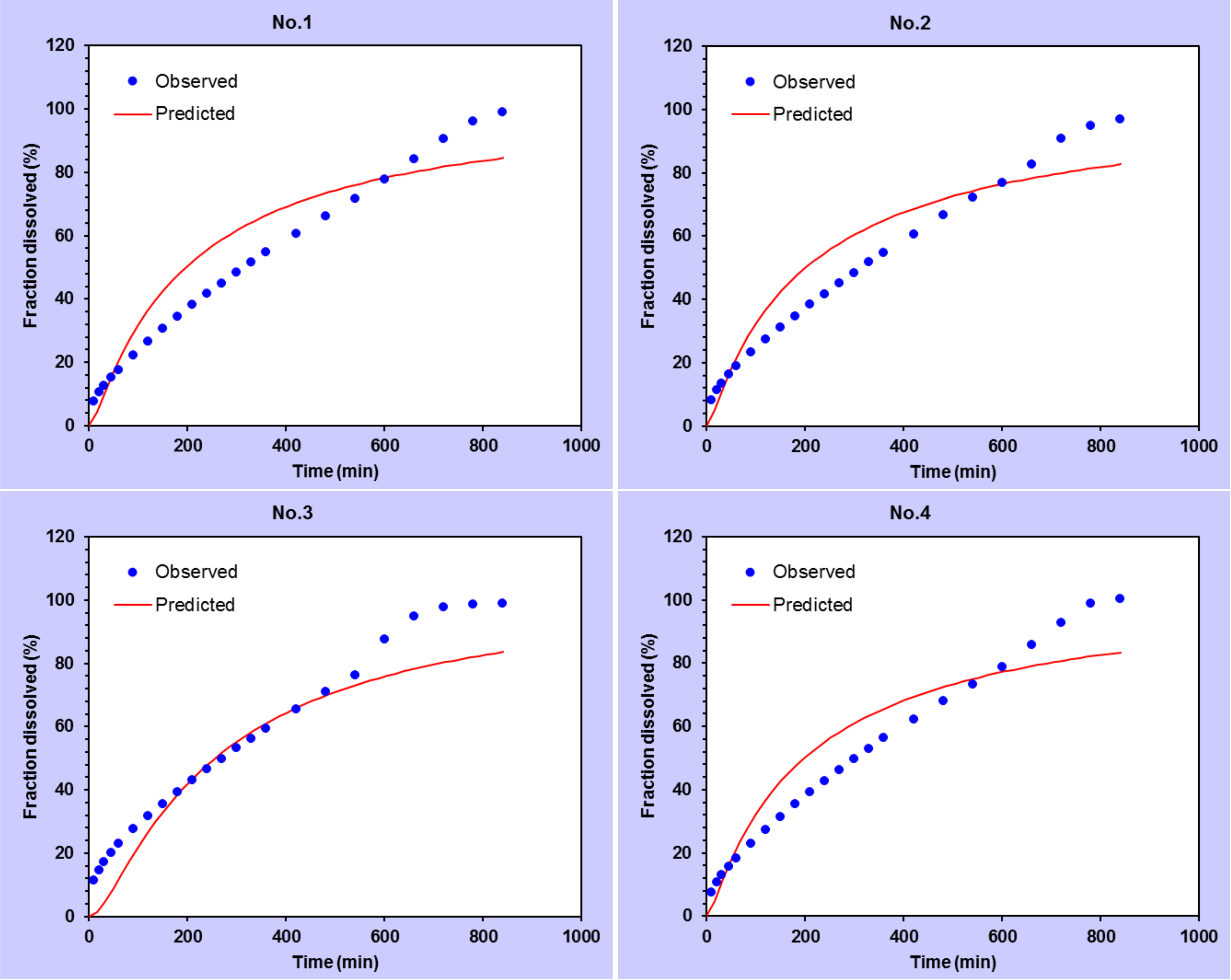

Supplementary materials – Model fitting summary – FlowLac® 100 (modelling the entire relevant dissolution data)

Model: **Probit_2**

Model equation: $F = F_{max} \cdot \phi[\alpha + \beta \cdot \log(t)]$

Fitted model parameters per tested tablet (N = 4) with statistics – mean, standard deviation (SD), and relative standard deviation expressed in % (RSD%) (output from DDSolver):

Parameter	No.1	No.2	No.3	No.4	Mean	SD	RSD(%)
α	-3.475	-3.414	-3.334	-3.509	-3.433	0.077	-2.235
β	1.474	1.466	1.490	1.496	1.481	0.014	0.940
F_{max}	104.053	101.773	103.988	105.401	103.804	1.502	1.447

Number of dissolution data points (N), degrees of freedom (df), and selected goodness of fit criteria – Pearson correlation coefficient (R), coefficient of determination (R^2), adjusted coefficient of determination ($R^2_{adjusted}$), and residual sum of squares (RSS) (manual calculation in MS Excel):

Parameter	No.1	No.2	No.3	No.4
N	23	23	23	23
df	20	20	20	20
R	0.953326535	0.95097734	0.94012289	0.953890935
R^2	0.908831482	0.904357901	0.883831048	0.909907915
$R^2_{adjusted}$	0.89971463	0.894793691	0.872214153	0.900898707
RSS	1836.487489	1829.326293	2400.15804	1893.189897

Graphical abstract of model fit presented as mean ± 1 SD of the fraction % of released carvedilol:

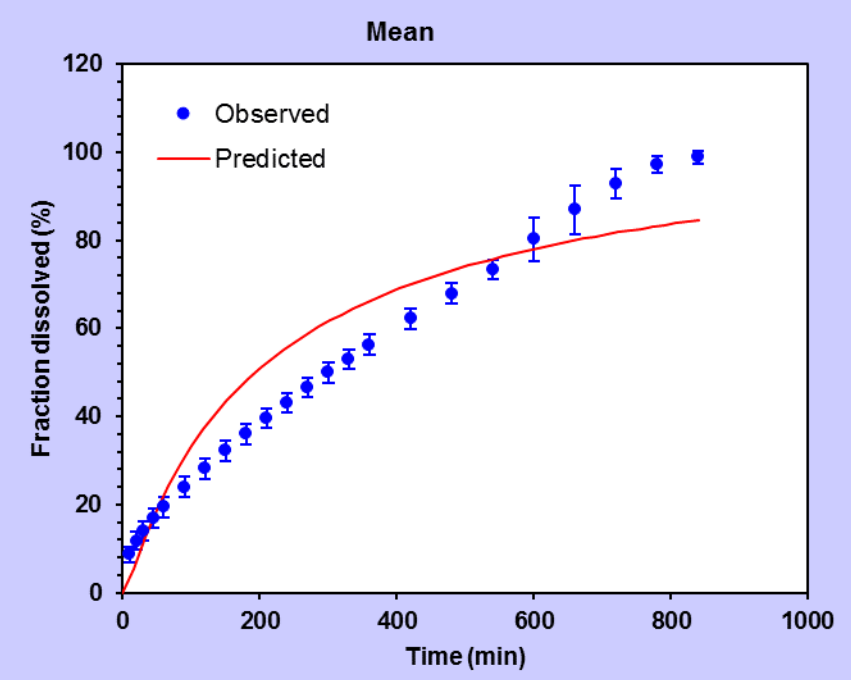

Supplementary materials – Model fitting summary – FlowLac® 100 (modelling the entire relevant dissolution data)

Graphical abstract of model fit presented as the fraction % of released carvedilol per tested tablet:

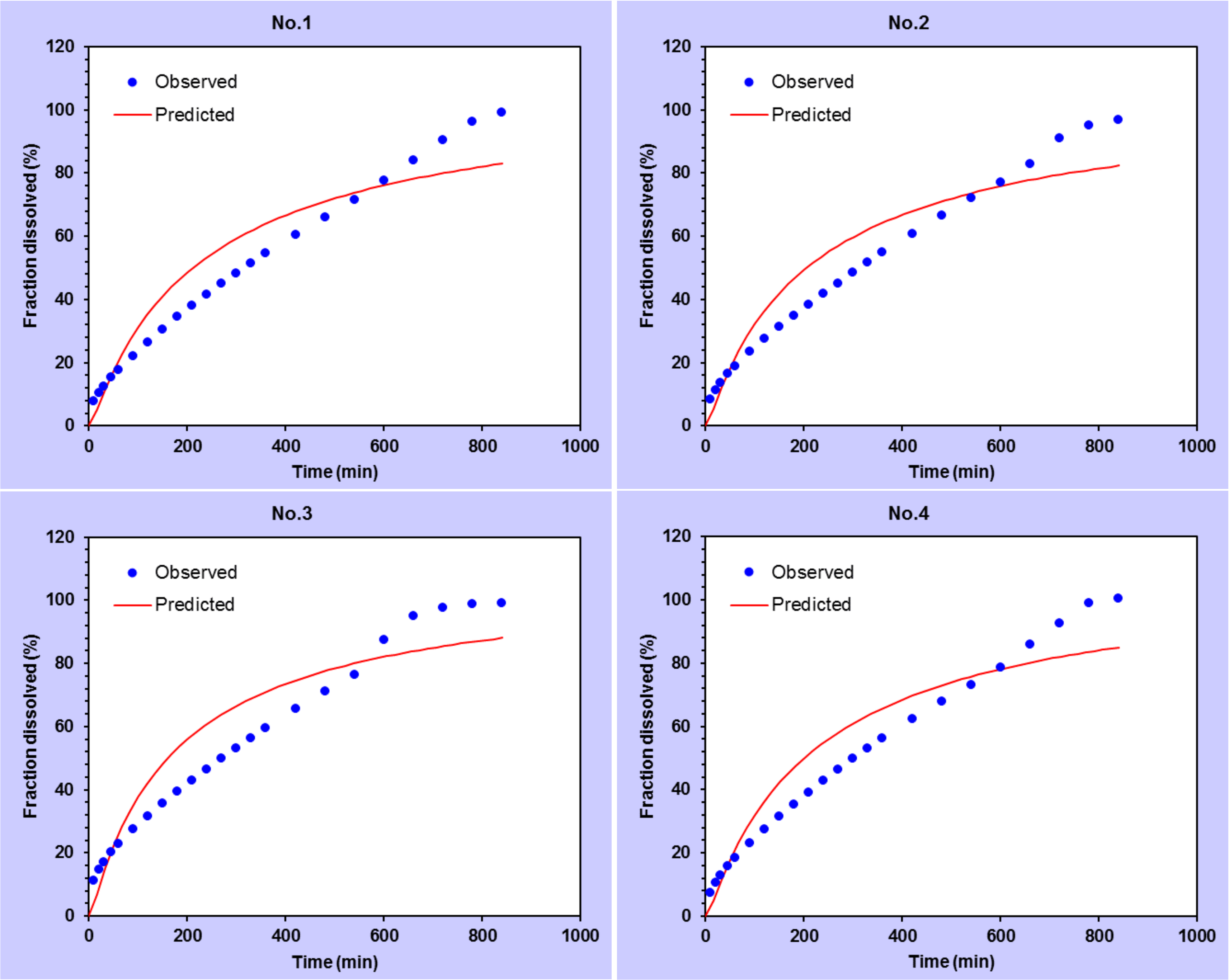

Supplementary materials – Model fitting summary – FlowLac® 100 (modelling up to app. 60 % of carvedilol released)

Model: **Zero–order**

Model equation: $F = k_0 \cdot t$

Fitted model parameters per tested tablet (N = 4) with statistics – mean, standard deviation (SD), and relative standard deviation expressed in % (RSD%) (output from DDSolver):

Parameter	No.1	No.2	No.3	No.4	Mean	SD	RSD(%)
k_0	0.164	0.165	0.182	0.169	0.170	0.008	4.895

Number of dissolution data points (N), degrees of freedom (df), and selected goodness of fit criteria – Pearson correlation coefficient (R), coefficient of determination (R^2), adjusted coefficient of determination ($R^2_{adjusted}$), and residual sum of squares (RSS) (manual calculation in MS Excel):

Parameter	No.1	No.2	No.3	No.4
N	16	16	16	16
df	15	15	15	15
R	0.995673407	0.995119905	0.993915845	0.995146261
R^2	0.991365533	0.990263624	0.987868707	0.990316081
$R^2_{adjusted}$	0.991365533	0.990263624	0.987868707	0.990316081
RSS	549.3447654	667.6997268	1169.3765	580.5234983

Graphical abstract of model fit presented as mean ± 1 SD of the fraction % of released carvedilol:

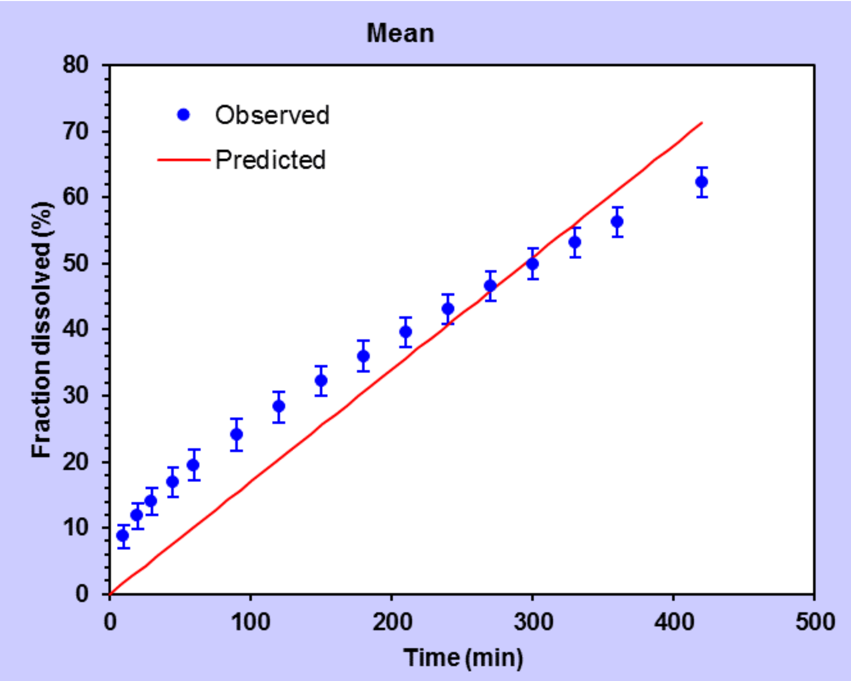

Supplementary materials – Model fitting summary – FlowLac® 100 (modelling up to app. 60 % of carvedilol released)

Graphical abstract of model fit presented as the fraction % of released carvedilol per tested tablet:

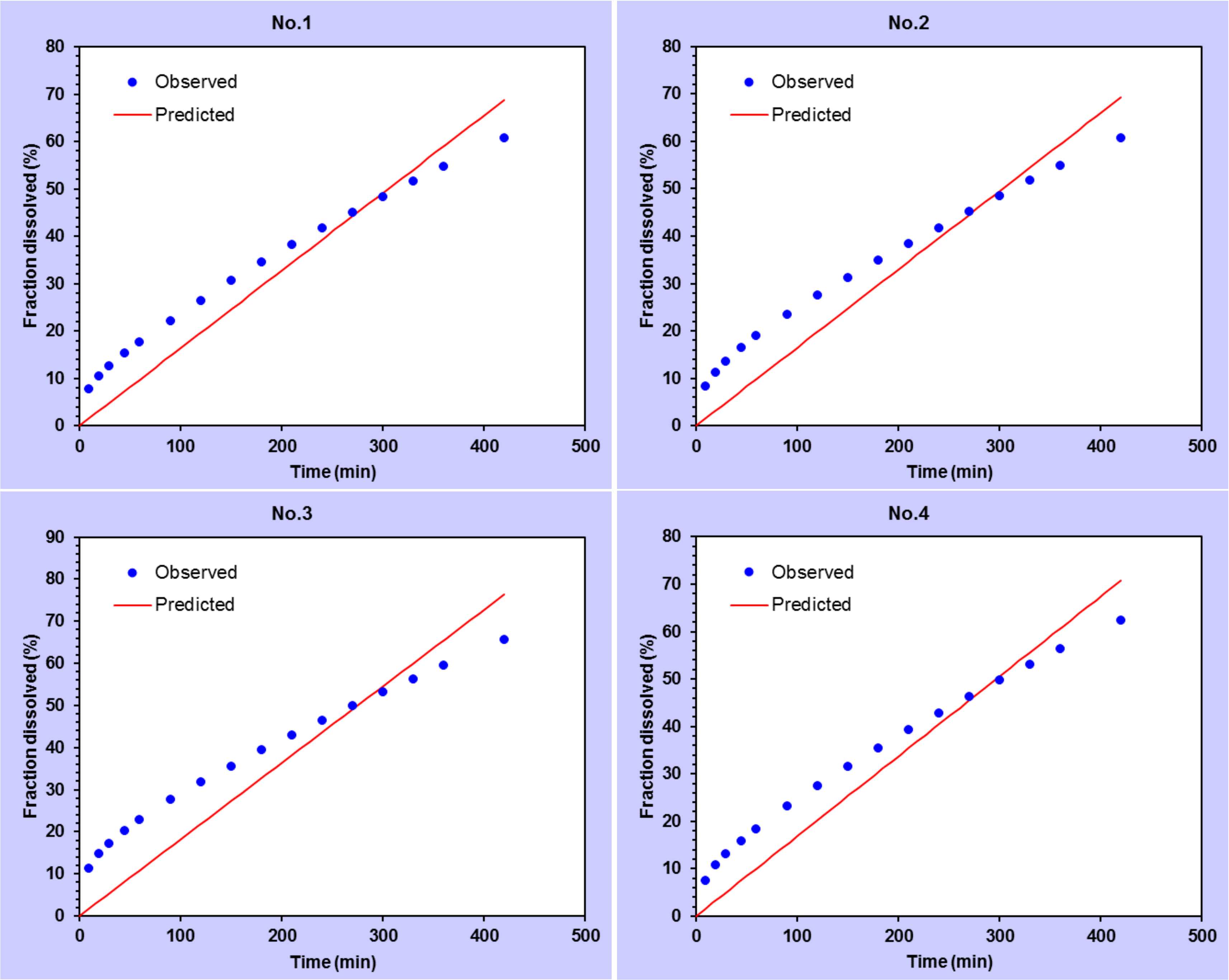

Supplementary materials – Model fitting summary – FlowLac® 100 (modelling up to app. 60 % of carvedilol released)

Model: **Zero–order with T_{lag}**

Model equation: $F = k_0 \cdot (t - T_{lag})$

Fitted model parameters per tested tablet (N = 4) with statistics – mean, standard deviation (SD), and relative standard deviation expressed in % (RSD%) (output from DDSolver):

Parameter	No.1	No.2	No.3	No.4	Mean	SD	RSD(%)
k_0	0.128	0.125	0.129	0.132	0.129	0.003	2.096
T_{lag}	-75.498	-85.366	-110.597	-75.008	-86.617	16.683	-19.261

Number of dissolution data points (N), degrees of freedom (df), and selected goodness of fit criteria – Pearson correlation coefficient (R), coefficient of determination (R^2), adjusted coefficient of determination ($R^2_{adjusted}$), and residual sum of squares (RSS) (manual calculation in MS Excel):

Parameter	No.1	No.2	No.3	No.4
N	16	16	16	16
df	14	14	14	14
R	0.995673407	0.995119905	0.993915845	0.995146261
R^2	0.991365533	0.990263624	0.987868707	0.990316081
$R^2_{adjusted}$	0.990748785	0.989568169	0.987002186	0.989624372
RSS	37.35274394	40.40897821	53.49035817	44.4825394

Graphical abstract of model fit presented as mean ± 1 SD of the fraction % of released carvedilol:

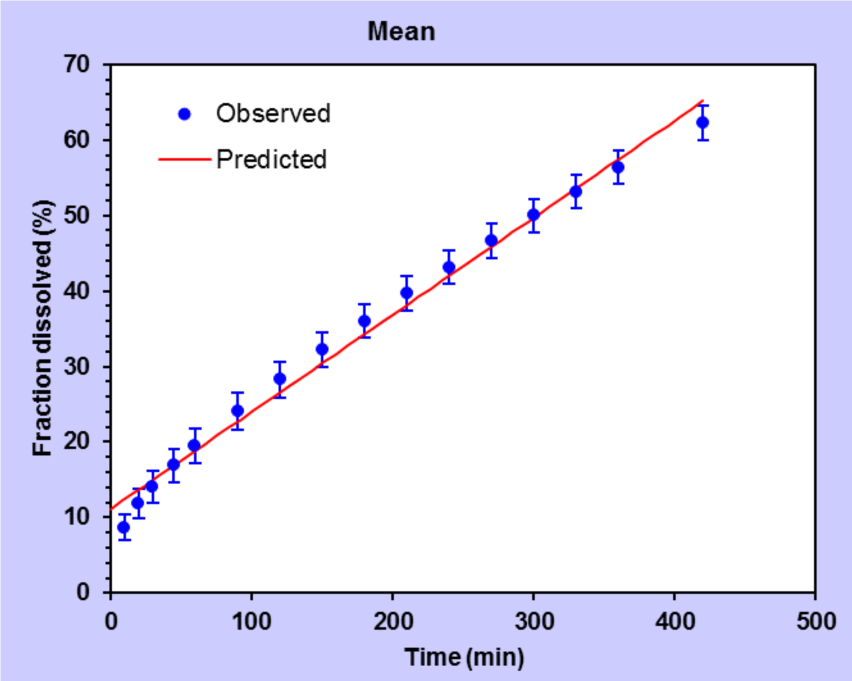

Supplementary materials – Model fitting summary – FlowLac® 100 (modelling up to app. 60 % of carvedilol released)

Graphical abstract of model fit presented as the fraction % of released carvedilol per tested tablet:

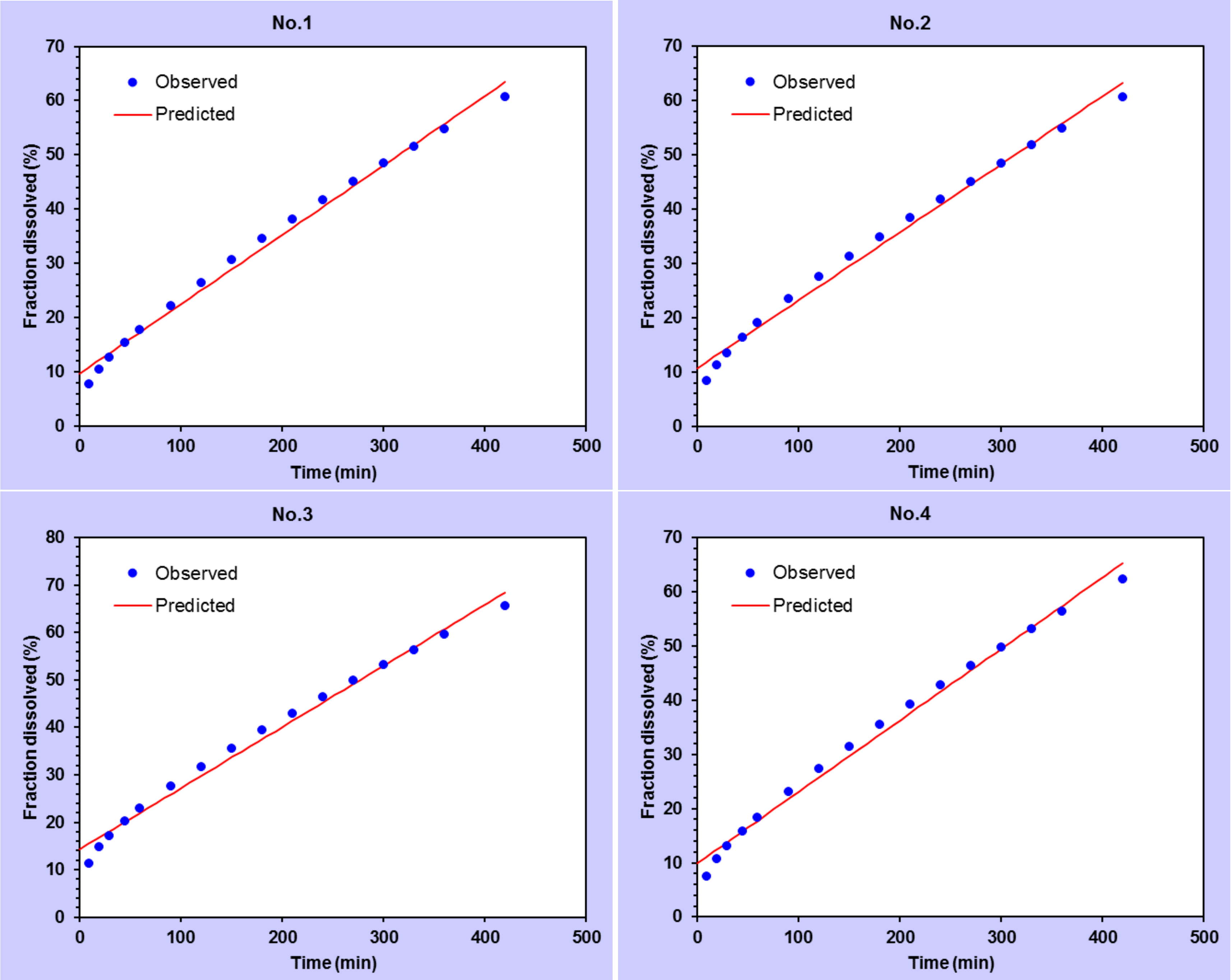

Supplementary materials – Model fitting summary – FlowLac® 100 (modelling up to app. 60 % of carvedilol released)

Model: **Zero–order with F_0**

Model equation: $F = F_0 + k_0 \cdot t$

Fitted model parameters per tested tablet (N = 4) with statistics – mean, standard deviation (SD), and relative standard deviation expressed in % (RSD%) (output from DDSolver):

Parameter	No.1	No.2	No.3	No.4	Mean	SD	RSD(%)
k_0	0.128	0.125	0.129	0.132	0.129	0.003	2.096
F_0	9.667	10.700	14.271	9.891	11.132	2.139	19.215

Number of dissolution data points (N), degrees of freedom (df), and selected goodness of fit criteria – Pearson correlation coefficient (R), coefficient of determination (R^2), adjusted coefficient of determination ($R^2_{adjusted}$), and residual sum of squares (RSS) (manual calculation in MS Excel):

Parameter	No.1	No.2	No.3	No.4
N	16	16	16	16
df	14	14	14	14
R	0.995673407	0.995119905	0.993915845	0.995146261
R^2	0.991365533	0.990263624	0.987868707	0.990316081
$R^2_{adjusted}$	0.990748785	0.989568169	0.987002186	0.989624372
RSS	37.35274394	40.40897821	53.49035817	44.4825394

Graphical abstract of model fit presented as mean ± 1 SD of the fraction % of released carvedilol:

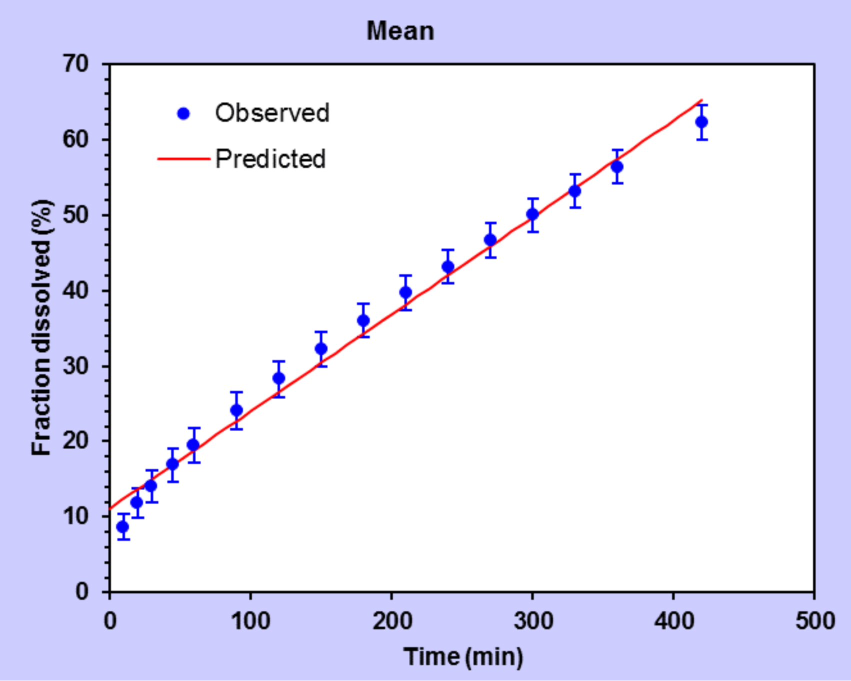

Supplementary materials – Model fitting summary – FlowLac® 100 (modelling up to app. 60 % of carvedilol released)

Graphical abstract of model fit presented as the fraction % of released carvedilol per tested tablet:

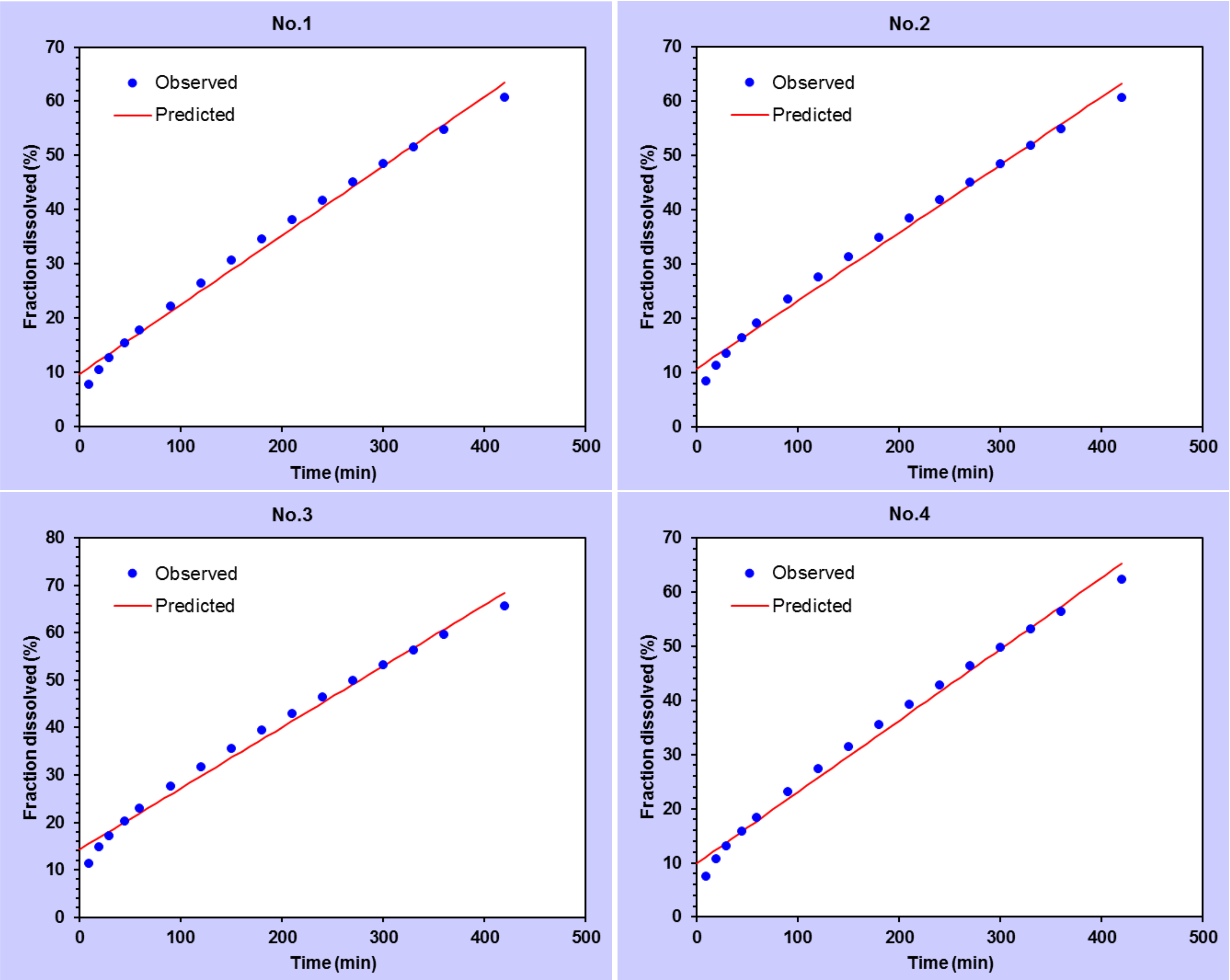

Supplementary materials – Model fitting summary – FlowLac® 100 (modelling up to app. 60 % of carvedilol released)

Model: **First–order**

Model equation: $F = 100 \cdot (1 - e^{-k_1 \cdot t})$

Fitted model parameters per tested tablet (N = 4) with statistics – mean, standard deviation (SD), and relative standard deviation expressed in % (RSD%) (output from DDSolver):

Parameter	No.1	No.2	No.3	No.4	Mean	SD	RSD(%)
k_1	0.002	0.002	0.003	0.002	0.002	0.000	6.955

Number of dissolution data points (N), degrees of freedom (df), and selected goodness of fit criteria – Pearson correlation coefficient (R), coefficient of determination (R^2), adjusted coefficient of determination ($R^2_{adjusted}$), and residual sum of squares (RSS) (manual calculation in MS Excel):

Parameter	No.1	No.2	No.3	No.4
N	16	16	16	16
df	15	15	15	15
R	0.9993187	0.998855181	0.998538763	0.999019535
R^2	0.998637863	0.997711672	0.997079661	0.998040031
$R^2_{adjusted}$	0.998637863	0.997711672	0.997079661	0.998040031
RSS	192.2663808	264.3396011	500.0232106	192.7216082

Graphical abstract of model fit presented as mean ± 1 SD of the fraction % of released carvedilol:

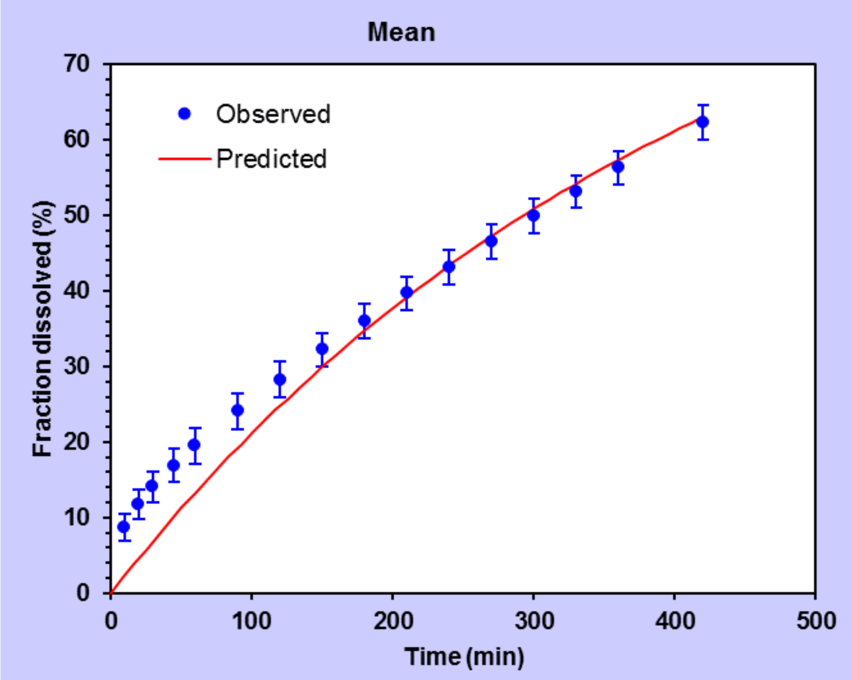

Supplementary materials – Model fitting summary – FlowLac® 100 (modelling up to app. 60 % of carvedilol released)

Graphical abstract of model fit presented as the fraction % of released carvedilol per tested tablet:

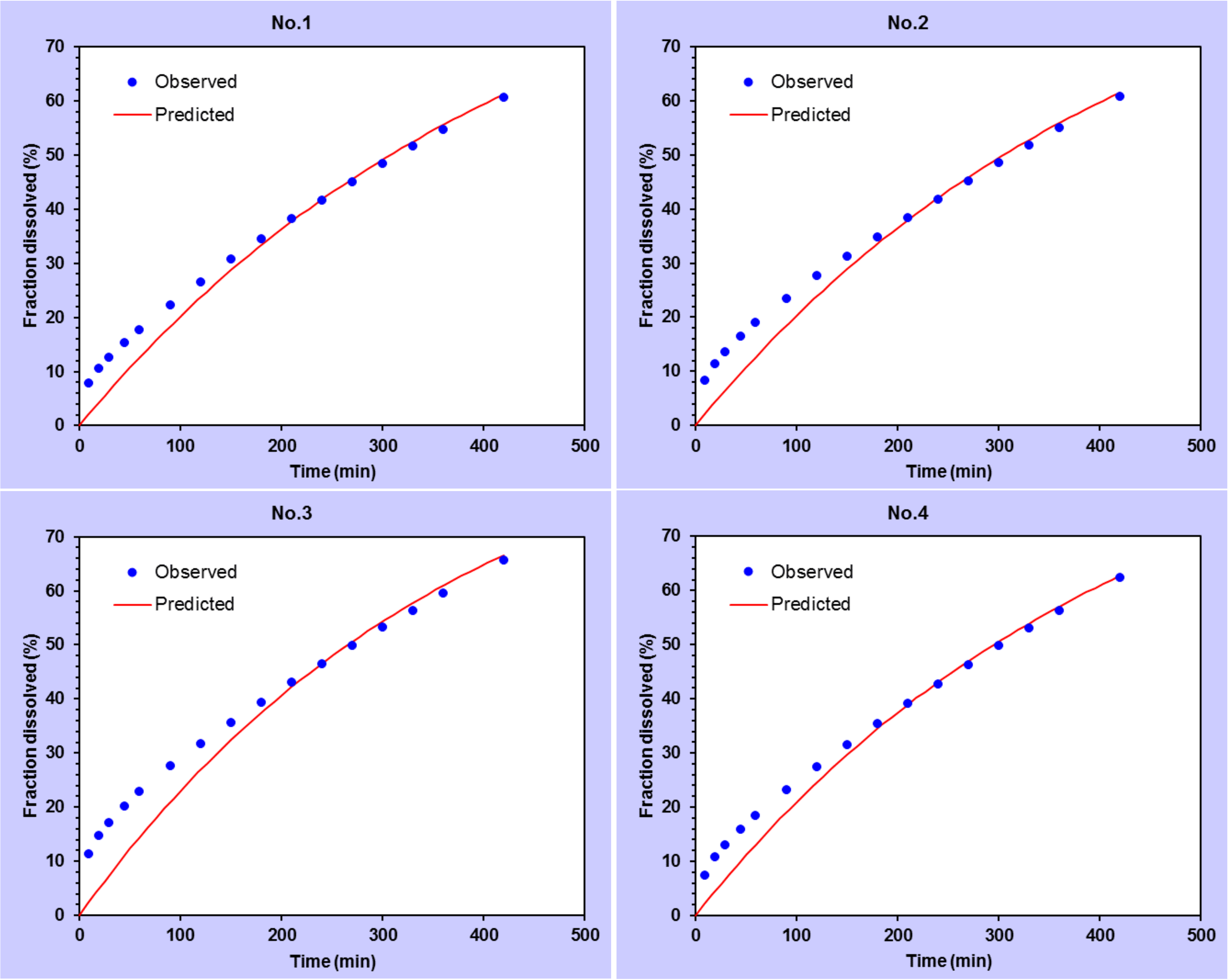

Supplementary materials – Model fitting summary – FlowLac® 100 (modelling up to app. 60 % of carvedilol released)

Model: **First–order with T_{lag}**

Model equation: $F = 100 \cdot \left[1 - e^{-k_1 \cdot (t - T_{lag})}\right]$

Fitted model parameters per tested tablet (N = 4) with statistics – mean, standard deviation (SD), and relative standard deviation expressed in % (RSD%) (output from DDSolver):

Parameter	No.1	No.2	No.3	No.4	Mean	SD	RSD(%)
k_1	0.002	0.002	0.002	0.002	0.002	0.000	4.667
T_{lag}	-33.667	-40.402	-52.187	-31.845	-39.525	9.209	-23.298

Number of dissolution data points (N), degrees of freedom (df), and selected goodness of fit criteria – Pearson correlation coefficient (R), coefficient of determination (R^2), adjusted coefficient of determination ($R^2_{adjusted}$), and residual sum of squares (RSS) (manual calculation in MS Excel):

Parameter	No.1	No.2	No.3	No.4
N	16	16	16	16
df	14	14	14	14
R	0.999595995	0.999150335	0.999003549	0.999309533
R^2	0.999192154	0.998301391	0.998008091	0.998619543
$R^2_{adjusted}$	0.999134451	0.998180062	0.997865812	0.998520939
RSS	3.676472359	7.20203711	9.000130533	6.614293647

Graphical abstract of model fit presented as mean ± 1 SD of the fraction % of released carvedilol:

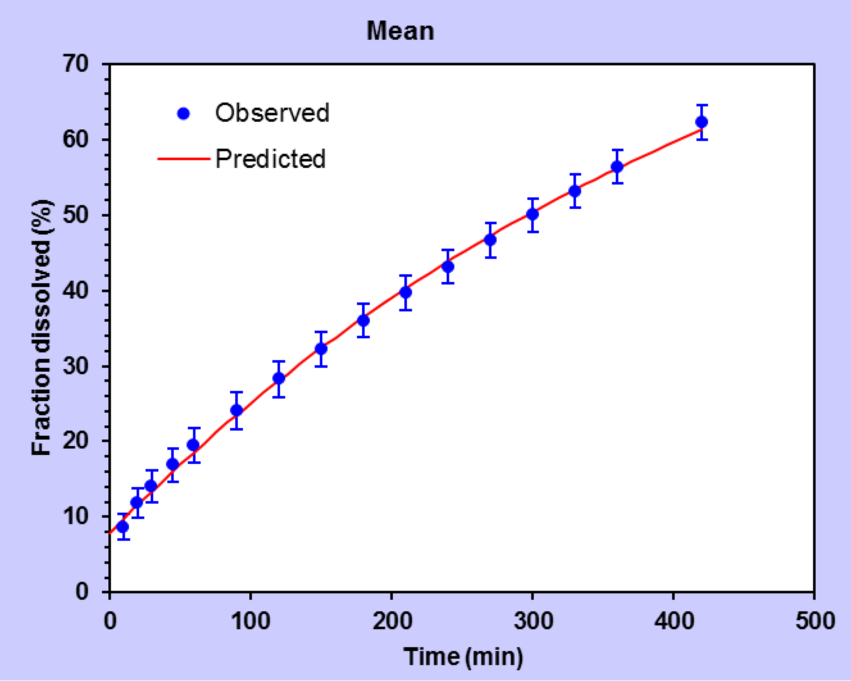

Supplementary materials – Model fitting summary – FlowLac® 100 (modelling up to app. 60 % of carvedilol released)

Graphical abstract of model fit presented as the fraction % of released carvedilol per tested tablet:

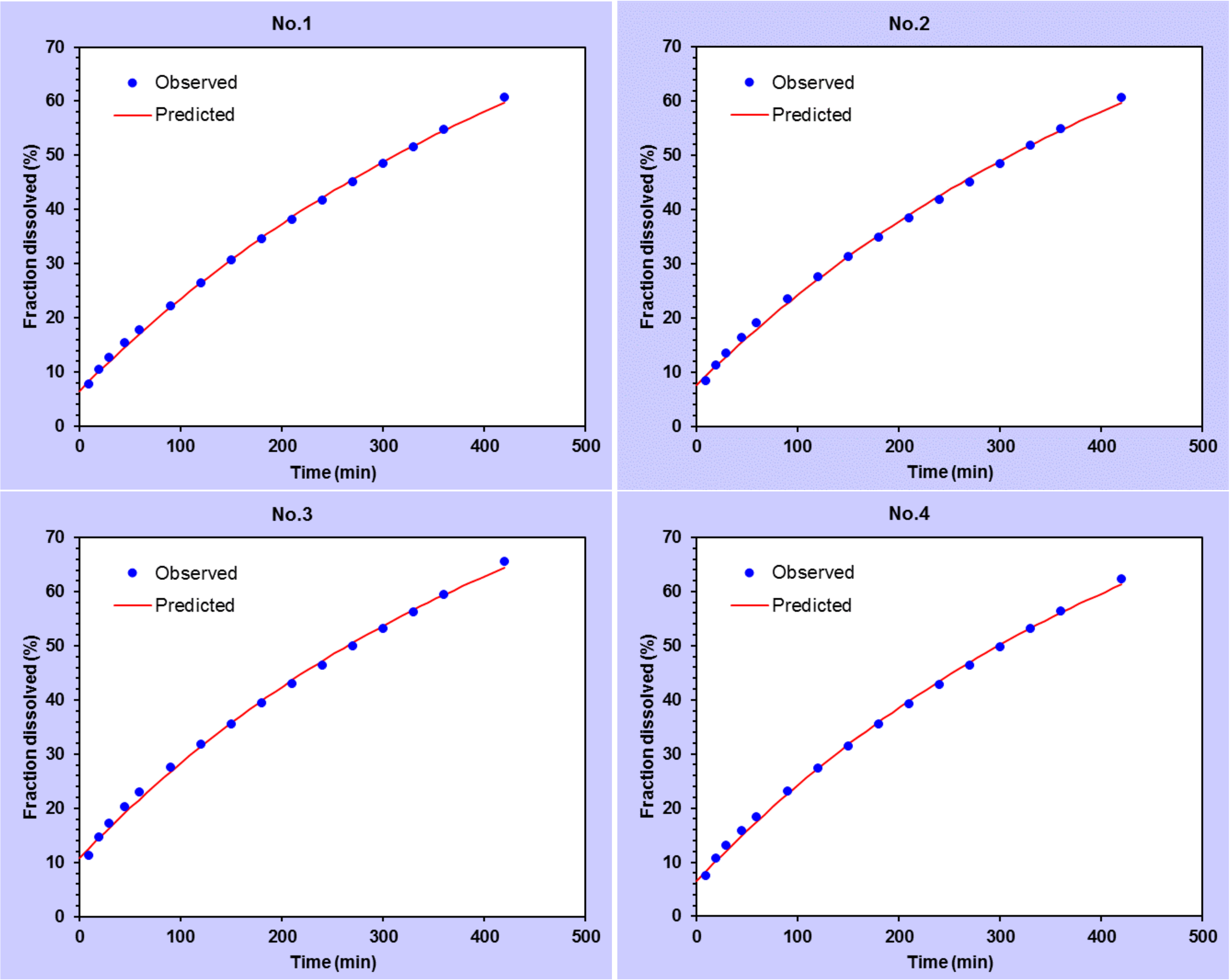

Supplementary materials – Model fitting summary – FlowLac® 100 (modelling up to app. 60 % of carvedilol released)

Model: **First–order with F_{max}**

Model equation: $F = F_{max} \cdot (1 - e^{-k_1 \cdot t})$

Fitted model parameters per tested tablet (N = 4) with statistics – mean, standard deviation (SD), and relative standard deviation expressed in % (RSD%) (output from DDSolver):

Parameter	No.1	No.2	No.3	No.4	Mean	SD	RSD(%)
k_1	0.005	0.005	0.006	0.005	0.005	0.000	1.566
F_{max}	63.635	63.739	68.834	65.408	65.404	2.427	3.710

Number of dissolution data points (N), degrees of freedom (df), and selected goodness of fit criteria – Pearson correlation coefficient (R), coefficient of determination (R^2), adjusted coefficient of determination ($R^2_{adjusted}$), and residual sum of squares (RSS) (manual calculation in MS Excel):

Parameter	No.1	No.2	No.3	No.4
N	16	16	16	16
df	14	14	14	14
R	0.984729637	0.984610947	0.985359564	0.985119491
R^2	0.969692459	0.969458717	0.970933471	0.970460411
$R^2_{adjusted}$	0.967527634	0.967277197	0.96885729	0.968350441
RSS	198.1906351	207.4824677	297.3860696	204.8055771

Graphical abstract of model fit presented as mean ± 1 SD of the fraction % of released carvedilol:

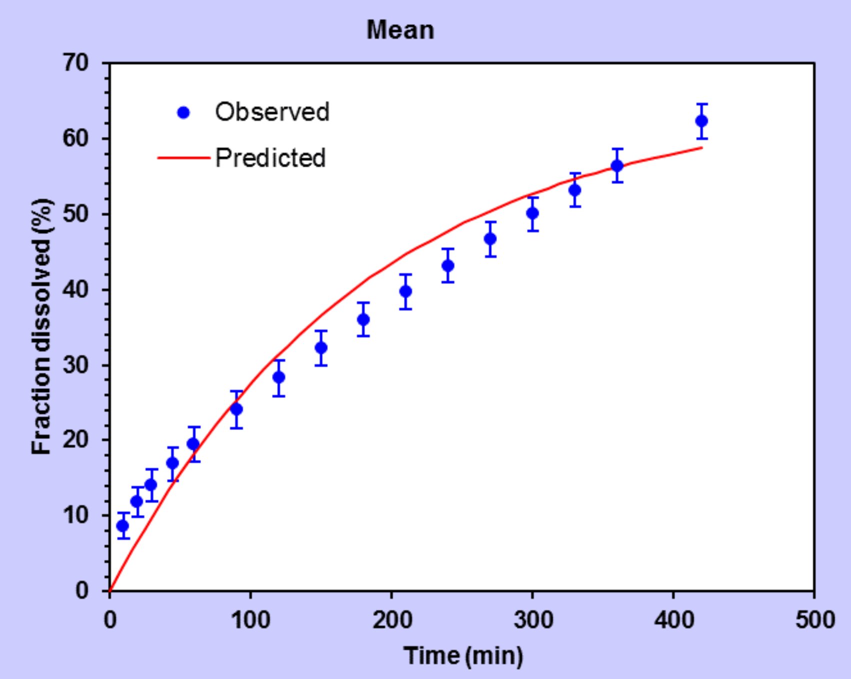

Supplementary materials – Model fitting summary – FlowLac® 100 (modelling up to app. 60 % of carvedilol released)

Graphical abstract of model fit presented as the fraction % of released carvedilol per tested tablet:

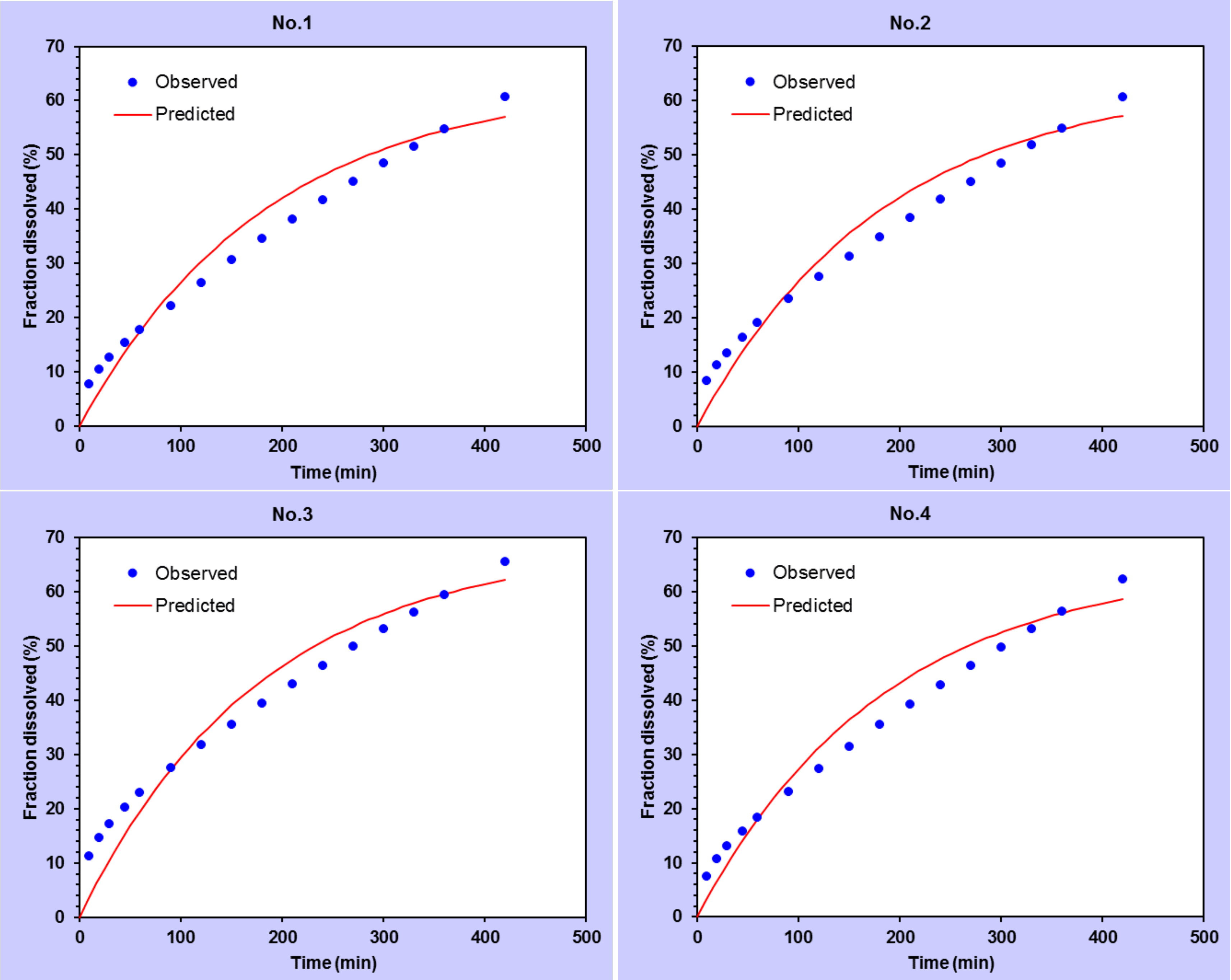

Supplementary materials – Model fitting summary – FlowLac® 100 (modelling up to app. 60 % of carvedilol released)

Model: **First–order with T_{lag} and F_{max}**

Model equation: $F = F_{max} \cdot \left[1 - e^{-k_1 \cdot (t - T_{lag})}\right]$

Fitted model parameters per tested tablet (N = 4) with statistics – mean, standard deviation (SD), and relative standard deviation expressed in % (RSD%) (output from DDSolver):

Parameter	No.1	No.2	No.3	No.4	Mean	SD	RSD(%)
k_1	0.006	0.006	0.006	0.006	0.006	0.000	0.953
T_{lag}	15.140	11.426	1.561	15.296	10.856	6.450	59.412
F_{max}	63.635	63.739	68.834	65.408	65.404	2.427	3.710

Number of dissolution data points (N), degrees of freedom (df), and selected goodness of fit criteria – Pearson correlation coefficient (R), coefficient of determination (R^2), adjusted coefficient of determination ($R^2_{adjusted}$), and residual sum of squares (RSS) (manual calculation in MS Excel):

Parameter	No.1	No.2	No.3	No.4
N	16	16	16	16
df	13	13	13	13
R	0.982345151	0.982885992	0.985140038	0.98277063
R^2	0.965001996	0.966064873	0.970500895	0.965838111
$R^2_{adjusted}$	0.959617688	0.960844084	0.965962571	0.960582436
RSS	377.426909	353.0748336	323.7438141	393.4465381

Graphical abstract of model fit presented as mean ± 1 SD of the fraction % of released carvedilol:

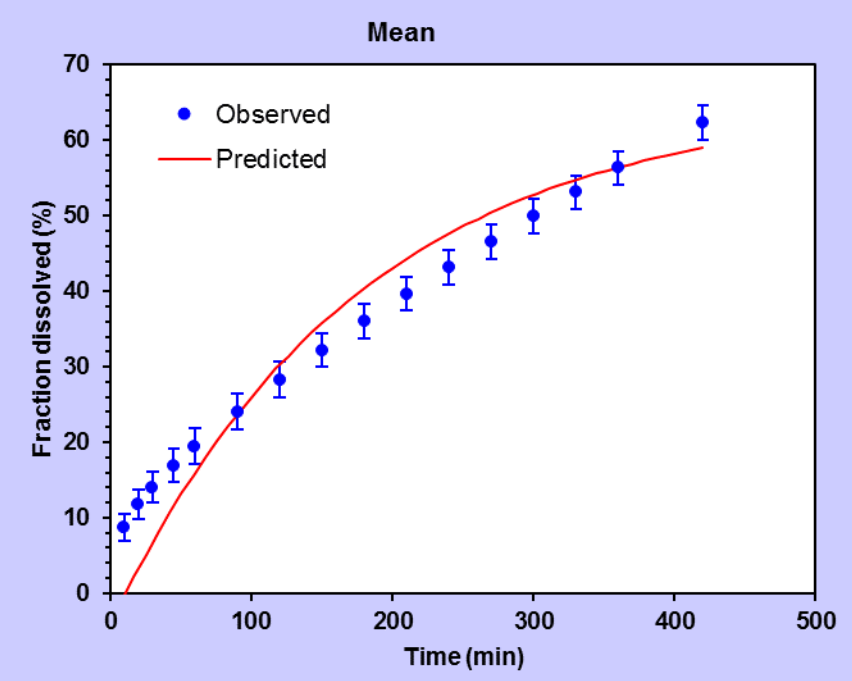

Supplementary materials – Model fitting summary – FlowLac® 100 (modelling up to app. 60 % of carvedilol released)

Graphical abstract of model fit presented as the fraction % of released carvedilol per tested tablet:

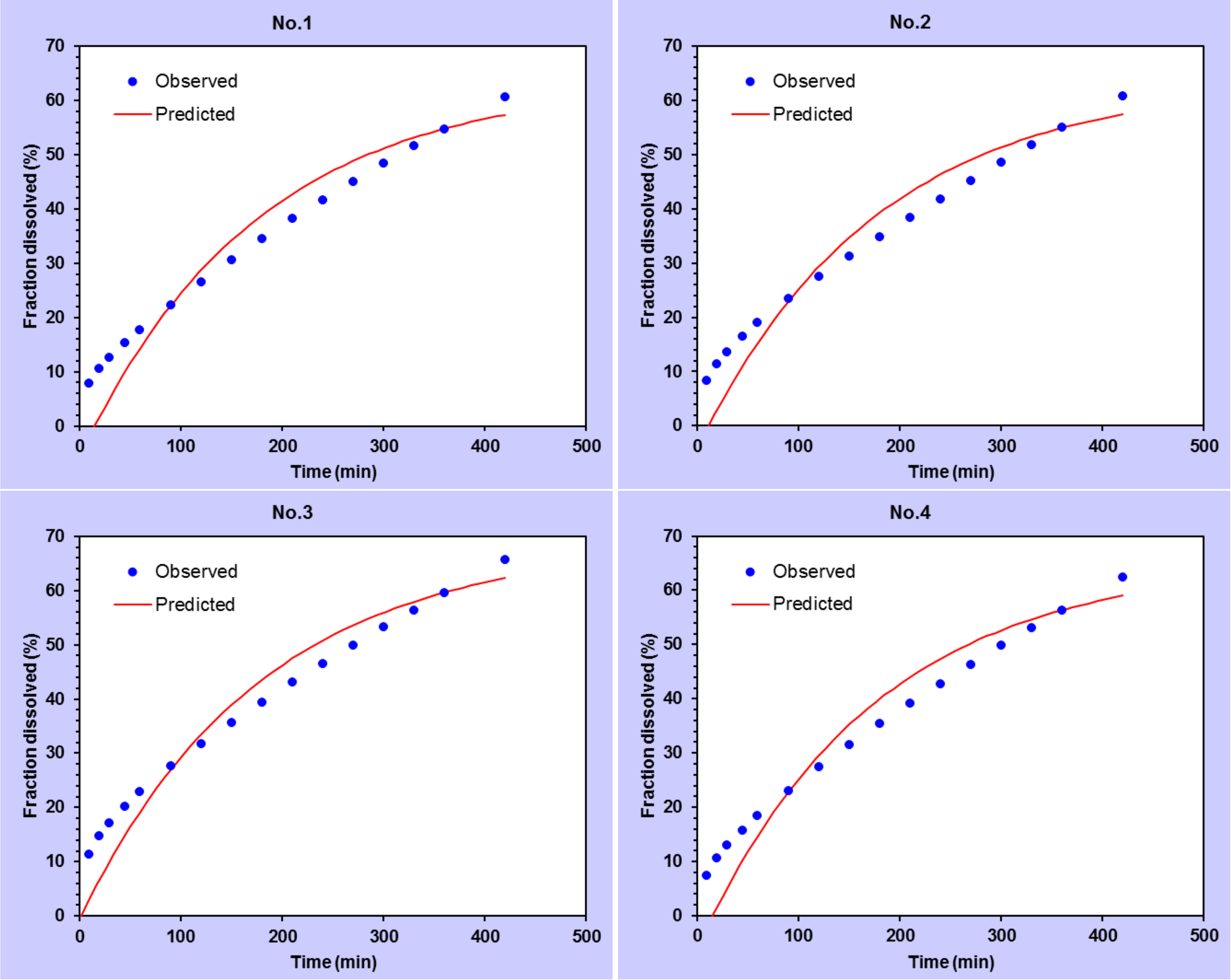

Supplementary materials – Model fitting summary – FlowLac® 100 (modelling up to app. 60 % of carvedilol released)

Model: **Higuchi**

Model equation: $F = k_H \cdot t^{0.5}$

Fitted model parameters per tested tablet (N = 4) with statistics – mean, standard deviation (SD), and relative standard deviation expressed in % (RSD%) (output from DDSolver):

Parameter	No.1	No.2	No.3	No.4	Mean	SD	RSD(%)
k_H	2.720	2.748	3.054	2.797	2.830	0.153	5.406

Number of dissolution data points (N), degrees of freedom (df), and selected goodness of fit criteria – Pearson correlation coefficient (R), coefficient of determination (R^2), adjusted coefficient of determination ($R^2_{adjusted}$), and residual sum of squares (RSS) (manual calculation in MS Excel):

Parameter	No.1	No.2	No.3	No.4
N	16	16	16	16
df	15	15	15	15
R	0.995082549	0.995676774	0.996678523	0.995643132
R^2	0.990189279	0.991372239	0.993368078	0.991305246
$R^2_{adjusted}$	0.990189279	0.991372239	0.993368078	0.991305246
RSS	105.6491944	70.18547972	30.12376082	109.8185164

Graphical abstract of model fit presented as mean ± 1 SD of the fraction % of released carvedilol:

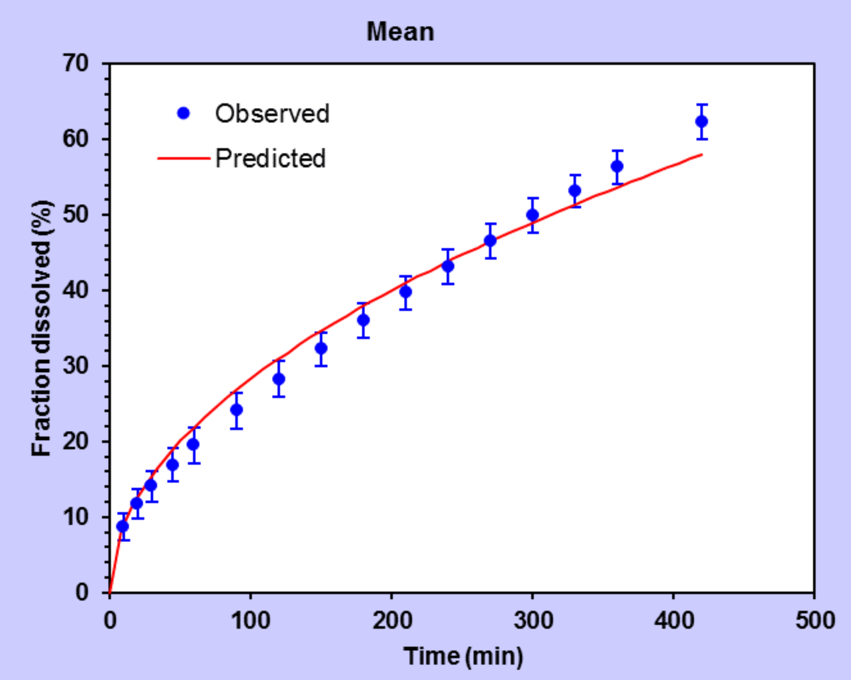

Supplementary materials – Model fitting summary – FlowLac® 100 (modelling up to app. 60 % of carvedilol released)

Graphical abstract of model fit presented as the fraction % of released carvedilol per tested tablet:

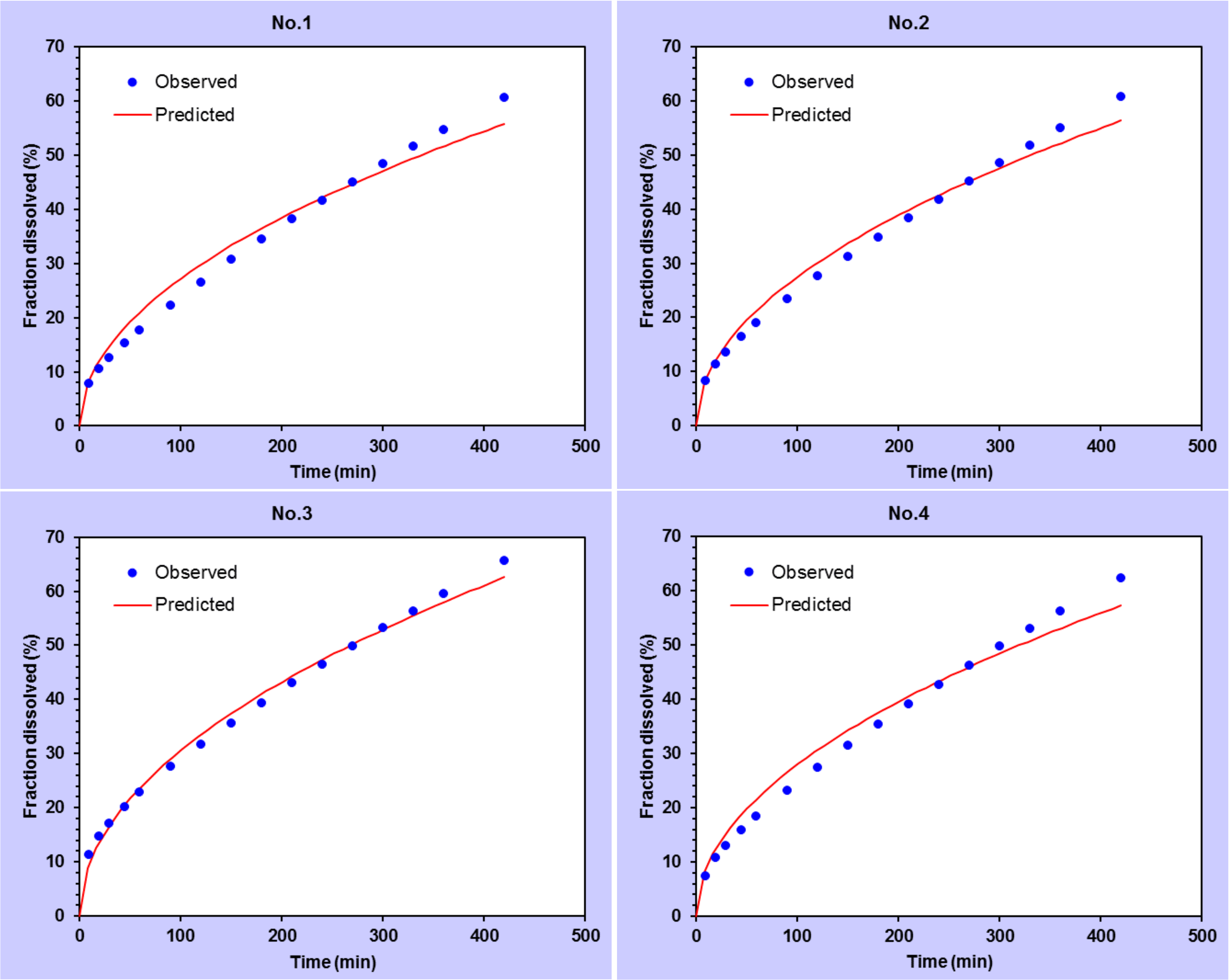

Supplementary materials – Model fitting summary – FlowLac® 100 (modelling up to app. 60 % of carvedilol released)

Model: **Higuchi with T_{lag}**

Model equation: $F = k_H \cdot (t - T_{lag})^{0.5}$

Fitted model parameters per tested tablet (N = 4) with statistics – mean, standard deviation (SD), and relative standard deviation expressed in % (RSD%) (output from DDSolver):

Parameter	No.1	No.2	No.3	No.4	Mean	SD	RSD(%)
k_H	2.935	2.926	3.142	3.018	3.005	0.100	3.334
T_{lag}	24.279	20.362	9.583	24.219	19.611	6.932	35.347

Number of dissolution data points (N), degrees of freedom (df), and selected goodness of fit criteria – Pearson correlation coefficient (R), coefficient of determination (R^2), adjusted coefficient of determination ($R^2_{adjusted}$), and residual sum of squares (RSS) (manual calculation in MS Excel):

Parameter	No.1	No.2	No.3	No.4
N	16	16	16	16
df	14	14	14	14
R	0.985150477	0.984843295	0.991428654	0.985956969
R^2	0.970521461	0.969916315	0.982930777	0.972111144
$R^2_{adjusted}$	0.968415852	0.96776748	0.981711547	0.970119083
RSS	235.6677804	246.0390606	133.8166838	237.6868109

Graphical abstract of model fit presented as mean ± 1 SD of the fraction % of released carvedilol:

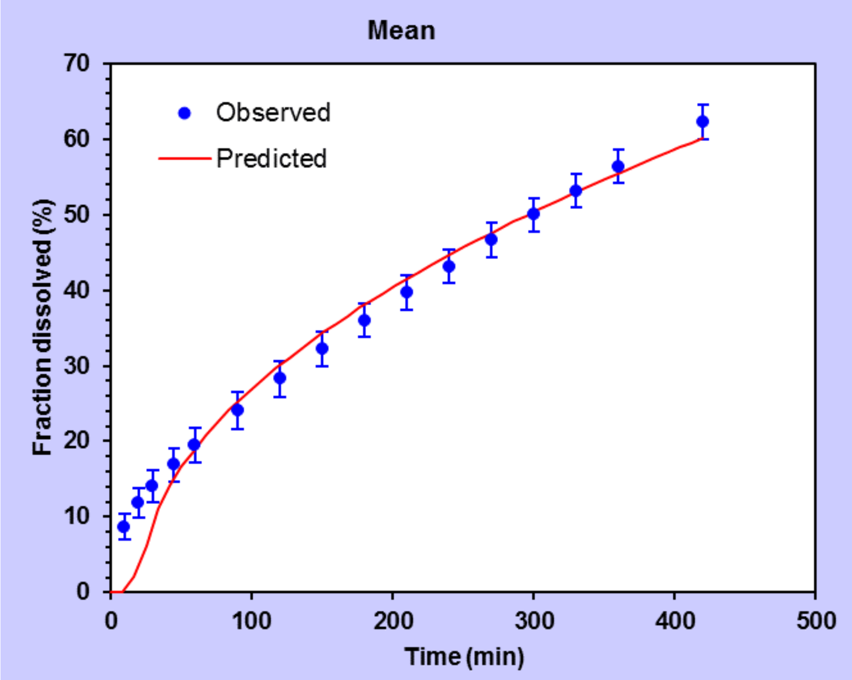

Supplementary materials – Model fitting summary – FlowLac® 100 (modelling up to app. 60 % of carvedilol released)

Graphical abstract of model fit presented as the fraction % of released carvedilol per tested tablet:

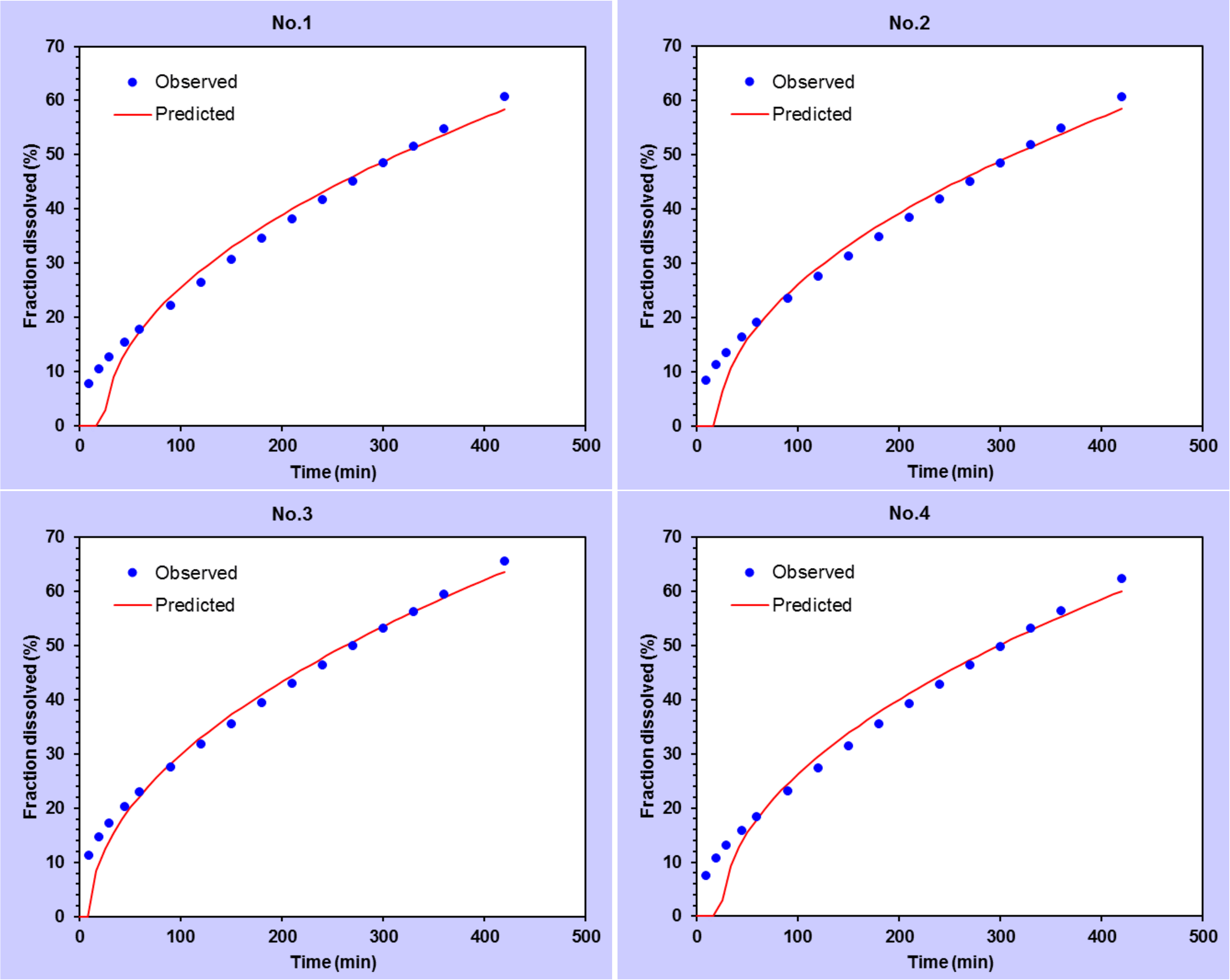

Supplementary materials – Model fitting summary – FlowLac® 100 (modelling up to app. 60 % of carvedilol released)

Model: **Higuchi with F_0**

Model equation: $F = F_0 + k_H \cdot t^{0.5}$

Fitted model parameters per tested tablet (N = 4) with statistics – mean, standard deviation (SD), and relative standard deviation expressed in % (RSD%) (output from DDSolver):

Parameter	No.1	No.2	No.3	No.4	Mean	SD	RSD(%)
k_H	3.060	2.999	3.094	3.155	3.077	0.065	2.118
F_0	-4.948	-3.649	-0.584	-5.203	-3.596	2.120	-58.951

Number of dissolution data points (N), degrees of freedom (df), and selected goodness of fit criteria – Pearson correlation coefficient (R), coefficient of determination (R^2), adjusted coefficient of determination ($R^2_{adjusted}$), and residual sum of squares (RSS) (manual calculation in MS Excel):

Parameter	No.1	No.2	No.3	No.4
N	16	16	16	16
df	14	14	14	14
R	0.995082549	0.995676774	0.996678523	0.995643132
R^2	0.990189279	0.991372239	0.993368078	0.991305246
$R^2_{adjusted}$	0.989488513	0.99075597	0.992894369	0.990684192
RSS	42.44122429	35.80788535	29.24205146	39.93886418

Graphical abstract of model fit presented as mean ± 1 SD of the fraction % of released carvedilol:

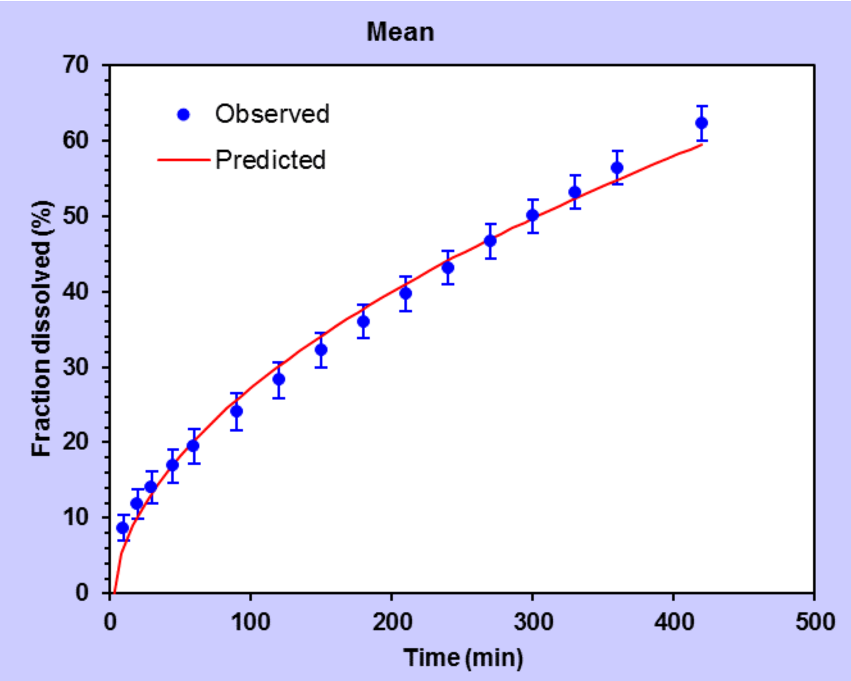

Supplementary materials – Model fitting summary – FlowLac® 100 (modelling up to app. 60 % of carvedilol released)

Graphical abstract of model fit presented as the fraction % of released carvedilol per tested tablet:

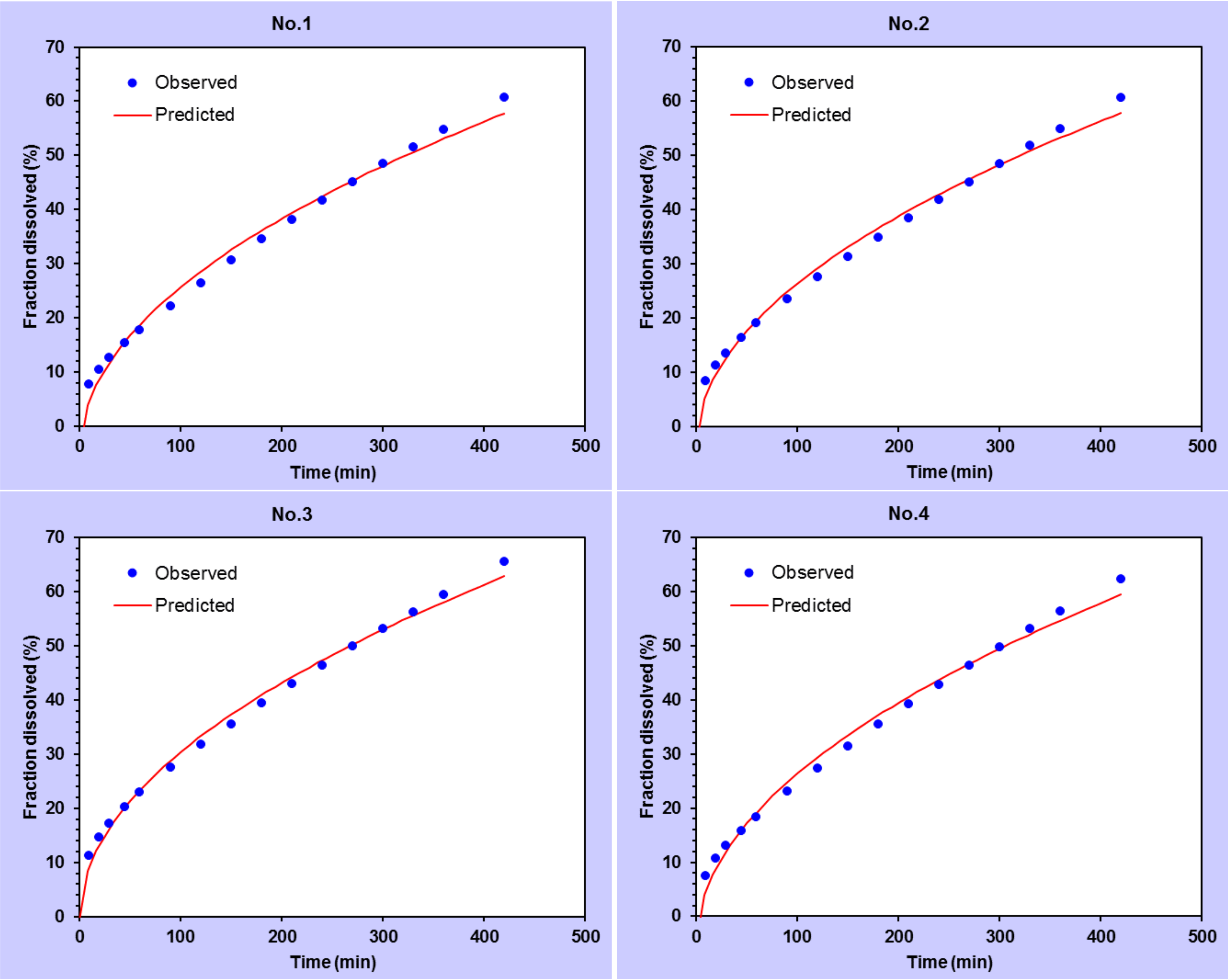

Supplementary materials – Model fitting summary – FlowLac® 100 (modelling up to app. 60 % of carvedilol released)

Model: **Korsmeyer–Peppas**

Model equation: $F = k_{KP} \cdot t^n$

Fitted model parameters per tested tablet (N = 4) with statistics – mean, standard deviation (SD), and relative standard deviation expressed in % (RSD%) (output from DDSolver):

Parameter	No.1	No.2	No.3	No.4	Mean	SD	RSD(%)
k_{KP}	1.891	2.191	3.425	1.847	2.338	0.740	31.665
n	0.563	0.539	0.476	0.573	0.538	0.044	8.095

Number of dissolution data points (N), degrees of freedom (df), and selected goodness of fit criteria – Pearson correlation coefficient (R), coefficient of determination (R^2), adjusted coefficient of determination ($R^2_{adjusted}$), and residual sum of squares (RSS) (manual calculation in MS Excel):

Parameter	No.1	No.2	No.3	No.4
N	16	16	16	16
df	14	14	14	14
R	0.997368729	0.997037729	0.995829128	0.998013563
R^2	0.994744382	0.994084233	0.991675652	0.996031073
$R^2_{adjusted}$	0.99436898	0.993661679	0.991081056	0.995747578
RSS	37.86108895	36.54120205	51.34480444	28.47332017

Graphical abstract of model fit presented as mean ± 1 SD of the fraction % of released carvedilol:

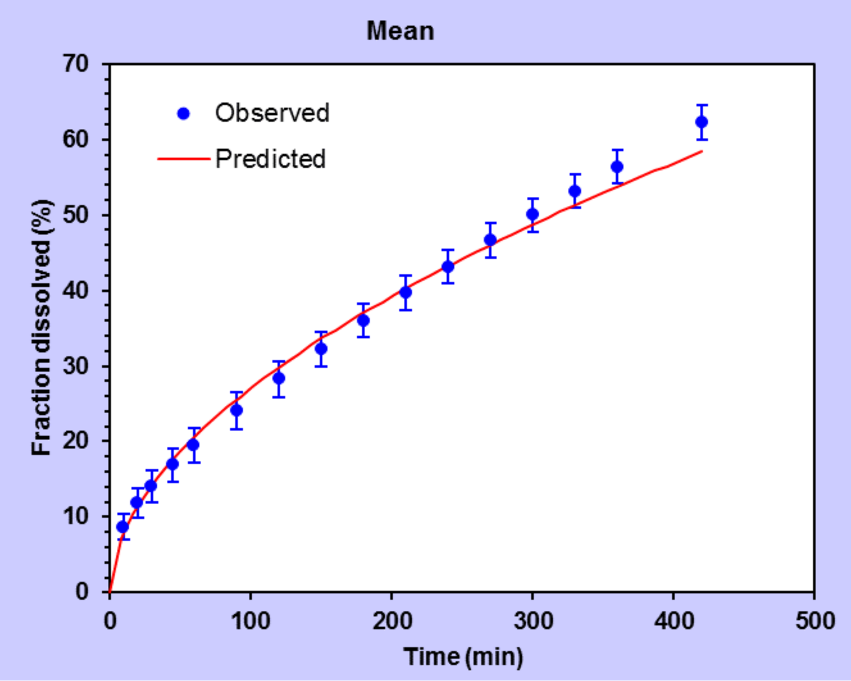

Supplementary materials – Model fitting summary – FlowLac® 100 (modelling up to app. 60 % of carvedilol released)

Graphical abstract of model fit presented as the fraction % of released carvedilol per tested tablet:

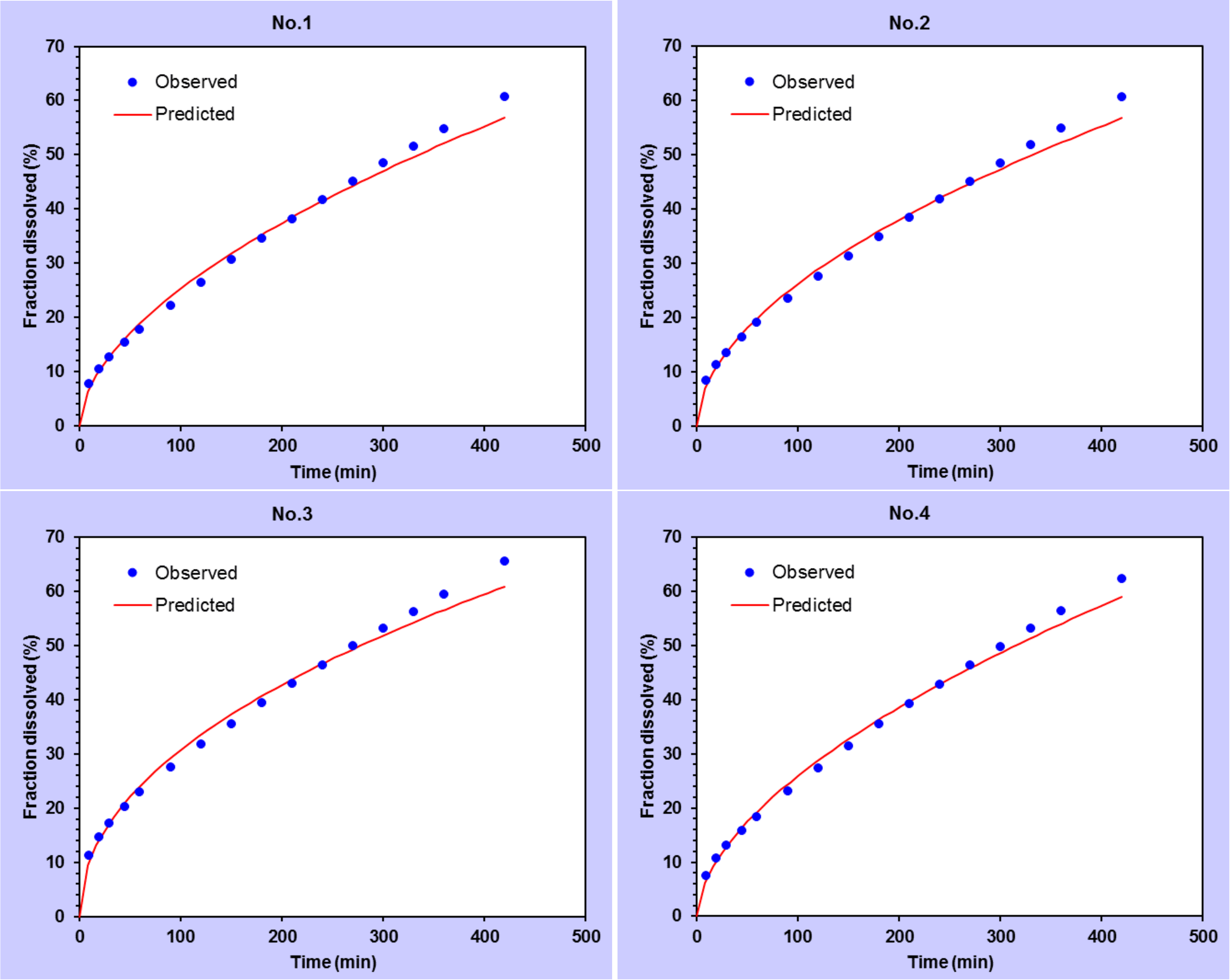

Supplementary materials – Model fitting summary – FlowLac® 100 (modelling up to app. 60 % of carvedilol released)

Model: **Korsmeyer–Peppas with T_{lag}**

Model equation: $F = k_{KP} \cdot (t - T_{lag})^n$

Fitted model parameters per tested tablet (N = 4) with statistics – mean, standard deviation (SD), and relative standard deviation expressed in % (RSD%) (output from DDSolver):

Parameter	No.1	No.2	No.3	No.4	Mean	SD	RSD(%)
k_{KP}	2.542	2.903	4.399	2.487	3.082	0.897	29.091
n	0.510	0.488	0.431	0.519	0.487	0.040	8.134
T_{lag}	4.000	4.000	4.000	4.000	4.000	0.000	0.000

Number of dissolution data points (N), degrees of freedom (df), and selected goodness of fit criteria – Pearson correlation coefficient (R), coefficient of determination (R^2), adjusted coefficient of determination ($R^2_{adjusted}$), and residual sum of squares (RSS) (manual calculation in MS Excel):

Parameter	No.1	No.2	No.3	No.4
N	16	16	16	16
df	13	13	13	13
R	0.994173801	0.993829971	0.992034428	0.995290897
R^2	0.988381547	0.987698012	0.984132306	0.990603971
$R^2_{adjusted}$	0.986594093	0.985805398	0.981691122	0.989158428
RSS	87.67909034	81.63393484	103.0654589	73.21592129

Graphical abstract of model fit presented as mean ± 1 SD of the fraction % of released carvedilol:

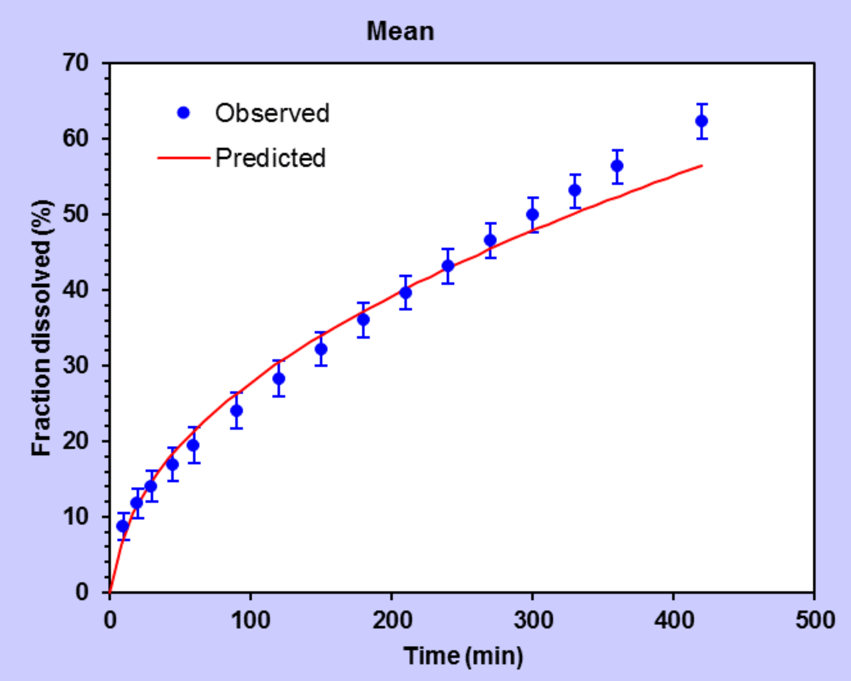

Supplementary materials – Model fitting summary – FlowLac® 100 (modelling up to app. 60 % of carvedilol released)

Graphical abstract of model fit presented as the fraction % of released carvedilol per tested tablet:

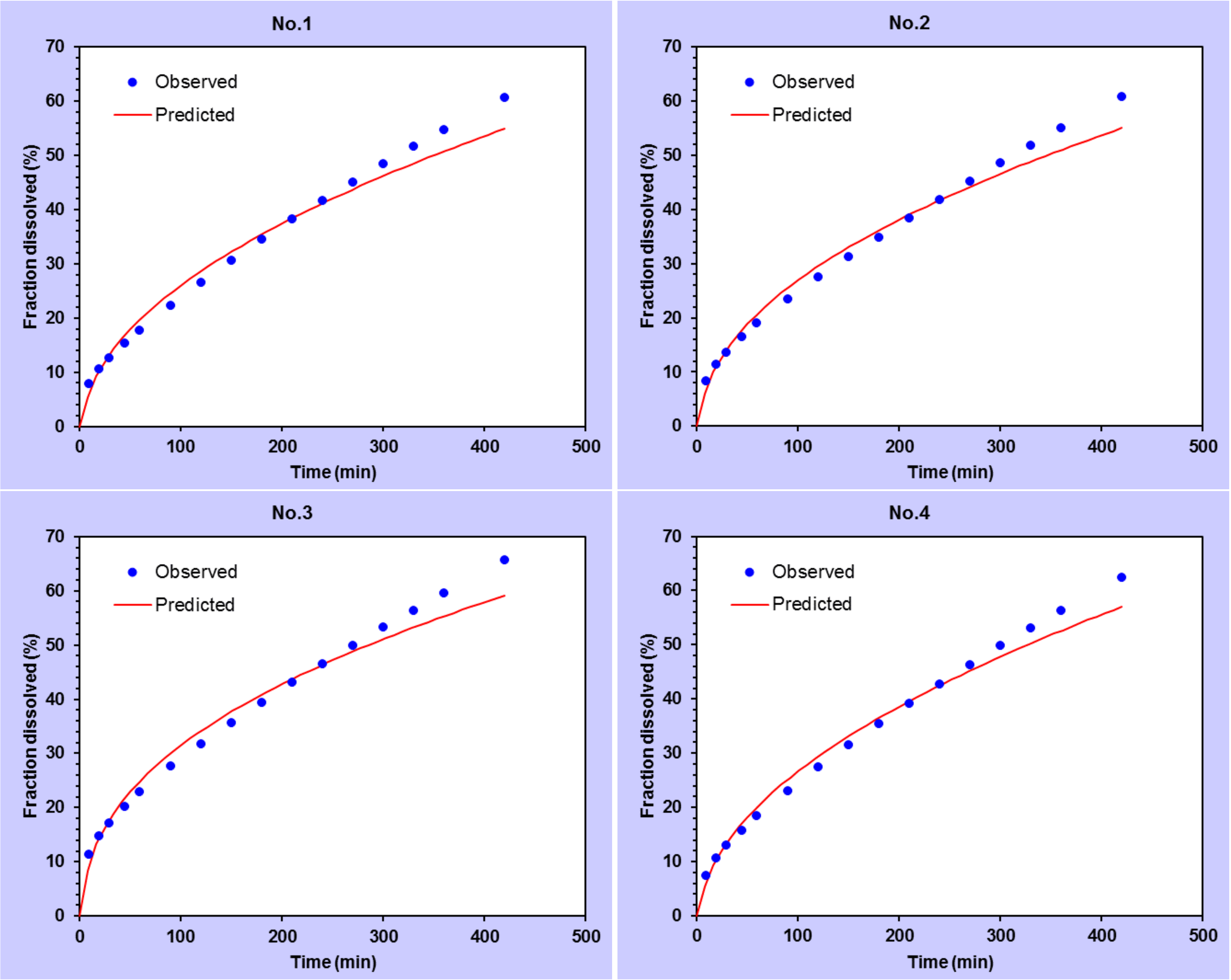

Supplementary materials – Model fitting summary – FlowLac® 100 (modelling up to app. 60 % of carvedilol released)

Model: **Korsmeyer–Peppas with F_0**

Model equation: $F = F_0 + k_{KP} \cdot t^n$

Fitted model parameters per tested tablet (N = 4) with statistics – mean, standard deviation (SD), and relative standard deviation expressed in % (RSD%) (output from DDSolver):

Parameter	No.1	No.2	No.3	No.4	Mean	SD	RSD(%)
k_{KP}	0.954	1.005	1.342	0.873	1.044	0.206	19.753
n	0.674	0.670	0.626	0.697	0.667	0.030	4.451
F_0	3.120	2.544	5.226	3.469	3.590	1.156	32.191

Number of dissolution data points (N), degrees of freedom (df), and selected goodness of fit criteria – Pearson correlation coefficient (R), coefficient of determination (R^2), adjusted coefficient of determination ($R^2_{adjusted}$), and residual sum of squares (RSS) (manual calculation in MS Excel):

Parameter	No.1	No.2	No.3	No.4
N	16	16	16	16
df	13	13	13	13
R	0.999604208	0.999631033	0.999480807	0.999849493
R^2	0.999208573	0.999262203	0.998961883	0.999699008
$R^2_{adjusted}$	0.999086815	0.999148695	0.998802173	0.999652701
RSS	6.321909011	6.904130894	5.27944619	2.867638319

Graphical abstract of model fit presented as mean ± 1 SD of the fraction % of released carvedilol:

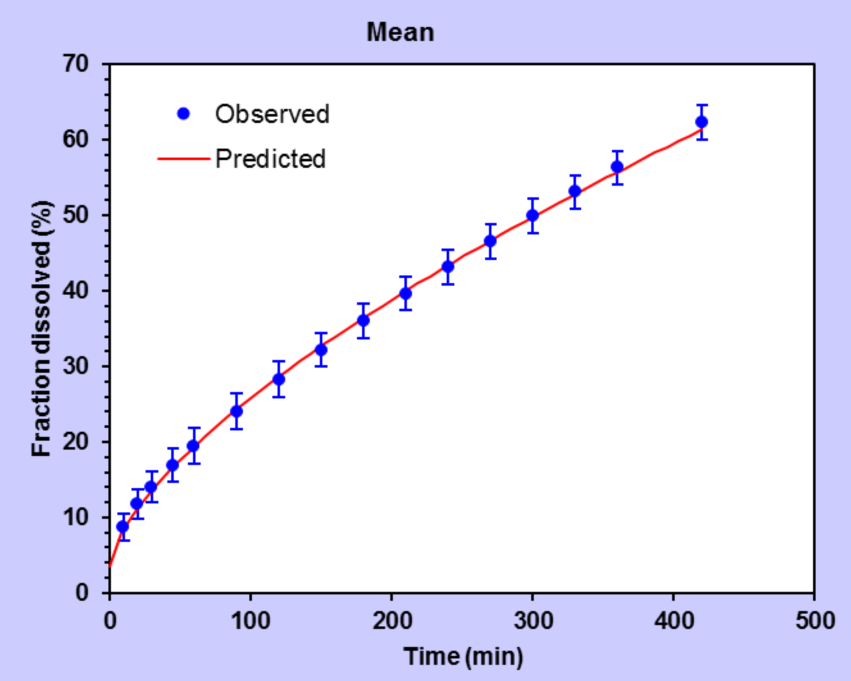

Supplementary materials – Model fitting summary – FlowLac® 100 (modelling up to app. 60 % of carvedilol released)

Graphical abstract of model fit presented as the fraction % of released carvedilol per tested tablet:

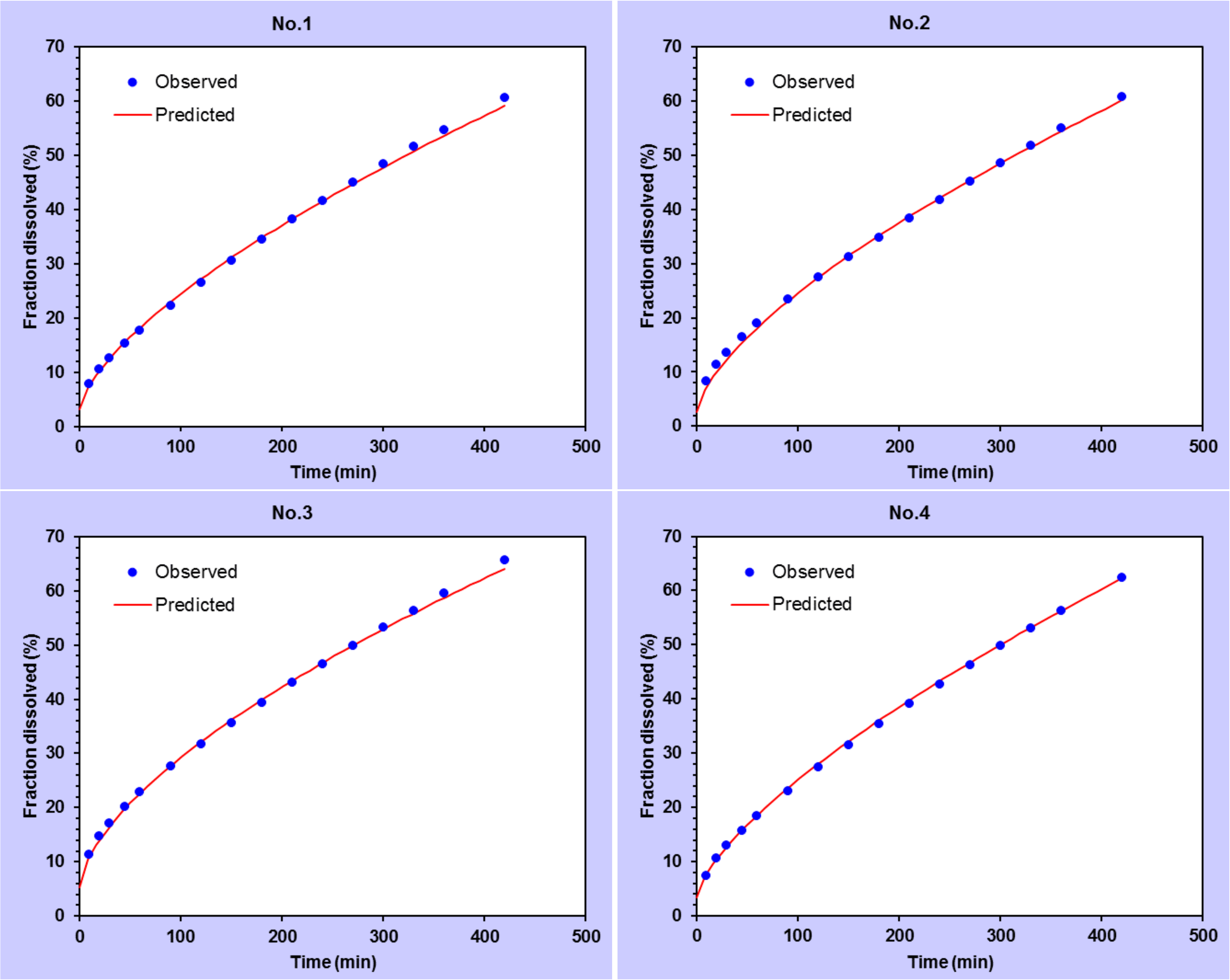

Supplementary materials – Model fitting summary – FlowLac® 100 (modelling up to app. 60 % of carvedilol released)

Model: **Hixson–Crowell**

Model equation: $F = 100 \cdot [1 - (1 - k_{HC} \cdot t)^3]$

Fitted model parameters per tested tablet (N = 4) with statistics – mean, standard deviation (SD), and relative standard deviation expressed in % (RSD%) (output from DDSolver):

Parameter	No.1	No.2	No.3	No.4	Mean	SD	RSD(%)
k_{HC}	0.001	0.001	0.001	0.001	0.001	0.000	6.189

Number of dissolution data points (N), degrees of freedom (df), and selected goodness of fit criteria – Pearson correlation coefficient (R), coefficient of determination (R^2), adjusted coefficient of determination ($R^2_{adjusted}$), and residual sum of squares (RSS) (manual calculation in MS Excel):

Parameter	No.1	No.2	No.3	No.4
N	16	16	16	16
df	15	15	15	15
R	0.999647068	0.999121329	0.998938803	0.999369509
R^2	0.99929426	0.998243429	0.997878731	0.998739416
$R^2_{adjusted}$	0.99929426	0.998243429	0.997878731	0.998739416
RSS	273.7352458	360.5284151	663.7487043	279.6777986

Graphical abstract of model fit presented as mean ± 1 SD of the fraction % of released carvedilol:

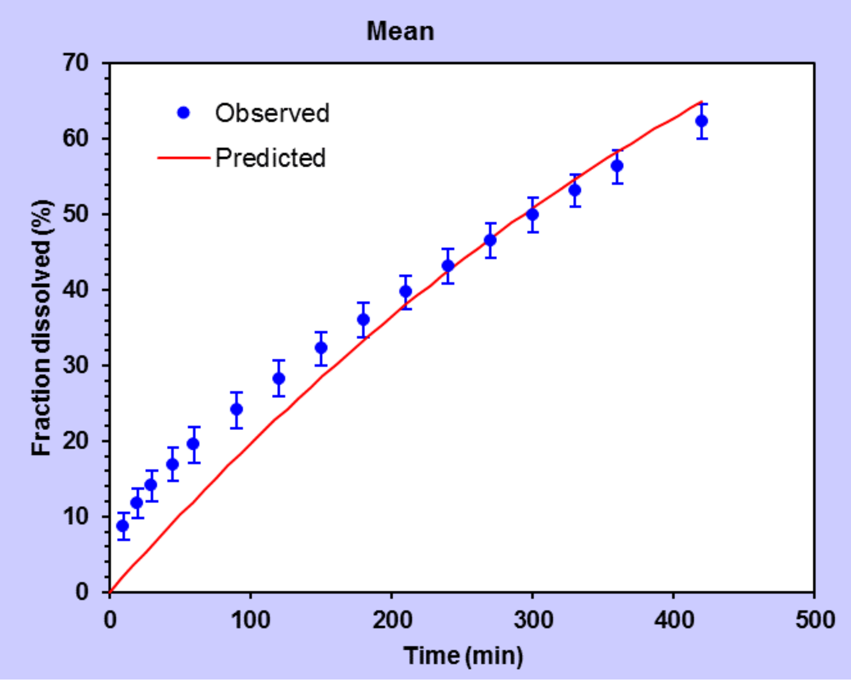

Supplementary materials – Model fitting summary – FlowLac® 100 (modelling up to app. 60 % of carvedilol released)

Graphical abstract of model fit presented as the fraction % of released carvedilol per tested tablet:

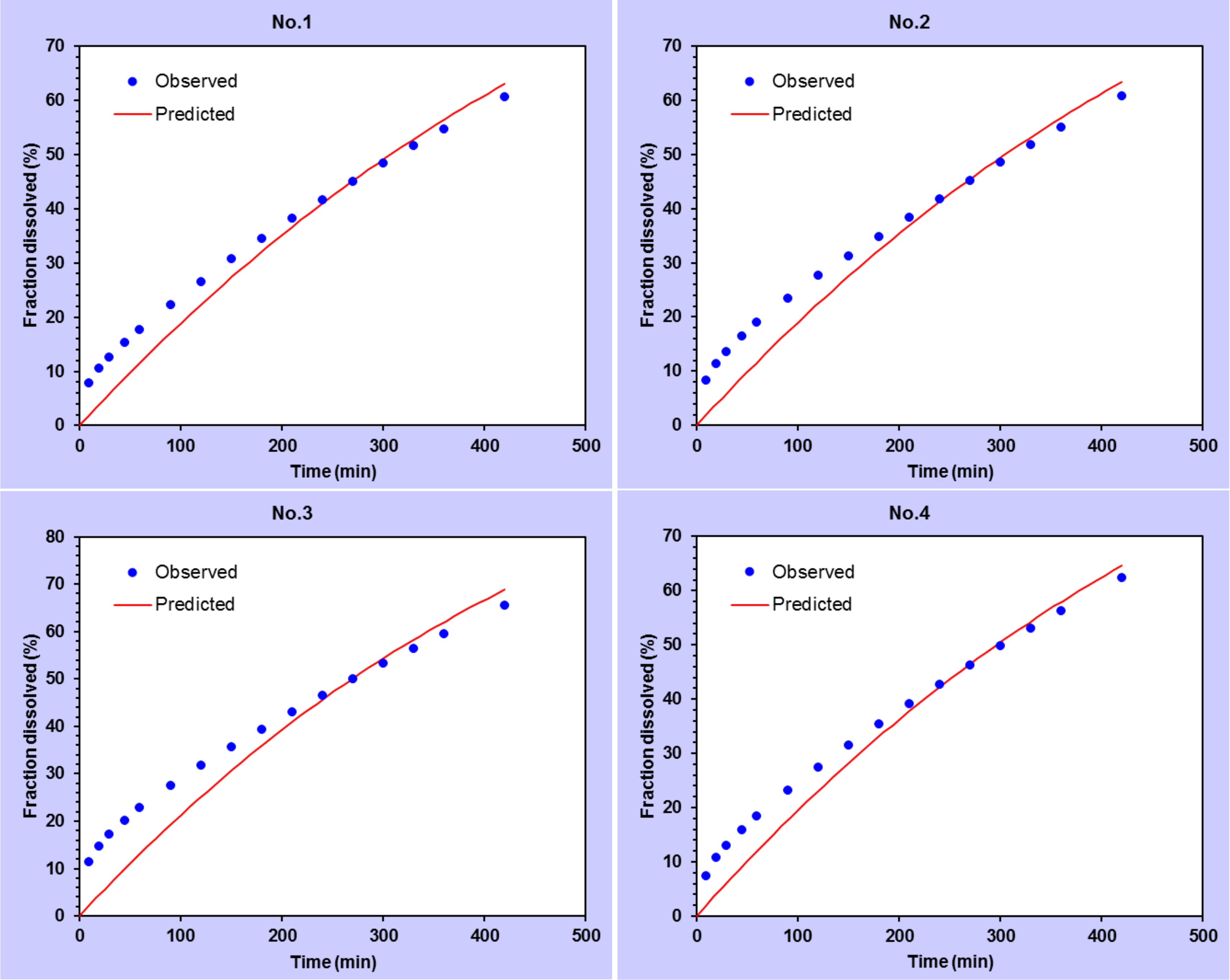

Supplementary materials – Model fitting summary – FlowLac® 100 (modelling up to app. 60 % of carvedilol released)

Model: **Hixson–Crowell with T_{lag}**

Model equation: $F = 100 \cdot \left\{1 - \left[1 - k_{HC} \cdot \left(t - T_{lag}\right)\right]^3\right\}$

Fitted model parameters per tested tablet (N = 4) with statistics – mean, standard deviation (SD), and relative standard deviation expressed in % (RSD%) (output from DDSolver):

Parameter	No.1	No.2	No.3	No.4	Mean	SD	RSD(%)
k_{HC}	0.001	0.001	0.001	0.001	0.001	0.000	3.540
T_{lag}	-46.679	-54.349	-70.012	-45.251	-54.073	11.353	-20.995

Number of dissolution data points (N), degrees of freedom (df), and selected goodness of fit criteria – Pearson correlation coefficient (R), coefficient of determination (R^2), adjusted coefficient of determination ($R^2_{adjusted}$), and residual sum of squares (RSS) (manual calculation in MS Excel):

Parameter	No.1	No.2	No.3	No.4
N	16	16	16	16
df	14	14	14	14
R	0.999512493	0.998957322	0.998693806	0.999239956
R^2	0.999025224	0.997915732	0.997389319	0.998480489
$R^2_{adjusted}$	0.998955597	0.997766856	0.997202842	0.998371952
RSS	4.268955869	8.702716628	11.60395377	7.033089417

Graphical abstract of model fit presented as mean ± 1 SD of the fraction % of released carvedilol:

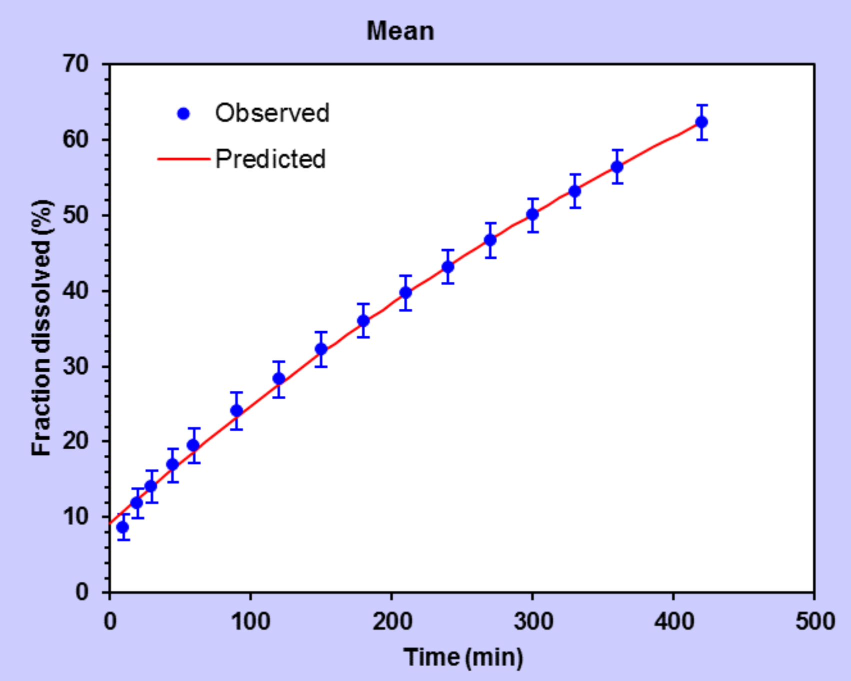

Supplementary materials – Model fitting summary – FlowLac® 100 (modelling up to app. 60 % of carvedilol released)

Graphical abstract of model fit presented as the fraction % of released carvedilol per tested tablet:

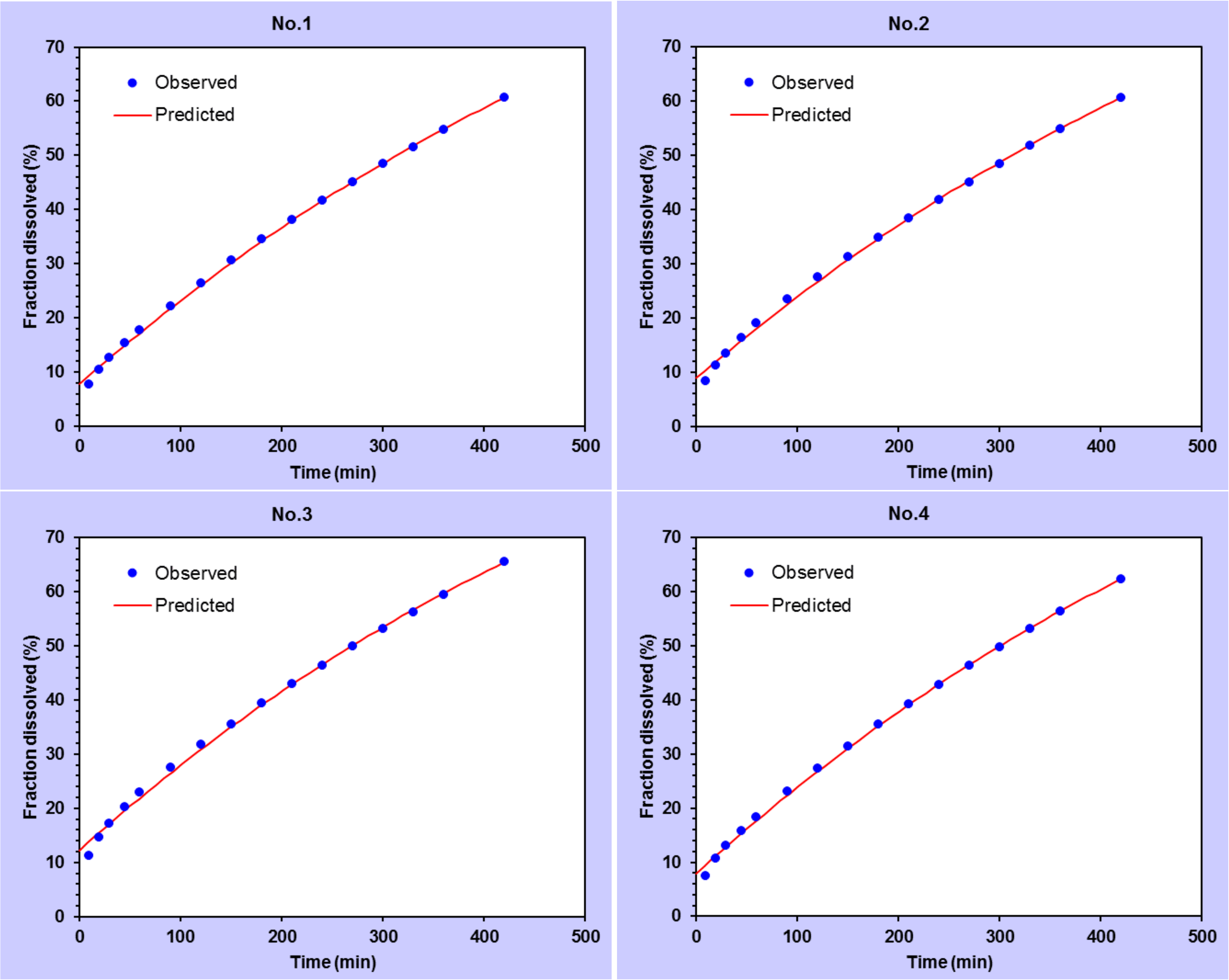

Supplementary materials – Model fitting summary – FlowLac® 100 (modelling up to app. 60 % of carvedilol released)

Model: **Hopfenberg**

Model equation: $F = 100 \cdot [1 - (1 - k_{HB} \cdot t)^n]$

Fitted model parameters per tested tablet (N = 4) with statistics – mean, standard deviation (SD), and relative standard deviation expressed in % (RSD%) (output from DDSolver):

Parameter	No.1	No.2	No.3	No.4	Mean	SD	RSD(%)
k_{HB}	0.001	0.001	0.001	0.001	0.001	0.000	8.342
n	3.000	3.000	4.125	3.000	3.281	0.563	17.143

Number of dissolution data points (N), degrees of freedom (df), and selected goodness of fit criteria – Pearson correlation coefficient (R), coefficient of determination (R^2), adjusted coefficient of determination ($R^2_{adjusted}$), and residual sum of squares (RSS) (manual calculation in MS Excel):

Parameter	No.1	No.2	No.3	No.4
N	16	16	16	16
df	14	14	14	14
R	0.999647068	0.999121329	0.998977333	0.999369509
R^2	0.99929426	0.998243429	0.997955713	0.998739416
$R^2_{adjusted}$	0.99924385	0.99811796	0.997809692	0.998649375
RSS	273.7352458	360.5284151	618.8827268	279.6777986

Graphical abstract of model fit presented as mean ± 1 SD of the fraction % of released carvedilol:

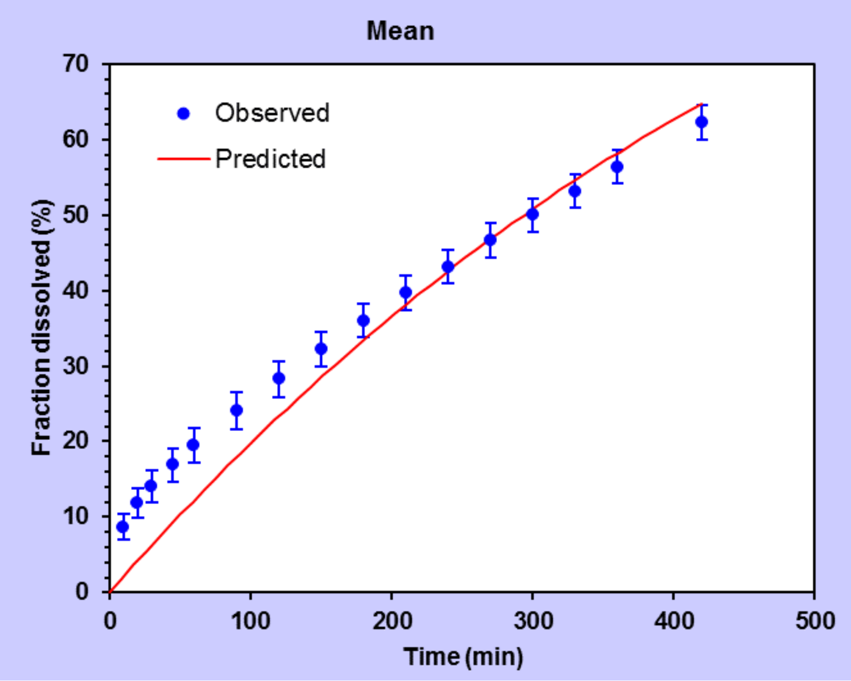

Supplementary materials – Model fitting summary – FlowLac® 100 (modelling up to app. 60 % of carvedilol released)

Graphical abstract of model fit presented as the fraction % of released carvedilol per tested tablet:

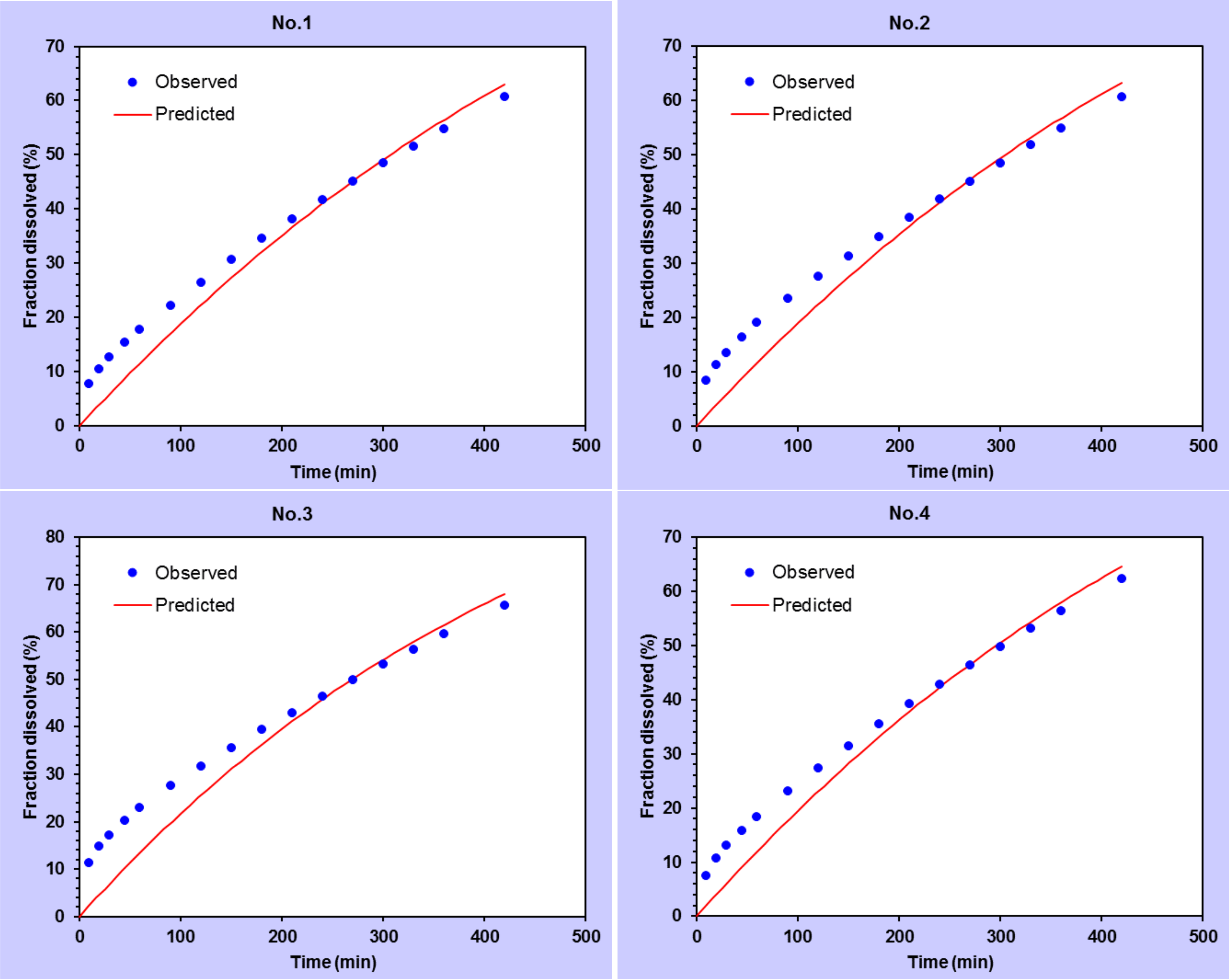

Supplementary materials – Model fitting summary – FlowLac® 100 (modelling up to app. 60 % of carvedilol released)

Model: **Hopfenberg with T_{lag}**

Model equation: $F = 100 \cdot \{1 - [1 - k_{HB} \cdot (t - T_{lag})]^n\}$

Fitted model parameters per tested tablet (N = 4) with statistics – mean, standard deviation (SD), and relative standard deviation expressed in % (RSD%) (output from DDSolver):

Parameter	No.1	No.2	No.3	No.4	Mean	SD	RSD(%)
k_{HB}	0.001	0.001	0.001	0.001	0.001	0.000	3.540
n	3.000	3.000	3.000	3.000	3.000	0.000	0.000
T_{lag}	-46.679	-54.349	-70.012	-45.251	-54.073	11.353	-20.995

Number of dissolution data points (N), degrees of freedom (df), and selected goodness of fit criteria – Pearson correlation coefficient (R), coefficient of determination (R^2), adjusted coefficient of determination ($R^2_{adjusted}$), and residual sum of squares (RSS) (manual calculation in MS Excel):

Parameter	No.1	No.2	No.3	No.4
N	16	16	16	16
df	13	13	13	13
R	0.999512493	0.998957322	0.998693806	0.999239956
R^2	0.999025224	0.997915732	0.997389319	0.998480489
$R^2_{adjusted}$	0.998875258	0.997595075	0.996987676	0.998246718
RSS	4.268955869	8.702716628	11.60395377	7.033089417

Graphical abstract of model fit presented as mean ± 1 SD of the fraction % of released carvedilol:

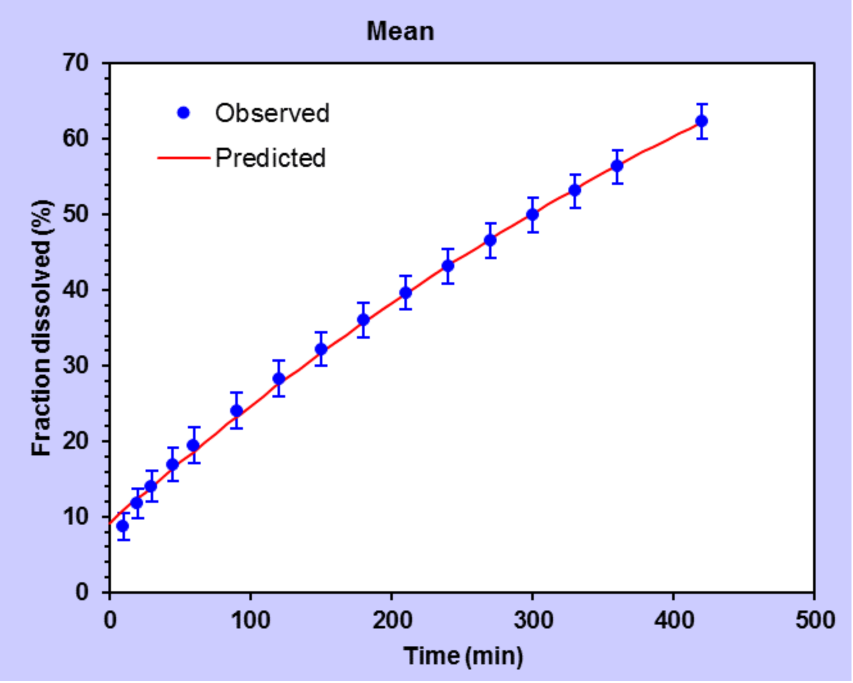

Supplementary materials – Model fitting summary – FlowLac® 100 (modelling up to app. 60 % of carvedilol released)

Graphical abstract of model fit presented as the fraction % of released carvedilol per tested tablet:

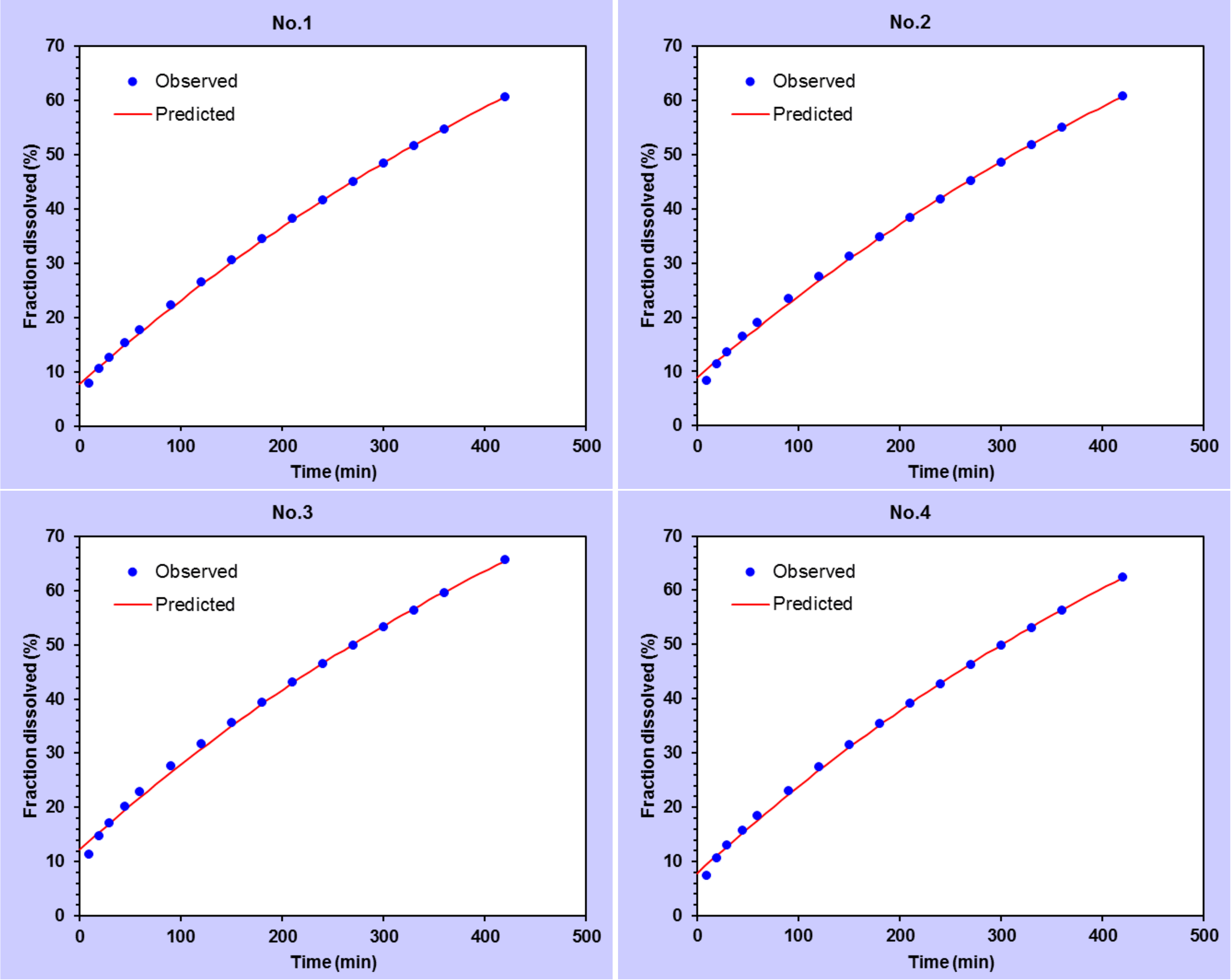

Supplementary materials – Model fitting summary – FlowLac® 100 (modelling up to app. 60 % of carvedilol released)

Model: **Baker–Lonsdale**

Model equation: $\frac{3}{2}\cdot\left[1-\left(1-\frac{F}{100}\right)^{\frac{2}{3}}\right]-\frac{F}{100}=k_{BL}\cdot t$

Fitted model parameters per tested tablet (N = 4) with statistics – mean, standard deviation (SD), and relative standard deviation expressed in % (RSD%) (output from DDSolver):

Parameter	No.1	No.2	No.3	No.4	Mean	SD	RSD(%)
k_{BL}	0.0001	0.0001	0.0002	0.0002	0.0002	0.0000	9.0088

Number of dissolution data points (N), degrees of freedom (df), and selected goodness of fit criteria – Pearson correlation coefficient (R), coefficient of determination (R^2), adjusted coefficient of determination ($R^2_{adjusted}$), and residual sum of squares (RSS) (manual calculation in MS Excel):

Parameter	No.1	No.2	No.3	No.4
N	16	16	16	16
df	15	15	15	15
R	0.990725408	0.991548727	0.992422231	0.99128461
R^2	0.981536834	0.983168878	0.984901885	0.982645178
$R^2_{adjusted}$	0.981536834	0.983168878	0.984901885	0.982645178
RSS	211.5726341	170.0207652	159.1193172	225.6547378

Graphical abstract of model fit presented as mean ± 1 SD of the fraction % of released carvedilol:

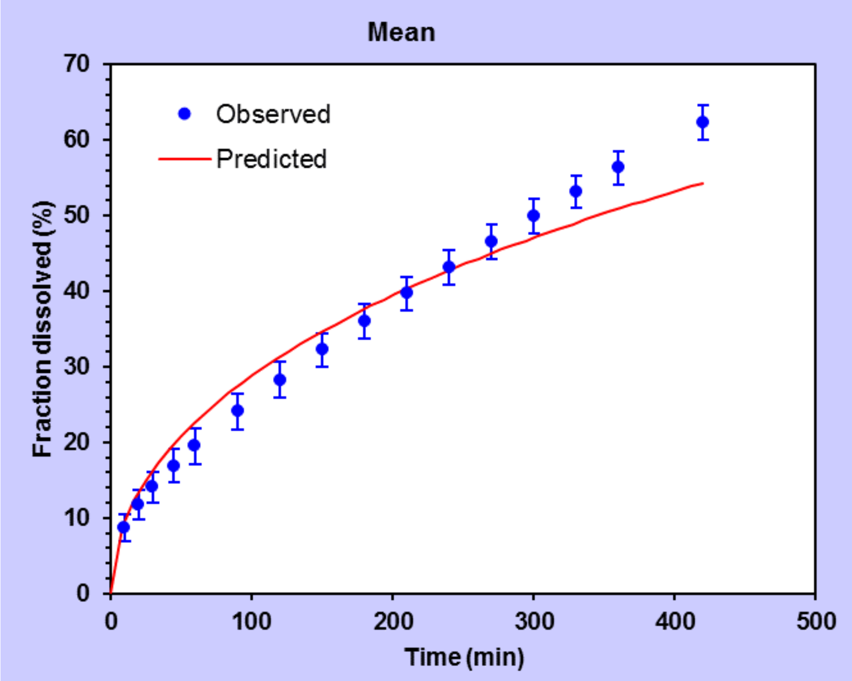

Supplementary materials – Model fitting summary – FlowLac® 100 (modelling up to app. 60 % of carvedilol released)

Graphical abstract of model fit presented as the fraction % of released carvedilol per tested tablet:

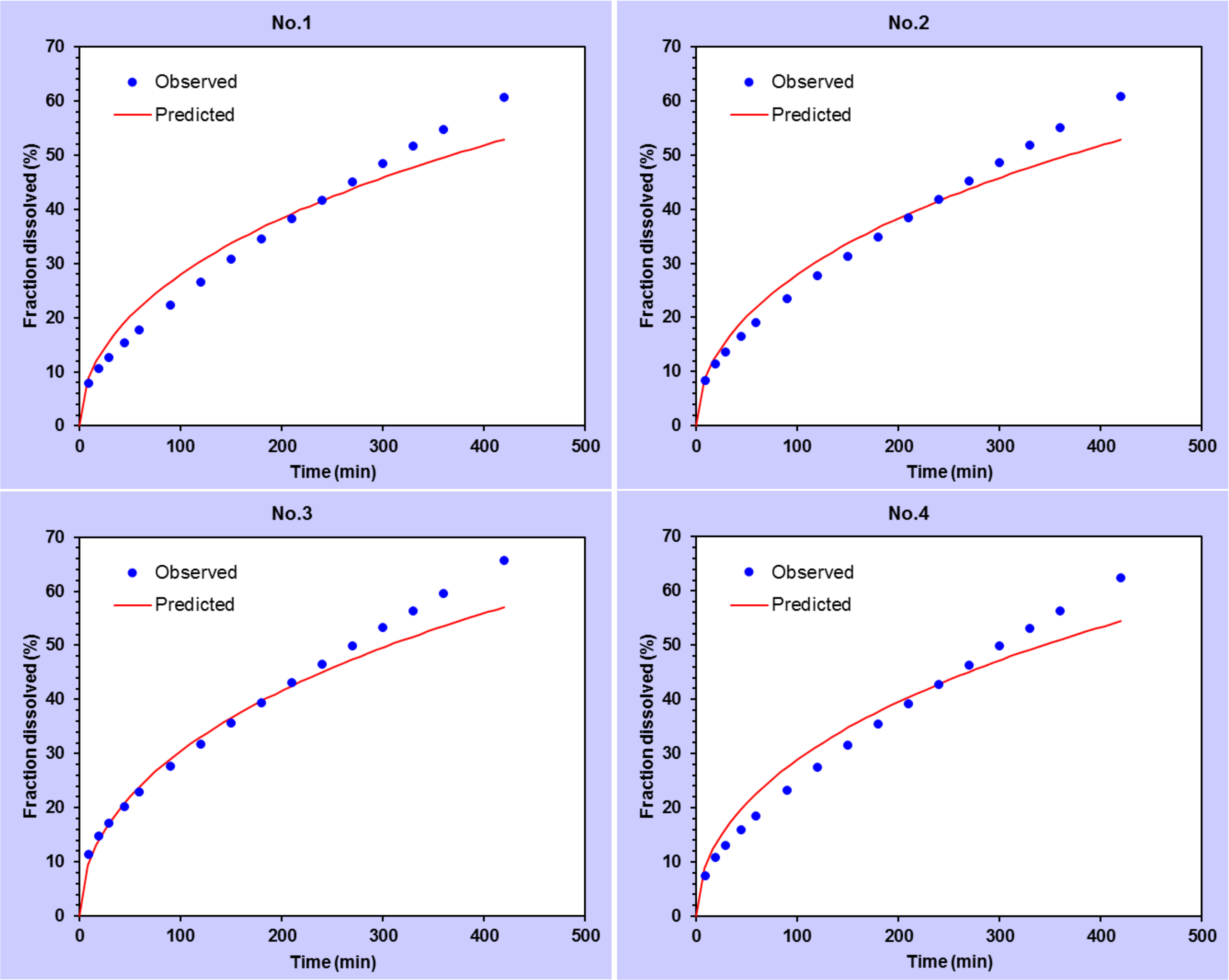

Supplementary materials – Model fitting summary – FlowLac® 100 (modelling up to app. 60 % of carvedilol released)

Model: **Baker–Lonsdale with T_{lag}**

Model equation: $\frac{3}{2} \cdot \left[1 - \left(1 - \frac{F}{100}\right)^{\frac{2}{3}}\right] - \frac{F}{100} = k_{BL} \cdot (t - T_{lag})$

Fitted model parameters per tested tablet (N = 4) with statistics – mean, standard deviation (SD), and relative standard deviation expressed in % (RSD%) (output from DDSolver):

Parameter	No.1	No.2	No.3	No.4	Mean	SD	RSD(%)
k_{BL}	0.0002	0.0002	0.0002	0.0002	0.0002	0.0000	9.0088
T_{lag}	34.8048	31.6596	24.1527	35.1820	31.4498	5.1146	16.2627

Number of dissolution data points (N), degrees of freedom (df), and selected goodness of fit criteria – Pearson correlation coefficient (R), coefficient of determination (R^2), adjusted coefficient of determination ($R^2_{adjusted}$), and residual sum of squares (RSS) (manual calculation in MS Excel):

Parameter	No.1	No.2	No.3	No.4
N	16	16	16	16
df	14	14	14	14
R	0.976480861	0.975525774	0.977758528	0.97685188
R^2	0.953514872	0.951650535	0.95601174	0.954239595
$R^2_{adjusted}$	0.950194506	0.948197002	0.952869721	0.950970995
RSS	416.9810975	457.4303876	473.320757	434.8646818

Graphical abstract of model fit presented as mean ± 1 SD of the fraction % of released carvedilol:

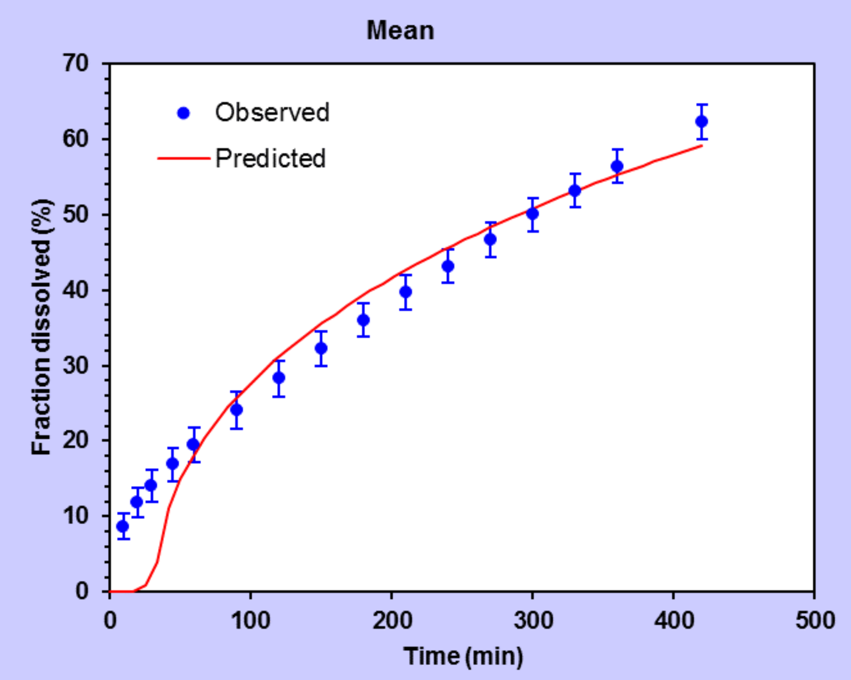

Supplementary materials – Model fitting summary – FlowLac® 100 (modelling up to app. 60 % of carvedilol released)

Graphical abstract of model fit presented as the fraction % of released carvedilol per tested tablet:

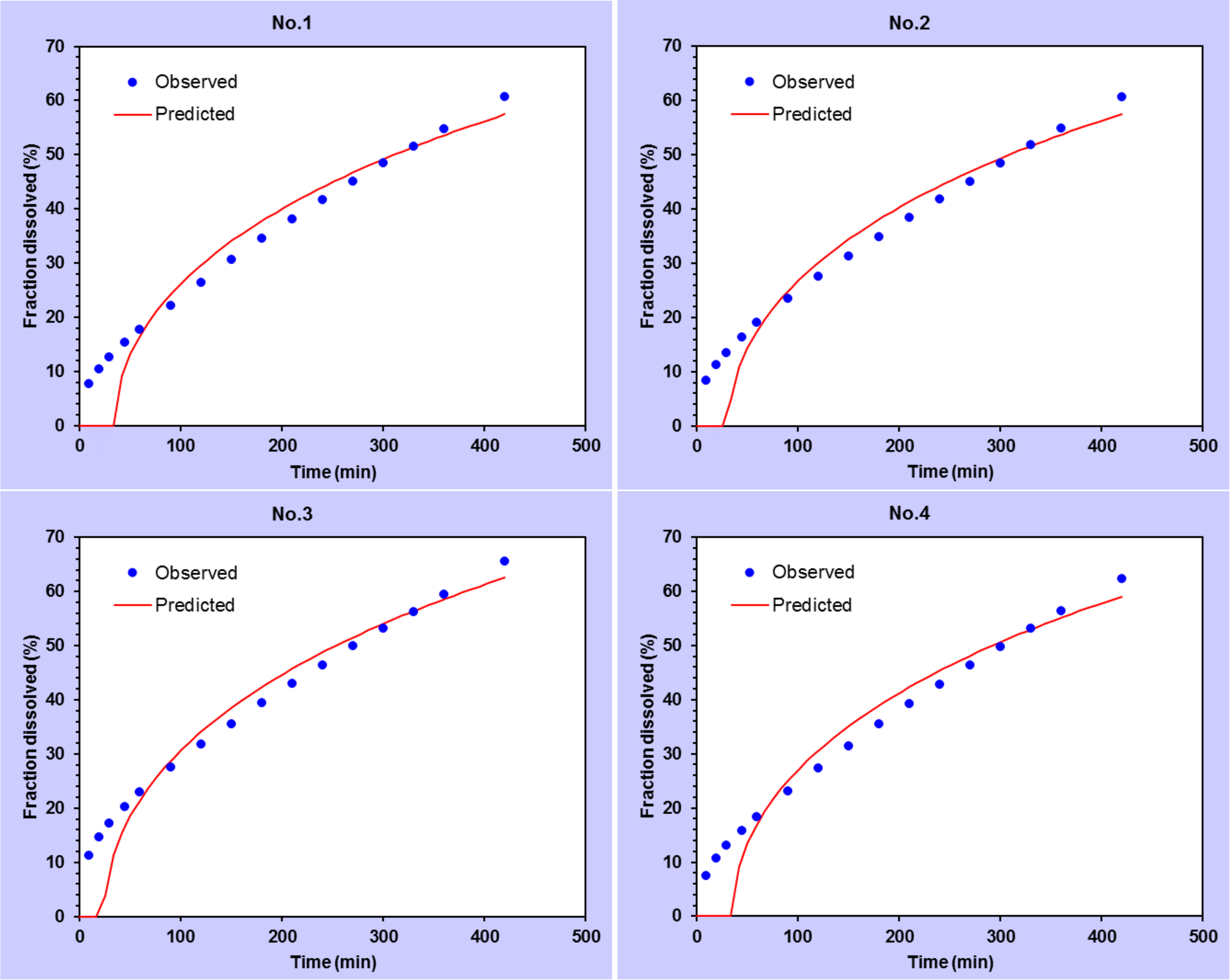

Supplementary materials – Model fitting summary – FlowLac® 100 (modelling up to app. 60 % of carvedilol released)

Model: **Makoid–Banakar**

Model equation: $F = k_{MB} \cdot t^n \cdot e^{-k \cdot t}$

Fitted model parameters per tested tablet (N = 4) with statistics – mean, standard deviation (SD), and relative standard deviation expressed in % (RSD%) (output from DDSolver):

Parameter	No.1	No.2	No.3	No.4	Mean	SD	RSD(%)
k_{MB}	2.484	2.869	4.577	2.339	3.067	1.031	33.621
n	0.482	0.452	0.383	0.497	0.453	0.051	11.218
k	-0.001	-0.001	-0.001	-0.001	-0.001	0.000	-12.206

Number of dissolution data points (N), degrees of freedom (df), and selected goodness of fit criteria – Pearson correlation coefficient (R), coefficient of determination (R^2), adjusted coefficient of determination ($R^2_{adjusted}$), and residual sum of squares (RSS) (manual calculation in MS Excel):

Parameter	No.1	No.2	No.3	No.4
N	16	16	16	16
df	13	13	13	13
R	0.999611774	0.999782764	0.999720052	0.999701969
R^2	0.999223698	0.999565576	0.999440182	0.999404026
$R^2_{adjusted}$	0.999104267	0.999498742	0.999354056	0.999312338
RSS	5.570575493	1.834779211	2.505248691	2.772180764

Graphical abstract of model fit presented as mean ± 1 SD of the fraction % of released carvedilol:

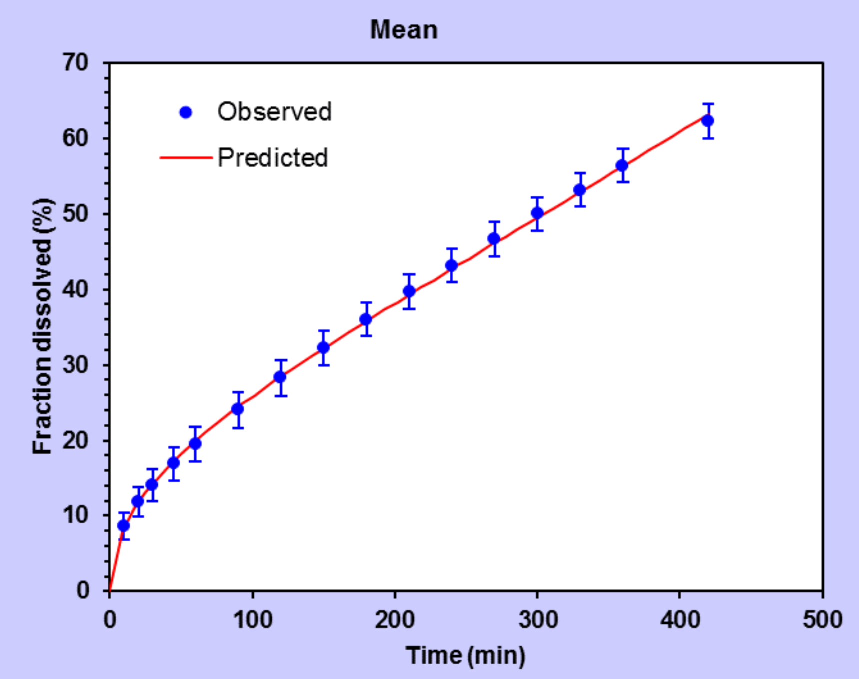

Supplementary materials – Model fitting summary – FlowLac® 100 (modelling up to app. 60 % of carvedilol released)

Graphical abstract of model fit presented as the fraction % of released carvedilol per tested tablet:

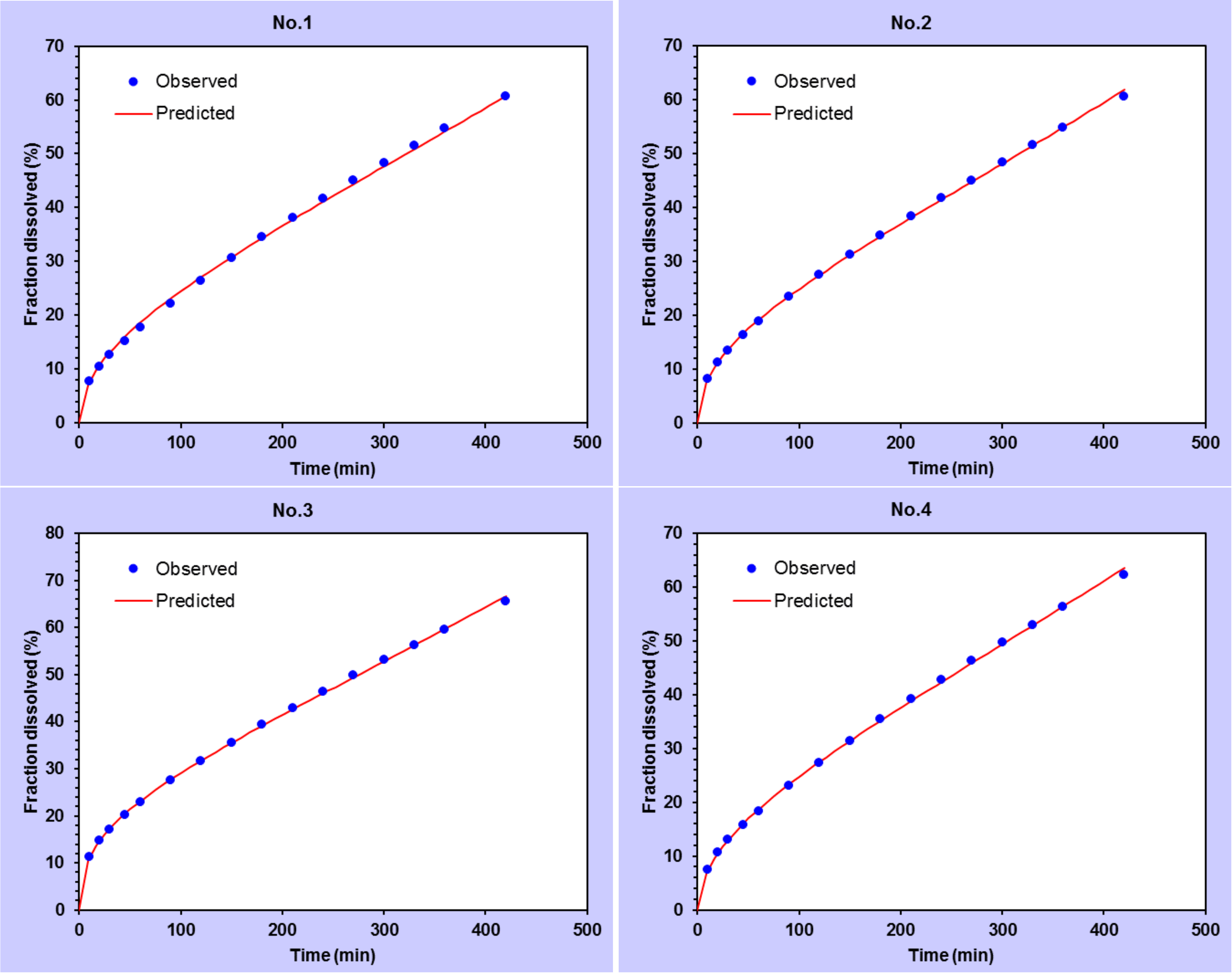

Supplementary materials – Model fitting summary – FlowLac® 100 (modelling up to app. 60 % of carvedilol released)

Model: **Makoid–Banakar with T_{lag}**

Model equation: $F = k_{MB} \cdot (t - T_{lag})^n \cdot e^{-k \cdot (t - T_{lag})}$

Fitted model parameters per tested tablet (N = 4) with statistics – mean, standard deviation (SD), and relative standard deviation expressed in % (RSD%) (output from DDSolver):

Parameter	No.1	No.2	No.3	No.4	Mean	SD	RSD(%)
k_{MB}	3.656	4.028	6.498	3.388	4.392	1.428	32.513
n	0.382	0.373	0.306	0.411	0.368	0.044	12.002
k	-0.001	-0.001	-0.001	-0.001	-0.001	0.000	-7.536
T_{lag}	4.000	4.000	5.028	4.000	4.257	0.514	12.073

Number of dissolution data points (N), degrees of freedom (df), and selected goodness of fit criteria – Pearson correlation coefficient (R), coefficient of determination (R^2), adjusted coefficient of determination ($R^2_{adjusted}$), and residual sum of squares (RSS) (manual calculation in MS Excel):

Parameter	No.1	No.2	No.3	No.4
N	16	16	16	16
df	12	12	12	12
R	0.997833496	0.999000477	0.999145718	0.998833583
R^2	0.995671686	0.998001954	0.998292165	0.997668526
$R^2_{adjusted}$	0.994589607	0.997502442	0.997865206	0.997085658
RSS	19.65714182	8.622391692	9.309309329	11.10215246

Graphical abstract of model fit presented as mean ± 1 SD of the fraction % of released carvedilol:

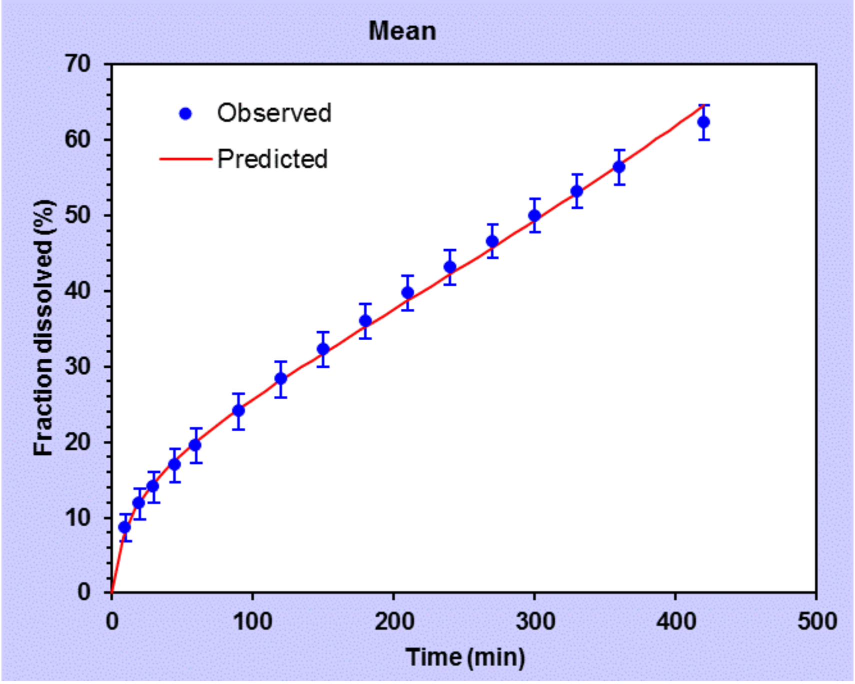

Supplementary materials – Model fitting summary – FlowLac® 100 (modelling up to app. 60 % of carvedilol released)

Graphical abstract of model fit presented as the fraction % of released carvedilol per tested tablet:

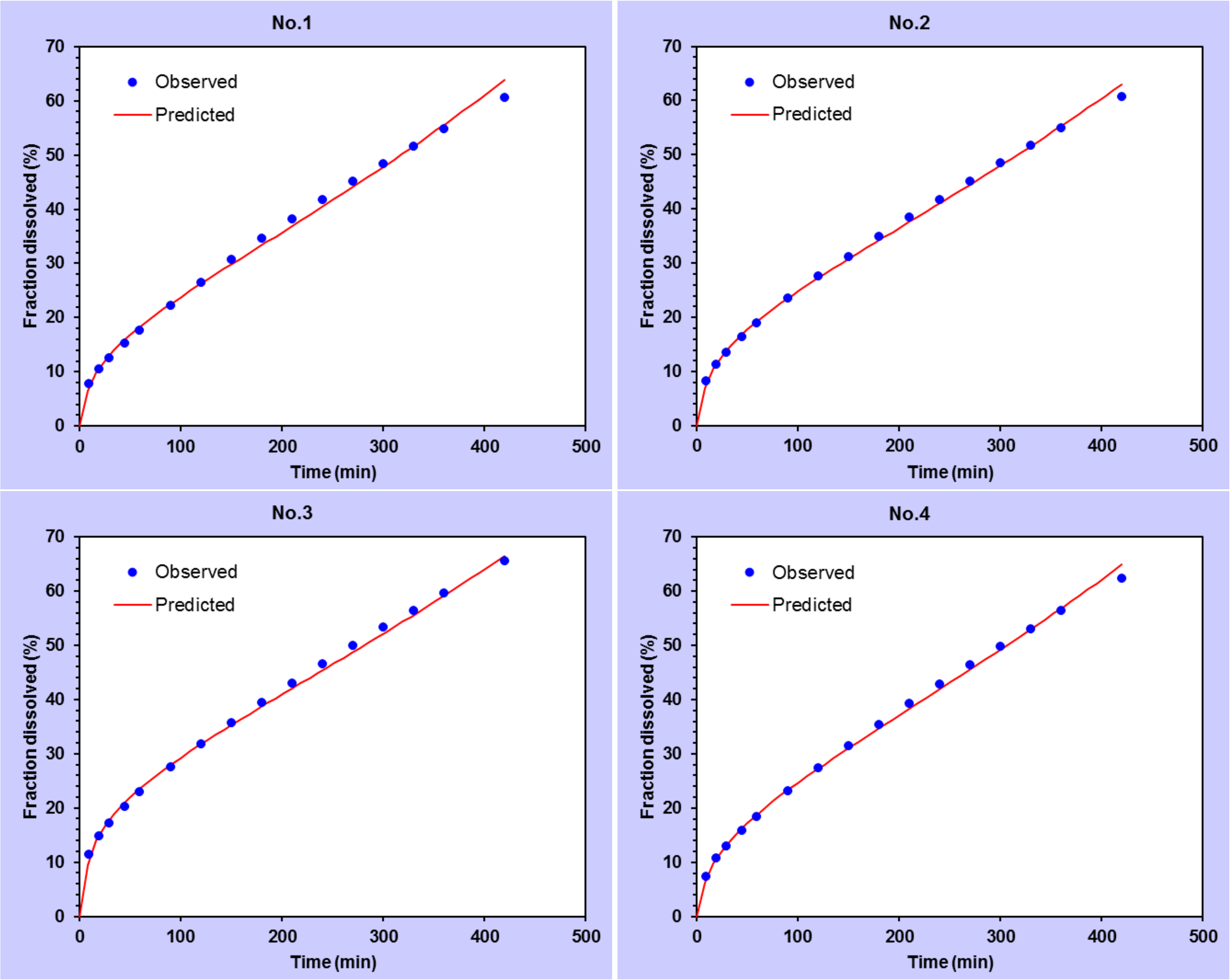

Supplementary materials – Model fitting summary – FlowLac® 100 (modelling up to app. 60 % of carvedilol released)

Model: **Peppas–Sahlin_1**

Model equation: $F = k_1 \cdot t^m + k_2 \cdot t^{2m}$

Fitted model parameters per tested tablet (N = 4) with statistics – mean, standard deviation (SD), and relative standard deviation expressed in % (RSD%) (output from DDSolver):

Parameter	No.1	No.2	No.3	No.4	Mean	SD	RSD(%)
k_1	2.008	2.271	3.131	2.066	2.369	0.520	21.965
k_2	0.130	0.112	0.074	0.134	0.112	0.028	24.688
m	0.450	0.450	0.450	0.450	0.450	0.000	0.000

Number of dissolution data points (N), degrees of freedom (df), and selected goodness of fit criteria – Pearson correlation coefficient (R), coefficient of determination (R^2), adjusted coefficient of determination ($R^2_{adjusted}$), and residual sum of squares (RSS) (manual calculation in MS Excel):

Parameter	No.1	No.2	No.3	No.4
N	16	16	16	16
df	13	13	13	13
R	0.999671822	0.999512744	0.99876202	0.999799055
R^2	0.999343752	0.999025726	0.997525573	0.999598151
$R^2_{adjusted}$	0.999242791	0.998875838	0.997144892	0.999536328
RSS	3.21327903	4.63422587	12.90715913	2.066819555

Graphical abstract of model fit presented as mean ± 1 SD of the fraction % of released carvedilol:

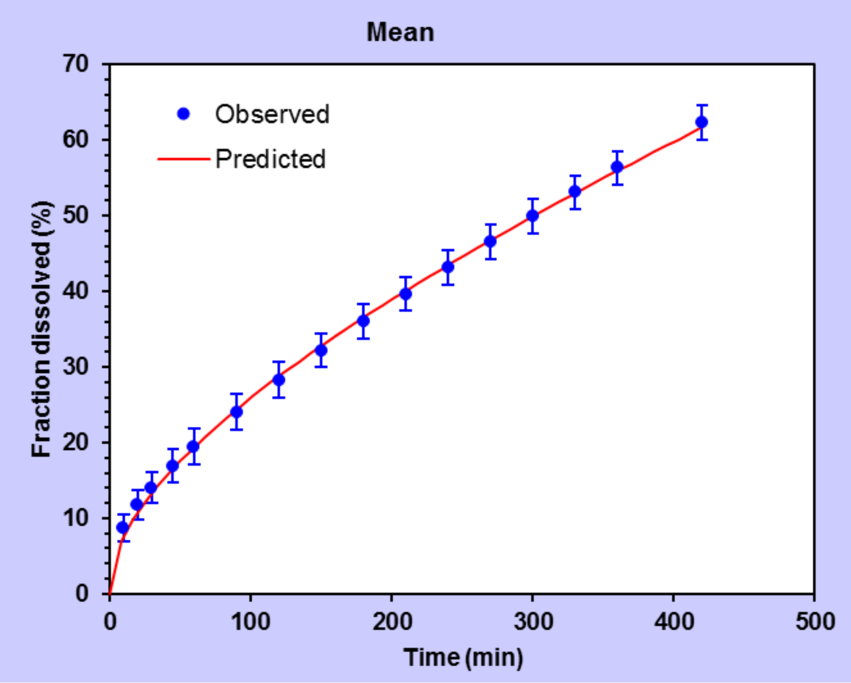

Supplementary materials – Model fitting summary – FlowLac® 100 (modelling up to app. 60 % of carvedilol released)

Graphical abstract of model fit presented as the fraction % of released carvedilol per tested tablet:

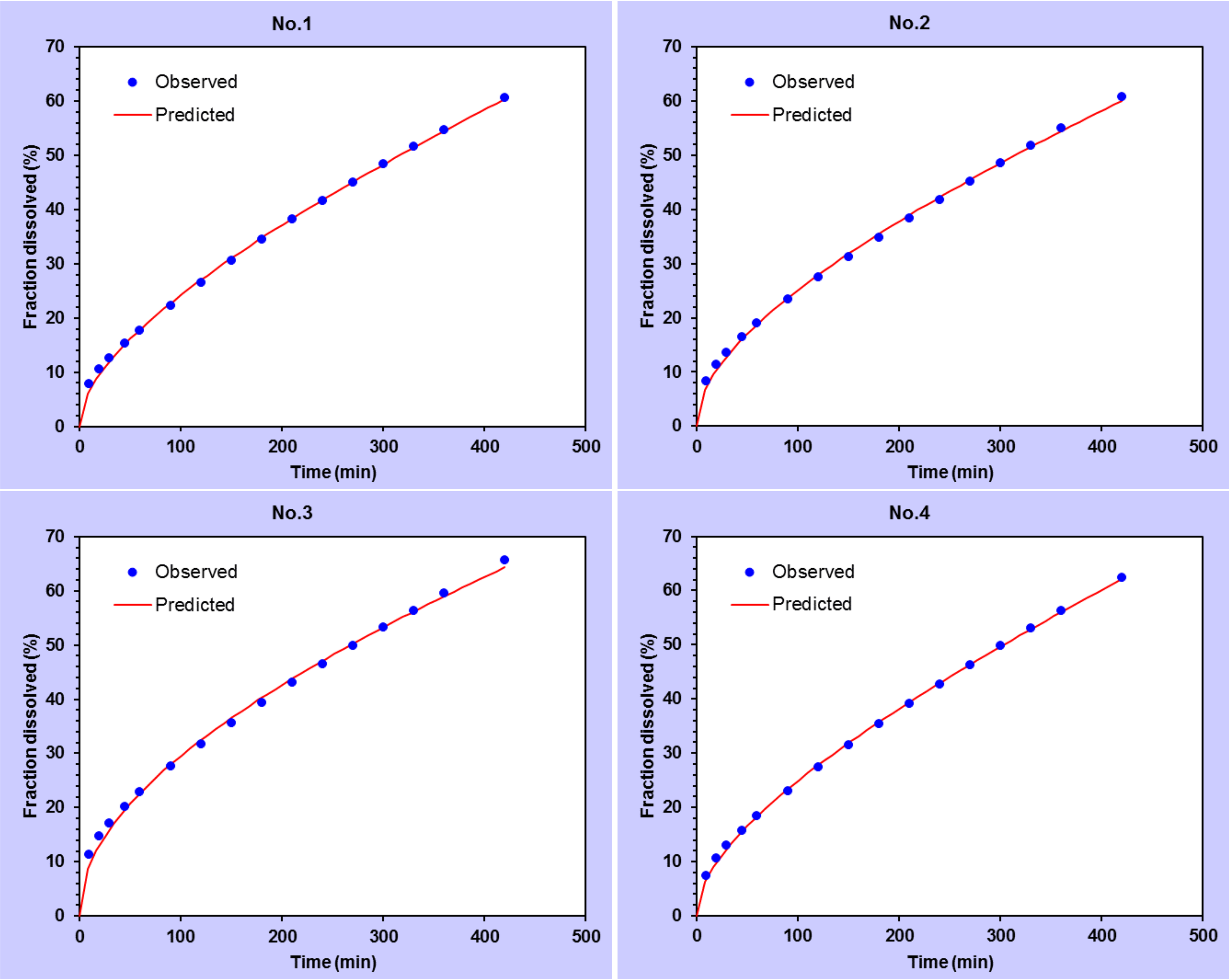

Supplementary materials – Model fitting summary – FlowLac® 100 (modelling up to app. 60 % of carvedilol released)

Model: **Peppas–Sahlin_1 with T_{lag}**

Model equation: $F = k_1 \cdot (t - T_{lag})^m + k_2 \cdot (t - T_{lag})^{2m}$

Fitted model parameters per tested tablet (N = 4) with statistics – mean, standard deviation (SD), and relative standard deviation expressed in % (RSD%) (output from DDSolver):

Parameter	No.1	No.2	No.3	No.4	Mean	SD	RSD(%)
k_1	2.211	2.480	3.366	2.278	2.584	0.534	20.660
k_2	0.117	0.098	0.058	0.120	0.098	0.029	29.310
m	0.450	0.450	0.450	0.450	0.450	0.000	0.000
T_{lag}	4.000	4.000	4.000	4.000	4.000	0.000	0.000

Number of dissolution data points (N), degrees of freedom (df), and selected goodness of fit criteria – Pearson correlation coefficient (R), coefficient of determination (R^2), adjusted coefficient of determination ($R^2_{adjusted}$), and residual sum of squares (RSS) (manual calculation in MS Excel):

Parameter	No.1	No.2	No.3	No.4
N	16	16	16	16
df	12	12	12	12
R	0.999021649	0.99873568	0.997361941	0.999296562
R^2	0.998044255	0.997472958	0.994730842	0.998593619
$R^2_{adjusted}$	0.997555319	0.996841198	0.993413552	0.998242024
RSS	10.1065421	12.75143325	29.18839667	7.73559815

Graphical abstract of model fit presented as mean ± 1 SD of the fraction % of released carvedilol:

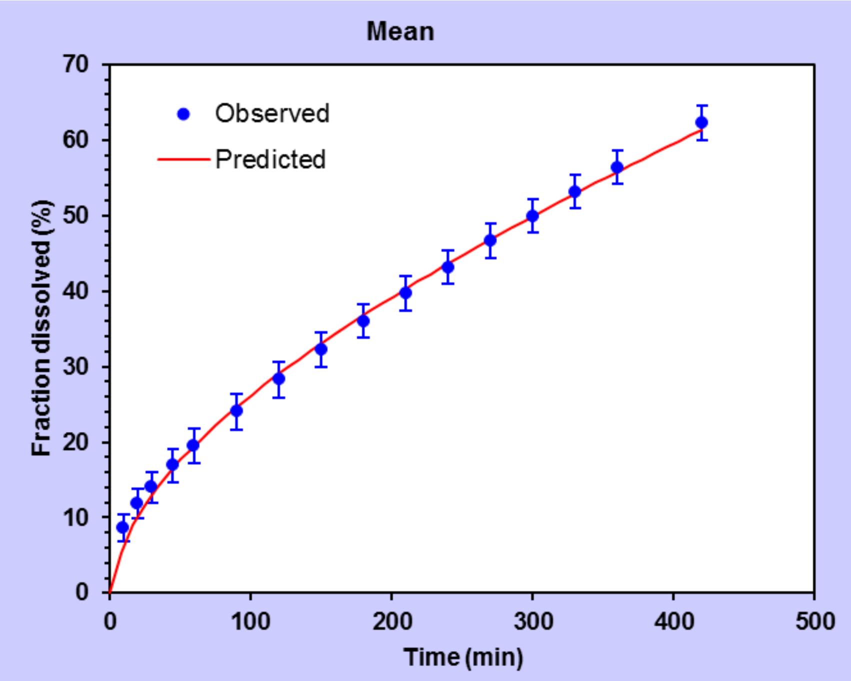

Supplementary materials – Model fitting summary – FlowLac® 100 (modelling up to app. 60 % of carvedilol released)

Graphical abstract of model fit presented as the fraction % of released carvedilol per tested tablet:

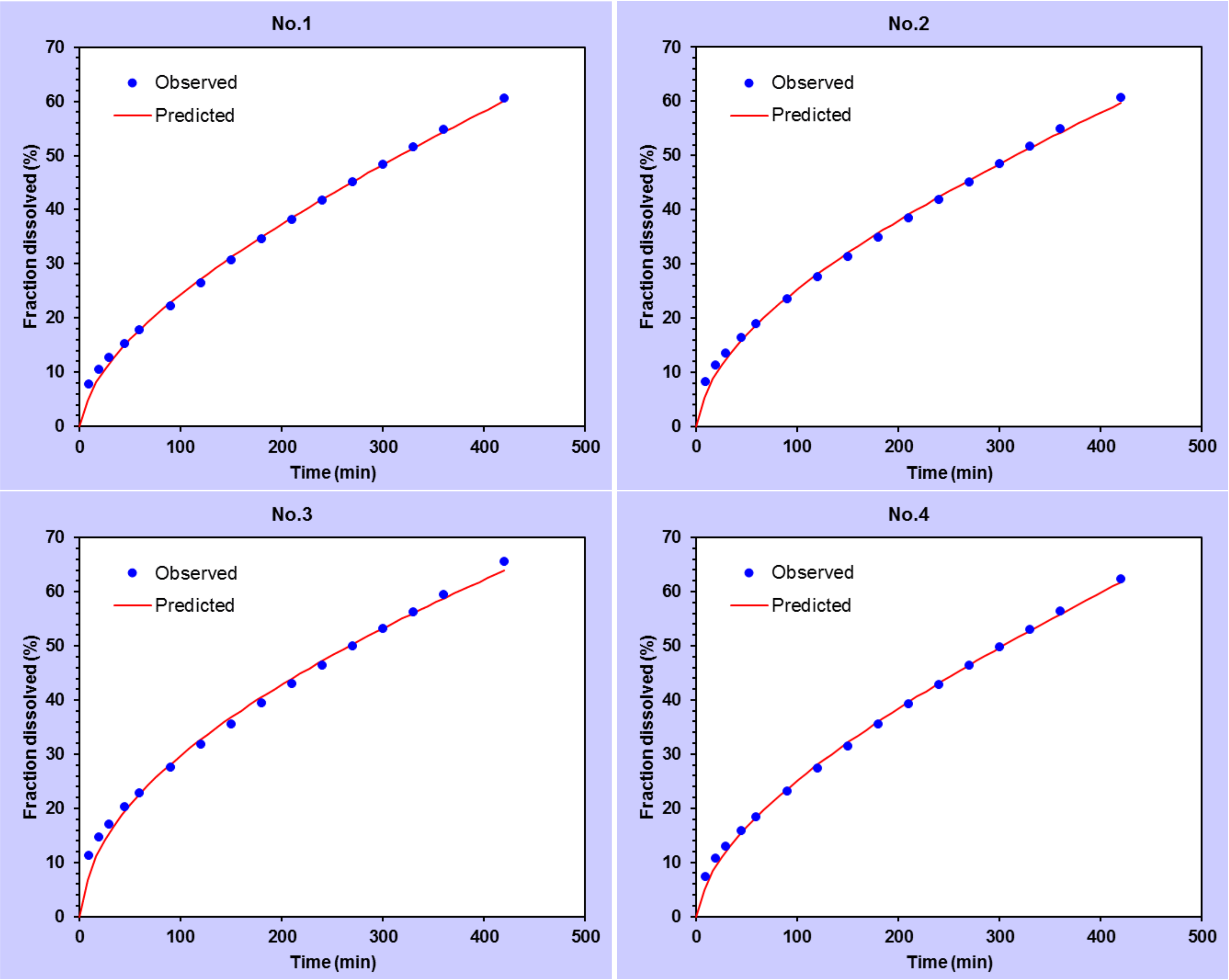

Supplementary materials – Model fitting summary – FlowLac® 100 (modelling up to app. 60 % of carvedilol released)

Model: **Peppas–Sahlin_2**

Model equation: $F = k_1 \cdot t^{0.5} + k_2 \cdot t$

Fitted model parameters per tested tablet (N = 4) with statistics – mean, standard deviation (SD), and relative standard deviation expressed in % (RSD%) (output from DDSolver):

Parameter	No.1	No.2	No.3	No.4	Mean	SD	RSD(%)
k_1	1.914	2.108	2.781	1.971	2.194	0.400	18.228
k_2	0.050	0.040	0.017	0.052	0.040	0.016	40.219

Number of dissolution data points (N), degrees of freedom (df), and selected goodness of fit criteria – Pearson correlation coefficient (R), coefficient of determination (R^2), adjusted coefficient of determination ($R^2_{adjusted}$), and residual sum of squares (RSS) (manual calculation in MS Excel):

Parameter	No.1	No.2	No.3	No.4
N	16	16	16	16
df	14	14	14	14
R	0.99963141	0.99941478	0.998357255	0.99976995
R^2	0.999262956	0.998829903	0.996717209	0.999539952
$R^2_{adjusted}$	0.99921031	0.998746325	0.996482724	0.999507092
RSS	3.78060593	5.925886224	18.37674777	2.491999949

Graphical abstract of model fit presented as mean ± 1 SD of the fraction % of released carvedilol:

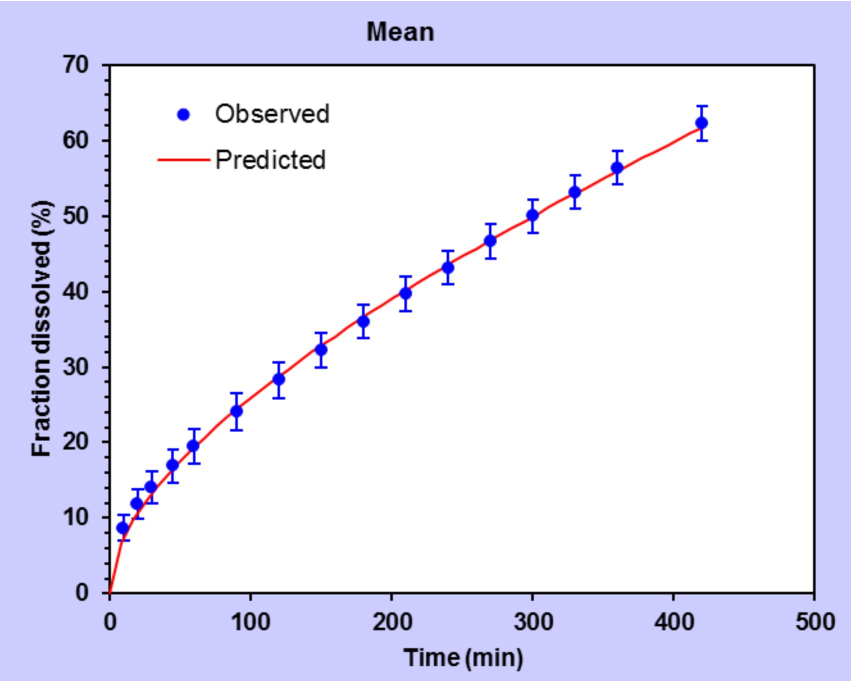

Supplementary materials – Model fitting summary – FlowLac® 100 (modelling up to app. 60 % of carvedilol released)

Graphical abstract of model fit presented as the fraction % of released carvedilol per tested tablet:

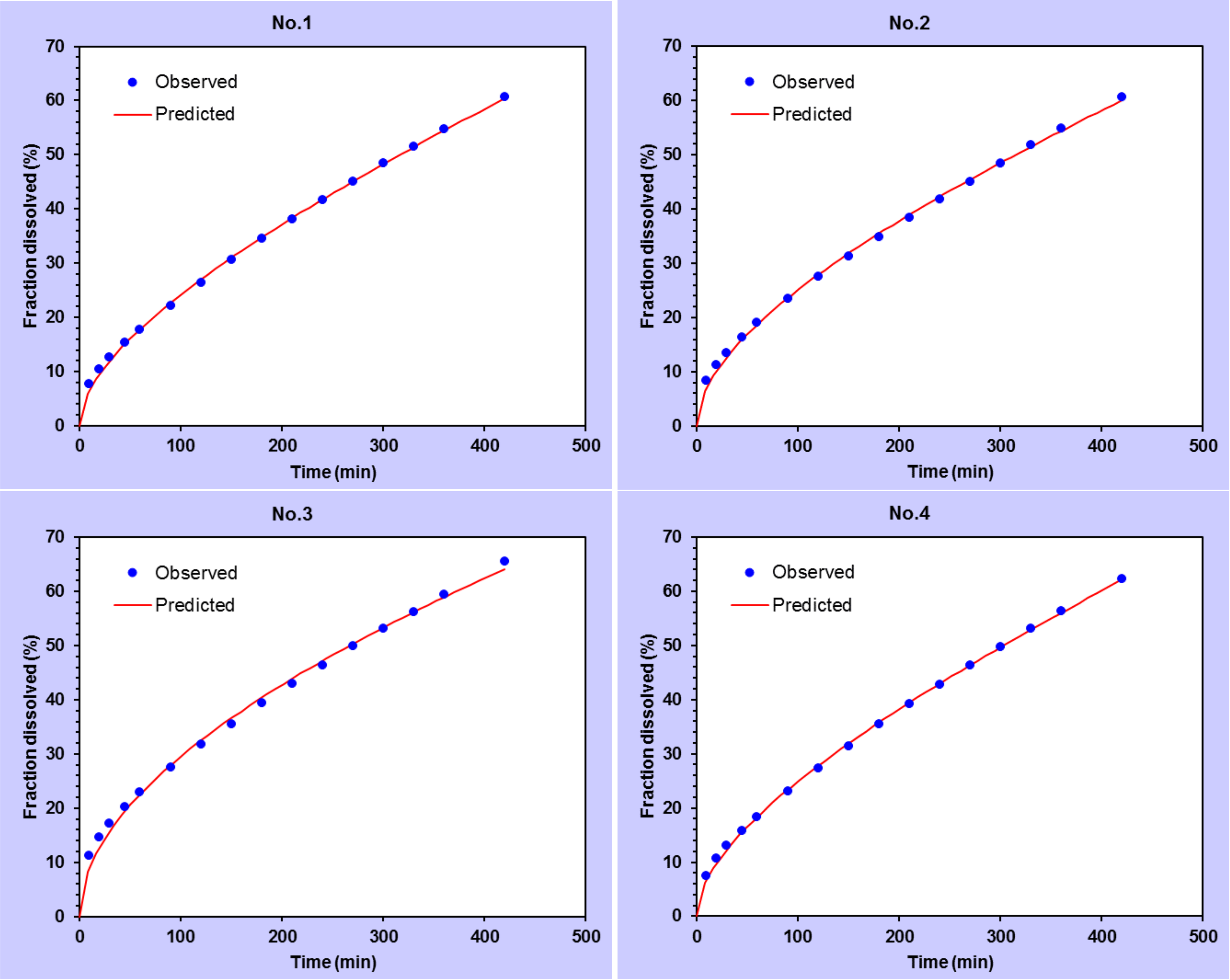

Supplementary materials – Model fitting summary – FlowLac® 100 (modelling up to app. 60 % of carvedilol released)

Model: **Peppas–Sahlin_2 with T_{lag}**

Model equation: $F = k_1 \cdot (t - T_{lag})^{0.5} + k_2 \cdot (t - T_{lag})$

Fitted model parameters per tested tablet (N = 4) with statistics – mean, standard deviation (SD), and relative standard deviation expressed in % (RSD%) (output from DDSolver):

Parameter	No.1	No.2	No.3	No.4	Mean	SD	RSD(%)
k_1	2.062	2.260	2.950	2.125	2.349	0.409	17.406
k_2	0.043	0.033	0.009	0.044	0.032	0.017	51.356
T_{lag}	4.000	4.000	4.000	4.000	4.000	0.000	0.000

Number of dissolution data points (N), degrees of freedom (df), and selected goodness of fit criteria – Pearson correlation coefficient (R), coefficient of determination (R^2), adjusted coefficient of determination ($R^2_{adjusted}$), and residual sum of squares (RSS) (manual calculation in MS Excel):

Parameter	No.1	No.2	No.3	No.4
N	16	16	16	16
df	13	13	13	13
R	0.998910881	0.998522226	0.99673842	0.999195781
R^2	0.997822948	0.997046635	0.993487478	0.998392209
$R^2_{adjusted}$	0.997488016	0.996592271	0.992485551	0.998144857
RSS	11.97315214	16.0510272	39.22646779	9.45461358

Graphical abstract of model fit presented as mean ± 1 SD of the fraction % of released carvedilol:

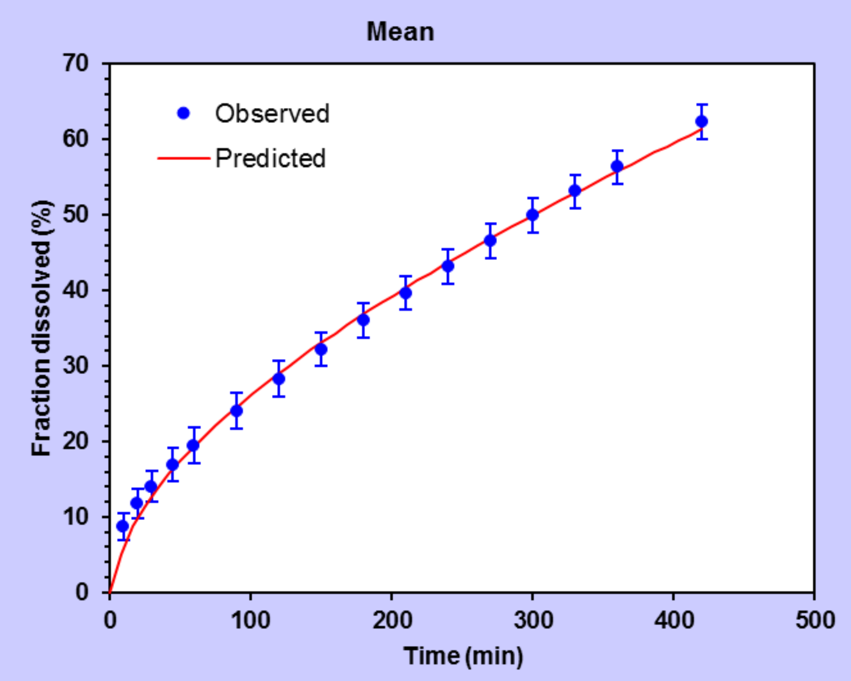

Supplementary materials – Model fitting summary – FlowLac® 100 (modelling up to app. 60 % of carvedilol released)

Graphical abstract of model fit presented as the fraction % of released carvedilol per tested tablet:

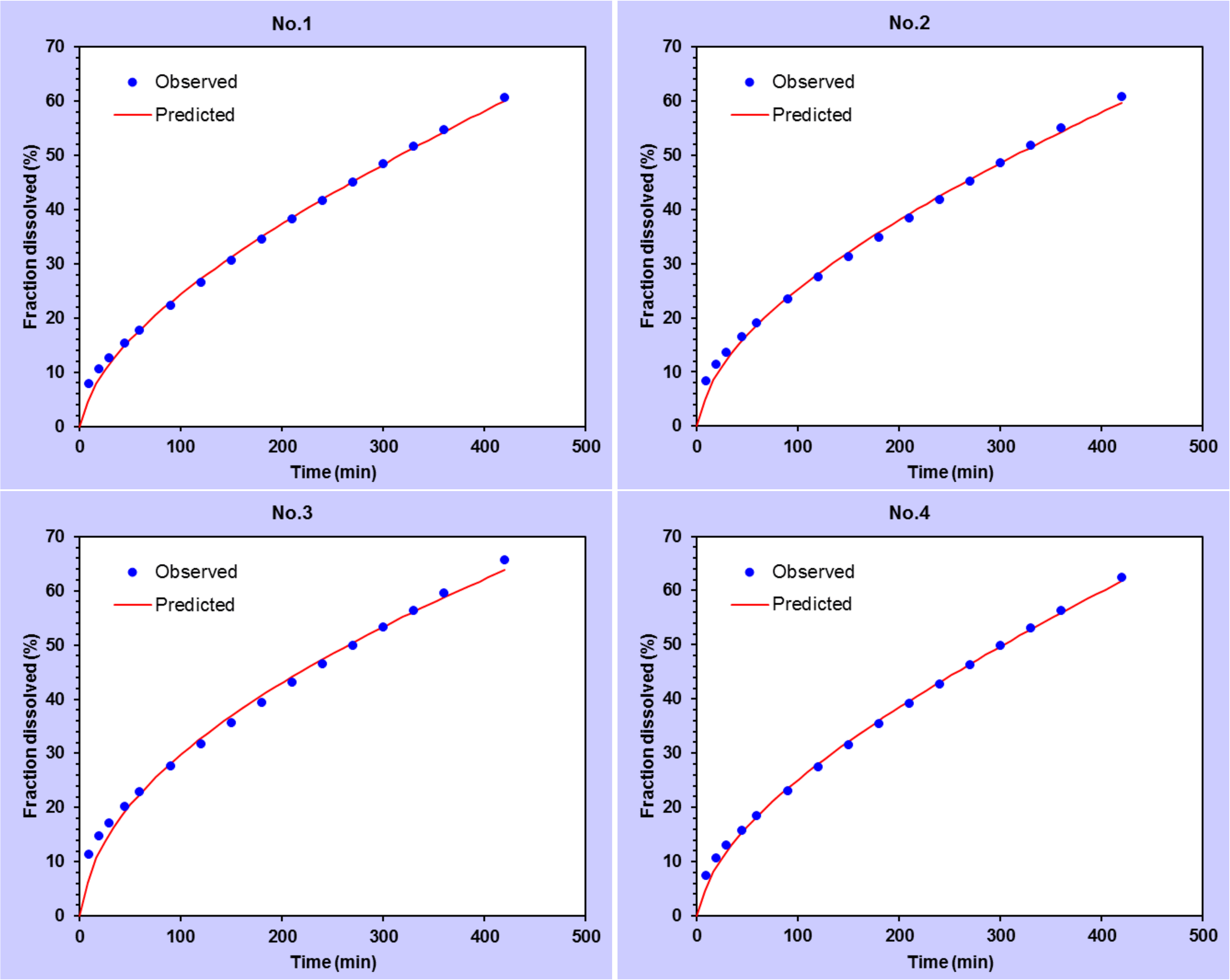

Supplementary materials – Model fitting summary – FlowLac® 100 (modelling up to app. 60 % of carvedilol released)

Model: **Quadratic**

Model equation: $F = 100 \cdot (k_1 \cdot t^2 + k_2 \cdot t)$

Fitted model parameters per tested tablet (N = 4) with statistics – mean, standard deviation (SD), and relative standard deviation expressed in % (RSD%) (output from DDSolver):

Parameter	No.1	No.2	No.3	No.4	Mean	SD	RSD(%)
k_1	-0.000003	-0.000003	-0.000004	-0.000003	-0.000003	0.000000	-16.346553
k_2	0.002437	0.002511	0.002937	0.002509	0.002598	0.000228	8.779716

Number of dissolution data points (N), degrees of freedom (df), and selected goodness of fit criteria – Pearson correlation coefficient (R), coefficient of determination (R^2), adjusted coefficient of determination ($R^2_{adjusted}$), and residual sum of squares (RSS) (manual calculation in MS Excel):

Parameter	No.1	No.2	No.3	No.4
N	16	16	16	16
df	14	14	14	14
R	0.993971184	0.991633645	0.986981482	0.993880751
R^2	0.987978714	0.983337286	0.974132447	0.987798947
$R^2_{adjusted}$	0.987120051	0.982147092	0.972284764	0.986927444
RSS	166.1631192	221.8079793	418.878333	173.0252614

Graphical abstract of model fit presented as mean ± 1 SD of the fraction % of released carvedilol:

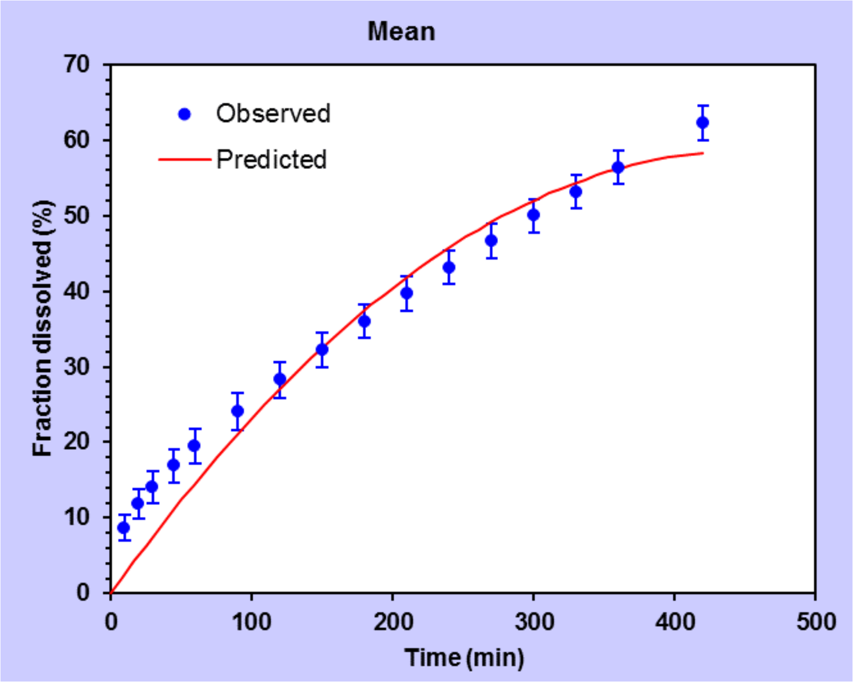

Supplementary materials – Model fitting summary – FlowLac® 100 (modelling up to app. 60 % of carvedilol released)

Graphical abstract of model fit presented as the fraction % of released carvedilol per tested tablet:

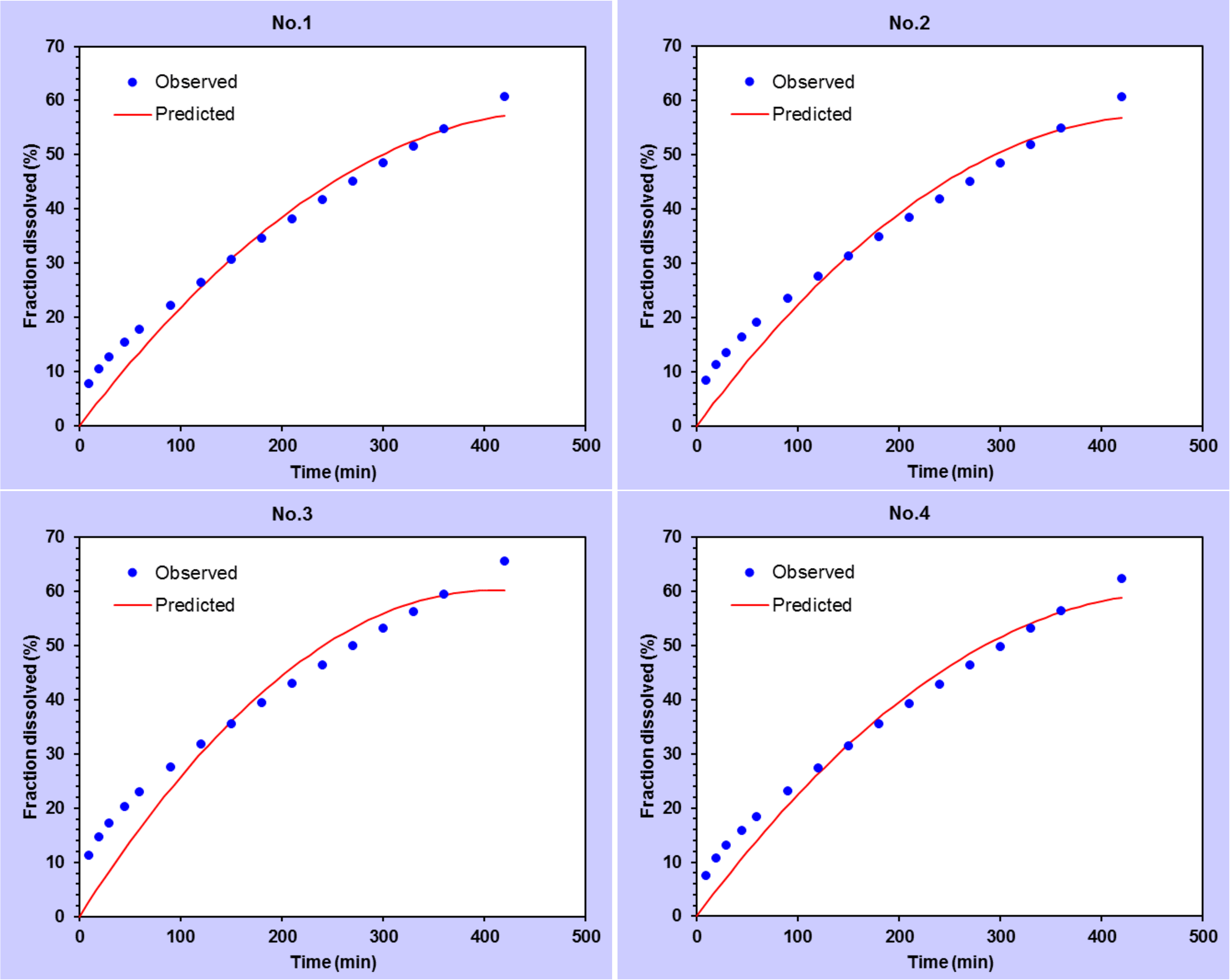

Supplementary materials – Model fitting summary – FlowLac® 100 (modelling up to app. 60 % of carvedilol released)

Model: **Quadratic with T_{lag}**

Model equation: $F = 100 \cdot \left[k_1 \cdot (t - T_{lag})^2 + k_2 \cdot (t - T_{lag})\right]$

Fitted model parameters per tested tablet (N = 4) with statistics – mean, standard deviation (SD), and relative standard deviation expressed in % (RSD%) (output from DDSolver):

Parameter	No.1	No.2	No.3	No.4	Mean	SD	RSD(%)
k_1	0.000	0.000	0.000	0.000	0.000	0.000	-15.677
k_2	0.002	0.003	0.003	0.003	0.003	0.000	8.612
T_{lag}	4.000	4.000	4.000	4.000	4.000	0.000	0.000

Number of dissolution data points (N), degrees of freedom (df), and selected goodness of fit criteria – Pearson correlation coefficient (R), coefficient of determination (R^2), adjusted coefficient of determination ($R^2_{adjusted}$), and residual sum of squares (RSS) (manual calculation in MS Excel):

Parameter	No.1	No.2	No.3	No.4
N	16	16	16	16
df	13	13	13	13
R	0.99308311	0.990686068	0.985980856	0.99300131
R^2	0.986214062	0.981458885	0.972158249	0.986051602
$R^2_{adjusted}$	0.984093149	0.978606406	0.967874903	0.983905695
RSS	213.5883563	277.4373453	507.8665337	222.4827091

Graphical abstract of model fit presented as mean ± 1 SD of the fraction % of released carvedilol:

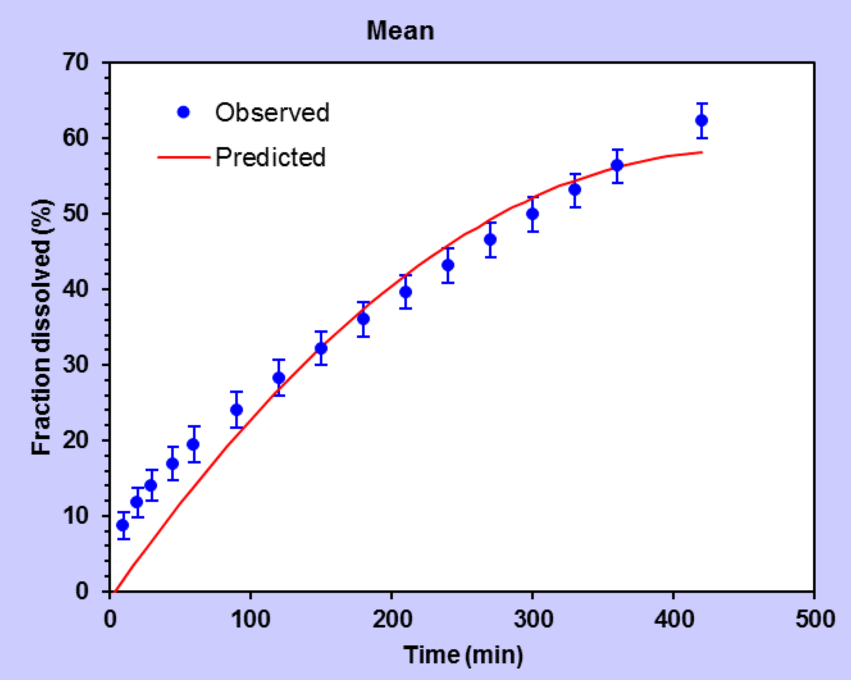

Supplementary materials – Model fitting summary – FlowLac® 100 (modelling up to app. 60 % of carvedilol released)

Graphical abstract of model fit presented as the fraction % of released carvedilol per tested tablet:

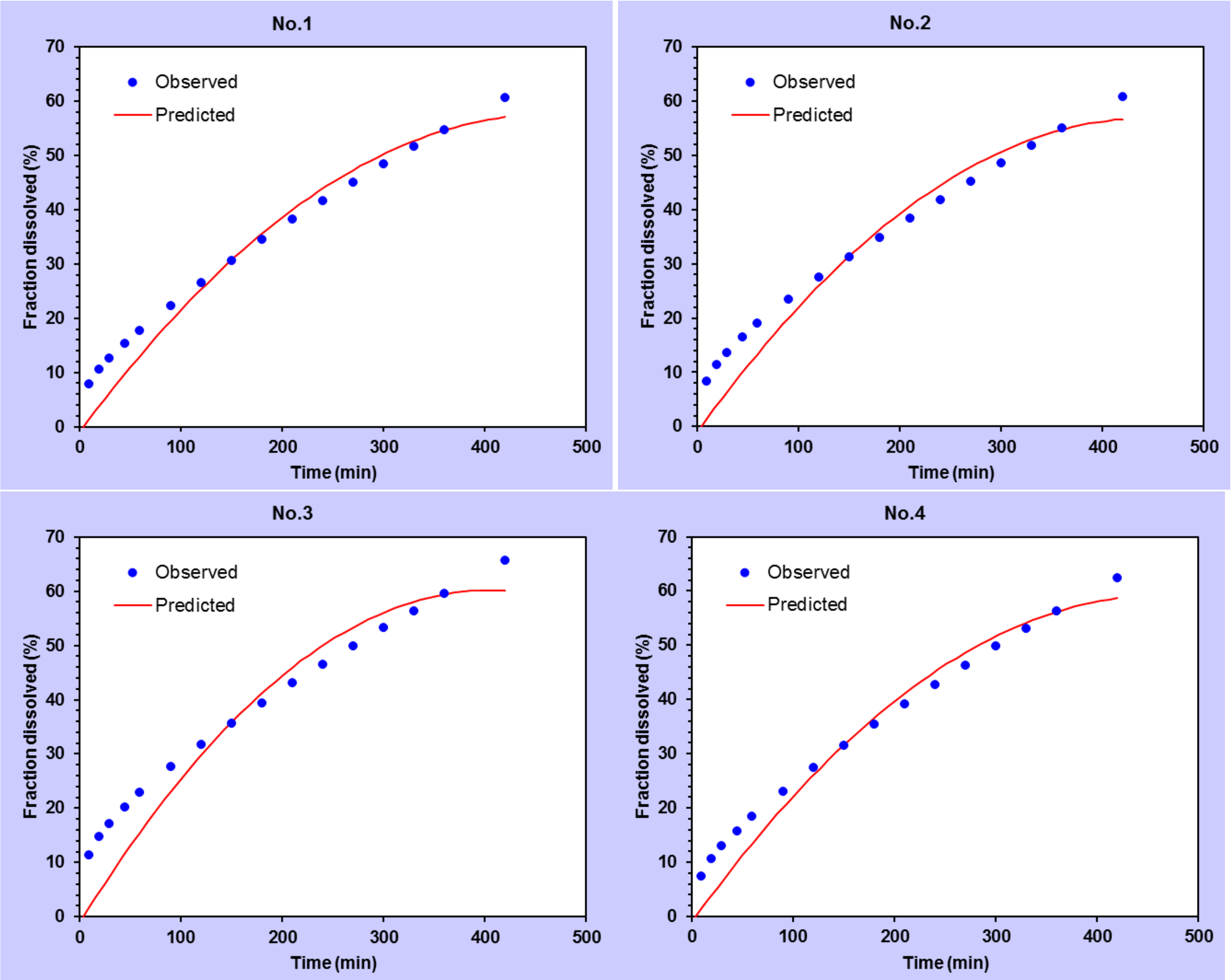

Supplementary materials – Model fitting summary – FlowLac® 100 (modelling up to app. 60 % of carvedilol released)

Model: **Weibull_1**

Model equation: $F = 100 \cdot \left[1 - e^{-\frac{(t-T_i)^{\beta}}{\alpha}}\right]$

Fitted model parameters per tested tablet (N = 4) with statistics – mean, standard deviation (SD), and relative standard deviation expressed in % (RSD%) (output from DDSolver):

Parameter	No.1	No.2	No.3	No.4	Mean	SD	RSD(%)
α	48.612	42.219	28.010	50.263	42.276	10.124	23.946
β	0.598	0.575	0.526	0.612	0.578	0.037	6.484
T_i	4.000	4.000	4.000	4.000	4.000	0.000	0.000

Number of dissolution data points (N), degrees of freedom (df), and selected goodness of fit criteria – Pearson correlation coefficient (R), coefficient of determination (R^2), adjusted coefficient of determination ($R^2_{adjusted}$), and residual sum of squares (RSS) (manual calculation in MS Excel):

Parameter	No.1	No.2	No.3	No.4
N	16	16	16	16
df	13	13	13	13
R	0.987565048	0.987005105	0.983750614	0.988620312
R^2	0.975284724	0.974179077	0.967765271	0.97737012
$R^2_{adjusted}$	0.971482374	0.970206628	0.962806082	0.9738886
RSS	148.1086679	141.0594976	174.5286503	140.3197065

Graphical abstract of model fit presented as mean ± 1 SD of the fraction % of released carvedilol:

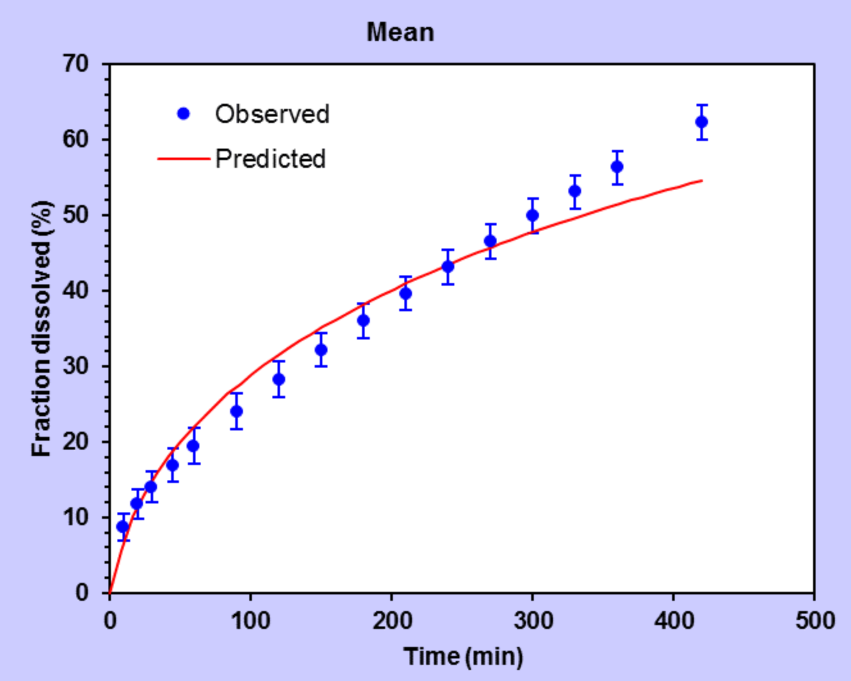

Supplementary materials – Model fitting summary – FlowLac® 100 (modelling up to app. 60 % of carvedilol released)

Graphical abstract of model fit presented as the fraction % of released carvedilol per tested tablet:

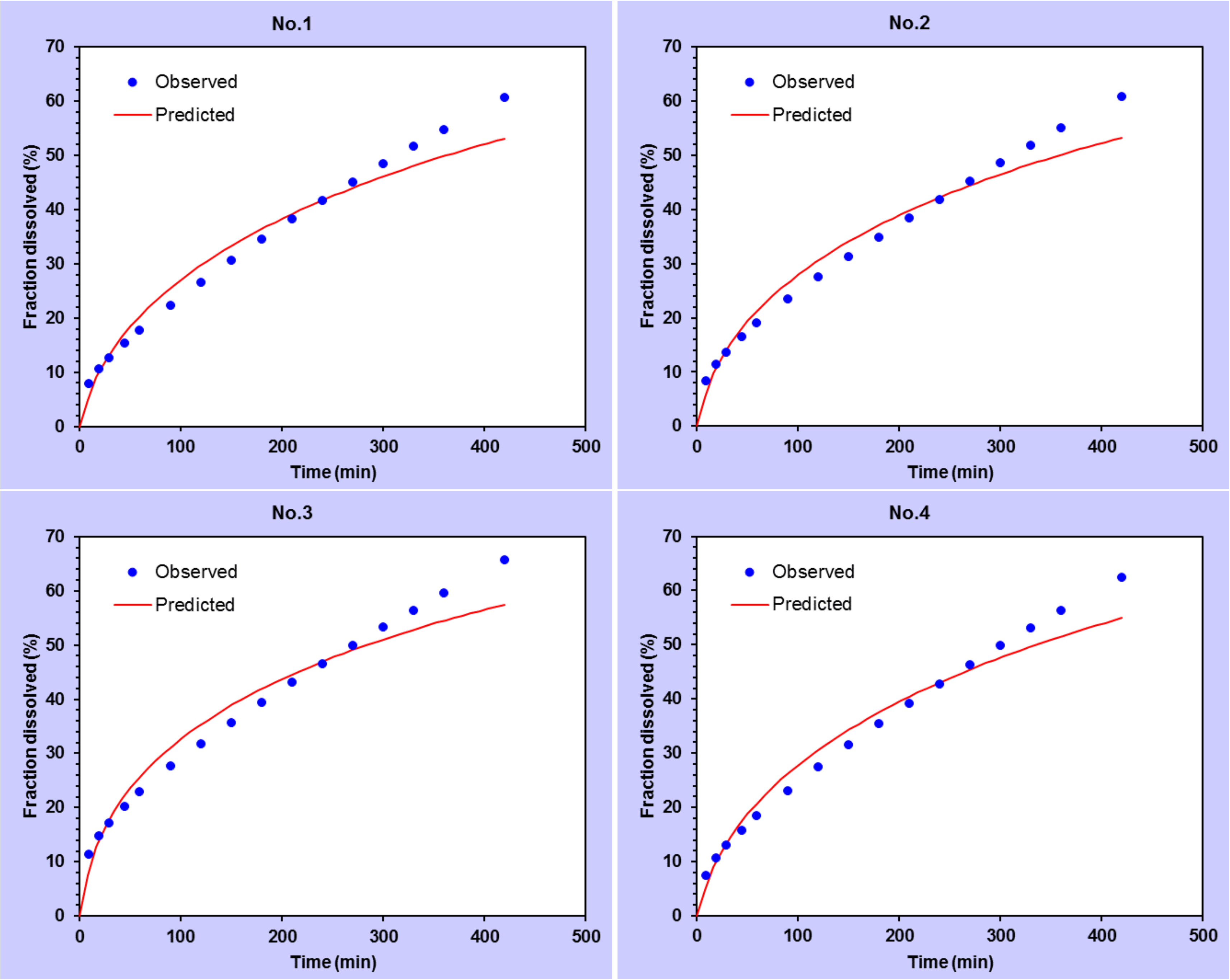

Supplementary materials – Model fitting summary – FlowLac® 100 (modelling up to app. 60 % of carvedilol released)

Model: **Weibull_2**

Model equation: $F = 100 \cdot \left(1 - e^{-\frac{t^{\beta}}{\alpha}}\right)$

Fitted model parameters per tested tablet (N = 4) with statistics – mean, standard deviation (SD), and relative standard deviation expressed in % (RSD%) (output from DDSolver):

Parameter	No.1	No.2	No.3	No.4	Mean	SD	RSD(%)
α	69.239	59.206	38.275	71.880	59.650	15.260	25.582
β	0.663	0.637	0.583	0.677	0.640	0.041	6.434

Number of dissolution data points (N), degrees of freedom (df), and selected goodness of fit criteria – Pearson correlation coefficient (R), coefficient of determination (R^2), adjusted coefficient of determination ($R^2_{adjusted}$), and residual sum of squares (RSS) (manual calculation in MS Excel):

Parameter	No.1	No.2	No.3	No.4
N	16	16	16	16
df	14	14	14	14
R	0.992147033	0.991546946	0.989018716	0.99279314
R^2	0.984355736	0.983165346	0.978158021	0.985638218
$R^2_{adjusted}$	0.983238289	0.98196287	0.976597879	0.984612377
RSS	89.45960793	87.41880271	114.0242517	84.10694651

Graphical abstract of model fit presented as mean ± 1 SD of the fraction % of released carvedilol:

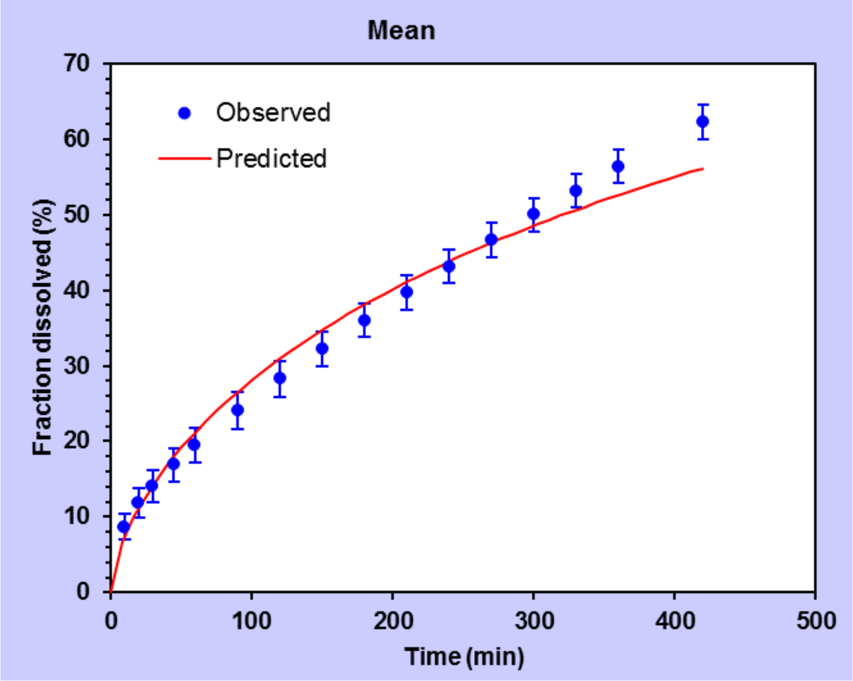

Supplementary materials – Model fitting summary – FlowLac® 100 (modelling up to app. 60 % of carvedilol released)

Graphical abstract of model fit presented as the fraction % of released carvedilol per tested tablet:

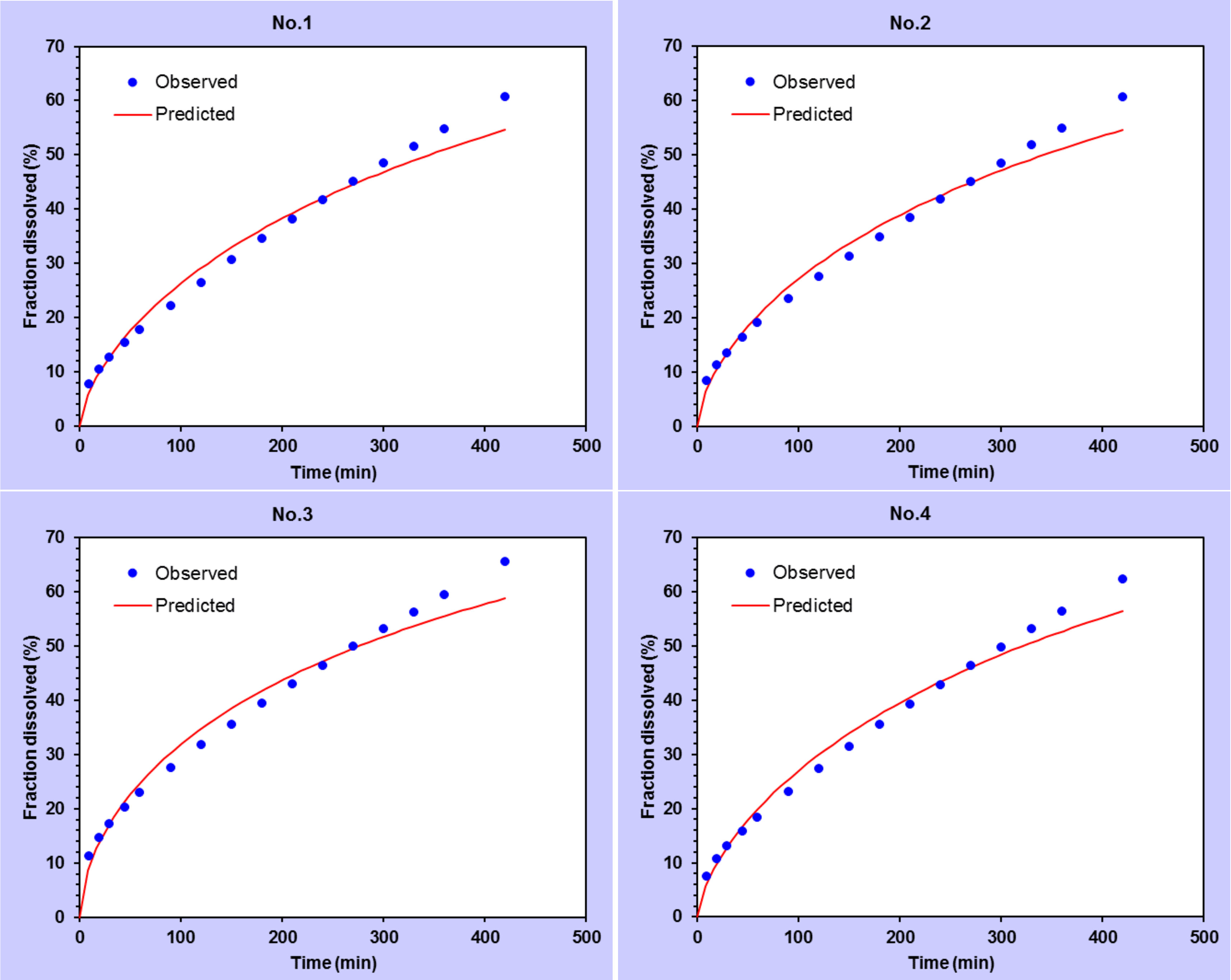

Supplementary materials – Model fitting summary – FlowLac® 100 (modelling up to app. 60 % of carvedilol released)

Model: **Weibull_3**

Model equation: $F = F_{max} \cdot \left(1 - e^{-\frac{t^\beta}{\alpha}}\right)$

Fitted model parameters per tested tablet (N = 4) with statistics – mean, standard deviation (SD), and relative standard deviation expressed in % (RSD%) (output from DDSolver):

Parameter	No.1	No.2	No.3	No.4	Mean	SD	RSD(%)
α	66.900	56.914	38.089	70.650	58.138	14.569	25.060
β	0.794	0.767	0.702	0.804	0.767	0.046	5.990
F_{max}	63.635	63.739	68.834	65.408	65.404	2.427	3.710

Number of dissolution data points (N), degrees of freedom (df), and selected goodness of fit criteria – Pearson correlation coefficient (R), coefficient of determination (R^2), adjusted coefficient of determination ($R^2_{adjusted}$), and residual sum of squares (RSS) (manual calculation in MS Excel):

Parameter	No.1	No.2	No.3	No.4
N	16	16	16	16
df	13	13	13	13
R	0.981342469	0.980387893	0.978025397	0.982435398
R^2	0.963033041	0.961160421	0.956533676	0.965179311
$R^2_{adjusted}$	0.957345816	0.955185102	0.94984655	0.959822282
RSS	175.43183	173.8433377	203.5283728	174.139167

Graphical abstract of model fit presented as mean ± 1 SD of the fraction % of released carvedilol:

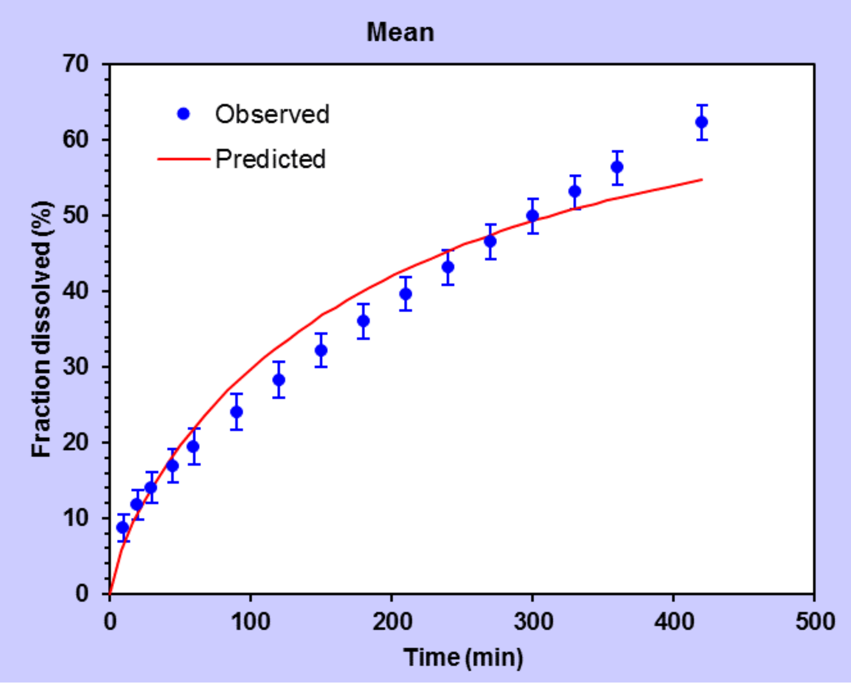

Supplementary materials – Model fitting summary – FlowLac® 100 (modelling up to app. 60 % of carvedilol released)

Graphical abstract of model fit presented as the fraction % of released carvedilol per tested tablet:

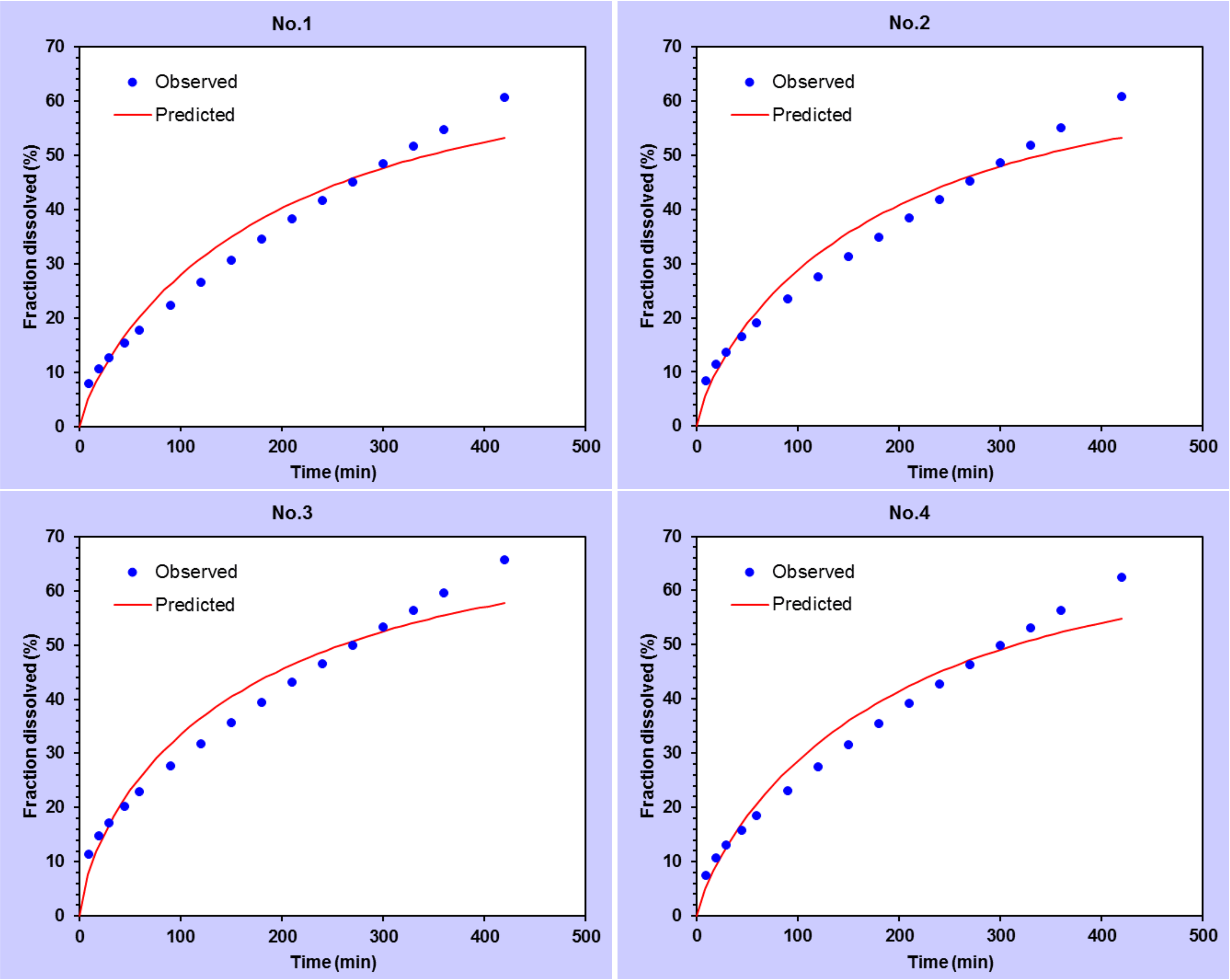

Supplementary materials – Model fitting summary – FlowLac® 100 (modelling up to app. 60 % of carvedilol released)

Model: **Weibull_4**

Model equation: $F = F_{max} \cdot \left[1 - e^{-\frac{(t-T_i)^\beta}{\alpha}}\right]$

Fitted model parameters per tested tablet (N = 4) with statistics – mean, standard deviation (SD), and relative standard deviation expressed in % (RSD%) (output from DDSolver):

Parameter	No.1	No.2	No.3	No.4	Mean	SD	RSD(%)
α	43.343	37.478	25.924	45.693	38.109	8.828	23.165
β	0.714	0.690	0.632	0.725	0.690	0.042	6.035
T_i	6.000	6.000	4.000	6.000	5.500	1.000	18.182
F_{max}	63.635	63.739	68.834	65.408	65.404	2.427	3.710

Number of dissolution data points (N), degrees of freedom (df), and selected goodness of fit criteria – Pearson correlation coefficient (R), coefficient of determination (R^2), adjusted coefficient of determination ($R^2_{adjusted}$), and residual sum of squares (RSS) (manual calculation in MS Excel):

Parameter	No.1	No.2	No.3	No.4
N	16	16	16	16
df	12	12	12	12
R	0.974469359	0.973453763	0.971786182	0.976151739
R^2	0.949590531	0.947612229	0.944368384	0.952872218
$R^2_{adjusted}$	0.936988164	0.934515287	0.93046048	0.941090273
RSS	229.926212	225.3785283	264.9275369	226.8690507

Graphical abstract of model fit presented as mean ± 1 SD of the fraction % of released carvedilol:

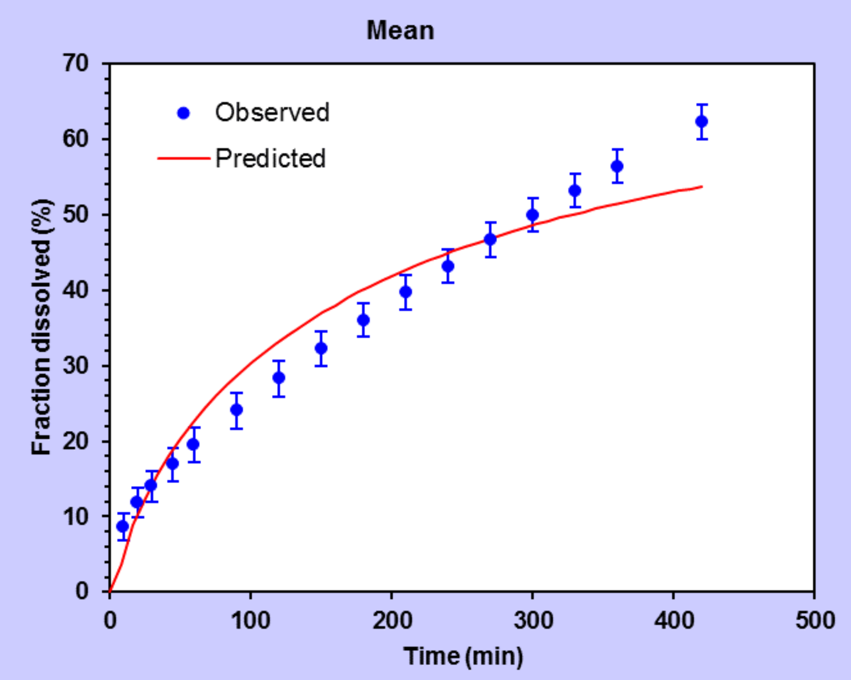

Supplementary materials – Model fitting summary – FlowLac® 100 (modelling up to app. 60 % of carvedilol released)

Graphical abstract of model fit presented as the fraction % of released carvedilol per tested tablet:

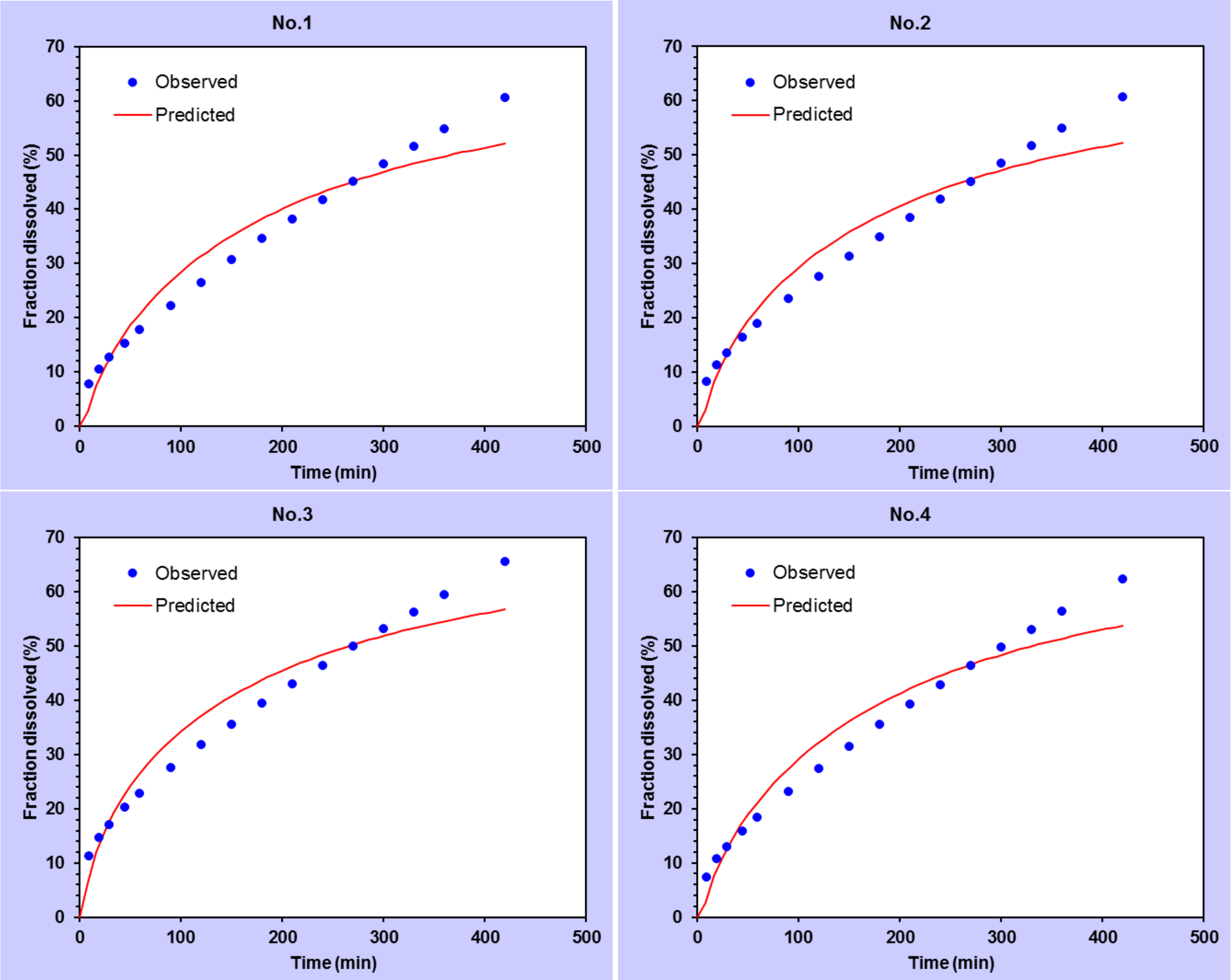

Supplementary materials – Model fitting summary – FlowLac® 100 (modelling up to app. 60 % of carvedilol released)

Model: **Logistic_1**

Model equation: $F = 100 \cdot \frac{e^{\alpha+\beta \cdot \log(t)}}{1+e^{\alpha+\beta \cdot \log(t)}}$

Fitted model parameters per tested tablet (N = 4) with statistics – mean, standard deviation (SD), and relative standard deviation expressed in % (RSD%) (output from DDSolver):

Parameter	No.1	No.2	No.3	No.4	Mean	SD	RSD(%)
α	-4.558	-4.392	-3.980	-4.614	-4.386	0.286	-6.529
β	1.788	1.726	1.635	1.835	1.746	0.087	4.964

Number of dissolution data points (N), degrees of freedom (df), and selected goodness of fit criteria – Pearson correlation coefficient (R), coefficient of determination (R^2), adjusted coefficient of determination ($R^2_{adjusted}$), and residual sum of squares (RSS) (manual calculation in MS Excel):

Parameter	No.1	No.2	No.3	No.4
N	16	16	16	16
df	14	14	14	14
R	0.985892477	0.985213428	0.981447357	0.986407771
R^2	0.971983977	0.970645498	0.963238915	0.973000291
$R^2_{adjusted}$	0.969982833	0.968548748	0.960613123	0.971071741
RSS	143.5020556	139.7055972	177.5474347	143.6693379

Graphical abstract of model fit presented as mean ± 1 SD of the fraction % of released carvedilol:

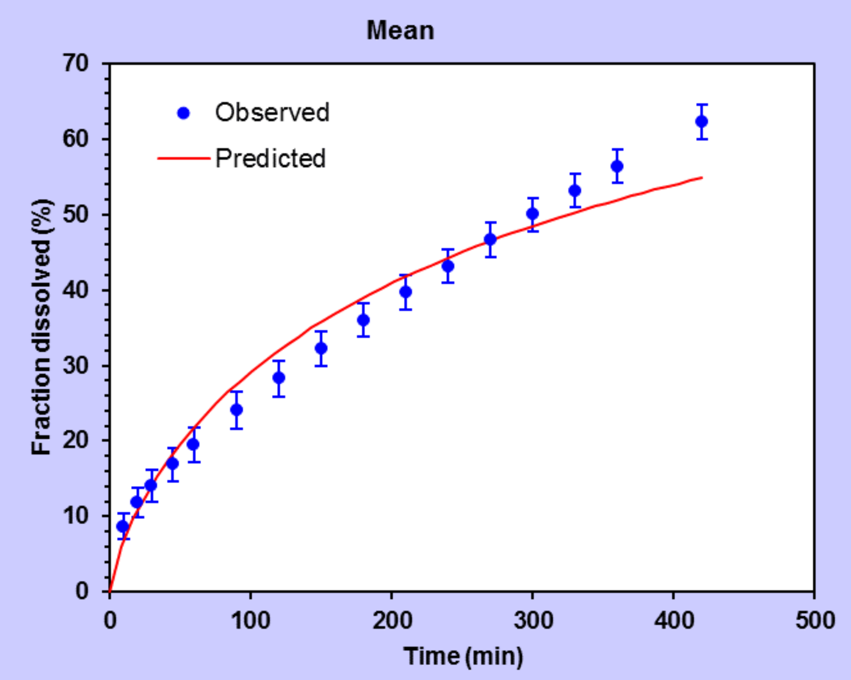

Supplementary materials – Model fitting summary – FlowLac® 100 (modelling up to app. 60 % of carvedilol released)

Graphical abstract of model fit presented as the fraction % of released carvedilol per tested tablet:

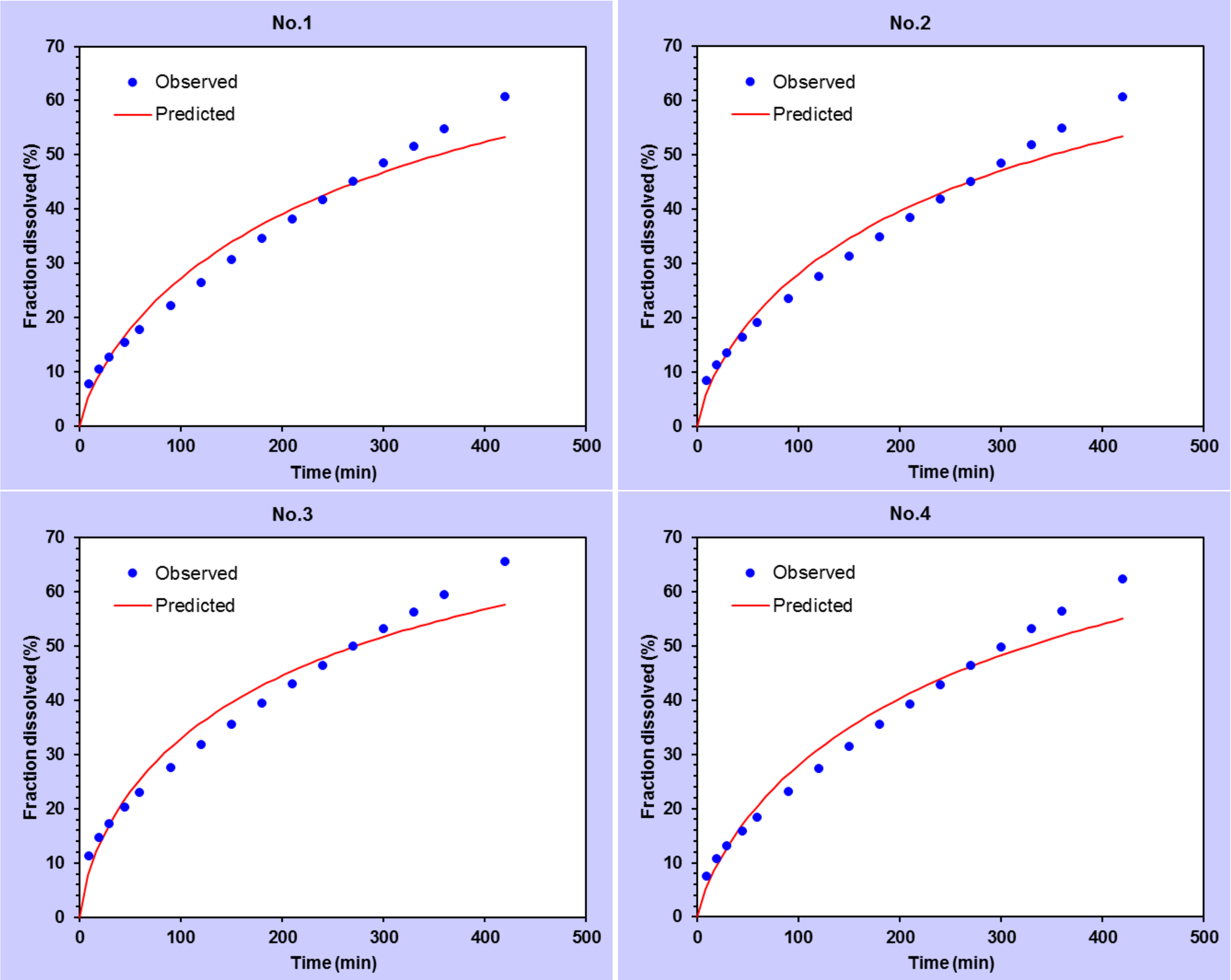

Supplementary materials – Model fitting summary – FlowLac® 100 (modelling up to app. 60 % of carvedilol released)

Model: **Logistic_2**

Model equation: $F = F_{max} \cdot \frac{e^{\alpha+\beta\cdot\log(t)}}{1+e^{\alpha+\beta\cdot\log(t)}}$

Fitted model parameters per tested tablet (N = 4) with statistics – mean, standard deviation (SD), and relative standard deviation expressed in % (RSD%) (output from DDSolver):

Parameter	No.1	No.2	No.3	No.4	Mean	SD	RSD(%)
α	-5.247	-5.069	-4.641	-5.305	-5.065	0.300	-5.932
β	2.587	2.521	2.371	2.614	2.523	0.109	4.305
F_{max}	63.635	63.739	68.834	65.408	65.404	2.427	3.710

Number of dissolution data points (N), degrees of freedom (df), and selected goodness of fit criteria – Pearson correlation coefficient (R), coefficient of determination (R^2), adjusted coefficient of determination ($R^2_{adjusted}$), and residual sum of squares (RSS) (manual calculation in MS Excel):

Parameter	No.1	No.2	No.3	No.4
N	16	16	16	16
df	13	13	13	13
R	0.961742067	0.960867474	0.957810551	0.963241138
R^2	0.924947803	0.923266303	0.917401052	0.927833489
$R^2_{adjusted}$	0.913401311	0.911461119	0.904693522	0.916730949
RSS	363.6387872	355.723446	403.8277424	371.9612195

Graphical abstract of model fit presented as mean ± 1 SD of the fraction % of released carvedilol:

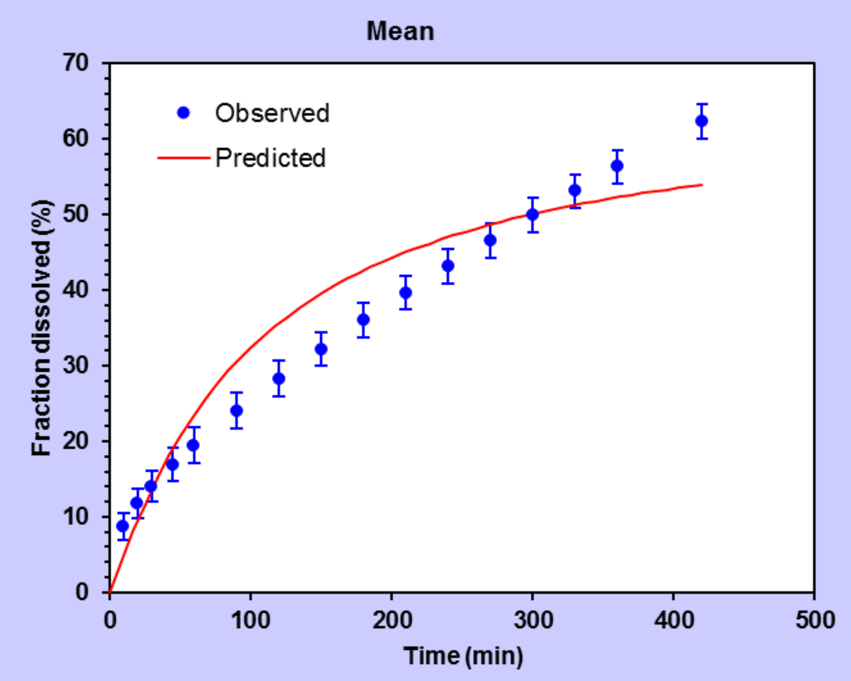

Supplementary materials – Model fitting summary – FlowLac® 100 (modelling up to app. 60 % of carvedilol released)

Graphical abstract of model fit presented as the fraction % of released carvedilol per tested tablet:

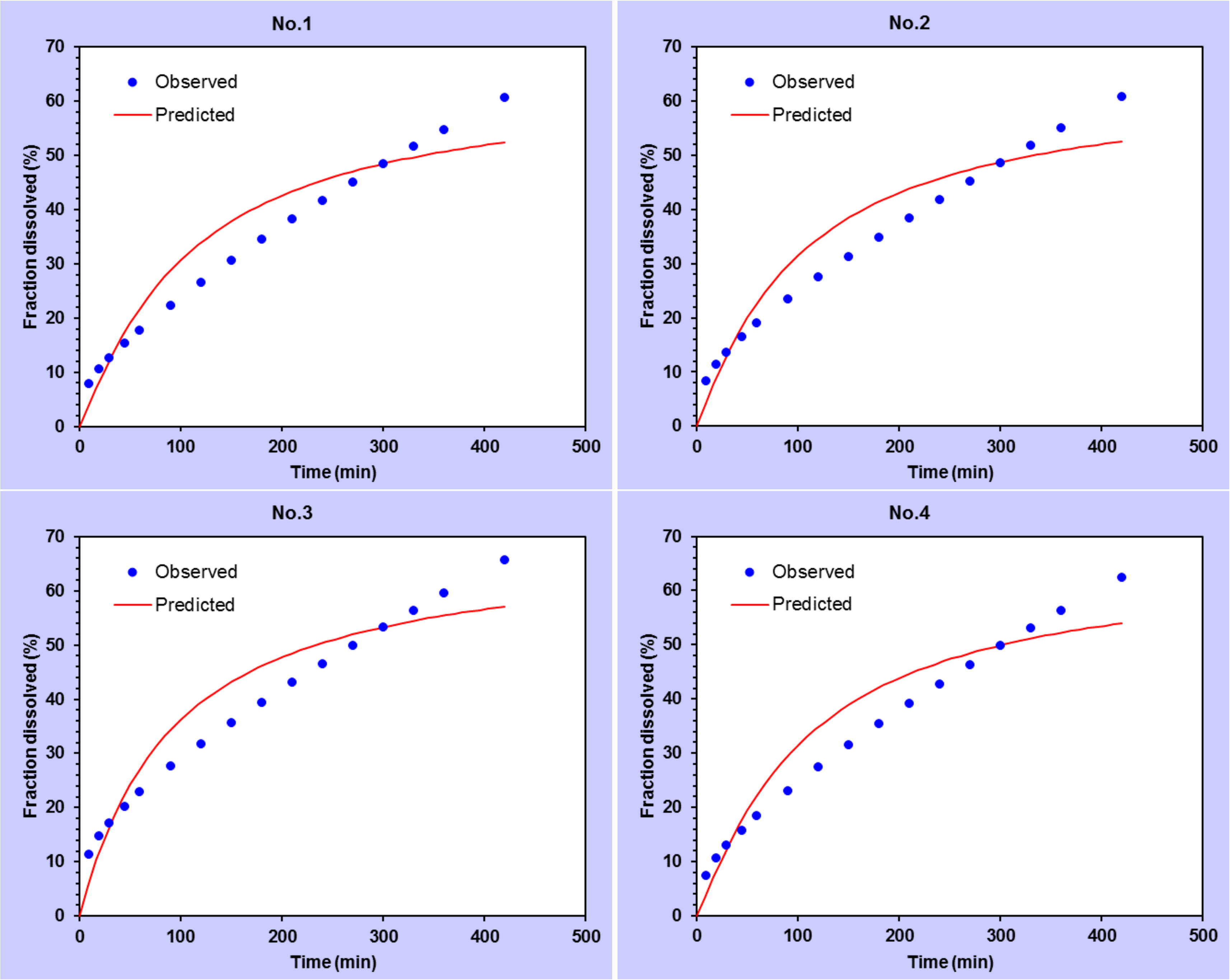

Supplementary materials – Model fitting summary – FlowLac® 100 (modelling up to app. 60 % of carvedilol released)

Model: **Logistic_3**

Model equation: $F = F_{max} \cdot \frac{1}{1+e^{-k\cdot(t-\gamma)}}$

Fitted model parameters per tested tablet (N = 4) with statistics – mean, standard deviation (SD), and relative standard deviation expressed in % (RSD%) (output from DDSolver):

Parameter	No.1	No.2	No.3	No.4	Mean	SD	RSD(%)
k	0.010	0.010	0.009	0.010	0.010	0.000	3.869
γ	168.865	164.452	151.114	169.155	163.396	8.466	5.182
F_{max}	63.635	63.739	68.834	65.408	65.404	2.427	3.710

Number of dissolution data points (N), degrees of freedom (df), and selected goodness of fit criteria – Pearson correlation coefficient (R), coefficient of determination (R^2), adjusted coefficient of determination ($R^2_{adjusted}$), and residual sum of squares (RSS) (manual calculation in MS Excel):

Parameter	No.1	No.2	No.3	No.4
N	16	16	16	16
df	13	13	13	13
R	0.995114396	0.994044998	0.994531634	0.994291613
R^2	0.99025266	0.988125457	0.989093171	0.988615812
$R^2_{adjusted}$	0.988753069	0.986298604	0.987415197	0.986864399
RSS	47.69545343	55.66544565	54.03075249	58.91926744

Graphical abstract of model fit presented as mean ± 1 SD of the fraction % of released carvedilol:

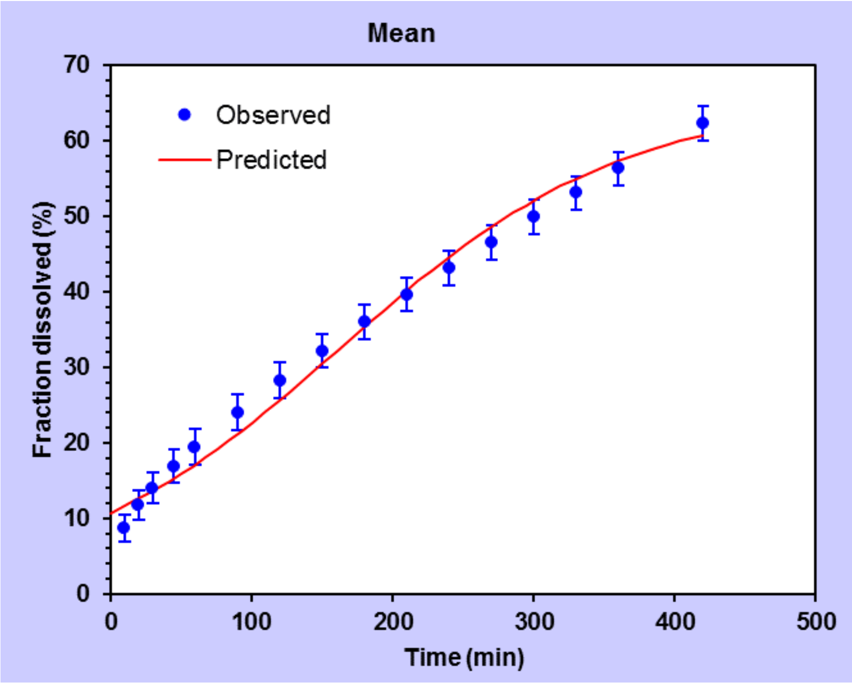

Supplementary materials – Model fitting summary – FlowLac® 100 (modelling up to app. 60 % of carvedilol released)

Graphical abstract of model fit presented as the fraction % of released carvedilol per tested tablet:

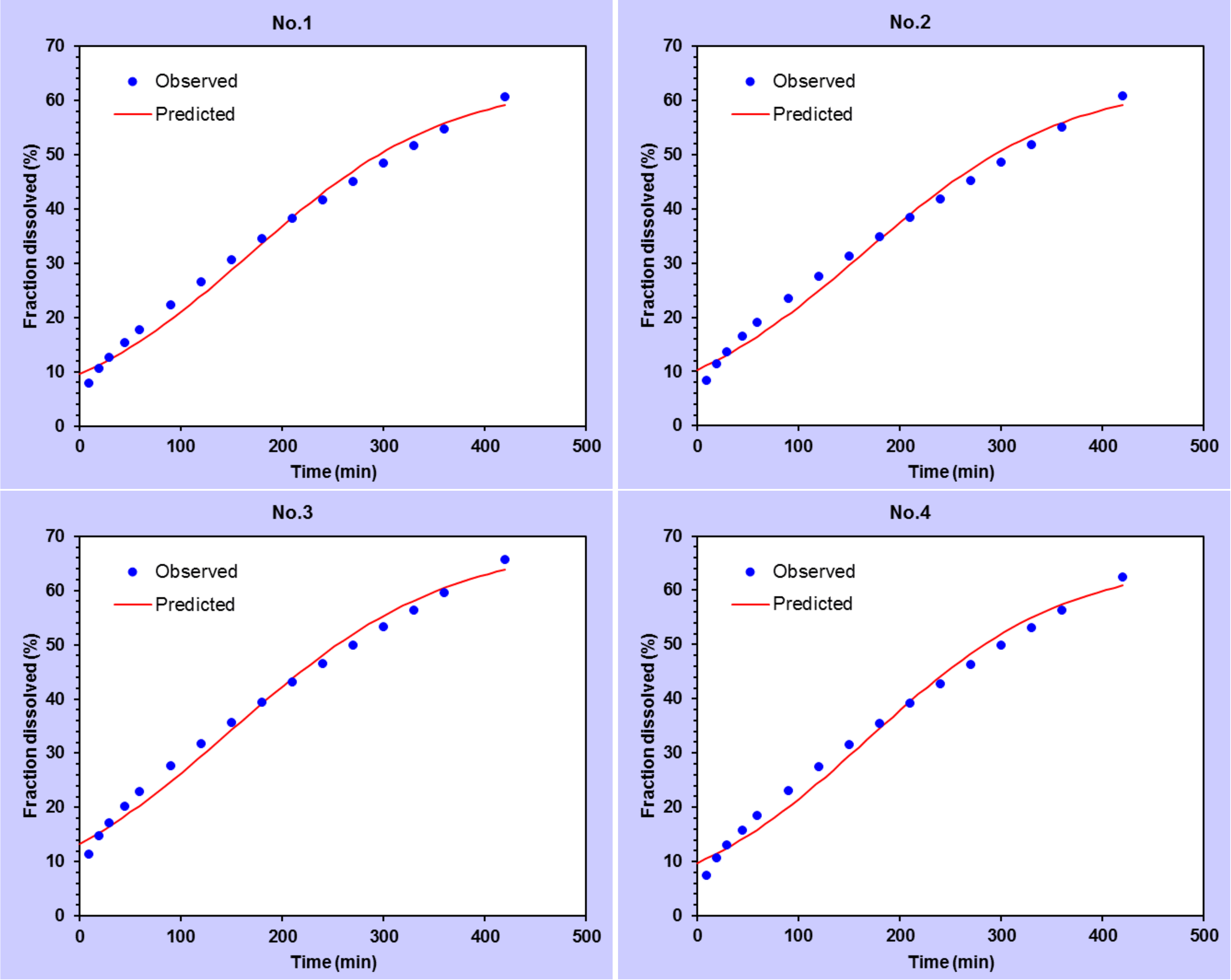

Supplementary materials – Model fitting summary – FlowLac® 100 (modelling up to app. 60 % of carvedilol released)

Model: **Gompertz_1**

Model equation: $F = 100 \cdot e^{-\alpha \cdot e^{-\beta \cdot \log(t)}}$

Fitted model parameters per tested tablet (N = 4) with statistics – mean, standard deviation (SD), and relative standard deviation expressed in % (RSD%) (output from DDSolver):

Parameter	No.1	No.2	No.3	No.4	Mean	SD	RSD(%)
α	8.788	8.211	7.384	9.168	8.388	0.776	9.253
β	0.986	0.962	0.973	1.019	0.985	0.025	2.516

Number of dissolution data points (N), degrees of freedom (df), and selected goodness of fit criteria – Pearson correlation coefficient (R), coefficient of determination (R^2), adjusted coefficient of determination ($R^2_{adjusted}$), and residual sum of squares (RSS) (manual calculation in MS Excel):

Parameter	No.1	No.2	No.3	No.4
N	16	16	16	16
df	14	14	14	14
R	0.968053031	0.968049663	0.964074503	0.968803544
R^2	0.937126671	0.93712015	0.929439647	0.938580306
$R^2_{adjusted}$	0.932635719	0.932628732	0.924399622	0.934193186
RSS	288.175669	273.38377	318.8196779	297.6386563

Graphical abstract of model fit presented as mean ± 1 SD of the fraction % of released carvedilol:

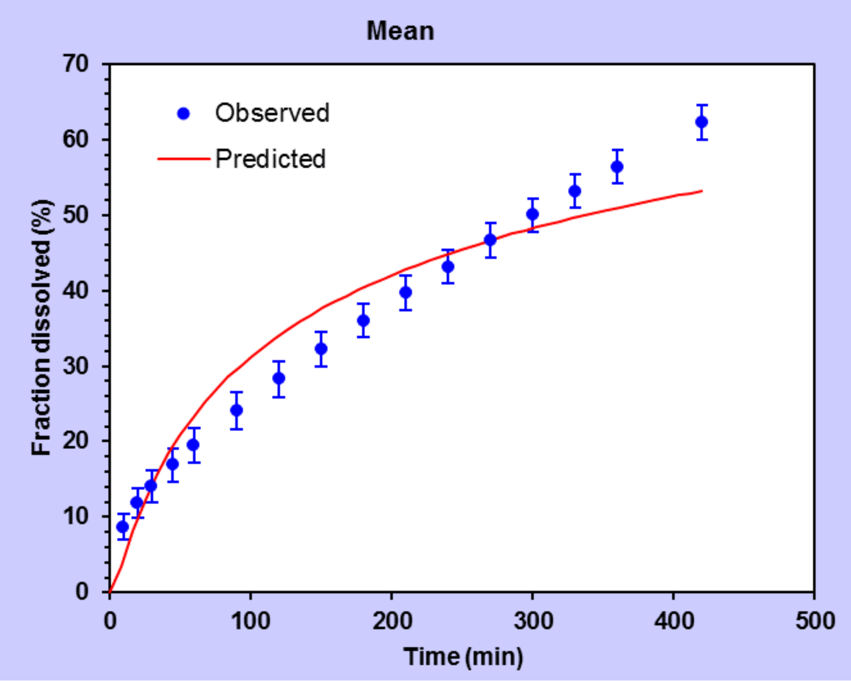

Supplementary materials – Model fitting summary – FlowLac® 100 (modelling up to app. 60 % of carvedilol released)

Graphical abstract of model fit presented as the fraction % of released carvedilol per tested tablet:

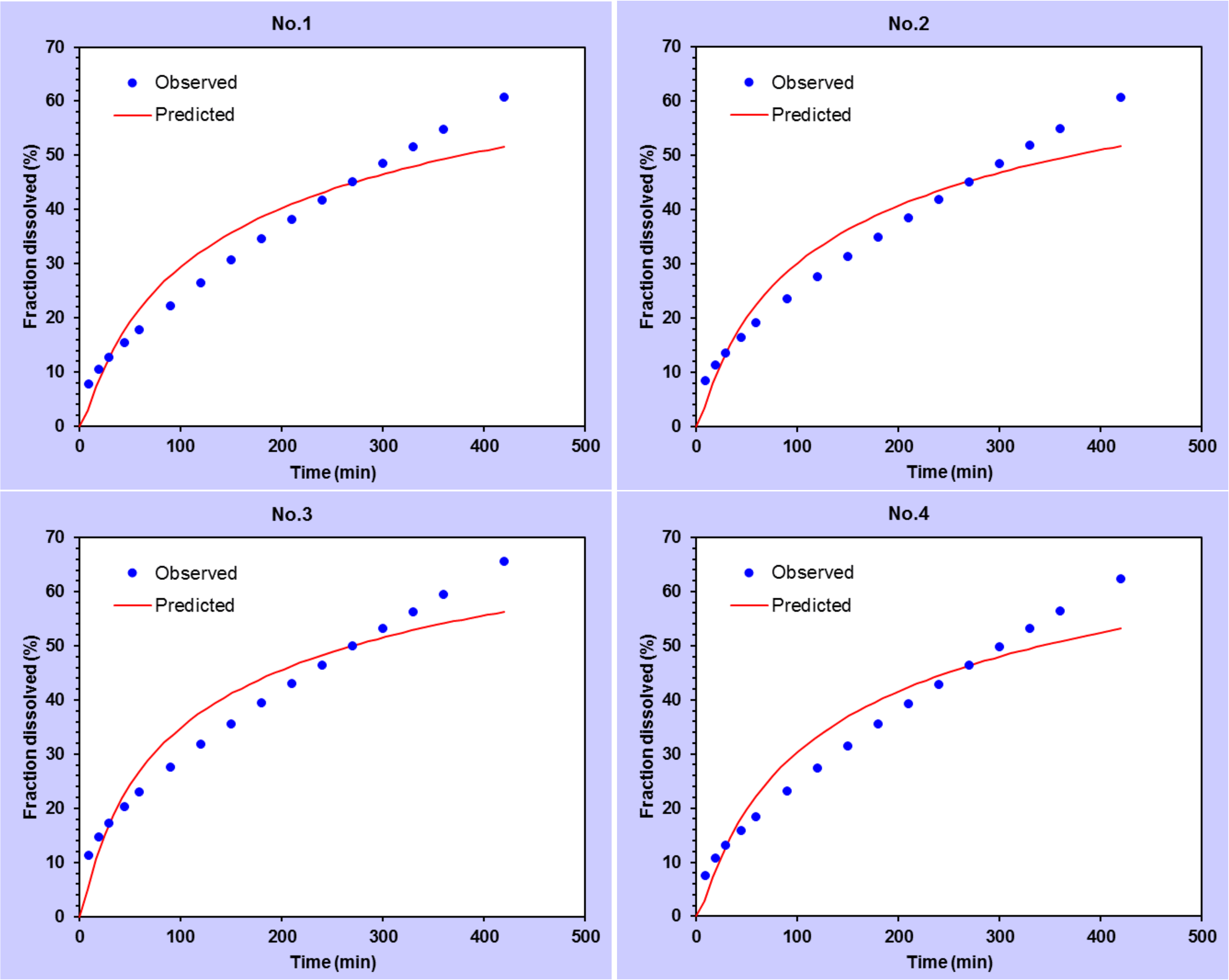

Supplementary materials – Model fitting summary – FlowLac® 100 (modelling up to app. 60 % of carvedilol released)

Model: **Gompertz_2**

Model equation: $F = F_{max} \cdot e^{-\alpha \cdot e^{-\beta \cdot \log(t)}}$

Fitted model parameters per tested tablet (N = 4) with statistics – mean, standard deviation (SD), and relative standard deviation expressed in % (RSD%) (output from DDSolver):

Parameter	No.1	No.2	No.3	No.4	Mean	SD	RSD(%)
α	25.007	22.956	18.921	25.630	23.129	3.029	13.095
β	1.829	1.799	1.742	1.841	1.803	0.044	2.451
F_{max}	63.635	63.739	68.834	65.408	65.404	2.427	3.710

Number of dissolution data points (N), degrees of freedom (df), and selected goodness of fit criteria – Pearson correlation coefficient (R), coefficient of determination (R^2), adjusted coefficient of determination ($R^2_{adjusted}$), and residual sum of squares (RSS) (manual calculation in MS Excel):

Parameter	No.1	No.2	No.3	No.4
N	16	16	16	16
df	13	13	13	13
R	0.935883207	0.93599335	0.933293663	0.938056045
R^2	0.875877378	0.876083551	0.871037062	0.879949144
$R^2_{adjusted}$	0.85678159	0.857019482	0.85119661	0.861479781
RSS	620.4400146	595.8961629	660.1808702	639.0384926

Graphical abstract of model fit presented as mean ± 1 SD of the fraction % of released carvedilol:

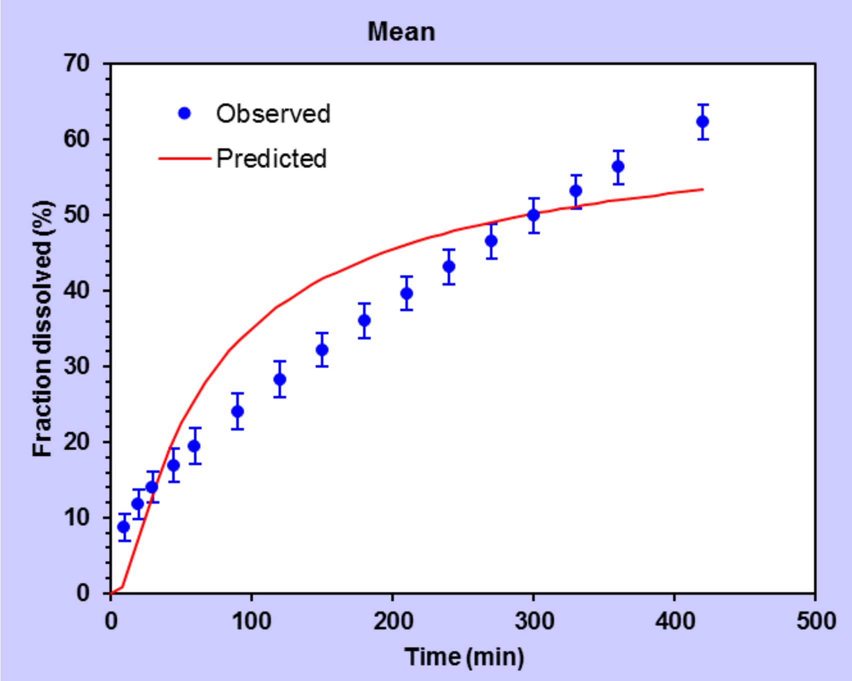

Supplementary materials – Model fitting summary – FlowLac® 100 (modelling up to app. 60 % of carvedilol released)

Graphical abstract of model fit presented as the fraction % of released carvedilol per tested tablet:

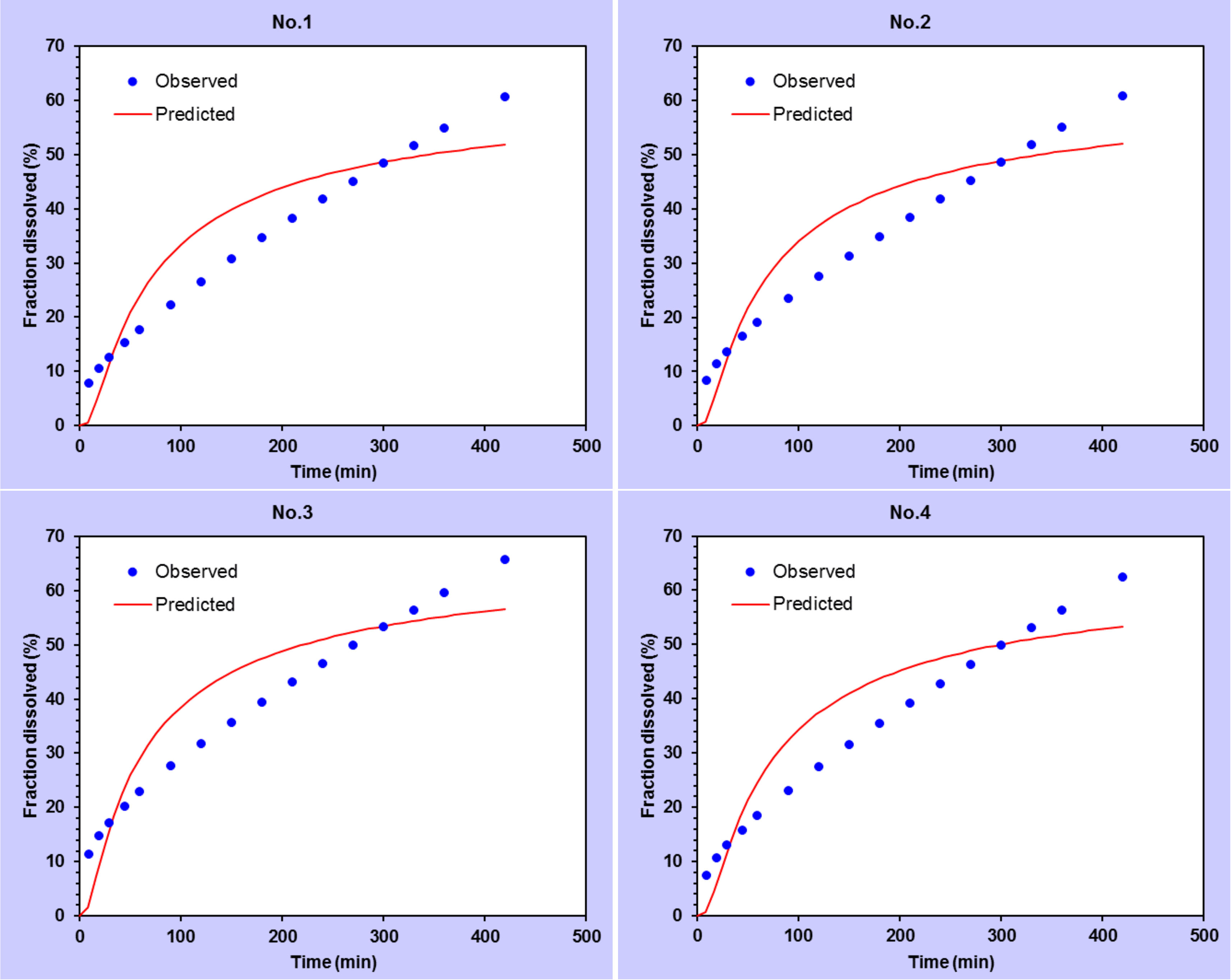

Supplementary materials – Model fitting summary – FlowLac® 100 (modelling up to app. 60 % of carvedilol released)

Model: **Gompertz_3**

Model equation: $F = F_{max} \cdot e^{-e^{-k\cdot(t-\gamma)}}$

Fitted model parameters per tested tablet (N = 4) with statistics – mean, standard deviation (SD), and relative standard deviation expressed in % (RSD%) (output from DDSolver):

Parameter	No.1	No.2	No.3	No.4	Mean	SD	RSD(%)
k	0.008	0.008	0.007	0.008	0.008	0.000	2.353
γ	105.238	100.788	88.035	105.489	99.887	8.192	8.201
F_{max}	63.635	63.739	68.834	65.408	65.404	2.427	3.710

Number of dissolution data points (N), degrees of freedom (df), and selected goodness of fit criteria – Pearson correlation coefficient (R), coefficient of determination (R^2), adjusted coefficient of determination ($R^2_{adjusted}$), and residual sum of squares (RSS) (manual calculation in MS Excel):

Parameter	No.1	No.2	No.3	No.4
N	16	16	16	16
df	13	13	13	13
R	0.995427095	0.994262697	0.994590981	0.994913922
R^2	0.990875101	0.988558312	0.989211219	0.989853712
$R^2_{adjusted}$	0.98947127	0.986798052	0.987551407	0.988292744
RSS	58.59823441	68.41570743	69.62865692	67.04698398

Graphical abstract of model fit presented as mean ± 1 SD of the fraction % of released carvedilol:

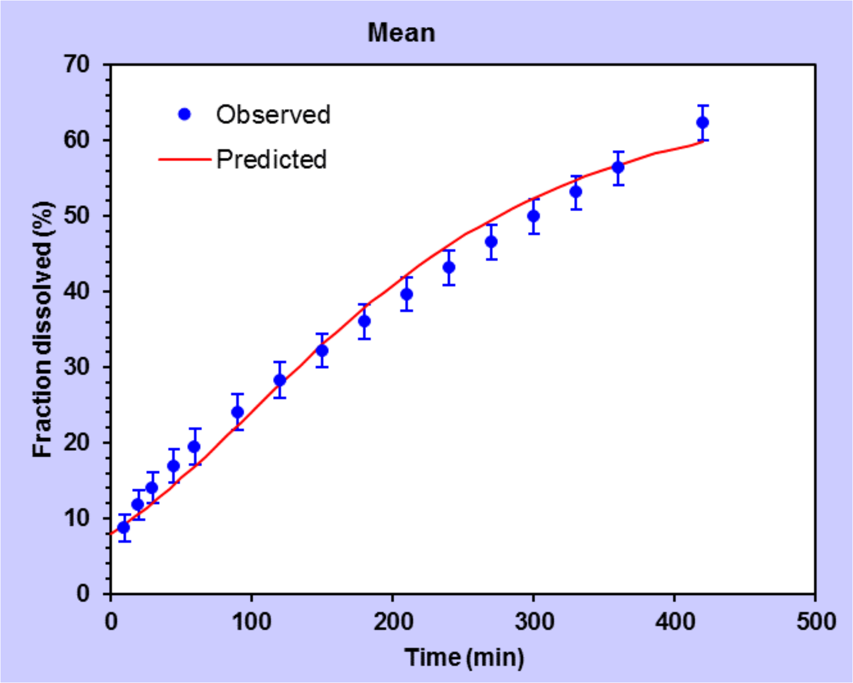

Supplementary materials – Model fitting summary – FlowLac® 100 (modelling up to app. 60 % of carvedilol released)

Graphical abstract of model fit presented as the fraction % of released carvedilol per tested tablet:

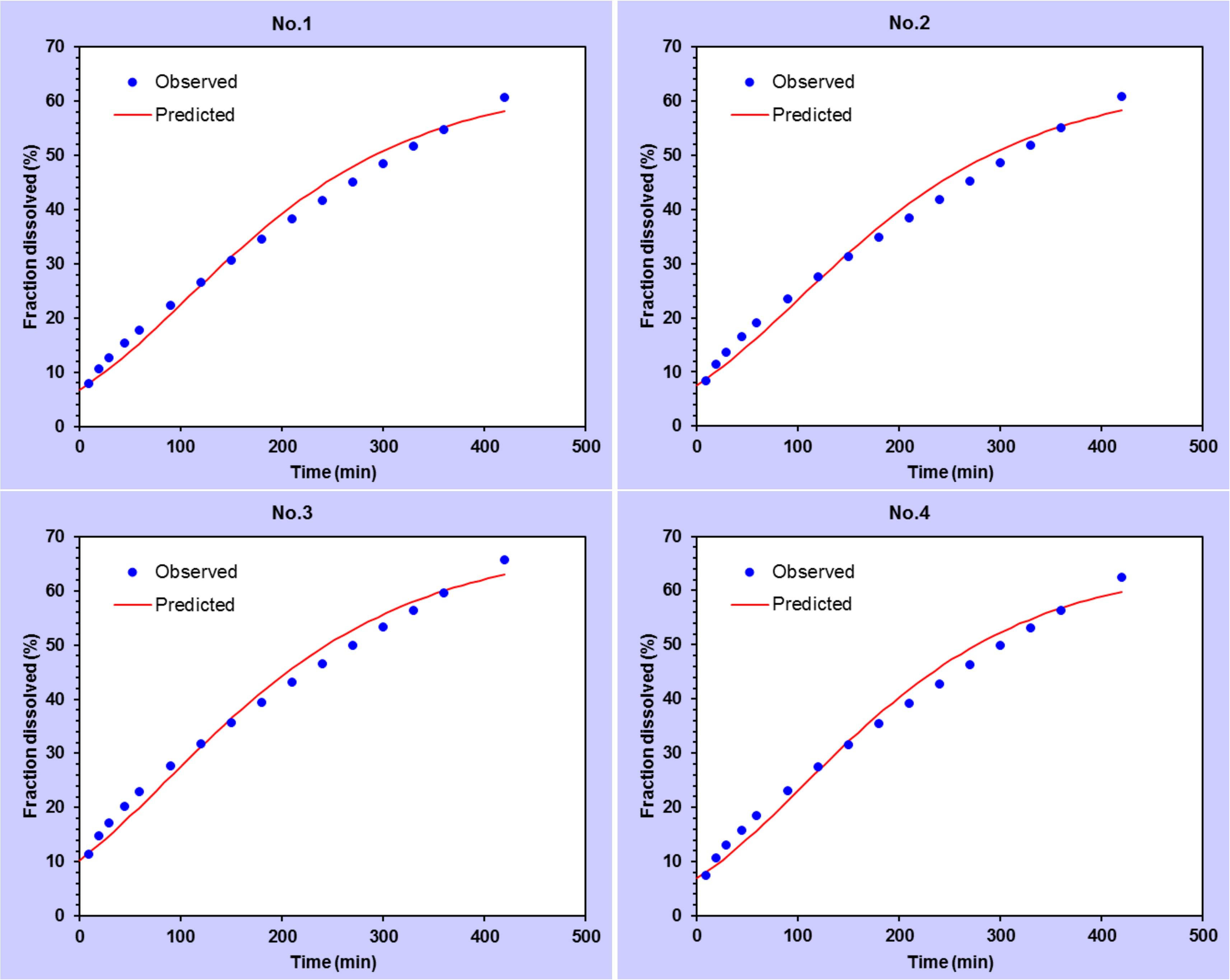

Supplementary materials – Model fitting summary – FlowLac® 100 (modelling up to app. 60 % of carvedilol released)

Model: **Gompertz_4**

Model equation: $F = F_{max} \cdot e^{-\beta \cdot e^{-k \cdot t}}$

Fitted model parameters per tested tablet (N = 4) with statistics – mean, standard deviation (SD), and relative standard deviation expressed in % (RSD%) (output from DDSolver):

Parameter	No.1	No.2	No.3	No.4	Mean	SD	RSD(%)
k	0.008	0.008	0.007	0.008	0.008	0.000	2.353
β	2.239	2.136	1.900	2.249	2.131	0.162	7.594
F_{max}	63.635	63.739	68.834	65.408	65.404	2.427	3.710

Number of dissolution data points (N), degrees of freedom (df), and selected goodness of fit criteria – Pearson correlation coefficient (R), coefficient of determination (R^2), adjusted coefficient of determination ($R^2_{adjusted}$), and residual sum of squares (RSS) (manual calculation in MS Excel):

Parameter	No.1	No.2	No.3	No.4
N	16	16	16	16
df	13	13	13	13
R	0.995427095	0.994262697	0.994590981	0.994913922
R^2	0.990875101	0.988558312	0.989211219	0.989853712
$R^2_{adjusted}$	0.98947127	0.986798052	0.987551407	0.988292744
RSS	58.59823441	68.41570743	69.62865692	67.04698398

Graphical abstract of model fit presented as mean ± 1 SD of the fraction % of released carvedilol:

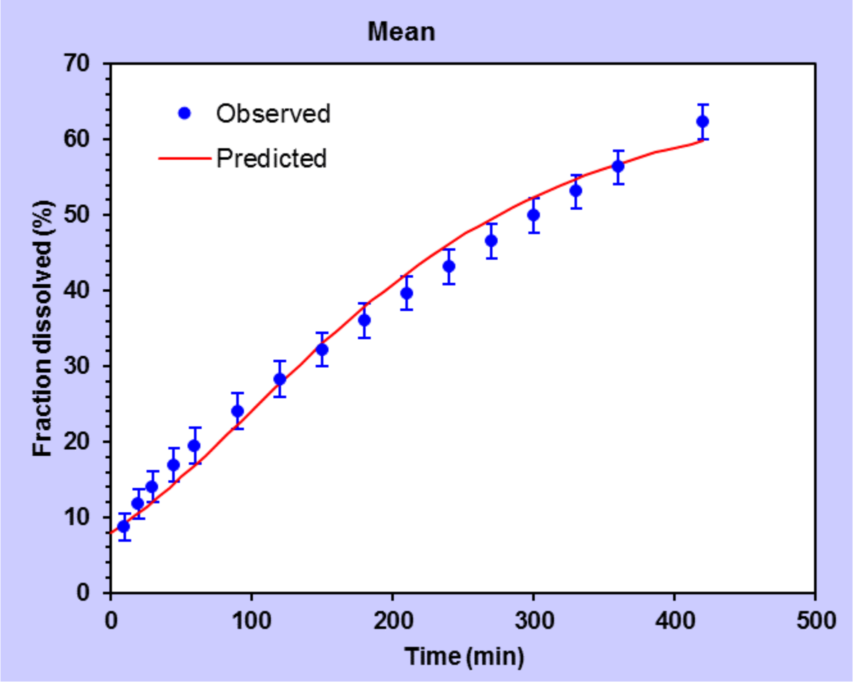

Supplementary materials – Model fitting summary – FlowLac® 100 (modelling up to app. 60 % of carvedilol released)

Graphical abstract of model fit presented as the fraction % of released carvedilol per tested tablet:

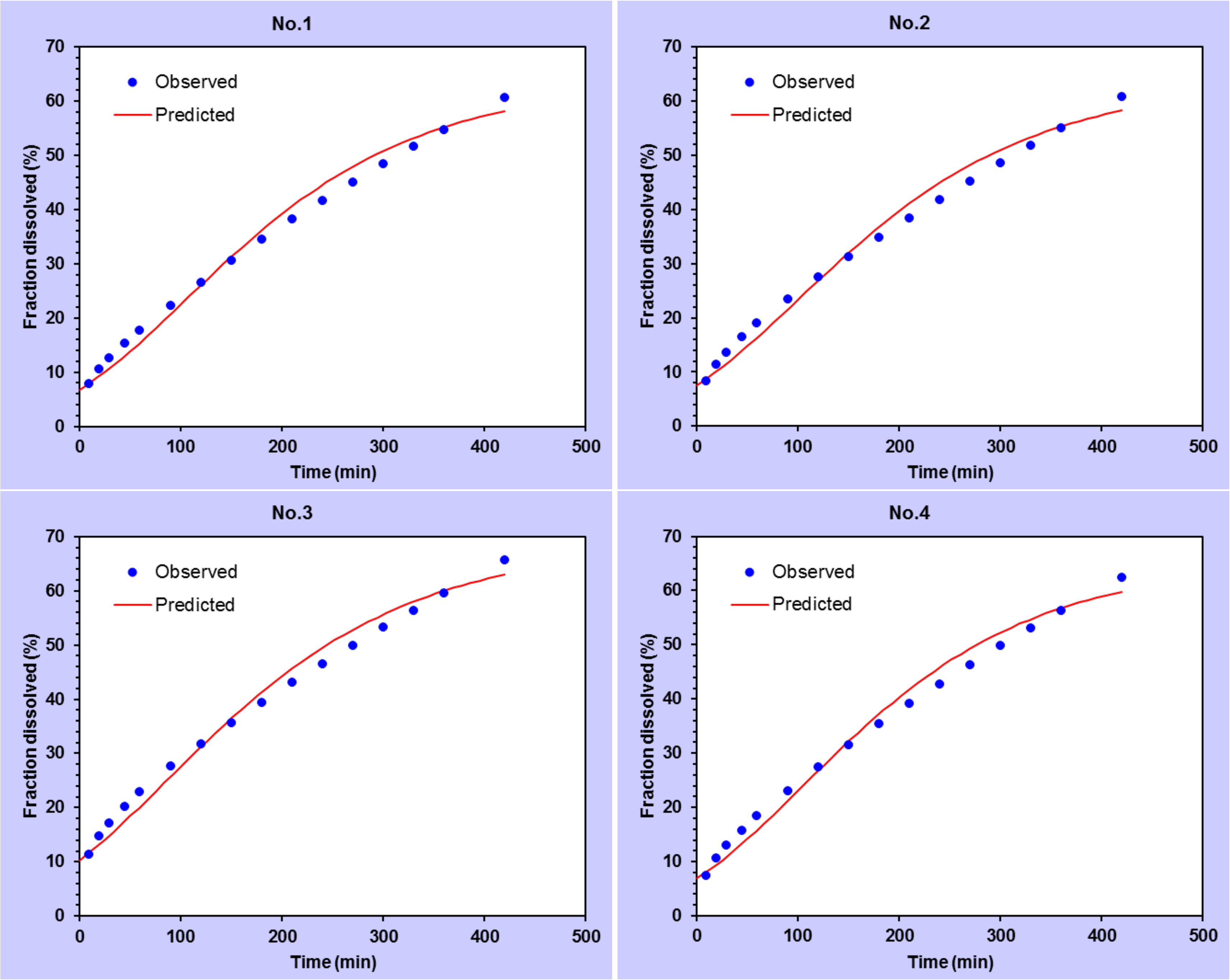

Supplementary materials – Model fitting summary – FlowLac® 100 (modelling up to app. 60 % of carvedilol released)

Model: **Probit_1**

Model equation: $F = 100 \cdot \phi[\alpha + \beta \cdot \log(t)]$

Fitted model parameters per tested tablet (N = 4) with statistics – mean, standard deviation (SD), and relative standard deviation expressed in % (RSD%) (output from DDSolver):

Parameter	No.1	No.2	No.3	No.4	Mean	SD	RSD(%)
α	-2.673	-2.585	-2.384	-2.707	-2.587	0.145	-5.587
β	1.044	1.012	0.978	1.073	1.027	0.041	3.975

Number of dissolution data points (N), degrees of freedom (df), and selected goodness of fit criteria – Pearson correlation coefficient (R), coefficient of determination (R^2), adjusted coefficient of determination ($R^2_{adjusted}$), and residual sum of squares (RSS) (manual calculation in MS Excel):

Parameter	No.1	No.2	No.3	No.4
N	16	16	16	16
df	14	14	14	14
R	0.980893801	0.980534585	0.977428133	0.98166949
R^2	0.962152649	0.961448072	0.955365756	0.963674987
$R^2_{adjusted}$	0.959449267	0.958694363	0.952177595	0.961080344
RSS	186.8088329	178.4550955	211.3468801	188.1155255

Graphical abstract of model fit presented as mean ± 1 SD of the fraction % of released carvedilol:

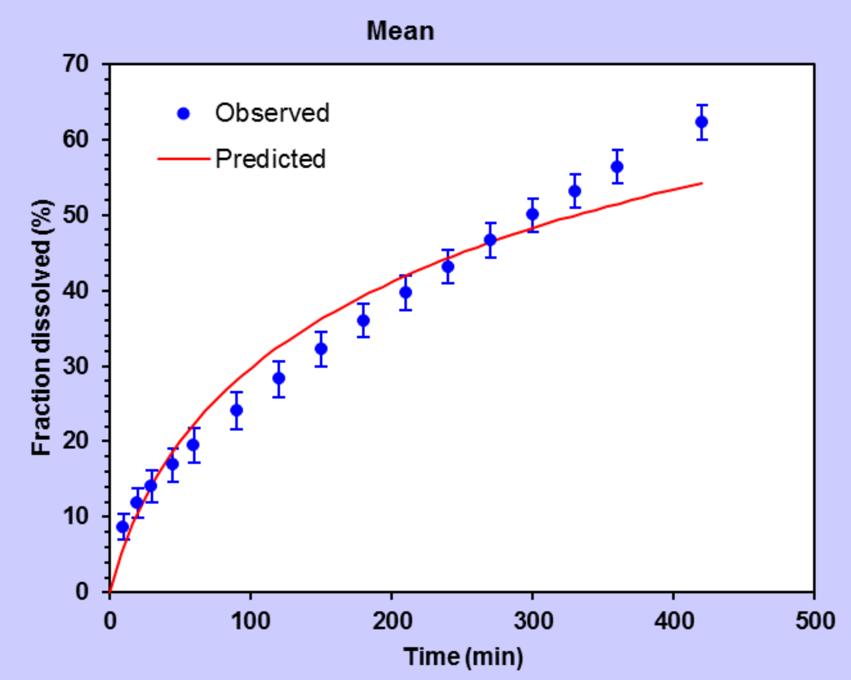

Supplementary materials – Model fitting summary – FlowLac® 100 (modelling up to app. 60 % of carvedilol released)

Graphical abstract of model fit presented as the fraction % of released carvedilol per tested tablet:

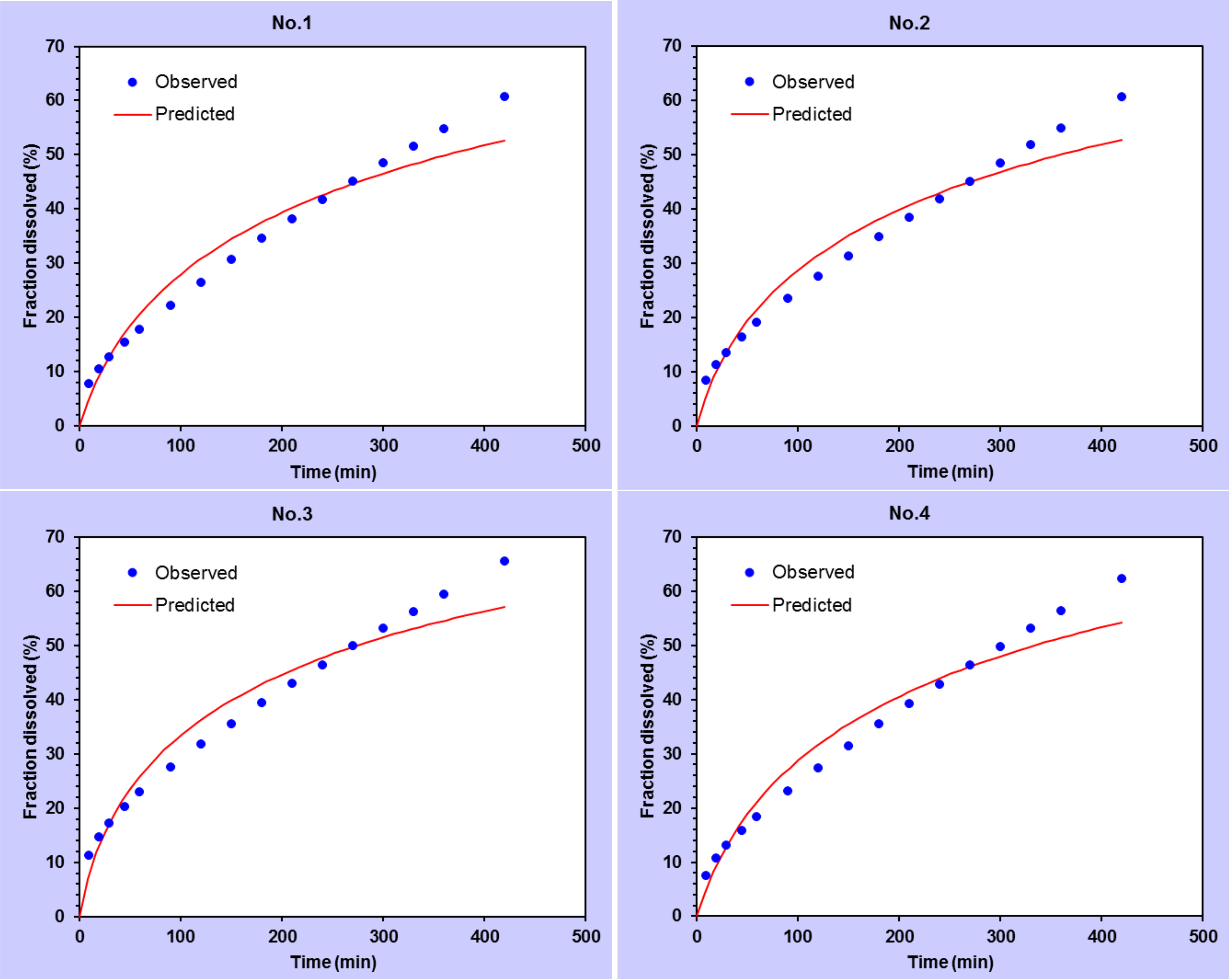

Supplementary materials – Model fitting summary – FlowLac® 100 (modelling up to app. 60 % of carvedilol released)

Model: **Probit_2**

Model equation: $F = F_{max} \cdot \phi[\alpha + \beta \cdot \log(t)]$

Fitted model parameters per tested tablet (N = 4) with statistics – mean, standard deviation (SD), and relative standard deviation expressed in % (RSD%) (output from DDSolver):

Parameter	No.1	No.2	No.3	No.4	Mean	SD	RSD(%)
α	-3.124	-3.023	-2.780	-3.154	-3.020	0.170	-5.622
β	1.537	1.500	1.416	1.551	1.501	0.060	4.028
F_{max}	63.635	63.739	68.834	65.408	65.404	2.427	3.710

Number of dissolution data points (N), degrees of freedom (df), and selected goodness of fit criteria – Pearson correlation coefficient (R), coefficient of determination (R^2), adjusted coefficient of determination ($R^2_{adjusted}$), and residual sum of squares (RSS) (manual calculation in MS Excel):

Parameter	No.1	No.2	No.3	No.4
N	16	16	16	16
df	13	13	13	13
R	0.96124563	0.960809788	0.958499134	0.962880784
R^2	0.923993161	0.923155449	0.91872059	0.927139404
$R^2_{adjusted}$	0.912299802	0.911333211	0.906216065	0.915930081
RSS	354.5479743	342.4992585	382.0130331	361.327003

Graphical abstract of model fit presented as mean ± 1 SD of the fraction % of released carvedilol:

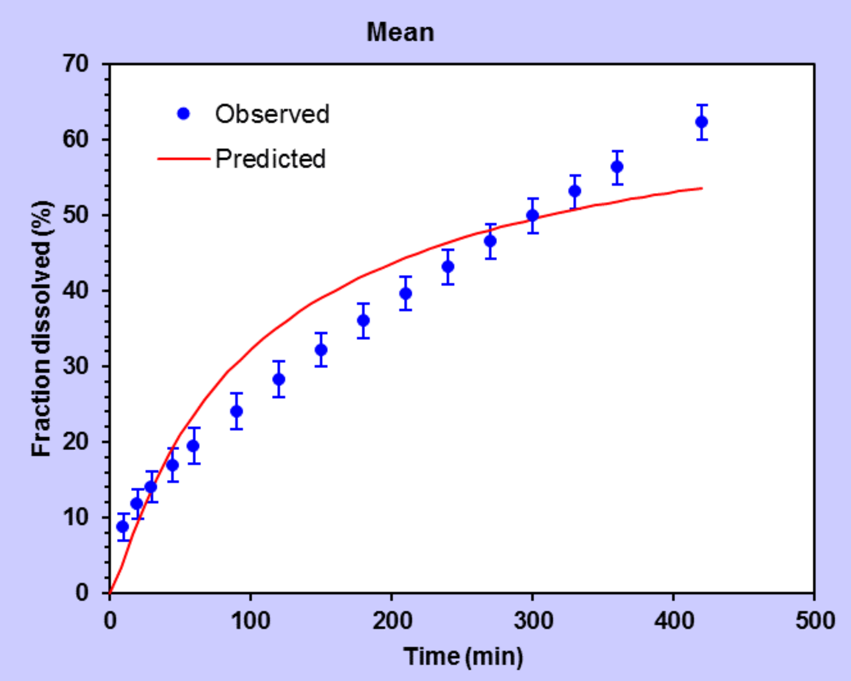

Supplementary materials – Model fitting summary – FlowLac® 100 (modelling up to app. 60 % of carvedilol released)

Graphical abstract of model fit presented as the fraction % of released carvedilol per tested tablet:

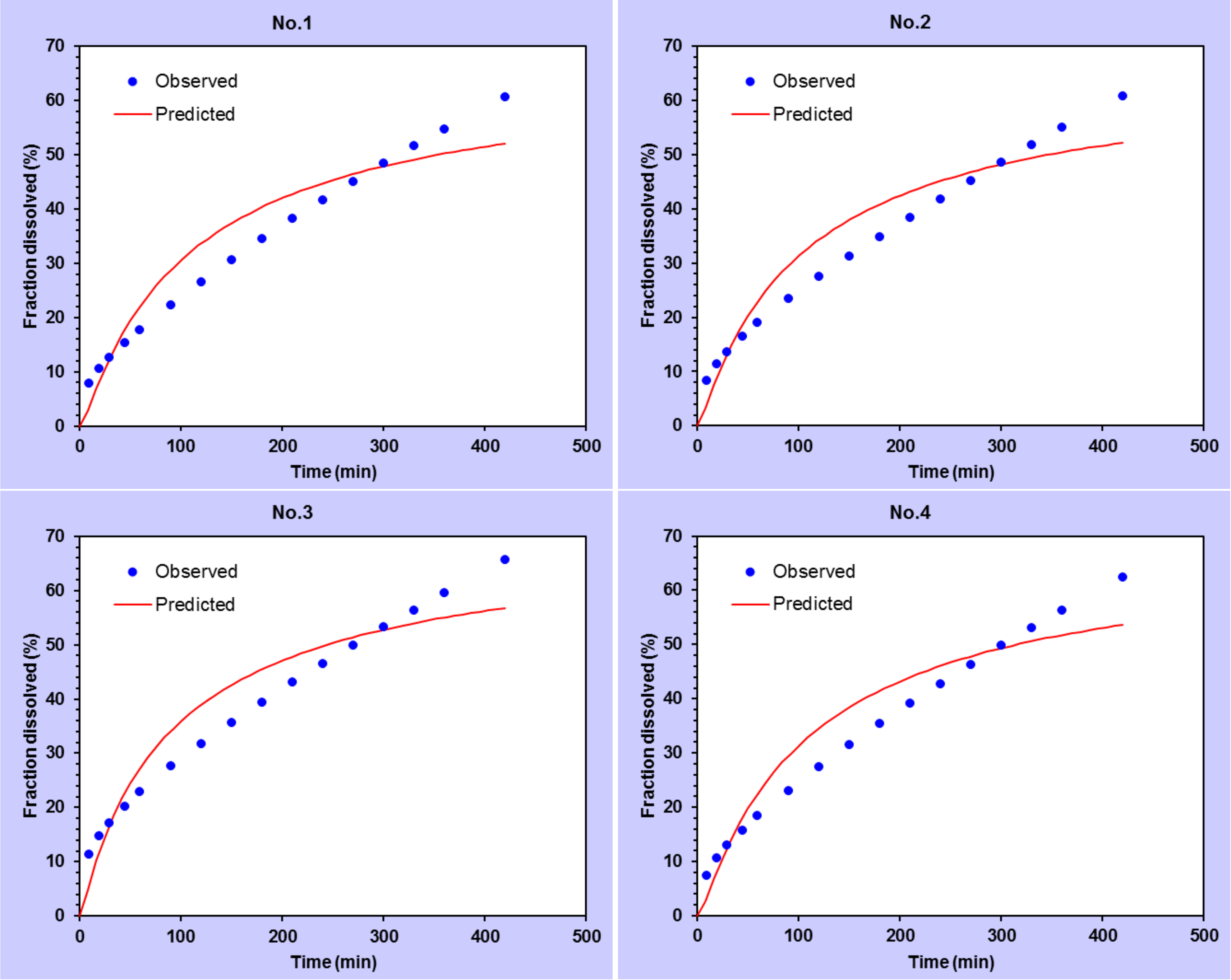

Supplement: Supplementary file 1 [file pharmaceutics-16-00498-s001.zip › Supplementary materials_Model fitting summary_FlowLac® 100.pdf]
