# Supplementary material for: Comparative Fitting of Mathematical Models to Carvedilol Release Profiles Obtained from Hypromellose Matrix Tablets
Source: Pharmaceutics. 2024 Apr 4;16(4):498. doi: 10.3390/pharmaceutics16040498 (PMC11053526; doi:10.3390/pharmaceutics16040498)

Supplementary materials – Model fitting summary – Glucidex® 19D (modelling the entire relevant dissolution data)

Model: **Zero–order**

Model equation: $F = k_0 \cdot t$

Fitted model parameters per tested tablet (N = 4) with statistics – mean, standard deviation (SD), and relative standard deviation expressed in % (RSD%) (output from DDSolver):

Parameter	No.1	No.2	No.3	No.4	Mean	SD	RSD(%)
k_0	0.094	0.094	0.092	0.101	0.095	0.004	3.945

Number of dissolution data points (N), degrees of freedom (df), and selected goodness of fit criteria – Pearson correlation coefficient (R), coefficient of determination (R^2), adjusted coefficient of determination ($R^2_{adjusted}$), and residual sum of squares (RSS) (manual calculation in MS Excel):

Parameter	No.1	No.2	No.3	No.4
N	31	31	31	31
df	30	30	30	30
R	0.973237822	0.972117433	0.979100512	0.962816084
R^2	0.947191858	0.945012304	0.958637813	0.927014811
$R^2_{adjusted}$	0.947191858	0.945012304	0.958637813	0.927014811
RSS	4189.013724	3791.191682	3141.741812	5274.039393

Graphical abstract of model fit presented as mean ± 1 SD of the fraction % of released carvedilol:

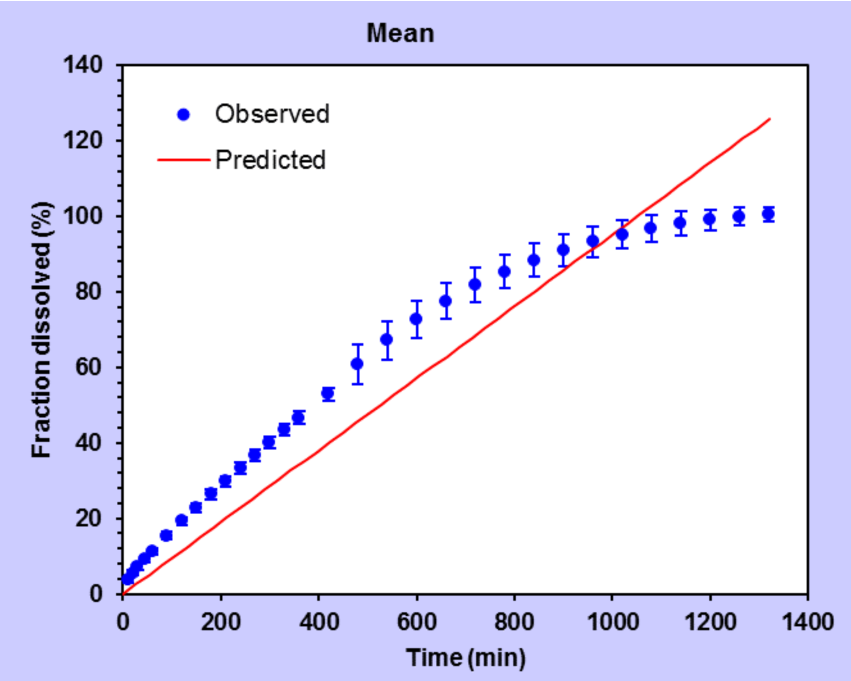

Supplementary materials – Model fitting summary – Glucidex® 19D (modelling the entire relevant dissolution data)

Graphical abstract of model fit presented as the fraction % of released carvedilol per tested tablet:

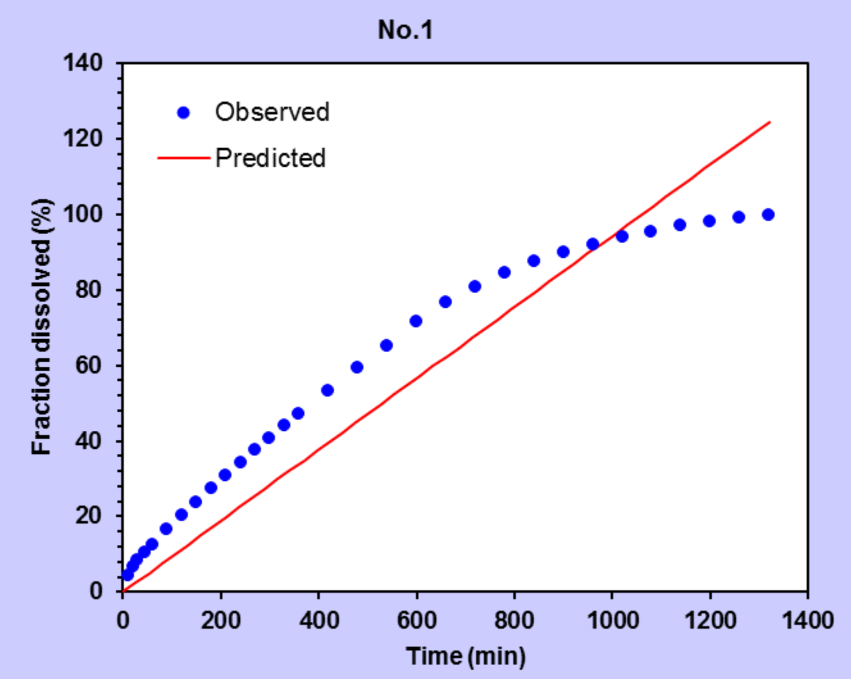

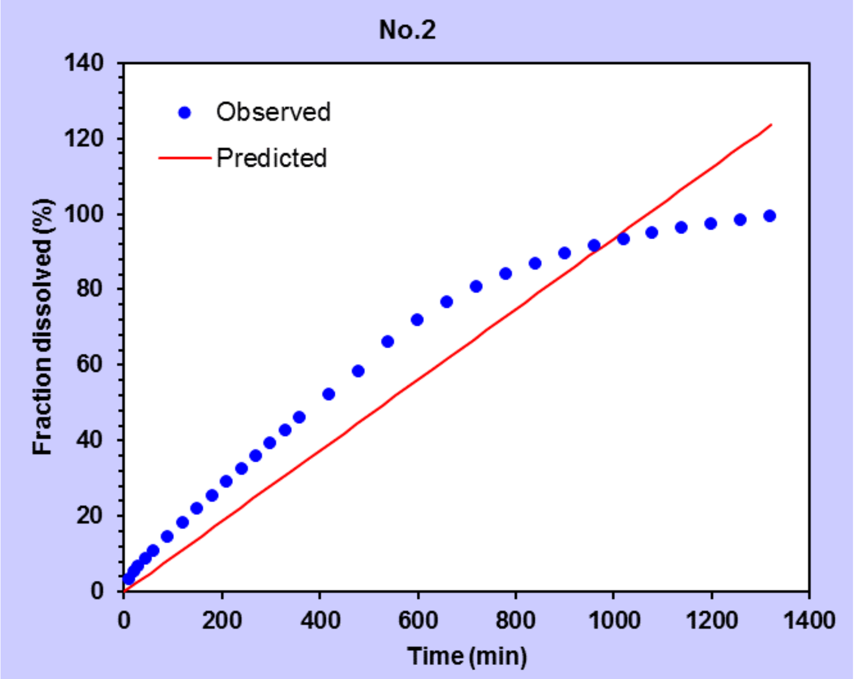

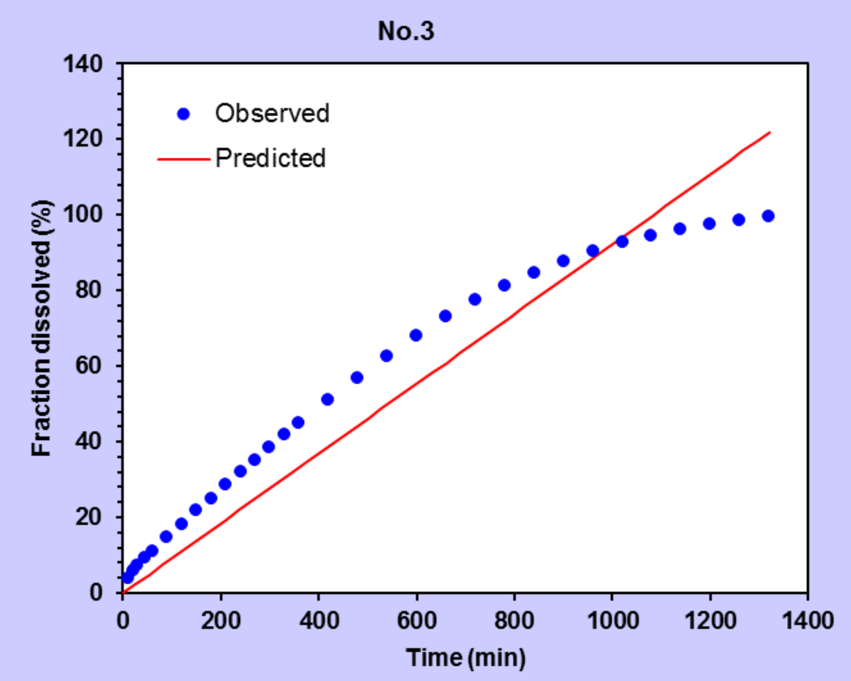

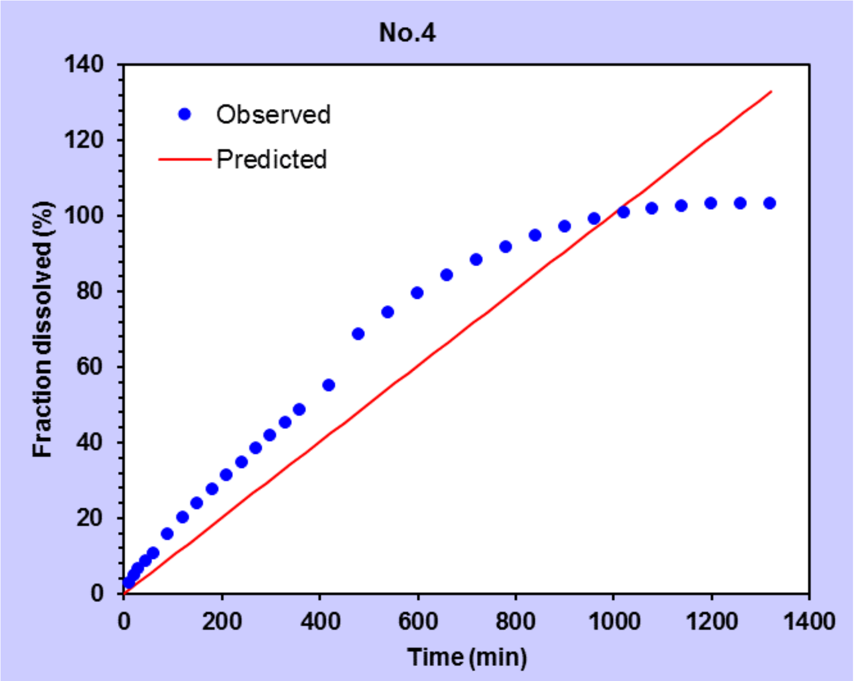

Supplementary materials – Model fitting summary – Glucidex® 19D (modelling the entire relevant dissolution data)

Model: **Zero–order with T_{lag}**

Model equation: $F = k_0 \cdot (t - T_{lag})$

Fitted model parameters per tested tablet (N = 4) with statistics – mean, standard deviation (SD), and relative standard deviation expressed in % (RSD%) (output from DDSolver):

Parameter	No.1	No.2	No.3	No.4	Mean	SD	RSD(%)
k_0	0.077	0.079	0.078	0.084	0.080	0.003	3.965
T_{lag}	-184.134	-159.750	-155.634	-165.273	-166.198	12.593	-7.577

Number of dissolution data points (N), degrees of freedom (df), and selected goodness of fit criteria – Pearson correlation coefficient (R), coefficient of determination (R^2), adjusted coefficient of determination ($R^2_{adjusted}$), and residual sum of squares (RSS) (manual calculation in MS Excel):

Parameter	No.1	No.2	No.3	No.4
N	31	31	31	31
df	29	29	29	29
R	0.973237822	0.972117433	0.979100512	0.962816084
R^2	0.947191858	0.945012304	0.958637813	0.927014811
$R^2_{adjusted}$	0.945370888	0.943116176	0.957211531	0.92449808
RSS	1779.409838	1918.041586	1396.309602	2975.419725

Graphical abstract of model fit presented as mean ± 1 SD of the fraction % of released carvedilol:

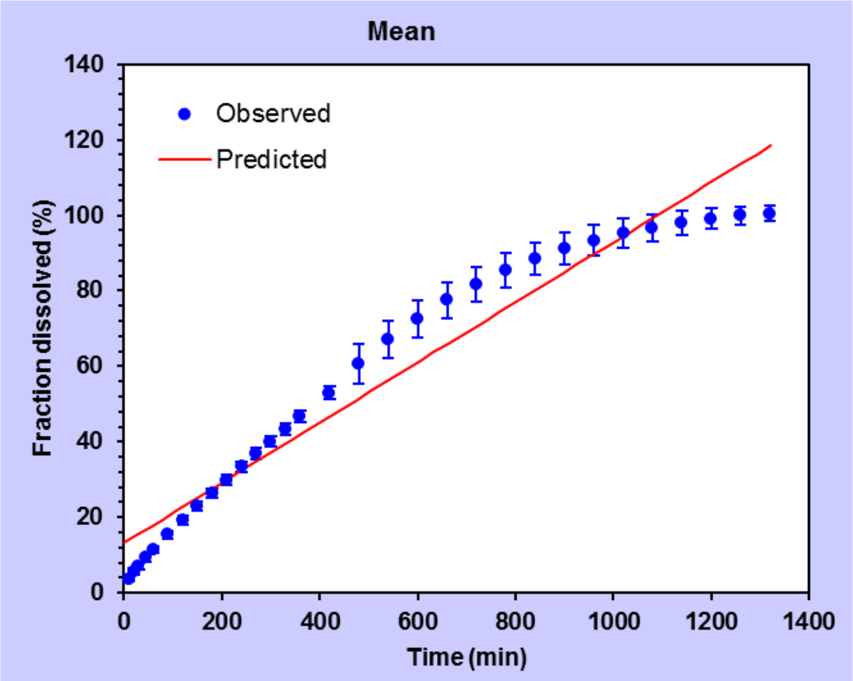

Supplementary materials – Model fitting summary – Glucidex® 19D (modelling the entire relevant dissolution data)

Graphical abstract of model fit presented as the fraction % of released carvedilol per tested tablet:

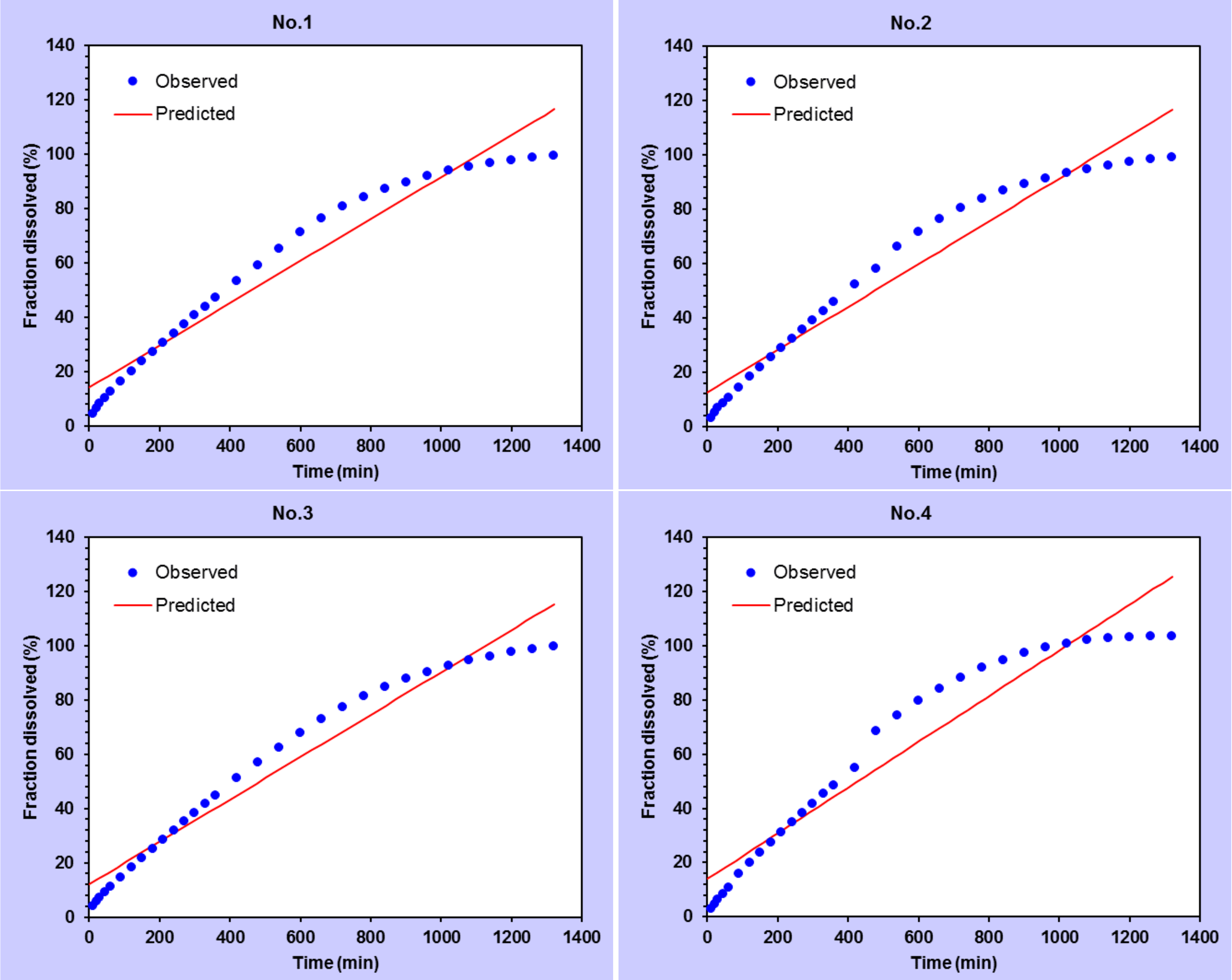

Supplementary materials – Model fitting summary – Glucidex® 19D (modelling the entire relevant dissolution data)

Model: **Zero–order with F_0**

Model equation: $F = F_0 + k_0 \cdot t$

Fitted model parameters per tested tablet (N = 4) with statistics – mean, standard deviation (SD), and relative standard deviation expressed in % (RSD%) (output from DDSolver):

Parameter	No.1	No.2	No.3	No.4	Mean	SD	RSD(%)
k_0	0.077	0.079	0.078	0.084	0.080	0.003	3.965
F_0	14.270	12.581	12.145	13.937	13.233	1.029	7.779

Number of dissolution data points (N), degrees of freedom (df), and selected goodness of fit criteria – Pearson correlation coefficient (R), coefficient of determination (R^2), adjusted coefficient of determination ($R^2_{adjusted}$), and residual sum of squares (RSS) (manual calculation in MS Excel):

Parameter	No.1	No.2	No.3	No.4
N	31	31	31	31
df	29	29	29	29
R	0.973237822	0.972117433	0.979100512	0.962816084
R^2	0.947191858	0.945012304	0.958637813	0.927014811
$R^2_{adjusted}$	0.945370888	0.943116176	0.957211531	0.92449808
RSS	1779.409838	1918.041586	1396.309602	2975.419725

Graphical abstract of model fit presented as mean ± 1 SD of the fraction % of released carvedilol:

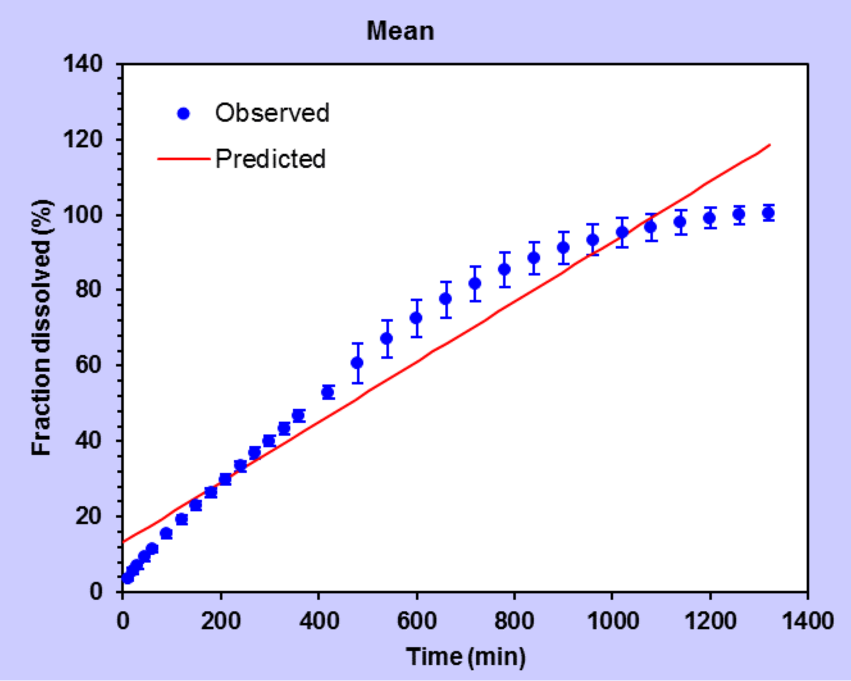

Supplementary materials – Model fitting summary – Glucidex® 19D (modelling the entire relevant dissolution data)

Graphical abstract of model fit presented as the fraction % of released carvedilol per tested tablet:

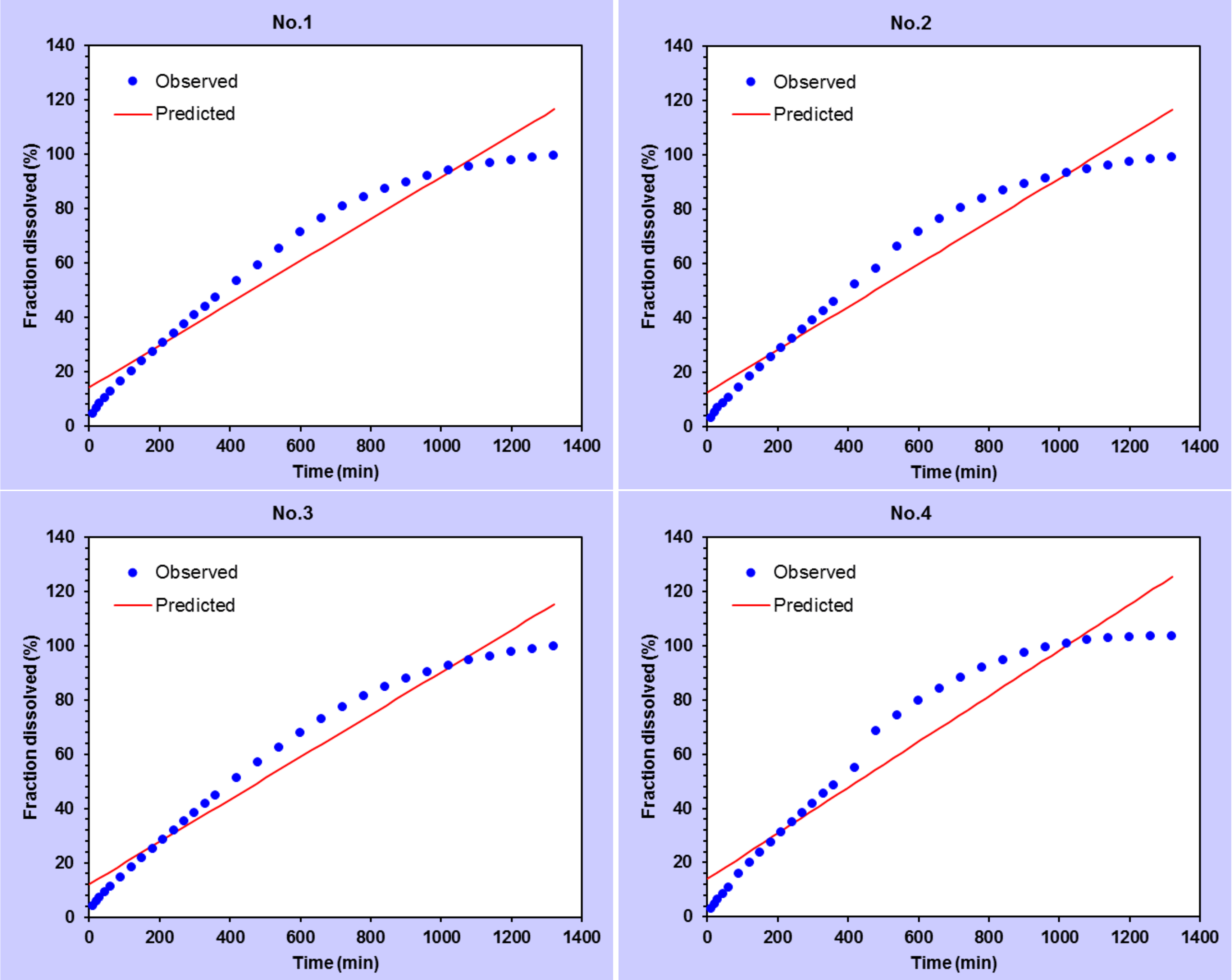

Supplementary materials – Model fitting summary – Glucidex® 19D (modelling the entire relevant dissolution data)

Model: **First–order**

Model equation: $F = 100 \cdot (1 - e^{-k_1 \cdot t})$

Fitted model parameters per tested tablet (N = 4) with statistics – mean, standard deviation (SD), and relative standard deviation expressed in % (RSD%) (output from DDSolver):

Parameter	No.1	No.2	No.3	No.4	Mean	SD	RSD(%)
k_1	0.001	0.002	0.001	0.003	0.002	0.001	27.673

Number of dissolution data points (N), degrees of freedom (df), and selected goodness of fit criteria – Pearson correlation coefficient (R), coefficient of determination (R^2), adjusted coefficient of determination ($R^2_{adjusted}$), and residual sum of squares (RSS) (manual calculation in MS Excel):

Parameter	No.1	No.2	No.3	No.4
N	31	31	31	31
df	30	30	30	30
R	0.998456372	0.99320336	0.998581517	0.987574435
R^2	0.996915126	0.986452913	0.997165046	0.975303265
$R^2_{adjusted}$	0.996915126	0.986452913	0.997165046	0.975303265
RSS	3476.18338	708.4197199	3296.411195	1618.419461

Graphical abstract of model fit presented as mean ± 1 SD of the fraction % of released carvedilol:

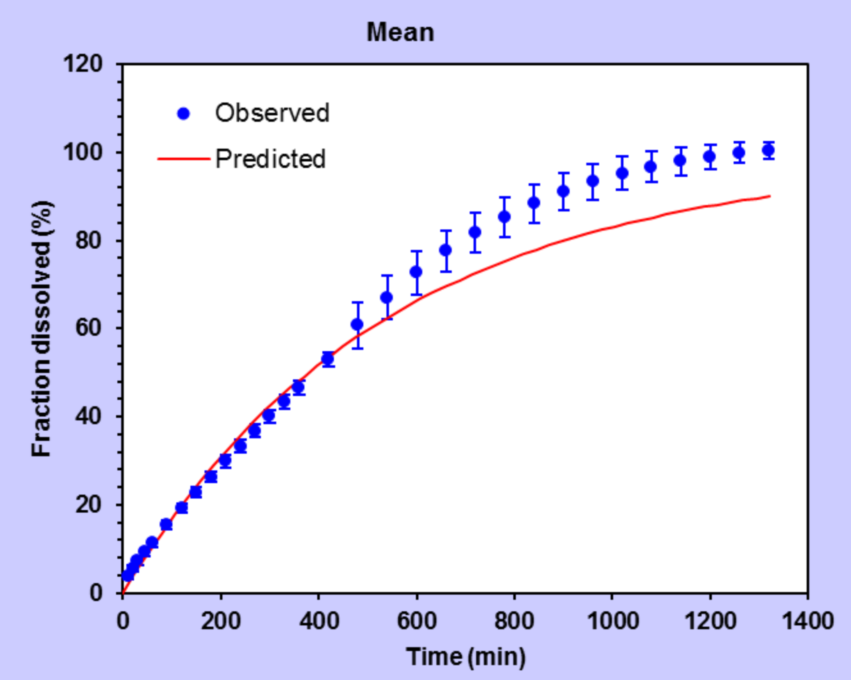

Supplementary materials – Model fitting summary – Glucidex® 19D (modelling the entire relevant dissolution data)

Graphical abstract of model fit presented as the fraction % of released carvedilol per tested tablet:

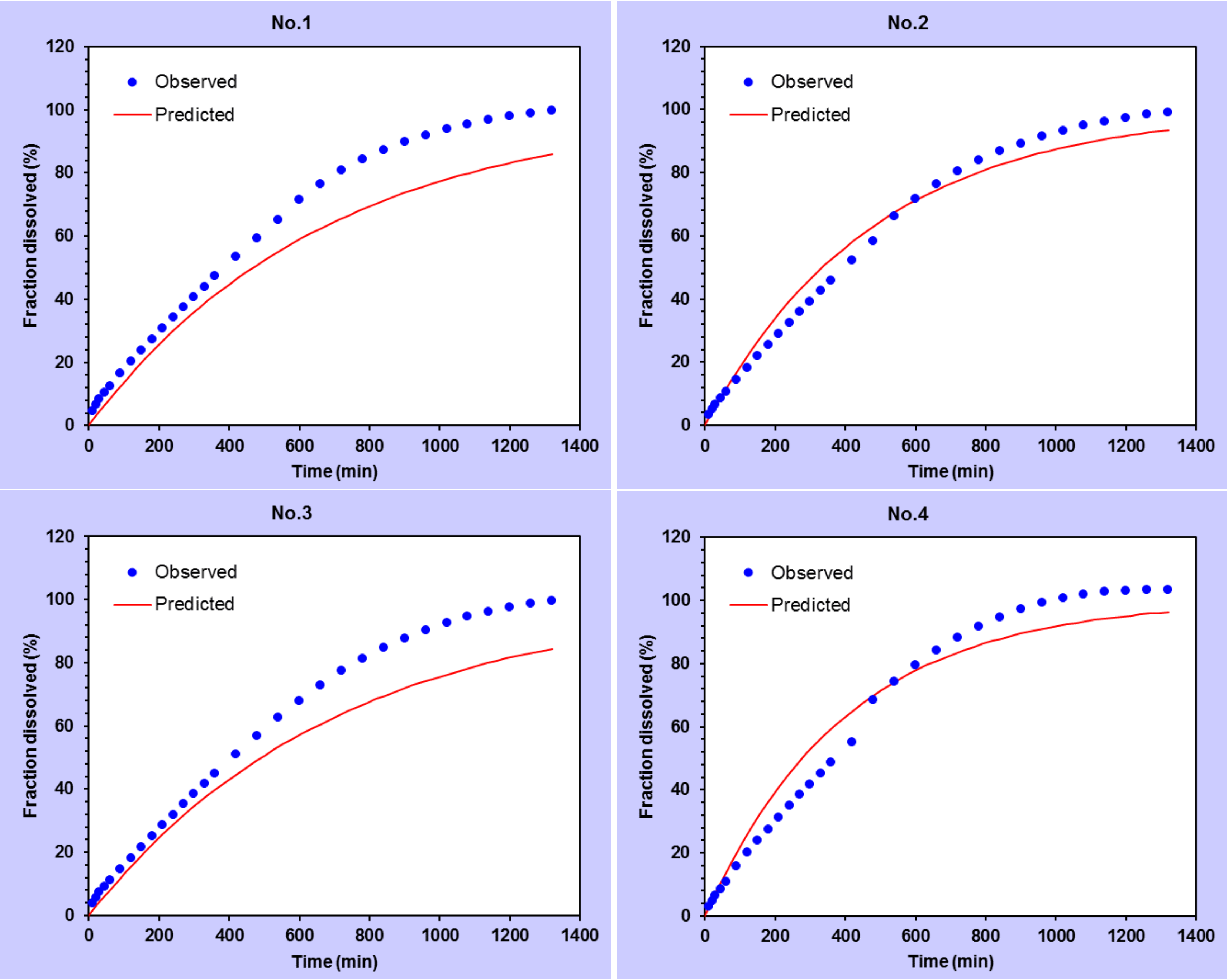

Supplementary materials – Model fitting summary – Glucidex® 19D (modelling the entire relevant dissolution data)

Model: **First–order with T_{lag}**

Model equation: $F = 100 \cdot \left[1 - e^{-k_1 \cdot (t - T_{lag})}\right]$

Fitted model parameters per tested tablet (N = 4) with statistics – mean, standard deviation (SD), and relative standard deviation expressed in % (RSD%) (output from DDSolver):

Parameter	No.1	No.2	No.3	No.4	Mean	SD	RSD(%)
k_1	/	/	/	/	/	/	/
T_{lag}	/	/	/	/	/	/	/

Number of dissolution data points (N), degrees of freedom (df), and selected goodness of fit criteria – Pearson correlation coefficient (R), coefficient of determination (R^2), adjusted coefficient of determination ($R^2_{adjusted}$), and residual sum of squares (RSS) (manual calculation in MS Excel):

Parameter	No.1	No.2	No.3	No.4
N	/	/	/	/
df	/	/	/	/
R	/	/	/	/
R^2	/	/	/	/
$R^2_{adjusted}$	/	/	/	/
RSS	/	/	/	/

Graphical abstract of model fit presented as mean ± 1 SD of the fraction % of released carvedilol: /

Graphical abstract of model fit presented as the fraction % of released carvedilol per tested tablet: /

Note: the model could not be fitted

Supplementary materials – Model fitting summary – Glucidex® 19D (modelling the entire relevant dissolution data)

Model: **First–order with F_{max}**

Model equation: $F = F_{max} \cdot (1 - e^{-k_1 \cdot t})$

Fitted model parameters per tested tablet (N = 4) with statistics – mean, standard deviation (SD), and relative standard deviation expressed in % (RSD%) (output from DDSolver):

Parameter	No.1	No.2	No.3	No.4	Mean	SD	RSD(%)
k_1	0.002	0.002	0.002	0.002	0.002	0.000	7.002
F_{max}	104.713	104.198	96.561	108.518	103.497	5.010	4.841

Number of dissolution data points (N), degrees of freedom (df), and selected goodness of fit criteria – Pearson correlation coefficient (R), coefficient of determination (R^2), adjusted coefficient of determination ($R^2_{adjusted}$), and residual sum of squares (RSS) (manual calculation in MS Excel):

Parameter	No.1	No.2	No.3	No.4
N	31	31	31	31
df	29	29	29	29
R	0.992009541	0.991871217	0.993394755	0.990821674
R^2	0.984082929	0.98380851	0.98683314	0.98172759
$R^2_{adjusted}$	0.983534064	0.983250183	0.986379111	0.981097507
RSS	836.8310907	1042.335999	1039.46618	1245.298817

Graphical abstract of model fit presented as mean ± 1 SD of the fraction % of released carvedilol:

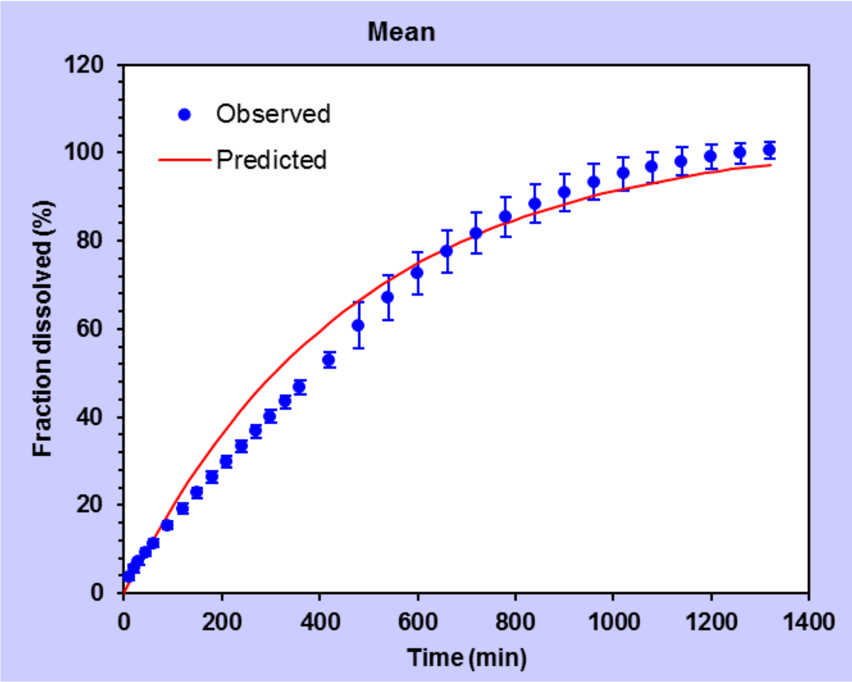

Supplementary materials – Model fitting summary – Glucidex® 19D (modelling the entire relevant dissolution data)

Graphical abstract of model fit presented as the fraction % of released carvedilol per tested tablet:

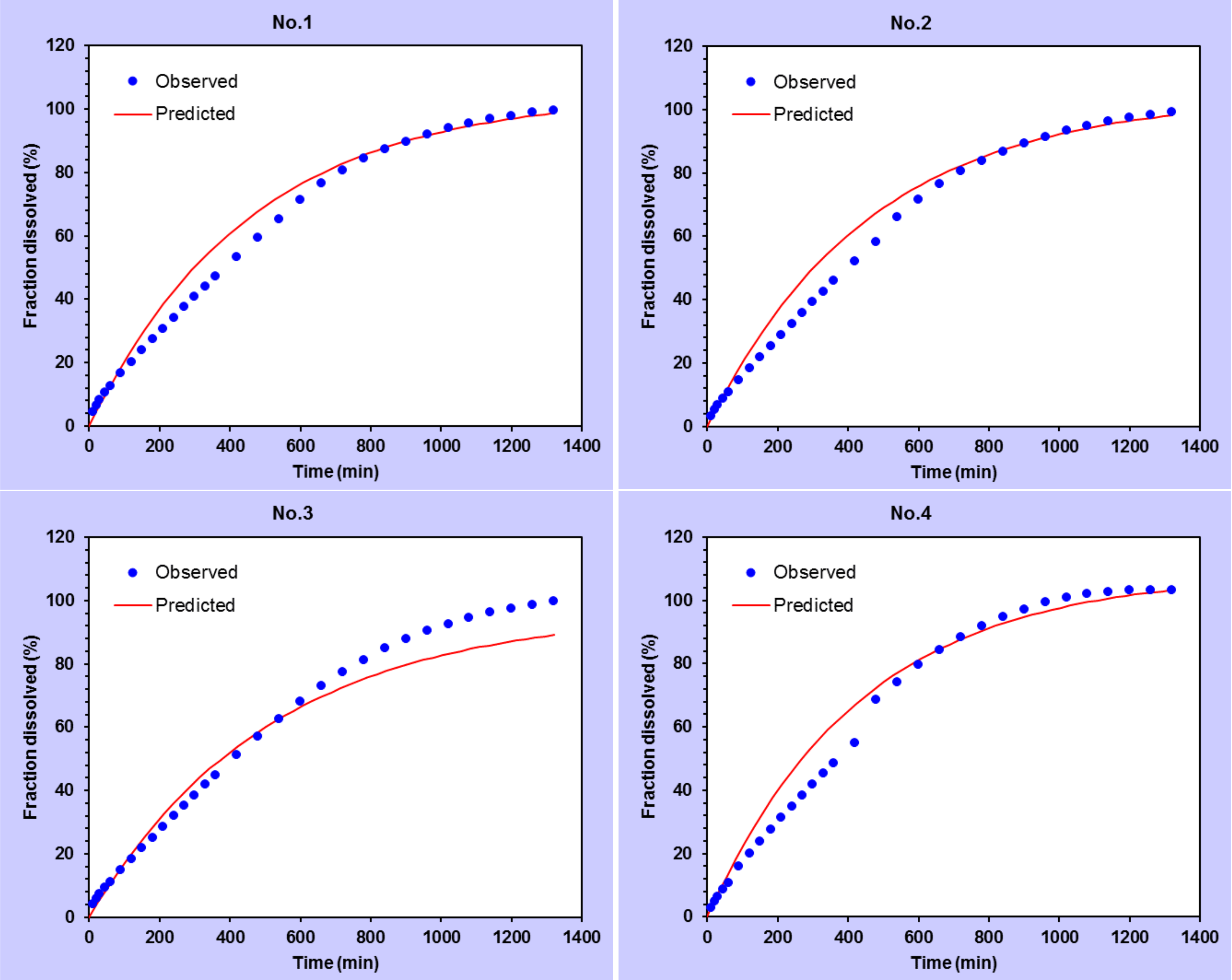

Supplementary materials – Model fitting summary – Glucidex® 19D (modelling the entire relevant dissolution data)

Model: **First–order with T_{lag} and F_{max}**

Model equation: $F = F_{max} \cdot \left[1 - e^{-k_1 \cdot (t - T_{lag})}\right]$

Fitted model parameters per tested tablet (N = 4) with statistics – mean, standard deviation (SD), and relative standard deviation expressed in % (RSD%) (output from DDSolver):

Parameter	No.1	No.2	No.3	No.4	Mean	SD	RSD(%)
k_1	0.002	0.002	0.002	0.003	0.002	0.000	6.307
T_{lag}	49.923	57.475	62.126	58.273	56.949	5.105	8.964
F_{max}	104.713	104.198	104.744	108.518	105.543	1.999	1.894

Number of dissolution data points (N), degrees of freedom (df), and selected goodness of fit criteria – Pearson correlation coefficient (R), coefficient of determination (R^2), adjusted coefficient of determination ($R^2_{adjusted}$), and residual sum of squares (RSS) (manual calculation in MS Excel):

Parameter	No.1	No.2	No.3	No.4
N	31	31	31	31
df	28	28	28	28
R	0.989782836	0.989297168	0.988038007	0.986154497
R^2	0.979670062	0.978708886	0.976219103	0.972500692
$R^2_{adjusted}$	0.978217923	0.977188092	0.974520468	0.970536455
RSS	1185.363414	1235.425224	1524.788263	1626.985659

Graphical abstract of model fit presented as mean ± 1 SD of the fraction % of released carvedilol:

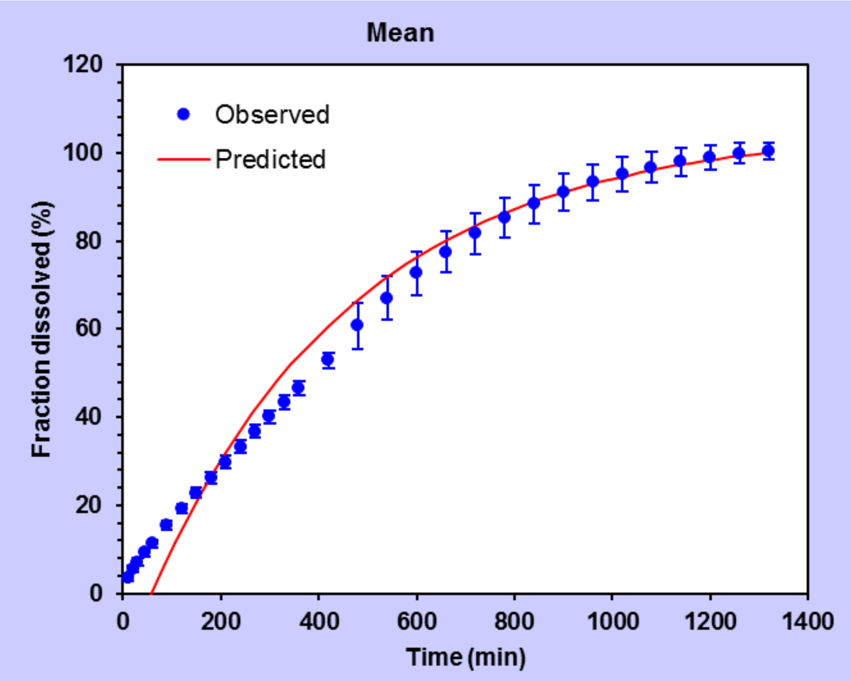

Supplementary materials – Model fitting summary – Glucidex® 19D (modelling the entire relevant dissolution data)

Graphical abstract of model fit presented as the fraction % of released carvedilol per tested tablet:

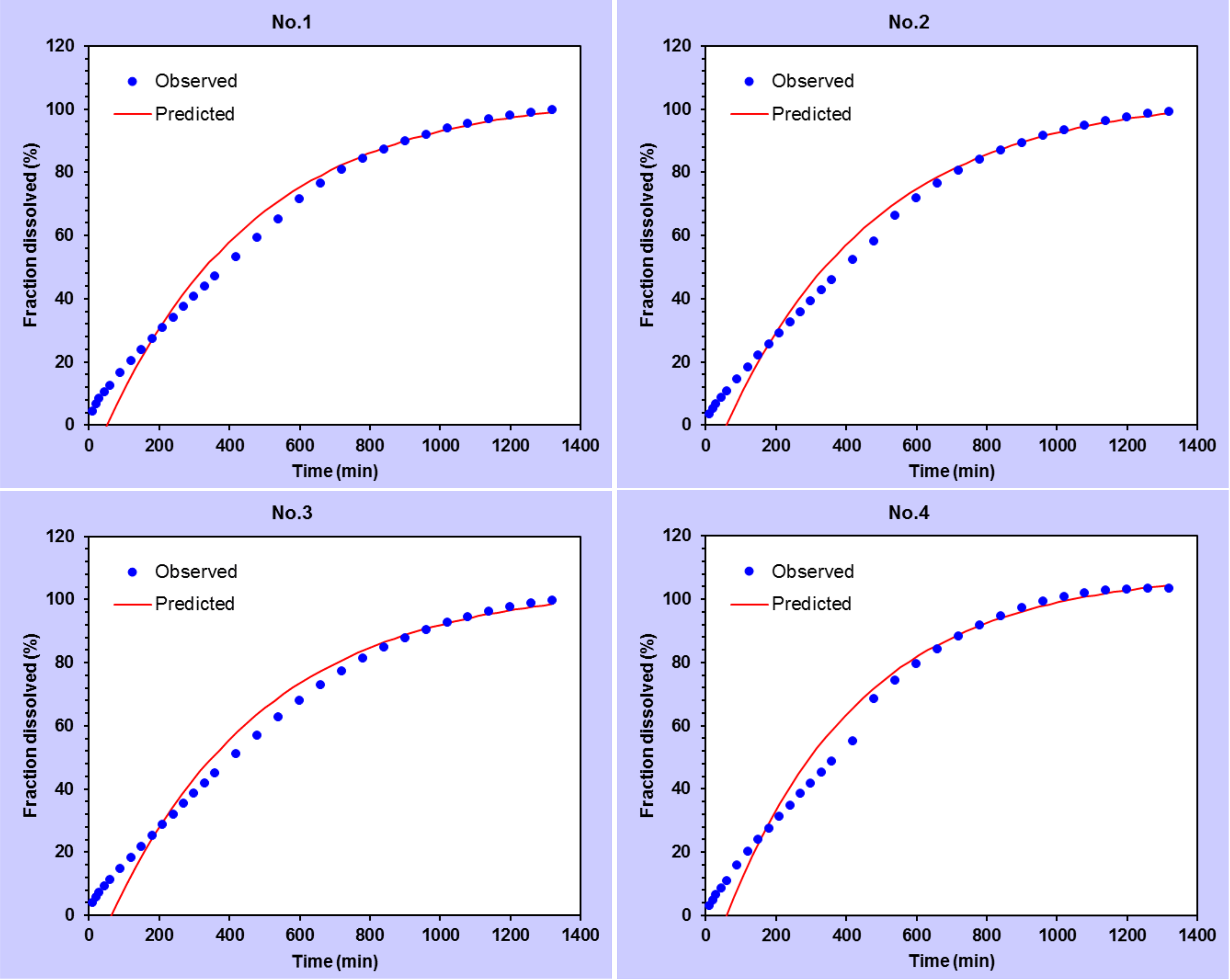

Supplementary materials – Model fitting summary – Glucidex® 19D (modelling the entire relevant dissolution data)

Model: **Higuchi**

Model equation: $F = k_H \cdot t^{0.5}$

Fitted model parameters per tested tablet (N = 4) with statistics – mean, standard deviation (SD), and relative standard deviation expressed in % (RSD%) (output from DDSolver):

Parameter	No.1	No.2	No.3	No.4	Mean	SD	RSD(%)
k_H	2.783	2.753	2.712	2.970	2.804	0.114	4.072

Number of dissolution data points (N), degrees of freedom (df), and selected goodness of fit criteria – Pearson correlation coefficient (R), coefficient of determination (R^2), adjusted coefficient of determination ($R^2_{adjusted}$), and residual sum of squares (RSS) (manual calculation in MS Excel):

Parameter	No.1	No.2	No.3	No.4
N	31	31	31	31
df	30	30	30	30
R	0.993628982	0.992570905	0.994168939	0.98889557
R^2	0.987298553	0.985197001	0.988371879	0.977914449
$R^2_{adjusted}$	0.987298553	0.985197001	0.988371879	0.977914449
RSS	1331.65408	1757.8572	1602.117528	2318.068633

Graphical abstract of model fit presented as mean ± 1 SD of the fraction % of released carvedilol:

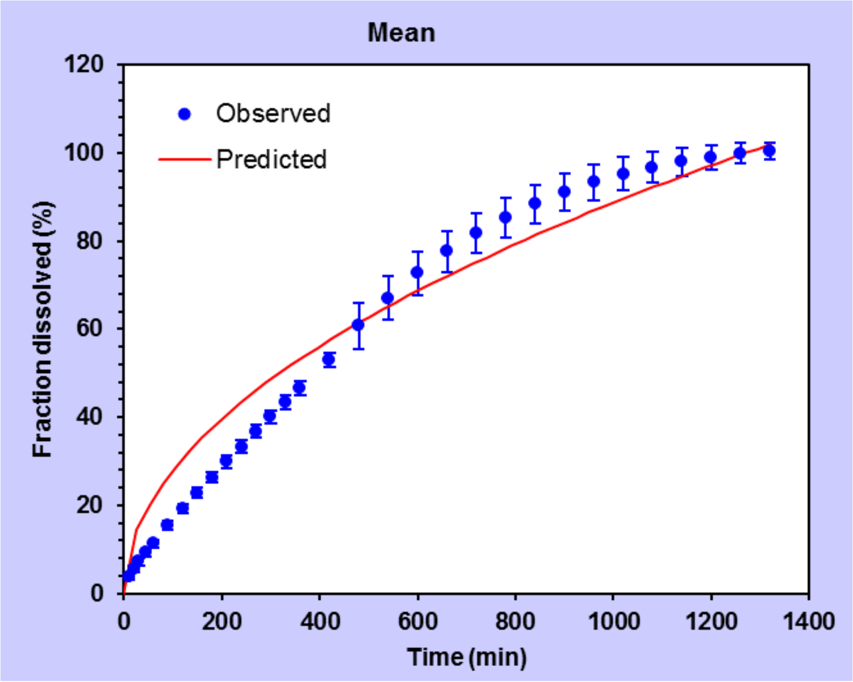

Supplementary materials – Model fitting summary – Glucidex® 19D (modelling the entire relevant dissolution data)

Graphical abstract of model fit presented as the fraction % of released carvedilol per tested tablet:

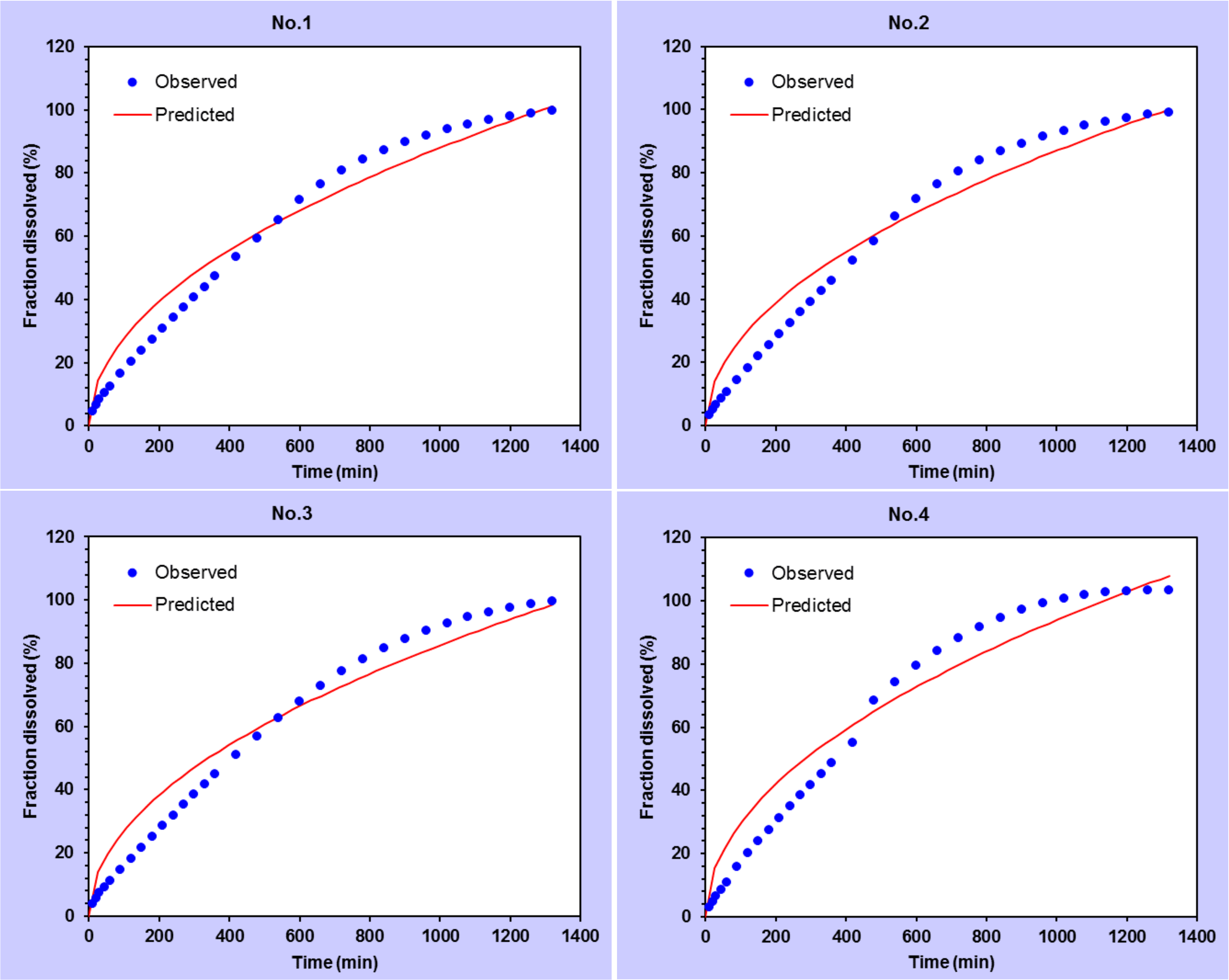

Supplementary materials – Model fitting summary – Glucidex® 19D (modelling the entire relevant dissolution data)

Model: **Higuchi with T_{lag}**

Model equation: $F = k_H \cdot (t - T_{lag})^{0.5}$

Fitted model parameters per tested tablet (N = 4) with statistics – mean, standard deviation (SD), and relative standard deviation expressed in % (RSD%) (output from DDSolver):

Parameter	No.1	No.2	No.3	No.4	Mean	SD	RSD(%)
k_H	2.957	2.952	2.936	3.151	2.999	0.102	3.400
T_{lag}	55.238	62.167	71.425	77.083	66.478	9.693	14.581

Number of dissolution data points (N), degrees of freedom (df), and selected goodness of fit criteria – Pearson correlation coefficient (R), coefficient of determination (R^2), adjusted coefficient of determination ($R^2_{adjusted}$), and residual sum of squares (RSS) (manual calculation in MS Excel):

Parameter	No.1	No.2	No.3	No.4
N	31	31	31	31
df	29	29	29	29
R	0.989876292	0.988685693	0.990569689	0.988119979
R^2	0.979855074	0.977499399	0.981228309	0.976381093
$R^2_{adjusted}$	0.979160421	0.976723517	0.980581009	0.975566648
RSS	706.3795709	801.9715402	671.9173491	1058.084195

Graphical abstract of model fit presented as mean ± 1 SD of the fraction % of released carvedilol:

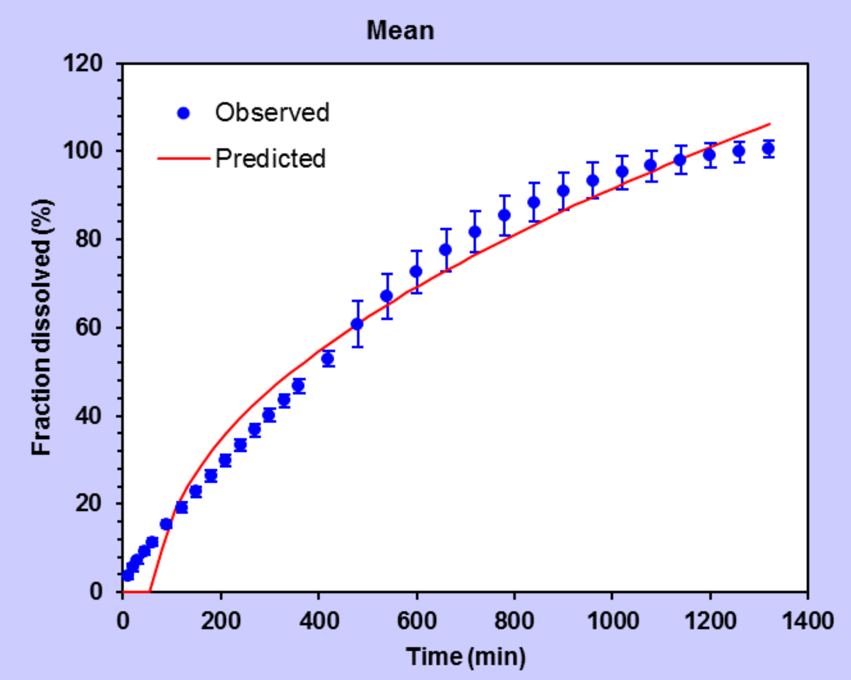

Supplementary materials – Model fitting summary – Glucidex® 19D (modelling the entire relevant dissolution data)

Graphical abstract of model fit presented as the fraction % of released carvedilol per tested tablet:

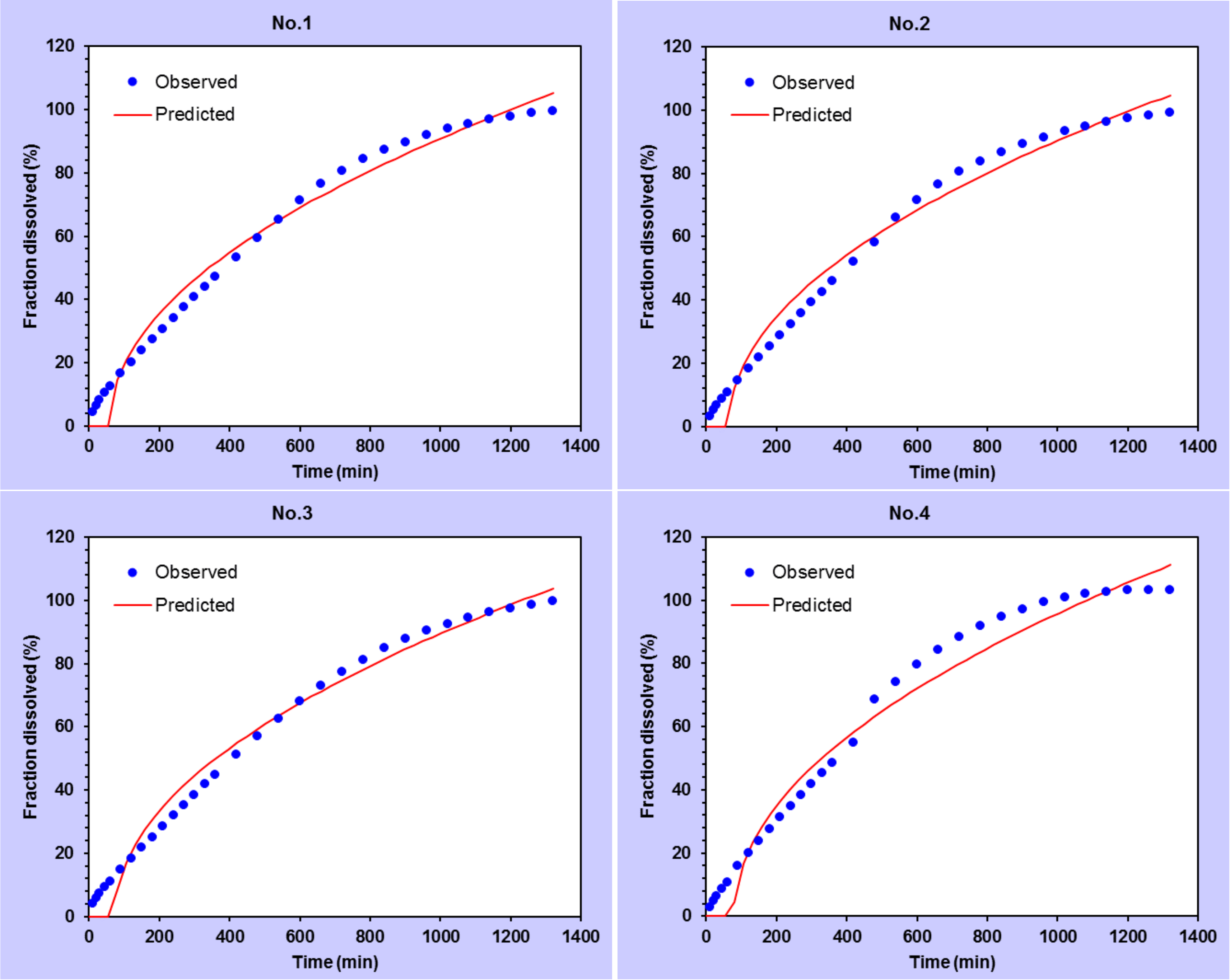

Supplementary materials – Model fitting summary – Glucidex® 19D (modelling the entire relevant dissolution data)

Model: **Higuchi with F_0**

Model equation: $F = F_0 + k_H \cdot t^{0.5}$

Fitted model parameters per tested tablet (N = 4) with statistics – mean, standard deviation (SD), and relative standard deviation expressed in % (RSD%) (output from DDSolver):

Parameter	No.1	No.2	No.3	No.4	Mean	SD	RSD(%)
k_H	3.267	3.321	3.272	3.577	3.359	0.147	4.376
F_0	-12.361	-14.488	-14.301	-15.482	-14.158	1.305	-9.221

Number of dissolution data points (N), degrees of freedom (df), and selected goodness of fit criteria – Pearson correlation coefficient (R), coefficient of determination (R^2), adjusted coefficient of determination ($R^2_{adjusted}$), and residual sum of squares (RSS) (manual calculation in MS Excel):

Parameter	No.1	No.2	No.3	No.4
N	31	31	31	31
df	29	29	29	29
R	0.993628982	0.992570905	0.994168939	0.98889557
R^2	0.987298553	0.985197001	0.988371879	0.977914449
$R^2_{adjusted}$	0.986860572	0.984686553	0.98797091	0.977152878
RSS	427.9847479	516.3476466	392.5434701	900.3714954

Graphical abstract of model fit presented as mean ± 1 SD of the fraction % of released carvedilol:

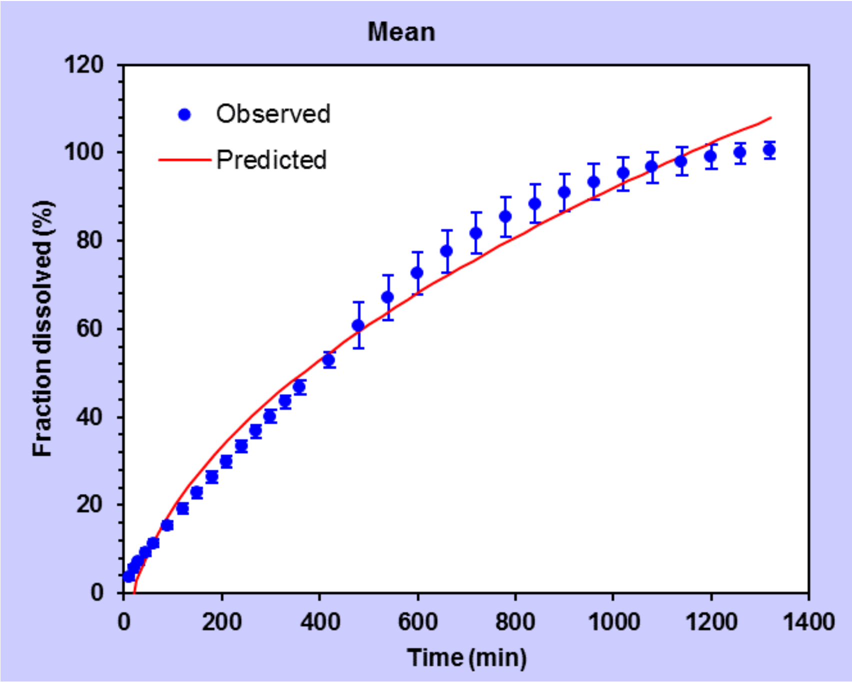

Supplementary materials – Model fitting summary – Glucidex® 19D (modelling the entire relevant dissolution data)

Graphical abstract of model fit presented as the fraction % of released carvedilol per tested tablet:

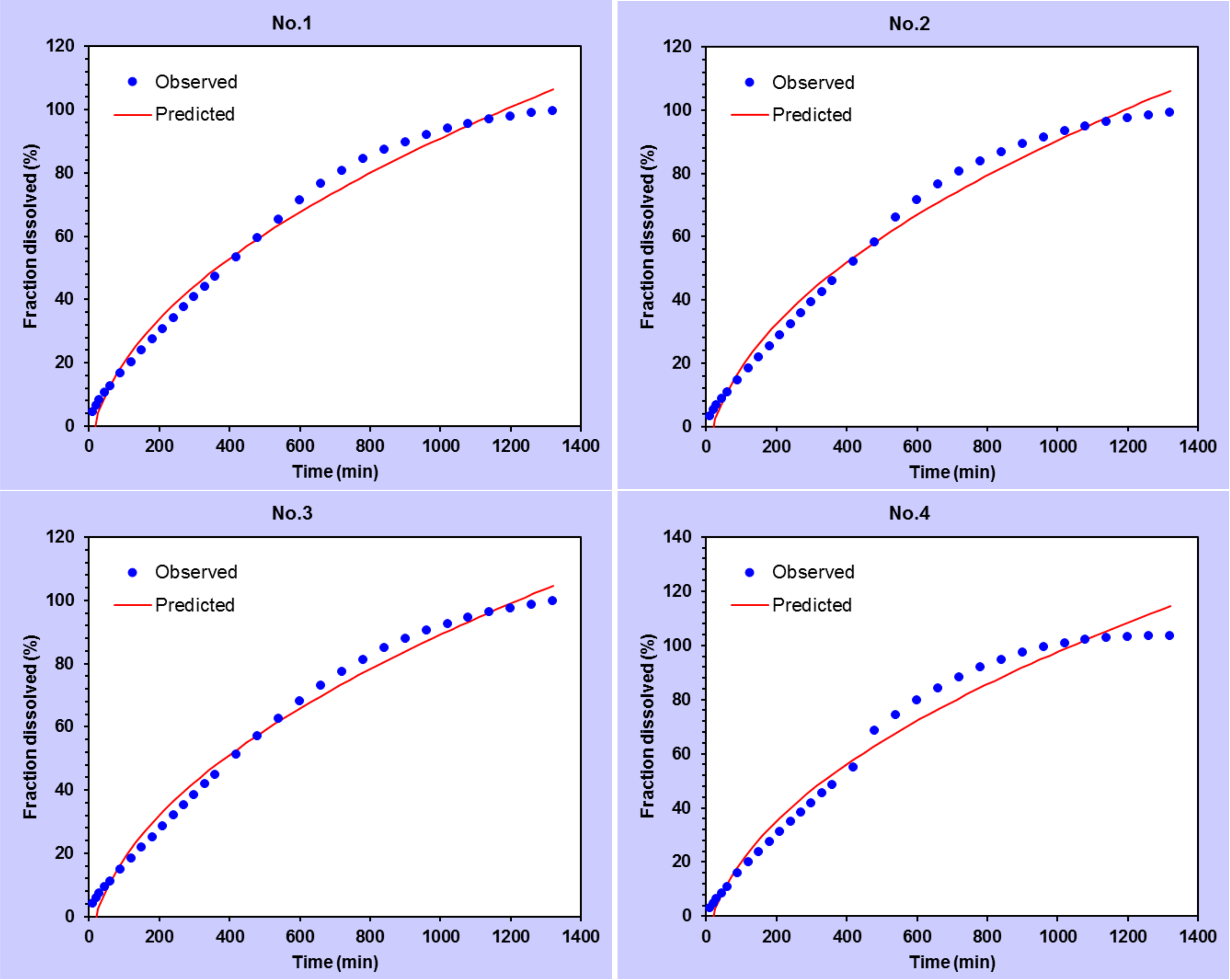

Supplementary materials – Model fitting summary – Glucidex® 19D (modelling the entire relevant dissolution data)

Model: **Korsmeyer–Peppas**

Model equation: $F = k_{KP} \cdot t^n$

Fitted model parameters per tested tablet (N = 4) with statistics – mean, standard deviation (SD), and relative standard deviation expressed in % (RSD%) (output from DDSolver):

Parameter	No.1	No.2	No.3	No.4	Mean	SD	RSD(%)
k_{KP}	0.823	0.548	0.654	0.559	0.646	0.127	19.684
n	0.684	0.745	0.714	0.760	0.726	0.034	4.628

Number of dissolution data points (N), degrees of freedom (df), and selected goodness of fit criteria – Pearson correlation coefficient (R), coefficient of determination (R^2), adjusted coefficient of determination ($R^2_{adjusted}$), and residual sum of squares (RSS) (manual calculation in MS Excel):

Parameter	No.1	No.2	No.3	No.4
N	31	31	31	31
df	29	29	29	29
R	0.991715422	0.988353277	0.99363519	0.980961318
R^2	0.983499479	0.976842199	0.98731089	0.962285108
$R^2_{adjusted}$	0.982930495	0.976043654	0.986873334	0.960984595
RSS	578.3980001	922.9680268	439.2533105	2353.780357

Graphical abstract of model fit presented as mean ± 1 SD of the fraction % of released carvedilol:

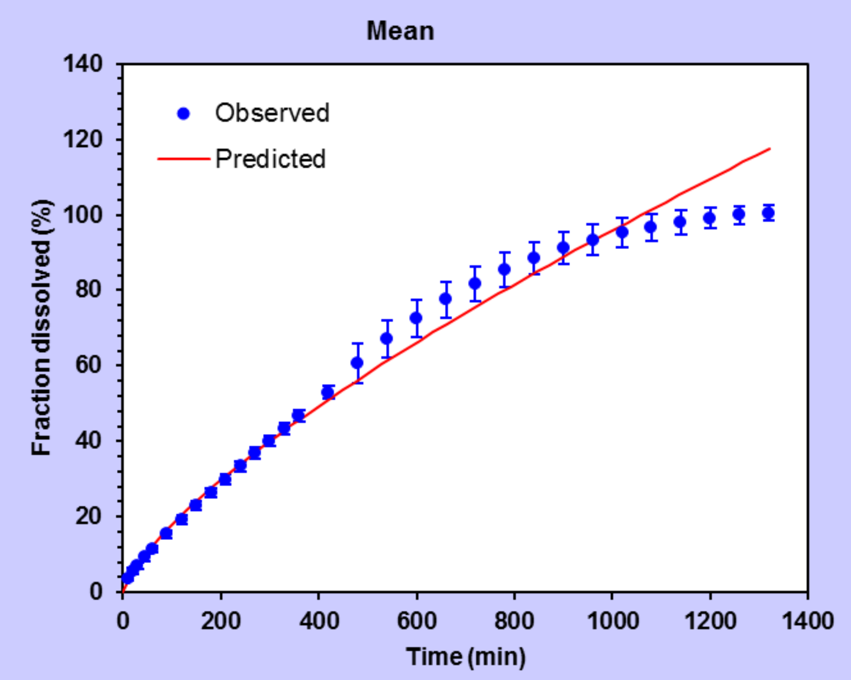

Supplementary materials – Model fitting summary – Glucidex® 19D (modelling the entire relevant dissolution data)

Graphical abstract of model fit presented as the fraction % of released carvedilol per tested tablet:

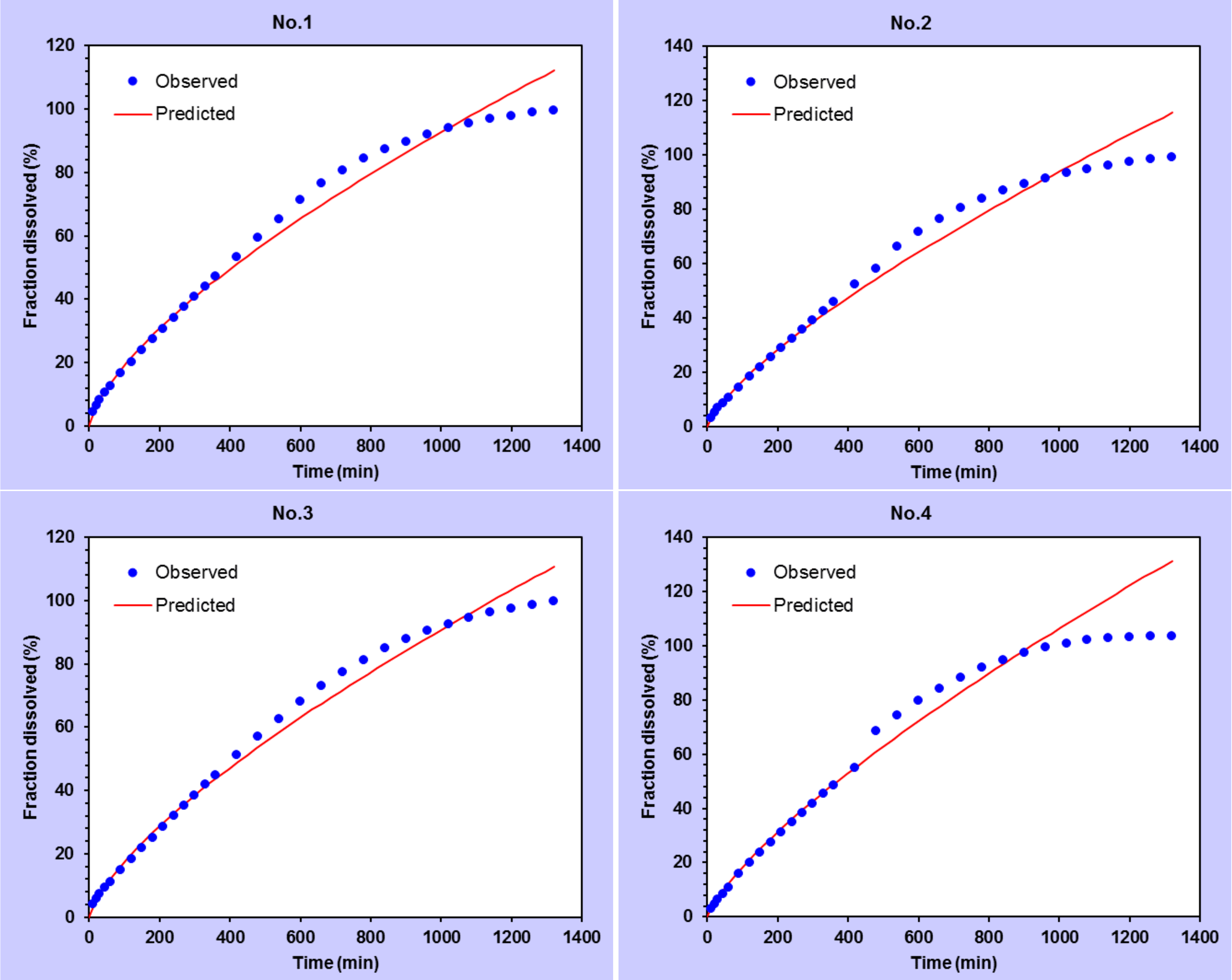

Supplementary materials – Model fitting summary – Glucidex® 19D (modelling the entire relevant dissolution data)

Model: **Korsmeyer–Peppas with T_{lag}**

Model equation: $F = k_{KP} \cdot (t - T_{lag})^n$

Fitted model parameters per tested tablet (N = 4) with statistics – mean, standard deviation (SD), and relative standard deviation expressed in % (RSD%) (output from DDSolver):

Parameter	No.1	No.2	No.3	No.4	Mean	SD	RSD(%)
k_{KP}	1.064	0.723	0.856	0.643	0.821	0.184	22.380
n	0.644	0.702	0.672	0.732	0.687	0.038	5.536
T_{lag}	4.000	4.000	4.000	4.000	4.000	0.000	0.000

Number of dissolution data points (N), degrees of freedom (df), and selected goodness of fit criteria – Pearson correlation coefficient (R), coefficient of determination (R^2), adjusted coefficient of determination ($R^2_{adjusted}$), and residual sum of squares (RSS) (manual calculation in MS Excel):

Parameter	No.1	No.2	No.3	No.4
N	31	31	31	31
df	28	28	28	28
R	0.992911521	0.990227728	0.994658019	0.982688955
R^2	0.985873289	0.980550953	0.989344574	0.965677583
$R^2_{adjusted}$	0.984864238	0.979161736	0.988583473	0.963225982
RSS	490.2351429	691.1833331	389.7878773	1538.569036

Graphical abstract of model fit presented as mean ± 1 SD of the fraction % of released carvedilol:

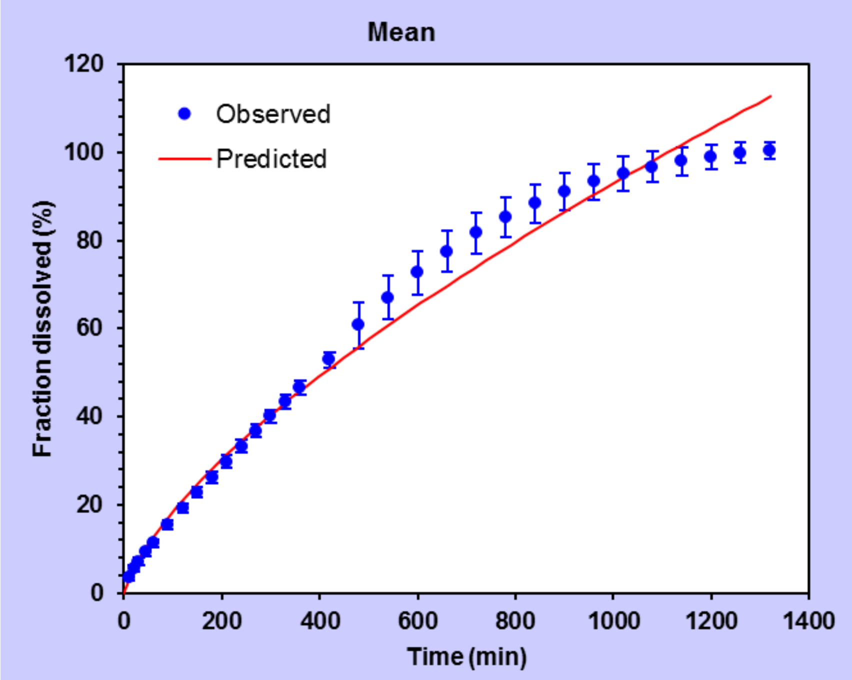

Supplementary materials – Model fitting summary – Glucidex® 19D (modelling the entire relevant dissolution data)

Graphical abstract of model fit presented as the fraction % of released carvedilol per tested tablet:

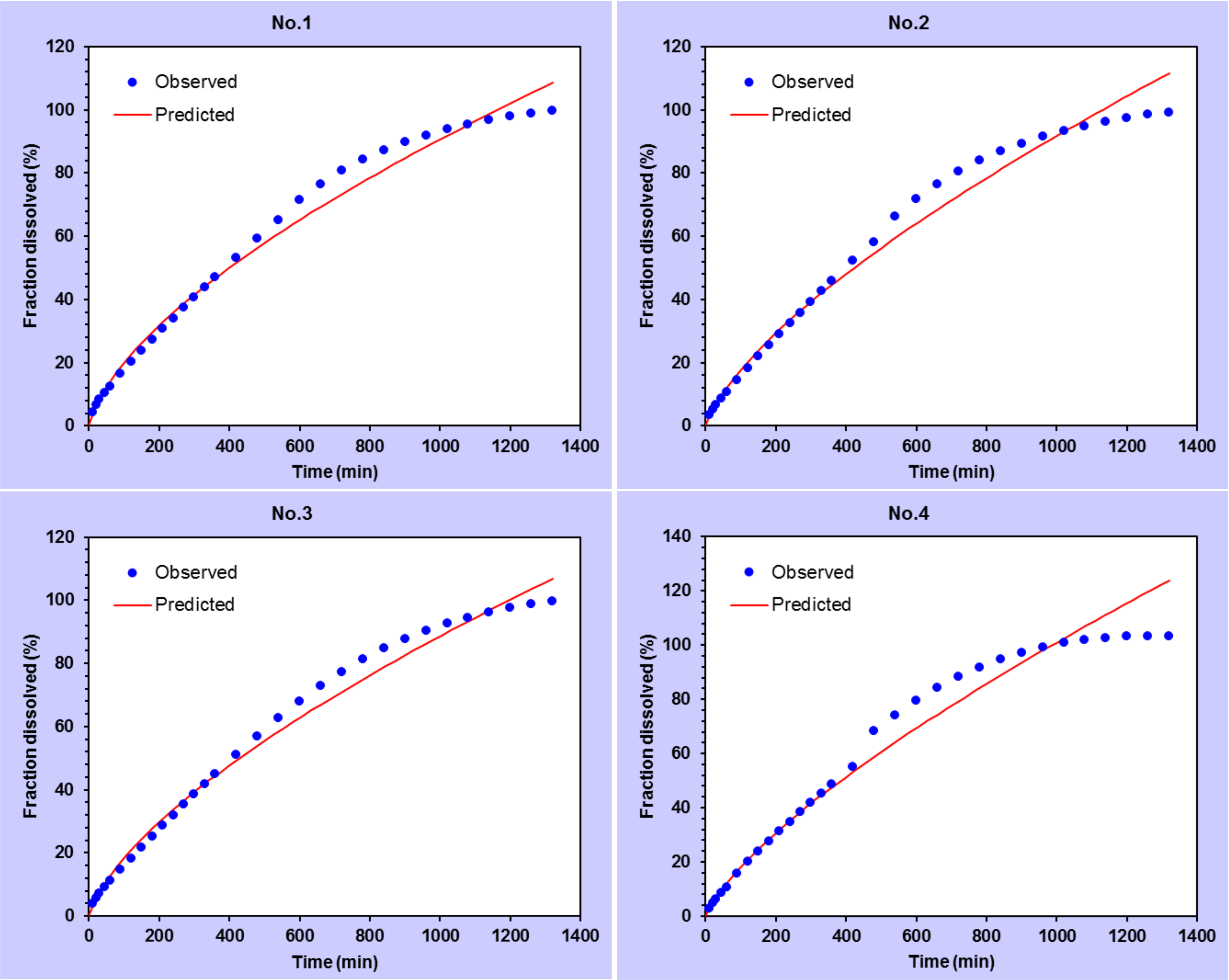

Supplementary materials – Model fitting summary – Glucidex® 19D (modelling the entire relevant dissolution data)

Model: **Korsmeyer–Peppas with F_0**

Model equation: $F = F_0 + k_{KP} \cdot t^n$

Fitted model parameters per tested tablet (N = 4) with statistics – mean, standard deviation (SD), and relative standard deviation expressed in % (RSD%) (output from DDSolver):

Parameter	No.1	No.2	No.3	No.4	Mean	SD	RSD(%)
k_{KP}	0.500	0.344	0.395	0.312	0.388	0.082	21.266
n	0.757	0.814	0.789	0.843	0.801	0.037	4.558
F_0	1.800	1.320	1.600	1.160	1.470	0.285	19.416

Number of dissolution data points (N), degrees of freedom (df), and selected goodness of fit criteria – Pearson correlation coefficient (R), coefficient of determination (R^2), adjusted coefficient of determination ($R^2_{adjusted}$), and residual sum of squares (RSS) (manual calculation in MS Excel):

Parameter	No.1	No.2	No.3	No.4
N	31	31	31	31
df	28	28	28	28
R	0.988771708	0.984806206	0.991024857	0.97550436
R^2	0.97766949	0.969843264	0.982130268	0.951608757
$R^2_{adjusted}$	0.976074454	0.967689212	0.980853859	0.948152239
RSS	965.7349299	1511.960134	773.4280199	3064.049477

Graphical abstract of model fit presented as mean ± 1 SD of the fraction % of released carvedilol:

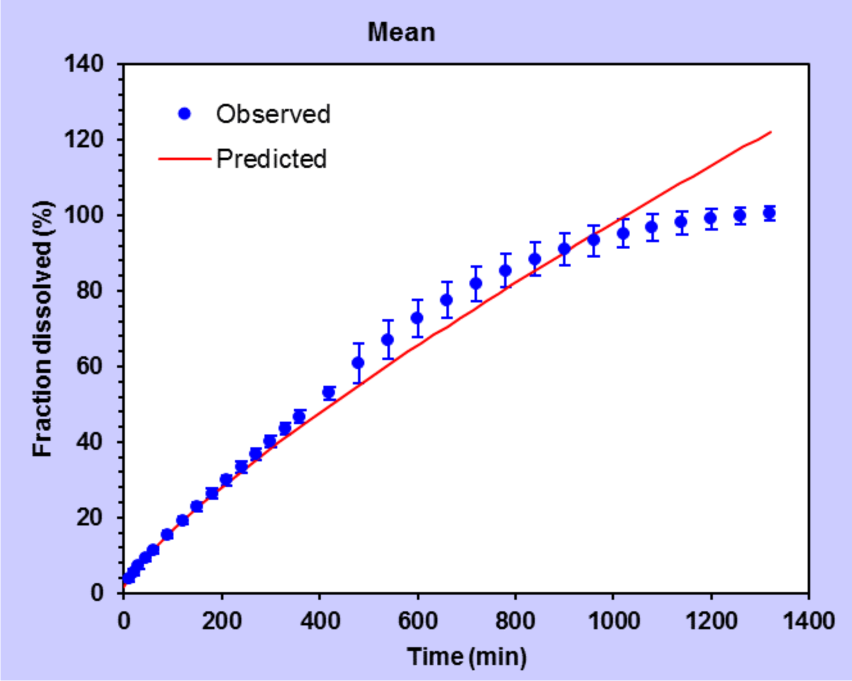

Supplementary materials – Model fitting summary – Glucidex® 19D (modelling the entire relevant dissolution data)

Graphical abstract of model fit presented as the fraction % of released carvedilol per tested tablet:

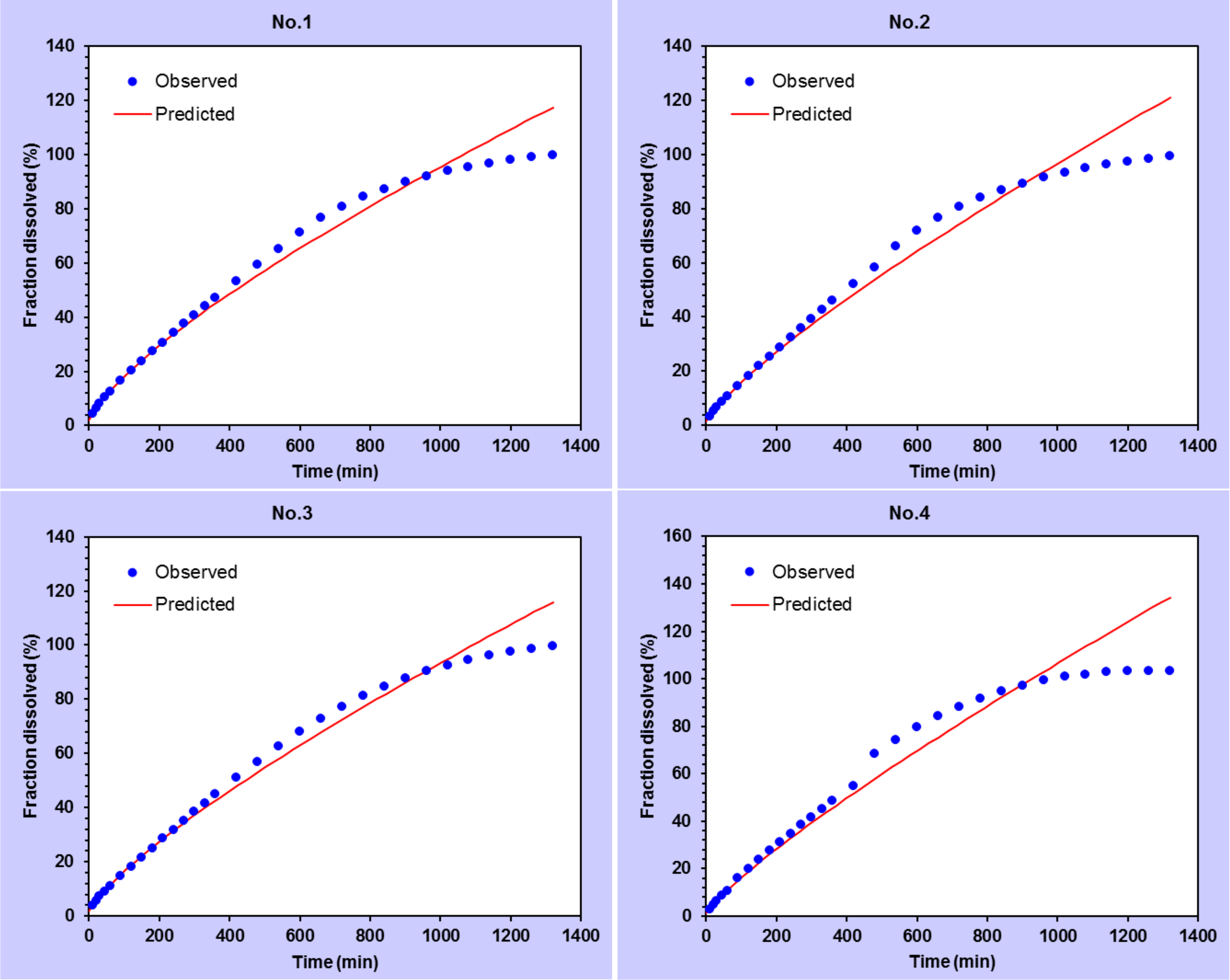

Supplementary materials – Model fitting summary – Glucidex® 19D (modelling the entire relevant dissolution data)

Model: **Hixson–Crowell**

Model equation: $F = 100 \cdot [1 - (1 - k_{HC} \cdot t)^3]$

Fitted model parameters per tested tablet (N = 4) with statistics – mean, standard deviation (SD), and relative standard deviation expressed in % (RSD%) (output from DDSolver):

Parameter	No.1	No.2	No.3	No.4	Mean	SD	RSD(%)
k_{HC}	0.001	0.001	0.001	0.001	0.001	0.000	9.160

Number of dissolution data points (N), degrees of freedom (df), and selected goodness of fit criteria – Pearson correlation coefficient (R), coefficient of determination (R^2), adjusted coefficient of determination ($R^2_{adjusted}$), and residual sum of squares (RSS) (manual calculation in MS Excel):

Parameter	No.1	No.2	No.3	No.4
N	31	31	31	31
df	30	30	30	30
R	0.997630716	0.998011251	0.999138918	0.996739838
R^2	0.995267046	0.996026458	0.998278578	0.993490304
$R^2_{adjusted}$	0.995267046	0.996026458	0.998278578	0.993490304
RSS	196.8199254	182.9489448	203.7001771	476.7170595

Graphical abstract of model fit presented as mean ± 1 SD of the fraction % of released carvedilol:

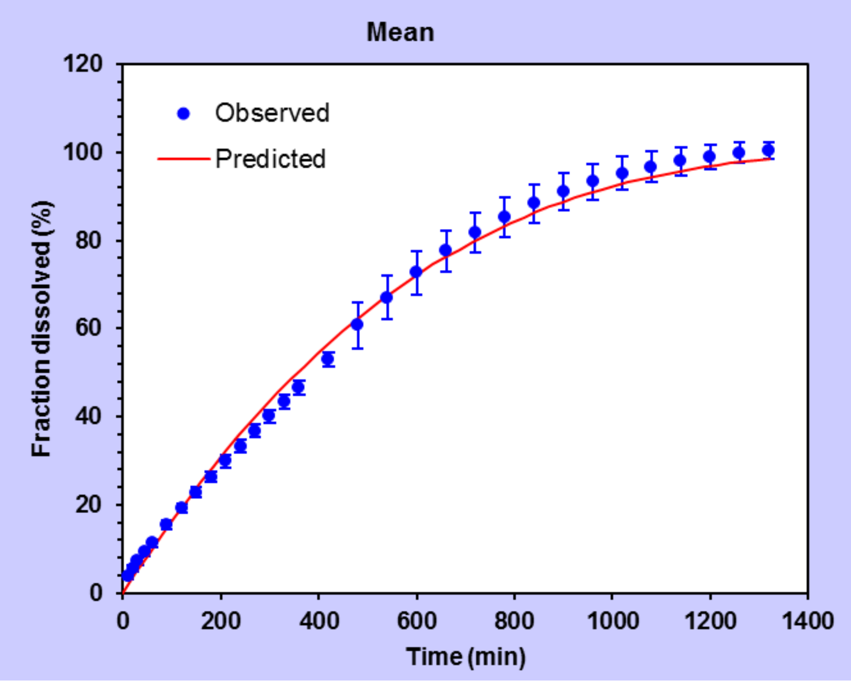

Supplementary materials – Model fitting summary – Glucidex® 19D (modelling the entire relevant dissolution data)

Graphical abstract of model fit presented as the fraction % of released carvedilol per tested tablet:

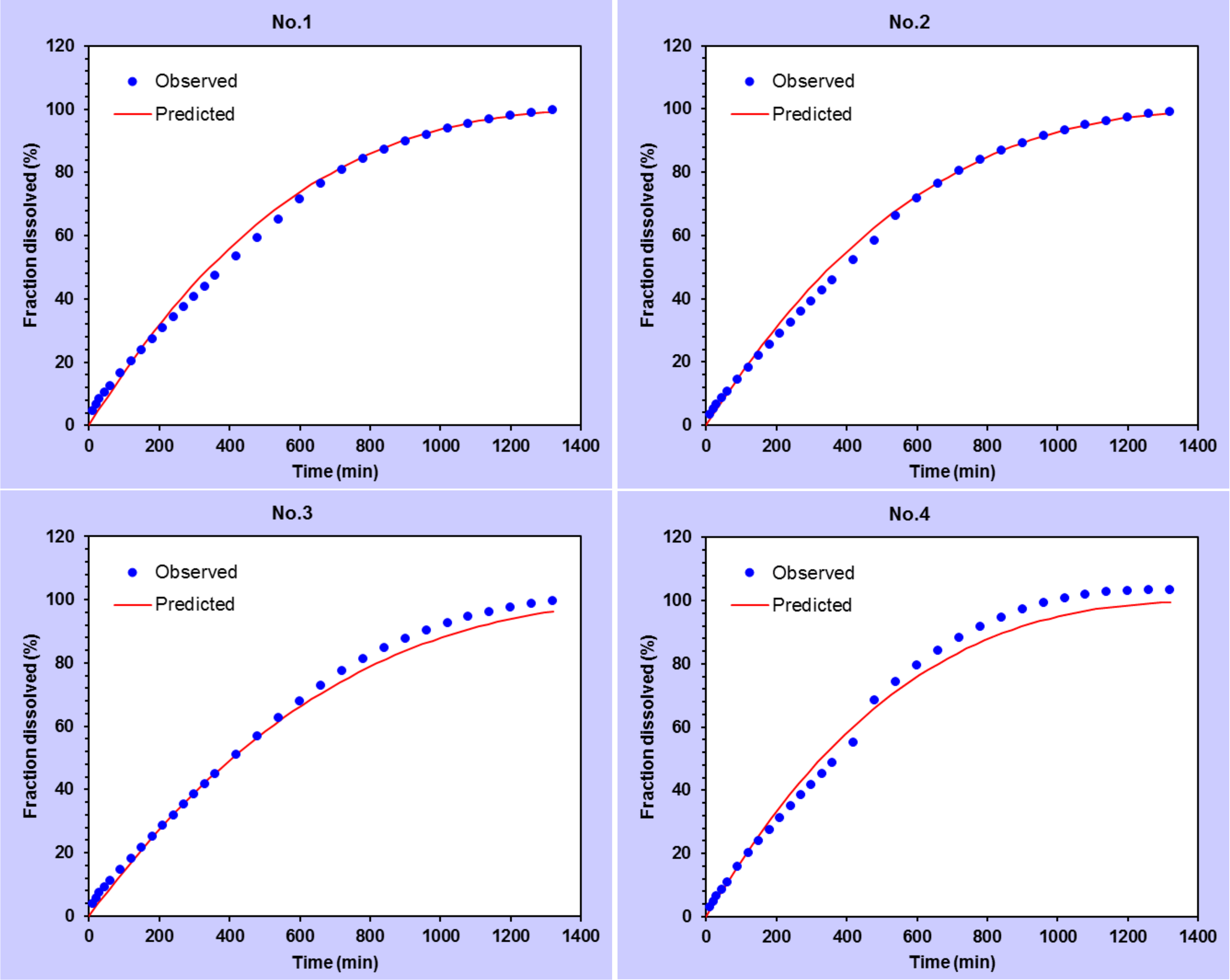

Supplementary materials – Model fitting summary – Glucidex® 19D (modelling the entire relevant dissolution data)

Model: **Hixson–Crowell with T_{lag}**

Model equation: $F = 100 \cdot \left\{1 - \left[1 - k_{HC} \cdot \left(t - T_{lag}\right)\right]^3\right\}$

Fitted model parameters per tested tablet (N = 4) with statistics – mean, standard deviation (SD), and relative standard deviation expressed in % (RSD%) (output from DDSolver):

Parameter	No.1	No.2	No.3	No.4	Mean	SD	RSD(%)
k_{HC}	0.001	0.001	0.001	0.001	0.001	0.000	13.511
T_{lag}	17.039	19.900	30.597	44.500	28.009	12.447	44.439

Number of dissolution data points (N), degrees of freedom (df), and selected goodness of fit criteria – Pearson correlation coefficient (R), coefficient of determination (R^2), adjusted coefficient of determination ($R^2_{adjusted}$), and residual sum of squares (RSS) (manual calculation in MS Excel):

Parameter	No.1	No.2	No.3	No.4
N	31	31	31	31
df	29	29	29	29
R	0.997418795	0.99781827	0.996605373	0.99073337
R^2	0.994844253	0.9956413	0.993222269	0.98155261
$R^2_{adjusted}$	0.994666469	0.995491	0.992988554	0.980916493
RSS	266.2637498	190.5148261	400.3037057	899.2697471

Graphical abstract of model fit presented as mean ± 1 SD of the fraction % of released carvedilol:

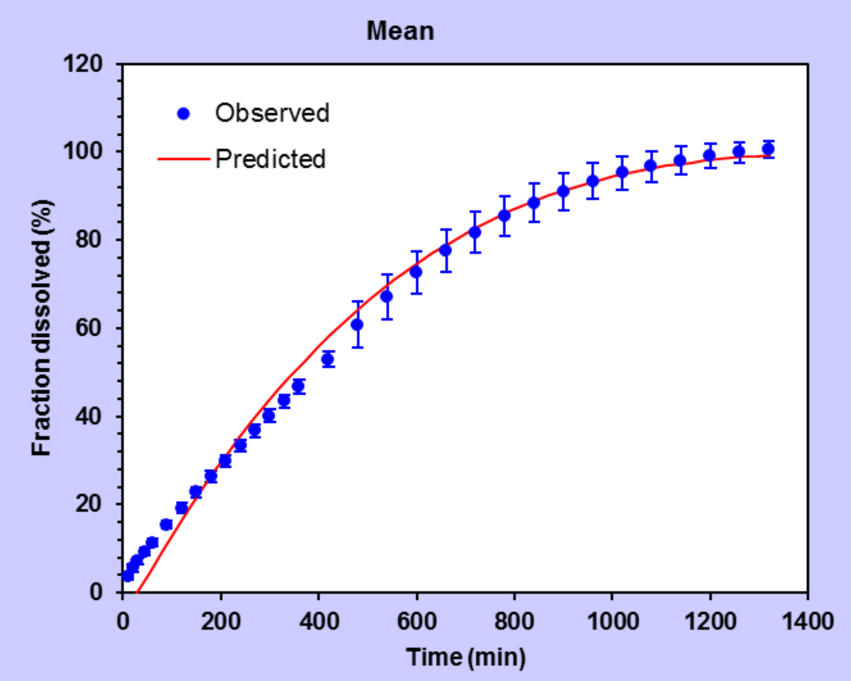

Supplementary materials – Model fitting summary – Glucidex® 19D (modelling the entire relevant dissolution data)

Graphical abstract of model fit presented as the fraction % of released carvedilol per tested tablet:

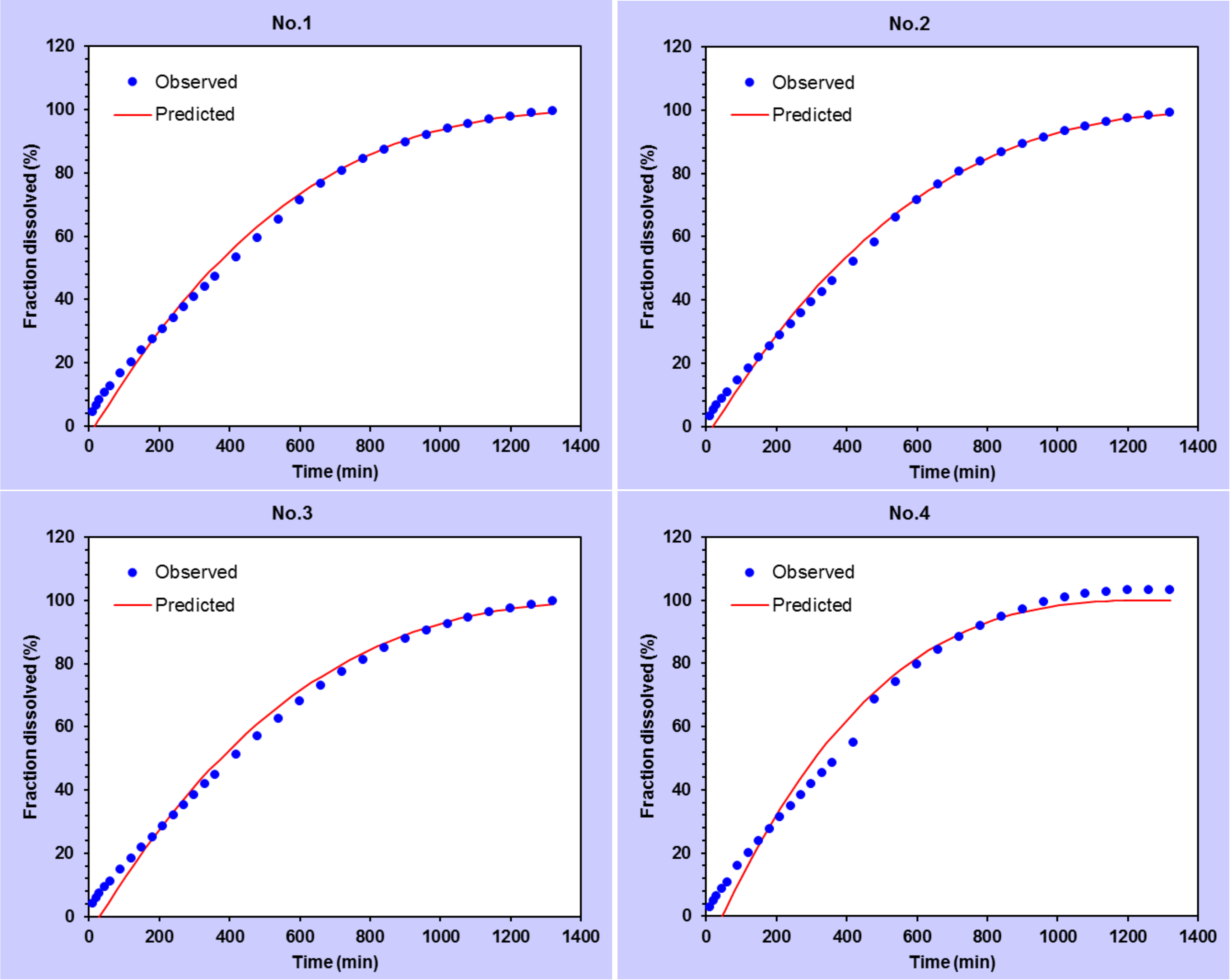

Supplementary materials – Model fitting summary – Glucidex® 19D (modelling the entire relevant dissolution data)

Model: **Hopfenberg**

Model equation: $F = 100 \cdot [1 - (1 - k_{HB} \cdot t)^n]$

Fitted model parameters per tested tablet (N = 4) with statistics – mean, standard deviation (SD), and relative standard deviation expressed in % (RSD%) (output from DDSolver):

Parameter	No.1	No.2	No.3	No.4	Mean	SD	RSD(%)
k_{HB}	0.001	0.001	0.001	0.001	0.001	0.000	14.075
n	2.000	2.000	2.268	2.000	2.067	0.134	6.473

Number of dissolution data points (N), degrees of freedom (df), and selected goodness of fit criteria – Pearson correlation coefficient (R), coefficient of determination (R^2), adjusted coefficient of determination ($R^2_{adjusted}$), and residual sum of squares (RSS) (manual calculation in MS Excel):

Parameter	No.1	No.2	No.3	No.4
N	31	31	31	31
df	29	29	29	29
R	0.999687073	0.999340173	0.999545853	0.997397063
R^2	0.999374244	0.998680781	0.999091913	0.994800901
$R^2_{adjusted}$	0.999352666	0.998635291	0.999060599	0.994621622
RSS	156.4885135	100.4286471	52.76811398	247.1988675

Graphical abstract of model fit presented as mean ± 1 SD of the fraction % of released carvedilol:

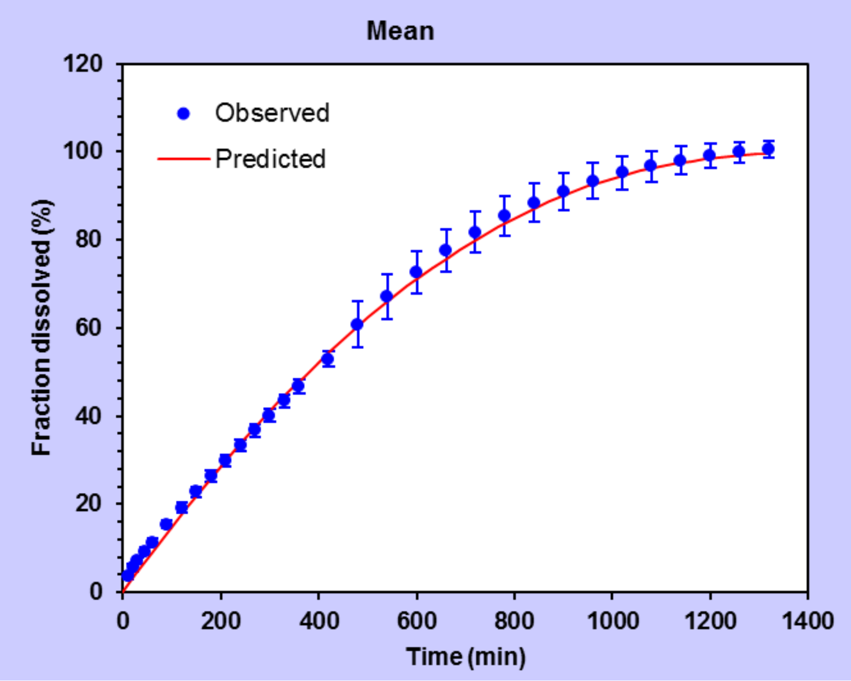

Supplementary materials – Model fitting summary – Glucidex® 19D (modelling the entire relevant dissolution data)

Graphical abstract of model fit presented as the fraction % of released carvedilol per tested tablet:

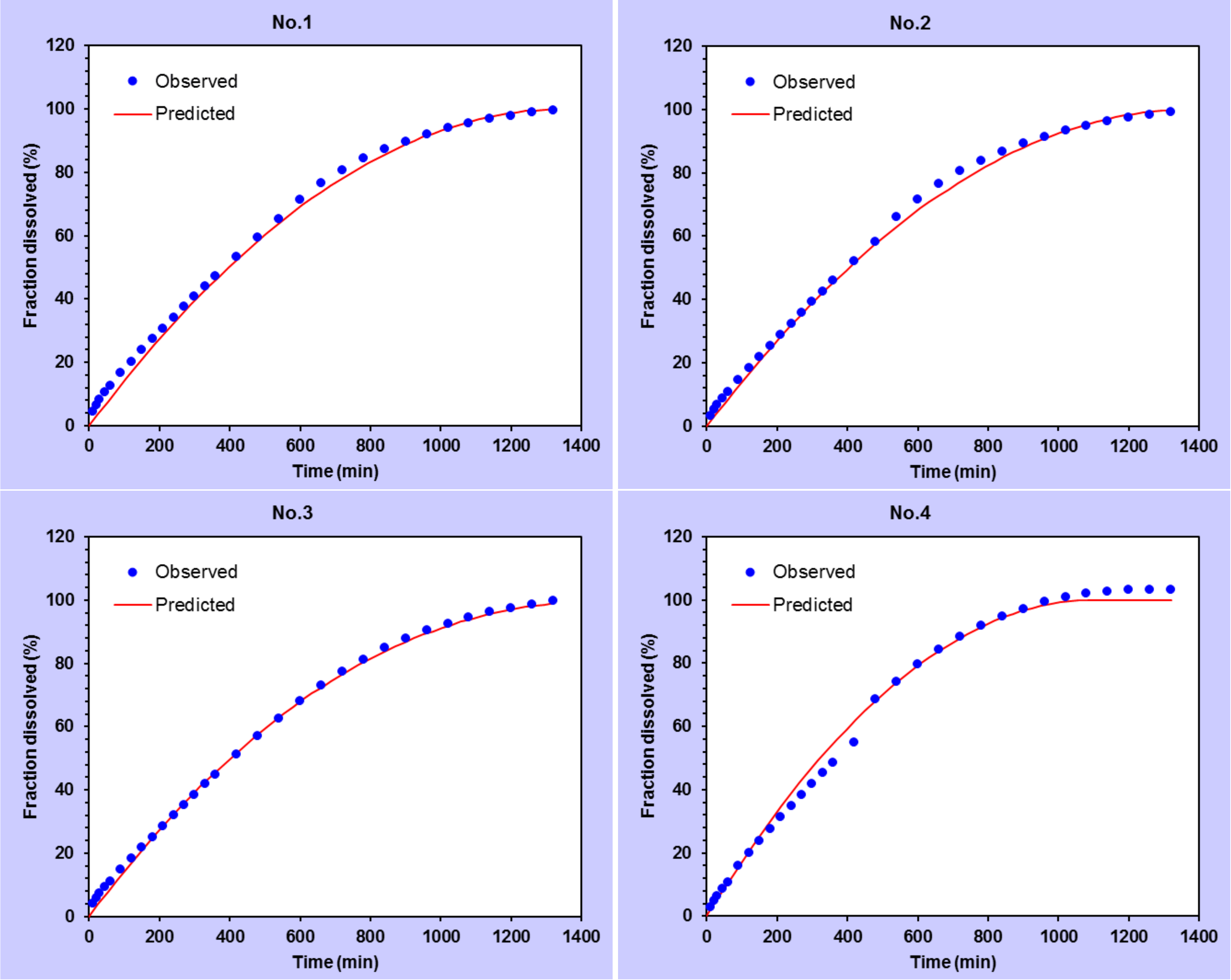

Supplementary materials – Model fitting summary – Glucidex® 19D (modelling the entire relevant dissolution data)

Model: **Hopfenberg with T_{lag}**

Model equation: $F = 100 \cdot \{1 - [1 - k_{HB} \cdot (t - T_{lag})]^n\}$

Fitted model parameters per tested tablet (N = 4) with statistics – mean, standard deviation (SD), and relative standard deviation expressed in % (RSD%) (output from DDSolver):

Parameter	No.1	No.2	No.3	No.4	Mean	SD	RSD(%)
k_{HB}	0.001	0.001	0.001	0.001	0.001	0.000	14.795
n	2.000	2.000	2.000	2.000	2.000	0.000	0.000
T_{lag}	-32.278	-23.858	-15.595	16.839	-13.723	21.483	-156.550

Number of dissolution data points (N), degrees of freedom (df), and selected goodness of fit criteria – Pearson correlation coefficient (R), coefficient of determination (R^2), adjusted coefficient of determination ($R^2_{adjusted}$), and residual sum of squares (RSS) (manual calculation in MS Excel):

Parameter	No.1	No.2	No.3	No.4
N	31	31	31	31
df	28	28	28	28
R	0.999634943	0.999259771	0.99982953	0.99704829
R^2	0.99927002	0.99852009	0.999659089	0.994105293
$R^2_{adjusted}$	0.999217879	0.998414382	0.999634738	0.993684242
RSS	31.80847294	70.24138726	11.57473552	248.9367949

Graphical abstract of model fit presented as mean ± 1 SD of the fraction % of released carvedilol:

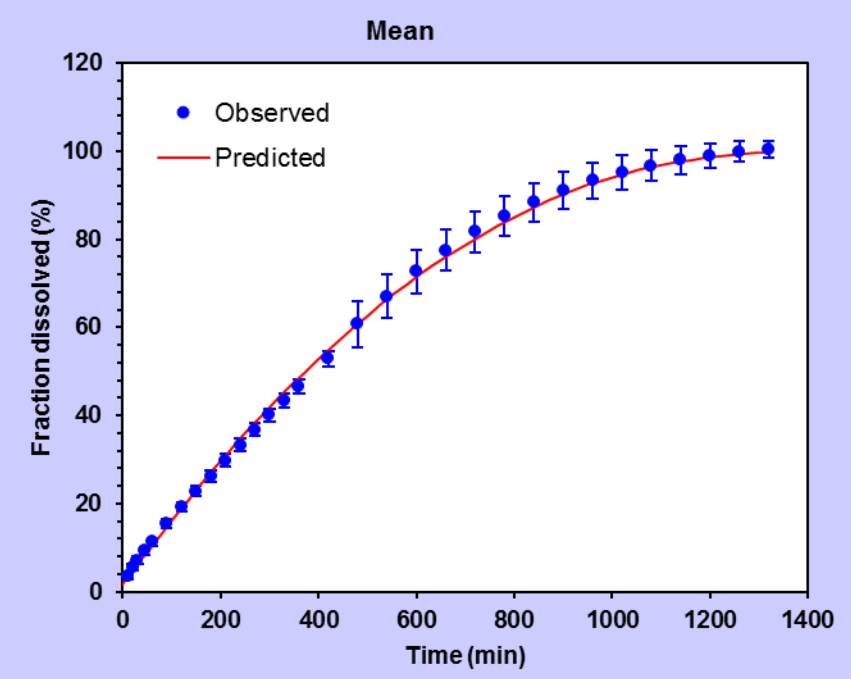

Supplementary materials – Model fitting summary – Glucidex® 19D (modelling the entire relevant dissolution data)

Graphical abstract of model fit presented as the fraction % of released carvedilol per tested tablet:

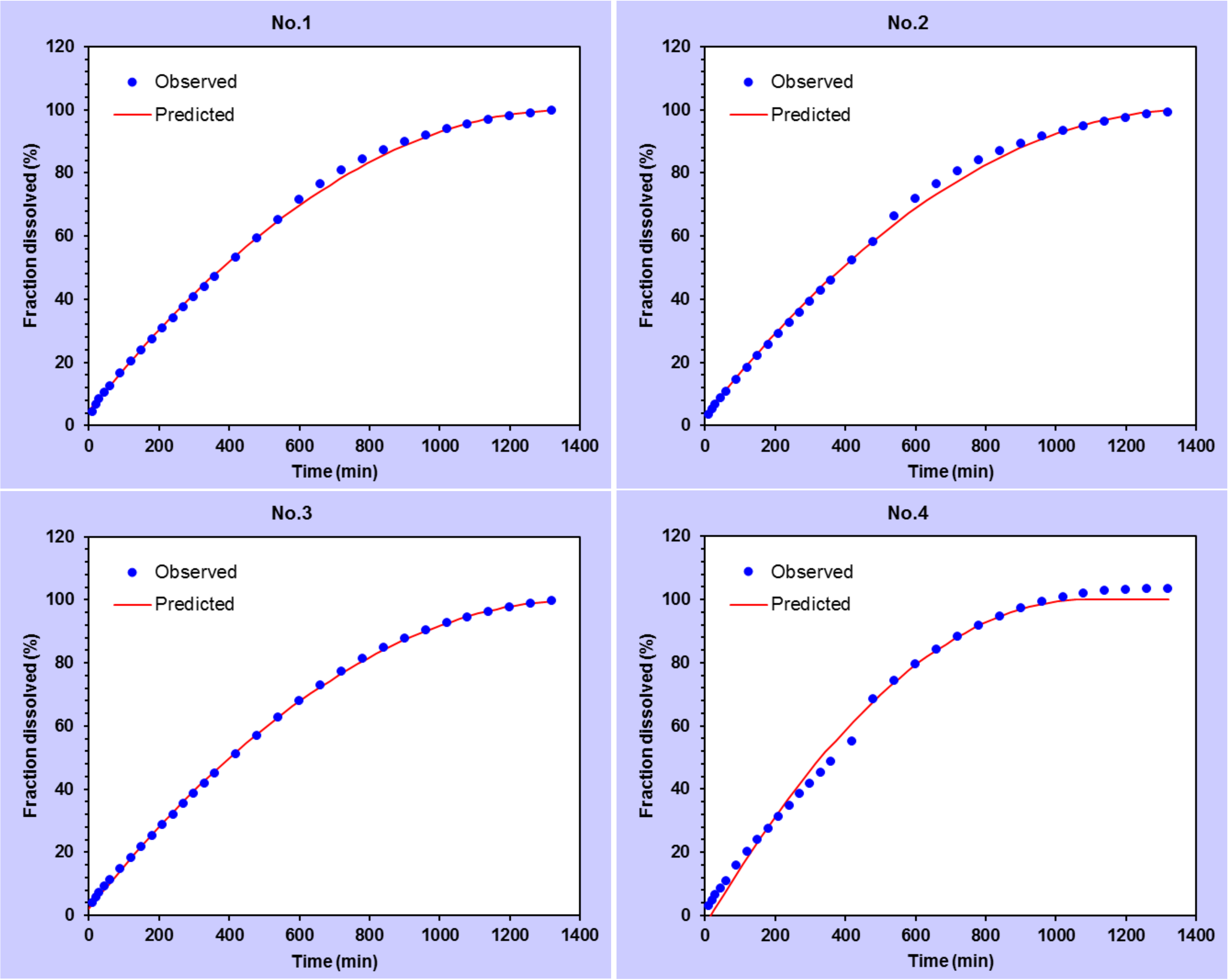

Supplementary materials – Model fitting summary – Glucidex® 19D (modelling the entire relevant dissolution data)

Model: **Baker–Lonsdale**

Model equation: $\frac{3}{2} \cdot \left[1 - \left(1 - \frac{F}{100}\right)^{\frac{2}{3}}\right] - \frac{F}{100} = k_{BL} \cdot t$

Fitted model parameters per tested tablet (N = 4) with statistics – mean, standard deviation (SD), and relative standard deviation expressed in % (RSD%) (output from DDSolver):

Parameter	No.1	No.2	No.3	No.4	Mean	SD	RSD(%)
k_{BL}	/	/	/	/	/	/	/

Number of dissolution data points (N), degrees of freedom (df), and selected goodness of fit criteria – Pearson correlation coefficient (R), coefficient of determination (R^2), adjusted coefficient of determination ($R^2_{adjusted}$), and residual sum of squares (RSS) (manual calculation in MS Excel):

Parameter	No.1	No.2	No.3	No.4
N	/	/	/	/
df	/	/	/	/
R	/	/	/	/
R^2	/	/	/	/
$R^2_{adjusted}$	/	/	/	/
RSS	/	/	/	/

Graphical abstract of model fit presented as mean ± 1 SD of the fraction % of released carvedilol:

Graphical abstract of model fit presented as the fraction % of released carvedilol per tested tablet:

Note: the model could not be fitted

Supplementary materials – Model fitting summary – Glucidex® 19D (modelling the entire relevant dissolution data)

Model: **Baker–Lonsdale with T_{lag}**

Model equation: $\frac{3}{2}\cdot\left[1-\left(1-\frac{F}{100}\right)^{\frac{2}{3}}\right]-\frac{F}{100}=k_{BL}\cdot\left(t-T_{lag}\right)$

Fitted model parameters per tested tablet (N = 4) with statistics – mean, standard deviation (SD), and relative standard deviation expressed in % (RSD%) (output from DDSolver):

Parameter	No.1	No.2	No.3	No.4	Mean	SD	RSD(%)
k_{BL}	/	/	/	/	/	/	/
T_{lag}	/	/	/	/	/	/	/

Number of dissolution data points (N), degrees of freedom (df), and selected goodness of fit criteria – Pearson correlation coefficient (R), coefficient of determination (R^2), adjusted coefficient of determination ($R^2_{adjusted}$), and residual sum of squares (RSS) (manual calculation in MS Excel):

Parameter	No.1	No.2	No.3	No.4
N	/	/	/	/
df	/	/	/	/
R	/	/	/	/
R^2	/	/	/	/
$R^2_{adjusted}$	/	/	/	/
RSS	/	/	/	/

Graphical abstract of model fit presented as mean ± 1 SD of the fraction % of released carvedilol:

Graphical abstract of model fit presented as the fraction % of released carvedilol per tested tablet:

Note: the model could not be fitted

Supplementary materials – Model fitting summary – Glucidex® 19D (modelling the entire relevant dissolution data)

Model: **Makoid–Banakar**

Model equation: $F = k_{MB} \cdot t^n \cdot e^{-k \cdot t}$

Fitted model parameters per tested tablet (N = 4) with statistics – mean, standard deviation (SD), and relative standard deviation expressed in % (RSD%) (output from DDSolver):

Parameter	No.1	No.2	No.3	No.4	Mean	SD	RSD(%)
k_{MB}	0.787009	0.470725	0.652657	0.412781	0.580793	0.171302	29.494429
n	0.695676	0.784402	0.714503	0.846490	0.760268	0.069000	9.075804
k	0.000063	0.000143	0.000002	0.000341	0.000137	0.000147	107.527482

Number of dissolution data points (N), degrees of freedom (df), and selected goodness of fit criteria – Pearson correlation coefficient (R), coefficient of determination (R^2), adjusted coefficient of determination ($R^2_{adjusted}$), and residual sum of squares (RSS) (manual calculation in MS Excel):

Parameter	No.1	No.2	No.3	No.4
N	31	31	31	31
df	28	28	28	28
R	0.99380576	0.99334578	0.993694086	0.994129338
R^2	0.987649889	0.986735839	0.987427937	0.988293141
$R^2_{adjusted}$	0.986767738	0.985788399	0.986529932	0.987456937
RSS	460.1468258	469.0734168	434.2485125	620.1704442

Graphical abstract of model fit presented as mean ± 1 SD of the fraction % of released carvedilol:

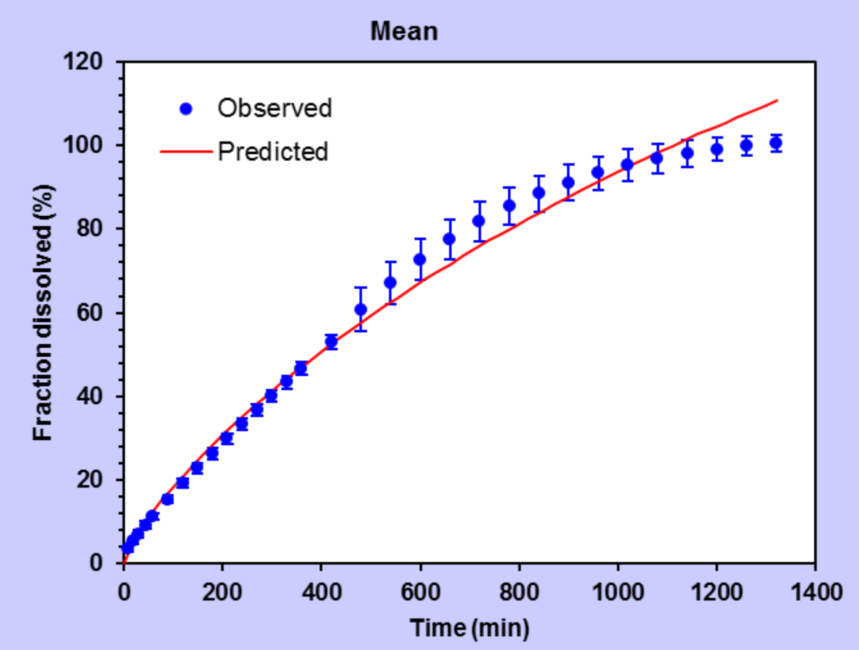

Supplementary materials – Model fitting summary – Glucidex® 19D (modelling the entire relevant dissolution data)

Graphical abstract of model fit presented as the fraction % of released carvedilol per tested tablet:

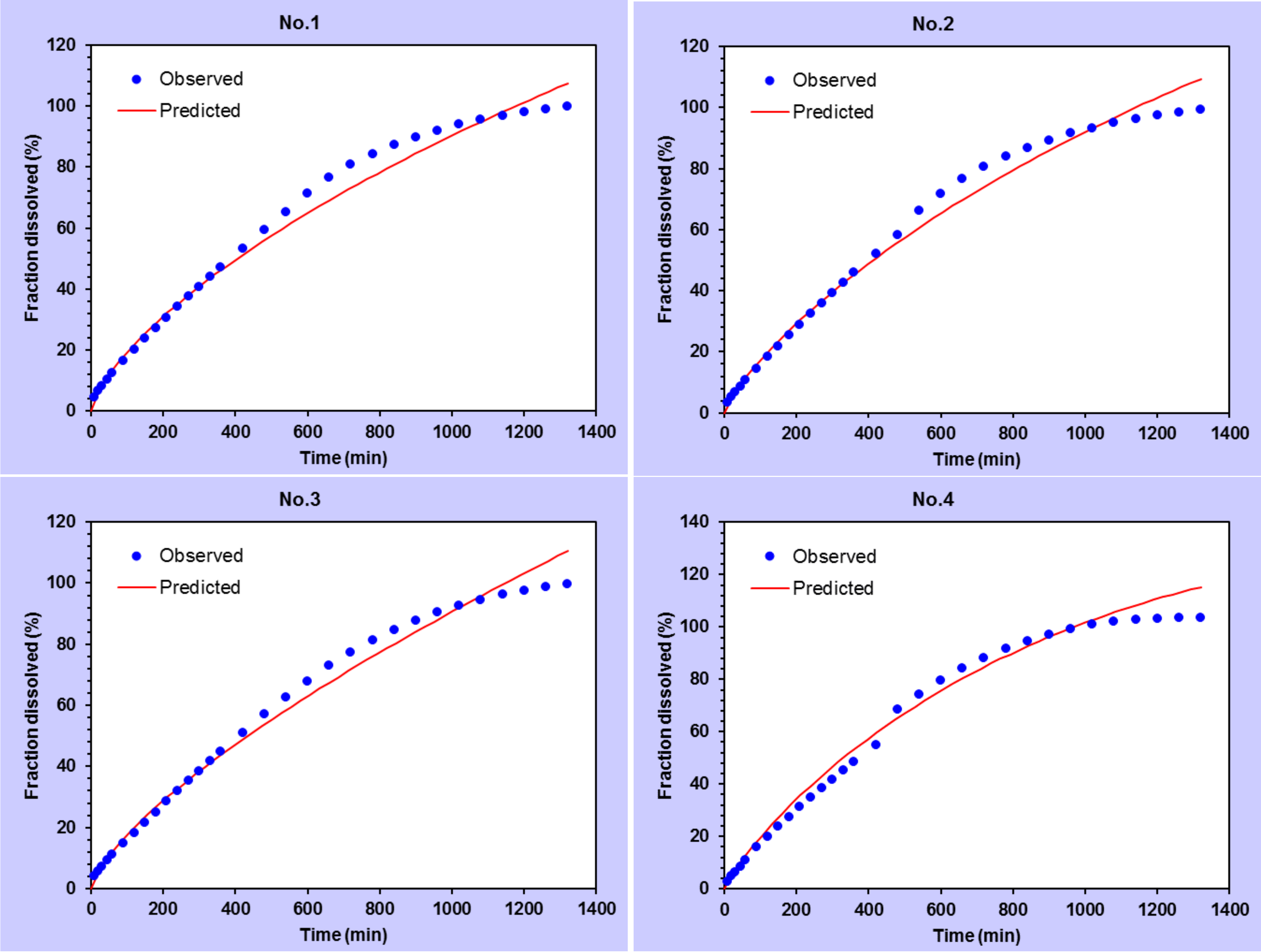

Supplementary materials – Model fitting summary – Glucidex® 19D (modelling the entire relevant dissolution data)

Model: **Makoid–Banakar with T_{lag}**

Model equation: $F = k_{MB} \cdot (t - T_{lag})^n \cdot e^{-k \cdot (t - T_{lag})}$

Fitted model parameters per tested tablet (N = 4) with statistics – mean, standard deviation (SD), and relative standard deviation expressed in % (RSD%) (output from DDSolver):

Parameter	No.1	No.2	No.3	No.4	Mean	SD	RSD(%)
k_{MB}	1.17757	0.73991	0.98941	0.57609	0.87075	0.26593	30.54065
n	0.61618	0.69518	0.63238	0.76261	0.67659	0.06670	9.85904
k	-0.00011	-0.00002	-0.00015	0.00012	-0.00004	0.00012	-281.43621
T_{lag}	4.00000	4.00000	4.00000	4.00000	4.00000	0.00000	0.00000

Number of dissolution data points (N), degrees of freedom (df), and selected goodness of fit criteria – Pearson correlation coefficient (R), coefficient of determination (R^2), adjusted coefficient of determination ($R^2_{adjusted}$), and residual sum of squares (RSS) (manual calculation in MS Excel):

Parameter	No.1	No.2	No.3	No.4
N	31	31	31	31
df	27	27	27	27
R	0.989304964	0.989305884	0.989983973	0.987936158
R^2	0.978724311	0.978726131	0.980068267	0.976017853
$R^2_{adjusted}$	0.976360346	0.976362368	0.97785363	0.97335317
RSS	737.9840886	765.387443	704.1474429	994.8957287

Graphical abstract of model fit presented as mean ± 1 SD of the fraction % of released carvedilol:

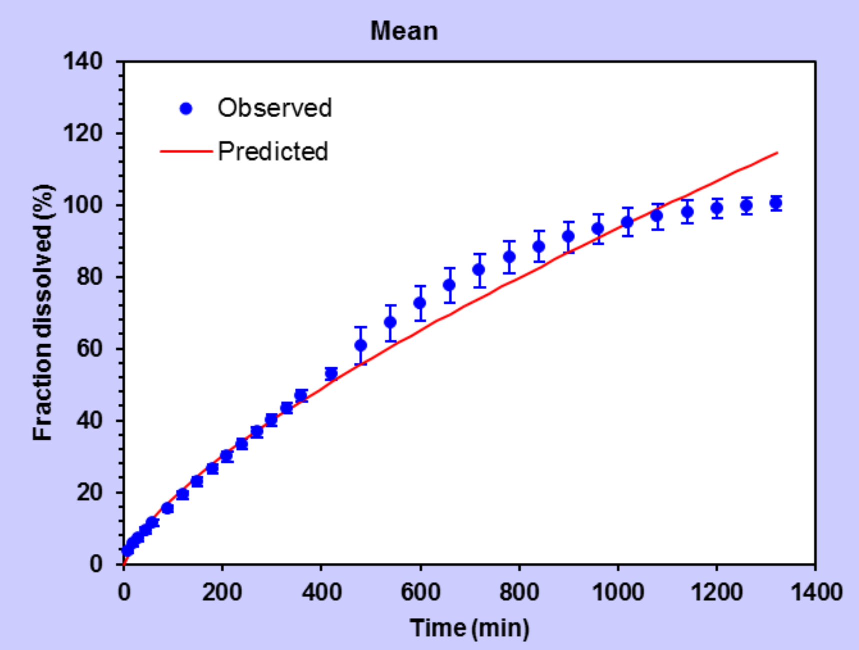

Supplementary materials – Model fitting summary – Glucidex® 19D (modelling the entire relevant dissolution data)

Graphical abstract of model fit presented as the fraction % of released carvedilol per tested tablet:

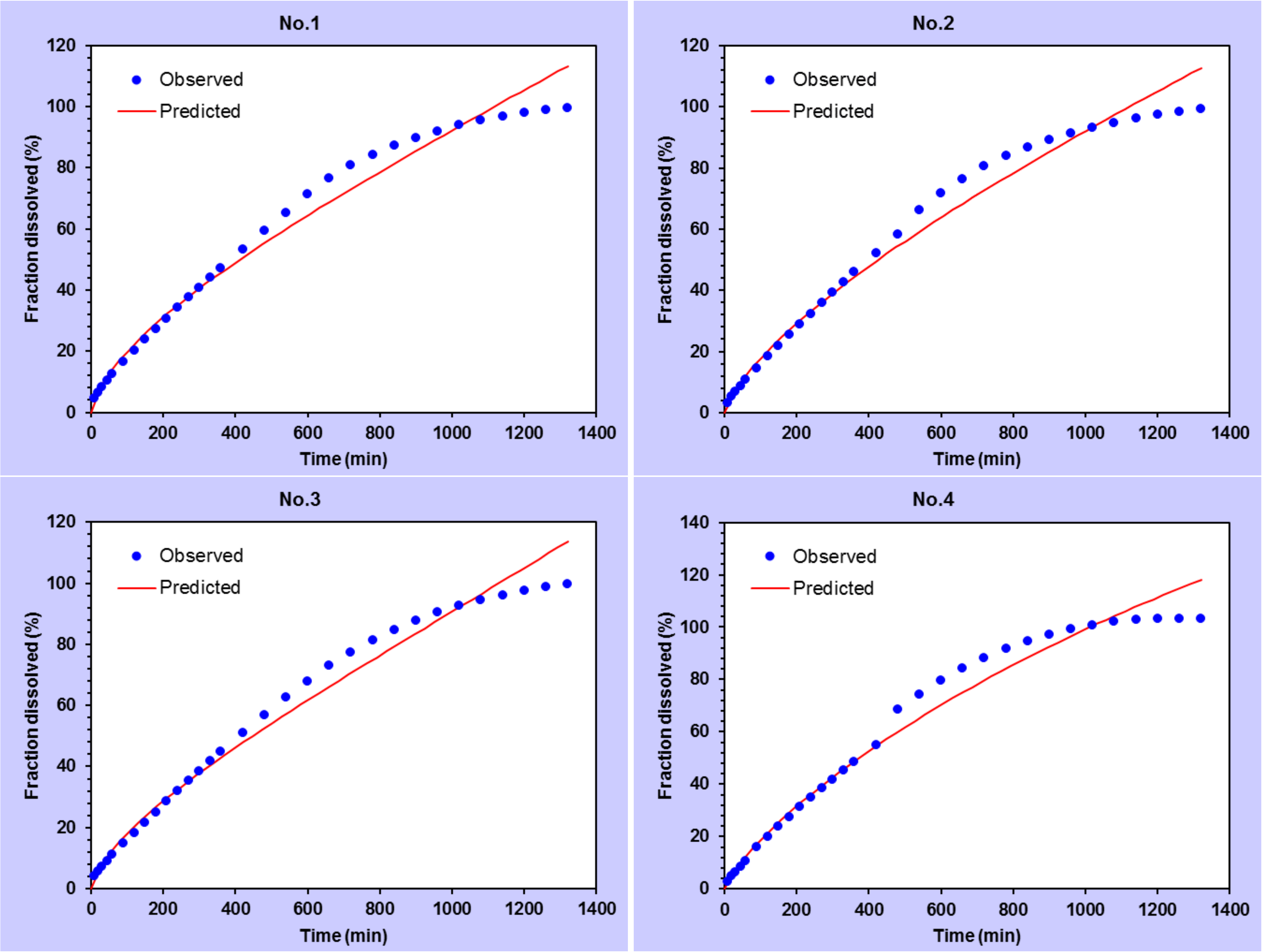

Supplementary materials – Model fitting summary – Glucidex® 19D (modelling the entire relevant dissolution data)

Model: **Peppas–Sahlin_1**

Model equation: $F = k_1 \cdot t^m + k_2 \cdot t^{2m}$

Fitted model parameters per tested tablet (N = 4) with statistics – mean, standard deviation (SD), and relative standard deviation expressed in % (RSD%) (output from DDSolver):

Parameter	No.1	No.2	No.3	No.4	Mean	SD	RSD(%)
k_1	2.081	1.838	1.706	2.115	1.935	0.196	10.152
k_2	0.089	0.099	0.103	0.100	0.098	0.006	6.181
m	0.450	0.450	0.450	0.450	0.450	0.000	0.000

Number of dissolution data points (N), degrees of freedom (df), and selected goodness of fit criteria – Pearson correlation coefficient (R), coefficient of determination (R^2), adjusted coefficient of determination ($R^2_{adjusted}$), and residual sum of squares (RSS) (manual calculation in MS Excel):

Parameter	No.1	No.2	No.3	No.4
N	31	31	31	31
df	28	28	28	28
R	0.990931488	0.988977905	0.992747049	0.983345197
R^2	0.981945213	0.978077296	0.985546703	0.966967776
$R^2_{adjusted}$	0.980655585	0.976511388	0.984514325	0.964608332
RSS	645.7777354	814.1123896	515.6007788	1436.719855

Graphical abstract of model fit presented as mean ± 1 SD of the fraction % of released carvedilol:

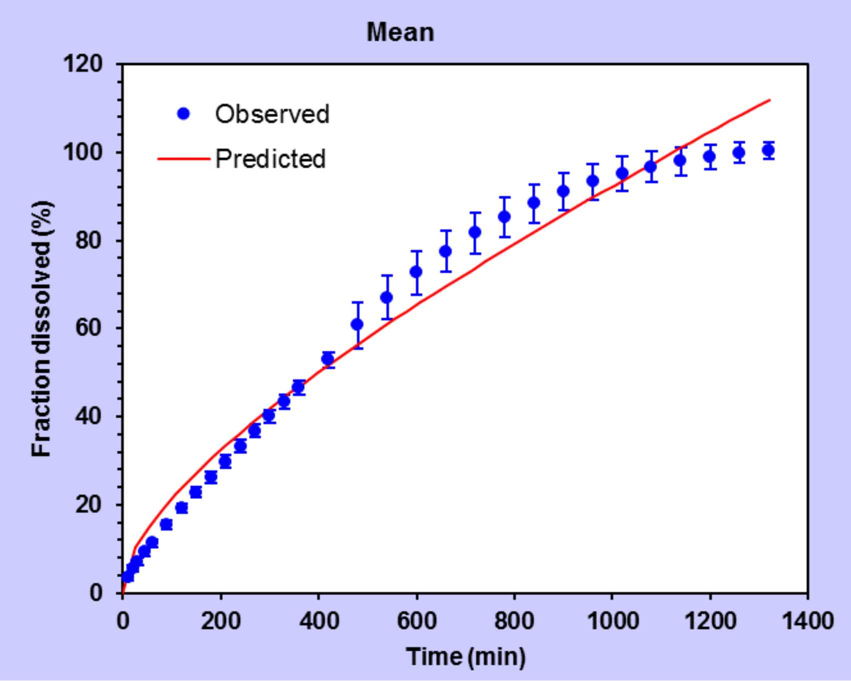

Supplementary materials – Model fitting summary – Glucidex® 19D (modelling the entire relevant dissolution data)

Graphical abstract of model fit presented as the fraction % of released carvedilol per tested tablet:

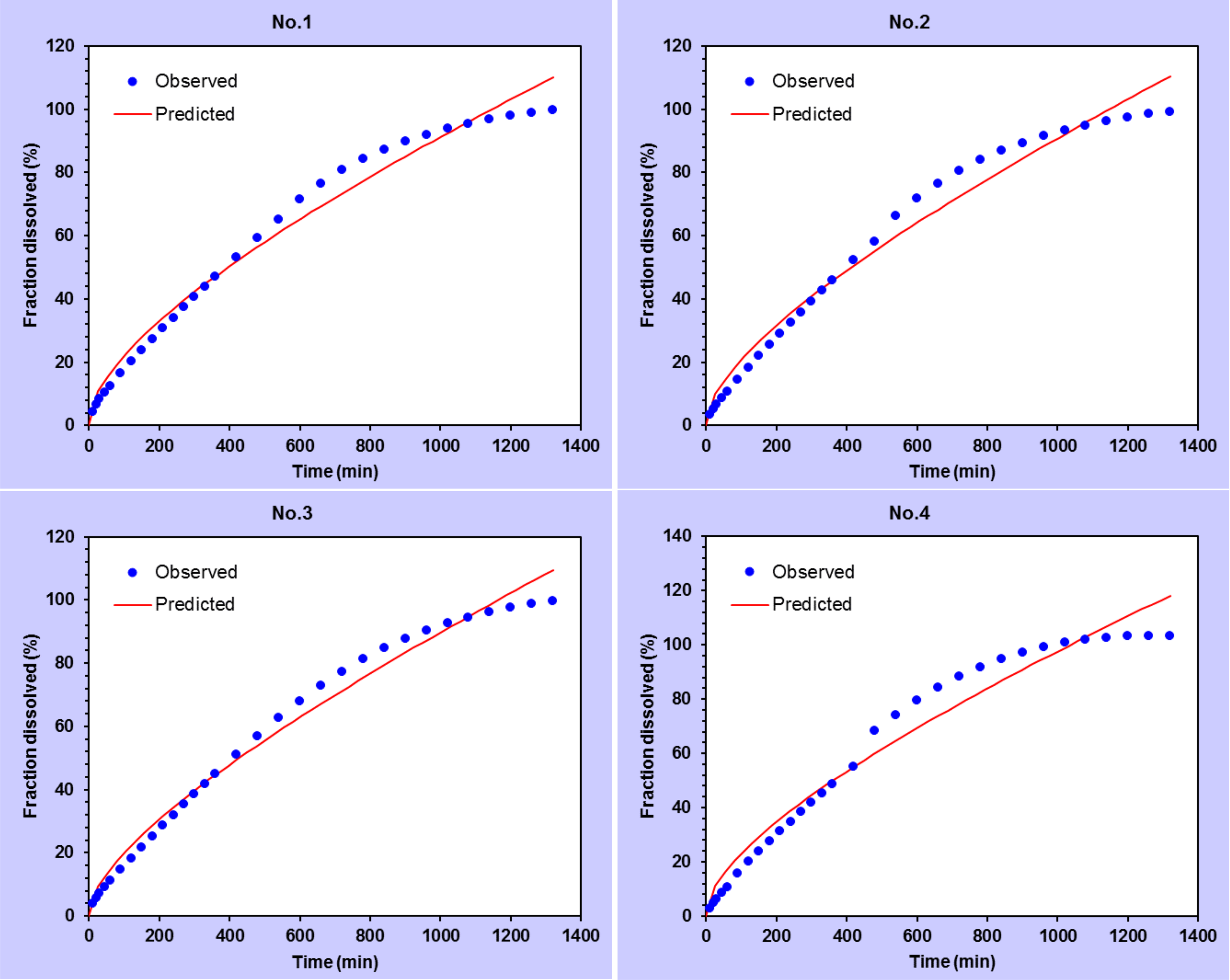

Supplementary materials – Model fitting summary – Glucidex® 19D (modelling the entire relevant dissolution data)

Model: **Peppas–Sahlin_1 with T_{lag}**

Model equation: $F = k_1 \cdot (t - T_{lag})^m + k_2 \cdot (t - T_{lag})^{2m}$

Fitted model parameters per tested tablet (N = 4) with statistics – mean, standard deviation (SD), and relative standard deviation expressed in % (RSD%) (output from DDSolver):

Parameter	No.1	No.2	No.3	No.4	Mean	SD	RSD(%)
k_1	2.167	1.923	1.786	2.211	2.022	0.202	9.977
k_2	0.086	0.096	0.100	0.096	0.094	0.006	6.429
m	0.450	0.450	0.450	0.450	0.450	0.000	0.000
T_{lag}	6.000	6.000	6.000	6.000	6.000	0.000	0.000

Number of dissolution data points (N), degrees of freedom (df), and selected goodness of fit criteria – Pearson correlation coefficient (R), coefficient of determination (R^2), adjusted coefficient of determination ($R^2_{adjusted}$), and residual sum of squares (RSS) (manual calculation in MS Excel):

Parameter	No.1	No.2	No.3	No.4
N	31	31	31	31
df	27	27	27	27
R	0.991337436	0.989468068	0.993072906	0.984013919
R^2	0.982749912	0.979047058	0.986193797	0.968283393
$R^2_{adjusted}$	0.980833236	0.976718954	0.984659775	0.964759326
RSS	601.2669603	761.6881782	480.4970791	1355.5355

Graphical abstract of model fit presented as mean ± 1 SD of the fraction % of released carvedilol:

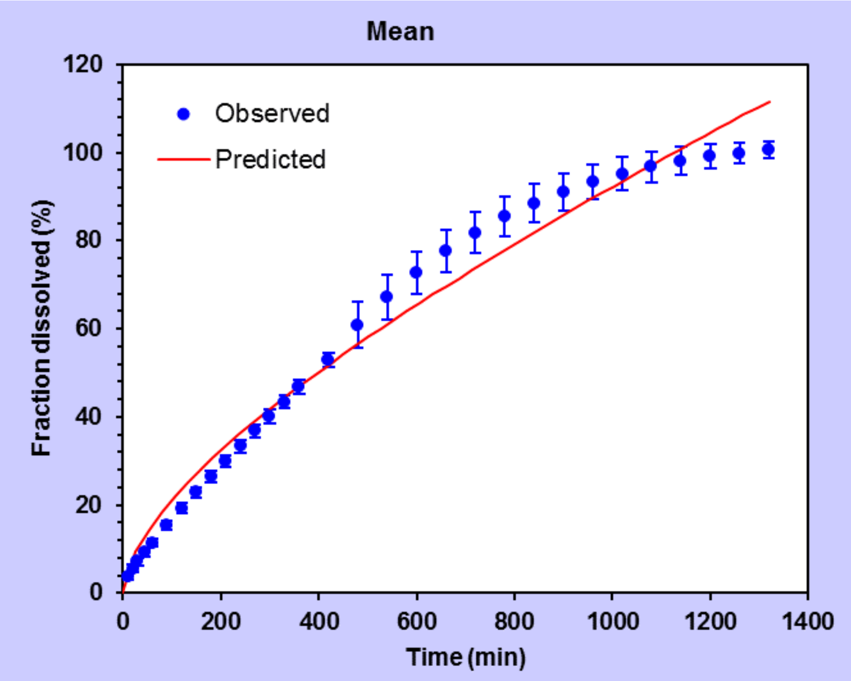

Supplementary materials – Model fitting summary – Glucidex® 19D (modelling the entire relevant dissolution data)

Graphical abstract of model fit presented as the fraction % of released carvedilol per tested tablet:

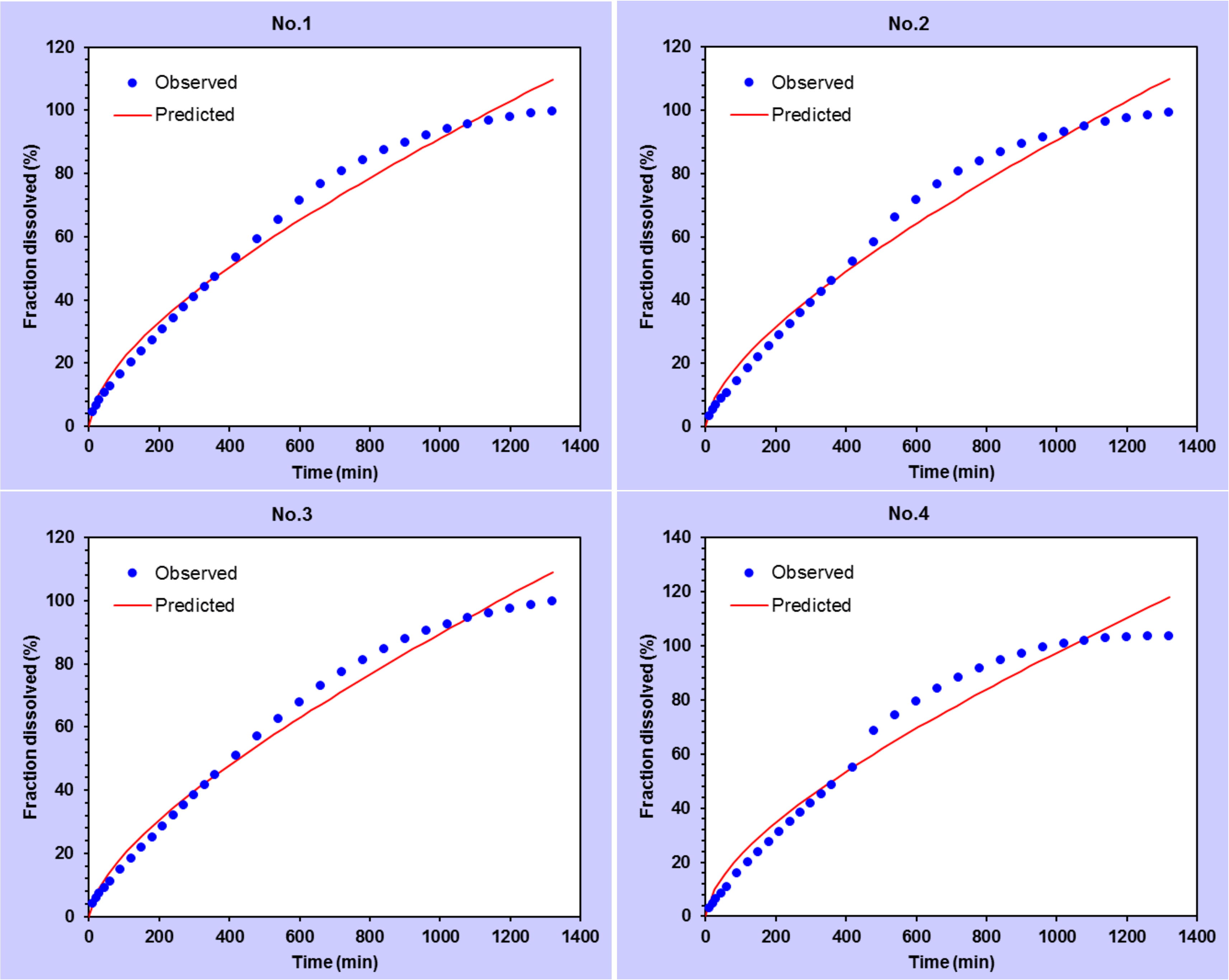

Supplementary materials – Model fitting summary – Glucidex® 19D (modelling the entire relevant dissolution data)

Model: **Peppas–Sahlin_2**

Model equation: $F = k_1 \cdot t^{0.5} + k_2 \cdot t$

Fitted model parameters per tested tablet (N = 4) with statistics – mean, standard deviation (SD), and relative standard deviation expressed in % (RSD%) (output from DDSolver):

Parameter	No.1	No.2	No.3	No.4	Mean	SD	RSD(%)
k_1	1.948	1.782	1.671	2.026	1.857	0.160	8.626
k_2	0.029	0.034	0.037	0.033	0.033	0.003	9.001

Number of dissolution data points (N), degrees of freedom (df), and selected goodness of fit criteria – Pearson correlation coefficient (R), coefficient of determination (R^2), adjusted coefficient of determination ($R^2_{adjusted}$), and residual sum of squares (RSS) (manual calculation in MS Excel):

Parameter	No.1	No.2	No.3	No.4
N	31	31	31	31
df	29	29	29	29
R	0.991242158	0.989190358	0.992759429	0.983859514
R^2	0.982561015	0.978497565	0.985571284	0.967979544
$R^2_{adjusted}$	0.981959671	0.977756102	0.985073742	0.96687539
RSS	639.6914002	821.6672955	528.3095735	1433.921178

Graphical abstract of model fit presented as mean ± 1 SD of the fraction % of released carvedilol:

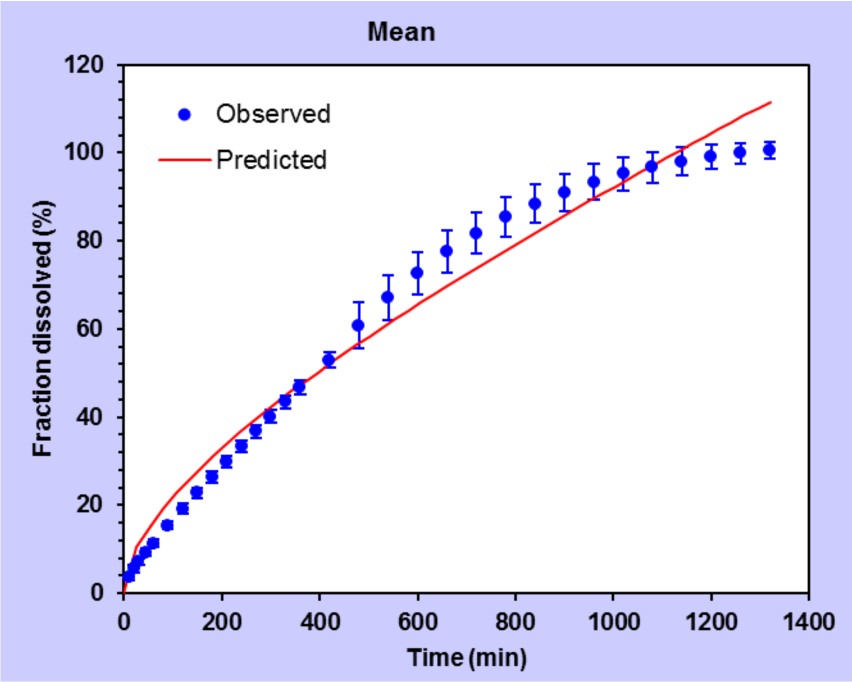

Supplementary materials – Model fitting summary – Glucidex® 19D (modelling the entire relevant dissolution data)

Graphical abstract of model fit presented as the fraction % of released carvedilol per tested tablet:

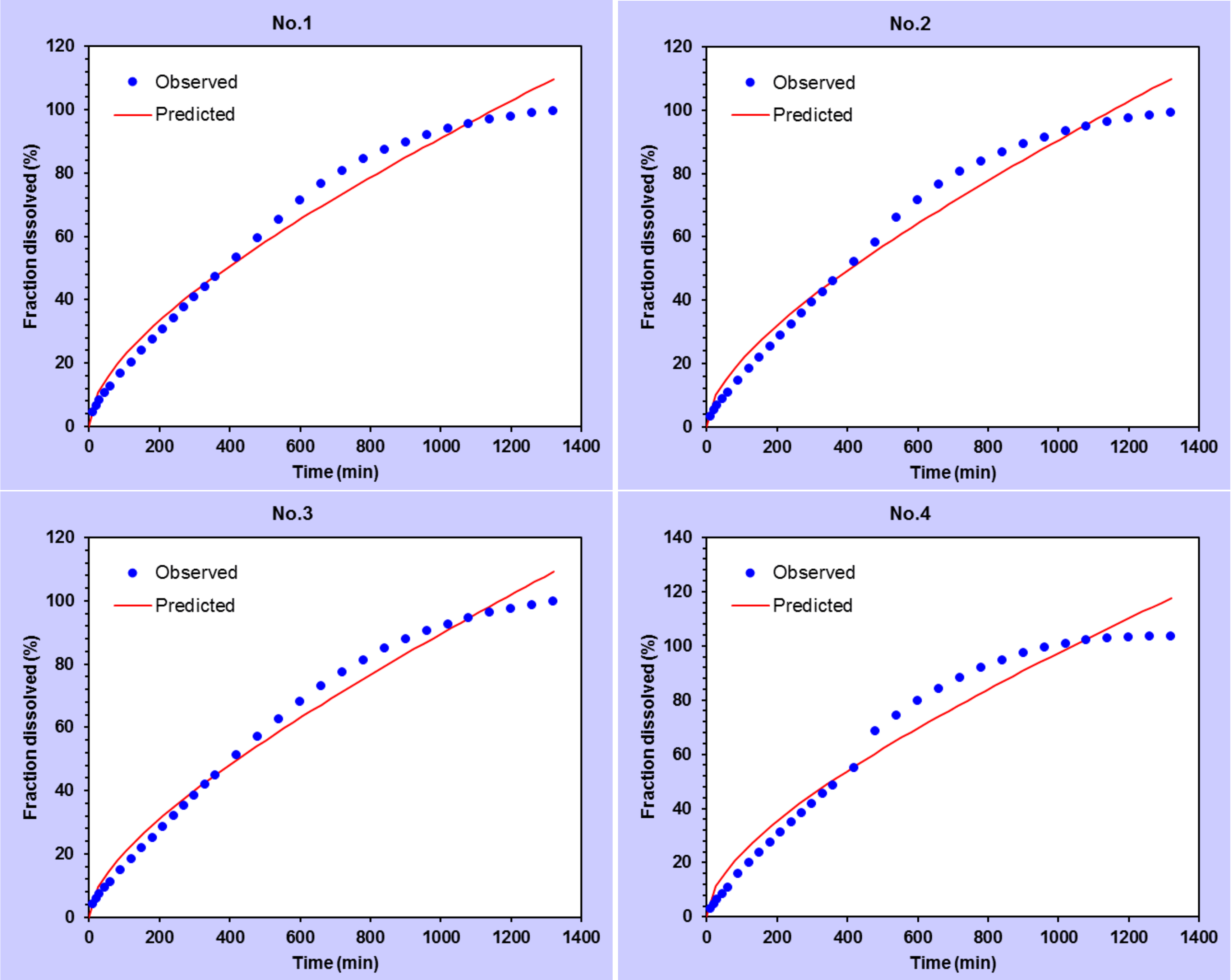

Supplementary materials – Model fitting summary – Glucidex® 19D (modelling the entire relevant dissolution data)

Model: **Peppas–Sahlin_2 with T_{lag}**

Model equation: $F = k_1 \cdot (t - T_{lag})^{0.5} + k_2 \cdot (t - T_{lag})$

Fitted model parameters per tested tablet (N = 4) with statistics – mean, standard deviation (SD), and relative standard deviation expressed in % (RSD%) (output from DDSolver):

Parameter	No.1	No.2	No.3	No.4	Mean	SD	RSD(%)
k_1	2.007	1.841	1.727	2.093	1.917	0.164	8.561
k_2	0.028	0.033	0.035	0.031	0.032	0.003	9.630
T_{lag}	6.000	6.000	6.000	6.000	6.000	0.000	0.000

Number of dissolution data points (N), degrees of freedom (df), and selected goodness of fit criteria – Pearson correlation coefficient (R), coefficient of determination (R^2), adjusted coefficient of determination ($R^2_{adjusted}$), and residual sum of squares (RSS) (manual calculation in MS Excel):

Parameter	No.1	No.2	No.3	No.4
N	31	31	31	31
df	28	28	28	28
R	0.991625222	0.989684248	0.993090168	0.984528969
R^2	0.983320581	0.97947491	0.986228082	0.96929729
$R^2_{adjusted}$	0.982129194	0.978008832	0.985244374	0.96710424
RSS	590.8223588	762.1154263	487.7445371	1342.553681

Graphical abstract of model fit presented as mean ± 1 SD of the fraction % of released carvedilol:

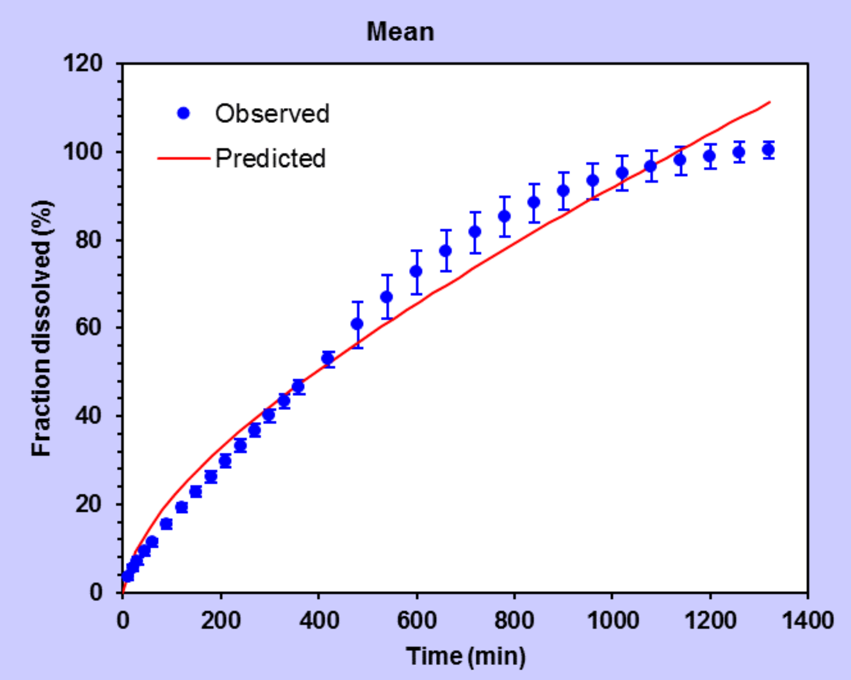

Supplementary materials – Model fitting summary – Glucidex® 19D (modelling the entire relevant dissolution data)

Graphical abstract of model fit presented as the fraction % of released carvedilol per tested tablet:

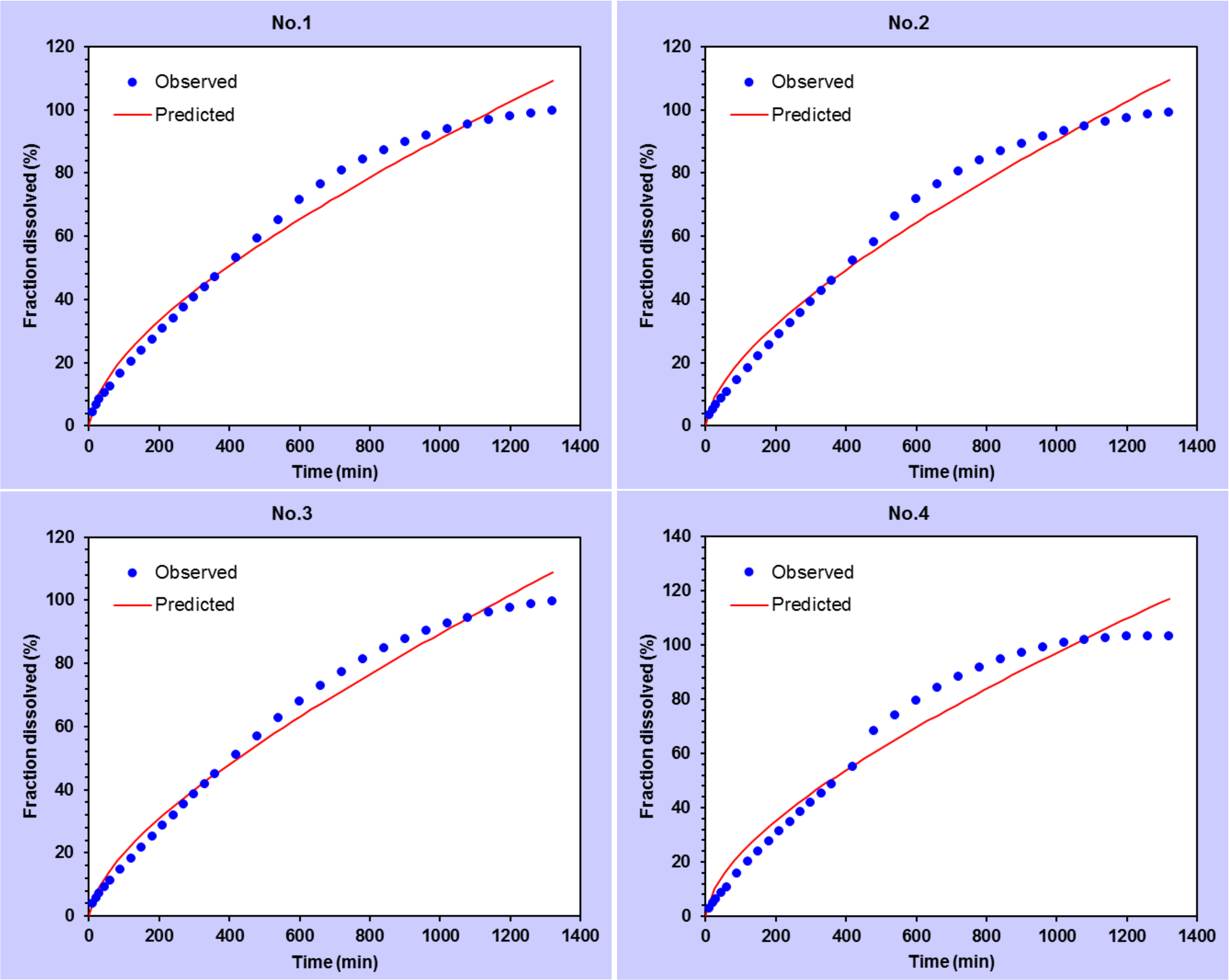

Supplementary materials – Model fitting summary – Glucidex® 19D (modelling the entire relevant dissolution data)

Model: **Quadratic**

Model equation: $F = 100 \cdot (k_1 \cdot t^2 + k_2 \cdot t)$

Fitted model parameters per tested tablet (N = 4) with statistics – mean, standard deviation (SD), and relative standard deviation expressed in % (RSD%) (output from DDSolver):

Parameter	No.1	No.2	No.3	No.4	Mean	SD	RSD(%)
k_1	-0.000001	-0.000001	-0.000001	-0.000001	-0.000001	0.000000	-10.723182
k_2	0.001559	0.001525	0.001458	0.001699	0.001560	0.000102	6.507698

Number of dissolution data points (N), degrees of freedom (df), and selected goodness of fit criteria – Pearson correlation coefficient (R), coefficient of determination (R^2), adjusted coefficient of determination ($R^2_{adjusted}$), and residual sum of squares (RSS) (manual calculation in MS Excel):

Parameter	No.1	No.2	No.3	No.4
N	31	31	31	31
df	29	29	29	29
R	0.999443093	0.999468004	0.999683917	0.998693693
R^2	0.998886495	0.99893629	0.999367934	0.997389093
$R^2_{adjusted}$	0.998848099	0.998899611	0.999346139	0.997299062
RSS	91.63147417	44.72502953	49.38547834	106.4502343

Graphical abstract of model fit presented as mean ± 1 SD of the fraction % of released carvedilol:

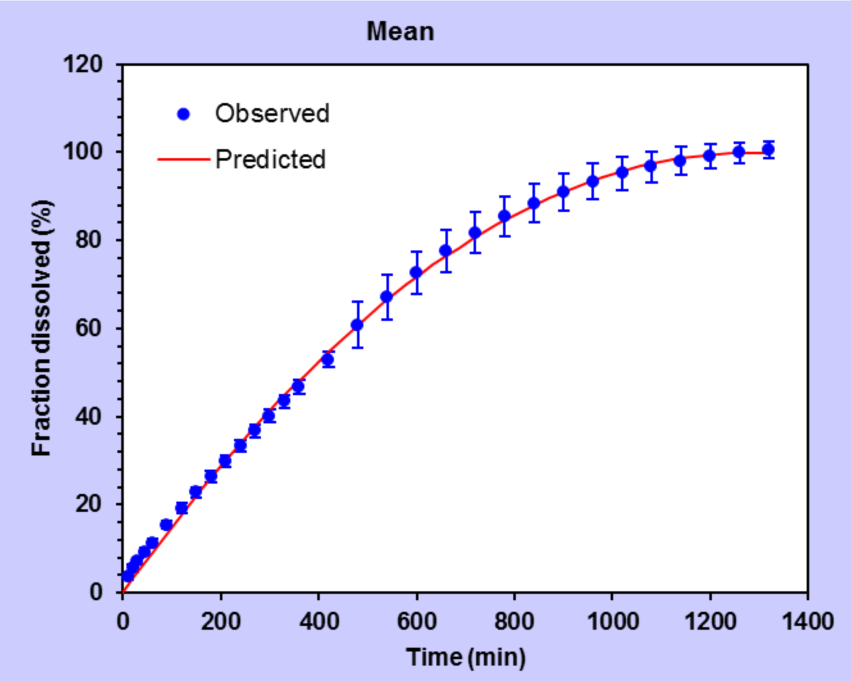

Supplementary materials – Model fitting summary – Glucidex® 19D (modelling the entire relevant dissolution data)

Graphical abstract of model fit presented as the fraction % of released carvedilol per tested tablet:

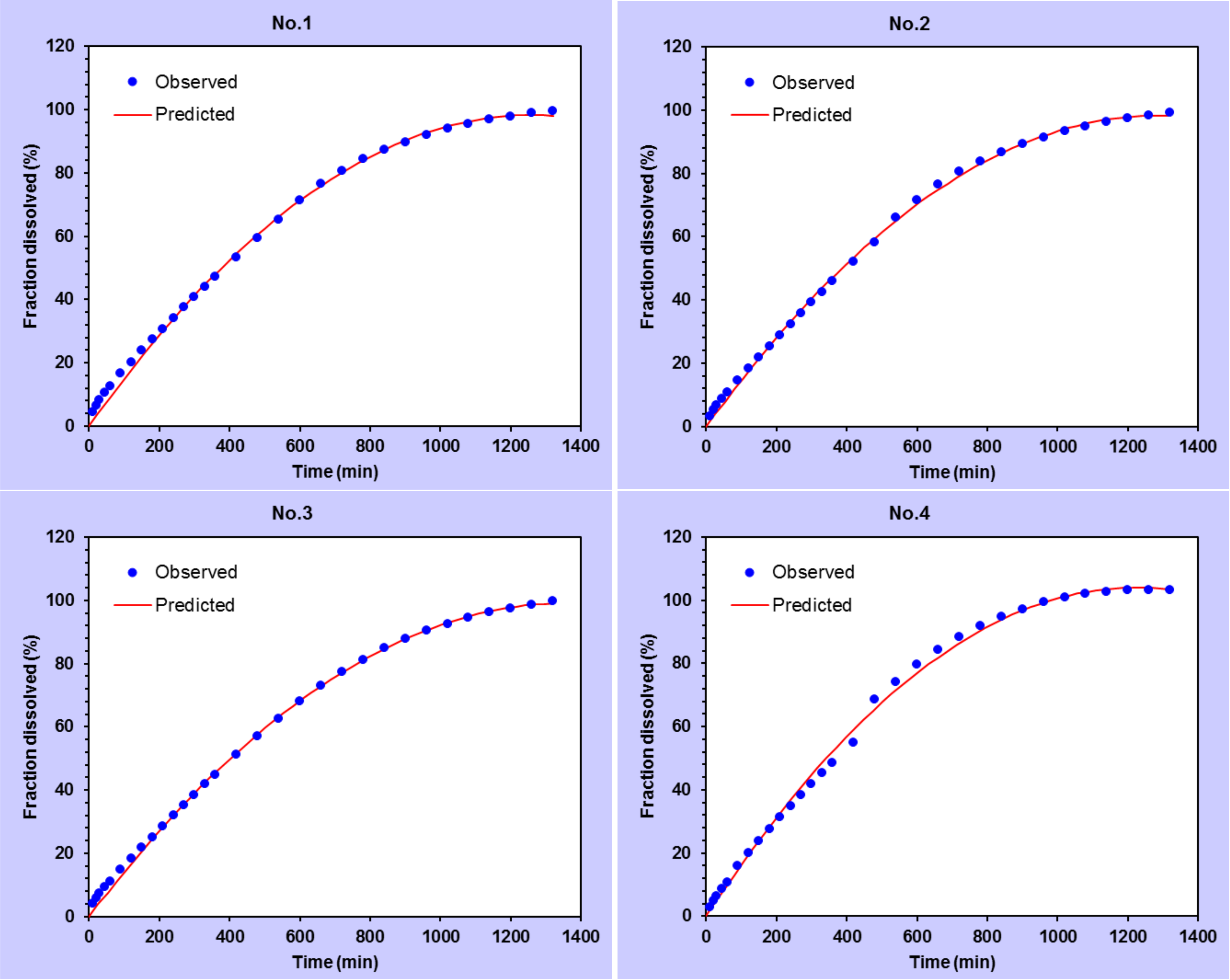

Supplementary materials – Model fitting summary – Glucidex® 19D (modelling the entire relevant dissolution data)

Model: **Quadratic with T_{lag}**

Model equation: $F = 100 \cdot \left[k_1 \cdot (t - T_{lag})^2 + k_2 \cdot (t - T_{lag})\right]$

Fitted model parameters per tested tablet (N = 4) with statistics – mean, standard deviation (SD), and relative standard deviation expressed in % (RSD%) (output from DDSolver):

Parameter	No.1	No.2	No.3	No.4	Mean	SD	RSD(%)
k_1	-0.000001	-0.000001	-0.000001	-0.000001	-0.000001	0.000000	-10.672098
k_2	0.001574	0.001540	0.001473	0.001717	0.001576	0.000103	6.513528
T_{lag}	4.000000	4.000000	4.000000	4.000000	4.000000	0.000000	0.000000

Number of dissolution data points (N), degrees of freedom (df), and selected goodness of fit criteria – Pearson correlation coefficient (R), coefficient of determination (R^2), adjusted coefficient of determination ($R^2_{adjusted}$), and residual sum of squares (RSS) (manual calculation in MS Excel):

Parameter	No.1	No.2	No.3	No.4
N	31	31	31	31
df	28	28	28	28
R	0.999347975	0.999425018	0.999608403	0.998686152
R^2	0.998696375	0.998850367	0.99921696	0.997374031
$R^2_{adjusted}$	0.998603259	0.998768251	0.999161029	0.997186462
RSS	121.6689592	57.34317286	70.44782151	109.1412615

Graphical abstract of model fit presented as mean ± 1 SD of the fraction % of released carvedilol:

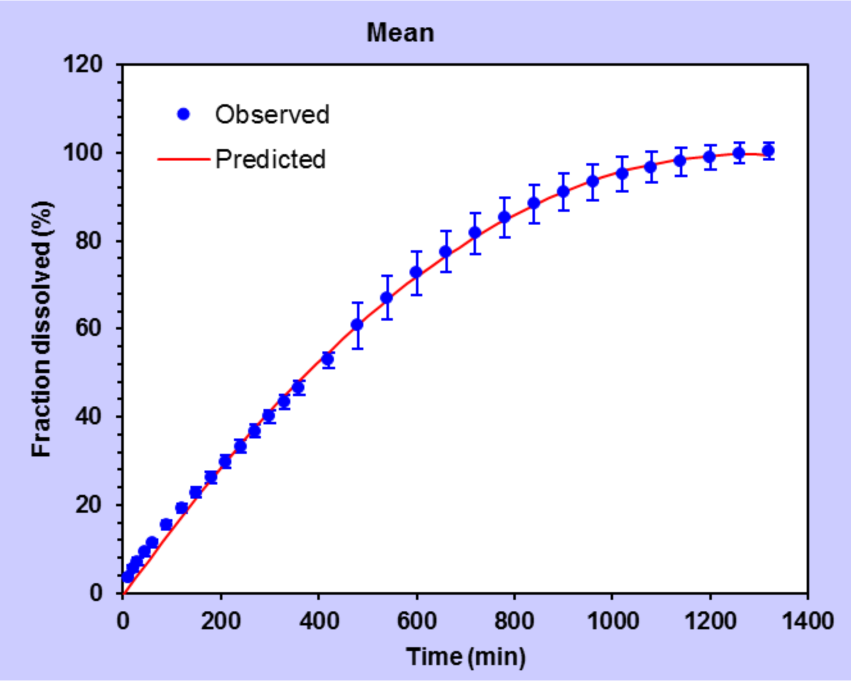

Supplementary materials – Model fitting summary – Glucidex® 19D (modelling the entire relevant dissolution data)

Graphical abstract of model fit presented as the fraction % of released carvedilol per tested tablet:

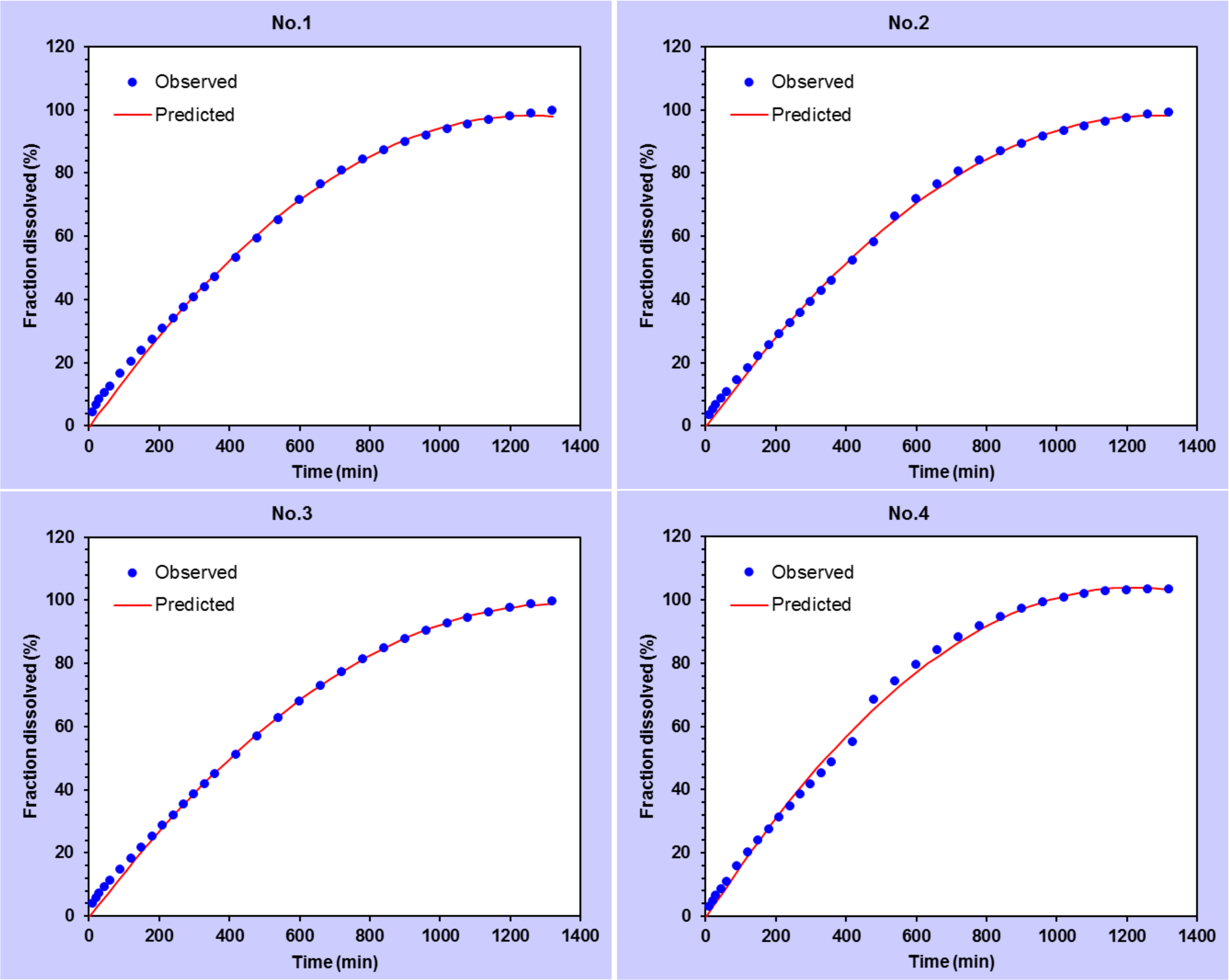

Supplementary materials – Model fitting summary – Glucidex® 19D (modelling the entire relevant dissolution data)

Model: **Weibull_1**

Model equation: $F = 100 \cdot \left[1 - e^{-\frac{(t-T_i)^{\beta}}{\alpha}}\right]$

Fitted model parameters per tested tablet (N = 4) with statistics – mean, standard deviation (SD), and relative standard deviation expressed in % (RSD%) (output from DDSolver):

Parameter	No.1	No.2	No.3	No.4	Mean	SD	RSD(%)
α	274.806	499.567	330.988	426.352	382.928	99.797	26.062
β	0.930	0.989	0.948	1.008	0.969	0.036	3.710
T_i	6.000	4.431	6.000	6.000	5.608	0.785	13.994

Number of dissolution data points (N), degrees of freedom (df), and selected goodness of fit criteria – Pearson correlation coefficient (R), coefficient of determination (R^2), adjusted coefficient of determination ($R^2_{adjusted}$), and residual sum of squares (RSS) (manual calculation in MS Excel):

Parameter	No.1	No.2	No.3	No.4
N	31	31	31	31
df	28	28	28	28
R	0.986287471	0.995411878	0.987319091	0.988434227
R^2	0.972762976	0.990844806	0.974798987	0.977002222
$R^2_{adjusted}$	0.970817474	0.990190864	0.972998915	0.975359523
RSS	1186.535975	897.3692149	1121.508866	1410.899972

Graphical abstract of model fit presented as mean ± 1 SD of the fraction % of released carvedilol:

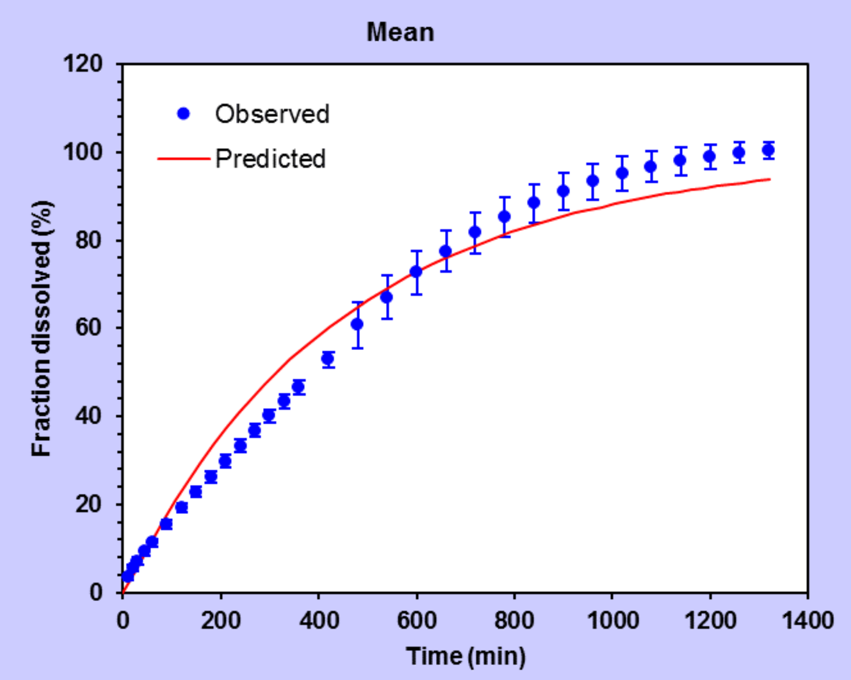

Supplementary materials – Model fitting summary – Glucidex® 19D (modelling the entire relevant dissolution data)

Graphical abstract of model fit presented as the fraction % of released carvedilol per tested tablet:

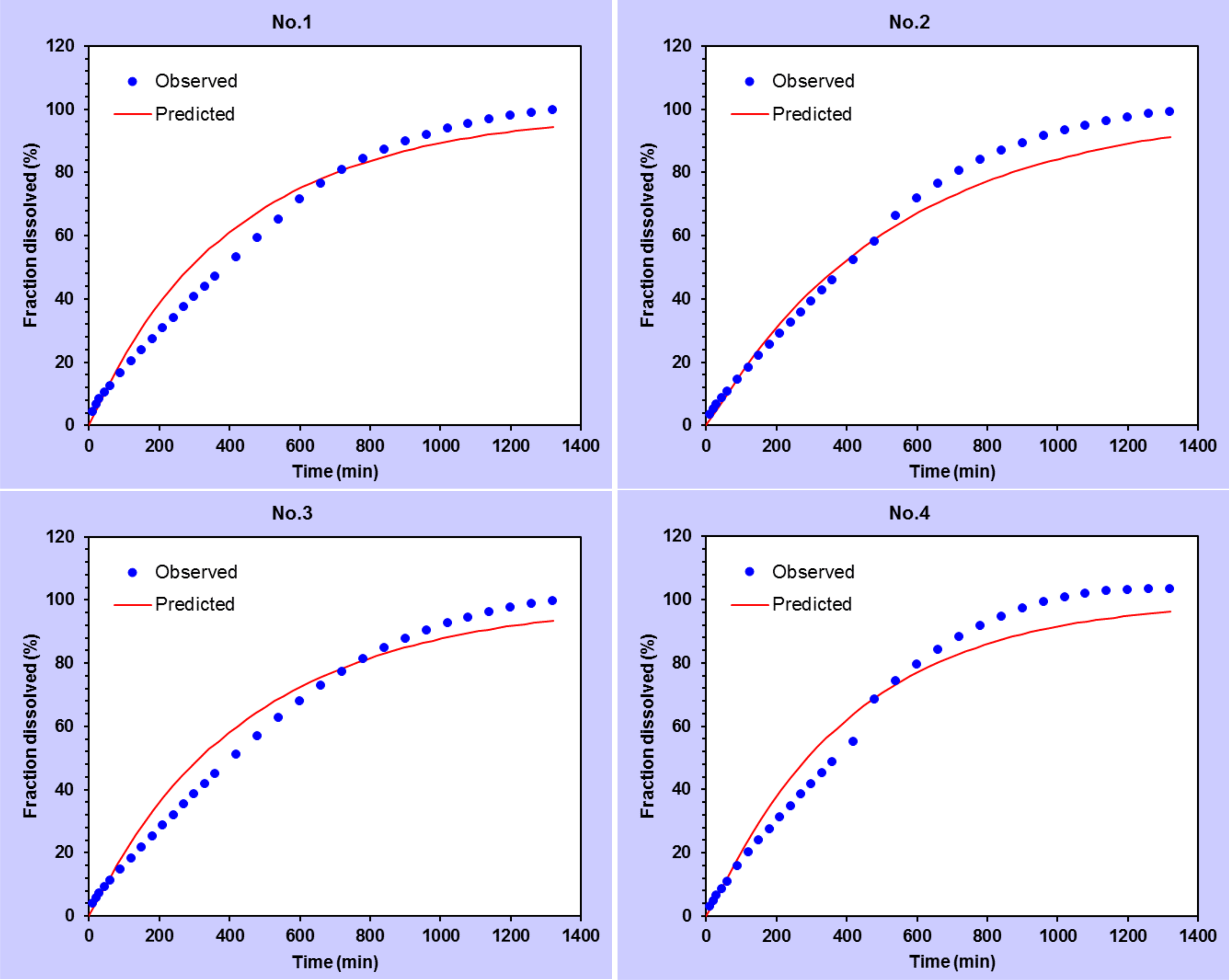

Supplementary materials – Model fitting summary – Glucidex® 19D (modelling the entire relevant dissolution data)

Model: **Weibull_2**

Model equation: $F = 100 \cdot \left(1 - e^{-\frac{t^{\beta}}{\alpha}}\right)$

Fitted model parameters per tested tablet (N = 4) with statistics – mean, standard deviation (SD), and relative standard deviation expressed in % (RSD%) (output from DDSolver):

Parameter	No.1	No.2	No.3	No.4	Mean	SD	RSD(%)
α	411.228	776.759	651.520	687.447	631.738	156.152	24.718
β	0.994	1.061	1.028	1.087	1.042	0.040	3.866

Number of dissolution data points (N), degrees of freedom (df), and selected goodness of fit criteria – Pearson correlation coefficient (R), coefficient of determination (R^2), adjusted coefficient of determination ($R^2_{adjusted}$), and residual sum of squares (RSS) (manual calculation in MS Excel):

Parameter	No.1	No.2	No.3	No.4
N	31	31	31	31
df	29	29	29	29
R	0.989048409	0.996788968	0.995447041	0.990324053
R^2	0.978216756	0.993588246	0.990914812	0.980741731
$R^2_{adjusted}$	0.97746561	0.993367151	0.99060153	0.980077653
RSS	970.5203707	587.672709	645.6818251	1116.480296

Graphical abstract of model fit presented as mean ± 1 SD of the fraction % of released carvedilol:

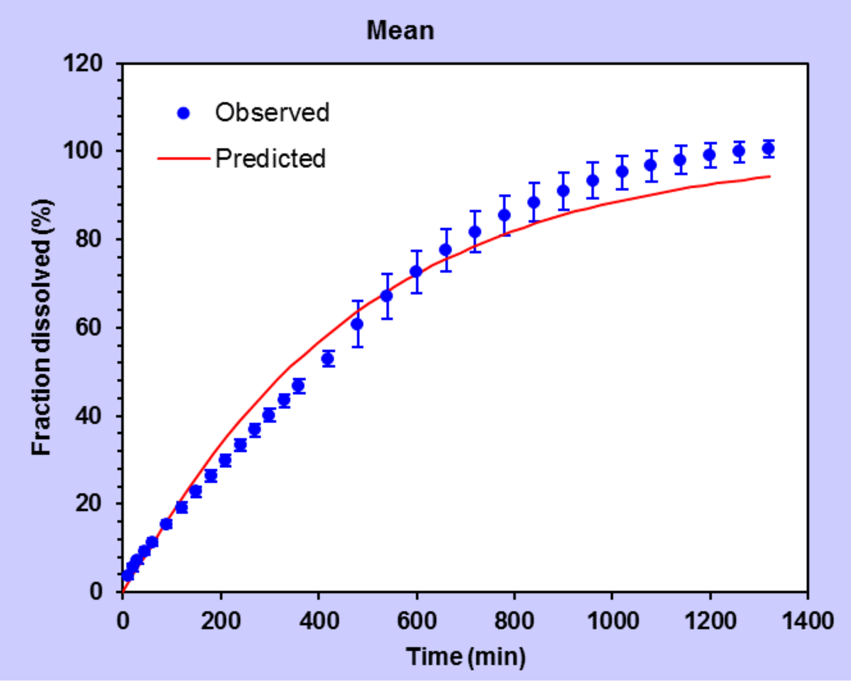

Supplementary materials – Model fitting summary – Glucidex® 19D (modelling the entire relevant dissolution data)

Graphical abstract of model fit presented as the fraction % of released carvedilol per tested tablet:

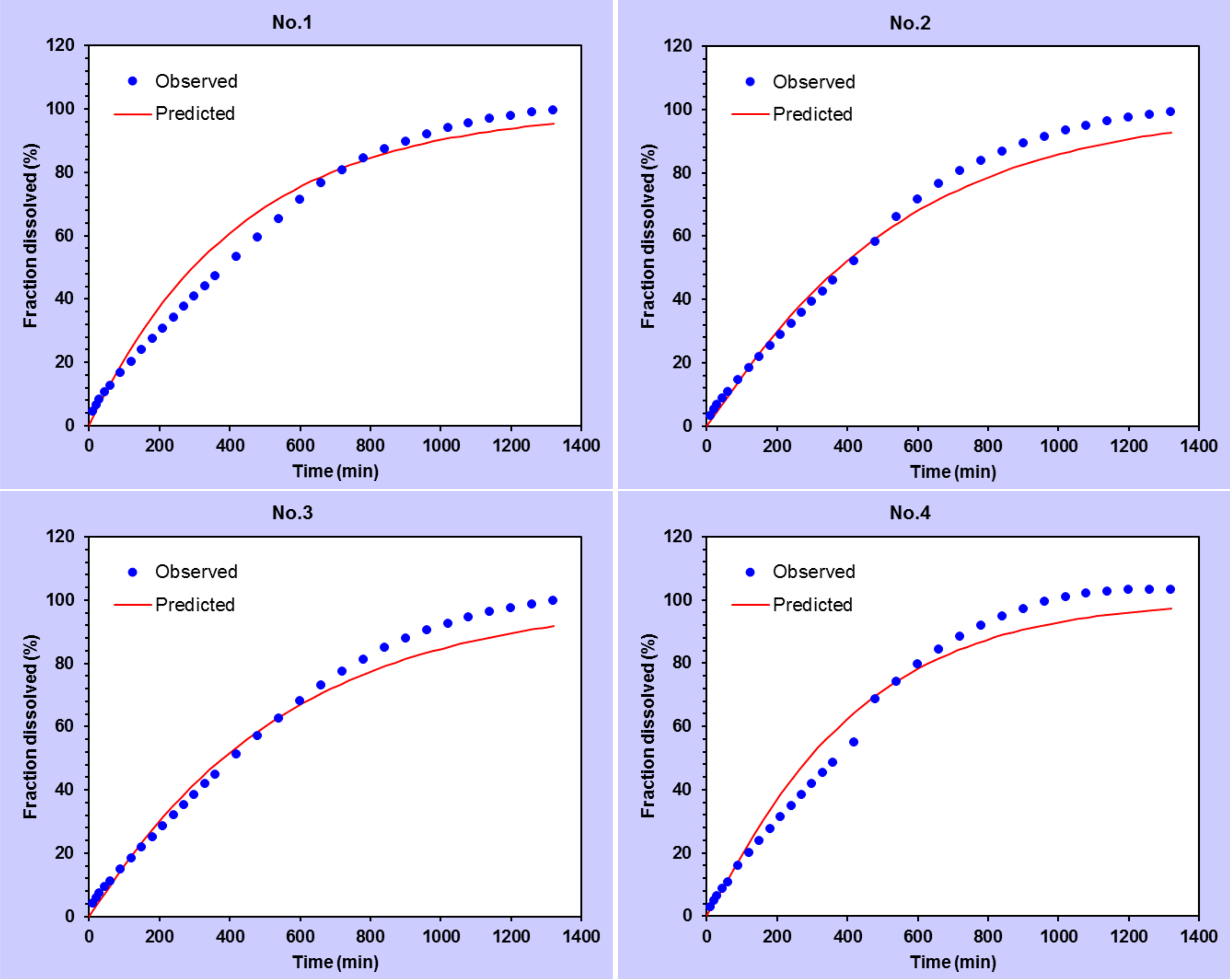

Supplementary materials – Model fitting summary – Glucidex® 19D (modelling the entire relevant dissolution data)

Model: **Weibull_3**

Model equation: $F = F_{max} \cdot \left(1 - e^{-\frac{t^{\beta}}{\alpha}}\right)$

Fitted model parameters per tested tablet (N = 4) with statistics – mean, standard deviation (SD), and relative standard deviation expressed in % (RSD%) (output from DDSolver):

Parameter	No.1	No.2	No.3	No.4	Mean	SD	RSD(%)
α	375.746	574.178	465.622	743.527	539.768	158.222	29.313
β	0.921	0.989	0.949	1.039	0.974	0.051	5.253
F_{max}	118.903	117.570	118.185	123.224	119.470	2.561	2.144

Number of dissolution data points (N), degrees of freedom (df), and selected goodness of fit criteria – Pearson correlation coefficient (R), coefficient of determination (R^2), adjusted coefficient of determination ($R^2_{adjusted}$), and residual sum of squares (RSS) (manual calculation in MS Excel):

Parameter	No.1	No.2	No.3	No.4
N	31	31	31	31
df	28	28	28	28
R	0.996473012	0.997354506	0.996670554	0.996559646
R^2	0.992958463	0.994716011	0.993352194	0.993131128
$R^2_{adjusted}$	0.992455496	0.994338583	0.992877351	0.992640494
RSS	408.9000542	414.2674434	437.6566459	511.6026827

Graphical abstract of model fit presented as mean ± 1 SD of the fraction % of released carvedilol:

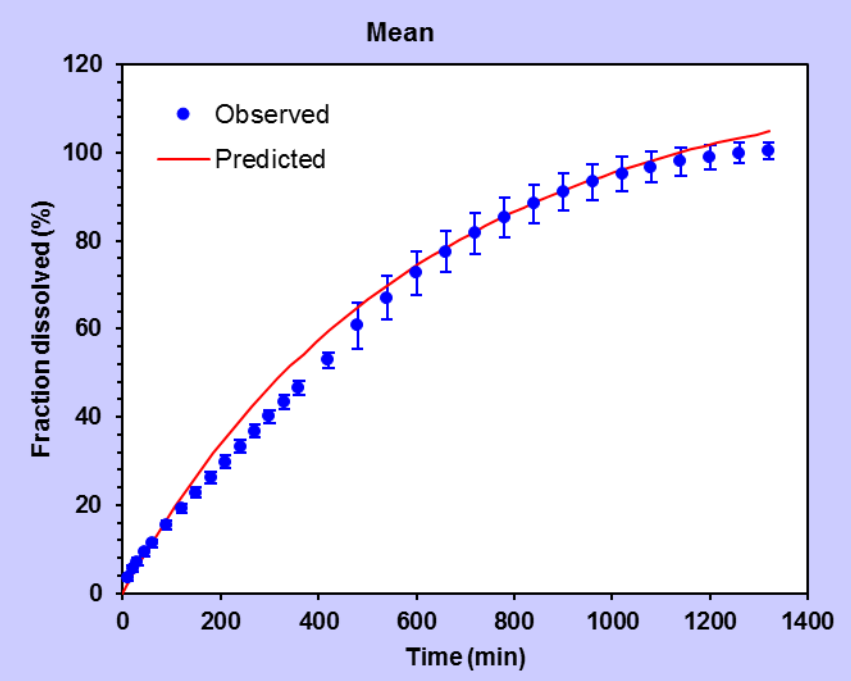

Supplementary materials – Model fitting summary – Glucidex® 19D (modelling the entire relevant dissolution data)

Graphical abstract of model fit presented as the fraction % of released carvedilol per tested tablet:

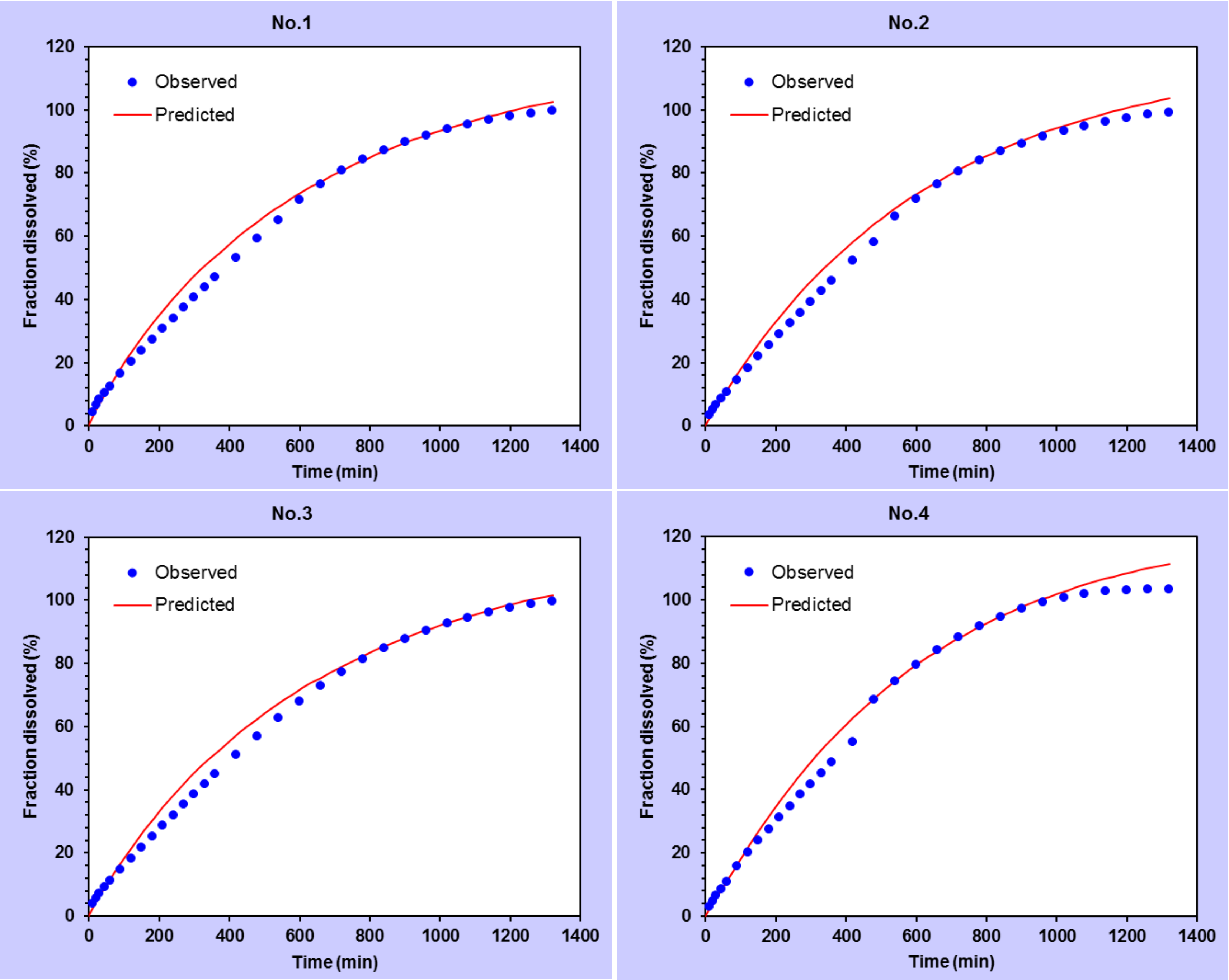

Supplementary materials – Model fitting summary – Glucidex® 19D (modelling the entire relevant dissolution data)

Model: **Weibull_4**

Model equation: $F = F_{max} \cdot \left[1 - e^{-\frac{(t-T_i)^\beta}{\alpha}}\right]$

Fitted model parameters per tested tablet (N = 4) with statistics – mean, standard deviation (SD), and relative standard deviation expressed in % (RSD%) (output from DDSolver):

Parameter	No.1	No.2	No.3	No.4	Mean	SD	RSD(%)
α	226.906	369.032	277.132	431.667	326.184	91.697	28.112
β	0.873	0.924	0.894	0.986	0.919	0.049	5.355
T_i	6.000	4.582	6.000	6.000	5.646	0.709	12.555
F_{max}	104.713	117.429	104.744	108.518	108.851	5.991	5.504

Number of dissolution data points (N), degrees of freedom (df), and selected goodness of fit criteria – Pearson correlation coefficient (R), coefficient of determination (R^2), adjusted coefficient of determination ($R^2_{adjusted}$), and residual sum of squares (RSS) (manual calculation in MS Excel):

Parameter	No.1	No.2	No.3	No.4
N	31	31	31	31
df	27	27	27	27
R	0.990310773	0.995524318	0.990985061	0.992518184
R^2	0.980715428	0.991068669	0.982051392	0.985092346
$R^2_{adjusted}$	0.978572698	0.990076298	0.980057102	0.98343594
RSS	879.4557027	645.7559103	861.6500266	813.5923423

Graphical abstract of model fit presented as mean ± 1 SD of the fraction % of released carvedilol:

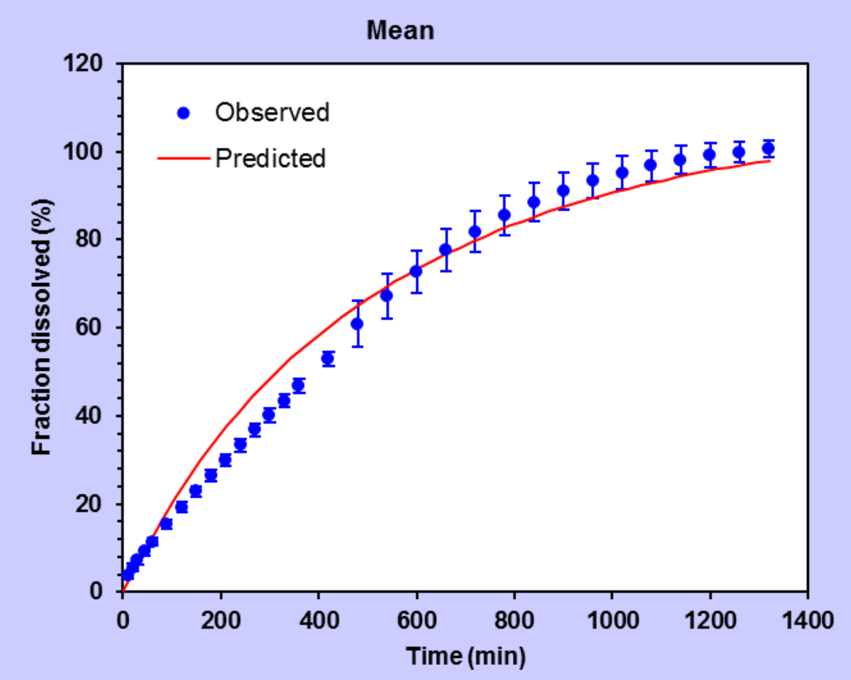

Supplementary materials – Model fitting summary – Glucidex® 19D (modelling the entire relevant dissolution data)

Graphical abstract of model fit presented as the fraction % of released carvedilol per tested tablet:

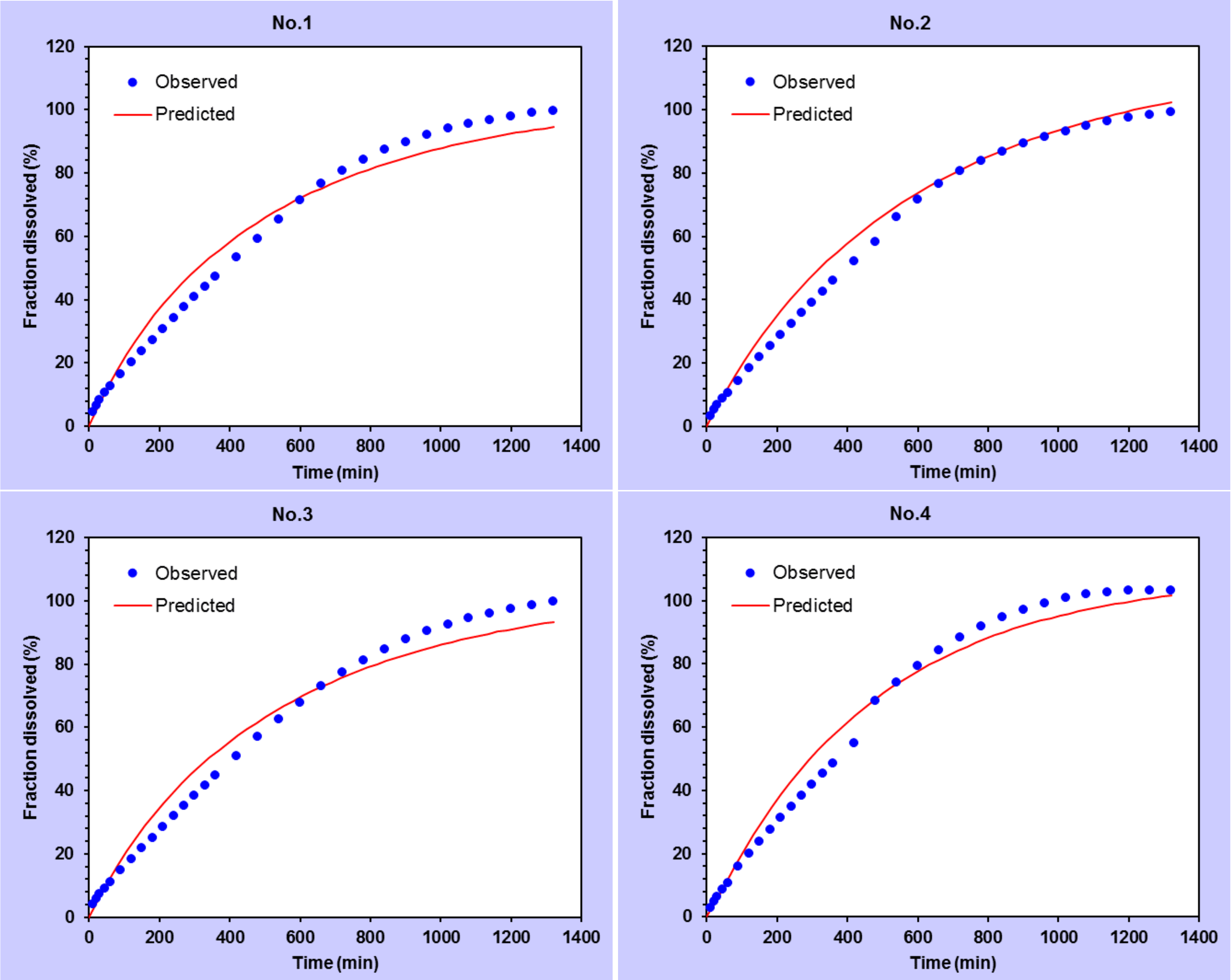

Supplementary materials – Model fitting summary – Glucidex® 19D (modelling the entire relevant dissolution data)

Model: **Logistic_1**

Model equation: $F = 100 \cdot \frac{e^{\alpha+\beta\cdot\log(t)}}{1+e^{\alpha+\beta\cdot\log(t)}}$

Fitted model parameters per tested tablet (N = 4) with statistics – mean, standard deviation (SD), and relative standard deviation expressed in % (RSD%) (output from DDSolver):

Parameter	No.1	No.2	No.3	No.4	Mean	SD	RSD(%)
α	-10.332	-10.564	-10.449	-8.318	-9.916	1.070	-10.788
β	3.984	4.003	3.964	3.542	3.873	0.221	5.718

Number of dissolution data points (N), degrees of freedom (df), and selected goodness of fit criteria – Pearson correlation coefficient (R), coefficient of determination (R^2), adjusted coefficient of determination ($R^2_{adjusted}$), and residual sum of squares (RSS) (manual calculation in MS Excel):

Parameter	No.1	No.2	No.3	No.4
N	31	31	31	31
df	29	29	29	29
R	0.995286493	0.998078405	0.996441767	0.960764307
R^2	0.990595204	0.996160502	0.992896195	0.923068053
$R^2_{adjusted}$	0.990270901	0.996028106	0.992651236	0.920415228
RSS	1642.217576	2315.496648	1922.283281	3683.849967

Graphical abstract of model fit presented as mean ± 1 SD of the fraction % of released carvedilol:

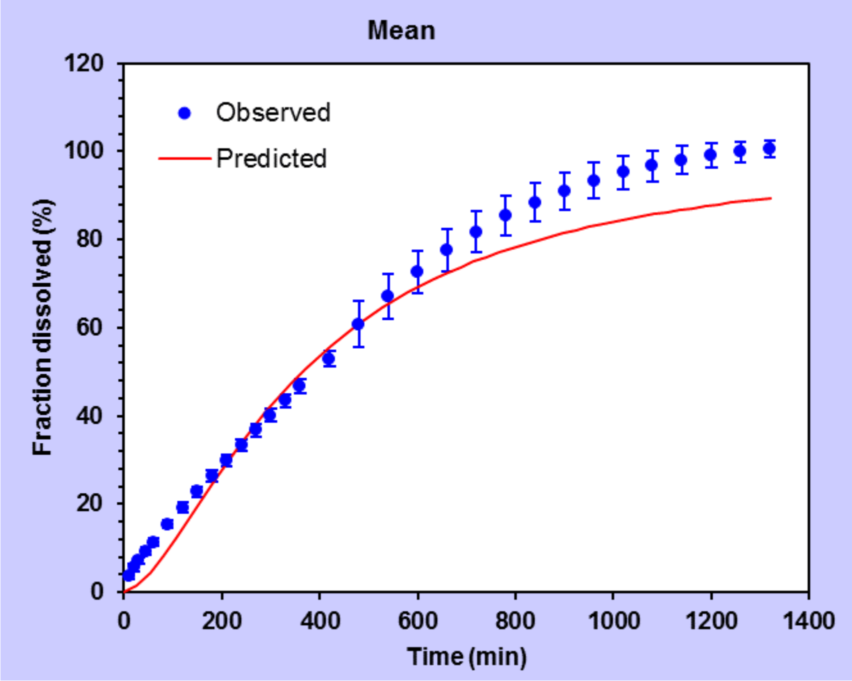

Supplementary materials – Model fitting summary – Glucidex® 19D (modelling the entire relevant dissolution data)

Graphical abstract of model fit presented as the fraction % of released carvedilol per tested tablet:

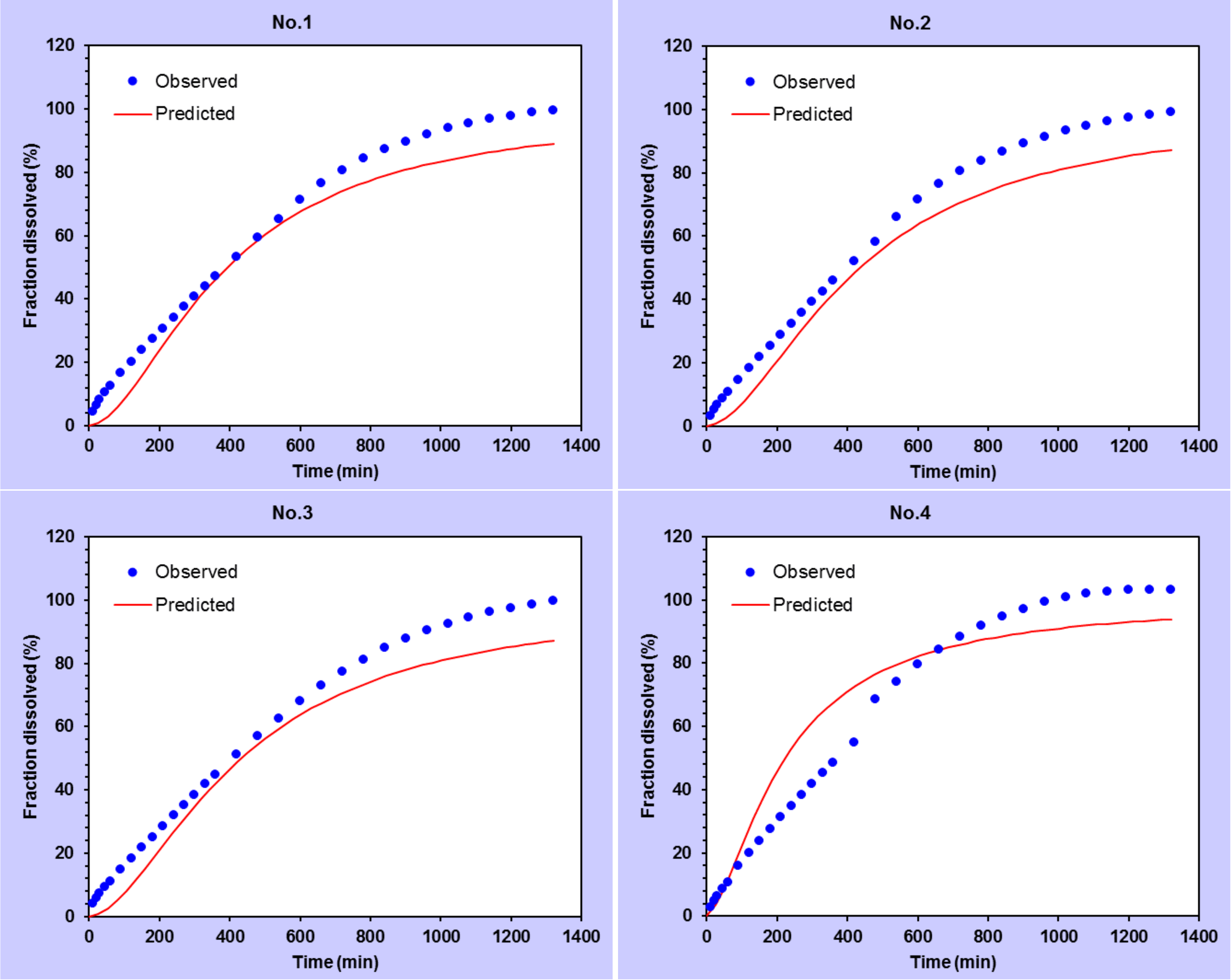

Supplementary materials – Model fitting summary – Glucidex® 19D (modelling the entire relevant dissolution data)

Model: **Logistic_2**

Model equation: $F = F_{max} \cdot \frac{e^{\alpha+\beta \cdot \log(t)}}{1+e^{\alpha+\beta \cdot \log(t)}}$

Fitted model parameters per tested tablet (N = 4) with statistics – mean, standard deviation (SD), and relative standard deviation expressed in % (RSD%) (output from DDSolver):

Parameter	No.1	No.2	No.3	No.4	Mean	SD	RSD(%)
α	-7.262	-8.895	-8.571	-8.170	-8.225	0.707	-8.596
β	2.991	3.342	3.200	3.386	3.229	0.178	5.503
F_{max}	104.713	113.032	113.624	108.518	109.972	4.182	3.803

Number of dissolution data points (N), degrees of freedom (df), and selected goodness of fit criteria – Pearson correlation coefficient (R), coefficient of determination (R^2), adjusted coefficient of determination ($R^2_{adjusted}$), and residual sum of squares (RSS) (manual calculation in MS Excel):

Parameter	No.1	No.2	No.3	No.4
N	31	31	31	31
df	28	28	28	28
R	0.971778267	0.997365647	0.996252298	0.973224857
R^2	0.944353001	0.994738234	0.992518641	0.947166623
$R^2_{adjusted}$	0.940378215	0.994362394	0.991984258	0.943392811
RSS	2363.084405	742.5928319	838.6237694	2779.748652

Graphical abstract of model fit presented as mean ± 1 SD of the fraction % of released carvedilol:

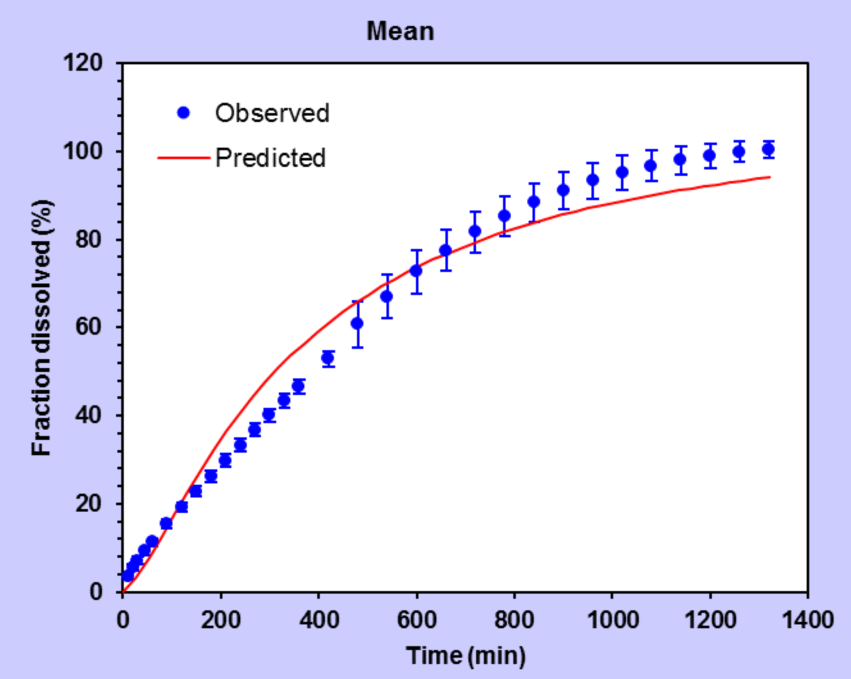

Supplementary materials – Model fitting summary – Glucidex® 19D (modelling the entire relevant dissolution data)

Graphical abstract of model fit presented as the fraction % of released carvedilol per tested tablet:

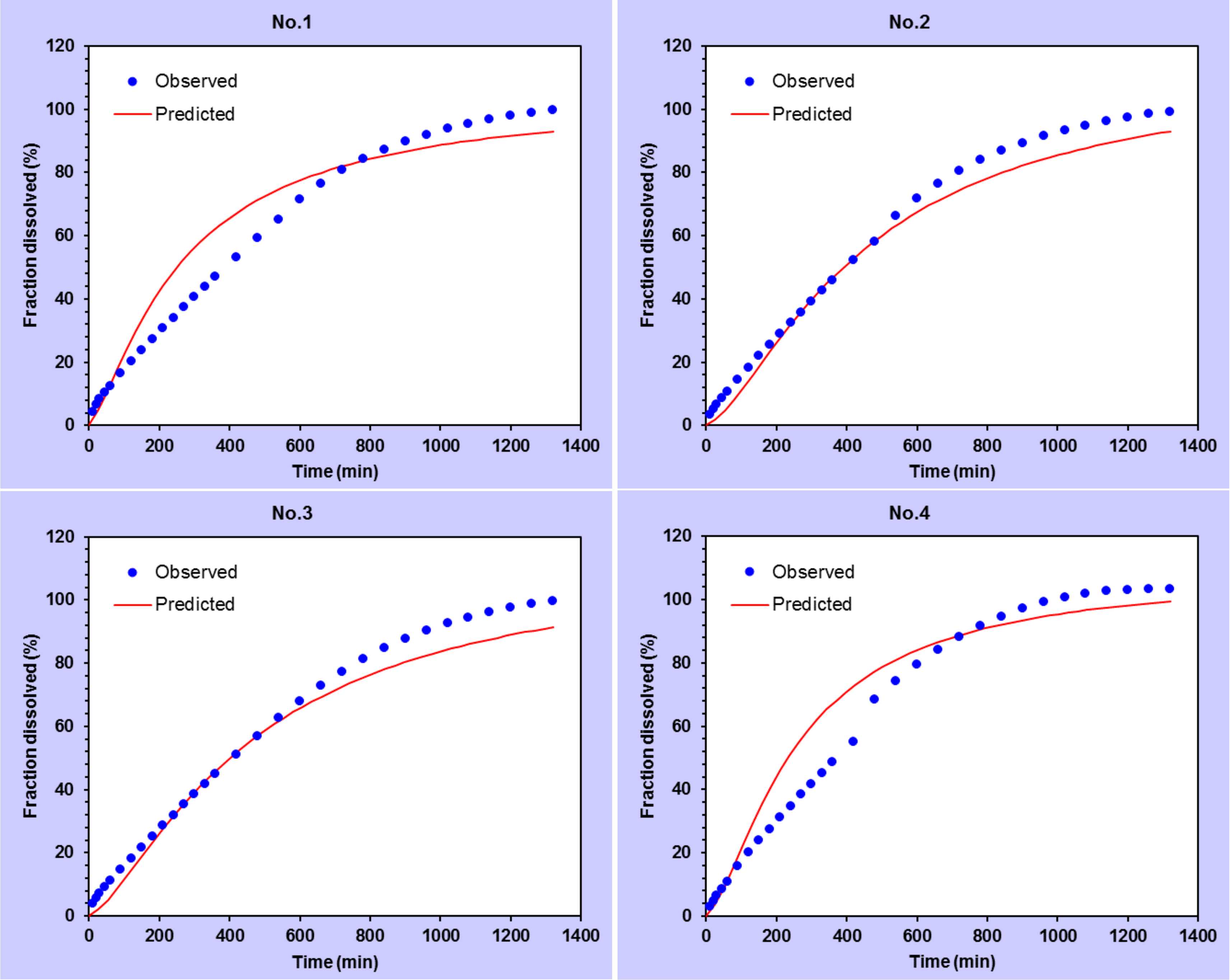

Supplementary materials – Model fitting summary – Glucidex® 19D (modelling the entire relevant dissolution data)

Model: **Logistic_3**

Model equation: $F = F_{max} \cdot \frac{1}{1+e^{-k\cdot(t-\gamma)}}$

Fitted model parameters per tested tablet (N = 4) with statistics – mean, standard deviation (SD), and relative standard deviation expressed in % (RSD%) (output from DDSolver):

Parameter	No.1	No.2	No.3	No.4	Mean	SD	RSD(%)
k	0.006	0.006	0.004	0.005	0.005	0.001	15.751
γ	377.942	390.735	408.684	475.082	413.111	43.195	10.456
F_{max}	94.128	93.666	99.461	108.518	98.943	6.904	6.978

Number of dissolution data points (N), degrees of freedom (df), and selected goodness of fit criteria – Pearson correlation coefficient (R), coefficient of determination (R^2), adjusted coefficient of determination ($R^2_{adjusted}$), and residual sum of squares (RSS) (manual calculation in MS Excel):

Parameter	No.1	No.2	No.3	No.4
N	31	31	31	31
df	28	28	28	28
R	0.995350519	0.995544599	0.997100961	0.987746061
R^2	0.990722655	0.991109049	0.994210327	0.975642282
$R^2_{adjusted}$	0.990059988	0.990473981	0.993796779	0.973902445
RSS	364.2492332	378.8969673	409.828058	1265.867902

Graphical abstract of model fit presented as mean ± 1 SD of the fraction % of released carvedilol:

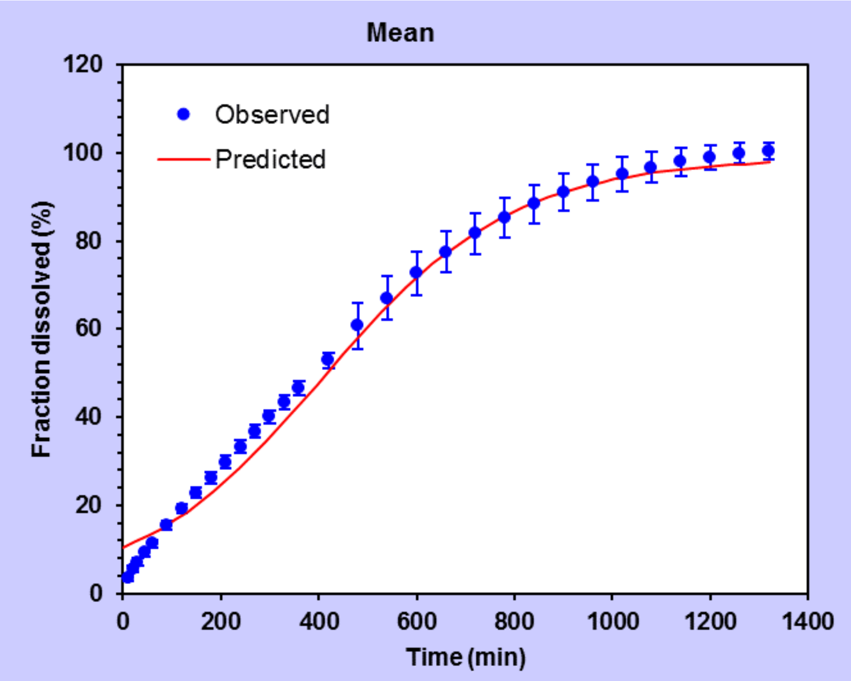

Supplementary materials – Model fitting summary – Glucidex® 19D (modelling the entire relevant dissolution data)

Graphical abstract of model fit presented as the fraction % of released carvedilol per tested tablet:

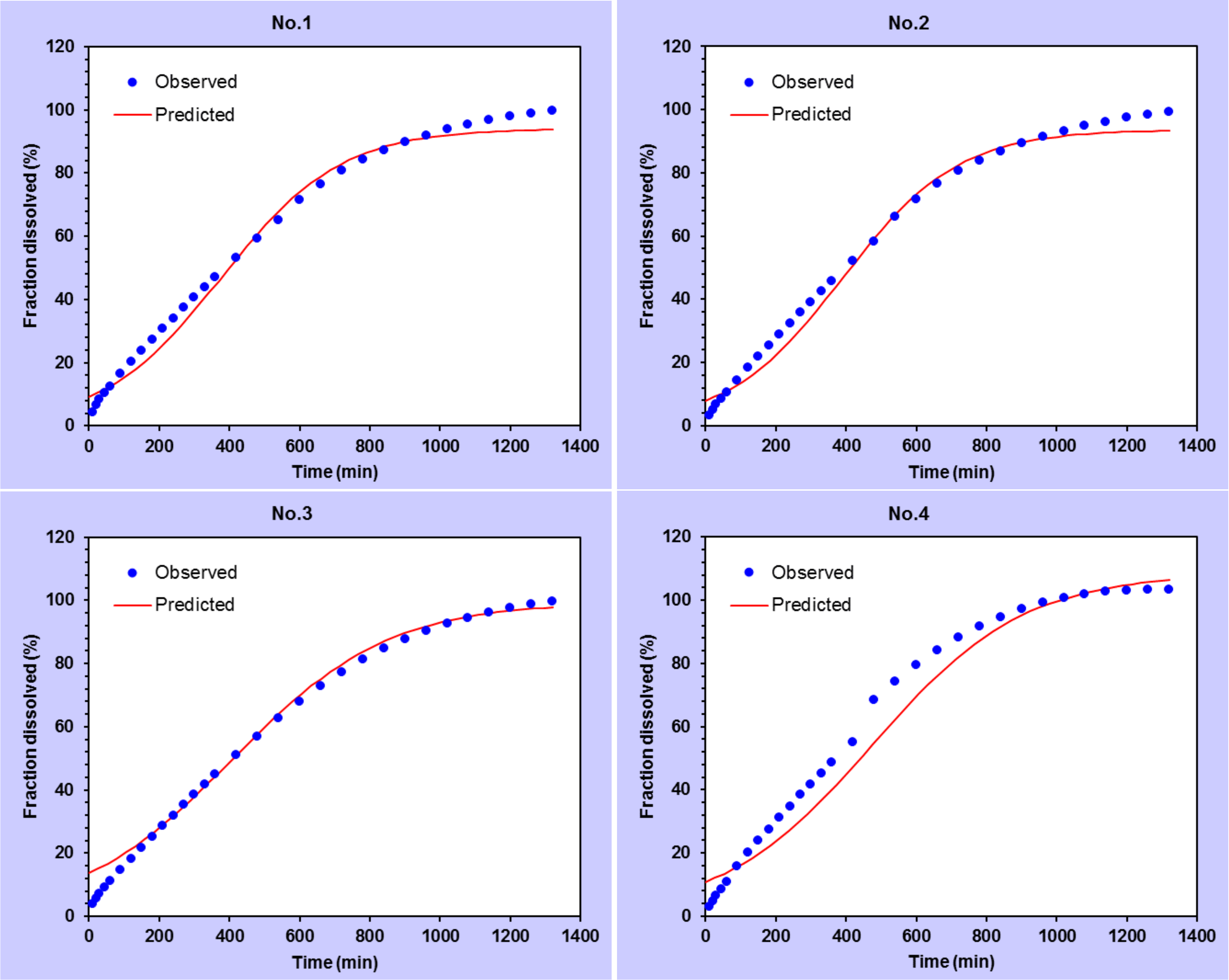

Supplementary materials – Model fitting summary – Glucidex® 19D (modelling the entire relevant dissolution data)

Model: **Gompertz_1**

Model equation: $F = 100 \cdot e^{-\alpha \cdot e^{-\beta \cdot \log(t)}}$

Fitted model parameters per tested tablet (N = 4) with statistics – mean, standard deviation (SD), and relative standard deviation expressed in % (RSD%) (output from DDSolver):

Parameter	No.1	No.2	No.3	No.4	Mean	SD	RSD(%)
α	331.601	256.846	310.274	214.381	278.276	52.944	19.026
β	2.591	2.353	2.523	2.385	2.463	0.113	4.576

Number of dissolution data points (N), degrees of freedom (df), and selected goodness of fit criteria – Pearson correlation coefficient (R), coefficient of determination (R^2), adjusted coefficient of determination ($R^2_{adjusted}$), and residual sum of squares (RSS) (manual calculation in MS Excel):

Parameter	No.1	No.2	No.3	No.4
N	31	31	31	31
df	29	29	29	29
R	0.953771031	0.97672897	0.956207821	0.960520917
R^2	0.90967918	0.953999481	0.914333396	0.922600432
$R^2_{adjusted}$	0.906564669	0.952413256	0.911379375	0.919931481
RSS	3178.610377	2331.91977	2991.929461	3901.077115

Graphical abstract of model fit presented as mean ± 1 SD of the fraction % of released carvedilol:

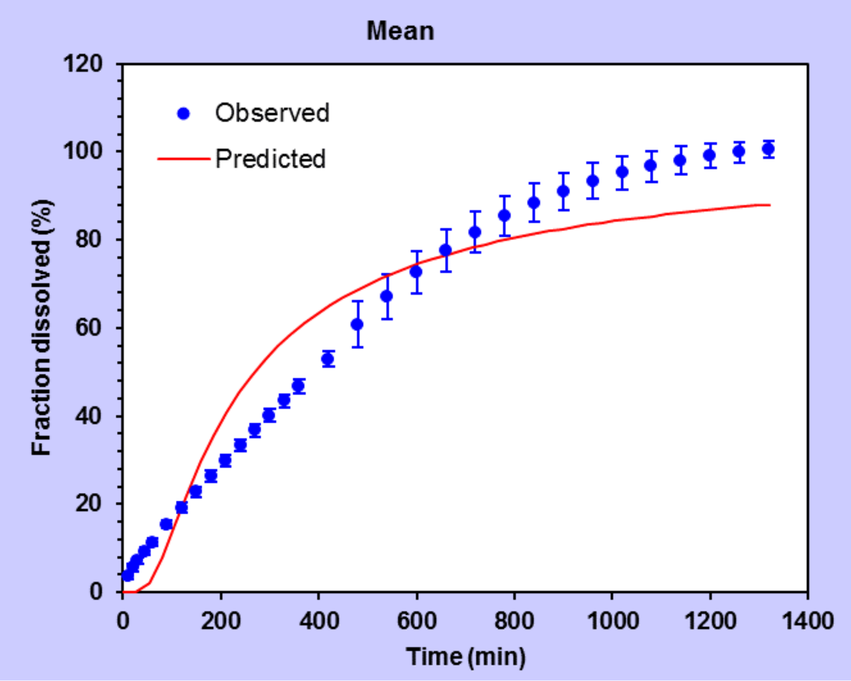

Supplementary materials – Model fitting summary – Glucidex® 19D (modelling the entire relevant dissolution data)

Graphical abstract of model fit presented as the fraction % of released carvedilol per tested tablet:

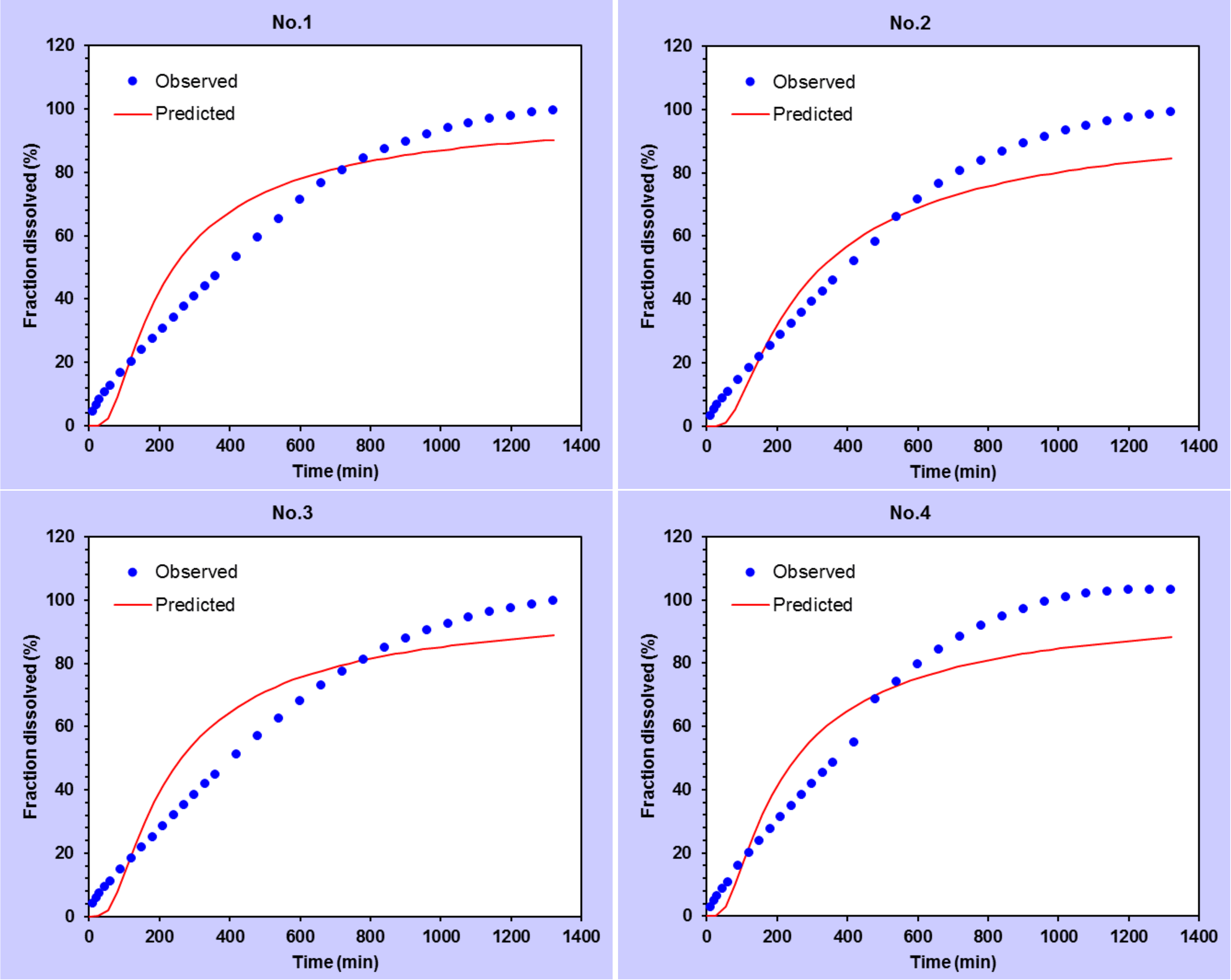

Supplementary materials – Model fitting summary – Glucidex® 19D (modelling the entire relevant dissolution data)

Model: **Gompertz_2**

Model equation: $F = F_{max} \cdot e^{-\alpha \cdot e^{-\beta \cdot \log(t)}}$

Fitted model parameters per tested tablet (N = 4) with statistics – mean, standard deviation (SD), and relative standard deviation expressed in % (RSD%) (output from DDSolver):

Parameter	No.1	No.2	No.3	No.4	Mean	SD	RSD(%)
α	116.781	136.612	116.259	160.189	132.460	20.772	15.682
β	2.035	2.087	2.001	2.035	2.039	0.035	1.737
F_{max}	104.713	104.198	104.744	124.591	109.561	10.023	9.148

Number of dissolution data points (N), degrees of freedom (df), and selected goodness of fit criteria – Pearson correlation coefficient (R), coefficient of determination (R^2), adjusted coefficient of determination ($R^2_{adjusted}$), and residual sum of squares (RSS) (manual calculation in MS Excel):

Parameter	No.1	No.2	No.3	No.4
N	31	31	31	31
df	28	28	28	28
R	0.972844633	0.975480157	0.973940306	0.991444315
R^2	0.94642668	0.951561537	0.948559719	0.982961829
$R^2_{adjusted}$	0.942600015	0.948101646	0.944885413	0.981744817
RSS	2411.289705	2335.444444	2495.077201	2288.459633

Graphical abstract of model fit presented as mean ± 1 SD of the fraction % of released carvedilol:

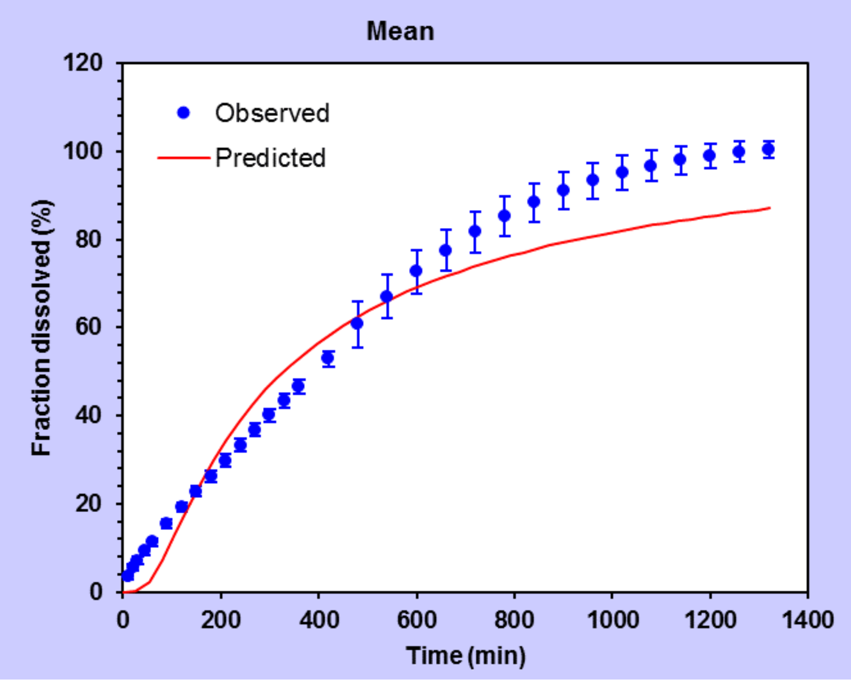

Supplementary materials – Model fitting summary – Glucidex® 19D (modelling the entire relevant dissolution data)

Graphical abstract of model fit presented as the fraction % of released carvedilol per tested tablet:

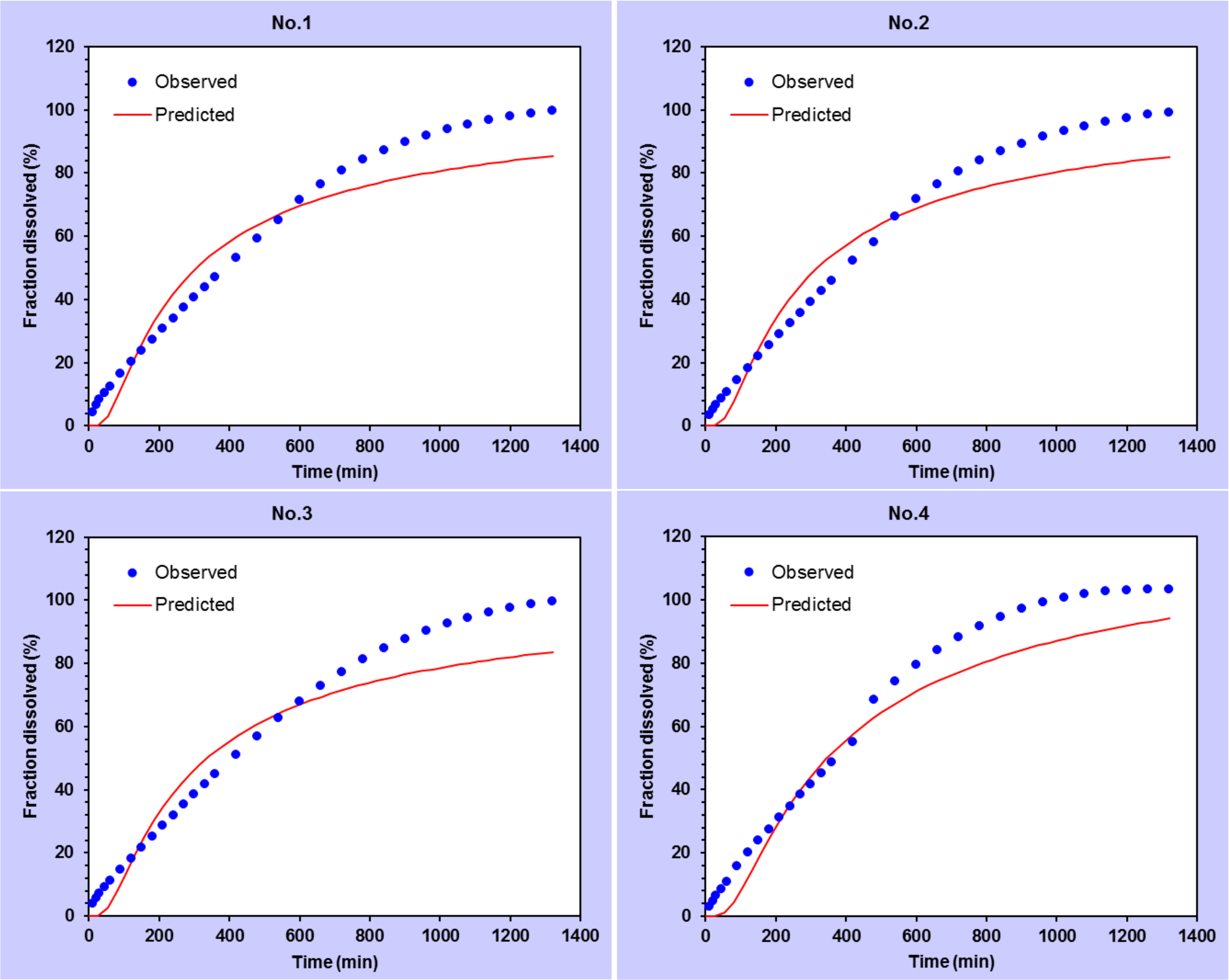

Supplementary materials – Model fitting summary – Glucidex® 19D (modelling the entire relevant dissolution data)

Model: **Gompertz_3**

Model equation: $F = F_{max} \cdot e^{-e^{-k\cdot(t-\gamma)}}$

Fitted model parameters per tested tablet (N = 4) with statistics – mean, standard deviation (SD), and relative standard deviation expressed in % (RSD%) (output from DDSolver):

Parameter	No.1	No.2	No.3	No.4	Mean	SD	RSD(%)
k	0.003	0.003	0.003	0.003	0.003	0.000	6.844
γ	298.555	289.799	322.288	291.181	300.456	15.054	5.010
F_{max}	104.713	102.502	104.744	108.518	105.119	2.497	2.376

Number of dissolution data points (N), degrees of freedom (df), and selected goodness of fit criteria – Pearson correlation coefficient (R), coefficient of determination (R^2), adjusted coefficient of determination ($R^2_{adjusted}$), and residual sum of squares (RSS) (manual calculation in MS Excel):

Parameter	No.1	No.2	No.3	No.4
N	31	31	31	31
df	28	28	28	28
R	0.998718939	0.999225294	0.998787865	0.99830952
R^2	0.99743952	0.998451188	0.9975772	0.996621898
$R^2_{adjusted}$	0.997256628	0.998340558	0.997404143	0.996380605
RSS	92.76853487	73.48007762	88.15665711	160.1544537

Graphical abstract of model fit presented as mean ± 1 SD of the fraction % of released carvedilol:

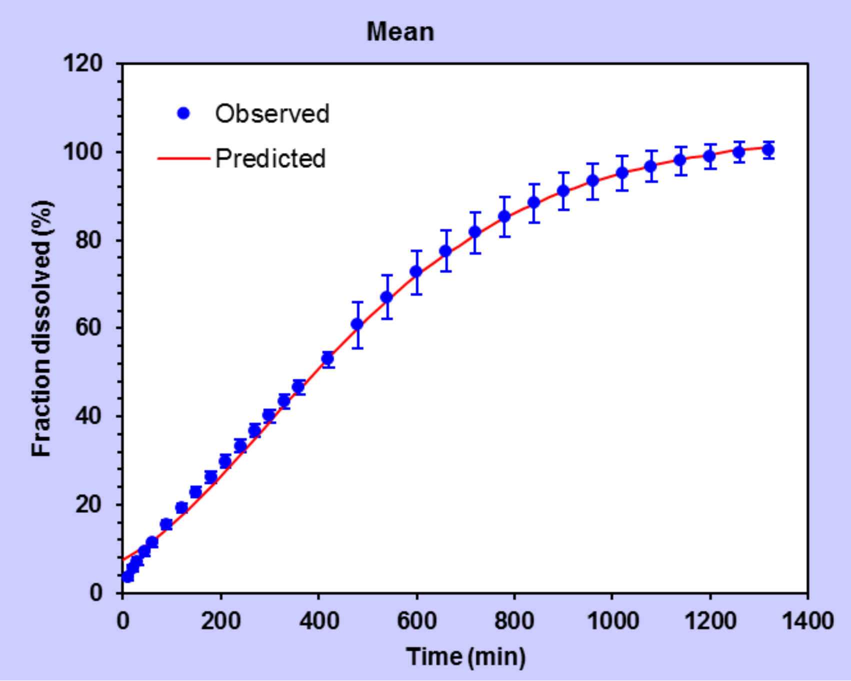

Supplementary materials – Model fitting summary – Glucidex® 19D (modelling the entire relevant dissolution data)

Graphical abstract of model fit presented as the fraction % of released carvedilol per tested tablet:

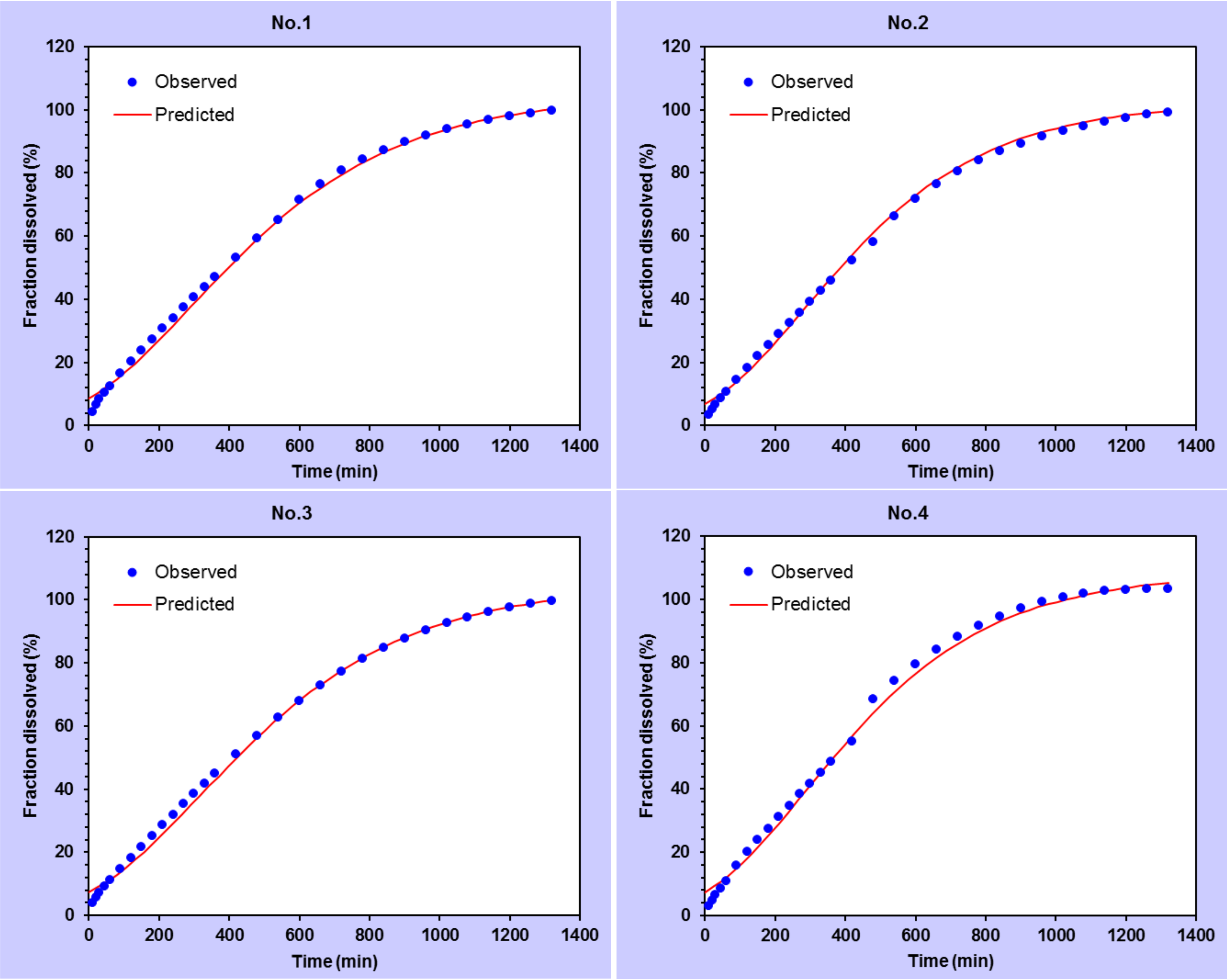

Supplementary materials – Model fitting summary – Glucidex® 19D (modelling the entire relevant dissolution data)

Model: **Gompertz_4**

Model equation: $F = F_{max} \cdot e^{-\beta \cdot e^{-k \cdot t}}$

Fitted model parameters per tested tablet (N = 4) with statistics – mean, standard deviation (SD), and relative standard deviation expressed in % (RSD%) (output from DDSolver):

Parameter	No.1	No.2	No.3	No.4	Mean	SD	RSD(%)
k	0.003	0.003	0.003	0.004	0.003	0.000	14.902
β	2.499	2.653	2.658	2.973	2.696	0.199	7.387
F_{max}	104.713	104.198	104.744	105.740	104.849	0.645	0.615

Number of dissolution data points (N), degrees of freedom (df), and selected goodness of fit criteria – Pearson correlation coefficient (R), coefficient of determination (R^2), adjusted coefficient of determination ($R^2_{adjusted}$), and residual sum of squares (RSS) (manual calculation in MS Excel):

Parameter	No.1	No.2	No.3	No.4
N	31	31	31	31
df	28	28	28	28
R	0.998718939	0.998542226	0.998787865	0.998498183
R^2	0.99743952	0.997086578	0.9975772	0.996998621
$R^2_{adjusted}$	0.997256628	0.996878476	0.997404143	0.996784237
RSS	92.76853487	113.6203312	88.15665711	146.6197384

Graphical abstract of model fit presented as mean ± 1 SD of the fraction % of released carvedilol:

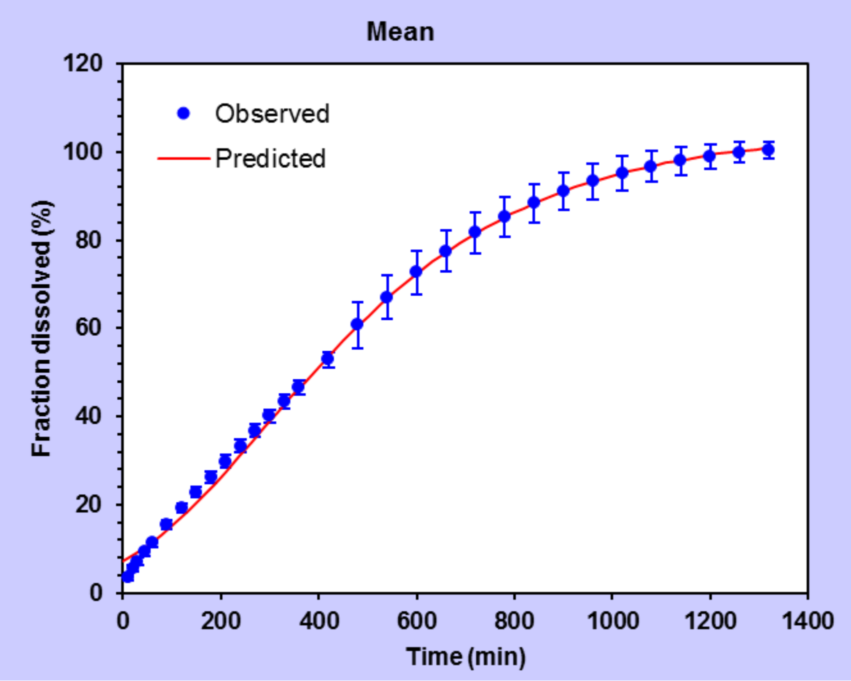

Supplementary materials – Model fitting summary – Glucidex® 19D (modelling the entire relevant dissolution data)

Graphical abstract of model fit presented as the fraction % of released carvedilol per tested tablet:

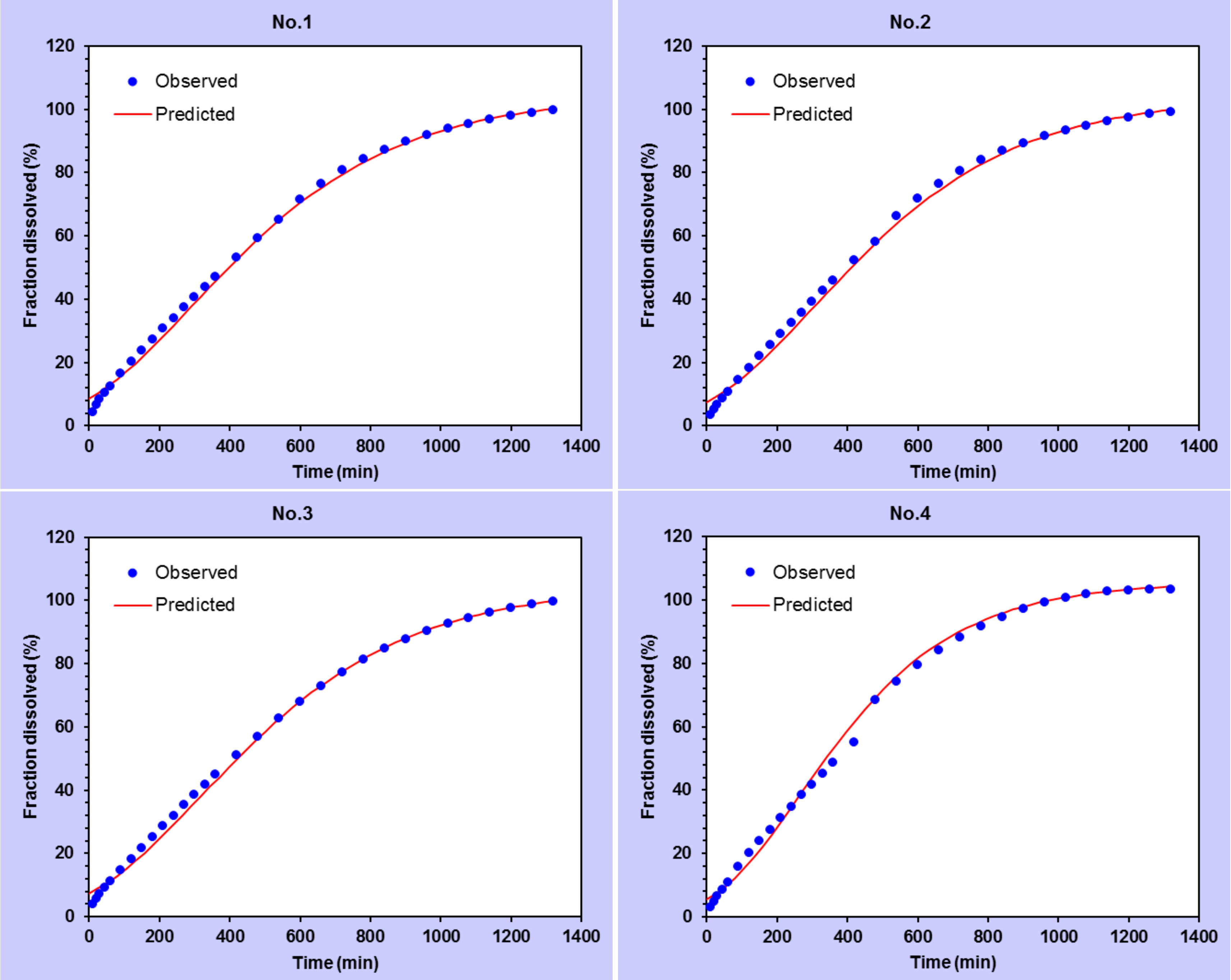

Supplementary materials – Model fitting summary – Glucidex® 19D (modelling the entire relevant dissolution data)

Model: **Probit_1**

Model equation: $F = 100 \cdot \phi[\alpha + \beta \cdot \log(t)]$

Fitted model parameters per tested tablet (N = 4) with statistics – mean, standard deviation (SD), and relative standard deviation expressed in % (RSD%) (output from DDSolver):

Parameter	No.1	No.2	No.3	No.4	Mean	SD	RSD(%)
α	-5.772	-5.923	-5.835	-4.653	-5.546	0.598	-10.789
β	2.211	2.236	2.199	1.973	2.155	0.122	5.661

Number of dissolution data points (N), degrees of freedom (df), and selected goodness of fit criteria – Pearson correlation coefficient (R), coefficient of determination (R^2), adjusted coefficient of determination ($R^2_{adjusted}$), and residual sum of squares (RSS) (manual calculation in MS Excel):

Parameter	No.1	No.2	No.3	No.4
N	31	31	31	31
df	29	29	29	29
R	0.995119209	0.997303378	0.99600404	0.964008868
R^2	0.99026224	0.994614027	0.992024048	0.929313098
$R^2_{adjusted}$	0.989926455	0.994428304	0.991749015	0.926875618
RSS	2427.604671	3092.868475	2805.121129	3469.526222

Graphical abstract of model fit presented as mean ± 1 SD of the fraction % of released carvedilol:

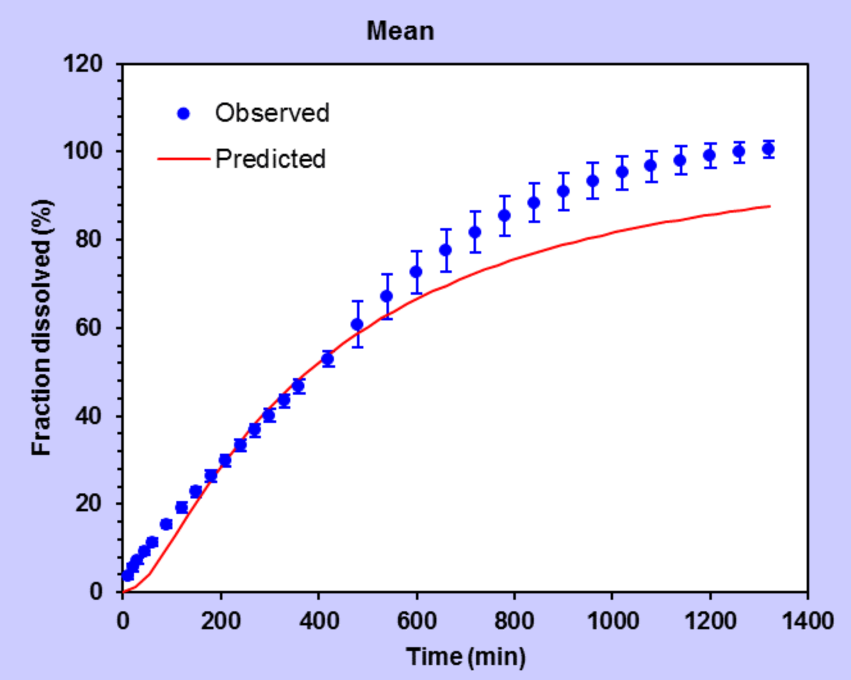

Supplementary materials – Model fitting summary – Glucidex® 19D (modelling the entire relevant dissolution data)

Graphical abstract of model fit presented as the fraction % of released carvedilol per tested tablet:

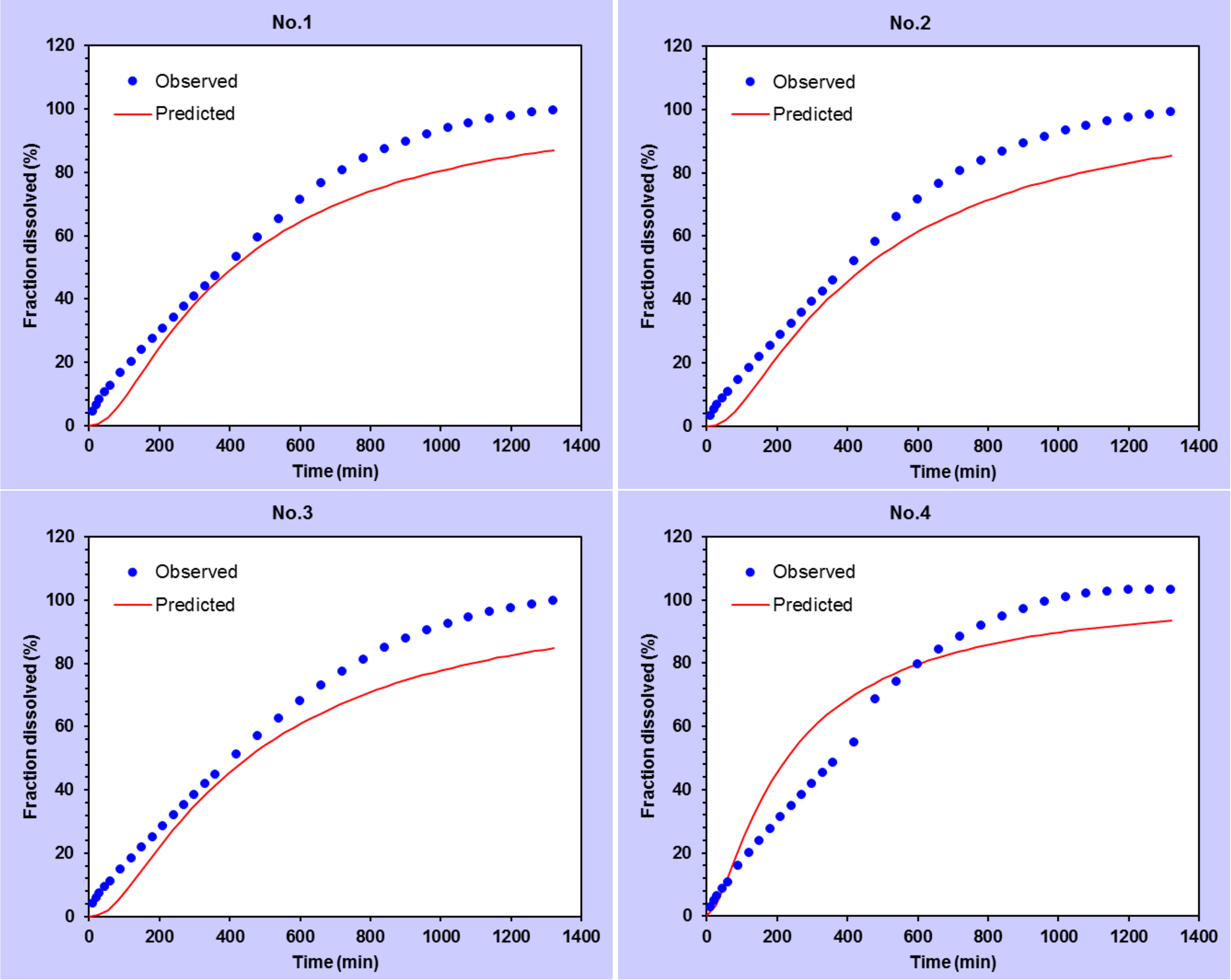

Supplementary materials – Model fitting summary – Glucidex® 19D (modelling the entire relevant dissolution data)

Model: **Probit_2**

Model equation: $F = F_{max} \cdot \phi[\alpha + \beta \cdot \log(t)]$

Fitted model parameters per tested tablet (N = 4) with statistics – mean, standard deviation (SD), and relative standard deviation expressed in % (RSD%) (output from DDSolver):

Parameter	No.1	No.2	No.3	No.4	Mean	SD	RSD(%)
α	-4.189	-5.087	-4.927	-4.633	-4.709	0.395	-8.378
β	1.724	1.912	1.839	1.920	1.849	0.091	4.899
F_{max}	104.713	113.032	113.624	108.518	109.972	4.182	3.803

Number of dissolution data points (N), degrees of freedom (df), and selected goodness of fit criteria – Pearson correlation coefficient (R), coefficient of determination (R^2), adjusted coefficient of determination ($R^2_{adjusted}$), and residual sum of squares (RSS) (manual calculation in MS Excel):

Parameter	No.1	No.2	No.3	No.4
N	31	31	31	31
df	28	28	28	28
R	0.970635144	0.995152575	0.993766794	0.972494428
R^2	0.942132582	0.990328648	0.98757244	0.945745412
$R^2_{adjusted}$	0.937999195	0.989637837	0.986684757	0.941870084
RSS	2389.565576	1030.305908	1151.507734	2792.51439

Graphical abstract of model fit presented as mean ± 1 SD of the fraction % of released carvedilol:

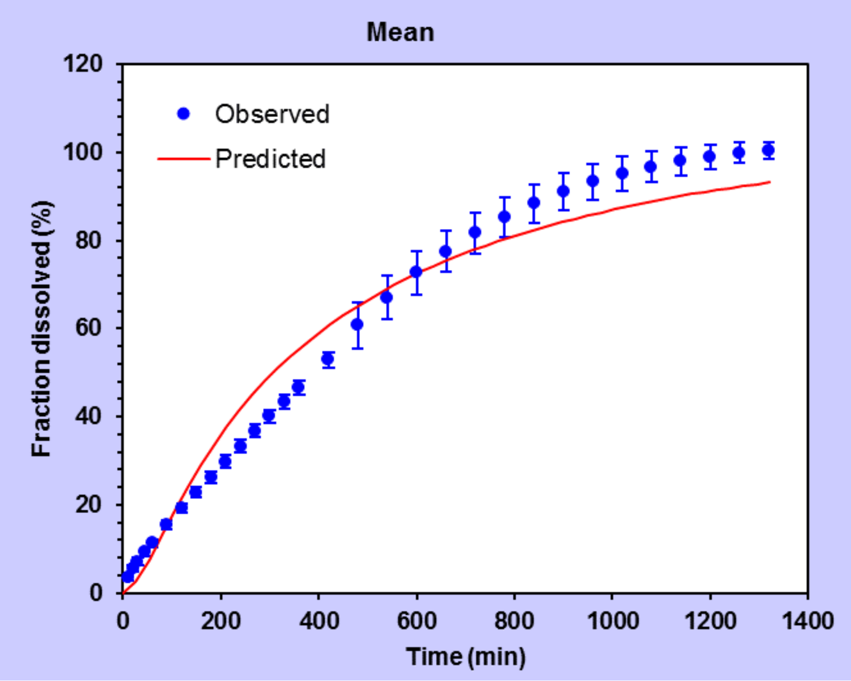

Supplementary materials – Model fitting summary – Glucidex® 19D (modelling the entire relevant dissolution data)

Graphical abstract of model fit presented as the fraction % of released carvedilol per tested tablet:

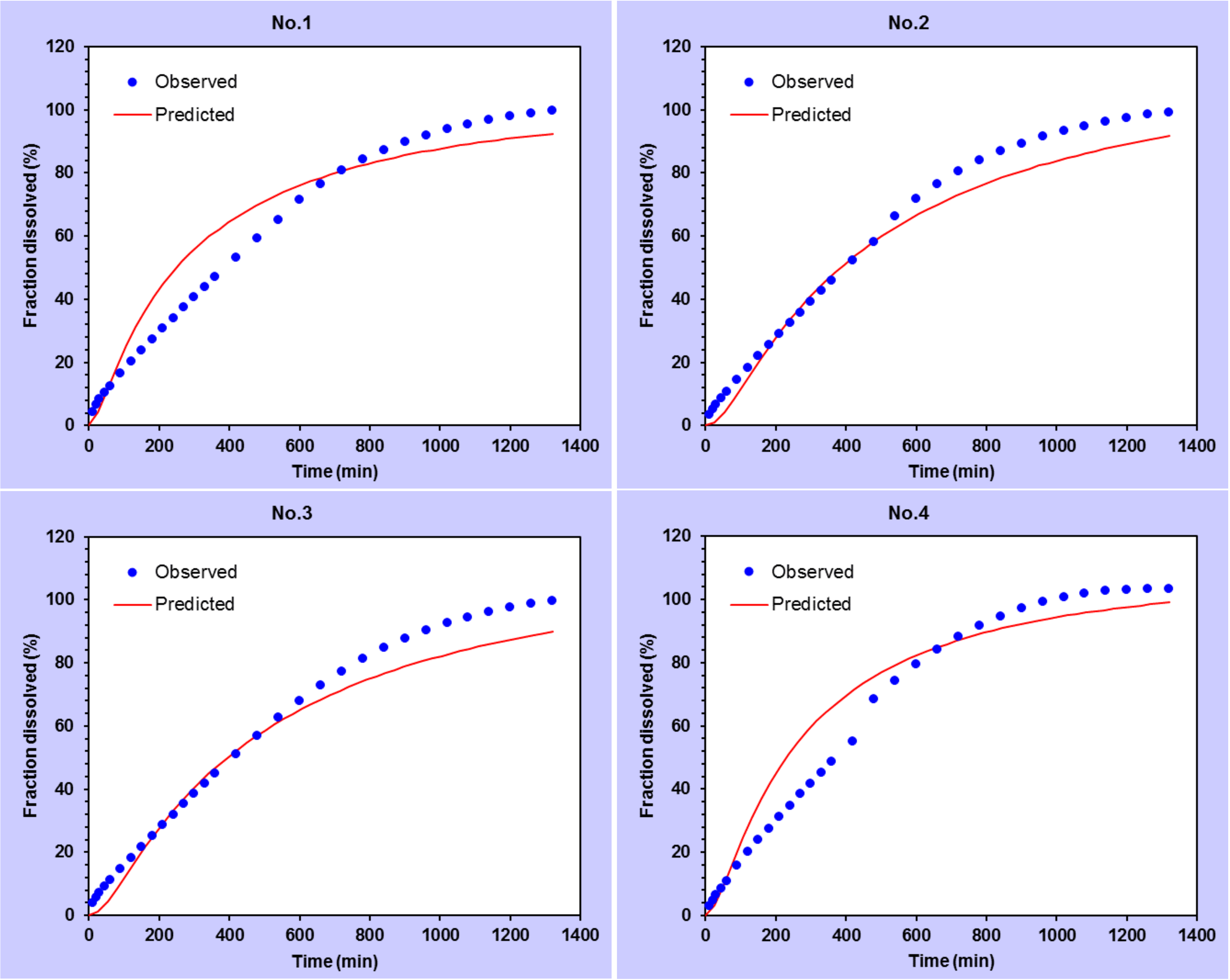

Supplementary materials – Model fitting summary – Glucidex® 19D (modelling up to app. 60 % of carvedilol released)

Model: **Zero–order**

Model equation: $F = k_0 \cdot t$

Fitted model parameters per tested tablet (N = 4) with statistics – mean, standard deviation (SD), and relative standard deviation expressed in % (RSD%) (output from DDSolver):

Parameter	No.1	No.2	No.3	No.4	Mean	SD	RSD(%)
k_0	0.134	0.129	0.127	0.141	0.133	0.006	4.615

Number of dissolution data points (N), degrees of freedom (df), and selected goodness of fit criteria – Pearson correlation coefficient (R), coefficient of determination (R^2), adjusted coefficient of determination ($R^2_{adjusted}$), and residual sum of squares (RSS) (manual calculation in MS Excel):

Parameter	No.1	No.2	No.3	No.4
N	17	17	17	17
df	16	16	16	16
R	0.998309714	0.999048015	0.999185538	0.997630328
R^2	0.996622284	0.998096936	0.998371739	0.995266272
$R^2_{adjusted}$	0.996622284	0.998096936	0.998371739	0.995266272
RSS	193.4351329	87.97575267	115.8307947	81.72517466

Graphical abstract of model fit presented as mean ± 1 SD of the fraction % of released carvedilol:

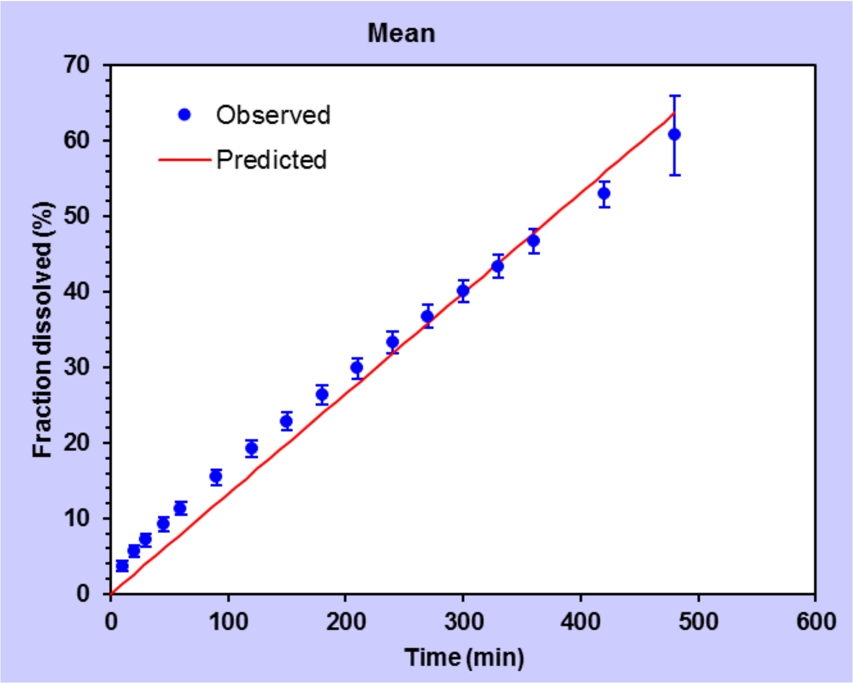

Supplementary materials – Model fitting summary – Glucidex® 19D (modelling up to app. 60 % of carvedilol released)

Graphical abstract of model fit presented as the fraction % of released carvedilol per tested tablet:

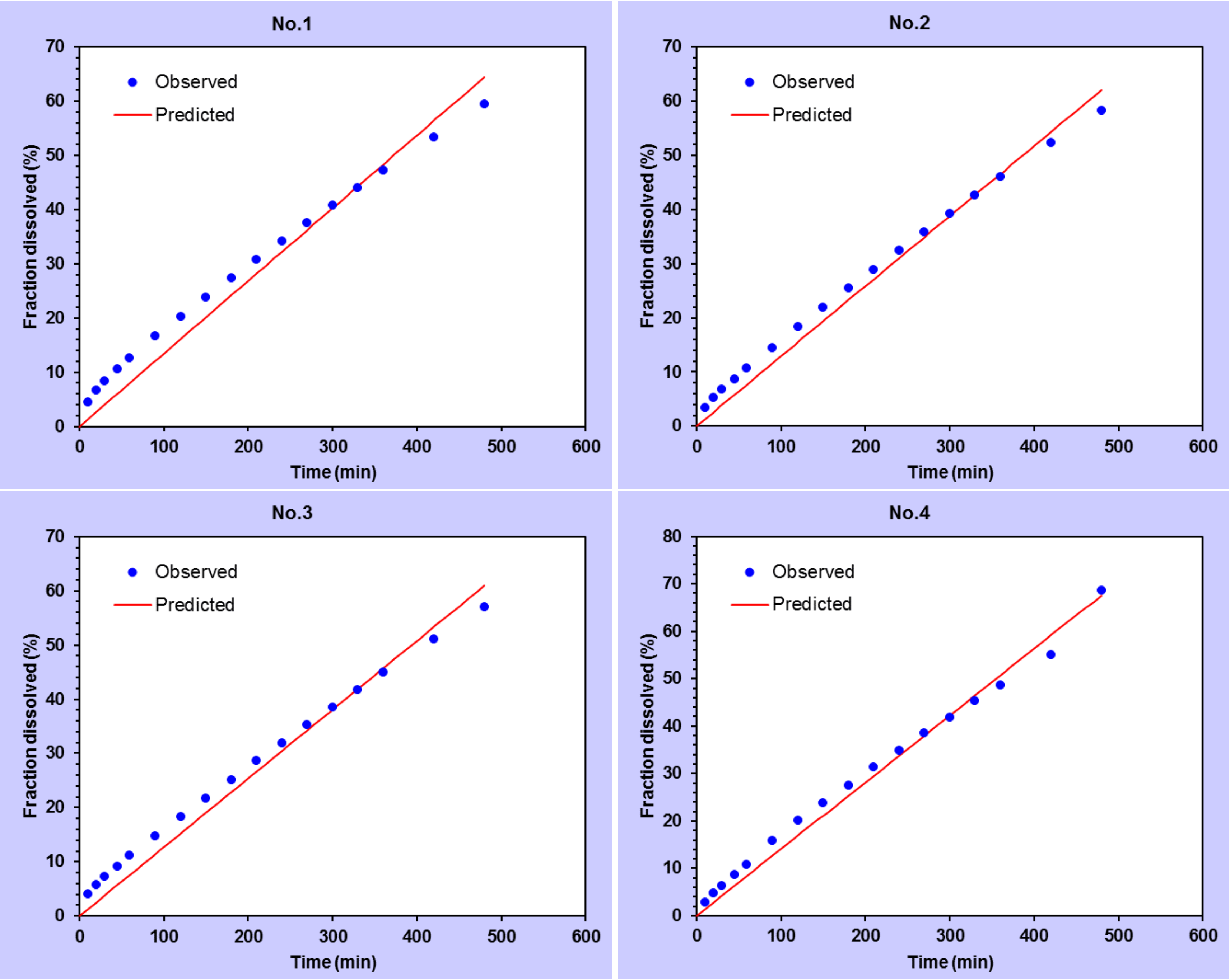

Supplementary materials – Model fitting summary – Glucidex® 19D (modelling up to app. 60 % of carvedilol released)

Model: **Zero–order with T_{lag}**

Model equation: $F = k_0 \cdot (t - T_{lag})$

Fitted model parameters per tested tablet (N = 4) with statistics – mean, standard deviation (SD), and relative standard deviation expressed in % (RSD%) (output from DDSolver):

Parameter	No.1	No.2	No.3	No.4	Mean	SD	RSD(%)
k_0	0.116	0.117	0.113	0.131	0.119	0.008	6.696
T_{lag}	-47.074	-31.088	-37.907	-22.904	-34.743	10.257	-29.521

Number of dissolution data points (N), degrees of freedom (df), and selected goodness of fit criteria – Pearson correlation coefficient (R), coefficient of determination (R^2), adjusted coefficient of determination ($R^2_{adjusted}$), and residual sum of squares (RSS) (manual calculation in MS Excel):

Parameter	No.1	No.2	No.3	No.4
N	17	17	17	17
df	15	15	15	15
R	0.998309714	0.999048015	0.999185538	0.997630328
R^2	0.996622284	0.998096936	0.998371739	0.995266272
$R^2_{adjusted}$	0.996397103	0.997970065	0.998263189	0.99495069
RSS	15.88301761	9.101294617	7.210772009	28.32093964

Graphical abstract of model fit presented as mean ± 1 SD of the fraction % of released carvedilol:

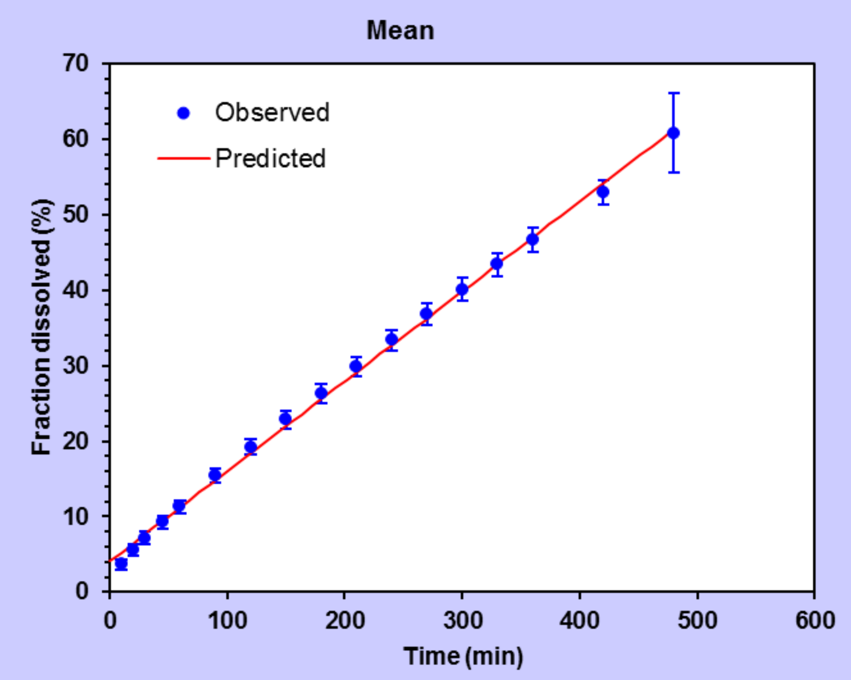

Supplementary materials – Model fitting summary – Glucidex® 19D (modelling up to app. 60 % of carvedilol released)

Graphical abstract of model fit presented as the fraction % of released carvedilol per tested tablet:

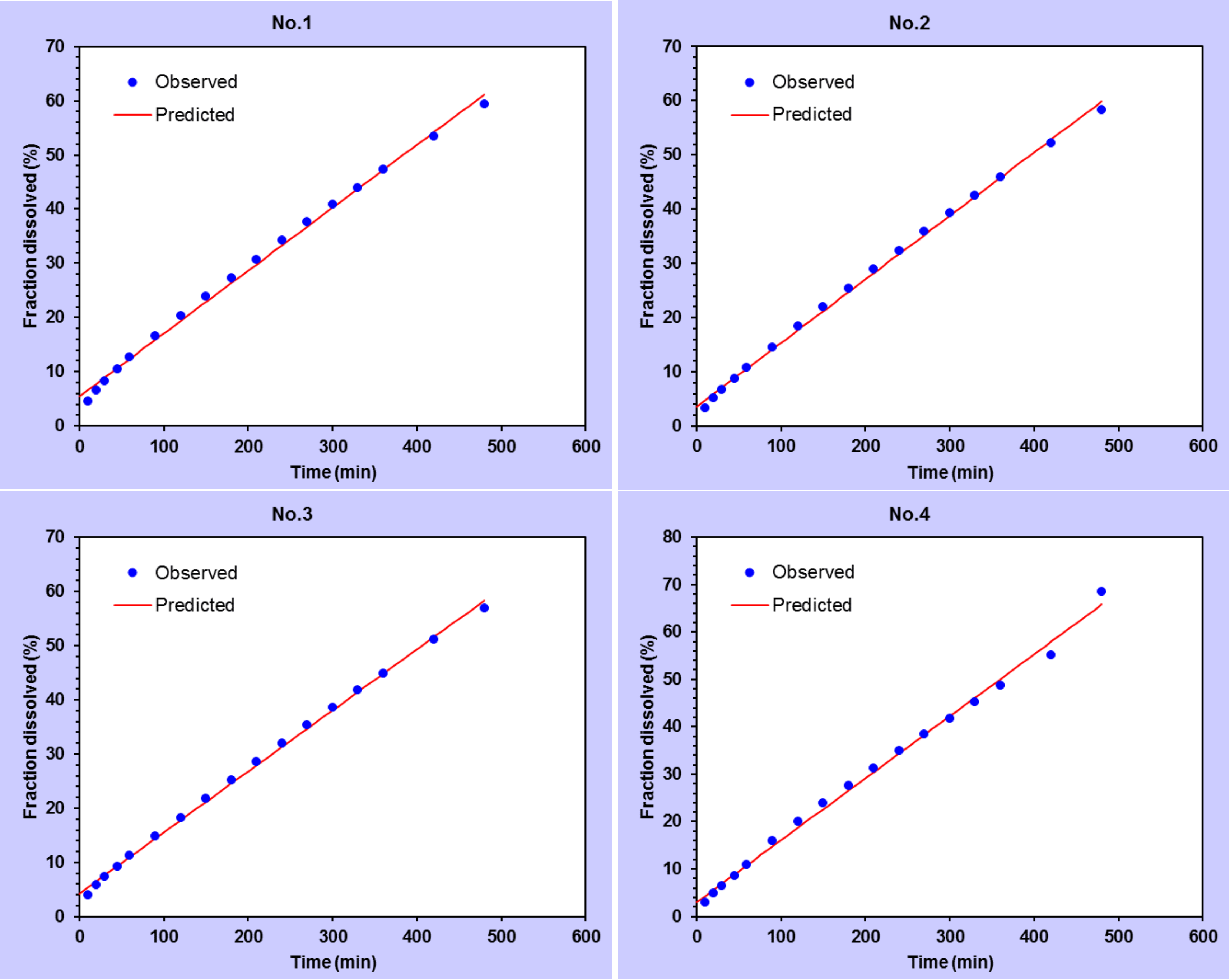

Supplementary materials – Model fitting summary – Glucidex® 19D (modelling up to app. 60 % of carvedilol released)

Model: **Zero–order with F_0**

Model equation: $F = F_0 + k_0 \cdot t$

Fitted model parameters per tested tablet (N = 4) with statistics – mean, standard deviation (SD), and relative standard deviation expressed in % (RSD%) (output from DDSolver):

Parameter	No.1	No.2	No.3	No.4	Mean	SD	RSD(%)
k_0	0.116	0.117	0.113	0.131	0.119	0.008	6.696
F_0	5.464	3.642	4.273	2.996	4.094	1.052	25.687

Number of dissolution data points (N), degrees of freedom (df), and selected goodness of fit criteria – Pearson correlation coefficient (R), coefficient of determination (R^2), adjusted coefficient of determination ($R^2_{adjusted}$), and residual sum of squares (RSS) (manual calculation in MS Excel):

Parameter	No.1	No.2	No.3	No.4
N	17	17	17	17
df	15	15	15	15
R	0.998309714	0.999048015	0.999185538	0.997630328
R^2	0.996622284	0.998096936	0.998371739	0.995266272
$R^2_{adjusted}$	0.996397103	0.997970065	0.998263189	0.99495069
RSS	15.88301761	9.101294617	7.210772009	28.32093964

Graphical abstract of model fit presented as mean ± 1 SD of the fraction % of released carvedilol:

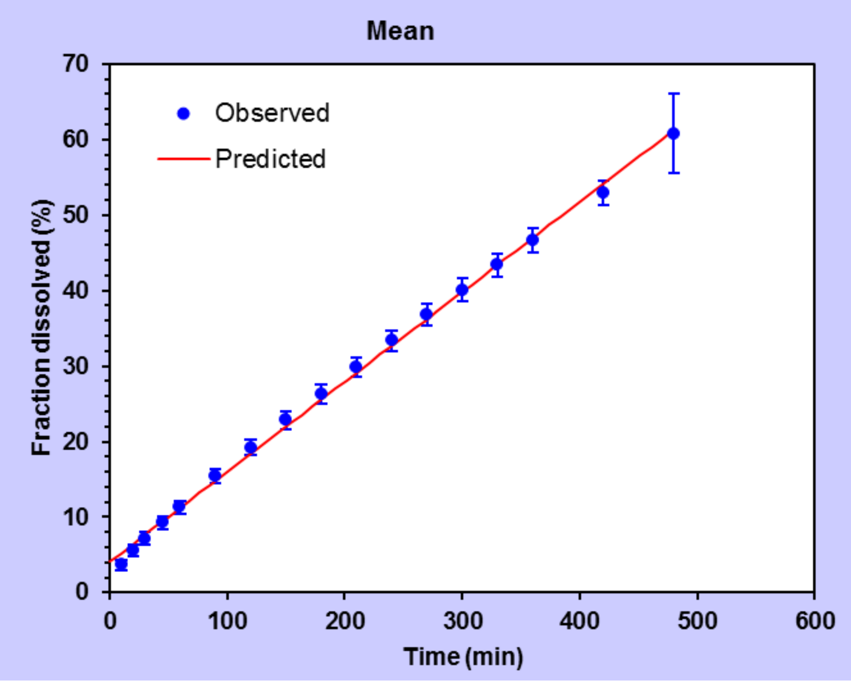

Supplementary materials – Model fitting summary – Glucidex® 19D (modelling up to app. 60 % of carvedilol released)

Graphical abstract of model fit presented as the fraction % of released carvedilol per tested tablet:

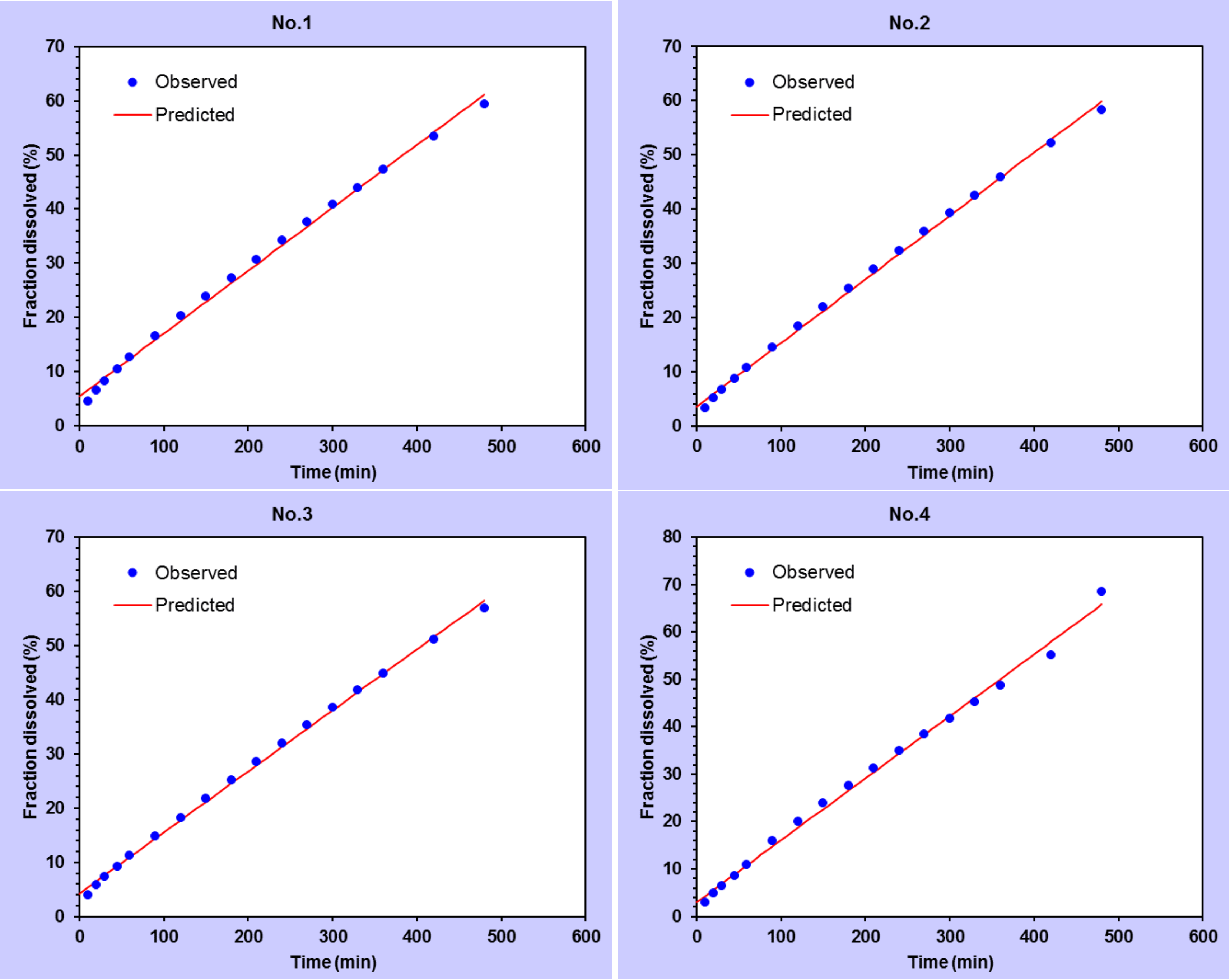

Supplementary materials – Model fitting summary – Glucidex® 19D (modelling up to app. 60 % of carvedilol released)

Model: **First–order**

Model equation: $F = 100 \cdot (1 - e^{-k_1 \cdot t})$

Fitted model parameters per tested tablet (N = 4) with statistics – mean, standard deviation (SD), and relative standard deviation expressed in % (RSD%) (output from DDSolver):

Parameter	No.1	No.2	No.3	No.4	Mean	SD	RSD(%)
k_1	0.002	0.002	0.002	0.002	0.002	0.000	7.212

Number of dissolution data points (N), degrees of freedom (df), and selected goodness of fit criteria – Pearson correlation coefficient (R), coefficient of determination (R^2), adjusted coefficient of determination ($R^2_{adjusted}$), and residual sum of squares (RSS) (manual calculation in MS Excel):

Parameter	No.1	No.2	No.3	No.4
N	17	17	17	17
df	16	16	16	16
R	0.998100711	0.997699462	0.997640004	0.991709073
R^2	0.99620503	0.995404216	0.995285577	0.983486885
$R^2_{adjusted}$	0.99620503	0.995404216	0.995285577	0.983486885
RSS	48.66458192	25.75728011	34.25621567	108.9210423

Graphical abstract of model fit presented as mean ± 1 SD of the fraction % of released carvedilol:

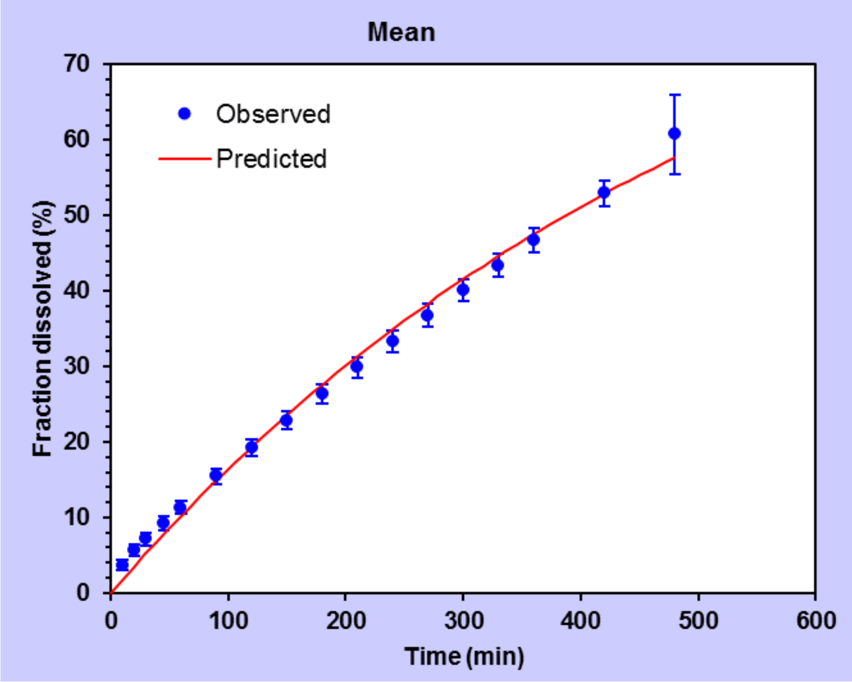

Supplementary materials – Model fitting summary – Glucidex® 19D (modelling up to app. 60 % of carvedilol released)

Graphical abstract of model fit presented as the fraction % of released carvedilol per tested tablet:

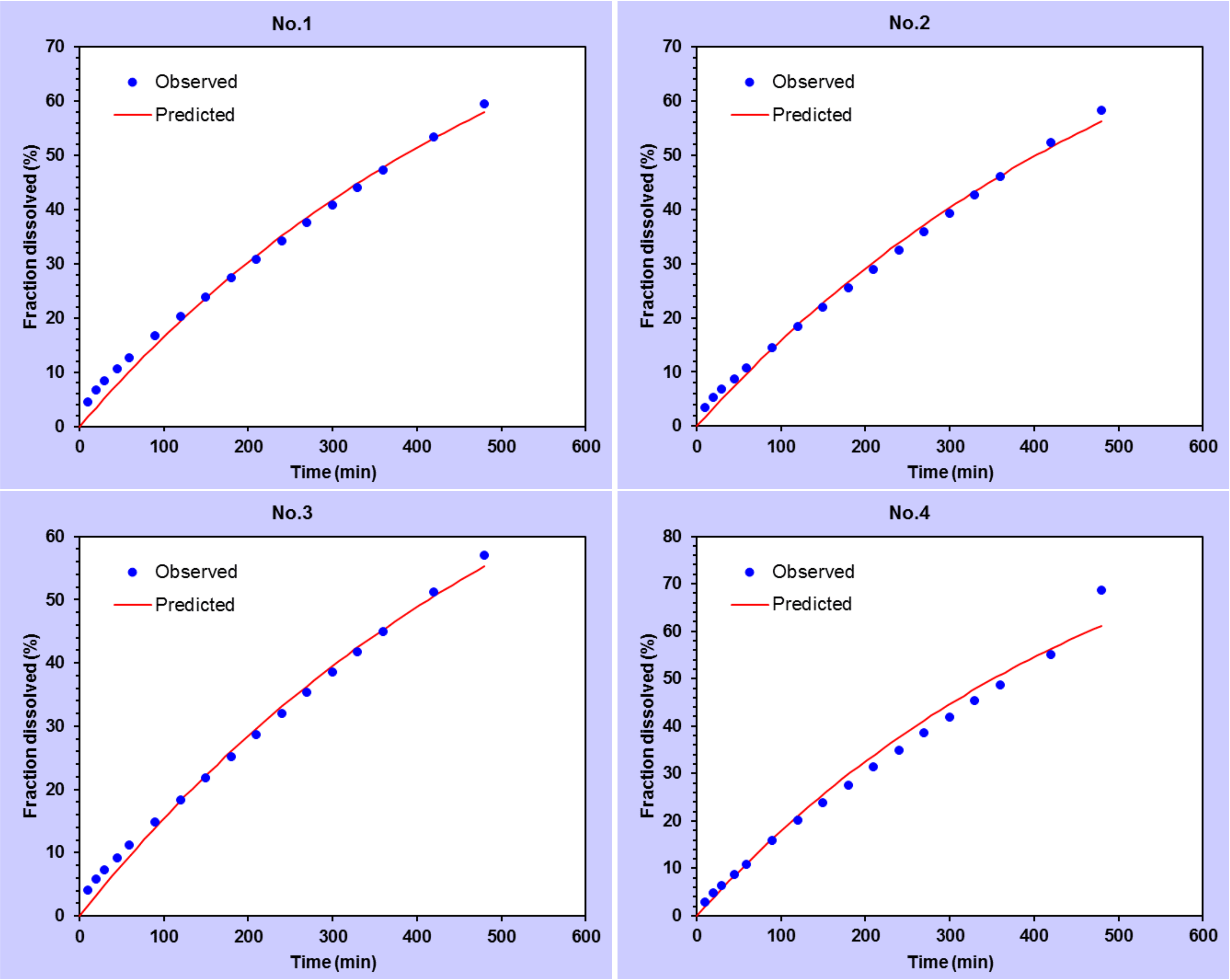

Supplementary materials – Model fitting summary – Glucidex® 19D (modelling up to app. 60 % of carvedilol released)

Model: **First–order with T_{lag}**

Model equation: $F = 100 \cdot \left[1 - e^{-k_1 \cdot (t - T_{lag})}\right]$

Fitted model parameters per tested tablet (N = 4) with statistics – mean, standard deviation (SD), and relative standard deviation expressed in % (RSD%) (output from DDSolver):

Parameter	No.1	No.2	No.3	No.4	Mean	SD	RSD(%)
k_1	0.002	0.002	0.002	0.002	0.002	0.000	10.097
T_{lag}	-11.934	-1.521	-6.765	11.369	-2.213	10.003	-452.070

Number of dissolution data points (N), degrees of freedom (df), and selected goodness of fit criteria – Pearson correlation coefficient (R), coefficient of determination (R^2), adjusted coefficient of determination ($R^2_{adjusted}$), and residual sum of squares (RSS) (manual calculation in MS Excel):

Parameter	No.1	No.2	No.3	No.4
N	17	17	17	17
df	15	15	15	15
R	0.998335421	0.997722931	0.997789972	0.991211385
R^2	0.996673612	0.995451047	0.995584829	0.98250001
$R^2_{adjusted}$	0.996451853	0.995147783	0.995290484	0.981333344
RSS	17.54221098	24.2456325	21.71358997	124.221705

Graphical abstract of model fit presented as mean ± 1 SD of the fraction % of released carvedilol:

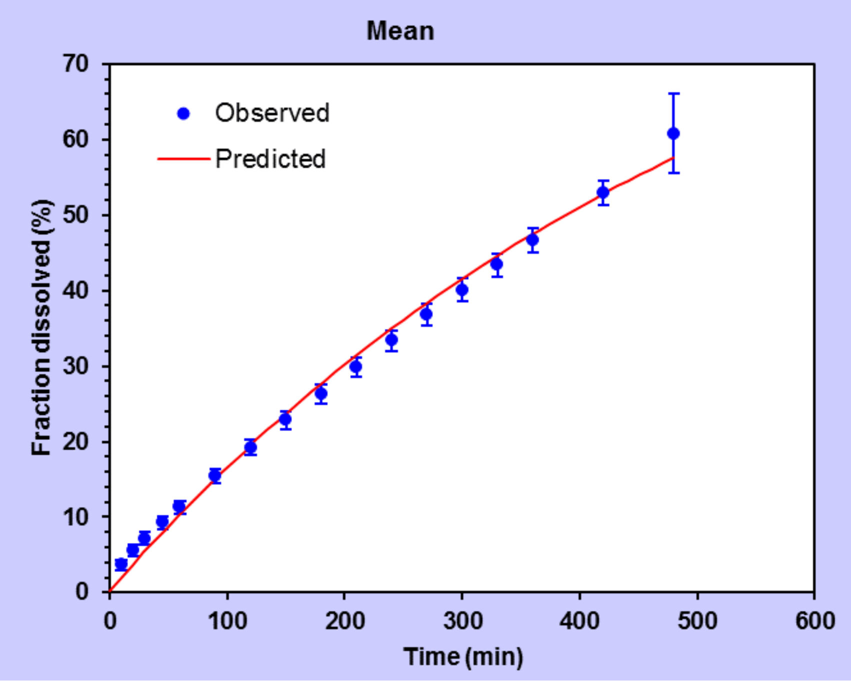

Supplementary materials – Model fitting summary – Glucidex® 19D (modelling up to app. 60 % of carvedilol released)

Graphical abstract of model fit presented as the fraction % of released carvedilol per tested tablet:

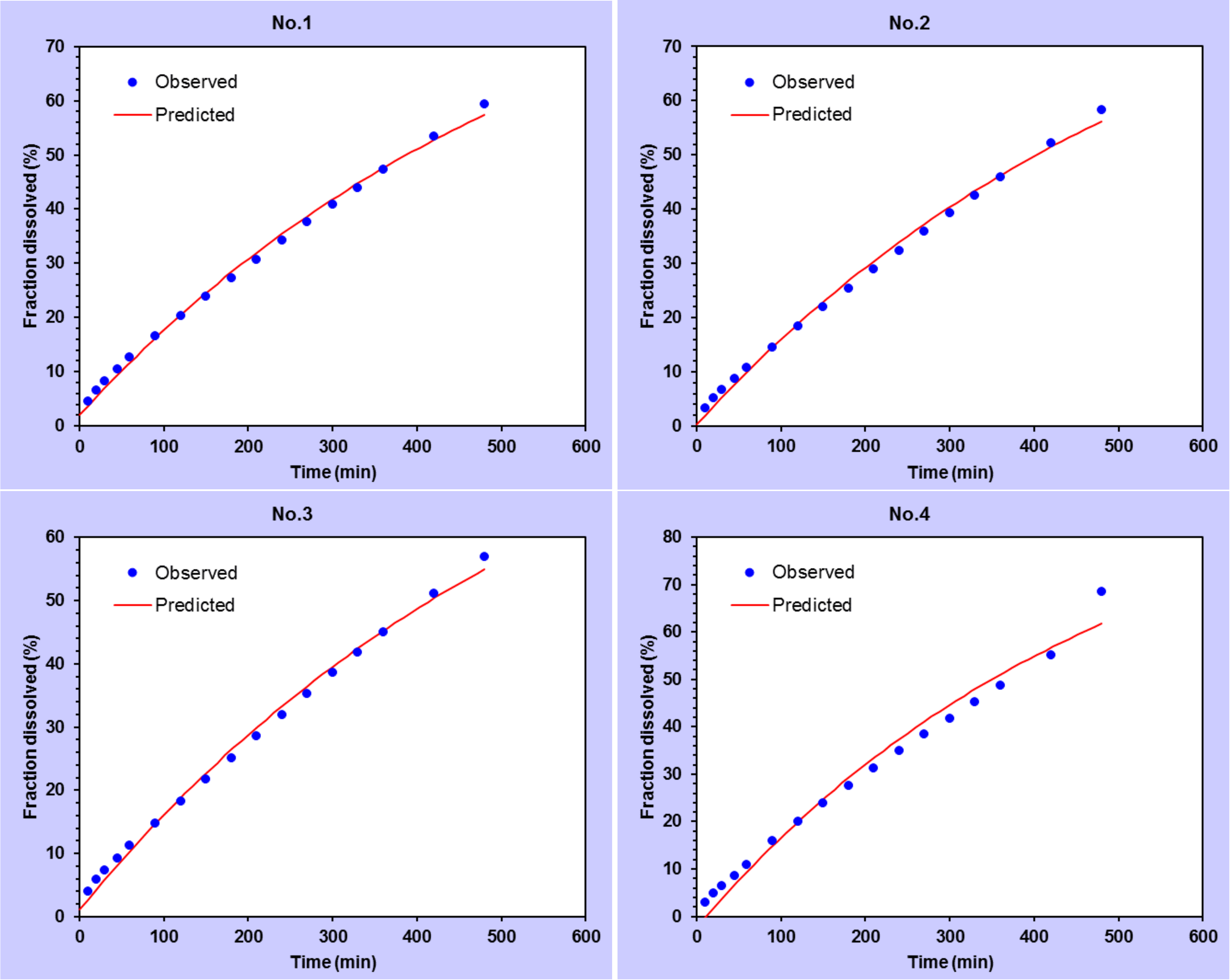

Supplementary materials – Model fitting summary – Glucidex® 19D (modelling up to app. 60 % of carvedilol released)

Model: **First–order with F_{max}**

Model equation: $F = F_{max} \cdot (1 - e^{-k_1 \cdot t})$

Fitted model parameters per tested tablet (N = 4) with statistics – mean, standard deviation (SD), and relative standard deviation expressed in % (RSD%) (output from DDSolver):

Parameter	No.1	No.2	No.3	No.4	Mean	SD	RSD(%)
k_1	0.004	0.004	0.004	0.004	0.004	0.000	6.981
F_{max}	62.325	49.707	59.808	71.913	60.938	9.125	14.974

Number of dissolution data points (N), degrees of freedom (df), and selected goodness of fit criteria – Pearson correlation coefficient (R), coefficient of determination (R^2), adjusted coefficient of determination ($R^2_{adjusted}$), and residual sum of squares (RSS) (manual calculation in MS Excel):

Parameter	No.1	No.2	No.3	No.4
N	17	17	17	17
df	15	15	15	15
R	0.979553529	0.976296964	0.976754709	0.975953389
R^2	0.959525117	0.953155762	0.954049762	0.952485018
$R^2_{adjusted}$	0.956826792	0.950032813	0.950986412	0.949317353
RSS	322.8397269	403.2360752	361.8548574	588.4285796

Graphical abstract of model fit presented as mean ± 1 SD of the fraction % of released carvedilol:

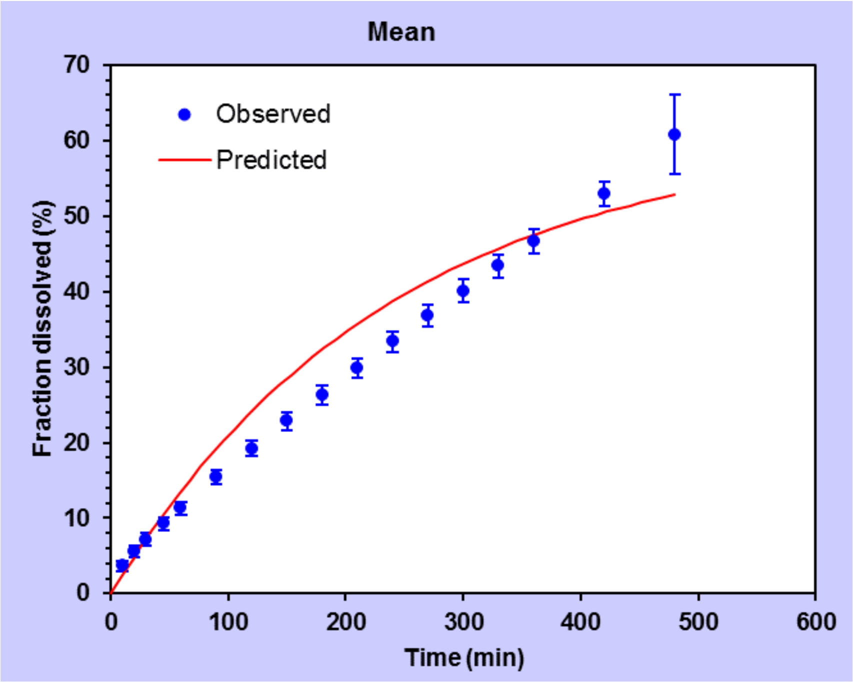

Supplementary materials – Model fitting summary – Glucidex® 19D (modelling up to app. 60 % of carvedilol released)

Graphical abstract of model fit presented as the fraction % of released carvedilol per tested tablet:

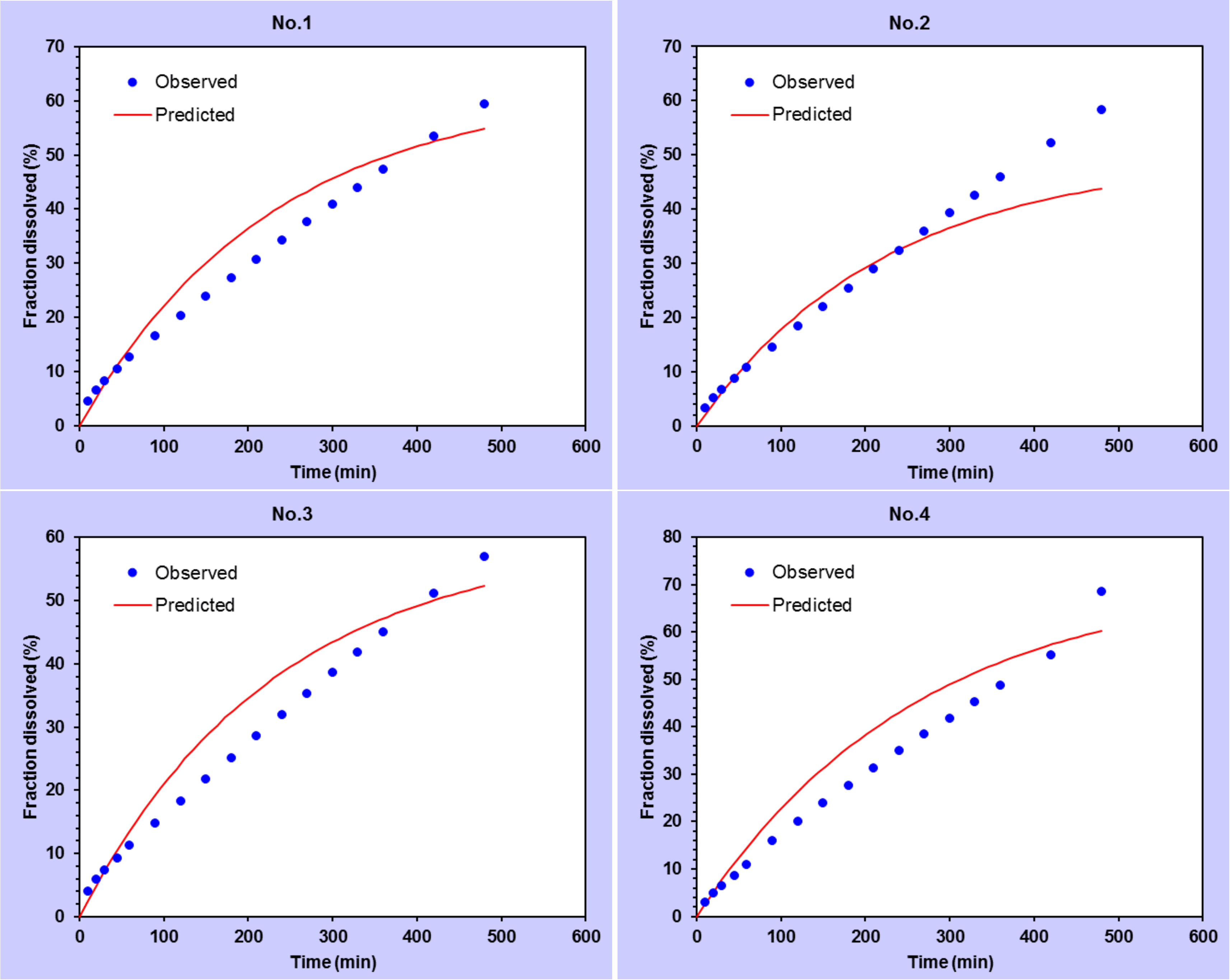

Supplementary materials – Model fitting summary – Glucidex® 19D (modelling up to app. 60 % of carvedilol released)

Model: **First–order with T_{lag} and F_{max}**

Model equation: $F = F_{max} \cdot \left[1 - e^{-k_1 \cdot (t - T_{lag})}\right]$

Fitted model parameters per tested tablet (N = 4) with statistics – mean, standard deviation (SD), and relative standard deviation expressed in % (RSD%) (output from DDSolver):

Parameter	No.1	No.2	No.3	No.4	Mean	SD	RSD(%)
k_1	0.005	0.005	0.005	0.004	0.005	0.000	5.241
T_{lag}	33.584	40.567	37.876	43.859	38.971	4.346	11.151
F_{max}	62.325	61.178	59.808	71.913	63.806	5.502	8.623

Number of dissolution data points (N), degrees of freedom (df), and selected goodness of fit criteria – Pearson correlation coefficient (R), coefficient of determination (R^2), adjusted coefficient of determination ($R^2_{adjusted}$), and residual sum of squares (RSS) (manual calculation in MS Excel):

Parameter	No.1	No.2	No.3	No.4
N	17	17	17	17
df	14	14	14	14
R	0.973751422	0.97023646	0.96967801	0.968755312
R^2	0.948191833	0.941358787	0.940275444	0.938486855
$R^2_{adjusted}$	0.940790666	0.932981471	0.931743364	0.929699263
RSS	626.2921066	714.6847825	667.4570771	969.5823831

Graphical abstract of model fit presented as mean ± 1 SD of the fraction % of released carvedilol:

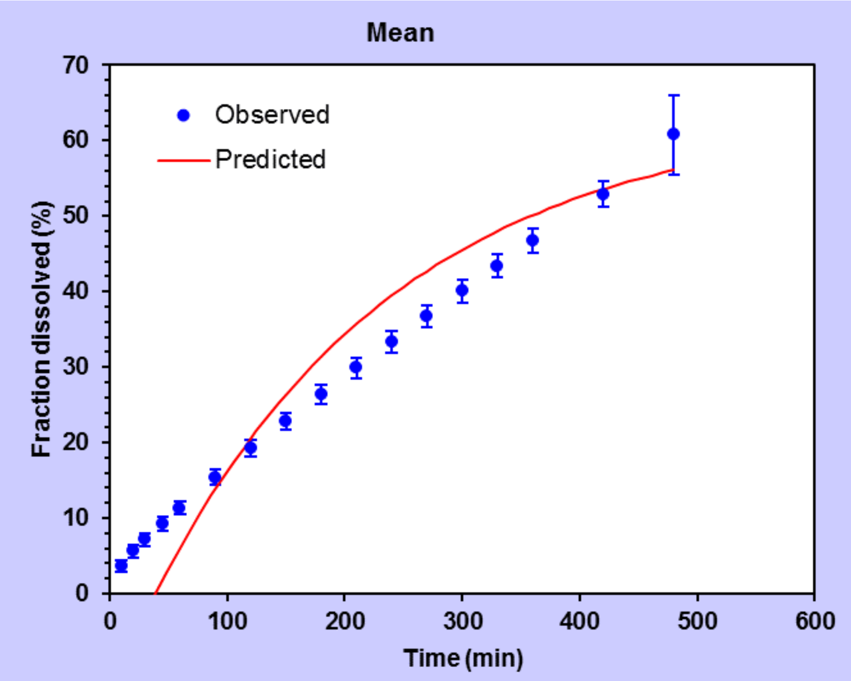

Supplementary materials – Model fitting summary – Glucidex® 19D (modelling up to app. 60 % of carvedilol released)

Graphical abstract of model fit presented as the fraction % of released carvedilol per tested tablet:

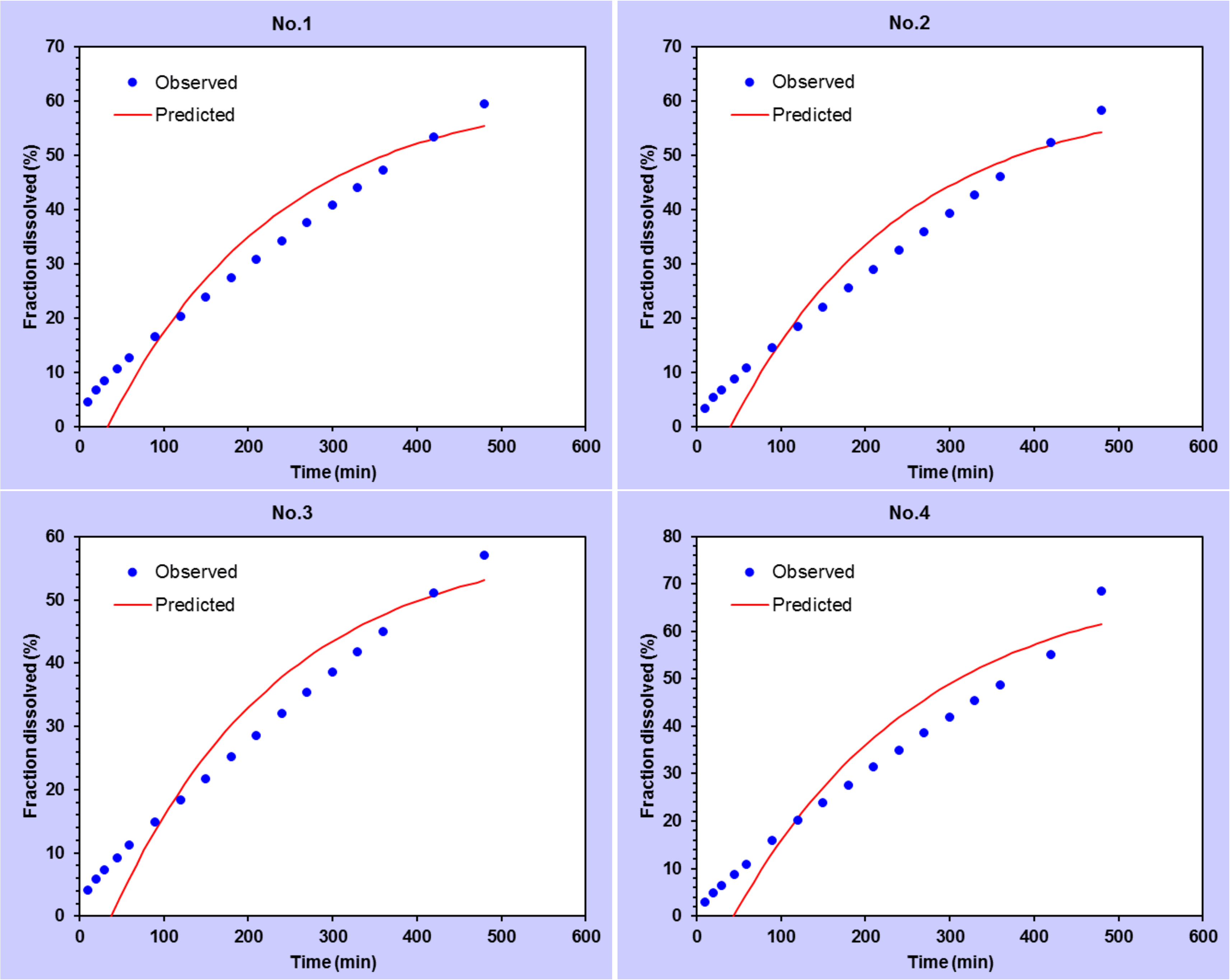

Supplementary materials – Model fitting summary – Glucidex® 19D (modelling up to app. 60 % of carvedilol released)

Model: **Higuchi**

Model equation: $F = k_H \cdot t^{0.5}$

Fitted model parameters per tested tablet (N = 4) with statistics – mean, standard deviation (SD), and relative standard deviation expressed in % (RSD%) (output from DDSolver):

Parameter	No.1	No.2	No.3	No.4	Mean	SD	RSD(%)
k_H	2.317	2.215	2.181	2.402	2.279	0.100	4.401

Number of dissolution data points (N), degrees of freedom (df), and selected goodness of fit criteria – Pearson correlation coefficient (R), coefficient of determination (R^2), adjusted coefficient of determination ($R^2_{adjusted}$), and residual sum of squares (RSS) (manual calculation in MS Excel):

Parameter	No.1	No.2	No.3	No.4
N	17	17	17	17
df	16	16	16	16
R	0.989812105	0.9875083	0.986970954	0.982825441
R^2	0.979728003	0.975172643	0.974111665	0.965945847
$R^2_{adjusted}$	0.979728003	0.975172643	0.974111665	0.965945847
RSS	320.3421942	445.677005	373.0313232	677.0380833

Graphical abstract of model fit presented as mean ± 1 SD of the fraction % of released carvedilol:

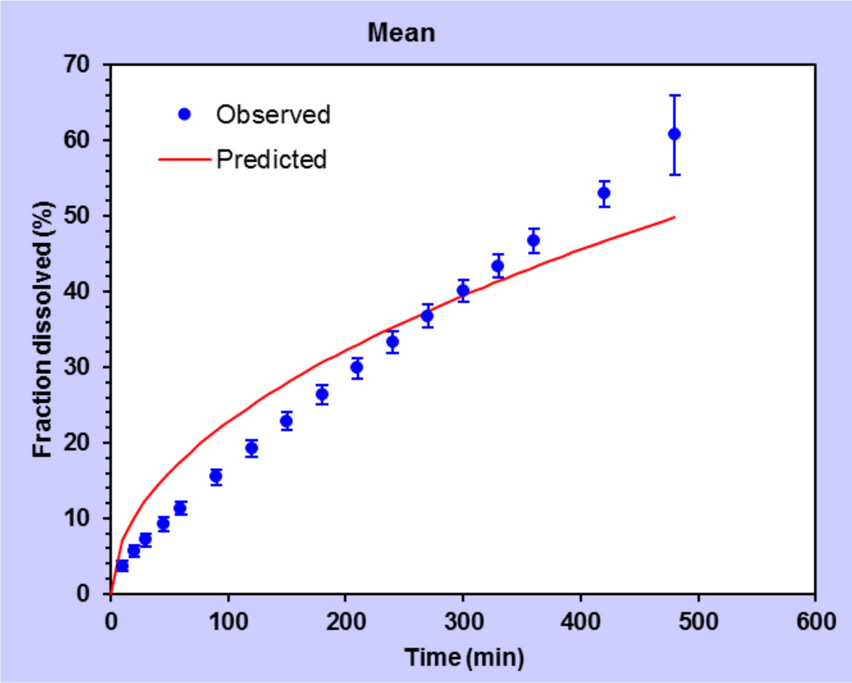

Supplementary materials – Model fitting summary – Glucidex® 19D (modelling up to app. 60 % of carvedilol released)

Graphical abstract of model fit presented as the fraction % of released carvedilol per tested tablet:

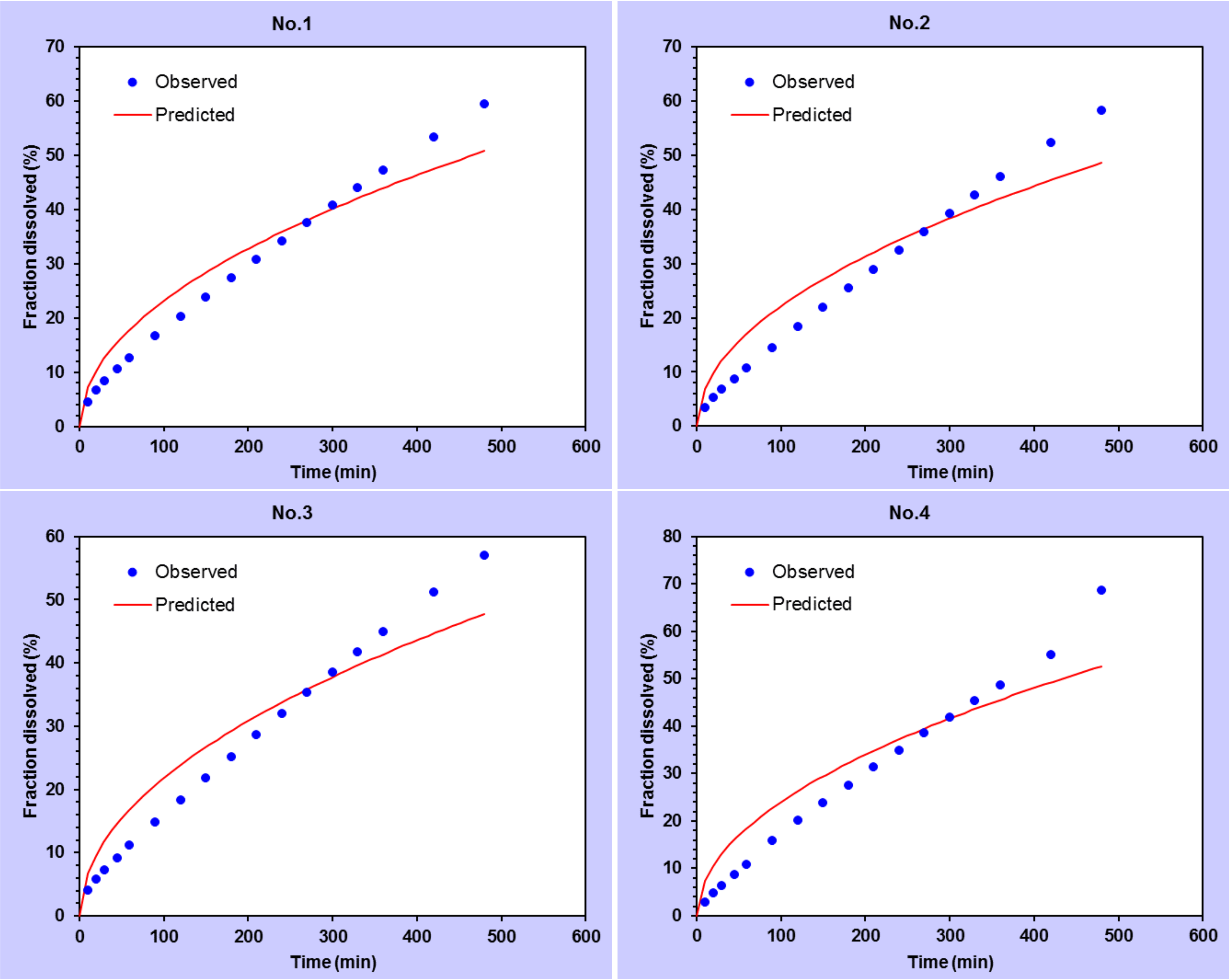

Supplementary materials – Model fitting summary – Glucidex® 19D (modelling up to app. 60 % of carvedilol released)

Model: **Higuchi with T_{lag}**

Model equation: $F = k_H \cdot (t - T_{lag})^{0.5}$

Fitted model parameters per tested tablet (N = 4) with statistics – mean, standard deviation (SD), and relative standard deviation expressed in % (RSD%) (output from DDSolver):

Parameter	No.1	No.2	No.3	No.4	Mean	SD	RSD(%)
k_H	2.661	2.612	2.550	2.895	2.680	0.151	5.628
T_{lag}	44.491	50.940	48.927	56.069	50.107	4.801	9.583

Number of dissolution data points (N), degrees of freedom (df), and selected goodness of fit criteria – Pearson correlation coefficient (R), coefficient of determination (R^2), adjusted coefficient of determination ($R^2_{adjusted}$), and residual sum of squares (RSS) (manual calculation in MS Excel):

Parameter	No.1	No.2	No.3	No.4
N	17	17	17	17
df	15	15	15	15
R	0.979458114	0.978175661	0.976475666	0.974859132
R^2	0.959338198	0.956827624	0.953504727	0.950350328
$R^2_{adjusted}$	0.956627411	0.953949466	0.950405042	0.94704035
RSS	295.9114905	278.6464838	296.706126	379.0424257

Graphical abstract of model fit presented as mean ± 1 SD of the fraction % of released carvedilol:

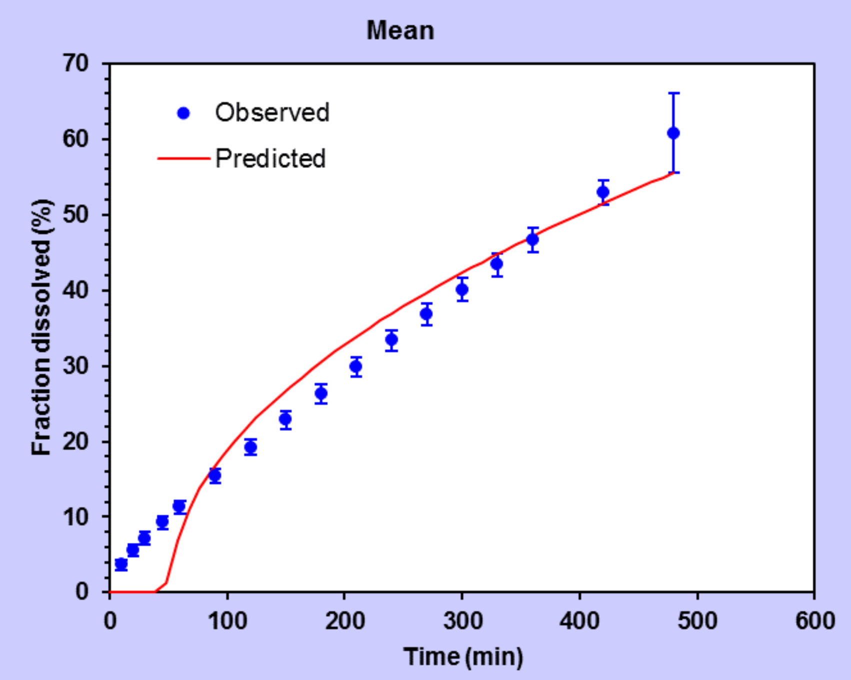

Supplementary materials – Model fitting summary – Glucidex® 19D (modelling up to app. 60 % of carvedilol released)

Graphical abstract of model fit presented as the fraction % of released carvedilol per tested tablet:

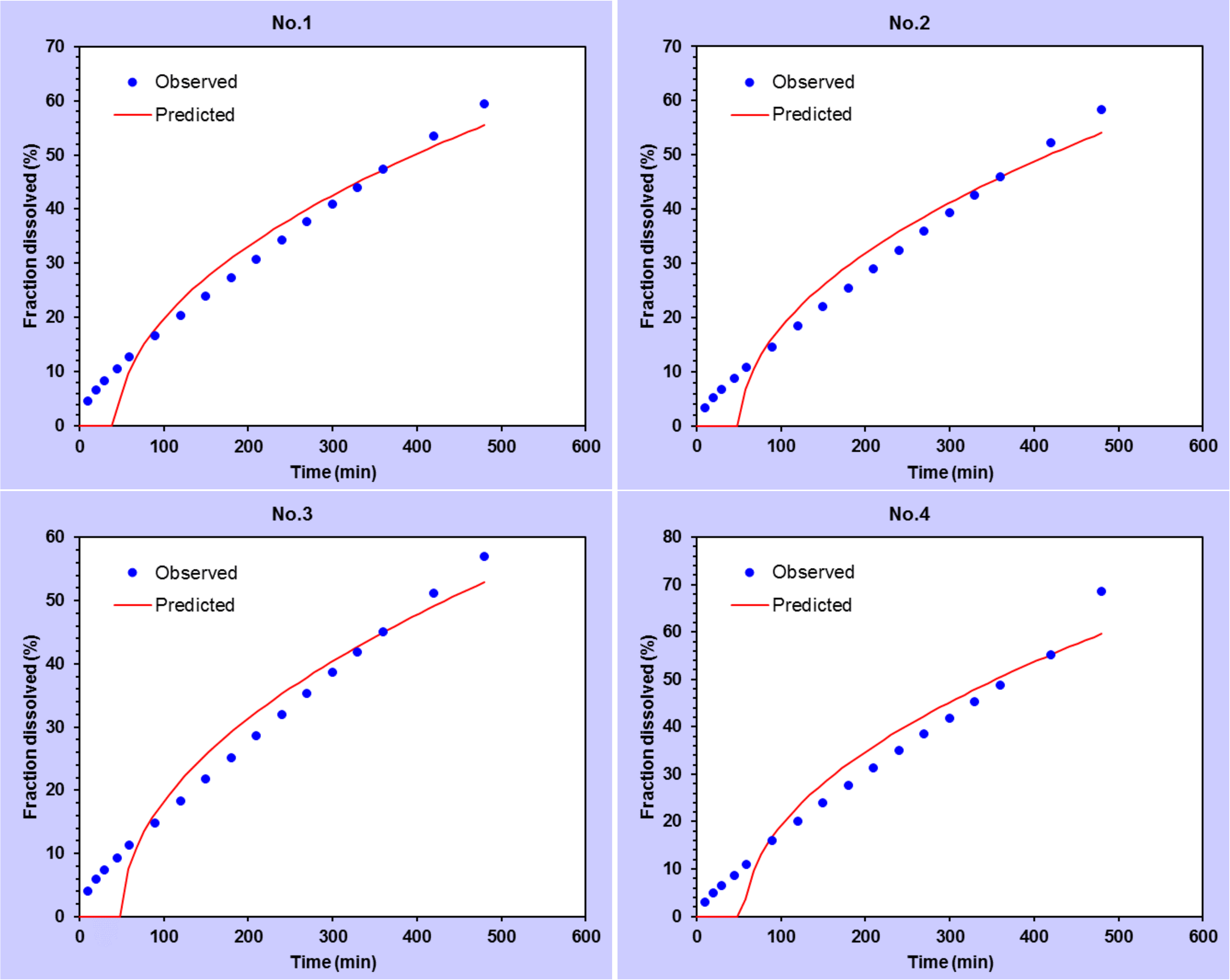

Supplementary materials – Model fitting summary – Glucidex® 19D (modelling up to app. 60 % of carvedilol released)

Model: **Higuchi with F_0**

Model equation: $F = F_0 + k_H \cdot t^{0.5}$

Fitted model parameters per tested tablet (N = 4) with statistics – mean, standard deviation (SD), and relative standard deviation expressed in % (RSD%) (output from DDSolver):

Parameter	No.1	No.2	No.3	No.4	Mean	SD	RSD(%)
k_H	2.904	2.922	2.810	3.252	2.972	0.193	6.502
F_0	-8.962	-10.802	-9.603	-12.997	-10.591	1.776	-16.771

Number of dissolution data points (N), degrees of freedom (df), and selected goodness of fit criteria – Pearson correlation coefficient (R), coefficient of determination (R^2), adjusted coefficient of determination ($R^2_{adjusted}$), and residual sum of squares (RSS) (manual calculation in MS Excel):

Parameter	No.1	No.2	No.3	No.4
N	17	17	17	17
df	15	15	15	15
R	0.989812105	0.9875083	0.986970954	0.982825441
R^2	0.979728003	0.975172643	0.974111665	0.965945847
$R^2_{adjusted}$	0.978376537	0.973517486	0.972385776	0.96367557
RSS	95.32491716	118.7354259	114.6467969	203.7391348

Graphical abstract of model fit presented as mean ± 1 SD of the fraction % of released carvedilol:

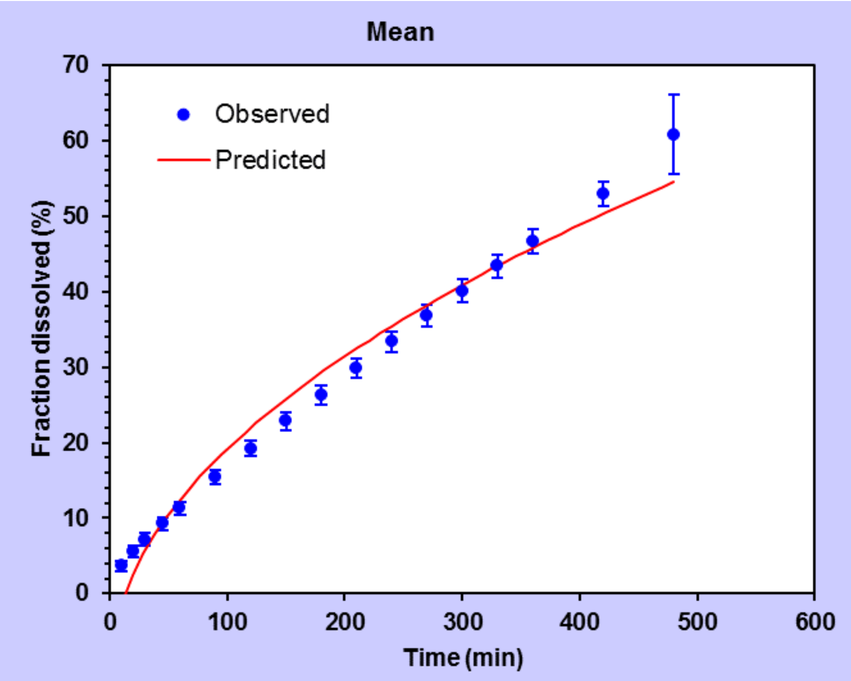

Supplementary materials – Model fitting summary – Glucidex® 19D (modelling up to app. 60 % of carvedilol released)

Graphical abstract of model fit presented as the fraction % of released carvedilol per tested tablet:

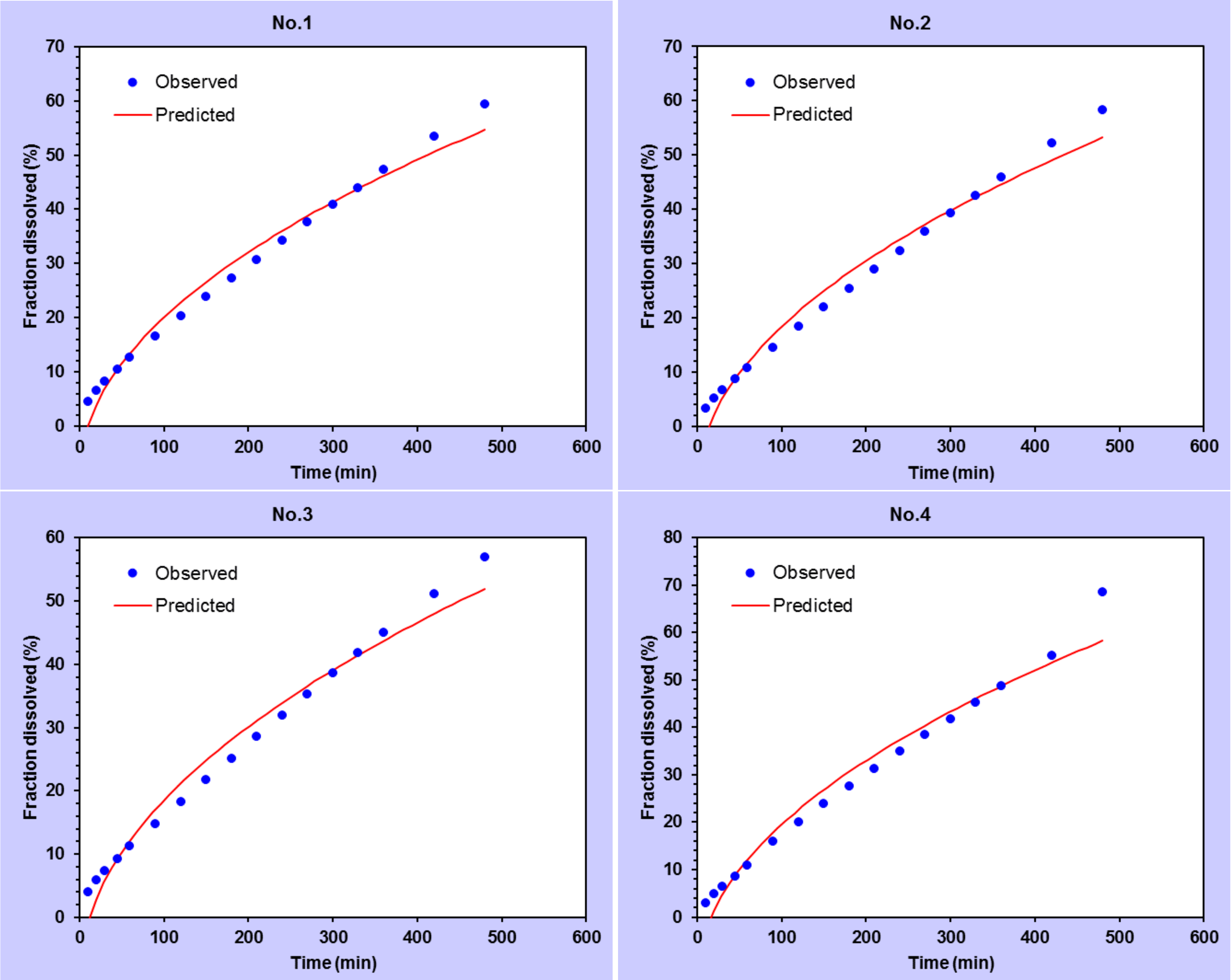

Supplementary materials – Model fitting summary – Glucidex® 19D (modelling up to app. 60 % of carvedilol released)

Model: **Korsmeyer–Peppas**

Model equation: $F = k_{KP} \cdot t^n$

Fitted model parameters per tested tablet (N = 4) with statistics – mean, standard deviation (SD), and relative standard deviation expressed in % (RSD%) (output from DDSolver):

Parameter	No.1	No.2	No.3	No.4	Mean	SD	RSD(%)
k_{KP}	0.836	0.521	0.676	0.417	0.612	0.183	29.959
n	0.678	0.755	0.704	0.809	0.737	0.058	7.850

Number of dissolution data points (N), degrees of freedom (df), and selected goodness of fit criteria – Pearson correlation coefficient (R), coefficient of determination (R^2), adjusted coefficient of determination ($R^2_{adjusted}$), and residual sum of squares (RSS) (manual calculation in MS Excel):

Parameter	No.1	No.2	No.3	No.4
N	17	17	17	17
df	15	15	15	15
R	0.997788901	0.998715542	0.997232552	0.996741534
R^2	0.995582692	0.997432735	0.994472763	0.993493685
$R^2_{adjusted}$	0.995288204	0.997261584	0.994104281	0.993059931
RSS	39.75752415	27.39170388	50.33297207	48.4891748

Graphical abstract of model fit presented as mean ± 1 SD of the fraction % of released carvedilol:

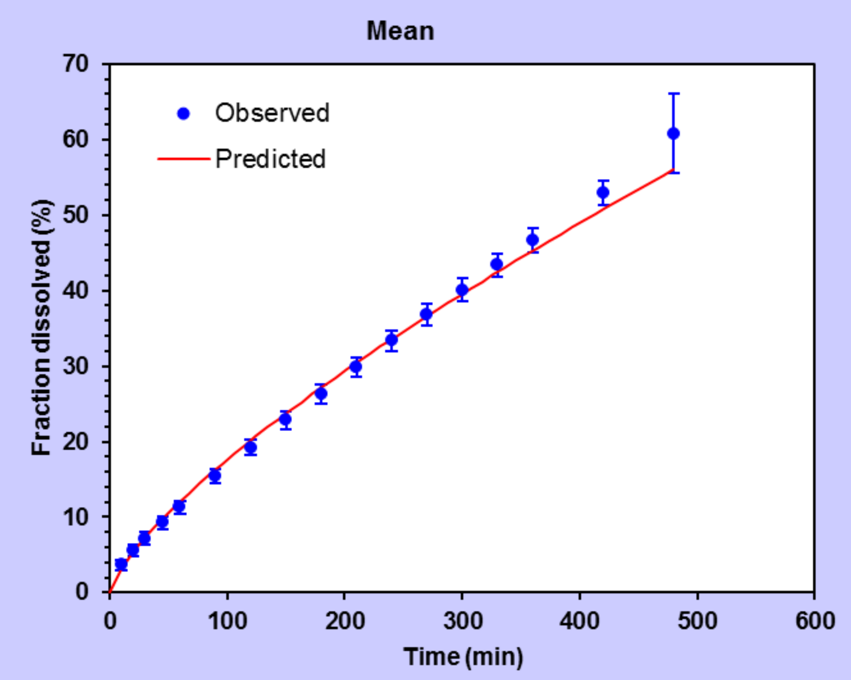

Supplementary materials – Model fitting summary – Glucidex® 19D (modelling up to app. 60 % of carvedilol released)

Graphical abstract of model fit presented as the fraction % of released carvedilol per tested tablet:

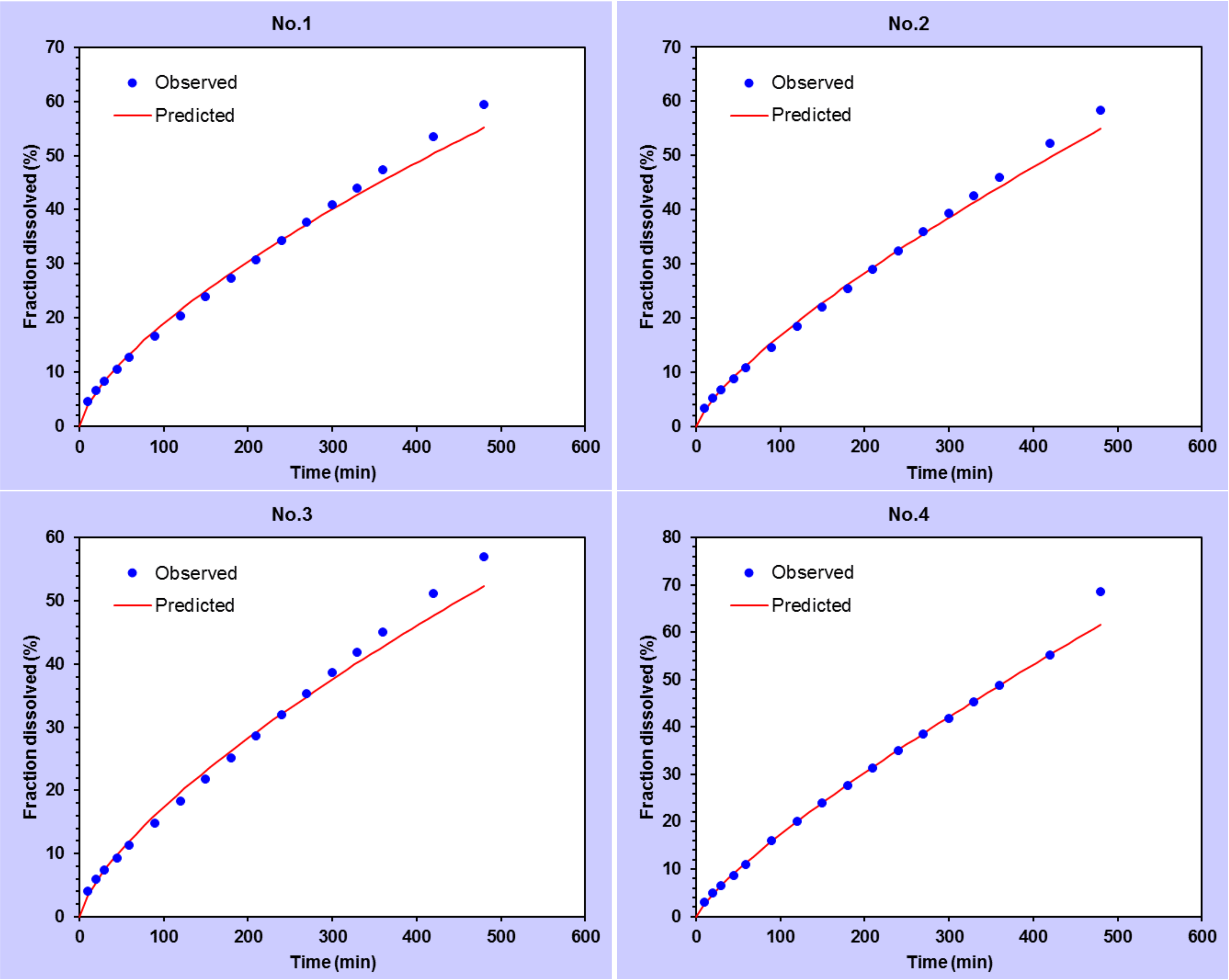

Supplementary materials – Model fitting summary – Glucidex® 19D (modelling up to app. 60 % of carvedilol released)

Model: **Korsmeyer–Peppas with T_{lag}**

Model equation: $F = k_{KP} \cdot (t - T_{lag})^n$

Fitted model parameters per tested tablet (N = 4) with statistics – mean, standard deviation (SD), and relative standard deviation expressed in % (RSD%) (output from DDSolver):

Parameter	No.1	No.2	No.3	No.4	Mean	SD	RSD(%)
k_{KP}	1.190	0.762	0.970	0.625	0.887	0.247	27.845
n	0.630	0.686	0.640	0.737	0.673	0.049	7.261
T_{lag}	4.939	4.000	4.000	4.000	4.235	0.470	11.092

Number of dissolution data points (N), degrees of freedom (df), and selected goodness of fit criteria – Pearson correlation coefficient (R), coefficient of determination (R^2), adjusted coefficient of determination ($R^2_{adjusted}$), and residual sum of squares (RSS) (manual calculation in MS Excel):

Parameter	No.1	No.2	No.3	No.4
N	17	17	17	17
df	14	14	14	14
R	0.99537102	0.996433259	0.994112618	0.99468885
R^2	0.990763467	0.99287924	0.988259897	0.989405908
$R^2_{adjusted}$	0.989443963	0.991861988	0.986582739	0.987892466
RSS	93.82546011	81.4426537	112.6370456	107.0821443

Graphical abstract of model fit presented as mean ± 1 SD of the fraction % of released carvedilol:

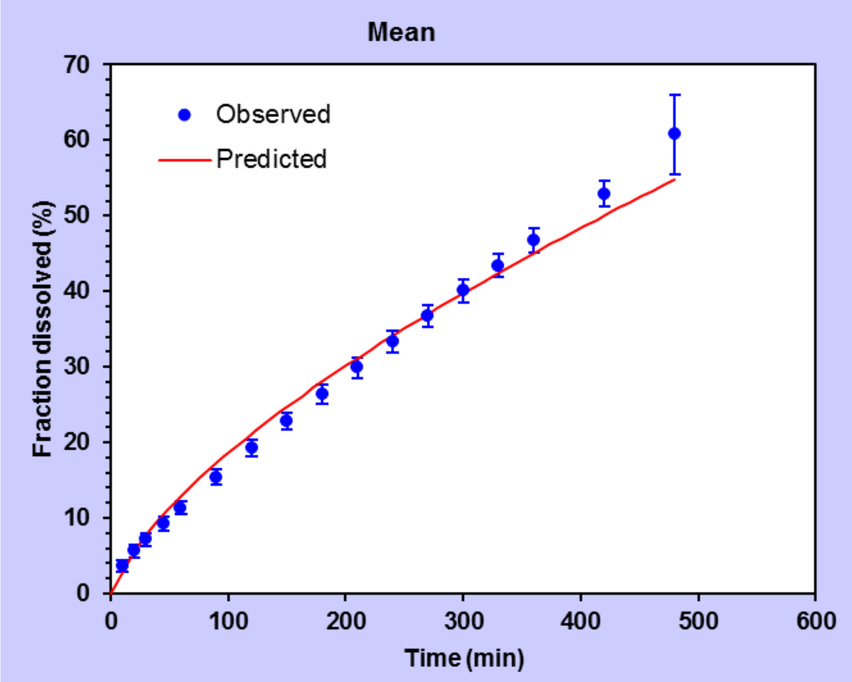

Supplementary materials – Model fitting summary – Glucidex® 19D (modelling up to app. 60 % of carvedilol released)

Graphical abstract of model fit presented as the fraction % of released carvedilol per tested tablet:

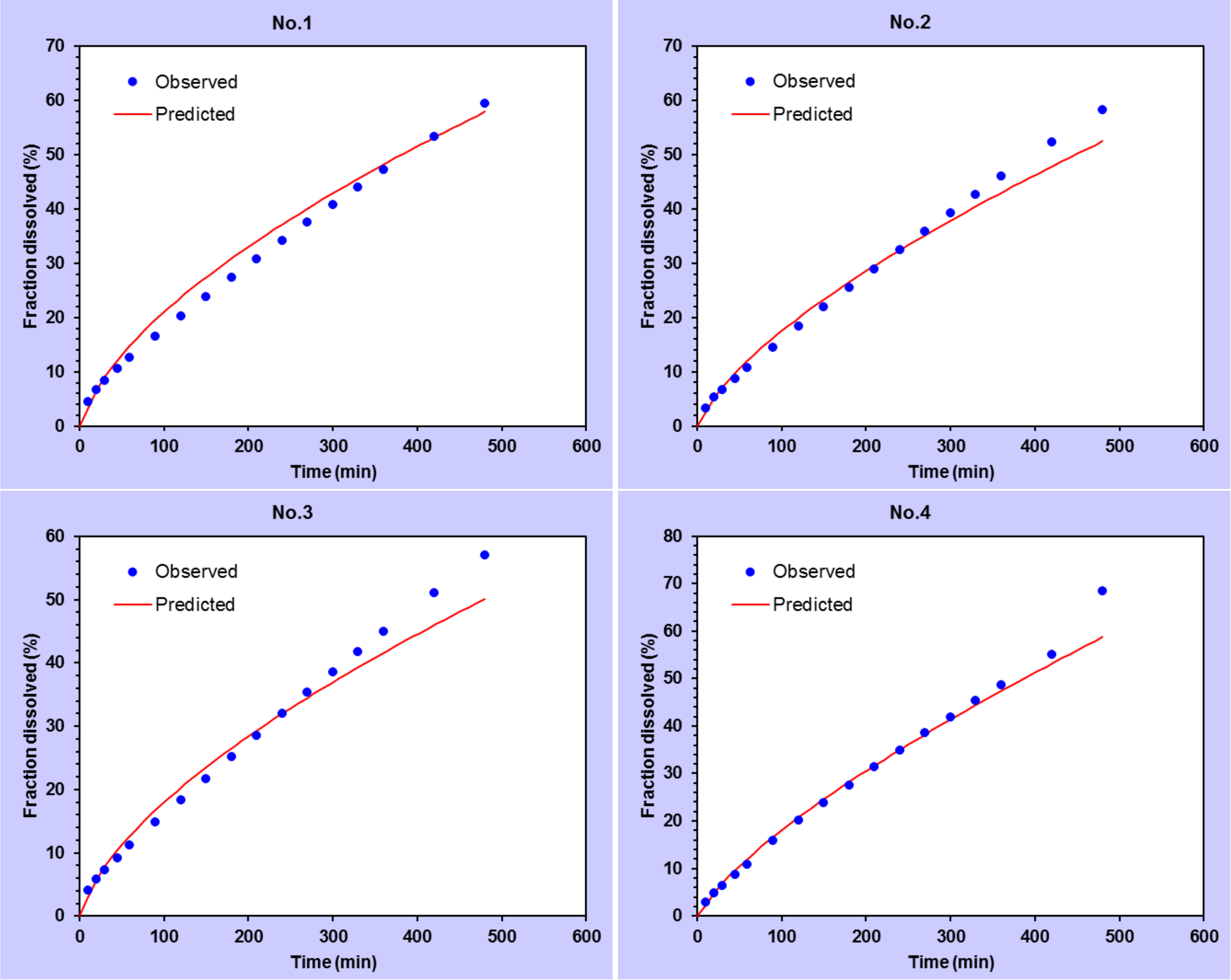

Supplementary materials – Model fitting summary – Glucidex® 19D (modelling up to app. 60 % of carvedilol released)

Model: **Korsmeyer–Peppas with F_0**

Model equation: $F = F_0 + k_{KP} \cdot t^n$

Fitted model parameters per tested tablet (N = 4) with statistics – mean, standard deviation (SD), and relative standard deviation expressed in % (RSD%) (output from DDSolver):

Parameter	No.1	No.2	No.3	No.4	Mean	SD	RSD(%)
k_{KP}	0.442	0.293	0.355	0.231	0.330	0.090	27.326
n	0.784	0.854	0.812	0.911	0.840	0.055	6.548
F_0	1.800	0.945	1.600	1.160	1.376	0.393	28.533

Number of dissolution data points (N), degrees of freedom (df), and selected goodness of fit criteria – Pearson correlation coefficient (R), coefficient of determination (R^2), adjusted coefficient of determination ($R^2_{adjusted}$), and residual sum of squares (RSS) (manual calculation in MS Excel):

Parameter	No.1	No.2	No.3	No.4
N	17	17	17	17
df	14	14	14	14
R	0.999701317	0.99991351	0.999489766	0.997821125
R^2	0.999402724	0.999827028	0.998979792	0.995646998
$R^2_{adjusted}$	0.999317399	0.999802318	0.998834047	0.99502514
RSS	4.894668236	0.90342791	9.744102758	26.85436481

Graphical abstract of model fit presented as mean ± 1 SD of the fraction % of released carvedilol:

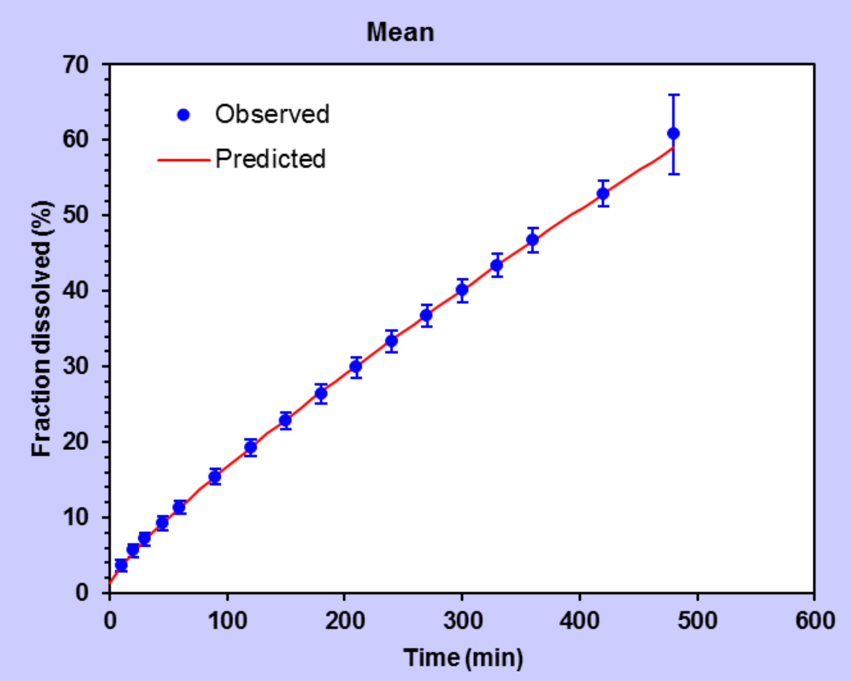

Supplementary materials – Model fitting summary – Glucidex® 19D (modelling up to app. 60 % of carvedilol released)

Graphical abstract of model fit presented as the fraction % of released carvedilol per tested tablet:

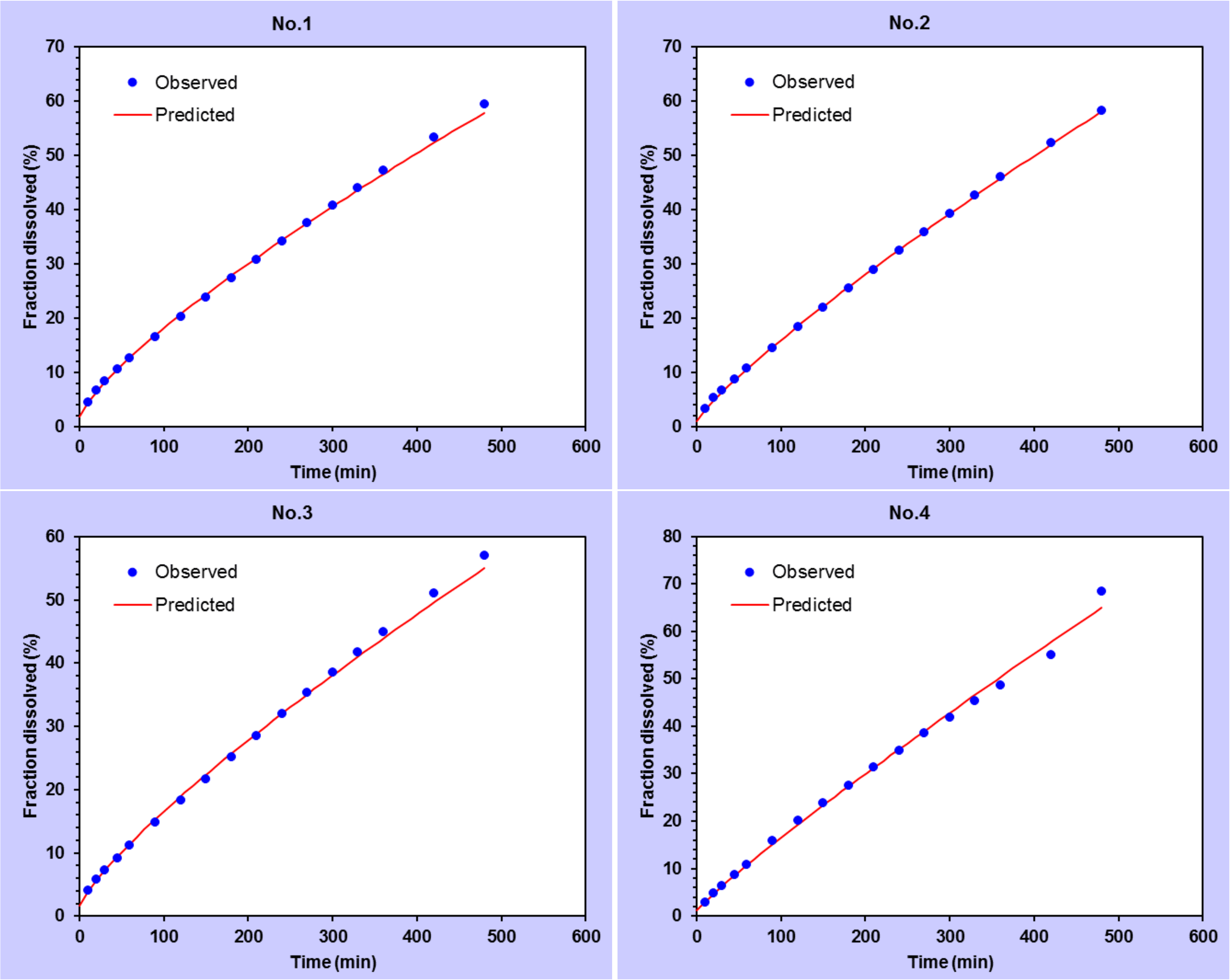

Supplementary materials – Model fitting summary – Glucidex® 19D (modelling up to app. 60 % of carvedilol released)

Model: **Hixson–Crowell**

Model equation: $F = 100 \cdot [1 - (1 - k_{HC} \cdot t)^3]$

Fitted model parameters per tested tablet (N = 4) with statistics – mean, standard deviation (SD), and relative standard deviation expressed in % (RSD%) (output from DDSolver):

Parameter	No.1	No.2	No.3	No.4	Mean	SD	RSD(%)
k_{HC}	0.001	0.001	0.001	0.001	0.001	0.000	6.195

Number of dissolution data points (N), degrees of freedom (df), and selected goodness of fit criteria – Pearson correlation coefficient (R), coefficient of determination (R^2), adjusted coefficient of determination ($R^2_{adjusted}$), and residual sum of squares (RSS) (manual calculation in MS Excel):

Parameter	No.1	No.2	No.3	No.4
N	17	17	17	17
df	16	16	16	16
R	0.999491719	0.999357606	0.99930093	0.99489443
R^2	0.998983697	0.998715624	0.998602349	0.989814926
$R^2_{adjusted}$	0.998983697	0.998715624	0.998602349	0.989814926
RSS	68.01720273	21.87495987	38.96751969	64.4066257

Graphical abstract of model fit presented as mean ± 1 SD of the fraction % of released carvedilol:

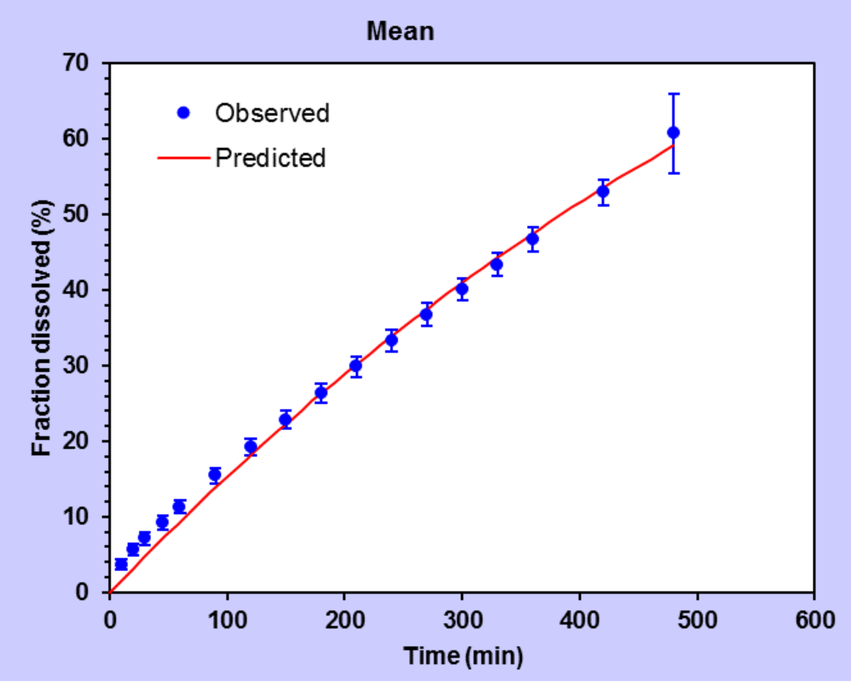

Supplementary materials – Model fitting summary – Glucidex® 19D (modelling up to app. 60 % of carvedilol released)

Graphical abstract of model fit presented as the fraction % of released carvedilol per tested tablet:

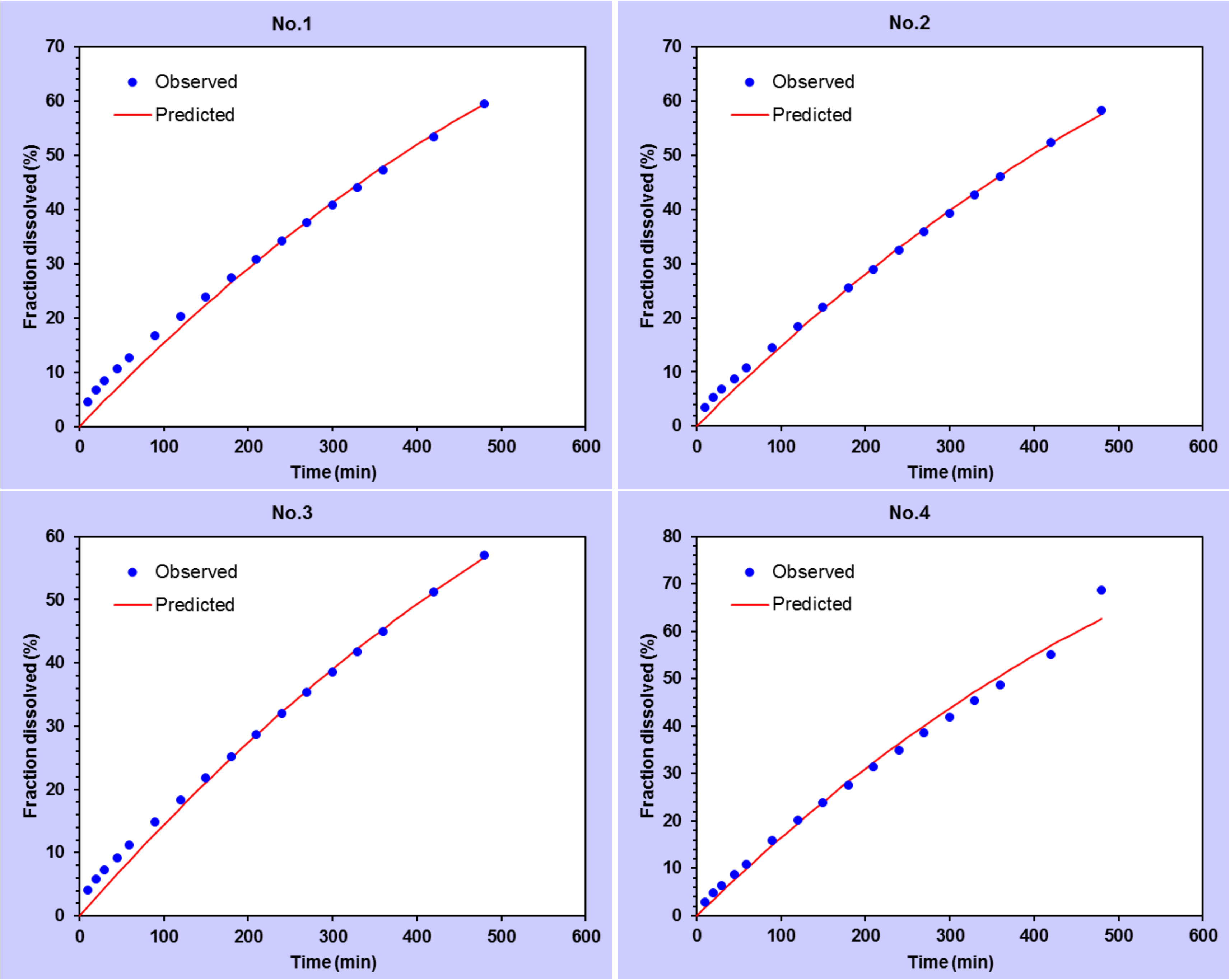

Supplementary materials – Model fitting summary – Glucidex® 19D (modelling up to app. 60 % of carvedilol released)

Model: **Hixson–Crowell with T_{lag}**

Model equation: $F = 100 \cdot \left\{1 - \left[1 - k_{HC} \cdot \left(t - T_{lag}\right)\right]^3\right\}$

Fitted model parameters per tested tablet (N = 4) with statistics – mean, standard deviation (SD), and relative standard deviation expressed in % (RSD%) (output from DDSolver):

Parameter	No.1	No.2	No.3	No.4	Mean	SD	RSD(%)
k_{HC}	0.001	0.001	0.000	0.001	0.001	0.000	8.767
T_{lag}	-23.073	-10.615	-16.682	0.330	-12.510	9.957	-79.595

Number of dissolution data points (N), degrees of freedom (df), and selected goodness of fit criteria – Pearson correlation coefficient (R), coefficient of determination (R^2), adjusted coefficient of determination ($R^2_{adjusted}$), and residual sum of squares (RSS) (manual calculation in MS Excel):

Parameter	No.1	No.2	No.3	No.4
N	17	17	17	17
df	15	15	15	15
R	0.999609647	0.999436976	0.999424094	0.994887994
R^2	0.999219446	0.99887427	0.998848519	0.989802122
$R^2_{adjusted}$	0.999167409	0.998799221	0.998771754	0.989122263
RSS	3.787254258	5.651797105	5.327687941	64.84028676

Graphical abstract of model fit presented as mean ± 1 SD of the fraction % of released carvedilol:

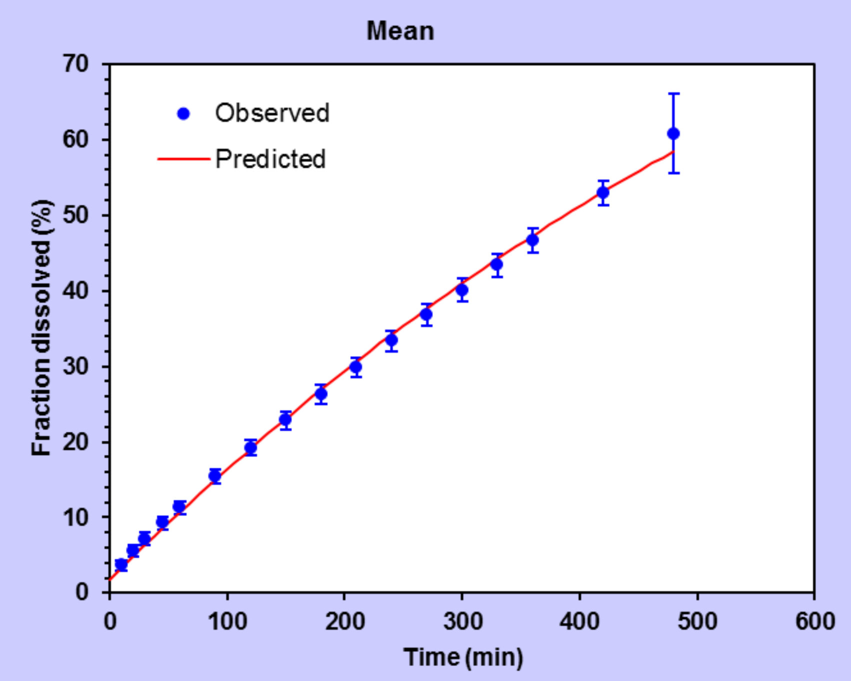

Supplementary materials – Model fitting summary – Glucidex® 19D (modelling up to app. 60 % of carvedilol released)

Graphical abstract of model fit presented as the fraction % of released carvedilol per tested tablet:

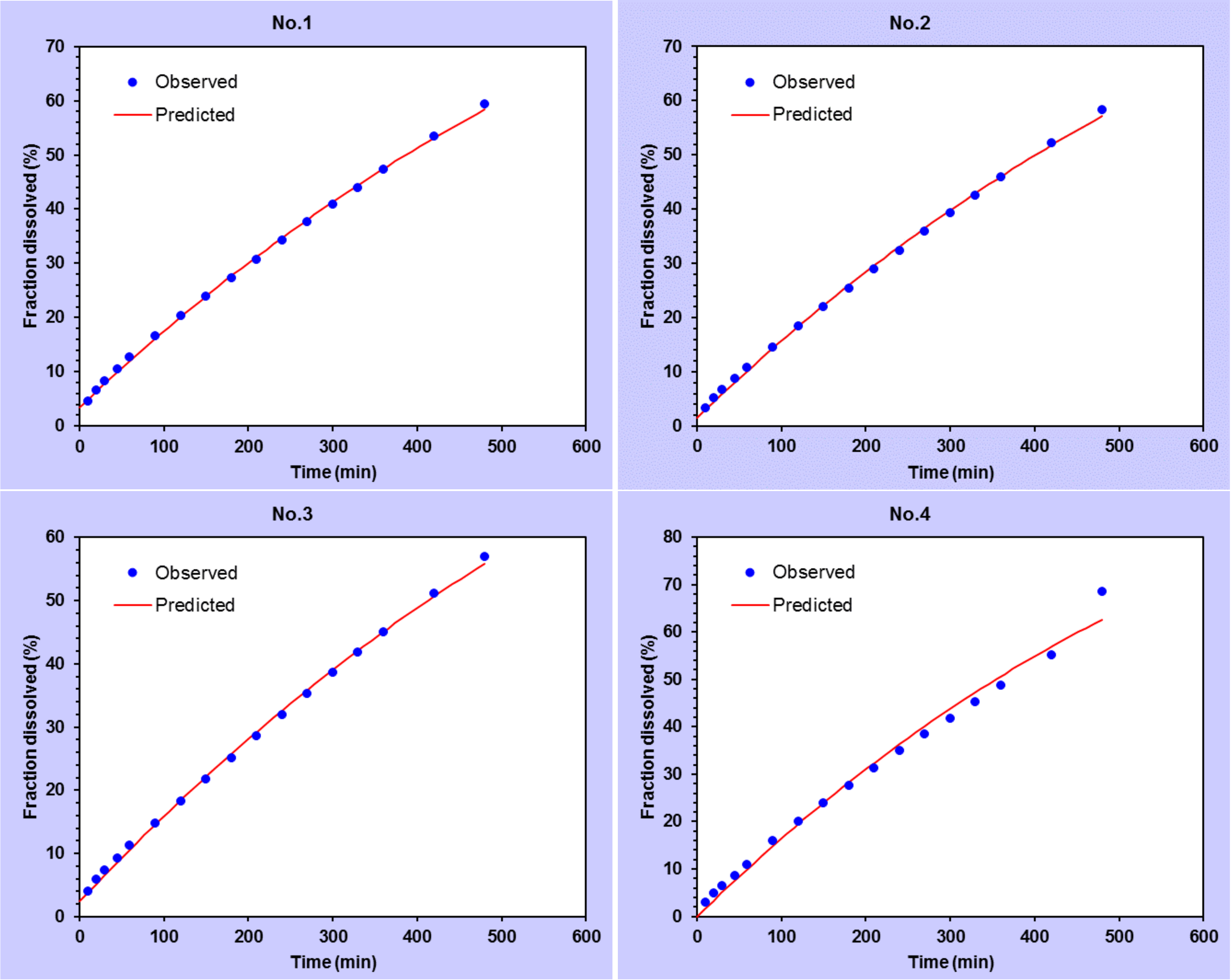

Supplementary materials – Model fitting summary – Glucidex® 19D (modelling up to app. 60 % of carvedilol released)

Model: **Hopfenberg**

Model equation: $F = 100 \cdot [1 - (1 - k_{HB} \cdot t)^n]$

Fitted model parameters per tested tablet (N = 4) with statistics – mean, standard deviation (SD), and relative standard deviation expressed in % (RSD%) (output from DDSolver):

Parameter	No.1	No.2	No.3	No.4	Mean	SD	RSD(%)
k_{HB}	0.001	0.001	0.001	0.001	0.001	0.000	25.423
n	3.000	3.000	3.000	2.000	2.750	0.500	18.182

Number of dissolution data points (N), degrees of freedom (df), and selected goodness of fit criteria – Pearson correlation coefficient (R), coefficient of determination (R^2), adjusted coefficient of determination ($R^2_{adjusted}$), and residual sum of squares (RSS) (manual calculation in MS Excel):

Parameter	No.1	No.2	No.3	No.4
N	17	17	17	17
df	15	15	15	15
R	0.999491719	0.999357606	0.99930093	0.996082187
R^2	0.998983697	0.998715624	0.998602349	0.992179723
$R^2_{adjusted}$	0.998915943	0.998629999	0.998509172	0.991658371
RSS	68.01720273	21.87495987	38.96751969	55.03677357

Graphical abstract of model fit presented as mean ± 1 SD of the fraction % of released carvedilol:

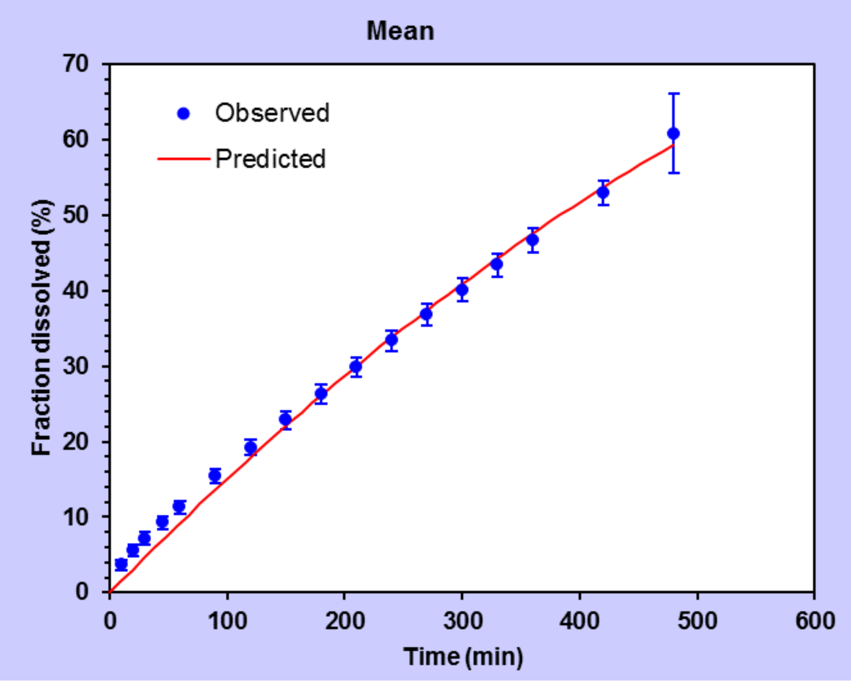

Supplementary materials – Model fitting summary – Glucidex® 19D (modelling up to app. 60 % of carvedilol released)

Graphical abstract of model fit presented as the fraction % of released carvedilol per tested tablet:

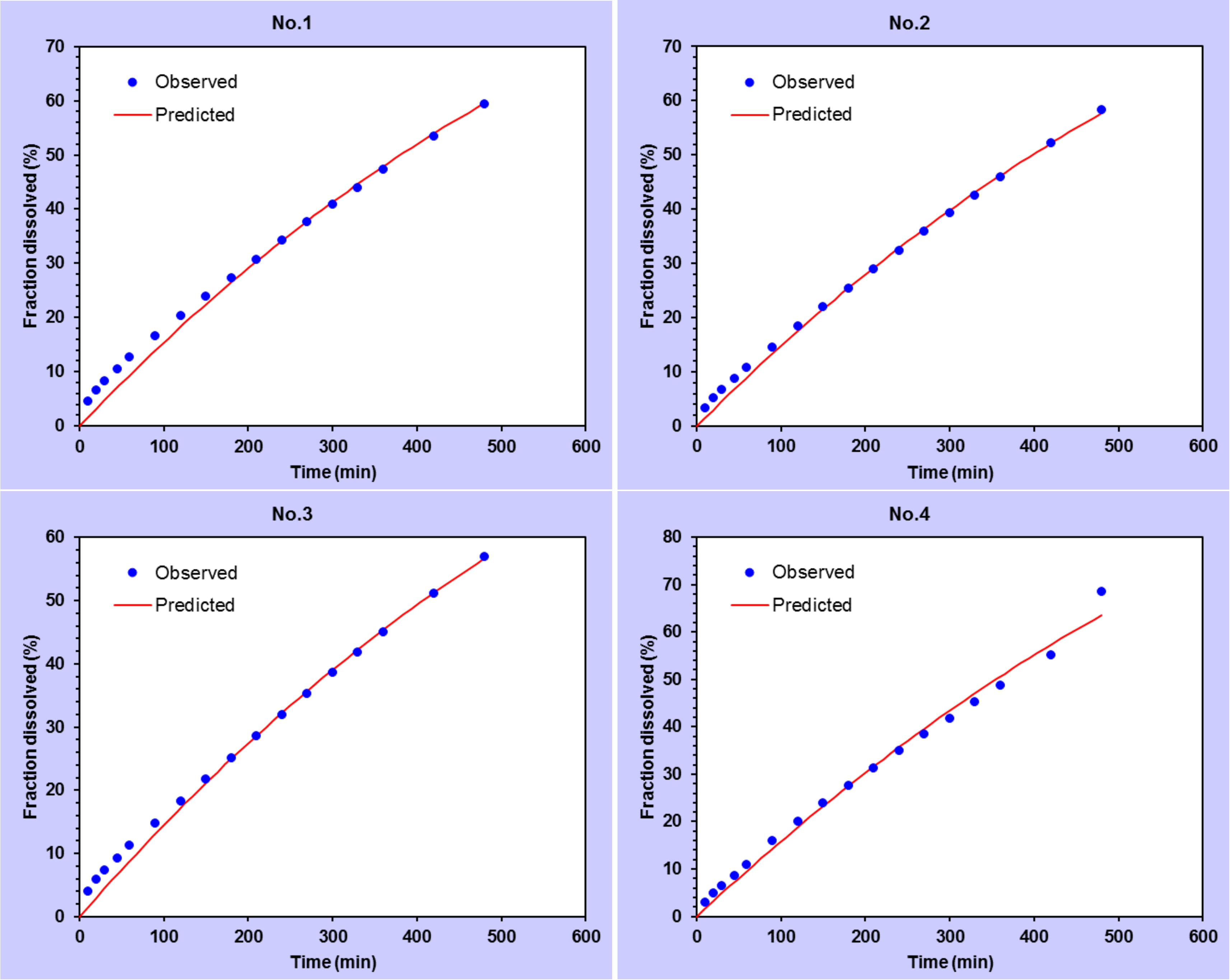

Supplementary materials – Model fitting summary – Glucidex® 19D (modelling up to app. 60 % of carvedilol released)

Model: **Hopfenberg with T_{lag}**

Model equation: $F = 100 \cdot \{1 - [1 - k_{HB} \cdot (t - T_{lag})]^n\}$

Fitted model parameters per tested tablet (N = 4) with statistics – mean, standard deviation (SD), and relative standard deviation expressed in % (RSD%) (output from DDSolver):

Parameter	No.1	No.2	No.3	No.4	Mean	SD	RSD(%)
k_{HB}	0.001	0.001	0.001	0.001	0.001	0.000	34.305
n	2.000	1.768	2.000	1.000	1.692	0.474	28.021
T_{lag}	-28.858	-17.871	-21.816	-22.904	-22.862	4.545	-19.879

Number of dissolution data points (N), degrees of freedom (df), and selected goodness of fit criteria – Pearson correlation coefficient (R), coefficient of determination (R^2), adjusted coefficient of determination ($R^2_{adjusted}$), and residual sum of squares (RSS) (manual calculation in MS Excel):

Parameter	No.1	No.2	No.3	No.4
N	17	17	17	17
df	14	14	14	14
R	0.999784664	0.999904079	0.999817045	0.997630328
R^2	0.999569374	0.999808168	0.999634123	0.995266272
$R^2_{adjusted}$	0.999507856	0.999780763	0.999581855	0.994590025
RSS	2.026382566	0.983614324	1.648849363	28.32093964

Graphical abstract of model fit presented as mean ± 1 SD of the fraction % of released carvedilol:

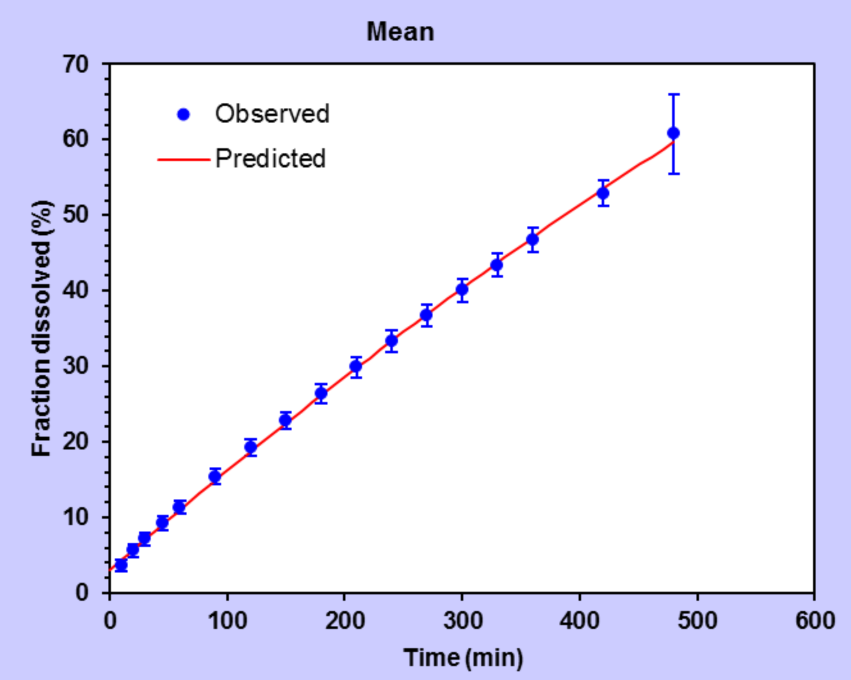

Supplementary materials – Model fitting summary – Glucidex® 19D (modelling up to app. 60 % of carvedilol released)

Graphical abstract of model fit presented as the fraction % of released carvedilol per tested tablet:

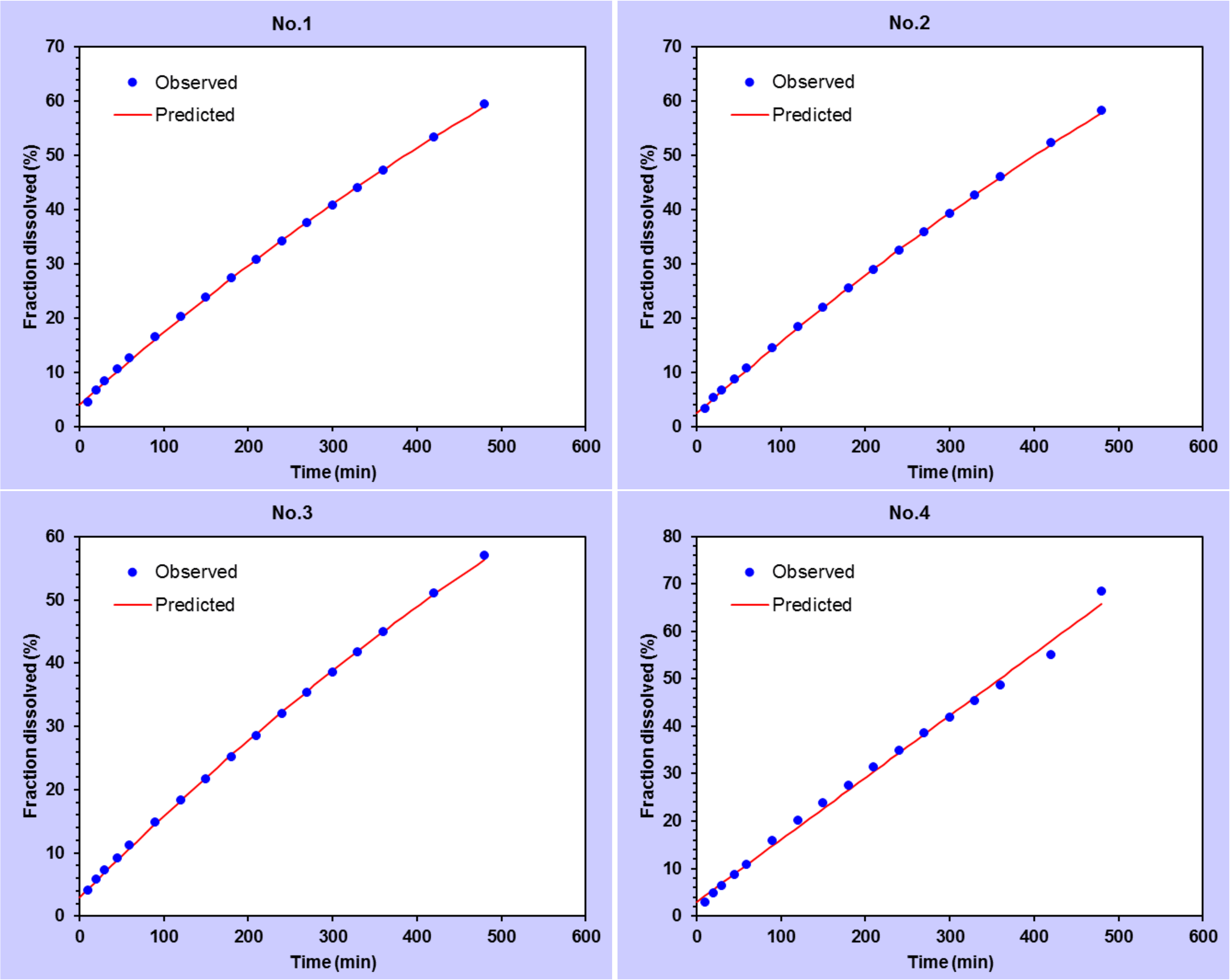

Supplementary materials – Model fitting summary – Glucidex® 19D (modelling up to app. 60 % of carvedilol released)

Model: **Baker–Lonsdale**

Model equation: $\frac{3}{2}\cdot\left[1-\left(1-\frac{F}{100}\right)^{\frac{2}{3}}\right]-\frac{F}{100}=k_{BL}\cdot t$

Fitted model parameters per tested tablet (N = 4) with statistics – mean, standard deviation (SD), and relative standard deviation expressed in % (RSD%) (output from DDSolver):

Parameter	No.1	No.2	No.3	No.4	Mean	SD	RSD(%)
k_{BL}	0.0001	0.0002	0.0001	0.0002	0.0001	0.0000	33.9201

Number of dissolution data points (N), degrees of freedom (df), and selected goodness of fit criteria – Pearson correlation coefficient (R), coefficient of determination (R^2), adjusted coefficient of determination ($R^2_{adjusted}$), and residual sum of squares (RSS) (manual calculation in MS Excel):

Parameter	No.1	No.2	No.3	No.4
N	17	17	17	17
df	16	16	16	16
R	0.985320343	0.979951811	0.979565459	0.972880053
R^2	0.970856178	0.960305551	0.959548488	0.946495598
$R^2_{adjusted}$	0.970856178	0.960305551	0.959548488	0.946495598
RSS	724.0564958	1401.145468	1205.44921	2244.363672

Graphical abstract of model fit presented as mean ± 1 SD of the fraction % of released carvedilol:

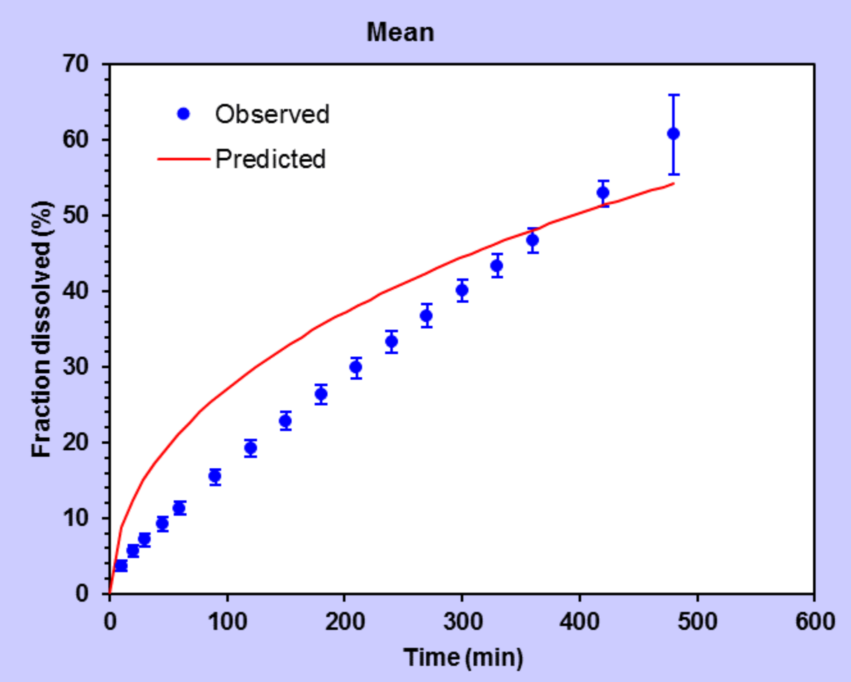

Supplementary materials – Model fitting summary – Glucidex® 19D (modelling up to app. 60 % of carvedilol released)

Graphical abstract of model fit presented as the fraction % of released carvedilol per tested tablet:

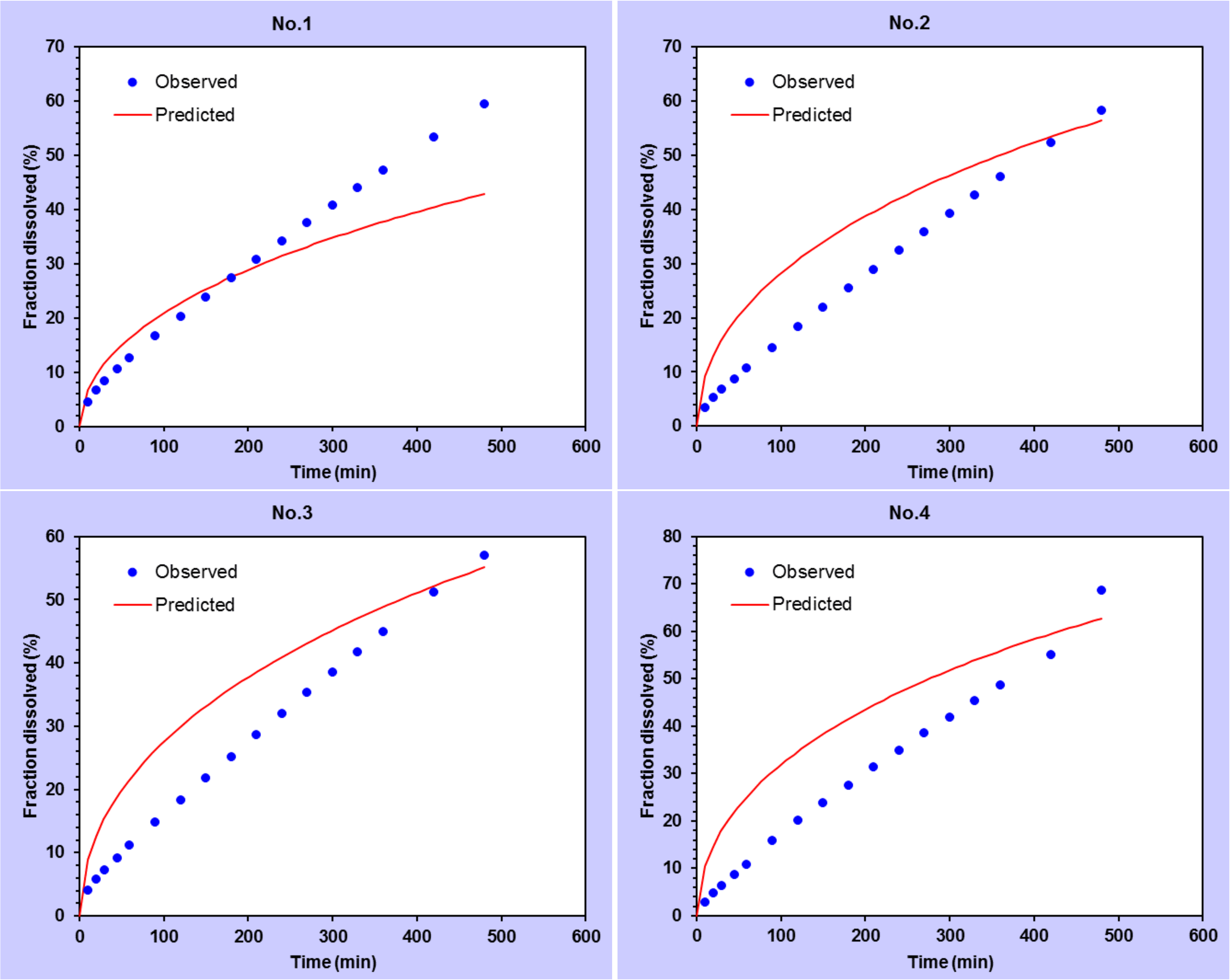

Supplementary materials – Model fitting summary – Glucidex® 19D (modelling up to app. 60 % of carvedilol released)

Model: **Baker–Lonsdale with T_{lag}**

Model equation: $\frac{3}{2}\cdot\left[1-\left(1-\frac{F}{100}\right)^{\frac{2}{3}}\right]-\frac{F}{100}=k_{BL}\cdot(t-T_{lag})$

Fitted model parameters per tested tablet (N = 4) with statistics – mean, standard deviation (SD), and relative standard deviation expressed in % (RSD%) (output from DDSolver):

Parameter	No.1	No.2	No.3	No.4	Mean	SD	RSD(%)
k_{BL}	0.0002	0.0002	0.0001	0.0002	0.0002	0.0000	14.6618
T_{lag}	53.9043	59.2128	57.2724	66.2694	59.1647	5.2196	8.8221

Number of dissolution data points (N), degrees of freedom (df), and selected goodness of fit criteria – Pearson correlation coefficient (R), coefficient of determination (R^2), adjusted coefficient of determination ($R^2_{adjusted}$), and residual sum of squares (RSS) (manual calculation in MS Excel):

Parameter	No.1	No.2	No.3	No.4
N	17	17	17	17
df	15	15	15	15
R	0.972278512	0.970462519	0.9702316	0.964433748
R^2	0.945325505	0.941797501	0.941349357	0.930132455
$R^2_{adjusted}$	0.941680538	0.937917335	0.937439315	0.925474619
RSS	411.4437538	398.9810285	386.9832987	586.6247912

Graphical abstract of model fit presented as mean ± 1 SD of the fraction % of released carvedilol:

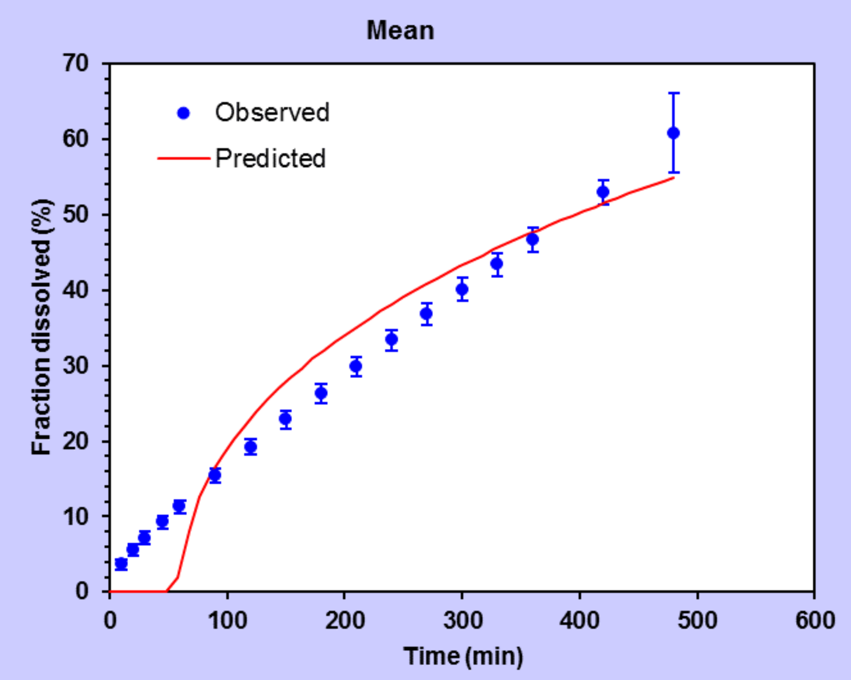

Supplementary materials – Model fitting summary – Glucidex® 19D (modelling up to app. 60 % of carvedilol released)

Graphical abstract of model fit presented as the fraction % of released carvedilol per tested tablet:

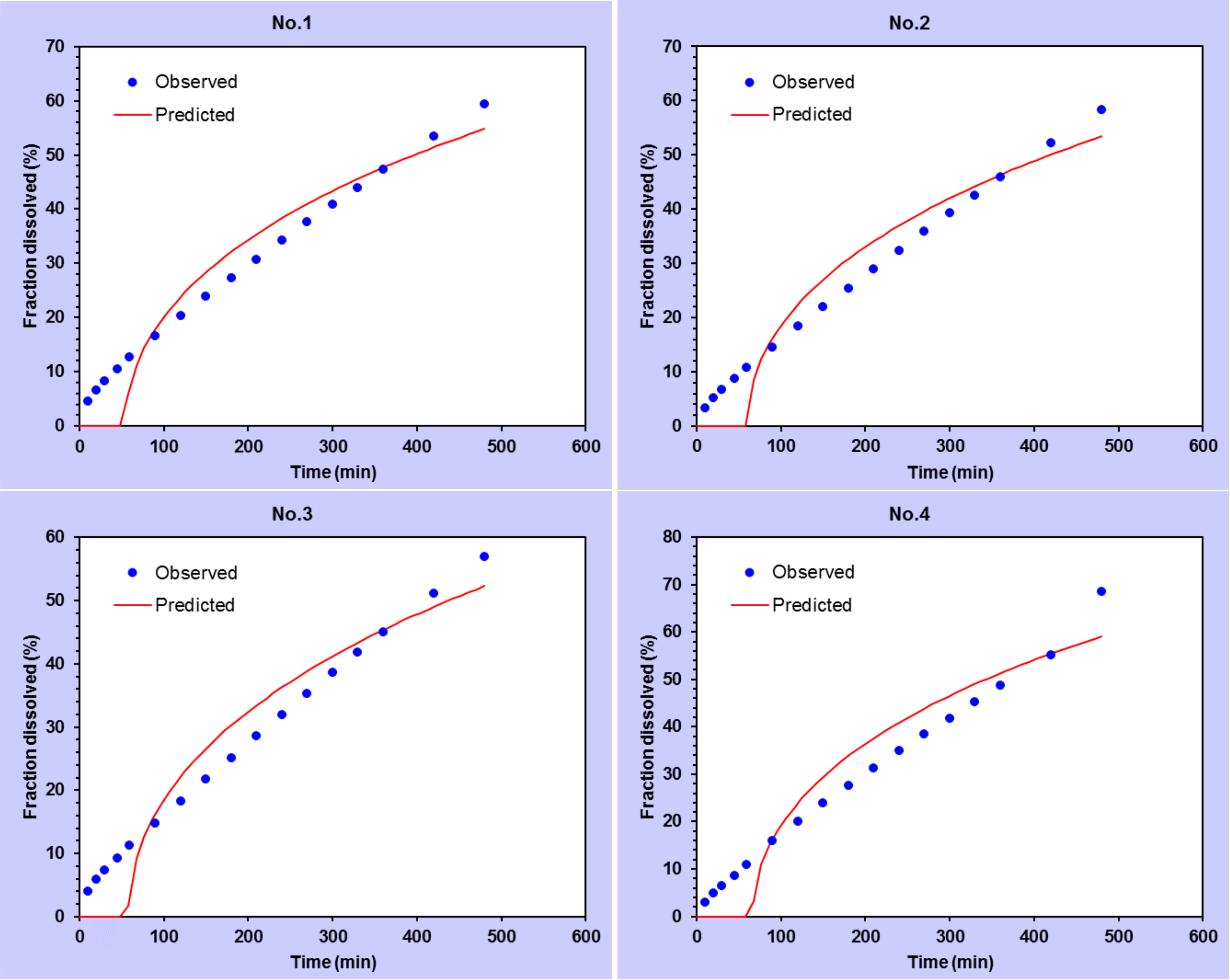

Supplementary materials – Model fitting summary – Glucidex® 19D (modelling up to app. 60 % of carvedilol released)

Model: **Makoid–Banakar**

Model equation: $F = k_{MB} \cdot t^n \cdot e^{-k \cdot t}$

Fitted model parameters per tested tablet (N = 4) with statistics – mean, standard deviation (SD), and relative standard deviation expressed in % (RSD%) (output from DDSolver):

Parameter	No.1	No.2	No.3	No.4	Mean	SD	RSD(%)
k_{MB}	1.1289	0.6815	0.9777	0.4924	0.8201	0.2868	34.9702
n	0.5834	0.6693	0.5877	0.7563	0.6492	0.0816	12.5750
k	-0.0008	-0.0007	-0.0010	-0.0005	-0.0008	0.0002	-30.2722

Number of dissolution data points (N), degrees of freedom (df), and selected goodness of fit criteria – Pearson correlation coefficient (R), coefficient of determination (R^2), adjusted coefficient of determination ($R^2_{adjusted}$), and residual sum of squares (RSS) (manual calculation in MS Excel):

Parameter	No.1	No.2	No.3	No.4
N	17	17	17	17
df	14	14	14	14
R	0.999412479	0.999333137	0.99885896	0.998389708
R^2	0.998825303	0.998666719	0.997719222	0.99678201
$R^2_{adjusted}$	0.998657489	0.99847625	0.997393396	0.996322297
RSS	5.814220755	6.778184772	10.80378429	19.26162676

Graphical abstract of model fit presented as mean ± 1 SD of the fraction % of released carvedilol:

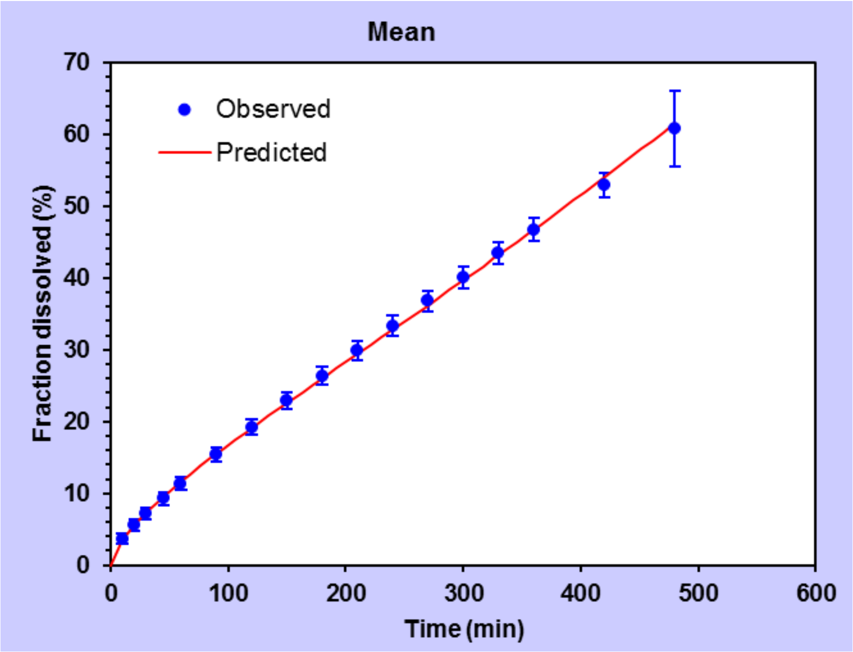

Supplementary materials – Model fitting summary – Glucidex® 19D (modelling up to app. 60 % of carvedilol released)

Graphical abstract of model fit presented as the fraction % of released carvedilol per tested tablet:

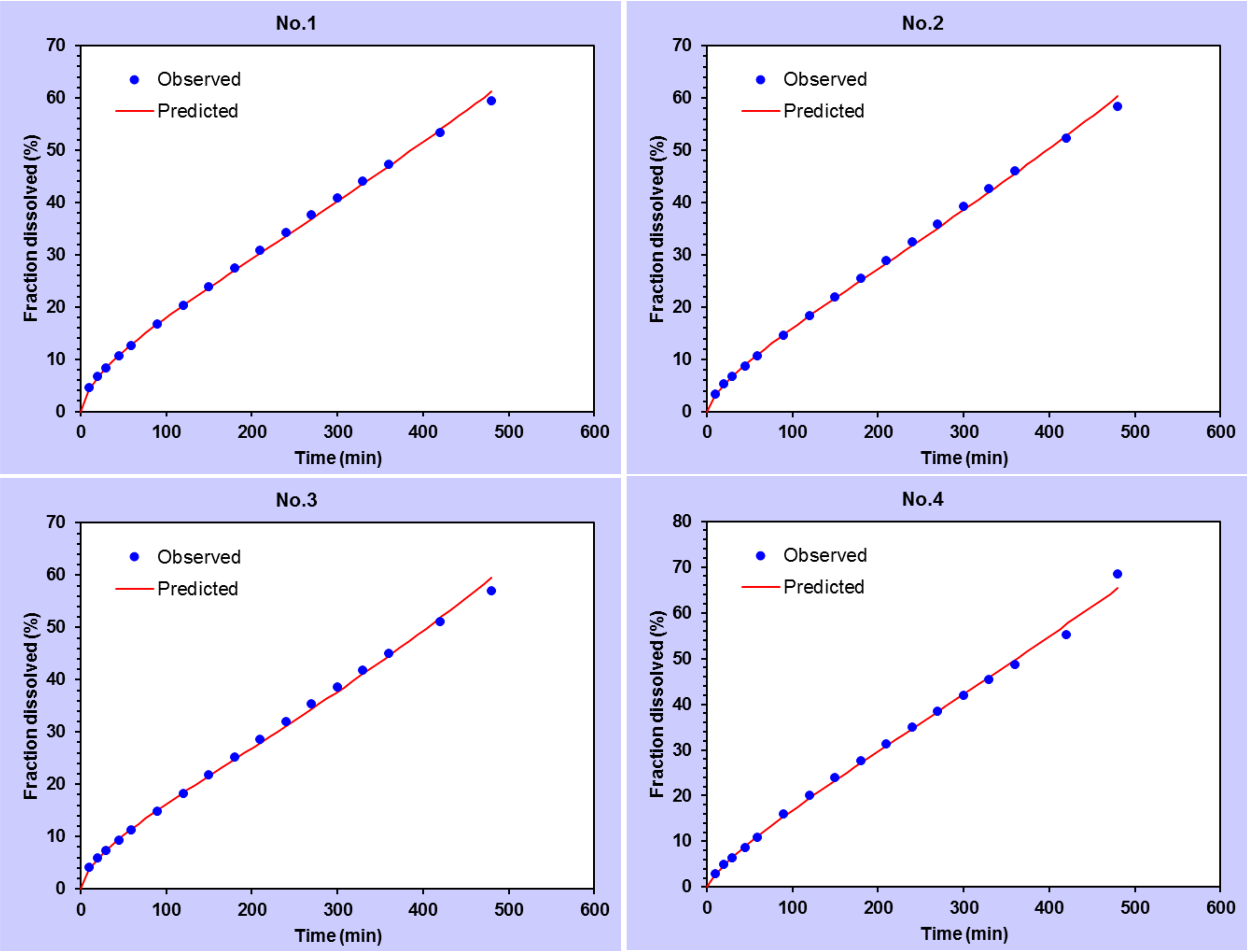

Supplementary materials – Model fitting summary – Glucidex® 19D (modelling up to app. 60 % of carvedilol released)

Model: **Makoid–Banakar with T_{lag}**

Model equation: $F = k_{MB} \cdot (t - T_{lag})^{n} \cdot e^{-k \cdot (t - T_{lag})}$

Fitted model parameters per tested tablet (N = 4) with statistics – mean, standard deviation (SD), and relative standard deviation expressed in % (RSD%) (output from DDSolver):

Parameter	No.1	No.2	No.3	No.4	Mean	SD	RSD(%)
k_{MB}	1.7272	1.1082	1.5723	0.8496	1.3143	0.4064	30.9198
n	0.4864	0.5583	0.4849	0.6317	0.5403	0.0699	12.9340
k	-0.0013	-0.0012	-0.0013	-0.0010	-0.0012	0.0001	-10.7384
T_{lag}	4.0000	4.0000	5.1302	4.0000	4.2826	0.5651	13.1958

Number of dissolution data points (N), degrees of freedom (df), and selected goodness of fit criteria – Pearson correlation coefficient (R), coefficient of determination (R^2), adjusted coefficient of determination ($R^2_{adjusted}$), and residual sum of squares (RSS) (manual calculation in MS Excel):

Parameter	No.1	No.2	No.3	No.4
N	17	17	17	17
df	13	13	13	13
R	0.998050086	0.997763063	0.998211285	0.998449863
R^2	0.996103973	0.995531129	0.99642577	0.99690213
$R^2_{adjusted}$	0.99520489	0.994499851	0.995600948	0.996187237
RSS	20.02520366	23.78719891	27.36795015	19.29592629

Graphical abstract of model fit presented as mean ± 1 SD of the fraction % of released carvedilol:

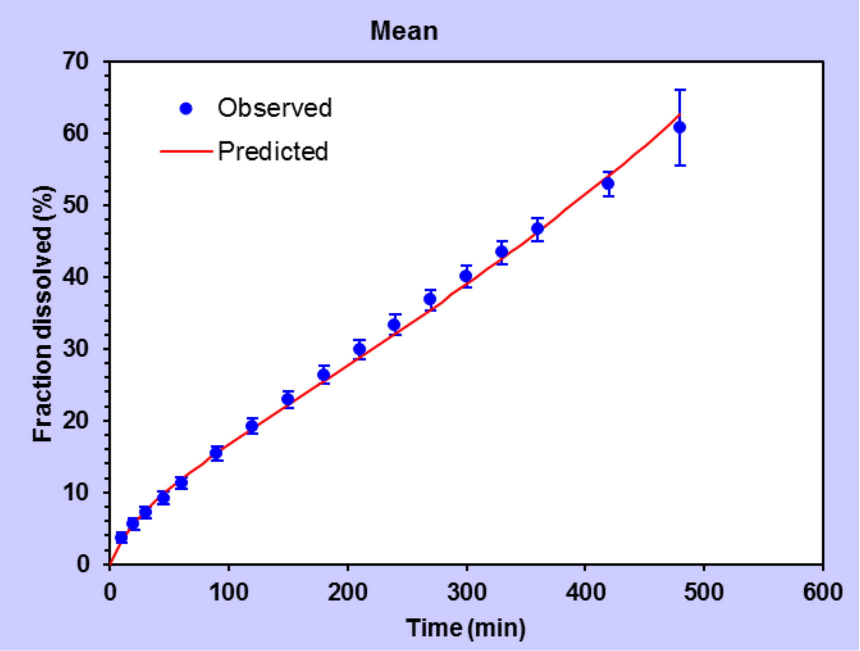

Supplementary materials – Model fitting summary – Glucidex® 19D (modelling up to app. 60 % of carvedilol released)

Graphical abstract of model fit presented as the fraction % of released carvedilol per tested tablet:

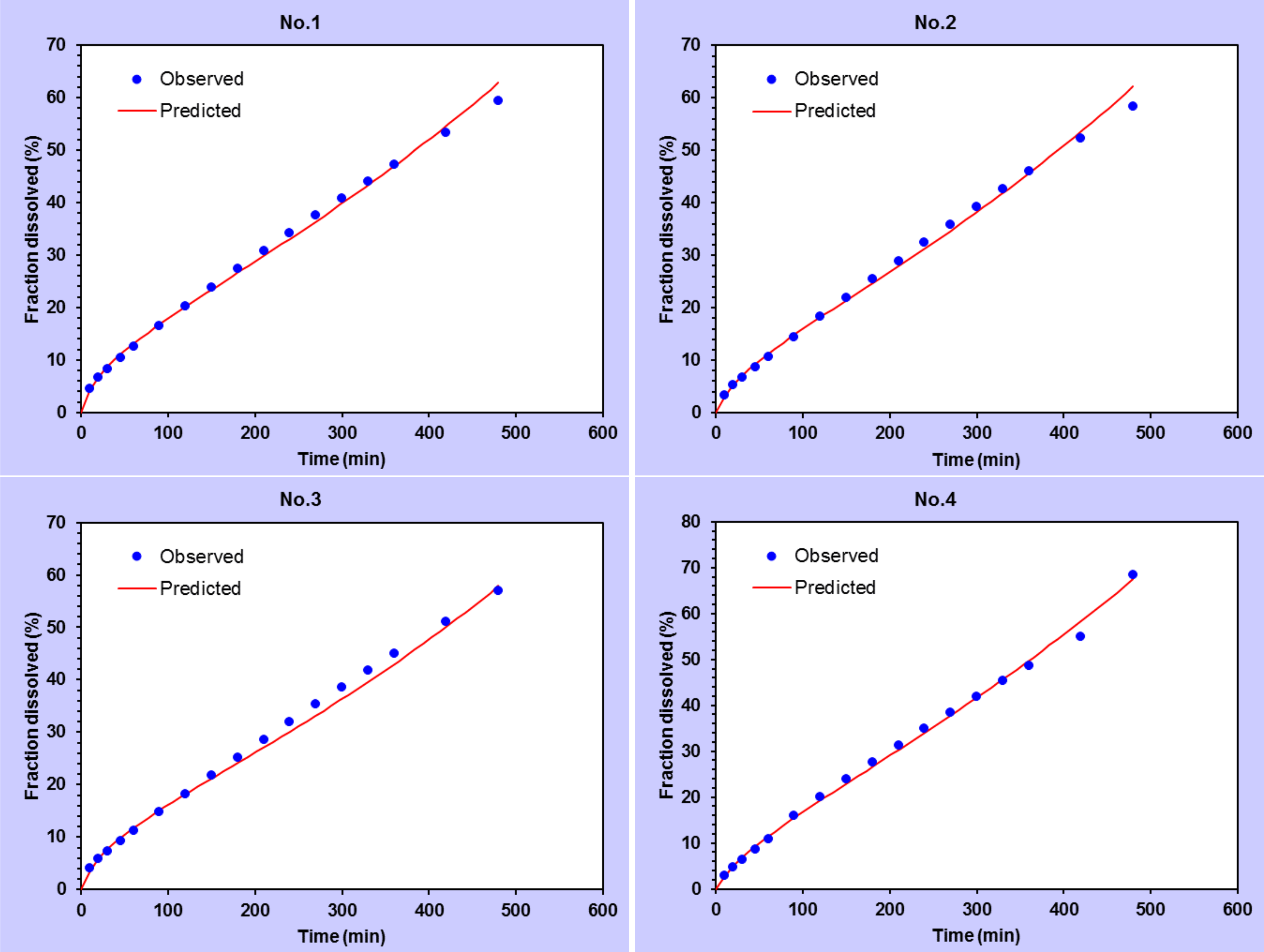

Supplementary materials – Model fitting summary – Glucidex® 19D (modelling up to app. 60 % of carvedilol released)

Model: **Peppas–Sahlin_1**

Model equation: $F = k_1 \cdot t^m + k_2 \cdot t^{2m}$

Fitted model parameters per tested tablet (N = 4) with statistics – mean, standard deviation (SD), and relative standard deviation expressed in % (RSD%) (output from DDSolver):

Parameter	No.1	No.2	No.3	No.4	Mean	SD	RSD(%)
k_1	0.889	0.448	0.597	0.229	0.541	0.277	51.185
k_2	0.173	0.197	0.182	0.234	0.196	0.027	13.780
m	0.450	0.450	0.450	0.450	0.450	0.000	0.000

Number of dissolution data points (N), degrees of freedom (df), and selected goodness of fit criteria – Pearson correlation coefficient (R), coefficient of determination (R^2), adjusted coefficient of determination ($R^2_{adjusted}$), and residual sum of squares (RSS) (manual calculation in MS Excel):

Parameter	No.1	No.2	No.3	No.4
N	17	17	17	17
df	14	14	14	14
R	0.999824585	0.999882874	0.999671083	0.99765529
R^2	0.999649201	0.999765761	0.999342275	0.995316078
$R^2_{adjusted}$	0.999599087	0.999732298	0.999248314	0.994646946
RSS	1.847714029	1.223538566	3.236249655	28.2334479

Graphical abstract of model fit presented as mean ± 1 SD of the fraction % of released carvedilol:

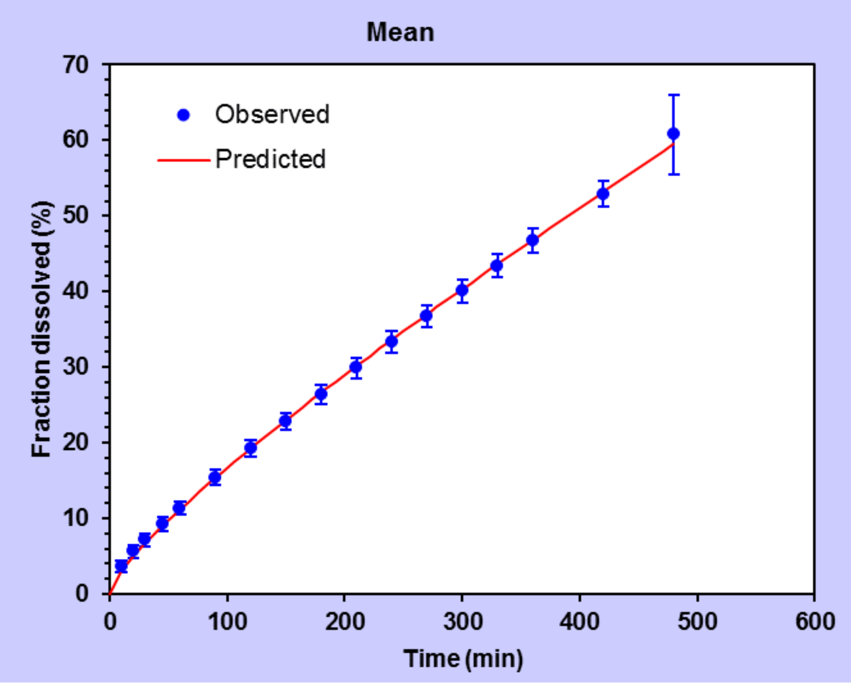

Supplementary materials – Model fitting summary – Glucidex® 19D (modelling up to app. 60 % of carvedilol released)

Graphical abstract of model fit presented as the fraction % of released carvedilol per tested tablet:

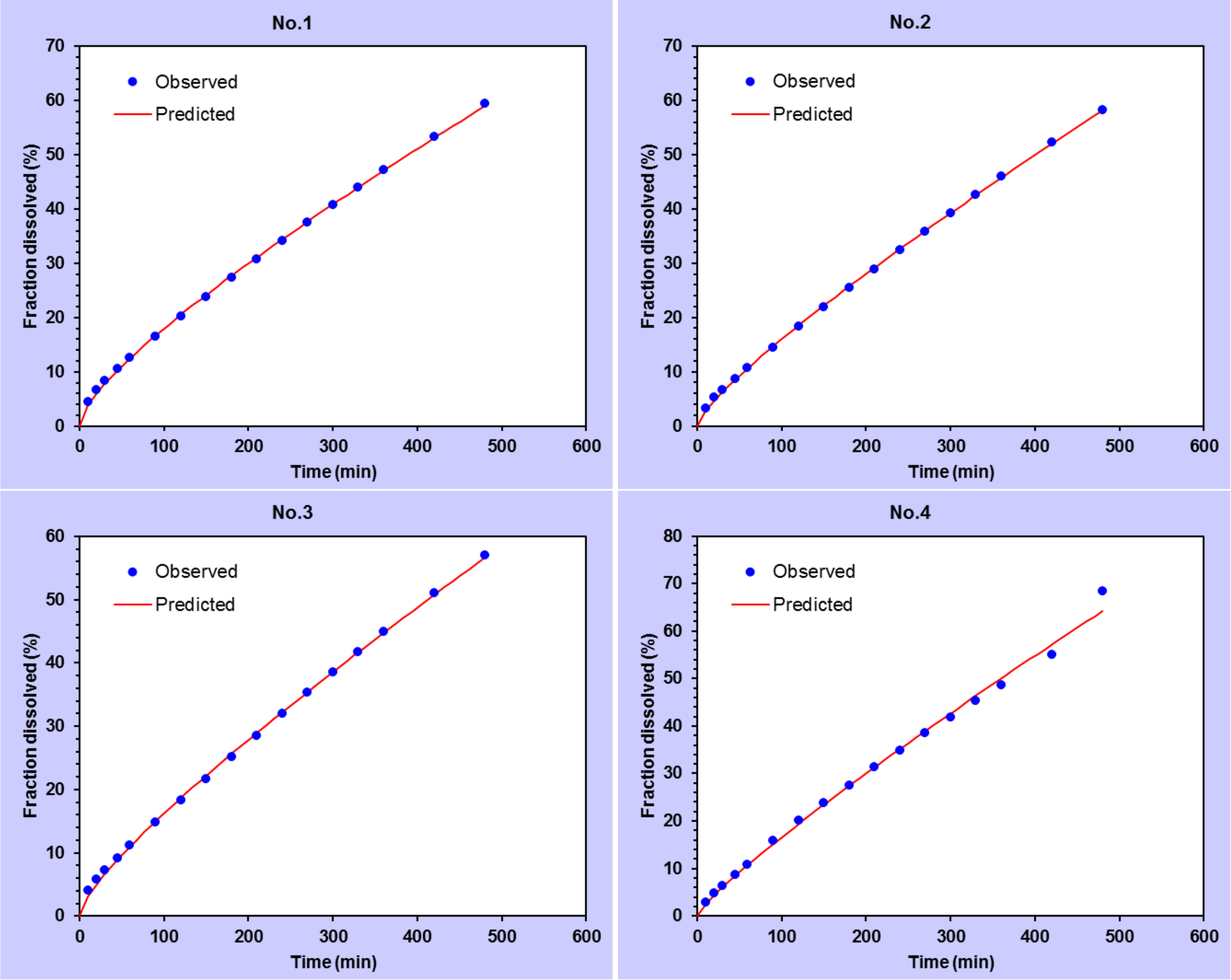

Supplementary materials – Model fitting summary – Glucidex® 19D (modelling up to app. 60 % of carvedilol released)

Model: **Peppas–Sahlin_1 with T_{lag}**

Model equation: $F = k_1 \cdot (t - T_{lag})^m + k_2 \cdot (t - T_{lag})^{2m}$

Fitted model parameters per tested tablet (N = 4) with statistics – mean, standard deviation (SD), and relative standard deviation expressed in % (RSD%) (output from DDSolver):

Parameter	No.1	No.2	No.3	No.4	Mean	SD	RSD(%)
k_1	1.035	0.583	0.730	0.373	0.680	0.278	40.869
k_2	0.164	0.189	0.174	0.226	0.188	0.027	14.407
m	0.450	0.450	0.450	0.450	0.450	0.000	0.000
T_{lag}	4.000	4.000	4.000	4.000	4.000	0.000	0.000

Number of dissolution data points (N), degrees of freedom (df), and selected goodness of fit criteria – Pearson correlation coefficient (R), coefficient of determination (R^2), adjusted coefficient of determination ($R^2_{adjusted}$), and residual sum of squares (RSS) (manual calculation in MS Excel):

Parameter	No.1	No.2	No.3	No.4
N	17	17	17	17
df	13	13	13	13
R	0.999533909	0.999699432	0.99935598	0.997483734
R^2	0.999068036	0.999398954	0.998712375	0.994973799
$R^2_{adjusted}$	0.998852968	0.999260251	0.998415231	0.993813907
RSS	5.126207437	3.285779125	6.584838925	30.65141023

Graphical abstract of model fit presented as mean ± 1 SD of the fraction % of released carvedilol:

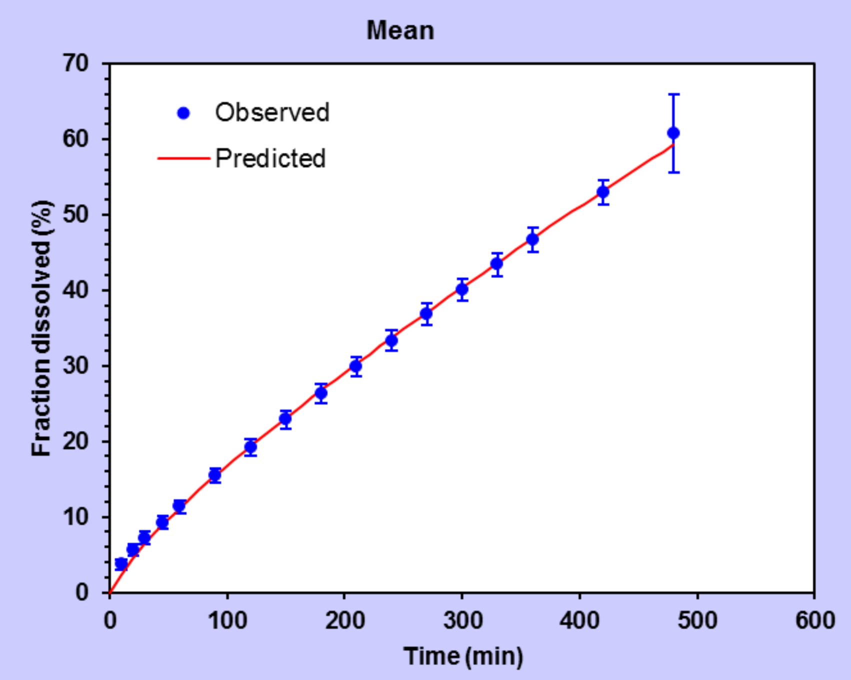

Supplementary materials – Model fitting summary – Glucidex® 19D (modelling up to app. 60 % of carvedilol released)

Graphical abstract of model fit presented as the fraction % of released carvedilol per tested tablet:

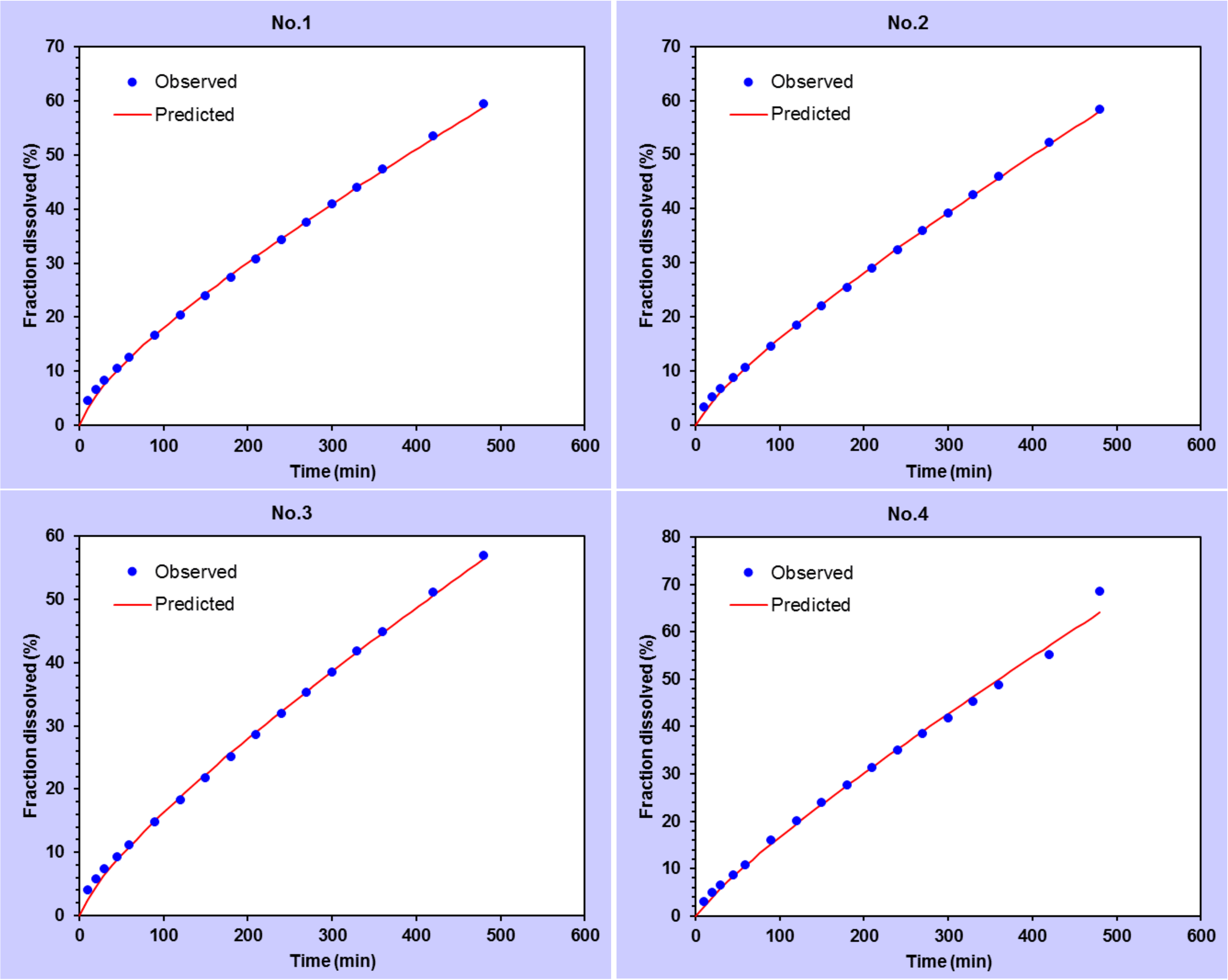

Supplementary materials – Model fitting summary – Glucidex® 19D (modelling up to app. 60 % of carvedilol released)

Model: **Peppas–Sahlin_2**

Model equation: $F = k_1 \cdot t^{0.5} + k_2 \cdot t$

Fitted model parameters per tested tablet (N = 4) with statistics – mean, standard deviation (SD), and relative standard deviation expressed in % (RSD%) (output from DDSolver):

Parameter	No.1	No.2	No.3	No.4	Mean	SD	RSD(%)
k_1	1.029	0.694	0.792	0.560	0.769	0.198	25.721
k_2	0.077	0.090	0.083	0.109	0.090	0.014	15.931

Number of dissolution data points (N), degrees of freedom (df), and selected goodness of fit criteria – Pearson correlation coefficient (R), coefficient of determination (R^2), adjusted coefficient of determination ($R^2_{adjusted}$), and residual sum of squares (RSS) (manual calculation in MS Excel):

Parameter	No.1	No.2	No.3	No.4
N	17	17	17	17
df	15	15	15	15
R	0.999915919	0.999950961	0.999832128	0.997944579
R^2	0.999831845	0.999901924	0.999664285	0.995893383
$R^2_{adjusted}$	0.999820635	0.999895386	0.999641904	0.995619609
RSS	0.914201943	0.482025616	1.684529964	24.62320248

Graphical abstract of model fit presented as mean ± 1 SD of the fraction % of released carvedilol:

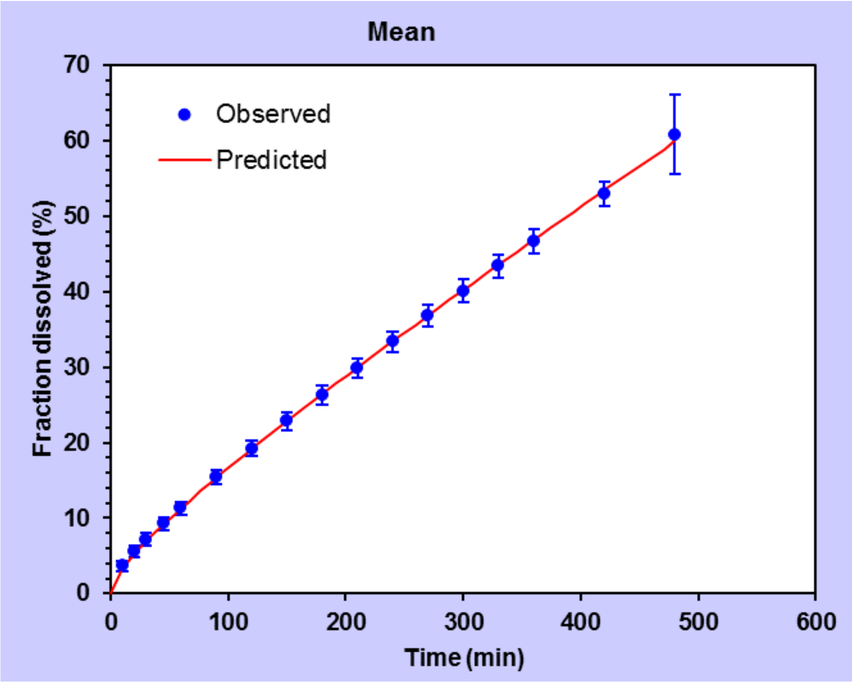

Supplementary materials – Model fitting summary – Glucidex® 19D (modelling up to app. 60 % of carvedilol released)

Graphical abstract of model fit presented as the fraction % of released carvedilol per tested tablet:

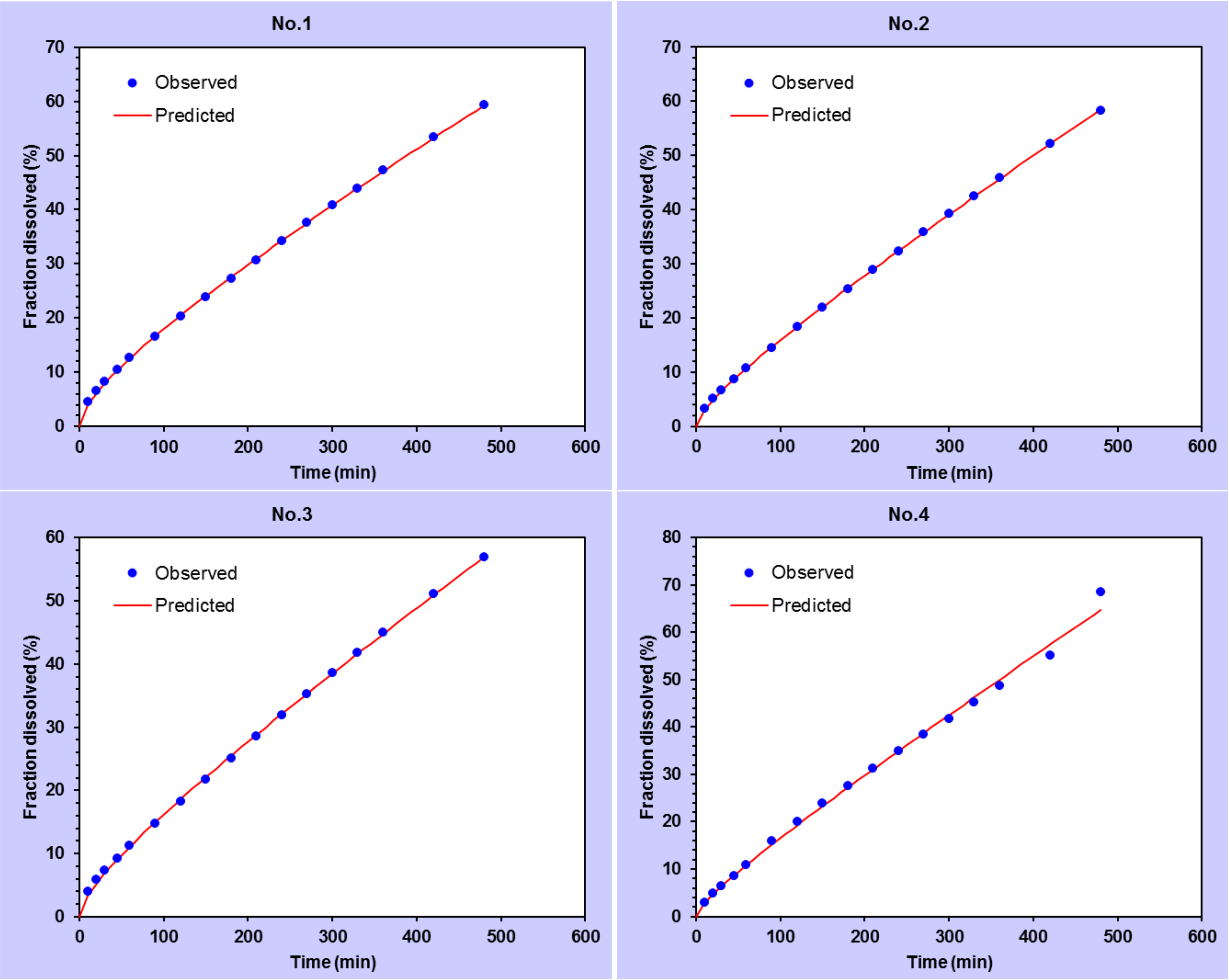

Supplementary materials – Model fitting summary – Glucidex® 19D (modelling up to app. 60 % of carvedilol released)

Model: **Peppas–Sahlin_2 with T_{lag}**

Model equation: $F = k_1 \cdot (t - T_{lag})^{0.5} + k_2 \cdot (t - T_{lag})$

Fitted model parameters per tested tablet (N = 4) with statistics – mean, standard deviation (SD), and relative standard deviation expressed in % (RSD%) (output from DDSolver):

Parameter	No.1	No.2	No.3	No.4	Mean	SD	RSD(%)
k_1	1.135	0.792	0.889	0.665	0.870	0.199	22.825
k_2	0.072	0.086	0.079	0.105	0.085	0.014	16.775
T_{lag}	4.000	4.000	4.000	4.000	4.000	0.000	0.000

Number of dissolution data points (N), degrees of freedom (df), and selected goodness of fit criteria – Pearson correlation coefficient (R), coefficient of determination (R^2), adjusted coefficient of determination ($R^2_{adjusted}$), and residual sum of squares (RSS) (manual calculation in MS Excel):

Parameter	No.1	No.2	No.3	No.4
N	17	17	17	17
df	14	14	14	14
R	0.999667913	0.999839056	0.999573341	0.99784761
R^2	0.999335937	0.999678138	0.999146864	0.995699852
$R^2_{adjusted}$	0.999241071	0.999632158	0.999024987	0.995085545
RSS	3.850634428	1.779610281	4.553352566	26.06848663

Graphical abstract of model fit presented as mean ± 1 SD of the fraction % of released carvedilol:

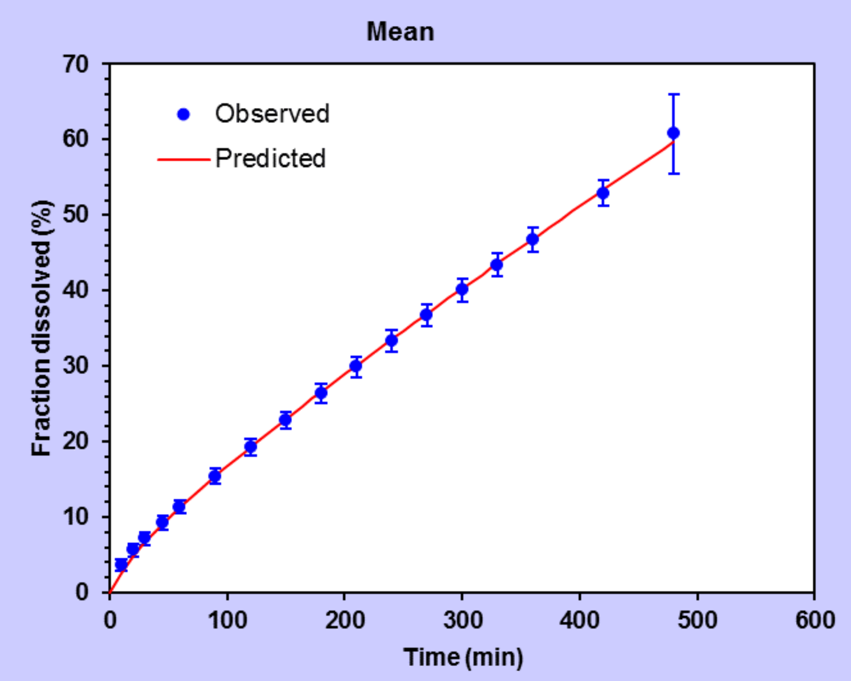

Supplementary materials – Model fitting summary – Glucidex® 19D (modelling up to app. 60 % of carvedilol released)

Graphical abstract of model fit presented as the fraction % of released carvedilol per tested tablet:

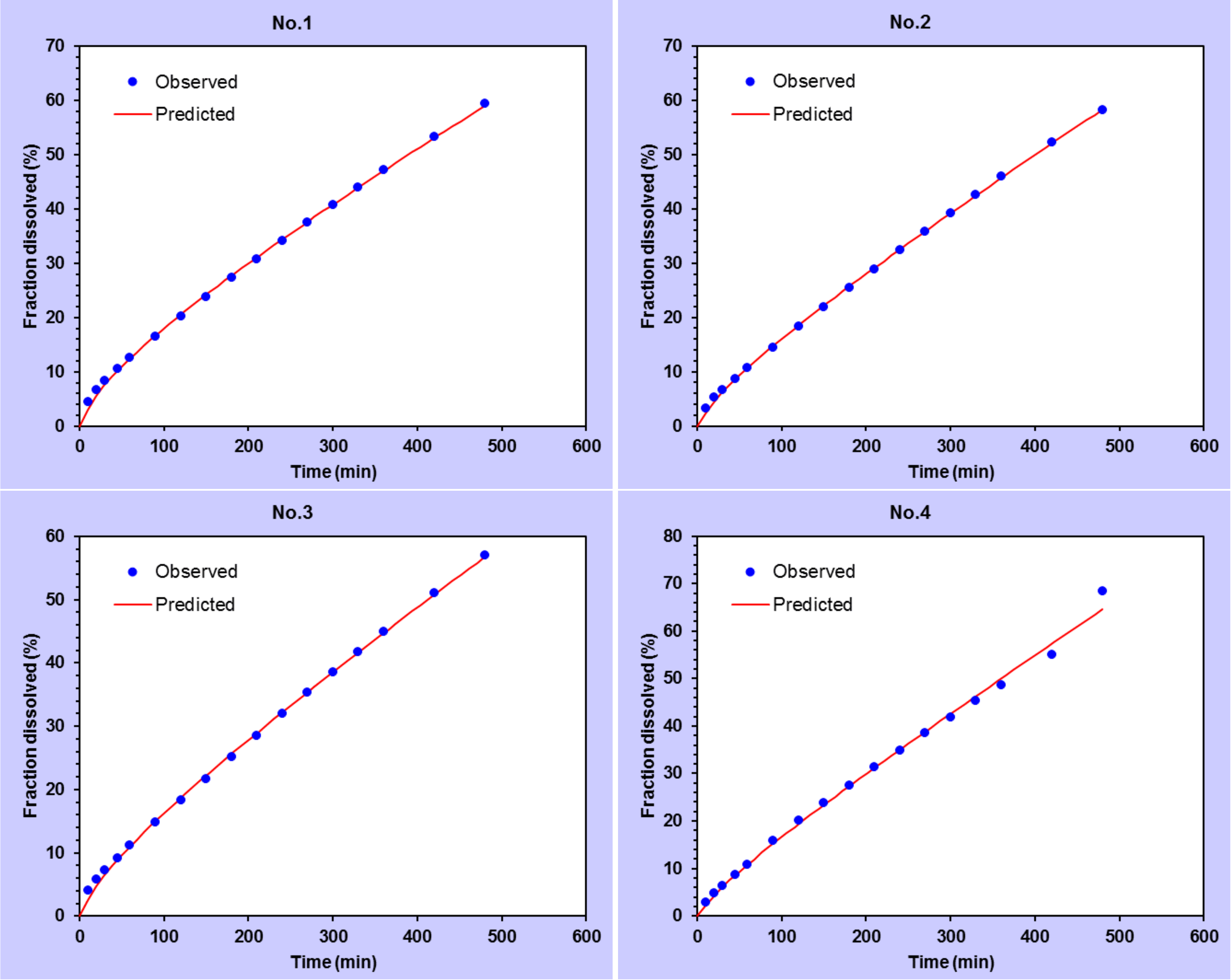

Supplementary materials – Model fitting summary – Glucidex® 19D (modelling up to app. 60 % of carvedilol released)

Model: **Quadratic**

Model equation: $F = 100 \cdot (k_1 \cdot t^2 + k_2 \cdot t)$

Fitted model parameters per tested tablet (N = 4) with statistics – mean, standard deviation (SD), and relative standard deviation expressed in % (RSD%) (output from DDSolver):

Parameter	No.1	No.2	No.3	No.4	Mean	SD	RSD(%)
k_1	-0.0000011	-0.0000008	-0.0000009	-0.0000005	-0.0000008	0.0000002	-30.0551547
k_2	0.0017357	0.0015651	0.0015683	0.0015916	0.0016152	0.0000812	5.0284801

Number of dissolution data points (N), degrees of freedom (df), and selected goodness of fit criteria – Pearson correlation coefficient (R), coefficient of determination (R^2), adjusted coefficient of determination ($R^2_{adjusted}$), and residual sum of squares (RSS) (manual calculation in MS Excel):

Parameter	No.1	No.2	No.3	No.4
N	17	17	17	17
df	15	15	15	15
R	0.997724921	0.999061751	0.998395791	0.996855262
R^2	0.995455017	0.998124382	0.996794155	0.993720414
$R^2_{adjusted}$	0.995152018	0.997999341	0.996580432	0.993301775
RSS	57.47069013	22.64199407	37.36836431	52.09212811

Graphical abstract of model fit presented as mean ± 1 SD of the fraction % of released carvedilol:

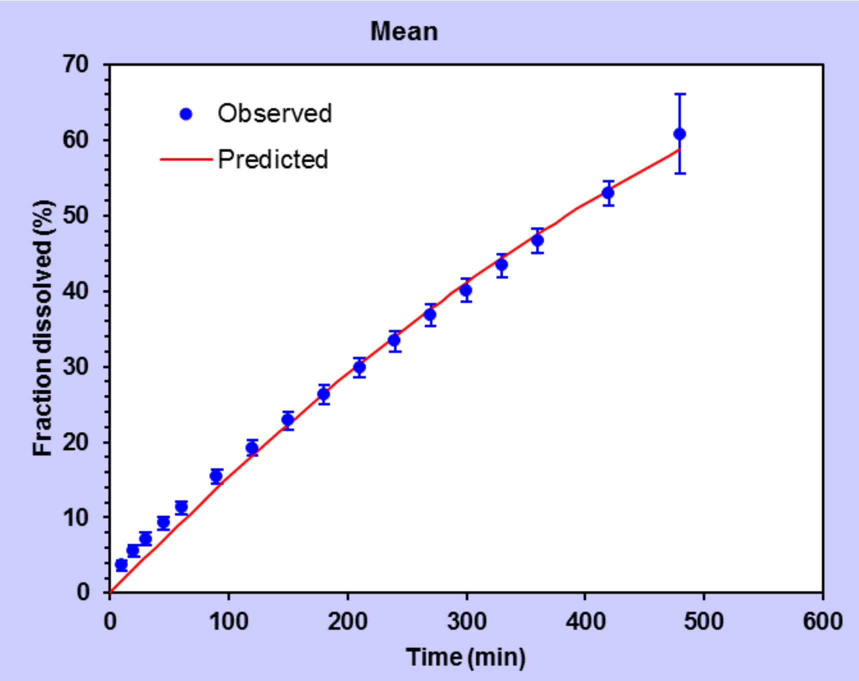

Supplementary materials – Model fitting summary – Glucidex® 19D (modelling up to app. 60 % of carvedilol released)

Graphical abstract of model fit presented as the fraction % of released carvedilol per tested tablet:

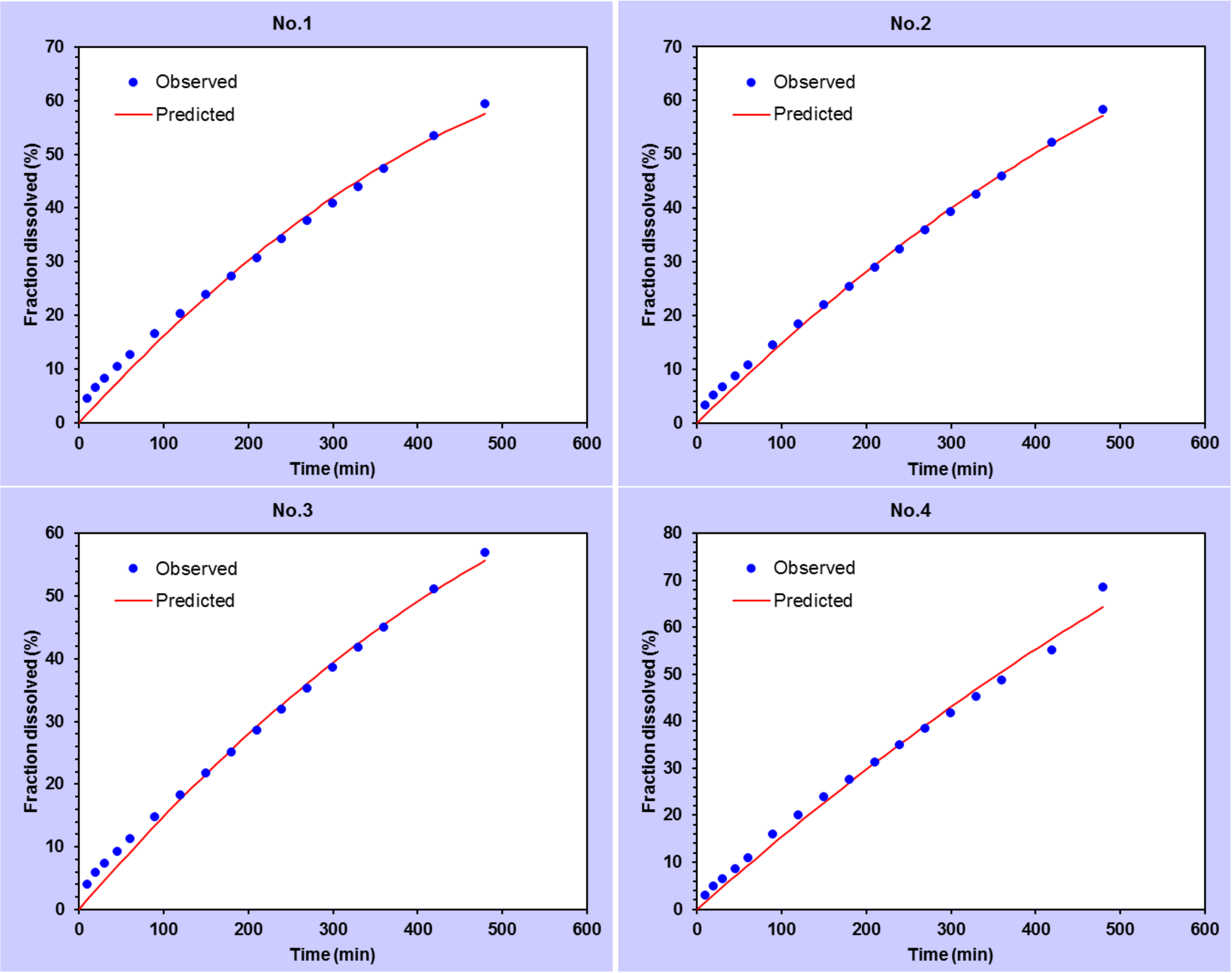

Supplementary materials – Model fitting summary – Glucidex® 19D (modelling up to app. 60 % of carvedilol released)

Model: **Quadratic with T_{lag}**

Model equation: $F = 100 \cdot \left[k_1 \cdot (t - T_{lag})^2 + k_2 \cdot (t - T_{lag})\right]$

Fitted model parameters per tested tablet (N = 4) with statistics – mean, standard deviation (SD), and relative standard deviation expressed in % (RSD%) (output from DDSolver):

Parameter	No.1	No.2	No.3	No.4	Mean	SD	RSD(%)
k_1	-0.0000012	-0.0000009	-0.0000009	-0.0000006	-0.0000009	0.0000002	-27.2717989
k_2	0.0017810	0.0016097	0.0016111	0.0016389	0.0016602	0.0000816	4.9172438
T_{lag}	4.0000000	4.0000000	4.0000000	4.0000000	4.0000000	0.0000000	0.0000000

Number of dissolution data points (N), degrees of freedom (df), and selected goodness of fit criteria – Pearson correlation coefficient (R), coefficient of determination (R^2), adjusted coefficient of determination ($R^2_{adjusted}$), and residual sum of squares (RSS) (manual calculation in MS Excel):

Parameter	No.1	No.2	No.3	No.4
N	17	17	17	17
df	14	14	14	14
R	0.997179242	0.998681176	0.997919408	0.99650333
R^2	0.994366441	0.997364092	0.995843144	0.993018886
$R^2_{adjusted}$	0.993561647	0.996987534	0.995249308	0.992021584
RSS	78.54973413	35.03284119	53.0129884	65.32275109

Graphical abstract of model fit presented as mean ± 1 SD of the fraction % of released carvedilol:

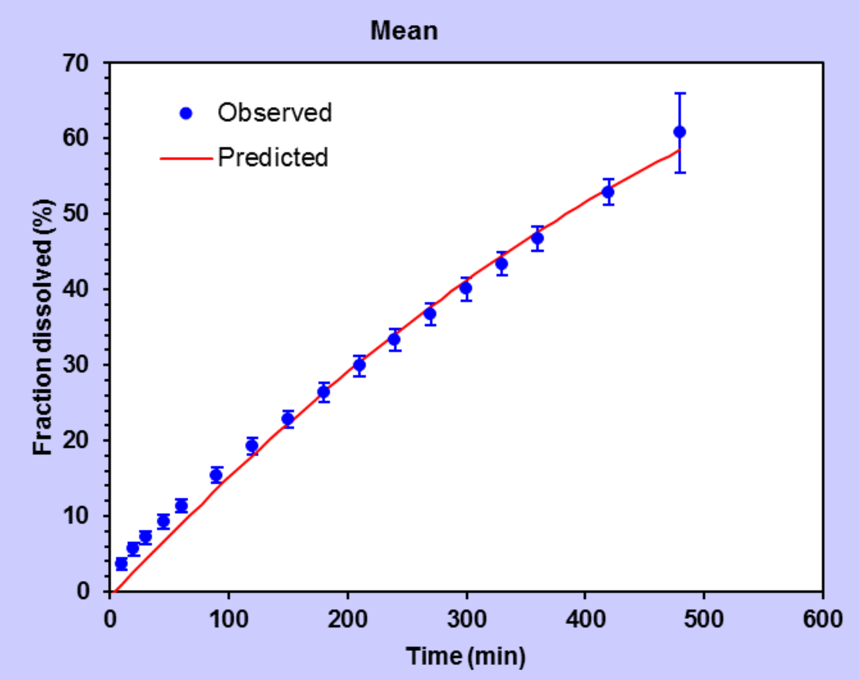

Supplementary materials – Model fitting summary – Glucidex® 19D (modelling up to app. 60 % of carvedilol released)

Graphical abstract of model fit presented as the fraction % of released carvedilol per tested tablet:

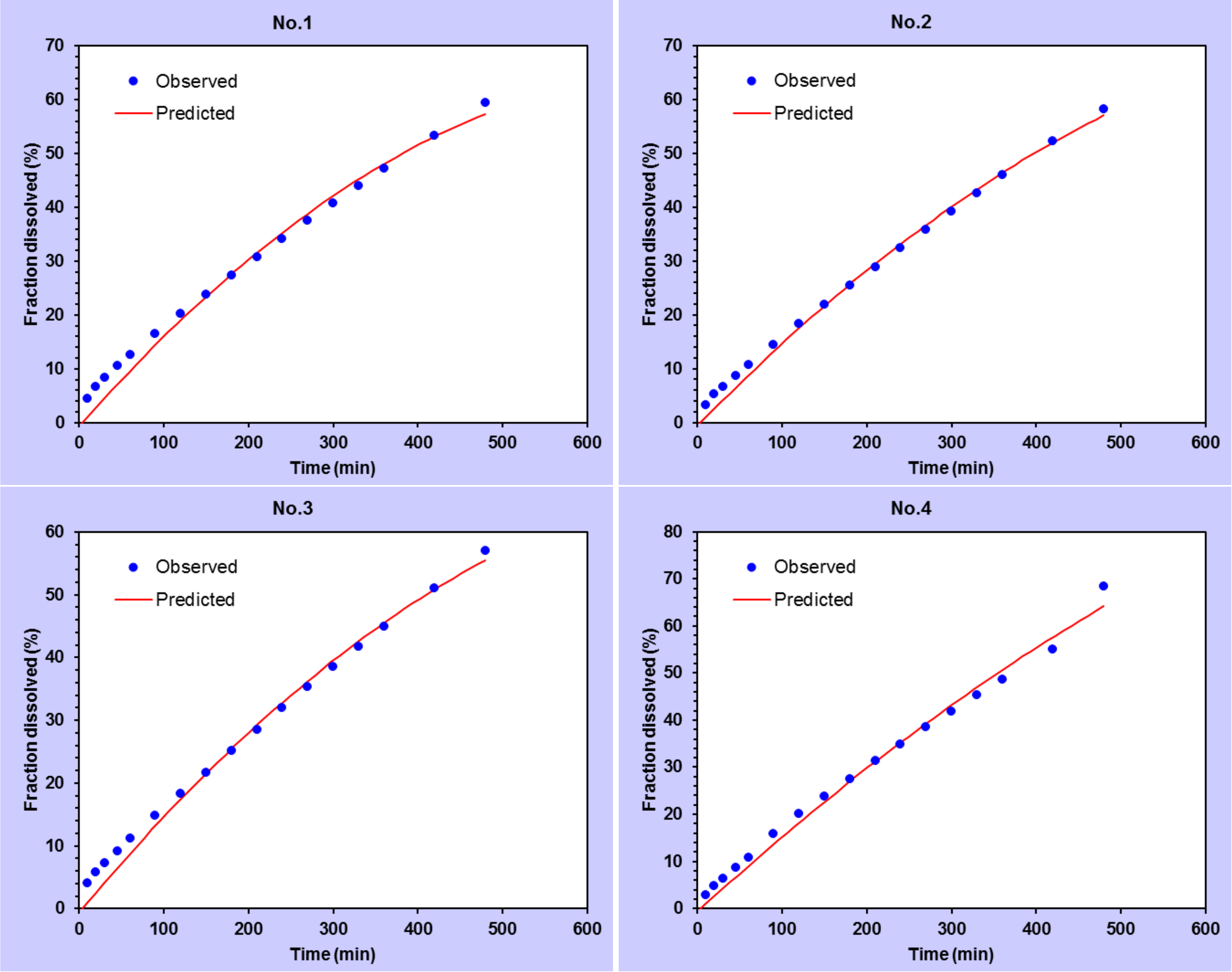

Supplementary materials – Model fitting summary – Glucidex® 19D (modelling up to app. 60 % of carvedilol released)

Model: **Weibull_1**

Model equation: $F = 100 \cdot \left[1 - e^{-\frac{(t-T_i)^{\beta}}{\alpha}}\right]$

Fitted model parameters per tested tablet (N = 4) with statistics – mean, standard deviation (SD), and relative standard deviation expressed in % (RSD%) (output from DDSolver):

Parameter	No.1	No.2	No.3	No.4	Mean	SD	RSD(%)
α	105.904	164.791	127.752	209.948	152.099	45.587	29.972
β	0.700	0.768	0.718	0.831	0.754	0.059	7.816
T_i	4.000	4.000	4.000	6.000	4.500	1.000	22.222

Number of dissolution data points (N), degrees of freedom (df), and selected goodness of fit criteria – Pearson correlation coefficient (R), coefficient of determination (R^2), adjusted coefficient of determination ($R^2_{adjusted}$), and residual sum of squares (RSS) (manual calculation in MS Excel):

Parameter	No.1	No.2	No.3	No.4
N	17	17	17	17
df	14	14	14	14
R	0.988326487	0.990483355	0.987623588	0.986582538
R^2	0.976789246	0.981057277	0.975400353	0.973345105
$R^2_{adjusted}$	0.973473424	0.978351174	0.971886117	0.969537263
RSS	169.3663001	153.5949061	178.5353471	222.5521392

Graphical abstract of model fit presented as mean ± 1 SD of the fraction % of released carvedilol:

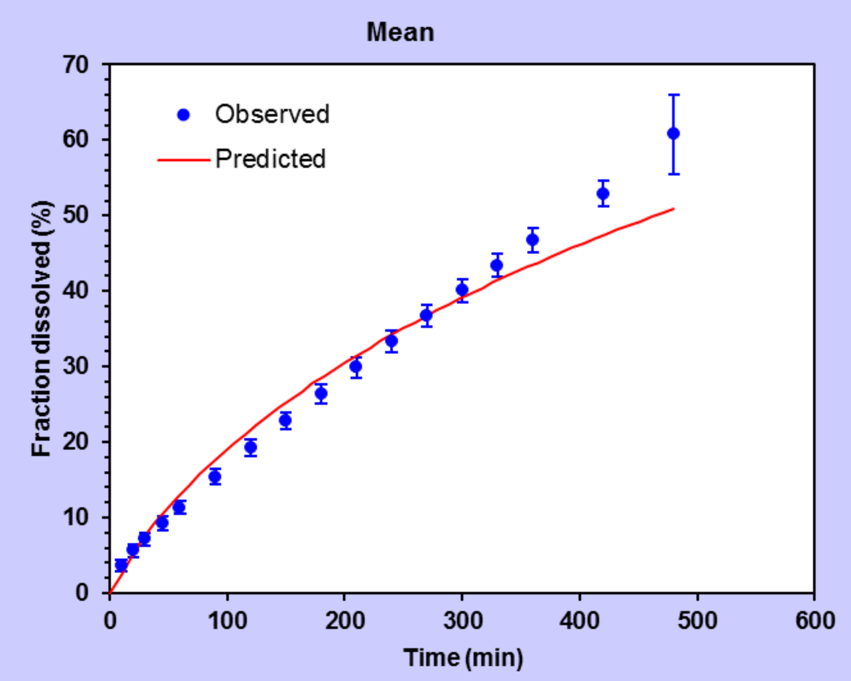

Supplementary materials – Model fitting summary – Glucidex® 19D (modelling up to app. 60 % of carvedilol released)

Graphical abstract of model fit presented as the fraction % of released carvedilol per tested tablet:

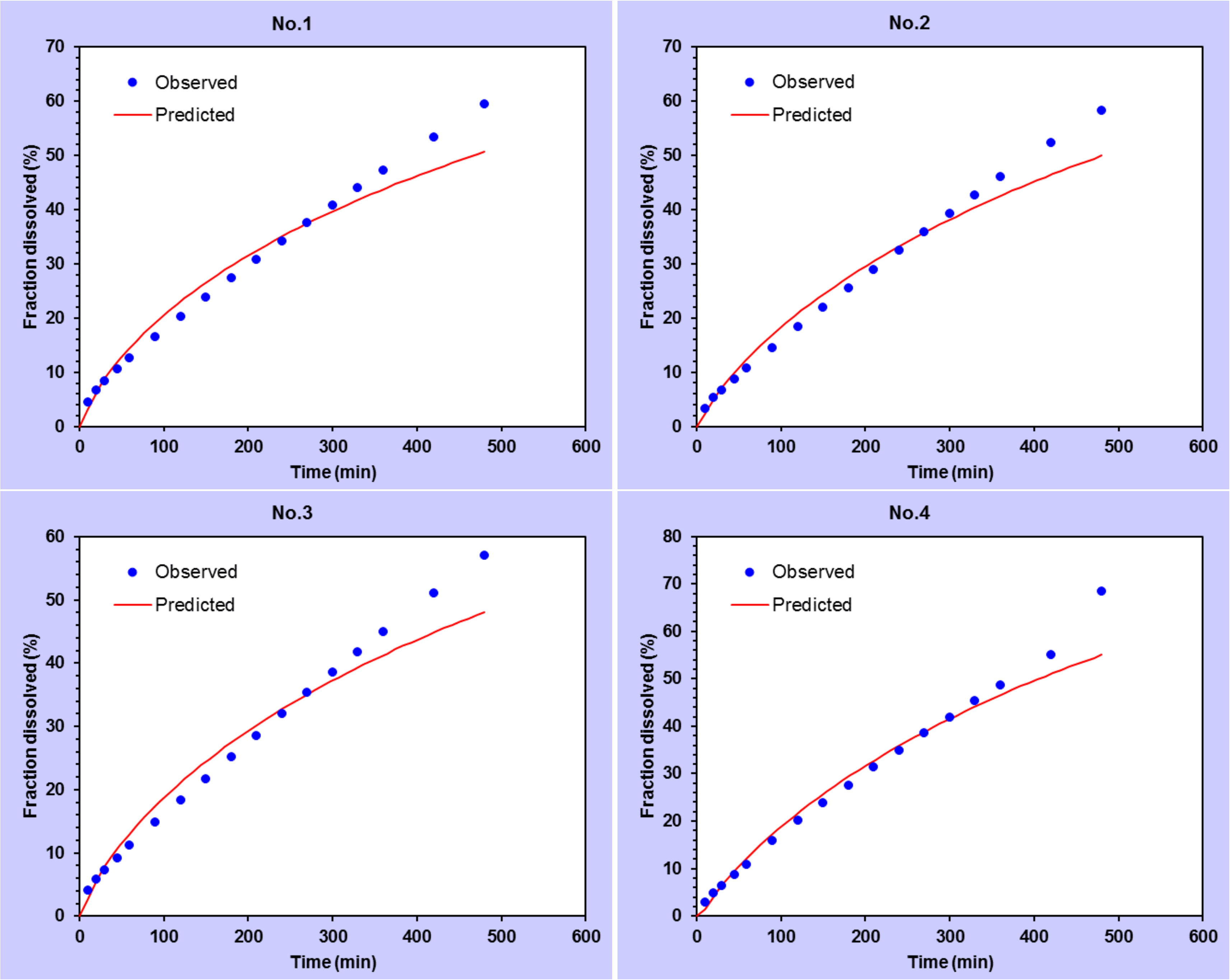

Supplementary materials – Model fitting summary – Glucidex® 19D (modelling up to app. 60 % of carvedilol released)

Model: **Weibull_2**

Model equation: $F = 100 \cdot \left(1 - e^{-\frac{t^{\beta}}{\alpha}}\right)$

Fitted model parameters per tested tablet (N = 4) with statistics – mean, standard deviation (SD), and relative standard deviation expressed in % (RSD%) (output from DDSolver):

Parameter	No.1	No.2	No.3	No.4	Mean	SD	RSD(%)
α	157.720	254.499	192.689	334.933	234.960	77.737	33.085
β	0.771	0.846	0.792	0.915	0.831	0.064	7.735

Number of dissolution data points (N), degrees of freedom (df), and selected goodness of fit criteria – Pearson correlation coefficient (R), coefficient of determination (R^2), adjusted coefficient of determination ($R^2_{adjusted}$), and residual sum of squares (RSS) (manual calculation in MS Excel):

Parameter	No.1	No.2	No.3	No.4
N	17	17	17	17
df	15	15	15	15
R	0.992427631	0.994119009	0.991939833	0.99019616
R^2	0.984912602	0.988272604	0.983944633	0.980488436
$R^2_{adjusted}$	0.983906775	0.987490778	0.982874275	0.979187665
RSS	102.857164	87.60612947	109.5193587	147.5016158

Graphical abstract of model fit presented as mean ± 1 SD of the fraction % of released carvedilol:

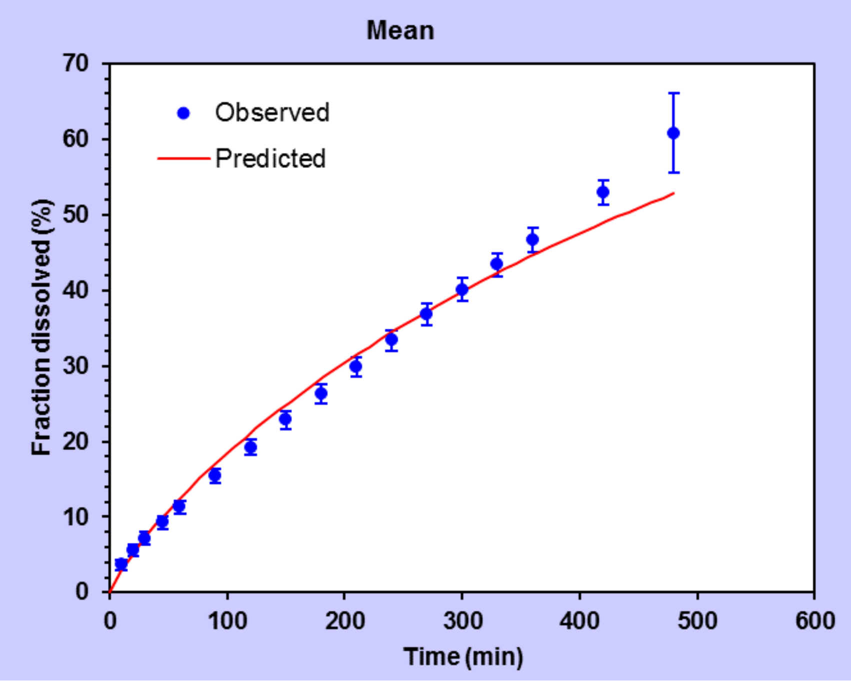

Supplementary materials – Model fitting summary – Glucidex® 19D (modelling up to app. 60 % of carvedilol released)

Graphical abstract of model fit presented as the fraction % of released carvedilol per tested tablet:

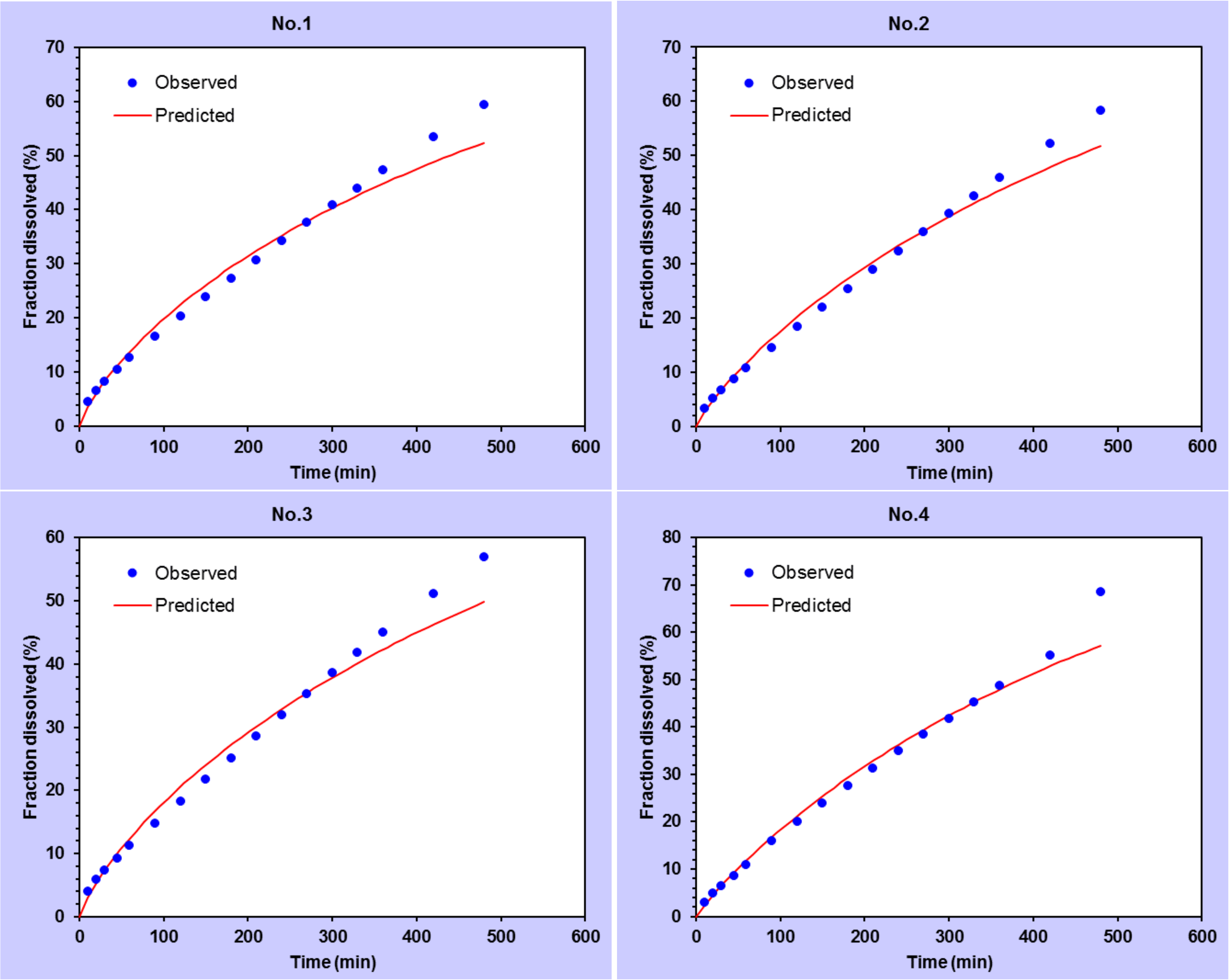

Supplementary materials – Model fitting summary – Glucidex® 19D (modelling up to app. 60 % of carvedilol released)

Model: **Weibull_3**

Model equation: $F = F_{max} \cdot \left(1 - e^{-\frac{t^{\beta}}{\alpha}}\right)$

Fitted model parameters per tested tablet (N = 4) with statistics – mean, standard deviation (SD), and relative standard deviation expressed in % (RSD%) (output from DDSolver):

Parameter	No.1	No.2	No.3	No.4	Mean	SD	RSD(%)
α	147.914	237.078	176.177	325.188	221.589	78.450	35.403
β	0.893	0.970	0.918	1.002	0.946	0.049	5.220
F_{max}	62.325	61.178	59.808	71.913	63.806	5.502	8.623

Number of dissolution data points (N), degrees of freedom (df), and selected goodness of fit criteria – Pearson correlation coefficient (R), coefficient of determination (R^2), adjusted coefficient of determination ($R^2_{adjusted}$), and residual sum of squares (RSS) (manual calculation in MS Excel):

Parameter	No.1	No.2	No.3	No.4
N	17	17	17	17
df	14	14	14	14
R	0.98185645	0.983970494	0.980862047	0.982844862
R^2	0.964042088	0.968197933	0.962090354	0.965984023
$R^2_{adjusted}$	0.958905243	0.96365478	0.956674691	0.961124597
RSS	192.8301321	177.6881358	195.3164451	227.8726192

Graphical abstract of model fit presented as mean ± 1 SD of the fraction % of released carvedilol:

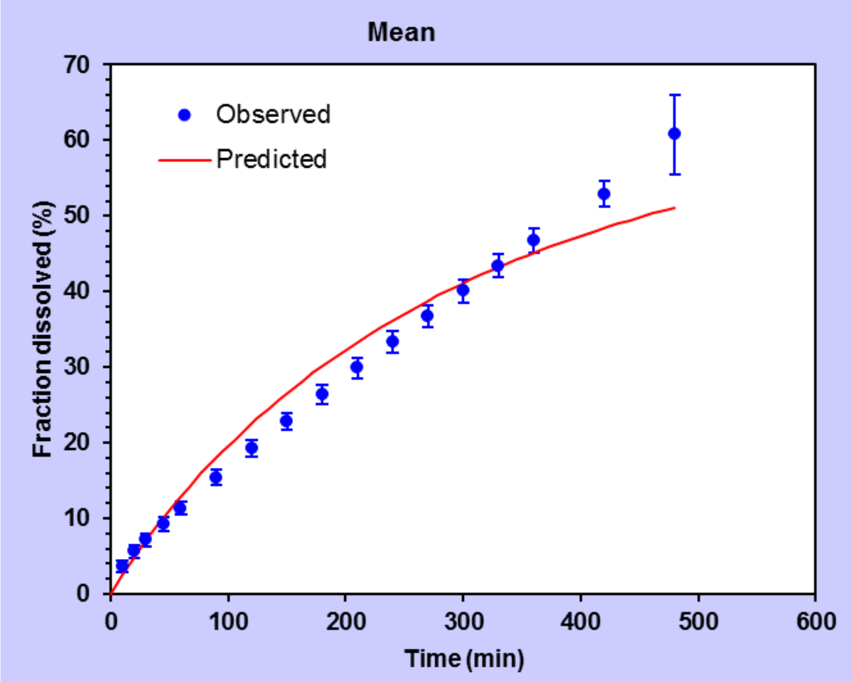

Supplementary materials – Model fitting summary – Glucidex® 19D (modelling up to app. 60 % of carvedilol released)

Graphical abstract of model fit presented as the fraction % of released carvedilol per tested tablet:

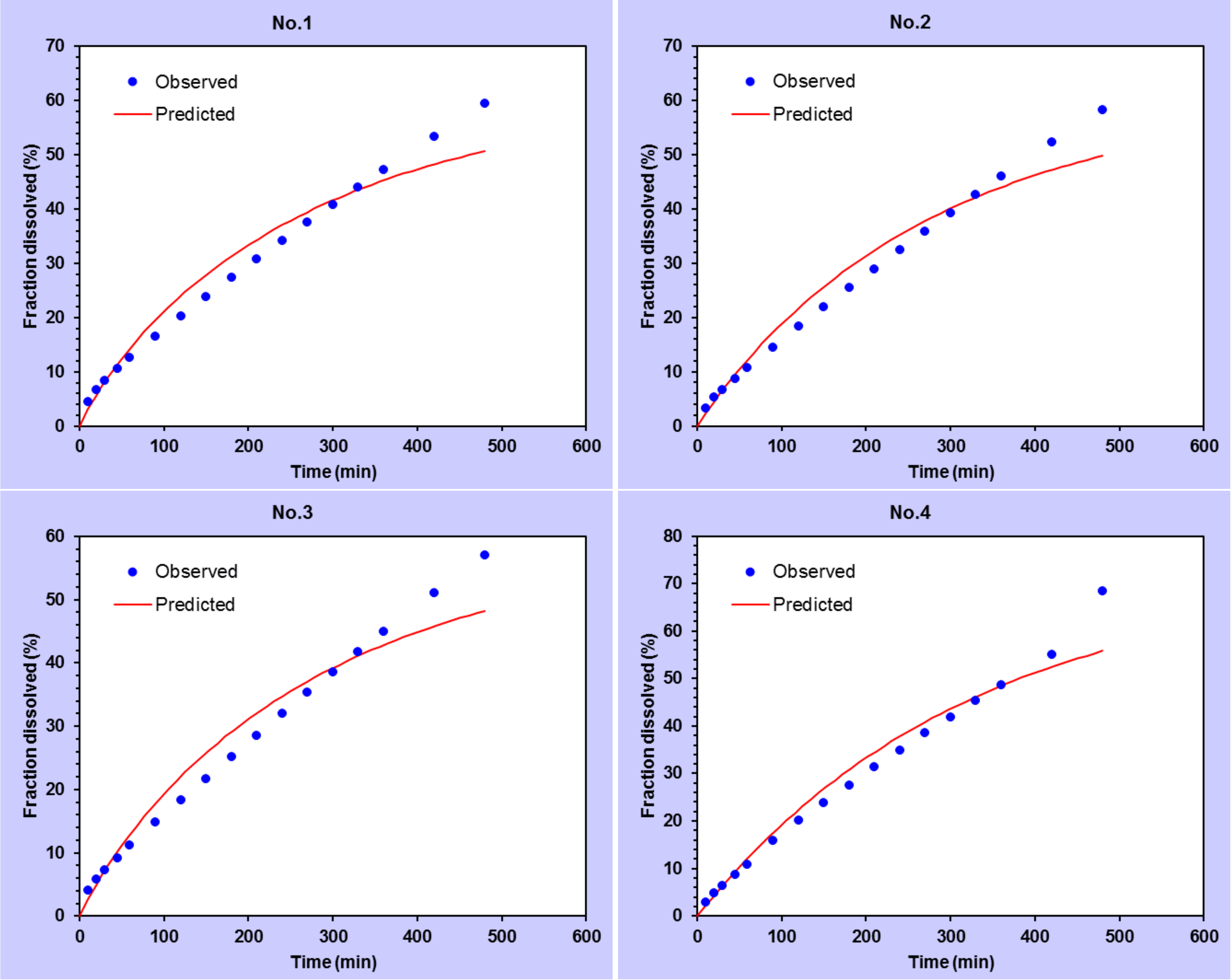

Supplementary materials – Model fitting summary – Glucidex® 19D (modelling up to app. 60 % of carvedilol released)

Model: **Weibull_4**

Model equation: $F = F_{max} \cdot \left[1 - e^{-\frac{(t-T_i)^\beta}{\alpha}}\right]$

Fitted model parameters per tested tablet (N = 4) with statistics – mean, standard deviation (SD), and relative standard deviation expressed in % (RSD%) (output from DDSolver):

Parameter	No.1	No.2	No.3	No.4	Mean	SD	RSD(%)
α	92.245	142.387	108.203	193.265	134.025	44.690	33.345
β	0.808	0.878	0.830	0.909	0.856	0.046	5.328
T_i	6.000	6.000	6.000	6.000	6.000	0.000	0.000
F_{max}	62.325	61.178	59.808	71.913	63.806	5.502	8.623

Number of dissolution data points (N), degrees of freedom (df), and selected goodness of fit criteria – Pearson correlation coefficient (R), coefficient of determination (R^2), adjusted coefficient of determination ($R^2_{adjusted}$), and residual sum of squares (RSS) (manual calculation in MS Excel):

Parameter	No.1	No.2	No.3	No.4
N	17	17	17	17
df	13	13	13	13
R	0.976424386	0.979243114	0.975305998	0.978957906
R^2	0.953404581	0.958917076	0.95122179	0.958358583
$R^2_{adjusted}$	0.942651792	0.949436402	0.93996528	0.948749025
RSS	247.4841463	231.0417527	250.5771327	287.8656205

Graphical abstract of model fit presented as mean ± 1 SD of the fraction % of released carvedilol:

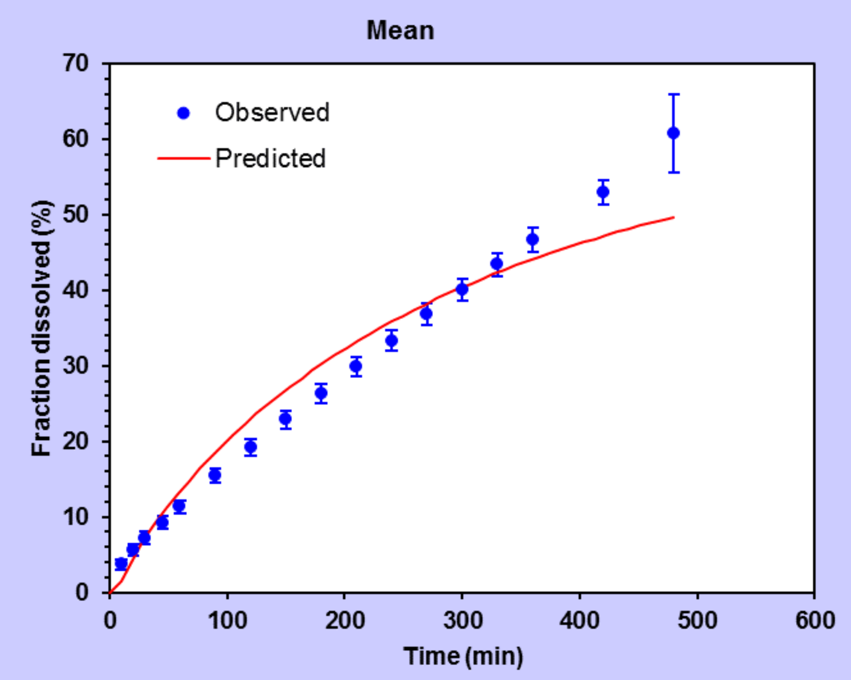

Supplementary materials – Model fitting summary – Glucidex® 19D (modelling up to app. 60 % of carvedilol released)

Graphical abstract of model fit presented as the fraction % of released carvedilol per tested tablet:

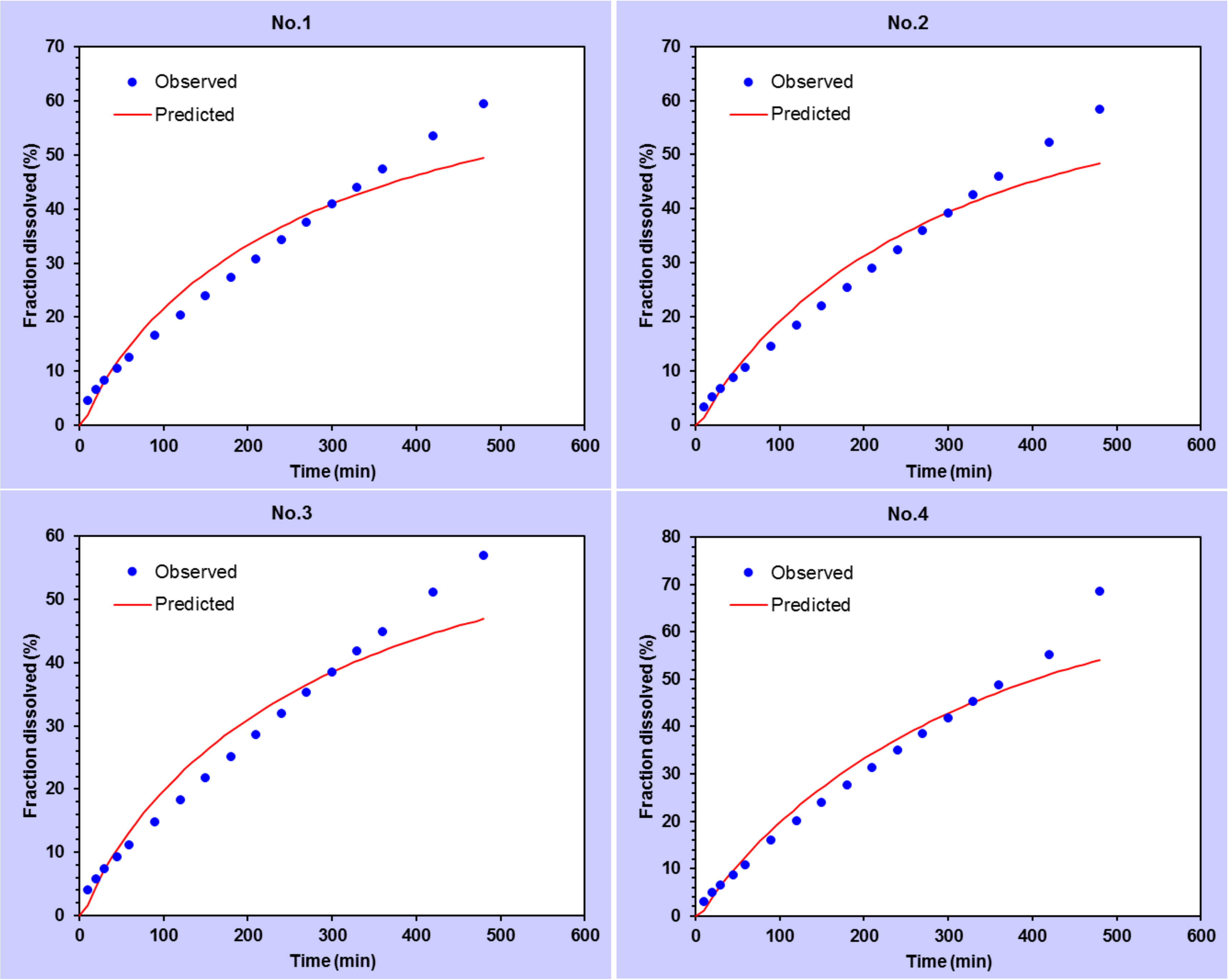

Supplementary materials – Model fitting summary – Glucidex® 19D (modelling up to app. 60 % of carvedilol released)

Model: **Logistic_1**

Model equation: $F = 100 \cdot \frac{e^{\alpha+\beta\cdot\log(t)}}{1+e^{\alpha+\beta\cdot\log(t)}}$

Fitted model parameters per tested tablet (N = 4) with statistics – mean, standard deviation (SD), and relative standard deviation expressed in % (RSD%) (output from DDSolver):

Parameter	No.1	No.2	No.3	No.4	Mean	SD	RSD(%)
α	-5.382	-5.862	-5.564	-6.204	-5.753	0.360	-6.260
β	2.018	2.186	2.051	2.388	2.161	0.168	7.771

Number of dissolution data points (N), degrees of freedom (df), and selected goodness of fit criteria – Pearson correlation coefficient (R), coefficient of determination (R^2), adjusted coefficient of determination ($R^2_{adjusted}$), and residual sum of squares (RSS) (manual calculation in MS Excel):

Parameter	No.1	No.2	No.3	No.4
N	17	17	17	17
df	15	15	15	15
R	0.985945026	0.988100415	0.98562822	0.982553598
R^2	0.972087593	0.976342431	0.971462987	0.965411573
$R^2_{adjusted}$	0.970226766	0.974765259	0.96956052	0.963105678
RSS	167.0150618	151.6121012	168.3813879	246.0187738

Graphical abstract of model fit presented as mean ± 1 SD of the fraction % of released carvedilol:

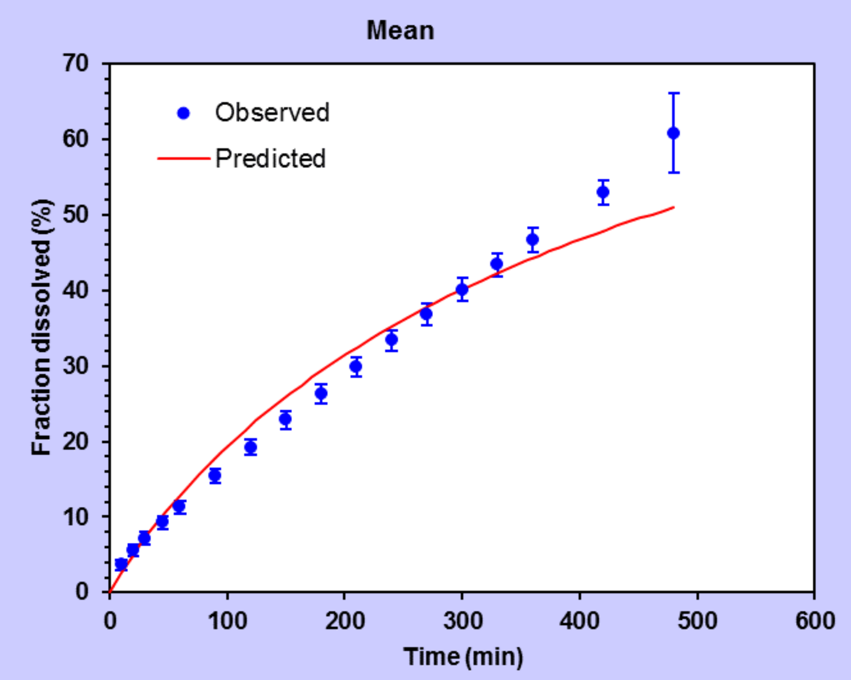

Supplementary materials – Model fitting summary – Glucidex® 19D (modelling up to app. 60 % of carvedilol released)

Graphical abstract of model fit presented as the fraction % of released carvedilol per tested tablet:

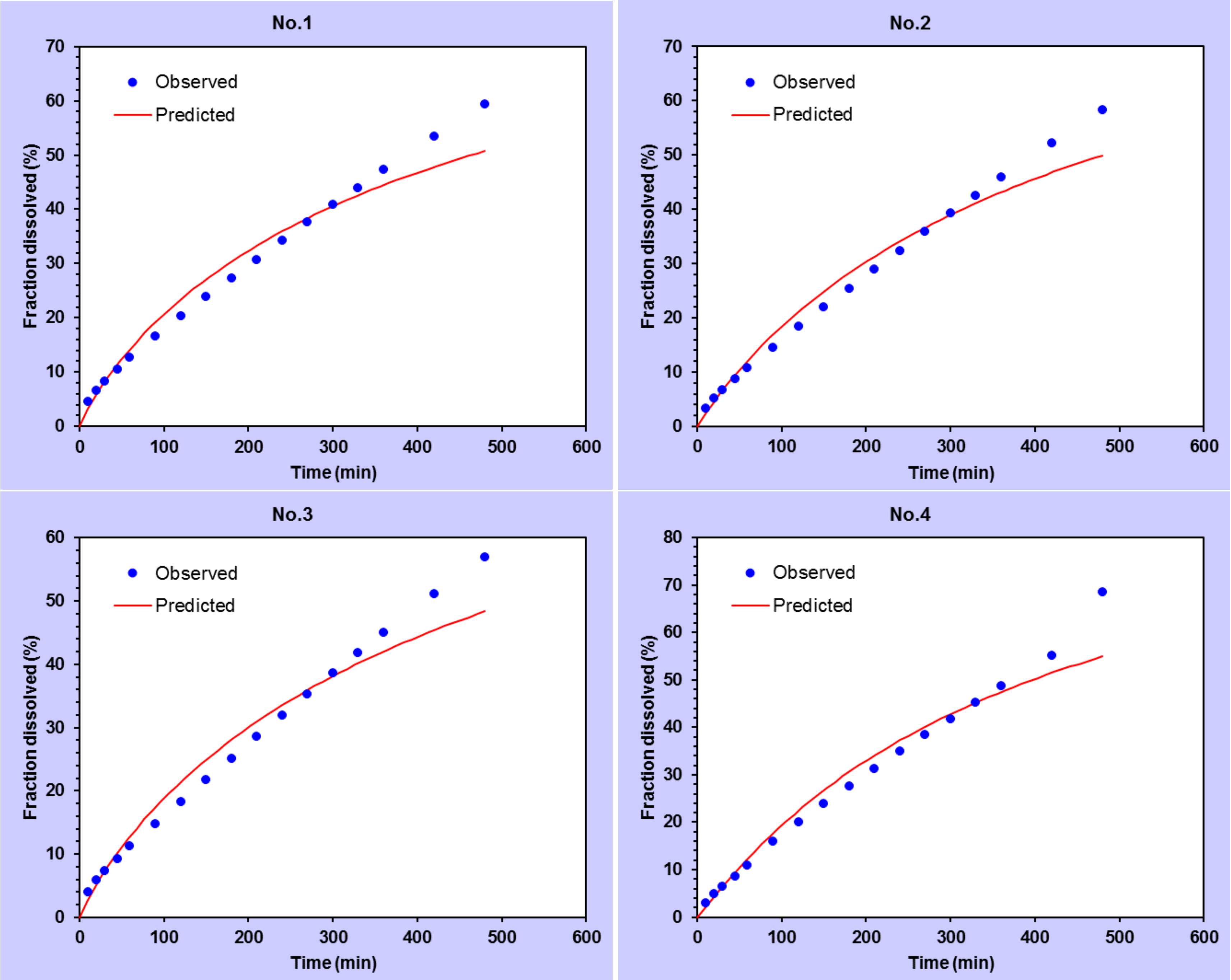

Supplementary materials – Model fitting summary – Glucidex® 19D (modelling up to app. 60 % of carvedilol released)

Model: **Logistic_2**

Model equation: $F = F_{max} \cdot \frac{e^{\alpha+\beta\cdot\log(t)}}{1+e^{\alpha+\beta\cdot\log(t)}}$

Fitted model parameters per tested tablet (N = 4) with statistics – mean, standard deviation (SD), and relative standard deviation expressed in % (RSD%) (output from DDSolver):

Parameter	No.1	No.2	No.3	No.4	Mean	SD	RSD(%)
α	-6.914	-7.472	-7.116	-6.685	-7.046	0.334	-4.734
β	2.930	3.116	2.985	2.915	2.986	0.091	3.057
F_{max}	68.244	66.989	65.488	71.913	68.159	2.745	4.027

Number of dissolution data points (N), degrees of freedom (df), and selected goodness of fit criteria – Pearson correlation coefficient (R), coefficient of determination (R^2), adjusted coefficient of determination ($R^2_{adjusted}$), and residual sum of squares (RSS) (manual calculation in MS Excel):

Parameter	No.1	No.2	No.3	No.4
N	17	17	17	17
df	14	14	14	14
R	0.983296711	0.986394361	0.983077139	0.966576614
R^2	0.966872422	0.972973835	0.966440662	0.93427035
$R^2_{adjusted}$	0.962139911	0.969112954	0.96164647	0.9248804
RSS	224.7260529	243.7371335	232.4286437	443.6560735

Graphical abstract of model fit presented as mean ± 1 SD of the fraction % of released carvedilol:

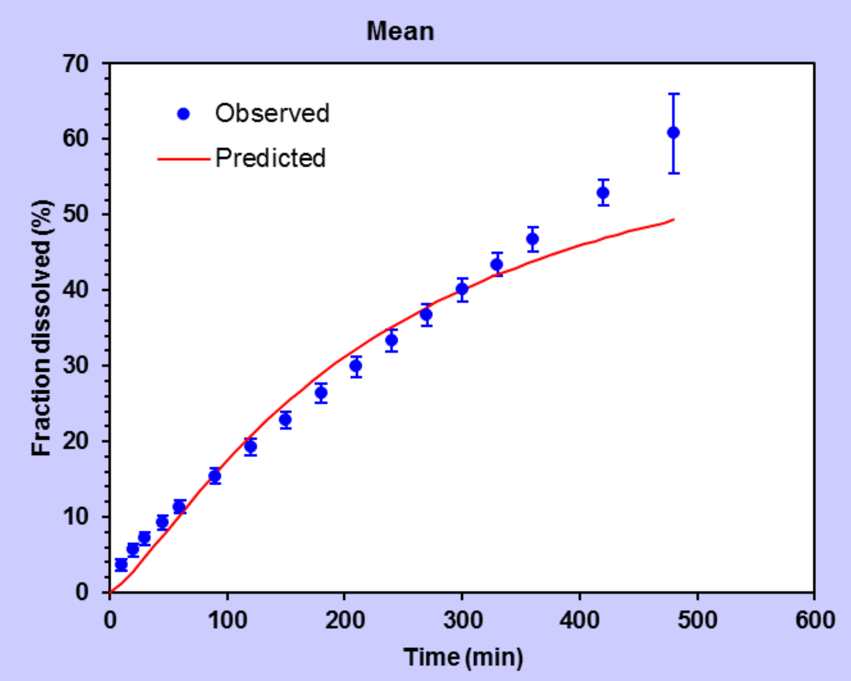

Supplementary materials – Model fitting summary – Glucidex® 19D (modelling up to app. 60 % of carvedilol released)

Graphical abstract of model fit presented as the fraction % of released carvedilol per tested tablet:

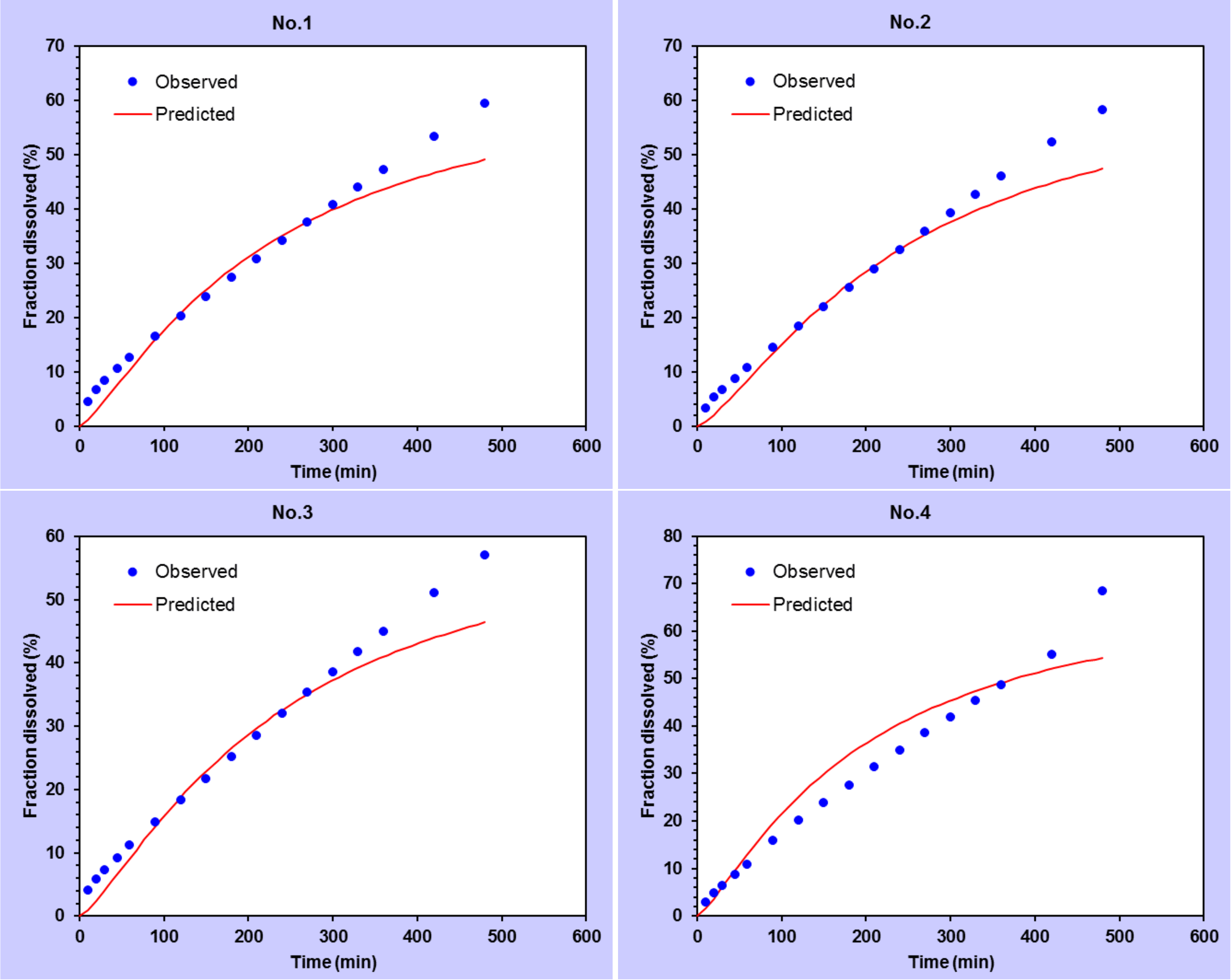

Supplementary materials – Model fitting summary – Glucidex® 19D (modelling up to app. 60 % of carvedilol released)

Model: **Logistic_3**

Model equation: $F = F_{max} \cdot \frac{1}{1+e^{-k\cdot(t-\gamma)}}$

Fitted model parameters per tested tablet (N = 4) with statistics – mean, standard deviation (SD), and relative standard deviation expressed in % (RSD%) (output from DDSolver):

Parameter	No.1	No.2	No.3	No.4	Mean	SD	RSD(%)
k	0.010	0.010	0.010	0.010	0.010	0.000	2.275
γ	218.778	228.853	224.910	250.575	230.779	13.833	5.994
F_{max}	62.325	61.178	59.808	71.913	63.806	5.502	8.623

Number of dissolution data points (N), degrees of freedom (df), and selected goodness of fit criteria – Pearson correlation coefficient (R), coefficient of determination (R^2), adjusted coefficient of determination ($R^2_{adjusted}$), and residual sum of squares (RSS) (manual calculation in MS Excel):

Parameter	No.1	No.2	No.3	No.4
N	17	17	17	17
df	14	14	14	14
R	0.993689808	0.993484214	0.994423461	0.985732895
R^2	0.987419435	0.987010883	0.98887802	0.97166934
$R^2_{adjusted}$	0.985622212	0.985155295	0.987289165	0.967622103
RSS	69.82411865	74.83076277	59.13918783	212.9838502

Graphical abstract of model fit presented as mean ± 1 SD of the fraction % of released carvedilol:

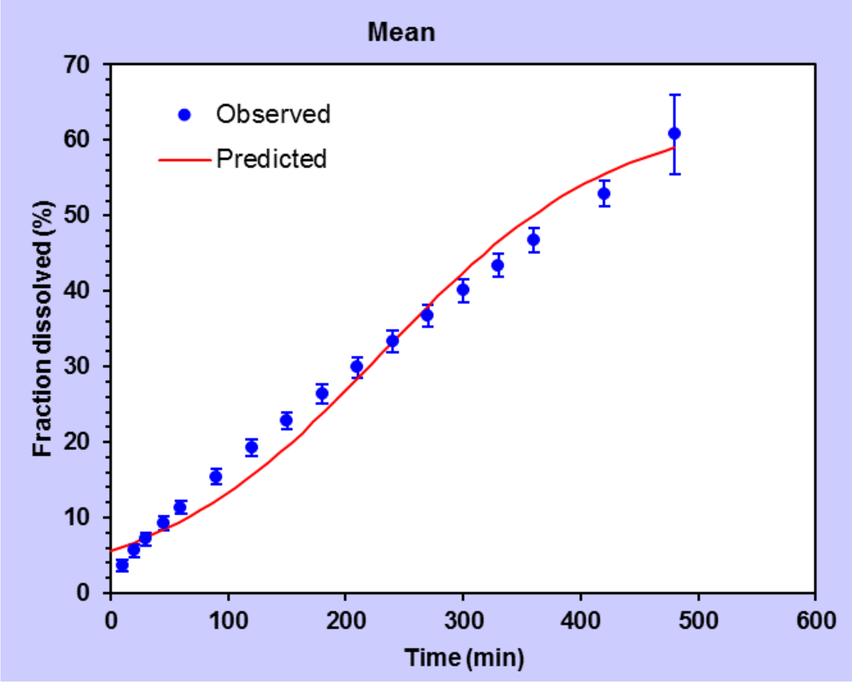

Supplementary materials – Model fitting summary – Glucidex® 19D (modelling up to app. 60 % of carvedilol released)

Graphical abstract of model fit presented as the fraction % of released carvedilol per tested tablet:

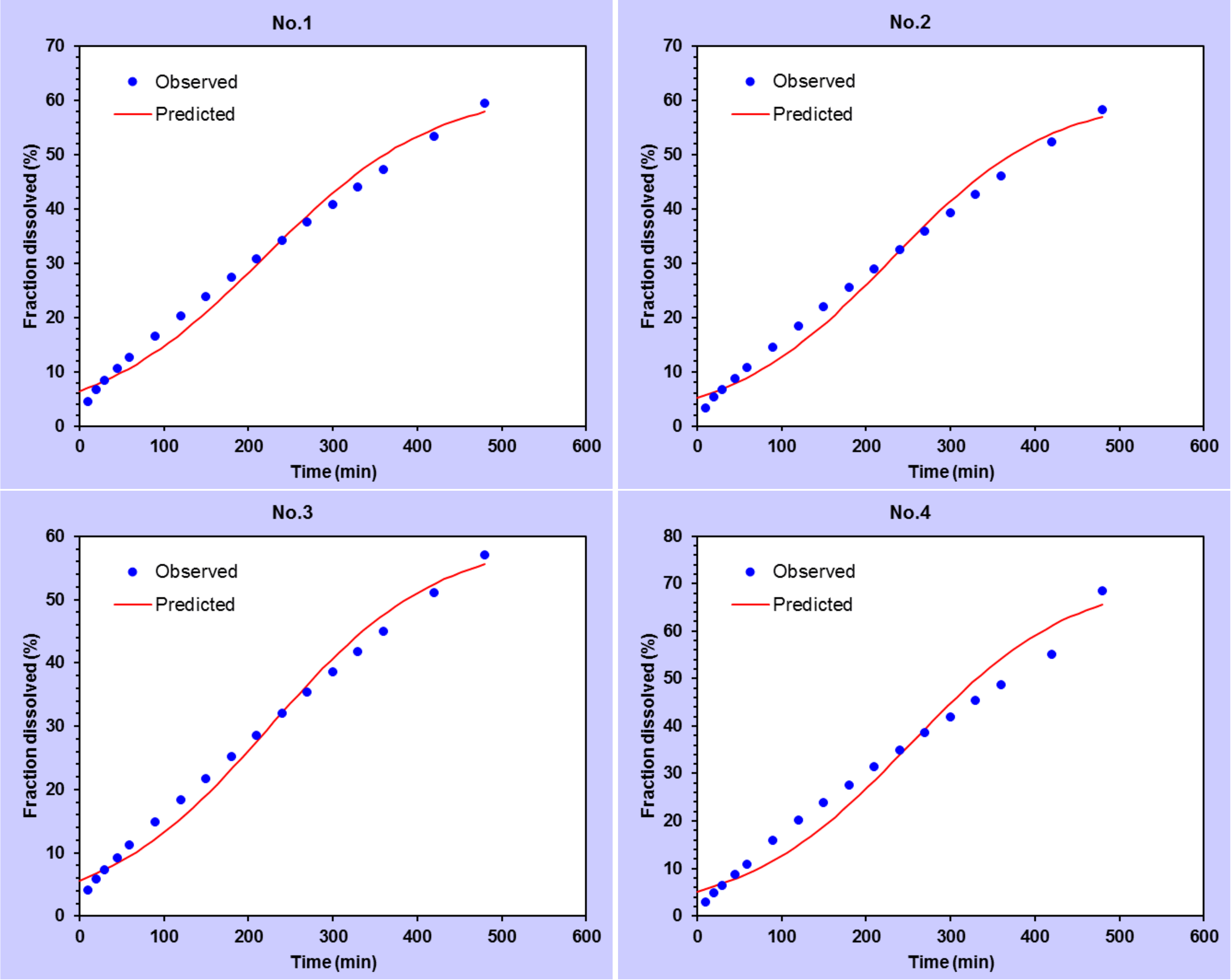

Supplementary materials – Model fitting summary – Glucidex® 19D (modelling up to app. 60 % of carvedilol released)

Model: **Gompertz_1**

Model equation: $F = 100 \cdot e^{-\alpha \cdot e^{-\beta \cdot \log(t)}}$

Fitted model parameters per tested tablet (N = 4) with statistics – mean, standard deviation (SD), and relative standard deviation expressed in % (RSD%) (output from DDSolver):

Parameter	No.1	No.2	No.3	No.4	Mean	SD	RSD(%)
α	11.298	12.995	11.607	15.799	12.925	2.053	15.887
β	1.022	1.061	1.007	1.182	1.068	0.080	7.447

Number of dissolution data points (N), degrees of freedom (df), and selected goodness of fit criteria – Pearson correlation coefficient (R), coefficient of determination (R^2), adjusted coefficient of determination ($R^2_{adjusted}$), and residual sum of squares (RSS) (manual calculation in MS Excel):

Parameter	No.1	No.2	No.3	No.4
N	17	17	17	17
df	15	15	15	15
R	0.964847875	0.966243558	0.963528545	0.959229928
R^2	0.930931422	0.933626614	0.928387257	0.920122055
$R^2_{adjusted}$	0.92632685	0.929201721	0.923613074	0.914796859
RSS	359.9721918	362.6620874	358.2735665	531.1112979

Graphical abstract of model fit presented as mean ± 1 SD of the fraction % of released carvedilol:

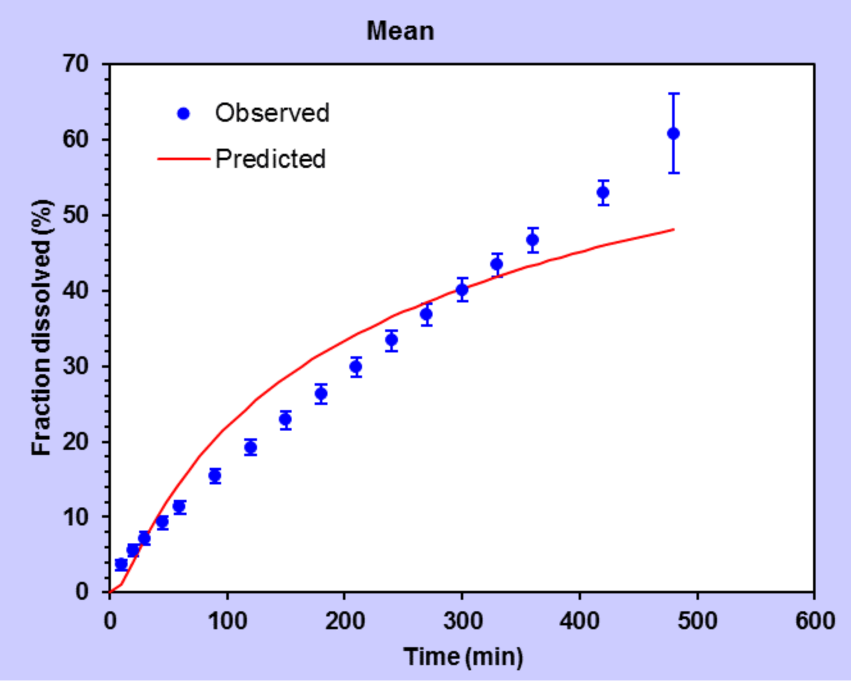

Supplementary materials – Model fitting summary – Glucidex® 19D (modelling up to app. 60 % of carvedilol released)

Graphical abstract of model fit presented as the fraction % of released carvedilol per tested tablet:

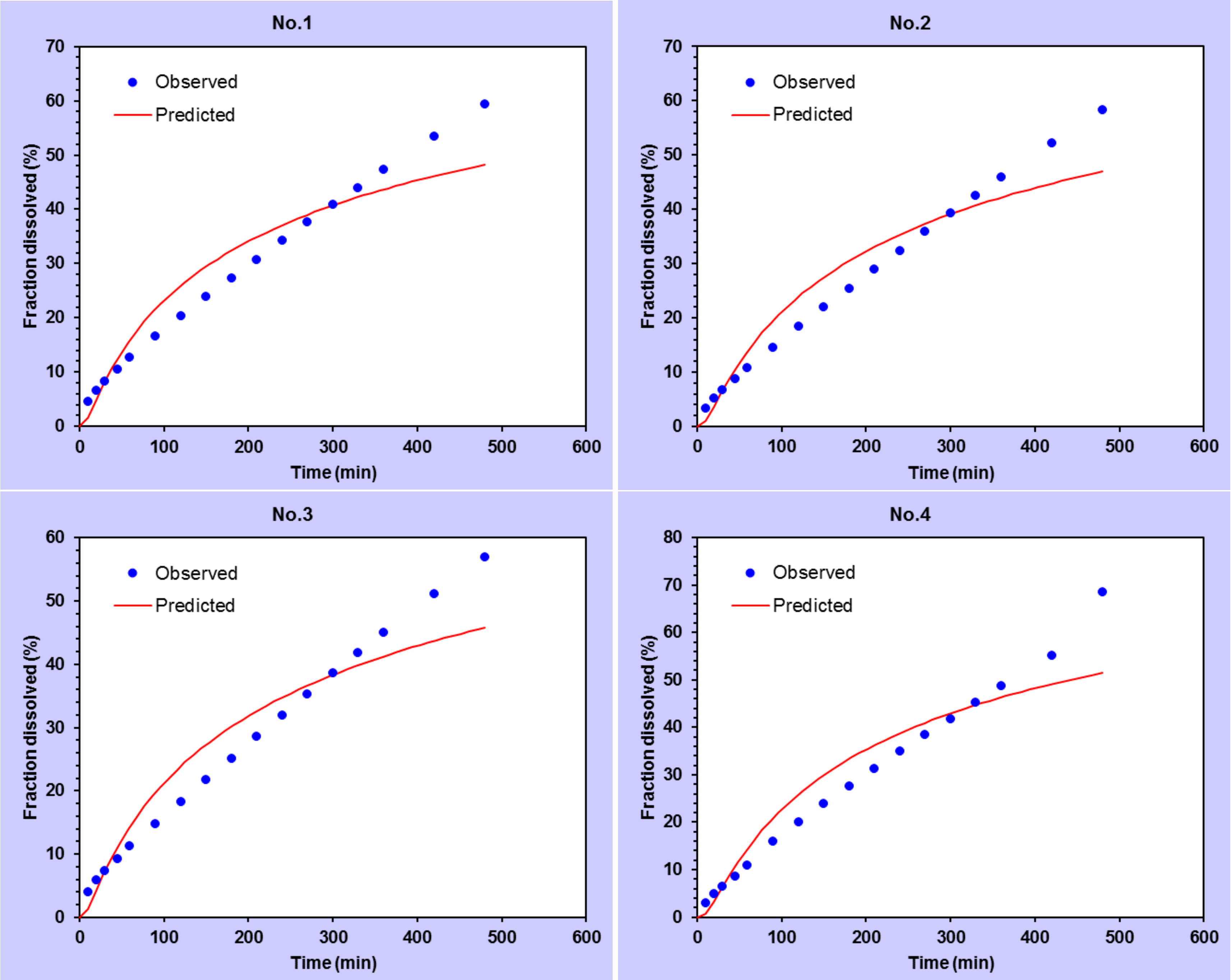

Supplementary materials – Model fitting summary – Glucidex® 19D (modelling up to app. 60 % of carvedilol released)

Model: **Gompertz_2**

Model equation: $F = F_{max} \cdot e^{-\alpha \cdot e^{-\beta \cdot \log(t)}}$

Fitted model parameters per tested tablet (N = 4) with statistics – mean, standard deviation (SD), and relative standard deviation expressed in % (RSD%) (output from DDSolver):

Parameter	No.1	No.2	No.3	No.4	Mean	SD	RSD(%)
α	46.953	55.831	49.838	34.224	46.711	9.109	19.501
β	1.808	1.861	1.818	1.754	1.810	0.044	2.433
F_{max}	62.325	61.178	59.808	71.913	63.806	5.502	8.623

Number of dissolution data points (N), degrees of freedom (df), and selected goodness of fit criteria – Pearson correlation coefficient (R), coefficient of determination (R^2), adjusted coefficient of determination ($R^2_{adjusted}$), and residual sum of squares (RSS) (manual calculation in MS Excel):

Parameter	No.1	No.2	No.3	No.4
N	17	17	17	17
df	14	14	14	14
R	0.96544994	0.966861826	0.963760116	0.93803262
R^2	0.932093587	0.934821791	0.928833562	0.879905196
$R^2_{adjusted}$	0.922392671	0.925510619	0.918666928	0.862748795
RSS	656.835199	630.5433384	623.470079	851.2217381

Graphical abstract of model fit presented as mean ± 1 SD of the fraction % of released carvedilol:

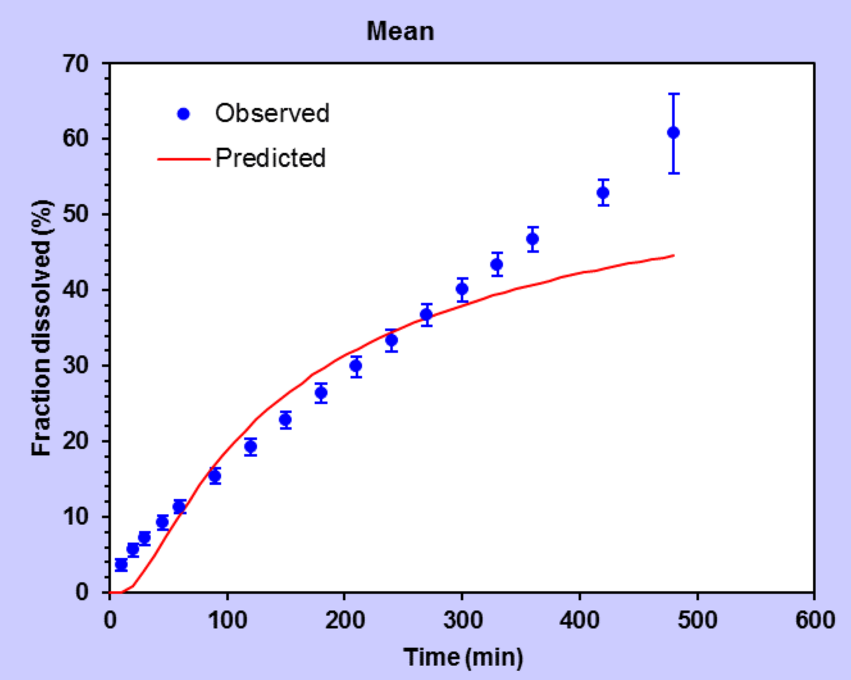

Supplementary materials – Model fitting summary – Glucidex® 19D (modelling up to app. 60 % of carvedilol released)

Graphical abstract of model fit presented as the fraction % of released carvedilol per tested tablet:

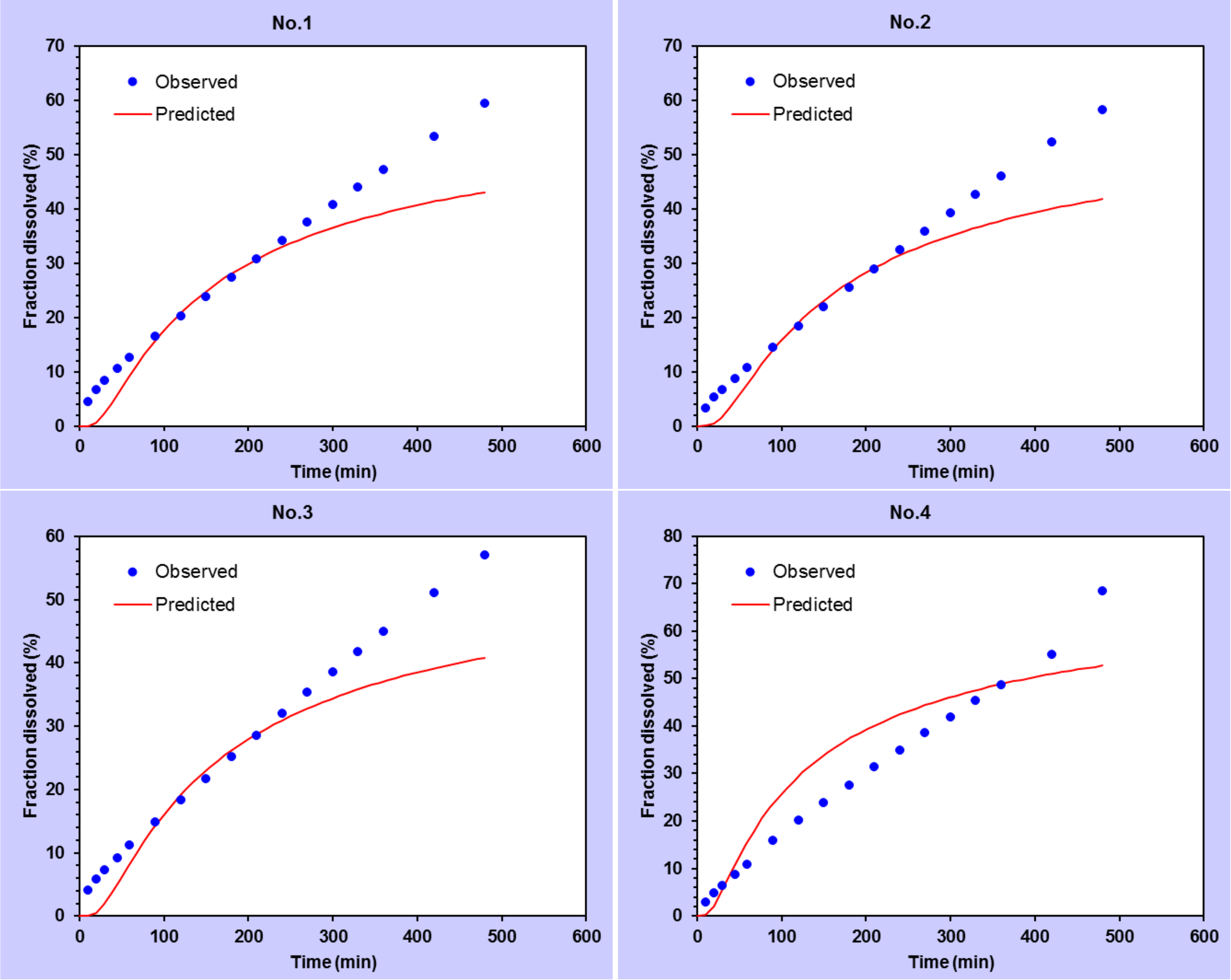

Supplementary materials – Model fitting summary – Glucidex® 19D (modelling up to app. 60 % of carvedilol released)

Model: **Gompertz_3**

Model equation: $F = F_{max} \cdot e^{-e^{-k\cdot(t-\gamma)}}$

Fitted model parameters per tested tablet (N = 4) with statistics – mean, standard deviation (SD), and relative standard deviation expressed in % (RSD%) (output from DDSolver):

Parameter	No.1	No.2	No.3	No.4	Mean	SD	RSD(%)
k	0.007	0.007	0.007	0.007	0.007	0.000	2.726
γ	144.965	154.960	150.969	173.254	156.037	12.191	7.813
F_{max}	62.325	61.178	59.808	71.913	63.806	5.502	8.623

Number of dissolution data points (N), degrees of freedom (df), and selected goodness of fit criteria – Pearson correlation coefficient (R), coefficient of determination (R^2), adjusted coefficient of determination ($R^2_{adjusted}$), and residual sum of squares (RSS) (manual calculation in MS Excel):

Parameter	No.1	No.2	No.3	No.4
N	17	17	17	17
df	14	14	14	14
R	0.993737588	0.993768536	0.993559728	0.988671903
R^2	0.987514393	0.987575903	0.987160933	0.977472132
$R^2_{adjusted}$	0.985730735	0.985801032	0.98532678	0.974253865
RSS	86.13107801	86.55547514	83.91155666	193.0521589

Graphical abstract of model fit presented as mean ± 1 SD of the fraction % of released carvedilol:

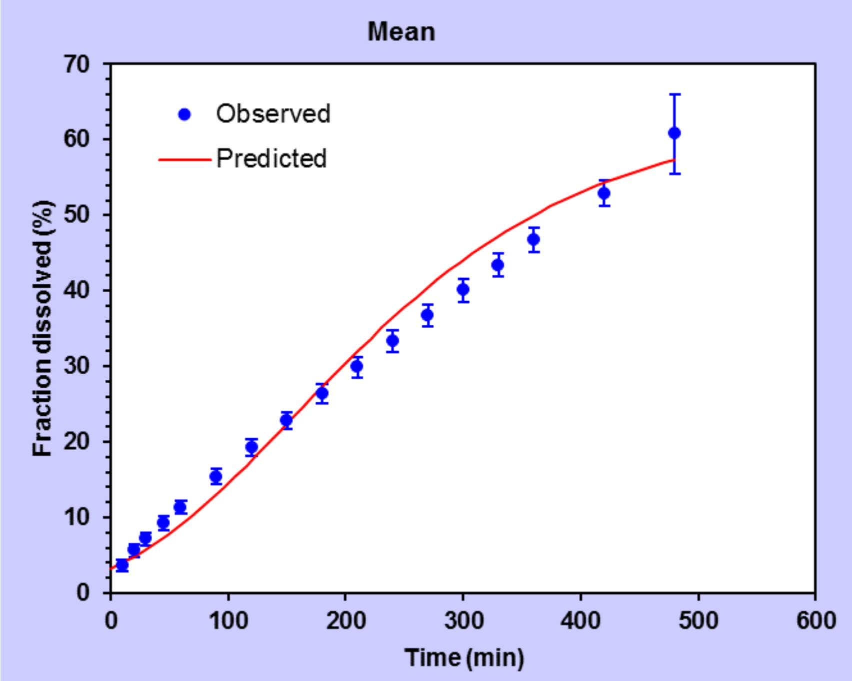

Supplementary materials – Model fitting summary – Glucidex® 19D (modelling up to app. 60 % of carvedilol released)

Graphical abstract of model fit presented as the fraction % of released carvedilol per tested tablet:

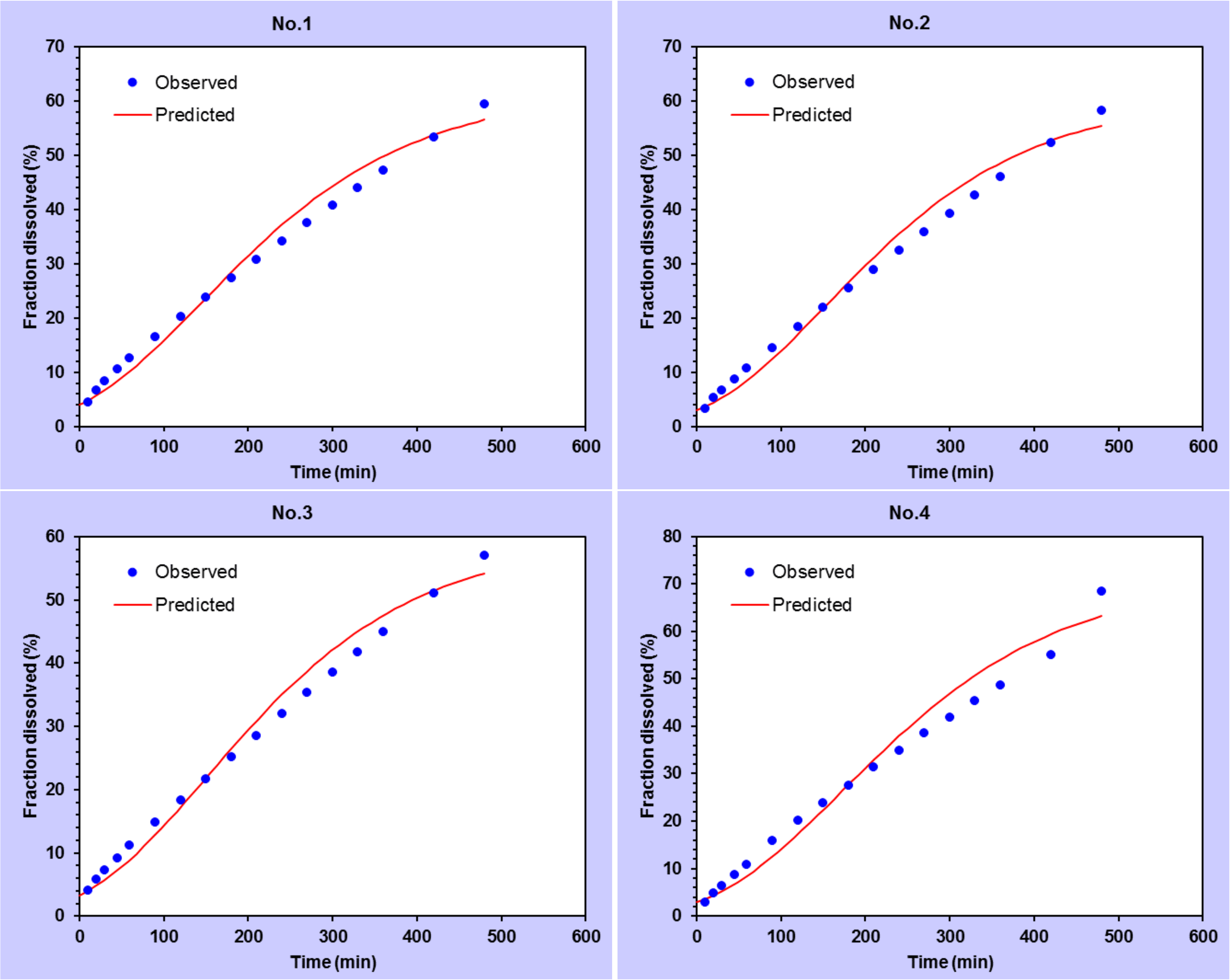

Supplementary materials – Model fitting summary – Glucidex® 19D (modelling up to app. 60 % of carvedilol released)

Model: **Gompertz_4**

Model equation: $F = F_{max} \cdot e^{-\beta \cdot e^{-k \cdot t}}$

Fitted model parameters per tested tablet (N = 4) with statistics – mean, standard deviation (SD), and relative standard deviation expressed in % (RSD%) (output from DDSolver):

Parameter	No.1	No.2	No.3	No.4	Mean	SD	RSD(%)
k	0.007	0.007	0.007	0.007	0.007	0.000	2.726
β	2.744	3.032	2.892	3.197	2.966	0.194	6.532
F_{max}	62.325	61.178	59.808	71.913	63.806	5.502	8.623

Number of dissolution data points (N), degrees of freedom (df), and selected goodness of fit criteria – Pearson correlation coefficient (R), coefficient of determination (R^2), adjusted coefficient of determination ($R^2_{adjusted}$), and residual sum of squares (RSS) (manual calculation in MS Excel):

Parameter	No.1	No.2	No.3	No.4
N	17	17	17	17
df	14	14	14	14
R	0.993737588	0.993768536	0.993559728	0.988671903
R^2	0.987514393	0.987575903	0.987160933	0.977472132
$R^2_{adjusted}$	0.985730735	0.985801032	0.98532678	0.974253865
RSS	86.13107801	86.55547514	83.91155666	193.0521589

Graphical abstract of model fit presented as mean ± 1 SD of the fraction % of released carvedilol:

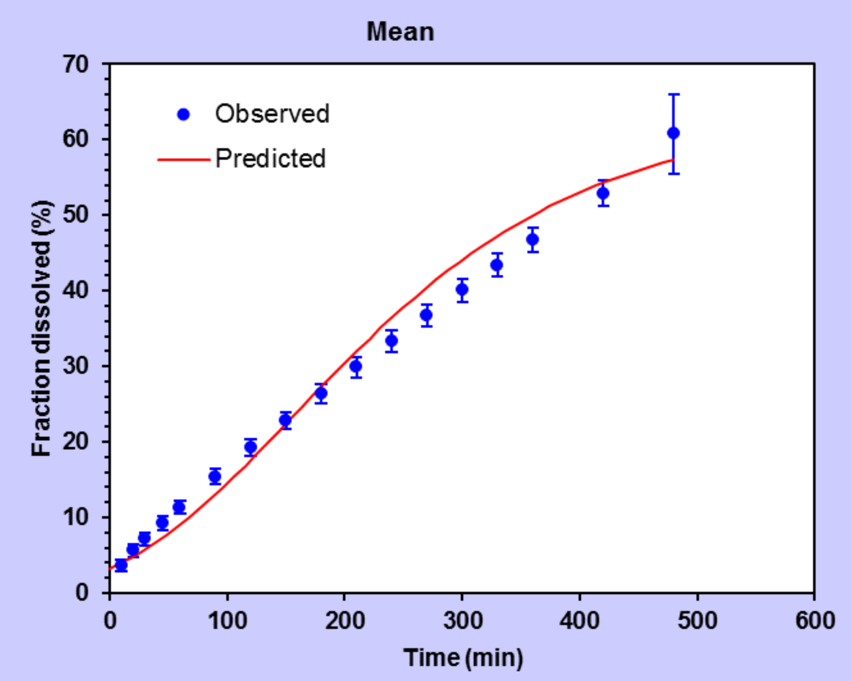

Supplementary materials – Model fitting summary – Glucidex® 19D (modelling up to app. 60 % of carvedilol released)

Graphical abstract of model fit presented as the fraction % of released carvedilol per tested tablet:

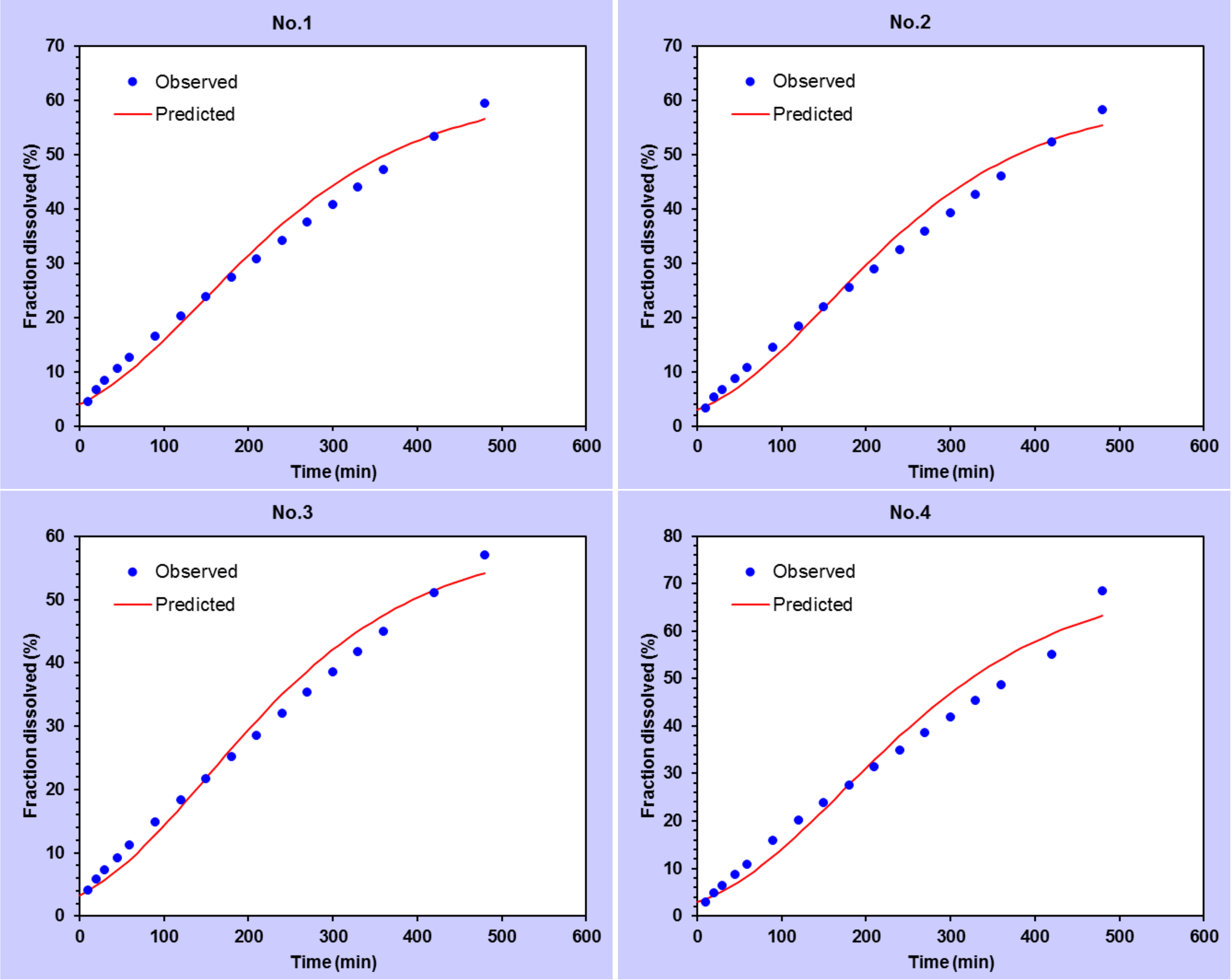

Supplementary materials – Model fitting summary – Glucidex® 19D (modelling up to app. 60 % of carvedilol released)

Model: **Probit_1**

Model equation: $F = 100 \cdot \phi[\alpha + \beta \cdot \log(t)]$

Fitted model parameters per tested tablet (N = 4) with statistics – mean, standard deviation (SD), and relative standard deviation expressed in % (RSD%) (output from DDSolver):

Parameter	No.1	No.2	No.3	No.4	Mean	SD	RSD(%)
α	-3.386	-3.628	-3.468	-3.470	-3.488	0.101	-2.901
β	1.307	1.390	1.313	1.324	1.334	0.038	2.856

Number of dissolution data points (N), degrees of freedom (df), and selected goodness of fit criteria – Pearson correlation coefficient (R), coefficient of determination (R^2), adjusted coefficient of determination ($R^2_{adjusted}$), and residual sum of squares (RSS) (manual calculation in MS Excel):

Parameter	No.1	No.2	No.3	No.4
N	17	17	17	17
df	15	15	15	15
R	0.9818452	0.983601082	0.981401183	0.975357856
R^2	0.964019996	0.967471088	0.963148282	0.951322947
$R^2_{adjusted}$	0.961621329	0.965302494	0.960691501	0.94807781
RSS	234.8233341	220.4842636	210.6265642	346.6759632

Graphical abstract of model fit presented as mean ± 1 SD of the fraction % of released carvedilol:

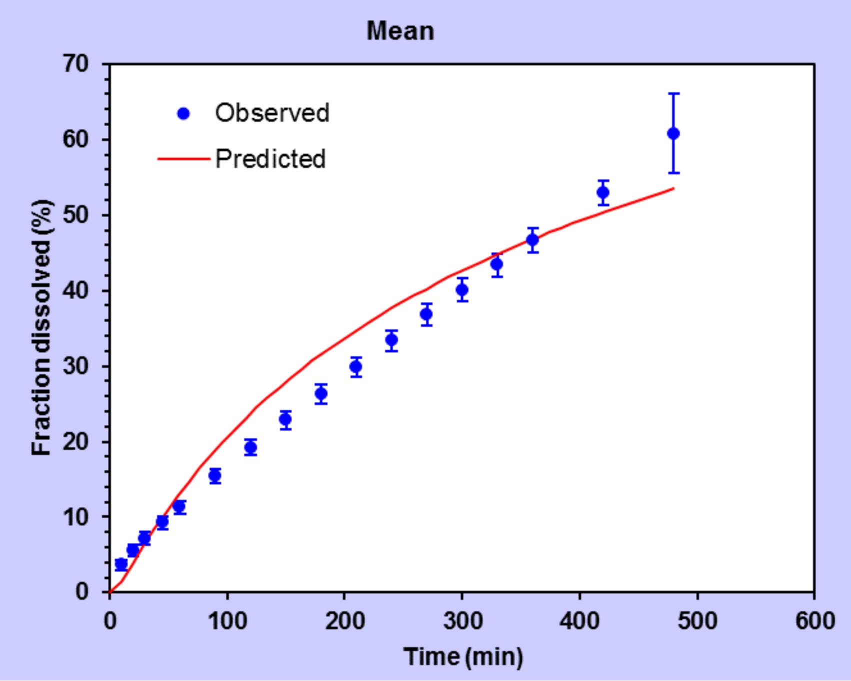

Supplementary materials – Model fitting summary – Glucidex® 19D (modelling up to app. 60 % of carvedilol released)

Graphical abstract of model fit presented as the fraction % of released carvedilol per tested tablet:

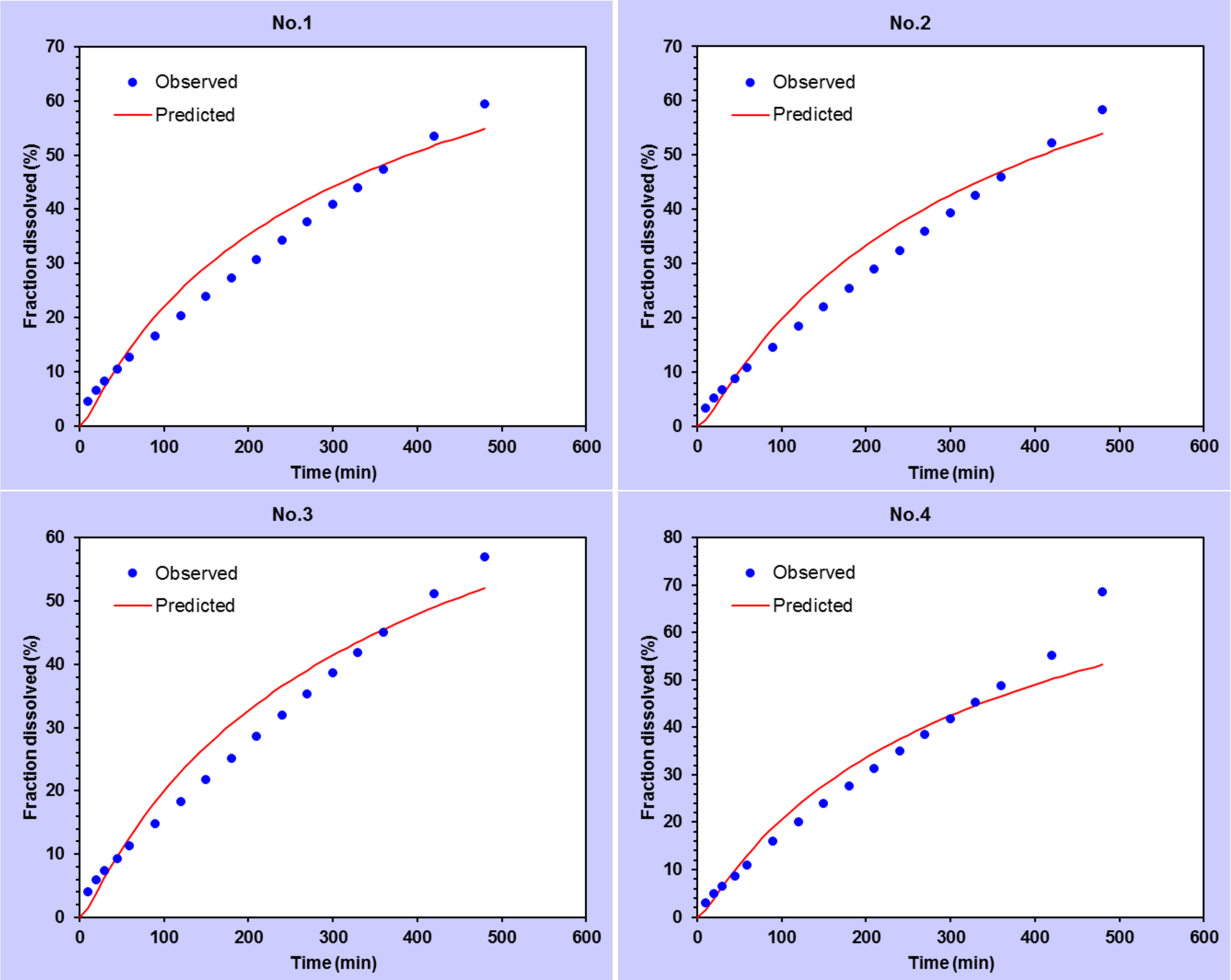

Supplementary materials – Model fitting summary – Glucidex® 19D (modelling up to app. 60 % of carvedilol released)

Model: **Probit_2**

Model equation: $F = F_{max} \cdot \phi[\alpha + \beta \cdot \log(t)]$

Fitted model parameters per tested tablet (N = 4) with statistics – mean, standard deviation (SD), and relative standard deviation expressed in % (RSD%) (output from DDSolver):

Parameter	No.1	No.2	No.3	No.4	Mean	SD	RSD(%)
α	-4.051	-4.332	-4.154	-4.156	-4.173	0.117	-2.797
β	1.714	1.805	1.740	1.733	1.748	0.039	2.252
F_{max}	68.244	66.989	65.488	72.606	68.332	3.064	4.484

Number of dissolution data points (N), degrees of freedom (df), and selected goodness of fit criteria – Pearson correlation coefficient (R), coefficient of determination (R^2), adjusted coefficient of determination ($R^2_{adjusted}$), and residual sum of squares (RSS) (manual calculation in MS Excel):

Parameter	No.1	No.2	No.3	No.4
N	17	17	17	17
df	14	14	14	14
R	0.979622447	0.982271742	0.978853185	0.973627362
R^2	0.959660139	0.964857775	0.958153557	0.94795024
$R^2_{adjusted}$	0.953897302	0.959837457	0.952175494	0.94051456
RSS	275.8680161	297.7408487	285.1960448	462.5105821

Graphical abstract of model fit presented as mean ± 1 SD of the fraction % of released carvedilol:

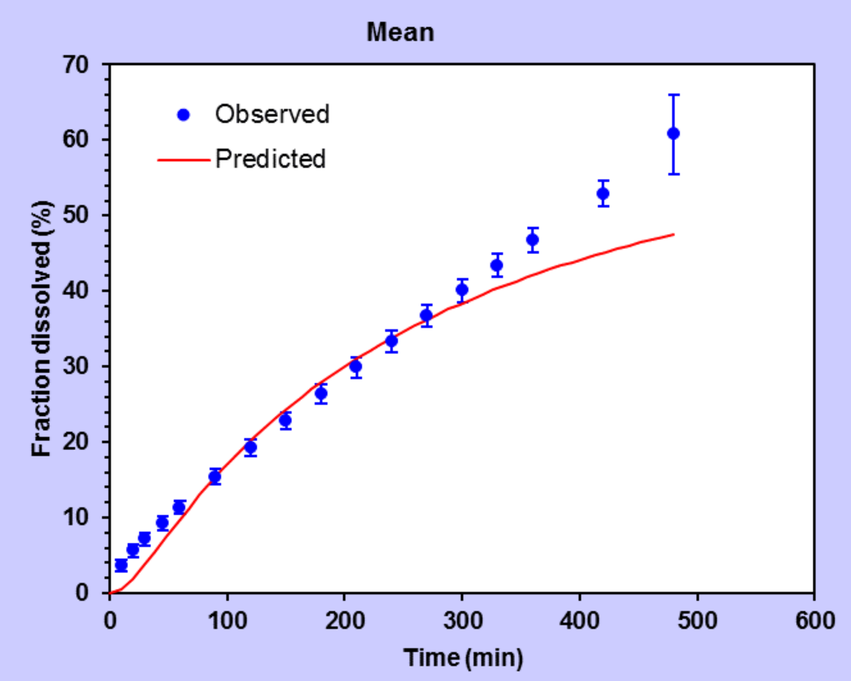

Supplementary materials – Model fitting summary – Glucidex® 19D (modelling up to app. 60 % of carvedilol released)

Graphical abstract of model fit presented as the fraction % of released carvedilol per tested tablet:

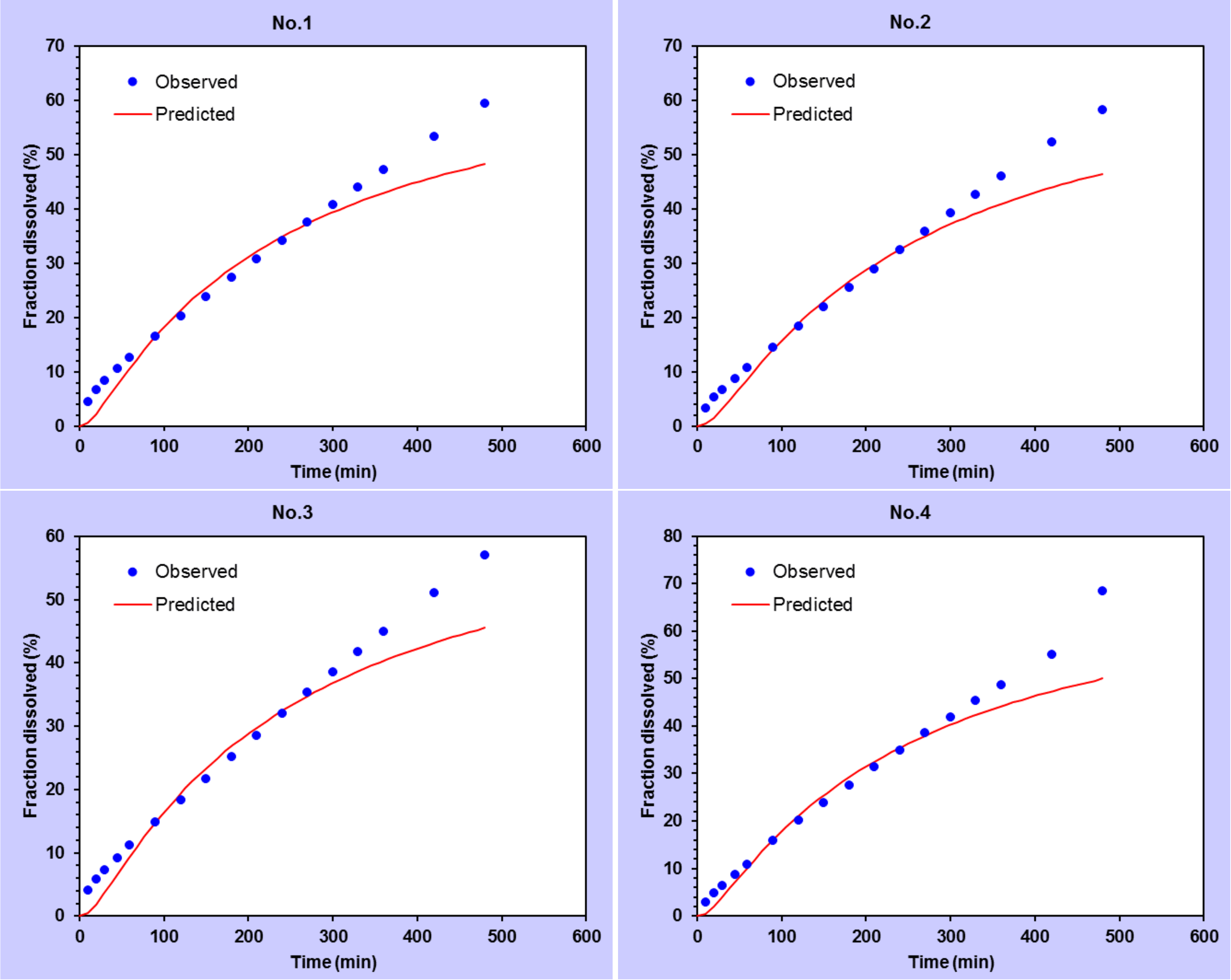

Supplement: Supplementary file 1 [file pharmaceutics-16-00498-s001.zip › Supplementary materials_Model fitting summary_Glucidex® 19D.pdf]
