# Supplementary material for: Comparative Fitting of Mathematical Models to Carvedilol Release Profiles Obtained from Hypromellose Matrix Tablets
Source: Pharmaceutics. 2024 Apr 4;16(4):498. doi: 10.3390/pharmaceutics16040498 (PMC11053526; doi:10.3390/pharmaceutics16040498)

Supplementary materials – Model fitting summary – Granulated sugar N°1 600 (modelling the entire relevant dissolution data)

Model: **Zero–order**

Model equation: $F = k_0 \cdot t$

Fitted model parameters per tested tablet (N = 4) with statistics – mean, standard deviation (SD), and relative standard deviation expressed in % (RSD%) (output from DDSolver):

Parameter	No.1	No.2	No.3	No.4	Mean	SD	RSD(%)
k_0	0.096	0.097	0.093	0.100	0.096	0.003	3.103

Number of dissolution data points (N), degrees of freedom (df), and selected goodness of fit criteria – Pearson correlation coefficient (R), coefficient of determination (R^2), adjusted coefficient of determination ($R^2_{adjusted}$), and residual sum of squares (RSS) (manual calculation in MS Excel):

Parameter	No.1	No.2	No.3	No.4
N	29	29	29	29
df	28	28	28	28
R	0.969965835	0.969424636	0.97259202	0.970295724
R^2	0.94083372	0.939784124	0.945935237	0.941473791
$R^2_{adjusted}$	0.94083372	0.939784124	0.945935237	0.941473791
RSS	3707.464981	3782.512122	3166.959062	4021.257071

Graphical abstract of model fit presented as mean ± 1 SD of the fraction % of released carvedilol:

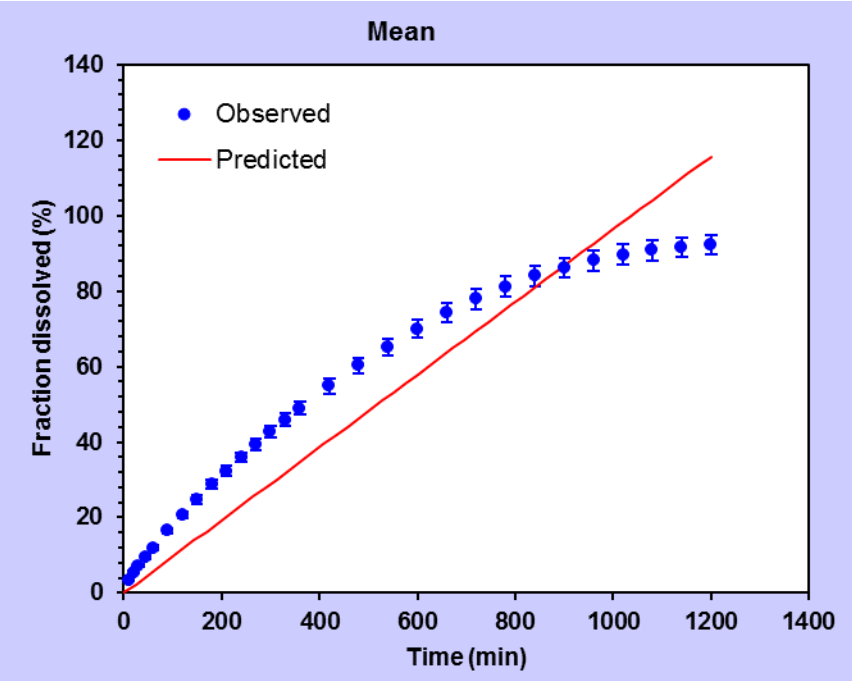

Supplementary materials – Model fitting summary – Granulated sugar N°1 600 (modelling the entire relevant dissolution data)

Graphical abstract of model fit presented as the fraction % of released carvedilol per tested tablet:

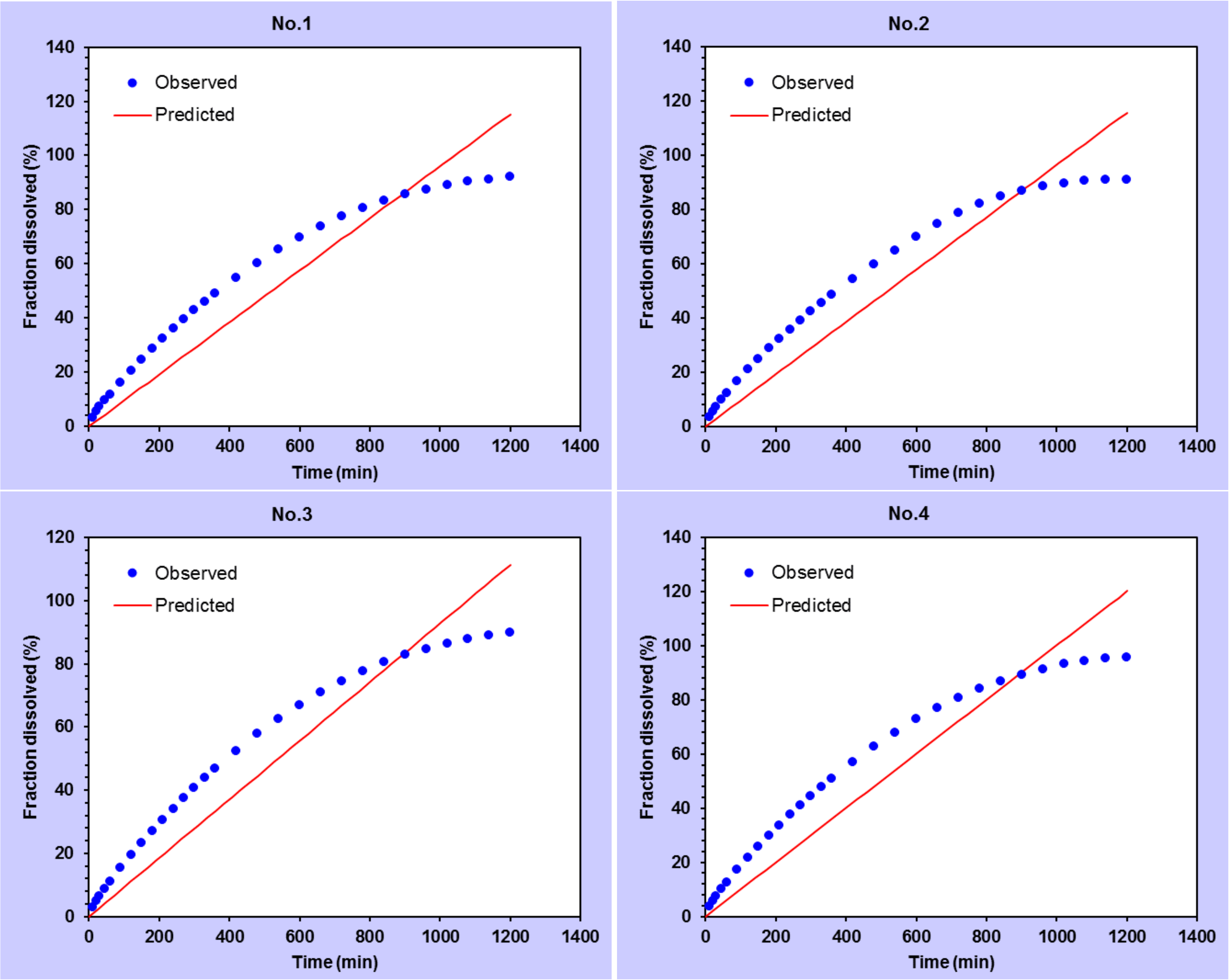

Supplementary materials – Model fitting summary – Granulated sugar N°1 600 (modelling the entire relevant dissolution data)

Model: **Zero–order with T_{lag}**

Model equation: $F = k_0 \cdot (t - T_{lag})$

Fitted model parameters per tested tablet (N = 4) with statistics – mean, standard deviation (SD), and relative standard deviation expressed in % (RSD%) (output from DDSolver):

Parameter	No.1	No.2	No.3	No.4	Mean	SD	RSD(%)
k_0	0.078	0.078	0.076	0.081	0.078	0.002	2.653
T_{lag}	-178.223	-178.515	-167.313	-178.687	-175.684	5.584	-3.178

Number of dissolution data points (N), degrees of freedom (df), and selected goodness of fit criteria – Pearson correlation coefficient (R), coefficient of determination (R^2), adjusted coefficient of determination ($R^2_{adjusted}$), and residual sum of squares (RSS) (manual calculation in MS Excel):

Parameter	No.1	No.2	No.3	No.4
N	29	29	29	29
df	27	27	27	27
R	0.969965835	0.969424636	0.97259202	0.970295724
R^2	0.94083372	0.939784124	0.945935237	0.941473791
$R^2_{adjusted}$	0.938642376	0.937553907	0.943932839	0.939306154
RSS	1555.867535	1601.592096	1352.819362	1671.15904

Graphical abstract of model fit presented as mean ± 1 SD of the fraction % of released carvedilol:

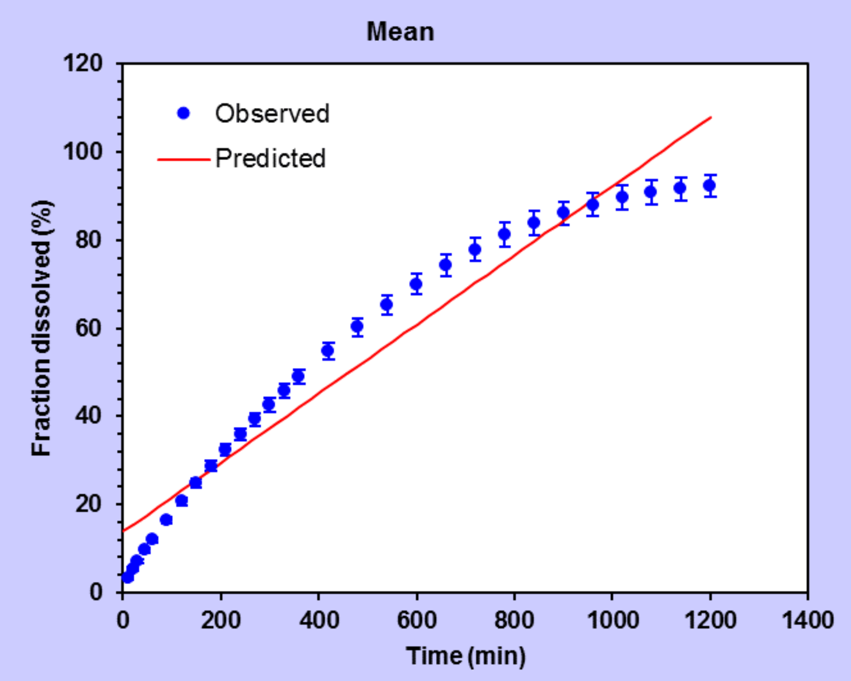

Supplementary materials – Model fitting summary – Granulated sugar N°1 600 (modelling the entire relevant dissolution data)

Graphical abstract of model fit presented as the fraction % of released carvedilol per tested tablet:

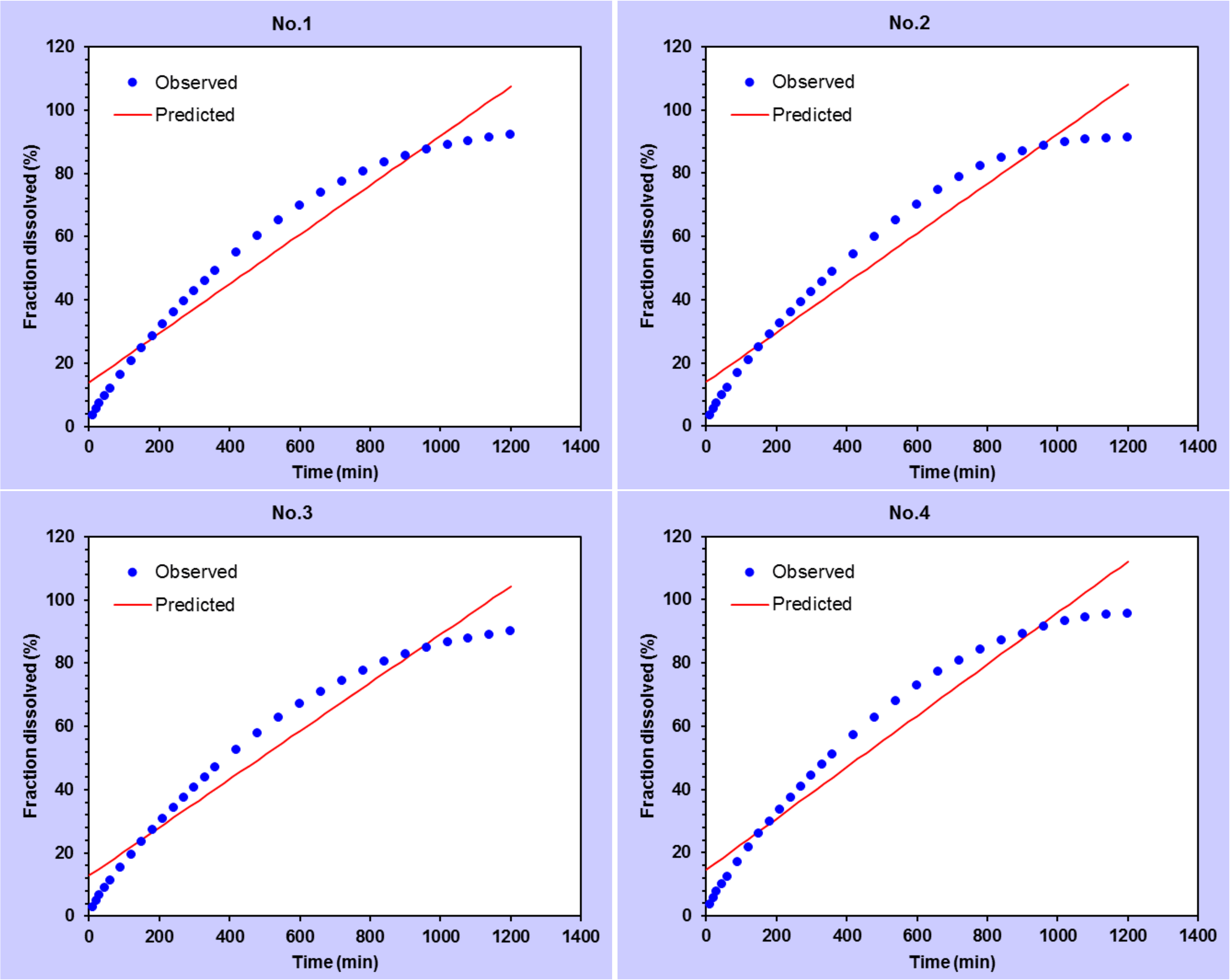

Supplementary materials – Model fitting summary – Granulated sugar N°1 600 (modelling the entire relevant dissolution data)

Model: **Zero–order with F_0**

Model equation: $F = F_0 + k_0 \cdot t$

Fitted model parameters per tested tablet (N = 4) with statistics – mean, standard deviation (SD), and relative standard deviation expressed in % (RSD%) (output from DDSolver):

Parameter	No.1	No.2	No.3	No.4	Mean	SD	RSD(%)
k_0	0.078	0.078	0.076	0.081	0.078	0.002	2.653
F_0	13.899	13.994	12.763	14.526	13.795	0.742	5.377

Number of dissolution data points (N), degrees of freedom (df), and selected goodness of fit criteria – Pearson correlation coefficient (R), coefficient of determination (R^2), adjusted coefficient of determination ($R^2_{adjusted}$), and residual sum of squares (RSS) (manual calculation in MS Excel):

Parameter	No.1	No.2	No.3	No.4
N	29	29	29	29
df	27	27	27	27
R	0.969965835	0.969424636	0.97259202	0.970295724
R^2	0.94083372	0.939784124	0.945935237	0.941473791
$R^2_{adjusted}$	0.938642376	0.937553907	0.943932839	0.939306154
RSS	1555.867535	1601.592096	1352.819362	1671.15904

Graphical abstract of model fit presented as mean ± 1 SD of the fraction % of released carvedilol:

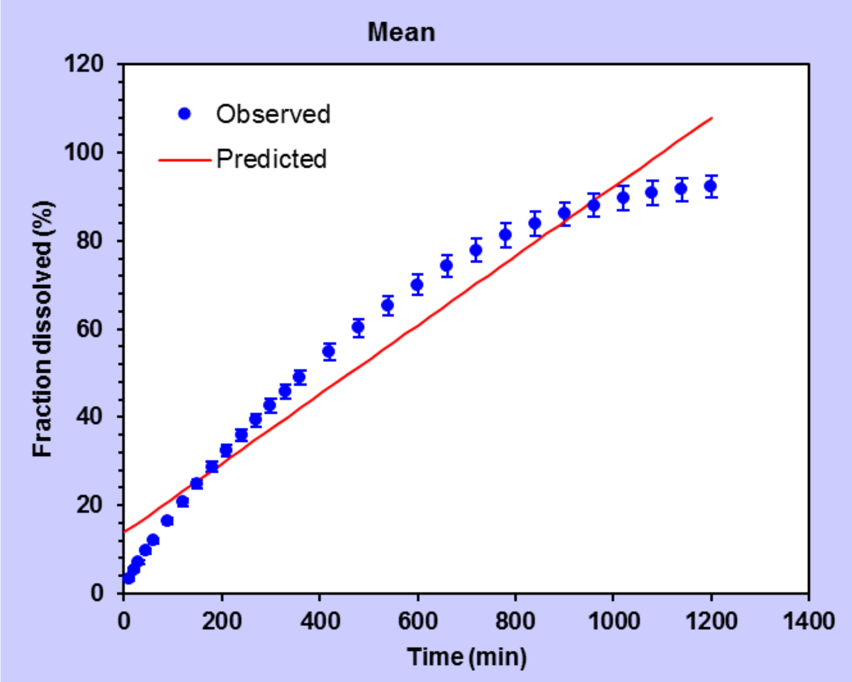

Supplementary materials – Model fitting summary – Granulated sugar N°1 600 (modelling the entire relevant dissolution data)

Graphical abstract of model fit presented as the fraction % of released carvedilol per tested tablet:

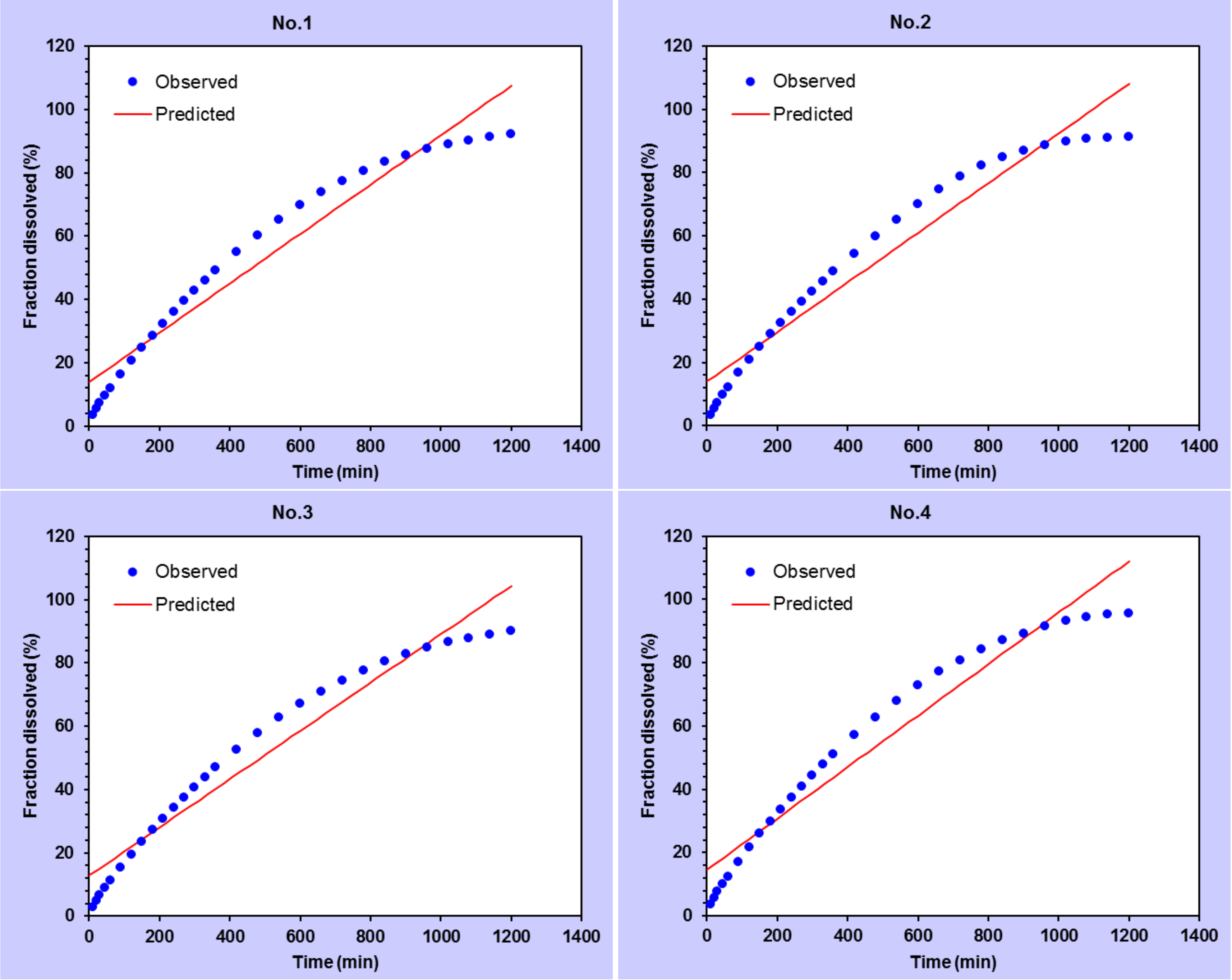

Supplementary materials – Model fitting summary – Granulated sugar N°1 600 (modelling the entire relevant dissolution data)

Model: **First–order**

Model equation: $F = 100 \cdot (1 - e^{-k_1 \cdot t})$

Fitted model parameters per tested tablet (N = 4) with statistics – mean, standard deviation (SD), and relative standard deviation expressed in % (RSD%) (output from DDSolver):

Parameter	No.1	No.2	No.3	No.4	Mean	SD	RSD(%)
k_1	0.002	0.002	0.002	0.002	0.002	0.000	8.291

Number of dissolution data points (N), degrees of freedom (df), and selected goodness of fit criteria – Pearson correlation coefficient (R), coefficient of determination (R^2), adjusted coefficient of determination ($R^2_{adjusted}$), and residual sum of squares (RSS) (manual calculation in MS Excel):

Parameter	No.1	No.2	No.3	No.4
N	29	29	29	29
df	28	28	28	28
R	0.998011669	0.996952388	0.999358606	0.997347463
R^2	0.996027292	0.993914063	0.998717624	0.994701963
$R^2_{adjusted}$	0.996027292	0.993914063	0.998717624	0.994701963
RSS	148.9493037	212.6171916	63.09555405	194.8054245

Graphical abstract of model fit presented as mean ± 1 SD of the fraction % of released carvedilol:

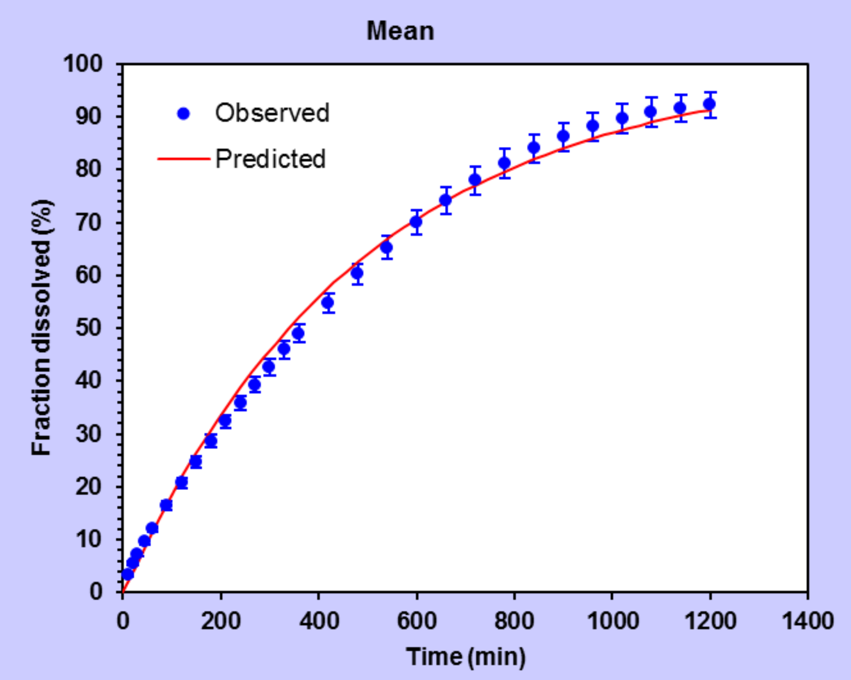

Supplementary materials – Model fitting summary – Granulated sugar N°1 600 (modelling the entire relevant dissolution data)

Graphical abstract of model fit presented as the fraction % of released carvedilol per tested tablet:

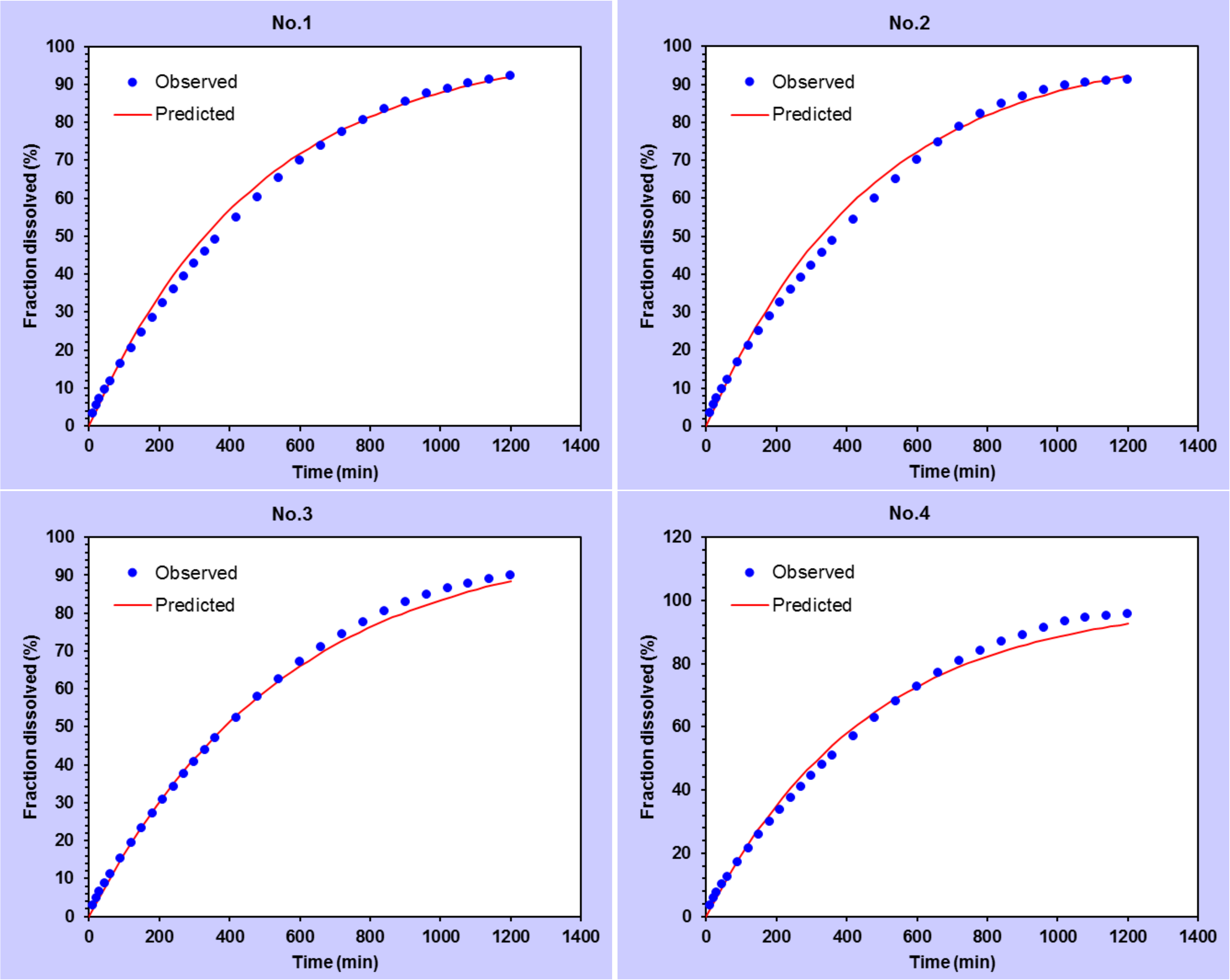

Supplementary materials – Model fitting summary – Granulated sugar N°1 600 (modelling the entire relevant dissolution data)

Model: **First–order with T_{lag}**

Model equation: $F = 100 \cdot \left[1 - e^{-k_1 \cdot (t - T_{lag})}\right]$

Fitted model parameters per tested tablet (N = 4) with statistics – mean, standard deviation (SD), and relative standard deviation expressed in % (RSD%) (output from DDSolver):

Parameter	No.1	No.2	No.3	No.4	Mean	SD	RSD(%)
k_1	0.002	0.002	0.002	0.003	0.002	0.000	12.754
T_{lag}	21.463	20.977	15.271	47.404	26.279	14.361	54.650

Number of dissolution data points (N), degrees of freedom (df), and selected goodness of fit criteria – Pearson correlation coefficient (R), coefficient of determination (R^2), adjusted coefficient of determination ($R^2_{adjusted}$), and residual sum of squares (RSS) (manual calculation in MS Excel):

Parameter	No.1	No.2	No.3	No.4
N	29	29	29	29
df	27	27	27	27
R	0.997542433	0.996432334	0.998561541	0.991854184
R^2	0.995090905	0.992877397	0.997125152	0.983774723
$R^2_{adjusted}$	0.994909086	0.992613597	0.997018676	0.983173787
RSS	196.3520184	250.0630322	99.70787816	984.9589761

Graphical abstract of model fit presented as mean ± 1 SD of the fraction % of released carvedilol:

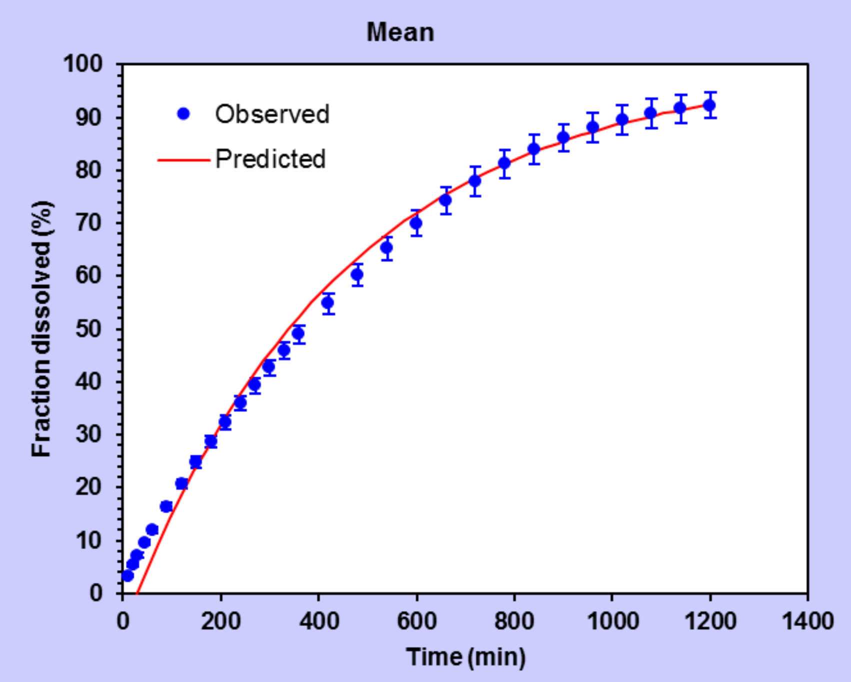

Supplementary materials – Model fitting summary – Granulated sugar N°1 600 (modelling the entire relevant dissolution data)

Graphical abstract of model fit presented as the fraction % of released carvedilol per tested tablet:

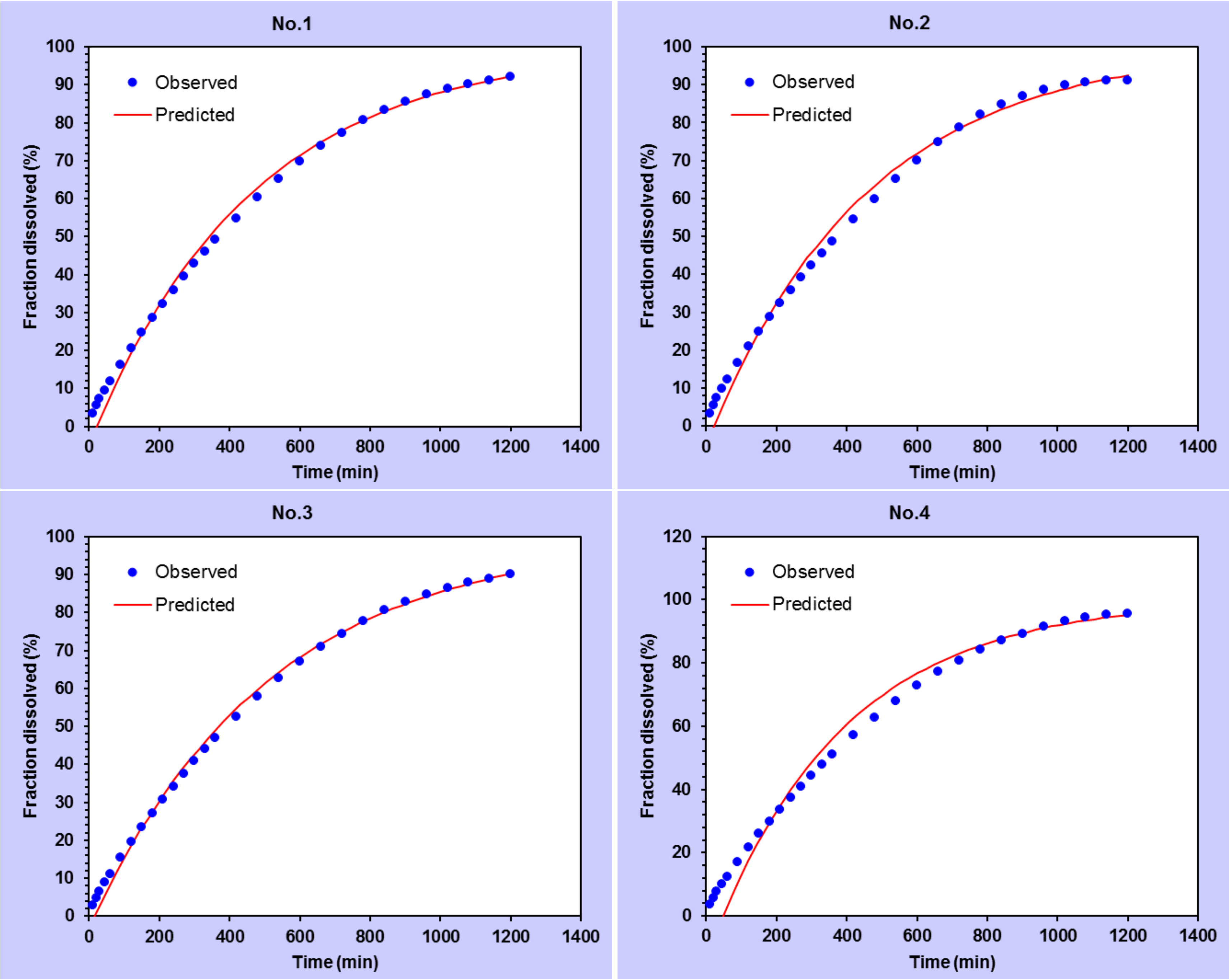

Supplementary materials – Model fitting summary – Granulated sugar N°1 600 (modelling the entire relevant dissolution data)

Model: **First–order with F_{max}**

Model equation: $F = F_{max} \cdot (1 - e^{-k_1 \cdot t})$

Fitted model parameters per tested tablet (N = 4) with statistics – mean, standard deviation (SD), and relative standard deviation expressed in % (RSD%) (output from DDSolver):

Parameter	No.1	No.2	No.3	No.4	Mean	SD	RSD(%)
k_1	0.002	0.003	0.002	0.002	0.002	0.000	3.600
F_{max}	96.850	95.810	94.561	100.491	96.928	2.553	2.634

Number of dissolution data points (N), degrees of freedom (df), and selected goodness of fit criteria – Pearson correlation coefficient (R), coefficient of determination (R^2), adjusted coefficient of determination ($R^2_{adjusted}$), and residual sum of squares (RSS) (manual calculation in MS Excel):

Parameter	No.1	No.2	No.3	No.4
N	29	29	29	29
df	27	27	27	27
R	0.995579524	0.992733396	0.995309008	0.994725358
R^2	0.991178589	0.985519596	0.990640022	0.989478537
$R^2_{adjusted}$	0.99085187	0.984983285	0.990293356	0.989088854
RSS	408.7618632	661.6196432	438.4628081	530.1037142

Graphical abstract of model fit presented as mean ± 1 SD of the fraction % of released carvedilol:

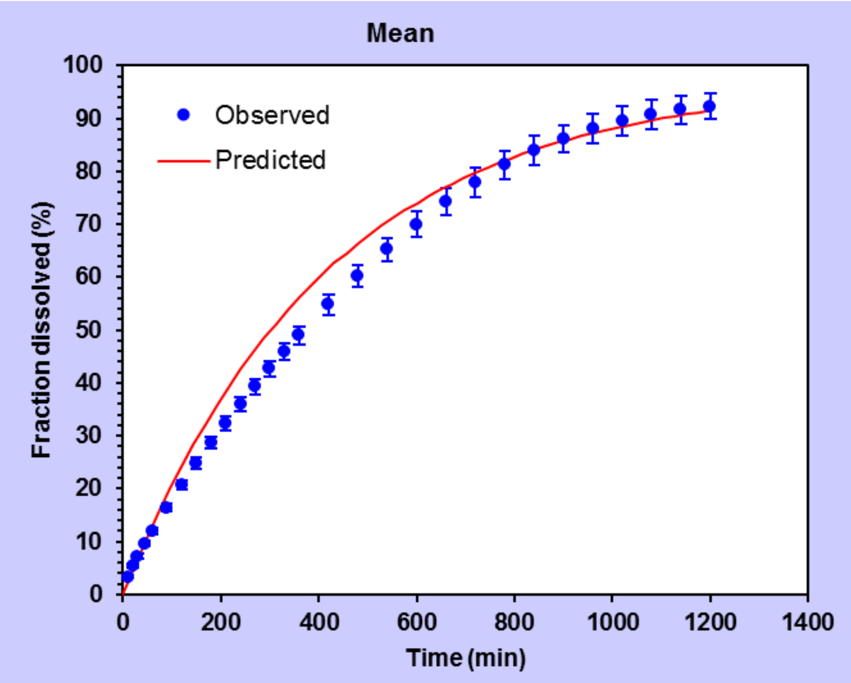

Supplementary materials – Model fitting summary – Granulated sugar N°1 600 (modelling the entire relevant dissolution data)

Graphical abstract of model fit presented as the fraction % of released carvedilol per tested tablet:

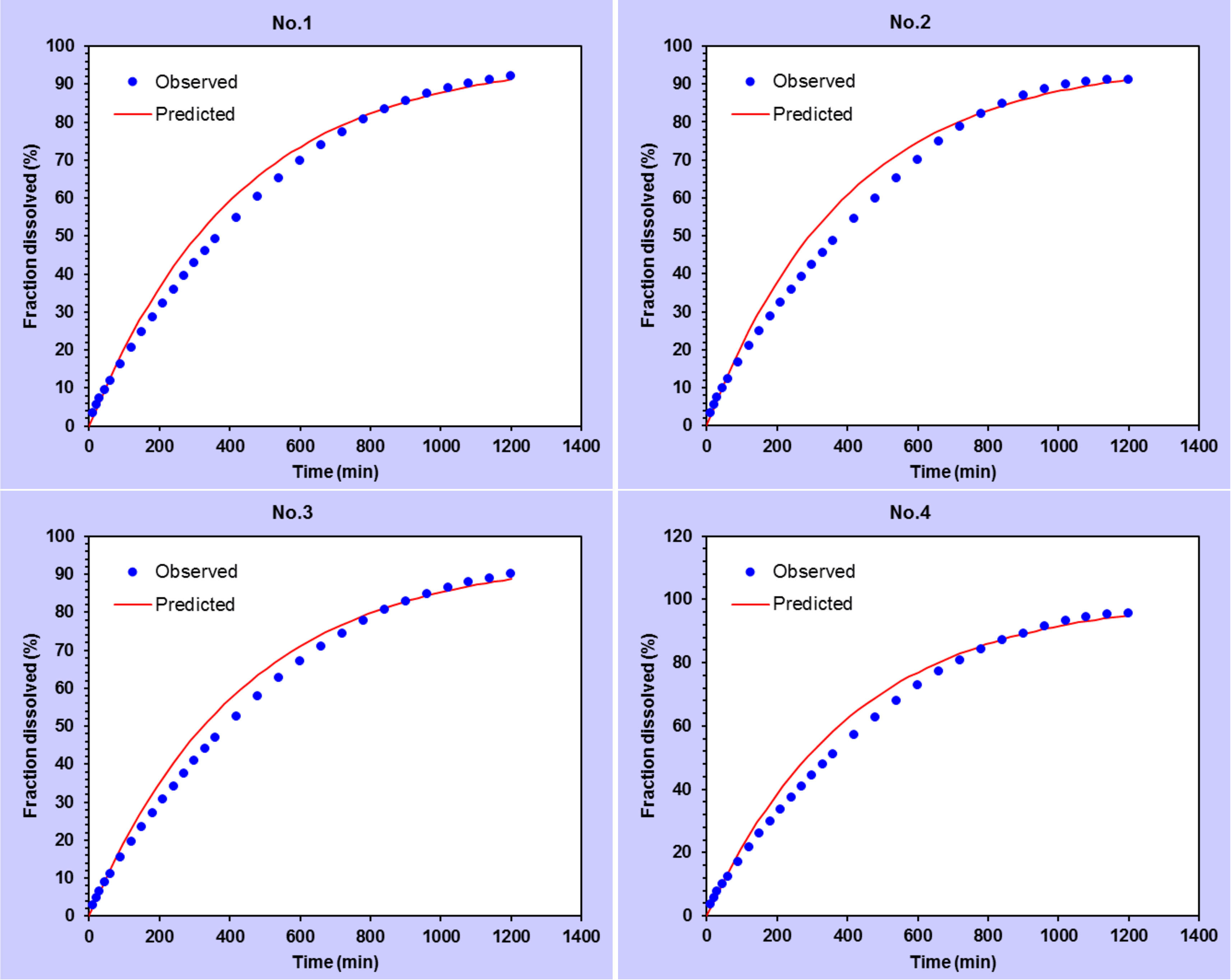

Supplementary materials – Model fitting summary – Granulated sugar N°1 600 (modelling the entire relevant dissolution data)

Model: **First–order with T_{lag} and F_{max}**

Model equation: $F = F_{max} \cdot \left[1 - e^{-k_1 \cdot (t - T_{lag})}\right]$

Fitted model parameters per tested tablet (N = 4) with statistics – mean, standard deviation (SD), and relative standard deviation expressed in % (RSD%) (output from DDSolver):

Parameter	No.1	No.2	No.3	No.4	Mean	SD	RSD(%)
k_1	0.003	0.003	0.002	0.003	0.003	0.000	3.831
T_{lag}	40.529	46.888	44.660	43.721	43.950	2.639	6.005
F_{max}	96.850	95.810	94.561	100.491	96.928	2.553	2.634

Number of dissolution data points (N), degrees of freedom (df), and selected goodness of fit criteria – Pearson correlation coefficient (R), coefficient of determination (R^2), adjusted coefficient of determination ($R^2_{adjusted}$), and residual sum of squares (RSS) (manual calculation in MS Excel):

Parameter	No.1	No.2	No.3	No.4
N	29	29	29	29
df	26	26	26	26
R	0.994014375	0.990372586	0.993526885	0.992849676
R^2	0.988064578	0.98083786	0.987095672	0.98575048
$R^2_{adjusted}$	0.987146469	0.979363849	0.986103032	0.984654363
RSS	620.7700512	965.9929011	663.164121	824.0261205

Graphical abstract of model fit presented as mean ± 1 SD of the fraction % of released carvedilol:

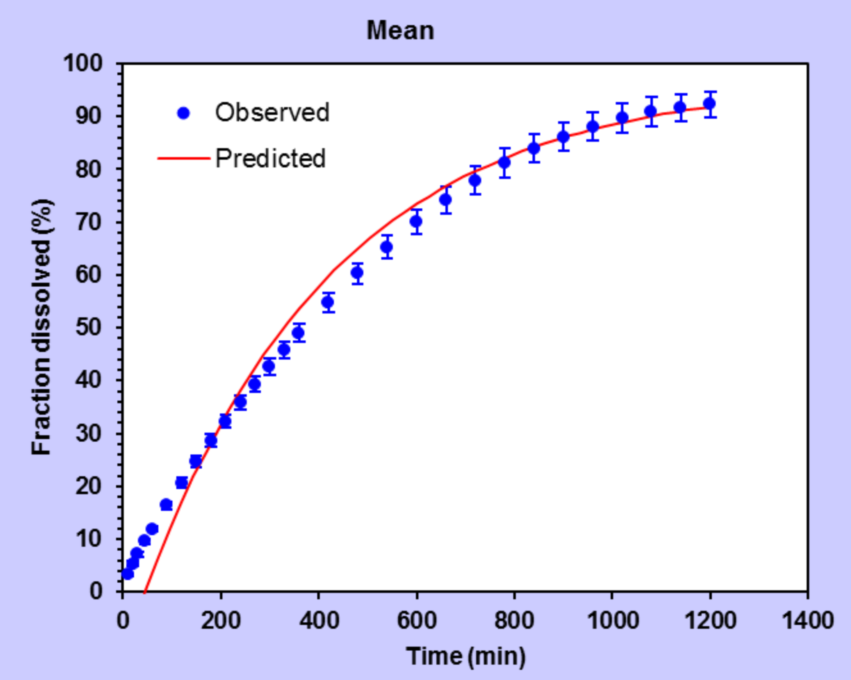

Supplementary materials – Model fitting summary – Granulated sugar N°1 600 (modelling the entire relevant dissolution data)

Graphical abstract of model fit presented as the fraction % of released carvedilol per tested tablet:

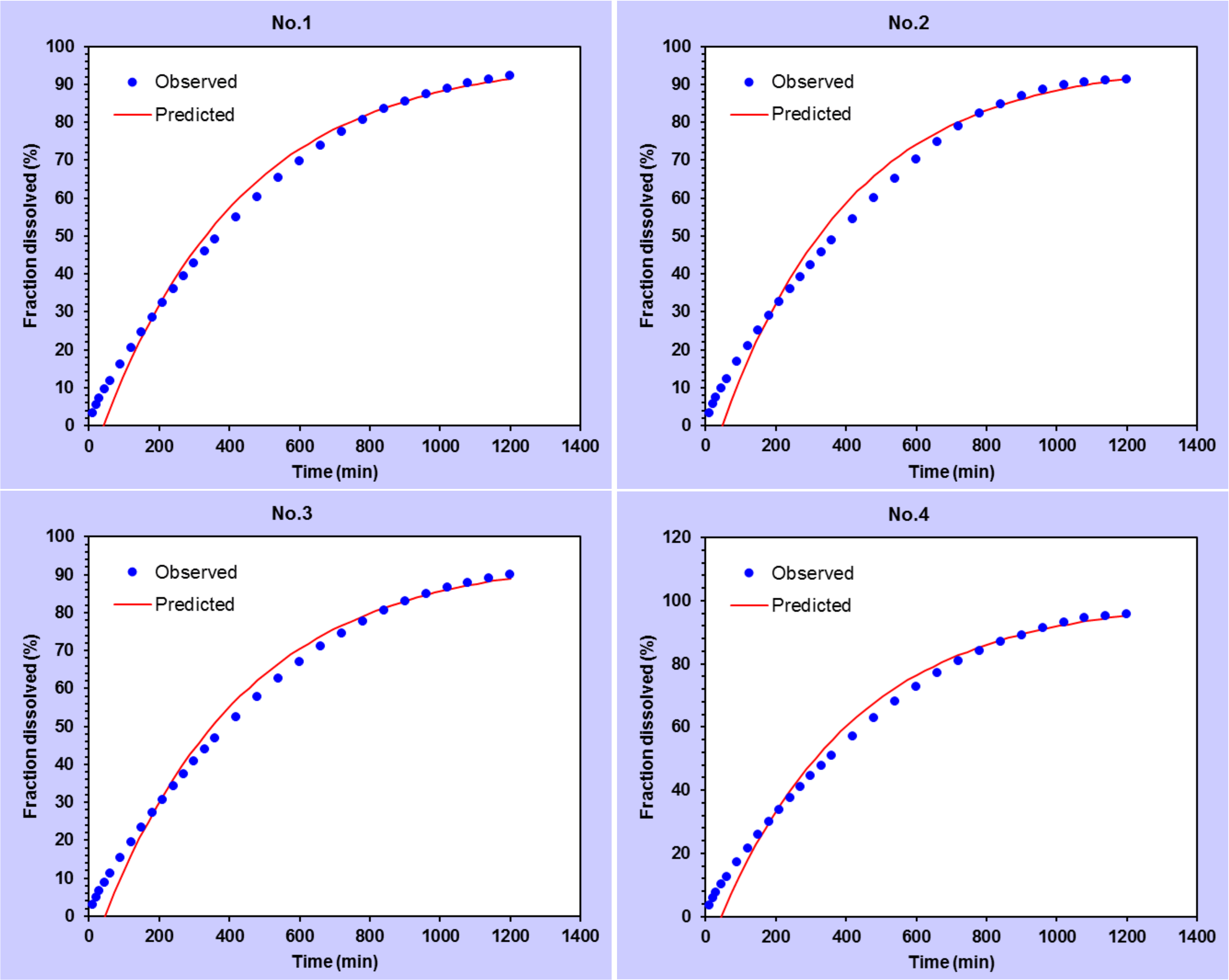

Supplementary materials – Model fitting summary – Granulated sugar N°1 600 (modelling the entire relevant dissolution data)

Model: **Higuchi**

Model equation: $F = k_H \cdot t^{0.5}$

Fitted model parameters per tested tablet (N = 4) with statistics – mean, standard deviation (SD), and relative standard deviation expressed in % (RSD%) (output from DDSolver):

Parameter	No.1	No.2	No.3	No.4	Mean	SD	RSD(%)
k_H	2.703	2.718	2.608	2.819	2.712	0.086	3.181

Number of dissolution data points (N), degrees of freedom (df), and selected goodness of fit criteria – Pearson correlation coefficient (R), coefficient of determination (R^2), adjusted coefficient of determination ($R^2_{adjusted}$), and residual sum of squares (RSS) (manual calculation in MS Excel):

Parameter	No.1	No.2	No.3	No.4
N	29	29	29	29
df	28	28	28	28
R	0.995850128	0.994896617	0.996358806	0.995836682
R^2	0.991717478	0.989819279	0.99273087	0.991690698
$R^2_{adjusted}$	0.991717478	0.989819279	0.99273087	0.991690698
RSS	880.8495719	934.8791051	901.7301814	950.1072259

Graphical abstract of model fit presented as mean ± 1 SD of the fraction % of released carvedilol:

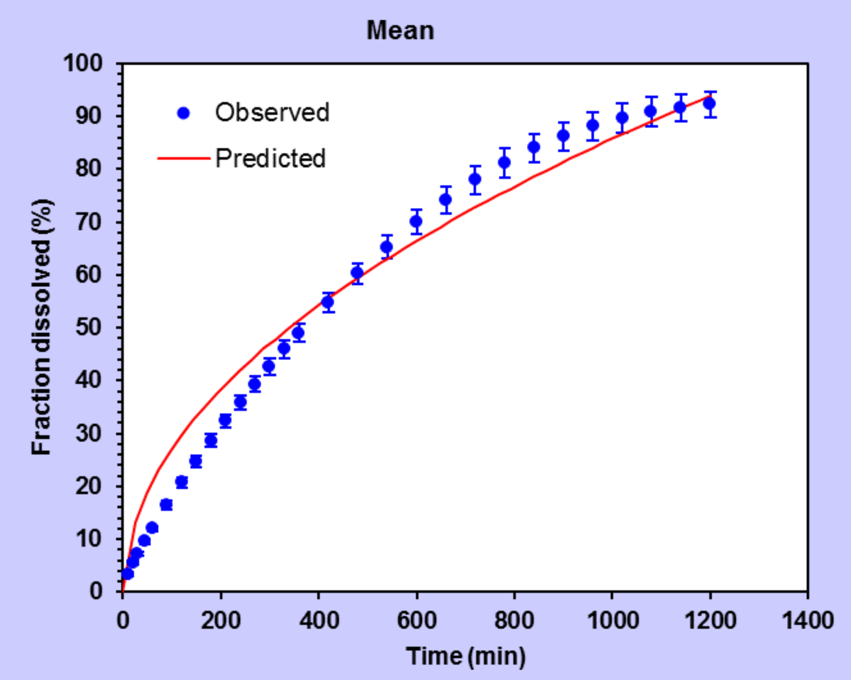

Supplementary materials – Model fitting summary – Granulated sugar N°1 600 (modelling the entire relevant dissolution data)

Graphical abstract of model fit presented as the fraction % of released carvedilol per tested tablet:

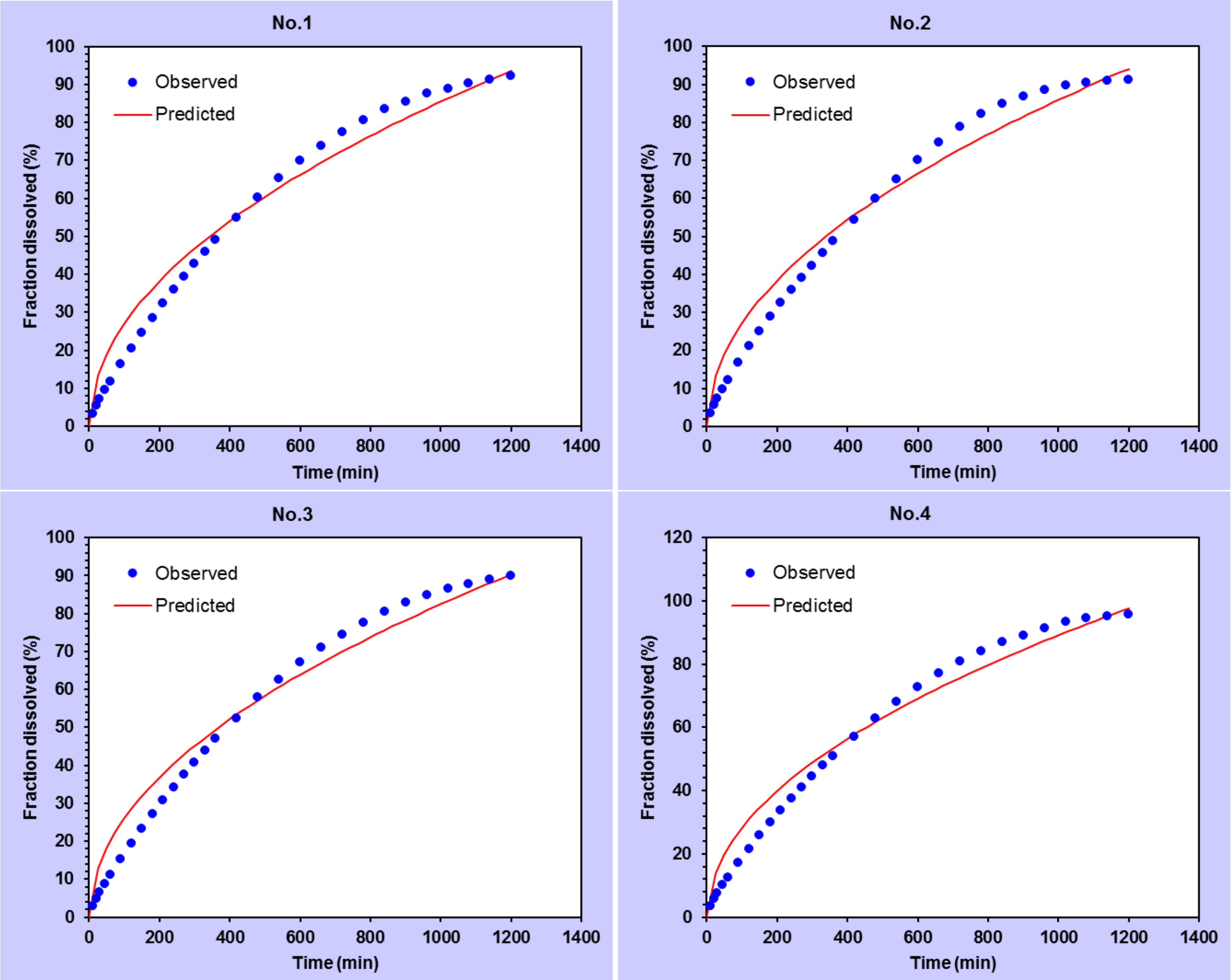

Supplementary materials – Model fitting summary – Granulated sugar N°1 600 (modelling the entire relevant dissolution data)

Model: **Higuchi with T_{lag}**

Model equation: $F = k_H \cdot (t - T_{lag})^{0.5}$

Fitted model parameters per tested tablet (N = 4) with statistics – mean, standard deviation (SD), and relative standard deviation expressed in % (RSD%) (output from DDSolver):

Parameter	No.1	No.2	No.3	No.4	Mean	SD	RSD(%)
k_H	2.847	2.864	2.769	2.970	2.862	0.083	2.896
T_{lag}	42.966	43.174	49.296	43.272	44.677	3.082	6.898

Number of dissolution data points (N), degrees of freedom (df), and selected goodness of fit criteria – Pearson correlation coefficient (R), coefficient of determination (R^2), adjusted coefficient of determination ($R^2_{adjusted}$), and residual sum of squares (RSS) (manual calculation in MS Excel):

Parameter	No.1	No.2	No.3	No.4
N	29	29	29	29
df	27	27	27	27
R	0.993362286	0.992148883	0.993256079	0.993247872
R^2	0.98676863	0.984359405	0.986557639	0.986541336
$R^2_{adjusted}$	0.98627858	0.983780124	0.986059774	0.986042867
RSS	354.2662498	424.0185529	350.8122747	393.0185174

Graphical abstract of model fit presented as mean ± 1 SD of the fraction % of released carvedilol:

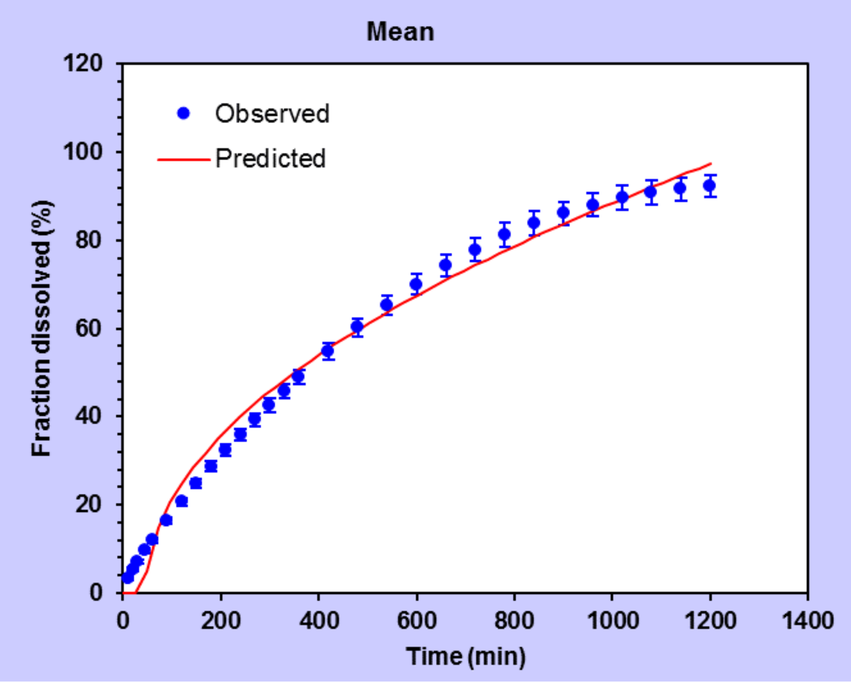

Supplementary materials – Model fitting summary – Granulated sugar N°1 600 (modelling the entire relevant dissolution data)

Graphical abstract of model fit presented as the fraction % of released carvedilol per tested tablet:

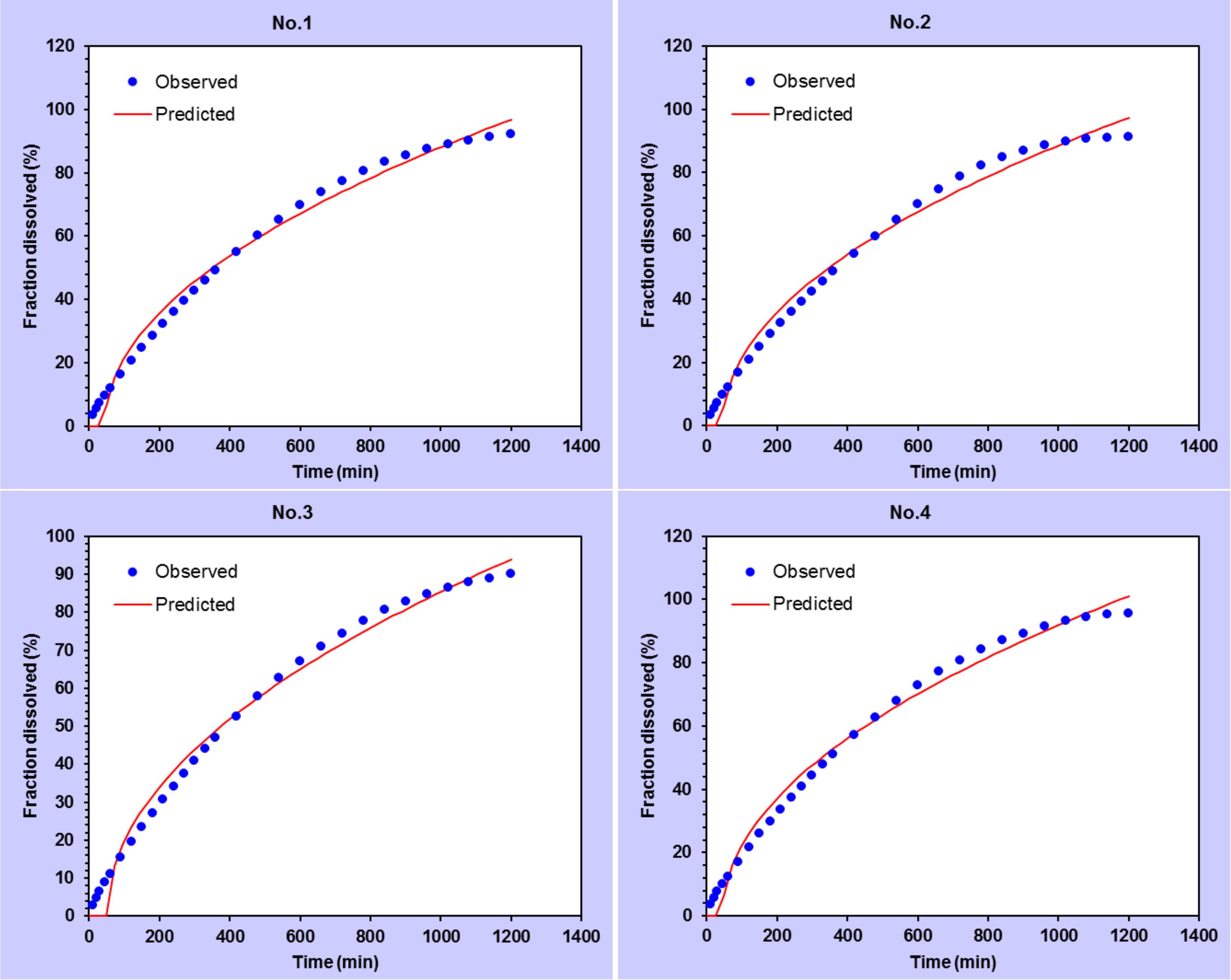

Supplementary materials – Model fitting summary – Granulated sugar N°1 600 (modelling the entire relevant dissolution data)

Model: **Higuchi with F_0**

Model equation: $F = F_0 + k_H \cdot t^{0.5}$

Fitted model parameters per tested tablet (N = 4) with statistics – mean, standard deviation (SD), and relative standard deviation expressed in % (RSD%) (output from DDSolver):

Parameter	No.1	No.2	No.3	No.4	Mean	SD	RSD(%)
k_H	3.156	3.171	3.080	3.289	3.174	0.086	2.715
F_0	-10.960	-10.969	-11.420	-11.364	-11.178	0.248	-2.218

Number of dissolution data points (N), degrees of freedom (df), and selected goodness of fit criteria – Pearson correlation coefficient (R), coefficient of determination (R^2), adjusted coefficient of determination ($R^2_{adjusted}$), and residual sum of squares (RSS) (manual calculation in MS Excel):

Parameter	No.1	No.2	No.3	No.4
N	29	29	29	29
df	27	27	27	27
R	0.995850128	0.994896617	0.996358806	0.995836682
R^2	0.991717478	0.989819279	0.99273087	0.991690698
$R^2_{adjusted}$	0.991410718	0.989442215	0.992461643	0.991382946
RSS	217.8015394	270.7817891	181.8896319	237.2640416

Graphical abstract of model fit presented as mean ± 1 SD of the fraction % of released carvedilol:

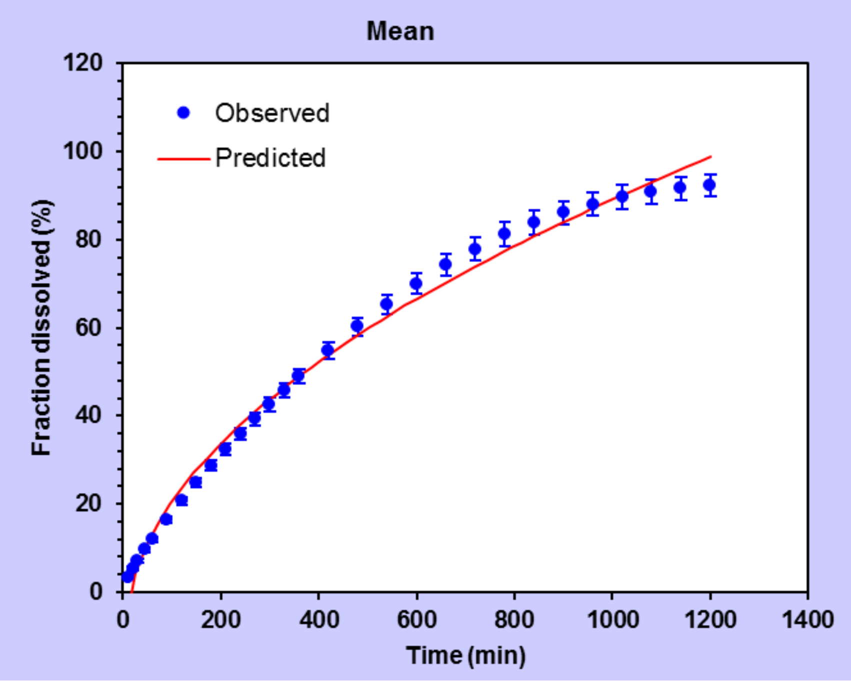

Supplementary materials – Model fitting summary – Granulated sugar N°1 600 (modelling the entire relevant dissolution data)

Graphical abstract of model fit presented as the fraction % of released carvedilol per tested tablet:

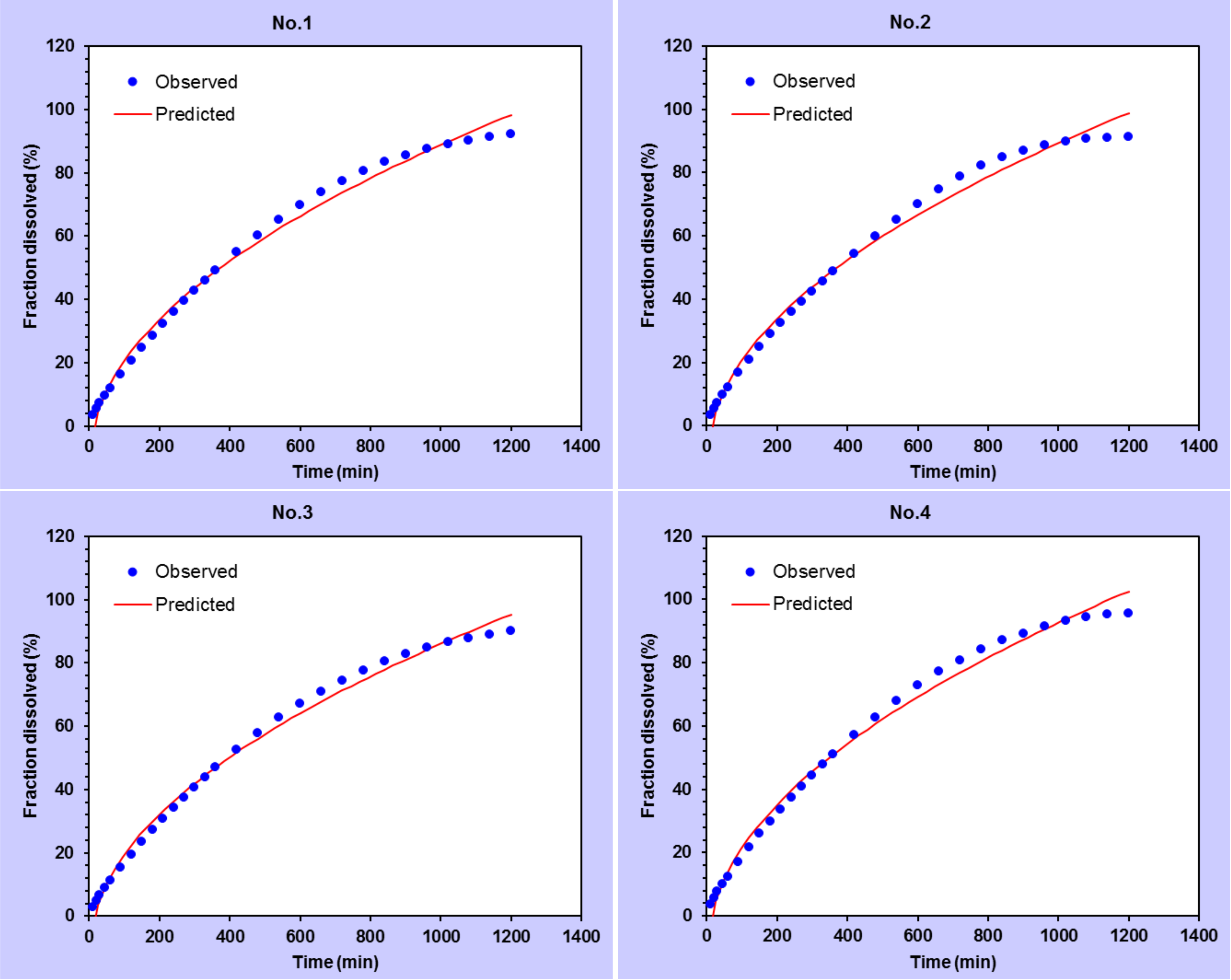

Supplementary materials – Model fitting summary – Granulated sugar N°1 600 (modelling the entire relevant dissolution data)

Model: **Korsmeyer–Peppas**

Model equation: $F = k_{KP} \cdot t^n$

Fitted model parameters per tested tablet (N = 4) with statistics – mean, standard deviation (SD), and relative standard deviation expressed in % (RSD%) (output from DDSolver):

Parameter	No.1	No.2	No.3	No.4	Mean	SD	RSD(%)
k_{KP}	0.661	0.683	0.562	0.705	0.653	0.063	9.676
n	0.719	0.715	0.739	0.716	0.722	0.011	1.570

Number of dissolution data points (N), degrees of freedom (df), and selected goodness of fit criteria – Pearson correlation coefficient (R), coefficient of determination (R^2), adjusted coefficient of determination ($R^2_{adjusted}$), and residual sum of squares (RSS) (manual calculation in MS Excel):

Parameter	No.1	No.2	No.3	No.4
N	29	29	29	29
df	27	27	27	27
R	0.989912431	0.989403213	0.99044274	0.990232711
R^2	0.97992662	0.978918718	0.980976821	0.980560823
$R^2_{adjusted}$	0.979183162	0.97813793	0.980272259	0.979840853
RSS	743.3813134	758.0771884	716.8944068	772.5171211

Graphical abstract of model fit presented as mean ± 1 SD of the fraction % of released carvedilol:

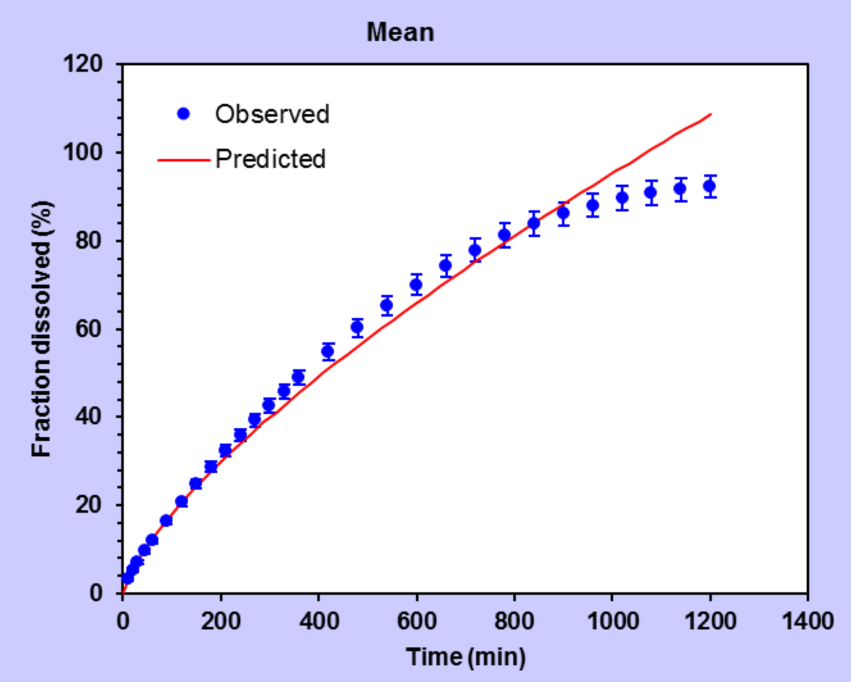

Supplementary materials – Model fitting summary – Granulated sugar N°1 600 (modelling the entire relevant dissolution data)

Graphical abstract of model fit presented as the fraction % of released carvedilol per tested tablet:

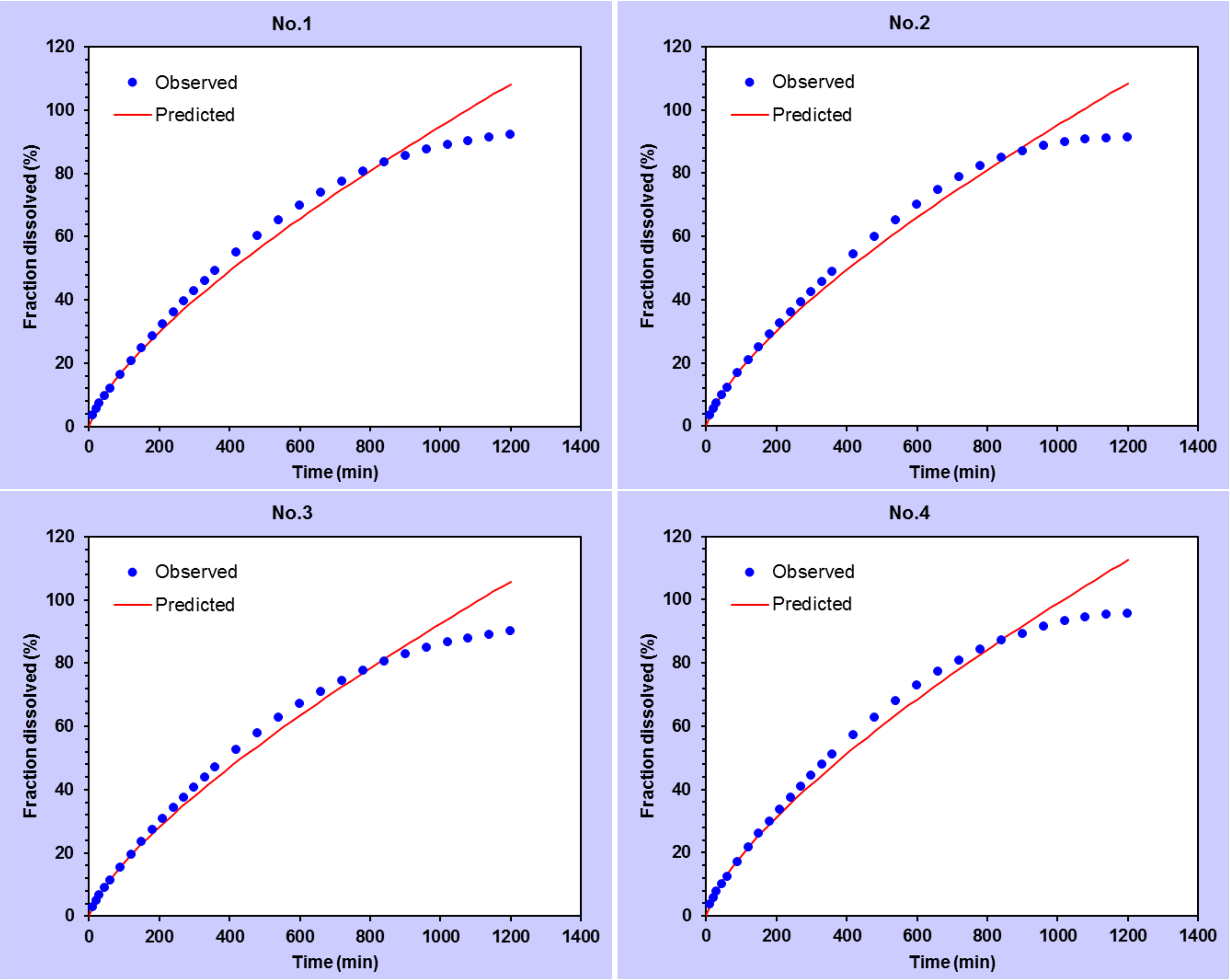

Supplementary materials – Model fitting summary – Granulated sugar N°1 600 (modelling the entire relevant dissolution data)

Model: **Korsmeyer–Peppas with T_{lag}**

Model equation: $F = k_{KP} \cdot \left(t - T_{lag}\right)^n$

Fitted model parameters per tested tablet (N = 4) with statistics – mean, standard deviation (SD), and relative standard deviation expressed in % (RSD%) (output from DDSolver):

Parameter	No.1	No.2	No.3	No.4	Mean	SD	RSD(%)
k_{KP}	0.866	0.892	0.740	0.922	0.855	0.080	9.367
n	0.677	0.673	0.696	0.673	0.680	0.011	1.589
T_{lag}	4.000	4.000	4.000	4.000	4.000	0.000	0.000

Number of dissolution data points (N), degrees of freedom (df), and selected goodness of fit criteria – Pearson correlation coefficient (R), coefficient of determination (R^2), adjusted coefficient of determination ($R^2_{adjusted}$), and residual sum of squares (RSS) (manual calculation in MS Excel):

Parameter	No.1	No.2	No.3	No.4
N	29	29	29	29
df	26	26	26	26
R	0.992137997	0.991527549	0.992666158	0.992385226
R^2	0.984337805	0.98312688	0.985386101	0.984828436
$R^2_{adjusted}$	0.983133021	0.981828948	0.984261955	0.983661393
RSS	471.5596421	500.2124559	440.0810216	490.2474532

Graphical abstract of model fit presented as mean ± 1 SD of the fraction % of released carvedilol:

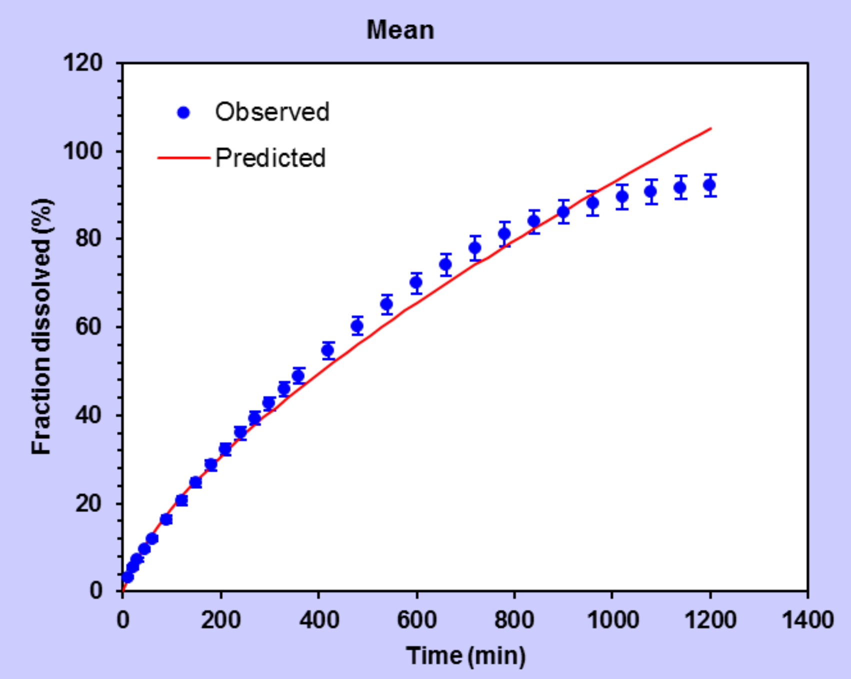

Supplementary materials – Model fitting summary – Granulated sugar N°1 600 (modelling the entire relevant dissolution data)

Graphical abstract of model fit presented as the fraction % of released carvedilol per tested tablet:

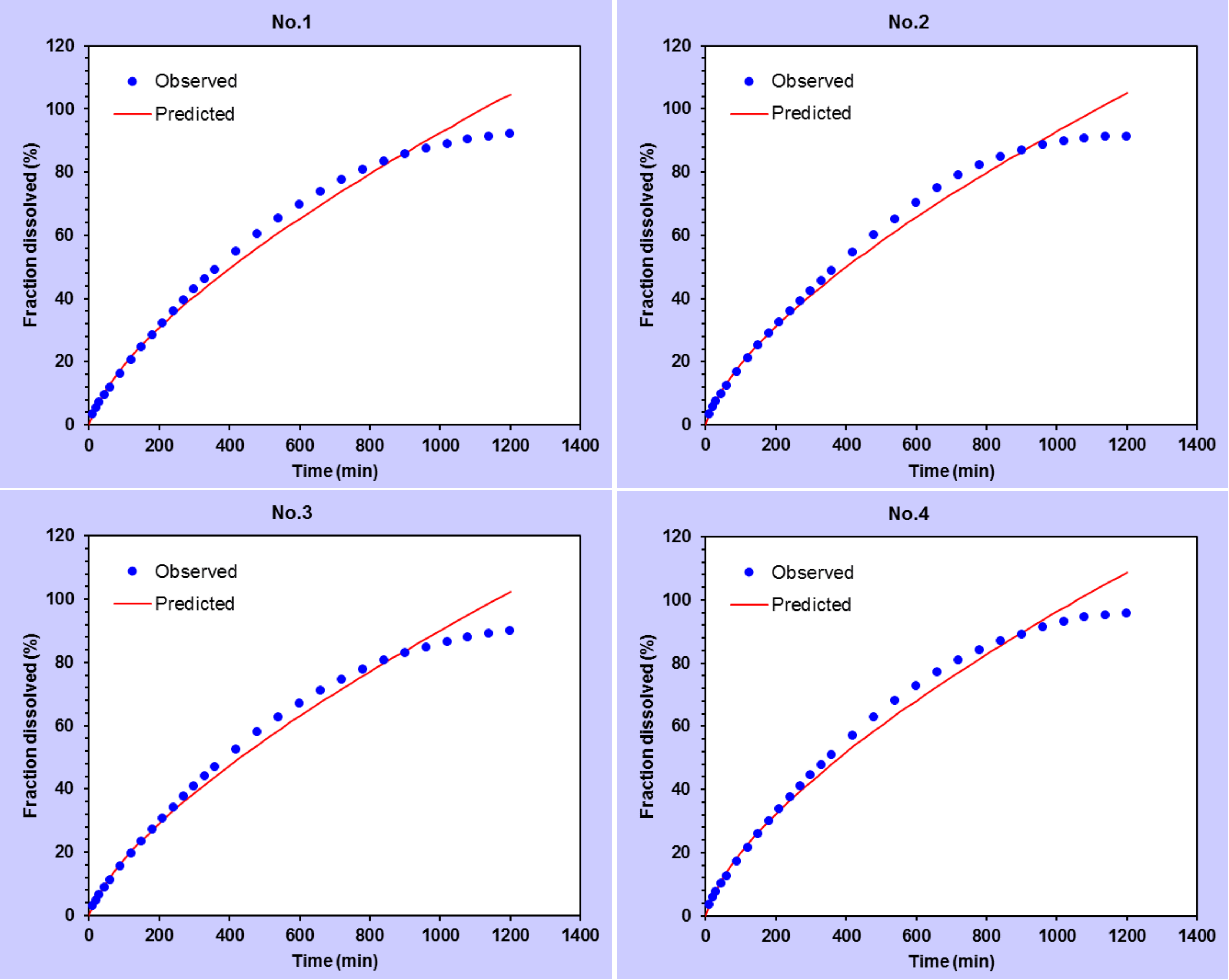

Supplementary materials – Model fitting summary – Granulated sugar N°1 600 (modelling the entire relevant dissolution data)

Model: **Korsmeyer–Peppas with F_0**

Model equation: $F = F_0 + k_{KP} \cdot t^n$

Fitted model parameters per tested tablet (N = 4) with statistics – mean, standard deviation (SD), and relative standard deviation expressed in % (RSD%) (output from DDSolver):

Parameter	No.1	No.2	No.3	No.4	Mean	SD	RSD(%)
k_{KP}	0.416	0.432	0.359	0.442	0.412	0.037	8.995
n	0.789	0.784	0.807	0.786	0.792	0.010	1.318
F_0	1.360	1.360	1.160	1.440	1.330	0.119	8.981

Number of dissolution data points (N), degrees of freedom (df), and selected goodness of fit criteria – Pearson correlation coefficient (R), coefficient of determination (R^2), adjusted coefficient of determination ($R^2_{adjusted}$), and residual sum of squares (RSS) (manual calculation in MS Excel):

Parameter	No.1	No.2	No.3	No.4
N	29	29	29	29
df	26	26	26	26
R	0.985892233	0.985554019	0.986633067	0.98629404
R^2	0.971983496	0.971316725	0.97344481	0.972775934
$R^2_{adjusted}$	0.96982838	0.969110319	0.971402103	0.970681775
RSS	1346.39883	1336.930489	1303.647932	1408.89619

Graphical abstract of model fit presented as mean ± 1 SD of the fraction % of released carvedilol:

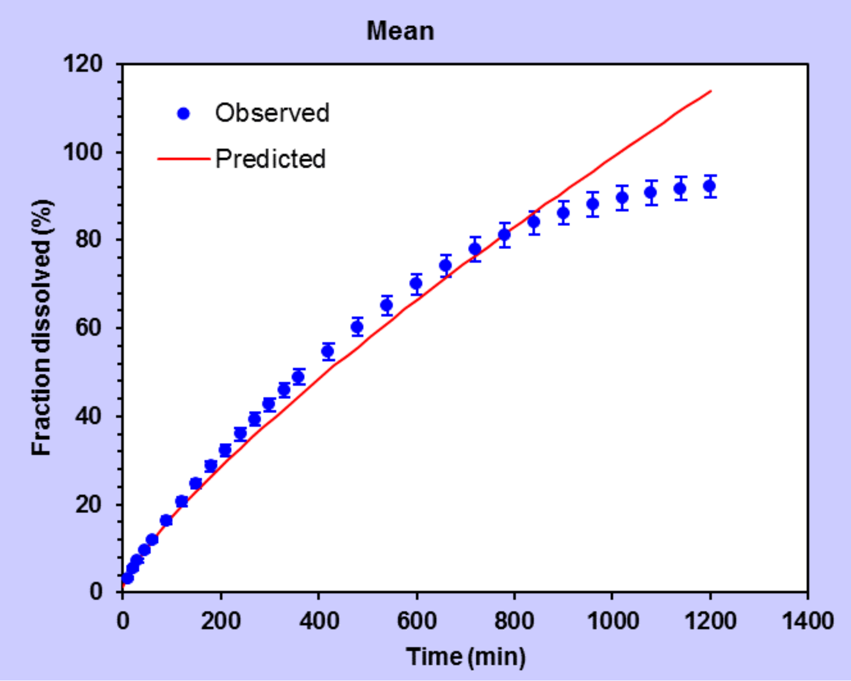

Supplementary materials – Model fitting summary – Granulated sugar N°1 600 (modelling the entire relevant dissolution data)

Graphical abstract of model fit presented as the fraction % of released carvedilol per tested tablet:

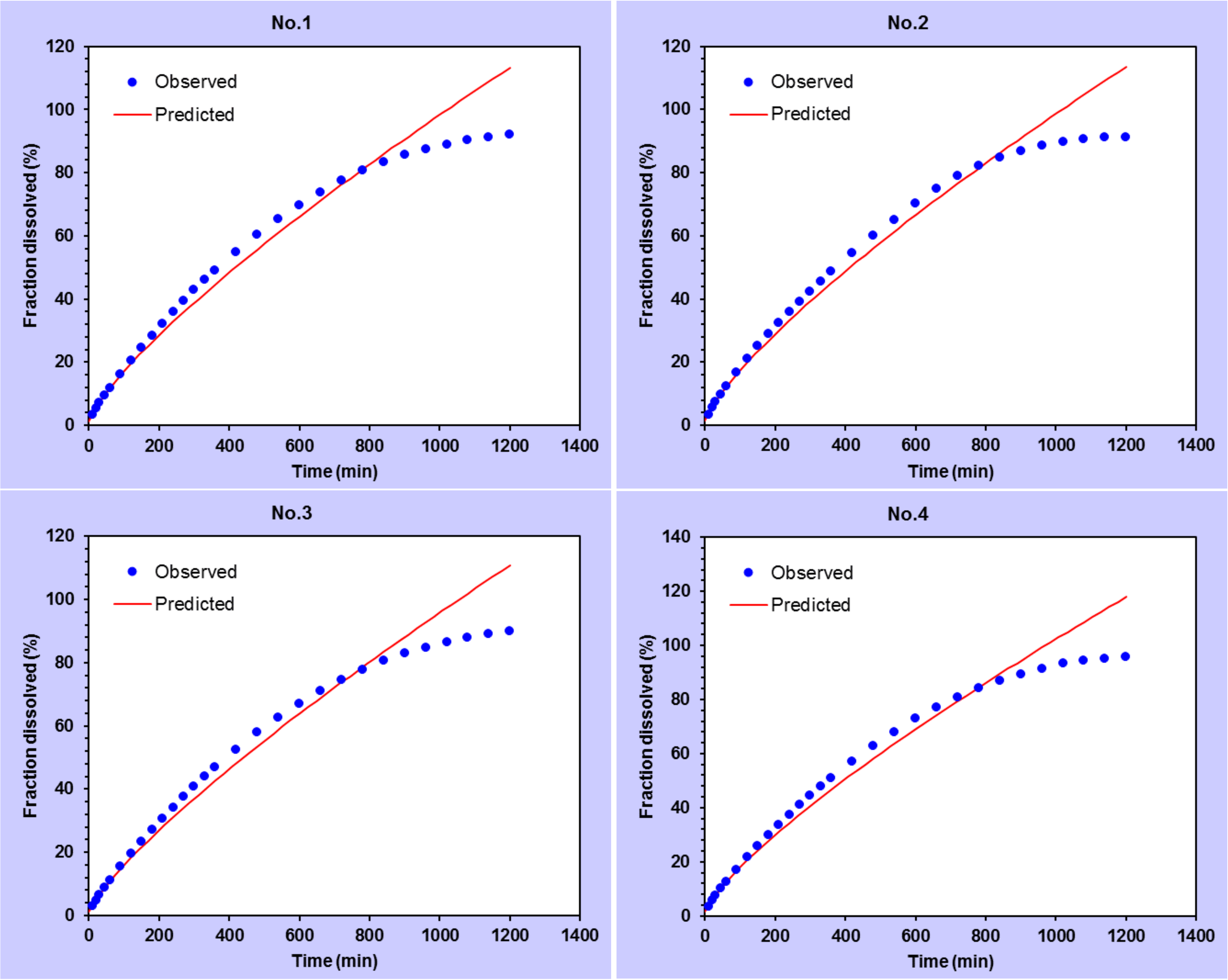

Supplementary materials – Model fitting summary – Granulated sugar N°1 600 (modelling the entire relevant dissolution data)

Model: **Hixson–Crowell**

Model equation: $F = 100 \cdot [1 - (1 - k_{HC} \cdot t)^3]$

Fitted model parameters per tested tablet (N = 4) with statistics – mean, standard deviation (SD), and relative standard deviation expressed in % (RSD%) (output from DDSolver):

Parameter	No.1	No.2	No.3	No.4	Mean	SD	RSD(%)
k_{HC}	0.001	0.001	0.000	0.001	0.001	0.000	7.069

Number of dissolution data points (N), degrees of freedom (df), and selected goodness of fit criteria – Pearson correlation coefficient (R), coefficient of determination (R^2), adjusted coefficient of determination ($R^2_{adjusted}$), and residual sum of squares (RSS) (manual calculation in MS Excel):

Parameter	No.1	No.2	No.3	No.4
N	29	29	29	29
df	28	28	28	28
R	0.999342006	0.999099812	0.999127221	0.999915566
R^2	0.998684446	0.998200434	0.998255203	0.99983114
$R^2_{adjusted}$	0.998684446	0.998200434	0.998255203	0.99983114
RSS	178.9473275	185.9839302	182.845969	66.51407845

Graphical abstract of model fit presented as mean ± 1 SD of the fraction % of released carvedilol:

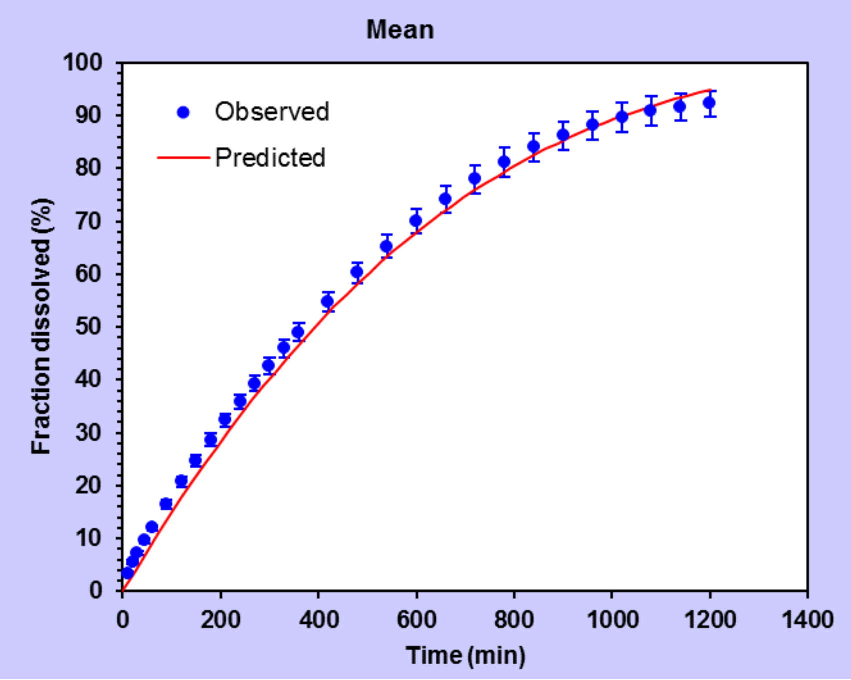

Supplementary materials – Model fitting summary – Granulated sugar N°1 600 (modelling the entire relevant dissolution data)

Graphical abstract of model fit presented as the fraction % of released carvedilol per tested tablet:

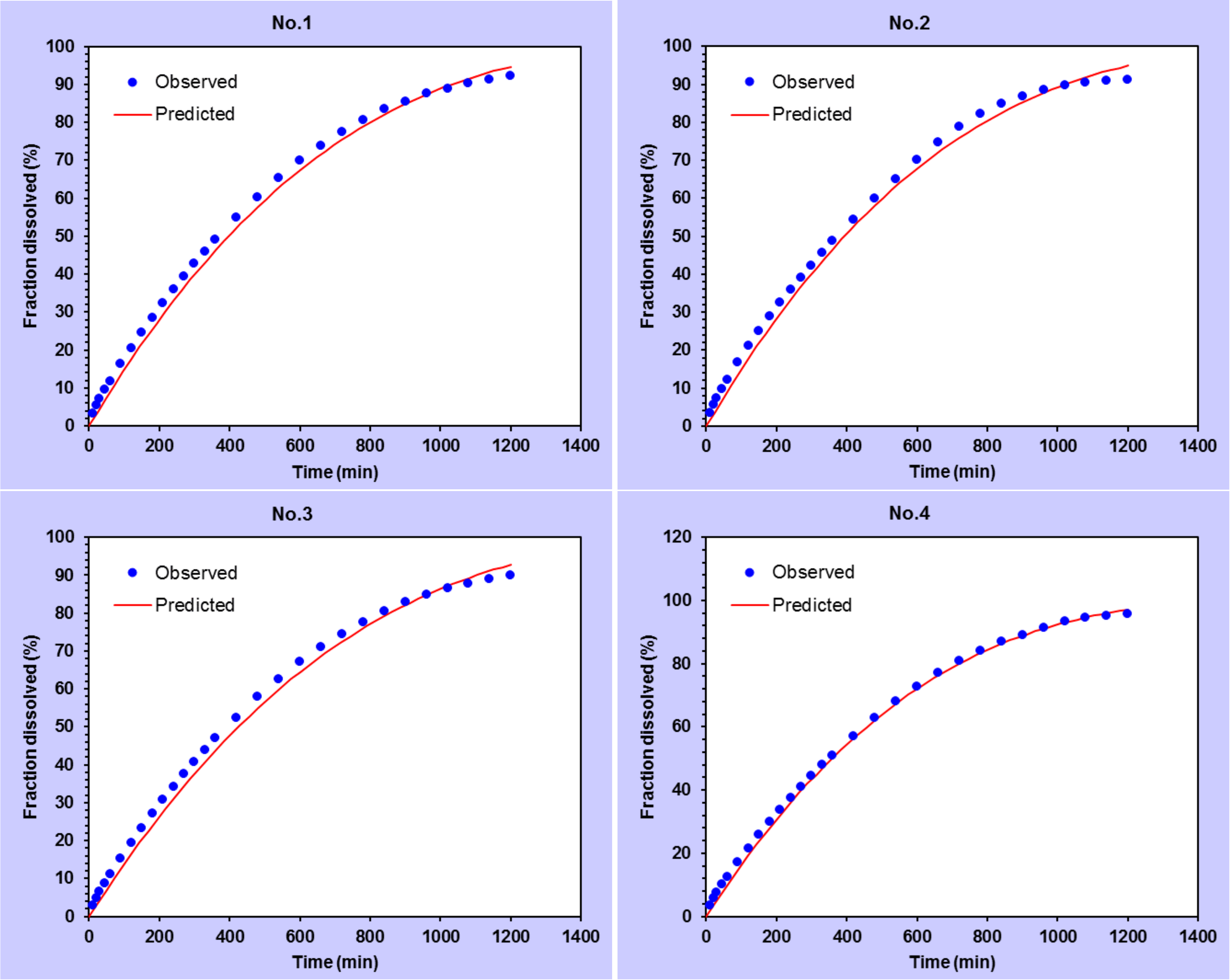

Supplementary materials – Model fitting summary – Granulated sugar N°1 600 (modelling the entire relevant dissolution data)

Model: **Hixson–Crowell with T_{lag}**

Model equation: $F = 100 \cdot \left\{1 - \left[1 - k_{HC} \cdot \left(t - T_{lag}\right)\right]^3\right\}$

Fitted model parameters per tested tablet (N = 4) with statistics – mean, standard deviation (SD), and relative standard deviation expressed in % (RSD%) (output from DDSolver):

Parameter	No.1	No.2	No.3	No.4	Mean	SD	RSD(%)
k_{HC}	0.000	0.000	0.000	0.001	0.001	0.000	8.203
T_{lag}	-38.424	-38.316	-39.808	-20.358	-34.226	9.271	-27.086

Number of dissolution data points (N), degrees of freedom (df), and selected goodness of fit criteria – Pearson correlation coefficient (R), coefficient of determination (R^2), adjusted coefficient of determination ($R^2_{adjusted}$), and residual sum of squares (RSS) (manual calculation in MS Excel):

Parameter	No.1	No.2	No.3	No.4
N	29	29	29	29
df	27	27	27	27
R	0.999043769	0.998825825	0.998790575	0.999888504
R^2	0.998088452	0.997653029	0.997582613	0.999777021
$R^2_{adjusted}$	0.998017654	0.997566104	0.99749308	0.999768763
RSS	75.57591683	91.38283176	84.23919303	9.938611082

Graphical abstract of model fit presented as mean ± 1 SD of the fraction % of released carvedilol:

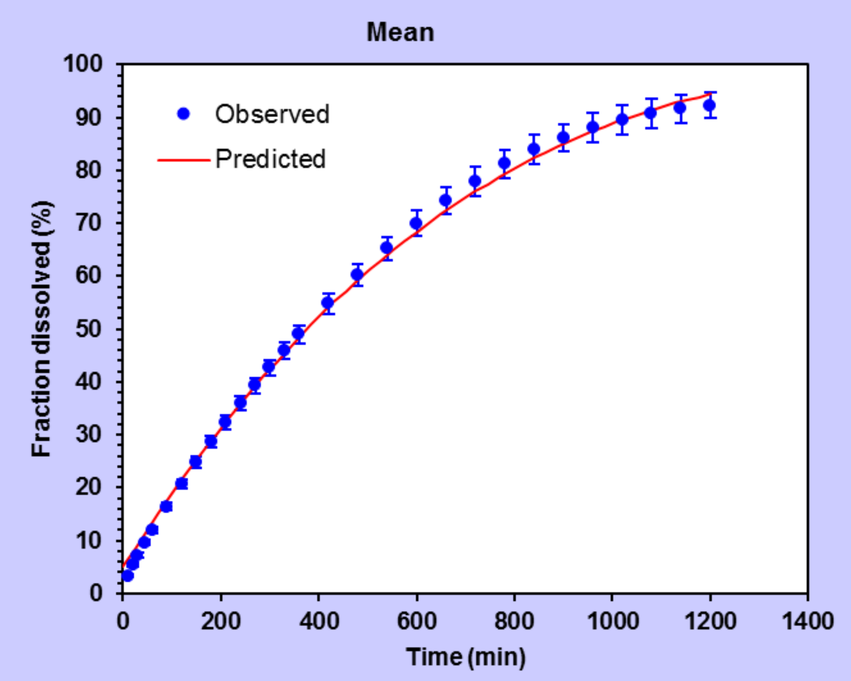

Supplementary materials – Model fitting summary – Granulated sugar N°1 600 (modelling the entire relevant dissolution data)

Graphical abstract of model fit presented as the fraction % of released carvedilol per tested tablet:

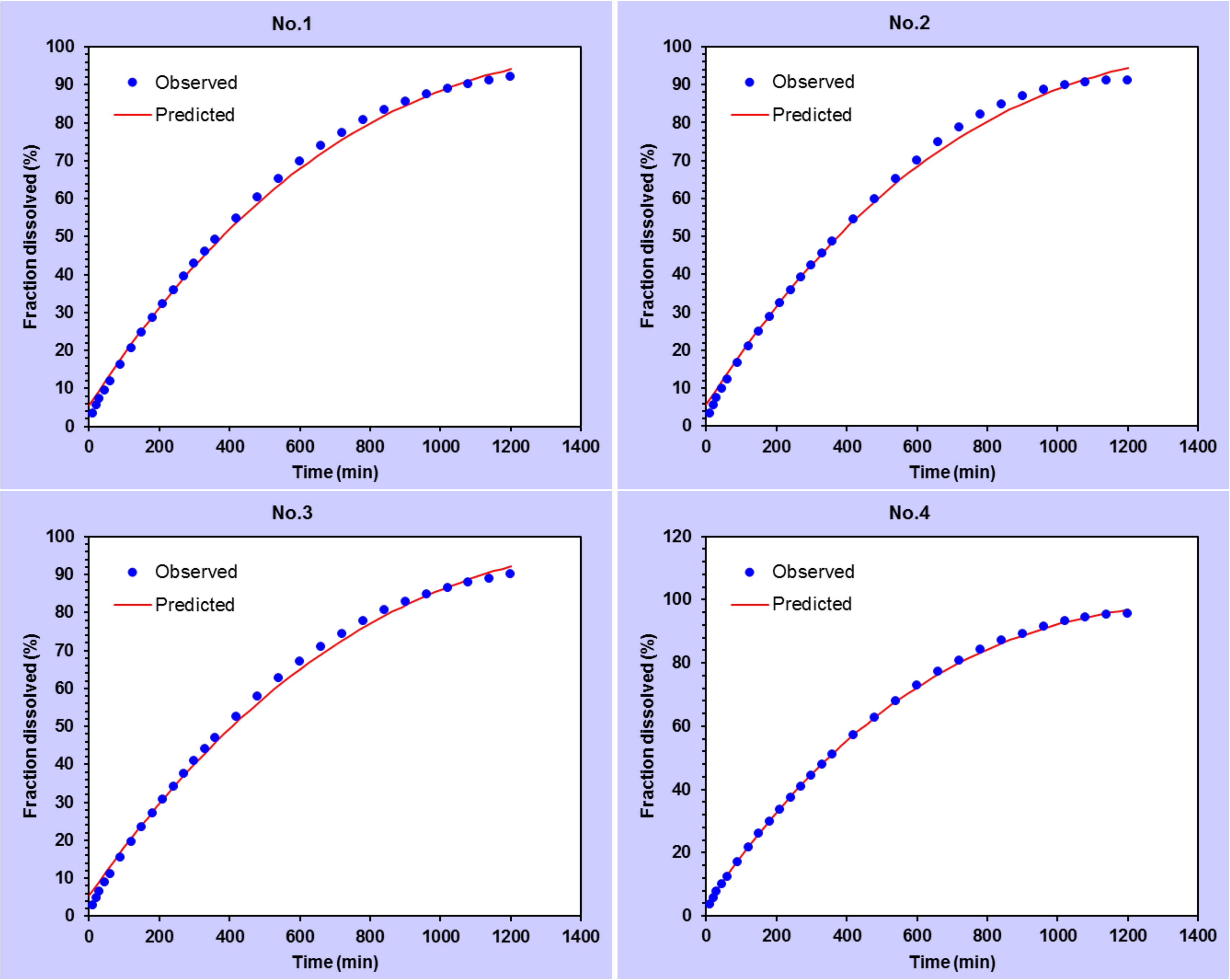

Supplementary materials – Model fitting summary – Granulated sugar N°1 600 (modelling the entire relevant dissolution data)

Model: **Hopfenberg**

Model equation: $F = 100 \cdot [1 - (1 - k_{HB} \cdot t)^n]$

Fitted model parameters per tested tablet (N = 4) with statistics – mean, standard deviation (SD), and relative standard deviation expressed in % (RSD%) (output from DDSolver):

Parameter	No.1	No.2	No.3	No.4	Mean	SD	RSD(%)
k_{HB}	0.001	0.001	0.000	0.001	0.001	0.000	7.069
n	3.000	3.000	3.000	3.000	3.000	0.000	0.000

Number of dissolution data points (N), degrees of freedom (df), and selected goodness of fit criteria – Pearson correlation coefficient (R), coefficient of determination (R^2), adjusted coefficient of determination ($R^2_{adjusted}$), and residual sum of squares (RSS) (manual calculation in MS Excel):

Parameter	No.1	No.2	No.3	No.4
N	29	29	29	29
df	27	27	27	27
R	0.999342006	0.999099812	0.999127221	0.999915566
R^2	0.998684446	0.998200434	0.998255203	0.99983114
$R^2_{adjusted}$	0.998635721	0.998133784	0.998190581	0.999824886
RSS	178.9473275	185.9839302	182.845969	66.51407845

Graphical abstract of model fit presented as mean ± 1 SD of the fraction % of released carvedilol:

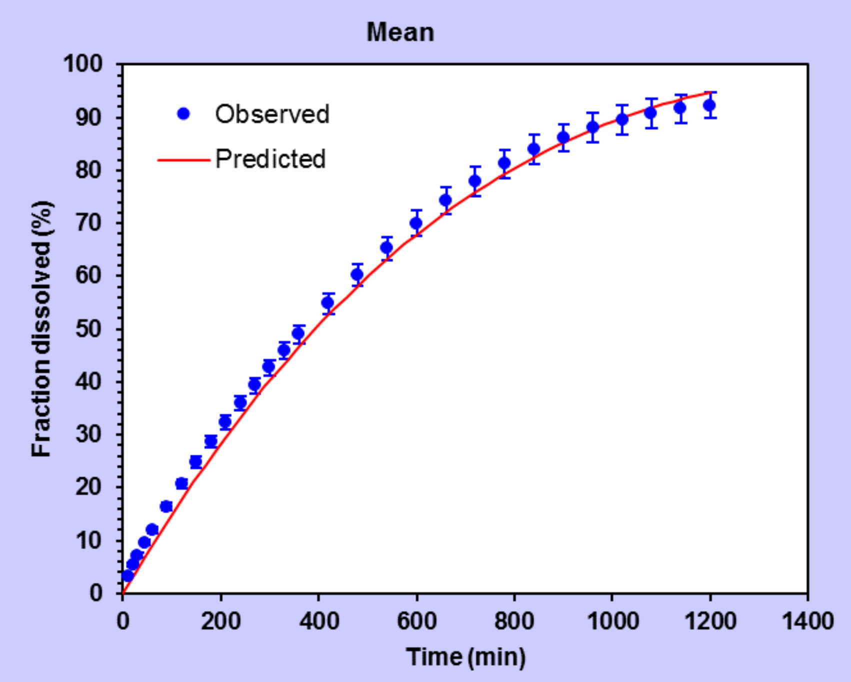

Supplementary materials – Model fitting summary – Granulated sugar N°1 600 (modelling the entire relevant dissolution data)

Graphical abstract of model fit presented as the fraction % of released carvedilol per tested tablet:

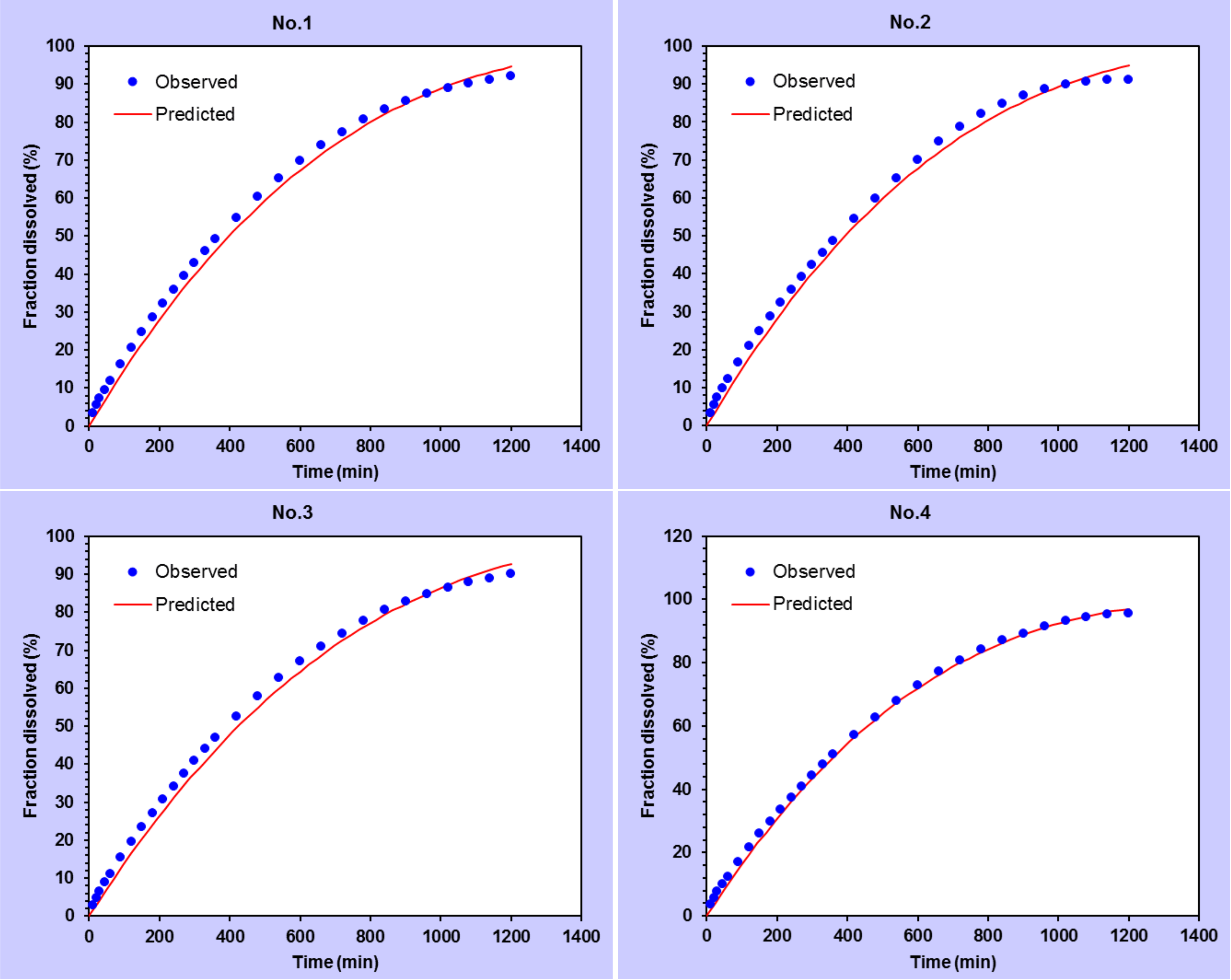

Supplementary materials – Model fitting summary – Granulated sugar N°1 600 (modelling the entire relevant dissolution data)

Model: **Hopfenberg with T_{lag}**

Model equation: $F = 100 \cdot \{1 - [1 - k_{HB} \cdot (t - T_{lag})]^n\}$

Fitted model parameters per tested tablet (N = 4) with statistics – mean, standard deviation (SD), and relative standard deviation expressed in % (RSD%) (output from DDSolver):

Parameter	No.1	No.2	No.3	No.4	Mean	SD	RSD(%)
k_{HB}	0.000	0.000	0.000	0.001	0.001	0.000	8.203
n	3.000	3.000	3.000	3.000	3.000	0.000	0.000
T_{lag}	-38.424	-38.316	-39.808	-20.358	-34.226	9.271	-27.086

Number of dissolution data points (N), degrees of freedom (df), and selected goodness of fit criteria – Pearson correlation coefficient (R), coefficient of determination (R^2), adjusted coefficient of determination ($R^2_{adjusted}$), and residual sum of squares (RSS) (manual calculation in MS Excel):

Parameter	No.1	No.2	No.3	No.4
N	29	29	29	29
df	26	26	26	26
R	0.999043769	0.998825825	0.998790575	0.999888504
R^2	0.998088452	0.997653029	0.997582613	0.999777021
$R^2_{adjusted}$	0.99794141	0.997472493	0.99739666	0.999759869
RSS	75.57591683	91.38283176	84.23919303	9.938611082

Graphical abstract of model fit presented as mean ± 1 SD of the fraction % of released carvedilol:

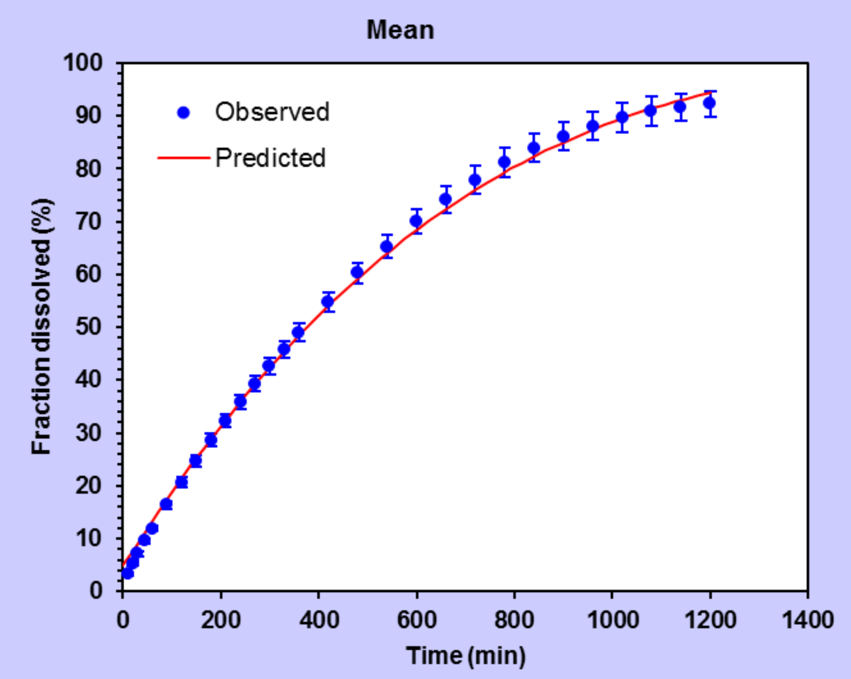

Supplementary materials – Model fitting summary – Granulated sugar N°1 600 (modelling the entire relevant dissolution data)

Graphical abstract of model fit presented as the fraction % of released carvedilol per tested tablet:

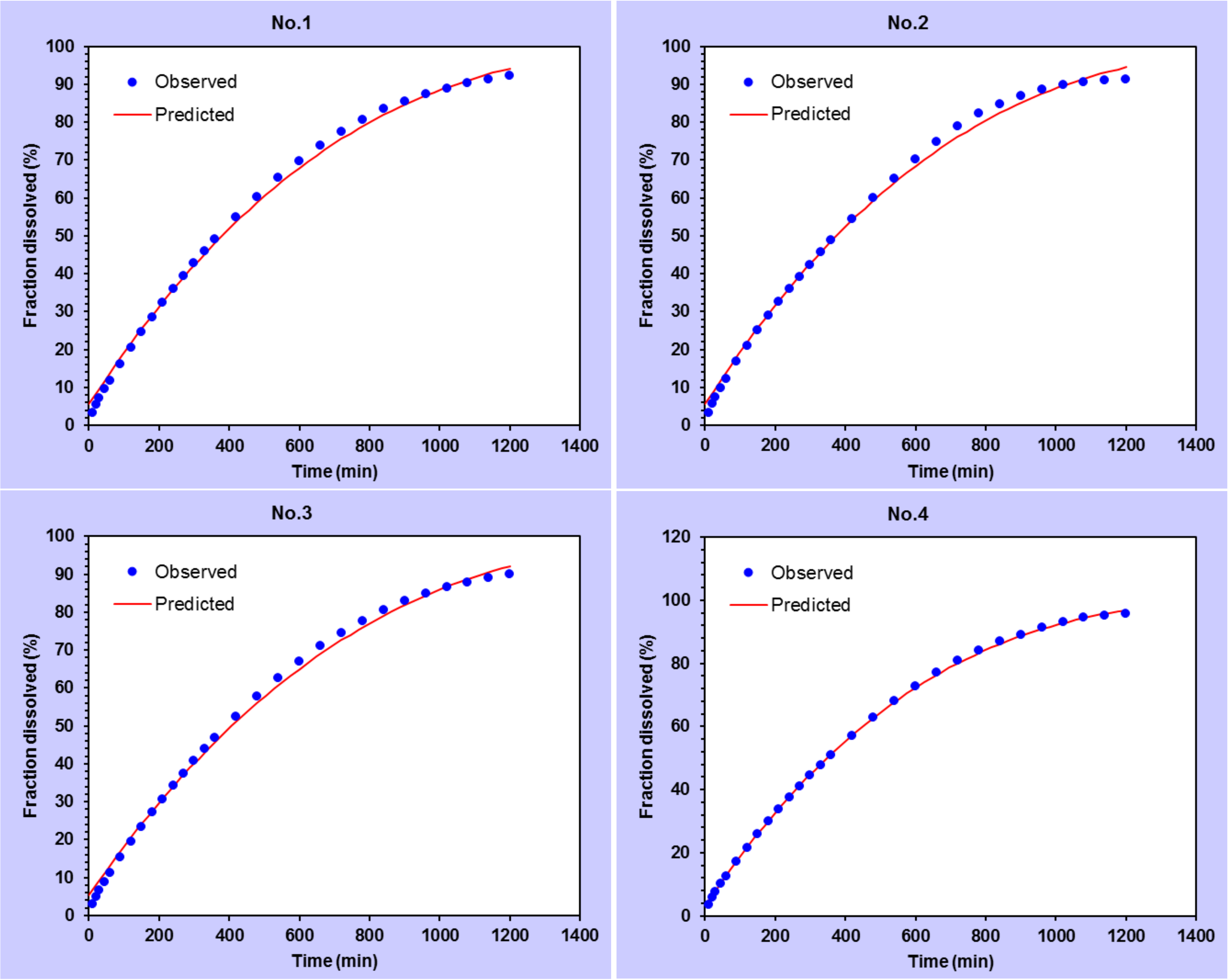

Supplementary materials – Model fitting summary – Granulated sugar N°1 600 (modelling the entire relevant dissolution data)

Model: **Baker–Lonsdale**

Model equation: $\frac{3}{2} \cdot \left[1 - \left(1 - \frac{F}{100}\right)^{\frac{2}{3}}\right] - \frac{F}{100} = k_{BL} \cdot t$

Fitted model parameters per tested tablet (N = 4) with statistics – mean, standard deviation (SD), and relative standard deviation expressed in % (RSD%) (output from DDSolver):

Parameter	No.1	No.2	No.3	No.4	Mean	SD	RSD(%)
k_{BL}	0.0003	0.0001	0.0002	0.0002	0.0002	0.0001	31.6908

Number of dissolution data points (N), degrees of freedom (df), and selected goodness of fit criteria – Pearson correlation coefficient (R), coefficient of determination (R^2), adjusted coefficient of determination ($R^2_{adjusted}$), and residual sum of squares (RSS) (manual calculation in MS Excel):

Parameter	No.1	No.2	No.3	No.4
N	29	29	29	29
df	28	28	28	28
R	0.988815326	0.992756507	0.989357472	0.993066878
R^2	0.977755749	0.985565482	0.978828207	0.986181824
$R^2_{adjusted}$	0.977755749	0.985565482	0.978828207	0.986181824
RSS	4641.507474	3341.170995	4423.593742	3230.99248

Graphical abstract of model fit presented as mean ± 1 SD of the fraction % of released carvedilol:

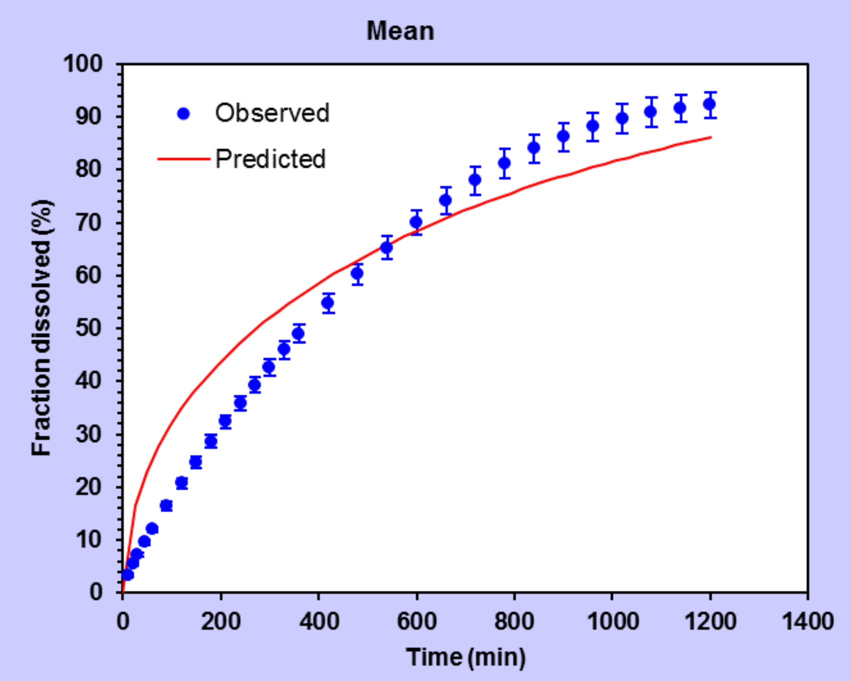

Supplementary materials – Model fitting summary – Granulated sugar N°1 600 (modelling the entire relevant dissolution data)

Graphical abstract of model fit presented as the fraction % of released carvedilol per tested tablet:

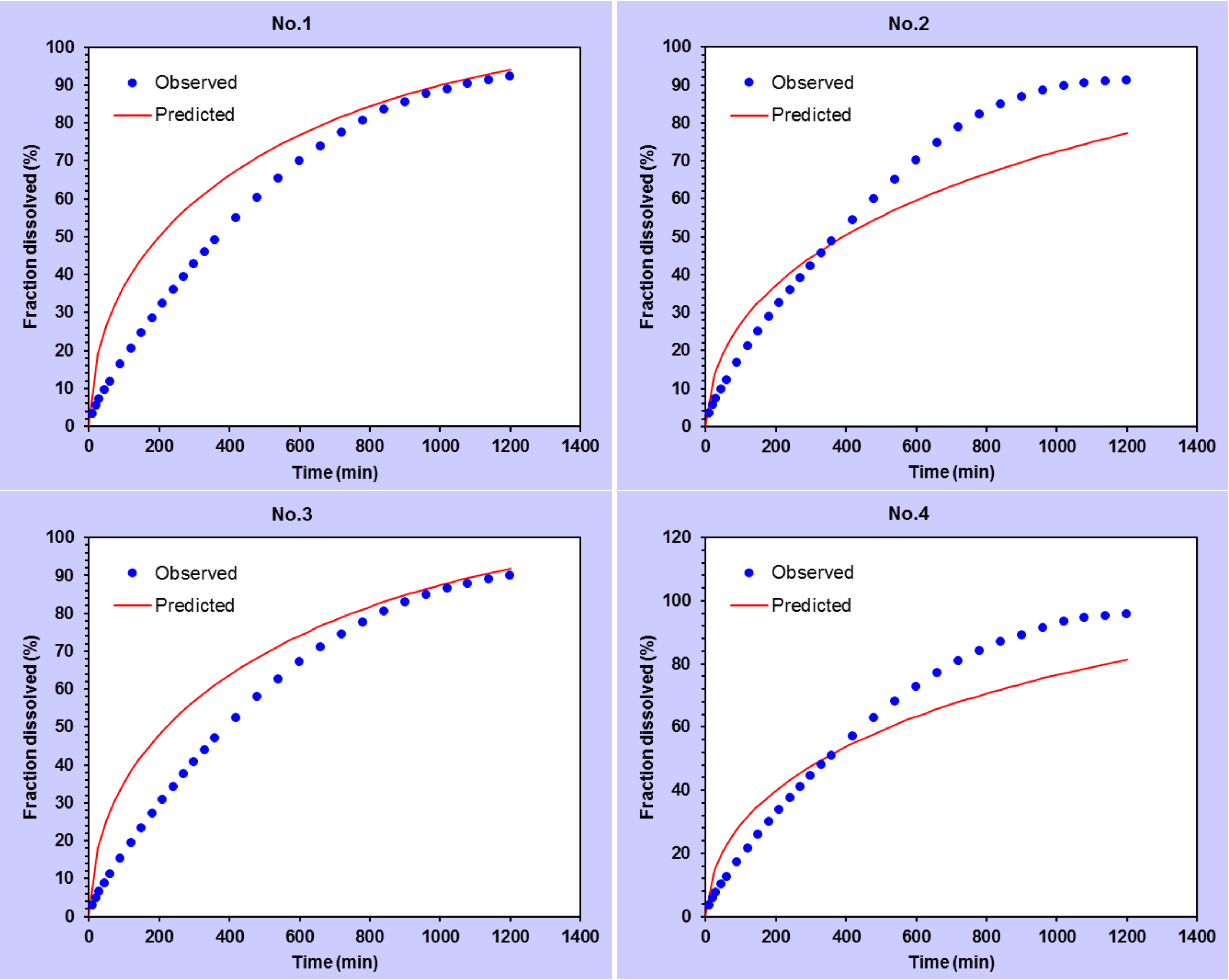

Supplementary materials – Model fitting summary – Granulated sugar N°1 600 (modelling the entire relevant dissolution data)

Model: **Baker–Lonsdale with T_{lag}**

Model equation: $\frac{3}{2}\cdot\left[1-\left(1-\frac{F}{100}\right)^{\frac{2}{3}}\right]-\frac{F}{100}=k_{BL}\cdot\left(t-T_{lag}\right)$

Fitted model parameters per tested tablet (N = 4) with statistics – mean, standard deviation (SD), and relative standard deviation expressed in % (RSD%) (output from DDSolver):

Parameter	No.1	No.2	No.3	No.4	Mean	SD	RSD(%)
k_{BL}	0.000	0.000	0.000	0.000	0.000	0.000	11.270
T_{lag}	99.924	99.089	101.378	107.779	102.042	3.939	3.860

Number of dissolution data points (N), degrees of freedom (df), and selected goodness of fit criteria – Pearson correlation coefficient (R), coefficient of determination (R^2), adjusted coefficient of determination ($R^2_{adjusted}$), and residual sum of squares (RSS) (manual calculation in MS Excel):

Parameter	No.1	No.2	No.3	No.4
N	29	29	29	29
df	27	27	27	27
R	0.985211343	0.982943741	0.985717942	0.983871959
R^2	0.97064139	0.966178398	0.971639862	0.968004031
$R^2_{adjusted}$	0.969554034	0.964925746	0.970589486	0.966818995
RSS	996.4579775	1131.396591	899.3826436	1221.87765

Graphical abstract of model fit presented as mean ± 1 SD of the fraction % of released carvedilol:

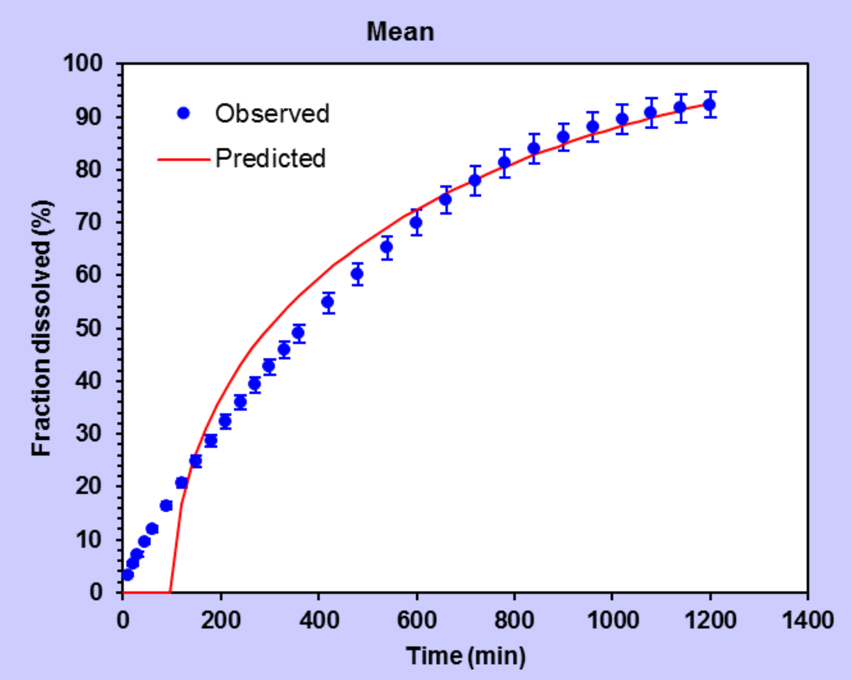

Supplementary materials – Model fitting summary – Granulated sugar N°1 600 (modelling the entire relevant dissolution data)

Graphical abstract of model fit presented as the fraction % of released carvedilol per tested tablet:

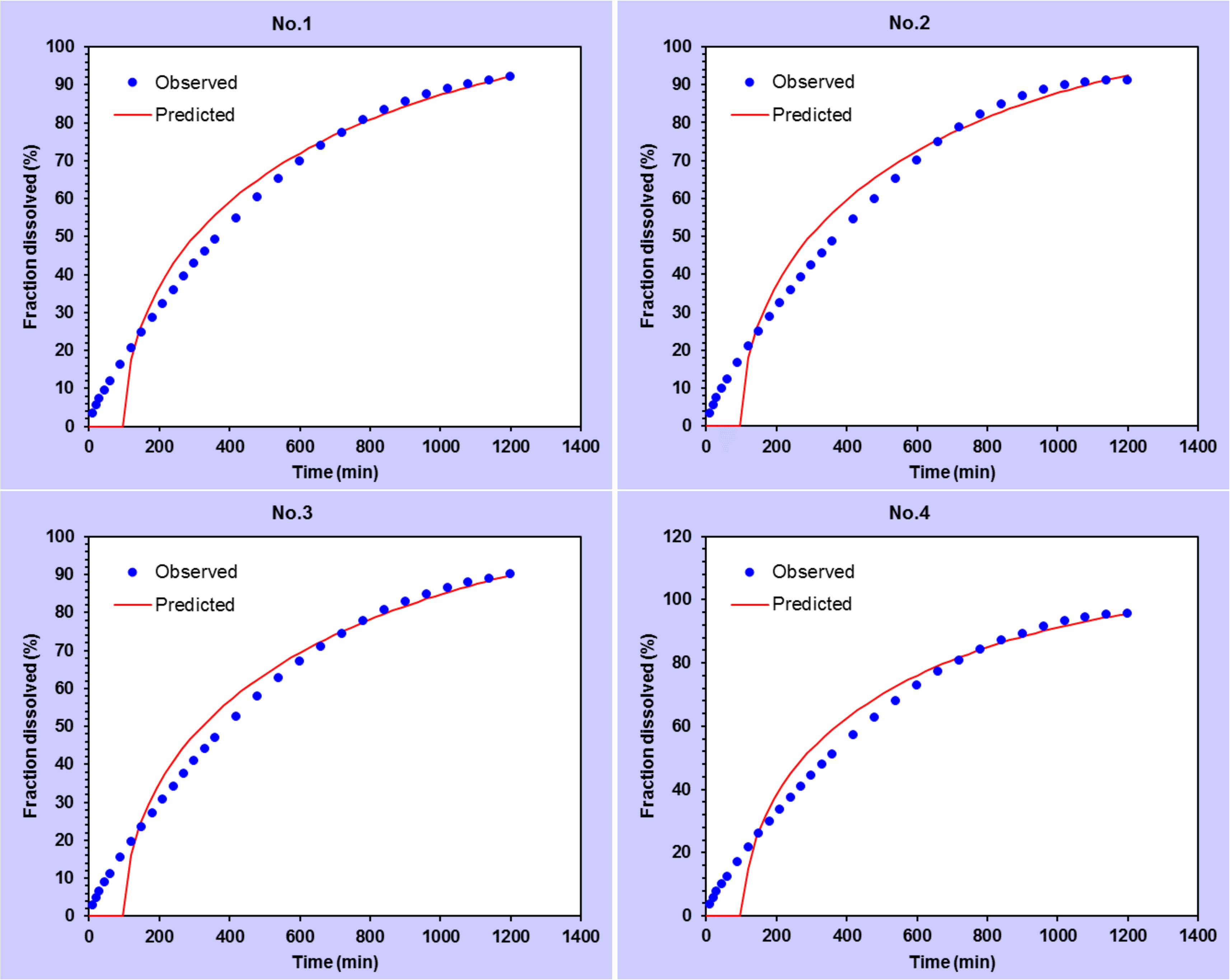

Supplementary materials – Model fitting summary – Granulated sugar N°1 600 (modelling the entire relevant dissolution data)

Model: **Makoid–Banakar**

Model equation: $F = k_{MB} \cdot t^n \cdot e^{-k \cdot t}$

Fitted model parameters per tested tablet (N = 4) with statistics – mean, standard deviation (SD), and relative standard deviation expressed in % (RSD%) (output from DDSolver):

Parameter	No.1	No.2	No.3	No.4	Mean	SD	RSD(%)
k_{MB}	0.5269	0.5152	0.4080	0.5325	0.4957	0.0589	11.8801
n	0.7910	0.7895	0.8237	0.7899	0.7985	0.0168	2.1012
k	0.0004	0.0003	0.0003	0.0003	0.0003	0.0000	9.8787

Number of dissolution data points (N), degrees of freedom (df), and selected goodness of fit criteria – Pearson correlation coefficient (R), coefficient of determination (R^2), adjusted coefficient of determination ($R^2_{adjusted}$), and residual sum of squares (RSS) (manual calculation in MS Excel):

Parameter	No.1	No.2	No.3	No.4
N	29	29	29	29
df	26	26	26	26
R	0.998970986	0.997427845	0.998768399	0.998062569
R^2	0.997943031	0.994862305	0.997538315	0.996128891
$R^2_{adjusted}$	0.997784802	0.994467098	0.997348954	0.995831113
RSS	84.67740157	137.1134869	61.99694105	111.268102

Graphical abstract of model fit presented as mean ± 1 SD of the fraction % of released carvedilol:

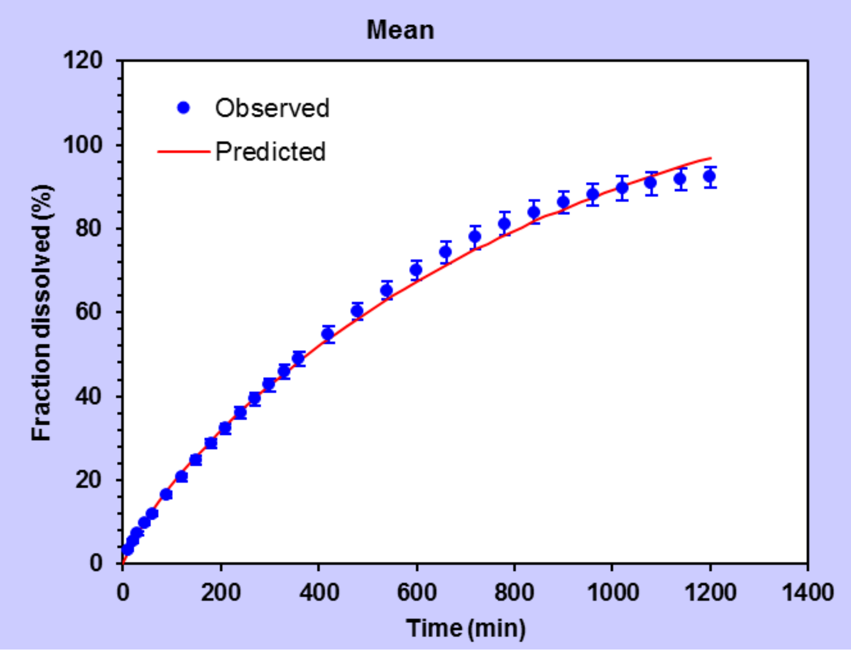

Supplementary materials – Model fitting summary – Granulated sugar N°1 600 (modelling the entire relevant dissolution data)

Graphical abstract of model fit presented as the fraction % of released carvedilol per tested tablet:

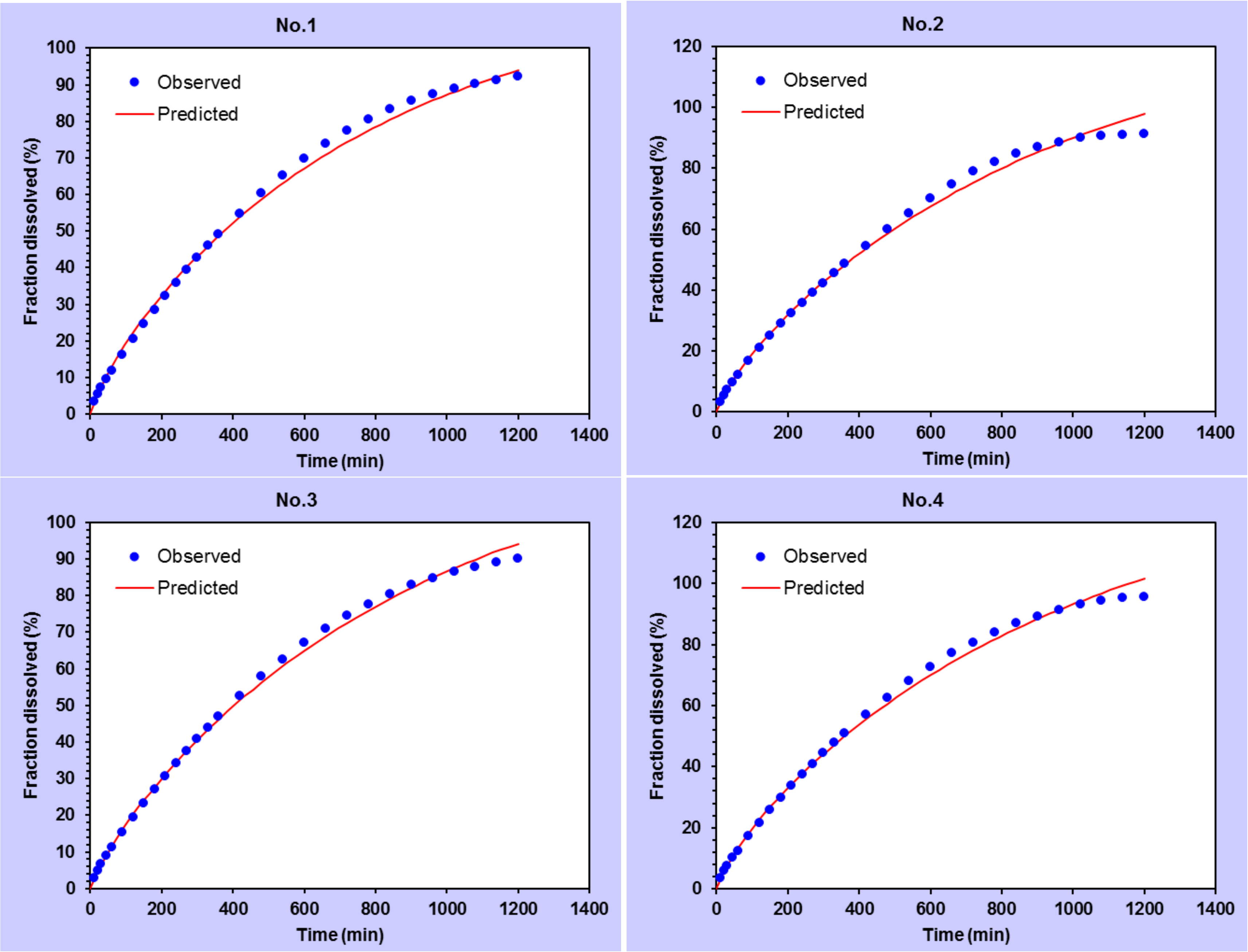

Supplementary materials – Model fitting summary – Granulated sugar N°1 600 (modelling the entire relevant dissolution data)

Model: **Makoid–Banakar with T_{lag}**

Model equation: $F = k_{MB} \cdot (t - T_{lag})^n \cdot e^{-k \cdot (t - T_{lag})}$

Fitted model parameters per tested tablet (N = 4) with statistics – mean, standard deviation (SD), and relative standard deviation expressed in % (RSD%) (output from DDSolver):

Parameter	No.1	No.2	No.3	No.4	Mean	SD	RSD(%)
k_{MB}	0.7862	0.8134	0.6563	0.8429	0.7747	0.0823	10.6173
n	0.7035	0.6984	0.7289	0.6982	0.7073	0.0146	2.0679
k	0.0001	0.0001	0.0001	0.0001	0.0001	0.0000	13.8509
T_{lag}	4.0000	4.0000	4.0000	4.0000	4.0000	0.0000	0.0000

Number of dissolution data points (N), degrees of freedom (df), and selected goodness of fit criteria – Pearson correlation coefficient (R), coefficient of determination (R^2), adjusted coefficient of determination ($R^2_{adjusted}$), and residual sum of squares (RSS) (manual calculation in MS Excel):

Parameter	No.1	No.2	No.3	No.4
N	29	29	29	29
df	25	25	25	25
R	0.995540012	0.994772041	0.996560117	0.995488431
R^2	0.991099916	0.989571414	0.993132067	0.990997216
$R^2_{adjusted}$	0.990031906	0.988319983	0.992307915	0.989916882
RSS	239.0510446	281.1903355	175.5555911	262.245181

Graphical abstract of model fit presented as mean ± 1 SD of the fraction % of released carvedilol:

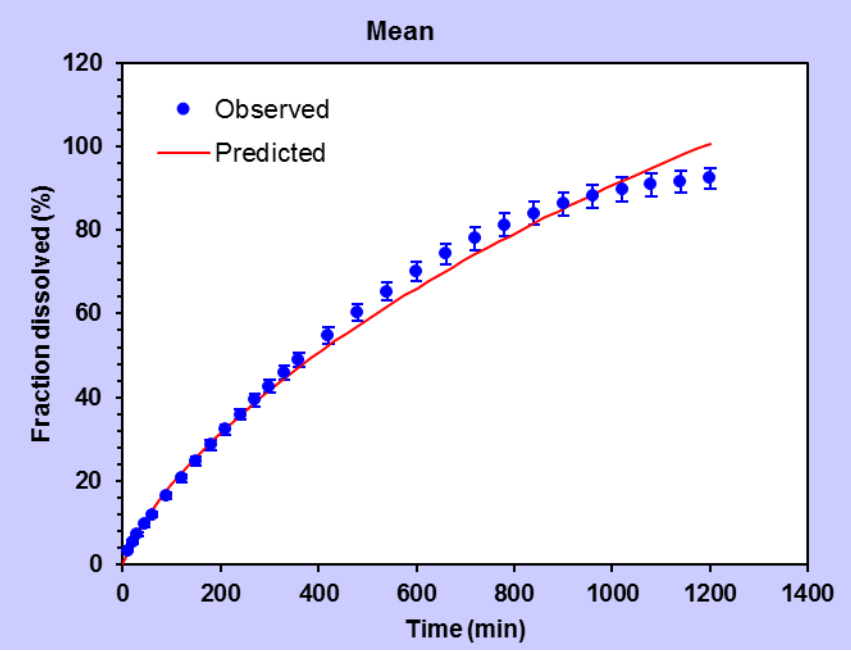

Supplementary materials – Model fitting summary – Granulated sugar N°1 600 (modelling the entire relevant dissolution data)

Graphical abstract of model fit presented as the fraction % of released carvedilol per tested tablet:

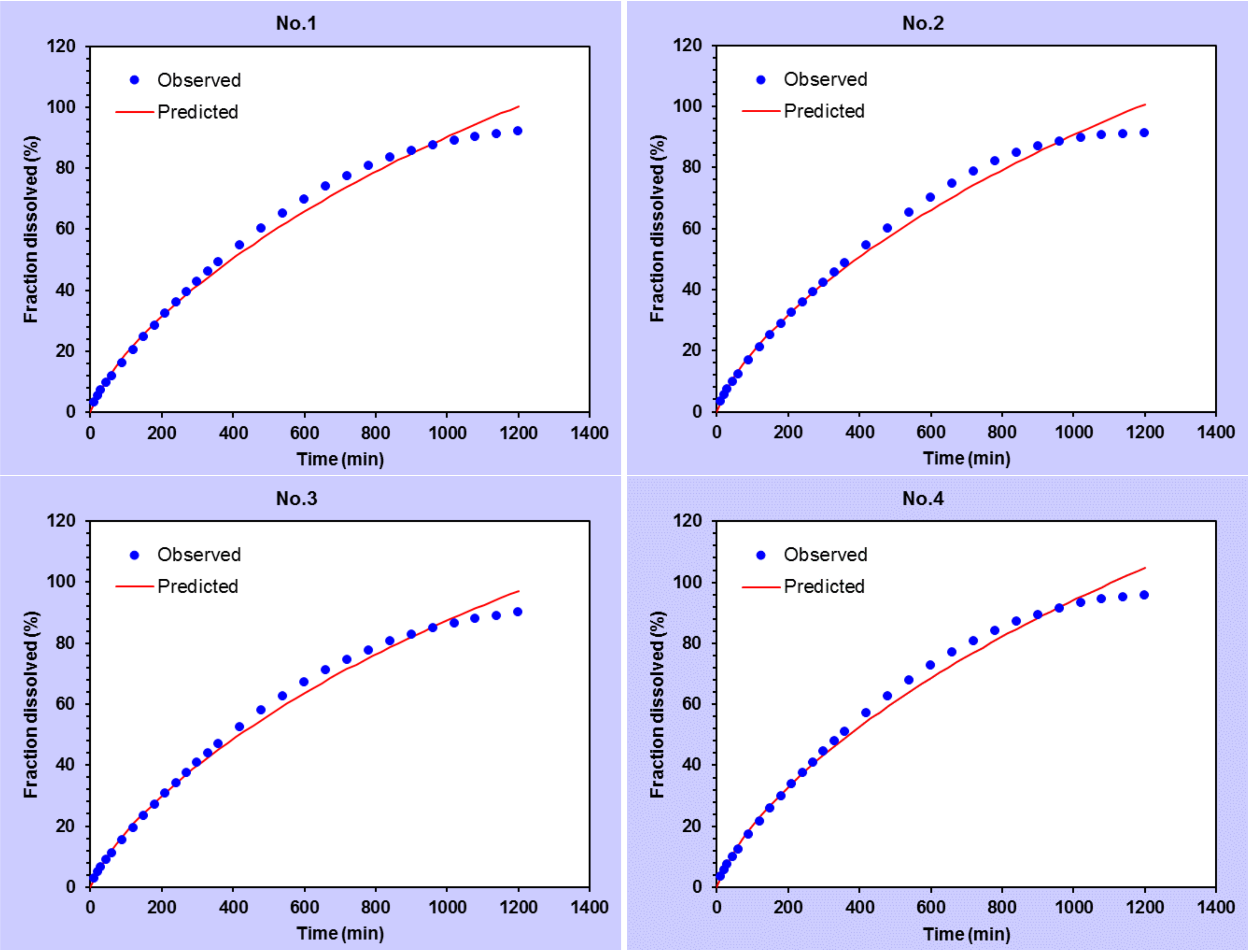

Supplementary materials – Model fitting summary – Granulated sugar N°1 600 (modelling the entire relevant dissolution data)

Model: **Peppas–Sahlin_1**

Model equation: $F = k_1 \cdot t^m + k_2 \cdot t^{2m}$

Fitted model parameters per tested tablet (N = 4) with statistics – mean, standard deviation (SD), and relative standard deviation expressed in % (RSD%) (output from DDSolver):

Parameter	No.1	No.2	No.3	No.4	Mean	SD	RSD(%)
k_1	2.169	2.178	1.972	2.263	2.146	0.123	5.739
k_2	0.082	0.082	0.085	0.085	0.084	0.002	2.216
m	0.450	0.450	0.450	0.450	0.450	0.000	0.000

Number of dissolution data points (N), degrees of freedom (df), and selected goodness of fit criteria – Pearson correlation coefficient (R), coefficient of determination (R^2), adjusted coefficient of determination ($R^2_{adjusted}$), and residual sum of squares (RSS) (manual calculation in MS Excel):

Parameter	No.1	No.2	No.3	No.4
N	29	29	29	29
df	26	26	26	26
R	0.991704566	0.990893264	0.992431765	0.991820721
R^2	0.983477946	0.981869461	0.984920807	0.983708343
$R^2_{adjusted}$	0.982207019	0.980474804	0.983760869	0.982455138
RSS	480.6977475	526.9008647	417.4533818	513.6065327

Graphical abstract of model fit presented as mean ± 1 SD of the fraction % of released carvedilol:

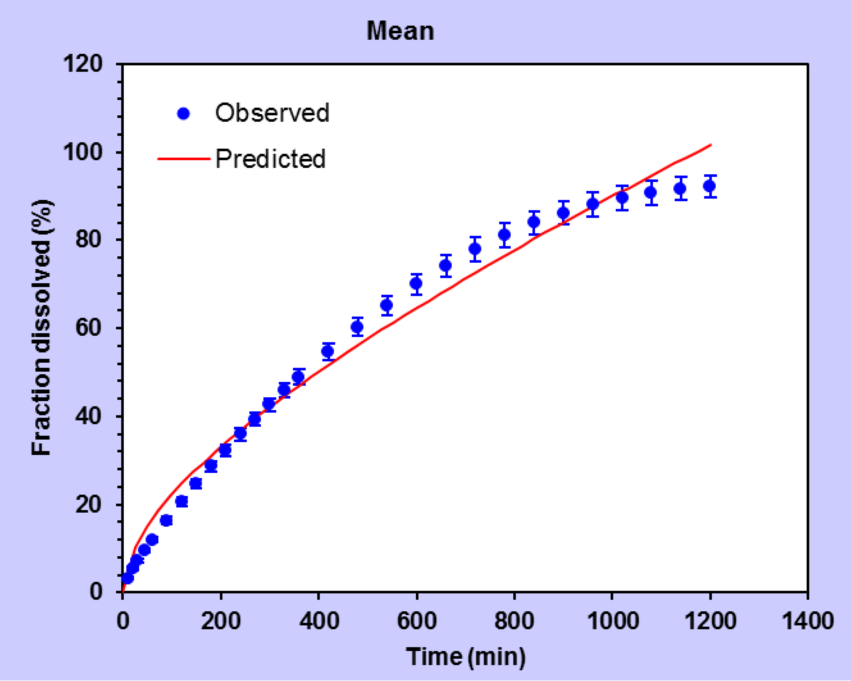

Supplementary materials – Model fitting summary – Granulated sugar N°1 600 (modelling the entire relevant dissolution data)

Graphical abstract of model fit presented as the fraction % of released carvedilol per tested tablet:

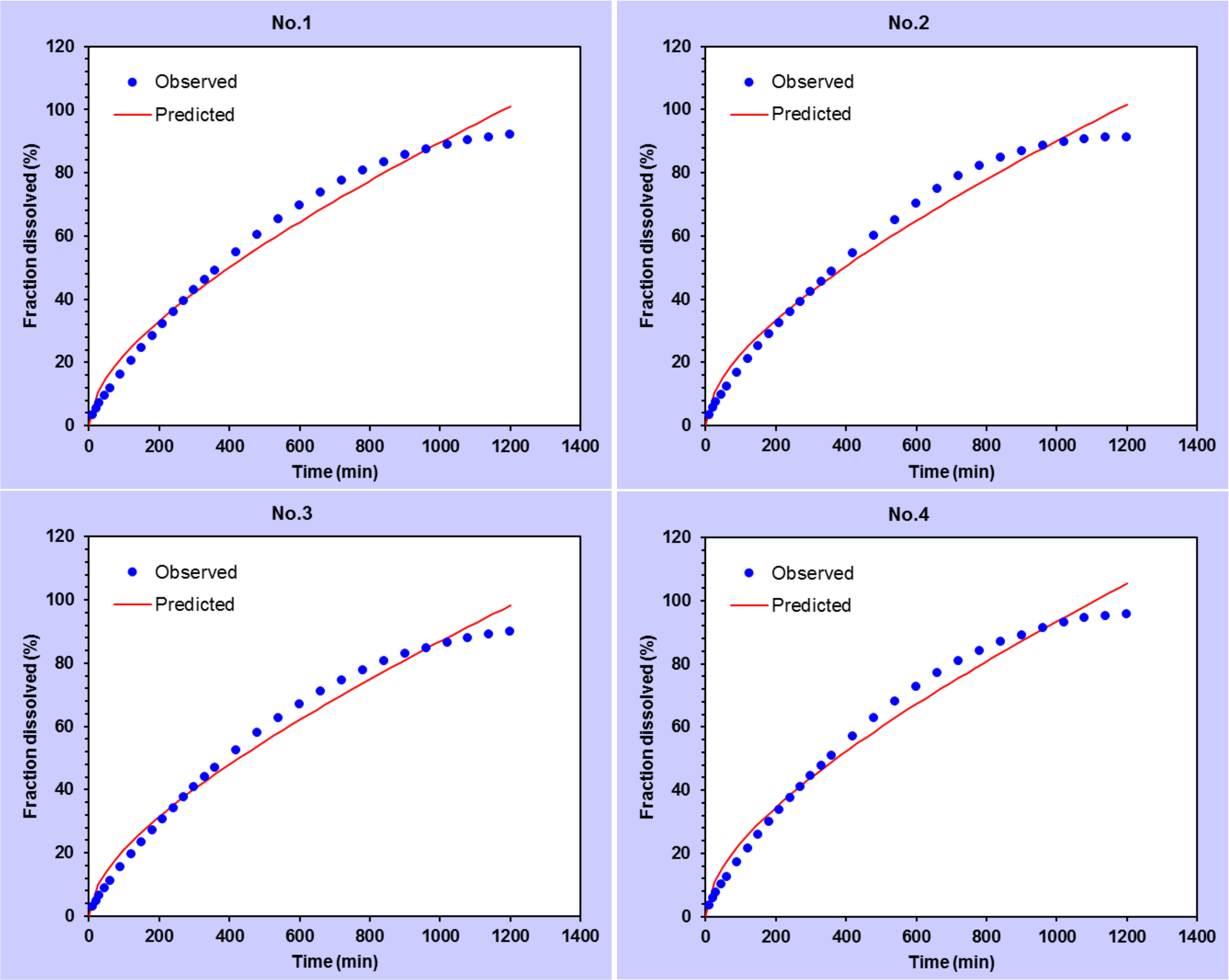

Supplementary materials – Model fitting summary – Granulated sugar N°1 600 (modelling the entire relevant dissolution data)

Model: **Peppas–Sahlin_1 with T_{lag}**

Model equation: $F = k_1 \cdot (t - T_{lag})^m + k_2 \cdot (t - T_{lag})^{2m}$

Fitted model parameters per tested tablet (N = 4) with statistics – mean, standard deviation (SD), and relative standard deviation expressed in % (RSD%) (output from DDSolver):

Parameter	No.1	No.2	No.3	No.4	Mean	SD	RSD(%)
k_1	2.264	2.273	2.063	2.361	2.240	0.126	5.640
k_2	0.078	0.078	0.082	0.081	0.080	0.002	2.346
m	0.450	0.450	0.450	0.450	0.450	0.000	0.000
T_{lag}	6.000	6.000	6.000	6.000	6.000	0.000	0.000

Number of dissolution data points (N), degrees of freedom (df), and selected goodness of fit criteria – Pearson correlation coefficient (R), coefficient of determination (R^2), adjusted coefficient of determination ($R^2_{adjusted}$), and residual sum of squares (RSS) (manual calculation in MS Excel):

Parameter	No.1	No.2	No.3	No.4
N	29	29	29	29
df	25	25	25	25
R	0.99242187	0.991587168	0.993118386	0.992522171
R^2	0.984901169	0.983245112	0.986284128	0.98510026
$R^2_{adjusted}$	0.983089309	0.981234525	0.984638223	0.983312291
RSS	424.3088651	471.5640525	366.9324468	453.6164012

Graphical abstract of model fit presented as mean ± 1 SD of the fraction % of released carvedilol:

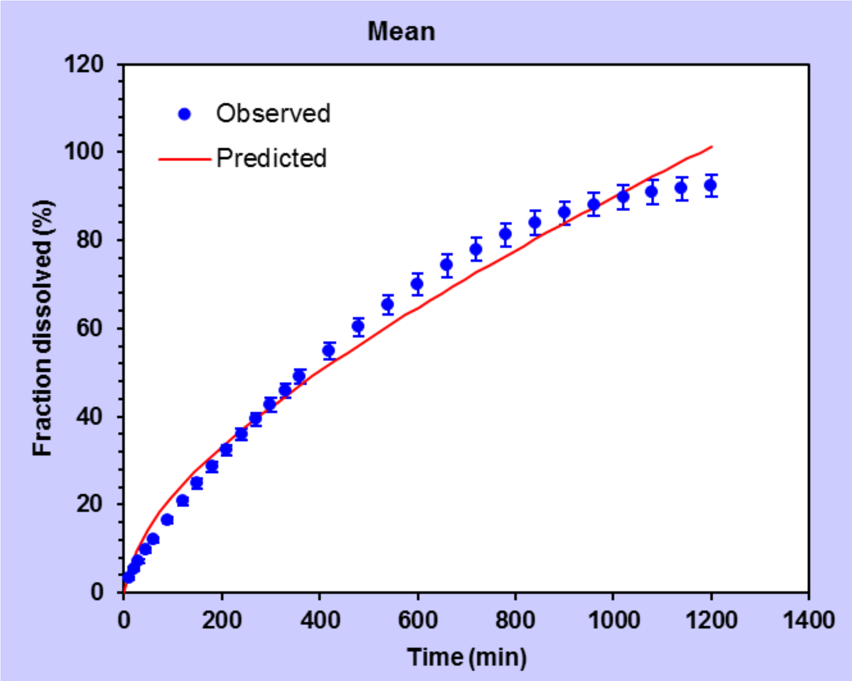

Supplementary materials – Model fitting summary – Granulated sugar N°1 600 (modelling the entire relevant dissolution data)

Graphical abstract of model fit presented as the fraction % of released carvedilol per tested tablet:

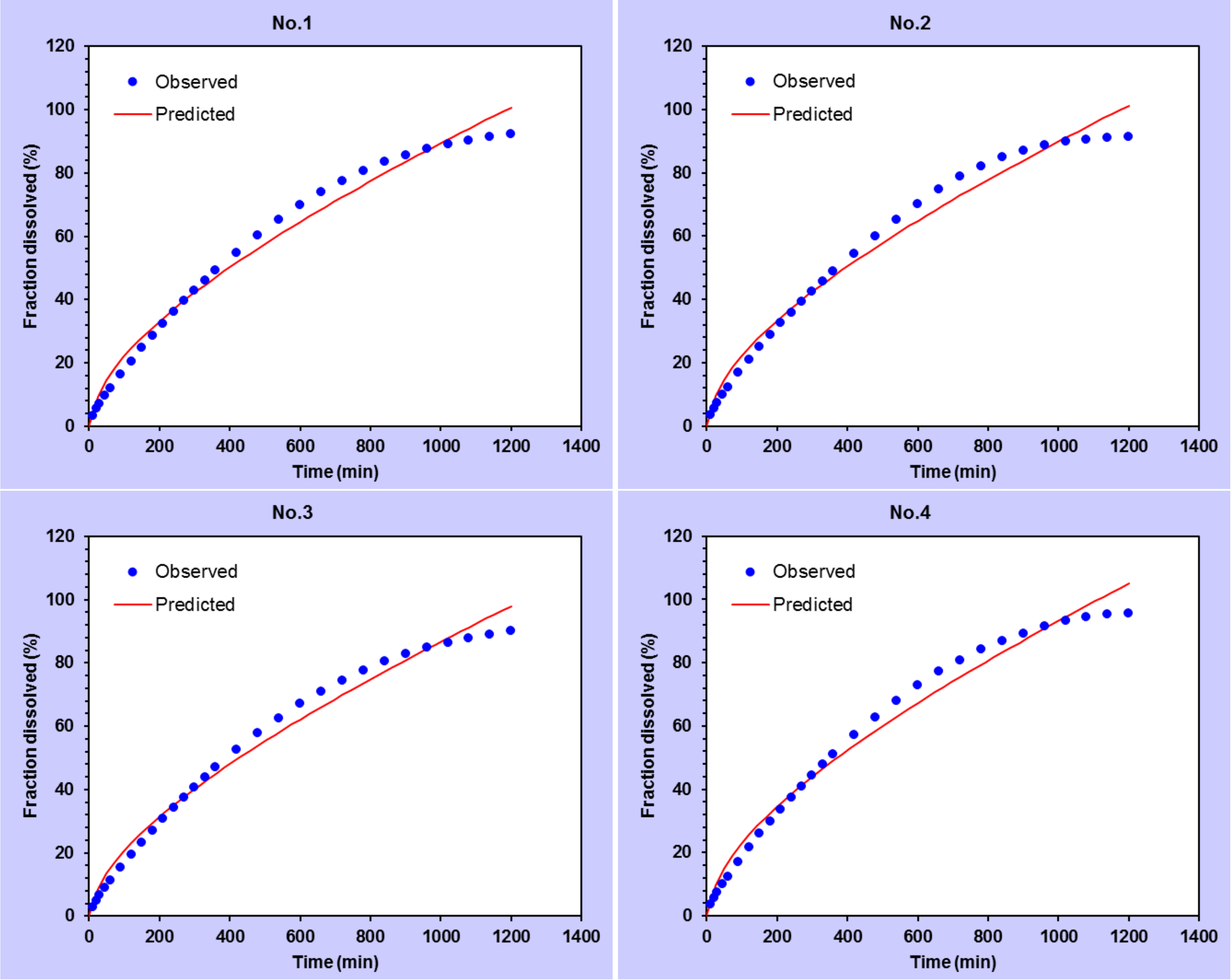

Supplementary materials – Model fitting summary – Granulated sugar N°1 600 (modelling the entire relevant dissolution data)

Model: **Peppas–Sahlin_2**

Model equation: $F = k_1 \cdot t^{0.5} + k_2 \cdot t$

Fitted model parameters per tested tablet (N = 4) with statistics – mean, standard deviation (SD), and relative standard deviation expressed in % (RSD%) (output from DDSolver):

Parameter	No.1	No.2	No.3	No.4	Mean	SD	RSD(%)
k_1	2.006	2.014	1.850	2.091	1.990	0.101	5.087
k_2	0.026	0.026	0.028	0.027	0.027	0.001	3.858

Number of dissolution data points (N), degrees of freedom (df), and selected goodness of fit criteria – Pearson correlation coefficient (R), coefficient of determination (R^2), adjusted coefficient of determination ($R^2_{adjusted}$), and residual sum of squares (RSS) (manual calculation in MS Excel):

Parameter	No.1	No.2	No.3	No.4
N	29	29	29	29
df	27	27	27	27
R	0.992313976	0.991478586	0.992894649	0.992411852
R^2	0.984687026	0.983029786	0.985839784	0.984881284
$R^2_{adjusted}$	0.984119879	0.982401259	0.985315332	0.984321332
RSS	463.8983599	510.8069735	408.4403738	495.7776605

Graphical abstract of model fit presented as mean ± 1 SD of the fraction % of released carvedilol:

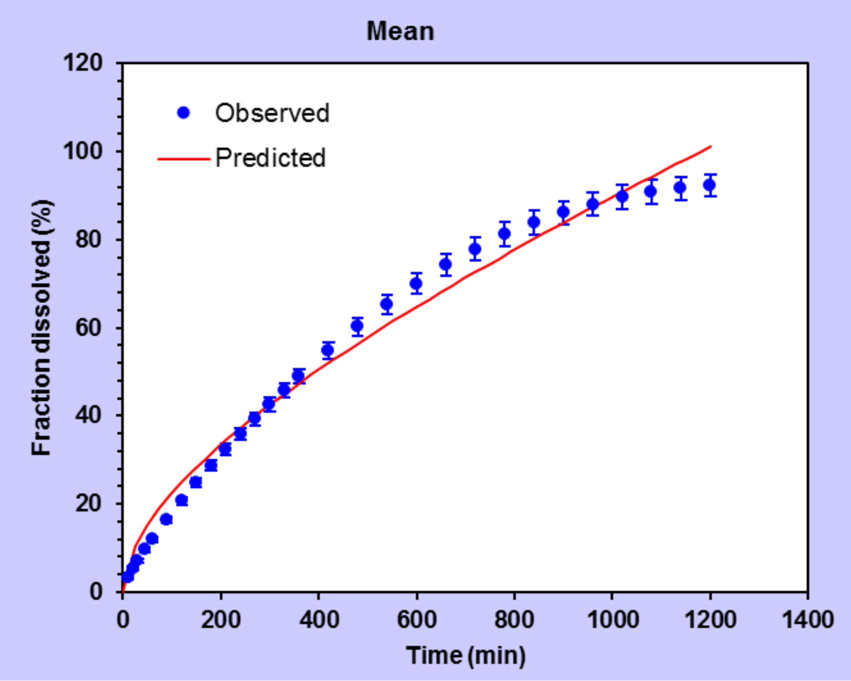

Supplementary materials – Model fitting summary – Granulated sugar N°1 600 (modelling the entire relevant dissolution data)

Graphical abstract of model fit presented as the fraction % of released carvedilol per tested tablet:

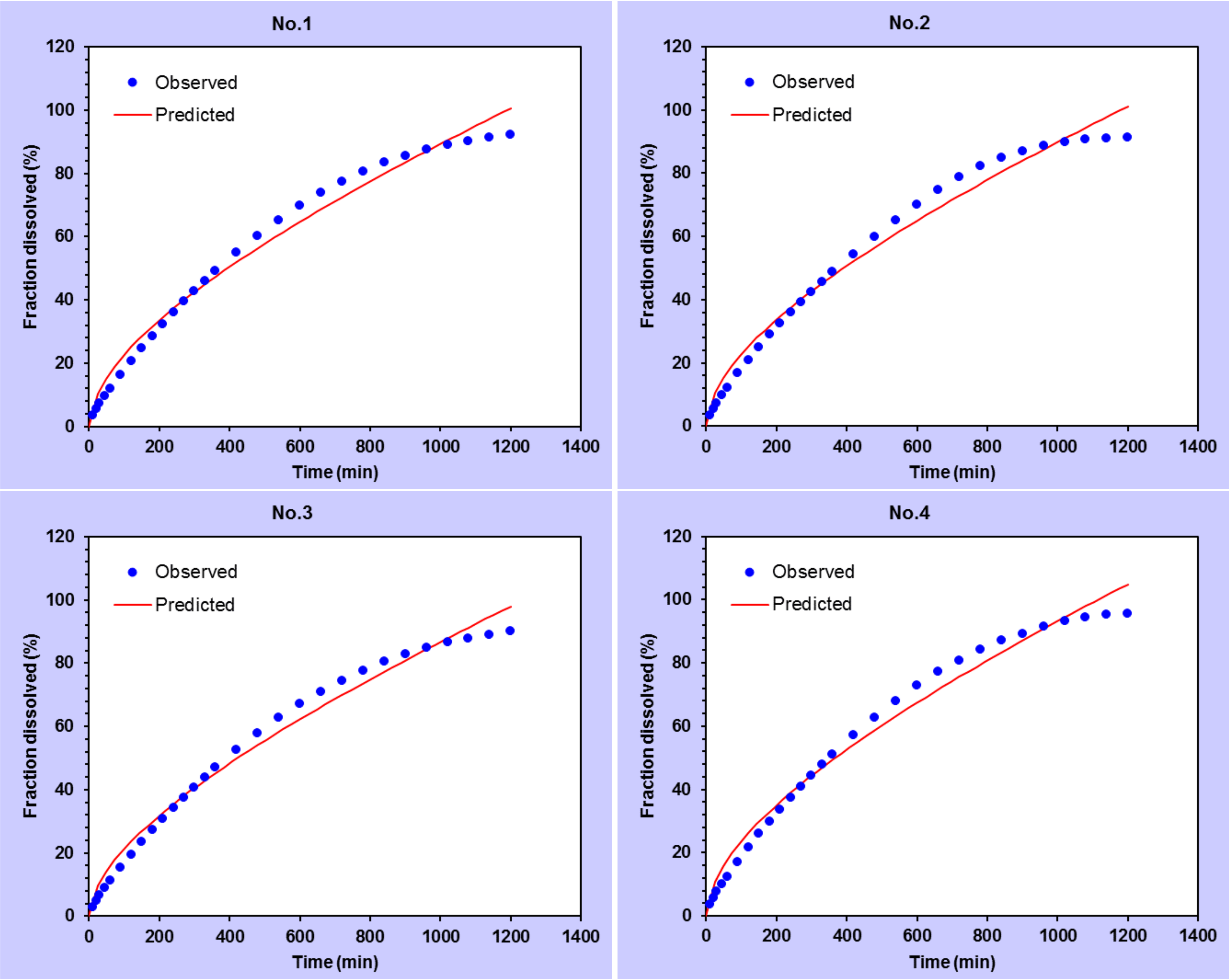

Supplementary materials – Model fitting summary – Granulated sugar N°1 600 (modelling the entire relevant dissolution data)

Model: **Peppas–Sahlin_2 with T_{lag}**

Model equation: $F = k_1 \cdot (t - T_{lag})^{0.5} + k_2 \cdot (t - T_{lag})$

Fitted model parameters per tested tablet (N = 4) with statistics – mean, standard deviation (SD), and relative standard deviation expressed in % (RSD%) (output from DDSolver):

Parameter	No.1	No.2	No.3	No.4	Mean	SD	RSD(%)
k_1	2.071	2.080	1.913	2.159	2.056	0.103	5.031
k_2	0.024	0.024	0.026	0.025	0.025	0.001	4.312
T_{lag}	6.000	6.000	6.000	6.000	6.000	0.000	0.000

Number of dissolution data points (N), degrees of freedom (df), and selected goodness of fit criteria – Pearson correlation coefficient (R), coefficient of determination (R^2), adjusted coefficient of determination ($R^2_{adjusted}$), and residual sum of squares (RSS) (manual calculation in MS Excel):

Parameter	No.1	No.2	No.3	No.4
N	29	29	29	29
df	26	26	26	26
R	0.992992527	0.992133777	0.993558812	0.993074043
R^2	0.986034159	0.984329431	0.987159114	0.986196055
$R^2_{adjusted}$	0.984959864	0.983124003	0.986171353	0.985134213
RSS	403.5981547	451.5903104	353.7073541	431.6994727

Graphical abstract of model fit presented as mean ± 1 SD of the fraction % of released carvedilol:

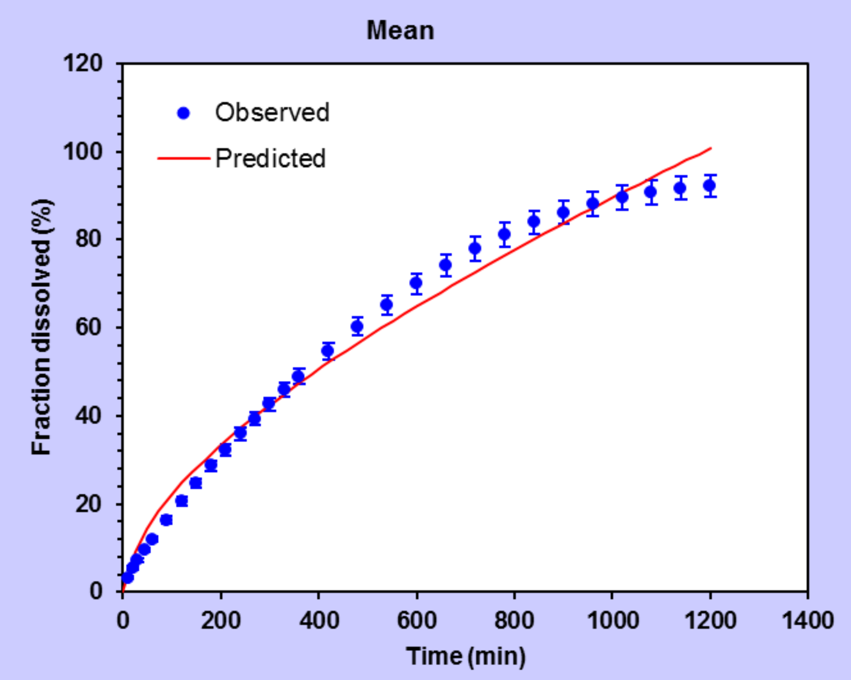

Supplementary materials – Model fitting summary – Granulated sugar N°1 600 (modelling the entire relevant dissolution data)

Graphical abstract of model fit presented as the fraction % of released carvedilol per tested tablet:

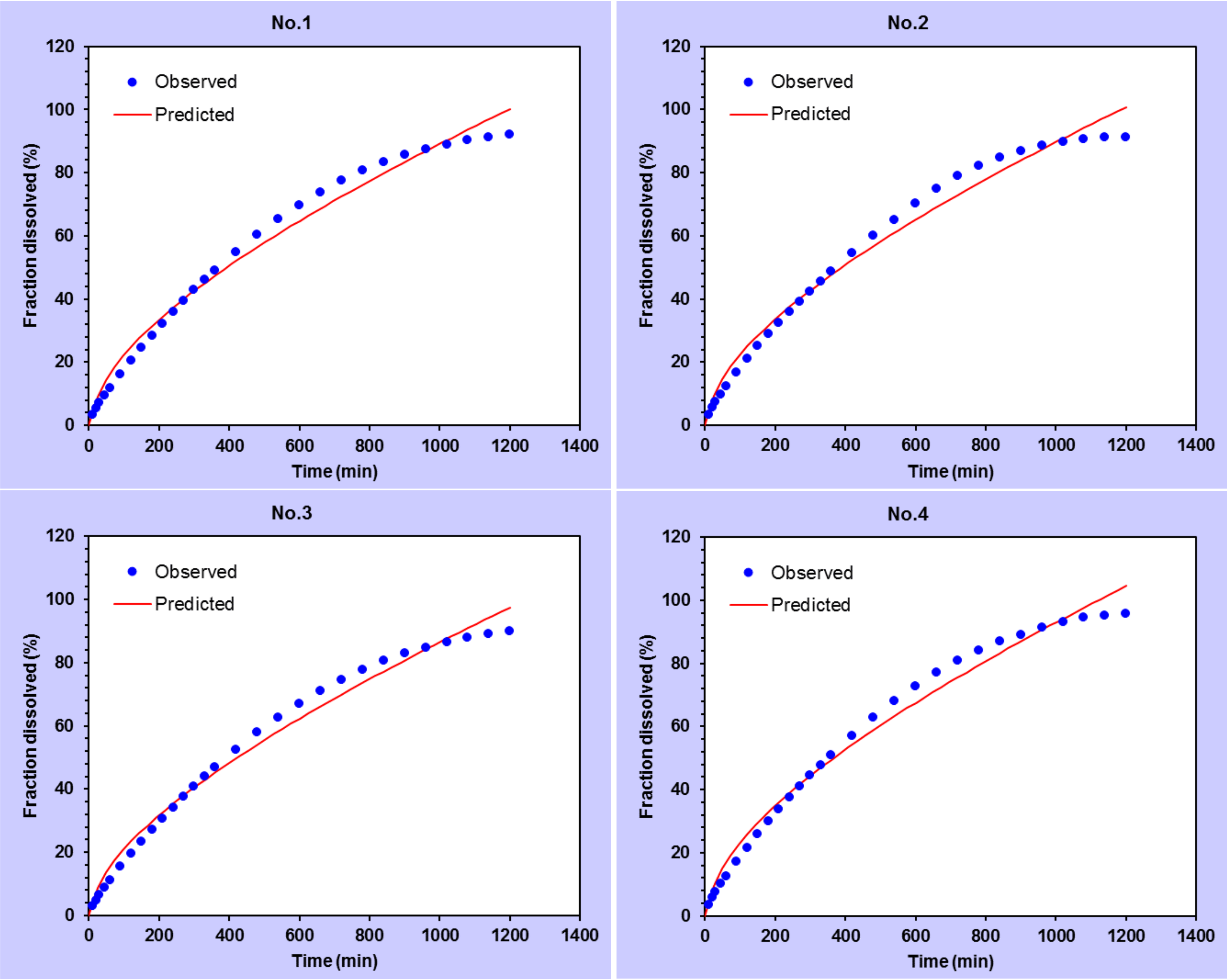

Supplementary materials – Model fitting summary – Granulated sugar N°1 600 (modelling the entire relevant dissolution data)

Model: **Quadratic**

Model equation: $F = 100 \cdot (k_1 \cdot t^2 + k_2 \cdot t)$

Fitted model parameters per tested tablet (N = 4) with statistics – mean, standard deviation (SD), and relative standard deviation expressed in % (RSD%) (output from DDSolver):

Parameter	No.1	No.2	No.3	No.4	Mean	SD	RSD(%)
k_1	-0.000001	-0.000001	-0.000001	-0.000001	-0.000001	0.000000	-4.959934
k_2	0.001616	0.001628	0.001535	0.001684	0.001616	0.000062	3.807809

Number of dissolution data points (N), degrees of freedom (df), and selected goodness of fit criteria – Pearson correlation coefficient (R), coefficient of determination (R^2), adjusted coefficient of determination ($R^2_{adjusted}$), and residual sum of squares (RSS) (manual calculation in MS Excel):

Parameter	No.1	No.2	No.3	No.4
N	29	29	29	29
df	27	27	27	27
R	0.999559246	0.999590941	0.999609674	0.999569937
R^2	0.999118685	0.99918205	0.999219501	0.999140059
$R^2_{adjusted}$	0.999086044	0.999151755	0.999190594	0.999108209
RSS	70.65041327	67.39611712	55.12076189	79.14904567

Graphical abstract of model fit presented as mean ± 1 SD of the fraction % of released carvedilol:

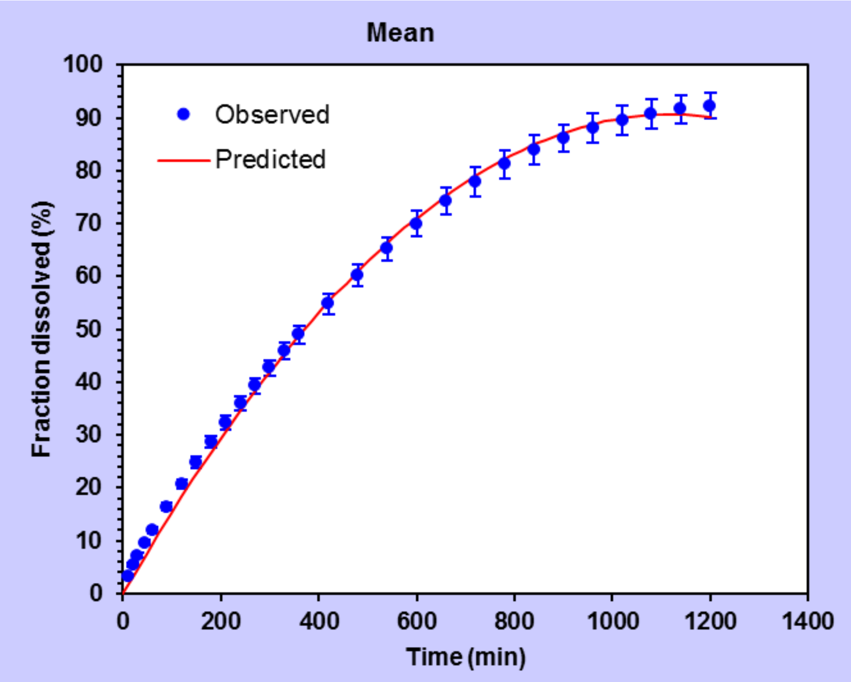

Supplementary materials – Model fitting summary – Granulated sugar N°1 600 (modelling the entire relevant dissolution data)

Graphical abstract of model fit presented as the fraction % of released carvedilol per tested tablet:

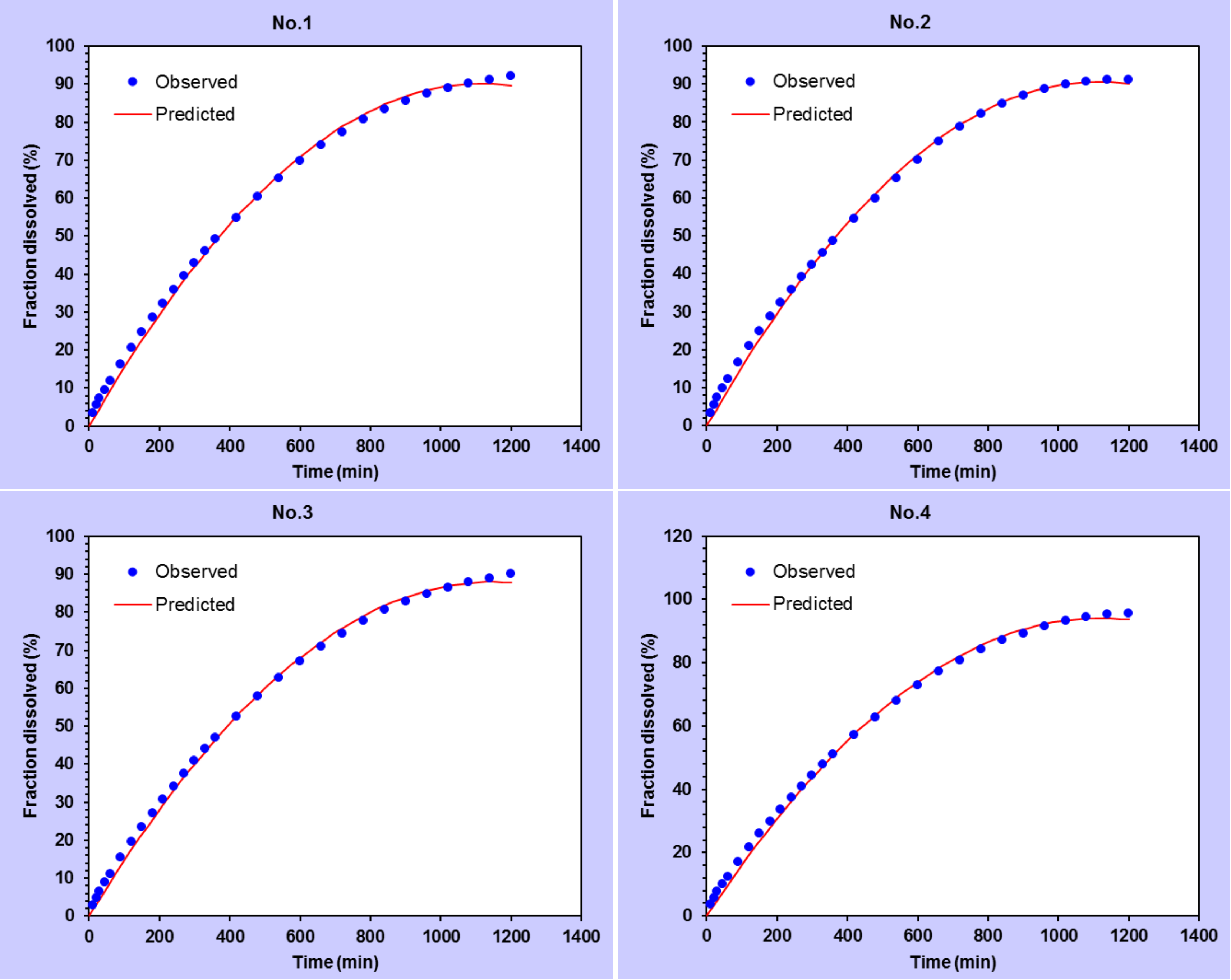

Supplementary materials – Model fitting summary – Granulated sugar N°1 600 (modelling the entire relevant dissolution data)

Model: **Quadratic with T_{lag}**

Model equation: $F = 100 \cdot \left[k_1 \cdot (t - T_{lag})^2 + k_2 \cdot (t - T_{lag})\right]$

Fitted model parameters per tested tablet (N = 4) with statistics – mean, standard deviation (SD), and relative standard deviation expressed in % (RSD%) (output from DDSolver):

Parameter	No.1	No.2	No.3	No.4	Mean	SD	RSD(%)
k_1	-0.000001	-0.000001	-0.000001	-0.000001	-0.000001	0.000000	-4.927101
k_2	0.001633	0.001646	0.001552	0.001702	0.001633	0.000062	3.801135
T_{lag}	4.000000	4.000000	4.000000	4.000000	4.000000	0.000000	0.000000

Number of dissolution data points (N), degrees of freedom (df), and selected goodness of fit criteria – Pearson correlation coefficient (R), coefficient of determination (R^2), adjusted coefficient of determination ($R^2_{adjusted}$), and residual sum of squares (RSS) (manual calculation in MS Excel):

Parameter	No.1	No.2	No.3	No.4
N	29	29	29	29
df	26	26	26	26
R	0.999448668	0.999482916	0.99950731	0.999456641
R^2	0.99889764	0.998966099	0.999014863	0.998913578
$R^2_{adjusted}$	0.998812843	0.998886569	0.998939084	0.998830007
RSS	98.9068036	95.36353836	78.70719662	110.6529227

Graphical abstract of model fit presented as mean ± 1 SD of the fraction % of released carvedilol:

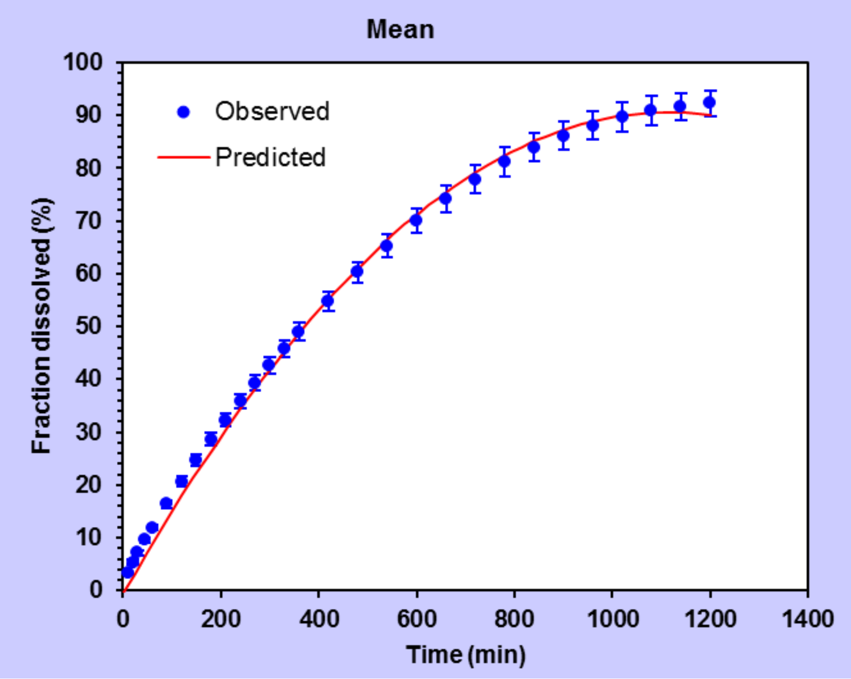

Supplementary materials – Model fitting summary – Granulated sugar N°1 600 (modelling the entire relevant dissolution data)

Graphical abstract of model fit presented as the fraction % of released carvedilol per tested tablet:

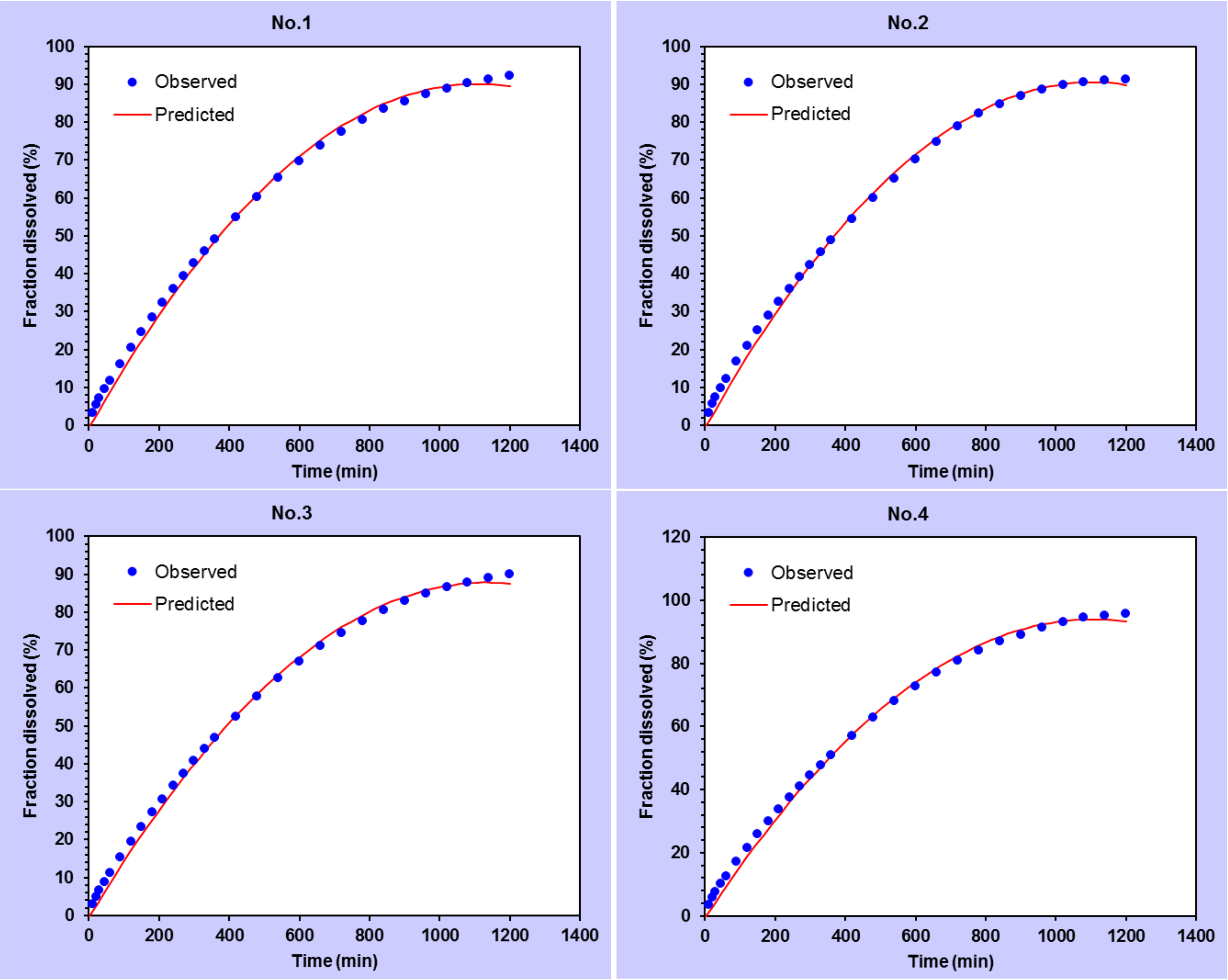

Supplementary materials – Model fitting summary – Granulated sugar N°1 600 (modelling the entire relevant dissolution data)

Model: **Weibull_1**

Model equation: $F = 100 \cdot \left[1 - e^{-\frac{(t-T_i)^{\beta}}{\alpha}}\right]$

Fitted model parameters per tested tablet (N = 4) with statistics – mean, standard deviation (SD), and relative standard deviation expressed in % (RSD%) (output from DDSolver):

Parameter	No.1	No.2	No.3	No.4	Mean	SD	RSD(%)
α	239.696	235.097	265.481	252.777	248.263	13.706	5.521
β	0.884	0.883	0.887	0.910	0.891	0.013	1.438
T_i	6.000	6.000	6.000	6.000	6.000	0.000	0.000

Number of dissolution data points (N), degrees of freedom (df), and selected goodness of fit criteria – Pearson correlation coefficient (R), coefficient of determination (R^2), adjusted coefficient of determination ($R^2_{adjusted}$), and residual sum of squares (RSS) (manual calculation in MS Excel):

Parameter	No.1	No.2	No.3	No.4
N	29	29	29	29
df	26	26	26	26
R	0.996125101	0.994857067	0.997057482	0.994126606
R^2	0.992265216	0.989740583	0.994123623	0.988287708
$R^2_{adjusted}$	0.991670233	0.988951397	0.993671594	0.987386762
RSS	295.1654864	370.8595021	225.2070282	441.1468626

Graphical abstract of model fit presented as mean ± 1 SD of the fraction % of released carvedilol:

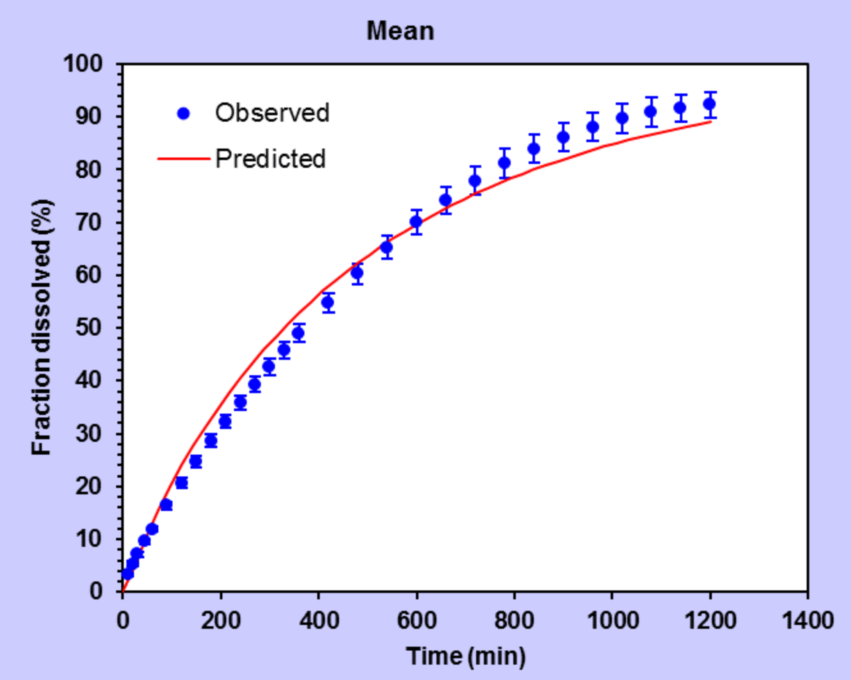

Supplementary materials – Model fitting summary – Granulated sugar N°1 600 (modelling the entire relevant dissolution data)

Graphical abstract of model fit presented as the fraction % of released carvedilol per tested tablet:

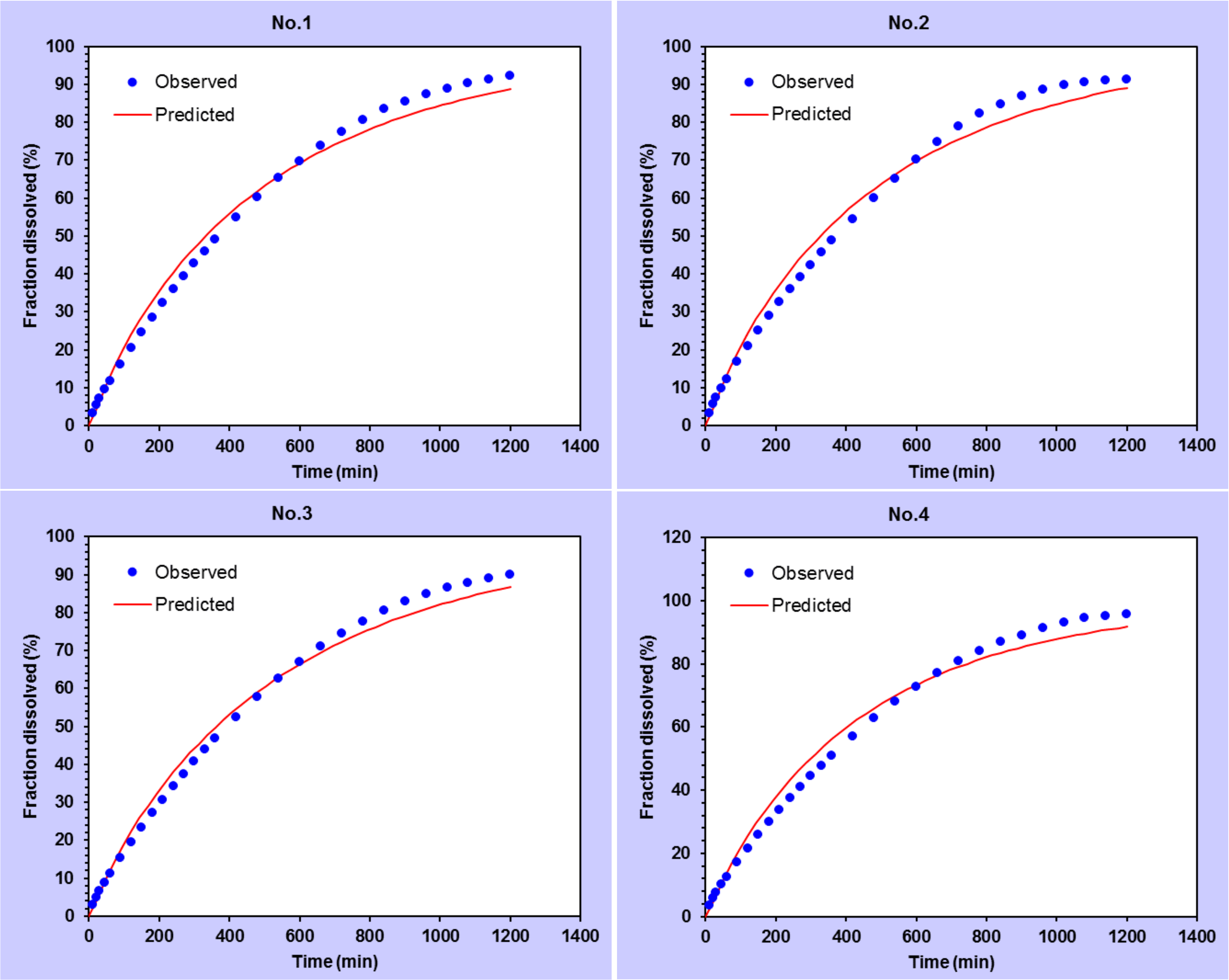

Supplementary materials – Model fitting summary – Granulated sugar N°1 600 (modelling the entire relevant dissolution data)

Model: **Weibull_2**

Model equation: $F = 100 \cdot \left(1 - e^{-\frac{t^{\beta}}{\alpha}}\right)$

Fitted model parameters per tested tablet (N = 4) with statistics – mean, standard deviation (SD), and relative standard deviation expressed in % (RSD%) (output from DDSolver):

Parameter	No.1	No.2	No.3	No.4	Mean	SD	RSD(%)
α	413.839	405.544	385.657	373.661	394.675	18.333	4.645
β	0.977	0.975	0.947	0.972	0.968	0.014	1.465

Number of dissolution data points (N), degrees of freedom (df), and selected goodness of fit criteria – Pearson correlation coefficient (R), coefficient of determination (R^2), adjusted coefficient of determination ($R^2_{adjusted}$), and residual sum of squares (RSS) (manual calculation in MS Excel):

Parameter	No.1	No.2	No.3	No.4
N	29	29	29	29
df	27	27	27	27
R	0.997753721	0.996663951	0.998413198	0.996015073
R^2	0.995512488	0.99333903	0.996828914	0.992046025
$R^2_{adjusted}$	0.995346284	0.993092328	0.996711466	0.991751433
RSS	165.0325694	229.7500164	119.4211073	301.5517411

Graphical abstract of model fit presented as mean ± 1 SD of the fraction % of released carvedilol:

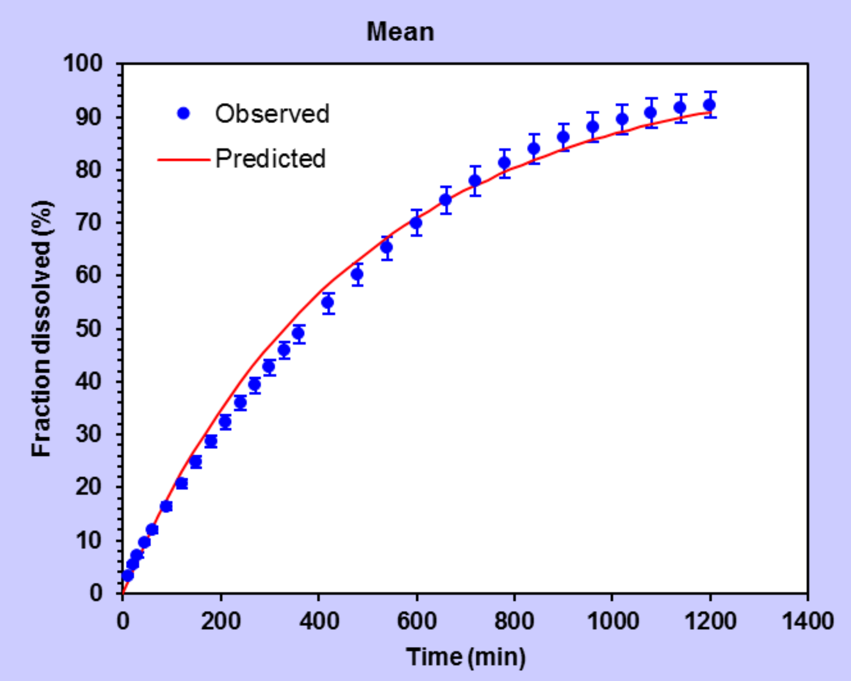

Supplementary materials – Model fitting summary – Granulated sugar N°1 600 (modelling the entire relevant dissolution data)

Graphical abstract of model fit presented as the fraction % of released carvedilol per tested tablet:

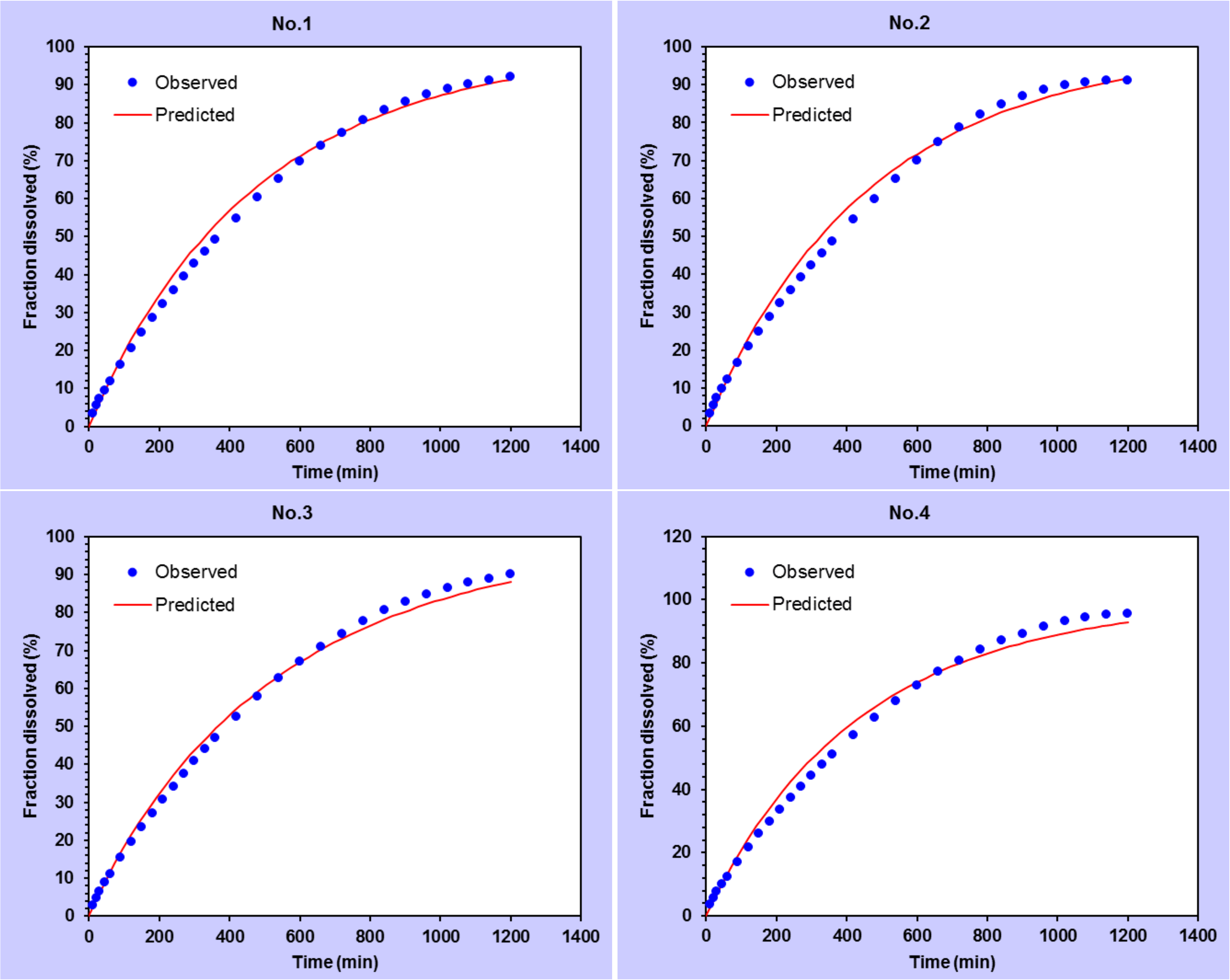

Supplementary materials – Model fitting summary – Granulated sugar N°1 600 (modelling the entire relevant dissolution data)

Model: **Weibull_3**

Model equation: $F = F_{max} \cdot \left(1 - e^{-\frac{t^{\beta}}{\alpha}}\right)$

Fitted model parameters per tested tablet (N = 4) with statistics – mean, standard deviation (SD), and relative standard deviation expressed in % (RSD%) (output from DDSolver):

Parameter	No.1	No.2	No.3	No.4	Mean	SD	RSD(%)
α	424.642	429.656	482.523	420.769	439.398	28.980	6.595
β	0.956	0.965	0.971	0.956	0.962	0.007	0.774
F_{max}	109.974	108.793	107.376	114.109	110.063	2.899	2.634

Number of dissolution data points (N), degrees of freedom (df), and selected goodness of fit criteria – Pearson correlation coefficient (R), coefficient of determination (R^2), adjusted coefficient of determination ($R^2_{adjusted}$), and residual sum of squares (RSS) (manual calculation in MS Excel):

Parameter	No.1	No.2	No.3	No.4
N	29	29	29	29
df	26	26	26	26
R	0.999148173	0.998159443	0.999325305	0.998936853
R^2	0.998297072	0.996322273	0.998651065	0.997874836
$R^2_{adjusted}$	0.998166078	0.996039371	0.9985473	0.997711362
RSS	174.3253613	278.454553	142.4433652	219.9525872

Graphical abstract of model fit presented as mean ± 1 SD of the fraction % of released carvedilol:

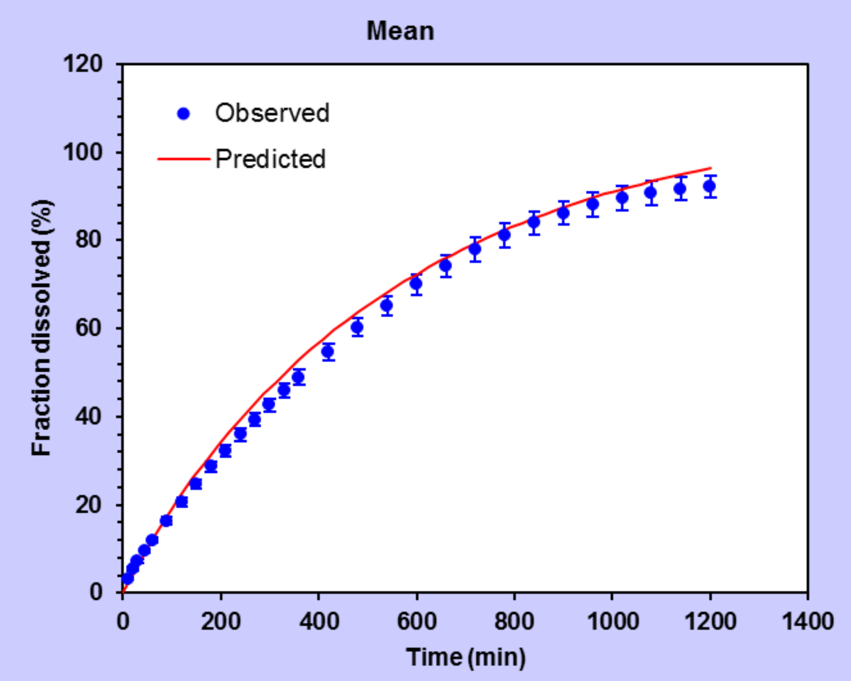

Supplementary materials – Model fitting summary – Granulated sugar N°1 600 (modelling the entire relevant dissolution data)

Graphical abstract of model fit presented as the fraction % of released carvedilol per tested tablet:

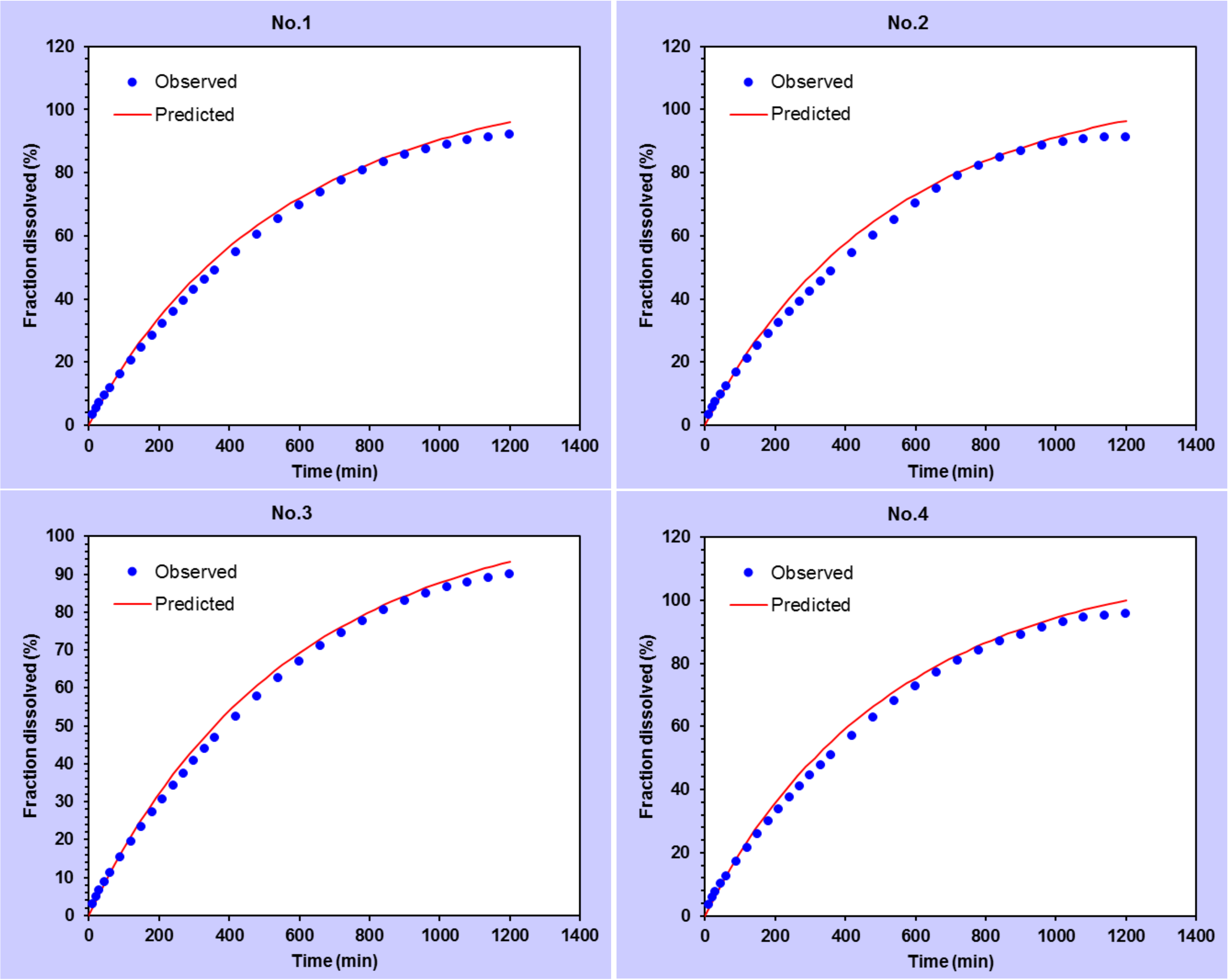

Supplementary materials – Model fitting summary – Granulated sugar N°1 600 (modelling the entire relevant dissolution data)

Model: **Weibull_4**

Model equation: $F = F_{max} \cdot \left[1 - e^{-\frac{(t-T_i)^{\beta}}{\alpha}}\right]$

Fitted model parameters per tested tablet (N = 4) with statistics – mean, standard deviation (SD), and relative standard deviation expressed in % (RSD%) (output from DDSolver):

Parameter	No.1	No.2	No.3	No.4	Mean	SD	RSD(%)
α	274.947	276.989	285.738	272.167	277.460	5.862	2.113
β	0.892	0.900	0.920	0.893	0.901	0.013	1.469
T_i	4.591	4.591	6.000	4.591	4.943	0.704	14.252
F_{max}	109.413	108.238	94.561	113.526	106.435	8.234	7.736

Number of dissolution data points (N), degrees of freedom (df), and selected goodness of fit criteria – Pearson correlation coefficient (R), coefficient of determination (R^2), adjusted coefficient of determination ($R^2_{adjusted}$), and residual sum of squares (RSS) (manual calculation in MS Excel):

Parameter	No.1	No.2	No.3	No.4
N	29	29	29	29
df	25	25	25	25
R	0.997728126	0.996390901	0.995345008	0.997385048
R^2	0.995461413	0.992794828	0.990711685	0.994776934
$R^2_{adjusted}$	0.994916783	0.991930207	0.989597088	0.994150166
RSS	325.2832392	460.4748079	315.6691838	396.2269611

Graphical abstract of model fit presented as mean ± 1 SD of the fraction % of released carvedilol:

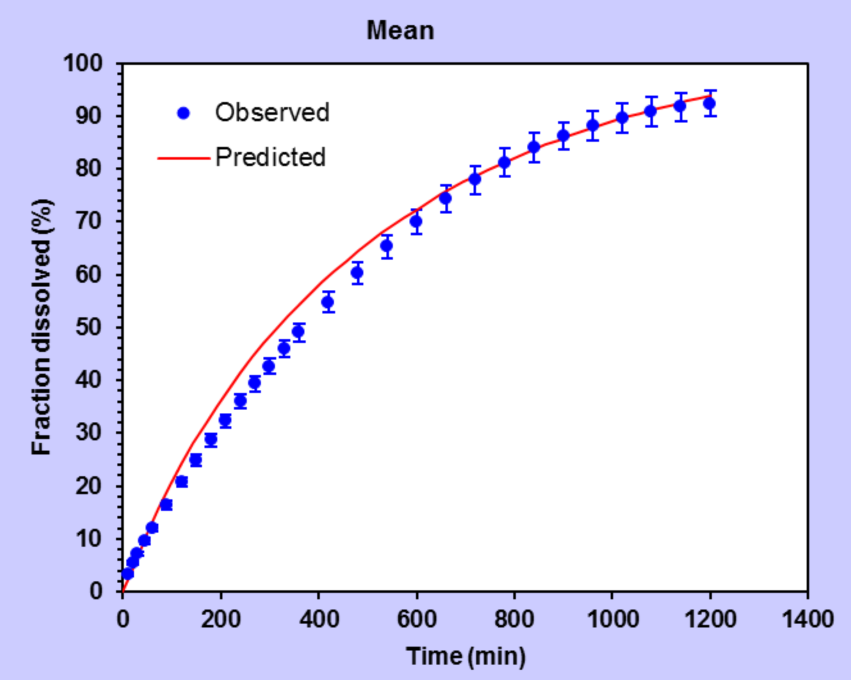

Supplementary materials – Model fitting summary – Granulated sugar N°1 600 (modelling the entire relevant dissolution data)

Graphical abstract of model fit presented as the fraction % of released carvedilol per tested tablet:

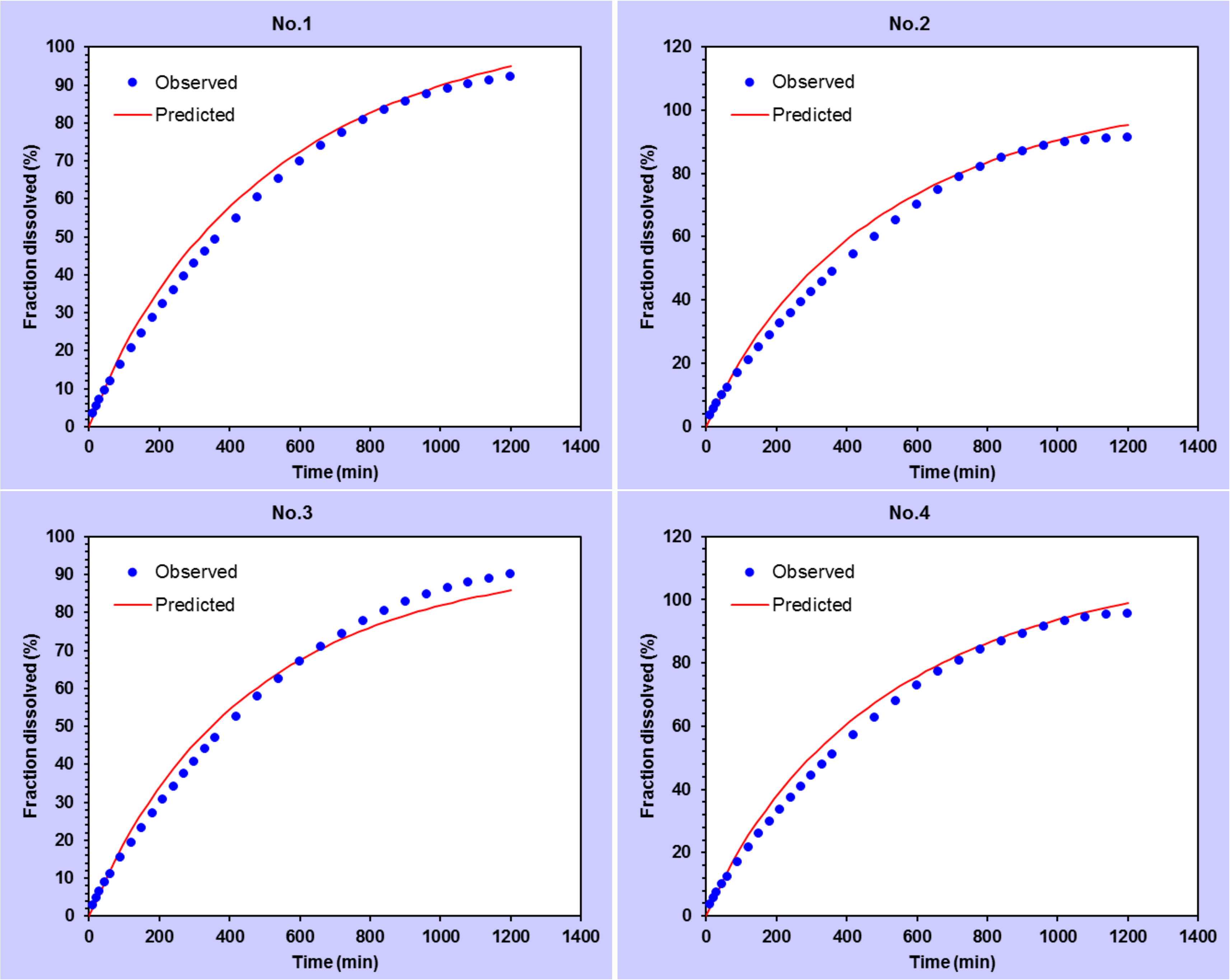

Supplementary materials – Model fitting summary – Granulated sugar N°1 600 (modelling the entire relevant dissolution data)

Model: **Logistic_1**

Model equation: $F = 100 \cdot \frac{e^{\alpha+\beta \cdot \log(t)}}{1+e^{\alpha+\beta \cdot \log(t)}}$

Fitted model parameters per tested tablet (N = 4) with statistics – mean, standard deviation (SD), and relative standard deviation expressed in % (RSD%) (output from DDSolver):

Parameter	No.1	No.2	No.3	No.4	Mean	SD	RSD(%)
α	-8.157	-8.159	-8.095	-7.441	-7.963	0.350	-4.390
β	3.168	3.179	3.133	3.127	3.152	0.025	0.806

Number of dissolution data points (N), degrees of freedom (df), and selected goodness of fit criteria – Pearson correlation coefficient (R), coefficient of determination (R^2), adjusted coefficient of determination ($R^2_{adjusted}$), and residual sum of squares (RSS) (manual calculation in MS Excel):

Parameter	No.1	No.2	No.3	No.4
N	29	29	29	29
df	27	27	27	27
R	0.996724368	0.995258435	0.996285952	0.976895306
R^2	0.993459465	0.990539352	0.992585699	0.954324438
$R^2_{adjusted}$	0.993217223	0.990188957	0.992311095	0.952632751
RSS	852.2782293	885.3178325	502.7218769	1675.691347

Graphical abstract of model fit presented as mean ± 1 SD of the fraction % of released carvedilol:

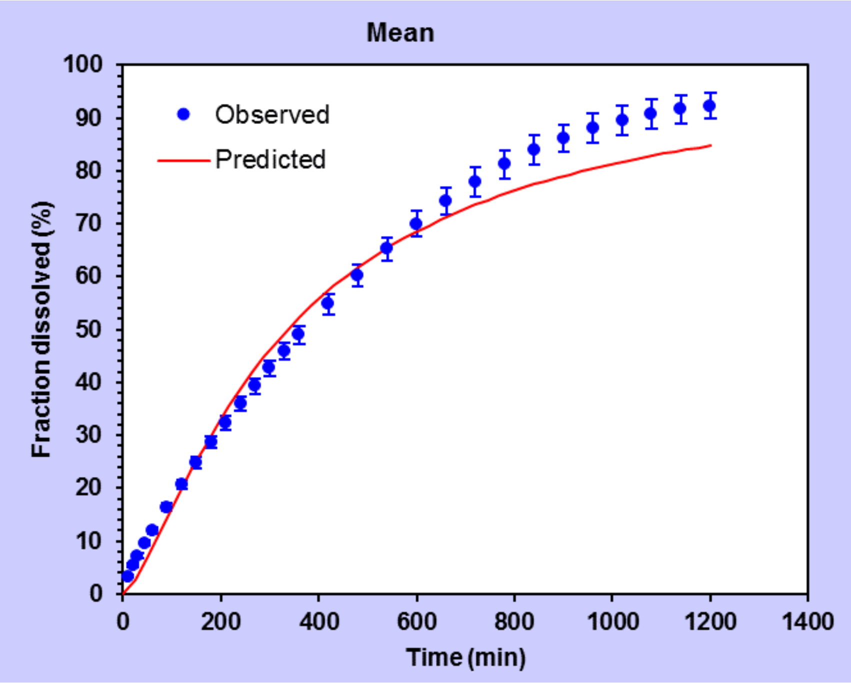

Supplementary materials – Model fitting summary – Granulated sugar N°1 600 (modelling the entire relevant dissolution data)

Graphical abstract of model fit presented as the fraction % of released carvedilol per tested tablet:

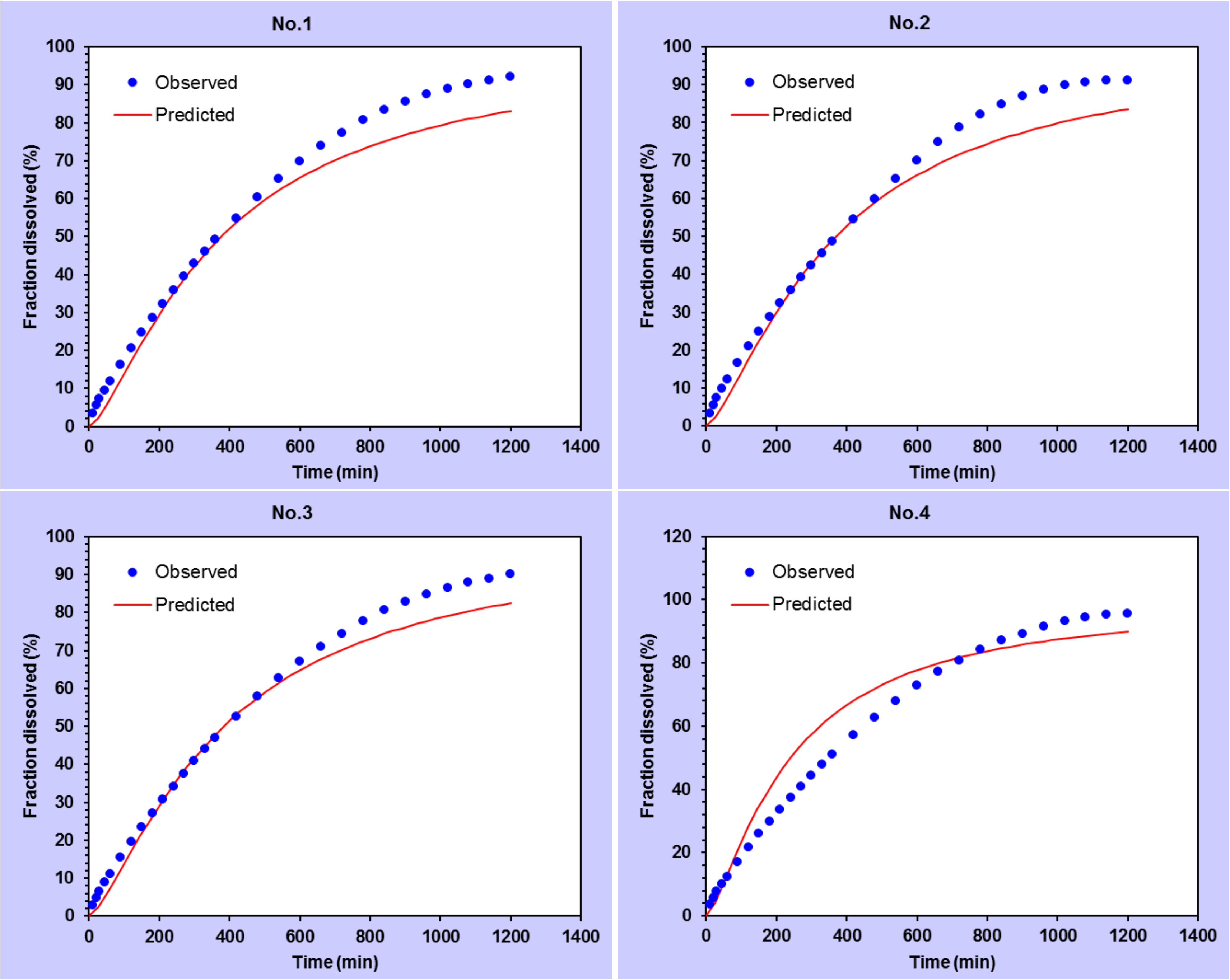

Supplementary materials – Model fitting summary – Granulated sugar N°1 600 (modelling the entire relevant dissolution data)

Model: **Logistic_2**

Model equation: $F = F_{max} \cdot \frac{e^{\alpha+\beta\cdot\log(t)}}{1+e^{\alpha+\beta\cdot\log(t)}}$

Fitted model parameters per tested tablet (N = 4) with statistics – mean, standard deviation (SD), and relative standard deviation expressed in % (RSD%) (output from DDSolver):

Parameter	No.1	No.2	No.3	No.4	Mean	SD	RSD(%)
α	α	-8.493	-7.483	-8.607	-8.530	-8.278	0.532
β	β	3.264	3.156	3.284	3.291	3.249	0.063
F_{max}	Fmax	105.061	95.810	102.578	109.010	103.115	5.544

Number of dissolution data points (N), degrees of freedom (df), and selected goodness of fit criteria – Pearson correlation coefficient (R), coefficient of determination (R^2), adjusted coefficient of determination ($R^2_{adjusted}$), and residual sum of squares (RSS) (manual calculation in MS Excel):

Parameter	No.1	No.2	No.3	No.4
N	29	29	29	29
df	26	26	26	26
R	0.998158655	0.97443965	0.998366143	0.997627167
R^2	0.996320701	0.949532632	0.996734955	0.995259965
$R^2_{adjusted}$	0.996037678	0.945650527	0.996483798	0.994895347
RSS	471.5290516	1709.859318	521.526984	475.2574507

Graphical abstract of model fit presented as mean ± 1 SD of the fraction % of released carvedilol:

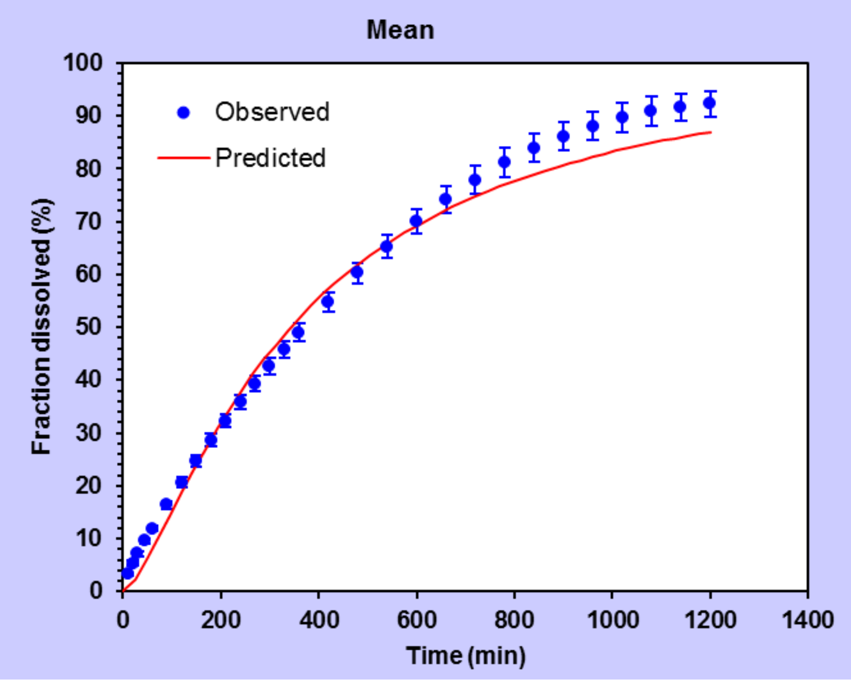

Supplementary materials – Model fitting summary – Granulated sugar N°1 600 (modelling the entire relevant dissolution data)

Graphical abstract of model fit presented as the fraction % of released carvedilol per tested tablet:

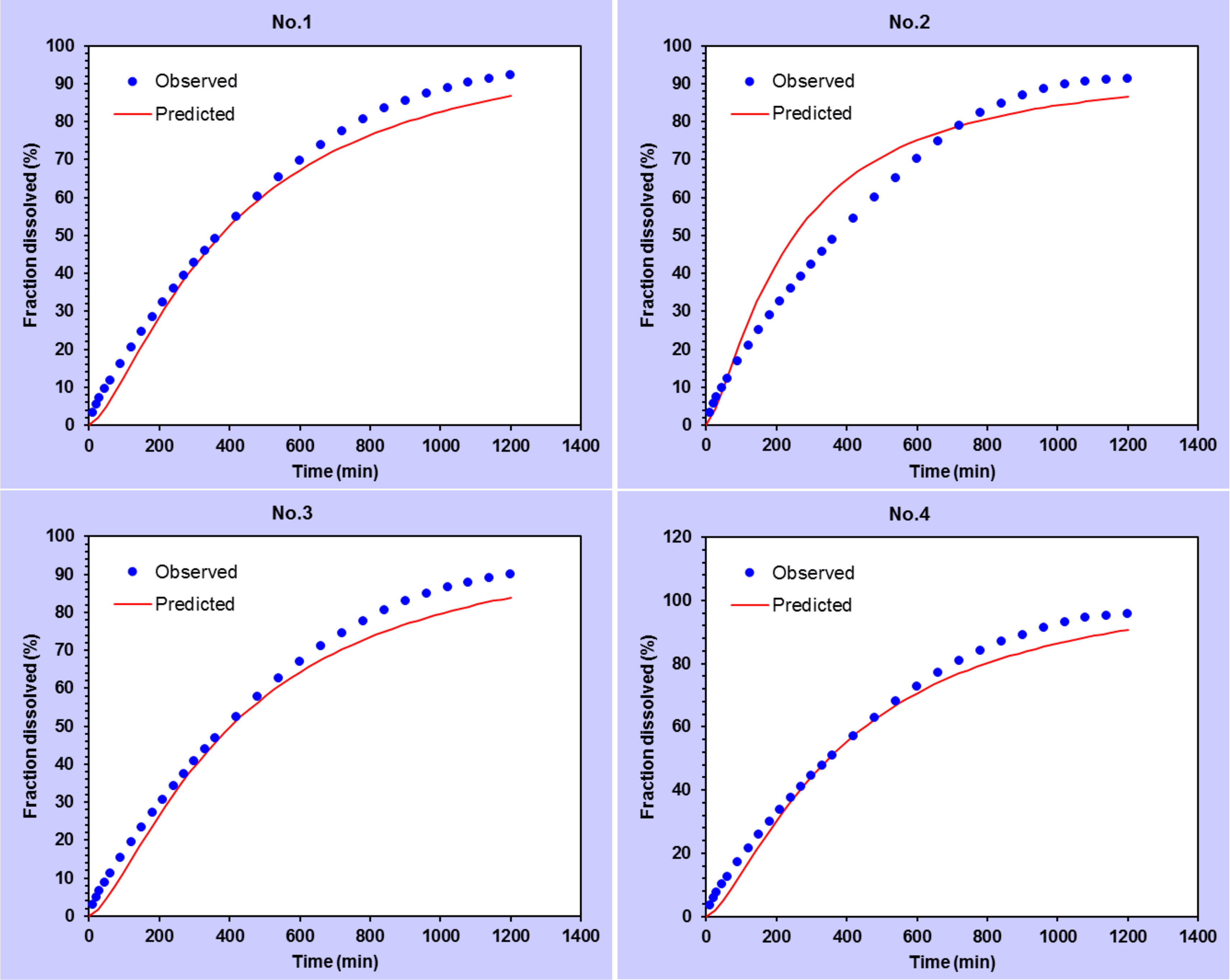

Supplementary materials – Model fitting summary – Granulated sugar N°1 600 (modelling the entire relevant dissolution data)

Model: **Logistic_3**

Model equation: $F = F_{max} \cdot \frac{1}{1+e^{-k\cdot(t-\gamma)}}$

Fitted model parameters per tested tablet (N = 4) with statistics – mean, standard deviation (SD), and relative standard deviation expressed in % (RSD%) (output from DDSolver):

Parameter	No.1	No.2	No.3	No.4	Mean	SD	RSD(%)
k	0.005	0.007	0.005	0.005	0.005	0.001	20.572
γ	443.996	332.231	456.111	438.350	417.672	57.441	13.753
F_{max}	96.850	86.125	94.561	100.491	94.507	6.098	6.452

Number of dissolution data points (N), degrees of freedom (df), and selected goodness of fit criteria – Pearson correlation coefficient (R), coefficient of determination (R^2), adjusted coefficient of determination ($R^2_{adjusted}$), and residual sum of squares (RSS) (manual calculation in MS Excel):

Parameter	No.1	No.2	No.3	No.4
N	29	29	29	29
df	26	26	26	26
R	0.984356004	0.992947227	0.984162477	0.985524525
R^2	0.968956743	0.985944195	0.968575782	0.971258589
$R^2_{adjusted}$	0.9665688	0.98486298	0.966158534	0.969047711
RSS	989.1106783	445.3300993	965.7778778	994.4941857

Graphical abstract of model fit presented as mean ± 1 SD of the fraction % of released carvedilol:

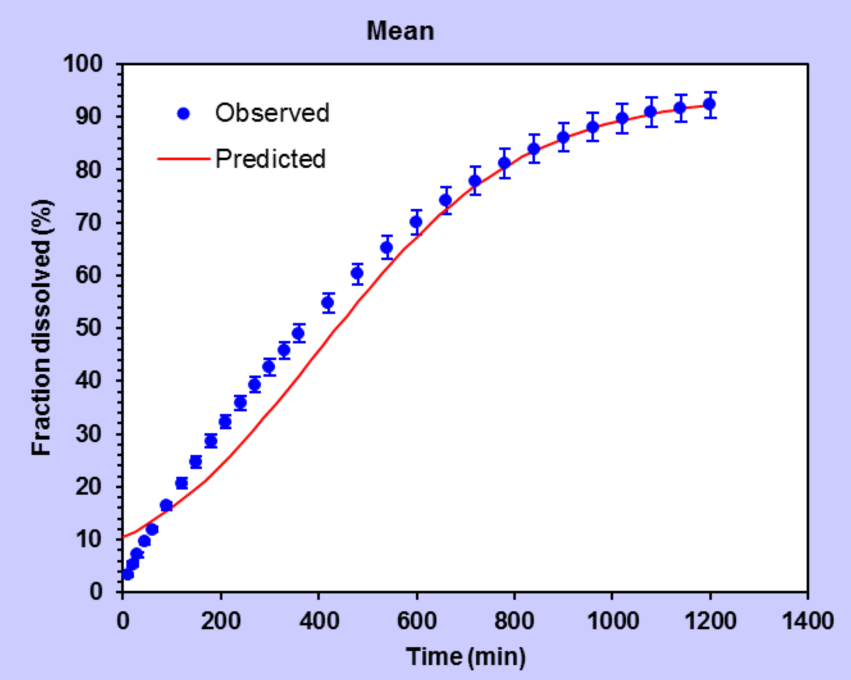

Supplementary materials – Model fitting summary – Granulated sugar N°1 600 (modelling the entire relevant dissolution data)

Graphical abstract of model fit presented as the fraction % of released carvedilol per tested tablet:

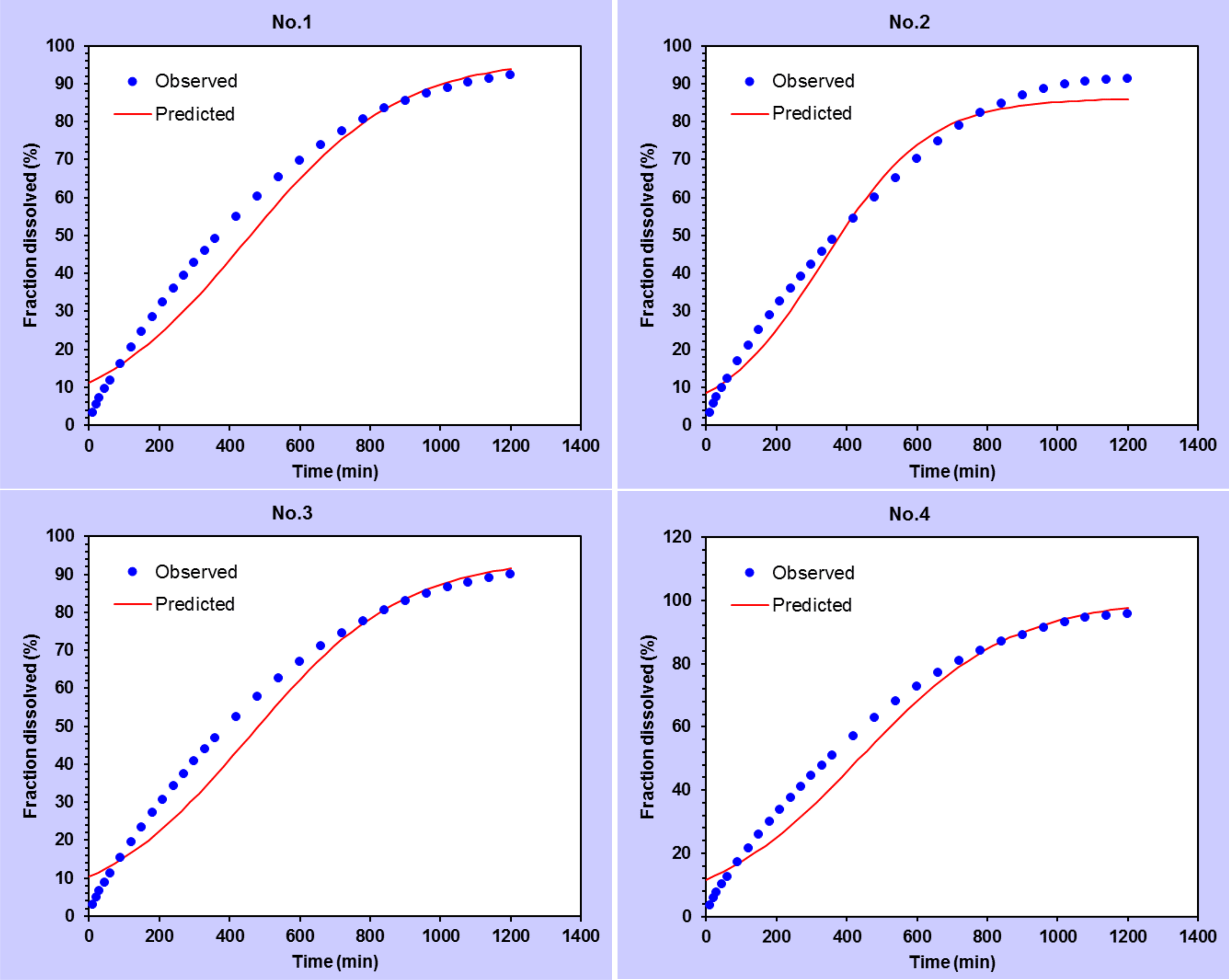

Supplementary materials – Model fitting summary – Granulated sugar N°1 600 (modelling the entire relevant dissolution data)

Model: **Gompertz_1**

Model equation: $F = 100 \cdot e^{-\alpha \cdot e^{-\beta \cdot \log(t)}}$

Fitted model parameters per tested tablet (N = 4) with statistics – mean, standard deviation (SD), and relative standard deviation expressed in % (RSD%) (output from DDSolver):

Parameter	No.1	No.2	No.3	No.4	Mean	SD	RSD(%)
α	85.005	87.084	73.057	128.531	93.419	24.210	25.916
β	1.885	1.902	1.781	2.121	1.922	0.143	7.441

Number of dissolution data points (N), degrees of freedom (df), and selected goodness of fit criteria – Pearson correlation coefficient (R), coefficient of determination (R^2), adjusted coefficient of determination ($R^2_{adjusted}$), and residual sum of squares (RSS) (manual calculation in MS Excel):

Parameter	No.1	No.2	No.3	No.4
N	29	29	29	29
df	27	27	27	27
R	0.98490964	0.982381427	0.987640491	0.977405016
R^2	0.970047	0.965073268	0.975433739	0.955320565
$R^2_{adjusted}$	0.968937629	0.963779685	0.974523878	0.953665771
RSS	1977.088917	2030.743375	2294.755904	1683.494036

Graphical abstract of model fit presented as mean ± 1 SD of the fraction % of released carvedilol:

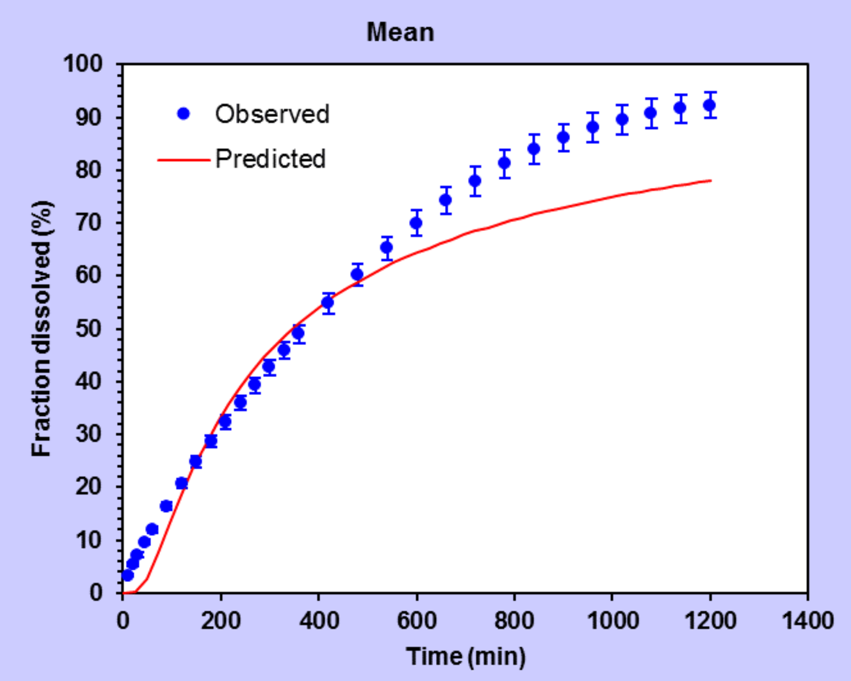

Supplementary materials – Model fitting summary – Granulated sugar N°1 600 (modelling the entire relevant dissolution data)

Graphical abstract of model fit presented as the fraction % of released carvedilol per tested tablet:

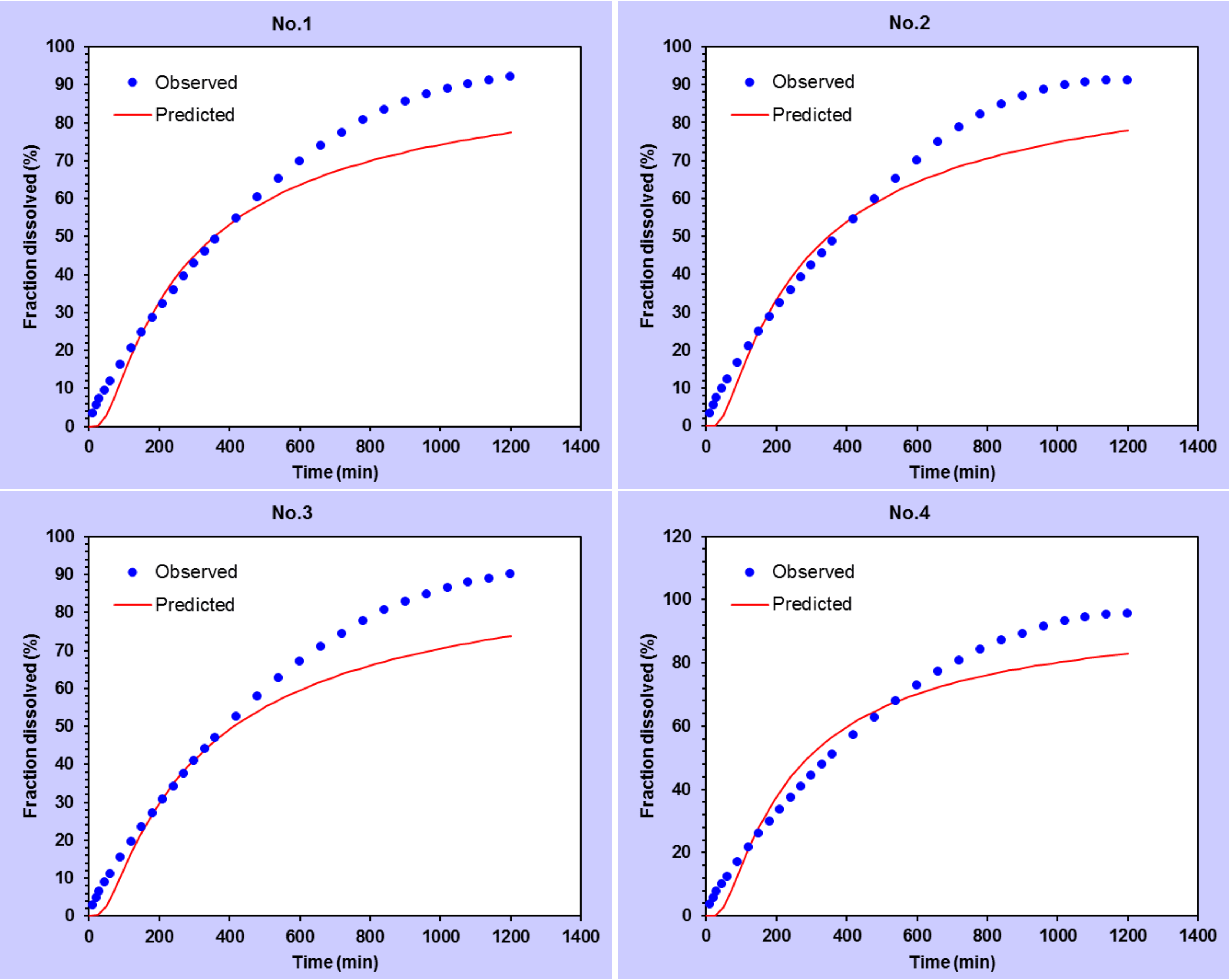

Supplementary materials – Model fitting summary – Granulated sugar N°1 600 (modelling the entire relevant dissolution data)

Model: **Gompertz_2**

Model equation: $F = F_{max} \cdot e^{-\alpha \cdot e^{-\beta \cdot \log(t)}}$

Fitted model parameters per tested tablet (N = 4) with statistics – mean, standard deviation (SD), and relative standard deviation expressed in % (RSD%) (output from DDSolver):

Parameter	No.1	No.2	No.3	No.4	Mean	SD	RSD(%)
α	94.060	111.664	114.630	99.712	105.017	9.743	9.278
β	1.892	1.972	2.040	1.912	1.954	0.067	3.424
F_{max}	108.396	106.038	94.561	111.219	105.054	7.309	6.957

Number of dissolution data points (N), degrees of freedom (df), and selected goodness of fit criteria – Pearson correlation coefficient (R), coefficient of determination (R^2), adjusted coefficient of determination ($R^2_{adjusted}$), and residual sum of squares (RSS) (manual calculation in MS Excel):

Parameter	No.1	No.2	No.3	No.4
N	29	29	29	29
df	26	26	26	26
R	0.989248974	0.987180386	0.980506608	0.989375238
R^2	0.978613533	0.974525115	0.961393209	0.978863361
$R^2_{adjusted}$	0.97696842	0.972565508	0.958423455	0.977237465
RSS	1109.816975	1304.056017	1552.791219	1495.493876

Graphical abstract of model fit presented as mean ± 1 SD of the fraction % of released carvedilol:

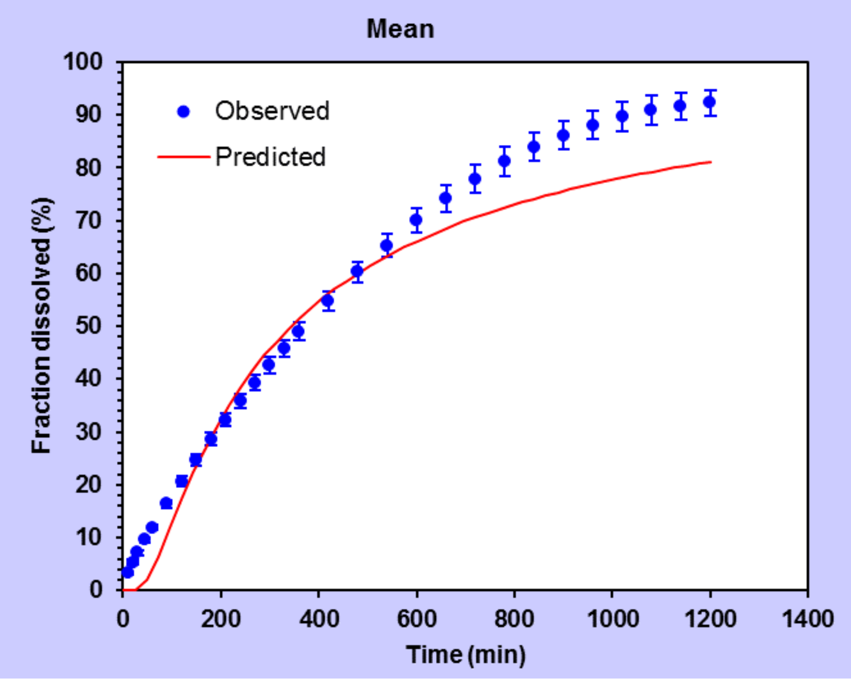

Supplementary materials – Model fitting summary – Granulated sugar N°1 600 (modelling the entire relevant dissolution data)

Graphical abstract of model fit presented as the fraction % of released carvedilol per tested tablet:

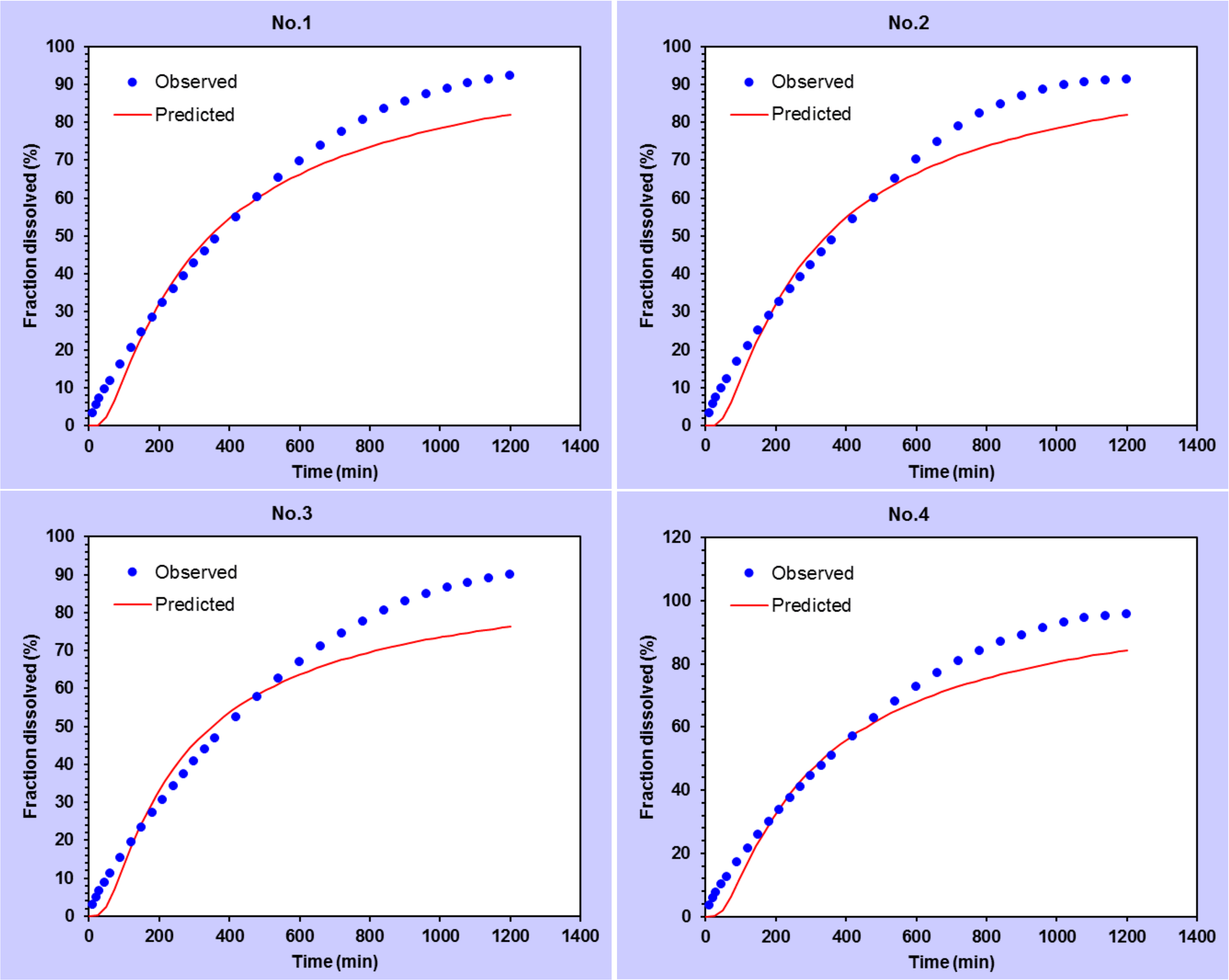

Supplementary materials – Model fitting summary – Granulated sugar N°1 600 (modelling the entire relevant dissolution data)

Model: **Gompertz_3**

Model equation: $F = F_{max} \cdot e^{-e^{-k\cdot(t-\gamma)}}$

Fitted model parameters per tested tablet (N = 4) with statistics – mean, standard deviation (SD), and relative standard deviation expressed in % (RSD%) (output from DDSolver):

Parameter	No.1	No.2	No.3	No.4	Mean	SD	RSD(%)
k	0.003	0.004	0.003	0.003	0.003	0.000	2.734
γ	270.486	261.089	280.292	267.496	269.841	7.994	2.963
F_{max}	96.850	95.810	94.561	100.491	96.928	2.553	2.634

Number of dissolution data points (N), degrees of freedom (df), and selected goodness of fit criteria – Pearson correlation coefficient (R), coefficient of determination (R^2), adjusted coefficient of determination ($R^2_{adjusted}$), and residual sum of squares (RSS) (manual calculation in MS Excel):

Parameter	No.1	No.2	No.3	No.4
N	29	29	29	29
df	26	26	26	26
R	0.996855279	0.997698589	0.996735352	0.997190168
R^2	0.993720448	0.995402474	0.993481361	0.994388232
$R^2_{adjusted}$	0.993237405	0.995048818	0.992979928	0.993956557
RSS	177.7503311	128.8602362	176.3666391	171.3433908

Graphical abstract of model fit presented as mean ± 1 SD of the fraction % of released carvedilol:

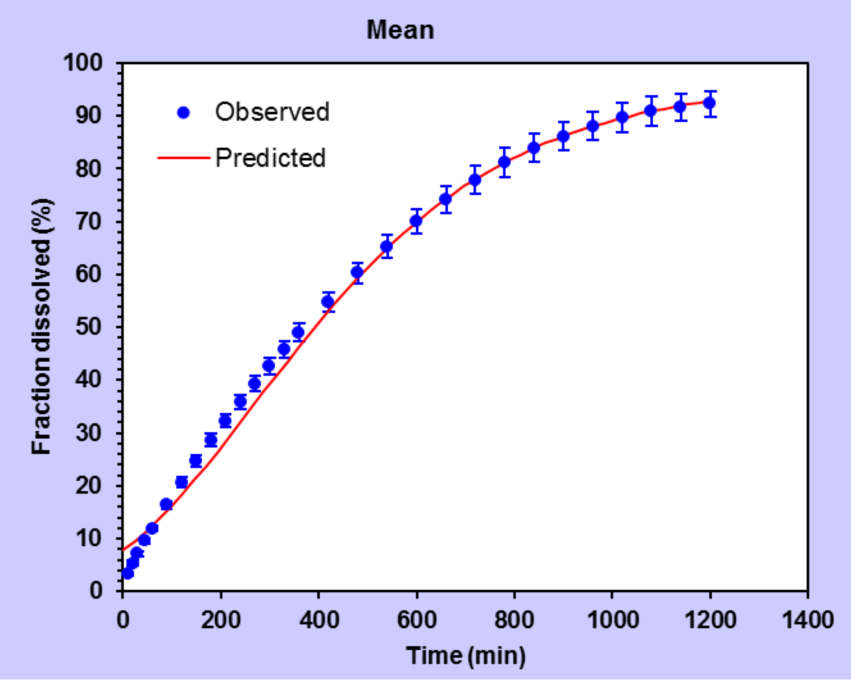

Supplementary materials – Model fitting summary – Granulated sugar N°1 600 (modelling the entire relevant dissolution data)

Graphical abstract of model fit presented as the fraction % of released carvedilol per tested tablet:

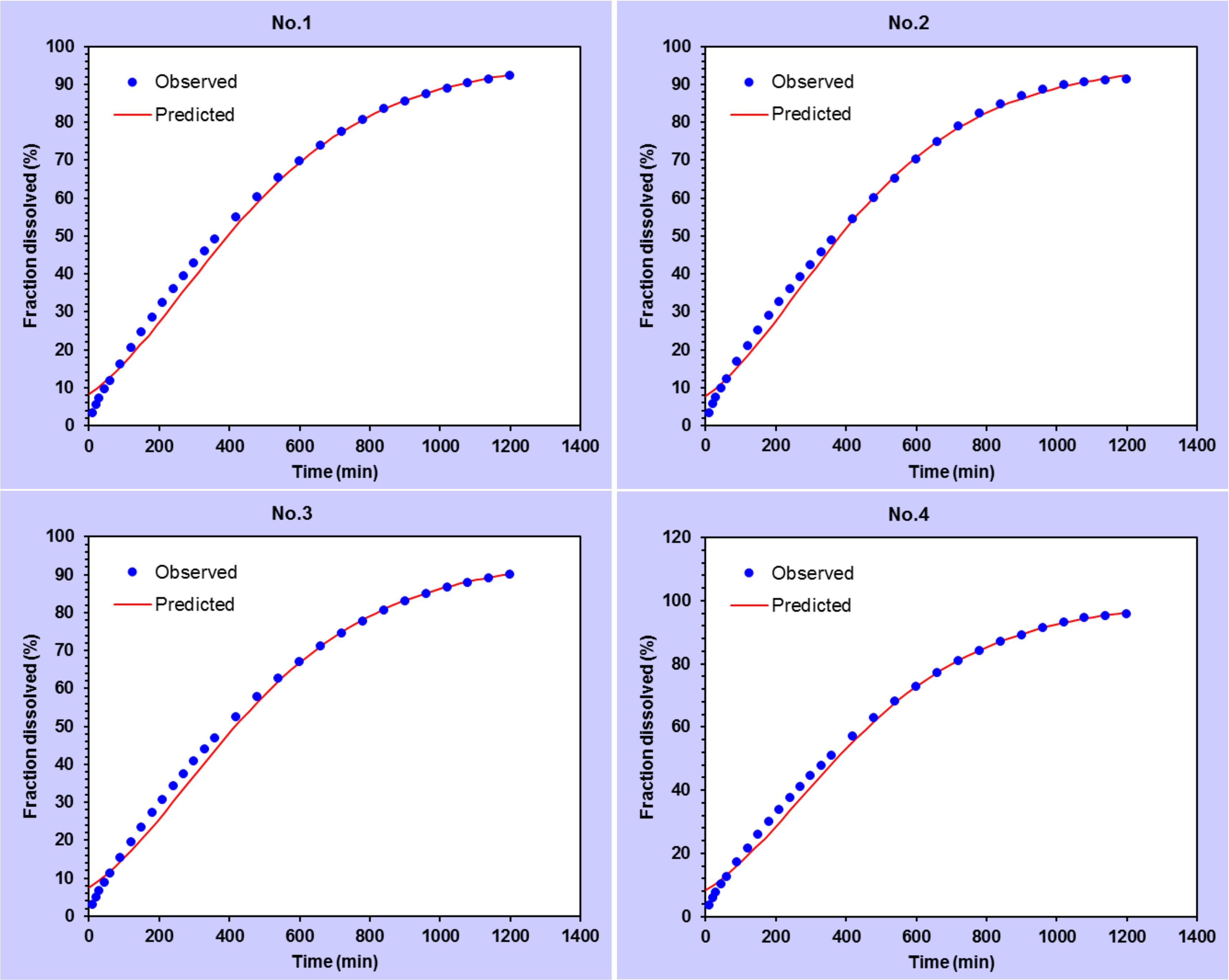

Supplementary materials – Model fitting summary – Granulated sugar N°1 600 (modelling the entire relevant dissolution data)

Model: **Gompertz_4**

Model equation: $F = F_{max} \cdot e^{-\beta \cdot e^{-k \cdot t}}$

Fitted model parameters per tested tablet (N = 4) with statistics – mean, standard deviation (SD), and relative standard deviation expressed in % (RSD%) (output from DDSolver):

Parameter	No.1	No.2	No.3	No.4	Mean	SD	RSD(%)
k	0.003	0.004	0.003	0.003	0.003	0.000	2.734
β	2.468	2.509	2.533	2.486	2.499	0.028	1.127
F_{max}	96.850	95.810	94.561	100.491	96.928	2.553	2.634

Number of dissolution data points (N), degrees of freedom (df), and selected goodness of fit criteria – Pearson correlation coefficient (R), coefficient of determination (R^2), adjusted coefficient of determination ($R^2_{adjusted}$), and residual sum of squares (RSS) (manual calculation in MS Excel):

Parameter	No.1	No.2	No.3	No.4
N	29	29	29	29
df	26	26	26	26
R	0.996855279	0.997698589	0.996735352	0.997190168
R^2	0.993720448	0.995402474	0.993481361	0.994388232
$R^2_{adjusted}$	0.993237405	0.995048818	0.992979928	0.993956557
RSS	177.7503311	128.8602362	176.3666391	171.3433908

Graphical abstract of model fit presented as mean ± 1 SD of the fraction % of released carvedilol:

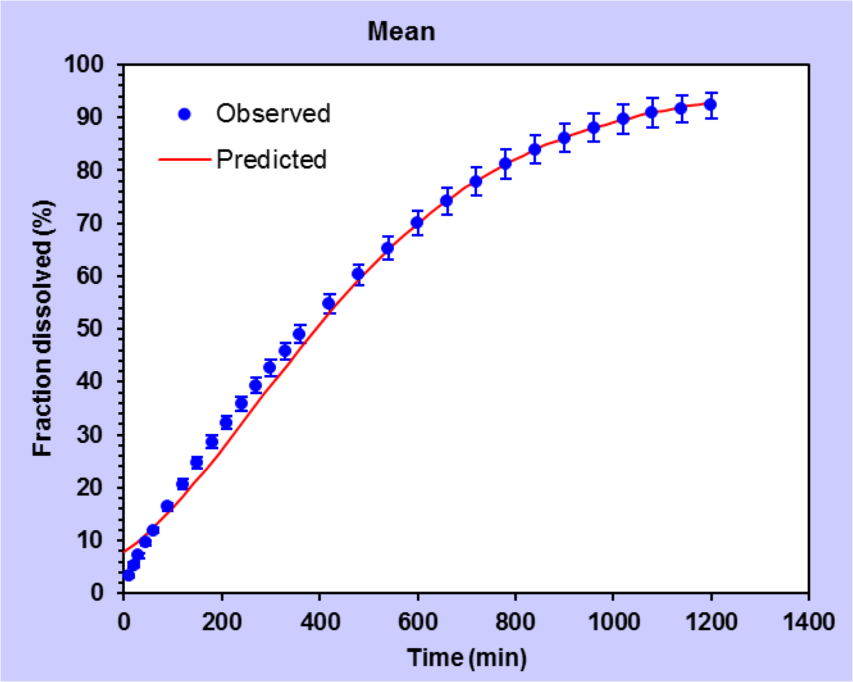

Supplementary materials – Model fitting summary – Granulated sugar N°1 600 (modelling the entire relevant dissolution data)

Graphical abstract of model fit presented as the fraction % of released carvedilol per tested tablet:

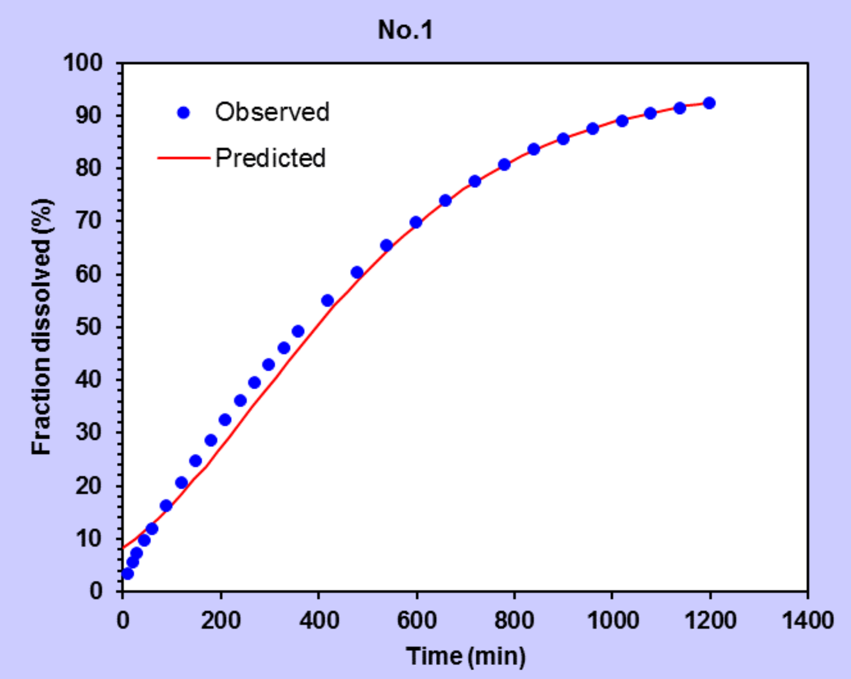

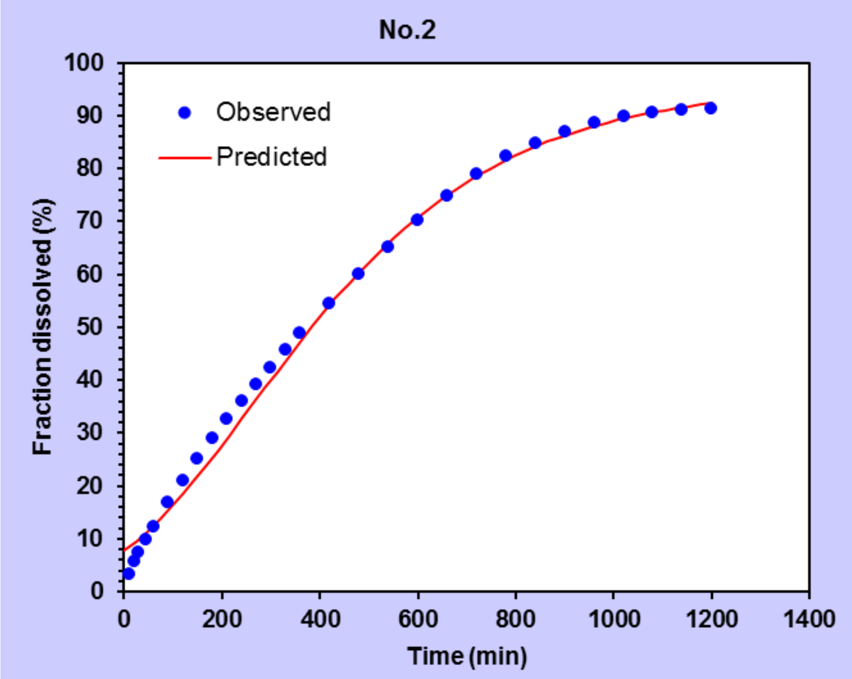

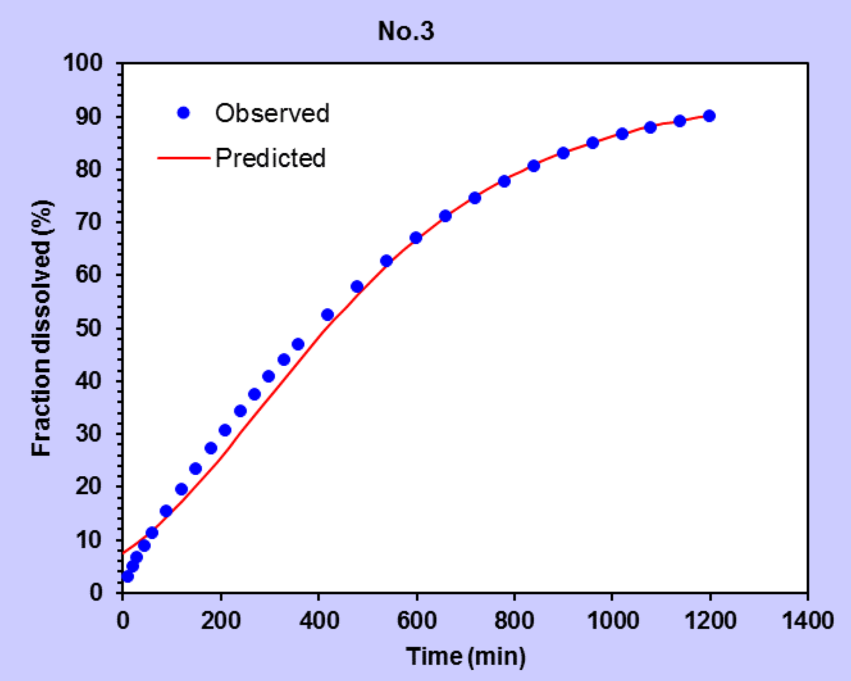

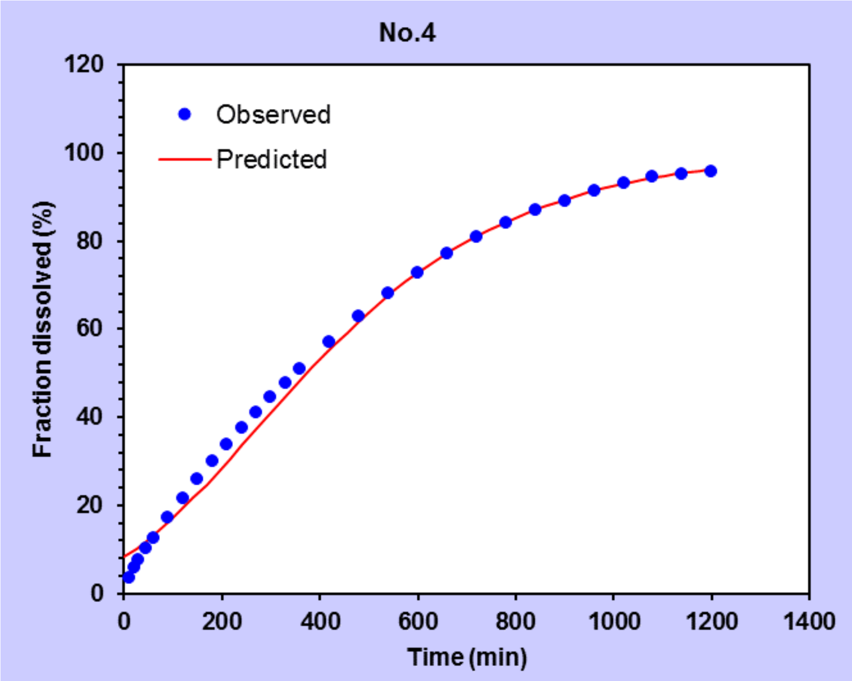

Supplementary materials – Model fitting summary – Granulated sugar N°1 600 (modelling the entire relevant dissolution data)

Model: **Probit_1**

Model equation: $F = 100 \cdot \phi[\alpha + \beta \cdot \log(t)]$

Fitted model parameters per tested tablet (N = 4) with statistics – mean, standard deviation (SD), and relative standard deviation expressed in % (RSD%) (output from DDSolver):

Parameter	No.1	No.2	No.3	No.4	Mean	SD	RSD(%)
α	-4.702	-4.705	-4.052	-4.262	-4.430	0.327	-7.374
β	1.829	1.836	1.634	1.791	1.772	0.095	5.338

Number of dissolution data points (N), degrees of freedom (df), and selected goodness of fit criteria – Pearson correlation coefficient (R), coefficient of determination (R^2), adjusted coefficient of determination ($R^2_{adjusted}$), and residual sum of squares (RSS) (manual calculation in MS Excel):

Parameter	No.1	No.2	No.3	No.4
N	29	29	29	29
df	27	27	27	27
R	0.994796025	0.993183882	0.984119785	0.976471257
R^2	0.989619132	0.986414224	0.968491751	0.953496116
$R^2_{adjusted}$	0.989234655	0.985911047	0.967324779	0.95177375
RSS	1020.682015	1068.698441	983.0733112	1650.790716

Graphical abstract of model fit presented as mean ± 1 SD of the fraction % of released carvedilol:

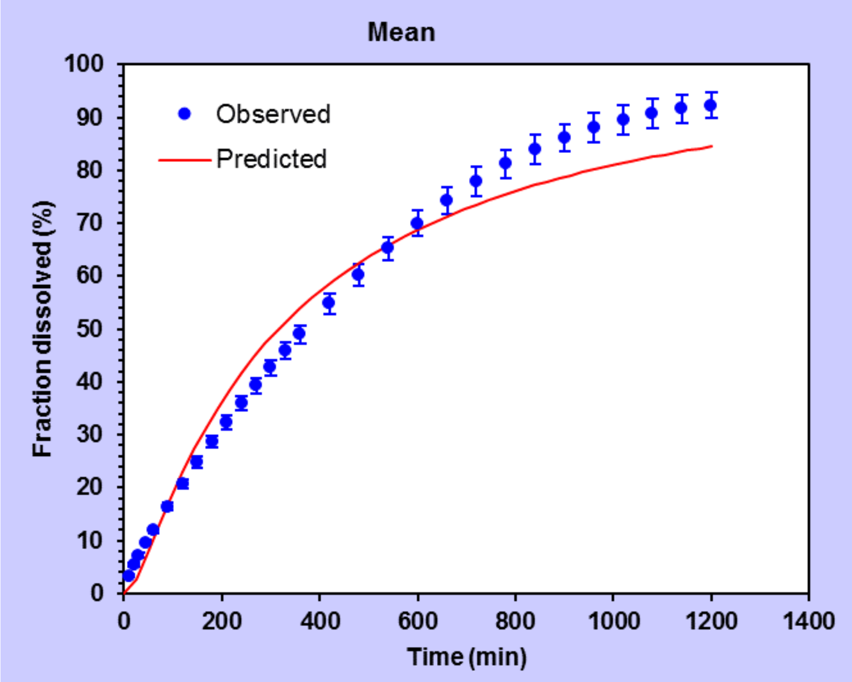

Supplementary materials – Model fitting summary – Granulated sugar N°1 600 (modelling the entire relevant dissolution data)

Graphical abstract of model fit presented as the fraction % of released carvedilol per tested tablet:

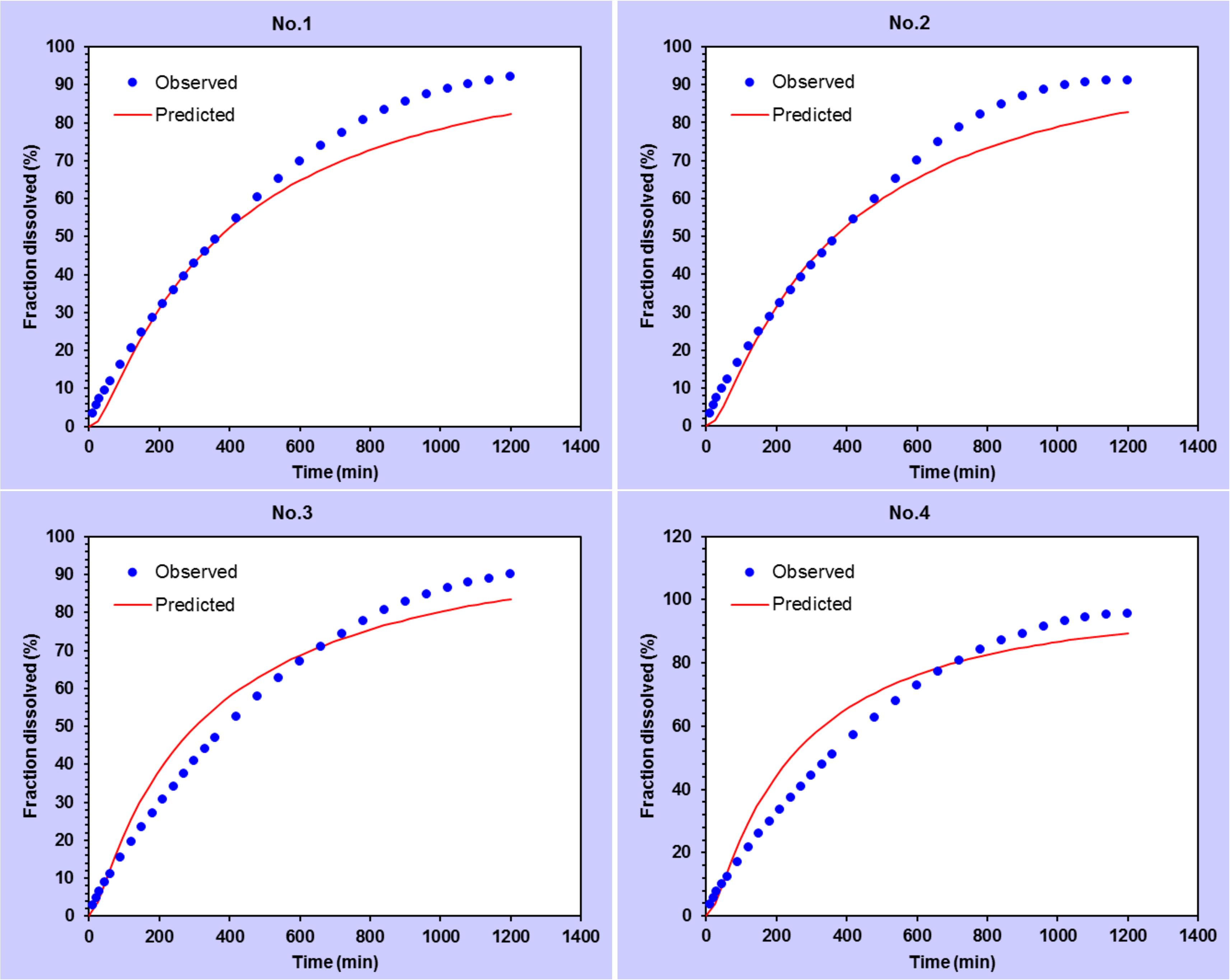

Supplementary materials – Model fitting summary – Granulated sugar N°1 600 (modelling the entire relevant dissolution data)

Model: **Probit_2**

Model equation: $F = F_{max} \cdot \phi[\alpha + \beta \cdot \log(t)]$

Fitted model parameters per tested tablet (N = 4) with statistics – mean, standard deviation (SD), and relative standard deviation expressed in % (RSD%) (output from DDSolver):

Parameter	No.1	No.2	No.3	No.4	Mean	SD	RSD(%)
α	-4.874	-4.285	-4.925	-4.892	-4.744	0.307	-6.468
β	1.874	1.806	1.881	1.887	1.862	0.038	2.018
F_{max}	105.061	95.810	102.578	109.010	103.115	5.544	5.376

Number of dissolution data points (N), degrees of freedom (df), and selected goodness of fit criteria – Pearson correlation coefficient (R), coefficient of determination (R^2), adjusted coefficient of determination ($R^2_{adjusted}$), and residual sum of squares (RSS) (manual calculation in MS Excel):

Parameter	No.1	No.2	No.3	No.4
N	29	29	29	29
df	26	26	26	26
R	0.996721963	0.974339968	0.996933109	0.996180975
R^2	0.993454671	0.949338373	0.993875624	0.992376535
$R^2_{adjusted}$	0.992951184	0.945441324	0.993404518	0.991790115
RSS	624.5242245	1662.1312	662.4886876	647.5310661

Graphical abstract of model fit presented as mean ± 1 SD of the fraction % of released carvedilol:

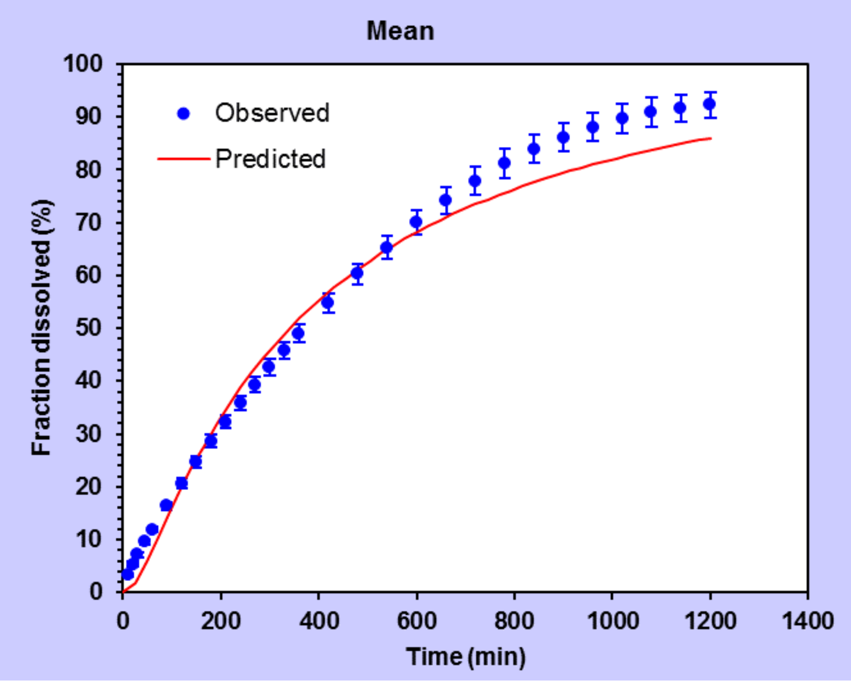

Supplementary materials – Model fitting summary – Granulated sugar N°1 600 (modelling the entire relevant dissolution data)

Graphical abstract of model fit presented as the fraction % of released carvedilol per tested tablet:

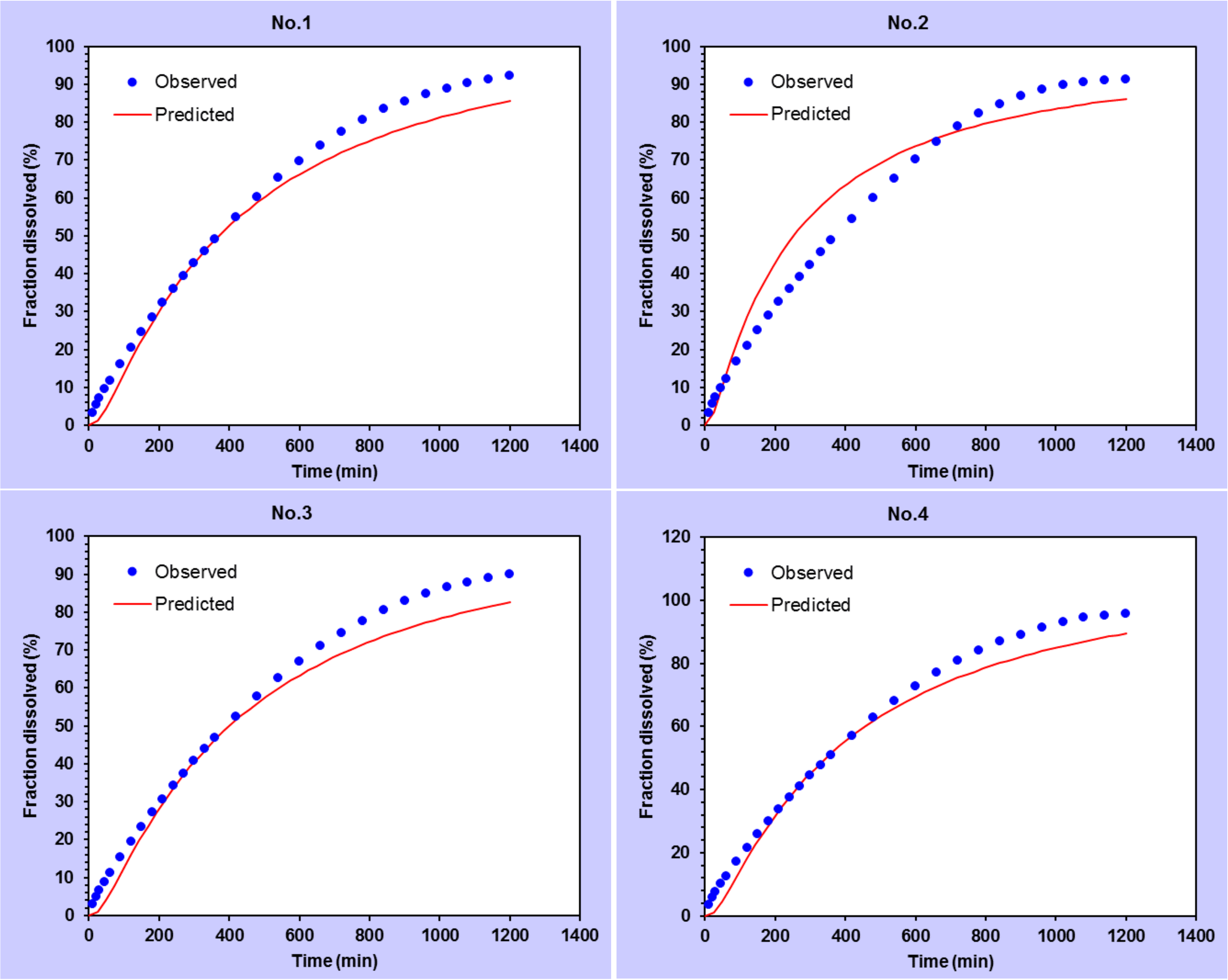

Supplementary materials – Model fitting summary – Granulated sugar N°1 600 (modelling up to app. 60 % of carvedilol released)

Model: **Zero–order**

Model equation: $F = k_0 \cdot t$

Fitted model parameters per tested tablet (N = 4) with statistics – mean, standard deviation (SD), and relative standard deviation expressed in % (RSD%) (output from DDSolver):

Parameter	No.1	No.2	No.3	No.4	Mean	SD	RSD(%)
k_0	0.139	0.138	0.133	0.145	0.139	0.005	3.557

Number of dissolution data points (N), degrees of freedom (df), and selected goodness of fit criteria – Pearson correlation coefficient (R), coefficient of determination (R^2), adjusted coefficient of determination ($R^2_{adjusted}$), and residual sum of squares (RSS) (manual calculation in MS Excel):

Parameter	No.1	No.2	No.3	No.4
N	17	17	17	17
df	16	16	16	16
R	0.995988204	0.995373125	0.996166949	0.99579743
R^2	0.991992502	0.990767658	0.99234859	0.991612523
$R^2_{adjusted}$	0.991992502	0.990767658	0.99234859	0.991612523
RSS	176.5717181	205.241252	143.2547843	203.8598418

Graphical abstract of model fit presented as mean ± 1 SD of the fraction % of released carvedilol:

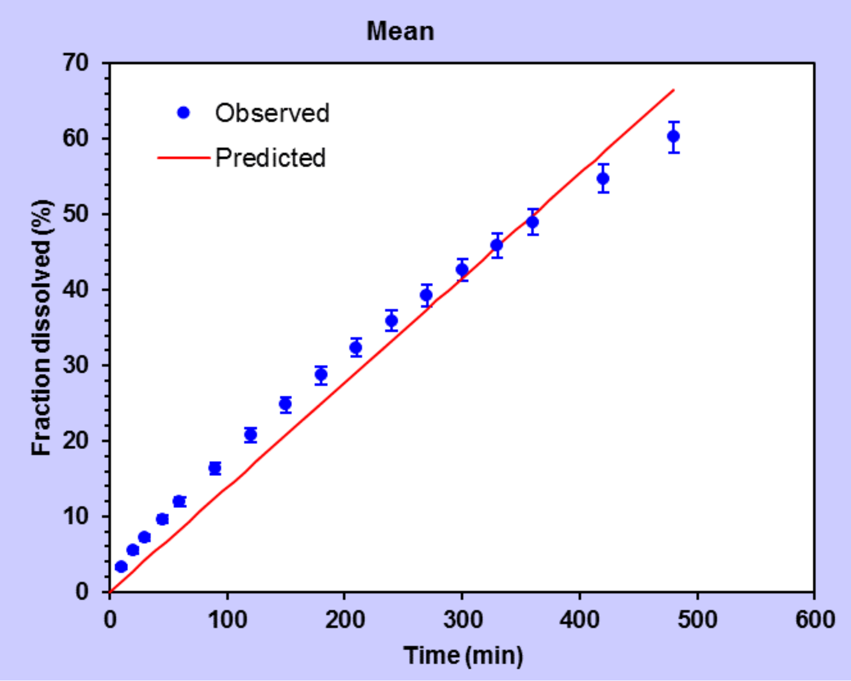

Supplementary materials – Model fitting summary – Granulated sugar N°1 600 (modelling up to app. 60 % of carvedilol released)

Graphical abstract of model fit presented as the fraction % of released carvedilol per tested tablet:

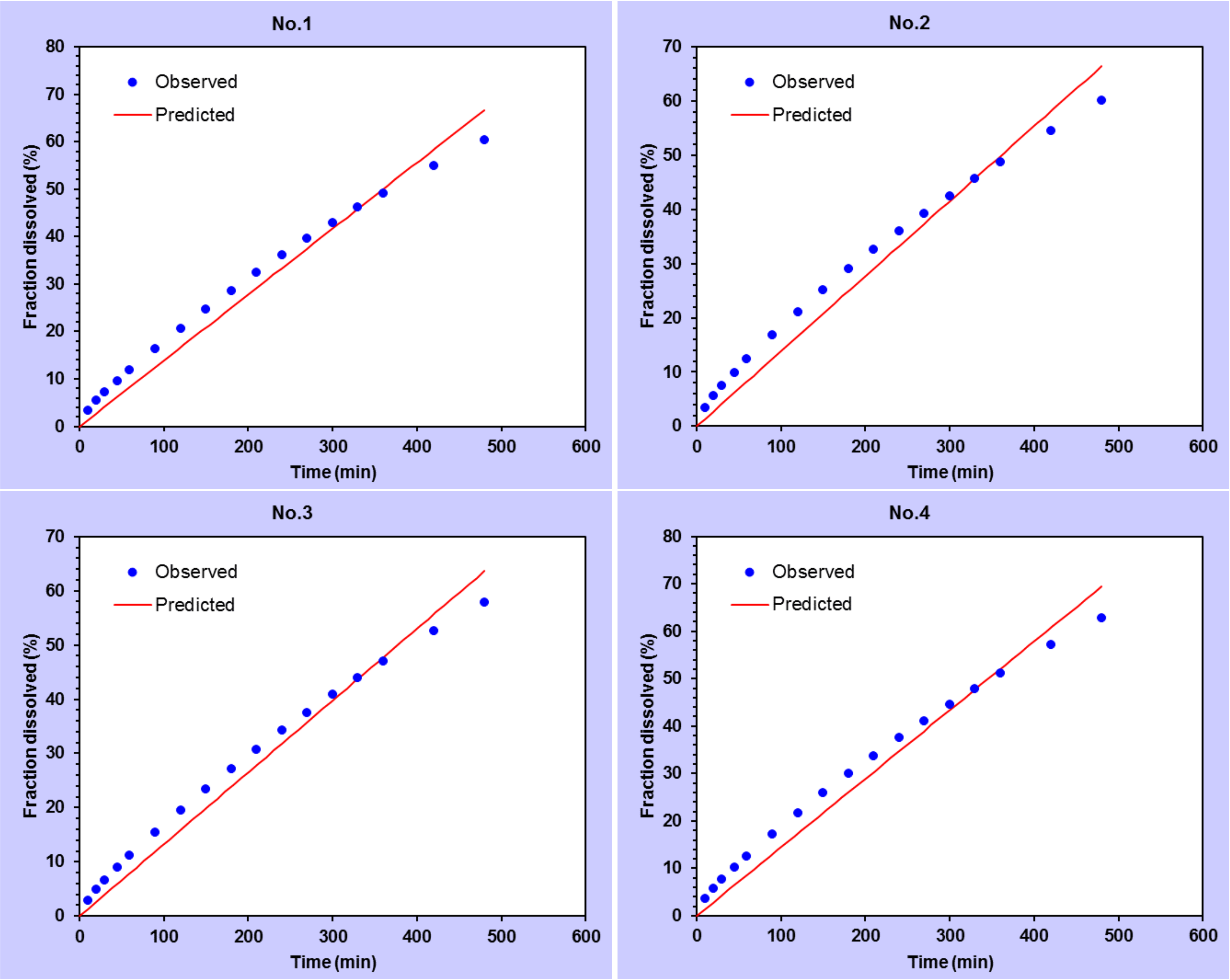

Supplementary materials – Model fitting summary – Granulated sugar N°1 600 (modelling up to app. 60 % of carvedilol released)

Model: **Zero–order with T_{lag}**

Model equation: $F = k_0 \cdot (t - T_{lag})$

Fitted model parameters per tested tablet (N = 4) with statistics – mean, standard deviation (SD), and relative standard deviation expressed in % (RSD%) (output from DDSolver):

Parameter	No.1	No.2	No.3	No.4	Mean	SD	RSD(%)
k_0	0.123	0.121	0.119	0.128	0.123	0.004	3.101
T_{lag}	-38.525	-42.437	-35.538	-40.135	-39.159	2.899	-7.403

Number of dissolution data points (N), degrees of freedom (df), and selected goodness of fit criteria – Pearson correlation coefficient (R), coefficient of determination (R^2), adjusted coefficient of determination ($R^2_{adjusted}$), and residual sum of squares (RSS) (manual calculation in MS Excel):

Parameter	No.1	No.2	No.3	No.4
N	17	17	17	17
df	15	15	15	15
R	0.995988204	0.995373125	0.996166949	0.99579743
R^2	0.991992502	0.990767658	0.99234859	0.991612523
$R^2_{adjusted}$	0.991458669	0.990152169	0.991838496	0.991053357
RSS	42.6138173	47.68335841	37.69373054	47.89923049

Graphical abstract of model fit presented as mean ± 1 SD of the fraction % of released carvedilol:

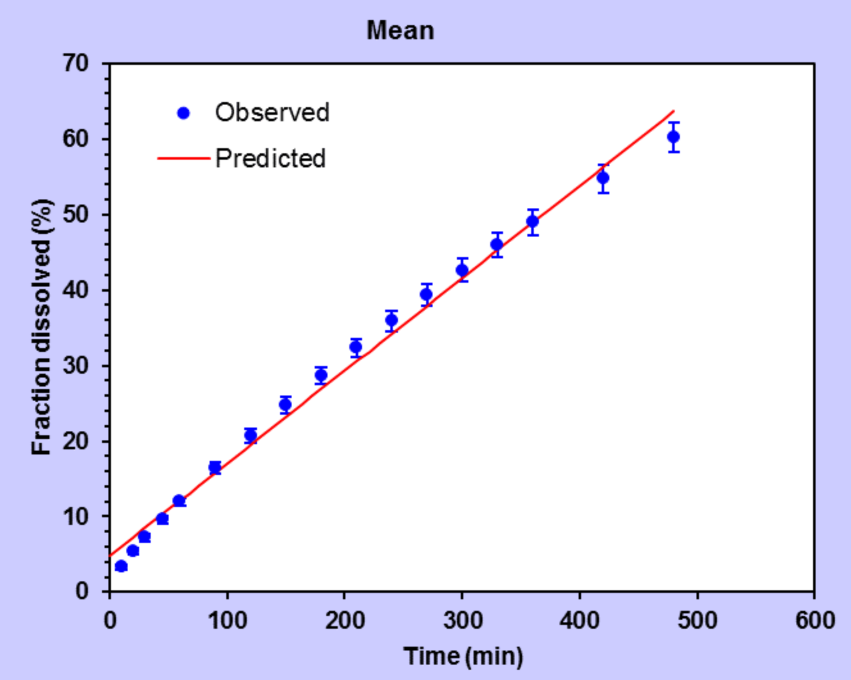

Supplementary materials – Model fitting summary – Granulated sugar N°1 600 (modelling up to app. 60 % of carvedilol released)

Graphical abstract of model fit presented as the fraction % of released carvedilol per tested tablet:

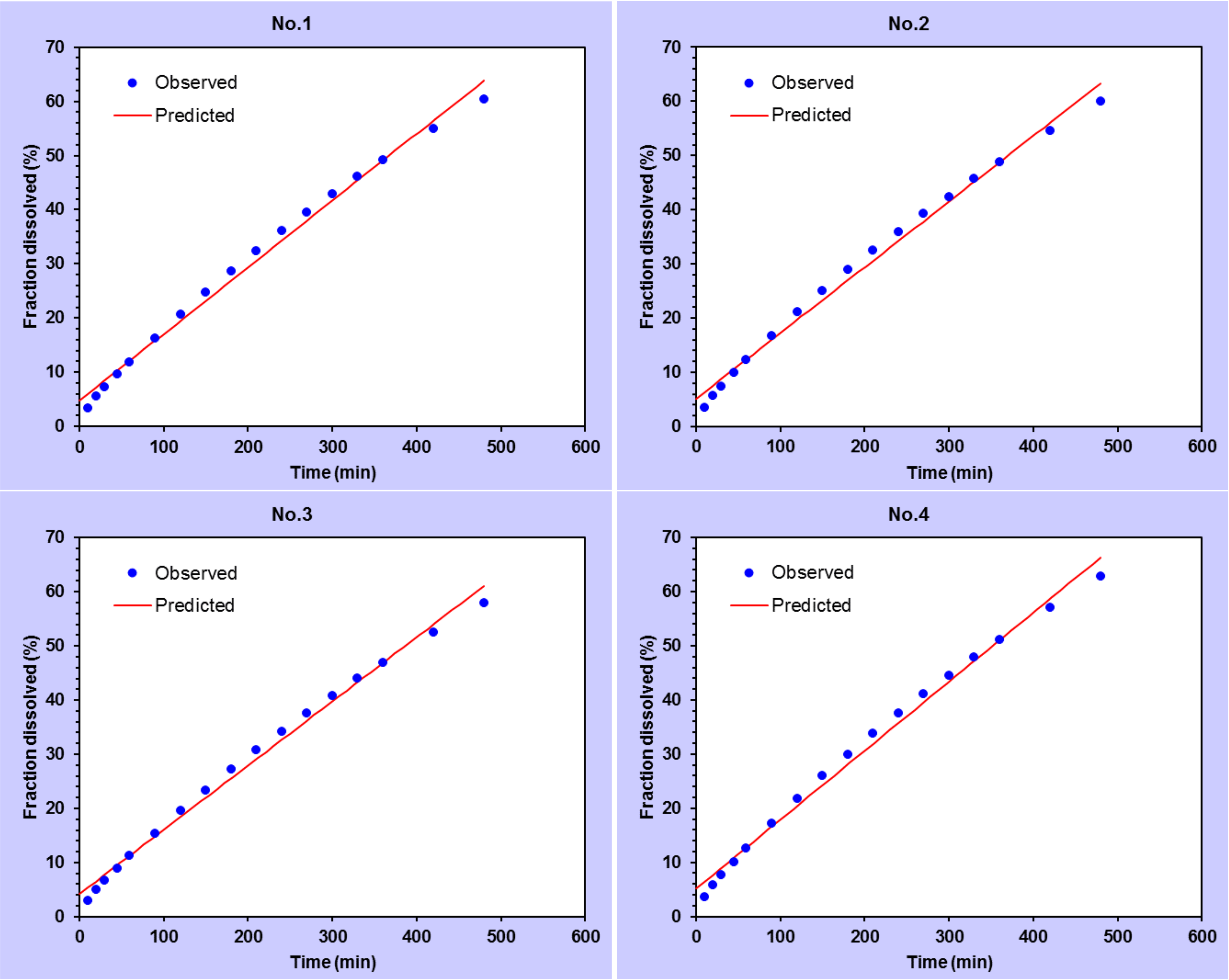

Supplementary materials – Model fitting summary – Granulated sugar N°1 600 (modelling up to app. 60 % of carvedilol released)

Model: **Zero–order with F_0**

Model equation: $F = F_0 + k_0 \cdot t$

Fitted model parameters per tested tablet (N = 4) with statistics – mean, standard deviation (SD), and relative standard deviation expressed in % (RSD%) (output from DDSolver):

Parameter	No.1	No.2	No.3	No.4	Mean	SD	RSD(%)
k_0	0.123	0.121	0.119	0.128	0.123	0.004	3.101
F_0	4.746	5.147	4.213	5.121	4.806	0.436	9.074

Number of dissolution data points (N), degrees of freedom (df), and selected goodness of fit criteria – Pearson correlation coefficient (R), coefficient of determination (R^2), adjusted coefficient of determination ($R^2_{adjusted}$), and residual sum of squares (RSS) (manual calculation in MS Excel):

Parameter	No.1	No.2	No.3	No.4
N	17	17	17	17
df	15	15	15	15
R	0.995988204	0.995373125	0.996166949	0.99579743
R^2	0.991992502	0.990767658	0.99234859	0.991612523
$R^2_{adjusted}$	0.991458669	0.990152169	0.991838496	0.991053357
RSS	42.6138173	47.68335841	37.69373054	47.89923049

Graphical abstract of model fit presented as mean ± 1 SD of the fraction % of released carvedilol:

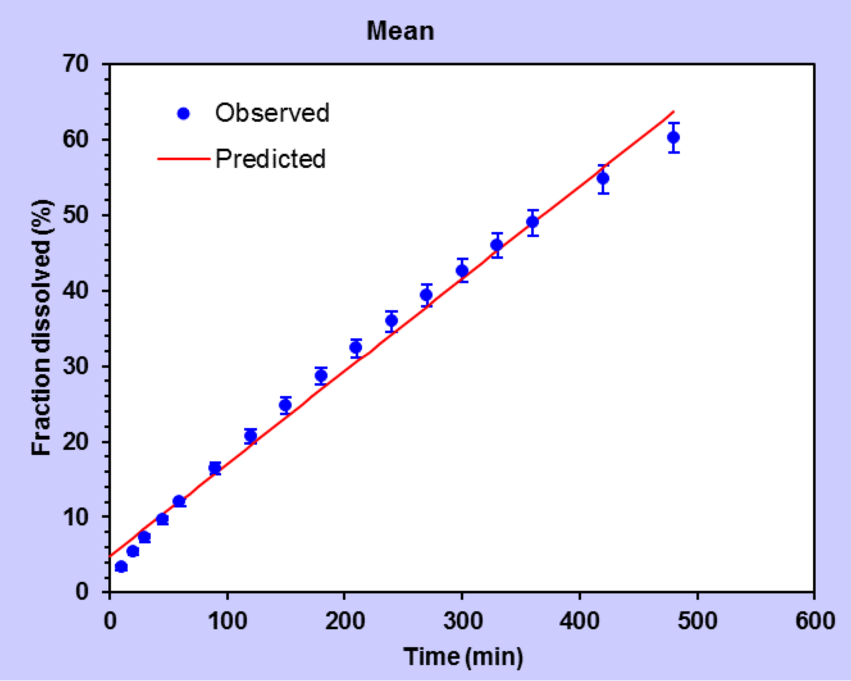

Supplementary materials – Model fitting summary – Granulated sugar N°1 600 (modelling up to app. 60 % of carvedilol released)

Graphical abstract of model fit presented as the fraction % of released carvedilol per tested tablet:

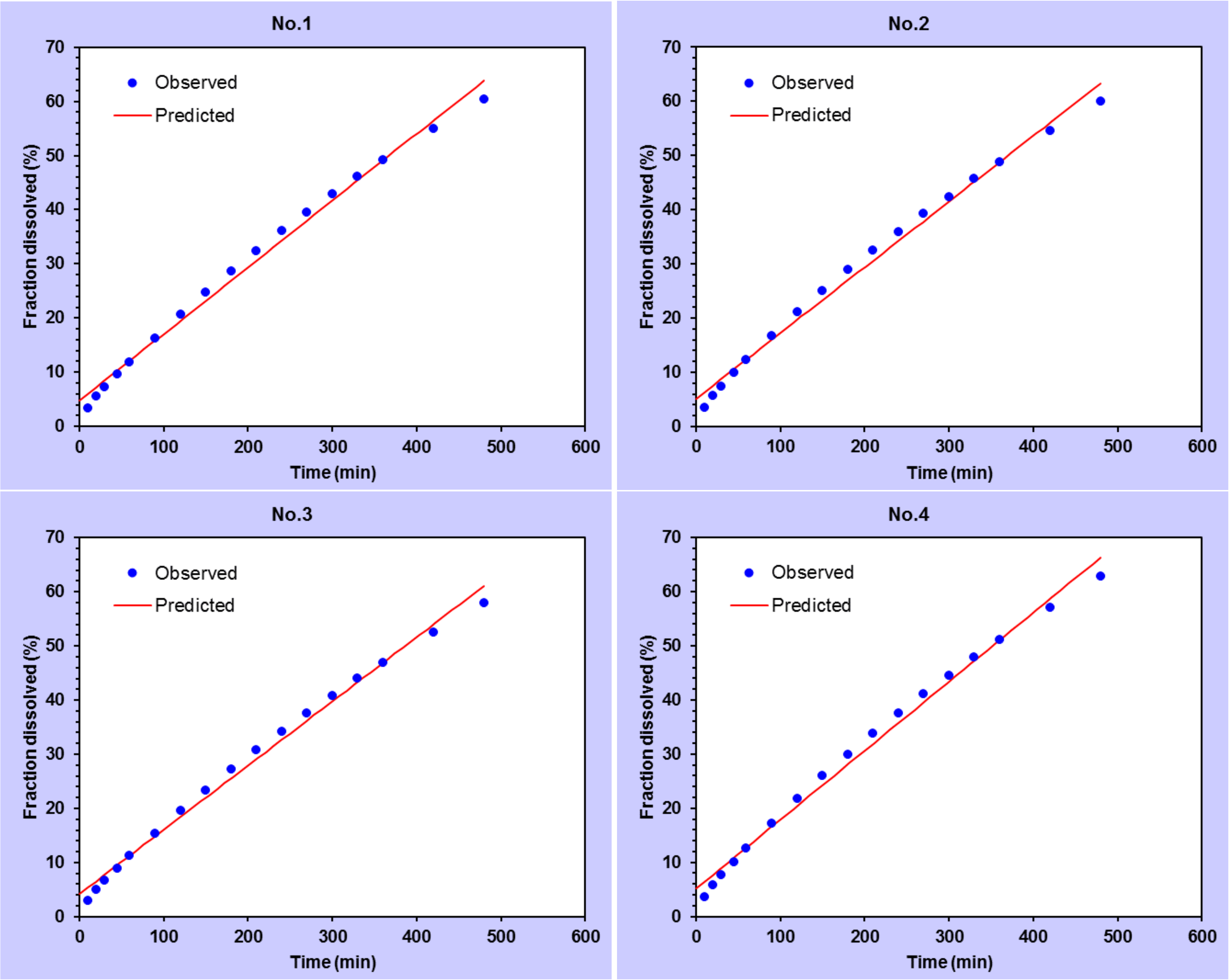

Supplementary materials – Model fitting summary – Granulated sugar N°1 600 (modelling up to app. 60 % of carvedilol released)

Model: **First–order**

Model equation: $F = 100 \cdot (1 - e^{-k_1 \cdot t})$

Fitted model parameters per tested tablet (N = 4) with statistics – mean, standard deviation (SD), and relative standard deviation expressed in % (RSD%) (output from DDSolver):

Parameter	No.1	No.2	No.3	No.4	Mean	SD	RSD(%)
k_1	0.002	0.002	0.002	0.002	0.002	0.000	5.023

Number of dissolution data points (N), degrees of freedom (df), and selected goodness of fit criteria – Pearson correlation coefficient (R), coefficient of determination (R^2), adjusted coefficient of determination ($R^2_{adjusted}$), and residual sum of squares (RSS) (manual calculation in MS Excel):

Parameter	No.1	No.2	No.3	No.4
N	17	17	17	17
df	16	16	16	16
R	0.99951629	0.999605774	0.999644857	0.999351185
R^2	0.999032815	0.999211703	0.99928984	0.998702791
$R^2_{adjusted}$	0.999032815	0.999211703	0.99928984	0.998702791
RSS	13.81147523	19.88260613	9.820906328	16.65676912

Graphical abstract of model fit presented as mean ± 1 SD of the fraction % of released carvedilol:

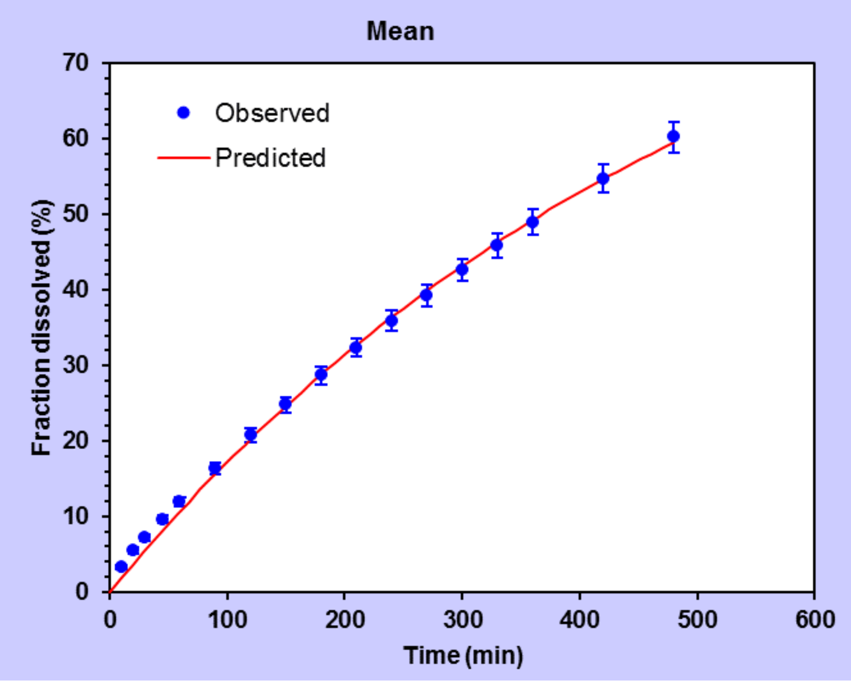

Supplementary materials – Model fitting summary – Granulated sugar N°1 600 (modelling up to app. 60 % of carvedilol released)

Graphical abstract of model fit presented as the fraction % of released carvedilol per tested tablet:

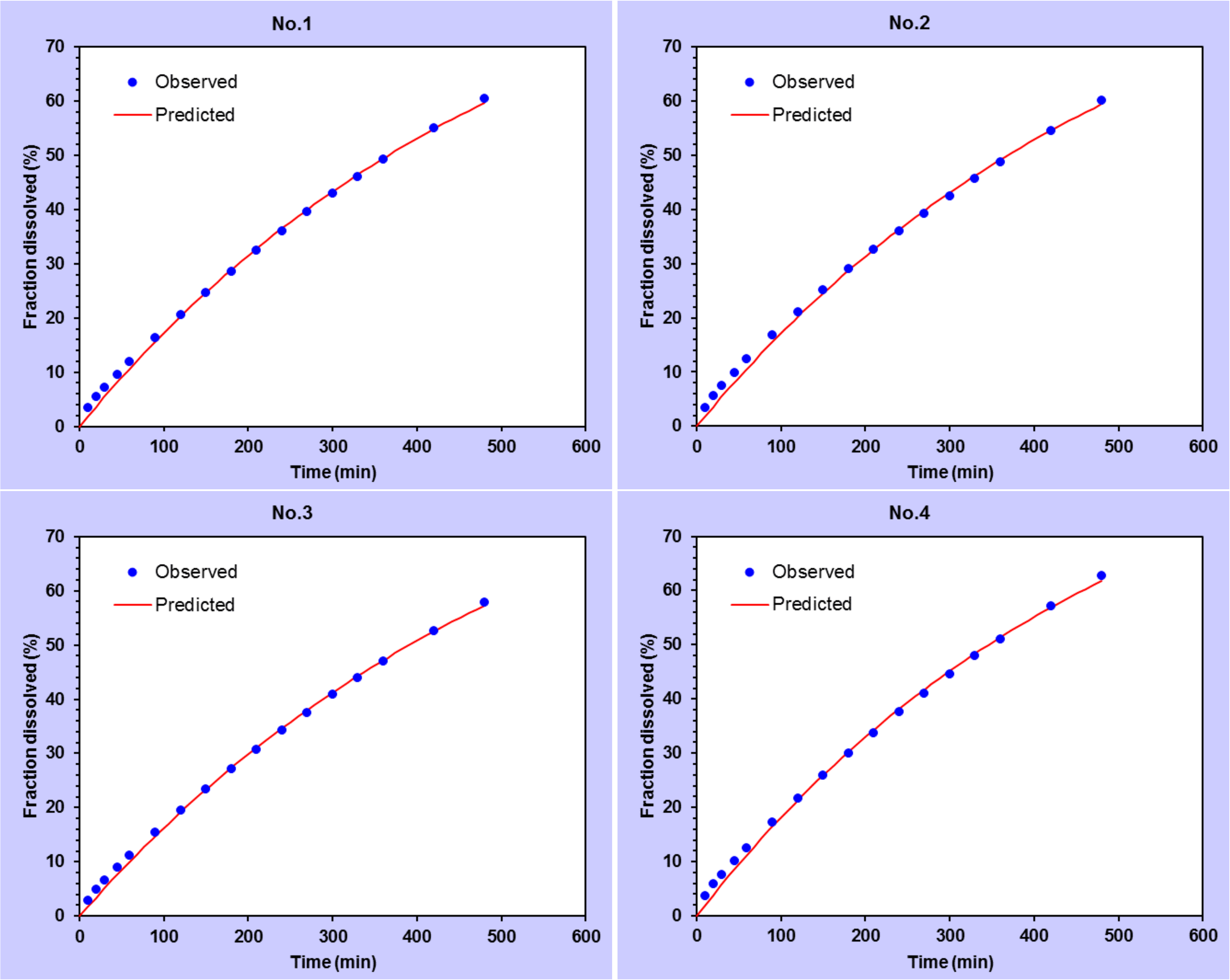

Supplementary materials – Model fitting summary – Granulated sugar N°1 600 (modelling up to app. 60 % of carvedilol released)

Model: **First–order with T_{lag}**

Model equation: $F = 100 \cdot \left[1 - e^{-k_1 \cdot (t - T_{lag})}\right]$

Fitted model parameters per tested tablet (N = 4) with statistics – mean, standard deviation (SD), and relative standard deviation expressed in % (RSD%) (output from DDSolver):

Parameter	No.1	No.2	No.3	No.4	Mean	SD	RSD(%)
k_1	0.002	0.002	0.002	0.002	0.002	0.000	5.138
T_{lag}	-5.792	-9.048	-5.425	-5.180	-6.361	1.809	-28.437

Number of dissolution data points (N), degrees of freedom (df), and selected goodness of fit criteria – Pearson correlation coefficient (R), coefficient of determination (R^2), adjusted coefficient of determination ($R^2_{adjusted}$), and residual sum of squares (RSS) (manual calculation in MS Excel):

Parameter	No.1	No.2	No.3	No.4
N	17	17	17	17
df	15	15	15	15
R	0.999579363	0.99967608	0.999690709	0.999419613
R^2	0.999158903	0.999352266	0.999381514	0.998839564
$R^2_{adjusted}$	0.99910283	0.999309083	0.999340282	0.998762201
RSS	5.0934725	3.698904393	3.400094964	7.698150922

Graphical abstract of model fit presented as mean ± 1 SD of the fraction % of released carvedilol:

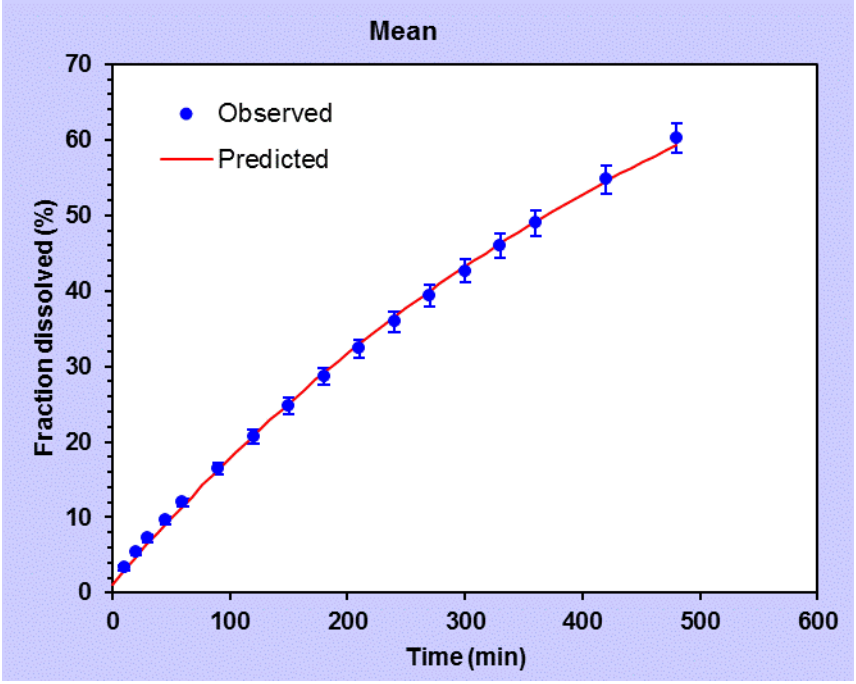

Supplementary materials – Model fitting summary – Granulated sugar N°1 600 (modelling up to app. 60 % of carvedilol released)

Graphical abstract of model fit presented as the fraction % of released carvedilol per tested tablet:

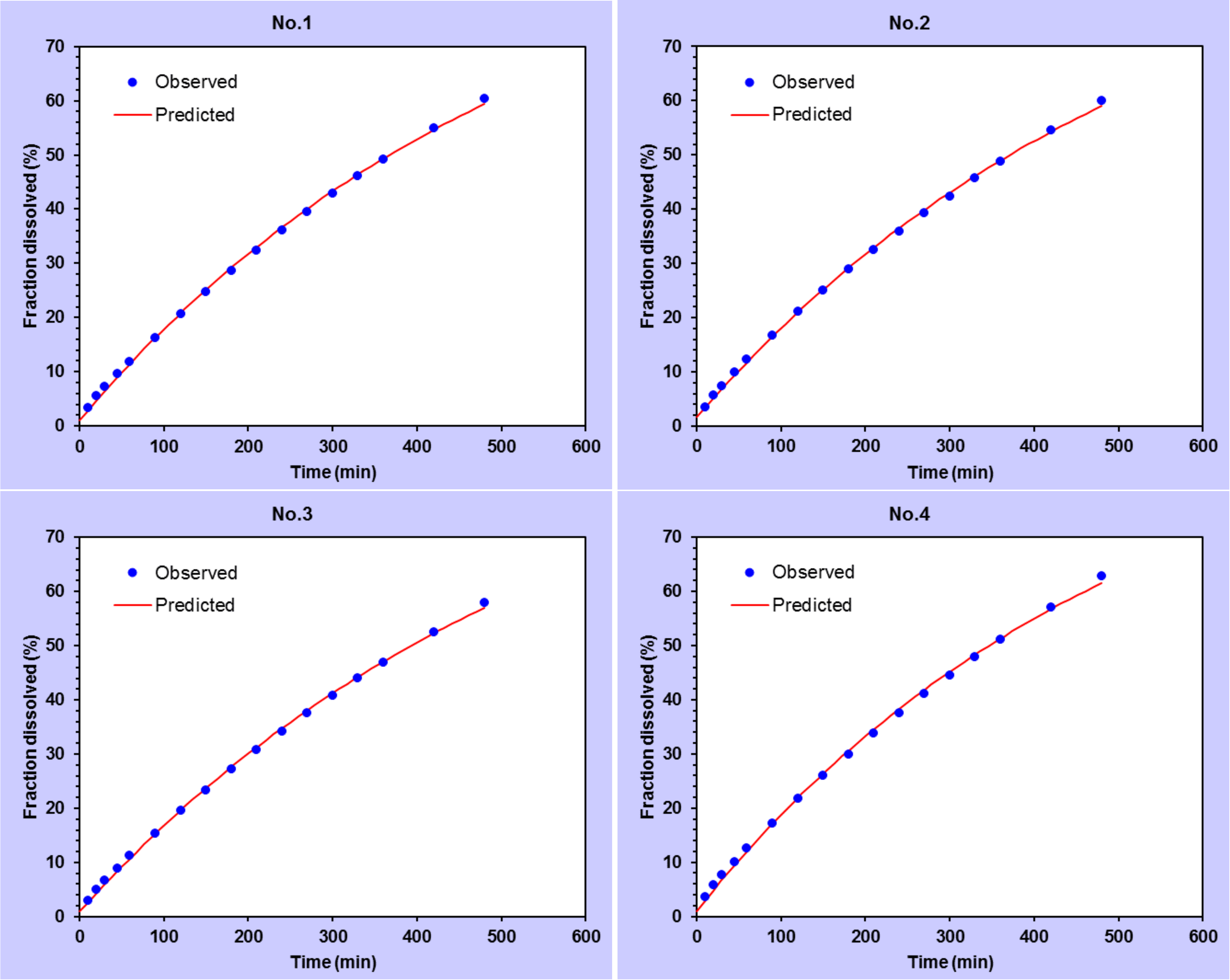

Supplementary materials – Model fitting summary – Granulated sugar N°1 600 (modelling up to app. 60 % of carvedilol released)

Model: **First–order with F_{max}**

Model equation: $F = F_{max} \cdot (1 - e^{-k_1 \cdot t})$

Fitted model parameters per tested tablet (N = 4) with statistics – mean, standard deviation (SD), and relative standard deviation expressed in % (RSD%) (output from DDSolver):

Parameter	No.1	No.2	No.3	No.4	Mean	SD	RSD(%)
k_1	0.005	0.005	0.005	0.005	0.005	0.000	0.392
F_{max}	63.394	62.976	60.779	65.905	63.264	2.102	3.322

Number of dissolution data points (N), degrees of freedom (df), and selected goodness of fit criteria – Pearson correlation coefficient (R), coefficient of determination (R^2), adjusted coefficient of determination ($R^2_{adjusted}$), and residual sum of squares (RSS) (manual calculation in MS Excel):

Parameter	No.1	No.2	No.3	No.4
N	17	17	17	17
df	15	15	15	15
R	0.984442801	0.985605243	0.984419229	0.984869518
R^2	0.969127629	0.971417694	0.969081219	0.969967967
$R^2_{adjusted}$	0.967069471	0.969512207	0.967019967	0.967965832
RSS	322.6497343	284.5624431	311.0481043	334.0471171

Graphical abstract of model fit presented as mean ± 1 SD of the fraction % of released carvedilol:

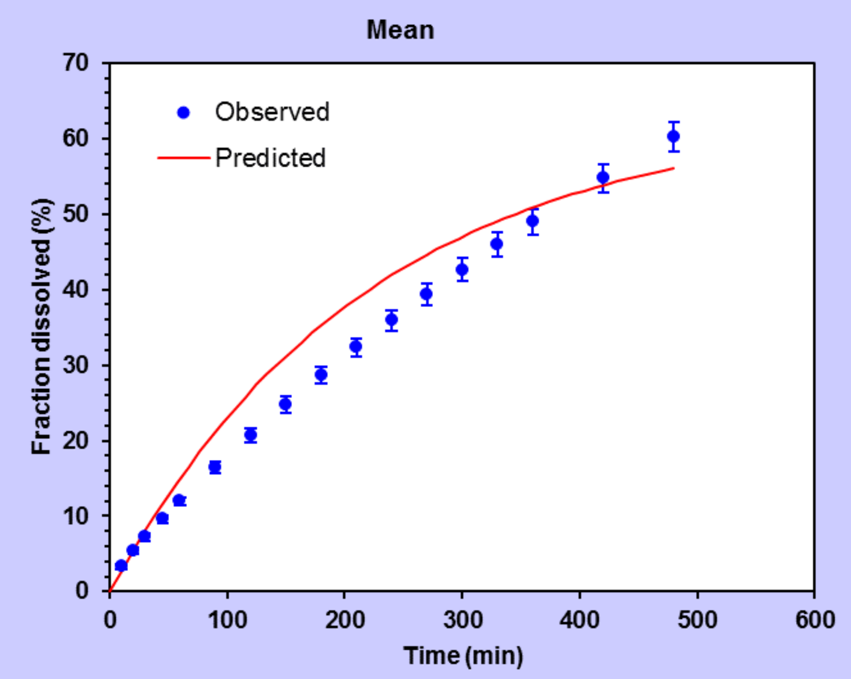

Supplementary materials – Model fitting summary – Granulated sugar N°1 600 (modelling up to app. 60 % of carvedilol released)

Graphical abstract of model fit presented as the fraction % of released carvedilol per tested tablet:

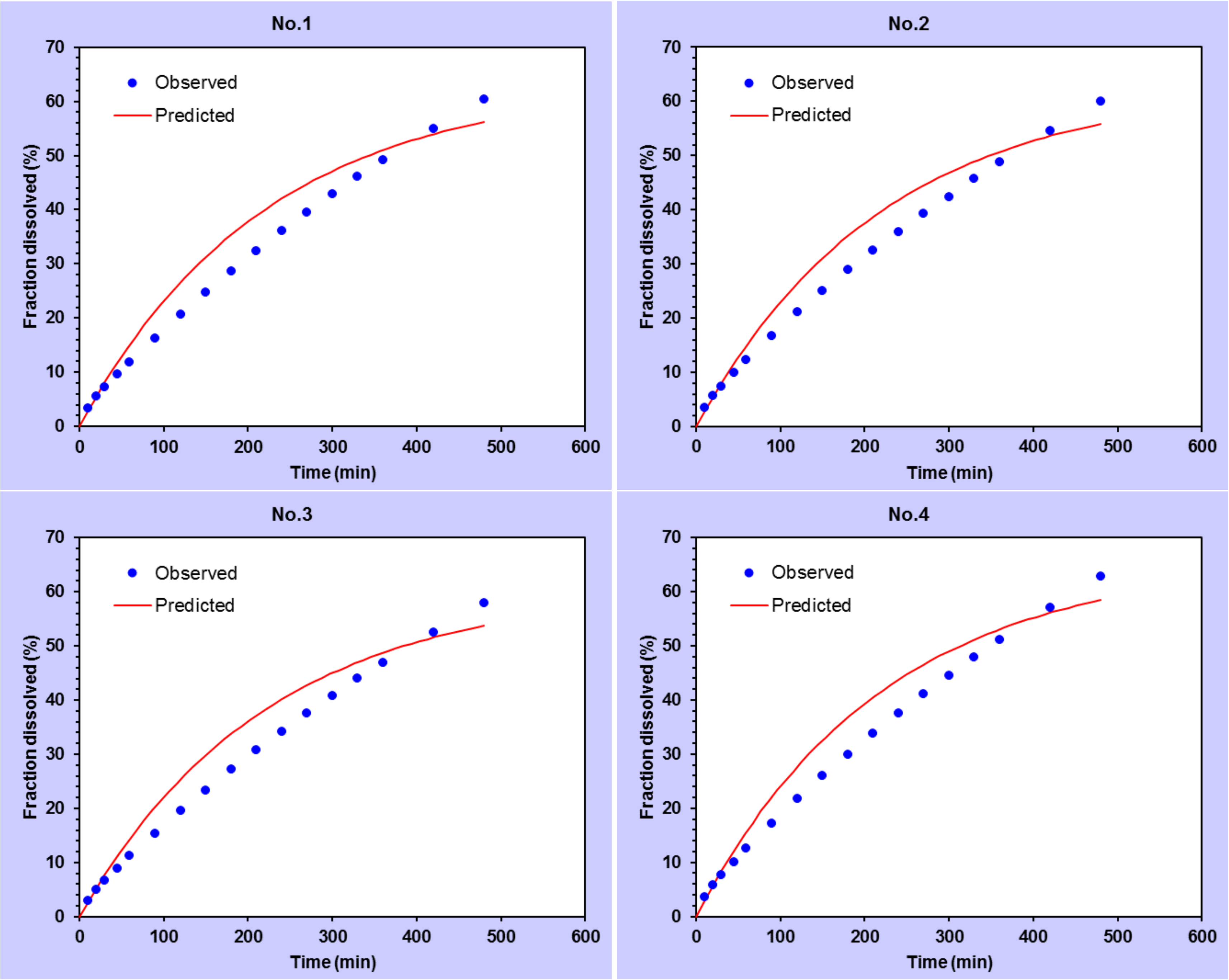

Supplementary materials – Model fitting summary – Granulated sugar N°1 600 (modelling up to app. 60 % of carvedilol released)

Model: **First–order with T_{lag} and F_{max}**

Model equation: $F = F_{max} \cdot \left[1 - e^{-k_1 \cdot (t - T_{lag})}\right]$

Fitted model parameters per tested tablet (N = 4) with statistics – mean, standard deviation (SD), and relative standard deviation expressed in % (RSD%) (output from DDSolver):

Parameter	No.1	No.2	No.3	No.4	Mean	SD	RSD(%)
k_1	0.005	0.005	0.005	0.005	0.005	0.000	0.238
T_{lag}	35.949	34.171	37.219	35.253	35.648	1.277	3.583
F_{max}	63.394	62.976	60.779	65.905	63.264	2.102	3.322

Number of dissolution data points (N), degrees of freedom (df), and selected goodness of fit criteria – Pearson correlation coefficient (R), coefficient of determination (R^2), adjusted coefficient of determination ($R^2_{adjusted}$), and residual sum of squares (RSS) (manual calculation in MS Excel):

Parameter	No.1	No.2	No.3	No.4
N	17	17	17	17
df	14	14	14	14
R	0.97868297	0.980431439	0.978458411	0.979334225
R^2	0.957820357	0.961245807	0.957380862	0.959095523
$R^2_{adjusted}$	0.951794693	0.955709493	0.951292414	0.953252027
RSS	585.8452252	535.6251178	551.2034599	616.081507

Graphical abstract of model fit presented as mean ± 1 SD of the fraction % of released carvedilol:

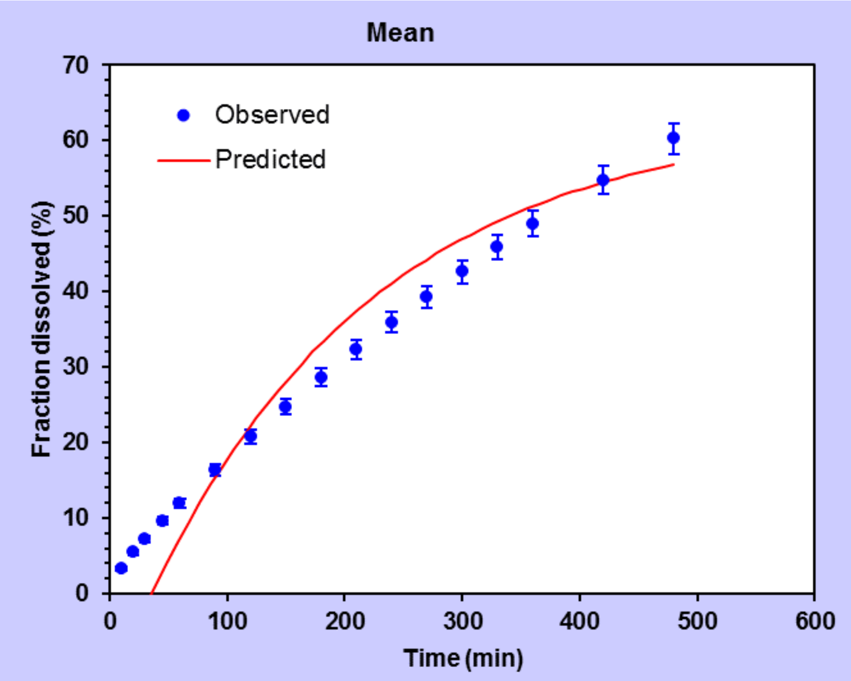

Supplementary materials – Model fitting summary – Granulated sugar N°1 600 (modelling up to app. 60 % of carvedilol released)

Graphical abstract of model fit presented as the fraction % of released carvedilol per tested tablet:

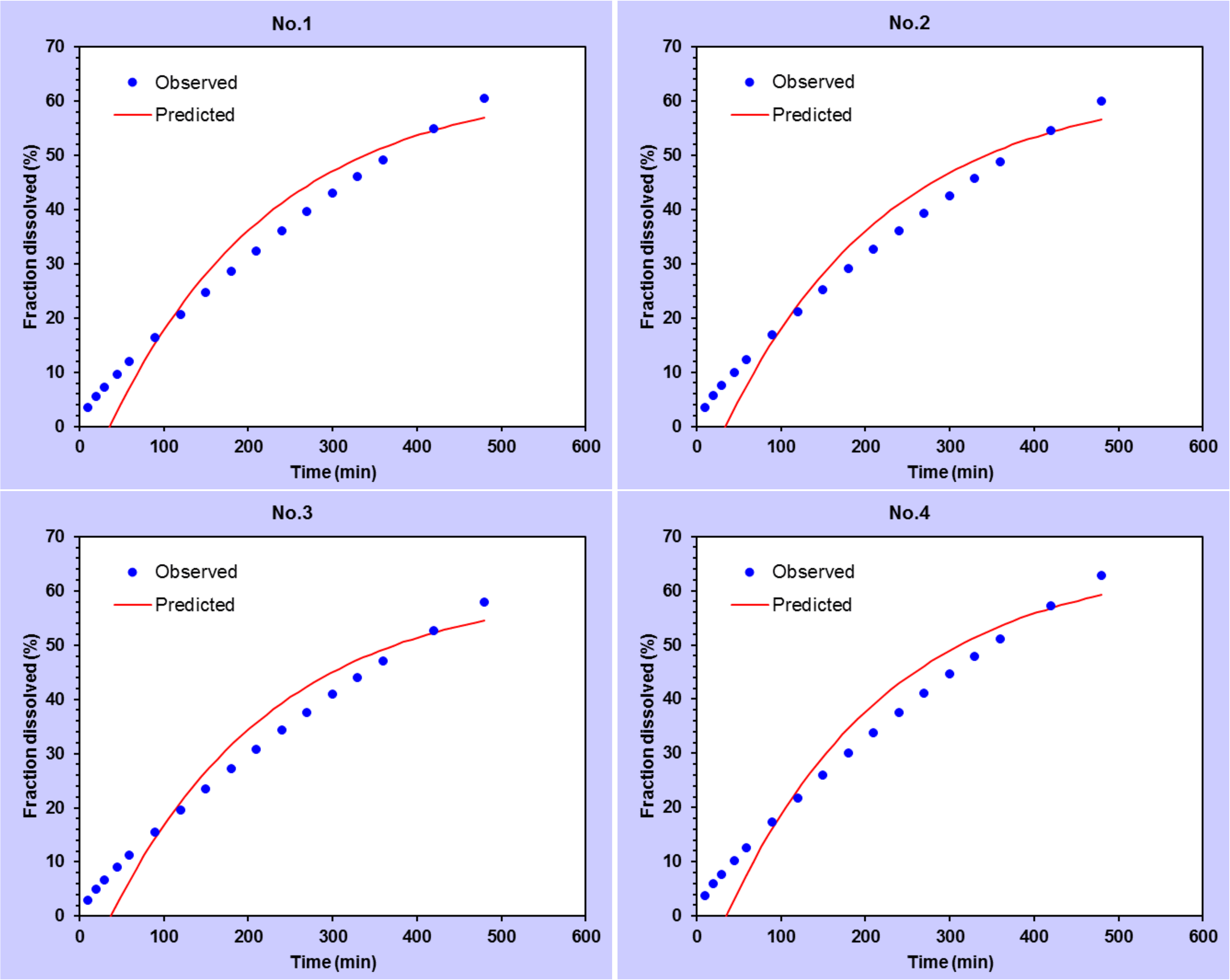

Supplementary materials – Model fitting summary – Granulated sugar N°1 600 (modelling up to app. 60 % of carvedilol released)

Model: **Higuchi**

Model equation: $F = k_H \cdot t^{0.5}$

Fitted model parameters per tested tablet (N = 4) with statistics – mean, standard deviation (SD), and relative standard deviation expressed in % (RSD%) (output from DDSolver):

Parameter	No.1	No.2	No.3	No.4	Mean	SD	RSD(%)
k_H	2.393	2.388	2.280	2.493	2.389	0.087	3.639

Number of dissolution data points (N), degrees of freedom (df), and selected goodness of fit criteria – Pearson correlation coefficient (R), coefficient of determination (R^2), adjusted coefficient of determination ($R^2_{adjusted}$), and residual sum of squares (RSS) (manual calculation in MS Excel):

Parameter	No.1	No.2	No.3	No.4
N	17	17	17	17
df	16	16	16	16
R	0.993076561	0.994208525	0.993023927	0.993490846
R^2	0.986201056	0.988450592	0.986096519	0.98702406
$R^2_{adjusted}$	0.986201056	0.988450592	0.986096519	0.98702406
RSS	399.4106157	351.9264194	390.7741287	412.5675758

Graphical abstract of model fit presented as mean ± 1 SD of the fraction % of released carvedilol:

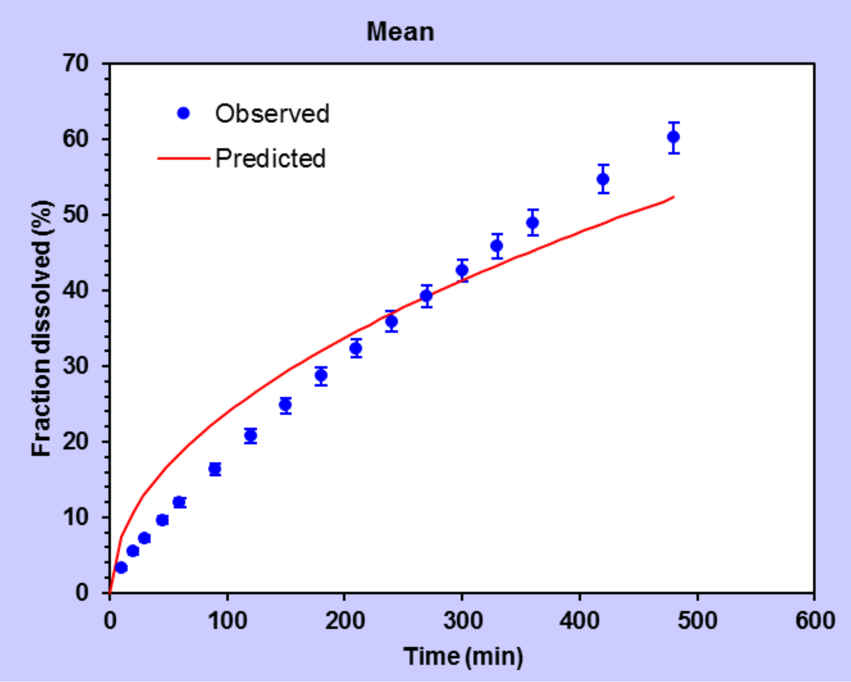

Supplementary materials – Model fitting summary – Granulated sugar N°1 600 (modelling up to app. 60 % of carvedilol released)

Graphical abstract of model fit presented as the fraction % of released carvedilol per tested tablet:

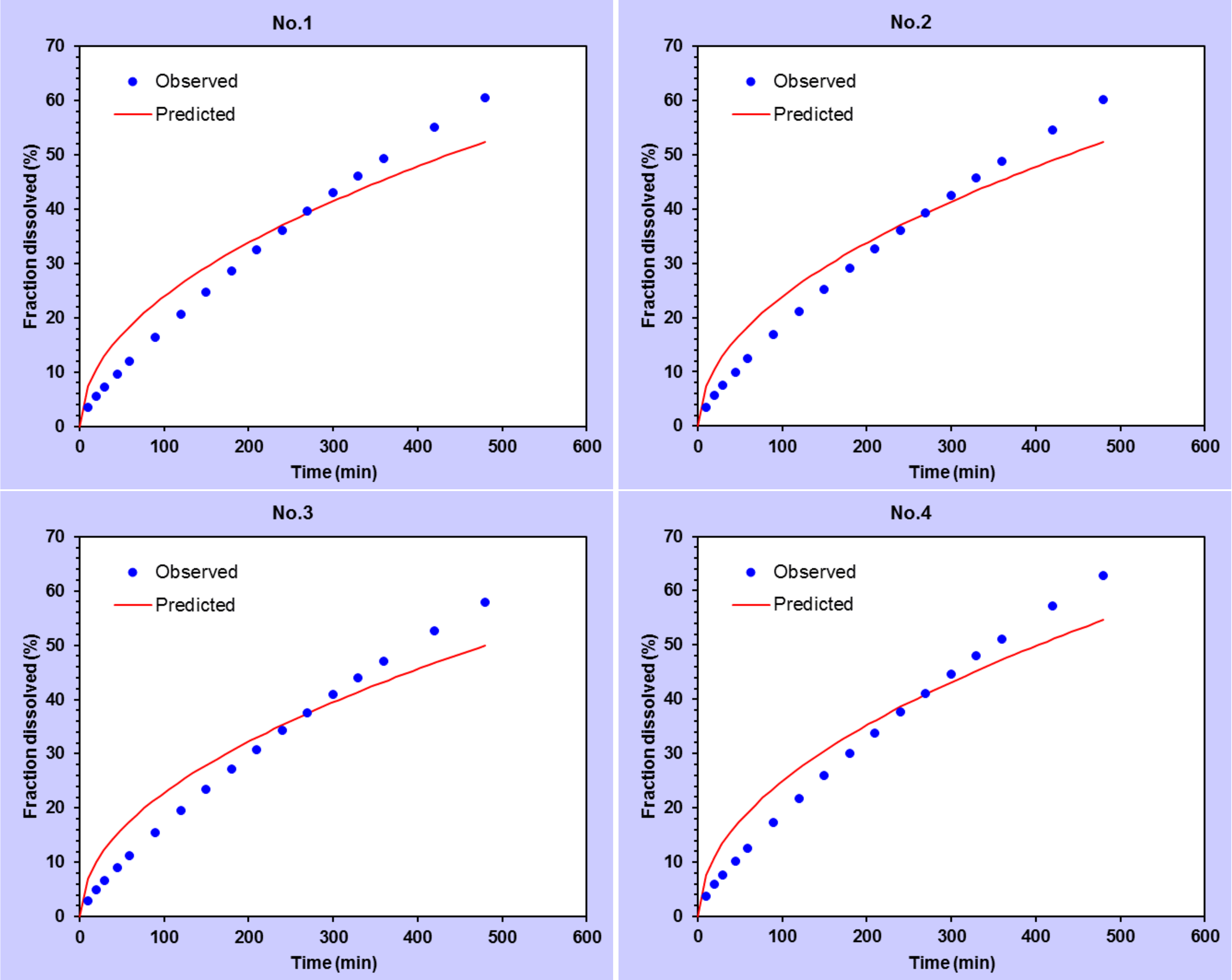

Supplementary materials – Model fitting summary – Granulated sugar N°1 600 (modelling up to app. 60 % of carvedilol released)

Model: **Higuchi with T_{lag}**

Model equation: $F = k_H \cdot (t - T_{lag})^{0.5}$

Fitted model parameters per tested tablet (N = 4) with statistics – mean, standard deviation (SD), and relative standard deviation expressed in % (RSD%) (output from DDSolver):

Parameter	No.1	No.2	No.3	No.4	Mean	SD	RSD(%)
k_H	2.754	2.729	2.636	2.860	2.745	0.092	3.361
T_{lag}	44.663	42.851	45.830	43.956	44.325	1.250	2.820

Number of dissolution data points (N), degrees of freedom (df), and selected goodness of fit criteria – Pearson correlation coefficient (R), coefficient of determination (R^2), adjusted coefficient of determination ($R^2_{adjusted}$), and residual sum of squares (RSS) (manual calculation in MS Excel):

Parameter	No.1	No.2	No.3	No.4
N	17	17	17	17
df	15	15	15	15
R	0.984437637	0.986712115	0.98329658	0.985627792
R^2	0.969117461	0.973600797	0.966872163	0.971462145
$R^2_{adjusted}$	0.967058625	0.97184085	0.964663641	0.969559621
RSS	231.2531461	194.4746533	227.5355182	230.0692262

Graphical abstract of model fit presented as mean ± 1 SD of the fraction % of released carvedilol:

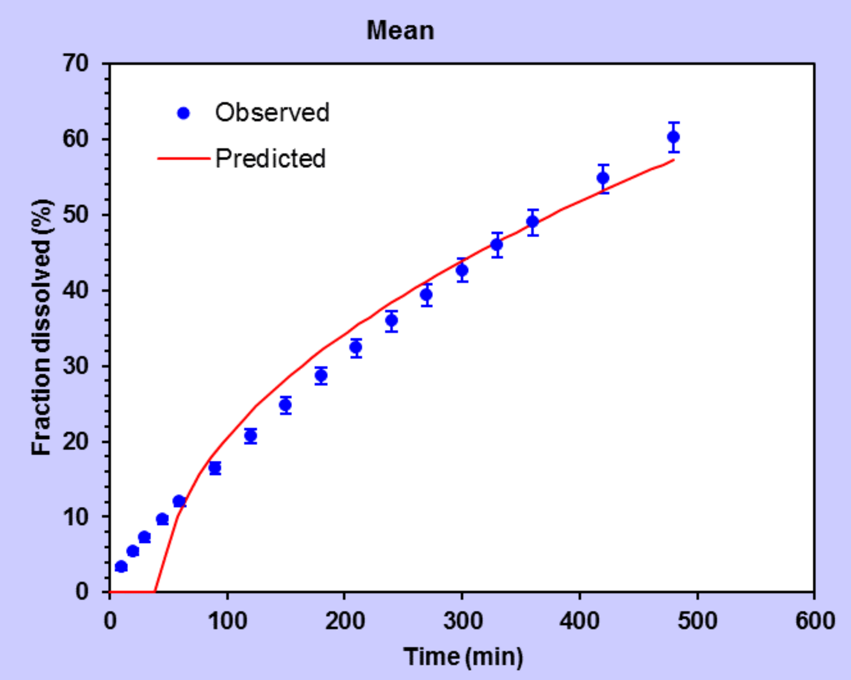

Supplementary materials – Model fitting summary – Granulated sugar N°1 600 (modelling up to app. 60 % of carvedilol released)

Graphical abstract of model fit presented as the fraction % of released carvedilol per tested tablet:

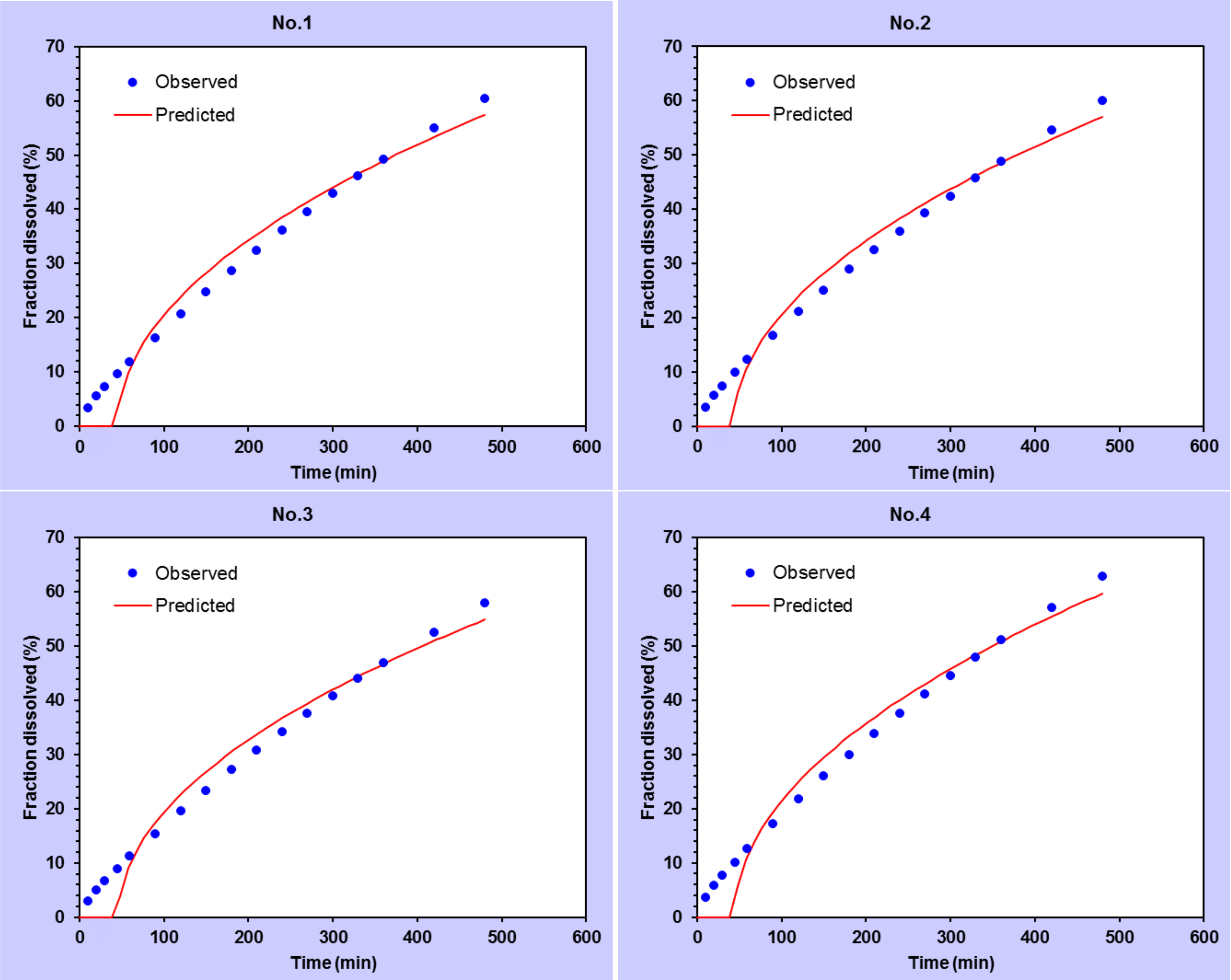

Supplementary materials – Model fitting summary – Granulated sugar N°1 600 (modelling up to app. 60 % of carvedilol released)

Model: **Higuchi with F_0**

Model equation: $F = F_0 + k_H \cdot t^{0.5}$

Fitted model parameters per tested tablet (N = 4) with statistics – mean, standard deviation (SD), and relative standard deviation expressed in % (RSD%) (output from DDSolver):

Parameter	No.1	No.2	No.3	No.4	Mean	SD	RSD(%)
k_H	3.099	3.057	2.982	3.212	3.087	0.096	3.115
F_0	-10.786	-10.213	-10.725	-10.991	-10.679	0.330	-3.095

Number of dissolution data points (N), degrees of freedom (df), and selected goodness of fit criteria – Pearson correlation coefficient (R), coefficient of determination (R^2), adjusted coefficient of determination ($R^2_{adjusted}$), and residual sum of squares (RSS) (manual calculation in MS Excel):

Parameter	No.1	No.2	No.3	No.4
N	17	17	17	17
df	15	15	15	15
R	0.993076561	0.994208525	0.993023927	0.993490846
R^2	0.986201056	0.988450592	0.986096519	0.98702406
$R^2_{adjusted}$	0.985281126	0.987680631	0.98516962	0.986158998
RSS	73.43438435	59.65058411	68.49378669	74.1030333

Graphical abstract of model fit presented as mean ± 1 SD of the fraction % of released carvedilol:

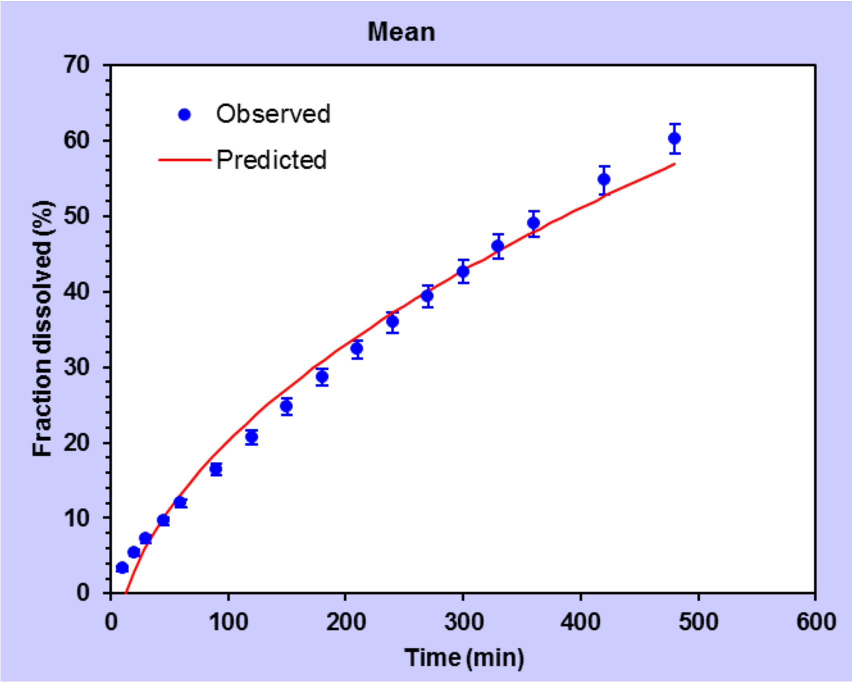

Supplementary materials – Model fitting summary – Granulated sugar N°1 600 (modelling up to app. 60 % of carvedilol released)

Graphical abstract of model fit presented as the fraction % of released carvedilol per tested tablet:

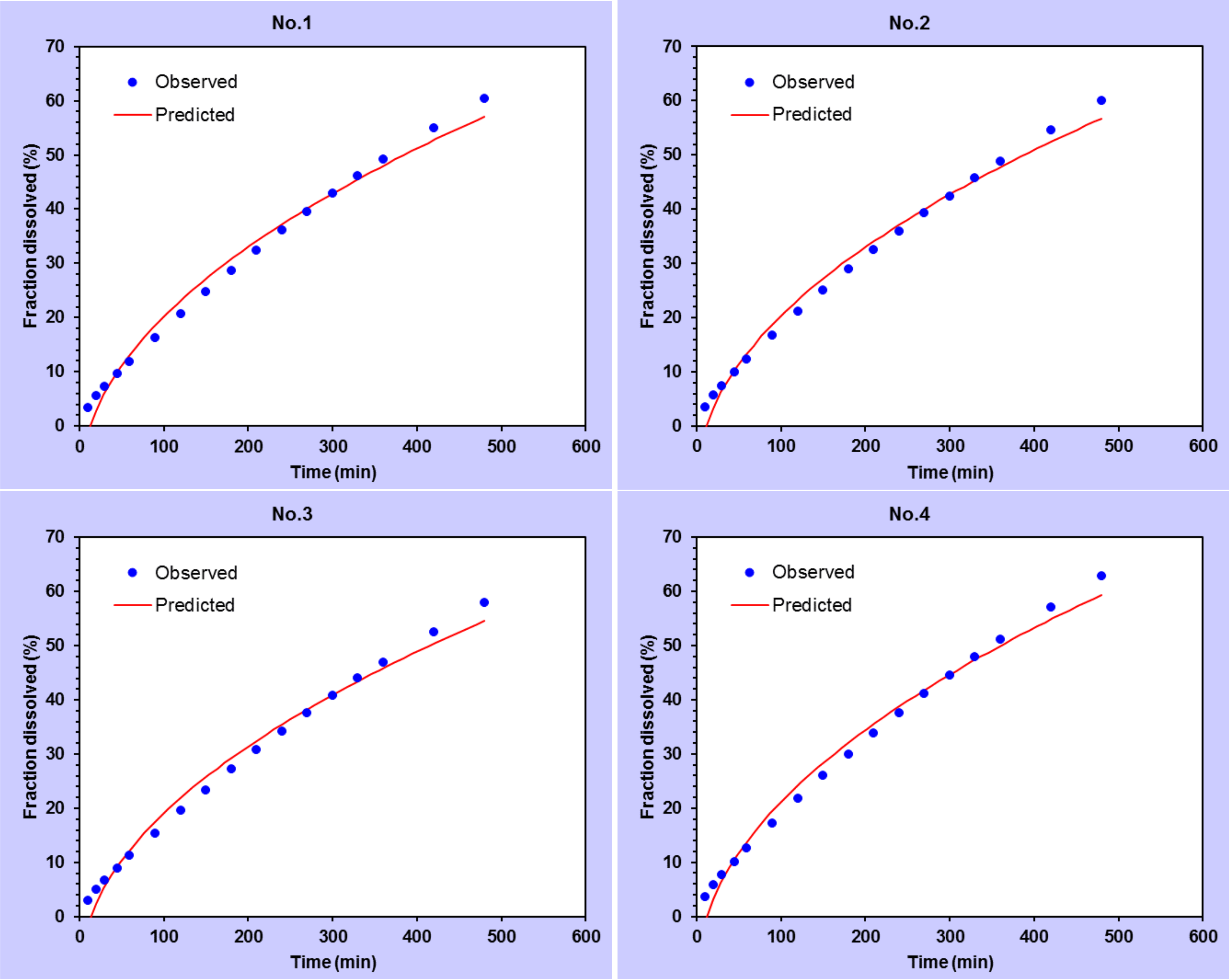

Supplementary materials – Model fitting summary – Granulated sugar N°1 600 (modelling up to app. 60 % of carvedilol released)

Model: **Korsmeyer–Peppas**

Model equation: $F = k_{KP} \cdot t^n$

Fitted model parameters per tested tablet (N = 4) with statistics – mean, standard deviation (SD), and relative standard deviation expressed in % (RSD%) (output from DDSolver):

Parameter	No.1	No.2	No.3	No.4	Mean	SD	RSD(%)
k_{KP}	0.555	0.584	0.463	0.597	0.550	0.060	10.958
n	0.759	0.751	0.783	0.754	0.762	0.015	1.949

Number of dissolution data points (N), degrees of freedom (df), and selected goodness of fit criteria – Pearson correlation coefficient (R), coefficient of determination (R^2), adjusted coefficient of determination ($R^2_{adjusted}$), and residual sum of squares (RSS) (manual calculation in MS Excel):

Parameter	No.1	No.2	No.3	No.4
N	17	17	17	17
df	15	15	15	15
R	0.99981512	0.999945687	0.999876698	0.99987466
R^2	0.999630275	0.999891376	0.999753412	0.999749335
$R^2_{adjusted}$	0.999605627	0.999884134	0.999736972	0.999732624
RSS	3.623002567	0.829936761	1.372374268	2.832829225

Graphical abstract of model fit presented as mean ± 1 SD of the fraction % of released carvedilol:

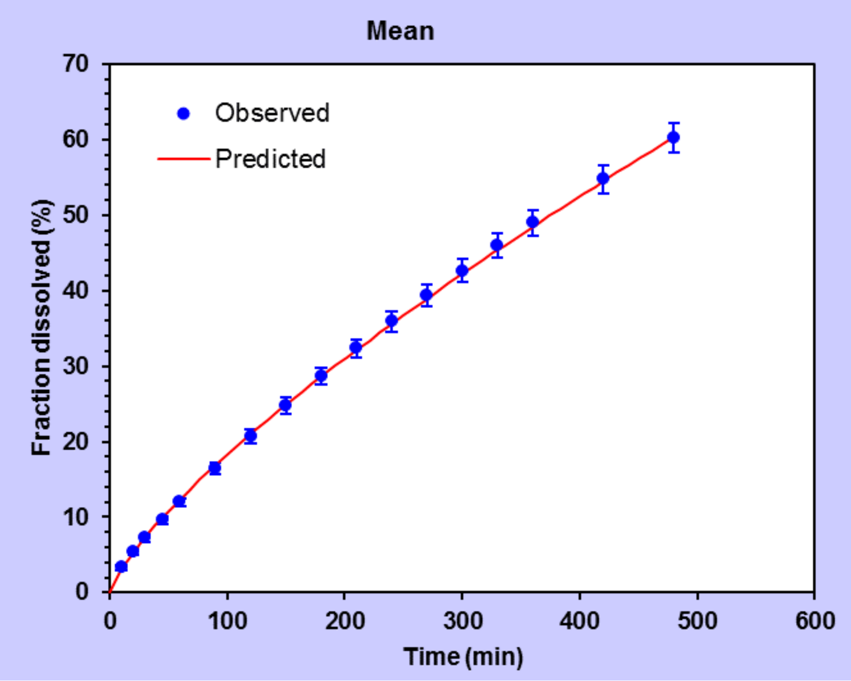

Supplementary materials – Model fitting summary – Granulated sugar N°1 600 (modelling up to app. 60 % of carvedilol released)

Graphical abstract of model fit presented as the fraction % of released carvedilol per tested tablet:

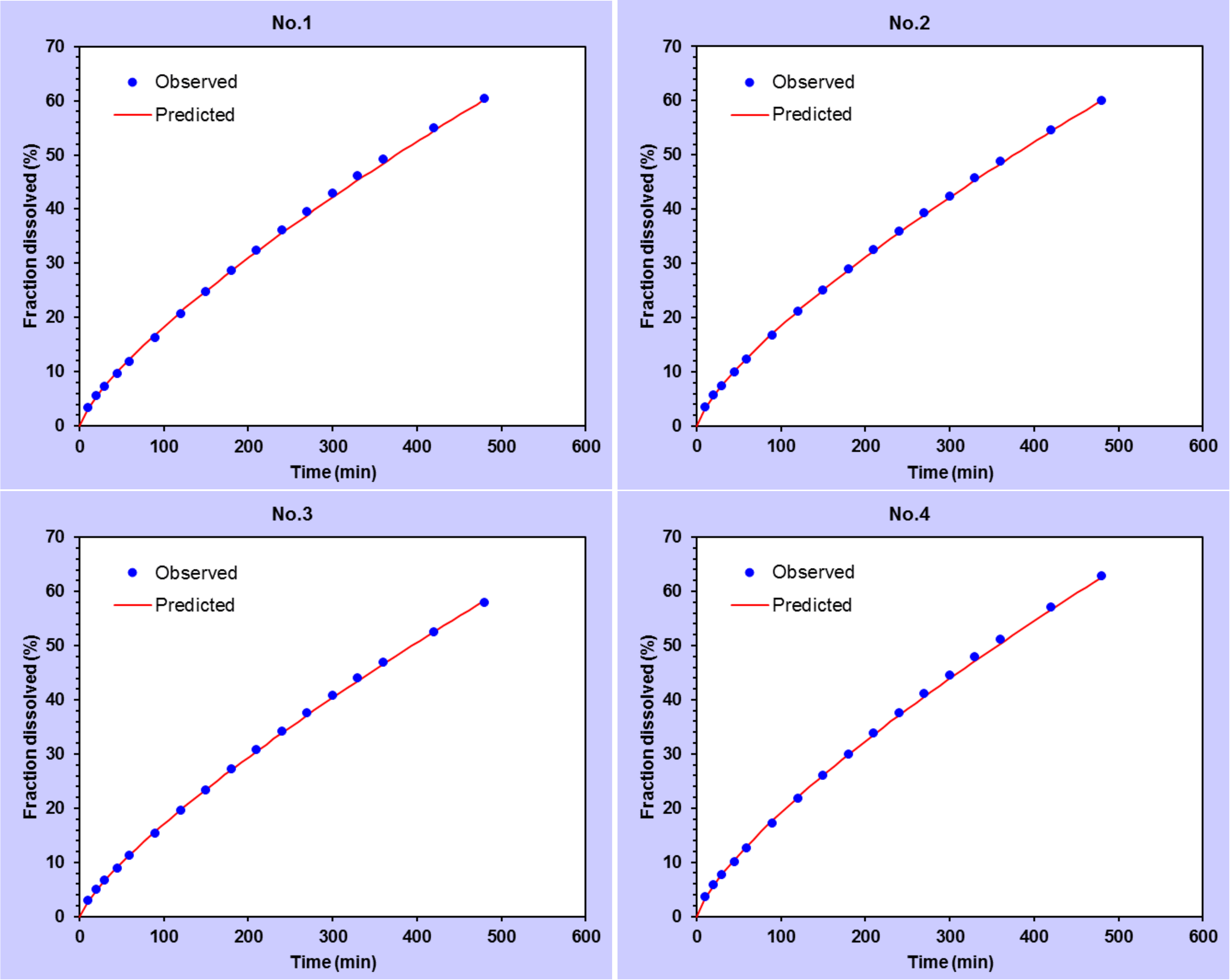

Supplementary materials – Model fitting summary – Granulated sugar N°1 600 (modelling up to app. 60 % of carvedilol released)

Model: **Korsmeyer–Peppas with T_{lag}**

Model equation: $F = k_{KP} \cdot \left(t - T_{lag}\right)^{n}$

Fitted model parameters per tested tablet (N = 4) with statistics – mean, standard deviation (SD), and relative standard deviation expressed in % (RSD%) (output from DDSolver):

Parameter	No.1	No.2	No.3	No.4	Mean	SD	RSD(%)
k_{KP}	0.812	0.930	0.684	0.870	0.824	0.105	12.775
n	0.691	0.674	0.714	0.686	0.691	0.017	2.401
T_{lag}	4.000	4.834	4.000	4.000	4.209	0.417	9.914

Number of dissolution data points (N), degrees of freedom (df), and selected goodness of fit criteria – Pearson correlation coefficient (R), coefficient of determination (R^2), adjusted coefficient of determination ($R^2_{adjusted}$), and residual sum of squares (RSS) (manual calculation in MS Excel):

Parameter	No.1	No.2	No.3	No.4
N	17	17	17	17
df	14	14	14	14
R	0.999025249	0.999122255	0.999397972	0.999114679
R^2	0.998051448	0.99824528	0.998796306	0.998230142
$R^2_{adjusted}$	0.997773083	0.997994605	0.99862435	0.997977306
RSS	31.38388488	19.73109161	19.15935768	31.08603481

Graphical abstract of model fit presented as mean ± 1 SD of the fraction % of released carvedilol:

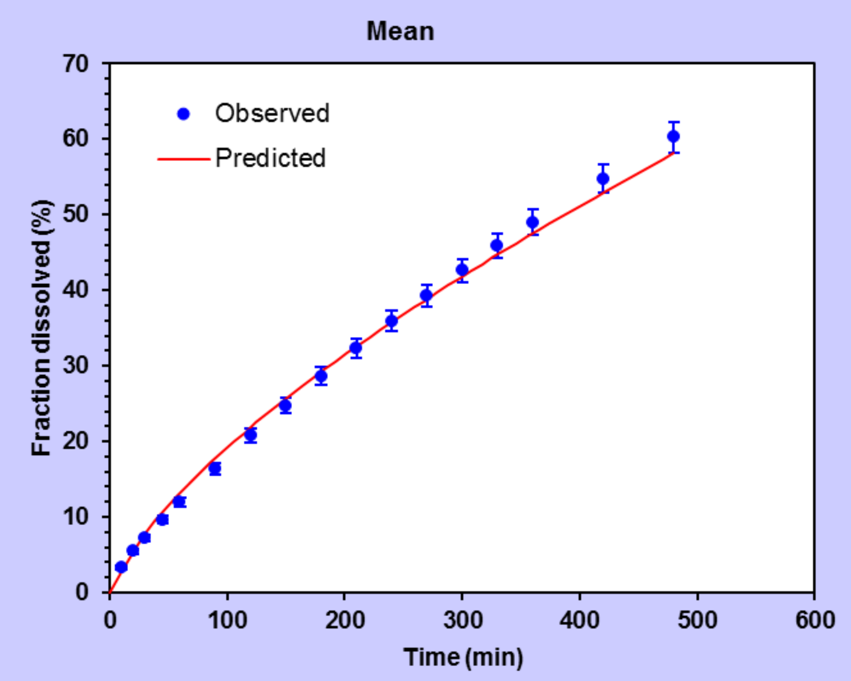

Supplementary materials – Model fitting summary – Granulated sugar N°1 600 (modelling up to app. 60 % of carvedilol released)

Graphical abstract of model fit presented as the fraction % of released carvedilol per tested tablet:

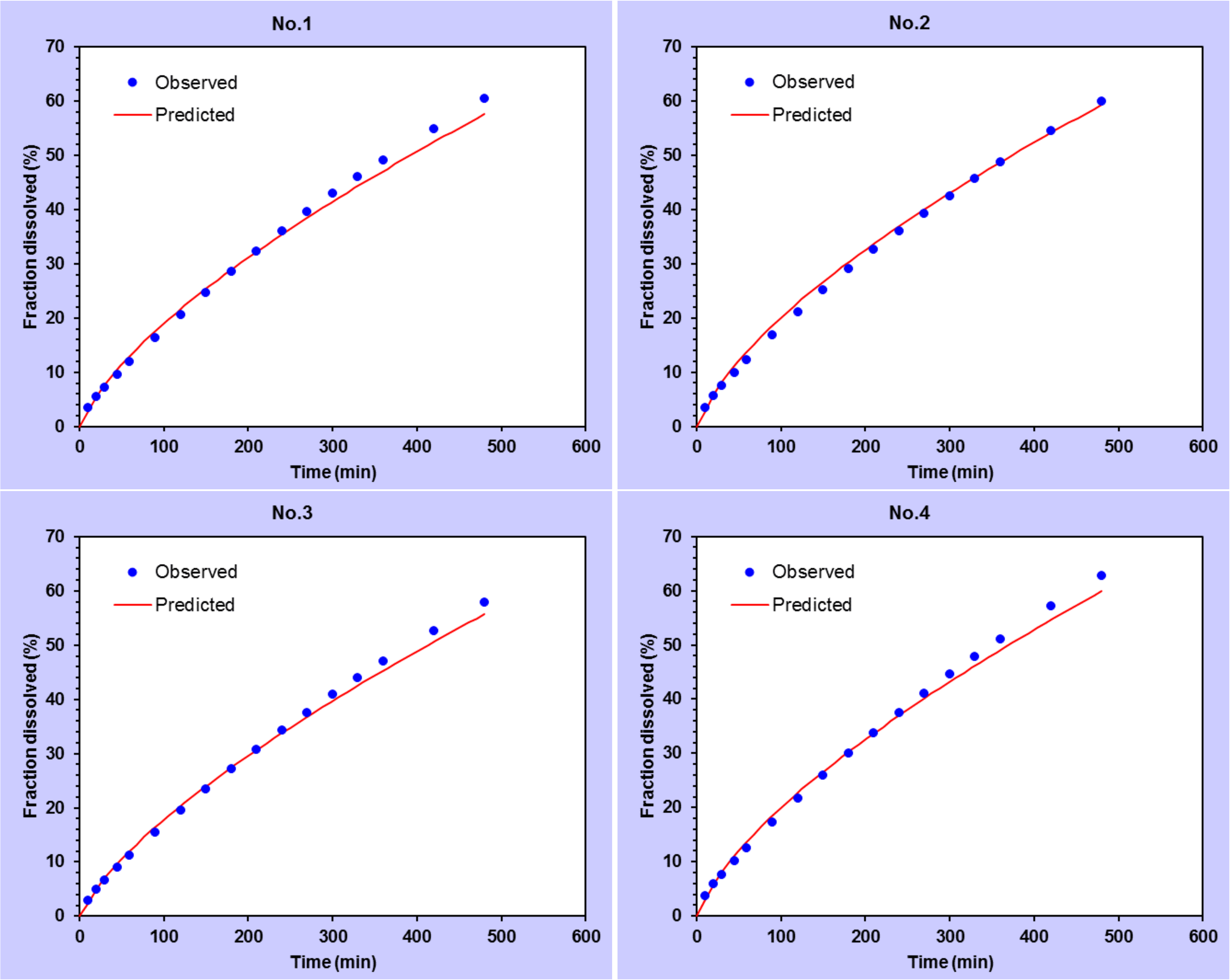

Supplementary materials – Model fitting summary – Granulated sugar N°1 600 (modelling up to app. 60 % of carvedilol released)

Model: **Korsmeyer–Peppas with F_0**

Model equation: $F = F_0 + k_{KP} \cdot t^n$

Fitted model parameters per tested tablet (N = 4) with statistics – mean, standard deviation (SD), and relative standard deviation expressed in % (RSD%) (output from DDSolver):

Parameter	No.1	No.2	No.3	No.4	Mean	SD	RSD(%)
k_{KP}	0.327	0.324	0.260	0.351	0.315	0.039	12.405
n	0.843	0.851	0.883	0.837	0.853	0.020	2.380
F_0	1.667	1.360	1.160	1.766	1.488	0.279	18.738

Number of dissolution data points (N), degrees of freedom (df), and selected goodness of fit criteria – Pearson correlation coefficient (R), coefficient of determination (R^2), adjusted coefficient of determination ($R^2_{adjusted}$), and residual sum of squares (RSS) (manual calculation in MS Excel):

Parameter	No.1	No.2	No.3	No.4
N	17	17	17	17
df	14	14	14	14
R	0.999424271	0.999148662	0.998976749	0.999456922
R^2	0.998848873	0.99829805	0.997954544	0.998914139
$R^2_{adjusted}$	0.998684426	0.998054914	0.997662336	0.998759016
RSS	10.52367174	19.97463222	22.1388032	10.07296759

Graphical abstract of model fit presented as mean ± 1 SD of the fraction % of released carvedilol:

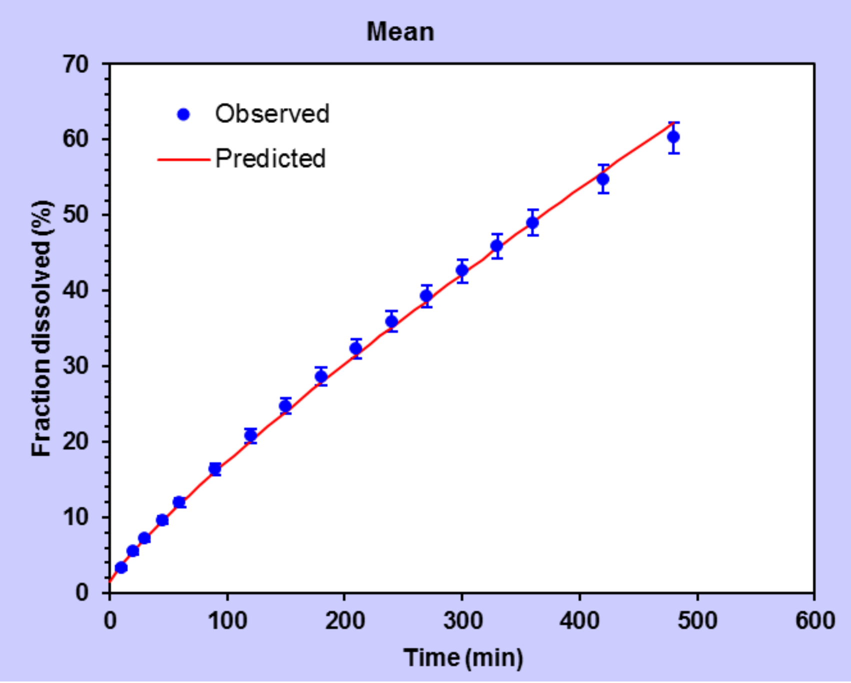

Supplementary materials – Model fitting summary – Granulated sugar N°1 600 (modelling up to app. 60 % of carvedilol released)

Graphical abstract of model fit presented as the fraction % of released carvedilol per tested tablet:

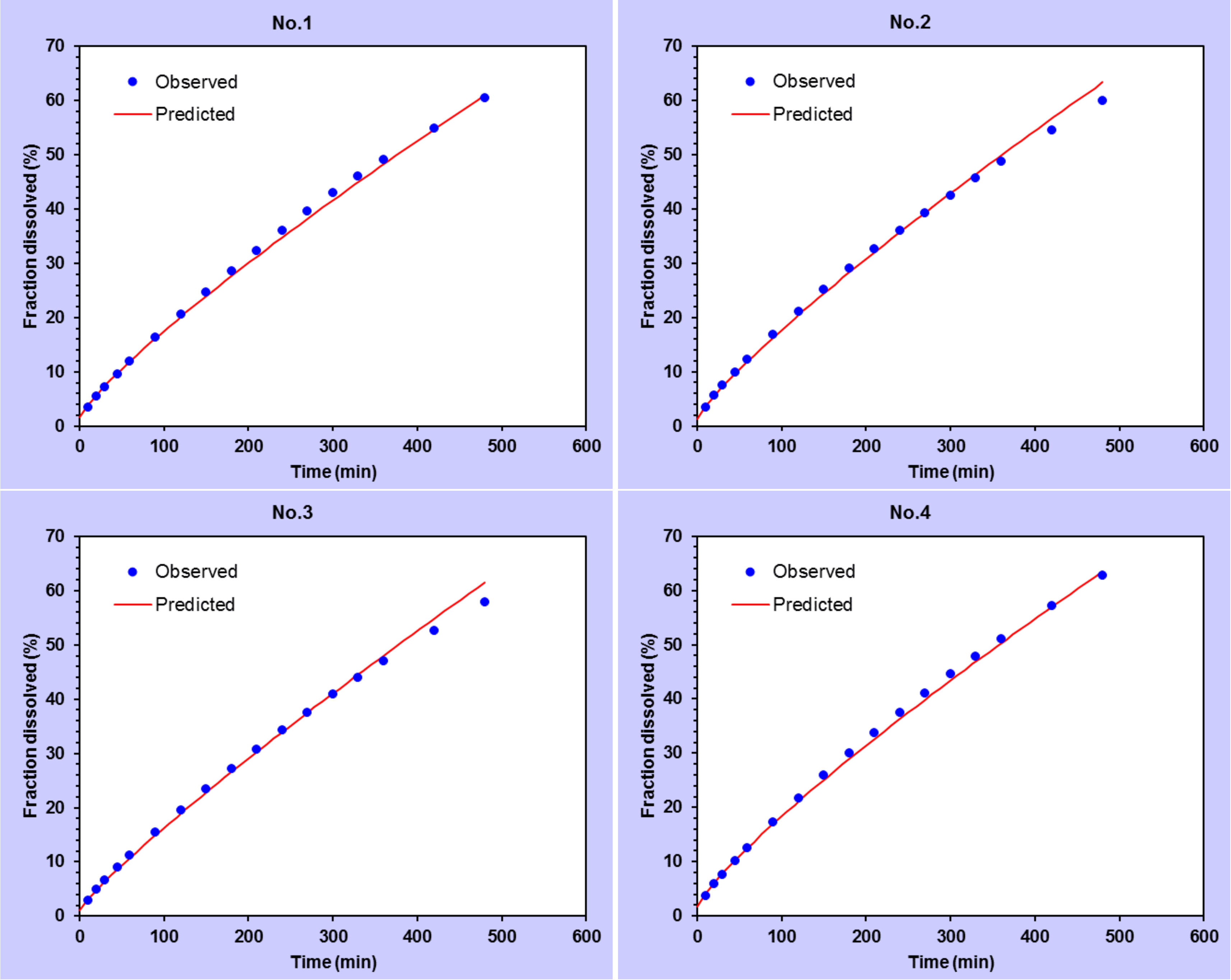

Supplementary materials – Model fitting summary – Granulated sugar N°1 600 (modelling up to app. 60 % of carvedilol released)

Model: **Hixson–Crowell**

Model equation: $F = 100 \cdot [1 - (1 - k_{HC} \cdot t)^3]$

Fitted model parameters per tested tablet (N = 4) with statistics – mean, standard deviation (SD), and relative standard deviation expressed in % (RSD%) (output from DDSolver):

Parameter	No.1	No.2	No.3	No.4	Mean	SD	RSD(%)
k_{HC}	0.001	0.001	0.001	0.001	0.001	0.000	4.478

Number of dissolution data points (N), degrees of freedom (df), and selected goodness of fit criteria – Pearson correlation coefficient (R), coefficient of determination (R^2), adjusted coefficient of determination ($R^2_{adjusted}$), and residual sum of squares (RSS) (manual calculation in MS Excel):

Parameter	No.1	No.2	No.3	No.4
N	17	17	17	17
df	16	16	16	16
R	0.999946457	0.999756655	0.999902346	0.999925215
R^2	0.999892917	0.99951337	0.999804702	0.999850436
$R^2_{adjusted}$	0.999892917	0.99951337	0.999804702	0.999850436
RSS	32.07170871	46.62034699	25.21785442	36.22645213

Graphical abstract of model fit presented as mean ± 1 SD of the fraction % of released carvedilol:

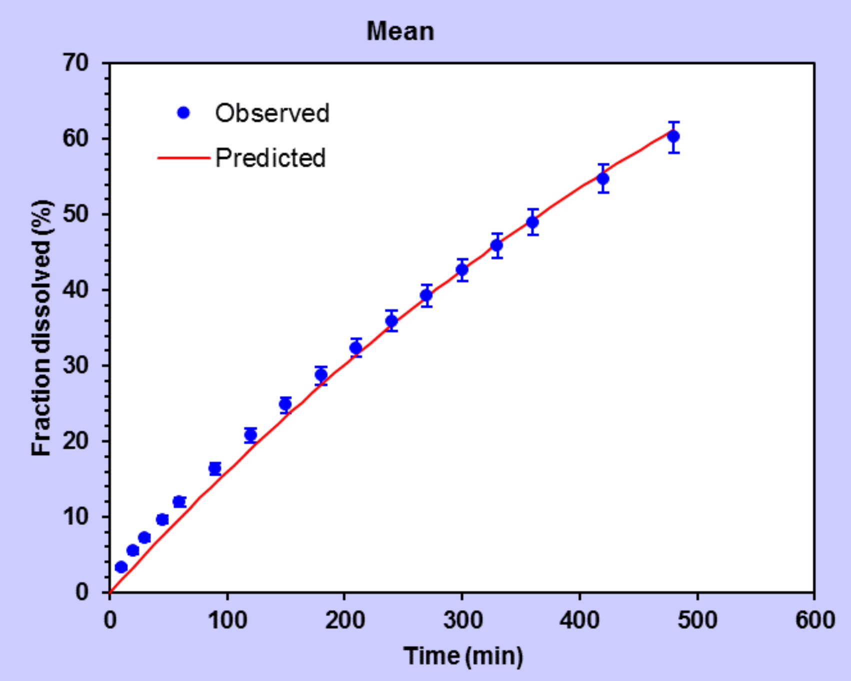

Supplementary materials – Model fitting summary – Granulated sugar N°1 600 (modelling up to app. 60 % of carvedilol released)

Graphical abstract of model fit presented as the fraction % of released carvedilol per tested tablet:

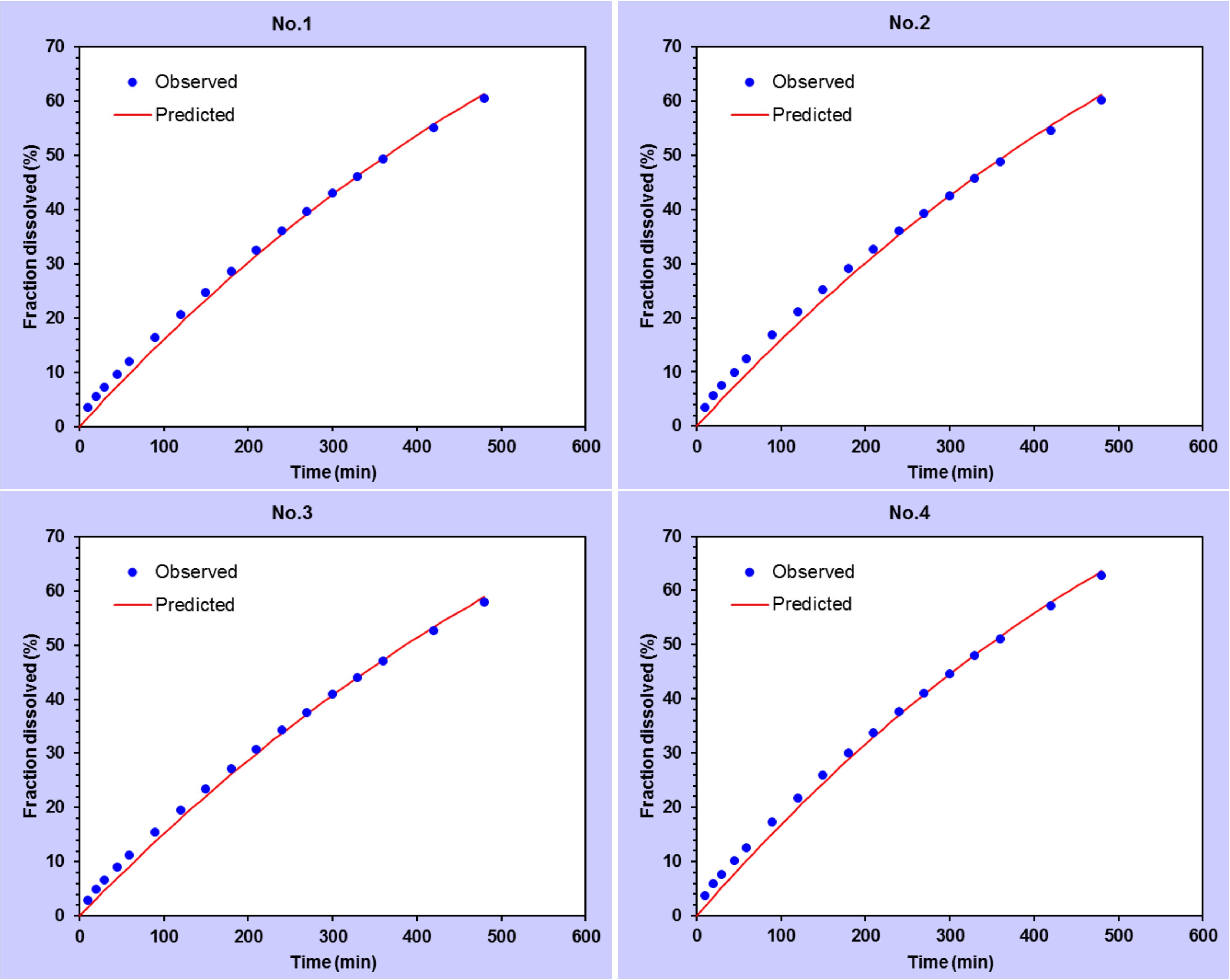

Supplementary materials – Model fitting summary – Granulated sugar N°1 600 (modelling up to app. 60 % of carvedilol released)

Model: **Hixson–Crowell with T_{lag}**

Model equation: $F = 100 \cdot \left\{1 - \left[1 - k_{HC} \cdot \left(t - T_{lag}\right)\right]^3\right\}$

Fitted model parameters per tested tablet (N = 4) with statistics – mean, standard deviation (SD), and relative standard deviation expressed in % (RSD%) (output from DDSolver):

Parameter	No.1	No.2	No.3	No.4	Mean	SD	RSD(%)
k_{HC}	0.001	0.001	0.001	0.001	0.001	0.000	4.404
T_{lag}	-16.202	-19.666	-15.038	-16.273	-16.795	1.996	-11.885

Number of dissolution data points (N), degrees of freedom (df), and selected goodness of fit criteria – Pearson correlation coefficient (R), coefficient of determination (R^2), adjusted coefficient of determination ($R^2_{adjusted}$), and residual sum of squares (RSS) (manual calculation in MS Excel):

Parameter	No.1	No.2	No.3	No.4
N	17	17	17	17
df	15	15	15	15
R	0.999916315	0.999690104	0.999866024	0.99990553
R^2	0.999832637	0.999380303	0.999732067	0.999811068
$R^2_{adjusted}$	0.999821479	0.99933899	0.999714205	0.999798473
RSS	0.917403471	3.266832952	1.354660802	1.091503238

Graphical abstract of model fit presented as mean ± 1 SD of the fraction % of released carvedilol:

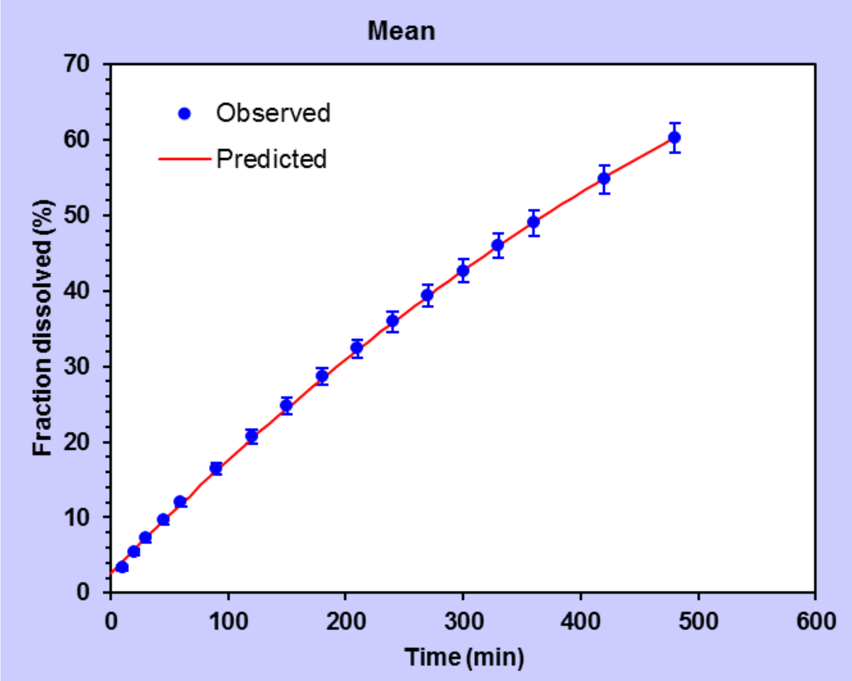

Supplementary materials – Model fitting summary – Granulated sugar N°1 600 (modelling up to app. 60 % of carvedilol released)

Graphical abstract of model fit presented as the fraction % of released carvedilol per tested tablet:

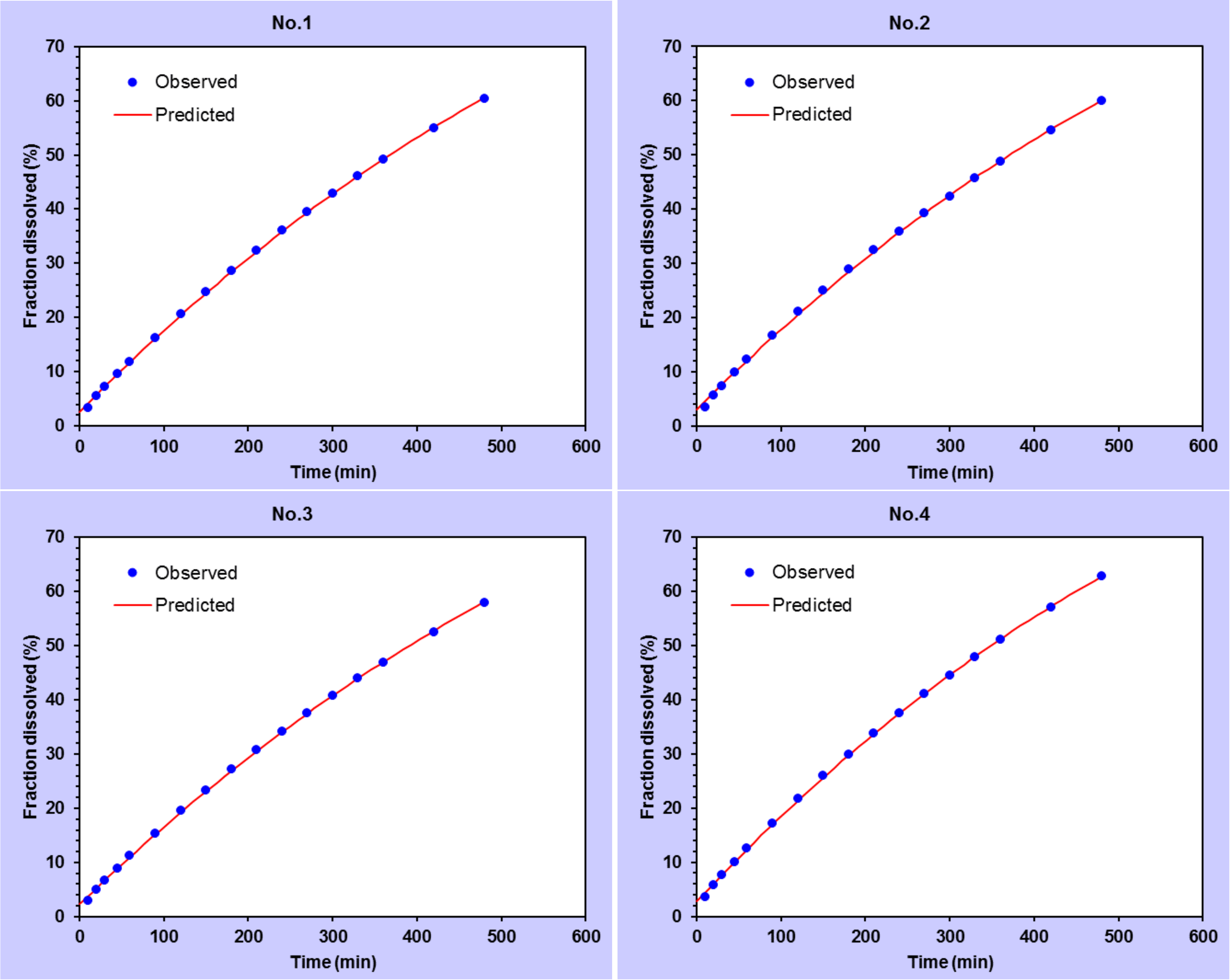

Supplementary materials – Model fitting summary – Granulated sugar N°1 600 (modelling up to app. 60 % of carvedilol released)

Model: **Hopfenberg**

Model equation: $F = 100 \cdot [1 - (1 - k_{HB} \cdot t)^n]$

Fitted model parameters per tested tablet (N = 4) with statistics – mean, standard deviation (SD), and relative standard deviation expressed in % (RSD%) (output from DDSolver):

Parameter	No.1	No.2	No.3	No.4	Mean	SD	RSD(%)
k_{HB}	0.001	0.001	0.001	0.001	0.001	0.000	4.478
n	3.000	3.000	3.000	3.000	3.000	0.000	0.000

Number of dissolution data points (N), degrees of freedom (df), and selected goodness of fit criteria – Pearson correlation coefficient (R), coefficient of determination (R^2), adjusted coefficient of determination ($R^2_{adjusted}$), and residual sum of squares (RSS) (manual calculation in MS Excel):

Parameter	No.1	No.2	No.3	No.4
N	17	17	17	17
df	15	15	15	15
R	0.999946457	0.999756655	0.999902346	0.999925215
R^2	0.999892917	0.99951337	0.999804702	0.999850436
$R^2_{adjusted}$	0.999885779	0.999480927	0.999791683	0.999840465
RSS	32.07170871	46.62034699	25.21785442	36.22645213

Graphical abstract of model fit presented as mean ± 1 SD of the fraction % of released carvedilol:

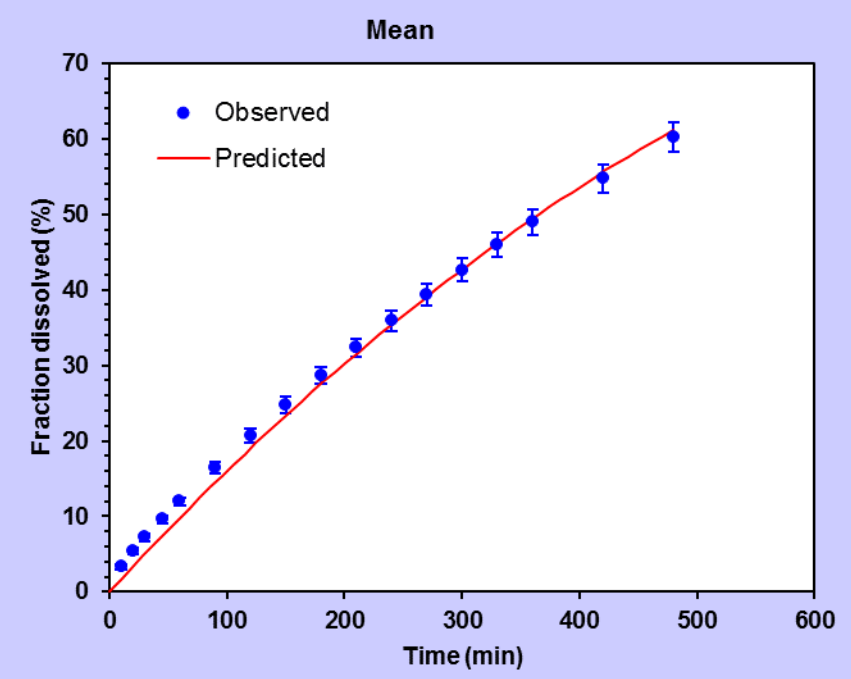

Supplementary materials – Model fitting summary – Granulated sugar N°1 600 (modelling up to app. 60 % of carvedilol released)

Graphical abstract of model fit presented as the fraction % of released carvedilol per tested tablet:

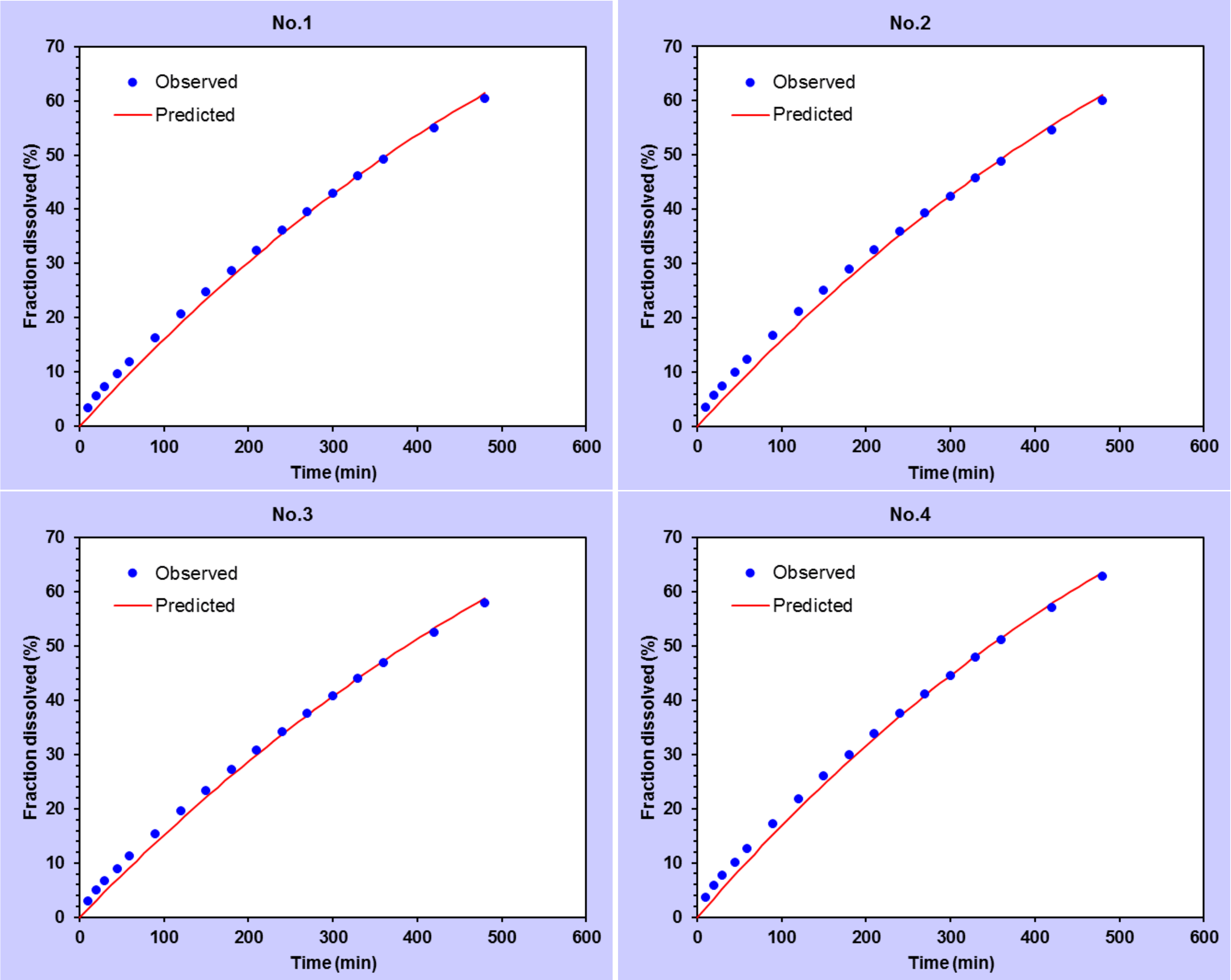

Supplementary materials – Model fitting summary – Granulated sugar N°1 600 (modelling up to app. 60 % of carvedilol released)

Model: **Hopfenberg with T_{lag}**

Model equation: $F = 100 \cdot \{1 - [1 - k_{HB} \cdot (t - T_{lag})]^n\}$

Fitted model parameters per tested tablet (N = 4) with statistics – mean, standard deviation (SD), and relative standard deviation expressed in % (RSD%) (output from DDSolver):

Parameter	No.1	No.2	No.3	No.4	Mean	SD	RSD(%)
k_{HB}	0.001	0.001	0.001	0.001	0.001	0.000	4.404
n	3.000	3.000	3.000	3.000	3.000	0.000	0.000
T_{lag}	-16.202	-19.666	-15.038	-16.273	-16.795	1.996	-11.885

Number of dissolution data points (N), degrees of freedom (df), and selected goodness of fit criteria – Pearson correlation coefficient (R), coefficient of determination (R^2), adjusted coefficient of determination ($R^2_{adjusted}$), and residual sum of squares (RSS) (manual calculation in MS Excel):

Parameter	No.1	No.2	No.3	No.4
N	17	17	17	17
df	14	14	14	14
R	0.999916315	0.999690104	0.999866024	0.99990553
R^2	0.999832637	0.999380303	0.999732067	0.999811068
$R^2_{adjusted}$	0.999808728	0.999291775	0.999693791	0.999784078
RSS	0.917403471	3.266832952	1.354660802	1.091503238

Graphical abstract of model fit presented as mean ± 1 SD of the fraction % of released carvedilol:

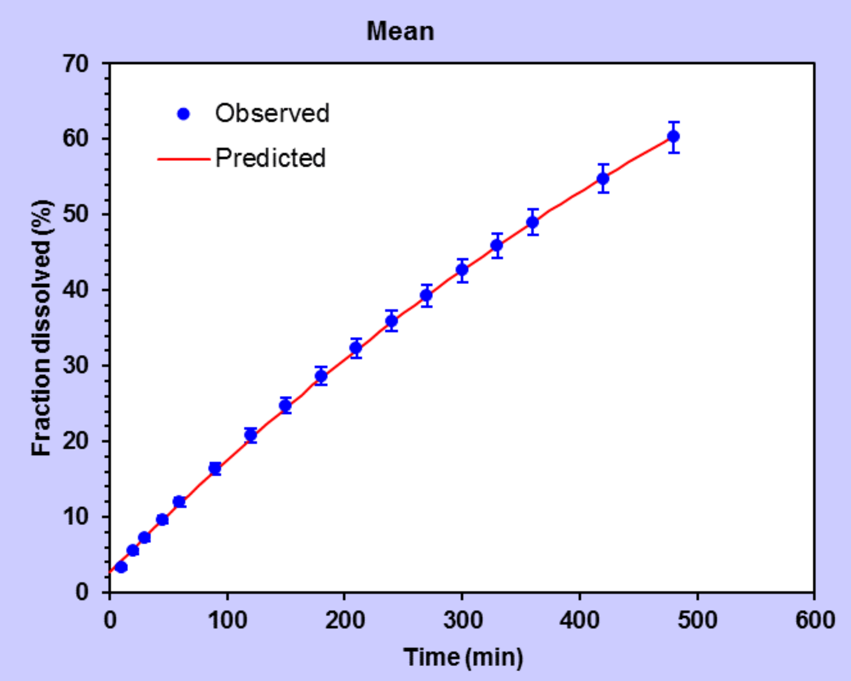

Supplementary materials – Model fitting summary – Granulated sugar N°1 600 (modelling up to app. 60 % of carvedilol released)

Graphical abstract of model fit presented as the fraction % of released carvedilol per tested tablet:

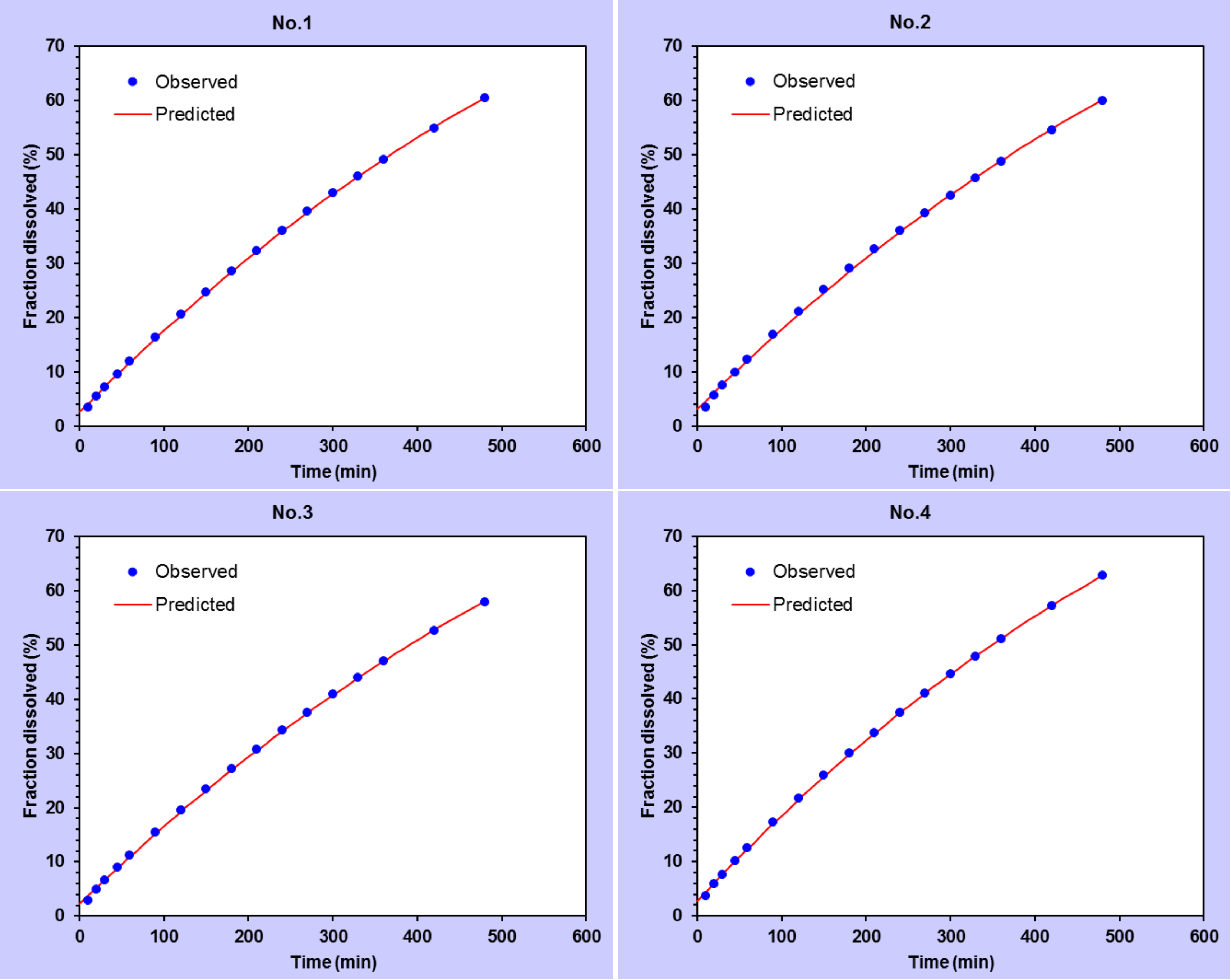

Supplementary materials – Model fitting summary – Granulated sugar N°1 600 (modelling up to app. 60 % of carvedilol released)

Model: **Baker–Lonsdale**

Model equation: $\frac{3}{2}\cdot\left[1-\left(1-\frac{F}{100}\right)^{\frac{2}{3}}\right]-\frac{F}{100}=k_{BL}\cdot t$

Fitted model parameters per tested tablet (N = 4) with statistics – mean, standard deviation (SD), and relative standard deviation expressed in % (RSD%) (output from DDSolver):

Parameter	No.1	No.2	No.3	No.4	Mean	SD	RSD(%)
k_{BL}	0.0001	0.0001	0.0002	0.0002	0.0001	0.0001	40.4320

Number of dissolution data points (N), degrees of freedom (df), and selected goodness of fit criteria – Pearson correlation coefficient (R), coefficient of determination (R^2), adjusted coefficient of determination ($R^2_{adjusted}$), and residual sum of squares (RSS) (manual calculation in MS Excel):

Parameter	No.1	No.2	No.3	No.4
N	17	17	17	17
df	16	16	16	16
R	0.989189488	0.990629925	0.987146783	0.986879307
R^2	0.978495843	0.981347648	0.974458771	0.973930766
$R^2_{adjusted}$	0.978495843	0.981347648	0.974458771	0.973930766
RSS	831.0947721	805.1198094	1210.52206	1378.310017

Graphical abstract of model fit presented as mean ± 1 SD of the fraction % of released carvedilol:

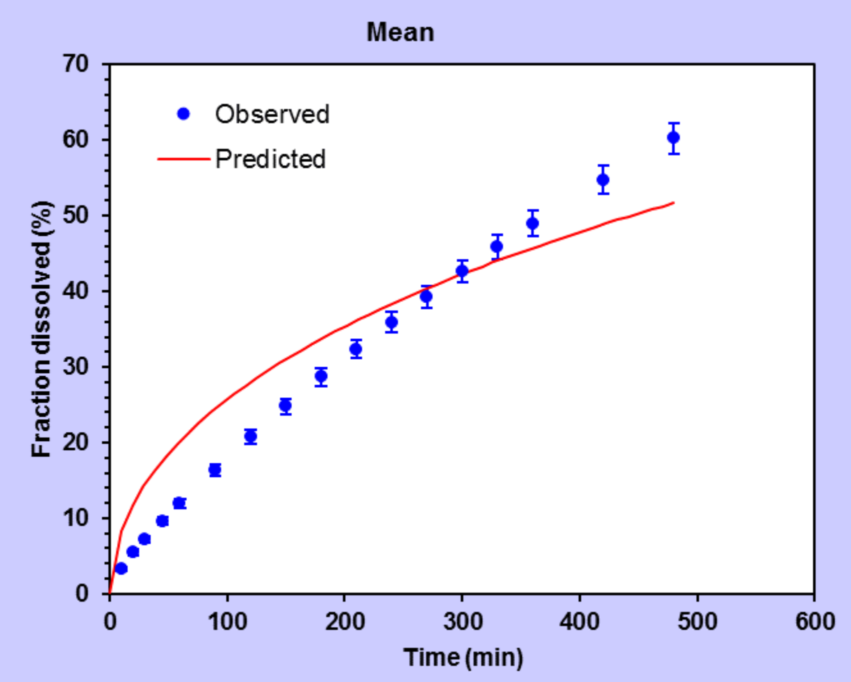

Supplementary materials – Model fitting summary – Granulated sugar N°1 600 (modelling up to app. 60 % of carvedilol released)

Graphical abstract of model fit presented as the fraction % of released carvedilol per tested tablet:

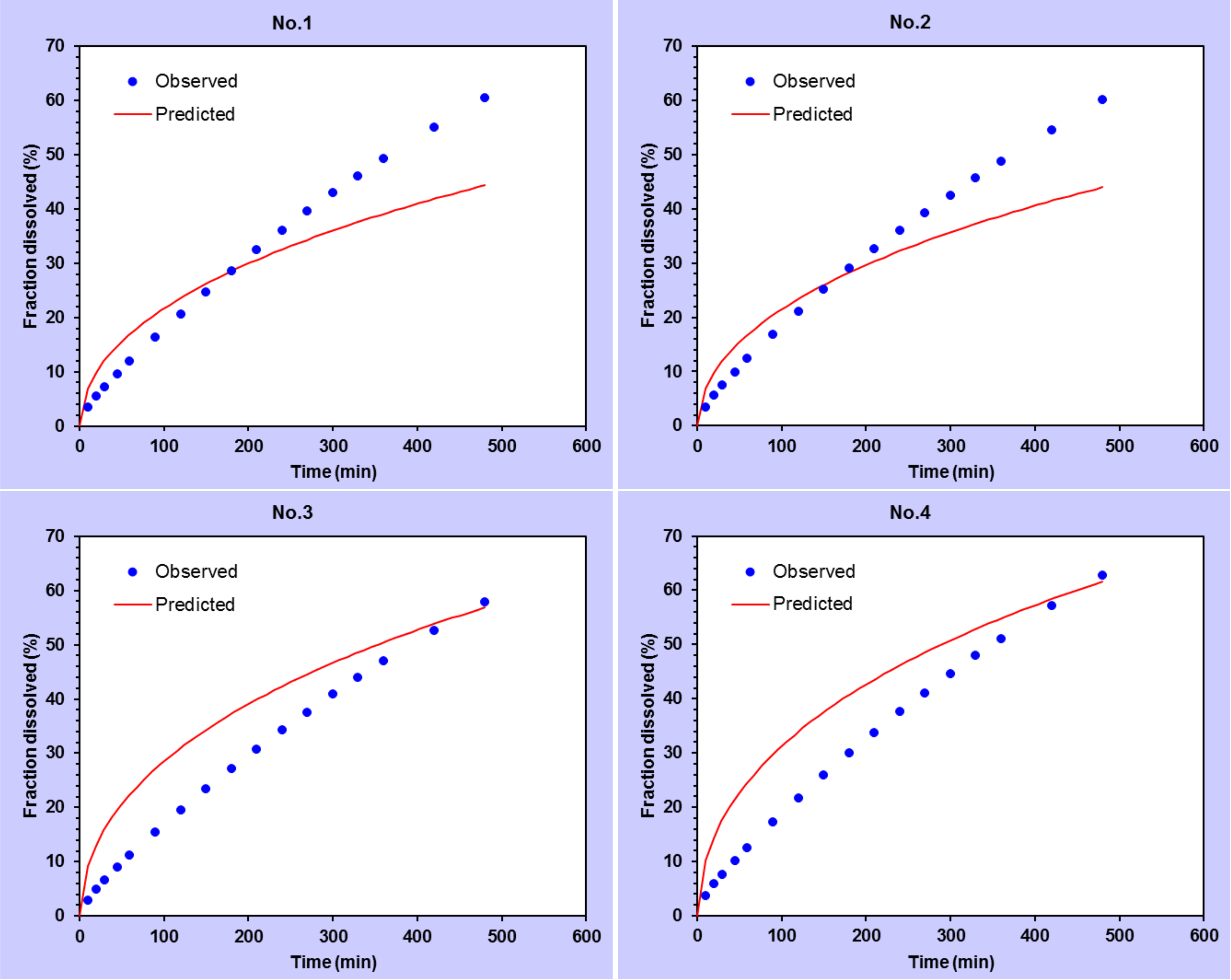

Supplementary materials – Model fitting summary – Granulated sugar N°1 600 (modelling up to app. 60 % of carvedilol released)

Model: **Baker–Lonsdale with T_{lag}**

Model equation: $\frac{3}{2}\cdot\left[1-\left(1-\frac{F}{100}\right)^{\frac{2}{3}}\right]-\frac{F}{100}=k_{BL}\cdot(t-T_{lag})$

Fitted model parameters per tested tablet (N = 4) with statistics – mean, standard deviation (SD), and relative standard deviation expressed in % (RSD%) (output from DDSolver):

Parameter	No.1	No.2	No.3	No.4	Mean	SD	RSD(%)
k_{BL}	0.0002	0.0002	0.0002	0.0002	0.0002	0.0000	8.2038
T_{lag}	53.7785	52.1779	54.2822	53.7359	53.4936	0.9115	1.7040

Number of dissolution data points (N), degrees of freedom (df), and selected goodness of fit criteria – Pearson correlation coefficient (R), coefficient of determination (R^2), adjusted coefficient of determination ($R^2_{adjusted}$), and residual sum of squares (RSS) (manual calculation in MS Excel):

Parameter	No.1	No.2	No.3	No.4
N	17	17	17	17
df	15	15	15	15
R	0.978268411	0.979145664	0.978696292	0.978364095
R^2	0.957009083	0.958726232	0.957846431	0.957196303
$R^2_{adjusted}$	0.954143022	0.955974647	0.955036193	0.954342723
RSS	332.362621	322.3045986	293.3815125	362.6656803

Graphical abstract of model fit presented as mean ± 1 SD of the fraction % of released carvedilol:

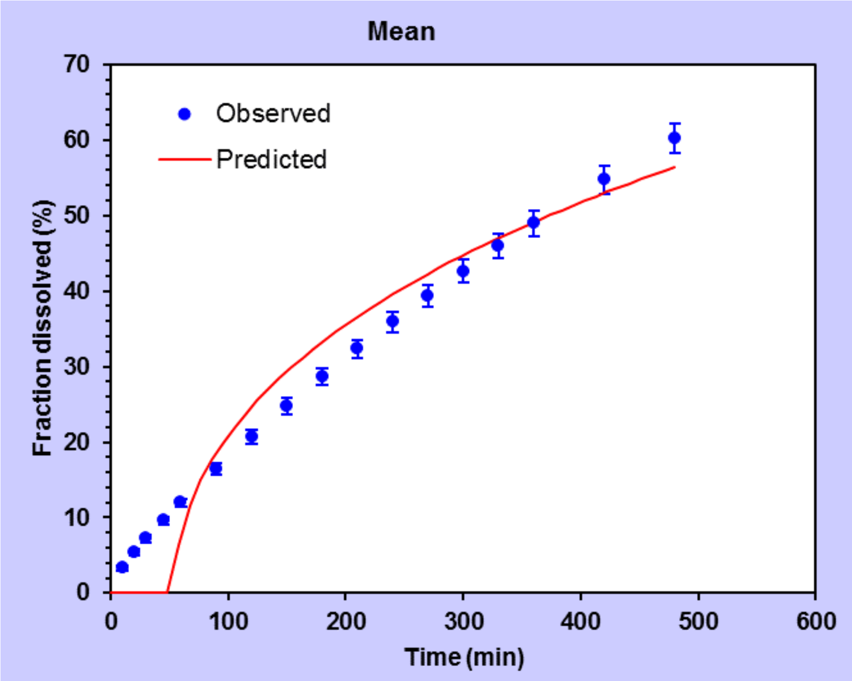

Supplementary materials – Model fitting summary – Granulated sugar N°1 600 (modelling up to app. 60 % of carvedilol released)

Graphical abstract of model fit presented as the fraction % of released carvedilol per tested tablet:

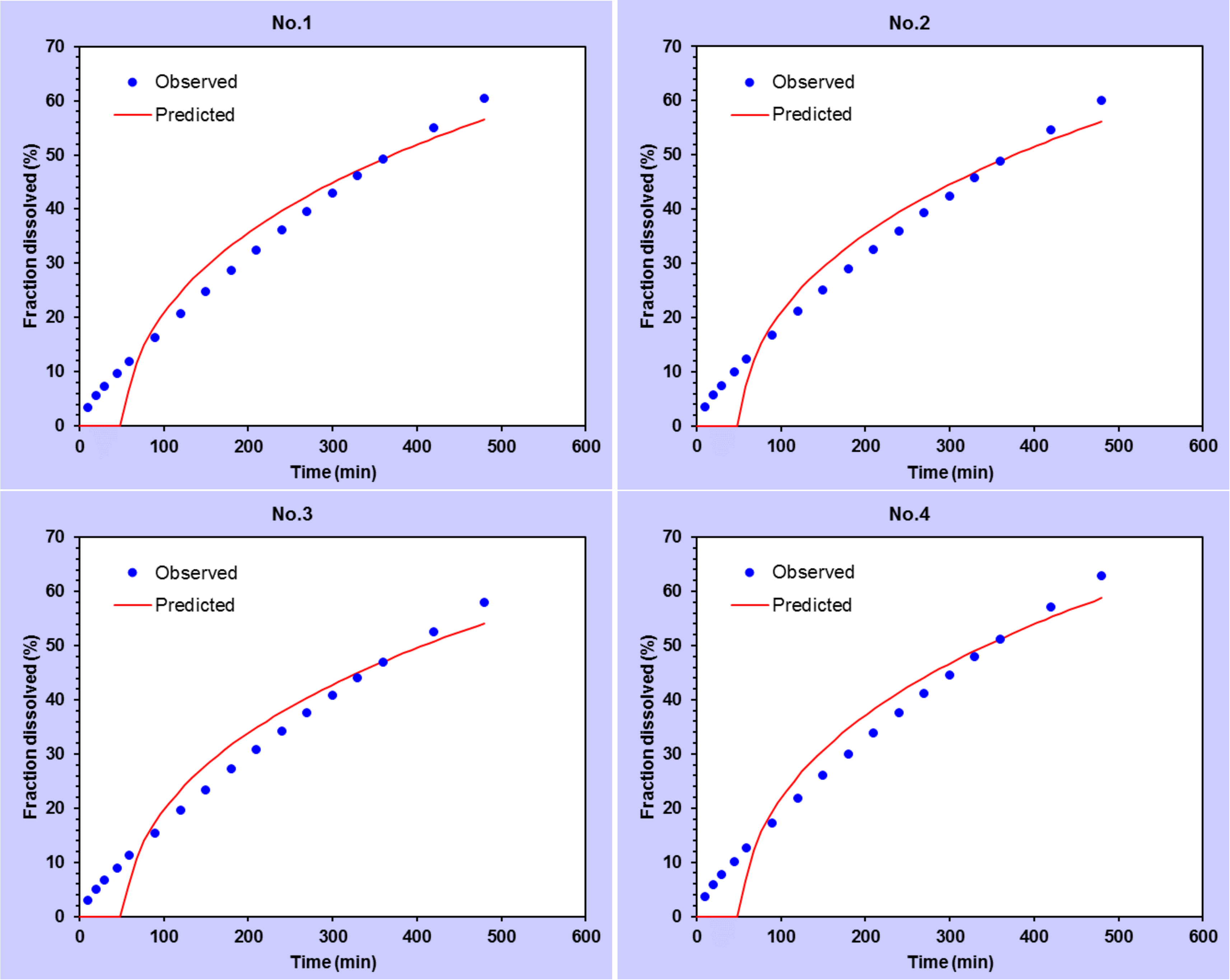

Supplementary materials – Model fitting summary – Granulated sugar N°1 600 (modelling up to app. 60 % of carvedilol released)

Model: **Makoid–Banakar**

Model equation: $F = k_{MB} \cdot t^n \cdot e^{-k \cdot t}$

Fitted model parameters per tested tablet (N = 4) with statistics – mean, standard deviation (SD), and relative standard deviation expressed in % (RSD%) (output from DDSolver):

Parameter	No.1	No.2	No.3	No.4	Mean	SD	RSD(%)
k_{MB}	0.6137	0.6103	0.4821	0.7063	0.6031	0.0921	15.2720
n	0.7275	0.7365	0.7707	0.7107	0.7364	0.0253	3.4300
k	-0.0003	-0.0001	-0.0001	-0.0002	-0.0002	0.0001	-44.2911

Number of dissolution data points (N), degrees of freedom (df), and selected goodness of fit criteria – Pearson correlation coefficient (R), coefficient of determination (R^2), adjusted coefficient of determination ($R^2_{adjusted}$), and residual sum of squares (RSS) (manual calculation in MS Excel):

Parameter	No.1	No.2	No.3	No.4
N	17	17	17	17
df	14	14	14	14
R	0.99933523	0.999811995	0.999712602	0.999632157
R^2	0.998670902	0.999624026	0.999425286	0.99926445
$R^2_{adjusted}$	0.998481031	0.999570315	0.999343184	0.999159371
RSS	7.360476935	2.014763513	2.92637069	6.053048818

Graphical abstract of model fit presented as mean ± 1 SD of the fraction % of released carvedilol:

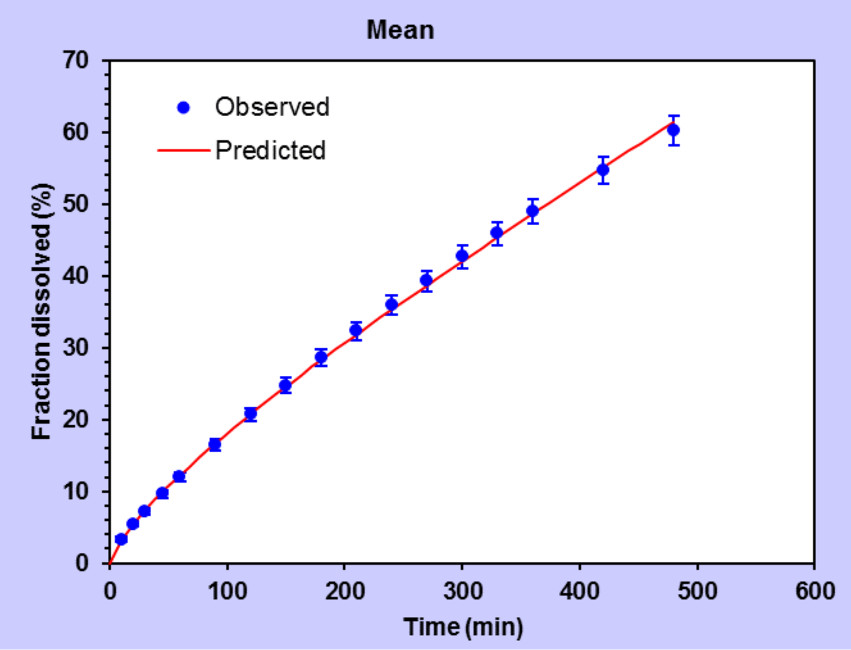

Supplementary materials – Model fitting summary – Granulated sugar N°1 600 (modelling up to app. 60 % of carvedilol released)

Graphical abstract of model fit presented as the fraction % of released carvedilol per tested tablet:

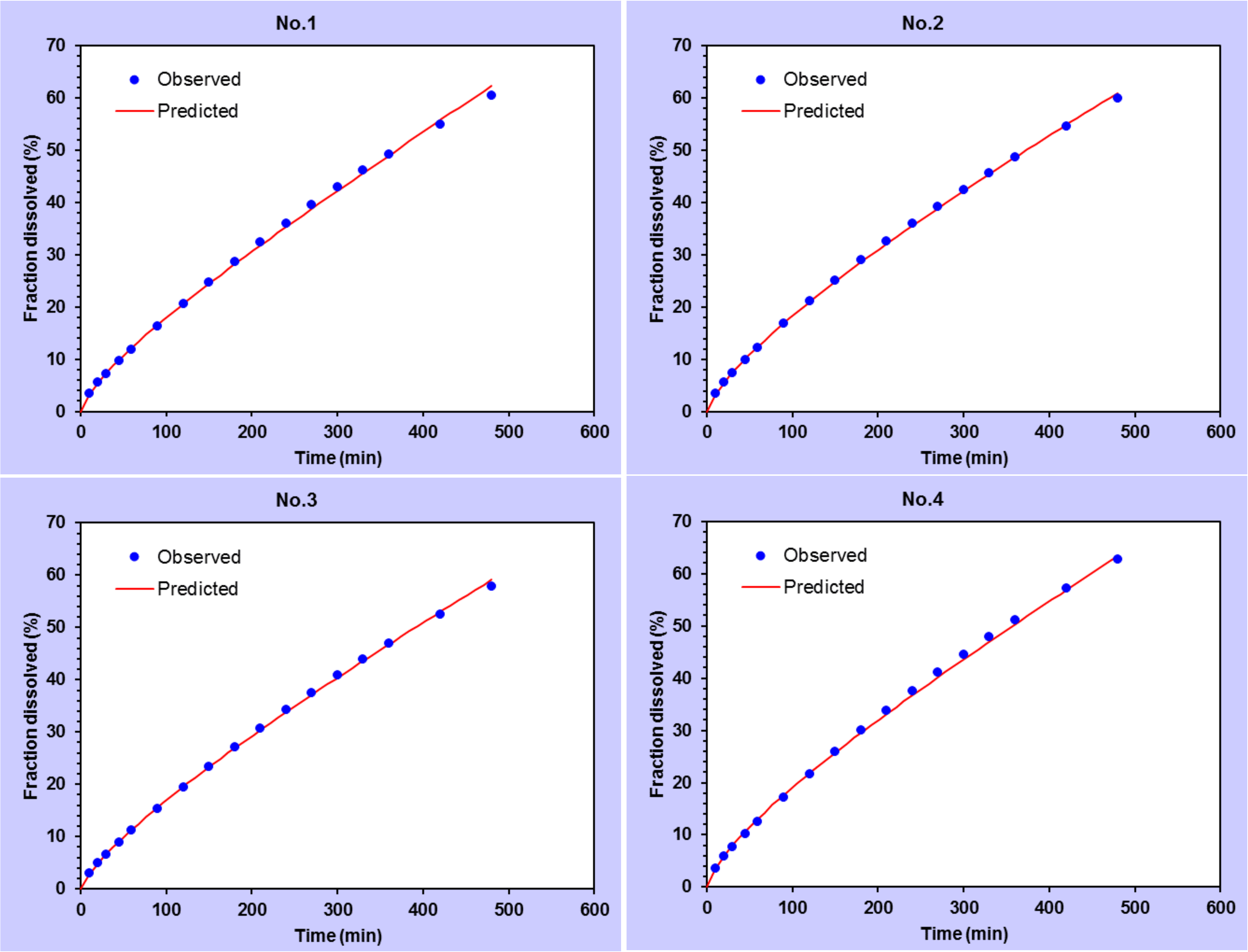

Supplementary materials – Model fitting summary – Granulated sugar N°1 600 (modelling up to app. 60 % of carvedilol released)

Model: **Makoid–Banakar with T_{lag}**

Model equation: $F = k_{MB} \cdot (t - T_{lag})^n \cdot e^{-k \cdot (t - T_{lag})}$

Fitted model parameters per tested tablet (N = 4) with statistics – mean, standard deviation (SD), and relative standard deviation expressed in % (RSD%) (output from DDSolver):

Parameter	No.1	No.2	No.3	No.4	Mean	SD	RSD(%)
k_{MB}	1.0391	1.0365	0.8387	1.1037	1.0045	0.1148	11.4275
n	0.6068	0.6152	0.6439	0.6049	0.6177	0.0180	2.9155
k	-0.0008	-0.0007	-0.0007	-0.0008	-0.0007	0.0001	-10.4685
T_{lag}	4.0000	4.0000	4.0000	4.0000	4.0000	0.0000	0.0000

Number of dissolution data points (N), degrees of freedom (df), and selected goodness of fit criteria – Pearson correlation coefficient (R), coefficient of determination (R^2), adjusted coefficient of determination ($R^2_{adjusted}$), and residual sum of squares (RSS) (manual calculation in MS Excel):

Parameter	No.1	No.2	No.3	No.4
N	17	17	17	17
df	13	13	13	13
R	0.997503968	0.998534731	0.998251645	0.99779412
R^2	0.995014166	0.997071609	0.996506347	0.995593107
$R^2_{adjusted}$	0.993863589	0.996395826	0.99570012	0.994576131
RSS	28.65871225	16.2468647	18.54669138	27.20439917

Graphical abstract of model fit presented as mean ± 1 SD of the fraction % of released carvedilol:

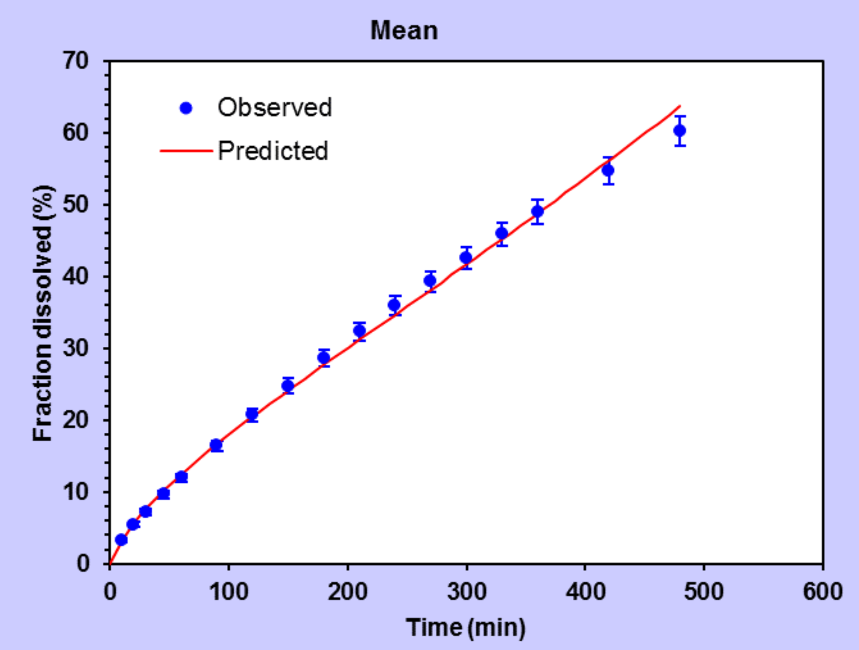

Supplementary materials – Model fitting summary – Granulated sugar N°1 600 (modelling up to app. 60 % of carvedilol released)

Graphical abstract of model fit presented as the fraction % of released carvedilol per tested tablet:

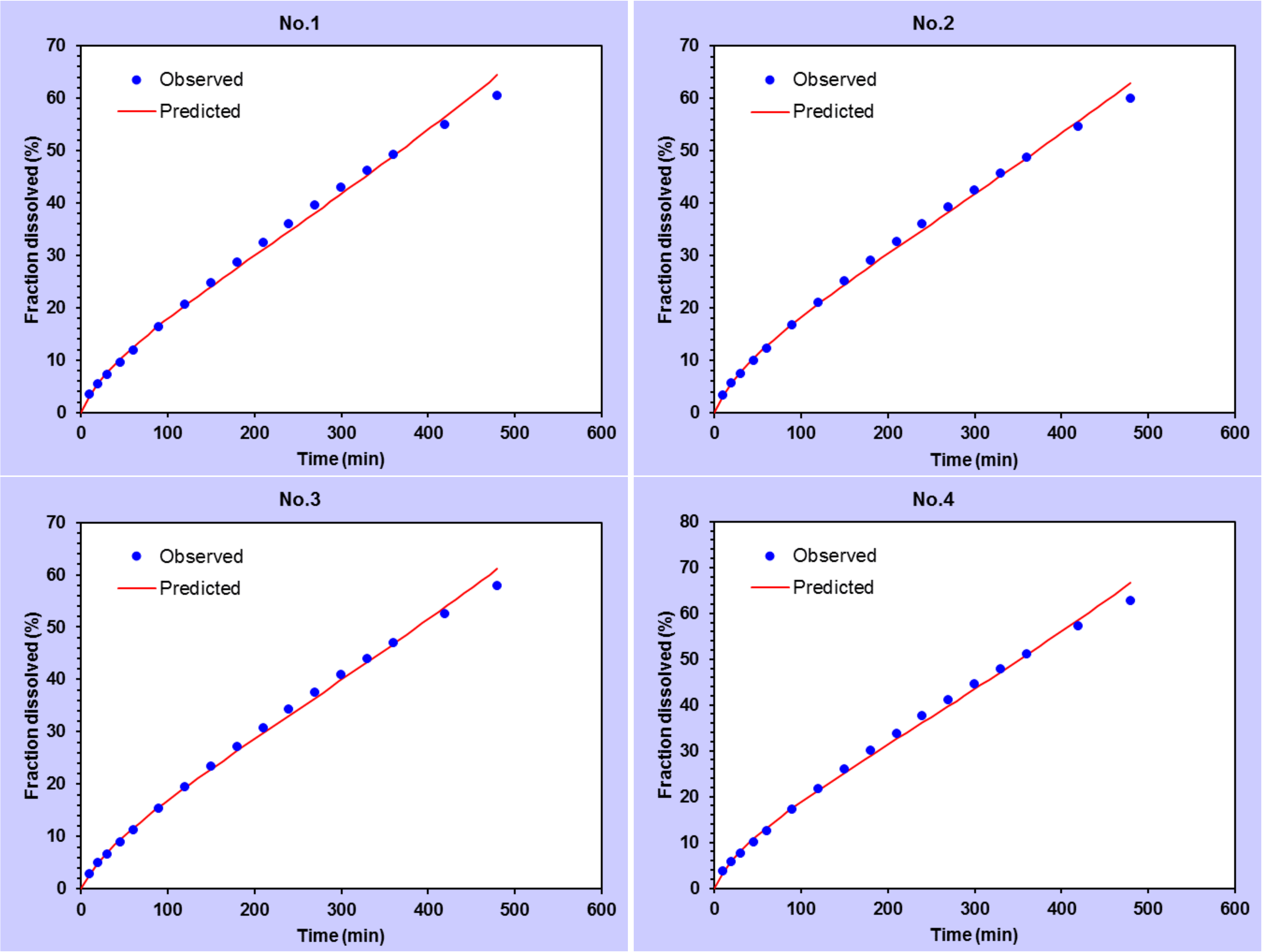

Supplementary materials – Model fitting summary – Granulated sugar N°1 600 (modelling up to app. 60 % of carvedilol released)

Model: **Peppas–Sahlin_1**

Model equation: $F = k_1 \cdot t^m + k_2 \cdot t^{2m}$

Fitted model parameters per tested tablet (N = 4) with statistics – mean, standard deviation (SD), and relative standard deviation expressed in % (RSD%) (output from DDSolver):

Parameter	No.1	No.2	No.3	No.4	Mean	SD	RSD(%)
k_1	0.763	0.876	0.653	0.843	0.784	0.099	12.657
k_2	0.191	0.181	0.188	0.195	0.189	0.006	3.042
m	0.450	0.450	0.450	0.450	0.450	0.000	0.000

Number of dissolution data points (N), degrees of freedom (df), and selected goodness of fit criteria – Pearson correlation coefficient (R), coefficient of determination (R^2), adjusted coefficient of determination ($R^2_{adjusted}$), and residual sum of squares (RSS) (manual calculation in MS Excel):

Parameter	No.1	No.2	No.3	No.4
N	17	17	17	17
df	14	14	14	14
R	0.999594171	0.999674408	0.999623597	0.999641887
R^2	0.999188508	0.999348922	0.999247336	0.999283903
$R^2_{adjusted}$	0.99907258	0.999255911	0.999139812	0.999181603
RSS	4.495748525	3.628453233	3.922958807	4.305452339

Graphical abstract of model fit presented as mean ± 1 SD of the fraction % of released carvedilol:

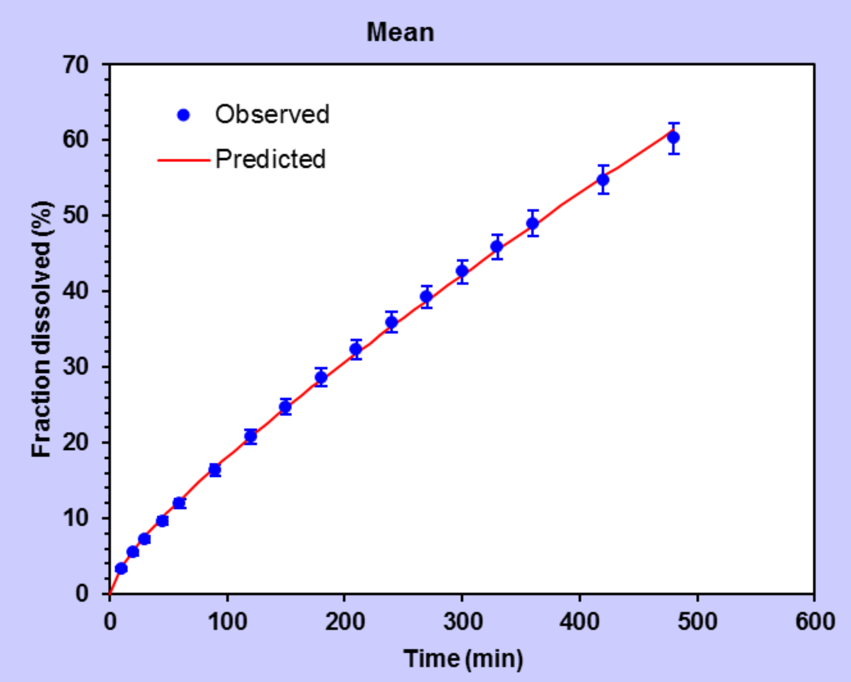

Supplementary materials – Model fitting summary – Granulated sugar N°1 600 (modelling up to app. 60 % of carvedilol released)

Graphical abstract of model fit presented as the fraction % of released carvedilol per tested tablet:

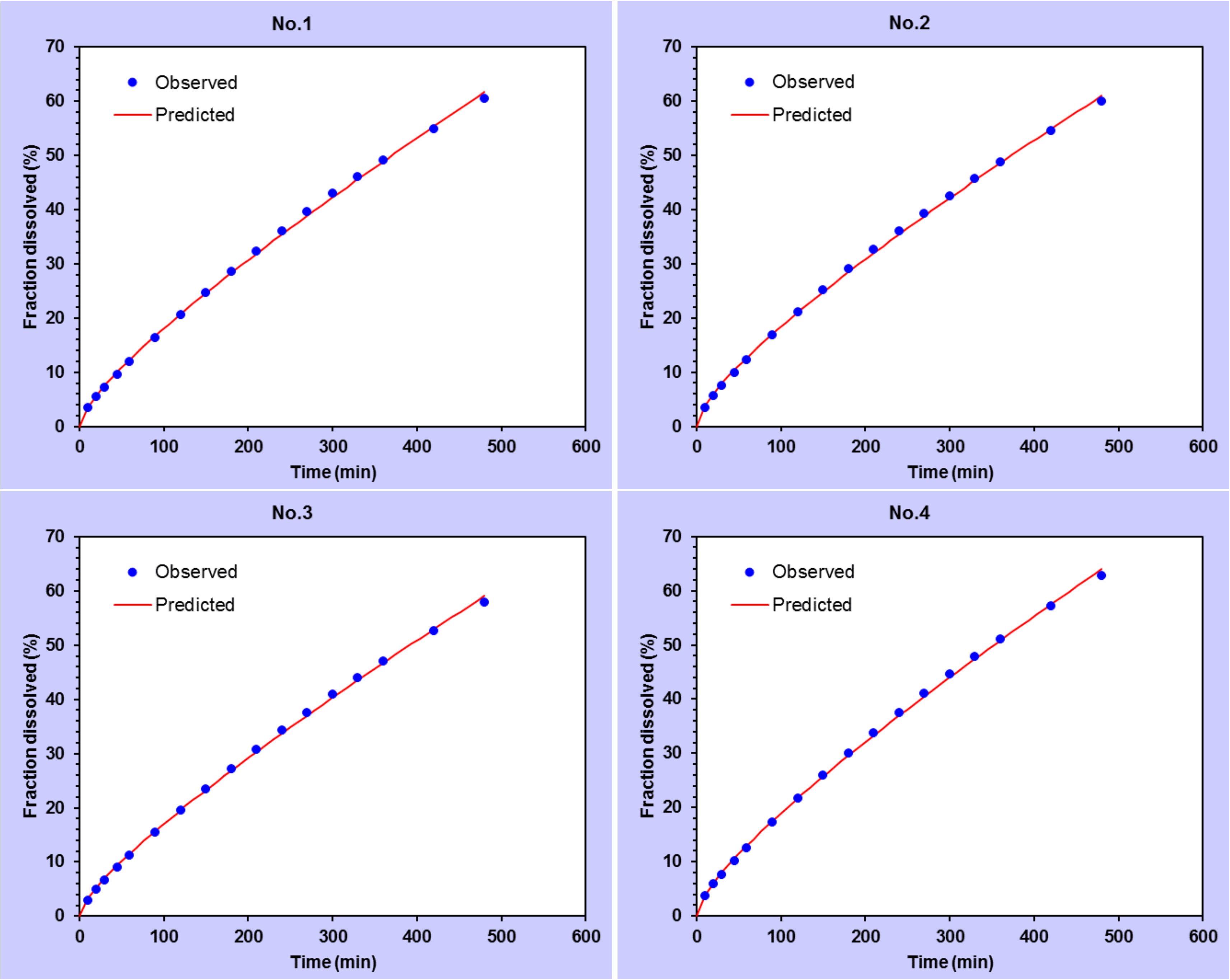

Supplementary materials – Model fitting summary – Granulated sugar N°1 600 (modelling up to app. 60 % of carvedilol released)

Model: **Peppas–Sahlin_1 with T_{lag}**

Model equation: $F = k_1 \cdot (t - T_{lag})^m + k_2 \cdot (t - T_{lag})^{2m}$

Fitted model parameters per tested tablet (N = 4) with statistics – mean, standard deviation (SD), and relative standard deviation expressed in % (RSD%) (output from DDSolver):

Parameter	No.1	No.2	No.3	No.4	Mean	SD	RSD(%)
k_1	0.919	1.035	0.802	1.007	0.941	0.105	11.133
k_2	0.182	0.172	0.179	0.185	0.179	0.006	3.153
m	0.450	0.450	0.450	0.450	0.450	0.000	0.000
T_{lag}	4.000	4.000	4.000	4.000	4.000	0.000	0.000

Number of dissolution data points (N), degrees of freedom (df), and selected goodness of fit criteria – Pearson correlation coefficient (R), coefficient of determination (R^2), adjusted coefficient of determination ($R^2_{adjusted}$), and residual sum of squares (RSS) (manual calculation in MS Excel):

Parameter	No.1	No.2	No.3	No.4
N	17	17	17	17
df	13	13	13	13
R	0.999676414	0.999809241	0.999740768	0.999736747
R^2	0.999352933	0.999618518	0.999481603	0.999473562
$R^2_{adjusted}$	0.99920361	0.999530484	0.999361973	0.999352077
RSS	3.454847847	2.009244129	2.58885668	3.024026072

Graphical abstract of model fit presented as mean ± 1 SD of the fraction % of released carvedilol:

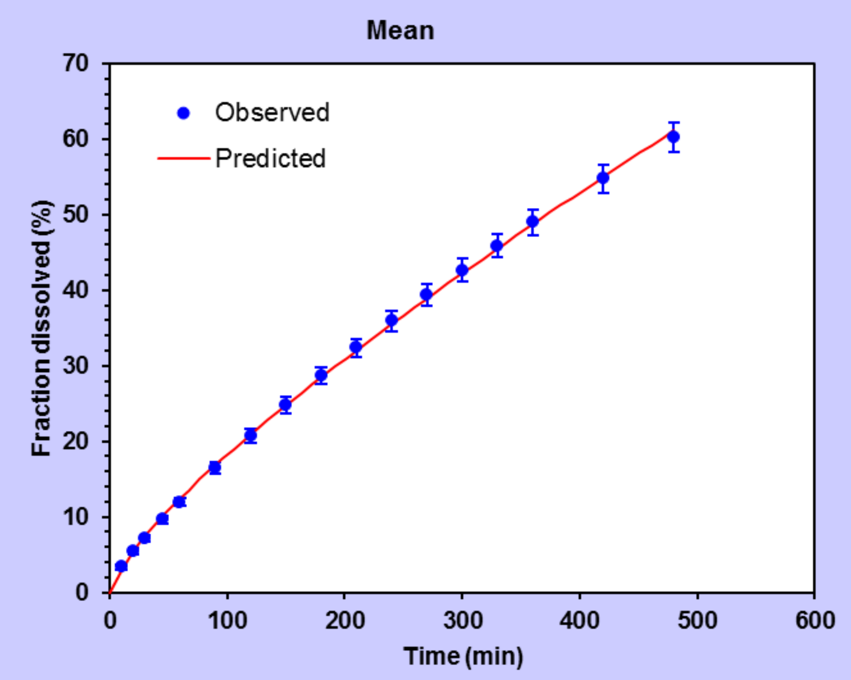

Supplementary materials – Model fitting summary – Granulated sugar N°1 600 (modelling up to app. 60 % of carvedilol released)

Graphical abstract of model fit presented as the fraction % of released carvedilol per tested tablet:

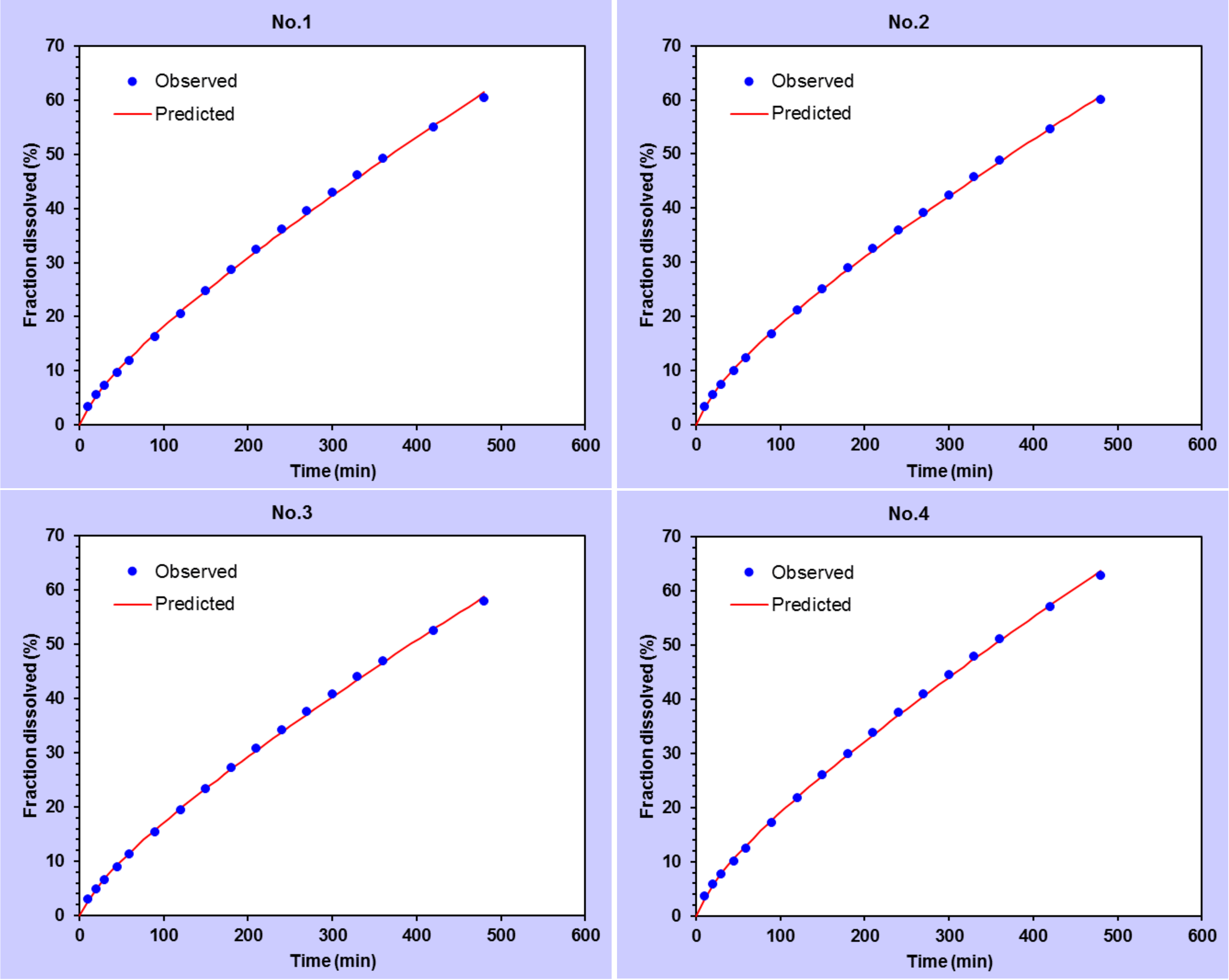

Supplementary materials – Model fitting summary – Granulated sugar N°1 600 (modelling up to app. 60 % of carvedilol released)

Model: **Peppas–Sahlin_2**

Model equation: $F = k_1 \cdot t^{0.5} + k_2 \cdot t$

Fitted model parameters per tested tablet (N = 4) with statistics – mean, standard deviation (SD), and relative standard deviation expressed in % (RSD%) (output from DDSolver):

Parameter	No.1	No.2	No.3	No.4	Mean	SD	RSD(%)
k_1	0.965	1.047	0.867	1.041	0.980	0.084	8.577
k_2	0.085	0.080	0.084	0.086	0.084	0.003	3.386

Number of dissolution data points (N), degrees of freedom (df), and selected goodness of fit criteria – Pearson correlation coefficient (R), coefficient of determination (R^2), adjusted coefficient of determination ($R^2_{adjusted}$), and residual sum of squares (RSS) (manual calculation in MS Excel):

Parameter	No.1	No.2	No.3	No.4
N	17	17	17	17
df	15	15	15	15
R	0.999388068	0.999500674	0.999395141	0.999449551
R^2	0.99877651	0.999001598	0.998790648	0.998899405
$R^2_{adjusted}$	0.998694944	0.998935037	0.998710025	0.998826032
RSS	7.050166986	5.809604549	6.577765282	6.895088853

Graphical abstract of model fit presented as mean ± 1 SD of the fraction % of released carvedilol:

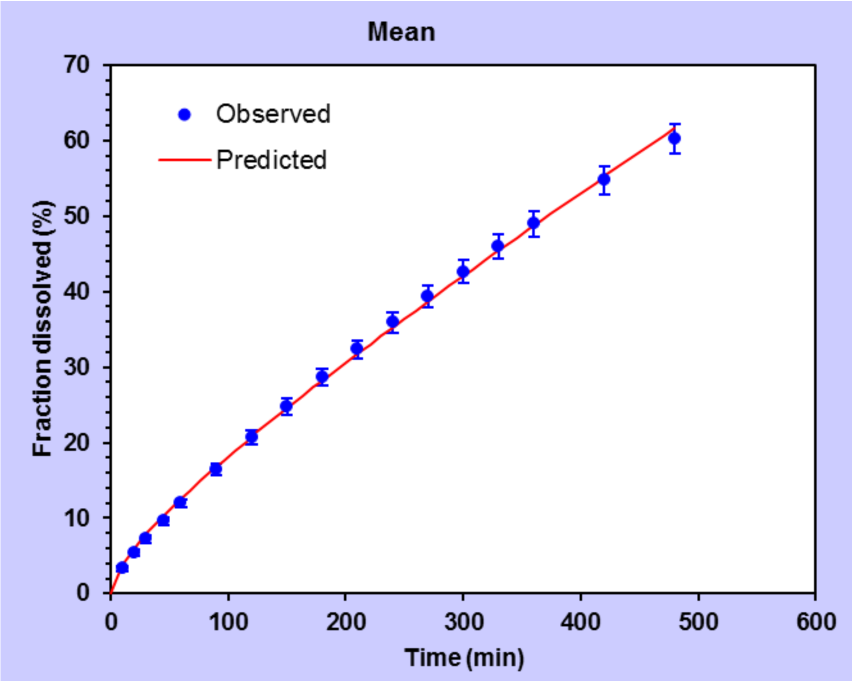

Supplementary materials – Model fitting summary – Granulated sugar N°1 600 (modelling up to app. 60 % of carvedilol released)

Graphical abstract of model fit presented as the fraction % of released carvedilol per tested tablet:

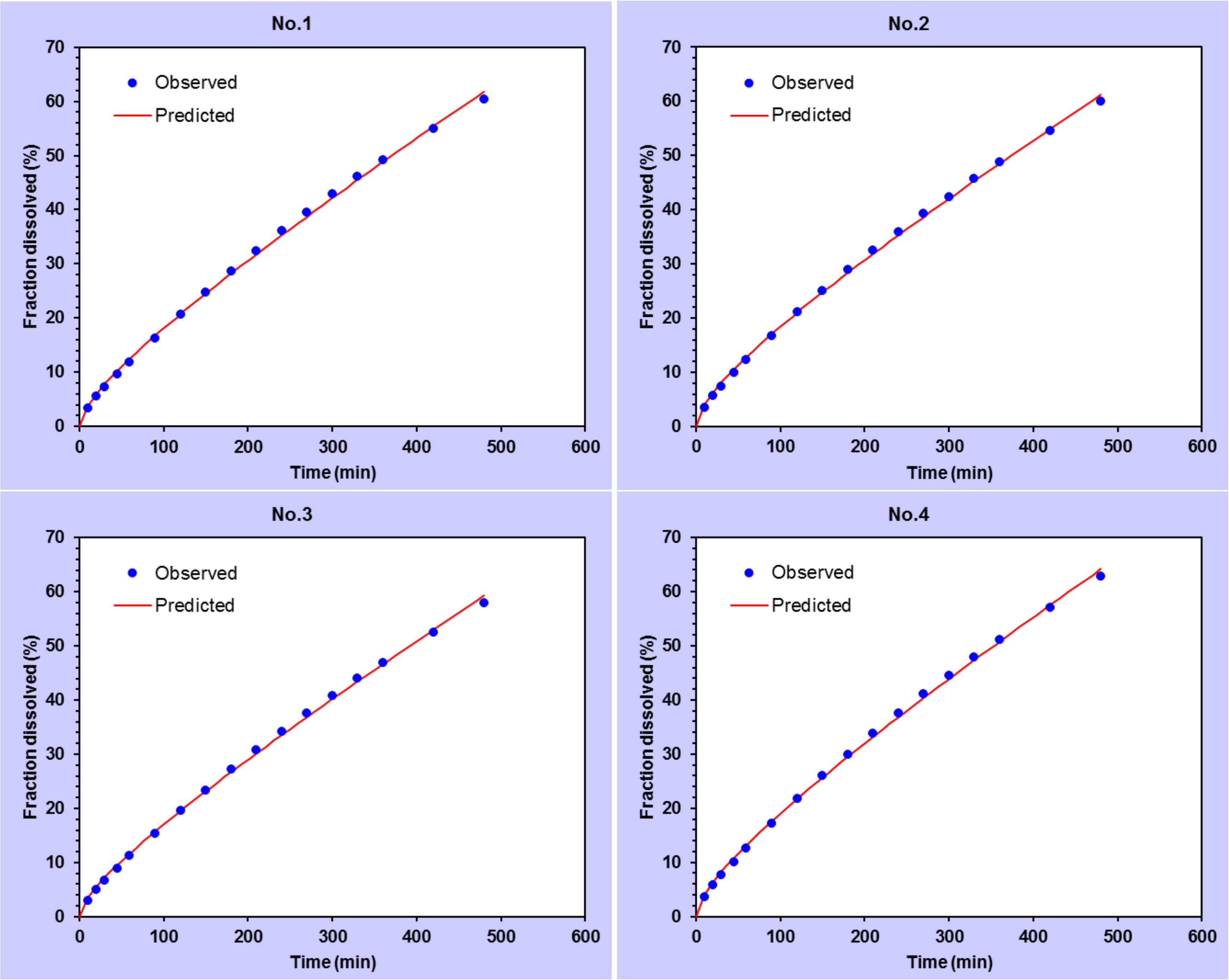

Supplementary materials – Model fitting summary – Granulated sugar N°1 600 (modelling up to app. 60 % of carvedilol released)

Model: **Peppas–Sahlin_2 with T_{lag}**

Model equation: $F = k_1 \cdot (t - T_{lag})^{0.5} + k_2 \cdot (t - T_{lag})$

Fitted model parameters per tested tablet (N = 4) with statistics – mean, standard deviation (SD), and relative standard deviation expressed in % (RSD%) (output from DDSolver):

Parameter	No.1	No.2	No.3	No.4	Mean	SD	RSD(%)
k_1	1.080	1.164	0.976	1.161	1.095	0.088	8.053
k_2	0.080	0.075	0.079	0.081	0.079	0.003	3.597
T_{lag}	4.000	4.000	4.000	4.000	4.000	0.000	0.000

Number of dissolution data points (N), degrees of freedom (df), and selected goodness of fit criteria – Pearson correlation coefficient (R), coefficient of determination (R^2), adjusted coefficient of determination ($R^2_{adjusted}$), and residual sum of squares (RSS) (manual calculation in MS Excel):

Parameter	No.1	No.2	No.3	No.4
N	17	17	17	17
df	14	14	14	14
R	0.999551844	0.999711019	0.999601684	0.999623122
R^2	0.999103889	0.999422122	0.999203527	0.999246386
$R^2_{adjusted}$	0.998975873	0.999339568	0.999089745	0.999138726
RSS	4.88793128	3.151010257	4.121057785	4.439302101

Graphical abstract of model fit presented as mean ± 1 SD of the fraction % of released carvedilol:

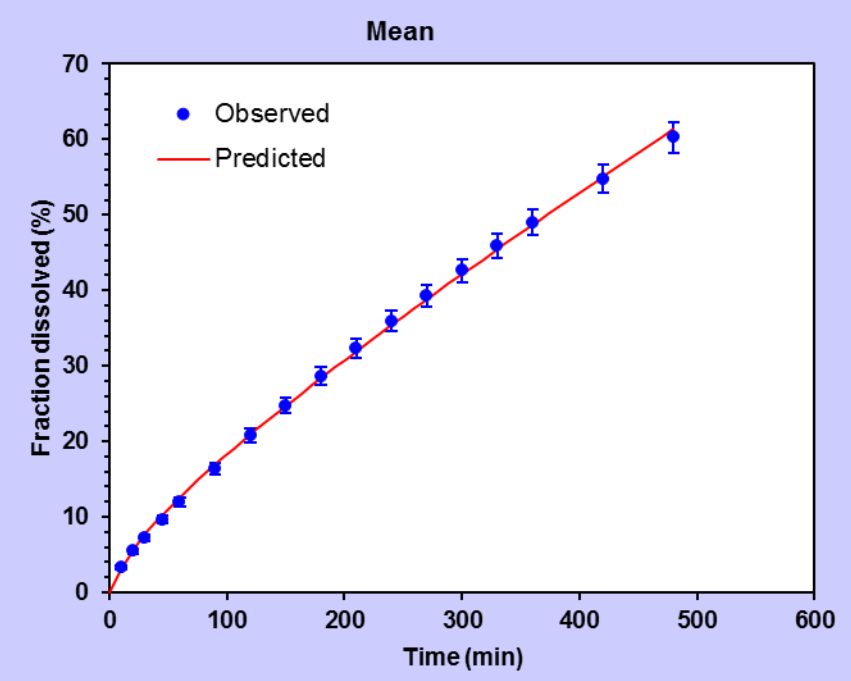

Supplementary materials – Model fitting summary – Granulated sugar N°1 600 (modelling up to app. 60 % of carvedilol released)

Graphical abstract of model fit presented as the fraction % of released carvedilol per tested tablet:

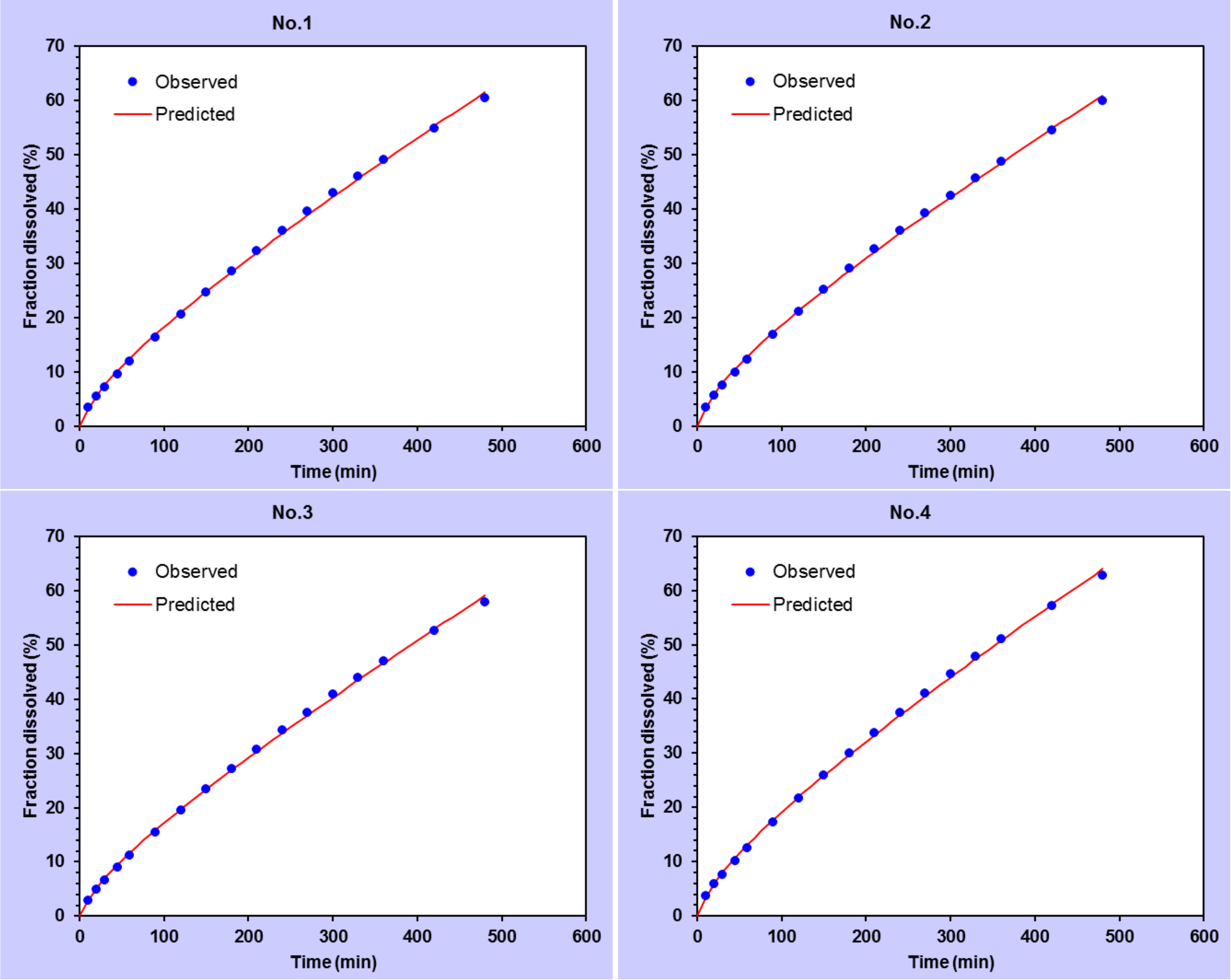

Supplementary materials – Model fitting summary – Granulated sugar N°1 600 (modelling up to app. 60 % of carvedilol released)

Model: **Quadratic**

Model equation: $F = 100 \cdot (k_1 \cdot t^2 + k_2 \cdot t)$

Fitted model parameters per tested tablet (N = 4) with statistics – mean, standard deviation (SD), and relative standard deviation expressed in % (RSD%) (output from DDSolver):

Parameter	No.1	No.2	No.3	No.4	Mean	SD	RSD(%)
k_1	-0.000001	-0.000001	-0.000001	-0.000001	-0.000001	0.000000	-7.572449
k_2	0.001813	0.001836	0.001709	0.001899	0.001814	0.000079	4.344953

Number of dissolution data points (N), degrees of freedom (df), and selected goodness of fit criteria – Pearson correlation coefficient (R), coefficient of determination (R^2), adjusted coefficient of determination ($R^2_{adjusted}$), and residual sum of squares (RSS) (manual calculation in MS Excel):

Parameter	No.1	No.2	No.3	No.4
N	17	17	17	17
df	15	15	15	15
R	0.999343972	0.998999698	0.99945481	0.999222844
R^2	0.998688374	0.998000396	0.998909917	0.998446292
$R^2_{adjusted}$	0.998600932	0.997867089	0.998837245	0.998342711
RSS	19.11230059	26.01246792	13.82902536	23.71277198

Graphical abstract of model fit presented as mean ± 1 SD of the fraction % of released carvedilol:

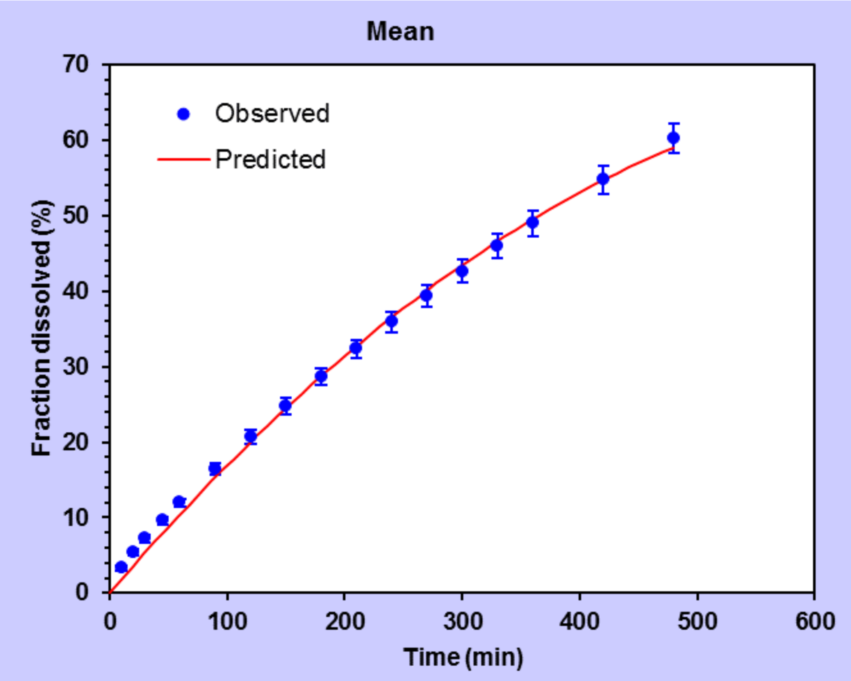

Supplementary materials – Model fitting summary – Granulated sugar N°1 600 (modelling up to app. 60 % of carvedilol released)

Graphical abstract of model fit presented as the fraction % of released carvedilol per tested tablet:

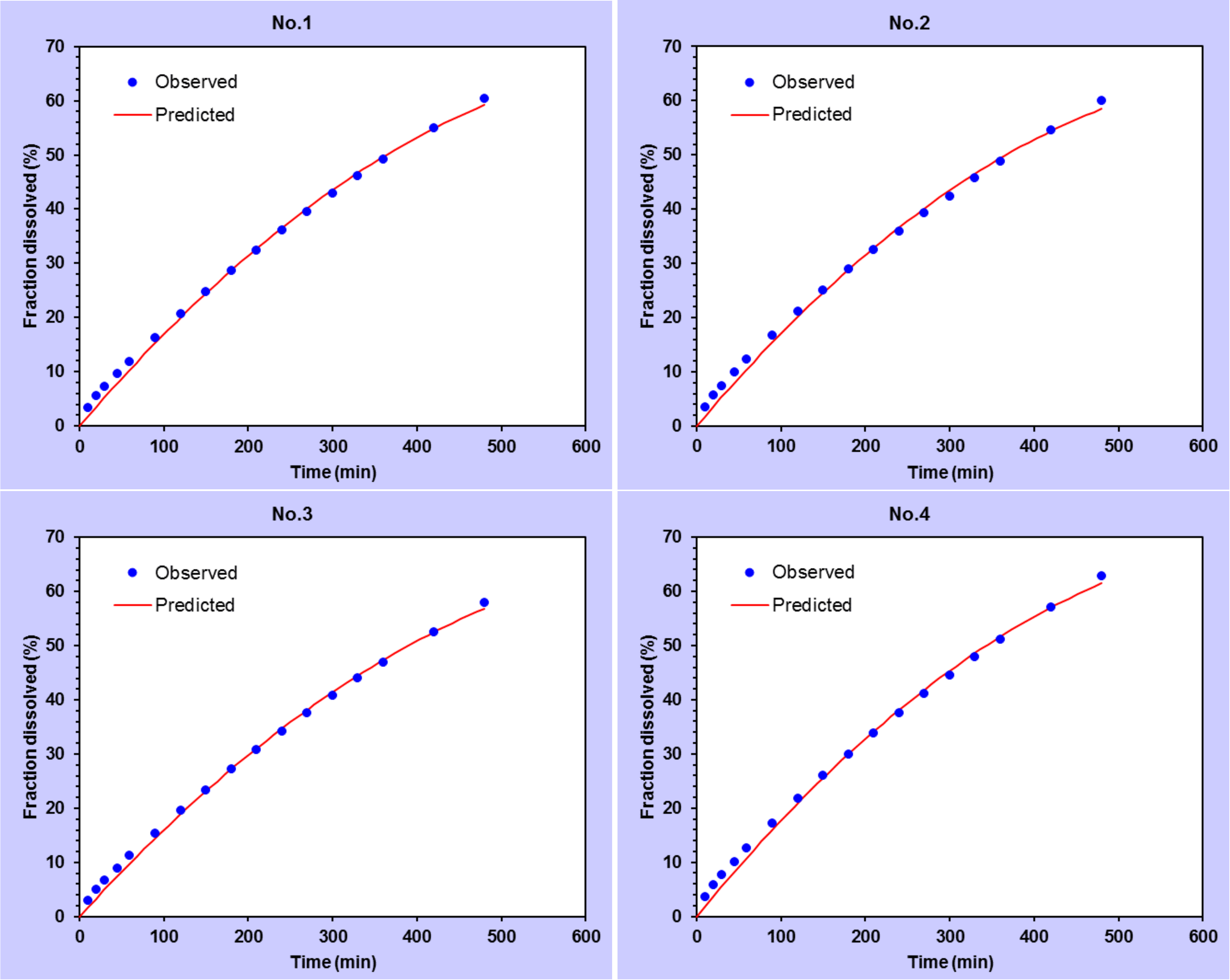

Supplementary materials – Model fitting summary – Granulated sugar N°1 600 (modelling up to app. 60 % of carvedilol released)

Model: **Quadratic with T_{lag}**

Model equation: $F = 100 \cdot \left[k_1 \cdot \left(t - T_{lag}\right)^2 + k_2 \cdot \left(t - T_{lag}\right)\right]$

Fitted model parameters per tested tablet (N = 4) with statistics – mean, standard deviation (SD), and relative standard deviation expressed in % (RSD%) (output from DDSolver):

Parameter	No.1	No.2	No.3	No.4	Mean	SD	RSD(%)
k_1	-0.000001	-0.000001	-0.000001	-0.000001	-0.000001	0.000000	-7.230850
k_2	0.001863	0.001885	0.001757	0.001951	0.001864	0.000080	4.313557
T_{lag}	4.000000	4.000000	4.000000	4.000000	4.000000	0.000000	0.000000

Number of dissolution data points (N), degrees of freedom (df), and selected goodness of fit criteria – Pearson correlation coefficient (R), coefficient of determination (R^2), adjusted coefficient of determination ($R^2_{adjusted}$), and residual sum of squares (RSS) (manual calculation in MS Excel):

Parameter	No.1	No.2	No.3	No.4
N	17	17	17	17
df	14	14	14	14
R	0.998994048	0.998611183	0.99913989	0.998855653
R^2	0.997989107	0.997224295	0.99828052	0.997712616
$R^2_{adjusted}$	0.997701837	0.996827766	0.99803488	0.997385847
RSS	32.67412566	41.30930269	24.73359971	39.27656689

Graphical abstract of model fit presented as mean ± 1 SD of the fraction % of released carvedilol:

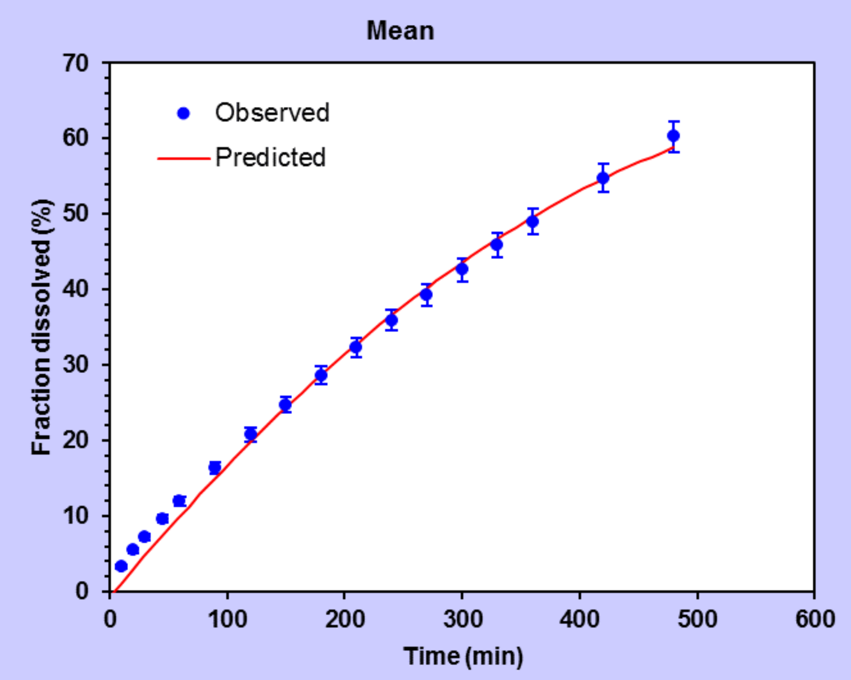

Supplementary materials – Model fitting summary – Granulated sugar N°1 600 (modelling up to app. 60 % of carvedilol released)

Graphical abstract of model fit presented as the fraction % of released carvedilol per tested tablet:

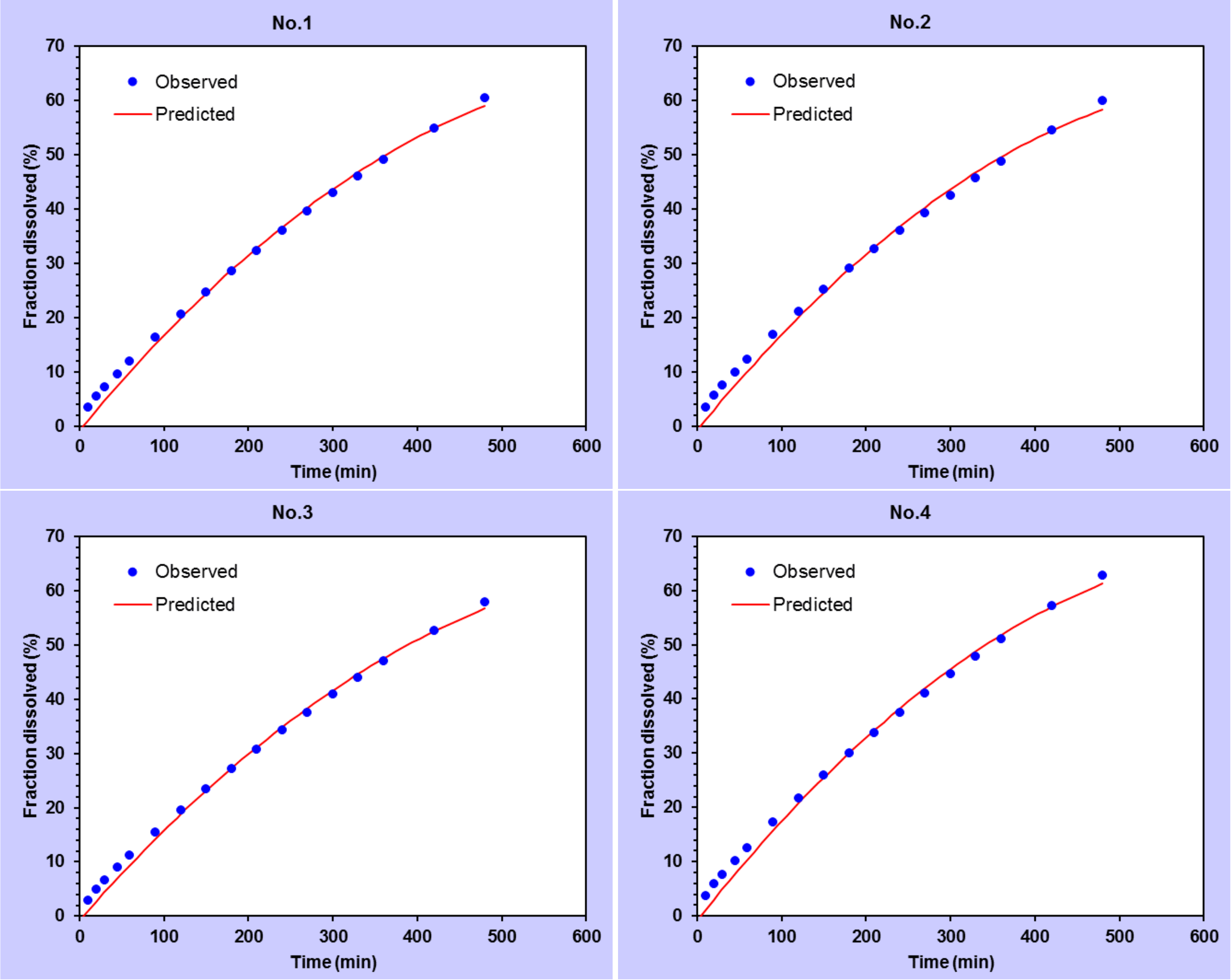

Supplementary materials – Model fitting summary – Granulated sugar N°1 600 (modelling up to app. 60 % of carvedilol released)

Model: **Weibull_1**

Model equation: $F = 100 \cdot \left[1 - e^{-\frac{(t-T_i)^{\beta}}{\alpha}}\right]$

Fitted model parameters per tested tablet (N = 4) with statistics – mean, standard deviation (SD), and relative standard deviation expressed in % (RSD%) (output from DDSolver):

Parameter	No.1	No.2	No.3	No.4	Mean	SD	RSD(%)
α	157.888	150.380	185.058	149.302	160.657	16.709	10.401
β	0.781	0.772	0.798	0.781	0.783	0.011	1.397
T_i	4.000	4.000	4.000	6.000	4.500	1.000	22.222

Number of dissolution data points (N), degrees of freedom (df), and selected goodness of fit criteria – Pearson correlation coefficient (R), coefficient of determination (R^2), adjusted coefficient of determination ($R^2_{adjusted}$), and residual sum of squares (RSS) (manual calculation in MS Excel):

Parameter	No.1	No.2	No.3	No.4
N	17	17	17	17
df	14	14	14	14
R	0.994999566	0.995546289	0.996030781	0.994444413
R^2	0.990024137	0.991112414	0.992077316	0.98891969
$R^2_{adjusted}$	0.988599014	0.989842759	0.990945504	0.987336789
RSS	95.77415479	79.481734	72.02132864	104.0250177

Graphical abstract of model fit presented as mean ± 1 SD of the fraction % of released carvedilol:

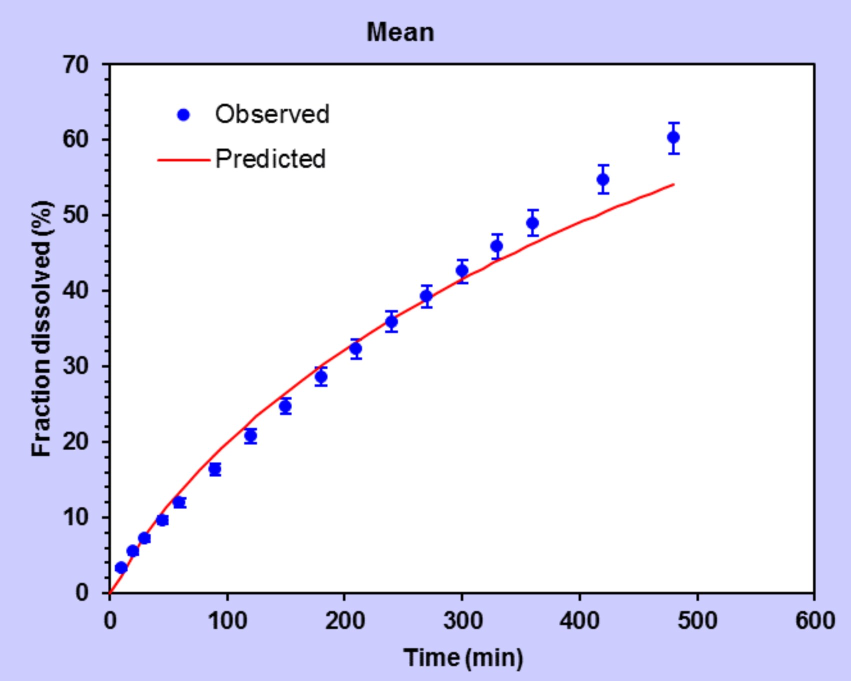

Supplementary materials – Model fitting summary – Granulated sugar N°1 600 (modelling up to app. 60 % of carvedilol released)

Graphical abstract of model fit presented as the fraction % of released carvedilol per tested tablet:

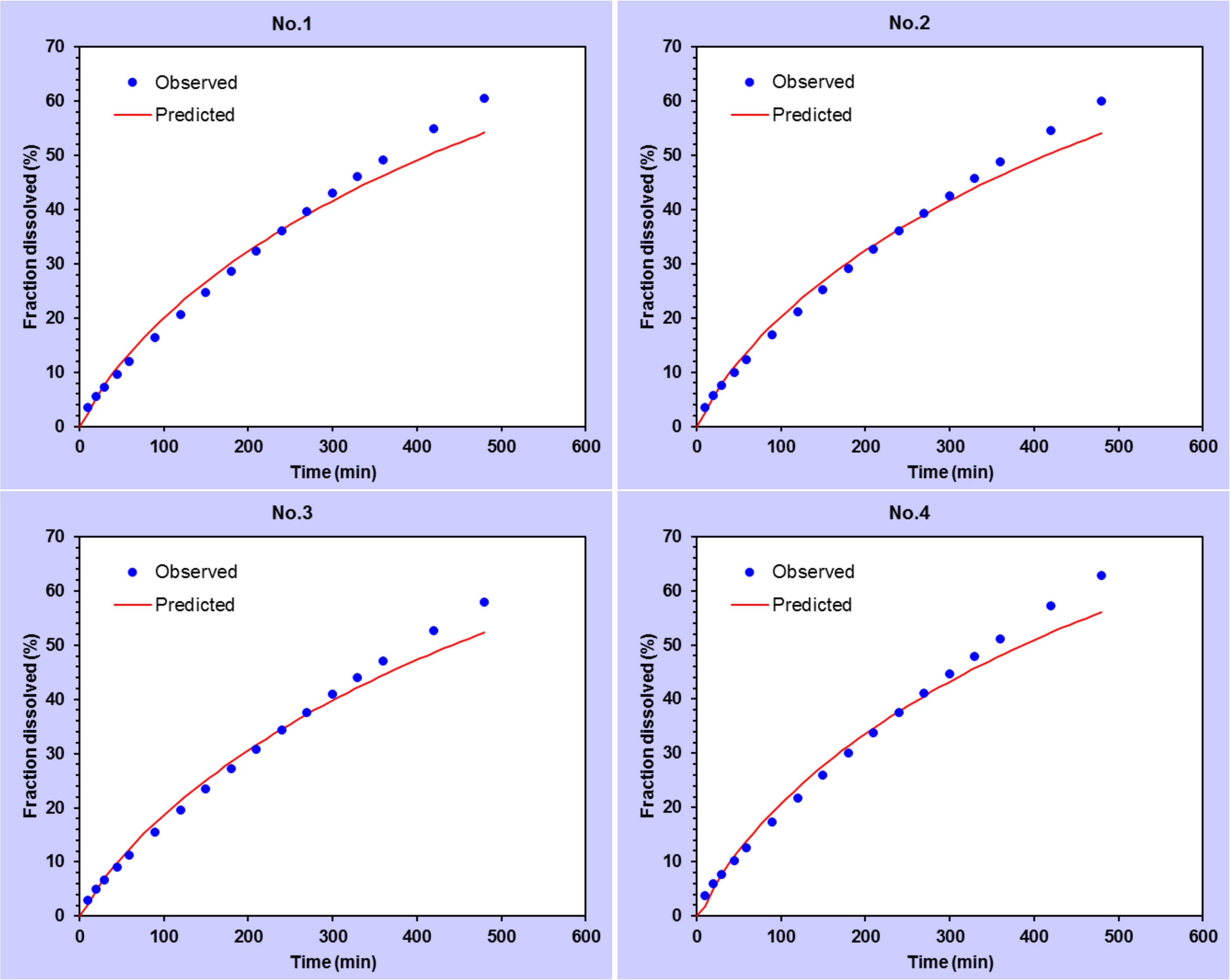

Supplementary materials – Model fitting summary – Granulated sugar N°1 600 (modelling up to app. 60 % of carvedilol released)

Model: **Weibull_2**

Model equation: $F = 100 \cdot \left(1 - e^{-\frac{t^{\beta}}{\alpha}}\right)$

Fitted model parameters per tested tablet (N = 4) with statistics – mean, standard deviation (SD), and relative standard deviation expressed in % (RSD%) (output from DDSolver):

Parameter	No.1	No.2	No.3	No.4	Mean	SD	RSD(%)
α	244.467	267.659	334.740	231.208	269.519	46.017	17.074
β	0.859	0.873	0.910	0.859	0.875	0.024	2.761

Number of dissolution data points (N), degrees of freedom (df), and selected goodness of fit criteria – Pearson correlation coefficient (R), coefficient of determination (R^2), adjusted coefficient of determination ($R^2_{adjusted}$), and residual sum of squares (RSS) (manual calculation in MS Excel):

Parameter	No.1	No.2	No.3	No.4
N	17	17	17	17
df	15	15	15	15
R	0.997553765	0.998396141	0.998597393	0.997345394
R^2	0.995113514	0.996794854	0.997196752	0.994697835
$R^2_{adjusted}$	0.994787748	0.996581178	0.997009869	0.994344357
RSS	42.61320929	32.5986773	17.19728763	47.69093022

Graphical abstract of model fit presented as mean ± 1 SD of the fraction % of released carvedilol:

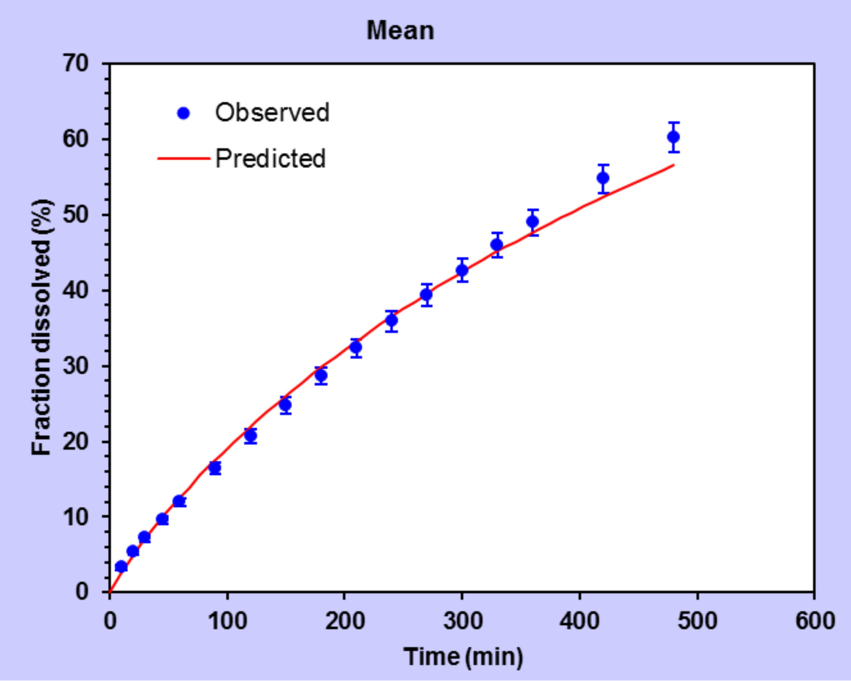

Supplementary materials – Model fitting summary – Granulated sugar N°1 600 (modelling up to app. 60 % of carvedilol released)

Graphical abstract of model fit presented as the fraction % of released carvedilol per tested tablet:

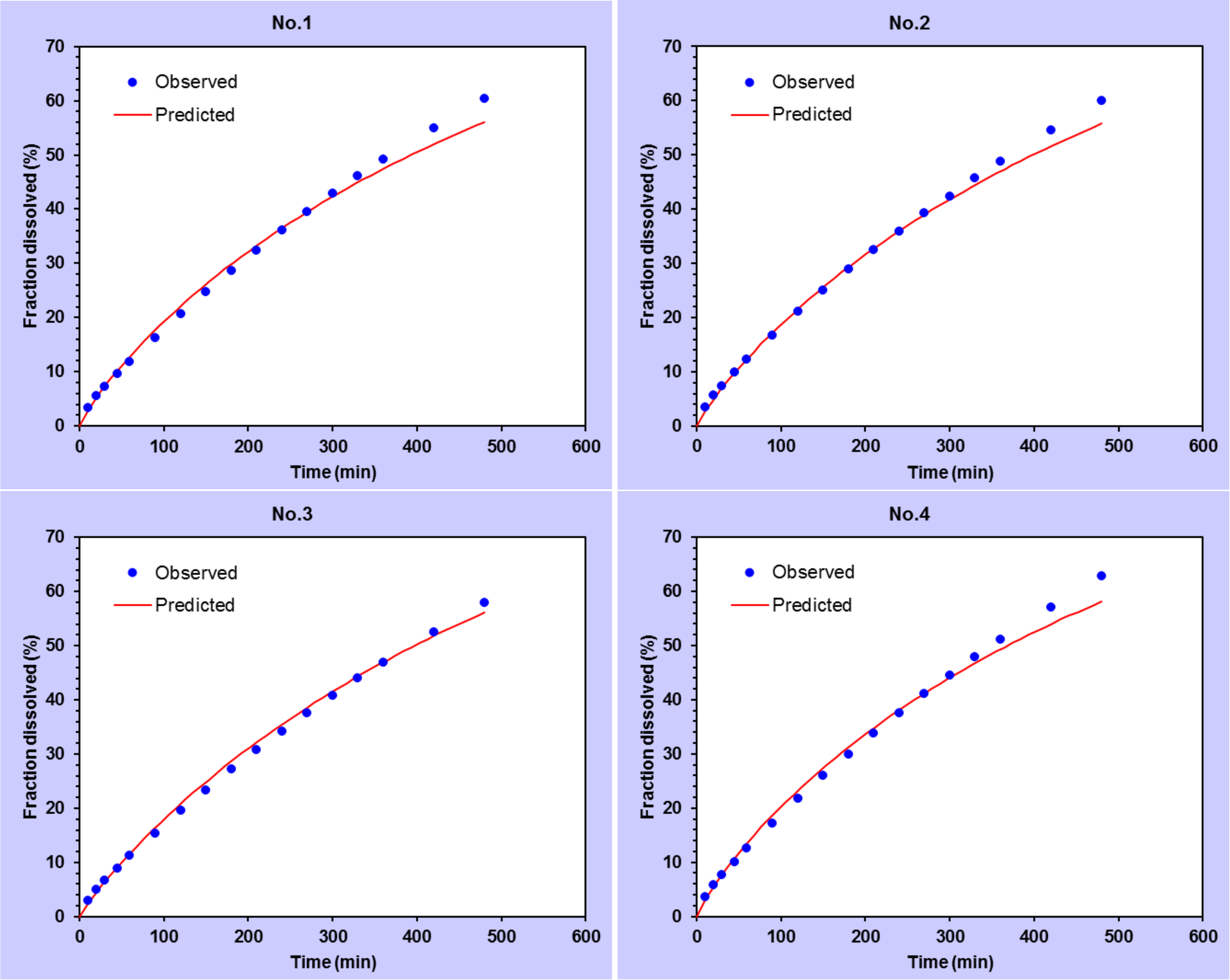

Supplementary materials – Model fitting summary – Granulated sugar N°1 600 (modelling up to app. 60 % of carvedilol released)

Model: **Weibull_3**

Model equation: $F = F_{max} \cdot \left(1 - e^{-\frac{t^{\beta}}{\alpha}}\right)$

Fitted model parameters per tested tablet (N = 4) with statistics – mean, standard deviation (SD), and relative standard deviation expressed in % (RSD%) (output from DDSolver):

Parameter	No.1	No.2	No.3	No.4	Mean	SD	RSD(%)
α	237.289	222.867	272.988	228.861	240.501	22.451	9.335
β	0.985	0.975	1.008	0.979	0.987	0.015	1.506
F_{max}	63.394	62.976	60.779	65.905	63.264	2.102	3.322

Number of dissolution data points (N), degrees of freedom (df), and selected goodness of fit criteria – Pearson correlation coefficient (R), coefficient of determination (R^2), adjusted coefficient of determination ($R^2_{adjusted}$), and residual sum of squares (RSS) (manual calculation in MS Excel):

Parameter	No.1	No.2	No.3	No.4
N	17	17	17	17
df	14	14	14	14
R	0.989381944	0.989773364	0.990071512	0.989458368
R^2	0.978876632	0.979651312	0.980241599	0.979027861
$R^2_{adjusted}$	0.975859008	0.976744357	0.97741897	0.976031842
RSS	131.3373321	120.2687399	112.6813842	139.1301301

Graphical abstract of model fit presented as mean ± 1 SD of the fraction % of released carvedilol:

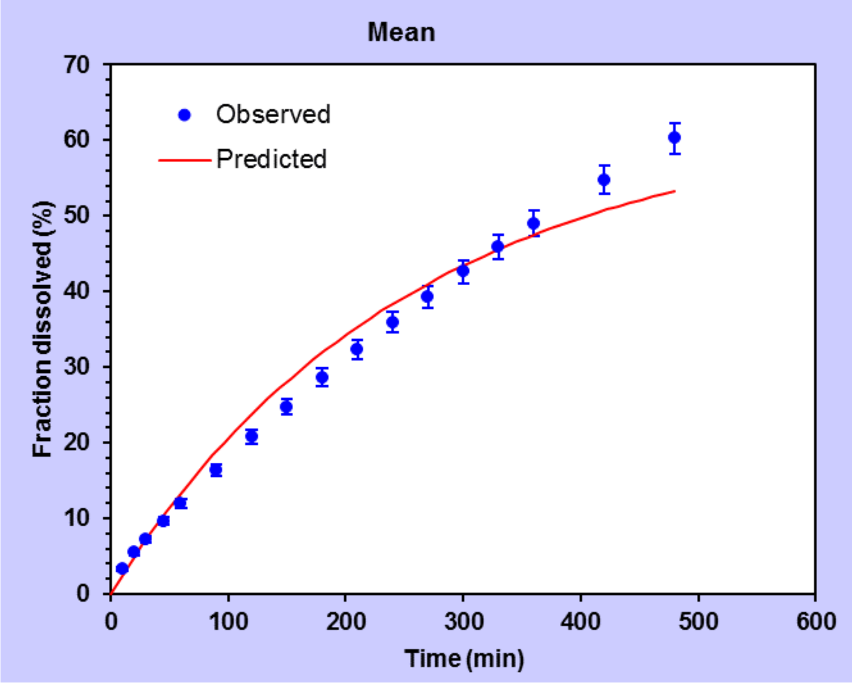

Supplementary materials – Model fitting summary – Granulated sugar N°1 600 (modelling up to app. 60 % of carvedilol released)

Graphical abstract of model fit presented as the fraction % of released carvedilol per tested tablet:

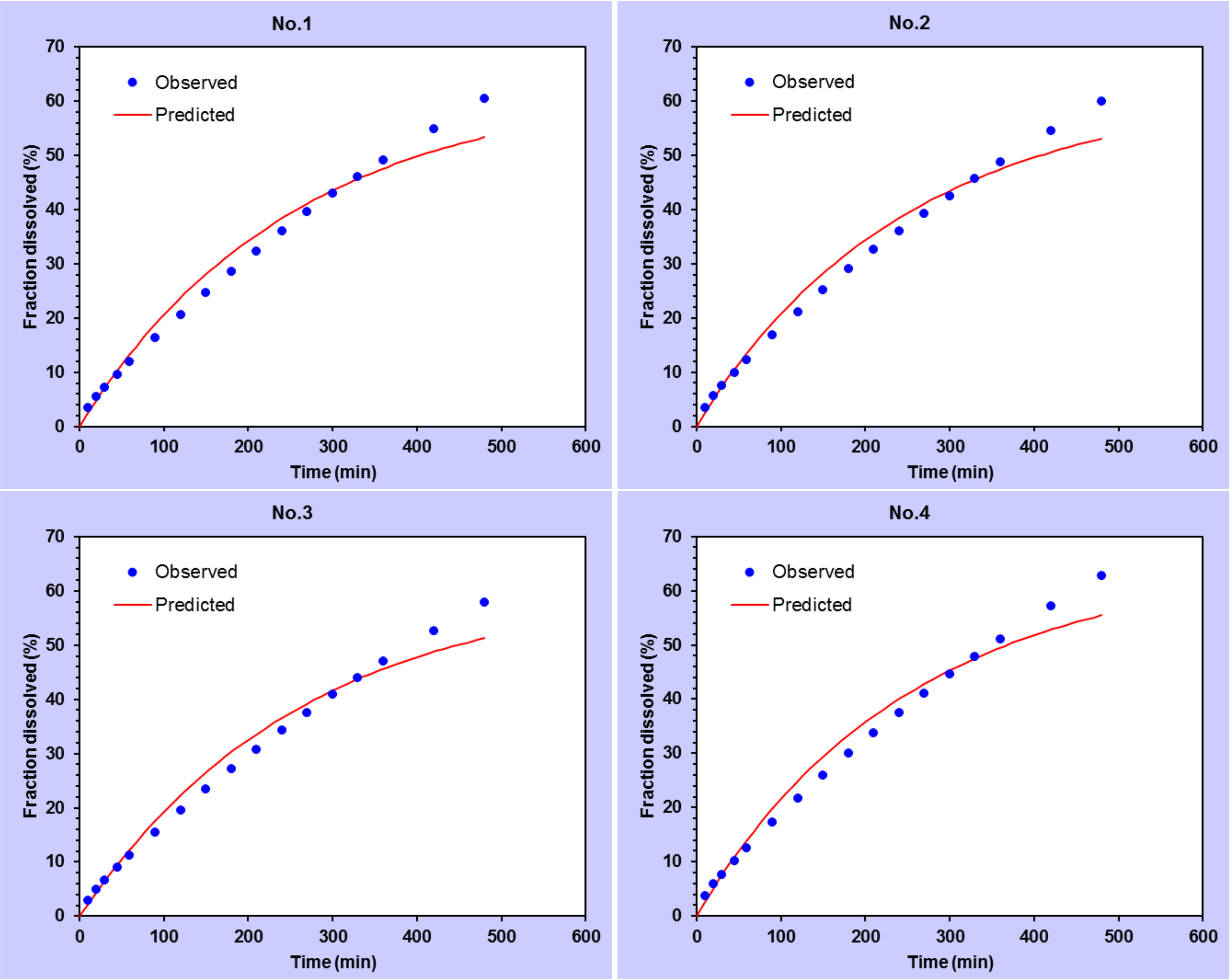

Supplementary materials – Model fitting summary – Granulated sugar N°1 600 (modelling up to app. 60 % of carvedilol released)

Model: **Weibull_4**

Model equation: $F = F_{max} \cdot \left[1 - e^{-\frac{(t-T_i)^{\beta}}{\alpha}}\right]$

Fitted model parameters per tested tablet (N = 4) with statistics – mean, standard deviation (SD), and relative standard deviation expressed in % (RSD%) (output from DDSolver):

Parameter	No.1	No.2	No.3	No.4	Mean	SD	RSD(%)
α	142.018	134.372	161.906	137.428	143.931	12.388	8.607
β	0.892	0.884	0.915	0.887	0.895	0.014	1.526
T_i	6.000	6.000	6.000	6.000	6.000	0.000	0.000
F_{max}	63.394	62.976	60.779	65.905	63.264	2.102	3.322

Number of dissolution data points (N), degrees of freedom (df), and selected goodness of fit criteria – Pearson correlation coefficient (R), coefficient of determination (R^2), adjusted coefficient of determination ($R^2_{adjusted}$), and residual sum of squares (RSS) (manual calculation in MS Excel):

Parameter	No.1	No.2	No.3	No.4
N	17	17	17	17
df	13	13	13	13
R	0.985499787	0.986099901	0.986523878	0.985616109
R^2	0.97120983	0.972393016	0.973229363	0.971439114
$R^2_{adjusted}$	0.964565945	0.966022173	0.967051523	0.96484814
RSS	178.8884806	162.7589684	153.5195182	189.1717718

Graphical abstract of model fit presented as mean ± 1 SD of the fraction % of released carvedilol:

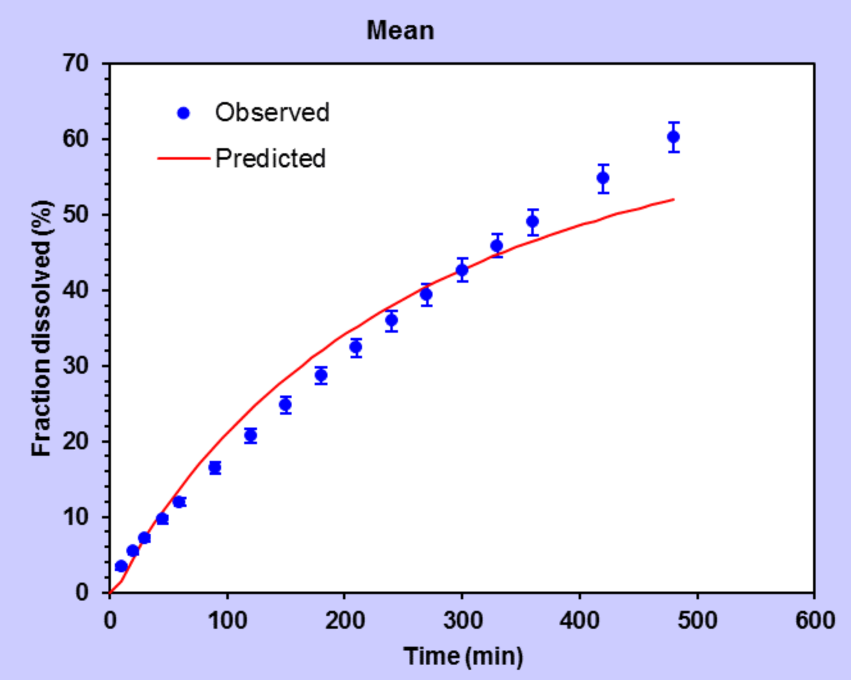

Supplementary materials – Model fitting summary – Granulated sugar N°1 600 (modelling up to app. 60 % of carvedilol released)

Graphical abstract of model fit presented as the fraction % of released carvedilol per tested tablet:

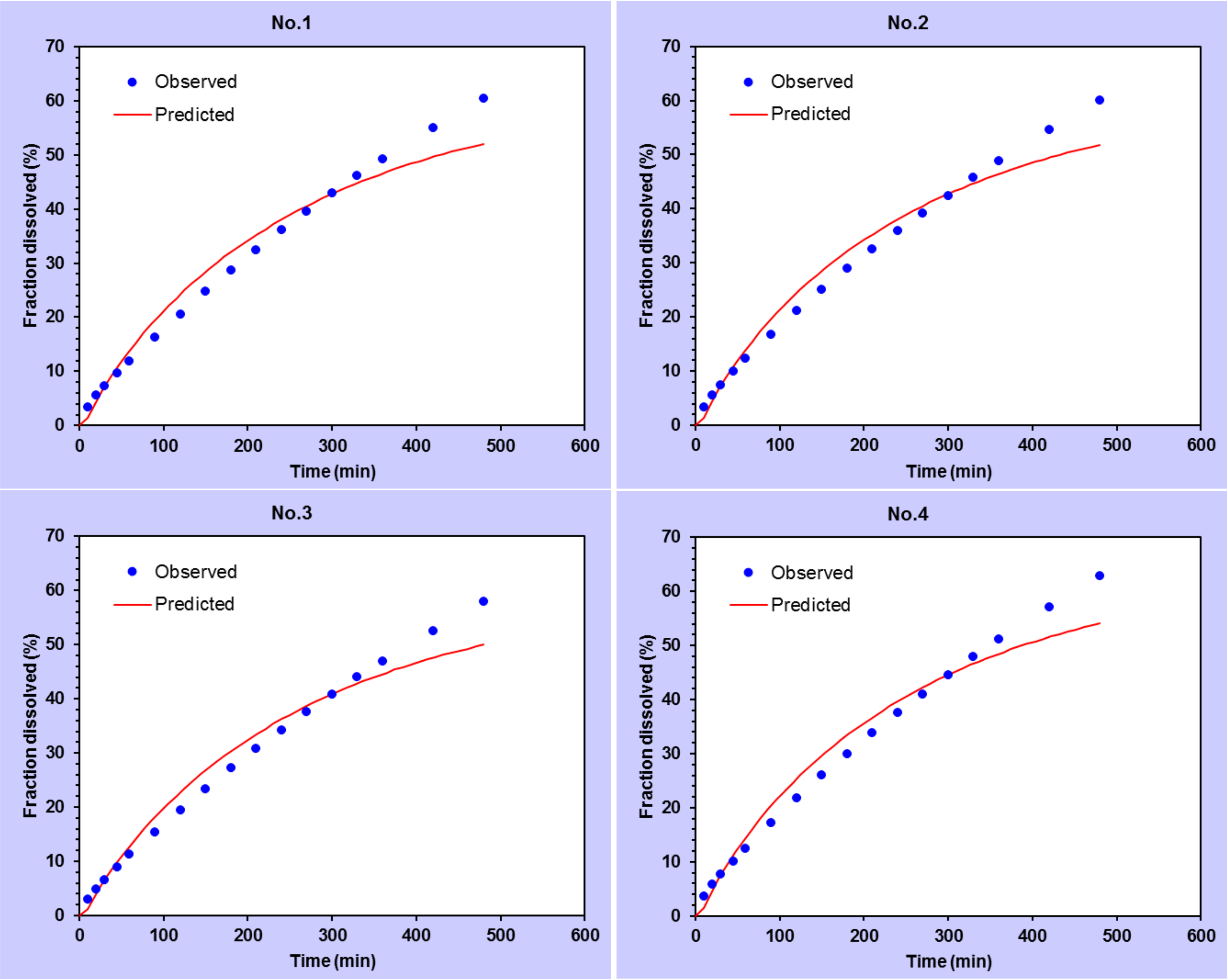

Supplementary materials – Model fitting summary – Granulated sugar N°1 600 (modelling up to app. 60 % of carvedilol released)

Model: **Logistic_1**

Model equation: $F = 100 \cdot \frac{e^{\alpha+\beta \cdot \log(t)}}{1+e^{\alpha+\beta \cdot \log(t)}}$

Fitted model parameters per tested tablet (N = 4) with statistics – mean, standard deviation (SD), and relative standard deviation expressed in % (RSD%) (output from DDSolver):

Parameter	No.1	No.2	No.3	No.4	Mean	SD	RSD(%)
α	-6.336	-6.268	-5.996	-5.820	-6.105	0.240	-3.932
β	2.417	2.389	2.266	2.257	2.332	0.083	3.549

Number of dissolution data points (N), degrees of freedom (df), and selected goodness of fit criteria – Pearson correlation coefficient (R), coefficient of determination (R^2), adjusted coefficient of determination ($R^2_{adjusted}$), and residual sum of squares (RSS) (manual calculation in MS Excel):

Parameter	No.1	No.2	No.3	No.4
N	17	17	17	17
df	15	15	15	15
R	0.99554423	0.995890887	0.994079392	0.99212801
R^2	0.991108314	0.991798658	0.988193837	0.984317988
$R^2_{adjusted}$	0.990515535	0.991251902	0.987406759	0.98327252
RSS	94.44687098	83.98717992	78.86372901	117.2337352

Graphical abstract of model fit presented as mean ± 1 SD of the fraction % of released carvedilol:

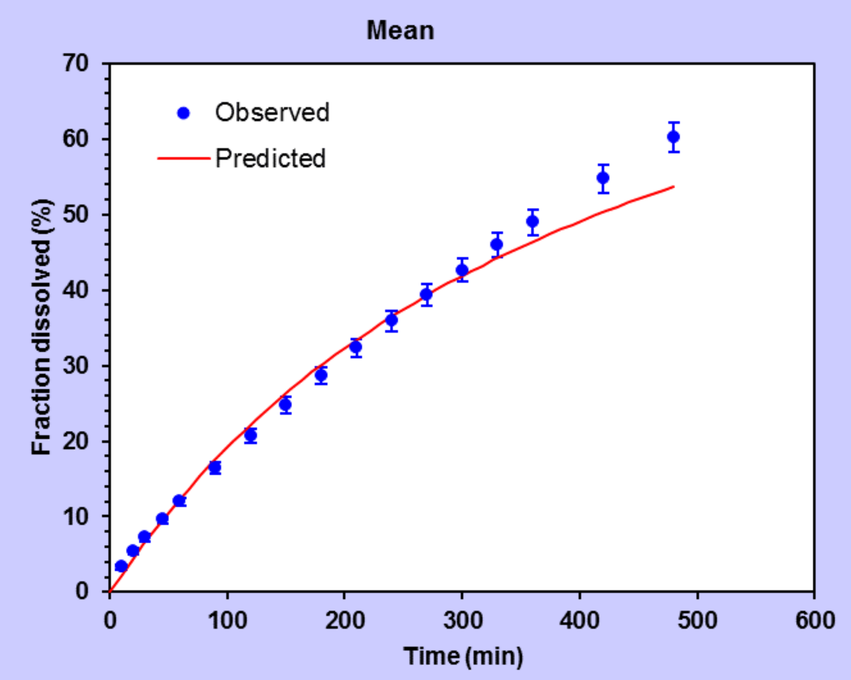

Supplementary materials – Model fitting summary – Granulated sugar N°1 600 (modelling up to app. 60 % of carvedilol released)

Graphical abstract of model fit presented as the fraction % of released carvedilol per tested tablet:

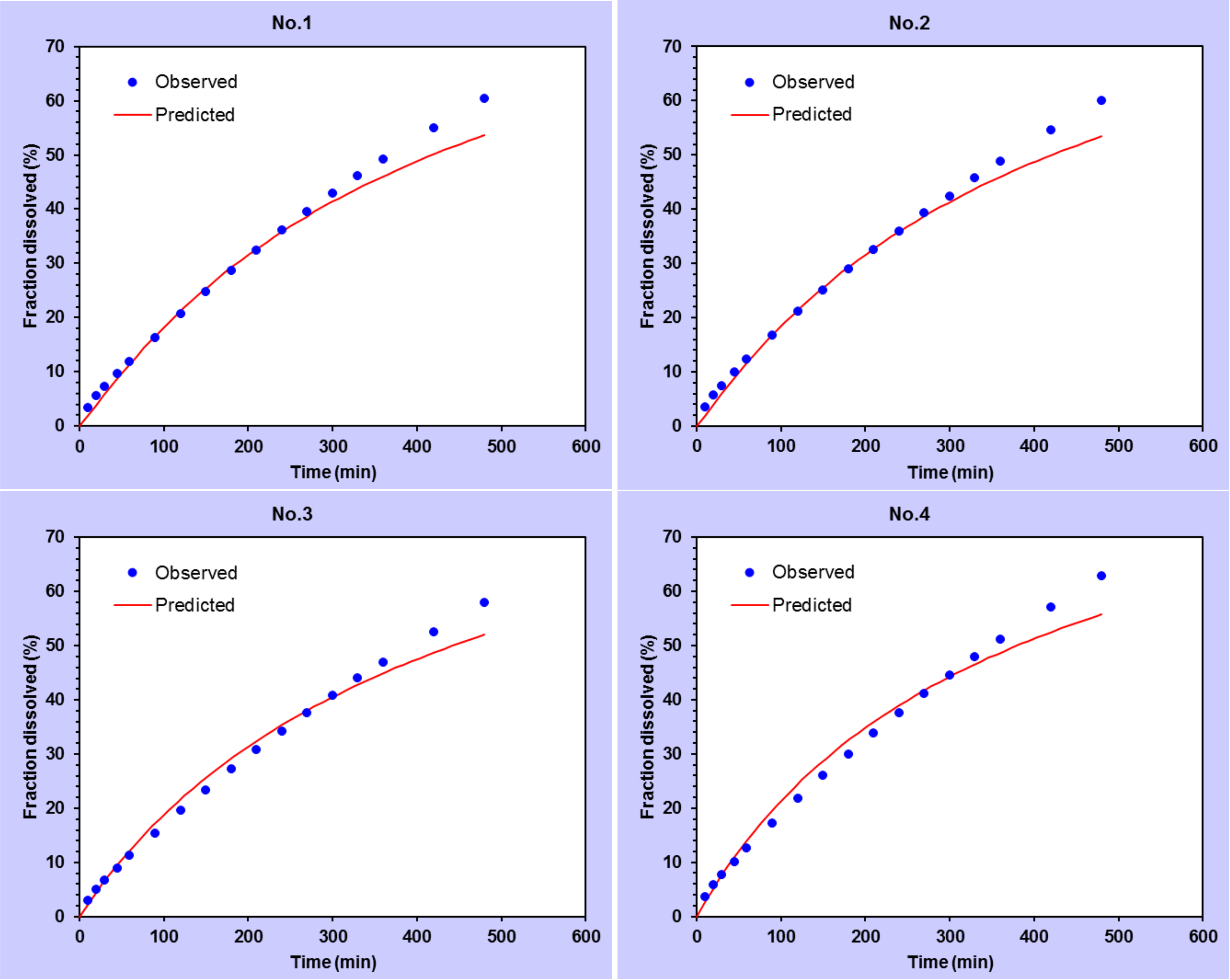

Supplementary materials – Model fitting summary – Granulated sugar N°1 600 (modelling up to app. 60 % of carvedilol released)

Model: **Logistic_2**

Model equation: $F = F_{max} \cdot \frac{e^{\alpha+\beta\cdot\log(t)}}{1+e^{\alpha+\beta\cdot\log(t)}}$

Fitted model parameters per tested tablet (N = 4) with statistics – mean, standard deviation (SD), and relative standard deviation expressed in % (RSD%) (output from DDSolver):

Parameter	No.1	No.2	No.3	No.4	Mean	SD	RSD(%)
α	-7.553	-7.472	-7.713	-7.508	-7.561	0.106	-1.407
β	3.188	3.162	3.242	3.172	3.191	0.036	1.119
F_{max}	68.769	68.315	65.932	71.492	68.627	2.280	3.322

Number of dissolution data points (N), degrees of freedom (df), and selected goodness of fit criteria – Pearson correlation coefficient (R), coefficient of determination (R^2), adjusted coefficient of determination ($R^2_{adjusted}$), and residual sum of squares (RSS) (manual calculation in MS Excel):

Parameter	No.1	No.2	No.3	No.4
N	17	17	17	17
df	14	14	14	14
R	0.991733409	0.991713736	0.992401133	0.991668428
R^2	0.983535154	0.983496133	0.984860009	0.983406271
$R^2_{adjusted}$	0.981183033	0.981138438	0.982697153	0.981035739
RSS	257.6508705	242.8964546	248.1263644	272.8697899

Graphical abstract of model fit presented as mean ± 1 SD of the fraction % of released carvedilol:

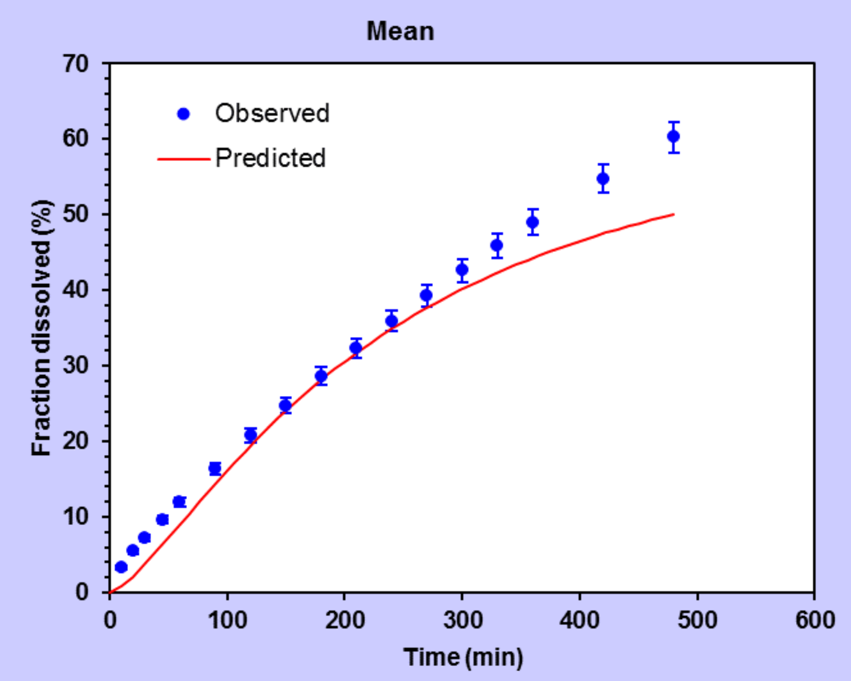

Supplementary materials – Model fitting summary – Granulated sugar N°1 600 (modelling up to app. 60 % of carvedilol released)

Graphical abstract of model fit presented as the fraction % of released carvedilol per tested tablet:

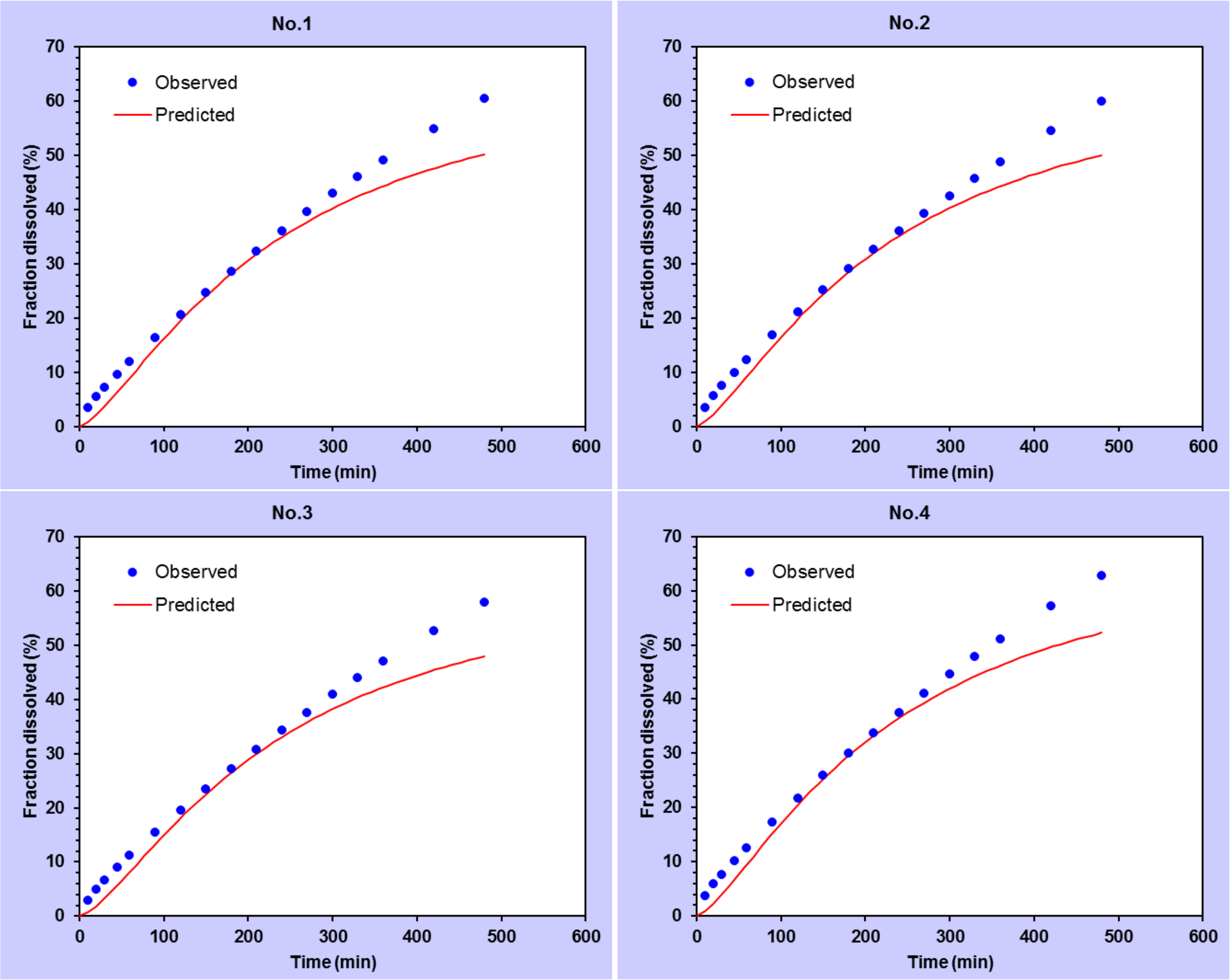

Supplementary materials – Model fitting summary – Granulated sugar N°1 600 (modelling up to app. 60 % of carvedilol released)

Model: **Logistic_3**

Model equation: $F = F_{max} \cdot \frac{1}{1+e^{-k\cdot(t-\gamma)}}$

Fitted model parameters per tested tablet (N = 4) with statistics – mean, standard deviation (SD), and relative standard deviation expressed in % (RSD%) (output from DDSolver):

Parameter	No.1	No.2	No.3	No.4	Mean	SD	RSD(%)
k	0.011	0.010	0.011	0.010	0.011	0.000	0.988
γ	218.827	217.211	221.443	218.105	218.896	1.822	0.832
F_{max}	63.394	62.976	60.779	65.905	63.264	2.102	3.322

Number of dissolution data points (N), degrees of freedom (df), and selected goodness of fit criteria – Pearson correlation coefficient (R), coefficient of determination (R^2), adjusted coefficient of determination ($R^2_{adjusted}$), and residual sum of squares (RSS) (manual calculation in MS Excel):

Parameter	No.1	No.2	No.3	No.4
N	17	17	17	17
df	14	14	14	14
R	0.992550543	0.991297575	0.991850361	0.992088426
R^2	0.985156581	0.982670882	0.983767139	0.984239445
$R^2_{adjusted}$	0.983036092	0.980195294	0.981448159	0.981987937
RSS	92.37918642	104.3737388	94.76781208	104.9291438

Graphical abstract of model fit presented as mean ± 1 SD of the fraction % of released carvedilol:

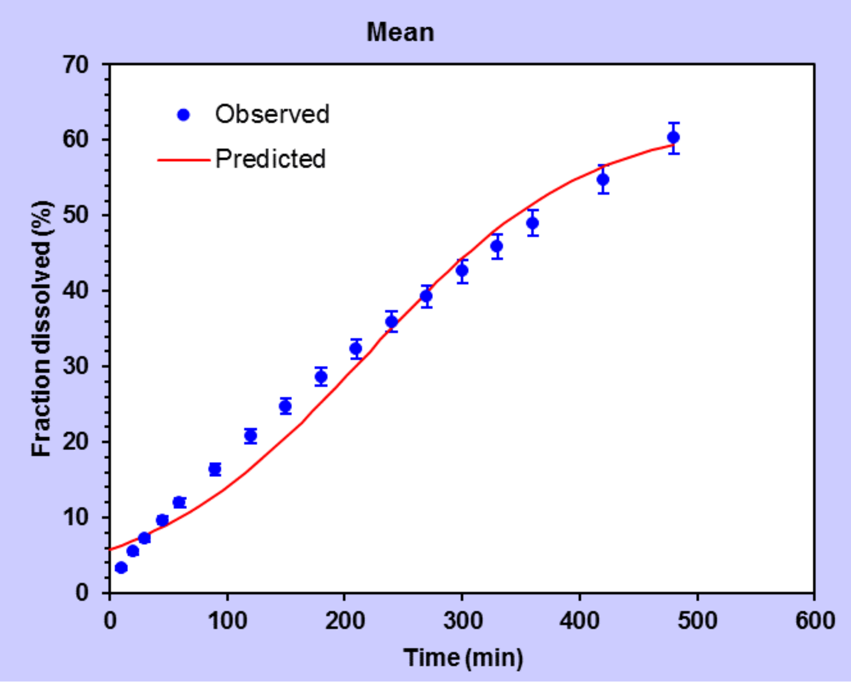

Supplementary materials – Model fitting summary – Granulated sugar N°1 600 (modelling up to app. 60 % of carvedilol released)

Graphical abstract of model fit presented as the fraction % of released carvedilol per tested tablet:

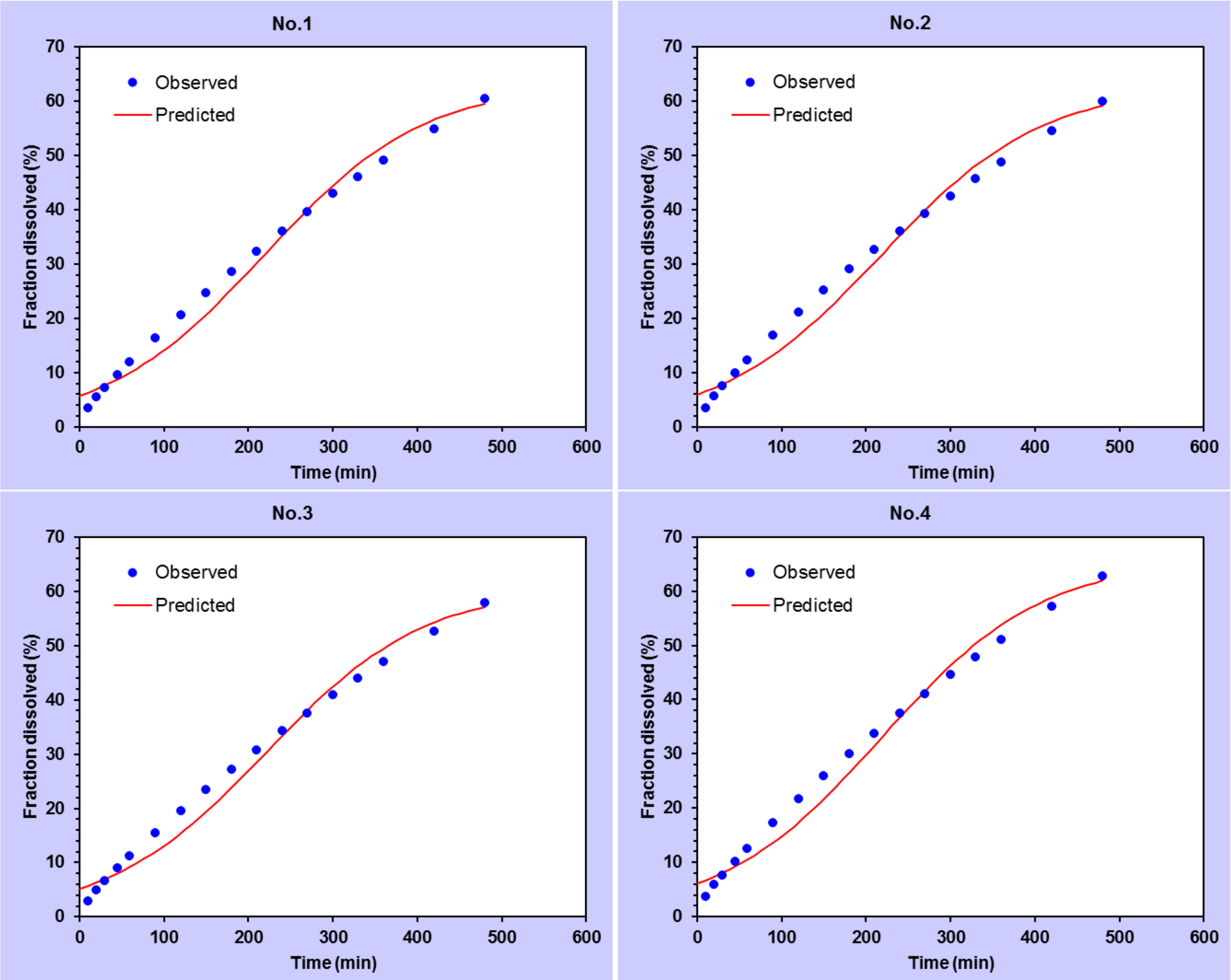

Supplementary materials – Model fitting summary – Granulated sugar N°1 600 (modelling up to app. 60 % of carvedilol released)

Model: **Gompertz_1**

Model equation: $F = 100 \cdot e^{-\alpha \cdot e^{-\beta \cdot \log(t)}}$

Fitted model parameters per tested tablet (N = 4) with statistics – mean, standard deviation (SD), and relative standard deviation expressed in % (RSD%) (output from DDSolver):

Parameter	No.1	No.2	No.3	No.4	Mean	SD	RSD(%)
α	13.616	13.209	13.677	13.954	13.614	0.308	2.260
β	1.116	1.103	1.096	1.146	1.115	0.022	2.004

Number of dissolution data points (N), degrees of freedom (df), and selected goodness of fit criteria – Pearson correlation coefficient (R), coefficient of determination (R^2), adjusted coefficient of determination ($R^2_{adjusted}$), and residual sum of squares (RSS) (manual calculation in MS Excel):

Parameter	No.1	No.2	No.3	No.4
N	17	17	17	17
df	15	15	15	15
R	0.973873063	0.975427759	0.975969076	0.973019676
R^2	0.948428742	0.951459313	0.952515638	0.94676729
$R^2_{adjusted}$	0.944990659	0.948223267	0.949350014	0.943218443
RSS	314.4691904	285.4908273	271.5512322	344.727003

Graphical abstract of model fit presented as mean ± 1 SD of the fraction % of released carvedilol:

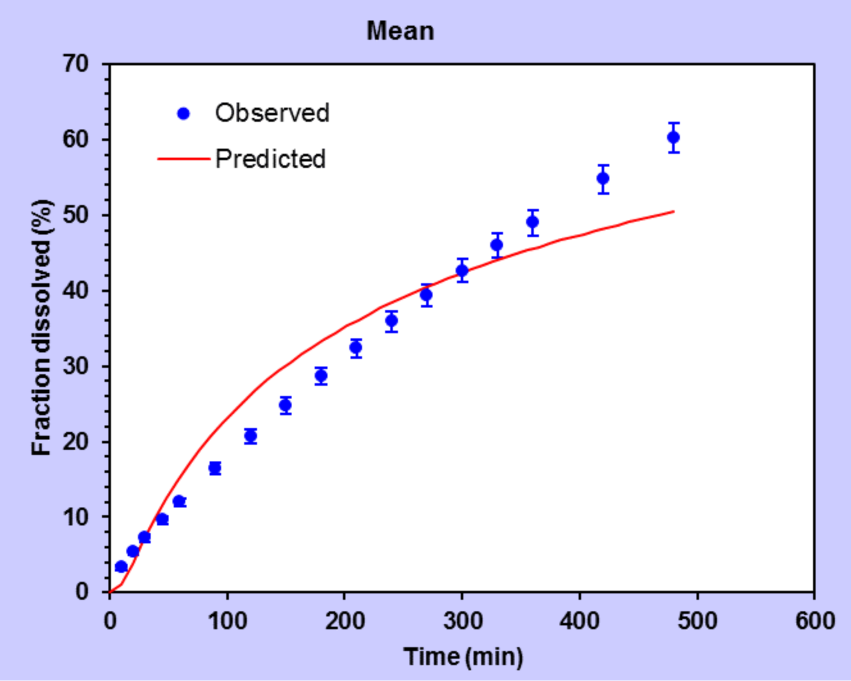

Supplementary materials – Model fitting summary – Granulated sugar N°1 600 (modelling up to app. 60 % of carvedilol released)

Graphical abstract of model fit presented as the fraction % of released carvedilol per tested tablet:

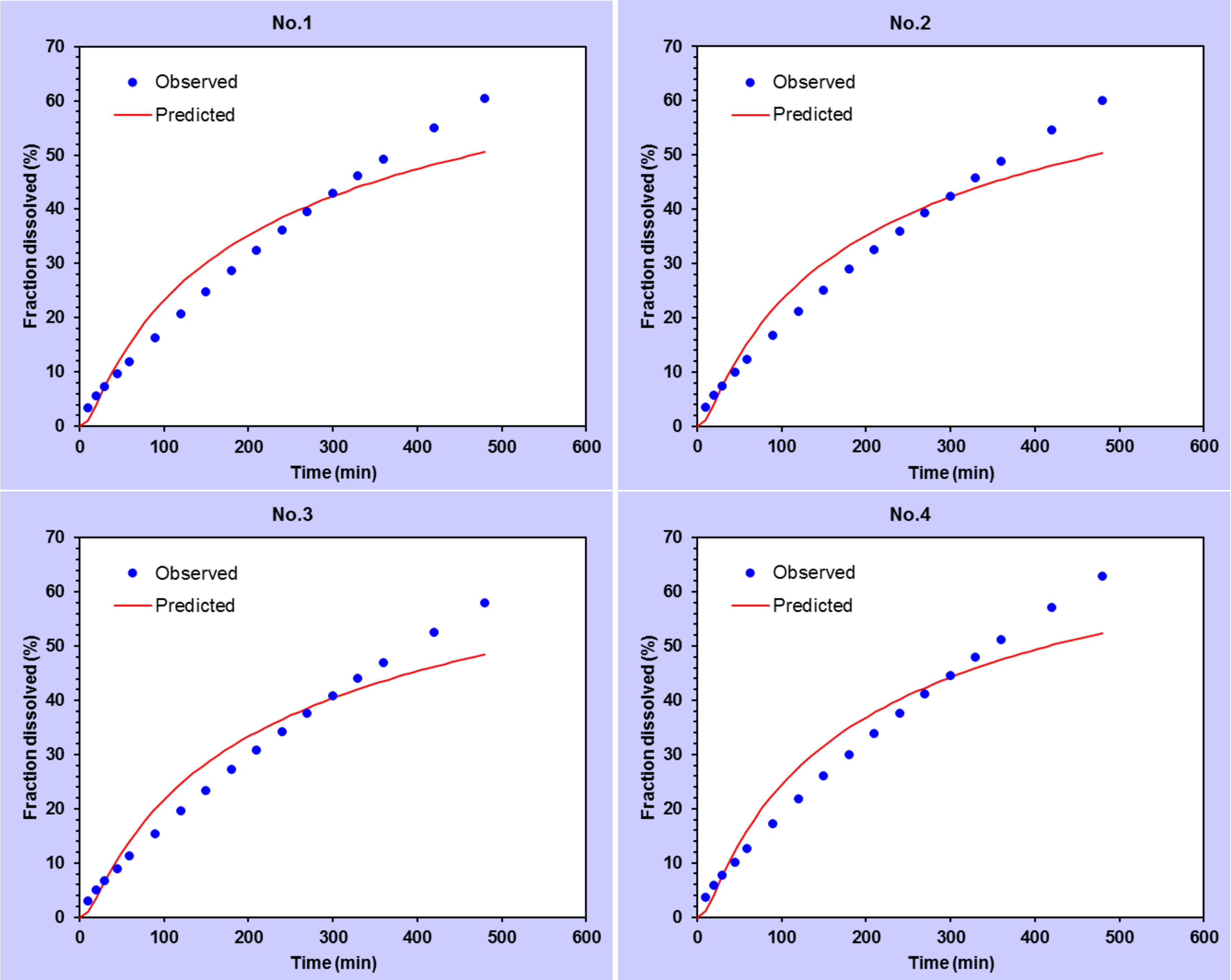

Supplementary materials – Model fitting summary – Granulated sugar N°1 600 (modelling up to app. 60 % of carvedilol released)

Model: **Gompertz_2**

Model equation: $F = F_{max} \cdot e^{-\alpha \cdot e^{-\beta \cdot \log(t)}}$

Fitted model parameters per tested tablet (N = 4) with statistics – mean, standard deviation (SD), and relative standard deviation expressed in % (RSD%) (output from DDSolver):

Parameter	No.1	No.2	No.3	No.4	Mean	SD	RSD(%)
α	60.079	58.150	62.608	59.108	59.986	1.917	3.196
β	1.930	1.919	1.942	1.924	1.929	0.010	0.505
F_{max}	63.394	62.976	60.779	65.905	63.264	2.102	3.322

Number of dissolution data points (N), degrees of freedom (df), and selected goodness of fit criteria – Pearson correlation coefficient (R), coefficient of determination (R^2), adjusted coefficient of determination ($R^2_{adjusted}$), and residual sum of squares (RSS) (manual calculation in MS Excel):

Parameter	No.1	No.2	No.3	No.4
N	17	17	17	17
df	14	14	14	14
R	0.973830623	0.974768734	0.97449485	0.974171115
R^2	0.948346082	0.950174085	0.949640214	0.949009362
$R^2_{adjusted}$	0.94096695	0.943056097	0.942445958	0.941724985
RSS	595.3690161	578.6660036	543.9725982	639.6274789

Graphical abstract of model fit presented as mean ± 1 SD of the fraction % of released carvedilol:

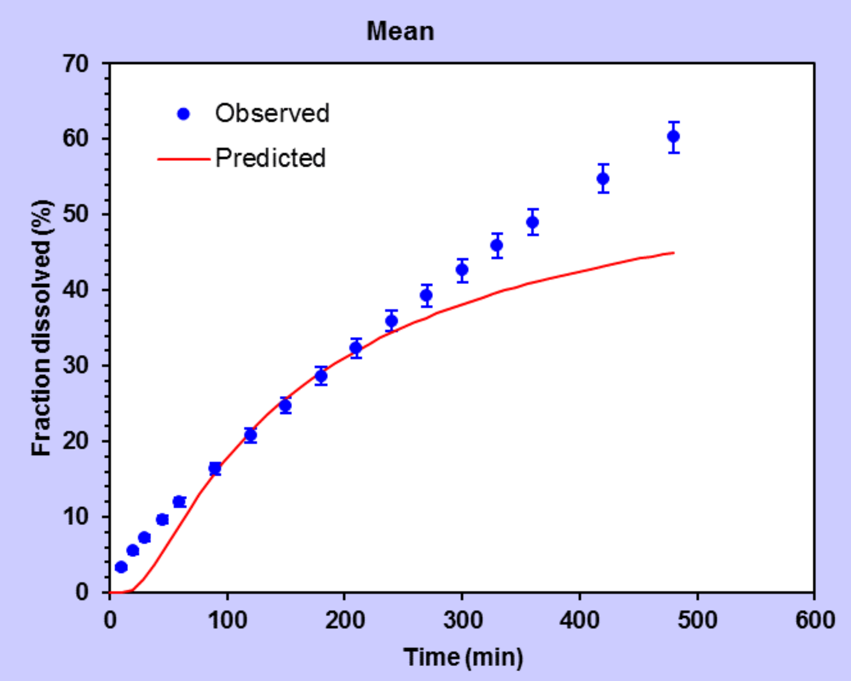

Supplementary materials – Model fitting summary – Granulated sugar N°1 600 (modelling up to app. 60 % of carvedilol released)

Graphical abstract of model fit presented as the fraction % of released carvedilol per tested tablet:

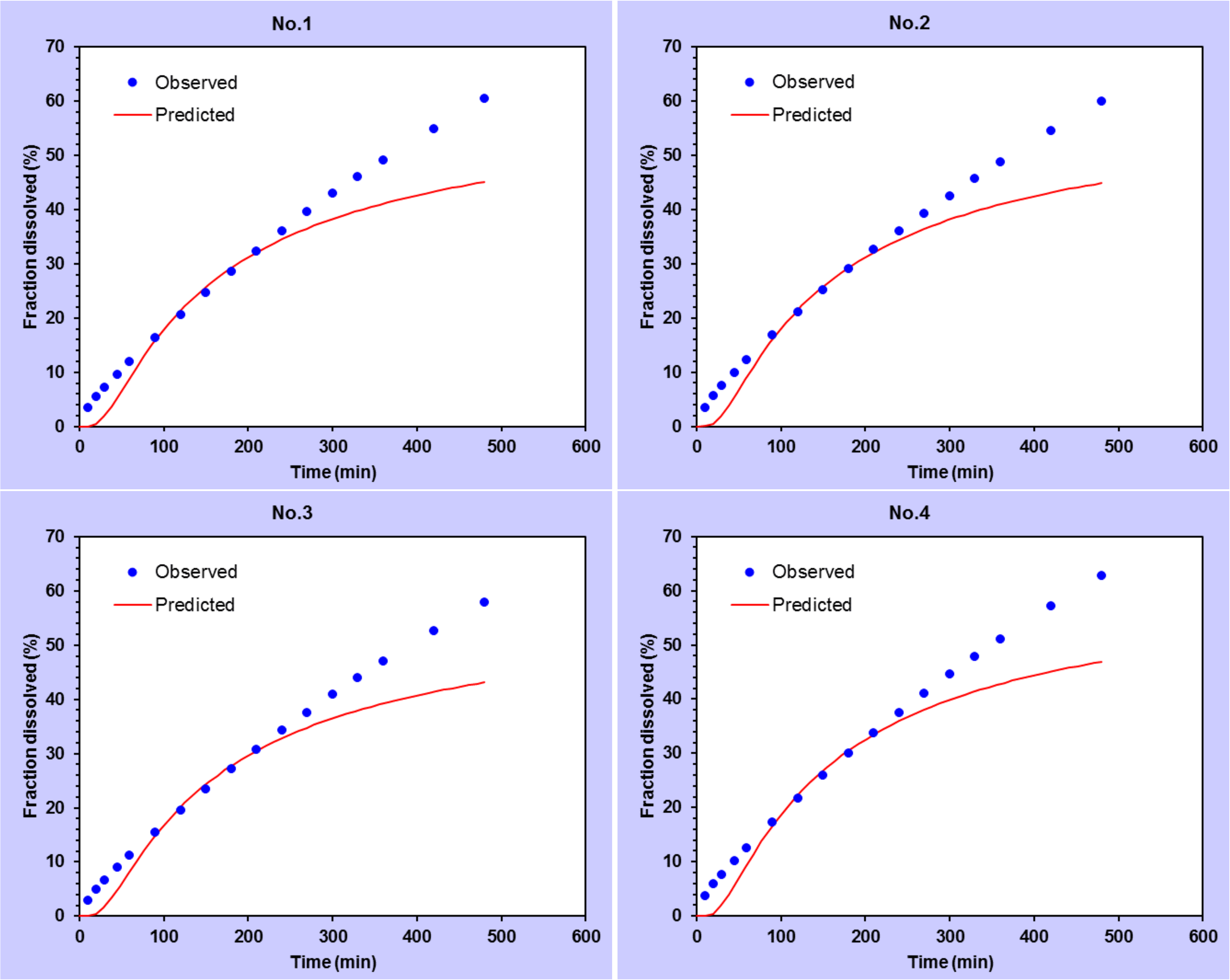

Supplementary materials – Model fitting summary – Granulated sugar N°1 600 (modelling up to app. 60 % of carvedilol released)

Model: **Gompertz_3**

Model equation: $F = F_{max} \cdot e^{-e^{-k\cdot(t-\gamma)}}$

Fitted model parameters per tested tablet (N = 4) with statistics – mean, standard deviation (SD), and relative standard deviation expressed in % (RSD%) (output from DDSolver):

Parameter	No.1	No.2	No.3	No.4	Mean	SD	RSD(%)
k	0.006	0.007	0.006	0.006	0.006	0.001	14.440
γ	168.390	144.228	171.156	167.497	162.818	12.490	7.671
F_{max}	68.299	62.976	65.481	71.004	66.940	3.474	5.189

Number of dissolution data points (N), degrees of freedom (df), and selected goodness of fit criteria – Pearson correlation coefficient (R), coefficient of determination (R^2), adjusted coefficient of determination ($R^2_{adjusted}$), and residual sum of squares (RSS) (manual calculation in MS Excel):

Parameter	No.1	No.2	No.3	No.4
N	17	17	17	17
df	14	14	14	14
R	0.997906499	0.995308539	0.997628737	0.997635335
R^2	0.99581738	0.990639088	0.995263098	0.995276262
$R^2_{adjusted}$	0.995219863	0.989301814	0.994586397	0.994601443
RSS	45.50925772	67.31116848	45.33423391	51.5665609

Graphical abstract of model fit presented as mean ± 1 SD of the fraction % of released carvedilol:

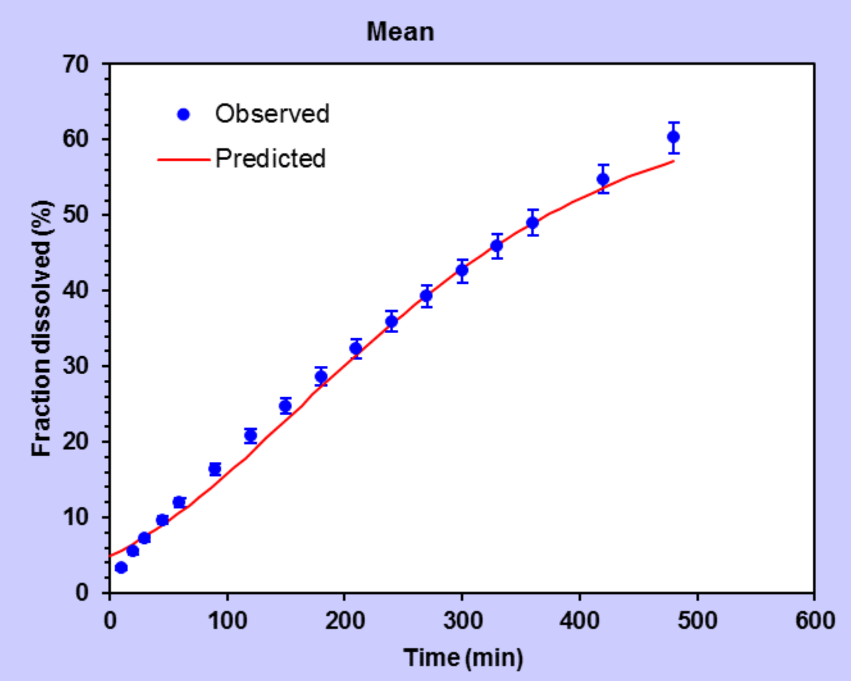

Supplementary materials – Model fitting summary – Granulated sugar N°1 600 (modelling up to app. 60 % of carvedilol released)

Graphical abstract of model fit presented as the fraction % of released carvedilol per tested tablet:

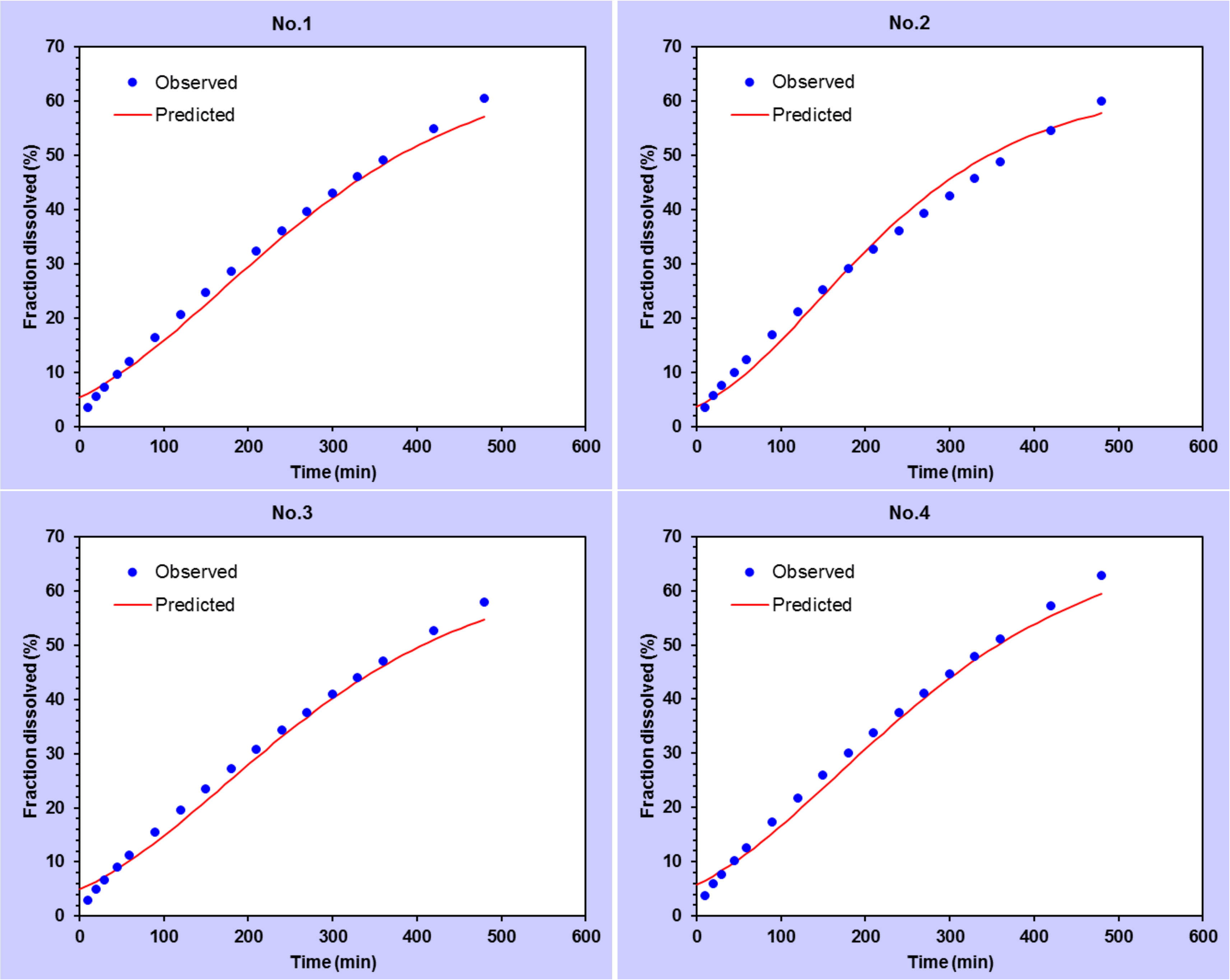

Supplementary materials – Model fitting summary – Granulated sugar N°1 600 (modelling up to app. 60 % of carvedilol released)

Model: **Gompertz_4**

Model equation: $F = F_{max} \cdot e^{-\beta \cdot e^{-k \cdot t}}$

Fitted model parameters per tested tablet (N = 4) with statistics – mean, standard deviation (SD), and relative standard deviation expressed in % (RSD%) (output from DDSolver):

Parameter	No.1	No.2	No.3	No.4	Mean	SD	RSD(%)
k	0.007	0.007	0.007	0.007	0.007	0.000	0.527
β	2.914	2.850	2.978	2.886	2.907	0.054	1.861
F_{max}	63.394	62.976	60.779	65.905	63.264	2.102	3.322

Number of dissolution data points (N), degrees of freedom (df), and selected goodness of fit criteria – Pearson correlation coefficient (R), coefficient of determination (R^2), adjusted coefficient of determination ($R^2_{adjusted}$), and residual sum of squares (RSS) (manual calculation in MS Excel):

Parameter	No.1	No.2	No.3	No.4
N	17	17	17	17
df	14	14	14	14
R	0.99593522	0.995308539	0.995608941	0.995693506
R^2	0.991886963	0.990639088	0.991237163	0.991405558
$R^2_{adjusted}$	0.990727958	0.989301814	0.989985329	0.99017778
RSS	61.55908768	67.31116848	60.84190278	69.37443356

Graphical abstract of model fit presented as mean ± 1 SD of the fraction % of released carvedilol:

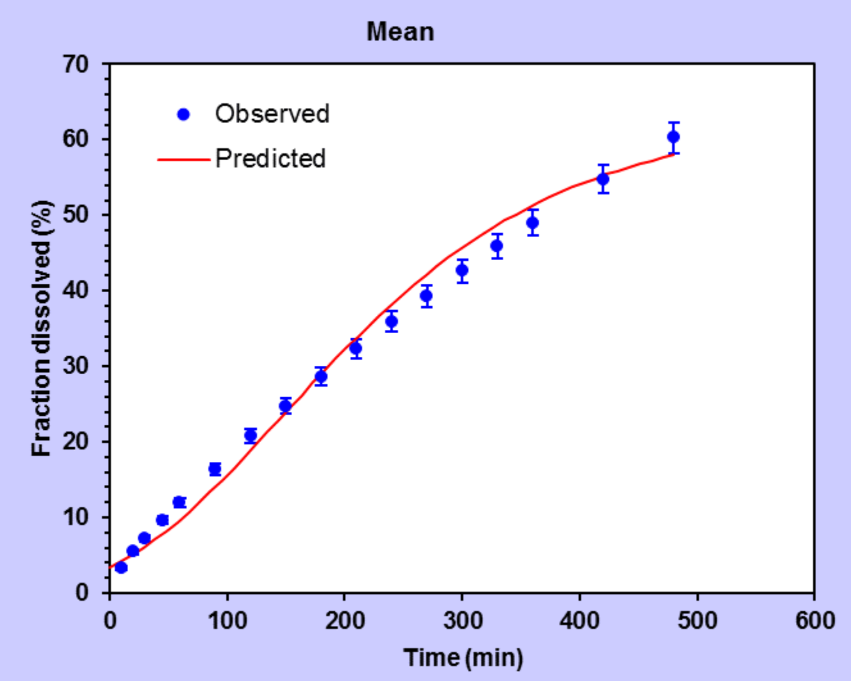

Supplementary materials – Model fitting summary – Granulated sugar N°1 600 (modelling up to app. 60 % of carvedilol released)

Graphical abstract of model fit presented as the fraction % of released carvedilol per tested tablet:

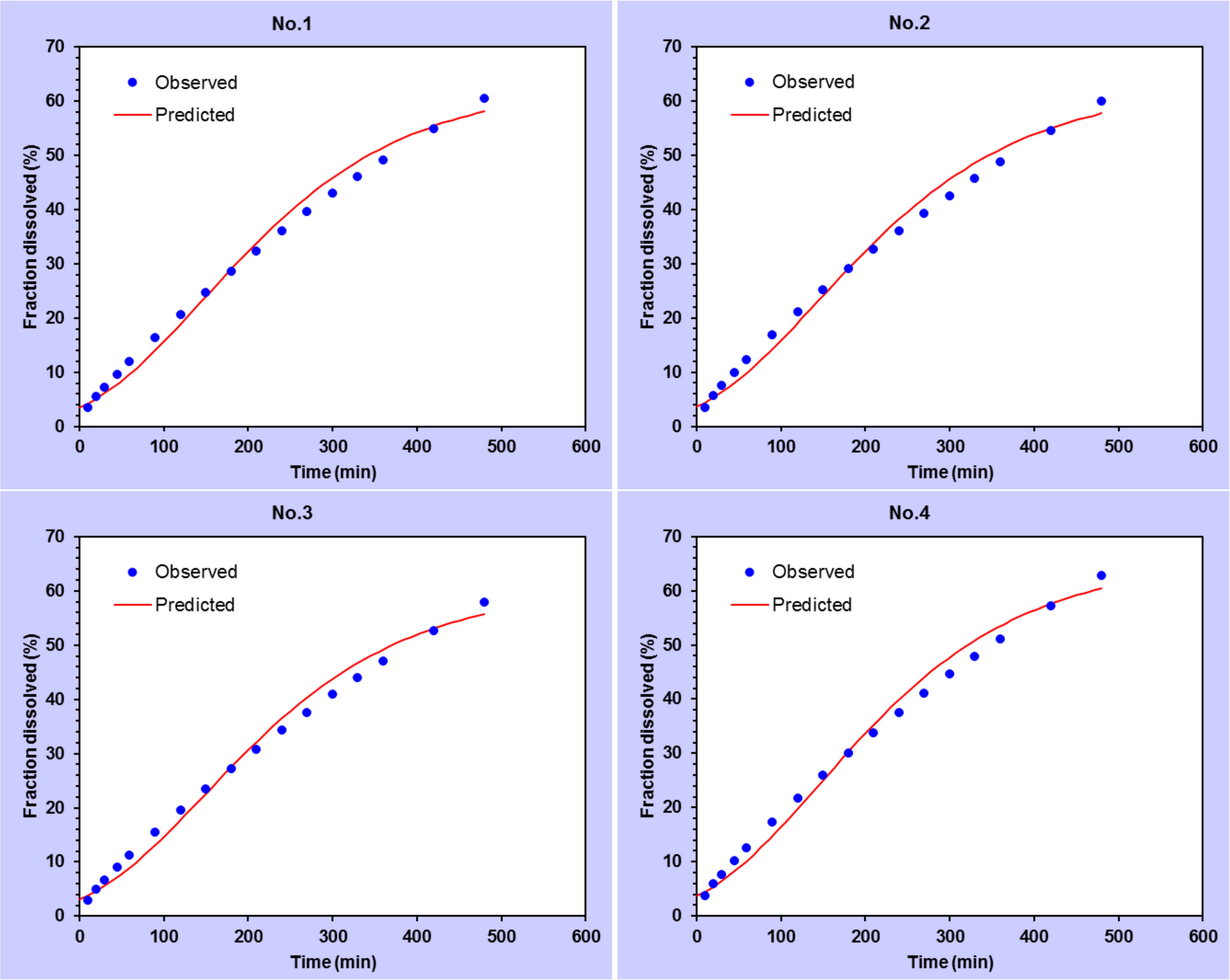

Supplementary materials – Model fitting summary – Granulated sugar N°1 600 (modelling up to app. 60 % of carvedilol released)

Model: **Probit_1**

Model equation: $F = 100 \cdot \phi[\alpha + \beta \cdot \log(t)]$

Fitted model parameters per tested tablet (N = 4) with statistics – mean, standard deviation (SD), and relative standard deviation expressed in % (RSD%) (output from DDSolver):

Parameter	No.1	No.2	No.3	No.4	Mean	SD	RSD(%)
α	-3.297	-3.263	-3.346	-3.295	-3.300	0.034	-1.042
β	1.251	1.237	1.251	1.269	1.252	0.013	1.042

Number of dissolution data points (N), degrees of freedom (df), and selected goodness of fit criteria – Pearson correlation coefficient (R), coefficient of determination (R^2), adjusted coefficient of determination ($R^2_{adjusted}$), and residual sum of squares (RSS) (manual calculation in MS Excel):

Parameter	No.1	No.2	No.3	No.4
N	17	17	17	17
df	15	15	15	15
R	0.987058837	0.988007794	0.988491067	0.986566079
R^2	0.974285148	0.976159401	0.97711459	0.973312628
$R^2_{adjusted}$	0.972570824	0.974570027	0.975588896	0.97153347
RSS	175.571766	155.4989079	146.6427139	192.1331939

Graphical abstract of model fit presented as mean ± 1 SD of the fraction % of released carvedilol:

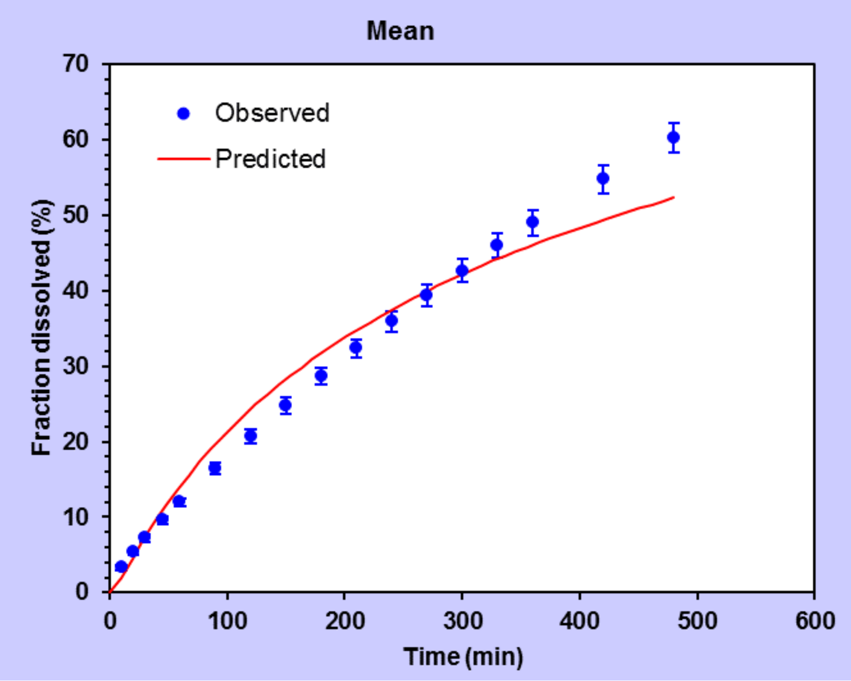

Supplementary materials – Model fitting summary – Granulated sugar N°1 600 (modelling up to app. 60 % of carvedilol released)

Graphical abstract of model fit presented as the fraction % of released carvedilol per tested tablet:

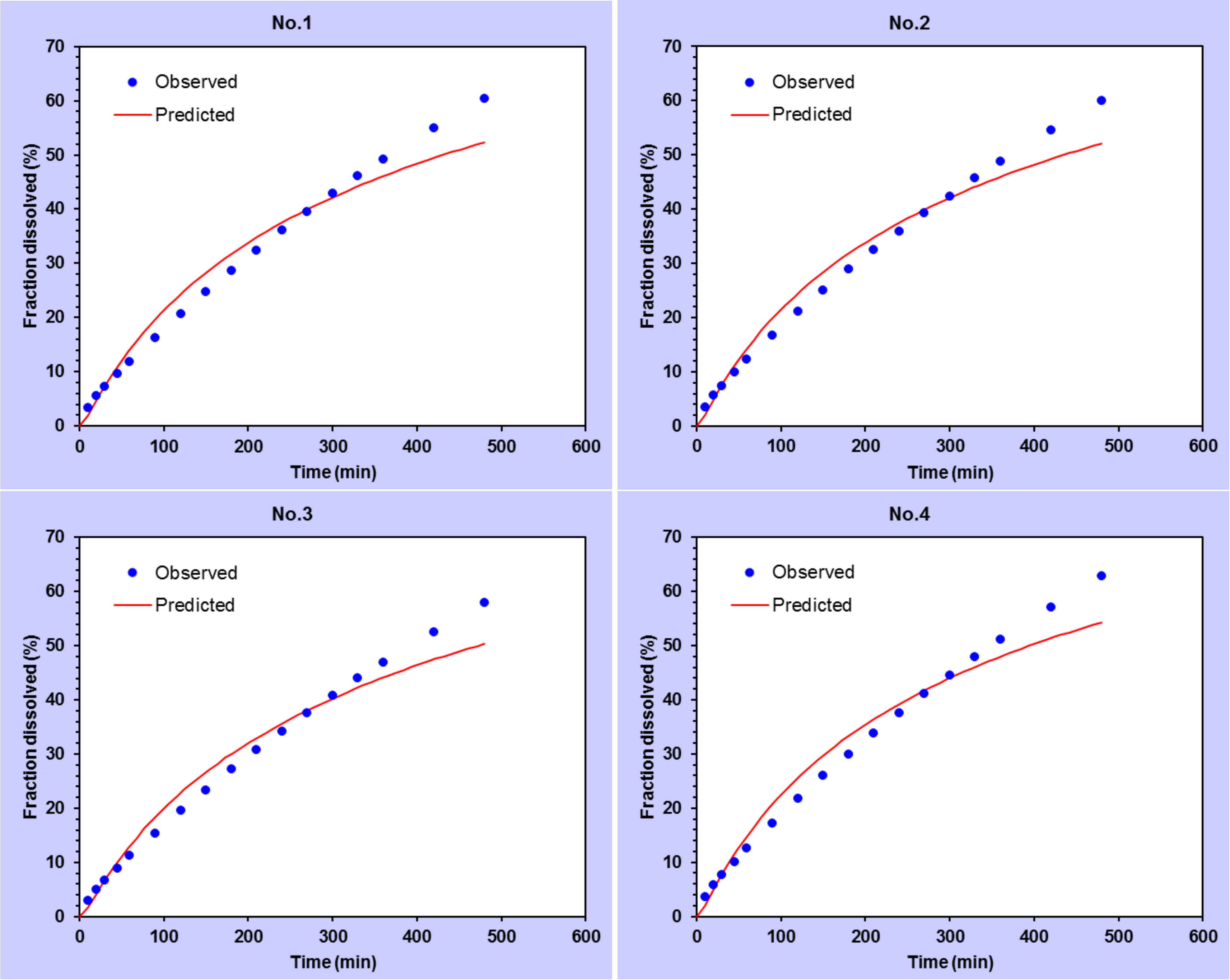

Supplementary materials – Model fitting summary – Granulated sugar N°1 600 (modelling up to app. 60 % of carvedilol released)

Model: **Probit_2**

Model equation: $F = F_{max} \cdot \phi[\alpha + \beta \cdot \log(t)]$

Fitted model parameters per tested tablet (N = 4) with statistics – mean, standard deviation (SD), and relative standard deviation expressed in % (RSD%) (output from DDSolver):

Parameter	No.1	No.2	No.3	No.4	Mean	SD	RSD(%)
α	-4.371	-4.327	-4.461	-4.347	-4.376	0.059	-1.346
β	1.853	1.839	1.875	1.845	1.853	0.016	0.841
F_{max}	69.415	68.957	65.932	72.164	69.117	2.552	3.693

Number of dissolution data points (N), degrees of freedom (df), and selected goodness of fit criteria – Pearson correlation coefficient (R), coefficient of determination (R^2), adjusted coefficient of determination ($R^2_{adjusted}$), and residual sum of squares (RSS) (manual calculation in MS Excel):

Parameter	No.1	No.2	No.3	No.4
N	17	17	17	17
df	14	14	14	14
R	0.987975744	0.988280626	0.989423389	0.988034531
R^2	0.976096071	0.976698596	0.978958643	0.976212235
$R^2_{adjusted}$	0.972681224	0.973369824	0.975952735	0.972813982
RSS	242.3714656	226.5309704	292.5135273	256.2777718

Graphical abstract of model fit presented as mean ± 1 SD of the fraction % of released carvedilol:

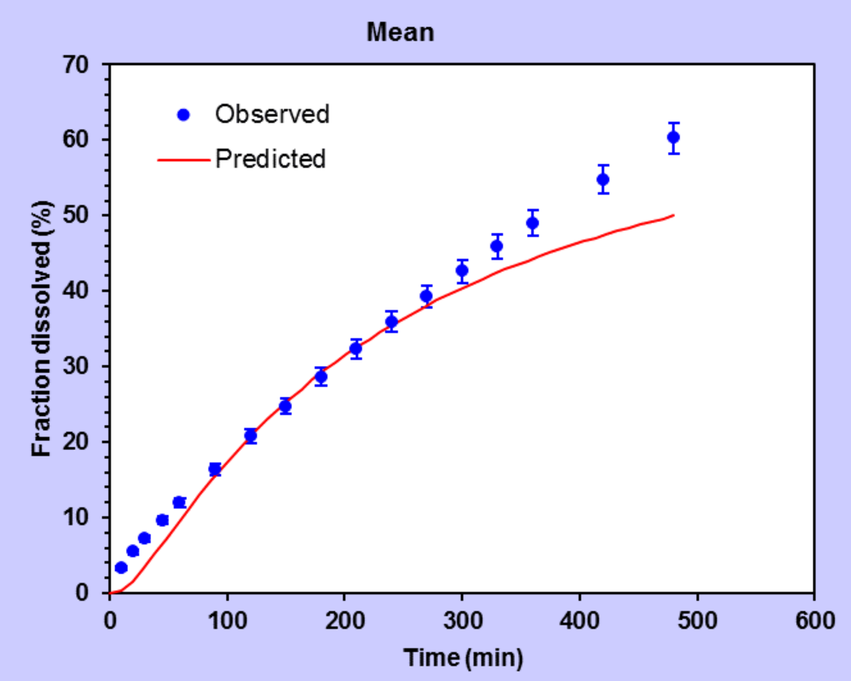

Supplementary materials – Model fitting summary – Granulated sugar N°1 600 (modelling up to app. 60 % of carvedilol released)

Graphical abstract of model fit presented as the fraction % of released carvedilol per tested tablet:

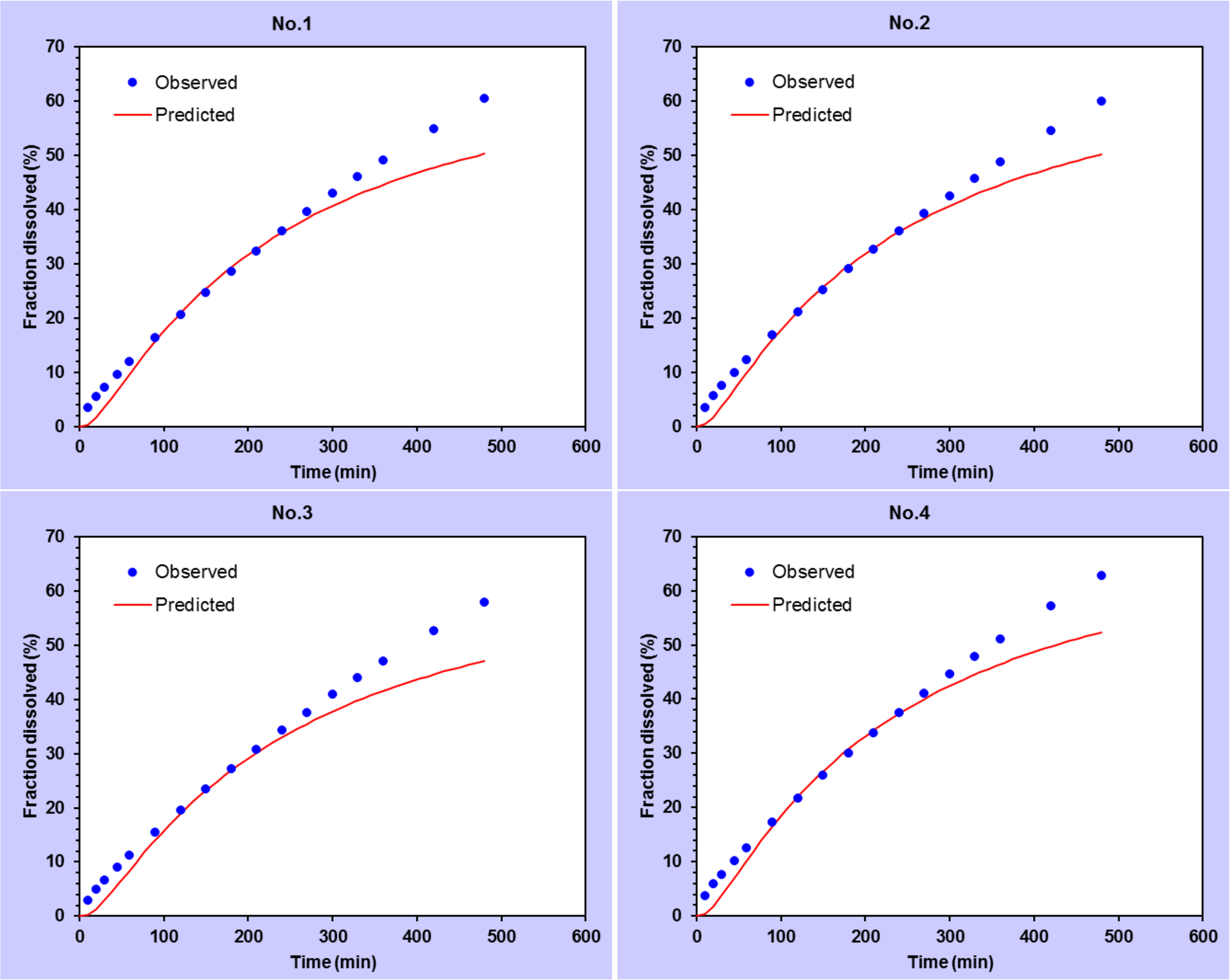

Supplement: Supplementary file 1 [file pharmaceutics-16-00498-s001.zip › Supplementary materials_Model fitting summary_Granulated sugar N°1 600.pdf]
