# Supplementary material for: Comparative Fitting of Mathematical Models to Carvedilol Release Profiles Obtained from Hypromellose Matrix Tablets
Source: Pharmaceutics. 2024 Apr 4;16(4):498. doi: 10.3390/pharmaceutics16040498 (PMC11053526; doi:10.3390/pharmaceutics16040498)

Supplementary materials – Model fitting summary – Kollidon® 25 (modelling the entire relevant dissolution data)

Model: **Zero–order**

Model equation: $F = k_0 \cdot t$

Fitted model parameters per tested tablet (N = 4) with statistics – mean, standard deviation (SD), and relative standard deviation expressed in % (RSD%) (output from DDSolver):

Parameter	No.1	No.2	No.3	No.4	Mean	SD	RSD(%)
k_0	0.224	0.248	0.210	0.240	0.230	0.017	7.209

Number of dissolution data points (N), degrees of freedom (df), and selected goodness of fit criteria – Pearson correlation coefficient (R), coefficient of determination (R^2), adjusted coefficient of determination ($R^2_{adjusted}$), and residual sum of squares (RSS) (manual calculation in MS Excel):

Parameter	No.1	No.2	No.3	No.4
N	20	20	20	20
df	19	19	19	19
R	0.915076966	0.672556835	0.941339455	0.626835756
R^2	0.837365854	0.452332696	0.88611997	0.392923065
$R^2_{adjusted}$	0.837365854	0.452332696	0.88611997	0.392923065
RSS	12301.14616	38376.89939	10324.12941	37627.27278

Graphical abstract of model fit presented as mean ± 1 SD of the fraction % of released carvedilol:

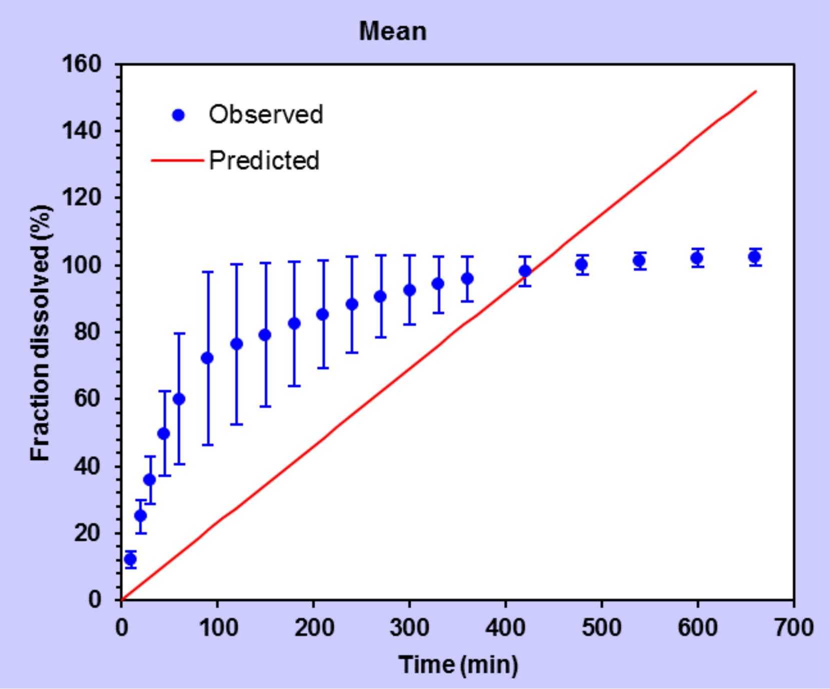

Supplementary materials – Model fitting summary – Kollidon® 25 (modelling the entire relevant dissolution data)

Graphical abstract of model fit presented as the fraction % of released carvedilol per tested tablet:

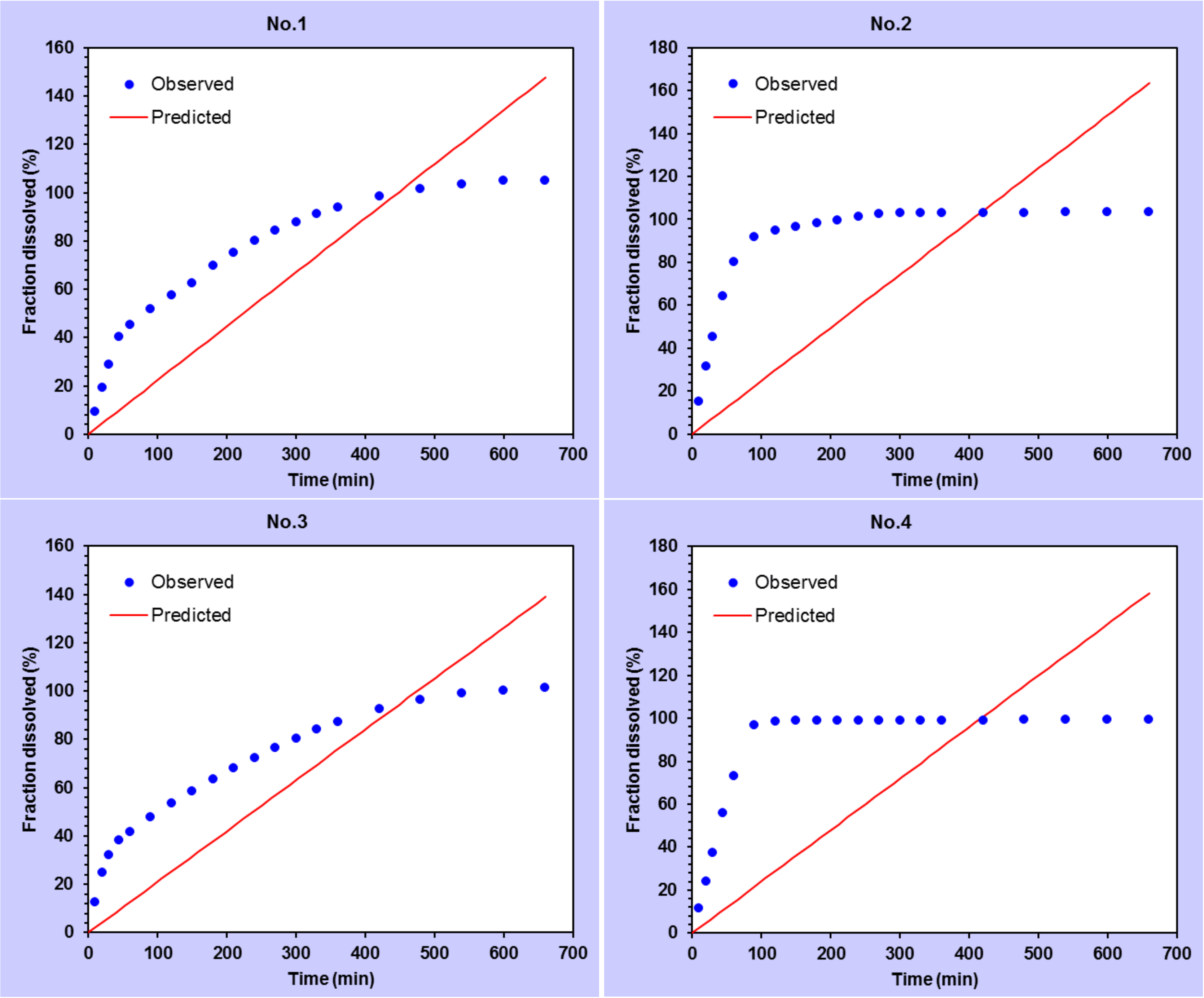

Supplementary materials – Model fitting summary – Kollidon® 25 (modelling the entire relevant dissolution data)

Model: **Zero–order with T_{lag}**

Model equation: $F = k_0 \cdot (t - T_{lag})$

Fitted model parameters per tested tablet (N = 4) with statistics – mean, standard deviation (SD), and relative standard deviation expressed in % (RSD%) (output from DDSolver):

Parameter	No.1	No.2	No.3	No.4	Mean	SD	RSD(%)
k_0	0.136	0.089	0.126	0.088	0.110	0.025	22.637
T_{lag}	-263.870	-725.875	-271.945	-702.177	-490.967	257.769	-52.502

Number of dissolution data points (N), degrees of freedom (df), and selected goodness of fit criteria – Pearson correlation coefficient (R), coefficient of determination (R^2), adjusted coefficient of determination ($R^2_{adjusted}$), and residual sum of squares (RSS) (manual calculation in MS Excel):

Parameter	No.1	No.2	No.3	No.4
N	20	20	20	20
df	18	18	18	18
R	0.915076966	0.672556835	0.941339455	0.626835756
R^2	0.837365854	0.452332696	0.88611997	0.392923065
$R^2_{adjusted}$	0.828330624	0.421906735	0.879793302	0.359196568
RSS	2767.689273	7406.789264	1581.247906	9253.600875

Graphical abstract of model fit presented as mean ± 1 SD of the fraction % of released carvedilol:

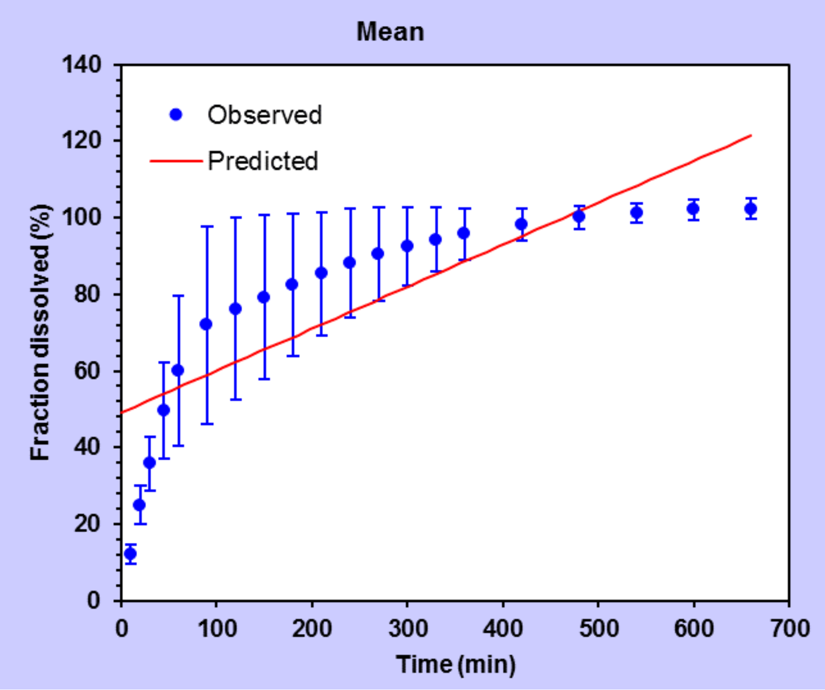

Supplementary materials – Model fitting summary – Kollidon® 25 (modelling the entire relevant dissolution data)

Graphical abstract of model fit presented as the fraction % of released carvedilol per tested tablet:

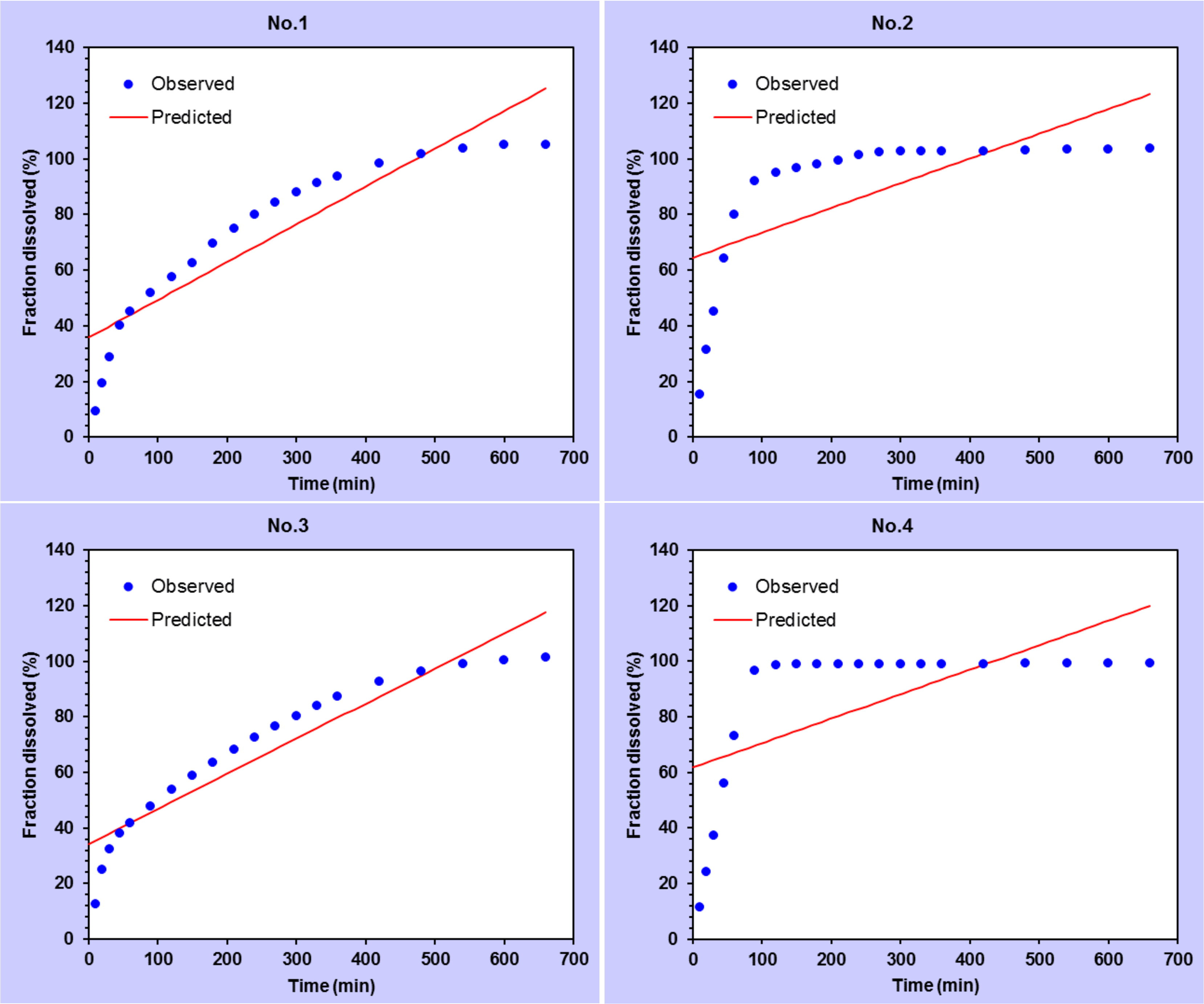

Supplementary materials – Model fitting summary – Kollidon® 25 (modelling the entire relevant dissolution data)

Model: **Zero–order with F_0**

Model equation: $F = F_0 + k_0 \cdot t$

Fitted model parameters per tested tablet (N = 4) with statistics – mean, standard deviation (SD), and relative standard deviation expressed in % (RSD%) (output from DDSolver):

Parameter	No.1	No.2	No.3	No.4	Mean	SD	RSD(%)
k_0	0.136	0.089	0.126	0.088	0.110	0.025	22.637
F_0	35.819	64.559	34.301	61.793	49.118	16.284	33.152

Number of dissolution data points (N), degrees of freedom (df), and selected goodness of fit criteria – Pearson correlation coefficient (R), coefficient of determination (R^2), adjusted coefficient of determination ($R^2_{adjusted}$), and residual sum of squares (RSS) (manual calculation in MS Excel):

Parameter	No.1	No.2	No.3	No.4
N	20	20	20	20
df	18	18	18	18
R	0.915076966	0.672556835	0.941339455	0.626835756
R^2	0.837365854	0.452332696	0.88611997	0.392923065
$R^2_{adjusted}$	0.828330624	0.421906735	0.879793302	0.359196568
RSS	2767.689273	7406.789264	1581.247906	9253.600875

Graphical abstract of model fit presented as mean ± 1 SD of the fraction % of released carvedilol:

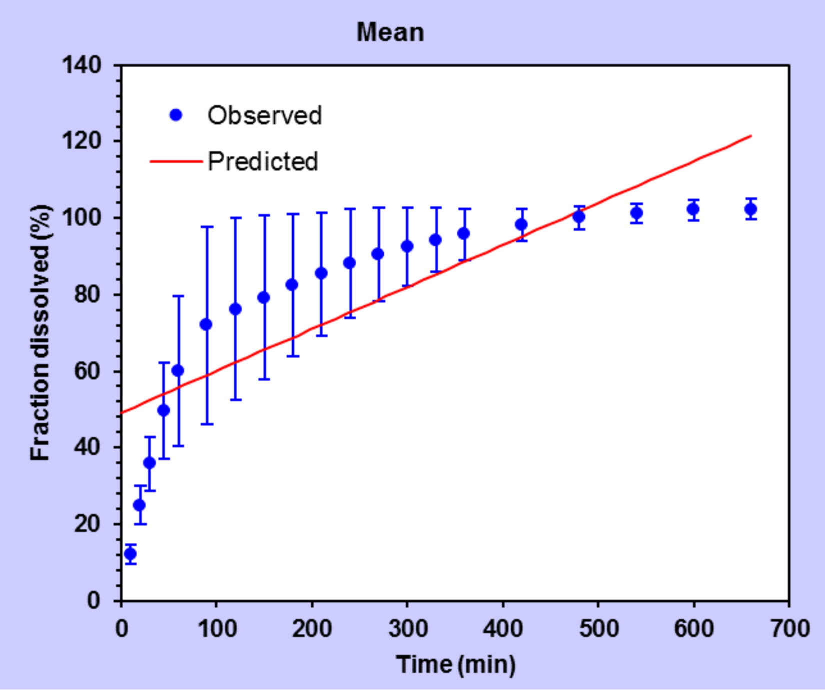

Supplementary materials – Model fitting summary – Kollidon® 25 (modelling the entire relevant dissolution data)

Graphical abstract of model fit presented as the fraction % of released carvedilol per tested tablet:

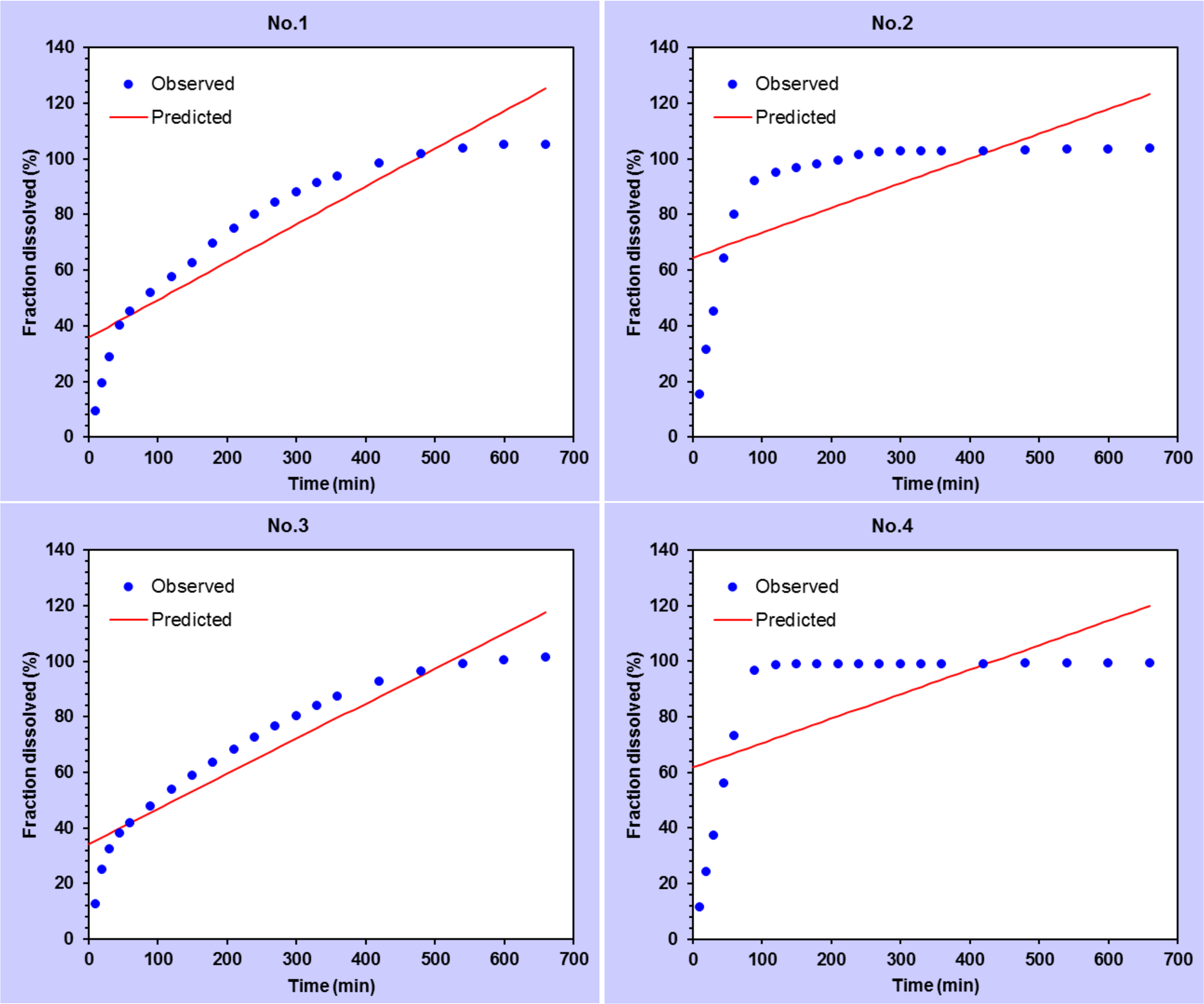

Supplementary materials – Model fitting summary – Kollidon® 25 (modelling the entire relevant dissolution data)

Model: **First–order**

Model equation: $F = 100 \cdot (1 - e^{-k_1 \cdot t})$

Fitted model parameters per tested tablet (N = 4) with statistics – mean, standard deviation (SD), and relative standard deviation expressed in % (RSD%) (output from DDSolver):

Parameter	No.1	No.2	No.3	No.4	Mean	SD	RSD(%)
k_1	0.008	0.024	0.007	0.023	0.015	0.009	61.340

Number of dissolution data points (N), degrees of freedom (df), and selected goodness of fit criteria – Pearson correlation coefficient (R), coefficient of determination (R^2), adjusted coefficient of determination ($R^2_{adjusted}$), and residual sum of squares (RSS) (manual calculation in MS Excel):

Parameter	No.1	No.2	No.3	No.4
N	20	20	20	20
df	19	19	19	19
R	0.986818213	0.997740842	0.984363724	0.992375358
R^2	0.973810185	0.995486789	0.968971941	0.984808851
$R^2_{adjusted}$	0.973810185	0.995486789	0.968971941	0.984808851
RSS	541.190565	232.3688376	912.2724542	585.2103023

Graphical abstract of model fit presented as mean ± 1 SD of the fraction % of released carvedilol:

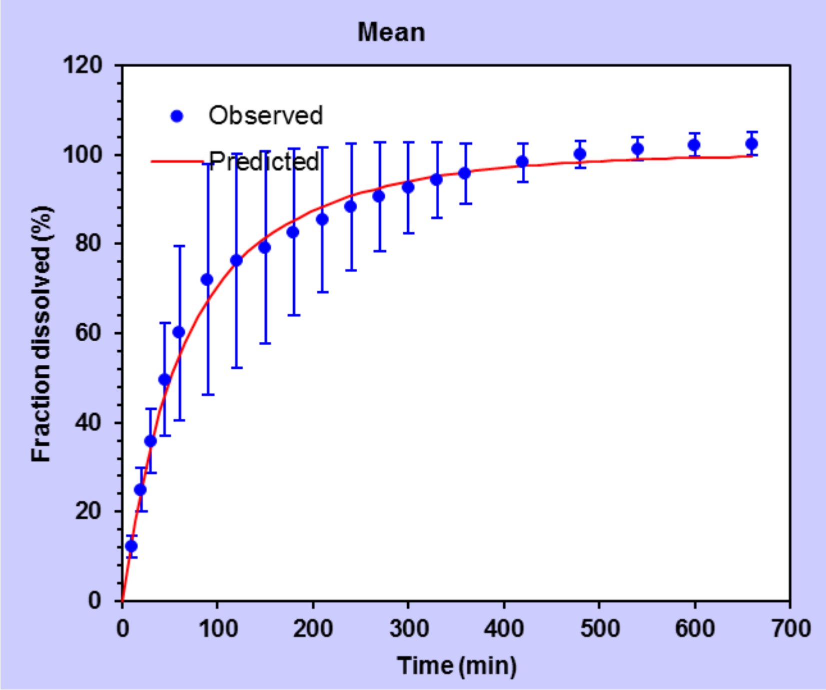

Supplementary materials – Model fitting summary – Kollidon® 25 (modelling the entire relevant dissolution data)

Graphical abstract of model fit presented as the fraction % of released carvedilol per tested tablet:

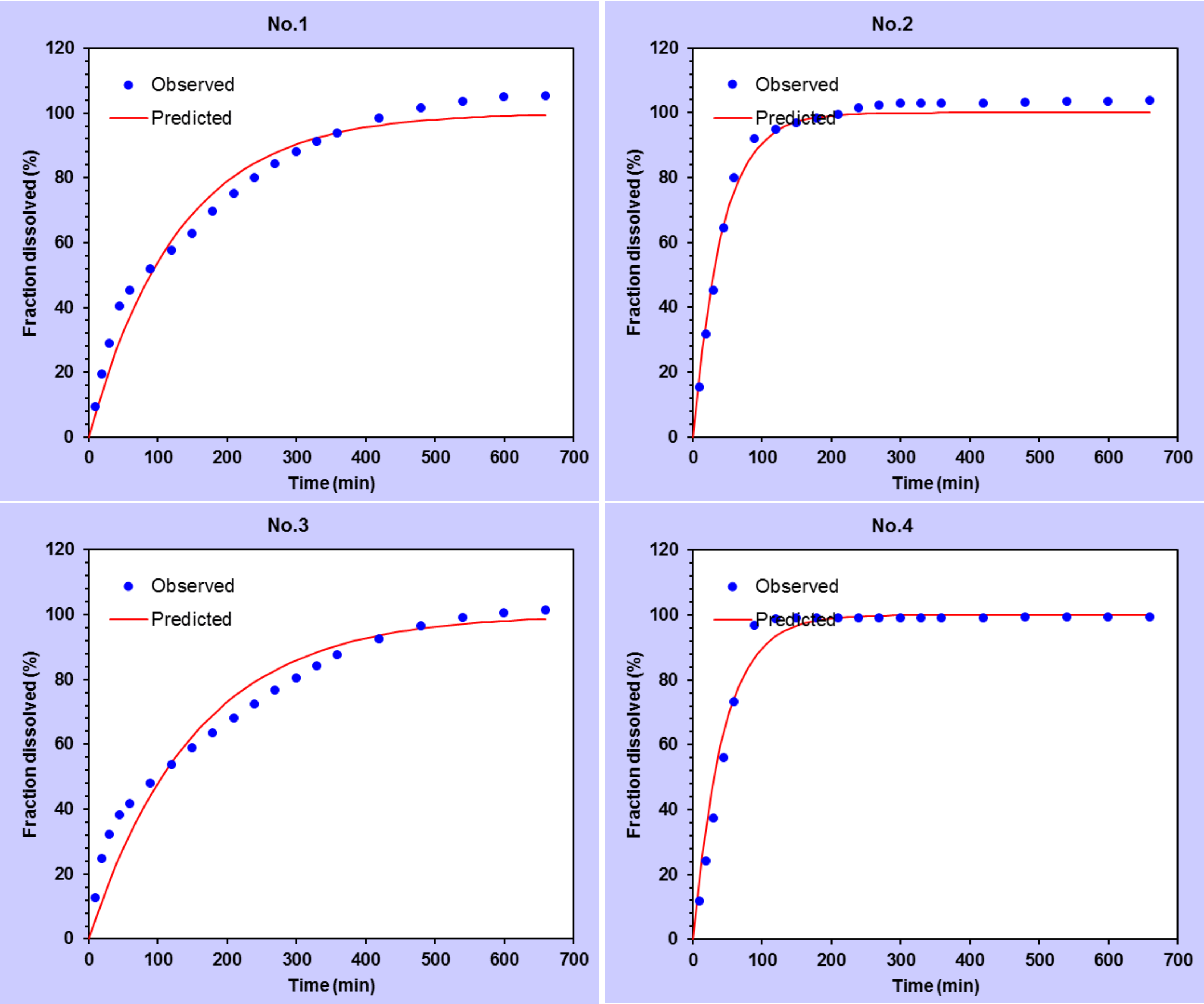

Supplementary materials – Model fitting summary – Kollidon® 25 (modelling the entire relevant dissolution data)

Model: **First–order with T_{lag}**

Model equation: $F = 100 \cdot \left[1 - e^{-k_1 \cdot (t - T_{lag})}\right]$

Fitted model parameters per tested tablet (N = 4) with statistics – mean, standard deviation (SD), and relative standard deviation expressed in % (RSD%) (output from DDSolver):

Parameter	No.1	No.2	No.3	No.4	Mean	SD	RSD(%)
k_1	/	/	/	/	/	/	/
T_{lag}	/	/	/	/	/	/	/

Number of dissolution data points (N), degrees of freedom (df), and selected goodness of fit criteria – Pearson correlation coefficient (R), coefficient of determination (R^2), adjusted coefficient of determination ($R^2_{adjusted}$), and residual sum of squares (RSS) (manual calculation in MS Excel):

Parameter	No.1	No.2	No.3	No.4
N	/	/	/	/
df	/	/	/	/
R	/	/	/	/
R^2	/	/	/	/
$R^2_{adjusted}$	/	/	/	/
RSS	/	/	/	/

Graphical abstract of model fit presented as mean ± 1 SD of the fraction % of released carvedilol: /

Graphical abstract of model fit presented as the fraction % of released carvedilol per tested tablet: /

Note: the model could not be fitted

Supplementary materials – Model fitting summary – Kollidon® 25 (modelling the entire relevant dissolution data)

Model: **First–order with F_{max}**

Model equation: $F = F_{max} \cdot (1 - e^{-k_1 \cdot t})$

Fitted model parameters per tested tablet (N = 4) with statistics – mean, standard deviation (SD), and relative standard deviation expressed in % (RSD%) (output from DDSolver):

Parameter	No.1	No.2	No.3	No.4	Mean	SD	RSD(%)
k_1	0.005	0.021	0.005	0.011	0.011	0.008	71.903
F_{max}	110.460	108.780	106.365	104.370	107.494	2.676	2.490

Number of dissolution data points (N), degrees of freedom (df), and selected goodness of fit criteria – Pearson correlation coefficient (R), coefficient of determination (R^2), adjusted coefficient of determination ($R^2_{adjusted}$), and residual sum of squares (RSS) (manual calculation in MS Excel):

Parameter	No.1	No.2	No.3	No.4
N	20	20	20	20
df	18	18	18	18
R	0.994010452	0.996412602	0.992976967	0.937339928
R^2	0.988056778	0.992838074	0.986003258	0.878606141
$R^2_{adjusted}$	0.987393266	0.992440189	0.985225661	0.871862038
RSS	844.1055036	673.2943238	1251.130824	2937.295199

Graphical abstract of model fit presented as mean ± 1 SD of the fraction % of released carvedilol:

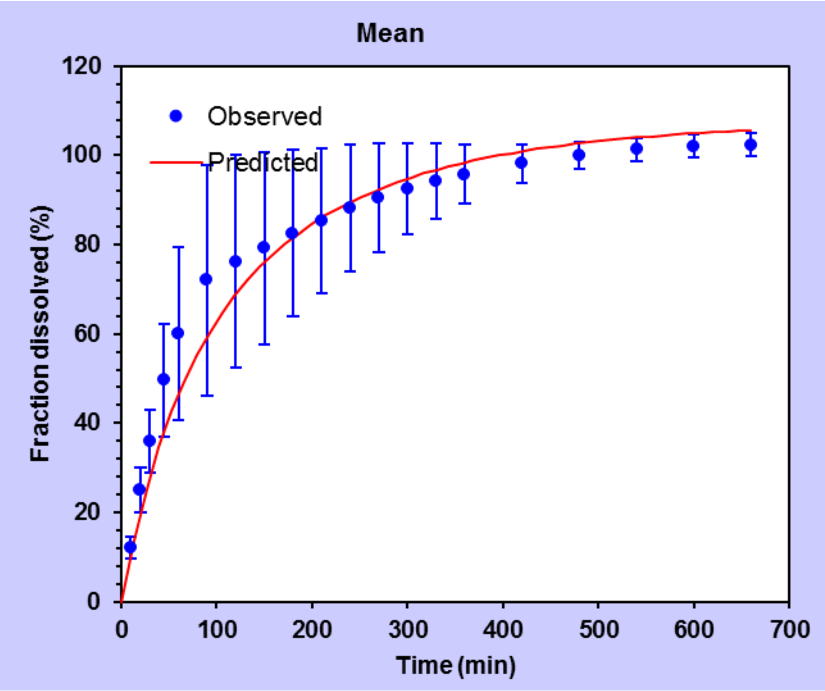

Supplementary materials – Model fitting summary – Kollidon® 25 (modelling the entire relevant dissolution data)

Graphical abstract of model fit presented as the fraction % of released carvedilol per tested tablet:

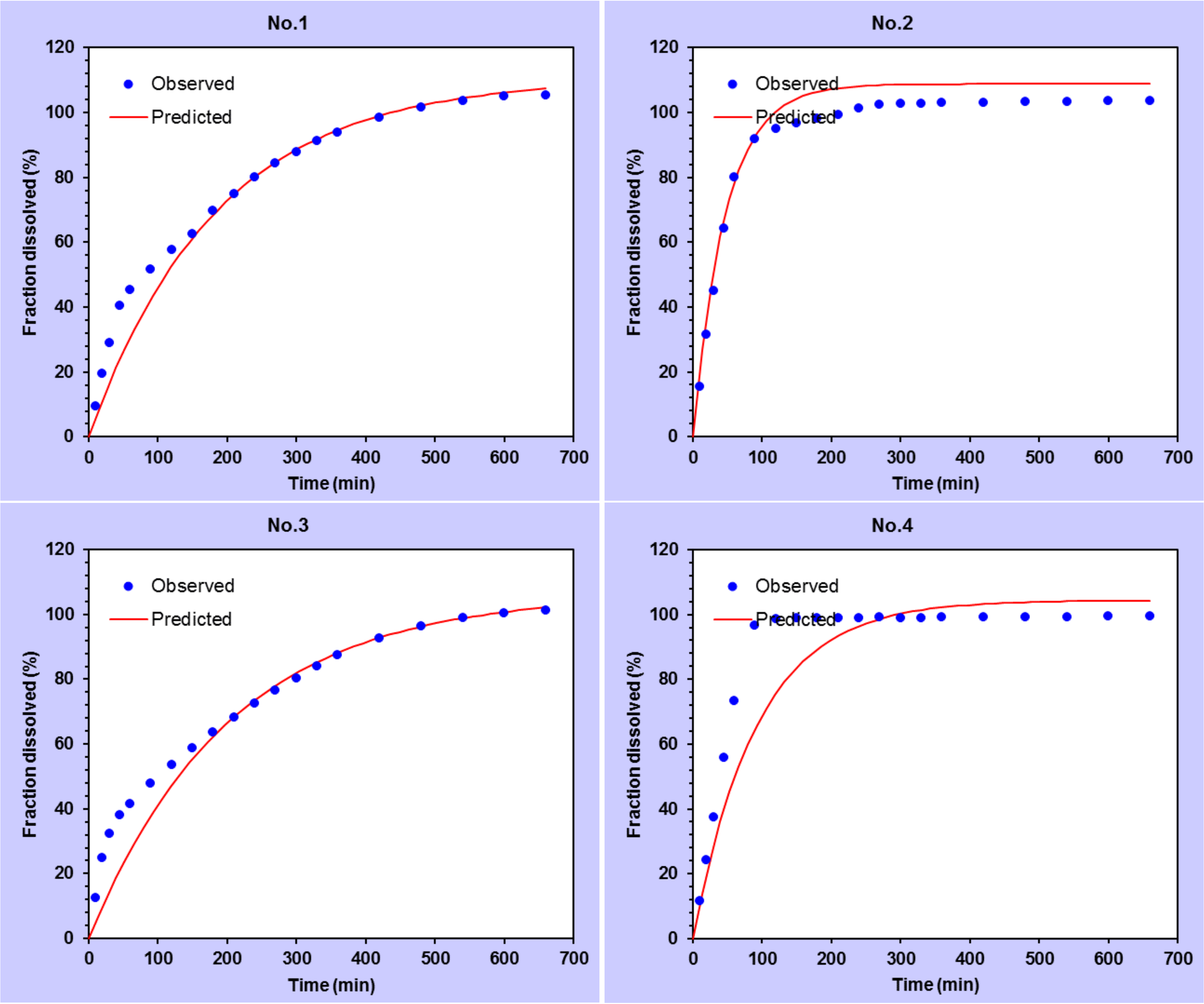

Supplementary materials – Model fitting summary – Kollidon® 25 (modelling the entire relevant dissolution data)

Model: **First–order with T_{lag} and F_{max}**

Model equation: $F = F_{max} \cdot \left[1 - e^{-k_1 \cdot (t - T_{lag})}\right]$

Fitted model parameters per tested tablet (N = 4) with statistics – mean, standard deviation (SD), and relative standard deviation expressed in % (RSD%) (output from DDSolver):

Parameter	No.1	No.2	No.3	No.4	Mean	SD	RSD(%)
k_1	0.005	0.004	0.004	0.004	0.004	0.000	10.270
T_{lag}	-36.562	-282.147	-34.468	-378.716	-182.973	174.777	-95.520
F_{max}	110.460	108.780	106.365	104.370	107.494	2.676	2.490

Number of dissolution data points (N), degrees of freedom (df), and selected goodness of fit criteria – Pearson correlation coefficient (R), coefficient of determination (R^2), adjusted coefficient of determination ($R^2_{adjusted}$), and residual sum of squares (RSS) (manual calculation in MS Excel):

Parameter	No.1	No.2	No.3	No.4
N	20	20	20	20
df	17	17	17	17
R	0.99321857	0.837549709	0.994000352	0.790870846
R^2	0.986483127	0.701489516	0.9880367	0.625476695
$R^2_{adjusted}$	0.984892907	0.666370635	0.986629253	0.58141513
RSS	256.1433791	7285.762153	184.5609697	11101.88591

Graphical abstract of model fit presented as mean ± 1 SD of the fraction % of released carvedilol:

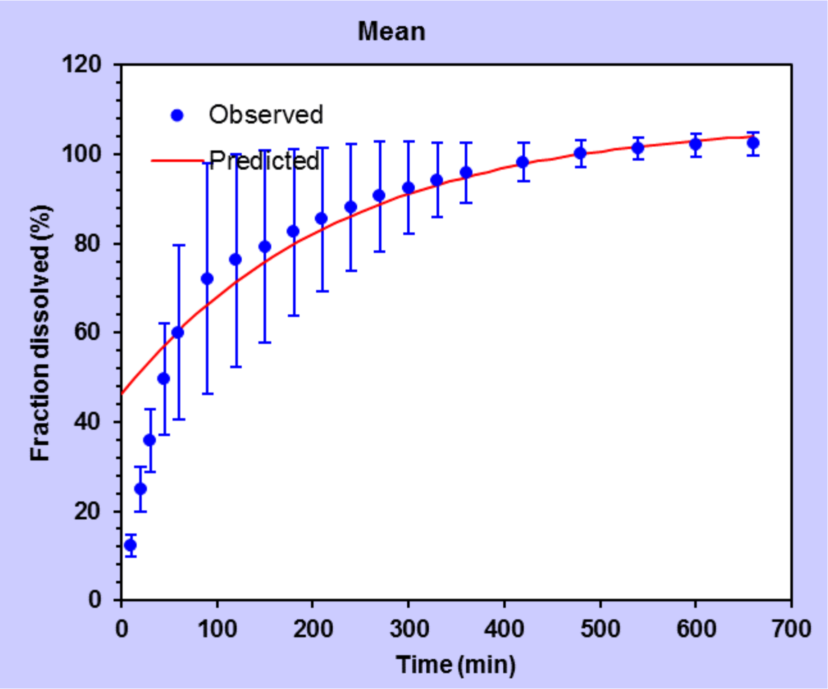

Supplementary materials – Model fitting summary – Kollidon® 25 (modelling the entire relevant dissolution data)

Graphical abstract of model fit presented as the fraction % of released carvedilol per tested tablet:

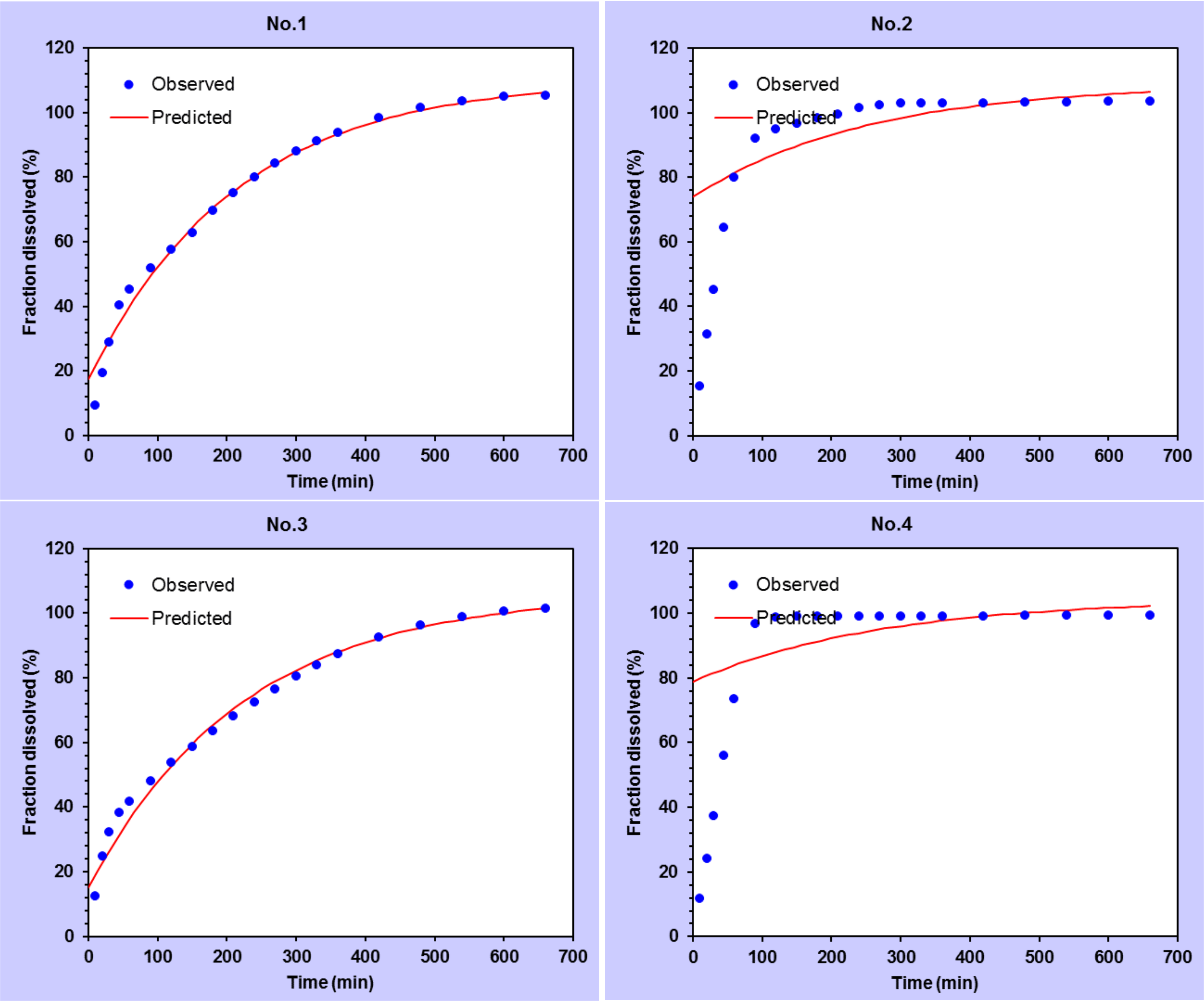

Supplementary materials – Model fitting summary – Kollidon® 25 (modelling the entire relevant dissolution data)

Model: **Higuchi**

Model equation: $F = k_H \cdot t^{0.5}$

Fitted model parameters per tested tablet (N = 4) with statistics – mean, standard deviation (SD), and relative standard deviation expressed in % (RSD%) (output from DDSolver):

Parameter	No.1	No.2	No.3	No.4	Mean	SD	RSD(%)
k_H	4.753	5.506	4.463	5.341	5.016	0.490	9.773

Number of dissolution data points (N), degrees of freedom (df), and selected goodness of fit criteria – Pearson correlation coefficient (R), coefficient of determination (R^2), adjusted coefficient of determination ($R^2_{adjusted}$), and residual sum of squares (RSS) (manual calculation in MS Excel):

Parameter	No.1	No.2	No.3	No.4
N	20	20	20	20
df	19	19	19	19
R	0.97917479	0.806065647	0.990171999	0.766925231
R^2	0.95878327	0.649741827	0.980440588	0.588174311
$R^2_{adjusted}$	0.95878327	0.649741827	0.980440588	0.588174311
RSS	966.8930753	10915.17027	628.0163556	11477.01044

Graphical abstract of model fit presented as mean ± 1 SD of the fraction % of released carvedilol:

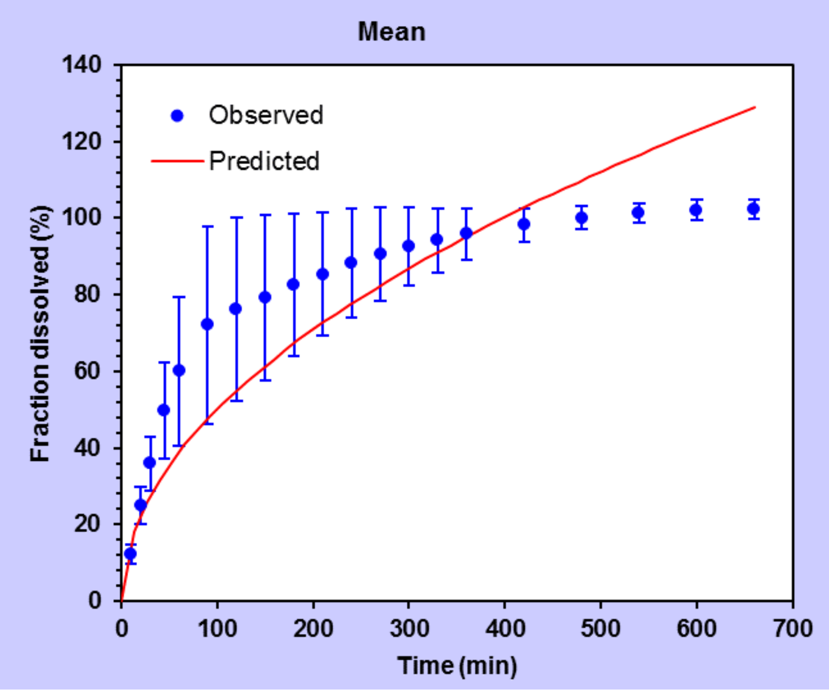

Supplementary materials – Model fitting summary – Kollidon® 25 (modelling the entire relevant dissolution data)

Graphical abstract of model fit presented as the fraction % of released carvedilol per tested tablet:

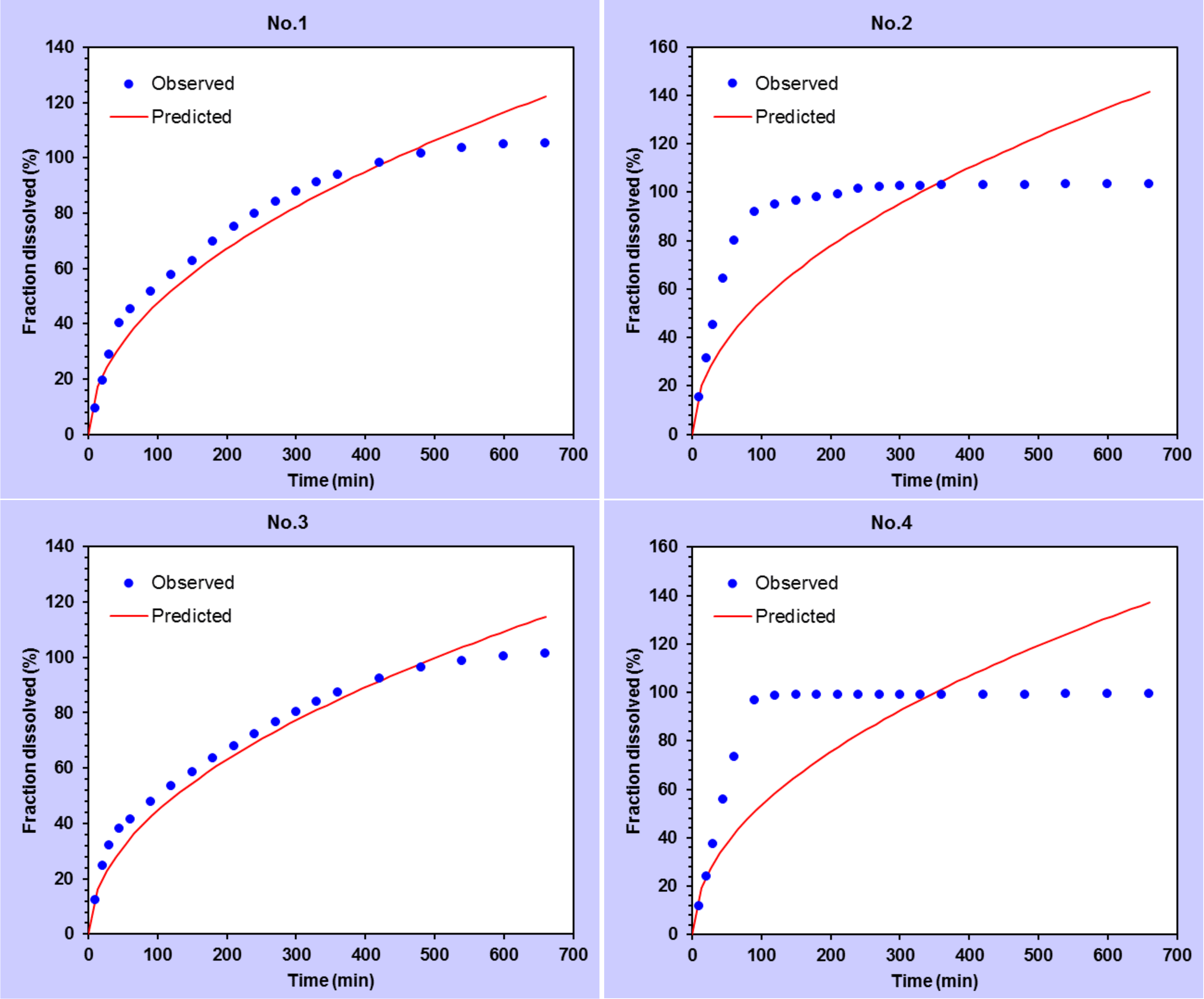

Supplementary materials – Model fitting summary – Kollidon® 25 (modelling the entire relevant dissolution data)

Model: **Higuchi with T_{lag}**

Model equation: $F = k_H \cdot (t - T_{lag})^{0.5}$

Fitted model parameters per tested tablet (N = 4) with statistics – mean, standard deviation (SD), and relative standard deviation expressed in % (RSD%) (output from DDSolver):

Parameter	No.1	No.2	No.3	No.4	Mean	SD	RSD(%)
k_H	4.274	3.571	4.064	3.392	3.825	0.413	10.788
T_{lag}	-63.173	-394.983	-54.475	-428.124	-235.189	204.128	-86.793

Number of dissolution data points (N), degrees of freedom (df), and selected goodness of fit criteria – Pearson correlation coefficient (R), coefficient of determination (R^2), adjusted coefficient of determination ($R^2_{adjusted}$), and residual sum of squares (RSS) (manual calculation in MS Excel):

Parameter	No.1	No.2	No.3	No.4
N	20	20	20	20
df	18	18	18	18
R	0.962562136	0.710891547	0.979797081	0.664500629
R^2	0.926525866	0.505366791	0.960002321	0.441561085
$R^2_{adjusted}$	0.922443969	0.477887169	0.957780227	0.410536701
RSS	1659.088446	7335.887667	714.944527	9435.213367

Graphical abstract of model fit presented as mean ± 1 SD of the fraction % of released carvedilol:

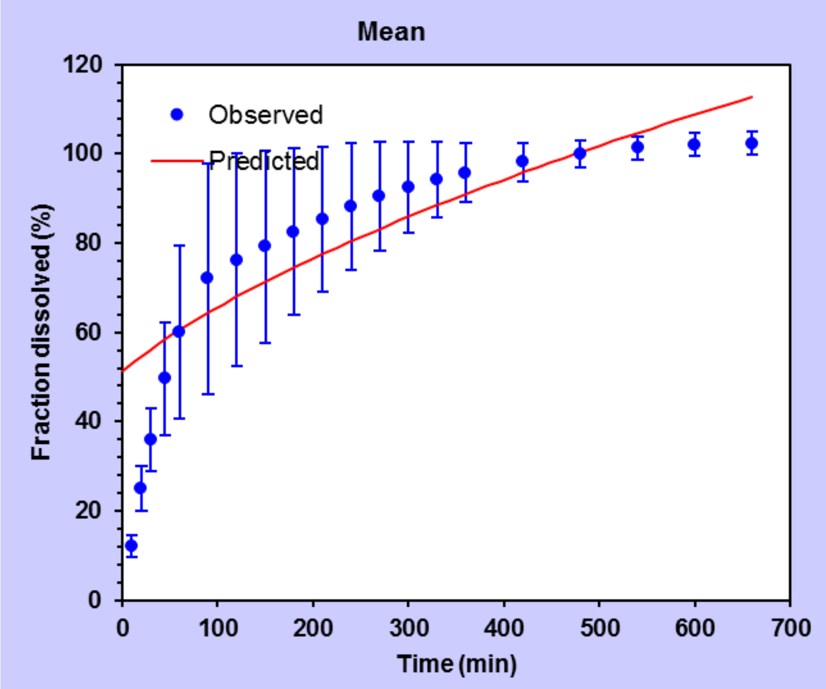

Supplementary materials – Model fitting summary – Kollidon® 25 (modelling the entire relevant dissolution data)

Graphical abstract of model fit presented as the fraction % of released carvedilol per tested tablet:

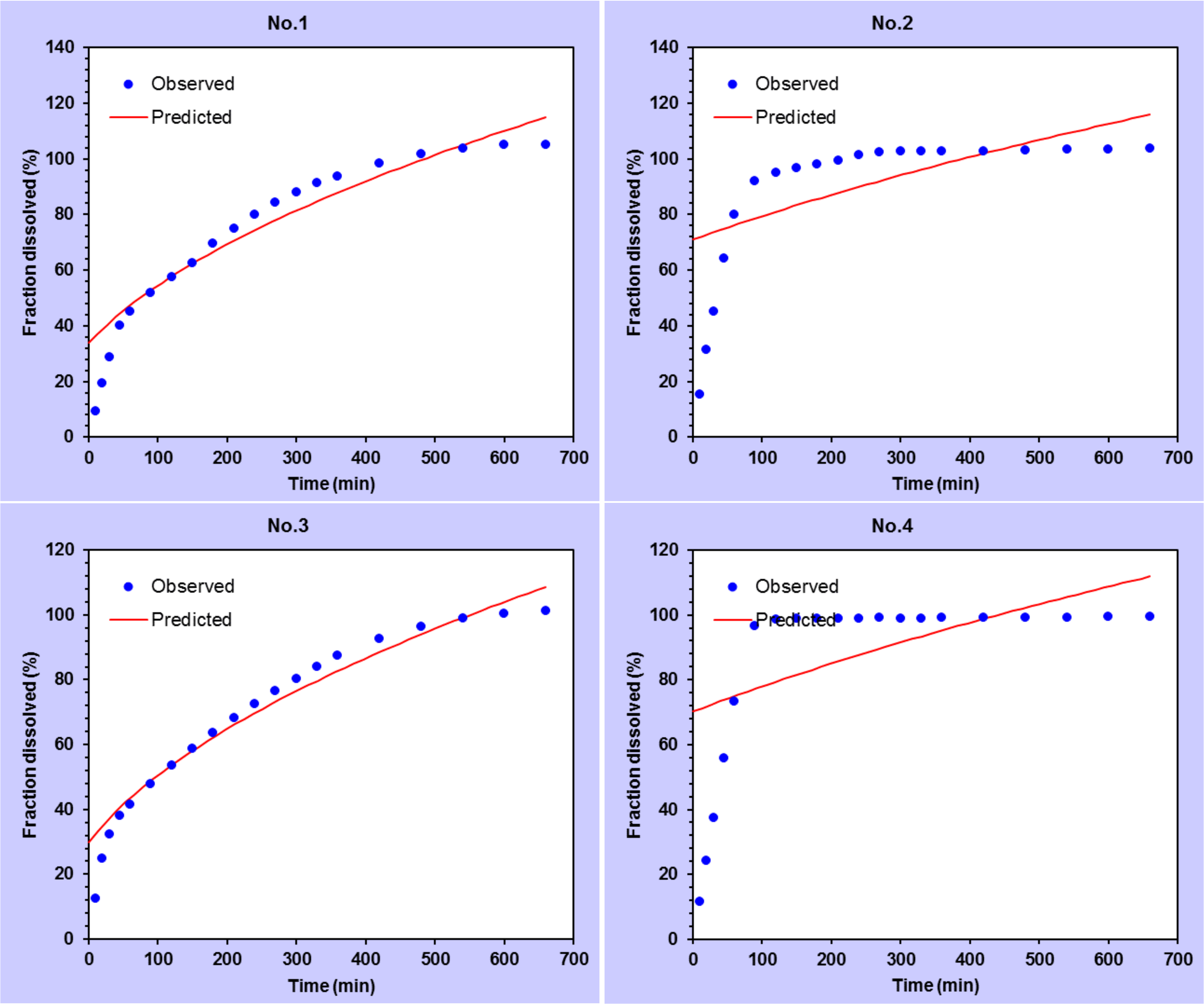

Supplementary materials – Model fitting summary – Kollidon® 25 (modelling the entire relevant dissolution data)

Model: **Higuchi with F_0**

Model equation: $F = F_0 + k_H \cdot t^{0.5}$

Fitted model parameters per tested tablet (N = 4) with statistics – mean, standard deviation (SD), and relative standard deviation expressed in % (RSD%) (output from DDSolver):

Parameter	No.1	No.2	No.3	No.4	Mean	SD	RSD(%)
k_H	4.260	3.126	3.891	3.158	3.609	0.560	15.512
F_0	8.689	41.919	10.069	38.456	24.783	17.852	72.034

Number of dissolution data points (N), degrees of freedom (df), and selected goodness of fit criteria – Pearson correlation coefficient (R), coefficient of determination (R^2), adjusted coefficient of determination ($R^2_{adjusted}$), and residual sum of squares (RSS) (manual calculation in MS Excel):

Parameter	No.1	No.2	No.3	No.4
N	20	20	20	20
df	18	18	18	18
R	0.97917479	0.806065647	0.990171999	0.766925231
R^2	0.95878327	0.649741827	0.980440588	0.588174311
$R^2_{adjusted}$	0.956493451	0.63028304	0.979353954	0.565295106
RSS	701.4215995	4736.978922	271.5865049	6277.409564

Graphical abstract of model fit presented as mean ± 1 SD of the fraction % of released carvedilol:

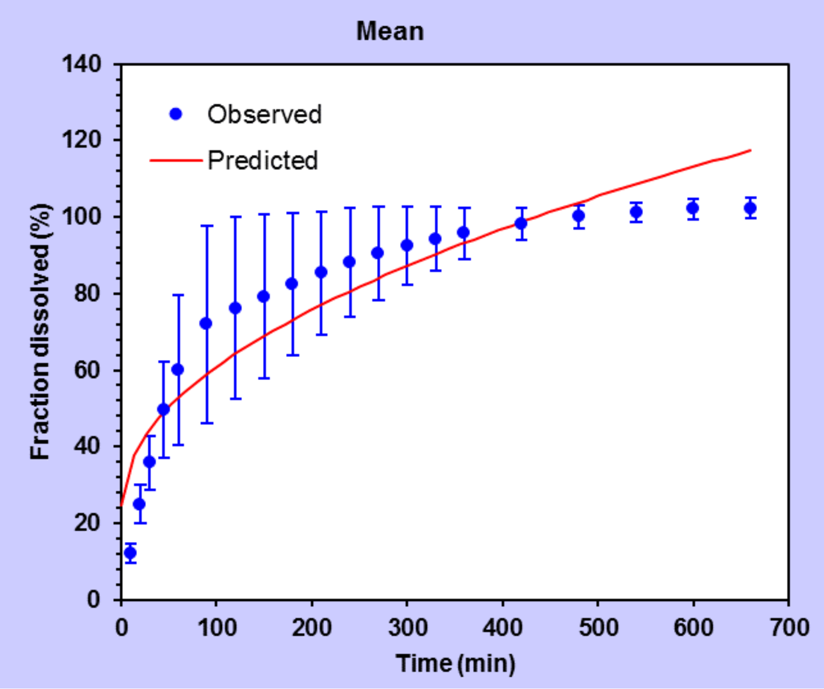

Supplementary materials – Model fitting summary – Kollidon® 25 (modelling the entire relevant dissolution data)

Graphical abstract of model fit presented as the fraction % of released carvedilol per tested tablet:

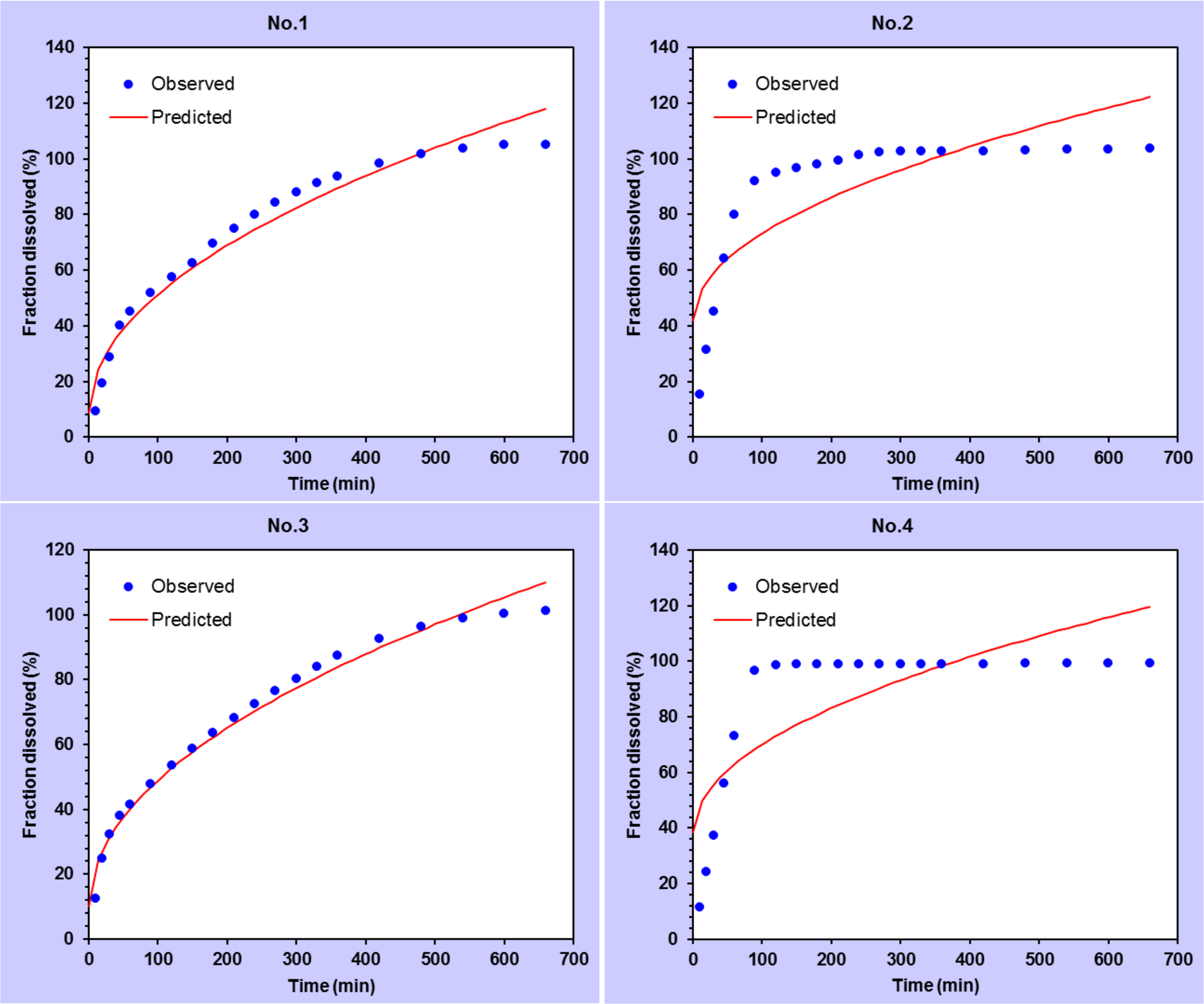

Supplementary materials – Model fitting summary – Kollidon® 25 (modelling the entire relevant dissolution data)

Model: **Korsmeyer–Peppas**

Model equation: $F = k_{KP} \cdot t^n$

Fitted model parameters per tested tablet (N = 4) with statistics – mean, standard deviation (SD), and relative standard deviation expressed in % (RSD%) (output from DDSolver):

Parameter	No.1	No.2	No.3	No.4	Mean	SD	RSD(%)
k_{KP}	5.103	8.225	5.791	12.257	7.844	3.233	41.209
n	0.492	0.294	0.462	0.369	0.404	0.090	22.368

Number of dissolution data points (N), degrees of freedom (df), and selected goodness of fit criteria – Pearson correlation coefficient (R), coefficient of determination (R^2), adjusted coefficient of determination ($R^2_{adjusted}$), and residual sum of squares (RSS) (manual calculation in MS Excel):

Parameter	No.1	No.2	No.3	No.4
N	20	20	20	20
df	18	18	18	18
R	0.979895748	0.862329041	0.99224043	0.805408536
R^2	0.960195677	0.743611376	0.98454107	0.648682911
$R^2_{adjusted}$	0.957984326	0.729367563	0.983682241	0.629165294
RSS	967.9886176	53701.56341	491.1404119	7023.364461

Graphical abstract of model fit presented as mean ± 1 SD of the fraction % of released carvedilol:

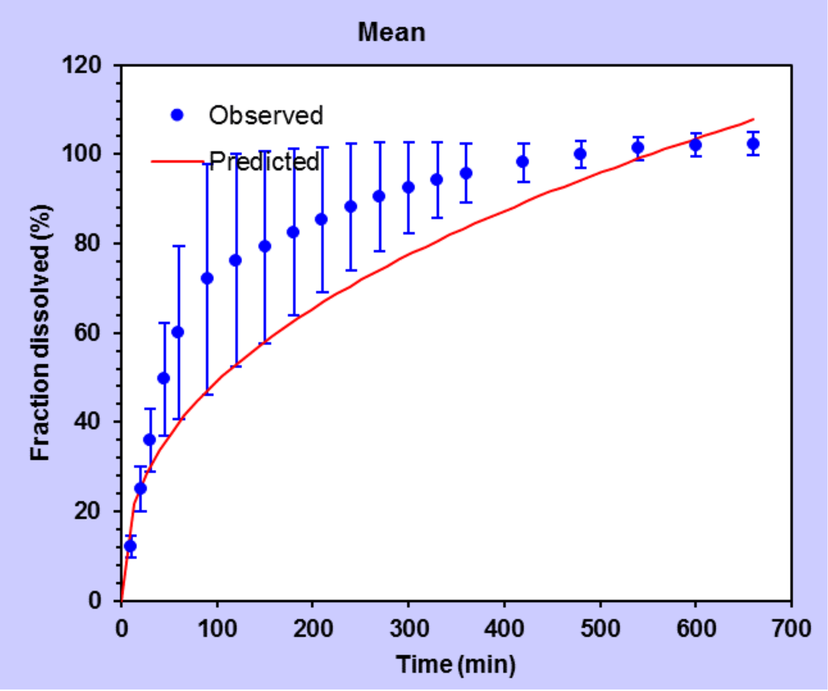

Supplementary materials – Model fitting summary – Kollidon® 25 (modelling the entire relevant dissolution data)

Graphical abstract of model fit presented as the fraction % of released carvedilol per tested tablet:

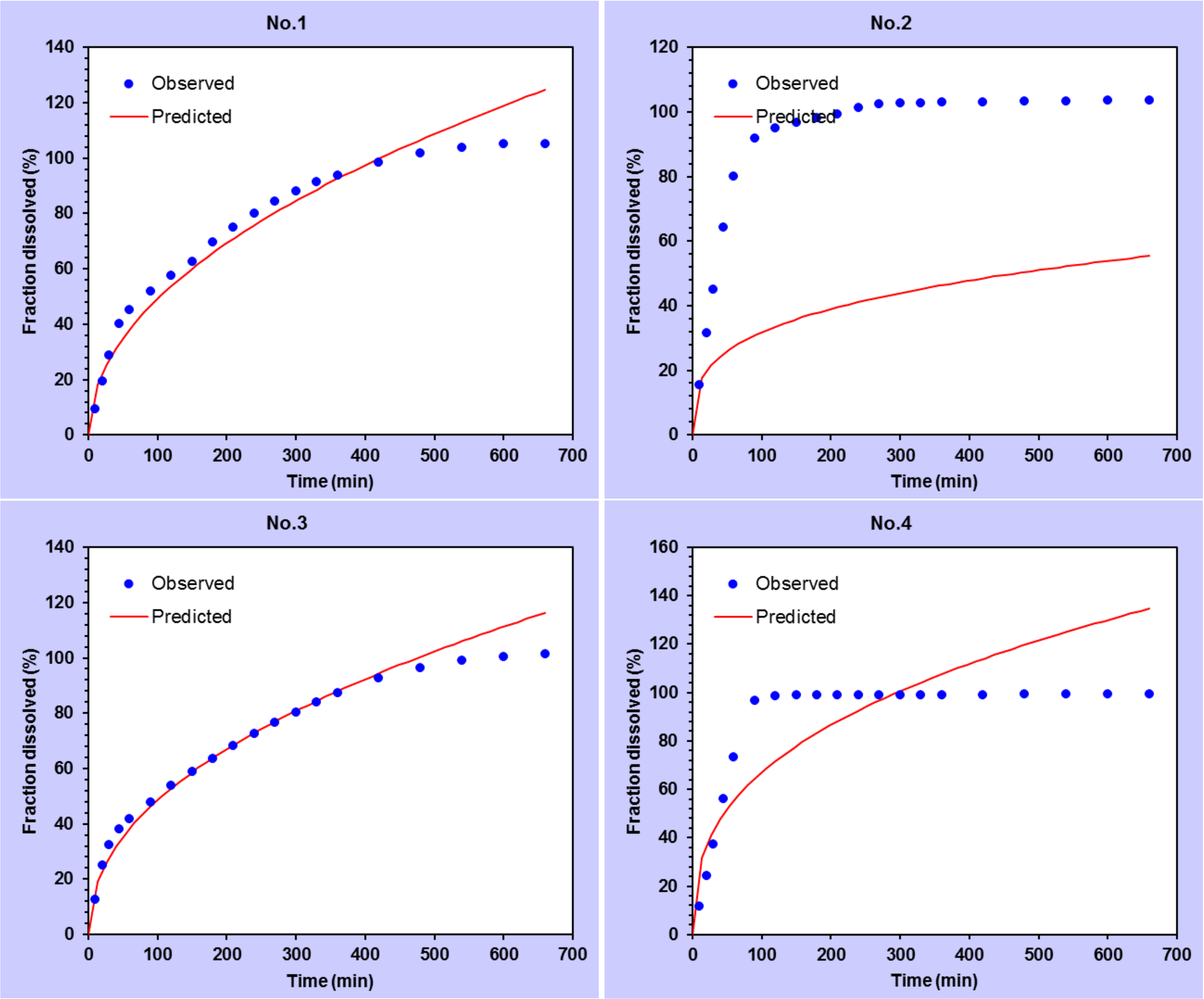

Supplementary materials – Model fitting summary – Kollidon® 25 (modelling the entire relevant dissolution data)

Model: **Korsmeyer–Peppas with T_{lag}**

Model equation: $F = k_{KP} \cdot (t - T_{lag})^n$

Fitted model parameters per tested tablet (N = 4) with statistics – mean, standard deviation (SD), and relative standard deviation expressed in % (RSD%) (output from DDSolver):

Parameter	No.1	No.2	No.3	No.4	Mean	SD	RSD(%)
k_{KP}	5.467	14.131	7.347	10.260	9.301	3.775	40.591
n	0.483	0.347	0.418	0.399	0.412	0.056	13.612
T_{lag}	4.000	4.000	4.000	4.000	4.000	0.000	0.000

Number of dissolution data points (N), degrees of freedom (df), and selected goodness of fit criteria – Pearson correlation coefficient (R), coefficient of determination (R^2), adjusted coefficient of determination ($R^2_{adjusted}$), and residual sum of squares (RSS) (manual calculation in MS Excel):

Parameter	No.1	No.2	No.3	No.4
N	20	20	20	20
df	17	17	17	17
R	0.982854646	0.857813689	0.995392538	0.805748397
R^2	0.966003255	0.735844326	0.990806305	0.64923048
$R^2_{adjusted}$	0.962003638	0.704767187	0.989724694	0.607963477
RSS	983.2007717	5172.20602	176.728776	7974.737406

Graphical abstract of model fit presented as mean ± 1 SD of the fraction % of released carvedilol:

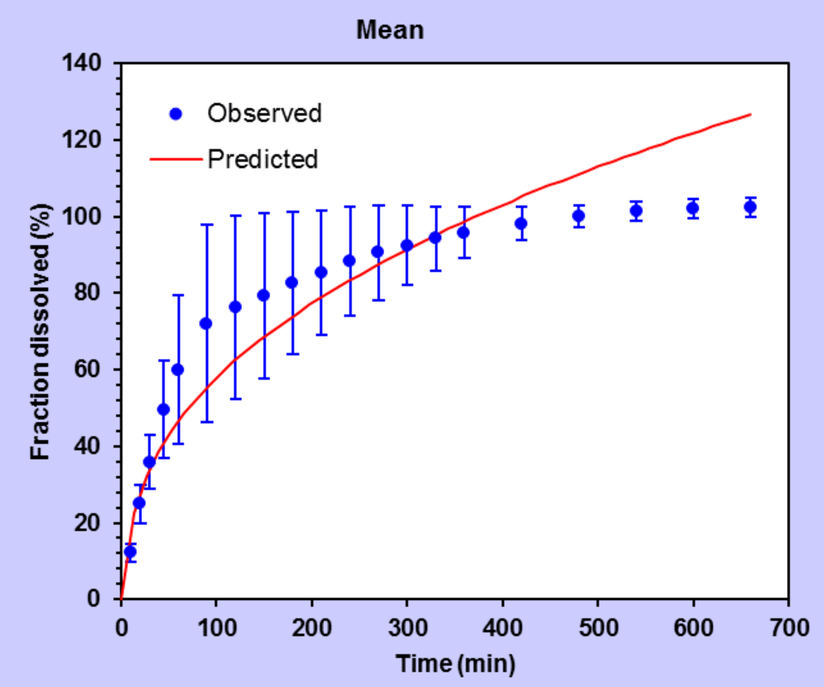

Supplementary materials – Model fitting summary – Kollidon® 25 (modelling the entire relevant dissolution data)

Graphical abstract of model fit presented as the fraction % of released carvedilol per tested tablet:

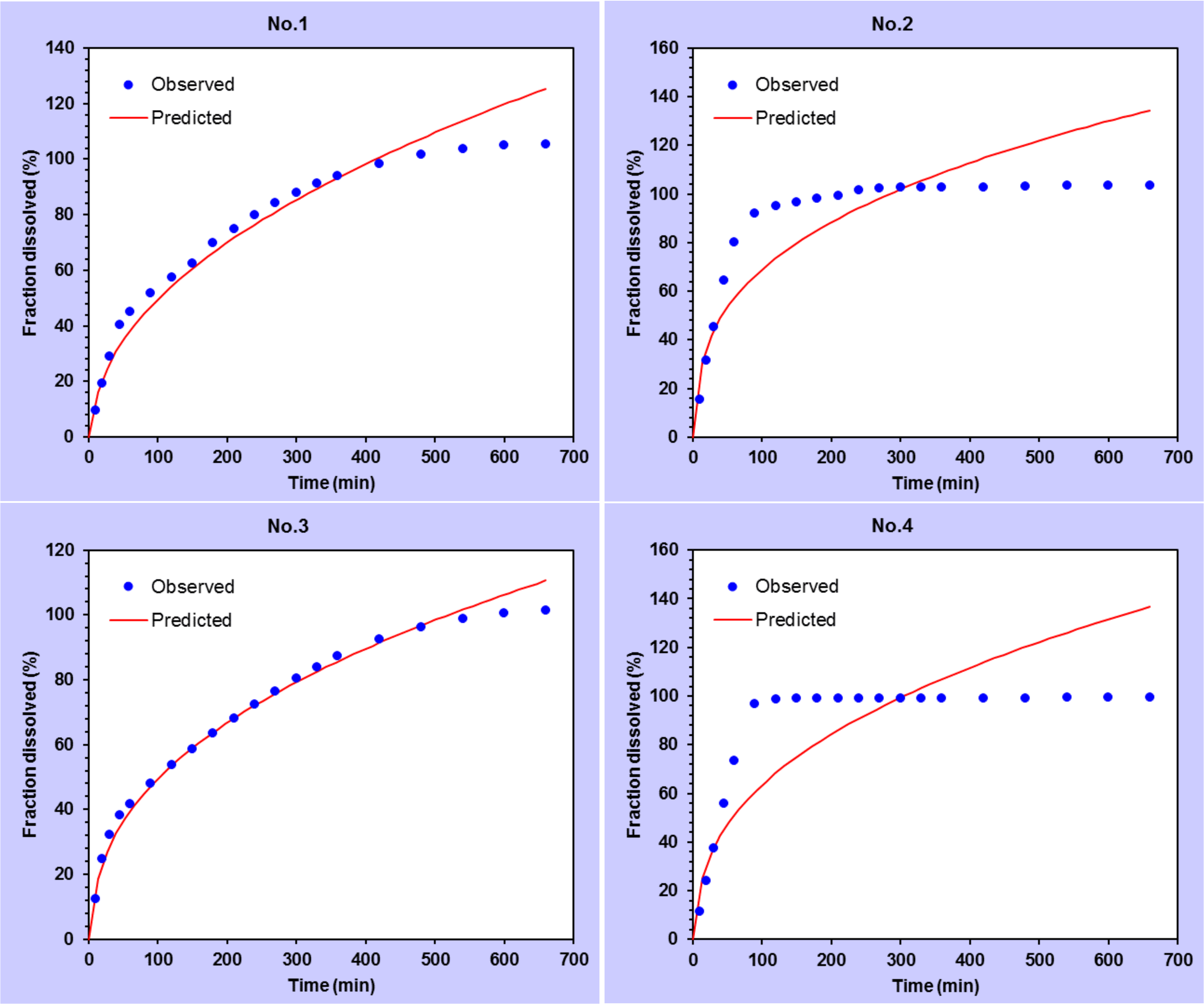

Supplementary materials – Model fitting summary – Kollidon® 25 (modelling the entire relevant dissolution data)

Model: **Korsmeyer–Peppas with F_0**

Model equation: $F = F_0 + k_{KP} \cdot t^n$

Fitted model parameters per tested tablet (N = 4) with statistics – mean, standard deviation (SD), and relative standard deviation expressed in % (RSD%) (output from DDSolver):

Parameter	No.1	No.2	No.3	No.4	Mean	SD	RSD(%)
k_{KP}	2.817	8.137	3.757	5.823	5.133	2.363	46.041
n	0.590	0.432	0.523	0.489	0.509	0.066	13.006
F_0	3.760	6.120	5.000	4.640	4.880	0.977	20.022

Number of dissolution data points (N), degrees of freedom (df), and selected goodness of fit criteria – Pearson correlation coefficient (R), coefficient of determination (R^2), adjusted coefficient of determination ($R^2_{adjusted}$), and residual sum of squares (RSS) (manual calculation in MS Excel):

Parameter	No.1	No.2	No.3	No.4
N	20	20	20	20
df	17	17	17	17
R	0.96991793	0.824886767	0.988746391	0.770217068
R^2	0.940740791	0.680438179	0.977619427	0.593234331
$R^2_{adjusted}$	0.93376912	0.64284267	0.974986418	0.545379547
RSS	2124.477585	7131.785848	589.4120706	10571.38191

Graphical abstract of model fit presented as mean ± 1 SD of the fraction % of released carvedilol:

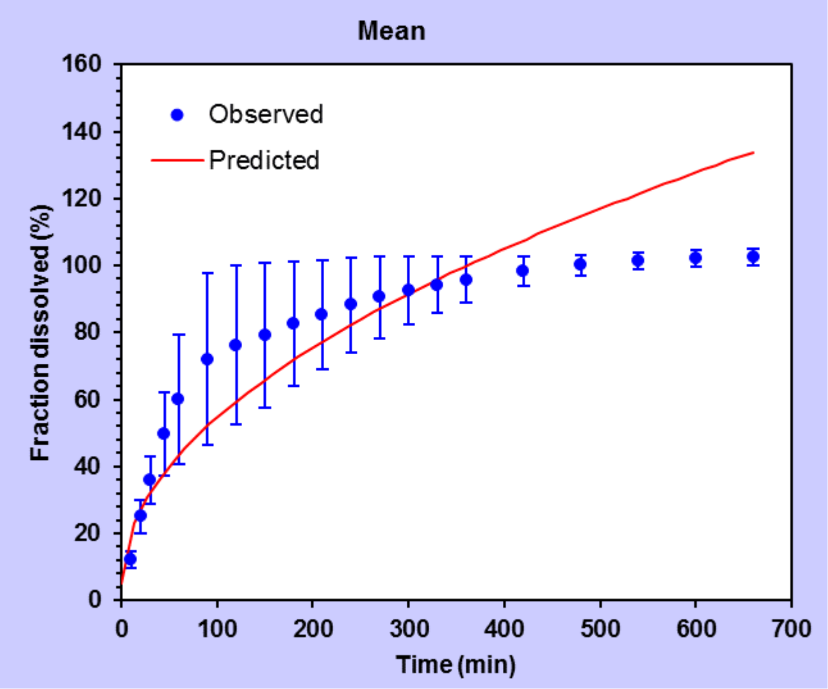

Supplementary materials – Model fitting summary – Kollidon® 25 (modelling the entire relevant dissolution data)

Graphical abstract of model fit presented as the fraction % of released carvedilol per tested tablet:

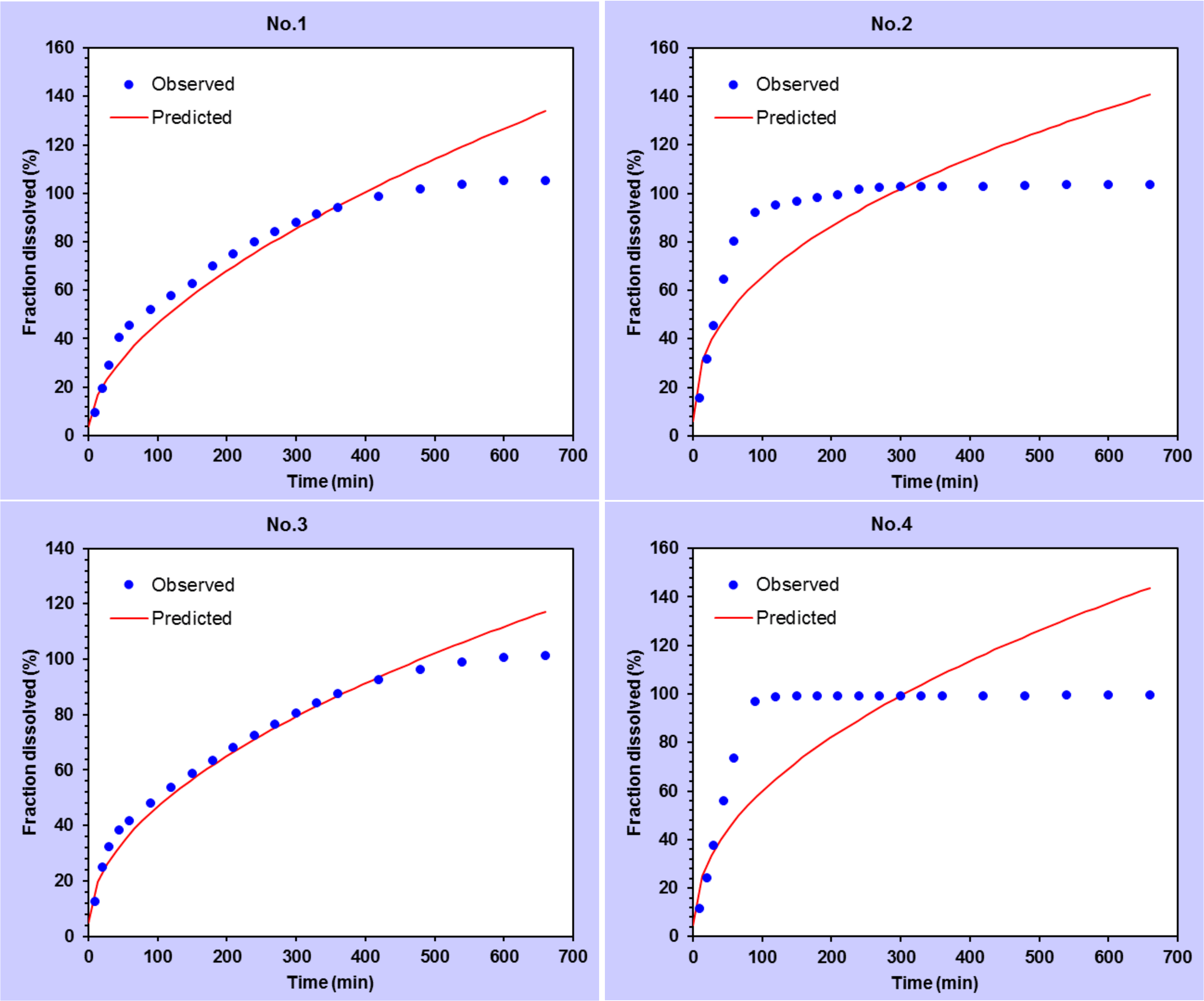

Supplementary materials – Model fitting summary – Kollidon® 25 (modelling the entire relevant dissolution data)

Model: **Hixson–Crowell**

Model equation: $F = 100 \cdot [1 - (1 - k_{HC} \cdot t)^3]$

Fitted model parameters per tested tablet (N = 4) with statistics – mean, standard deviation (SD), and relative standard deviation expressed in % (RSD%) (output from DDSolver):

Parameter	No.1	No.2	No.3	No.4	Mean	SD	RSD(%)
k_{HC}	0.002	0.002	0.001	0.003	0.002	0.001	29.989

Number of dissolution data points (N), degrees of freedom (df), and selected goodness of fit criteria – Pearson correlation coefficient (R), coefficient of determination (R^2), adjusted coefficient of determination ($R^2_{adjusted}$), and residual sum of squares (RSS) (manual calculation in MS Excel):

Parameter	No.1	No.2	No.3	No.4
N	20	20	20	20
df	19	19	19	19
R	0.989942015	0.908295408	0.991797564	0.8226958
R^2	0.979985193	0.825000548	0.983662407	0.67682838
$R^2_{adjusted}$	0.979985193	0.825000548	0.983662407	0.67682838
RSS	1176.629813	8981.942031	1826.800999	11106.76785

Graphical abstract of model fit presented as mean ± 1 SD of the fraction % of released carvedilol:

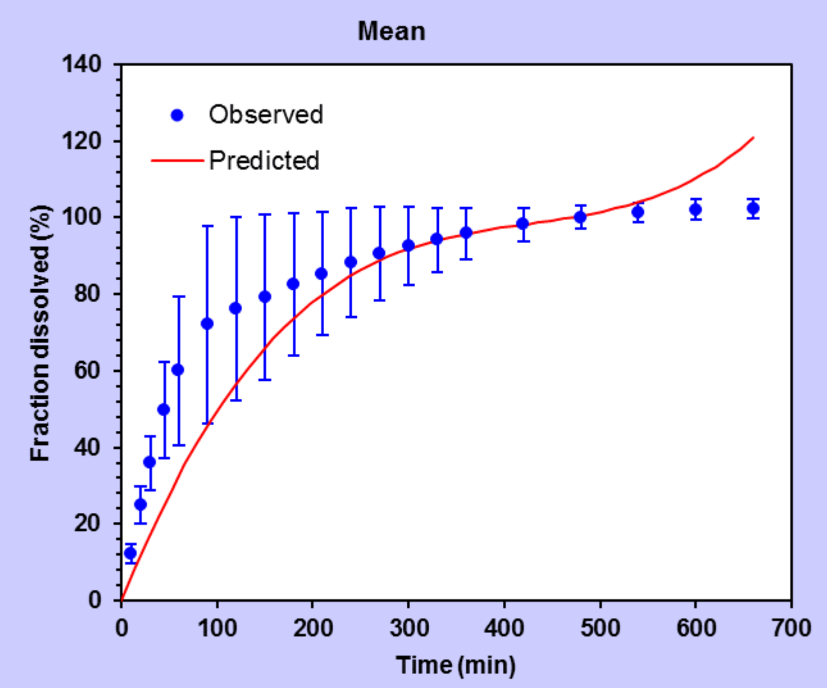

Supplementary materials – Model fitting summary – Kollidon® 25 (modelling the entire relevant dissolution data)

Graphical abstract of model fit presented as the fraction % of released carvedilol per tested tablet:

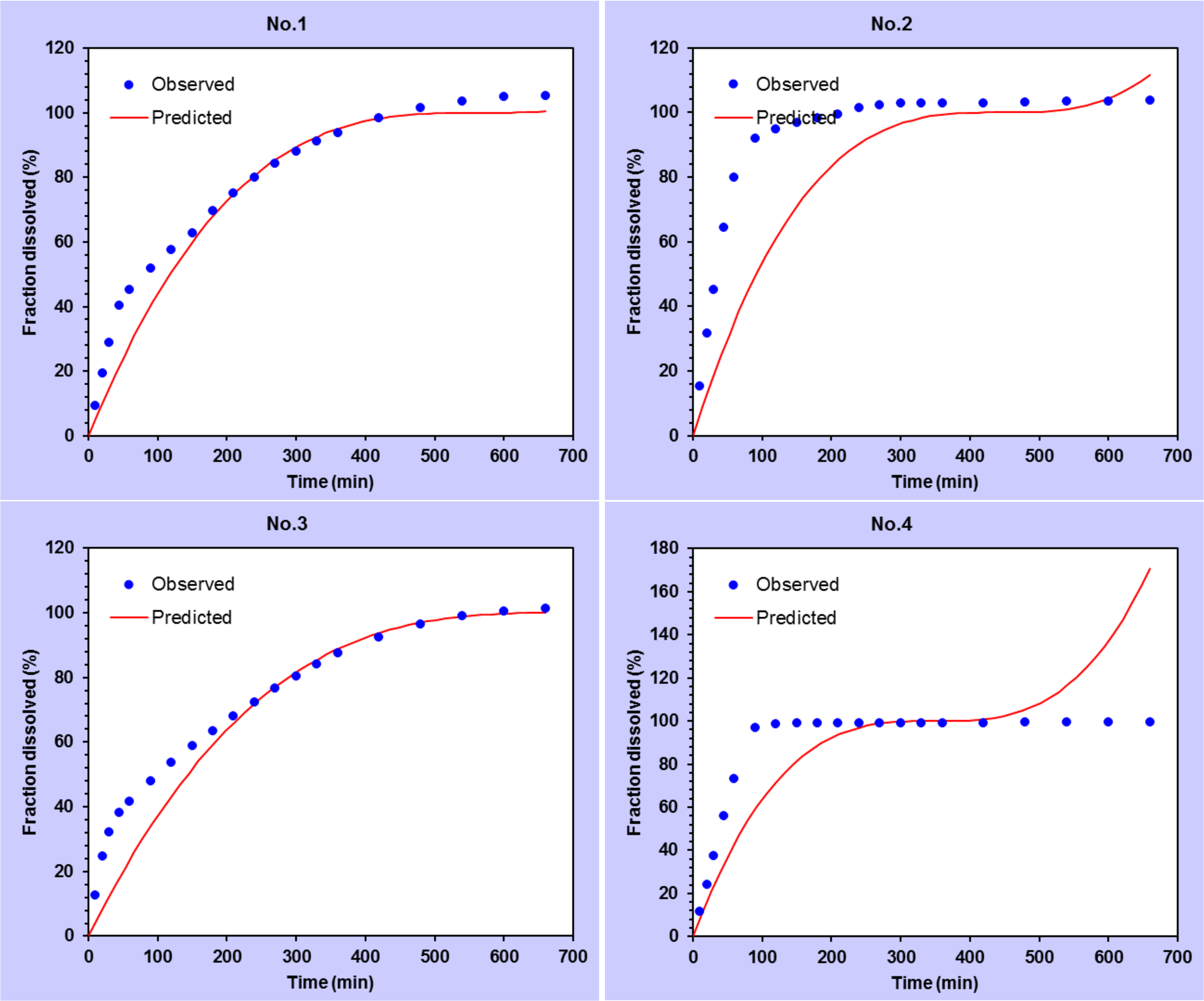

Supplementary materials – Model fitting summary – Kollidon® 25 (modelling the entire relevant dissolution data)

Model: **Hixson–Crowell with T_{lag}**

Model equation: $F = 100 \cdot \left\{1 - \left[1 - k_{HC} \cdot \left(t - T_{lag}\right)\right]^3\right\}$

Fitted model parameters per tested tablet (N = 4) with statistics – mean, standard deviation (SD), and relative standard deviation expressed in % (RSD%) (output from DDSolver):

Parameter	No.1	No.2	No.3	No.4	Mean	SD	RSD(%)
k_{HC}	0.002	0.000	0.001	0.001	0.001	0.001	71.308
T_{lag}	-35.443	-48.862	-54.179	-394.936	-133.355	174.565	-130.903

Number of dissolution data points (N), degrees of freedom (df), and selected goodness of fit criteria – Pearson correlation coefficient (R), coefficient of determination (R^2), adjusted coefficient of determination ($R^2_{adjusted}$), and residual sum of squares (RSS) (manual calculation in MS Excel):

Parameter	No.1	No.2	No.3	No.4
N	20	20	20	20
df	18	18	18	18
R	0.991160412	0.694109079	0.992918577	0.827136372
R^2	0.982398963	0.481787413	0.985887301	0.684154578
$R^2_{adjusted}$	0.981421127	0.452997825	0.985103262	0.66660761
RSS	327.1812215	165967.51	199.0357911	10326.72463

Graphical abstract of model fit presented as mean ± 1 SD of the fraction % of released carvedilol:

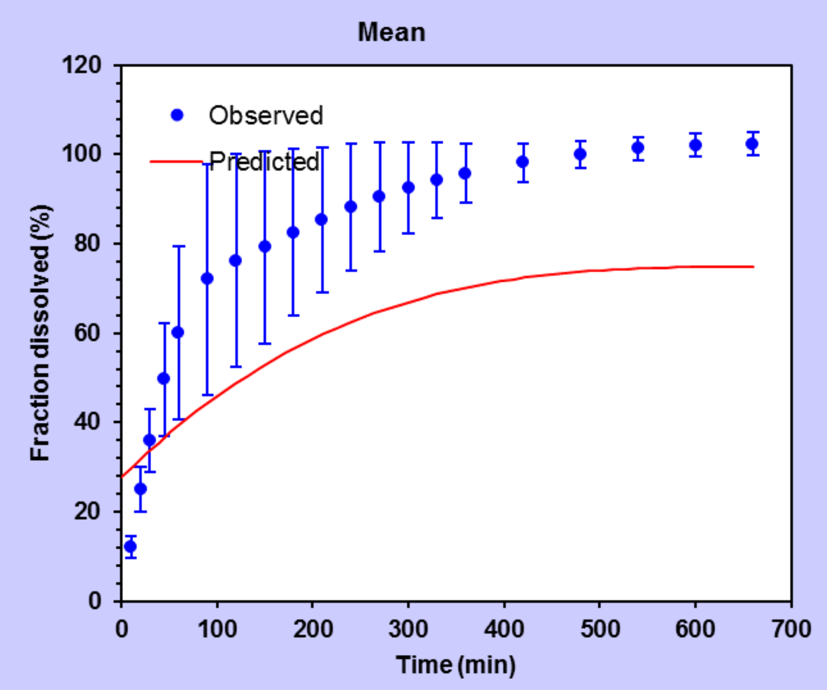

Supplementary materials – Model fitting summary – Kollidon® 25 (modelling the entire relevant dissolution data)

Graphical abstract of model fit presented as the fraction % of released carvedilol per tested tablet:

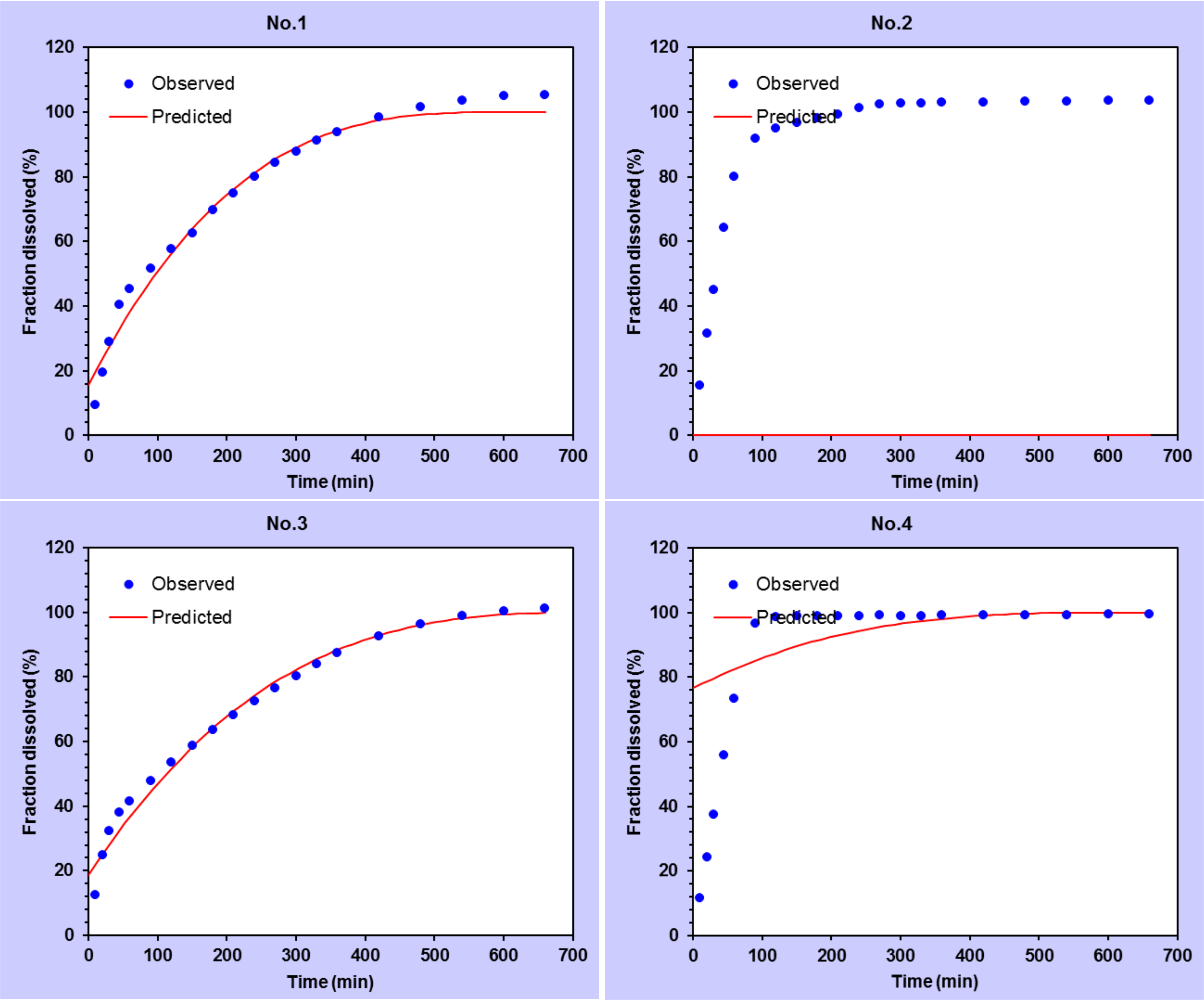

Supplementary materials – Model fitting summary – Kollidon® 25 (modelling the entire relevant dissolution data)

Model: **Hopfenberg**

Model equation: $F = 100 \cdot [1 - (1 - k_{HB} \cdot t)^n]$

Fitted model parameters per tested tablet (N = 4) with statistics – mean, standard deviation (SD), and relative standard deviation expressed in % (RSD%) (output from DDSolver):

Parameter	No.1	No.2	No.3	No.4	Mean	SD	RSD(%)
k_{HB}	0.002	0.005	0.001	0.003	0.003	0.001	52.485
n	4.500	4.500	4.500	3.000	4.125	0.750	18.182

Number of dissolution data points (N), degrees of freedom (df), and selected goodness of fit criteria – Pearson correlation coefficient (R), coefficient of determination (R^2), adjusted coefficient of determination ($R^2_{adjusted}$), and residual sum of squares (RSS) (manual calculation in MS Excel):

Parameter	No.1	No.2	No.3	No.4
N	20	20	20	20
df	18	18	18	18
R	0.975029625	0.996825753	0.975948861	0.930199732
R^2	0.950682769	0.993661582	0.95247618	0.865271541
$R^2_{adjusted}$	0.947942923	0.993309447	0.949835967	0.857786626
RSS	1166.70916	151.5855093	1636.097508	4229.555303

Graphical abstract of model fit presented as mean ± 1 SD of the fraction % of released carvedilol:

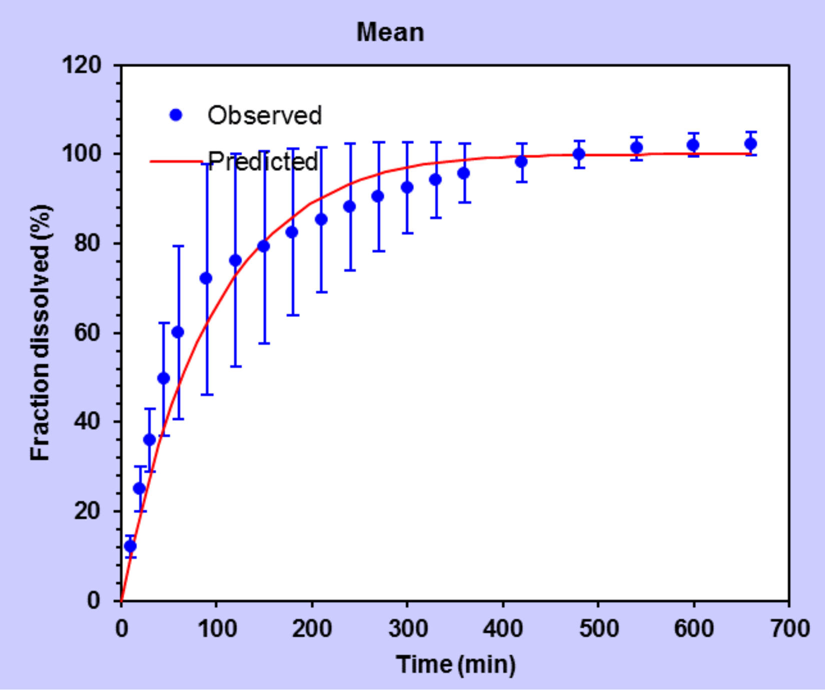

Supplementary materials – Model fitting summary – Kollidon® 25 (modelling the entire relevant dissolution data)

Graphical abstract of model fit presented as the fraction % of released carvedilol per tested tablet:

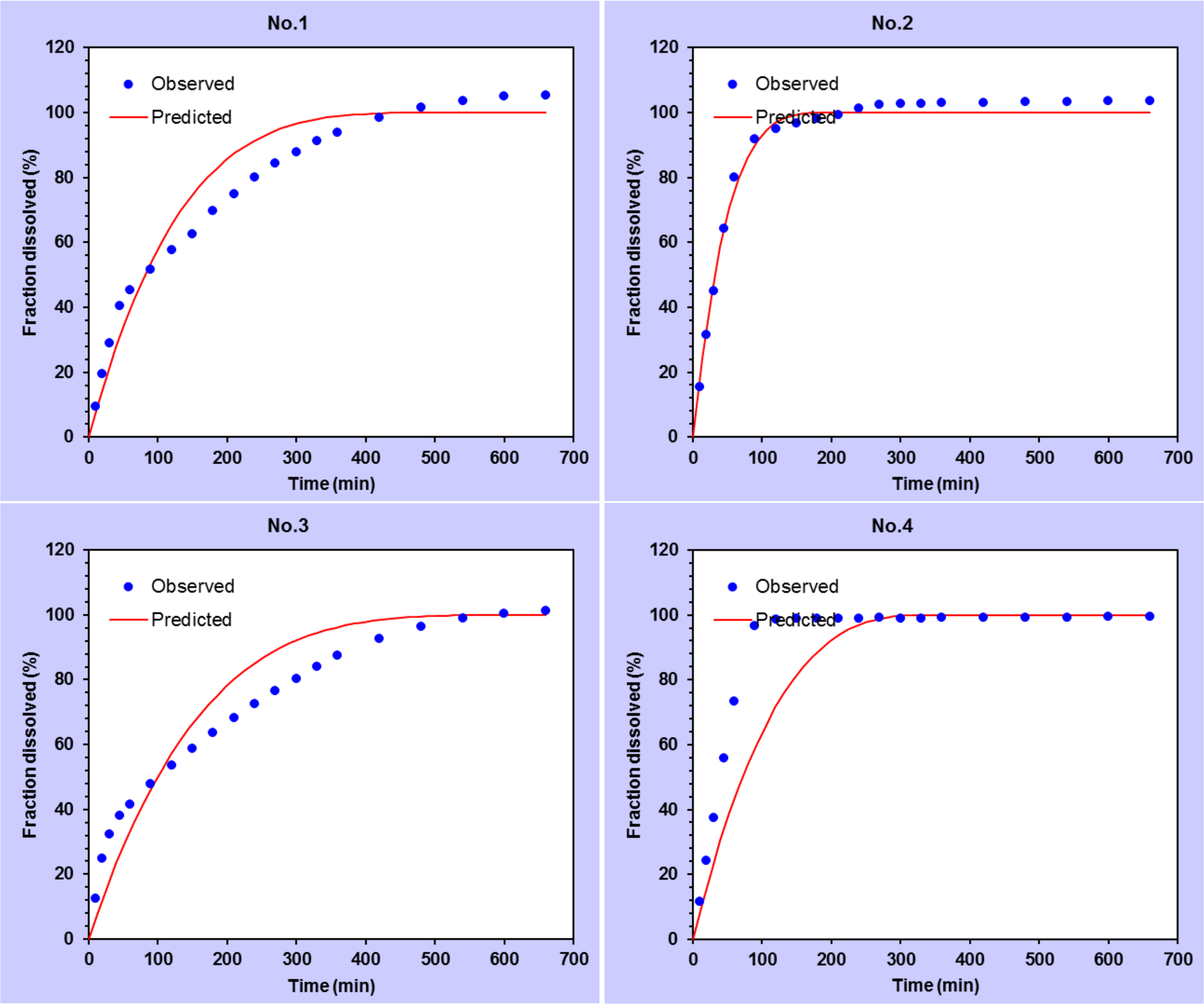

Supplementary materials – Model fitting summary – Kollidon® 25 (modelling the entire relevant dissolution data)

Model: **Hopfenberg with T_{lag}**

Model equation: $F = 100 \cdot \{1 - [1 - k_{HB} \cdot (t - T_{lag})]^n\}$

Fitted model parameters per tested tablet (N = 4) with statistics – mean, standard deviation (SD), and relative standard deviation expressed in % (RSD%) (output from DDSolver):

Parameter	No.1	No.2	No.3	No.4	Mean	SD	RSD(%)
k_{HB}	0.002	0.004	0.001	0.001	0.002	0.001	69.898
n	3.000	3.000	3.000	1.000	2.500	1.000	40.000
T_{lag}	-35.443	-27.921	-54.179	-702.177	-204.930	331.682	-161.851

Number of dissolution data points (N), degrees of freedom (df), and selected goodness of fit criteria – Pearson correlation coefficient (R), coefficient of determination (R^2), adjusted coefficient of determination ($R^2_{adjusted}$), and residual sum of squares (RSS) (manual calculation in MS Excel):

Parameter	No.1	No.2	No.3	No.4
N	20	20	20	20
df	17	17	17	17
R	0.991150047	0.980826035	0.992918577	0.705196784
R^2	0.982378415	0.962019712	0.985887301	0.497302505
$R^2_{adjusted}$	0.980305287	0.957551442	0.984226983	0.438161623
RSS	327.6910104	989.4809554	199.0357911	8481.671068

Graphical abstract of model fit presented as mean ± 1 SD of the fraction % of released carvedilol:

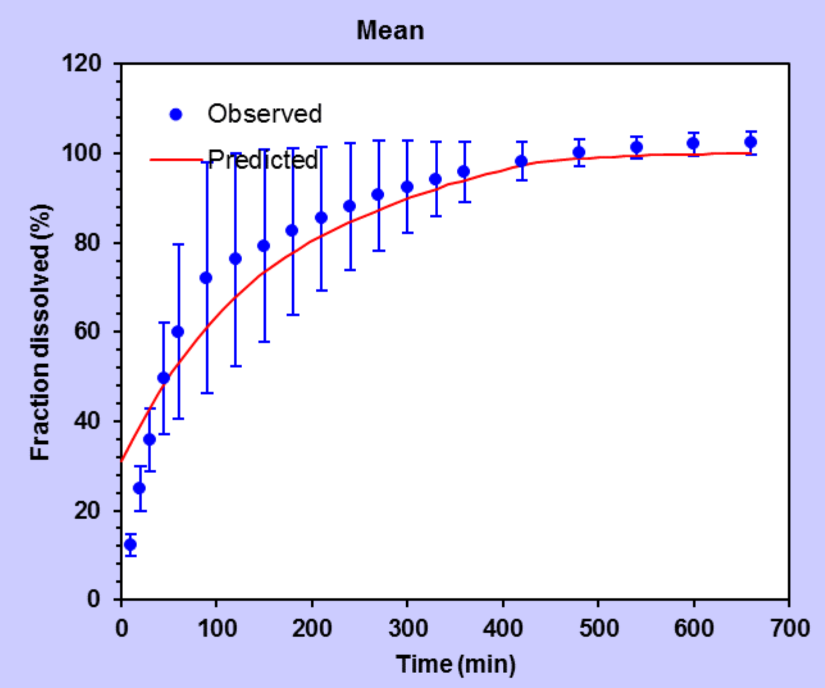

Supplementary materials – Model fitting summary – Kollidon® 25 (modelling the entire relevant dissolution data)

Graphical abstract of model fit presented as the fraction % of released carvedilol per tested tablet:

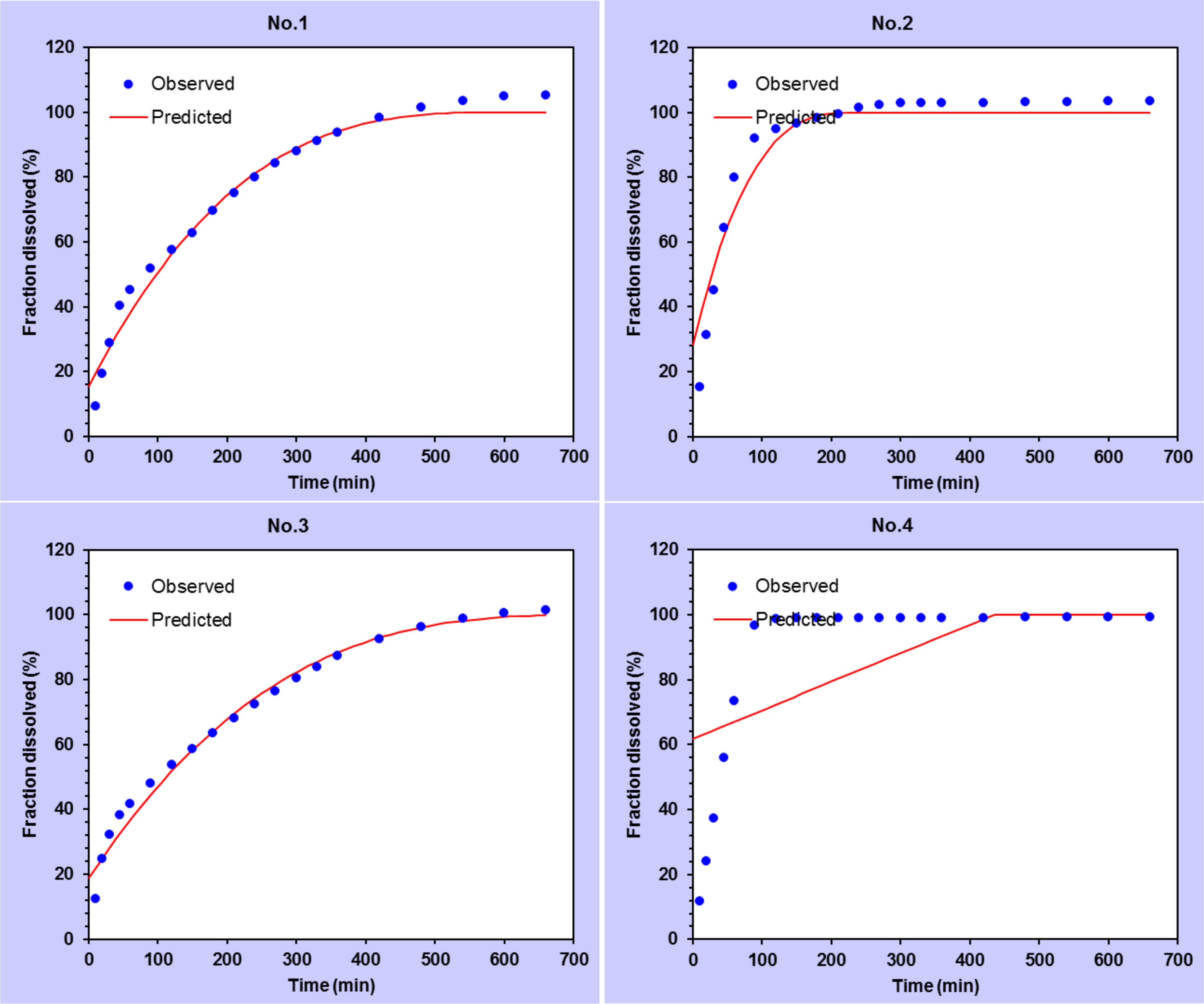

Supplementary materials – Model fitting summary – Kollidon® 25 (modelling the entire relevant dissolution data)

Model: **Baker–Lonsdale**

Model equation: $\frac{3}{2}\cdot\left[1-\left(1-\frac{F}{100}\right)^{\frac{2}{3}}\right]-\frac{F}{100}=k_{BL}\cdot t$

Fitted model parameters per tested tablet (N = 4) with statistics – mean, standard deviation (SD), and relative standard deviation expressed in % (RSD%) (output from DDSolver):

Parameter	No.1	No.2	No.3	No.4	Mean	SD	RSD(%)
k_{BL}	0.001	0.002	0.000	0.001	0.001	0.001	102.876

Number of dissolution data points (N), degrees of freedom (df), and selected goodness of fit criteria – Pearson correlation coefficient (R), coefficient of determination (R^2), adjusted coefficient of determination ($R^2_{adjusted}$), and residual sum of squares (RSS) (manual calculation in MS Excel):

Parameter	No.1	No.2	No.3	No.4
N	20	20	20	20
df	19	19	19	19
R	-0.32094385	-0.57977927	0.04359815	0.84720415
R^2	0.103004953	0.336144001	0.001900798	0.717754872
$R^2_{adjusted}$	0.103004953	0.336144001	0.001900798	0.717754872
RSS	397576.9192	2420092.77	102328.2339	11133.40613

Graphical abstract of model fit presented as mean ± 1 SD of the fraction % of released carvedilol:

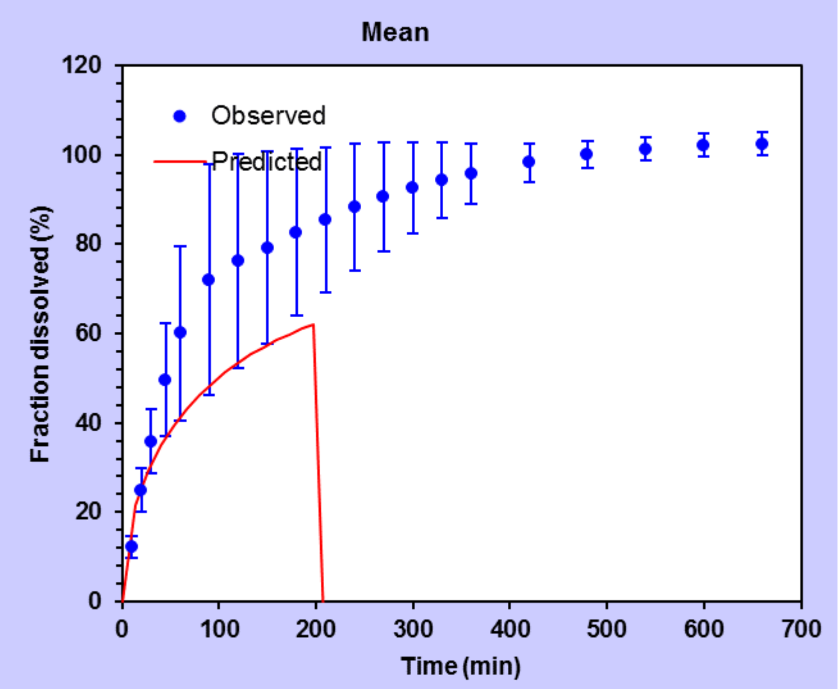

Supplementary materials – Model fitting summary – Kollidon® 25 (modelling the entire relevant dissolution data)

Graphical abstract of model fit presented as the fraction % of released carvedilol per tested tablet:

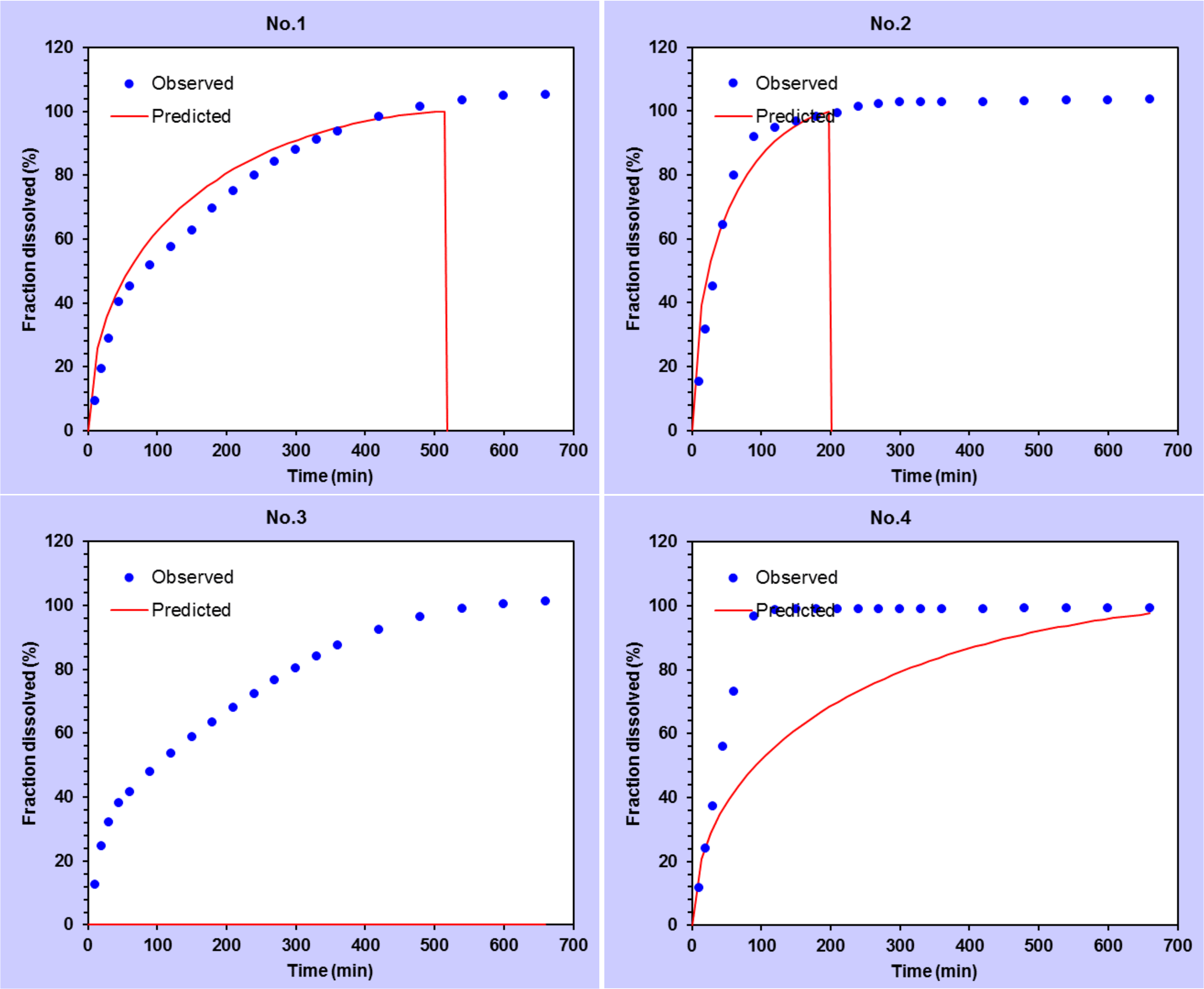

Supplementary materials – Model fitting summary – Kollidon® 25 (modelling the entire relevant dissolution data)

Model: **Baker–Lonsdale with T_{lag}**

Model equation: $\frac{3}{2}\cdot\left[1-\left(1-\frac{F}{100}\right)^{\frac{2}{3}}\right]-\frac{F}{100}=k_{BL}\cdot\left(t-T_{lag}\right)$

Fitted model parameters per tested tablet (N = 4) with statistics – mean, standard deviation (SD), and relative standard deviation expressed in % (RSD%) (output from DDSolver):

Parameter	No.1	No.2	No.3	No.4	Mean	SD	RSD(%)
k_{BL}	0.000	0.002	0.001	0.000	0.001	0.001	142.512
T_{lag}	44.925	-2.480	28.291	-542.110	-117.844	283.525	-240.595

Number of dissolution data points (N), degrees of freedom (df), and selected goodness of fit criteria – Pearson correlation coefficient (R), coefficient of determination (R^2), adjusted coefficient of determination ($R^2_{adjusted}$), and residual sum of squares (RSS) (manual calculation in MS Excel):

Parameter	No.1	No.2	No.3	No.4
N	20	20	20	20
df	18	18	18	18
R	0.738805025	-0.58292327	0.980553758	0.308893491
R^2	0.545832864	0.339799538	0.961485672	0.095415189
$R^2_{adjusted}$	0.520601357	0.303121735	0.959345987	0.045160477
RSS	116357.3045	2435536.454	1610.959418	157167.9313

Graphical abstract of model fit presented as mean ± 1 SD of the fraction % of released carvedilol:

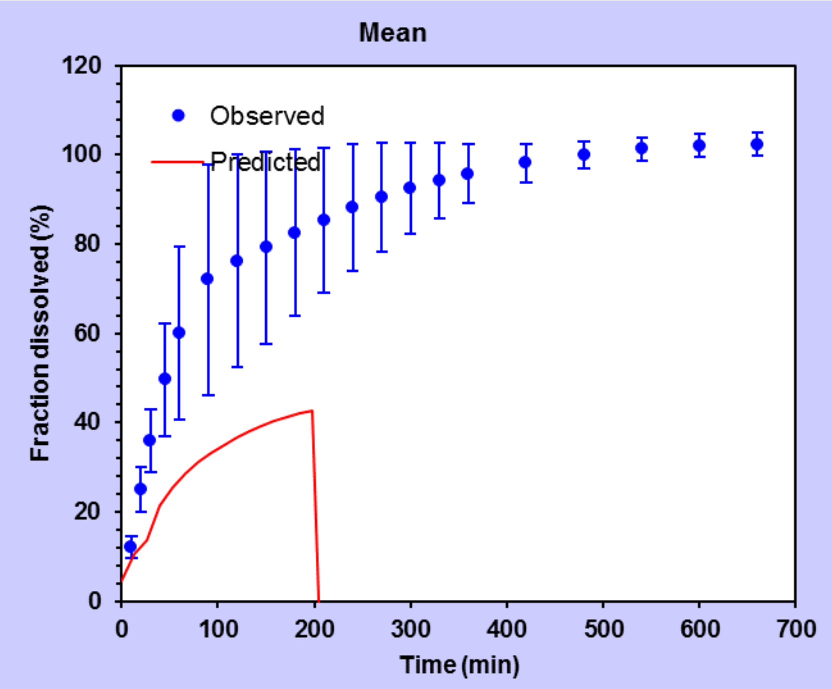

Supplementary materials – Model fitting summary – Kollidon® 25 (modelling the entire relevant dissolution data)

Graphical abstract of model fit presented as the fraction % of released carvedilol per tested tablet:

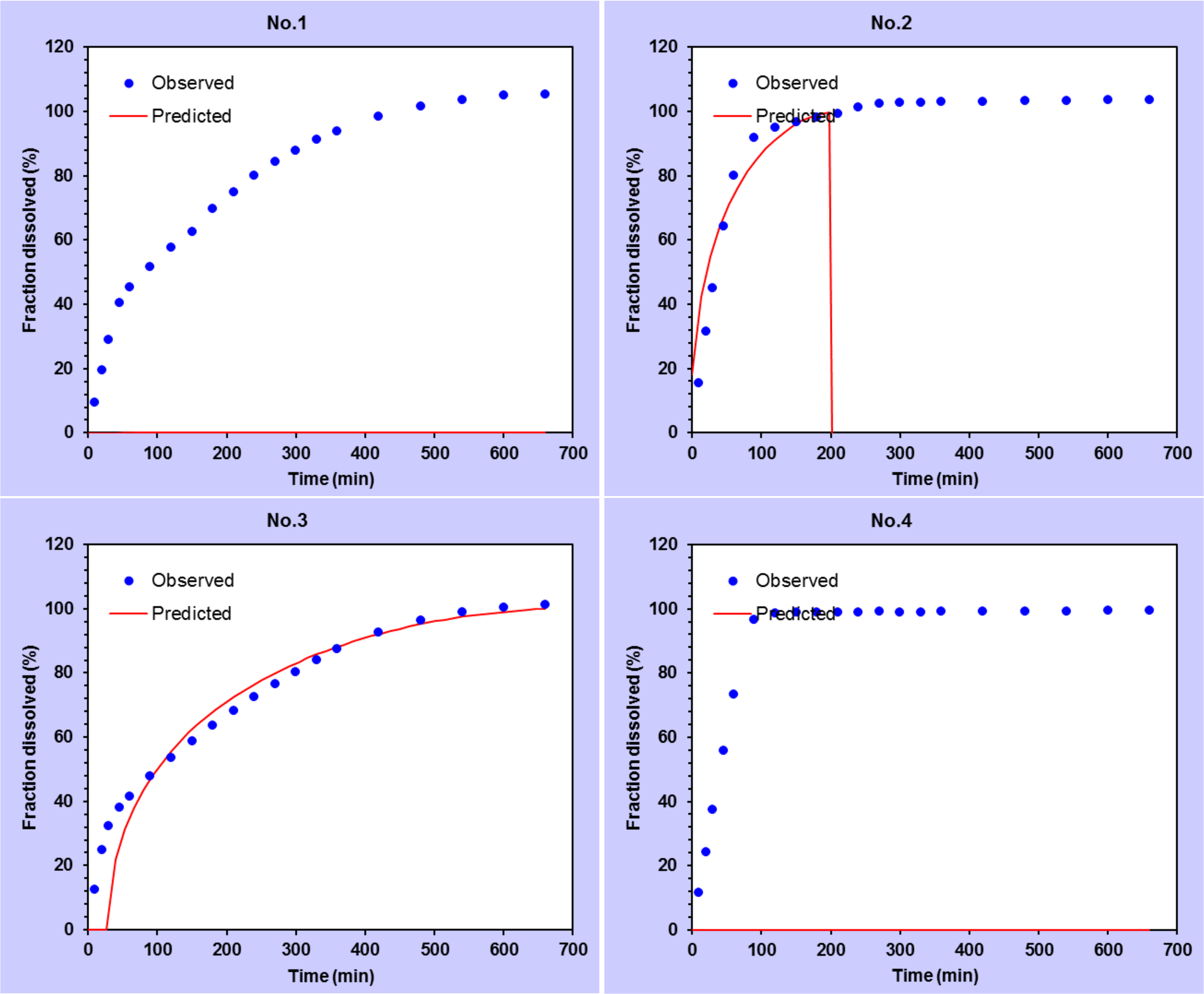

Supplementary materials – Model fitting summary – Kollidon® 25 (modelling the entire relevant dissolution data)

Model: **Makoid–Banakar**

Model equation: $F = k_{MB} \cdot t^n \cdot e^{-k \cdot t}$

Fitted model parameters per tested tablet (N = 4) with statistics – mean, standard deviation (SD), and relative standard deviation expressed in % (RSD%) (output from DDSolver):

Parameter	No.1	No.2	No.3	No.4	Mean	SD	RSD(%)
k_{MB}	2.341	4.114	4.485	2.263	3.301	1.163	35.240
n	0.709	0.699	0.541	0.831	0.695	0.119	17.096
k	0.001	0.002	0.001	0.003	0.002	0.001	55.527

Number of dissolution data points (N), degrees of freedom (df), and selected goodness of fit criteria – Pearson correlation coefficient (R), coefficient of determination (R^2), adjusted coefficient of determination ($R^2_{adjusted}$), and residual sum of squares (RSS) (manual calculation in MS Excel):

Parameter	No.1	No.2	No.3	No.4
N	20	20	20	20
df	17	17	17	17
R	0.992709304	0.949496905	0.996030005	0.931066236
R^2	0.985471762	0.901544372	0.992075771	0.866884336
$R^2_{adjusted}$	0.983762558	0.889961357	0.991143509	0.851223669
RSS	261.2213073	1688.500618	111.2549635	2564.293864

Graphical abstract of model fit presented as mean ± 1 SD of the fraction % of released carvedilol:

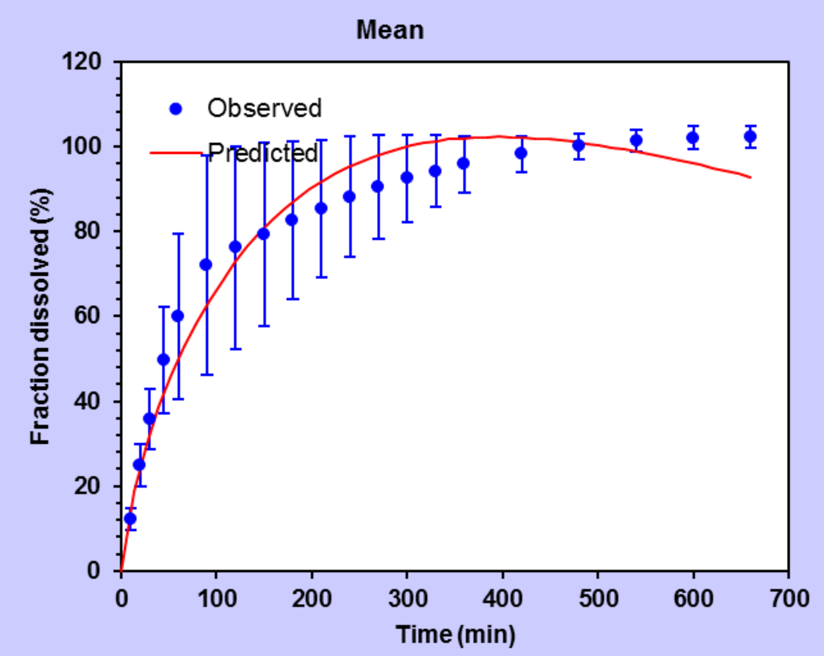

Supplementary materials – Model fitting summary – Kollidon® 25 (modelling the entire relevant dissolution data)

Graphical abstract of model fit presented as the fraction % of released carvedilol per tested tablet:

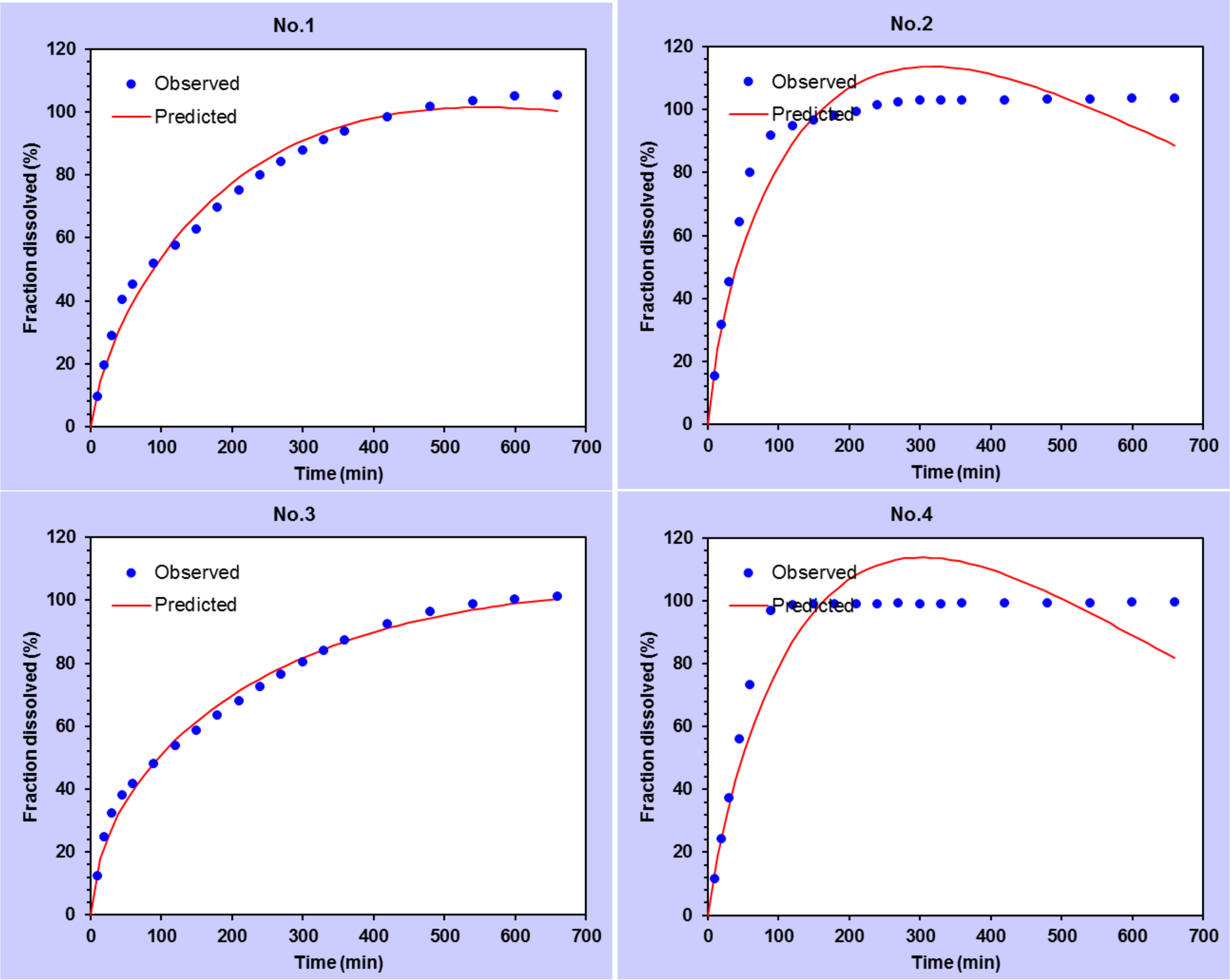

Supplementary materials – Model fitting summary – Kollidon® 25 (modelling the entire relevant dissolution data)

Model: **Makoid–Banakar with T_{lag}**

Model equation: $F = k_{MB} \cdot (t - T_{lag})^n \cdot e^{-k \cdot (t - T_{lag})}$

Fitted model parameters per tested tablet (N = 4) with statistics – mean, standard deviation (SD), and relative standard deviation expressed in % (RSD%) (output from DDSolver):

Parameter	No.1	No.2	No.3	No.4	Mean	SD	RSD(%)
k_{MB}	3.589	6.173	6.214	3.695	4.918	1.474	29.962
n	0.617	0.611	0.472	0.725	0.606	0.104	17.133
k	0.001	0.002	0.000	0.002	0.001	0.001	63.426
T_{lag}	4.000	4.000	4.000	4.000	4.000	0.000	0.000

Number of dissolution data points (N), degrees of freedom (df), and selected goodness of fit criteria – Pearson correlation coefficient (R), coefficient of determination (R^2), adjusted coefficient of determination ($R^2_{adjusted}$), and residual sum of squares (RSS) (manual calculation in MS Excel):

Parameter	No.1	No.2	No.3	No.4
N	20	20	20	20
df	16	16	16	16
R	0.995525087	0.960013951	0.997206436	0.942940243
R^2	0.991070199	0.921626787	0.994420677	0.889136302
$R^2_{adjusted}$	0.989395861	0.906931809	0.993374553	0.868349359
RSS	156.7088363	1291.991981	77.70040025	2024.517234

Graphical abstract of model fit presented as mean ± 1 SD of the fraction % of released carvedilol:

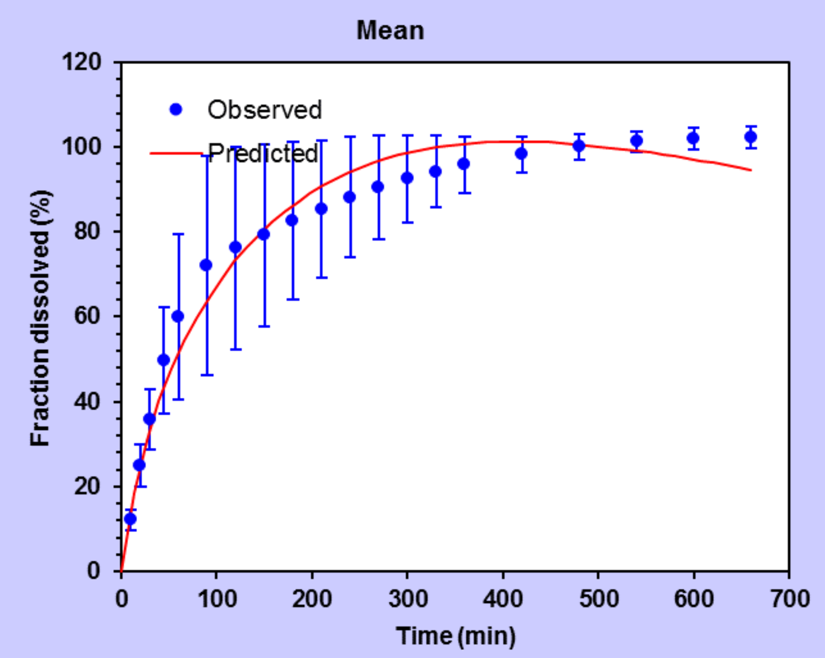

Supplementary materials – Model fitting summary – Kollidon® 25 (modelling the entire relevant dissolution data)

Graphical abstract of model fit presented as the fraction % of released carvedilol per tested tablet:

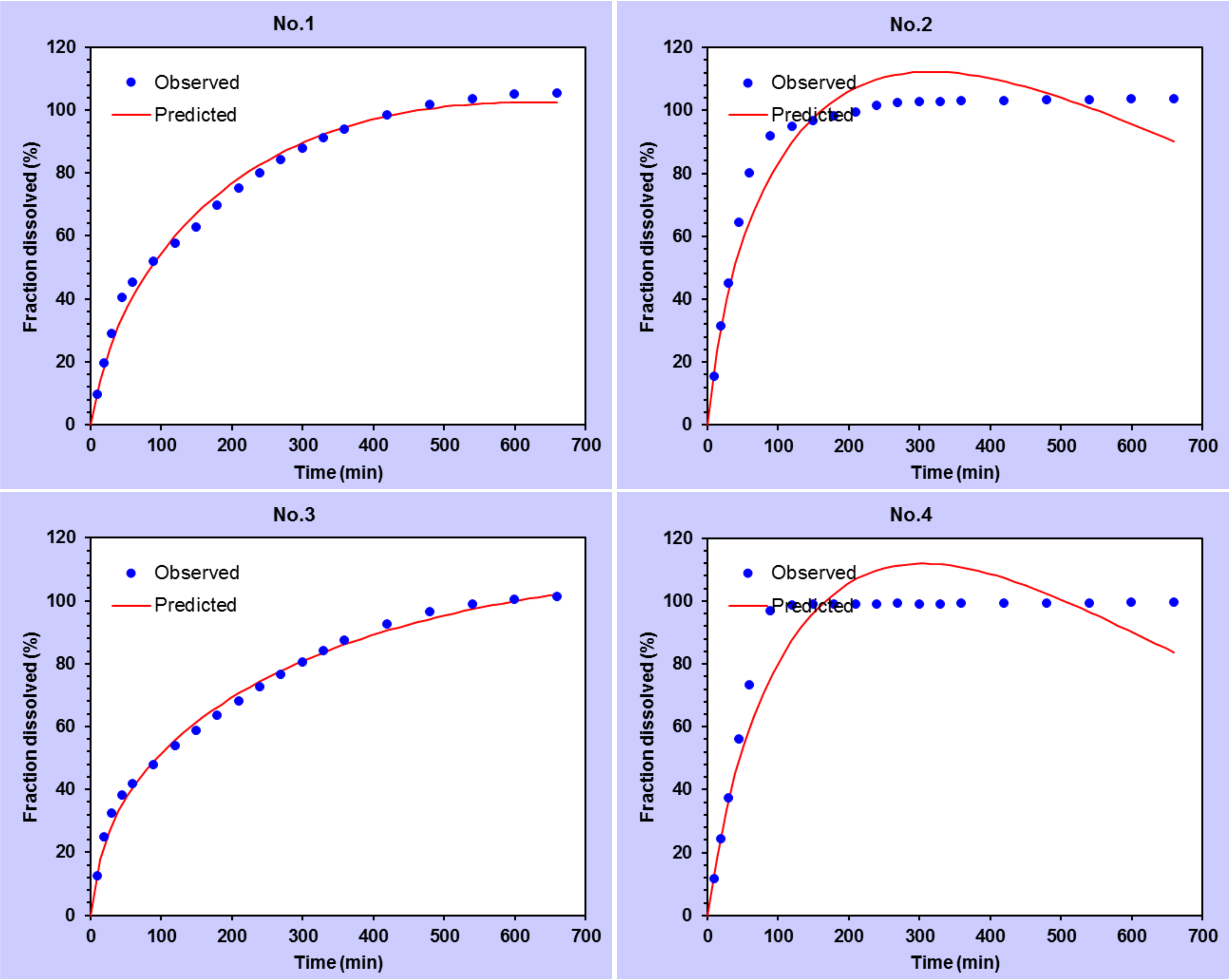

Supplementary materials – Model fitting summary – Kollidon® 25 (modelling the entire relevant dissolution data)

Model: **Peppas–Sahlin_1**

Model equation: $F = k_1 \cdot t^m + k_2 \cdot t^{2m}$

Fitted model parameters per tested tablet (N = 4) with statistics – mean, standard deviation (SD), and relative standard deviation expressed in % (RSD%) (output from DDSolver):

Parameter	No.1	No.2	No.3	No.4	Mean	SD	RSD(%)
k_1	7.551	14.063	6.989	13.730	10.583	3.835	36.238
k_2	-0.082	-0.465	-0.070	-0.458	-0.269	0.222	-82.714
m	0.450	0.450	0.450	0.450	0.450	0.000	0.000

Number of dissolution data points (N), degrees of freedom (df), and selected goodness of fit criteria – Pearson correlation coefficient (R), coefficient of determination (R^2), adjusted coefficient of determination ($R^2_{adjusted}$), and residual sum of squares (RSS) (manual calculation in MS Excel):

Parameter	No.1	No.2	No.3	No.4
N	20	20	20	20
df	17	17	17	17
R	0.992151332	0.967447005	0.996600069	0.950860038
R^2	0.984364266	0.935953708	0.993211697	0.904134812
$R^2_{adjusted}$	0.982524768	0.928418851	0.992413073	0.892856555
RSS	345.6756736	1158.373525	102.8217822	2120.338465

Graphical abstract of model fit presented as mean ± 1 SD of the fraction % of released carvedilol:

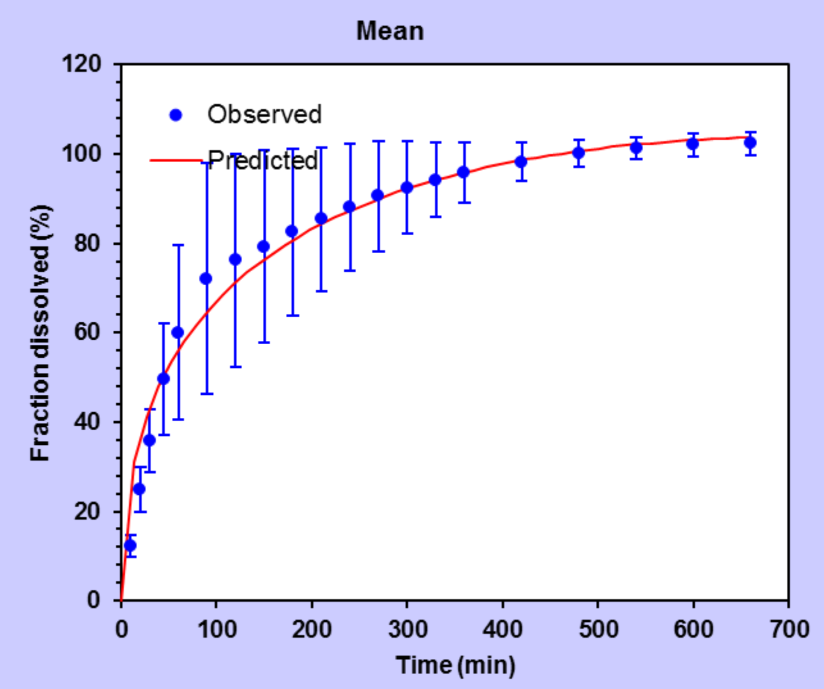

Supplementary materials – Model fitting summary – Kollidon® 25 (modelling the entire relevant dissolution data)

Graphical abstract of model fit presented as the fraction % of released carvedilol per tested tablet:

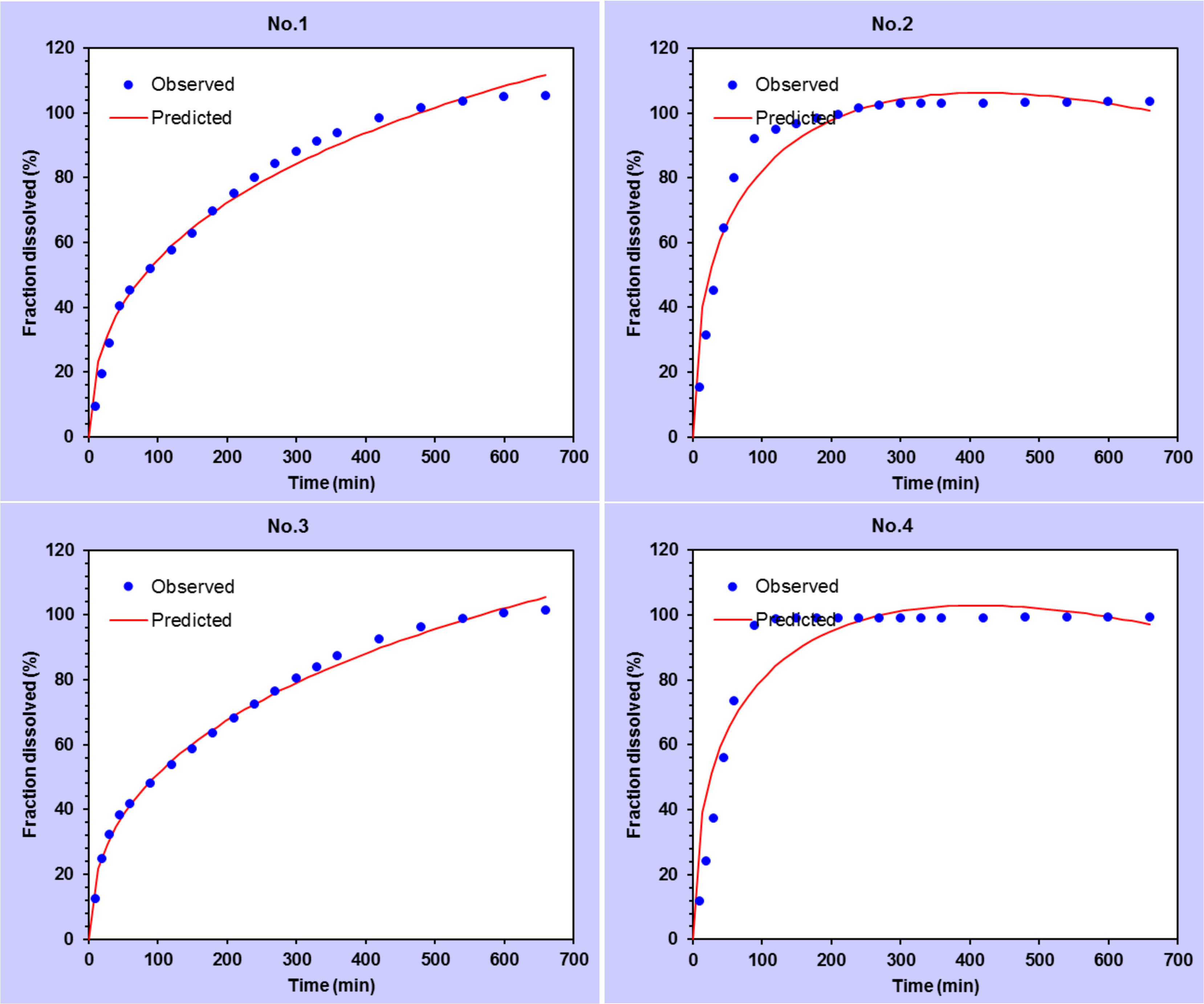

Supplementary materials – Model fitting summary – Kollidon® 25 (modelling the entire relevant dissolution data)

Model: **Peppas–Sahlin_1 with T_{lag}**

Model equation: $F = k_1 \cdot (t - T_{lag})^m + k_2 \cdot (t - T_{lag})^{2m}$

Fitted model parameters per tested tablet (N = 4) with statistics – mean, standard deviation (SD), and relative standard deviation expressed in % (RSD%) (output from DDSolver):

Parameter	No.1	No.2	No.3	No.4	Mean	SD	RSD(%)
k_1	7.879	14.519	7.273	14.200	10.968	3.926	35.797
k_2	-0.102	-0.495	-0.087	-0.488	-0.293	0.229	-78.197
m	0.450	0.450	0.450	0.450	0.450	0.000	0.000
T_{lag}	6.000	6.000	6.000	6.000	6.000	0.000	0.000

Number of dissolution data points (N), degrees of freedom (df), and selected goodness of fit criteria – Pearson correlation coefficient (R), coefficient of determination (R^2), adjusted coefficient of determination ($R^2_{adjusted}$), and residual sum of squares (RSS) (manual calculation in MS Excel):

Parameter	No.1	No.2	No.3	No.4
N	20	20	20	20
df	16	16	16	16
R	0.99563607	0.977100574	0.997670192	0.961488008
R^2	0.991291183	0.954725531	0.995345813	0.92445919
$R^2_{adjusted}$	0.98965828	0.946236568	0.994473152	0.910295289
RSS	168.3060233	671.188066	69.62897374	1430.160906

Graphical abstract of model fit presented as mean ± 1 SD of the fraction % of released carvedilol:

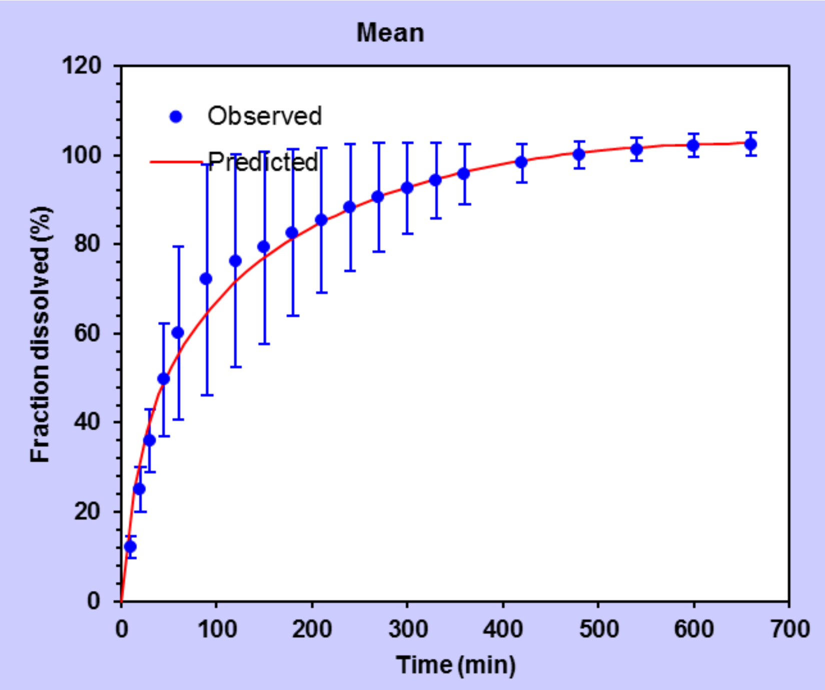

Supplementary materials – Model fitting summary – Kollidon® 25 (modelling the entire relevant dissolution data)

Graphical abstract of model fit presented as the fraction % of released carvedilol per tested tablet:

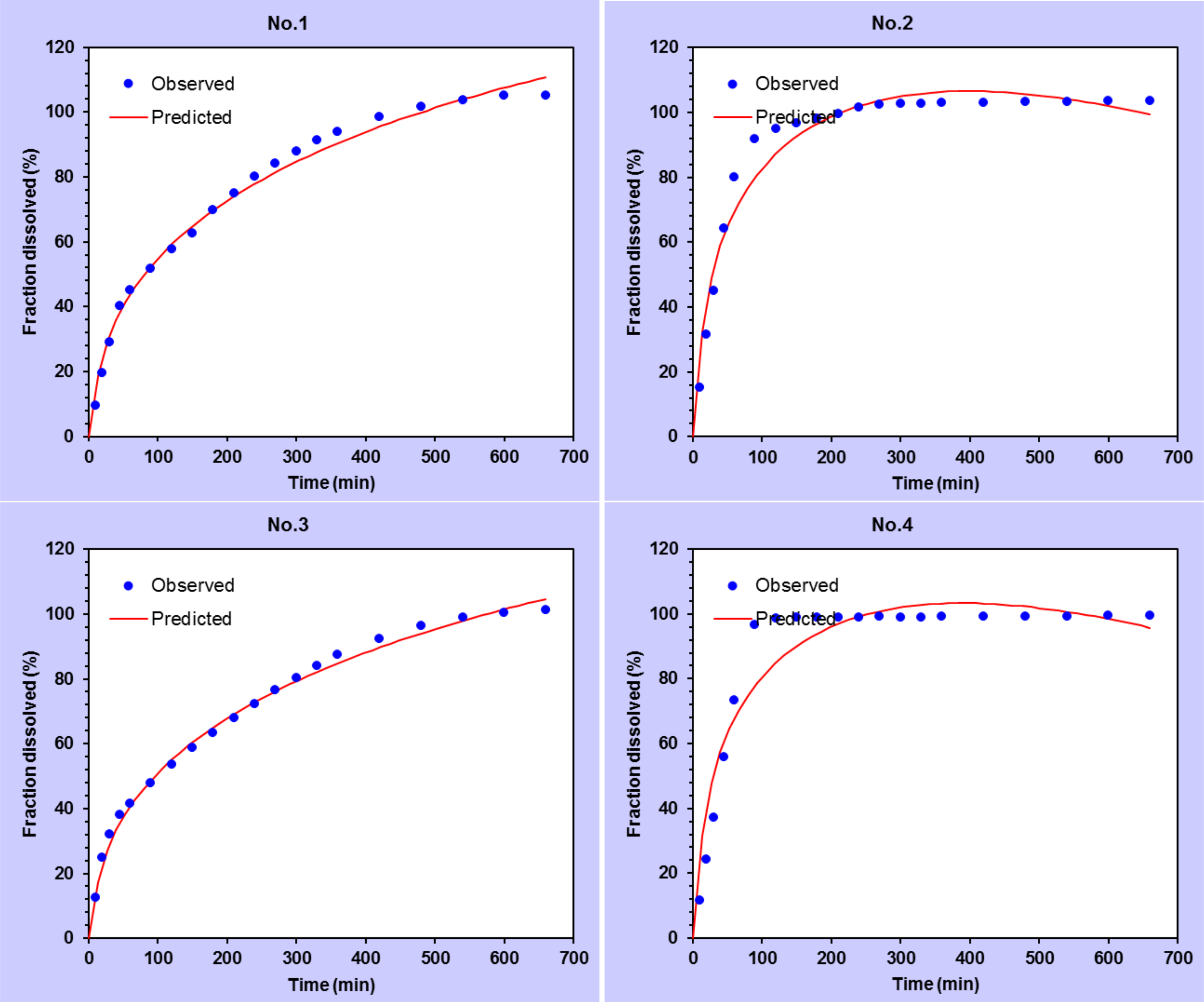

Supplementary materials – Model fitting summary – Kollidon® 25 (modelling the entire relevant dissolution data)

Model: **Peppas–Sahlin_2**

Model equation: $F = k_1 \cdot t^{0.5} + k_2 \cdot t$

Fitted model parameters per tested tablet (N = 4) with statistics – mean, standard deviation (SD), and relative standard deviation expressed in % (RSD%) (output from DDSolver):

Parameter	No.1	No.2	No.3	No.4	Mean	SD	RSD(%)
k_1	6.247	10.993	5.756	10.764	8.440	2.824	33.464
k_2	-0.076	-0.281	-0.066	-0.277	-0.175	0.120	-68.516

Number of dissolution data points (N), degrees of freedom (df), and selected goodness of fit criteria – Pearson correlation coefficient (R), coefficient of determination (R^2), adjusted coefficient of determination ($R^2_{adjusted}$), and residual sum of squares (RSS) (manual calculation in MS Excel):

Parameter	No.1	No.2	No.3	No.4
N	20	20	20	20
df	18	18	18	18
R	0.99493268	0.965498975	0.997267881	0.951169709
R^2	0.989891038	0.932188271	0.994543226	0.904723815
$R^2_{adjusted}$	0.989329429	0.928420953	0.994240072	0.899430693
RSS	232.0783694	1003.374219	77.04552618	1795.278509

Graphical abstract of model fit presented as mean ± 1 SD of the fraction % of released carvedilol:

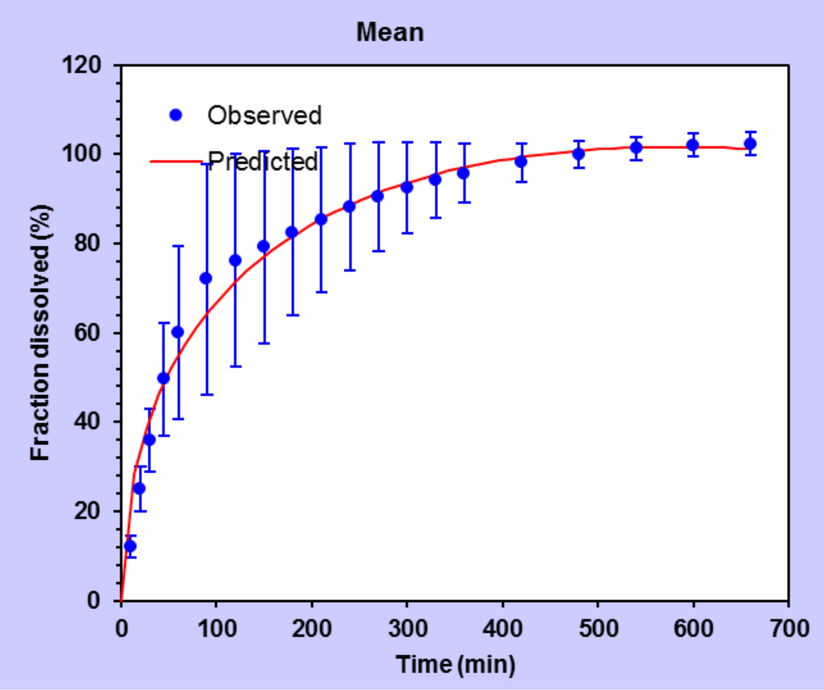

Supplementary materials – Model fitting summary – Kollidon® 25 (modelling the entire relevant dissolution data)

Graphical abstract of model fit presented as the fraction % of released carvedilol per tested tablet:

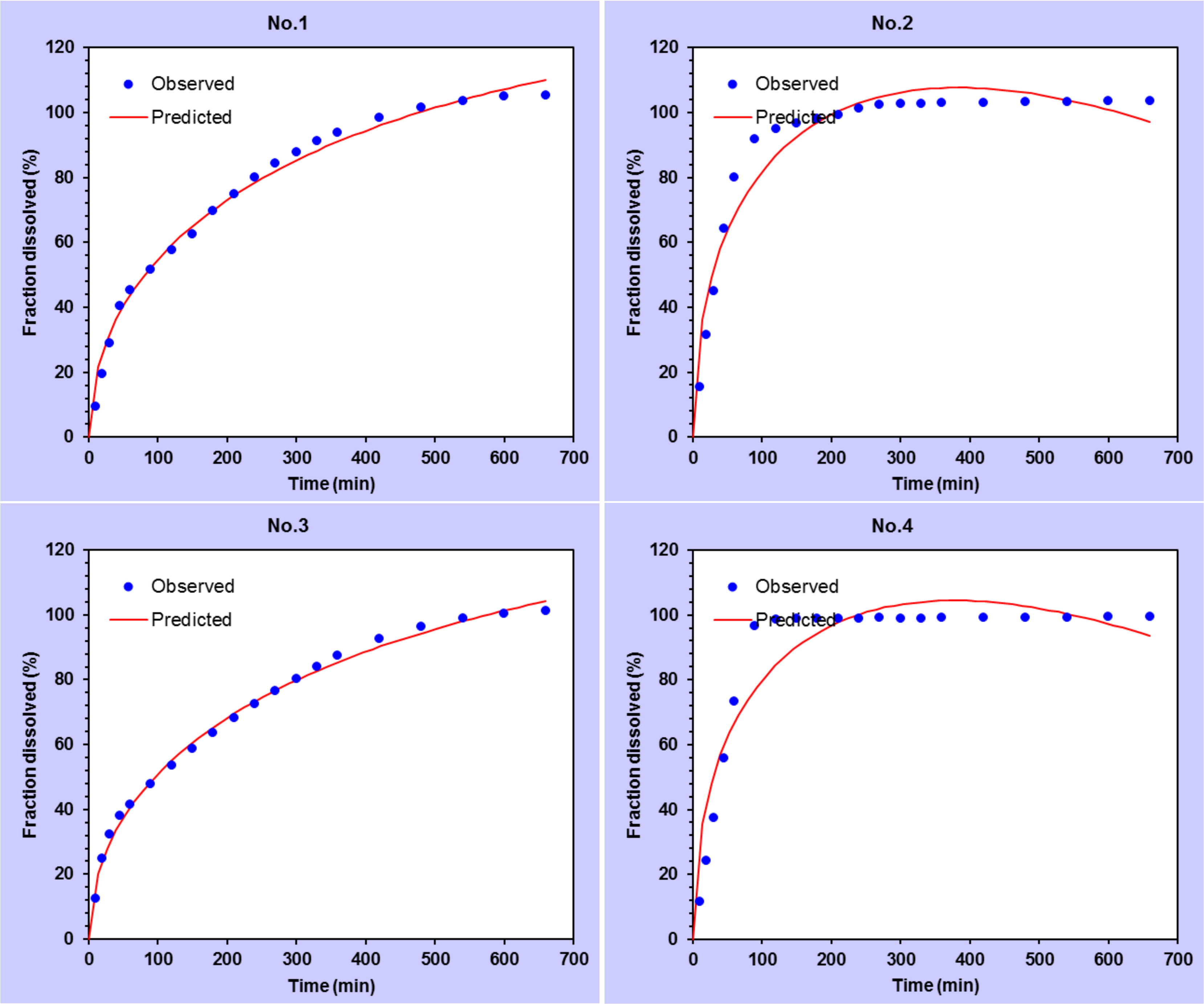

Supplementary materials – Model fitting summary – Kollidon® 25 (modelling the entire relevant dissolution data)

Model: **Peppas–Sahlin_2 with T_{lag}**

Model equation: $F = k_1 \cdot (t - T_{lag})^{0.5} + k_2 \cdot (t - T_{lag})$

Fitted model parameters per tested tablet (N = 4) with statistics – mean, standard deviation (SD), and relative standard deviation expressed in % (RSD%) (output from DDSolver):

Parameter	No.1	No.2	No.3	No.4	Mean	SD	RSD(%)
k_1	6.476	11.302	5.952	11.084	8.703	2.884	33.138
k_2	-0.086	-0.295	-0.075	-0.292	-0.187	0.123	-65.852
T_{lag}	6.000	6.000	4.000	6.000	5.500	1.000	18.182

Number of dissolution data points (N), degrees of freedom (df), and selected goodness of fit criteria – Pearson correlation coefficient (R), coefficient of determination (R^2), adjusted coefficient of determination ($R^2_{adjusted}$), and residual sum of squares (RSS) (manual calculation in MS Excel):

Parameter	No.1	No.2	No.3	No.4
N	20	20	20	20
df	17	17	17	17
R	0.99728799	0.973008506	0.997491366	0.95914821
R^2	0.994583334	0.946745553	0.994989026	0.919965288
$R^2_{adjusted}$	0.993946079	0.940480323	0.9943995	0.91054944
RSS	99.04674975	724.4952507	71.84214166	1290.219705

Graphical abstract of model fit presented as mean ± 1 SD of the fraction % of released carvedilol:

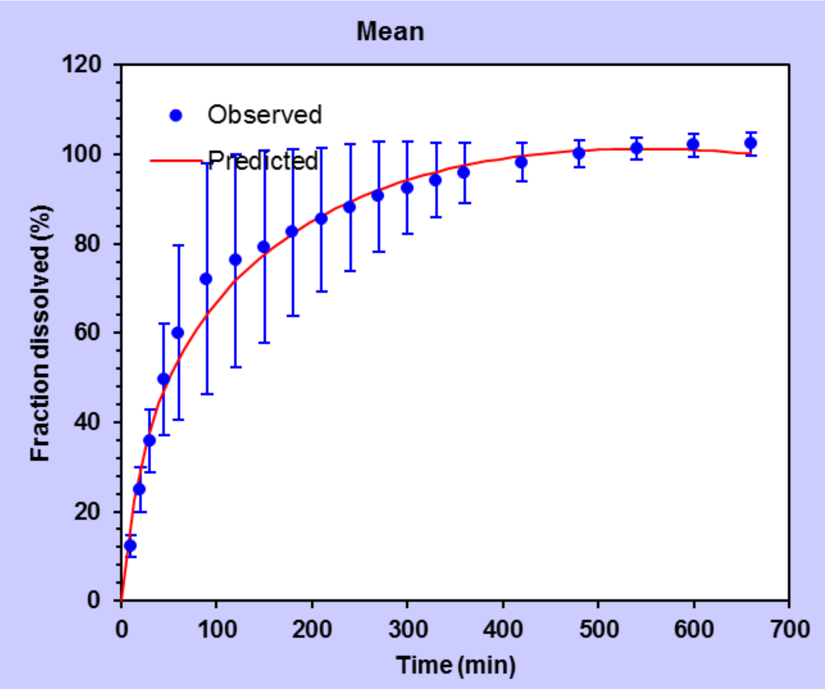

Supplementary materials – Model fitting summary – Kollidon® 25 (modelling the entire relevant dissolution data)

Graphical abstract of model fit presented as the fraction % of released carvedilol per tested tablet:

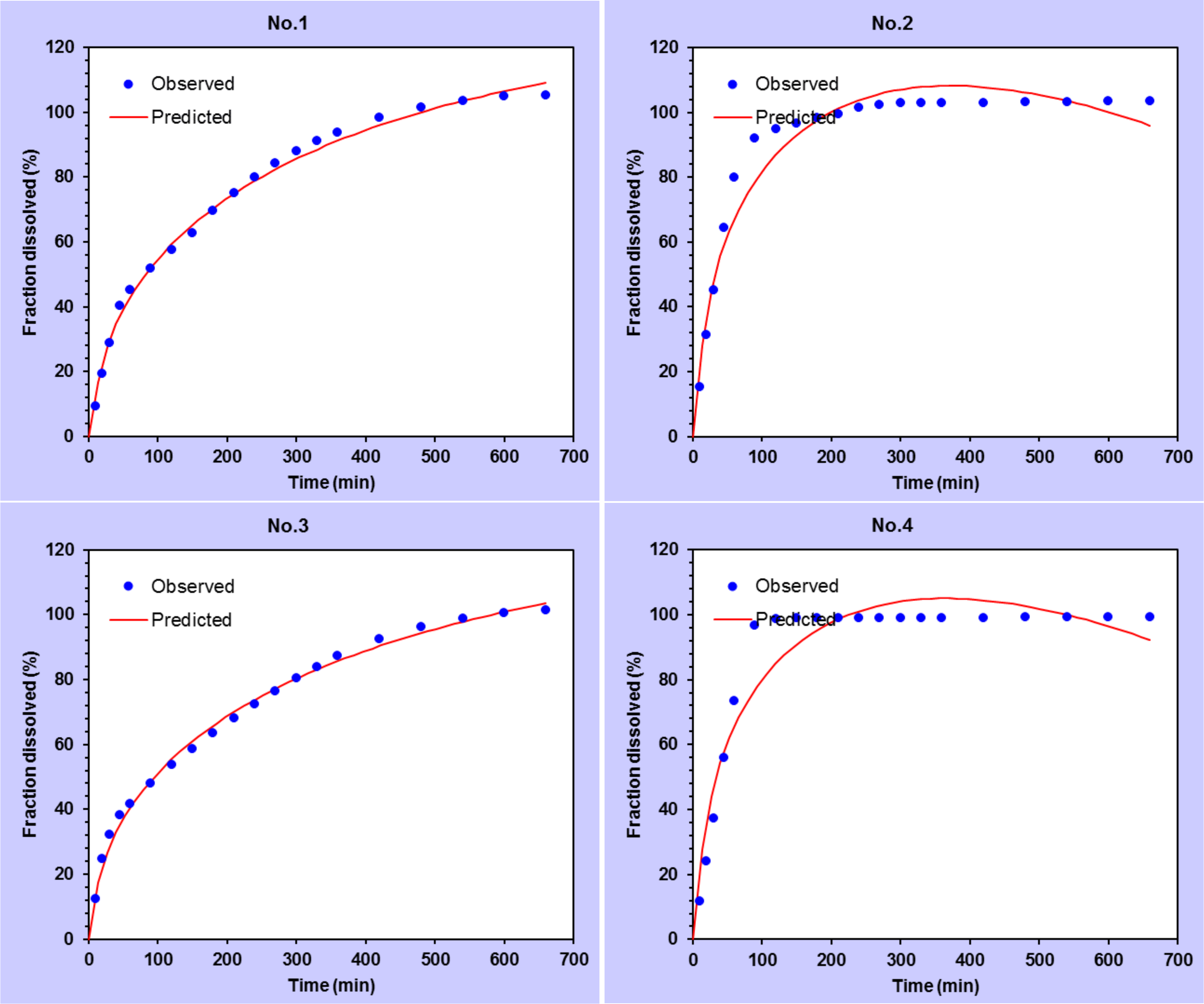

Supplementary materials – Model fitting summary – Kollidon® 25 (modelling the entire relevant dissolution data)

Model: **Quadratic**

Model equation: $F = 100 \cdot (k_1 \cdot t^2 + k_2 \cdot t)$

Fitted model parameters per tested tablet (N = 4) with statistics – mean, standard deviation (SD), and relative standard deviation expressed in % (RSD%) (output from DDSolver):

Parameter	No.1	No.2	No.3	No.4	Mean	SD	RSD(%)
k_1	0.000	0.000	0.000	0.000	0.000	0.000	-31.003
k_2	0.004	0.006	0.004	0.006	0.005	0.001	20.359

Number of dissolution data points (N), degrees of freedom (df), and selected goodness of fit criteria – Pearson correlation coefficient (R), coefficient of determination (R^2), adjusted coefficient of determination ($R^2_{adjusted}$), and residual sum of squares (RSS) (manual calculation in MS Excel):

Parameter	No.1	No.2	No.3	No.4
N	20	20	20	20
df	18	18	18	18
R	0.982394354	0.867329815	0.982450834	0.842369397
R^2	0.965098667	0.752261008	0.965209642	0.709586201
$R^2_{adjusted}$	0.963159704	0.738497731	0.963276844	0.693452101
RSS	2007.884522	10880.49679	2227.210673	10450.46898

Graphical abstract of model fit presented as mean ± 1 SD of the fraction % of released carvedilol:

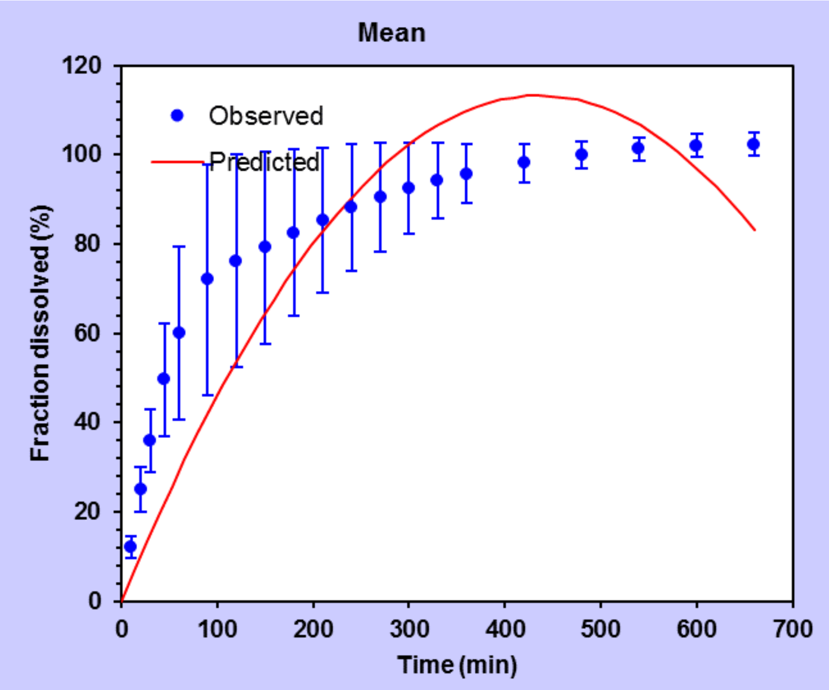

Supplementary materials – Model fitting summary – Kollidon® 25 (modelling the entire relevant dissolution data)

Graphical abstract of model fit presented as the fraction % of released carvedilol per tested tablet:

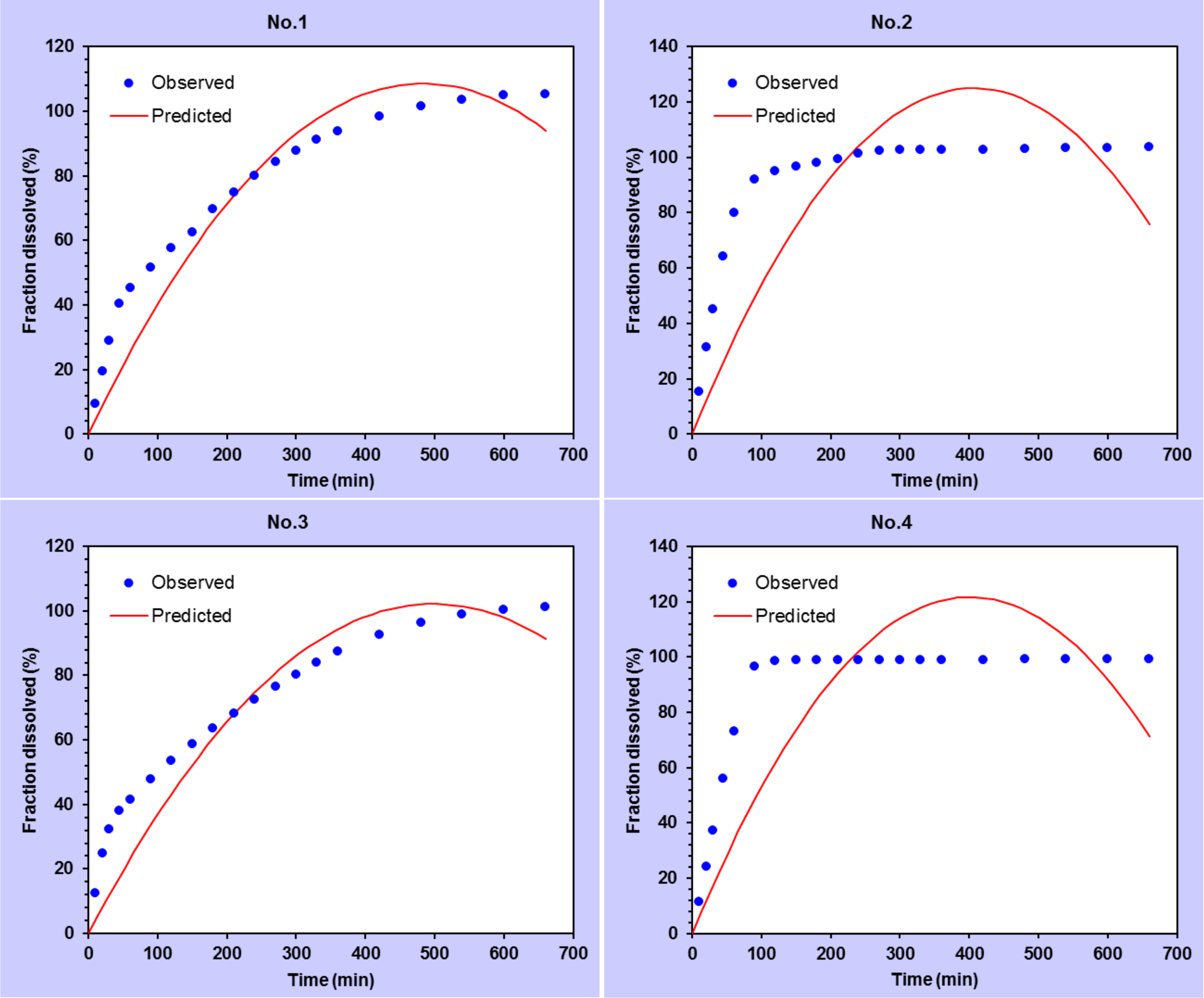

Supplementary materials – Model fitting summary – Kollidon® 25 (modelling the entire relevant dissolution data)

Model: **Quadratic with T_{lag}**

Model equation: $F = 100 \cdot \left[k_1 \cdot \left(t - T_{lag}\right)^2 + k_2 \cdot \left(t - T_{lag}\right)\right]$

Fitted model parameters per tested tablet (N = 4) with statistics – mean, standard deviation (SD), and relative standard deviation expressed in % (RSD%) (output from DDSolver):

Parameter	No.1	No.2	No.3	No.4	Mean	SD	RSD(%)
k_1	0.000	0.000	0.000	0.000	0.000	0.000	-30.646
k_2	0.005	0.006	0.004	0.006	0.005	0.001	20.174
T_{lag}	4.000	4.000	4.000	4.000	4.000	0.000	0.000

Number of dissolution data points (N), degrees of freedom (df), and selected goodness of fit criteria – Pearson correlation coefficient (R), coefficient of determination (R^2), adjusted coefficient of determination ($R^2_{adjusted}$), and residual sum of squares (RSS) (manual calculation in MS Excel):

Parameter	No.1	No.2	No.3	No.4
N	20	20	20	20
df	17	17	17	17
R	0.982064146	0.867621991	0.982053781	0.842552123
R^2	0.964449987	0.752767919	0.964429629	0.709894081
$R^2_{adjusted}$	0.960267633	0.723681792	0.960244879	0.675763972
RSS	2315.298293	11787.57973	2539.56799	11250.07844

Graphical abstract of model fit presented as mean ± 1 SD of the fraction % of released carvedilol:

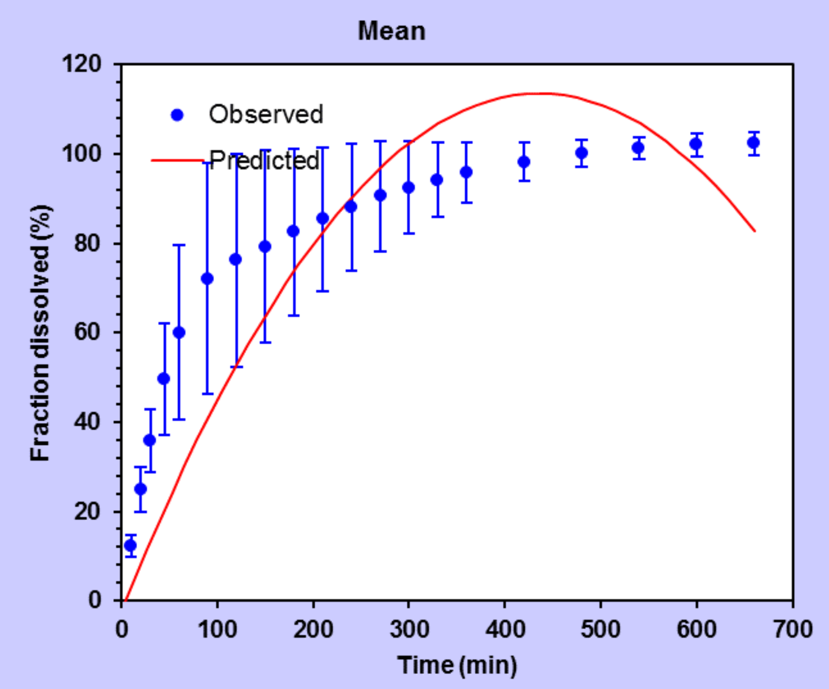

Supplementary materials – Model fitting summary – Kollidon® 25 (modelling the entire relevant dissolution data)

Graphical abstract of model fit presented as the fraction % of released carvedilol per tested tablet:

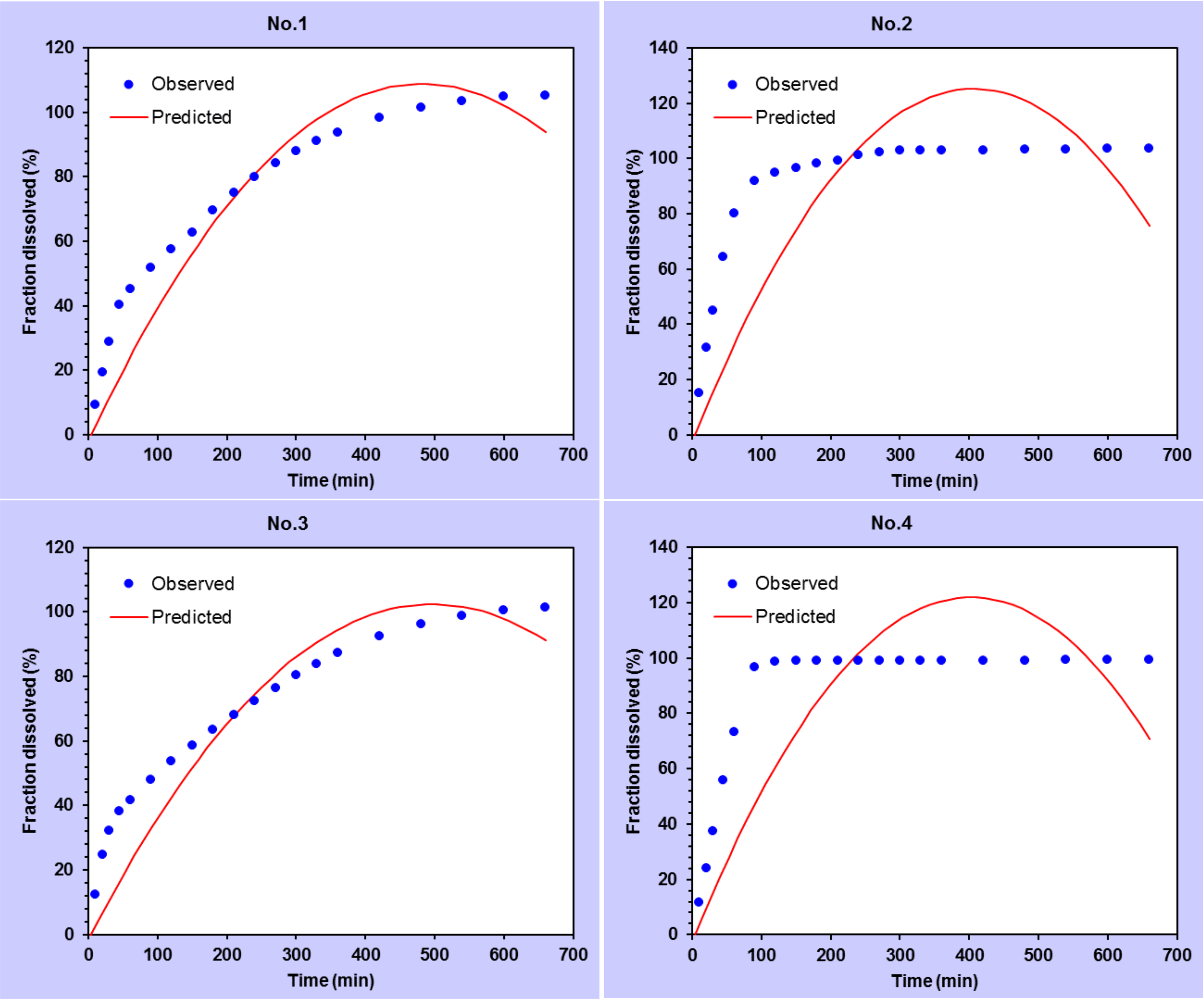

Supplementary materials – Model fitting summary – Kollidon® 25 (modelling the entire relevant dissolution data)

Model: **Weibull_1**

Model equation: $F = 100 \cdot \left[1 - e^{-\frac{(t-T_i)^\beta}{\alpha}}\right]$

Fitted model parameters per tested tablet (N = 4) with statistics – mean, standard deviation (SD), and relative standard deviation expressed in % (RSD%) (output from DDSolver):

Parameter	No.1	No.2	No.3	No.4	Mean	SD	RSD(%)
α	42.402	37.051	28.170	22.578	32.550	8.869	27.246
β	0.791	0.984	0.688	0.812	0.819	0.123	15.010
T_i	4.000	4.000	4.000	6.000	4.500	1.000	22.222

Number of dissolution data points (N), degrees of freedom (df), and selected goodness of fit criteria – Pearson correlation coefficient (R), coefficient of determination (R^2), adjusted coefficient of determination ($R^2_{adjusted}$), and residual sum of squares (RSS) (manual calculation in MS Excel):

Parameter	No.1	No.2	No.3	No.4
N	20	20	20	20
df	17	17	17	17
R	0.988365175	0.997641776	0.983115119	0.982480497
R^2	0.97686572	0.995289113	0.966515337	0.965267926
$R^2_{adjusted}$	0.97414404	0.994734891	0.962575965	0.9611818
RSS	422.2386376	137.9886523	477.3882538	622.1360107

Graphical abstract of model fit presented as mean ± 1 SD of the fraction % of released carvedilol:

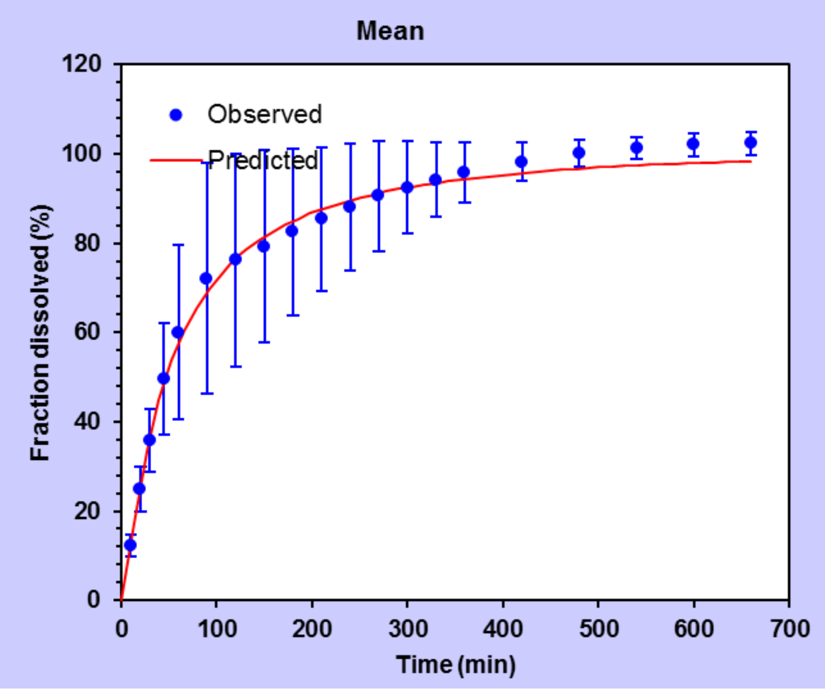

Supplementary materials – Model fitting summary – Kollidon® 25 (modelling the entire relevant dissolution data)

Graphical abstract of model fit presented as the fraction % of released carvedilol per tested tablet:

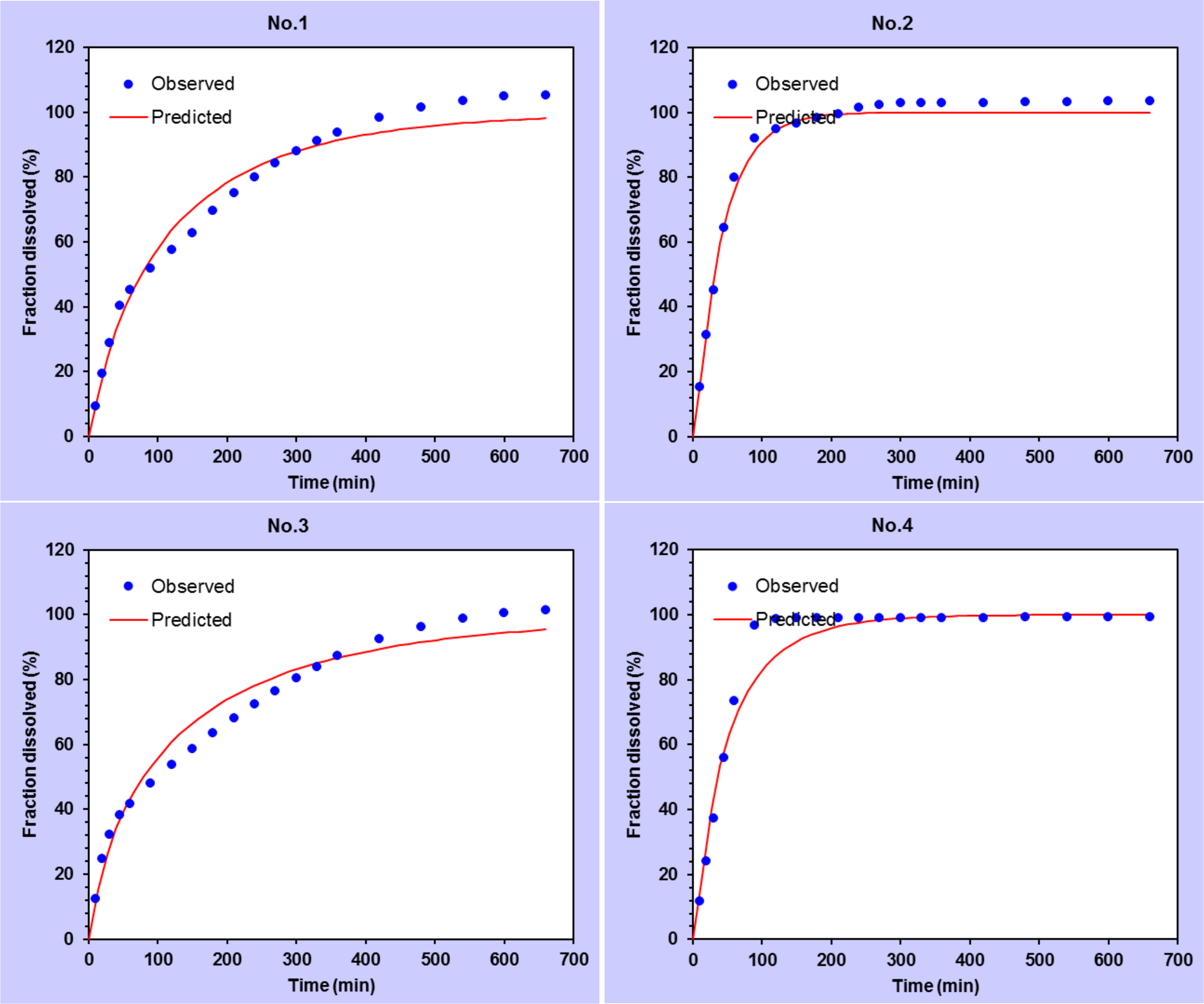

Supplementary materials – Model fitting summary – Kollidon® 25 (modelling the entire relevant dissolution data)

Model: **Weibull_2**

Model equation: $F = 100 \cdot \left(1 - e^{-\frac{t^{\beta}}{\alpha}}\right)$

Fitted model parameters per tested tablet (N = 4) with statistics – mean, standard deviation (SD), and relative standard deviation expressed in % (RSD%) (output from DDSolver):

Parameter	No.1	No.2	No.3	No.4	Mean	SD	RSD(%)
α	65.578	83.960	40.714	40.580	57.708	21.081	36.531
β	0.869	1.194	0.753	0.922	0.935	0.187	20.000

Number of dissolution data points (N), degrees of freedom (df), and selected goodness of fit criteria – Pearson correlation coefficient (R), coefficient of determination (R^2), adjusted coefficient of determination ($R^2_{adjusted}$), and residual sum of squares (RSS) (manual calculation in MS Excel):

Parameter	No.1	No.2	No.3	No.4
N	20	20	20	20
df	18	18	18	18
R	0.989145261	0.996774049	0.985524839	0.979823813
R^2	0.978408348	0.993558505	0.971259209	0.960054704
$R^2_{adjusted}$	0.977208812	0.993200645	0.969662498	0.957835521
RSS	384.0403027	148.1770323	406.5485417	730.5162458

Graphical abstract of model fit presented as mean ± 1 SD of the fraction % of released carvedilol:

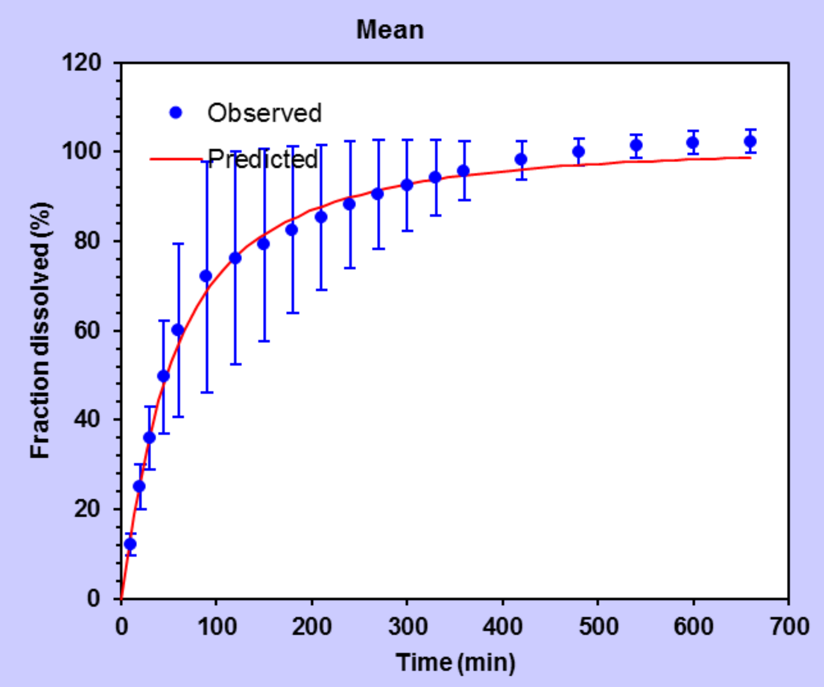

Supplementary materials – Model fitting summary – Kollidon® 25 (modelling the entire relevant dissolution data)

Graphical abstract of model fit presented as the fraction % of released carvedilol per tested tablet:

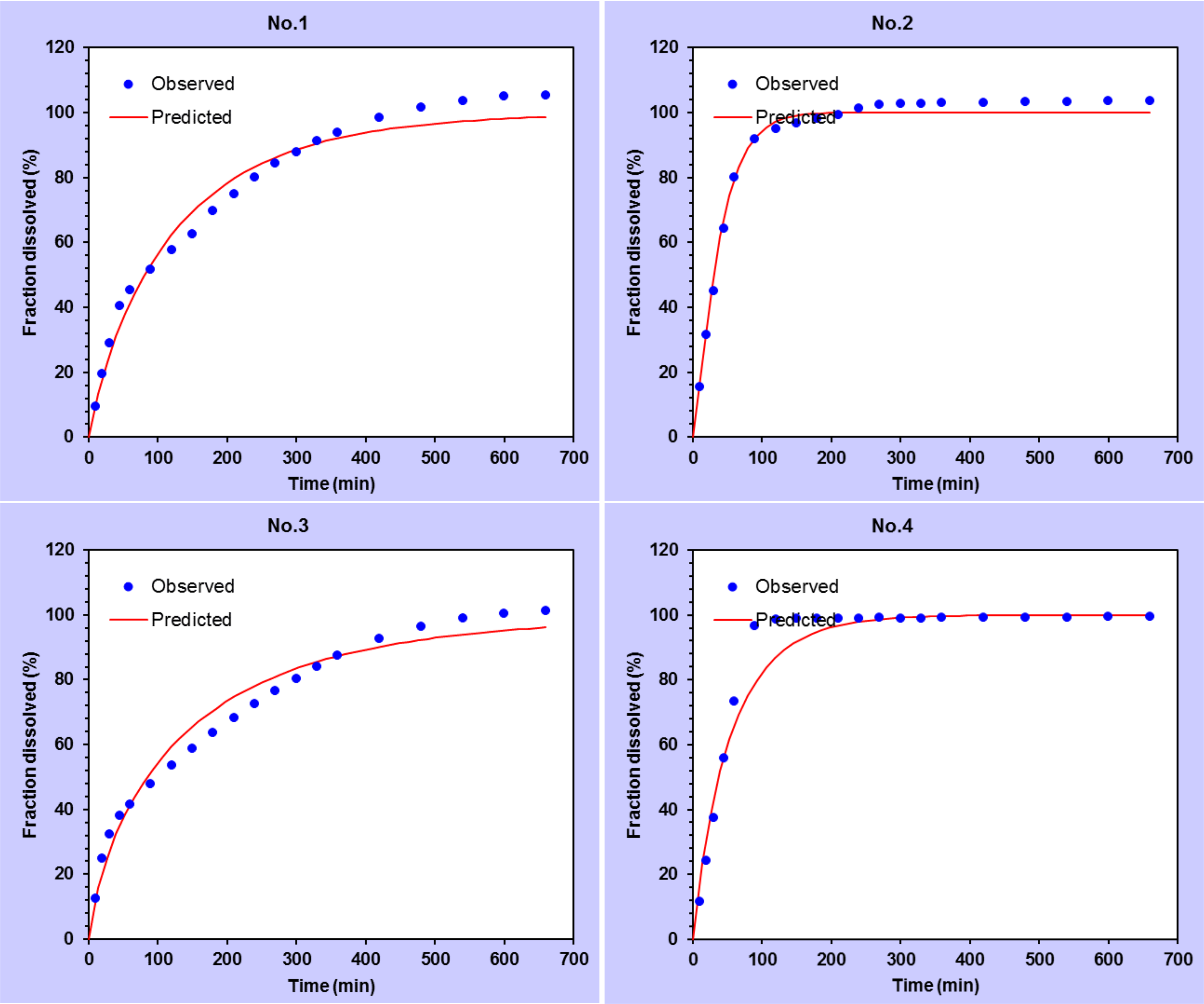

Supplementary materials – Model fitting summary – Kollidon® 25 (modelling the entire relevant dissolution data)

Model: **Weibull_3**

Model equation: $F = F_{max} \cdot \left(1 - e^{-\frac{t^{\beta}}{\alpha}}\right)$

Fitted model parameters per tested tablet (N = 4) with statistics – mean, standard deviation (SD), and relative standard deviation expressed in % (RSD%) (output from DDSolver):

Parameter	No.1	No.2	No.3	No.4	Mean	SD	RSD(%)
α	57.202	16.388	37.255	22.043	33.222	18.254	54.946
β	0.796	0.662	0.708	0.728	0.724	0.056	7.691
F_{max}	110.460	108.780	106.365	104.370	107.494	2.676	2.490

Number of dissolution data points (N), degrees of freedom (df), and selected goodness of fit criteria – Pearson correlation coefficient (R), coefficient of determination (R^2), adjusted coefficient of determination ($R^2_{adjusted}$), and residual sum of squares (RSS) (manual calculation in MS Excel):

Parameter	No.1	No.2	No.3	No.4
N	20	20	20	20
df	17	17	17	17
R	0.995672135	0.964617098	0.991047764	0.948342304
R^2	0.991363	0.930486146	0.982175671	0.899353125
$R^2_{adjusted}$	0.990346882	0.922308046	0.980078692	0.887512316
RSS	156.4939455	1015.513087	258.8321082	1657.186483

Graphical abstract of model fit presented as mean ± 1 SD of the fraction % of released carvedilol:

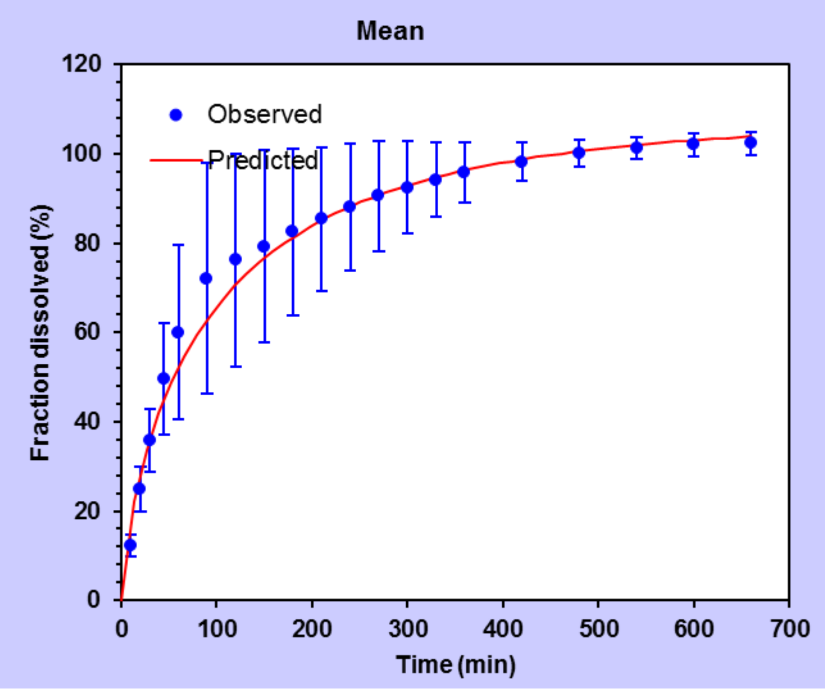

Supplementary materials – Model fitting summary – Kollidon® 25 (modelling the entire relevant dissolution data)

Graphical abstract of model fit presented as the fraction % of released carvedilol per tested tablet:

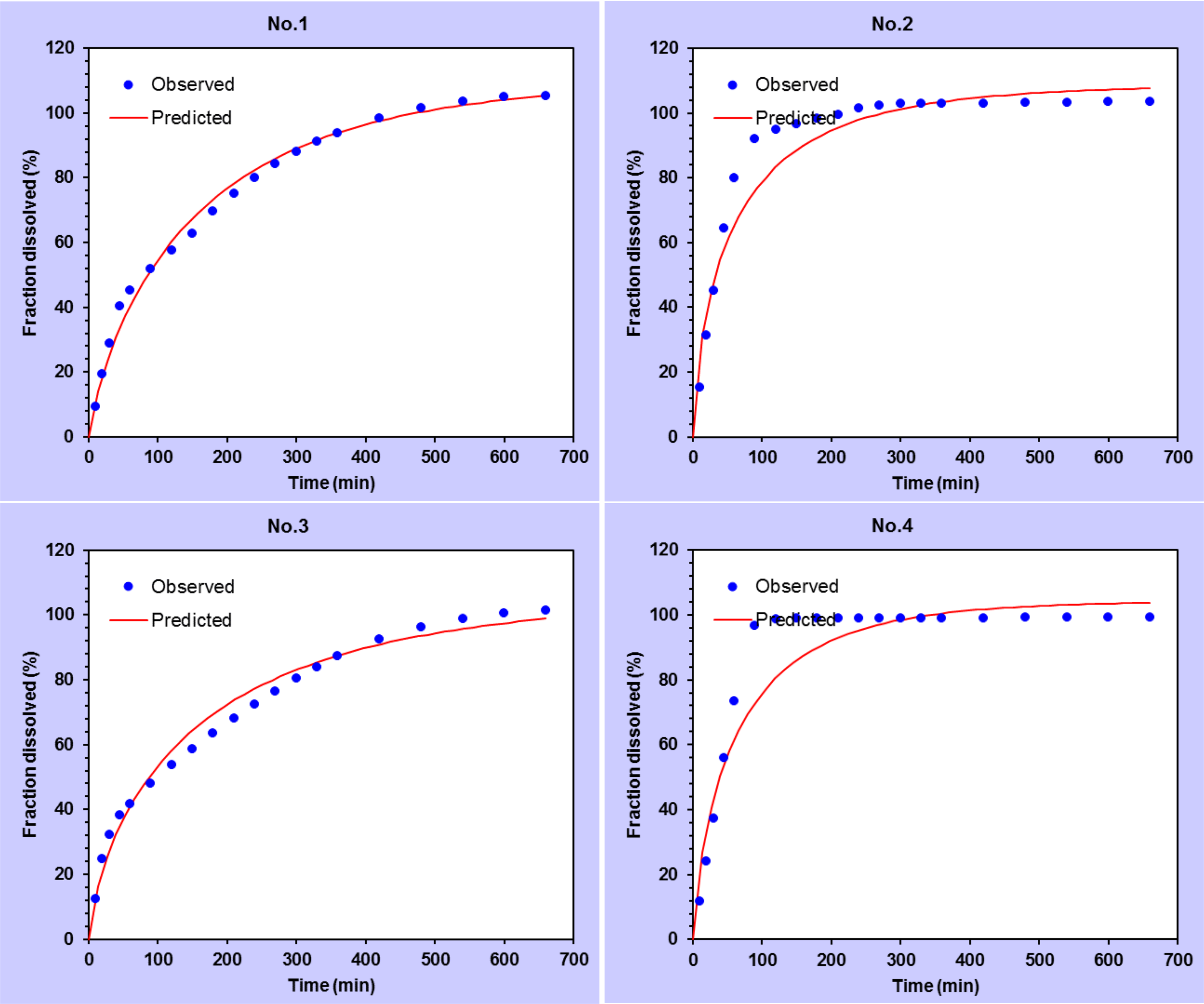

Supplementary materials – Model fitting summary – Kollidon® 25 (modelling the entire relevant dissolution data)

Model: **Weibull_4**

Model equation: $F = F_{max} \cdot \left[1 - e^{-\frac{(t-T_i)^{\beta}}{\alpha}}\right]$

Fitted model parameters per tested tablet (N = 4) with statistics – mean, standard deviation (SD), and relative standard deviation expressed in % (RSD%) (output from DDSolver):

Parameter	No.1	No.2	No.3	No.4	Mean	SD	RSD(%)
α	40.170	12.702	28.995	16.808	24.669	12.437	50.414
β	0.736	0.620	0.630	0.683	0.667	0.053	7.993
T_i	4.000	6.000	4.833	6.000	5.208	0.975	18.728
F_{max}	110.460	108.780	110.593	104.370	108.551	2.907	2.678

Number of dissolution data points (N), degrees of freedom (df), and selected goodness of fit criteria – Pearson correlation coefficient (R), coefficient of determination (R^2), adjusted coefficient of determination ($R^2_{adjusted}$), and residual sum of squares (RSS) (manual calculation in MS Excel):

Parameter	No.1	No.2	No.3	No.4
N	20	20	20	20
df	16	16	16	16
R	0.995330331	0.974875351	0.993535734	0.958146869
R^2	0.990682467	0.95038195	0.987113256	0.918045423
$R^2_{adjusted}$	0.98893543	0.941078565	0.984696991	0.90267894
RSS	164.5622657	774.2575524	320.3808507	1374.488532

Graphical abstract of model fit presented as mean ± 1 SD of the fraction % of released carvedilol:

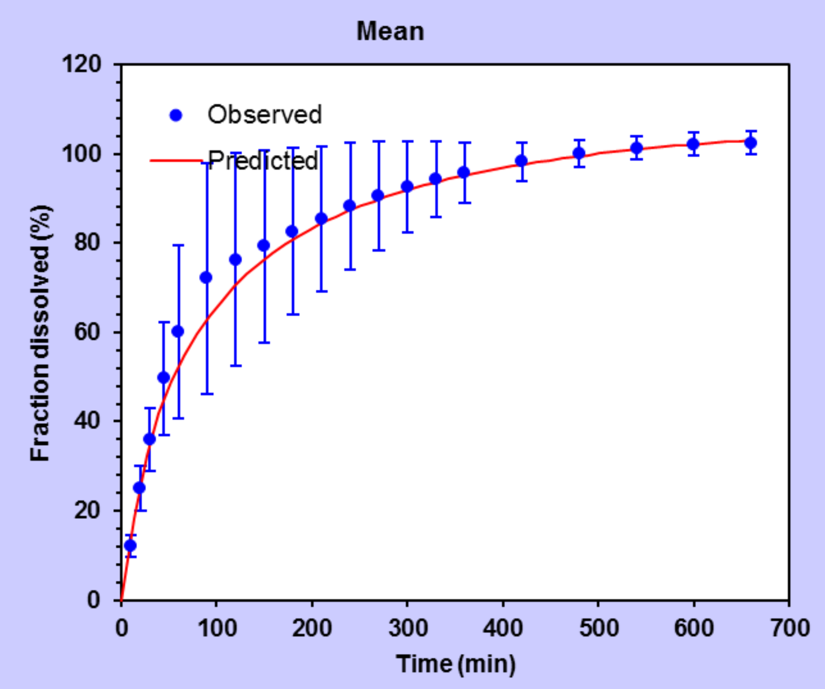

Supplementary materials – Model fitting summary – Kollidon® 25 (modelling the entire relevant dissolution data)

Graphical abstract of model fit presented as the fraction % of released carvedilol per tested tablet:

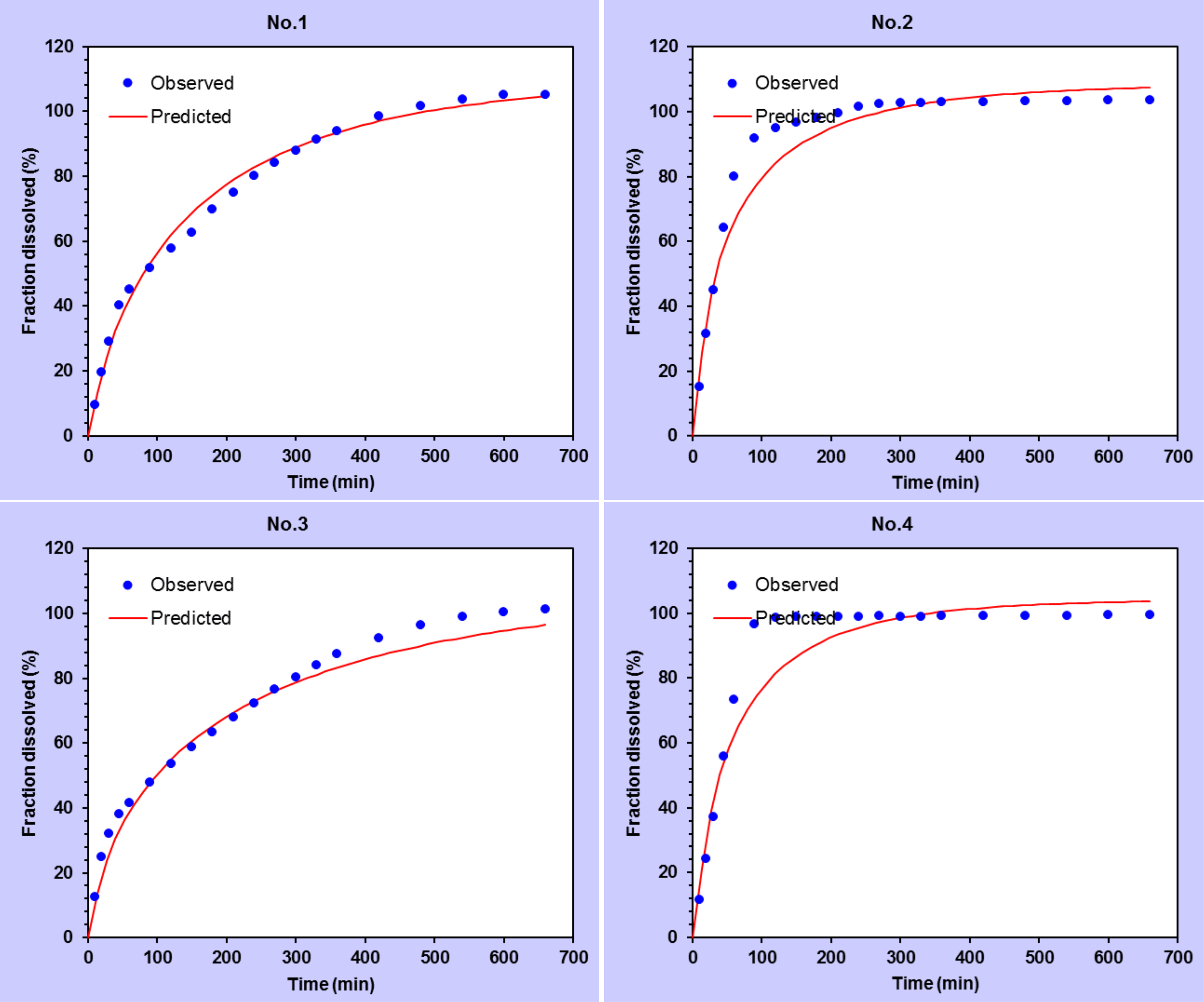

Supplementary materials – Model fitting summary – Kollidon® 25 (modelling the entire relevant dissolution data)

Model: **Logistic_1**

Model equation: $F = 100 \cdot \frac{e^{\alpha+\beta\cdot\log(t)}}{1+e^{\alpha+\beta\cdot\log(t)}}$

Fitted model parameters per tested tablet (N = 4) with statistics – mean, standard deviation (SD), and relative standard deviation expressed in % (RSD%) (output from DDSolver):

Parameter	No.1	No.2	No.3	No.4	Mean	SD	RSD(%)
α	-5.748	-7.252	-5.304	-7.612	-6.479	1.125	-17.361
β	3.148	4.981	2.865	4.818	3.953	1.101	27.861

Number of dissolution data points (N), degrees of freedom (df), and selected goodness of fit criteria – Pearson correlation coefficient (R), coefficient of determination (R^2), adjusted coefficient of determination ($R^2_{adjusted}$), and residual sum of squares (RSS) (manual calculation in MS Excel):

Parameter	No.1	No.2	No.3	No.4
N	20	20	20	20
df	18	18	18	18
R	0.971454838	0.990351662	0.961091929	0.99389096
R^2	0.943724502	0.980796415	0.923697695	0.98781924
$R^2_{adjusted}$	0.940598085	0.97972955	0.919458679	0.987142531
RSS	960.7500943	287.0284623	1182.213487	251.380242

Graphical abstract of model fit presented as mean ± 1 SD of the fraction % of released carvedilol:

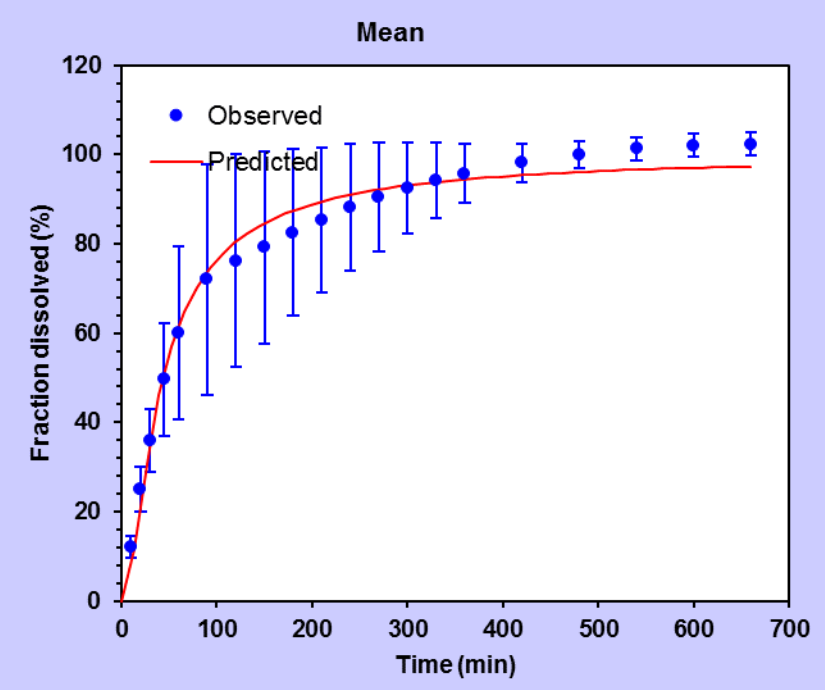

Supplementary materials – Model fitting summary – Kollidon® 25 (modelling the entire relevant dissolution data)

Graphical abstract of model fit presented as the fraction % of released carvedilol per tested tablet:

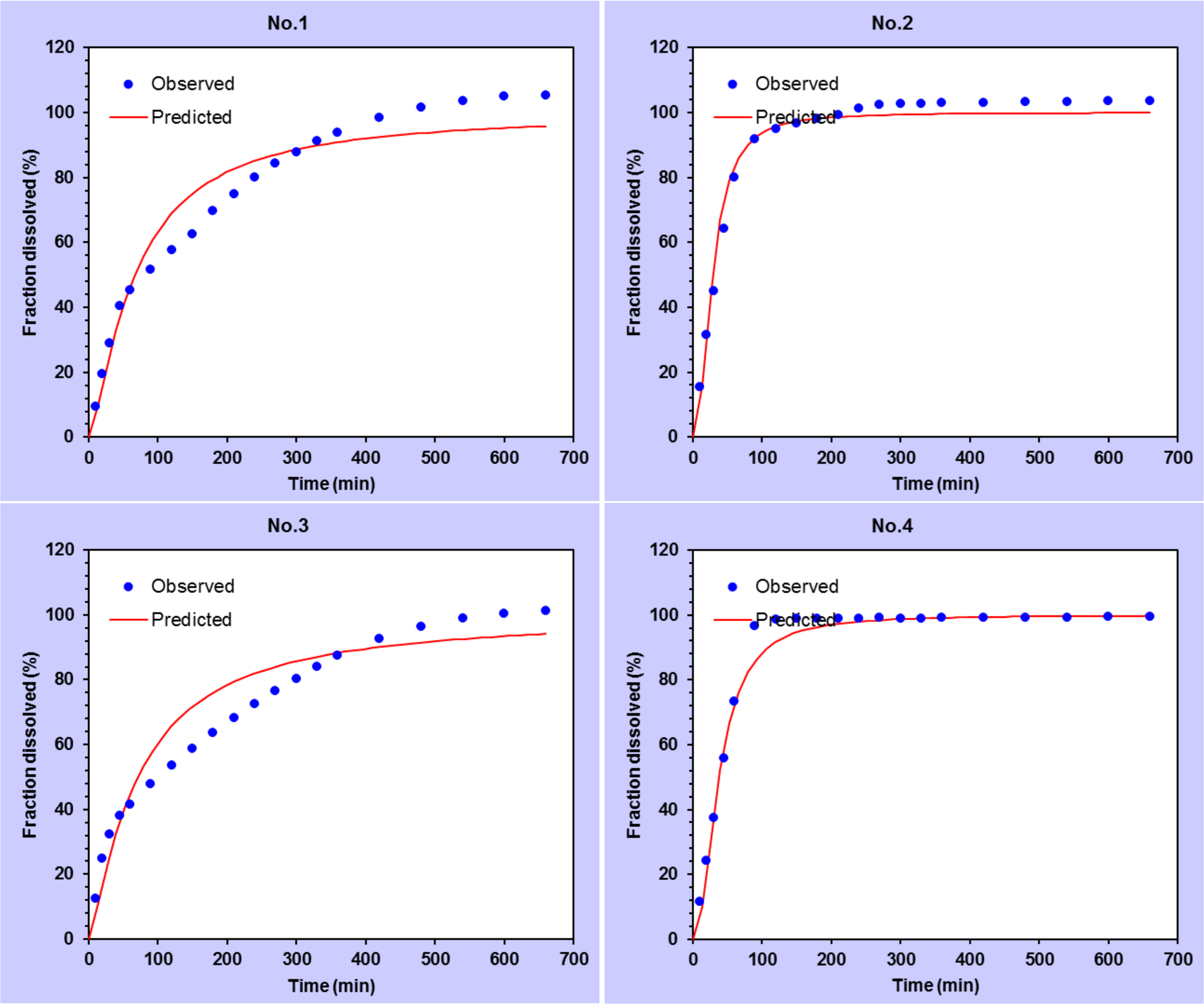

Supplementary materials – Model fitting summary – Kollidon® 25 (modelling the entire relevant dissolution data)

Model: **Logistic_2**

Model equation: $F = F_{max} \cdot \frac{e^{\alpha+\beta\cdot\log(t)}}{1+e^{\alpha+\beta\cdot\log(t)}}$

Fitted model parameters per tested tablet (N = 4) with statistics – mean, standard deviation (SD), and relative standard deviation expressed in % (RSD%) (output from DDSolver):

Parameter	No.1	No.2	No.3	No.4	Mean	SD	RSD(%)
α	-5.459	-4.081	-4.903	-4.322	-4.691	0.617	-13.157
β	2.836	2.702	2.551	2.889	2.745	0.151	5.513
F_{max}	110.460	108.780	106.365	104.370	107.494	2.676	2.490

Number of dissolution data points (N), degrees of freedom (df), and selected goodness of fit criteria – Pearson correlation coefficient (R), coefficient of determination (R^2), adjusted coefficient of determination ($R^2_{adjusted}$), and residual sum of squares (RSS) (manual calculation in MS Excel):

Parameter	No.1	No.2	No.3	No.4
N	20	20	20	20
df	17	17	17	17
R	0.984238655	0.990491996	0.973233276	0.978870894
R^2	0.968725731	0.981074394	0.94718301	0.958188227
$R^2_{adjusted}$	0.965046405	0.978847853	0.940969247	0.953269195
RSS	617.6690834	338.2867768	853.1713236	942.8907706

Graphical abstract of model fit presented as mean ± 1 SD of the fraction % of released carvedilol:

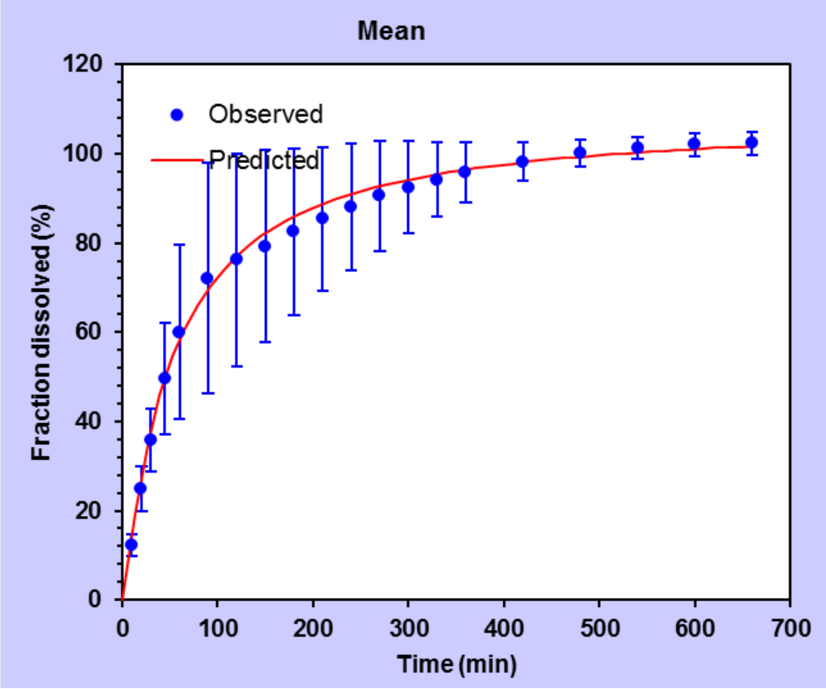

Supplementary materials – Model fitting summary – Kollidon® 25 (modelling the entire relevant dissolution data)

Graphical abstract of model fit presented as the fraction % of released carvedilol per tested tablet:

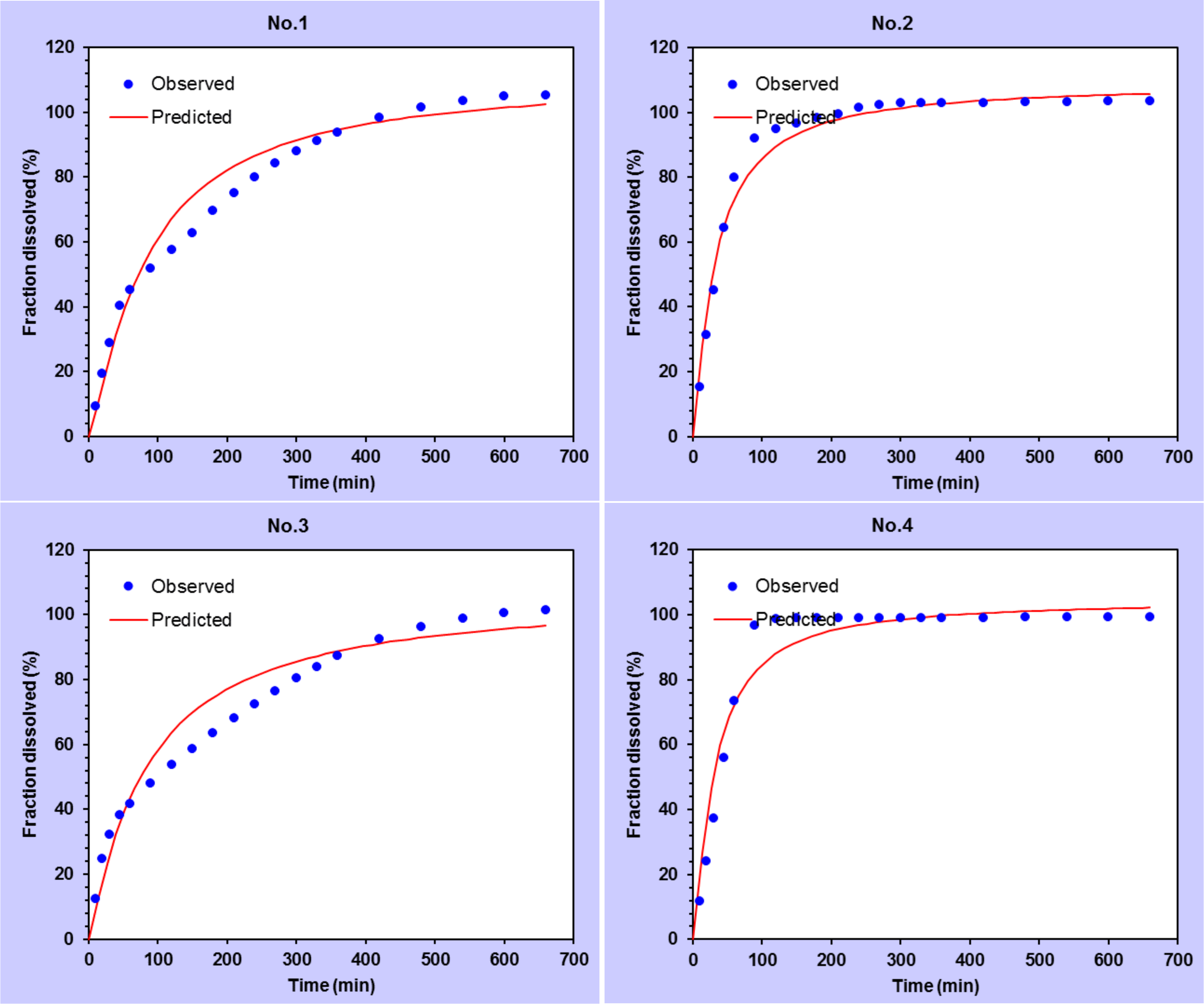

Supplementary materials – Model fitting summary – Kollidon® 25 (modelling the entire relevant dissolution data)

Model: **Logistic_3**

Model equation: $F = F_{max} \cdot \frac{1}{1+e^{-k\cdot(t-\gamma)}}$

Fitted model parameters per tested tablet (N = 4) with statistics – mean, standard deviation (SD), and relative standard deviation expressed in % (RSD%) (output from DDSolver):

Parameter	No.1	No.2	No.3	No.4	Mean	SD	RSD(%)
k	0.011	0.005	0.007	0.005	0.007	0.003	35.542
γ	146.529	-83.559	149.793	-124.064	22.175	146.419	660.296
F_{max}	110.460	108.780	106.365	104.370	107.494	2.676	2.490

Number of dissolution data points (N), degrees of freedom (df), and selected goodness of fit criteria – Pearson correlation coefficient (R), coefficient of determination (R^2), adjusted coefficient of determination ($R^2_{adjusted}$), and residual sum of squares (RSS) (manual calculation in MS Excel):

Parameter	No.1	No.2	No.3	No.4
N	20	20	20	20
df	17	17	17	17
R	0.978556132	0.822658245	0.981206078	0.790484349
R^2	0.957572104	0.676766589	0.962765368	0.624865507
$R^2_{adjusted}$	0.952580586	0.638739129	0.958384823	0.580732037
RSS	1090.799194	5850.729227	551.3843252	8401.472977

Graphical abstract of model fit presented as mean ± 1 SD of the fraction % of released carvedilol:

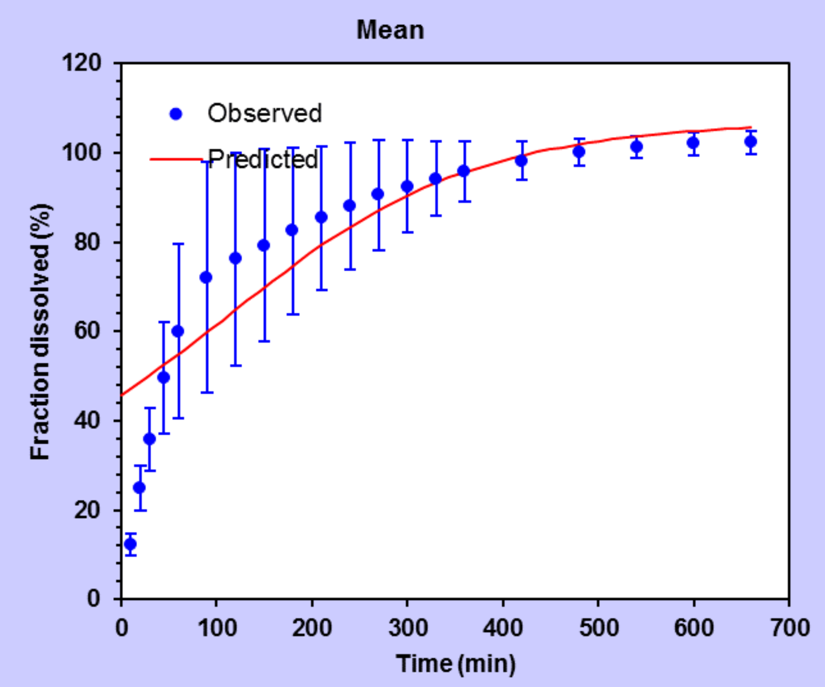

Supplementary materials – Model fitting summary – Kollidon® 25 (modelling the entire relevant dissolution data)

Graphical abstract of model fit presented as the fraction % of released carvedilol per tested tablet:

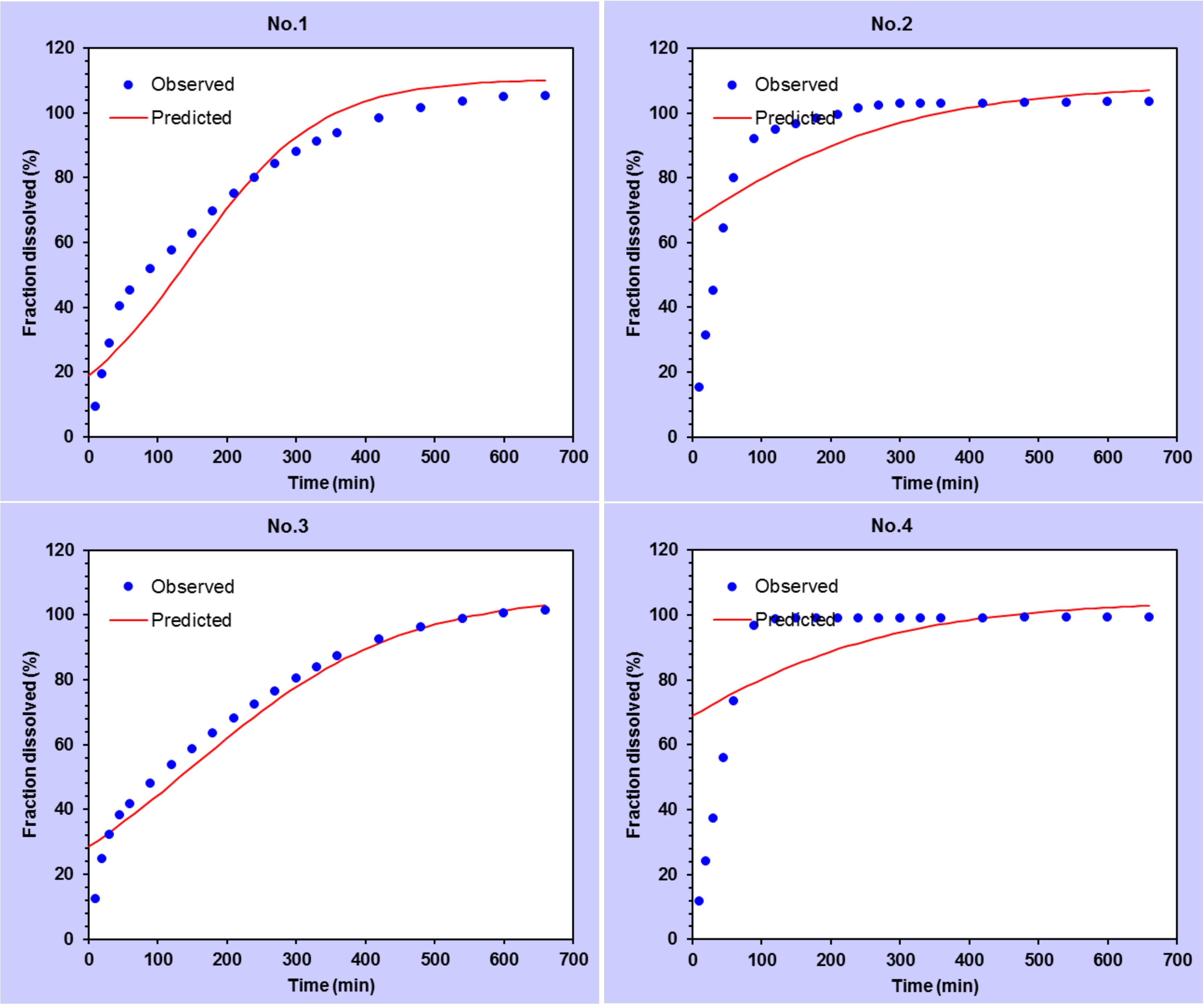

Supplementary materials – Model fitting summary – Kollidon® 25 (modelling the entire relevant dissolution data)

Model: **Gompertz_1**

Model equation: $F = 100 \cdot e^{-\alpha \cdot e^{-\beta \cdot \log(t)}}$

Fitted model parameters per tested tablet (N = 4) with statistics – mean, standard deviation (SD), and relative standard deviation expressed in % (RSD%) (output from DDSolver):

Parameter	No.1	No.2	No.3	No.4	Mean	SD	RSD(%)
α	47.200	413.963	40.208	168.324	167.424	174.566	104.266
β	2.396	4.218	2.258	3.732	3.151	0.974	30.903

Number of dissolution data points (N), degrees of freedom (df), and selected goodness of fit criteria – Pearson correlation coefficient (R), coefficient of determination (R^2), adjusted coefficient of determination ($R^2_{adjusted}$), and residual sum of squares (RSS) (manual calculation in MS Excel):

Parameter	No.1	No.2	No.3	No.4
N	20	20	20	20
df	18	18	18	18
R	0.955799519	0.993004346	0.94110599	0.978465684
R^2	0.913552721	0.986057631	0.885680485	0.957395094
$R^2_{adjusted}$	0.908750095	0.985283055	0.879329401	0.955028155
RSS	1475.498541	572.4981929	1879.214255	654.4897631

Graphical abstract of model fit presented as mean ± 1 SD of the fraction % of released carvedilol:

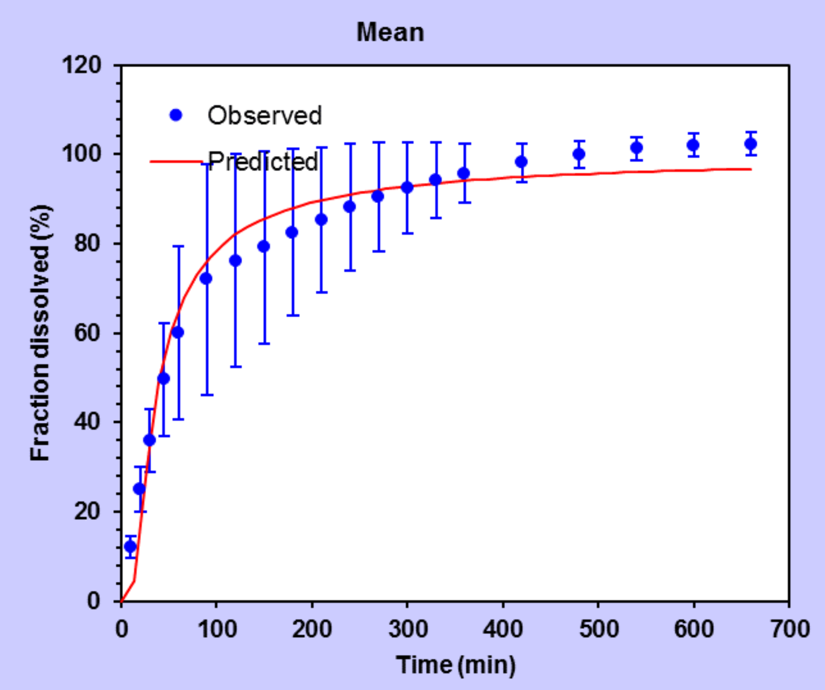

Supplementary materials – Model fitting summary – Kollidon® 25 (modelling the entire relevant dissolution data)

Graphical abstract of model fit presented as the fraction % of released carvedilol per tested tablet:

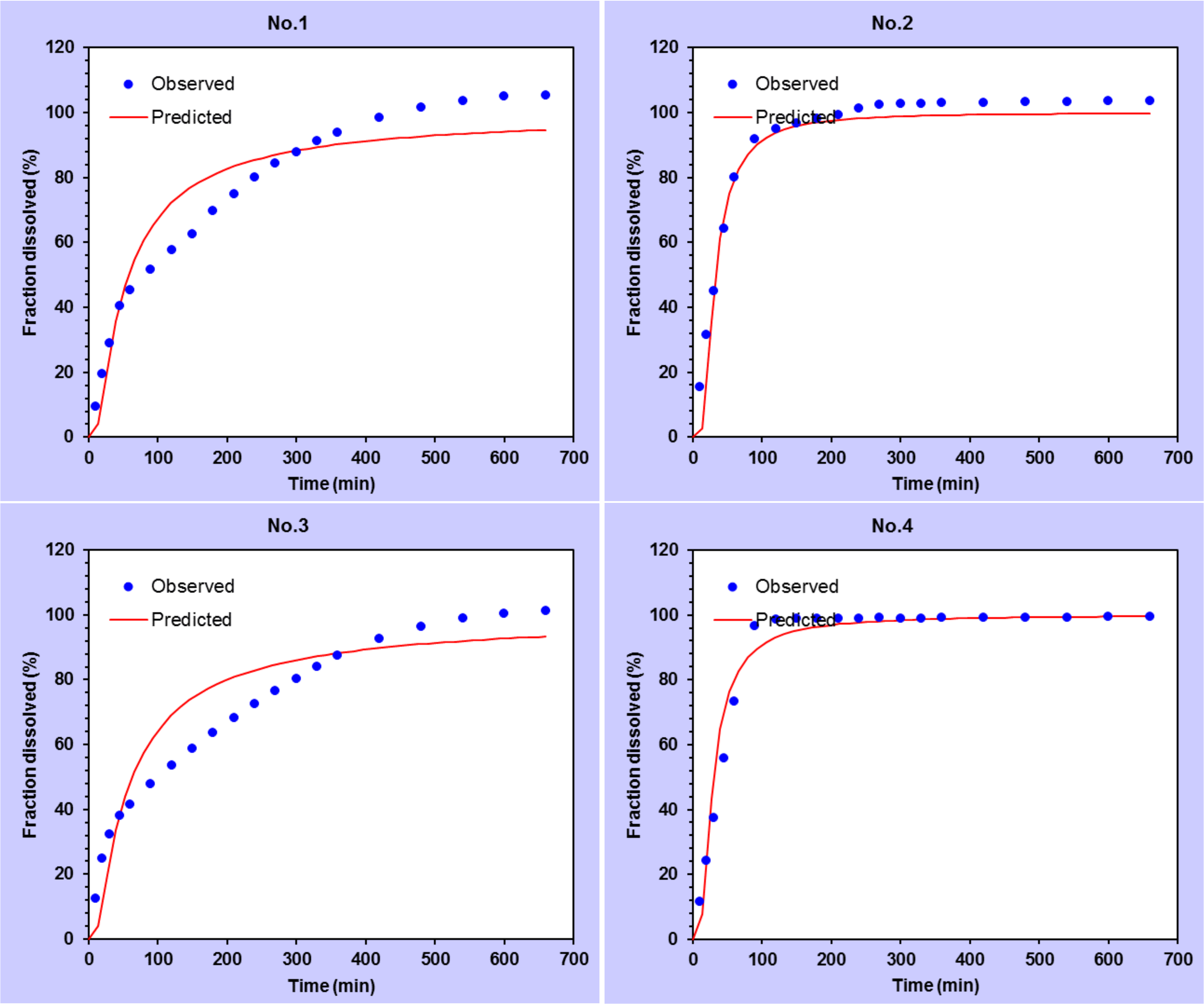

Supplementary materials – Model fitting summary – Kollidon® 25 (modelling the entire relevant dissolution data)

Model: **Gompertz_2**

Model equation: $F = F_{max} \cdot e^{-\alpha \cdot e^{-\beta \cdot \log(t)}}$

Fitted model parameters per tested tablet (N = 4) with statistics – mean, standard deviation (SD), and relative standard deviation expressed in % (RSD%) (output from DDSolver):

Parameter	No.1	No.2	No.3	No.4	Mean	SD	RSD(%)
α	37.013	23.228	26.662	23.451	27.589	6.476	23.472
β	2.144	2.269	1.958	2.170	2.135	0.130	6.074
F_{max}	110.460	109.838	106.365	109.464	109.032	1.825	1.673

Number of dissolution data points (N), degrees of freedom (df), and selected goodness of fit criteria – Pearson correlation coefficient (R), coefficient of determination (R^2), adjusted coefficient of determination ($R^2_{adjusted}$), and residual sum of squares (RSS) (manual calculation in MS Excel):

Parameter	No.1	No.2	No.3	No.4
N	20	20	20	20
df	17	17	17	17
R	0.970269545	0.992804314	0.954771464	0.973588003
R^2	0.941422991	0.985660405	0.911588548	0.947873599
$R^2_{adjusted}$	0.934531578	0.983973394	0.9011872	0.941741081
RSS	1220.885767	250.3794448	1521.977626	894.2895926

Graphical abstract of model fit presented as mean ± 1 SD of the fraction % of released carvedilol:

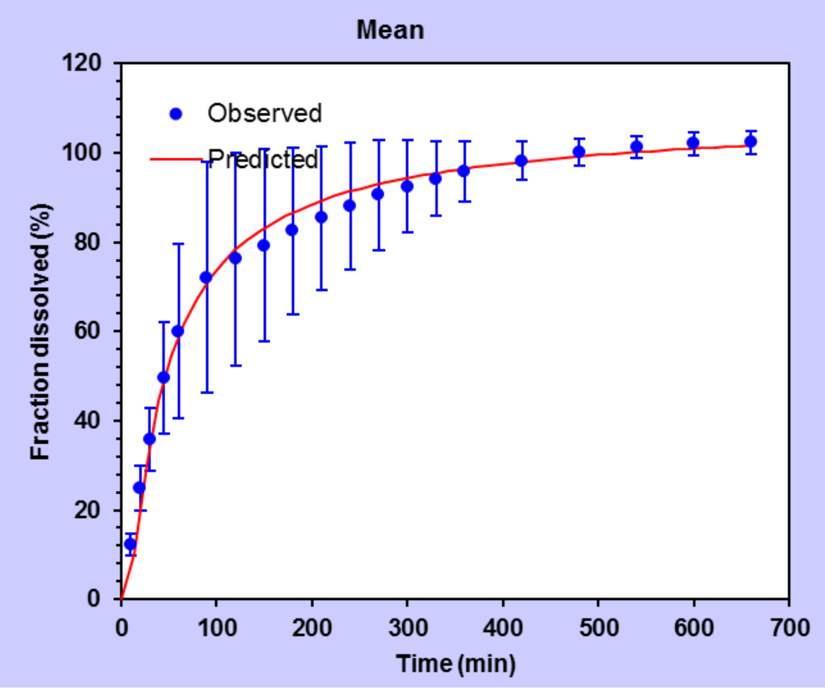

Supplementary materials – Model fitting summary – Kollidon® 25 (modelling the entire relevant dissolution data)

Graphical abstract of model fit presented as the fraction % of released carvedilol per tested tablet:

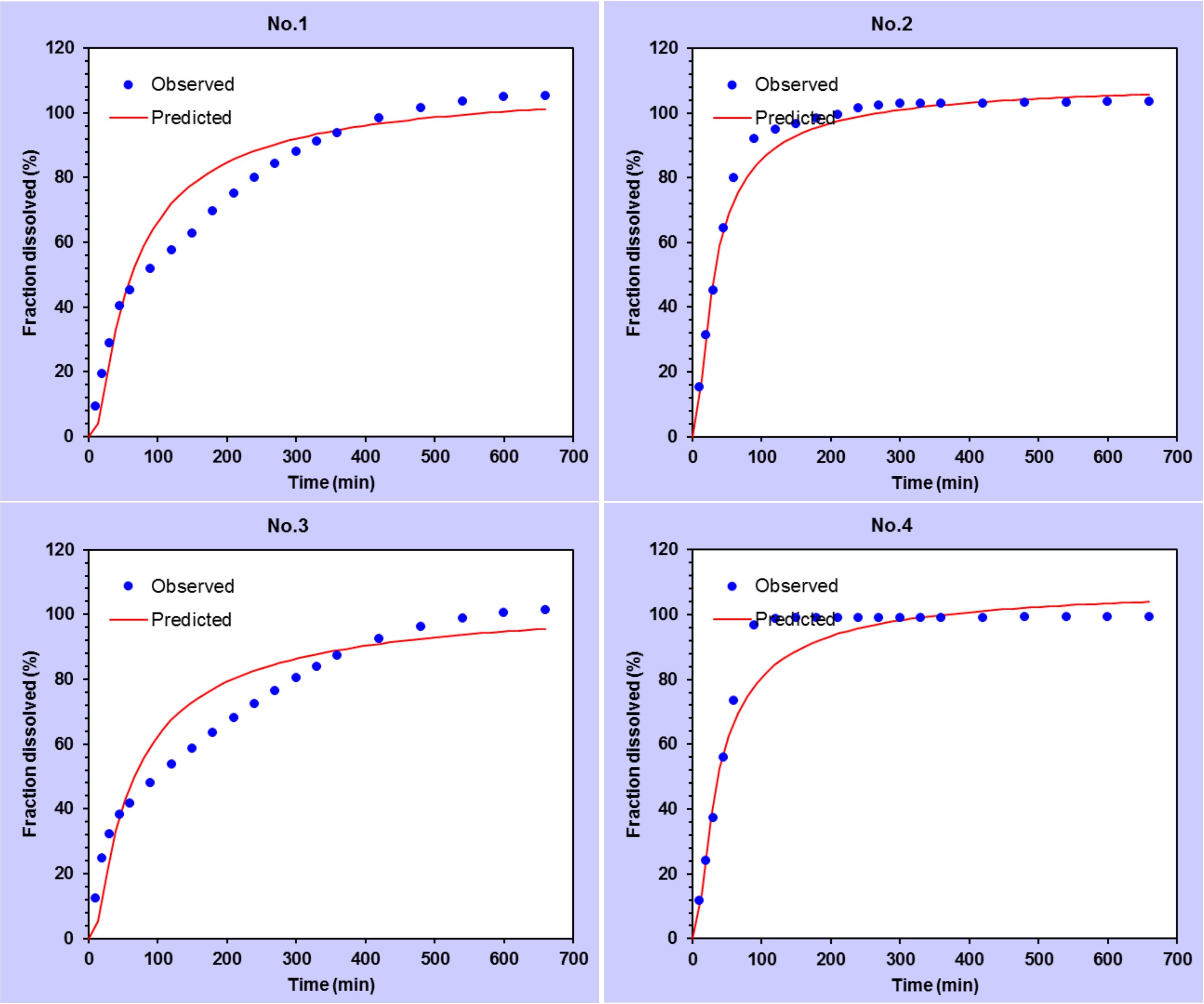

Supplementary materials – Model fitting summary – Kollidon® 25 (modelling the entire relevant dissolution data)

Model: **Gompertz_3**

Model equation: $F = F_{max} \cdot e^{-e^{-k \cdot (t-\gamma)}}$

Fitted model parameters per tested tablet (N = 4) with statistics – mean, standard deviation (SD), and relative standard deviation expressed in % (RSD%) (output from DDSolver):

Parameter	No.1	No.2	No.3	No.4	Mean	SD	RSD(%)
k	0.006	0.005	0.005	0.004	0.005	0.001	12.269
γ	63.544	-177.971	67.054	-243.956	-72.832	161.765	-222.107
F_{max}	110.460	108.780	106.365	104.370	107.494	2.676	2.490

Number of dissolution data points (N), degrees of freedom (df), and selected goodness of fit criteria – Pearson correlation coefficient (R), coefficient of determination (R^2), adjusted coefficient of determination ($R^2_{adjusted}$), and residual sum of squares (RSS) (manual calculation in MS Excel):

Parameter	No.1	No.2	No.3	No.4
N	20	20	20	20
df	17	17	17	17
R	0.982943796	0.831744036	0.989292281	0.793253052
R^2	0.966178505	0.691798141	0.978699217	0.629250405
$R^2_{adjusted}$	0.962199506	0.655539099	0.976193242	0.585632805
RSS	648.4911533	6500.73204	305.4541389	9750.446472

Graphical abstract of model fit presented as mean ± 1 SD of the fraction % of released carvedilol:

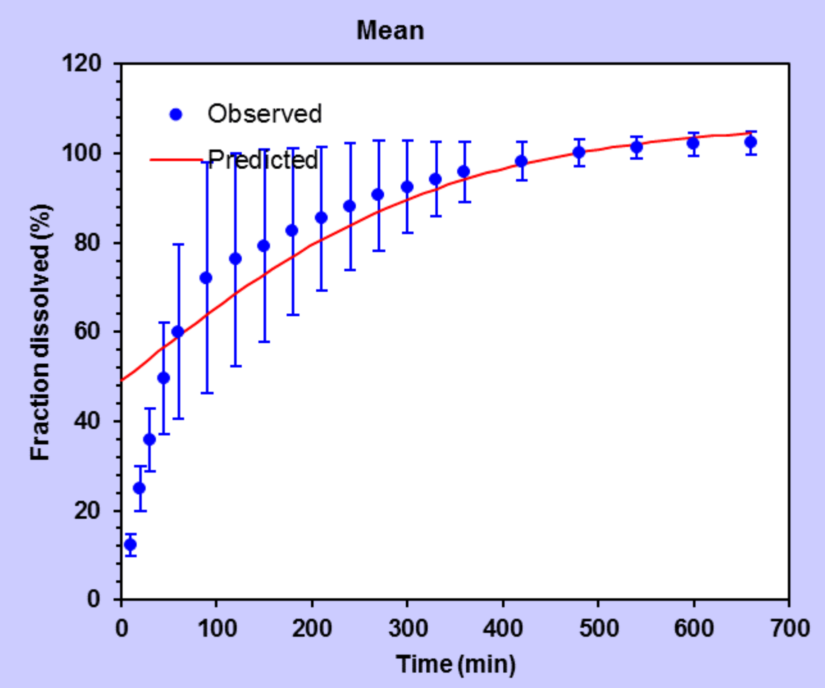

Supplementary materials – Model fitting summary – Kollidon® 25 (modelling the entire relevant dissolution data)

Graphical abstract of model fit presented as the fraction % of released carvedilol per tested tablet:

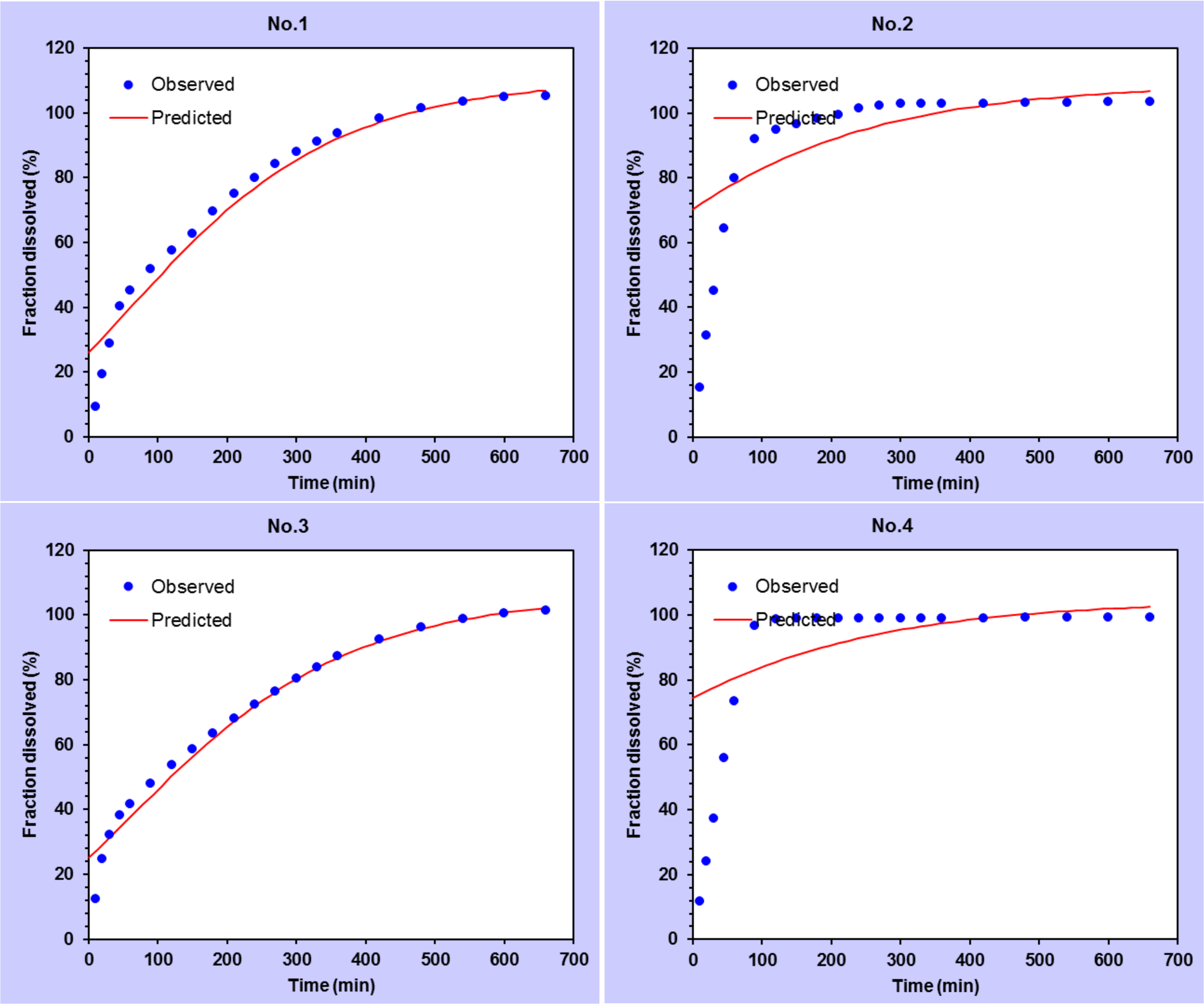

Supplementary materials – Model fitting summary – Kollidon® 25 (modelling the entire relevant dissolution data)

Model: **Gompertz_4**

Model equation: $F = F_{max} \cdot e^{-\beta \cdot e^{-k \cdot t}}$

Fitted model parameters per tested tablet (N = 4) with statistics – mean, standard deviation (SD), and relative standard deviation expressed in % (RSD%) (output from DDSolver):

Parameter	No.1	No.2	No.3	No.4	Mean	SD	RSD(%)
k	0.006	0.005	0.005	0.004	0.005	0.001	12.269
β	1.441	0.652	1.439	0.509	1.011	0.500	49.444
F_{max}	110.460	108.780	106.365	104.370	107.494	2.676	2.490

Number of dissolution data points (N), degrees of freedom (df), and selected goodness of fit criteria – Pearson correlation coefficient (R), coefficient of determination (R^2), adjusted coefficient of determination ($R^2_{adjusted}$), and residual sum of squares (RSS) (manual calculation in MS Excel):

Parameter	No.1	No.2	No.3	No.4
N	20	20	20	20
df	17	17	17	17
R	0.982943796	0.819179949	0.989292281	0.782433718
R^2	0.966178505	0.671055789	0.978699217	0.612202523
$R^2_{adjusted}$	0.962199506	0.63235647	0.976193242	0.56657929
RSS	648.4911533	5307.474555	305.4541389	7617.045553

Graphical abstract of model fit presented as mean ± 1 SD of the fraction % of released carvedilol:

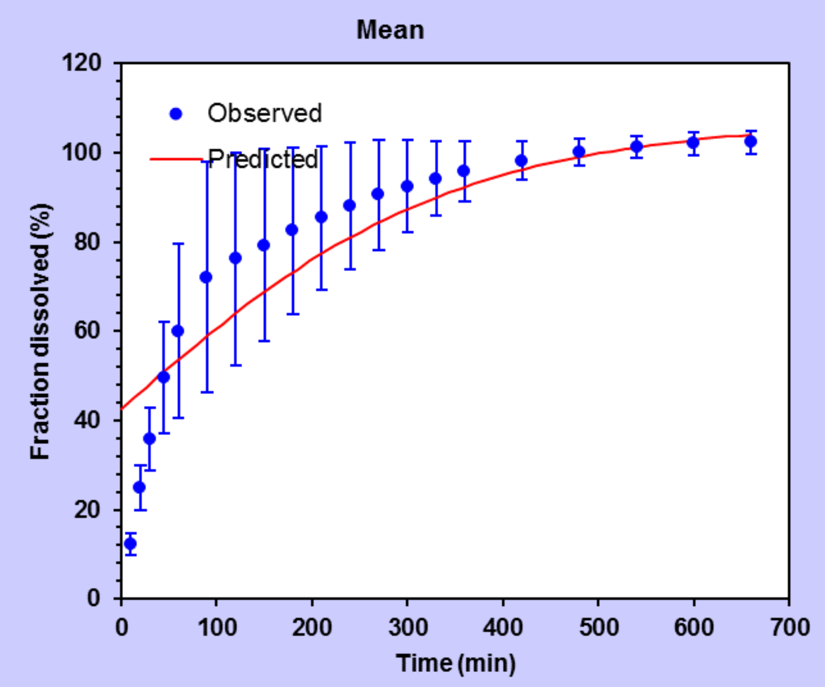

Supplementary materials – Model fitting summary – Kollidon® 25 (modelling the entire relevant dissolution data)

Graphical abstract of model fit presented as the fraction % of released carvedilol per tested tablet:

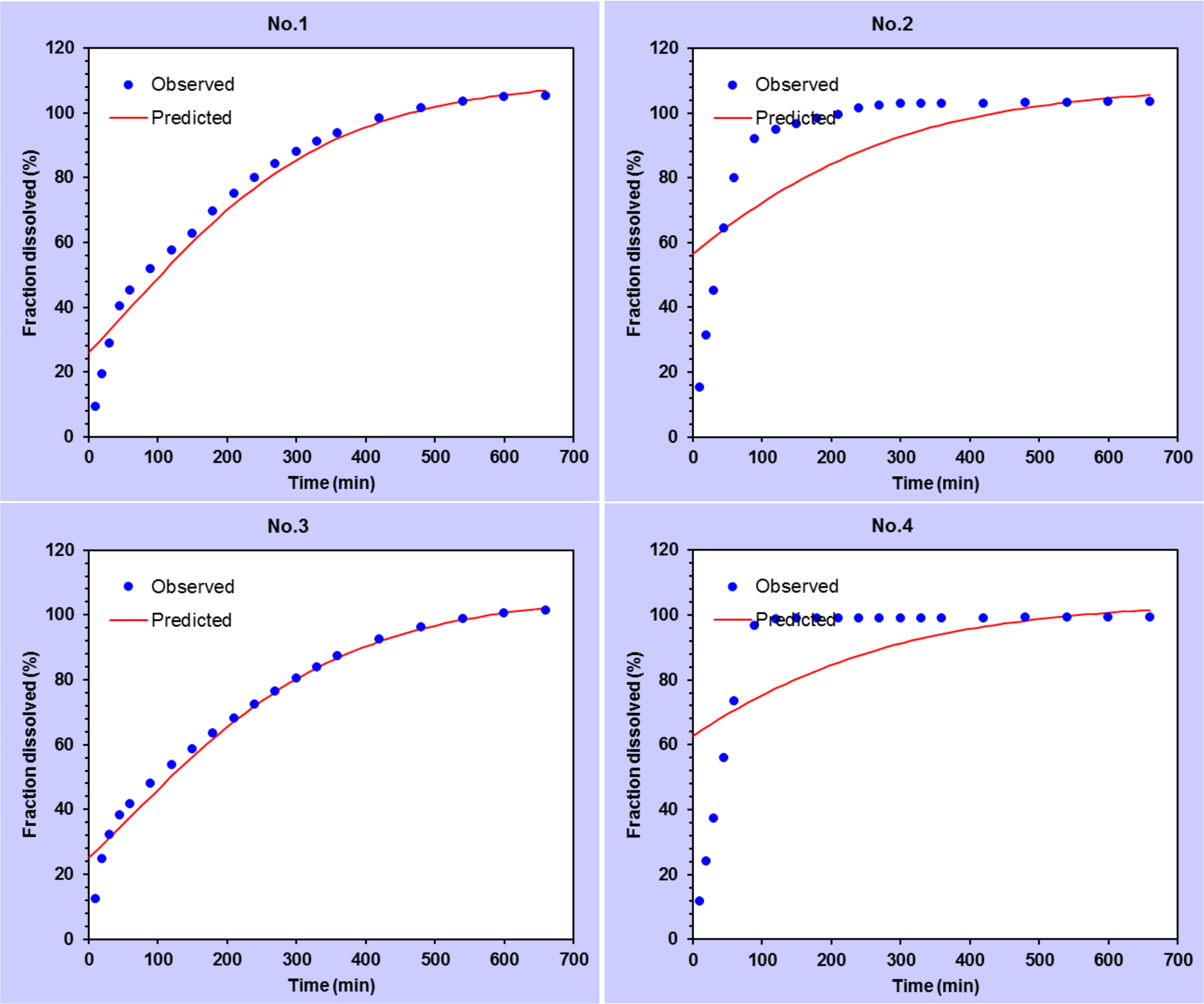

Supplementary materials – Model fitting summary – Kollidon® 25 (modelling the entire relevant dissolution data)

Model: **Probit_1**

Model equation: $F = 100 \cdot \phi[\alpha + \beta \cdot \log(t)]$

Fitted model parameters per tested tablet (N = 4) with statistics – mean, standard deviation (SD), and relative standard deviation expressed in % (RSD%) (output from DDSolver):

Parameter	No.1	No.2	No.3	No.4	Mean	SD	RSD(%)
α	-3.319	-3.918	-3.045	-3.931	-3.553	0.443	-12.469
β	1.813	2.681	1.638	2.464	2.149	0.502	23.351

Number of dissolution data points (N), degrees of freedom (df), and selected goodness of fit criteria – Pearson correlation coefficient (R), coefficient of determination (R^2), adjusted coefficient of determination ($R^2_{adjusted}$), and residual sum of squares (RSS) (manual calculation in MS Excel):

Parameter	No.1	No.2	No.3	No.4
N	20	20	20	20
df	18	18	18	18
R	0.977443367	0.994465922	0.968294896	0.988541249
R^2	0.955395535	0.98896247	0.937595006	0.977213801
$R^2_{adjusted}$	0.952917509	0.988349274	0.934128062	0.975947901
RSS	774.2652508	194.0775042	915.0079376	477.629334

Graphical abstract of model fit presented as mean ± 1 SD of the fraction % of released carvedilol:

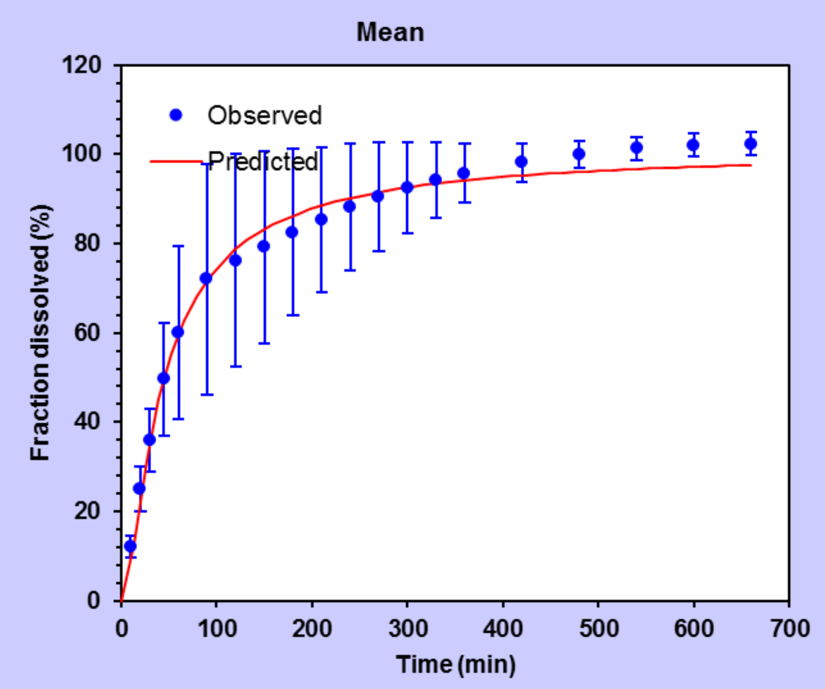

Supplementary materials – Model fitting summary – Kollidon® 25 (modelling the entire relevant dissolution data)

Graphical abstract of model fit presented as the fraction % of released carvedilol per tested tablet:

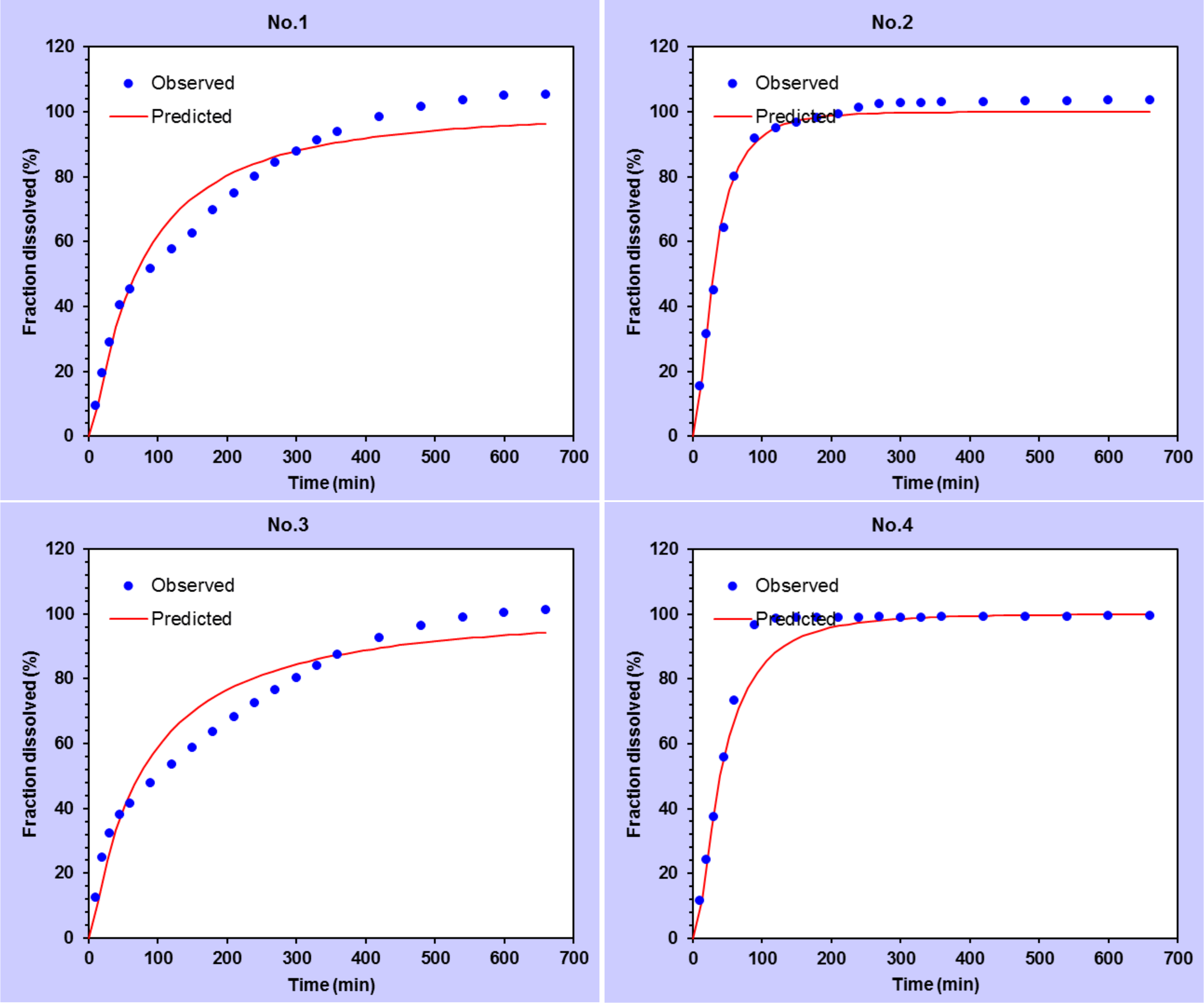

Supplementary materials – Model fitting summary – Kollidon® 25 (modelling the entire relevant dissolution data)

Model: **Probit_2**

Model equation: $F = F_{max} \cdot \phi[\alpha + \beta \cdot \log(t)]$

Fitted model parameters per tested tablet (N = 4) with statistics – mean, standard deviation (SD), and relative standard deviation expressed in % (RSD%) (output from DDSolver):

Parameter	No.1	No.2	No.3	No.4	Mean	SD	RSD(%)
α	-3.179	-2.310	-2.881	-2.465	-2.709	0.395	-14.589
β	1.650	1.530	1.496	1.636	1.578	0.077	4.858
F_{max}	110.460	108.780	106.365	104.370	107.494	2.676	2.490

Number of dissolution data points (N), degrees of freedom (df), and selected goodness of fit criteria – Pearson correlation coefficient (R), coefficient of determination (R^2), adjusted coefficient of determination ($R^2_{adjusted}$), and residual sum of squares (RSS) (manual calculation in MS Excel):

Parameter	No.1	No.2	No.3	No.4
N	20	20	20	20
df	17	17	17	17
R	0.987525941	0.984911788	0.977106245	0.97184005
R^2	0.975207485	0.970051229	0.954736614	0.944473083
$R^2_{adjusted}$	0.972290718	0.966527845	0.949411509	0.937940505
RSS	466.80762	500.1534439	695.7546594	1116.634876

Graphical abstract of model fit presented as mean ± 1 SD of the fraction % of released carvedilol:

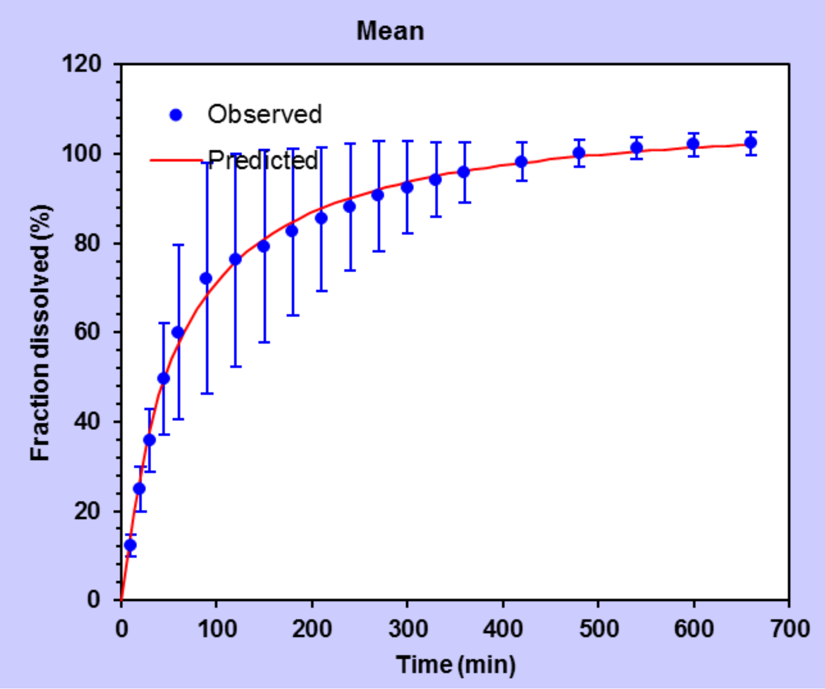

Supplementary materials – Model fitting summary – Kollidon® 25 (modelling the entire relevant dissolution data)

Graphical abstract of model fit presented as the fraction % of released carvedilol per tested tablet:

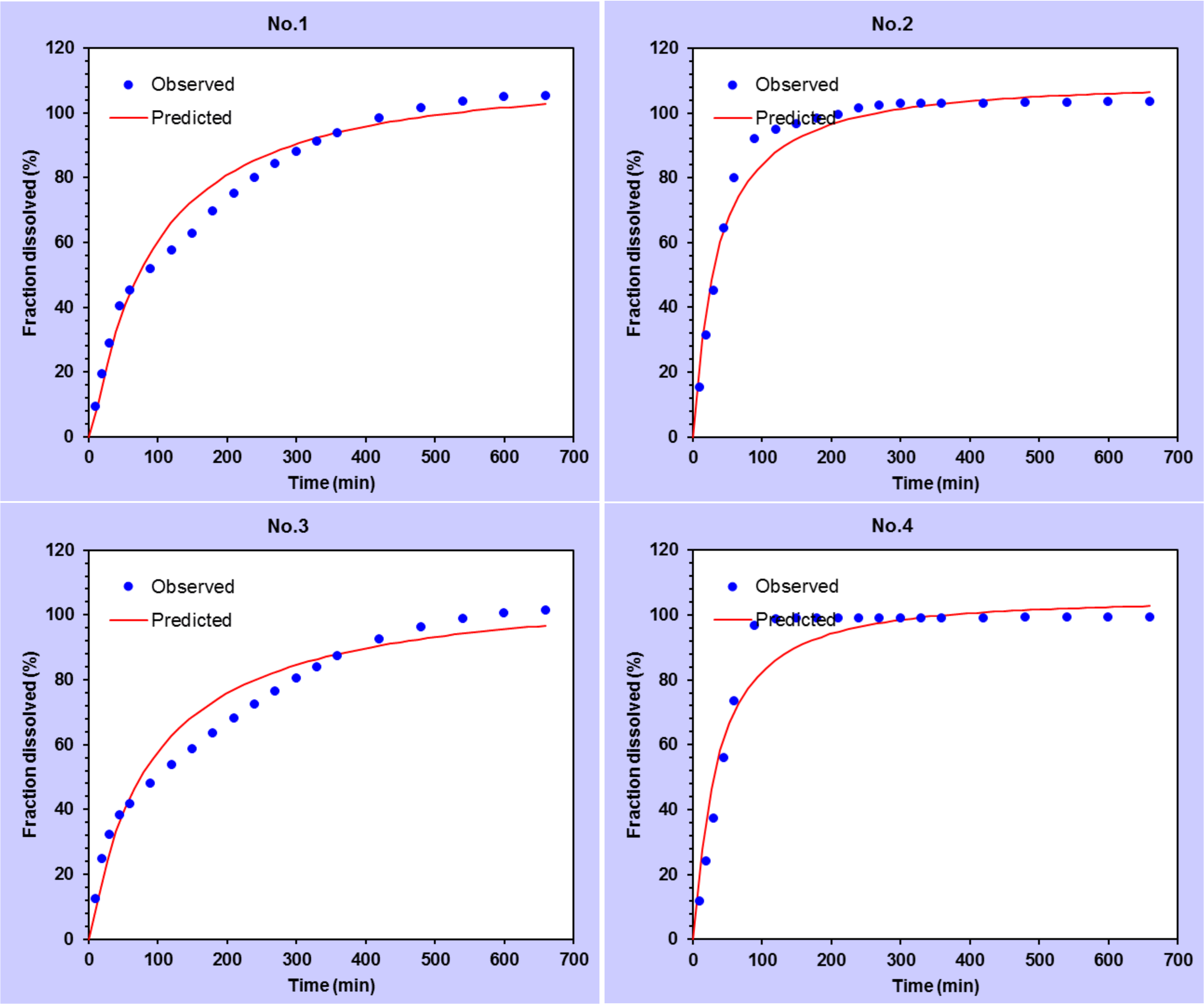

Supplementary materials – Model fitting summary – Kollidon® 25 (modelling up to app. 60 % of carvedilol released)

Model: **Zero–order**

Model equation: $F = k_0 \cdot t$

Fitted model parameters per tested tablet (N = 4) with statistics – mean, standard deviation (SD), and relative standard deviation expressed in % (RSD%) (output from DDSolver):

Parameter	No.1	No.2	No.3	No.4	Mean	SD	RSD(%)
k_0	0.836	1.399	0.825	1.229	1.072	0.288	26.818

Number of dissolution data points (N), degrees of freedom (df), and selected goodness of fit criteria – Pearson correlation coefficient (R), coefficient of determination (R^2), adjusted coefficient of determination ($R^2_{adjusted}$), and residual sum of squares (RSS) (manual calculation in MS Excel):

Parameter	No.1	No.2	No.3	No.4
N	5	5	5	5
df	4	4	4	4
R	0.982294892	0.996964941	0.947703854	0.999759117
R^2	0.964903254	0.993939093	0.898142594	0.999518293
$R^2_{adjusted}$	0.964903254	0.993939093	0.898142594	0.999518293
RSS	54.64003559	41.23569395	205.8151246	1.352811388

Graphical abstract of model fit presented as mean ± 1 SD of the fraction % of released carvedilol:

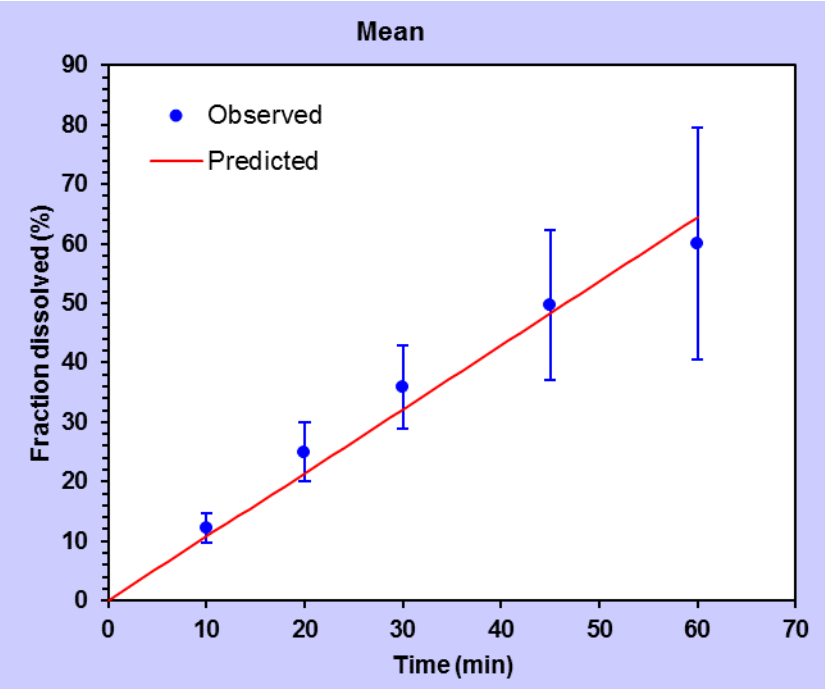

Supplementary materials – Model fitting summary – Kollidon® 25 (modelling up to app. 60 % of carvedilol released)

Graphical abstract of model fit presented as the fraction % of released carvedilol per tested tablet:

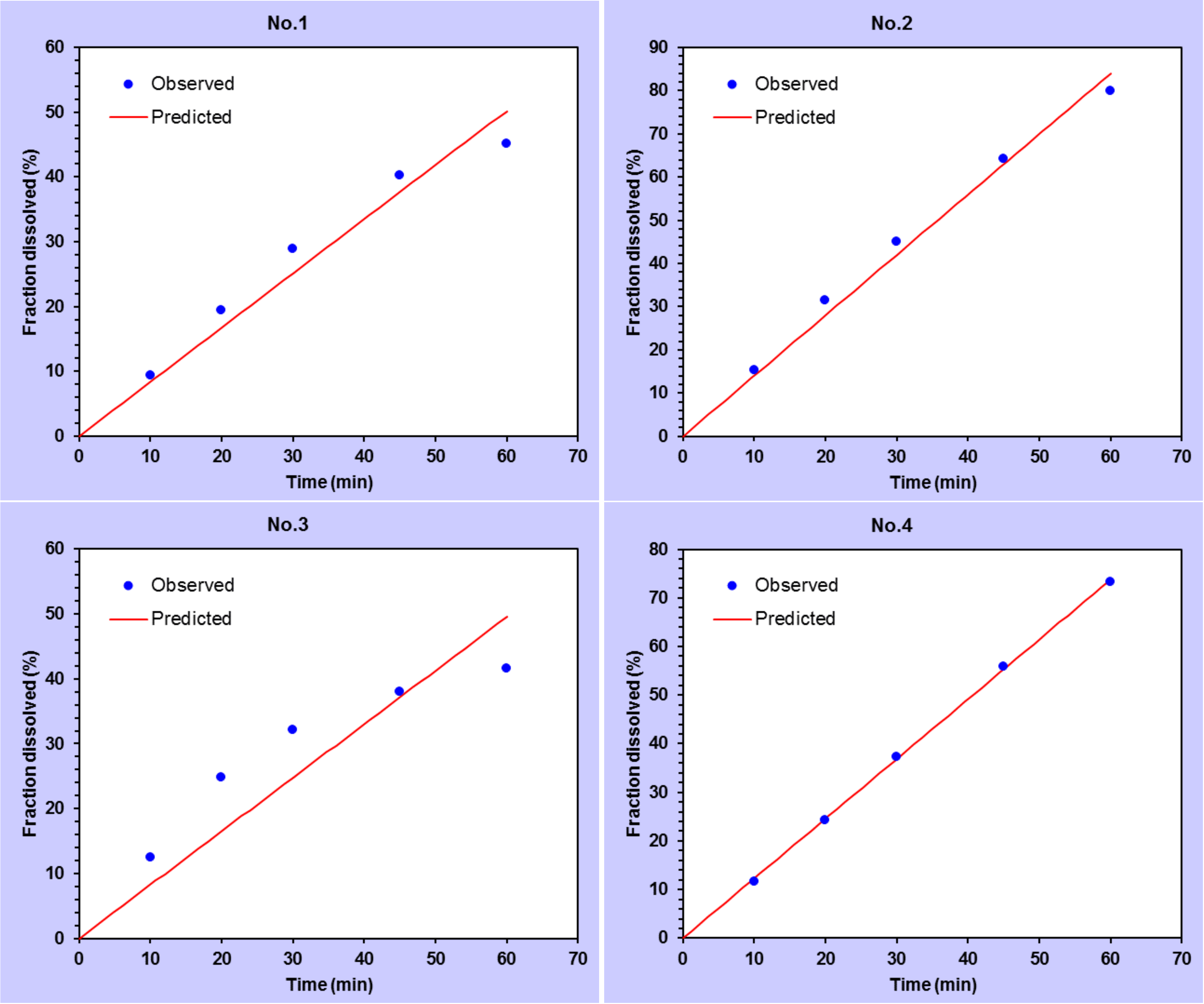

Supplementary materials – Model fitting summary – Kollidon® 25 (modelling up to app. 60 % of carvedilol released)

Model: **Zero–order with T_{lag}**

Model equation: $F = k_0 \cdot (t - T_{lag})$

Fitted model parameters per tested tablet (N = 4) with statistics – mean, standard deviation (SD), and relative standard deviation expressed in % (RSD%) (output from DDSolver):

Parameter	No.1	No.2	No.3	No.4	Mean	SD	RSD(%)
k_0	0.727	1.288	0.553	1.238	0.952	0.367	38.587
T_{lag}	-6.387	-3.680	-20.950	0.328	-7.672	9.272	-120.850

Number of dissolution data points (N), degrees of freedom (df), and selected goodness of fit criteria – Pearson correlation coefficient (R), coefficient of determination (R^2), adjusted coefficient of determination ($R^2_{adjusted}$), and residual sum of squares (RSS) (manual calculation in MS Excel):

Parameter	No.1	No.2	No.3	No.4
N	5	5	5	5
df	3	3	3	3
R	0.982294892	0.996964941	0.947703854	0.999759117
R^2	0.964903254	0.993939093	0.898142594	0.999518293
$R^2_{adjusted}$	0.953204339	0.991918791	0.864190126	0.999357724
RSS	30.38718354	15.98110759	54.8168038	1.167721519

Graphical abstract of model fit presented as mean ± 1 SD of the fraction % of released carvedilol:

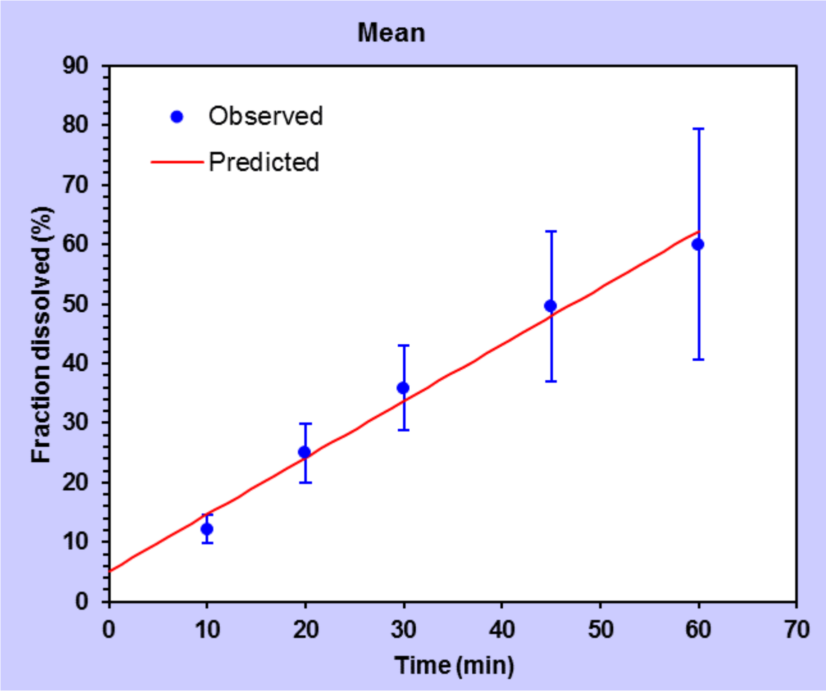

Supplementary materials – Model fitting summary – Kollidon® 25 (modelling up to app. 60 % of carvedilol released)

Graphical abstract of model fit presented as the fraction % of released carvedilol per tested tablet:

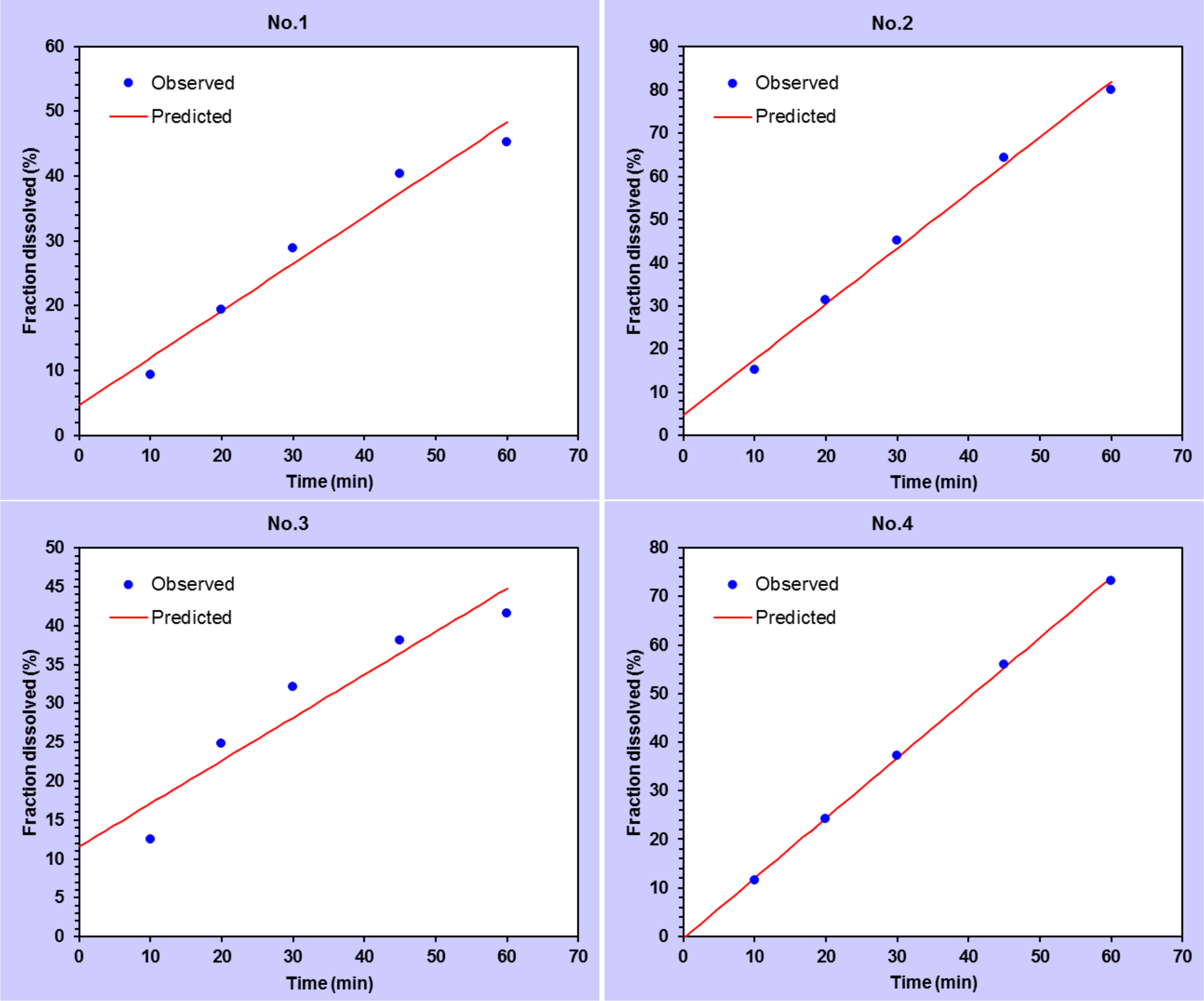

Supplementary materials – Model fitting summary – Kollidon® 25 (modelling up to app. 60 % of carvedilol released)

Model: **Zero–order with F_0**

Model equation: $F = F_0 + k_0 \cdot t$

Fitted model parameters per tested tablet (N = 4) with statistics – mean, standard deviation (SD), and relative standard deviation expressed in % (RSD%) (output from DDSolver):

Parameter	No.1	No.2	No.3	No.4	Mean	SD	RSD(%)
k_0	0.727	1.288	0.553	1.238	0.952	0.367	38.587
F_0	4.644	4.739	11.588	-0.406	5.141	4.924	95.773

Number of dissolution data points (N), degrees of freedom (df), and selected goodness of fit criteria – Pearson correlation coefficient (R), coefficient of determination (R^2), adjusted coefficient of determination ($R^2_{adjusted}$), and residual sum of squares (RSS) (manual calculation in MS Excel):

Parameter	No.1	No.2	No.3	No.4
N	5	5	5	5
df	3	3	3	3
R	0.982294892	0.996964941	0.947703854	0.999759117
R^2	0.964903254	0.993939093	0.898142594	0.999518293
$R^2_{adjusted}$	0.953204339	0.991918791	0.864190126	0.999357724
RSS	30.38718354	15.98110759	54.8168038	1.167721519

Graphical abstract of model fit presented as mean ± 1 SD of the fraction % of released carvedilol:

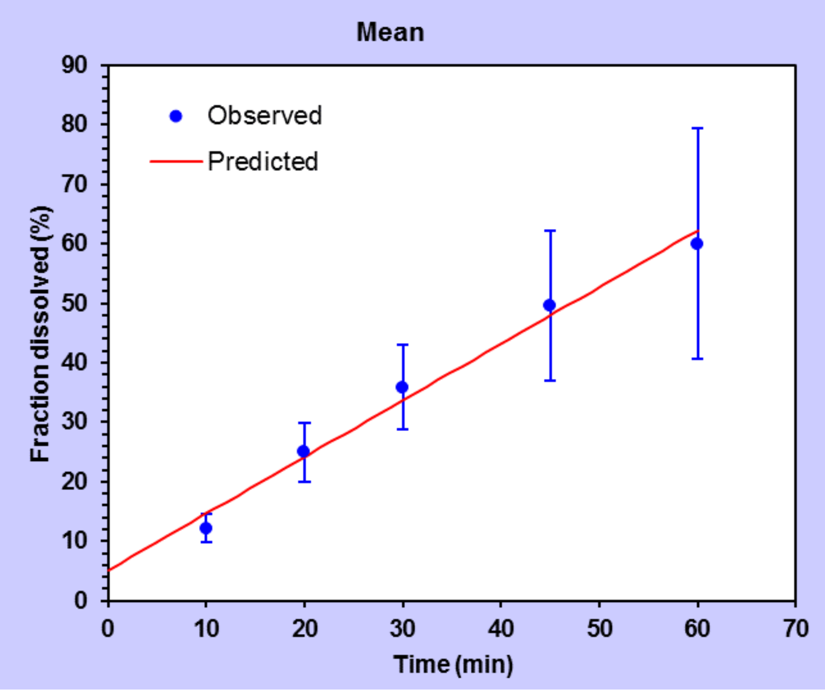

Supplementary materials – Model fitting summary – Kollidon® 25 (modelling up to app. 60 % of carvedilol released)

Graphical abstract of model fit presented as the fraction % of released carvedilol per tested tablet:

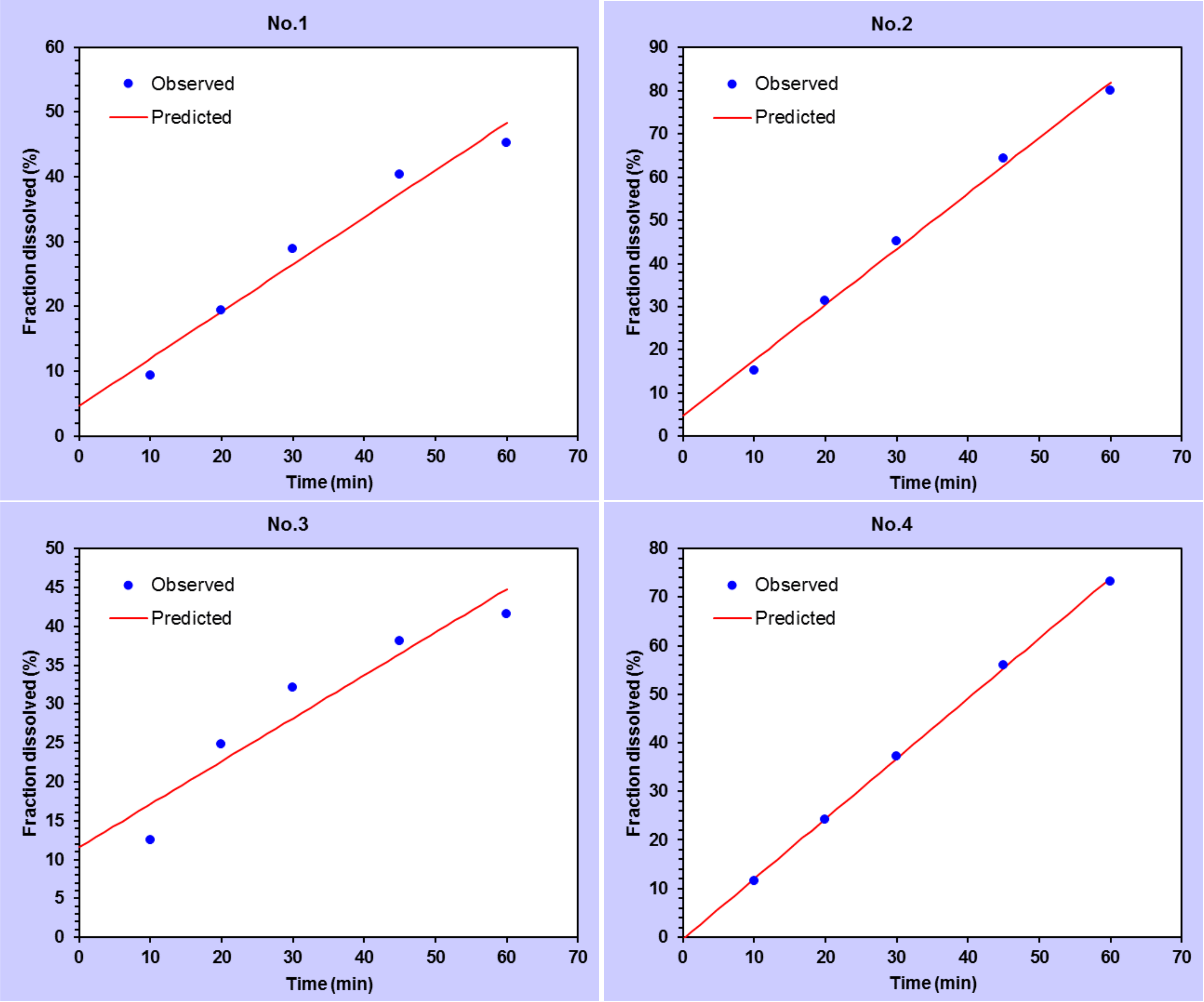

Supplementary materials – Model fitting summary – Kollidon® 25 (modelling up to app. 60 % of carvedilol released)

Model: **First–order**

Model equation: $F = 100 \cdot (1 - e^{-k_1 \cdot t})$

Fitted model parameters per tested tablet (N = 4) with statistics – mean, standard deviation (SD), and relative standard deviation expressed in % (RSD%) (output from DDSolver):

Parameter	No.1	No.2	No.3	No.4	Mean	SD	RSD(%)
k_1	0.011	0.021	0.010	0.017	0.015	0.005	35.550

Number of dissolution data points (N), degrees of freedom (df), and selected goodness of fit criteria – Pearson correlation coefficient (R), coefficient of determination (R^2), adjusted coefficient of determination ($R^2_{adjusted}$), and residual sum of squares (RSS) (manual calculation in MS Excel):

Parameter	No.1	No.2	No.3	No.4
N	5	5	5	5
df	4	4	4	4
R	0.993288888	0.997154391	0.968384398	0.994634302
R^2	0.986622815	0.99431688	0.937768342	0.989297395
$R^2_{adjusted}$	0.986622815	0.99431688	0.937768342	0.989297395
RSS	11.95318387	100.1865637	97.70020652	137.2574403

Graphical abstract of model fit presented as mean ± 1 SD of the fraction % of released carvedilol:

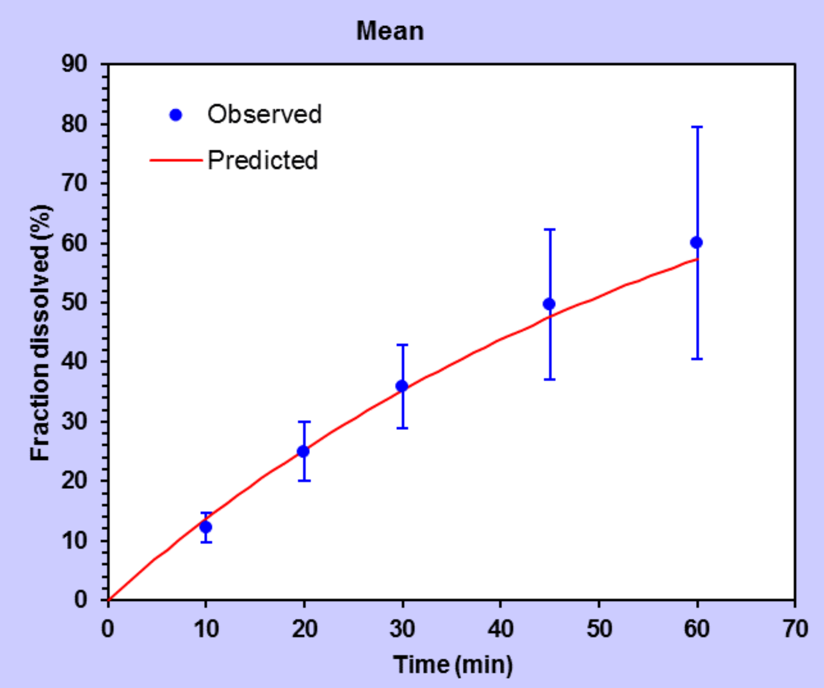

Supplementary materials – Model fitting summary – Kollidon® 25 (modelling up to app. 60 % of carvedilol released)

Graphical abstract of model fit presented as the fraction % of released carvedilol per tested tablet:

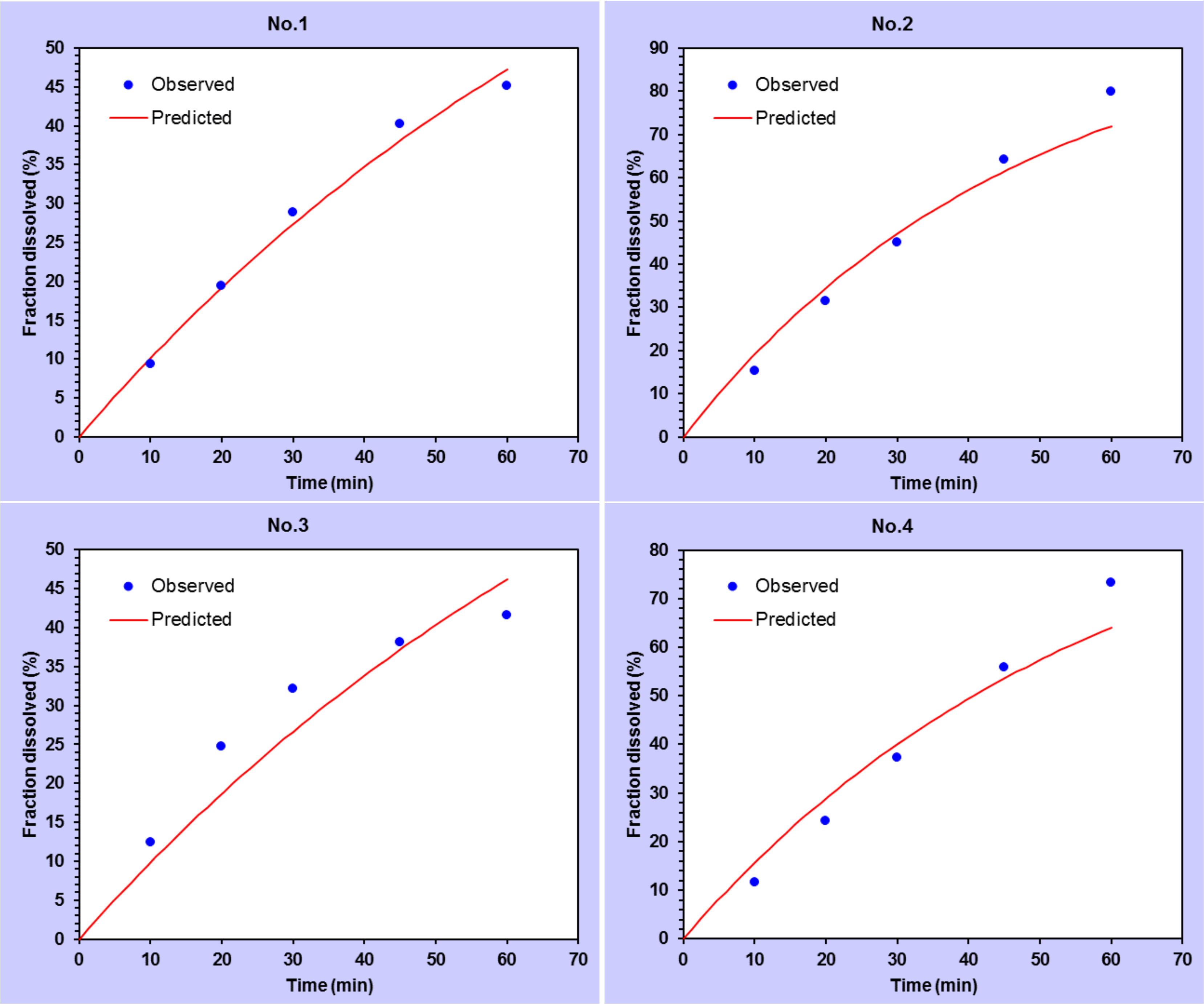

Supplementary materials – Model fitting summary – Kollidon® 25 (modelling up to app. 60 % of carvedilol released)

Model: **First–order with T_{lag}**

Model equation: $F = 100 \cdot \left[1 - e^{-k_1 \cdot (t - T_{lag})}\right]$

Fitted model parameters per tested tablet (N = 4) with statistics – mean, standard deviation (SD), and relative standard deviation expressed in % (RSD%) (output from DDSolver):

Parameter	No.1	No.2	No.3	No.4	Mean	SD	RSD(%)
k_1	0.010	0.029	0.008	0.024	0.018	0.010	57.539
T_{lag}	-1.300	6.595	-13.742	7.760	-0.172	9.902	-5766.457

Number of dissolution data points (N), degrees of freedom (df), and selected goodness of fit criteria – Pearson correlation coefficient (R), coefficient of determination (R^2), adjusted coefficient of determination ($R^2_{adjusted}$), and residual sum of squares (RSS) (manual calculation in MS Excel):

Parameter	No.1	No.2	No.3	No.4
N	5	5	5	5
df	3	3	3	3
R	0.993053541	0.991962304	0.963800169	0.988561336
R^2	0.986155335	0.983989212	0.928910766	0.977253515
$R^2_{adjusted}$	0.981540446	0.978652282	0.905214354	0.969671354
RSS	12.49214808	58.9945118	39.35864847	69.82124249

Graphical abstract of model fit presented as mean ± 1 SD of the fraction % of released carvedilol:

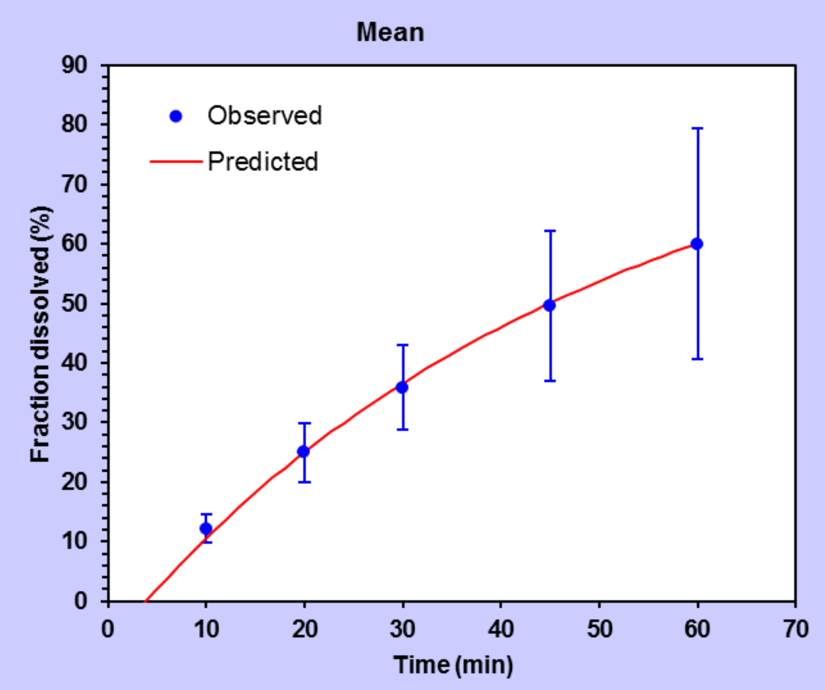

Supplementary materials – Model fitting summary – Kollidon® 25 (modelling up to app. 60 % of carvedilol released)

Graphical abstract of model fit presented as the fraction % of released carvedilol per tested tablet:

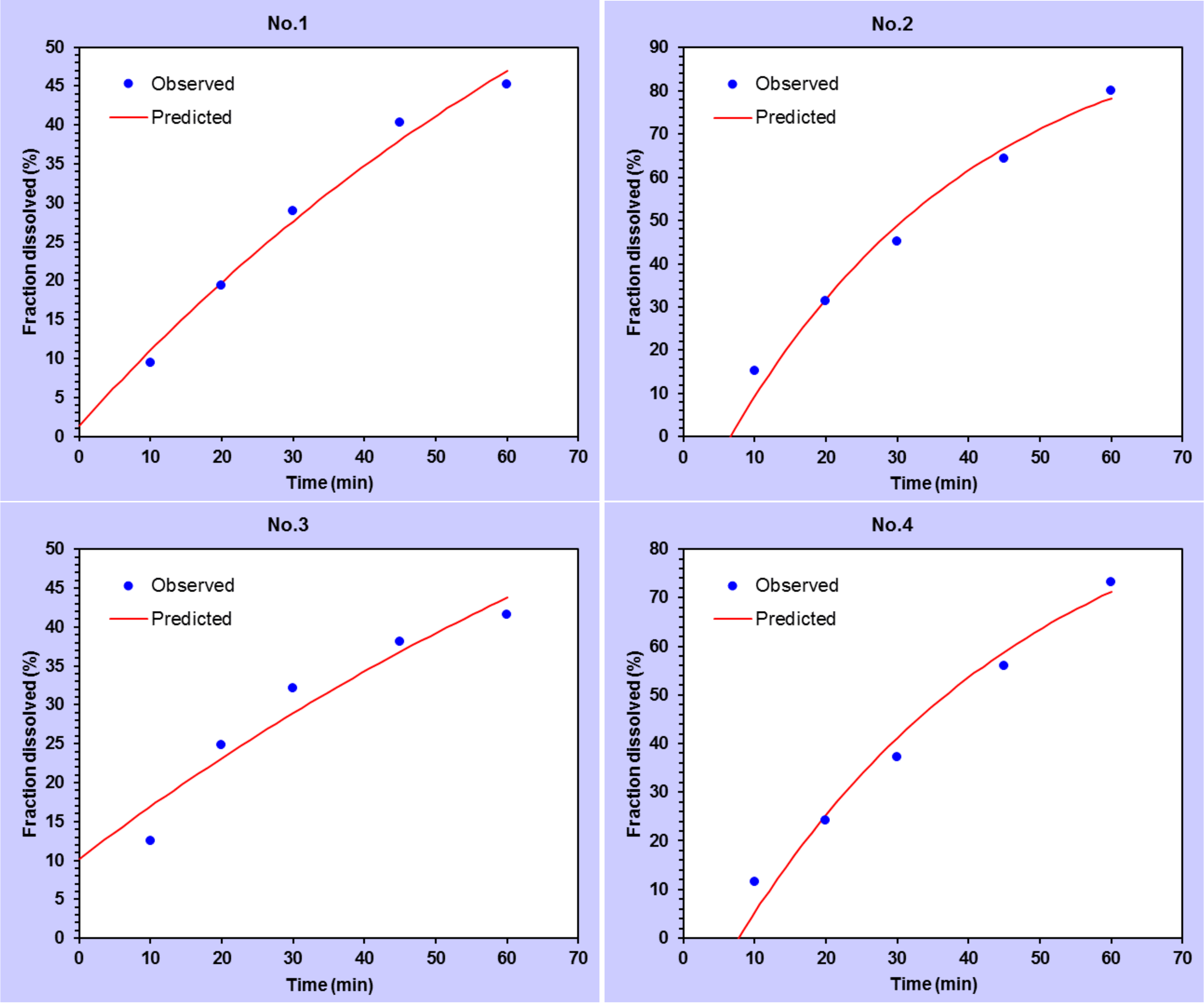

Supplementary materials – Model fitting summary – Kollidon® 25 (modelling up to app. 60 % of carvedilol released)

Model: **First–order with F_{max}**

Model equation: $F = F_{max} \cdot (1 - e^{-k_1 \cdot t})$

Fitted model parameters per tested tablet (N = 4) with statistics – mean, standard deviation (SD), and relative standard deviation expressed in % (RSD%) (output from DDSolver):

Parameter	No.1	No.2	No.3	No.4	Mean	SD	RSD(%)
k_1	0.044	0.041	0.048	0.038	0.043	0.004	9.191
F_{max}	47.460	68.250	43.680	76.965	59.089	16.085	27.221

Number of dissolution data points (N), degrees of freedom (df), and selected goodness of fit criteria – Pearson correlation coefficient (R), coefficient of determination (R^2), adjusted coefficient of determination ($R^2_{adjusted}$), and residual sum of squares (RSS) (manual calculation in MS Excel):

Parameter	No.1	No.2	No.3	No.4
N	5	5	5	5
df	3	3	3	3
R	0.989026477	0.977896873	0.999868688	0.969038067
R^2	0.978173373	0.956282294	0.999737392	0.939034776
$R^2_{adjusted}$	0.97089783	0.941709726	0.999649856	0.918713035
RSS	161.5692789	469.715515	22.51798317	767.422396

Graphical abstract of model fit presented as mean ± 1 SD of the fraction % of released carvedilol:

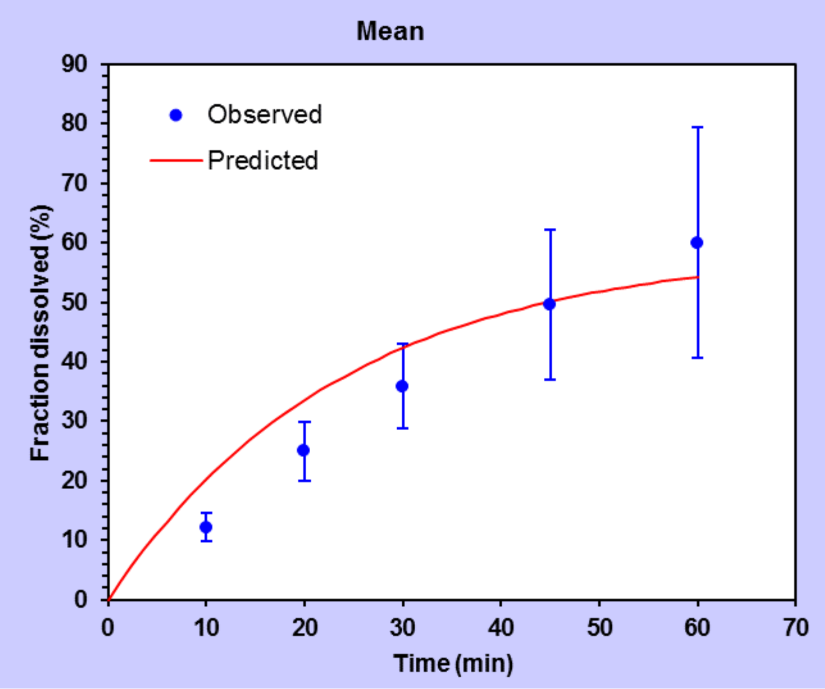

Supplementary materials – Model fitting summary – Kollidon® 25 (modelling up to app. 60 % of carvedilol released)

Graphical abstract of model fit presented as the fraction % of released carvedilol per tested tablet:

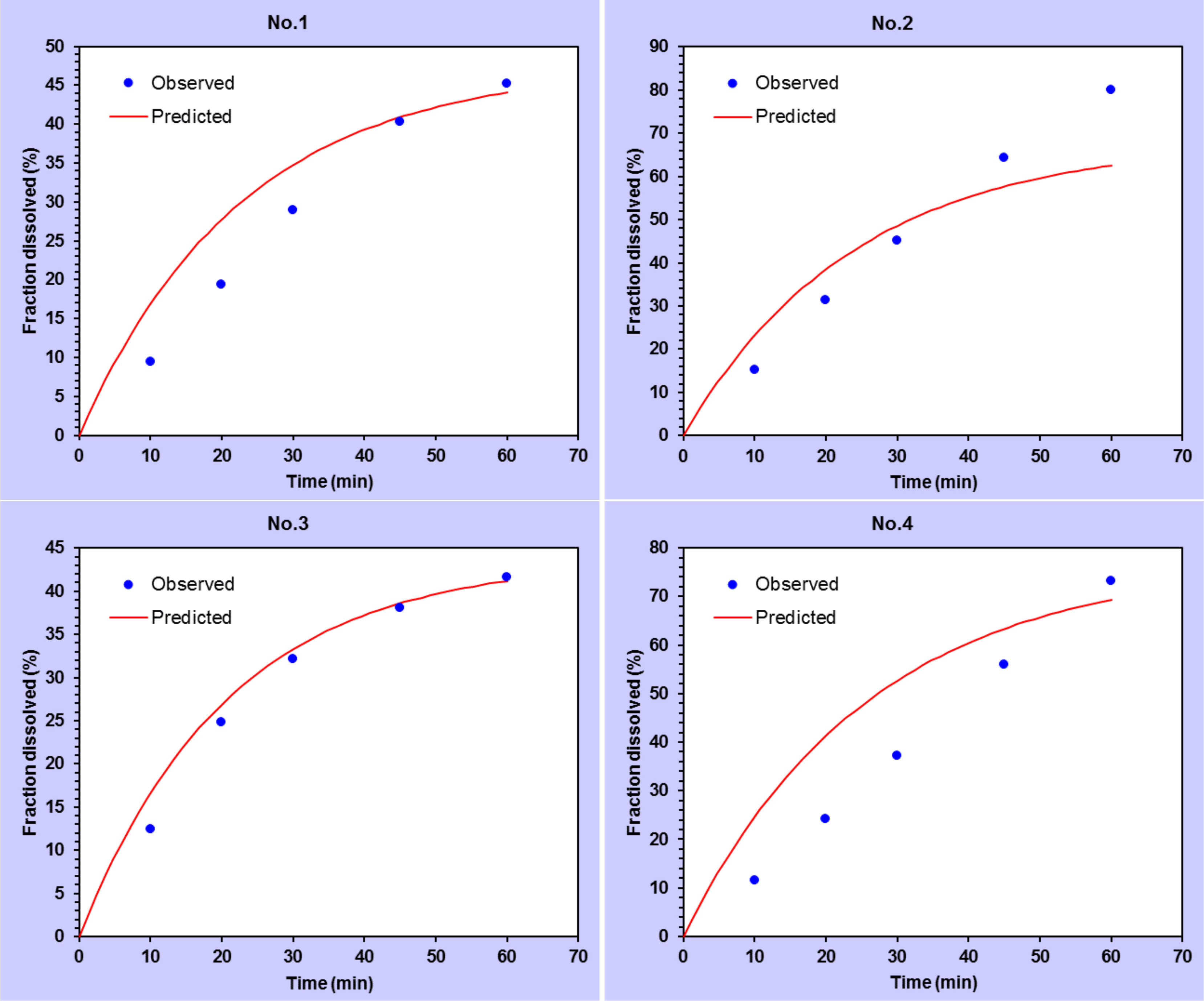

Supplementary materials – Model fitting summary – Kollidon® 25 (modelling up to app. 60 % of carvedilol released)

Model: **First–order with T_{lag} and F_{max}**

Model equation: $F = F_{max} \cdot \left[1 - e^{-k_1 \cdot (t - T_{lag})}\right]$

Fitted model parameters per tested tablet (N = 4) with statistics – mean, standard deviation (SD), and relative standard deviation expressed in % (RSD%) (output from DDSolver):

Parameter	No.1	No.2	No.3	No.4	Mean	SD	RSD(%)
k_1	0.057	0.082	0.053	0.086	0.070	0.017	24.070
T_{lag}	9.797	7.554	4.433	9.306	7.773	2.426	31.210
F_{max}	47.460	70.000	43.680	51.310	53.113	11.681	21.994

Number of dissolution data points (N), degrees of freedom (df), and selected goodness of fit criteria – Pearson correlation coefficient (R), coefficient of determination (R^2), adjusted coefficient of determination ($R^2_{adjusted}$), and residual sum of squares (RSS) (manual calculation in MS Excel):

Parameter	No.1	No.2	No.3	No.4
N	5	5	5	5
df	2	2	2	2
R	0.975005878	0.910898247	0.999408017	0.87850771
R^2	0.950636463	0.829735617	0.998816384	0.771775797
$R^2_{adjusted}$	0.901272926	0.659471234	0.997632769	0.543551594
RSS	94.42484365	501.3341204	2.107280324	708.4791209

Graphical abstract of model fit presented as mean ± 1 SD of the fraction % of released carvedilol:

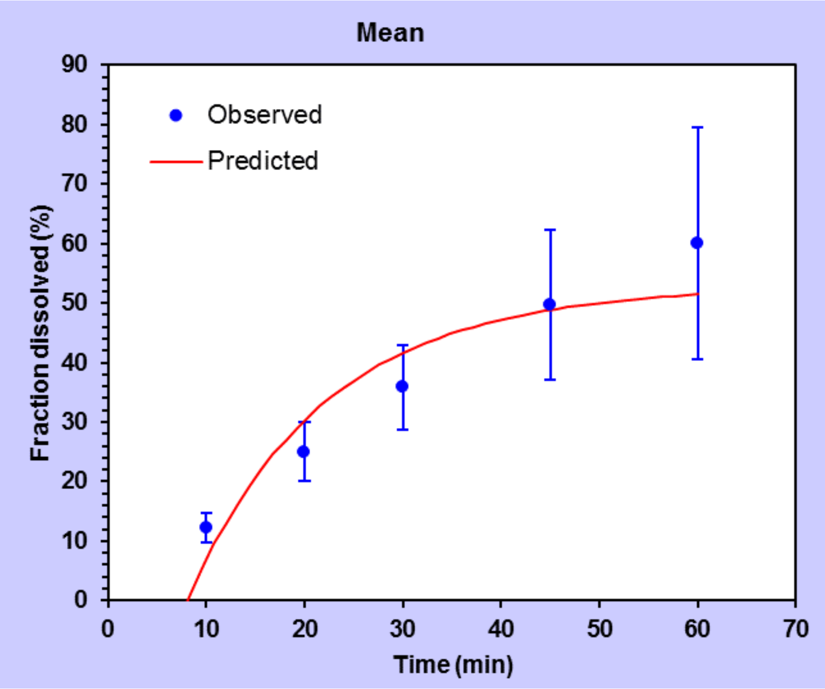

Supplementary materials – Model fitting summary – Kollidon® 25 (modelling up to app. 60 % of carvedilol released)

Graphical abstract of model fit presented as the fraction % of released carvedilol per tested tablet:

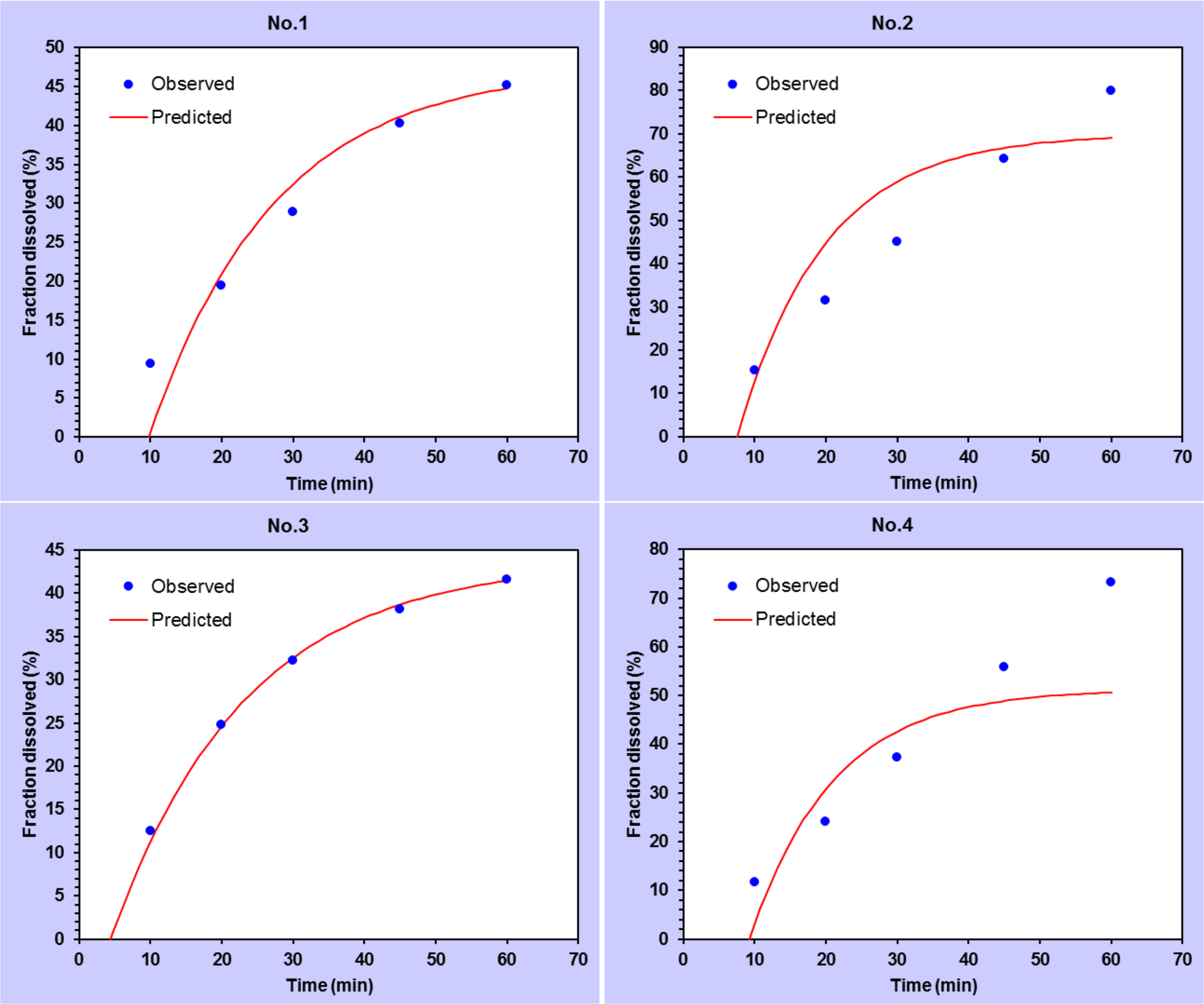

Supplementary materials – Model fitting summary – Kollidon® 25 (modelling up to app. 60 % of carvedilol released)

Model: **Higuchi**

Model equation: $F = k_H \cdot t^{0.5}$

Fitted model parameters per tested tablet (N = 4) with statistics – mean, standard deviation (SD), and relative standard deviation expressed in % (RSD%) (output from DDSolver):

Parameter	No.1	No.2	No.3	No.4	Mean	SD	RSD(%)
k_H	5.426	9.014	5.483	7.830	6.938	1.781	25.663

Number of dissolution data points (N), degrees of freedom (df), and selected goodness of fit criteria – Pearson correlation coefficient (R), coefficient of determination (R^2), adjusted coefficient of determination ($R^2_{adjusted}$), and residual sum of squares (RSS) (manual calculation in MS Excel):

Parameter	No.1	No.2	No.3	No.4
N	5	5	5	5
df	4	4	4	4
R	0.995810521	0.998715054	0.980059703	0.994207637
R^2	0.991638594	0.997431759	0.960517021	0.988448825
$R^2_{adjusted}$	0.991638594	0.997431759	0.960517021	0.988448825
RSS	109.8135227	388.5367939	30.69190012	492.8052013

Graphical abstract of model fit presented as mean ± 1 SD of the fraction % of released carvedilol:

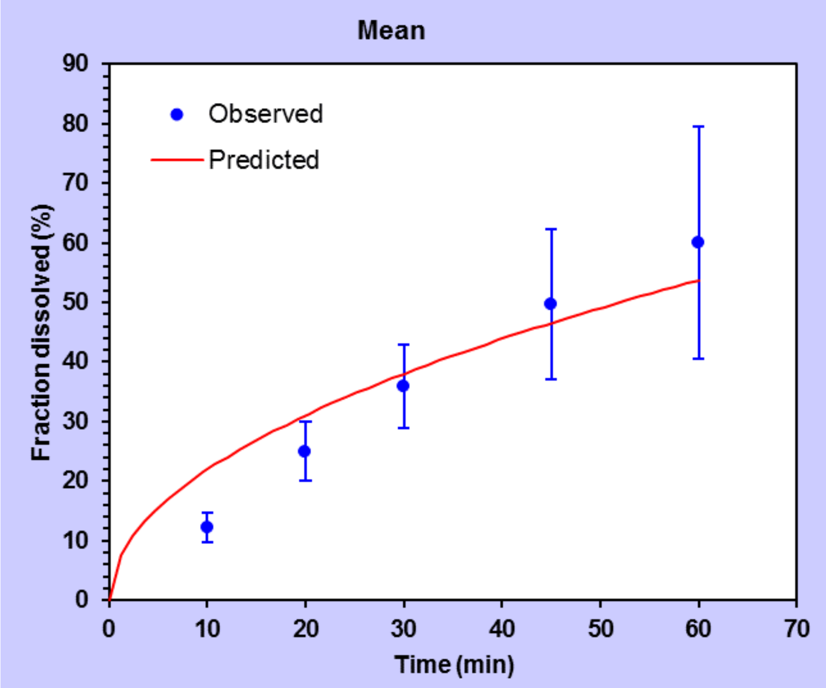

Supplementary materials – Model fitting summary – Kollidon® 25 (modelling up to app. 60 % of carvedilol released)

Graphical abstract of model fit presented as the fraction % of released carvedilol per tested tablet:

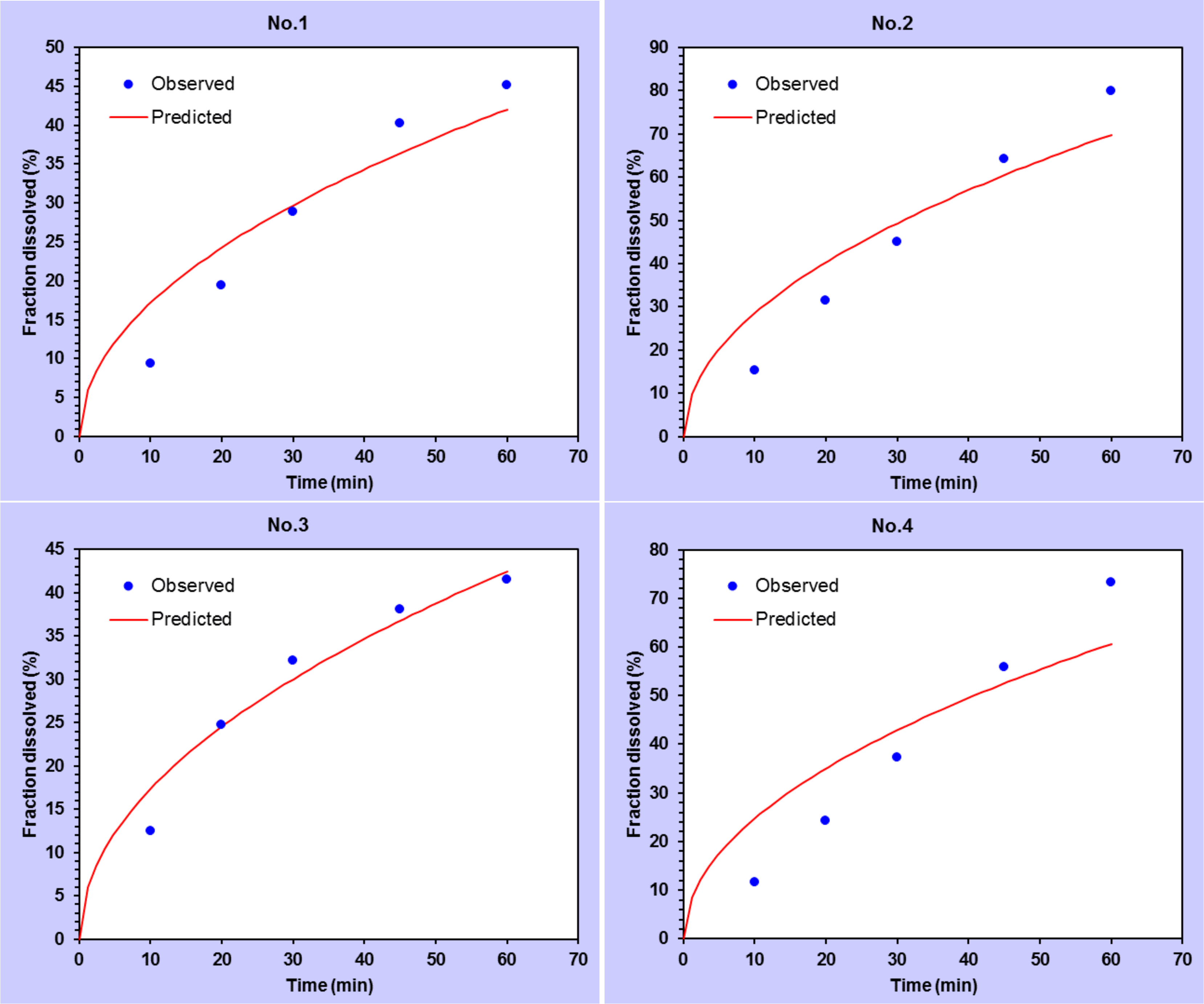

Supplementary materials – Model fitting summary – Kollidon® 25 (modelling up to app. 60 % of carvedilol released)

Model: **Higuchi with T_{lag}**

Model equation: $F = k_H \cdot (t - T_{lag})^{0.5}$

Fitted model parameters per tested tablet (N = 4) with statistics – mean, standard deviation (SD), and relative standard deviation expressed in % (RSD%) (output from DDSolver):

Parameter	No.1	No.2	No.3	No.4	Mean	SD	RSD(%)
k_H	6.425	11.195	5.594	10.302	8.379	2.781	33.191
T_{lag}	8.934	10.987	1.661	13.007	8.647	4.946	57.193

Number of dissolution data points (N), degrees of freedom (df), and selected goodness of fit criteria – Pearson correlation coefficient (R), coefficient of determination (R^2), adjusted coefficient of determination ($R^2_{adjusted}$), and residual sum of squares (RSS) (manual calculation in MS Excel):

Parameter	No.1	No.2	No.3	No.4
N	5	5	5	5
df	3	3	3	3
R	0.992368929	0.976531836	0.981931621	0.973151199
R^2	0.984796091	0.953614426	0.964189709	0.947023256
$R^2_{adjusted}$	0.979728122	0.938152569	0.952252945	0.929364342
RSS	15.3435254	255.9633961	22.81546698	183.3101411

Graphical abstract of model fit presented as mean ± 1 SD of the fraction % of released carvedilol:

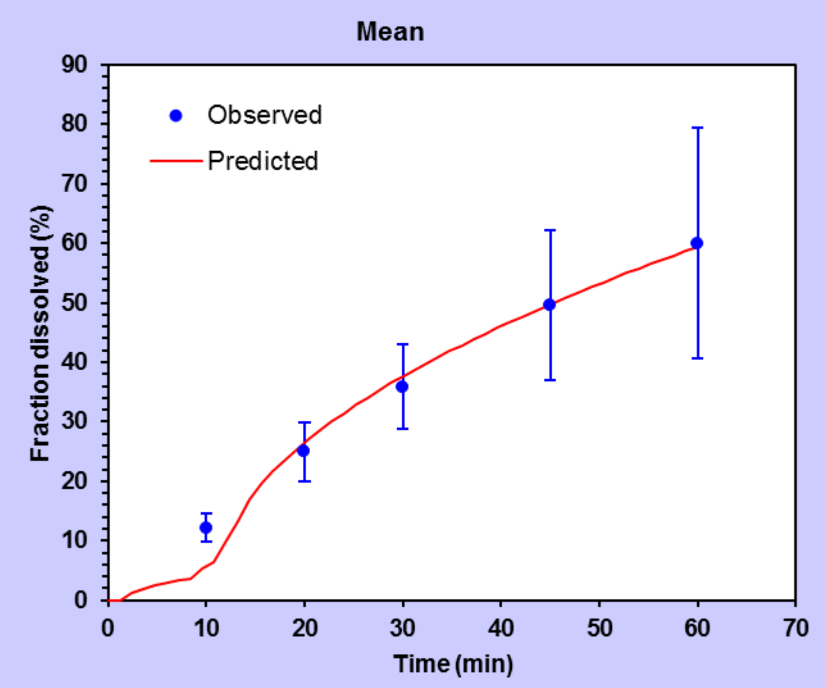

Supplementary materials – Model fitting summary – Kollidon® 25 (modelling up to app. 60 % of carvedilol released)

Graphical abstract of model fit presented as the fraction % of released carvedilol per tested tablet:

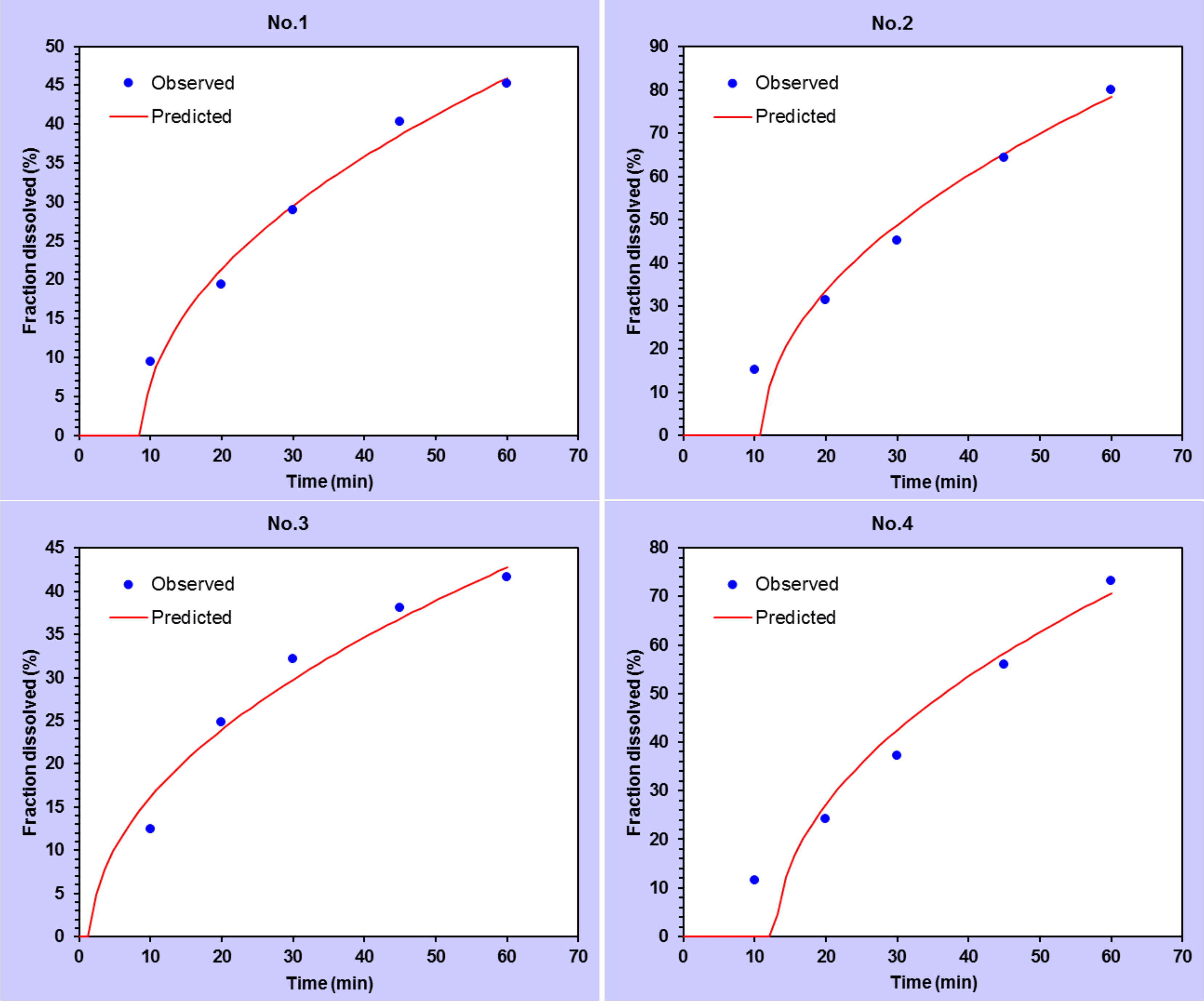

Supplementary materials – Model fitting summary – Kollidon® 25 (modelling up to app. 60 % of carvedilol released)

Model: **Higuchi with F_0**

Model equation: $F = F_0 + k_H \cdot t^{0.5}$

Fitted model parameters per tested tablet (N = 4) with statistics – mean, standard deviation (SD), and relative standard deviation expressed in % (RSD%) (output from DDSolver):

Parameter	No.1	No.2	No.3	No.4	Mean	SD	RSD(%)
k_H	8.119	14.210	6.300	13.563	10.548	3.935	37.304
F_0	-16.121	-31.100	-4.891	-34.316	-21.607	13.677	-63.297

Number of dissolution data points (N), degrees of freedom (df), and selected goodness of fit criteria – Pearson correlation coefficient (R), coefficient of determination (R^2), adjusted coefficient of determination ($R^2_{adjusted}$), and residual sum of squares (RSS) (manual calculation in MS Excel):

Parameter	No.1	No.2	No.3	No.4
N	5	5	5	5
df	3	3	3	3
R	0.995810521	0.998715054	0.980059703	0.994207637
R^2	0.991638594	0.997431759	0.960517021	0.988448825
$R^2_{adjusted}$	0.988851459	0.996575678	0.947356028	0.984598434
RSS	7.239405493	6.771815793	21.24863396	28.00157182

Graphical abstract of model fit presented as mean ± 1 SD of the fraction % of released carvedilol:

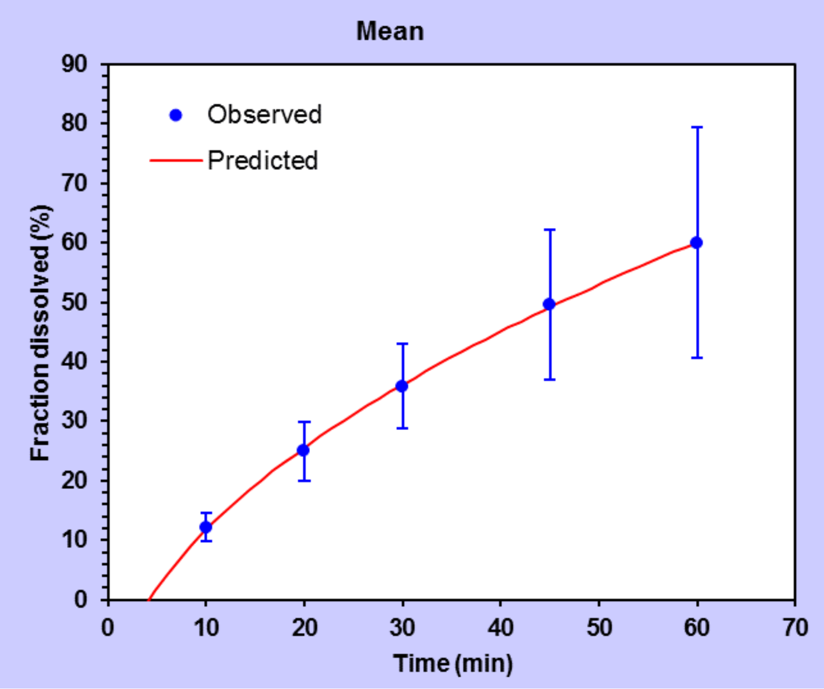

Supplementary materials – Model fitting summary – Kollidon® 25 (modelling up to app. 60 % of carvedilol released)

Graphical abstract of model fit presented as the fraction % of released carvedilol per tested tablet:

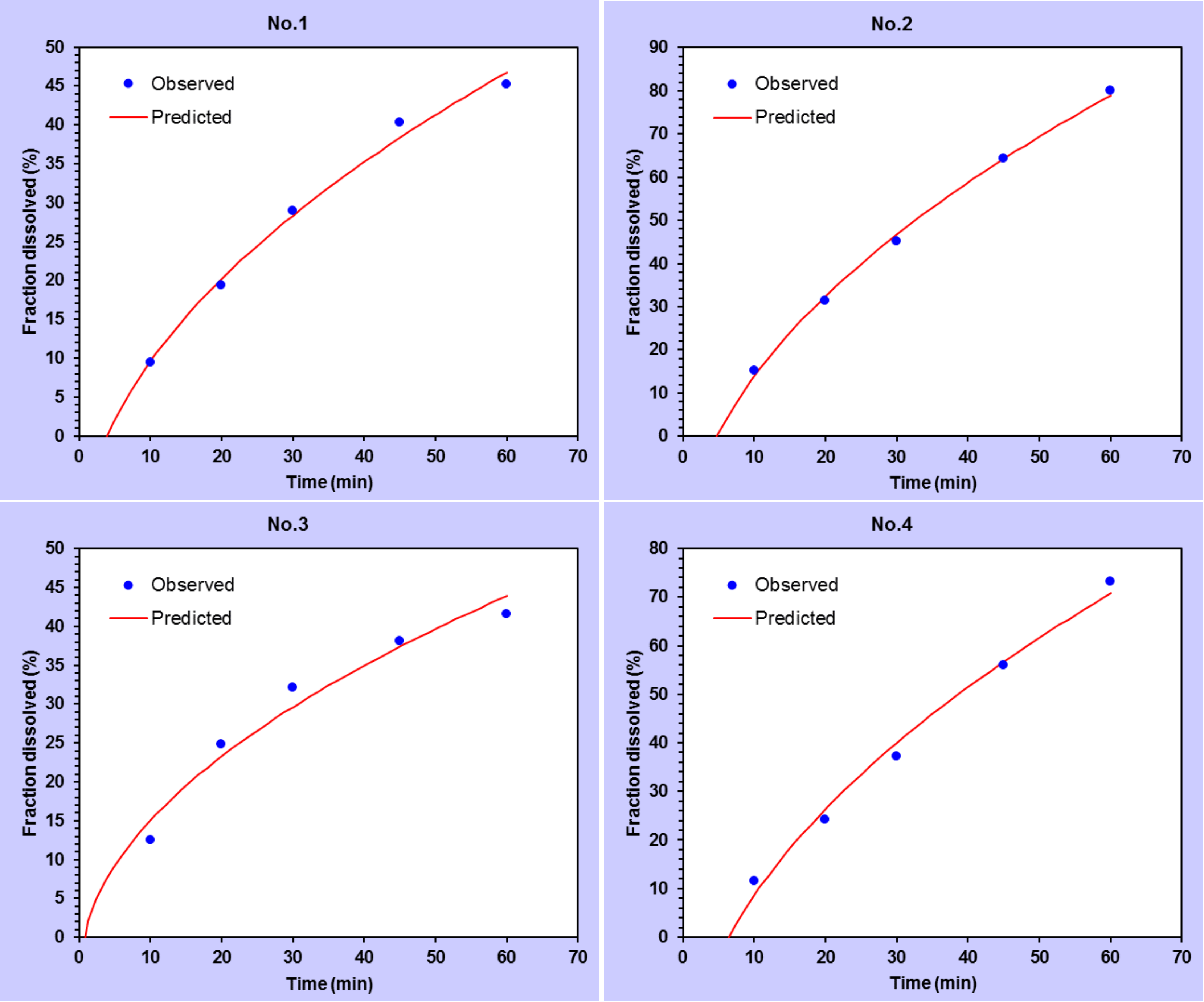

Supplementary materials – Model fitting summary – Kollidon® 25 (modelling up to app. 60 % of carvedilol released)

Model: **Korsmeyer–Peppas**

Model equation: $F = k_{KP} \cdot t^n$

Fitted model parameters per tested tablet (N = 4) with statistics – mean, standard deviation (SD), and relative standard deviation expressed in % (RSD%) (output from DDSolver):

Parameter	No.1	No.2	No.3	No.4	Mean	SD	RSD(%)
k_{KP}	1.265	1.894	2.983	1.089	1.808	0.856	47.353
n	0.898	0.924	0.669	1.033	0.881	0.153	17.336

Number of dissolution data points (N), degrees of freedom (df), and selected goodness of fit criteria – Pearson correlation coefficient (R), coefficient of determination (R^2), adjusted coefficient of determination ($R^2_{adjusted}$), and residual sum of squares (RSS) (manual calculation in MS Excel):

Parameter	No.1	No.2	No.3	No.4
N	5	5	5	5
df	3	3	3	3
R	0.986266972	0.998195188	0.970426583	0.999582342
R^2	0.97272254	0.996393634	0.941727752	0.999164858
$R^2_{adjusted}$	0.963630053	0.995191512	0.92230367	0.998886478
RSS	30.68383313	14.45503211	40.28862071	2.938084956

Graphical abstract of model fit presented as mean ± 1 SD of the fraction % of released carvedilol:

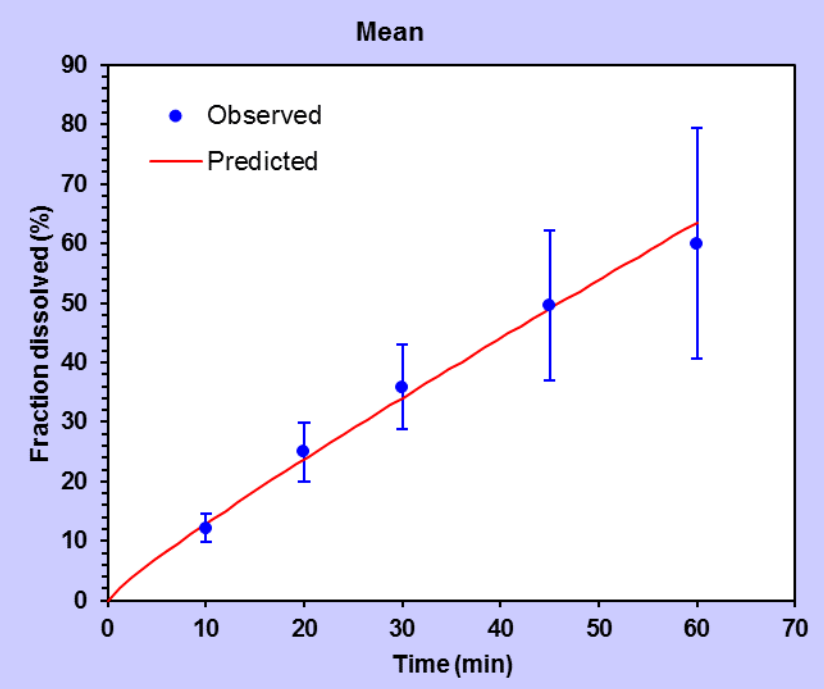

Supplementary materials – Model fitting summary – Kollidon® 25 (modelling up to app. 60 % of carvedilol released)

Graphical abstract of model fit presented as the fraction % of released carvedilol per tested tablet:

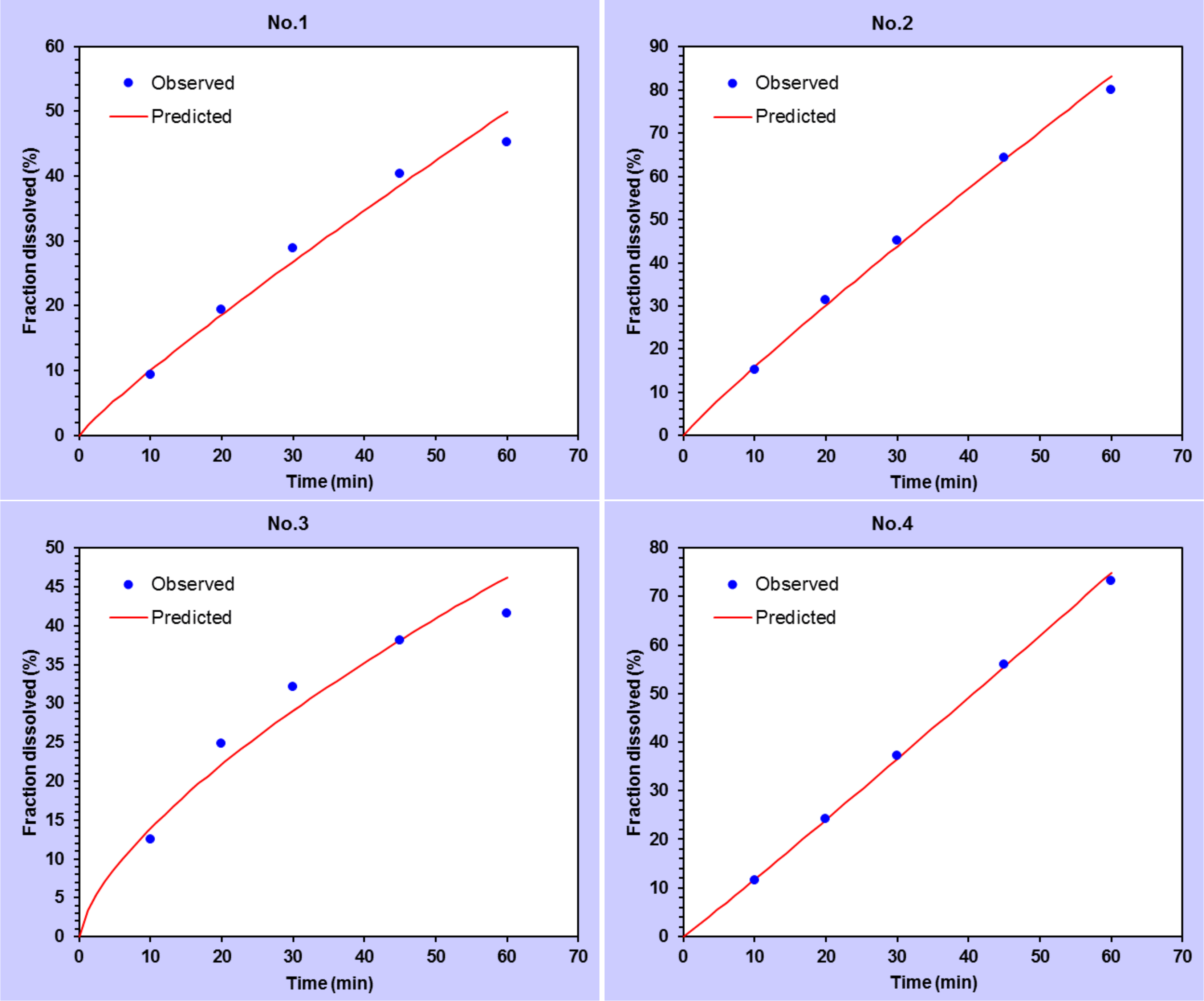

Supplementary materials – Model fitting summary – Kollidon® 25 (modelling up to app. 60 % of carvedilol released)

Model: **Korsmeyer–Peppas with T_{lag}**

Model equation: $F = k_{KP} \cdot (t - T_{lag})^n$

Fitted model parameters per tested tablet (N = 4) with statistics – mean, standard deviation (SD), and relative standard deviation expressed in % (RSD%) (output from DDSolver):

Parameter	No.1	No.2	No.3	No.4	Mean	SD	RSD(%)
k_{KP}	2.617	4.028	5.064	2.548	3.564	1.210	33.960
n	0.724	0.743	0.544	0.829	0.710	0.120	16.858
T_{lag}	4.000	4.000	4.000	4.000	4.000	0.000	0.000

Number of dissolution data points (N), degrees of freedom (df), and selected goodness of fit criteria – Pearson correlation coefficient (R), coefficient of determination (R^2), adjusted coefficient of determination ($R^2_{adjusted}$), and residual sum of squares (RSS) (manual calculation in MS Excel):

Parameter	No.1	No.2	No.3	No.4
N	5	5	5	5
df	2	2	2	2
R	0.992817811	0.999891835	0.982509946	0.999438662
R^2	0.985687207	0.999783683	0.965325794	0.99887764
$R^2_{adjusted}$	0.971374414	0.999567365	0.930651589	0.99775528
RSS	14.06760444	0.575831612	23.54341385	4.935620392

Graphical abstract of model fit presented as mean ± 1 SD of the fraction % of released carvedilol:

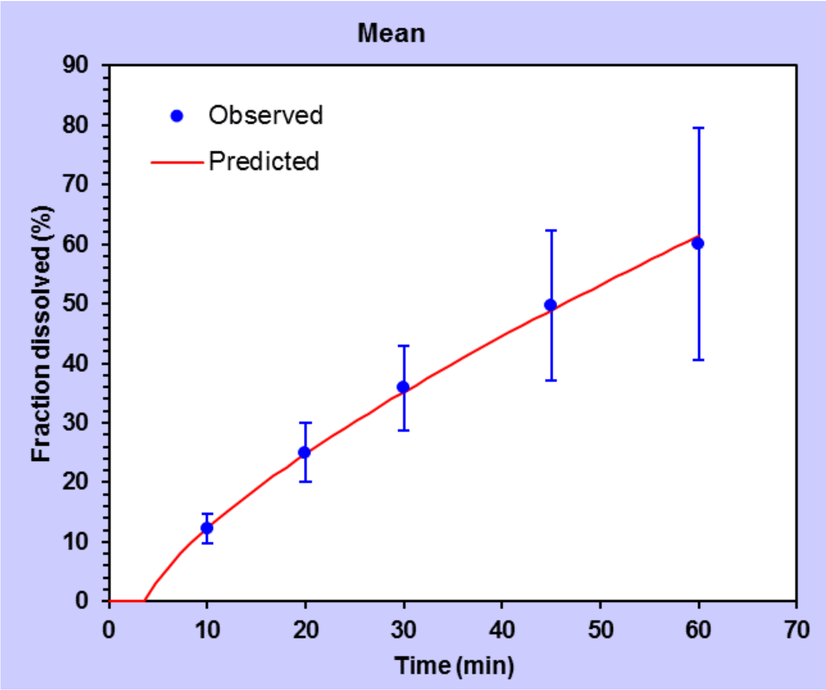

Supplementary materials – Model fitting summary – Kollidon® 25 (modelling up to app. 60 % of carvedilol released)

Graphical abstract of model fit presented as the fraction % of released carvedilol per tested tablet:

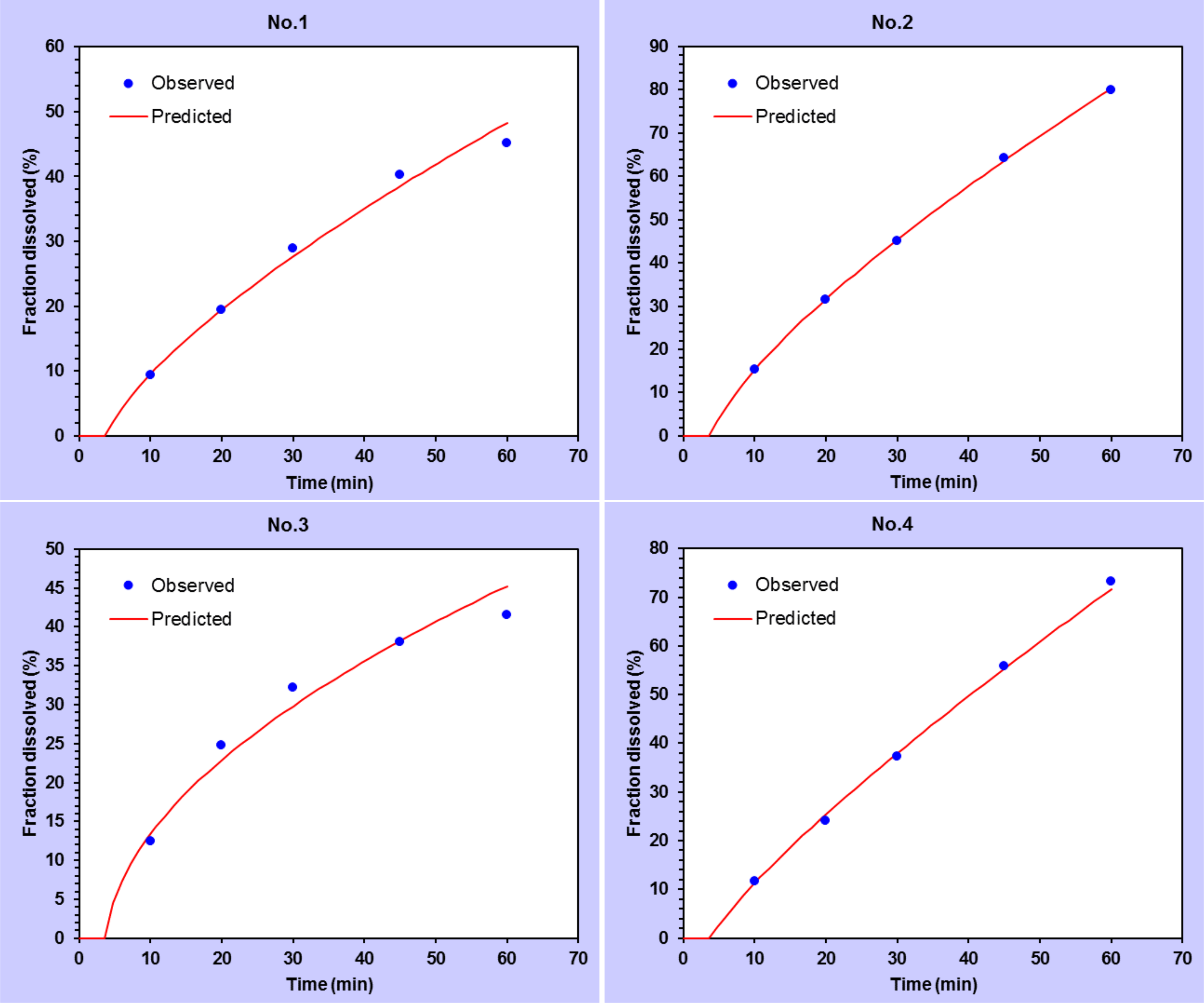

Supplementary materials – Model fitting summary – Kollidon® 25 (modelling up to app. 60 % of carvedilol released)

Model: **Korsmeyer–Peppas with F_0**

Model equation: $F = F_0 + k_{KP} \cdot t^n$

Fitted model parameters per tested tablet (N = 4) with statistics – mean, standard deviation (SD), and relative standard deviation expressed in % (RSD%) (output from DDSolver):

Parameter	No.1	No.2	No.3	No.4	Mean	SD	RSD(%)
k_{KP}	0.472	0.783	1.184	0.436	0.719	0.347	48.298
n	1.131	1.110	0.877	1.231	1.087	0.150	13.778
F_0	3.760	7.427	5.000	5.644	5.458	1.528	27.996

Number of dissolution data points (N), degrees of freedom (df), and selected goodness of fit criteria – Pearson correlation coefficient (R), coefficient of determination (R^2), adjusted coefficient of determination ($R^2_{adjusted}$), and residual sum of squares (RSS) (manual calculation in MS Excel):

Parameter	No.1	No.2	No.3	No.4
N	5	5	5	5
df	2	2	2	2
R	0.976473405	0.99467637	0.95667327	0.997350551
R^2	0.953500311	0.989381082	0.915223746	0.994708121
$R^2_{adjusted}$	0.907000622	0.978762163	0.830447492	0.989416242
RSS	64.24725083	35.83808565	68.47117589	21.26680504

Graphical abstract of model fit presented as mean ± 1 SD of the fraction % of released carvedilol:

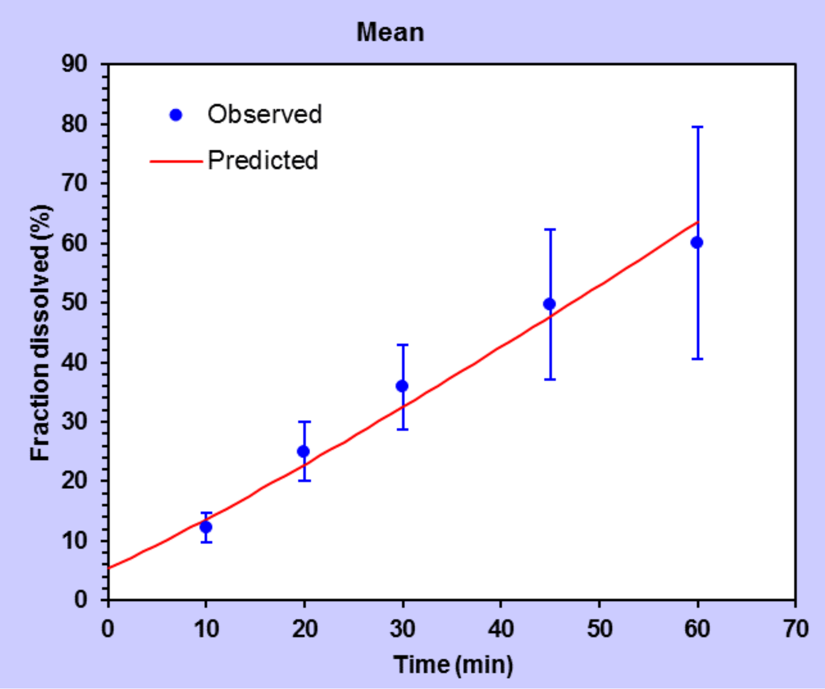

Supplementary materials – Model fitting summary – Kollidon® 25 (modelling up to app. 60 % of carvedilol released)

Graphical abstract of model fit presented as the fraction % of released carvedilol per tested tablet:

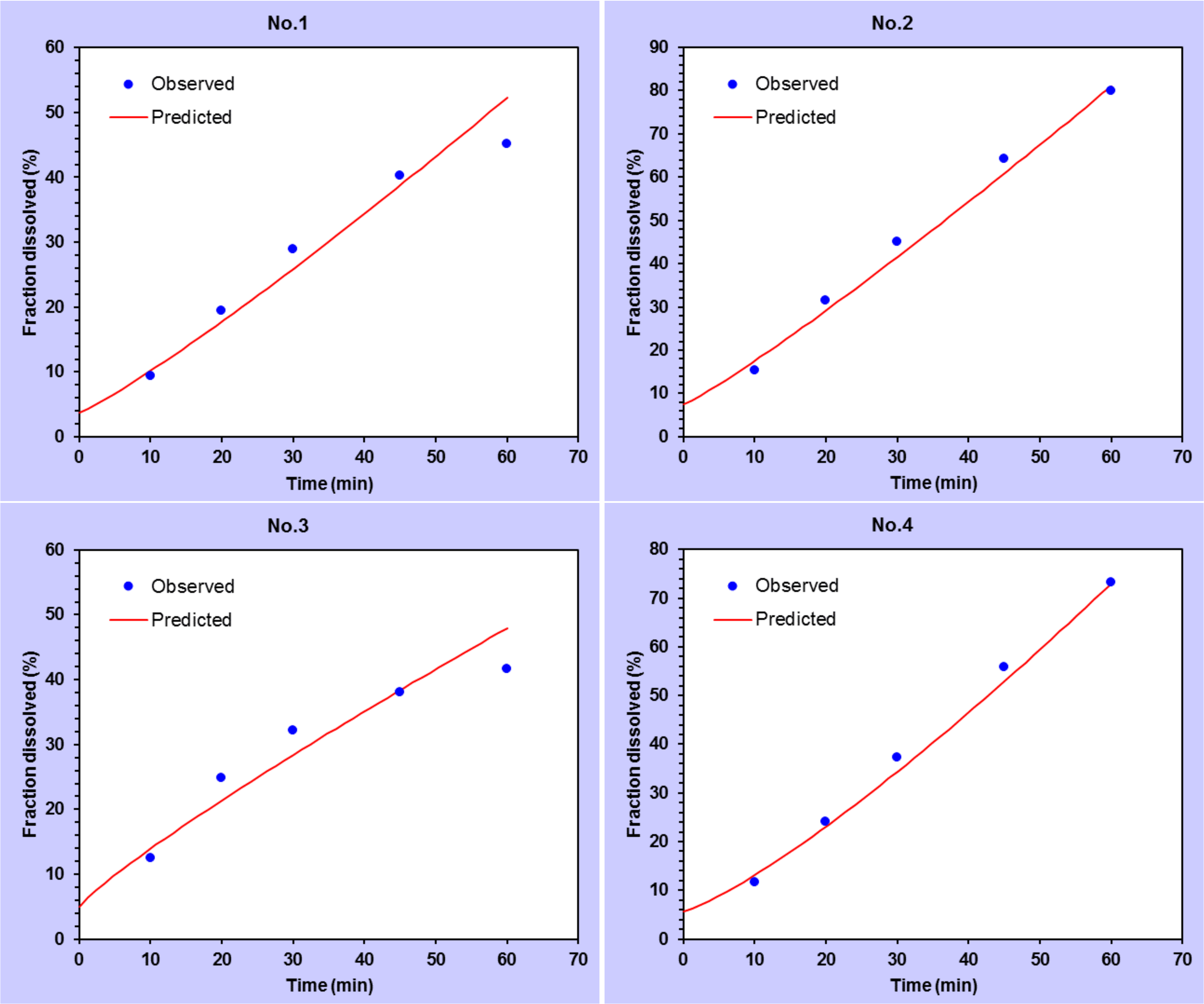

Supplementary materials – Model fitting summary – Kollidon® 25 (modelling up to app. 60 % of carvedilol released)

Model: **Hixson–Crowell**

Model equation: $F = 100 \cdot [1 - (1 - k_{HC} \cdot t)^3]$

Fitted model parameters per tested tablet (N = 4) with statistics – mean, standard deviation (SD), and relative standard deviation expressed in % (RSD%) (output from DDSolver):

Parameter	No.1	No.2	No.3	No.4	Mean	SD	RSD(%)
k_{HC}	0.003	0.007	0.003	0.005	0.005	0.002	36.421

Number of dissolution data points (N), degrees of freedom (df), and selected goodness of fit criteria – Pearson correlation coefficient (R), coefficient of determination (R^2), adjusted coefficient of determination ($R^2_{adjusted}$), and residual sum of squares (RSS) (manual calculation in MS Excel):

Parameter	No.1	No.2	No.3	No.4
N	5	5	5	5
df	4	4	4	4
R	0.990597891	0.998933078	0.962517517	0.996941653
R^2	0.981284182	0.997867295	0.926439971	0.993892659
$R^2_{adjusted}$	0.981284182	0.997867295	0.926439971	0.993892659
RSS	19.951604	35.73543381	126.8981201	77.4165844

Graphical abstract of model fit presented as mean ± 1 SD of the fraction % of released carvedilol:

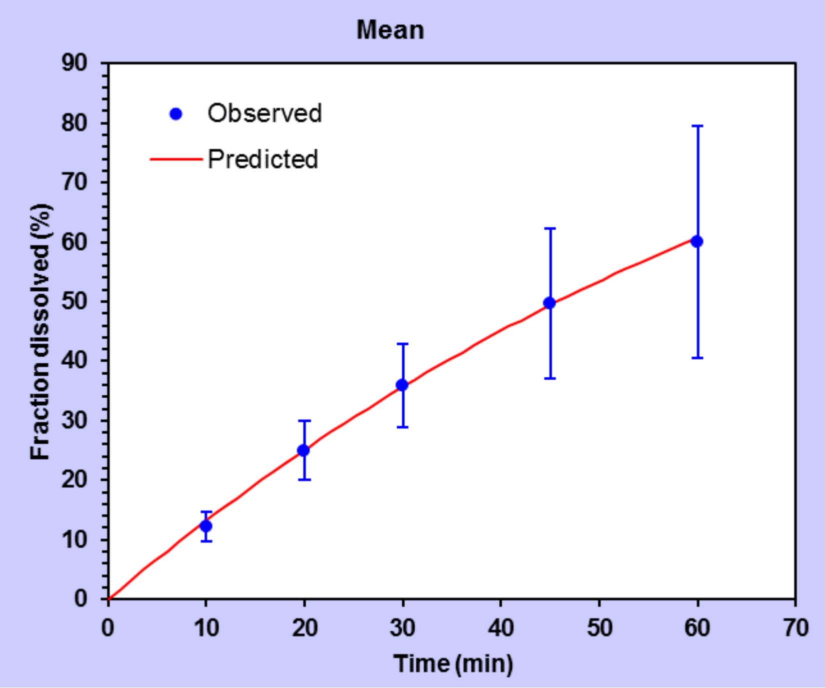

Supplementary materials – Model fitting summary – Kollidon® 25 (modelling up to app. 60 % of carvedilol released)

Graphical abstract of model fit presented as the fraction % of released carvedilol per tested tablet:

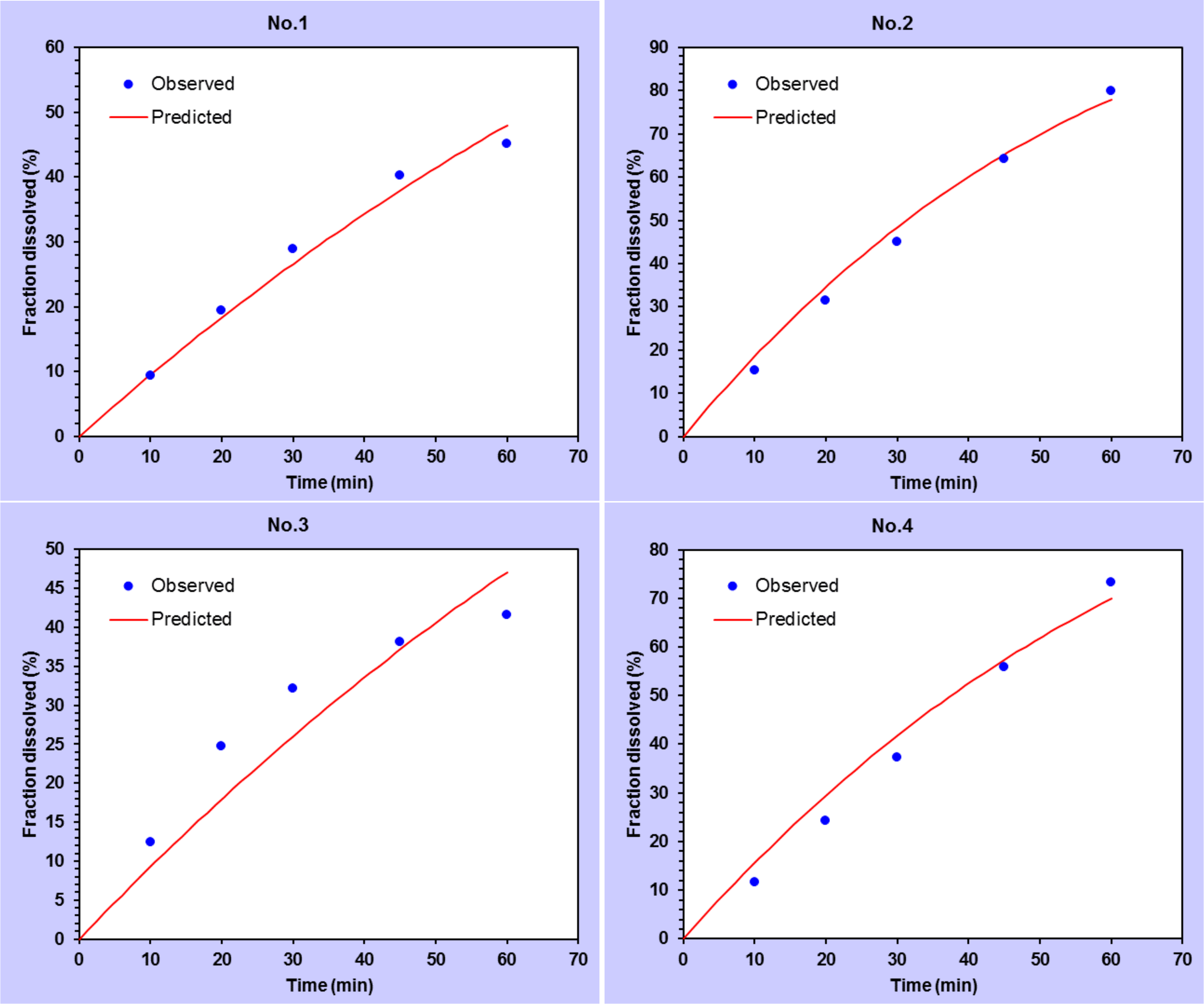

Supplementary materials – Model fitting summary – Kollidon® 25 (modelling up to app. 60 % of carvedilol released)

Model: **Hixson–Crowell with T_{lag}**

Model equation: $F = 100 \cdot \left\{1 - \left[1 - k_{HC} \cdot \left(t - T_{lag}\right)\right]^3\right\}$

Fitted model parameters per tested tablet (N = 4) with statistics – mean, standard deviation (SD), and relative standard deviation expressed in % (RSD%) (output from DDSolver):

Parameter	No.1	No.2	No.3	No.4	Mean	SD	RSD(%)
k_{HC}	0.003	0.007	0.002	0.006	0.005	0.002	50.741
T_{lag}	-2.892	3.573	-15.993	5.518	-2.449	9.719	-396.887

Number of dissolution data points (N), degrees of freedom (df), and selected goodness of fit criteria – Pearson correlation coefficient (R), coefficient of determination (R^2), adjusted coefficient of determination ($R^2_{adjusted}$), and residual sum of squares (RSS) (manual calculation in MS Excel):

Parameter	No.1	No.2	No.3	No.4
N	5	5	5	5
df	3	3	3	3
R	0.990168718	0.998425959	0.958800977	0.995807651
R^2	0.980434091	0.996854395	0.919299314	0.991632877
$R^2_{adjusted}$	0.973912121	0.99580586	0.892399085	0.988843836
RSS	17.27505608	9.650489008	43.9893273	22.55632809

Graphical abstract of model fit presented as mean ± 1 SD of the fraction % of released carvedilol:

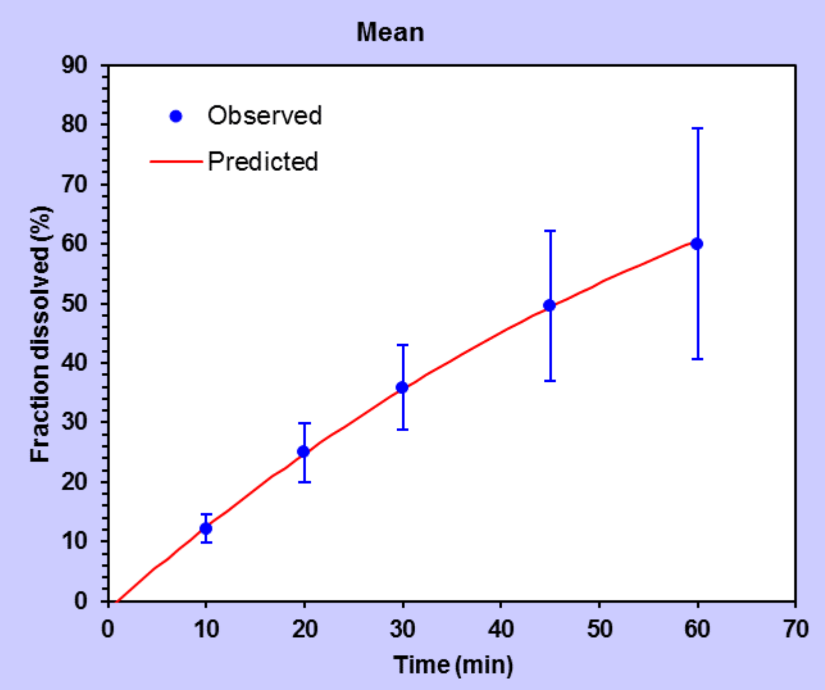

Supplementary materials – Model fitting summary – Kollidon® 25 (modelling up to app. 60 % of carvedilol released)

Graphical abstract of model fit presented as the fraction % of released carvedilol per tested tablet:

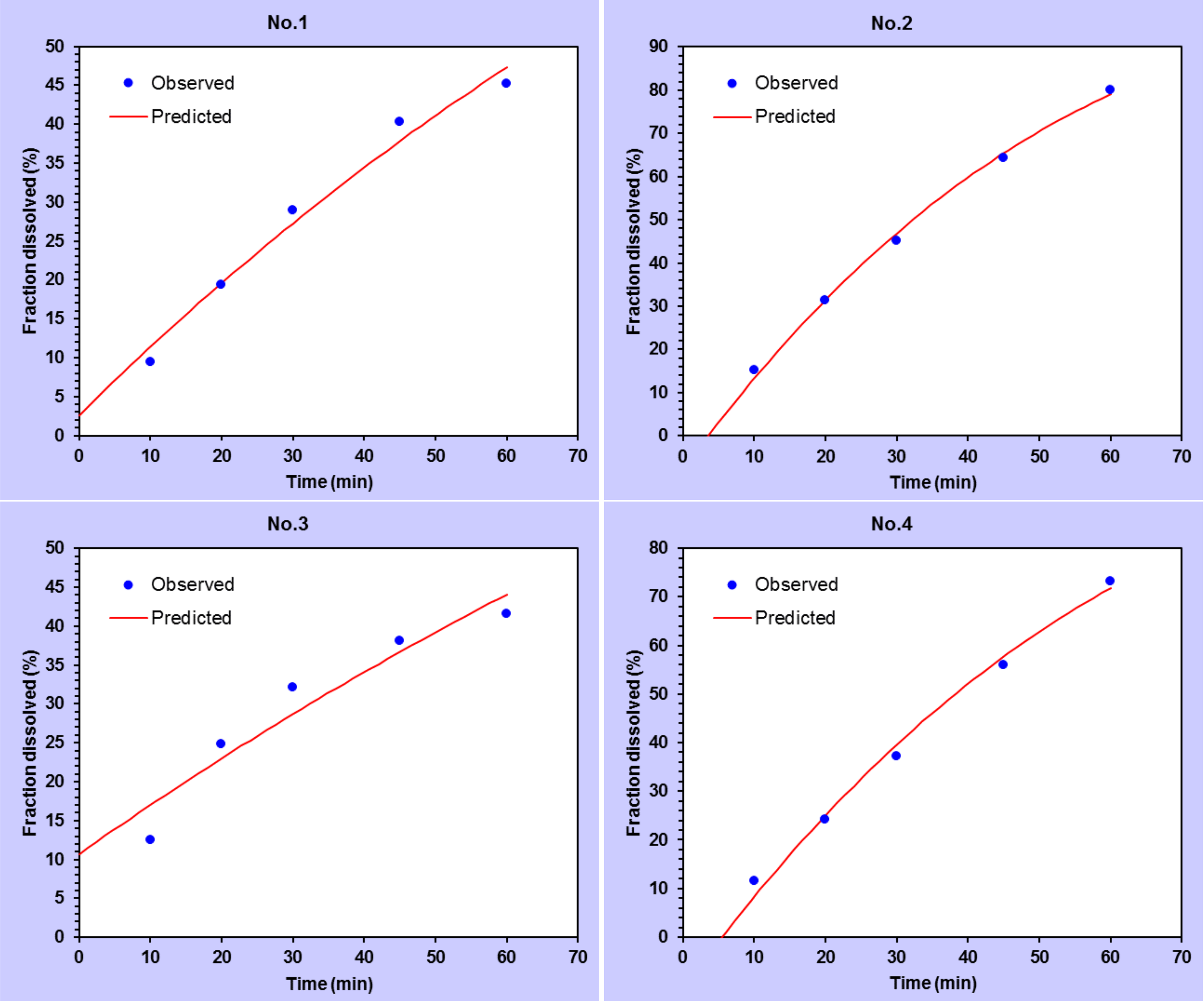

Supplementary materials – Model fitting summary – Kollidon® 25 (modelling up to app. 60 % of carvedilol released)

Model: **Hopfenberg**

Model equation: $F = 100 \cdot [1 - (1 - k_{HB} \cdot t)^n]$

Fitted model parameters per tested tablet (N = 4) with statistics – mean, standard deviation (SD), and relative standard deviation expressed in % (RSD%) (output from DDSolver):

Parameter	No.1	No.2	No.3	No.4	Mean	SD	RSD(%)
k_{HB}	0.003	0.009	0.003	0.012	0.007	0.004	64.712
n	3.000	2.000	3.000	1.000	2.250	0.957	42.552

Number of dissolution data points (N), degrees of freedom (df), and selected goodness of fit criteria – Pearson correlation coefficient (R), coefficient of determination (R^2), adjusted coefficient of determination ($R^2_{adjusted}$), and residual sum of squares (RSS) (manual calculation in MS Excel):

Parameter	No.1	No.2	No.3	No.4
N	5	5	5	5
df	3	3	3	3
R	0.990597891	0.999790957	0.962517517	0.999759117
R^2	0.981284182	0.999581958	0.926439971	0.999518293
$R^2_{adjusted}$	0.975045576	0.99944261	0.901919962	0.999357724
RSS	19.951604	9.39177121	126.8981201	1.352811388

Graphical abstract of model fit presented as mean ± 1 SD of the fraction % of released carvedilol:

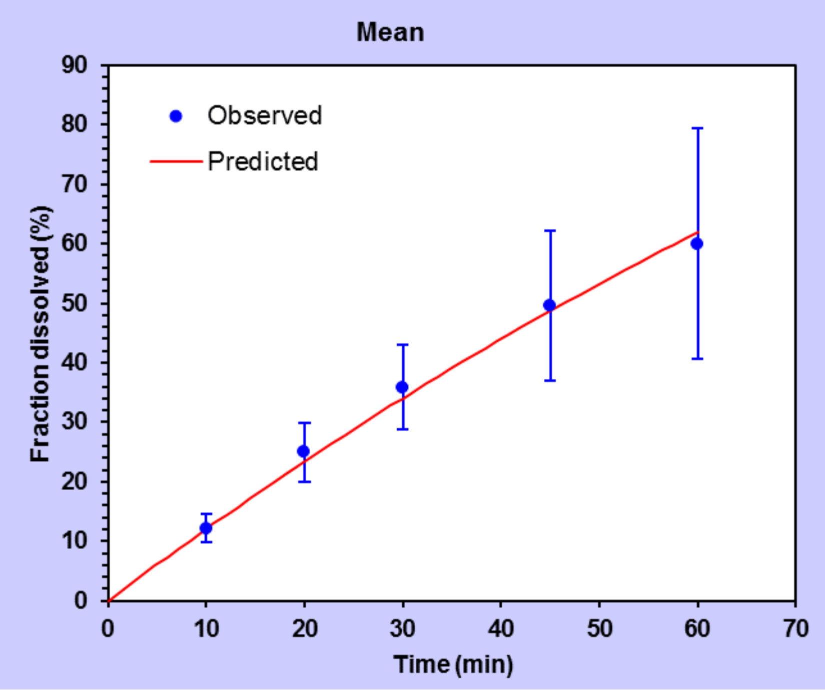

Supplementary materials – Model fitting summary – Kollidon® 25 (modelling up to app. 60 % of carvedilol released)

Graphical abstract of model fit presented as the fraction % of released carvedilol per tested tablet:

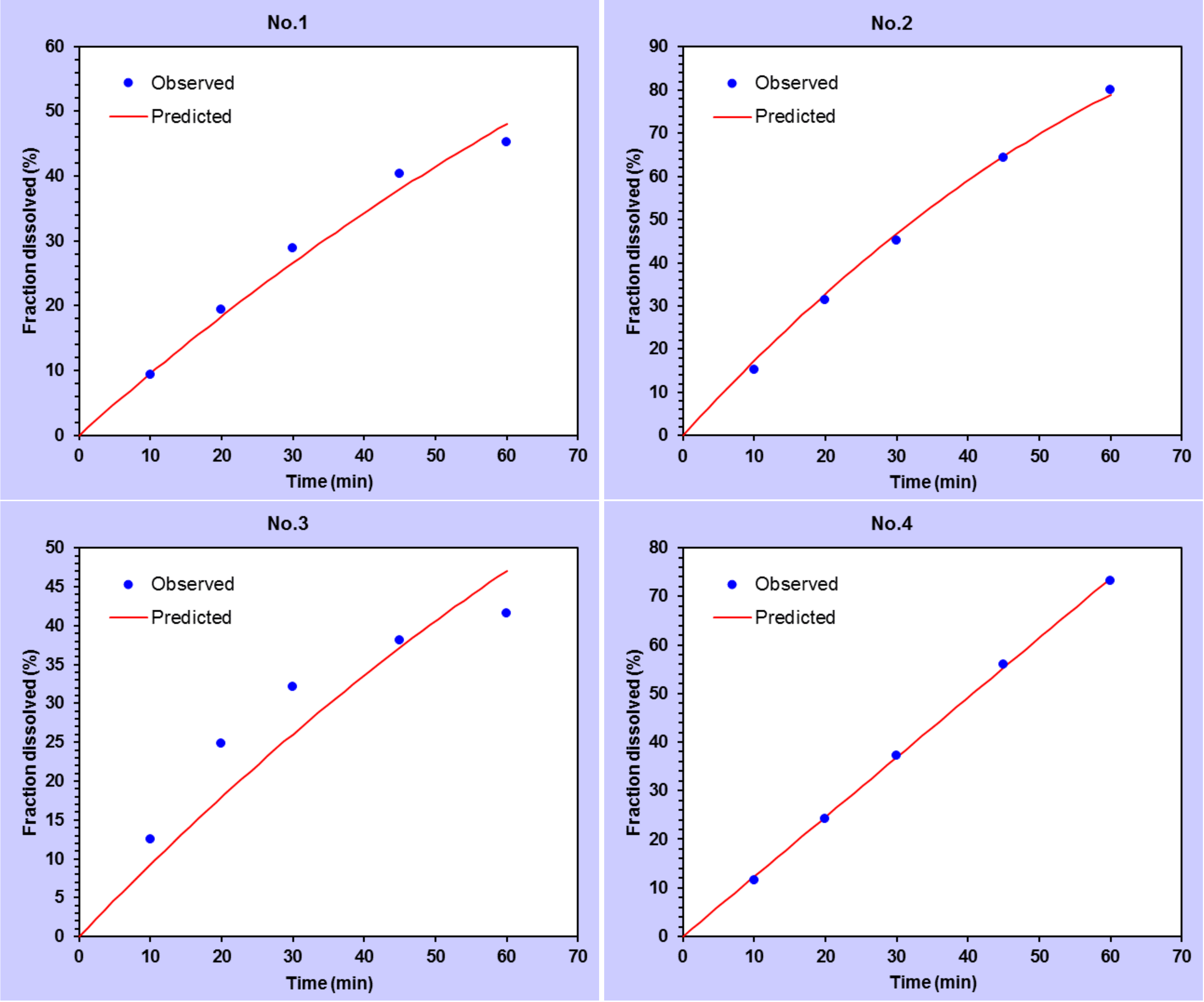

Supplementary materials – Model fitting summary – Kollidon® 25 (modelling up to app. 60 % of carvedilol released)

Model: **Hopfenberg with T_{lag}**

Model equation: $F = 100 \cdot \{1 - [1 - k_{HB} \cdot (t - T_{lag})]^n\}$

Fitted model parameters per tested tablet (N = 4) with statistics – mean, standard deviation (SD), and relative standard deviation expressed in % (RSD%) (output from DDSolver):

Parameter	No.1	No.2	No.3	No.4	Mean	SD	RSD(%)
k_{HB}	0.003	0.009	0.002	0.012	0.007	0.005	72.169
n	3.000	2.000	3.000	1.000	2.250	0.957	42.552
T_{lag}	-2.892	1.912	-15.993	0.328	-4.162	8.137	-195.529

Number of dissolution data points (N), degrees of freedom (df), and selected goodness of fit criteria – Pearson correlation coefficient (R), coefficient of determination (R^2), adjusted coefficient of determination ($R^2_{adjusted}$), and residual sum of squares (RSS) (manual calculation in MS Excel):

Parameter	No.1	No.2	No.3	No.4
N	5	5	5	5
df	2	2	2	2
R	0.990168718	0.99971181	0.958800977	0.999759117
R^2	0.980434091	0.999423703	0.919299314	0.999518293
$R^2_{adjusted}$	0.960868181	0.998847407	0.838598627	0.999036586
RSS	17.27505608	1.641081172	43.9893273	1.167721519

Graphical abstract of model fit presented as mean ± 1 SD of the fraction % of released carvedilol:

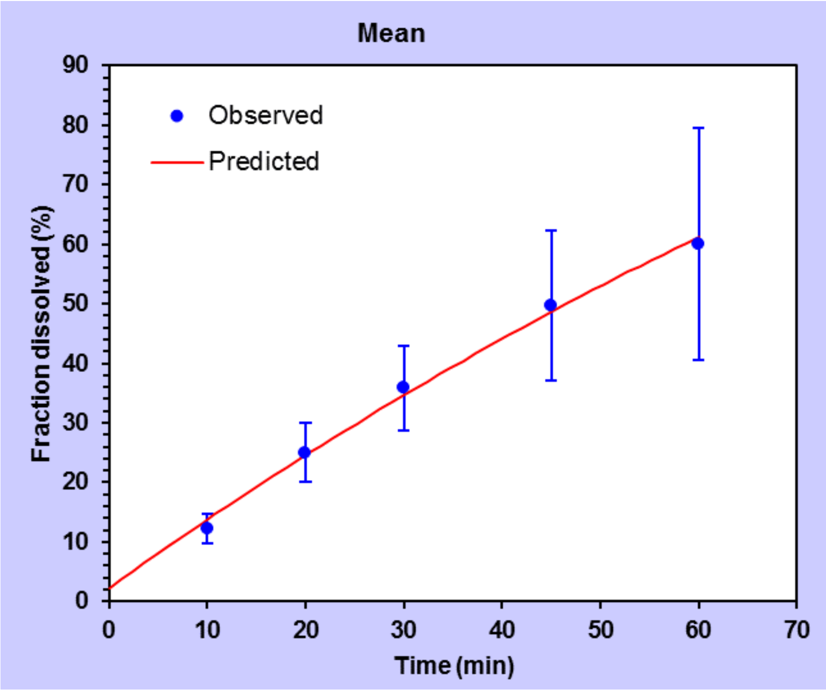

Supplementary materials – Model fitting summary – Kollidon® 25 (modelling up to app. 60 % of carvedilol released)

Graphical abstract of model fit presented as the fraction % of released carvedilol per tested tablet:

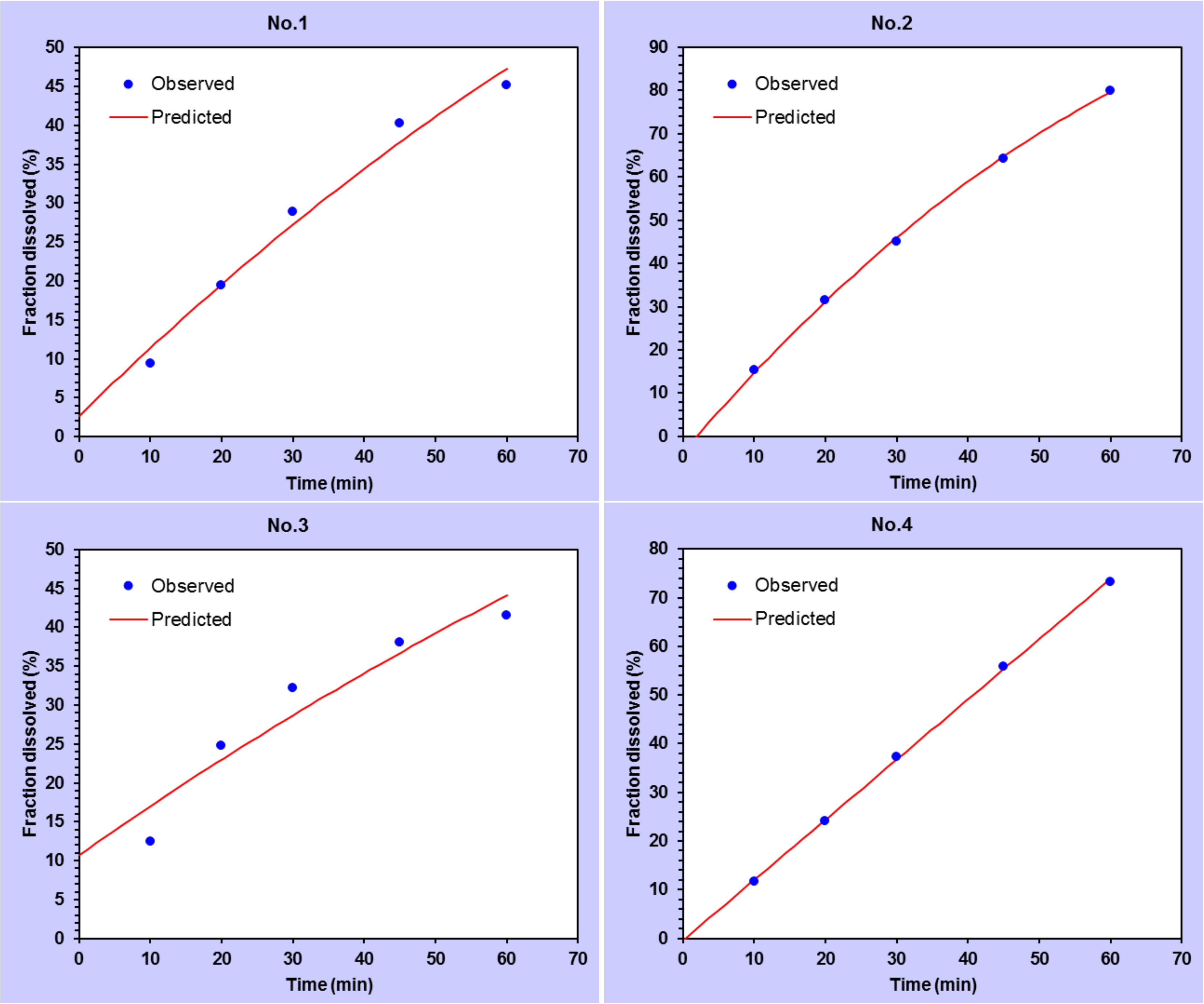

Supplementary materials – Model fitting summary – Kollidon® 25 (modelling up to app. 60 % of carvedilol released)

Model: **Baker–Lonsdale**

Model equation: $\frac{3}{2}\cdot\left[1-\left(1-\frac{F}{100}\right)^{\frac{2}{3}}\right]-\frac{F}{100}=k_{BL}\cdot t$

Fitted model parameters per tested tablet (N = 4) with statistics – mean, standard deviation (SD), and relative standard deviation expressed in % (RSD%) (output from DDSolver):

Parameter	No.1	No.2	No.3	No.4	Mean	SD	RSD(%)
k_{BL}	0.000	0.004	0.001	0.003	0.002	0.002	83.875

Number of dissolution data points (N), degrees of freedom (df), and selected goodness of fit criteria – Pearson correlation coefficient (R), coefficient of determination (R^2), adjusted coefficient of determination ($R^2_{adjusted}$), and residual sum of squares (RSS) (manual calculation in MS Excel):

Parameter	No.1	No.2	No.3	No.4
N	5	5	5	5
df	4	4	4	4
R	0.996496795	0.991563385	0.984465782	0.985205253
R^2	0.993005862	0.983197946	0.969172875	0.97062939
$R^2_{adjusted}$	0.993005862	0.983197946	0.969172875	0.97062939
RSS	199.6516229	1944.453817	49.99010118	2103.649384

Graphical abstract of model fit presented as mean ± 1 SD of the fraction % of released carvedilol:

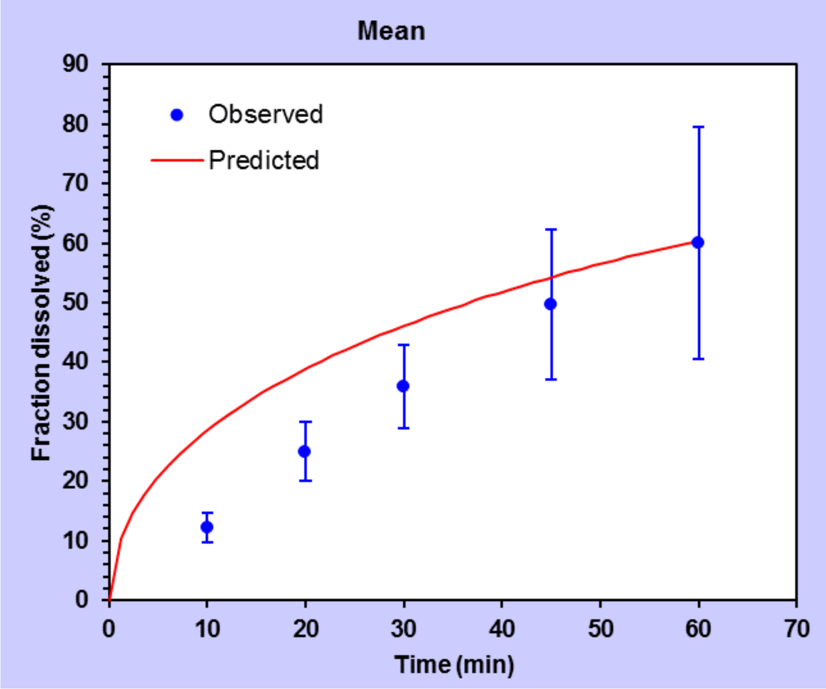

Supplementary materials – Model fitting summary – Kollidon® 25 (modelling up to app. 60 % of carvedilol released)

Graphical abstract of model fit presented as the fraction % of released carvedilol per tested tablet:

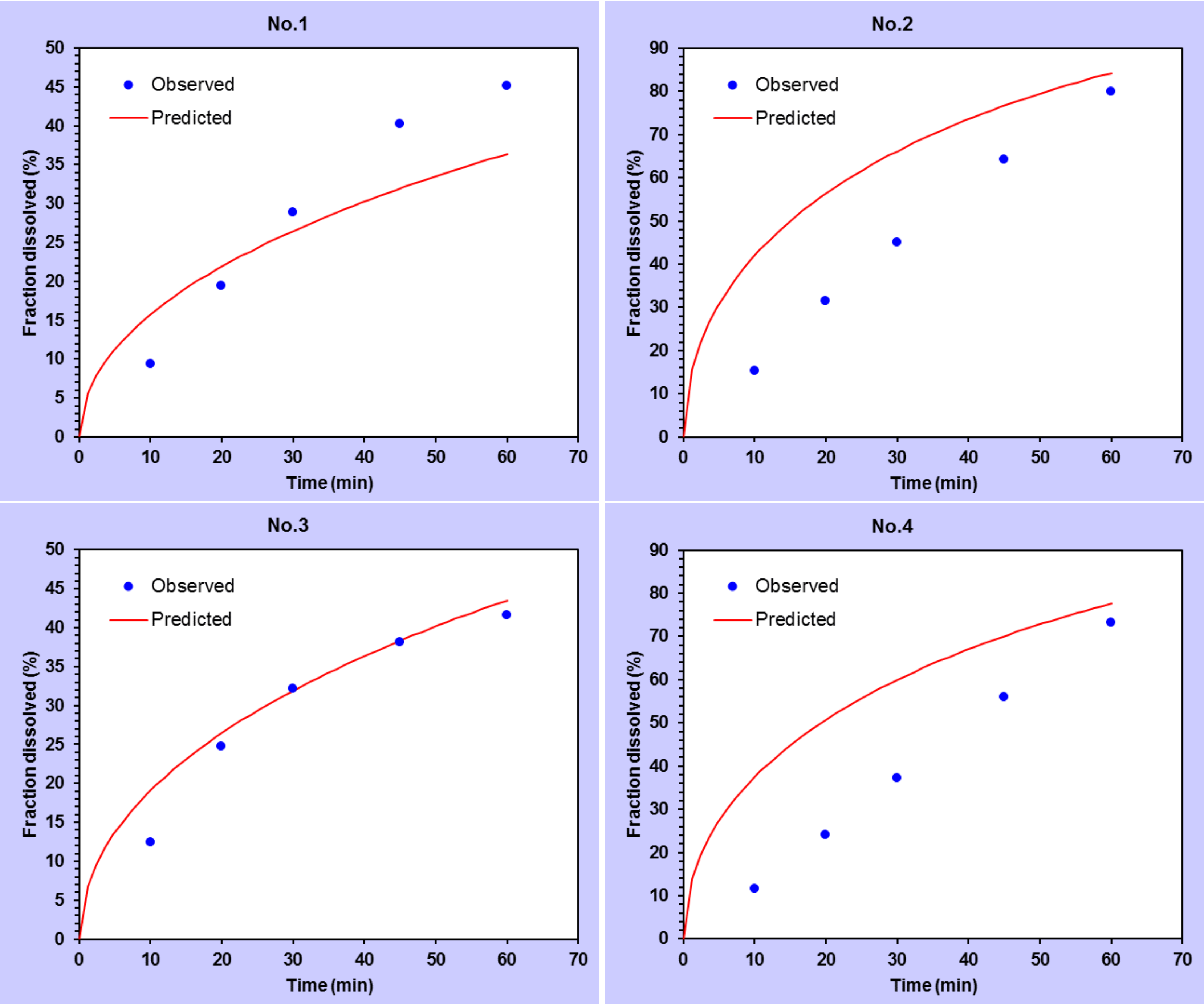

Supplementary materials – Model fitting summary – Kollidon® 25 (modelling up to app. 60 % of carvedilol released)

Model: **Baker–Lonsdale with T_{lag}**

Model equation: $\frac{3}{2}\cdot\left[1-\left(1-\frac{F}{100}\right)^{\frac{2}{3}}\right]-\frac{F}{100}=k_{BL}\cdot\left(t-T_{lag}\right)$

Fitted model parameters per tested tablet (N = 4) with statistics – mean, standard deviation (SD), and relative standard deviation expressed in % (RSD%) (output from DDSolver):

Parameter	No.1	No.2	No.3	No.4	Mean	SD	RSD(%)
k_{BL}	0.001	0.004	0.001	0.003	0.002	0.002	77.504
T_{lag}	10.147	16.989	4.307	14.839	11.570	5.622	48.590

Number of dissolution data points (N), degrees of freedom (df), and selected goodness of fit criteria – Pearson correlation coefficient (R), coefficient of determination (R^2), adjusted coefficient of determination ($R^2_{adjusted}$), and residual sum of squares (RSS) (manual calculation in MS Excel):

Parameter	No.1	No.2	No.3	No.4
N	5	5	5	5
df	3	3	3	3
R	0.974768358	0.984266352	0.989544795	0.964539851
R^2	0.950173352	0.968780251	0.9791989	0.930337124
$R^2_{adjusted}$	0.933564469	0.958373669	0.972265201	0.907116166
RSS	97.45120436	299.2426659	14.2004915	233.9412235

Graphical abstract of model fit presented as mean ± 1 SD of the fraction % of released carvedilol:

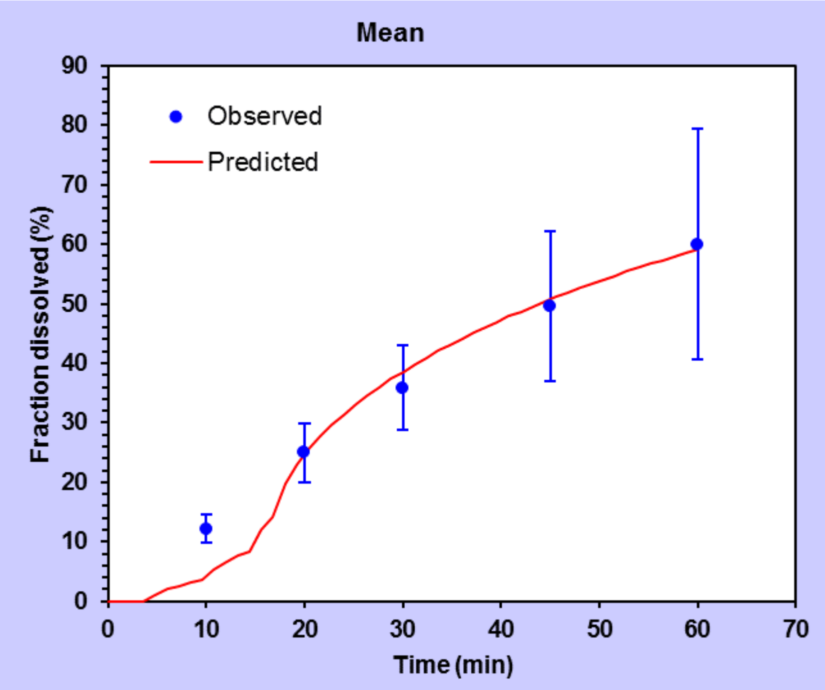

Supplementary materials – Model fitting summary – Kollidon® 25 (modelling up to app. 60 % of carvedilol released)

Graphical abstract of model fit presented as the fraction % of released carvedilol per tested tablet:

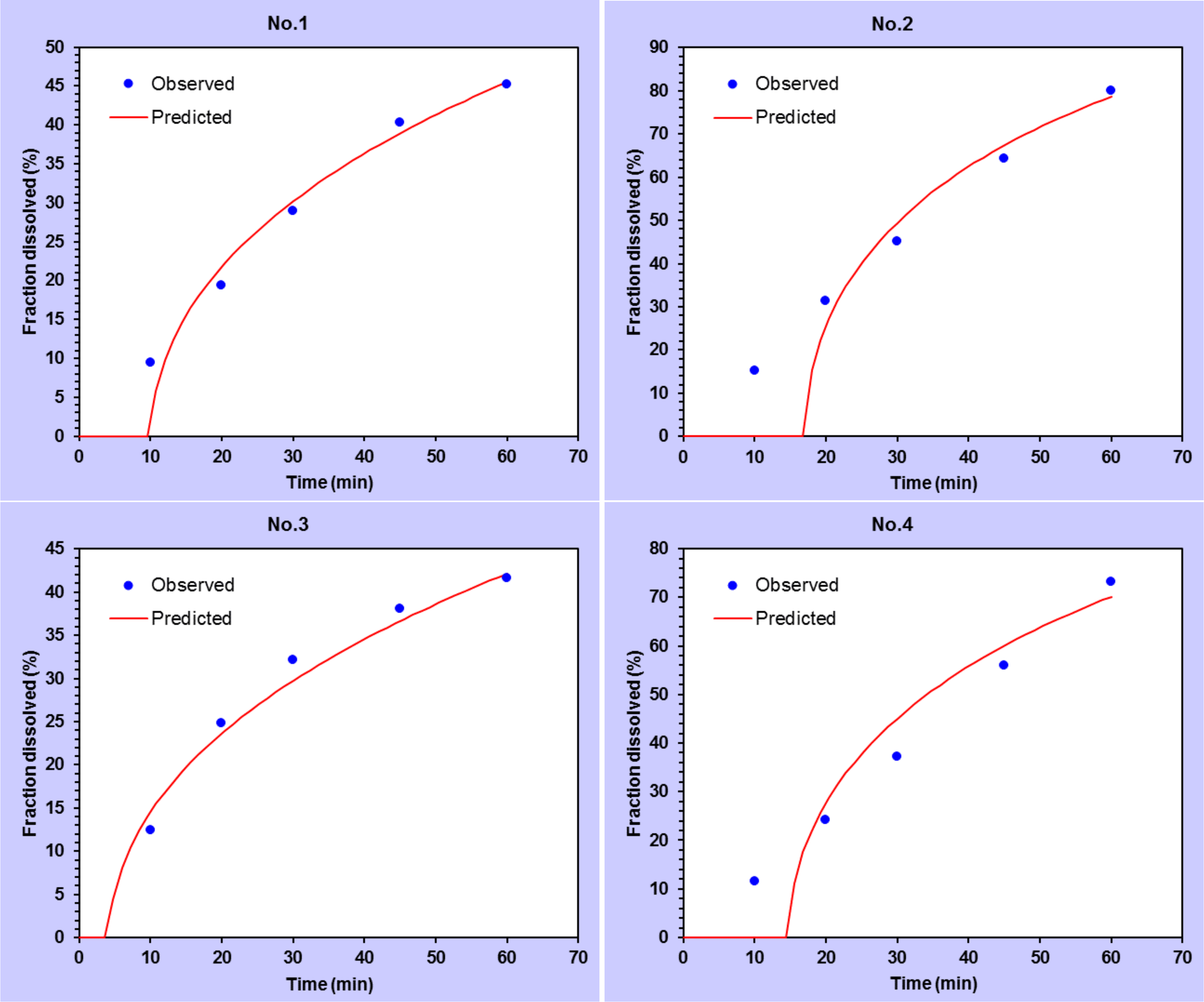

Supplementary materials – Model fitting summary – Kollidon® 25 (modelling up to app. 60 % of carvedilol released)

Model: **Makoid–Banakar**

Model equation: $F = k_{MB} \cdot t^n \cdot e^{-k \cdot t}$

Fitted model parameters per tested tablet (N = 4) with statistics – mean, standard deviation (SD), and relative standard deviation expressed in % (RSD%) (output from DDSolver):

Parameter	No.1	No.2	No.3	No.4	Mean	SD	RSD(%)
k_{MB}	0.549	1.247	0.905	0.911	0.903	0.285	31.541
n	1.293	1.122	1.234	1.118	1.192	0.086	7.257
k	0.014	0.007	0.021	0.003	0.011	0.008	68.406

Number of dissolution data points (N), degrees of freedom (df), and selected goodness of fit criteria – Pearson correlation coefficient (R), coefficient of determination (R^2), adjusted coefficient of determination ($R^2_{adjusted}$), and residual sum of squares (RSS) (manual calculation in MS Excel):

Parameter	No.1	No.2	No.3	No.4
N	5	5	5	5
df	2	2	2	2
R	0.999076154	0.999913856	0.998153684	0.999983377
R^2	0.998153162	0.999827719	0.996310777	0.999966755
$R^2_{adjusted}$	0.996306323	0.999655439	0.992621555	0.99993351
RSS	1.603638016	0.454819359	2.023758967	0.081433656

Graphical abstract of model fit presented as mean ± 1 SD of the fraction % of released carvedilol:

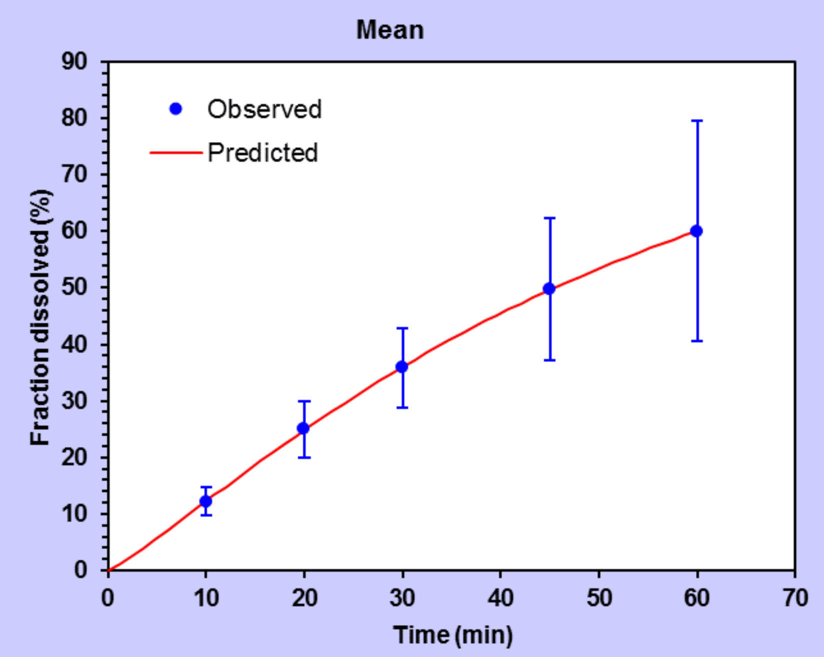

Supplementary materials – Model fitting summary – Kollidon® 25 (modelling up to app. 60 % of carvedilol released)

Graphical abstract of model fit presented as the fraction % of released carvedilol per tested tablet:

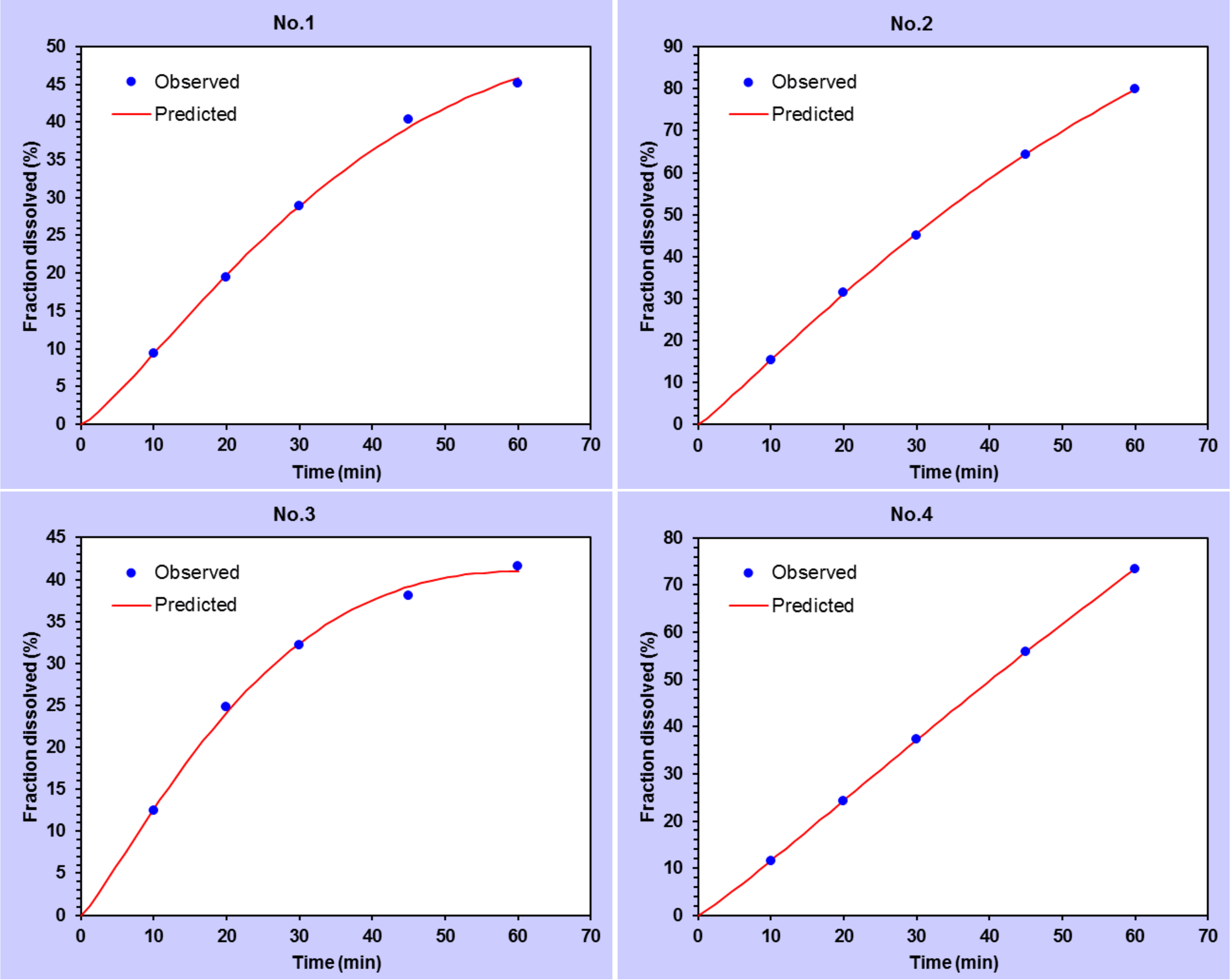

Supplementary materials – Model fitting summary – Kollidon® 25 (modelling up to app. 60 % of carvedilol released)

Model: **Makoid–Banakar with T_{lag}**

Model equation: $F = k_{MB} \cdot (t - T_{lag})^n \cdot e^{-k \cdot (t - T_{lag})}$

Fitted model parameters per tested tablet (N = 4) with statistics – mean, standard deviation (SD), and relative standard deviation expressed in % (RSD%) (output from DDSolver):

Parameter	No.1	No.2	No.3	No.4	Mean	SD	RSD(%)
k_{MB}	2.116	4.070	3.146	3.023	3.089	0.800	25.886
n	0.844	0.737	0.814	0.732	0.782	0.056	7.161
k	0.006	0.000	0.013	-0.005	0.003	0.007	221.657
T_{lag}	4.000	4.000	4.000	4.000	4.000	0.000	0.000

Number of dissolution data points (N), degrees of freedom (df), and selected goodness of fit criteria – Pearson correlation coefficient (R), coefficient of determination (R^2), adjusted coefficient of determination ($R^2_{adjusted}$), and residual sum of squares (RSS) (manual calculation in MS Excel):

Parameter	No.1	No.2	No.3	No.4
N	5	5	5	5
df	1	1	1	1
R	0.997487187	0.999875467	0.999582528	0.999664669
R^2	0.994980689	0.999750949	0.99916523	0.999329451
$R^2_{adjusted}$	0.979922755	0.999003795	0.996660919	0.997317805
RSS	4.348581242	0.657874896	0.453283027	1.657335908

Graphical abstract of model fit presented as mean ± 1 SD of the fraction % of released carvedilol:

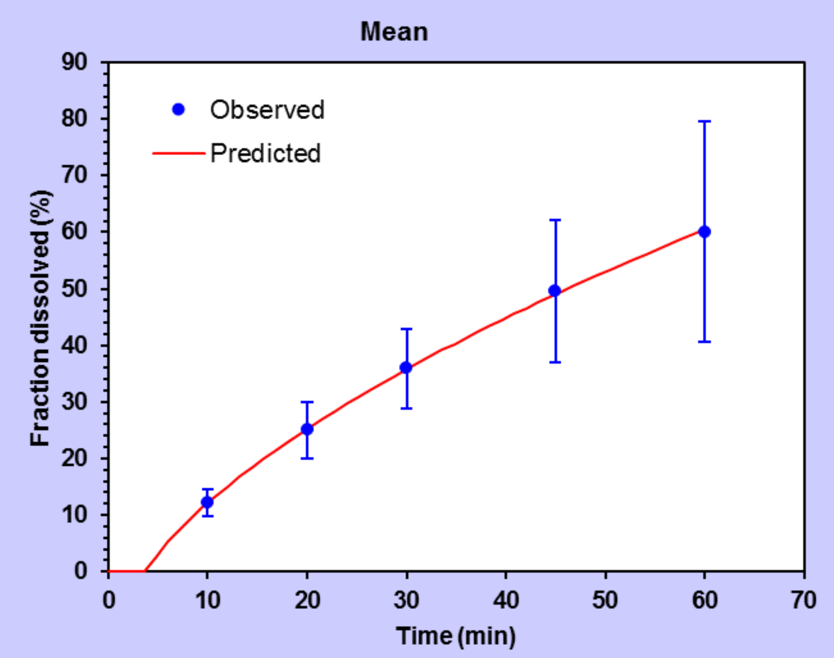

Supplementary materials – Model fitting summary – Kollidon® 25 (modelling up to app. 60 % of carvedilol released)

Graphical abstract of model fit presented as the fraction % of released carvedilol per tested tablet:

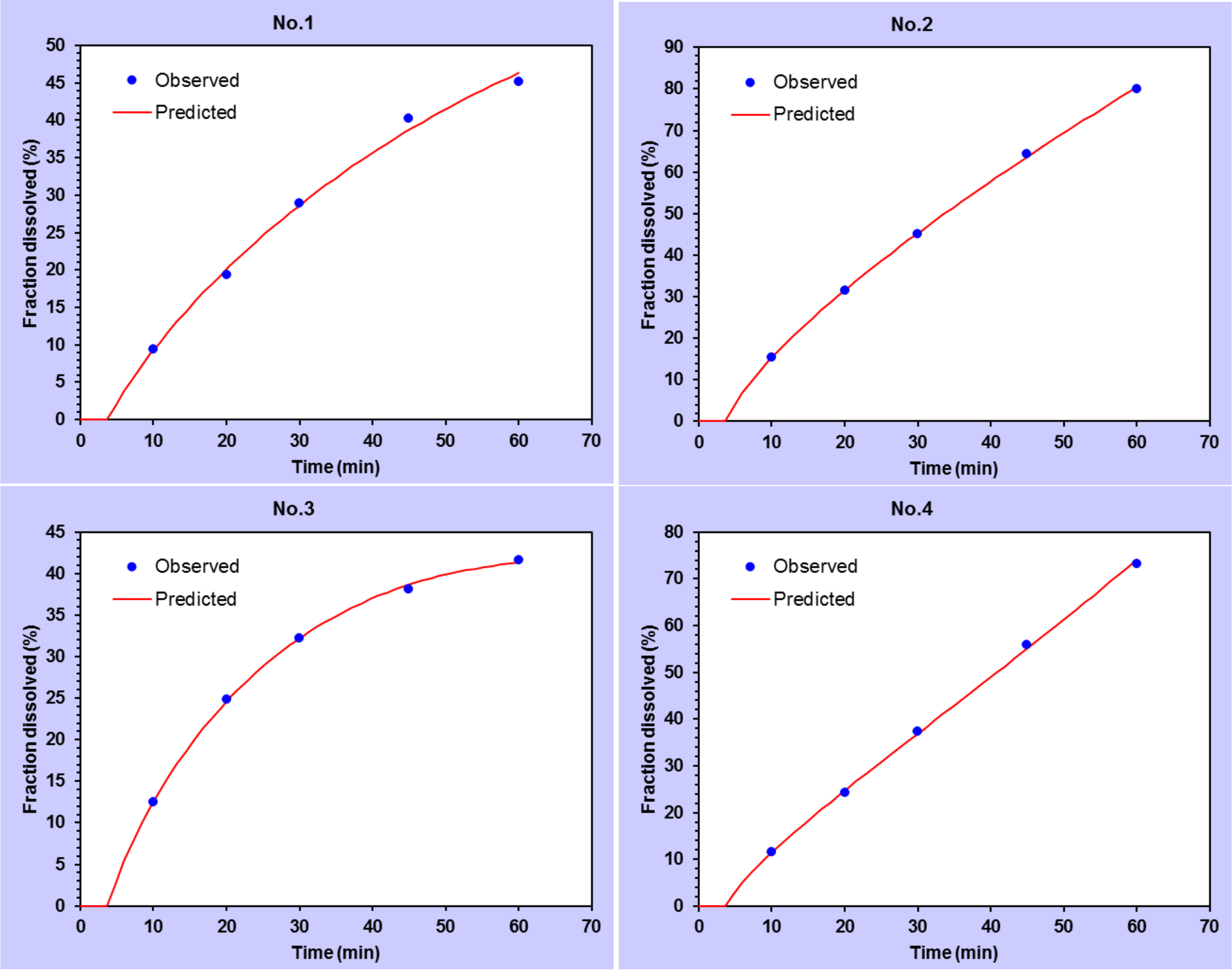

Supplementary materials – Model fitting summary – Kollidon® 25 (modelling up to app. 60 % of carvedilol released)

Model: **Peppas–Sahlin_1**

Model equation: $F = k_1 \cdot t^m + k_2 \cdot t^{2m}$

Fitted model parameters per tested tablet (N = 4) with statistics – mean, standard deviation (SD), and relative standard deviation expressed in % (RSD%) (output from DDSolver):

Parameter	No.1	No.2	No.3	No.4	Mean	SD	RSD(%)
k_1	1.346	0.520	4.898	-1.924	1.210	2.823	233.357
k_2	0.982	1.957	0.319	2.152	1.353	0.858	63.465
m	0.450	0.450	0.450	0.450	0.450	0.000	0.000

Number of dissolution data points (N), degrees of freedom (df), and selected goodness of fit criteria – Pearson correlation coefficient (R), coefficient of determination (R^2), adjusted coefficient of determination ($R^2_{adjusted}$), and residual sum of squares (RSS) (manual calculation in MS Excel):

Parameter	No.1	No.2	No.3	No.4
N	5	5	5	5
df	2	2	2	2
R	0.988211871	0.998682427	0.973745885	0.999920535
R^2	0.976562701	0.997366591	0.948181048	0.999841076
$R^2_{adjusted}$	0.953125402	0.994733181	0.896362096	0.999682152
RSS	20.67221605	7.087559673	29.18372269	0.386968852

Graphical abstract of model fit presented as mean ± 1 SD of the fraction % of released carvedilol:

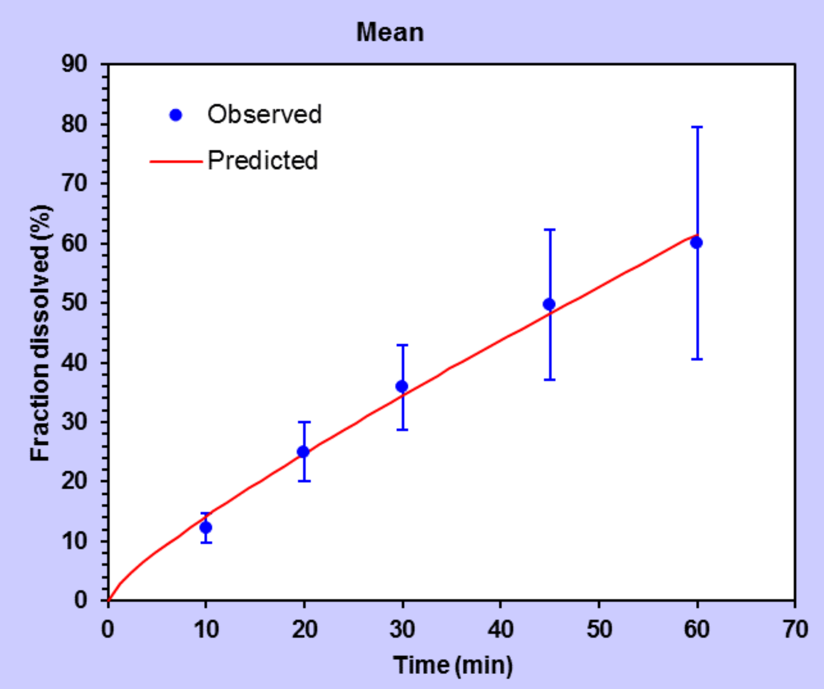

Supplementary materials – Model fitting summary – Kollidon® 25 (modelling up to app. 60 % of carvedilol released)

Graphical abstract of model fit presented as the fraction % of released carvedilol per tested tablet:

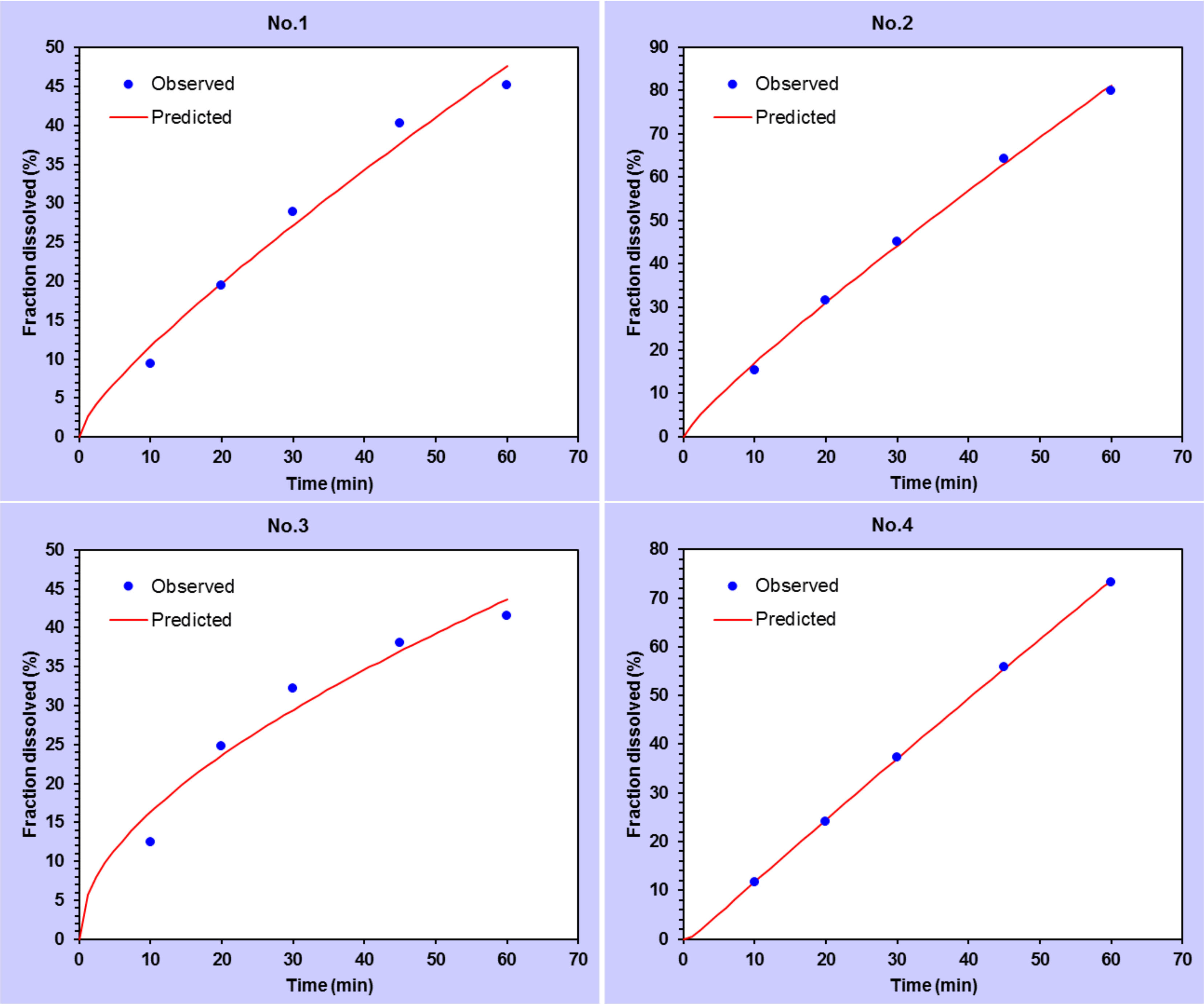

Supplementary materials – Model fitting summary – Kollidon® 25 (modelling up to app. 60 % of carvedilol released)

Model: **Peppas–Sahlin_1 with T_{lag}**

Model equation: $F = k_1 \cdot (t - T_{lag})^m + k_2 \cdot (t - T_{lag})^{2m}$

Fitted model parameters per tested tablet (N = 4) with statistics – mean, standard deviation (SD), and relative standard deviation expressed in % (RSD%) (output from DDSolver):

Parameter	No.1	No.2	No.3	No.4	Mean	SD	RSD(%)
k_1	3.215	3.572	6.790	0.711	3.572	2.494	69.830
k_2	0.735	1.571	0.041	1.838	1.046	0.819	78.255
m	0.450	0.450	0.450	0.450	0.450	0.000	0.000
T_{lag}	4.000	4.000	4.000	4.000	4.000	0.000	0.000

Number of dissolution data points (N), degrees of freedom (df), and selected goodness of fit criteria – Pearson correlation coefficient (R), coefficient of determination (R^2), adjusted coefficient of determination ($R^2_{adjusted}$), and residual sum of squares (RSS) (manual calculation in MS Excel):

Parameter	No.1	No.2	No.3	No.4
N	5	5	5	5
df	1	1	1	1
R	0.992184039	0.999708613	0.986630522	0.999789206
R^2	0.984429166	0.999417311	0.973439786	0.999578457
$R^2_{adjusted}$	0.937716665	0.997669244	0.893759145	0.998313826
RSS	13.89886376	1.579359275	15.85848131	1.053086204

Graphical abstract of model fit presented as mean ± 1 SD of the fraction % of released carvedilol:

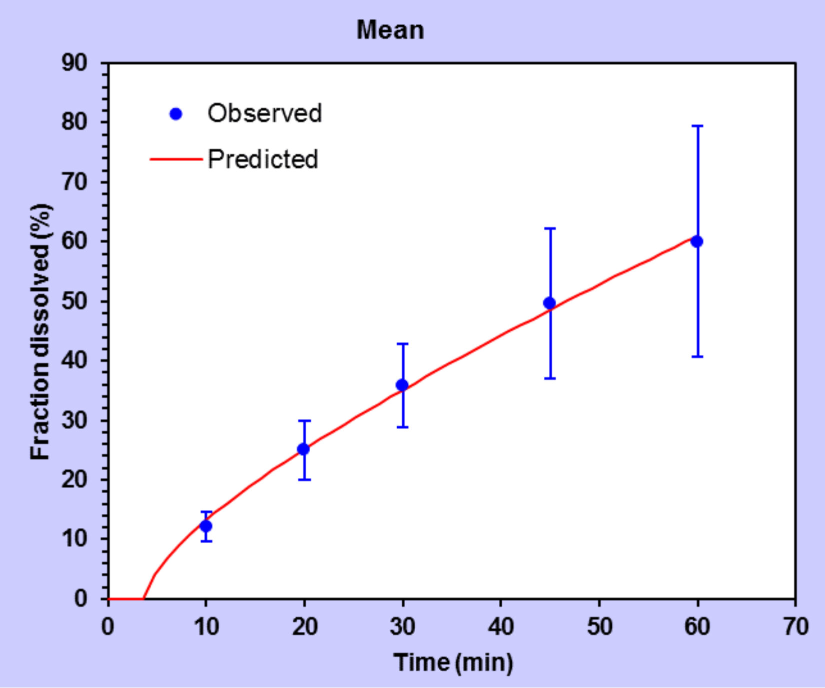

Supplementary materials – Model fitting summary – Kollidon® 25 (modelling up to app. 60 % of carvedilol released)

Graphical abstract of model fit presented as the fraction % of released carvedilol per tested tablet:

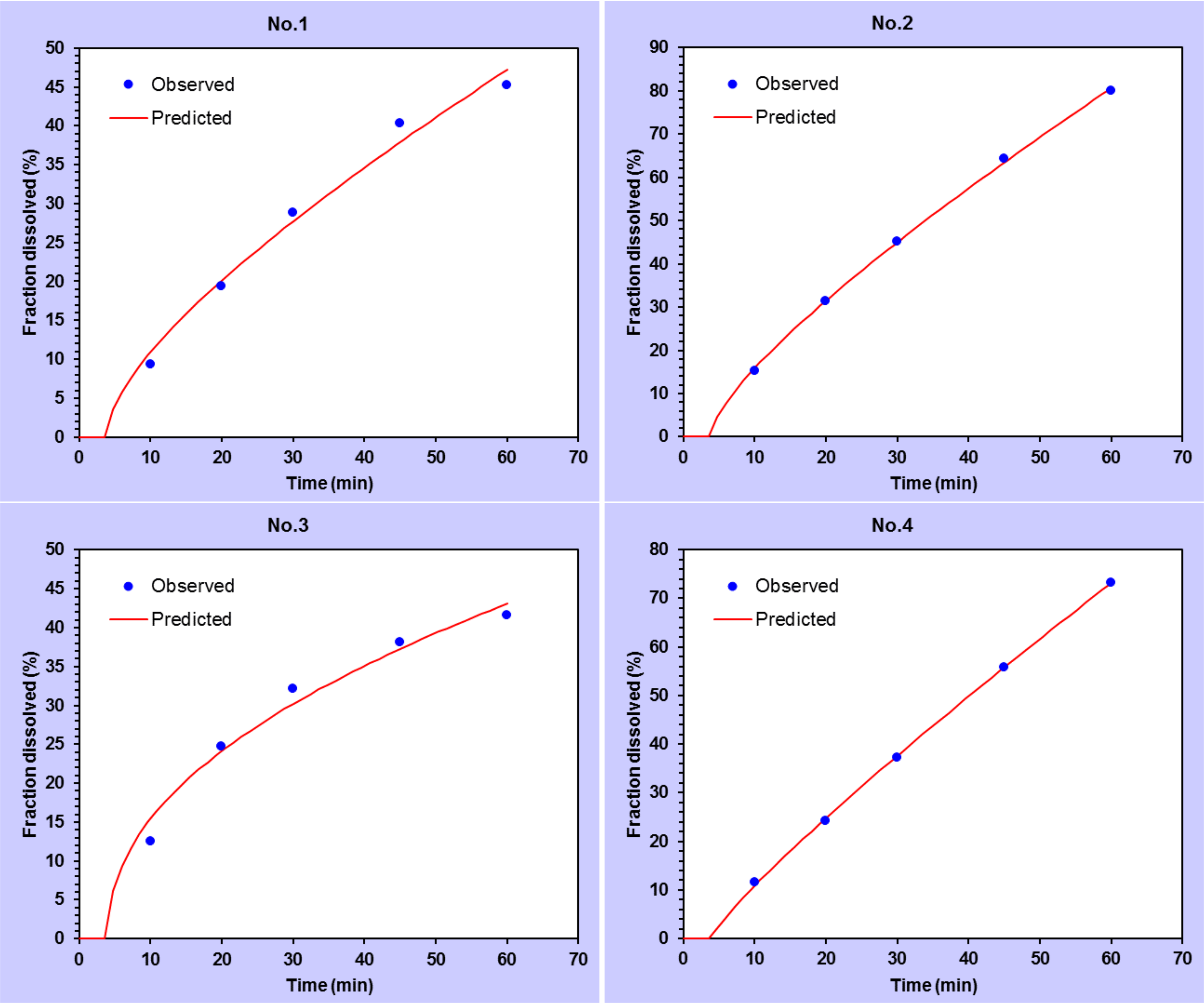

Supplementary materials – Model fitting summary – Kollidon® 25 (modelling up to app. 60 % of carvedilol released)

Model: **Peppas–Sahlin_2**

Model equation: $F = k_1 \cdot t^{0.5} + k_2 \cdot t$

Fitted model parameters per tested tablet (N = 4) with statistics – mean, standard deviation (SD), and relative standard deviation expressed in % (RSD%) (output from DDSolver):

Parameter	No.1	No.2	No.3	No.4	Mean	SD	RSD(%)
k_1	2.080	2.052	4.884	-0.102	2.228	2.044	91.728
k_2	0.525	1.092	0.094	1.244	0.739	0.530	71.711

Number of dissolution data points (N), degrees of freedom (df), and selected goodness of fit criteria – Pearson correlation coefficient (R), coefficient of determination (R^2), adjusted coefficient of determination ($R^2_{adjusted}$), and residual sum of squares (RSS) (manual calculation in MS Excel):

Parameter	No.1	No.2	No.3	No.4
N	5	5	5	5
df	3	3	3	3
R	0.987372025	0.998189819	0.975504021	0.999741855
R^2	0.974903517	0.996382915	0.951608095	0.999483776
$R^2_{adjusted}$	0.966538022	0.99517722	0.93547746	0.999311701
RSS	22.3672663	9.839701743	27.8923674	1.275464018

Graphical abstract of model fit presented as mean ± 1 SD of the fraction % of released carvedilol:

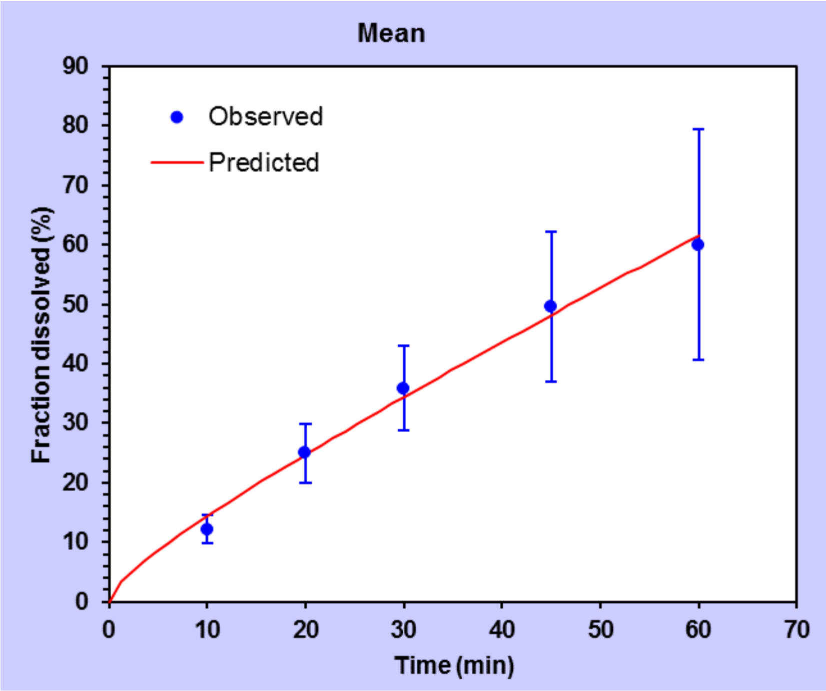

Supplementary materials – Model fitting summary – Kollidon® 25 (modelling up to app. 60 % of carvedilol released)

Graphical abstract of model fit presented as the fraction % of released carvedilol per tested tablet:

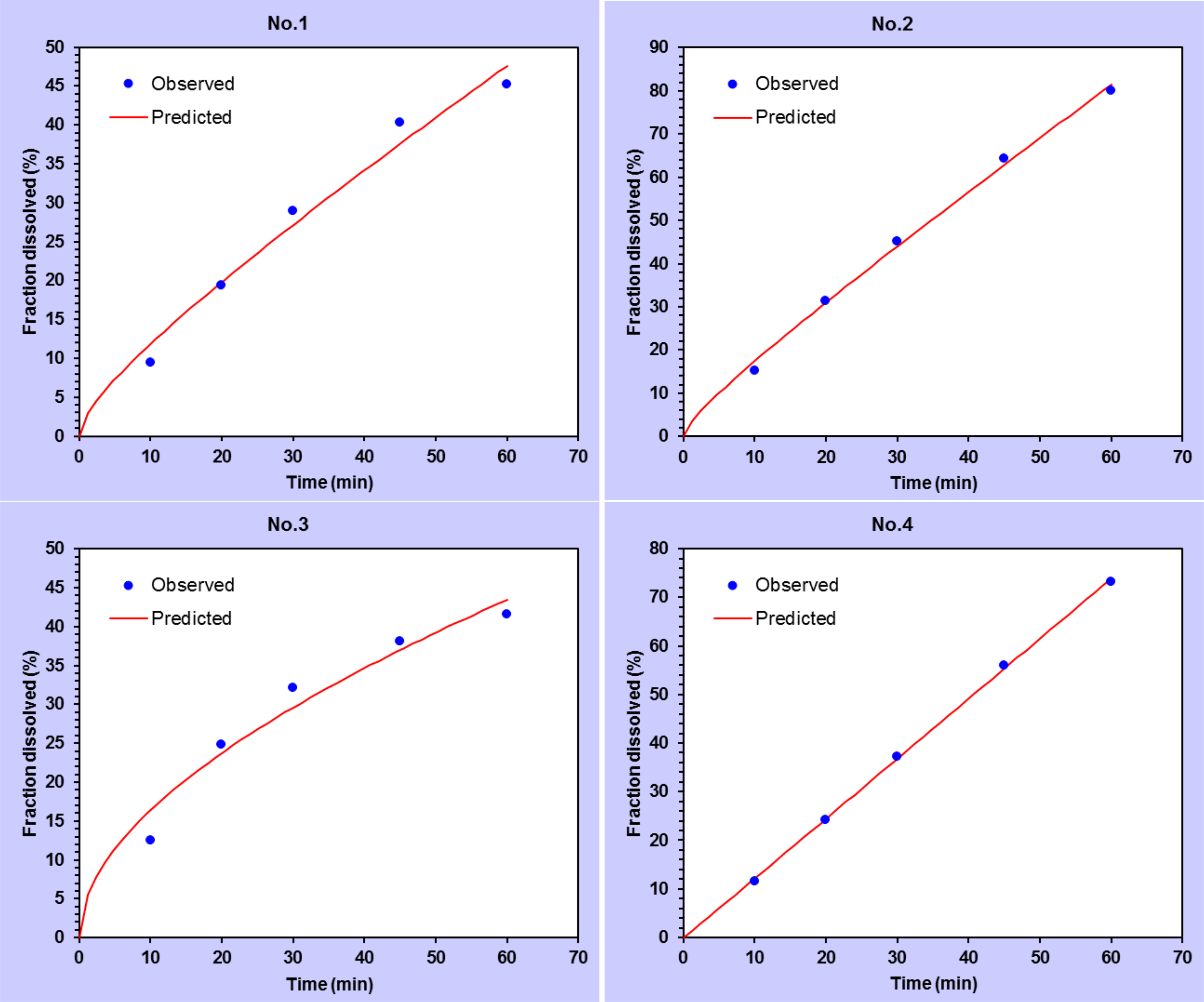

Supplementary materials – Model fitting summary – Kollidon® 25 (modelling up to app. 60 % of carvedilol released)

Model: **Peppas–Sahlin_2 with T_{lag}**

Model equation: $F = k_1 \cdot (t - T_{lag})^{0.5} + k_2 \cdot (t - T_{lag})$

Fitted model parameters per tested tablet (N = 4) with statistics – mean, standard deviation (SD), and relative standard deviation expressed in % (RSD%) (output from DDSolver):

Parameter	No.1	No.2	No.3	No.4	Mean	SD	RSD(%)
k_1	3.615	5.095	6.476	2.003	4.297	1.925	44.787
k_2	0.358	0.781	-0.101	1.043	0.521	0.501	96.242
T_{lag}	4.000	4.697	4.000	4.000	4.174	0.348	8.348

Number of dissolution data points (N), degrees of freedom (df), and selected goodness of fit criteria – Pearson correlation coefficient (R), coefficient of determination (R^2), adjusted coefficient of determination ($R^2_{adjusted}$), and residual sum of squares (RSS) (manual calculation in MS Excel):

Parameter	No.1	No.2	No.3	No.4
N	5	5	5	5
df	2	2	2	2
R	0.992108283	0.999759286	0.989457524	0.999888622
R^2	0.984278845	0.999518629	0.979026191	0.999777257
$R^2_{adjusted}$	0.96855769	0.999037258	0.958052383	0.999554514
RSS	14.26698105	1.994015447	13.17234445	0.547051894

Graphical abstract of model fit presented as mean ± 1 SD of the fraction % of released carvedilol:

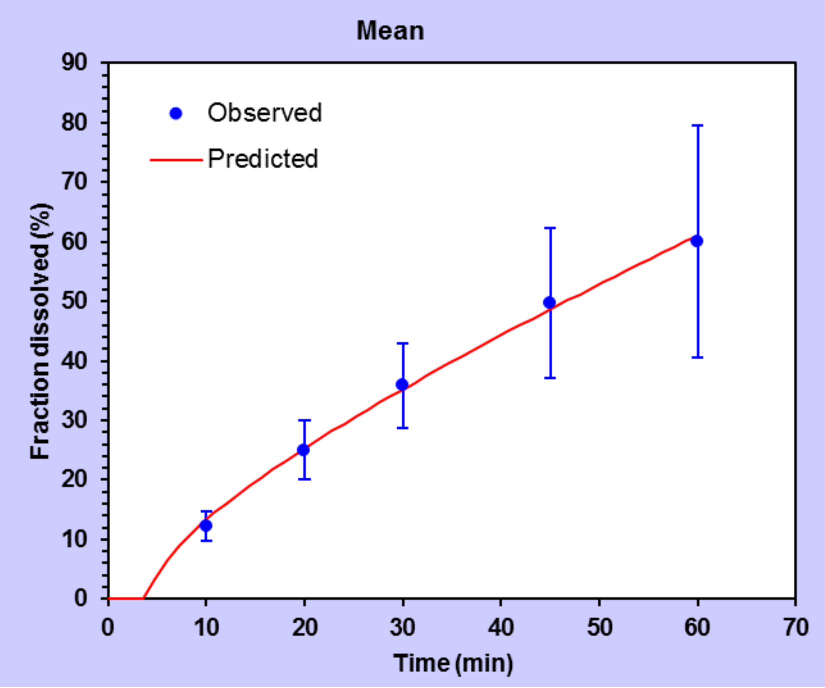

Supplementary materials – Model fitting summary – Kollidon® 25 (modelling up to app. 60 % of carvedilol released)

Graphical abstract of model fit presented as the fraction % of released carvedilol per tested tablet:

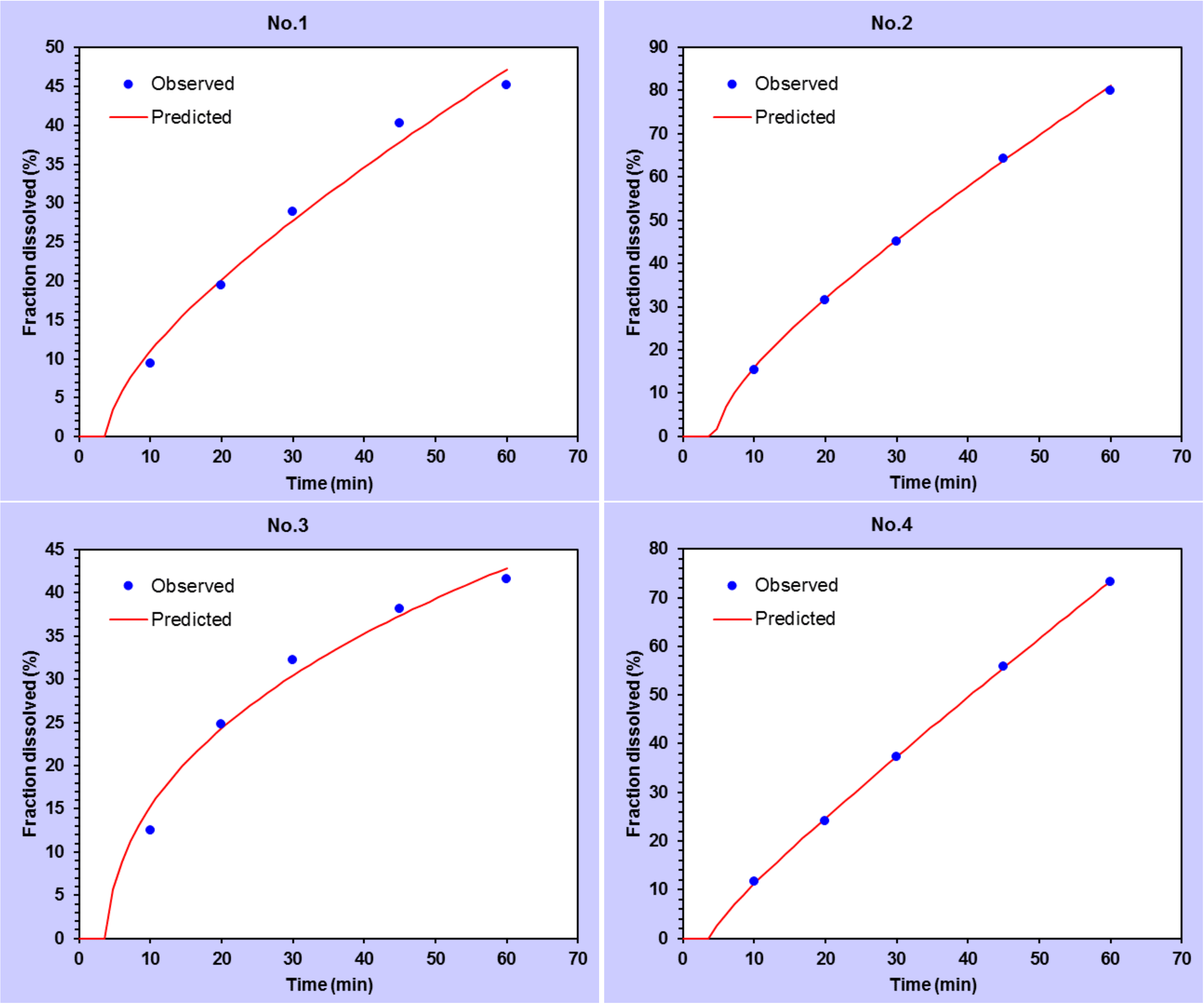

Supplementary materials – Model fitting summary – Kollidon® 25 (modelling up to app. 60 % of carvedilol released)

Model: **Quadratic**

Model equation: $F = 100 \cdot (k_1 \cdot t^2 + k_2 \cdot t)$

Fitted model parameters per tested tablet (N = 4) with statistics – mean, standard deviation (SD), and relative standard deviation expressed in % (RSD%) (output from DDSolver):

Parameter	No.1	No.2	No.3	No.4	Mean	SD	RSD(%)
k_1	0.000	0.000	0.000	0.000	0.000	0.000	-83.347
k_2	0.011	0.017	0.014	0.012	0.014	0.002	17.316

Number of dissolution data points (N), degrees of freedom (df), and selected goodness of fit criteria – Pearson correlation coefficient (R), coefficient of determination (R^2), adjusted coefficient of determination ($R^2_{adjusted}$), and residual sum of squares (RSS) (manual calculation in MS Excel):

Parameter	No.1	No.2	No.3	No.4
N	5	5	5	5
df	3	3	3	3
R	0.997476612	0.999851847	0.996912046	0.999786682
R^2	0.994959591	0.999703715	0.993833628	0.99957341
$R^2_{adjusted}$	0.993279454	0.999604954	0.991778171	0.999431214
RSS	6.013389695	1.01173849	3.32021561	1.33447087

Graphical abstract of model fit presented as mean ± 1 SD of the fraction % of released carvedilol:

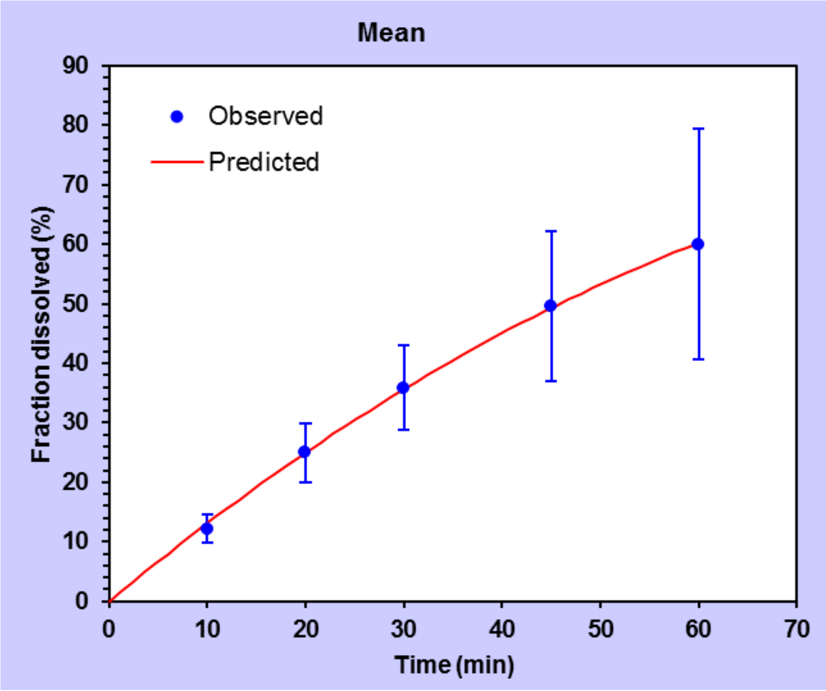

Supplementary materials – Model fitting summary – Kollidon® 25 (modelling up to app. 60 % of carvedilol released)

Graphical abstract of model fit presented as the fraction % of released carvedilol per tested tablet:

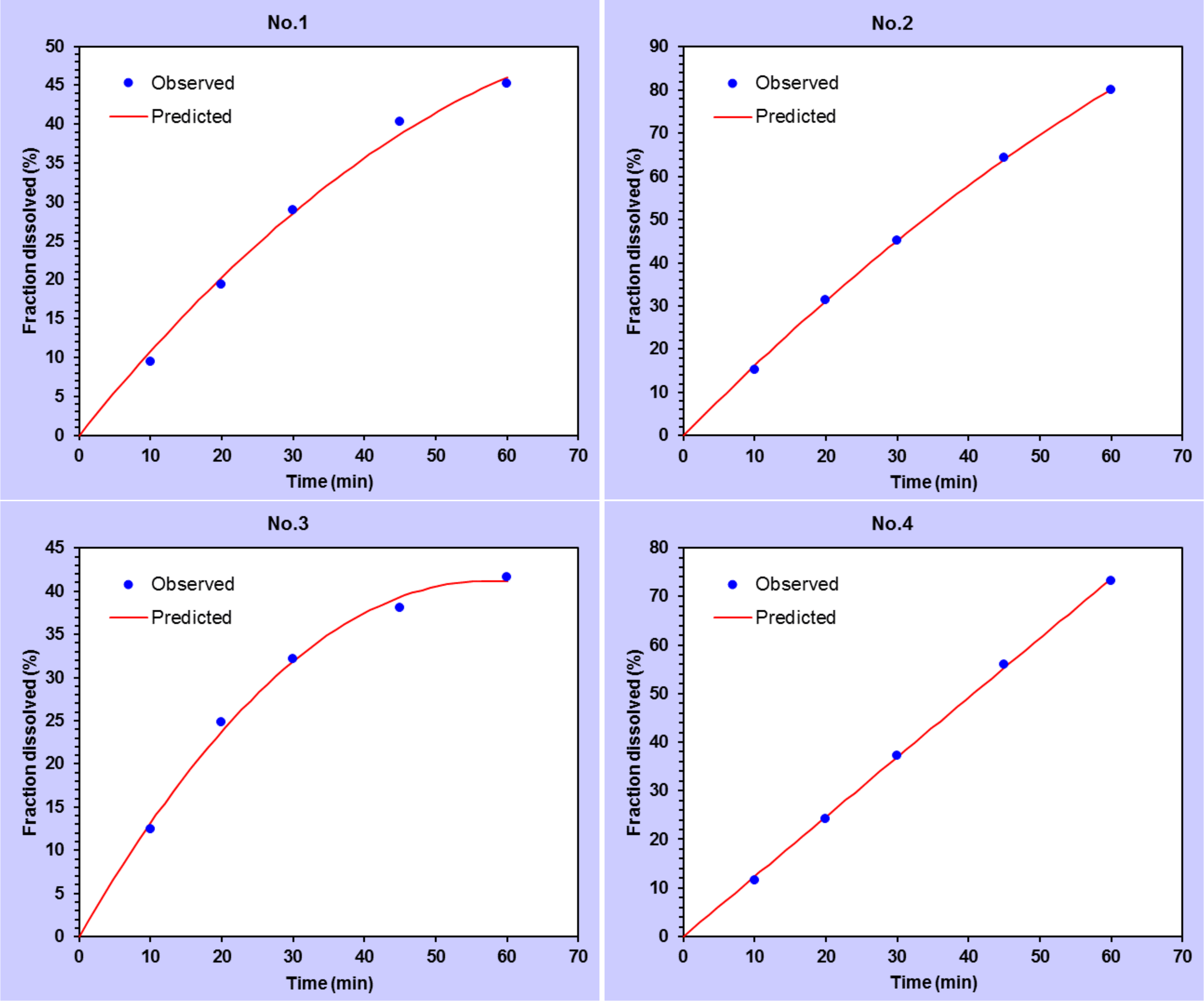

Supplementary materials – Model fitting summary – Kollidon® 25 (modelling up to app. 60 % of carvedilol released)

Model: **Quadratic with T_{lag}**

Model equation: $F = 100 \cdot \left[k_1 \cdot (t - T_{lag})^2 + k_2 \cdot (t - T_{lag})\right]$

Fitted model parameters per tested tablet (N = 4) with statistics – mean, standard deviation (SD), and relative standard deviation expressed in % (RSD%) (output from DDSolver):

Parameter	No.1	No.2	No.3	No.4	Mean	SD	RSD(%)
k_1	0.000	0.000	0.000	0.000	0.000	0.000	-46.170
k_2	0.014	0.021	0.017	0.016	0.017	0.003	17.034
T_{lag}	4.000	4.000	4.000	4.000	4.000	0.000	0.000

Number of dissolution data points (N), degrees of freedom (df), and selected goodness of fit criteria – Pearson correlation coefficient (R), coefficient of determination (R^2), adjusted coefficient of determination ($R^2_{adjusted}$), and residual sum of squares (RSS) (manual calculation in MS Excel):

Parameter	No.1	No.2	No.3	No.4
N	5	5	5	5
df	2	2	2	2
R	0.998888008	0.998523346	0.992783076	0.999263971
R^2	0.997777253	0.997048872	0.985618236	0.998528484
$R^2_{adjusted}$	0.995554506	0.994097744	0.971236472	0.997056967
RSS	2.473355595	14.54131158	18.97805146	5.921675909

Graphical abstract of model fit presented as mean ± 1 SD of the fraction % of released carvedilol:

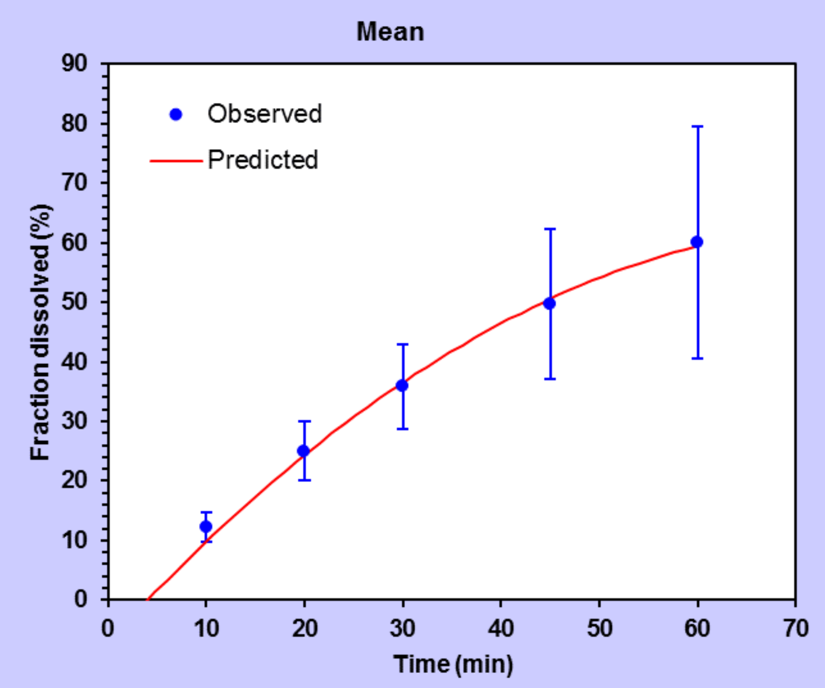

Supplementary materials – Model fitting summary – Kollidon® 25 (modelling up to app. 60 % of carvedilol released)

Graphical abstract of model fit presented as the fraction % of released carvedilol per tested tablet:

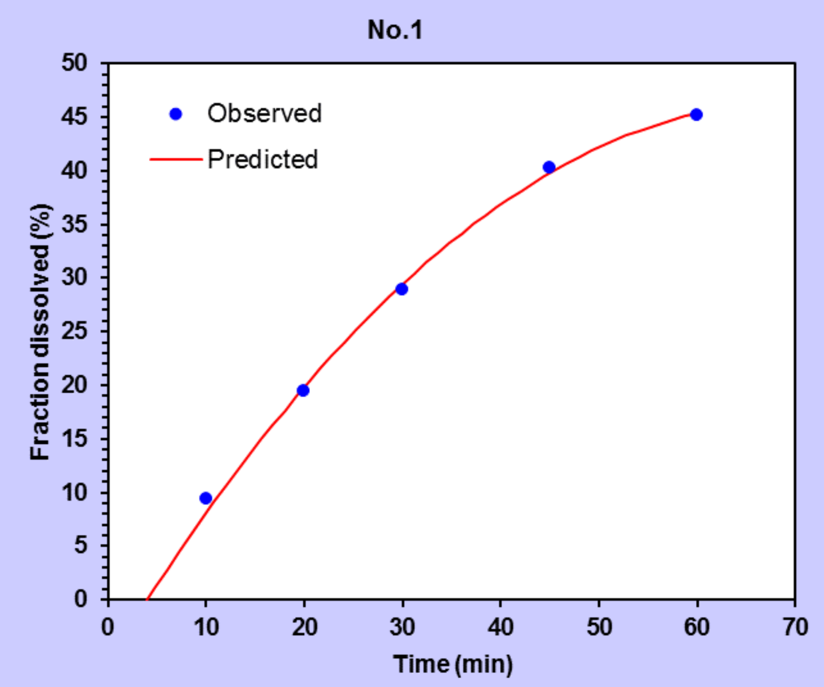

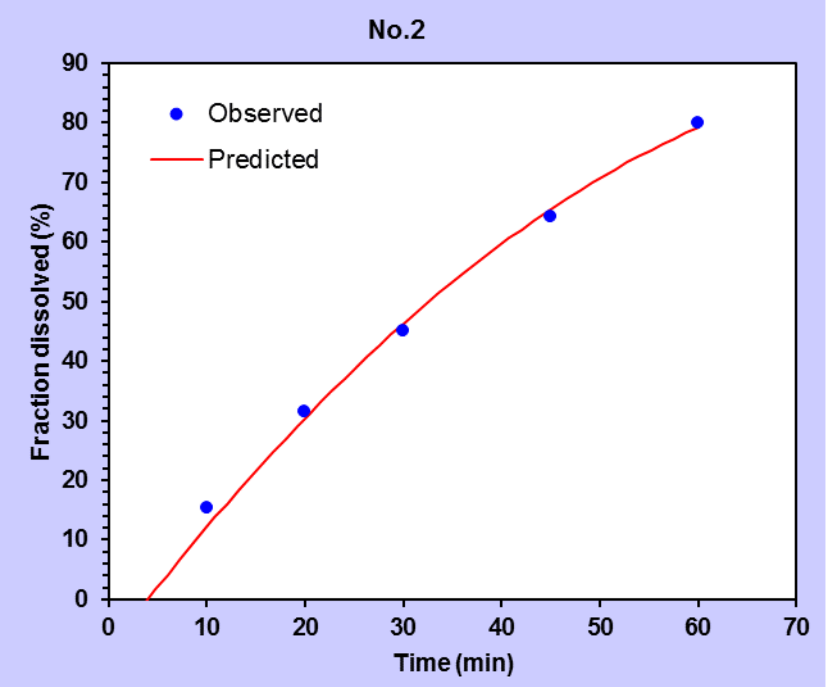

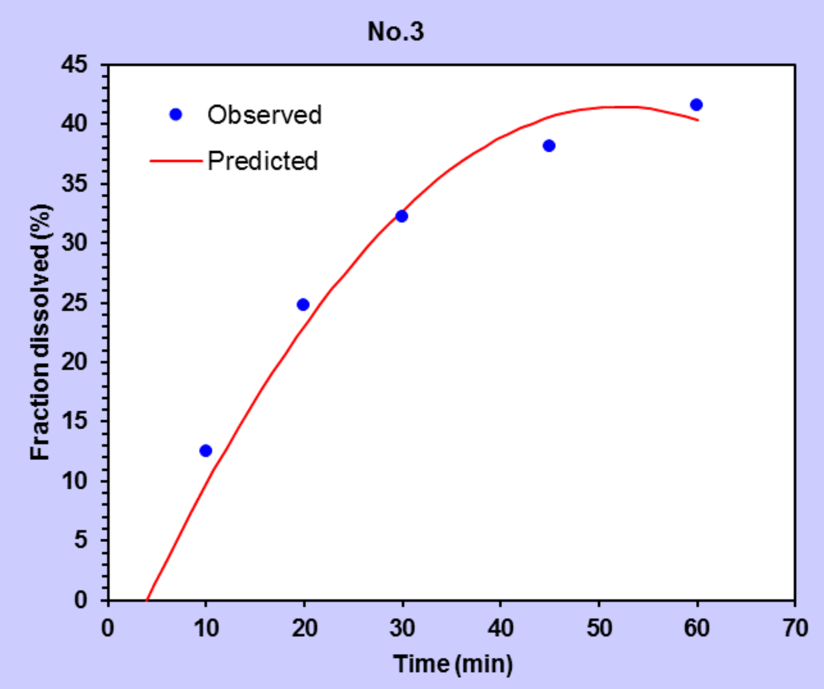

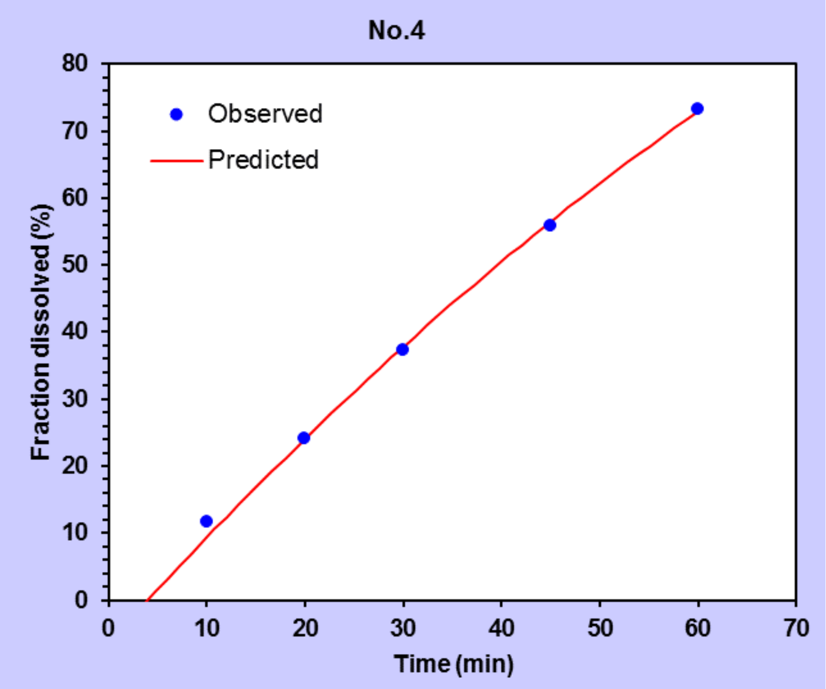

Supplementary materials – Model fitting summary – Kollidon® 25 (modelling up to app. 60 % of carvedilol released)

Model: **Weibull_1**

Model equation: $F = 100 \cdot \left[1 - e^{-\frac{(t-T_i)^\beta}{\alpha}}\right]$

Fitted model parameters per tested tablet (N = 4) with statistics – mean, standard deviation (SD), and relative standard deviation expressed in % (RSD%) (output from DDSolver):

Parameter	No.1	No.2	No.3	No.4	Mean	SD	RSD(%)
α	45.115	39.256	21.673	58.729	41.193	15.359	37.285
β	0.832	1.001	0.631	1.048	0.878	0.189	21.542
T_i	4.000	4.000	4.000	4.000	4.000	0.000	0.000

Number of dissolution data points (N), degrees of freedom (df), and selected goodness of fit criteria – Pearson correlation coefficient (R), coefficient of determination (R^2), adjusted coefficient of determination ($R^2_{adjusted}$), and residual sum of squares (RSS) (manual calculation in MS Excel):

Parameter	No.1	No.2	No.3	No.4
N	5	5	5	5
df	2	2	2	2
R	0.996431869	0.994430708	0.988947455	0.993214582
R^2	0.992876469	0.988892432	0.978017069	0.986475206
$R^2_{adjusted}$	0.985752938	0.977784865	0.956034138	0.972950411
RSS	6.247732412	32.73750116	13.74840669	40.38043882

Graphical abstract of model fit presented as mean ± 1 SD of the fraction % of released carvedilol:

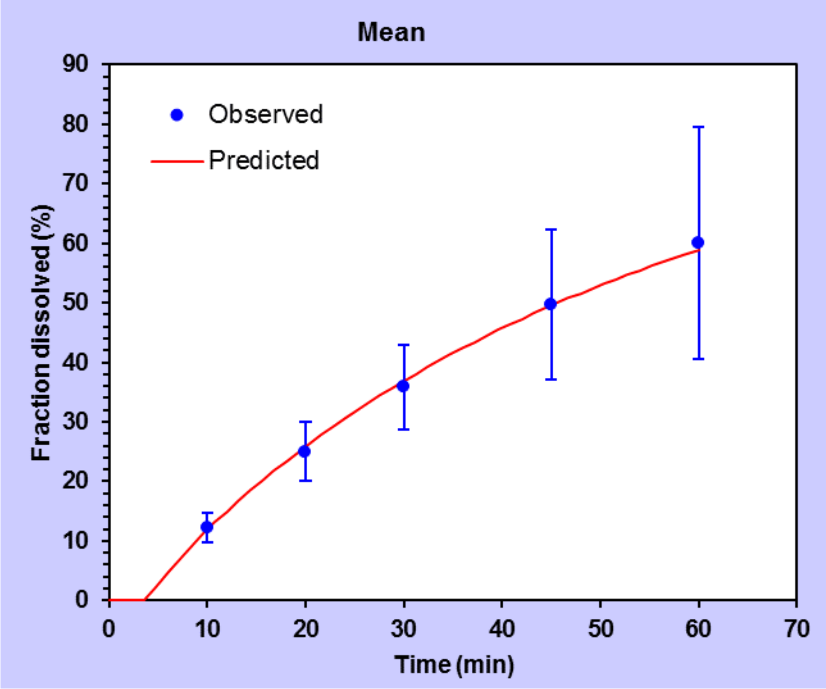

Supplementary materials – Model fitting summary – Kollidon® 25 (modelling up to app. 60 % of carvedilol released)

Graphical abstract of model fit presented as the fraction % of released carvedilol per tested tablet:

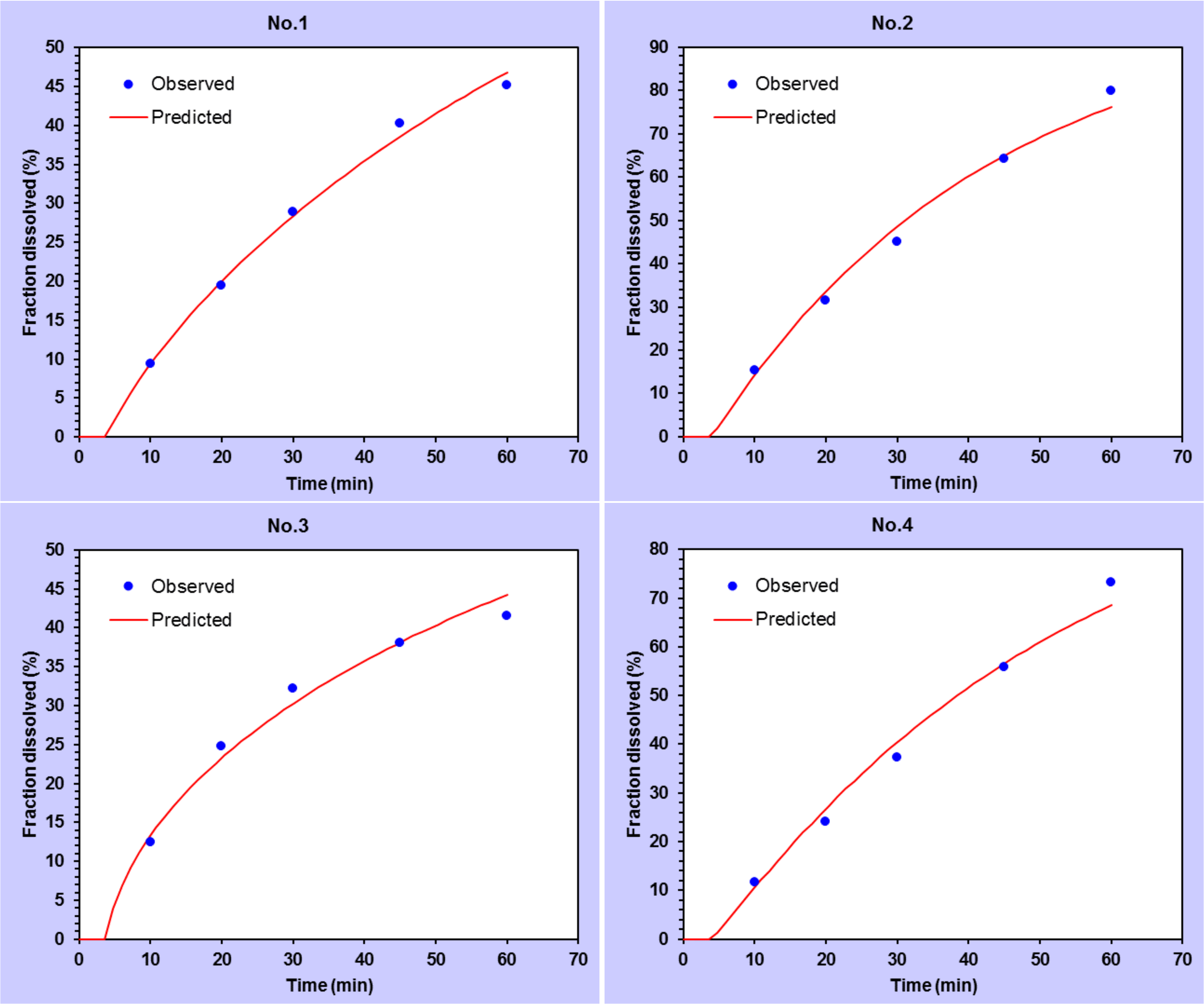

Supplementary materials – Model fitting summary – Kollidon® 25 (modelling up to app. 60 % of carvedilol released)

Model: **Weibull_2**

Model equation: $F = 100 \cdot \left(1 - e^{-\frac{t^{\beta}}{\alpha}}\right)$

Fitted model parameters per tested tablet (N = 4) with statistics – mean, standard deviation (SD), and relative standard deviation expressed in % (RSD%) (output from DDSolver):

Parameter	No.1	No.2	No.3	No.4	Mean	SD	RSD(%)
α	104.803	111.290	40.218	175.626	107.984	55.343	51.251
β	1.034	1.252	0.777	1.312	1.094	0.243	22.175

Number of dissolution data points (N), degrees of freedom (df), and selected goodness of fit criteria – Pearson correlation coefficient (R), coefficient of determination (R^2), adjusted coefficient of determination ($R^2_{adjusted}$), and residual sum of squares (RSS) (manual calculation in MS Excel):

Parameter	No.1	No.2	No.3	No.4
N	5	5	5	5
df	3	3	3	3
R	0.992839368	0.998417752	0.978989703	0.997912995
R^2	0.985730011	0.996838007	0.958420838	0.995830346
$R^2_{adjusted}$	0.980973349	0.99578401	0.944561117	0.994440461
RSS	14.06595716	8.908460448	26.33407273	11.97551104

Graphical abstract of model fit presented as mean ± 1 SD of the fraction % of released carvedilol:

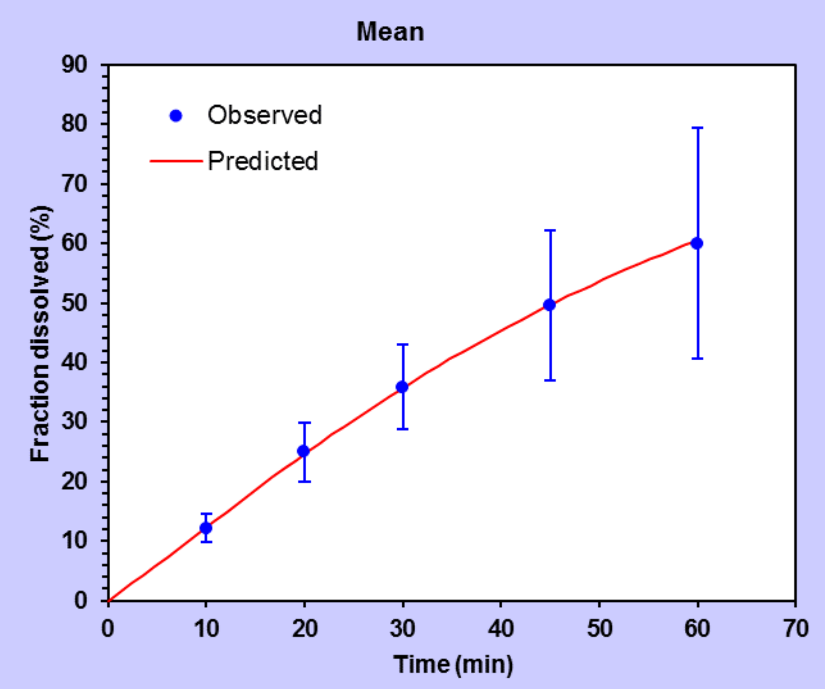

Supplementary materials – Model fitting summary – Kollidon® 25 (modelling up to app. 60 % of carvedilol released)

Graphical abstract of model fit presented as the fraction % of released carvedilol per tested tablet:

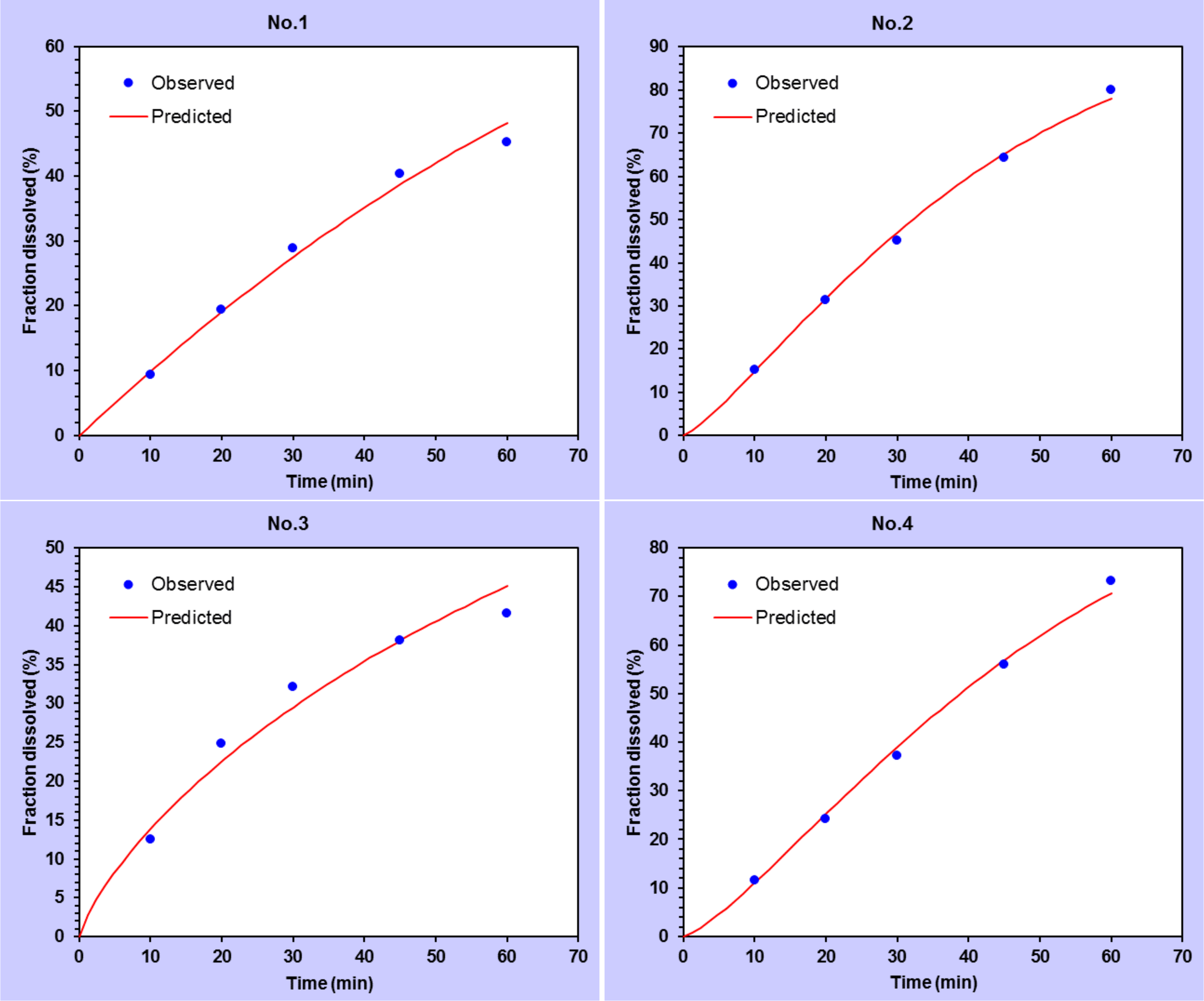

Supplementary materials – Model fitting summary – Kollidon® 25 (modelling up to app. 60 % of carvedilol released)

Model: **Weibull_3**

Model equation: $F = F_{max} \cdot \left(1 - e^{-\frac{t^{\beta}}{\alpha}}\right)$

Fitted model parameters per tested tablet (N = 4) with statistics – mean, standard deviation (SD), and relative standard deviation expressed in % (RSD%) (output from DDSolver):

Parameter	No.1	No.2	No.3	No.4	Mean	SD	RSD(%)
α	143.974	184.198	46.773	347.553	180.625	125.349	69.398
β	1.469	1.435	1.210	1.583	1.424	0.156	10.973
F_{max}	47.460	95.189	43.680	79.363	66.423	24.978	37.605

Number of dissolution data points (N), degrees of freedom (df), and selected goodness of fit criteria – Pearson correlation coefficient (R), coefficient of determination (R^2), adjusted coefficient of determination ($R^2_{adjusted}$), and residual sum of squares (RSS) (manual calculation in MS Excel):

Parameter	No.1	No.2	No.3	No.4
N	5	5	5	5
df	2	2	2	2
R	0.997708293	0.997385127	0.999213153	0.995777384
R^2	0.995421838	0.994777092	0.998426926	0.991572598
$R^2_{adjusted}$	0.990843676	0.989554183	0.996853851	0.983145197
RSS	4.326717502	39.72125277	0.86834593	51.33116455

Graphical abstract of model fit presented as mean ± 1 SD of the fraction % of released carvedilol:

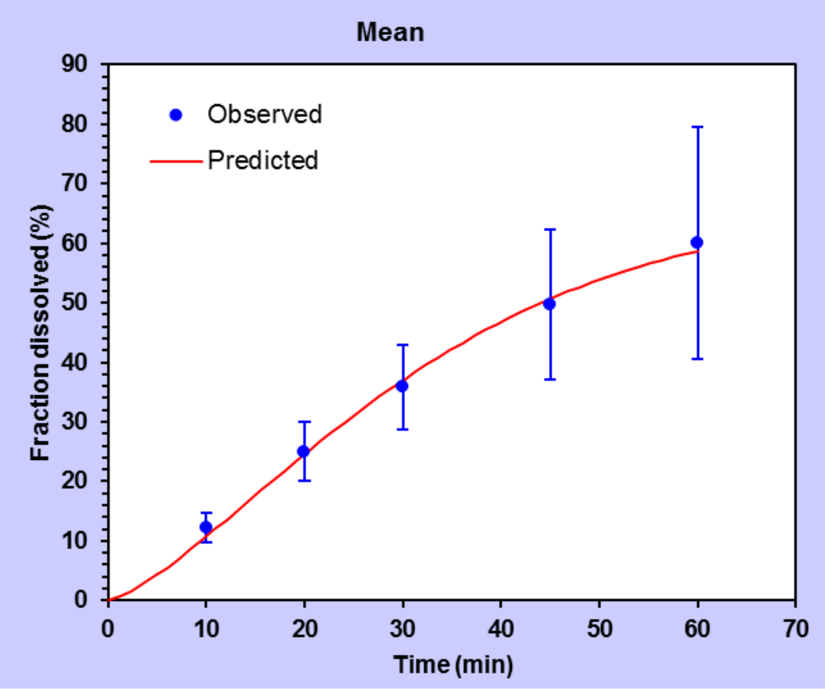

Supplementary materials – Model fitting summary – Kollidon® 25 (modelling up to app. 60 % of carvedilol released)

Graphical abstract of model fit presented as the fraction % of released carvedilol per tested tablet:

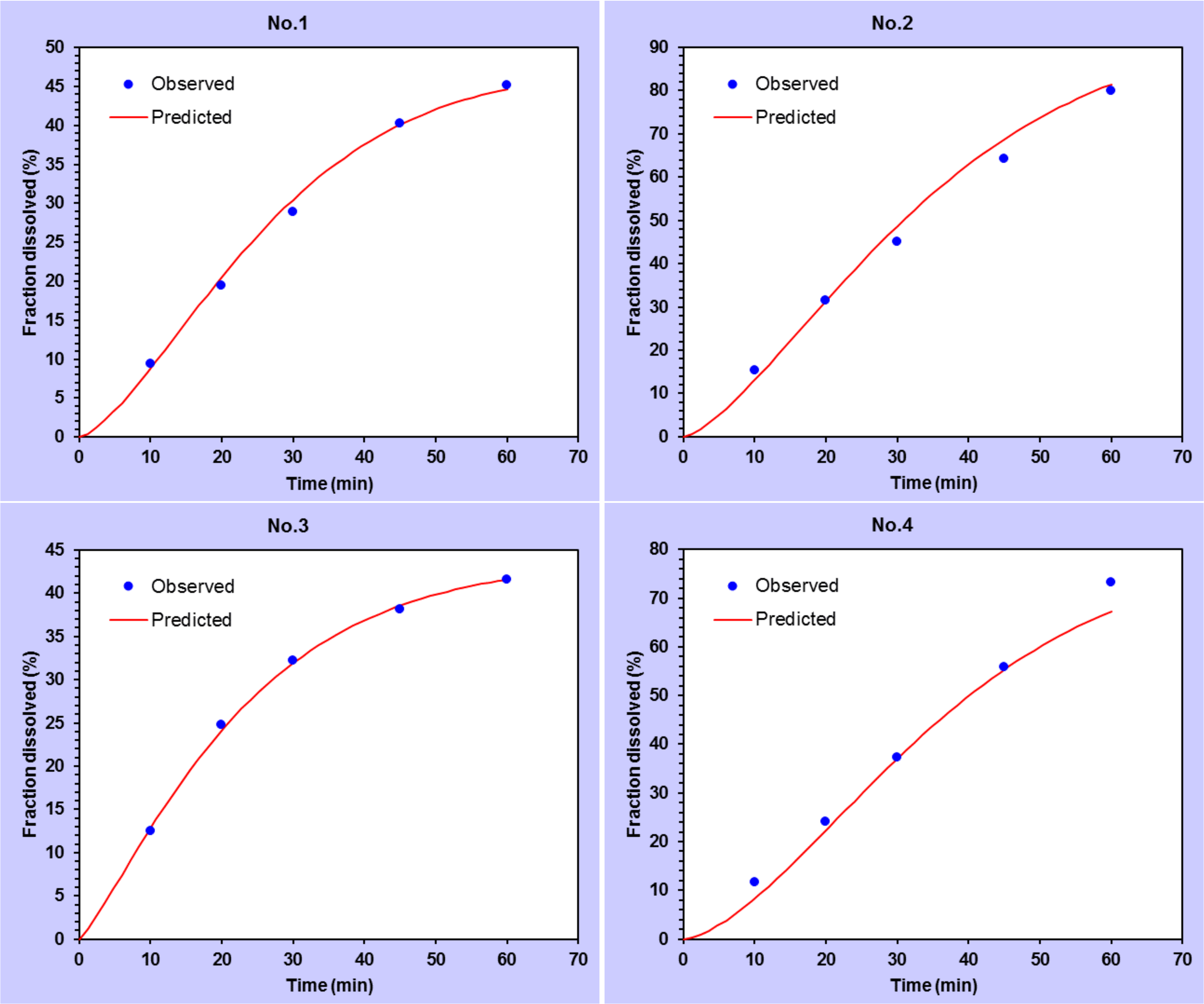

Supplementary materials – Model fitting summary – Kollidon® 25 (modelling up to app. 60 % of carvedilol released)

Model: **Weibull_4**

Model equation: $F = F_{max} \cdot \left[1 - e^{-\frac{(t-T_i)^{\beta}}{\alpha}}\right]$

Fitted model parameters per tested tablet (N = 4) with statistics – mean, standard deviation (SD), and relative standard deviation expressed in % (RSD%) (output from DDSolver):

Parameter	No.1	No.2	No.3	No.4	Mean	SD	RSD(%)
α	46.060	46.821	17.314	69.851	45.011	21.514	47.796
β	1.133	1.163	0.972	1.247	1.129	0.115	10.218
T_i	4.609	4.000	4.000	6.000	4.652	0.943	20.275
F_{max}	53.881	84.000	43.680	76.965	64.631	18.989	29.381

Number of dissolution data points (N), degrees of freedom (df), and selected goodness of fit criteria – Pearson correlation coefficient (R), coefficient of determination (R^2), adjusted coefficient of determination ($R^2_{adjusted}$), and residual sum of squares (RSS) (manual calculation in MS Excel):

Parameter	No.1	No.2	No.3	No.4
N	5	5	5	5
df	1	1	1	1
R	0.99744459	0.985832562	0.999531789	0.984509861
R^2	0.99489571	0.97186584	0.999063797	0.969259666
$R^2_{adjusted}$	0.979582838	0.887463362	0.99625519	0.877038662
RSS	12.59283902	81.50419595	0.562282126	83.03010022

Graphical abstract of model fit presented as mean ± 1 SD of the fraction % of released carvedilol:

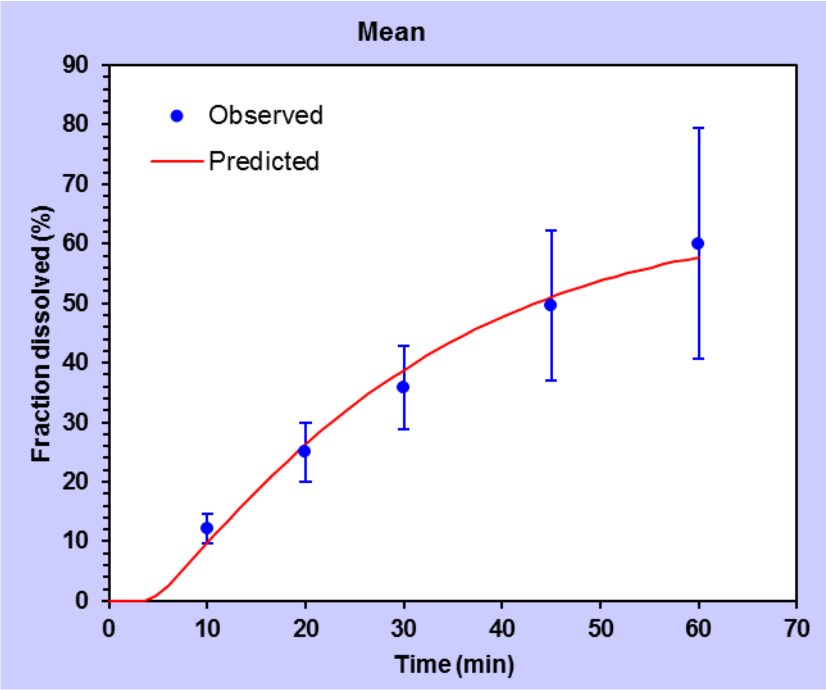

Supplementary materials – Model fitting summary – Kollidon® 25 (modelling up to app. 60 % of carvedilol released)

Graphical abstract of model fit presented as the fraction % of released carvedilol per tested tablet:

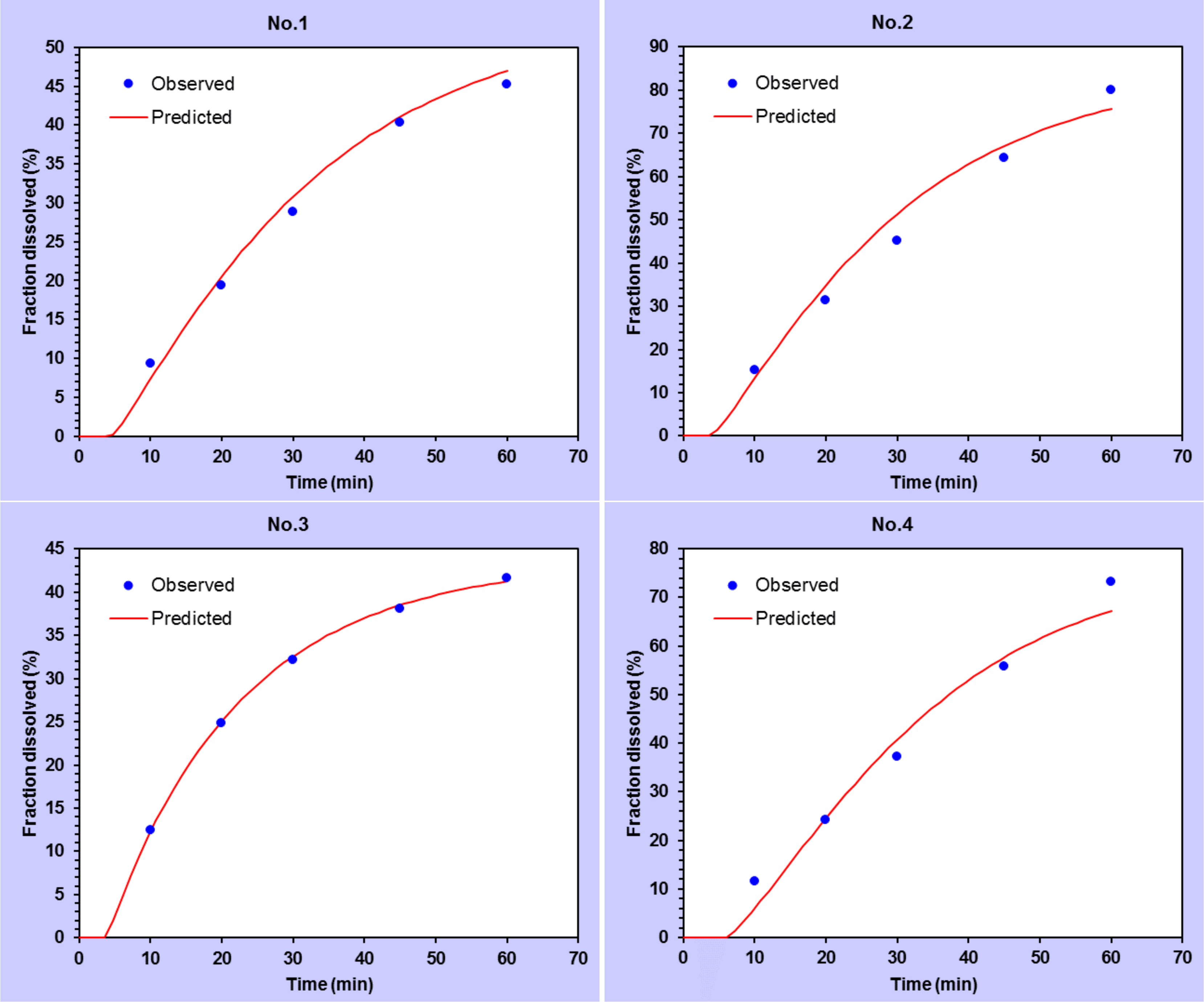

Supplementary materials – Model fitting summary – Kollidon® 25 (modelling up to app. 60 % of carvedilol released)

Model: **Logistic_1**

Model equation: $F = 100 \cdot \frac{e^{\alpha+\beta\cdot\log(t)}}{1+e^{\alpha+\beta\cdot\log(t)}}$

Fitted model parameters per tested tablet (N = 4) with statistics – mean, standard deviation (SD), and relative standard deviation expressed in % (RSD%) (output from DDSolver):

Parameter	No.1	No.2	No.3	No.4	Mean	SD	RSD(%)
α	-4.976	-5.728	-3.906	-6.000	-5.152	0.937	-18.188
β	2.733	3.874	2.067	3.822	3.124	0.879	28.143

Number of dissolution data points (N), degrees of freedom (df), and selected goodness of fit criteria – Pearson correlation coefficient (R), coefficient of determination (R^2), adjusted coefficient of determination ($R^2_{adjusted}$), and residual sum of squares (RSS) (manual calculation in MS Excel):

Parameter	No.1	No.2	No.3	No.4
N	5	5	5	5
df	3	3	3	3
R	0.996308232	0.991211136	0.985049556	0.991340678
R^2	0.992630094	0.982499517	0.970322628	0.982756341
$R^2_{adjusted}$	0.990173458	0.976666023	0.96043017	0.977008454
RSS	6.657084087	48.43016584	17.73763912	45.37971084

Graphical abstract of model fit presented as mean ± 1 SD of the fraction % of released carvedilol:

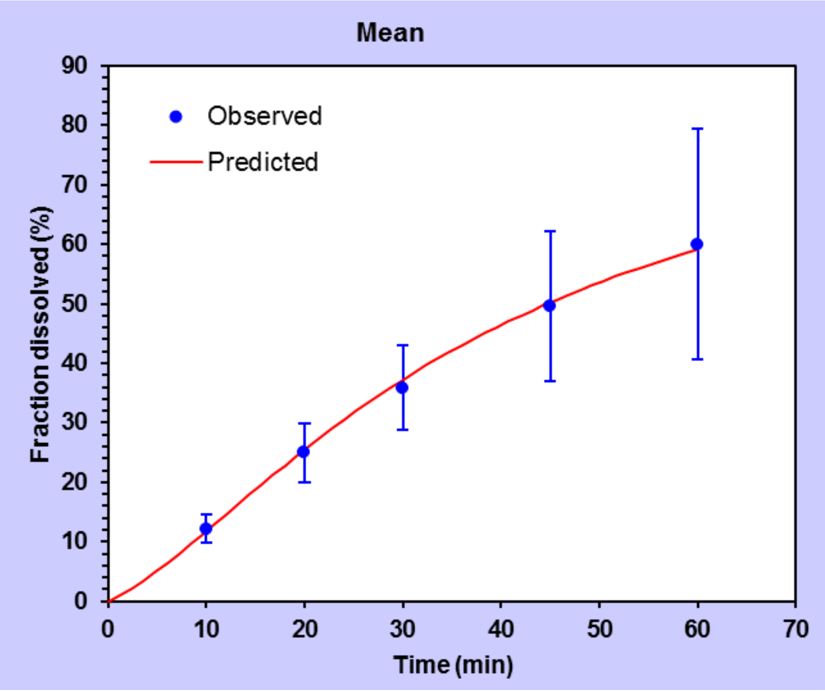

Supplementary materials – Model fitting summary – Kollidon® 25 (modelling up to app. 60 % of carvedilol released)

Graphical abstract of model fit presented as the fraction % of released carvedilol per tested tablet:

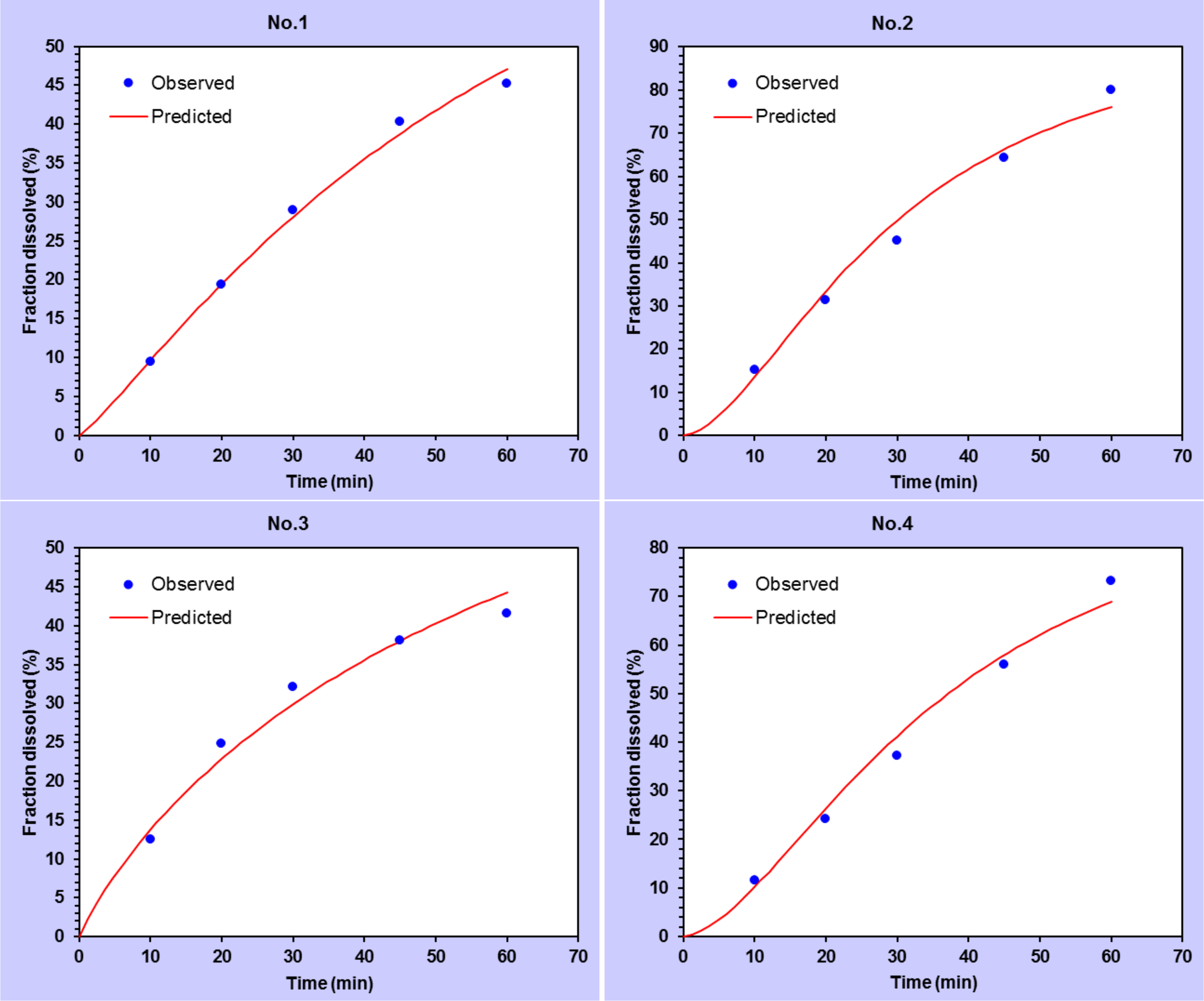

Supplementary materials – Model fitting summary – Kollidon® 25 (modelling up to app. 60 % of carvedilol released)

Model: **Logistic_2**

Model equation: $F = F_{max} \cdot \frac{e^{\alpha+\beta\cdot\log(t)}}{1+e^{\alpha+\beta\cdot\log(t)}}$

Fitted model parameters per tested tablet (N = 4) with statistics – mean, standard deviation (SD), and relative standard deviation expressed in % (RSD%) (output from DDSolver):

Parameter	No.1	No.2	No.3	No.4	Mean	SD	RSD(%)
α	-7.253	-8.357	-5.935	-7.757	-7.326	1.031	-14.077
β	5.501	5.677	4.852	5.574	5.401	0.373	6.909
F_{max}	47.460	91.464	43.680	76.965	64.892	23.135	35.651

Number of dissolution data points (N), degrees of freedom (df), and selected goodness of fit criteria – Pearson correlation coefficient (R), coefficient of determination (R^2), adjusted coefficient of determination ($R^2_{adjusted}$), and residual sum of squares (RSS) (manual calculation in MS Excel):

Parameter	No.1	No.2	No.3	No.4
N	5	5	5	5
df	2	2	2	2
R	0.981003896	0.992270655	0.995060276	0.966332325
R^2	0.962368644	0.984601052	0.990144953	0.933798163
$R^2_{adjusted}$	0.924737288	0.969202105	0.980289906	0.867596326
RSS	38.90504731	145.6655983	7.016591715	202.446175

Graphical abstract of model fit presented as mean ± 1 SD of the fraction % of released carvedilol:

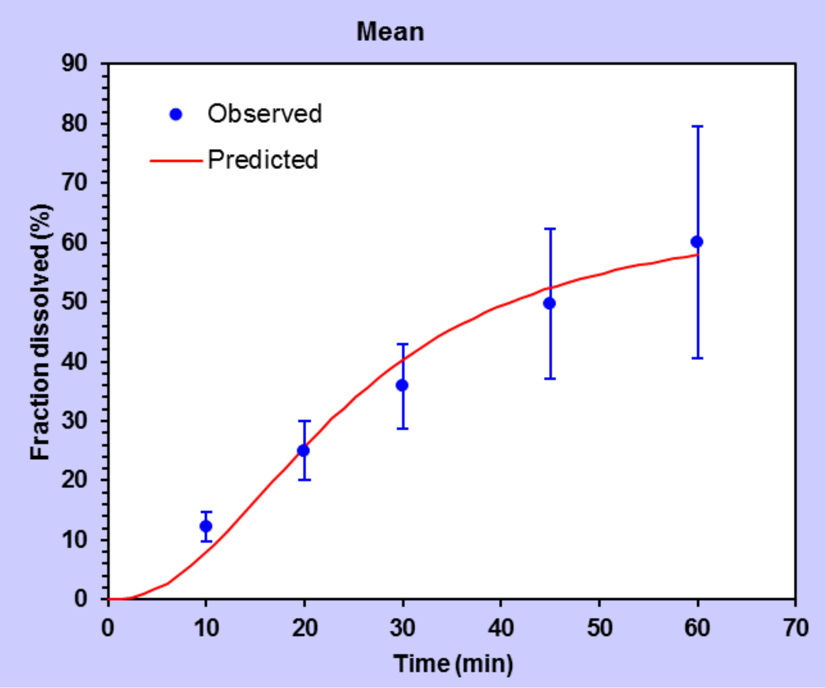

Supplementary materials – Model fitting summary – Kollidon® 25 (modelling up to app. 60 % of carvedilol released)

Graphical abstract of model fit presented as the fraction % of released carvedilol per tested tablet:

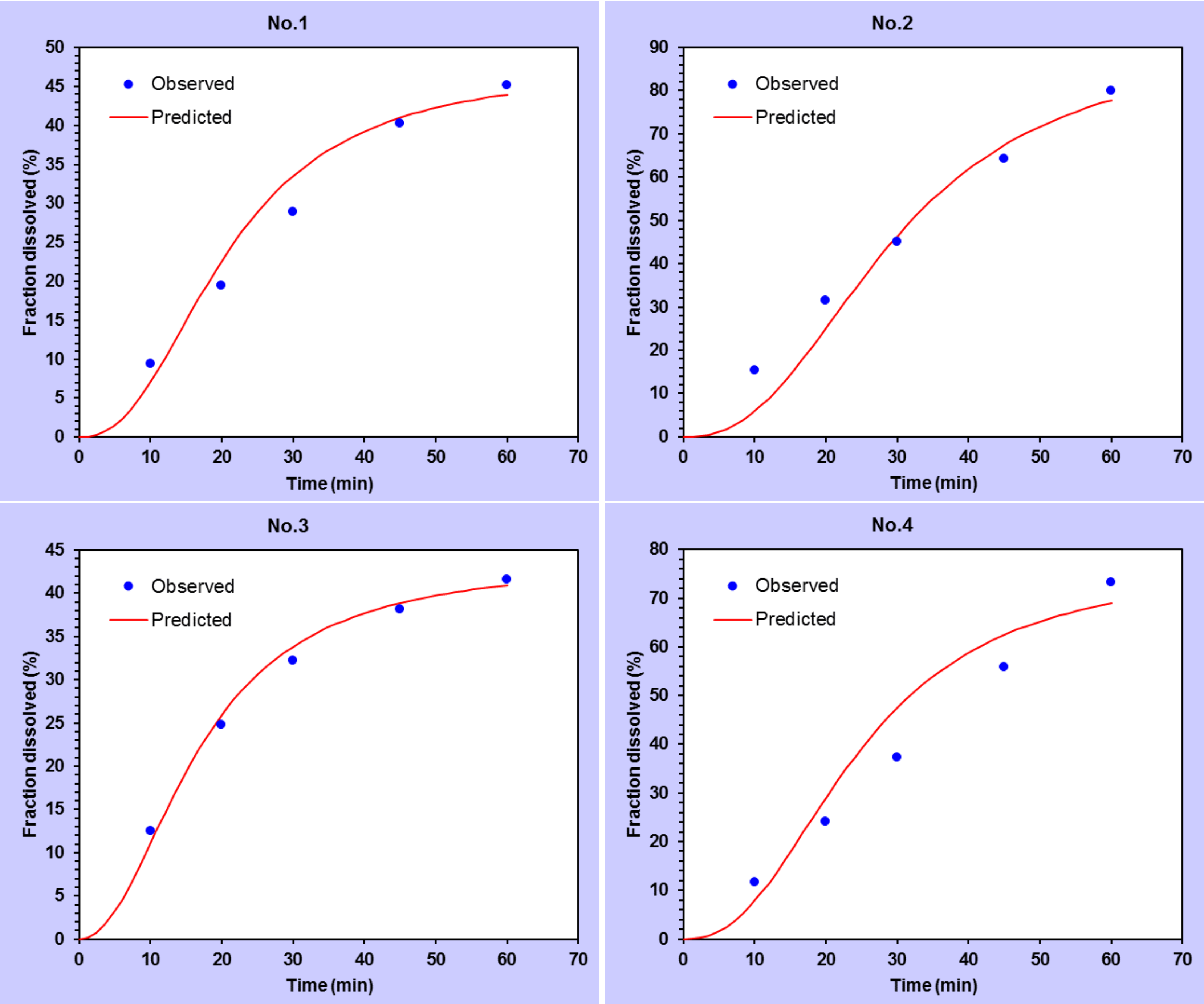

Supplementary materials – Model fitting summary – Kollidon® 25 (modelling up to app. 60 % of carvedilol released)

Model: **Logistic_3**

Model equation: $F = F_{max} \cdot \frac{1}{1+e^{-k\cdot(t-\gamma)}}$

Fitted model parameters per tested tablet (N = 4) with statistics – mean, standard deviation (SD), and relative standard deviation expressed in % (RSD%) (output from DDSolver):

Parameter	No.1	No.2	No.3	No.4	Mean	SD	RSD(%)
k	0.087	0.086	0.075	0.090	0.085	0.007	7.905
γ	25.175	27.617	18.827	29.896	25.378	4.774	18.812
F_{max}	47.460	84.000	43.680	76.965	63.026	20.419	32.397

Number of dissolution data points (N), degrees of freedom (df), and selected goodness of fit criteria – Pearson correlation coefficient (R), coefficient of determination (R^2), adjusted coefficient of determination ($R^2_{adjusted}$), and residual sum of squares (RSS) (manual calculation in MS Excel):

Parameter	No.1	No.2	No.3	No.4
N	5	5	5	5
df	2	2	2	2
R	0.999284531	0.995629643	0.989057497	0.994199691
R^2	0.998569573	0.991278387	0.978234732	0.988433026
$R^2_{adjusted}$	0.997139146	0.982556774	0.956469464	0.976866051
RSS	1.299051207	28.47879564	12.66487863	36.03242651

Graphical abstract of model fit presented as mean ± 1 SD of the fraction % of released carvedilol:

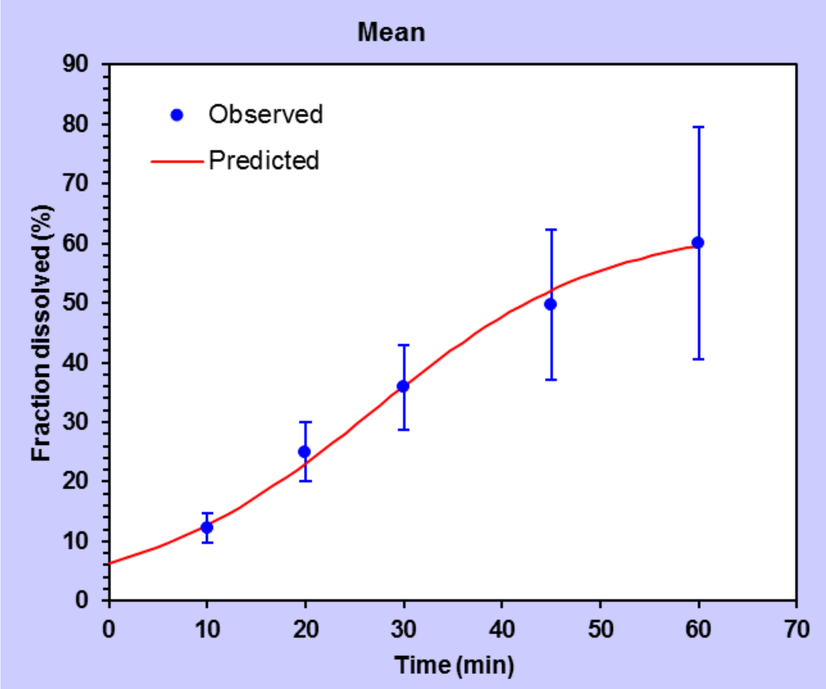

Supplementary materials – Model fitting summary – Kollidon® 25 (modelling up to app. 60 % of carvedilol released)

Graphical abstract of model fit presented as the fraction % of released carvedilol per tested tablet:

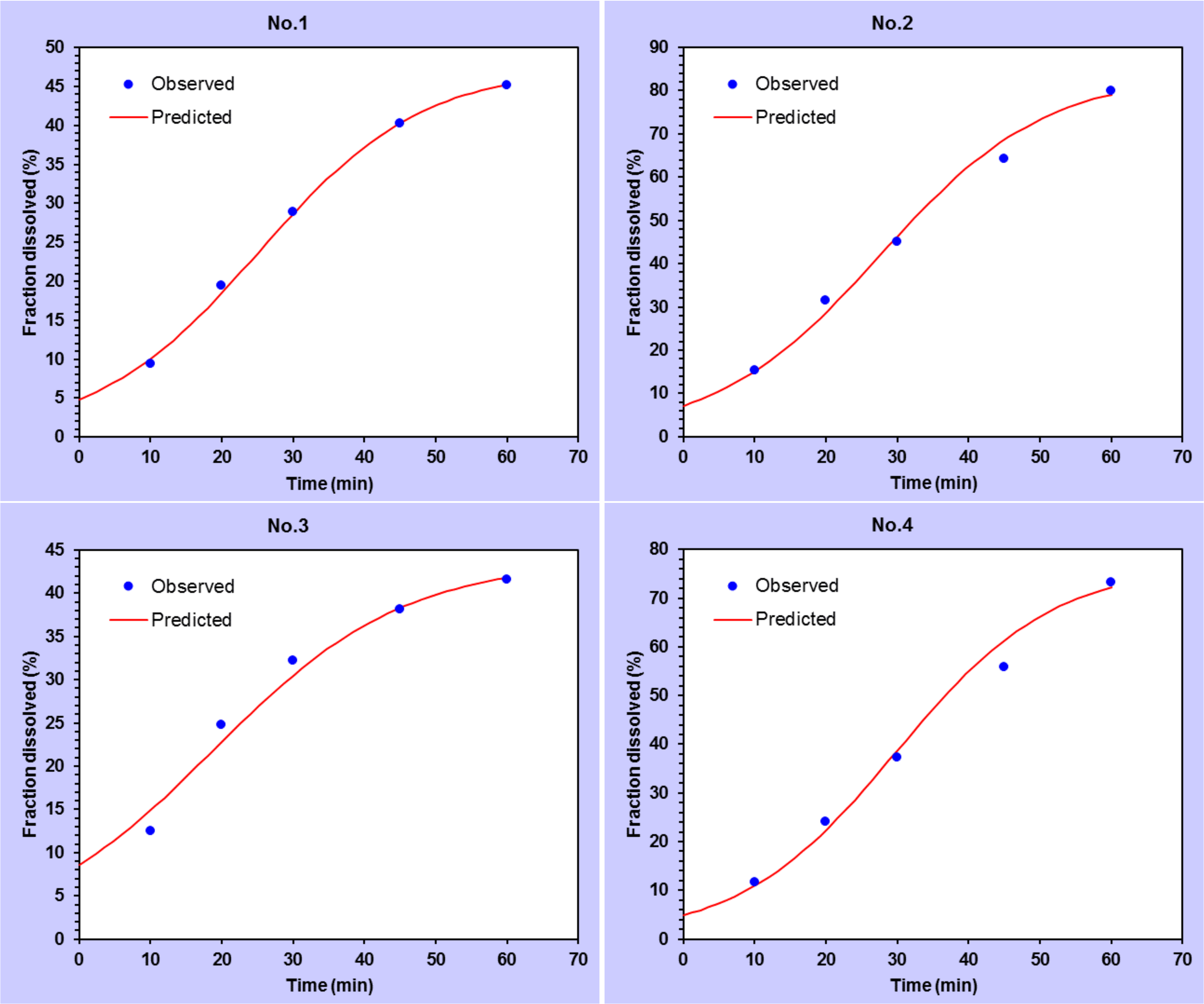

Supplementary materials – Model fitting summary – Kollidon® 25 (modelling up to app. 60 % of carvedilol released)

Model: **Gompertz_1**

Model equation: $F = 100 \cdot e^{-\alpha \cdot e^{-\beta \cdot \log(t)}}$

Fitted model parameters per tested tablet (N = 4) with statistics – mean, standard deviation (SD), and relative standard deviation expressed in % (RSD%) (output from DDSolver):

Parameter	No.1	No.2	No.3	No.4	Mean	SD	RSD(%)
α	10.286	31.114	6.136	28.057	18.898	12.519	66.243
β	1.444	2.630	1.116	2.397	1.897	0.731	38.524

Number of dissolution data points (N), degrees of freedom (df), and selected goodness of fit criteria – Pearson correlation coefficient (R), coefficient of determination (R^2), adjusted coefficient of determination ($R^2_{adjusted}$), and residual sum of squares (RSS) (manual calculation in MS Excel):

Parameter	No.1	No.2	No.3	No.4
N	5	5	5	5
df	3	3	3	3
R	0.997323529	0.975240274	0.994444115	0.973958878
R^2	0.994654222	0.951093592	0.988919099	0.948595896
$R^2_{adjusted}$	0.992872296	0.934791456	0.985225465	0.931461195
RSS	4.753944635	135.6358228	6.085853098	129.1148945

Graphical abstract of model fit presented as mean ± 1 SD of the fraction % of released carvedilol:

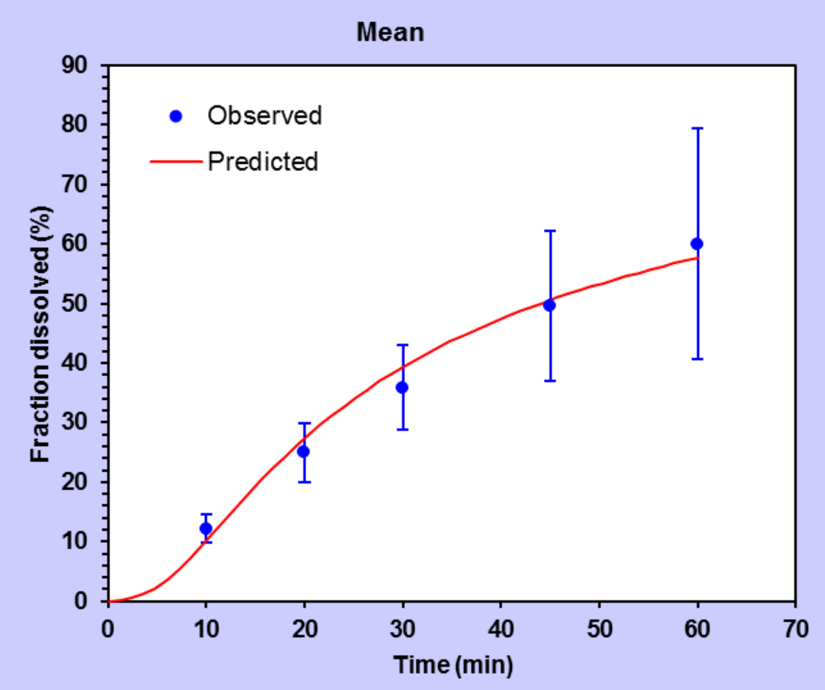

Supplementary materials – Model fitting summary – Kollidon® 25 (modelling up to app. 60 % of carvedilol released)

Graphical abstract of model fit presented as the fraction % of released carvedilol per tested tablet:

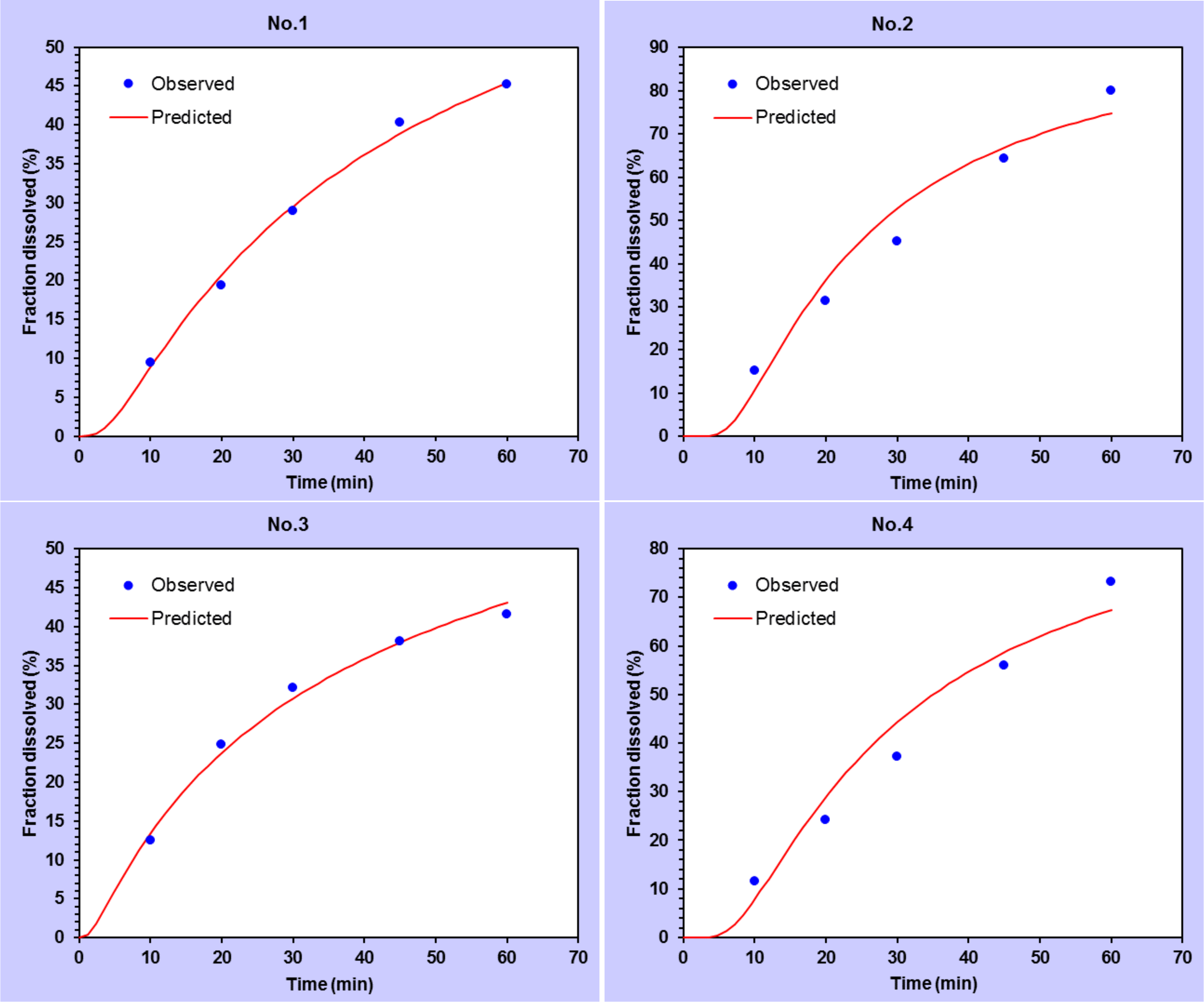

Supplementary materials – Model fitting summary – Kollidon® 25 (modelling up to app. 60 % of carvedilol released)

Model: **Gompertz_2**

Model equation: $F = F_{max} \cdot e^{-\alpha \cdot e^{-\beta \cdot \log(t)}}$

Fitted model parameters per tested tablet (N = 4) with statistics – mean, standard deviation (SD), and relative standard deviation expressed in % (RSD%) (output from DDSolver):

Parameter	No.1	No.2	No.3	No.4	Mean	SD	RSD(%)
α	275.352	243.505	86.550	304.326	227.433	97.151	42.716
β	4.331	4.126	4.001	4.198	4.164	0.138	3.308
F_{max}	47.460	84.000	43.680	76.965	63.026	20.419	32.397

Number of dissolution data points (N), degrees of freedom (df), and selected goodness of fit criteria – Pearson correlation coefficient (R), coefficient of determination (R^2), adjusted coefficient of determination ($R^2_{adjusted}$), and residual sum of squares (RSS) (manual calculation in MS Excel):

Parameter	No.1	No.2	No.3	No.4
N	5	5	5	5
df	2	2	2	2
R	0.983682014	0.974903388	0.984884571	0.970462495
R^2	0.967630305	0.950436617	0.969997618	0.941797455
$R^2_{adjusted}$	0.93526061	0.900873234	0.939995236	0.88359491
RSS	84.84854144	287.303153	25.01279558	220.9504493

Graphical abstract of model fit presented as mean ± 1 SD of the fraction % of released carvedilol:

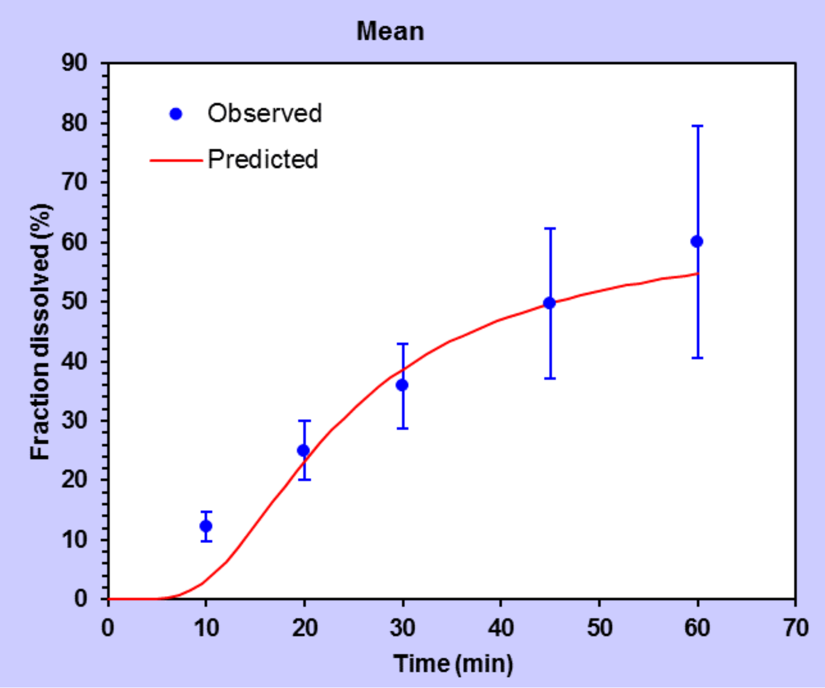

Supplementary materials – Model fitting summary – Kollidon® 25 (modelling up to app. 60 % of carvedilol released)

Graphical abstract of model fit presented as the fraction % of released carvedilol per tested tablet:

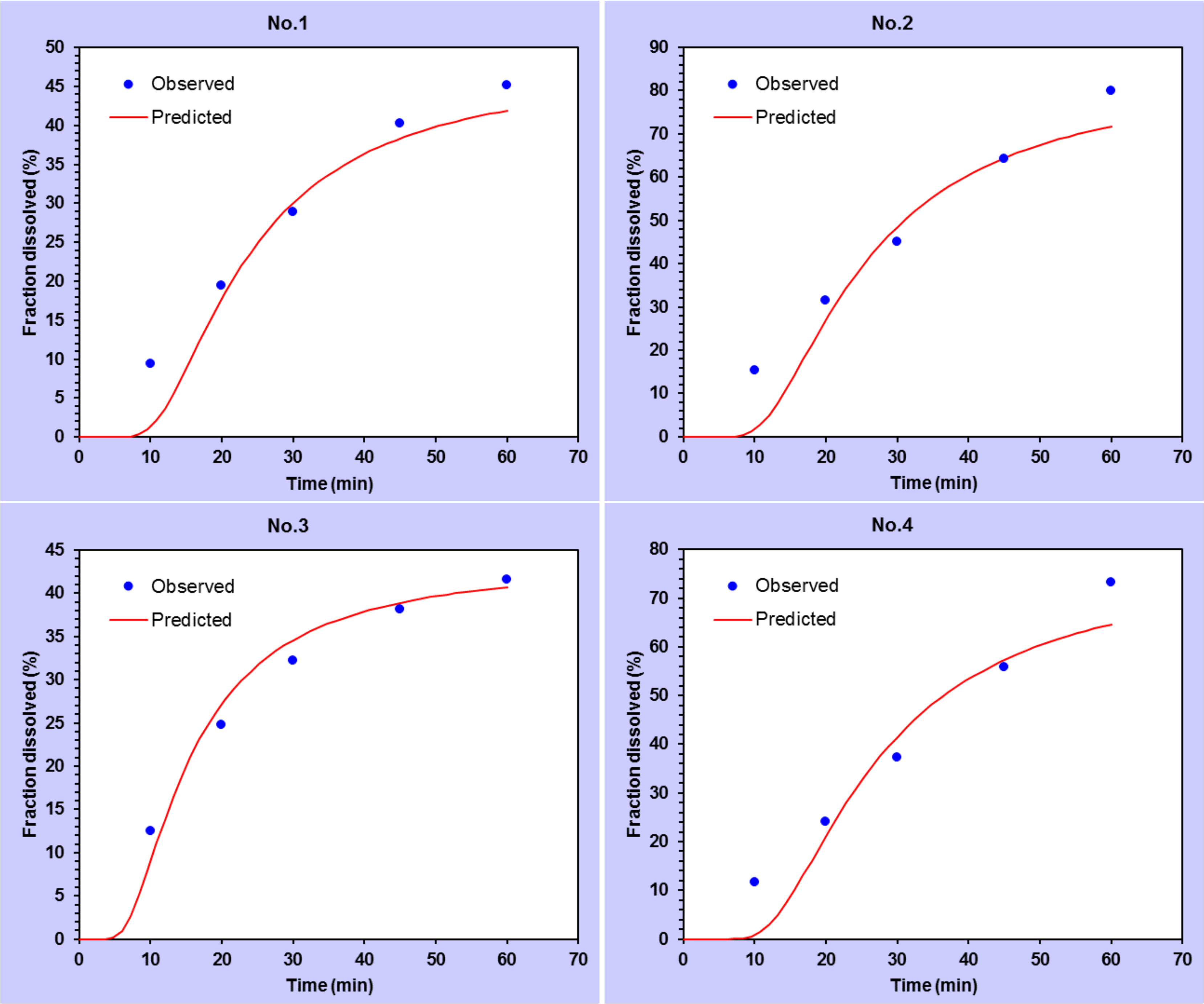

Supplementary materials – Model fitting summary – Kollidon® 25 (modelling up to app. 60 % of carvedilol released)

Model: **Gompertz_3**

Model equation: $F = F_{max} \cdot e^{-e^{-k \cdot (t-\gamma)}}$

Fitted model parameters per tested tablet (N = 4) with statistics – mean, standard deviation (SD), and relative standard deviation expressed in % (RSD%) (output from DDSolver):

Parameter	No.1	No.2	No.3	No.4	Mean	SD	RSD(%)
k	0.060	0.046	0.075	0.070	0.063	0.013	20.134
γ	17.601	20.534	12.721	22.452	18.327	4.237	23.116
F_{max}	47.267	92.296	42.049	76.965	64.644	24.007	37.137

Number of dissolution data points (N), degrees of freedom (df), and selected goodness of fit criteria – Pearson correlation coefficient (R), coefficient of determination (R^2), adjusted coefficient of determination ($R^2_{adjusted}$), and residual sum of squares (RSS) (manual calculation in MS Excel):

Parameter	No.1	No.2	No.3	No.4
N	5	5	5	5
df	2	2	2	2
R	0.999262147	0.998228068	0.998628606	0.98579261
R^2	0.998524838	0.996459275	0.997259093	0.97178707
$R^2_{adjusted}$	0.997049677	0.99291855	0.994518185	0.943574139
RSS	4.532277055	30.30324468	2.276311431	99.25467604

Graphical abstract of model fit presented as mean ± 1 SD of the fraction % of released carvedilol:

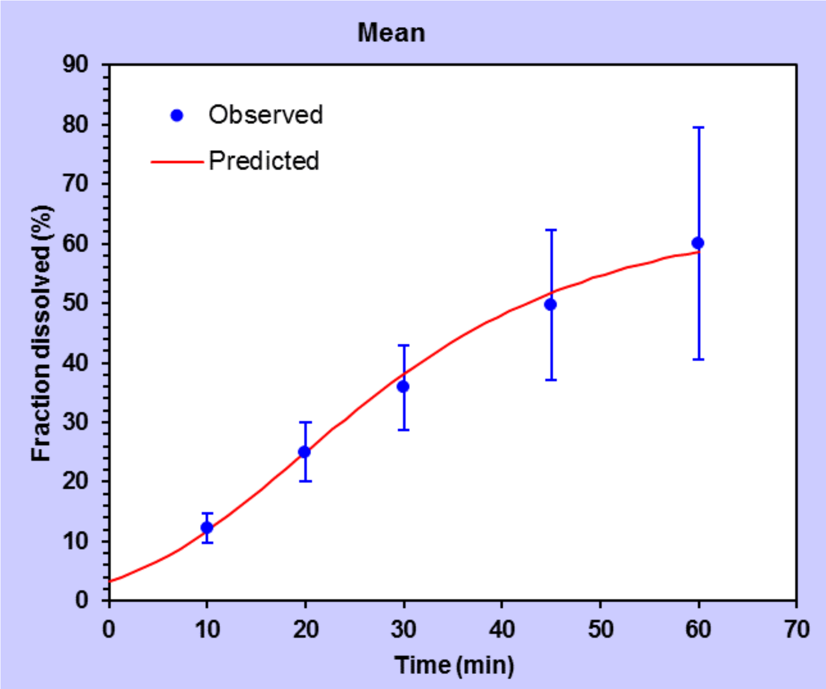

Supplementary materials – Model fitting summary – Kollidon® 25 (modelling up to app. 60 % of carvedilol released)

Graphical abstract of model fit presented as the fraction % of released carvedilol per tested tablet:

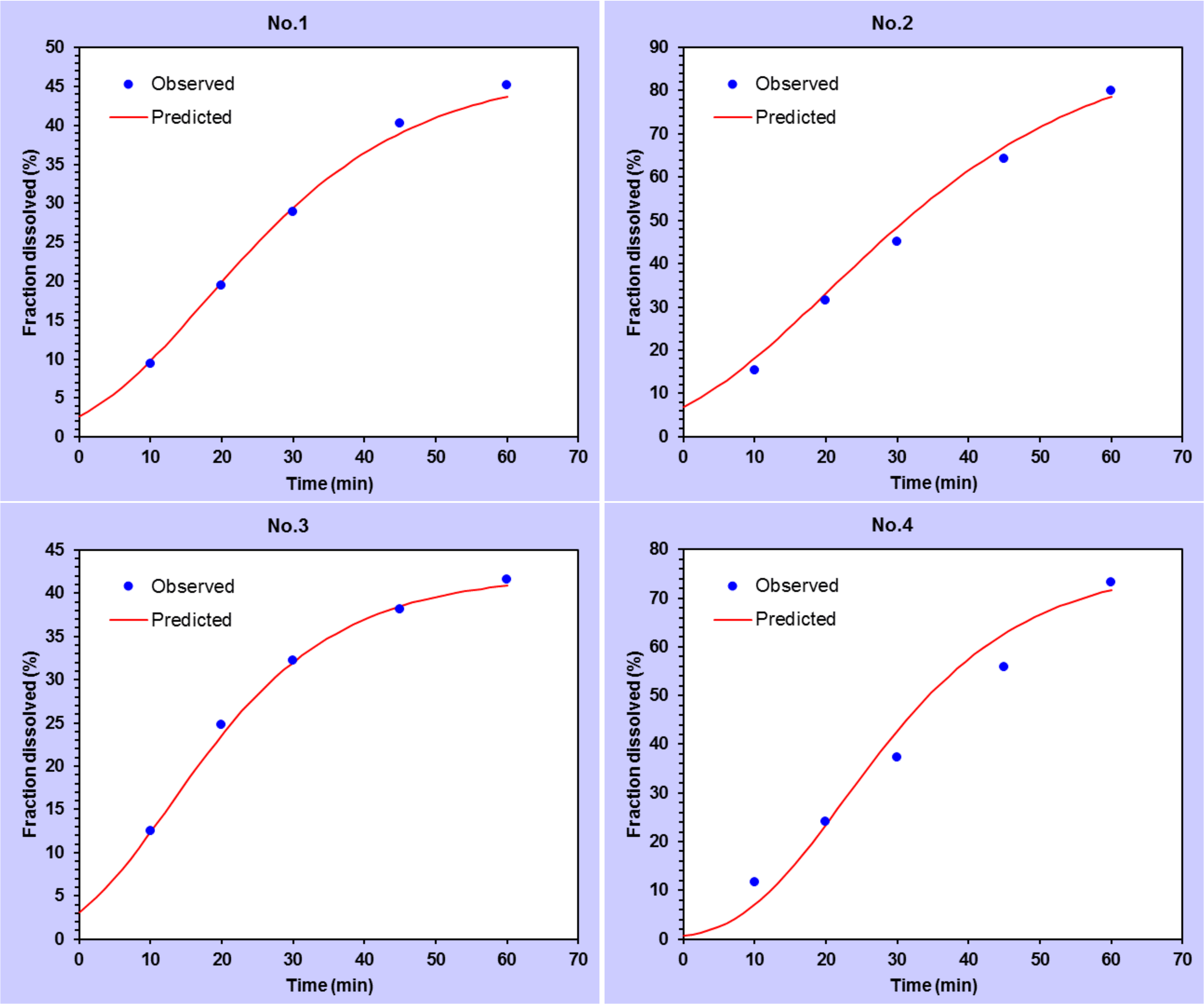

Supplementary materials – Model fitting summary – Kollidon® 25 (modelling up to app. 60 % of carvedilol released)

Model: **Gompertz_4**

Model equation: $F = F_{max} \cdot e^{-\beta \cdot e^{-k \cdot t}}$

Fitted model parameters per tested tablet (N = 4) with statistics – mean, standard deviation (SD), and relative standard deviation expressed in % (RSD%) (output from DDSolver):

Parameter	No.1	No.2	No.3	No.4	Mean	SD	RSD(%)
k	0.070	0.068	0.075	0.070	0.071	0.003	3.913
β	3.604	4.032	2.417	4.830	3.721	1.007	27.054
F_{max}	47.460	84.000	42.707	76.965	62.783	20.730	33.018

Number of dissolution data points (N), degrees of freedom (df), and selected goodness of fit criteria – Pearson correlation coefficient (R), coefficient of determination (R^2), adjusted coefficient of determination ($R^2_{adjusted}$), and residual sum of squares (RSS) (manual calculation in MS Excel):

Parameter	No.1	No.2	No.3	No.4
N	5	5	5	5
df	2	2	2	2
R	0.997734499	0.990351521	0.998808329	0.98579261
R^2	0.99547413	0.980796136	0.997618078	0.97178707
$R^2_{adjusted}$	0.990948261	0.961592271	0.995236157	0.943574139
RSS	5.171198865	76.13783751	3.354908348	99.25467604

Graphical abstract of model fit presented as mean ± 1 SD of the fraction % of released carvedilol:

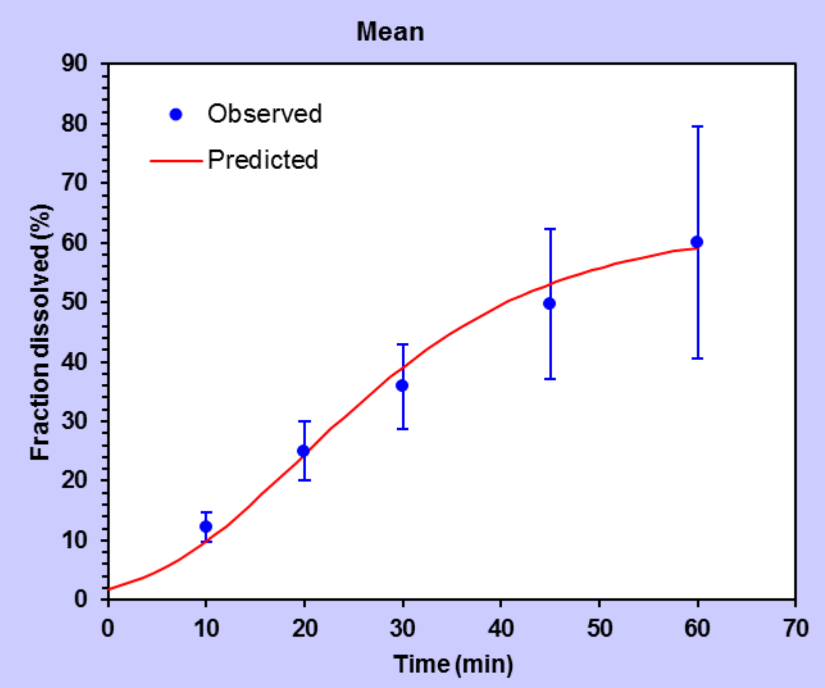

Supplementary materials – Model fitting summary – Kollidon® 25 (modelling up to app. 60 % of carvedilol released)

Graphical abstract of model fit presented as the fraction % of released carvedilol per tested tablet:

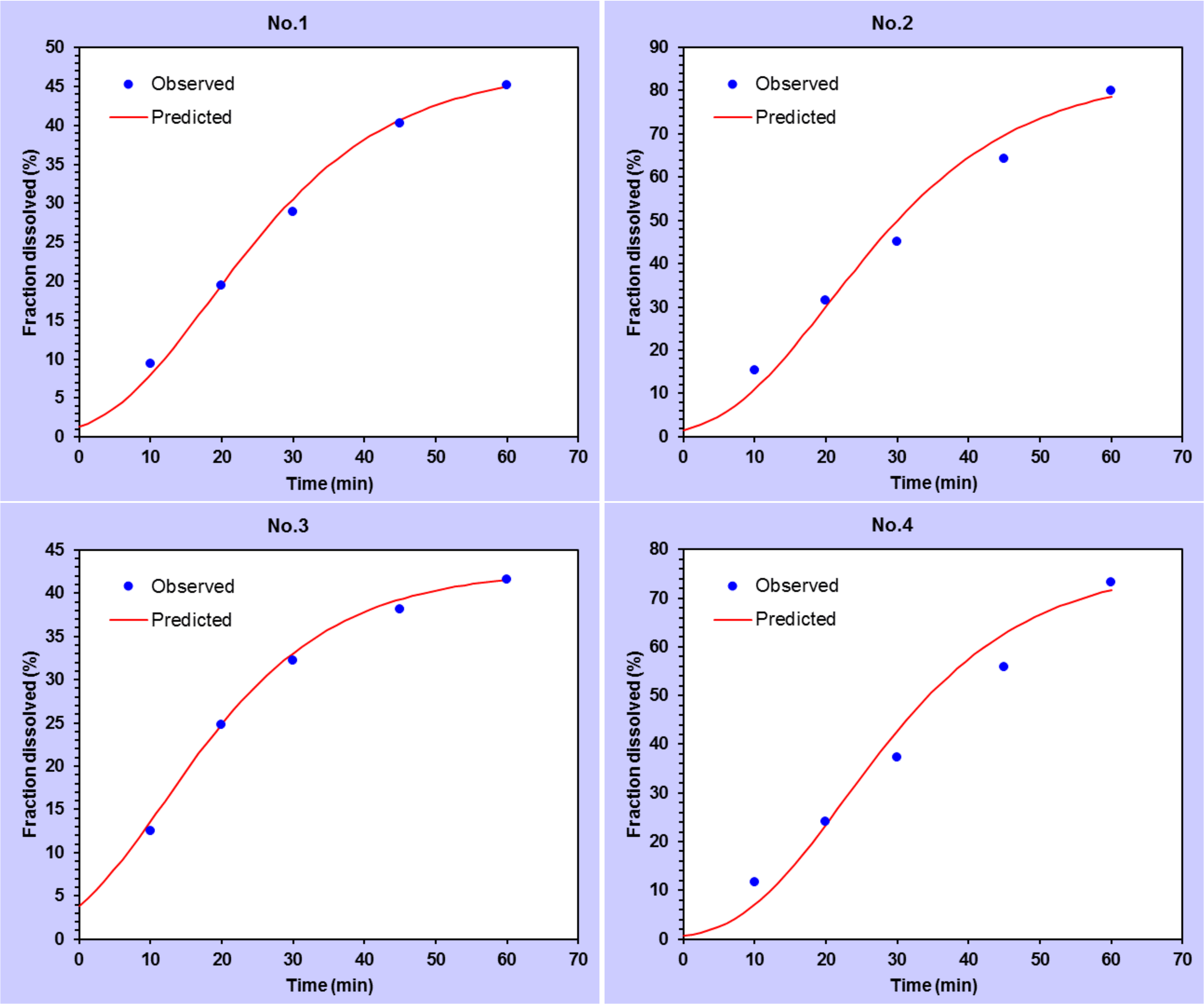

Supplementary materials – Model fitting summary – Kollidon® 25 (modelling up to app. 60 % of carvedilol released)

Model: **Probit_1**

Model equation: $F = 100 \cdot \phi[\alpha + \beta \cdot \log(t)]$

Fitted model parameters per tested tablet (N = 4) with statistics – mean, standard deviation (SD), and relative standard deviation expressed in % (RSD%) (output from DDSolver):

Parameter	No.1	No.2	No.3	No.4	Mean	SD	RSD(%)
α	-2.900	-3.462	-2.306	-3.589	-3.064	0.587	-19.164
β	1.581	2.343	1.210	2.288	1.855	0.553	29.788

Number of dissolution data points (N), degrees of freedom (df), and selected goodness of fit criteria – Pearson correlation coefficient (R), coefficient of determination (R^2), adjusted coefficient of determination ($R^2_{adjusted}$), and residual sum of squares (RSS) (manual calculation in MS Excel):

Parameter	No.1	No.2	No.3	No.4
N	5	5	5	5
df	3	3	3	3
R	0.997432842	0.990196474	0.988672298	0.988730635
R^2	0.994872274	0.980489058	0.977472913	0.977588268
$R^2_{adjusted}$	0.993163032	0.973985411	0.969963884	0.970117691
RSS	4.448505587	53.34940041	12.98539243	58.17780113

Graphical abstract of model fit presented as mean ± 1 SD of the fraction % of released carvedilol:

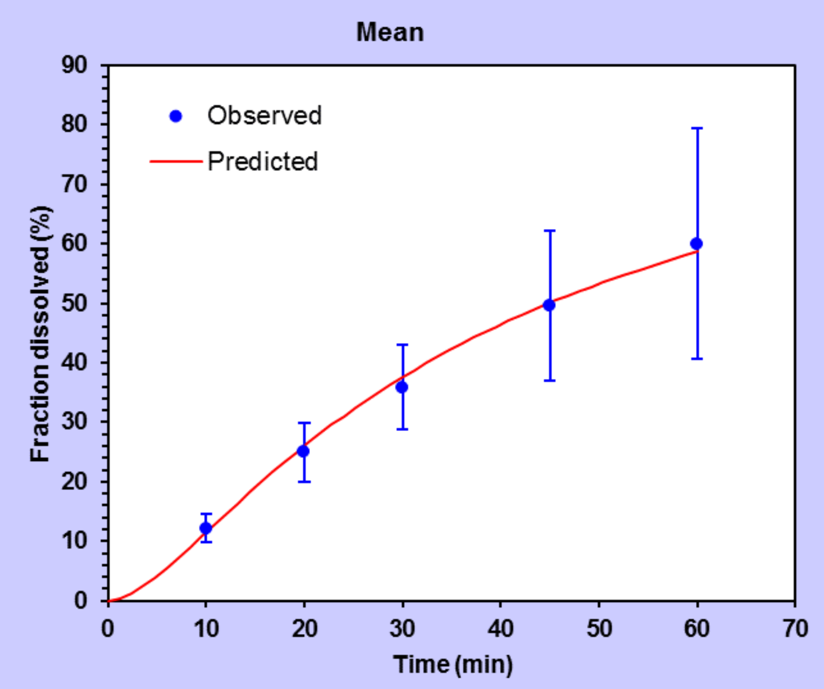

Supplementary materials – Model fitting summary – Kollidon® 25 (modelling up to app. 60 % of carvedilol released)

Graphical abstract of model fit presented as the fraction % of released carvedilol per tested tablet:

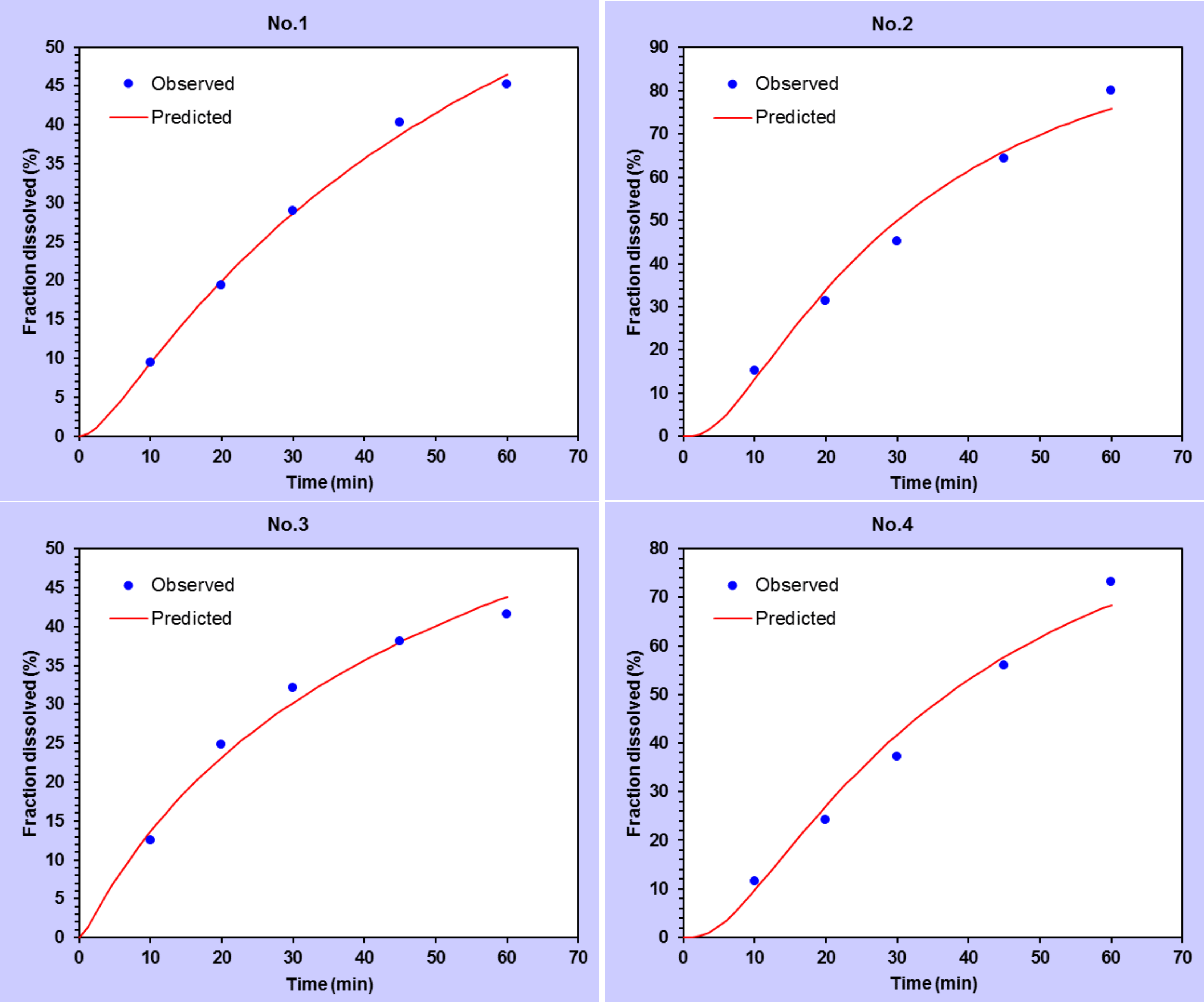

Supplementary materials – Model fitting summary – Kollidon® 25 (modelling up to app. 60 % of carvedilol released)

Model: **Probit_2**

Model equation: $F = F_{max} \cdot \phi[\alpha + \beta \cdot \log(t)]$

Fitted model parameters per tested tablet (N = 4) with statistics – mean, standard deviation (SD), and relative standard deviation expressed in % (RSD%) (output from DDSolver):

Parameter	No.1	No.2	No.3	No.4	Mean	SD	RSD(%)
α	-4.232	-4.231	-3.434	-5.230	-4.282	0.735	-17.175
β	3.198	3.109	2.804	3.446	3.139	0.265	8.436
F_{max}	47.460	84.000	43.680	83.490	64.658	22.095	34.173

Number of dissolution data points (N), degrees of freedom (df), and selected goodness of fit criteria – Pearson correlation coefficient (R), coefficient of determination (R^2), adjusted coefficient of determination ($R^2_{adjusted}$), and residual sum of squares (RSS) (manual calculation in MS Excel):

Parameter	No.1	No.2	No.3	No.4
N	5	5	5	5
df	2	2	2	2
R	0.984602301	0.975278868	0.99765034	0.991426336
R^2	0.969441692	0.951168871	0.9953062	0.982926179
$R^2_{adjusted}$	0.938883384	0.902337742	0.9906124	0.965852359
RSS	29.8424097	150.6232212	3.135150233	127.5999154

Graphical abstract of model fit presented as mean ± 1 SD of the fraction % of released carvedilol:

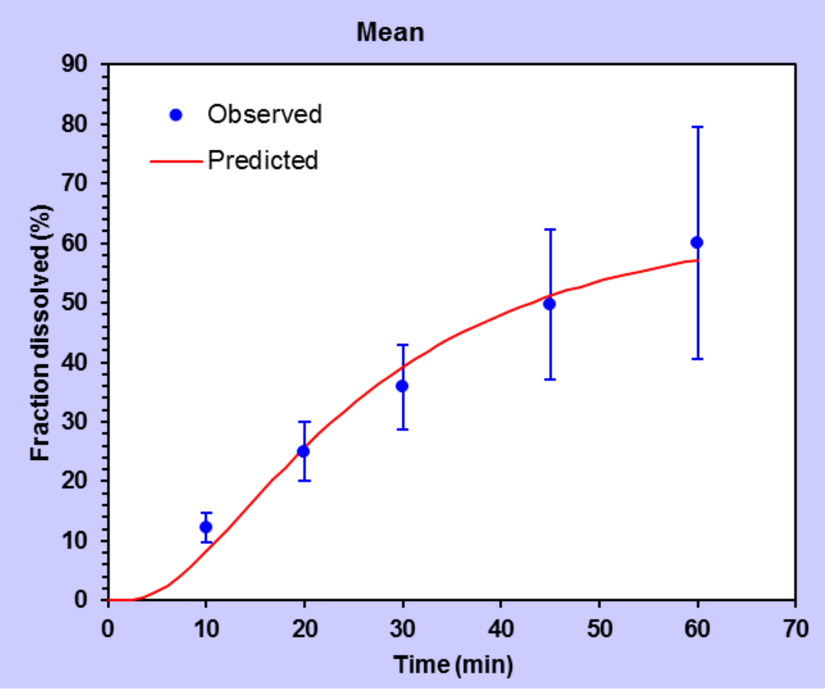

Supplementary materials – Model fitting summary – Kollidon® 25 (modelling up to app. 60 % of carvedilol released)

Graphical abstract of model fit presented as the fraction % of released carvedilol per tested tablet:

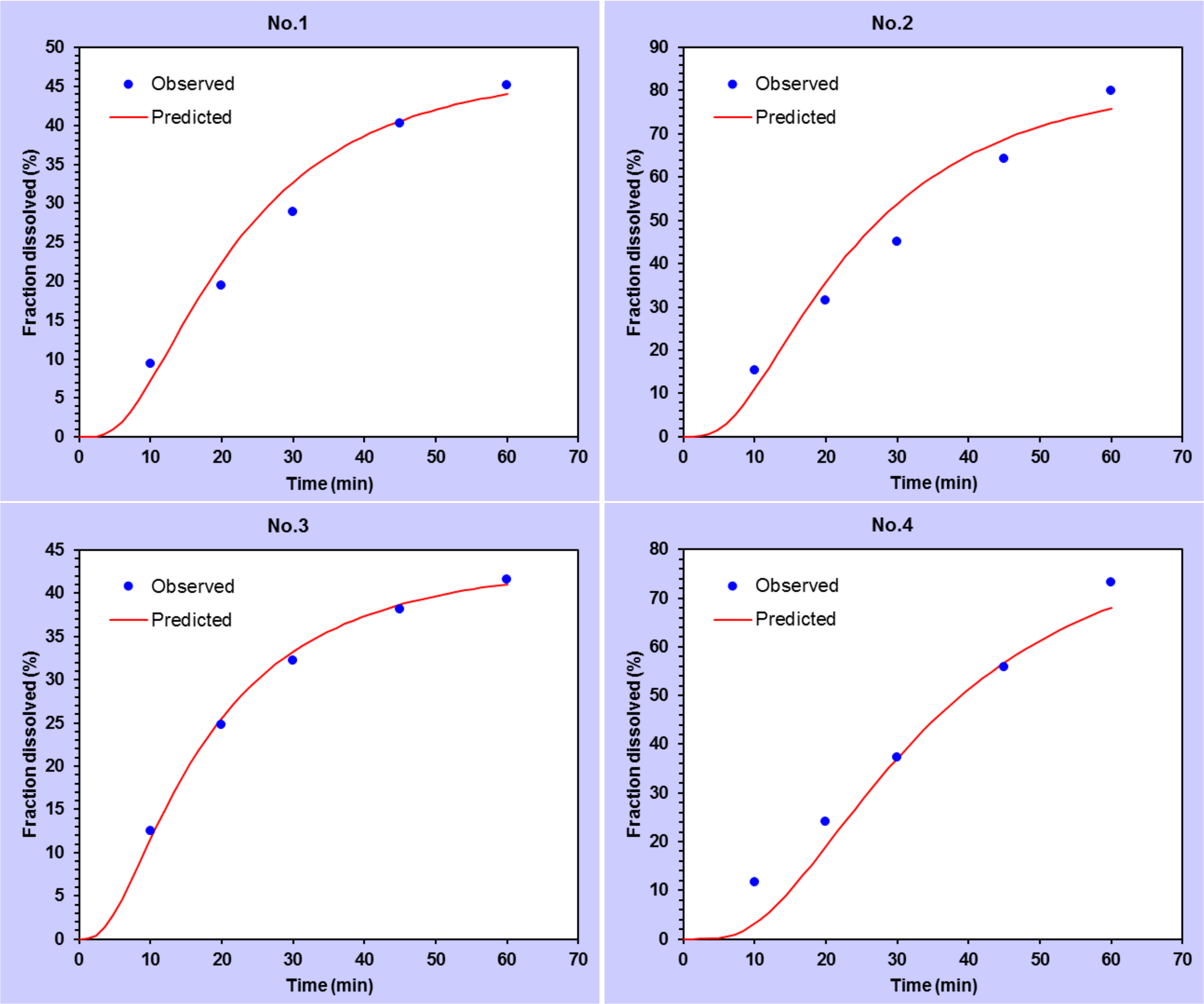

Supplement: Supplementary file 1 [file pharmaceutics-16-00498-s001.zip › Supplementary materials_Model fitting summary_Kollidon® 25.pdf]
