# Supplementary material for: Comparative Fitting of Mathematical Models to Carvedilol Release Profiles Obtained from Hypromellose Matrix Tablets
Source: Pharmaceutics. 2024 Apr 4;16(4):498. doi: 10.3390/pharmaceutics16040498 (PMC11053526; doi:10.3390/pharmaceutics16040498)

Supplementary materials – Model fitting summary – Kollidon® 90 F (modelling the entire relevant dissolution data)

Model: **Zero–order**

Model equation: $F = k_0 \cdot t$

Fitted model parameters per tested tablet (N = 4) with statistics – mean, standard deviation (SD), and relative standard deviation expressed in % (RSD%) (output from DDSolver):

Parameter	No.1	No.2	No.3	No.4	Mean	SD	RSD(%)
k_0	0.173	0.182	0.194	0.193	0.186	0.010	5.350

Number of dissolution data points (N), degrees of freedom (df), and selected goodness of fit criteria – Pearson correlation coefficient (R), coefficient of determination (R^2), adjusted coefficient of determination ($R^2_{adjusted}$), and residual sum of squares (RSS) (manual calculation in MS Excel):

Parameter	No.1	No.2	No.3	No.4
N	20	20	20	20
df	19	19	19	19
R	0.988730303	0.974173201	0.947824752	0.967269623
R^2	0.977587612	0.949013426	0.898371761	0.935610523
$R^2_{adjusted}$	0.977587612	0.949013426	0.898371761	0.935610523
RSS	549.9656784	1496.81032	3674.965726	1926.110321

Graphical abstract of model fit presented as mean ± 1 SD of the fraction % of released carvedilol:

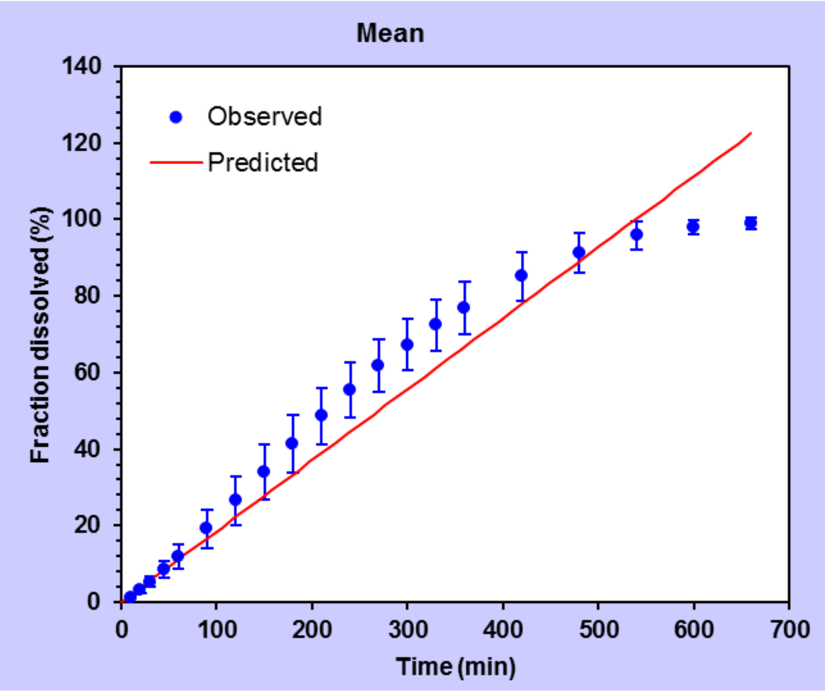

Supplementary materials – Model fitting summary – Kollidon® 90 F (modelling the entire relevant dissolution data)

Graphical abstract of model fit presented as the fraction % of released carvedilol per tested tablet:

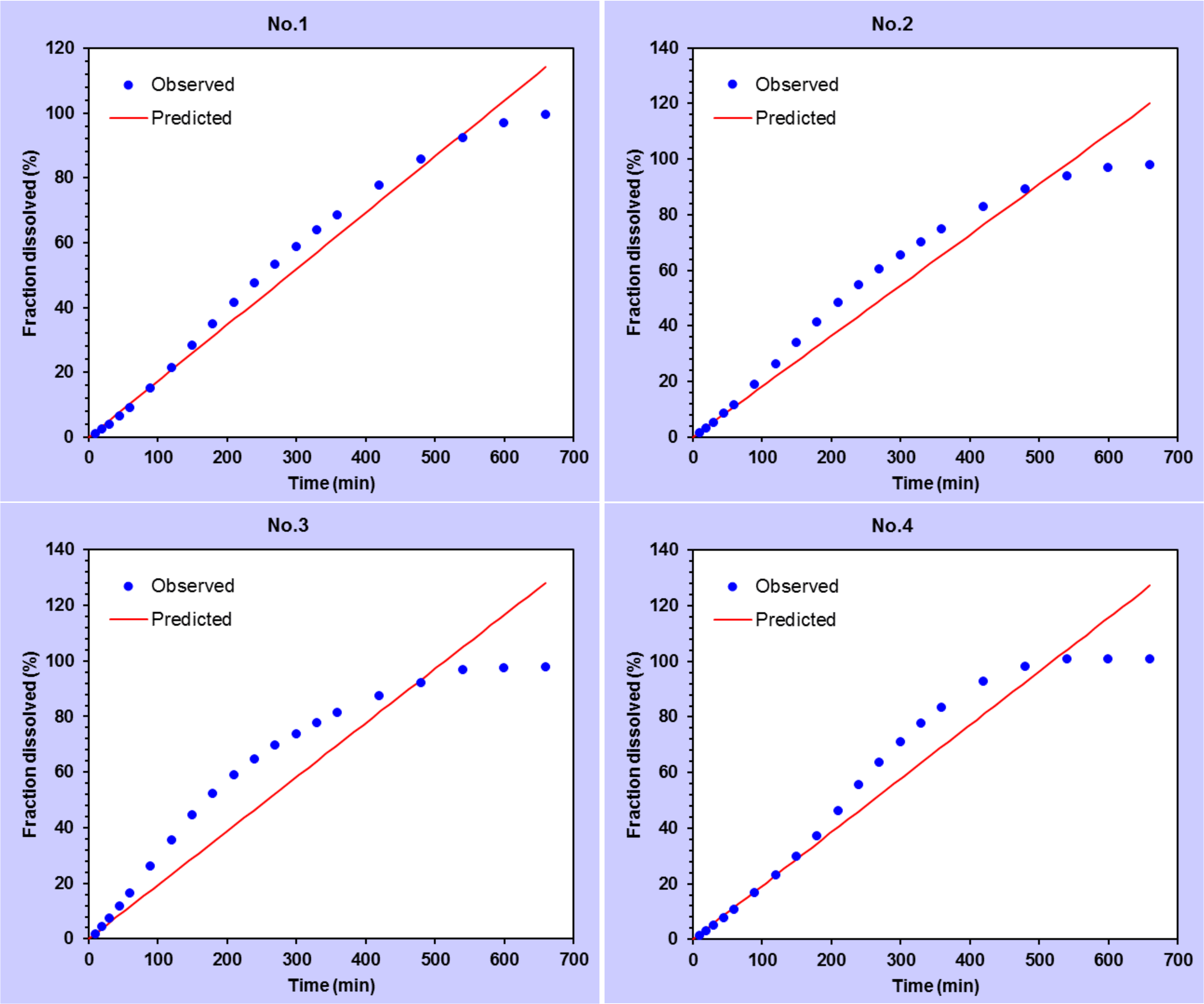

Supplementary materials – Model fitting summary – Kollidon® 90 F (modelling the entire relevant dissolution data)

Model: **Zero–order with T_{lag}**

Model equation: $F = k_0 \cdot (t - T_{lag})$

Fitted model parameters per tested tablet (N = 4) with statistics – mean, standard deviation (SD), and relative standard deviation expressed in % (RSD%) (output from DDSolver):

Parameter	No.1	No.2	No.3	No.4	Mean	SD	RSD(%)
k_0	0.166	0.164	0.160	0.180	0.168	0.009	5.120
T_{lag}	-17.200	-43.039	-86.658	-28.936	-43.958	30.364	-69.073

Number of dissolution data points (N), degrees of freedom (df), and selected goodness of fit criteria – Pearson correlation coefficient (R), coefficient of determination (R^2), adjusted coefficient of determination ($R^2_{adjusted}$), and residual sum of squares (RSS) (manual calculation in MS Excel):

Parameter	No.1	No.2	No.3	No.4
N	20	20	20	20
df	18	18	18	18
R	0.988730303	0.974173201	0.947824752	0.967269623
R^2	0.977587612	0.949013426	0.898371761	0.935610523
$R^2_{adjusted}$	0.976342479	0.946180839	0.892725747	0.93203333
RSS	489.301041	1124.339609	2243.787767	1724.515442

Graphical abstract of model fit presented as mean ± 1 SD of the fraction % of released carvedilol:

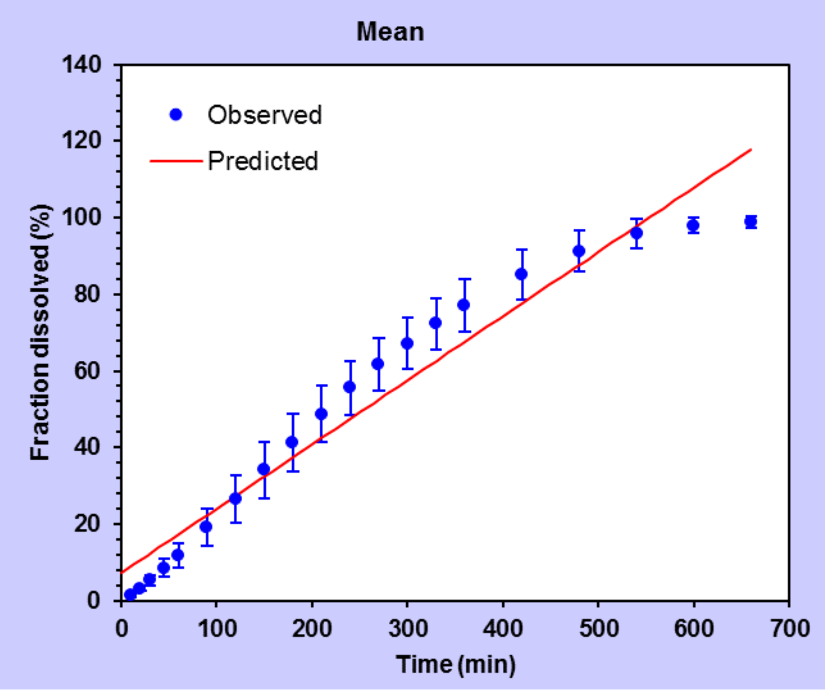

Supplementary materials – Model fitting summary – Kollidon® 90 F (modelling the entire relevant dissolution data)

Graphical abstract of model fit presented as the fraction % of released carvedilol per tested tablet:

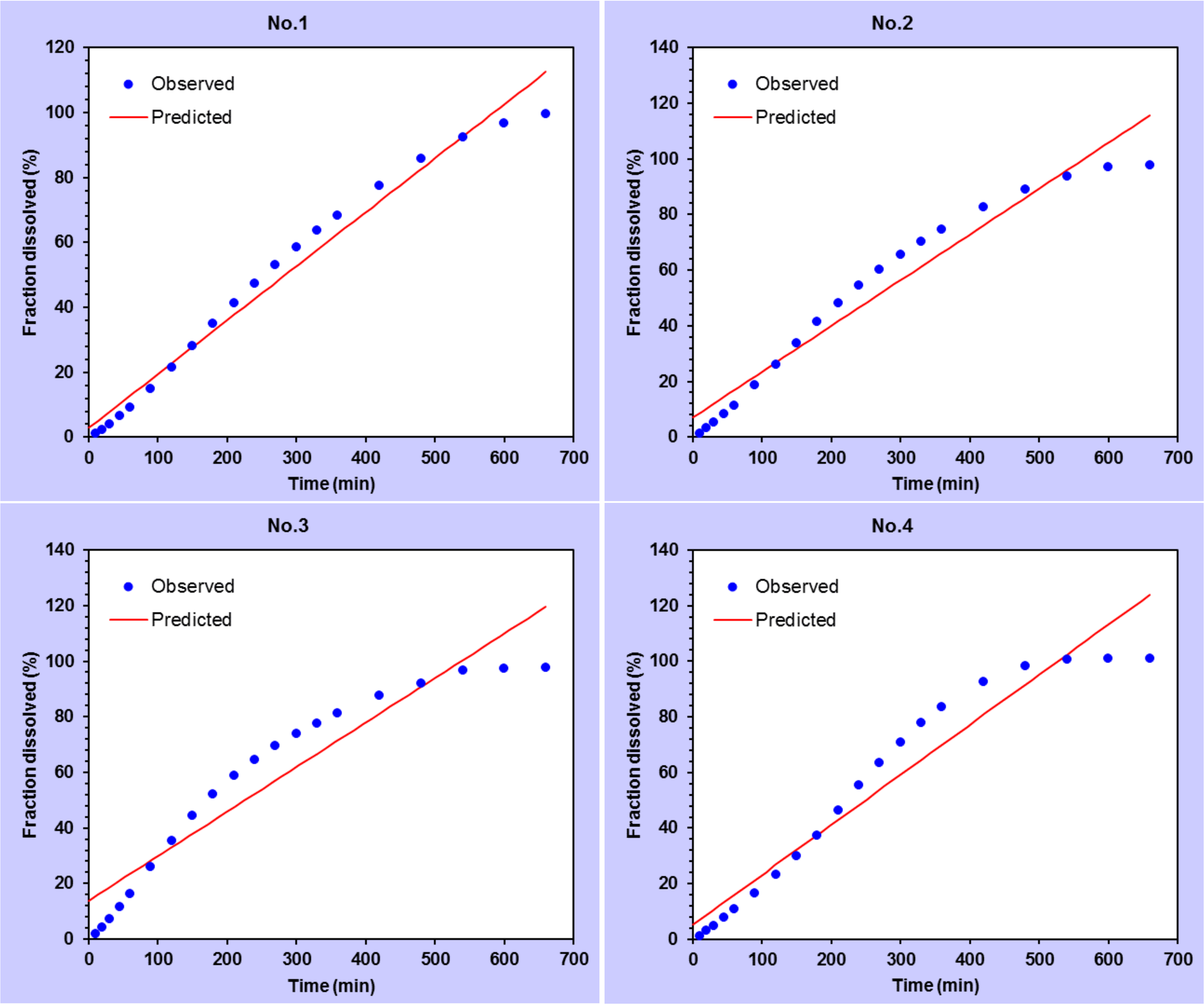

Supplementary materials – Model fitting summary – Kollidon® 90 F (modelling the entire relevant dissolution data)

Model: **Zero–order with F_0**

Model equation: $F = F_0 + k_0 \cdot t$

Fitted model parameters per tested tablet (N = 4) with statistics – mean, standard deviation (SD), and relative standard deviation expressed in % (RSD%) (output from DDSolver):

Parameter	No.1	No.2	No.3	No.4	Mean	SD	RSD(%)
k_0	0.166	0.164	0.160	0.180	0.168	0.009	5.120
F_0	2.857	7.080	13.878	5.209	7.256	4.741	65.336

Number of dissolution data points (N), degrees of freedom (df), and selected goodness of fit criteria – Pearson correlation coefficient (R), coefficient of determination (R^2), adjusted coefficient of determination ($R^2_{adjusted}$), and residual sum of squares (RSS) (manual calculation in MS Excel):

Parameter	No.1	No.2	No.3	No.4
N	20	20	20	20
df	18	18	18	18
R	0.988730303	0.974173201	0.947824752	0.967269623
R^2	0.977587612	0.949013426	0.898371761	0.935610523
$R^2_{adjusted}$	0.976342479	0.946180839	0.892725747	0.93203333
RSS	489.301041	1124.339609	2243.787767	1724.515442

Graphical abstract of model fit presented as mean ± 1 SD of the fraction % of released carvedilol:

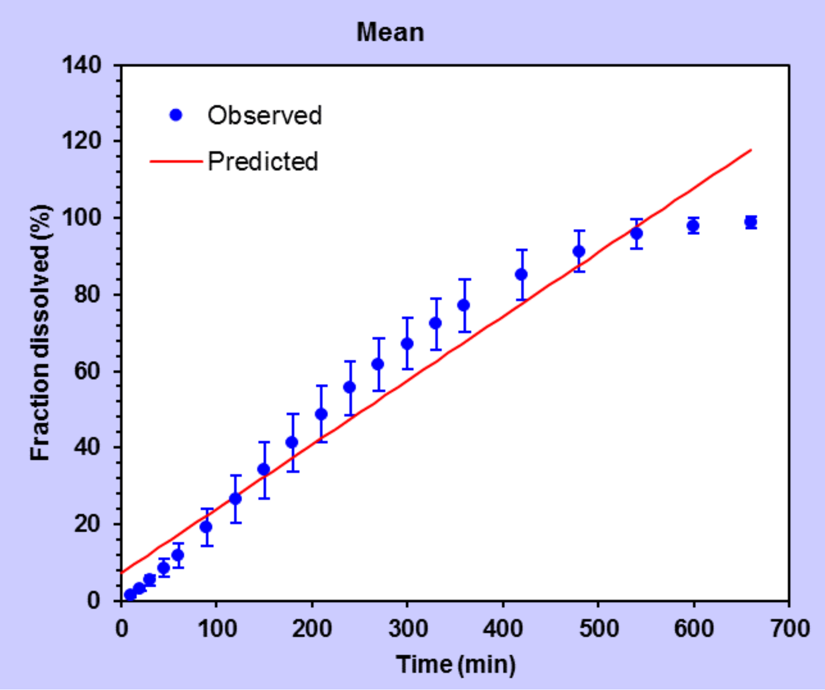

Supplementary materials – Model fitting summary – Kollidon® 90 F (modelling the entire relevant dissolution data)

Graphical abstract of model fit presented as the fraction % of released carvedilol per tested tablet:

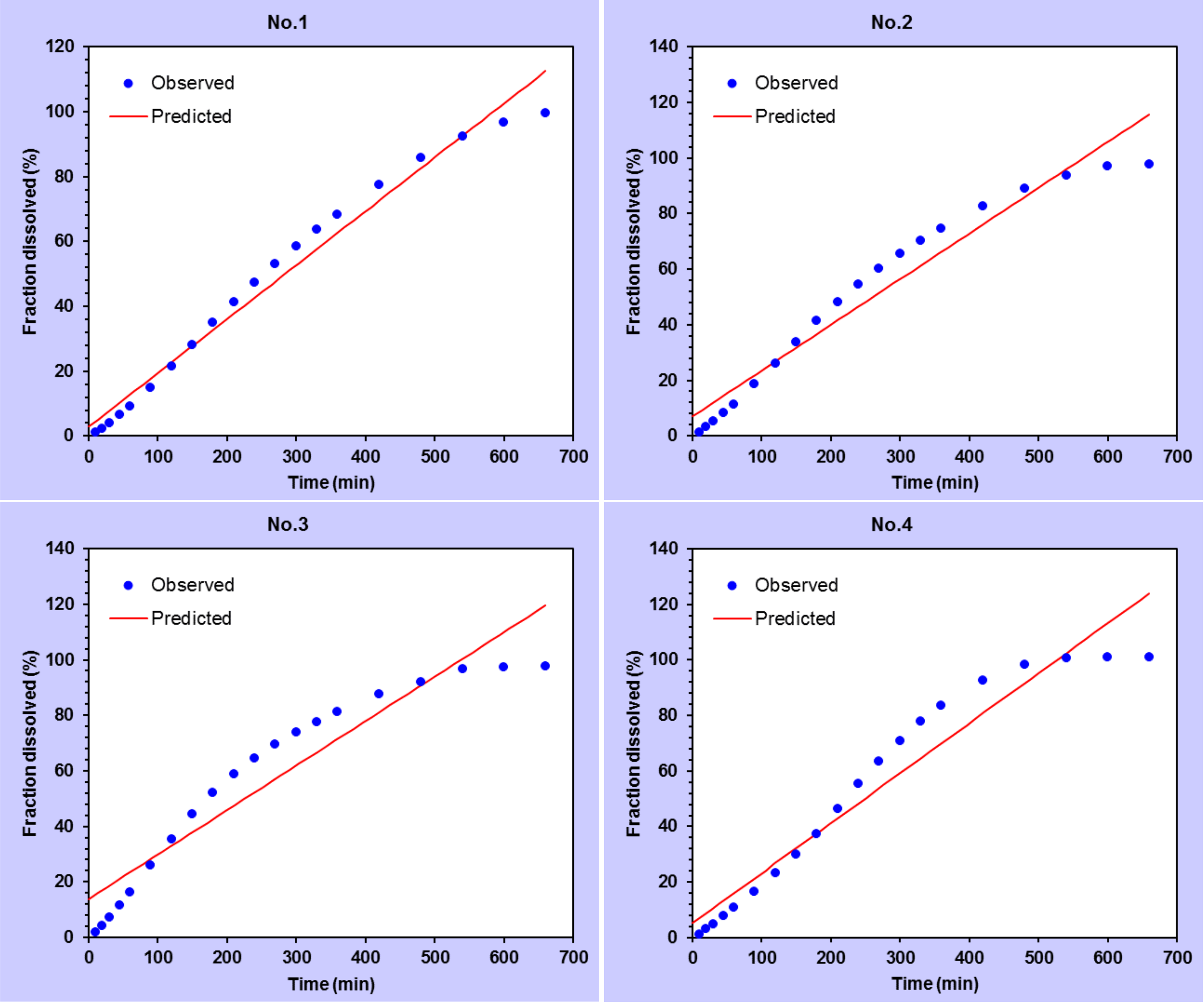

Supplementary materials – Model fitting summary – Kollidon® 90 F (modelling the entire relevant dissolution data)

Model: **First–order**

Model equation: $F = 100 \cdot (1 - e^{-k_1 \cdot t})$

Fitted model parameters per tested tablet (N = 4) with statistics – mean, standard deviation (SD), and relative standard deviation expressed in % (RSD%) (output from DDSolver):

Parameter	No.1	No.2	No.3	No.4	Mean	SD	RSD(%)
k_1	0.005	0.004	0.004	0.005	0.004	0.001	18.176

Number of dissolution data points (N), degrees of freedom (df), and selected goodness of fit criteria – Pearson correlation coefficient (R), coefficient of determination (R^2), adjusted coefficient of determination ($R^2_{adjusted}$), and residual sum of squares (RSS) (manual calculation in MS Excel):

Parameter	No.1	No.2	No.3	No.4
N	20	20	20	20
df	19	19	19	19
R	0.965288968	0.994605084	0.99906521	0.966000626
R^2	0.931782791	0.989239274	0.998131293	0.93315721
$R^2_{adjusted}$	0.931782791	0.989239274	0.998131293	0.93315721
RSS	5119.263065	709.3927791	386.1062519	4119.744343

Graphical abstract of model fit presented as mean ± 1 SD of the fraction % of released carvedilol:

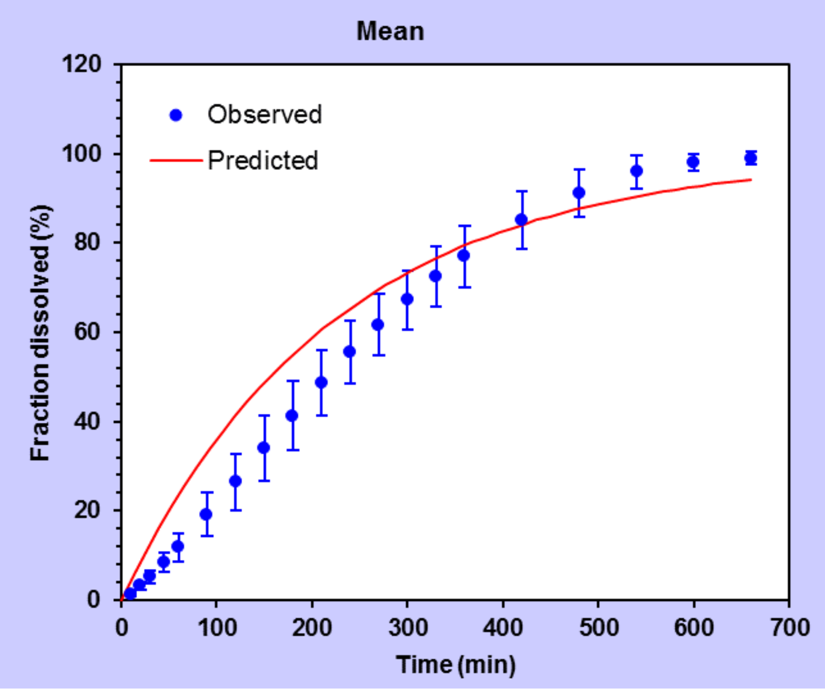

Supplementary materials – Model fitting summary – Kollidon® 90 F (modelling the entire relevant dissolution data)

Graphical abstract of model fit presented as the fraction % of released carvedilol per tested tablet:

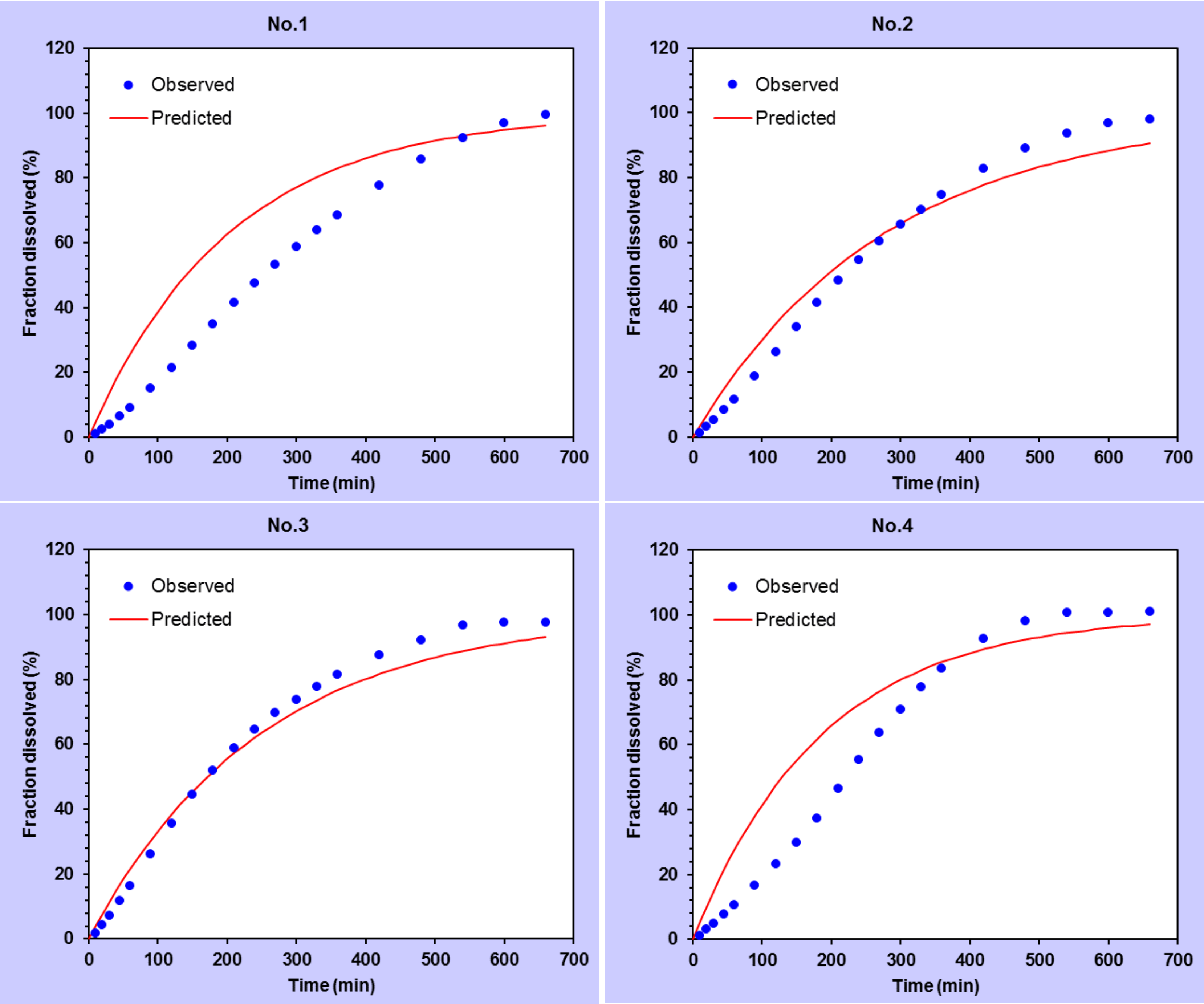

Supplementary materials – Model fitting summary – Kollidon® 90 F (modelling the entire relevant dissolution data)

Model: **First–order with T_{lag}**

Model equation: $F = 100 \cdot \left[1 - e^{-k_1 \cdot (t - T_{lag})}\right]$

Fitted model parameters per tested tablet (N = 4) with statistics – mean, standard deviation (SD), and relative standard deviation expressed in % (RSD%) (output from DDSolver):

Parameter	No.1	No.2	No.3	No.4	Mean	SD	RSD(%)
k_1	/	/	/	/	/	/	/
T_{lag}	/	/	/	/	/	/	/

Number of dissolution data points (N), degrees of freedom (df), and selected goodness of fit criteria – Pearson correlation coefficient (R), coefficient of determination (R^2), adjusted coefficient of determination ($R^2_{adjusted}$), and residual sum of squares (RSS) (manual calculation in MS Excel):

Parameter	No.1	No.2	No.3	No.4
N	/	/	/	/
df	/	/	/	/
R	/	/	/	/
R^2	/	/	/	/
$R^2_{adjusted}$	/	/	/	/
RSS	/	/	/	/

Graphical abstract of model fit presented as mean ± 1 SD of the fraction % of released carvedilol: /

Graphical abstract of model fit presented as the fraction % of released carvedilol per tested tablet: /

Note: the model could not be fitted

Supplementary materials – Model fitting summary – Kollidon® 90 F (modelling the entire relevant dissolution data)

Model: **First–order with F_{max}**

Model equation: $F = F_{max} \cdot (1 - e^{-k_1 \cdot t})$

Fitted model parameters per tested tablet (N = 4) with statistics – mean, standard deviation (SD), and relative standard deviation expressed in % (RSD%) (output from DDSolver):

Parameter	No.1	No.2	No.3	No.4	Mean	SD	RSD(%)
k_1	0.004	0.004	0.005	0.004	0.004	0.000	8.455
F_{max}	84.933	102.787	102.579	105.928	99.057	9.540	9.630

Number of dissolution data points (N), degrees of freedom (df), and selected goodness of fit criteria – Pearson correlation coefficient (R), coefficient of determination (R^2), adjusted coefficient of determination ($R^2_{adjusted}$), and residual sum of squares (RSS) (manual calculation in MS Excel):

Parameter	No.1	No.2	No.3	No.4
N	20	20	20	20
df	18	18	18	18
R	0.981977425	0.990284781	0.997543915	0.980721398
R^2	0.964279663	0.980663948	0.995093862	0.96181446
$R^2_{adjusted}$	0.9622952	0.979589723	0.994821299	0.959693041
RSS	2078.672021	1460.005231	546.2843065	2379.326906

Graphical abstract of model fit presented as mean ± 1 SD of the fraction % of released carvedilol:

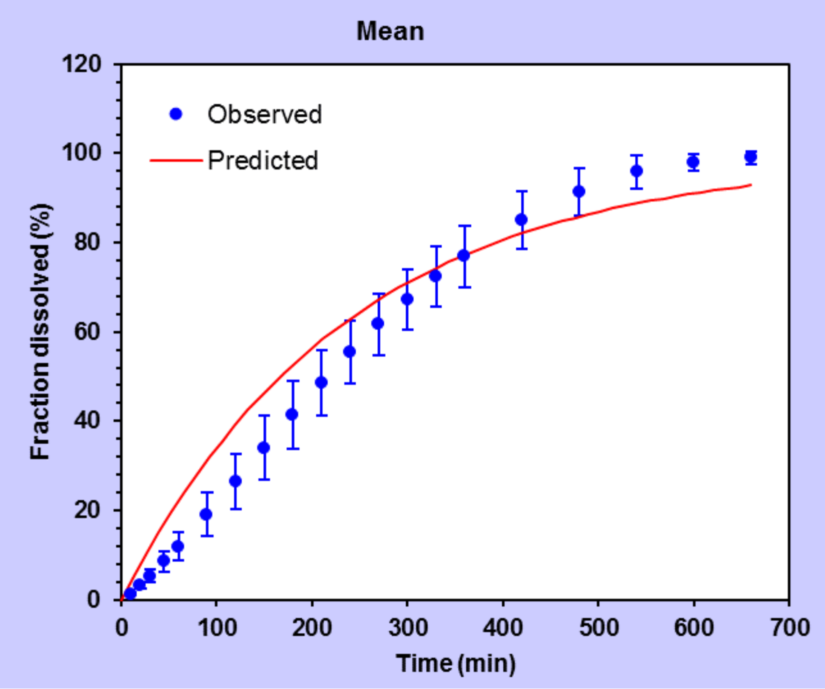

Supplementary materials – Model fitting summary – Kollidon® 90 F (modelling the entire relevant dissolution data)

Graphical abstract of model fit presented as the fraction % of released carvedilol per tested tablet:

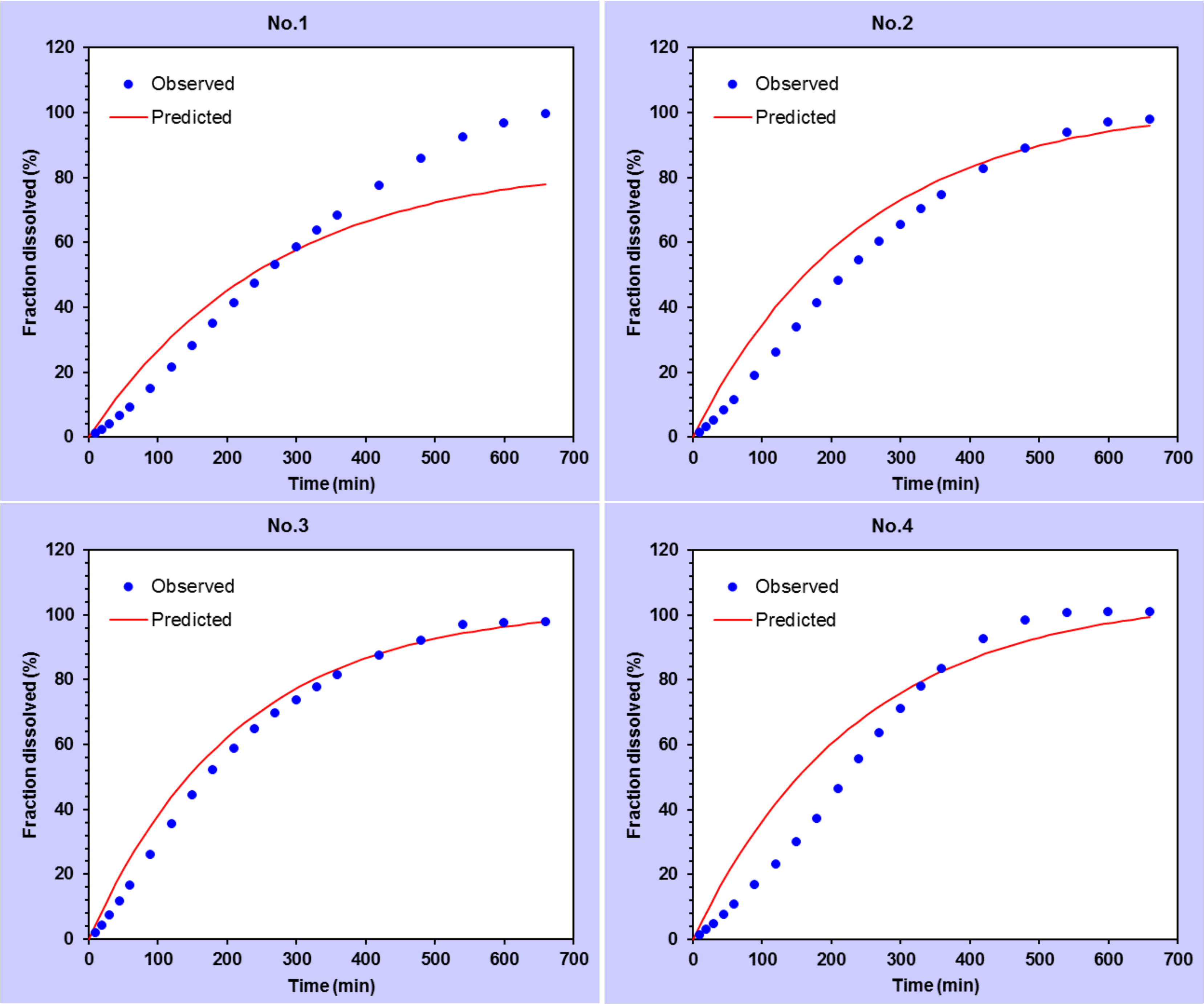

Supplementary materials – Model fitting summary – Kollidon® 90 F (modelling the entire relevant dissolution data)

Model: **First–order with T_{lag} and F_{max}**

Model equation: $F = F_{max} \cdot \left[1 - e^{-k_1 \cdot (t - T_{lag})}\right]$

Fitted model parameters per tested tablet (N = 4) with statistics – mean, standard deviation (SD), and relative standard deviation expressed in % (RSD%) (output from DDSolver):

Parameter	No.1	No.2	No.3	No.4	Mean	SD	RSD(%)
k_1	0.004	0.005	0.005	0.005	0.005	0.000	8.935
T_{lag}	62.518	49.744	28.950	56.227	49.360	14.572	29.522
F_{max}	104.533	102.787	102.579	105.928	103.957	1.579	1.519

Number of dissolution data points (N), degrees of freedom (df), and selected goodness of fit criteria – Pearson correlation coefficient (R), coefficient of determination (R^2), adjusted coefficient of determination ($R^2_{adjusted}$), and residual sum of squares (RSS) (manual calculation in MS Excel):

Parameter	No.1	No.2	No.3	No.4
N	20	20	20	20
df	17	17	17	17
R	0.974162417	0.984512496	0.995958477	0.965966848
R^2	0.948992414	0.969264854	0.991933287	0.933091952
$R^2_{adjusted}$	0.942991522	0.965648954	0.990984262	0.925220417
RSS	2607.812022	1509.24849	342.1631527	3205.050977

Graphical abstract of model fit presented as mean ± 1 SD of the fraction % of released carvedilol:

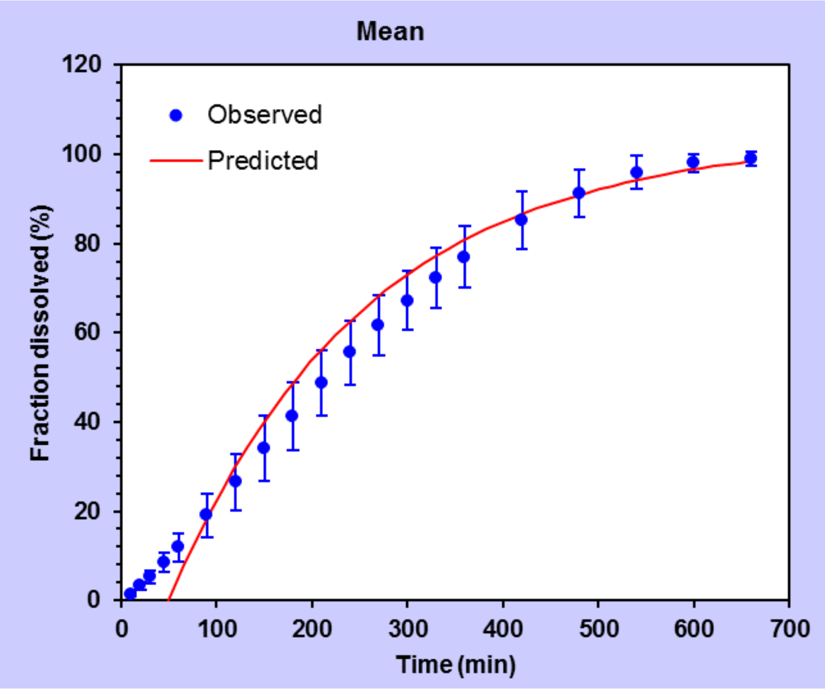

Supplementary materials – Model fitting summary – Kollidon® 90 F (modelling the entire relevant dissolution data)

Graphical abstract of model fit presented as the fraction % of released carvedilol per tested tablet:

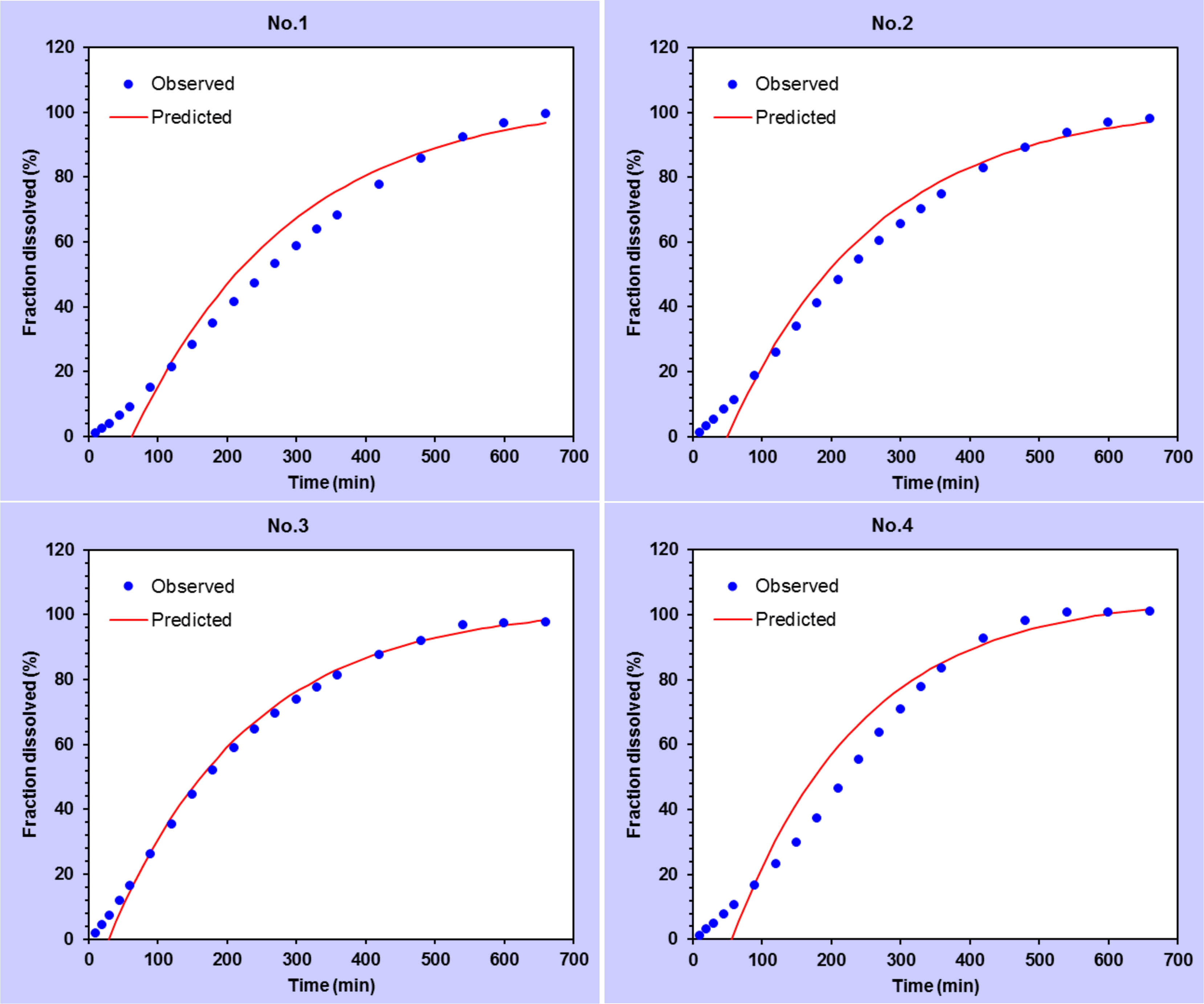

Supplementary materials – Model fitting summary – Kollidon® 90 F (modelling the entire relevant dissolution data)

Model: **Higuchi**

Model equation: $F = k_H \cdot t^{0.5}$

Fitted model parameters per tested tablet (N = 4) with statistics – mean, standard deviation (SD), and relative standard deviation expressed in % (RSD%) (output from DDSolver):

Parameter	No.1	No.2	No.3	No.4	Mean	SD	RSD(%)
k_H	3.432	3.655	3.975	3.852	3.729	0.238	6.372

Number of dissolution data points (N), degrees of freedom (df), and selected goodness of fit criteria – Pearson correlation coefficient (R), coefficient of determination (R^2), adjusted coefficient of determination ($R^2_{adjusted}$), and residual sum of squares (RSS) (manual calculation in MS Excel):

Parameter	No.1	No.2	No.3	No.4
N	20	20	20	20
df	19	19	19	19
R	0.990834068	0.99331536	0.990037648	0.983007849
R^2	0.98175215	0.986675405	0.980174544	0.966304431
$R^2_{adjusted}$	0.98175215	0.986675405	0.980174544	0.966304431
RSS	2691.725897	2037.048714	1382.096556	3398.605991

Graphical abstract of model fit presented as mean ± 1 SD of the fraction % of released carvedilol:

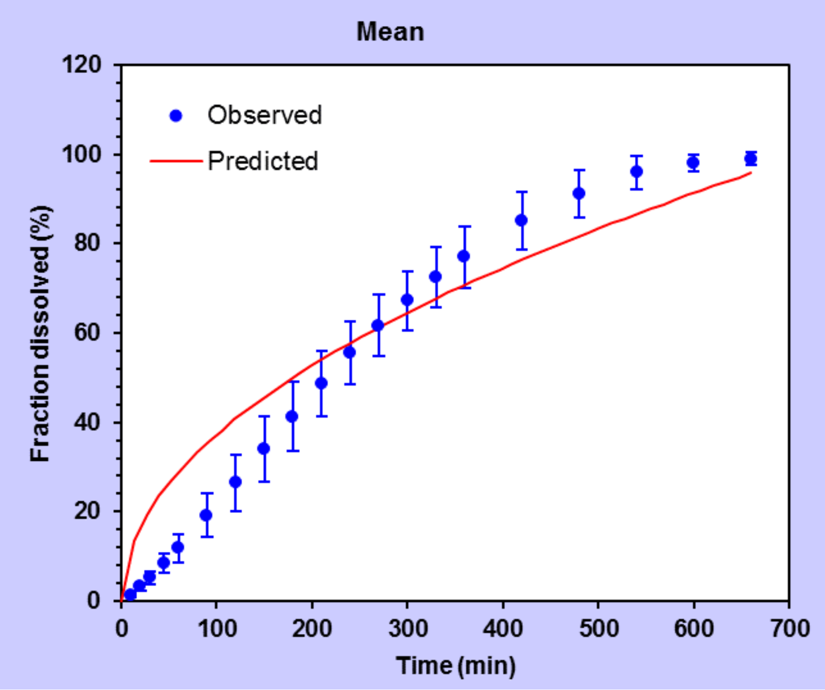

Supplementary materials – Model fitting summary – Kollidon® 90 F (modelling the entire relevant dissolution data)

Graphical abstract of model fit presented as the fraction % of released carvedilol per tested tablet:

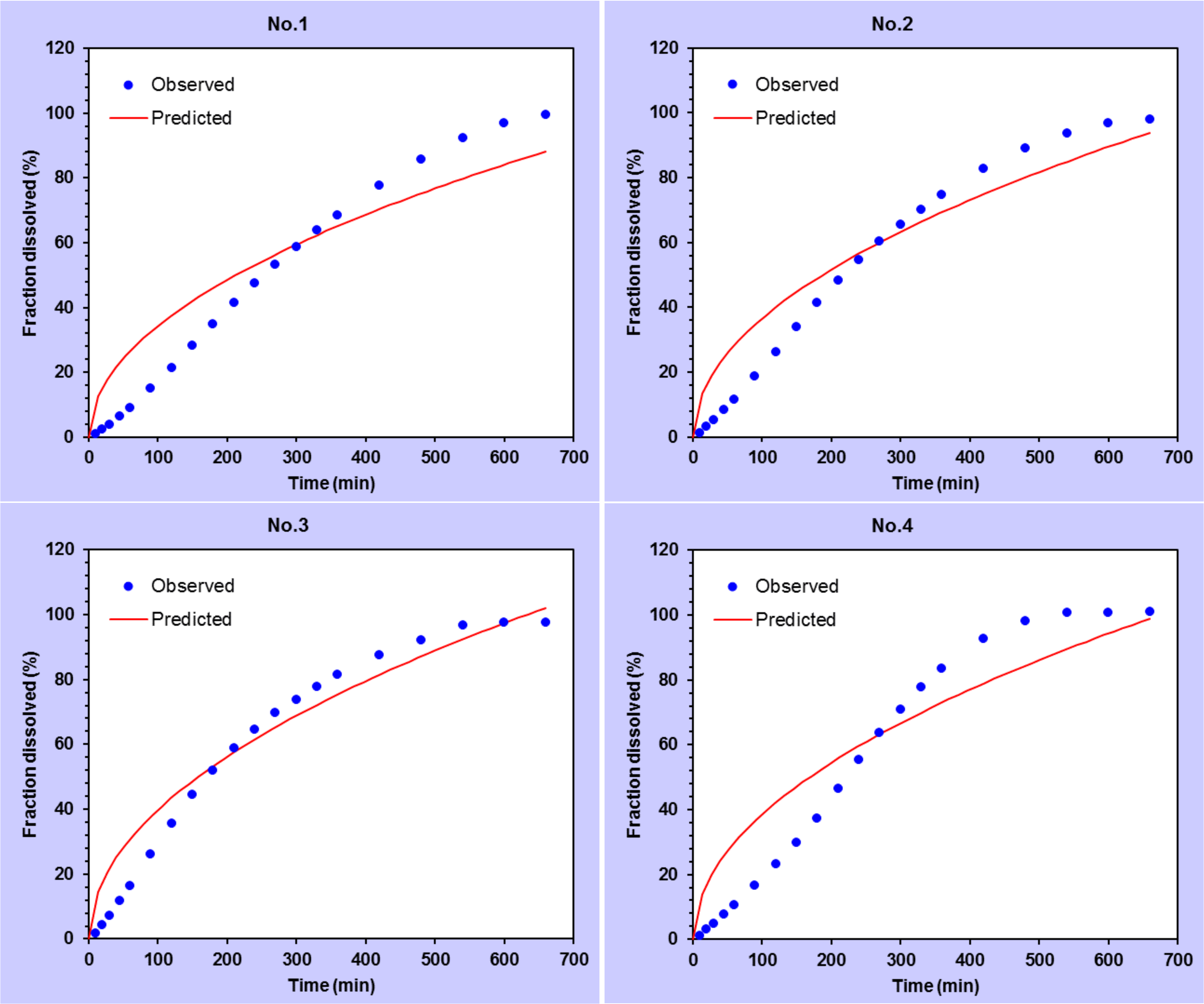

Supplementary materials – Model fitting summary – Kollidon® 90 F (modelling the entire relevant dissolution data)

Model: **Higuchi with T_{lag}**

Model equation: $F = k_H \cdot (t - T_{lag})^{0.5}$

Fitted model parameters per tested tablet (N = 4) with statistics – mean, standard deviation (SD), and relative standard deviation expressed in % (RSD%) (output from DDSolver):

Parameter	No.1	No.2	No.3	No.4	Mean	SD	RSD(%)
k_H	4.076	4.114	4.159	4.407	4.189	0.149	3.558
T_{lag}	99.452	71.771	27.175	77.331	68.932	30.297	43.952

Number of dissolution data points (N), degrees of freedom (df), and selected goodness of fit criteria – Pearson correlation coefficient (R), coefficient of determination (R^2), adjusted coefficient of determination ($R^2_{adjusted}$), and residual sum of squares (RSS) (manual calculation in MS Excel):

Parameter	No.1	No.2	No.3	No.4
N	20	20	20	20
df	18	18	18	18
R	0.99291541	0.994444043	0.991194351	0.986409523
R^2	0.985881012	0.988918954	0.982466242	0.973003748
$R^2_{adjusted}$	0.985096623	0.988303341	0.981492144	0.971503956
RSS	542.5491371	413.3399837	410.660483	839.8743655

Graphical abstract of model fit presented as mean ± 1 SD of the fraction % of released carvedilol:

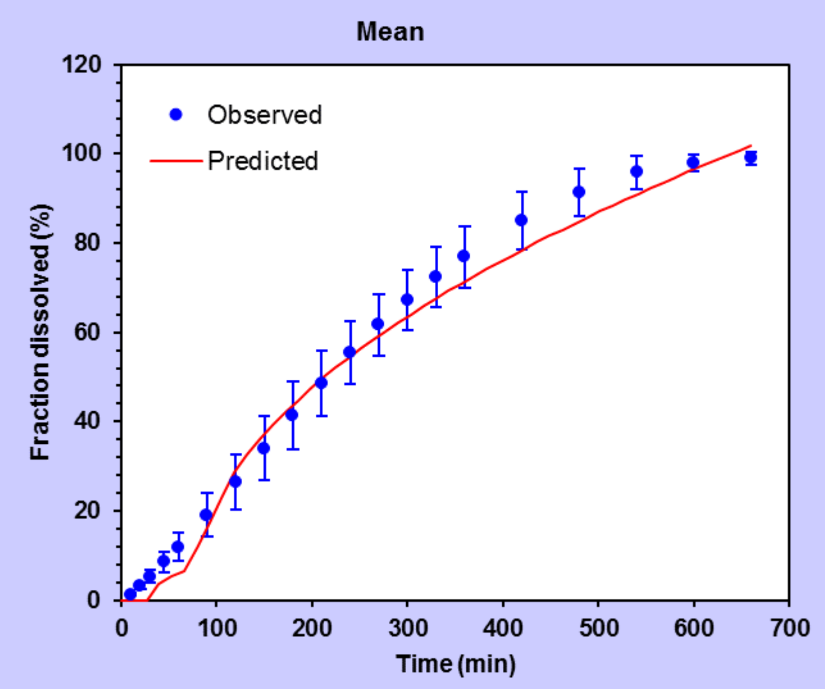

Supplementary materials – Model fitting summary – Kollidon® 90 F (modelling the entire relevant dissolution data)

Graphical abstract of model fit presented as the fraction % of released carvedilol per tested tablet:

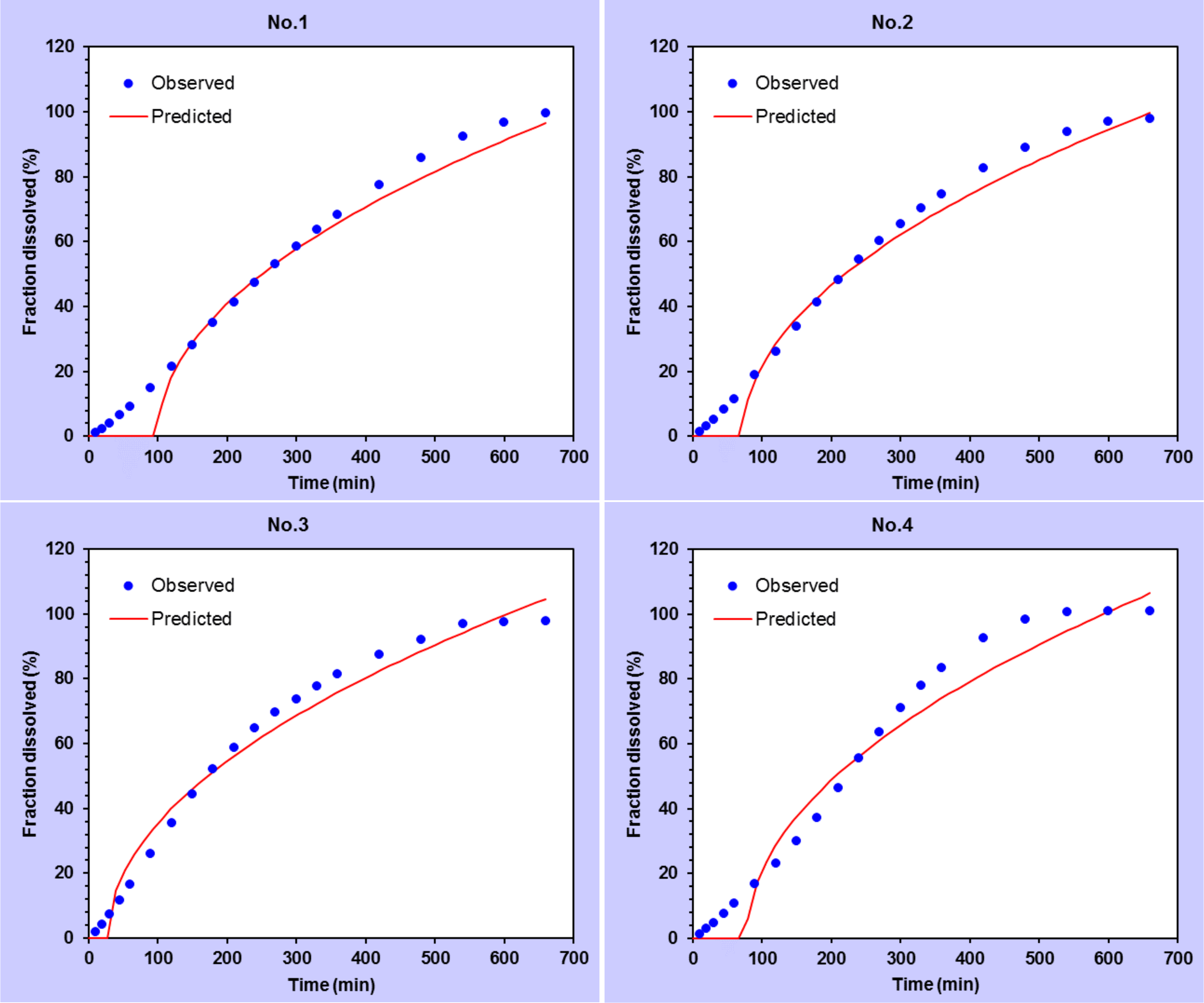

Supplementary materials – Model fitting summary – Kollidon® 90 F (modelling the entire relevant dissolution data)

Model: **Higuchi with F_0**

Model equation: $F = F_0 + k_H \cdot t^{0.5}$

Fitted model parameters per tested tablet (N = 4) with statistics – mean, standard deviation (SD), and relative standard deviation expressed in % (RSD%) (output from DDSolver):

Parameter	No.1	No.2	No.3	No.4	Mean	SD	RSD(%)
k_H	4.882	4.919	4.906	5.365	5.018	0.232	4.618
F_0	-25.540	-22.267	-16.389	-26.645	-22.710	4.606	-20.281

Number of dissolution data points (N), degrees of freedom (df), and selected goodness of fit criteria – Pearson correlation coefficient (R), coefficient of determination (R^2), adjusted coefficient of determination ($R^2_{adjusted}$), and residual sum of squares (RSS) (manual calculation in MS Excel):

Parameter	No.1	No.2	No.3	No.4
N	20	20	20	20
df	18	18	18	18
R	0.990834068	0.99331536	0.990037648	0.983007849
R^2	0.98175215	0.986675405	0.980174544	0.966304431
$R^2_{adjusted}$	0.980738381	0.98593515	0.97907313	0.964432455
RSS	398.3819968	293.8297003	437.7141148	902.4538068

Graphical abstract of model fit presented as mean ± 1 SD of the fraction % of released carvedilol:

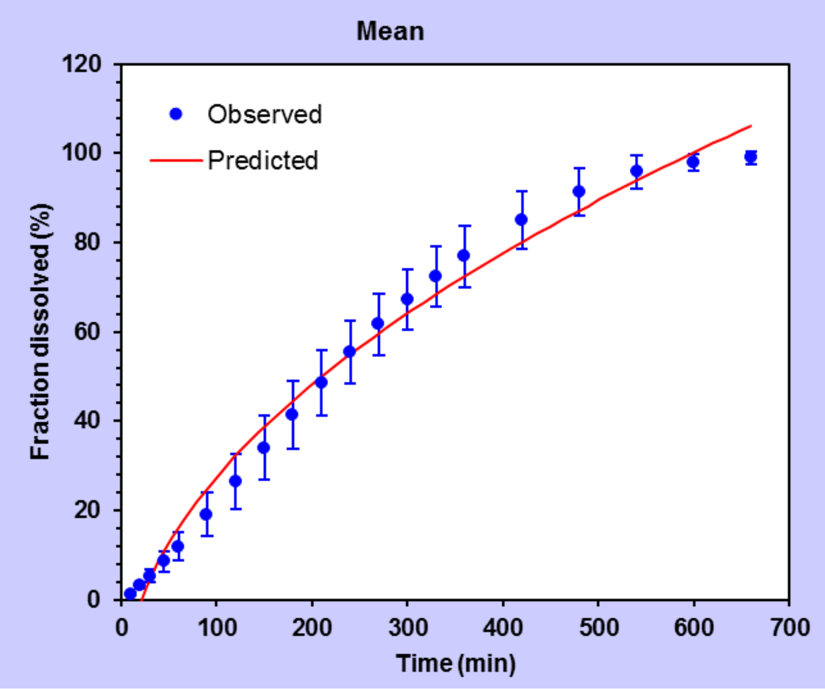

Supplementary materials – Model fitting summary – Kollidon® 90 F (modelling the entire relevant dissolution data)

Graphical abstract of model fit presented as the fraction % of released carvedilol per tested tablet:

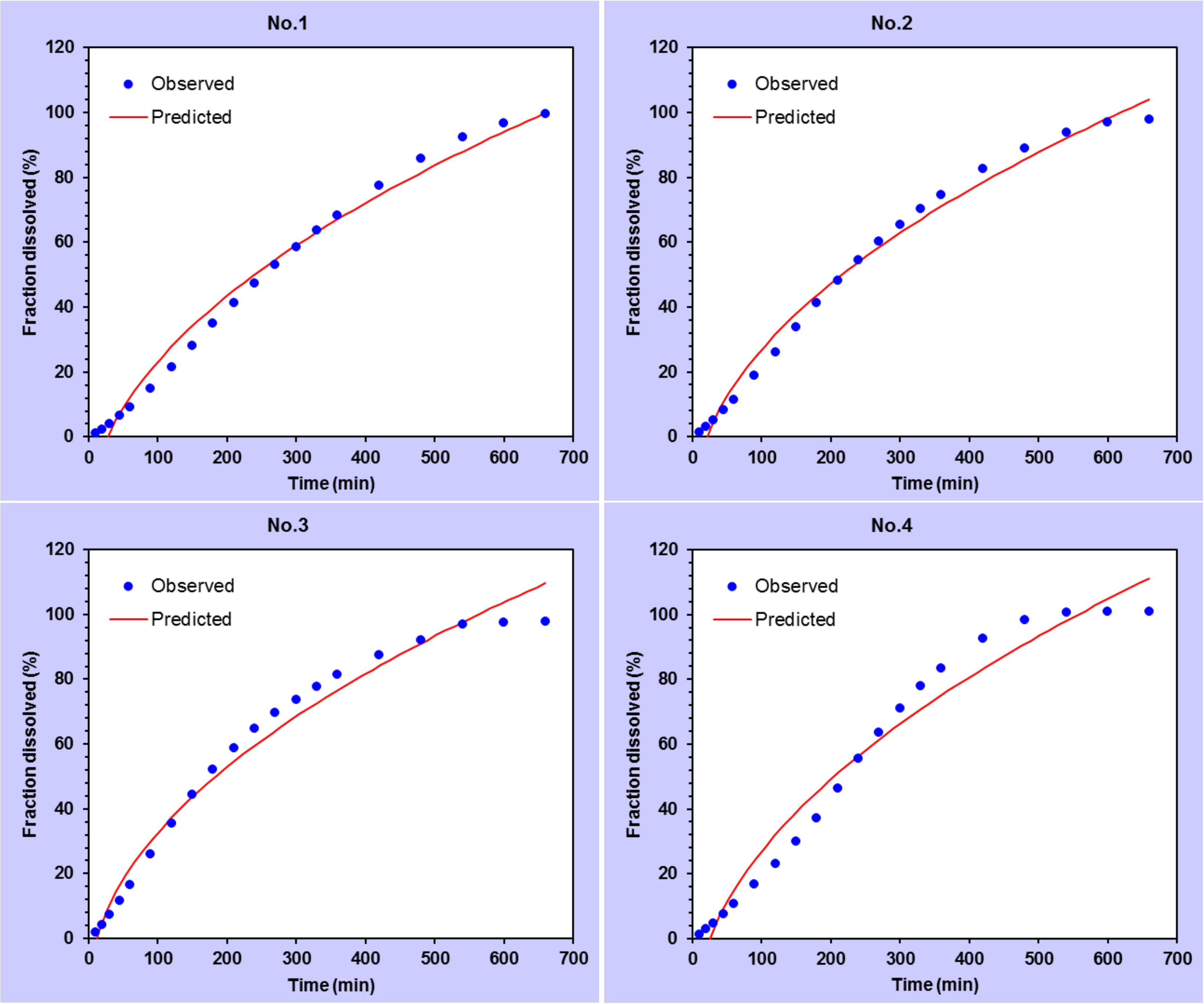

Supplementary materials – Model fitting summary – Kollidon® 90 F (modelling the entire relevant dissolution data)

Model: **Korsmeyer–Peppas**

Model equation: $F = k_{KP} \cdot t^n$

Fitted model parameters per tested tablet (N = 4) with statistics – mean, standard deviation (SD), and relative standard deviation expressed in % (RSD%) (output from DDSolver):

Parameter	No.1	No.2	No.3	No.4	Mean	SD	RSD(%)
k_{KP}	0.090	0.187	0.392	0.092	0.190	0.142	74.642
n	1.122	0.974	0.895	1.157	1.037	0.123	11.882

Number of dissolution data points (N), degrees of freedom (df), and selected goodness of fit criteria – Pearson correlation coefficient (R), coefficient of determination (R^2), adjusted coefficient of determination ($R^2_{adjusted}$), and residual sum of squares (RSS) (manual calculation in MS Excel):

Parameter	No.1	No.2	No.3	No.4
N	20	20	20	20
df	18	18	18	18
R	0.981825575	0.976191439	0.959732912	0.953812624
R^2	0.96398146	0.952949725	0.921087263	0.909758522
$R^2_{adjusted}$	0.96198043	0.950335821	0.916703222	0.904745107
RSS	1763.82919	2338.727034	2689.647686	8319.116376

Graphical abstract of model fit presented as mean ± 1 SD of the fraction % of released carvedilol:

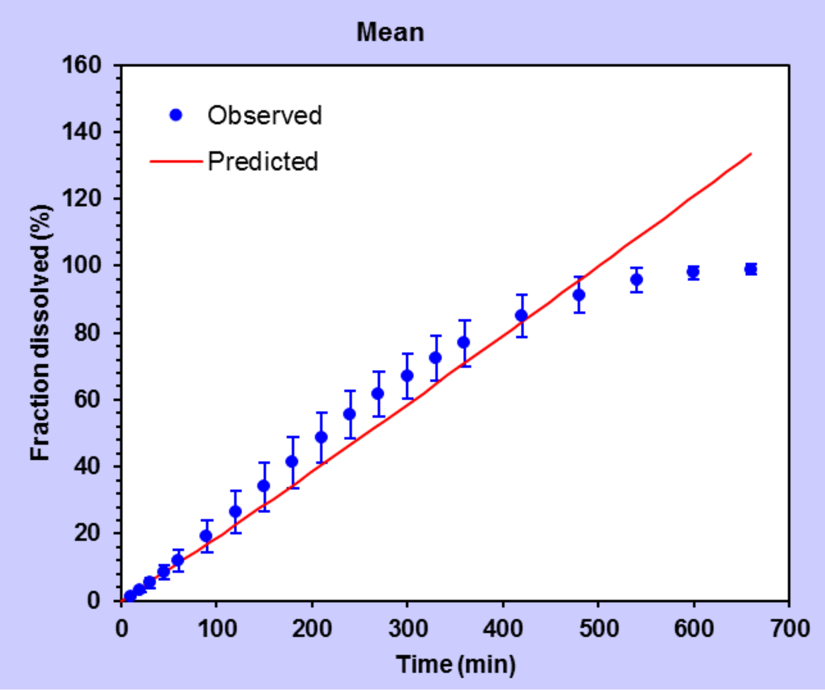

Supplementary materials – Model fitting summary – Kollidon® 90 F (modelling the entire relevant dissolution data)

Graphical abstract of model fit presented as the fraction % of released carvedilol per tested tablet:

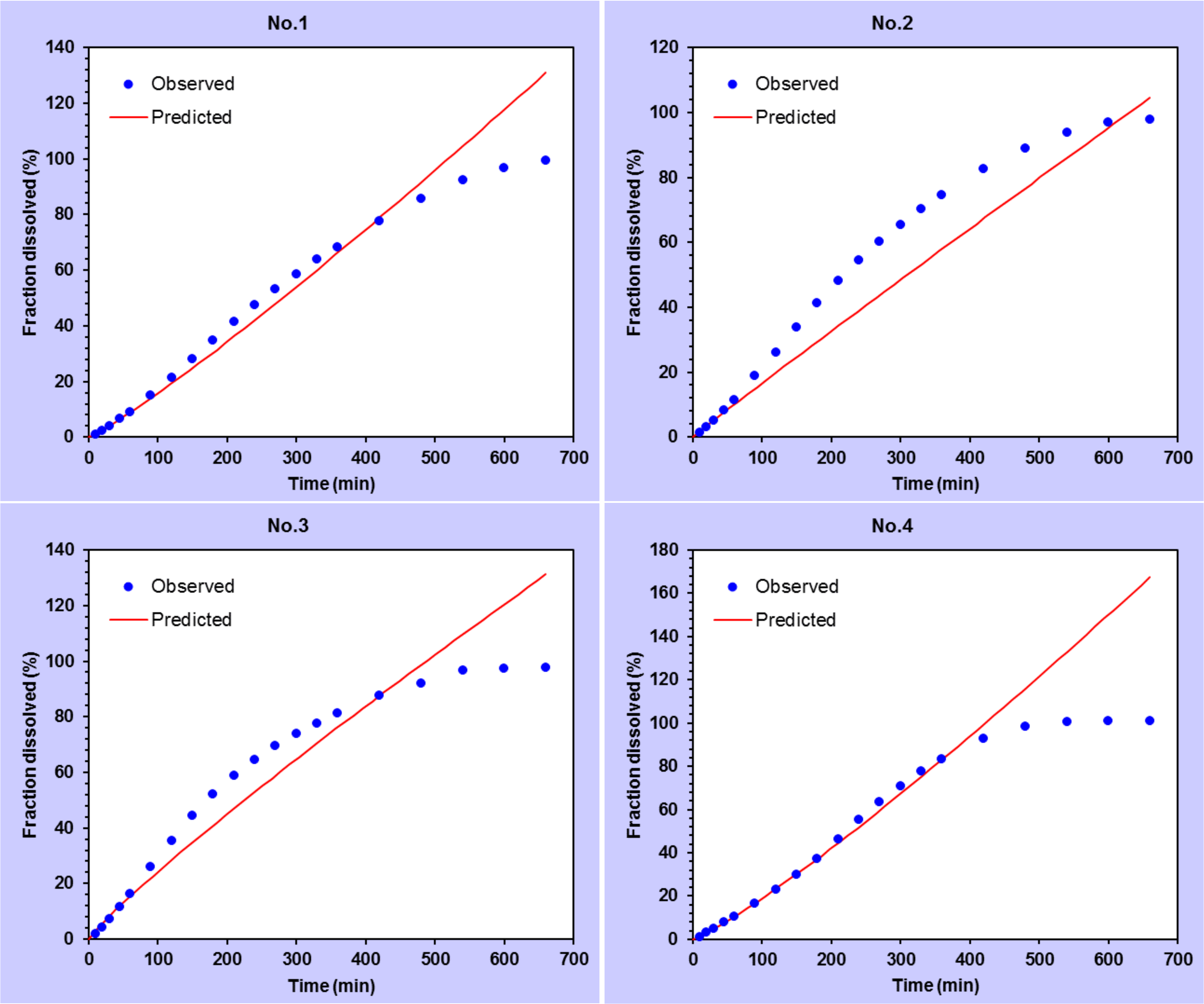

Supplementary materials – Model fitting summary – Kollidon® 90 F (modelling the entire relevant dissolution data)

Model: **Korsmeyer–Peppas with T_{lag}**

Model equation: $F = k_{KP} \cdot \left(t - T_{lag}\right)^n$

Fitted model parameters per tested tablet (N = 4) with statistics – mean, standard deviation (SD), and relative standard deviation expressed in % (RSD%) (output from DDSolver):

Parameter	No.1	No.2	No.3	No.4	Mean	SD	RSD(%)
k_{KP}	0.163	0.239	0.437	0.189	0.257	0.124	48.338
n	0.999	0.970	0.886	1.016	0.968	0.057	5.930
T_{lag}	4.866	6.000	6.000	6.000	5.716	0.567	9.922

Number of dissolution data points (N), degrees of freedom (df), and selected goodness of fit criteria – Pearson correlation coefficient (R), coefficient of determination (R^2), adjusted coefficient of determination ($R^2_{adjusted}$), and residual sum of squares (RSS) (manual calculation in MS Excel):

Parameter	No.1	No.2	No.3	No.4
N	20	20	20	20
df	17	17	17	17
R	0.988776892	0.976594805	0.961222834	0.966013035
R^2	0.977679743	0.953737414	0.923949337	0.933181184
$R^2_{adjusted}$	0.97505383	0.948294756	0.9150022	0.925320147
RSS	924.6232743	1811.203059	3344.308424	2452.102114

Graphical abstract of model fit presented as mean ± 1 SD of the fraction % of released carvedilol:

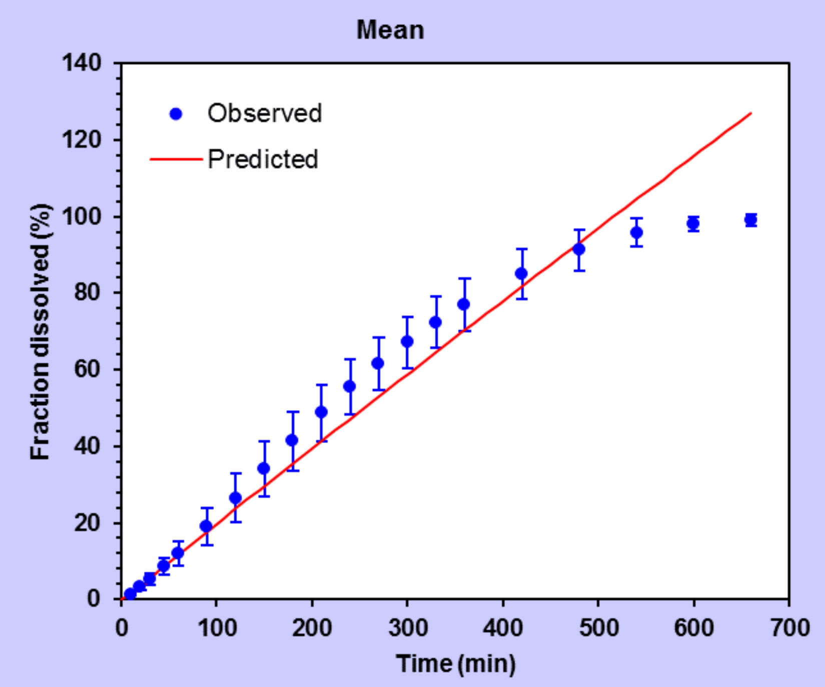

Supplementary materials – Model fitting summary – Kollidon® 90 F (modelling the entire relevant dissolution data)

Graphical abstract of model fit presented as the fraction % of released carvedilol per tested tablet:

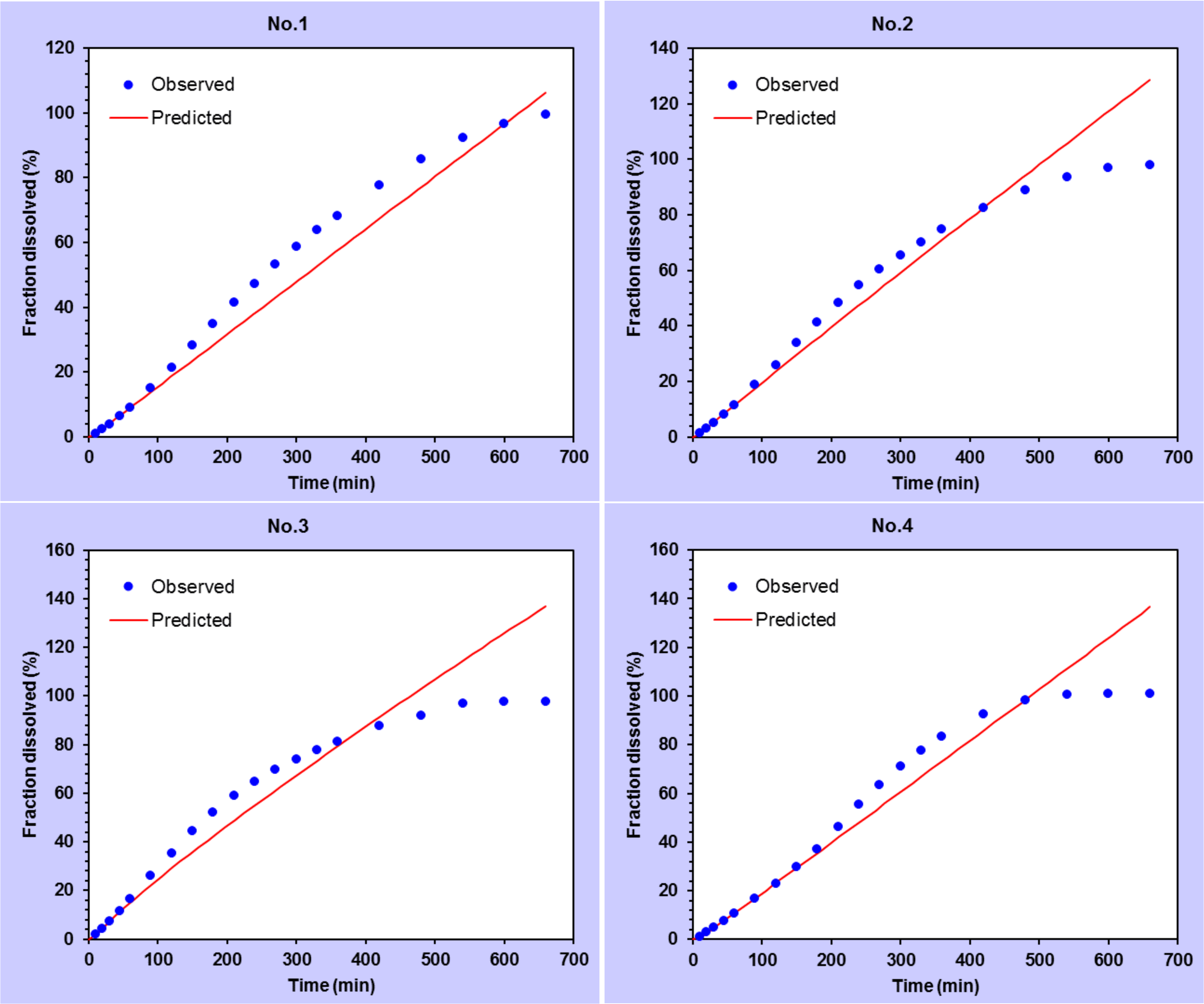

Supplementary materials – Model fitting summary – Kollidon® 90 F (modelling the entire relevant dissolution data)

Model: **Korsmeyer–Peppas with F_0**

Model equation: $F = F_0 + k_{KP} \cdot t^n$

Fitted model parameters per tested tablet (N = 4) with statistics – mean, standard deviation (SD), and relative standard deviation expressed in % (RSD%) (output from DDSolver):

Parameter	No.1	No.2	No.3	No.4	Mean	SD	RSD(%)
k_{KP}	0.058	0.127	0.190	0.075	0.113	0.059	52.471
n	1.197	1.076	1.030	1.173	1.119	0.079	7.103
F_0	0.400	0.585	0.720	0.480	0.546	0.138	25.338

Number of dissolution data points (N), degrees of freedom (df), and selected goodness of fit criteria – Pearson correlation coefficient (R), coefficient of determination (R^2), adjusted coefficient of determination ($R^2_{adjusted}$), and residual sum of squares (RSS) (manual calculation in MS Excel):

Parameter	No.1	No.2	No.3	No.4
N	20	20	20	20
df	17	17	17	17
R	0.976766214	0.96780415	0.944288833	0.952269809
R^2	0.954072237	0.936644873	0.891681401	0.90681779
$R^2_{adjusted}$	0.948668971	0.929191328	0.878938036	0.895855177
RSS	2845.594745	3049.156009	6470.312447	5122.561888

Graphical abstract of model fit presented as mean ± 1 SD of the fraction % of released carvedilol:

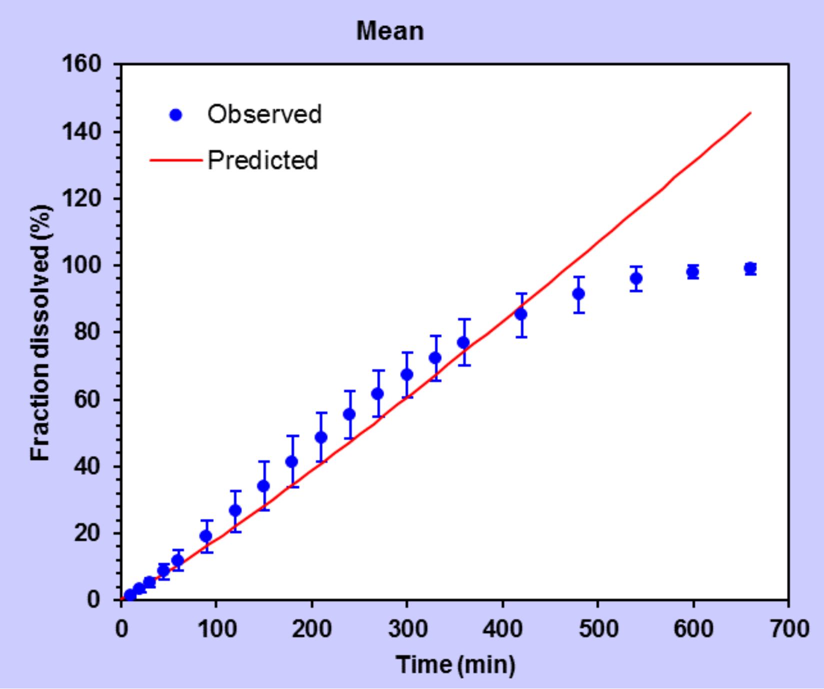

Supplementary materials – Model fitting summary – Kollidon® 90 F (modelling the entire relevant dissolution data)

Graphical abstract of model fit presented as the fraction % of released carvedilol per tested tablet:

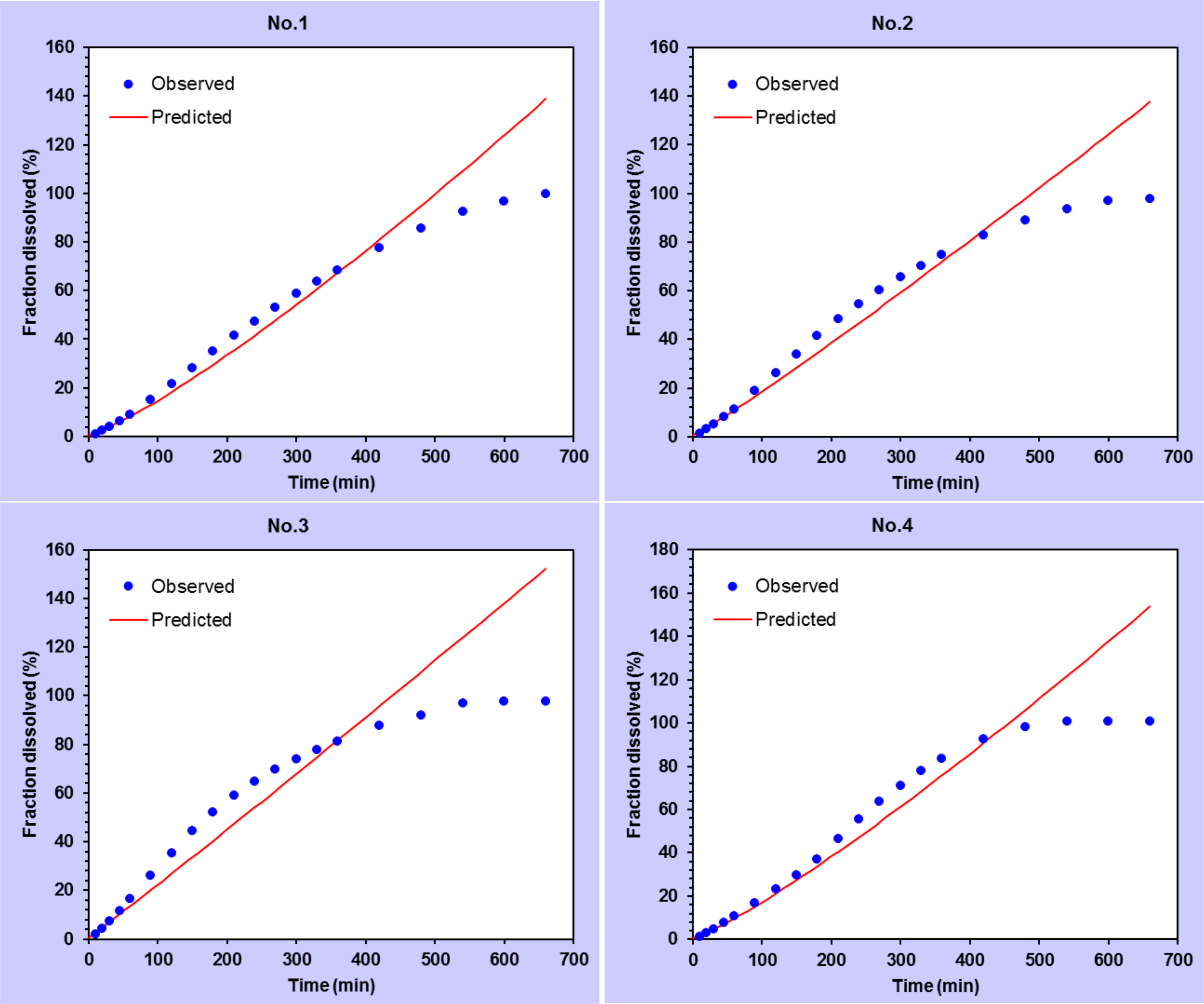

Supplementary materials – Model fitting summary – Kollidon® 90 F (modelling the entire relevant dissolution data)

Model: **Hixson–Crowell**

Model equation: $F = 100 \cdot [1 - (1 - k_{HC} \cdot t)^3]$

Fitted model parameters per tested tablet (N = 4) with statistics – mean, standard deviation (SD), and relative standard deviation expressed in % (RSD%) (output from DDSolver):

Parameter	No.1	No.2	No.3	No.4	Mean	SD	RSD(%)
k_{HC}	0.001	0.001	0.001	0.001	0.001	0.000	17.497

Number of dissolution data points (N), degrees of freedom (df), and selected goodness of fit criteria – Pearson correlation coefficient (R), coefficient of determination (R^2), adjusted coefficient of determination ($R^2_{adjusted}$), and residual sum of squares (RSS) (manual calculation in MS Excel):

Parameter	No.1	No.2	No.3	No.4
N	20	20	20	20
df	19	19	19	19
R	0.998191549	0.999047373	0.999386649	0.993076303
R^2	0.996386368	0.998095654	0.998773673	0.986200545
$R^2_{adjusted}$	0.996386368	0.998095654	0.998773673	0.986200545
RSS	773.5686104	271.2043455	62.16166551	1411.396115

Graphical abstract of model fit presented as mean ± 1 SD of the fraction % of released carvedilol:

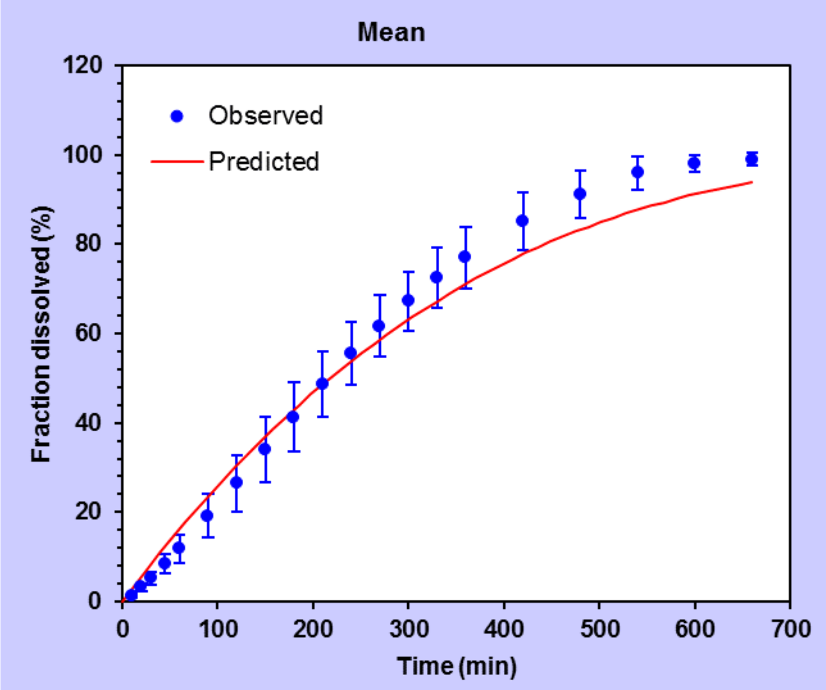

Supplementary materials – Model fitting summary – Kollidon® 90 F (modelling the entire relevant dissolution data)

Graphical abstract of model fit presented as the fraction % of released carvedilol per tested tablet:

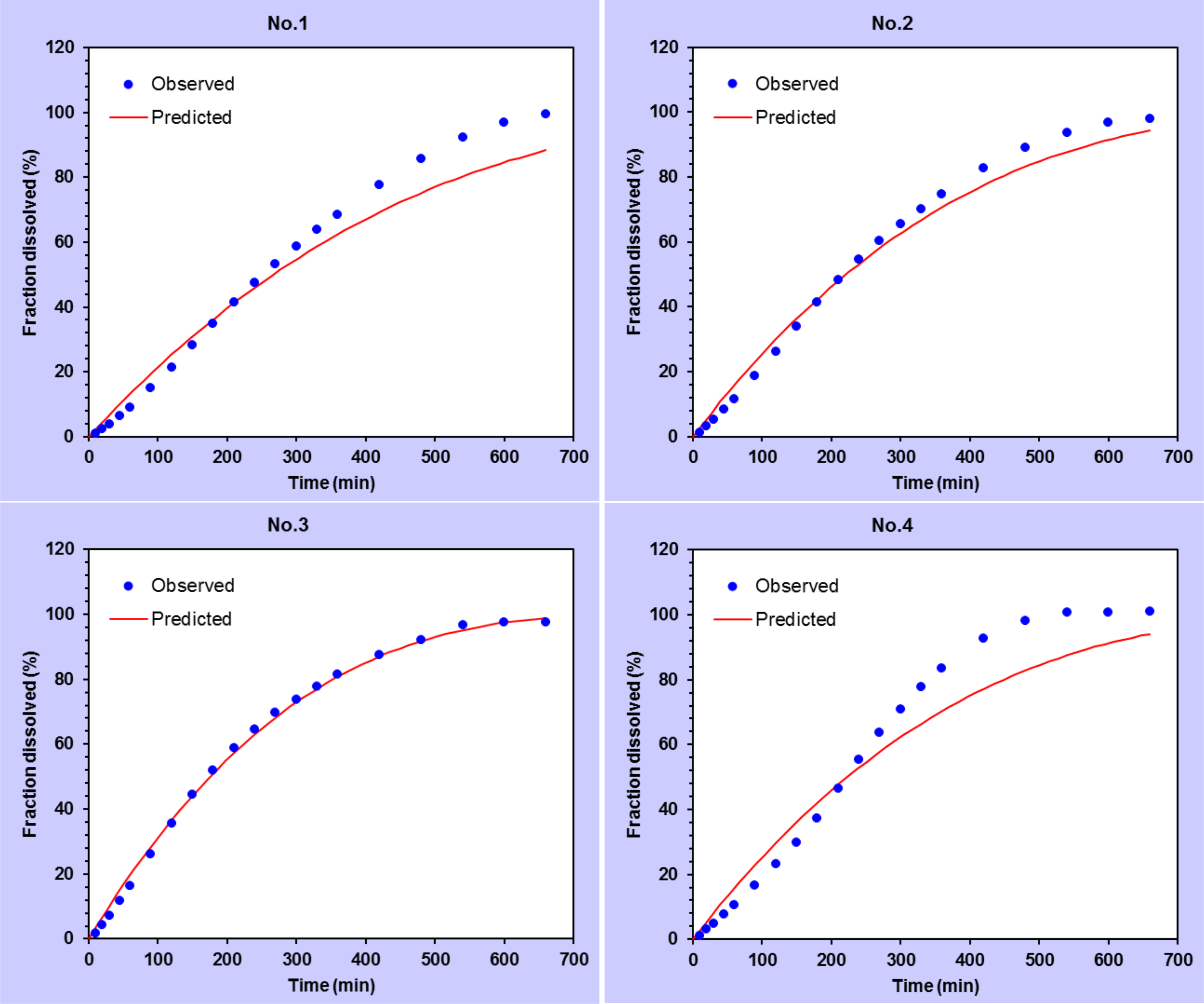

Supplementary materials – Model fitting summary – Kollidon® 90 F (modelling the entire relevant dissolution data)

Model: **Hixson–Crowell with T_{lag}**

Model equation: $F = 100 \cdot \left\{1 - \left[1 - k_{HC} \cdot \left(t - T_{lag}\right)\right]^3\right\}$

Fitted model parameters per tested tablet (N = 4) with statistics – mean, standard deviation (SD), and relative standard deviation expressed in % (RSD%) (output from DDSolver):

Parameter	No.1	No.2	No.3	No.4	Mean	SD	RSD(%)
k_{HC}	0.001	0.001	0.001	0.001	0.001	0.000	11.603
T_{lag}	49.538	27.696	2.944	45.079	31.314	21.131	67.480

Number of dissolution data points (N), degrees of freedom (df), and selected goodness of fit criteria – Pearson correlation coefficient (R), coefficient of determination (R^2), adjusted coefficient of determination ($R^2_{adjusted}$), and residual sum of squares (RSS) (manual calculation in MS Excel):

Parameter	No.1	No.2	No.3	No.4
N	20	20	20	20
df	18	18	18	18
R	0.989196769	0.997363807	0.999398954	0.982614107
R^2	0.978510247	0.994734563	0.998798269	0.965530484
$R^2_{adjusted}$	0.977316372	0.994442038	0.998731506	0.96361551
RSS	906.4516015	160.6963435	42.79836223	1198.274305

Graphical abstract of model fit presented as mean ± 1 SD of the fraction % of released carvedilol:

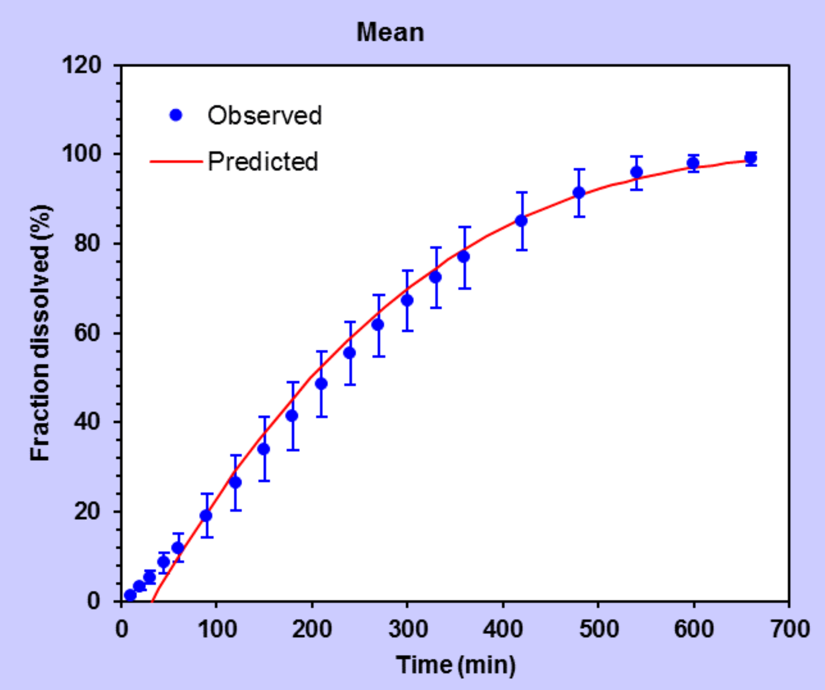

Supplementary materials – Model fitting summary – Kollidon® 90 F (modelling the entire relevant dissolution data)

Graphical abstract of model fit presented as the fraction % of released carvedilol per tested tablet:

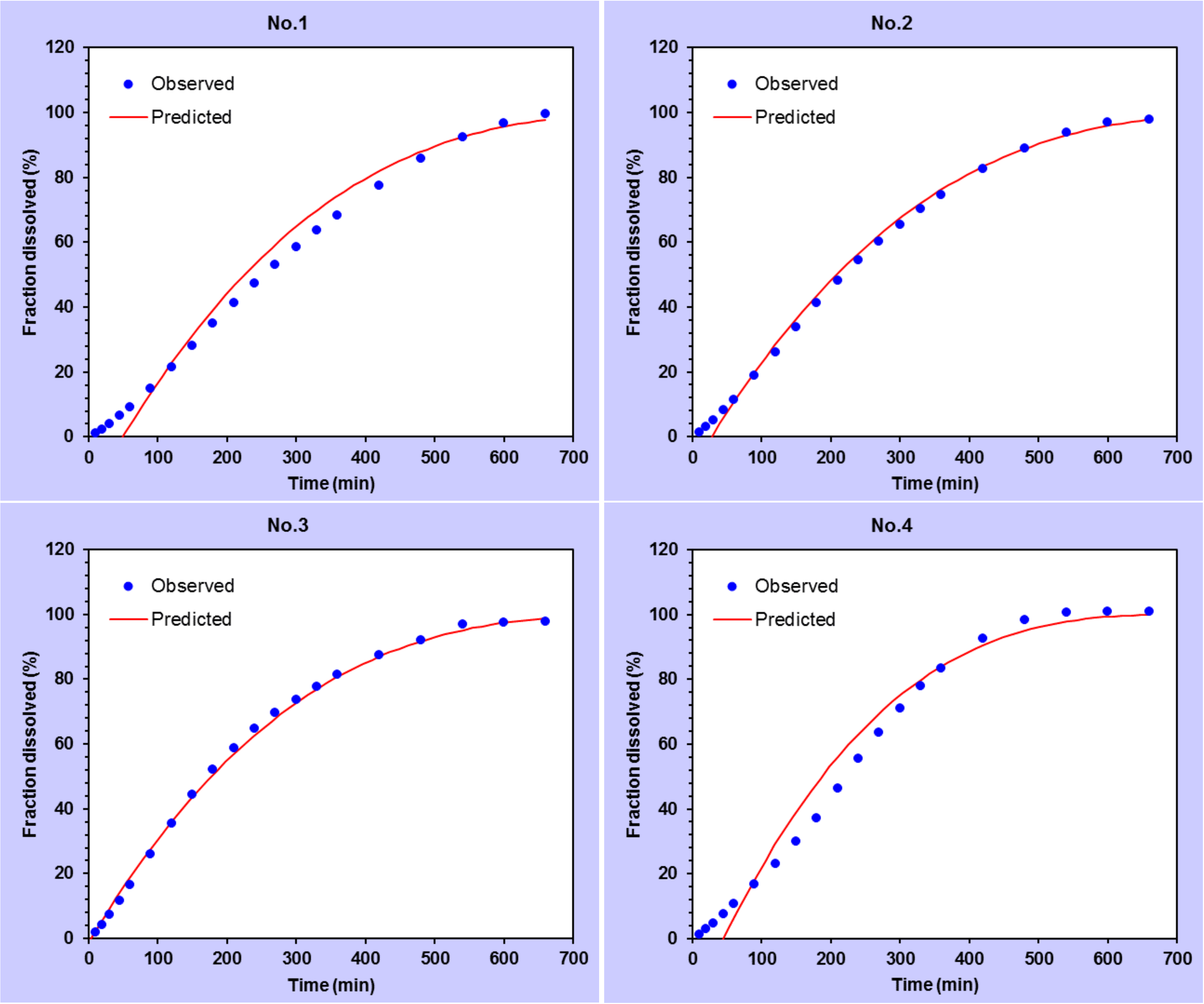

Supplementary materials – Model fitting summary – Kollidon® 90 F (modelling the entire relevant dissolution data)

Model: **Hopfenberg**

Model equation: $F = 100 \cdot [1 - (1 - k_{HB} \cdot t)^n]$

Fitted model parameters per tested tablet (N = 4) with statistics – mean, standard deviation (SD), and relative standard deviation expressed in % (RSD%) (output from DDSolver):

Parameter	No.1	No.2	No.3	No.4	Mean	SD	RSD(%)
k_{HB}	0.002	0.001	0.001	0.002	0.002	0.000	28.931
n	1.313	2.000	3.000	1.000	1.828	0.886	48.460

Number of dissolution data points (N), degrees of freedom (df), and selected goodness of fit criteria – Pearson correlation coefficient (R), coefficient of determination (R^2), adjusted coefficient of determination ($R^2_{adjusted}$), and residual sum of squares (RSS) (manual calculation in MS Excel):

Parameter	No.1	No.2	No.3	No.4
N	20	20	20	20
df	18	18	18	18
R	0.998513623	0.99939439	0.999386649	0.99809606
R^2	0.997029454	0.998789146	0.998773673	0.996195745
$R^2_{adjusted}$	0.996864424	0.998721876	0.998705544	0.995984397
RSS	138.9711052	93.38076732	62.16166551	143.155868

Graphical abstract of model fit presented as mean ± 1 SD of the fraction % of released carvedilol:

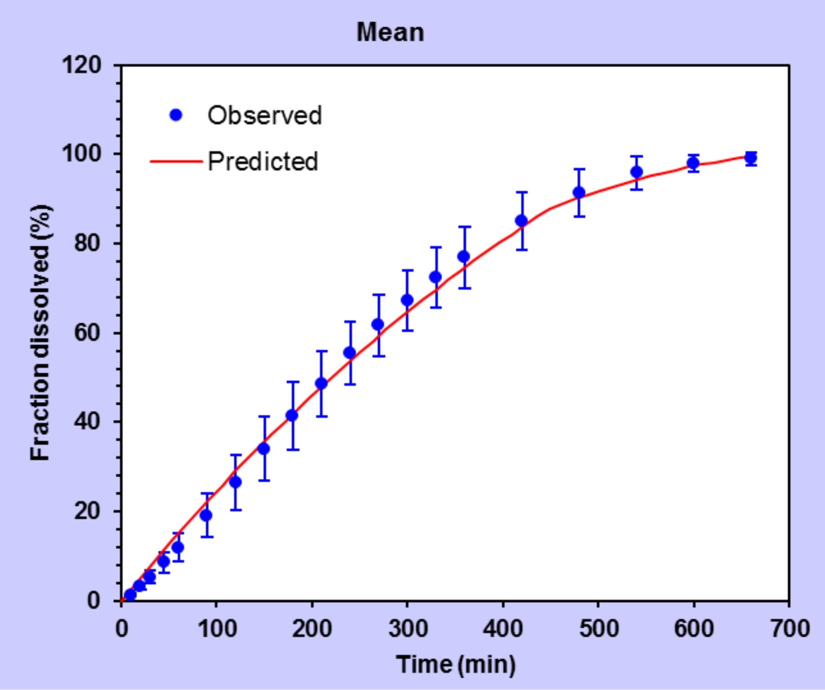

Supplementary materials – Model fitting summary – Kollidon® 90 F (modelling the entire relevant dissolution data)

Graphical abstract of model fit presented as the fraction % of released carvedilol per tested tablet:

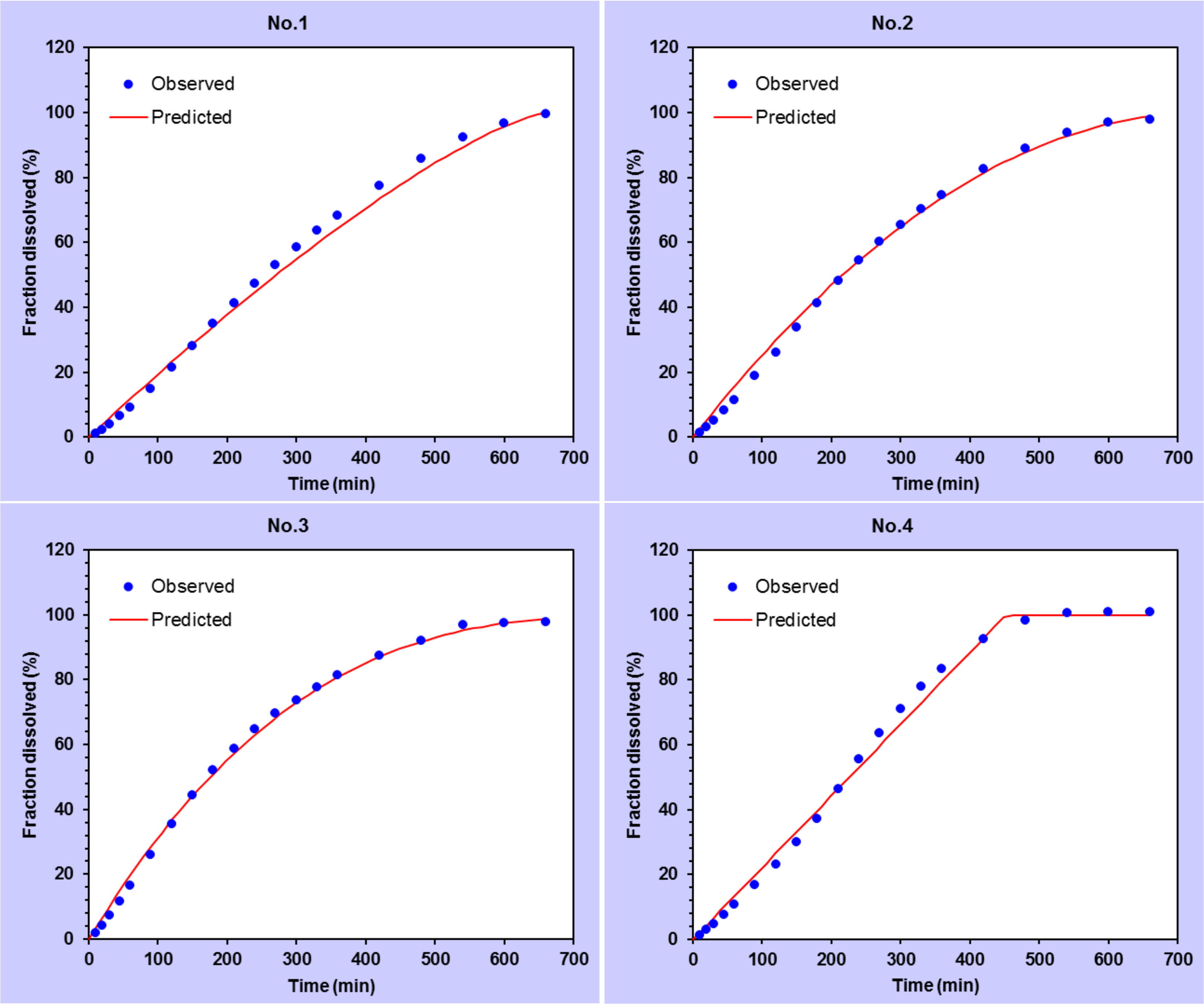

Supplementary materials – Model fitting summary – Kollidon® 90 F (modelling the entire relevant dissolution data)

Model: **Hopfenberg with T_{lag}**

Model equation: $F = 100 \cdot \{1 - [1 - k_{HB} \cdot (t - T_{lag})]^n\}$

Fitted model parameters per tested tablet (N = 4) with statistics – mean, standard deviation (SD), and relative standard deviation expressed in % (RSD%) (output from DDSolver):

Parameter	No.1	No.2	No.3	No.4	Mean	SD	RSD(%)
k_{HB}	0.001	0.001	0.001	0.002	0.002	0.000	30.917
n	2.000	2.000	3.000	1.000	2.000	0.816	40.825
T_{lag}	31.968	9.963	2.944	7.813	13.172	12.870	97.709

Number of dissolution data points (N), degrees of freedom (df), and selected goodness of fit criteria – Pearson correlation coefficient (R), coefficient of determination (R^2), adjusted coefficient of determination ($R^2_{adjusted}$), and residual sum of squares (RSS) (manual calculation in MS Excel):

Parameter	No.1	No.2	No.3	No.4
N	20	20	20	20
df	17	17	17	17
R	0.997120454	0.999444438	0.999398954	0.998307612
R^2	0.994249199	0.998889184	0.998798269	0.996618089
$R^2_{adjusted}$	0.993572634	0.9987585	0.998656889	0.996220217
RSS	175.3787216	29.14626817	42.79836223	106.4637843

Graphical abstract of model fit presented as mean ± 1 SD of the fraction % of released carvedilol:

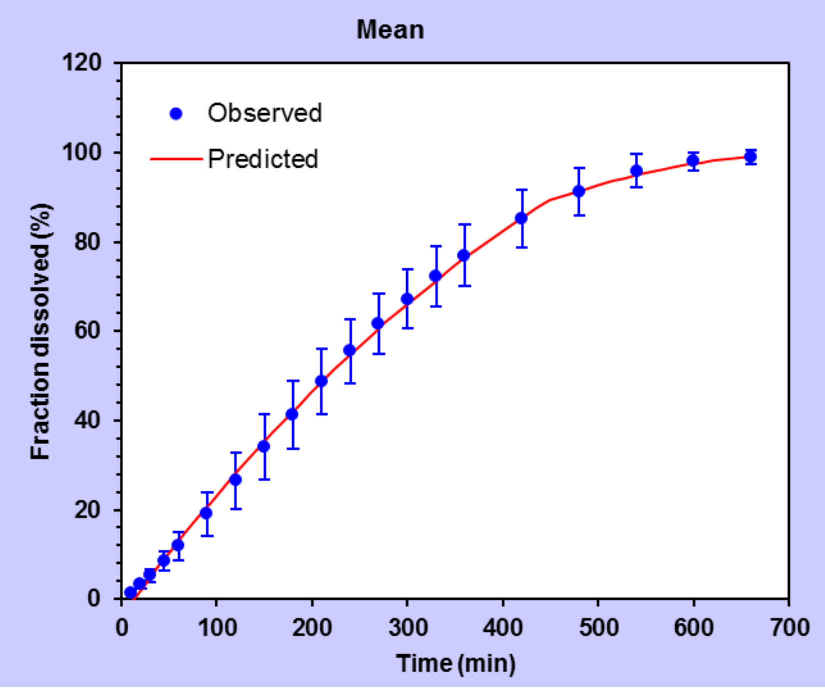

Supplementary materials – Model fitting summary – Kollidon® 90 F (modelling the entire relevant dissolution data)

Graphical abstract of model fit presented as the fraction % of released carvedilol per tested tablet:

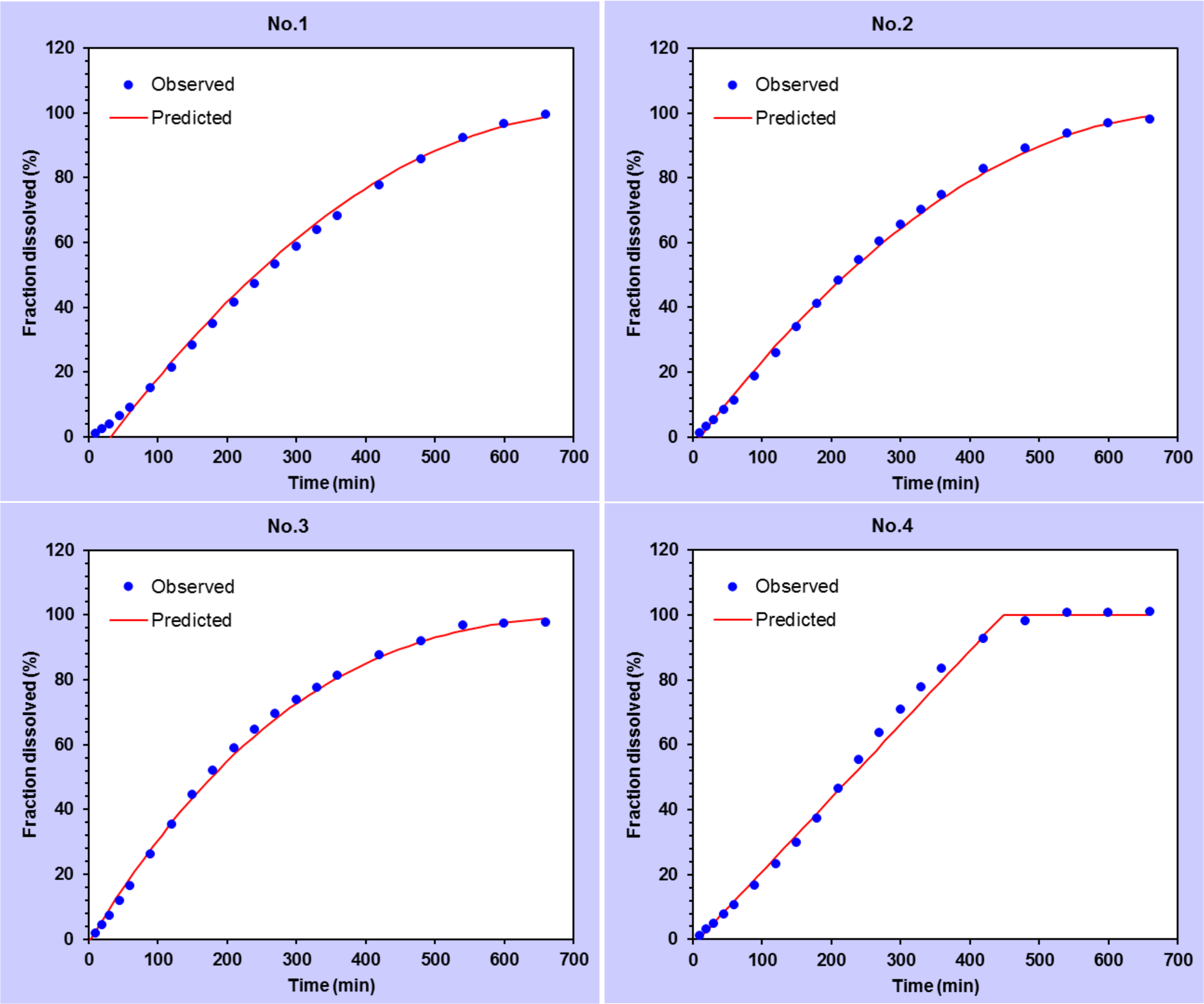

Supplementary materials – Model fitting summary – Kollidon® 90 F (modelling the entire relevant dissolution data)

Model: **Baker–Lonsdale**

Model equation: $\frac{3}{2}\cdot\left[1-\left(1-\frac{F}{100}\right)^{\frac{2}{3}}\right]-\frac{F}{100}=k_{BL}\cdot t$

Fitted model parameters per tested tablet (N = 4) with statistics – mean, standard deviation (SD), and relative standard deviation expressed in % (RSD%) (output from DDSolver):

Parameter	No.1	No.2	No.3	No.4	Mean	SD	RSD(%)
k_{BL}	0.0003	0.0003	0.0003	0.0000	0.0002	0.0002	66.7966

Number of dissolution data points (N), degrees of freedom (df), and selected goodness of fit criteria – Pearson correlation coefficient (R), coefficient of determination (R^2), adjusted coefficient of determination ($R^2_{adjusted}$), and residual sum of squares (RSS) (manual calculation in MS Excel):

Parameter	No.1	No.2	No.3	No.4
N	20	20	20	20
df	19	19	19	19
R	0.977365588	0.987278998	0.994098861	0.093193901
R^2	0.955243492	0.97471982	0.988232546	0.008685103
$R^2_{adjusted}$	0.955243492	0.97471982	0.988232546	0.008685103
RSS				

Graphical abstract of model fit presented as mean ± 1 SD of the fraction % of released carvedilol:

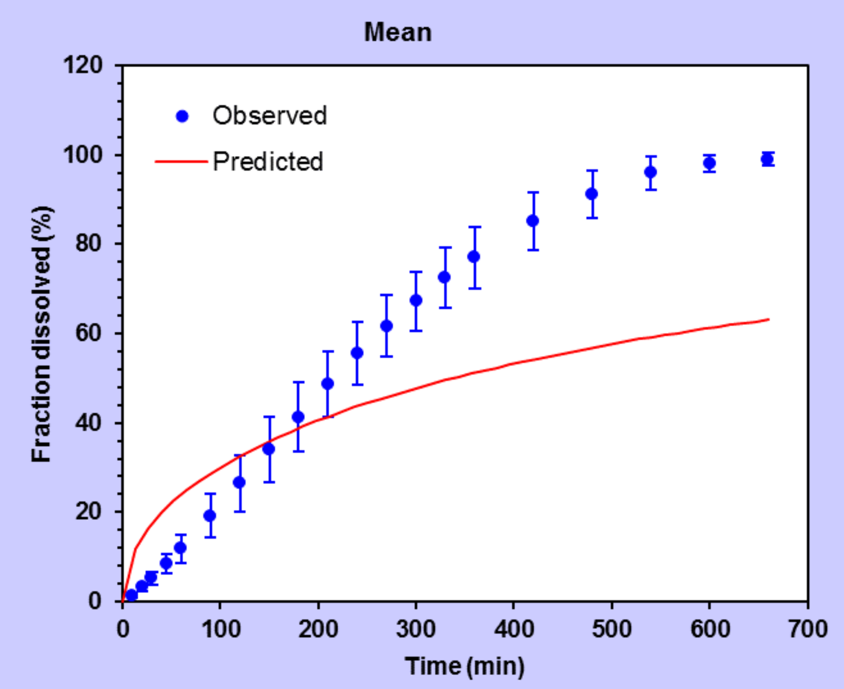

Supplementary materials – Model fitting summary – Kollidon® 90 F (modelling the entire relevant dissolution data)

Graphical abstract of model fit presented as the fraction % of released carvedilol per tested tablet:

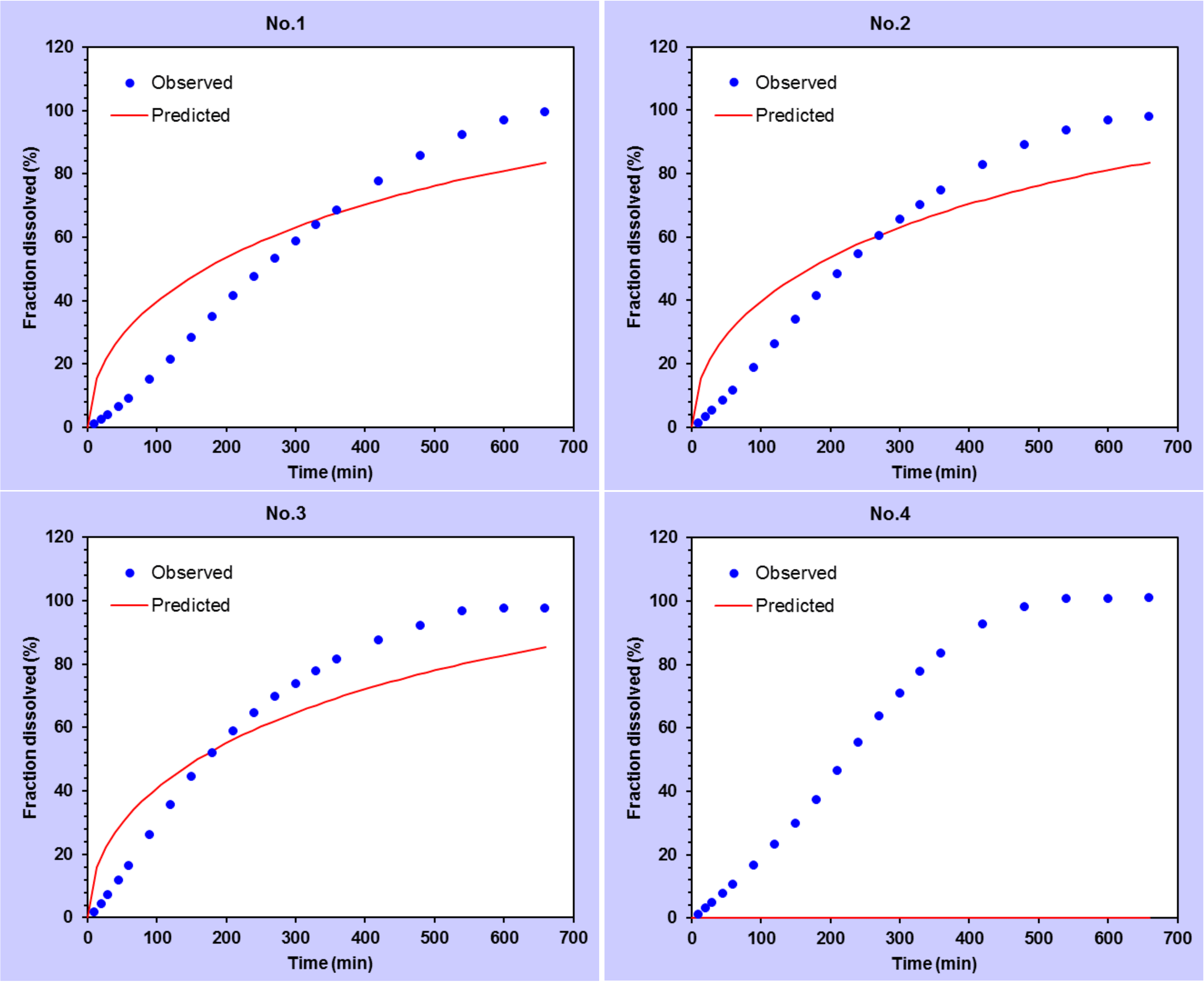

Supplementary materials – Model fitting summary – Kollidon® 90 F (modelling the entire relevant dissolution data)

Model: **Baker–Lonsdale with T_{lag}**

Model equation: $\frac{3}{2}\cdot\left[1-\left(1-\frac{F}{100}\right)^{\frac{2}{3}}\right]-\frac{F}{100}=k_{BL}\cdot(t-T_{lag})$

Fitted model parameters per tested tablet (N = 4) with statistics – mean, standard deviation (SD), and relative standard deviation expressed in % (RSD%) (output from DDSolver):

Parameter	No.1	No.2	No.3	No.4	Mean	SD	RSD(%)
k_{BL}	0.001	0.001	0.001	0.001	0.001	0.000	8.476
T_{lag}	145.923	81.299	60.398	118.635	101.564	38.142	37.554

Number of dissolution data points (N), degrees of freedom (df), and selected goodness of fit criteria – Pearson correlation coefficient (R), coefficient of determination (R^2), adjusted coefficient of determination ($R^2_{adjusted}$), and residual sum of squares (RSS) (manual calculation in MS Excel):

Parameter	No.1	No.2	No.3	No.4
N	20	20	20	20
df	18	18	18	18
R	0.981631942	0.982166068	0.9860754	0.982191309
R^2	0.96360127	0.964650184	0.972344695	0.964699767
$R^2_{adjusted}$	0.961579119	0.962686306	0.970808289	0.962738643
RSS	1288.606159	1028.515602	807.5346995	1189.535922

Graphical abstract of model fit presented as mean ± 1 SD of the fraction % of released carvedilol:

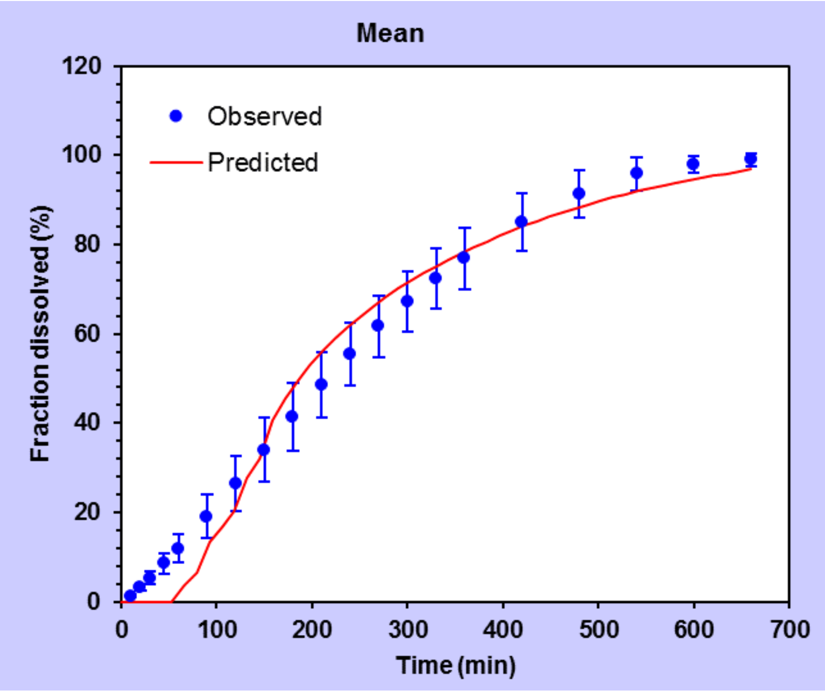

Supplementary materials – Model fitting summary – Kollidon® 90 F (modelling the entire relevant dissolution data)

Graphical abstract of model fit presented as the fraction % of released carvedilol per tested tablet:

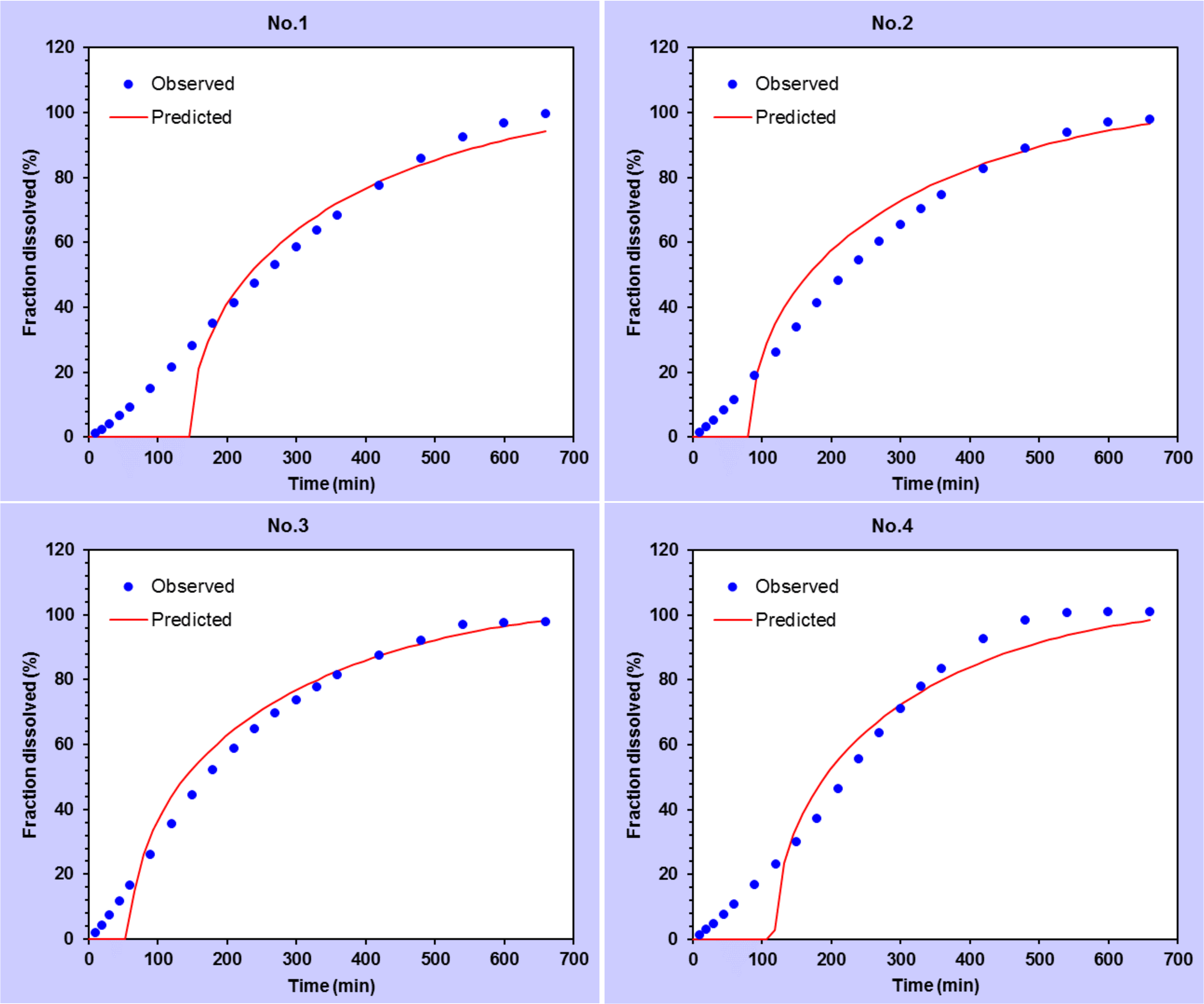

Supplementary materials – Model fitting summary – Kollidon® 90 F (modelling the entire relevant dissolution data)

Model: **Makoid–Banakar**

Model equation: $F = k_{MB} \cdot t^n \cdot e^{-k \cdot t}$

Fitted model parameters per tested tablet (N = 4) with statistics – mean, standard deviation (SD), and relative standard deviation expressed in % (RSD%) (output from DDSolver):

Parameter	No.1	No.2	No.3	No.4	Mean	SD	RSD(%)
k_{MB}	0.048	0.067	0.098	0.059	0.068	0.022	31.805
n	1.311	1.290	1.280	1.301	1.296	0.013	1.042
k	0.001	0.002	0.002	0.001	0.002	0.000	25.493

Number of dissolution data points (N), degrees of freedom (df), and selected goodness of fit criteria – Pearson correlation coefficient (R), coefficient of determination (R^2), adjusted coefficient of determination ($R^2_{adjusted}$), and residual sum of squares (RSS) (manual calculation in MS Excel):

Parameter	No.1	No.2	No.3	No.4
N	20	20	20	20
df	17	17	17	17
R	0.999617895	0.999782059	0.999104786	0.99270872
R^2	0.999235935	0.999564166	0.998210374	0.985470604
$R^2_{adjusted}$	0.999146045	0.999512892	0.99799983	0.983761263
RSS	17.24214809	9.719633754	40.50844747	391.8981825

Graphical abstract of model fit presented as mean ± 1 SD of the fraction % of released carvedilol:

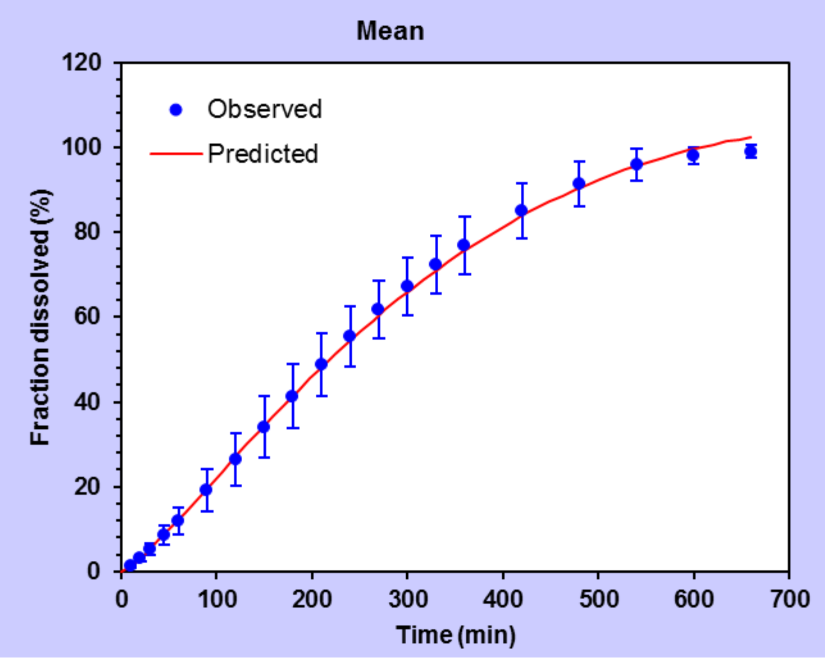

Supplementary materials – Model fitting summary – Kollidon® 90 F (modelling the entire relevant dissolution data)

Graphical abstract of model fit presented as the fraction % of released carvedilol per tested tablet:

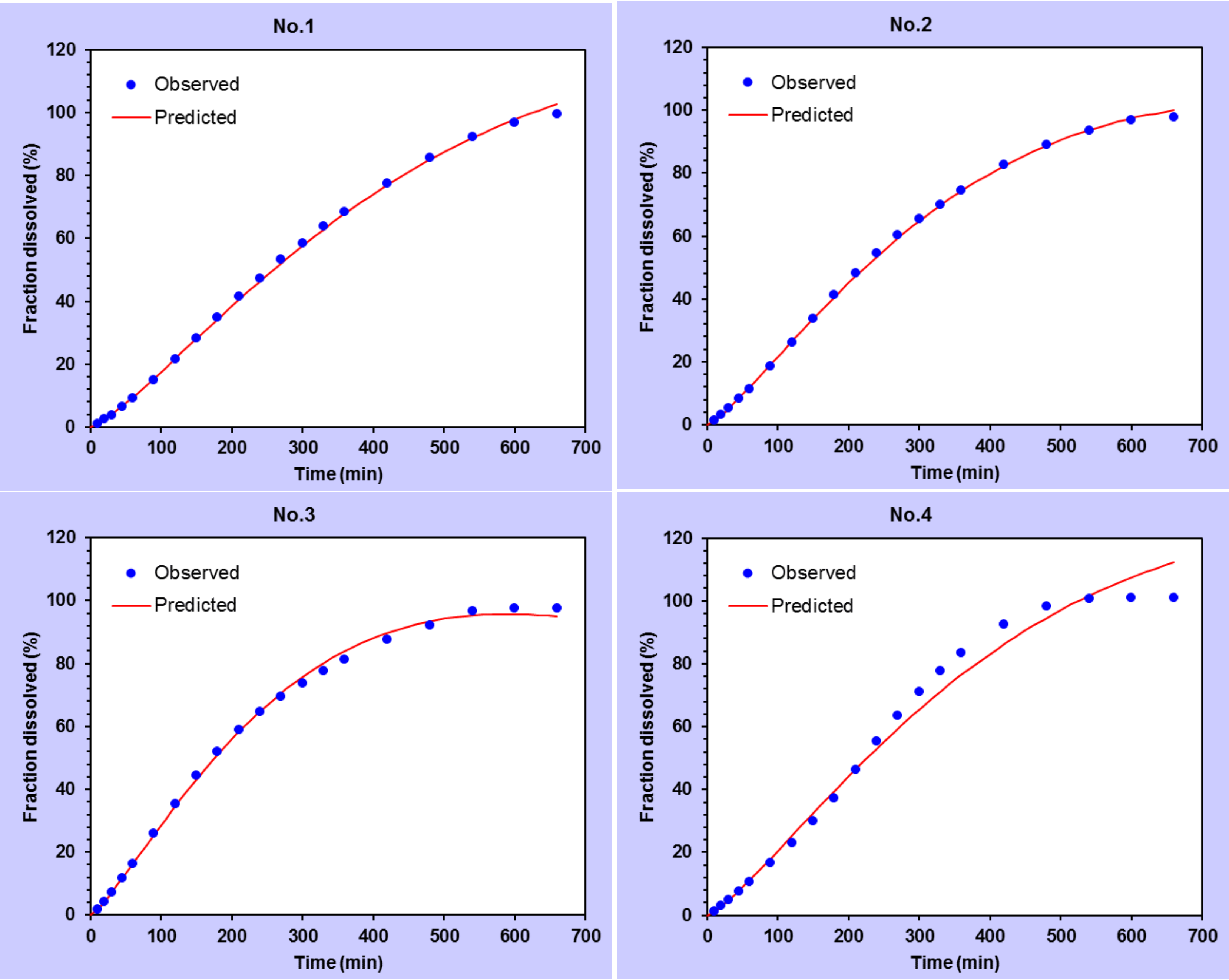

Supplementary materials – Model fitting summary – Kollidon® 90 F (modelling the entire relevant dissolution data)

Model: **Makoid–Banakar with T_{lag}**

Model equation: $F = k_{MB} \cdot (t - T_{lag})^n \cdot e^{-k \cdot (t - T_{lag})}$

Fitted model parameters per tested tablet (N = 4) with statistics – mean, standard deviation (SD), and relative standard deviation expressed in % (RSD%) (output from DDSolver):

Parameter	No.1	No.2	No.3	No.4	Mean	SD	RSD(%)
k_{MB}	0.113	0.156	0.224	0.139	0.158	0.048	30.173
n	1.123	1.106	1.099	1.113	1.110	0.010	0.931
k	0.001	0.001	0.002	0.001	0.001	0.000	42.937
T_{lag}	4.000	4.000	4.000	4.000	4.000	0.000	0.000

Number of dissolution data points (N), degrees of freedom (df), and selected goodness of fit criteria – Pearson correlation coefficient (R), coefficient of determination (R^2), adjusted coefficient of determination ($R^2_{adjusted}$), and residual sum of squares (RSS) (manual calculation in MS Excel):

Parameter	No.1	No.2	No.3	No.4
N	20	20	20	20
df	16	16	16	16
R	0.996997283	0.997403582	0.999432106	0.985687318
R^2	0.994003583	0.994813906	0.998864534	0.971579488
$R^2_{adjusted}$	0.992879255	0.993841513	0.998651634	0.966250642
RSS	143.2128035	119.019309	25.93393341	783.1155355

Graphical abstract of model fit presented as mean ± 1 SD of the fraction % of released carvedilol:

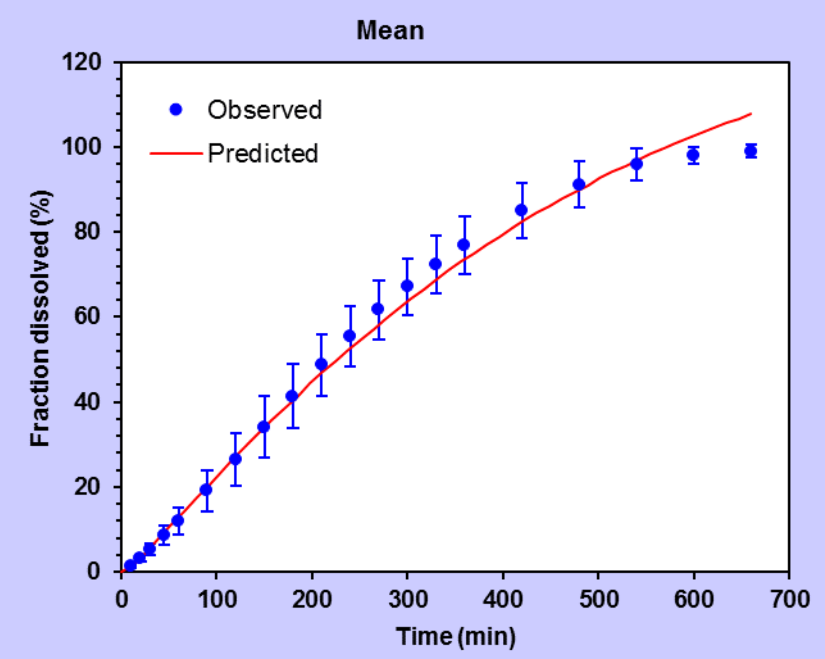

Supplementary materials – Model fitting summary – Kollidon® 90 F (modelling the entire relevant dissolution data)

Graphical abstract of model fit presented as the fraction % of released carvedilol per tested tablet:

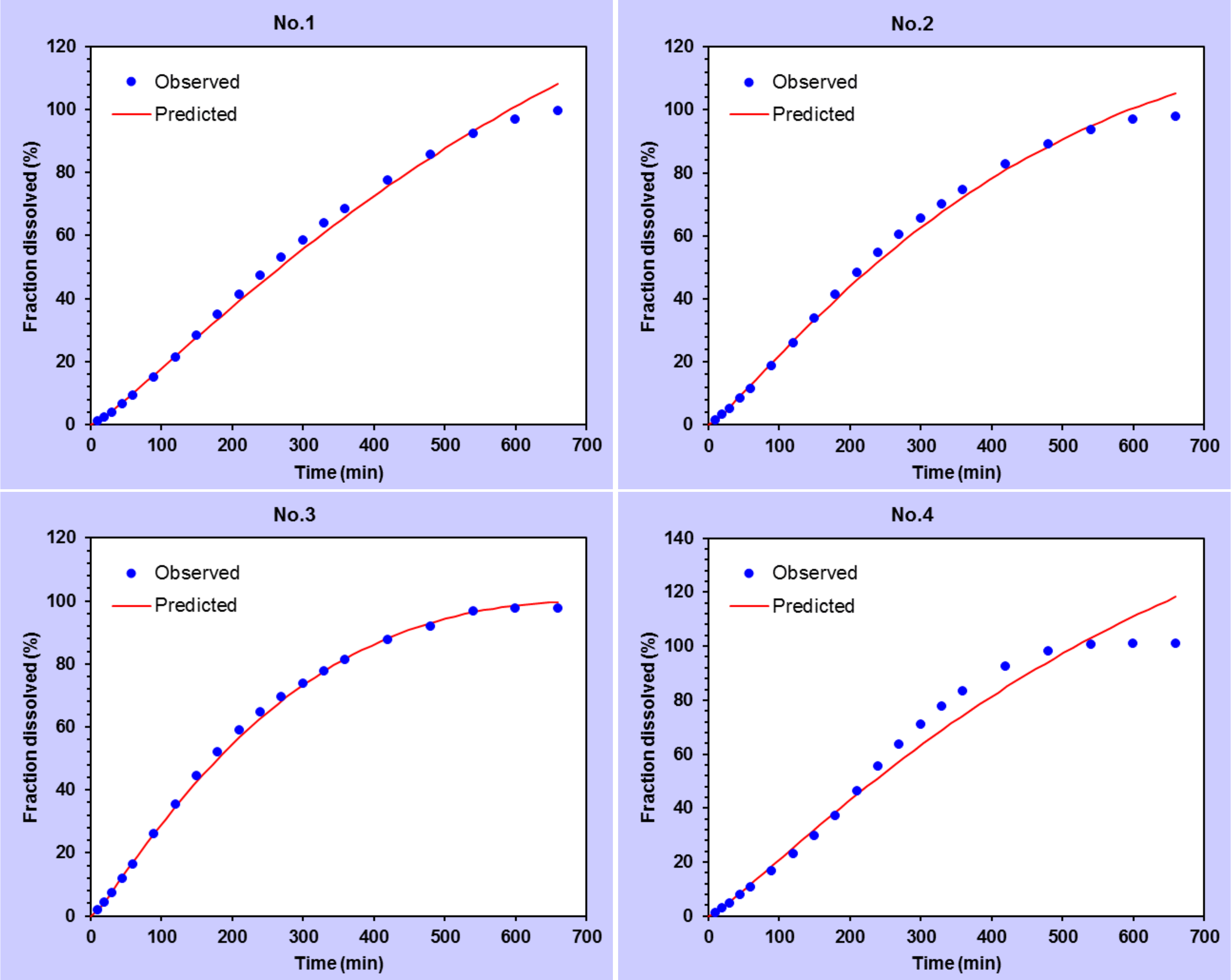

Supplementary materials – Model fitting summary – Kollidon® 90 F (modelling the entire relevant dissolution data)

Model: **Peppas–Sahlin_1**

Model equation: $F = k_1 \cdot t^m + k_2 \cdot t^{2m}$

Fitted model parameters per tested tablet (N = 4) with statistics – mean, standard deviation (SD), and relative standard deviation expressed in % (RSD%) (output from DDSolver):

Parameter	No.1	No.2	No.3	No.4	Mean	SD	RSD(%)
k_1	0.181	1.278	2.986	0.836	1.320	1.198	90.762
k_2	0.307	0.252	0.163	0.301	0.256	0.067	26.130
m	0.450	0.450	0.450	0.450	0.450	0.000	0.000

Number of dissolution data points (N), degrees of freedom (df), and selected goodness of fit criteria – Pearson correlation coefficient (R), coefficient of determination (R^2), adjusted coefficient of determination ($R^2_{adjusted}$), and residual sum of squares (RSS) (manual calculation in MS Excel):

Parameter	No.1	No.2	No.3	No.4
N	20	20	20	20
df	17	17	17	17
R	0.993359461	0.986635736	0.978937475	0.977061129
R^2	0.986763019	0.973450075	0.95831858	0.954648451
$R^2_{adjusted}$	0.985205727	0.970326554	0.953414883	0.949312974
RSS	302.5701389	629.0795423	1042.154526	1260.960763

Graphical abstract of model fit presented as mean ± 1 SD of the fraction % of released carvedilol:

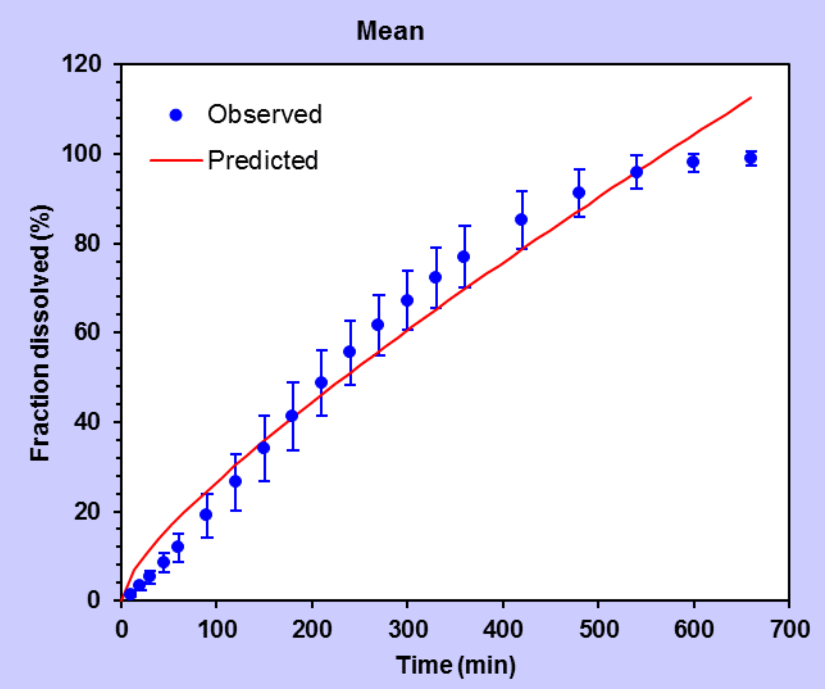

Supplementary materials – Model fitting summary – Kollidon® 90 F (modelling the entire relevant dissolution data)

Graphical abstract of model fit presented as the fraction % of released carvedilol per tested tablet:

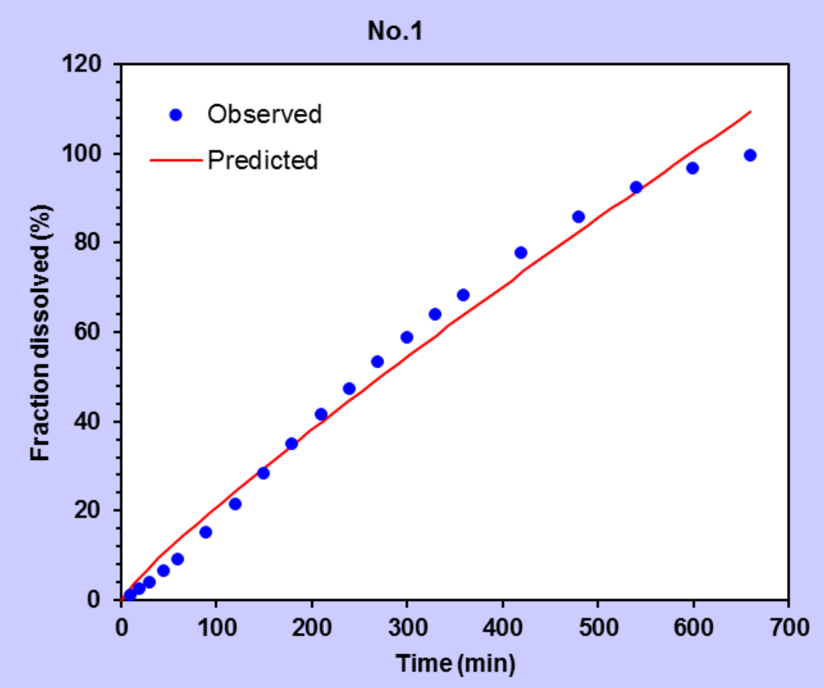

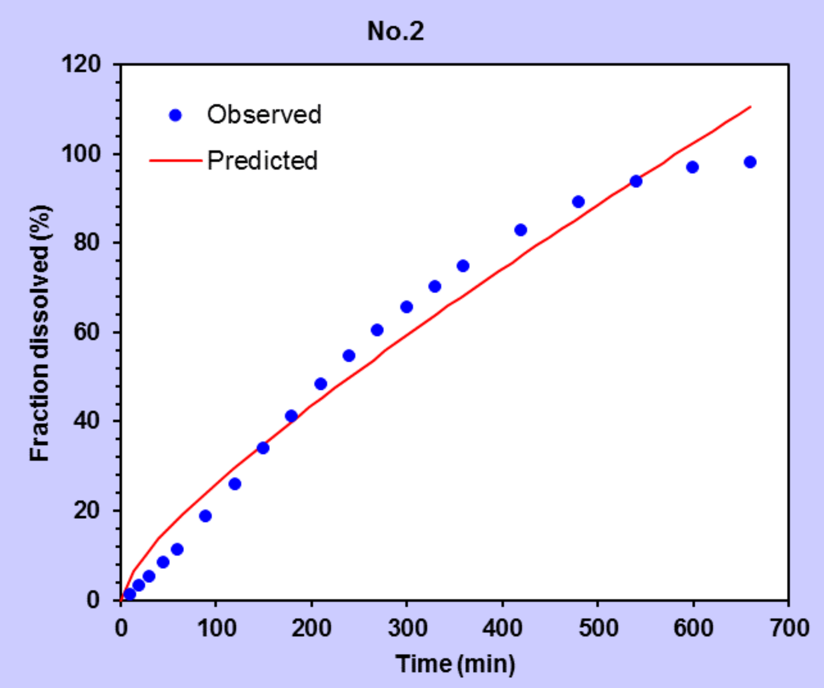

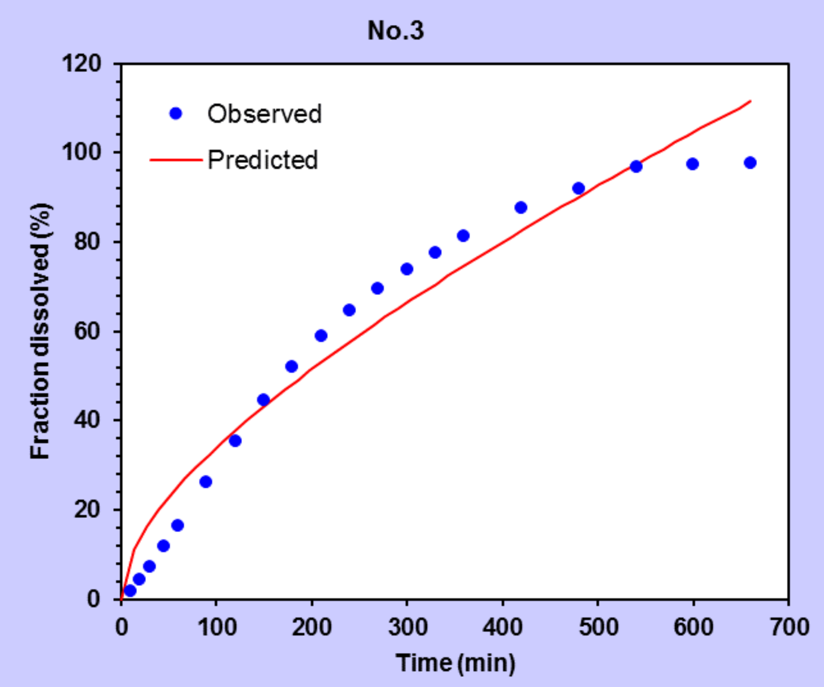

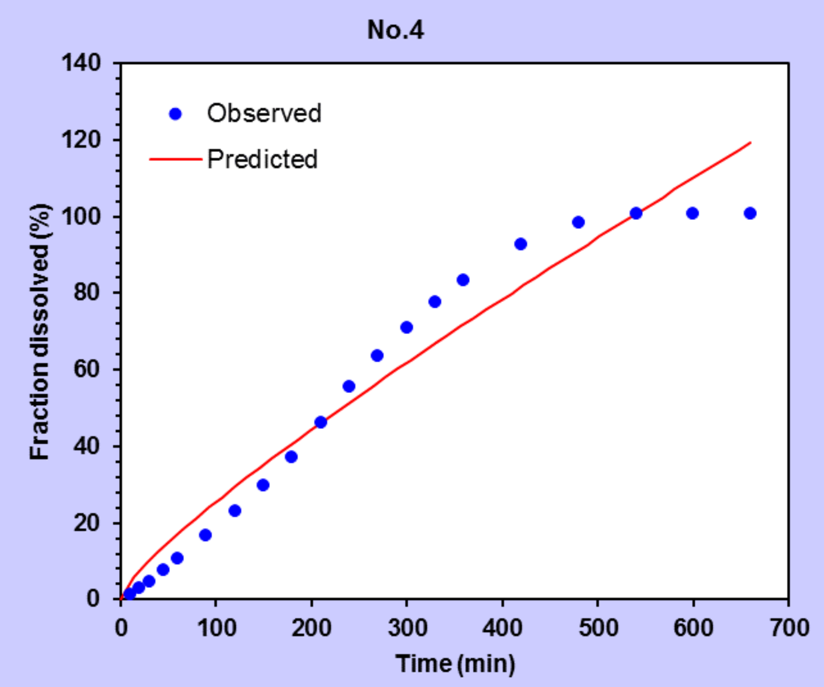

Supplementary materials – Model fitting summary – Kollidon® 90 F (modelling the entire relevant dissolution data)

Model: **Peppas–Sahlin_1 with T_{lag}**

Model equation: $F = k_1 \cdot (t - T_{lag})^m + k_2 \cdot (t - T_{lag})^{2m}$

Fitted model parameters per tested tablet (N = 4) with statistics – mean, standard deviation (SD), and relative standard deviation expressed in % (RSD%) (output from DDSolver):

Parameter	No.1	No.2	No.3	No.4	Mean	SD	RSD(%)
k_1	0.353	1.486	3.245	1.045	1.532	1.233	80.479
k_2	0.299	0.241	0.148	0.290	0.245	0.069	28.263
m	0.450	0.450	0.450	0.450	0.450	0.000	0.000
T_{lag}	6.000	6.000	6.000	6.000	6.000	0.000	0.000

Number of dissolution data points (N), degrees of freedom (df), and selected goodness of fit criteria – Pearson correlation coefficient (R), coefficient of determination (R^2), adjusted coefficient of determination ($R^2_{adjusted}$), and residual sum of squares (RSS) (manual calculation in MS Excel):

Parameter	No.1	No.2	No.3	No.4
N	20	20	20	20
df	16	16	16	16
R	0.993822541	0.987775921	0.981575577	0.977967835
R^2	0.987683243	0.97570127	0.963490614	0.956421086
$R^2_{adjusted}$	0.985373851	0.971145258	0.956645104	0.948250039
RSS	276.1590196	563.7472473	892.3639169	1197.694437

Graphical abstract of model fit presented as mean ± 1 SD of the fraction % of released carvedilol:

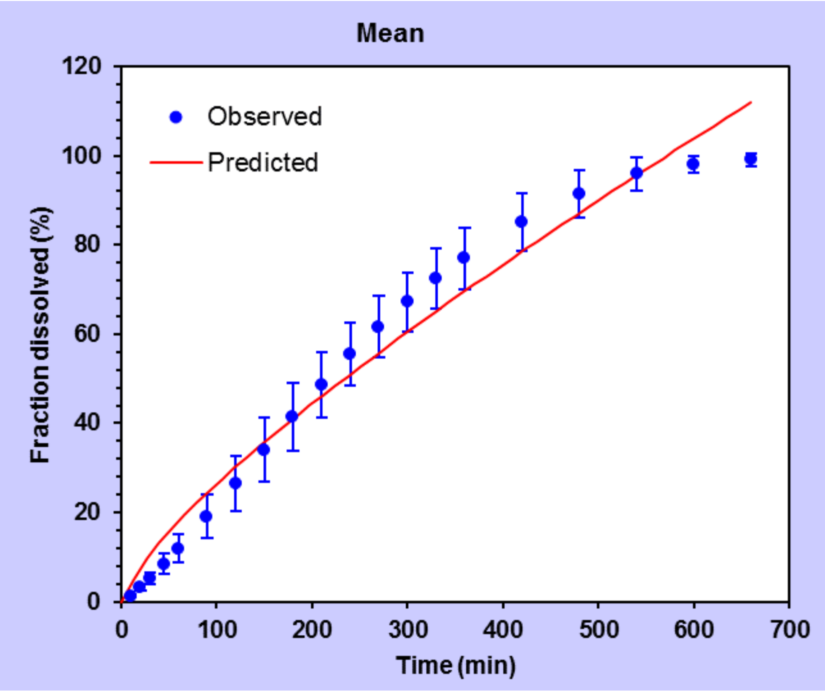

Supplementary materials – Model fitting summary – Kollidon® 90 F (modelling the entire relevant dissolution data)

Graphical abstract of model fit presented as the fraction % of released carvedilol per tested tablet:

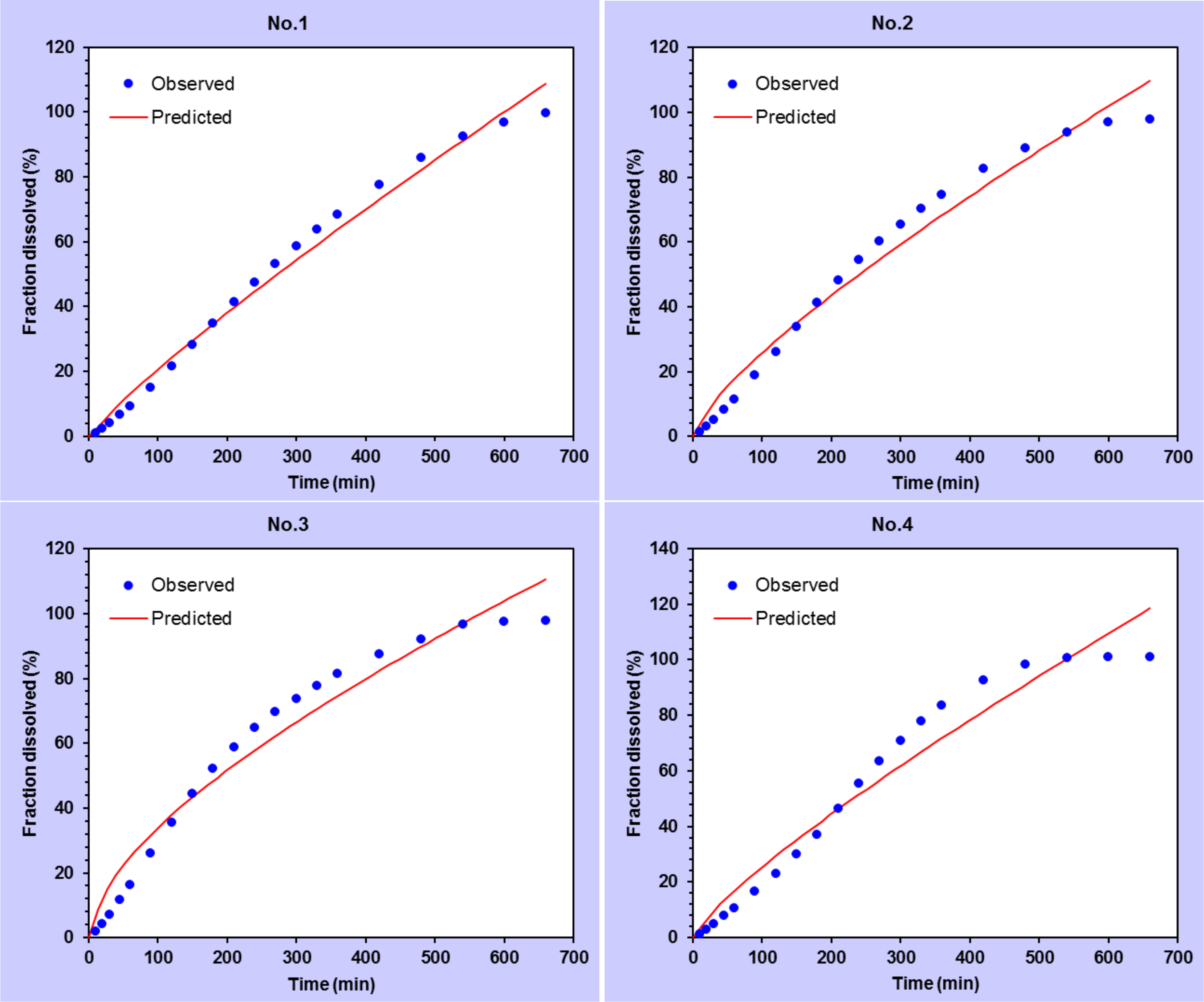

Supplementary materials – Model fitting summary – Kollidon® 90 F (modelling the entire relevant dissolution data)

Model: **Peppas–Sahlin_2**

Model equation: $F = k_1 \cdot t^{0.5} + k_2 \cdot t$

Fitted model parameters per tested tablet (N = 4) with statistics – mean, standard deviation (SD), and relative standard deviation expressed in % (RSD%) (output from DDSolver):

Parameter	No.1	No.2	No.3	No.4	Mean	SD	RSD(%)
k_1	0.774	1.625	2.929	1.360	1.672	0.911	54.461
k_2	0.136	0.104	0.054	0.127	0.105	0.037	35.214

Number of dissolution data points (N), degrees of freedom (df), and selected goodness of fit criteria – Pearson correlation coefficient (R), coefficient of determination (R^2), adjusted coefficient of determination ($R^2_{adjusted}$), and residual sum of squares (RSS) (manual calculation in MS Excel):

Parameter	No.1	No.2	No.3	No.4
N	20	20	20	20
df	18	18	18	18
R	0.992235241	0.986080507	0.980535415	0.975943562
R^2	0.984530774	0.972354765	0.9614497	0.952465837
$R^2_{adjusted}$	0.983671373	0.970818919	0.959308016	0.94982505
RSS	364.9161919	680.4183272	1021.770456	1354.166307

Graphical abstract of model fit presented as mean ± 1 SD of the fraction % of released carvedilol:

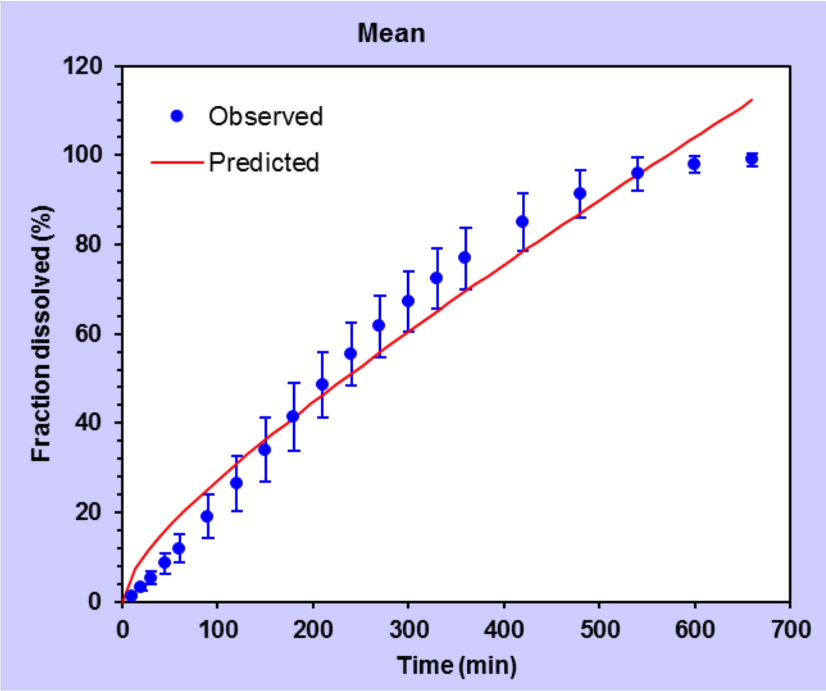

Supplementary materials – Model fitting summary – Kollidon® 90 F (modelling the entire relevant dissolution data)

Graphical abstract of model fit presented as the fraction % of released carvedilol per tested tablet:

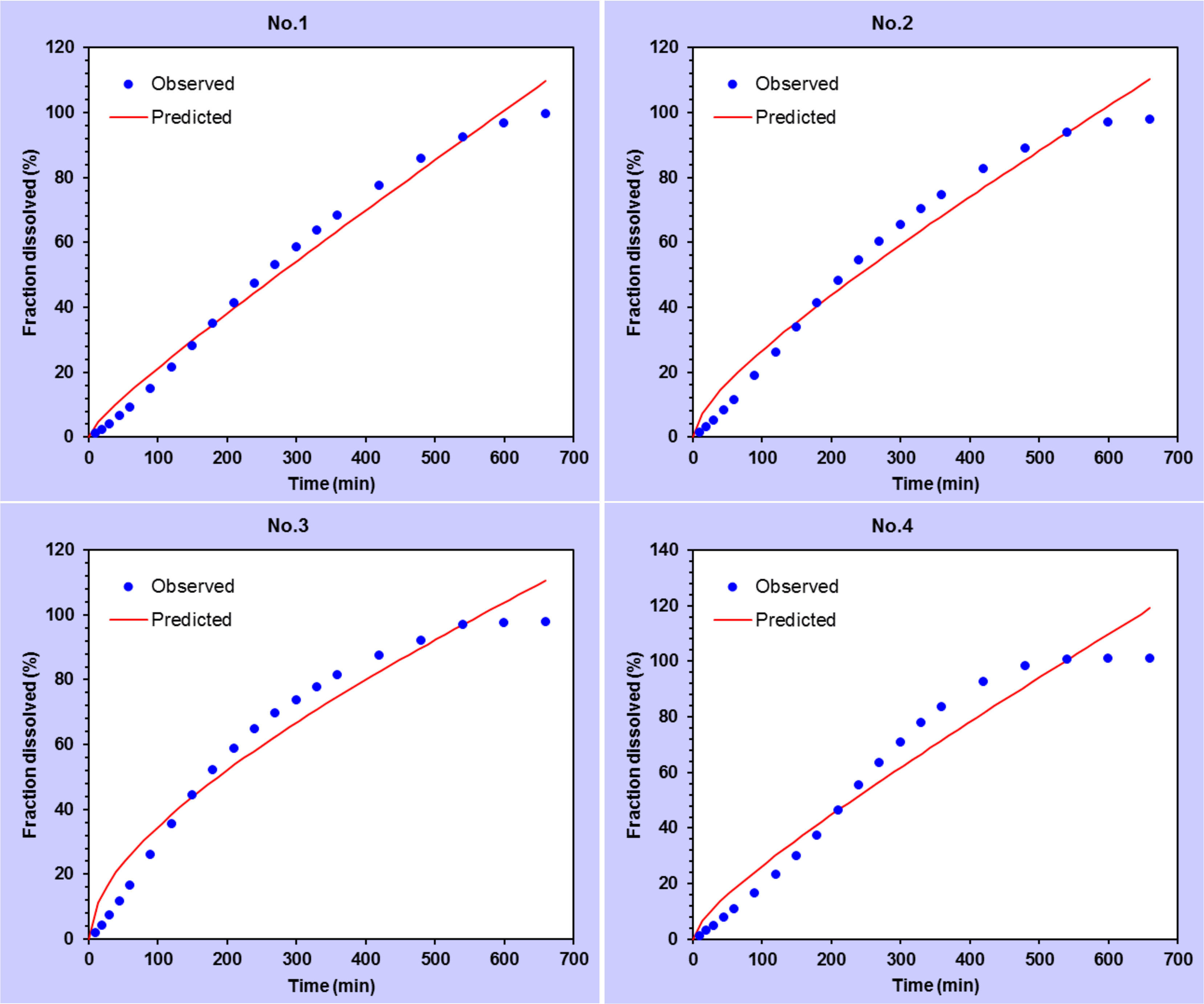

Supplementary materials – Model fitting summary – Kollidon® 90 F (modelling the entire relevant dissolution data)

Model: **Peppas–Sahlin_2 with T_{lag}**

Model equation: $F = k_1 \cdot (t - T_{lag})^{0.5} + k_2 \cdot (t - T_{lag})$

Fitted model parameters per tested tablet (N = 4) with statistics – mean, standard deviation (SD), and relative standard deviation expressed in % (RSD%) (output from DDSolver):

Parameter	No.1	No.2	No.3	No.4	Mean	SD	RSD(%)
k_1	0.901	1.777	3.117	1.515	1.827	0.935	51.156
k_2	0.131	0.098	0.046	0.122	0.099	0.038	38.546
T_{lag}	6.000	6.000	6.000	6.000	6.000	0.000	0.000

Number of dissolution data points (N), degrees of freedom (df), and selected goodness of fit criteria – Pearson correlation coefficient (R), coefficient of determination (R^2), adjusted coefficient of determination ($R^2_{adjusted}$), and residual sum of squares (RSS) (manual calculation in MS Excel):

Parameter	No.1	No.2	No.3	No.4
N	20	20	20	20
df	17	17	17	17
R	0.992856277	0.987411364	0.983248858	0.977061617
R^2	0.985763586	0.974981202	0.966778317	0.954649404
$R^2_{adjusted}$	0.984088714	0.972037814	0.962869883	0.94931404
RSS	327.4883095	599.6573627	854.0267941	1272.137818

Graphical abstract of model fit presented as mean ± 1 SD of the fraction % of released carvedilol:

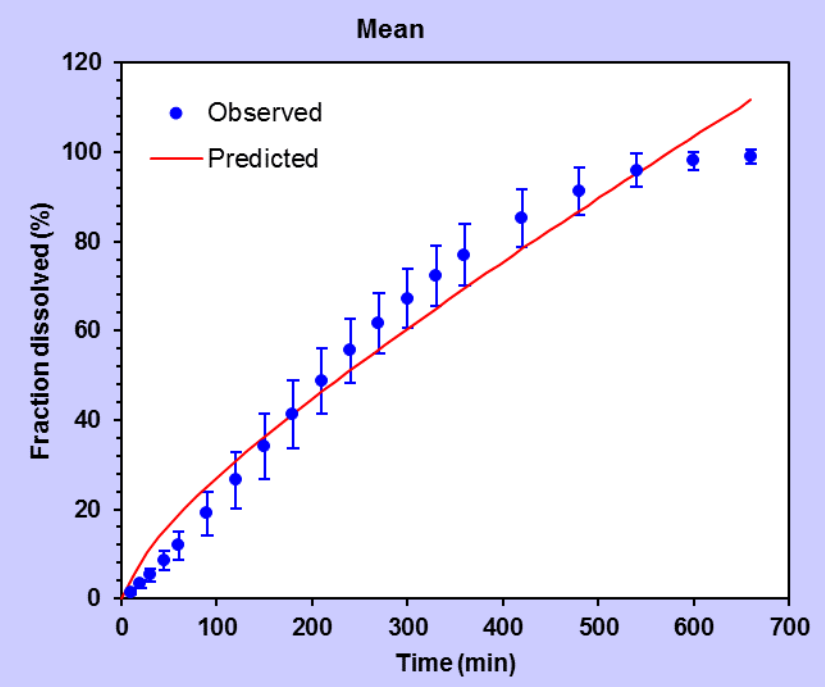

Supplementary materials – Model fitting summary – Kollidon® 90 F (modelling the entire relevant dissolution data)

Graphical abstract of model fit presented as the fraction % of released carvedilol per tested tablet:

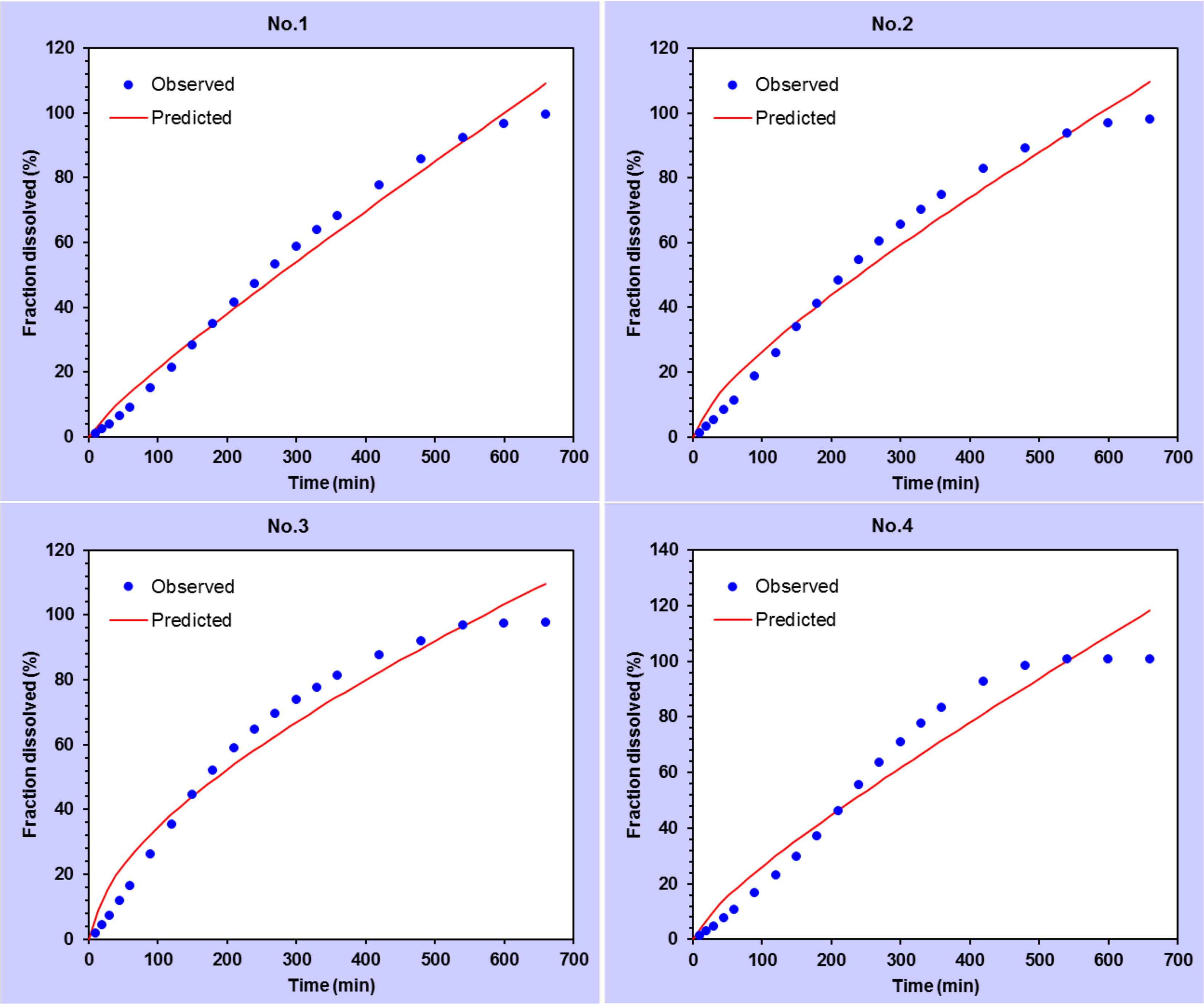

Supplementary materials – Model fitting summary – Kollidon® 90 F (modelling the entire relevant dissolution data)

Model: **Quadratic**

Model equation: $F = 100 \cdot (k_1 \cdot t^2 + k_2 \cdot t)$

Fitted model parameters per tested tablet (N = 4) with statistics – mean, standard deviation (SD), and relative standard deviation expressed in % (RSD%) (output from DDSolver):

Parameter	No.1	No.2	No.3	No.4	Mean	SD	RSD(%)
k_1	-0.000001	-0.000002	-0.000003	-0.000002	-0.000002	0.000001	-41.207870
k_2	0.002198	0.002653	0.003281	0.002764	0.002724	0.000445	16.326928

Number of dissolution data points (N), degrees of freedom (df), and selected goodness of fit criteria – Pearson correlation coefficient (R), coefficient of determination (R^2), adjusted coefficient of determination ($R^2_{adjusted}$), and residual sum of squares (RSS) (manual calculation in MS Excel):

Parameter	No.1	No.2	No.3	No.4
N	20	20	20	20
df	18	18	18	18
R	0.998545324	0.999063919	0.999111072	0.993389219
R^2	0.997092764	0.998128715	0.998222933	0.986822141
$R^2_{adjusted}$	0.99693125	0.998024755	0.998124208	0.986090038
RSS	109.8283479	86.83830327	49.51648235	509.5224812

Graphical abstract of model fit presented as mean ± 1 SD of the fraction % of released carvedilol:

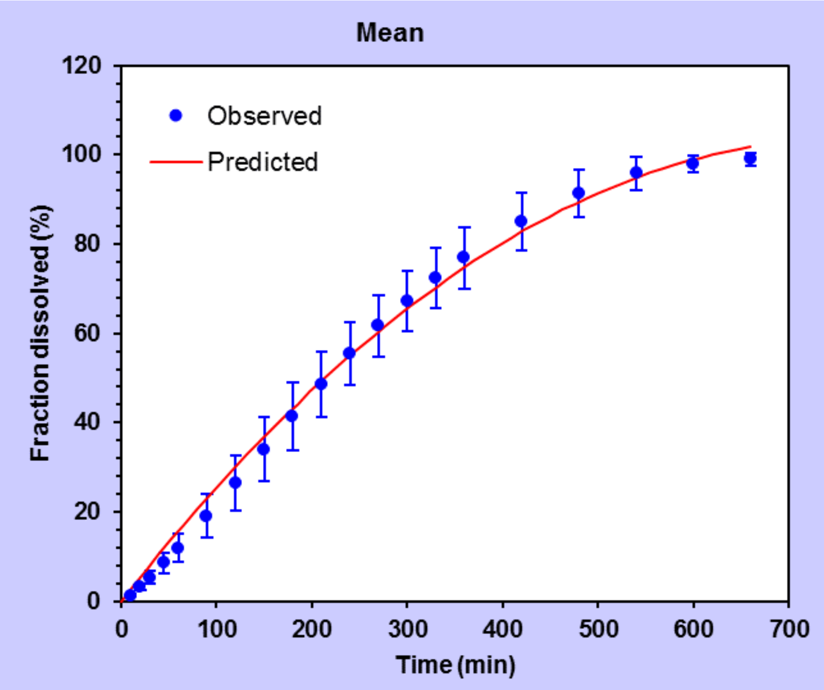

Supplementary materials – Model fitting summary – Kollidon® 90 F (modelling the entire relevant dissolution data)

Graphical abstract of model fit presented as the fraction % of released carvedilol per tested tablet:

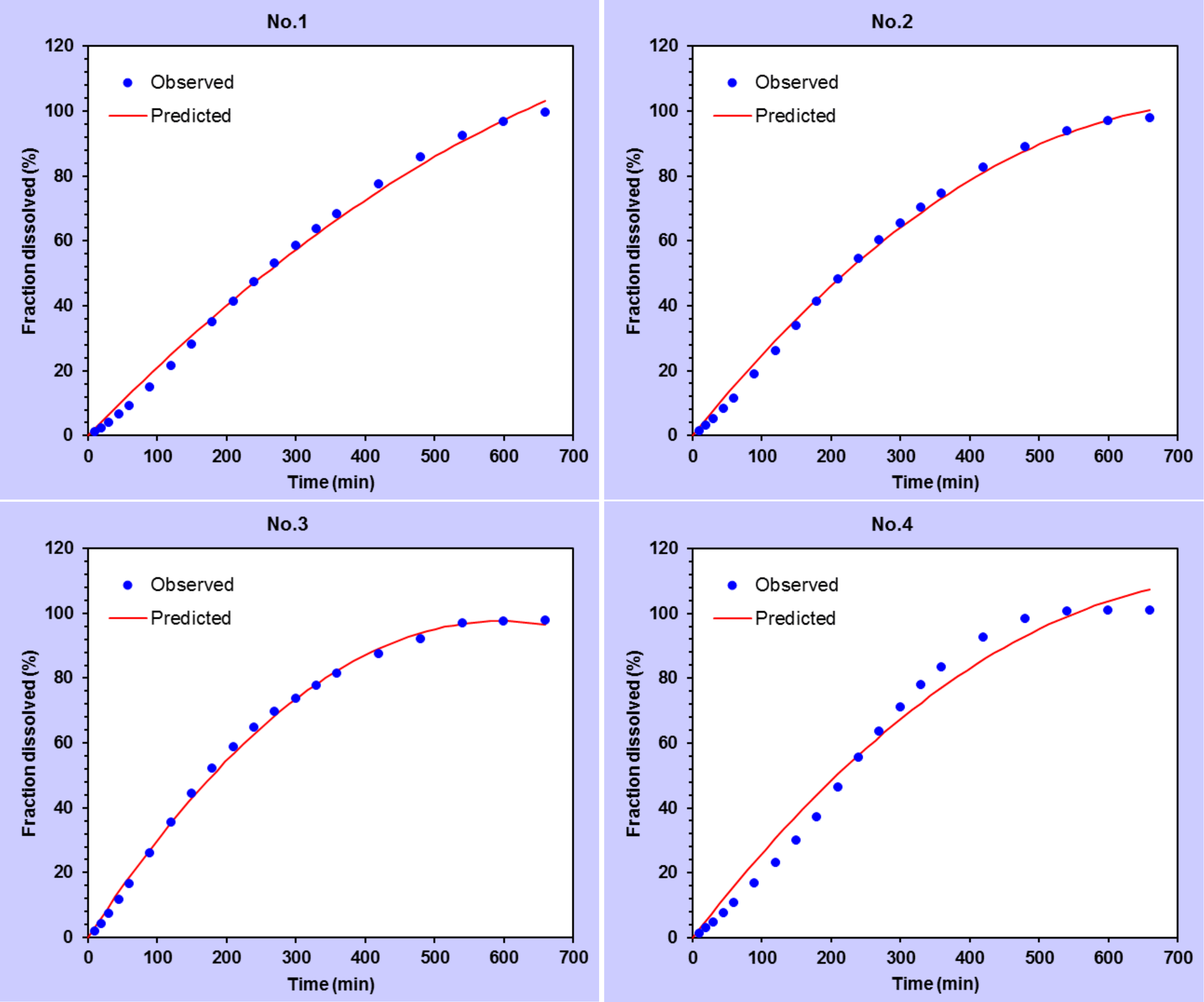

Supplementary materials – Model fitting summary – Kollidon® 90 F (modelling the entire relevant dissolution data)

Model: **Quadratic with T_{lag}**

Model equation: $F = 100 \cdot \left[k_1 \cdot \left(t - T_{lag}\right)^2 + k_2 \cdot \left(t - T_{lag}\right)\right]$

Fitted model parameters per tested tablet (N = 4) with statistics – mean, standard deviation (SD), and relative standard deviation expressed in % (RSD%) (output from DDSolver):

Parameter	No.1	No.2	No.3	No.4	Mean	SD	RSD(%)
k_1	-0.000001	-0.000002	-0.000003	-0.000002	-0.000002	0.000001	-39.722668
k_2	0.002251	0.002713	0.003346	0.002830	0.002785	0.000450	16.146179
T_{lag}	6.000000	6.000000	4.000000	6.000000	5.500000	1.000000	18.181818

Number of dissolution data points (N), degrees of freedom (df), and selected goodness of fit criteria – Pearson correlation coefficient (R), coefficient of determination (R^2), adjusted coefficient of determination ($R^2_{adjusted}$), and residual sum of squares (RSS) (manual calculation in MS Excel):

Parameter	No.1	No.2	No.3	No.4
N	20	20	20	20
df	17	17	17	17
R	0.998794465	0.999268561	0.999170414	0.99379189
R^2	0.997590383	0.998537657	0.998341516	0.987622322
$R^2_{adjusted}$	0.997306899	0.998365617	0.998146401	0.986166124
RSS	72.01822561	46.13268564	37.27772004	419.4605627

Graphical abstract of model fit presented as mean ± 1 SD of the fraction % of released carvedilol:

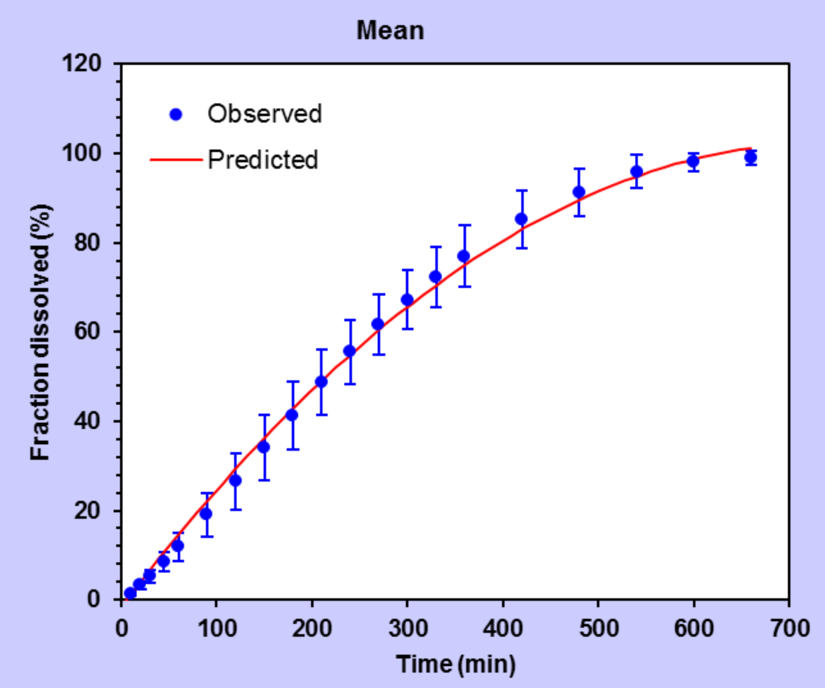

Supplementary materials – Model fitting summary – Kollidon® 90 F (modelling the entire relevant dissolution data)

Graphical abstract of model fit presented as the fraction % of released carvedilol per tested tablet:

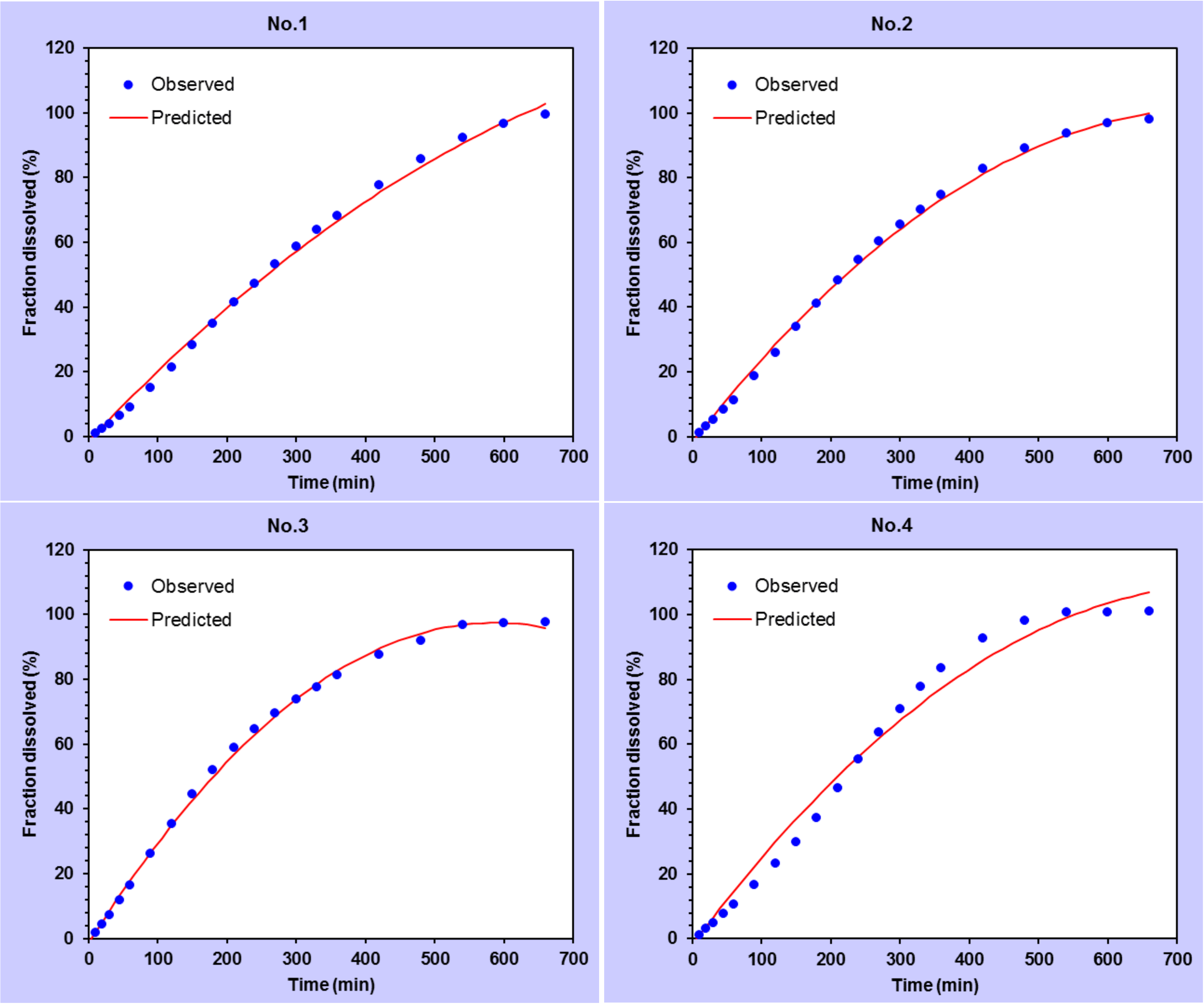

Supplementary materials – Model fitting summary – Kollidon® 90 F (modelling the entire relevant dissolution data)

Model: **Weibull_1**

Model equation: $F = 100 \cdot \left[1 - e^{-\frac{(t-T_i)^{\beta}}{\alpha}}\right]$

Fitted model parameters per tested tablet (N = 4) with statistics – mean, standard deviation (SD), and relative standard deviation expressed in % (RSD%) (output from DDSolver):

Parameter	No.1	No.2	No.3	No.4	Mean	SD	RSD(%)
α	1683.547	1020.704	588.931	1358.254	1162.859	468.650	40.302
β	1.304	1.238	1.180	1.299	1.255	0.059	4.661
T_i	6.000	6.000	6.000	6.000	6.000	0.000	0.000

Number of dissolution data points (N), degrees of freedom (df), and selected goodness of fit criteria – Pearson correlation coefficient (R), coefficient of determination (R^2), adjusted coefficient of determination ($R^2_{adjusted}$), and residual sum of squares (RSS) (manual calculation in MS Excel):

Parameter	No.1	No.2	No.3	No.4
N	20	20	20	20
df	17	17	17	17
R	0.995806518	0.99824477	0.999525845	0.993372361
R^2	0.991630622	0.99649262	0.999051914	0.986788647
$R^2_{adjusted}$	0.990645989	0.996079987	0.998940375	0.985234371
RSS	227.2883591	98.46418712	22.07488655	551.8115488

Graphical abstract of model fit presented as mean ± 1 SD of the fraction % of released carvedilol:

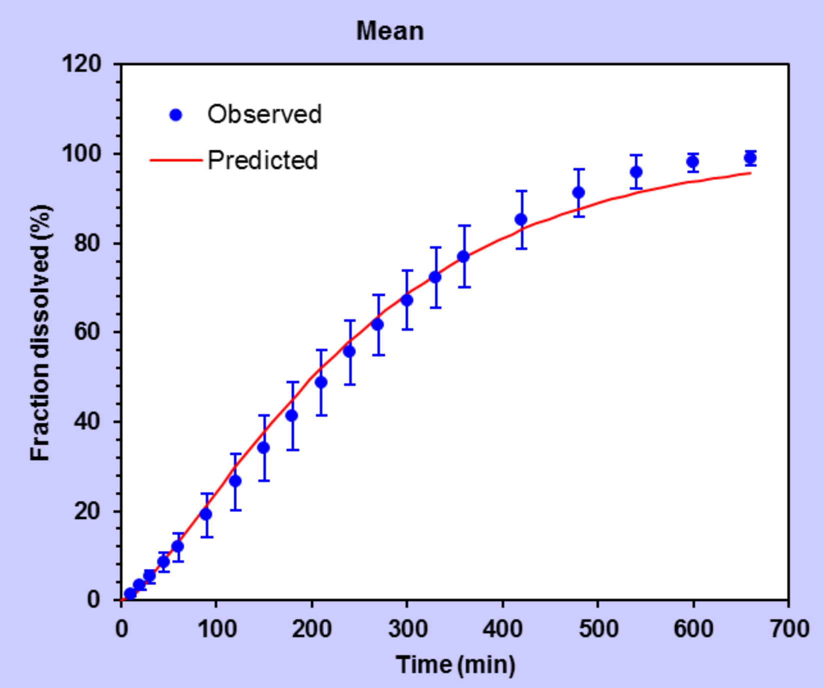

Supplementary materials – Model fitting summary – Kollidon® 90 F (modelling the entire relevant dissolution data)

Graphical abstract of model fit presented as the fraction % of released carvedilol per tested tablet:

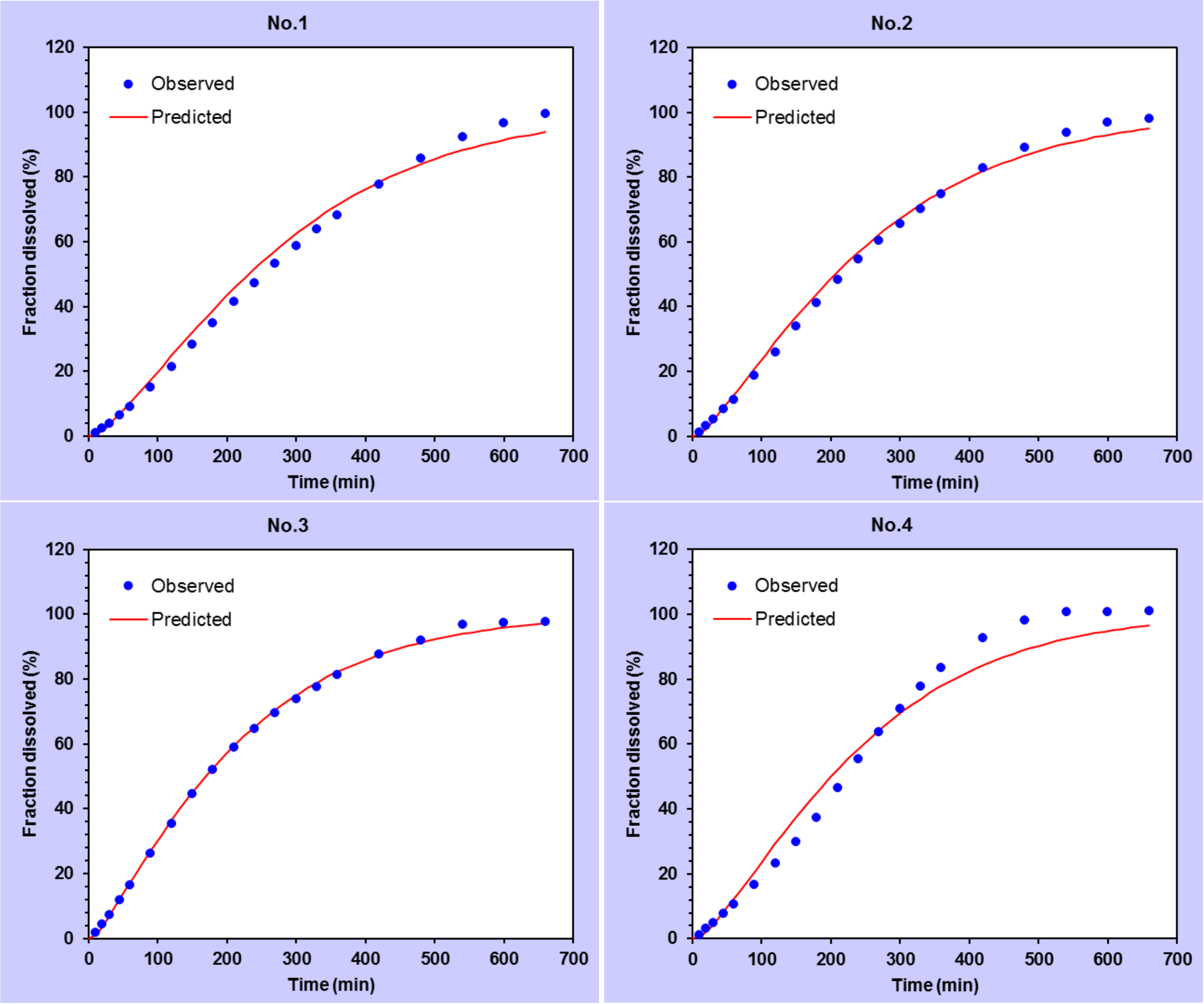

Supplementary materials – Model fitting summary – Kollidon® 90 F (modelling the entire relevant dissolution data)

Model: **Weibull_2**

Model equation: $F = 100 \cdot \left(1 - e^{-\frac{t^{\beta}}{\alpha}}\right)$

Fitted model parameters per tested tablet (N = 4) with statistics – mean, standard deviation (SD), and relative standard deviation expressed in % (RSD%) (output from DDSolver):

Parameter	No.1	No.2	No.3	No.4	Mean	SD	RSD(%)
α	3277.284	2275.808	1056.304	2820.830	2357.556	959.247	40.688
β	1.419	1.363	1.280	1.431	1.373	0.069	4.993

Number of dissolution data points (N), degrees of freedom (df), and selected goodness of fit criteria – Pearson correlation coefficient (R), coefficient of determination (R^2), adjusted coefficient of determination ($R^2_{adjusted}$), and residual sum of squares (RSS) (manual calculation in MS Excel):

Parameter	No.1	No.2	No.3	No.4
N	20	20	20	20
df	18	18	18	18
R	0.996777402	0.999773403	0.999674161	0.99487399
R^2	0.99356519	0.999546857	0.999348427	0.989774255
$R^2_{adjusted}$	0.9932077	0.999521683	0.999312229	0.989206158
RSS	181.9677729	47.09688557	15.97199764	378.2187817

Graphical abstract of model fit presented as mean ± 1 SD of the fraction % of released carvedilol:

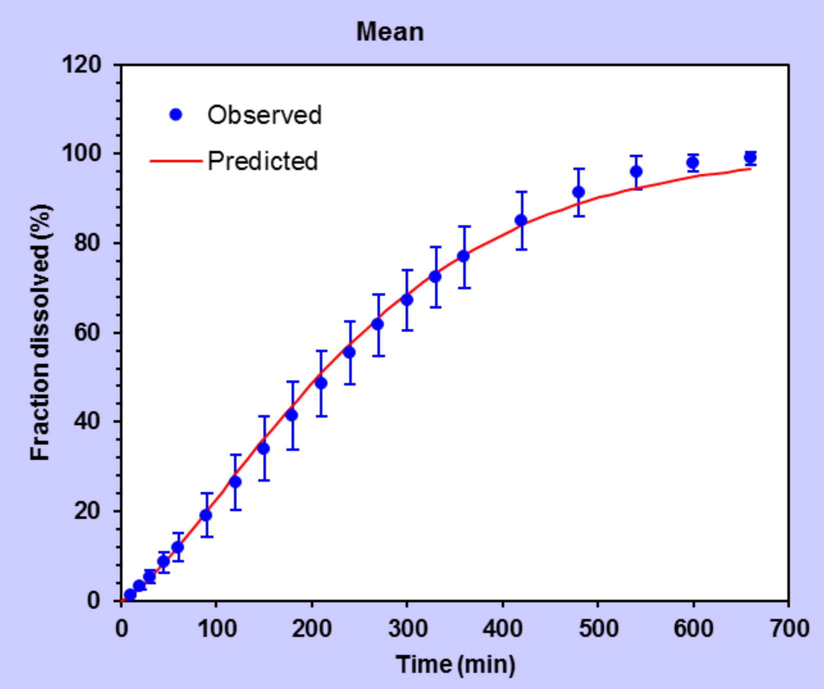

Supplementary materials – Model fitting summary – Kollidon® 90 F (modelling the entire relevant dissolution data)

Graphical abstract of model fit presented as the fraction % of released carvedilol per tested tablet:

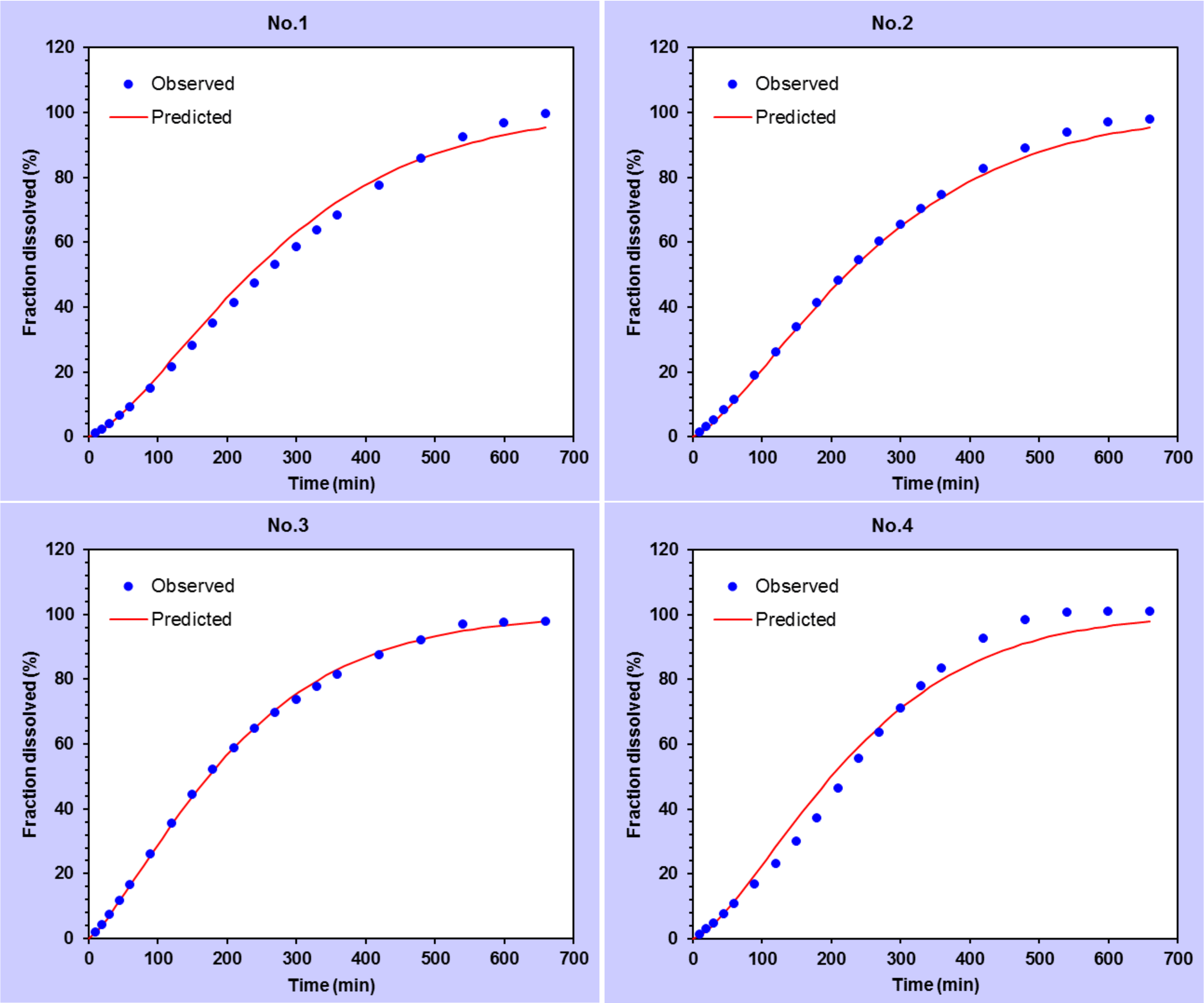

Supplementary materials – Model fitting summary – Kollidon® 90 F (modelling the entire relevant dissolution data)

Model: **Weibull_3**

Model equation: $F = F_{max} \cdot \left(1 - e^{-\frac{t^{\beta}}{\alpha}}\right)$

Fitted model parameters per tested tablet (N = 4) with statistics – mean, standard deviation (SD), and relative standard deviation expressed in % (RSD%) (output from DDSolver):

Parameter	No.1	No.2	No.3	No.4	Mean	SD	RSD(%)
α	2967.528	1958.426	952.091	2641.063	2129.777	890.607	41.817
β	1.357	1.314	1.246	1.396	1.328	0.064	4.830
F_{max}	110.122	109.456	102.579	105.928	107.021	3.486	3.258

Number of dissolution data points (N), degrees of freedom (df), and selected goodness of fit criteria – Pearson correlation coefficient (R), coefficient of determination (R^2), adjusted coefficient of determination ($R^2_{adjusted}$), and residual sum of squares (RSS) (manual calculation in MS Excel):

Parameter	No.1	No.2	No.3	No.4
N	20	20	20	20
df	17	17	17	17
R	0.999756414	0.999836763	0.99971802	0.996541739
R^2	0.999512888	0.999673552	0.99943612	0.993095438
$R^2_{adjusted}$	0.99945558	0.999635146	0.999369781	0.992283137
RSS	13.27904303	16.40345905	13.96503215	233.2019689

Graphical abstract of model fit presented as mean ± 1 SD of the fraction % of released carvedilol:

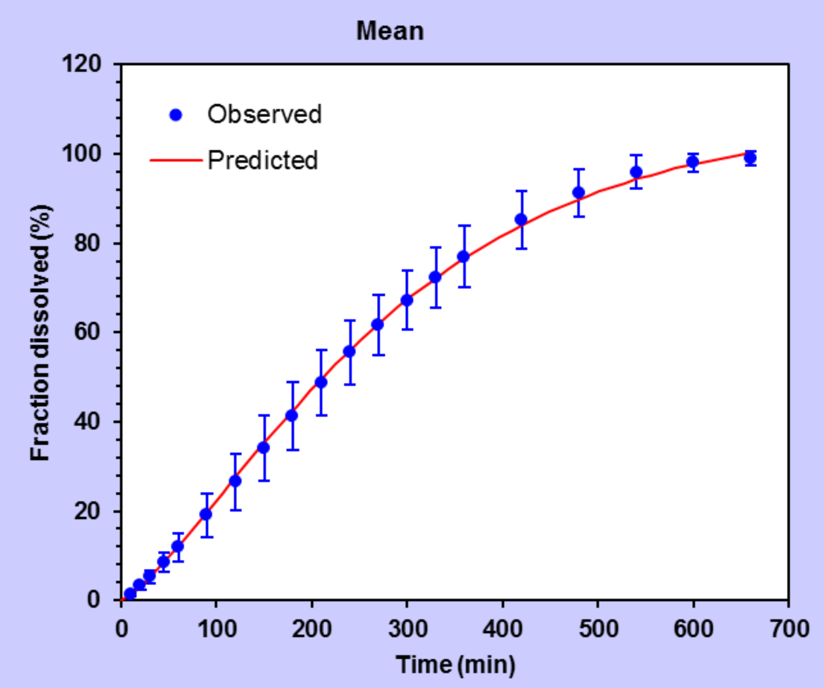

Supplementary materials – Model fitting summary – Kollidon® 90 F (modelling the entire relevant dissolution data)

Graphical abstract of model fit presented as the fraction % of released carvedilol per tested tablet:

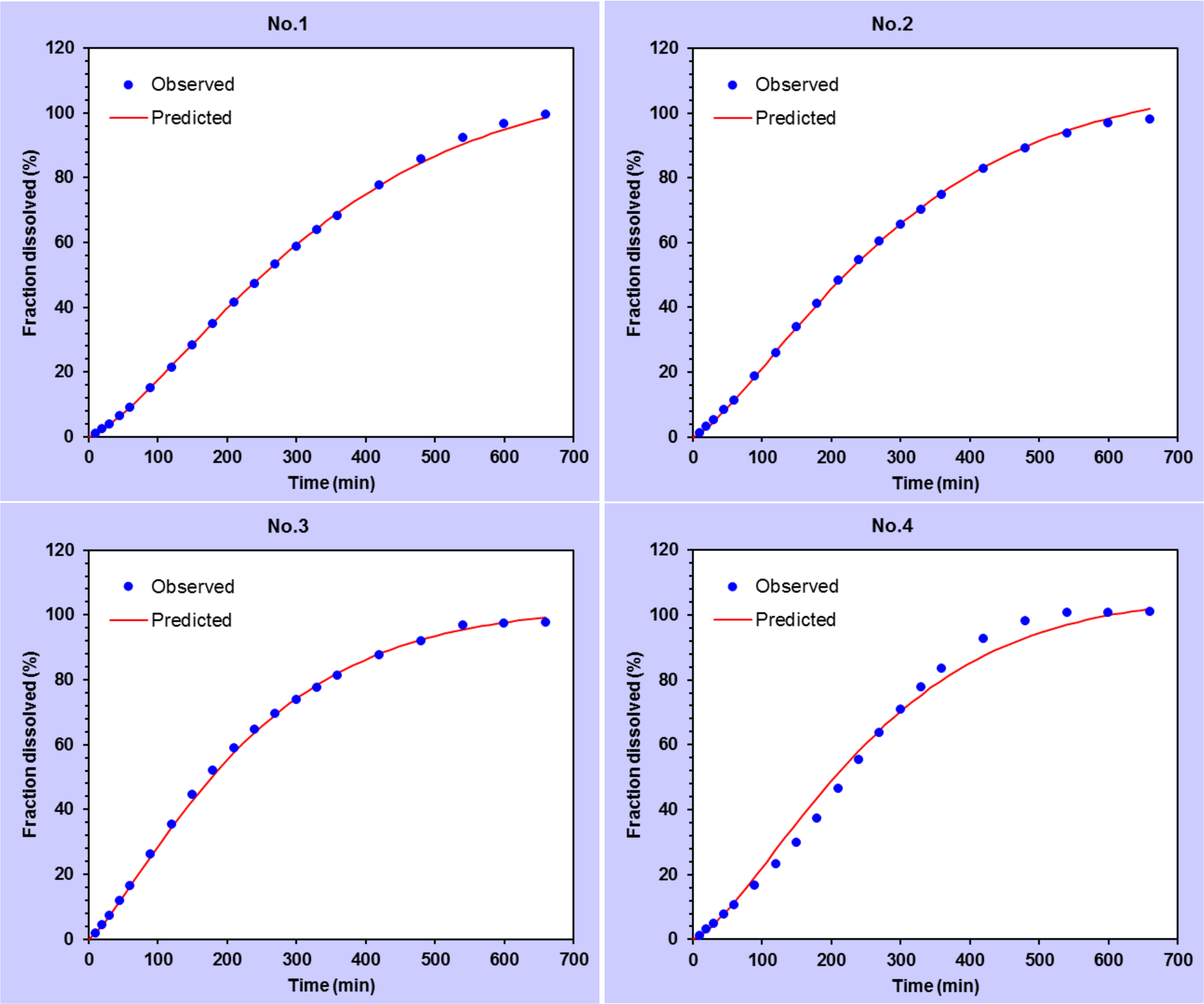

Supplementary materials – Model fitting summary – Kollidon® 90 F (modelling the entire relevant dissolution data)

Model: **Weibull_4**

Model equation: $F = F_{max} \cdot \left[1 - e^{-\frac{(t-T_i)^{\beta}}{\alpha}}\right]$

Fitted model parameters per tested tablet (N = 4) with statistics – mean, standard deviation (SD), and relative standard deviation expressed in % (RSD%) (output from DDSolver):

Parameter	No.1	No.2	No.3	No.4	Mean	SD	RSD(%)
α	1473.368	949.767	541.574	1374.547	1084.814	427.499	39.408
β	1.256	1.211	1.150	1.284	1.225	0.059	4.784
T_i	6.000	6.000	6.000	6.000	6.000	0.000	0.000
F_{max}	104.533	102.787	102.579	105.928	103.957	1.579	1.519

Number of dissolution data points (N), degrees of freedom (df), and selected goodness of fit criteria – Pearson correlation coefficient (R), coefficient of determination (R^2), adjusted coefficient of determination ($R^2_{adjusted}$), and residual sum of squares (RSS) (manual calculation in MS Excel):

Parameter	No.1	No.2	No.3	No.4
N	20	20	20	20
df	16	16	16	16
R	0.997967075	0.999061443	0.999803802	0.994977745
R^2	0.995938283	0.998123767	0.999607642	0.989980713
$R^2_{adjusted}$	0.995176711	0.997771973	0.999534075	0.988102097
RSS	133.1983359	61.92614999	11.69816159	357.8603266

Graphical abstract of model fit presented as mean ± 1 SD of the fraction % of released carvedilol:

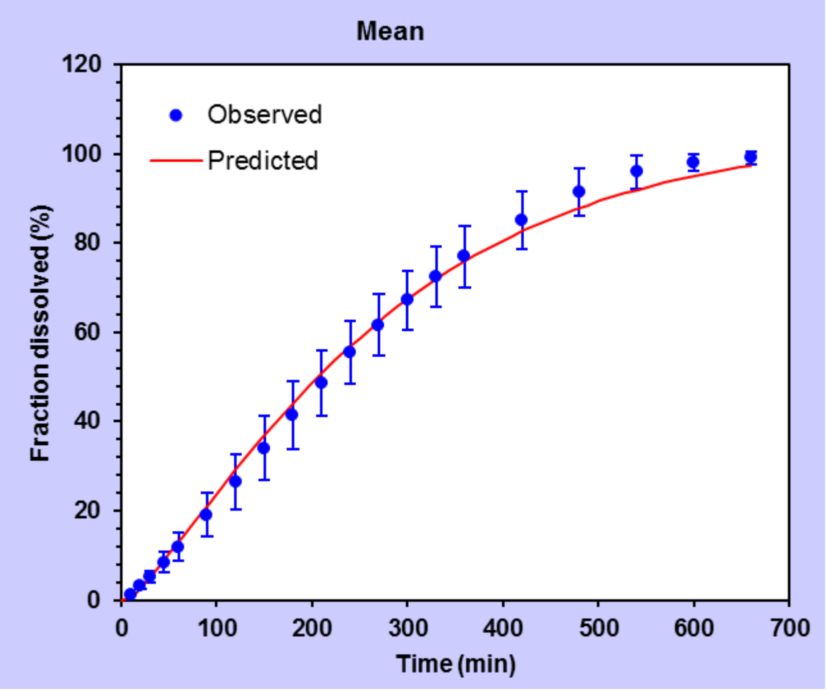

Supplementary materials – Model fitting summary – Kollidon® 90 F (modelling the entire relevant dissolution data)

Graphical abstract of model fit presented as the fraction % of released carvedilol per tested tablet:

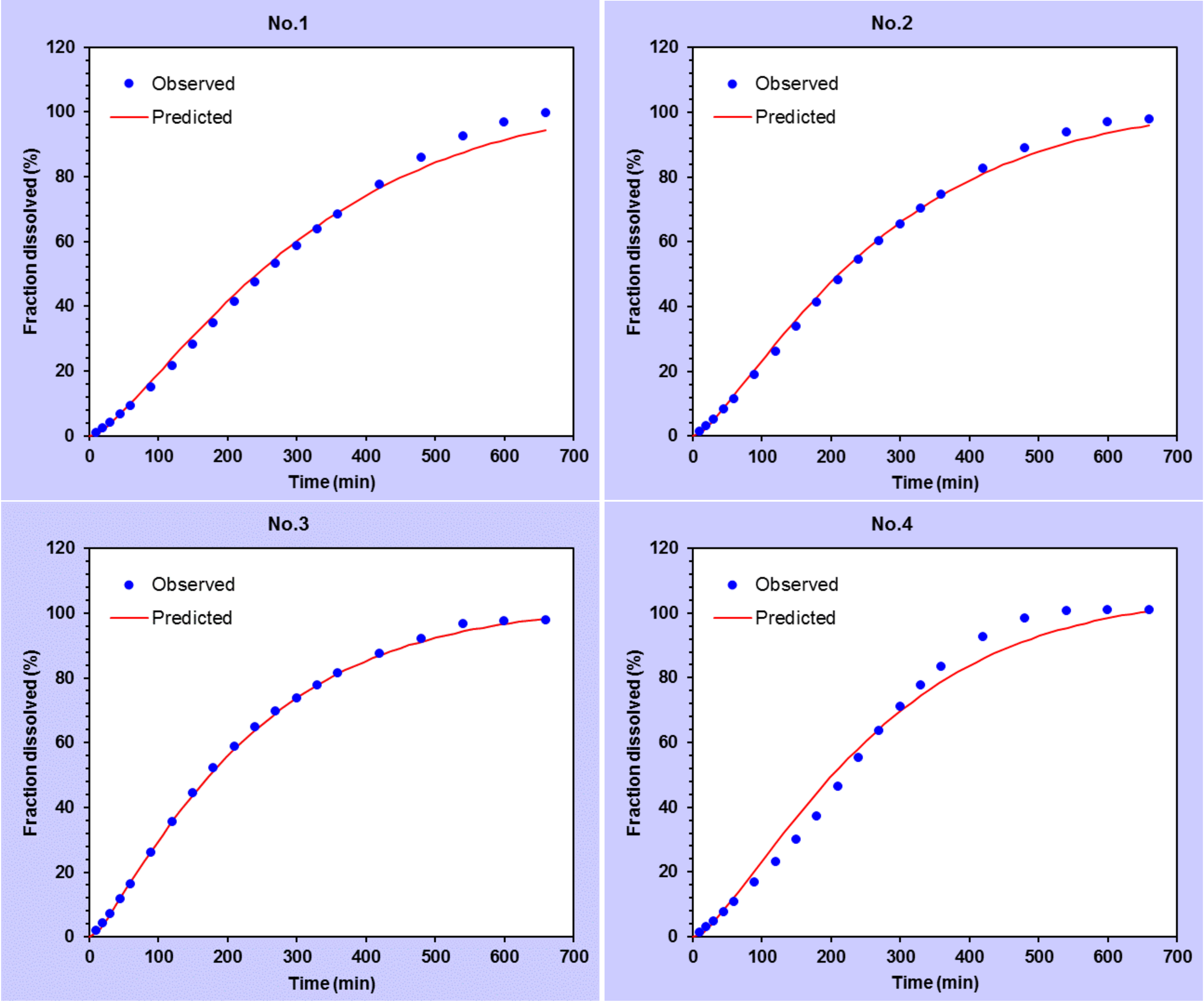

Supplementary materials – Model fitting summary – Kollidon® 90 F (modelling the entire relevant dissolution data)

Model: **Logistic_1**

Model equation: $F = 100 \cdot \frac{e^{\alpha+\beta \cdot \log(t)}}{1+e^{\alpha+\beta \cdot \log(t)}}$

Fitted model parameters per tested tablet (N = 4) with statistics – mean, standard deviation (SD), and relative standard deviation expressed in % (RSD%) (output from DDSolver):

Parameter	No.1	No.2	No.3	No.4	Mean	SD	RSD(%)
α	-9.978	-10.679	-10.206	-10.862	-10.431	0.410	-3.927
β	4.413	4.553	4.536	4.638	4.535	0.093	2.043

Number of dissolution data points (N), degrees of freedom (df), and selected goodness of fit criteria – Pearson correlation coefficient (R), coefficient of determination (R^2), adjusted coefficient of determination ($R^2_{adjusted}$), and residual sum of squares (RSS) (manual calculation in MS Excel):

Parameter	No.1	No.2	No.3	No.4
N	20	20	20	20
df	18	18	18	18
R	0.974066198	0.997386055	0.997921109	0.994246825
R^2	0.948804959	0.994778943	0.99584654	0.988526749
$R^2_{adjusted}$	0.94596079	0.994488885	0.995615792	0.987889346
RSS	1758.902813	390.9789249	280.7252252	1209.165692

Graphical abstract of model fit presented as mean ± 1 SD of the fraction % of released carvedilol:

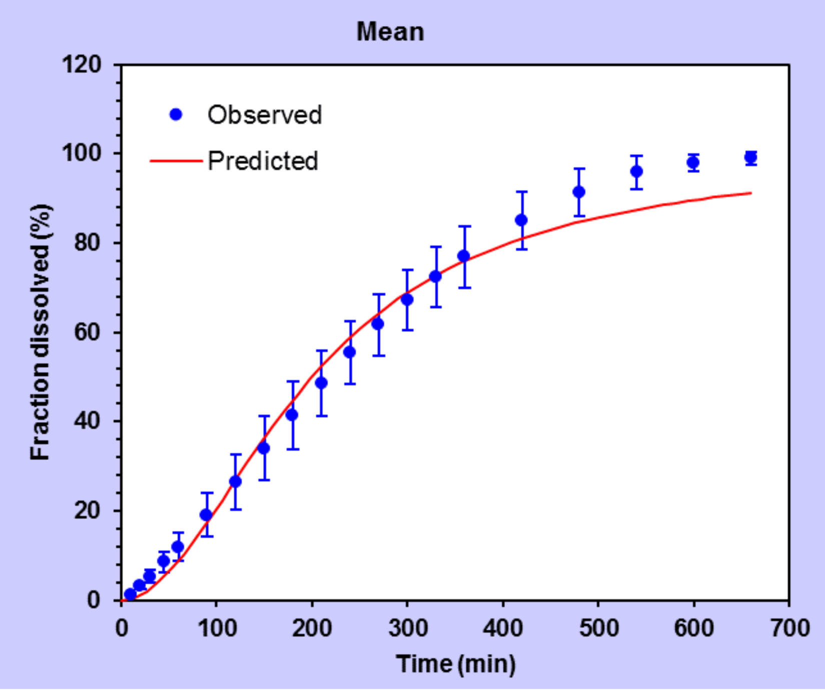

Supplementary materials – Model fitting summary – Kollidon® 90 F (modelling the entire relevant dissolution data)

Graphical abstract of model fit presented as the fraction % of released carvedilol per tested tablet:

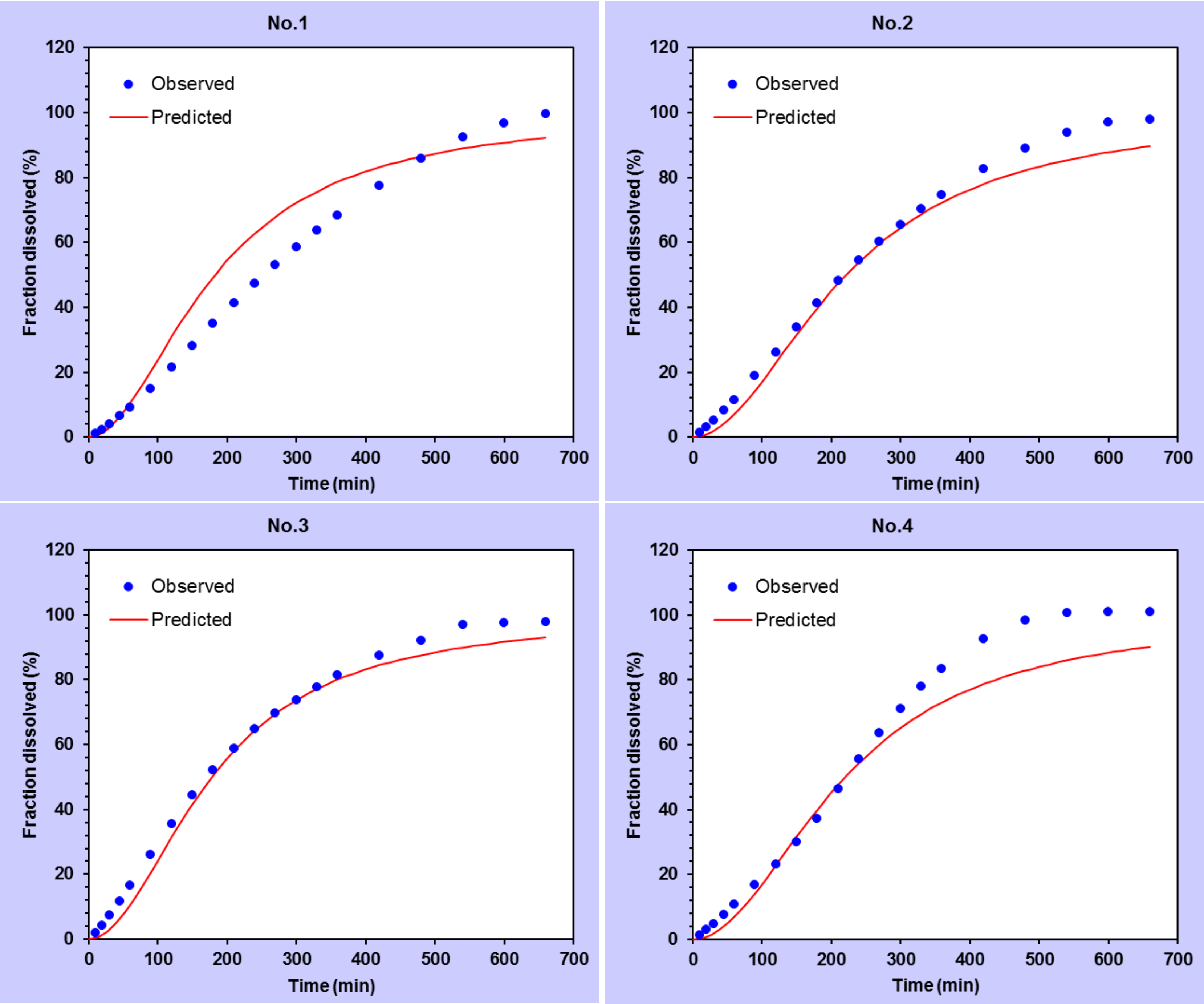

Supplementary materials – Model fitting summary – Kollidon® 90 F (modelling the entire relevant dissolution data)

Model: **Logistic_2**

Model equation: $F = F_{max} \cdot \frac{e^{\alpha+\beta\cdot\log(t)}}{1+e^{\alpha+\beta\cdot\log(t)}}$

Fitted model parameters per tested tablet (N = 4) with statistics – mean, standard deviation (SD), and relative standard deviation expressed in % (RSD%) (output from DDSolver):

Parameter	No.1	No.2	No.3	No.4	Mean	SD	RSD(%)
α	-9.205	-9.756	-8.943	-10.946	-9.712	0.890	-9.160
β	3.942	4.223	3.818	4.494	4.119	0.302	7.333
F_{max}	104.533	98.185	115.265	118.304	109.072	9.358	8.580

Number of dissolution data points (N), degrees of freedom (df), and selected goodness of fit criteria – Pearson correlation coefficient (R), coefficient of determination (R^2), adjusted coefficient of determination ($R^2_{adjusted}$), and residual sum of squares (RSS) (manual calculation in MS Excel):

Parameter	No.1	No.2	No.3	No.4
N	20	20	20	20
df	17	17	17	17
R	0.985573912	0.994431677	0.999276778	0.997691786
R^2	0.971355936	0.988894361	0.998554079	0.9953889
$R^2_{adjusted}$	0.967986046	0.987587815	0.99838397	0.994846418
RSS	785.3324779	435.4535195	175.2813998	556.6122692

Graphical abstract of model fit presented as mean ± 1 SD of the fraction % of released carvedilol:

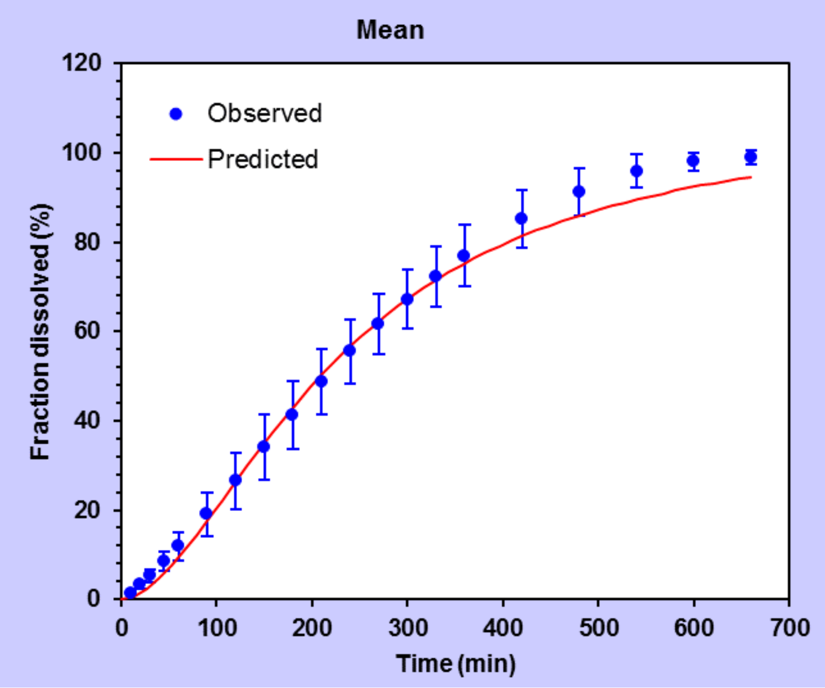

Supplementary materials – Model fitting summary – Kollidon® 90 F (modelling the entire relevant dissolution data)

Graphical abstract of model fit presented as the fraction % of released carvedilol per tested tablet:

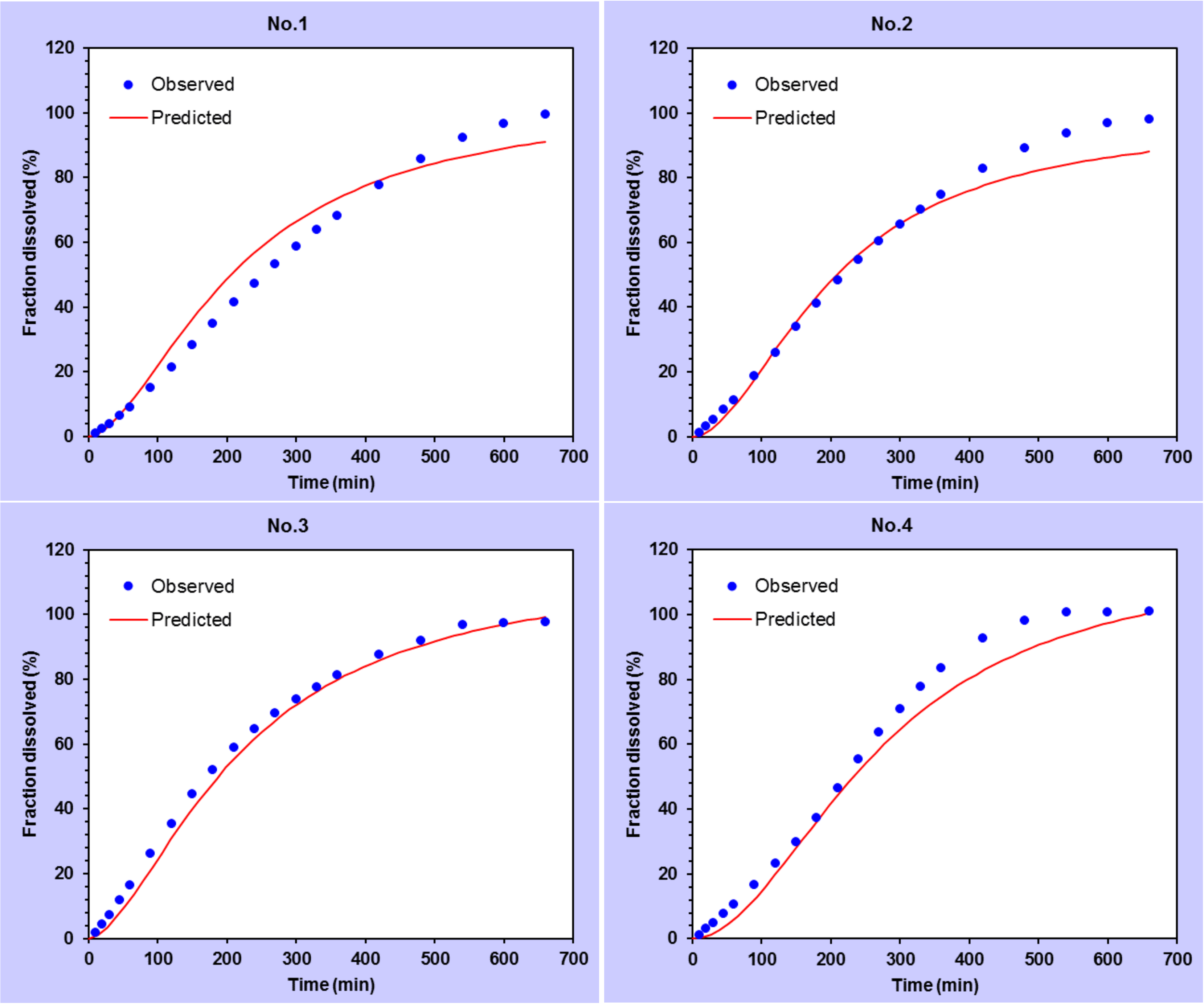

Supplementary materials – Model fitting summary – Kollidon® 90 F (modelling the entire relevant dissolution data)

Model: **Logistic_3**

Model equation: $F = F_{max} \cdot \frac{1}{1+e^{-k\cdot(t-\gamma)}}$

Fitted model parameters per tested tablet (N = 4) with statistics – mean, standard deviation (SD), and relative standard deviation expressed in % (RSD%) (output from DDSolver):

Parameter	No.1	No.2	No.3	No.4	Mean	SD	RSD(%)
k	0.010	0.010	0.010	0.010	0.010	0.000	2.663
γ	240.632	280.346	243.863	248.480	253.330	18.296	7.222
F_{max}	91.323	102.787	102.579	110.609	101.825	7.936	7.794

Number of dissolution data points (N), degrees of freedom (df), and selected goodness of fit criteria – Pearson correlation coefficient (R), coefficient of determination (R^2), adjusted coefficient of determination ($R^2_{adjusted}$), and residual sum of squares (RSS) (manual calculation in MS Excel):

Parameter	No.1	No.2	No.3	No.4
N	20	20	20	20
df	17	17	17	17
R	0.995971315	0.981510612	0.973465428	0.995630236
R^2	0.991958861	0.963363081	0.947634939	0.991279567
$R^2_{adjusted}$	0.991012845	0.959052855	0.941474343	0.990253633
RSS	378.7335252	1134.441052	1532.660783	337.4184623

Graphical abstract of model fit presented as mean ± 1 SD of the fraction % of released carvedilol:

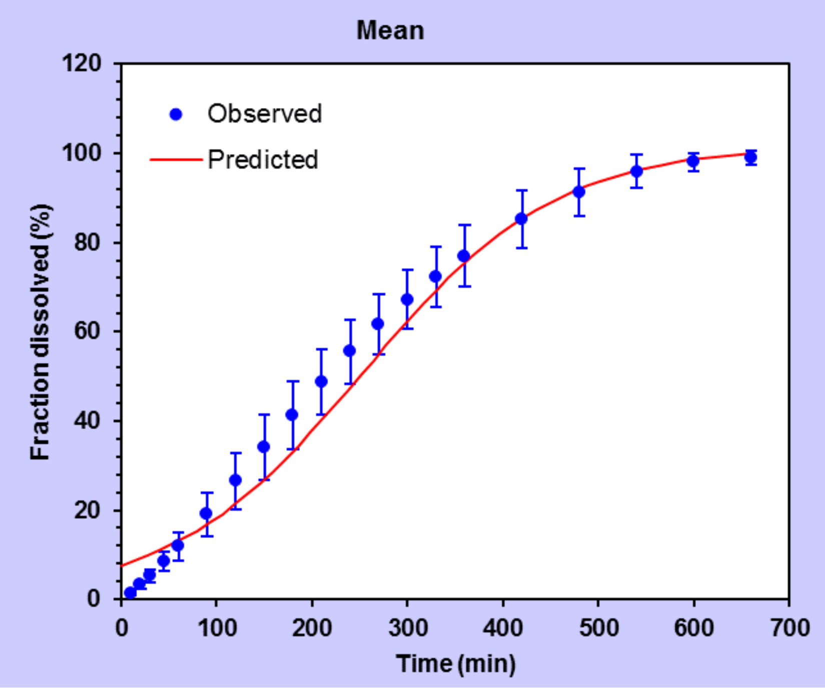

Supplementary materials – Model fitting summary – Kollidon® 90 F (modelling the entire relevant dissolution data)

Graphical abstract of model fit presented as the fraction % of released carvedilol per tested tablet:

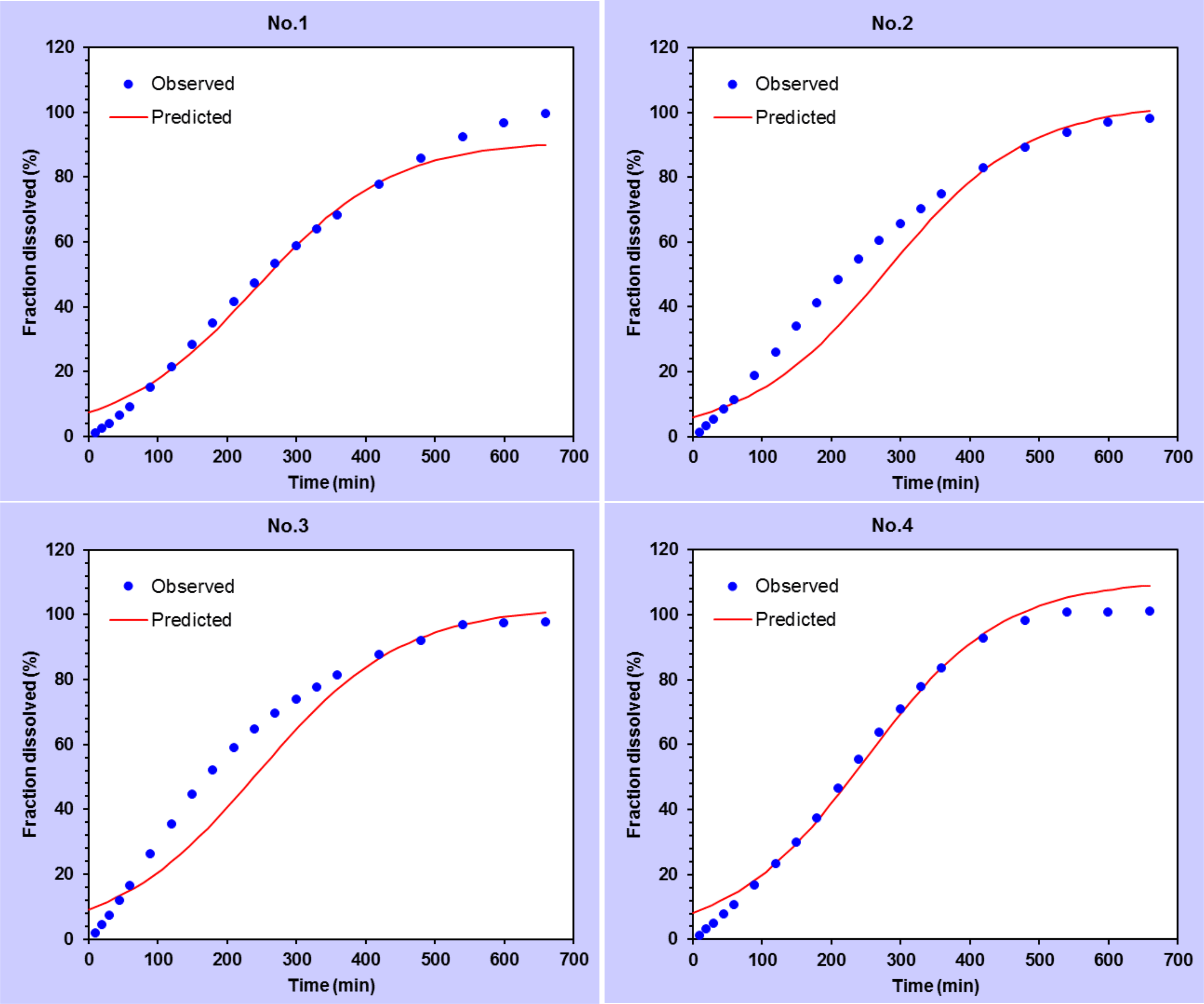

Supplementary materials – Model fitting summary – Kollidon® 90 F (modelling the entire relevant dissolution data)

Model: **Gompertz_1**

Model equation: $F = 100 \cdot e^{-\alpha \cdot e^{-\beta \cdot \log(t)}}$

Fitted model parameters per tested tablet (N = 4) with statistics – mean, standard deviation (SD), and relative standard deviation expressed in % (RSD%) (output from DDSolver):

Parameter	No.1	No.2	No.3	No.4	Mean	SD	RSD(%)
α	366.272	264.608	281.781	215.778	282.110	62.688	22.221
β	2.740	2.630	2.776	2.525	2.668	0.114	4.259

Number of dissolution data points (N), degrees of freedom (df), and selected goodness of fit criteria – Pearson correlation coefficient (R), coefficient of determination (R^2), adjusted coefficient of determination ($R^2_{adjusted}$), and residual sum of squares (RSS) (manual calculation in MS Excel):

Parameter	No.1	No.2	No.3	No.4
N	20	20	20	20
df	18	18	18	18
R	0.968826466	0.980168215	0.986844998	0.971001814
R^2	0.93862472	0.96072973	0.973863051	0.942844523
$R^2_{adjusted}$	0.935214983	0.958548049	0.972410998	0.939669219
RSS	1379.559284	964.3387655	626.4613721	2483.762895

Graphical abstract of model fit presented as mean ± 1 SD of the fraction % of released carvedilol:

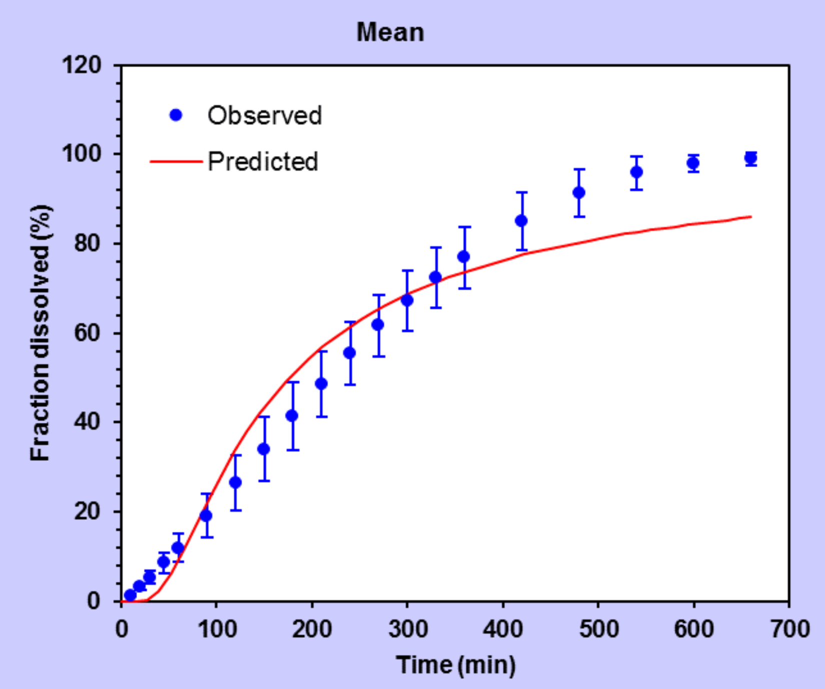

Supplementary materials – Model fitting summary – Kollidon® 90 F (modelling the entire relevant dissolution data)

Graphical abstract of model fit presented as the fraction % of released carvedilol per tested tablet:

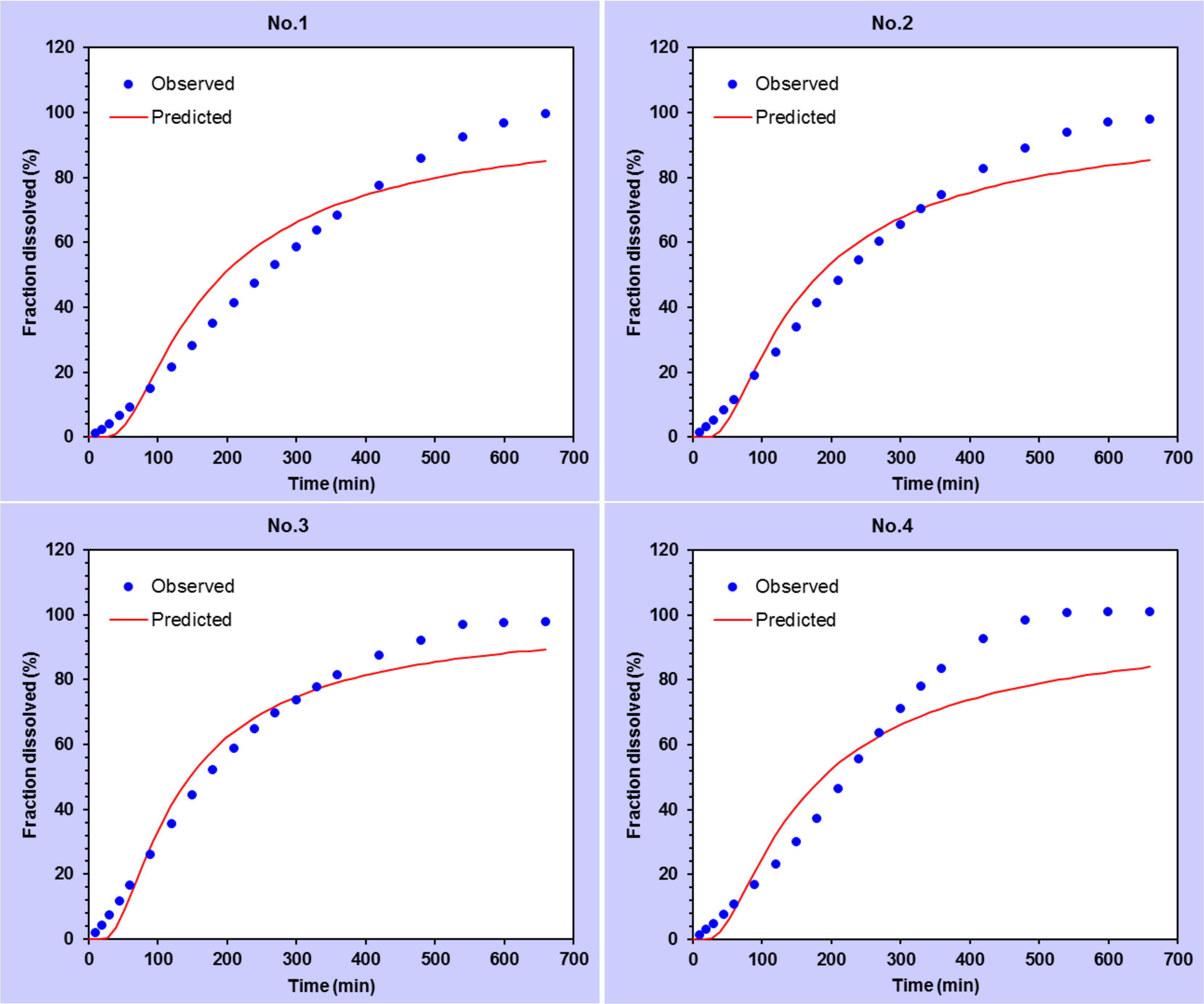

Supplementary materials – Model fitting summary – Kollidon® 90 F (modelling the entire relevant dissolution data)

Model: **Gompertz_2**

Model equation: $F = F_{max} \cdot e^{-\alpha \cdot e^{-\beta \cdot \log(t)}}$

Fitted model parameters per tested tablet (N = 4) with statistics – mean, standard deviation (SD), and relative standard deviation expressed in % (RSD%) (output from DDSolver):

Parameter	No.1	No.2	No.3	No.4	Mean	SD	RSD(%)
α	161.764	142.961	144.735	262.613	178.018	57.030	32.036
β	2.261	2.206	2.294	2.400	2.290	0.082	3.576
F_{max}	104.533	114.704	114.808	130.514	116.140	10.726	9.236

Number of dissolution data points (N), degrees of freedom (df), and selected goodness of fit criteria – Pearson correlation coefficient (R), coefficient of determination (R^2), adjusted coefficient of determination ($R^2_{adjusted}$), and residual sum of squares (RSS) (manual calculation in MS Excel):

Parameter	No.1	No.2	No.3	No.4
N	20	20	20	20
df	17	17	17	17
R	0.982320725	0.993303772	0.997586035	0.993834179
R^2	0.964954007	0.986652383	0.995177896	0.987706375
$R^2_{adjusted}$	0.960830949	0.985082075	0.99461059	0.986260067
RSS	1529.222537	807.8170259	548.8231355	857.8745843

Graphical abstract of model fit presented as mean ± 1 SD of the fraction % of released carvedilol:

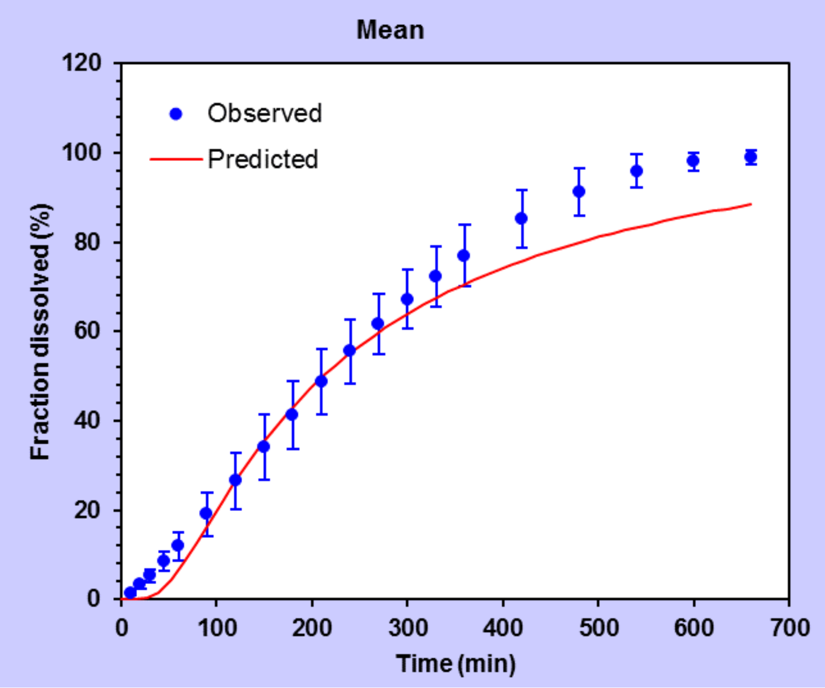

Supplementary materials – Model fitting summary – Kollidon® 90 F (modelling the entire relevant dissolution data)

Graphical abstract of model fit presented as the fraction % of released carvedilol per tested tablet:

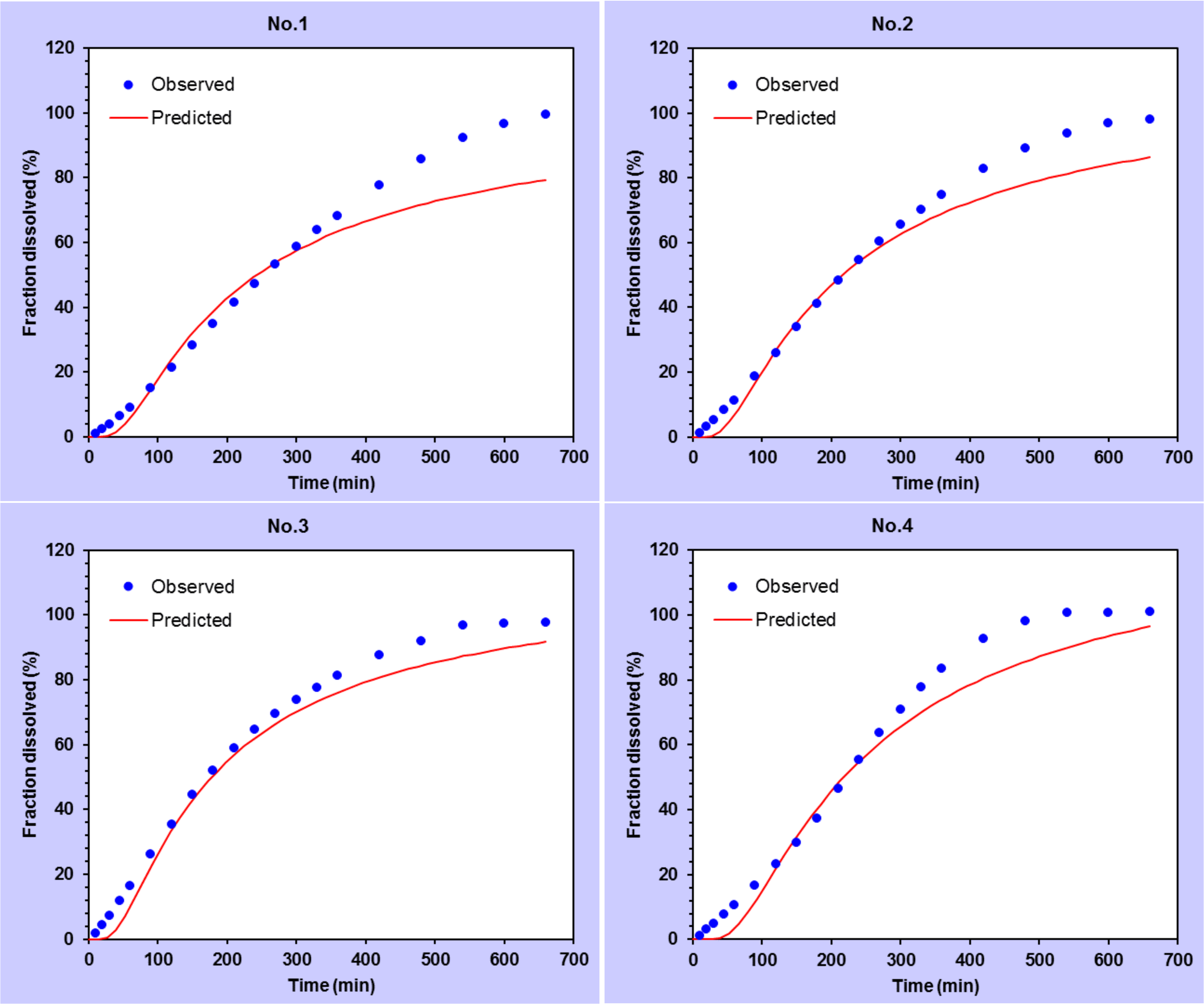

Supplementary materials – Model fitting summary – Kollidon® 90 F (modelling the entire relevant dissolution data)

Model: **Gompertz_3**

Model equation: $F = F_{max} \cdot e^{-e^{-k\cdot(t-\gamma)}}$

Fitted model parameters per tested tablet (N = 4) with statistics – mean, standard deviation (SD), and relative standard deviation expressed in % (RSD%) (output from DDSolver):

Parameter	No.1	No.2	No.3	No.4	Mean	SD	RSD(%)
k	0.006	0.007	0.010	0.007	0.008	0.001	18.913
γ	210.386	183.960	119.649	179.176	173.293	38.306	22.105
F_{max}	104.533	102.787	91.182	105.928	101.108	6.741	6.667

Number of dissolution data points (N), degrees of freedom (df), and selected goodness of fit criteria – Pearson correlation coefficient (R), coefficient of determination (R^2), adjusted coefficient of determination ($R^2_{adjusted}$), and residual sum of squares (RSS) (manual calculation in MS Excel):

Parameter	No.1	No.2	No.3	No.4
N	20	20	20	20
df	17	17	17	17
R	0.998040101	0.997917927	0.996252261	0.999195219
R^2	0.996084043	0.99584019	0.992518568	0.998391086
$R^2_{adjusted}$	0.995623342	0.995350801	0.9916384	0.998201802
RSS	105.8352767	108.2619931	220.2511458	47.7065461

Graphical abstract of model fit presented as mean ± 1 SD of the fraction % of released carvedilol:

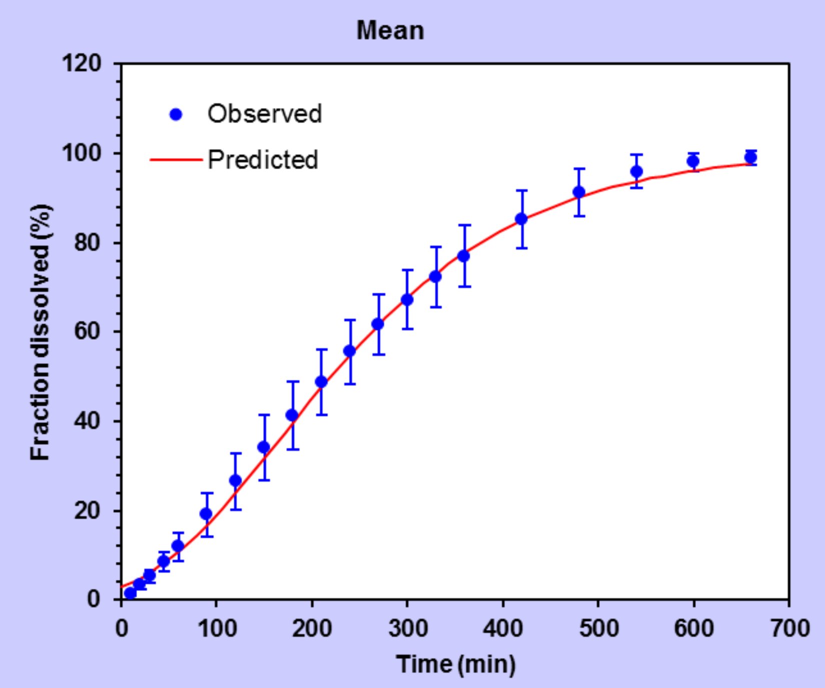

Supplementary materials – Model fitting summary – Kollidon® 90 F (modelling the entire relevant dissolution data)

Graphical abstract of model fit presented as the fraction % of released carvedilol per tested tablet:

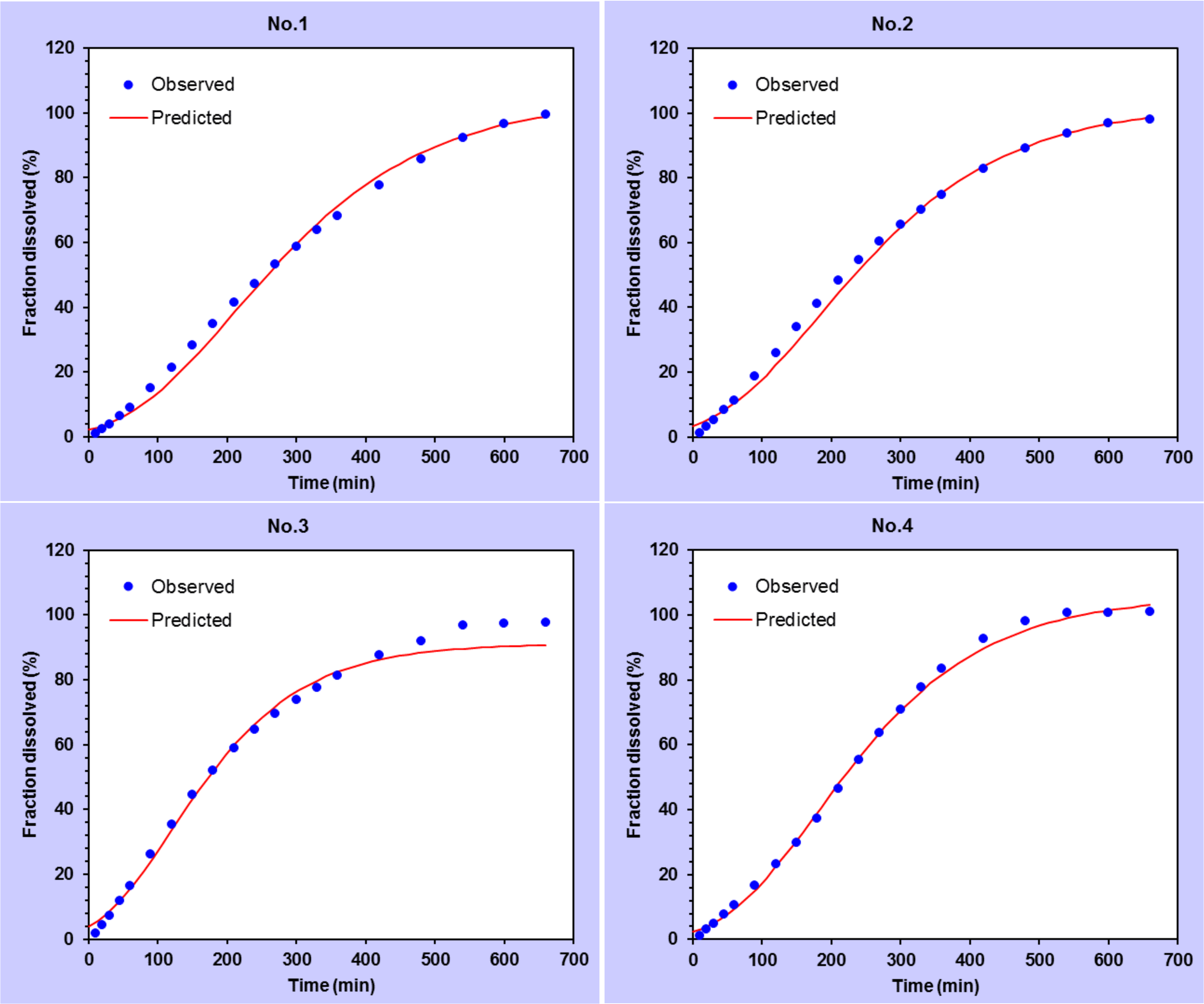

Supplementary materials – Model fitting summary – Kollidon® 90 F (modelling the entire relevant dissolution data)

Model: **Gompertz_4**

Model equation: $F = F_{max} \cdot e^{-\beta \cdot e^{-k \cdot t}}$

Fitted model parameters per tested tablet (N = 4) with statistics – mean, standard deviation (SD), and relative standard deviation expressed in % (RSD%) (output from DDSolver):

Parameter	No.1	No.2	No.3	No.4	Mean	SD	RSD(%)
k	0.006	0.007	0.007	0.007	0.007	0.000	6.340
β	3.874	3.411	2.777	3.799	3.465	0.502	14.478
F_{max}	104.533	102.787	102.579	105.928	103.957	1.579	1.519

Number of dissolution data points (N), degrees of freedom (df), and selected goodness of fit criteria – Pearson correlation coefficient (R), coefficient of determination (R^2), adjusted coefficient of determination ($R^2_{adjusted}$), and residual sum of squares (RSS) (manual calculation in MS Excel):

Parameter	No.1	No.2	No.3	No.4
N	20	20	20	20
df	17	17	17	17
R	0.998040101	0.997917927	0.994249875	0.999195219
R^2	0.996084043	0.99584019	0.988532813	0.998391086
$R^2_{adjusted}$	0.995623342	0.995350801	0.987183733	0.998201802
RSS	105.8352767	108.2619931	292.0463294	47.7065461

Graphical abstract of model fit presented as mean ± 1 SD of the fraction % of released carvedilol:

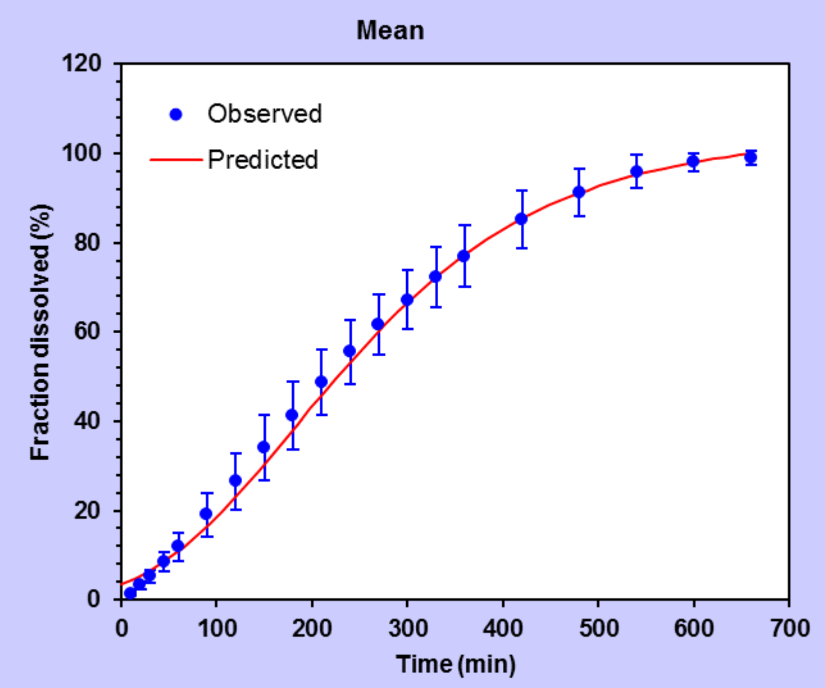

Supplementary materials – Model fitting summary – Kollidon® 90 F (modelling the entire relevant dissolution data)

Graphical abstract of model fit presented as the fraction % of released carvedilol per tested tablet:

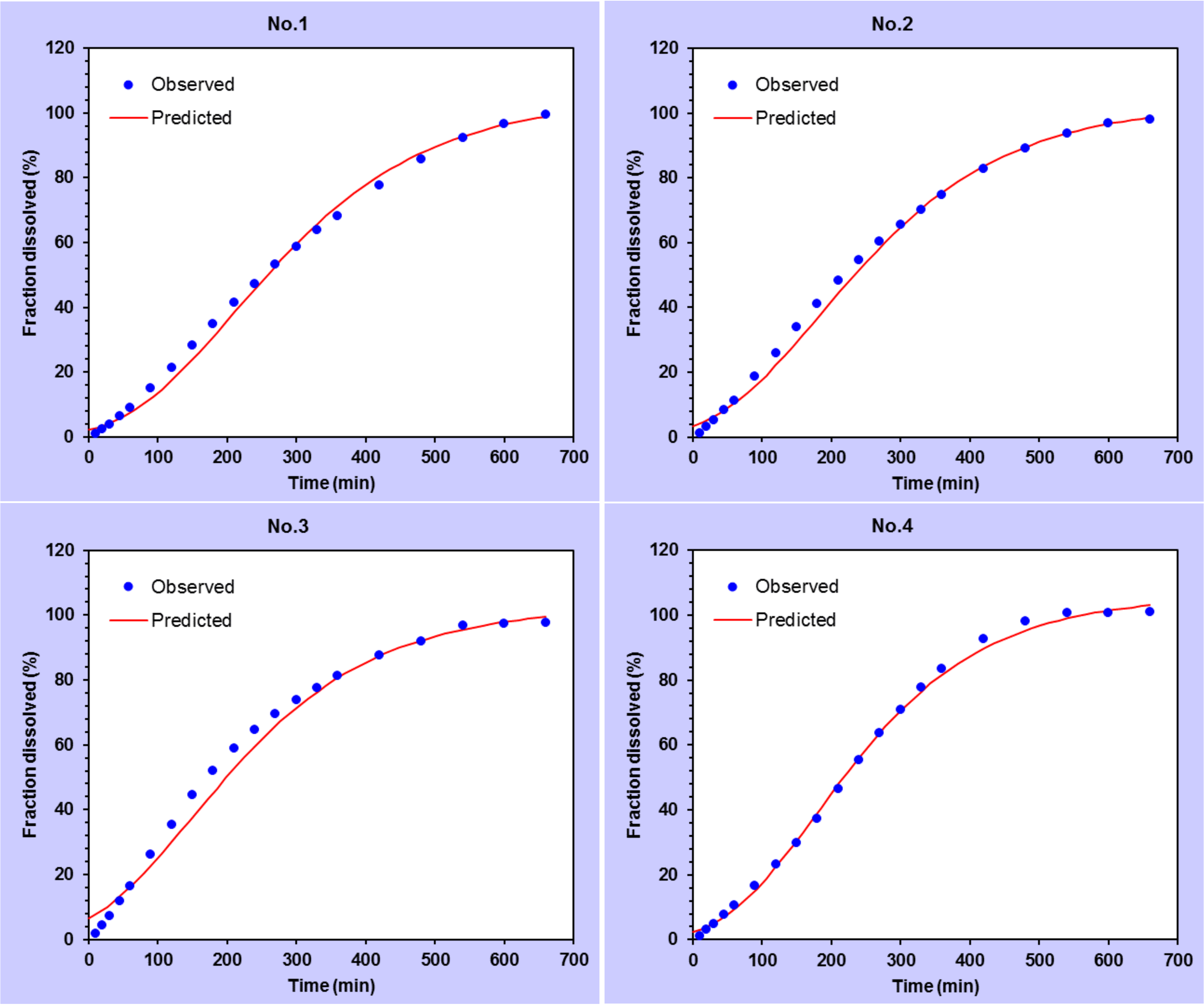

Supplementary materials – Model fitting summary – Kollidon® 90 F (modelling the entire relevant dissolution data)

Model: **Probit_1**

Model equation: $F = 100 \cdot \phi[\alpha + \beta \cdot \log(t)]$

Fitted model parameters per tested tablet (N = 4) with statistics – mean, standard deviation (SD), and relative standard deviation expressed in % (RSD%) (output from DDSolver):

Parameter	No.1	No.2	No.3	No.4	Mean	SD	RSD(%)
α	-5.366	-5.871	-5.657	-5.933	-5.707	0.256	-4.486
β	2.368	2.505	2.516	2.533	2.480	0.076	3.064

Number of dissolution data points (N), degrees of freedom (df), and selected goodness of fit criteria – Pearson correlation coefficient (R), coefficient of determination (R^2), adjusted coefficient of determination ($R^2_{adjusted}$), and residual sum of squares (RSS) (manual calculation in MS Excel):

Parameter	No.1	No.2	No.3	No.4
N	20	20	20	20
df	18	18	18	18
R	0.974457674	0.996666876	0.998505572	0.991401161
R^2	0.949567759	0.993344862	0.997013377	0.982876262
$R^2_{adjusted}$	0.946765968	0.992975132	0.996847453	0.981924943
RSS	1526.278685	530.9331481	318.1139996	1669.449644

Graphical abstract of model fit presented as mean ± 1 SD of the fraction % of released carvedilol:

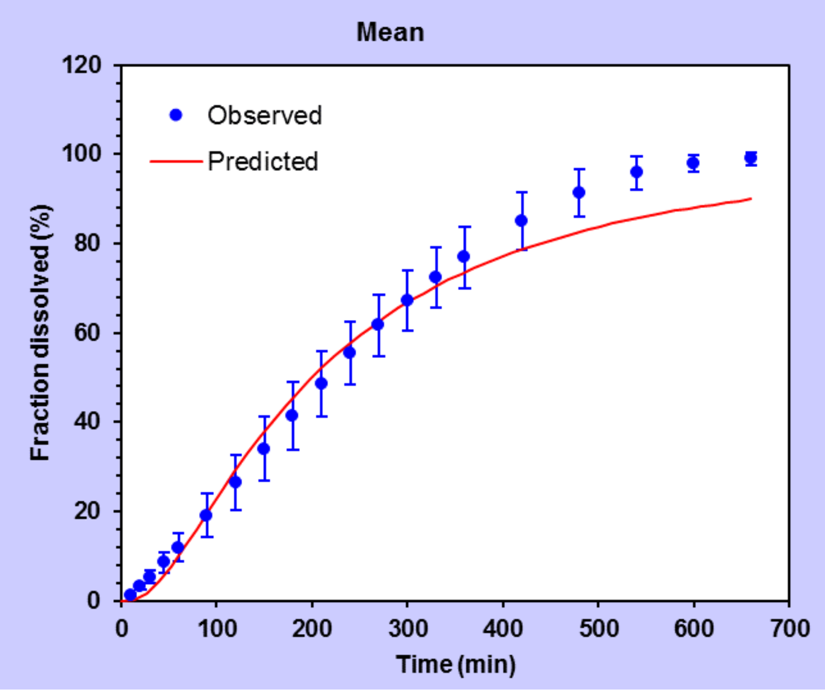

Supplementary materials – Model fitting summary – Kollidon® 90 F (modelling the entire relevant dissolution data)

Graphical abstract of model fit presented as the fraction % of released carvedilol per tested tablet:

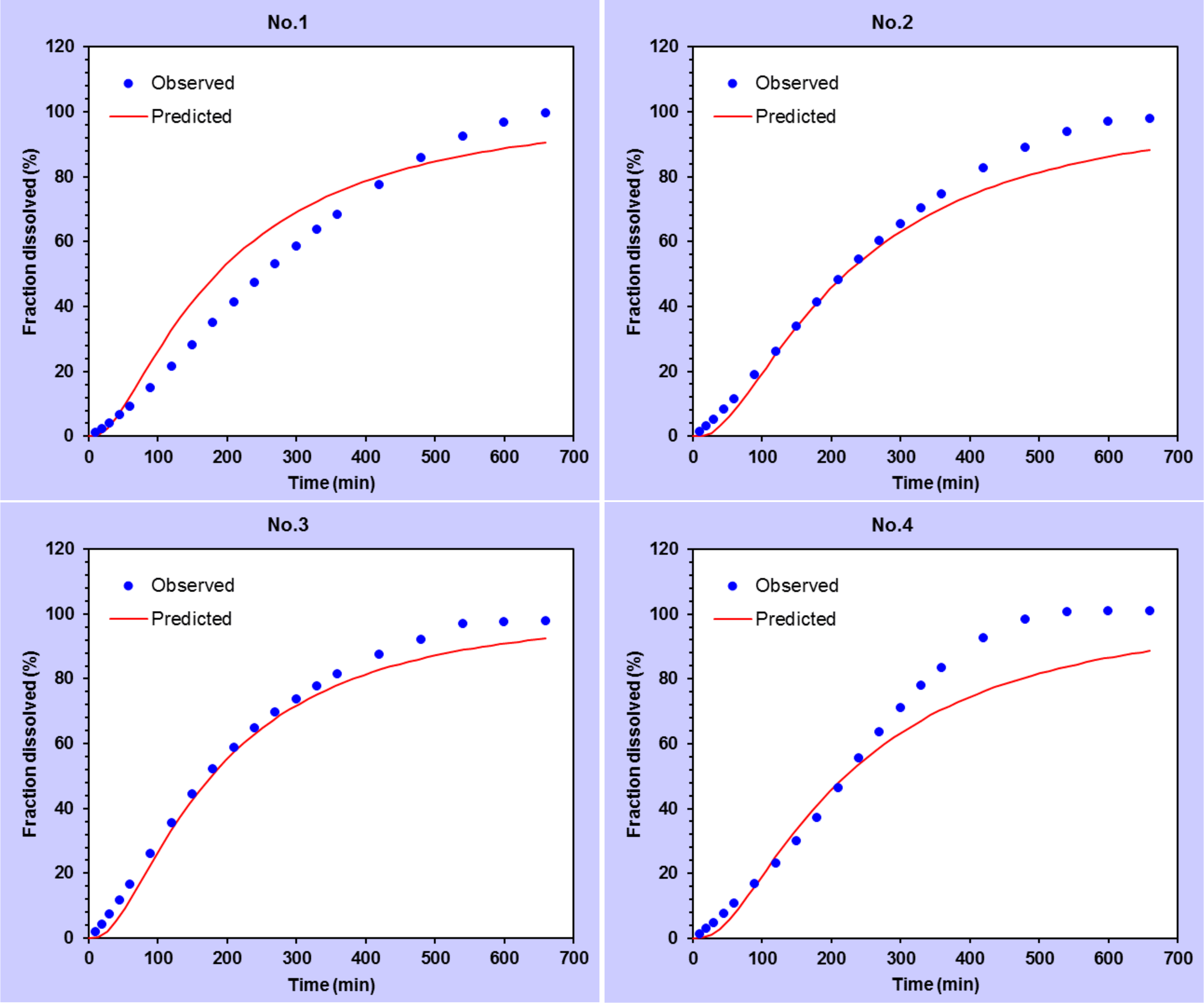

Supplementary materials – Model fitting summary – Kollidon® 90 F (modelling the entire relevant dissolution data)

Model: **Probit_2**

Model equation: $F = F_{max} \cdot \phi[\alpha + \beta \cdot \log(t)]$

Fitted model parameters per tested tablet (N = 4) with statistics – mean, standard deviation (SD), and relative standard deviation expressed in % (RSD%) (output from DDSolver):

Parameter	No.1	No.2	No.3	No.4	Mean	SD	RSD(%)
α	-5.028	-5.639	-5.304	-6.018	-5.497	0.428	-7.781
β	2.158	2.302	2.305	2.476	2.310	0.130	5.631
F_{max}	104.533	114.796	110.119	118.304	111.938	5.968	5.331

Number of dissolution data points (N), degrees of freedom (df), and selected goodness of fit criteria – Pearson correlation coefficient (R), coefficient of determination (R^2), adjusted coefficient of determination ($R^2_{adjusted}$), and residual sum of squares (RSS) (manual calculation in MS Excel):

Parameter	No.1	No.2	No.3	No.4
N	20	20	20	20
df	17	17	17	17
R	0.981708094	0.999547316	0.999569007	0.995813877
R^2	0.963750782	0.999094837	0.9991382	0.991645278
$R^2_{adjusted}$	0.959486168	0.998988347	0.999036812	0.990662369
RSS	994.516144	570.599553	130.3451678	700.8578044

Graphical abstract of model fit presented as mean ± 1 SD of the fraction % of released carvedilol:

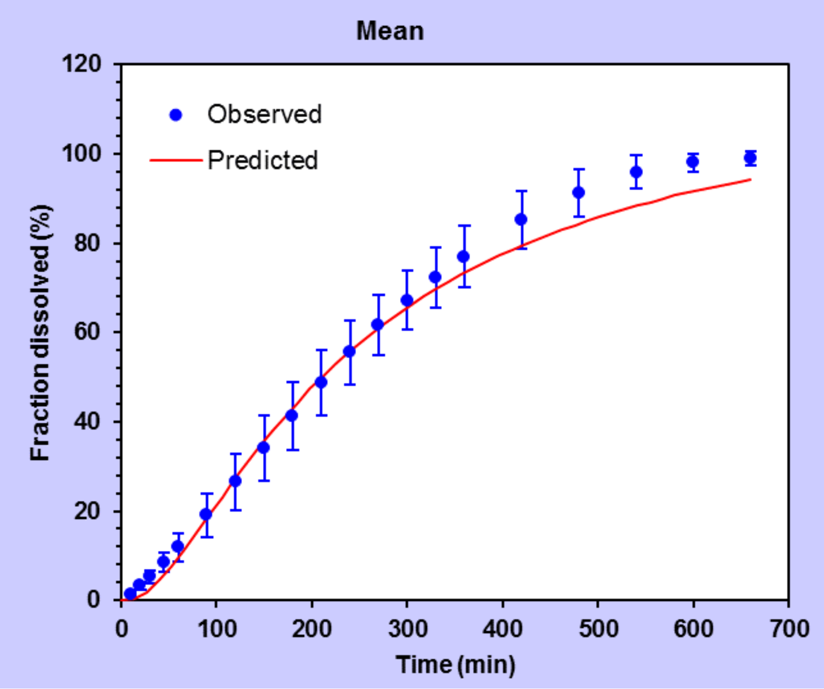

Supplementary materials – Model fitting summary – Kollidon® 90 F (modelling the entire relevant dissolution data)

Graphical abstract of model fit presented as the fraction % of released carvedilol per tested tablet:

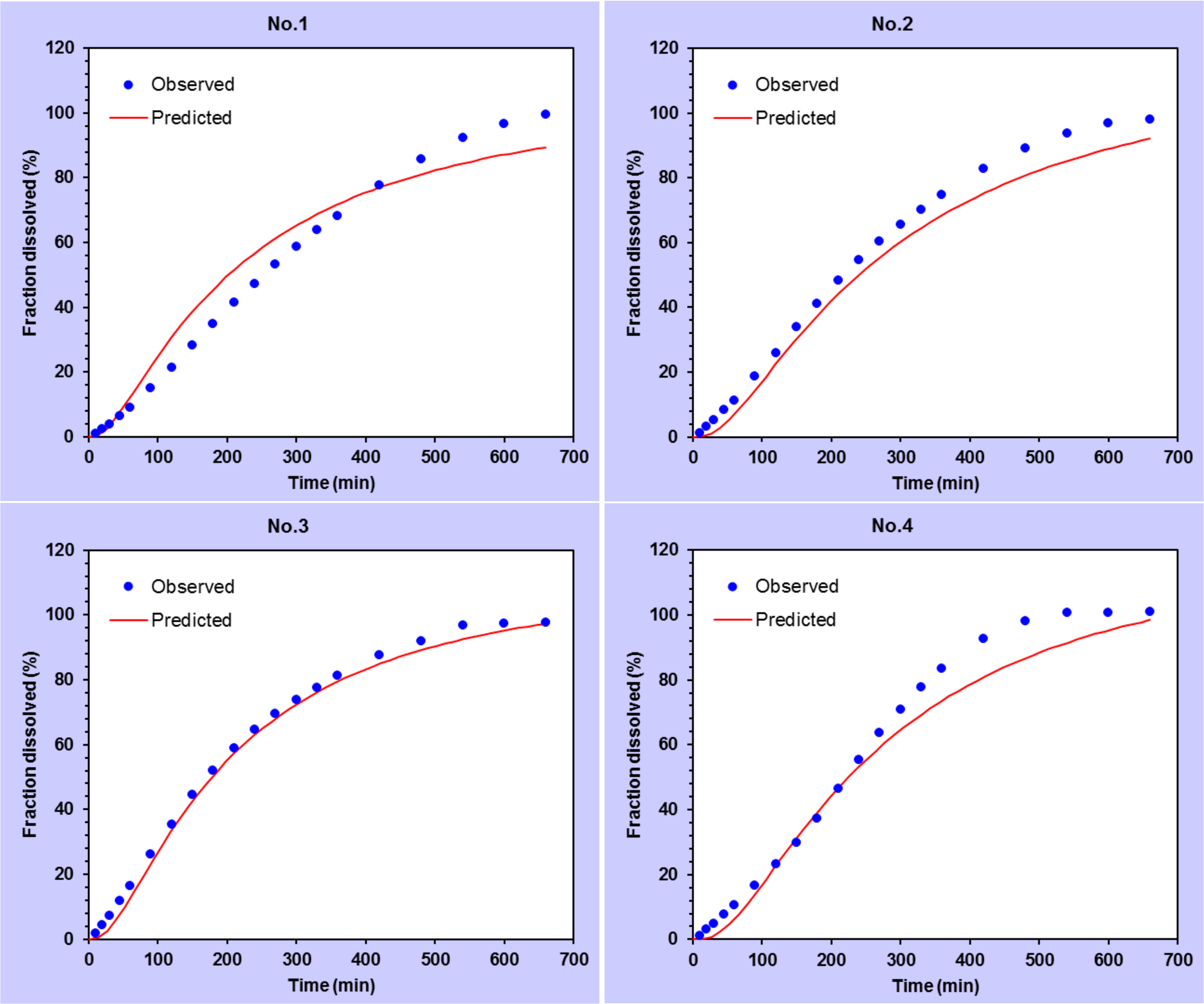

Supplementary materials – Model fitting summary – Kollidon® 90 F (modelling up to app. 60 % of carvedilol released)

Model: **Zero–order**

Model equation: $F = k_0 \cdot t$

Fitted model parameters per tested tablet (N = 4) with statistics – mean, standard deviation (SD), and relative standard deviation expressed in % (RSD%) (output from DDSolver):

Parameter	No.1	No.2	No.3	No.4	Mean	SD	RSD(%)
k_0	0.192	0.224	0.275	0.219	0.228	0.034	15.042

Number of dissolution data points (N), degrees of freedom (df), and selected goodness of fit criteria – Pearson correlation coefficient (R), coefficient of determination (R^2), adjusted coefficient of determination ($R^2_{adjusted}$), and residual sum of squares (RSS) (manual calculation in MS Excel):

Parameter	No.1	No.2	No.3	No.4
N	12	12	12	12
df	11	11	11	11
R	0.999239327	0.999494182	0.996130976	0.996426993
R^2	0.998479233	0.998988619	0.992276922	0.992866753
$R^2_{adjusted}$	0.998479233	0.998988619	0.992276922	0.992866753
RSS	29.71168527	17.93748584	52.428366	77.9146195

Graphical abstract of model fit presented as mean ± 1 SD of the fraction % of released carvedilol:

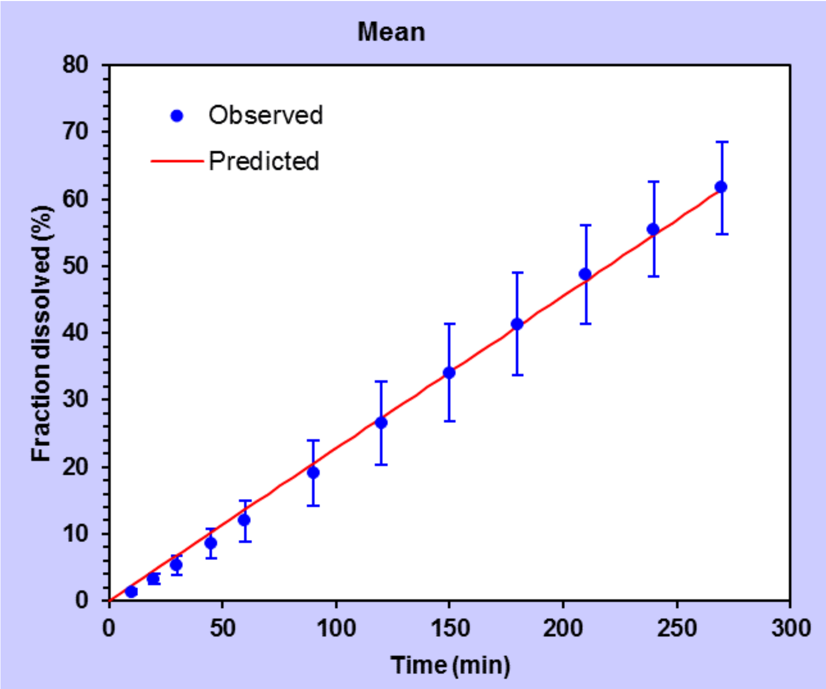

Supplementary materials – Model fitting summary – Kollidon® 90 F (modelling up to app. 60 % of carvedilol released)

Graphical abstract of model fit presented as the fraction % of released carvedilol per tested tablet:

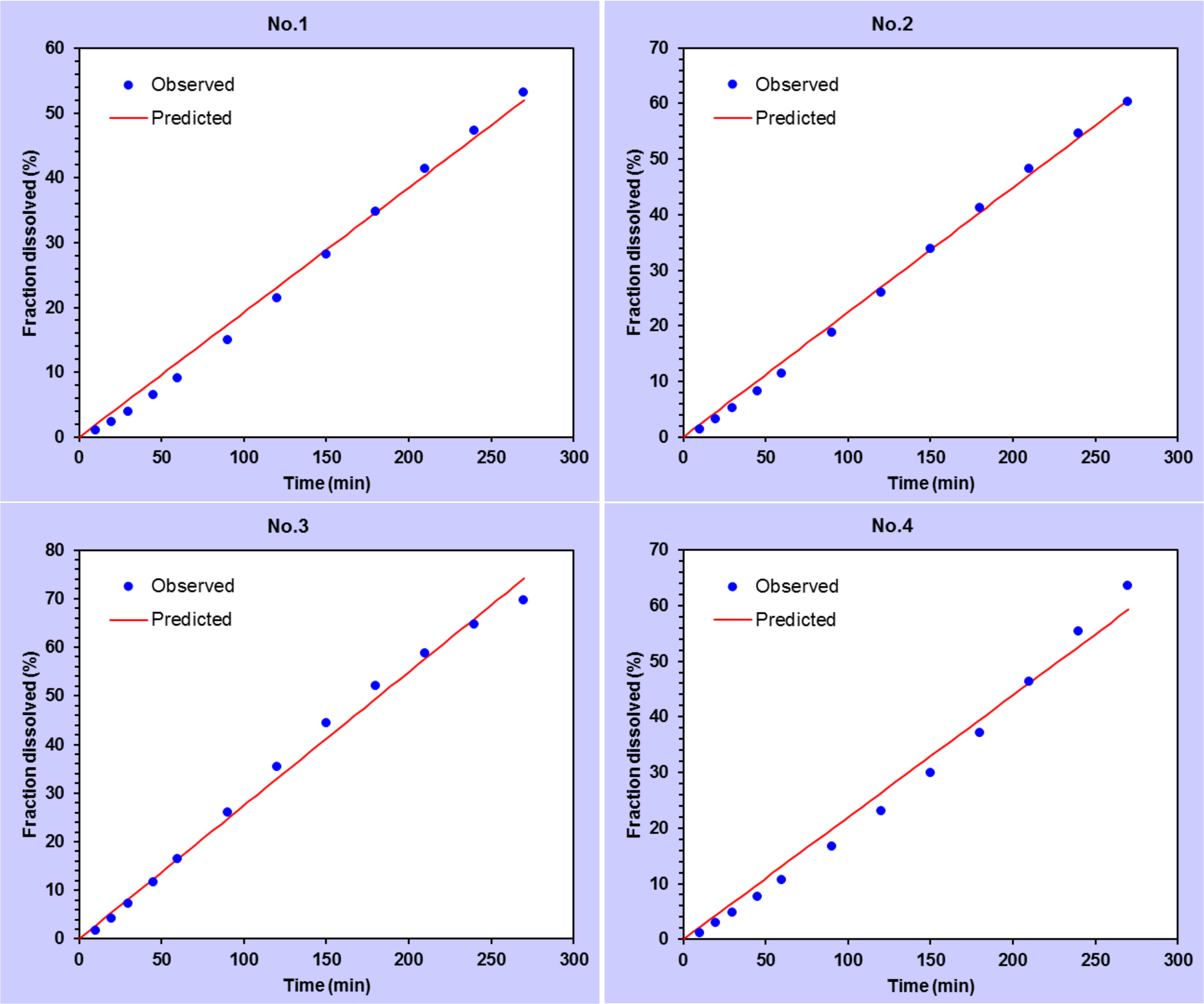

Supplementary materials – Model fitting summary – Kollidon® 90 F (modelling up to app. 60 % of carvedilol released)

Model: **Zero–order with T_{lag}**

Model equation: $F = k_0 \cdot (t - T_{lag})$

Fitted model parameters per tested tablet (N = 4) with statistics – mean, standard deviation (SD), and relative standard deviation expressed in % (RSD%) (output from DDSolver):

Parameter	No.1	No.2	No.3	No.4	Mean	SD	RSD(%)
k_0	0.206	0.234	0.273	0.237	0.237	0.028	11.641
T_{lag}	11.663	7.535	-1.008	13.373	7.891	6.419	81.346

Number of dissolution data points (N), degrees of freedom (df), and selected goodness of fit criteria – Pearson correlation coefficient (R), coefficient of determination (R^2), adjusted coefficient of determination ($R^2_{adjusted}$), and residual sum of squares (RSS) (manual calculation in MS Excel):

Parameter	No.1	No.2	No.3	No.4
N	12	12	12	12
df	10	10	10	10
R	0.999239327	0.999494182	0.996130976	0.996426993
R^2	0.998479233	0.998988619	0.992276922	0.992866753
$R^2_{adjusted}$	0.998327156	0.998887481	0.991504614	0.992153428
RSS	5.783501954	4.985086993	52.11304374	36.18512967

Graphical abstract of model fit presented as mean ± 1 SD of the fraction % of released carvedilol:

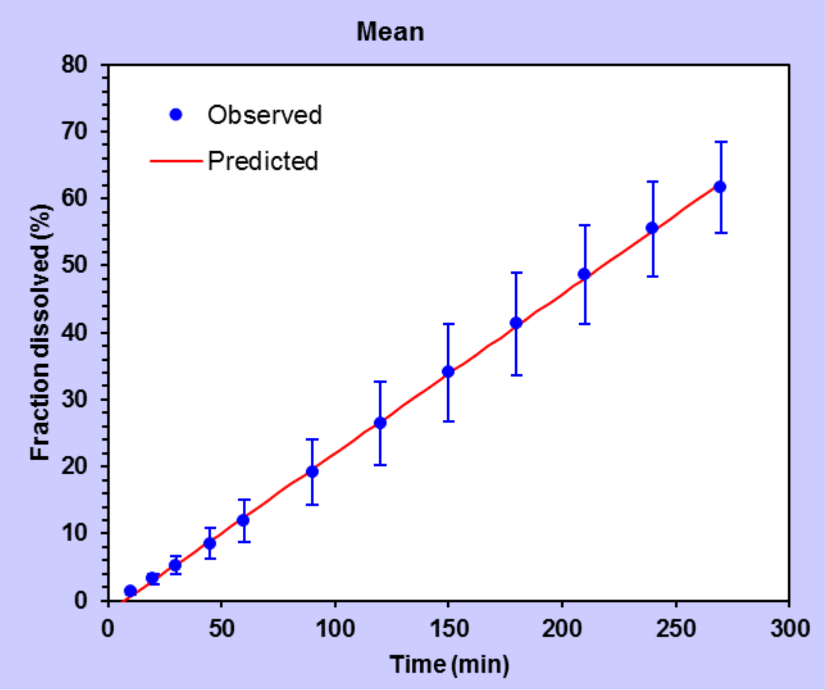

Supplementary materials – Model fitting summary – Kollidon® 90 F (modelling up to app. 60 % of carvedilol released)

Graphical abstract of model fit presented as the fraction % of released carvedilol per tested tablet:

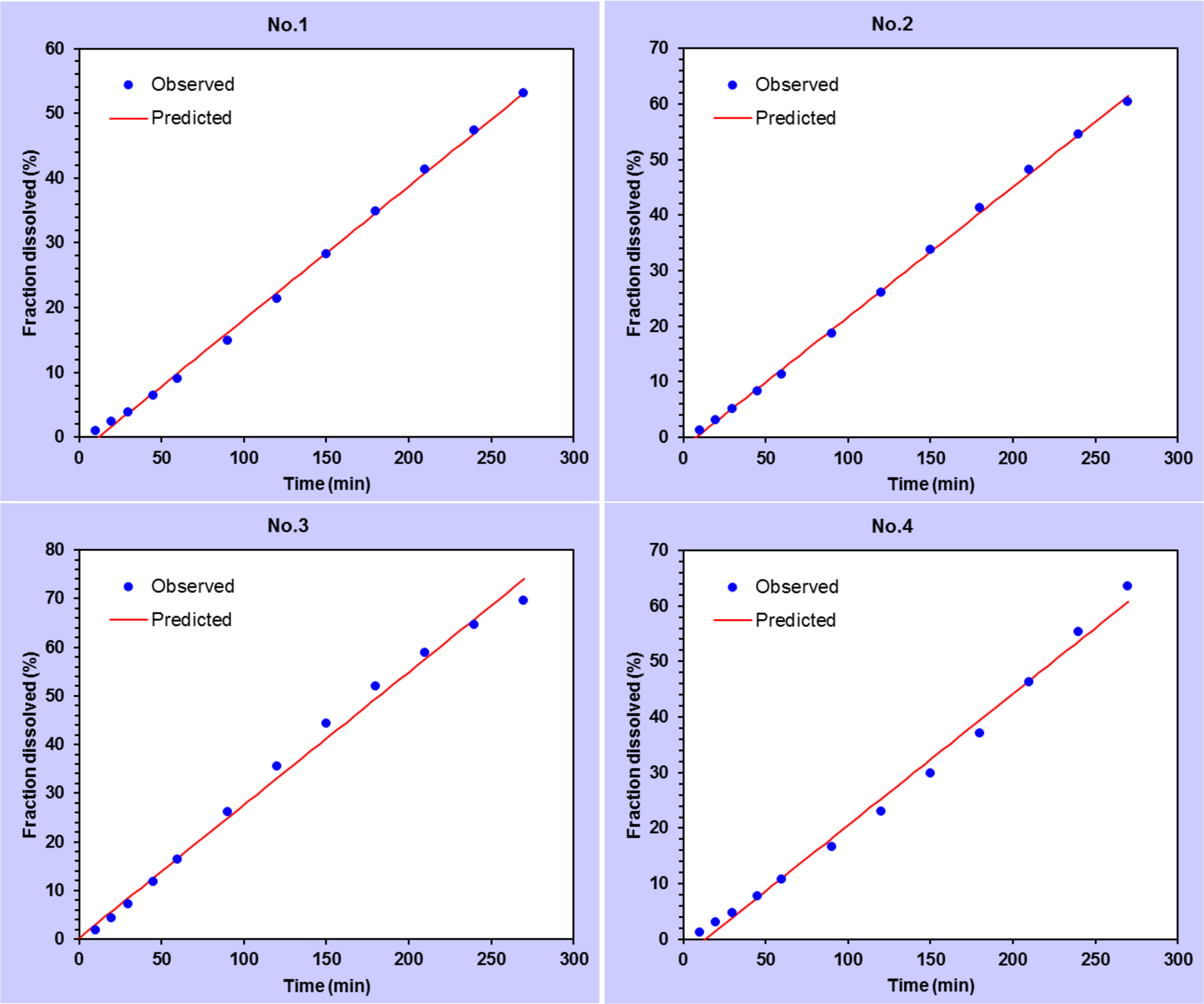

Supplementary materials – Model fitting summary – Kollidon® 90 F (modelling up to app. 60 % of carvedilol released)

Model: **Zero–order with F_0**

Model equation: $F = F_0 + k_0 \cdot t$

Fitted model parameters per tested tablet (N = 4) with statistics – mean, standard deviation (SD), and relative standard deviation expressed in % (RSD%) (output from DDSolver):

Parameter	No.1	No.2	No.3	No.4	Mean	SD	RSD(%)
k_0	0.206	0.234	0.273	0.237	0.237	0.028	11.641
F_0	-2.398	-1.764	0.275	-3.167	-1.764	1.475	-83.653

Number of dissolution data points (N), degrees of freedom (df), and selected goodness of fit criteria – Pearson correlation coefficient (R), coefficient of determination (R^2), adjusted coefficient of determination ($R^2_{adjusted}$), and residual sum of squares (RSS) (manual calculation in MS Excel):

Parameter	No.1	No.2	No.3	No.4
N	12	12	12	12
df	10	10	10	10
R	0.999239327	0.999494182	0.996130976	0.996426993
R^2	0.998479233	0.998988619	0.992276922	0.992866753
$R^2_{adjusted}$	0.998327156	0.998887481	0.991504614	0.992153428
RSS	5.783501954	4.985086993	52.11304374	36.18512967

Graphical abstract of model fit presented as mean ± 1 SD of the fraction % of released carvedilol:

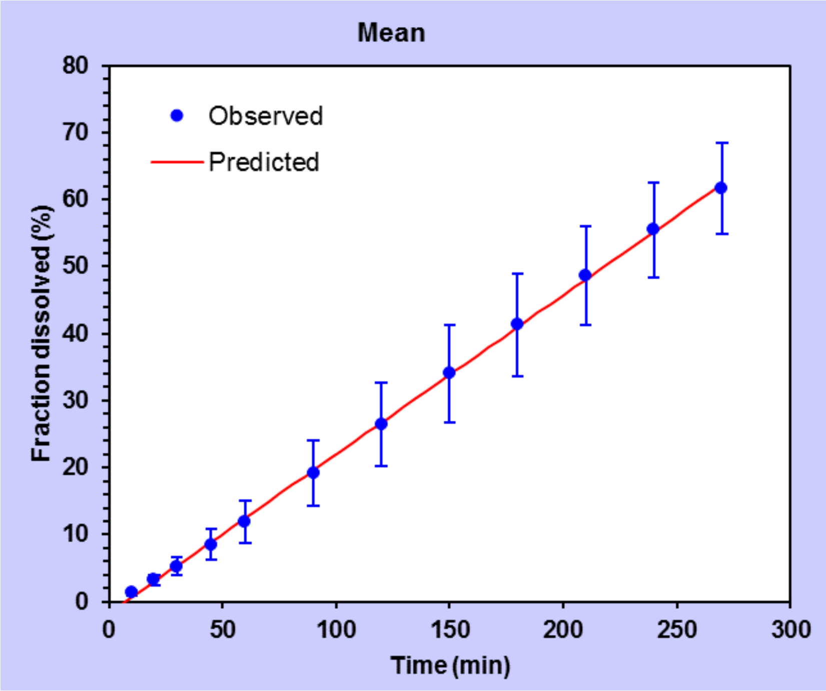

Supplementary materials – Model fitting summary – Kollidon® 90 F (modelling up to app. 60 % of carvedilol released)

Graphical abstract of model fit presented as the fraction % of released carvedilol per tested tablet:

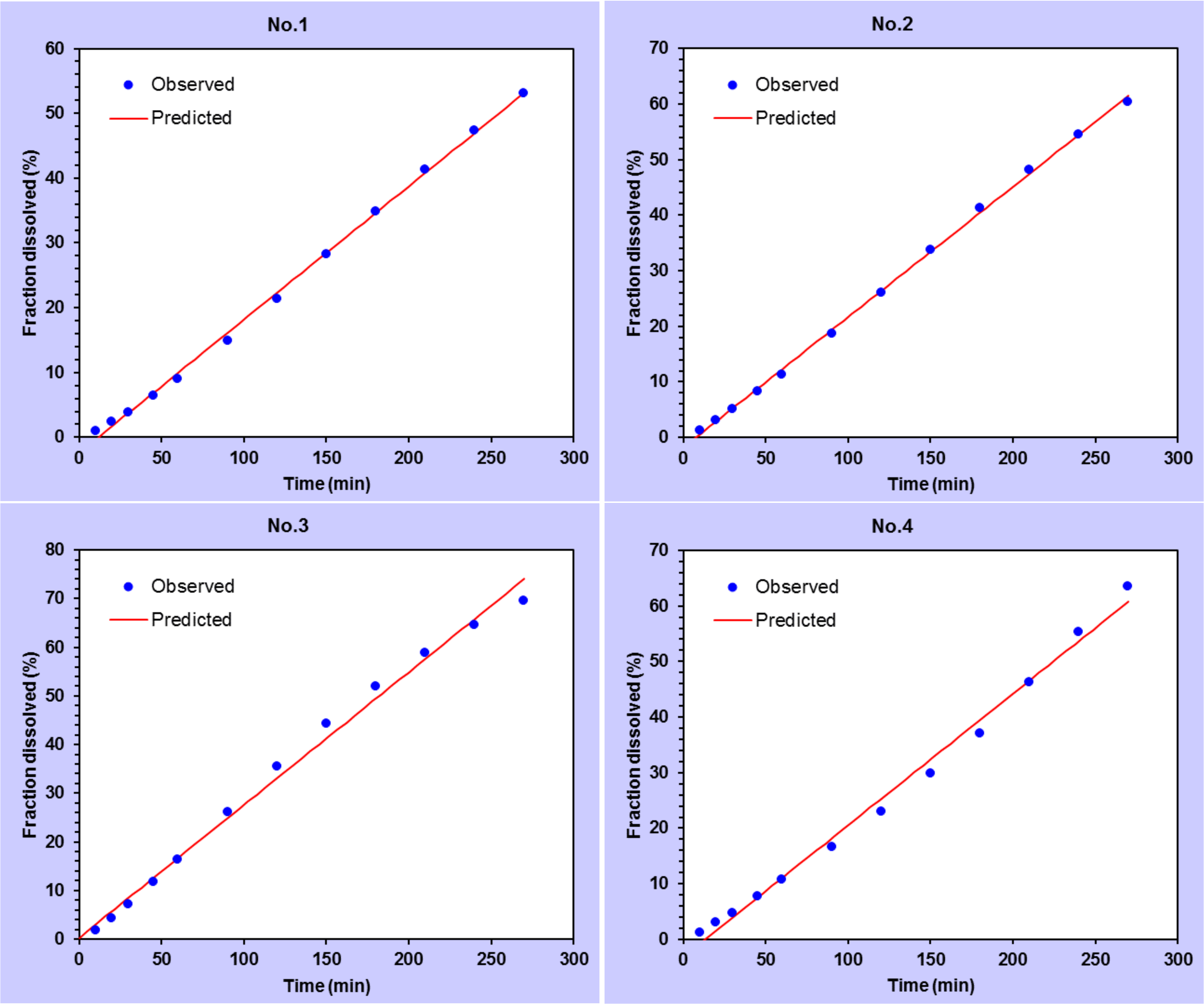

Supplementary materials – Model fitting summary – Kollidon® 90 F (modelling up to app. 60 % of carvedilol released)

Model: **First–order**

Model equation: $F = 100 \cdot (1 - e^{-k_1 \cdot t})$

Fitted model parameters per tested tablet (N = 4) with statistics – mean, standard deviation (SD), and relative standard deviation expressed in % (RSD%) (output from DDSolver):

Parameter	No.1	No.2	No.3	No.4	Mean	SD	RSD(%)
k_1	0.003	0.003	0.004	0.003	0.003	0.001	21.367

Number of dissolution data points (N), degrees of freedom (df), and selected goodness of fit criteria – Pearson correlation coefficient (R), coefficient of determination (R^2), adjusted coefficient of determination ($R^2_{adjusted}$), and residual sum of squares (RSS) (manual calculation in MS Excel):

Parameter	No.1	No.2	No.3	No.4
N	12	12	12	12
df	11	11	11	11
R	0.993594614	0.994561908	0.997709684	0.982985773
R^2	0.987230257	0.989153388	0.995424614	0.96626103
$R^2_{adjusted}$	0.987230257	0.989153388	0.995424614	0.96626103
RSS	143.3126062	166.4601285	153.7179079	348.7548002

Graphical abstract of model fit presented as mean ± 1 SD of the fraction % of released carvedilol:

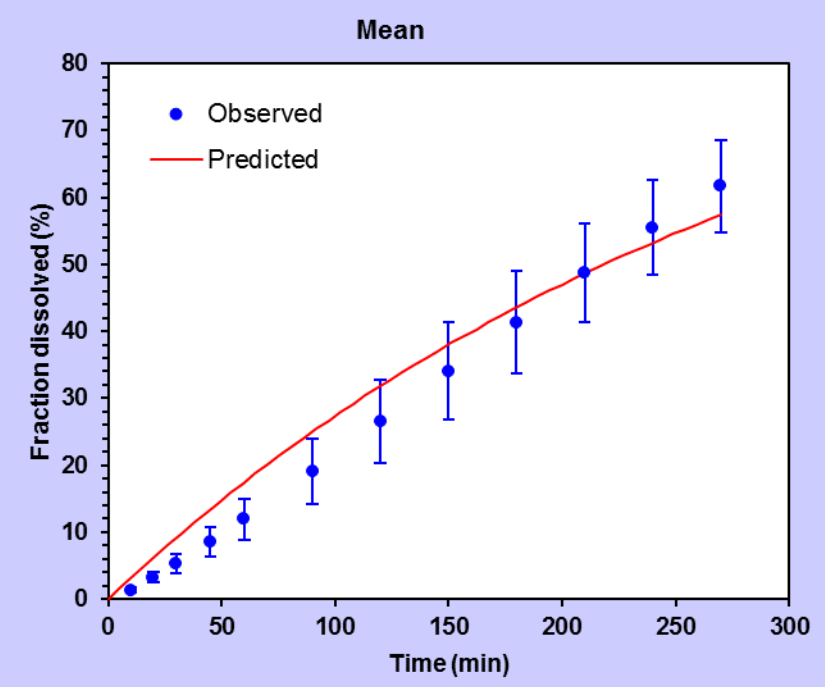

Supplementary materials – Model fitting summary – Kollidon® 90 F (modelling up to app. 60 % of carvedilol released)

Graphical abstract of model fit presented as the fraction % of released carvedilol per tested tablet:

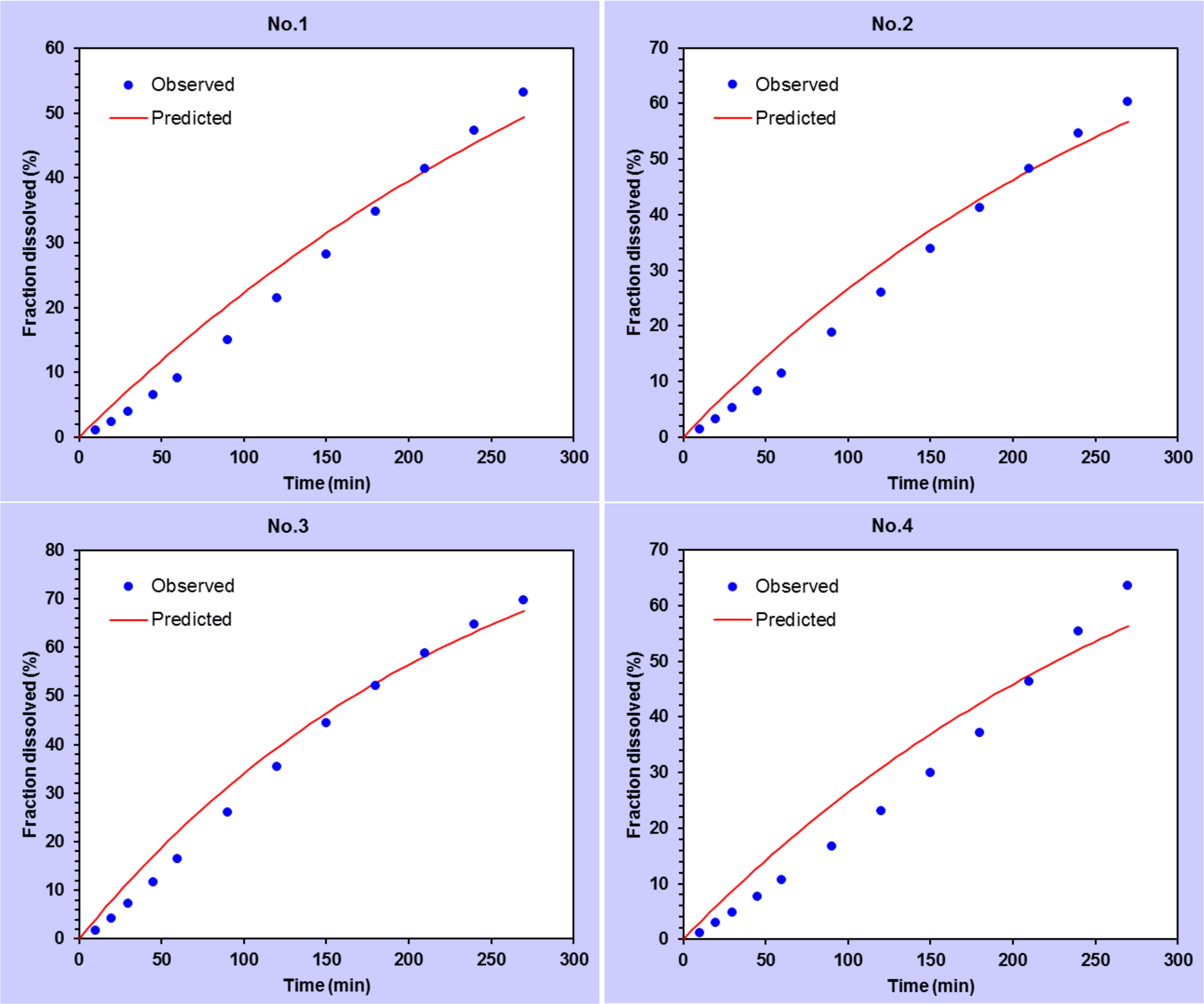

Supplementary materials – Model fitting summary – Kollidon® 90 F (modelling up to app. 60 % of carvedilol released)

Model: **First–order with T_{lag}**

Model equation: $F = 100 \cdot \left[1 - e^{-k_1 \cdot (t - T_{lag})}\right]$

Fitted model parameters per tested tablet (N = 4) with statistics – mean, standard deviation (SD), and relative standard deviation expressed in % (RSD%) (output from DDSolver):

Parameter	No.1	No.2	No.3	No.4	Mean	SD	RSD(%)
k_1	0.003	0.003	0.005	0.004	0.004	0.001	19.576
T_{lag}	21.254	19.825	16.122	25.696	20.724	3.958	19.096

Number of dissolution data points (N), degrees of freedom (df), and selected goodness of fit criteria – Pearson correlation coefficient (R), coefficient of determination (R^2), adjusted coefficient of determination ($R^2_{adjusted}$), and residual sum of squares (RSS) (manual calculation in MS Excel):

Parameter	No.1	No.2	No.3	No.4
N	12	12	12	12
df	10	10	10	10
R	0.992338437	0.993254257	0.99687314	0.979927757
R^2	0.984735574	0.98655402	0.993756057	0.96025841
$R^2_{adjusted}$	0.983209131	0.985209422	0.993131662	0.956284251
RSS	62.45005585	73.49844556	48.02872977	230.7397213

Graphical abstract of model fit presented as mean ± 1 SD of the fraction % of released carvedilol:

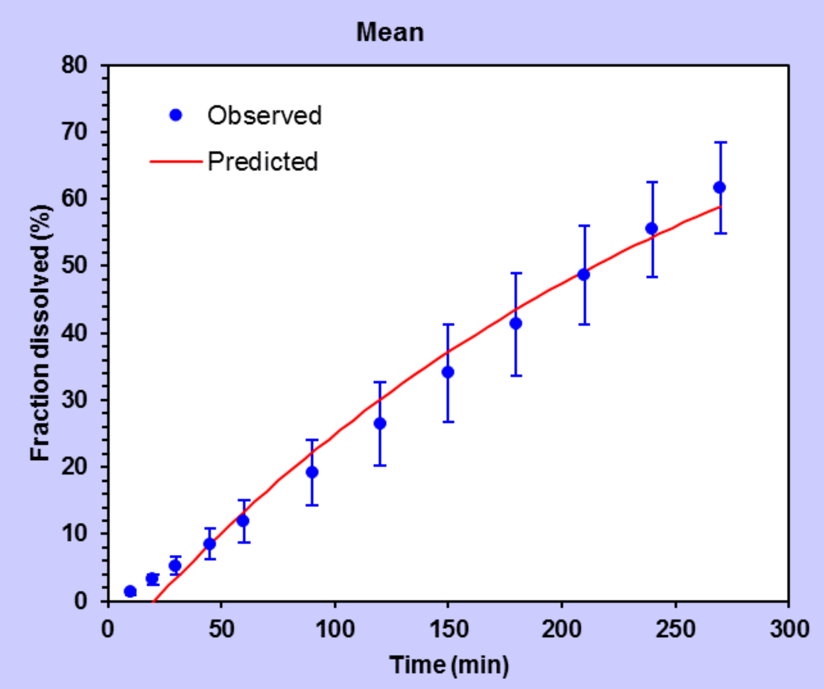

Supplementary materials – Model fitting summary – Kollidon® 90 F (modelling up to app. 60 % of carvedilol released)

Graphical abstract of model fit presented as the fraction % of released carvedilol per tested tablet:

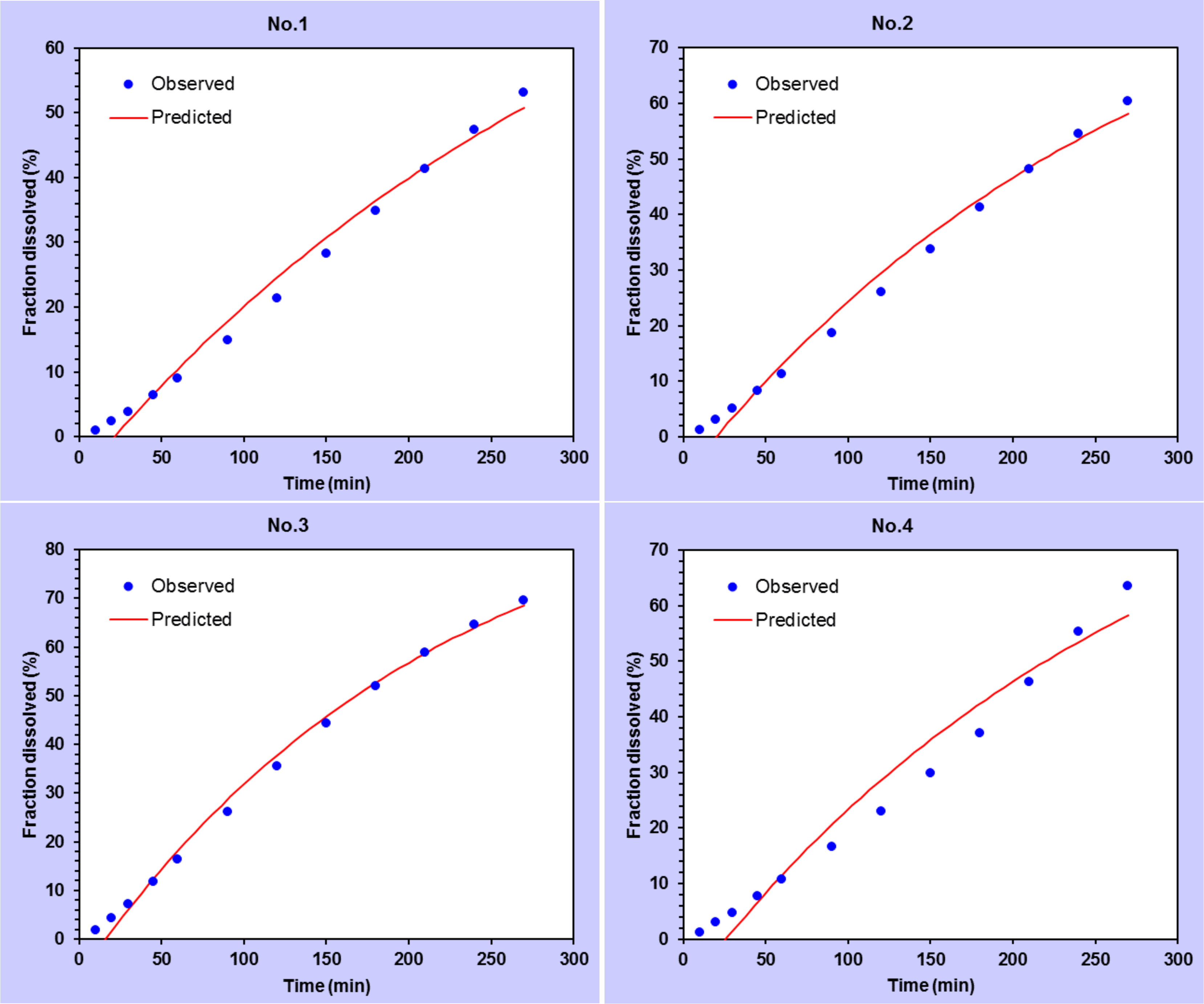

Supplementary materials – Model fitting summary – Kollidon® 90 F (modelling up to app. 60 % of carvedilol released)

Model: **First–order with F_{max}**

Model equation: $F = F_{max} \cdot (1 - e^{-k_1 \cdot t})$

Fitted model parameters per tested tablet (N = 4) with statistics – mean, standard deviation (SD), and relative standard deviation expressed in % (RSD%) (output from DDSolver):

Parameter	No.1	No.2	No.3	No.4	Mean	SD	RSD(%)
k_1	0.008	0.008	0.009	0.007	0.008	0.001	9.139
F_{max}	55.824	63.365	59.385	66.746	61.330	4.746	7.738

Number of dissolution data points (N), degrees of freedom (df), and selected goodness of fit criteria – Pearson correlation coefficient (R), coefficient of determination (R^2), adjusted coefficient of determination ($R^2_{adjusted}$), and residual sum of squares (RSS) (manual calculation in MS Excel):

Parameter	No.1	No.2	No.3	No.4
N	12	12	12	12
df	10	10	10	10
R	0.964918011	0.969995069	0.980305467	0.953941327
R^2	0.931066767	0.940890435	0.960998809	0.910004055
$R^2_{adjusted}$	0.924173444	0.934979478	0.957098689	0.90100446
RSS	764.7845119	870.4061673	762.1929296	1191.163001

Graphical abstract of model fit presented as mean ± 1 SD of the fraction % of released carvedilol:

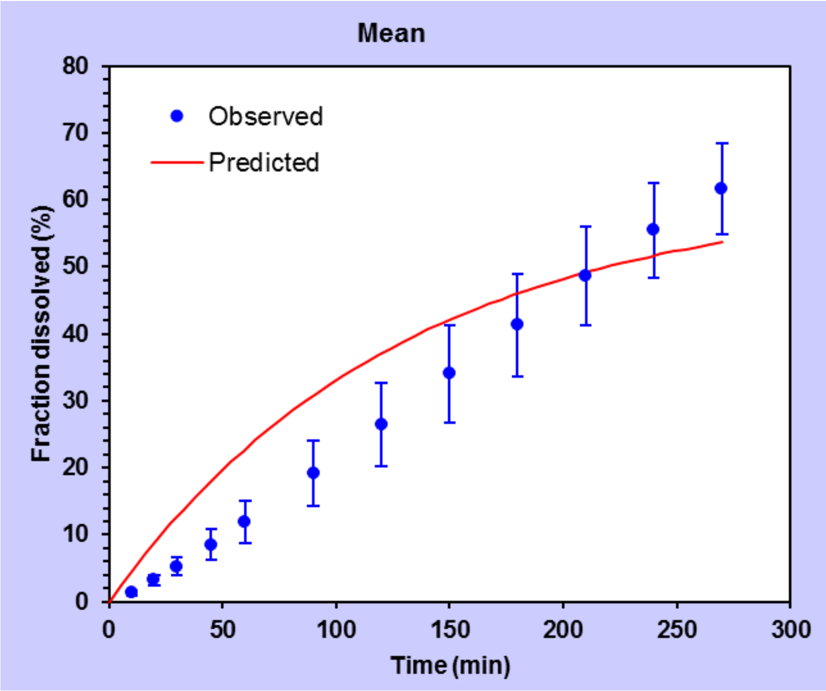

Supplementary materials – Model fitting summary – Kollidon® 90 F (modelling up to app. 60 % of carvedilol released)

Graphical abstract of model fit presented as the fraction % of released carvedilol per tested tablet:

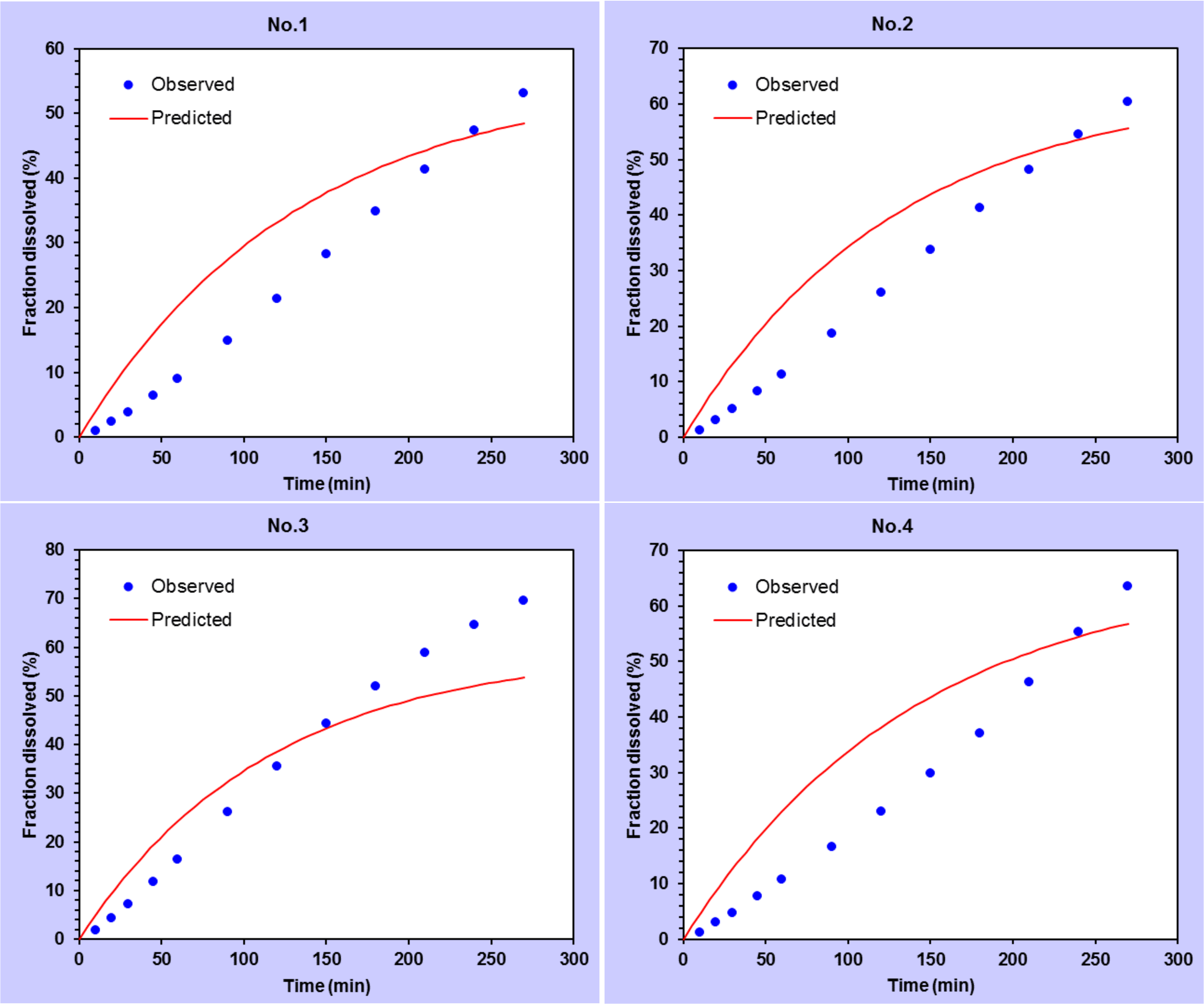

Supplementary materials – Model fitting summary – Kollidon® 90 F (modelling up to app. 60 % of carvedilol released)

Model: **First–order with T_{lag} and F_{max}**

Model equation: $F = F_{max} \cdot \left[1 - e^{-k_1 \cdot (t - T_{lag})}\right]$

Fitted model parameters per tested tablet (N = 4) with statistics – mean, standard deviation (SD), and relative standard deviation expressed in % (RSD%) (output from DDSolver):

Parameter	No.1	No.2	No.3	No.4	Mean	SD	RSD(%)
k_1	0.010	0.010	0.010	0.009	0.010	0.000	5.175
T_{lag}	38.125	36.031	31.329	39.859	36.336	3.687	10.147
F_{max}	55.824	63.365	73.089	66.746	64.756	7.191	11.104

Number of dissolution data points (N), degrees of freedom (df), and selected goodness of fit criteria – Pearson correlation coefficient (R), coefficient of determination (R^2), adjusted coefficient of determination ($R^2_{adjusted}$), and residual sum of squares (RSS) (manual calculation in MS Excel):

Parameter	No.1	No.2	No.3	No.4
N	12	12	12	12
df	9	9	9	9
R	0.949160553	0.95592497	0.97163066	0.936474641
R^2	0.900905754	0.913792548	0.944066139	0.876984754
$R^2_{adjusted}$	0.878884811	0.894635336	0.931636393	0.849648033
RSS	833.8510259	945.3361711	843.1819828	1397.133909

Graphical abstract of model fit presented as mean ± 1 SD of the fraction % of released carvedilol:

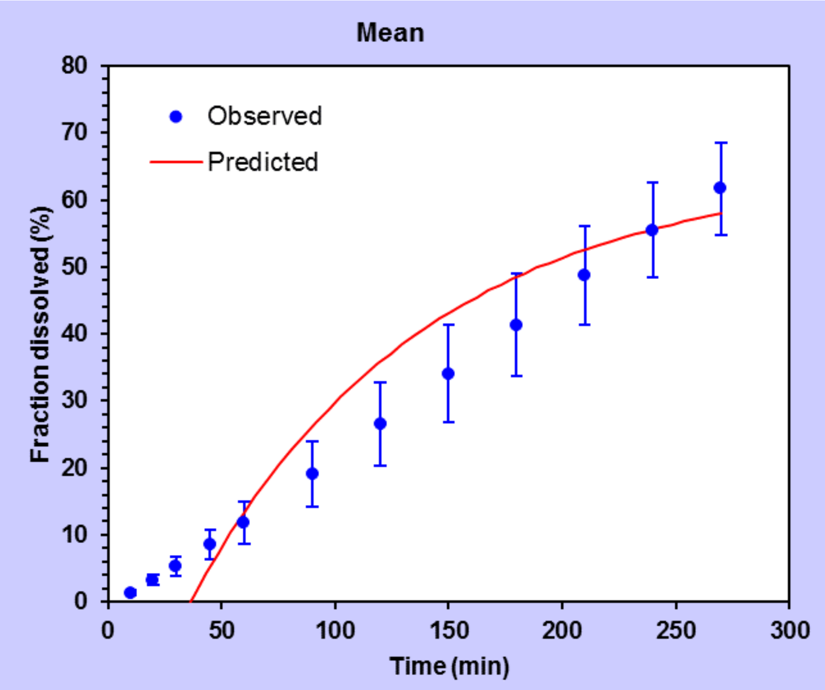

Supplementary materials – Model fitting summary – Kollidon® 90 F (modelling up to app. 60 % of carvedilol released)

Graphical abstract of model fit presented as the fraction % of released carvedilol per tested tablet:

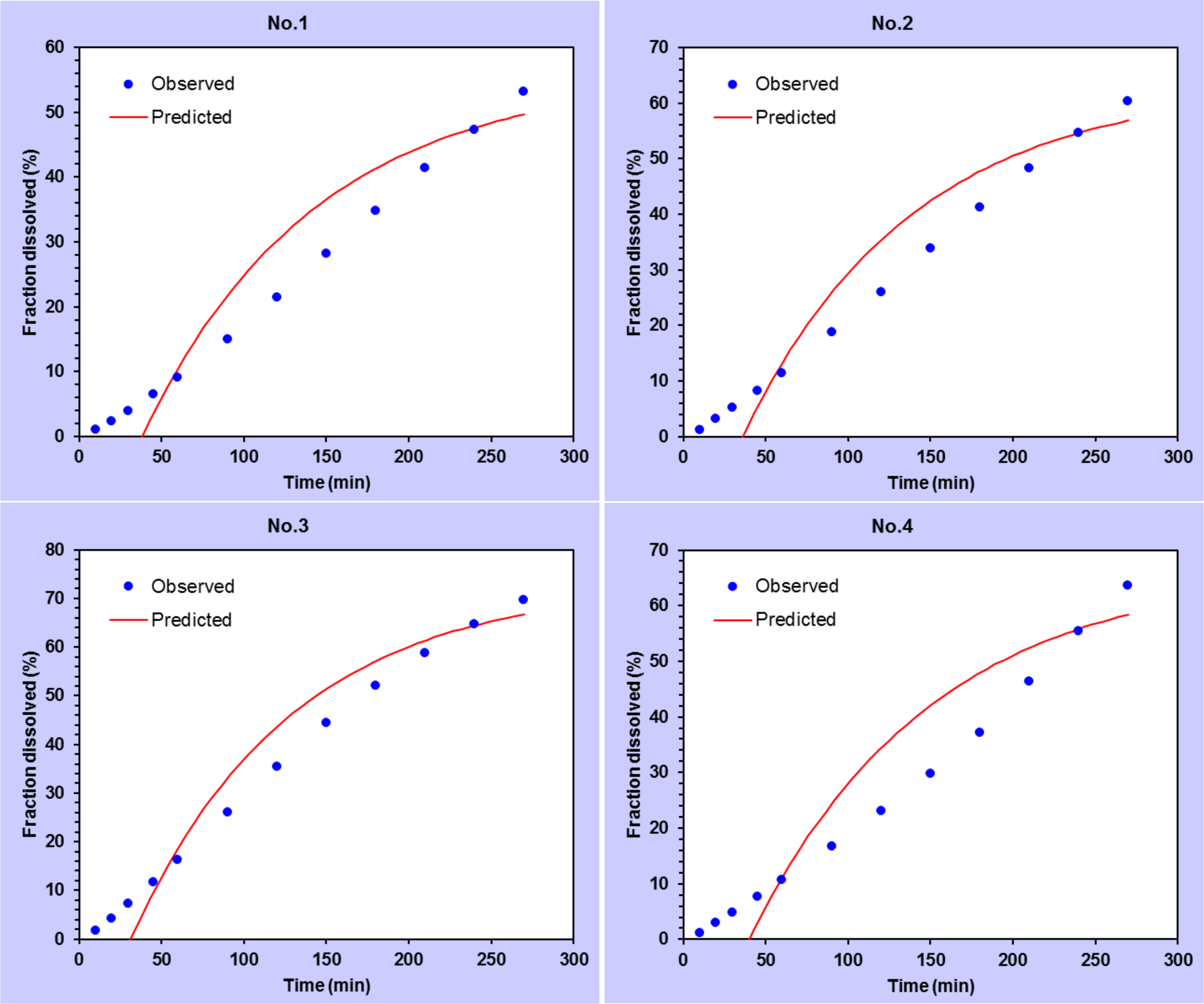

Supplementary materials – Model fitting summary – Kollidon® 90 F (modelling up to app. 60 % of carvedilol released)

Model: **Higuchi**

Model equation: $F = k_H \cdot t^{0.5}$

Fitted model parameters per tested tablet (N = 4) with statistics – mean, standard deviation (SD), and relative standard deviation expressed in % (RSD%) (output from DDSolver):

Parameter	No.1	No.2	No.3	No.4	Mean	SD	RSD(%)
k_H	2.488	2.919	3.610	2.826	2.961	0.471	15.904

Number of dissolution data points (N), degrees of freedom (df), and selected goodness of fit criteria – Pearson correlation coefficient (R), coefficient of determination (R^2), adjusted coefficient of determination ($R^2_{adjusted}$), and residual sum of squares (RSS) (manual calculation in MS Excel):

Parameter	No.1	No.2	No.3	No.4
N	12	12	12	12
df	11	11	11	11
R	0.978426046	0.983398806	0.992431595	0.968339402
R^2	0.957317528	0.967073212	0.984920471	0.937681197
$R^2_{adjusted}$	0.957317528	0.967073212	0.984920471	0.937681197
RSS	798.1347166	929.1990796	1003.253157	1163.862738

Graphical abstract of model fit presented as mean ± 1 SD of the fraction % of released carvedilol:

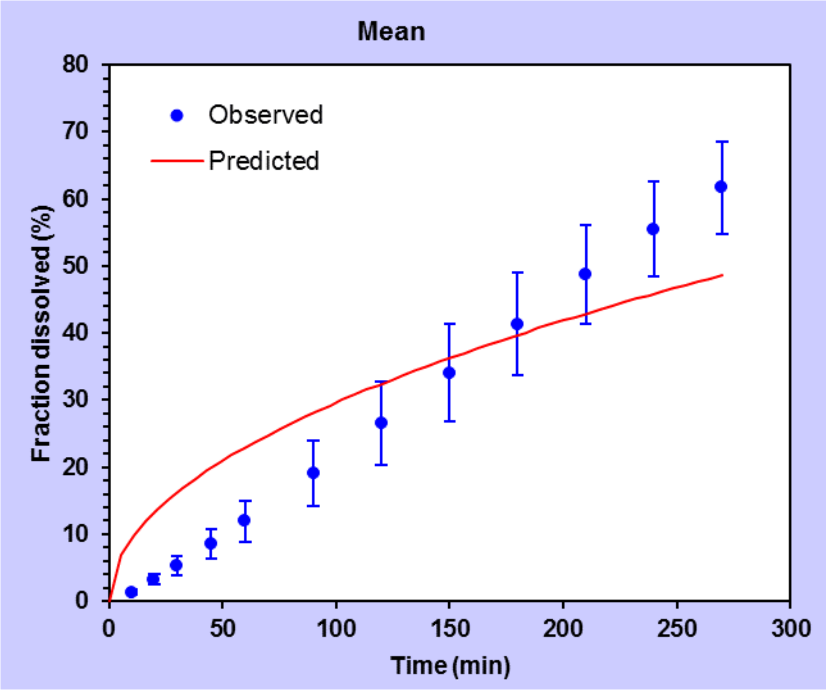

Supplementary materials – Model fitting summary – Kollidon® 90 F (modelling up to app. 60 % of carvedilol released)

Graphical abstract of model fit presented as the fraction % of released carvedilol per tested tablet:

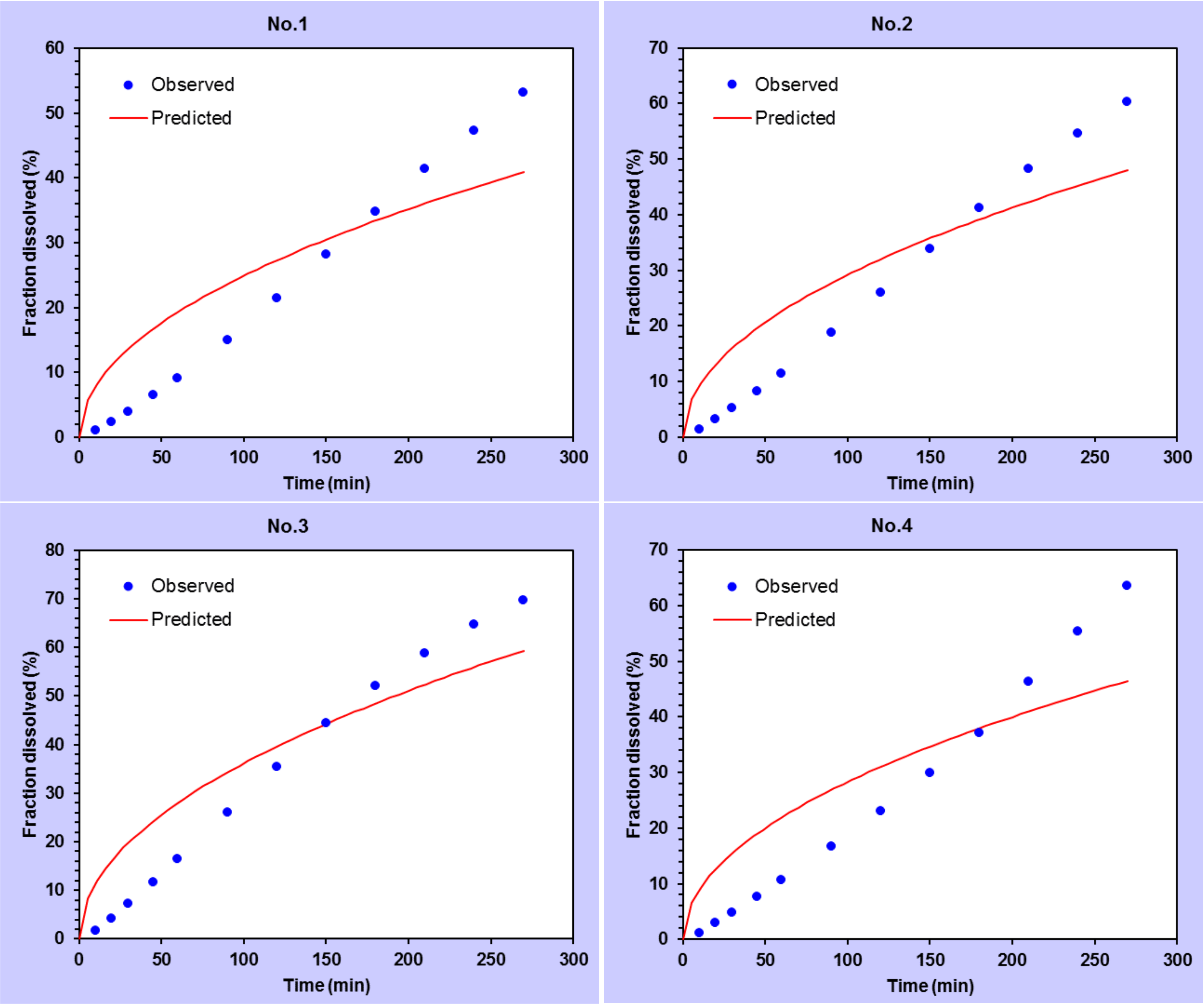

Supplementary materials – Model fitting summary – Kollidon® 90 F (modelling up to app. 60 % of carvedilol released)

Model: **Higuchi with T_{lag}**

Model equation: $F = k_H \cdot (t - T_{lag})^{0.5}$

Fitted model parameters per tested tablet (N = 4) with statistics – mean, standard deviation (SD), and relative standard deviation expressed in % (RSD%) (output from DDSolver):

Parameter	No.1	No.2	No.3	No.4	Mean	SD	RSD(%)
k_H	3.234	3.715	4.392	3.760	3.775	0.475	12.591
T_{lag}	42.065	39.835	34.167	67.227	45.824	14.651	31.973

Number of dissolution data points (N), degrees of freedom (df), and selected goodness of fit criteria – Pearson correlation coefficient (R), coefficient of determination (R^2), adjusted coefficient of determination ($R^2_{adjusted}$), and residual sum of squares (RSS) (manual calculation in MS Excel):

Parameter	No.1	No.2	No.3	No.4
N	12	12	12	12
df	10	10	10	10
R	0.97460823	0.978474979	0.985723477	0.970402032
R^2	0.949861202	0.957413285	0.971650773	0.941680103
$R^2_{adjusted}$	0.944847322	0.953154614	0.968815851	0.935848113
RSS	210.9749795	229.8010945	206.5782957	391.2429798

Graphical abstract of model fit presented as mean ± 1 SD of the fraction % of released carvedilol:

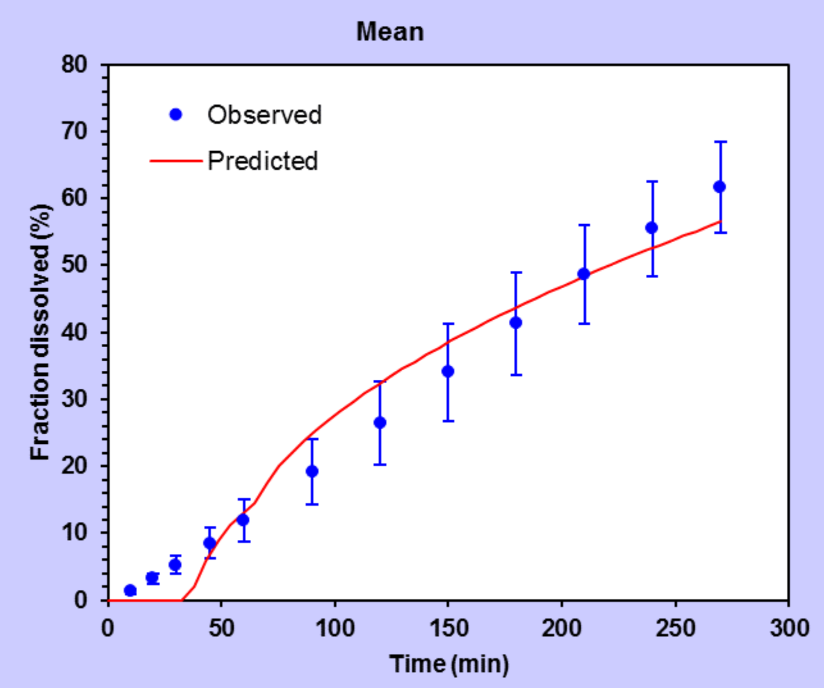

Supplementary materials – Model fitting summary – Kollidon® 90 F (modelling up to app. 60 % of carvedilol released)

Graphical abstract of model fit presented as the fraction % of released carvedilol per tested tablet:

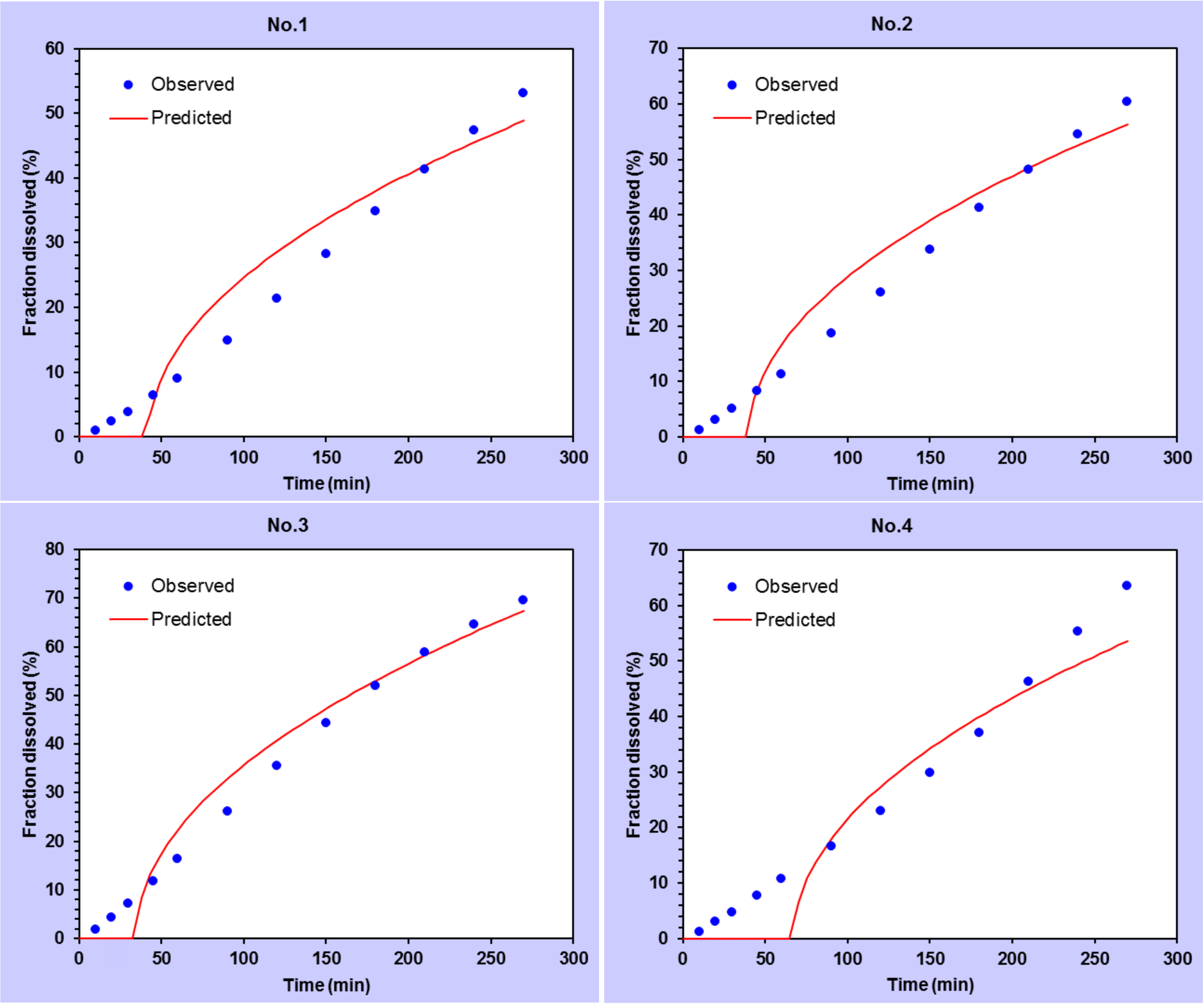

Supplementary materials – Model fitting summary – Kollidon® 90 F (modelling up to app. 60 % of carvedilol released)

Model: **Higuchi with F_0**

Model equation: $F = F_0 + k_H \cdot t^{0.5}$

Fitted model parameters per tested tablet (N = 4) with statistics – mean, standard deviation (SD), and relative standard deviation expressed in % (RSD%) (output from DDSolver):

Parameter	No.1	No.2	No.3	No.4	Mean	SD	RSD(%)
k_H	4.038	4.620	5.456	4.616	4.682	0.584	12.463
F_0	-18.389	-20.196	-21.896	-21.233	-20.429	1.529	-7.486

Number of dissolution data points (N), degrees of freedom (df), and selected goodness of fit criteria – Pearson correlation coefficient (R), coefficient of determination (R^2), adjusted coefficient of determination ($R^2_{adjusted}$), and residual sum of squares (RSS) (manual calculation in MS Excel):

Parameter	No.1	No.2	No.3	No.4
N	12	12	12	12
df	10	10	10	10
R	0.978426046	0.983398806	0.992431595	0.968339402
R^2	0.957317528	0.967073212	0.984920471	0.937681197
$R^2_{adjusted}$	0.953049281	0.963780534	0.983412519	0.931449316
RSS	162.3221214	162.2958889	101.7521918	316.1272686

Graphical abstract of model fit presented as mean ± 1 SD of the fraction % of released carvedilol:

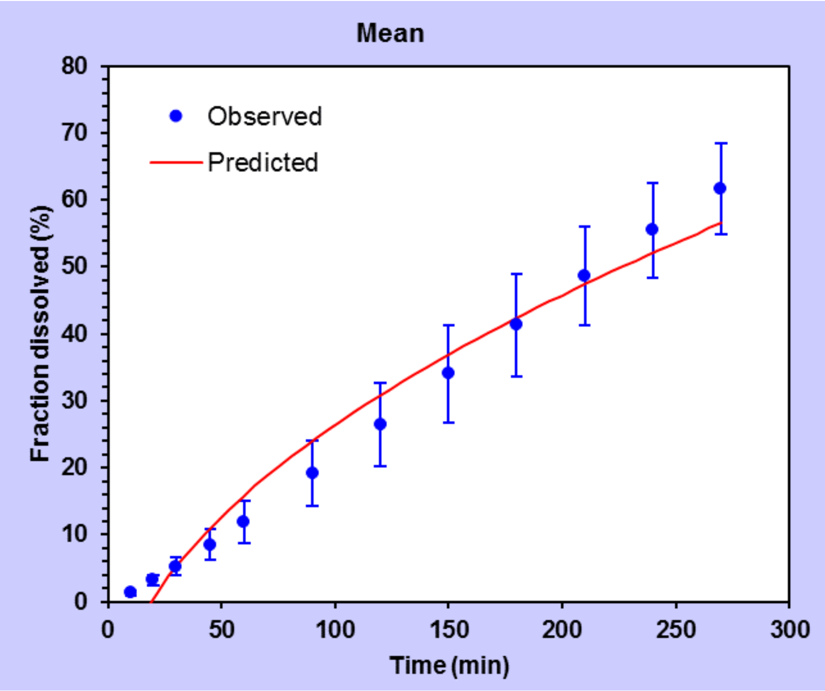

Supplementary materials – Model fitting summary – Kollidon® 90 F (modelling up to app. 60 % of carvedilol released)

Graphical abstract of model fit presented as the fraction % of released carvedilol per tested tablet:

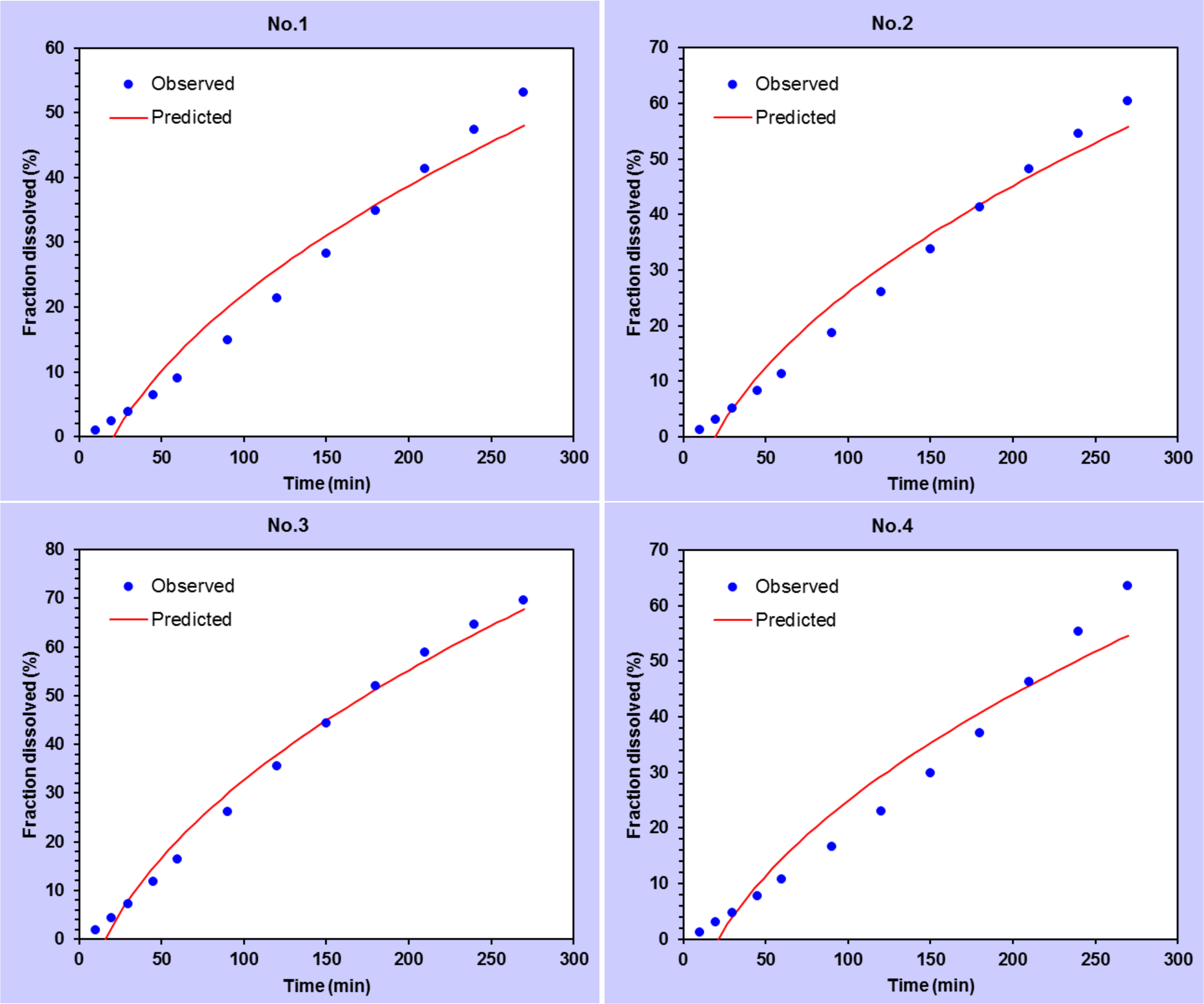

Supplementary materials – Model fitting summary – Kollidon® 90 F (modelling up to app. 60 % of carvedilol released)

Model: **Korsmeyer–Peppas**

Model equation: $F = k_{KP} \cdot t^n$

Fitted model parameters per tested tablet (N = 4) with statistics – mean, standard deviation (SD), and relative standard deviation expressed in % (RSD%) (output from DDSolver):

Parameter	No.1	No.2	No.3	No.4	Mean	SD	RSD(%)
k_{KP}	0.073	0.096	0.193	0.084	0.111	0.055	49.314
n	1.178	1.164	1.077	1.180	1.150	0.049	4.250

Number of dissolution data points (N), degrees of freedom (df), and selected goodness of fit criteria – Pearson correlation coefficient (R), coefficient of determination (R^2), adjusted coefficient of determination ($R^2_{adjusted}$), and residual sum of squares (RSS) (manual calculation in MS Excel):

Parameter	No.1	No.2	No.3	No.4
N	12	12	12	12
df	10	10	10	10
R	0.999375457	0.998315146	0.994098146	0.999300283
R^2	0.998751304	0.996633131	0.988231124	0.998601056
$R^2_{adjusted}$	0.998626434	0.996296444	0.987054237	0.998461162
RSS	9.649984514	29.02759087	163.5019079	8.111274101

Graphical abstract of model fit presented as mean ± 1 SD of the fraction % of released carvedilol:

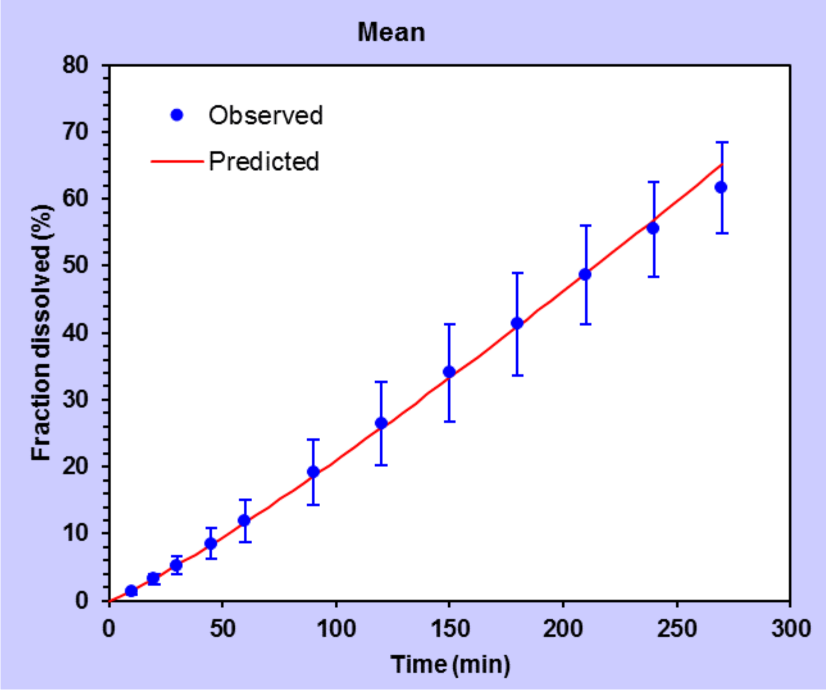

Supplementary materials – Model fitting summary – Kollidon® 90 F (modelling up to app. 60 % of carvedilol released)

Graphical abstract of model fit presented as the fraction % of released carvedilol per tested tablet:

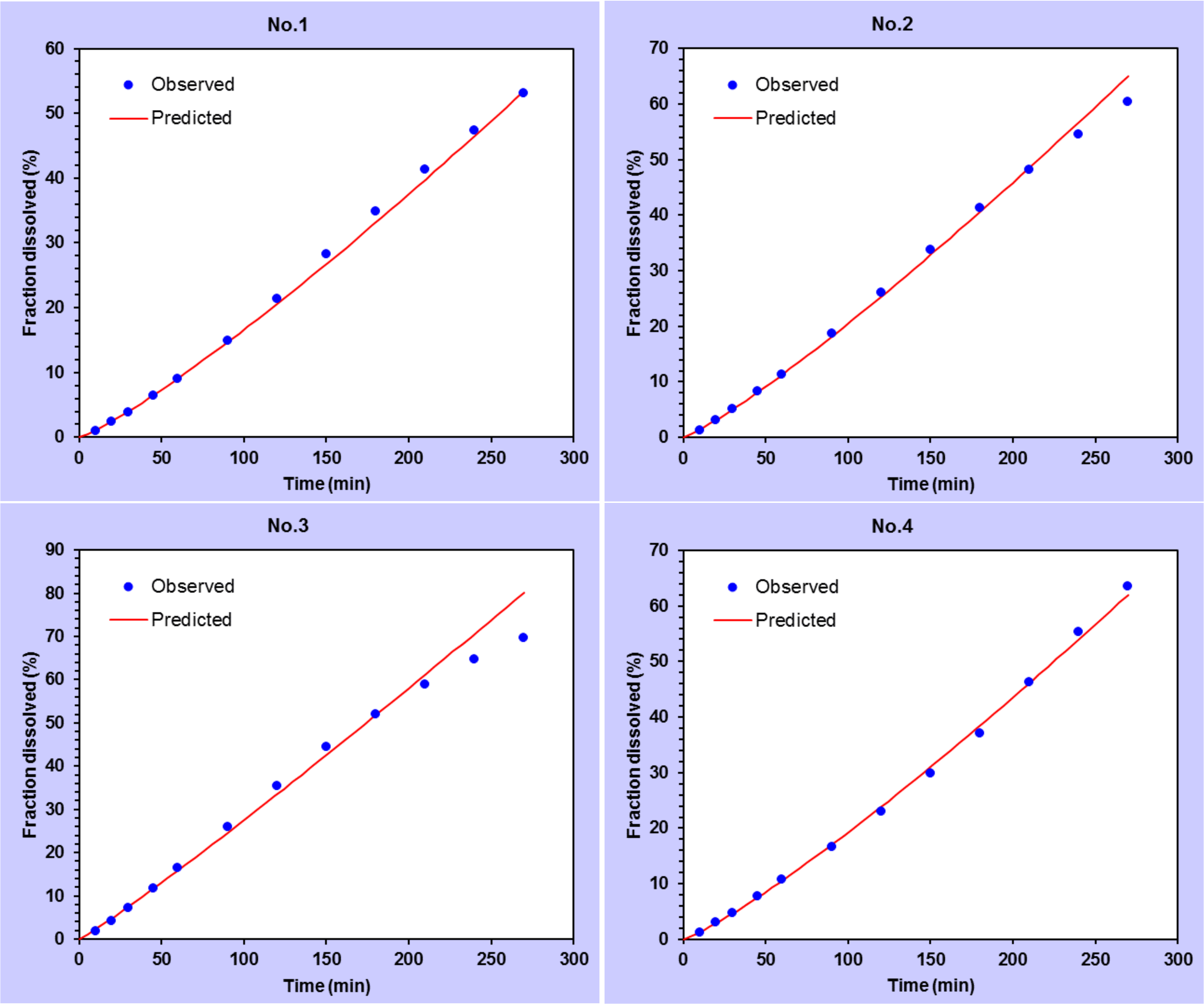

Supplementary materials – Model fitting summary – Kollidon® 90 F (modelling up to app. 60 % of carvedilol released)

Model: **Korsmeyer–Peppas with T_{lag}**

Model equation: $F = k_{KP} \cdot \left(t - T_{lag}\right)^n$

Fitted model parameters per tested tablet (N = 4) with statistics – mean, standard deviation (SD), and relative standard deviation expressed in % (RSD%) (output from DDSolver):

Parameter	No.1	No.2	No.3	No.4	Mean	SD	RSD(%)
k_{KP}	0.125	0.184	0.291	0.163	0.191	0.071	37.349
n	1.081	1.039	0.997	1.053	1.043	0.035	3.378
T_{lag}	4.000	4.000	4.000	4.000	4.000	0.000	0.000

Number of dissolution data points (N), degrees of freedom (df), and selected goodness of fit criteria – Pearson correlation coefficient (R), coefficient of determination (R^2), adjusted coefficient of determination ($R^2_{adjusted}$), and residual sum of squares (RSS) (manual calculation in MS Excel):

Parameter	No.1	No.2	No.3	No.4
N	12	12	12	12
df	9	9	9	9
R	0.999681012	0.999434546	0.996205121	0.997640871
R^2	0.999362126	0.998869412	0.992424643	0.995287308
$R^2_{adjusted}$	0.999220376	0.99861817	0.990741231	0.994240043
RSS	8.251985492	7.17593589	66.80814593	53.80468962

Graphical abstract of model fit presented as mean ± 1 SD of the fraction % of released carvedilol:

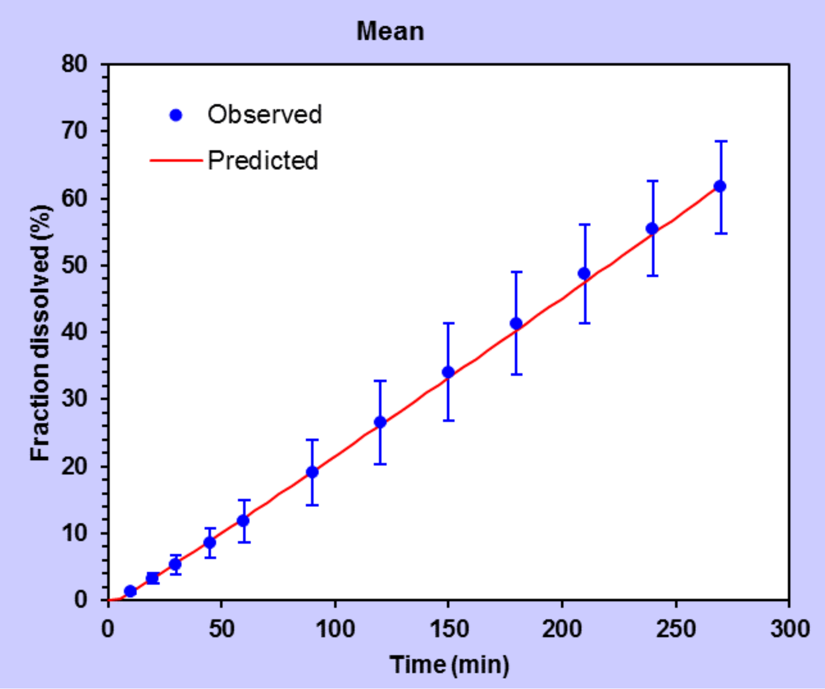

Supplementary materials – Model fitting summary – Kollidon® 90 F (modelling up to app. 60 % of carvedilol released)

Graphical abstract of model fit presented as the fraction % of released carvedilol per tested tablet:

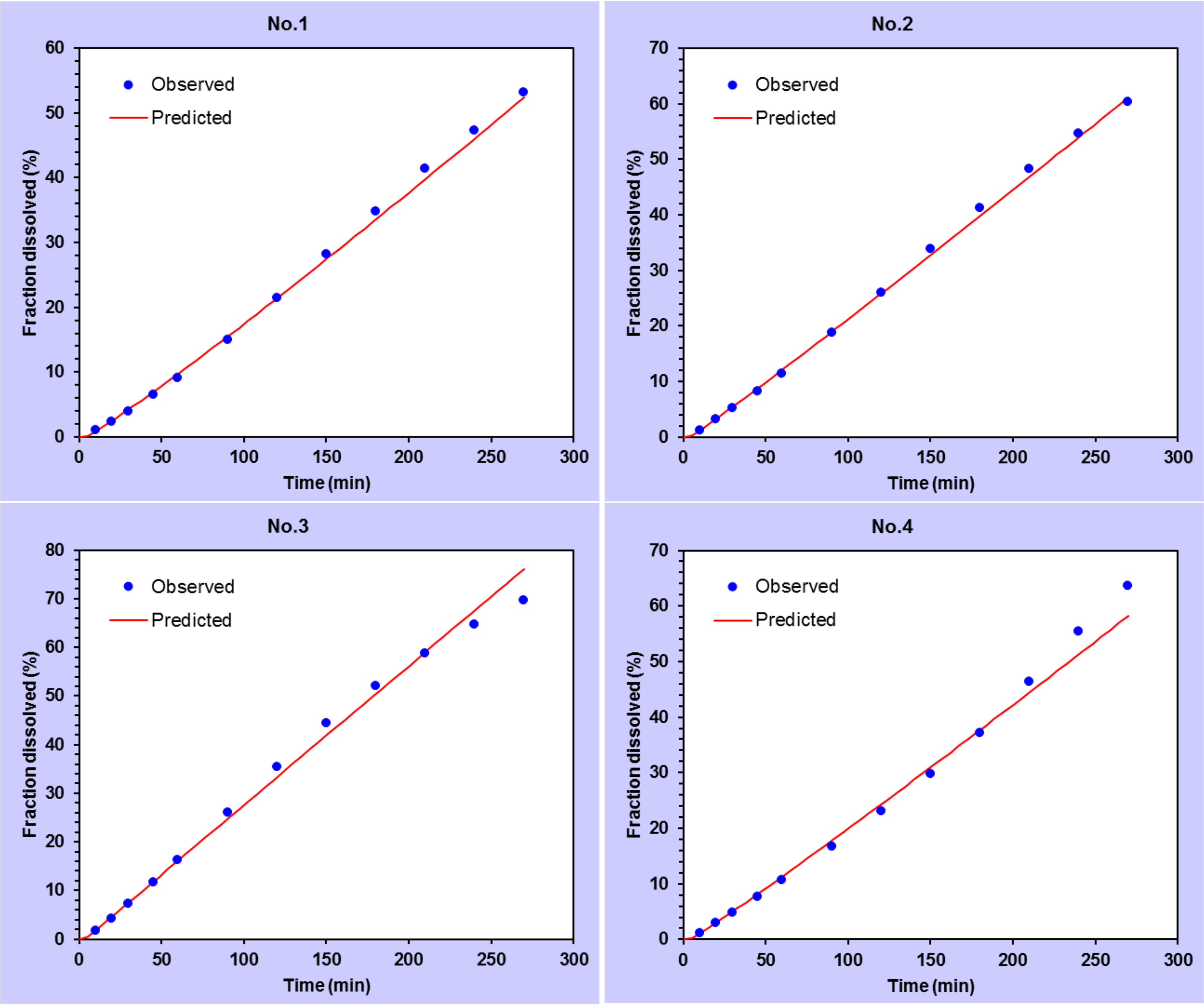

Supplementary materials – Model fitting summary – Kollidon® 90 F (modelling up to app. 60 % of carvedilol released)

Model: **Korsmeyer–Peppas with F_0**

Model equation: $F = F_0 + k_{KP} \cdot t^n$

Fitted model parameters per tested tablet (N = 4) with statistics – mean, standard deviation (SD), and relative standard deviation expressed in % (RSD%) (output from DDSolver):

Parameter	No.1	No.2	No.3	No.4	Mean	SD	RSD(%)
k_{KP}	0.035	0.061	0.089	0.046	0.058	0.023	40.174
n	1.325	1.229	1.226	1.289	1.267	0.048	3.800
F_0	0.400	0.627	0.720	0.609	0.589	0.135	22.936

Number of dissolution data points (N), degrees of freedom (df), and selected goodness of fit criteria – Pearson correlation coefficient (R), coefficient of determination (R^2), adjusted coefficient of determination ($R^2_{adjusted}$), and residual sum of squares (RSS) (manual calculation in MS Excel):

Parameter	No.1	No.2	No.3	No.4
N	12	12	12	12
df	9	9	9	9
R	0.997505677	0.997217449	0.988858685	0.99970074
R^2	0.995017575	0.99444264	0.977841499	0.99940157
$R^2_{adjusted}$	0.99391037	0.993207672	0.972917388	0.999268586
RSS	57.88222913	87.99551088	399.9590399	8.71284657

Graphical abstract of model fit presented as mean ± 1 SD of the fraction % of released carvedilol:

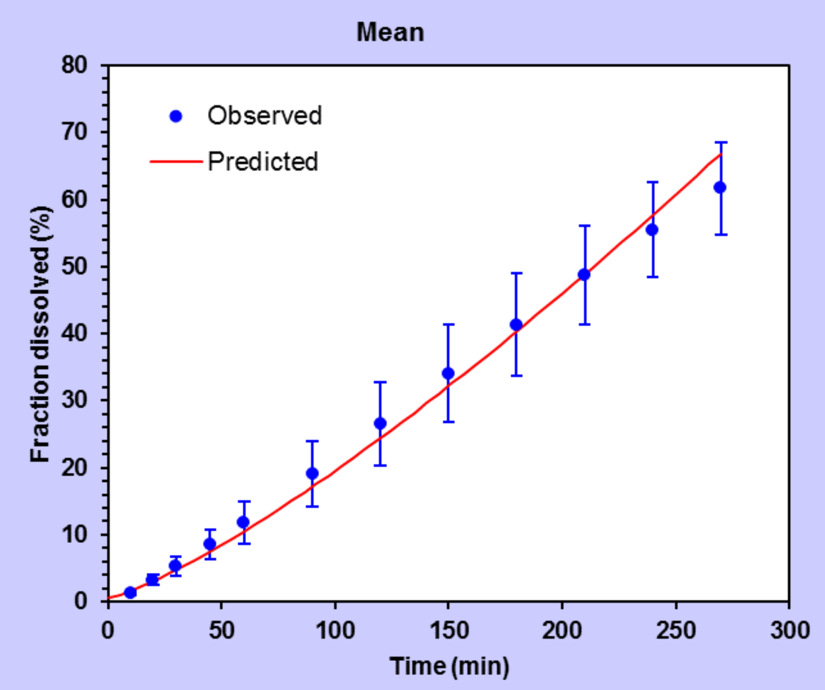

Supplementary materials – Model fitting summary – Kollidon® 90 F (modelling up to app. 60 % of carvedilol released)

Graphical abstract of model fit presented as the fraction % of released carvedilol per tested tablet:

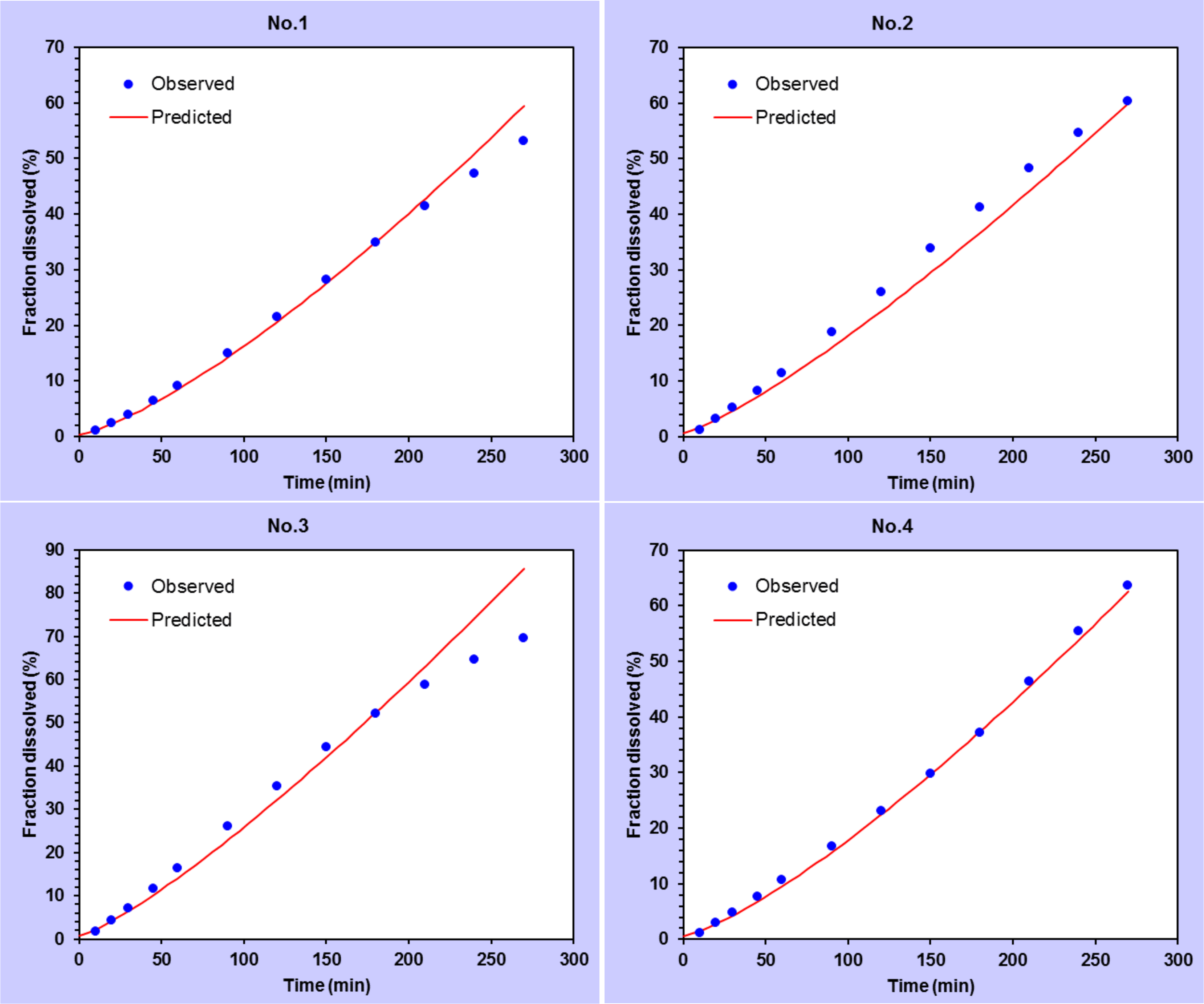

Supplementary materials – Model fitting summary – Kollidon® 90 F (modelling up to app. 60 % of carvedilol released)

Model: **Hixson–Crowell**

Model equation: $F = 100 \cdot [1 - (1 - k_{HC} \cdot t)^3]$

Fitted model parameters per tested tablet (N = 4) with statistics – mean, standard deviation (SD), and relative standard deviation expressed in % (RSD%) (output from DDSolver):

Parameter	No.1	No.2	No.3	No.4	Mean	SD	RSD(%)
k_{HC}	0.001	0.001	0.001	0.001	0.001	0.000	19.000

Number of dissolution data points (N), degrees of freedom (df), and selected goodness of fit criteria – Pearson correlation coefficient (R), coefficient of determination (R^2), adjusted coefficient of determination ($R^2_{adjusted}$), and residual sum of squares (RSS) (manual calculation in MS Excel):

Parameter	No.1	No.2	No.3	No.4
N	12	12	12	12
df	11	11	11	11
R	0.996205116	0.997339827	0.999384183	0.988346023
R^2	0.992424633	0.99468673	0.998768746	0.976827861
$R^2_{adjusted}$	0.992424633	0.99468673	0.998768746	0.976827861
RSS	94.10101497	93.89821883	59.89352222	234.6546687

Graphical abstract of model fit presented as mean ± 1 SD of the fraction % of released carvedilol:

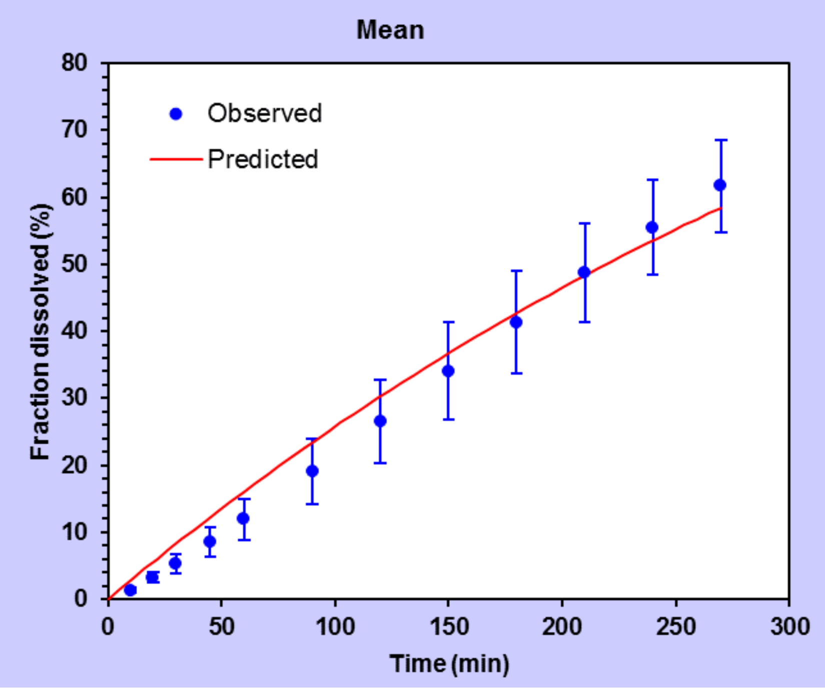

Supplementary materials – Model fitting summary – Kollidon® 90 F (modelling up to app. 60 % of carvedilol released)

Graphical abstract of model fit presented as the fraction % of released carvedilol per tested tablet:

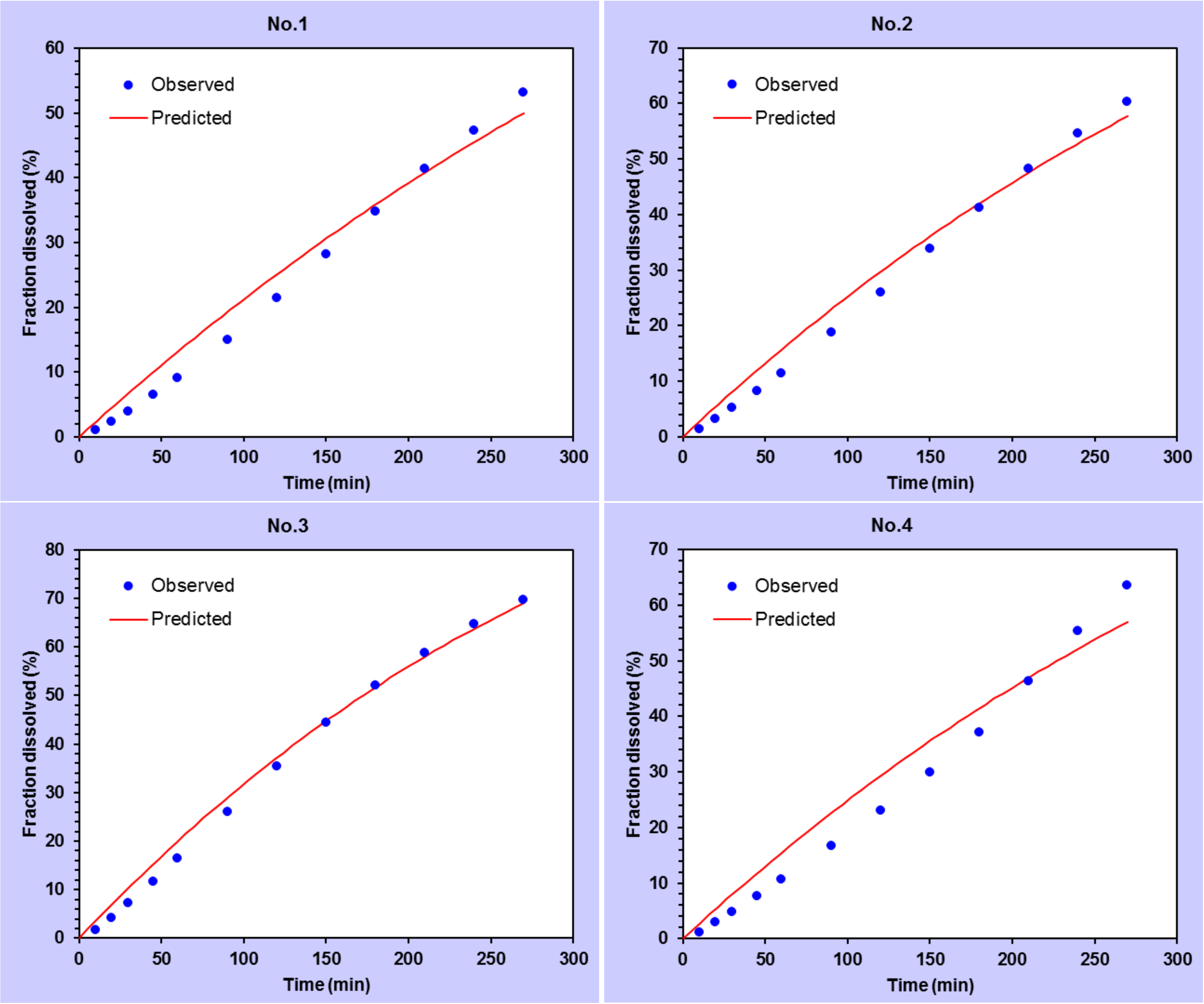

Supplementary materials – Model fitting summary – Kollidon® 90 F (modelling up to app. 60 % of carvedilol released)

Model: **Hixson–Crowell with T_{lag}**

Model equation: $F = 100 \cdot \left\{1 - \left[1 - k_{HC} \cdot \left(t - T_{lag}\right)\right]^3\right\}$

Fitted model parameters per tested tablet (N = 4) with statistics – mean, standard deviation (SD), and relative standard deviation expressed in % (RSD%) (output from DDSolver):

Parameter	No.1	No.2	No.3	No.4	Mean	SD	RSD(%)
k_{HC}	0.001	0.001	0.001	0.001	0.001	0.000	16.683
T_{lag}	18.155	15.884	10.693	21.735	16.617	4.626	27.837

Number of dissolution data points (N), degrees of freedom (df), and selected goodness of fit criteria – Pearson correlation coefficient (R), coefficient of determination (R^2), adjusted coefficient of determination ($R^2_{adjusted}$), and residual sum of squares (RSS) (manual calculation in MS Excel):

Parameter	No.1	No.2	No.3	No.4
N	12	12	12	12
df	10	10	10	10
R	0.995696812	0.996904472	0.999296536	0.98699639
R^2	0.991412142	0.993818526	0.998593568	0.974161873
$R^2_{adjusted}$	0.990553356	0.993200379	0.998452924	0.971578061
RSS	33.69449661	31.78277555	9.622600291	139.2739301

Graphical abstract of model fit presented as mean ± 1 SD of the fraction % of released carvedilol:

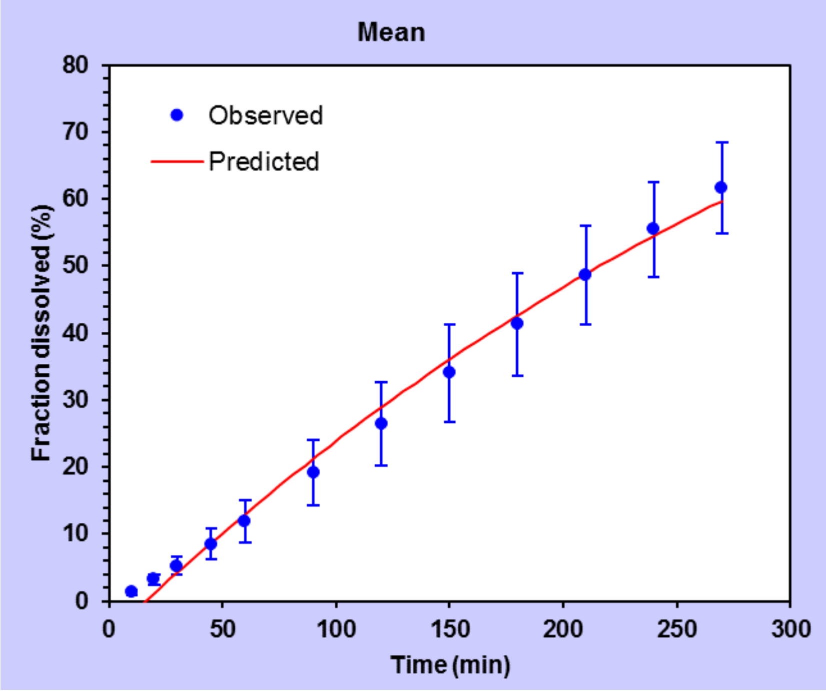

Supplementary materials – Model fitting summary – Kollidon® 90 F (modelling up to app. 60 % of carvedilol released)

Graphical abstract of model fit presented as the fraction % of released carvedilol per tested tablet:

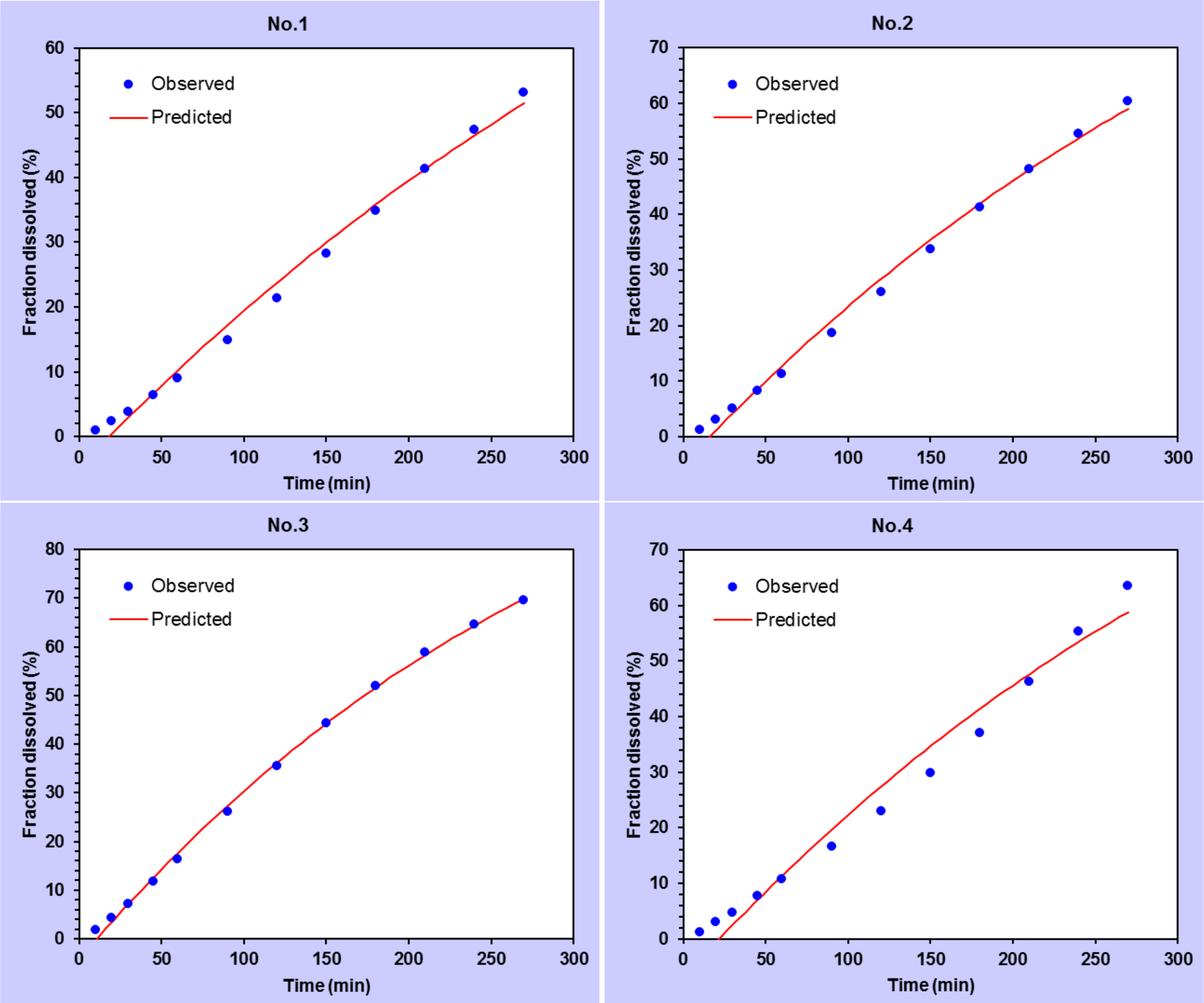

Supplementary materials – Model fitting summary – Kollidon® 90 F (modelling up to app. 60 % of carvedilol released)

Model: **Hopfenberg**

Model equation: $F = 100 \cdot [1 - (1 - k_{HB} \cdot t)^n]$

Fitted model parameters per tested tablet (N = 4) with statistics – mean, standard deviation (SD), and relative standard deviation expressed in % (RSD%) (output from DDSolver):

Parameter	No.1	No.2	No.3	No.4	Mean	SD	RSD(%)
k_{HB}	0.002	0.002	0.002	0.002	0.002	0.000	13.203
n	1.000	1.000	2.000	1.000	1.250	0.500	40.000

Number of dissolution data points (N), degrees of freedom (df), and selected goodness of fit criteria – Pearson correlation coefficient (R), coefficient of determination (R^2), adjusted coefficient of determination ($R^2_{adjusted}$), and residual sum of squares (RSS) (manual calculation in MS Excel):

Parameter	No.1	No.2	No.3	No.4
N	12	12	12	12
df	10	10	10	10
R	0.999239327	0.999494182	0.999493591	0.996426993
R^2	0.998479233	0.998988619	0.998987439	0.992866753
$R^2_{adjusted}$	0.998327156	0.998887481	0.998886183	0.992153428
RSS	29.71168527	17.93748584	34.88204279	77.9146195

Graphical abstract of model fit presented as mean ± 1 SD of the fraction % of released carvedilol:

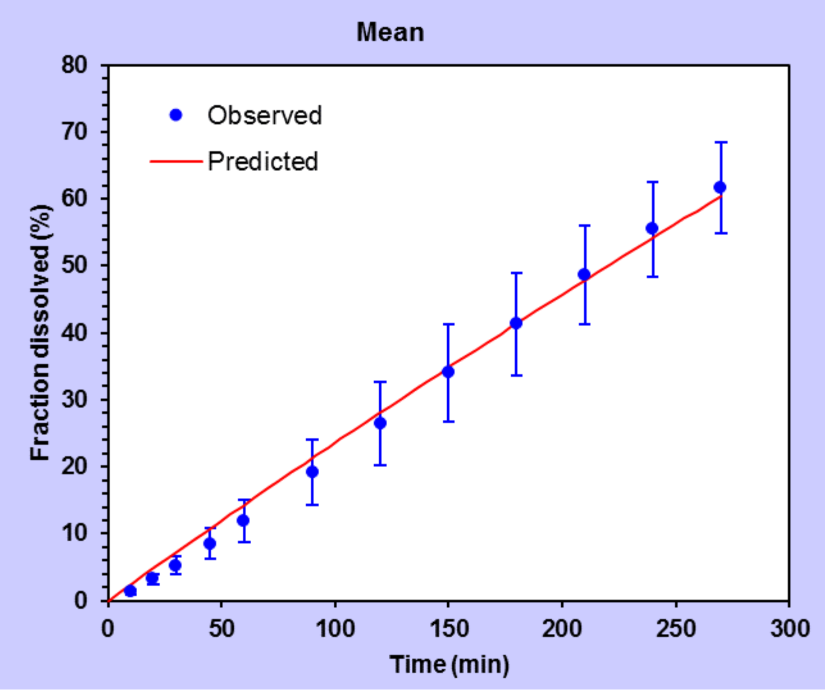

Supplementary materials – Model fitting summary – Kollidon® 90 F (modelling up to app. 60 % of carvedilol released)

Graphical abstract of model fit presented as the fraction % of released carvedilol per tested tablet:

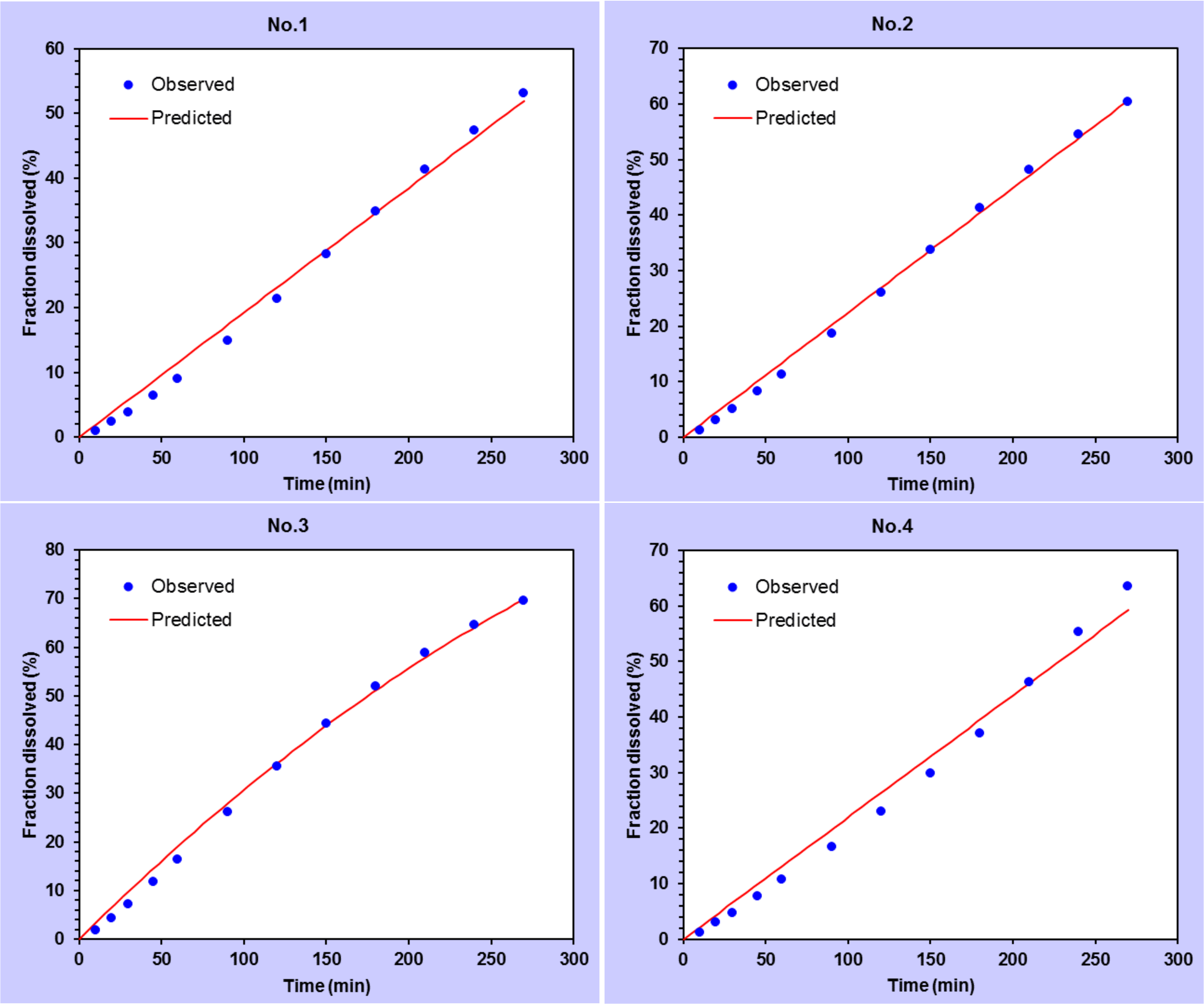

Supplementary materials – Model fitting summary – Kollidon® 90 F (modelling up to app. 60 % of carvedilol released)

Model: **Hopfenberg with T_{lag}**

Model equation: $F = 100 \cdot \{1 - [1 - k_{HB} \cdot (t - T_{lag})]^n\}$

Fitted model parameters per tested tablet (N = 4) with statistics – mean, standard deviation (SD), and relative standard deviation expressed in % (RSD%) (output from DDSolver):

Parameter	No.1	No.2	No.3	No.4	Mean	SD	RSD(%)
k_{HB}	0.002	0.002	0.002	0.002	0.002	0.000	13.666
n	1.000	1.000	2.000	1.000	1.250	0.500	40.000
T_{lag}	11.663	7.535	7.868	13.373	10.110	2.870	28.392

Number of dissolution data points (N), degrees of freedom (df), and selected goodness of fit criteria – Pearson correlation coefficient (R), coefficient of determination (R^2), adjusted coefficient of determination ($R^2_{adjusted}$), and residual sum of squares (RSS) (manual calculation in MS Excel):

Parameter	No.1	No.2	No.3	No.4
N	12	12	12	12
df	9	9	9	9
R	0.999239327	0.999494182	0.999518638	0.996426993
R^2	0.998479233	0.998988619	0.999037507	0.992866753
$R^2_{adjusted}$	0.998141285	0.998763868	0.99882362	0.991281587
RSS	5.783501954	4.985086993	6.531190252	36.18512967

Graphical abstract of model fit presented as mean ± 1 SD of the fraction % of released carvedilol:

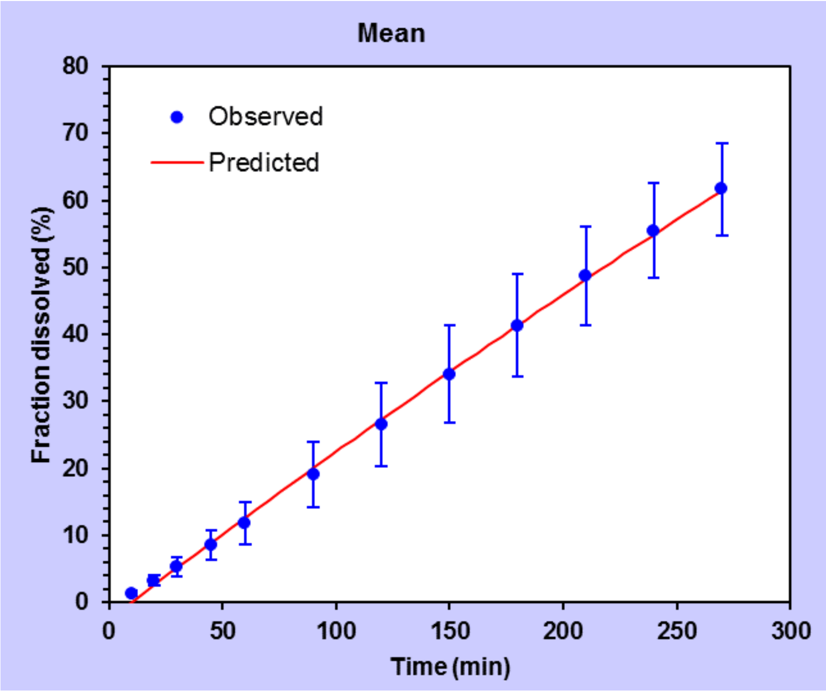

Supplementary materials – Model fitting summary – Kollidon® 90 F (modelling up to app. 60 % of carvedilol released)

Graphical abstract of model fit presented as the fraction % of released carvedilol per tested tablet:

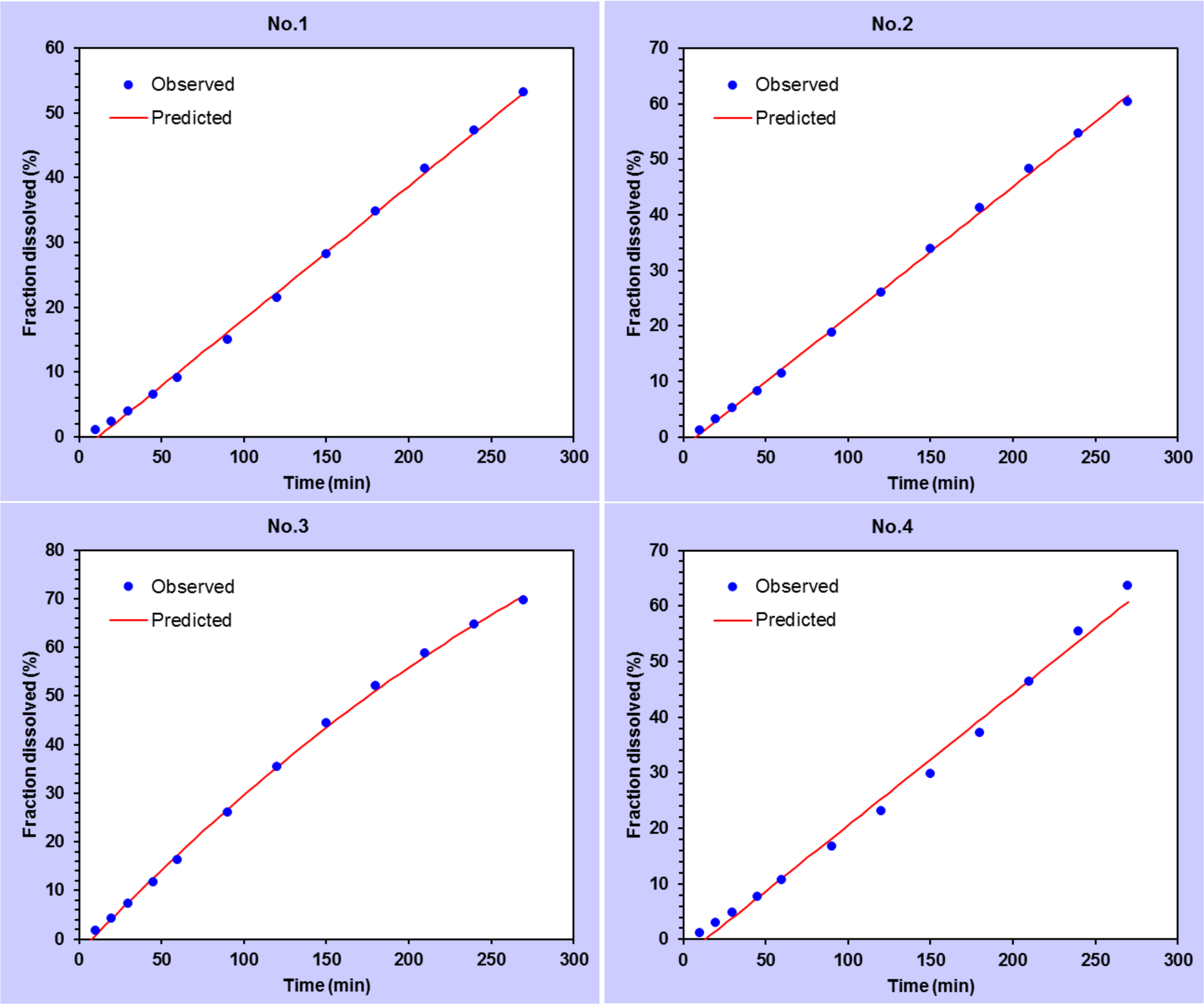

Supplementary materials – Model fitting summary – Kollidon® 90 F (modelling up to app. 60 % of carvedilol released)

Model: **Baker–Lonsdale**

Model equation: $\frac{3}{2}\cdot\left[1-\left(1-\frac{F}{100}\right)^{\frac{2}{3}}\right]-\frac{F}{100}=k_{BL}\cdot t$

Fitted model parameters per tested tablet (N = 4) with statistics – mean, standard deviation (SD), and relative standard deviation expressed in % (RSD%) (output from DDSolver):

Parameter	No.1	No.2	No.3	No.4	Mean	SD	RSD(%)
k_{BL}	0.0002	0.0003	0.0005	0.0003	0.0003	0.0001	30.9690

Number of dissolution data points (N), degrees of freedom (df), and selected goodness of fit criteria – Pearson correlation coefficient (R), coefficient of determination (R^2), adjusted coefficient of determination ($R^2_{adjusted}$), and residual sum of squares (RSS) (manual calculation in MS Excel):

Parameter	No.1	No.2	No.3	No.4
N	12	12	12	12
df	11	11	11	11
R	0.970523044	0.974642999	0.984521343	0.95627077
R^2	0.94191498	0.949928976	0.969282274	0.914453786
$R^2_{adjusted}$	0.94191498	0.949928976	0.969282274	0.914453786
RSS	1942.387723	2367.4792	2711.033577	3086.304511

Graphical abstract of model fit presented as mean ± 1 SD of the fraction % of released carvedilol:

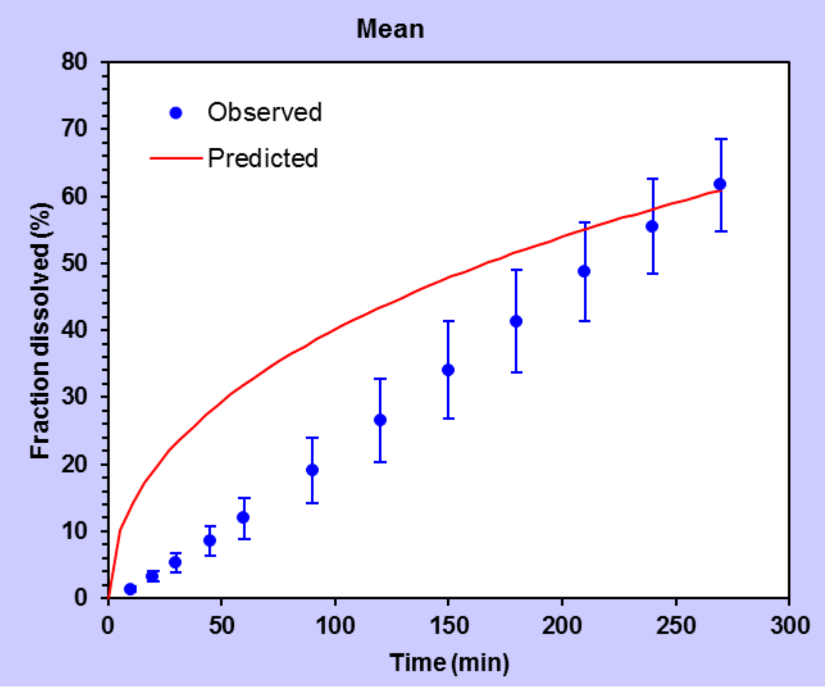

Supplementary materials – Model fitting summary – Kollidon® 90 F (modelling up to app. 60 % of carvedilol released)

Graphical abstract of model fit presented as the fraction % of released carvedilol per tested tablet:

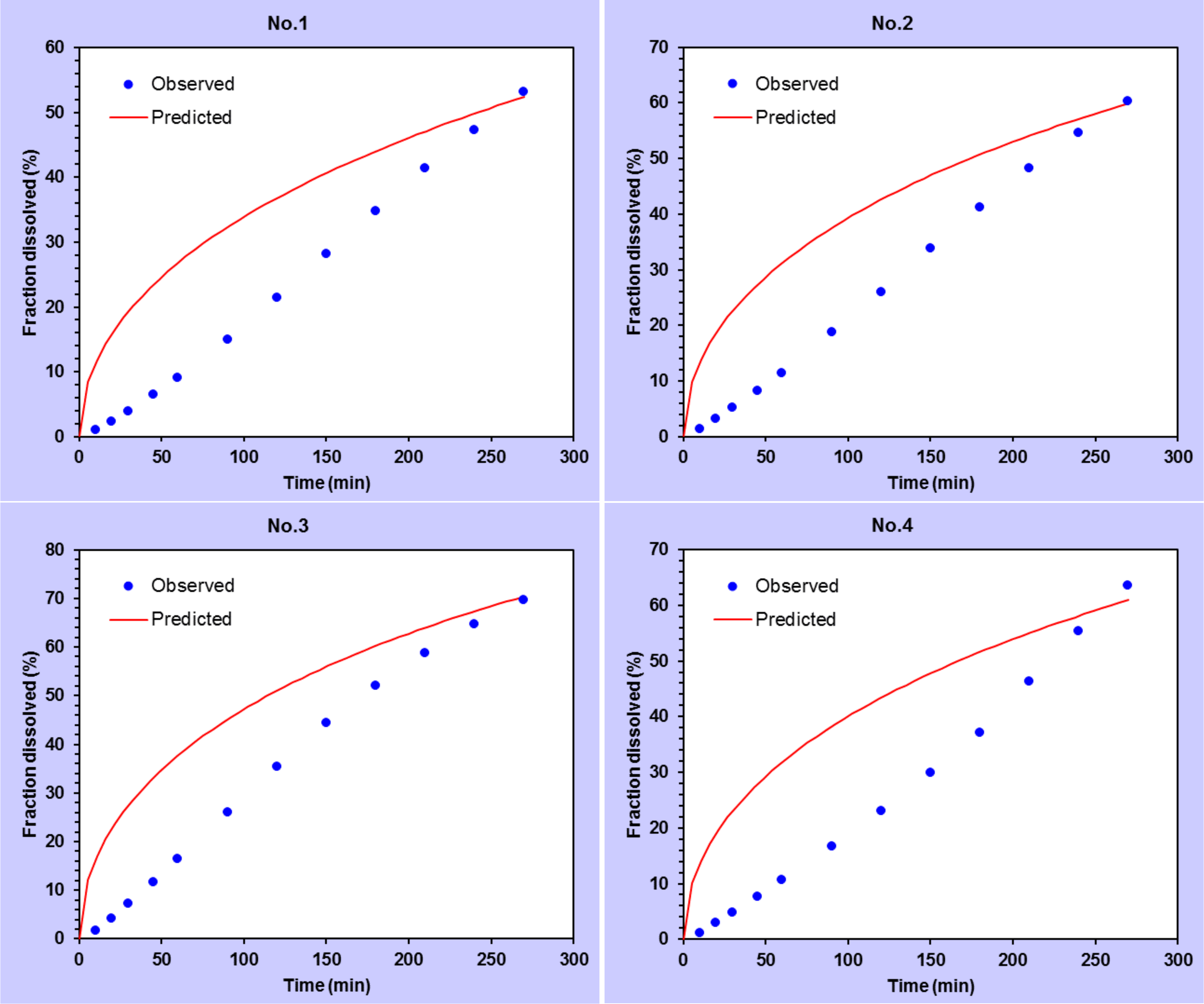

Supplementary materials – Model fitting summary – Kollidon® 90 F (modelling up to app. 60 % of carvedilol released)

Model: **Baker–Lonsdale with T_{lag}**

Model equation: $\frac{3}{2}\cdot\left[1-\left(1-\frac{F}{100}\right)^{\frac{2}{3}}\right]-\frac{F}{100}=k_{BL}\cdot\left(t-T_{lag}\right)$

Fitted model parameters per tested tablet (N = 4) with statistics – mean, standard deviation (SD), and relative standard deviation expressed in % (RSD%) (output from DDSolver):

Parameter	No.1	No.2	No.3	No.4	Mean	SD	RSD(%)
k_{BL}	0.0002	0.0003	0.0005	0.0003	0.0003	0.0001	30.9690
T_{lag}	67.0528	43.2082	38.9764	72.2132	55.3626	16.7017	30.1678

Number of dissolution data points (N), degrees of freedom (df), and selected goodness of fit criteria – Pearson correlation coefficient (R), coefficient of determination (R^2), adjusted coefficient of determination ($R^2_{adjusted}$), and residual sum of squares (RSS) (manual calculation in MS Excel):

Parameter	No.1	No.2	No.3	No.4
N	12	12	12	12
df	10	10	10	10
R	0.976807823	0.971225481	0.980148252	0.965942031
R^2	0.954153524	0.943278935	0.960690595	0.933044006
$R^2_{adjusted}$	0.949568876	0.937606829	0.956759655	0.926348407
RSS	239.9907417	322.2228865	304.5754631	418.0986875

Graphical abstract of model fit presented as mean ± 1 SD of the fraction % of released carvedilol:

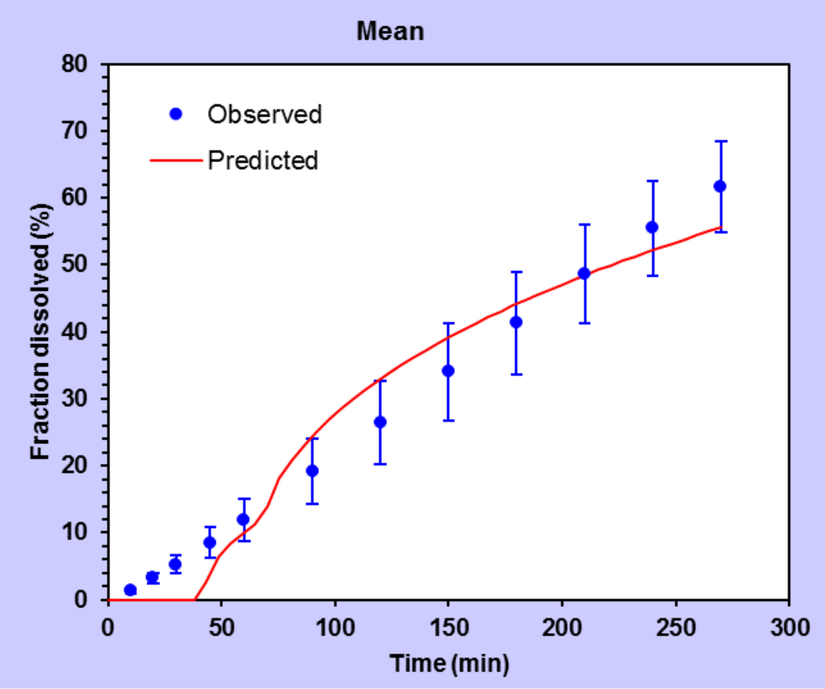

Supplementary materials – Model fitting summary – Kollidon® 90 F (modelling up to app. 60 % of carvedilol released)

Graphical abstract of model fit presented as the fraction % of released carvedilol per tested tablet:

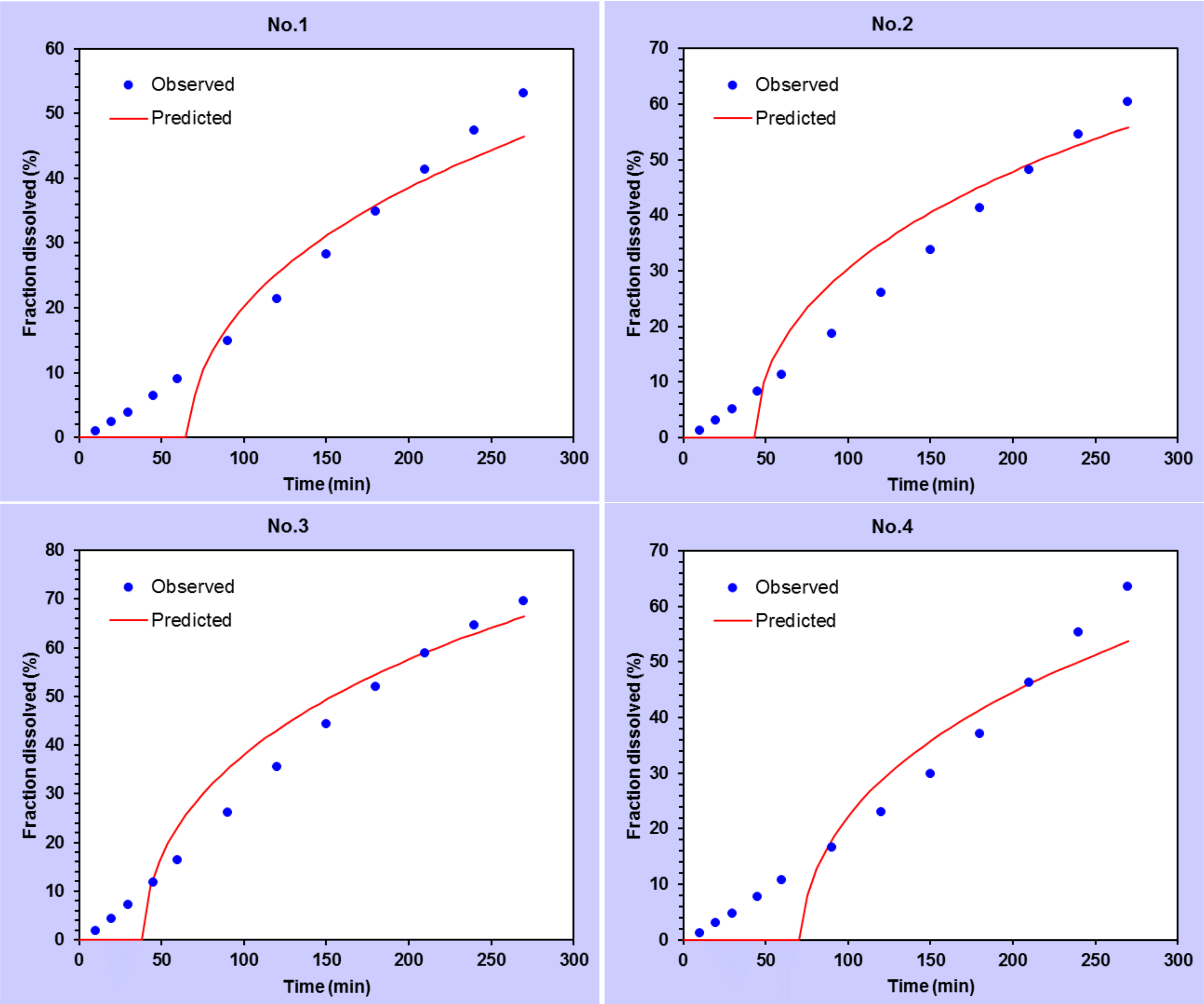

Supplementary materials – Model fitting summary – Kollidon® 90 F (modelling up to app. 60 % of carvedilol released)

Model: **Makoid–Banakar**

Model equation: $F = k_{MB} \cdot t^n \cdot e^{-k \cdot t}$

Fitted model parameters per tested tablet (N = 4) with statistics – mean, standard deviation (SD), and relative standard deviation expressed in % (RSD%) (output from DDSolver):

Parameter	No.1	No.2	No.3	No.4	Mean	SD	RSD(%)
k_{MB}	0.055	0.075	0.089	0.084	0.076	0.015	19.859
n	1.260	1.250	1.312	1.178	1.250	0.055	4.393
k	0.001	0.001	0.003	0.000	0.001	0.001	103.167

Number of dissolution data points (N), degrees of freedom (df), and selected goodness of fit criteria – Pearson correlation coefficient (R), coefficient of determination (R^2), adjusted coefficient of determination ($R^2_{adjusted}$), and residual sum of squares (RSS) (manual calculation in MS Excel):

Parameter	No.1	No.2	No.3	No.4
N	12	12	12	12
df	9	9	9	9
R	0.9997758	0.99983591	0.999938256	0.999346047
R^2	0.99955165	0.999671847	0.999876515	0.998692522
$R^2_{adjusted}$	0.999452017	0.999598925	0.999849074	0.998401972
RSS	1.765881264	1.622468595	0.842997025	7.208721772

Graphical abstract of model fit presented as mean ± 1 SD of the fraction % of released carvedilol:

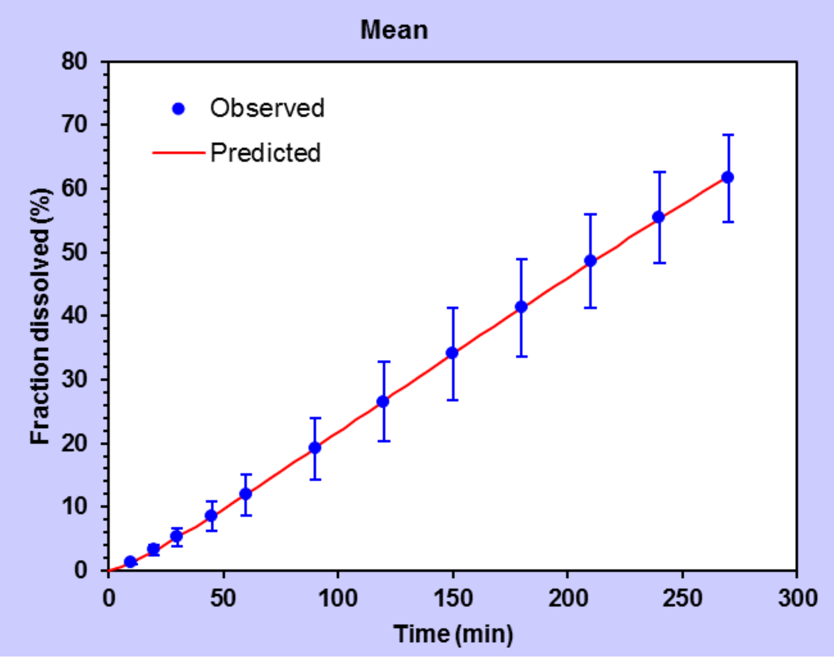

Supplementary materials – Model fitting summary – Kollidon® 90 F (modelling up to app. 60 % of carvedilol released)

Graphical abstract of model fit presented as the fraction % of released carvedilol per tested tablet:

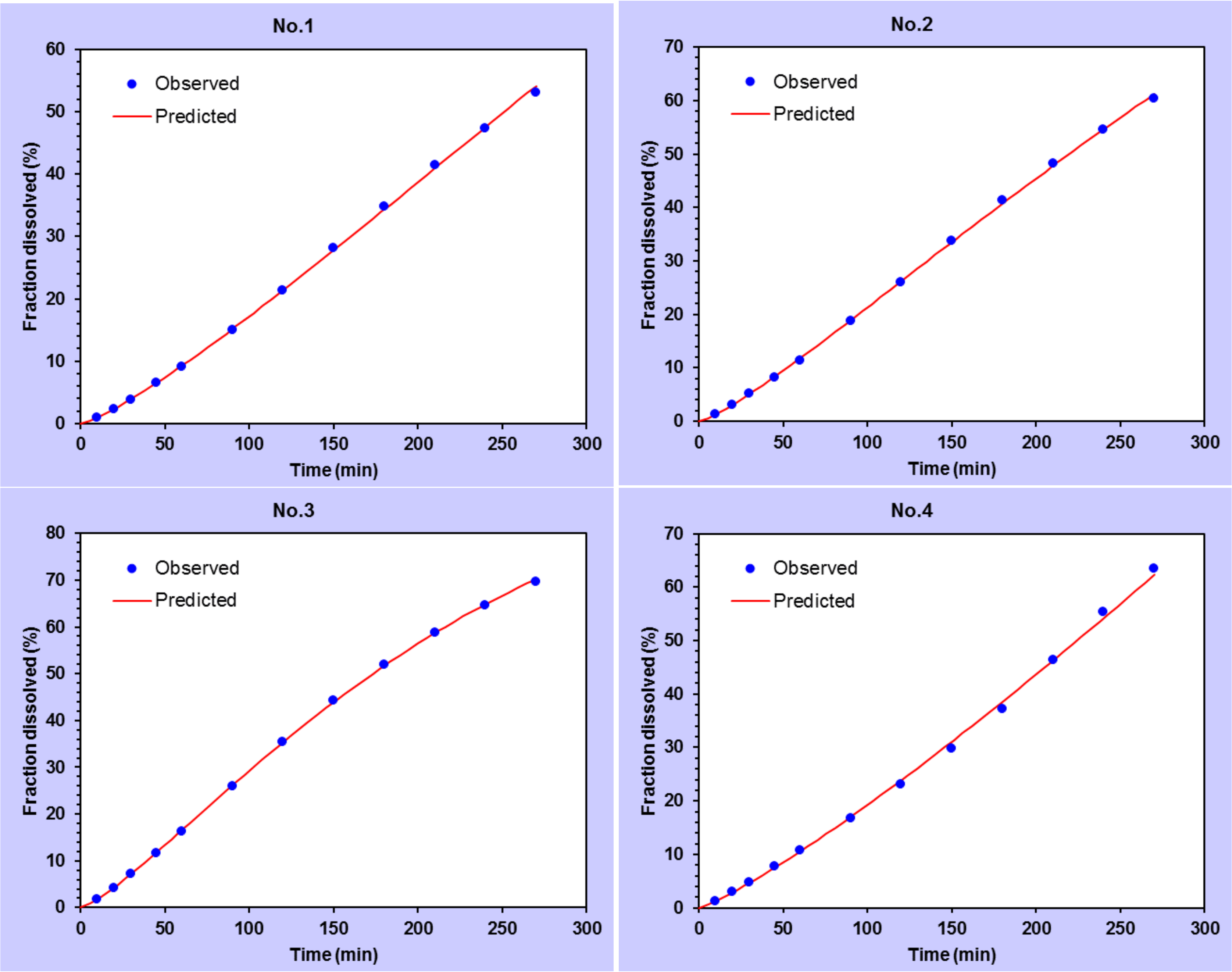

Supplementary materials – Model fitting summary – Kollidon® 90 F (modelling up to app. 60 % of carvedilol released)

Model: **Makoid–Banakar with T_{lag}**

Model equation: $F = k_{MB} \cdot (t - T_{lag})^n \cdot e^{-k \cdot (t - T_{lag})}$

Fitted model parameters per tested tablet (N = 4) with statistics – mean, standard deviation (SD), and relative standard deviation expressed in % (RSD%) (output from DDSolver):

Parameter	No.1	No.2	No.3	No.4	Mean	SD	RSD(%)
k_{MB}	0.154	0.208	0.259	0.218	0.210	0.043	20.567
n	1.000	0.994	1.041	0.941	0.994	0.041	4.142
k	-0.001	-0.001	0.001	-0.002	-0.001	0.001	-140.037
T_{lag}	4.000	4.000	4.000	4.000	4.000	0.000	0.000

Number of dissolution data points (N), degrees of freedom (df), and selected goodness of fit criteria – Pearson correlation coefficient (R), coefficient of determination (R^2), adjusted coefficient of determination ($R^2_{adjusted}$), and residual sum of squares (RSS) (manual calculation in MS Excel):

Parameter	No.1	No.2	No.3	No.4
N	12	12	12	12
df	8	8	8	8
R	0.998160095	0.998393338	0.998314022	0.999846216
R^2	0.996323576	0.996789258	0.996630887	0.999692455
$R^2_{adjusted}$	0.994944917	0.99558523	0.99536747	0.999577126
RSS	16.02253486	17.33825174	24.20330757	1.566953753

Graphical abstract of model fit presented as mean ± 1 SD of the fraction % of released carvedilol:

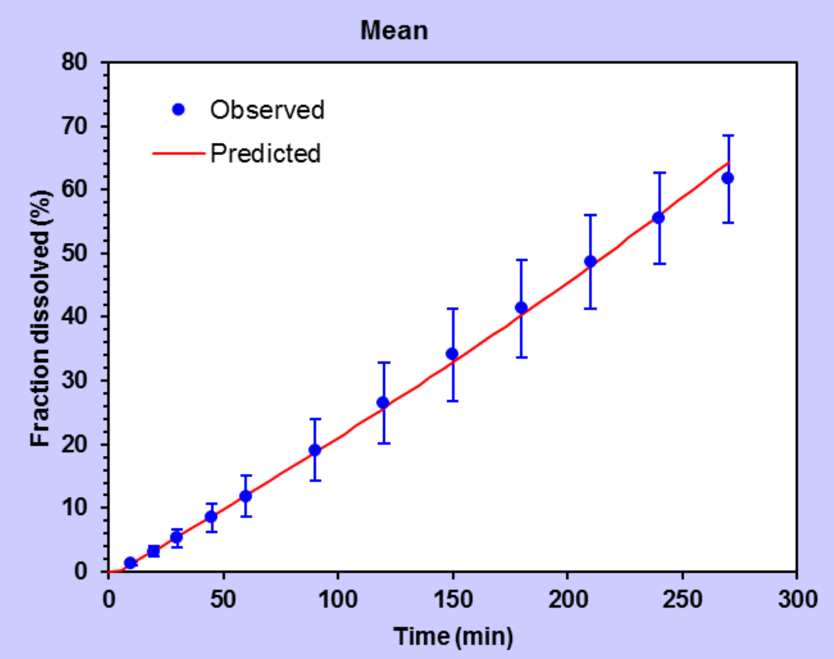

Supplementary materials – Model fitting summary – Kollidon® 90 F (modelling up to app. 60 % of carvedilol released)

Graphical abstract of model fit presented as the fraction % of released carvedilol per tested tablet:

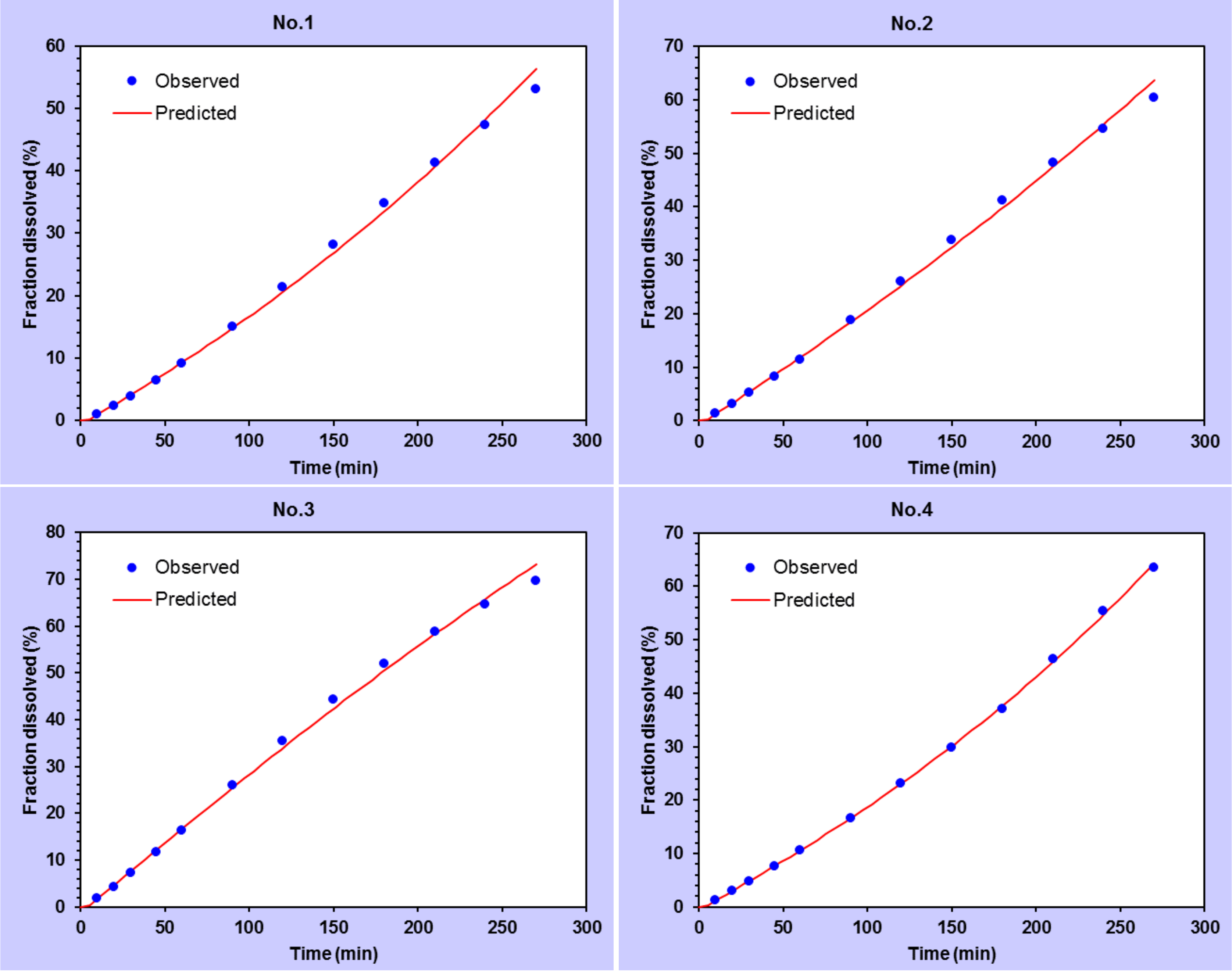

Supplementary materials – Model fitting summary – Kollidon® 90 F (modelling up to app. 60 % of carvedilol released)

Model: **Peppas–Sahlin_1**

Model equation: $F = k_1 \cdot t^m + k_2 \cdot t^{2m}$

Fitted model parameters per tested tablet (N = 4) with statistics – mean, standard deviation (SD), and relative standard deviation expressed in % (RSD%) (output from DDSolver):

Parameter	No.1	No.2	No.3	No.4	Mean	SD	RSD(%)
k_1	-1.325	-1.175	-0.523	-1.736	-1.190	0.504	-42.364
k_2	0.452	0.492	0.516	0.536	0.499	0.036	7.267
m	0.450	0.450	0.450	0.450	0.450	0.000	0.000

Number of dissolution data points (N), degrees of freedom (df), and selected goodness of fit criteria – Pearson correlation coefficient (R), coefficient of determination (R^2), adjusted coefficient of determination ($R^2_{adjusted}$), and residual sum of squares (RSS) (manual calculation in MS Excel):

Parameter	No.1	No.2	No.3	No.4
N	12	12	12	12
df	9	9	9	9
R	0.999469202	0.99959018	0.997496198	0.996945059
R^2	0.998938686	0.999180529	0.994998666	0.99389945
$R^2_{adjusted}$	0.998702838	0.998998424	0.993887258	0.992543772
RSS	4.19340208	4.060876266	34.58923603	32.01394163

Graphical abstract of model fit presented as mean ± 1 SD of the fraction % of released carvedilol:

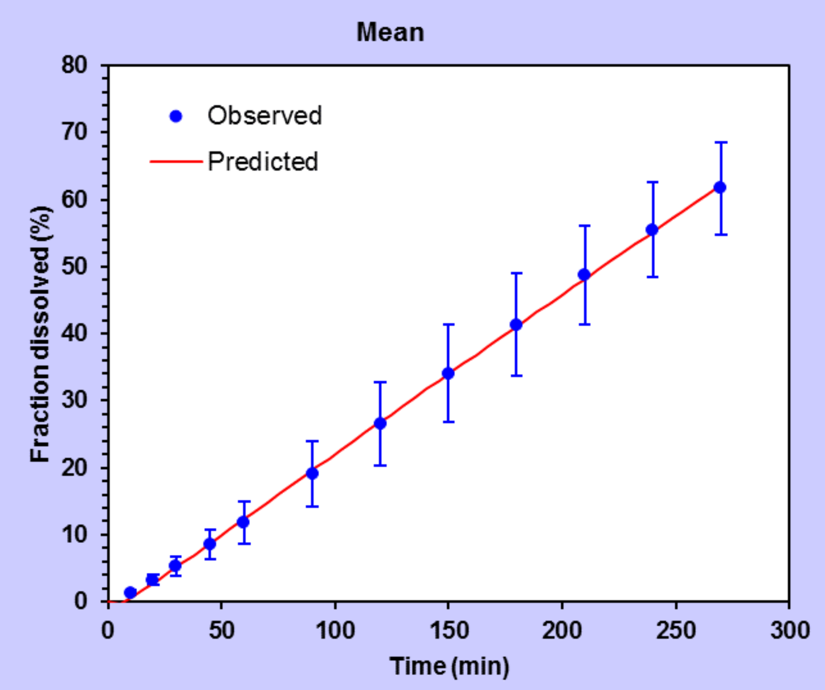

Supplementary materials – Model fitting summary – Kollidon® 90 F (modelling up to app. 60 % of carvedilol released)

Graphical abstract of model fit presented as the fraction % of released carvedilol per tested tablet:

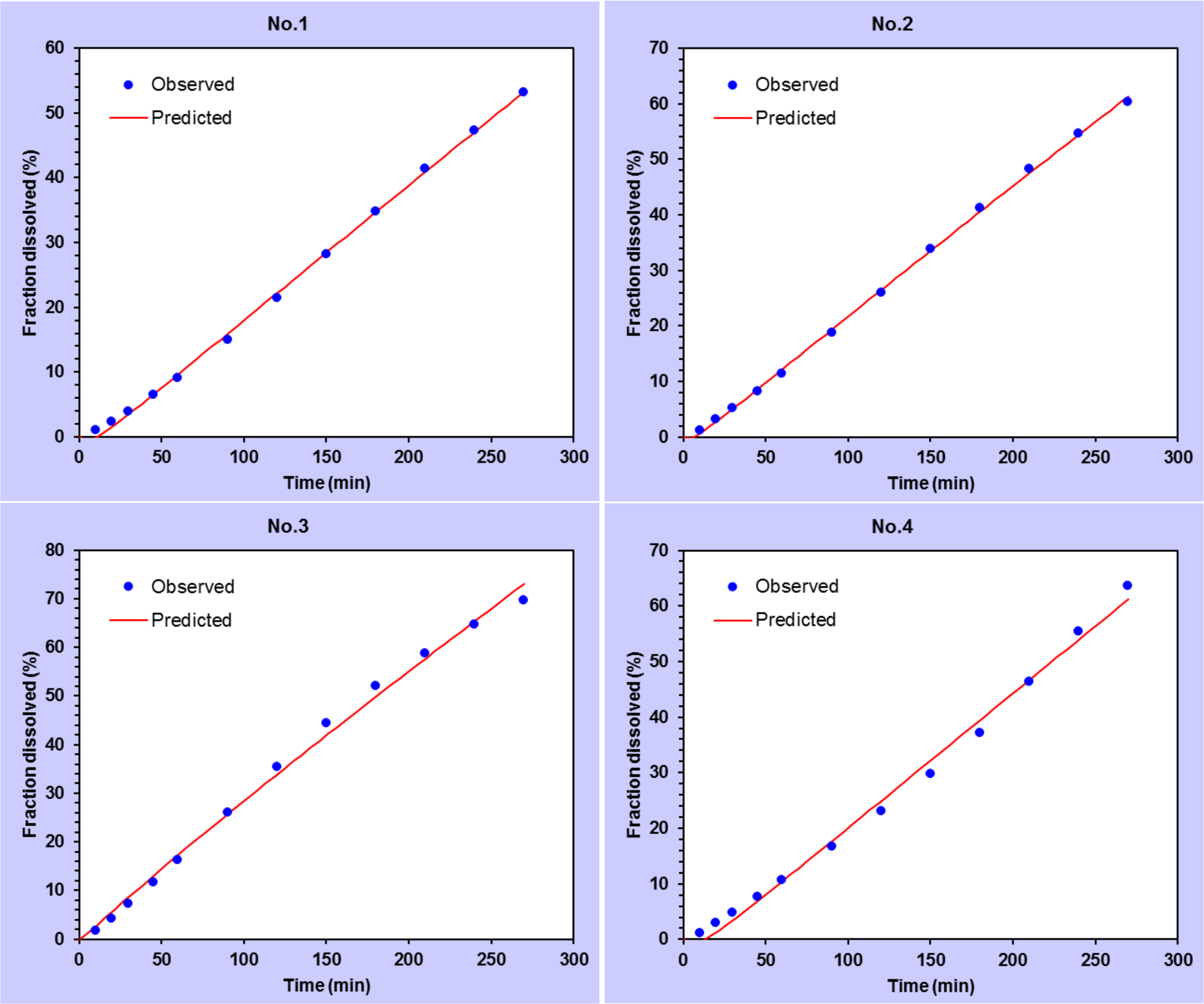

Supplementary materials – Model fitting summary – Kollidon® 90 F (modelling up to app. 60 % of carvedilol released)

Model: **Peppas–Sahlin_1 with T_{lag}**

Model equation: $F = k_1 \cdot (t - T_{lag})^m + k_2 \cdot (t - T_{lag})^{2m}$

Fitted model parameters per tested tablet (N = 4) with statistics – mean, standard deviation (SD), and relative standard deviation expressed in % (RSD%) (output from DDSolver):

Parameter	No.1	No.2	No.3	No.4	Mean	SD	RSD(%)
k_1	-1.116	-0.916	-0.164	-1.513	-0.928	0.566	-61.050
k_2	0.440	0.476	0.492	0.524	0.483	0.035	7.254
m	0.450	0.450	0.450	0.450	0.450	0.000	0.000
T_{lag}	4.000	4.000	4.000	4.000	4.000	0.000	0.000

Number of dissolution data points (N), degrees of freedom (df), and selected goodness of fit criteria – Pearson correlation coefficient (R), coefficient of determination (R^2), adjusted coefficient of determination ($R^2_{adjusted}$), and residual sum of squares (RSS) (manual calculation in MS Excel):

Parameter	No.1	No.2	No.3	No.4
N	12	12	12	12
df	8	8	8	8
R	0.999365534	0.999538059	0.99784379	0.996728147
R^2	0.99873147	0.999076332	0.99569223	0.993467
$R^2_{adjusted}$	0.998255771	0.998729957	0.994076816	0.991017124
RSS	5.120117394	4.643366085	29.73099368	34.71925506

Graphical abstract of model fit presented as mean ± 1 SD of the fraction % of released carvedilol:

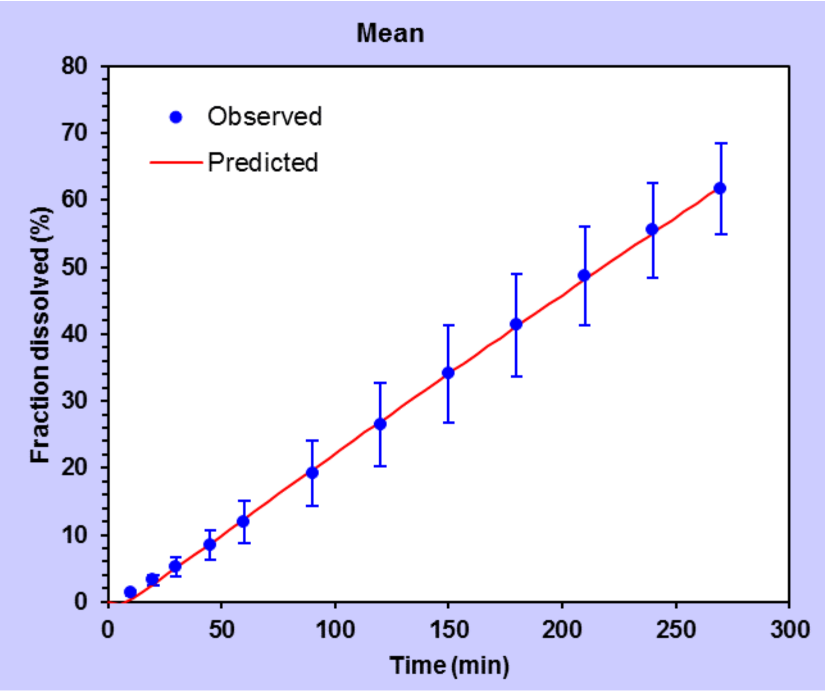

Supplementary materials – Model fitting summary – Kollidon® 90 F (modelling up to app. 60 % of carvedilol released)

Graphical abstract of model fit presented as the fraction % of released carvedilol per tested tablet:

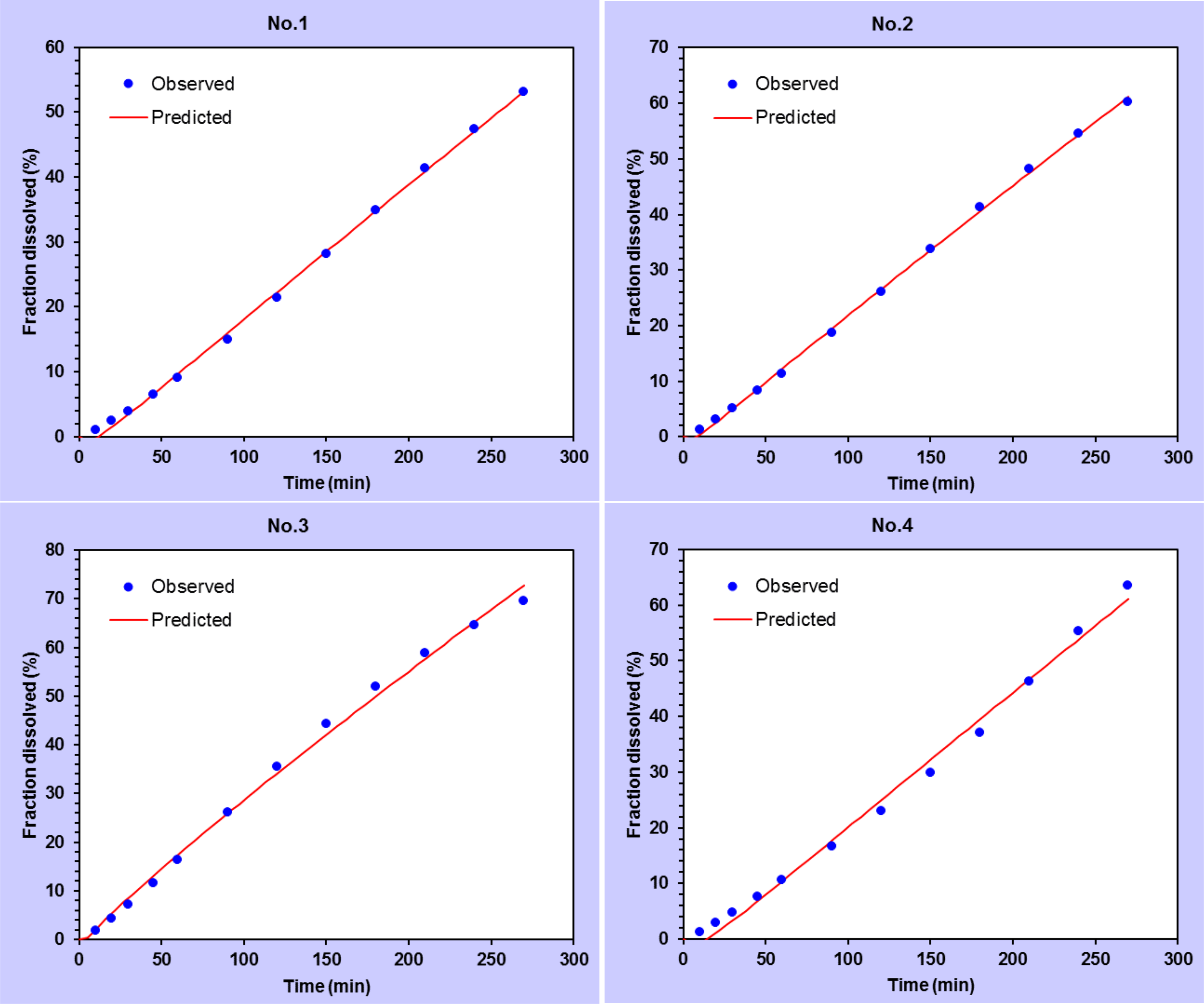

Supplementary materials – Model fitting summary – Kollidon® 90 F (modelling up to app. 60 % of carvedilol released)

Model: **Peppas–Sahlin_2**

Model equation: $F = k_1 \cdot t^{0.5} + k_2 \cdot t$

Fitted model parameters per tested tablet (N = 4) with statistics – mean, standard deviation (SD), and relative standard deviation expressed in % (RSD%) (output from DDSolver):

Parameter	No.1	No.2	No.3	No.4	Mean	SD	RSD(%)
k_1	-0.591	-0.399	0.237	-0.868	-0.405	0.470	-115.823
k_2	0.235	0.253	0.257	0.282	0.257	0.019	7.520

Number of dissolution data points (N), degrees of freedom (df), and selected goodness of fit criteria – Pearson correlation coefficient (R), coefficient of determination (R^2), adjusted coefficient of determination ($R^2_{adjusted}$), and residual sum of squares (RSS) (manual calculation in MS Excel):

Parameter	No.1	No.2	No.3	No.4
N	12	12	12	12
df	10	10	10	10
R	0.999740953	0.999464795	0.996603004	0.998282558
R^2	0.999481974	0.998929876	0.993217548	0.996568066
$R^2_{adjusted}$	0.999430171	0.998822864	0.992539302	0.996224872
RSS	1.98856422	5.311438045	47.97613664	18.14829195

Graphical abstract of model fit presented as mean ± 1 SD of the fraction % of released carvedilol:

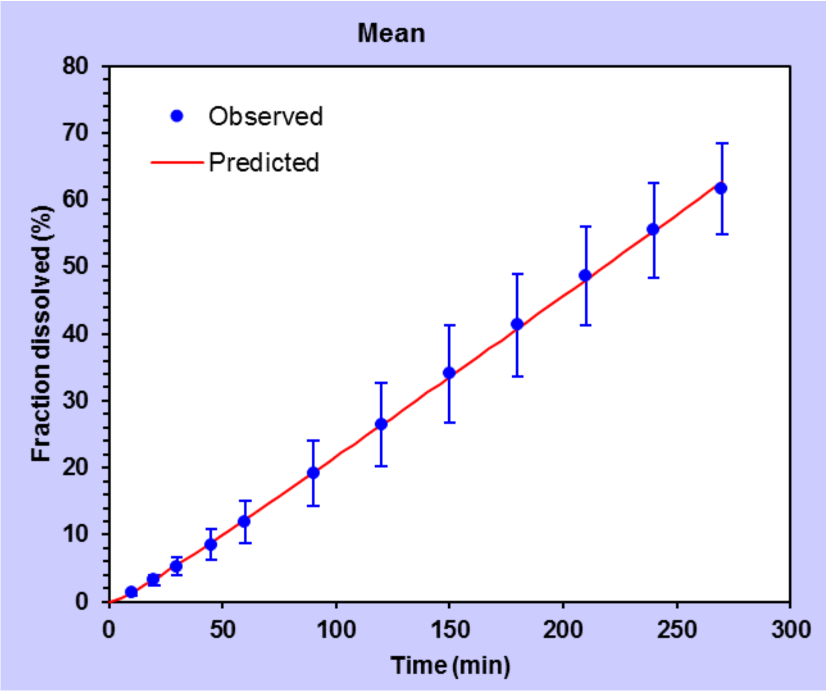

Supplementary materials – Model fitting summary – Kollidon® 90 F (modelling up to app. 60 % of carvedilol released)

Graphical abstract of model fit presented as the fraction % of released carvedilol per tested tablet:

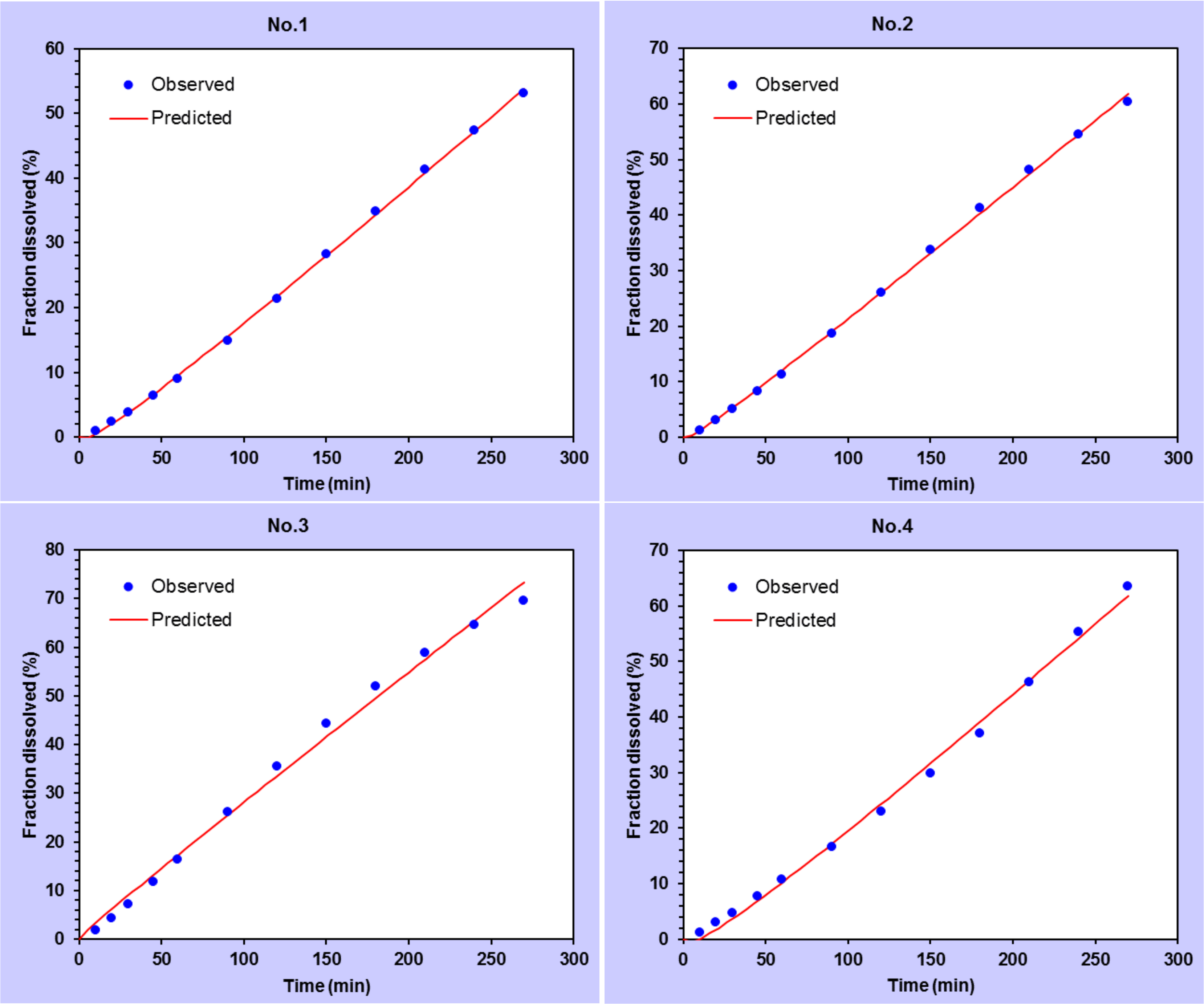

Supplementary materials – Model fitting summary – Kollidon® 90 F (modelling up to app. 60 % of carvedilol released)

Model: **Peppas–Sahlin_2 with T_{lag}**

Model equation: $F = k_1 \cdot (t - T_{lag})^{0.5} + k_2 \cdot (t - T_{lag})$

Fitted model parameters per tested tablet (N = 4) with statistics – mean, standard deviation (SD), and relative standard deviation expressed in % (RSD%) (output from DDSolver):

Parameter	No.1	No.2	No.3	No.4	Mean	SD	RSD(%)
k_1	-0.435	-0.204	0.511	-0.704	-0.208	0.521	-250.709
k_2	0.228	0.244	0.244	0.275	0.248	0.020	7.977
T_{lag}	4.000	4.000	6.000	4.000	4.500	1.000	22.222

Number of dissolution data points (N), degrees of freedom (df), and selected goodness of fit criteria – Pearson correlation coefficient (R), coefficient of determination (R^2), adjusted coefficient of determination ($R^2_{adjusted}$), and residual sum of squares (RSS) (manual calculation in MS Excel):

Parameter	No.1	No.2	No.3	No.4
N	12	12	12	12
df	9	9	9	9
R	0.999706019	0.999510691	0.997167899	0.998117849
R^2	0.999412124	0.999021622	0.994343819	0.99623924
$R^2_{adjusted}$	0.999281484	0.998804205	0.99308689	0.995403516
RSS	2.303223249	4.825630756	40.45689648	20.20003178

Graphical abstract of model fit presented as mean ± 1 SD of the fraction % of released carvedilol:

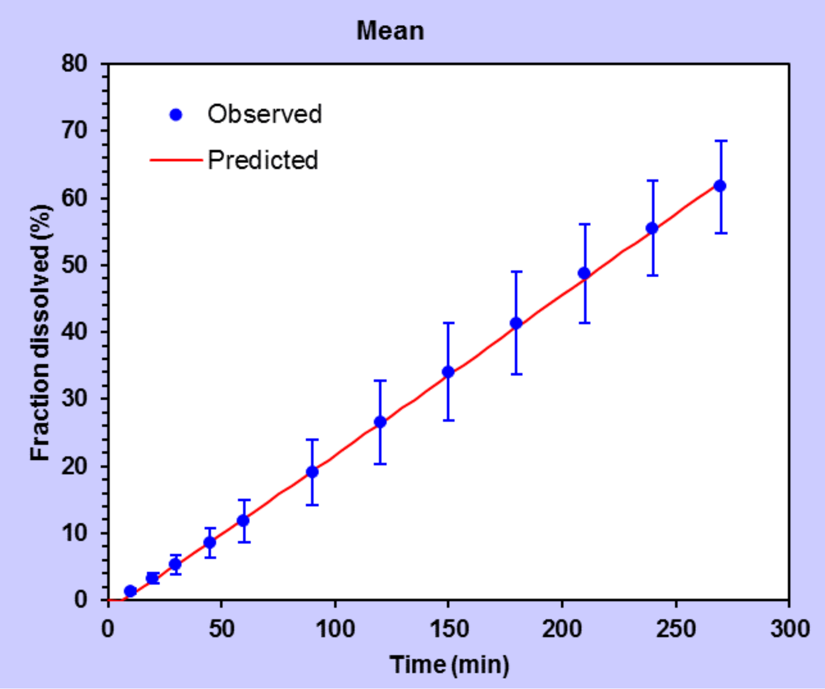

Supplementary materials – Model fitting summary – Kollidon® 90 F (modelling up to app. 60 % of carvedilol released)

Graphical abstract of model fit presented as the fraction % of released carvedilol per tested tablet:

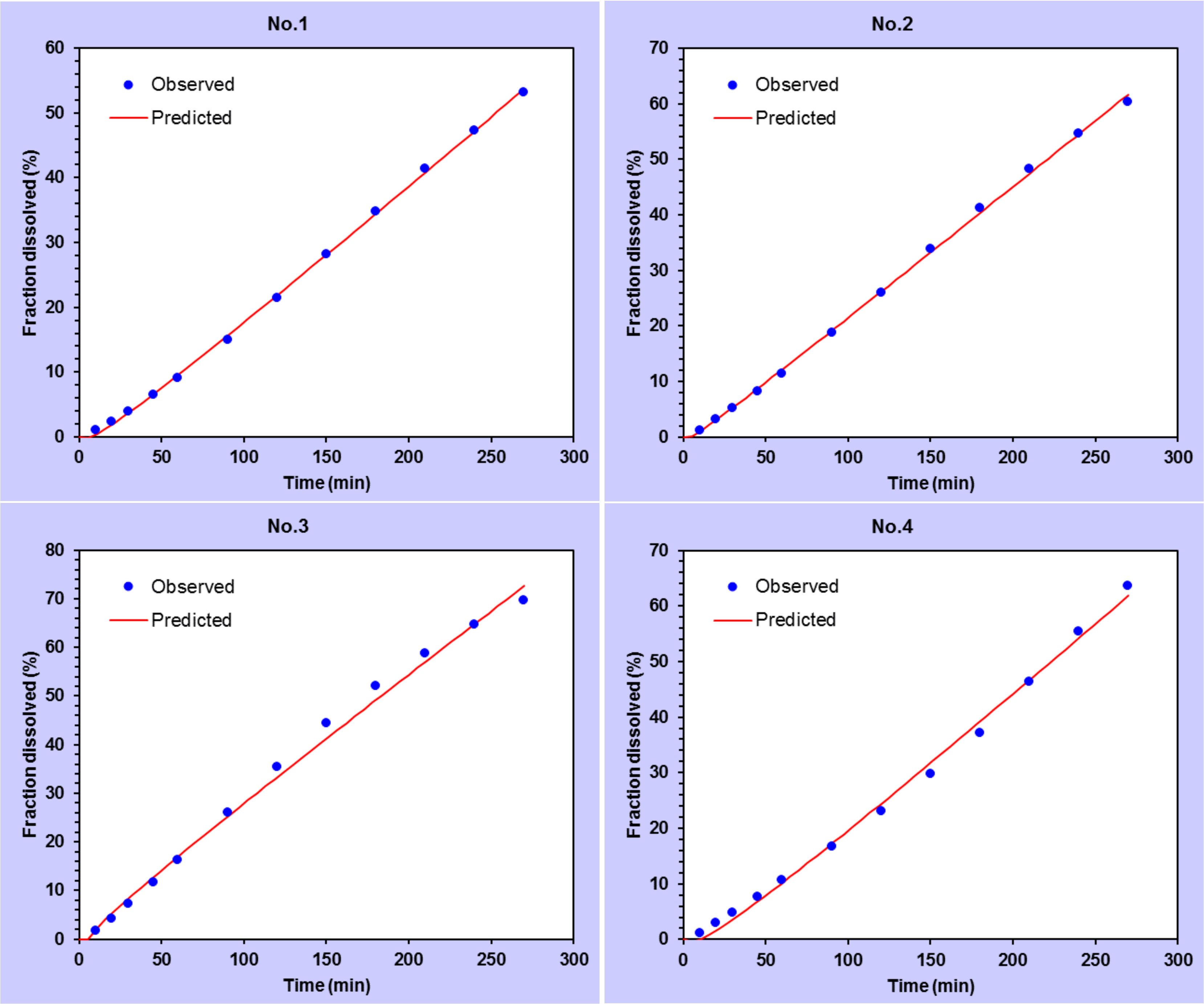

Supplementary materials – Model fitting summary – Kollidon® 90 F (modelling up to app. 60 % of carvedilol released)

Model: **Quadratic**

Model equation: $F = 100 \cdot (k_1 \cdot t^2 + k_2 \cdot t)$

Fitted model parameters per tested tablet (N = 4) with statistics – mean, standard deviation (SD), and relative standard deviation expressed in % (RSD%) (output from DDSolver):

Parameter	No.1	No.2	No.3	No.4	Mean	SD	RSD(%)
k_1	0.000002	0.000001	-0.000002	0.000003	0.000001	0.000002	222.521567
k_2	0.001593	0.002076	0.003120	0.001557	0.002087	0.000729	34.918132

Number of dissolution data points (N), degrees of freedom (df), and selected goodness of fit criteria – Pearson correlation coefficient (R), coefficient of determination (R^2), adjusted coefficient of determination ($R^2_{adjusted}$), and residual sum of squares (RSS) (manual calculation in MS Excel):

Parameter	No.1	No.2	No.3	No.4
N	12	12	12	12
df	10	10	10	10
R	0.999126139	0.999091496	0.998844164	0.99984553
R^2	0.998253041	0.998183817	0.997689664	0.999691084
$R^2_{adjusted}$	0.998078346	0.998002198	0.997458631	0.999660193
RSS	9.118272339	12.58252115	26.02342807	1.605189746

Graphical abstract of model fit presented as mean ± 1 SD of the fraction % of released carvedilol:

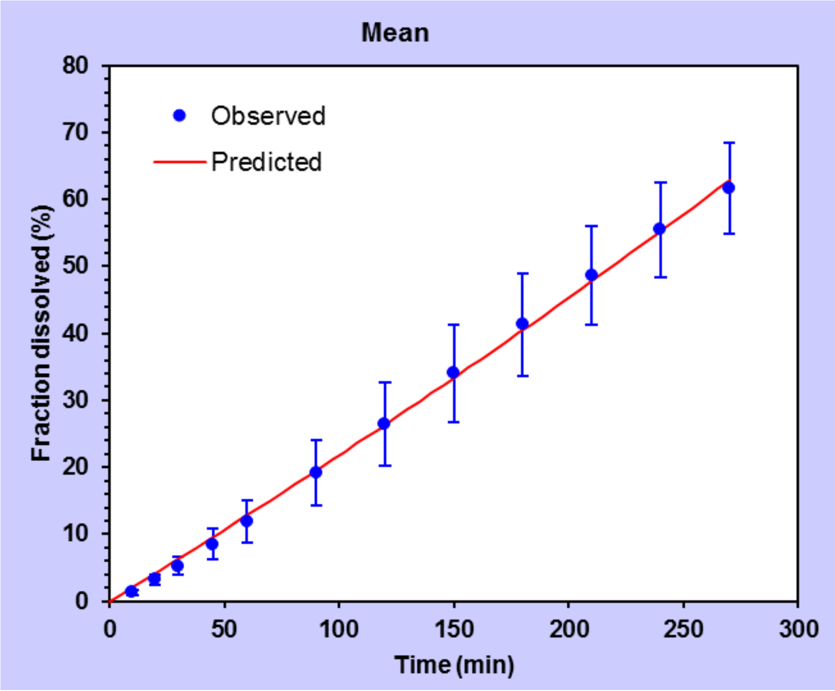

Supplementary materials – Model fitting summary – Kollidon® 90 F (modelling up to app. 60 % of carvedilol released)

Graphical abstract of model fit presented as the fraction % of released carvedilol per tested tablet:

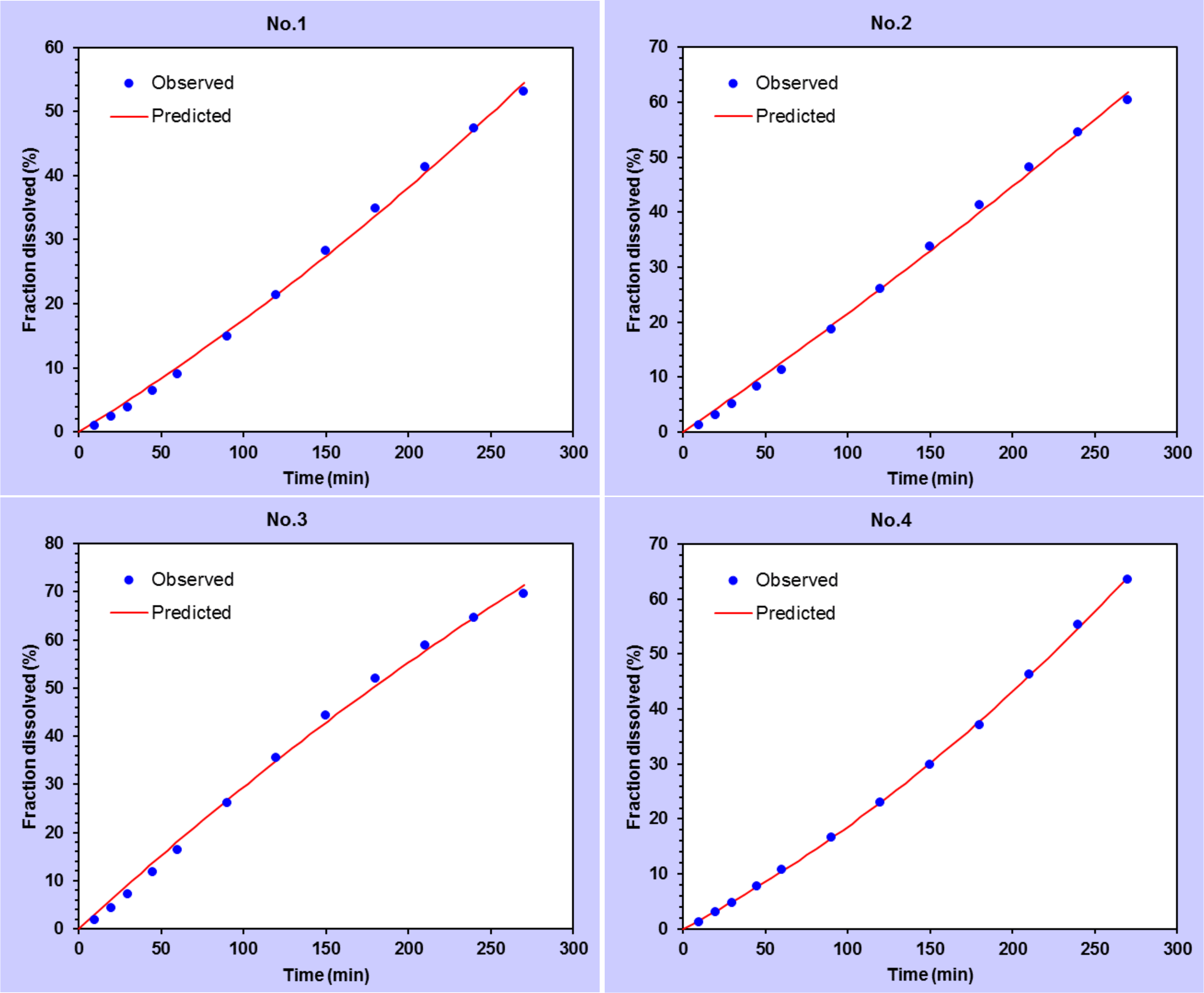

Supplementary materials – Model fitting summary – Kollidon® 90 F (modelling up to app. 60 % of carvedilol released)

Model: **Quadratic with T_{lag}**

Model equation: $F = 100 \cdot \left[k_1 \cdot \left(t - T_{lag}\right)^2 + k_2 \cdot \left(t - T_{lag}\right)\right]$

Fitted model parameters per tested tablet (N = 4) with statistics – mean, standard deviation (SD), and relative standard deviation expressed in % (RSD%) (output from DDSolver):

Parameter	No.1	No.2	No.3	No.4	Mean	SD	RSD(%)
k_1	0.000001	0.000000	-0.000002	0.000003	0.000000	0.000002	502.348859
k_2	0.001718	0.002227	0.003325	0.001684	0.002239	0.000765	34.189969
T_{lag}	4.000000	4.000000	6.000000	4.000000	4.500000	1.000000	22.222222

Number of dissolution data points (N), degrees of freedom (df), and selected goodness of fit criteria – Pearson correlation coefficient (R), coefficient of determination (R^2), adjusted coefficient of determination ($R^2_{adjusted}$), and residual sum of squares (RSS) (manual calculation in MS Excel):

Parameter	No.1	No.2	No.3	No.4
N	12	12	12	12
df	9	9	9	9
R	0.999404908	0.999397592	0.999336288	0.999828488
R^2	0.99881017	0.998795547	0.998673017	0.999657005
$R^2_{adjusted}$	0.998545764	0.998527891	0.998378132	0.999580784
RSS	5.476282271	7.13831971	11.26473684	1.941548067

Graphical abstract of model fit presented as mean ± 1 SD of the fraction % of released carvedilol:

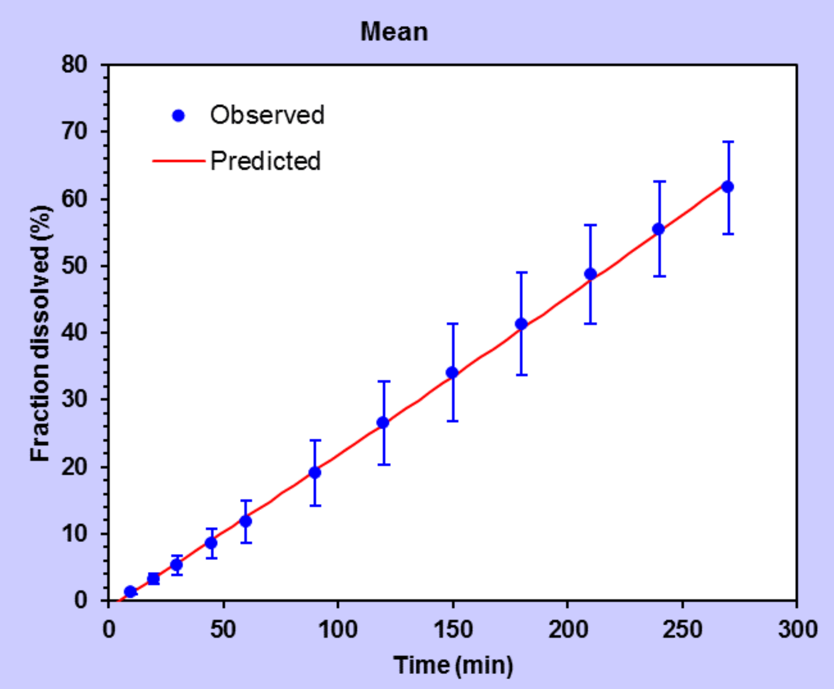

Supplementary materials – Model fitting summary – Kollidon® 90 F (modelling up to app. 60 % of carvedilol released)

Graphical abstract of model fit presented as the fraction % of released carvedilol per tested tablet:

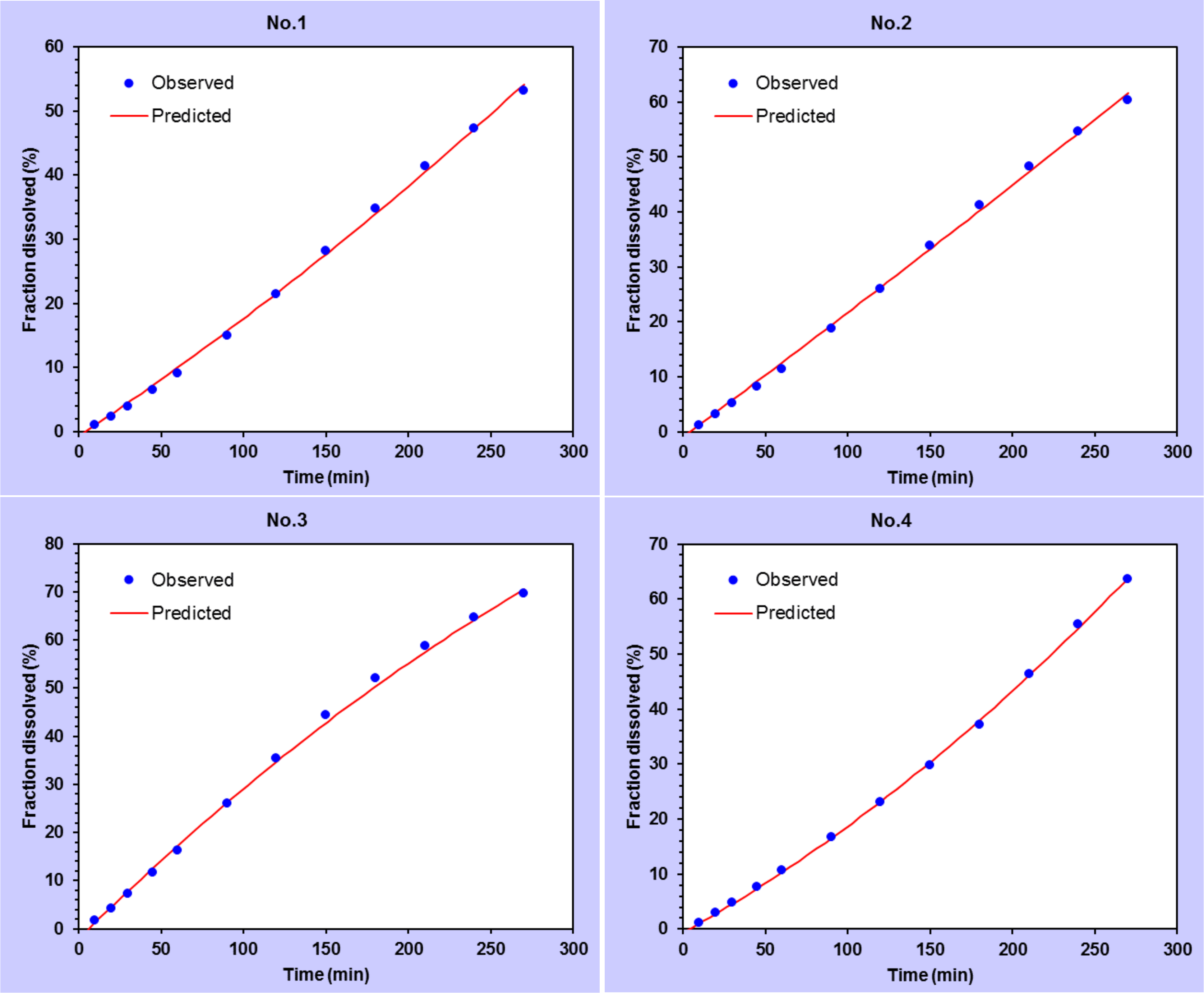

Supplementary materials – Model fitting summary – Kollidon® 90 F (modelling up to app. 60 % of carvedilol released)

Model: **Weibull_1**

Model equation: $F = 100 \cdot \left[1 - e^{-\frac{(t-T_i)^\beta}{\alpha}}\right]$

Fitted model parameters per tested tablet (N = 4) with statistics – mean, standard deviation (SD), and relative standard deviation expressed in % (RSD%) (output from DDSolver):

Parameter	No.1	No.2	No.3	No.4	Mean	SD	RSD(%)
α	1124.049	727.963	501.545	827.836	795.348	258.173	32.460
β	1.210	1.147	1.137	1.159	1.163	0.032	2.786
T_i	4.733	4.000	4.000	4.000	4.183	0.366	8.757

Number of dissolution data points (N), degrees of freedom (df), and selected goodness of fit criteria – Pearson correlation coefficient (R), coefficient of determination (R^2), adjusted coefficient of determination ($R^2_{adjusted}$), and residual sum of squares (RSS) (manual calculation in MS Excel):

Parameter	No.1	No.2	No.3	No.4
N	12	12	12	12
df	9	9	9	9
R	0.998379627	0.998379863	0.999657429	0.99096889
R^2	0.99676188	0.99676235	0.999314975	0.982019341
$R^2_{adjusted}$	0.996042298	0.996042872	0.999162748	0.978023639
RSS	31.35006106	39.70958669	13.83624008	150.4844543

Graphical abstract of model fit presented as mean ± 1 SD of the fraction % of released carvedilol:

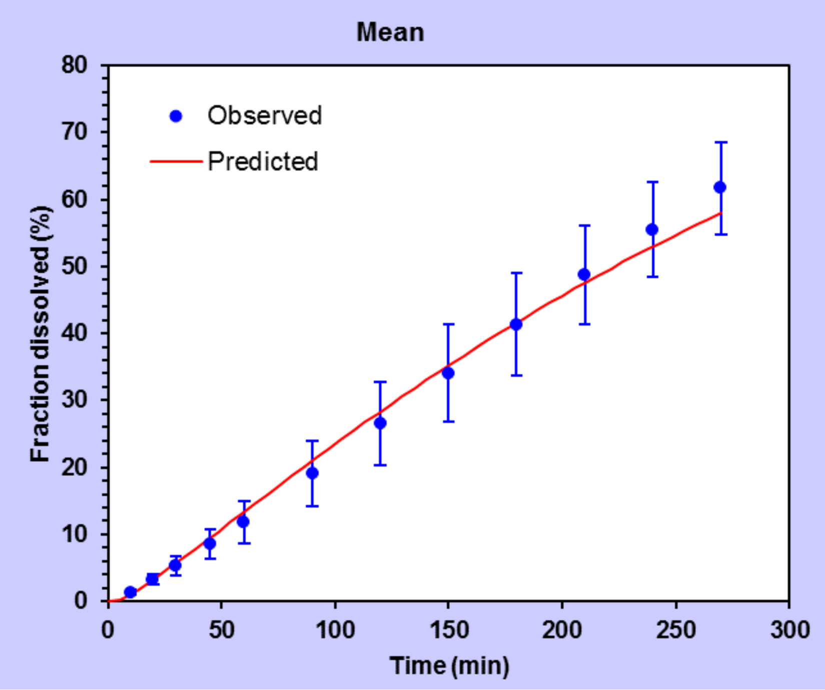

Supplementary materials – Model fitting summary – Kollidon® 90 F (modelling up to app. 60 % of carvedilol released)

Graphical abstract of model fit presented as the fraction % of released carvedilol per tested tablet:

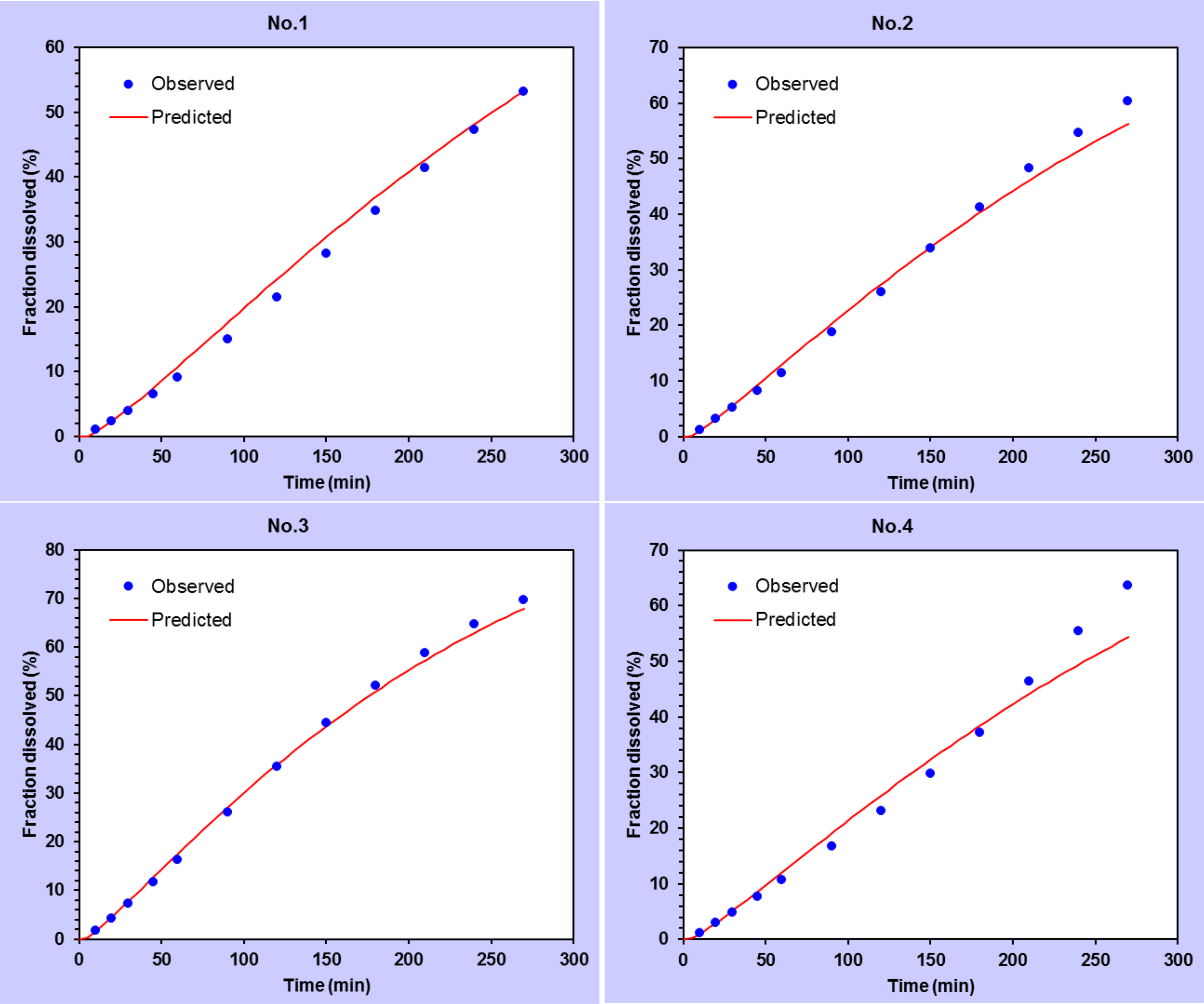

Supplementary materials – Model fitting summary – Kollidon® 90 F (modelling up to app. 60 % of carvedilol released)

Model: **Weibull_2**

Model equation: $F = 100 \cdot \left(1 - e^{-\frac{t^{\beta}}{\alpha}}\right)$

Fitted model parameters per tested tablet (N = 4) with statistics – mean, standard deviation (SD), and relative standard deviation expressed in % (RSD%) (output from DDSolver):

Parameter	No.1	No.2	No.3	No.4	Mean	SD	RSD(%)
α	2439.487	1707.421	1029.831	2065.488	1810.557	600.198	33.150
β	1.343	1.315	1.275	1.348	1.320	0.034	2.549

Number of dissolution data points (N), degrees of freedom (df), and selected goodness of fit criteria – Pearson correlation coefficient (R), coefficient of determination (R^2), adjusted coefficient of determination ($R^2_{adjusted}$), and residual sum of squares (RSS) (manual calculation in MS Excel):

Parameter	No.1	No.2	No.3	No.4
N	12	12	12	12
df	10	10	10	10
R	0.999748292	0.999762455	0.999905922	0.994422556
R^2	0.999496647	0.999524966	0.999811854	0.988876221
$R^2_{adjusted}$	0.999446312	0.999477463	0.999793039	0.987763843
RSS	2.894186697	3.494425624	1.395746239	67.45934088

Graphical abstract of model fit presented as mean ± 1 SD of the fraction % of released carvedilol:

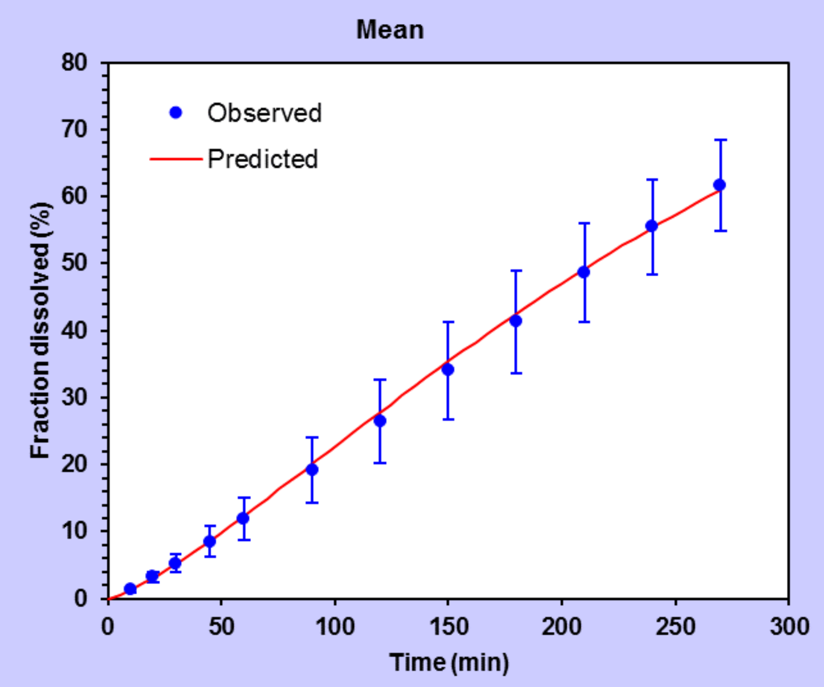

Supplementary materials – Model fitting summary – Kollidon® 90 F (modelling up to app. 60 % of carvedilol released)

Graphical abstract of model fit presented as the fraction % of released carvedilol per tested tablet:

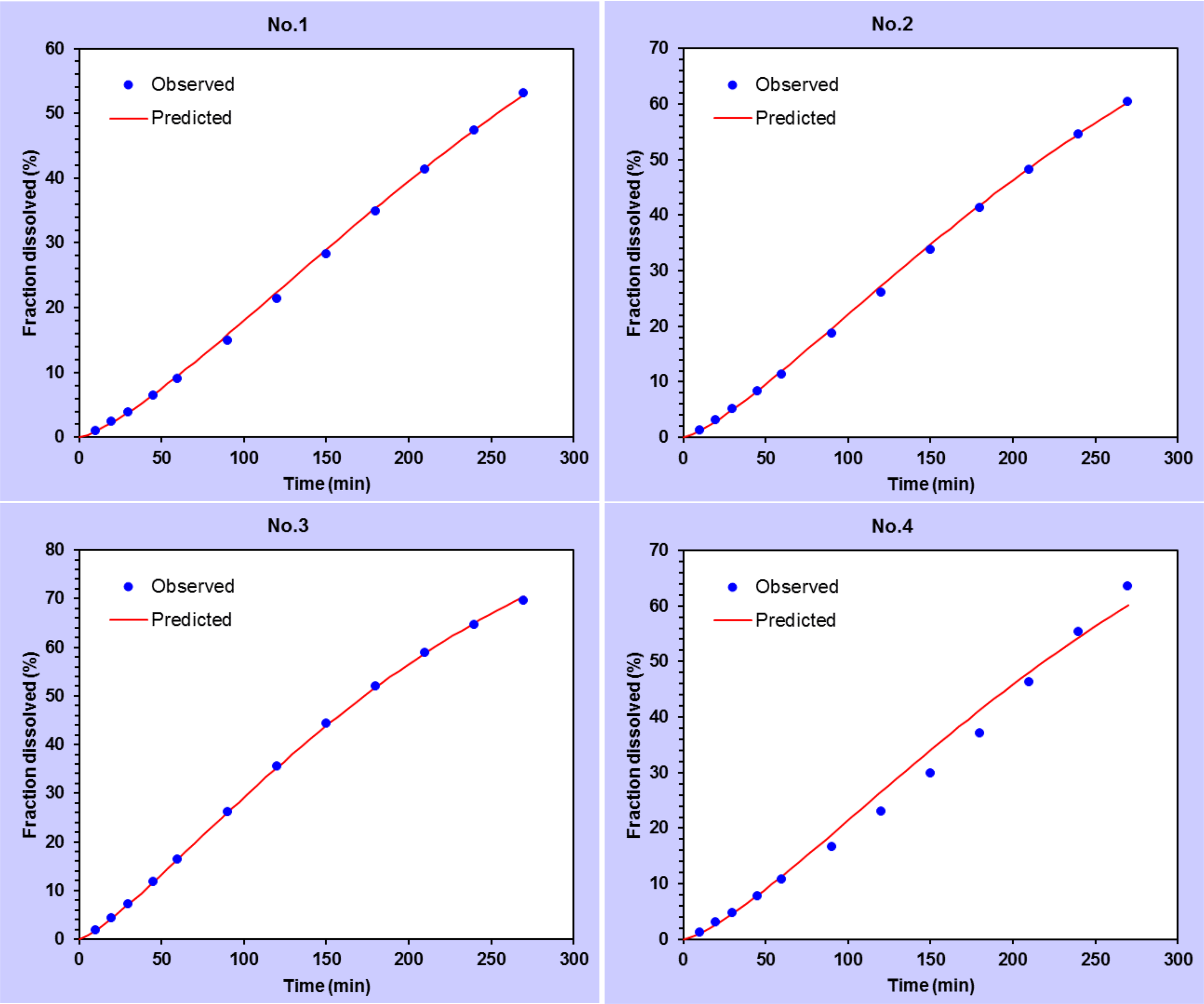

Supplementary materials – Model fitting summary – Kollidon® 90 F (modelling up to app. 60 % of carvedilol released)

Model: **Weibull_3**

Model equation: $F = F_{max} \cdot \left(1 - e^{-\frac{t^{\beta}}{\alpha}}\right)$

Fitted model parameters per tested tablet (N = 4) with statistics – mean, standard deviation (SD), and relative standard deviation expressed in % (RSD%) (output from DDSolver):

Parameter	No.1	No.2	No.3	No.4	Mean	SD	RSD(%)
α	2351.019	1816.097	1192.951	1815.329	1793.849	473.453	26.393
β	1.437	1.425	1.417	1.438	1.429	0.010	0.711
F_{max}	64.207	74.540	73.089	66.746	69.645	4.960	7.121

Number of dissolution data points (N), degrees of freedom (df), and selected goodness of fit criteria – Pearson correlation coefficient (R), coefficient of determination (R^2), adjusted coefficient of determination ($R^2_{adjusted}$), and residual sum of squares (RSS) (manual calculation in MS Excel):

Parameter	No.1	No.2	No.3	No.4
N	12	12	12	12
df	9	9	9	9
R	0.99786132	0.997508418	0.99749425	0.984768091
R^2	0.995727214	0.995023044	0.994994778	0.969768192
$R^2_{adjusted}$	0.994777706	0.993917054	0.993882507	0.963050013
RSS	61.7760285	47.06557868	38.60052835	171.7317203

Graphical abstract of model fit presented as mean ± 1 SD of the fraction % of released carvedilol:

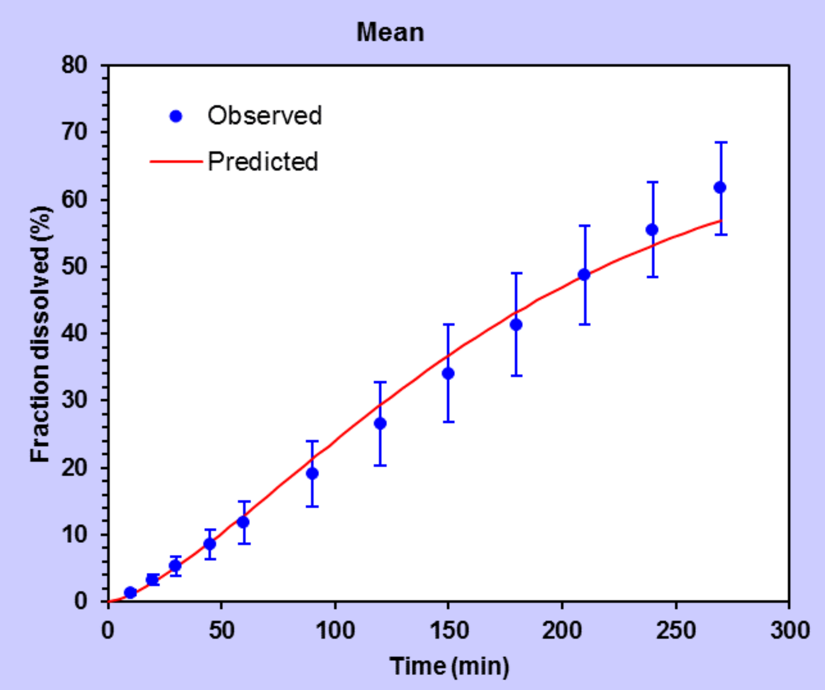

Supplementary materials – Model fitting summary – Kollidon® 90 F (modelling up to app. 60 % of carvedilol released)

Graphical abstract of model fit presented as the fraction % of released carvedilol per tested tablet:

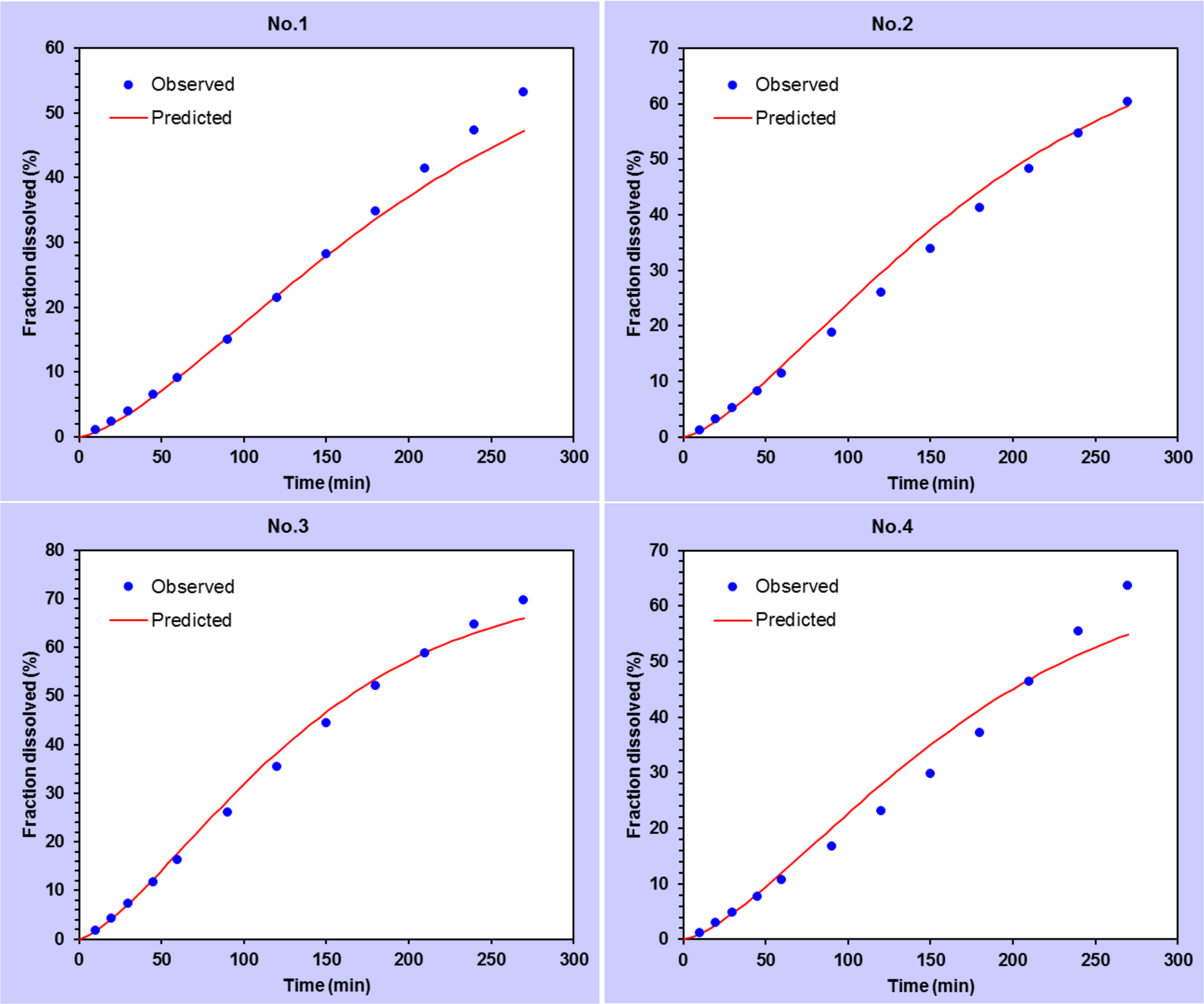

Supplementary materials – Model fitting summary – Kollidon® 90 F (modelling up to app. 60 % of carvedilol released)

Model: **Weibull_4**

Model equation: $F = F_{max} \cdot \left[1 - e^{-\frac{(t-T_i)^\beta}{\alpha}}\right]$

Fitted model parameters per tested tablet (N = 4) with statistics – mean, standard deviation (SD), and relative standard deviation expressed in % (RSD%) (output from DDSolver):

Parameter	No.1	No.2	No.3	No.4	Mean	SD	RSD(%)
α	891.601	695.445	576.042	785.891	737.244	134.072	18.186
β	1.320	1.285	1.247	1.277	1.282	0.030	2.334
T_i	6.000	6.000	4.582	6.000	5.646	0.709	12.555
F_{max}	55.824	63.365	82.370	66.746	67.076	11.171	16.655

Number of dissolution data points (N), degrees of freedom (df), and selected goodness of fit criteria – Pearson correlation coefficient (R), coefficient of determination (R^2), adjusted coefficient of determination ($R^2_{adjusted}$), and residual sum of squares (RSS) (manual calculation in MS Excel):

Parameter	No.1	No.2	No.3	No.4
N	12	12	12	12
df	8	8	8	8
R	0.990324645	0.991499667	0.998044078	0.981551848
R^2	0.980742902	0.98307159	0.996091982	0.963444031
$R^2_{adjusted}$	0.97352149	0.976723437	0.994626475	0.949735542
RSS	95.24735714	104.1055018	58.93154637	221.9579165

Graphical abstract of model fit presented as mean ± 1 SD of the fraction % of released carvedilol:

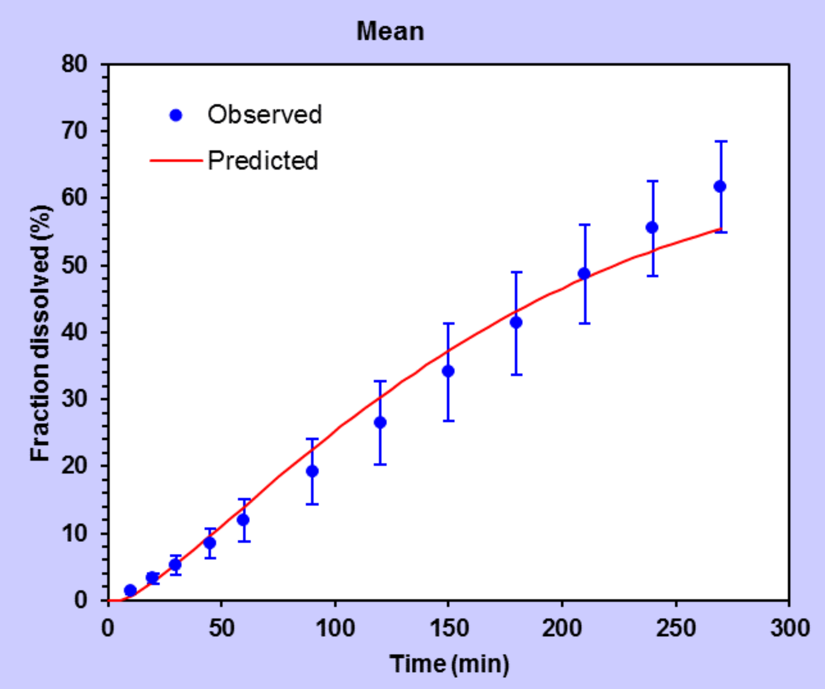

Supplementary materials – Model fitting summary – Kollidon® 90 F (modelling up to app. 60 % of carvedilol released)

Graphical abstract of model fit presented as the fraction % of released carvedilol per tested tablet:

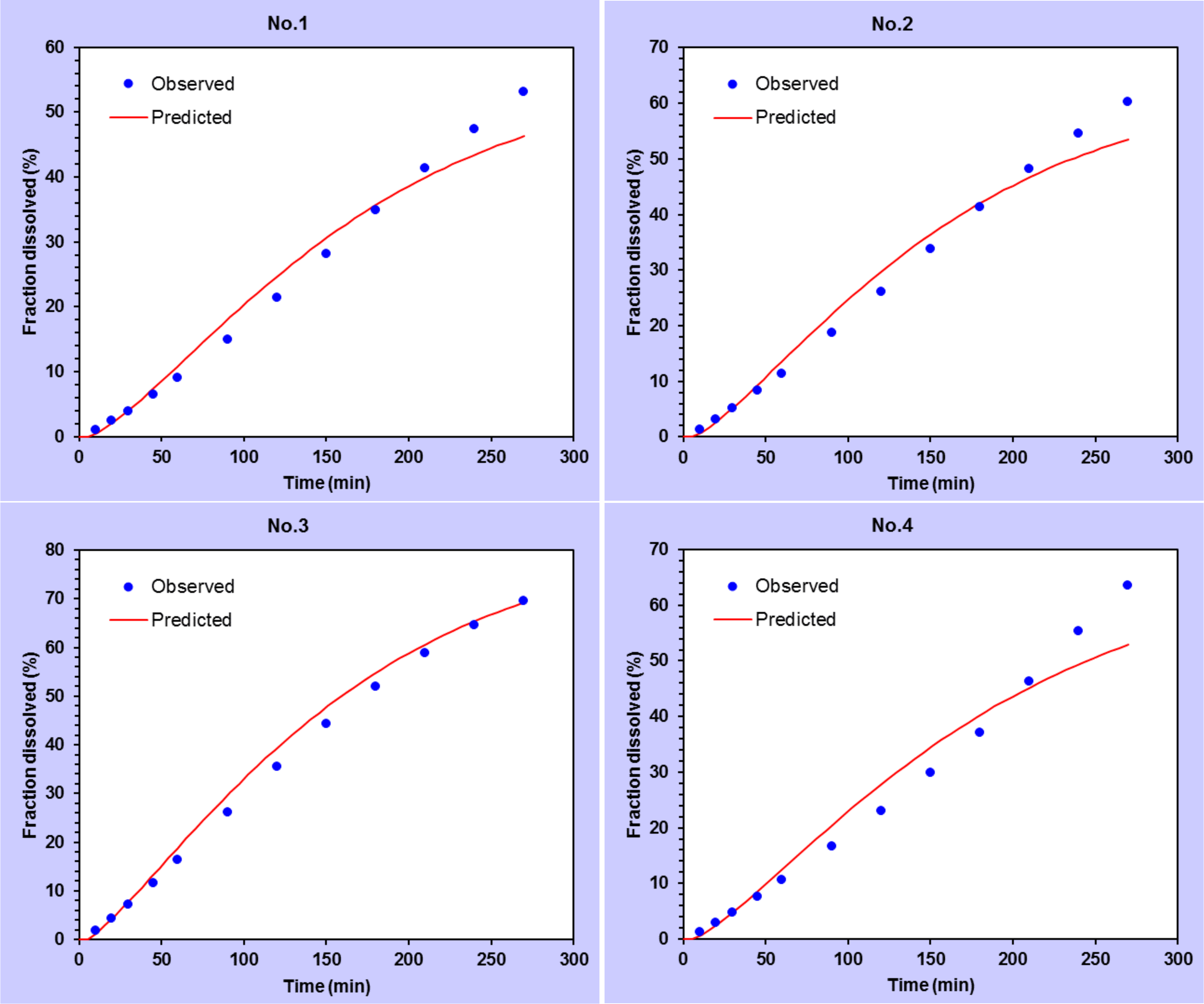

Supplementary materials – Model fitting summary – Kollidon® 90 F (modelling up to app. 60 % of carvedilol released)

Model: **Logistic_1**

Model equation: $F = 100 \cdot \frac{e^{\alpha+\beta\cdot\log(t)}}{1+e^{\alpha+\beta\cdot\log(t)}}$

Fitted model parameters per tested tablet (N = 4) with statistics – mean, standard deviation (SD), and relative standard deviation expressed in % (RSD%) (output from DDSolver):

Parameter	No.1	No.2	No.3	No.4	Mean	SD	RSD(%)
α	-8.616	-8.392	-7.517	-7.900	-8.106	0.494	-6.092
β	3.594	3.545	3.371	3.321	3.458	0.132	3.816

Number of dissolution data points (N), degrees of freedom (df), and selected goodness of fit criteria – Pearson correlation coefficient (R), coefficient of determination (R^2), adjusted coefficient of determination ($R^2_{adjusted}$), and residual sum of squares (RSS) (manual calculation in MS Excel):

Parameter	No.1	No.2	No.3	No.4
N	12	12	12	12
df	10	10	10	10
R	0.998035197	0.998600855	0.998292929	0.987138699
R^2	0.996074254	0.997203668	0.996588772	0.974442811
$R^2_{adjusted}$	0.995681679	0.996924034	0.996247649	0.971887092
RSS	32.24158798	43.38345137	30.66975396	162.3653548

Graphical abstract of model fit presented as mean ± 1 SD of the fraction % of released carvedilol:

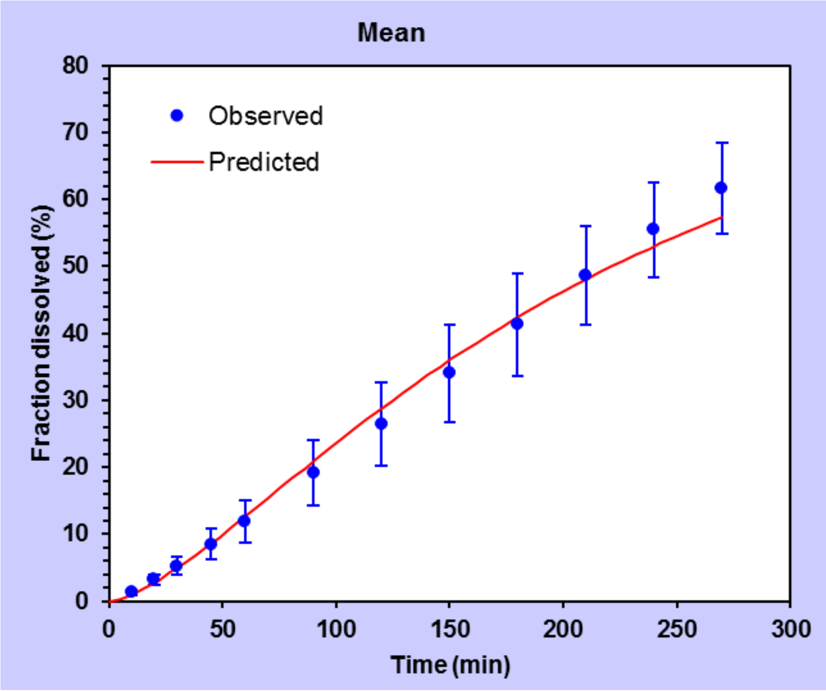

Supplementary materials – Model fitting summary – Kollidon® 90 F (modelling up to app. 60 % of carvedilol released)

Graphical abstract of model fit presented as the fraction % of released carvedilol per tested tablet:

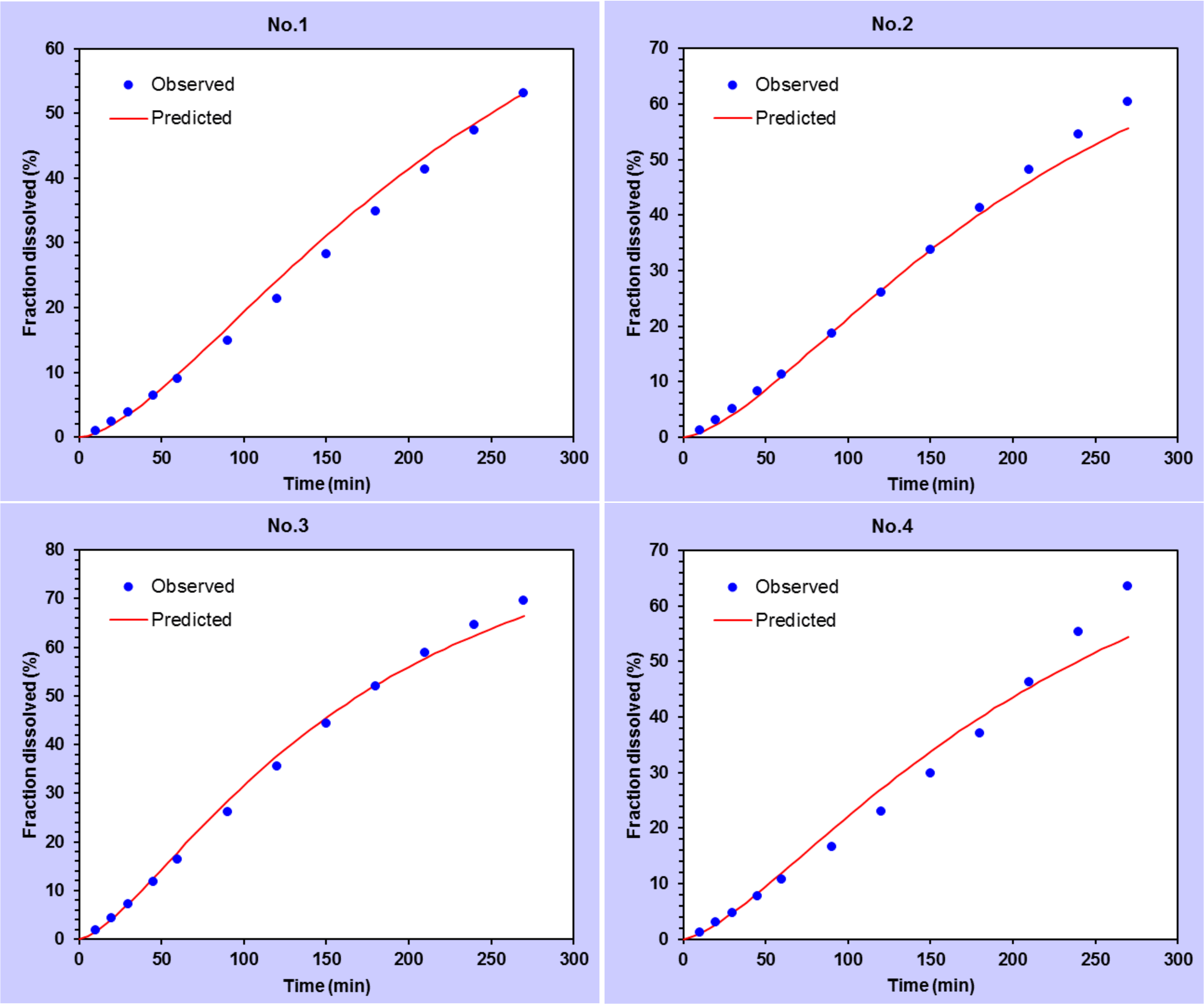

Supplementary materials – Model fitting summary – Kollidon® 90 F (modelling up to app. 60 % of carvedilol released)

Model: **Logistic_2**

Model equation: $F = F_{max} \cdot \frac{e^{\alpha+\beta \cdot \log(t)}}{1+e^{\alpha+\beta \cdot \log(t)}}$

Fitted model parameters per tested tablet (N = 4) with statistics – mean, standard deviation (SD), and relative standard deviation expressed in % (RSD%) (output from DDSolver):

Parameter	No.1	No.2	No.3	No.4	Mean	SD	RSD(%)
α	-8.932	-9.978	-9.736	-8.699	-9.336	0.617	-6.607
β	4.325	4.503	4.510	4.155	4.373	0.169	3.865
F_{max}	55.824	70.768	81.628	66.746	68.742	10.662	15.510

Number of dissolution data points (N), degrees of freedom (df), and selected goodness of fit criteria – Pearson correlation coefficient (R), coefficient of determination (R^2), adjusted coefficient of determination ($R^2_{adjusted}$), and residual sum of squares (RSS) (manual calculation in MS Excel):

Parameter	No.1	No.2	No.3	No.4
N	12	12	12	12
df	9	9	9	9
R	0.97524723	0.996753534	0.998626575	0.964216337
R^2	0.95110716	0.993517608	0.997255035	0.929713145
$R^2_{adjusted}$	0.940242085	0.992077077	0.996645043	0.914093844
RSS	218.9700392	192.8545214	155.7090652	408.1234788

Graphical abstract of model fit presented as mean ± 1 SD of the fraction % of released carvedilol:

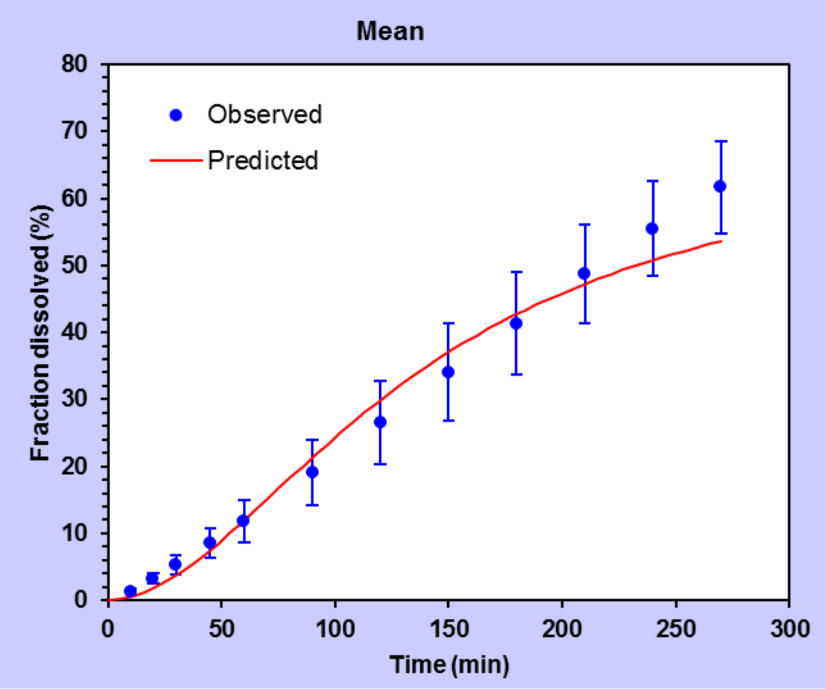

Supplementary materials – Model fitting summary – Kollidon® 90 F (modelling up to app. 60 % of carvedilol released)

Graphical abstract of model fit presented as the fraction % of released carvedilol per tested tablet:

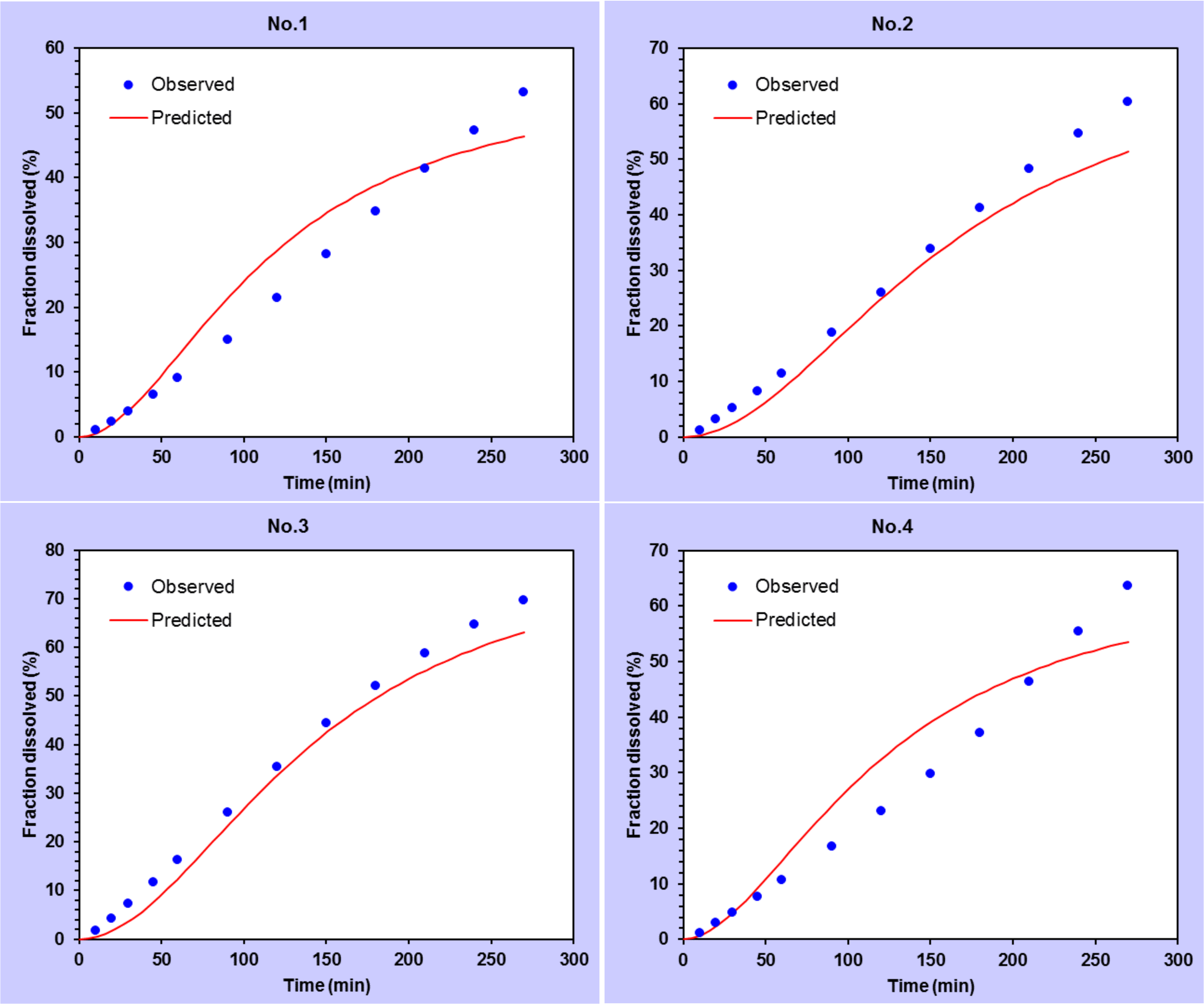

Supplementary materials – Model fitting summary – Kollidon® 90 F (modelling up to app. 60 % of carvedilol released)

Model: **Logistic_3**

Model equation: $F = F_{max} \cdot \frac{1}{1+e^{-k\cdot(t-\gamma)}}$

Fitted model parameters per tested tablet (N = 4) with statistics – mean, standard deviation (SD), and relative standard deviation expressed in % (RSD%) (output from DDSolver):

Parameter	No.1	No.2	No.3	No.4	Mean	SD	RSD(%)
k	0.023	0.022	0.020	0.015	0.020	0.003	16.791
γ	150.159	145.323	114.303	179.345	147.282	26.632	18.082
F_{max}	55.824	63.365	66.745	74.522	65.114	7.758	11.914

Number of dissolution data points (N), degrees of freedom (df), and selected goodness of fit criteria – Pearson correlation coefficient (R), coefficient of determination (R^2), adjusted coefficient of determination ($R^2_{adjusted}$), and residual sum of squares (RSS) (manual calculation in MS Excel):

Parameter	No.1	No.2	No.3	No.4
N	12	12	12	12
df	9	9	9	9
R	0.994989263	0.994989986	0.99740061	0.997534959
R^2	0.990003633	0.990005072	0.994807977	0.995075994
$R^2_{adjusted}$	0.987782219	0.987783977	0.993654194	0.993981771
RSS	51.038294	65.18361341	94.90643117	55.51067406

Graphical abstract of model fit presented as mean ± 1 SD of the fraction % of released carvedilol:

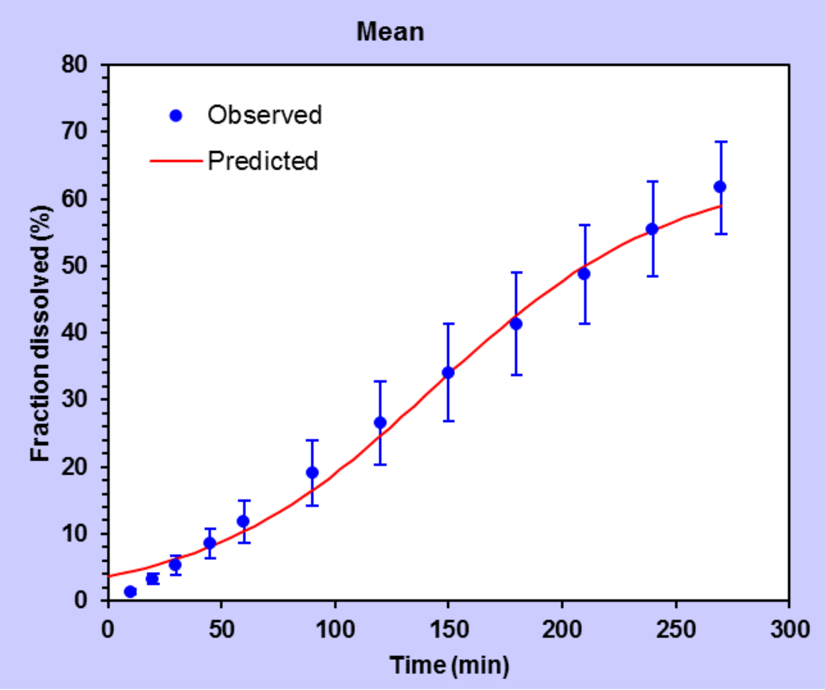

Supplementary materials – Model fitting summary – Kollidon® 90 F (modelling up to app. 60 % of carvedilol released)

Graphical abstract of model fit presented as the fraction % of released carvedilol per tested tablet:

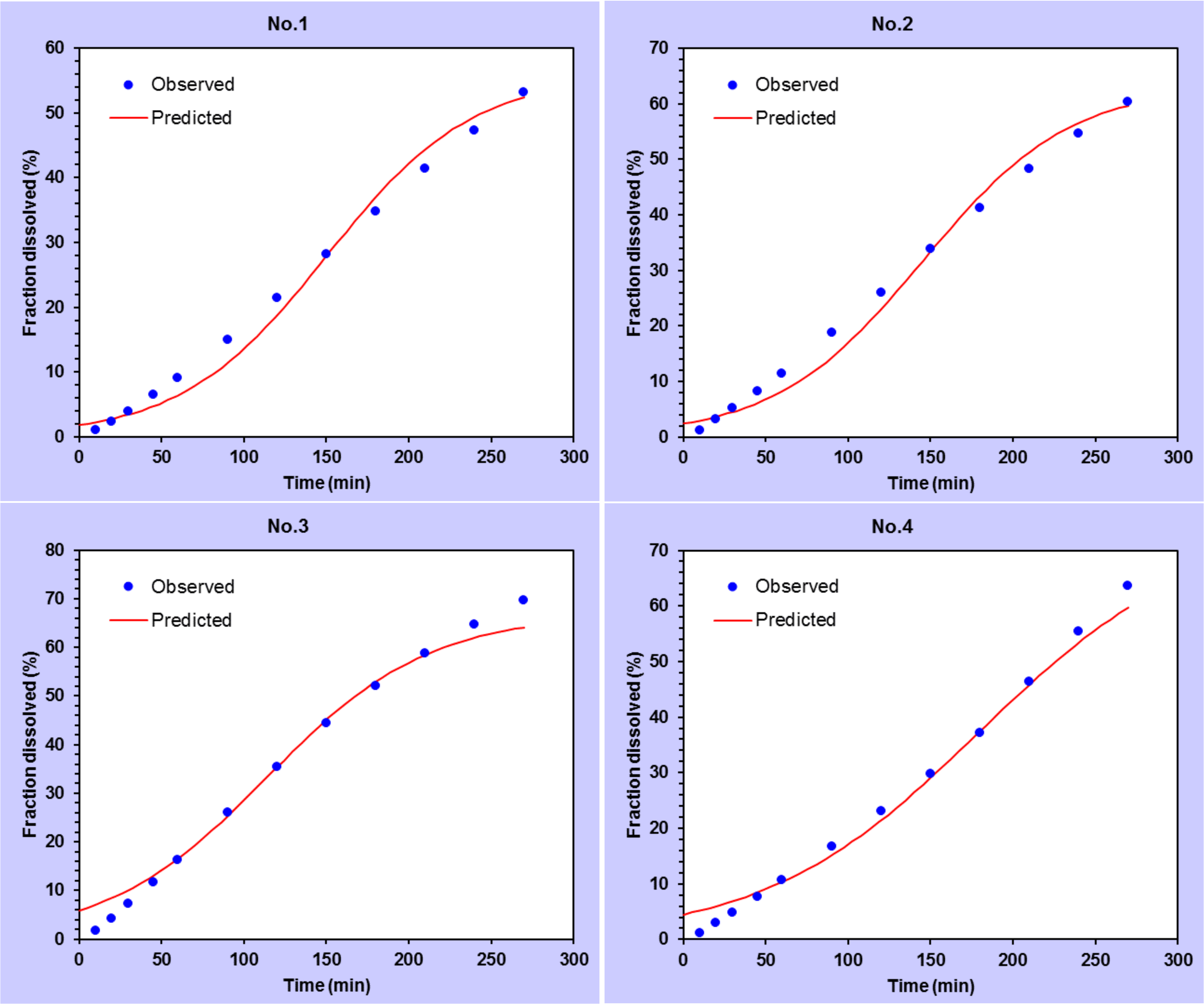

Supplementary materials – Model fitting summary – Kollidon® 90 F (modelling up to app. 60 % of carvedilol released)

Model: **Gompertz_1**

Model equation: $F = 100 \cdot e^{-\alpha \cdot e^{-\beta \cdot \log(t)}}$

Fitted model parameters per tested tablet (N = 4) with statistics – mean, standard deviation (SD), and relative standard deviation expressed in % (RSD%) (output from DDSolver):

Parameter	No.1	No.2	No.3	No.4	Mean	SD	RSD(%)
α	23.740	25.557	30.170	26.617	26.521	2.707	10.208
β	1.386	1.497	1.701	1.500	1.521	0.131	8.624

Number of dissolution data points (N), degrees of freedom (df), and selected goodness of fit criteria – Pearson correlation coefficient (R), coefficient of determination (R^2), adjusted coefficient of determination ($R^2_{adjusted}$), and residual sum of squares (RSS) (manual calculation in MS Excel):

Parameter	No.1	No.2	No.3	No.4
N	12	12	12	12
df	10	10	10	10
R	0.97827576	0.977849743	0.982787595	0.960371746
R^2	0.957023464	0.95619012	0.965871457	0.922313891
$R^2_{adjusted}$	0.95272581	0.951809132	0.962458603	0.91454528
RSS	211.5286612	266.7519168	278.7970437	459.5693544

Graphical abstract of model fit presented as mean ± 1 SD of the fraction % of released carvedilol:

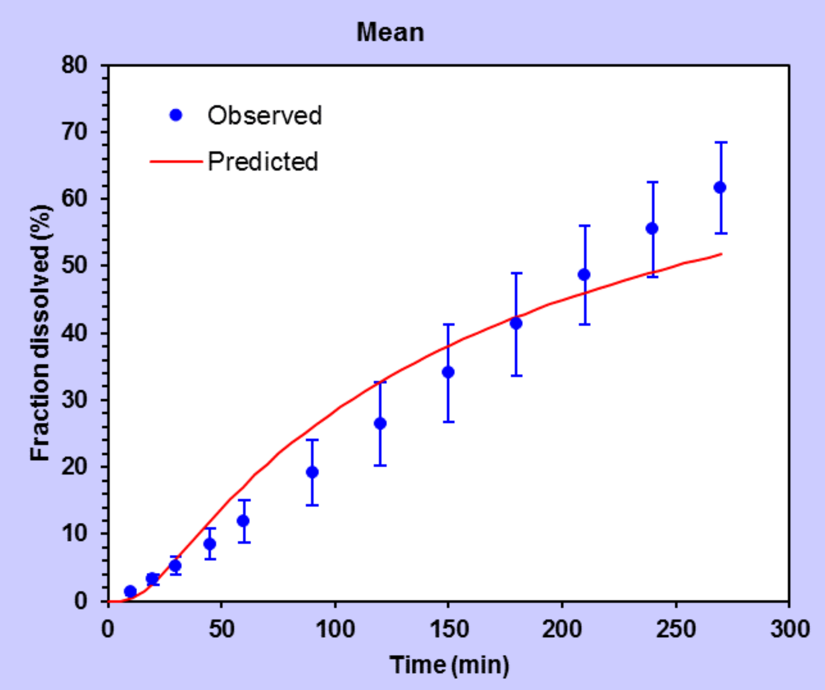

Supplementary materials – Model fitting summary – Kollidon® 90 F (modelling up to app. 60 % of carvedilol released)

Graphical abstract of model fit presented as the fraction % of released carvedilol per tested tablet:

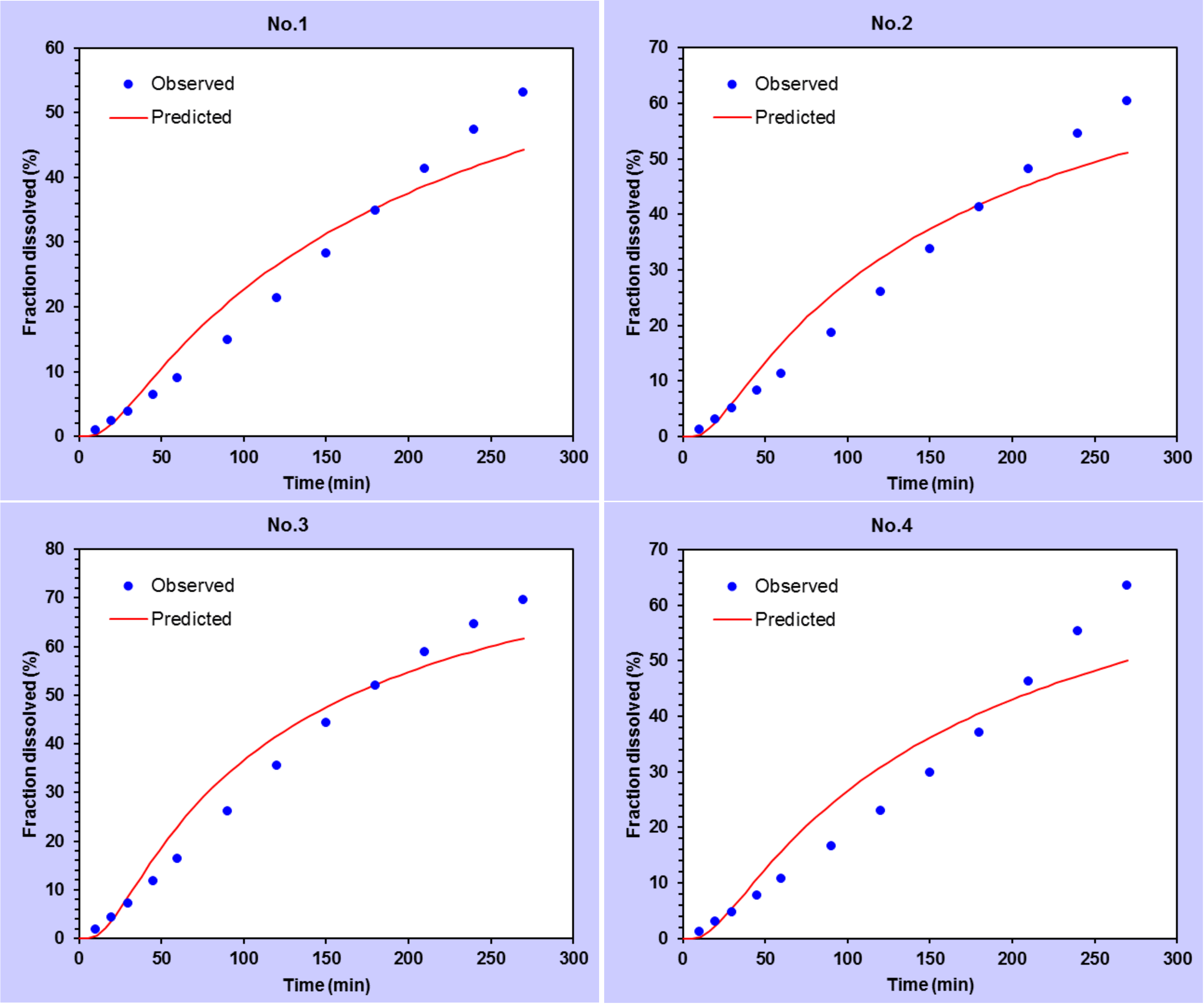

Supplementary materials – Model fitting summary – Kollidon® 90 F (modelling up to app. 60 % of carvedilol released)

Model: **Gompertz_2**

Model equation: $F = F_{max} \cdot e^{-\alpha \cdot e^{-\beta \cdot \log(t)}}$

Fitted model parameters per tested tablet (N = 4) with statistics – mean, standard deviation (SD), and relative standard deviation expressed in % (RSD%) (output from DDSolver):

Parameter	No.1	No.2	No.3	No.4	Mean	SD	RSD(%)
α	156.062	123.864	159.825	106.873	136.656	25.587	18.724
β	2.534	2.370	2.654	2.231	2.447	0.185	7.572
F_{max}	55.824	71.946	73.089	75.784	69.161	9.036	13.065

Number of dissolution data points (N), degrees of freedom (df), and selected goodness of fit criteria – Pearson correlation coefficient (R), coefficient of determination (R^2), adjusted coefficient of determination ($R^2_{adjusted}$), and residual sum of squares (RSS) (manual calculation in MS Excel):

Parameter	No.1	No.2	No.3	No.4
N	12	12	12	12
df	9	9	9	9
R	0.971996165	0.981902006	0.982759329	0.968527009
R^2	0.944776546	0.964131549	0.9658159	0.938044567
$R^2_{adjusted}$	0.932504667	0.956160782	0.958219433	0.924276693
RSS	321.5710149	276.1367767	377.8372538	459.8281053

Graphical abstract of model fit presented as mean ± 1 SD of the fraction % of released carvedilol:

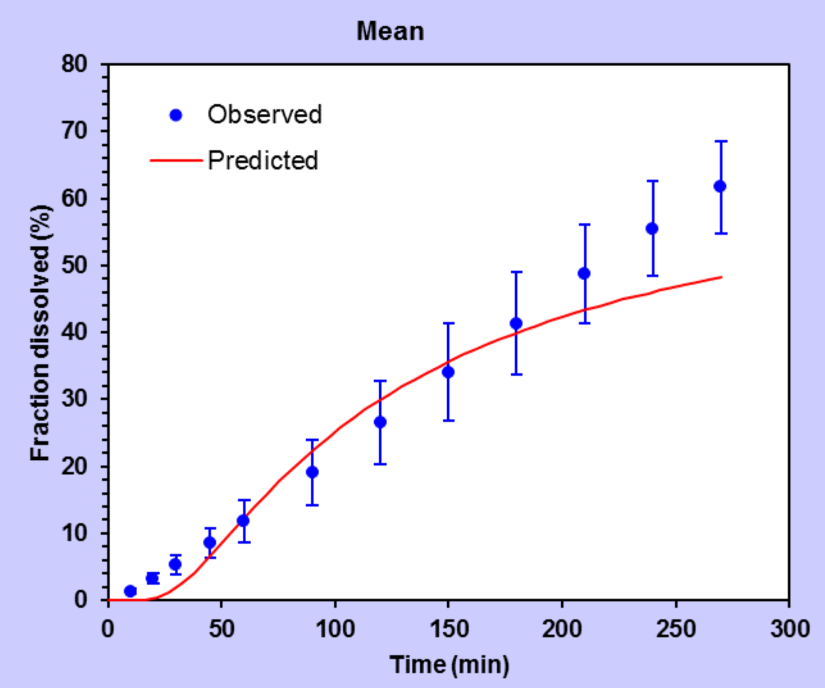

Supplementary materials – Model fitting summary – Kollidon® 90 F (modelling up to app. 60 % of carvedilol released)

Graphical abstract of model fit presented as the fraction % of released carvedilol per tested tablet:

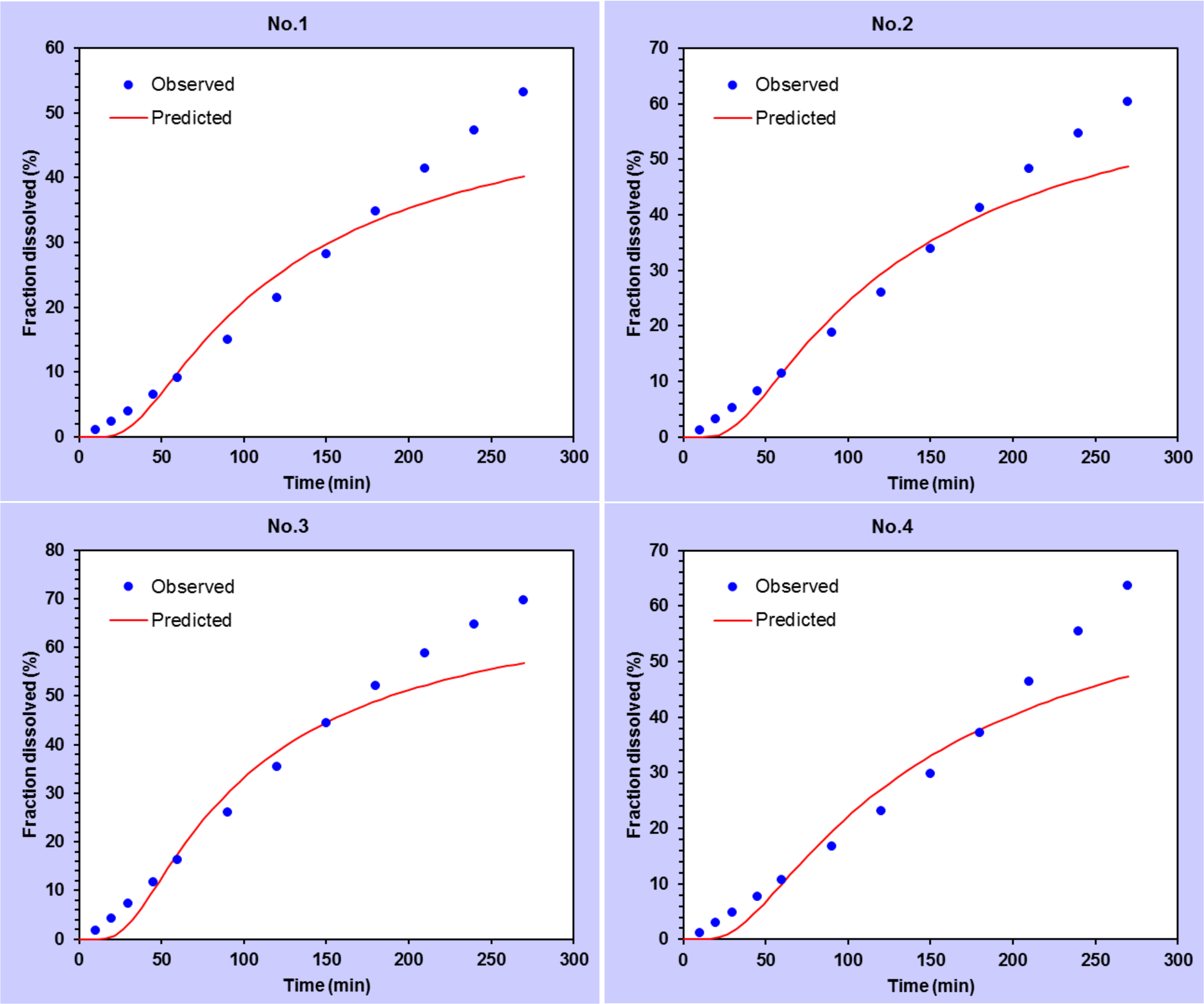

Supplementary materials – Model fitting summary – Kollidon® 90 F (modelling up to app. 60 % of carvedilol released)

Model: **Gompertz_3**

Model equation: $F = F_{max} \cdot e^{-e^{-k\cdot(t-\gamma)}}$

Fitted model parameters per tested tablet (N = 4) with statistics – mean, standard deviation (SD), and relative standard deviation expressed in % (RSD%) (output from DDSolver):

Parameter	No.1	No.2	No.3	No.4	Mean	SD	RSD(%)
k	0.014	0.014	0.012	0.014	0.014	0.001	9.696
γ	106.878	102.389	97.661	111.479	104.602	5.932	5.671
F_{max}	55.824	63.365	78.519	66.746	66.113	9.447	14.289

Number of dissolution data points (N), degrees of freedom (df), and selected goodness of fit criteria – Pearson correlation coefficient (R), coefficient of determination (R^2), adjusted coefficient of determination ($R^2_{adjusted}$), and residual sum of squares (RSS) (manual calculation in MS Excel):

Parameter	No.1	No.2	No.3	No.4
N	12	12	12	12
df	9	9	9	9
R	0.993121407	0.994461528	0.999386678	0.985591524
R^2	0.986290129	0.988953731	0.998773732	0.971390653
$R^2_{adjusted}$	0.983243491	0.986499004	0.998501228	0.96503302
RSS	71.88021589	74.79827606	24.35880582	196.5546735

Graphical abstract of model fit presented as mean ± 1 SD of the fraction % of released carvedilol:

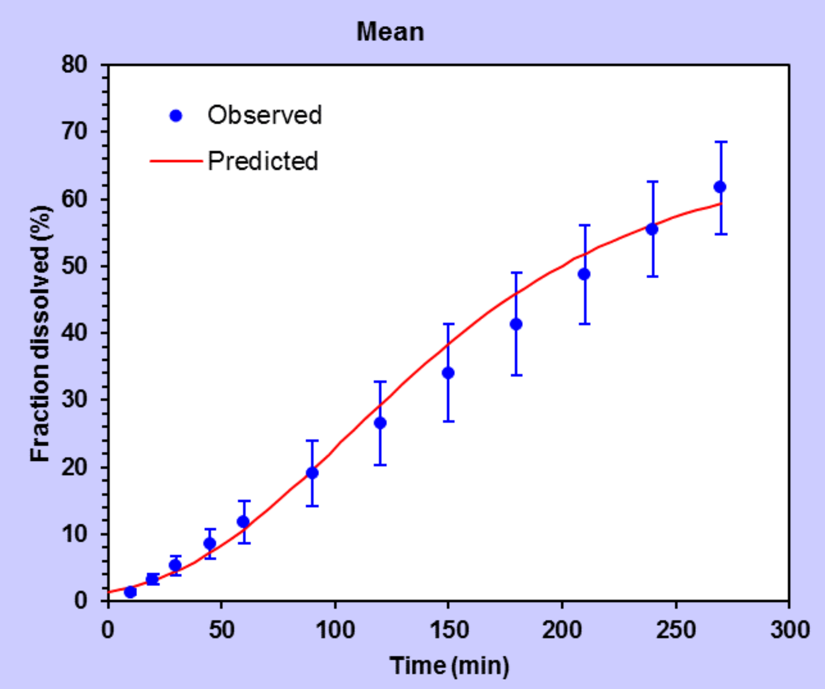

Supplementary materials – Model fitting summary – Kollidon® 90 F (modelling up to app. 60 % of carvedilol released)

Graphical abstract of model fit presented as the fraction % of released carvedilol per tested tablet:

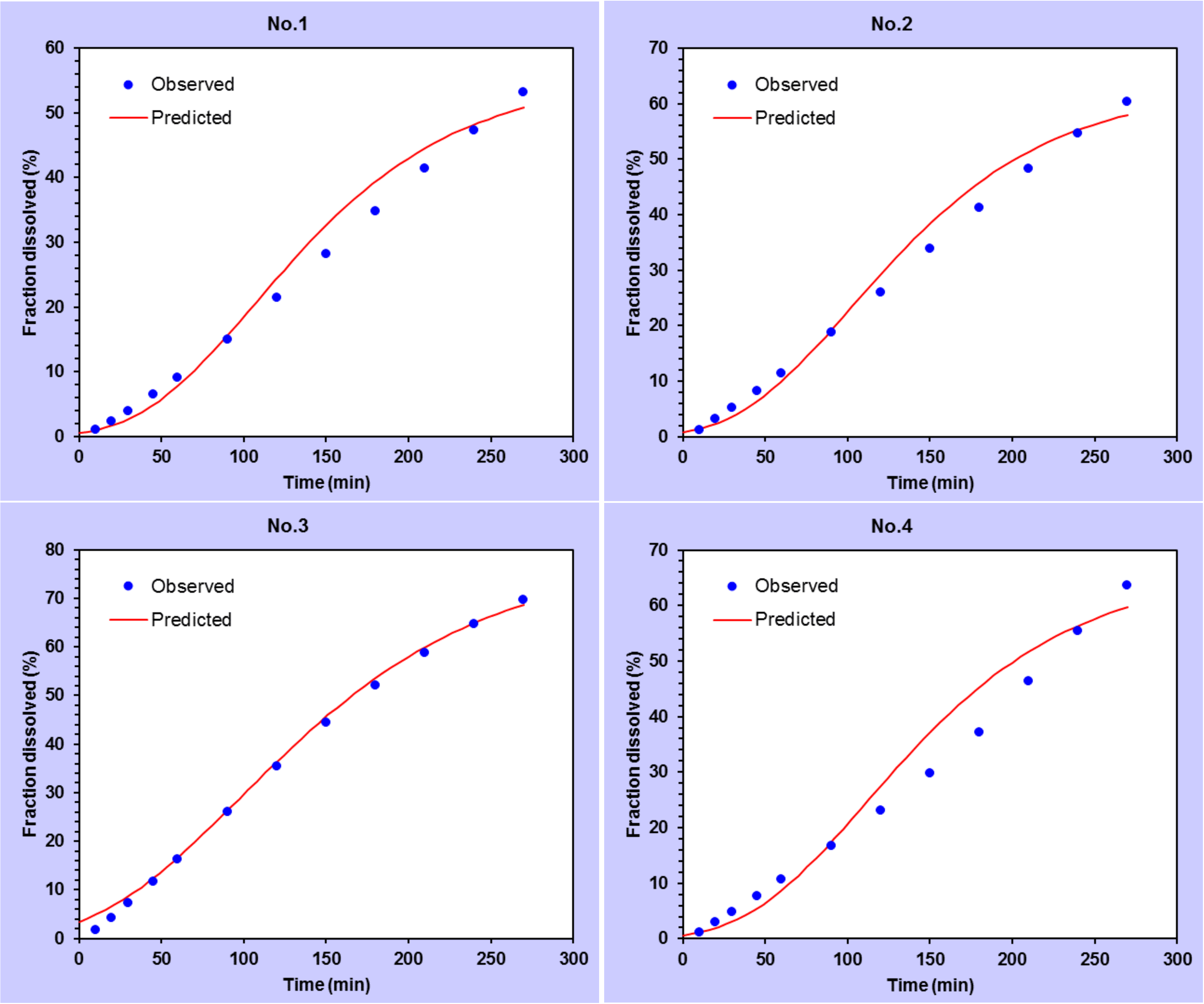

Supplementary materials – Model fitting summary – Kollidon® 90 F (modelling up to app. 60 % of carvedilol released)

Model: **Gompertz_4**

Model equation: $F = F_{max} \cdot e^{-\beta \cdot e^{-k \cdot t}}$

Fitted model parameters per tested tablet (N = 4) with statistics – mean, standard deviation (SD), and relative standard deviation expressed in % (RSD%) (output from DDSolver):

Parameter	No.1	No.2	No.3	No.4	Mean	SD	RSD(%)
k	0.014	0.014	0.015	0.014	0.014	0.000	2.756
β	4.667	4.400	3.974	4.676	4.429	0.329	7.439
F_{max}	55.824	63.365	73.089	66.746	64.756	7.191	11.104

Number of dissolution data points (N), degrees of freedom (df), and selected goodness of fit criteria – Pearson correlation coefficient (R), coefficient of determination (R^2), adjusted coefficient of determination ($R^2_{adjusted}$), and residual sum of squares (RSS) (manual calculation in MS Excel):

Parameter	No.1	No.2	No.3	No.4
N	12	12	12	12
df	9	9	9	9
R	0.993121407	0.994461528	0.997558984	0.985591524
R^2	0.986290129	0.988953731	0.995123926	0.971390653
$R^2_{adjusted}$	0.983243491	0.986499004	0.994040354	0.96503302
RSS	71.88021589	74.79827606	44.85670094	196.5546735

Graphical abstract of model fit presented as mean ± 1 SD of the fraction % of released carvedilol:

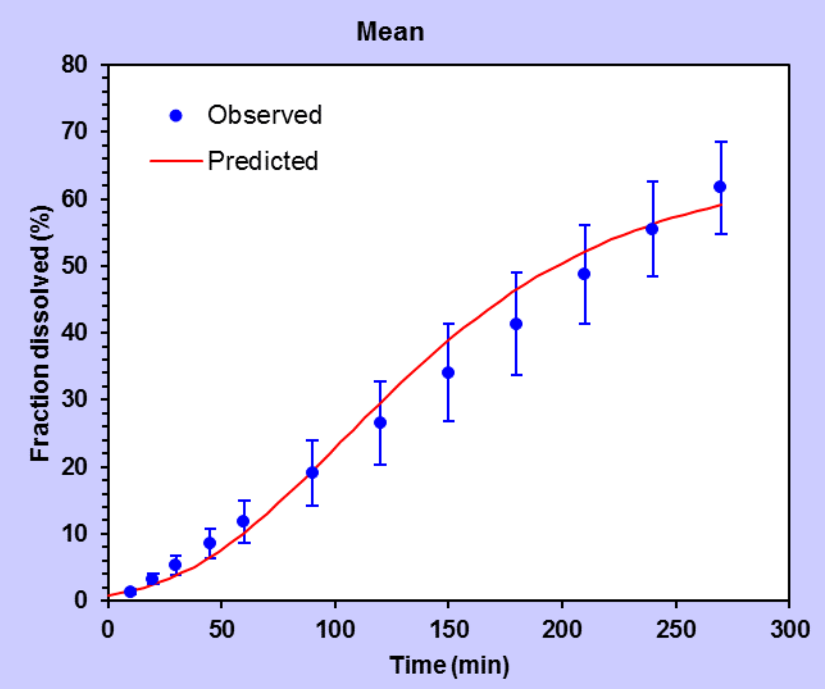

Supplementary materials – Model fitting summary – Kollidon® 90 F (modelling up to app. 60 % of carvedilol released)

Graphical abstract of model fit presented as the fraction % of released carvedilol per tested tablet:

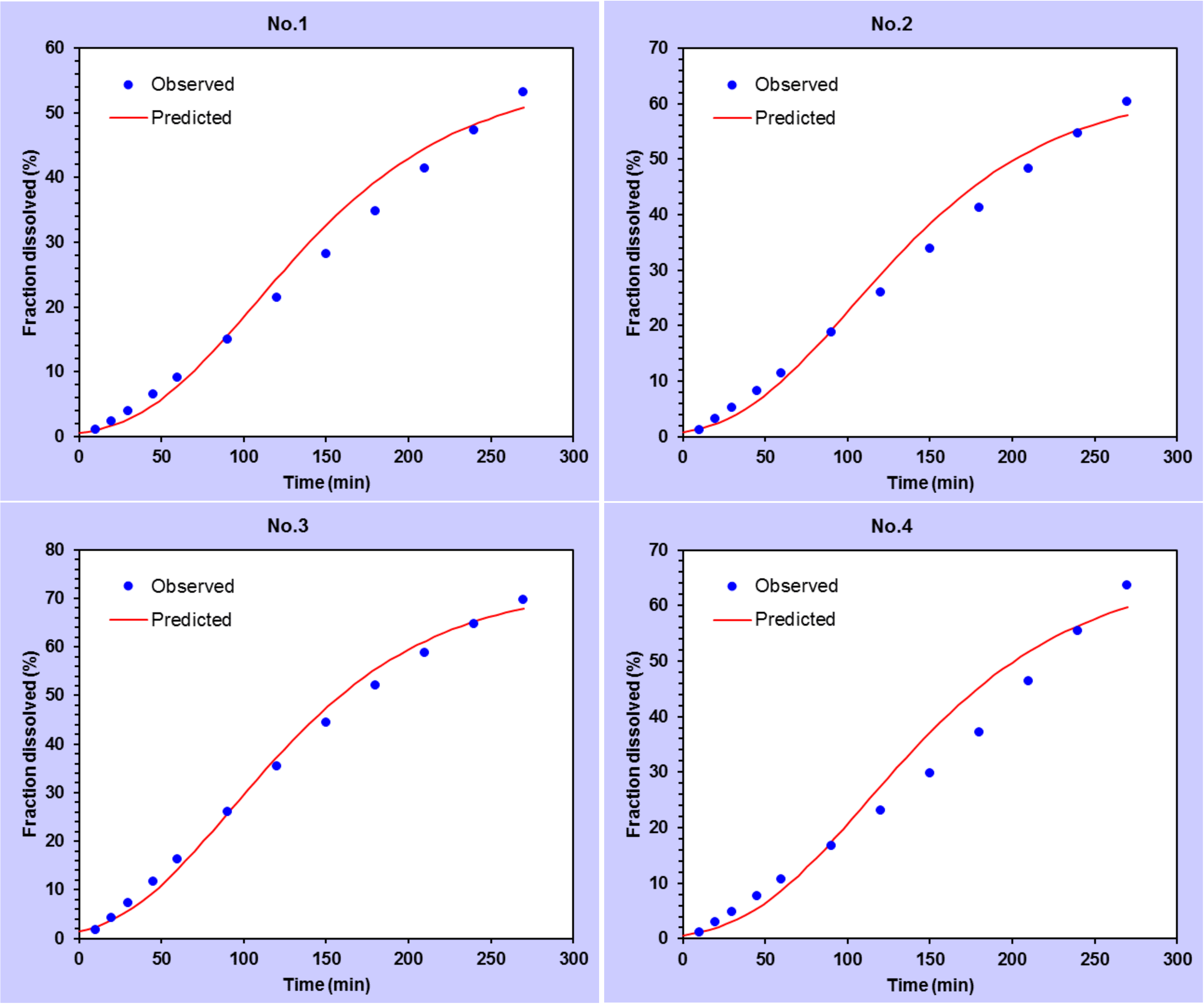

Supplementary materials – Model fitting summary – Kollidon® 90 F (modelling up to app. 60 % of carvedilol released)

Model: **Probit_1**

Model equation: $F = 100 \cdot \phi[\alpha + \beta \cdot \log(t)]$

Fitted model parameters per tested tablet (N = 4) with statistics – mean, standard deviation (SD), and relative standard deviation expressed in % (RSD%) (output from DDSolver):

Parameter	No.1	No.2	No.3	No.4	Mean	SD	RSD(%)
α	-4.218	-4.170	-4.492	-4.229	-4.278	0.146	-3.403
β	1.697	1.750	2.038	1.759	1.811	0.154	8.497

Number of dissolution data points (N), degrees of freedom (df), and selected goodness of fit criteria – Pearson correlation coefficient (R), coefficient of determination (R^2), adjusted coefficient of determination ($R^2_{adjusted}$), and residual sum of squares (RSS) (manual calculation in MS Excel):

Parameter	No.1	No.2	No.3	No.4
N	12	12	12	12
df	10	10	10	10
R	0.991060167	0.991177862	0.995971991	0.978239825
R^2	0.982200255	0.982433555	0.991960208	0.956953155
$R^2_{adjusted}$	0.98042028	0.980676911	0.991156228	0.952648471
RSS	104.26362	123.9673834	71.81579724	279.8091808

Graphical abstract of model fit presented as mean ± 1 SD of the fraction % of released carvedilol:

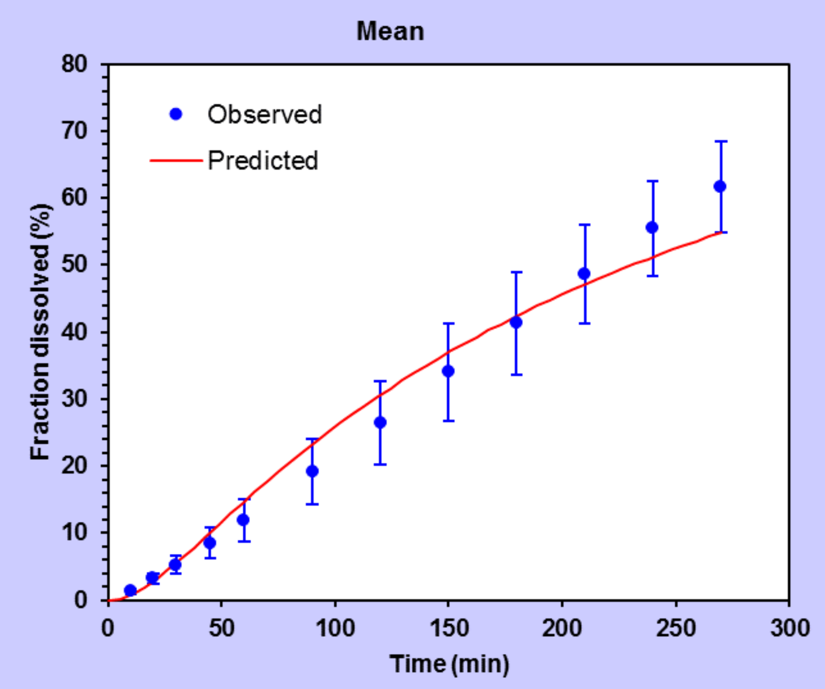

Supplementary materials – Model fitting summary – Kollidon® 90 F (modelling up to app. 60 % of carvedilol released)

Graphical abstract of model fit presented as the fraction % of released carvedilol per tested tablet:

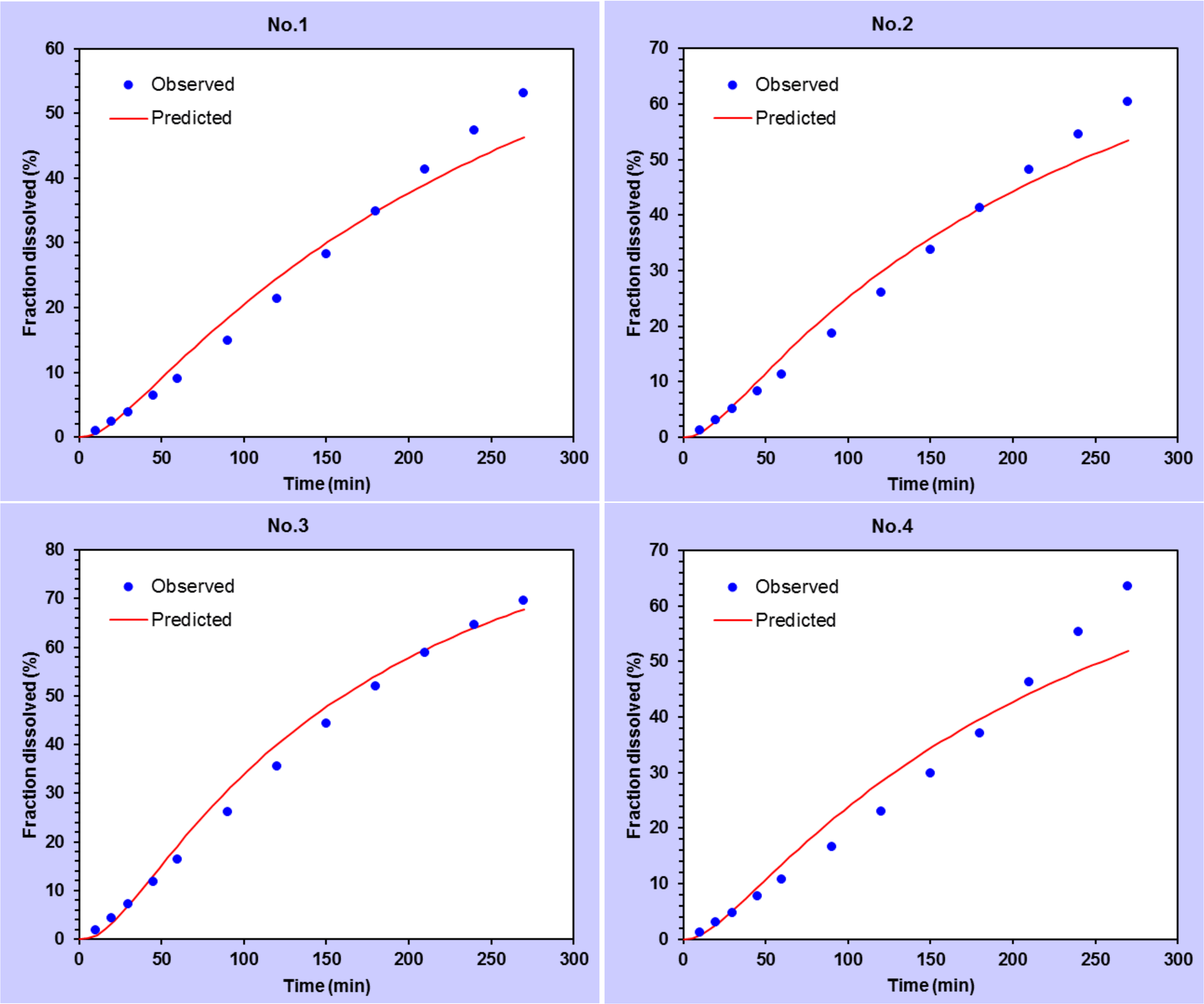

Supplementary materials – Model fitting summary – Kollidon® 90 F (modelling up to app. 60 % of carvedilol released)

Model: **Probit_2**

Model equation: $F = F_{max} \cdot \phi[\alpha + \beta \cdot \log(t)]$

Fitted model parameters per tested tablet (N = 4) with statistics – mean, standard deviation (SD), and relative standard deviation expressed in % (RSD%) (output from DDSolver):

Parameter	No.1	No.2	No.3	No.4	Mean	SD	RSD(%)
α	-4.953	-5.573	-5.486	-4.818	-5.208	0.378	-7.253
β	2.399	2.517	2.545	2.299	2.440	0.113	4.634
F_{max}	55.824	70.768	81.628	66.746	68.742	10.662	15.510

Number of dissolution data points (N), degrees of freedom (df), and selected goodness of fit criteria – Pearson correlation coefficient (R), coefficient of determination (R^2), adjusted coefficient of determination ($R^2_{adjusted}$), and residual sum of squares (RSS) (manual calculation in MS Excel):

Parameter	No.1	No.2	No.3	No.4
N	12	12	12	12
df	9	9	9	9
R	0.971223446	0.994019261	0.997655101	0.959515403
R^2	0.943274981	0.988074292	0.9953157	0.920669809
$R^2_{adjusted}$	0.930669422	0.985424135	0.994274745	0.903040878
RSS	252.0439829	231.1818493	181.1258137	454.7471252

Graphical abstract of model fit presented as mean ± 1 SD of the fraction % of released carvedilol:

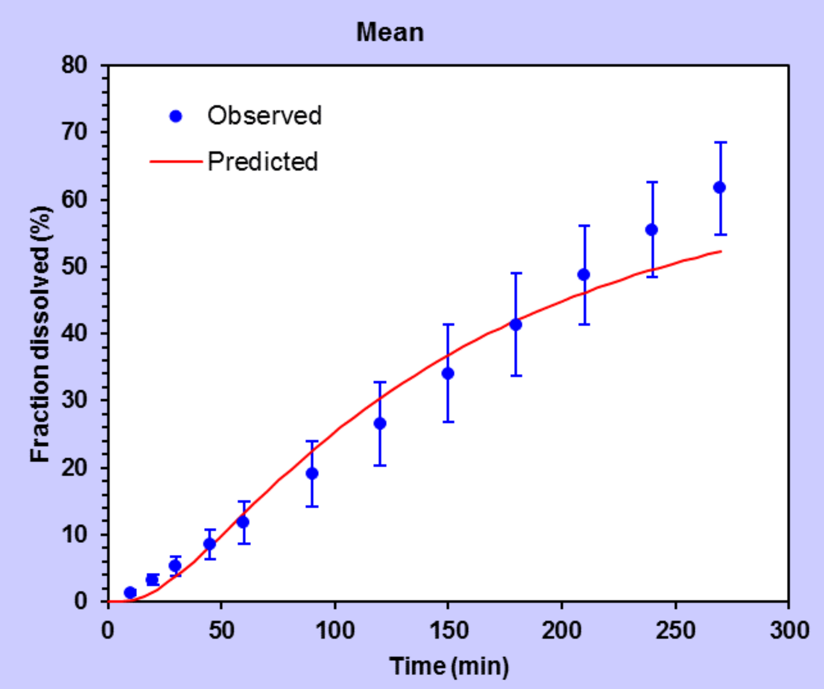

Supplementary materials – Model fitting summary – Kollidon® 90 F (modelling up to app. 60 % of carvedilol released)

Graphical abstract of model fit presented as the fraction % of released carvedilol per tested tablet:

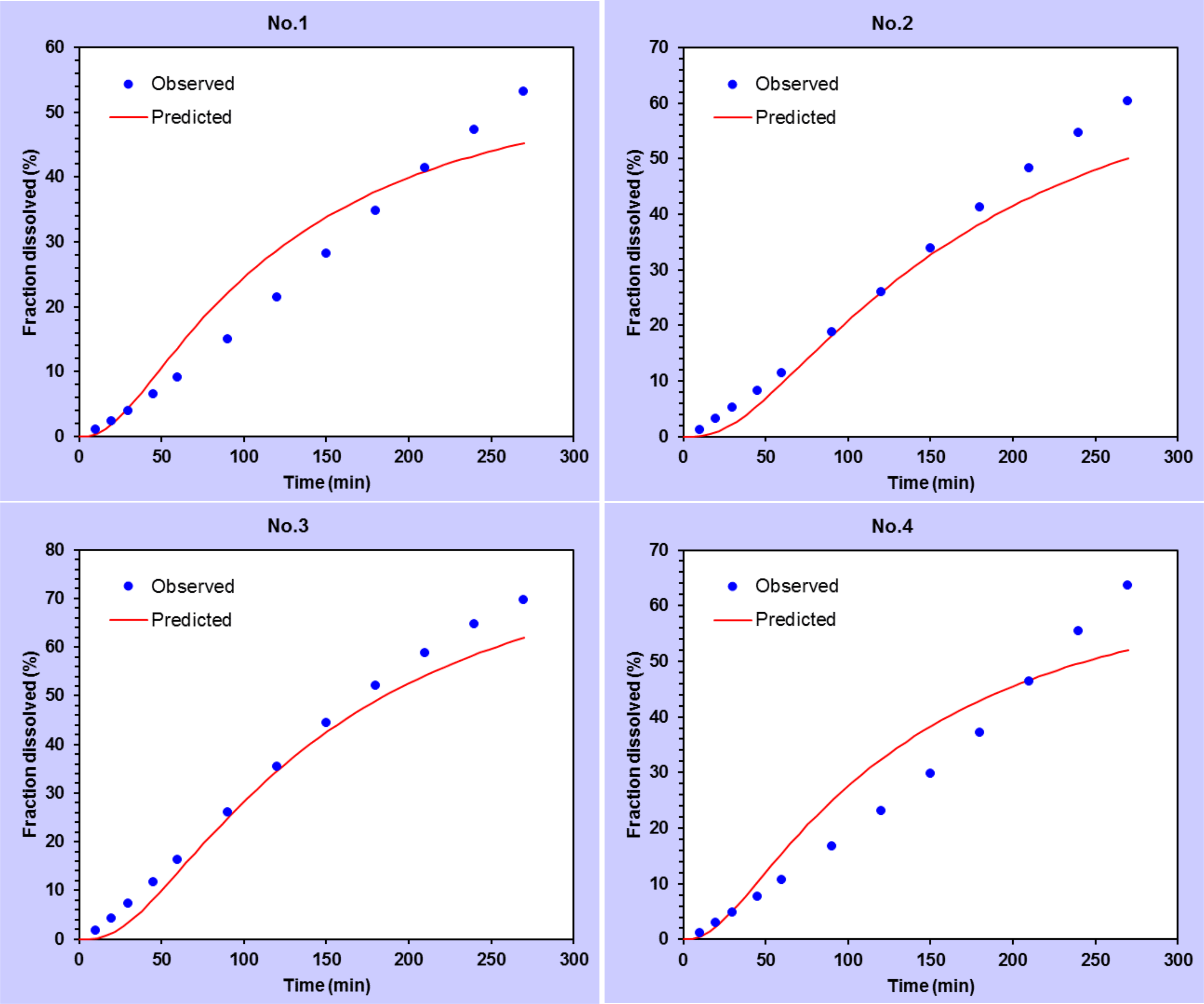

Supplement: Supplementary file 1 [file pharmaceutics-16-00498-s001.zip › Supplementary materials_Model fitting summary_Kollidon® 90 F.pdf]
