# Supplementary material for: Comparative Fitting of Mathematical Models to Carvedilol Release Profiles Obtained from Hypromellose Matrix Tablets
Source: Pharmaceutics. 2024 Apr 4;16(4):498. doi: 10.3390/pharmaceutics16040498 (PMC11053526; doi:10.3390/pharmaceutics16040498)

Supplementary materials – Model fitting summary – Lactochem® Crystals (modelling the entire relevant dissolution data)

Model: **Zero–order**

Model equation: $F = k_0 \cdot t$

Fitted model parameters per tested tablet (N = 4) with statistics – mean, standard deviation (SD), and relative standard deviation expressed in % (RSD%) (output from DDSolver):

Parameter	No.1	No.2	No.3	No.4	Mean	SD	RSD(%)
k_0	0.172	0.179	0.181	0.184	0.179	0.005	2.748

Number of dissolution data points (N), degrees of freedom (df), and selected goodness of fit criteria – Pearson correlation coefficient (R), coefficient of determination (R^2), adjusted coefficient of determination ($R^2_{adjusted}$), and residual sum of squares (RSS) (manual calculation in MS Excel):

Parameter	No.1	No.2	No.3	No.4
N	21	21	21	21
df	20	20	20	20
R	0.97911633	0.980159574	0.976857129	0.973126397
R^2	0.958668788	0.96071279	0.954249851	0.946974984
$R^2_{adjusted}$	0.958668788	0.96071279	0.954249851	0.946974984
RSS	7295.126895	9018.591073	8175.770666	10363.84546

Graphical abstract of model fit presented as mean ± 1 SD of the fraction % of released carvedilol:

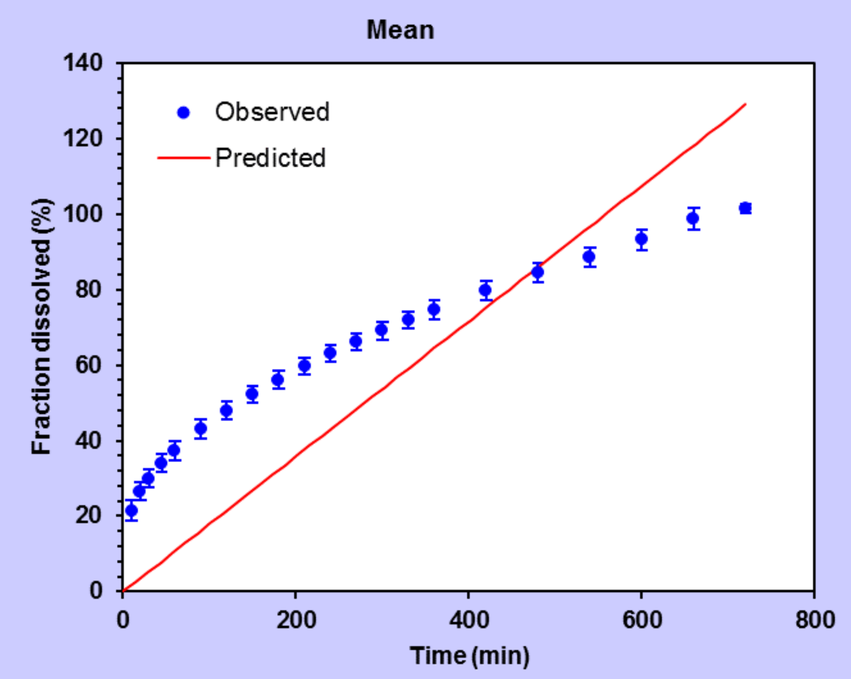

Supplementary materials – Model fitting summary – Lactochem® Crystals (modelling the entire relevant dissolution data)

Graphical abstract of model fit presented as the fraction % of released carvedilol per tested tablet:

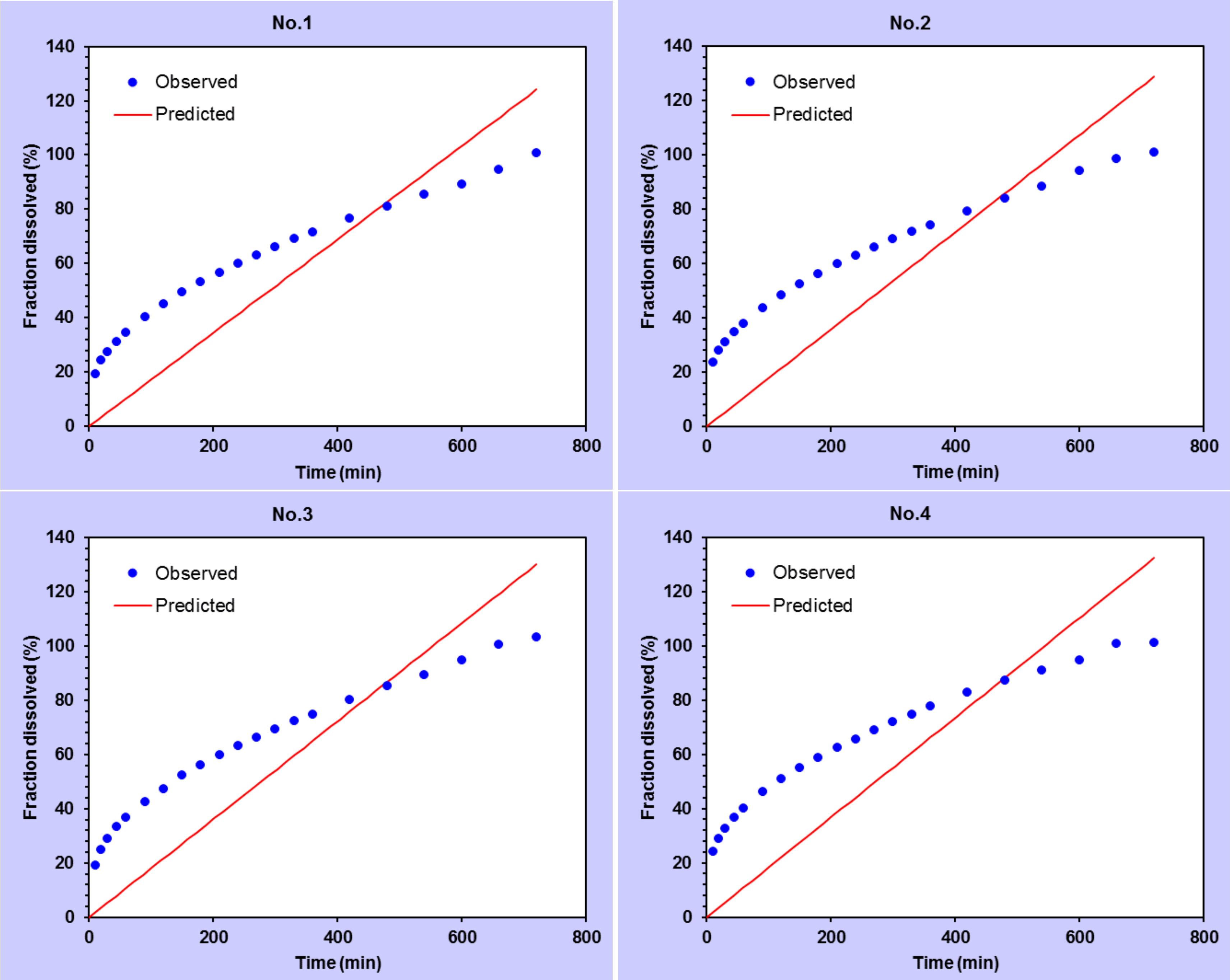

Supplementary materials – Model fitting summary – Lactochem® Crystals (modelling the entire relevant dissolution data)

Model: **Zero–order with T_{lag}**

Model equation: $F = k_0 \cdot (t - T_{lag})$

Fitted model parameters per tested tablet (N = 4) with statistics – mean, standard deviation (SD), and relative standard deviation expressed in % (RSD%) (output from DDSolver):

Parameter	No.1	No.2	No.3	No.4	Mean	SD	RSD(%)
k_0	0.106	0.105	0.111	0.105	0.107	0.003	2.754
T_{lag}	-275.881	-313.257	-278.383	-334.198	-300.430	28.246	-9.402

Number of dissolution data points (N), degrees of freedom (df), and selected goodness of fit criteria – Pearson correlation coefficient (R), coefficient of determination (R^2), adjusted coefficient of determination ($R^2_{adjusted}$), and residual sum of squares (RSS) (manual calculation in MS Excel):

Parameter	No.1	No.2	No.3	No.4
N	21	21	21	21
df	19	19	19	19
R	0.97911633	0.980159574	0.976857129	0.973126397
R^2	0.958668788	0.96071279	0.954249851	0.946974984
$R^2_{adjusted}$	0.956493461	0.958645042	0.951841948	0.944184194
RSS	478.0826498	442.4753921	581.6516116	605.6825665

Graphical abstract of model fit presented as mean ± 1 SD of the fraction % of released carvedilol:

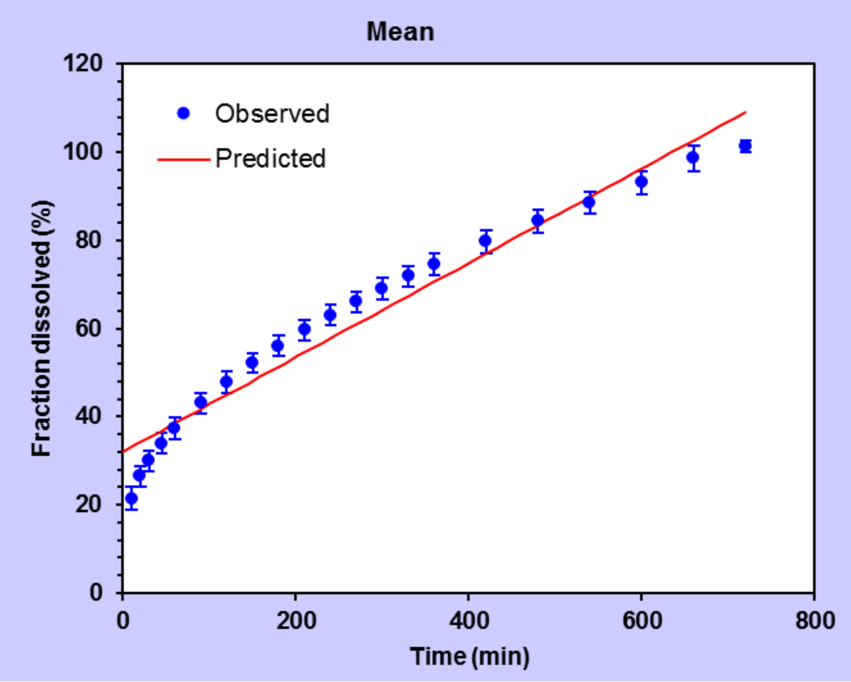

Supplementary materials – Model fitting summary – Lactochem® Crystals (modelling the entire relevant dissolution data)

Graphical abstract of model fit presented as the fraction % of released carvedilol per tested tablet:

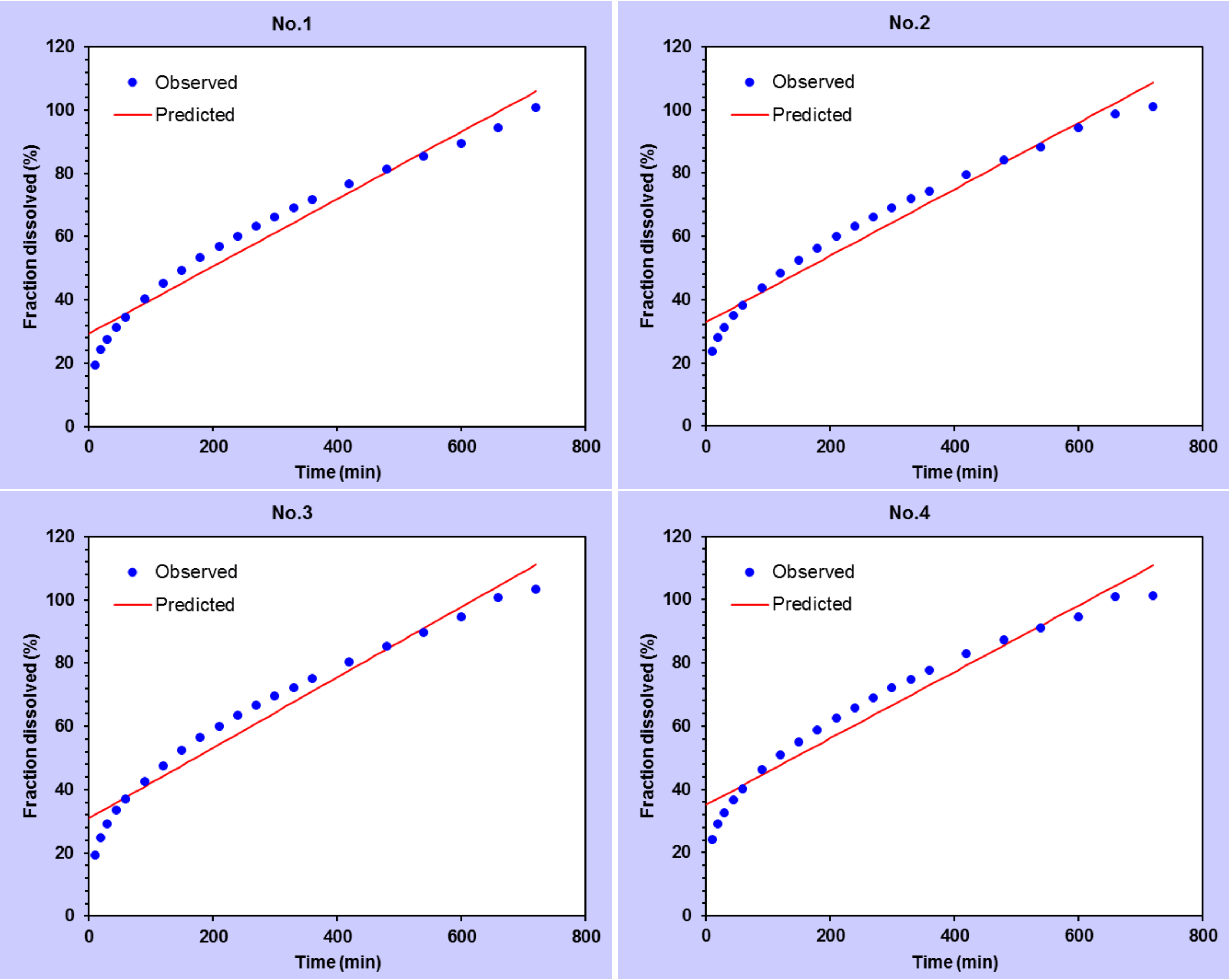

Supplementary materials – Model fitting summary – Lactochem® Crystals (modelling the entire relevant dissolution data)

Model: **Zero–order with F_0**

Model equation: $F = F_0 + k_0 \cdot t$

Fitted model parameters per tested tablet (N = 4) with statistics – mean, standard deviation (SD), and relative standard deviation expressed in % (RSD%) (output from DDSolver):

Parameter	No.1	No.2	No.3	No.4	Mean	SD	RSD(%)
k_0	0.106	0.105	0.111	0.105	0.107	0.003	2.754
F_0	29.367	32.939	30.996	35.135	32.109	2.490	7.756

Number of dissolution data points (N), degrees of freedom (df), and selected goodness of fit criteria – Pearson correlation coefficient (R), coefficient of determination (R^2), adjusted coefficient of determination ($R^2_{adjusted}$), and residual sum of squares (RSS) (manual calculation in MS Excel):

Parameter	No.1	No.2	No.3	No.4
N	21	21	21	21
df	19	19	19	19
R	0.97911633	0.980159574	0.976857129	0.973126397
R^2	0.958668788	0.96071279	0.954249851	0.946974984
$R^2_{adjusted}$	0.956493461	0.958645042	0.951841948	0.944184194
RSS	478.0826498	442.4753921	581.6516116	605.6825665

Graphical abstract of model fit presented as mean ± 1 SD of the fraction % of released carvedilol:

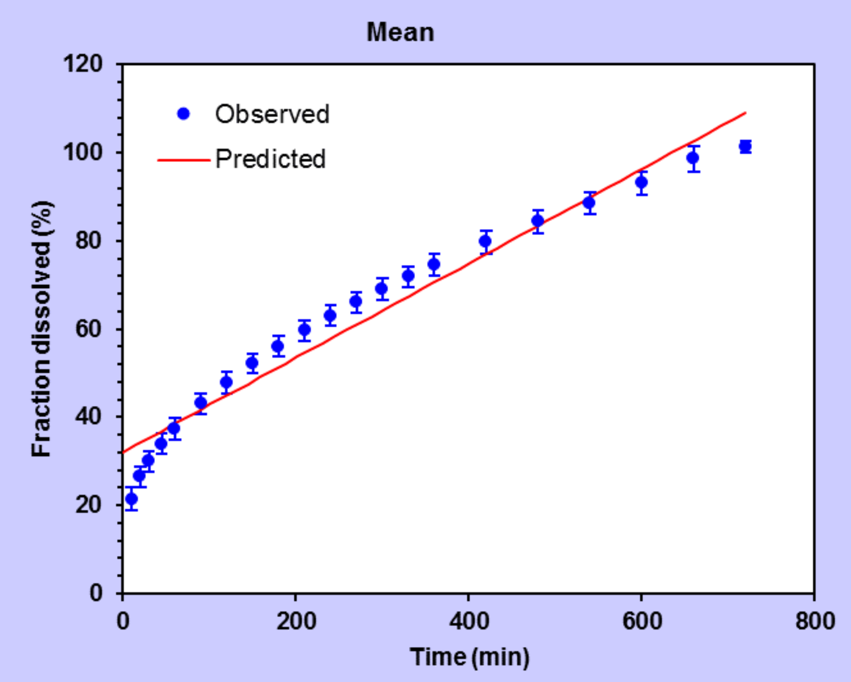

Supplementary materials – Model fitting summary – Lactochem® Crystals (modelling the entire relevant dissolution data)

Graphical abstract of model fit presented as the fraction % of released carvedilol per tested tablet:

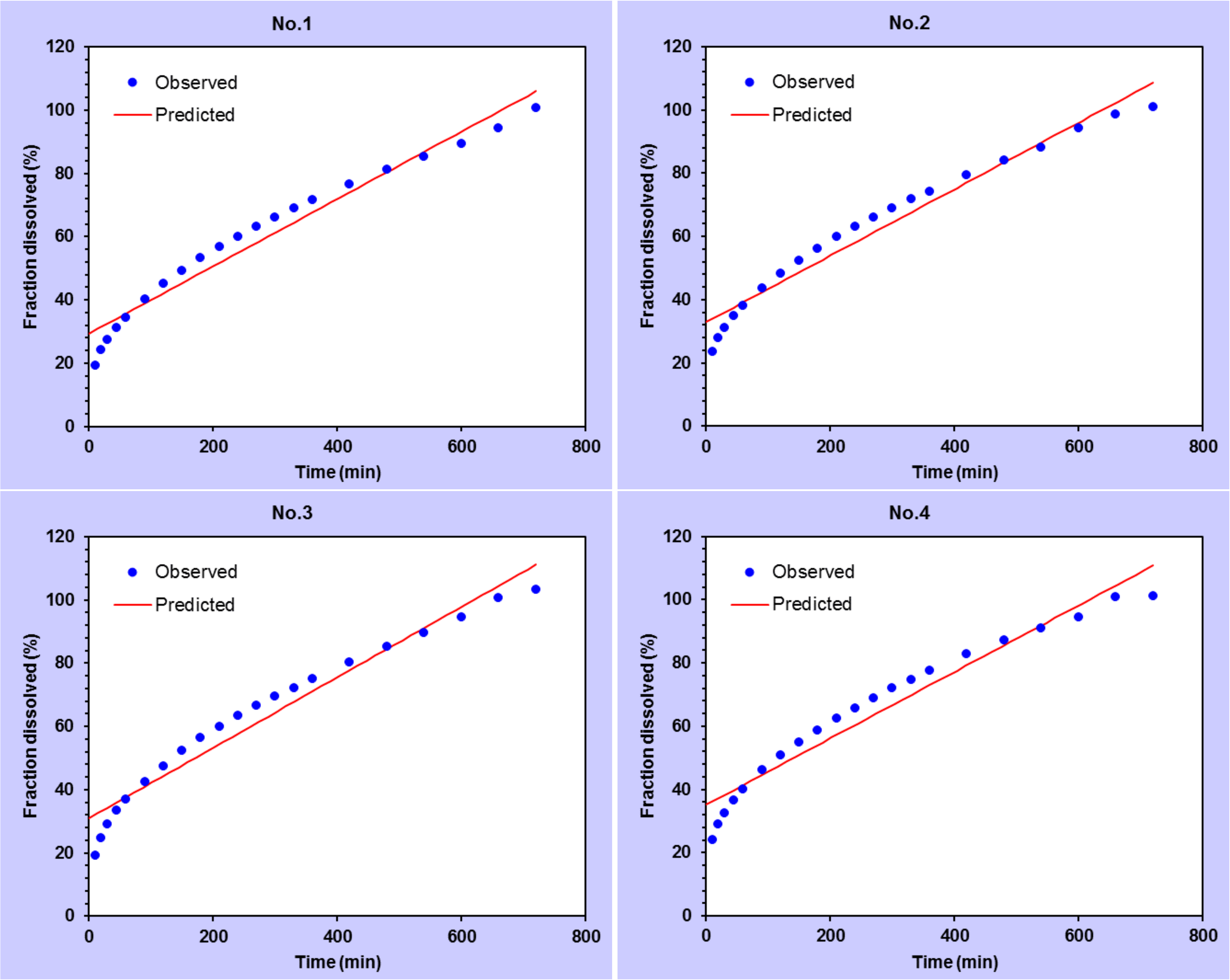

Supplementary materials – Model fitting summary – Lactochem® Crystals (modelling the entire relevant dissolution data)

Model: **First–order**

Model equation: $F = 100 \cdot (1 - e^{-k_1 \cdot t})$

Fitted model parameters per tested tablet (N = 4) with statistics – mean, standard deviation (SD), and relative standard deviation expressed in % (RSD%) (output from DDSolver):

Parameter	No.1	No.2	No.3	No.4	Mean	SD	RSD(%)
k_1	0.004	0.005	0.004	0.005	0.004	0.000	8.626

Number of dissolution data points (N), degrees of freedom (df), and selected goodness of fit criteria – Pearson correlation coefficient (R), coefficient of determination (R^2), adjusted coefficient of determination ($R^2_{adjusted}$), and residual sum of squares (RSS) (manual calculation in MS Excel):

Parameter	No.1	No.2	No.3	No.4
N	21	21	21	21
df	20	20	20	20
R	0.988579911	0.978888222	0.985053052	0.986170354
R^2	0.977290241	0.958222151	0.970329515	0.972531968
$R^2_{adjusted}$	0.977290241	0.958222151	0.970329515	0.972531968
RSS	1572.441181	1904.845629	1598.040929	2173.644884

Graphical abstract of model fit presented as mean ± 1 SD of the fraction % of released carvedilol:

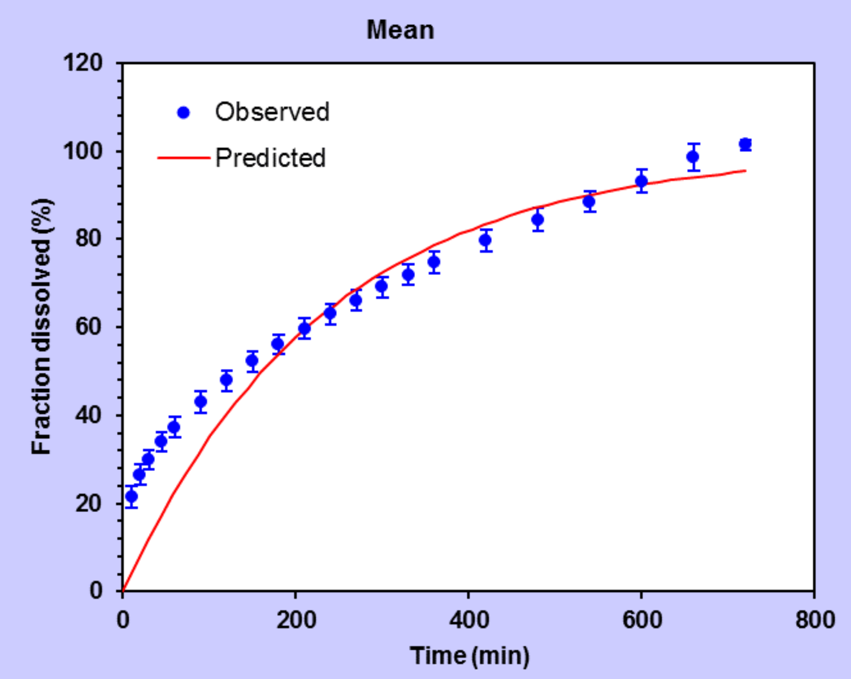

Supplementary materials – Model fitting summary – Lactochem® Crystals (modelling the entire relevant dissolution data)

Graphical abstract of model fit presented as the fraction % of released carvedilol per tested tablet:

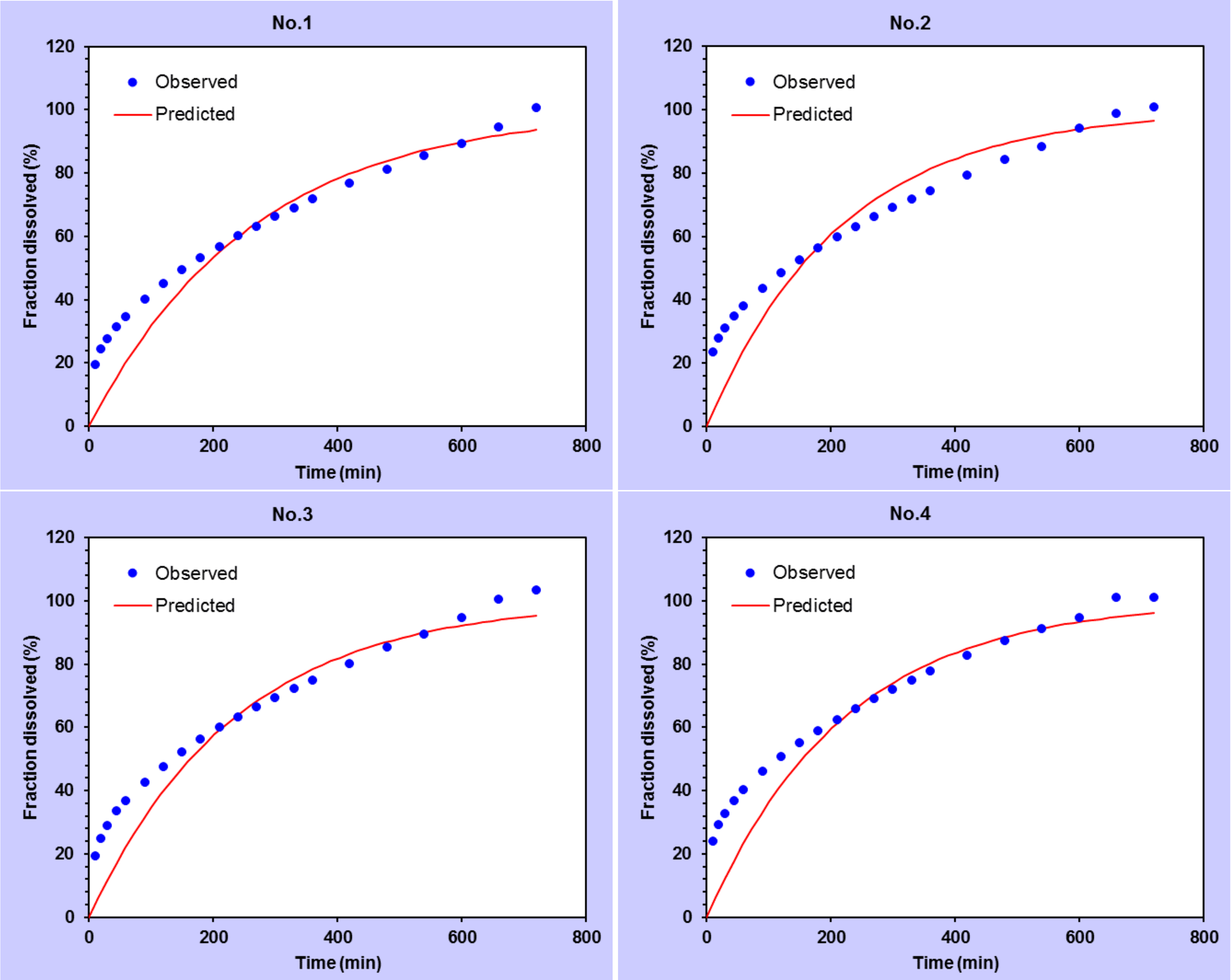

Supplementary materials – Model fitting summary – Lactochem® Crystals (modelling the entire relevant dissolution data)

Model: **First–order with T_{lag}**

Model equation: $F = 100 \cdot \left[1 - e^{-k_1 \cdot (t - T_{lag})}\right]$

Fitted model parameters per tested tablet (N = 4) with statistics – mean, standard deviation (SD), and relative standard deviation expressed in % (RSD%) (output from DDSolver):

Parameter	No.1	No.2	No.3	No.4	Mean	SD	RSD(%)
k_1	/	/	/	/	/	/	/
T_{lag}	/	/	/	/	/	/	/

Number of dissolution data points (N), degrees of freedom (df), and selected goodness of fit criteria – Pearson correlation coefficient (R), coefficient of determination (R^2), adjusted coefficient of determination ($R^2_{adjusted}$), and residual sum of squares (RSS) (manual calculation in MS Excel):

Parameter	No.1	No.2	No.3	No.4
N	/	/	/	/
df	/	/	/	/
R	/	/	/	/
R^2	/	/	/	/
$R^2_{adjusted}$	/	/	/	/
RSS	/	/	/	/

Graphical abstract of model fit presented as mean ± 1 SD of the fraction % of released carvedilol: /

Graphical abstract of model fit presented as the fraction % of released carvedilol per tested tablet: /

Note: the model could not be fitted

Supplementary materials – Model fitting summary – Lactochem® Crystals (modelling the entire relevant dissolution data)

Model: **First–order with F_{max}**

Model equation: $F = F_{max} \cdot (1 - e^{-k_1 \cdot t})$

Fitted model parameters per tested tablet (N = 4) with statistics – mean, standard deviation (SD), and relative standard deviation expressed in % (RSD%) (output from DDSolver):

Parameter	No.1	No.2	No.3	No.4	Mean	SD	RSD(%)
k_1	0.003	0.004	0.003	0.004	0.004	0.000	6.684
F_{max}	105.599	105.910	108.402	106.038	106.487	1.290	1.211

Number of dissolution data points (N), degrees of freedom (df), and selected goodness of fit criteria – Pearson correlation coefficient (R), coefficient of determination (R^2), adjusted coefficient of determination ($R^2_{adjusted}$), and residual sum of squares (RSS) (manual calculation in MS Excel):

Parameter	No.1	No.2	No.3	No.4
N	21	21	21	21
df	19	19	19	19
R	0.993087251	0.989931142	0.992458356	0.992521099
R^2	0.986222288	0.979963666	0.984973588	0.985098132
$R^2_{adjusted}$	0.985497146	0.978909122	0.984182725	0.984313824
RSS	1873.60923	2322.998511	1996.535473	2604.556985

Graphical abstract of model fit presented as mean ± 1 SD of the fraction % of released carvedilol:

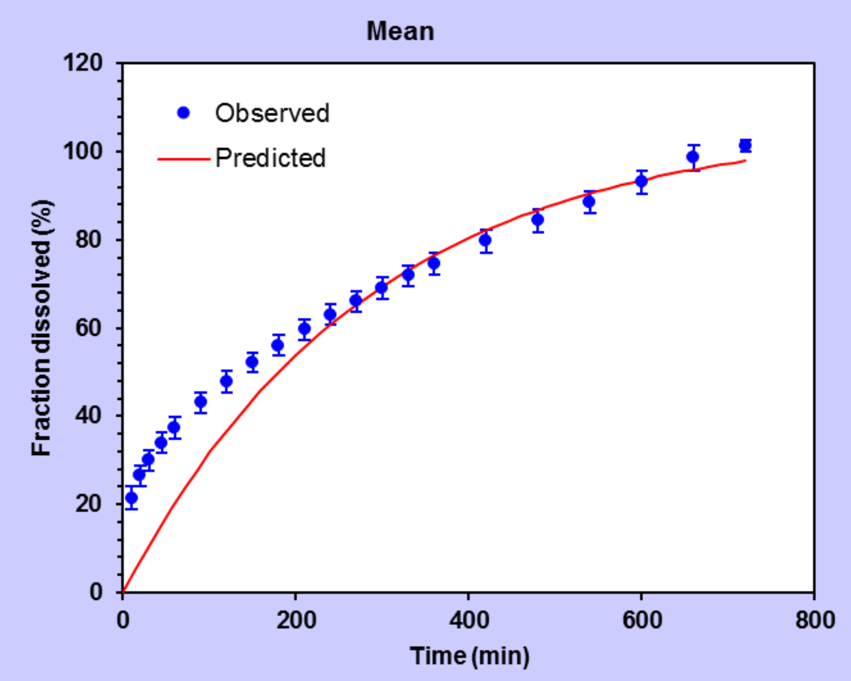

Supplementary materials – Model fitting summary – Lactochem® Crystals (modelling the entire relevant dissolution data)

Graphical abstract of model fit presented as the fraction % of released carvedilol per tested tablet:

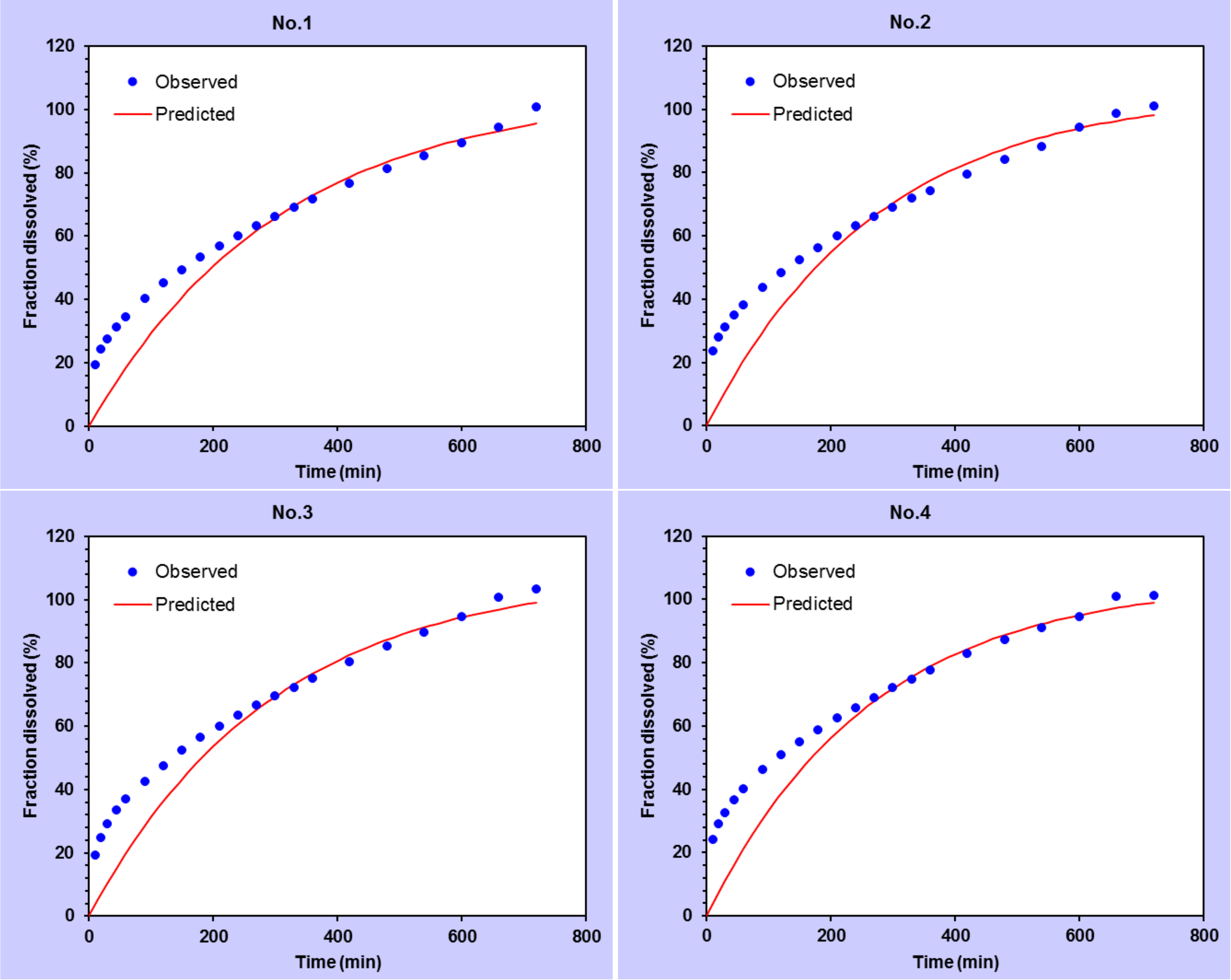

Supplementary materials – Model fitting summary – Lactochem® Crystals (modelling the entire relevant dissolution data)

Model: **First–order with T_{lag} and F_{max}**

Model equation: $F = F_{max} \cdot \left[1 - e^{-k_1 \cdot (t - T_{lag})}\right]$

Fitted model parameters per tested tablet (N = 4) with statistics – mean, standard deviation (SD), and relative standard deviation expressed in % (RSD%) (output from DDSolver):

Parameter	No.1	No.2	No.3	No.4	Mean	SD	RSD(%)
k_1	0.003	0.003	0.003	0.004	0.003	0.000	5.623
T_{lag}	-58.570	-62.527	-49.641	-66.946	-59.421	7.363	-12.391
F_{max}	105.599	105.910	108.402	106.038	106.487	1.290	1.211

Number of dissolution data points (N), degrees of freedom (df), and selected goodness of fit criteria – Pearson correlation coefficient (R), coefficient of determination (R^2), adjusted coefficient of determination ($R^2_{adjusted}$), and residual sum of squares (RSS) (manual calculation in MS Excel):

Parameter	No.1	No.2	No.3	No.4
N	21	21	21	21
df	18	18	18	18
R	0.993655557	0.991736731	0.992445483	0.993509489
R^2	0.987351366	0.983541743	0.984948037	0.987061104
$R^2_{adjusted}$	0.985945962	0.981713048	0.983275597	0.985623449
RSS	298.6062417	360.9034033	342.0802216	316.9307409

Graphical abstract of model fit presented as mean ± 1 SD of the fraction % of released carvedilol:

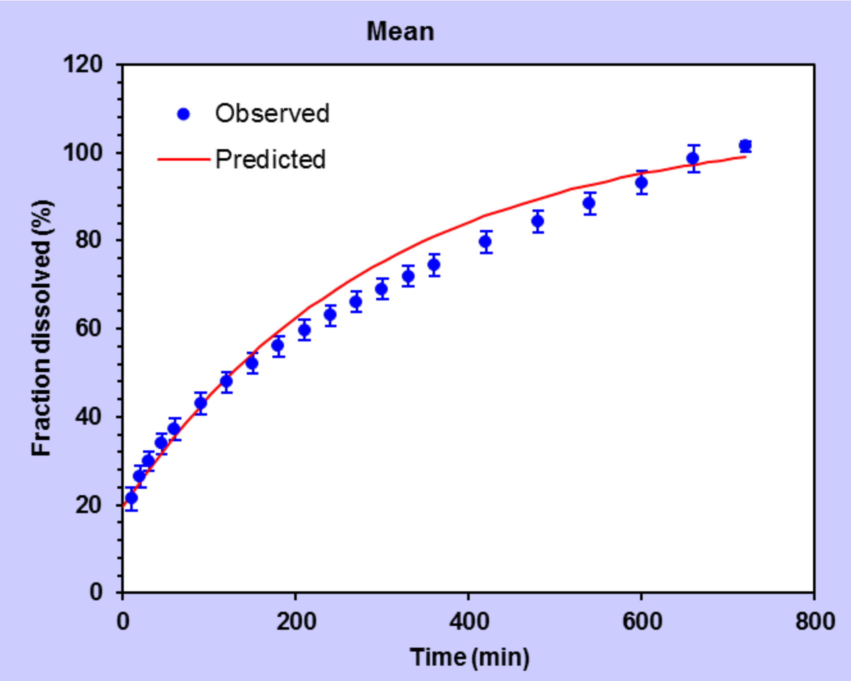

Supplementary materials – Model fitting summary – Lactochem® Crystals (modelling the entire relevant dissolution data)

Graphical abstract of model fit presented as the fraction % of released carvedilol per tested tablet:

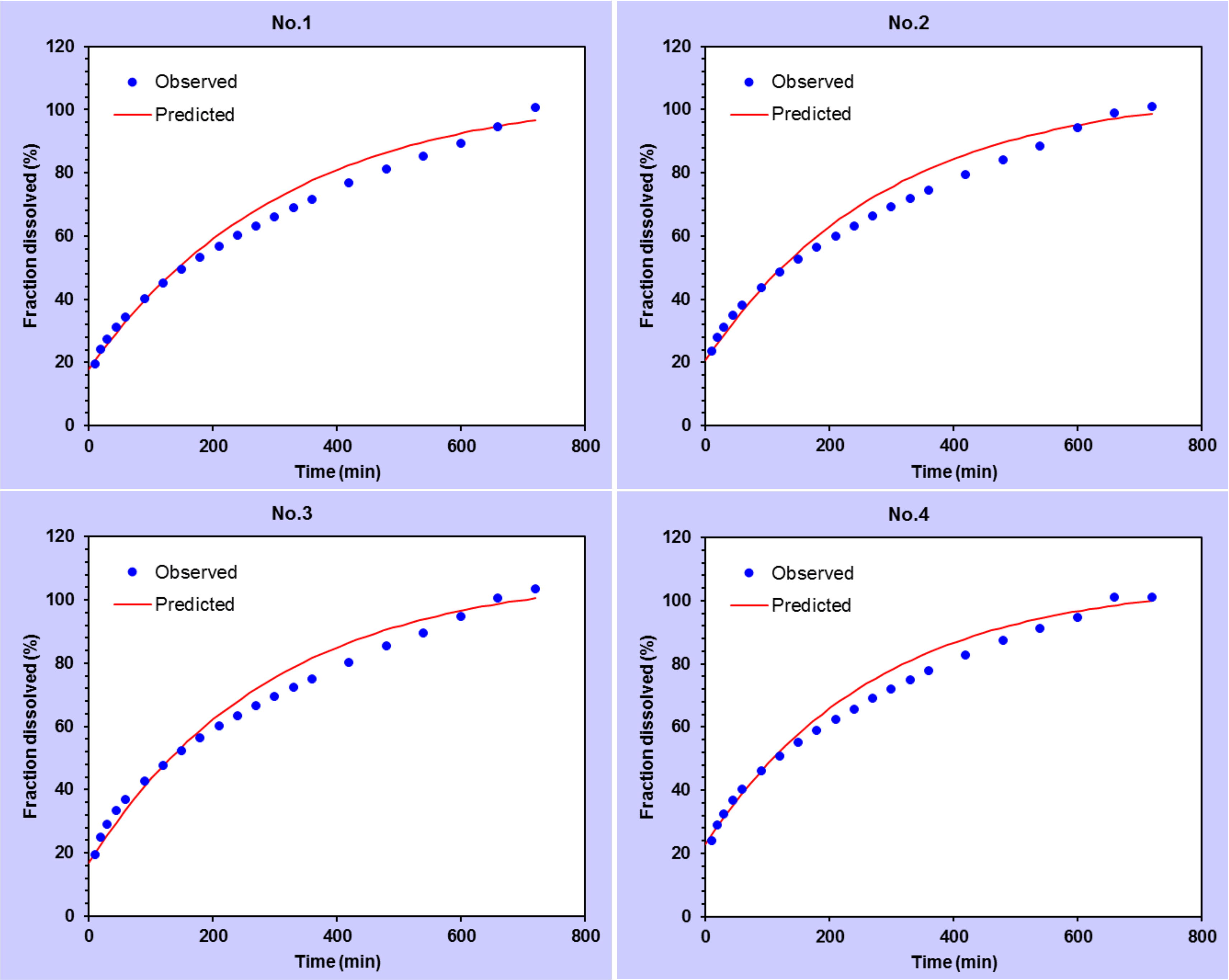

Supplementary materials – Model fitting summary – Lactochem® Crystals (modelling the entire relevant dissolution data)

Model: **Higuchi**

Model equation: $F = k_H \cdot t^{0.5}$

Fitted model parameters per tested tablet (N = 4) with statistics – mean, standard deviation (SD), and relative standard deviation expressed in % (RSD%) (output from DDSolver):

Parameter	No.1	No.2	No.3	No.4	Mean	SD	RSD(%)
k_H	3.800	3.966	3.991	4.090	3.962	0.120	3.040

Number of dissolution data points (N), degrees of freedom (df), and selected goodness of fit criteria – Pearson correlation coefficient (R), coefficient of determination (R^2), adjusted coefficient of determination ($R^2_{adjusted}$), and residual sum of squares (RSS) (manual calculation in MS Excel):

Parameter	No.1	No.2	No.3	No.4
N	21	21	21	21
df	20	20	20	20
R	0.999588591	0.999646315	0.999730759	0.999584092
R^2	0.999177351	0.999292756	0.99946159	0.999168356
$R^2_{adjusted}$	0.999177351	0.999292756	0.99946159	0.999168356
RSS	292.3294451	599.6832562	326.5356363	787.8962288

Graphical abstract of model fit presented as mean ± 1 SD of the fraction % of released carvedilol:

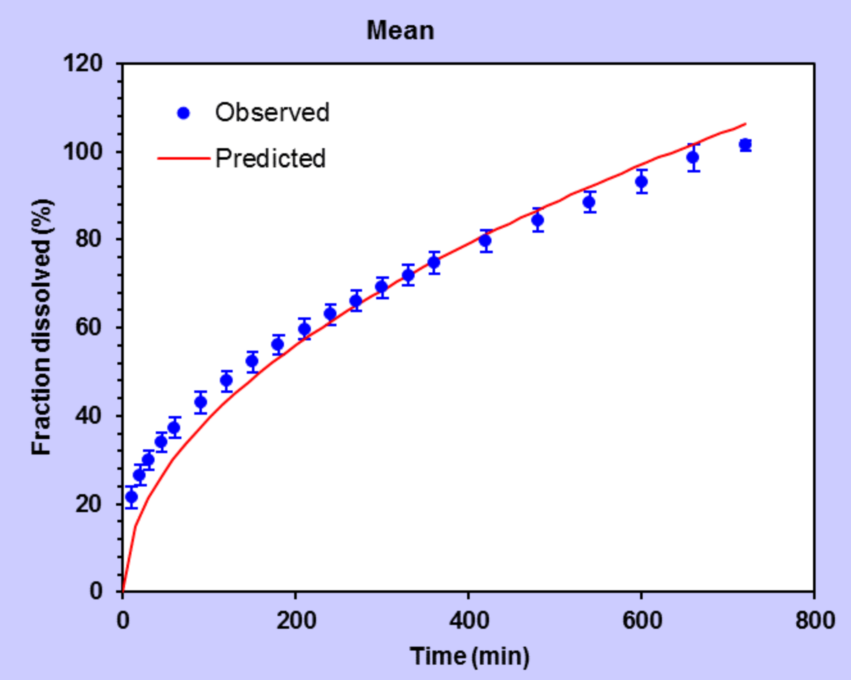

Supplementary materials – Model fitting summary – Lactochem® Crystals (modelling the entire relevant dissolution data)

Graphical abstract of model fit presented as the fraction % of released carvedilol per tested tablet:

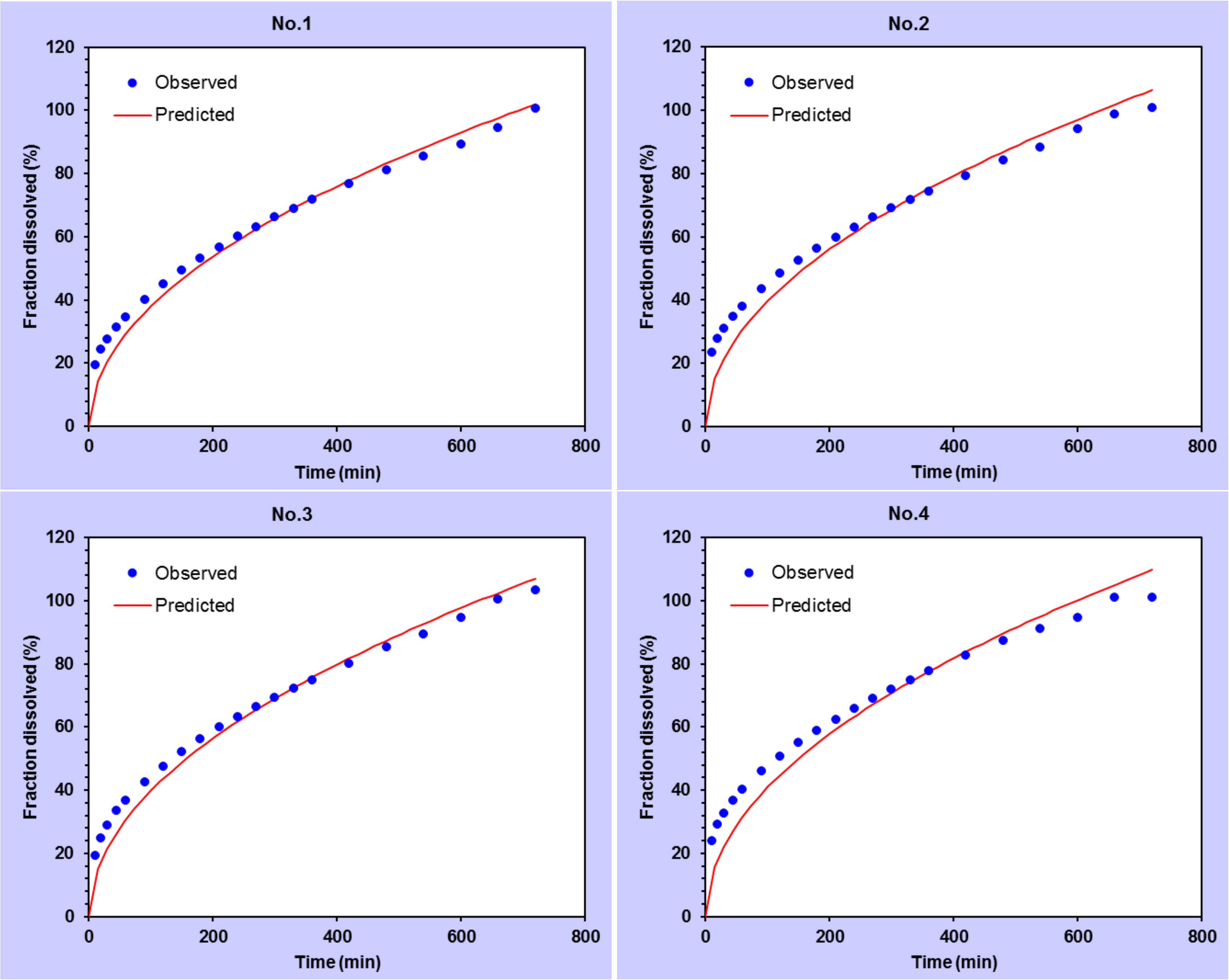

Supplementary materials – Model fitting summary – Lactochem® Crystals (modelling the entire relevant dissolution data)

Model: **Higuchi with T_{lag}**

Model equation: $F = k_H \cdot (t - T_{lag})^{0.5}$

Fitted model parameters per tested tablet (N = 4) with statistics – mean, standard deviation (SD), and relative standard deviation expressed in % (RSD%) (output from DDSolver):

Parameter	No.1	No.2	No.3	No.4	Mean	SD	RSD(%)
k_H	3.612	3.683	3.779	3.725	3.700	0.071	1.906
T_{lag}	-30.715	-46.462	-33.138	-59.768	-42.521	13.422	-31.565

Number of dissolution data points (N), degrees of freedom (df), and selected goodness of fit criteria – Pearson correlation coefficient (R), coefficient of determination (R^2), adjusted coefficient of determination ($R^2_{adjusted}$), and residual sum of squares (RSS) (manual calculation in MS Excel):

Parameter	No.1	No.2	No.3	No.4
N	21	21	21	21
df	19	19	19	19
R	0.998965961	0.998806778	0.998354269	0.996247864
R^2	0.997932991	0.99761498	0.996711246	0.992509807
$R^2_{adjusted}$	0.997824201	0.997489453	0.996538154	0.992115587
RSS	26.4274668	30.84723455	48.34692034	104.482846

Graphical abstract of model fit presented as mean ± 1 SD of the fraction % of released carvedilol:

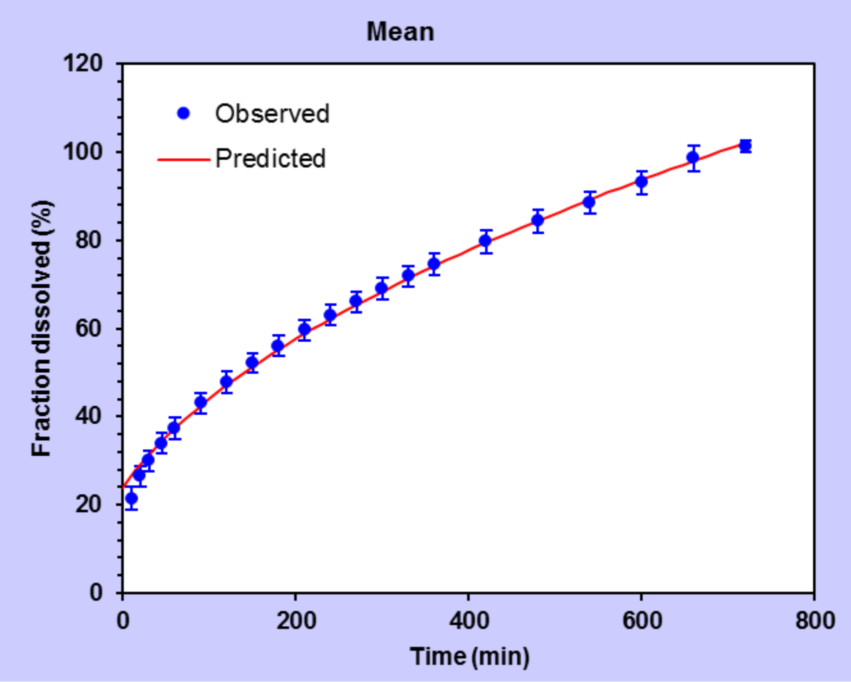

Supplementary materials – Model fitting summary – Lactochem® Crystals (modelling the entire relevant dissolution data)

Graphical abstract of model fit presented as the fraction % of released carvedilol per tested tablet:

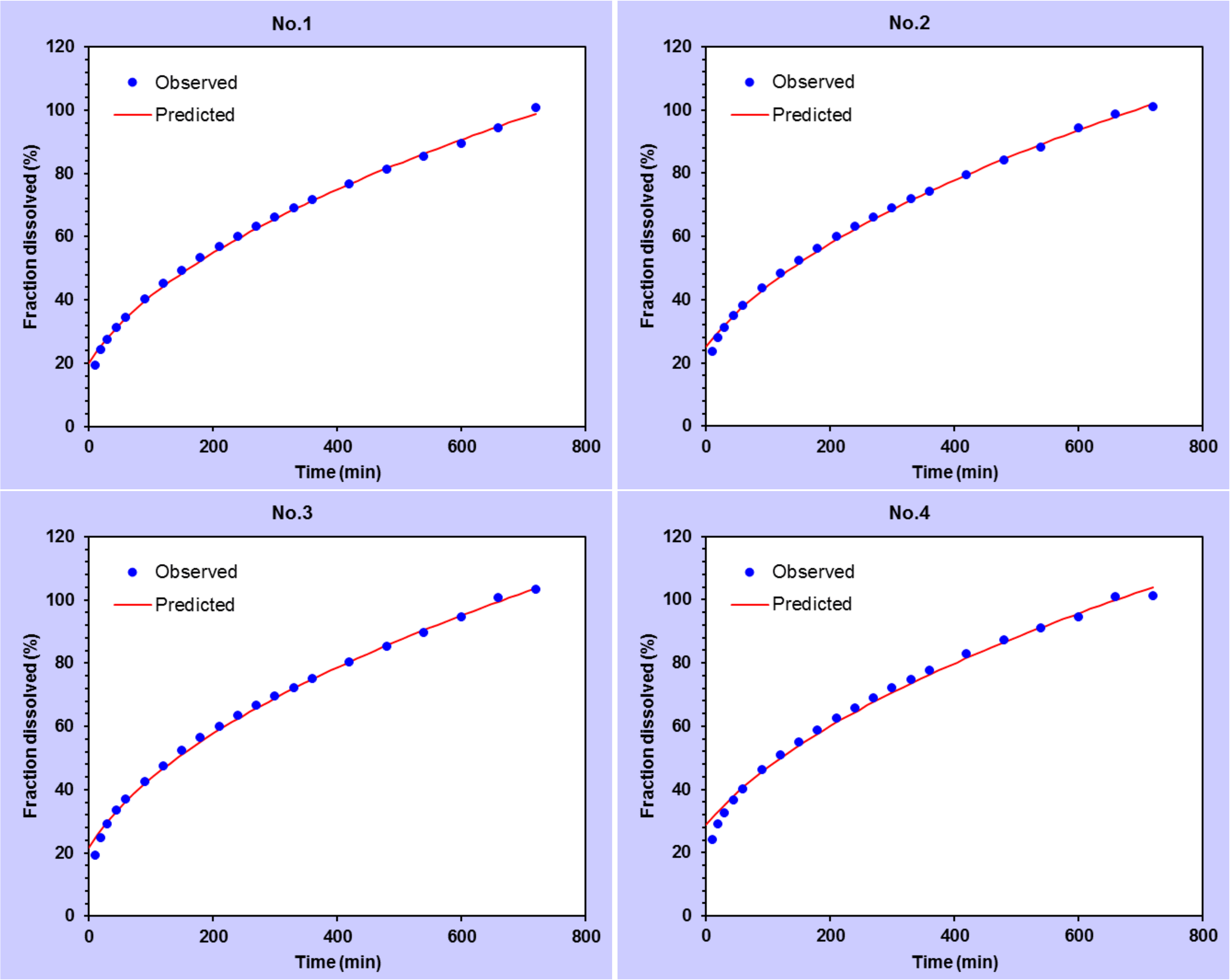

Supplementary materials – Model fitting summary – Lactochem® Crystals (modelling the entire relevant dissolution data)

Model: **Higuchi with F_0**

Model equation: $F = F_0 + k_H \cdot t^{0.5}$

Fitted model parameters per tested tablet (N = 4) with statistics – mean, standard deviation (SD), and relative standard deviation expressed in % (RSD%) (output from DDSolver):

Parameter	No.1	No.2	No.3	No.4	Mean	SD	RSD(%)
k_H	3.328	3.284	3.489	3.307	3.352	0.093	2.783
F_0	8.677	12.552	9.226	14.396	11.213	2.726	24.316

Number of dissolution data points (N), degrees of freedom (df), and selected goodness of fit criteria – Pearson correlation coefficient (R), coefficient of determination (R^2), adjusted coefficient of determination ($R^2_{adjusted}$), and residual sum of squares (RSS) (manual calculation in MS Excel):

Parameter	No.1	No.2	No.3	No.4
N	21	21	21	21
df	19	19	19	19
R	0.999588591	0.999646315	0.999730759	0.999584092
R^2	0.999177351	0.999292756	0.99946159	0.999168356
$R^2_{adjusted}$	0.999134054	0.999255533	0.999433252	0.999124585
RSS	9.51566806	7.965391915	6.845163365	9.49951912

Graphical abstract of model fit presented as mean ± 1 SD of the fraction % of released carvedilol:

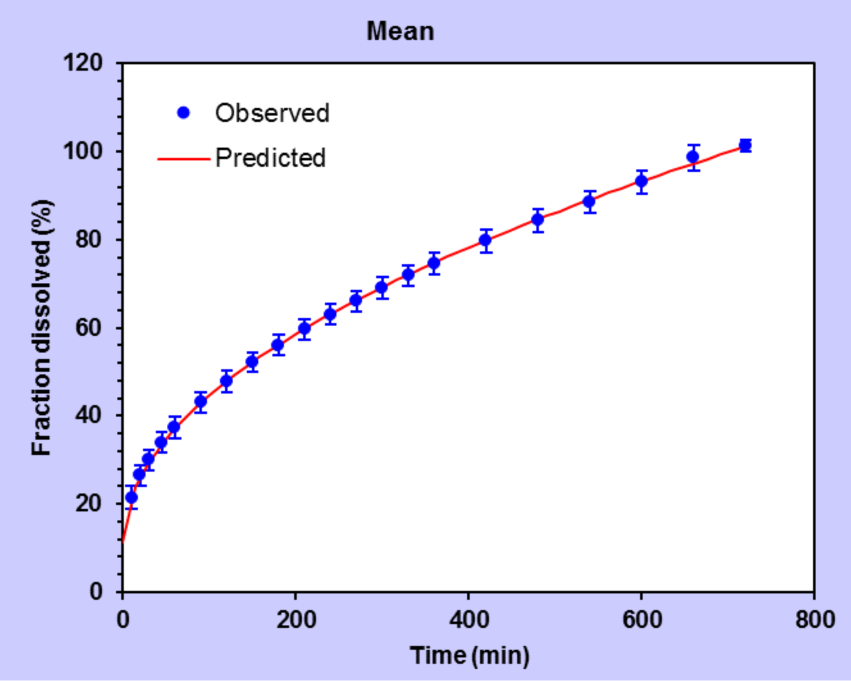

Supplementary materials – Model fitting summary – Lactochem® Crystals (modelling the entire relevant dissolution data)

Graphical abstract of model fit presented as the fraction % of released carvedilol per tested tablet:

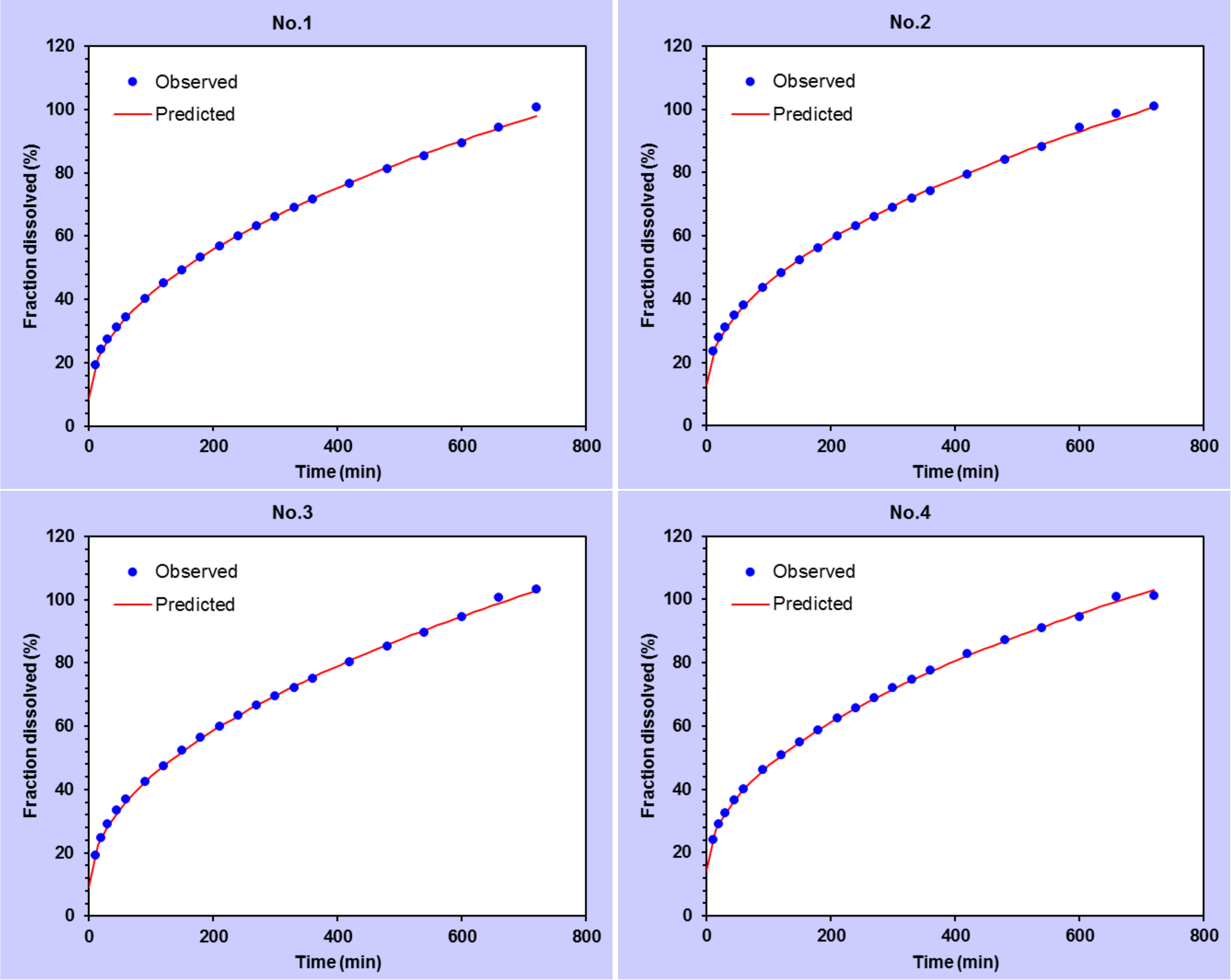

Supplementary materials – Model fitting summary – Lactochem® Crystals (modelling the entire relevant dissolution data)

Model: **Korsmeyer–Peppas**

Model equation: $F = k_{KP} \cdot t^n$

Fitted model parameters per tested tablet (N = 4) with statistics – mean, standard deviation (SD), and relative standard deviation expressed in % (RSD%) (output from DDSolver):

Parameter	No.1	No.2	No.3	No.4	Mean	SD	RSD(%)
k_{KP}	7.409	9.482	7.638	10.147	8.669	1.353	15.609
n	0.384	0.350	0.388	0.343	0.366	0.023	6.261

Number of dissolution data points (N), degrees of freedom (df), and selected goodness of fit criteria – Pearson correlation coefficient (R), coefficient of determination (R^2), adjusted coefficient of determination ($R^2_{adjusted}$), and residual sum of squares (RSS) (manual calculation in MS Excel):

Parameter	No.1	No.2	No.3	No.4
N	21	21	21	21
df	19	19	19	19
R	0.997045718	0.995164102	0.998022921	0.997238685
R^2	0.994100163	0.99035159	0.996049752	0.994484995
$R^2_{adjusted}$	0.993789645	0.989843779	0.995841844	0.994194731
RSS	111.1280911	162.7215842	90.92081478	108.8362387

Graphical abstract of model fit presented as mean ± 1 SD of the fraction % of released carvedilol:

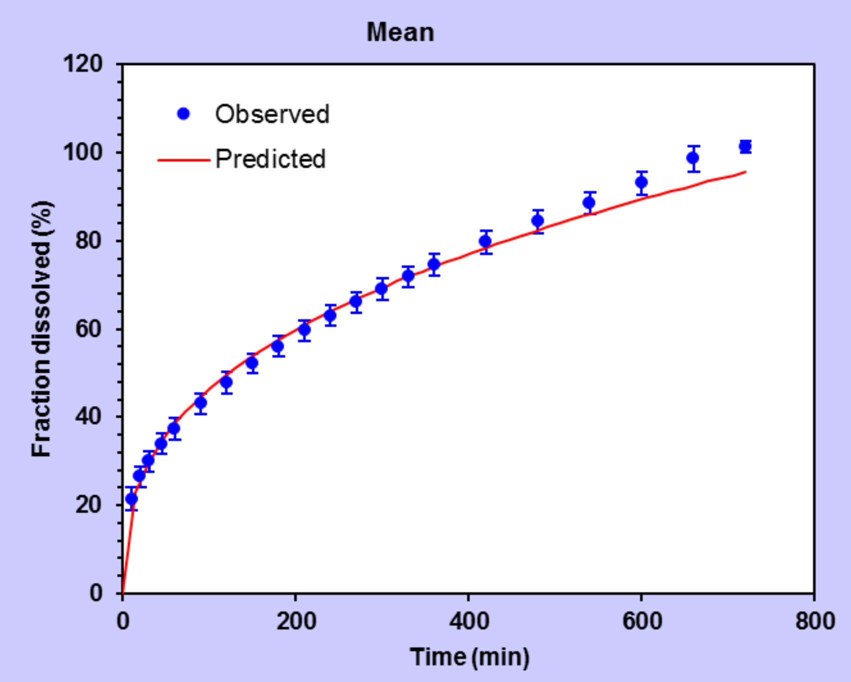

Supplementary materials – Model fitting summary – Lactochem® Crystals (modelling the entire relevant dissolution data)

Graphical abstract of model fit presented as the fraction % of released carvedilol per tested tablet:

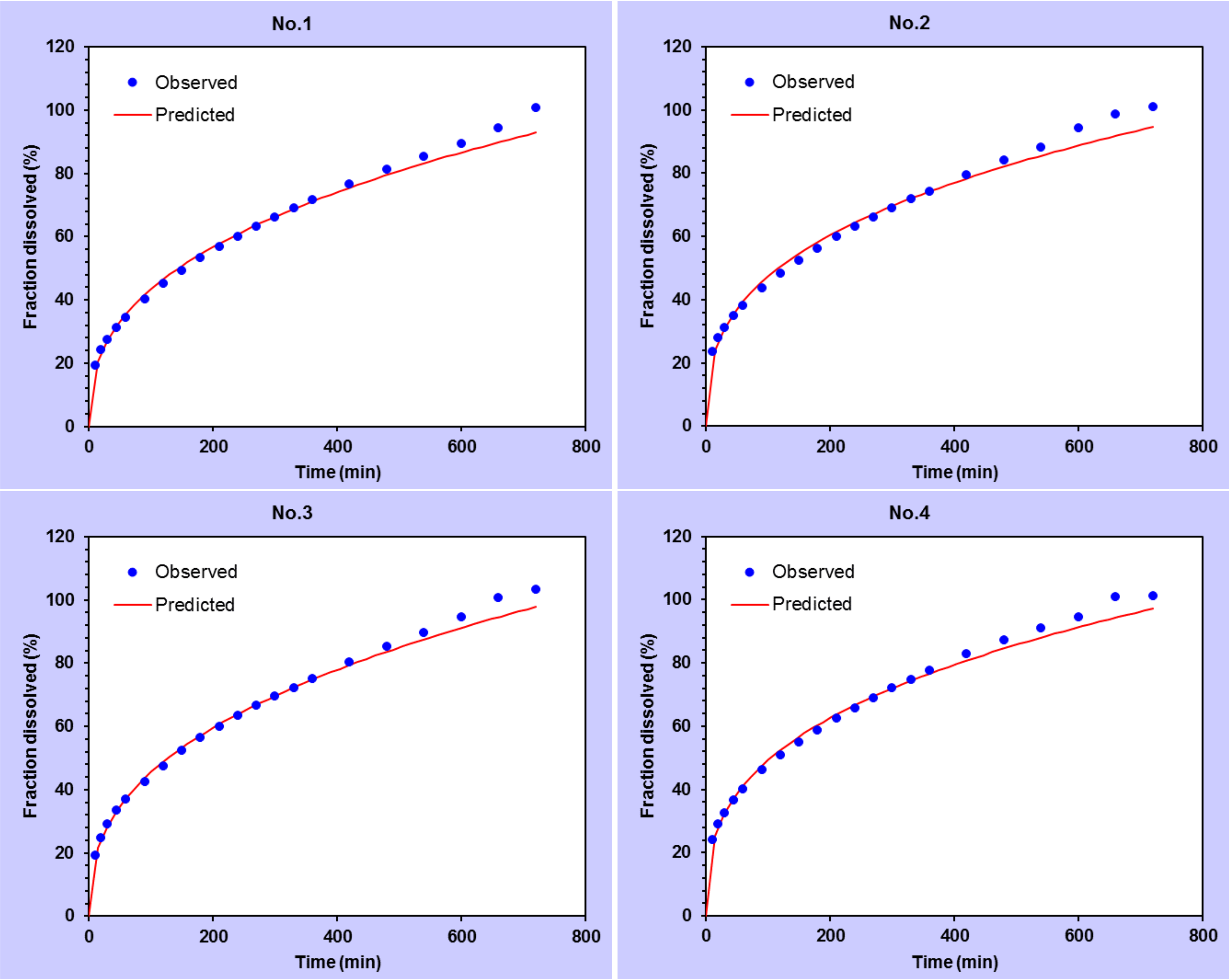

Supplementary materials – Model fitting summary – Lactochem® Crystals (modelling the entire relevant dissolution data)

Model: **Korsmeyer–Peppas with T_{lag}**

Model equation: $F = k_{KP} \cdot (t - T_{lag})^n$

Fitted model parameters per tested tablet (N = 4) with statistics – mean, standard deviation (SD), and relative standard deviation expressed in % (RSD%) (output from DDSolver):

Parameter	No.1	No.2	No.3	No.4	Mean	SD	RSD(%)
k_{KP}	8.715	11.032	8.925	11.670	10.086	1.487	14.744
n	0.358	0.325	0.363	0.321	0.342	0.022	6.360
T_{lag}	4.000	4.000	4.000	4.000	4.000	0.000	0.000

Number of dissolution data points (N), degrees of freedom (df), and selected goodness of fit criteria – Pearson correlation coefficient (R), coefficient of determination (R^2), adjusted coefficient of determination ($R^2_{adjusted}$), and residual sum of squares (RSS) (manual calculation in MS Excel):

Parameter	No.1	No.2	No.3	No.4
N	21	21	21	21
df	18	18	18	18
R	0.994331448	0.991540173	0.995921364	0.994519844
R^2	0.988695029	0.983151914	0.991859364	0.989069721
$R^2_{adjusted}$	0.987438921	0.981279904	0.990954848	0.987855245
RSS	188.7727624	262.0327086	147.424056	172.3950436

Graphical abstract of model fit presented as mean ± 1 SD of the fraction % of released carvedilol:

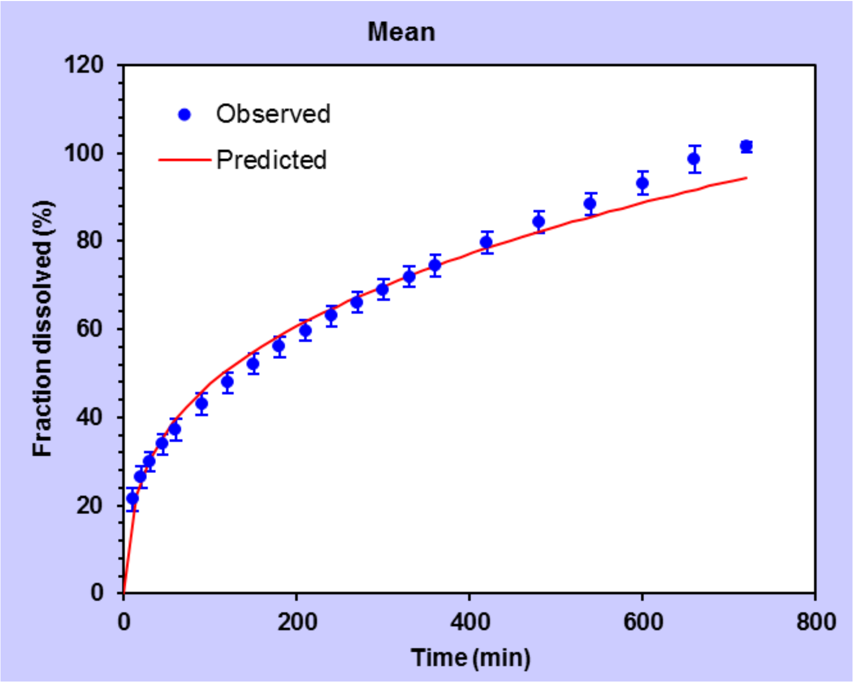

Supplementary materials – Model fitting summary – Lactochem® Crystals (modelling the entire relevant dissolution data)

Graphical abstract of model fit presented as the fraction % of released carvedilol per tested tablet:

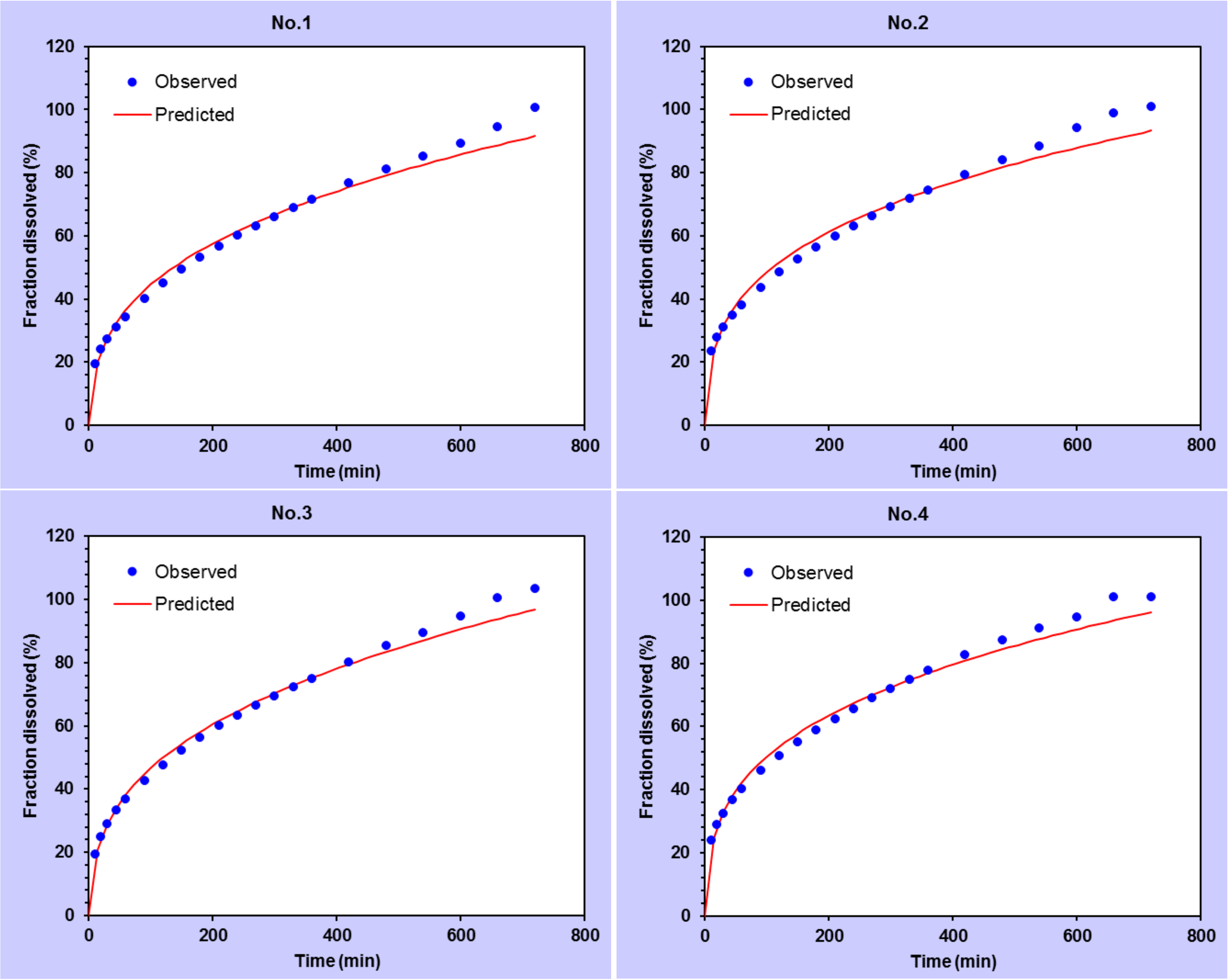

Supplementary materials – Model fitting summary – Lactochem® Crystals (modelling the entire relevant dissolution data)

Model: **Korsmeyer–Peppas with F_0**

Model equation: $F = F_0 + k_{KP} \cdot t^n$

Fitted model parameters per tested tablet (N = 4) with statistics – mean, standard deviation (SD), and relative standard deviation expressed in % (RSD%) (output from DDSolver):

Parameter	No.1	No.2	No.3	No.4	Mean	SD	RSD(%)
k_{KP}	3.588	4.766	3.994	5.138	4.371	0.707	16.176
n	0.489	0.444	0.481	0.438	0.463	0.026	5.562
F_0	8.114	9.399	7.679	9.598	8.697	0.945	10.868

Number of dissolution data points (N), degrees of freedom (df), and selected goodness of fit criteria – Pearson correlation coefficient (R), coefficient of determination (R^2), adjusted coefficient of determination ($R^2_{adjusted}$), and residual sum of squares (RSS) (manual calculation in MS Excel):

Parameter	No.1	No.2	No.3	No.4
N	21	21	21	21
df	18	18	18	18
R	0.999501693	0.998721206	0.999675723	0.999464894
R^2	0.999003634	0.997444047	0.999351551	0.998930075
$R^2_{adjusted}$	0.998892926	0.997160052	0.999279501	0.998811194
RSS	12.08774967	41.8141251	8.257029253	14.77968445

Graphical abstract of model fit presented as mean ± 1 SD of the fraction % of released carvedilol:

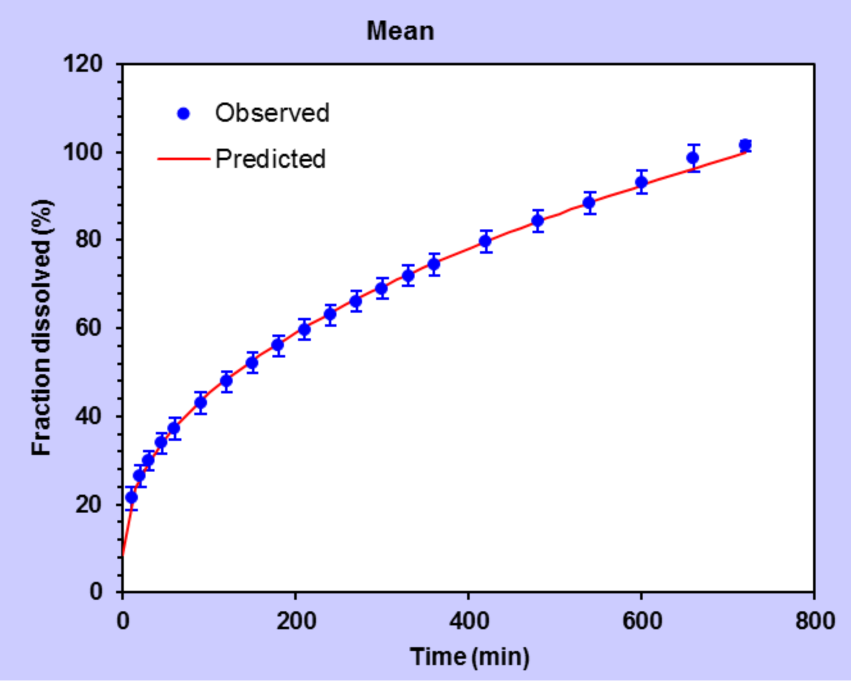

Supplementary materials – Model fitting summary – Lactochem® Crystals (modelling the entire relevant dissolution data)

Graphical abstract of model fit presented as the fraction % of released carvedilol per tested tablet:

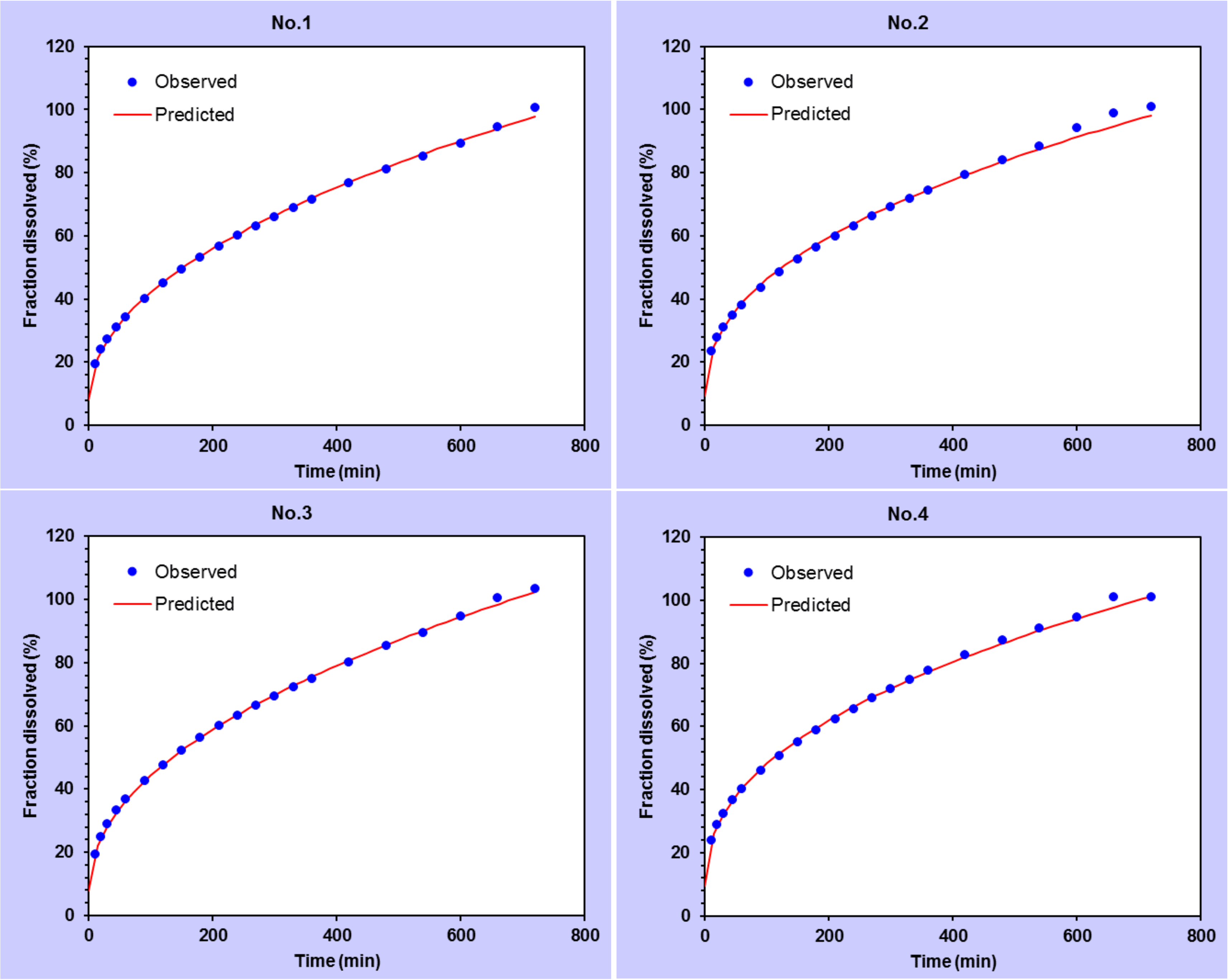

Supplementary materials – Model fitting summary – Lactochem® Crystals (modelling the entire relevant dissolution data)

Model: **Hixson–Crowell**

Model equation: $F = 100 \cdot [1 - (1 - k_{HC} \cdot t)^3]$

Fitted model parameters per tested tablet (N = 4) with statistics – mean, standard deviation (SD), and relative standard deviation expressed in % (RSD%) (output from DDSolver):

Parameter	No.1	No.2	No.3	No.4	Mean	SD	RSD(%)
k_{HC}	0.001	0.001	0.001	0.002	0.001	0.000	27.214

Number of dissolution data points (N), degrees of freedom (df), and selected goodness of fit criteria – Pearson correlation coefficient (R), coefficient of determination (R^2), adjusted coefficient of determination ($R^2_{adjusted}$), and residual sum of squares (RSS) (manual calculation in MS Excel):

Parameter	No.1	No.2	No.3	No.4
N	21	21	21	21
df	20	20	20	20
R	0.994693487	0.992102538	0.992222324	0.965928862
R^2	0.989415133	0.984267446	0.98450514	0.933018566
$R^2_{adjusted}$	0.989415133	0.984267446	0.98450514	0.933018566
RSS	2576.943201	3058.820847	2554.016749	3293.053559

Graphical abstract of model fit presented as mean ± 1 SD of the fraction % of released carvedilol:

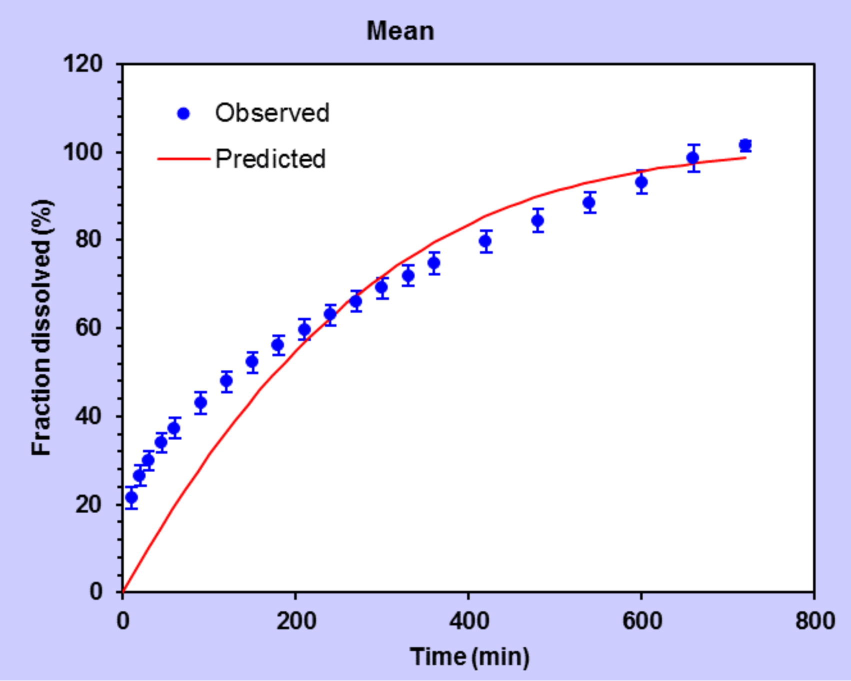

Supplementary materials – Model fitting summary – Lactochem® Crystals (modelling the entire relevant dissolution data)

Graphical abstract of model fit presented as the fraction % of released carvedilol per tested tablet:

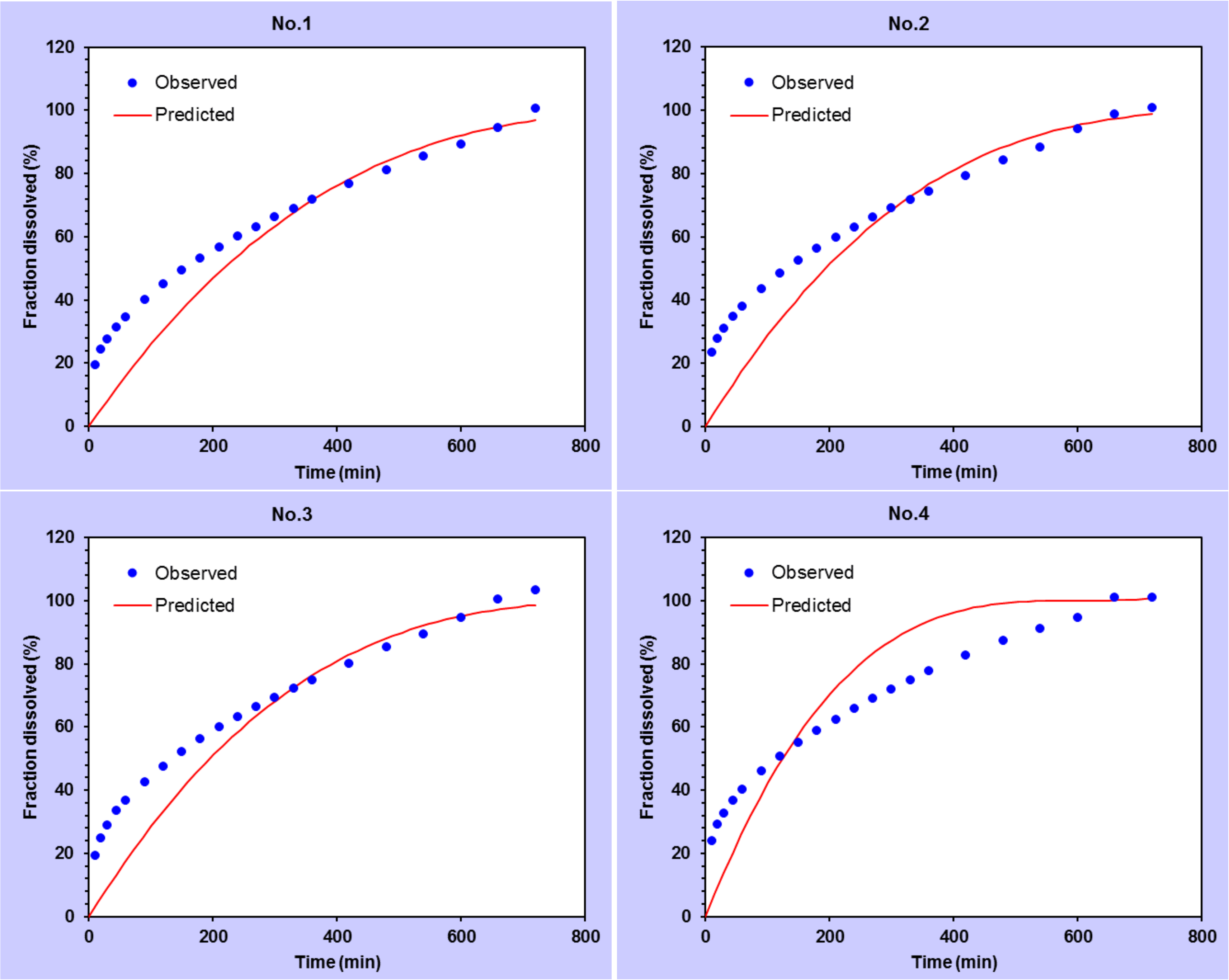

Supplementary materials – Model fitting summary – Lactochem® Crystals (modelling the entire relevant dissolution data)

Model: **Hixson–Crowell with T_{lag}**

Model equation: $F = 100 \cdot \left\{1 - \left[1 - k_{HC} \cdot \left(t - T_{lag}\right)\right]^3\right\}$

Fitted model parameters per tested tablet (N = 4) with statistics – mean, standard deviation (SD), and relative standard deviation expressed in % (RSD%) (output from DDSolver):

Parameter	No.1	No.2	No.3	No.4	Mean	SD	RSD(%)
k_{HC}	0.001	0.001	0.001	0.001	0.001	0.000	5.867
T_{lag}	-104.566	-119.134	-96.693	-116.528	-109.230	10.492	-9.606

Number of dissolution data points (N), degrees of freedom (df), and selected goodness of fit criteria – Pearson correlation coefficient (R), coefficient of determination (R^2), adjusted coefficient of determination ($R^2_{adjusted}$), and residual sum of squares (RSS) (manual calculation in MS Excel):

Parameter	No.1	No.2	No.3	No.4
N	21	21	21	21
df	19	19	19	19
R	0.996304444	0.996866977	0.995198543	0.996728369
R^2	0.992622545	0.99374377	0.990420141	0.993467442
$R^2_{adjusted}$	0.992234258	0.993414495	0.989915938	0.993123623
RSS	89.28226896	123.8050881	138.5133001	85.67827478

Graphical abstract of model fit presented as mean ± 1 SD of the fraction % of released carvedilol:

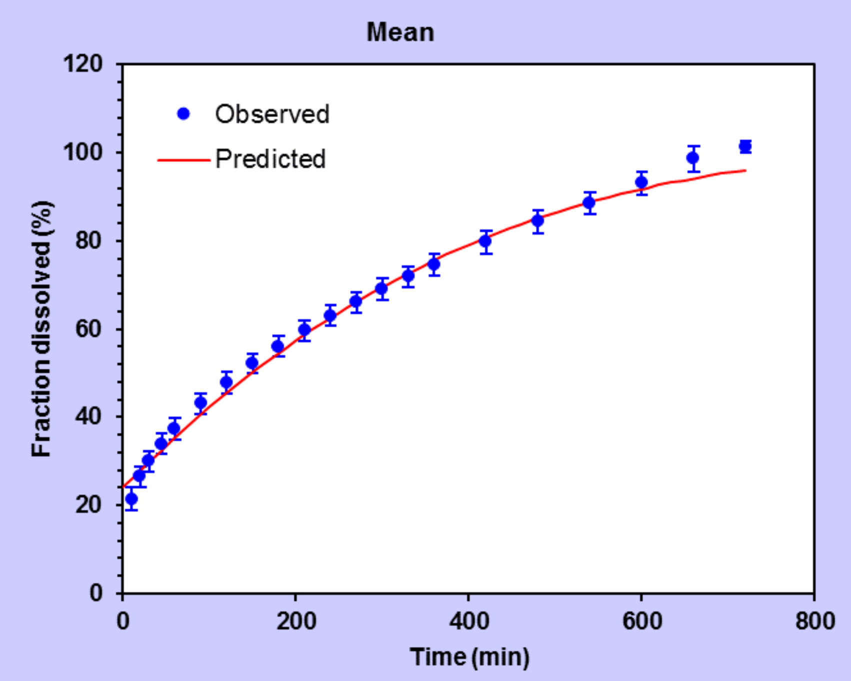

Supplementary materials – Model fitting summary – Lactochem® Crystals (modelling the entire relevant dissolution data)

Graphical abstract of model fit presented as the fraction % of released carvedilol per tested tablet:

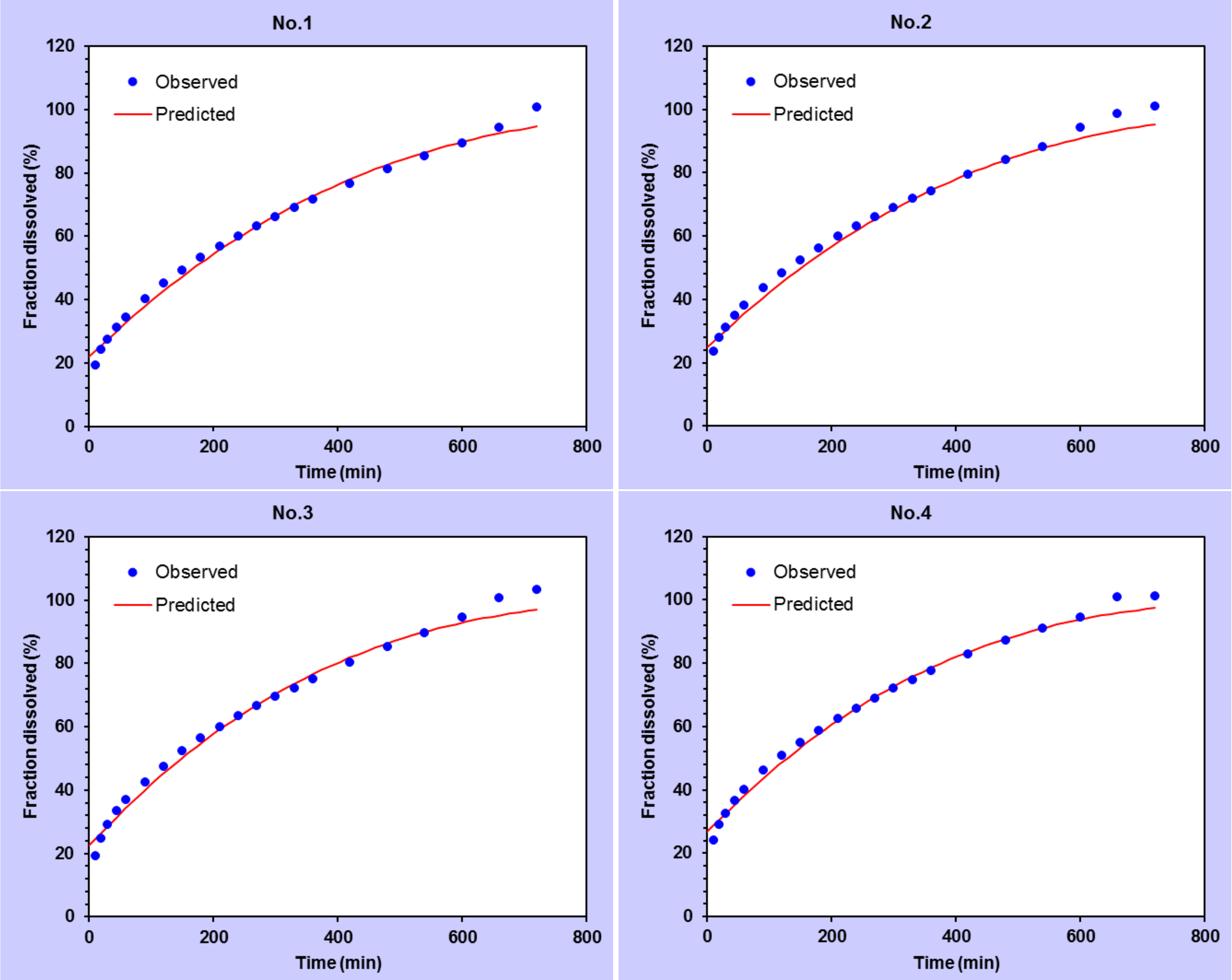

Supplementary materials – Model fitting summary – Lactochem® Crystals (modelling the entire relevant dissolution data)

Model: **Hopfenberg**

Model equation: $F = 100 \cdot [1 - (1 - k_{HB} \cdot t)^n]$

Fitted model parameters per tested tablet (N = 4) with statistics – mean, standard deviation (SD), and relative standard deviation expressed in % (RSD%) (output from DDSolver):

Parameter	No.1	No.2	No.3	No.4	Mean	SD	RSD(%)
k_{HB}	0.001	0.001	0.001	0.001	0.001	0.000	6.422
n	4.500	4.500	4.500	4.500	4.500	0.000	0.000

Number of dissolution data points (N), degrees of freedom (df), and selected goodness of fit criteria – Pearson correlation coefficient (R), coefficient of determination (R^2), adjusted coefficient of determination ($R^2_{adjusted}$), and residual sum of squares (RSS) (manual calculation in MS Excel):

Parameter	No.1	No.2	No.3	No.4
N	21	21	21	21
df	19	19	19	19
R	0.979348007	0.969620379	0.972377177	0.973457665
R^2	0.959122519	0.940163679	0.945517375	0.947619825
$R^2_{adjusted}$	0.956971072	0.937014399	0.942649869	0.944862973
RSS	2337.811303	2831.147776	2275.19111	2626.250045

Graphical abstract of model fit presented as mean ± 1 SD of the fraction % of released carvedilol:

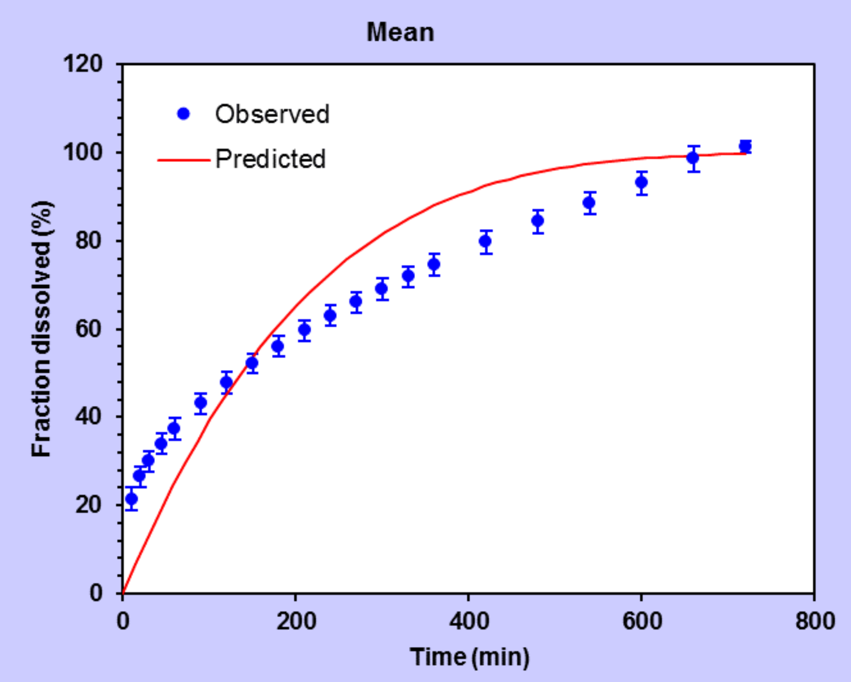

Supplementary materials – Model fitting summary – Lactochem® Crystals (modelling the entire relevant dissolution data)

Graphical abstract of model fit presented as the fraction % of released carvedilol per tested tablet:

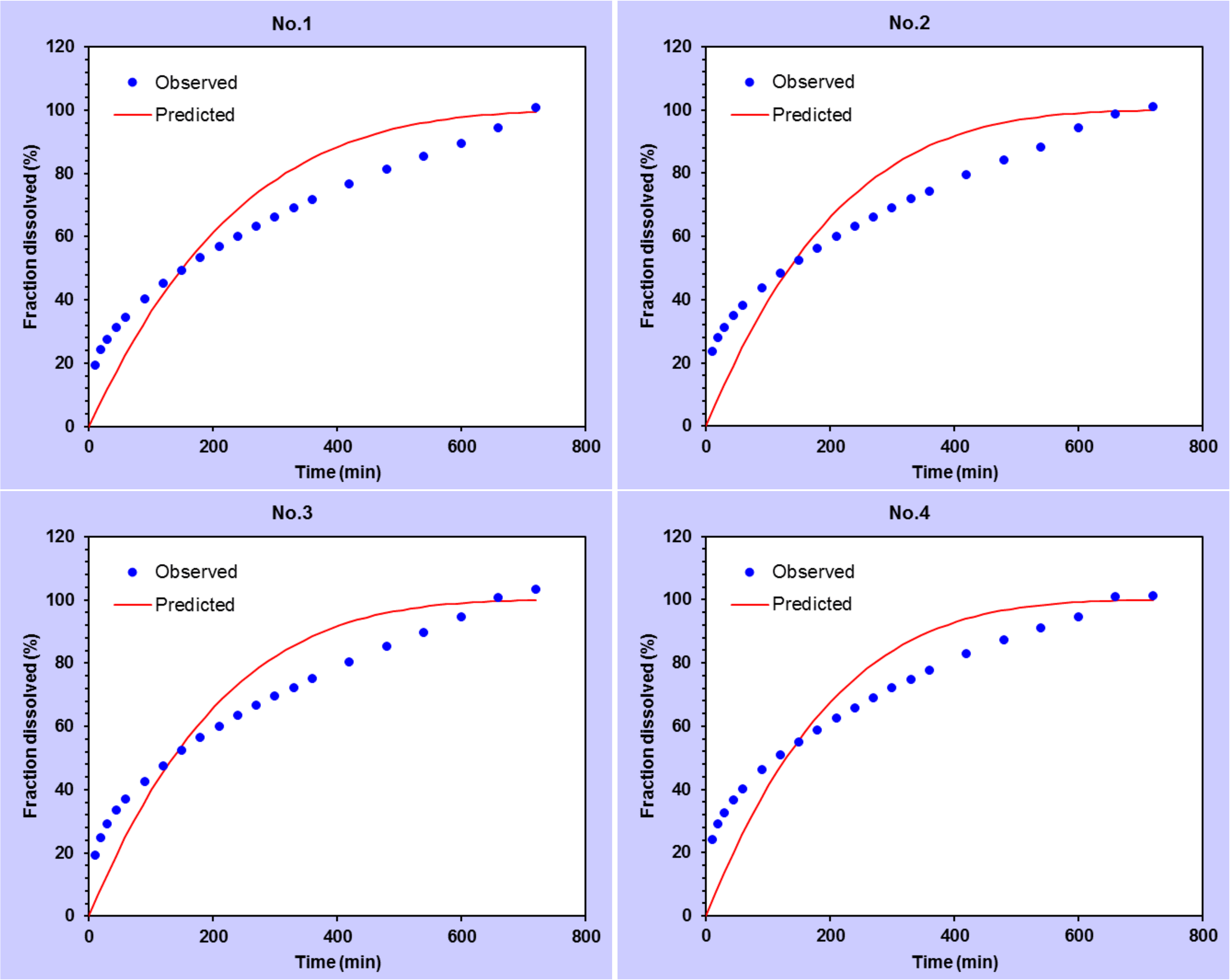

Supplementary materials – Model fitting summary – Lactochem® Crystals (modelling the entire relevant dissolution data)

Model: **Hopfenberg with T_{lag}**

Model equation: $F = 100 \cdot \{1 - [1 - k_{HB} \cdot (t - T_{lag})]^n\}$

Fitted model parameters per tested tablet (N = 4) with statistics – mean, standard deviation (SD), and relative standard deviation expressed in % (RSD%) (output from DDSolver):

Parameter	No.1	No.2	No.3	No.4	Mean	SD	RSD(%)
k_{HB}	0.001	0.001	0.001	0.001	0.001	0.000	13.199
n	3.000	2.000	3.000	3.000	2.750	0.500	18.182
T_{lag}	-104.566	-136.357	-96.693	-116.528	-113.536	17.262	-15.204

Number of dissolution data points (N), degrees of freedom (df), and selected goodness of fit criteria – Pearson correlation coefficient (R), coefficient of determination (R^2), adjusted coefficient of determination ($R^2_{adjusted}$), and residual sum of squares (RSS) (manual calculation in MS Excel):

Parameter	No.1	No.2	No.3	No.4
N	21	21	21	21
df	18	18	18	18
R	0.996304444	0.996483716	0.995198543	0.996728369
R^2	0.992622545	0.992979796	0.990420141	0.993467442
$R^2_{adjusted}$	0.991802827	0.992199773	0.989355712	0.992741602
RSS	89.28226896	79.46189575	138.5133001	85.67827478

Graphical abstract of model fit presented as mean ± 1 SD of the fraction % of released carvedilol:

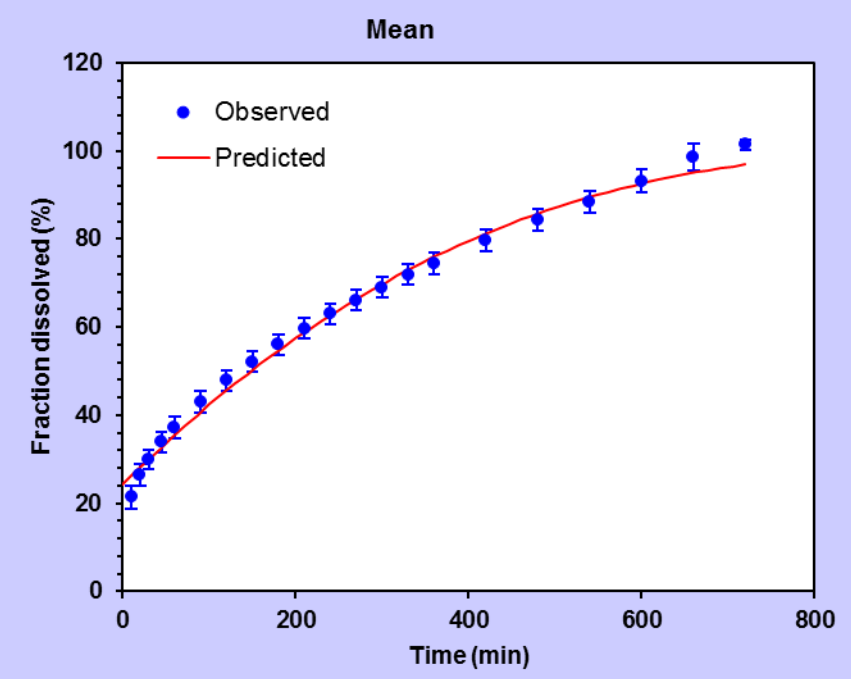

Supplementary materials – Model fitting summary – Lactochem® Crystals (modelling the entire relevant dissolution data)

Graphical abstract of model fit presented as the fraction % of released carvedilol per tested tablet:

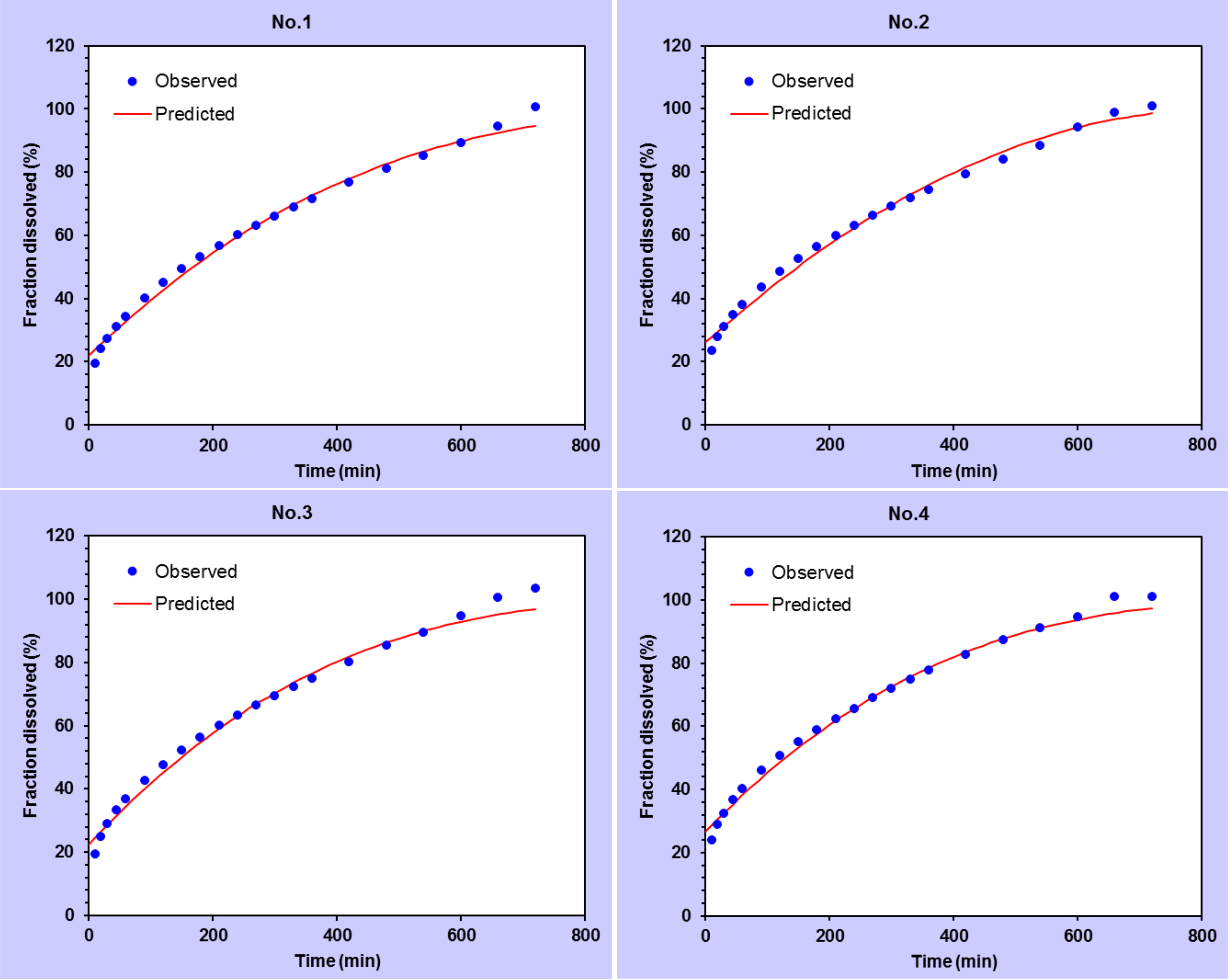

Supplementary materials – Model fitting summary – Lactochem® Crystals (modelling the entire relevant dissolution data)

Model: **Baker–Lonsdale**

Model equation: $\frac{3}{2} \cdot \left[1 - \left(1 - \frac{F}{100}\right)^{\frac{2}{3}}\right] - \frac{F}{100} = k_{BL} \cdot t$

Fitted model parameters per tested tablet (N = 4) with statistics – mean, standard deviation (SD), and relative standard deviation expressed in % (RSD%) (output from DDSolver):

Parameter	No.1	No.2	No.3	No.4	Mean	SD	RSD(%)
k_{BL}	0.0004	0.0004	0.0005	0.0005	0.0005	0.0001	14.2188

Number of dissolution data points (N), degrees of freedom (df), and selected goodness of fit criteria – Pearson correlation coefficient (R), coefficient of determination (R^2), adjusted coefficient of determination ($R^2_{adjusted}$), and residual sum of squares (RSS) (manual calculation in MS Excel):

Parameter	No.1	No.2	No.3	No.4
N	21	21	21	21
df	20	20	20	20
R	0.99058167	0.989711415	0.987973091	0.989725681
R^2	0.981252045	0.979528685	0.976090828	0.979556924
$R^2_{adjusted}$	0.981252045	0.979528685	0.976090828	0.979556924
RSS	224.4498867	430.0941359	369.1727486	258.0965261

Graphical abstract of model fit presented as mean ± 1 SD of the fraction % of released carvedilol:

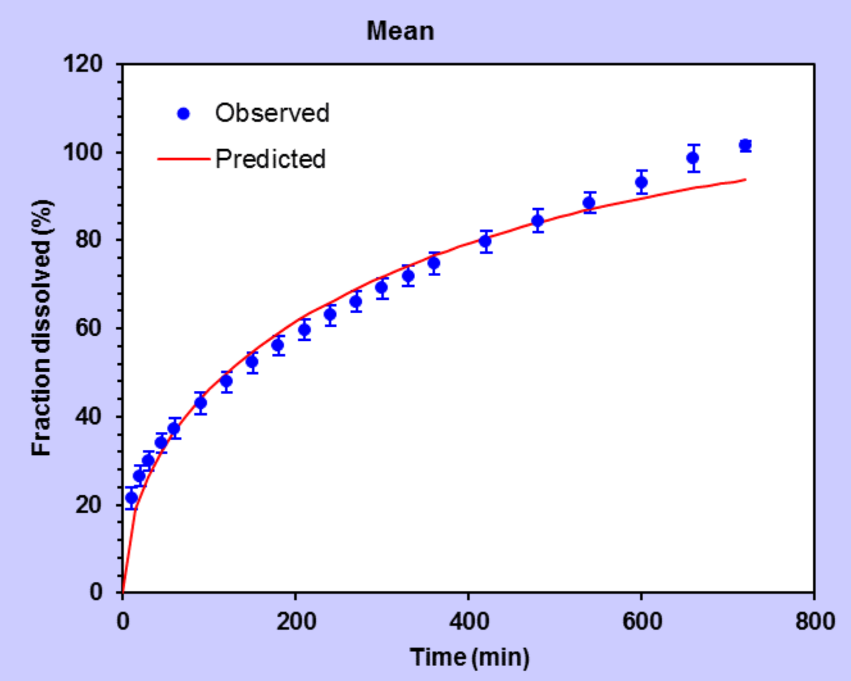

Supplementary materials – Model fitting summary – Lactochem® Crystals (modelling the entire relevant dissolution data)

Graphical abstract of model fit presented as the fraction % of released carvedilol per tested tablet:

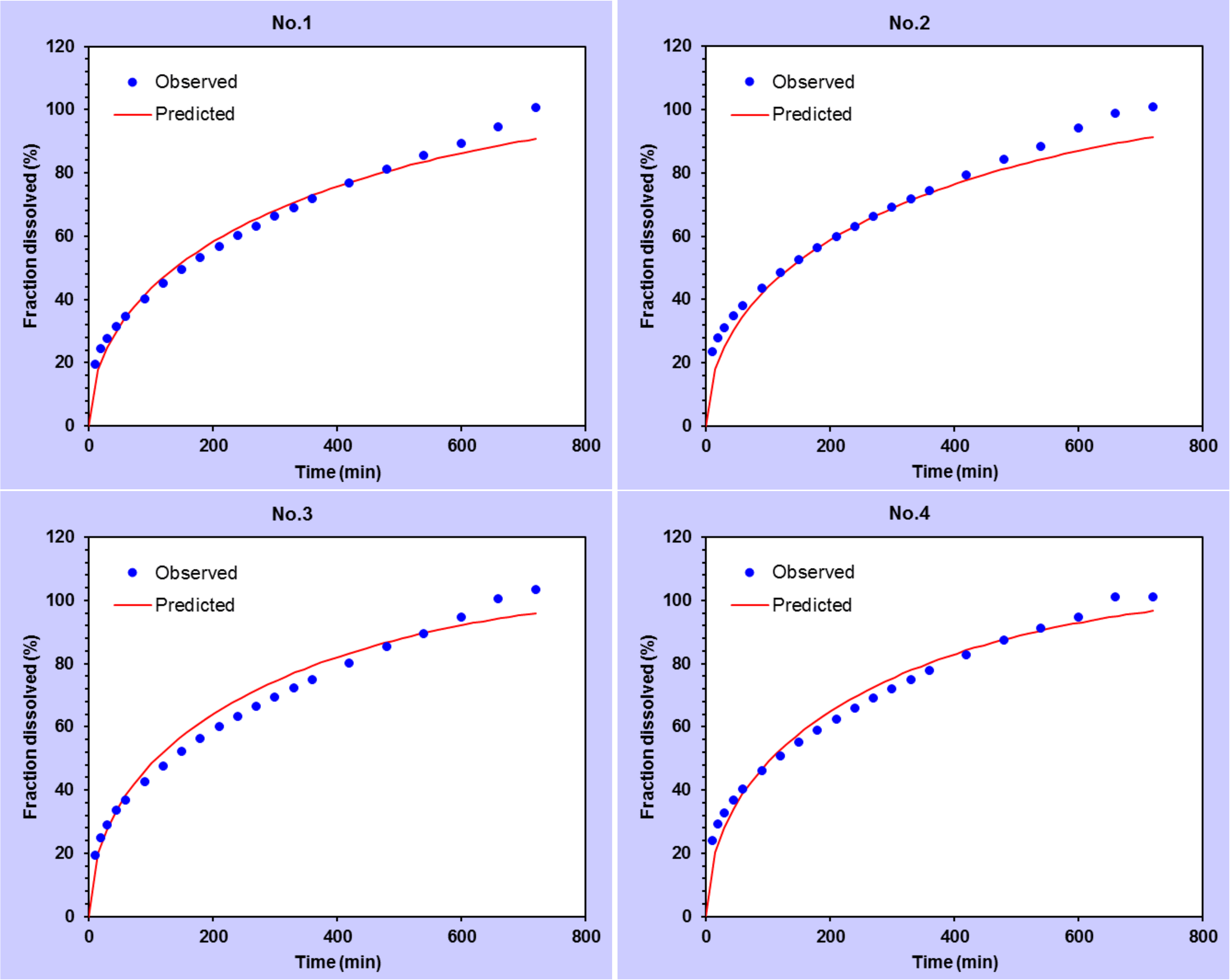

Supplementary materials – Model fitting summary – Lactochem® Crystals (modelling the entire relevant dissolution data)

Model: **Baker–Lonsdale with T_{lag}**

Model equation: $\frac{3}{2} \cdot \left[1 - \left(1 - \frac{F}{100}\right)^{\frac{2}{3}}\right] - \frac{F}{100} = k_{BL} \cdot (t - T_{lag})$

Fitted model parameters per tested tablet (N = 4) with statistics – mean, standard deviation (SD), and relative standard deviation expressed in % (RSD%) (output from DDSolver):

Parameter	No.1	No.2	No.3	No.4	Mean	SD	RSD(%)
k_{BL}	0.0005	0.0005	0.0005	0.0005	0.0005	0.0000	7.6292
T_{lag}	25.9581	31.2547	22.3208	10.3454	22.4697	8.8763	39.5034

Number of dissolution data points (N), degrees of freedom (df), and selected goodness of fit criteria – Pearson correlation coefficient (R), coefficient of determination (R^2), adjusted coefficient of determination ($R^2_{adjusted}$), and residual sum of squares (RSS) (manual calculation in MS Excel):

Parameter	No.1	No.2	No.3	No.4
N	21	21	21	21
df	19	19	19	19
R	0.975054807	0.965996948	0.973998624	0.979281757
R^2	0.950731877	0.933150104	0.94867332	0.958992759
$R^2_{adjusted}$	0.948138818	0.929631688	0.945971915	0.956834483
RSS	1522.084022	2793.652637	1472.829469	974.6546355

Graphical abstract of model fit presented as mean ± 1 SD of the fraction % of released carvedilol:

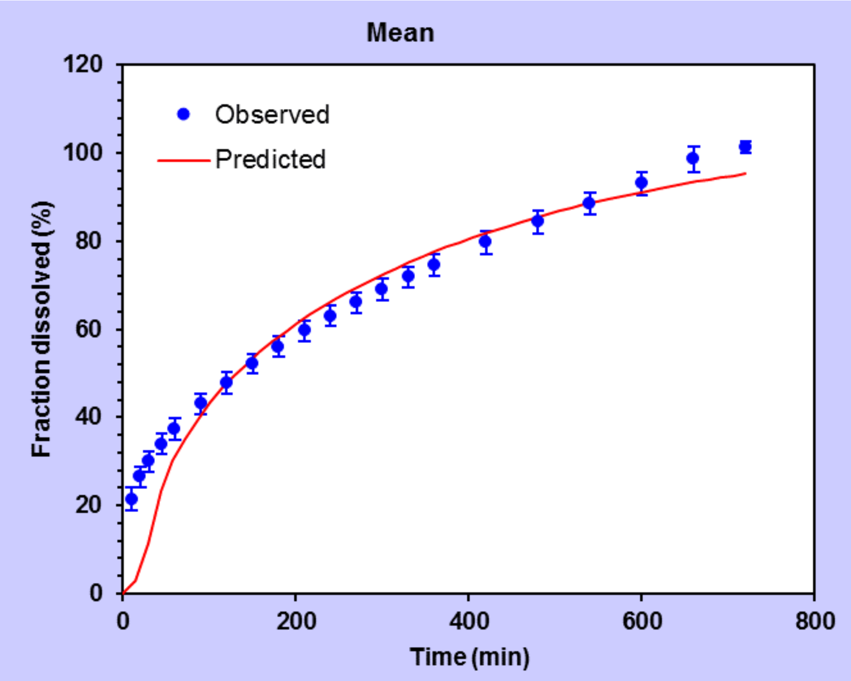

Supplementary materials – Model fitting summary – Lactochem® Crystals (modelling the entire relevant dissolution data)

Graphical abstract of model fit presented as the fraction % of released carvedilol per tested tablet:

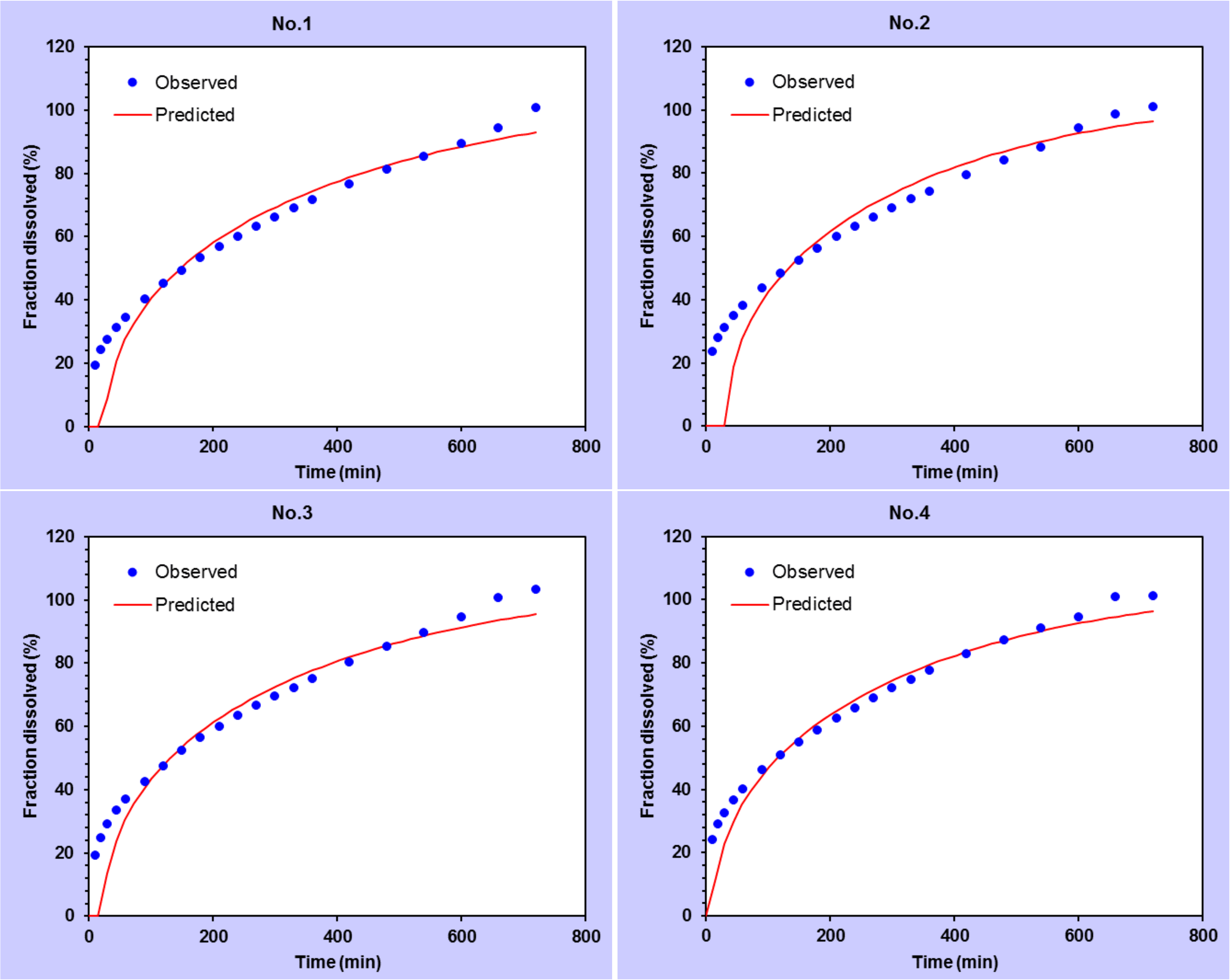

Supplementary materials – Model fitting summary – Lactochem® Crystals (modelling the entire relevant dissolution data)

Model: **Makoid–Banakar**

Model equation: $F = k_{MB} \cdot t^n \cdot e^{-k \cdot t}$

Fitted model parameters per tested tablet (N = 4) with statistics – mean, standard deviation (SD), and relative standard deviation expressed in % (RSD%) (output from DDSolver):

Parameter	No.1	No.2	No.3	No.4	Mean	SD	RSD(%)
k_{MB}	8.6524	11.6147	8.4592	11.5843	10.0777	1.7591	17.4554
n	0.3381	0.2896	0.3565	0.3036	0.3219	0.0308	9.5622
k	-0.0003	-0.0004	-0.0002	-0.0003	-0.0003	0.0001	-23.0751

Number of dissolution data points (N), degrees of freedom (df), and selected goodness of fit criteria – Pearson correlation coefficient (R), coefficient of determination (R^2), adjusted coefficient of determination ($R^2_{adjusted}$), and residual sum of squares (RSS) (manual calculation in MS Excel):

Parameter	No.1	No.2	No.3	No.4
N	21	21	21	21
df	18	18	18	18
R	0.999630359	0.999354628	0.999885505	0.999105752
R^2	0.999260856	0.998709672	0.999771022	0.998212303
$R^2_{adjusted}$	0.999178728	0.998566303	0.999745581	0.99801367
RSS	8.569748653	14.60235302	2.911377636	20.44522156

Graphical abstract of model fit presented as mean ± 1 SD of the fraction % of released carvedilol:

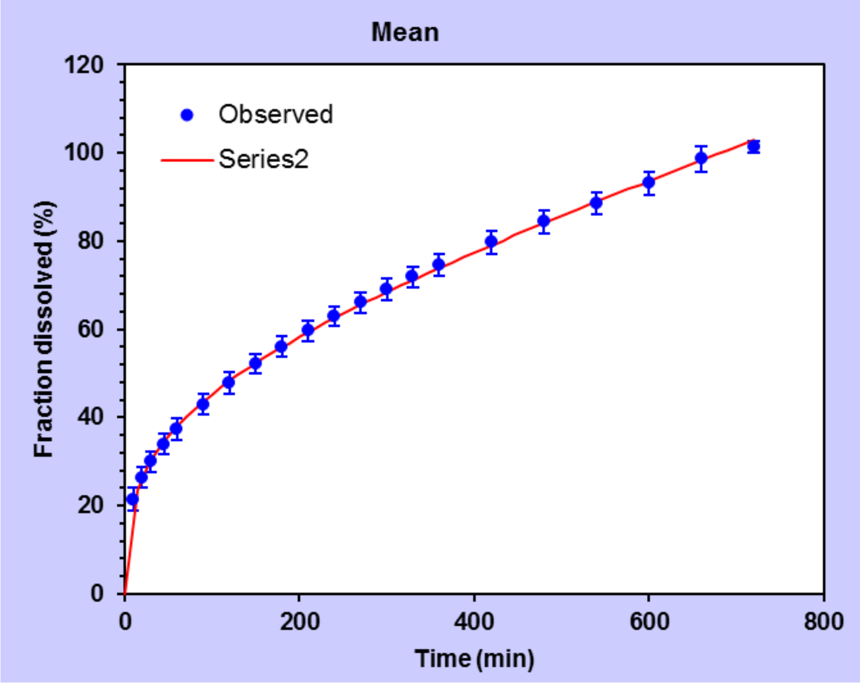

Supplementary materials – Model fitting summary – Lactochem® Crystals (modelling the entire relevant dissolution data)

Graphical abstract of model fit presented as the fraction % of released carvedilol per tested tablet:

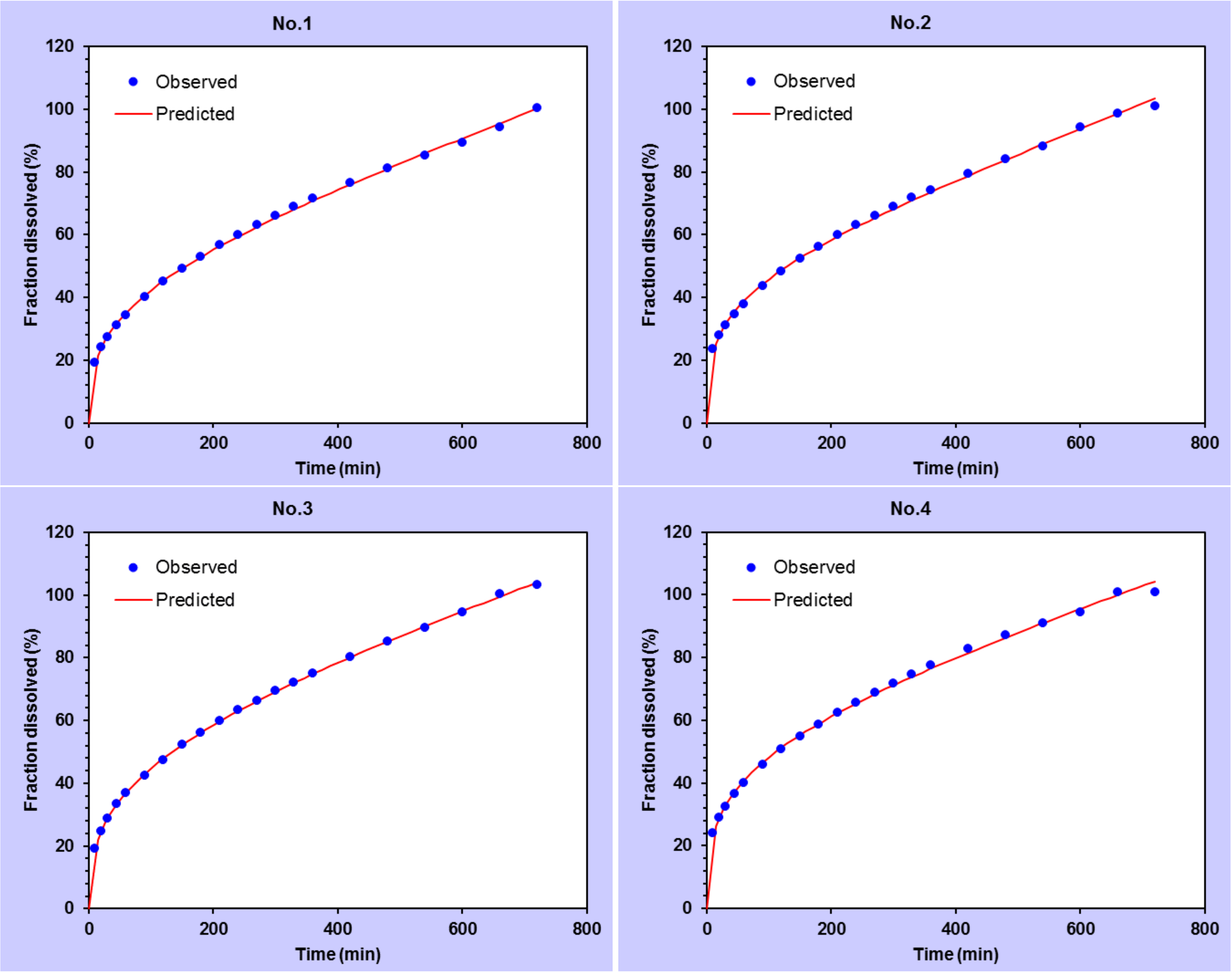

Supplementary materials – Model fitting summary – Lactochem® Crystals (modelling the entire relevant dissolution data)

Model: **Makoid–Banakar with T_{lag}**

Model equation: $F = k_{MB} \cdot (t - T_{lag})^n \cdot e^{-k\cdot(t-T_{lag})}$

Fitted model parameters per tested tablet (N = 4) with statistics – mean, standard deviation (SD), and relative standard deviation expressed in % (RSD%) (output from DDSolver):

Parameter	No.1	No.2	No.3	No.4	Mean	SD	RSD(%)
k_{MB}	10.8003	14.0802	10.6430	14.1503	12.4184	1.9605	15.7873
n	0.2908	0.2485	0.3077	0.2609	0.2770	0.0271	9.7911
k	-0.0005	-0.0005	-0.0004	-0.0004	-0.0004	0.0001	-14.1881
T_{lag}	4.0000	4.0000	4.0000	4.0000	4.0000	0.0000	0.0000

Number of dissolution data points (N), degrees of freedom (df), and selected goodness of fit criteria – Pearson correlation coefficient (R), coefficient of determination (R^2), adjusted coefficient of determination ($R^2_{adjusted}$), and residual sum of squares (RSS) (manual calculation in MS Excel):

Parameter	No.1	No.2	No.3	No.4
N	21	21	21	21
df	17	17	17	17
R	0.99893777	0.998413902	0.999526387	0.998086172
R^2	0.997876668	0.99683032	0.999052999	0.996176007
$R^2_{adjusted}$	0.997501963	0.996270965	0.998885881	0.995501185
RSS	24.80161459	36.10410422	12.11274815	43.89535713

Graphical abstract of model fit presented as mean ± 1 SD of the fraction % of released carvedilol:

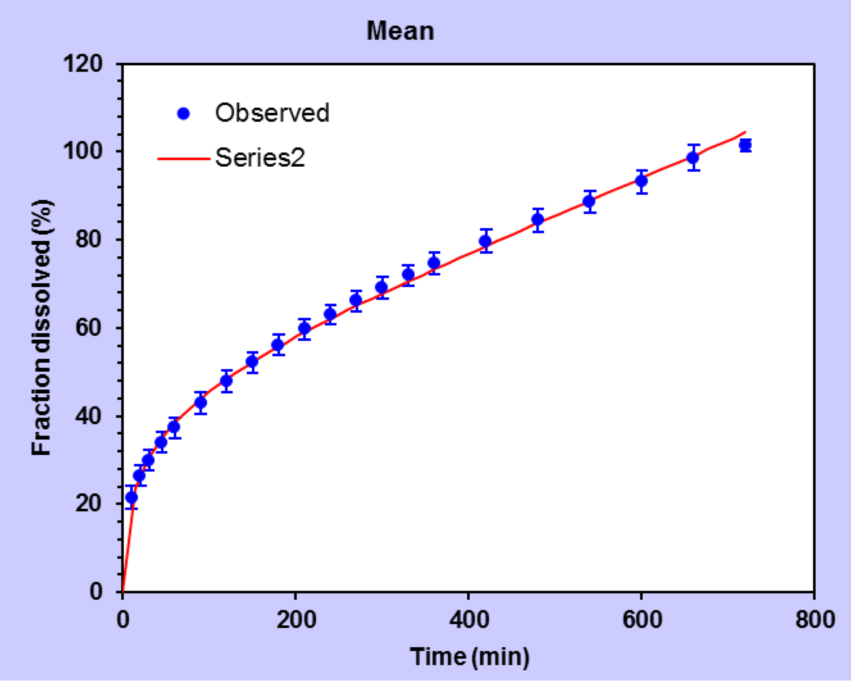

Supplementary materials – Model fitting summary – Lactochem® Crystals (modelling the entire relevant dissolution data)

Graphical abstract of model fit presented as the fraction % of released carvedilol per tested tablet:

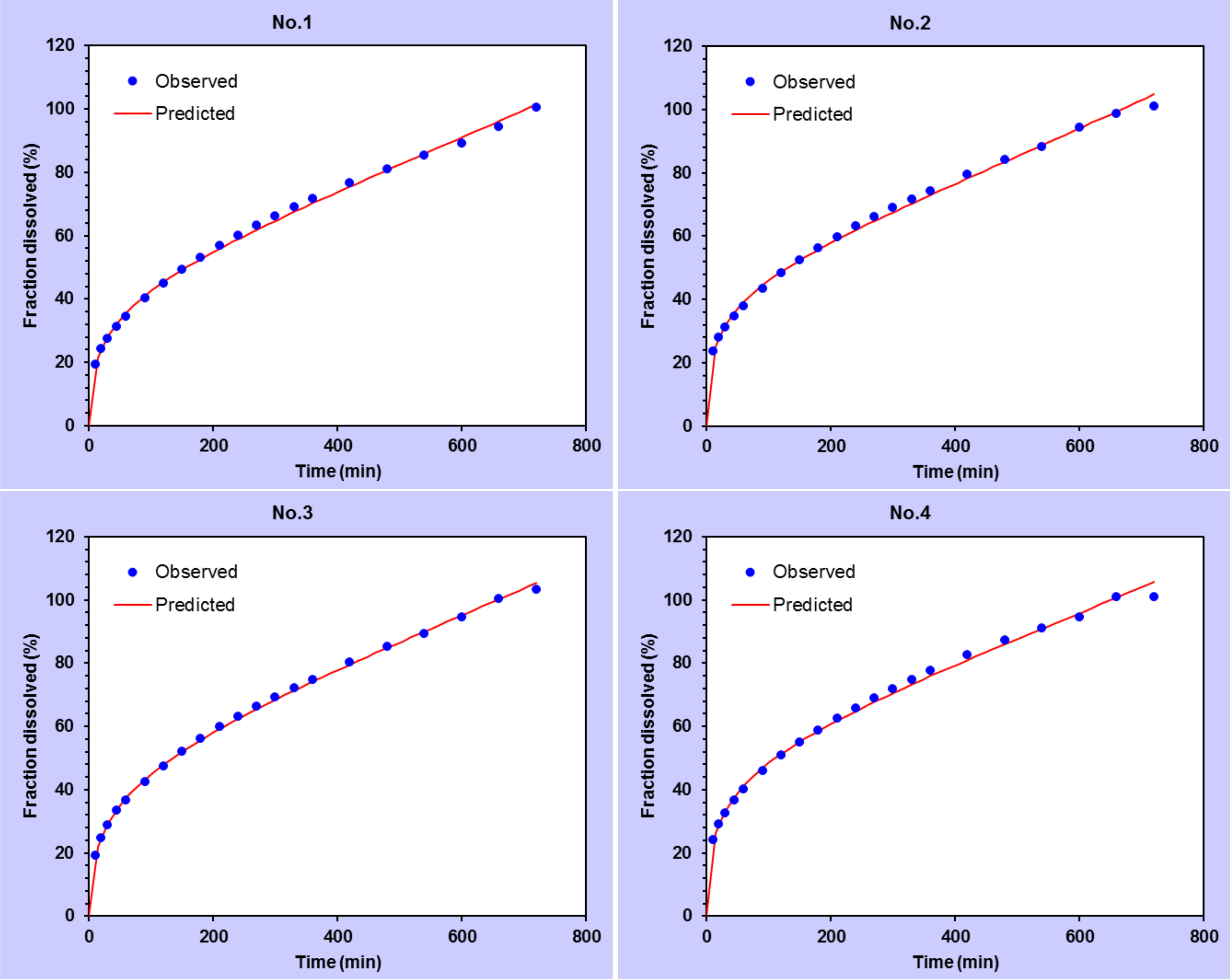

Supplementary materials – Model fitting summary – Lactochem® Crystals (modelling the entire relevant dissolution data)

Model: **Peppas–Sahlin_1**

Model equation: $F = k_1 \cdot t^m + k_2 \cdot t^{2m}$

Fitted model parameters per tested tablet (N = 4) with statistics – mean, standard deviation (SD), and relative standard deviation expressed in % (RSD%) (output from DDSolver):

Parameter	No.1	No.2	No.3	No.4	Mean	SD	RSD(%)
k_1	5.449	6.103	5.781	6.590	5.981	0.486	8.127
k_2	-0.022	-0.051	-0.027	-0.073	-0.043	0.023	-53.399
m	0.450	0.450	0.450	0.450	0.450	0.000	0.000

Number of dissolution data points (N), degrees of freedom (df), and selected goodness of fit criteria – Pearson correlation coefficient (R), coefficient of determination (R^2), adjusted coefficient of determination ($R^2_{adjusted}$), and residual sum of squares (RSS) (manual calculation in MS Excel):

Parameter	No.1	No.2	No.3	No.4
N	21	21	21	21
df	18	18	18	18
R	0.997884638	0.995612367	0.998391623	0.99644988
R^2	0.995773752	0.991243985	0.996785833	0.992912363
$R^2_{adjusted}$	0.995304169	0.990271094	0.996428704	0.992124847
RSS	64.30439692	137.1427277	53.93043212	112.9687599

Graphical abstract of model fit presented as mean ± 1 SD of the fraction % of released carvedilol:

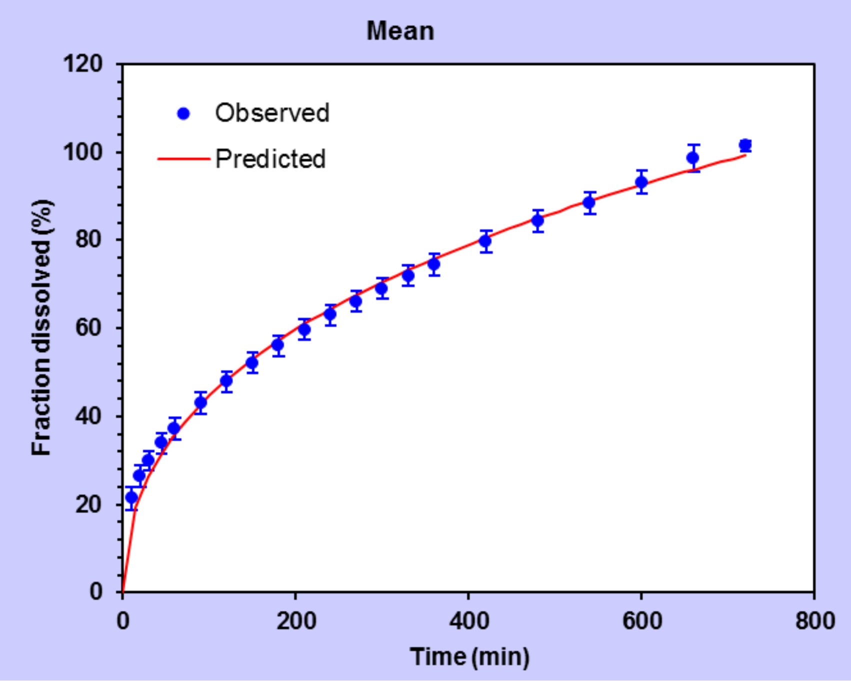

Supplementary materials – Model fitting summary – Lactochem® Crystals (modelling the entire relevant dissolution data)

Graphical abstract of model fit presented as the fraction % of released carvedilol per tested tablet:

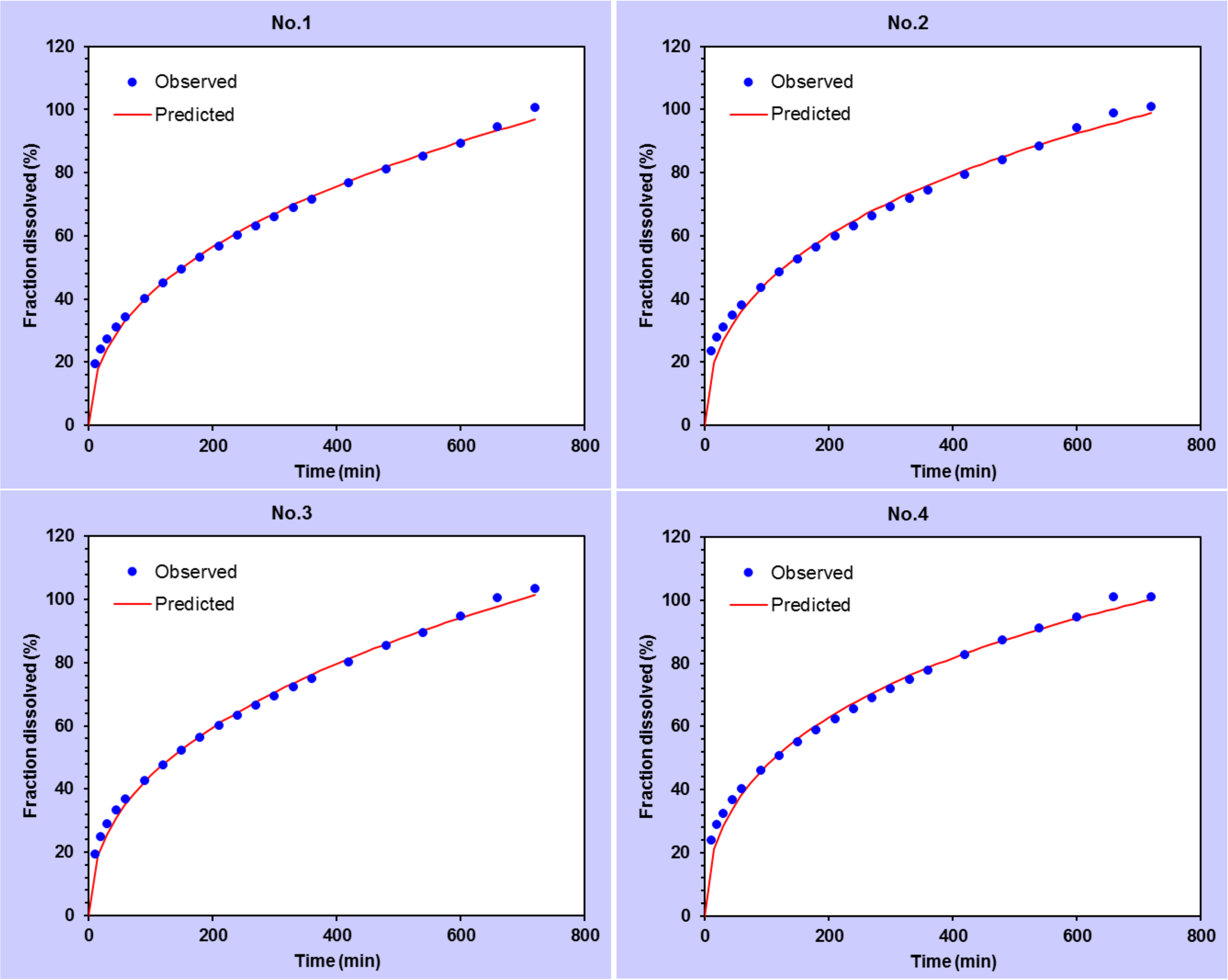

Supplementary materials – Model fitting summary – Lactochem® Crystals (modelling the entire relevant dissolution data)

Model: **Peppas–Sahlin_1 with T_{lag}**

Model equation: $F = k_1 \cdot (t - T_{lag})^m + k_2 \cdot (t - T_{lag})^{2m}$

Fitted model parameters per tested tablet (N = 4) with statistics – mean, standard deviation (SD), and relative standard deviation expressed in % (RSD%) (output from DDSolver):

Parameter	No.1	No.2	No.3	No.4	Mean	SD	RSD(%)
k_1	5.638	6.295	5.984	6.795	6.178	0.491	7.949
k_2	-0.033	-0.062	-0.039	-0.084	-0.054	0.023	-43.142
m	0.450	0.450	0.450	0.450	0.450	0.000	0.000
T_{lag}	4.000	4.000	4.000	4.000	4.000	0.000	0.000

Number of dissolution data points (N), degrees of freedom (df), and selected goodness of fit criteria – Pearson correlation coefficient (R), coefficient of determination (R^2), adjusted coefficient of determination ($R^2_{adjusted}$), and residual sum of squares (RSS) (manual calculation in MS Excel):

Parameter	No.1	No.2	No.3	No.4
N	21	21	21	21
df	17	17	17	17
R	0.99622339	0.993063089	0.996958211	0.994007684
R^2	0.992461043	0.986174298	0.993925674	0.988051276
$R^2_{adjusted}$	0.991130639	0.983734468	0.992853734	0.985942678
RSS	123.912691	232.4731919	111.077669	206.4327294

Graphical abstract of model fit presented as mean ± 1 SD of the fraction % of released carvedilol:

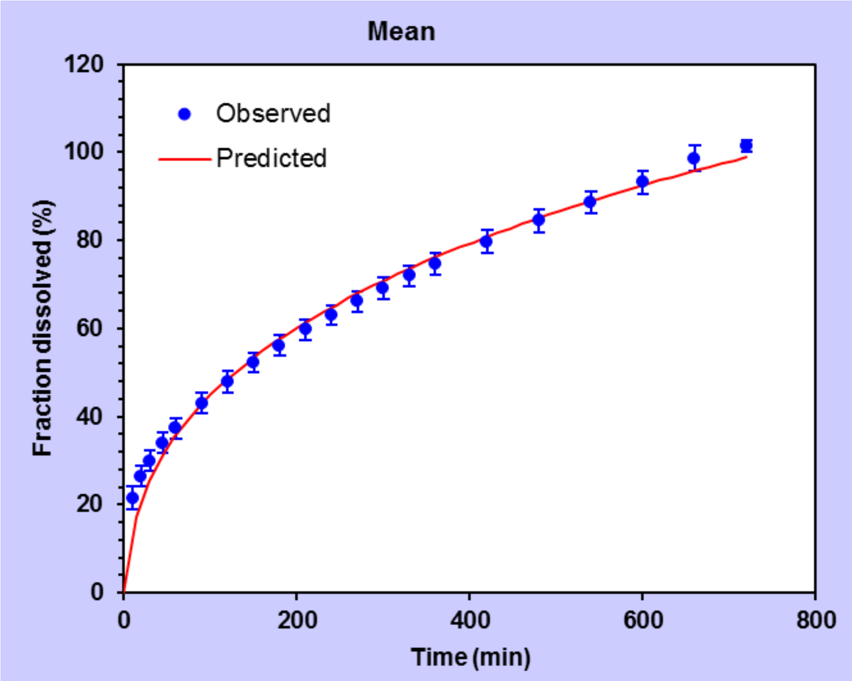

Supplementary materials – Model fitting summary – Lactochem® Crystals (modelling the entire relevant dissolution data)

Graphical abstract of model fit presented as the fraction % of released carvedilol per tested tablet:

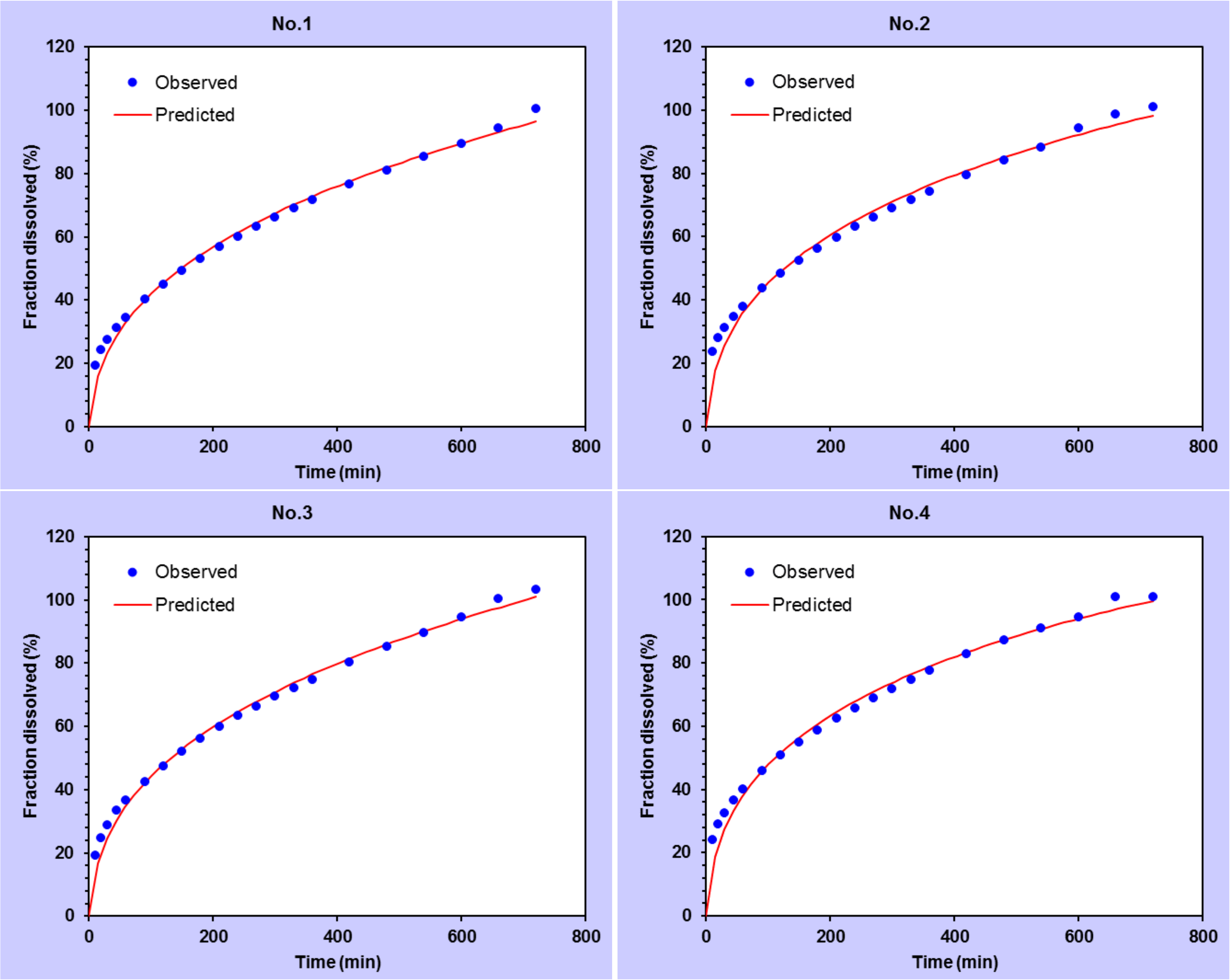

Supplementary materials – Model fitting summary – Lactochem® Crystals (modelling the entire relevant dissolution data)

Model: **Peppas–Sahlin_2**

Model equation: $F = k_1 \cdot t^{0.5} + k_2 \cdot t$

Fitted model parameters per tested tablet (N = 4) with statistics – mean, standard deviation (SD), and relative standard deviation expressed in % (RSD%) (output from DDSolver):

Parameter	No.1	No.2	No.3	No.4	Mean	SD	RSD(%)
k_1	4.482	4.960	4.751	5.332	4.881	0.359	7.351
k_2	-0.033	-0.049	-0.037	-0.061	-0.045	0.012	-27.489

Number of dissolution data points (N), degrees of freedom (df), and selected goodness of fit criteria – Pearson correlation coefficient (R), coefficient of determination (R^2), adjusted coefficient of determination ($R^2_{adjusted}$), and residual sum of squares (RSS) (manual calculation in MS Excel):

Parameter	No.1	No.2	No.3	No.4
N	21	21	21	21
df	19	19	19	19
R	0.996617776	0.99364219	0.997166224	0.994493871
R^2	0.993246991	0.987324801	0.994340479	0.989018059
$R^2_{adjusted}$	0.99289157	0.986657686	0.994042609	0.988440062
RSS	115.2206038	223.5932323	106.5338833	199.9754239

Graphical abstract of model fit presented as mean ± 1 SD of the fraction % of released carvedilol:

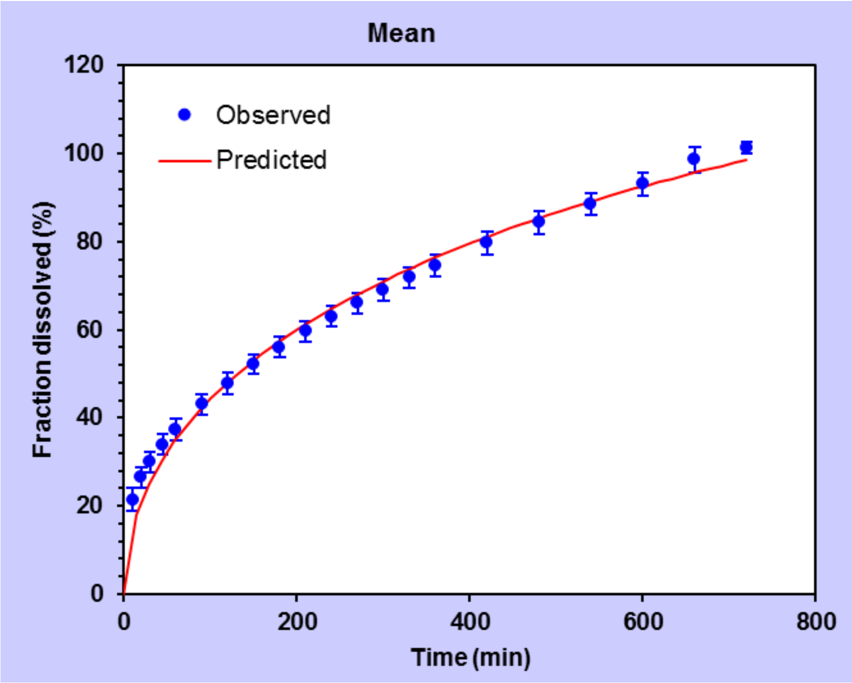

Supplementary materials – Model fitting summary – Lactochem® Crystals (modelling the entire relevant dissolution data)

Graphical abstract of model fit presented as the fraction % of released carvedilol per tested tablet:

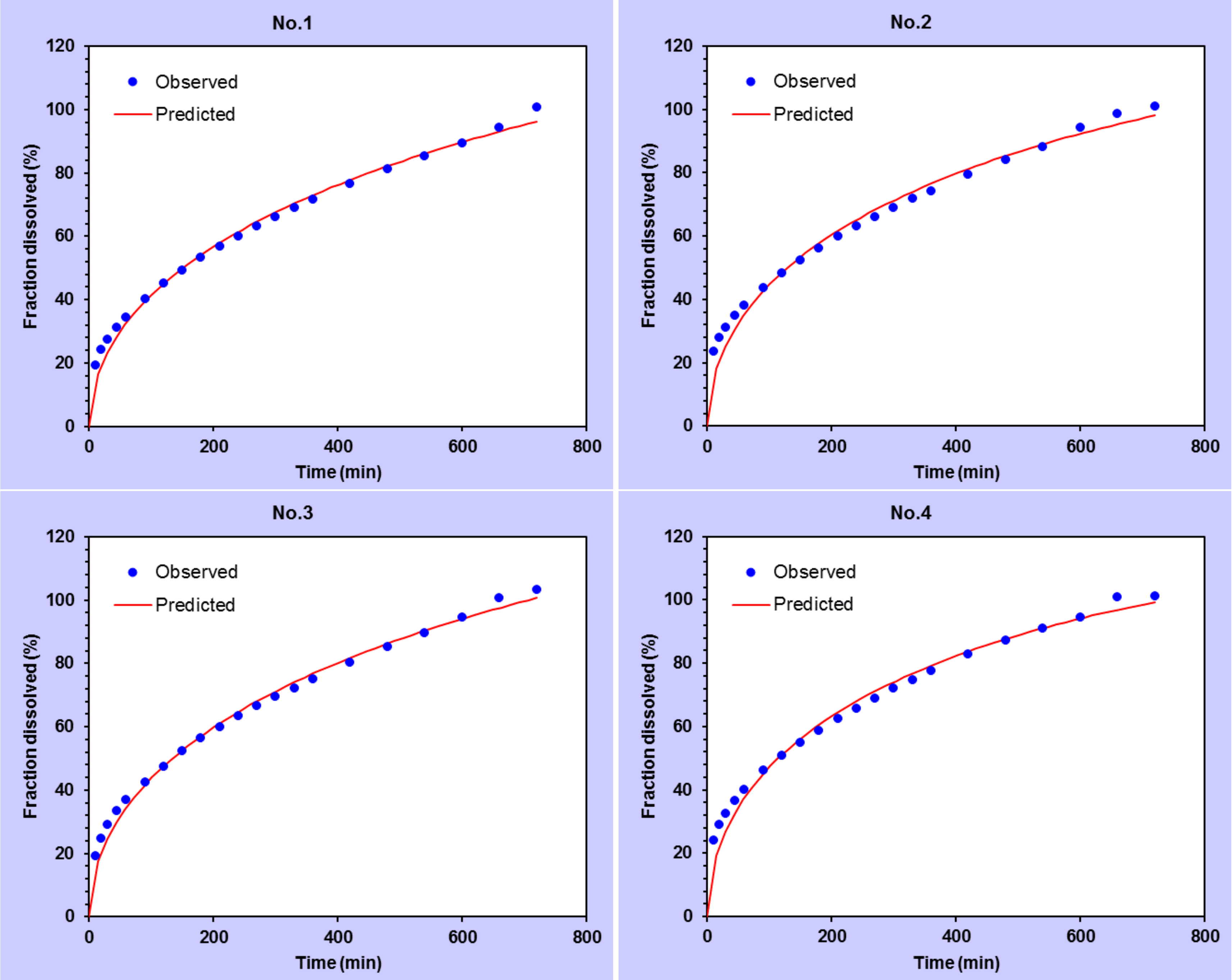

Supplementary materials – Model fitting summary – Lactochem® Crystals (modelling the entire relevant dissolution data)

Model: **Peppas–Sahlin_2 with T_{lag}**

Model equation: $F = k_1 \cdot (t - T_{lag})^{0.5} + k_2 \cdot (t - T_{lag})$

Fitted model parameters per tested tablet (N = 4) with statistics – mean, standard deviation (SD), and relative standard deviation expressed in % (RSD%) (output from DDSolver):

Parameter	No.1	No.2	No.3	No.4	Mean	SD	RSD(%)
k_1	4.610	5.089	4.888	5.470	5.014	0.362	7.216
k_2	-0.038	-0.054	-0.043	-0.066	-0.050	0.012	-24.834
T_{lag}	4.000	4.000	4.000	4.000	4.000	0.000	0.000

Number of dissolution data points (N), degrees of freedom (df), and selected goodness of fit criteria – Pearson correlation coefficient (R), coefficient of determination (R^2), adjusted coefficient of determination ($R^2_{adjusted}$), and residual sum of squares (RSS) (manual calculation in MS Excel):

Parameter	No.1	No.2	No.3	No.4
N	21	21	21	21
df	18	18	18	18
R	0.99469784	0.990889633	0.995440477	0.991792107
R^2	0.989423792	0.981862266	0.990901744	0.983651584
$R^2_{adjusted}$	0.988248658	0.979846962	0.989890827	0.981835093
RSS	196.5153588	347.1934471	187.9892979	325.665157

Graphical abstract of model fit presented as mean ± 1 SD of the fraction % of released carvedilol:

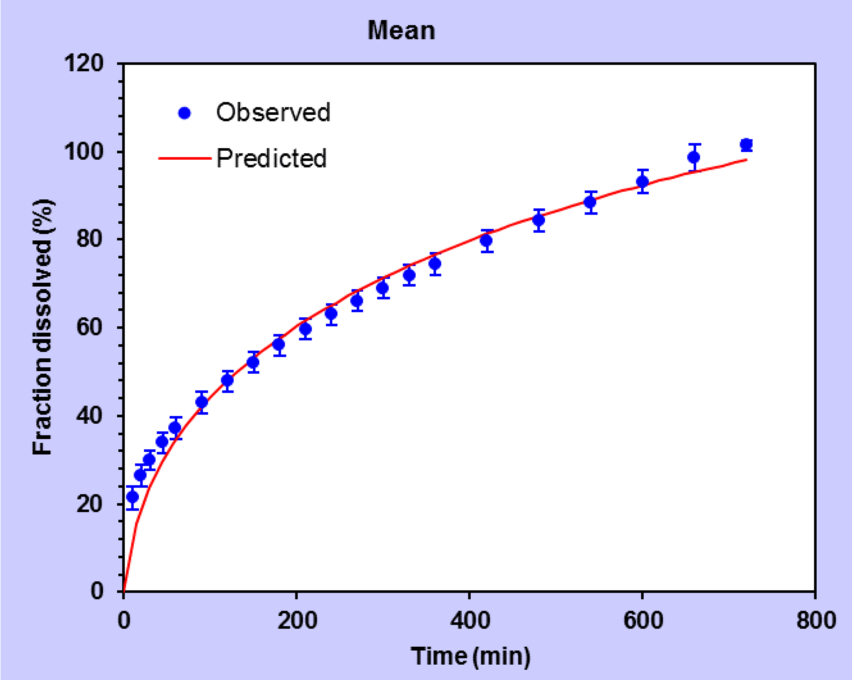

Supplementary materials – Model fitting summary – Lactochem® Crystals (modelling the entire relevant dissolution data)

Graphical abstract of model fit presented as the fraction % of released carvedilol per tested tablet:

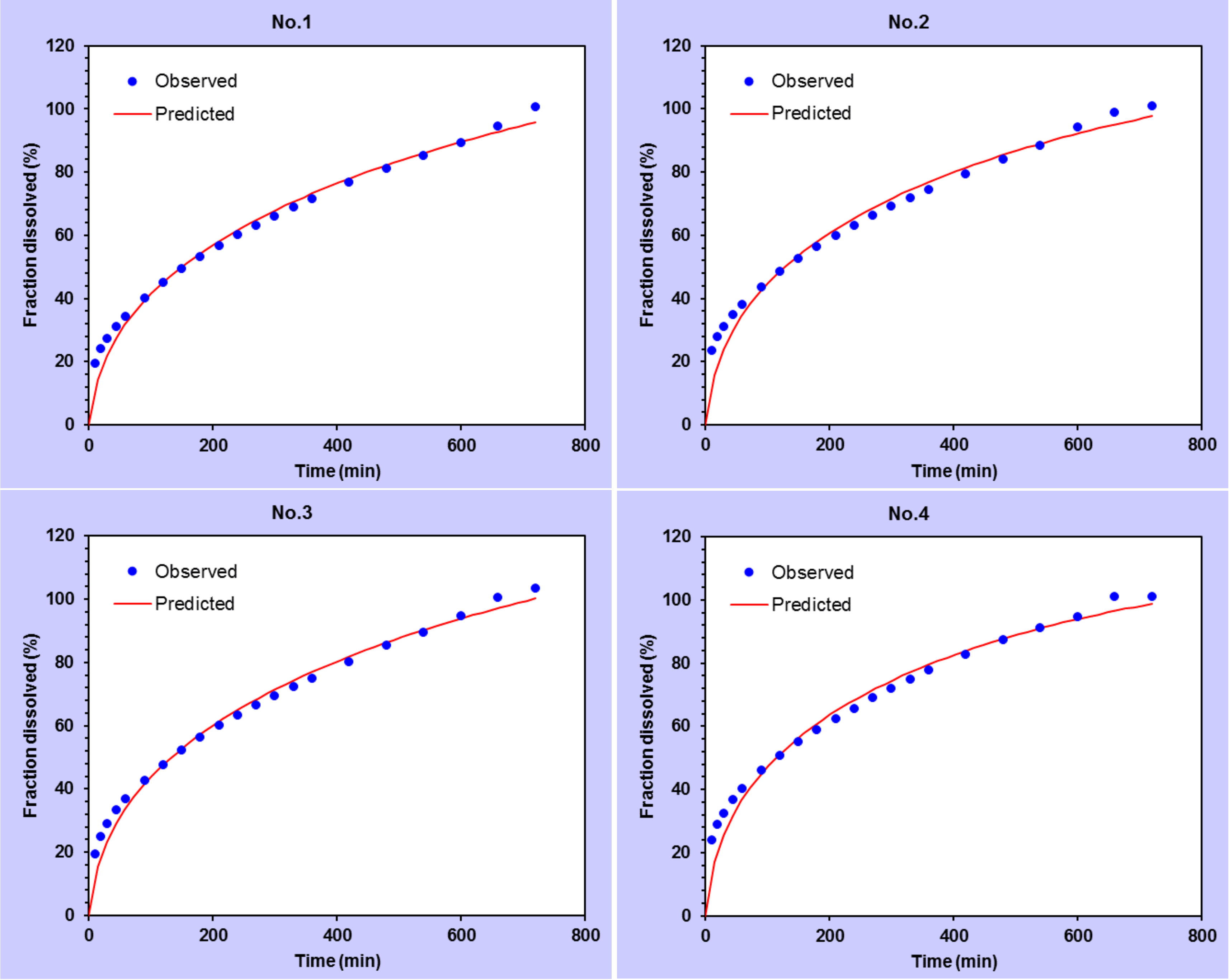

Supplementary materials – Model fitting summary – Lactochem® Crystals (modelling the entire relevant dissolution data)

Model: **Quadratic**

Model equation: $F = 100 \cdot (k_1 \cdot t^2 + k_2 \cdot t)$

Fitted model parameters per tested tablet (N = 4) with statistics – mean, standard deviation (SD), and relative standard deviation expressed in % (RSD%) (output from DDSolver):

Parameter	No.1	No.2	No.3	No.4	Mean	SD	RSD(%)
k_1	-0.000003	-0.000003	-0.000003	-0.000003	-0.000003	0.000000	-7.321823
k_2	0.003127	0.003326	0.003304	0.003512	0.003317	0.000157	4.747420

Number of dissolution data points (N), degrees of freedom (df), and selected goodness of fit criteria – Pearson correlation coefficient (R), coefficient of determination (R^2), adjusted coefficient of determination ($R^2_{adjusted}$), and residual sum of squares (RSS) (manual calculation in MS Excel):

Parameter	No.1	No.2	No.3	No.4
N	21	21	21	21
df	19	19	19	19
R	0.978044511	0.974388444	0.978063833	0.975089477
R^2	0.956571066	0.949432839	0.956608862	0.950799489
$R^2_{adjusted}$	0.954285332	0.94677141	0.954325117	0.948209988
RSS	2383.243184	3141.742548	2608.341351	3391.446016

Graphical abstract of model fit presented as mean ± 1 SD of the fraction % of released carvedilol:

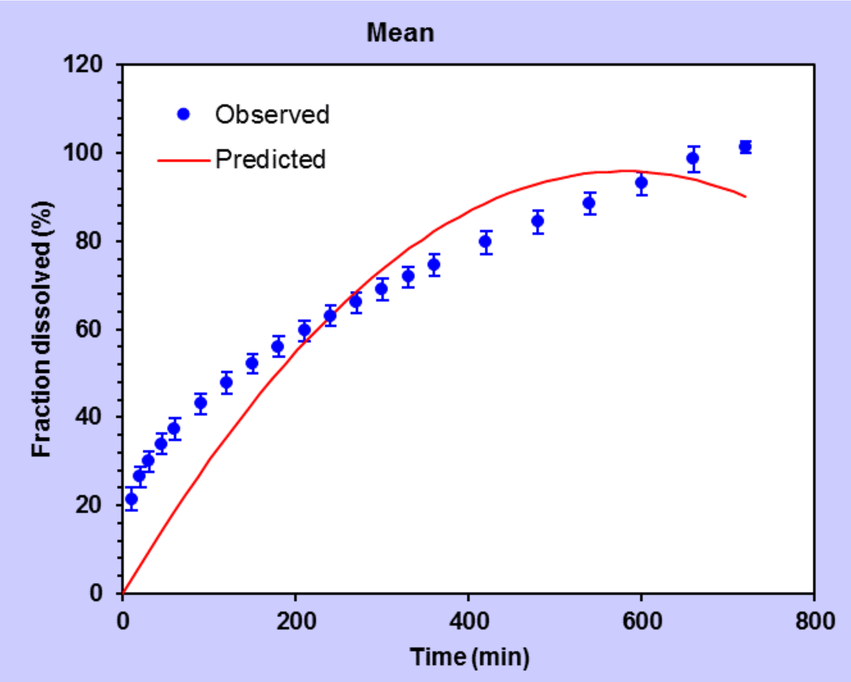

Supplementary materials – Model fitting summary – Lactochem® Crystals (modelling the entire relevant dissolution data)

Graphical abstract of model fit presented as the fraction % of released carvedilol per tested tablet:

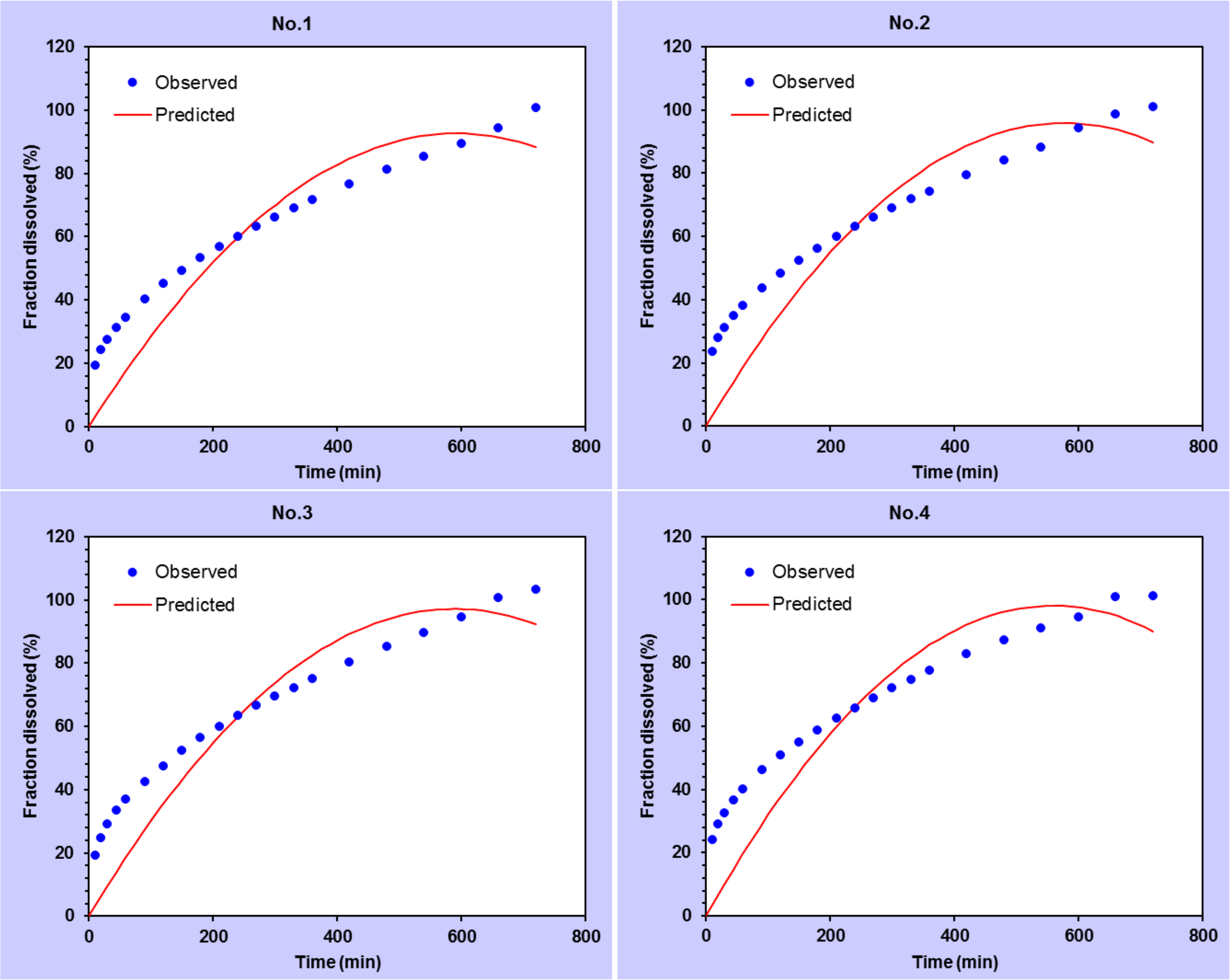

Supplementary materials – Model fitting summary – Lactochem® Crystals (modelling the entire relevant dissolution data)

Model: **Quadratic with T_{lag}**

Model equation: $F = 100 \cdot \left[k_1 \cdot \left(t - T_{lag}\right)^2 + k_2 \cdot \left(t - T_{lag}\right)\right]$

Fitted model parameters per tested tablet (N = 4) with statistics – mean, standard deviation (SD), and relative standard deviation expressed in % (RSD%) (output from DDSolver):

Parameter	No.1	No.2	No.3	No.4	Mean	SD	RSD(%)
k_1	-0.000003	-0.000003	-0.000003	-0.000003	-0.000003	0.000000	-7.228429
k_2	0.003164	0.003363	0.003342	0.003551	0.003355	0.000158	4.712390
T_{lag}	4.000000	4.000000	4.000000	4.000000	4.000000	0.000000	0.000000

Number of dissolution data points (N), degrees of freedom (df), and selected goodness of fit criteria – Pearson correlation coefficient (R), coefficient of determination (R^2), adjusted coefficient of determination ($R^2_{adjusted}$), and residual sum of squares (RSS) (manual calculation in MS Excel):

Parameter	No.1	No.2	No.3	No.4
N	21	21	21	21
df	18	18	18	18
R	0.977510384	0.973933635	0.977540656	0.974703068
R^2	0.955526551	0.948546725	0.955585734	0.950046071
$R^2_{adjusted}$	0.950585057	0.942829695	0.950650816	0.944495634
RSS	2642.441949	3460.435819	2894.050008	3740.923589

Graphical abstract of model fit presented as mean ± 1 SD of the fraction % of released carvedilol:

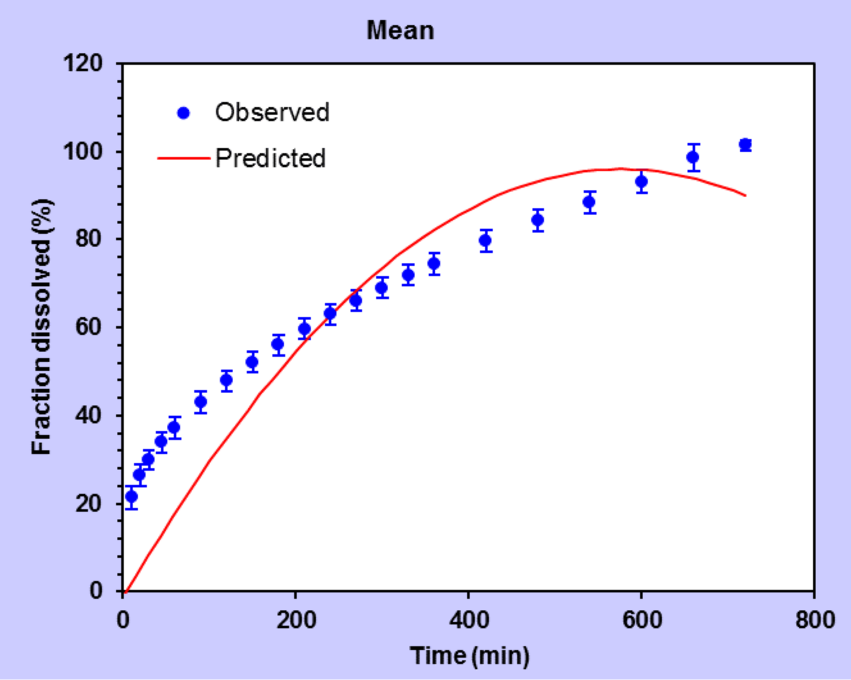

Supplementary materials – Model fitting summary – Lactochem® Crystals (modelling the entire relevant dissolution data)

Graphical abstract of model fit presented as the fraction % of released carvedilol per tested tablet:

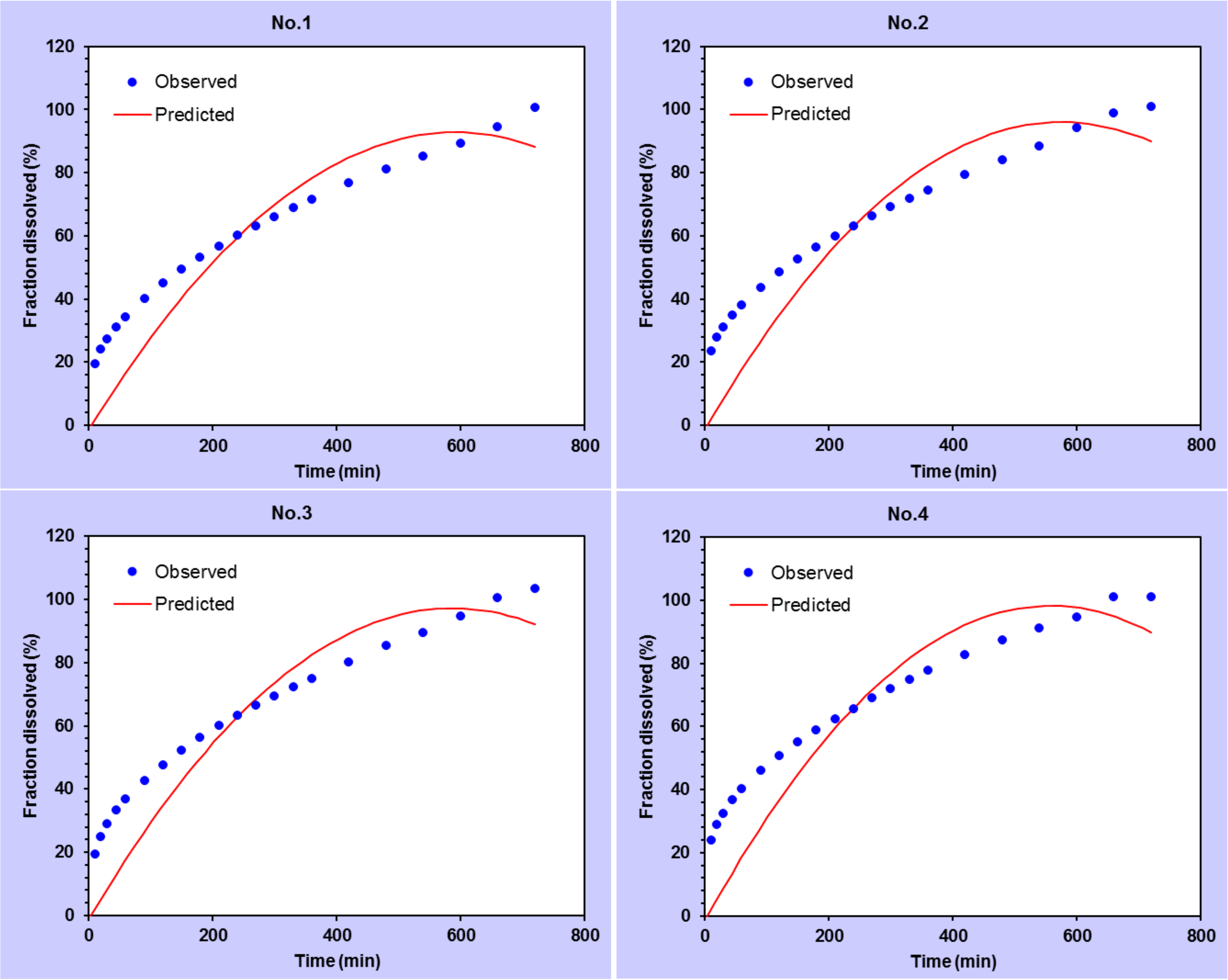

Supplementary materials – Model fitting summary – Lactochem® Crystals (modelling the entire relevant dissolution data)

Model: **Weibull_1**

Model equation: $F = 100 \cdot \left[1 - e^{-\frac{(t-T_i)^\beta}{\alpha}}\right]$

Fitted model parameters per tested tablet (N = 4) with statistics – mean, standard deviation (SD), and relative standard deviation expressed in % (RSD%) (output from DDSolver):

Parameter	No.1	No.2	No.3	No.4	Mean	SD	RSD(%)
α	17.790	15.297	17.183	12.937	15.802	2.185	13.827
β	0.535	0.531	0.542	0.504	0.528	0.017	3.185
T_i	4.000	4.000	4.000	4.000	4.000	0.000	0.000

Number of dissolution data points (N), degrees of freedom (df), and selected goodness of fit criteria – Pearson correlation coefficient (R), coefficient of determination (R^2), adjusted coefficient of determination ($R^2_{adjusted}$), and residual sum of squares (RSS) (manual calculation in MS Excel):

Parameter	No.1	No.2	No.3	No.4
N	21	21	21	21
df	18	18	18	18
R	0.970929537	0.961993323	0.970265223	0.967365136
R^2	0.942704166	0.925431153	0.941414603	0.935795306
$R^2_{adjusted}$	0.936337962	0.917145726	0.934905115	0.928661451
RSS	734.0307615	877.512858	882.0176602	828.7737989

Graphical abstract of model fit presented as mean ± 1 SD of the fraction % of released carvedilol:

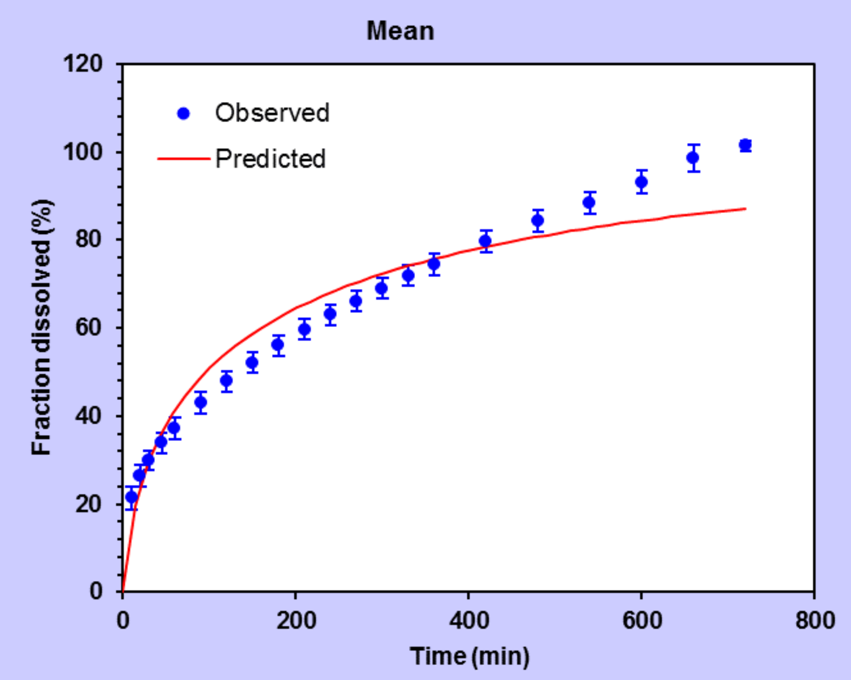

Supplementary materials – Model fitting summary – Lactochem® Crystals (modelling the entire relevant dissolution data)

Graphical abstract of model fit presented as the fraction % of released carvedilol per tested tablet:

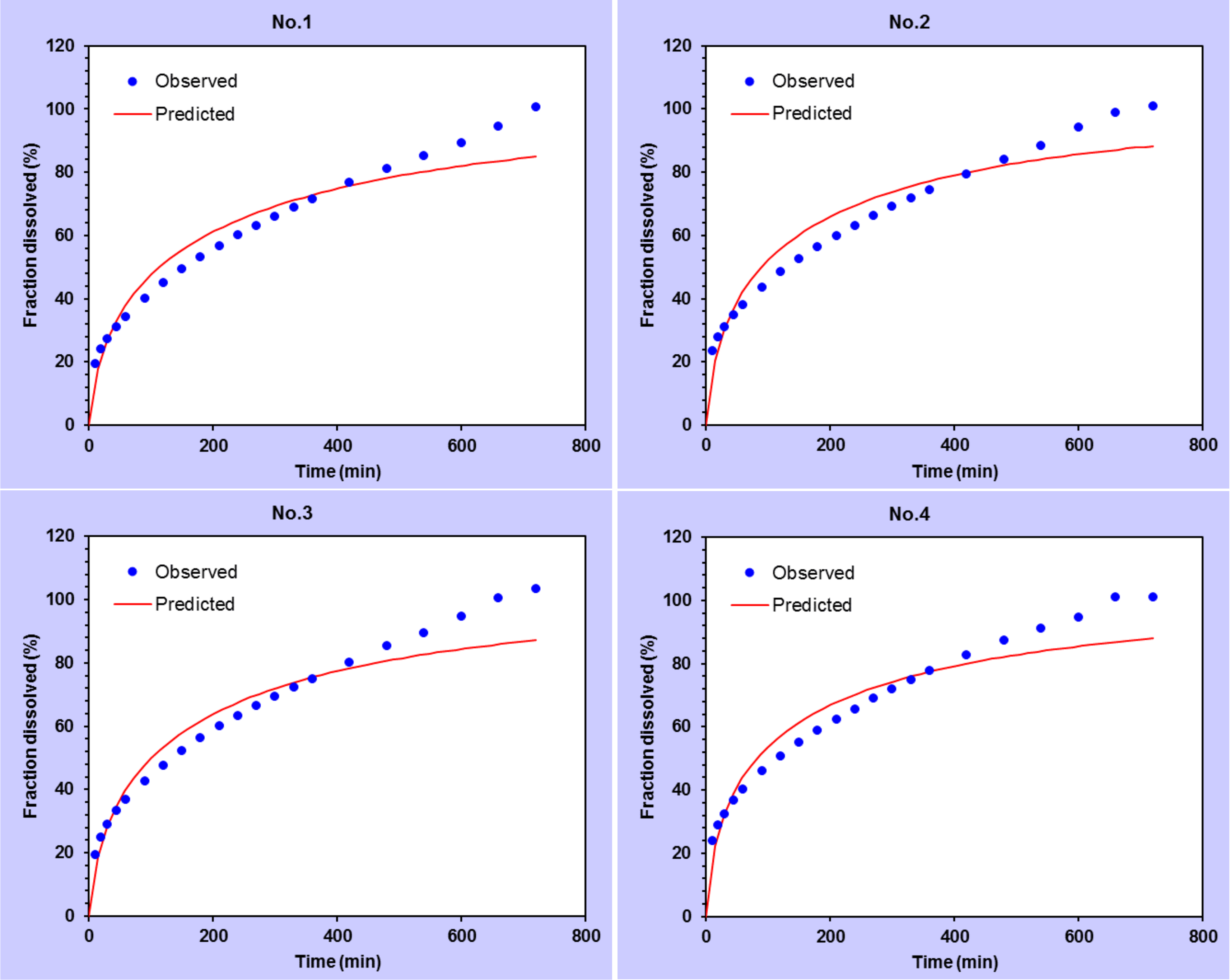

Supplementary materials – Model fitting summary – Lactochem® Crystals (modelling the entire relevant dissolution data)

Model: **Weibull_2**

Model equation: $F = 100 \cdot \left(1 - e^{-\frac{t^{\beta}}{\alpha}}\right)$

Fitted model parameters per tested tablet (N = 4) with statistics – mean, standard deviation (SD), and relative standard deviation expressed in % (RSD%) (output from DDSolver):

Parameter	No.1	No.2	No.3	No.4	Mean	SD	RSD(%)
α	23.761	20.546	23.140	17.143	21.147	3.011	14.239
β	0.585	0.582	0.594	0.553	0.579	0.018	3.077

Number of dissolution data points (N), degrees of freedom (df), and selected goodness of fit criteria – Pearson correlation coefficient (R), coefficient of determination (R^2), adjusted coefficient of determination ($R^2_{adjusted}$), and residual sum of squares (RSS) (manual calculation in MS Excel):

Parameter	No.1	No.2	No.3	No.4
N	21	21	21	21
df	19	19	19	19
R	0.976714846	0.968700554	0.975718236	0.973807817
R^2	0.953971891	0.938380764	0.952026076	0.948301665
$R^2_{adjusted}$	0.951549359	0.935137646	0.949501133	0.9455807
RSS	577.7804833	718.0560118	707.7593067	657.764027

Graphical abstract of model fit presented as mean ± 1 SD of the fraction % of released carvedilol:

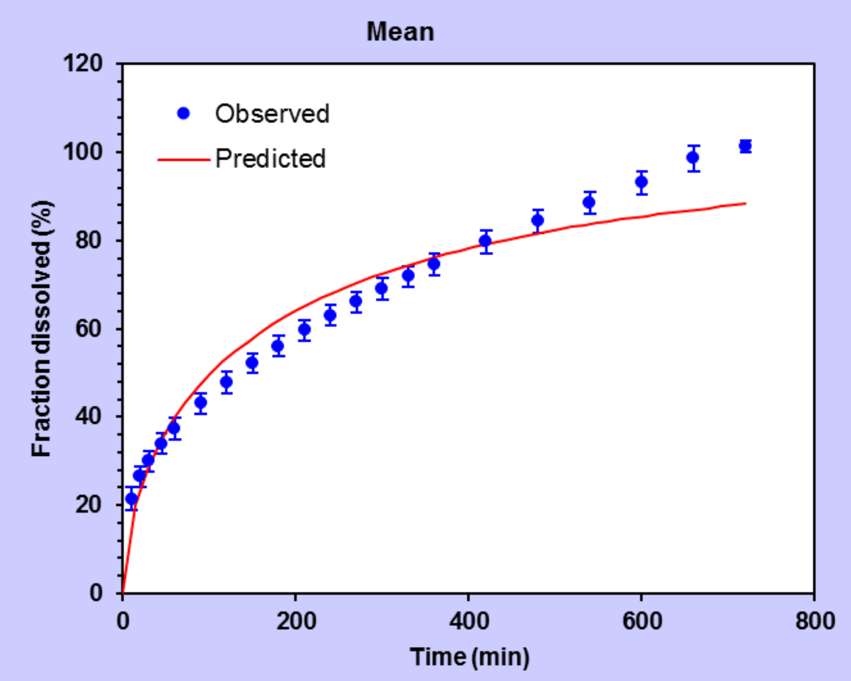

Supplementary materials – Model fitting summary – Lactochem® Crystals (modelling the entire relevant dissolution data)

Graphical abstract of model fit presented as the fraction % of released carvedilol per tested tablet:

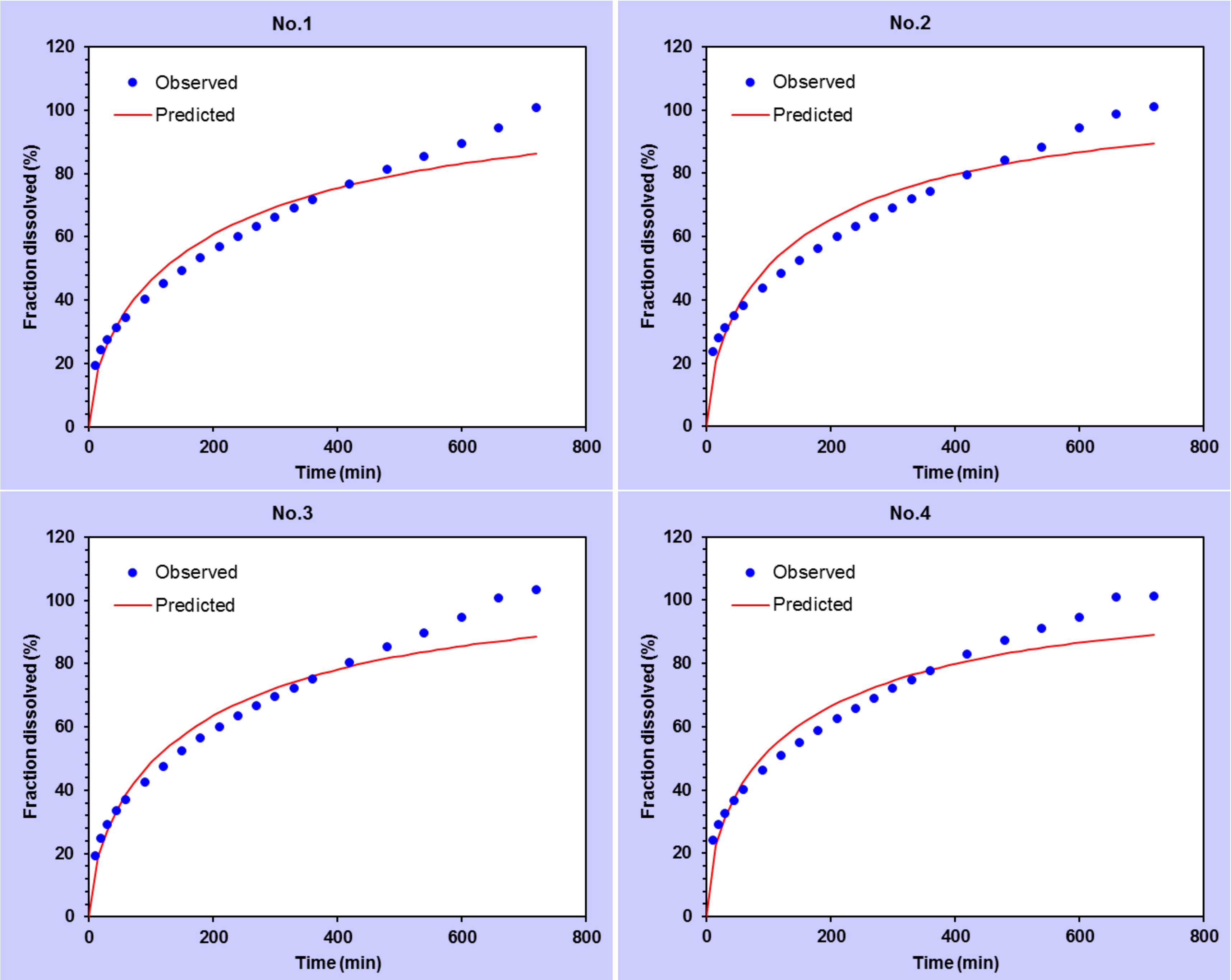

Supplementary materials – Model fitting summary – Lactochem® Crystals (modelling the entire relevant dissolution data)

Model: **Weibull_3**

Model equation: $F = F_{max} \cdot \left(1 - e^{-\frac{t^{\beta}}{\alpha}}\right)$

Fitted model parameters per tested tablet (N = 4) with statistics – mean, standard deviation (SD), and relative standard deviation expressed in % (RSD%) (output from DDSolver):

Parameter	No.1	No.2	No.3	No.4	Mean	SD	RSD(%)
α	25.623	26.422	30.733	25.652	27.108	2.445	9.020
β	0.585	0.561	0.596	0.568	0.578	0.016	2.742
F_{max}	105.599	114.327	122.506	114.465	114.224	6.906	6.046

Number of dissolution data points (N), degrees of freedom (df), and selected goodness of fit criteria – Pearson correlation coefficient (R), coefficient of determination (R^2), adjusted coefficient of determination ($R^2_{adjusted}$), and residual sum of squares (RSS) (manual calculation in MS Excel):

Parameter	No.1	No.2	No.3	No.4
N	21	21	21	21
df	18	18	18	18
R	0.980007913	0.984211274	0.986738303	0.987483188
R^2	0.96041551	0.968671832	0.973652479	0.975123047
$R^2_{adjusted}$	0.956017234	0.965190924	0.970724977	0.972358941
RSS	483.7407281	563.7295371	505.2034525	461.2821267

Graphical abstract of model fit presented as mean ± 1 SD of the fraction % of released carvedilol:

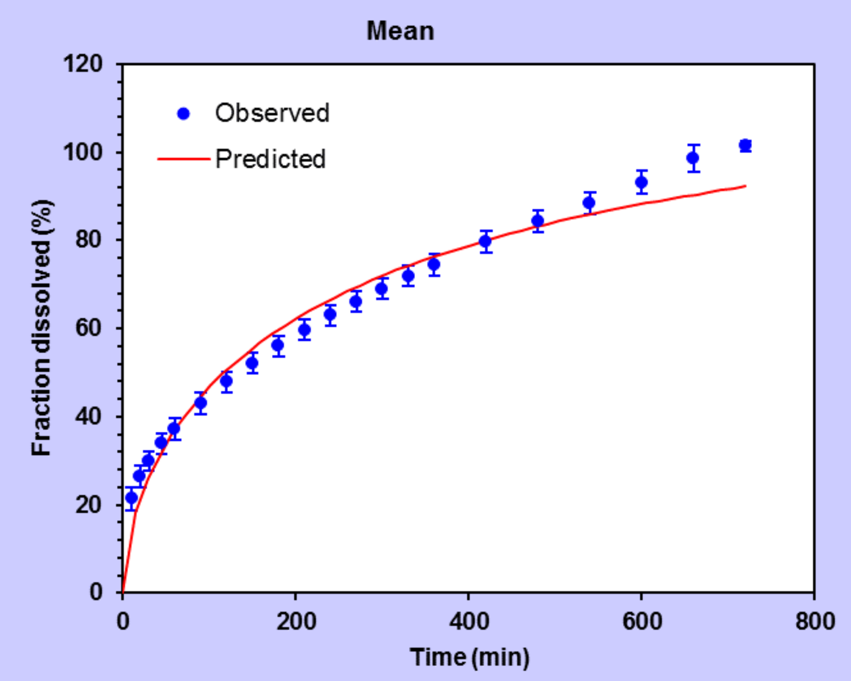

Supplementary materials – Model fitting summary – Lactochem® Crystals (modelling the entire relevant dissolution data)

Graphical abstract of model fit presented as the fraction % of released carvedilol per tested tablet:

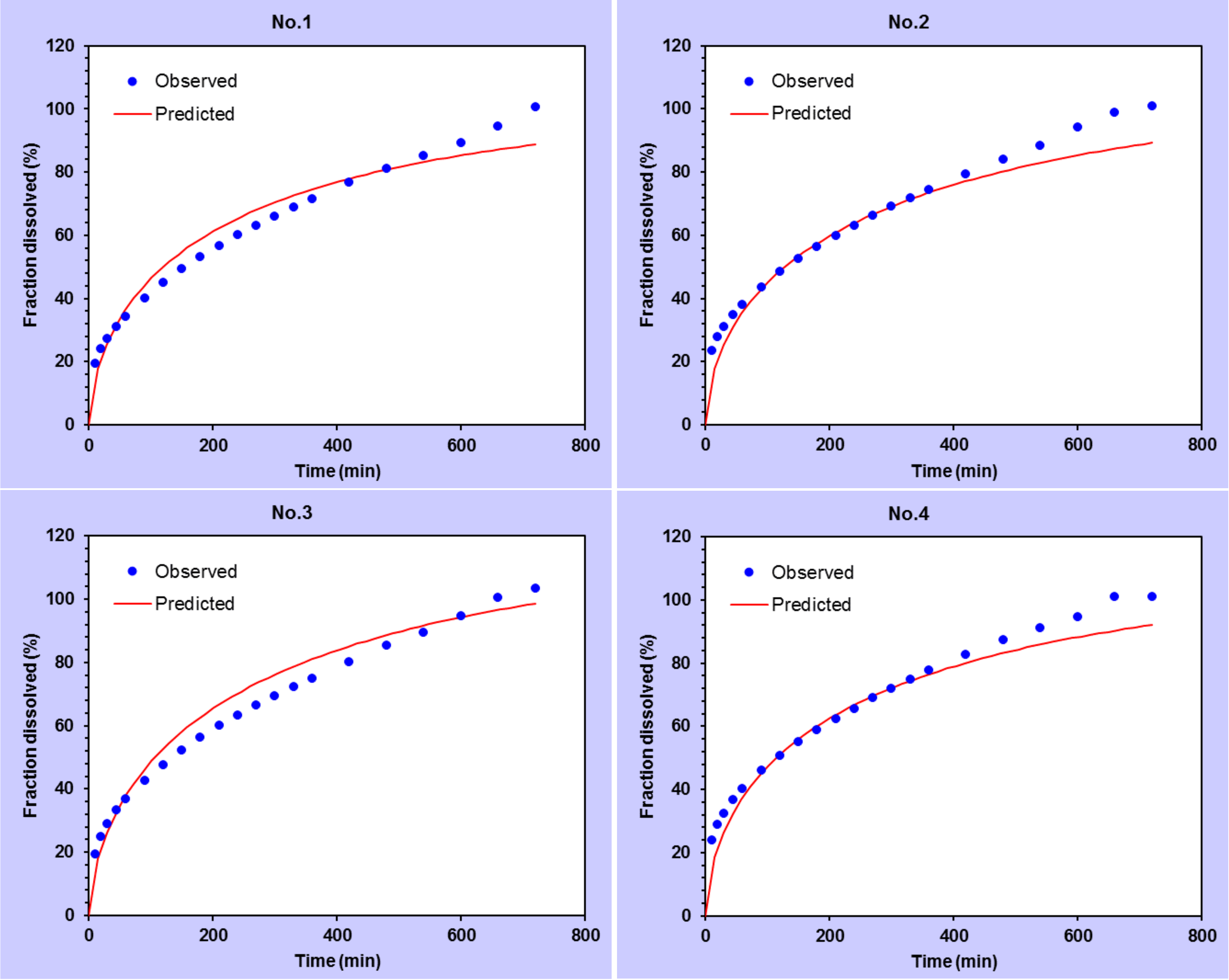

Supplementary materials – Model fitting summary – Lactochem® Crystals (modelling the entire relevant dissolution data)

Model: **Weibull_4**

Model equation: $F = F_{max} \cdot \left[1 - e^{-\frac{(t-T_i)^{\beta}}{\alpha}}\right]$

Fitted model parameters per tested tablet (N = 4) with statistics – mean, standard deviation (SD), and relative standard deviation expressed in % (RSD%) (output from DDSolver):

Parameter	No.1	No.2	No.3	No.4	Mean	SD	RSD(%)
α	19.299	15.503	20.035	15.029	17.466	2.566	14.690
β	0.537	0.513	0.552	0.520	0.531	0.018	3.298
T_i	4.000	4.000	4.000	4.000	4.000	0.000	0.000
F_{max}	105.599	105.910	108.402	106.038	106.487	1.290	1.211

Number of dissolution data points (N), degrees of freedom (df), and selected goodness of fit criteria – Pearson correlation coefficient (R), coefficient of determination (R^2), adjusted coefficient of determination ($R^2_{adjusted}$), and residual sum of squares (RSS) (manual calculation in MS Excel):

Parameter	No.1	No.2	No.3	No.4
N	21	21	21	21
df	17	17	17	17
R	0.974529724	0.967580708	0.975663449	0.971579112
R^2	0.949708182	0.936212426	0.951919166	0.94396597
$R^2_{adjusted}$	0.940833155	0.924955795	0.943434313	0.934077612
RSS	623.3218857	758.5834282	652.7278522	676.9685496

Graphical abstract of model fit presented as mean ± 1 SD of the fraction % of released carvedilol:

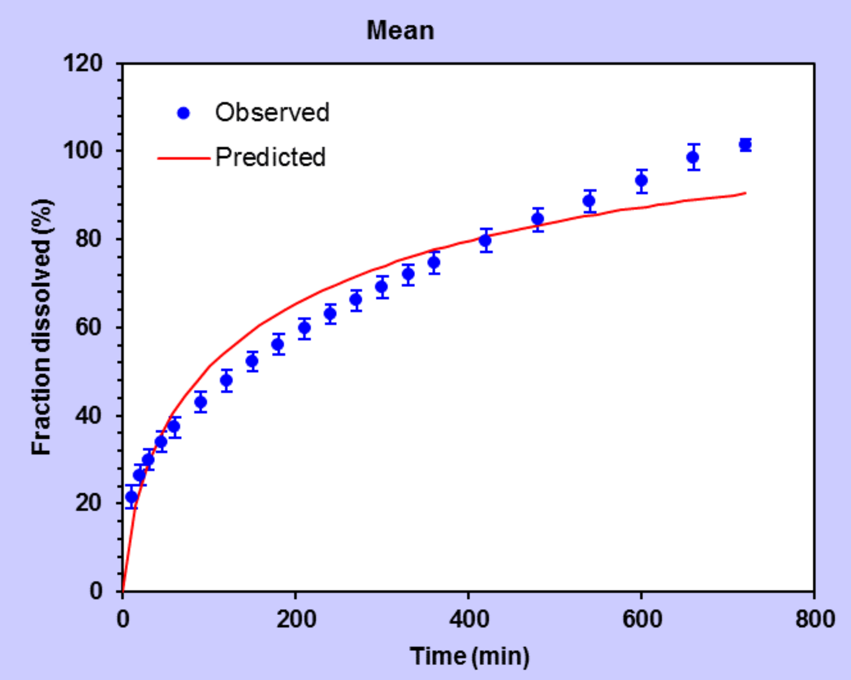

Supplementary materials – Model fitting summary – Lactochem® Crystals (modelling the entire relevant dissolution data)

Graphical abstract of model fit presented as the fraction % of released carvedilol per tested tablet:

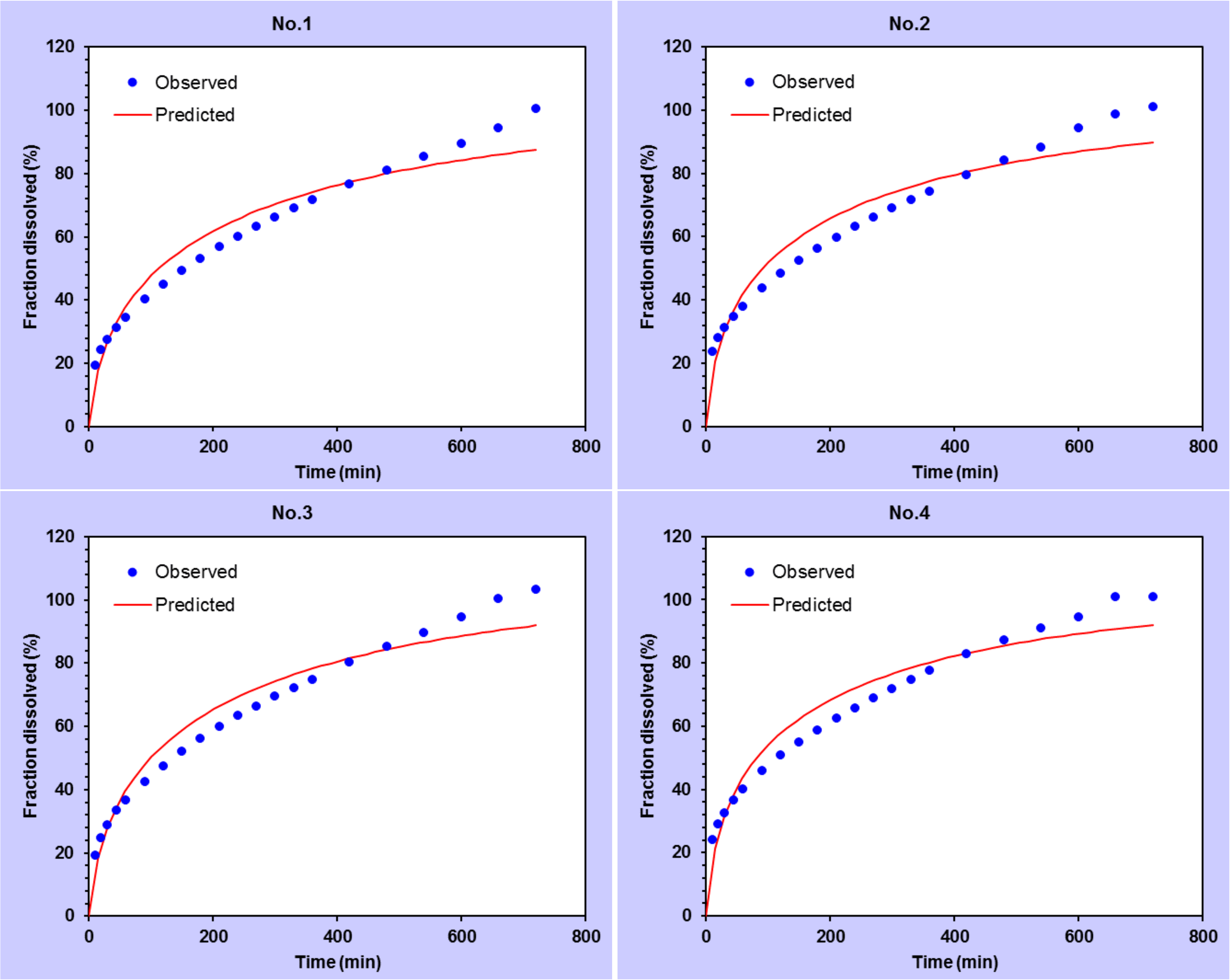

Supplementary materials – Model fitting summary – Lactochem® Crystals (modelling the entire relevant dissolution data)

Model: **Logistic_1**

Model equation: $F = 100 \cdot \frac{e^{\alpha+\beta\cdot\log(t)}}{1+e^{\alpha+\beta\cdot\log(t)}}$

Fitted model parameters per tested tablet (N = 4) with statistics – mean, standard deviation (SD), and relative standard deviation expressed in % (RSD%) (output from DDSolver):

Parameter	No.1	No.2	No.3	No.4	Mean	SD	RSD(%)
α	-4.074	-4.255	-4.063	-3.772	-4.041	0.200	-4.941
β	2.022	2.219	2.066	1.997	2.076	0.099	4.777

Number of dissolution data points (N), degrees of freedom (df), and selected goodness of fit criteria – Pearson correlation coefficient (R), coefficient of determination (R^2), adjusted coefficient of determination ($R^2_{adjusted}$), and residual sum of squares (RSS) (manual calculation in MS Excel):

Parameter	No.1	No.2	No.3	No.4
N	21	21	21	21
df	19	19	19	19
R	0.954215936	0.938803206	0.952080277	0.94851785
R^2	0.910528052	0.881351459	0.906456854	0.899686112
$R^2_{adjusted}$	0.905819002	0.875106799	0.901533531	0.894406434
RSS	1056.059601	1503.711414	1214.767297	1152.842455

Graphical abstract of model fit presented as mean ± 1 SD of the fraction % of released carvedilol:

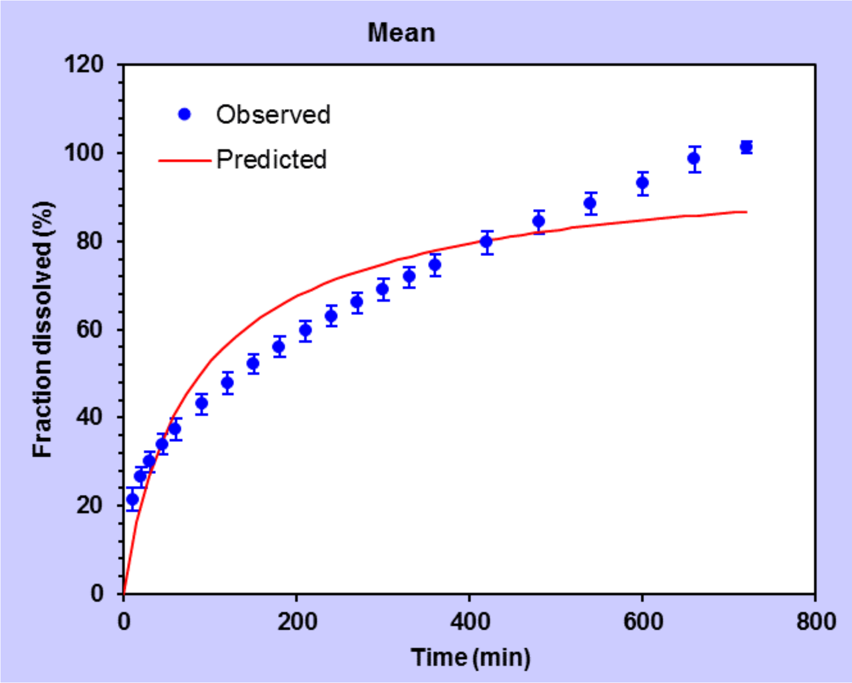

Supplementary materials – Model fitting summary – Lactochem® Crystals (modelling the entire relevant dissolution data)

Graphical abstract of model fit presented as the fraction % of released carvedilol per tested tablet:

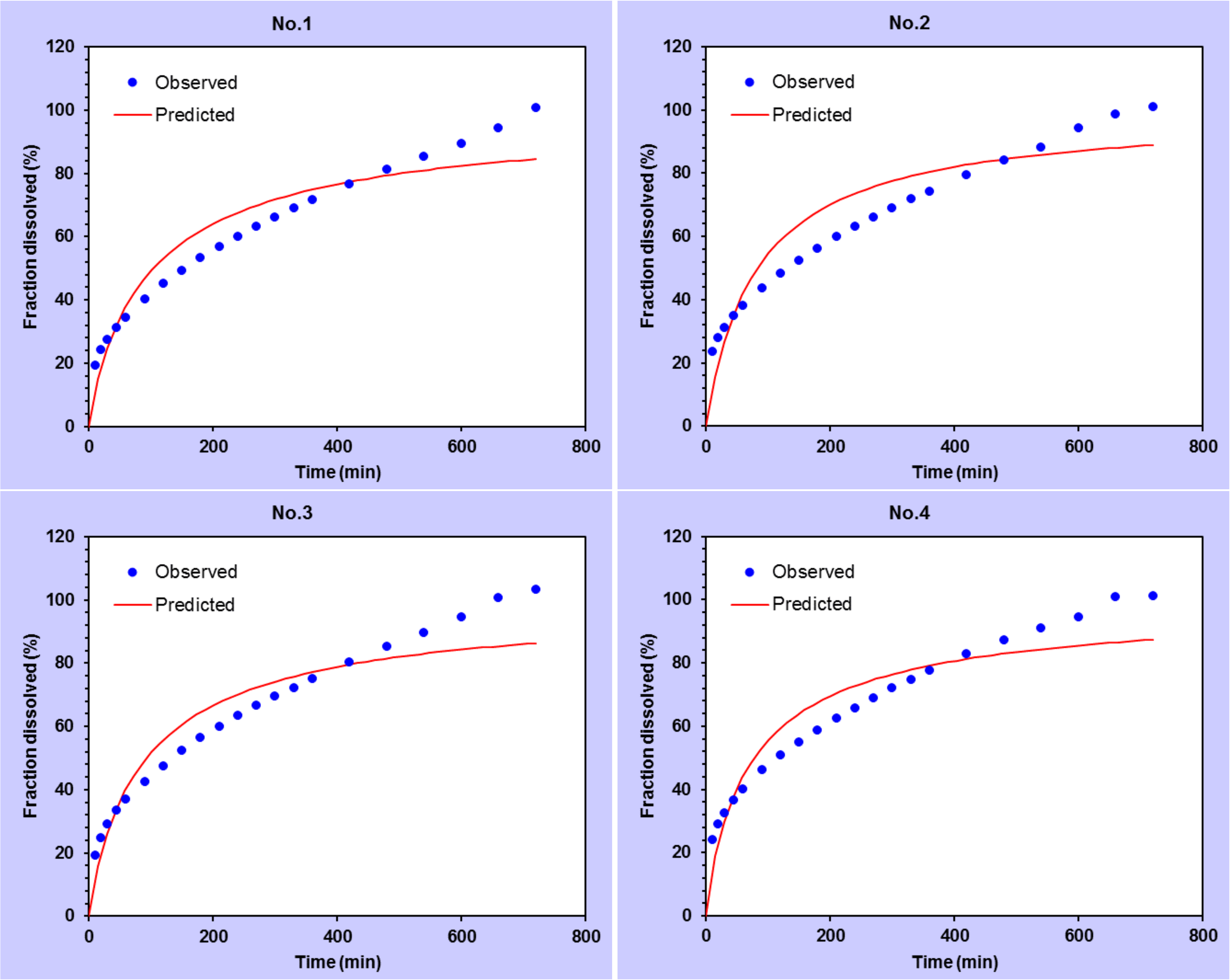

Supplementary materials – Model fitting summary – Lactochem® Crystals (modelling the entire relevant dissolution data)

Model: **Logistic_2**

Model equation: $F = F_{max} \cdot \frac{e^{\alpha+\beta \cdot \log(t)}}{1+e^{\alpha+\beta \cdot \log(t)}}$

Fitted model parameters per tested tablet (N = 4) with statistics – mean, standard deviation (SD), and relative standard deviation expressed in % (RSD%) (output from DDSolver):

Parameter	No.1	No.2	No.3	No.4	Mean	SD	RSD(%)
α	-4.446	-4.434	-4.286	-4.485	-4.413	0.087	-1.977
β	1.666	1.824	2.103	1.887	1.870	0.181	9.675
F_{max}	152.042	131.475	108.402	131.634	130.888	17.833	13.624

Number of dissolution data points (N), degrees of freedom (df), and selected goodness of fit criteria – Pearson correlation coefficient (R), coefficient of determination (R^2), adjusted coefficient of determination ($R^2_{adjusted}$), and residual sum of squares (RSS) (manual calculation in MS Excel):

Parameter	No.1	No.2	No.3	No.4
N	21	21	21	21
df	18	18	18	18
R	0.991077302	0.983671672	0.959368983	0.986844672
R^2	0.982234218	0.967609958	0.920388846	0.973862406
$R^2_{adjusted}$	0.980260242	0.964011065	0.911543162	0.970958229
RSS	804.1366399	1028.238558	1145.710493	941.6781438

Graphical abstract of model fit presented as mean ± 1 SD of the fraction % of released carvedilol:

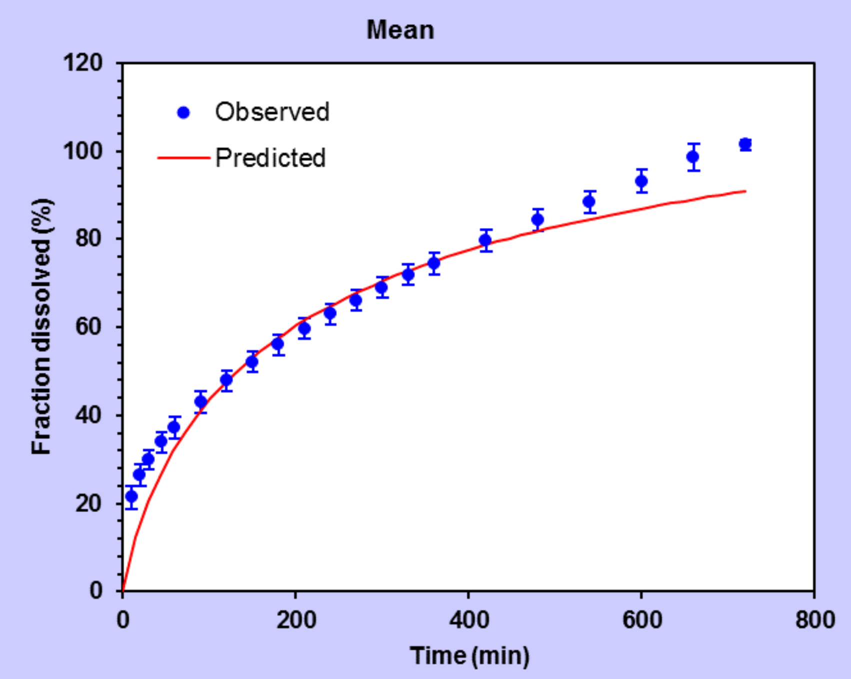

Supplementary materials – Model fitting summary – Lactochem® Crystals (modelling the entire relevant dissolution data)

Graphical abstract of model fit presented as the fraction % of released carvedilol per tested tablet:

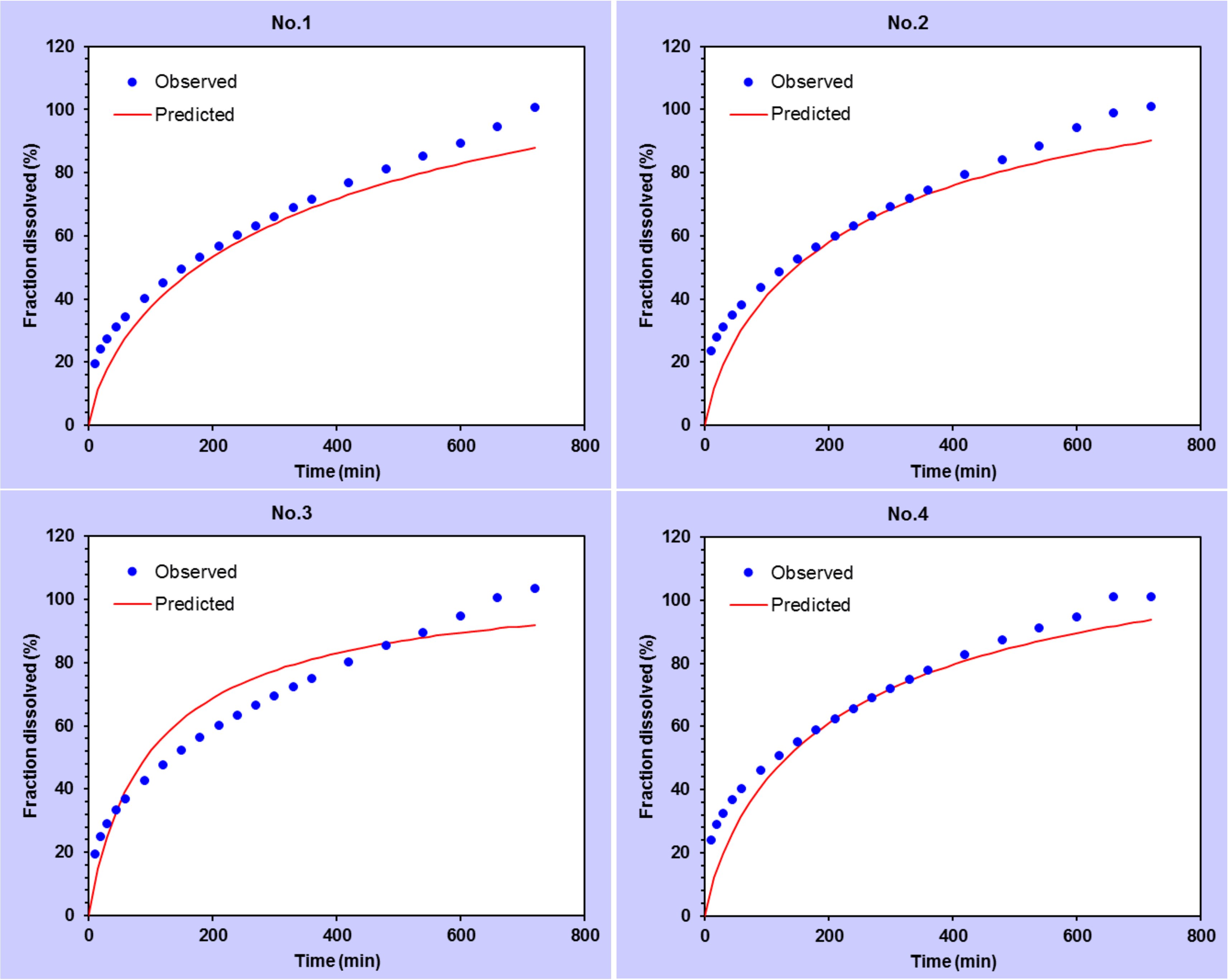

Supplementary materials – Model fitting summary – Lactochem® Crystals (modelling the entire relevant dissolution data)

Model: **Logistic_3**

Model equation: $F = F_{max} \cdot \frac{1}{1+e^{-k\cdot(t-\gamma)}}$

Fitted model parameters per tested tablet (N = 4) with statistics – mean, standard deviation (SD), and relative standard deviation expressed in % (RSD%) (output from DDSolver):

Parameter	No.1	No.2	No.3	No.4	Mean	SD	RSD(%)
k	0.005	0.005	0.005	0.005	0.005	0.000	2.342
γ	213.926	184.872	202.397	167.039	192.059	20.516	10.682
F_{max}	105.599	105.910	108.402	106.038	106.487	1.290	1.211

Number of dissolution data points (N), degrees of freedom (df), and selected goodness of fit criteria – Pearson correlation coefficient (R), coefficient of determination (R^2), adjusted coefficient of determination ($R^2_{adjusted}$), and residual sum of squares (RSS) (manual calculation in MS Excel):

Parameter	No.1	No.2	No.3	No.4
N	21	21	21	21
df	18	18	18	18
R	0.988608854	0.99202968	0.988382486	0.991525881
R^2	0.977347466	0.984122887	0.976899938	0.983123573
$R^2_{adjusted}$	0.974830517	0.982358763	0.974333264	0.981248414
RSS	267.6699602	182.4732223	300.0942197	194.7346541

Graphical abstract of model fit presented as mean ± 1 SD of the fraction % of released carvedilol:

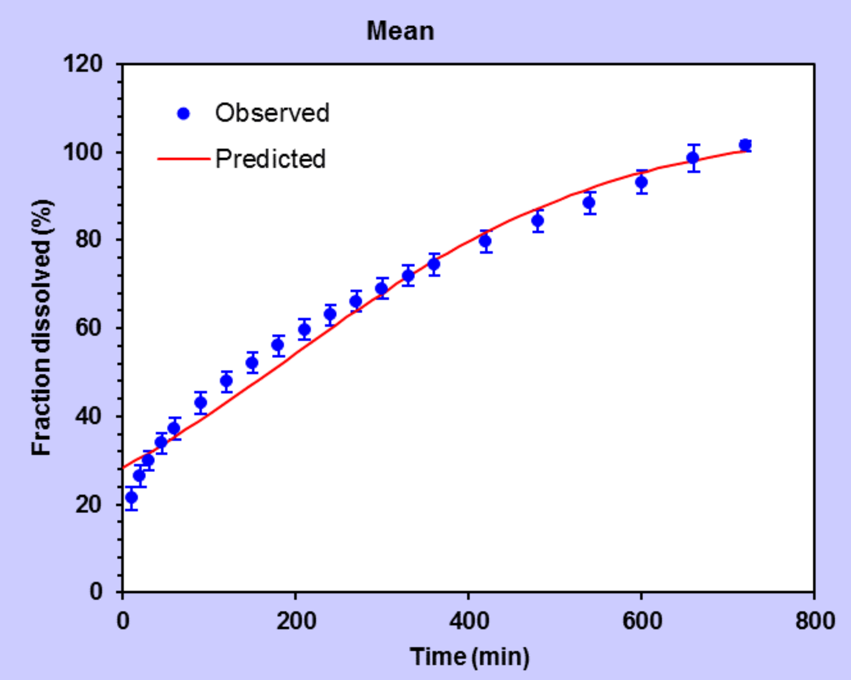

Supplementary materials – Model fitting summary – Lactochem® Crystals (modelling the entire relevant dissolution data)

Graphical abstract of model fit presented as the fraction % of released carvedilol per tested tablet:

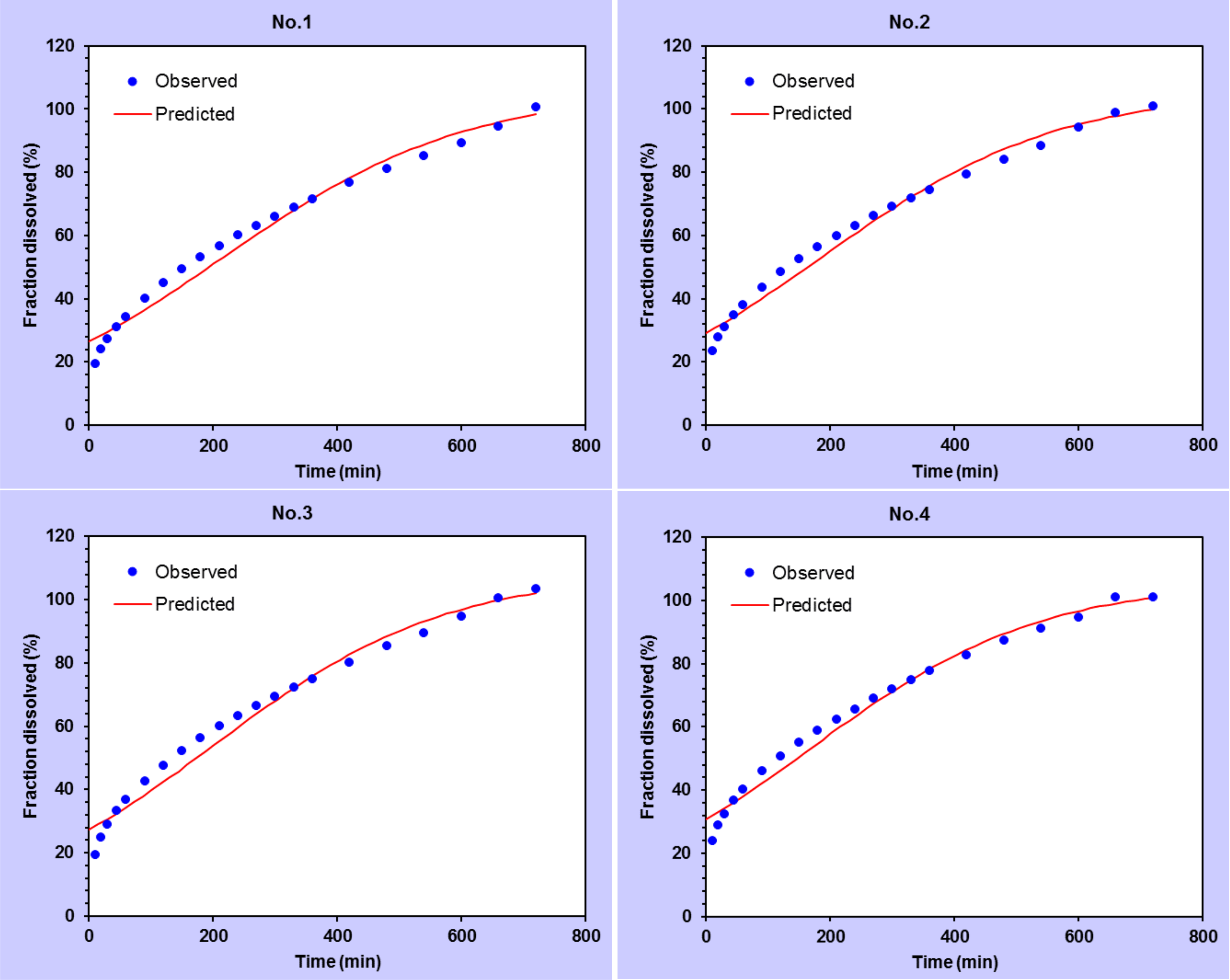

Supplementary materials – Model fitting summary – Lactochem® Crystals (modelling the entire relevant dissolution data)

Model: **Gompertz_1**

Model equation: $F = 100 \cdot e^{-\alpha \cdot e^{-\beta \cdot \log(t)}}$

Fitted model parameters per tested tablet (N = 4) with statistics – mean, standard deviation (SD), and relative standard deviation expressed in % (RSD%) (output from DDSolver):

Parameter	No.1	No.2	No.3	No.4	Mean	SD	RSD(%)
α	13.699	19.137	13.790	12.279	14.726	3.021	20.511
β	1.520	1.768	1.561	1.556	1.601	0.113	7.045

Number of dissolution data points (N), degrees of freedom (df), and selected goodness of fit criteria – Pearson correlation coefficient (R), coefficient of determination (R^2), adjusted coefficient of determination ($R^2_{adjusted}$), and residual sum of squares (RSS) (manual calculation in MS Excel):

Parameter	No.1	No.2	No.3	No.4
N	21	21	21	21
df	19	19	19	19
R	0.929451101	0.911896943	0.927863901	0.923840163
R^2	0.86387935	0.831556035	0.86093142	0.853480646
$R^2_{adjusted}$	0.856715105	0.822690563	0.853612021	0.845769101
RSS	1637.21844	2331.742187	1771.978847	1700.703215

Graphical abstract of model fit presented as mean ± 1 SD of the fraction % of released carvedilol:

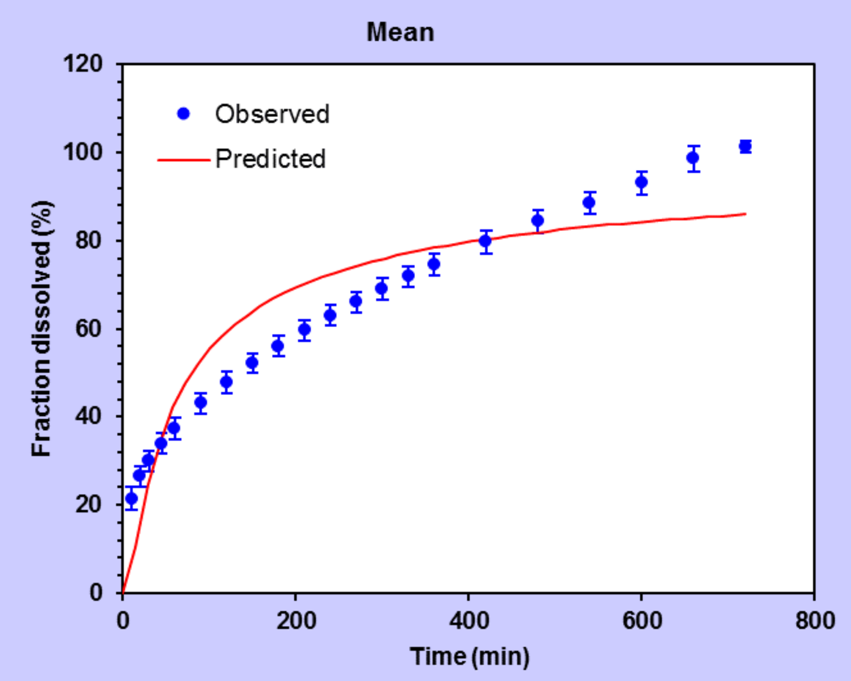

Supplementary materials – Model fitting summary – Lactochem® Crystals (modelling the entire relevant dissolution data)

Graphical abstract of model fit presented as the fraction % of released carvedilol per tested tablet:

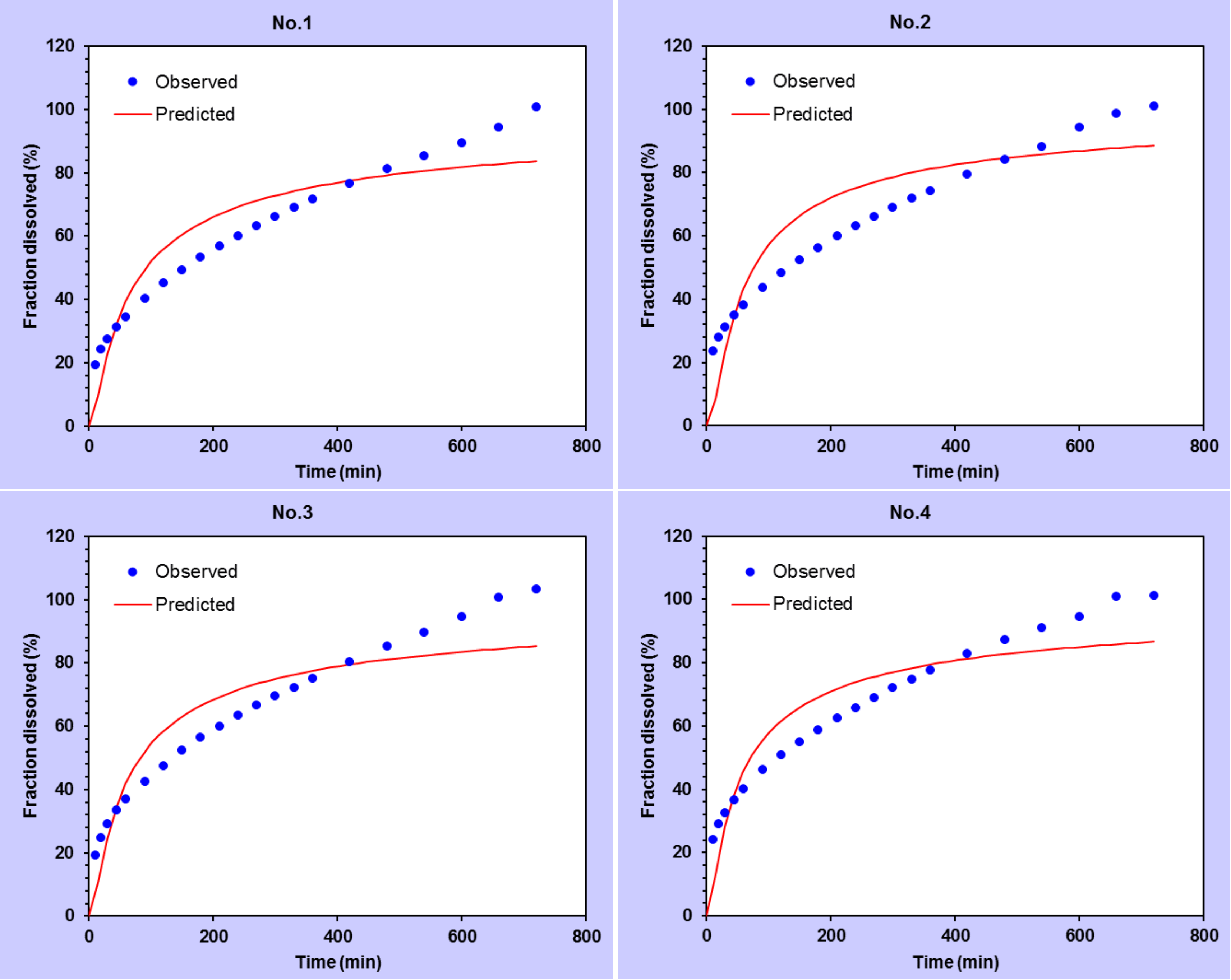

Supplementary materials – Model fitting summary – Lactochem® Crystals (modelling the entire relevant dissolution data)

Model: **Gompertz_2**

Model equation: $F = F_{max} \cdot e^{-\alpha \cdot e^{-\beta \cdot \log(t)}}$

Fitted model parameters per tested tablet (N = 4) with statistics – mean, standard deviation (SD), and relative standard deviation expressed in % (RSD%) (output from DDSolver):

Parameter	No.1	No.2	No.3	No.4	Mean	SD	RSD(%)
α	14.238	14.320	16.192	15.548	15.074	0.956	6.342
β	1.502	1.559	1.589	1.636	1.571	0.056	3.587
F_{max}	105.599	105.910	108.402	106.038	106.487	1.290	1.211

Number of dissolution data points (N), degrees of freedom (df), and selected goodness of fit criteria – Pearson correlation coefficient (R), coefficient of determination (R^2), adjusted coefficient of determination ($R^2_{adjusted}$), and residual sum of squares (RSS) (manual calculation in MS Excel):

Parameter	No.1	No.2	No.3	No.4
N	21	21	21	21
df	18	18	18	18
R	0.935478018	0.924423401	0.935713117	0.928860003
R^2	0.875119123	0.854558624	0.875559038	0.862780905
$R^2_{adjusted}$	0.86124347	0.838398471	0.861732265	0.847534339
RSS	1696.675753	2008.760103	1910.888295	1979.515714

Graphical abstract of model fit presented as mean ± 1 SD of the fraction % of released carvedilol:

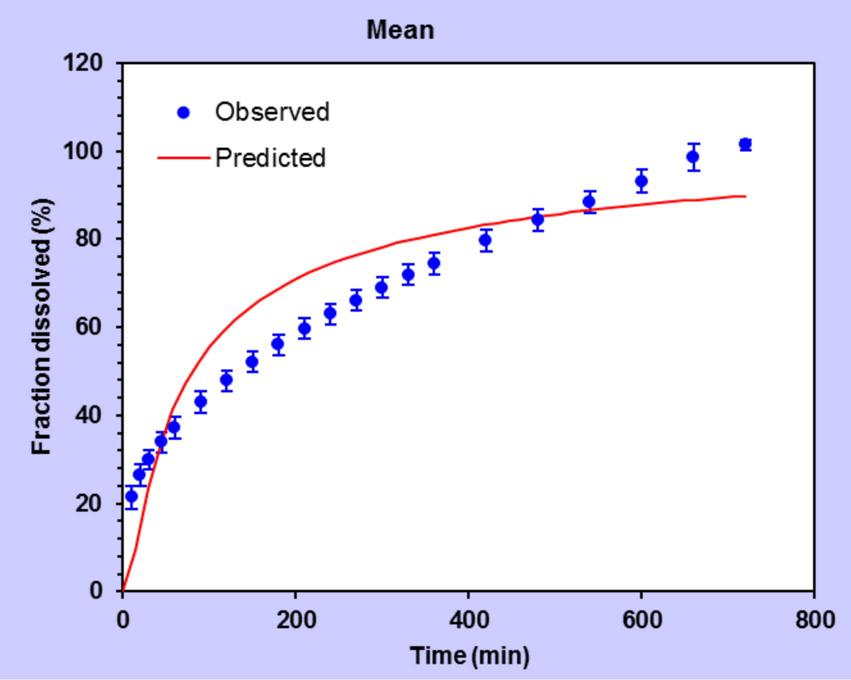

Supplementary materials – Model fitting summary – Lactochem® Crystals (modelling the entire relevant dissolution data)

Graphical abstract of model fit presented as the fraction % of released carvedilol per tested tablet:

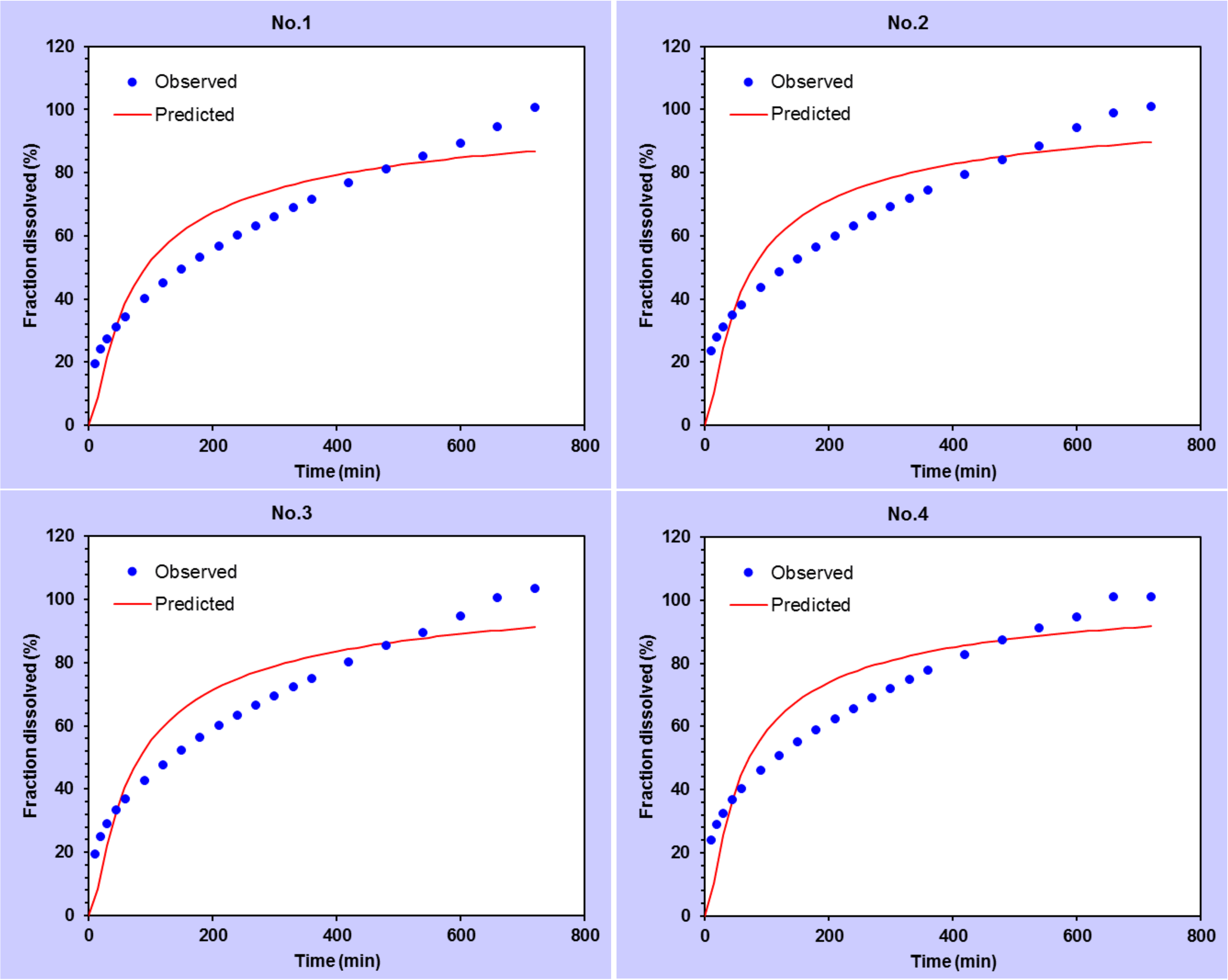

Supplementary materials – Model fitting summary – Lactochem® Crystals (modelling the entire relevant dissolution data)

Model: **Gompertz_3**

Model equation: $F = F_{max} \cdot e^{-e^{-k \cdot (t-\gamma)}}$

Fitted model parameters per tested tablet (N = 4) with statistics – mean, standard deviation (SD), and relative standard deviation expressed in % (RSD%) (output from DDSolver):

Parameter	No.1	No.2	No.3	No.4	Mean	SD	RSD(%)
k	0.004	0.003	0.004	0.004	0.004	0.000	11.711
γ	105.675	82.977	100.173	73.038	90.466	15.115	16.708
F_{max}	105.599	112.121	108.402	106.038	108.040	2.986	2.764

Number of dissolution data points (N), degrees of freedom (df), and selected goodness of fit criteria – Pearson correlation coefficient (R), coefficient of determination (R^2), adjusted coefficient of determination ($R^2_{adjusted}$), and residual sum of squares (RSS) (manual calculation in MS Excel):

Parameter	No.1	No.2	No.3	No.4
N	21	21	21	21
df	18	18	18	18
R	0.993973886	0.995190121	0.993260627	0.99520094
R^2	0.987984085	0.990403378	0.986566674	0.990424912
$R^2_{adjusted}$	0.986648984	0.989337086	0.985074082	0.989361013
RSS	154.5174566	126.0160817	191.0949361	125.3623748

Graphical abstract of model fit presented as mean ± 1 SD of the fraction % of released carvedilol:

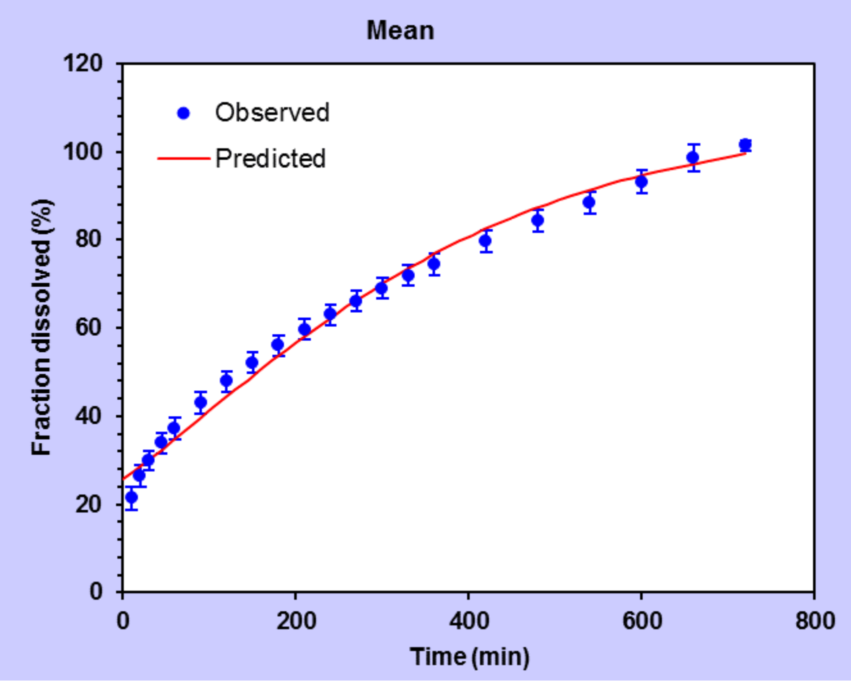

Supplementary materials – Model fitting summary – Lactochem® Crystals (modelling the entire relevant dissolution data)

Graphical abstract of model fit presented as the fraction % of released carvedilol per tested tablet:

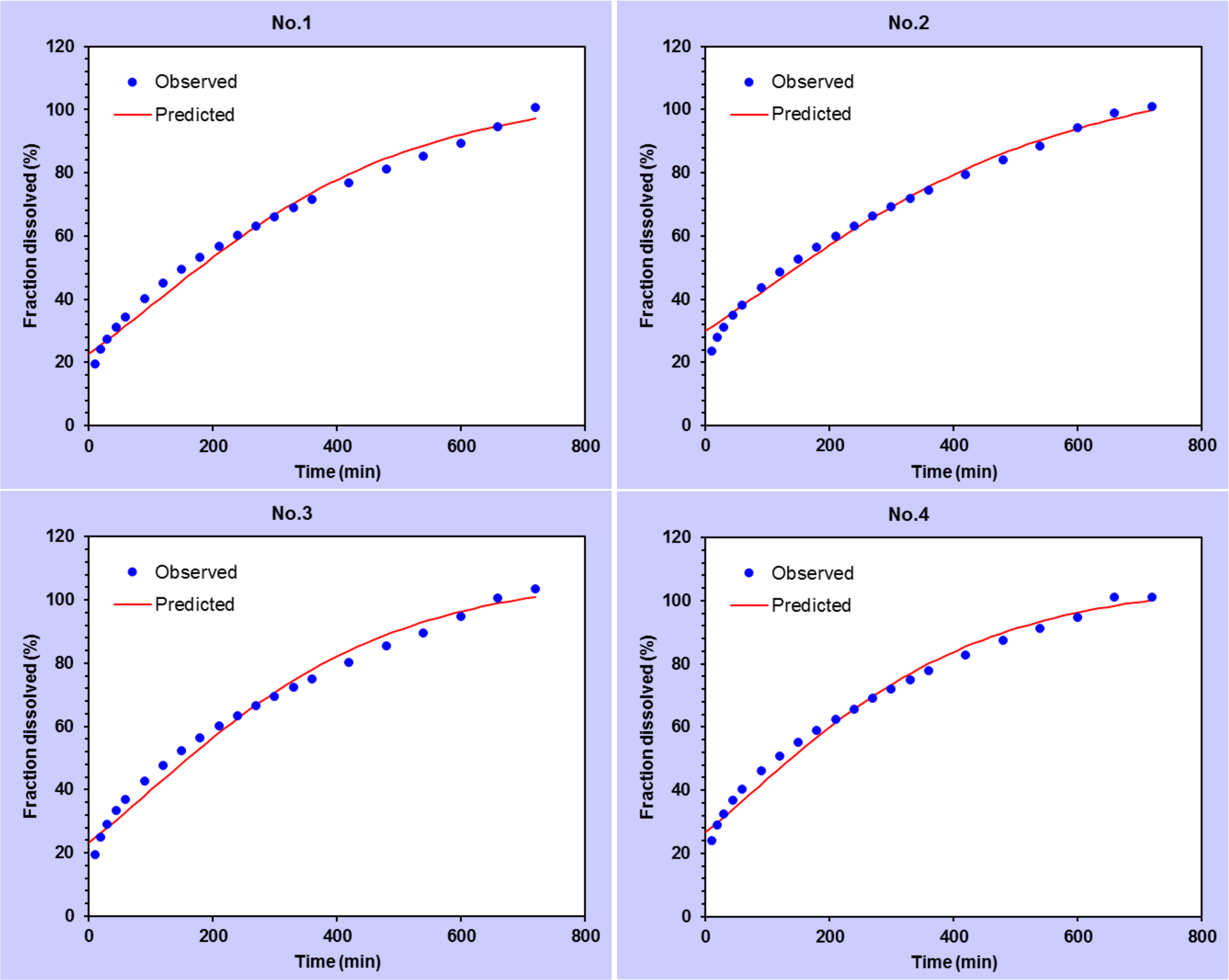

Supplementary materials – Model fitting summary – Lactochem® Crystals (modelling the entire relevant dissolution data)

Model: **Gompertz_4**

Model equation: $F = F_{max} \cdot e^{-\beta \cdot e^{-k \cdot t}}$

Fitted model parameters per tested tablet (N = 4) with statistics – mean, standard deviation (SD), and relative standard deviation expressed in % (RSD%) (output from DDSolver):

Parameter	No.1	No.2	No.3	No.4	Mean	SD	RSD(%)
k	0.004	0.003	0.004	0.004	0.004	0.000	10.875
β	1.531	1.353	1.535	1.382	1.450	0.096	6.639
F_{max}	105.599	111.821	108.402	106.038	107.965	2.850	2.640

Number of dissolution data points (N), degrees of freedom (df), and selected goodness of fit criteria – Pearson correlation coefficient (R), coefficient of determination (R^2), adjusted coefficient of determination ($R^2_{adjusted}$), and residual sum of squares (RSS) (manual calculation in MS Excel):

Parameter	No.1	No.2	No.3	No.4
N	21	21	21	21
df	18	18	18	18
R	0.993973886	0.995149195	0.993260627	0.99520094
R^2	0.987984085	0.990321921	0.986566674	0.990424912
$R^2_{adjusted}$	0.986648984	0.989246579	0.985074082	0.989361013
RSS	154.5174566	111.7716797	191.0949361	125.3623748

Graphical abstract of model fit presented as mean ± 1 SD of the fraction % of released carvedilol:

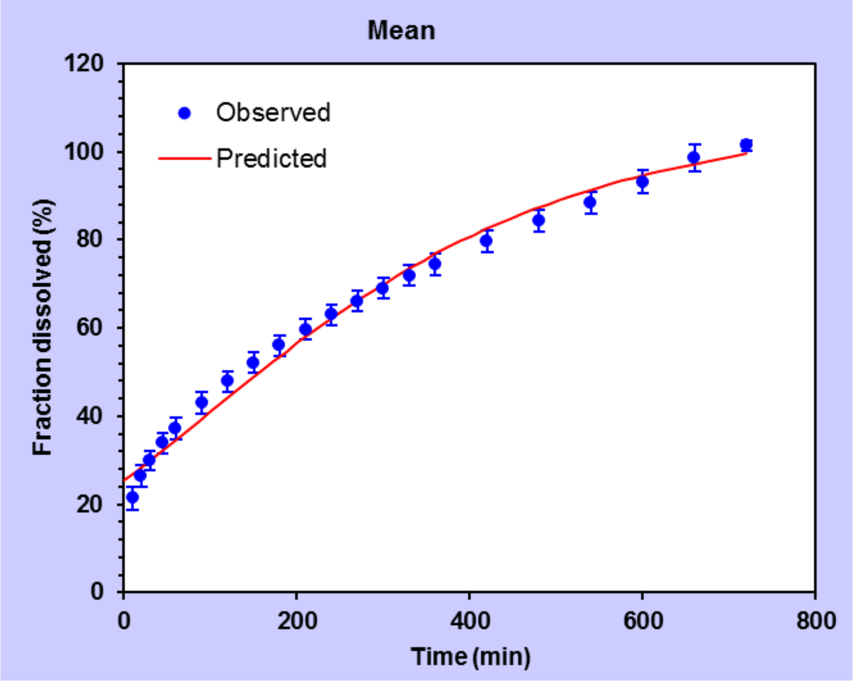

Supplementary materials – Model fitting summary – Lactochem® Crystals (modelling the entire relevant dissolution data)

Graphical abstract of model fit presented as the fraction % of released carvedilol per tested tablet:

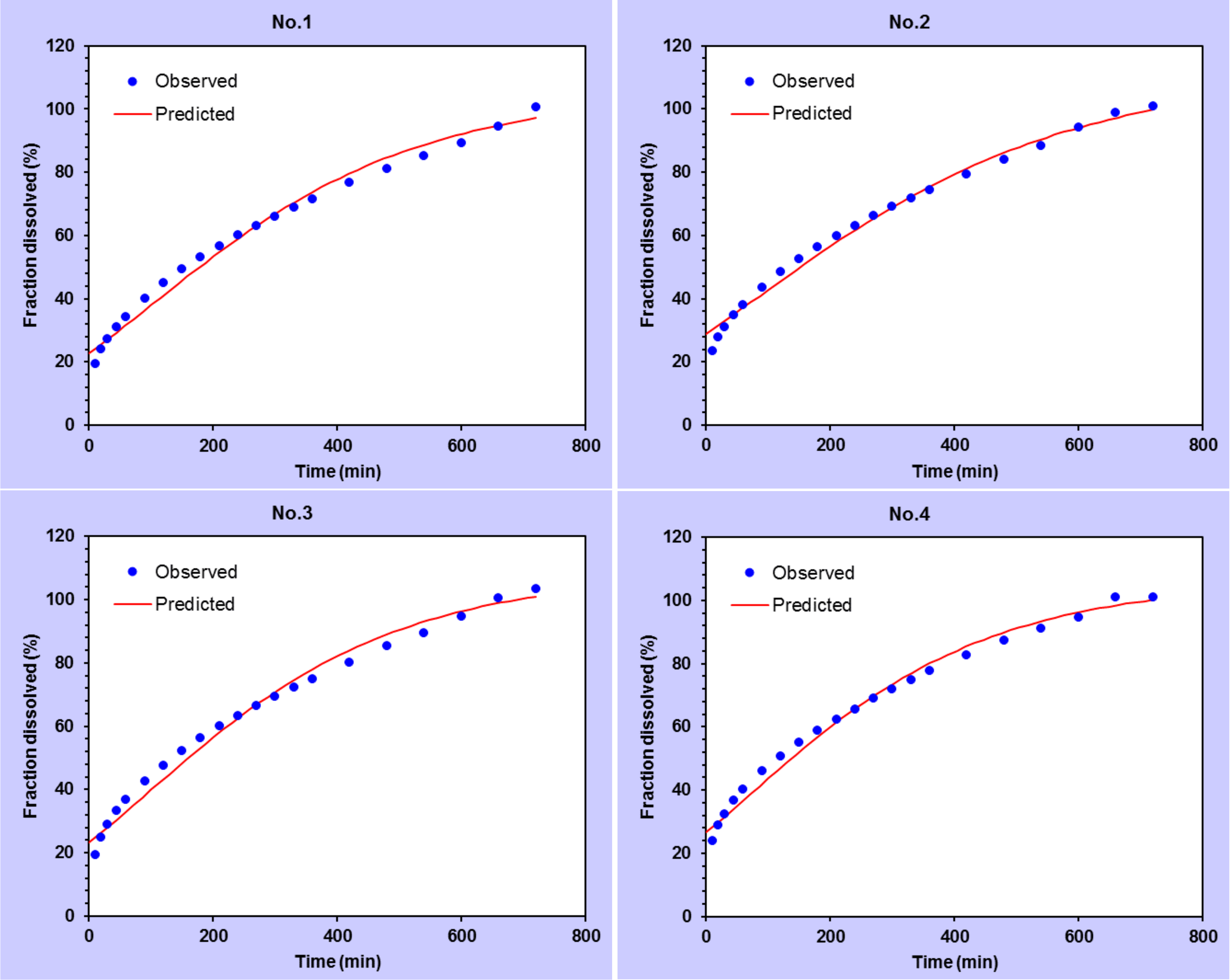

Supplementary materials – Model fitting summary – Lactochem® Crystals (modelling the entire relevant dissolution data)

Model: **Probit_1**

Model equation: $F = 100 \cdot \phi[\alpha + \beta \cdot \log(t)]$

Fitted model parameters per tested tablet (N = 4) with statistics – mean, standard deviation (SD), and relative standard deviation expressed in % (RSD%) (output from DDSolver):

Parameter	No.1	No.2	No.3	No.4	Mean	SD	RSD(%)
α	-2.450	-2.484	-2.439	-2.261	-2.408	0.100	-4.171
β	1.213	1.289	1.238	1.194	1.233	0.041	3.359

Number of dissolution data points (N), degrees of freedom (df), and selected goodness of fit criteria – Pearson correlation coefficient (R), coefficient of determination (R^2), adjusted coefficient of determination ($R^2_{adjusted}$), and residual sum of squares (RSS) (manual calculation in MS Excel):

Parameter	No.1	No.2	No.3	No.4
N	21	21	21	21
df	19	19	19	19
R	0.956325818	0.944619721	0.955197606	0.952679932
R^2	0.914559071	0.892306417	0.912402466	0.907599053
$R^2_{adjusted}$	0.910062179	0.886638334	0.90779207	0.902735845
RSS	1001.902256	1282.115047	1153.360023	1070.100986

Graphical abstract of model fit presented as mean ± 1 SD of the fraction % of released carvedilol:

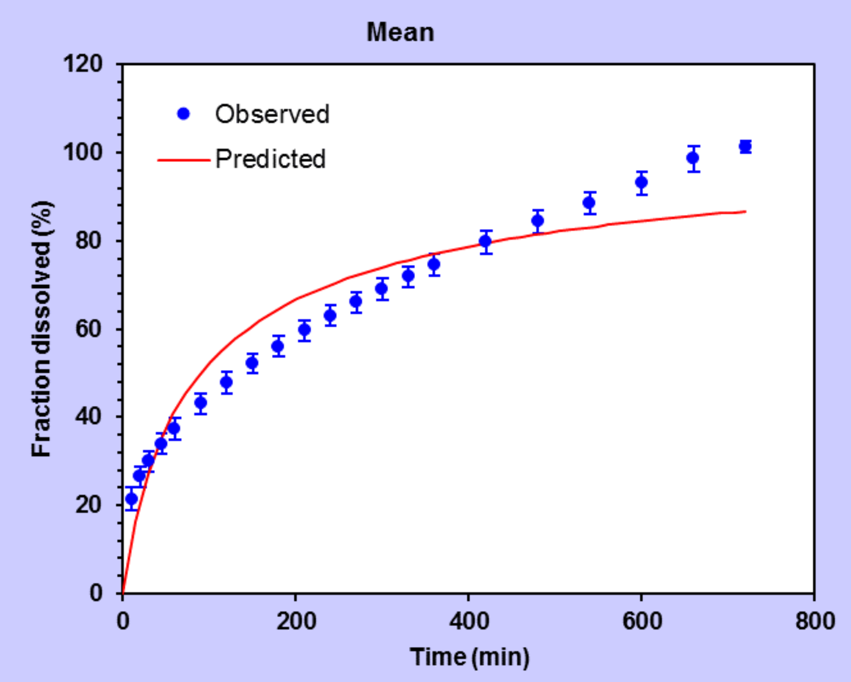

Supplementary materials – Model fitting summary – Lactochem® Crystals (modelling the entire relevant dissolution data)

Graphical abstract of model fit presented as the fraction % of released carvedilol per tested tablet:

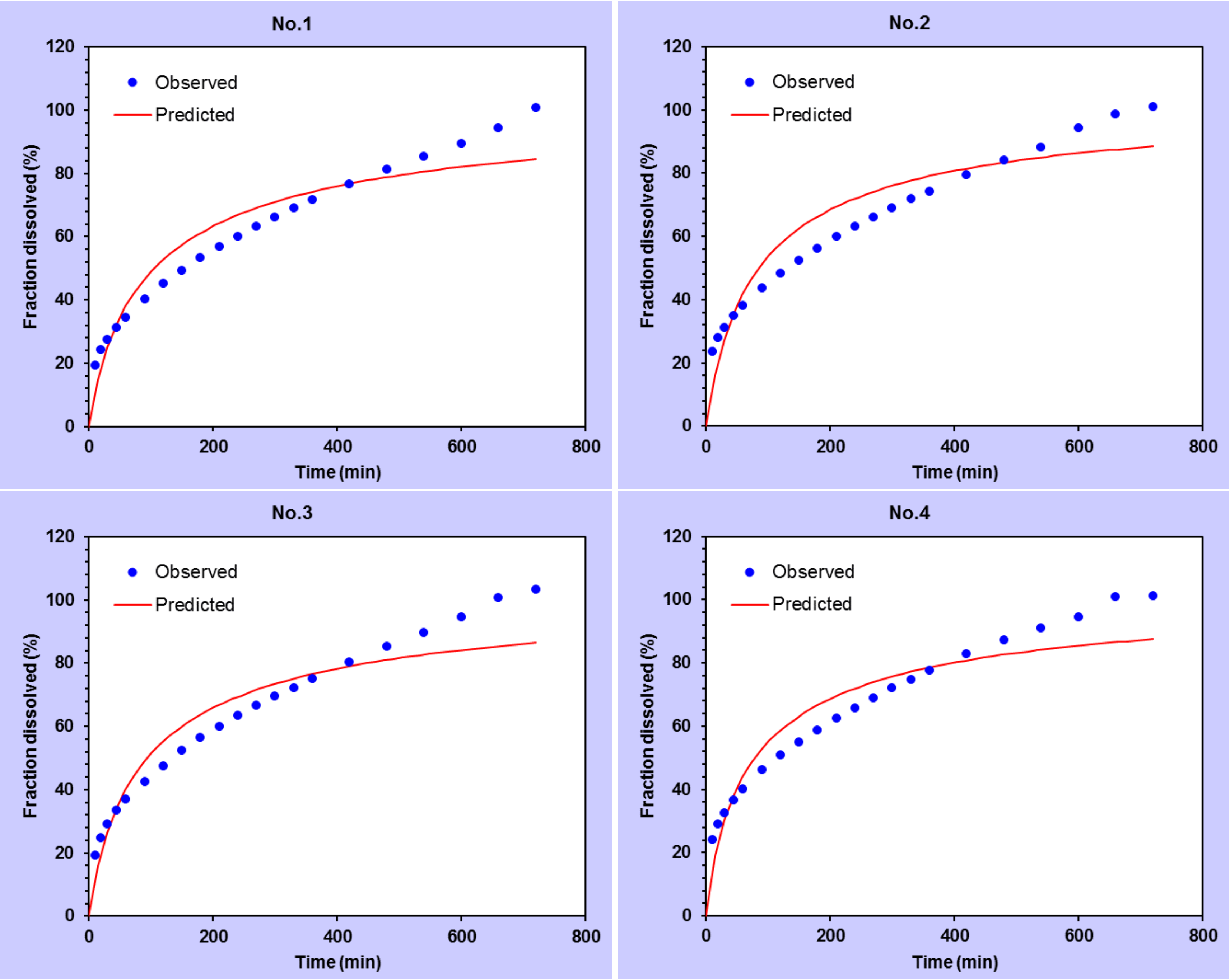

Supplementary materials – Model fitting summary – Lactochem® Crystals (modelling the entire relevant dissolution data)

Model: **Probit_2**

Model equation: $F = F_{max} \cdot \phi[\alpha + \beta \cdot \log(t)]$

Fitted model parameters per tested tablet (N = 4) with statistics – mean, standard deviation (SD), and relative standard deviation expressed in % (RSD%) (output from DDSolver):

Parameter	No.1	No.2	No.3	No.4	Mean	SD	RSD(%)
α	-2.495	-2.395	-2.561	-2.409	-2.465	0.078	-3.155
β	1.203	1.200	1.253	1.235	1.223	0.025	2.075
F_{max}	105.599	105.910	108.402	106.038	106.487	1.290	1.211

Number of dissolution data points (N), degrees of freedom (df), and selected goodness of fit criteria – Pearson correlation coefficient (R), coefficient of determination (R^2), adjusted coefficient of determination ($R^2_{adjusted}$), and residual sum of squares (RSS) (manual calculation in MS Excel):

Parameter	No.1	No.2	No.3	No.4
N	21	21	21	21
df	18	18	18	18
R	0.96083611	0.952757454	0.961474717	0.95707098
R^2	0.923206031	0.907746766	0.924433632	0.91598486
$R^2_{adjusted}$	0.914673367	0.897496406	0.916037368	0.906649845
RSS	945.229007	1121.538623	1037.252334	1052.347711

Graphical abstract of model fit presented as mean ± 1 SD of the fraction % of released carvedilol:

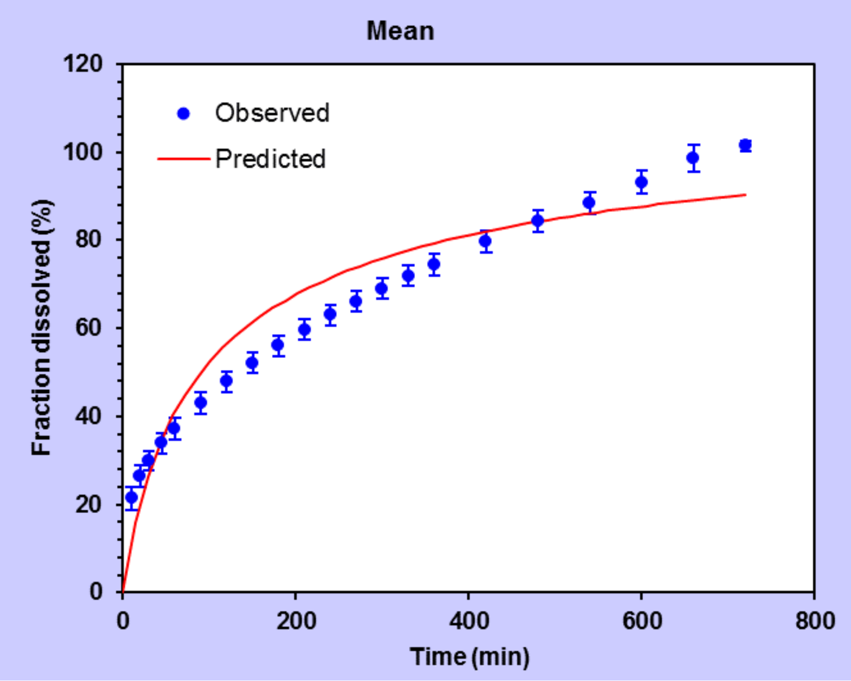

Supplementary materials – Model fitting summary – Lactochem® Crystals (modelling the entire relevant dissolution data)

Graphical abstract of model fit presented as the fraction % of released carvedilol per tested tablet:

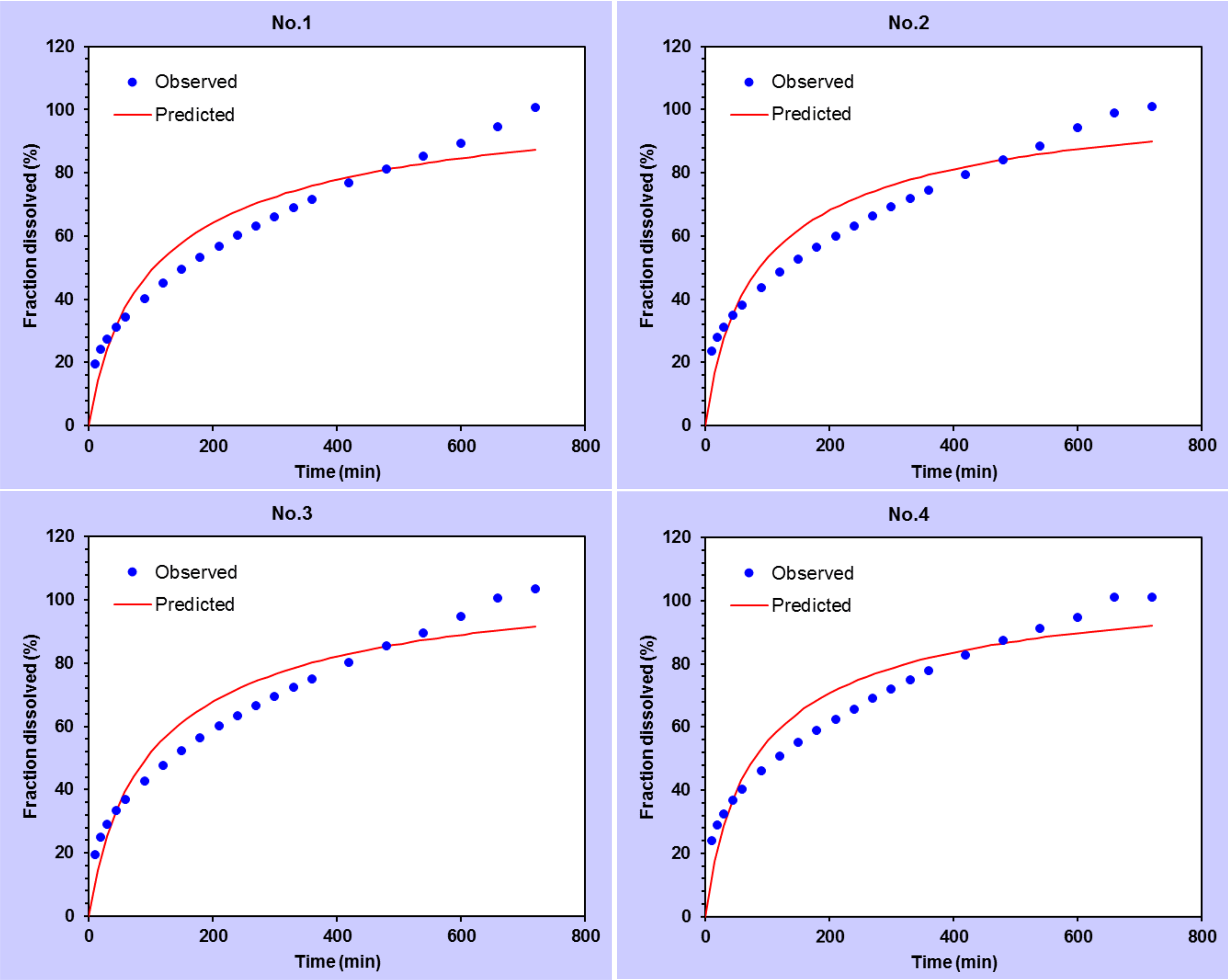

Supplementary materials – Model fitting summary – Lactochem® Crystals (modelling up to app. 60 % of carvedilol released)

Model: **Zero–order**

Model equation: $F = k_0 \cdot t$

Fitted model parameters per tested tablet (N = 4) with statistics – mean, standard deviation (SD), and relative standard deviation expressed in % (RSD%) (output from DDSolver):

Parameter	No.1	No.2	No.3	No.4	Mean	SD	RSD(%)
k_0	0.333	0.357	0.352	0.373	0.354	0.016	4.650

Number of dissolution data points (N), degrees of freedom (df), and selected goodness of fit criteria – Pearson correlation coefficient (R), coefficient of determination (R^2), adjusted coefficient of determination ($R^2_{adjusted}$), and residual sum of squares (RSS) (manual calculation in MS Excel):

Parameter	No.1	No.2	No.3	No.4
N	10	10	10	10
df	9	9	9	9
R	0.985928282	0.986755694	0.98138674	0.982805719
R^2	0.972054577	0.9736868	0.963119934	0.965907082
$R^2_{adjusted}$	0.972054577	0.9736868	0.963119934	0.965907082
RSS	1684.446311	2322.132601	1839.08039	2553.731103

Graphical abstract of model fit presented as mean ± 1 SD of the fraction % of released carvedilol:

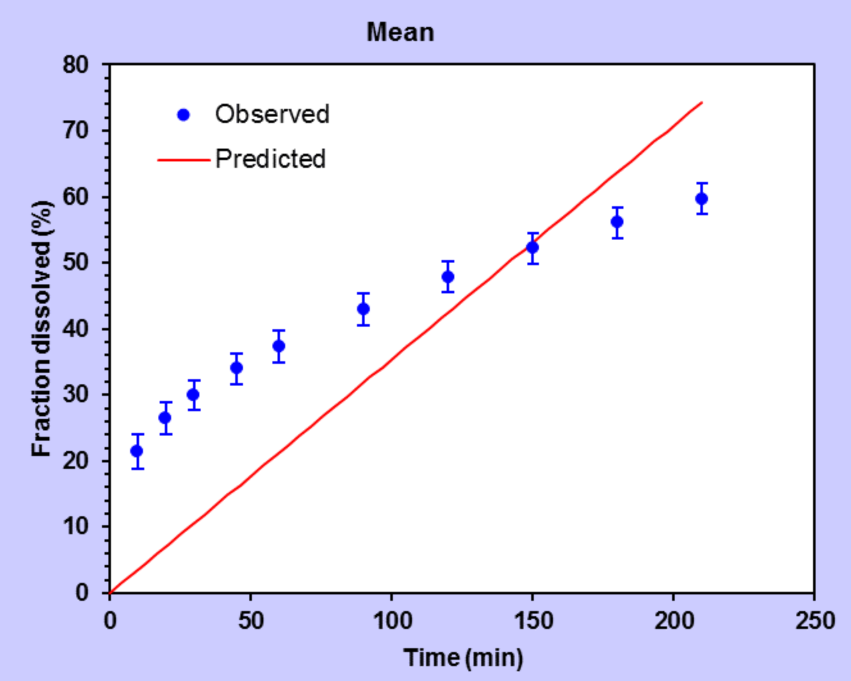

Supplementary materials – Model fitting summary – Lactochem® Crystals (modelling up to app. 60 % of carvedilol released)

Graphical abstract of model fit presented as the fraction % of released carvedilol per tested tablet:

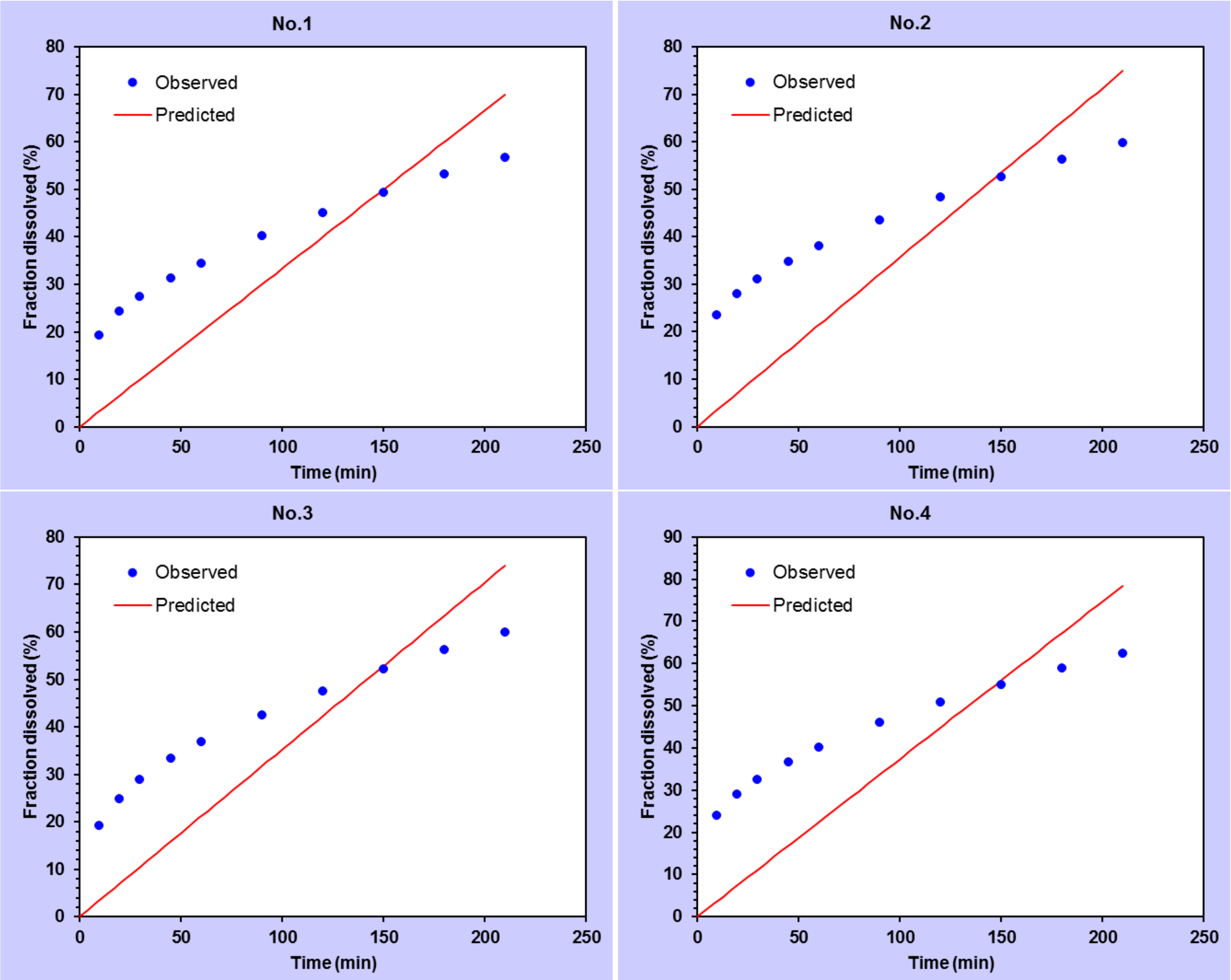

Supplementary materials – Model fitting summary – Lactochem® Crystals (modelling up to app. 60 % of carvedilol released)

Model: **Zero–order with T_{lag}**

Model equation: $F = k_0 \cdot (t - T_{lag})$

Fitted model parameters per tested tablet (N = 4) with statistics – mean, standard deviation (SD), and relative standard deviation expressed in % (RSD%) (output from DDSolver):

Parameter	No.1	No.2	No.3	No.4	Mean	SD	RSD(%)
k_0	0.179	0.174	0.192	0.183	0.182	0.007	4.073
T_{lag}	-121.418	-146.842	-117.692	-146.550	-133.125	15.744	-11.827

Number of dissolution data points (N), degrees of freedom (df), and selected goodness of fit criteria – Pearson correlation coefficient (R), coefficient of determination (R^2), adjusted coefficient of determination ($R^2_{adjusted}$), and residual sum of squares (RSS) (manual calculation in MS Excel):

Parameter	No.1	No.2	No.3	No.4
N	10	10	10	10
df	8	8	8	8
R	0.985928282	0.986755694	0.98138674	0.982805719
R^2	0.972054577	0.9736868	0.963119934	0.965907082
$R^2_{adjusted}$	0.968561399	0.97039765	0.958509926	0.961645467
RSS	41.18589009	36.81172194	63.10225653	52.82558831

Graphical abstract of model fit presented as mean ± 1 SD of the fraction % of released carvedilol:

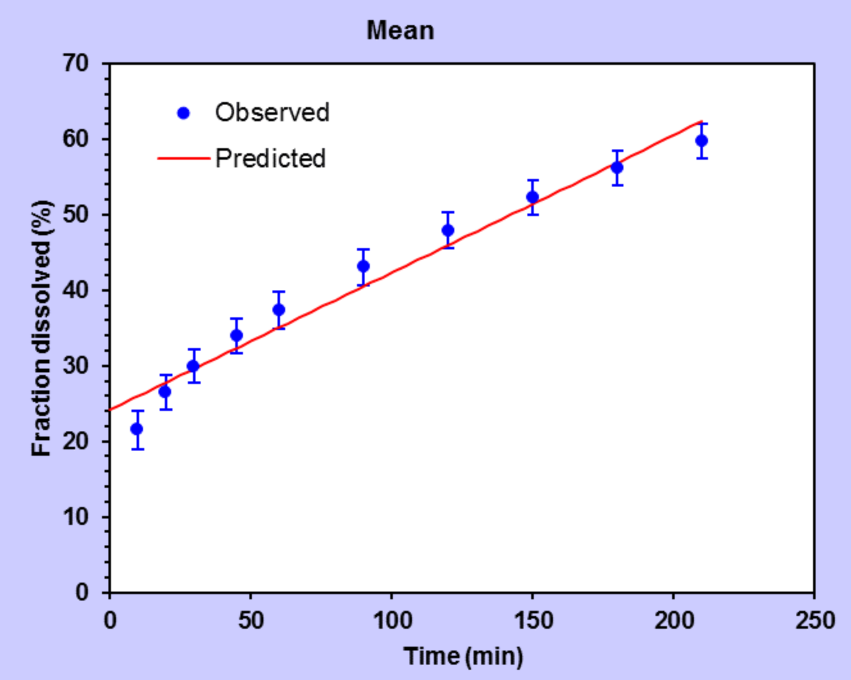

Supplementary materials – Model fitting summary – Lactochem® Crystals (modelling up to app. 60 % of carvedilol released)

Graphical abstract of model fit presented as the fraction % of released carvedilol per tested tablet:

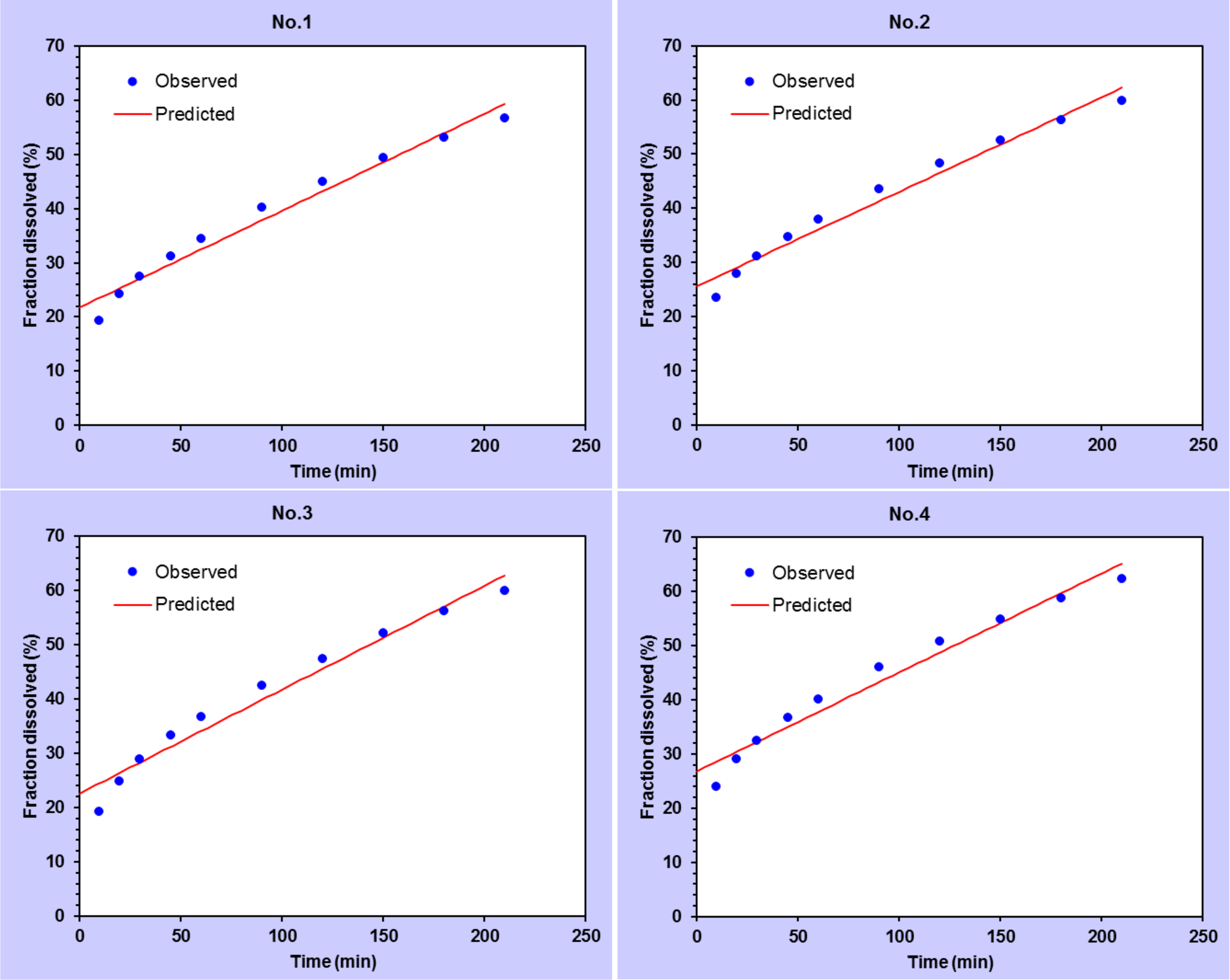

Supplementary materials – Model fitting summary – Lactochem® Crystals (modelling up to app. 60 % of carvedilol released)

Model: **Zero–order with F_0**

Model equation: $F = F_0 + k_0 \cdot t$

Fitted model parameters per tested tablet (N = 4) with statistics – mean, standard deviation (SD), and relative standard deviation expressed in % (RSD%) (output from DDSolver):

Parameter	No.1	No.2	No.3	No.4	Mean	SD	RSD(%)
k_0	0.179	0.174	0.192	0.183	0.182	0.007	4.073
F_0	21.712	25.604	22.572	26.785	24.168	2.415	9.992

Number of dissolution data points (N), degrees of freedom (df), and selected goodness of fit criteria – Pearson correlation coefficient (R), coefficient of determination (R^2), adjusted coefficient of determination ($R^2_{adjusted}$), and residual sum of squares (RSS) (manual calculation in MS Excel):

Parameter	No.1	No.2	No.3	No.4
N	10	10	10	10
df	8	8	8	8
R	0.985928282	0.986755694	0.98138674	0.982805719
R^2	0.972054577	0.9736868	0.963119934	0.965907082
$R^2_{adjusted}$	0.968561399	0.97039765	0.958509926	0.961645467
RSS	41.18589009	36.81172194	63.10225653	52.82558831

Graphical abstract of model fit presented as mean ± 1 SD of the fraction % of released carvedilol:

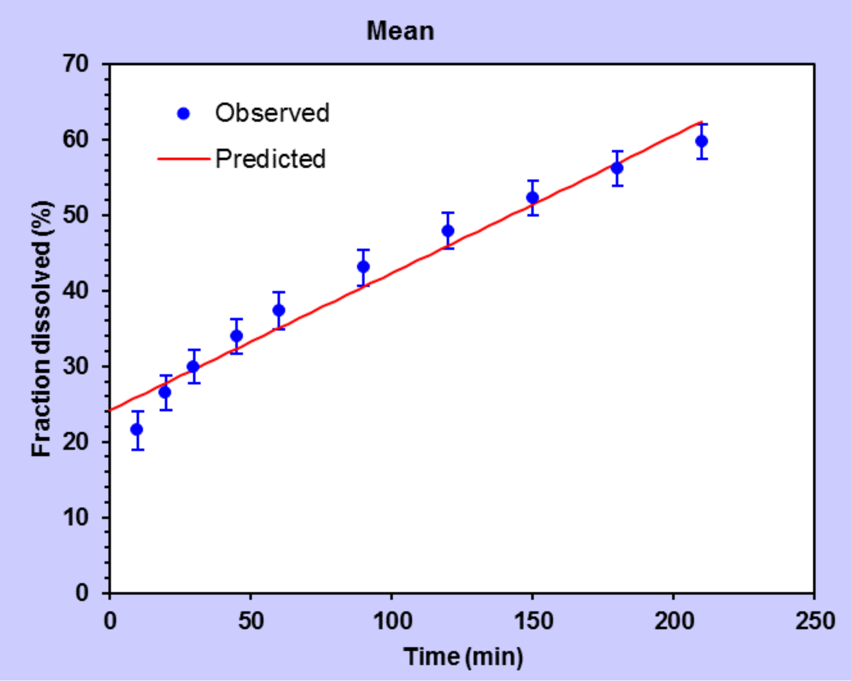

Supplementary materials – Model fitting summary – Lactochem® Crystals (modelling up to app. 60 % of carvedilol released)

Graphical abstract of model fit presented as the fraction % of released carvedilol per tested tablet:

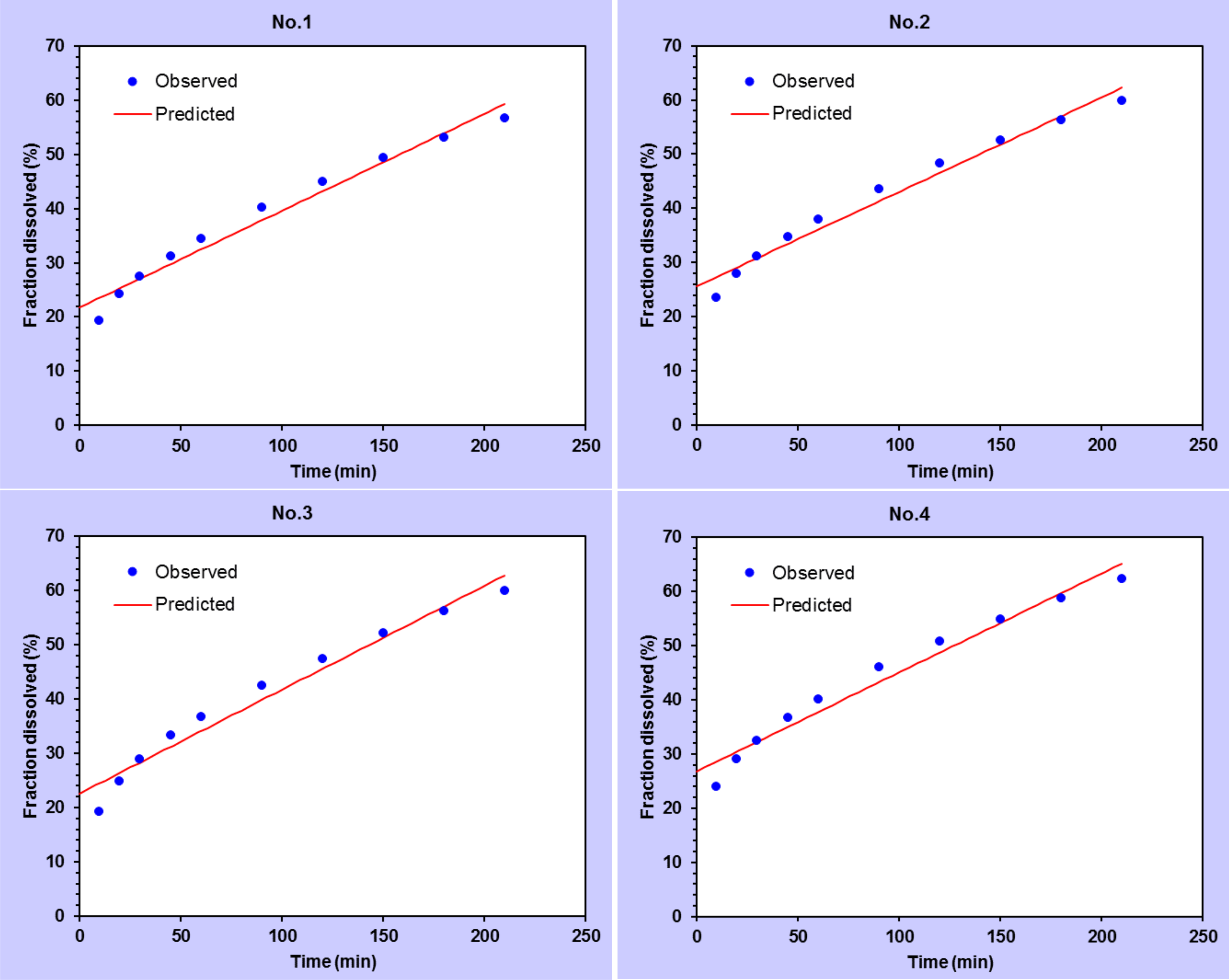

Supplementary materials – Model fitting summary – Lactochem® Crystals (modelling up to app. 60 % of carvedilol released)

Model: **First–order**

Model equation: $F = 100 \cdot (1 - e^{-k_1 \cdot t})$

Fitted model parameters per tested tablet (N = 4) with statistics – mean, standard deviation (SD), and relative standard deviation expressed in % (RSD%) (output from DDSolver):

Parameter	No.1	No.2	No.3	No.4	Mean	SD	RSD(%)
k_1	0.005	0.005	0.005	0.005	0.005	0.000	6.726

Number of dissolution data points (N), degrees of freedom (df), and selected goodness of fit criteria – Pearson correlation coefficient (R), coefficient of determination (R^2), adjusted coefficient of determination ($R^2_{adjusted}$), and residual sum of squares (RSS) (manual calculation in MS Excel):

Parameter	No.1	No.2	No.3	No.4
N	10	10	10	10
df	9	9	9	9
R	0.997561087	0.998382434	0.995764808	0.997495556
R^2	0.995128123	0.996767484	0.991547553	0.994997384
$R^2_{adjusted}$	0.995128123	0.996767484	0.991547553	0.994997384
RSS	1009.874107	1413.851344	1036.05623	1489.638287

Graphical abstract of model fit presented as mean ± 1 SD of the fraction % of released carvedilol:

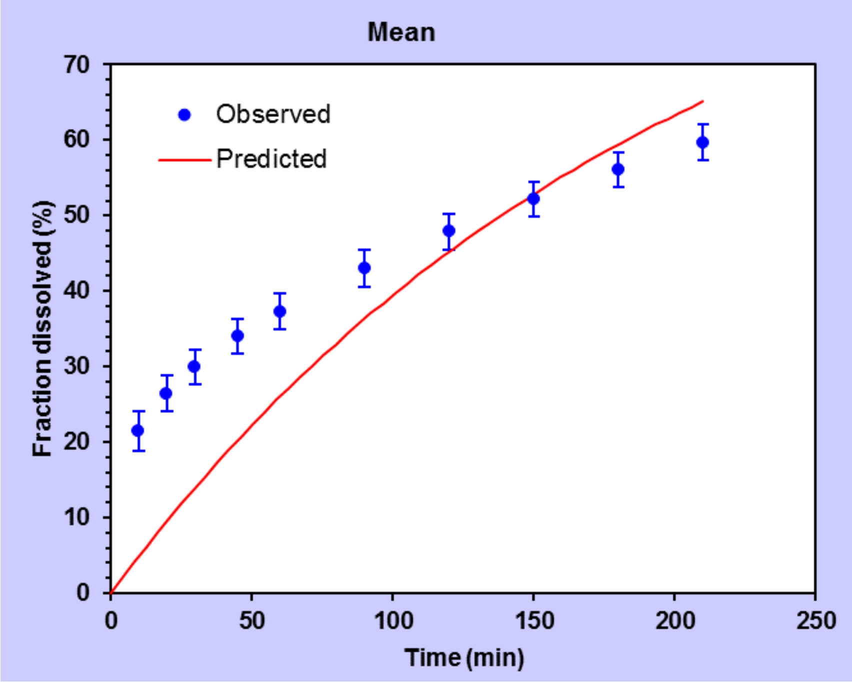

Supplementary materials – Model fitting summary – Lactochem® Crystals (modelling up to app. 60 % of carvedilol released)

Graphical abstract of model fit presented as the fraction % of released carvedilol per tested tablet:

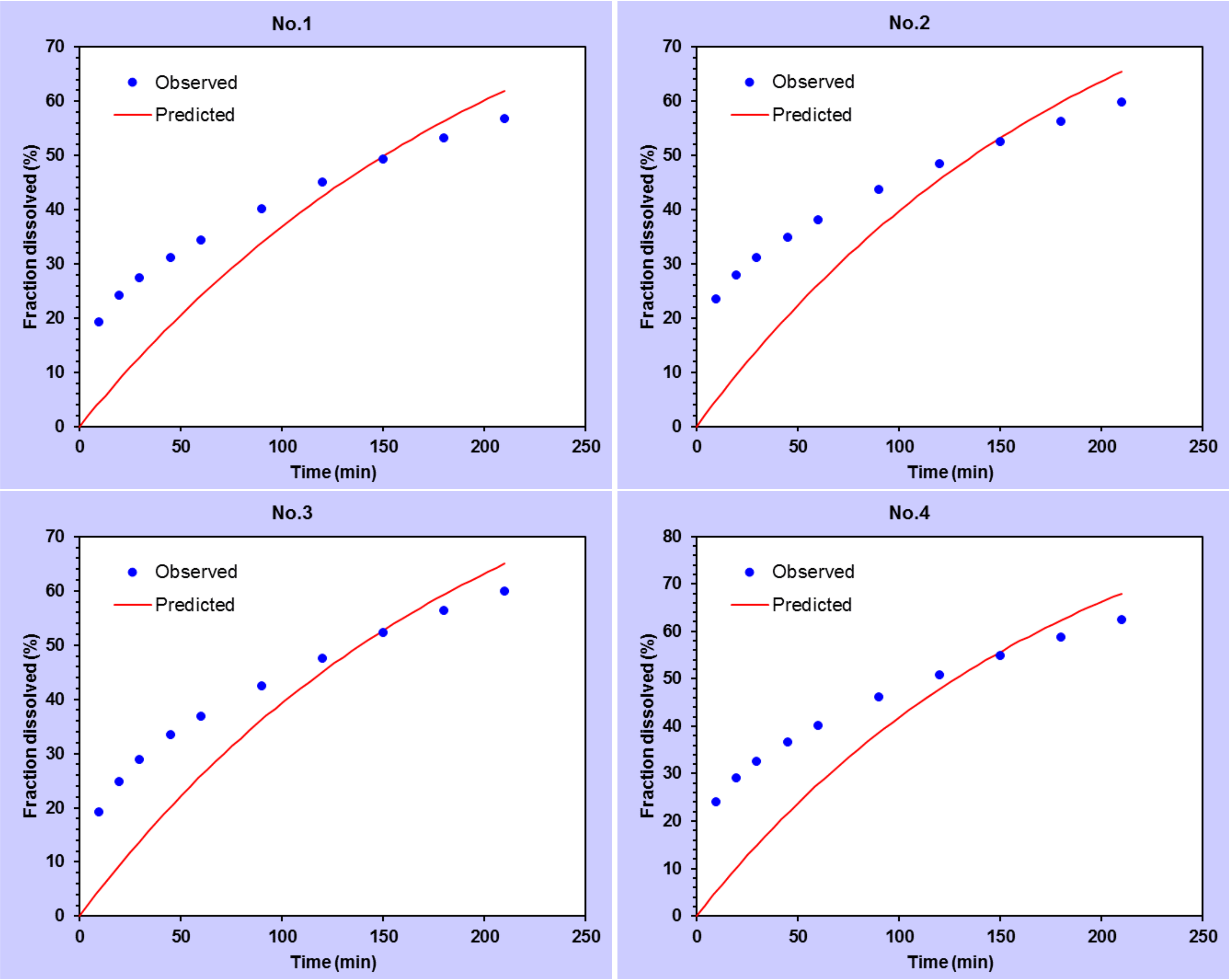

Supplementary materials – Model fitting summary – Lactochem® Crystals (modelling up to app. 60 % of carvedilol released)

Model: **First–order with T_{lag}**

Model equation: $F = 100 \cdot \left[1 - e^{-k_1 \cdot (t - T_{lag})}\right]$

Fitted model parameters per tested tablet (N = 4) with statistics – mean, standard deviation (SD), and relative standard deviation expressed in % (RSD%) (output from DDSolver):

Parameter	No.1	No.2	No.3	No.4	Mean	SD	RSD(%)
k_1	0.003	0.003	0.003	0.003	0.003	0.000	5.695
T_{lag}	-74.502	-87.744	-69.026	-84.589	-78.965	8.706	-11.026

Number of dissolution data points (N), degrees of freedom (df), and selected goodness of fit criteria – Pearson correlation coefficient (R), coefficient of determination (R^2), adjusted coefficient of determination ($R^2_{adjusted}$), and residual sum of squares (RSS) (manual calculation in MS Excel):

Parameter	No.1	No.2	No.3	No.4
N	10	10	10	10
df	8	8	8	8
R	0.995036024	0.995811062	0.992708987	0.994106229
R^2	0.99009669	0.991639672	0.985471132	0.988247194
$R^2_{adjusted}$	0.988858776	0.990594631	0.983655024	0.986778094
RSS	15.08763828	12.1179635	25.74882651	18.98694114

Graphical abstract of model fit presented as mean ± 1 SD of the fraction % of released carvedilol:

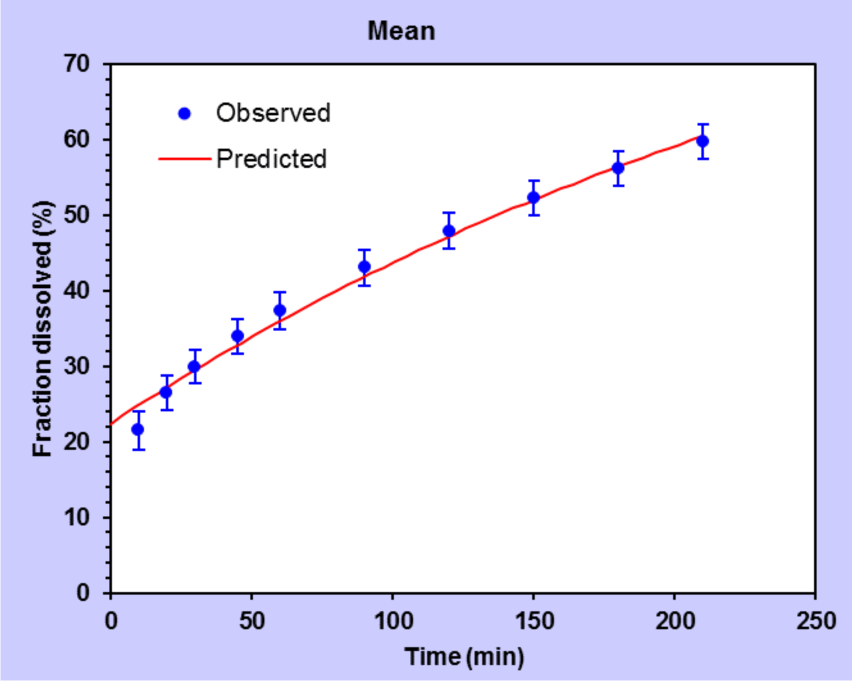

Supplementary materials – Model fitting summary – Lactochem® Crystals (modelling up to app. 60 % of carvedilol released)

Graphical abstract of model fit presented as the fraction % of released carvedilol per tested tablet:

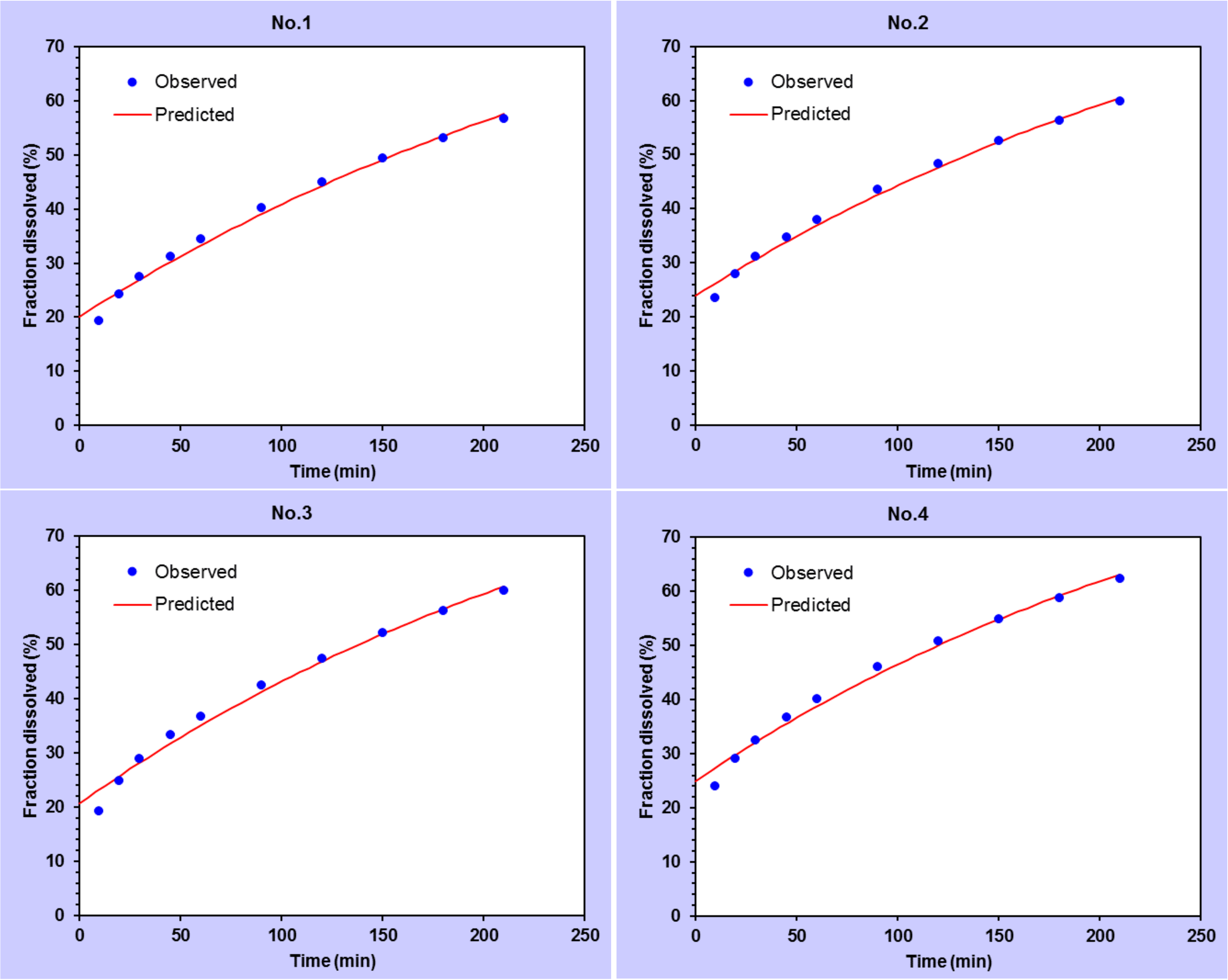

Supplementary materials – Model fitting summary – Lactochem® Crystals (modelling up to app. 60 % of carvedilol released)

Model: **First–order with F_{max}**

Model equation: $F = F_{max} \cdot (1 - e^{-k_1 \cdot t})$

Fitted model parameters per tested tablet (N = 4) with statistics – mean, standard deviation (SD), and relative standard deviation expressed in % (RSD%) (output from DDSolver):

Parameter	No.1	No.2	No.3	No.4	Mean	SD	RSD(%)
k_1	0.013	0.020	0.013	0.020	0.017	0.004	24.363
F_{max}	59.551	62.796	62.900	65.515	62.691	2.442	3.895

Number of dissolution data points (N), degrees of freedom (df), and selected goodness of fit criteria – Pearson correlation coefficient (R), coefficient of determination (R^2), adjusted coefficient of determination ($R^2_{adjusted}$), and residual sum of squares (RSS) (manual calculation in MS Excel):

Parameter	No.1	No.2	No.3	No.4
N	10	10	10	10
df	8	8	8	8
R	0.988576225	0.96244841	0.990601768	0.967817015
R^2	0.977282953	0.926306942	0.981291862	0.936669775
$R^2_{adjusted}$	0.974443323	0.91709531	0.978953345	0.928753497
RSS	350.516223	487.457212	349.9762181	491.5672938

Graphical abstract of model fit presented as mean ± 1 SD of the fraction % of released carvedilol:

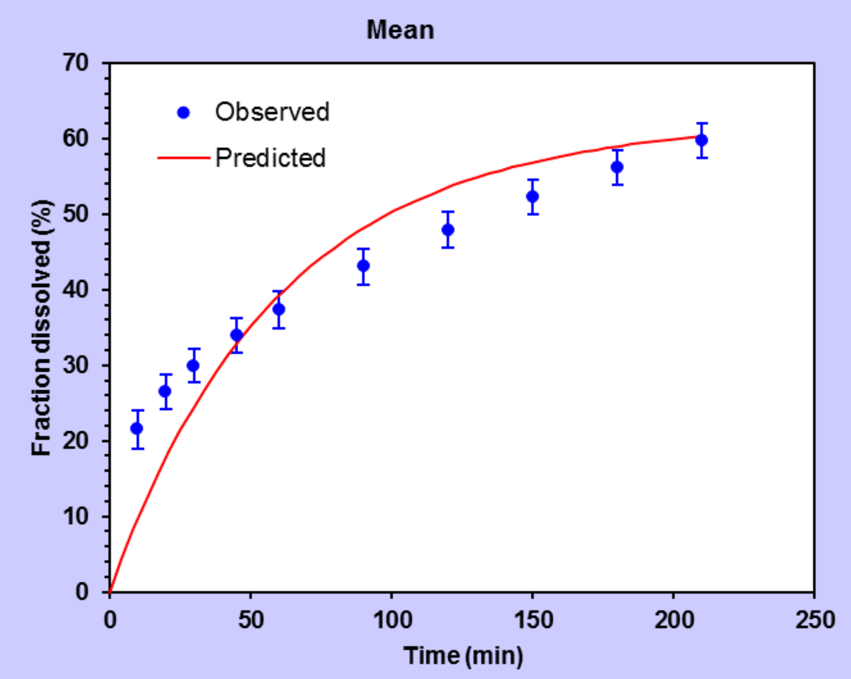

Supplementary materials – Model fitting summary – Lactochem® Crystals (modelling up to app. 60 % of carvedilol released)

Graphical abstract of model fit presented as the fraction % of released carvedilol per tested tablet:

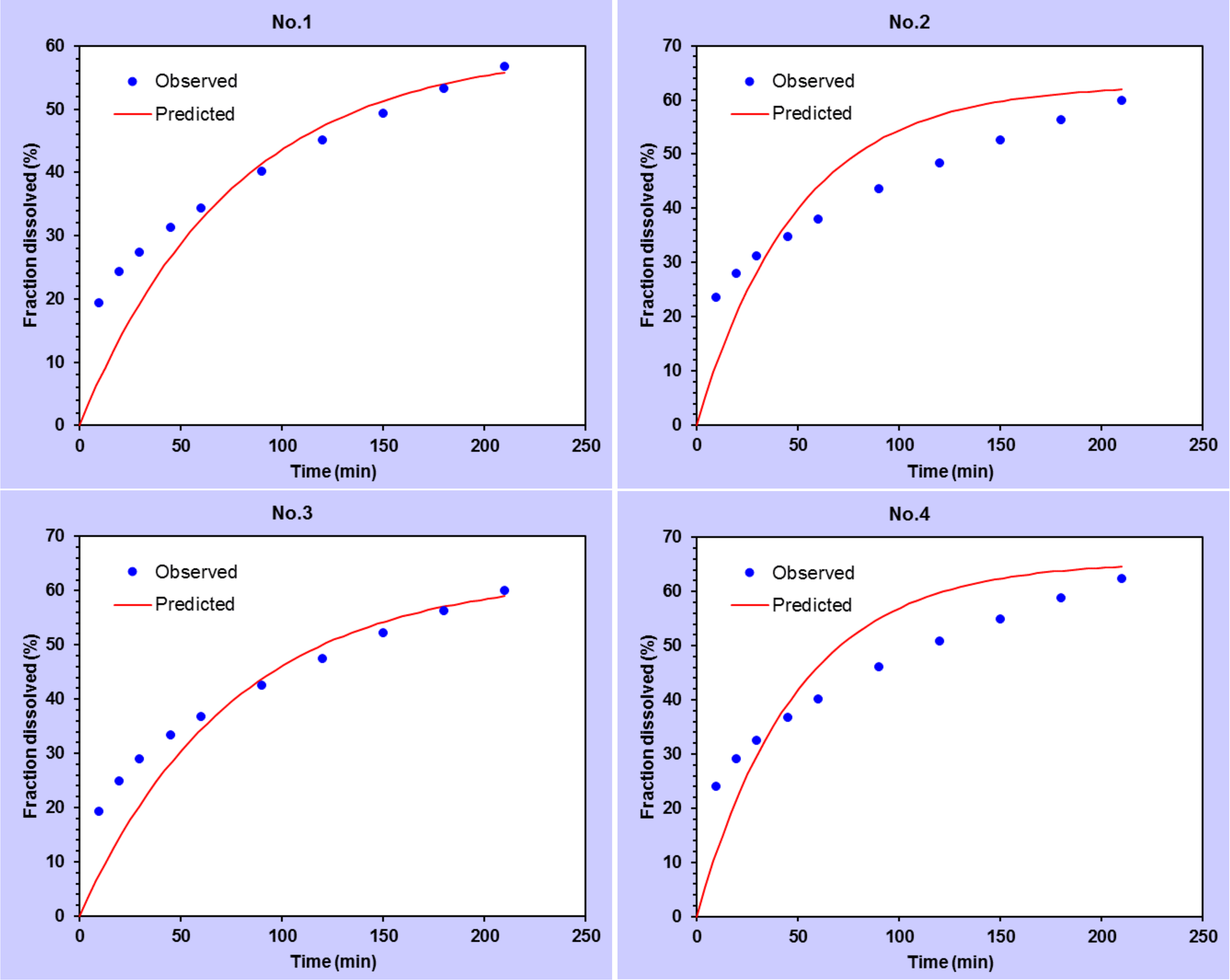

Supplementary materials – Model fitting summary – Lactochem® Crystals (modelling up to app. 60 % of carvedilol released)

Model: **First–order with T_{lag} and F_{max}**

Model equation: $F = F_{max} \cdot \left[1 - e^{-k_1 \cdot (t - T_{lag})}\right]$

Fitted model parameters per tested tablet (N = 4) with statistics – mean, standard deviation (SD), and relative standard deviation expressed in % (RSD%) (output from DDSolver):

Parameter	No.1	No.2	No.3	No.4	Mean	SD	RSD(%)
k_1	0.012	0.011	0.012	0.012	0.012	0.000	3.211
T_{lag}	-23.462	-29.871	-22.225	-23.391	-24.737	3.470	-14.026
F_{max}	59.551	59.525	62.900	65.515	61.873	2.900	4.686

Number of dissolution data points (N), degrees of freedom (df), and selected goodness of fit criteria – Pearson correlation coefficient (R), coefficient of determination (R^2), adjusted coefficient of determination ($R^2_{adjusted}$), and residual sum of squares (RSS) (manual calculation in MS Excel):

Parameter	No.1	No.2	No.3	No.4
N	10	10	10	10
df	7	7	7	7
R	0.991965938	0.993425027	0.993200258	0.994394136
R^2	0.983996421	0.986893283	0.986446753	0.988819698
$R^2_{adjusted}$	0.97942397	0.983148507	0.982574397	0.985625326
RSS	50.61526841	44.53825132	50.34983466	38.4353509

Graphical abstract of model fit presented as mean ± 1 SD of the fraction % of released carvedilol:

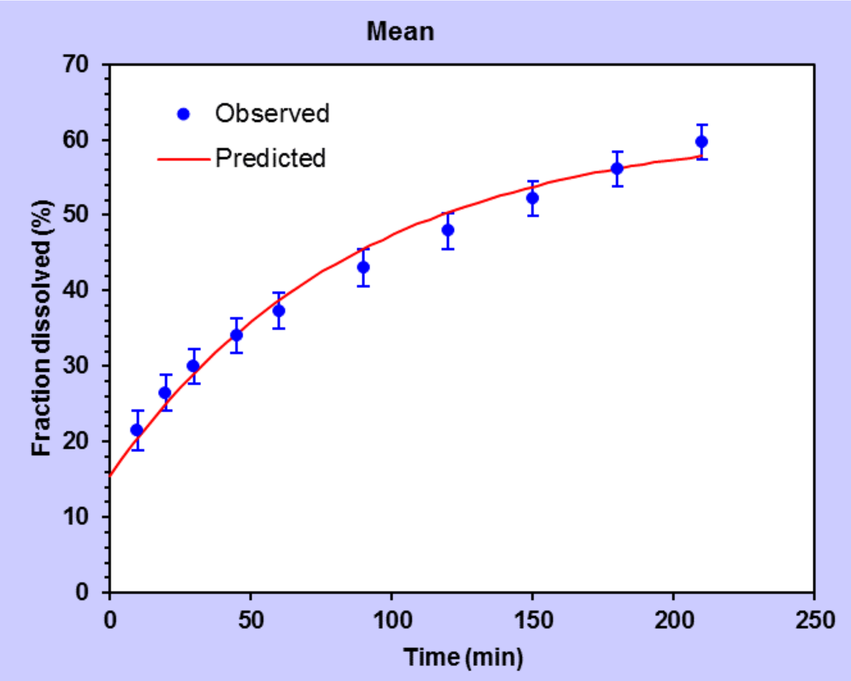

Supplementary materials – Model fitting summary – Lactochem® Crystals (modelling up to app. 60 % of carvedilol released)

Graphical abstract of model fit presented as the fraction % of released carvedilol per tested tablet:

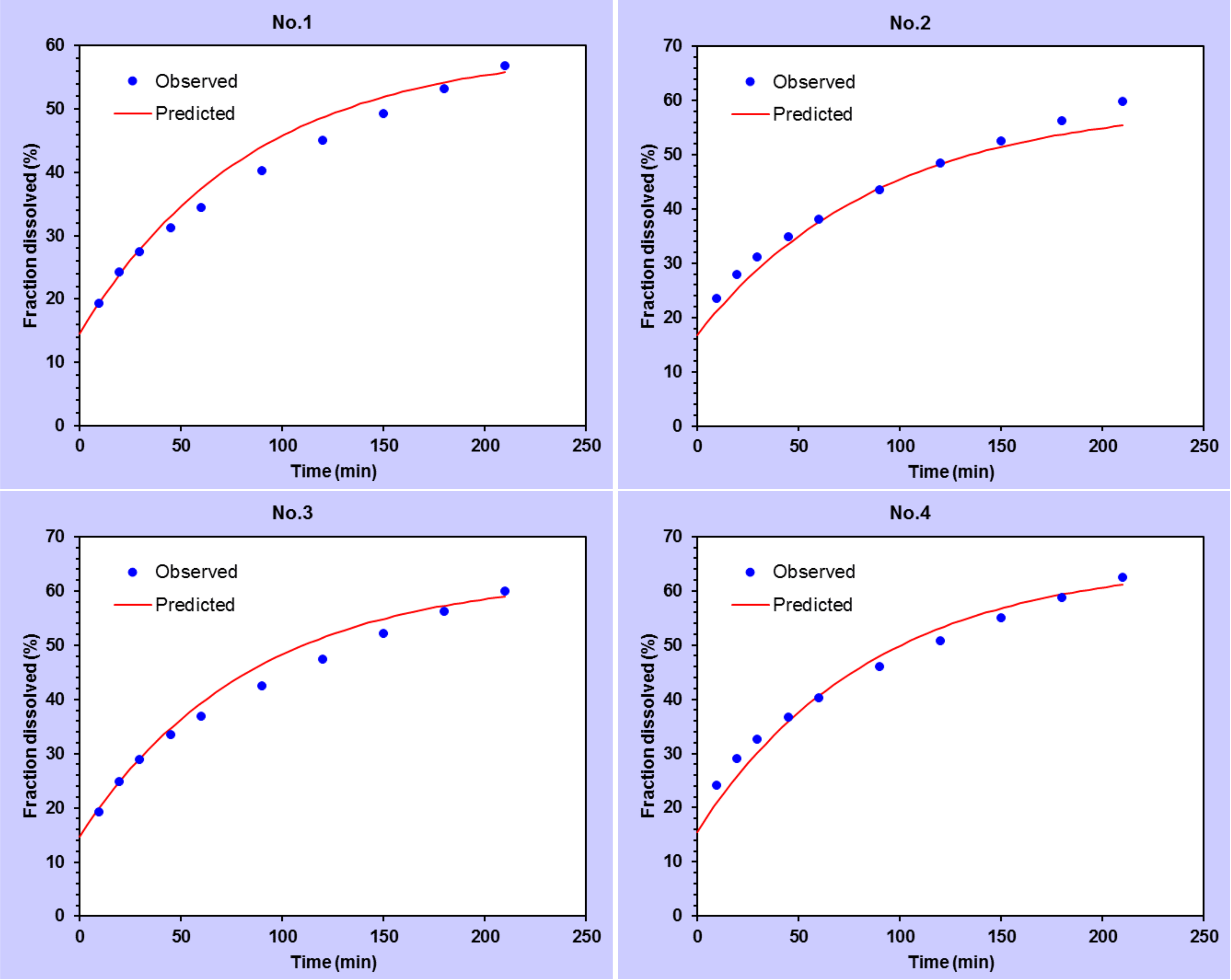

Supplementary materials – Model fitting summary – Lactochem® Crystals (modelling up to app. 60 % of carvedilol released)

Model: **Higuchi**

Model equation: $F = k_H \cdot t^{0.5}$

Fitted model parameters per tested tablet (N = 4) with statistics – mean, standard deviation (SD), and relative standard deviation expressed in % (RSD%) (output from DDSolver):

Parameter	No.1	No.2	No.3	No.4	Mean	SD	RSD(%)
k_H	4.161	4.484	4.396	4.697	4.434	0.222	5.004

Number of dissolution data points (N), degrees of freedom (df), and selected goodness of fit criteria – Pearson correlation coefficient (R), coefficient of determination (R^2), adjusted coefficient of determination ($R^2_{adjusted}$), and residual sum of squares (RSS) (manual calculation in MS Excel):

Parameter	No.1	No.2	No.3	No.4
N	10	10	10	10
df	9	9	9	9
R	0.999894794	0.999944898	0.999403423	0.999816551
R^2	0.9997896	0.999889798	0.998807202	0.999633136
$R^2_{adjusted}$	0.9997896	0.999889798	0.998807202	0.999633136
RSS	128.7881048	273.0338166	125.7634879	293.2854843

Graphical abstract of model fit presented as mean ± 1 SD of the fraction % of released carvedilol:

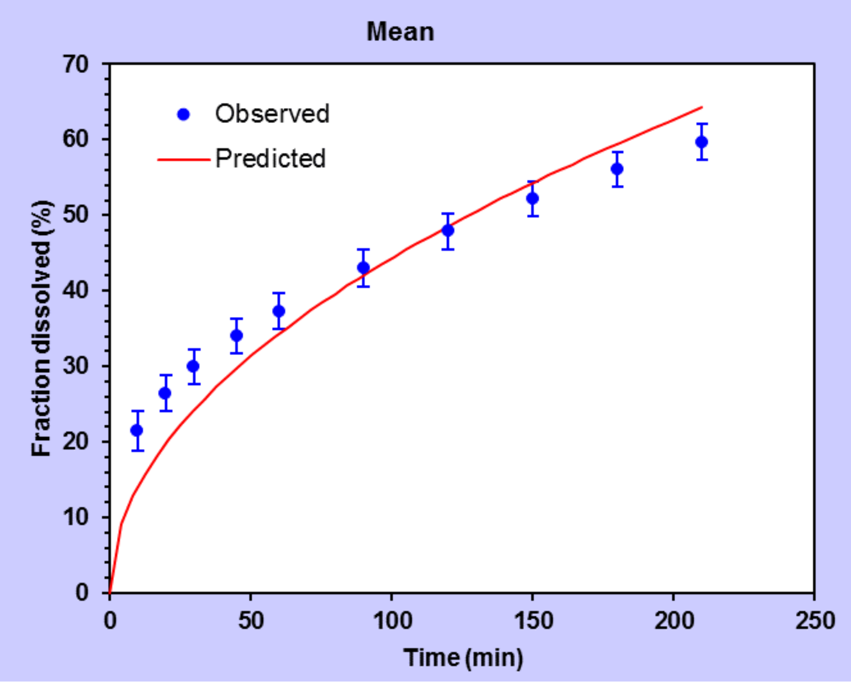

Supplementary materials – Model fitting summary – Lactochem® Crystals (modelling up to app. 60 % of carvedilol released)

Graphical abstract of model fit presented as the fraction % of released carvedilol per tested tablet:

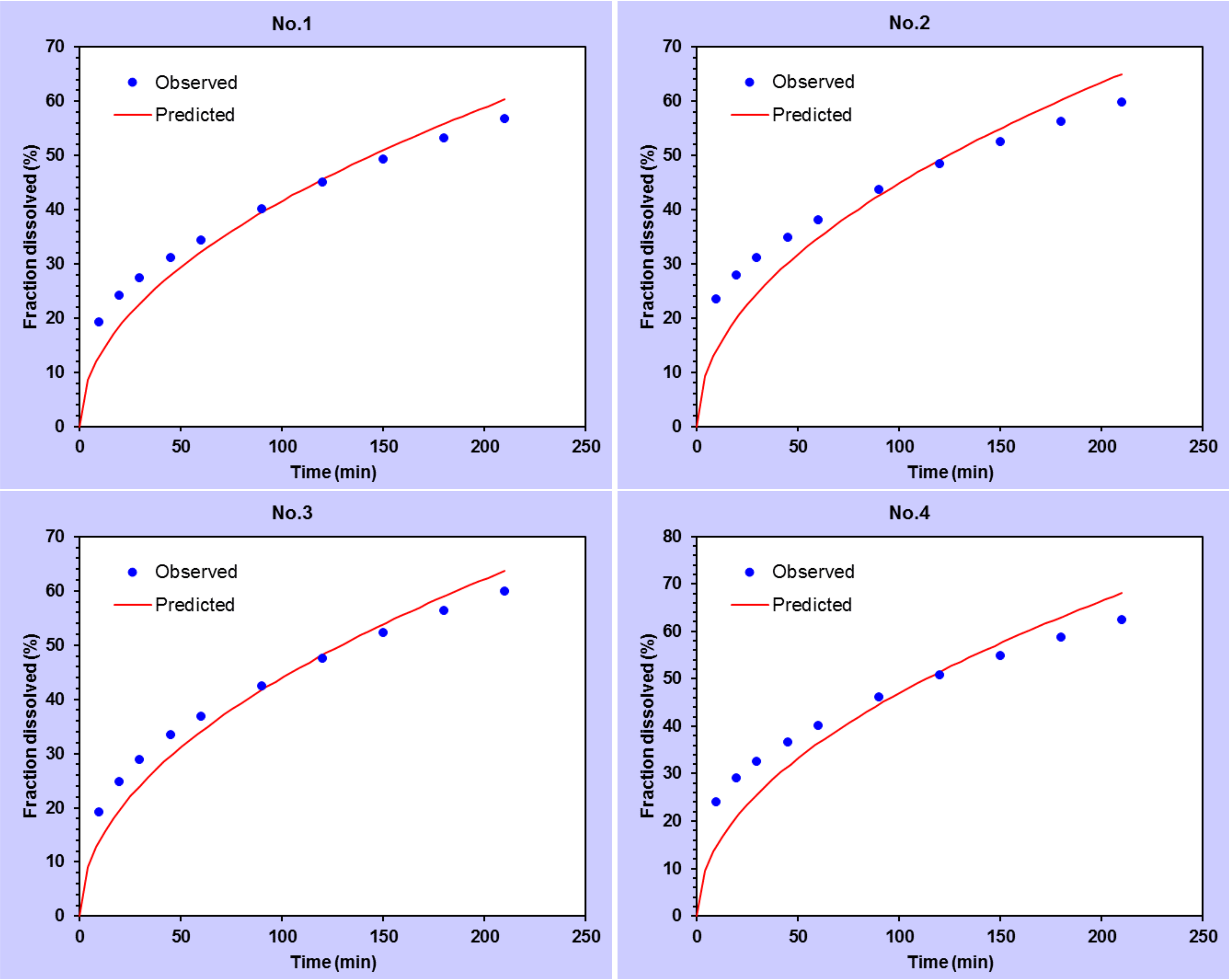

Supplementary materials – Model fitting summary – Lactochem® Crystals (modelling up to app. 60 % of carvedilol released)

Model: **Higuchi with T_{lag}**

Model equation: $F = k_H \cdot (t - T_{lag})^{0.5}$

Fitted model parameters per tested tablet (N = 4) with statistics – mean, standard deviation (SD), and relative standard deviation expressed in % (RSD%) (output from DDSolver):

Parameter	No.1	No.2	No.3	No.4	Mean	SD	RSD(%)
k_H	3.744	3.858	3.970	4.032	3.901	0.127	3.260
T_{lag}	-22.449	-33.931	-21.477	-34.474	-28.083	7.081	-25.215

Number of dissolution data points (N), degrees of freedom (df), and selected goodness of fit criteria – Pearson correlation coefficient (R), coefficient of determination (R^2), adjusted coefficient of determination ($R^2_{adjusted}$), and residual sum of squares (RSS) (manual calculation in MS Excel):

Parameter	No.1	No.2	No.3	No.4
N	10	10	10	10
df	8	8	8	8
R	0.998456751	0.998050248	0.996649693	0.99635231
R^2	0.996915883	0.996104298	0.993310611	0.992717926
$R^2_{adjusted}$	0.996530368	0.995617335	0.992474438	0.991807667
RSS	5.027484072	5.942181643	12.71734585	12.39404759

Graphical abstract of model fit presented as mean ± 1 SD of the fraction % of released carvedilol:

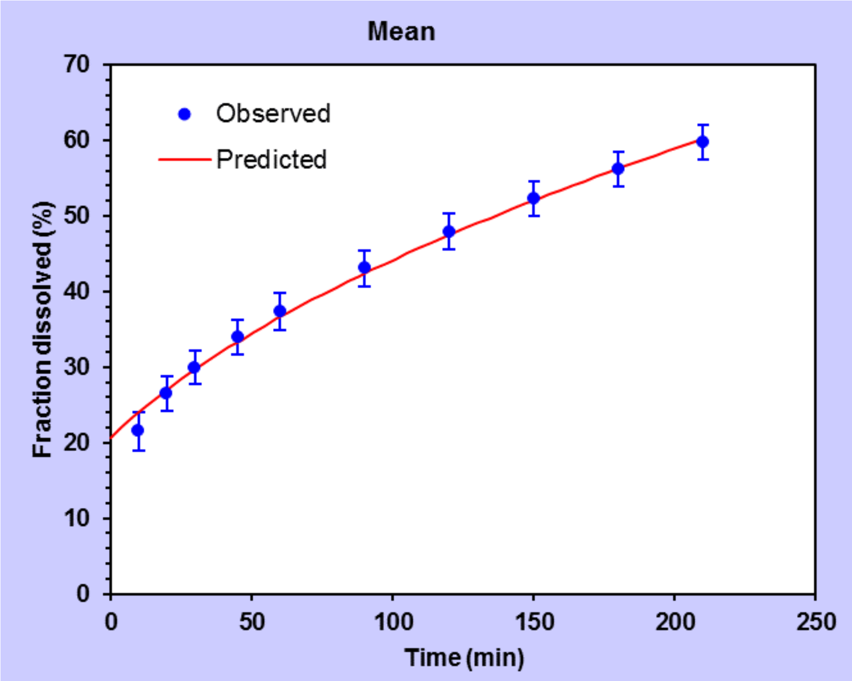

Supplementary materials – Model fitting summary – Lactochem® Crystals (modelling up to app. 60 % of carvedilol released)

Graphical abstract of model fit presented as the fraction % of released carvedilol per tested tablet:

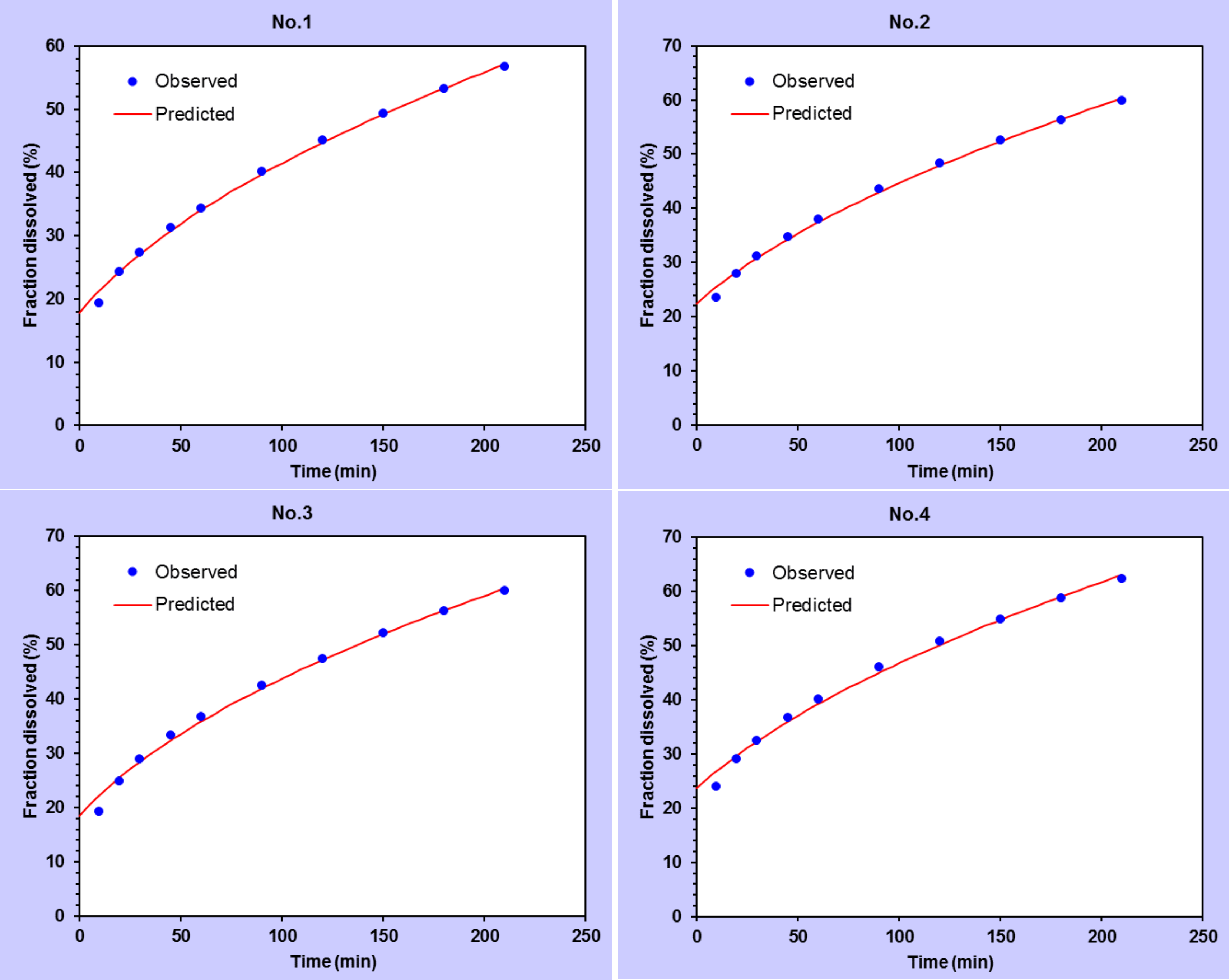

Supplementary materials – Model fitting summary – Lactochem® Crystals (modelling up to app. 60 % of carvedilol released)

Model: **Higuchi with F_0**

Model equation: $F = F_0 + k_H \cdot t^{0.5}$

Fitted model parameters per tested tablet (N = 4) with statistics – mean, standard deviation (SD), and relative standard deviation expressed in % (RSD%) (output from DDSolver):

Parameter	No.1	No.2	No.3	No.4	Mean	SD	RSD(%)
k_H	3.271	3.187	3.522	3.353	3.333	0.143	4.297
F_0	9.238	13.464	9.066	13.945	11.428	2.636	23.069

Number of dissolution data points (N), degrees of freedom (df), and selected goodness of fit criteria – Pearson correlation coefficient (R), coefficient of determination (R^2), adjusted coefficient of determination ($R^2_{adjusted}$), and residual sum of squares (RSS) (manual calculation in MS Excel):

Parameter	No.1	No.2	No.3	No.4
N	10	10	10	10
df	8	8	8	8
R	0.999894794	0.999944898	0.999403423	0.999816551
R^2	0.9997896	0.999889798	0.998807202	0.999633136
$R^2_{adjusted}$	0.9997633	0.999876023	0.998658102	0.999587279
RSS	0.310087378	0.15417057	2.04089242	0.568440068

Graphical abstract of model fit presented as mean ± 1 SD of the fraction % of released carvedilol:

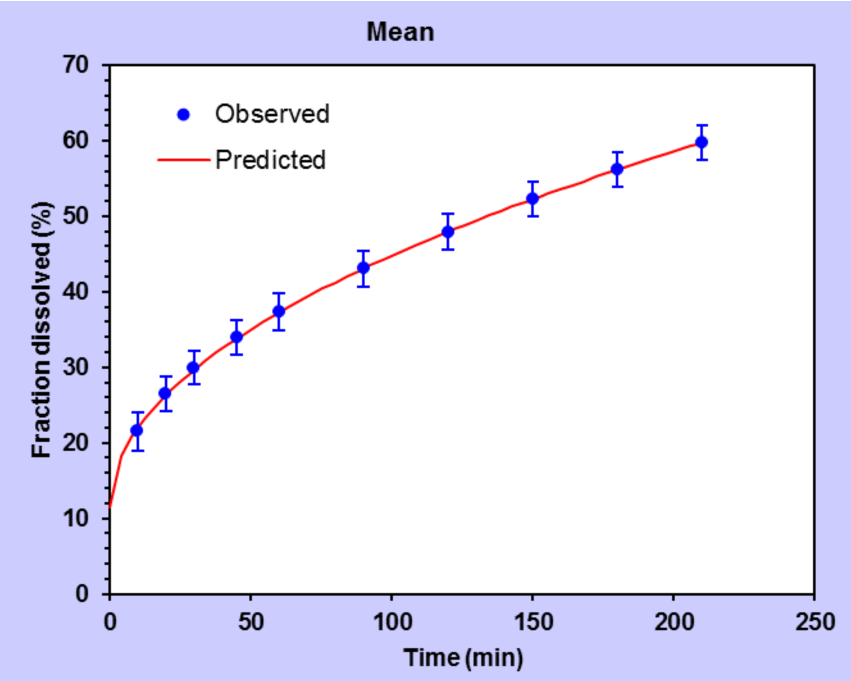

Supplementary materials – Model fitting summary – Lactochem® Crystals (modelling up to app. 60 % of carvedilol released)

Graphical abstract of model fit presented as the fraction % of released carvedilol per tested tablet:

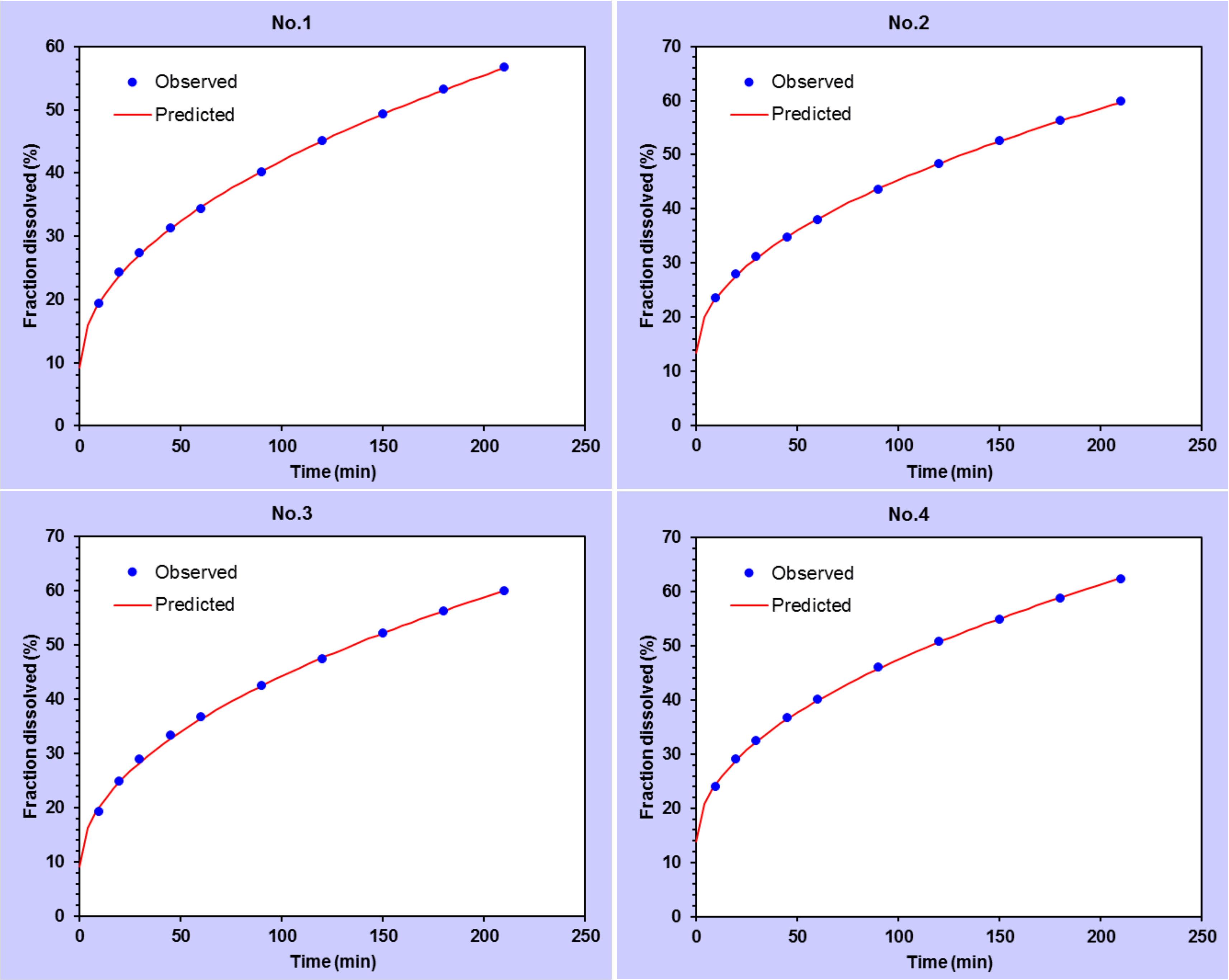

Supplementary materials – Model fitting summary – Lactochem® Crystals (modelling up to app. 60 % of carvedilol released)

Model: **Korsmeyer–Peppas**

Model equation: $F = k_{KP} \cdot t^n$

Fitted model parameters per tested tablet (N = 4) with statistics – mean, standard deviation (SD), and relative standard deviation expressed in % (RSD%) (output from DDSolver):

Parameter	No.1	No.2	No.3	No.4	Mean	SD	RSD(%)
k_{KP}	8.285	11.007	8.153	11.247	9.673	1.683	17.398
n	0.355	0.310	0.370	0.316	0.338	0.029	8.685

Number of dissolution data points (N), degrees of freedom (df), and selected goodness of fit criteria – Pearson correlation coefficient (R), coefficient of determination (R^2), adjusted coefficient of determination ($R^2_{adjusted}$), and residual sum of squares (RSS) (manual calculation in MS Excel):

Parameter	No.1	No.2	No.3	No.4
N	10	10	10	10
df	8	8	8	8
R	0.998306471	0.996808076	0.999458502	0.998507195
R^2	0.996615809	0.993626341	0.998917297	0.997016618
$R^2_{adjusted}$	0.996192786	0.992829634	0.99878196	0.996643695
RSS	5.884264868	10.28811078	1.986423245	5.352405627

Graphical abstract of model fit presented as mean ± 1 SD of the fraction % of released carvedilol:

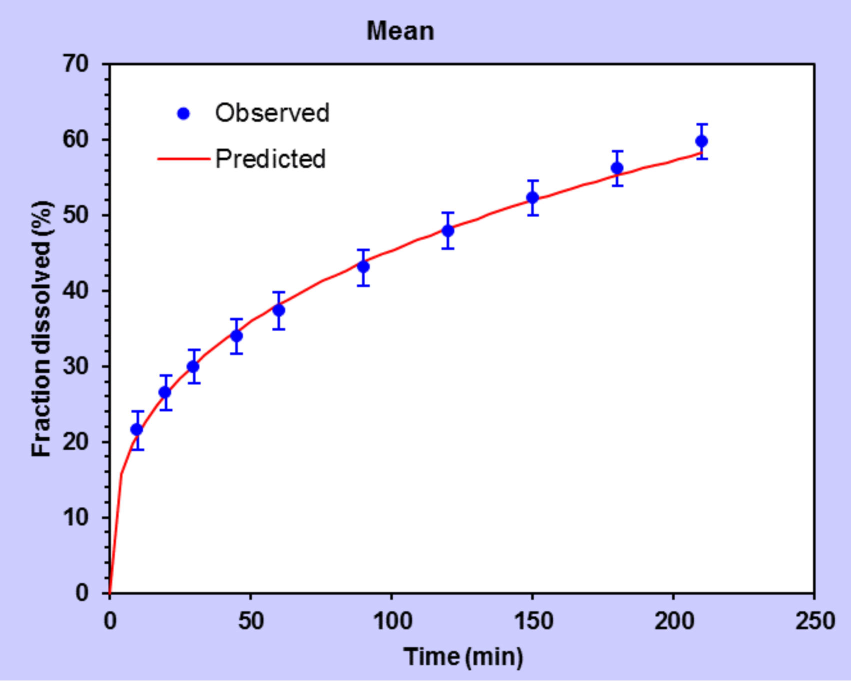

Supplementary materials – Model fitting summary – Lactochem® Crystals (modelling up to app. 60 % of carvedilol released)

Graphical abstract of model fit presented as the fraction % of released carvedilol per tested tablet:

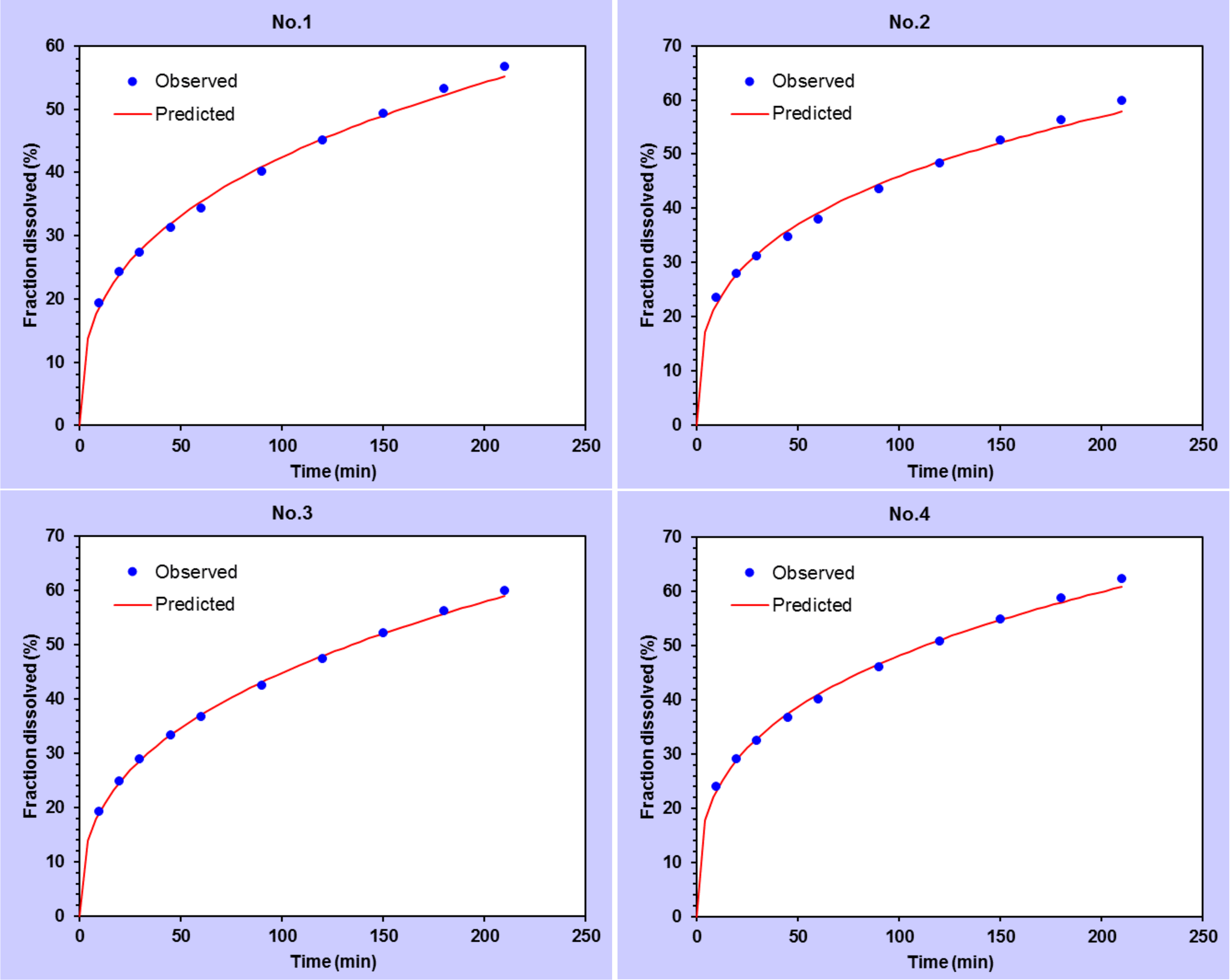

Supplementary materials – Model fitting summary – Lactochem® Crystals (modelling up to app. 60 % of carvedilol released)

Model: **Korsmeyer–Peppas with T_{lag}**

Model equation: $F = k_{KP} \cdot \left(t - T_{lag}\right)^n$

Fitted model parameters per tested tablet (N = 4) with statistics – mean, standard deviation (SD), and relative standard deviation expressed in % (RSD%) (output from DDSolver):

Parameter	No.1	No.2	No.3	No.4	Mean	SD	RSD(%)
k_{KP}	10.309	13.352	10.211	13.668	11.885	1.881	15.829
n	0.311	0.271	0.325	0.277	0.296	0.026	8.837
T_{lag}	4.000	4.000	4.000	4.000	4.000	0.000	0.000

Number of dissolution data points (N), degrees of freedom (df), and selected goodness of fit criteria – Pearson correlation coefficient (R), coefficient of determination (R^2), adjusted coefficient of determination ($R^2_{adjusted}$), and residual sum of squares (RSS) (manual calculation in MS Excel):

Parameter	No.1	No.2	No.3	No.4
N	10	10	10	10
df	7	7	7	7
R	0.994497728	0.99169029	0.997335329	0.99462673
R^2	0.98902573	0.983449631	0.994677759	0.989282333
$R^2_{adjusted}$	0.985890225	0.978720954	0.993157118	0.986220142
RSS	19.76278259	27.1535874	11.11325764	19.64403909

Graphical abstract of model fit presented as mean ± 1 SD of the fraction % of released carvedilol:

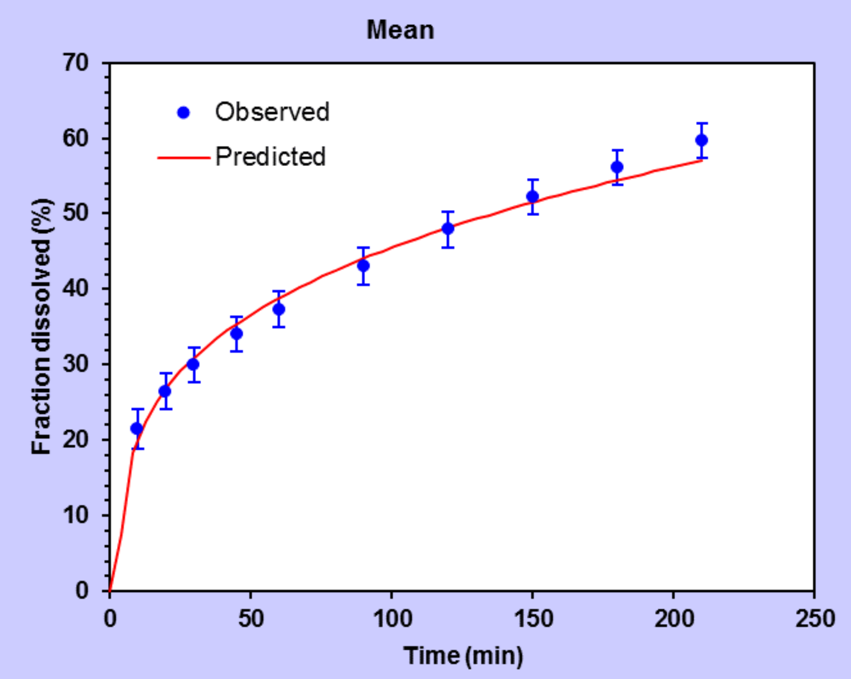

Supplementary materials – Model fitting summary – Lactochem® Crystals (modelling up to app. 60 % of carvedilol released)

Graphical abstract of model fit presented as the fraction % of released carvedilol per tested tablet:

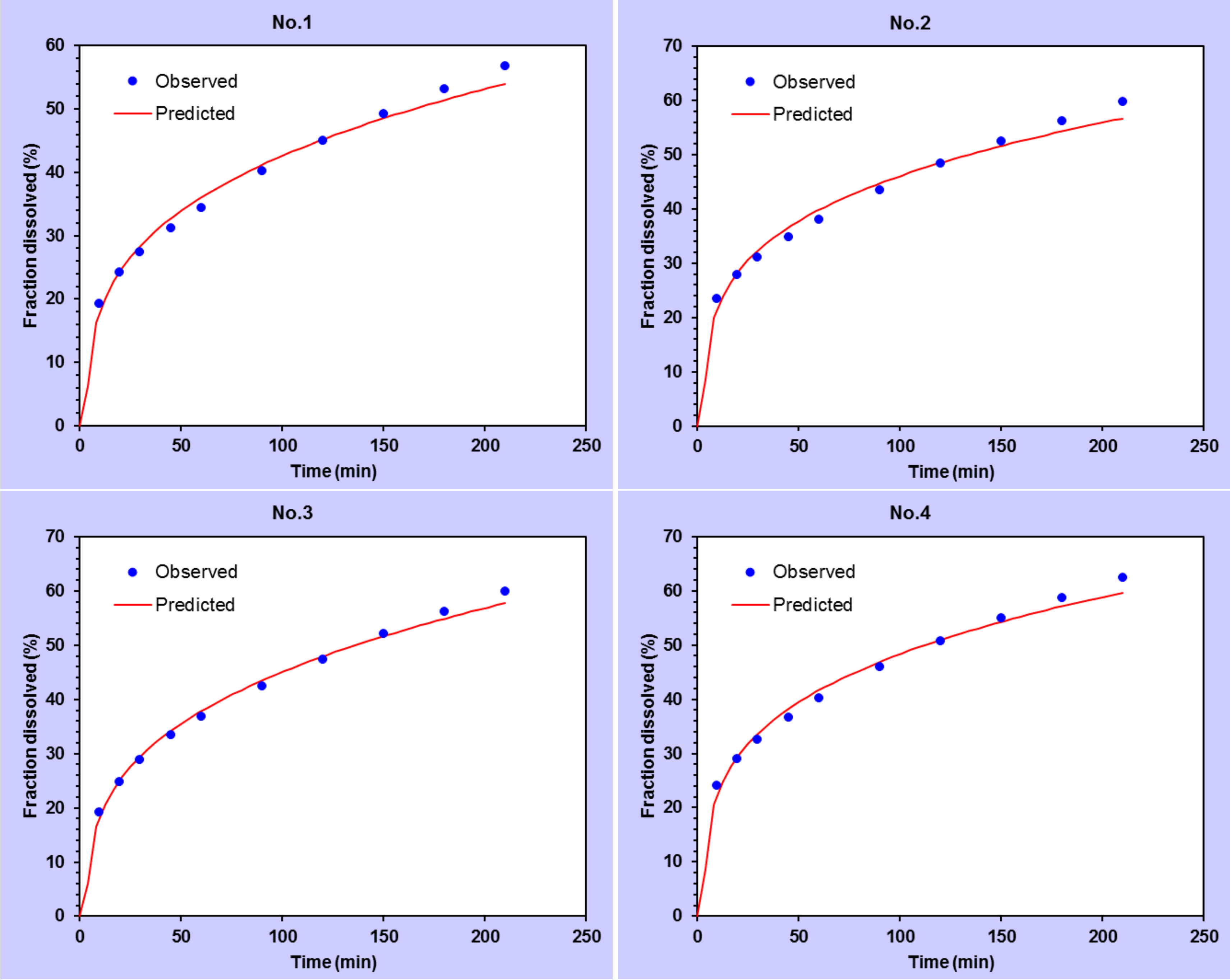

Supplementary materials – Model fitting summary – Lactochem® Crystals (modelling up to app. 60 % of carvedilol released)

Model: **Korsmeyer–Peppas with F_0**

Model equation: $F = F_0 + k_{KP} \cdot t^n$

Fitted model parameters per tested tablet (N = 4) with statistics – mean, standard deviation (SD), and relative standard deviation expressed in % (RSD%) (output from DDSolver):

Parameter	No.1	No.2	No.3	No.4	Mean	SD	RSD(%)
k_{KP}	3.967	4.948	3.952	5.398	4.566	0.724	15.864
n	0.469	0.434	0.485	0.425	0.453	0.028	6.234
F_0	7.719	9.345	7.679	9.598	8.585	1.029	11.982

Number of dissolution data points (N), degrees of freedom (df), and selected goodness of fit criteria – Pearson correlation coefficient (R), coefficient of determination (R^2), adjusted coefficient of determination ($R^2_{adjusted}$), and residual sum of squares (RSS) (manual calculation in MS Excel):

Parameter	No.1	No.2	No.3	No.4
N	10	10	10	10
df	7	7	7	7
R	0.999820327	0.999489871	0.999535881	0.999909532
R^2	0.999640686	0.998980002	0.999071978	0.999819073
$R^2_{adjusted}$	0.999538025	0.998688574	0.998806829	0.999767379
RSS	0.56352098	2.170194336	2.141473611	0.320083261

Graphical abstract of model fit presented as mean ± 1 SD of the fraction % of released carvedilol:

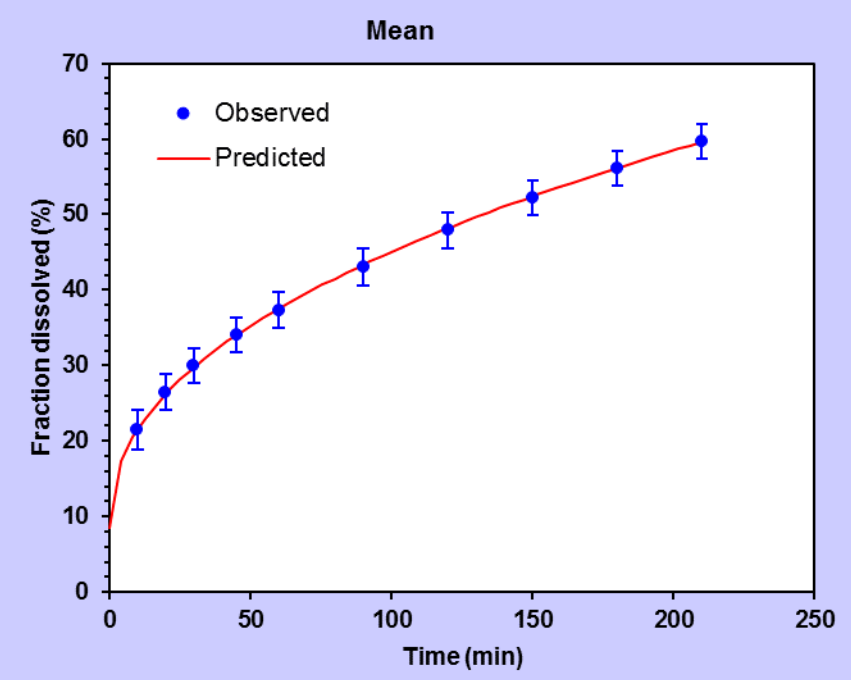

Supplementary materials – Model fitting summary – Lactochem® Crystals (modelling up to app. 60 % of carvedilol released)

Graphical abstract of model fit presented as the fraction % of released carvedilol per tested tablet:

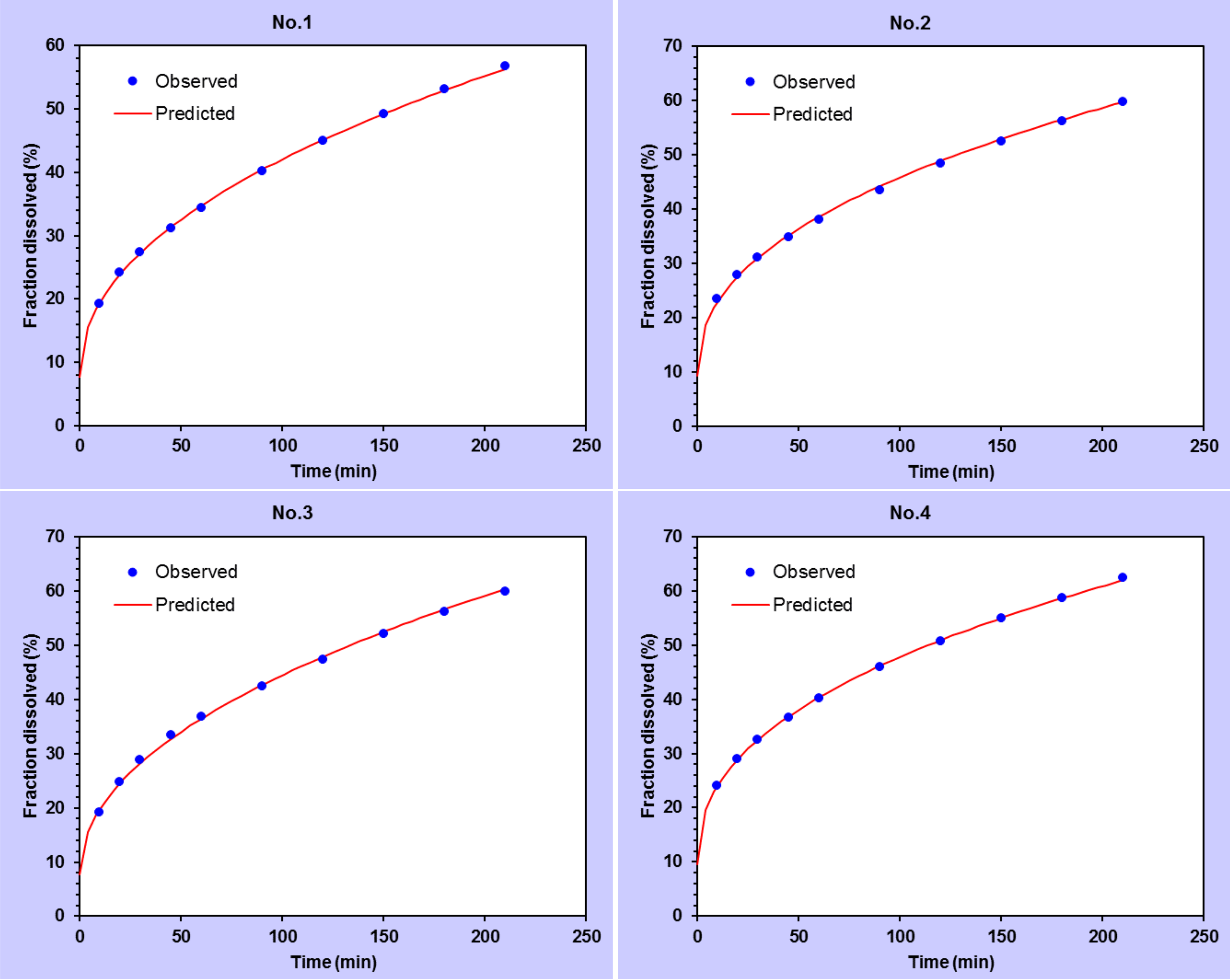

Supplementary materials – Model fitting summary – Lactochem® Crystals (modelling up to app. 60 % of carvedilol released)

Model: **Hixson–Crowell**

Model equation: $F = 100 \cdot [1 - (1 - k_{HC} \cdot t)^3]$

Fitted model parameters per tested tablet (N = 4) with statistics – mean, standard deviation (SD), and relative standard deviation expressed in % (RSD%) (output from DDSolver):

Parameter	No.1	No.2	No.3	No.4	Mean	SD	RSD(%)
k_{HC}	0.001	0.001	0.001	0.002	0.001	0.000	5.963

Number of dissolution data points (N), degrees of freedom (df), and selected goodness of fit criteria – Pearson correlation coefficient (R), coefficient of determination (R^2), adjusted coefficient of determination ($R^2_{adjusted}$), and residual sum of squares (RSS) (manual calculation in MS Excel):

Parameter	No.1	No.2	No.3	No.4
N	10	10	10	10
df	9	9	9	9
R	0.995324776	0.996441727	0.992846961	0.994823413
R^2	0.990671411	0.992896116	0.985745089	0.989673624
$R^2_{adjusted}$	0.990671411	0.992896116	0.985745089	0.989673624
RSS	1198.722379	1667.877973	1258.276212	1784.628004

Graphical abstract of model fit presented as mean ± 1 SD of the fraction % of released carvedilol:

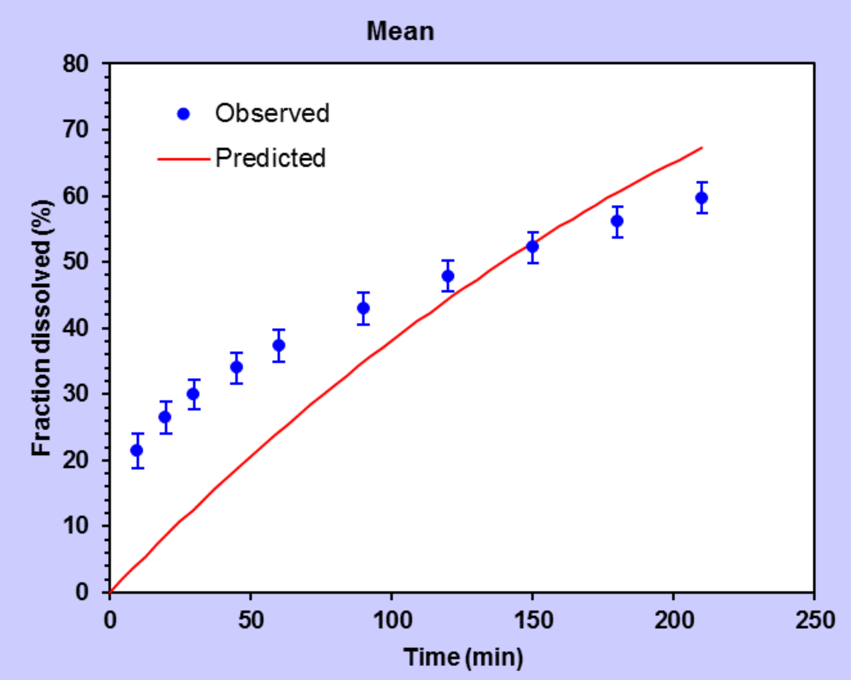

Supplementary materials – Model fitting summary – Lactochem® Crystals (modelling up to app. 60 % of carvedilol released)

Graphical abstract of model fit presented as the fraction % of released carvedilol per tested tablet:

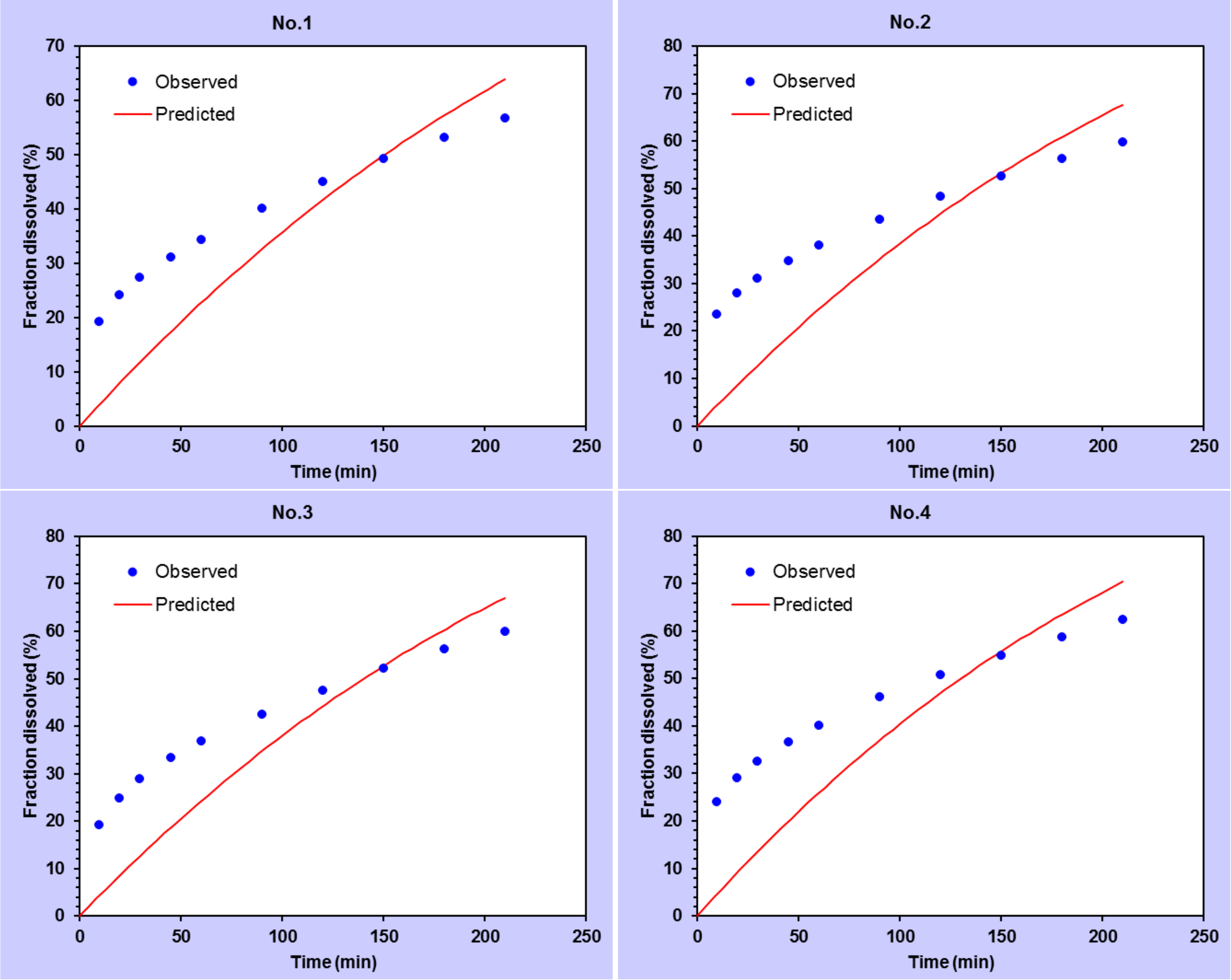

Supplementary materials – Model fitting summary – Lactochem® Crystals (modelling up to app. 60 % of carvedilol released)

Model: **Hixson–Crowell with T_{lag}**

Model equation: $F = 100 \cdot \left\{1 - \left[1 - k_{HC} \cdot \left(t - T_{lag}\right)\right]^3\right\}$

Fitted model parameters per tested tablet (N = 4) with statistics – mean, standard deviation (SD), and relative standard deviation expressed in % (RSD%) (output from DDSolver):

Parameter	No.1	No.2	No.3	No.4	Mean	SD	RSD(%)
k_{HC}	0.001	0.001	0.001	0.001	0.001	0.000	4.817
T_{lag}	-88.545	-105.153	-83.506	-102.714	-94.979	10.589	-11.149

Number of dissolution data points (N), degrees of freedom (df), and selected goodness of fit criteria – Pearson correlation coefficient (R), coefficient of determination (R^2), adjusted coefficient of determination ($R^2_{adjusted}$), and residual sum of squares (RSS) (manual calculation in MS Excel):

Parameter	No.1	No.2	No.3	No.4
N	10	10	10	10
df	8	8	8	8
R	0.992616722	0.993453961	0.989659483	0.991101869
R^2	0.985287957	0.986950773	0.979425892	0.982282916
$R^2_{adjusted}$	0.983448951	0.985319619	0.976854129	0.98006828
RSS	22.06607064	18.60548361	35.89107026	28.06465835

Graphical abstract of model fit presented as mean ± 1 SD of the fraction % of released carvedilol:

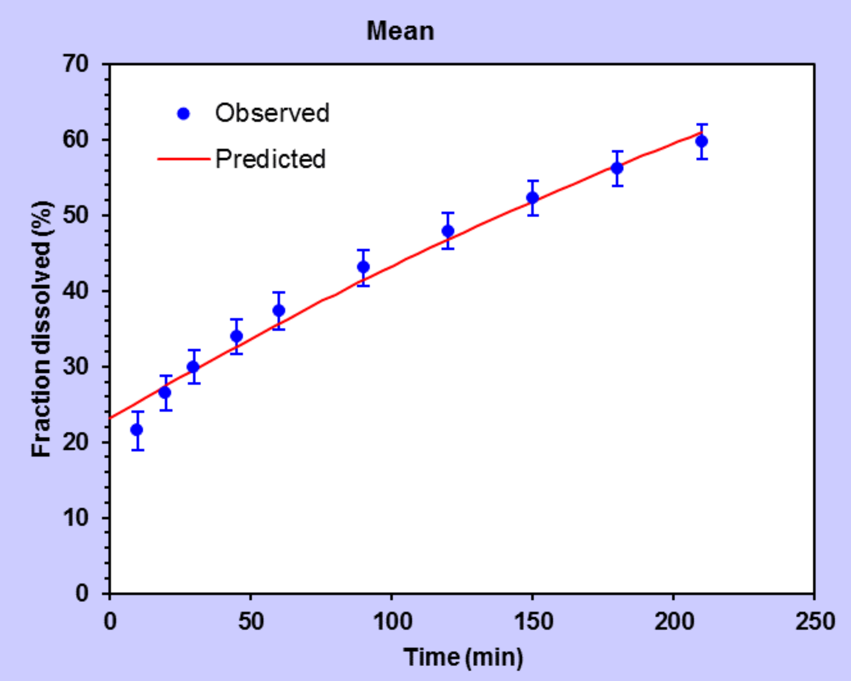

Supplementary materials – Model fitting summary – Lactochem® Crystals (modelling up to app. 60 % of carvedilol released)

Graphical abstract of model fit presented as the fraction % of released carvedilol per tested tablet:

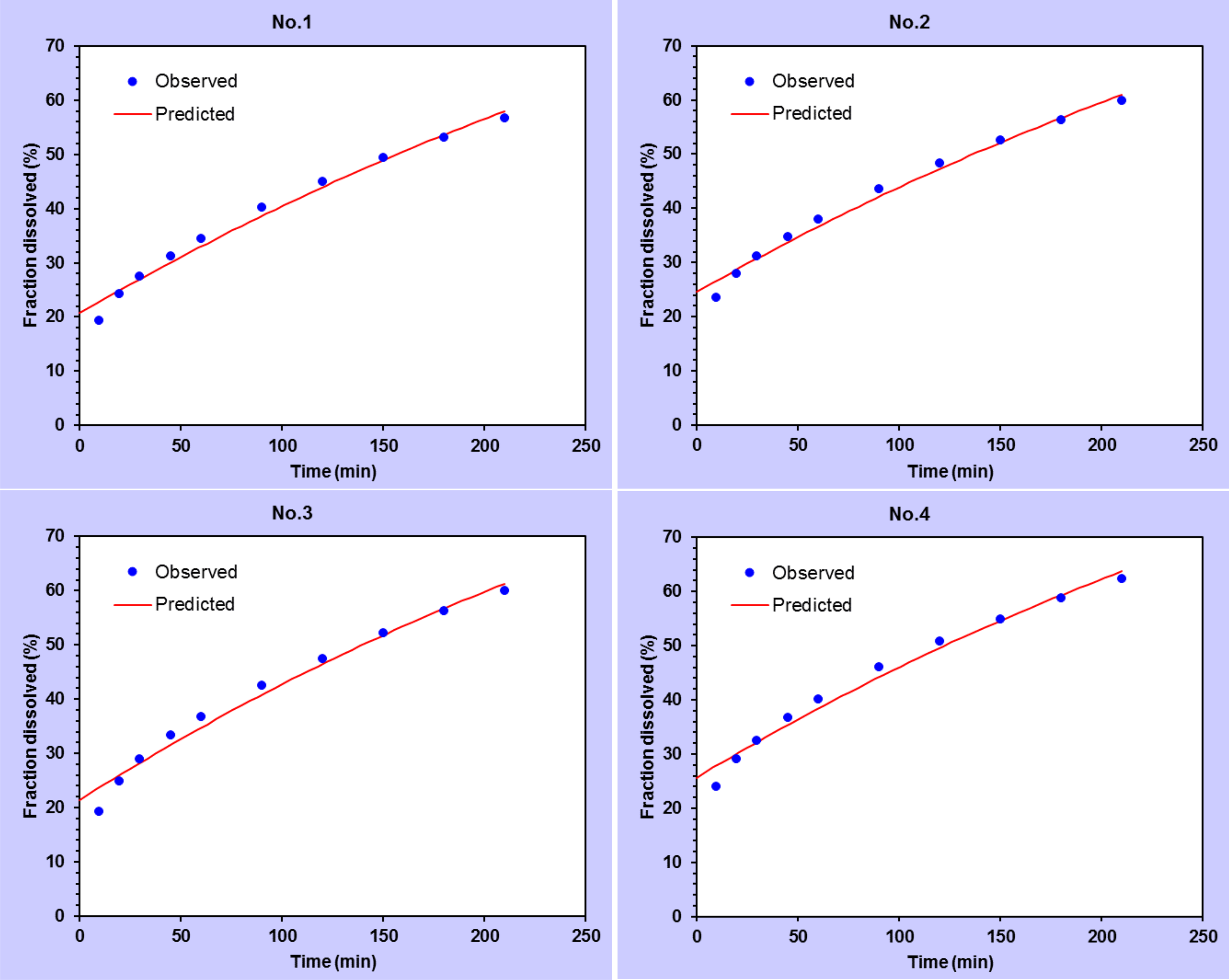

Supplementary materials – Model fitting summary – Lactochem® Crystals (modelling up to app. 60 % of carvedilol released)

Model: **Hopfenberg**

Model equation: $F = 100 \cdot [1 - (1 - k_{HB} \cdot t)^n]$

Fitted model parameters per tested tablet (N = 4) with statistics – mean, standard deviation (SD), and relative standard deviation expressed in % (RSD%) (output from DDSolver):

Parameter	No.1	No.2	No.3	No.4	Mean	SD	RSD(%)
k_{HB}	0.001	0.001	0.001	0.002	0.001	0.000	5.963
n	3.000	3.000	3.000	3.000	3.000	0.000	0.000

Number of dissolution data points (N), degrees of freedom (df), and selected goodness of fit criteria – Pearson correlation coefficient (R), coefficient of determination (R^2), adjusted coefficient of determination ($R^2_{adjusted}$), and residual sum of squares (RSS) (manual calculation in MS Excel):

Parameter	No.1	No.2	No.3	No.4
N	10	10	10	10
df	8	8	8	8
R	0.995324776	0.996441727	0.992846961	0.994823413
R^2	0.990671411	0.992896116	0.985745089	0.989673624
$R^2_{adjusted}$	0.989505337	0.992008131	0.983963225	0.988382827
RSS	1198.722379	1667.877973	1258.276212	1784.628004

Graphical abstract of model fit presented as mean ± 1 SD of the fraction % of released carvedilol:

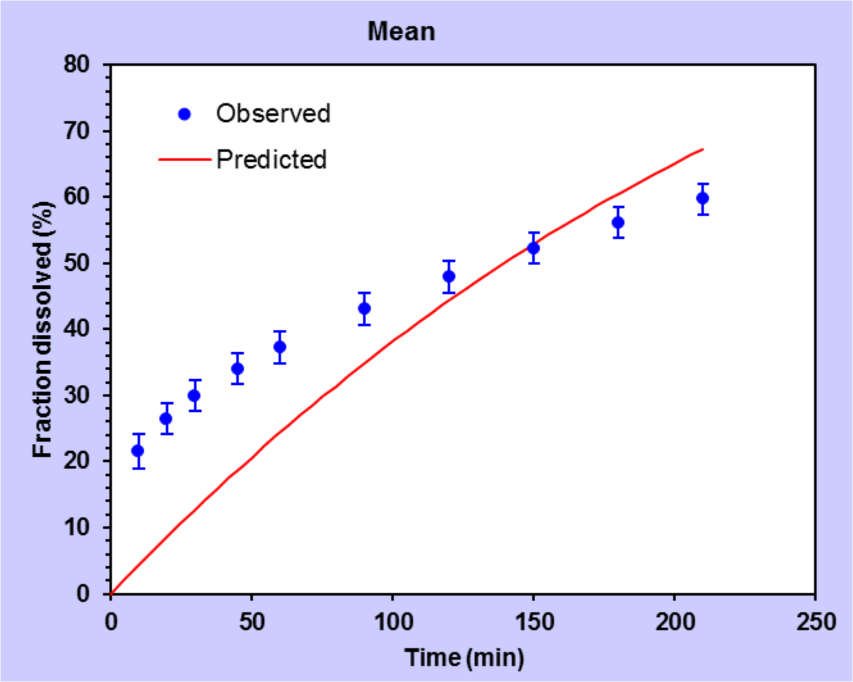

Supplementary materials – Model fitting summary – Lactochem® Crystals (modelling up to app. 60 % of carvedilol released)

Graphical abstract of model fit presented as the fraction % of released carvedilol per tested tablet:

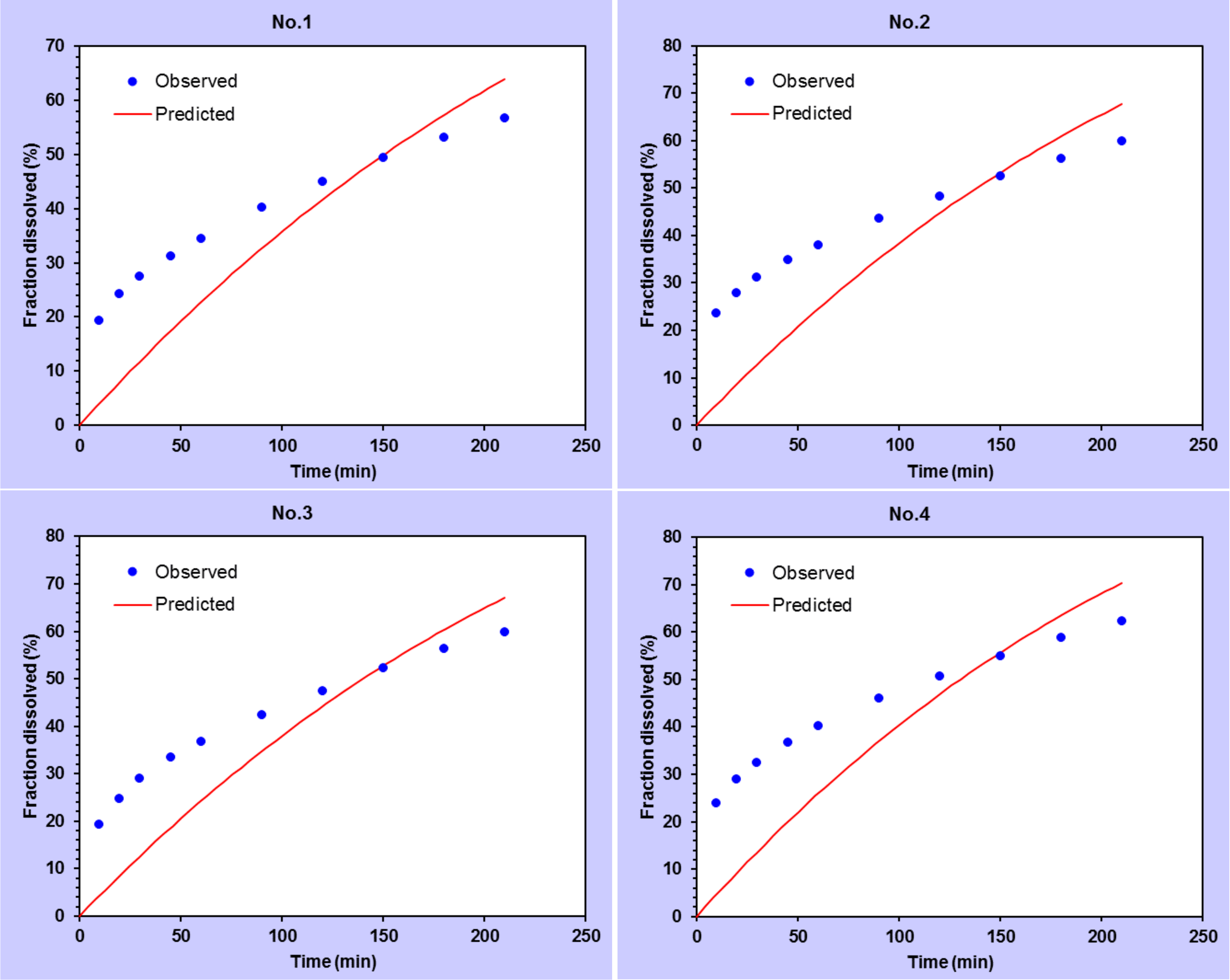

Supplementary materials – Model fitting summary – Lactochem® Crystals (modelling up to app. 60 % of carvedilol released)

Model: **Hopfenberg with T_{lag}**

Model equation: $F = 100 \cdot \{1 - [1 - k_{HB} \cdot (t - T_{lag})]^n\}$

Fitted model parameters per tested tablet (N = 4) with statistics – mean, standard deviation (SD), and relative standard deviation expressed in % (RSD%) (output from DDSolver):

Parameter	No.1	No.2	No.3	No.4	Mean	SD	RSD(%)
k_{HB}	0.001	0.001	0.001	0.001	0.001	0.000	4.817
n	3.000	3.000	3.000	3.000	3.000	0.000	0.000
T_{lag}	-88.545	-105.153	-83.506	-102.714	-94.979	10.589	-11.149

Number of dissolution data points (N), degrees of freedom (df), and selected goodness of fit criteria – Pearson correlation coefficient (R), coefficient of determination (R^2), adjusted coefficient of determination ($R^2_{adjusted}$), and residual sum of squares (RSS) (manual calculation in MS Excel):

Parameter	No.1	No.2	No.3	No.4
N	10	10	10	10
df	7	7	7	7
R	0.992616722	0.993453961	0.989659483	0.991101869
R2	0.985287957	0.986950773	0.979425892	0.982282916
R2adjusted	0.981084516	0.983222422	0.973547576	0.977220892
RSS	22.06607064	18.60548361	35.89107026	28.06465835

Graphical abstract of model fit presented as mean ± 1 SD of the fraction % of released carvedilol:

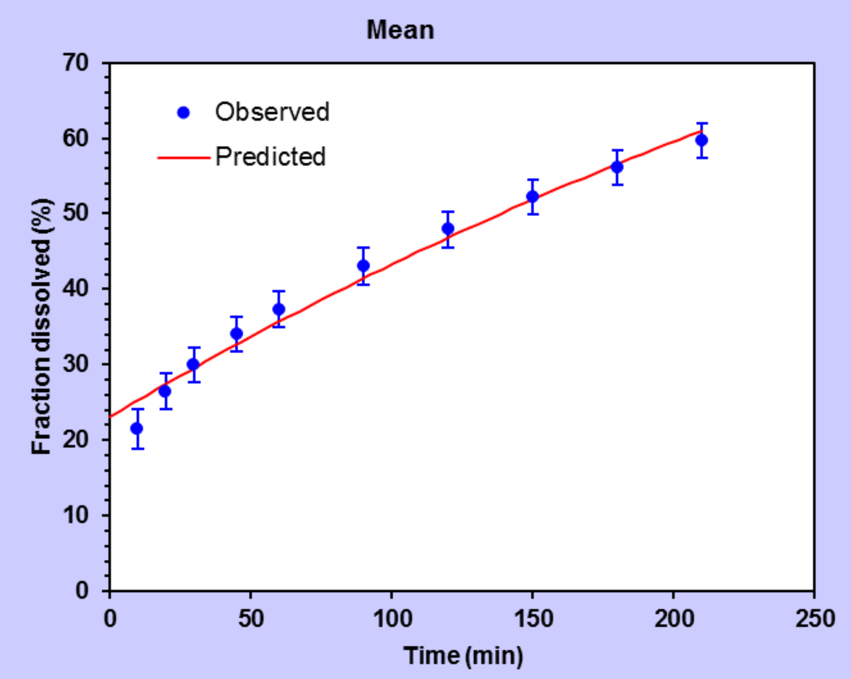

Supplementary materials – Model fitting summary – Lactochem® Crystals (modelling up to app. 60 % of carvedilol released)

Graphical abstract of model fit presented as the fraction % of released carvedilol per tested tablet:

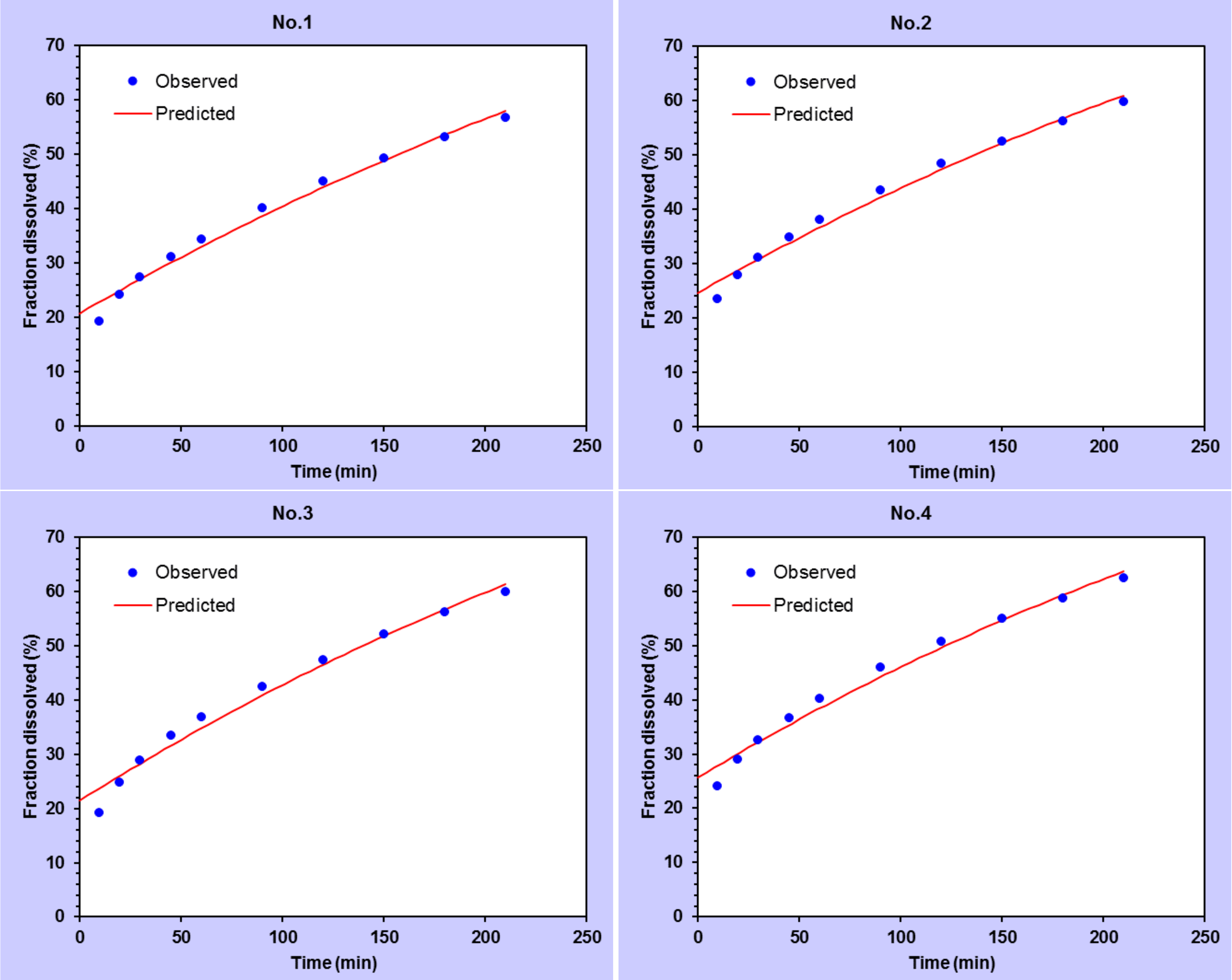

Supplementary materials – Model fitting summary – Lactochem® Crystals (modelling up to app. 60 % of carvedilol released)

Model: **Baker–Lonsdale**

Model equation: $\frac{3}{2}\cdot\left[1-\left(1-\frac{F}{100}\right)^{\frac{2}{3}}\right]-\frac{F}{100}=k_{BL}\cdot t$

Fitted model parameters per tested tablet (N = 4) with statistics – mean, standard deviation (SD), and relative standard deviation expressed in % (RSD%) (output from DDSolver):

Parameter	No.1	No.2	No.3	No.4	Mean	SD	RSD(%)
k_{BL}	0.0003	0.0005	0.0005	0.0006	0.0005	0.0001	24.4578

Number of dissolution data points (N), degrees of freedom (df), and selected goodness of fit criteria – Pearson correlation coefficient (R), coefficient of determination (R^2), adjusted coefficient of determination ($R^2_{adjusted}$), and residual sum of squares (RSS) (manual calculation in MS Excel):

Parameter	No.1	No.2	No.3	No.4
N	10	10	10	10
df	9	9	9	9
R	0.999097226	0.998184696	0.99930374	0.999094964
R^2	0.998195268	0.996372687	0.998607965	0.998190746
$R^2_{adjusted}$	0.998195268	0.996372687	0.998607965	0.998190746
RSS	102.709607	262.4039129	81.91065282	266.8009766

Graphical abstract of model fit presented as mean ± 1 SD of the fraction % of released carvedilol:

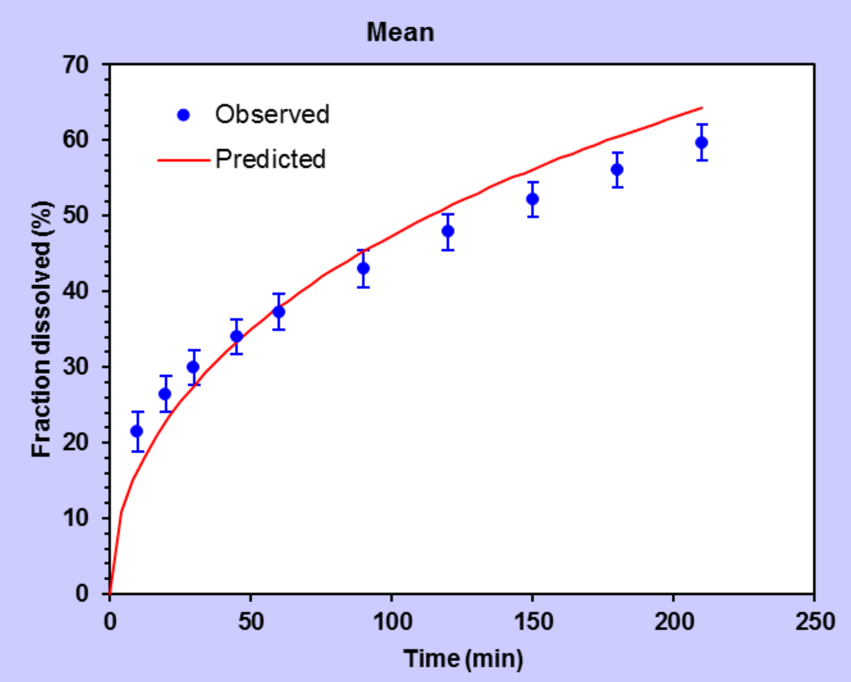

Supplementary materials – Model fitting summary – Lactochem® Crystals (modelling up to app. 60 % of carvedilol released)

Graphical abstract of model fit presented as the fraction % of released carvedilol per tested tablet:

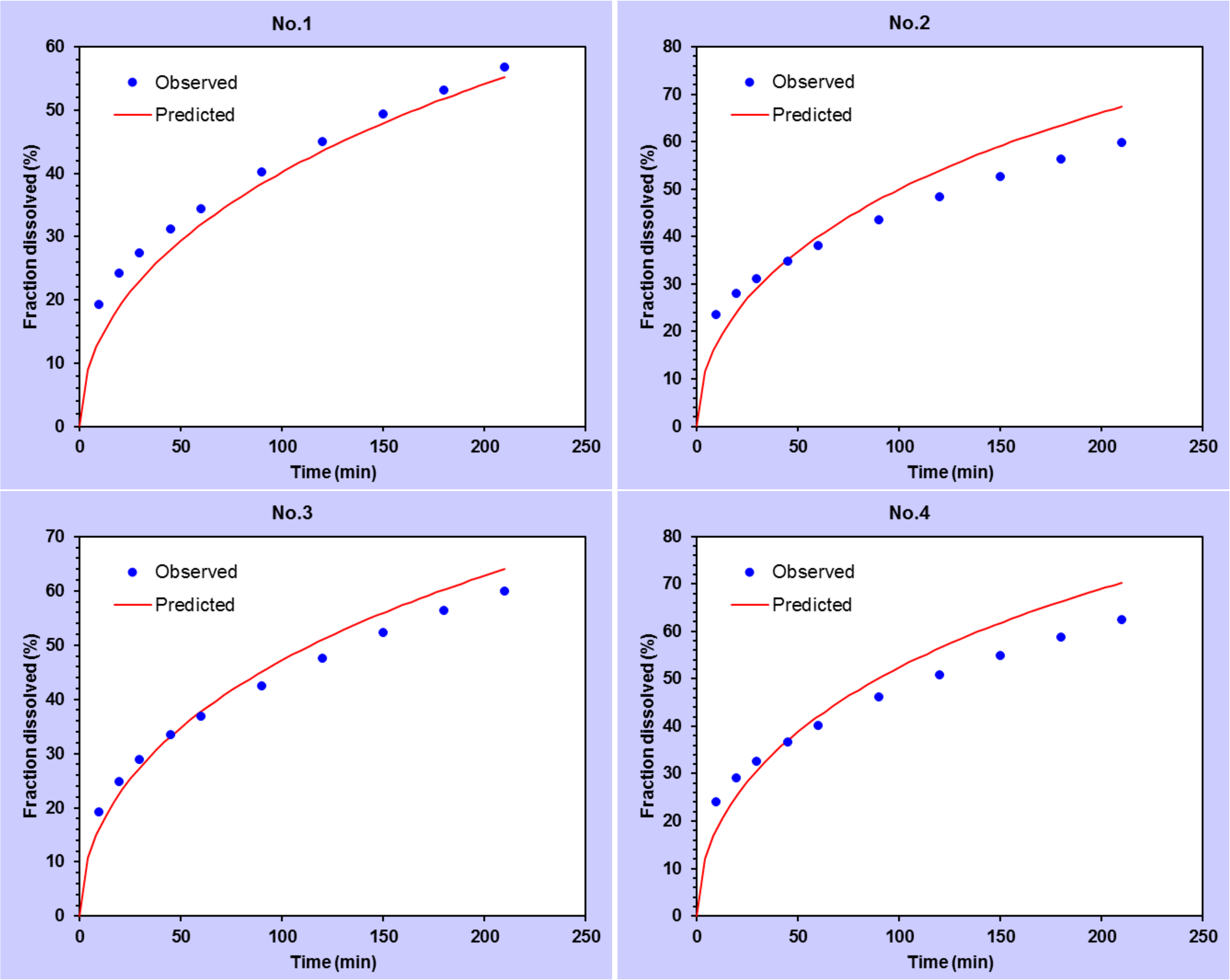

Supplementary materials – Model fitting summary – Lactochem® Crystals (modelling up to app. 60 % of carvedilol released)

Model: **Baker–Lonsdale with T_{lag}**

Model equation: $\frac{3}{2}\cdot\left[1-\left(1-\frac{F}{100}\right)^{\frac{2}{3}}\right]-\frac{F}{100}=k_{BL}\cdot\left(t-T_{lag}\right)$

Fitted model parameters per tested tablet (N = 4) with statistics – mean, standard deviation (SD), and relative standard deviation expressed in % (RSD%) (output from DDSolver):

Parameter	No.1	No.2	No.3	No.4	Mean	SD	RSD(%)
k_{BL}	0.0003	0.0004	0.0004	0.0004	0.0004	0.0000	8.6685
T_{lag}	-11.3013	-19.7661	-9.7556	-19.4925	-15.0789	5.2933	-35.1040

Number of dissolution data points (N), degrees of freedom (df), and selected goodness of fit criteria – Pearson correlation coefficient (R), coefficient of determination (R^2), adjusted coefficient of determination ($R^2_{adjusted}$), and residual sum of squares (RSS) (manual calculation in MS Excel):

Parameter	No.1	No.2	No.3	No.4
N	10	10	10	10
df	8	8	8	8
R	0.999652933	0.999690691	0.999201466	0.999332336
R^2	0.999305986	0.999381479	0.99840357	0.998665117
$R^2_{adjusted}$	0.999219234	0.999304163	0.998204016	0.998498256
RSS	1.068696584	0.865377443	2.745467447	2.213622446

Graphical abstract of model fit presented as mean ± 1 SD of the fraction % of released carvedilol:

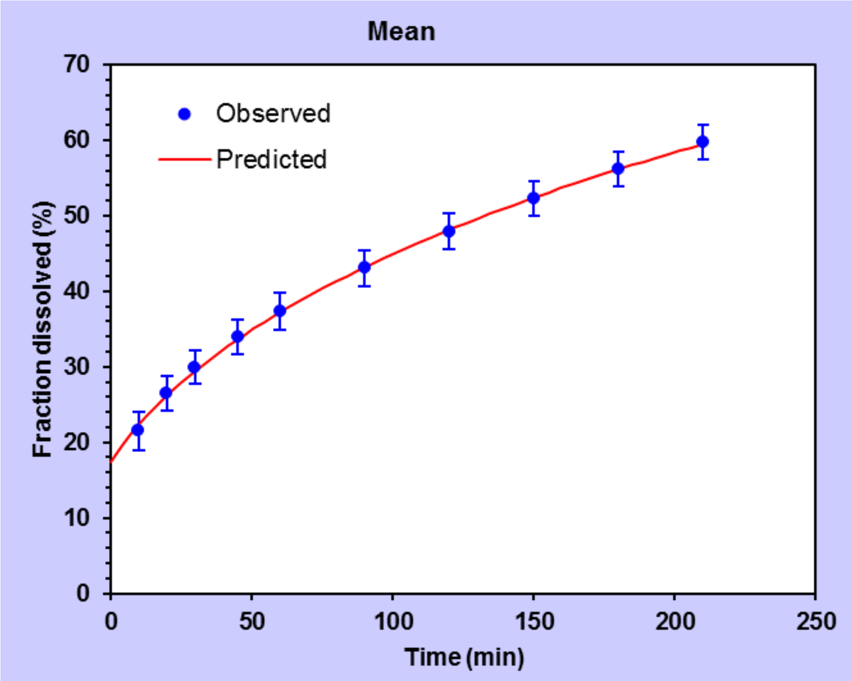

Supplementary materials – Model fitting summary – Lactochem® Crystals (modelling up to app. 60 % of carvedilol released)

Graphical abstract of model fit presented as the fraction % of released carvedilol per tested tablet:

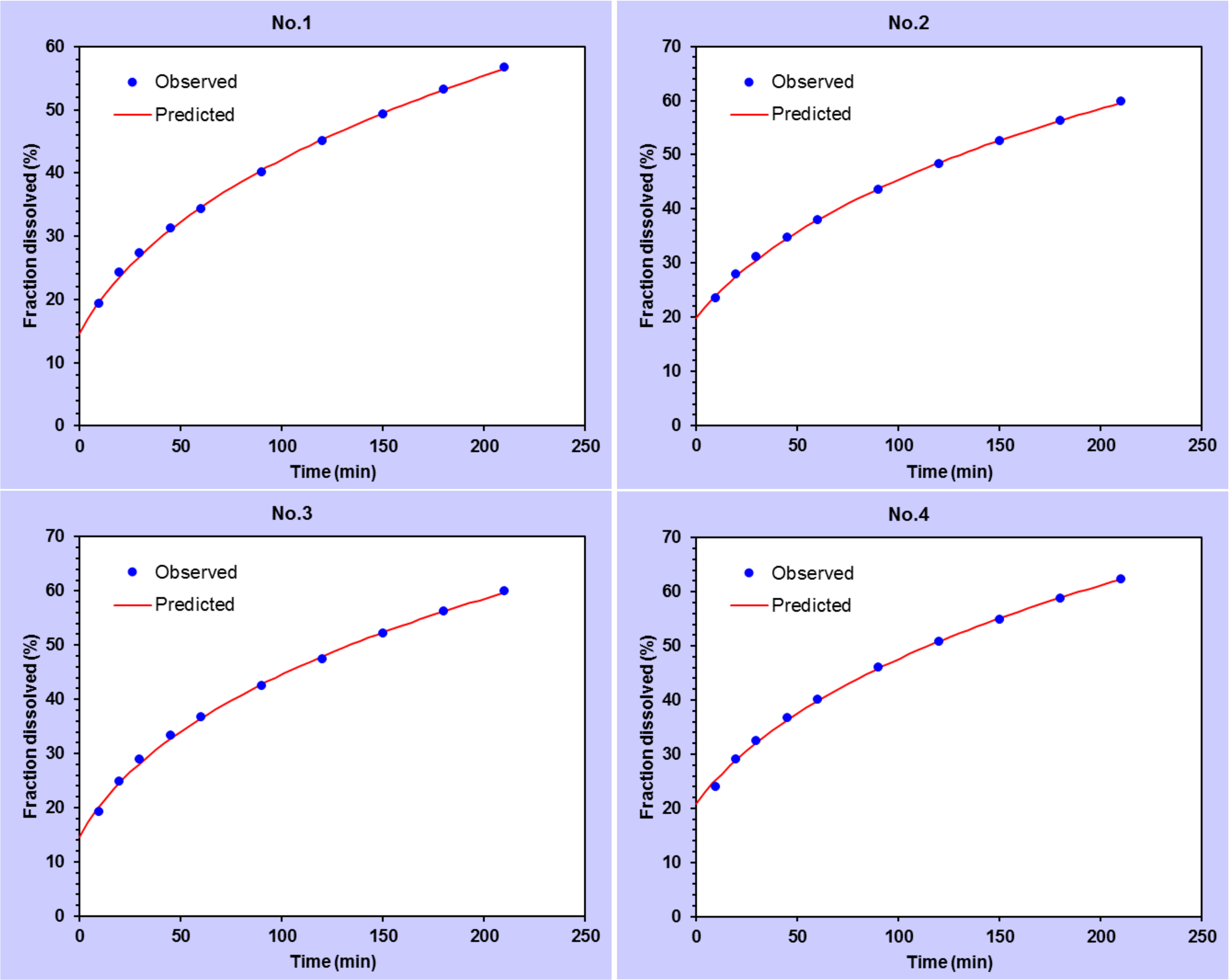

Supplementary materials – Model fitting summary – Lactochem® Crystals (modelling up to app. 60 % of carvedilol released)

Model: **Makoid–Banakar**

Model equation: $F = k_{MB} \cdot t^{n} \cdot e^{-k \cdot t}$

Fitted model parameters per tested tablet (N = 4) with statistics – mean, standard deviation (SD), and relative standard deviation expressed in % (RSD%) (output from DDSolver):

Parameter	No.1	No.2	No.3	No.4	Mean	SD	RSD(%)
k_{MB}	9.546	13.177	8.579	12.728	11.008	2.288	20.782
n	0.303	0.244	0.352	0.271	0.292	0.046	15.782
k	-0.001	-0.001	0.000	-0.001	-0.001	0.000	-43.581

Number of dissolution data points (N), degrees of freedom (df), and selected goodness of fit criteria – Pearson correlation coefficient (R), coefficient of determination (R^2), adjusted coefficient of determination ($R^2_{adjusted}$), and residual sum of squares (RSS) (manual calculation in MS Excel):

Parameter	No.1	No.2	No.3	No.4
N	10	10	10	10
df	7	7	7	7
R	0.99989939	0.999820939	0.999846847	0.999936327
R^2	0.99979879	0.99964191	0.999693718	0.999872659
$R^2_{adjusted}$	0.999741301	0.999539599	0.999606209	0.999836275
RSS	0.296642544	0.501506992	0.524061609	0.197370633

Graphical abstract of model fit presented as mean ± 1 SD of the fraction % of released carvedilol:

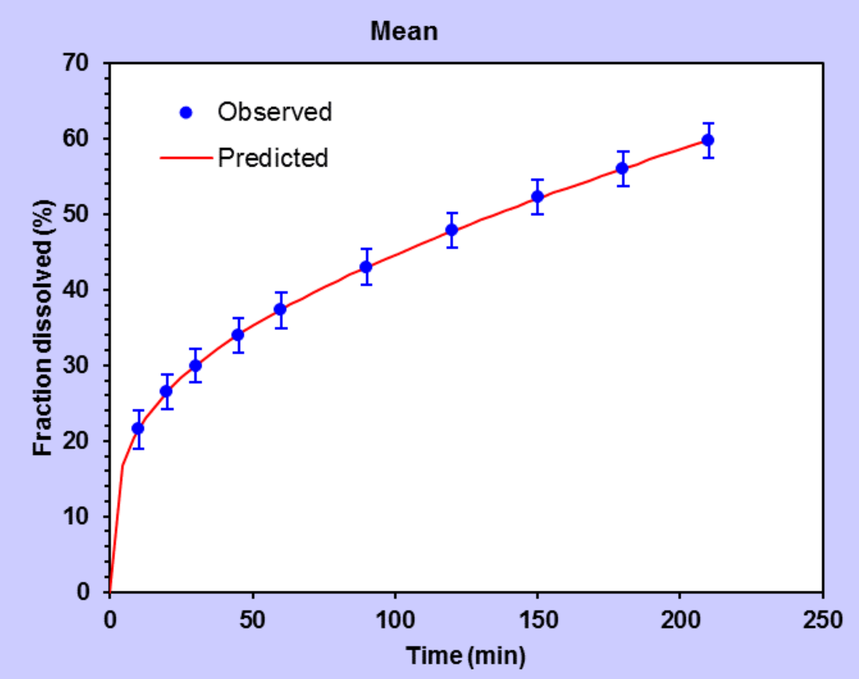

Supplementary materials – Model fitting summary – Lactochem® Crystals (modelling up to app. 60 % of carvedilol released)

Graphical abstract of model fit presented as the fraction % of released carvedilol per tested tablet:

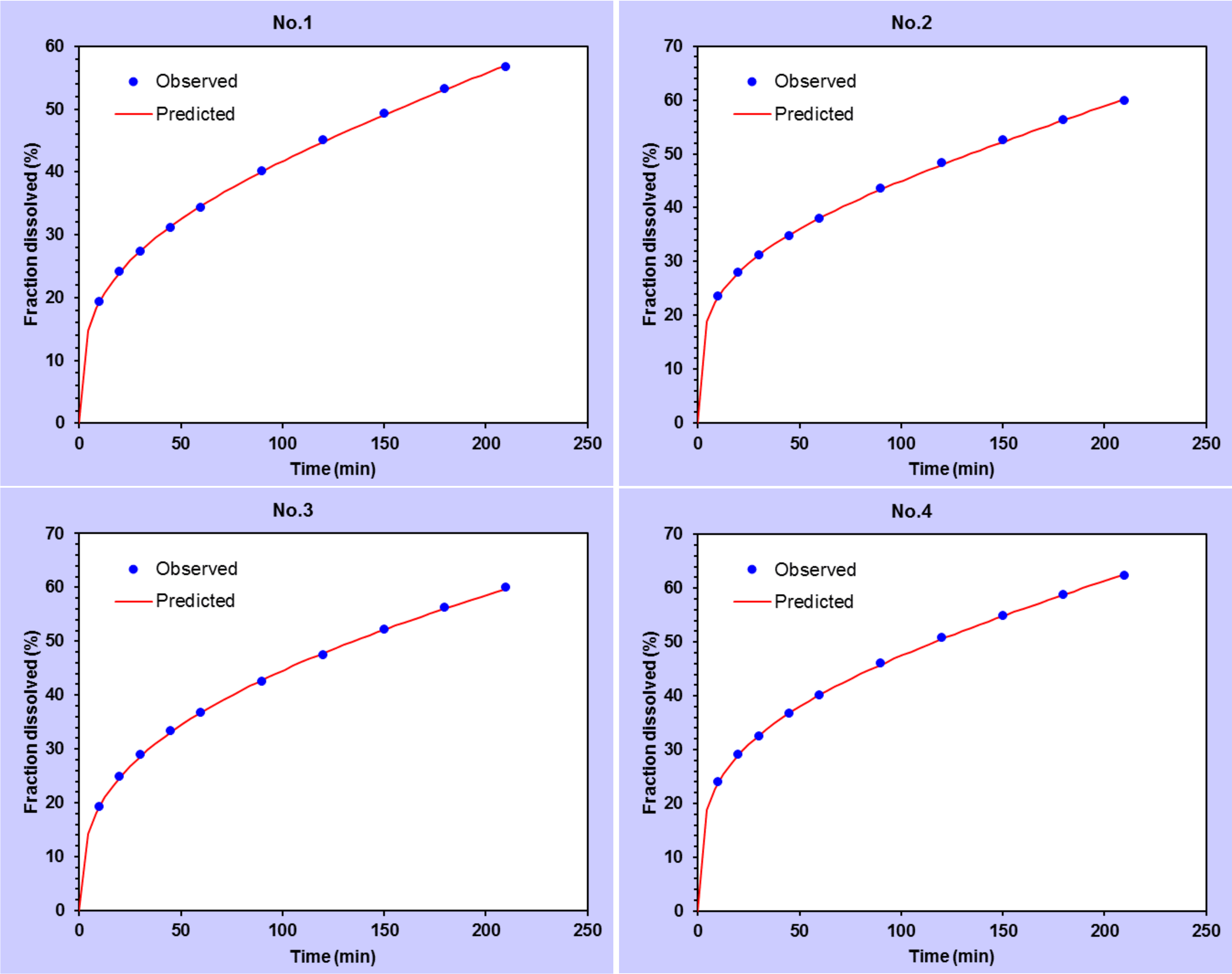

Supplementary materials – Model fitting summary – Lactochem® Crystals (modelling up to app. 60 % of carvedilol released)

Model: **Makoid–Banakar with T_{lag}**

Model equation: $F = k_{MB} \cdot (t - T_{lag})^{n} \cdot e^{-k \cdot (t - T_{lag})}$

Fitted model parameters per tested tablet (N = 4) with statistics – mean, standard deviation (SD), and relative standard deviation expressed in % (RSD%) (output from DDSolver):

Parameter	No.1	No.2	No.3	No.4	Mean	SD	RSD(%)
k_{MB}	12.451	16.383	11.623	16.159	14.154	2.469	17.447
n	0.234	0.188	0.273	0.209	0.226	0.036	16.086
k	-0.001	-0.001	-0.001	-0.001	-0.001	0.000	-18.832
T_{lag}	4.000	4.000	4.000	4.000	4.000	0.000	0.000

Number of dissolution data points (N), degrees of freedom (df), and selected goodness of fit criteria – Pearson correlation coefficient (R), coefficient of determination (R^2), adjusted coefficient of determination ($R^2_{adjusted}$), and residual sum of squares (RSS) (manual calculation in MS Excel):

Parameter	No.1	No.2	No.3	No.4
N	10	10	10	10
df	6	6	6	6
R	0.999486389	0.999250132	0.999881552	0.999428535
R2	0.998973042	0.998500827	0.999763118	0.998857396
R2adjusted	0.998459563	0.99775124	0.999644676	0.998286093
RSS	1.518261017	2.104898801	0.405603007	1.773926006

Graphical abstract of model fit presented as mean ± 1 SD of the fraction % of released carvedilol:

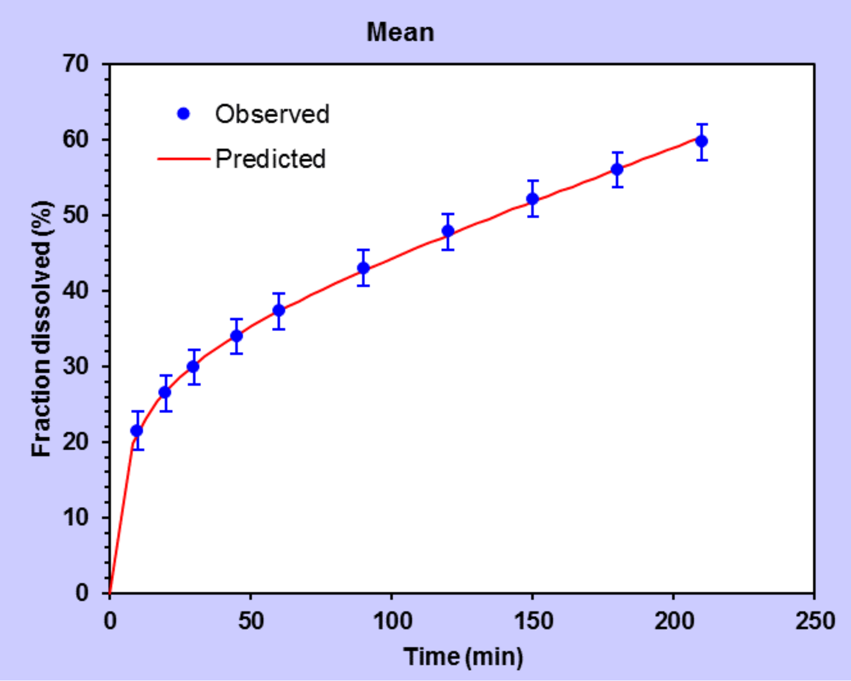

Supplementary materials – Model fitting summary – Lactochem® Crystals (modelling up to app. 60 % of carvedilol released)

Graphical abstract of model fit presented as the fraction % of released carvedilol per tested tablet:

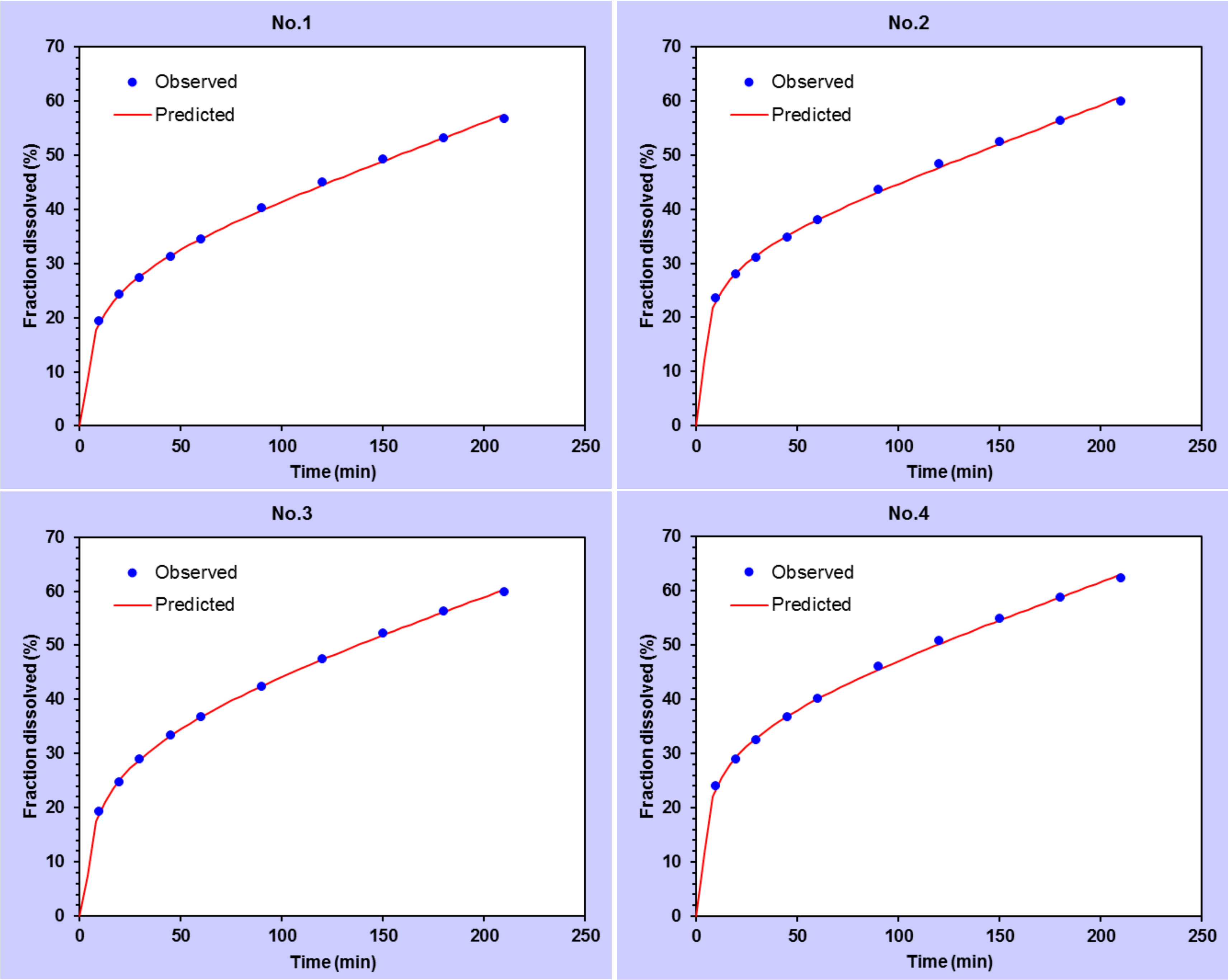

Supplementary materials – Model fitting summary – Lactochem® Crystals (modelling up to app. 60 % of carvedilol released)

Model: **Peppas–Sahlin_1**

Model equation: $F = k_1 \cdot t^m + k_2 \cdot t^{2m}$

Fitted model parameters per tested tablet (N = 4) with statistics – mean, standard deviation (SD), and relative standard deviation expressed in % (RSD%) (output from DDSolver):

Parameter	No.1	No.2	No.3	No.4	Mean	SD	RSD(%)
k_1	7.494	7.609	6.710	7.994	7.452	0.539	7.235
k_2	-0.112	-0.215	-0.126	-0.227	-0.170	0.059	-34.929
m	0.397	0.450	0.450	0.450	0.437	0.026	6.026

Number of dissolution data points (N), degrees of freedom (df), and selected goodness of fit criteria – Pearson correlation coefficient (R), coefficient of determination (R^2), adjusted coefficient of determination ($R^2_{adjusted}$), and residual sum of squares (RSS) (manual calculation in MS Excel):

Parameter	No.1	No.2	No.3	No.4
N	10	10	10	10
df	7	7	7	7
R	0.997560812	0.991703955	0.998347672	0.99421512
R^2	0.995127574	0.983476735	0.996698074	0.988463705
$R^2_{adjusted}$	0.993735452	0.978755802	0.995754666	0.985167621
RSS	8.422443559	29.30627704	6.657149159	22.68835396

Graphical abstract of model fit presented as mean ± 1 SD of the fraction % of released carvedilol:

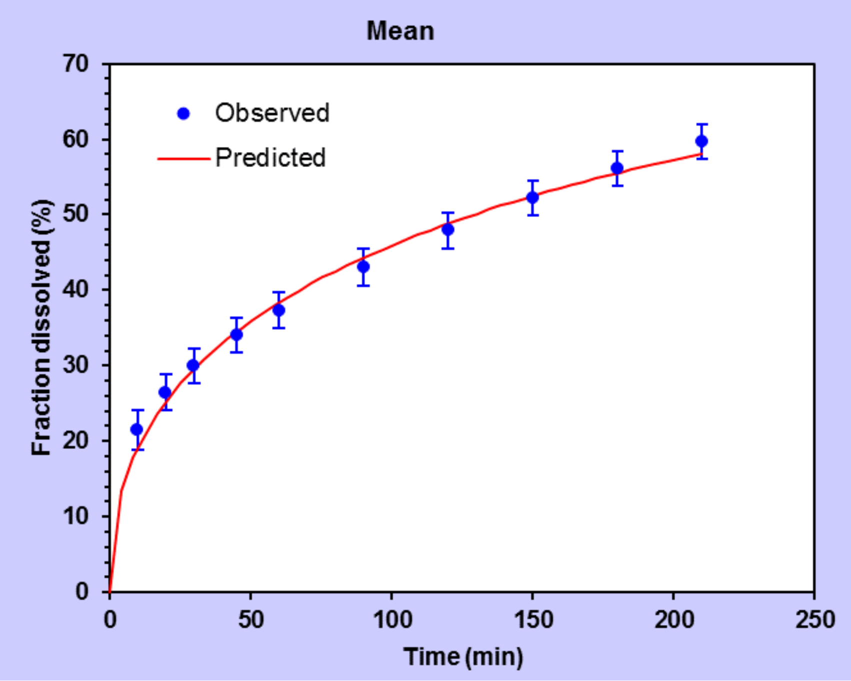

Supplementary materials – Model fitting summary – Lactochem® Crystals (modelling up to app. 60 % of carvedilol released)

Graphical abstract of model fit presented as the fraction % of released carvedilol per tested tablet:

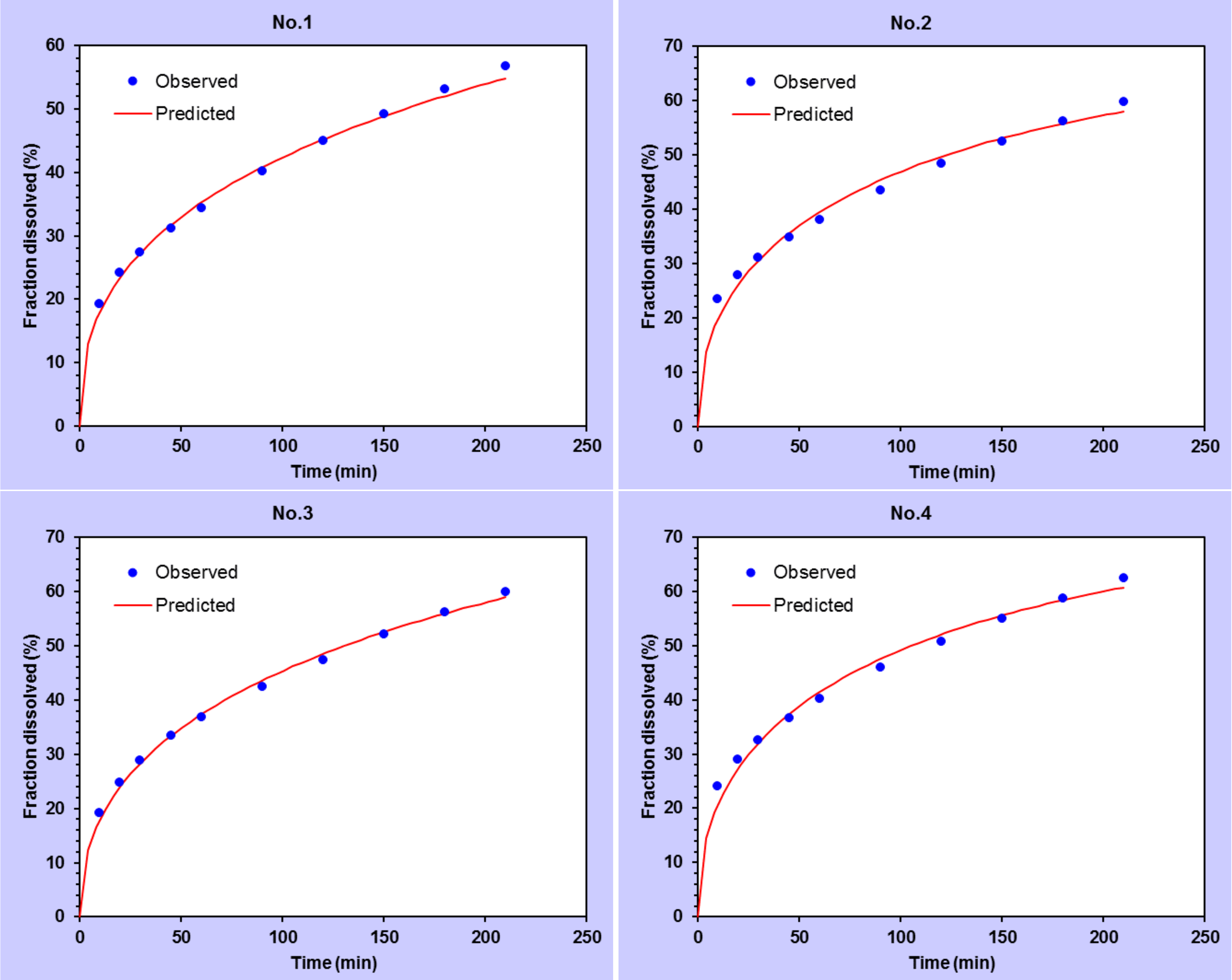

Supplementary materials – Model fitting summary – Lactochem® Crystals (modelling up to app. 60 % of carvedilol released)

Model: **Peppas–Sahlin_1 with T_{lag}**

Model equation: $F = k_1 \cdot (t - T_{lag})^m + k_2 \cdot (t - T_{lag})^{2m}$

Fitted model parameters per tested tablet (N = 4) with statistics – mean, standard deviation (SD), and relative standard deviation expressed in % (RSD%) (output from DDSolver):

Parameter	No.1	No.2	No.3	No.4	Mean	SD	RSD(%)
k_1	7.002	8.230	7.332	8.653	7.804	0.768	9.835
k_2	-0.182	-0.274	-0.185	-0.291	-0.233	0.057	-24.660
m	0.450	0.450	0.450	0.450	0.450	0.000	0.000
T_{lag}	4.000	4.000	4.000	4.000	4.000	0.000	0.000

Number of dissolution data points (N), degrees of freedom (df), and selected goodness of fit criteria – Pearson correlation coefficient (R), coefficient of determination (R^2), adjusted coefficient of determination ($R^2_{adjusted}$), and residual sum of squares (RSS) (manual calculation in MS Excel):

Parameter	No.1	No.2	No.3	No.4
N	10	10	10	10
df	6	6	6	6
R	0.990654107	0.982103136	0.994156678	0.985792973
R^2	0.981395559	0.964526569	0.988347501	0.971787785
$R^2_{adjusted}$	0.972093339	0.946789854	0.982521252	0.957681678
RSS	36.88636953	70.68257569	26.56829793	62.43693527

Graphical abstract of model fit presented as mean ± 1 SD of the fraction % of released carvedilol:

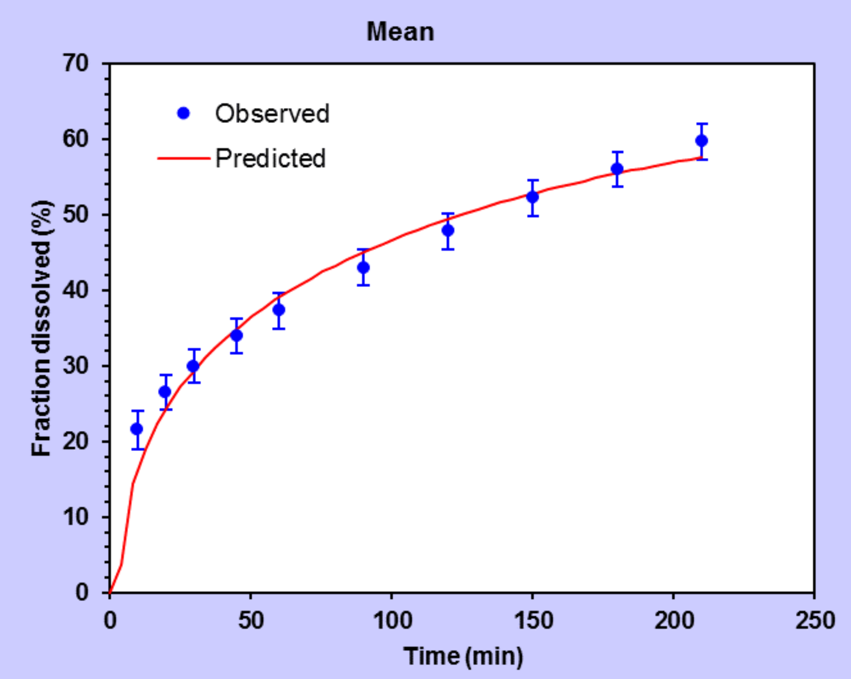

Supplementary materials – Model fitting summary – Lactochem® Crystals (modelling up to app. 60 % of carvedilol released)

Graphical abstract of model fit presented as the fraction % of released carvedilol per tested tablet:

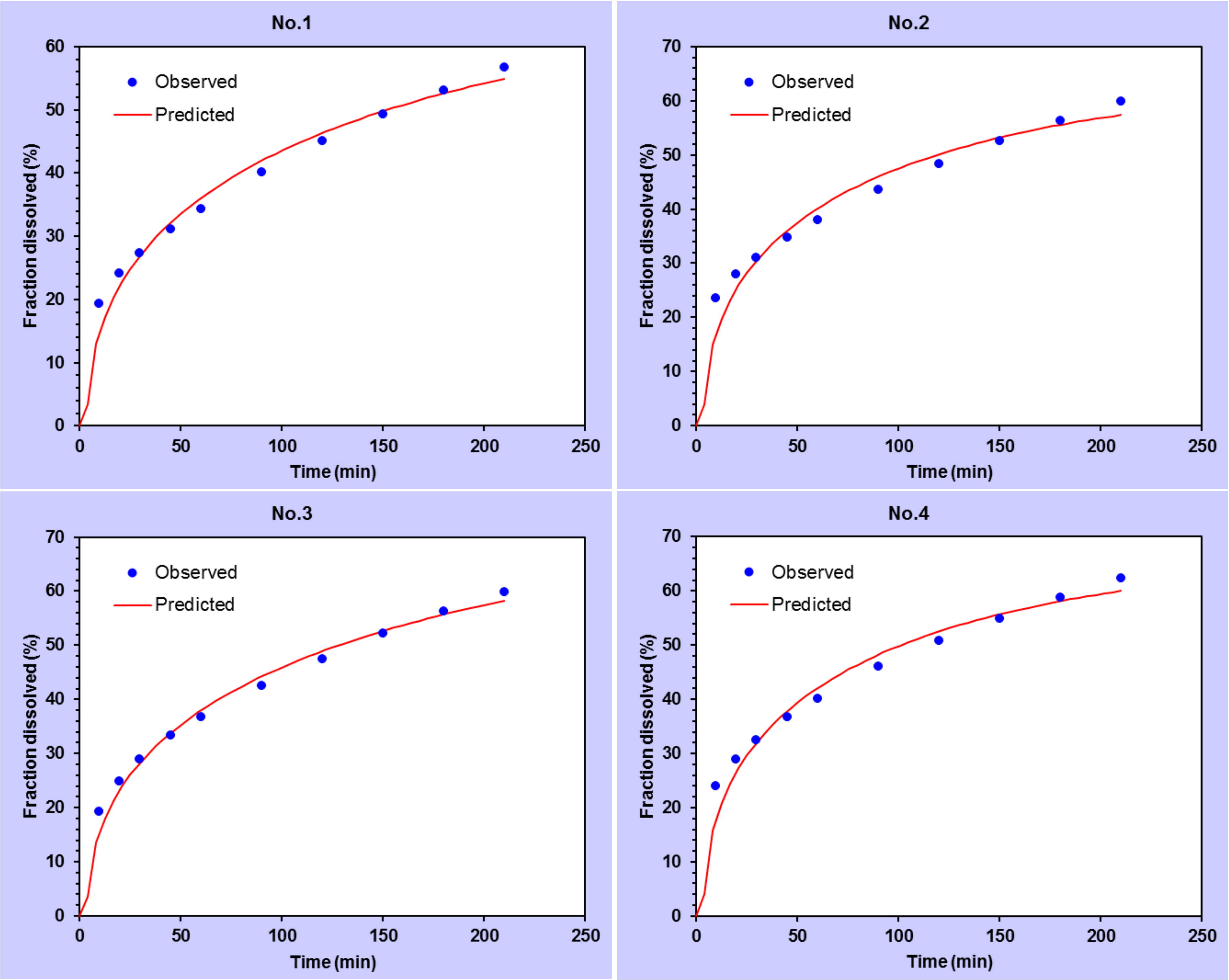

Supplementary materials – Model fitting summary – Lactochem® Crystals (modelling up to app. 60 % of carvedilol released)

Model: **Peppas–Sahlin_2**

Model equation: $F = k_1 \cdot t^{0.5} + k_2 \cdot t$

Fitted model parameters per tested tablet (N = 4) with statistics – mean, standard deviation (SD), and relative standard deviation expressed in % (RSD%) (output from DDSolver):

Parameter	No.1	No.2	No.3	No.4	Mean	SD	RSD(%)
k_1	5.516	6.451	5.779	6.782	6.132	0.586	9.551
k_2	-0.118	-0.171	-0.120	-0.181	-0.148	0.033	-22.545

Number of dissolution data points (N), degrees of freedom (df), and selected goodness of fit criteria – Pearson correlation coefficient (R), coefficient of determination (R^2), adjusted coefficient of determination ($R^2_{adjusted}$), and residual sum of squares (RSS) (manual calculation in MS Excel):

Parameter	No.1	No.2	No.3	No.4
N	10	10	10	10
df	8	8	8	8
R	0.994290846	0.987577766	0.996651979	0.990560247
R^2	0.988614287	0.975309843	0.993315168	0.981209604
$R^2_{adjusted}$	0.987191073	0.972223574	0.992479564	0.978860804
RSS	22.08568395	48.13316432	14.67908587	40.6577843

Graphical abstract of model fit presented as mean ± 1 SD of the fraction % of released carvedilol:

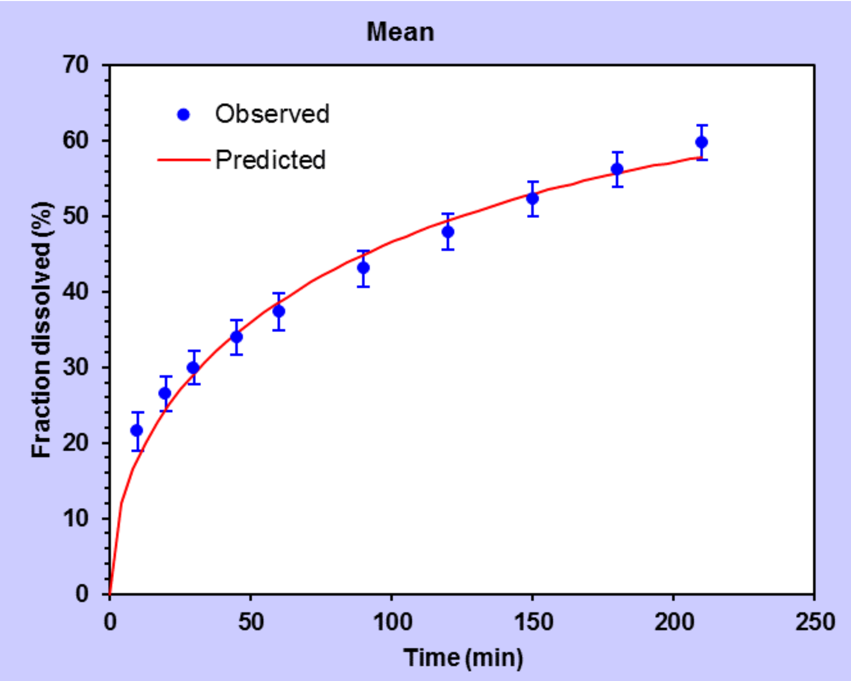

Supplementary materials – Model fitting summary – Lactochem® Crystals (modelling up to app. 60 % of carvedilol released)

Graphical abstract of model fit presented as the fraction % of released carvedilol per tested tablet:

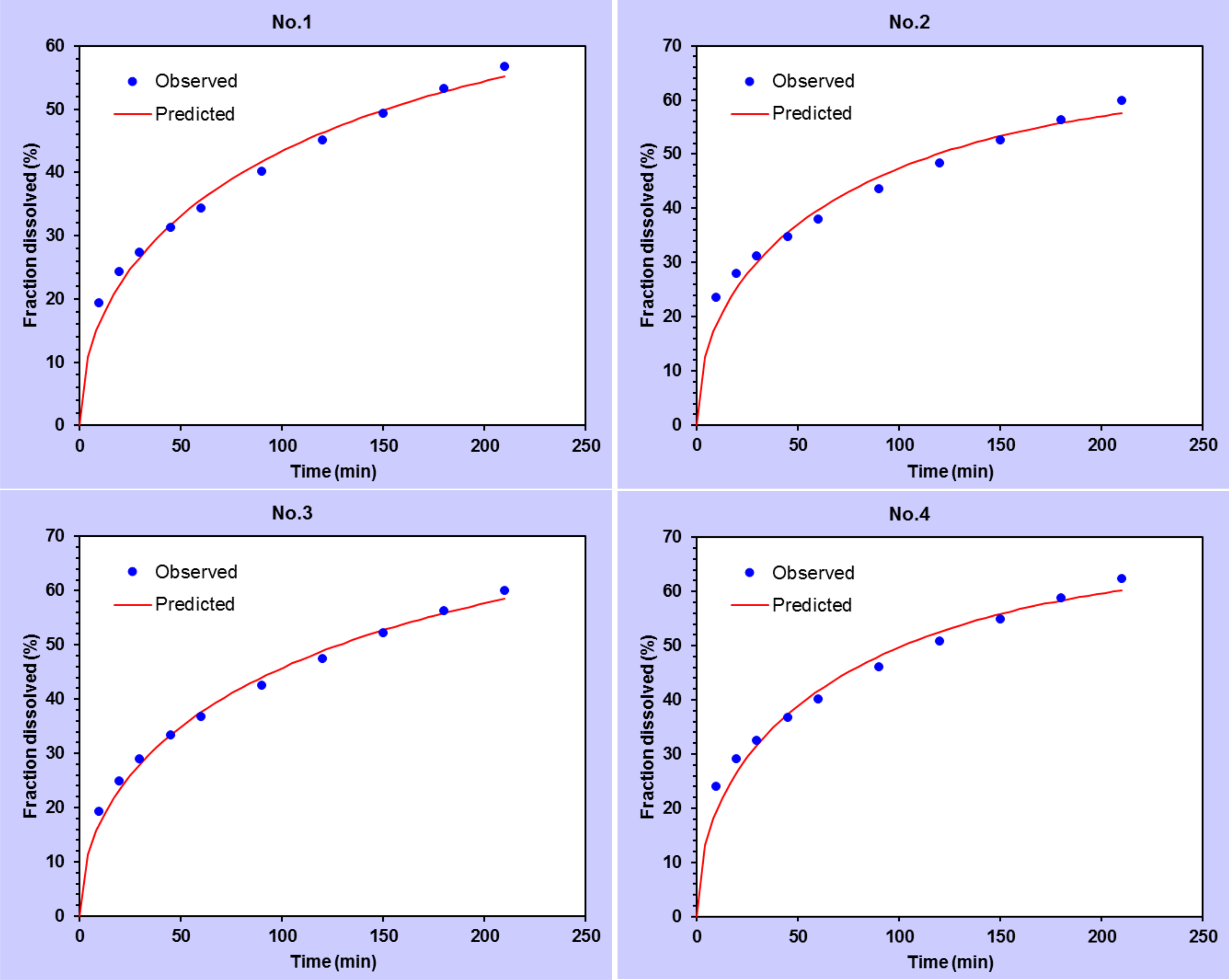

Supplementary materials – Model fitting summary – Lactochem® Crystals (modelling up to app. 60 % of carvedilol released)

Model: **Peppas–Sahlin_2 with T_{lag}**

Model equation: $F = k_1 \cdot (t - T_{lag})^{0.5} + k_2 \cdot (t - T_{lag})$

Fitted model parameters per tested tablet (N = 4) with statistics – mean, standard deviation (SD), and relative standard deviation expressed in % (RSD%) (output from DDSolver):

Parameter	No.1	No.2	No.3	No.4	Mean	SD	RSD(%)
k_1	5.962	6.928	6.259	7.289	6.609	0.607	9.183
k_2	-0.151	-0.207	-0.155	-0.219	-0.183	0.035	-19.147
T_{lag}	4.000	4.000	4.000	4.000	4.000	0.000	0.000

Number of dissolution data points (N), degrees of freedom (df), and selected goodness of fit criteria – Pearson correlation coefficient (R), coefficient of determination (R^2), adjusted coefficient of determination ($R^2_{adjusted}$), and residual sum of squares (RSS) (manual calculation in MS Excel):

Parameter	No.1	No.2	No.3	No.4
N	10	10	10	10
df	7	7	7	7
R	0.986984804	0.976504367	0.991048777	0.980559201
R^2	0.974139002	0.953560778	0.982177678	0.961496347
$R^2_{adjusted}$	0.966750146	0.940292429	0.977085585	0.950495303
RSS	57.08694701	104.6479287	45.19677444	96.55536289

Graphical abstract of model fit presented as mean ± 1 SD of the fraction % of released carvedilol:

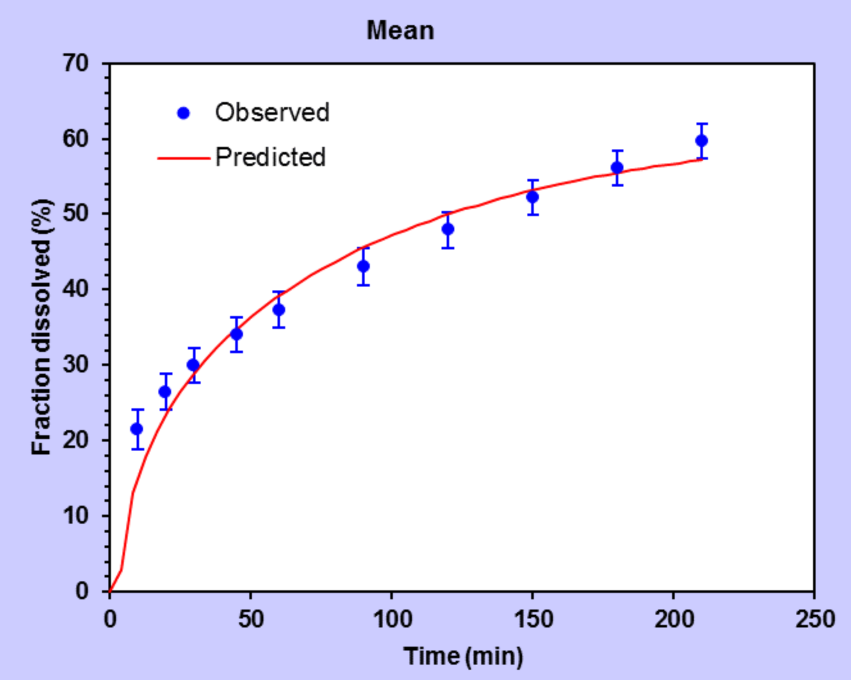

Supplementary materials – Model fitting summary – Lactochem® Crystals (modelling up to app. 60 % of carvedilol released)

Graphical abstract of model fit presented as the fraction % of released carvedilol per tested tablet:

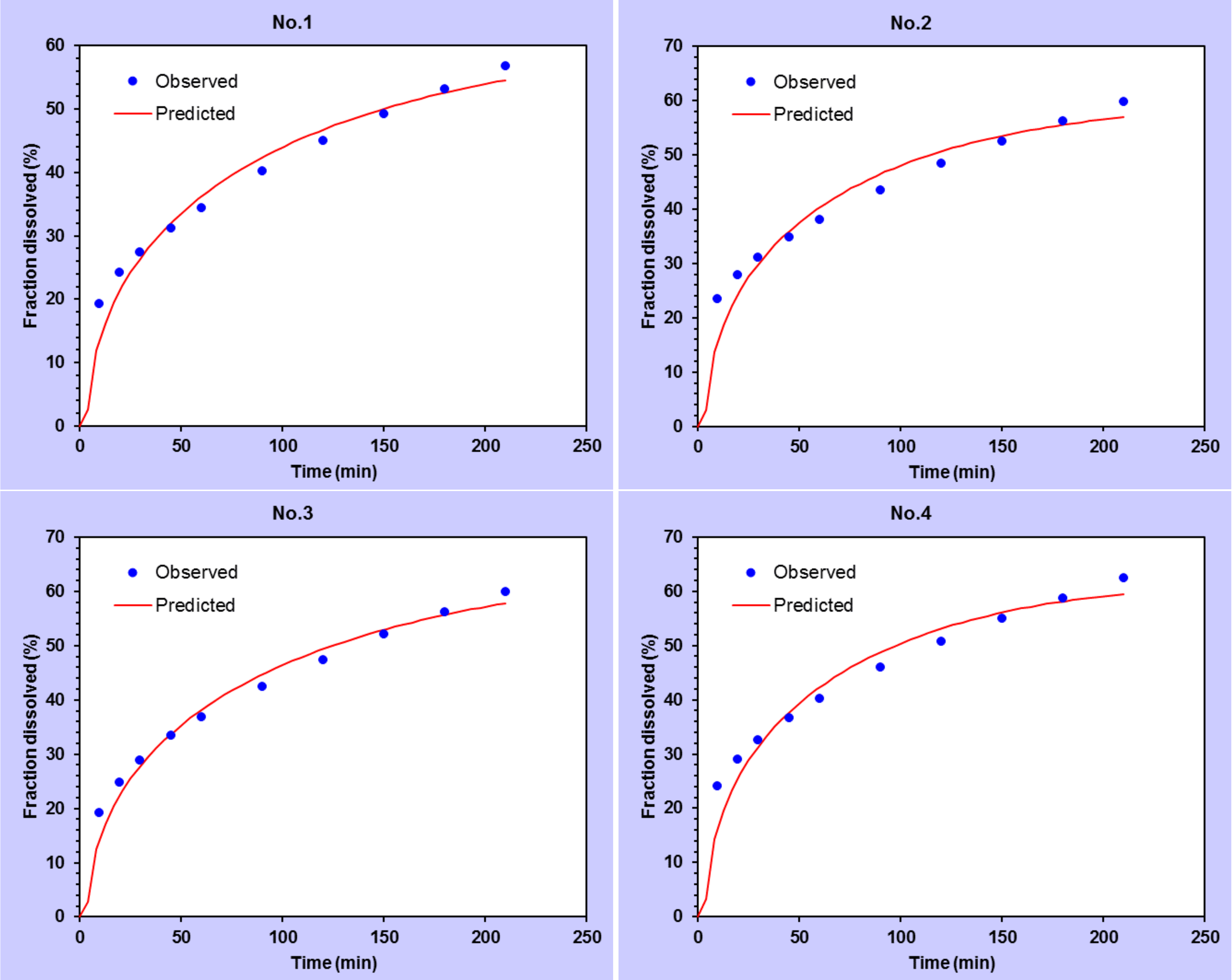

Supplementary materials – Model fitting summary – Lactochem® Crystals (modelling up to app. 60 % of carvedilol released)

Model: **Quadratic**

Model equation: $F = 100 \cdot (k_1 \cdot t^2 + k_2 \cdot t)$

Fitted model parameters per tested tablet (N = 4) with statistics – mean, standard deviation (SD), and relative standard deviation expressed in % (RSD%) (output from DDSolver):

Parameter	No.1	No.2	No.3	No.4	Mean	SD	RSD(%)
k_1	-0.00002	-0.00002	-0.00002	-0.00003	-0.00002	0.00000	-8.90870
k_2	0.00675	0.00750	0.00713	0.00790	0.00732	0.00050	6.77625

Number of dissolution data points (N), degrees of freedom (df), and selected goodness of fit criteria – Pearson correlation coefficient (R), coefficient of determination (R^2), adjusted coefficient of determination ($R^2_{adjusted}$), and residual sum of squares (RSS) (manual calculation in MS Excel):

Parameter	No.1	No.2	No.3	No.4
N	10	10	10	10
df	8	8	8	8
R	0.960903019	0.948088243	0.963588534	0.951154876
R^2	0.923334612	0.898871316	0.928502862	0.904695598
$R^2_{adjusted}$	0.913751438	0.88623023	0.91956572	0.892782548
RSS	525.612757	781.0570113	543.314443	823.539811

Graphical abstract of model fit presented as mean ± 1 SD of the fraction % of released carvedilol:

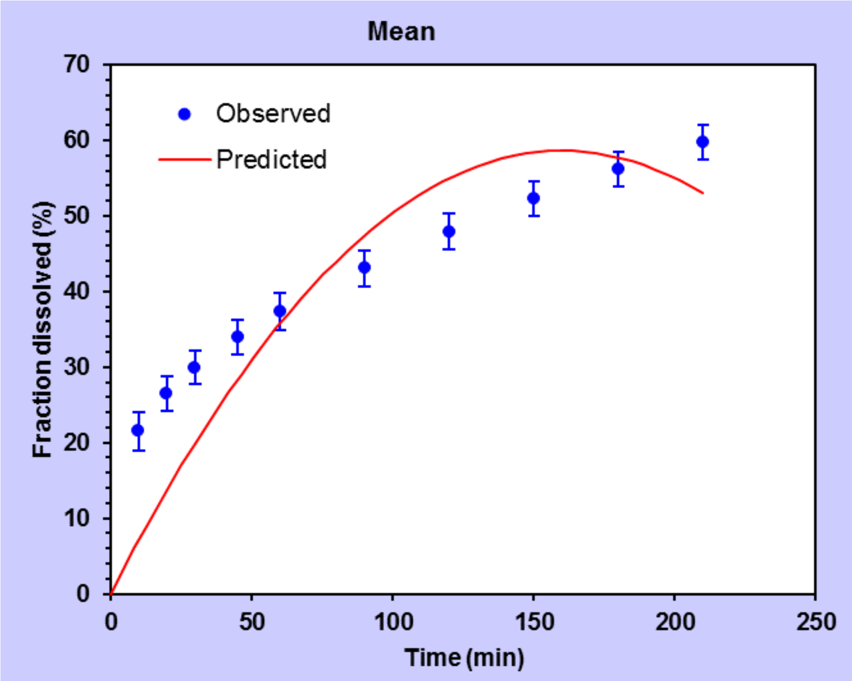

Supplementary materials – Model fitting summary – Lactochem® Crystals (modelling up to app. 60 % of carvedilol released)

Graphical abstract of model fit presented as the fraction % of released carvedilol per tested tablet:

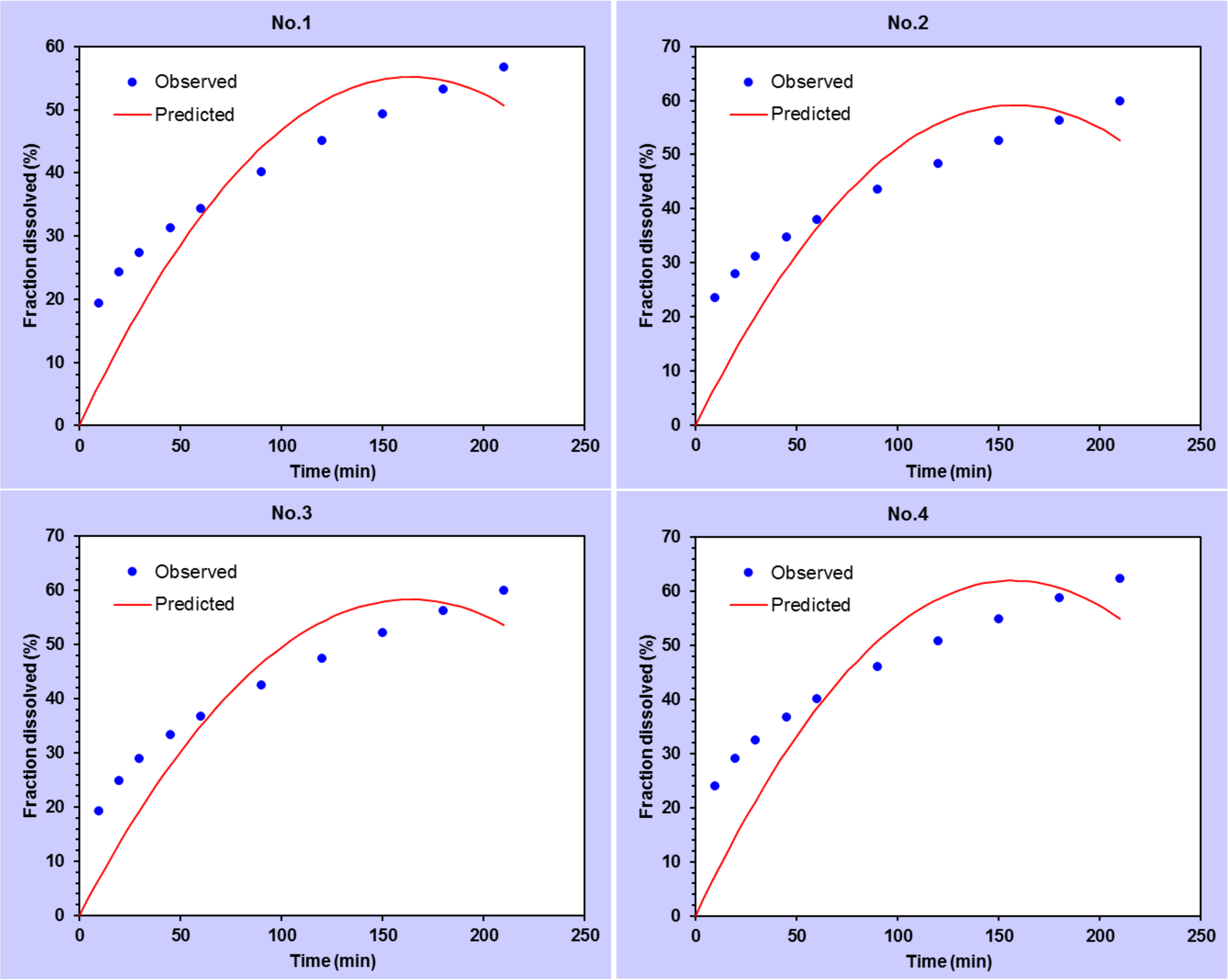

Supplementary materials – Model fitting summary – Lactochem® Crystals (modelling up to app. 60 % of carvedilol released)

Model: **Quadratic with T_{lag}**

Model equation: $F = 100 \cdot \left[k_1 \cdot \left(t - T_{lag}\right)^2 + k_2 \cdot \left(t - T_{lag}\right)\right]$

Fitted model parameters per tested tablet (N = 4) with statistics – mean, standard deviation (SD), and relative standard deviation expressed in % (RSD%) (output from DDSolver):

Parameter	No.1	No.2	No.3	No.4	Mean	SD	RSD(%)
k_1	-0.00002	-0.00003	-0.00002	-0.00003	-0.00002	0.00000	-8.59159
k_2	0.00701	0.00778	0.00742	0.00820	0.00760	0.00050	6.63720
T_{lag}	4.00000	4.00000	4.00000	4.00000	4.00000	0.00000	0.00000

Number of dissolution data points (N), degrees of freedom (df), and selected goodness of fit criteria – Pearson correlation coefficient (R), coefficient of determination (R^2), adjusted coefficient of determination ($R^2_{adjusted}$), and residual sum of squares (RSS) (manual calculation in MS Excel):

Parameter	No.1	No.2	No.3	No.4
N	10	10	10	10
df	7	7	7	7
R	0.9585508	0.946521523	0.961184626	0.949591329
R^2	0.918819637	0.895902994	0.923875886	0.901723692
$R^2_{adjusted}$	0.895625247	0.866160992	0.902126139	0.873644747
RSS	702.2059284	1019.173568	732.6421765	1081.253949

Graphical abstract of model fit presented as mean ± 1 SD of the fraction % of released carvedilol:

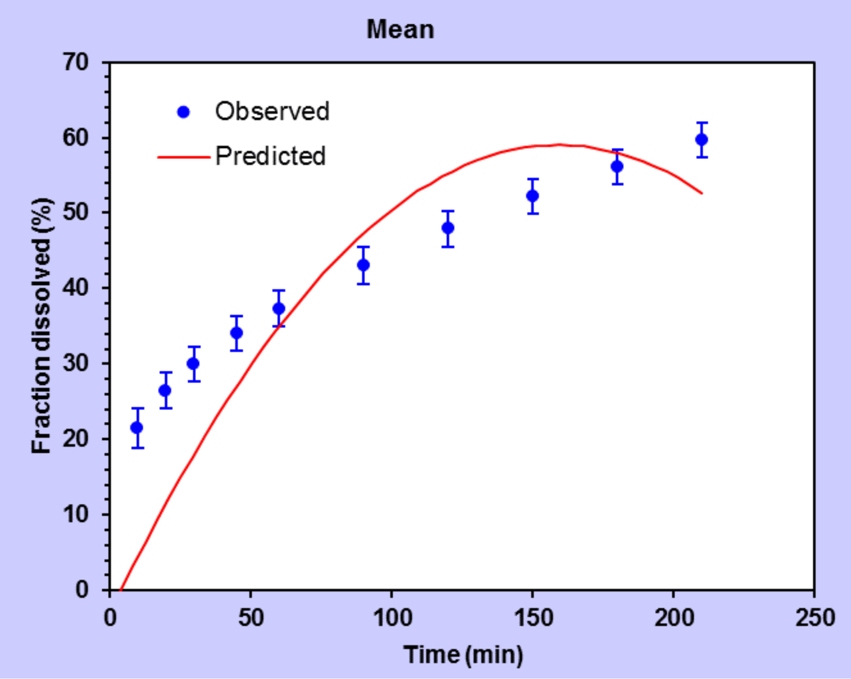

Supplementary materials – Model fitting summary – Lactochem® Crystals (modelling up to app. 60 % of carvedilol released)

Graphical abstract of model fit presented as the fraction % of released carvedilol per tested tablet:

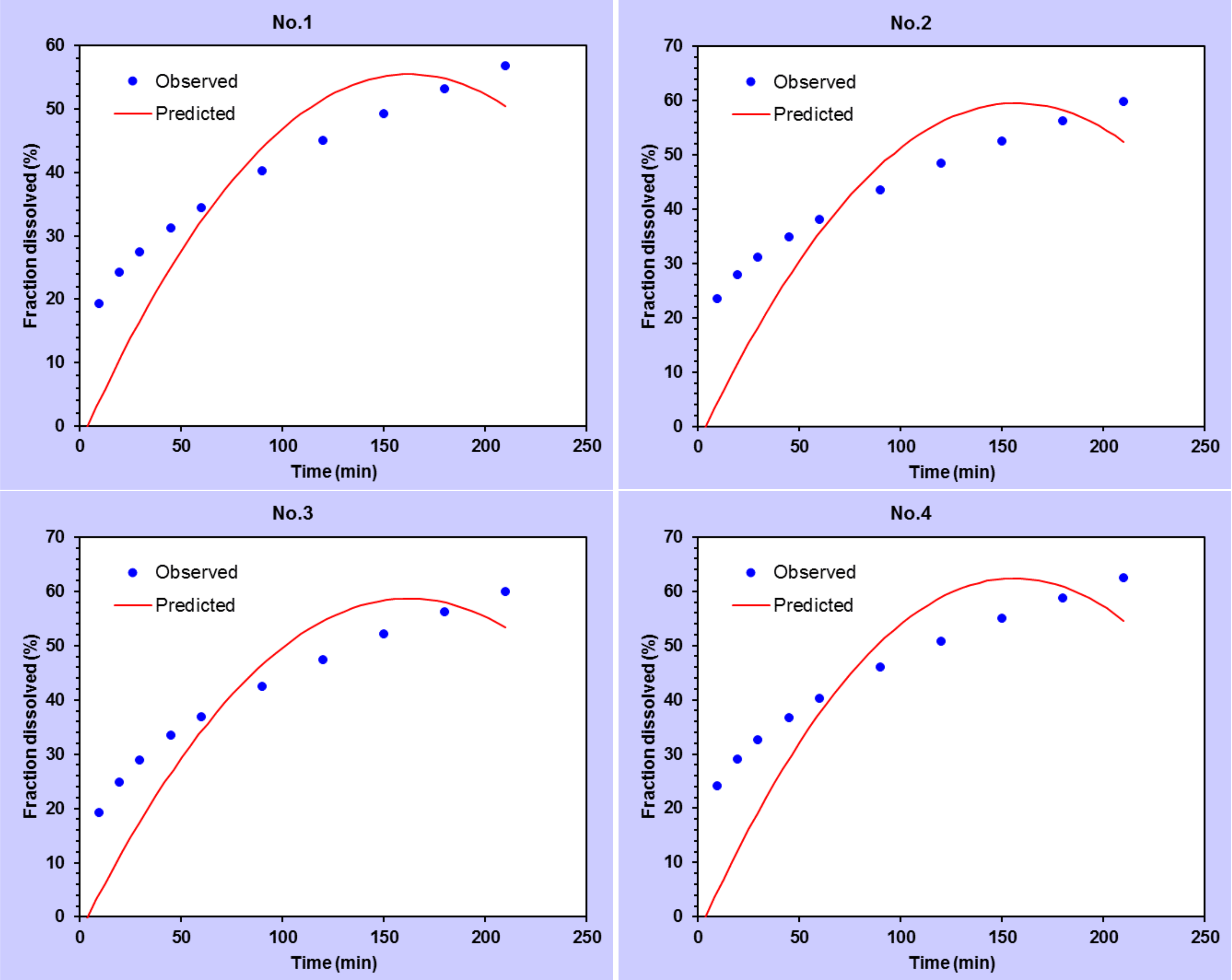

Supplementary materials – Model fitting summary – Lactochem® Crystals (modelling up to app. 60 % of carvedilol released)

Model: **Weibull_1**

Model equation: $F = 100 \cdot \left[1 - e^{-\frac{(t-T_i)^{\beta}}{\alpha}}\right]$

Fitted model parameters per tested tablet (N = 4) with statistics – mean, standard deviation (SD), and relative standard deviation expressed in % (RSD%) (output from DDSolver):

Parameter	No.1	No.2	No.3	No.4	Mean	SD	RSD(%)
α	10.565	7.996	10.876	7.904	9.335	1.605	17.193
β	0.391	0.353	0.415	0.366	0.381	0.027	7.142
T_i	4.000	4.000	4.000	4.000	4.000	0.000	0.000

Number of dissolution data points (N), degrees of freedom (df), and selected goodness of fit criteria – Pearson correlation coefficient (R), coefficient of determination (R^2), adjusted coefficient of determination ($R^2_{adjusted}$), and residual sum of squares (RSS) (manual calculation in MS Excel):

Parameter	No.1	No.2	No.3	No.4
N	10	10	10	10
df	7	7	7	7
R	0.98925557	0.985207769	0.992729381	0.988534351
R^2	0.978626583	0.970634349	0.985511623	0.977200163
$R^2_{adjusted}$	0.972519892	0.962244163	0.981372087	0.970685924
RSS	34.8717523	44.07653044	27.28932399	37.82403691

Graphical abstract of model fit presented as mean ± 1 SD of the fraction % of released carvedilol:

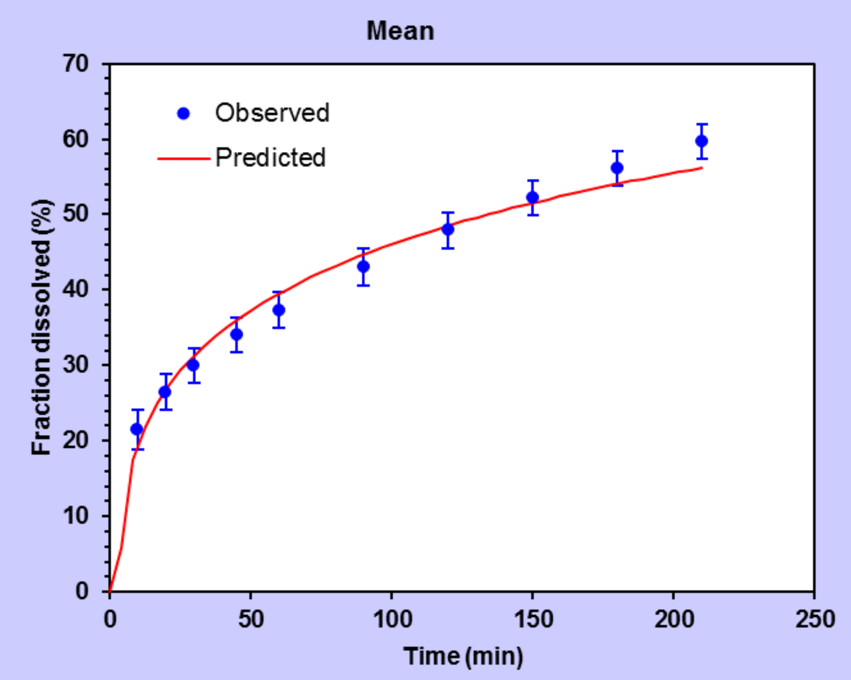

Supplementary materials – Model fitting summary – Lactochem® Crystals (modelling up to app. 60 % of carvedilol released)

Graphical abstract of model fit presented as the fraction % of released carvedilol per tested tablet:

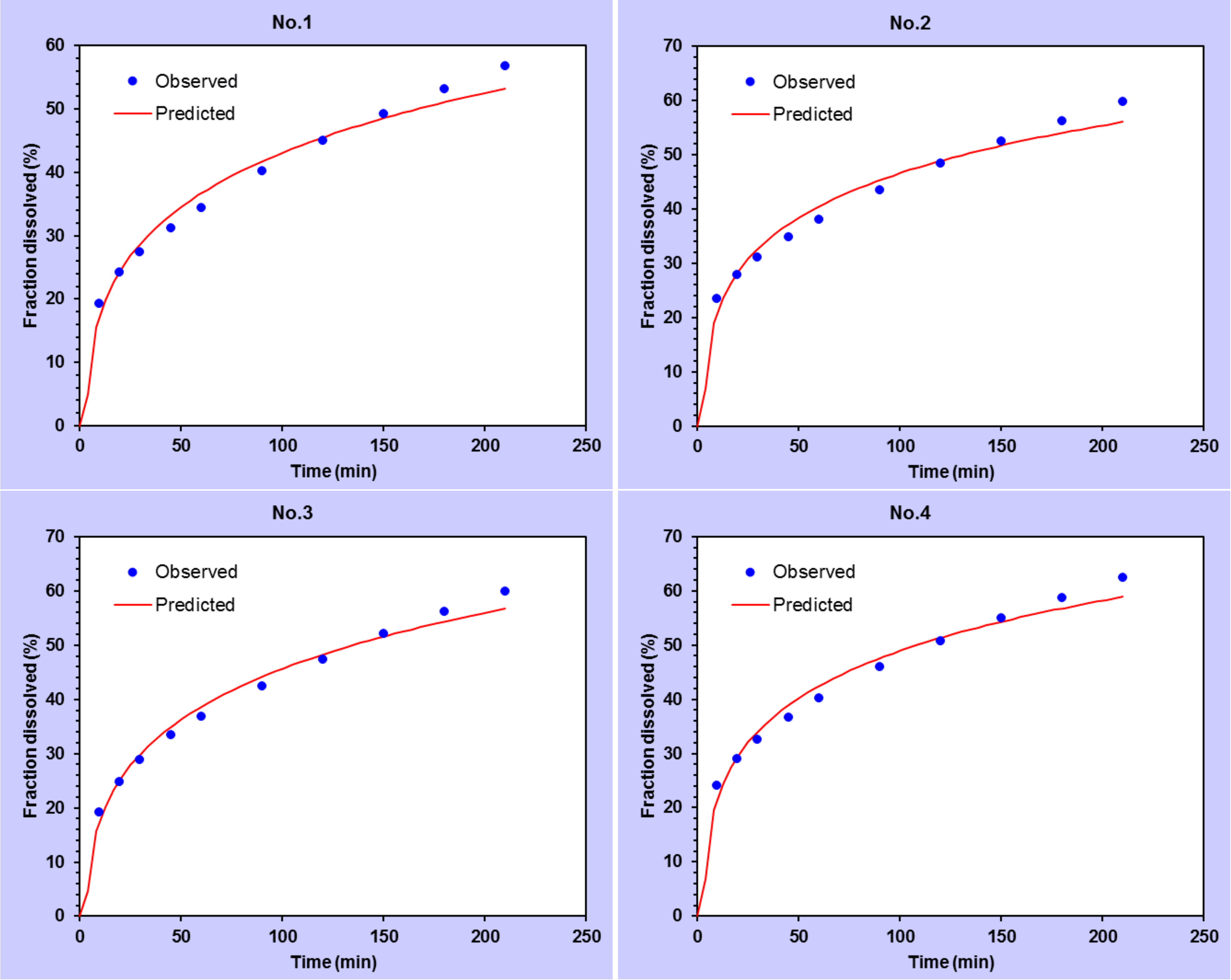

Supplementary materials – Model fitting summary – Lactochem® Crystals (modelling up to app. 60 % of carvedilol released)

Model: **Weibull_2**

Model equation: $F = 100 \cdot \left(1 - e^{-\frac{t^{\beta}}{\alpha}}\right)$

Fitted model parameters per tested tablet (N = 4) with statistics – mean, standard deviation (SD), and relative standard deviation expressed in % (RSD%) (output from DDSolver):

Parameter	No.1	No.2	No.3	No.4	Mean	SD	RSD(%)
α	13.971	10.323	14.569	10.274	12.284	2.306	18.770
β	0.447	0.405	0.473	0.419	0.436	0.030	6.988

Number of dissolution data points (N), degrees of freedom (df), and selected goodness of fit criteria – Pearson correlation coefficient (R), coefficient of determination (R^2), adjusted coefficient of determination ($R^2_{adjusted}$), and residual sum of squares (RSS) (manual calculation in MS Excel):

Parameter	No.1	No.2	No.3	No.4
N	10	10	10	10
df	8	8	8	8
R	0.994961539	0.992368622	0.996953248	0.994683303
R^2	0.989948465	0.984795481	0.993915778	0.989394873
$R^2_{adjusted}$	0.988692023	0.982894917	0.993155251	0.988069232
RSS	16.08823432	22.58061267	11.07865742	17.37769215

Graphical abstract of model fit presented as mean ± 1 SD of the fraction % of released carvedilol:

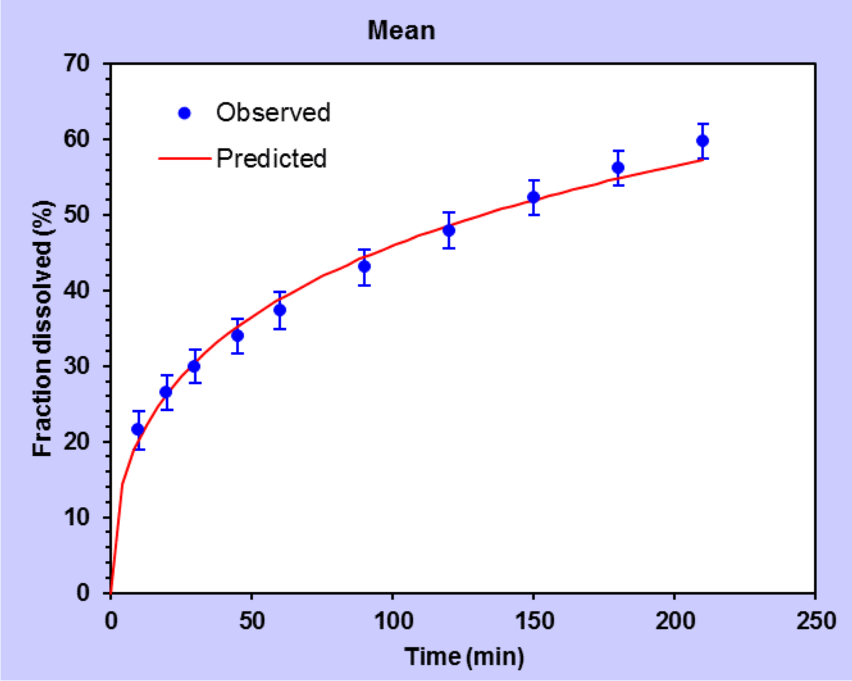

Supplementary materials – Model fitting summary – Lactochem® Crystals (modelling up to app. 60 % of carvedilol released)

Graphical abstract of model fit presented as the fraction % of released carvedilol per tested tablet:

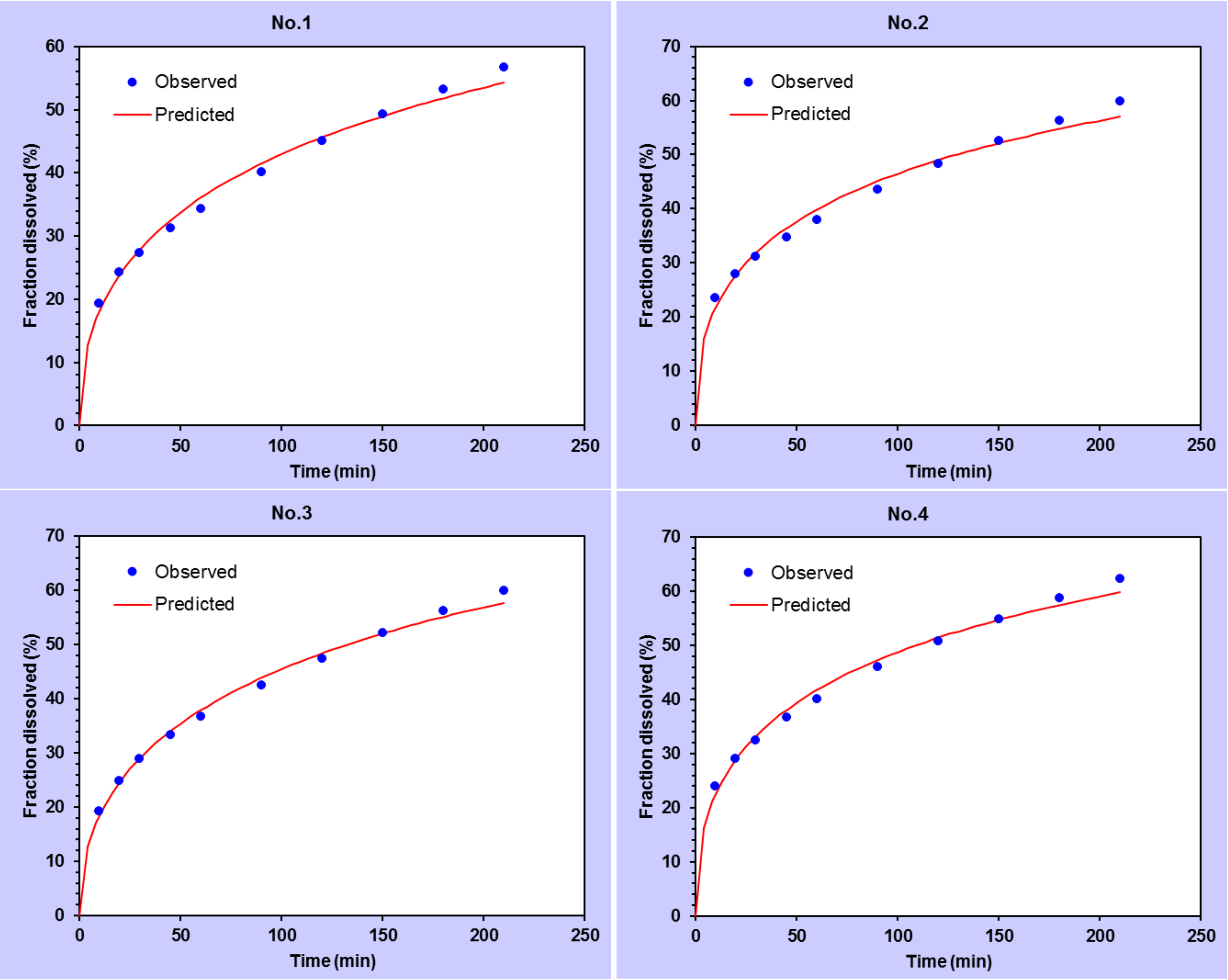

Supplementary materials – Model fitting summary – Lactochem® Crystals (modelling up to app. 60 % of carvedilol released)

Model: **Weibull_3**

Model equation: $F = F_{max} \cdot \left(1 - e^{-\frac{t^{\beta}}{\alpha}}\right)$

Fitted model parameters per tested tablet (N = 4) with statistics – mean, standard deviation (SD), and relative standard deviation expressed in % (RSD%) (output from DDSolver):

Parameter	No.1	No.2	No.3	No.4	Mean	SD	RSD(%)
α	13.459	10.048	14.704	10.349	12.140	2.302	18.962
β	0.637	0.584	0.657	0.592	0.618	0.035	5.729
F_{max}	59.551	62.796	62.900	65.515	62.691	2.442	3.895

Number of dissolution data points (N), degrees of freedom (df), and selected goodness of fit criteria – Pearson correlation coefficient (R), coefficient of determination (R^2), adjusted coefficient of determination ($R^2_{adjusted}$), and residual sum of squares (RSS) (manual calculation in MS Excel):

Parameter	No.1	No.2	No.3	No.4
N	10	10	10	10
df	7	7	7	7
R	0.98058353	0.976527107	0.985163107	0.980939799
R^2	0.961544059	0.953605191	0.970546348	0.962242888
$R^2_{adjusted}$	0.950556647	0.940349531	0.962131019	0.951455142
RSS	59.23622417	67.77964944	52.95573715	61.2656947

Graphical abstract of model fit presented as mean ± 1 SD of the fraction % of released carvedilol:

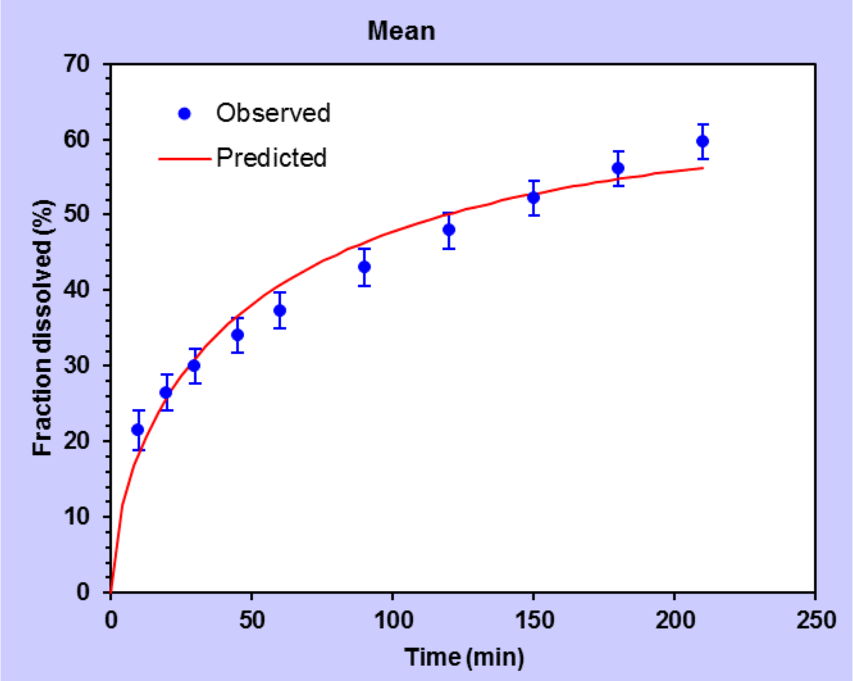

Supplementary materials – Model fitting summary – Lactochem® Crystals (modelling up to app. 60 % of carvedilol released)

Graphical abstract of model fit presented as the fraction % of released carvedilol per tested tablet:

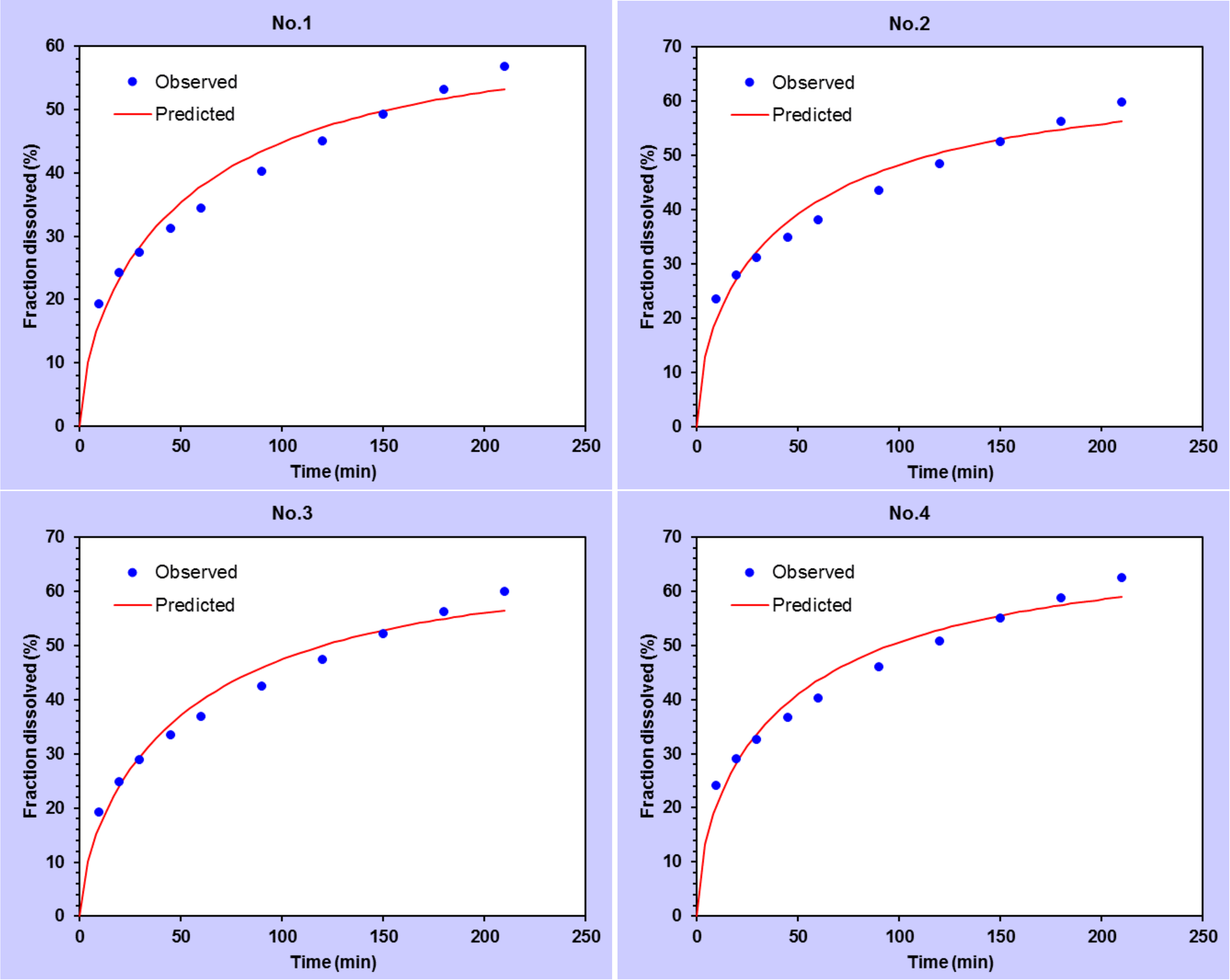

Supplementary materials – Model fitting summary – Lactochem® Crystals (modelling up to app. 60 % of carvedilol released)

Model: **Weibull_4**

Model equation: $F = F_{max} \cdot \left[1 - e^{-\frac{(t-T_i)^{\beta}}{\alpha}}\right]$

Fitted model parameters per tested tablet (N = 4) with statistics – mean, standard deviation (SD), and relative standard deviation expressed in % (RSD%) (output from DDSolver):

Parameter	No.1	No.2	No.3	No.4	Mean	SD	RSD(%)
α	8.921	6.875	9.665	7.060	8.130	1.379	16.959
β	0.554	0.507	0.572	0.515	0.537	0.031	5.853
T_i	4.000	4.000	4.000	4.000	4.000	0.000	0.000
F_{max}	59.551	62.796	62.900	65.515	62.691	2.442	3.895

Number of dissolution data points (N), degrees of freedom (df), and selected goodness of fit criteria – Pearson correlation coefficient (R), coefficient of determination (R^2), adjusted coefficient of determination ($R^2_{adjusted}$), and residual sum of squares (RSS) (manual calculation in MS Excel):

Parameter	No.1	No.2	No.3	No.4
N	10	10	10	10
df	6	6	6	6
R	0.97145457	0.965868471	0.977640519	0.971269303
R^2	0.943723982	0.932901903	0.955780984	0.943364058
$R^2_{adjusted}$	0.915585972	0.899352855	0.933671476	0.915046088
RSS	86.2393323	97.36625395	79.03645297	91.20731275

Graphical abstract of model fit presented as mean ± 1 SD of the fraction % of released carvedilol:

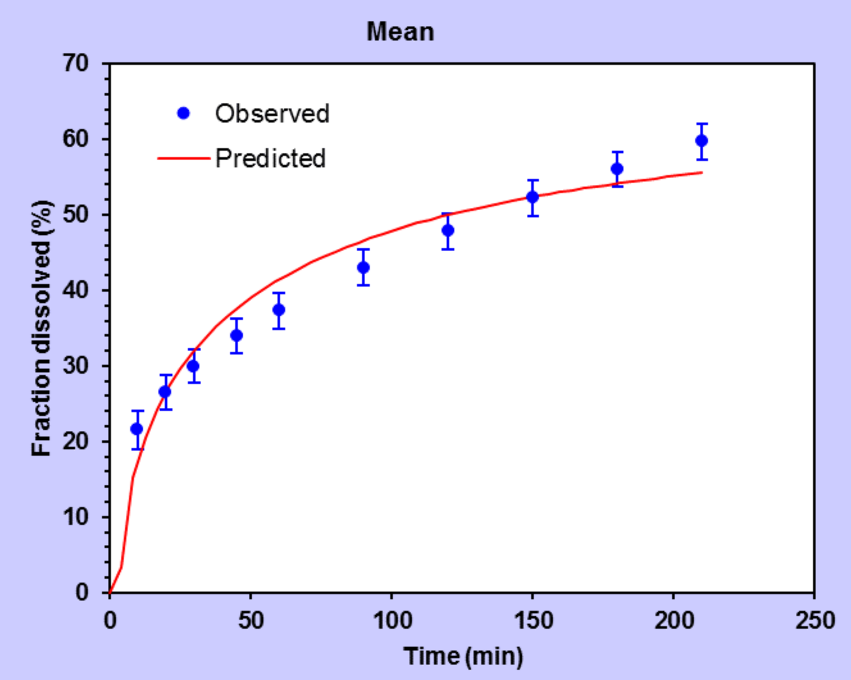

Supplementary materials – Model fitting summary – Lactochem® Crystals (modelling up to app. 60 % of carvedilol released)

Graphical abstract of model fit presented as the fraction % of released carvedilol per tested tablet:

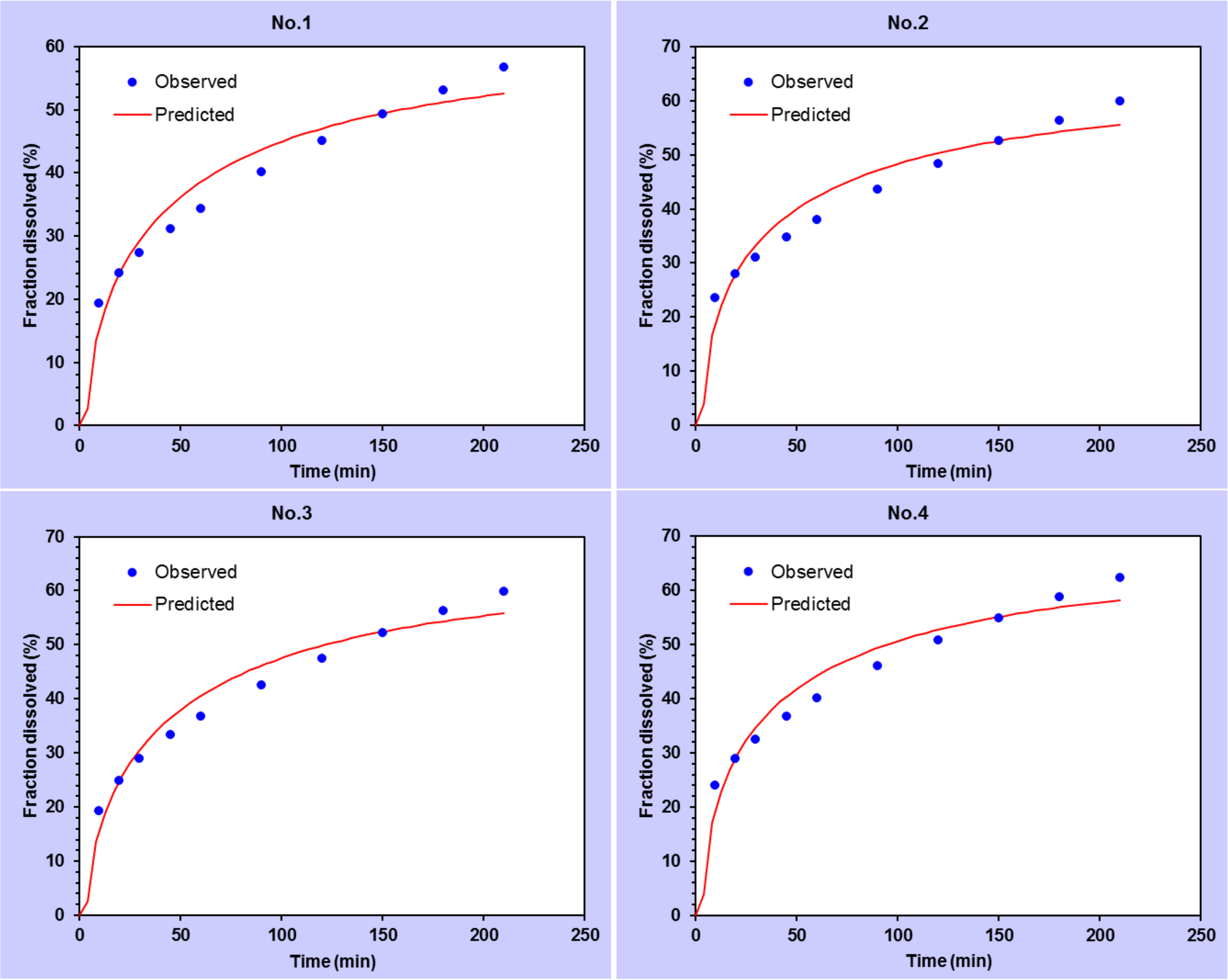

Supplementary materials – Model fitting summary – Lactochem® Crystals (modelling up to app. 60 % of carvedilol released)

Model: **Logistic_1**

Model equation: $F = 100 \cdot \frac{e^{\alpha+\beta\cdot\log(t)}}{1+e^{\alpha+\beta\cdot\log(t)}}$

Fitted model parameters per tested tablet (N = 4) with statistics – mean, standard deviation (SD), and relative standard deviation expressed in % (RSD%) (output from DDSolver):

Parameter	No.1	No.2	No.3	No.4	Mean	SD	RSD(%)
α	-2.829	-2.513	-2.906	-2.534	-2.695	0.201	-7.466
β	1.282	1.195	1.373	1.252	1.276	0.074	5.830

Number of dissolution data points (N), degrees of freedom (df), and selected goodness of fit criteria – Pearson correlation coefficient (R), coefficient of determination (R^2), adjusted coefficient of determination ($R^2_{adjusted}$), and residual sum of squares (RSS) (manual calculation in MS Excel):

Parameter	No.1	No.2	No.3	No.4
N	10	10	10	10
df	8	8	8	8
R	0.990836712	0.987280304	0.993292339	0.989881178
R^2	0.981757391	0.974722399	0.98662967	0.979864746
$R^2_{adjusted}$	0.979477065	0.971562699	0.984958378	0.977347839
RSS	27.92540203	36.12312201	23.55459629	31.76023075

Graphical abstract of model fit presented as mean ± 1 SD of the fraction % of released carvedilol:

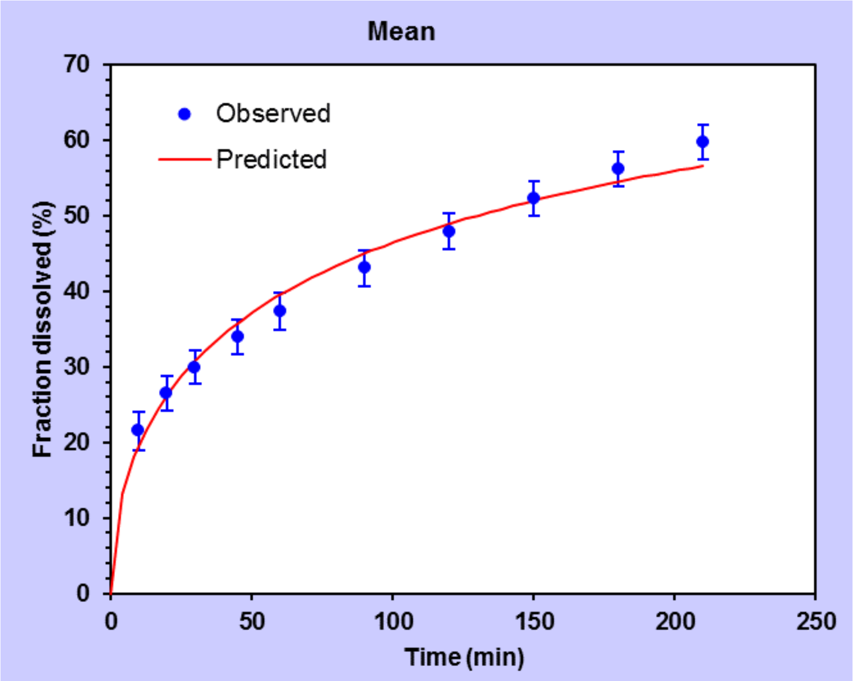

Supplementary materials – Model fitting summary – Lactochem® Crystals (modelling up to app. 60 % of carvedilol released)

Graphical abstract of model fit presented as the fraction % of released carvedilol per tested tablet:

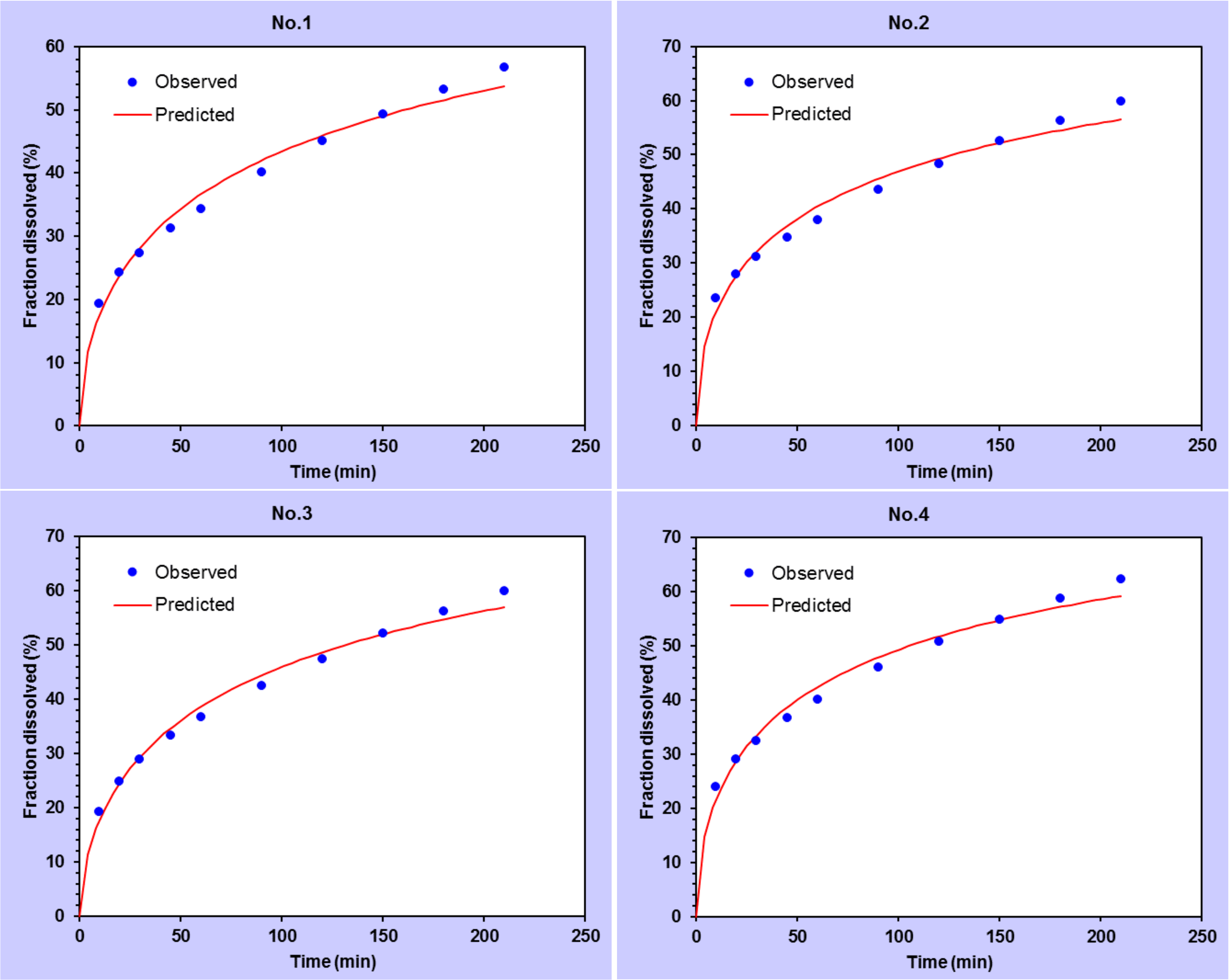

Supplementary materials – Model fitting summary – Lactochem® Crystals (modelling up to app. 60 % of carvedilol released)

Model: **Logistic_2**

Model equation: $F = F_{max} \cdot \frac{e^{\alpha+\beta\cdot\log(t)}}{1+e^{\alpha+\beta\cdot\log(t)}}$

Fitted model parameters per tested tablet (N = 4) with statistics – mean, standard deviation (SD), and relative standard deviation expressed in % (RSD%) (output from DDSolver):

Parameter	No.1	No.2	No.3	No.4	Mean	SD	RSD(%)
α	-3.744	-3.386	-3.855	-3.429	-3.603	0.231	-6.422
β	2.507	2.364	2.565	2.394	2.457	0.094	3.843
F_{max}	59.551	62.796	62.900	65.515	62.691	2.442	3.895

Number of dissolution data points (N), degrees of freedom (df), and selected goodness of fit criteria – Pearson correlation coefficient (R), coefficient of determination (R^2), adjusted coefficient of determination ($R^2_{adjusted}$), and residual sum of squares (RSS) (manual calculation in MS Excel):

Parameter	No.1	No.2	No.3	No.4
N	10	10	10	10
df	7	7	7	7
R	0.957118736	0.951171167	0.964456125	0.957407492
R^2	0.916076275	0.904726589	0.930175616	0.916629106
$R^2_{adjusted}$	0.892098068	0.877505615	0.910225793	0.89280885
RSS	146.6278611	159.9785052	142.5764268	155.5375862

Graphical abstract of model fit presented as mean ± 1 SD of the fraction % of released carvedilol:

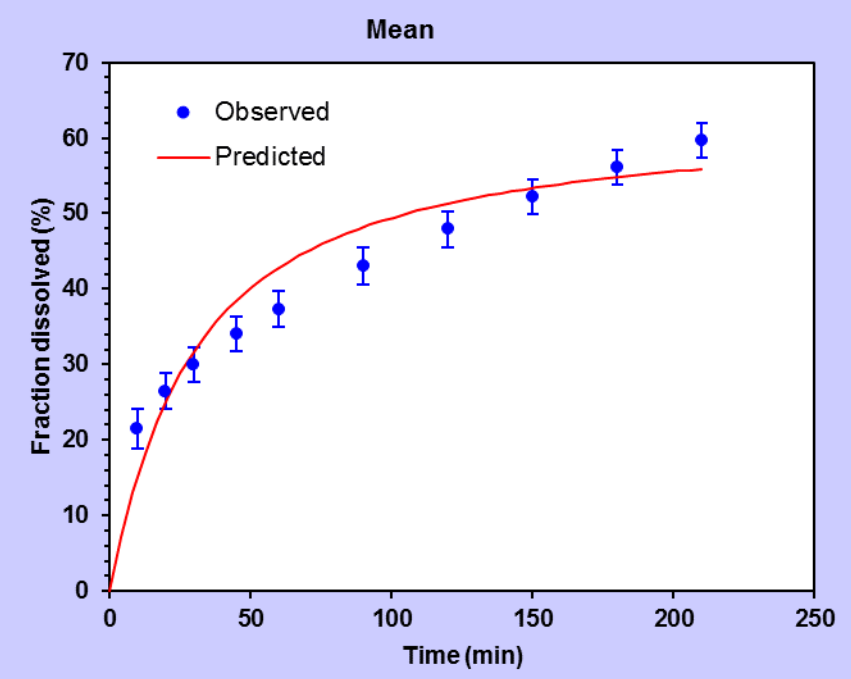

Supplementary materials – Model fitting summary – Lactochem® Crystals (modelling up to app. 60 % of carvedilol released)

Graphical abstract of model fit presented as the fraction % of released carvedilol per tested tablet:

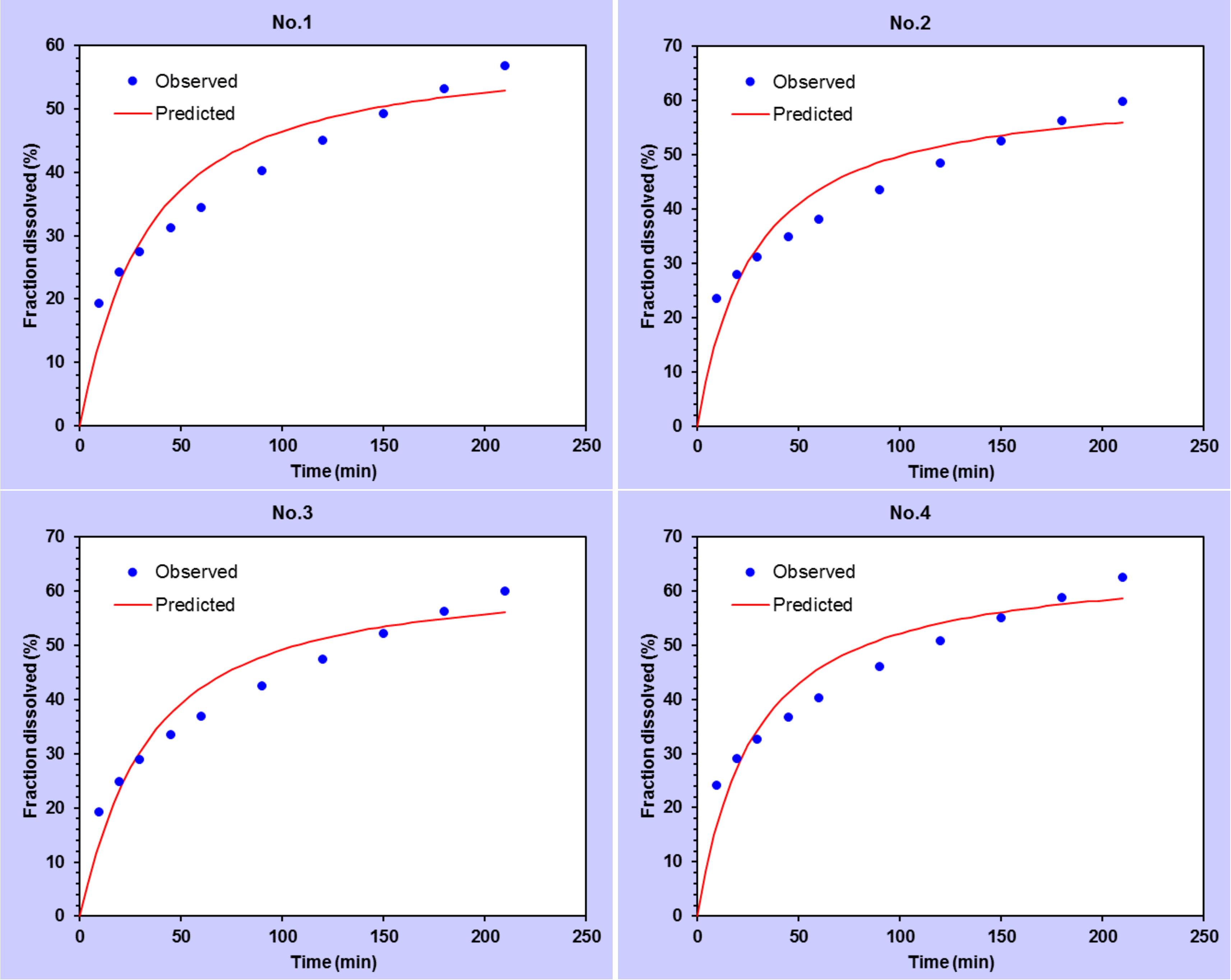

Supplementary materials – Model fitting summary – Lactochem® Crystals (modelling up to app. 60 % of carvedilol released)

Model: **Logistic_3**

Model equation: $F = F_{max} \cdot \frac{1}{1+e^{-k\cdot(t-\gamma)}}$

Fitted model parameters per tested tablet (N = 4) with statistics – mean, standard deviation (SD), and relative standard deviation expressed in % (RSD%) (output from DDSolver):

Parameter	No.1	No.2	No.3	No.4	Mean	SD	RSD(%)
k	0.017	0.016	0.017	0.016	0.016	0.001	3.378
γ	45.569	36.706	46.563	36.234	41.268	5.559	13.469
F_{max}	59.551	62.796	62.900	65.515	62.691	2.442	3.895

Number of dissolution data points (N), degrees of freedom (df), and selected goodness of fit criteria – Pearson correlation coefficient (R), coefficient of determination (R^2), adjusted coefficient of determination ($R^2_{adjusted}$), and residual sum of squares (RSS) (manual calculation in MS Excel):

Parameter	No.1	No.2	No.3	No.4
N	10	10	10	10
df	7	7	7	7
R	0.995547187	0.99641137	0.992928614	0.995501399
R^2	0.991114202	0.992835618	0.985907232	0.991023035
$R^2_{adjusted}$	0.988575403	0.990788651	0.981880726	0.988458187
RSS	14.07561647	11.12022374	24.92999836	14.56316307

Graphical abstract of model fit presented as mean ± 1 SD of the fraction % of released carvedilol:

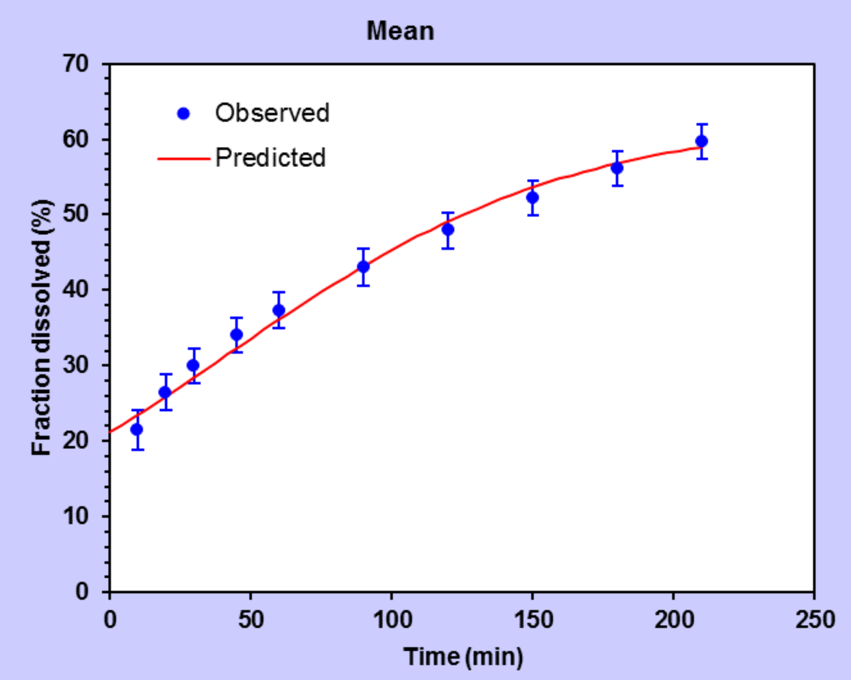

Supplementary materials – Model fitting summary – Lactochem® Crystals (modelling up to app. 60 % of carvedilol released)

Graphical abstract of model fit presented as the fraction % of released carvedilol per tested tablet:

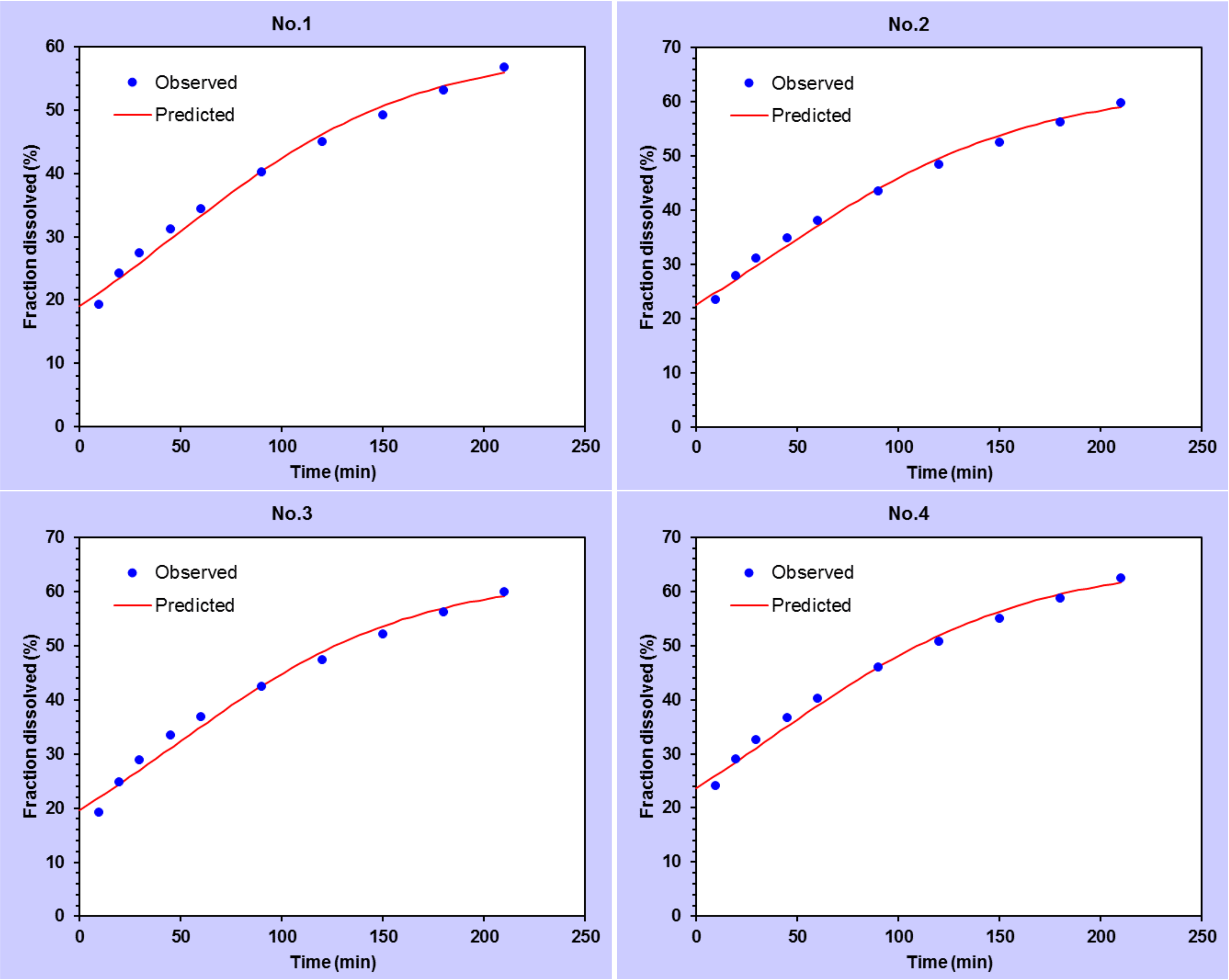

Supplementary materials – Model fitting summary – Lactochem® Crystals (modelling up to app. 60 % of carvedilol released)

Model: **Gompertz_1**

Model equation: $F = 100 \cdot e^{-\alpha \cdot e^{-\beta \cdot \log(t)}}$

Fitted model parameters per tested tablet (N = 4) with statistics – mean, standard deviation (SD), and relative standard deviation expressed in % (RSD%) (output from DDSolver):

Parameter	No.1	No.2	No.3	No.4	Mean	SD	RSD(%)
α	4.077	3.557	4.378	3.690	3.926	0.374	9.527
β	0.801	0.781	0.874	0.833	0.822	0.040	4.915

Number of dissolution data points (N), degrees of freedom (df), and selected goodness of fit criteria – Pearson correlation coefficient (R), coefficient of determination (R^2), adjusted coefficient of determination ($R^2_{adjusted}$), and residual sum of squares (RSS) (manual calculation in MS Excel):

Parameter	No.1	No.2	No.3	No.4
N	10	10	10	10
df	8	8	8	8
R	0.981382112	0.977373506	0.984855442	0.980624214
R^2	0.963110849	0.955258971	0.969940241	0.961623848
$R^2_{adjusted}$	0.958499706	0.949666342	0.966182771	0.956826829
RSS	54.57202091	62.73900853	51.62720379	59.67755042

Graphical abstract of model fit presented as mean ± 1 SD of the fraction % of released carvedilol:

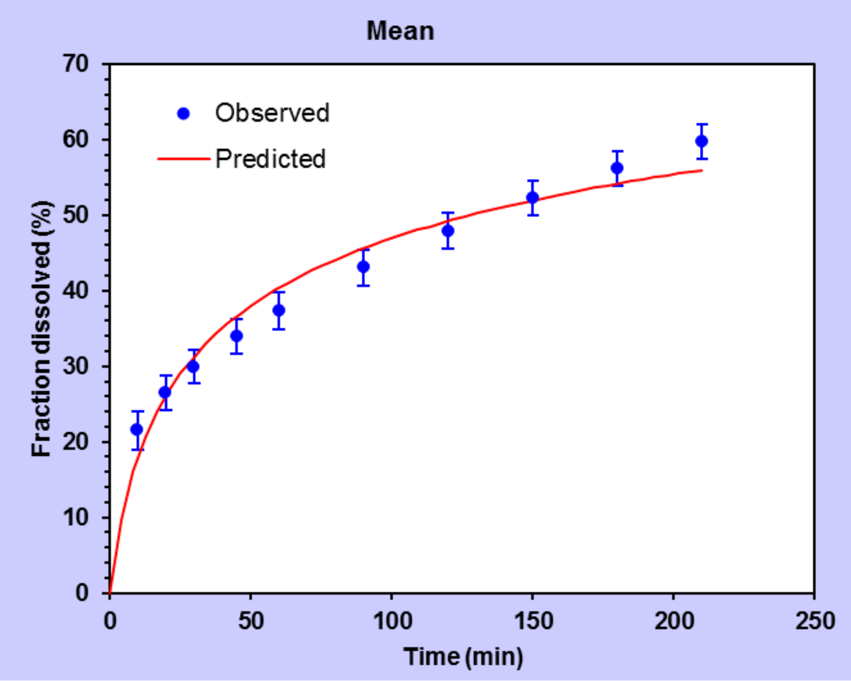

Supplementary materials – Model fitting summary – Lactochem® Crystals (modelling up to app. 60 % of carvedilol released)

Graphical abstract of model fit presented as the fraction % of released carvedilol per tested tablet:

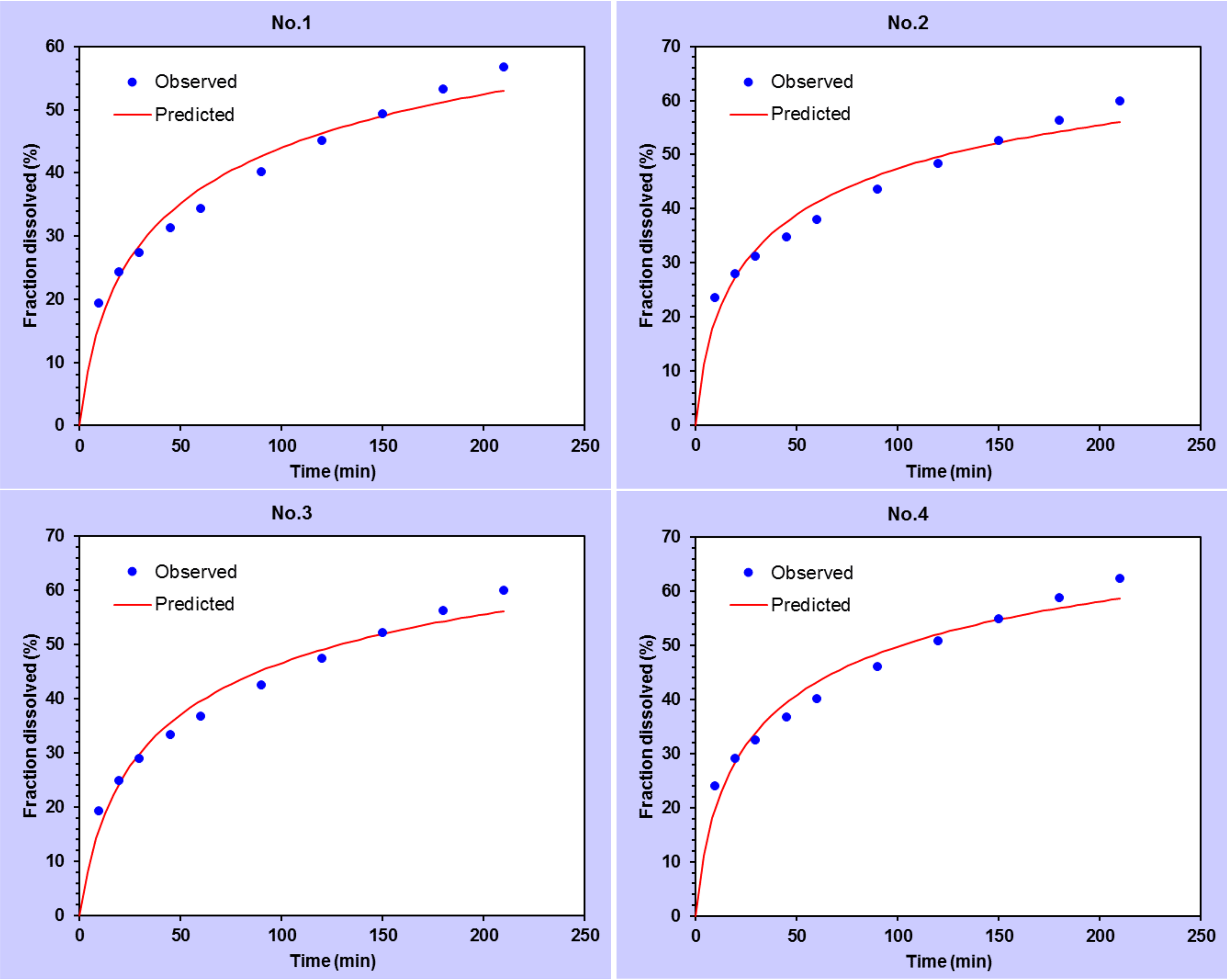

Supplementary materials – Model fitting summary – Lactochem® Crystals (modelling up to app. 60 % of carvedilol released)

Model: **Gompertz_2**

Model equation: $F = F_{max} \cdot e^{-\alpha \cdot e^{-\beta \cdot \log(t)}}$

Fitted model parameters per tested tablet (N = 4) with statistics – mean, standard deviation (SD), and relative standard deviation expressed in % (RSD%) (output from DDSolver):

Parameter	No.1	No.2	No.3	No.4	Mean	SD	RSD(%)
α	14.492	11.580	15.524	11.940	13.384	1.927	14.401
β	2.060	1.977	2.097	2.000	2.033	0.055	2.710
F_{max}	59.551	62.796	62.900	65.515	62.691	2.442	3.895

Number of dissolution data points (N), degrees of freedom (df), and selected goodness of fit criteria – Pearson correlation coefficient (R), coefficient of determination (R^2), adjusted coefficient of determination ($R^2_{adjusted}$), and residual sum of squares (RSS) (manual calculation in MS Excel):

Parameter	No.1	No.2	No.3	No.4
N	10	10	10	10
df	7	7	7	7
R	0.934082232	0.927971165	0.943431387	0.935377827
R^2	0.872509616	0.861130484	0.890062782	0.874931678
$R^2_{adjusted}$	0.836083793	0.821453479	0.858652148	0.839197872
RSS	252.2819409	267.1850754	253.741757	267.5730177

Graphical abstract of model fit presented as mean ± 1 SD of the fraction % of released carvedilol:

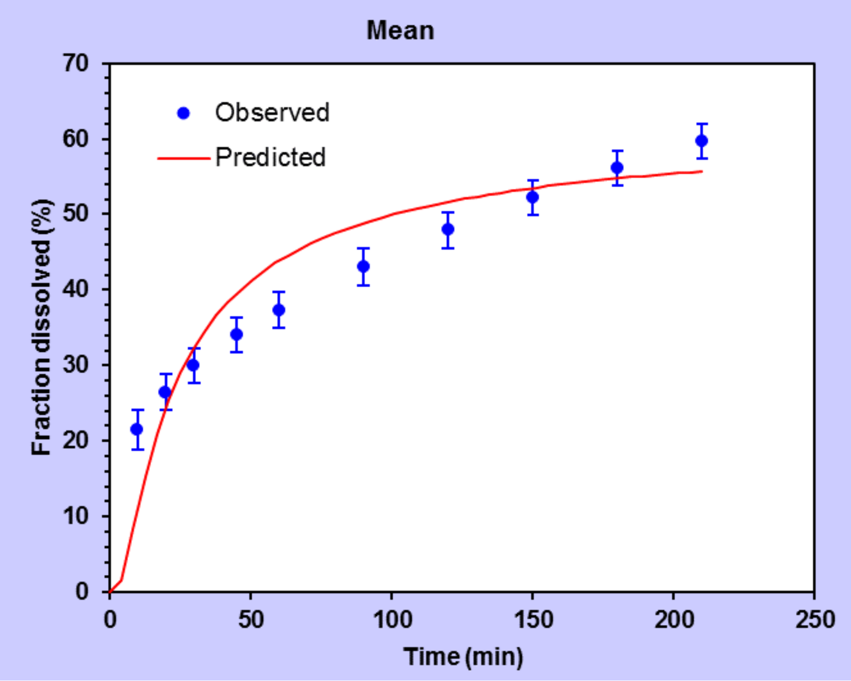

Supplementary materials – Model fitting summary – Lactochem® Crystals (modelling up to app. 60 % of carvedilol released)

Graphical abstract of model fit presented as the fraction % of released carvedilol per tested tablet:

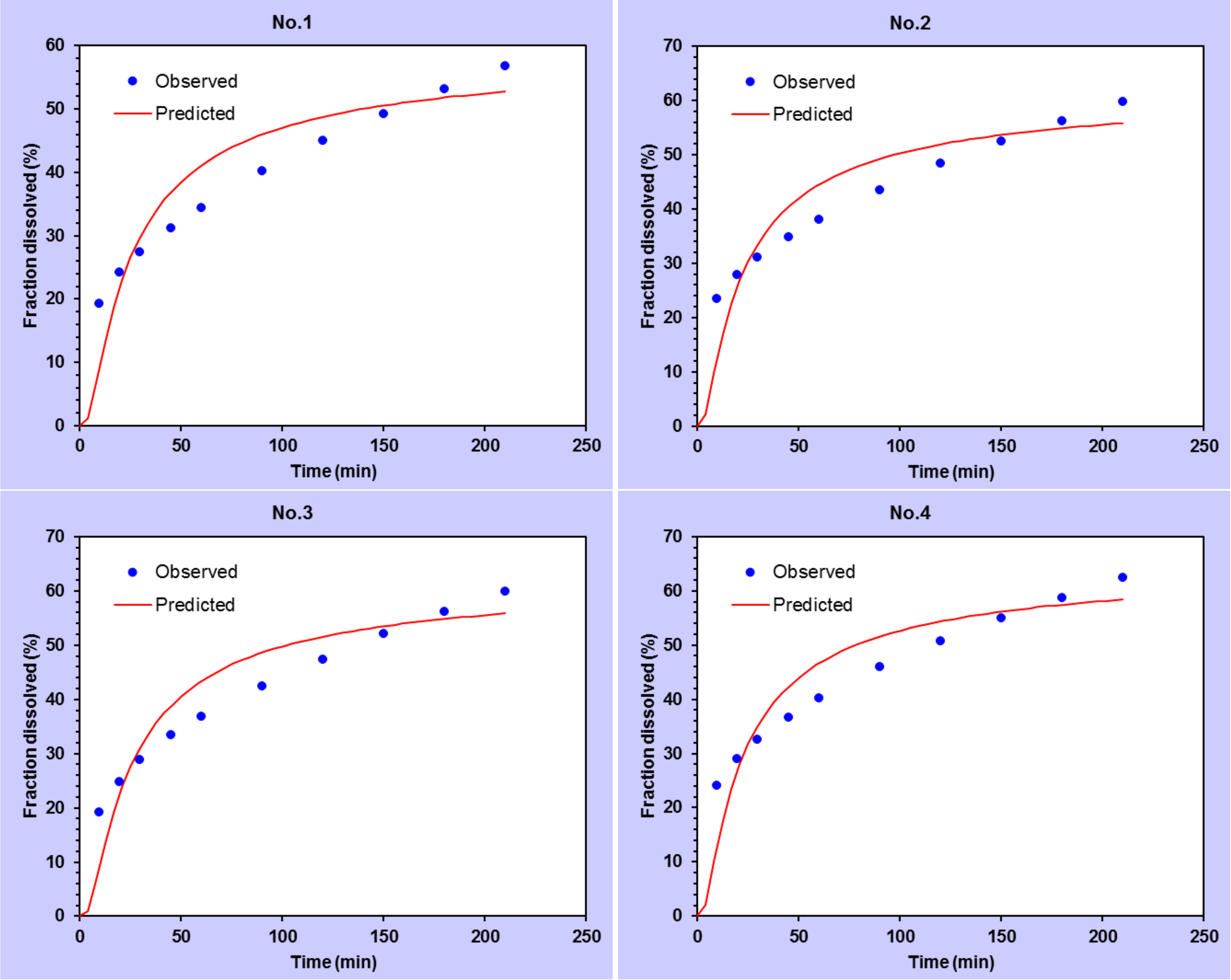

Supplementary materials – Model fitting summary – Lactochem® Crystals (modelling up to app. 60 % of carvedilol released)

Model: **Gompertz_3**

Model equation: $F = F_{max} \cdot e^{-e^{-k\cdot(t-\gamma)}}$

Fitted model parameters per tested tablet (N = 4) with statistics – mean, standard deviation (SD), and relative standard deviation expressed in % (RSD%) (output from DDSolver):

Parameter	No.1	No.2	No.3	No.4	Mean	SD	RSD(%)
k	0.014	0.014	0.014	0.014	0.014	0.000	2.473
γ	18.035	9.623	18.975	9.211	13.961	5.264	37.705
F_{max}	59.551	62.796	62.900	65.515	62.691	2.442	3.895

Number of dissolution data points (N), degrees of freedom (df), and selected goodness of fit criteria – Pearson correlation coefficient (R), coefficient of determination (R^2), adjusted coefficient of determination ($R^2_{adjusted}$), and residual sum of squares (RSS) (manual calculation in MS Excel):

Parameter	No.1	No.2	No.3	No.4
N	10	10	10	10
df	7	7	7	7
R	0.995941929	0.996284409	0.99483088	0.996553755
R^2	0.991900327	0.992582624	0.989688481	0.993119386
$R^2_{adjusted}$	0.989586134	0.990463374	0.986742332	0.991153497
RSS	16.83648429	15.38630047	21.85447518	14.37102914

Graphical abstract of model fit presented as mean ± 1 SD of the fraction % of released carvedilol:

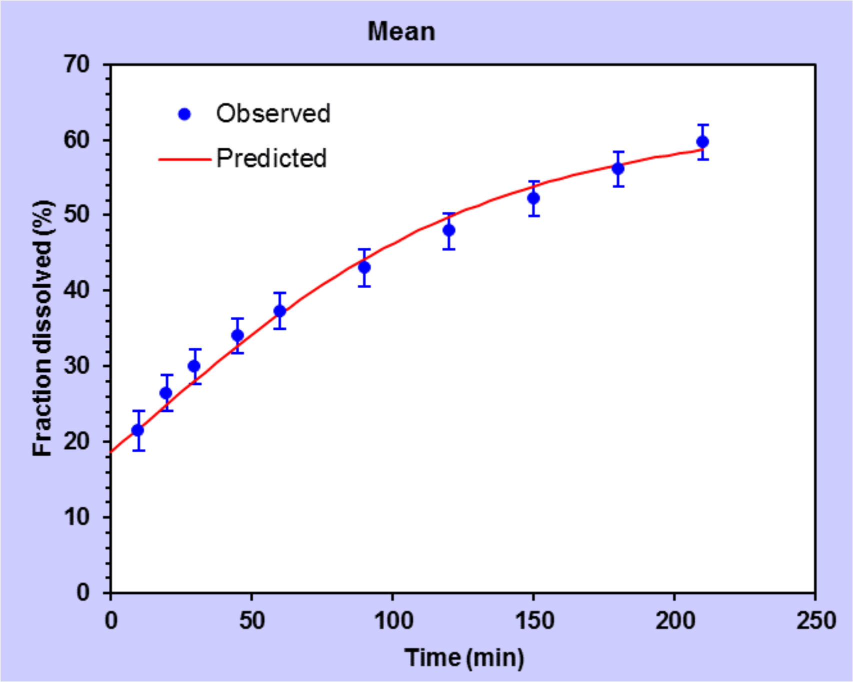

Supplementary materials – Model fitting summary – Lactochem® Crystals (modelling up to app. 60 % of carvedilol released)

Graphical abstract of model fit presented as the fraction % of released carvedilol per tested tablet:

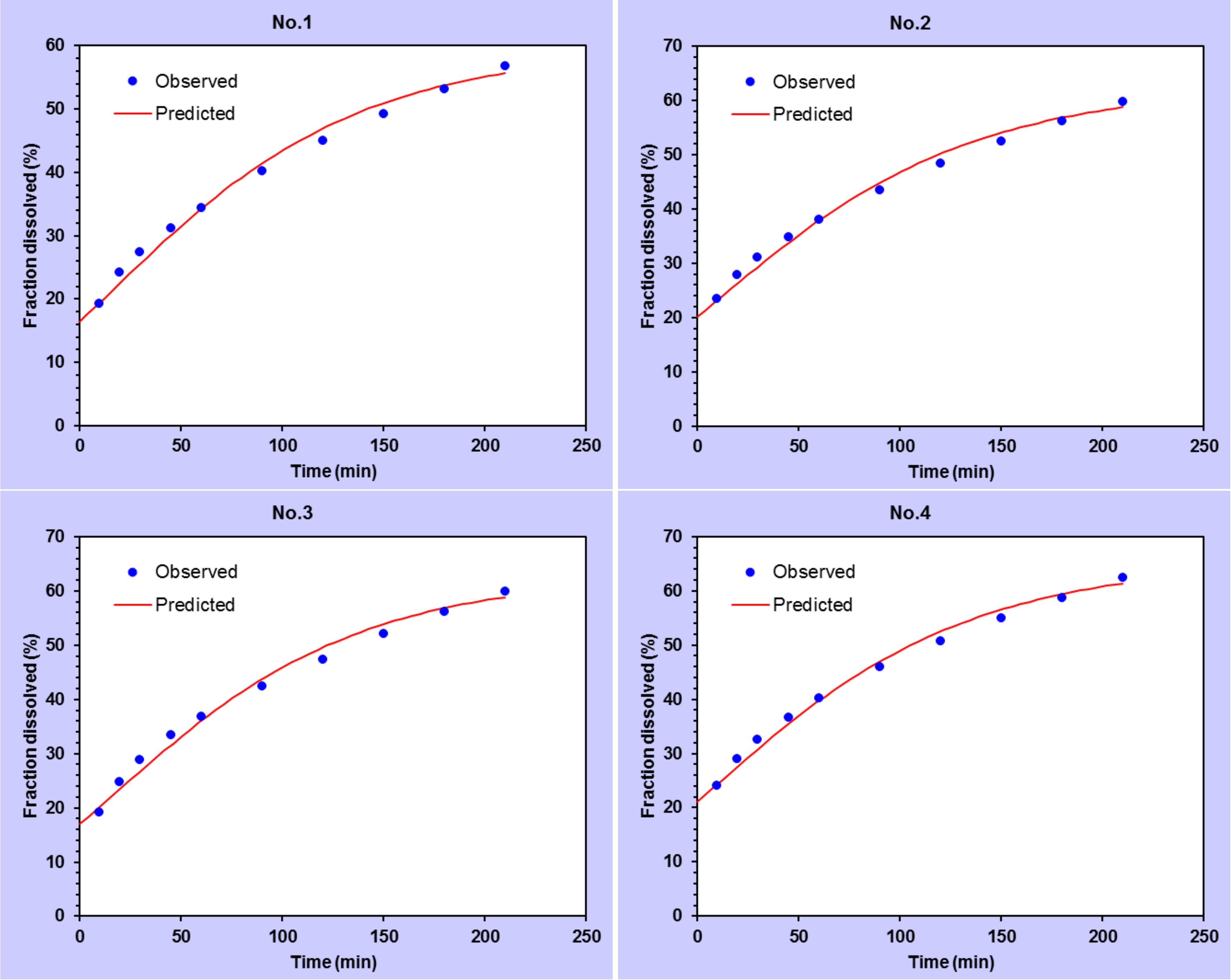

Supplementary materials – Model fitting summary – Lactochem® Crystals (modelling up to app. 60 % of carvedilol released)

Model: **Gompertz_4**

Model equation: $F = F_{max} \cdot e^{-\beta \cdot e^{-k \cdot t}}$

Fitted model parameters per tested tablet (N = 4) with statistics – mean, standard deviation (SD), and relative standard deviation expressed in % (RSD%) (output from DDSolver):

Parameter	No.1	No.2	No.3	No.4	Mean	SD	RSD(%)
k	0.014	0.014	0.014	0.014	0.014	0.000	2.473
β	1.289	1.139	1.310	1.133	1.218	0.095	7.765
F_{max}	59.551	62.796	62.900	65.515	62.691	2.442	3.895

Number of dissolution data points (N), degrees of freedom (df), and selected goodness of fit criteria – Pearson correlation coefficient (R), coefficient of determination (R^2), adjusted coefficient of determination ($R^2_{adjusted}$), and residual sum of squares (RSS) (manual calculation in MS Excel):

Parameter	No.1	No.2	No.3	No.4
N	10	10	10	10
df	7	7	7	7
R	0.995941929	0.996284409	0.99483088	0.996553755
R^2	0.991900327	0.992582624	0.989688481	0.993119386
$R^2_{adjusted}$	0.989586134	0.990463374	0.986742332	0.991153497
RSS	16.83648429	15.38630047	21.85447518	14.37102914

Graphical abstract of model fit presented as mean ± 1 SD of the fraction % of released carvedilol:

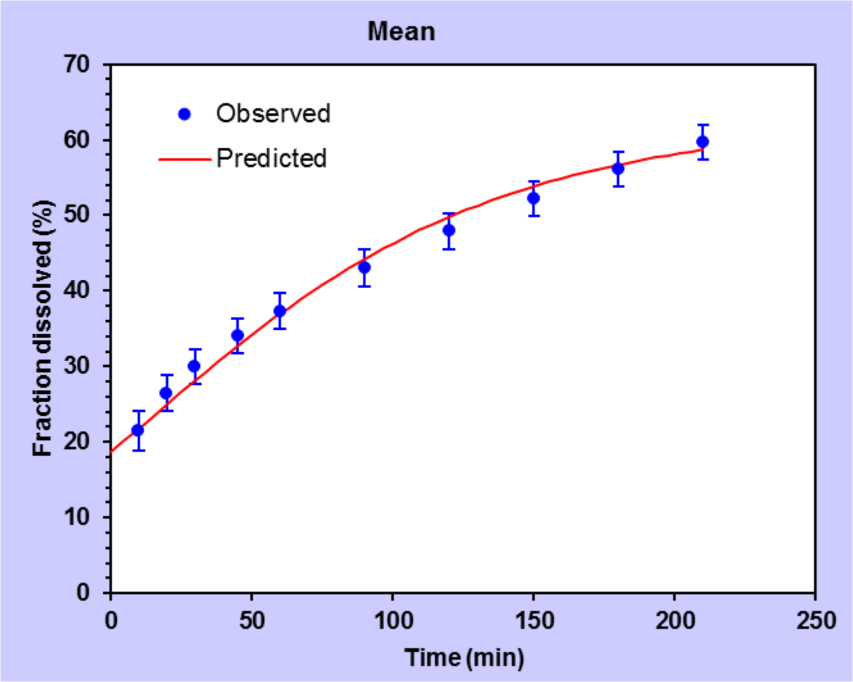

Supplementary materials – Model fitting summary – Lactochem® Crystals (modelling up to app. 60 % of carvedilol released)

Graphical abstract of model fit presented as the fraction % of released carvedilol per tested tablet:

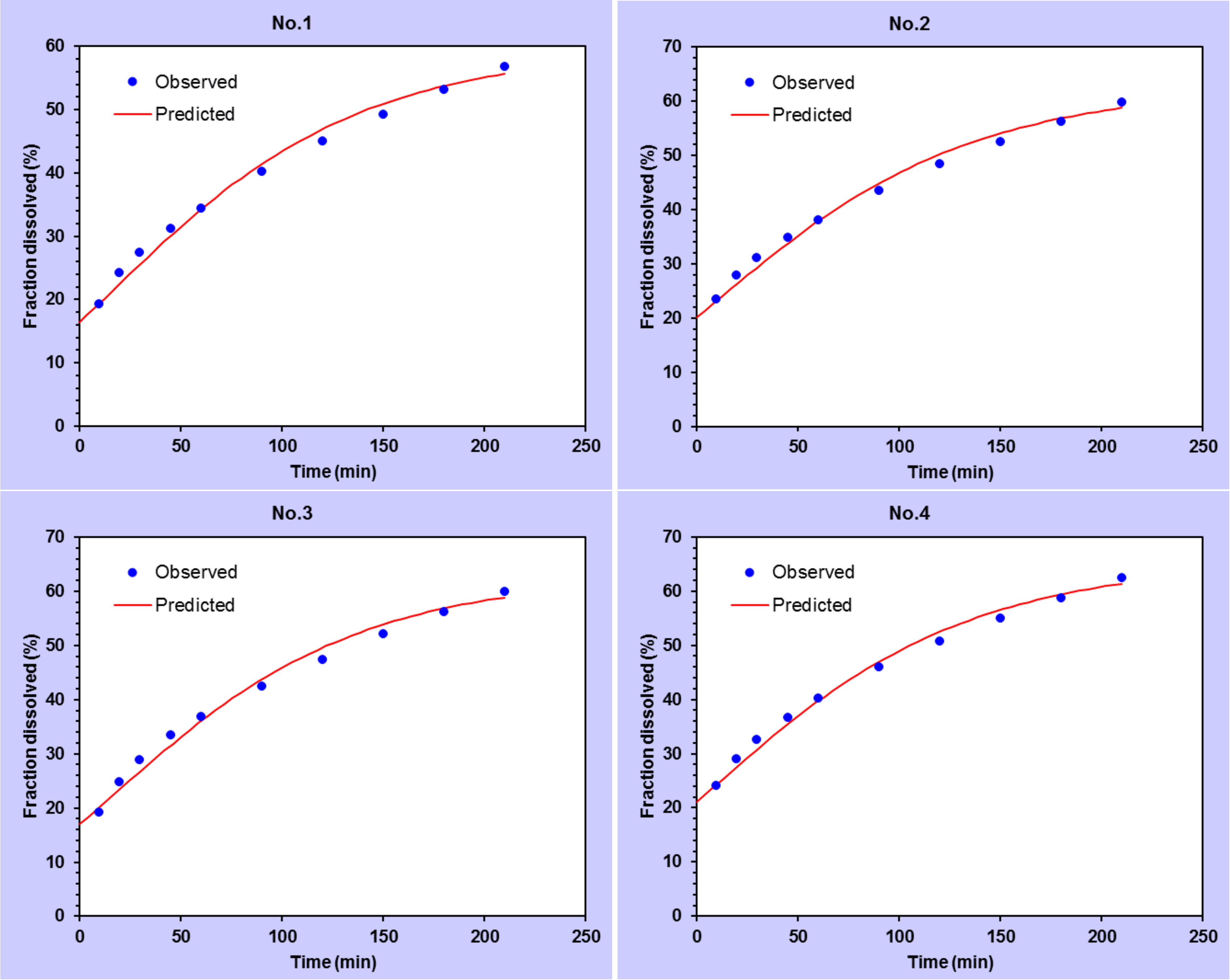

Supplementary materials – Model fitting summary – Lactochem® Crystals (modelling up to app. 60 % of carvedilol released)

Model: **Probit_1**

Model equation: $F = 100 \cdot \phi[\alpha + \beta \cdot \log(t)]$

Fitted model parameters per tested tablet (N = 4) with statistics – mean, standard deviation (SD), and relative standard deviation expressed in % (RSD%) (output from DDSolver):

Parameter	No.1	No.2	No.3	No.4	Mean	SD	RSD(%)
α	-1.729	-1.550	-1.780	-1.565	-1.656	0.116	-6.994
β	0.783	0.737	0.841	0.774	0.783	0.043	5.480

Number of dissolution data points (N), degrees of freedom (df), and selected goodness of fit criteria – Pearson correlation coefficient (R), coefficient of determination (R^2), adjusted coefficient of determination ($R^2_{adjusted}$), and residual sum of squares (RSS) (manual calculation in MS Excel):

Parameter	No.1	No.2	No.3	No.4
N	10	10	10	10
df	8	8	8	8
R	0.988995878	0.985706491	0.991919763	0.988647224
R^2	0.978112847	0.971617286	0.983904817	0.977423334
$R^2_{adjusted}$	0.975376953	0.968069446	0.981892919	0.974601251
RSS	33.12497337	40.28430219	28.14504006	35.41265101

Graphical abstract of model fit presented as mean ± 1 SD of the fraction % of released carvedilol:

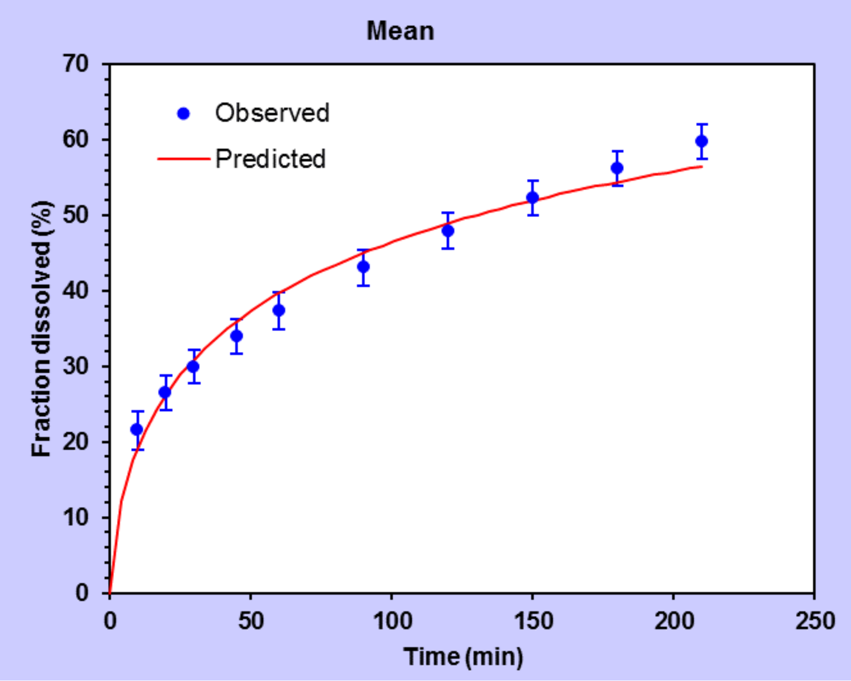

Supplementary materials – Model fitting summary – Lactochem® Crystals (modelling up to app. 60 % of carvedilol released)

Graphical abstract of model fit presented as the fraction % of released carvedilol per tested tablet:

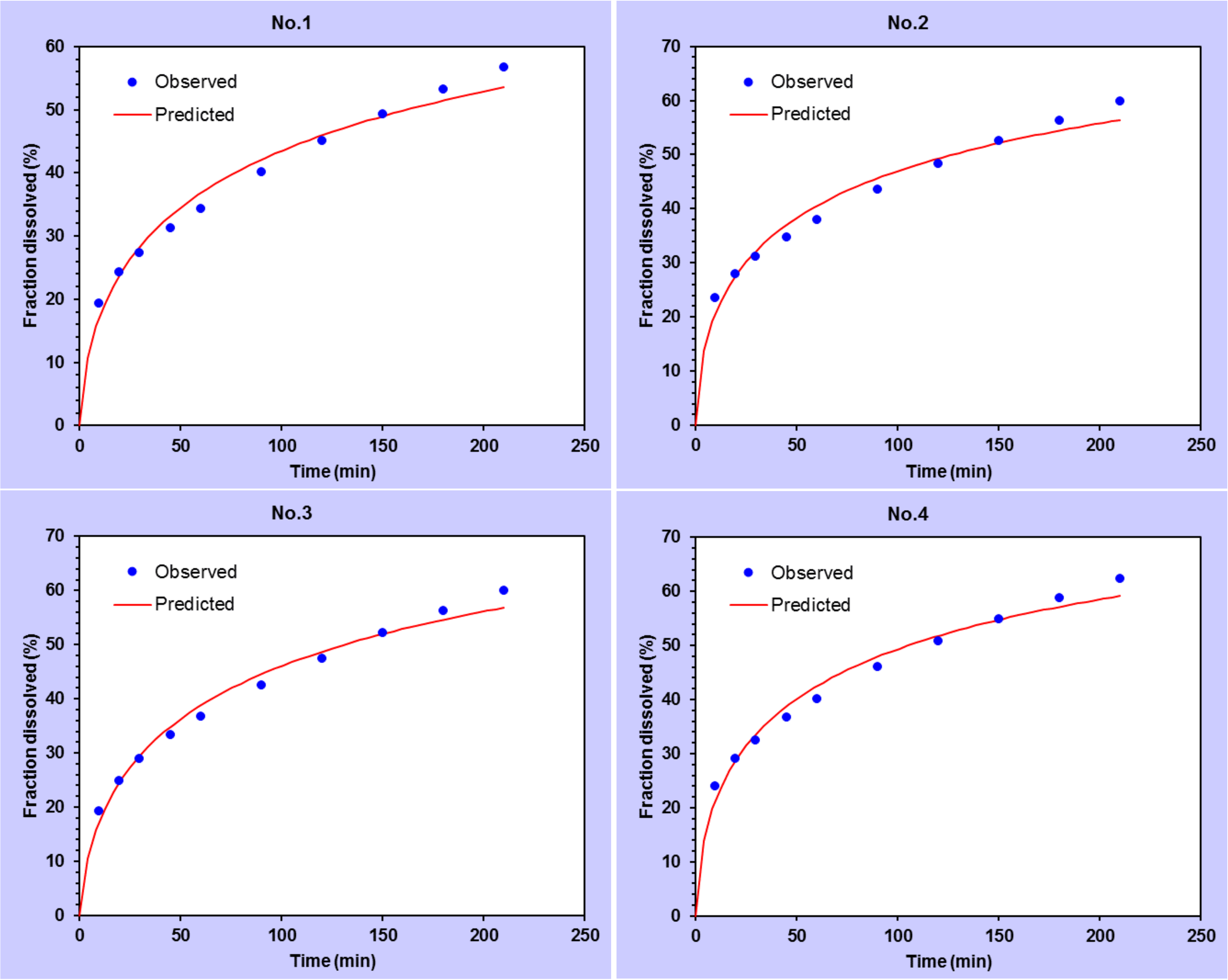

Supplementary materials – Model fitting summary – Lactochem® Crystals (modelling up to app. 60 % of carvedilol released)

Model: **Probit_2**

Model equation: $F = F_{max} \cdot \phi[\alpha + \beta \cdot \log(t)]$

Fitted model parameters per tested tablet (N = 4) with statistics – mean, standard deviation (SD), and relative standard deviation expressed in % (RSD%) (output from DDSolver):

Parameter	No.1	No.2	No.3	No.4	Mean	SD	RSD(%)
α	-2.203	-1.979	-2.270	-2.004	-2.114	0.145	-6.836
β	1.472	1.381	1.507	1.399	1.440	0.059	4.126
F_{max}	59.551	62.796	62.900	65.515	62.691	2.442	3.895

Number of dissolution data points (N), degrees of freedom (df), and selected goodness of fit criteria – Pearson correlation coefficient (R), coefficient of determination (R^2), adjusted coefficient of determination ($R^2_{adjusted}$), and residual sum of squares (RSS) (manual calculation in MS Excel):

Parameter	No.1	No.2	No.3	No.4
N	10	10	10	10
df	7	7	7	7
R	0.962698189	0.957884052	0.969453388	0.963719989
R^2	0.926787803	0.917541856	0.939839872	0.928756217
$R^2_{adjusted}$	0.905870032	0.893982387	0.922651264	0.90840085
RSS	121.1064925	130.634536	116.1576587	125.3571934

Graphical abstract of model fit presented as mean ± 1 SD of the fraction % of released carvedilol:

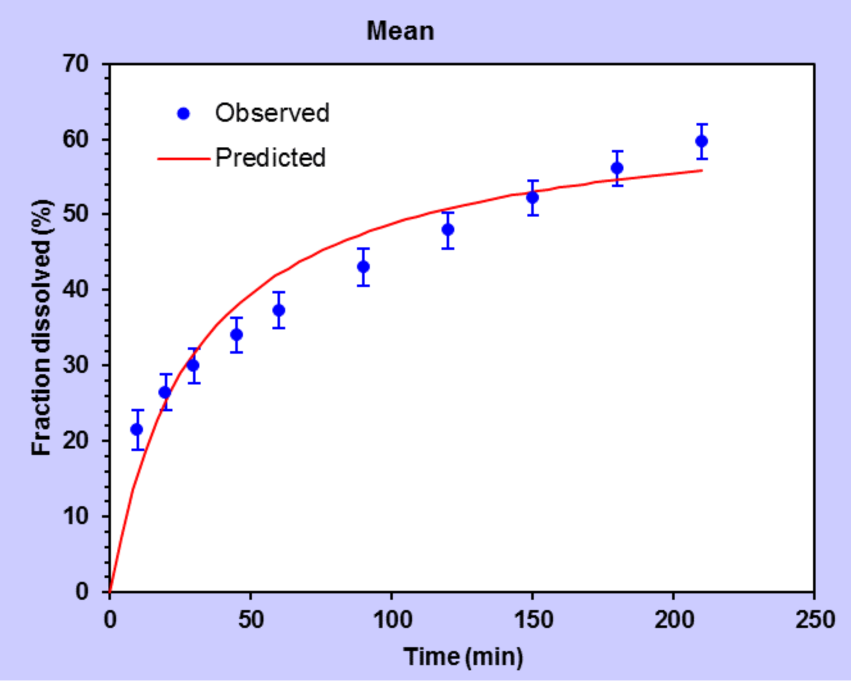

Supplementary materials – Model fitting summary – Lactochem® Crystals (modelling up to app. 60 % of carvedilol released)

Graphical abstract of model fit presented as the fraction % of released carvedilol per tested tablet:

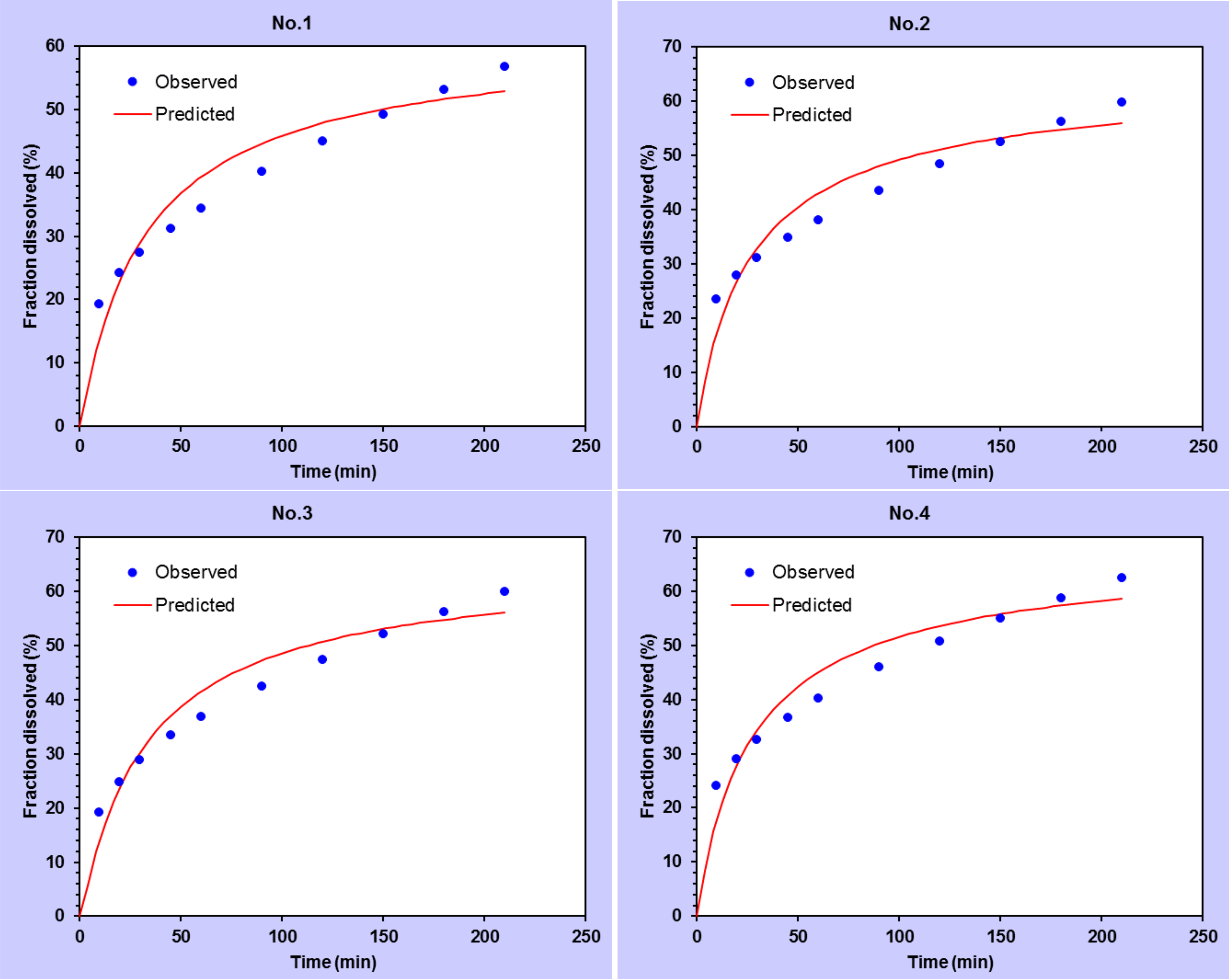

Supplement: Supplementary file 1 [file pharmaceutics-16-00498-s001.zip › Supplementary materials_Model fitting summary_Lactochem® Crystals.pdf]
