# Supplementary material for: Comparative Fitting of Mathematical Models to Carvedilol Release Profiles Obtained from Hypromellose Matrix Tablets
Source: Pharmaceutics. 2024 Apr 4;16(4):498. doi: 10.3390/pharmaceutics16040498 (PMC11053526; doi:10.3390/pharmaceutics16040498)

Supplementary materials – Model fitting summary – Lactochem® Fine Powder (modelling the entire relevant dissolution data)

Model: **Zero–order**

Model equation: $F = k_0 \cdot t$

Fitted model parameters per tested tablet (N = 4) with statistics – mean, standard deviation (SD), and relative standard deviation expressed in % (RSD%) (output from DDSolver):

Parameter	No.1	No.2	No.3	No.4	Mean	SD	RSD(%)
k_0	0.154	0.139	0.140	0.148	0.145	0.007	4.672

Number of dissolution data points (N), degrees of freedom (df), and selected goodness of fit criteria – Pearson correlation coefficient (R), coefficient of determination (R^2), adjusted coefficient of determination ($R^2_{adjusted}$), and residual sum of squares (RSS) (manual calculation in MS Excel):

Parameter	No.1	No.2	No.3	No.4
N	22	22	22	22
df	21	21	21	21
R	0.990587886	0.984505985	0.990610898	0.995427579
R^2	0.981264359	0.969252034	0.981309951	0.990876065
$R^2_{adjusted}$	0.981264359	0.969252034	0.981309951	0.990876065
RSS	2010.858425	2409.336739	2222.953456	1146.336119

Graphical abstract of model fit presented as mean ± 1 SD of the fraction % of released carvedilol:

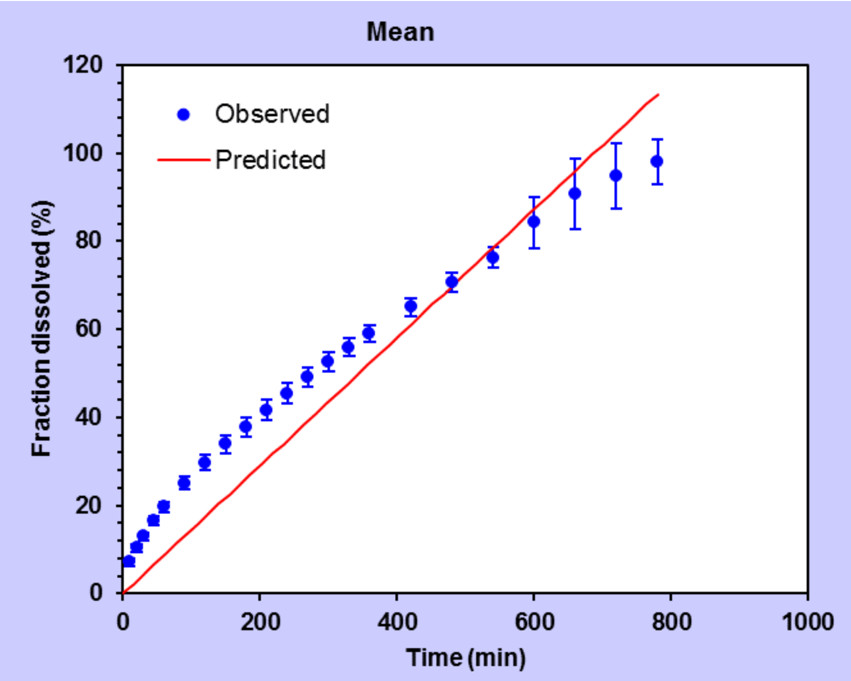

Supplementary materials – Model fitting summary – Lactochem® Fine Powder (modelling the entire relevant dissolution data)

Graphical abstract of model fit presented as the fraction % of released carvedilol per tested tablet:

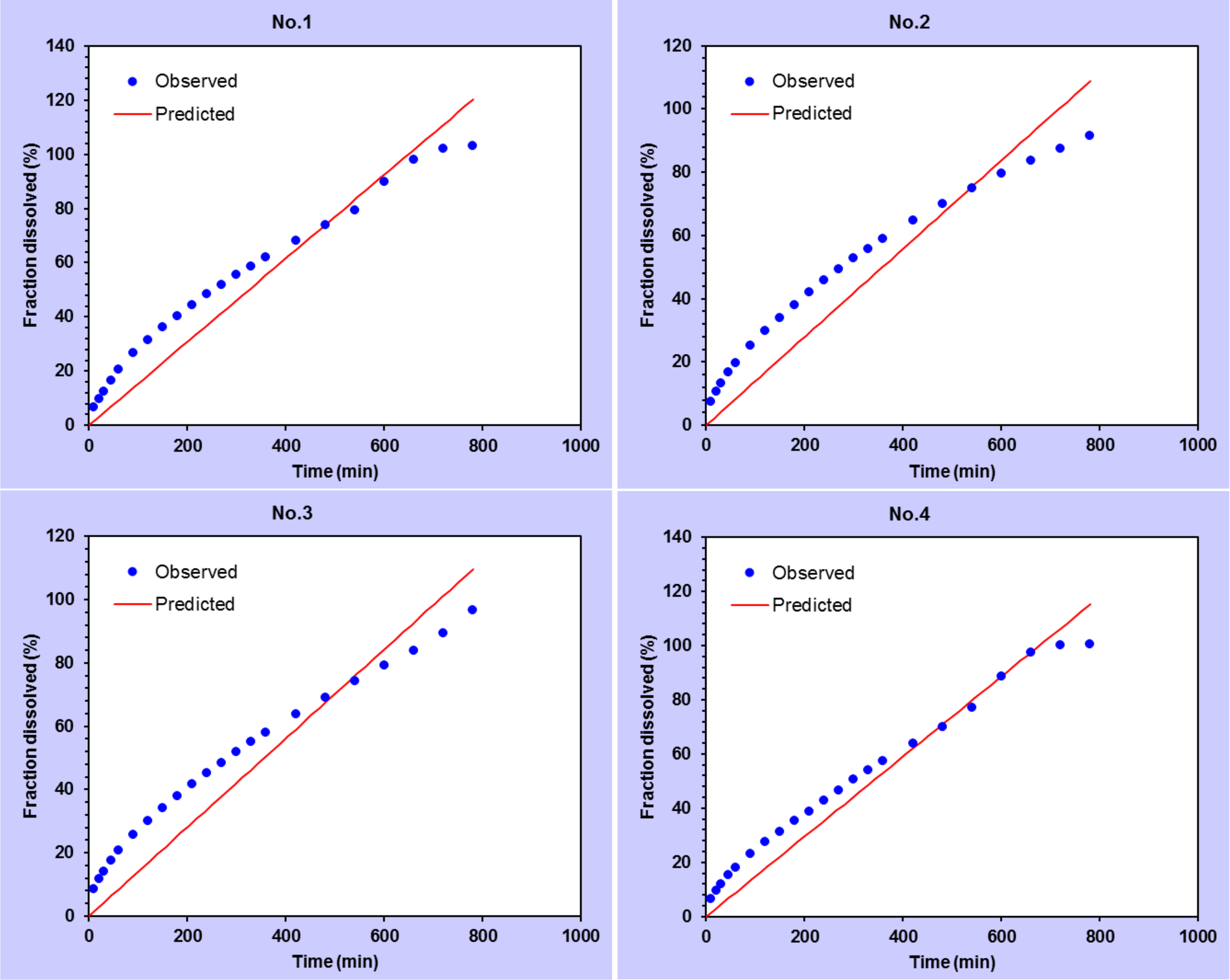

Supplementary materials – Model fitting summary – Lactochem® Fine Powder (modelling the entire relevant dissolution data)

Model: **Zero–order with T_{lag}**

Model equation: $F = k_0 \cdot (t - T_{lag})$

Fitted model parameters per tested tablet (N = 4) with statistics – mean, standard deviation (SD), and relative standard deviation expressed in % (RSD%) (output from DDSolver):

Parameter	No.1	No.2	No.3	No.4	Mean	SD	RSD(%)
k_0	0.125	0.108	0.109	0.125	0.117	0.010	8.304
T_{lag}	-112.221	-141.828	-140.036	-85.874	-119.990	26.476	-22.066

Number of dissolution data points (N), degrees of freedom (df), and selected goodness of fit criteria – Pearson correlation coefficient (R), coefficient of determination (R^2), adjusted coefficient of determination ($R^2_{adjusted}$), and residual sum of squares (RSS) (manual calculation in MS Excel):

Parameter	No.1	No.2	No.3	No.4
N	22	22	22	22
df	20	20	20	20
R	0.990587886	0.984505985	0.990610898	0.995427579
R^2	0.981264359	0.969252034	0.981309951	0.990876065
$R^2_{adjusted}$	0.980327577	0.967714635	0.980375449	0.990419868
RSS	364.1012456	450.5109642	275.7917489	176.5843736

Graphical abstract of model fit presented as mean ± 1 SD of the fraction % of released carvedilol:

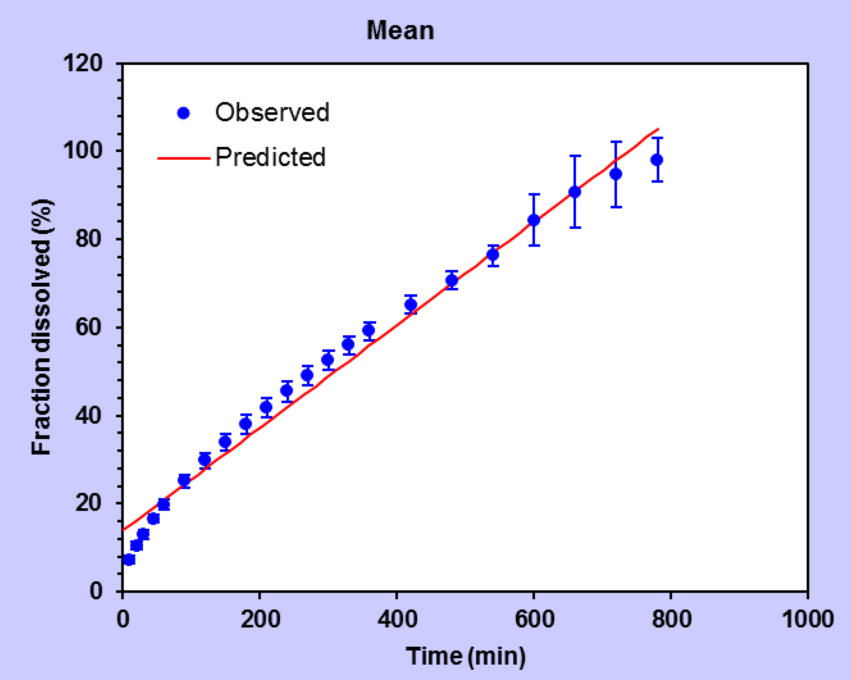

Supplementary materials – Model fitting summary – Lactochem® Fine Powder (modelling the entire relevant dissolution data)

Graphical abstract of model fit presented as the fraction % of released carvedilol per tested tablet:

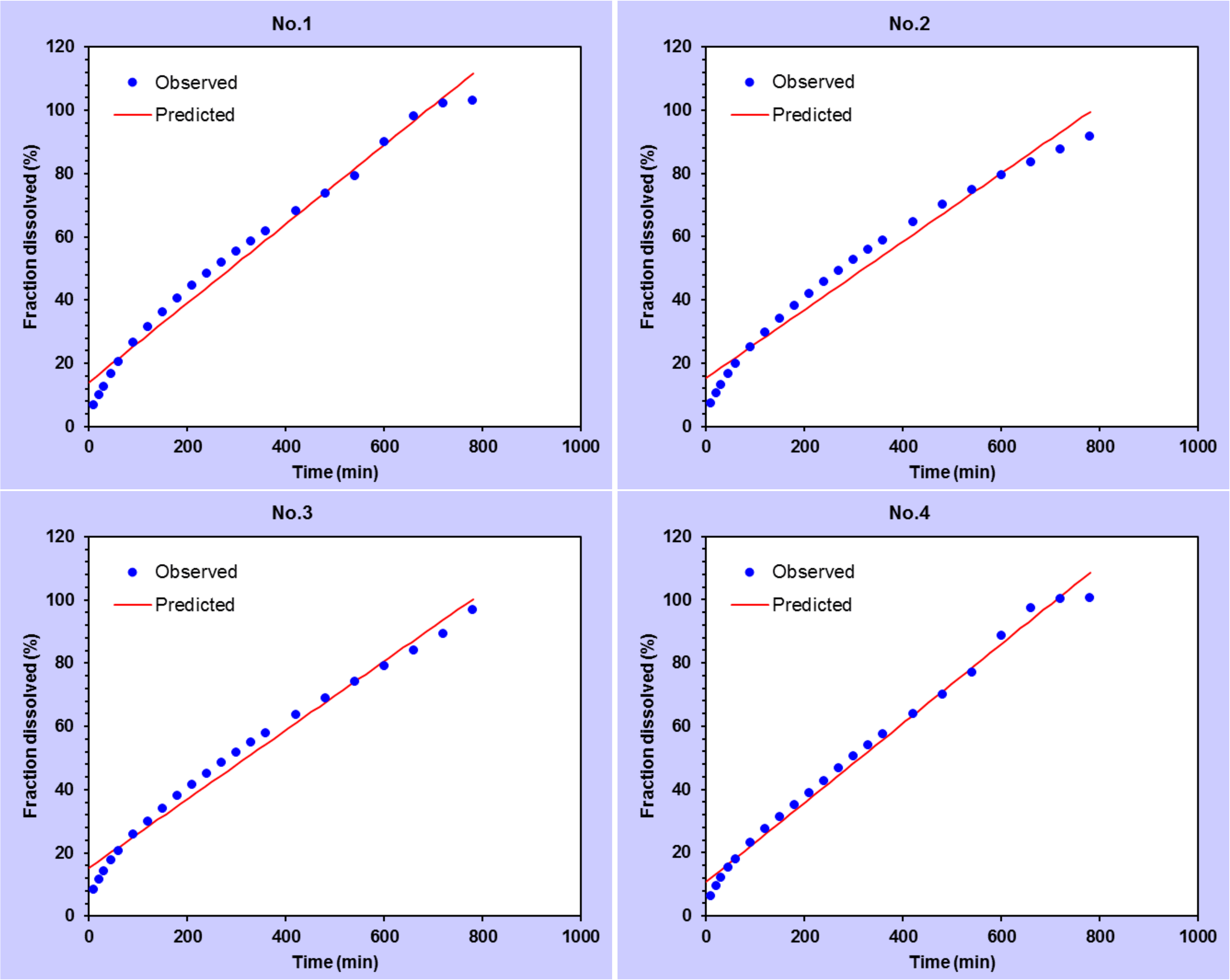

Supplementary materials – Model fitting summary – Lactochem® Fine Powder (modelling the entire relevant dissolution data)

Model: **Zero–order with F_0**

Model equation: $F = F_0 + k_0 \cdot t$

Fitted model parameters per tested tablet (N = 4) with statistics – mean, standard deviation (SD), and relative standard deviation expressed in % (RSD%) (output from DDSolver):

Parameter	No.1	No.2	No.3	No.4	Mean	SD	RSD(%)
k_0	0.125	0.108	0.109	0.125	0.117	0.010	8.304
F_0	14.034	15.306	15.261	10.770	13.843	2.132	15.400

Number of dissolution data points (N), degrees of freedom (df), and selected goodness of fit criteria – Pearson correlation coefficient (R), coefficient of determination (R^2), adjusted coefficient of determination ($R^2_{adjusted}$), and residual sum of squares (RSS) (manual calculation in MS Excel):

Parameter	No.1	No.2	No.3	No.4
N	22	22	22	22
df	20	20	20	20
R	0.990587886	0.984505985	0.990610898	0.995427579
R^2	0.981264359	0.969252034	0.981309951	0.990876065
$R^2_{adjusted}$	0.980327577	0.967714635	0.980375449	0.990419868
RSS	364.1012456	450.5109642	275.7917489	176.5843736

Graphical abstract of model fit presented as mean ± 1 SD of the fraction % of released carvedilol:

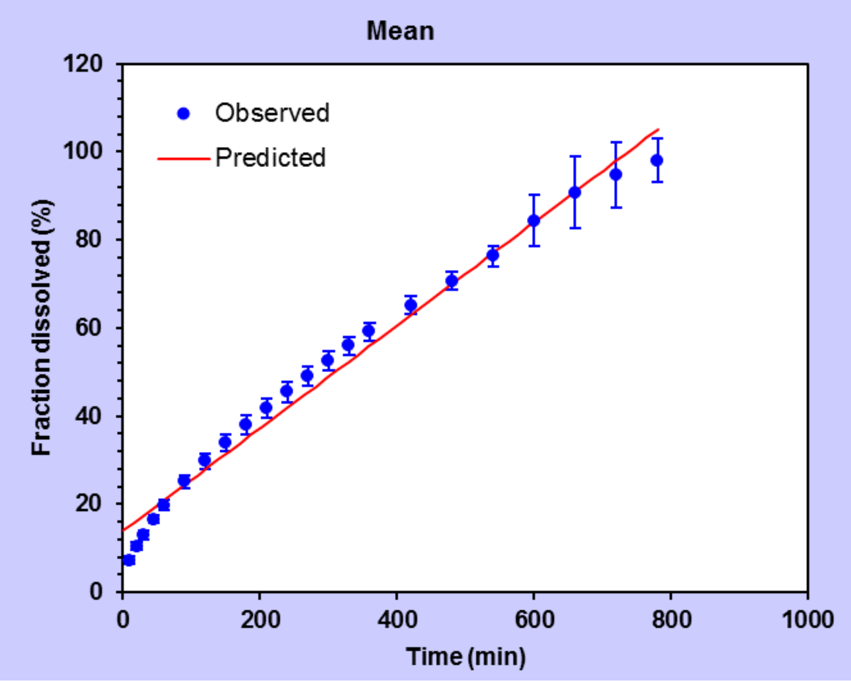

Supplementary materials – Model fitting summary – Lactochem® Fine Powder (modelling the entire relevant dissolution data)

Graphical abstract of model fit presented as the fraction % of released carvedilol per tested tablet:

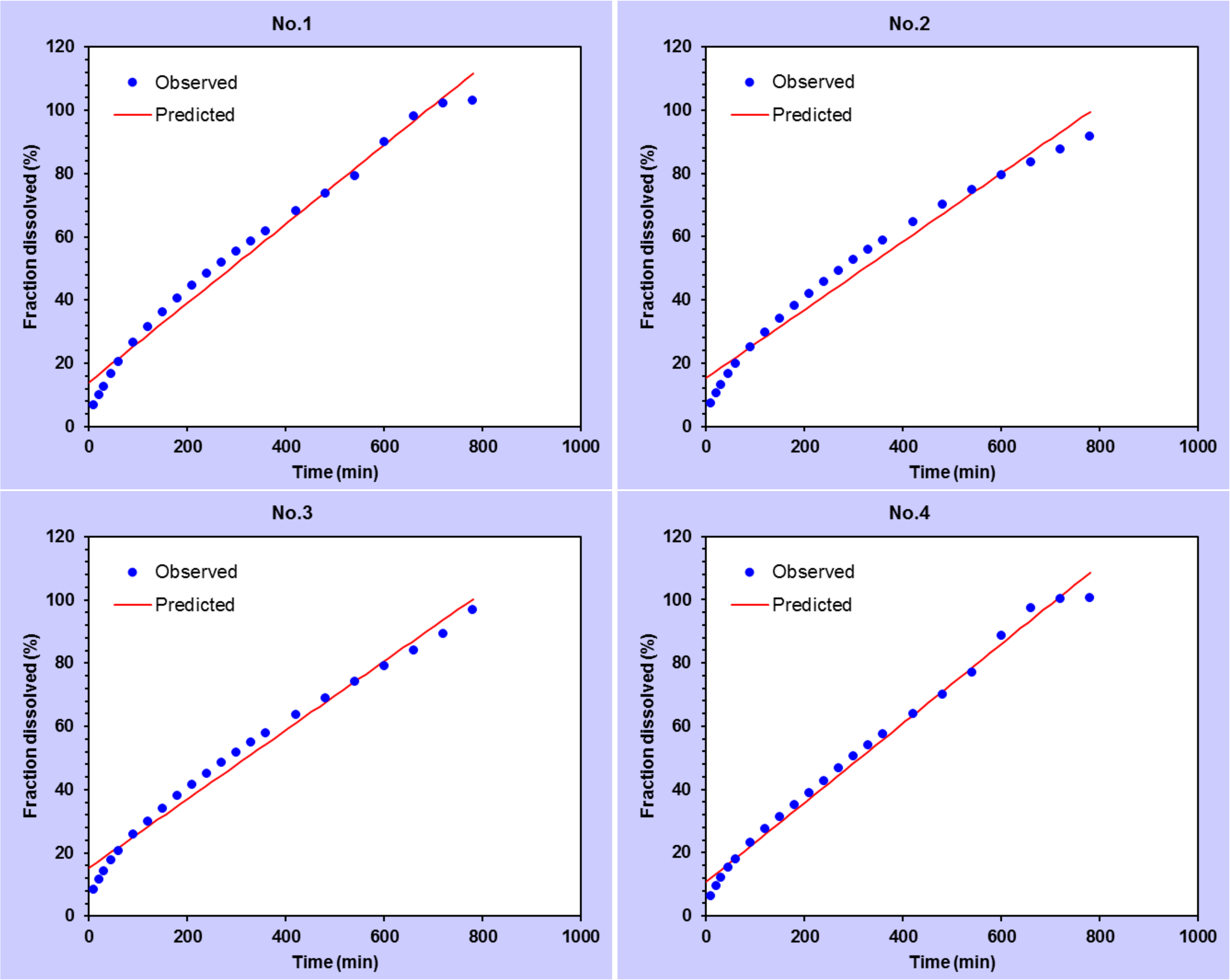

Supplementary materials – Model fitting summary – Lactochem® Fine Powder (modelling the entire relevant dissolution data)

Model: **First–order**

Model equation: $F = 100 \cdot (1 - e^{-k_1 \cdot t})$

Fitted model parameters per tested tablet (N = 4) with statistics – mean, standard deviation (SD), and relative standard deviation expressed in % (RSD%) (output from DDSolver):

Parameter	No.1	No.2	No.3	No.4	Mean	SD	RSD(%)
k_1	0.003	0.003	0.003	0.002	0.003	0.000	4.518

Number of dissolution data points (N), degrees of freedom (df), and selected goodness of fit criteria – Pearson correlation coefficient (R), coefficient of determination (R^2), adjusted coefficient of determination ($R^2_{adjusted}$), and residual sum of squares (RSS) (manual calculation in MS Excel):

Parameter	No.1	No.2	No.3	No.4
N	22	22	22	22
df	21	21	21	21
R	0.986962288	0.995063907	0.990490604	0.98201663
R^2	0.974094559	0.990152179	0.981071638	0.964356662
$R^2_{adjusted}$	0.974094559	0.990152179	0.981071638	0.964356662
RSS	945.0488823	248.2835765	404.8188849	1051.48614

Graphical abstract of model fit presented as mean ± 1 SD of the fraction % of released carvedilol:

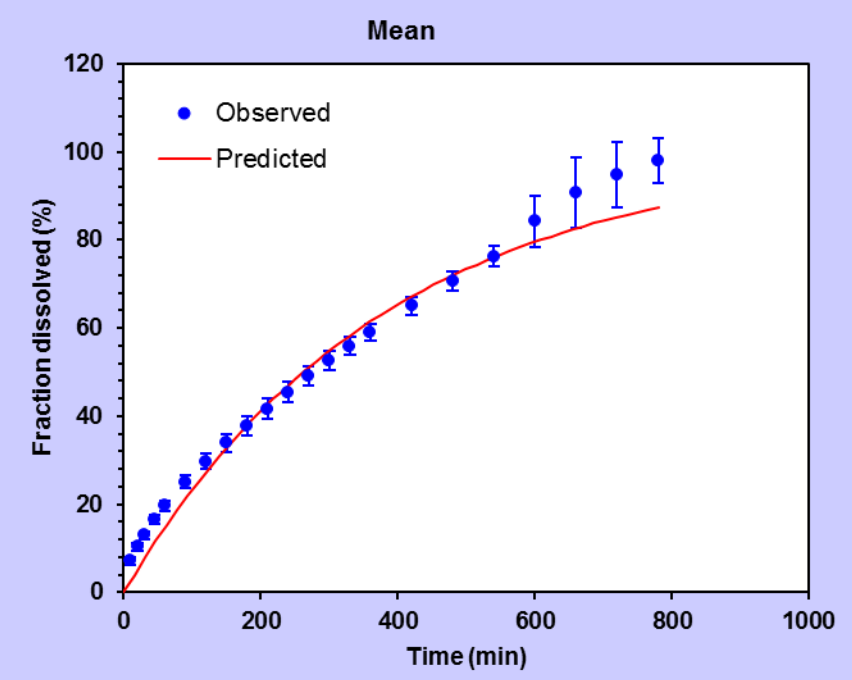

Supplementary materials – Model fitting summary – Lactochem® Fine Powder (modelling the entire relevant dissolution data)

Graphical abstract of model fit presented as the fraction % of released carvedilol per tested tablet:

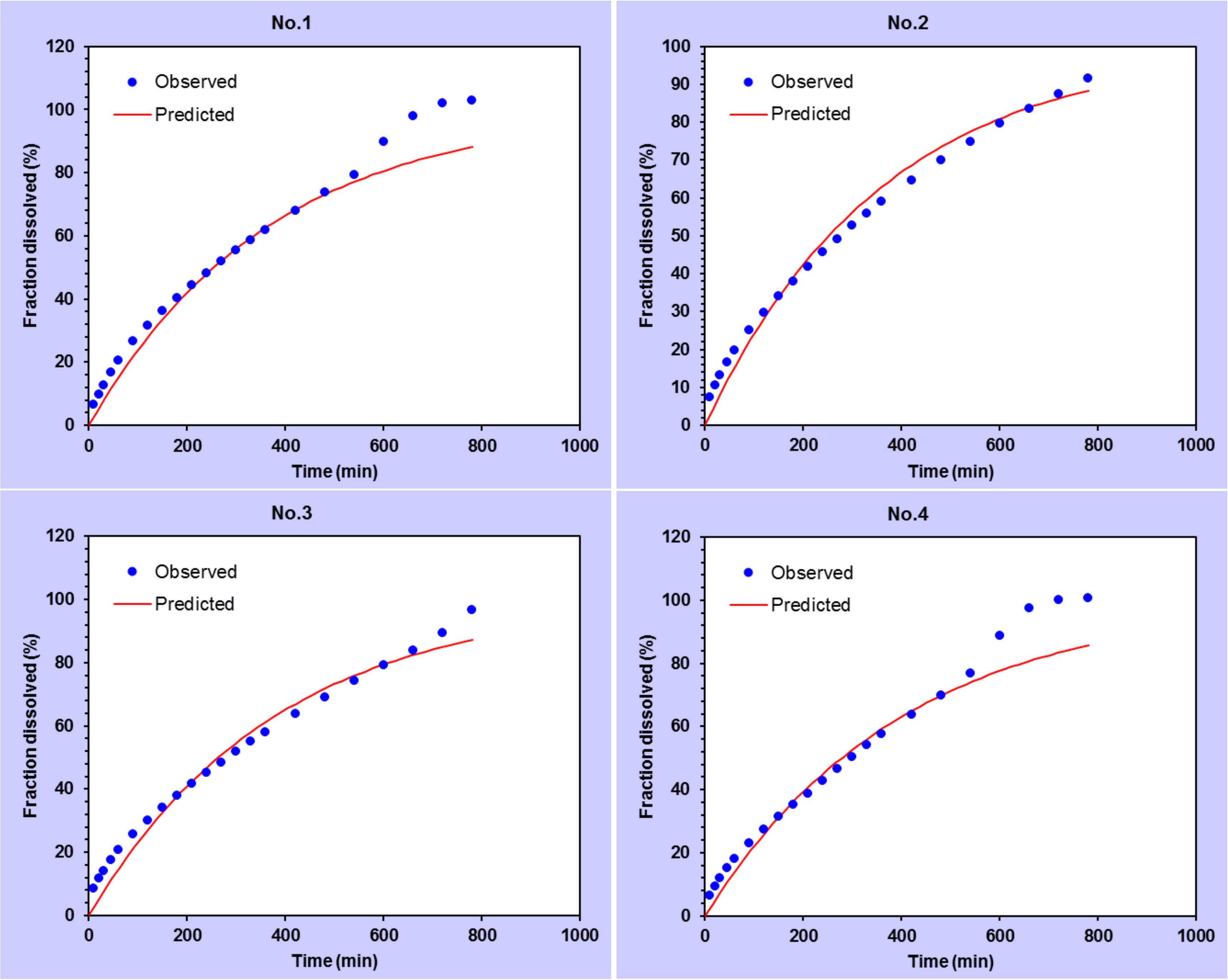

Supplementary materials – Model fitting summary – Lactochem® Fine Powder (modelling the entire relevant dissolution data)

Model: **First–order with T_{lag}**

Model equation: $F = 100 \cdot \left[1 - e^{-k_1 \cdot (t - T_{lag})}\right]$

Fitted model parameters per tested tablet (N = 4) with statistics – mean, standard deviation (SD), and relative standard deviation expressed in % (RSD%) (output from DDSolver):

Parameter	No.1	No.2	No.3	No.4	Mean	SD	RSD(%)
k_1	/	/	/	/	/	/	/
T_{lag}	/	/	/	/	/	/	/

Number of dissolution data points (N), degrees of freedom (df), and selected goodness of fit criteria – Pearson correlation coefficient (R), coefficient of determination (R^2), adjusted coefficient of determination ($R^2_{adjusted}$), and residual sum of squares (RSS) (manual calculation in MS Excel):

Parameter	No.1	No.2	No.3	No.4
N	/	/	/	/
df	/	/	/	/
R	/	/	/	/
R^2	/	/	/	/
$R^2_{adjusted}$	/	/	/	/
RSS	/	/	/	/

Graphical abstract of model fit presented as mean ± 1 SD of the fraction % of released carvedilol: /

Graphical abstract of model fit presented as the fraction % of released carvedilol per tested tablet: /

Note: the model could not be fitted

Supplementary materials – Model fitting summary – Lactochem® Fine Powder (modelling the entire relevant dissolution data)

Model: **First–order with F_{max}**

Model equation: $F = F_{max} \cdot (1 - e^{-k_1 \cdot t})$

Fitted model parameters per tested tablet (N = 4) with statistics – mean, standard deviation (SD), and relative standard deviation expressed in % (RSD%) (output from DDSolver):

Parameter	No.1	No.2	No.3	No.4	Mean	SD	RSD(%)
k_1	0.003	0.003	0.003	0.003	0.003	0.000	7.725
F_{max}	108.197	89.085	101.654	105.603	101.135	8.471	8.376

Number of dissolution data points (N), degrees of freedom (df), and selected goodness of fit criteria – Pearson correlation coefficient (R), coefficient of determination (R^2), adjusted coefficient of determination ($R^2_{adjusted}$), and residual sum of squares (RSS) (manual calculation in MS Excel):

Parameter	No.1	No.2	No.3	No.4
N	22	22	22	22
df	20	20	20	20
R	0.98655425	0.990631785	0.988258327	0.97793479
R^2	0.973289289	0.981351334	0.976654521	0.956356453
$R^2_{adjusted}$	0.971953753	0.9804189	0.975487247	0.954174276
RSS	534.7572466	340.8873999	558.5954581	909.8573478

Graphical abstract of model fit presented as mean ± 1 SD of the fraction % of released carvedilol:

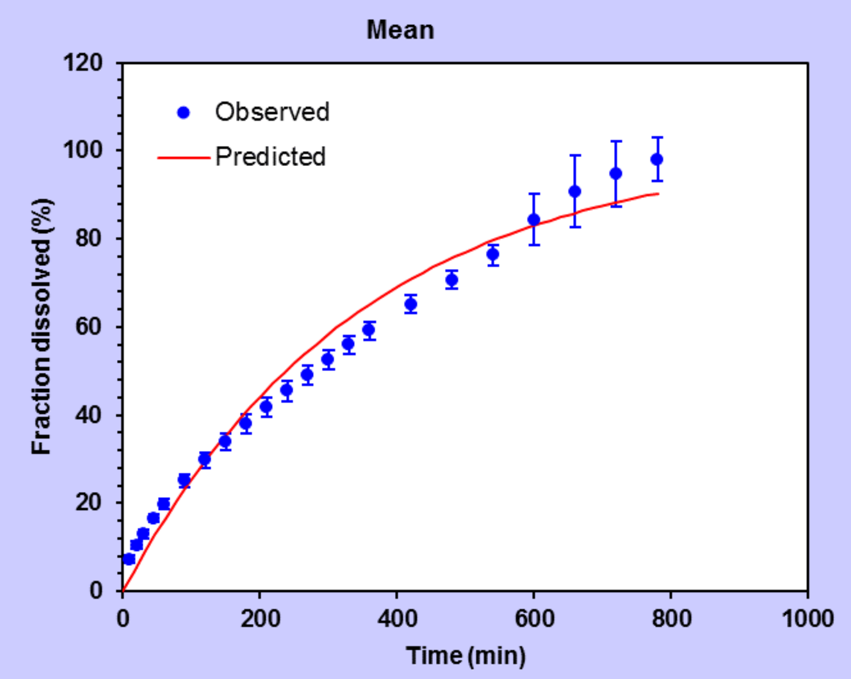

Supplementary materials – Model fitting summary – Lactochem® Fine Powder (modelling the entire relevant dissolution data)

Graphical abstract of model fit presented as the fraction % of released carvedilol per tested tablet:

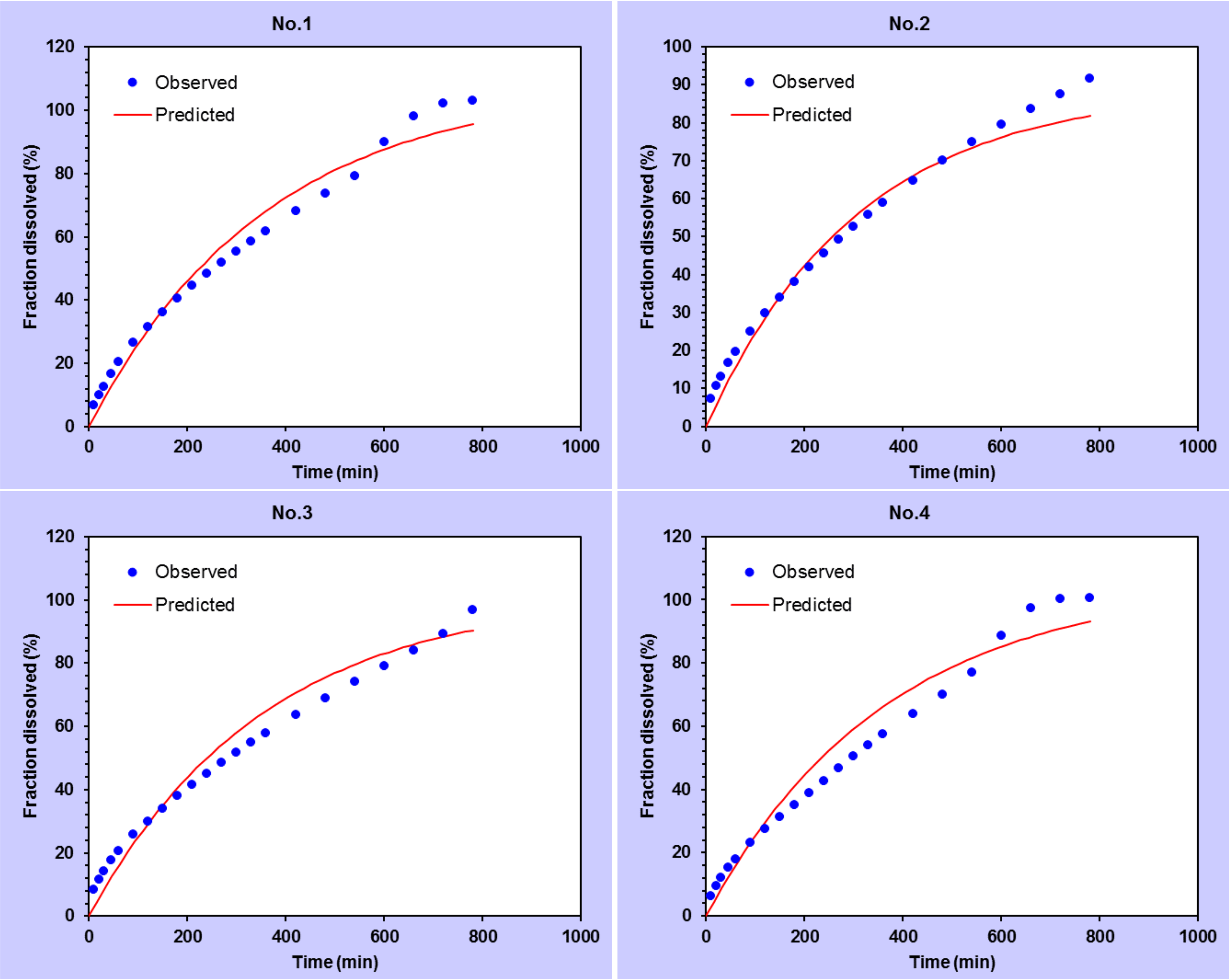

Supplementary materials – Model fitting summary – Lactochem® Fine Powder (modelling the entire relevant dissolution data)

Model: **First–order with T_{lag} and F_{max}**

Model equation: $F = F_{max} \cdot \left[1 - e^{-k_1 \cdot (t - T_{lag})}\right]$

Fitted model parameters per tested tablet (N = 4) with statistics – mean, standard deviation (SD), and relative standard deviation expressed in % (RSD%) (output from DDSolver):

Parameter	No.1	No.2	No.3	No.4	Mean	SD	RSD(%)
k_1	0.003	0.003	0.003	0.004	0.003	0.000	8.811
T_{lag}	41.980	19.704	18.597	56.388	34.167	18.316	53.606
F_{max}	108.197	96.227	101.654	105.603	102.920	5.210	5.062

Number of dissolution data points (N), degrees of freedom (df), and selected goodness of fit criteria – Pearson correlation coefficient (R), coefficient of determination (R^2), adjusted coefficient of determination ($R^2_{adjusted}$), and residual sum of squares (RSS) (manual calculation in MS Excel):

Parameter	No.1	No.2	No.3	No.4
N	22	22	22	22
df	19	19	19	19
R	0.976314678	0.990317689	0.986861771	0.961353671
R^2	0.953190351	0.980729126	0.973896155	0.924200881
$R^2_{adjusted}$	0.94826302	0.978700613	0.971148382	0.916222026
RSS	2348.538295	703.3861157	927.3450481	3706.594928

Graphical abstract of model fit presented as mean ± 1 SD of the fraction % of released carvedilol:

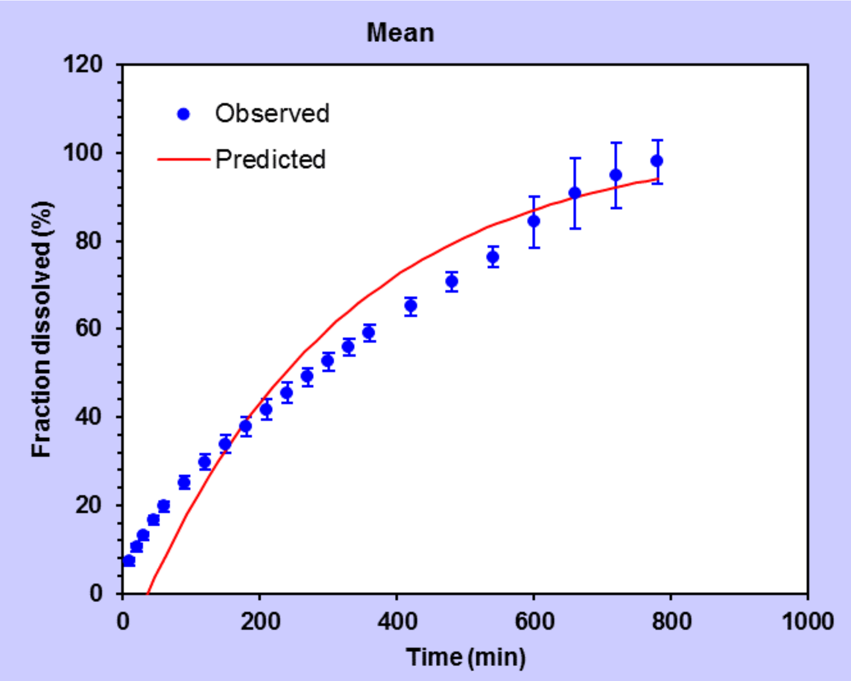

Supplementary materials – Model fitting summary – Lactochem® Fine Powder (modelling the entire relevant dissolution data)

Graphical abstract of model fit presented as the fraction % of released carvedilol per tested tablet:

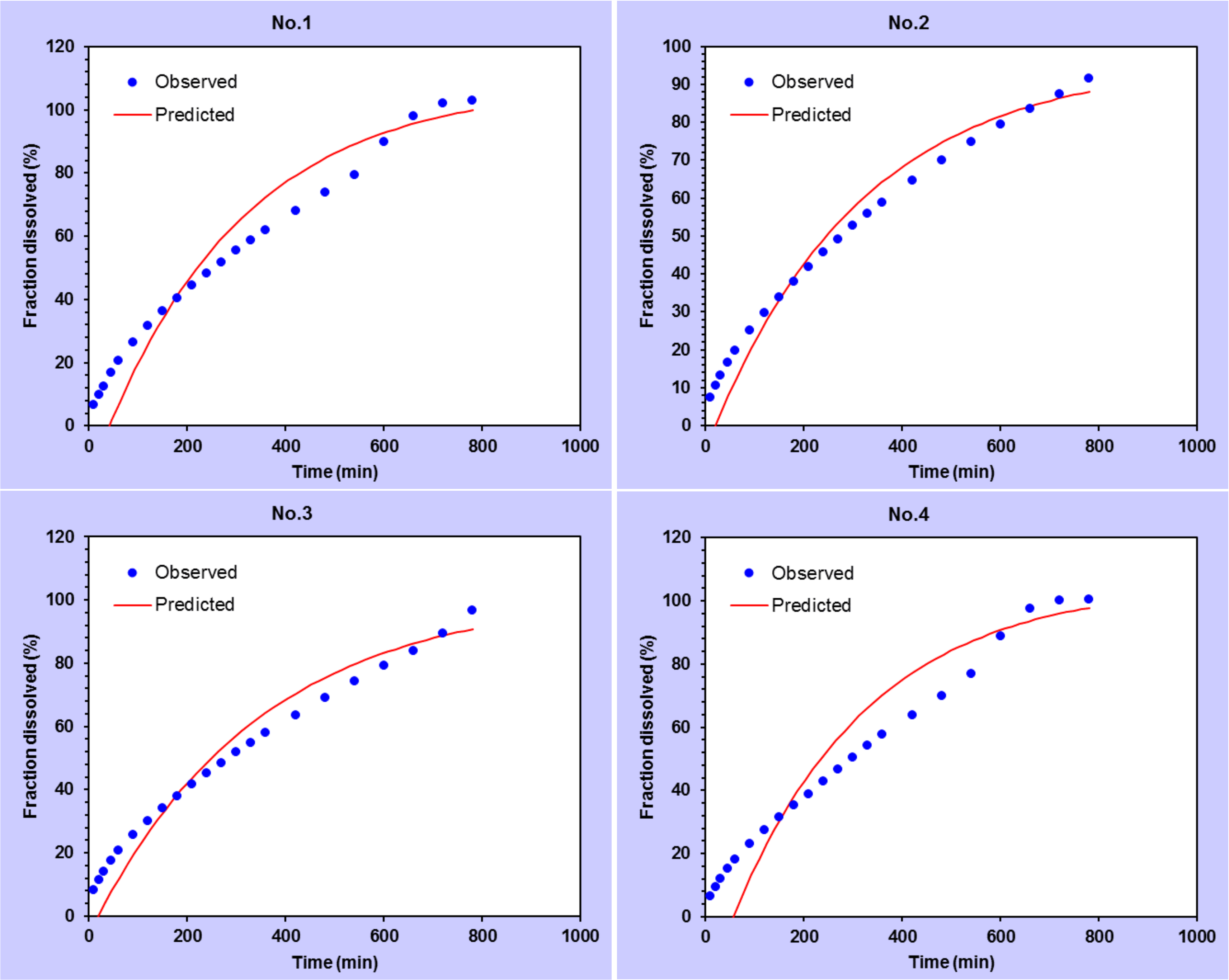

Supplementary materials – Model fitting summary – Lactochem® Fine Powder (modelling the entire relevant dissolution data)

Model: **Higuchi**

Model equation: $F = k_H \cdot t^{0.5}$

Fitted model parameters per tested tablet (N = 4) with statistics – mean, standard deviation (SD), and relative standard deviation expressed in % (RSD%) (output from DDSolver):

Parameter	No.1	No.2	No.3	No.4	Mean	SD	RSD(%)
k_H	3.420	3.125	3.140	3.249	3.234	0.136	4.217

Number of dissolution data points (N), degrees of freedom (df), and selected goodness of fit criteria – Pearson correlation coefficient (R), coefficient of determination (R^2), adjusted coefficient of determination ($R^2_{adjusted}$), and residual sum of squares (RSS) (manual calculation in MS Excel):

Parameter	No.1	No.2	No.3	No.4
N	22	22	22	22
df	21	21	21	21
R	0.994665651	0.998702596	0.996306355	0.987574065
R^2	0.989359757	0.997406876	0.992626353	0.975302533
$R^2_{adjusted}$	0.989359757	0.997406876	0.992626353	0.975302533
RSS	702.5750812	232.3503361	291.6988905	1230.264666

Graphical abstract of model fit presented as mean ± 1 SD of the fraction % of released carvedilol:

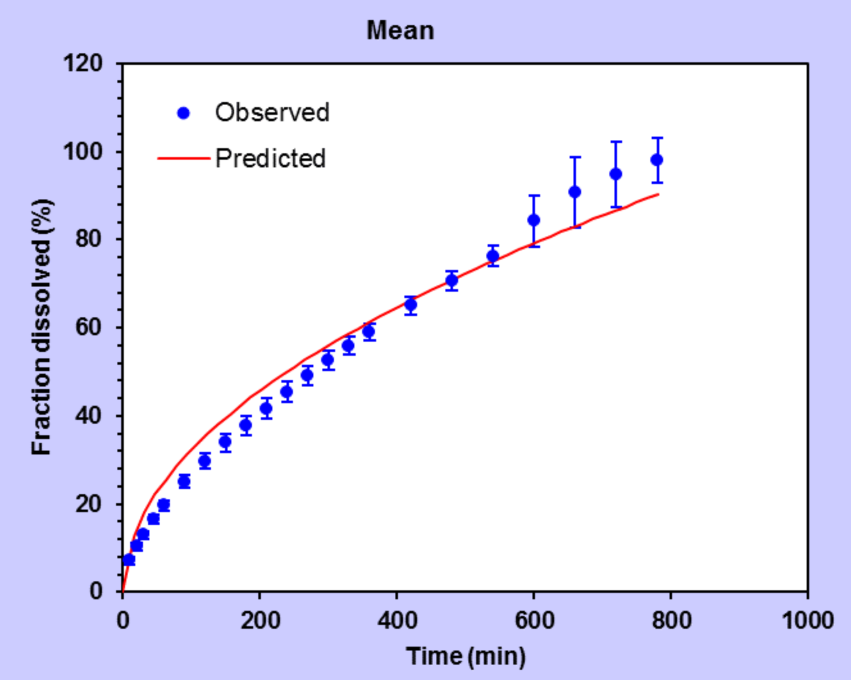

Supplementary materials – Model fitting summary – Lactochem® Fine Powder (modelling the entire relevant dissolution data)

Graphical abstract of model fit presented as the fraction % of released carvedilol per tested tablet:

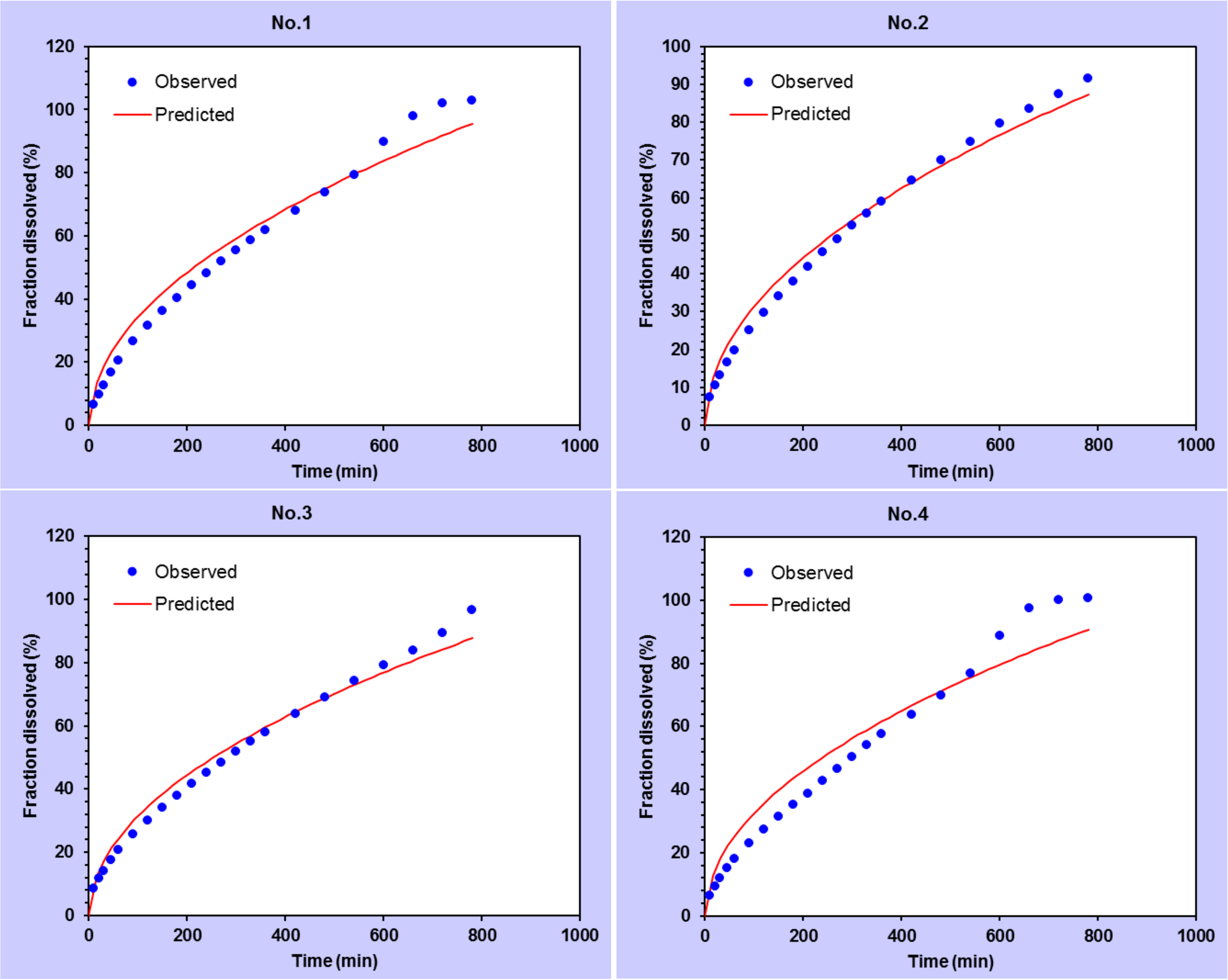

Supplementary materials – Model fitting summary – Lactochem® Fine Powder (modelling the entire relevant dissolution data)

Model: **Higuchi with T_{lag}**

Model equation: $F = k_H \cdot (t - T_{lag})^{0.5}$

Fitted model parameters per tested tablet (N = 4) with statistics – mean, standard deviation (SD), and relative standard deviation expressed in % (RSD%) (output from DDSolver):

Parameter	No.1	No.2	No.3	No.4	Mean	SD	RSD(%)
k_H	3.768	3.315	3.376	3.712	3.543	0.231	6.509
T_{lag}	50.631	32.468	39.372	66.335	47.202	14.790	31.333

Number of dissolution data points (N), degrees of freedom (df), and selected goodness of fit criteria – Pearson correlation coefficient (R), coefficient of determination (R^2), adjusted coefficient of determination ($R^2_{adjusted}$), and residual sum of squares (RSS) (manual calculation in MS Excel):

Parameter	No.1	No.2	No.3	No.4
N	22	22	22	22
df	20	20	20	20
R	0.986187691	0.992146324	0.988779513	0.976727265
R^2	0.972566162	0.984354328	0.977684925	0.953996151
$R^2_{adjusted}$	0.97119447	0.983572045	0.976569171	0.951695959
RSS	873.9624432	401.7070804	616.7062103	1276.491624

Graphical abstract of model fit presented as mean ± 1 SD of the fraction % of released carvedilol:

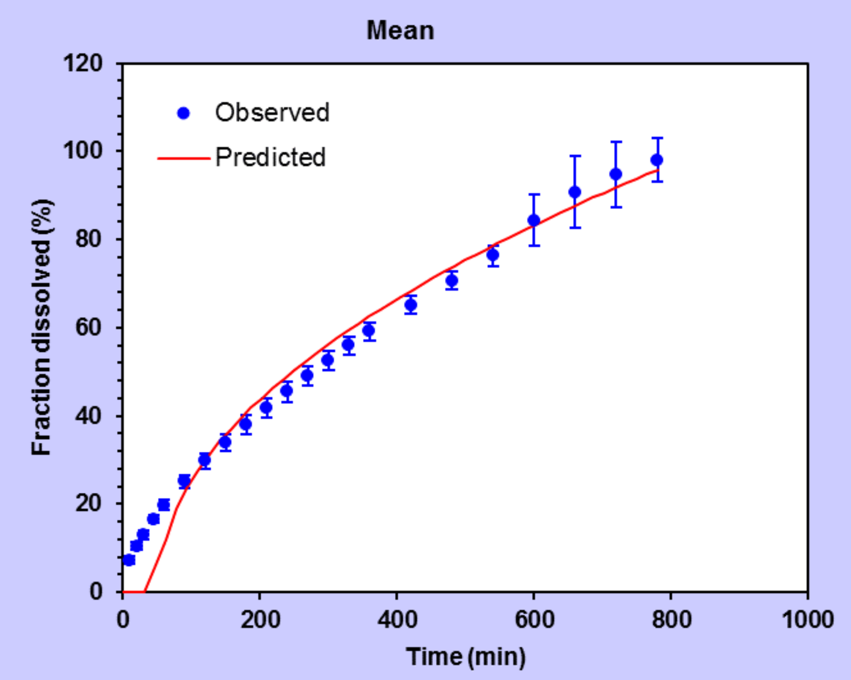

Supplementary materials – Model fitting summary – Lactochem® Fine Powder (modelling the entire relevant dissolution data)

Graphical abstract of model fit presented as the fraction % of released carvedilol per tested tablet:

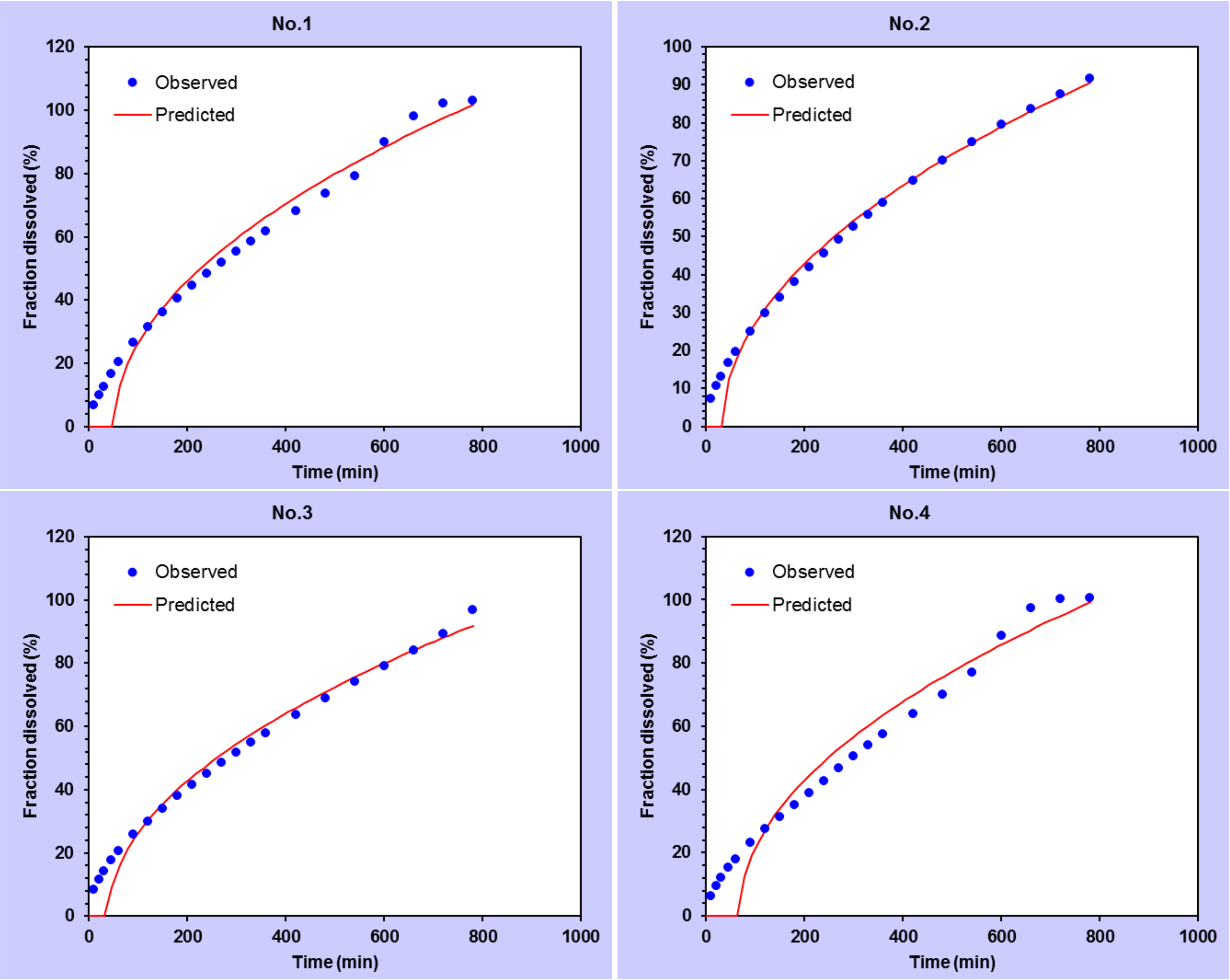

Supplementary materials – Model fitting summary – Lactochem® Fine Powder (modelling the entire relevant dissolution data)

Model: **Higuchi with F_0**

Model equation: $F = F_0 + k_H \cdot t^{0.5}$

Fitted model parameters per tested tablet (N = 4) with statistics – mean, standard deviation (SD), and relative standard deviation expressed in % (RSD%) (output from DDSolver):

Parameter	No.1	No.2	No.3	No.4	Mean	SD	RSD(%)
k_H	4.002	3.489	3.493	3.965	3.737	0.285	7.621
F_0	-11.143	-6.977	-6.768	-13.726	-9.653	3.381	-35.025

Number of dissolution data points (N), degrees of freedom (df), and selected goodness of fit criteria – Pearson correlation coefficient (R), coefficient of determination (R^2), adjusted coefficient of determination ($R^2_{adjusted}$), and residual sum of squares (RSS) (manual calculation in MS Excel):

Parameter	No.1	No.2	No.3	No.4
N	22	22	22	22
df	20	20	20	20
R	0.994665651	0.998702596	0.996306355	0.987574065
R^2	0.989359757	0.997406876	0.992626353	0.975302533
$R^2_{adjusted}$	0.988827745	0.997277219	0.992257671	0.97406766
RSS	206.7783914	37.99376415	108.80608	477.9940622

Graphical abstract of model fit presented as mean ± 1 SD of the fraction % of released carvedilol:

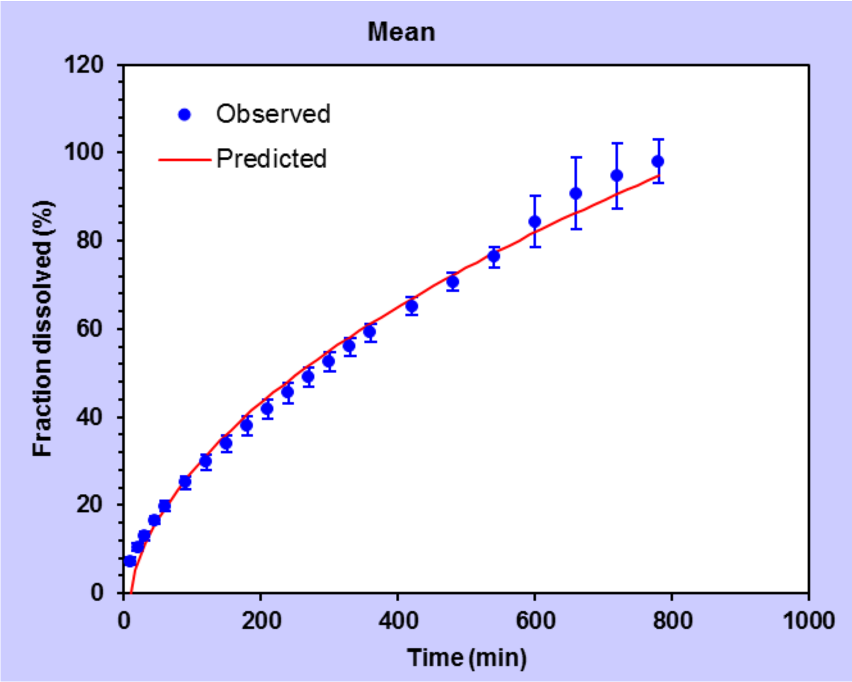

Supplementary materials – Model fitting summary – Lactochem® Fine Powder (modelling the entire relevant dissolution data)

Graphical abstract of model fit presented as the fraction % of released carvedilol per tested tablet:

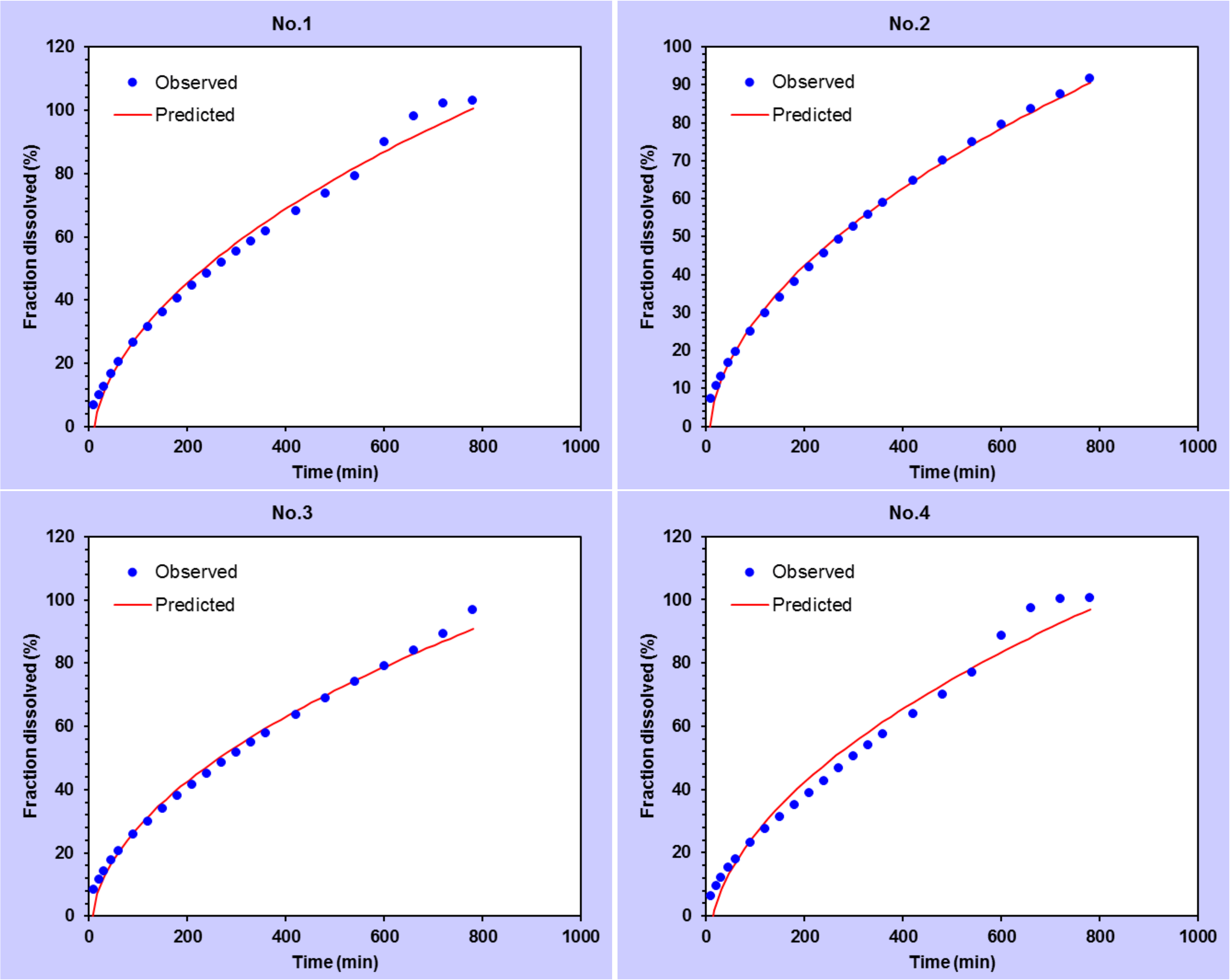

Supplementary materials – Model fitting summary – Lactochem® Fine Powder (modelling the entire relevant dissolution data)

Model: **Korsmeyer–Peppas**

Model equation: $F = k_{KP} \cdot t^n$

Fitted model parameters per tested tablet (N = 4) with statistics – mean, standard deviation (SD), and relative standard deviation expressed in % (RSD%) (output from DDSolver):

Parameter	No.1	No.2	No.3	No.4	Mean	SD	RSD(%)
k_{KP}	1.505	1.792	2.090	1.352	1.685	0.326	19.356
n	0.634	0.591	0.565	0.639	0.607	0.035	5.799

Number of dissolution data points (N), degrees of freedom (df), and selected goodness of fit criteria – Pearson correlation coefficient (R), coefficient of determination (R^2), adjusted coefficient of determination ($R^2_{adjusted}$), and residual sum of squares (RSS) (manual calculation in MS Excel):

Parameter	No.1	No.2	No.3	No.4
N	22	22	22	22
df	20	20	20	20
R	0.998360949	0.999815173	0.998444767	0.995004644
R^2	0.996724585	0.999630379	0.996891954	0.990034241
$R^2_{adjusted}$	0.996560814	0.999611898	0.996736551	0.989535953
RSS	75.80475613	7.048510332	75.62212239	371.9541107

Graphical abstract of model fit presented as mean ± 1 SD of the fraction % of released carvedilol:

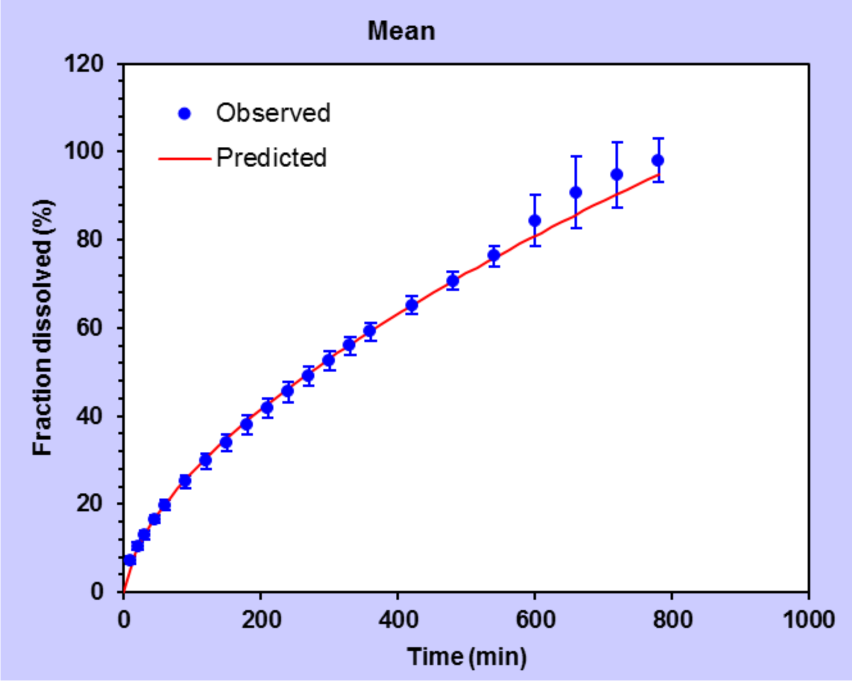

Supplementary materials – Model fitting summary – Lactochem® Fine Powder (modelling the entire relevant dissolution data)

Graphical abstract of model fit presented as the fraction % of released carvedilol per tested tablet:

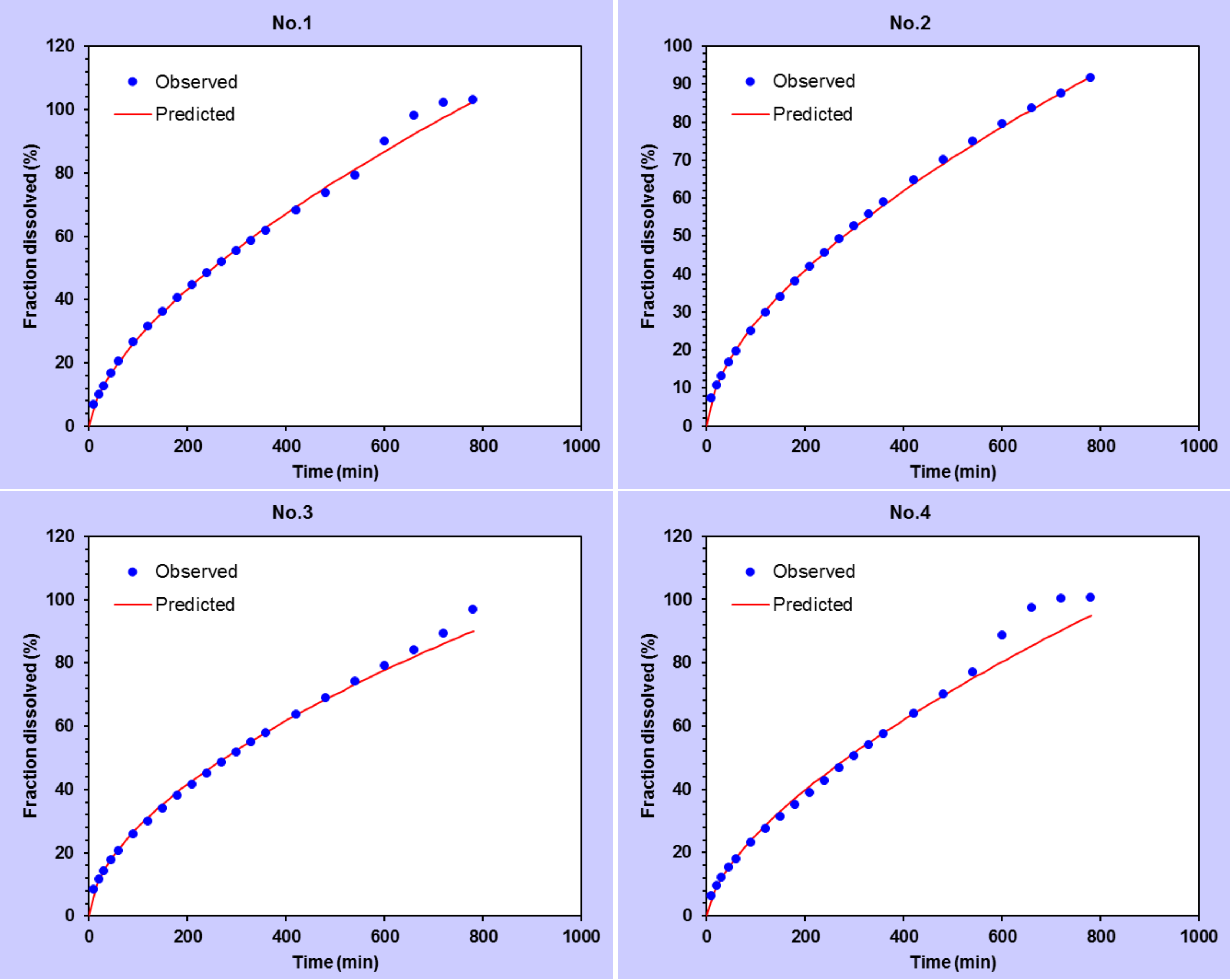

Supplementary materials – Model fitting summary – Lactochem® Fine Powder (modelling the entire relevant dissolution data)

Model: **Korsmeyer–Peppas with T_{lag}**

Model equation: $F = k_{KP} \cdot (t - T_{lag})^n$

Fitted model parameters per tested tablet (N = 4) with statistics – mean, standard deviation (SD), and relative standard deviation expressed in % (RSD%) (output from DDSolver):

Parameter	No.1	No.2	No.3	No.4	Mean	SD	RSD(%)
k_{KP}	1.969	2.324	2.687	1.763	2.186	0.407	18.597
n	0.589	0.548	0.523	0.611	0.568	0.040	6.992
T_{lag}	4.000	4.000	4.000	4.939	4.235	0.470	11.092

Number of dissolution data points (N), degrees of freedom (df), and selected goodness of fit criteria – Pearson correlation coefficient (R), coefficient of determination (R^2), adjusted coefficient of determination ($R^2_{adjusted}$), and residual sum of squares (RSS) (manual calculation in MS Excel):

Parameter	No.1	No.2	No.3	No.4
N	22	22	22	22
df	19	19	19	19
R	0.997296368	0.999245101	0.996496241	0.993149172
R^2	0.994600046	0.998490772	0.993004758	0.986345278
$R^2_{adjusted}$	0.994031629	0.998331906	0.992268417	0.984907939
RSS	165.0951007	56.31632003	197.2600018	485.6142085

Graphical abstract of model fit presented as mean ± 1 SD of the fraction % of released carvedilol:

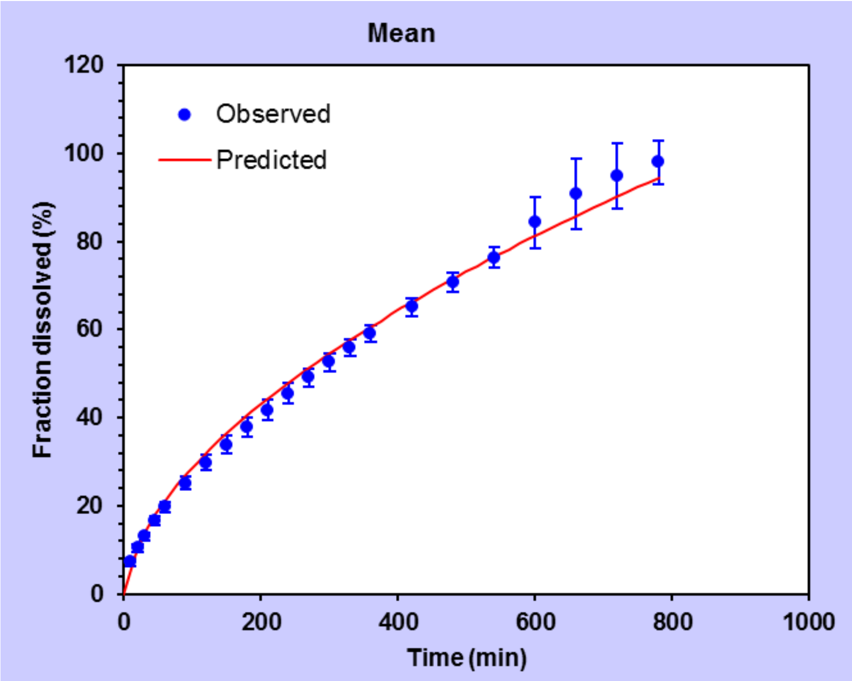

Supplementary materials – Model fitting summary – Lactochem® Fine Powder (modelling the entire relevant dissolution data)

Graphical abstract of model fit presented as the fraction % of released carvedilol per tested tablet:

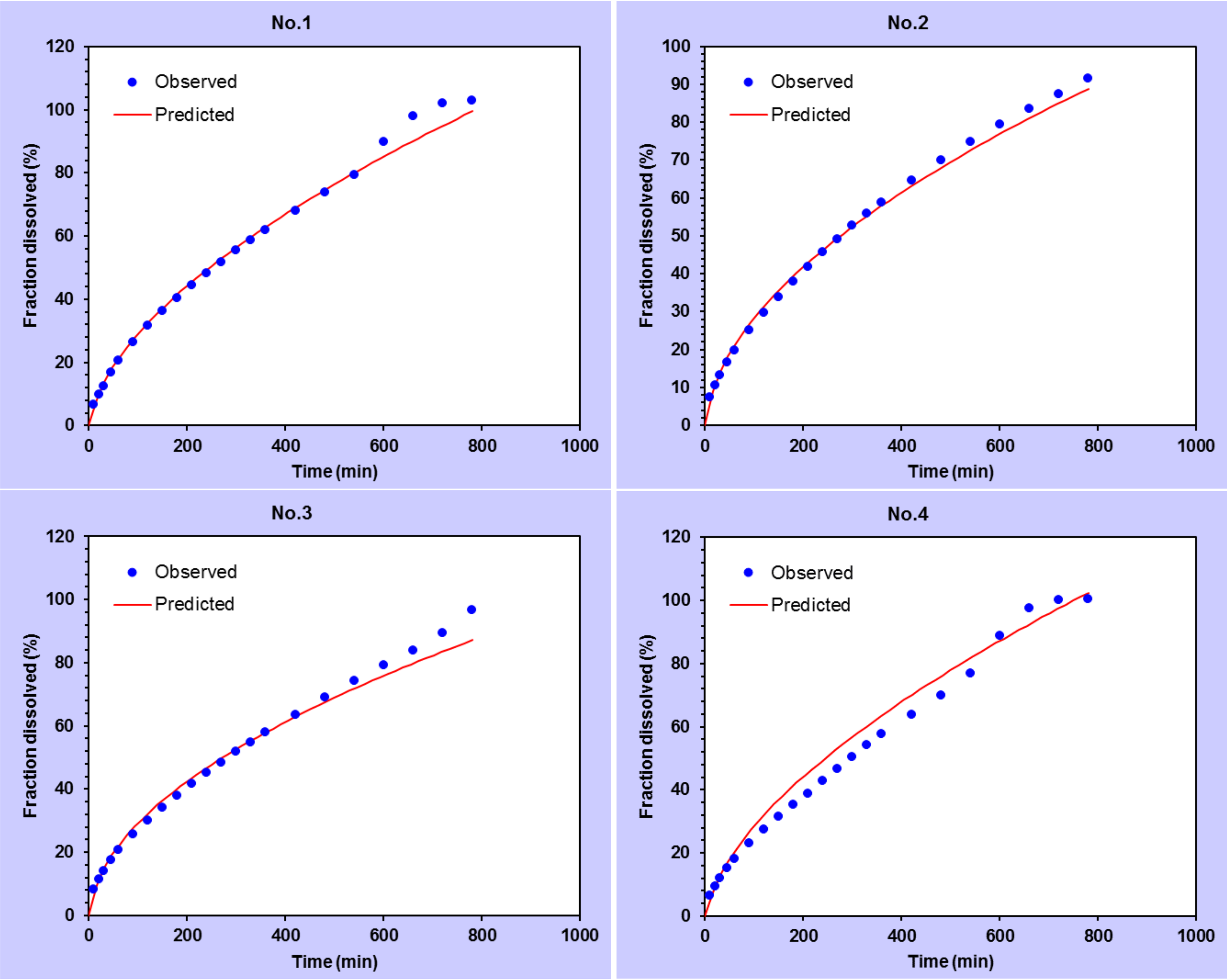

Supplementary materials – Model fitting summary – Lactochem® Fine Powder (modelling the entire relevant dissolution data)

Model: **Korsmeyer–Peppas with F_0**

Model equation: $F = F_0 + k_{KP} \cdot t^n$

Fitted model parameters per tested tablet (N = 4) with statistics – mean, standard deviation (SD), and relative standard deviation expressed in % (RSD%) (output from DDSolver):

Parameter	No.1	No.2	No.3	No.4	Mean	SD	RSD(%)
k_{KP}	0.855	1.008	1.155	0.745	0.941	0.179	19.004
n	0.723	0.680	0.656	0.734	0.698	0.037	5.268
F_0	2.680	2.960	3.360	2.560	2.890	0.355	12.299

Number of dissolution data points (N), degrees of freedom (df), and selected goodness of fit criteria – Pearson correlation coefficient (R), coefficient of determination (R^2), adjusted coefficient of determination ($R^2_{adjusted}$), and residual sum of squares (RSS) (manual calculation in MS Excel):

Parameter	No.1	No.2	No.3	No.4
N	22	22	22	22
df	19	19	19	19
R	0.998552916	0.998986888	0.999689337	0.997448131
R^2	0.997107927	0.997974802	0.999378771	0.994902773
$R^2_{adjusted}$	0.996803498	0.997761623	0.999313379	0.994366223
RSS	80.77096652	54.15056603	9.17943028	113.3122583

Graphical abstract of model fit presented as mean ± 1 SD of the fraction % of released carvedilol:

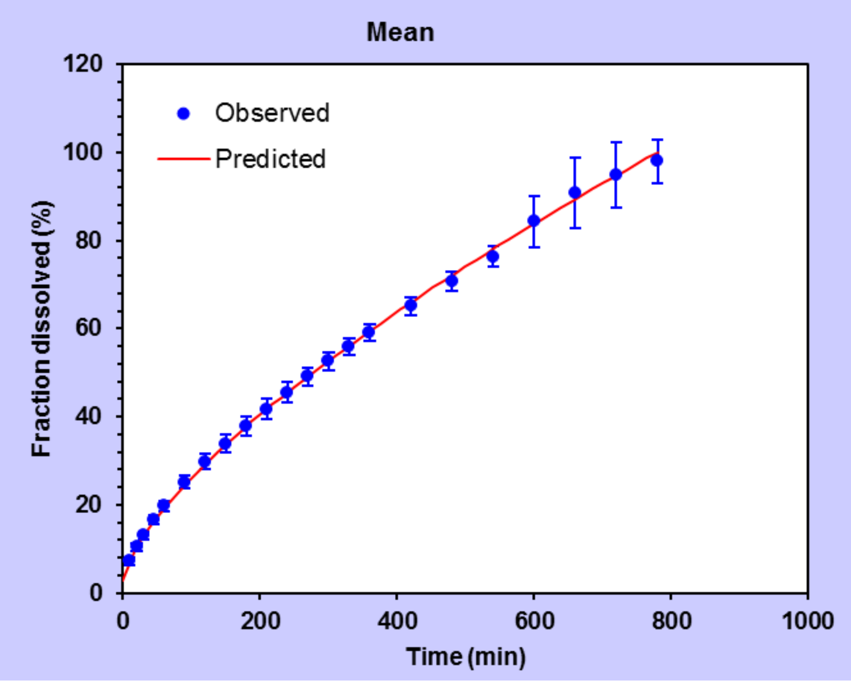

Supplementary materials – Model fitting summary – Lactochem® Fine Powder (modelling the entire relevant dissolution data)

Graphical abstract of model fit presented as the fraction % of released carvedilol per tested tablet:

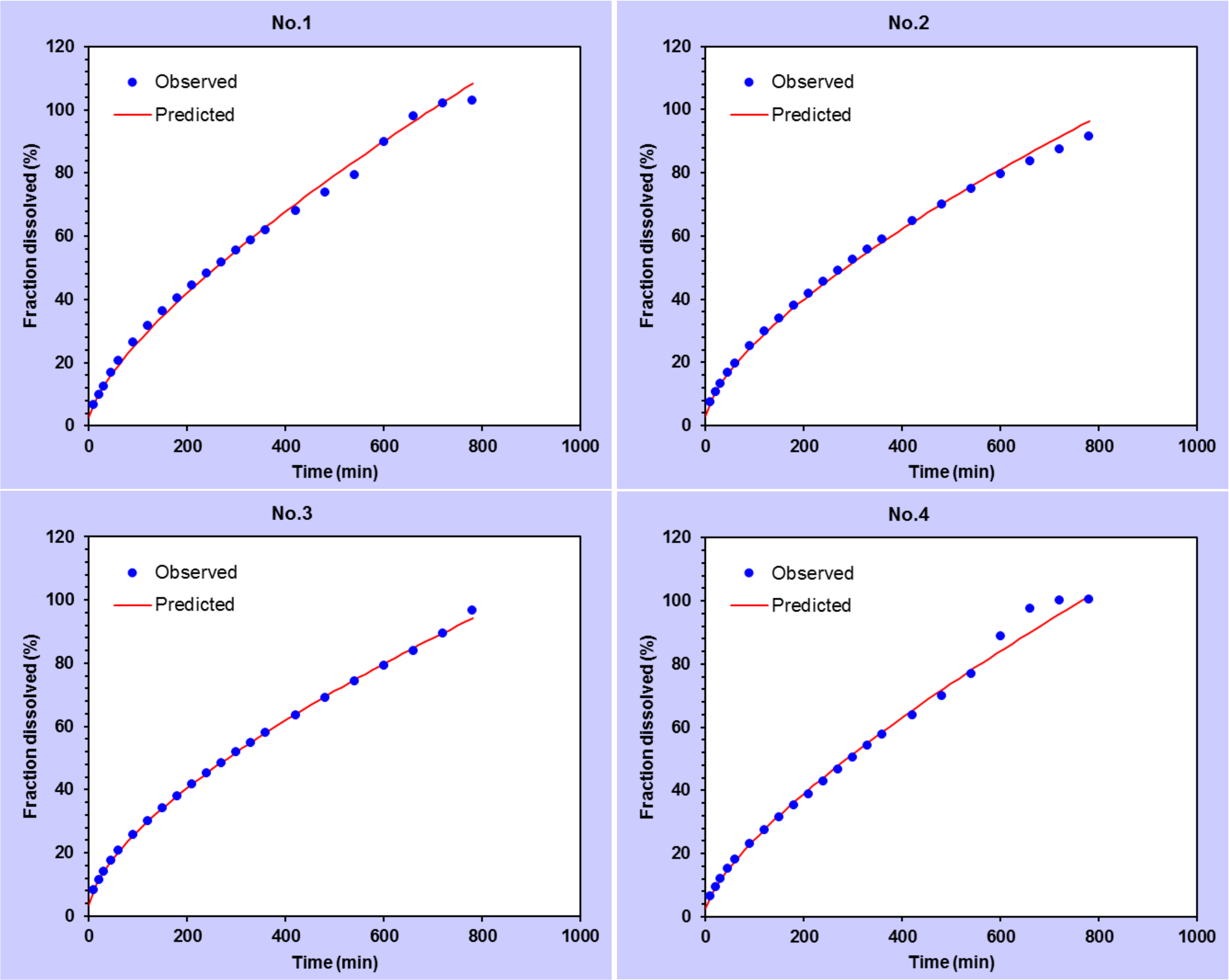

Supplementary materials – Model fitting summary – Lactochem® Fine Powder (modelling the entire relevant dissolution data)

Model: **Hixson–Crowell**

Model equation: $F = 100 \cdot [1 - (1 - k_{HC} \cdot t)^3]$

Fitted model parameters per tested tablet (N = 4) with statistics – mean, standard deviation (SD), and relative standard deviation expressed in % (RSD%) (output from DDSolver):

Parameter	No.1	No.2	No.3	No.4	Mean	SD	RSD(%)
k_{HC}	0.001	0.001	0.001	0.001	0.001	0.000	9.230

Number of dissolution data points (N), degrees of freedom (df), and selected goodness of fit criteria – Pearson correlation coefficient (R), coefficient of determination (R^2), adjusted coefficient of determination ($R^2_{adjusted}$), and residual sum of squares (RSS) (manual calculation in MS Excel):

Parameter	No.1	No.2	No.3	No.4
N	22	22	22	22
df	21	21	21	21
R	0.988969329	0.999077178	0.995442579	0.985349907
R^2	0.978060333	0.998155208	0.990905928	0.97091444
$R^2_{adjusted}$	0.978060333	0.998155208	0.990905928	0.97091444
RSS	501.916437	406.675733	491.2329483	594.3854276

Graphical abstract of model fit presented as mean ± 1 SD of the fraction % of released carvedilol:

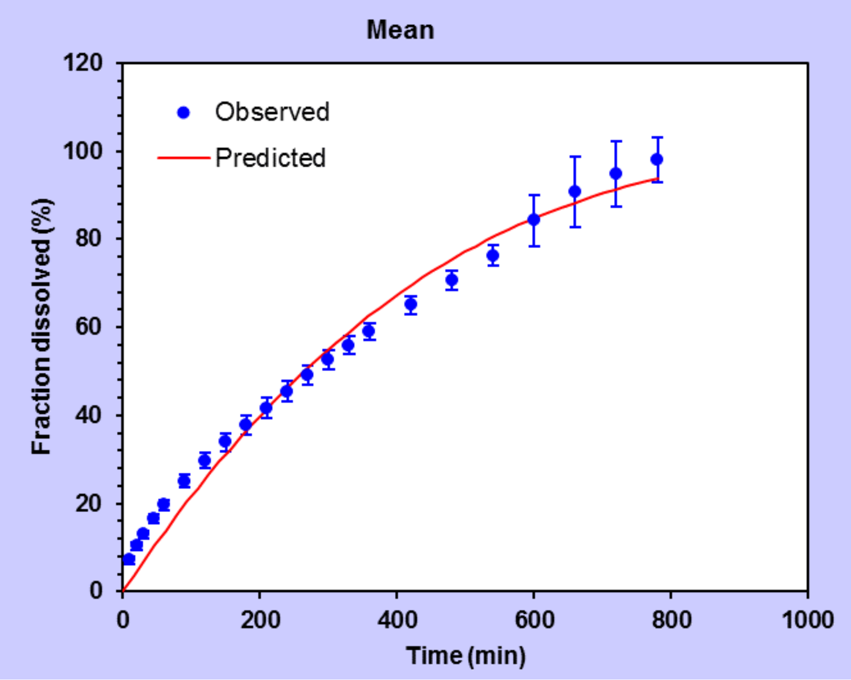

Supplementary materials – Model fitting summary – Lactochem® Fine Powder (modelling the entire relevant dissolution data)

Graphical abstract of model fit presented as the fraction % of released carvedilol per tested tablet:

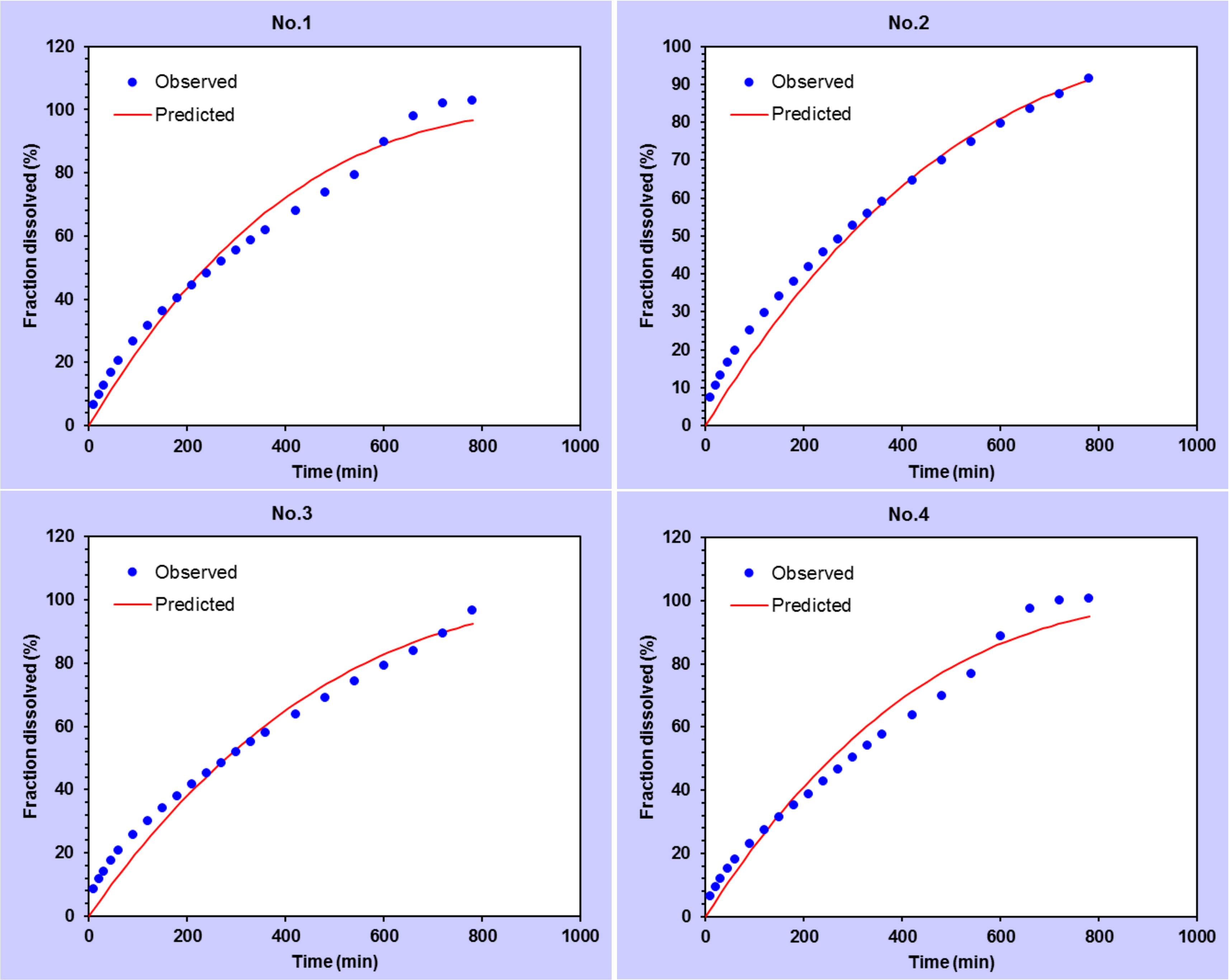

Supplementary materials – Model fitting summary – Lactochem® Fine Powder (modelling the entire relevant dissolution data)

Model: **Hixson–Crowell with T_{lag}**

Model equation: $F = 100 \cdot \left\{1 - \left[1 - k_{HC} \cdot \left(t - T_{lag}\right)\right]^3\right\}$

Fitted model parameters per tested tablet (N = 4) with statistics – mean, standard deviation (SD), and relative standard deviation expressed in % (RSD%) (output from DDSolver):

Parameter	No.1	No.2	No.3	No.4	Mean	SD	RSD(%)
k_{HC}	0.001	0.001	0.001	0.001	0.001	0.000	13.145
T_{lag}	-2.656	-39.776	-24.172	10.384	-14.055	22.295	-158.620

Number of dissolution data points (N), degrees of freedom (df), and selected goodness of fit criteria – Pearson correlation coefficient (R), coefficient of determination (R^2), adjusted coefficient of determination ($R^2_{adjusted}$), and residual sum of squares (RSS) (manual calculation in MS Excel):

Parameter	No.1	No.2	No.3	No.4
N	22	22	22	22
df	20	20	20	20
R	0.989049318	0.9992258	0.995982419	0.984562444
R^2	0.978218553	0.998452199	0.991980978	0.969363207
$R^2_{adjusted}$	0.977129481	0.998374809	0.991580027	0.967831367
RSS	475.1248345	24.07610943	184.1624894	699.8376538

Graphical abstract of model fit presented as mean ± 1 SD of the fraction % of released carvedilol:

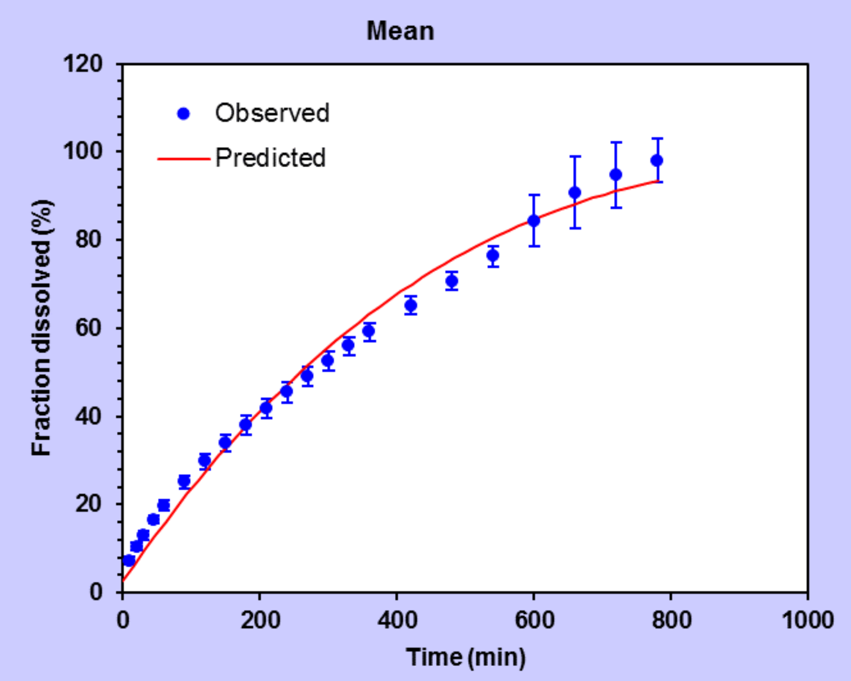

Supplementary materials – Model fitting summary – Lactochem® Fine Powder (modelling the entire relevant dissolution data)

Graphical abstract of model fit presented as the fraction % of released carvedilol per tested tablet:

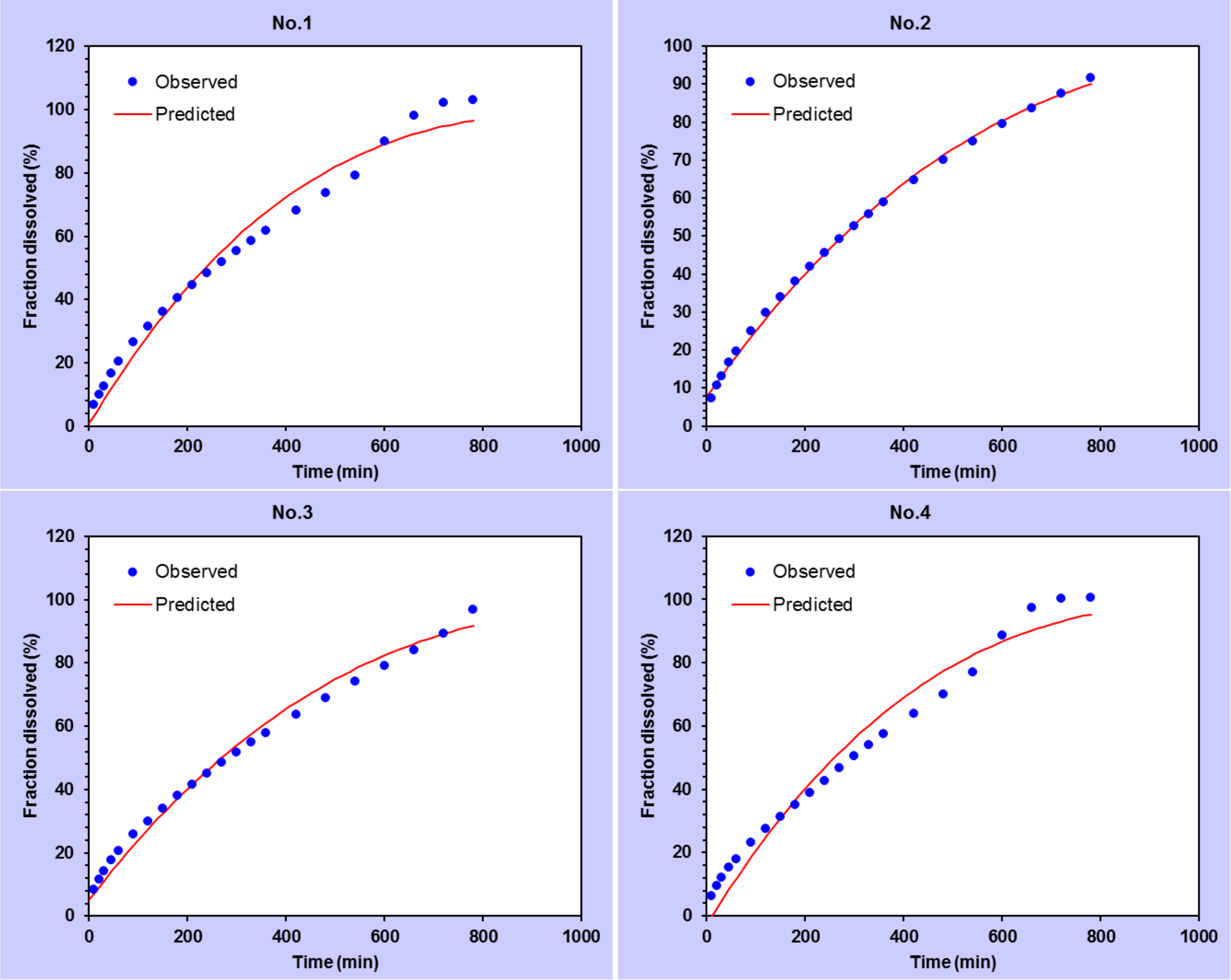

Supplementary materials – Model fitting summary – Lactochem® Fine Powder (modelling the entire relevant dissolution data)

Model: **Hopfenberg**

Model equation: $F = 100 \cdot [1 - (1 - k_{HB} \cdot t)^n]$

Fitted model parameters per tested tablet (N = 4) with statistics – mean, standard deviation (SD), and relative standard deviation expressed in % (RSD%) (output from DDSolver):

Parameter	No.1	No.2	No.3	No.4	Mean	SD	RSD(%)
k_{HB}	0.001	0.001	0.001	0.001	0.001	0.000	32.321
n	2.000	3.000	4.125	2.000	2.781	1.012	36.397

Number of dissolution data points (N), degrees of freedom (df), and selected goodness of fit criteria – Pearson correlation coefficient (R), coefficient of determination (R^2), adjusted coefficient of determination ($R^2_{adjusted}$), and residual sum of squares (RSS) (manual calculation in MS Excel):

Parameter	No.1	No.2	No.3	No.4
N	22	22	22	22
df	20	20	20	20
R	0.993163941	0.999077178	0.994667086	0.991290897
R^2	0.986374614	0.998155208	0.989362613	0.982657643
$R^2_{adjusted}$	0.985693345	0.998062968	0.988830743	0.981790525
RSS	487.7166701	406.675733	447.0321175	420.1696938

Graphical abstract of model fit presented as mean ± 1 SD of the fraction % of released carvedilol:

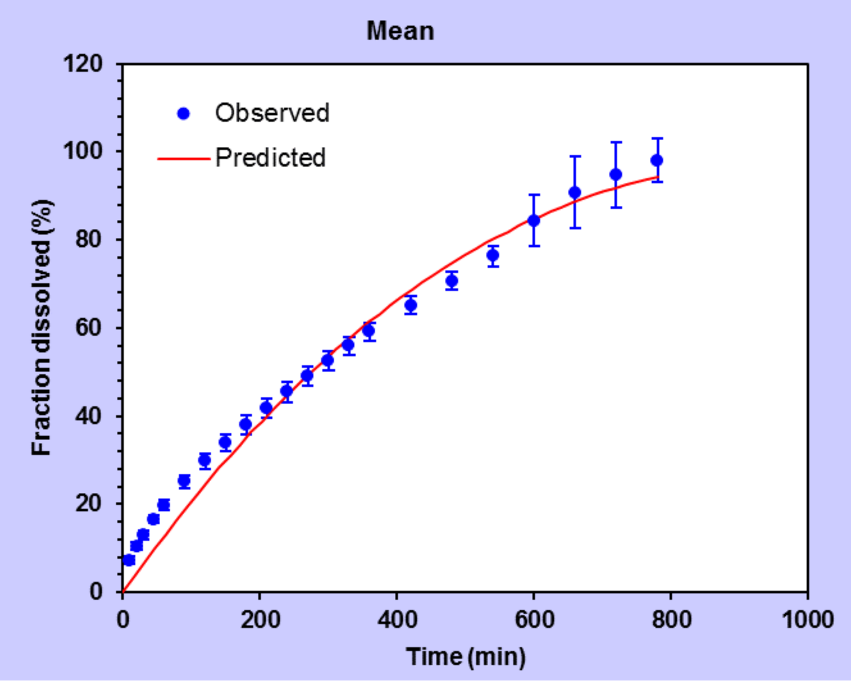

Supplementary materials – Model fitting summary – Lactochem® Fine Powder (modelling the entire relevant dissolution data)

Graphical abstract of model fit presented as the fraction % of released carvedilol per tested tablet:

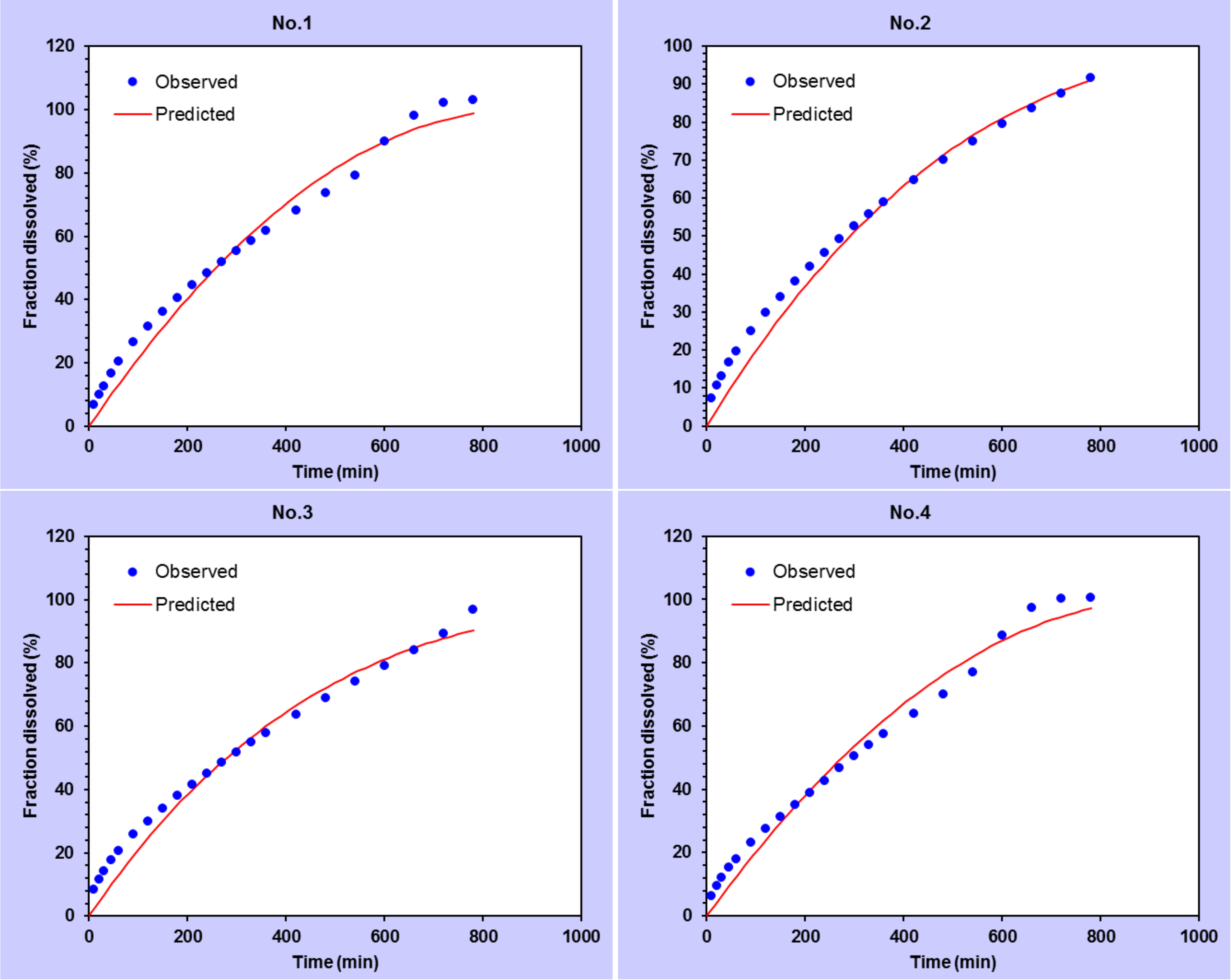

Supplementary materials – Model fitting summary – Lactochem® Fine Powder (modelling the entire relevant dissolution data)

Model: **Hopfenberg with T_{lag}**

Model equation: $F = 100 \cdot \{1 - [1 - k_{HB} \cdot (t - T_{lag})]^n\}$

Fitted model parameters per tested tablet (N = 4) with statistics – mean, standard deviation (SD), and relative standard deviation expressed in % (RSD%) (output from DDSolver):

Parameter	No.1	No.2	No.3	No.4	Mean	SD	RSD(%)
k_{HB}	0.001	0.001	0.001	0.001	0.001	0.000	29.576
n	1.545	3.000	2.000	1.000	1.886	0.848	44.939
T_{lag}	-33.044	-39.776	-51.787	-73.744	-49.588	17.873	-36.043

Number of dissolution data points (N), degrees of freedom (df), and selected goodness of fit criteria – Pearson correlation coefficient (R), coefficient of determination (R^2), adjusted coefficient of determination ($R^2_{adjusted}$), and residual sum of squares (RSS) (manual calculation in MS Excel):

Parameter	No.1	No.2	No.3	No.4
N	22	22	22	22
df	19	19	19	19
R	0.996275989	0.9992258	0.997699156	0.997924005
R^2	0.992565847	0.998452199	0.995403606	0.99585232
$R^2_{adjusted}$	0.991783304	0.998289272	0.994919775	0.995415722
RSS	232.8224472	24.07610943	76.42333697	80.37728248

Graphical abstract of model fit presented as mean ± 1 SD of the fraction % of released carvedilol:

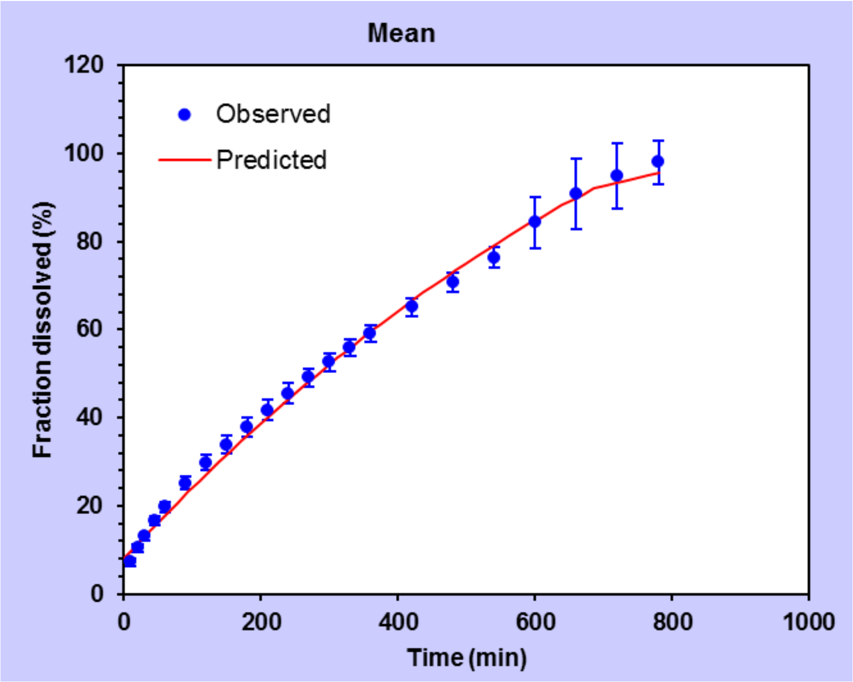

Supplementary materials – Model fitting summary – Lactochem® Fine Powder (modelling the entire relevant dissolution data)

Graphical abstract of model fit presented as the fraction % of released carvedilol per tested tablet:

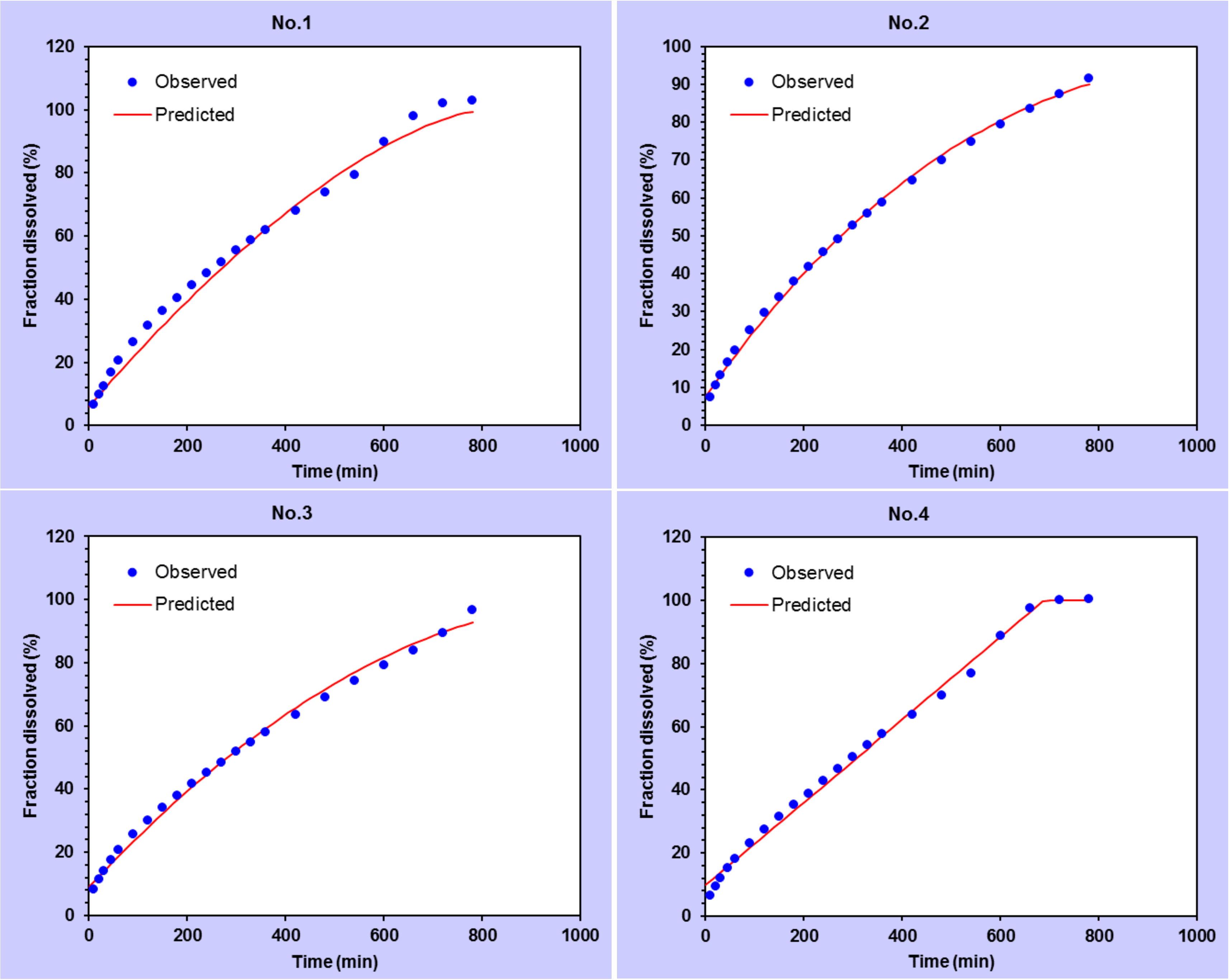

Supplementary materials – Model fitting summary – Lactochem® Fine Powder (modelling the entire relevant dissolution data)

Model: **Baker–Lonsdale**

Model equation: $\frac{3}{2}\cdot\left[1-\left(1-\frac{F}{100}\right)^{\frac{2}{3}}\right]-\frac{F}{100}=k_{BL}\cdot t$

Fitted model parameters per tested tablet (N = 4) with statistics – mean, standard deviation (SD), and relative standard deviation expressed in % (RSD%) (output from DDSolver):

Parameter	No.1	No.2	No.3	No.4	Mean	SD	RSD(%)
k_{BL}	0.0002	0.0002	0.0004	0.0002	0.0003	0.0001	36.2335

Number of dissolution data points (N), degrees of freedom (df), and selected goodness of fit criteria – Pearson correlation coefficient (R), coefficient of determination (R^2), adjusted coefficient of determination ($R^2_{adjusted}$), and residual sum of squares (RSS) (manual calculation in MS Excel):

Parameter	No.1	No.2	No.3	No.4
N	22	22	22	22
df	21	21	21	21
R	0.983292502	0.993239593	0.977608238	0.972526509
R^2	0.966864144	0.986524889	0.955717867	0.945807811
$R^2_{adjusted}$	0.966864144	0.986524889	0.955717867	0.945807811
RSS	2440.990389	1619.823835	3780.163952	2760.507663

Graphical abstract of model fit presented as mean ± 1 SD of the fraction % of released carvedilol:

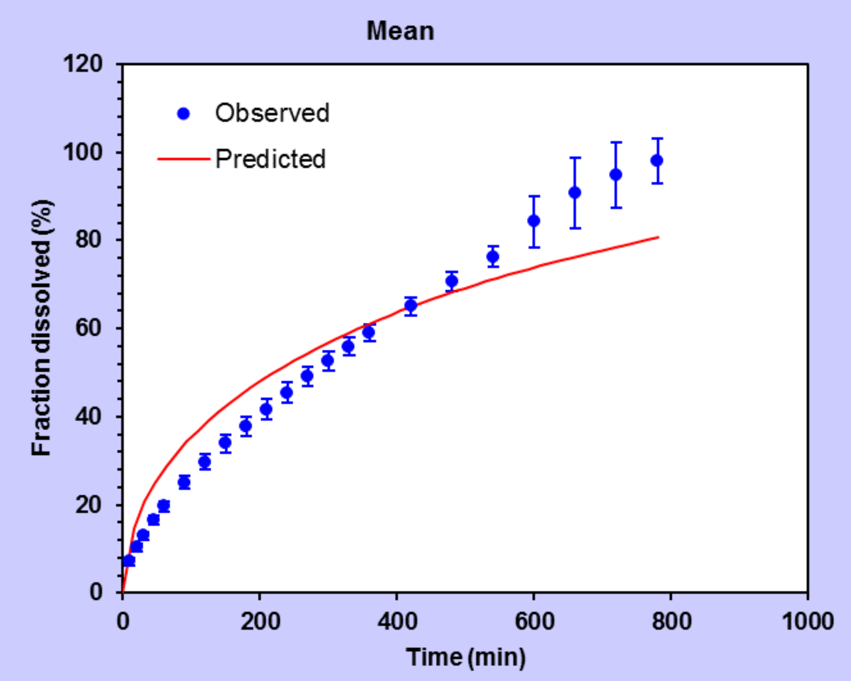

Supplementary materials – Model fitting summary – Lactochem® Fine Powder (modelling the entire relevant dissolution data)

Graphical abstract of model fit presented as the fraction % of released carvedilol per tested tablet:

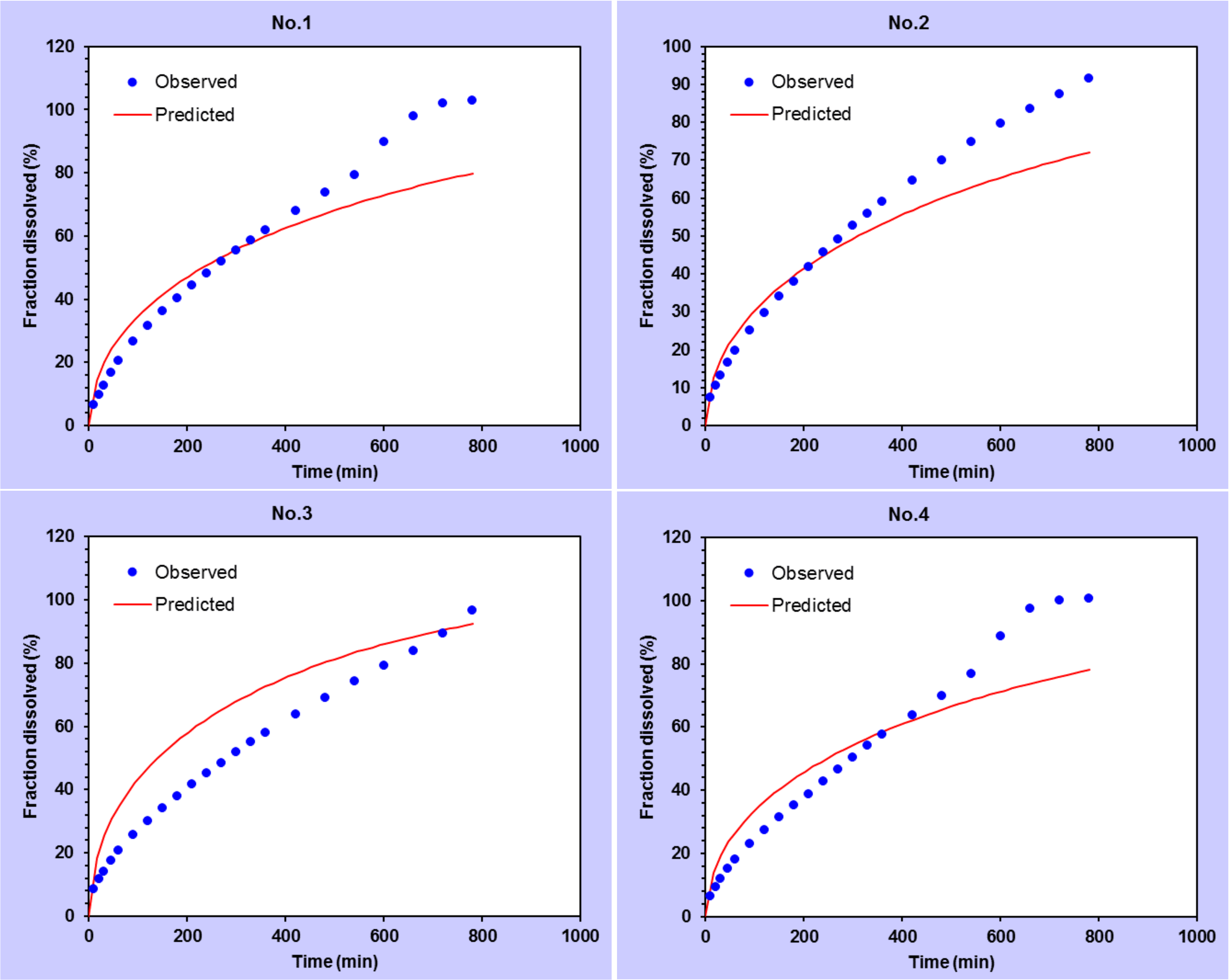

Supplementary materials – Model fitting summary – Lactochem® Fine Powder (modelling the entire relevant dissolution data)

Model: **Baker–Lonsdale with T_{lag}**

Model equation: $\frac{3}{2} \cdot \left[1 - \left(1 - \frac{F}{100}\right)^{\frac{2}{3}}\right] - \frac{F}{100} = k_{BL} \cdot (t - T_{lag})$

Fitted model parameters per tested tablet (N = 4) with statistics – mean, standard deviation (SD), and relative standard deviation expressed in % (RSD%) (output from DDSolver):

Parameter	No.1	No.2	No.3	No.4	Mean	SD	RSD(%)
k_{BL}	0.0005	0.0004	0.0004	0.0004	0.0004	0.0001	12.9809
T_{lag}	83.3649	72.9655	85.1096	93.4309	83.7177	8.4067	10.0417

Number of dissolution data points (N), degrees of freedom (df), and selected goodness of fit criteria – Pearson correlation coefficient (R), coefficient of determination (R^2), adjusted coefficient of determination ($R^2_{adjusted}$), and residual sum of squares (RSS) (manual calculation in MS Excel):

Parameter	No.1	No.2	No.3	No.4
N	22	22	22	22
df	20	20	20	20
R	0.961939656	0.977228824	0.969642827	0.94900972
R^2	0.925327903	0.954976174	0.940207211	0.900619449
$R^2_{adjusted}$	0.921594298	0.952724983	0.937217572	0.895650421
RSS	2060.833361	1336.63711	1913.430731	2610.519649

Graphical abstract of model fit presented as mean ± 1 SD of the fraction % of released carvedilol:

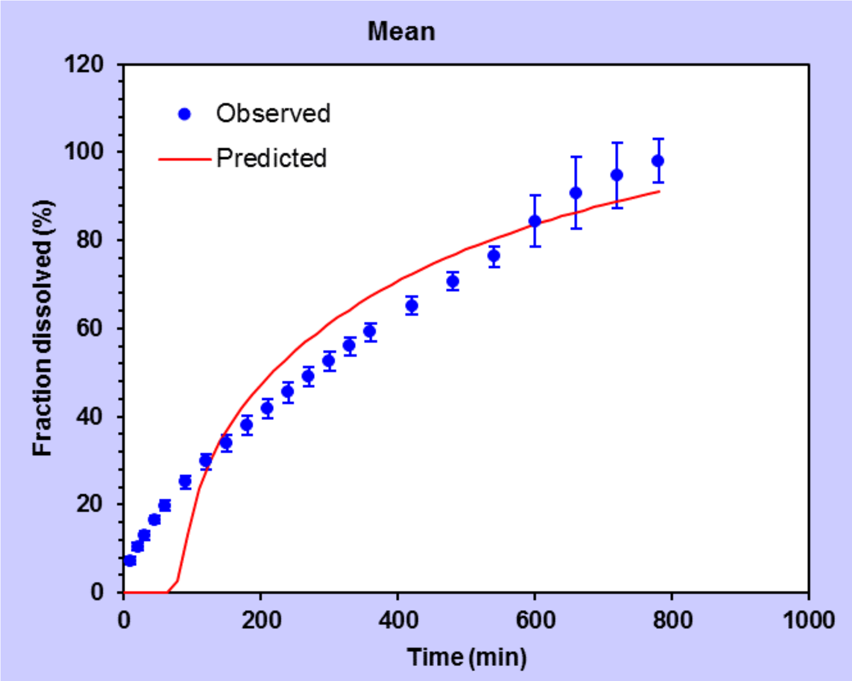

Supplementary materials – Model fitting summary – Lactochem® Fine Powder (modelling the entire relevant dissolution data)

Graphical abstract of model fit presented as the fraction % of released carvedilol per tested tablet:

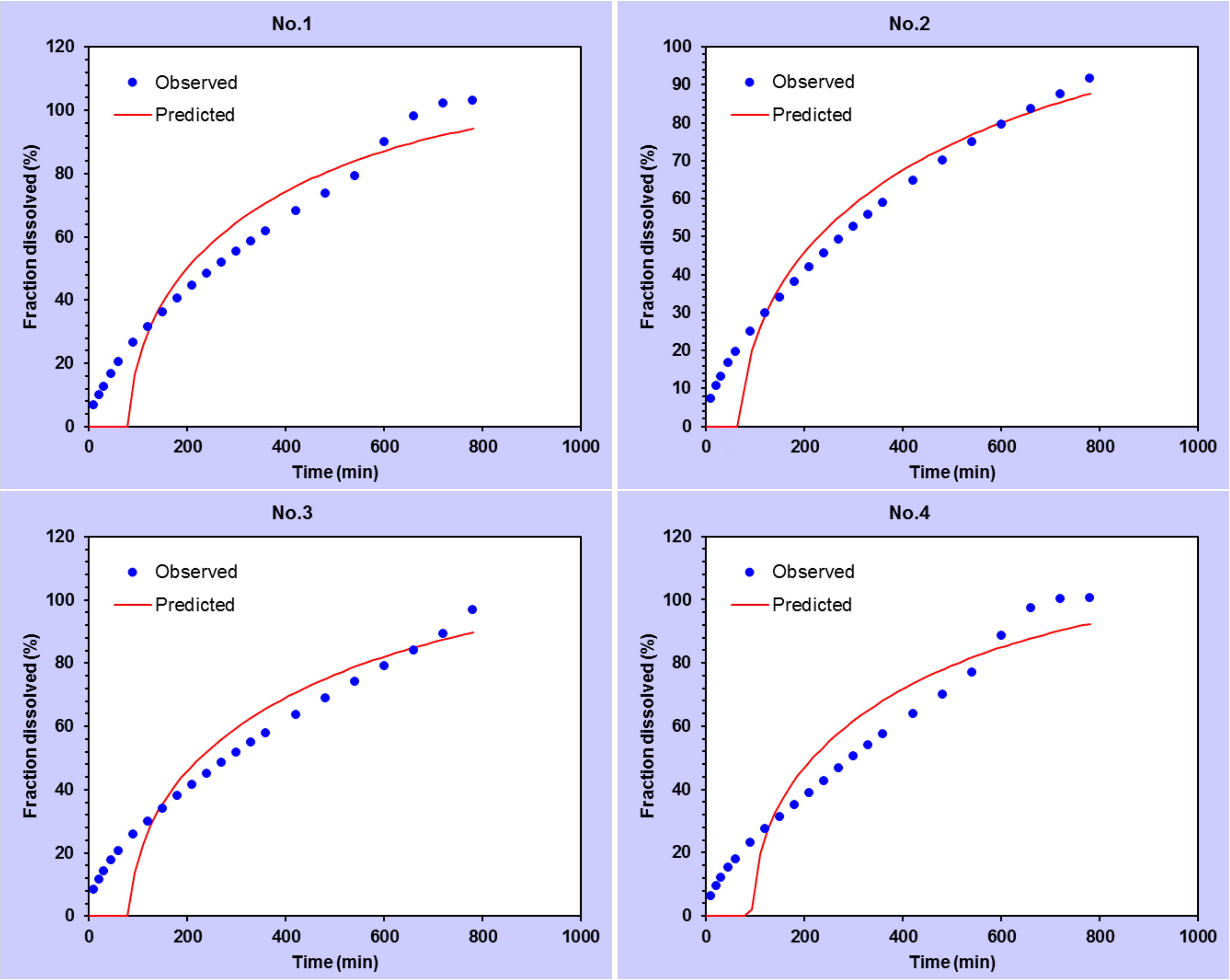

Supplementary materials – Model fitting summary – Lactochem® Fine Powder (modelling the entire relevant dissolution data)

Model: **Makoid–Banakar**

Model equation: $F = k_{MB} \cdot t^n \cdot e^{-k \cdot t}$

Fitted model parameters per tested tablet (N = 4) with statistics – mean, standard deviation (SD), and relative standard deviation expressed in % (RSD%) (output from DDSolver):

Parameter	No.1	No.2	No.3	No.4	Mean	SD	RSD(%)
k_{MB}	1.5758	1.8786	2.4754	1.7165	1.9116	0.3958	20.7037
n	0.6203	0.5777	0.5163	0.5681	0.5706	0.0427	7.4838
k	-0.0001	-0.0001	-0.0003	-0.0004	-0.0002	0.0002	-77.9337

Number of dissolution data points (N), degrees of freedom (df), and selected goodness of fit criteria – Pearson correlation coefficient (R), coefficient of determination (R^2), adjusted coefficient of determination ($R^2_{adjusted}$), and residual sum of squares (RSS) (manual calculation in MS Excel):

Parameter	No.1	No.2	No.3	No.4
N	22	22	22	22
df	19	19	19	19
R	0.998751612	0.999583495	0.999786039	0.997806284
R^2	0.997504783	0.999167163	0.999572124	0.99561738
$R^2_{adjusted}$	0.997242129	0.999079496	0.999527085	0.995156051
RSS	48.56491111	12.36237676	6.373396643	84.91092538

Graphical abstract of model fit presented as mean ± 1 SD of the fraction % of released carvedilol:

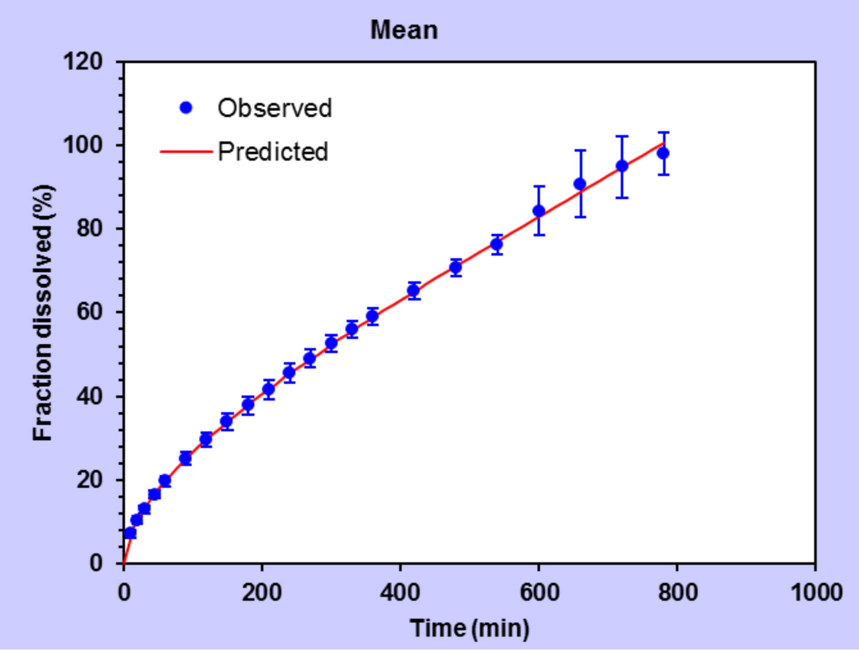

Supplementary materials – Model fitting summary – Lactochem® Fine Powder (modelling the entire relevant dissolution data)

Graphical abstract of model fit presented as the fraction % of released carvedilol per tested tablet:

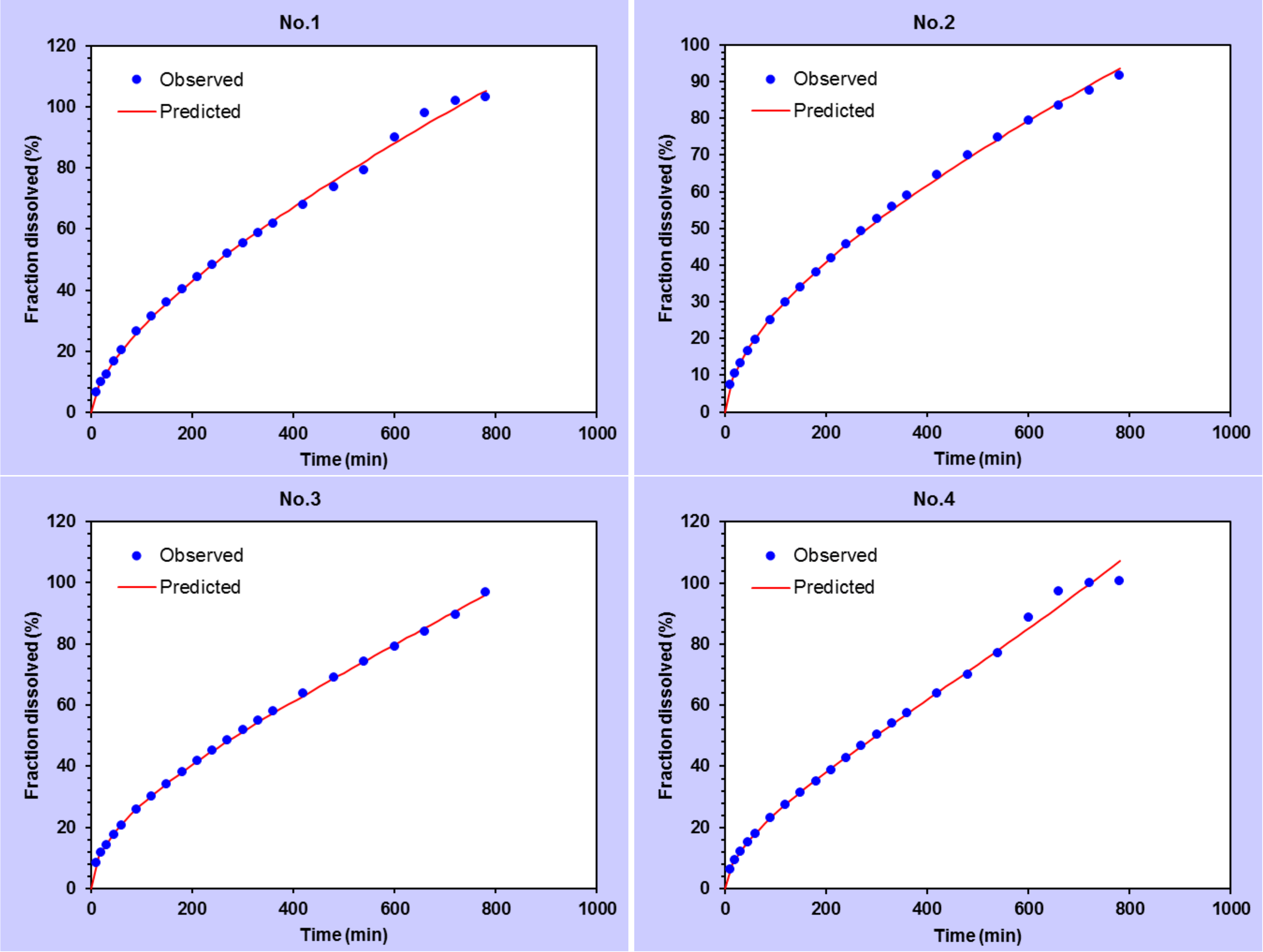

Supplementary materials – Model fitting summary – Lactochem® Fine Powder (modelling the entire relevant dissolution data)

Model: **Makoid–Banakar with T_{lag}**

Model equation: $F = k_{MB} \cdot (t - T_{lag})^{n} \cdot e^{-k \cdot (t - T_{lag})}$

Fitted model parameters per tested tablet (N = 4) with statistics – mean, standard deviation (SD), and relative standard deviation expressed in % (RSD%) (output from DDSolver):

Parameter	No.1	No.2	No.3	No.4	Mean	SD	RSD(%)
k_{MB}	2.3318	2.7188	3.4435	2.4609	2.7387	0.4966	18.1335
n	0.5376	0.4995	0.4467	0.4925	0.4941	0.0373	7.5476
k	-0.0003	-0.0003	-0.0005	-0.0007	-0.0004	0.0002	-37.2754
T_{lag}	4.0000	4.0000	4.0000	4.0000	4.0000	0.0000	0.0000

Number of dissolution data points (N), degrees of freedom (df), and selected goodness of fit criteria – Pearson correlation coefficient (R), coefficient of determination (R^2), adjusted coefficient of determination ($R^2_{adjusted}$), and residual sum of squares (RSS) (manual calculation in MS Excel):

Parameter	No.1	No.2	No.3	No.4
N	22	22	22	22
df	18	18	18	18
R	0.998699592	0.998383459	0.99920267	0.99702846
R^2	0.997400874	0.99676953	0.998405975	0.994065749
$R^2_{adjusted}$	0.996967687	0.996231119	0.998140304	0.993076707
RSS	50.92493587	48.8772626	24.59169316	117.1241969

Graphical abstract of model fit presented as mean ± 1 SD of the fraction % of released carvedilol:

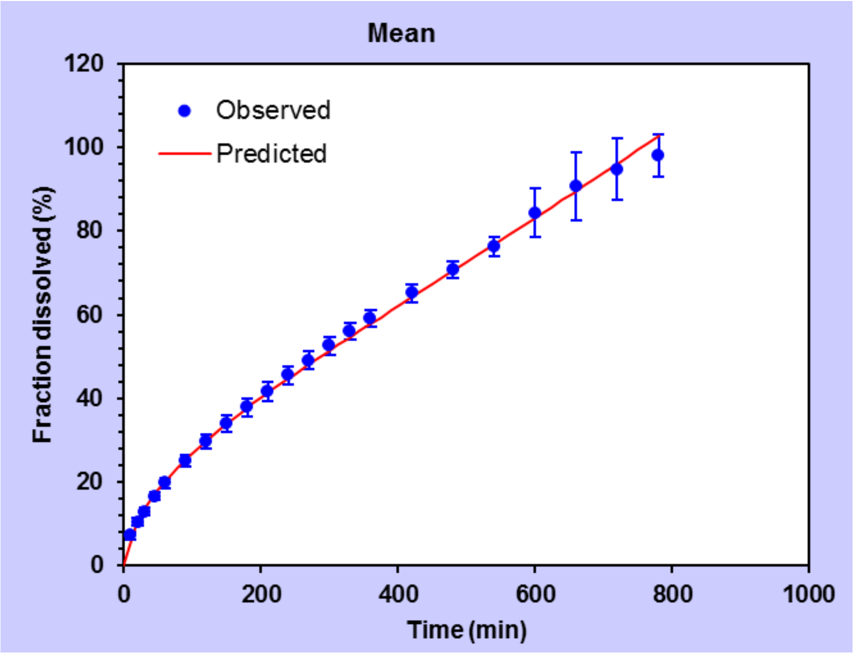

Supplementary materials – Model fitting summary – Lactochem® Fine Powder (modelling the entire relevant dissolution data)

Graphical abstract of model fit presented as the fraction % of released carvedilol per tested tablet:

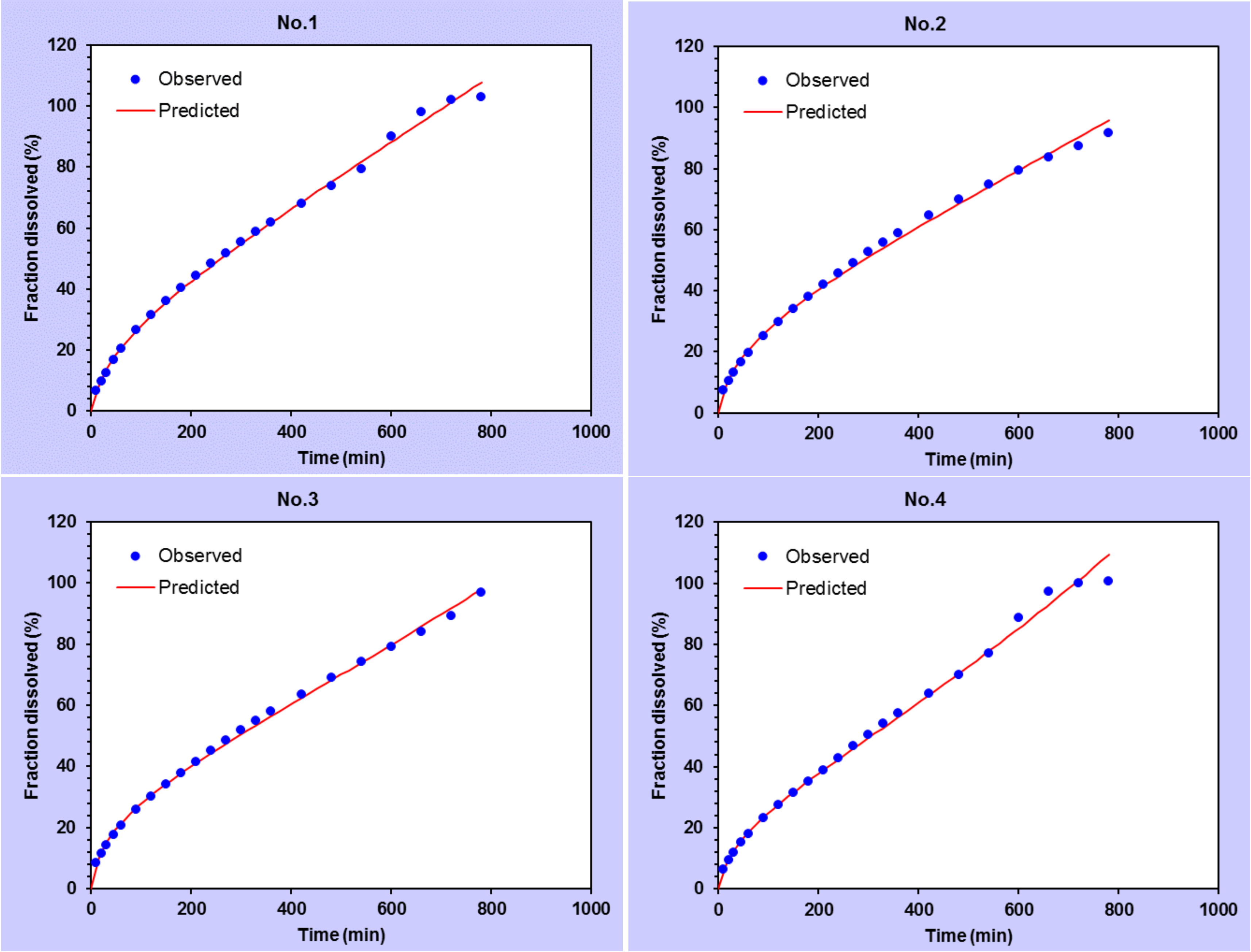

Supplementary materials – Model fitting summary – Lactochem® Fine Powder (modelling the entire relevant dissolution data)

Model: **Peppas–Sahlin_1**

Model equation: $F = k_1 \cdot t^m + k_2 \cdot t^{2m}$

Fitted model parameters per tested tablet (N = 4) with statistics – mean, standard deviation (SD), and relative standard deviation expressed in % (RSD%) (output from DDSolver):

Parameter	No.1	No.2	No.3	No.4	Mean	SD	RSD(%)
k_1	2.278	2.656	2.549	1.524	2.252	0.510	22.661
k_2	0.151	0.101	0.109	0.184	0.136	0.039	28.522
m	0.450	0.450	0.450	0.450	0.450	0.000	0.000

Number of dissolution data points (N), degrees of freedom (df), and selected goodness of fit criteria – Pearson correlation coefficient (R), coefficient of determination (R^2), adjusted coefficient of determination ($R^2_{adjusted}$), and residual sum of squares (RSS) (manual calculation in MS Excel):

Parameter	No.1	No.2	No.3	No.4
N	22	22	22	22
df	19	19	19	19
R	0.998735019	0.999335588	0.999776951	0.997955084
R^2	0.997471638	0.998671617	0.999553952	0.995914349
$R^2_{adjusted}$	0.997205495	0.998531787	0.999507	0.99548428
RSS	49.4965494	21.65580639	6.670511754	80.12636686

Graphical abstract of model fit presented as mean ± 1 SD of the fraction % of released carvedilol:

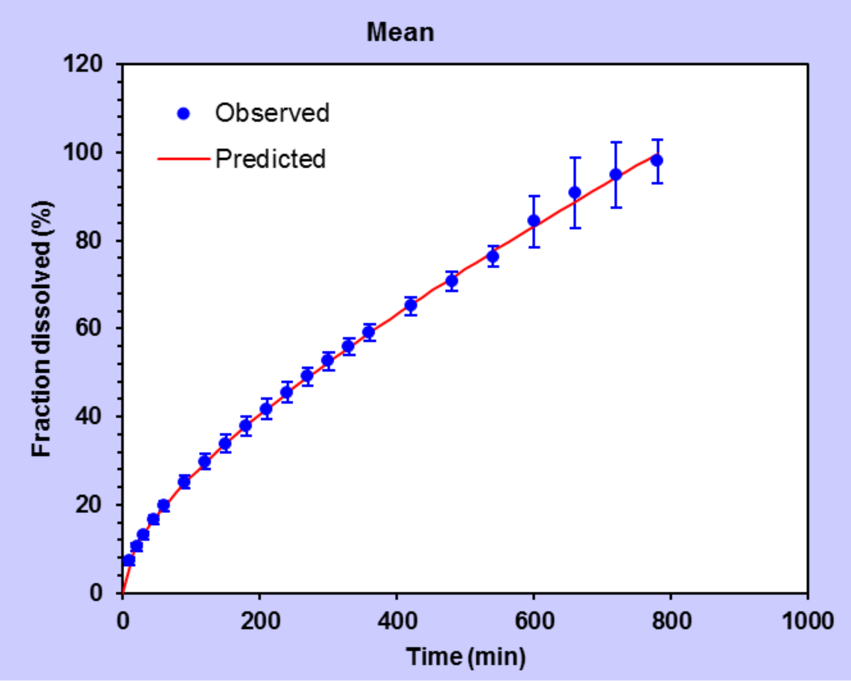

Supplementary materials – Model fitting summary – Lactochem® Fine Powder (modelling the entire relevant dissolution data)

Graphical abstract of model fit presented as the fraction % of released carvedilol per tested tablet:

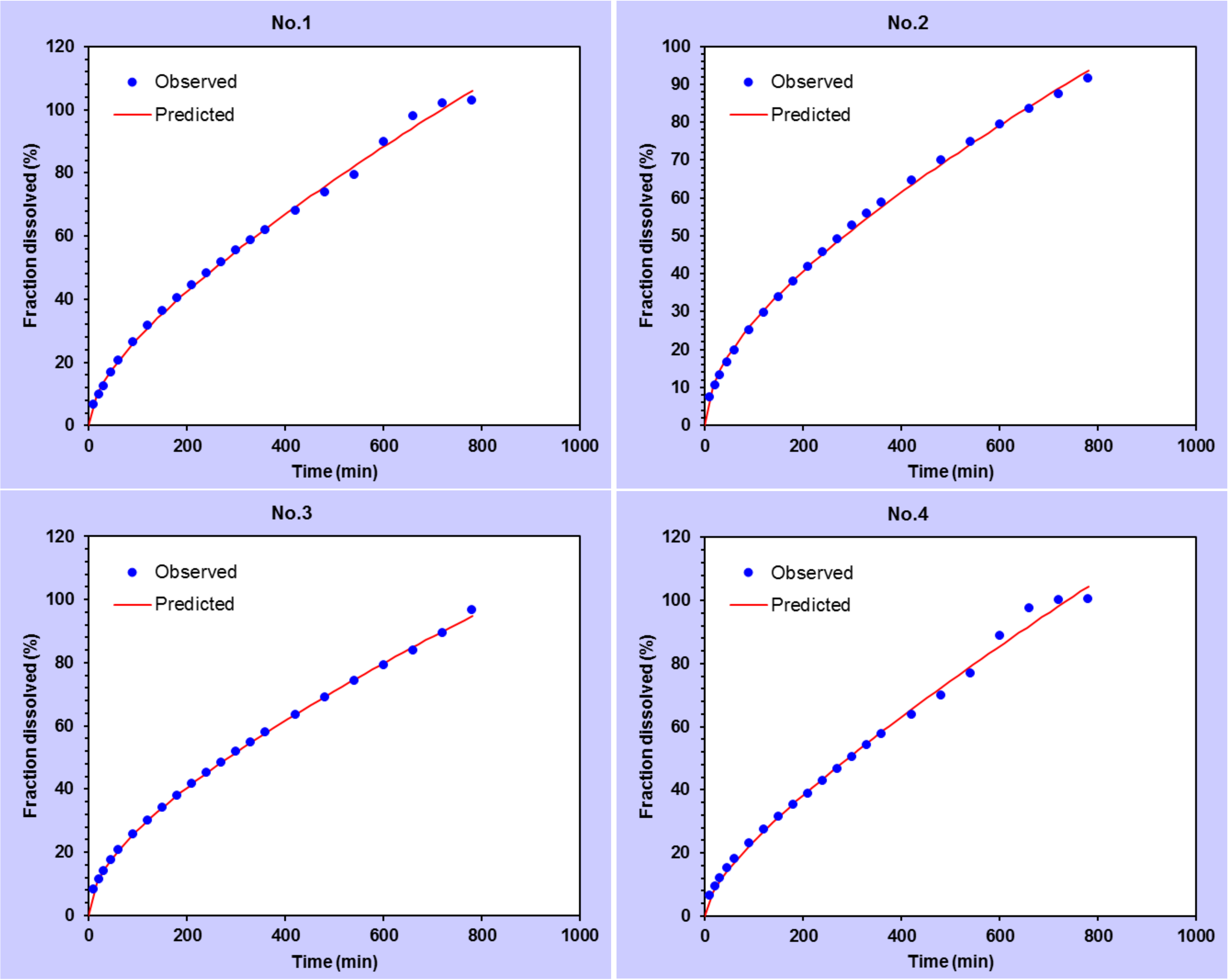

Supplementary materials – Model fitting summary – Lactochem® Fine Powder (modelling the entire relevant dissolution data)

Model: **Peppas–Sahlin_1 with T_{lag}**

Model equation: $F = k_1 \cdot (t - T_{lag})^m + k_2 \cdot (t - T_{lag})^{2m}$

Fitted model parameters per tested tablet (N = 4) with statistics – mean, standard deviation (SD), and relative standard deviation expressed in % (RSD%) (output from DDSolver):

Parameter	No.1	No.2	No.3	No.4	Mean	SD	RSD(%)
k_1	2.431	2.803	2.689	1.657	2.395	0.516	21.546
k_2	0.143	0.093	0.102	0.178	0.129	0.039	30.369
m	0.450	0.450	0.450	0.450	0.450	0.000	0.000
T_{lag}	4.000	4.000	4.000	4.000	4.000	0.000	0.000

Number of dissolution data points (N), degrees of freedom (df), and selected goodness of fit criteria – Pearson correlation coefficient (R), coefficient of determination (R^2), adjusted coefficient of determination ($R^2_{adjusted}$), and residual sum of squares (RSS) (manual calculation in MS Excel):

Parameter	No.1	No.2	No.3	No.4
N	22	22	22	22
df	18	18	18	18
R	0.998793556	0.99951333	0.999608298	0.997758674
R^2	0.997588567	0.999026897	0.99921675	0.995522373
$R^2_{adjusted}$	0.997186661	0.998864713	0.999086208	0.994776101
RSS	46.89024739	14.69804385	12.99952622	89.66217505

Graphical abstract of model fit presented as mean ± 1 SD of the fraction % of released carvedilol:

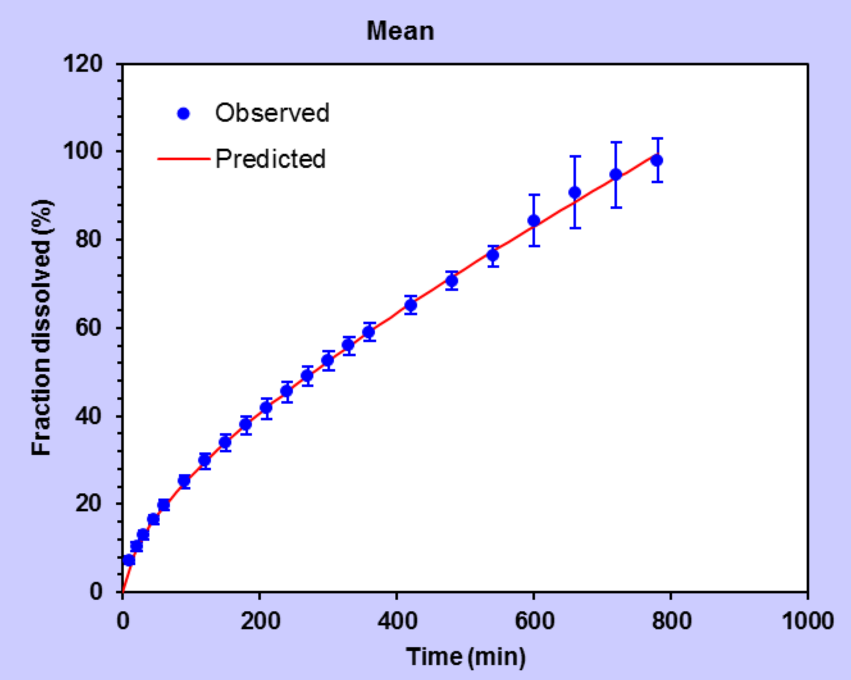

Supplementary materials – Model fitting summary – Lactochem® Fine Powder (modelling the entire relevant dissolution data)

Graphical abstract of model fit presented as the fraction % of released carvedilol per tested tablet:

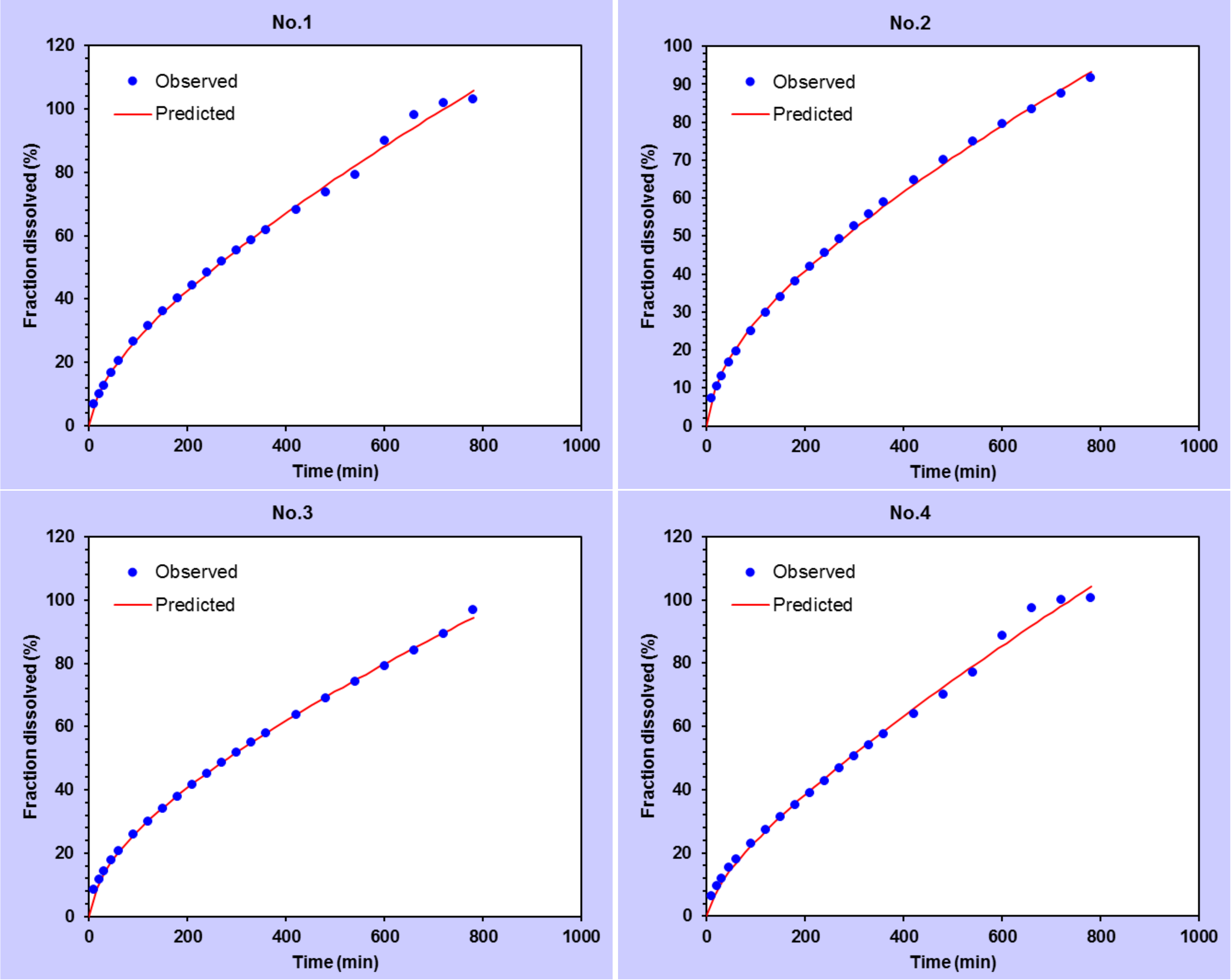

Supplementary materials – Model fitting summary – Lactochem® Fine Powder (modelling the entire relevant dissolution data)

Model: **Peppas–Sahlin_2**

Model equation: $F = k_1 \cdot t^{0.5} + k_2 \cdot t$

Fitted model parameters per tested tablet (N = 4) with statistics – mean, standard deviation (SD), and relative standard deviation expressed in % (RSD%) (output from DDSolver):

Parameter	No.1	No.2	No.3	No.4	Mean	SD	RSD(%)
k_1	2.194	2.422	2.331	1.620	2.142	0.360	16.828
k_2	0.058	0.033	0.038	0.076	0.051	0.020	38.801

Number of dissolution data points (N), degrees of freedom (df), and selected goodness of fit criteria – Pearson correlation coefficient (R), coefficient of determination (R^2), adjusted coefficient of determination ($R^2_{adjusted}$), and residual sum of squares (RSS) (manual calculation in MS Excel):

Parameter	No.1	No.2	No.3	No.4
N	22	22	22	22
df	20	20	20	20
R	0.99875641	0.999469424	0.999757528	0.998037995
R^2	0.997514367	0.99893913	0.999515114	0.99607984
$R^2_{adjusted}$	0.997390085	0.998886086	0.99949087	0.995883832
RSS	48.71427604	17.67113934	7.507630023	76.75473248

Graphical abstract of model fit presented as mean ± 1 SD of the fraction % of released carvedilol:

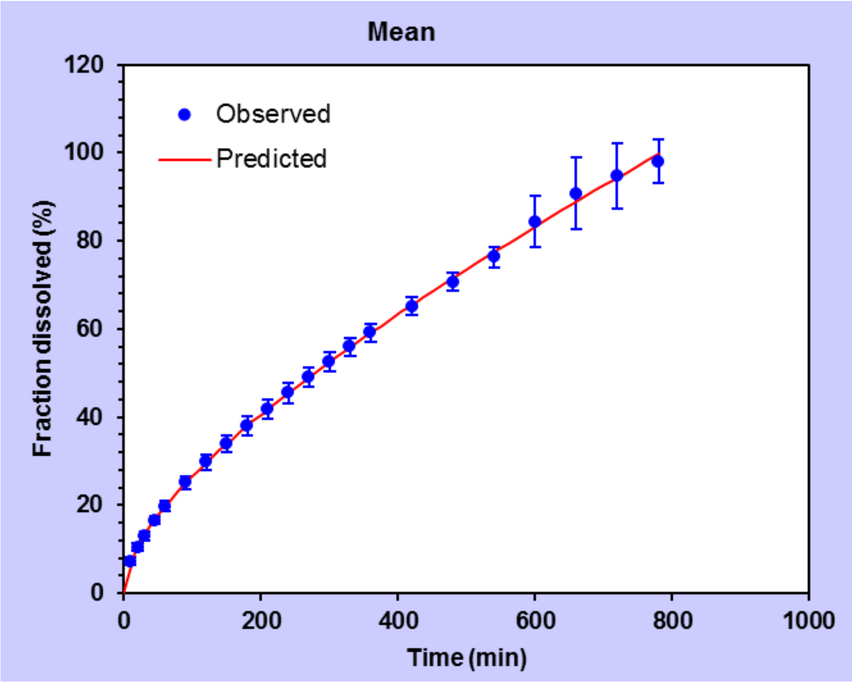

Supplementary materials – Model fitting summary – Lactochem® Fine Powder (modelling the entire relevant dissolution data)

Graphical abstract of model fit presented as the fraction % of released carvedilol per tested tablet:

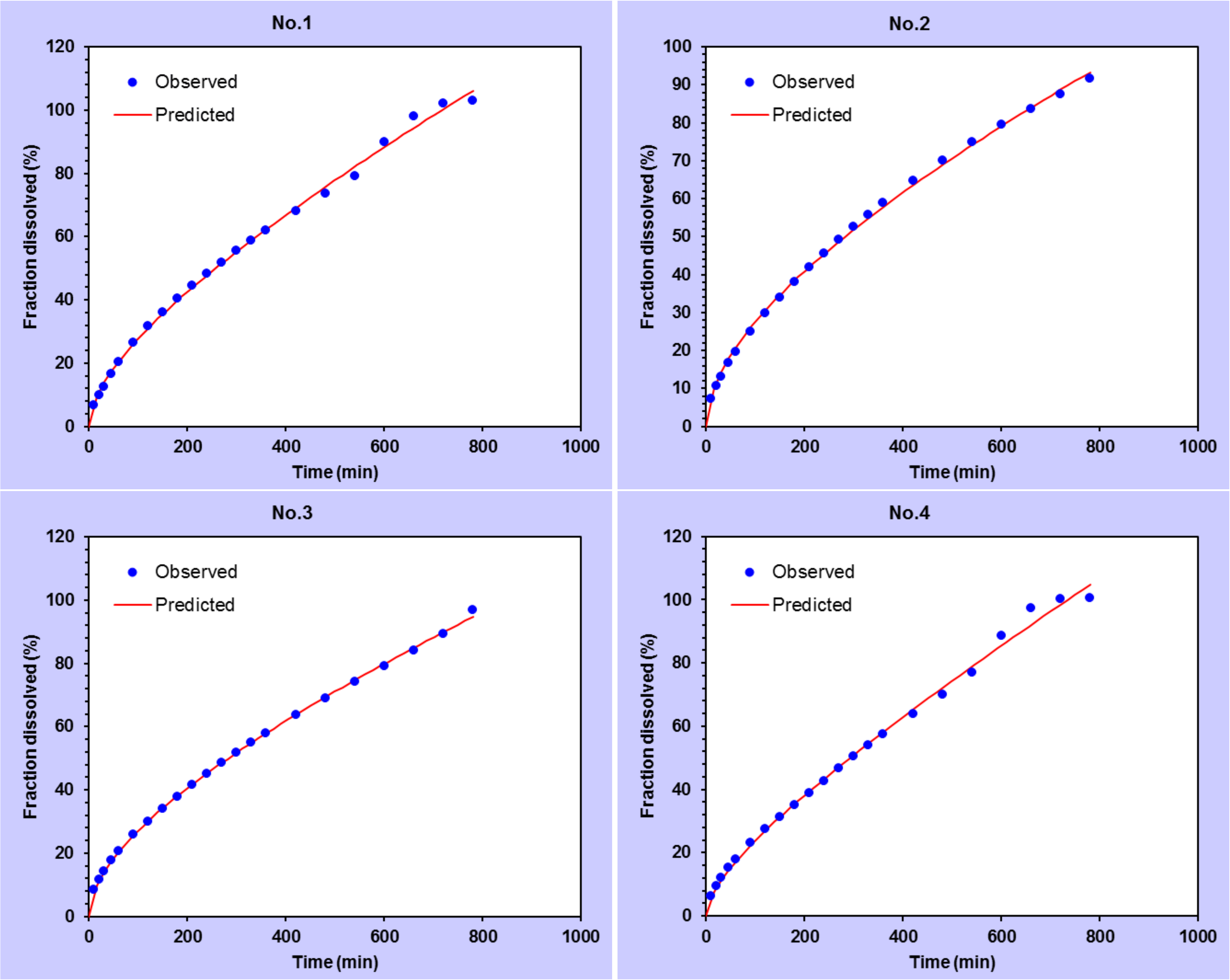

Supplementary materials – Model fitting summary – Lactochem® Fine Powder (modelling the entire relevant dissolution data)

Model: **Peppas–Sahlin_2 with T_{lag}**

Model equation: $F = k_1 \cdot (t - T_{lag})^{0.5} + k_2 \cdot (t - T_{lag})$

Fitted model parameters per tested tablet (N = 4) with statistics – mean, standard deviation (SD), and relative standard deviation expressed in % (RSD%) (output from DDSolver):

Parameter	No.1	No.2	No.3	No.4	Mean	SD	RSD(%)
k_1	2.301	2.524	2.428	1.713	2.242	0.364	16.252
k_2	0.054	0.029	0.034	0.073	0.048	0.020	42.076
T_{lag}	4.000	4.000	4.000	4.000	4.000	0.000	0.000

Number of dissolution data points (N), degrees of freedom (df), and selected goodness of fit criteria – Pearson correlation coefficient (R), coefficient of determination (R^2), adjusted coefficient of determination ($R^2_{adjusted}$), and residual sum of squares (RSS) (manual calculation in MS Excel):

Parameter	No.1	No.2	No.3	No.4
N	22	22	22	22
df	19	19	19	19
R	0.998800004	0.999578211	0.999531481	0.99786196
R^2	0.997601449	0.999156599	0.999063182	0.995728491
$R^2_{adjusted}$	0.99734897	0.99906782	0.998964569	0.995278858
RSS	46.71431072	12.5385727	16.71508631	85.85454262

Graphical abstract of model fit presented as mean ± 1 SD of the fraction % of released carvedilol:

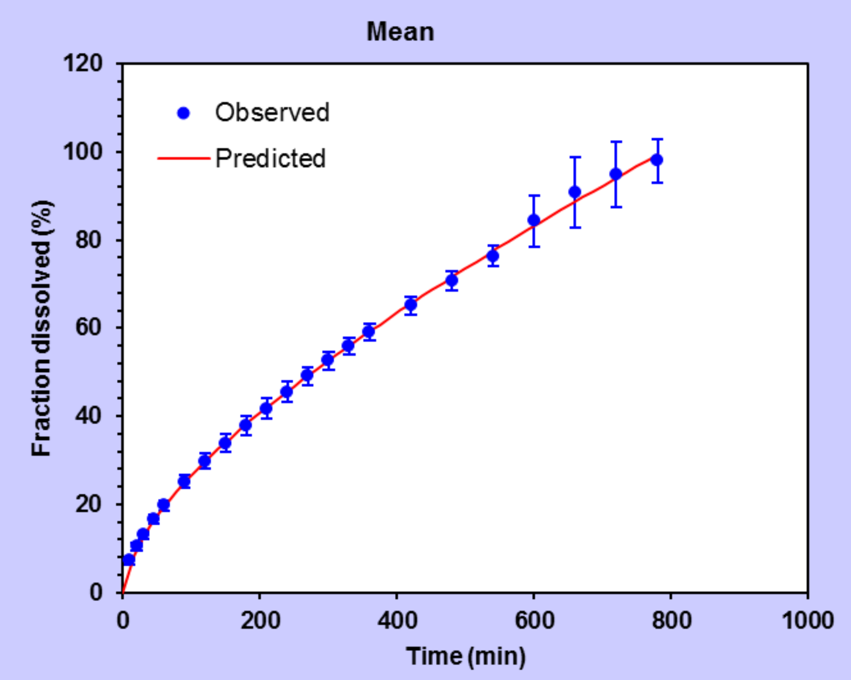

Supplementary materials – Model fitting summary – Lactochem® Fine Powder (modelling the entire relevant dissolution data)

Graphical abstract of model fit presented as the fraction % of released carvedilol per tested tablet:

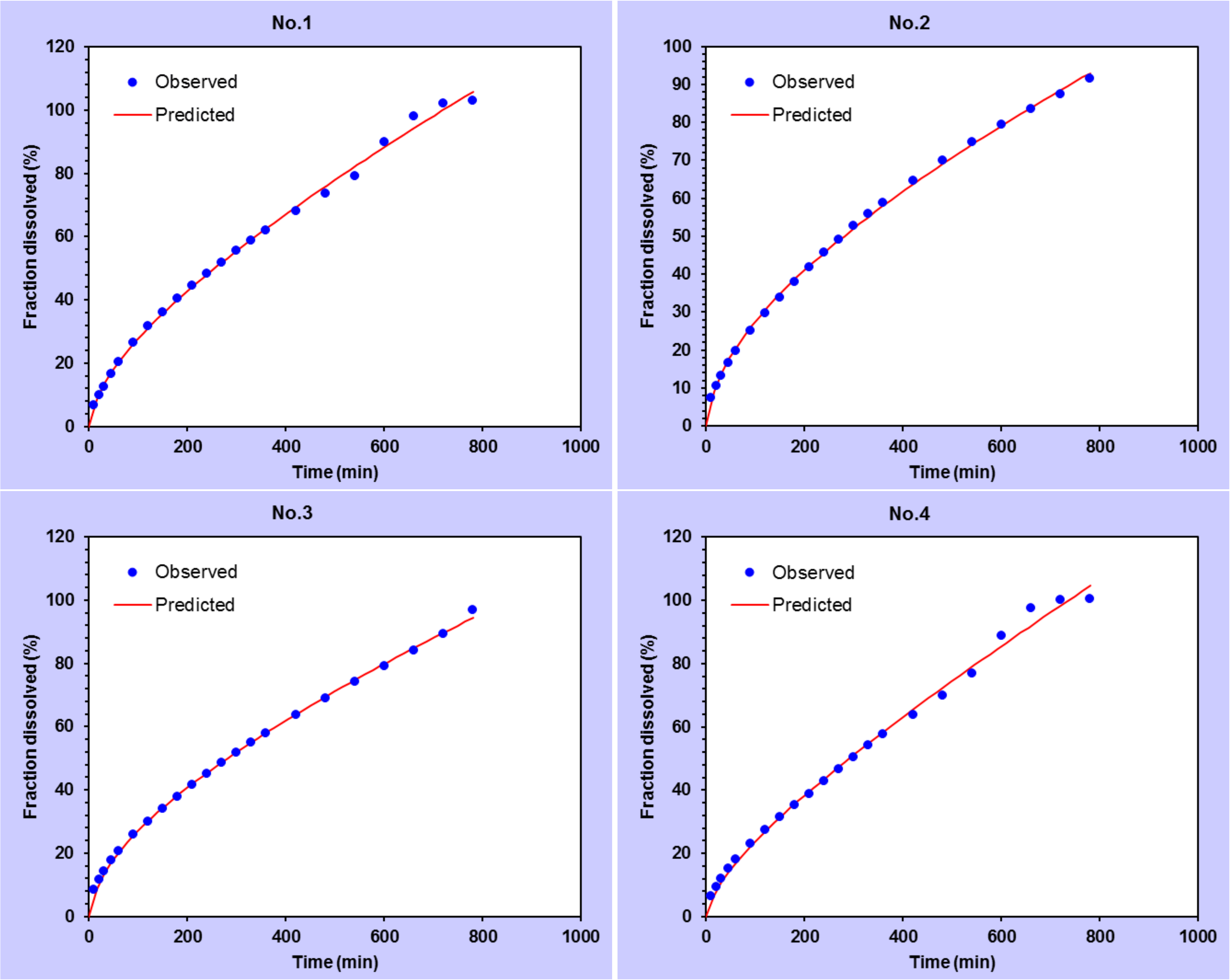

Supplementary materials – Model fitting summary – Lactochem® Fine Powder (modelling the entire relevant dissolution data)

Model: **Quadratic**

Model equation: $F = 100 \cdot (k_1 \cdot t^2 + k_2 \cdot t)$

Fitted model parameters per tested tablet (N = 4) with statistics – mean, standard deviation (SD), and relative standard deviation expressed in % (RSD%) (output from DDSolver):

Parameter	No.1	No.2	No.3	No.4	Mean	SD	RSD(%)
k_1	-0.0000012	-0.0000014	-0.0000013	-0.0000009	-0.0000012	0.0000002	-18.8533980
k_2	0.0022539	0.0022158	0.0021468	0.0019884	0.0021513	0.0001173	5.4504571

Number of dissolution data points (N), degrees of freedom (df), and selected goodness of fit criteria – Pearson correlation coefficient (R), coefficient of determination (R^2), adjusted coefficient of determination ($R^2_{adjusted}$), and residual sum of squares (RSS) (manual calculation in MS Excel):

Parameter	No.1	No.2	No.3	No.4
N	22	22	22	22
df	20	20	20	20
R	0.994193721	0.995756027	0.993083424	0.995322782
R^2	0.988421155	0.991530065	0.986214688	0.99066744
$R^2_{adjusted}$	0.987842212	0.991106568	0.985525422	0.990200812
RSS	477.3777139	381.4370266	564.838252	356.9501084

Graphical abstract of model fit presented as mean ± 1 SD of the fraction % of released carvedilol:

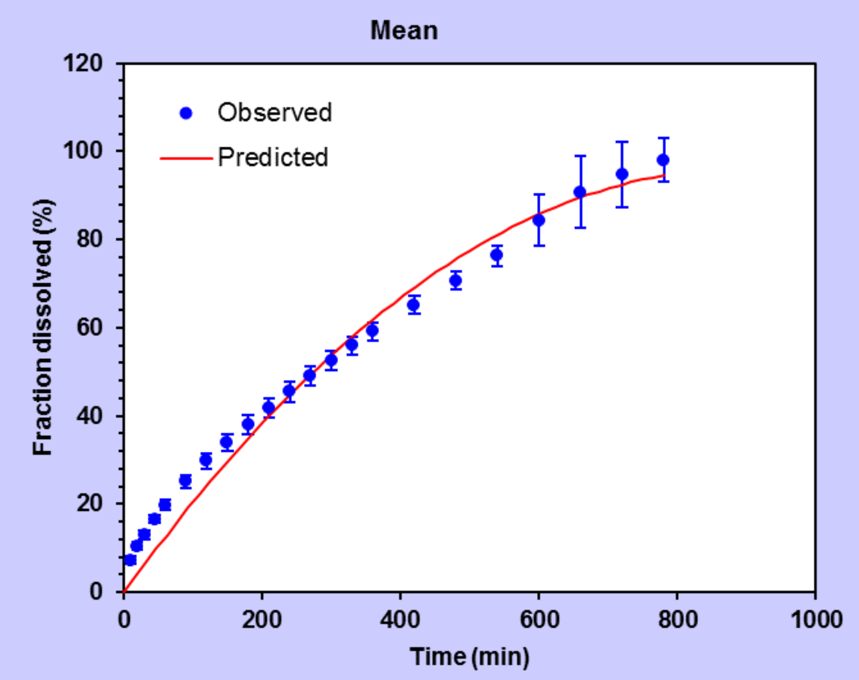

Supplementary materials – Model fitting summary – Lactochem® Fine Powder (modelling the entire relevant dissolution data)

Graphical abstract of model fit presented as the fraction % of released carvedilol per tested tablet:

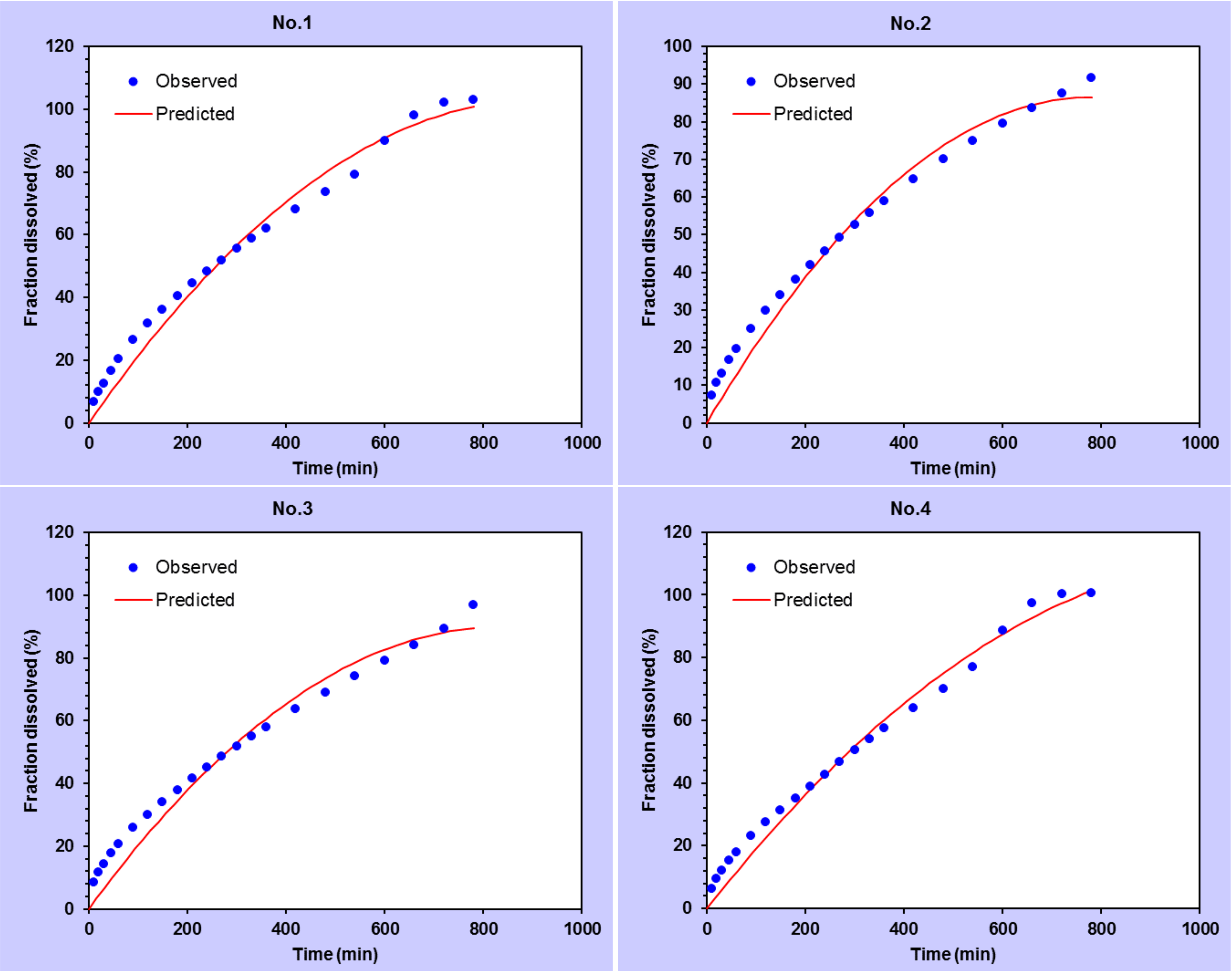

Supplementary materials – Model fitting summary – Lactochem® Fine Powder (modelling the entire relevant dissolution data)

Model: **Quadratic with T_{lag}**

Model equation: $F = 100 \cdot \left[k_1 \cdot (t - T_{lag})^2 + k_2 \cdot (t - T_{lag})\right]$

Fitted model parameters per tested tablet (N = 4) with statistics – mean, standard deviation (SD), and relative standard deviation expressed in % (RSD%) (output from DDSolver):

Parameter	No.1	No.2	No.3	No.4	Mean	SD	RSD(%)
k_1	-0.0000013	-0.0000015	-0.0000013	-0.0000009	-0.0000012	0.0000002	-18.3332922
k_2	0.0022888	0.0022485	0.0021778	0.0020209	0.0021840	0.0001180	5.4022105
T_{lag}	4.0000000	4.0000000	4.0000000	4.0000000	4.0000000	0.0000000	0.0000000

Number of dissolution data points (N), degrees of freedom (df), and selected goodness of fit criteria – Pearson correlation coefficient (R), coefficient of determination (R^2), adjusted coefficient of determination ($R^2_{adjusted}$), and residual sum of squares (RSS) (manual calculation in MS Excel):

Parameter	No.1	No.2	No.3	No.4
N	22	22	22	22
df	19	19	19	19
R	0.993771168	0.995317308	0.99257863	0.994939851
R^2	0.987581134	0.990656544	0.985212337	0.989905306
$R^2_{adjusted}$	0.986273885	0.989673022	0.983655741	0.988842707
RSS	555.8646986	456.9380706	651.9768079	416.7082159

Graphical abstract of model fit presented as mean ± 1 SD of the fraction % of released carvedilol:

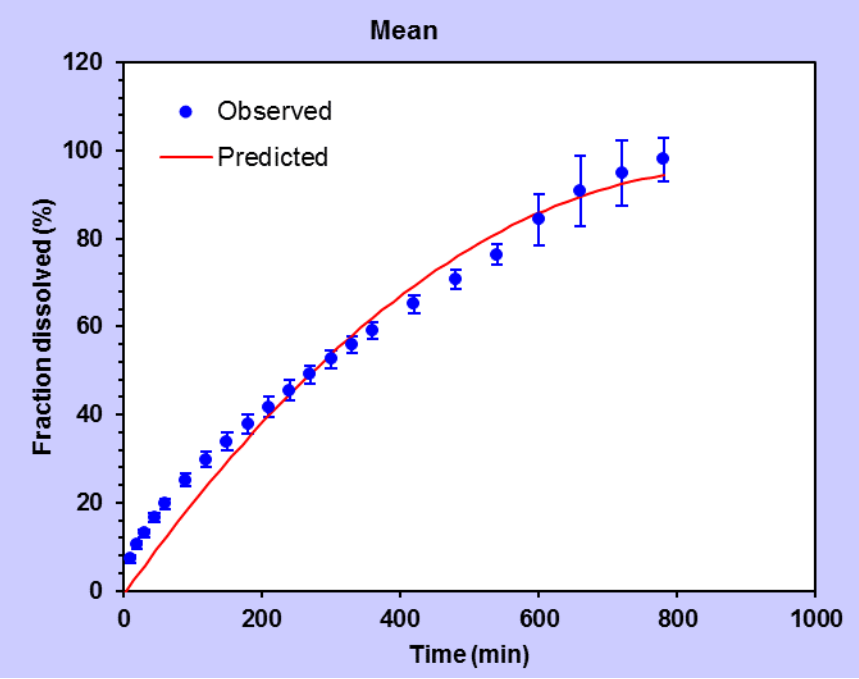

Supplementary materials – Model fitting summary – Lactochem® Fine Powder (modelling the entire relevant dissolution data)

Graphical abstract of model fit presented as the fraction % of released carvedilol per tested tablet:

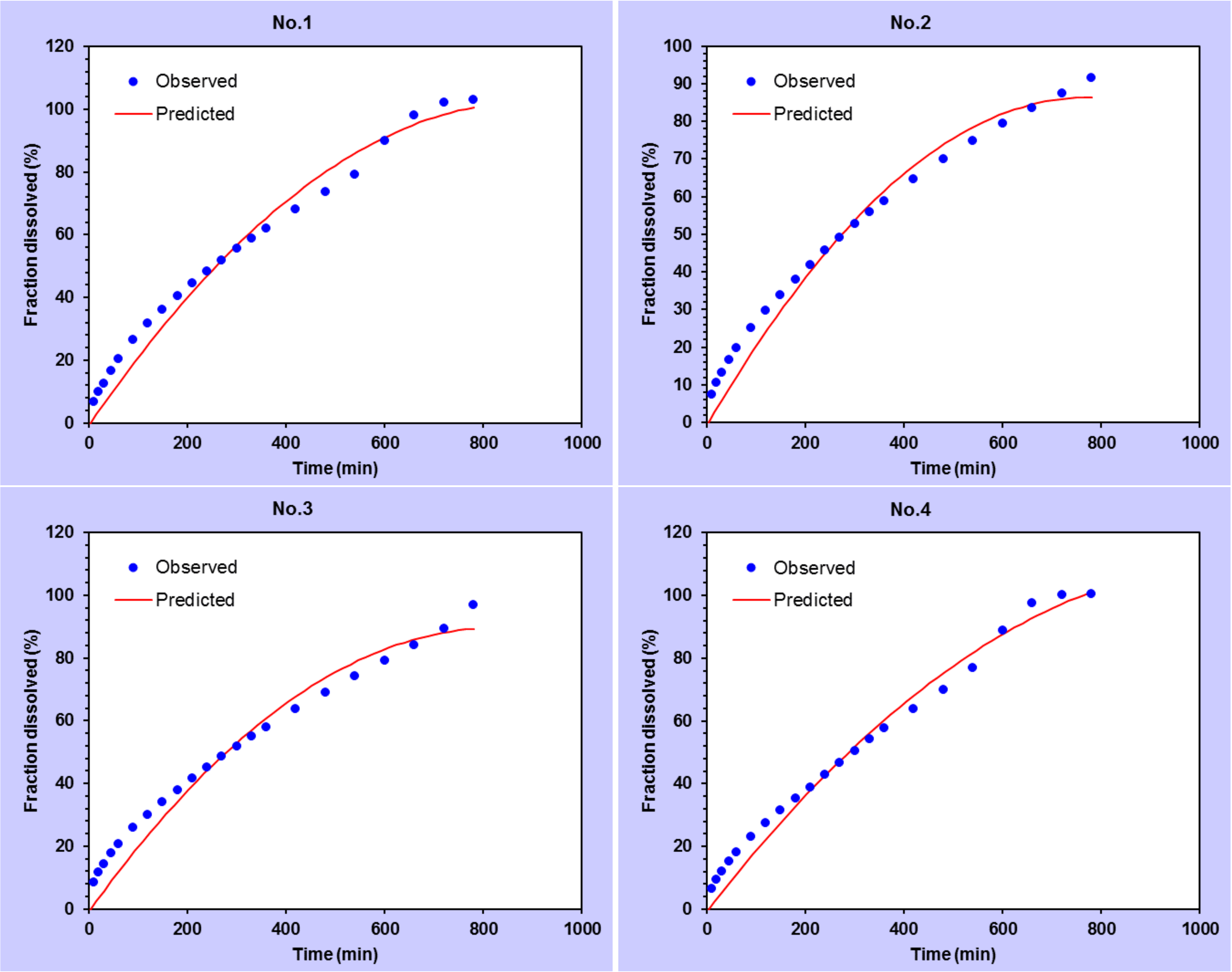

Supplementary materials – Model fitting summary – Lactochem® Fine Powder (modelling the entire relevant dissolution data)

Model: **Weibull_1**

Model equation: $F = 100 \cdot \left[1 - e^{-\frac{(t-T_i)^\beta}{\alpha}}\right]$

Fitted model parameters per tested tablet (N = 4) with statistics – mean, standard deviation (SD), and relative standard deviation expressed in % (RSD%) (output from DDSolver):

Parameter	No.1	No.2	No.3	No.4	Mean	SD	RSD(%)
α	90.643	73.826	66.420	96.675	81.891	14.137	17.263
β	0.779	0.724	0.709	0.768	0.745	0.034	4.519
T_i	6.000	6.000	6.000	6.000	6.000	0.000	0.000

Number of dissolution data points (N), degrees of freedom (df), and selected goodness of fit criteria – Pearson correlation coefficient (R), coefficient of determination (R^2), adjusted coefficient of determination ($R^2_{adjusted}$), and residual sum of squares (RSS) (manual calculation in MS Excel):

Parameter	No.1	No.2	No.3	No.4
N	22	22	22	22
df	19	19	19	19
R	0.974488886	0.986701428	0.977563668	0.967099654
R^2	0.949628588	0.973579708	0.955630725	0.93528174
$R^2_{adjusted}$	0.944326334	0.970798625	0.950960275	0.928469292
RSS	1224.611016	450.2659632	722.546263	1673.608927

Graphical abstract of model fit presented as mean ± 1 SD of the fraction % of released carvedilol:

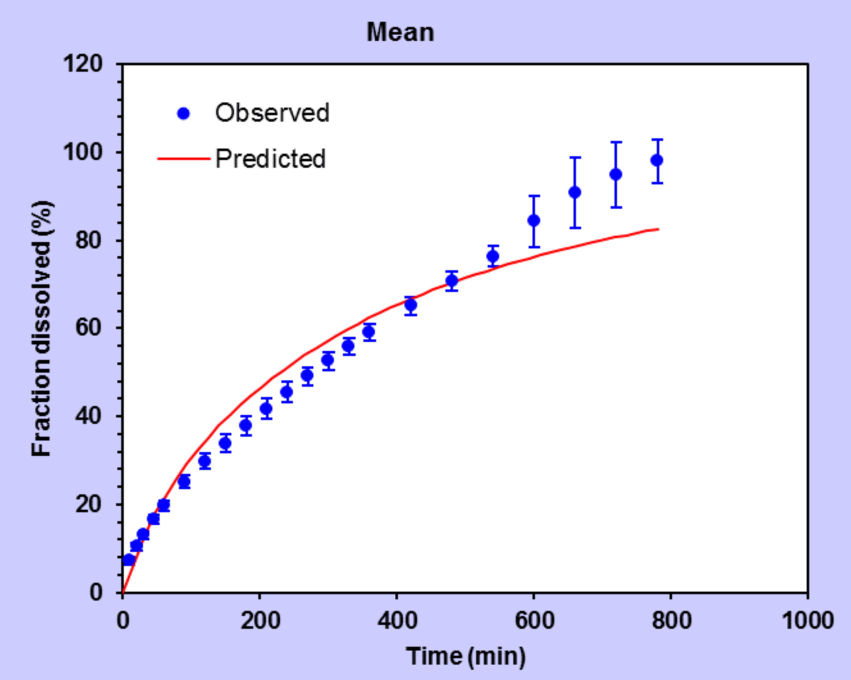

Supplementary materials – Model fitting summary – Lactochem® Fine Powder (modelling the entire relevant dissolution data)

Graphical abstract of model fit presented as the fraction % of released carvedilol per tested tablet:

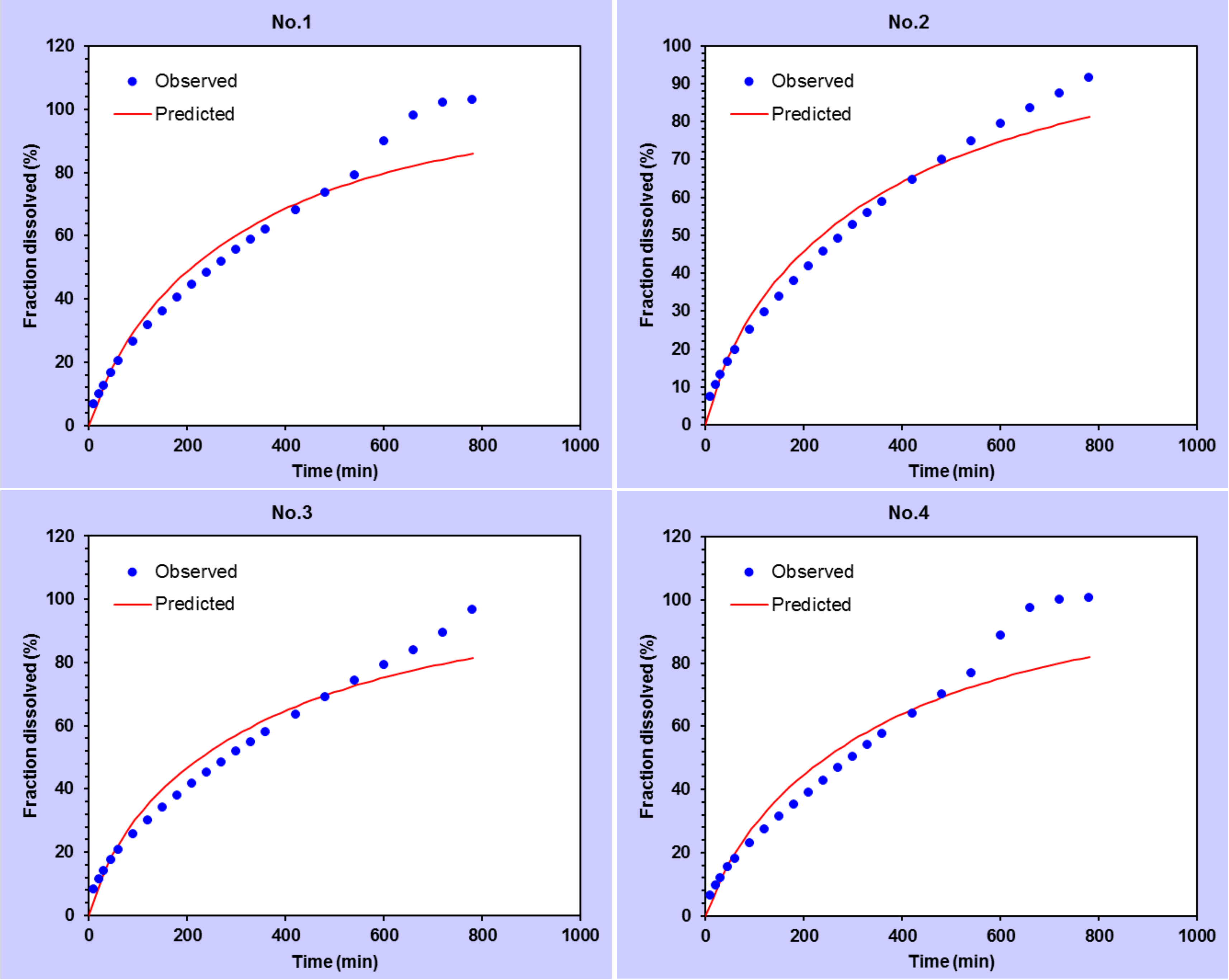

Supplementary materials – Model fitting summary – Lactochem® Fine Powder (modelling the entire relevant dissolution data)

Model: **Weibull_2**

Model equation: $F = 100 \cdot \left(1 - e^{-\frac{t^{\beta}}{\alpha}}\right)$

Fitted model parameters per tested tablet (N = 4) with statistics – mean, standard deviation (SD), and relative standard deviation expressed in % (RSD%) (output from DDSolver):

Parameter	No.1	No.2	No.3	No.4	Mean	SD	RSD(%)
α	136.698	106.054	95.291	145.795	120.959	24.122	19.942
β	0.850	0.785	0.770	0.839	0.811	0.039	4.843

Number of dissolution data points (N), degrees of freedom (df), and selected goodness of fit criteria – Pearson correlation coefficient (R), coefficient of determination (R^2), adjusted coefficient of determination ($R^2_{adjusted}$), and residual sum of squares (RSS) (manual calculation in MS Excel):

Parameter	No.1	No.2	No.3	No.4
N	22	22	22	22
df	20	20	20	20
R	0.978550386	0.990679995	0.982470667	0.972362229
R^2	0.957560857	0.981446852	0.965248611	0.945488305
$R^2_{adjusted}$	0.9554389	0.980519194	0.963511041	0.94276272
RSS	1000.721903	321.2828731	575.6726532	1370.415524

Graphical abstract of model fit presented as mean ± 1 SD of the fraction % of released carvedilol:

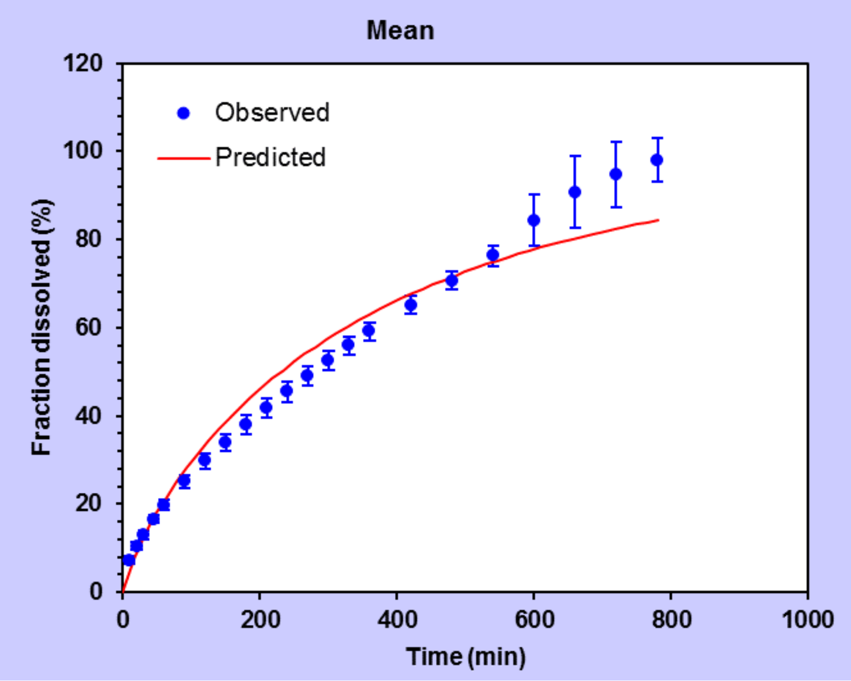

Supplementary materials – Model fitting summary – Lactochem® Fine Powder (modelling the entire relevant dissolution data)

Graphical abstract of model fit presented as the fraction % of released carvedilol per tested tablet:

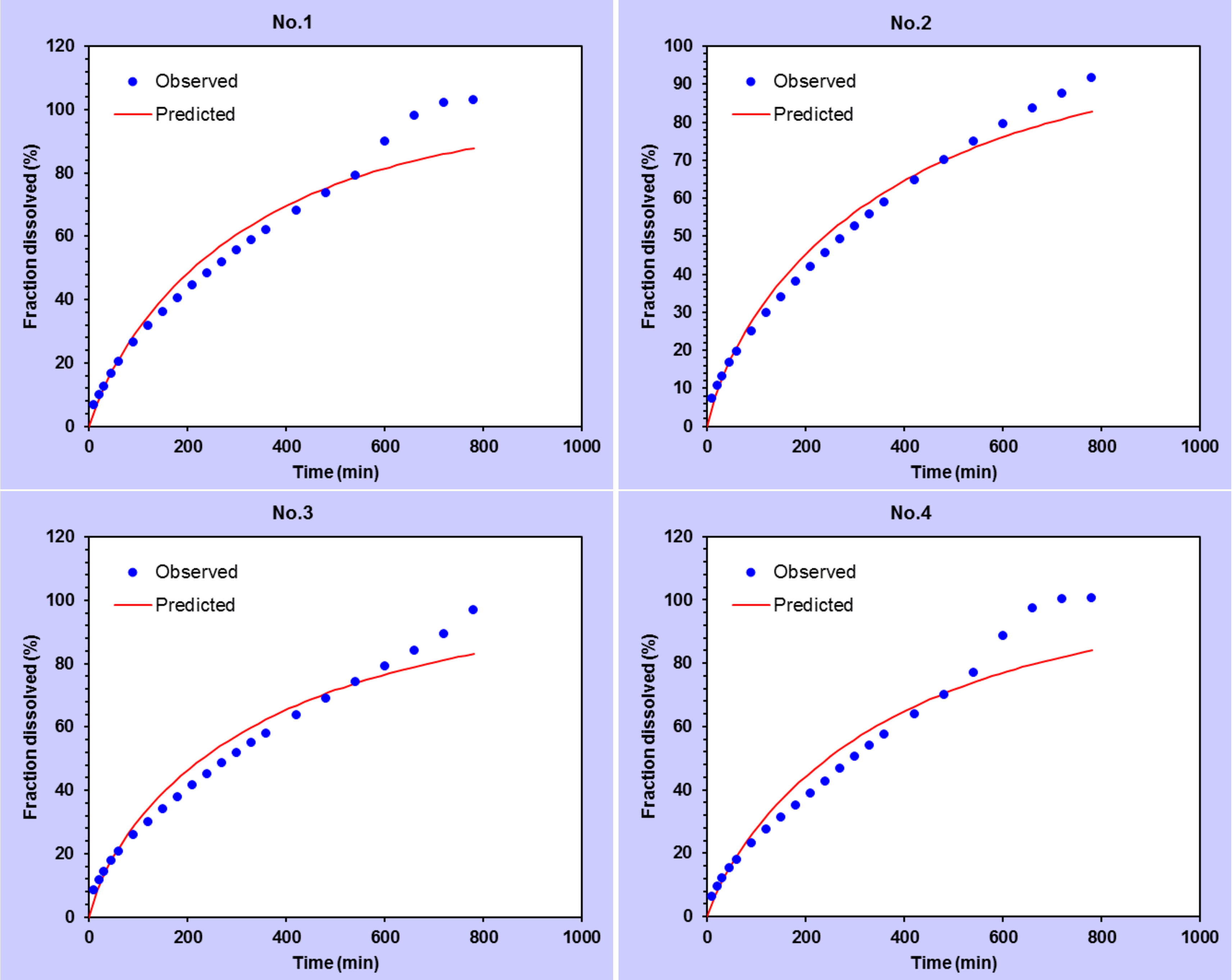

Supplementary materials – Model fitting summary – Lactochem® Fine Powder (modelling the entire relevant dissolution data)

Model: **Weibull_3**

Model equation: $F = F_{max} \cdot \left(1 - e^{-\frac{t^{\beta}}{\alpha}}\right)$

Fitted model parameters per tested tablet (N = 4) with statistics – mean, standard deviation (SD), and relative standard deviation expressed in % (RSD%) (output from DDSolver):

Parameter	No.1	No.2	No.3	No.4	Mean	SD	RSD(%)
α	155.848	124.342	104.997	230.274	153.865	55.082	35.799
β	0.858	0.785	0.747	0.874	0.816	0.060	7.364
F_{max}	108.197	114.454	119.822	111.287	113.440	4.963	4.375

Number of dissolution data points (N), degrees of freedom (df), and selected goodness of fit criteria – Pearson correlation coefficient (R), coefficient of determination (R^2), adjusted coefficient of determination ($R^2_{adjusted}$), and residual sum of squares (RSS) (manual calculation in MS Excel):

Parameter	No.1	No.2	No.3	No.4
N	22	22	22	22
df	19	19	19	19
R	0.982468822	0.994456864	0.988812169	0.983813227
R^2	0.965244985	0.988944455	0.977749506	0.967888466
$R^2_{adjusted}$	0.961586563	0.987780714	0.975407348	0.964508305
RSS	748.2227124	291.0670726	516.6570313	1076.807408

Graphical abstract of model fit presented as mean ± 1 SD of the fraction % of released carvedilol:

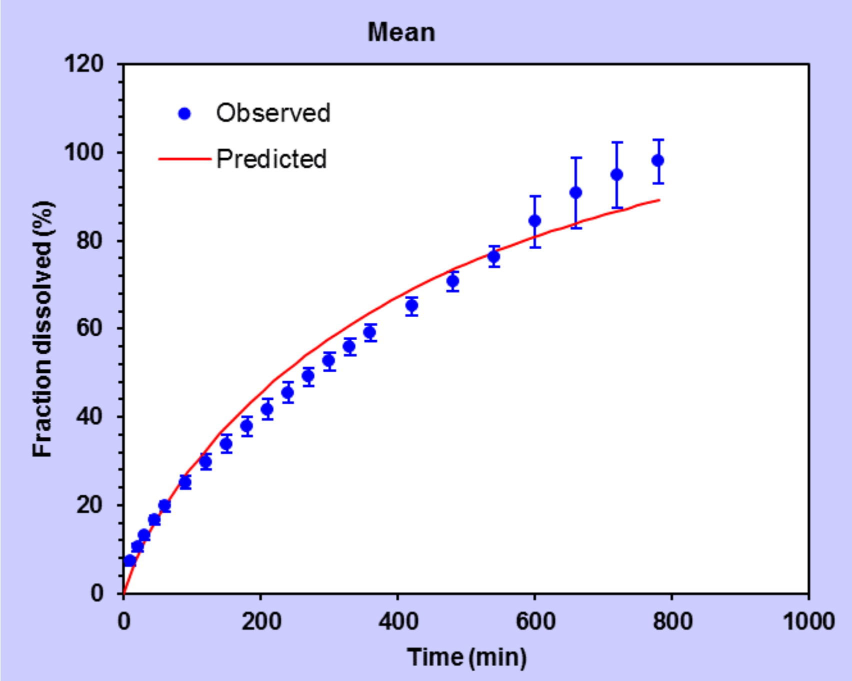

Supplementary materials – Model fitting summary – Lactochem® Fine Powder (modelling the entire relevant dissolution data)

Graphical abstract of model fit presented as the fraction % of released carvedilol per tested tablet:

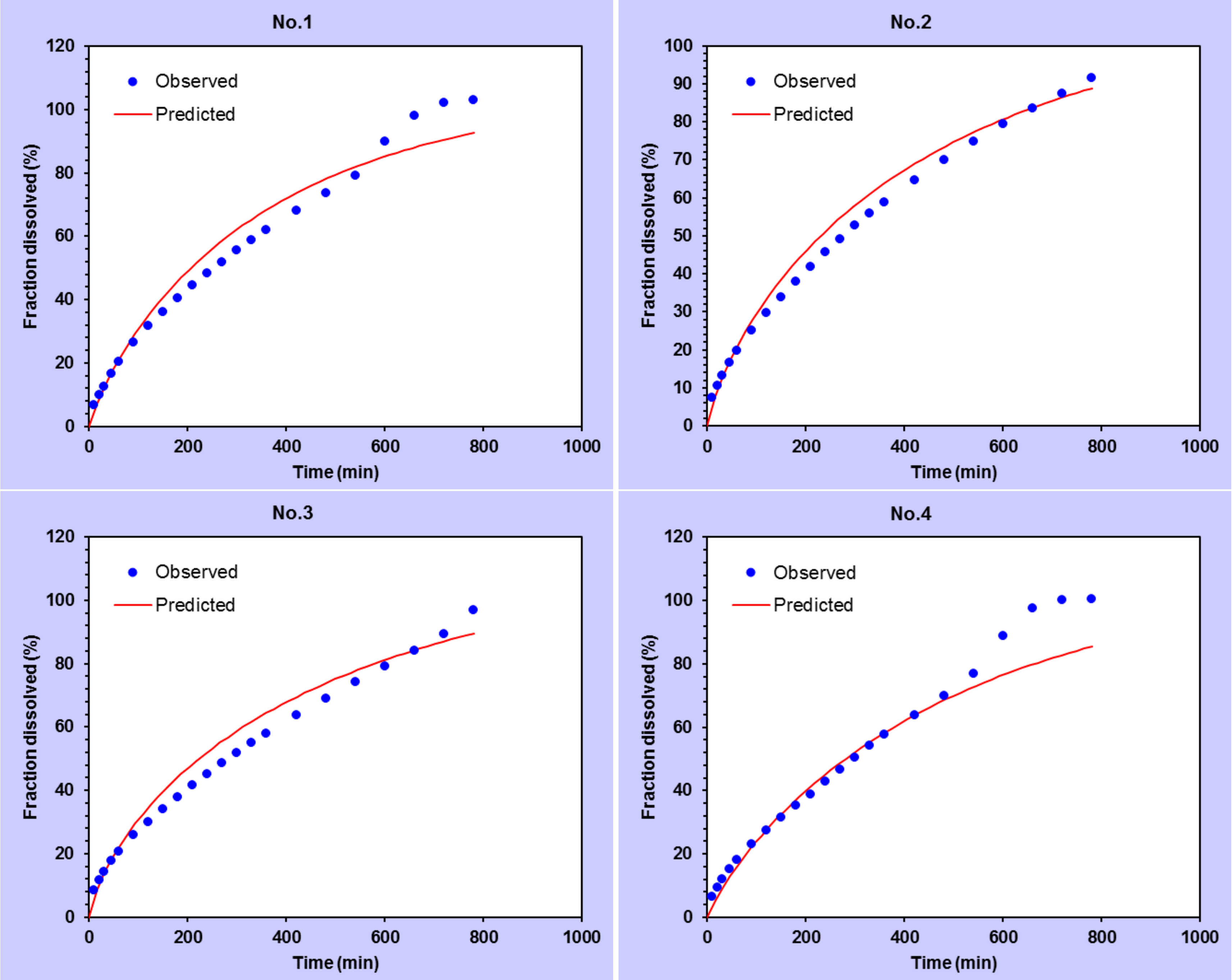

Supplementary materials – Model fitting summary – Lactochem® Fine Powder (modelling the entire relevant dissolution data)

Model: **Weibull_4**

Model equation: $F = F_{max} \cdot \left[1 - e^{-\frac{(t-T_i)^{\beta}}{\alpha}}\right]$

Fitted model parameters per tested tablet (N = 4) with statistics – mean, standard deviation (SD), and relative standard deviation expressed in % (RSD%) (output from DDSolver):

Parameter	No.1	No.2	No.3	No.4	Mean	SD	RSD(%)
α	104.793	76.172	65.275	117.313	90.888	24.250	26.681
β	0.790	0.743	0.700	0.800	0.758	0.046	6.081
T_i	6.000	6.000	6.000	6.000	6.000	0.000	0.000
F_{max}	108.197	96.227	101.654	105.603	102.920	5.210	5.062

Number of dissolution data points (N), degrees of freedom (df), and selected goodness of fit criteria – Pearson correlation coefficient (R), coefficient of determination (R^2), adjusted coefficient of determination ($R^2_{adjusted}$), and residual sum of squares (RSS) (manual calculation in MS Excel):

Parameter	No.1	No.2	No.3	No.4
N	22	22	22	22
df	18	18	18	18
R	0.978633062	0.984735451	0.978519938	0.969626692
R^2	0.95772267	0.969703908	0.957501269	0.940175922
$R^2_{adjusted}$	0.950676448	0.964654559	0.950418147	0.930205243
RSS	903.4574288	500.2741418	700.0797166	1309.228755

Graphical abstract of model fit presented as mean ± 1 SD of the fraction % of released carvedilol:

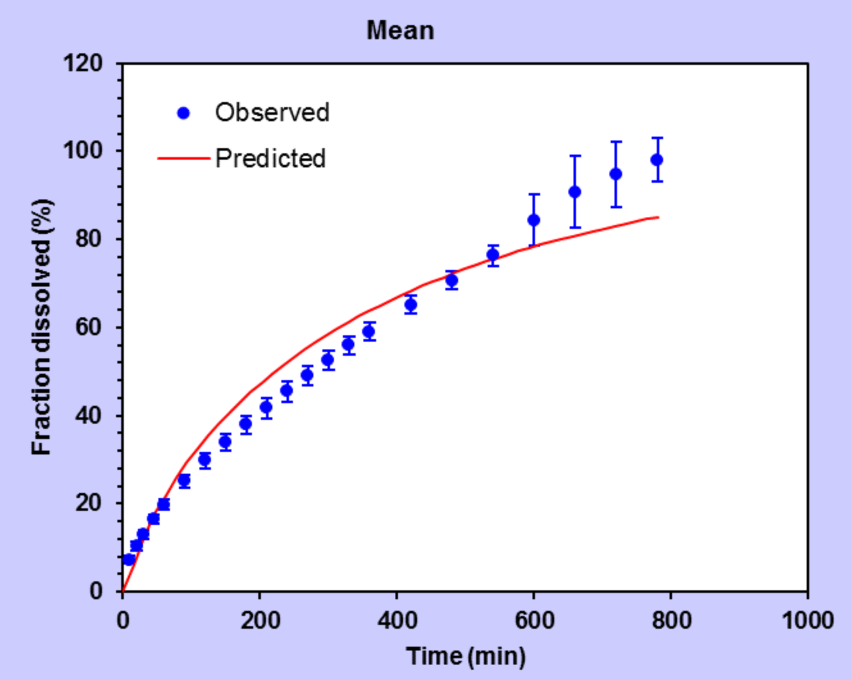

Supplementary materials – Model fitting summary – Lactochem® Fine Powder (modelling the entire relevant dissolution data)

Graphical abstract of model fit presented as the fraction % of released carvedilol per tested tablet:

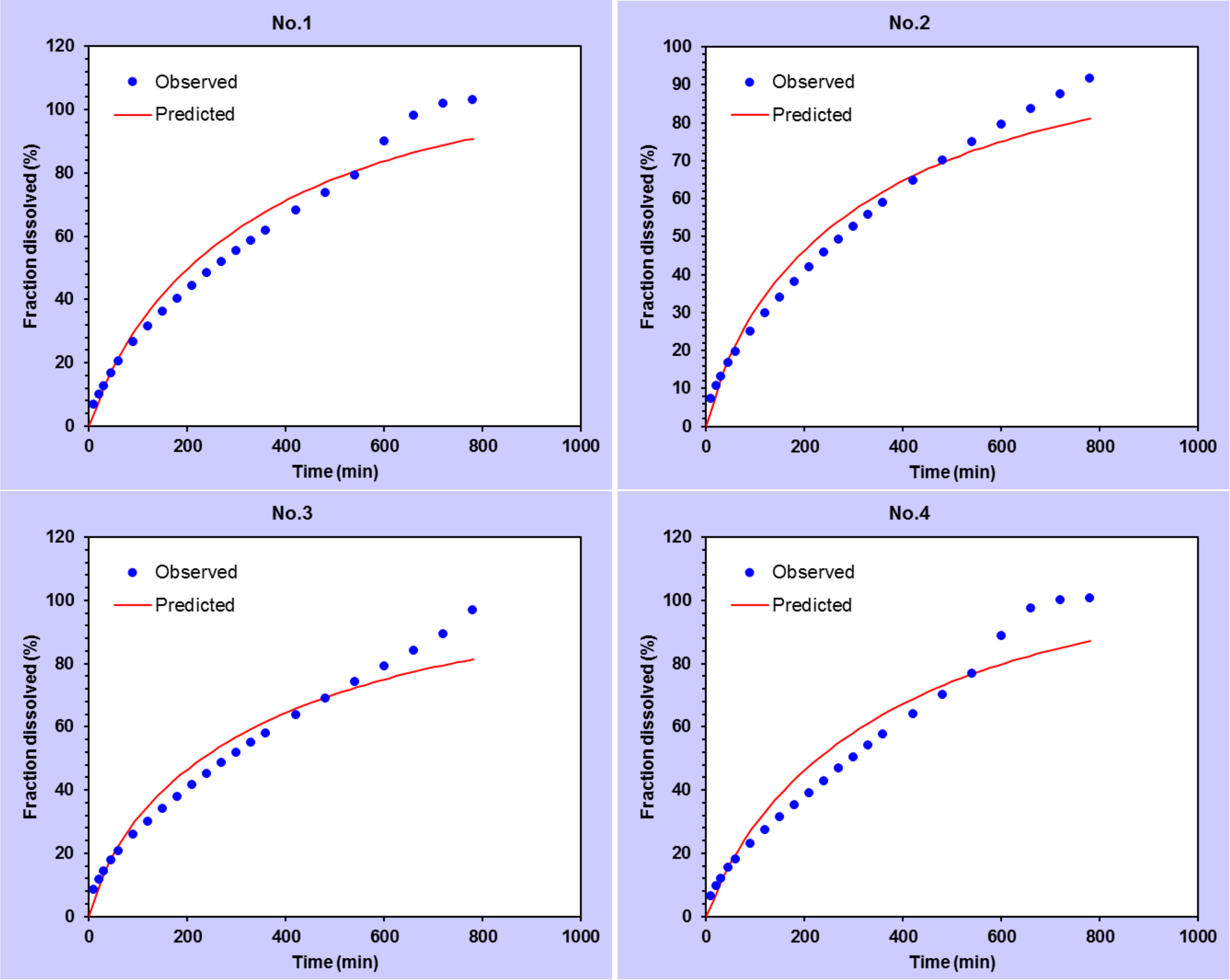

Supplementary materials – Model fitting summary – Lactochem® Fine Powder (modelling the entire relevant dissolution data)

Model: **Logistic_1**

Model equation: $F = 100 \cdot \frac{e^{\alpha+\beta \cdot \log(t)}}{1+e^{\alpha+\beta \cdot \log(t)}}$

Fitted model parameters per tested tablet (N = 4) with statistics – mean, standard deviation (SD), and relative standard deviation expressed in % (RSD%) (output from DDSolver):

Parameter	No.1	No.2	No.3	No.4	Mean	SD	RSD(%)
α	-6.060	-6.433	-5.611	-6.024	-6.032	0.336	-5.578
β	2.701	2.638	2.457	2.606	2.601	0.103	3.973

Number of dissolution data points (N), degrees of freedom (df), and selected goodness of fit criteria – Pearson correlation coefficient (R), coefficient of determination (R^2), adjusted coefficient of determination ($R^2_{adjusted}$), and residual sum of squares (RSS) (manual calculation in MS Excel):

Parameter	No.1	No.2	No.3	No.4
N	22	22	22	22
df	20	20	20	20
R	0.953717735	0.987762152	0.96140753	0.946038014
R^2	0.909577517	0.975674068	0.924304439	0.894987924
$R^2_{adjusted}$	0.905056393	0.974457772	0.920519661	0.88973732
RSS	1886.864058	788.3648873	1297.571698	2224.802885

Graphical abstract of model fit presented as mean ± 1 SD of the fraction % of released carvedilol:

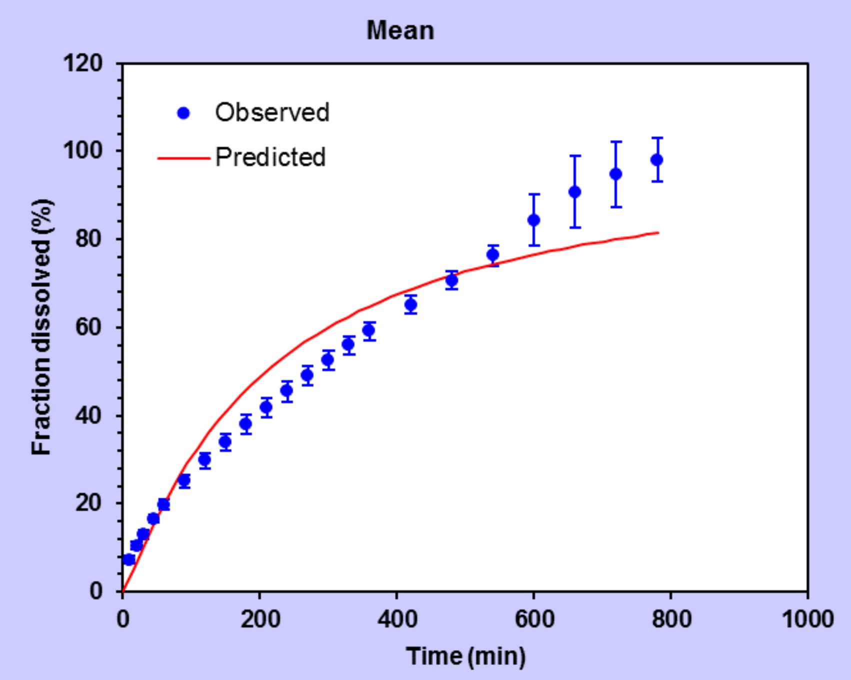

Supplementary materials – Model fitting summary – Lactochem® Fine Powder (modelling the entire relevant dissolution data)

Graphical abstract of model fit presented as the fraction % of released carvedilol per tested tablet:

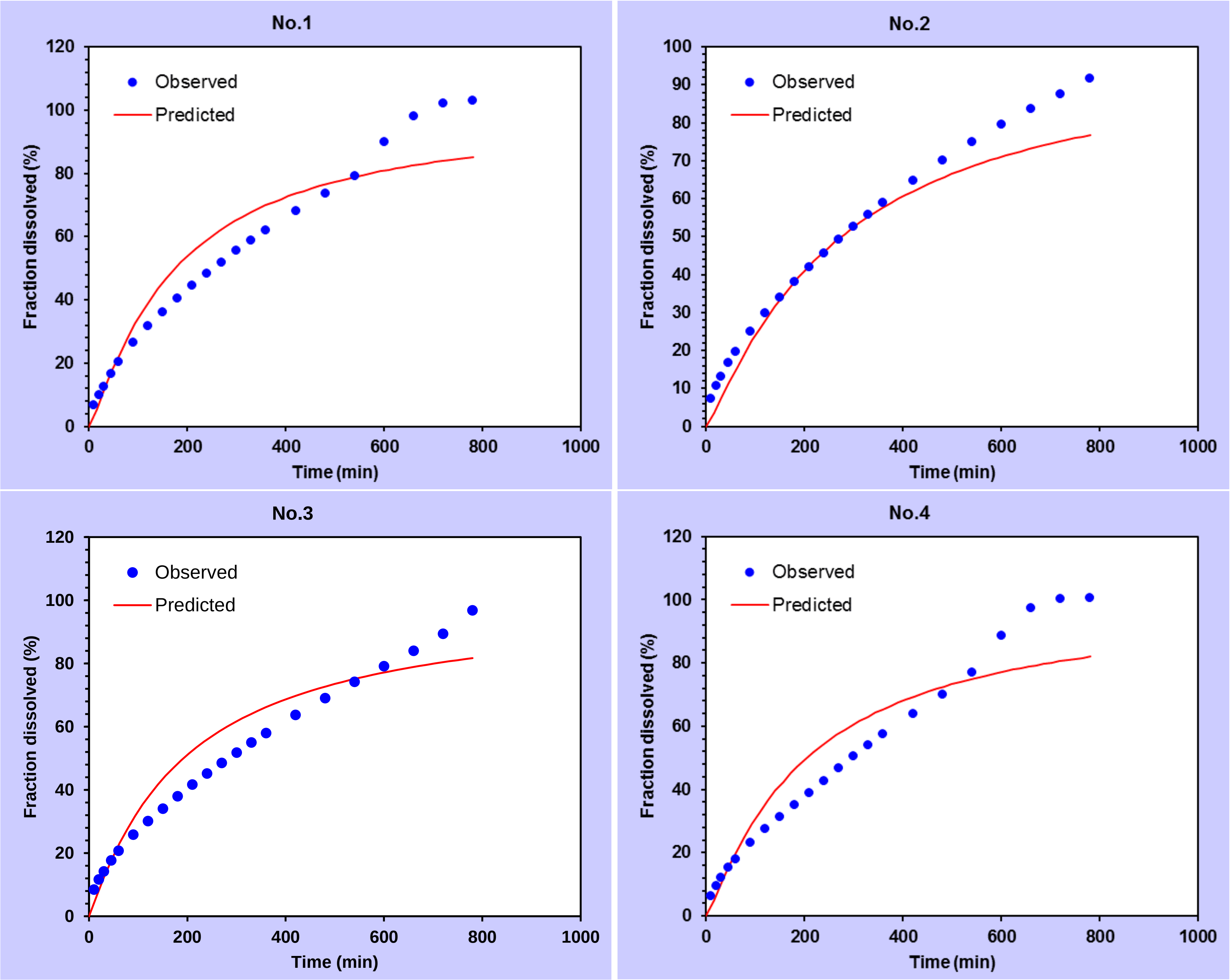

Supplementary materials – Model fitting summary – Lactochem® Fine Powder (modelling the entire relevant dissolution data)

Model: **Logistic_2**

Model equation: $F = F_{max} \cdot \frac{e^{\alpha+\beta\cdot\log(t)}}{1+e^{\alpha+\beta\cdot\log(t)}}$

Fitted model parameters per tested tablet (N = 4) with statistics – mean, standard deviation (SD), and relative standard deviation expressed in % (RSD%) (output from DDSolver):

Parameter	No.1	No.2	No.3	No.4	Mean	SD	RSD(%)
α	-6.222	-5.799	-6.330	-6.387	-6.184	0.266	-4.305
β	2.720	2.568	2.502	2.760	2.637	0.123	4.646
F_{max}	108.197	96.227	112.744	105.603	105.693	6.966	6.591

Number of dissolution data points (N), degrees of freedom (df), and selected goodness of fit criteria – Pearson correlation coefficient (R), coefficient of determination (R^2), adjusted coefficient of determination ($R^2_{adjusted}$), and residual sum of squares (RSS) (manual calculation in MS Excel):

Parameter	No.1	No.2	No.3	No.4
N	22	22	22	22
df	19	19	19	19
R	0.960020775	0.969376616	0.985727996	0.947672695
R^2	0.921639888	0.939691024	0.971659682	0.898083536
$R^2_{adjusted}$	0.913391455	0.93334271	0.96867649	0.887355488
RSS	1803.144967	1024.528607	739.901307	2338.709315

Graphical abstract of model fit presented as mean ± 1 SD of the fraction % of released carvedilol:

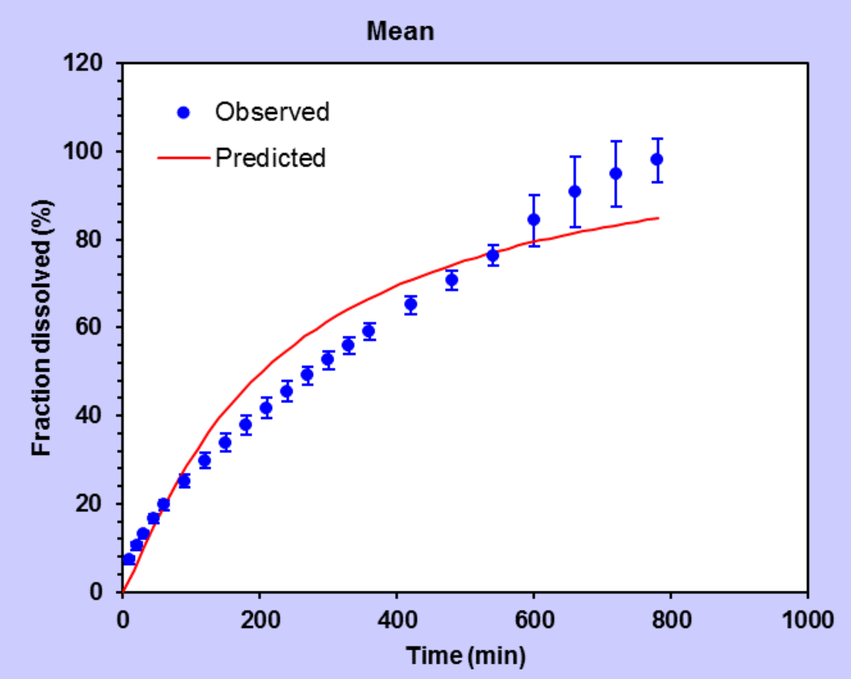

Supplementary materials – Model fitting summary – Lactochem® Fine Powder (modelling the entire relevant dissolution data)

Graphical abstract of model fit presented as the fraction % of released carvedilol per tested tablet:

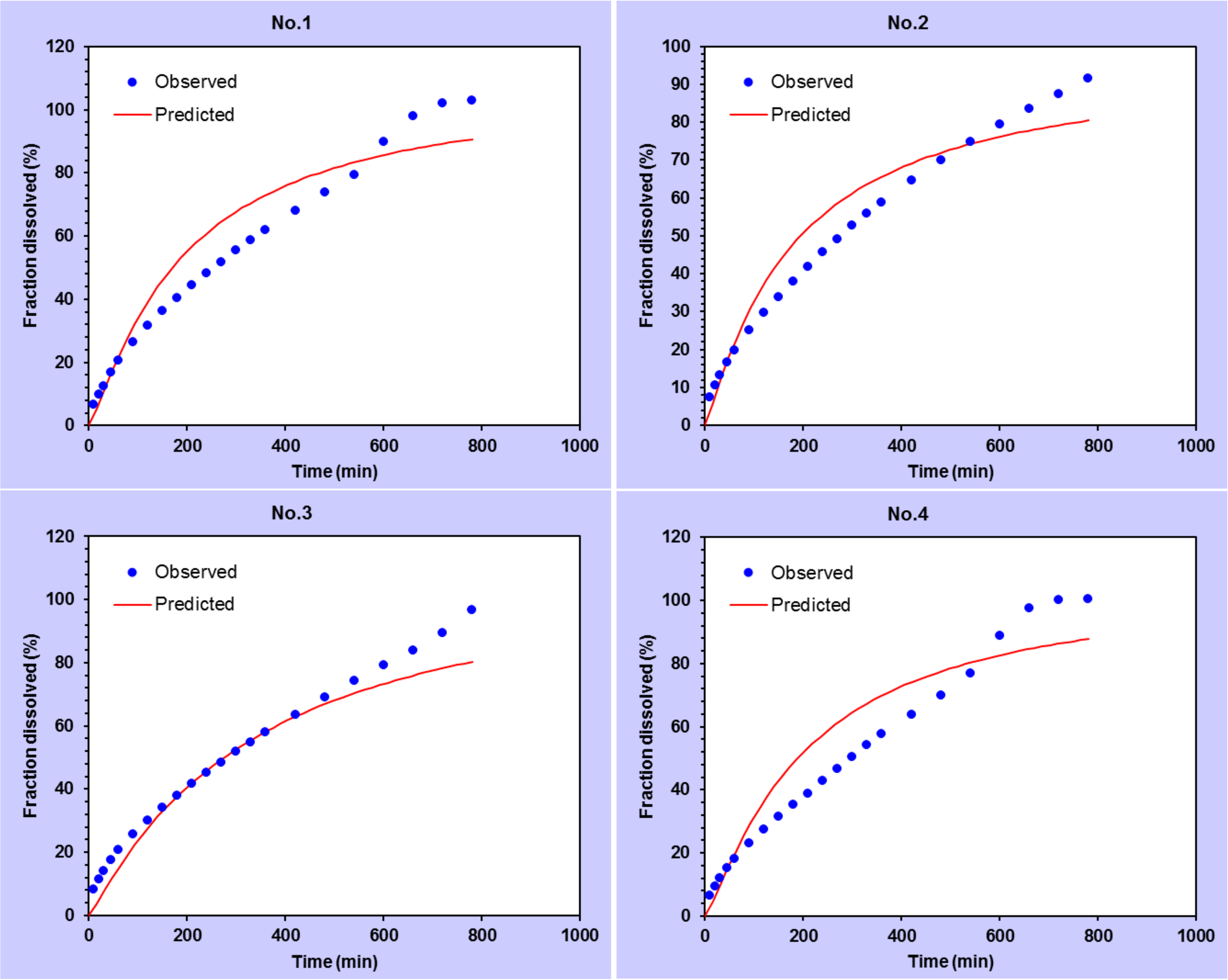

Supplementary materials – Model fitting summary – Lactochem® Fine Powder (modelling the entire relevant dissolution data)

Model: **Logistic_3**

Model equation: $F = F_{max} \cdot \frac{1}{1+e^{-k\cdot(t-\gamma)}}$

Fitted model parameters per tested tablet (N = 4) with statistics – mean, standard deviation (SD), and relative standard deviation expressed in % (RSD%) (output from DDSolver):

Parameter	No.1	No.2	No.3	No.4	Mean	SD	RSD(%)
k	0.006	0.006	0.006	0.007	0.006	0.000	7.715
γ	312.026	299.461	321.106	322.862	313.864	10.712	3.413
F_{max}	108.197	96.227	101.654	105.603	102.920	5.210	5.062

Number of dissolution data points (N), degrees of freedom (df), and selected goodness of fit criteria – Pearson correlation coefficient (R), coefficient of determination (R^2), adjusted coefficient of determination ($R^2_{adjusted}$), and residual sum of squares (RSS) (manual calculation in MS Excel):

Parameter	No.1	No.2	No.3	No.4
N	22	22	22	22
df	19	19	19	19
R	0.987060341	0.988616476	0.988323496	0.991060717
R^2	0.974288116	0.977362537	0.976783334	0.982201345
$R^2_{adjusted}$	0.971581602	0.974979646	0.974339474	0.980327803
RSS	561.8408634	364.0741217	377.239868	391.1422707

Graphical abstract of model fit presented as mean ± 1 SD of the fraction % of released carvedilol:

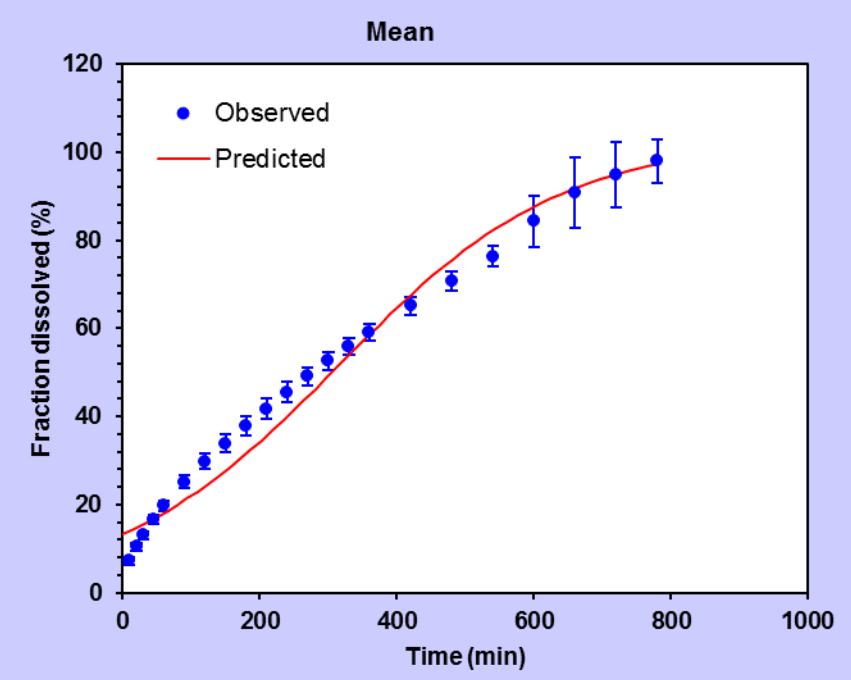

Supplementary materials – Model fitting summary – Lactochem® Fine Powder (modelling the entire relevant dissolution data)

Graphical abstract of model fit presented as the fraction % of released carvedilol per tested tablet:

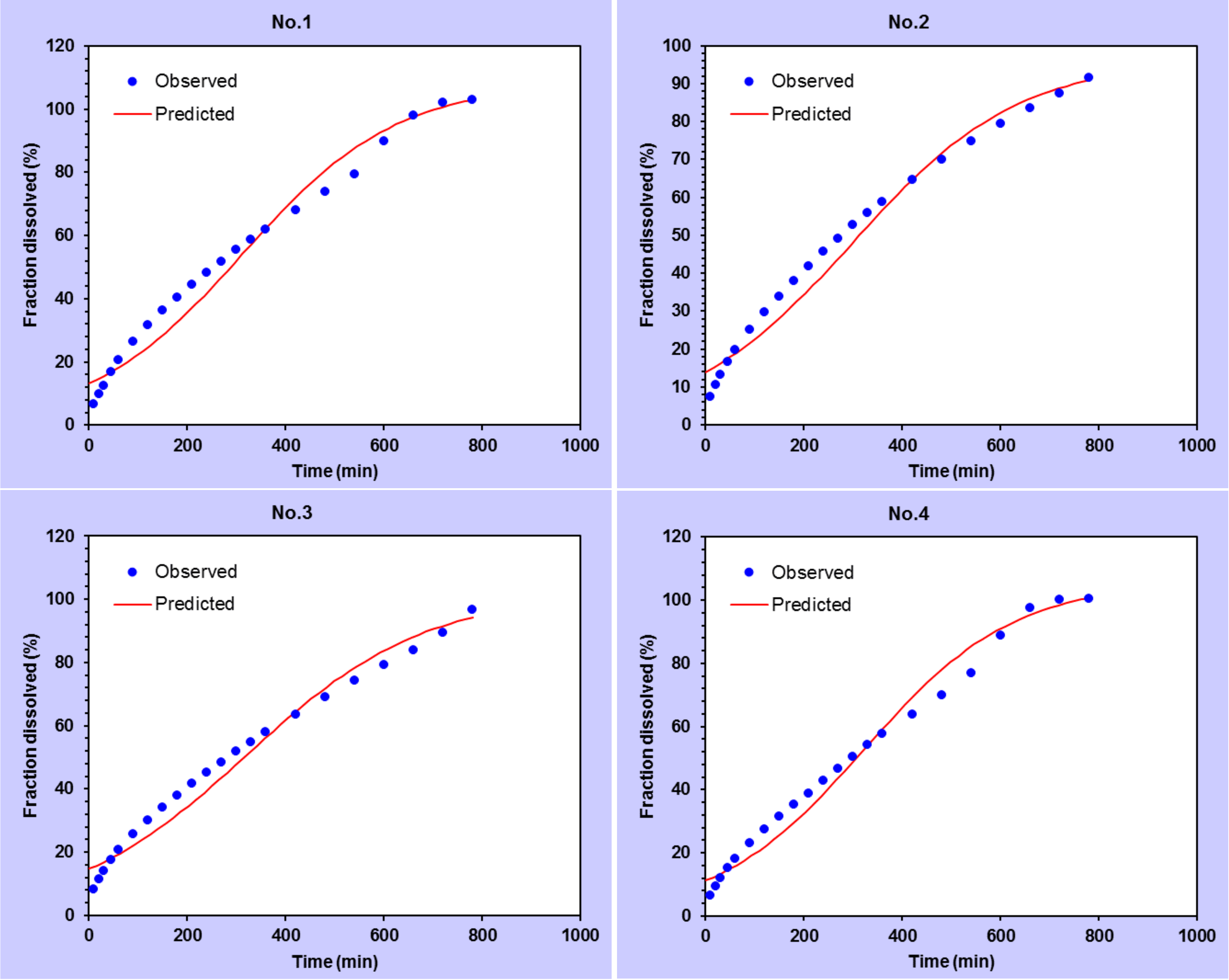

Supplementary materials – Model fitting summary – Lactochem® Fine Powder (modelling the entire relevant dissolution data)

Model: **Gompertz_1**

Model equation: $F = 100 \cdot e^{-\alpha \cdot e^{-\beta \cdot \log(t)}}$

Fitted model parameters per tested tablet (N = 4) with statistics – mean, standard deviation (SD), and relative standard deviation expressed in % (RSD%) (output from DDSolver):

Parameter	No.1	No.2	No.3	No.4	Mean	SD	RSD(%)
α	35.537	24.819	42.477	31.793	33.657	7.369	21.894
β	1.821	1.601	1.683	1.715	1.705	0.091	5.325

Number of dissolution data points (N), degrees of freedom (df), and selected goodness of fit criteria – Pearson correlation coefficient (R), coefficient of determination (R^2), adjusted coefficient of determination ($R^2_{adjusted}$), and residual sum of squares (RSS) (manual calculation in MS Excel):

Parameter	No.1	No.2	No.3	No.4
N	22	22	22	22
df	20	20	20	20
R	0.923588231	0.947861203	0.965359791	0.911387255
R^2	0.85301522	0.89844086	0.931919527	0.830626729
$R^2_{adjusted}$	0.845665981	0.893362903	0.928515503	0.822158066
RSS	3024.429044	1675.796065	1995.487102	3465.43175

Graphical abstract of model fit presented as mean ± 1 SD of the fraction % of released carvedilol:

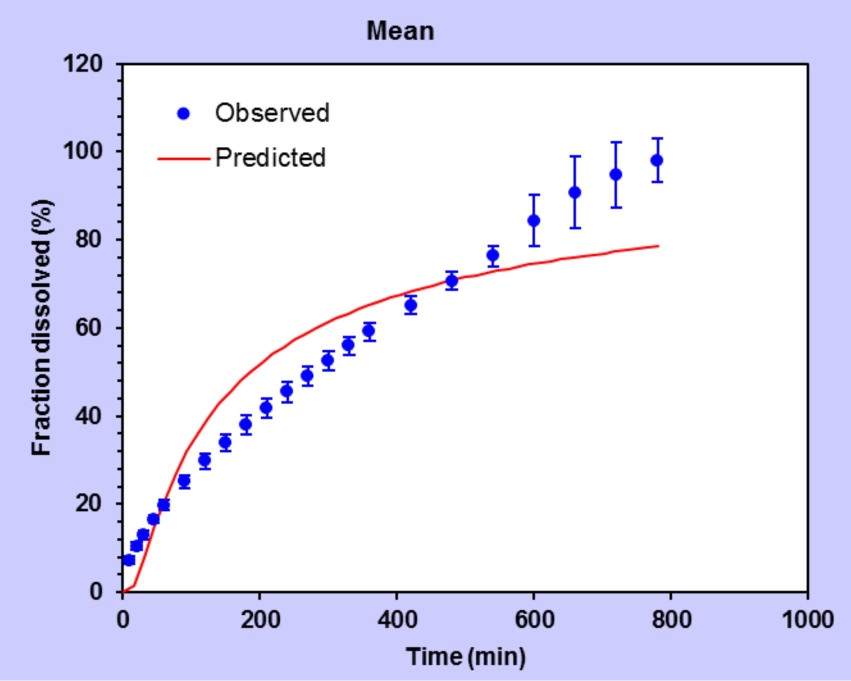

Supplementary materials – Model fitting summary – Lactochem® Fine Powder (modelling the entire relevant dissolution data)

Graphical abstract of model fit presented as the fraction % of released carvedilol per tested tablet:

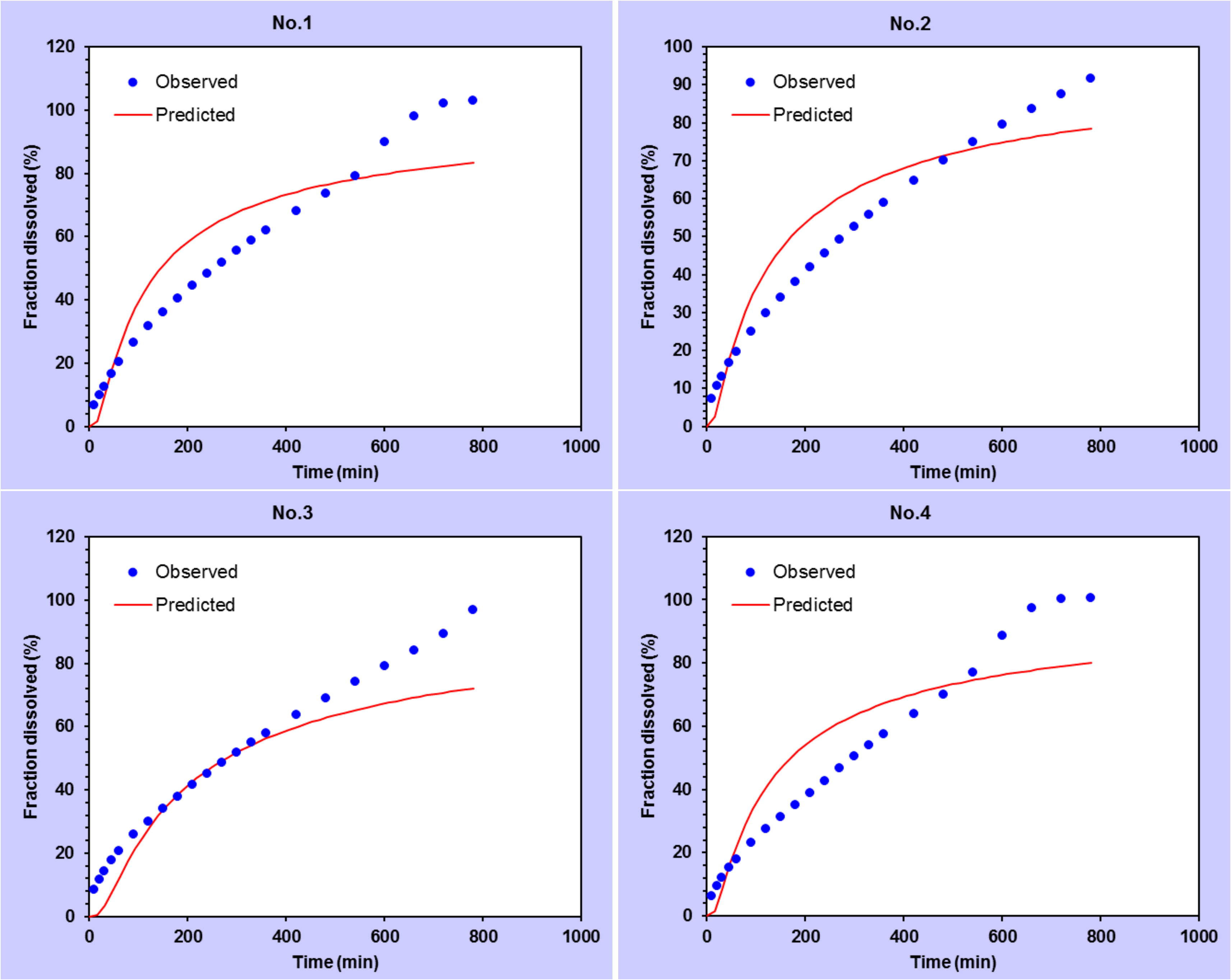

Supplementary materials – Model fitting summary – Lactochem® Fine Powder (modelling the entire relevant dissolution data)

Model: **Gompertz_2**

Model equation: $F = F_{max} \cdot e^{-\alpha \cdot e^{-\beta \cdot \log(t)}}$

Fitted model parameters per tested tablet (N = 4) with statistics – mean, standard deviation (SD), and relative standard deviation expressed in % (RSD%) (output from DDSolver):

Parameter	No.1	No.2	No.3	No.4	Mean	SD	RSD(%)
α	48.691	39.081	24.898	65.468	44.534	17.038	38.257
β	1.690	1.616	1.604	1.862	1.693	0.119	7.011
F_{max}	121.095	107.699	101.654	105.603	109.013	8.436	7.738

Number of dissolution data points (N), degrees of freedom (df), and selected goodness of fit criteria – Pearson correlation coefficient (R), coefficient of determination (R^2), adjusted coefficient of determination ($R^2_{adjusted}$), and residual sum of squares (RSS) (manual calculation in MS Excel):

Parameter	No.1	No.2	No.3	No.4
N	22	22	22	22
df	19	19	19	19
R	0.970266827	0.978609999	0.935500517	0.950495943
R^2	0.941417715	0.95767753	0.875161217	0.903442537
$R^2_{adjusted}$	0.935251159	0.953222533	0.862020292	0.893278594
RSS	1789.642308	1273.277039	2136.905708	2439.120355

Graphical abstract of model fit presented as mean ± 1 SD of the fraction % of released carvedilol:

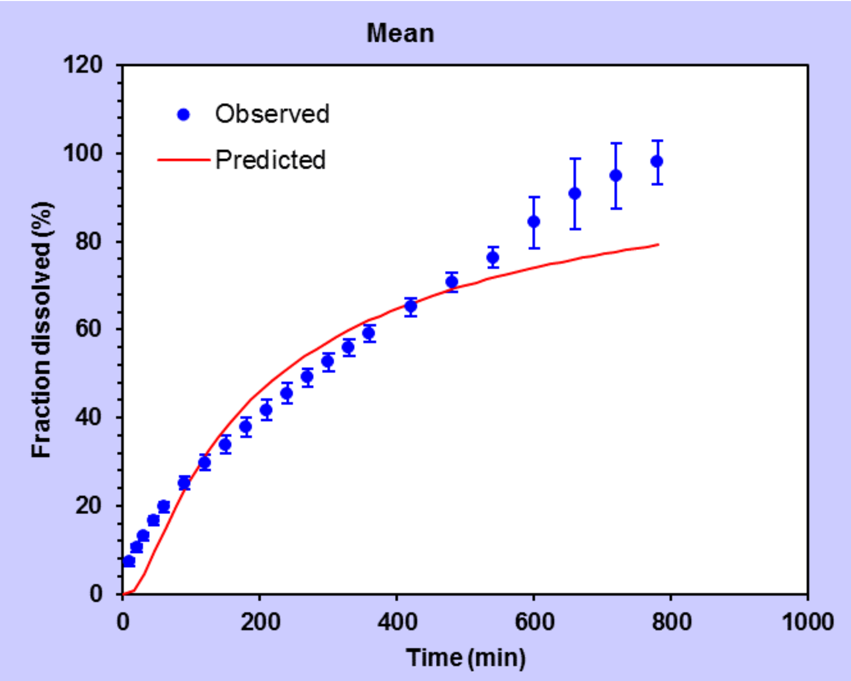

Supplementary materials – Model fitting summary – Lactochem® Fine Powder (modelling the entire relevant dissolution data)

Graphical abstract of model fit presented as the fraction % of released carvedilol per tested tablet:

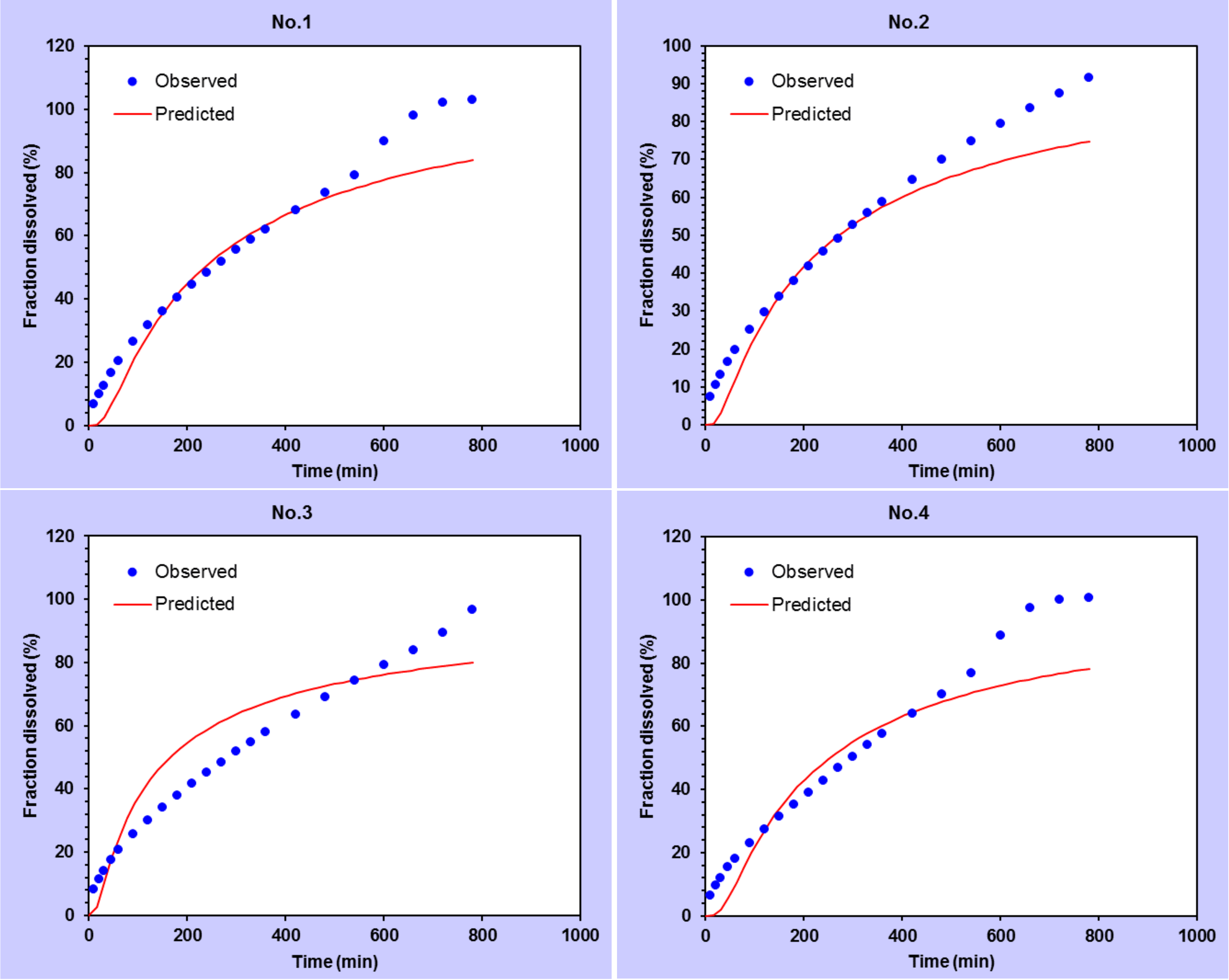

Supplementary materials – Model fitting summary – Lactochem® Fine Powder (modelling the entire relevant dissolution data)

Model: **Gompertz_3**

Model equation: $F = F_{max} \cdot e^{-e^{-k\cdot(t-\gamma)}}$

Fitted model parameters per tested tablet (N = 4) with statistics – mean, standard deviation (SD), and relative standard deviation expressed in % (RSD%) (output from DDSolver):

Parameter	No.1	No.2	No.3	No.4	Mean	SD	RSD(%)
k	0.005	0.004	0.004	0.005	0.004	0.000	7.995
γ	198.748	183.044	198.128	211.018	197.735	11.452	5.792
F_{max}	108.197	96.227	101.654	105.603	102.920	5.210	5.062

Number of dissolution data points (N), degrees of freedom (df), and selected goodness of fit criteria – Pearson correlation coefficient (R), coefficient of determination (R^2), adjusted coefficient of determination ($R^2_{adjusted}$), and residual sum of squares (RSS) (manual calculation in MS Excel):

Parameter	No.1	No.2	No.3	No.4
N	22	22	22	22
df	19	19	19	19
R	0.990428883	0.996037465	0.994135318	0.989108461
R^2	0.980949372	0.992090632	0.988305031	0.978335548
$R^2_{adjusted}$	0.978944043	0.991258067	0.987073982	0.976055079
RSS	465.447793	130.7235061	210.0525071	557.9250617

Graphical abstract of model fit presented as mean ± 1 SD of the fraction % of released carvedilol:

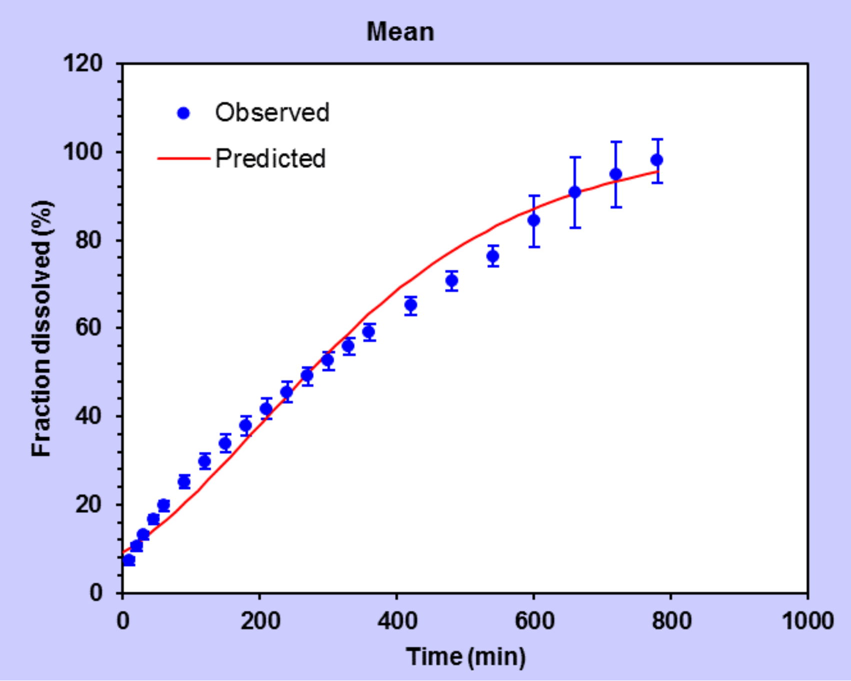

Supplementary materials – Model fitting summary – Lactochem® Fine Powder (modelling the entire relevant dissolution data)

Graphical abstract of model fit presented as the fraction % of released carvedilol per tested tablet:

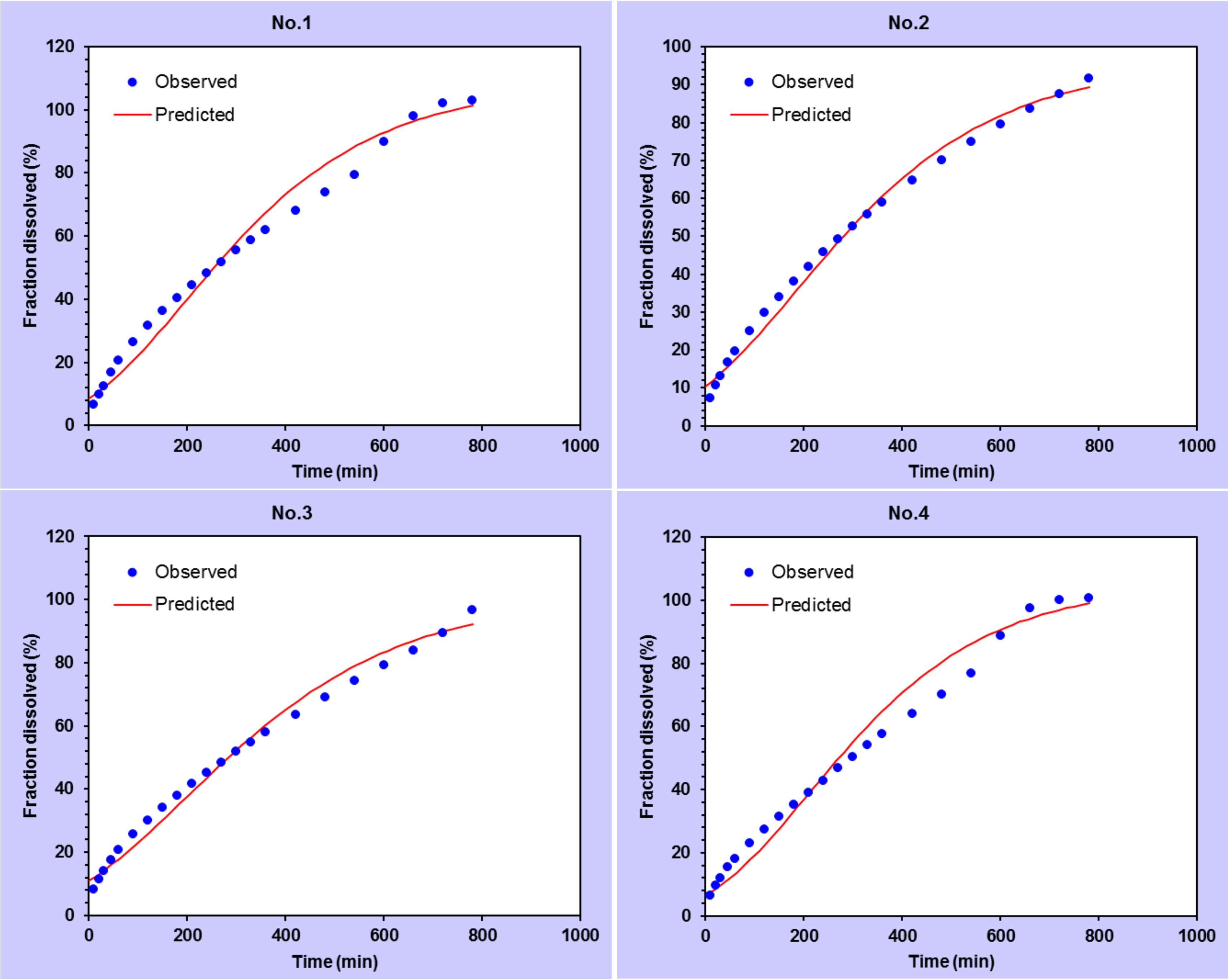

Supplementary materials – Model fitting summary – Lactochem® Fine Powder (modelling the entire relevant dissolution data)

Model: **Gompertz_4**

Model equation: $F = F_{max} \cdot e^{-\beta \cdot e^{-k \cdot t}}$

Fitted model parameters per tested tablet (N = 4) with statistics – mean, standard deviation (SD), and relative standard deviation expressed in % (RSD%) (output from DDSolver):

Parameter	No.1	No.2	No.3	No.4	Mean	SD	RSD(%)
k	0.005	0.004	0.004	0.005	0.004	0.000	7.995
β	2.528	2.222	2.219	2.775	2.436	0.268	11.010
F_{max}	108.197	96.227	101.654	105.603	102.920	5.210	5.062

Number of dissolution data points (N), degrees of freedom (df), and selected goodness of fit criteria – Pearson correlation coefficient (R), coefficient of determination (R^2), adjusted coefficient of determination ($R^2_{adjusted}$), and residual sum of squares (RSS) (manual calculation in MS Excel):

Parameter	No.1	No.2	No.3	No.4
N	22	22	22	22
df	19	19	19	19
R	0.990428883	0.996037465	0.994135318	0.989108461
R^2	0.980949372	0.992090632	0.988305031	0.978335548
$R^2_{adjusted}$	0.978944043	0.991258067	0.987073982	0.976055079
RSS	465.447793	130.7235061	210.0525071	557.9250617

Graphical abstract of model fit presented as mean ± 1 SD of the fraction % of released carvedilol:

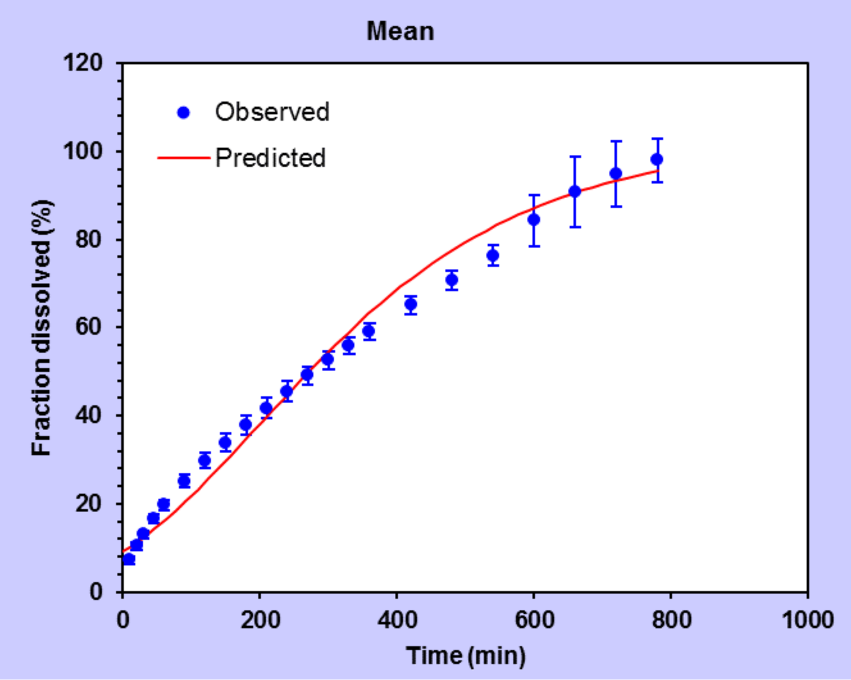

Supplementary materials – Model fitting summary – Lactochem® Fine Powder (modelling the entire relevant dissolution data)

Graphical abstract of model fit presented as the fraction % of released carvedilol per tested tablet:

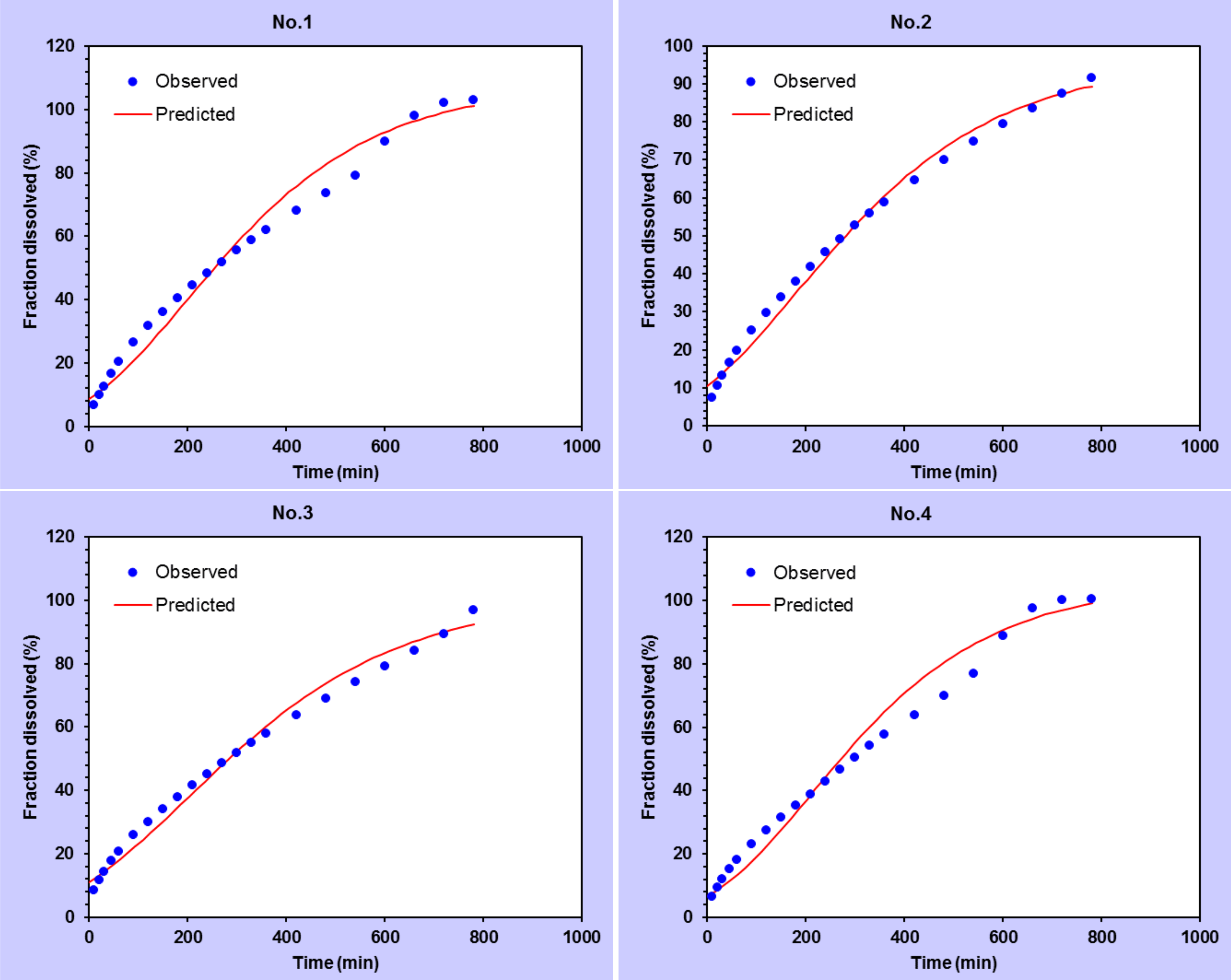

Supplementary materials – Model fitting summary – Lactochem® Fine Powder (modelling the entire relevant dissolution data)

Model: **Probit_1**

Model equation: $F = 100 \cdot \phi[\alpha + \beta \cdot \log(t)]$

Fitted model parameters per tested tablet (N = 4) with statistics – mean, standard deviation (SD), and relative standard deviation expressed in % (RSD%) (output from DDSolver):

Parameter	No.1	No.2	No.3	No.4	Mean	SD	RSD(%)
α	-3.515	-3.299	-3.300	-3.498	-3.403	0.120	-3.516
β	1.561	1.428	1.442	1.508	1.485	0.062	4.148

Number of dissolution data points (N), degrees of freedom (df), and selected goodness of fit criteria – Pearson correlation coefficient (R), coefficient of determination (R^2), adjusted coefficient of determination ($R^2_{adjusted}$), and residual sum of squares (RSS) (manual calculation in MS Excel):

Parameter	No.1	No.2	No.3	No.4
N	22	22	22	22
df	20	20	20	20
R	0.954356057	0.972120486	0.960298081	0.944563895
R^2	0.910795484	0.945018238	0.922172405	0.892200951
$R^2_{adjusted}$	0.906335259	0.94226915	0.918281025	0.886810999
RSS	34.91674035	38.41859667	52.71406293	37.59143067

Graphical abstract of model fit presented as mean ± 1 SD of the fraction % of released carvedilol:

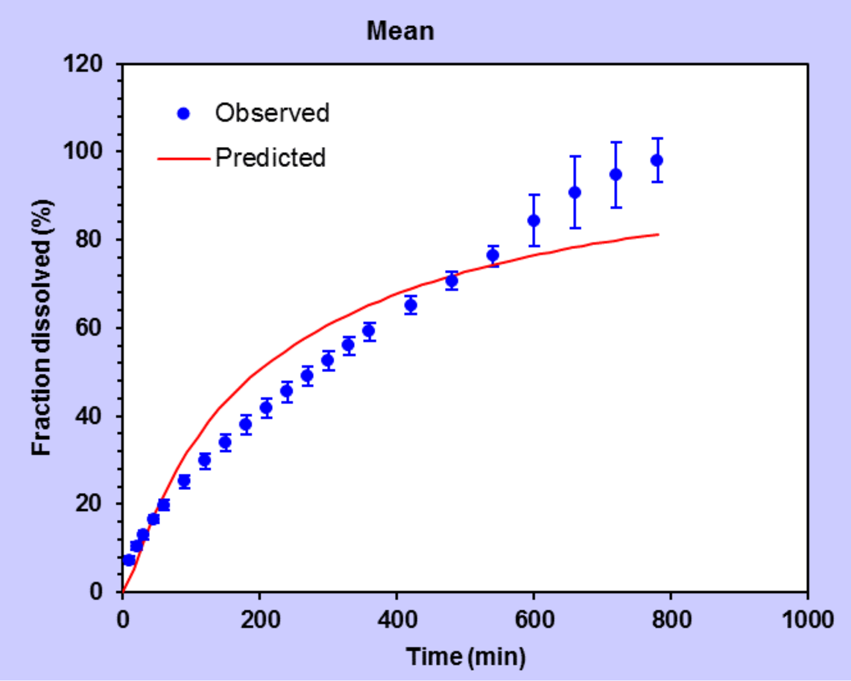

Supplementary materials – Model fitting summary – Lactochem® Fine Powder (modelling the entire relevant dissolution data)

Graphical abstract of model fit presented as the fraction % of released carvedilol per tested tablet:

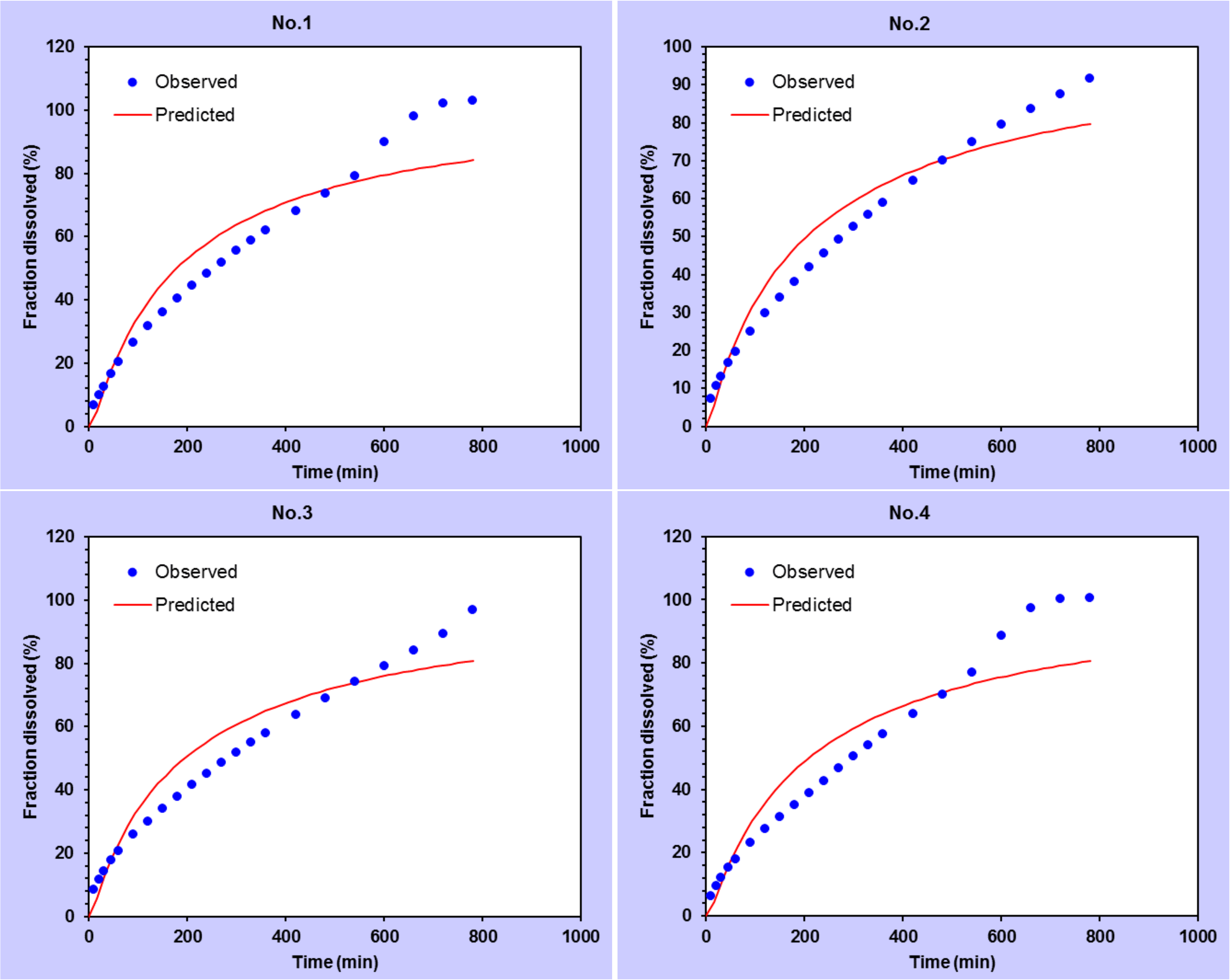

Supplementary materials – Model fitting summary – Lactochem® Fine Powder (modelling the entire relevant dissolution data)

Model: **Probit_2**

Model equation: $F = F_{max} \cdot \phi[\alpha + \beta \cdot \log(t)]$

Fitted model parameters per tested tablet (N = 4) with statistics – mean, standard deviation (SD), and relative standard deviation expressed in % (RSD%) (output from DDSolver):

Parameter	No.1	No.2	No.3	No.4	Mean	SD	RSD(%)
α	-3.621	-3.929	-3.438	-3.708	-3.674	0.204	-5.552
β	1.580	1.586	1.336	1.597	1.525	0.126	8.265
F_{max}	108.197	106.724	120.097	105.603	110.155	6.712	6.093

Number of dissolution data points (N), degrees of freedom (df), and selected goodness of fit criteria – Pearson correlation coefficient (R), coefficient of determination (R^2), adjusted coefficient of determination ($R^2_{adjusted}$), and residual sum of squares (RSS) (manual calculation in MS Excel):

Parameter	No.1	No.2	No.3	No.4
N	22	22	22	22
df	19	19	19	19
R	0.959493036	0.988393923	0.981203434	0.9464893
R^2	0.920626886	0.976922546	0.962760179	0.895841995
$R^2_{adjusted}$	0.912271822	0.974493341	0.958840198	0.884877995
RSS	1740.597386	629.8962432	732.529317	2278.253874

Graphical abstract of model fit presented as mean ± 1 SD of the fraction % of released carvedilol:

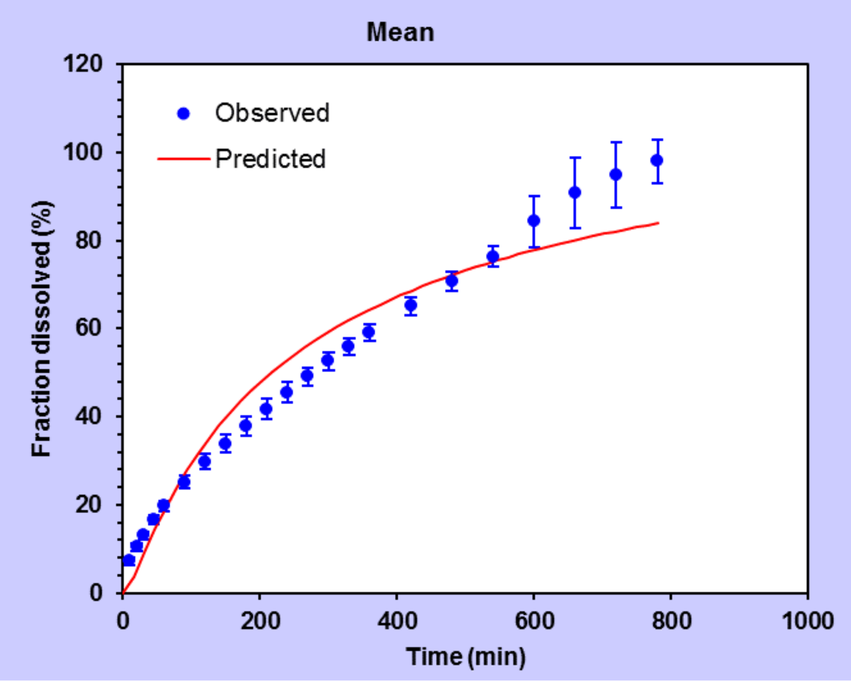

Supplementary materials – Model fitting summary – Lactochem® Fine Powder (modelling the entire relevant dissolution data)

Graphical abstract of model fit presented as the fraction % of released carvedilol per tested tablet:

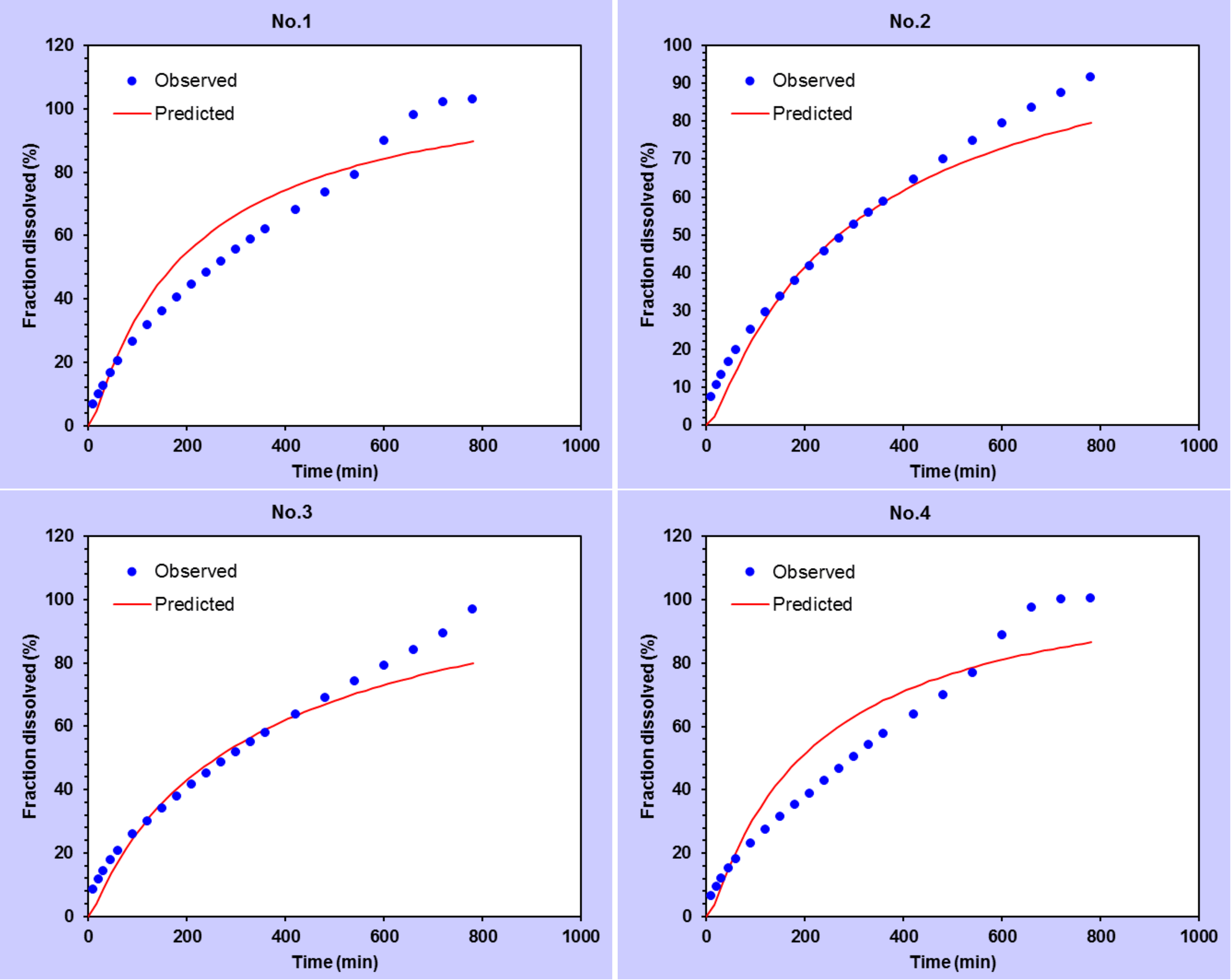

Supplementary materials – Model fitting summary – Lactochem® Fine Powder (modelling up to app. 60 % of carvedilol released)

Model: **Zero–order**

Model equation: $F = k_0 \cdot t$

Fitted model parameters per tested tablet (N = 4) with statistics – mean, standard deviation (SD), and relative standard deviation expressed in % (RSD%) (output from DDSolver):

Parameter	No.1	No.2	No.3	No.4	Mean	SD	RSD(%)
k_0	0.195	0.185	0.184	0.176	0.185	0.008	4.227

Number of dissolution data points (N), degrees of freedom (df), and selected goodness of fit criteria – Pearson correlation coefficient (R), coefficient of determination (R^2), adjusted coefficient of determination ($R^2_{adjusted}$), and residual sum of squares (RSS) (manual calculation in MS Excel):

Parameter	No.1	No.2	No.3	No.4
N	15	15	15	15
df	14	14	14	14
R	0.989866486	0.992736581	0.992174769	0.995659898
R^2	0.97983566	0.985525919	0.984410772	0.991338633
$R^2_{adjusted}$	0.97983566	0.985525919	0.984410772	0.991338633
RSS	595.4374532	575.6504007	701.6598579	402.2815947

Graphical abstract of model fit presented as mean ± 1 SD of the fraction % of released carvedilol:

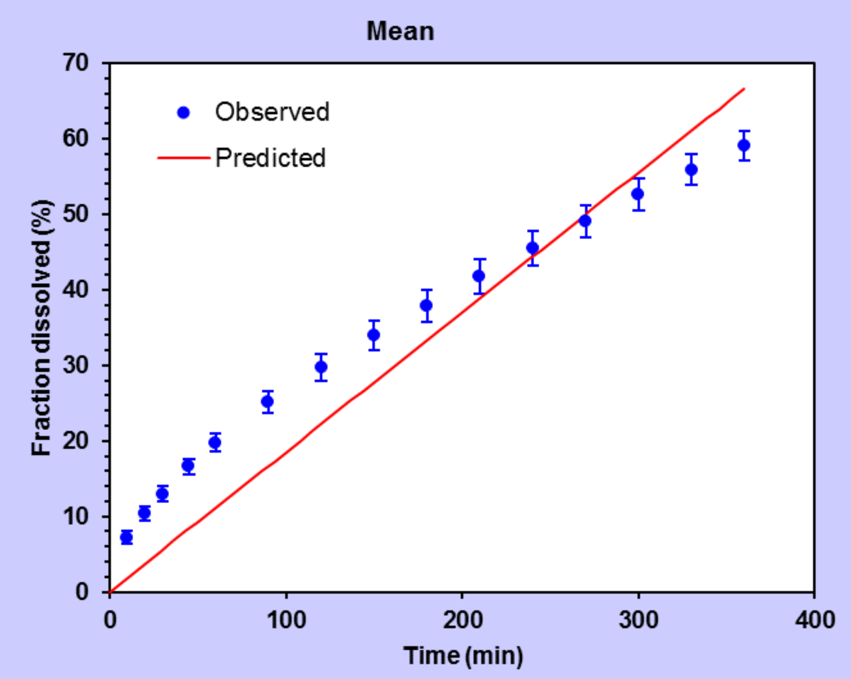

Supplementary materials – Model fitting summary – Lactochem® Fine Powder (modelling up to app. 60 % of carvedilol released)

Graphical abstract of model fit presented as the fraction % of released carvedilol per tested tablet:

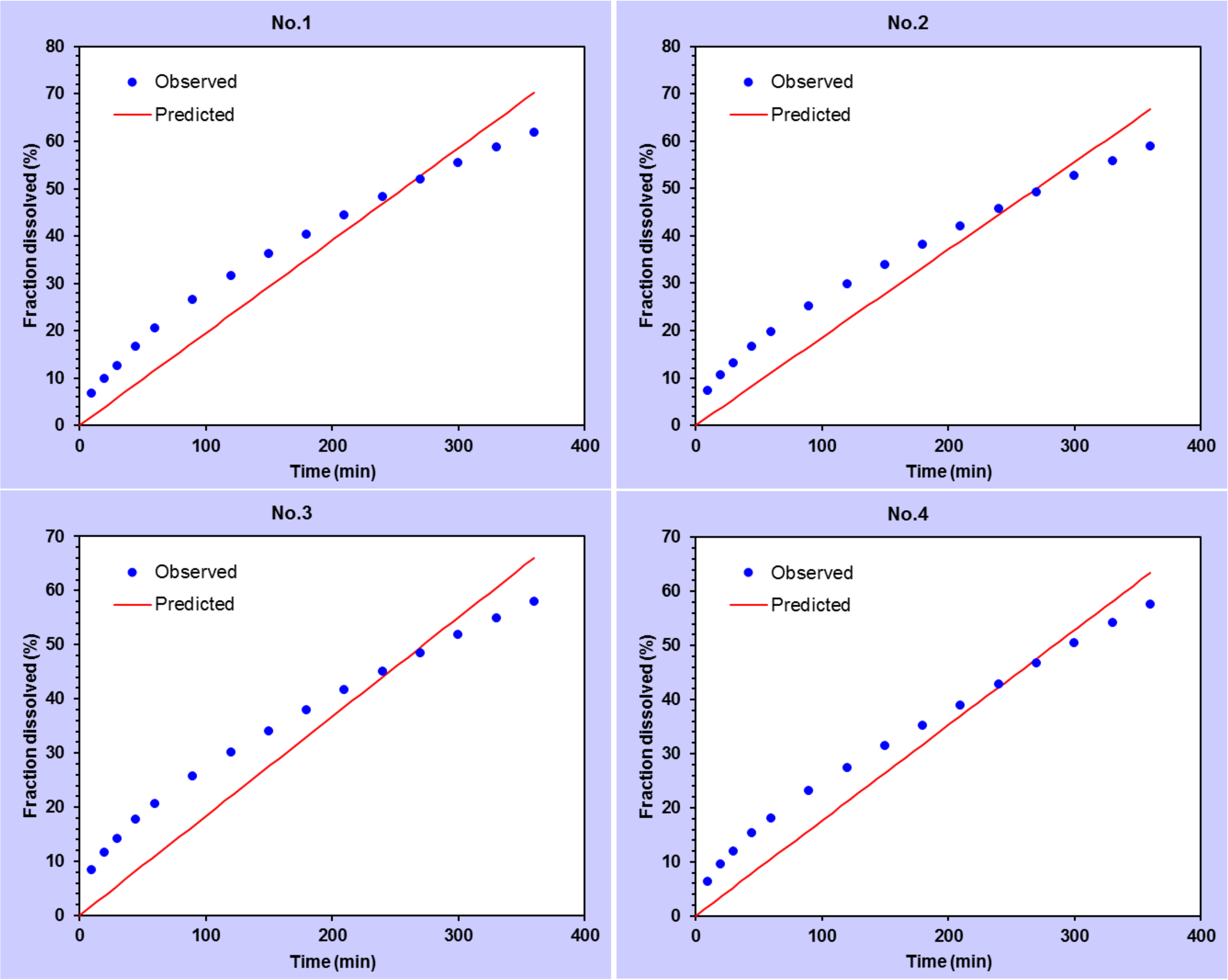

Supplementary materials – Model fitting summary – Lactochem® Fine Powder (modelling up to app. 60 % of carvedilol released)

Model: **Zero–order with T_{lag}**

Model equation: $F = k_0 \cdot (t - T_{lag})$

Fitted model parameters per tested tablet (N = 4) with statistics – mean, standard deviation (SD), and relative standard deviation expressed in % (RSD%) (output from DDSolver):

Parameter	No.1	No.2	No.3	No.4	Mean	SD	RSD(%)
k_0	0.155	0.144	0.137	0.141	0.144	0.007	5.114
T_{lag}	-64.090	-70.013	-81.953	-60.250	-69.077	9.477	-13.720

Number of dissolution data points (N), degrees of freedom (df), and selected goodness of fit criteria – Pearson correlation coefficient (R), coefficient of determination (R^2), adjusted coefficient of determination ($R^2_{adjusted}$), and residual sum of squares (RSS) (manual calculation in MS Excel):

Parameter	No.1	No.2	No.3	No.4
N	15	15	15	15
df	13	13	13	13
R	0.989866486	0.992736581	0.992174769	0.995659898
R^2	0.97983566	0.985525919	0.984410772	0.991338633
$R^2_{adjusted}$	0.978284557	0.984412529	0.9832116	0.990672374
RSS	97.67854181	60.45974053	59.32211893	34.65650559

Graphical abstract of model fit presented as mean ± 1 SD of the fraction % of released carvedilol:

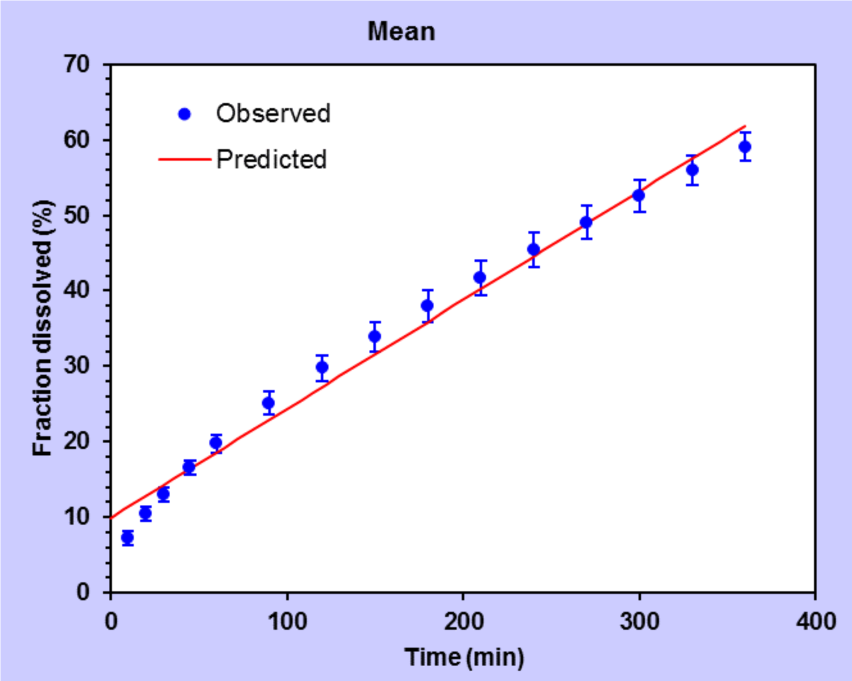

Supplementary materials – Model fitting summary – Lactochem® Fine Powder (modelling up to app. 60 % of carvedilol released)

Graphical abstract of model fit presented as the fraction % of released carvedilol per tested tablet:

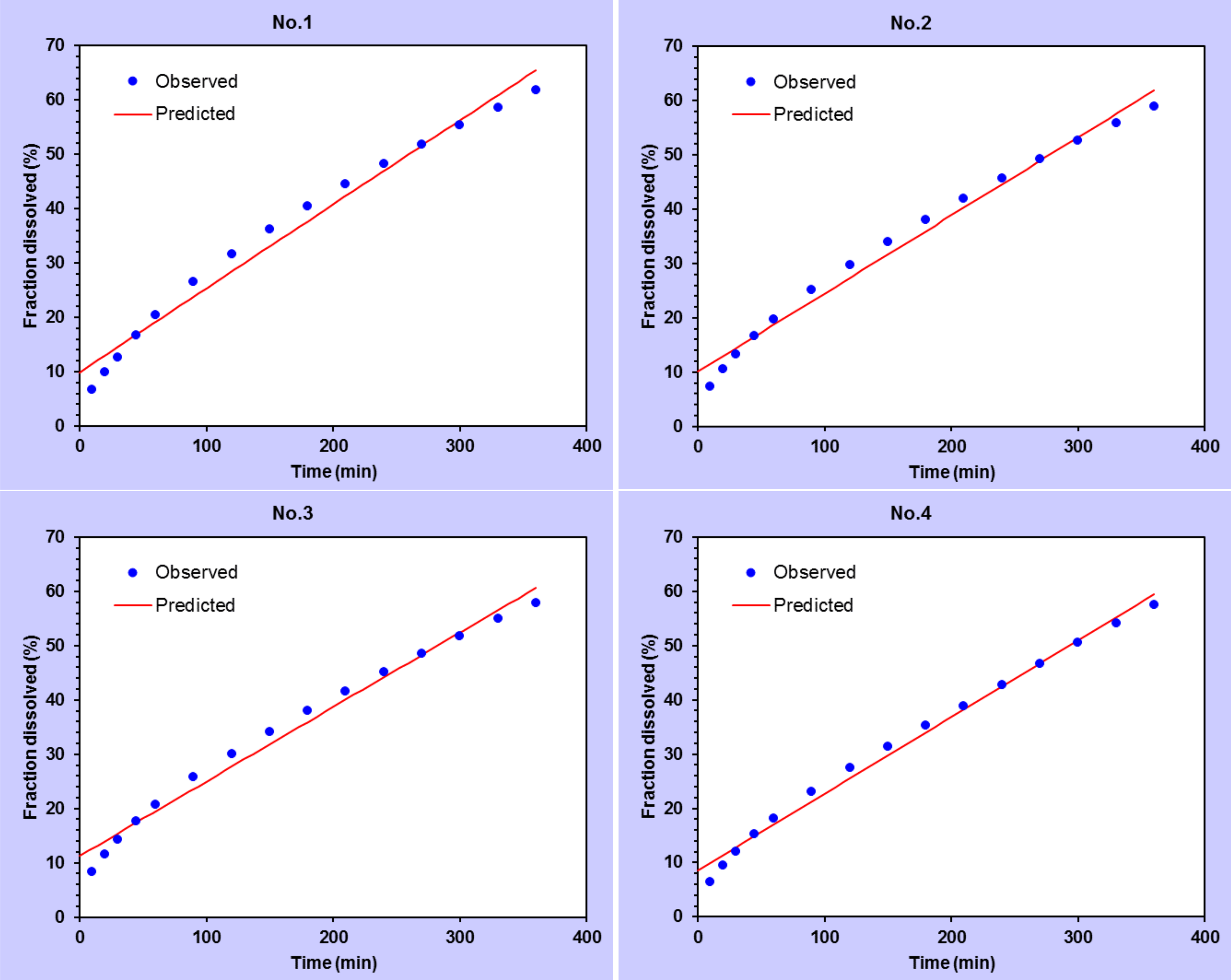

Supplementary materials – Model fitting summary – Lactochem® Fine Powder (modelling up to app. 60 % of carvedilol released)

Model: **Zero–order with F_0**

Model equation: $F = F_0 + k_0 \cdot t$

Fitted model parameters per tested tablet (N = 4) with statistics – mean, standard deviation (SD), and relative standard deviation expressed in % (RSD%) (output from DDSolver):

Parameter	No.1	No.2	No.3	No.4	Mean	SD	RSD(%)
k_0	0.155	0.144	0.137	0.141	0.144	0.007	5.114
F_0	9.905	10.077	11.252	8.513	9.937	1.122	11.296

Number of dissolution data points (N), degrees of freedom (df), and selected goodness of fit criteria – Pearson correlation coefficient (R), coefficient of determination (R^2), adjusted coefficient of determination ($R^2_{adjusted}$), and residual sum of squares (RSS) (manual calculation in MS Excel):

Parameter	No.1	No.2	No.3	No.4
N	15	15	15	15
df	13	13	13	13
R	0.989866486	0.992736581	0.992174769	0.995659898
R^2	0.97983566	0.985525919	0.984410772	0.991338633
$R^2_{adjusted}$	0.978284557	0.984412529	0.9832116	0.990672374
RSS	97.67854181	60.45974053	59.32211893	34.65650559

Graphical abstract of model fit presented as mean ± 1 SD of the fraction % of released carvedilol:

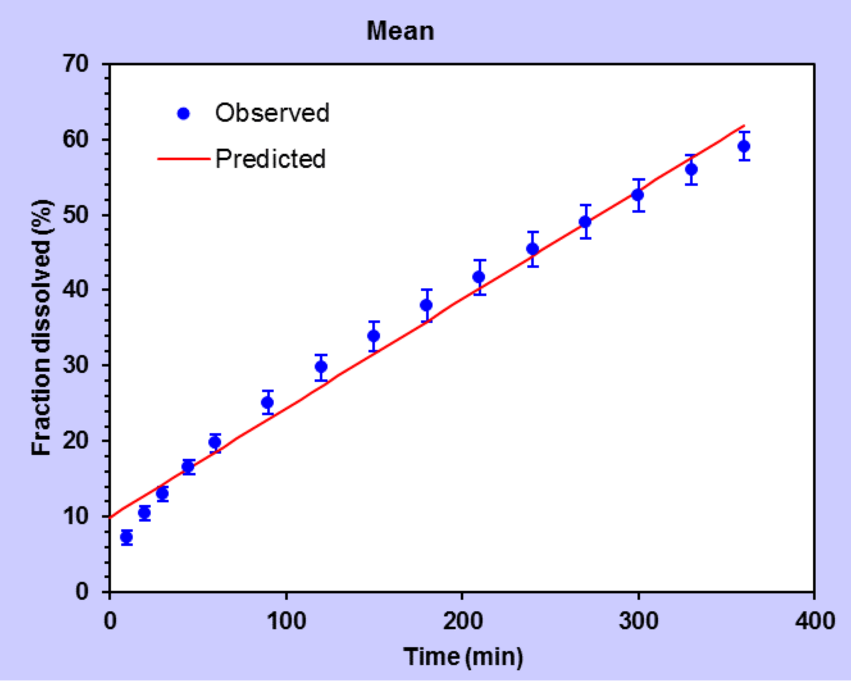

Supplementary materials – Model fitting summary – Lactochem® Fine Powder (modelling up to app. 60 % of carvedilol released)

Graphical abstract of model fit presented as the fraction % of released carvedilol per tested tablet:

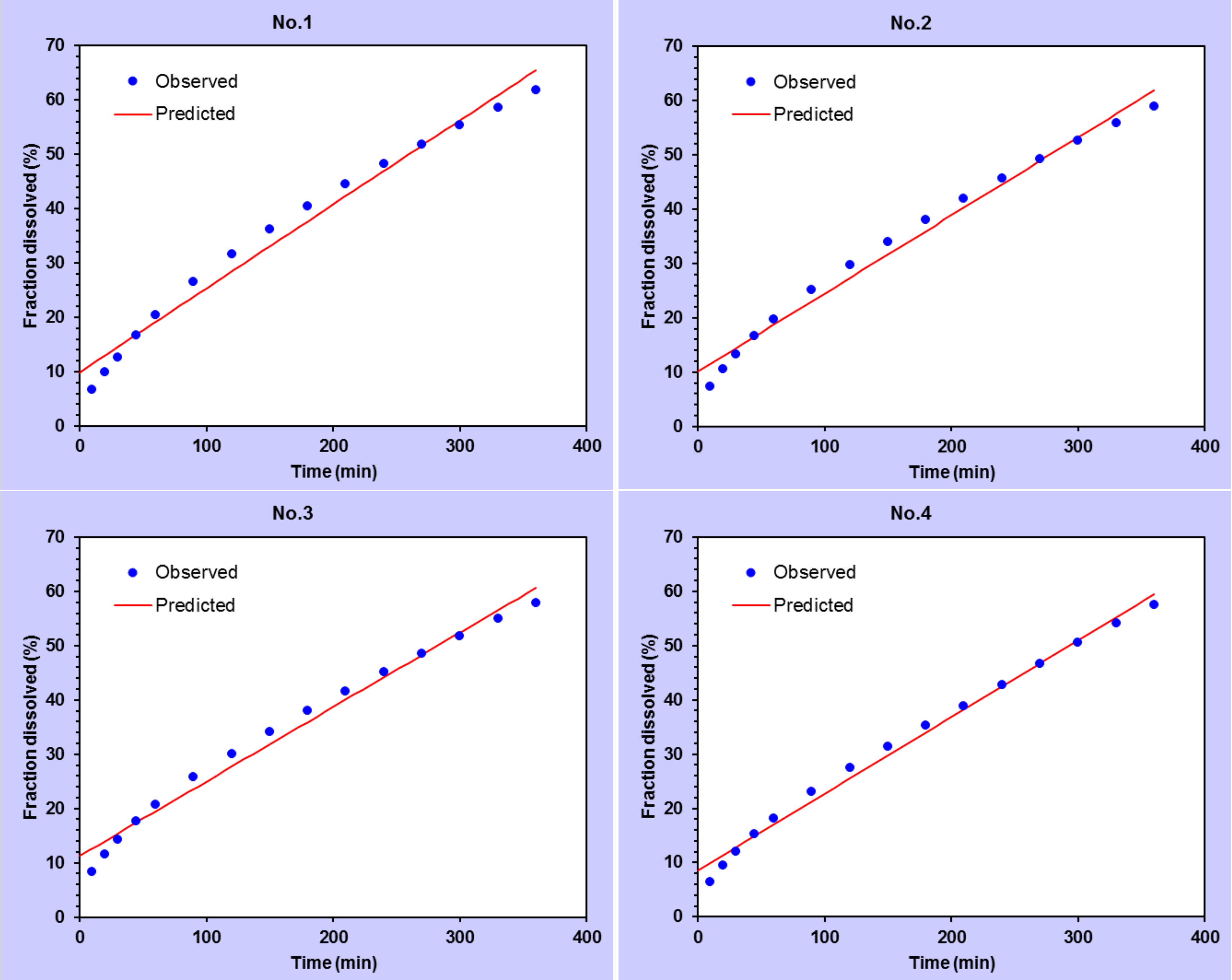

Supplementary materials – Model fitting summary – Lactochem® Fine Powder (modelling up to app. 60 % of carvedilol released)

Model: **First–order**

Model equation: $F = 100 \cdot (1 - e^{-k_1 \cdot t})$

Fitted model parameters per tested tablet (N = 4) with statistics – mean, standard deviation (SD), and relative standard deviation expressed in % (RSD%) (output from DDSolver):

Parameter	No.1	No.2	No.3	No.4	Mean	SD	RSD(%)
k_1	0.003	0.003	0.003	0.002	0.003	0.000	6.004

Number of dissolution data points (N), degrees of freedom (df), and selected goodness of fit criteria – Pearson correlation coefficient (R), coefficient of determination (R^2), adjusted coefficient of determination ($R^2_{adjusted}$), and residual sum of squares (RSS) (manual calculation in MS Excel):

Parameter	No.1	No.2	No.3	No.4
N	15	15	15	15
df	14	14	14	14
R	0.999224224	0.999410494	0.999089976	0.998724085
R^2	0.998449049	0.998821336	0.998180779	0.997449798
$R^2_{adjusted}$	0.998449049	0.998821336	0.998180779	0.997449798
RSS	152.9072656	193.9242531	283.9607381	137.4545954

Graphical abstract of model fit presented as mean ± 1 SD of the fraction % of released carvedilol:

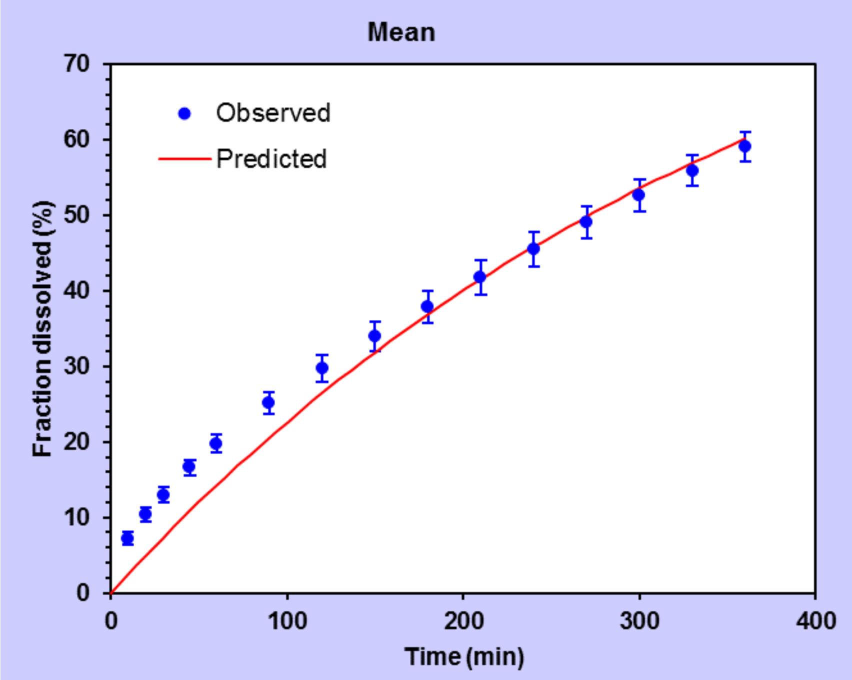

Supplementary materials – Model fitting summary – Lactochem® Fine Powder (modelling up to app. 60 % of carvedilol released)

Graphical abstract of model fit presented as the fraction % of released carvedilol per tested tablet:

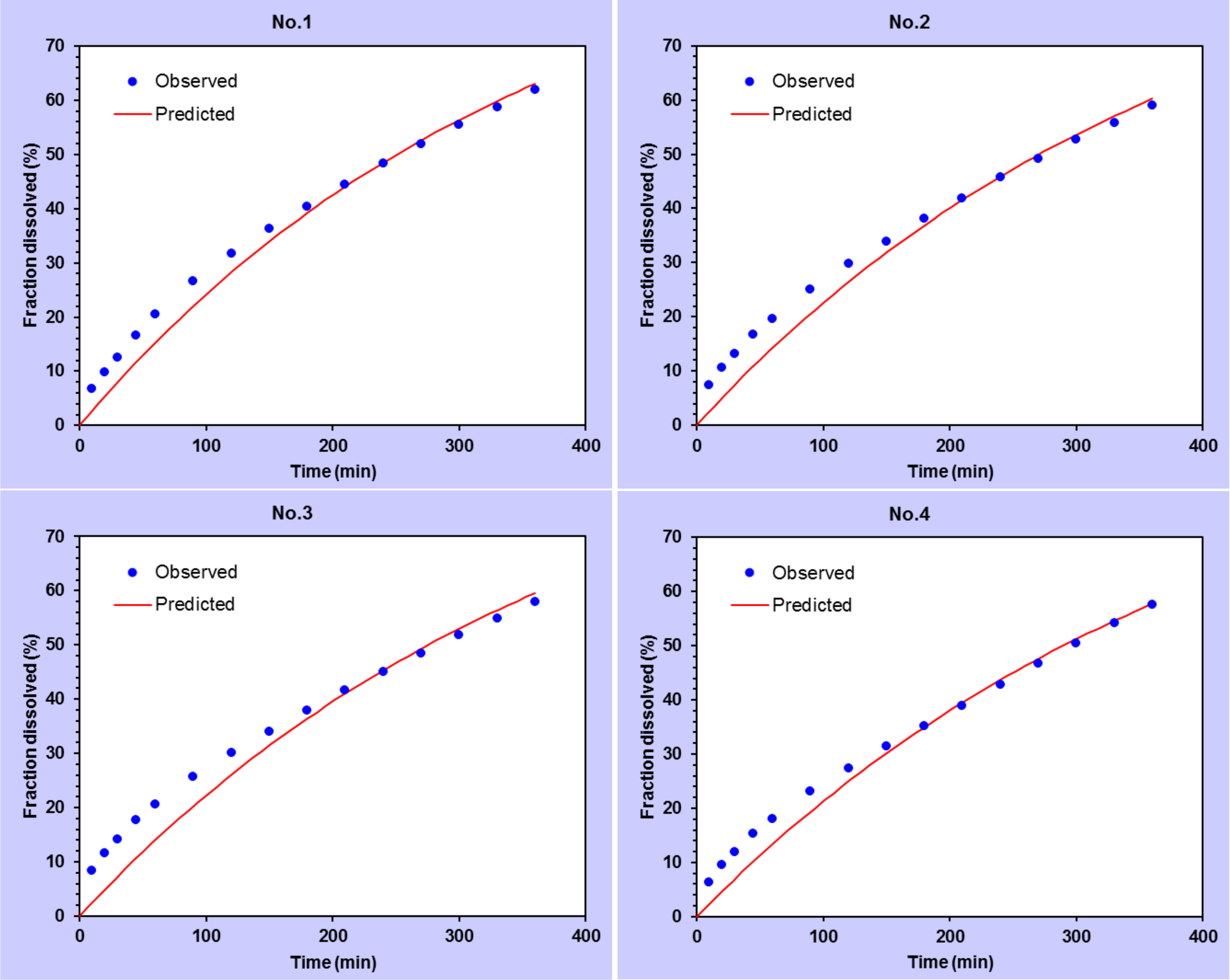

Supplementary materials – Model fitting summary – Lactochem® Fine Powder (modelling up to app. 60 % of carvedilol released)

Model: **First–order with T_{lag}**

Model equation: $F = 100 \cdot \left[1 - e^{-k_1 \cdot (t - T_{lag})}\right]$

Fitted model parameters per tested tablet (N = 4) with statistics – mean, standard deviation (SD), and relative standard deviation expressed in % (RSD%) (output from DDSolver):

Parameter	No.1	No.2	No.3	No.4	Mean	SD	RSD(%)
k_1	0.002	0.002	0.002	0.002	0.002	0.000	7.011
T_{lag}	-26.836	-32.736	-41.940	-26.548	-32.015	7.205	-22.506

Number of dissolution data points (N), degrees of freedom (df), and selected goodness of fit criteria – Pearson correlation coefficient (R), coefficient of determination (R^2), adjusted coefficient of determination ($R^2_{adjusted}$), and residual sum of squares (RSS) (manual calculation in MS Excel):

Parameter	No.1	No.2	No.3	No.4
N	15	15	15	15
df	13	13	13	13
R	0.998948122	0.999277363	0.99883277	0.998899001
R^2	0.99789735	0.998555248	0.997666902	0.997799215
$R^2_{adjusted}$	0.997735608	0.998444113	0.997487433	0.997629924
RSS	10.49663718	6.091400277	8.990755868	8.935912491

Graphical abstract of model fit presented as mean ± 1 SD of the fraction % of released carvedilol:

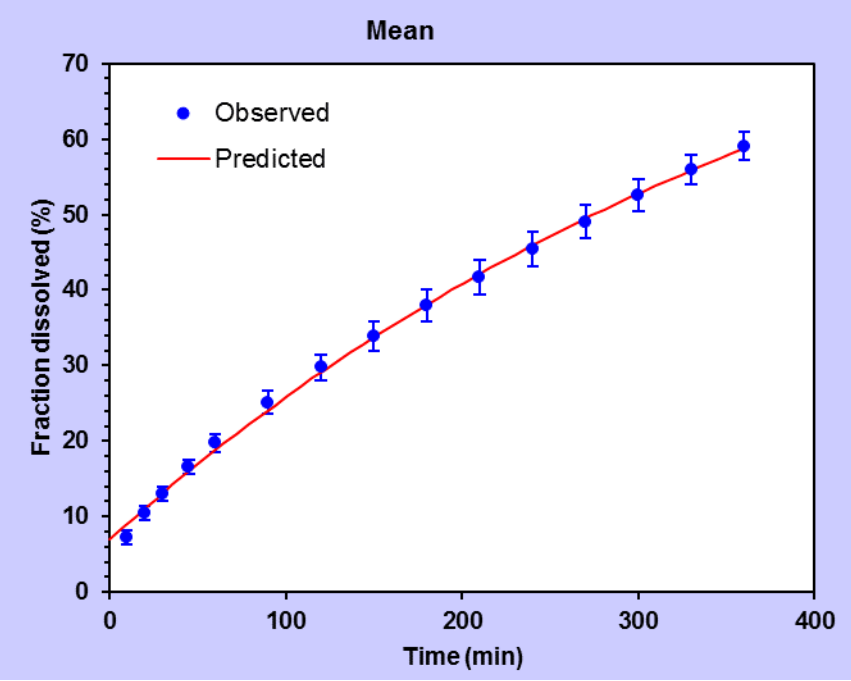

Supplementary materials – Model fitting summary – Lactochem® Fine Powder (modelling up to app. 60 % of carvedilol released)

Graphical abstract of model fit presented as the fraction % of released carvedilol per tested tablet:

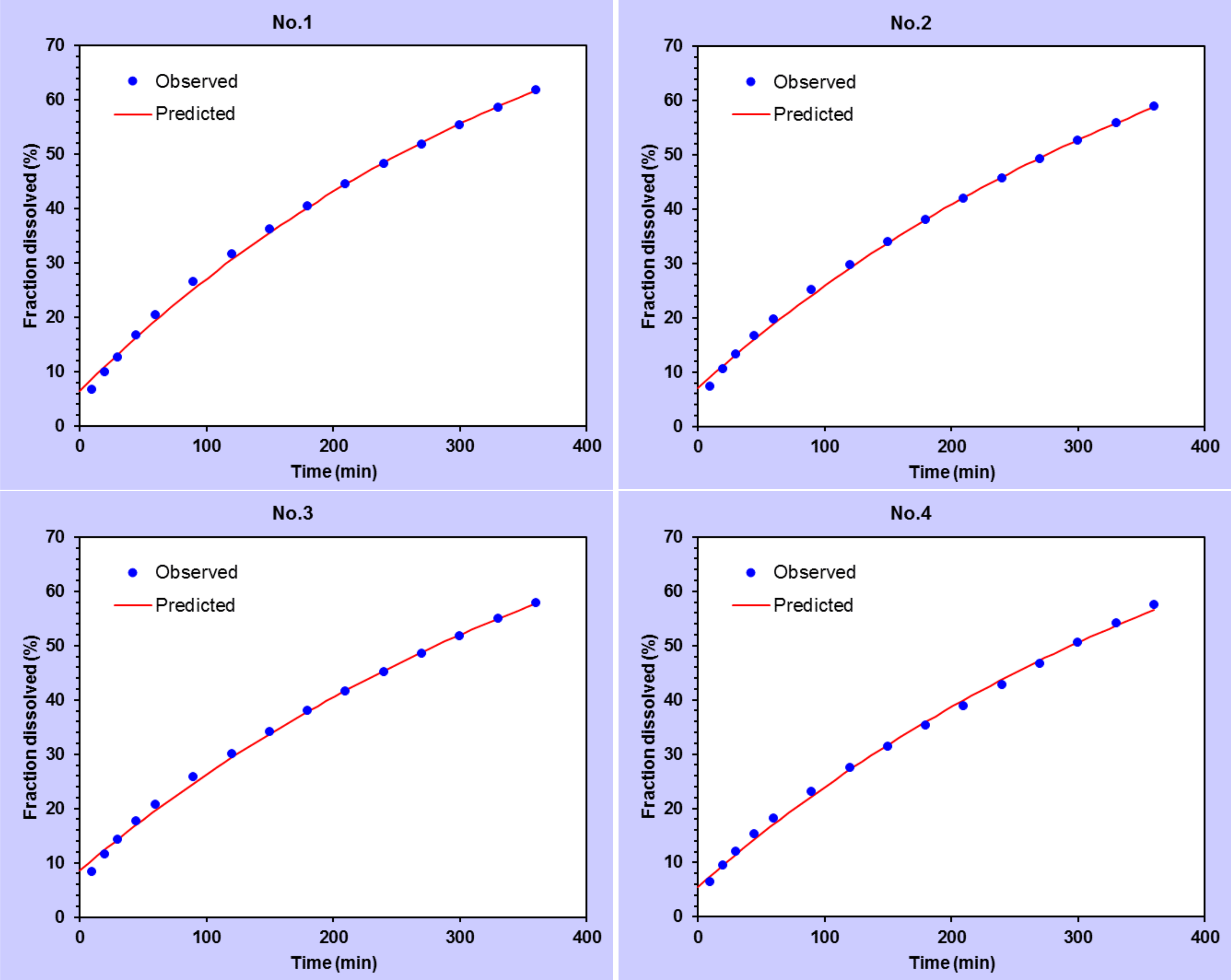

Supplementary materials – Model fitting summary – Lactochem® Fine Powder (modelling up to app. 60 % of carvedilol released)

Model: **First–order with F_{max}**

Model equation: $F = F_{max} \cdot (1 - e^{-k_1 \cdot t})$

Fitted model parameters per tested tablet (N = 4) with statistics – mean, standard deviation (SD), and relative standard deviation expressed in % (RSD%) (output from DDSolver):

Parameter	No.1	No.2	No.3	No.4	Mean	SD	RSD(%)
k_1	0.007	0.007	0.007	0.006	0.007	0.000	2.491
F_{max}	64.947	61.883	60.836	60.457	62.031	2.036	3.282

Number of dissolution data points (N), degrees of freedom (df), and selected goodness of fit criteria – Pearson correlation coefficient (R), coefficient of determination (R^2), adjusted coefficient of determination ($R^2_{adjusted}$), and residual sum of squares (RSS) (manual calculation in MS Excel):

Parameter	No.1	No.2	No.3	No.4
N	15	15	15	15
df	13	13	13	13
R	0.990555856	0.987539361	0.987549945	0.983548309
R^2	0.981200903	0.97523399	0.975254894	0.967367276
$R^2_{adjusted}$	0.979754819	0.973328912	0.973351424	0.964857066
RSS	131.7100173	148.0012209	148.657108	187.8334824

Graphical abstract of model fit presented as mean ± 1 SD of the fraction % of released carvedilol:

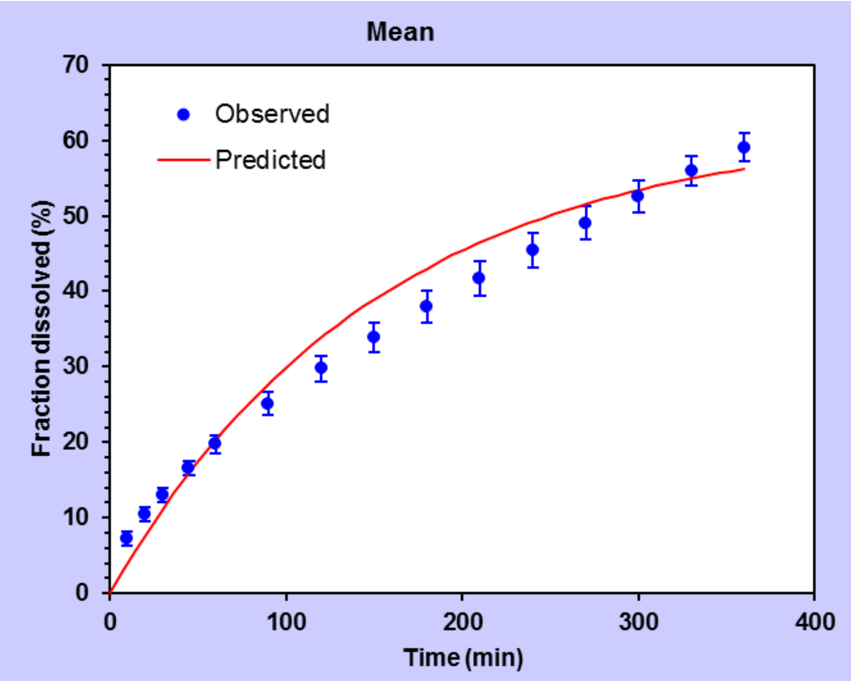

Supplementary materials – Model fitting summary – Lactochem® Fine Powder (modelling up to app. 60 % of carvedilol released)

Graphical abstract of model fit presented as the fraction % of released carvedilol per tested tablet:

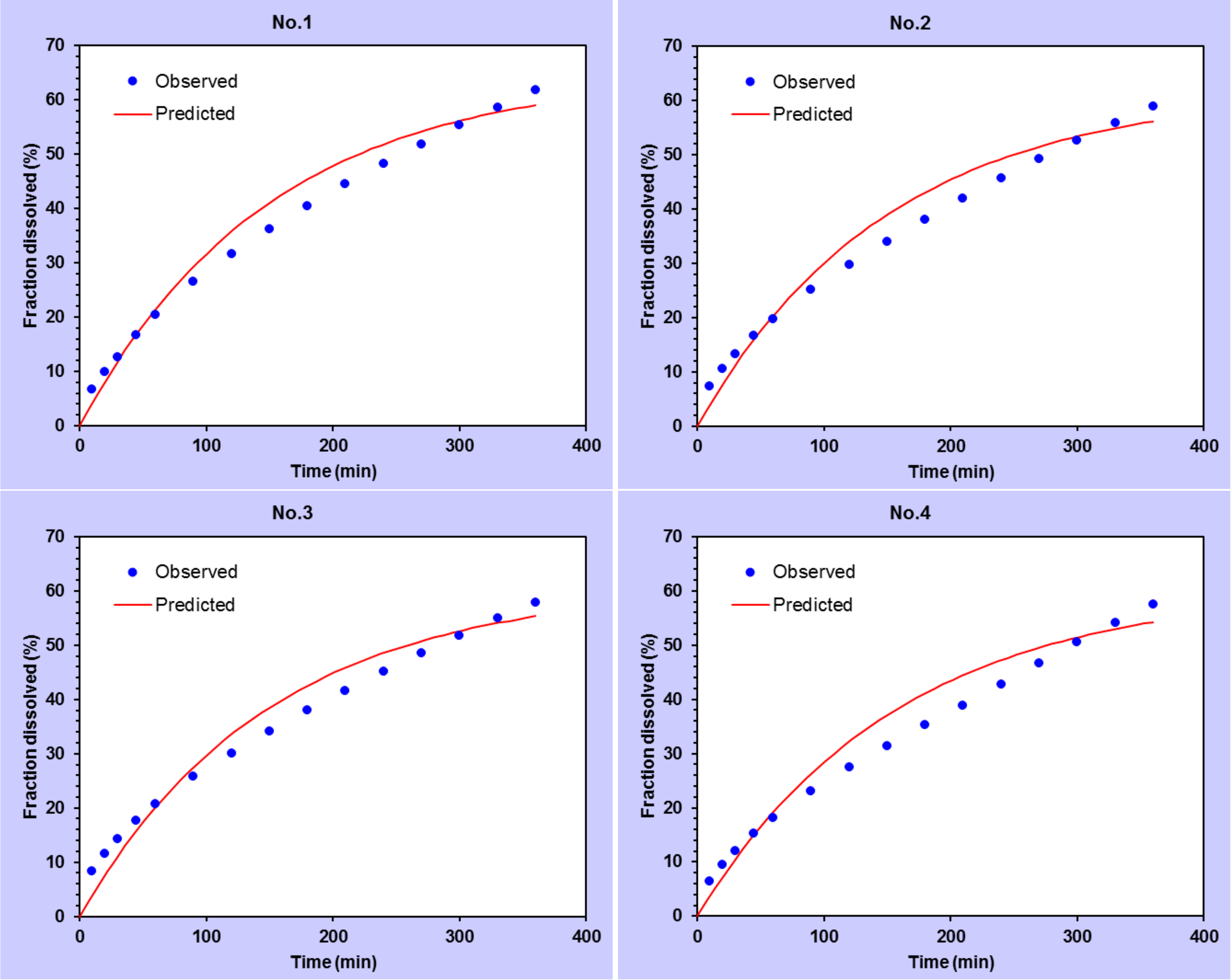

Supplementary materials – Model fitting summary – Lactochem® Fine Powder (modelling up to app. 60 % of carvedilol released)

Model: **First–order with T_{lag} and F_{max}**

Model equation: $F = F_{max} \cdot \left[1 - e^{-k_1 \cdot (t - T_{lag})}\right]$

Fitted model parameters per tested tablet (N = 4) with statistics – mean, standard deviation (SD), and relative standard deviation expressed in % (RSD%) (output from DDSolver):

Parameter	No.1	No.2	No.3	No.4	Mean	SD	RSD(%)
k_1	0.007	0.007	0.007	0.007	0.007	0.000	1.703
T_{lag}	14.848	13.323	8.891	17.575	13.659	3.633	26.600
F_{max}	64.947	61.883	60.836	60.457	62.031	2.036	3.282

Number of dissolution data points (N), degrees of freedom (df), and selected goodness of fit criteria – Pearson correlation coefficient (R), coefficient of determination (R^2), adjusted coefficient of determination ($R^2_{adjusted}$), and residual sum of squares (RSS) (manual calculation in MS Excel):

Parameter	No.1	No.2	No.3	No.4
N	15	15	15	15
df	12	12	12	12
R	0.98844173	0.985391389	0.986177365	0.980388085
R^2	0.977017054	0.970996189	0.972545795	0.961160798
$R^2_{adjusted}$	0.973186564	0.966162221	0.967970095	0.954687598
RSS	283.3112453	295.9417534	254.7503339	373.401654

Graphical abstract of model fit presented as mean ± 1 SD of the fraction % of released carvedilol:

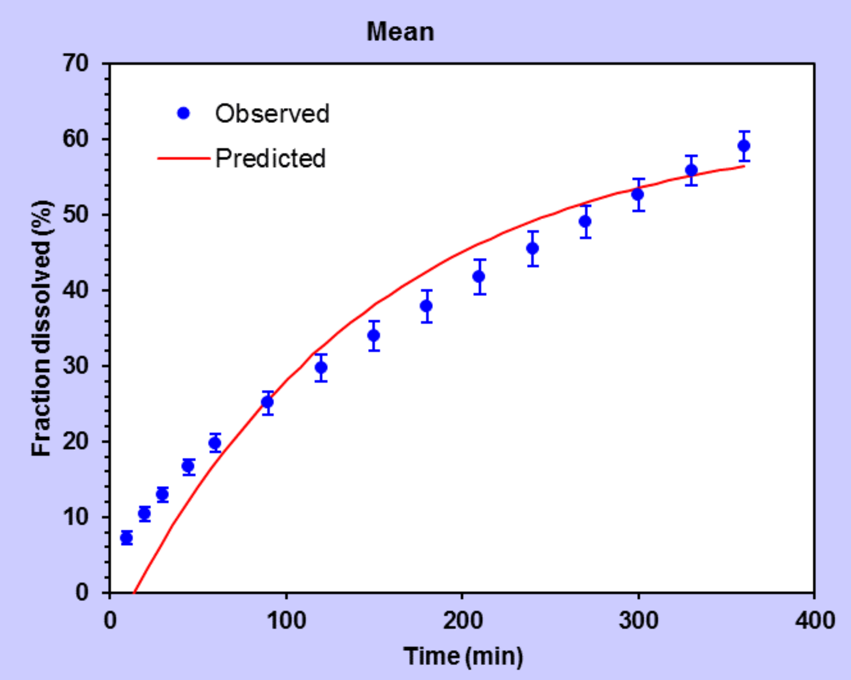

Supplementary materials – Model fitting summary – Lactochem® Fine Powder (modelling up to app. 60 % of carvedilol released)

Graphical abstract of model fit presented as the fraction % of released carvedilol per tested tablet:

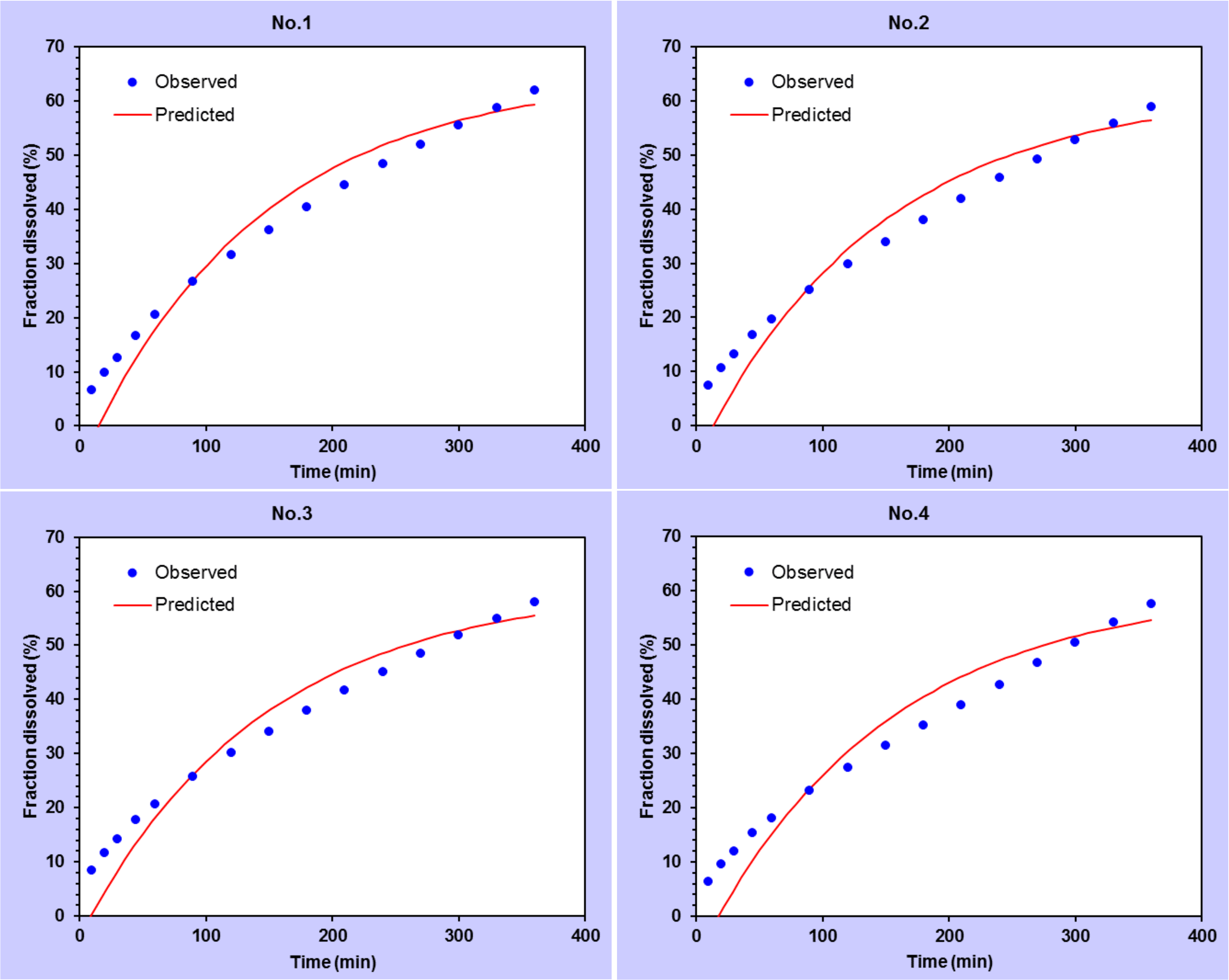

Supplementary materials – Model fitting summary – Lactochem® Fine Powder (modelling up to app. 60 % of carvedilol released)

Model: **Higuchi**

Model equation: $F = k_H \cdot t^{0.5}$

Fitted model parameters per tested tablet (N = 4) with statistics – mean, standard deviation (SD), and relative standard deviation expressed in % (RSD%) (output from DDSolver):

Parameter	No.1	No.2	No.3	No.4	Mean	SD	RSD(%)
k_H	3.079	2.927	2.911	2.770	2.922	0.126	4.313

Number of dissolution data points (N), degrees of freedom (df), and selected goodness of fit criteria – Pearson correlation coefficient (R), coefficient of determination (R^2), adjusted coefficient of determination ($R^2_{adjusted}$), and residual sum of squares (RSS) (manual calculation in MS Excel):

Parameter	No.1	No.2	No.3	No.4
N	15	15	15	15
df	14	14	14	14
R	0.99907224	0.998082863	0.998353259	0.99576646
R^2	0.998145341	0.996169401	0.99670923	0.991550842
$R^2_{adjusted}$	0.998145341	0.996169401	0.99670923	0.991550842
RSS	109.9720377	77.02953089	37.20225415	123.9345847

Graphical abstract of model fit presented as mean ± 1 SD of the fraction % of released carvedilol:

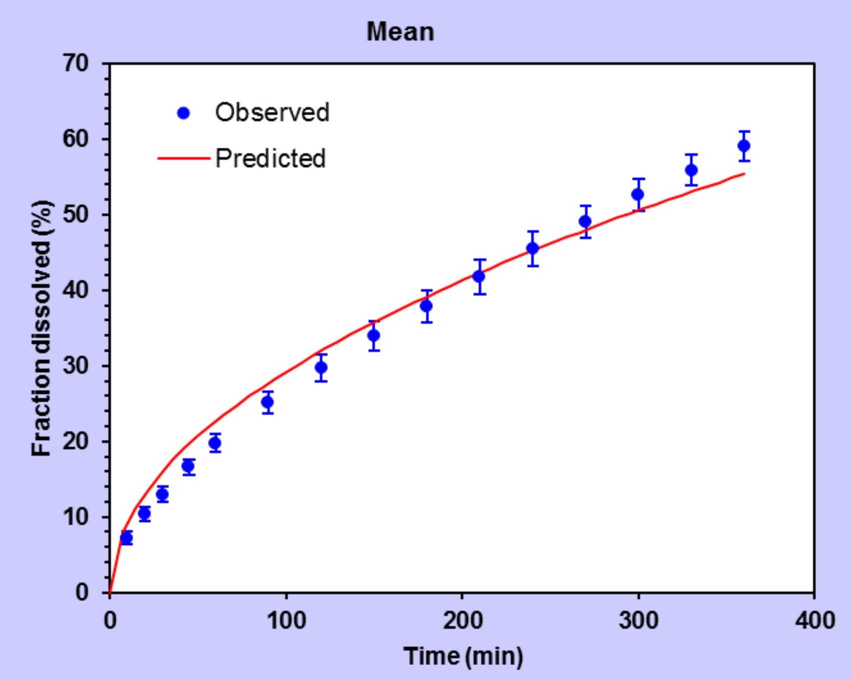

Supplementary materials – Model fitting summary – Lactochem® Fine Powder (modelling up to app. 60 % of carvedilol released)

Graphical abstract of model fit presented as the fraction % of released carvedilol per tested tablet:

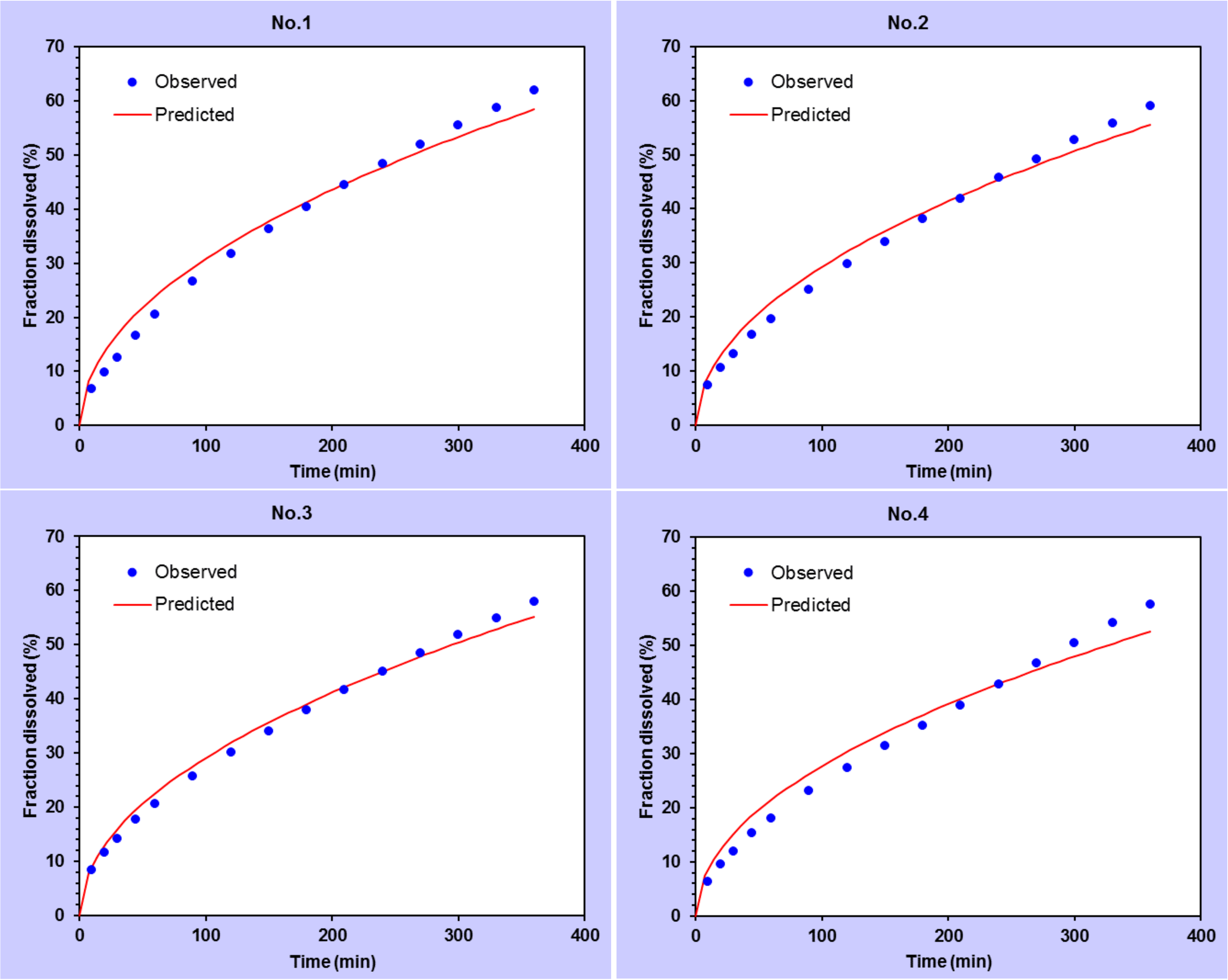

Supplementary materials – Model fitting summary – Lactochem® Fine Powder (modelling up to app. 60 % of carvedilol released)

Model: **Higuchi with T_{lag}**

Model equation: $F = k_H \cdot (t - T_{lag})^{0.5}$

Fitted model parameters per tested tablet (N = 4) with statistics – mean, standard deviation (SD), and relative standard deviation expressed in % (RSD%) (output from DDSolver):

Parameter	No.1	No.2	No.3	No.4	Mean	SD	RSD(%)
k_H	3.293	3.120	3.050	3.019	3.121	0.123	3.932
T_{lag}	19.631	18.844	14.075	24.515	19.266	4.274	22.184

Number of dissolution data points (N), degrees of freedom (df), and selected goodness of fit criteria – Pearson correlation coefficient (R), coefficient of determination (R^2), adjusted coefficient of determination ($R^2_{adjusted}$), and residual sum of squares (RSS) (manual calculation in MS Excel):

Parameter	No.1	No.2	No.3	No.4
N	15	15	15	15
df	13	13	13	13
R	0.992907923	0.991410096	0.992416552	0.986713887
R^2	0.985866144	0.982893979	0.984890613	0.973604294
$R^2_{adjusted}$	0.984778924	0.981578131	0.983728353	0.971573855
RSS	121.1066867	129.7737377	102.814608	190.826924

Graphical abstract of model fit presented as mean ± 1 SD of the fraction % of released carvedilol:

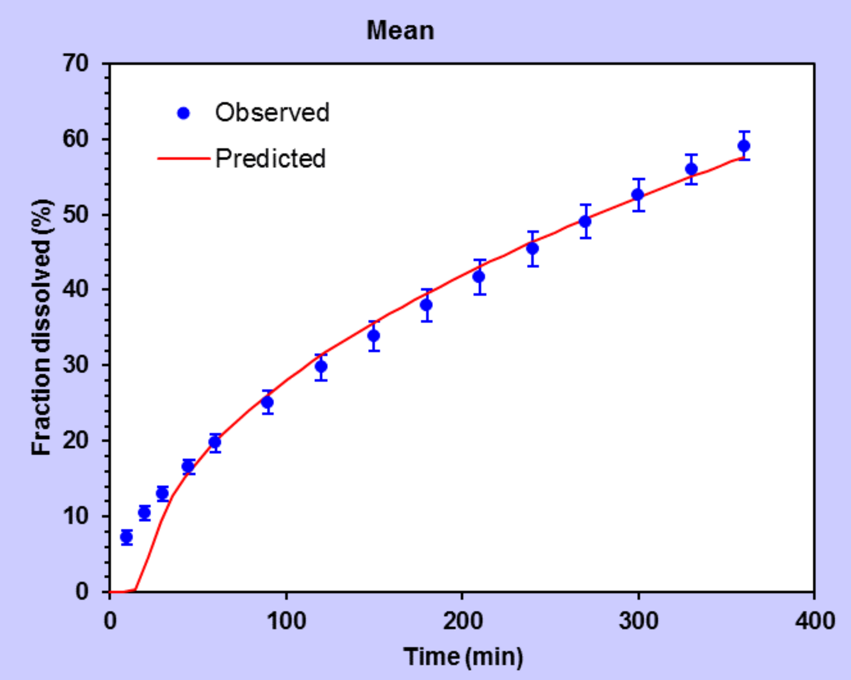

Supplementary materials – Model fitting summary – Lactochem® Fine Powder (modelling up to app. 60 % of carvedilol released)

Graphical abstract of model fit presented as the fraction % of released carvedilol per tested tablet:

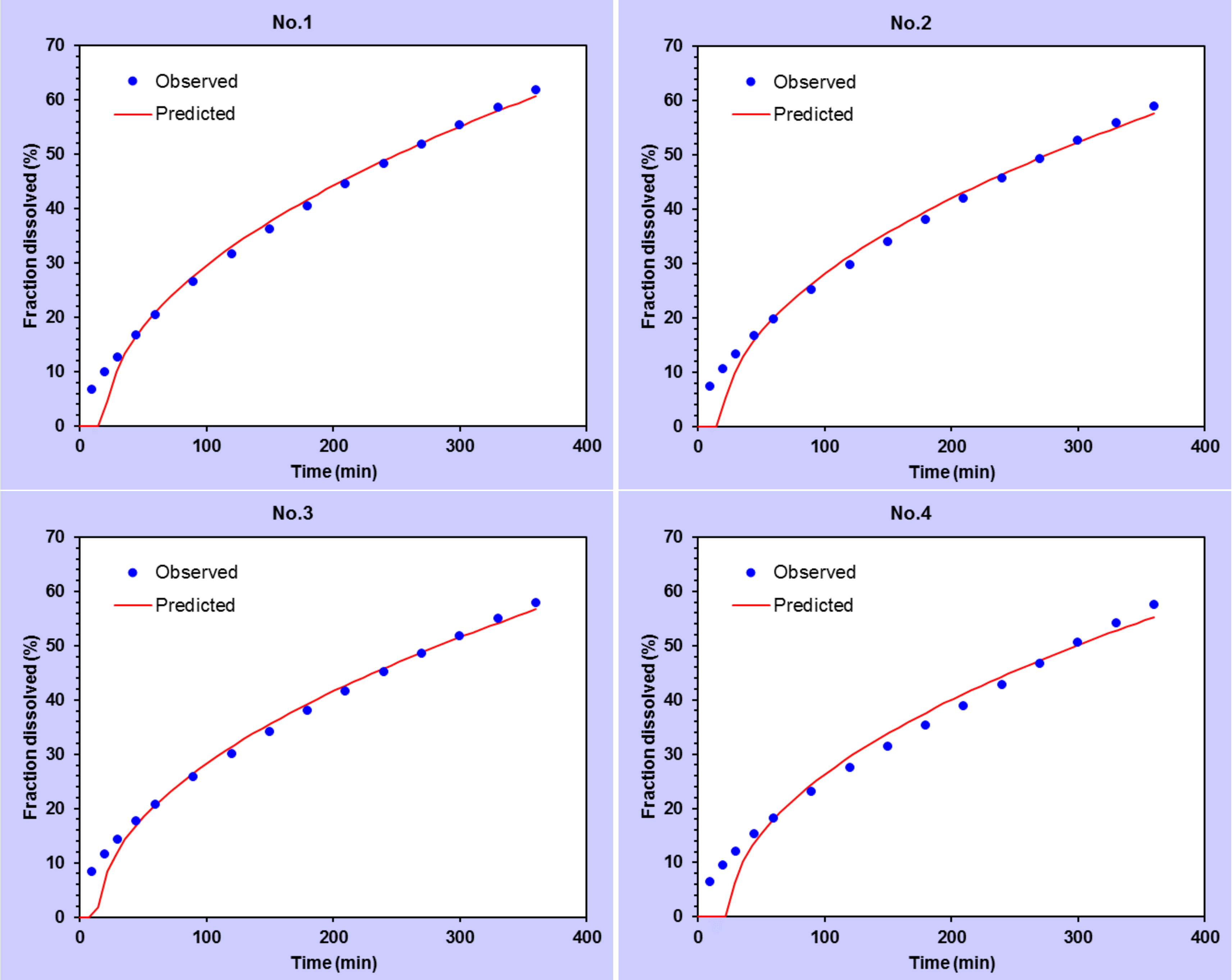

Supplementary materials – Model fitting summary – Lactochem® Fine Powder (modelling up to app. 60 % of carvedilol released)

Model: **Higuchi with F_0**

Model equation: $F = F_0 + k_H \cdot t^{0.5}$

Fitted model parameters per tested tablet (N = 4) with statistics – mean, standard deviation (SD), and relative standard deviation expressed in % (RSD%) (output from DDSolver):

Parameter	No.1	No.2	No.3	No.4	Mean	SD	RSD(%)
k_H	3.549	3.292	3.143	3.215	3.300	0.177	5.361
F_0	-6.508	-5.059	-3.217	-6.148	-5.233	1.478	-28.250

Number of dissolution data points (N), degrees of freedom (df), and selected goodness of fit criteria – Pearson correlation coefficient (R), coefficient of determination (R^2), adjusted coefficient of determination ($R^2_{adjusted}$), and residual sum of squares (RSS) (manual calculation in MS Excel):

Parameter	No.1	No.2	No.3	No.4
N	15	15	15	15
df	13	13	13	13
R	0.99907224	0.998082863	0.998353259	0.99576646
R^2	0.998145341	0.996169401	0.99670923	0.991550842
$R^2_{adjusted}$	0.998002675	0.99587474	0.996456093	0.990900907
RSS	8.984194023	16.00081002	12.52245912	33.8073965

Graphical abstract of model fit presented as mean ± 1 SD of the fraction % of released carvedilol:

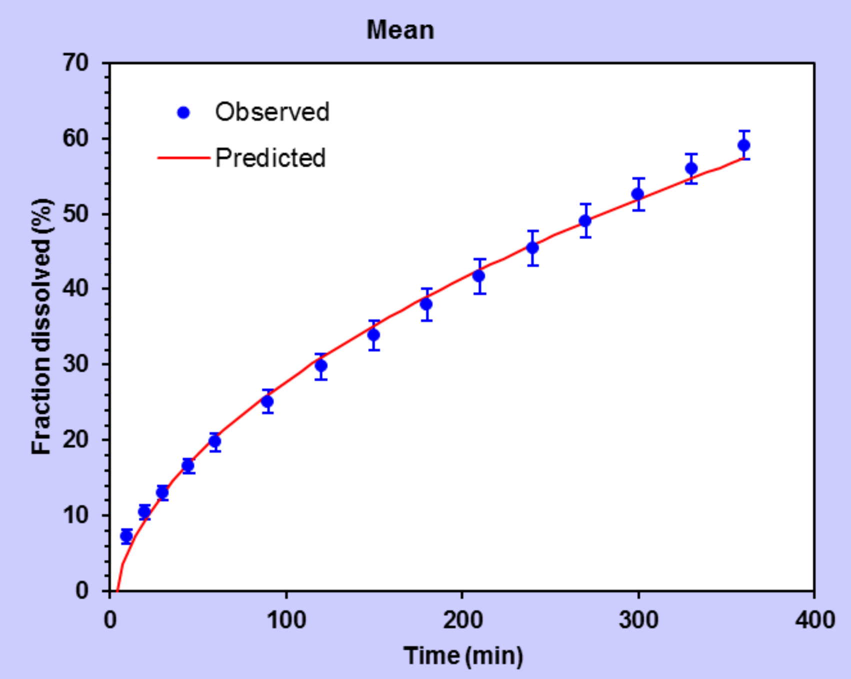

Supplementary materials – Model fitting summary – Lactochem® Fine Powder (modelling up to app. 60 % of carvedilol released)

Graphical abstract of model fit presented as the fraction % of released carvedilol per tested tablet:

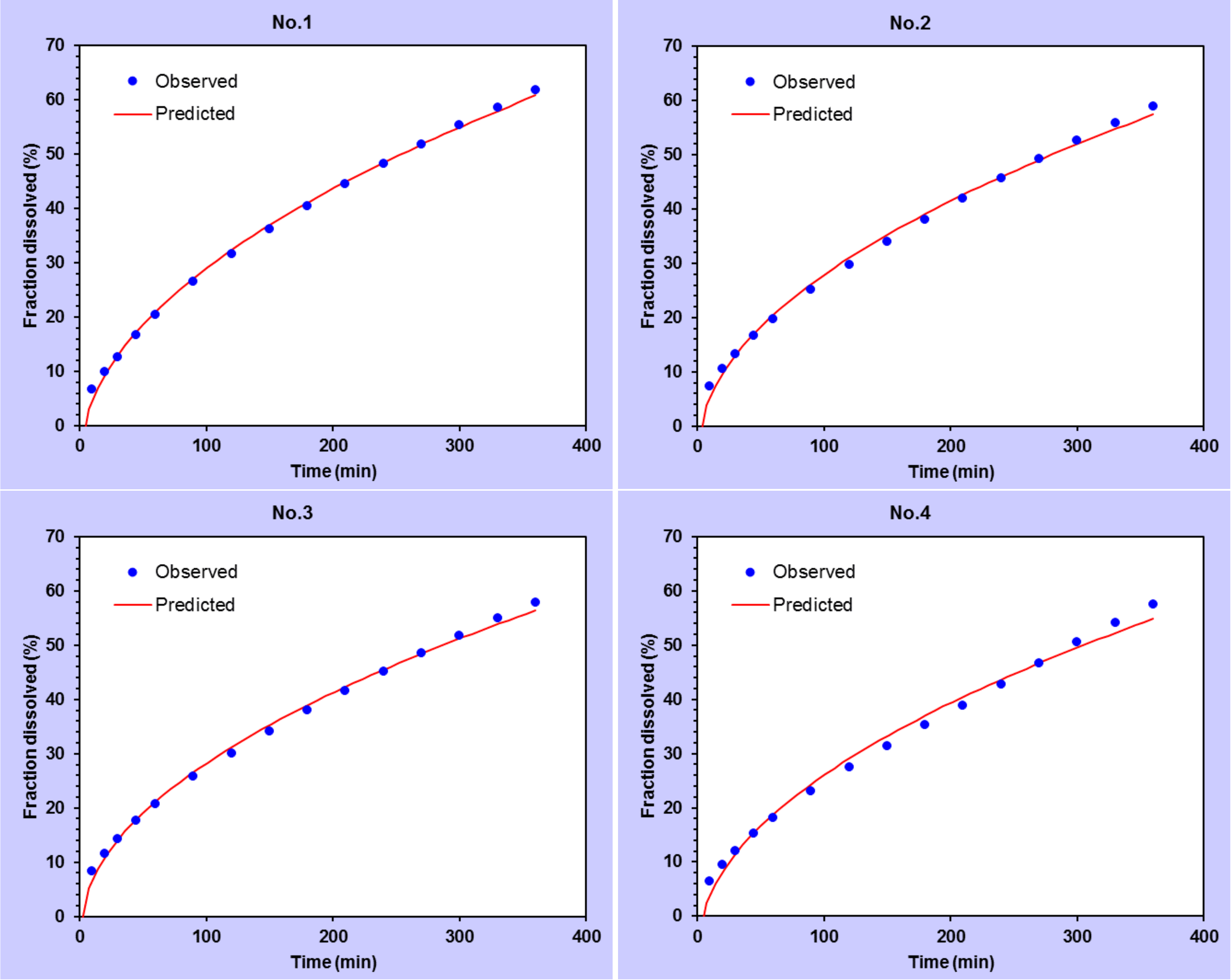

Supplementary materials – Model fitting summary – Lactochem® Fine Powder (modelling up to app. 60 % of carvedilol released)

Model: **Korsmeyer–Peppas**

Model equation: $F = k_{KP} \cdot t^n$

Fitted model parameters per tested tablet (N = 4) with statistics – mean, standard deviation (SD), and relative standard deviation expressed in % (RSD%) (output from DDSolver):

Parameter	No.1	No.2	No.3	No.4	Mean	SD	RSD(%)
k_{KP}	1.533	1.824	2.259	1.503	1.780	0.351	19.725
n	0.630	0.587	0.546	0.612	0.594	0.036	6.124

Number of dissolution data points (N), degrees of freedom (df), and selected goodness of fit criteria – Pearson correlation coefficient (R), coefficient of determination (R^2), adjusted coefficient of determination ($R^2_{adjusted}$), and residual sum of squares (RSS) (manual calculation in MS Excel):

Parameter	No.1	No.2	No.3	No.4
N	15	15	15	15
df	13	13	13	13
R	0.999875767	0.999627885	0.999228213	0.998718477
R^2	0.999751548	0.999255909	0.998457023	0.997438597
$R^2_{adjusted}$	0.999732437	0.999198671	0.998338332	0.997241566
RSS	1.318140963	5.153395104	8.725633562	13.95738272

Graphical abstract of model fit presented as mean ± 1 SD of the fraction % of released carvedilol:

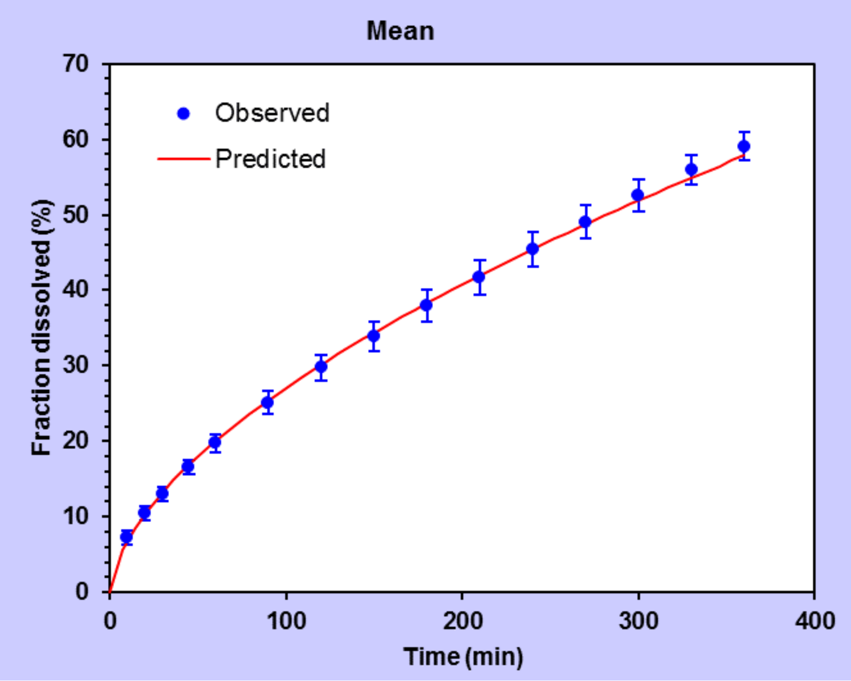

Supplementary materials – Model fitting summary – Lactochem® Fine Powder (modelling up to app. 60 % of carvedilol released)

Graphical abstract of model fit presented as the fraction % of released carvedilol per tested tablet:

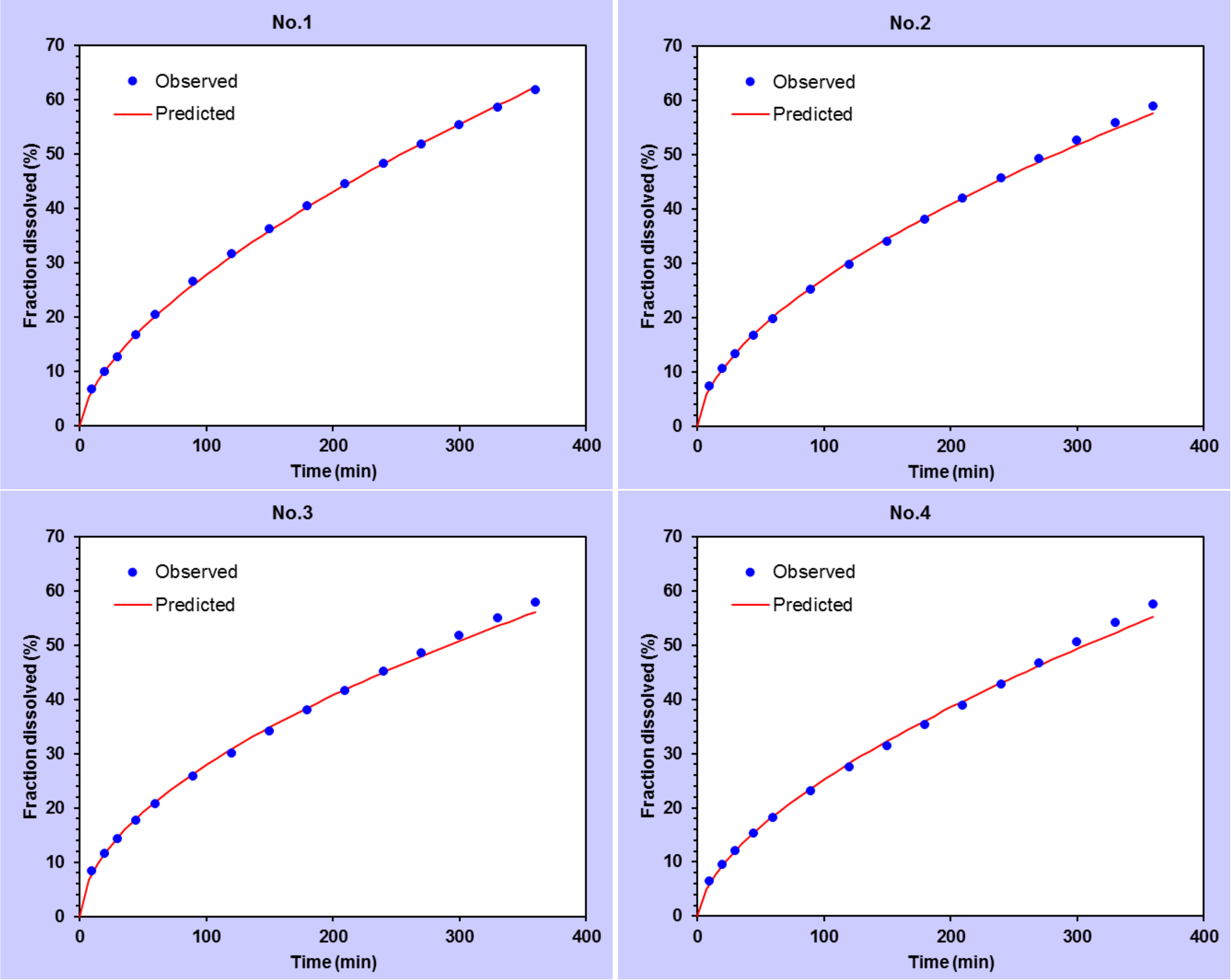

Supplementary materials – Model fitting summary – Lactochem® Fine Powder (modelling up to app. 60 % of carvedilol released)

Model: **Korsmeyer–Peppas with T_{lag}**

Model equation: $F = k_{KP} \cdot \left(t - T_{lag}\right)^{n}$

Fitted model parameters per tested tablet (N = 4) with statistics – mean, standard deviation (SD), and relative standard deviation expressed in % (RSD%) (output from DDSolver):

Parameter	No.1	No.2	No.3	No.4	Mean	SD	RSD(%)
k_{KP}	2.130	2.487	3.017	2.076	2.427	0.433	17.851
n	0.569	0.530	0.493	0.553	0.536	0.033	6.185
T_{lag}	4.000	4.000	4.000	4.000	4.000	0.000	0.000

Number of dissolution data points (N), degrees of freedom (df), and selected goodness of fit criteria – Pearson correlation coefficient (R), coefficient of determination (R^2), adjusted coefficient of determination ($R^2_{adjusted}$), and residual sum of squares (RSS) (manual calculation in MS Excel):

Parameter	No.1	No.2	No.3	No.4
N	15	15	15	15
df	12	12	12	12
R	0.999577069	0.998038579	0.997240864	0.99663576
R^2	0.999154316	0.996081006	0.99448934	0.993282838
$R^2_{adjusted}$	0.999013369	0.99542784	0.993570897	0.99216331
RSS	9.414417504	30.01504072	34.80336923	44.09712428

Graphical abstract of model fit presented as mean ± 1 SD of the fraction % of released carvedilol:

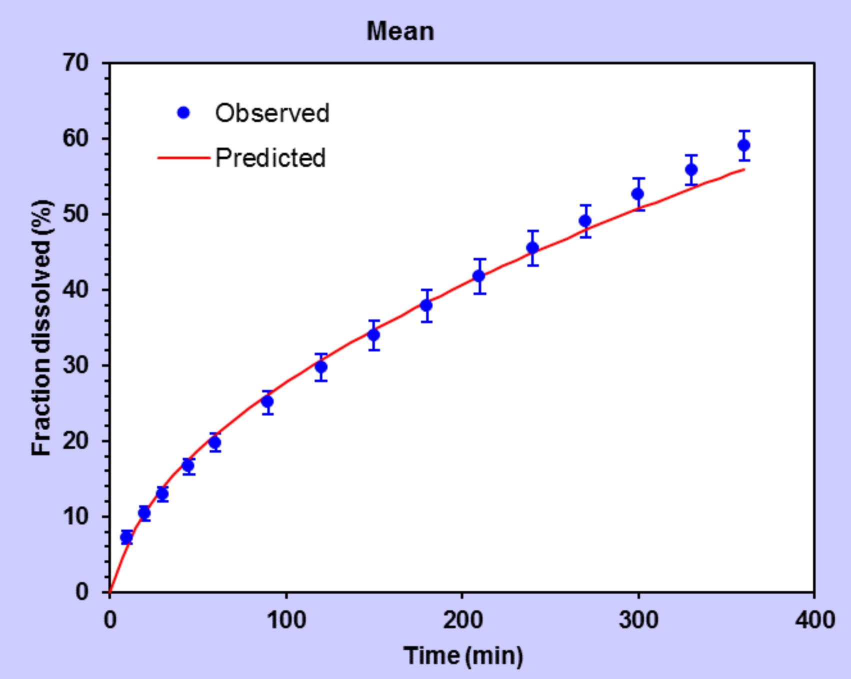

Supplementary materials – Model fitting summary – Lactochem® Fine Powder (modelling up to app. 60 % of carvedilol released)

Graphical abstract of model fit presented as the fraction % of released carvedilol per tested tablet:

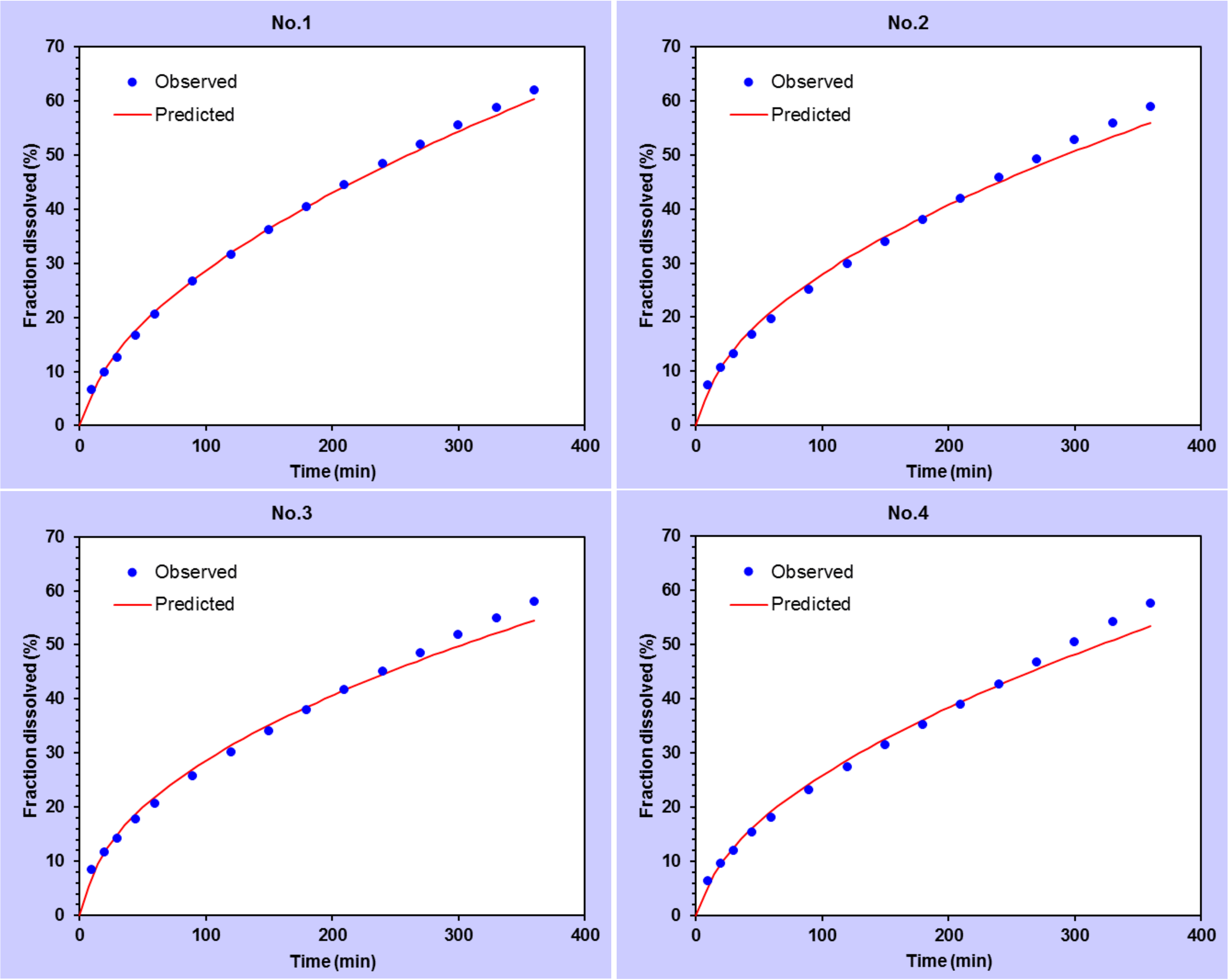

Supplementary materials – Model fitting summary – Lactochem® Fine Powder (modelling up to app. 60 % of carvedilol released)

Model: **Korsmeyer–Peppas with F_0**

Model equation: $F = F_0 + k_{KP} \cdot t^n$

Fitted model parameters per tested tablet (N = 4) with statistics – mean, standard deviation (SD), and relative standard deviation expressed in % (RSD%) (output from DDSolver):

Parameter	No.1	No.2	No.3	No.4	Mean	SD	RSD(%)
k_{KP}	0.782	0.913	1.152	0.785	0.908	0.174	19.141
n	0.734	0.697	0.656	0.721	0.702	0.034	4.870
F_0	3.393	3.607	3.360	2.560	3.230	0.460	14.242

Number of dissolution data points (N), degrees of freedom (df), and selected goodness of fit criteria – Pearson correlation coefficient (R), coefficient of determination (R^2), adjusted coefficient of determination ($R^2_{adjusted}$), and residual sum of squares (RSS) (manual calculation in MS Excel):

Parameter	No.1	No.2	No.3	No.4
N	15	15	15	15
df	12	12	12	12
R	0.998676233	0.999867571	0.999937023	0.999722617
R^2	0.997354218	0.99973516	0.999874049	0.999445312
$R^2_{adjusted}$	0.996913255	0.999691019	0.999853058	0.999352864
RSS	22.40138387	2.134850752	0.825568014	2.631733041

Graphical abstract of model fit presented as mean ± 1 SD of the fraction % of released carvedilol:

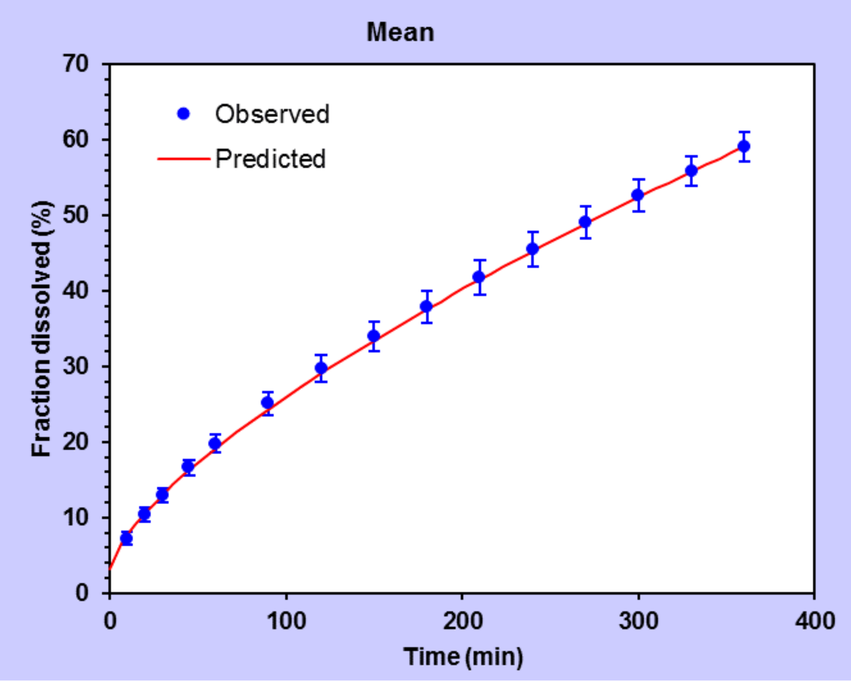

Supplementary materials – Model fitting summary – Lactochem® Fine Powder (modelling up to app. 60 % of carvedilol released)

Graphical abstract of model fit presented as the fraction % of released carvedilol per tested tablet:

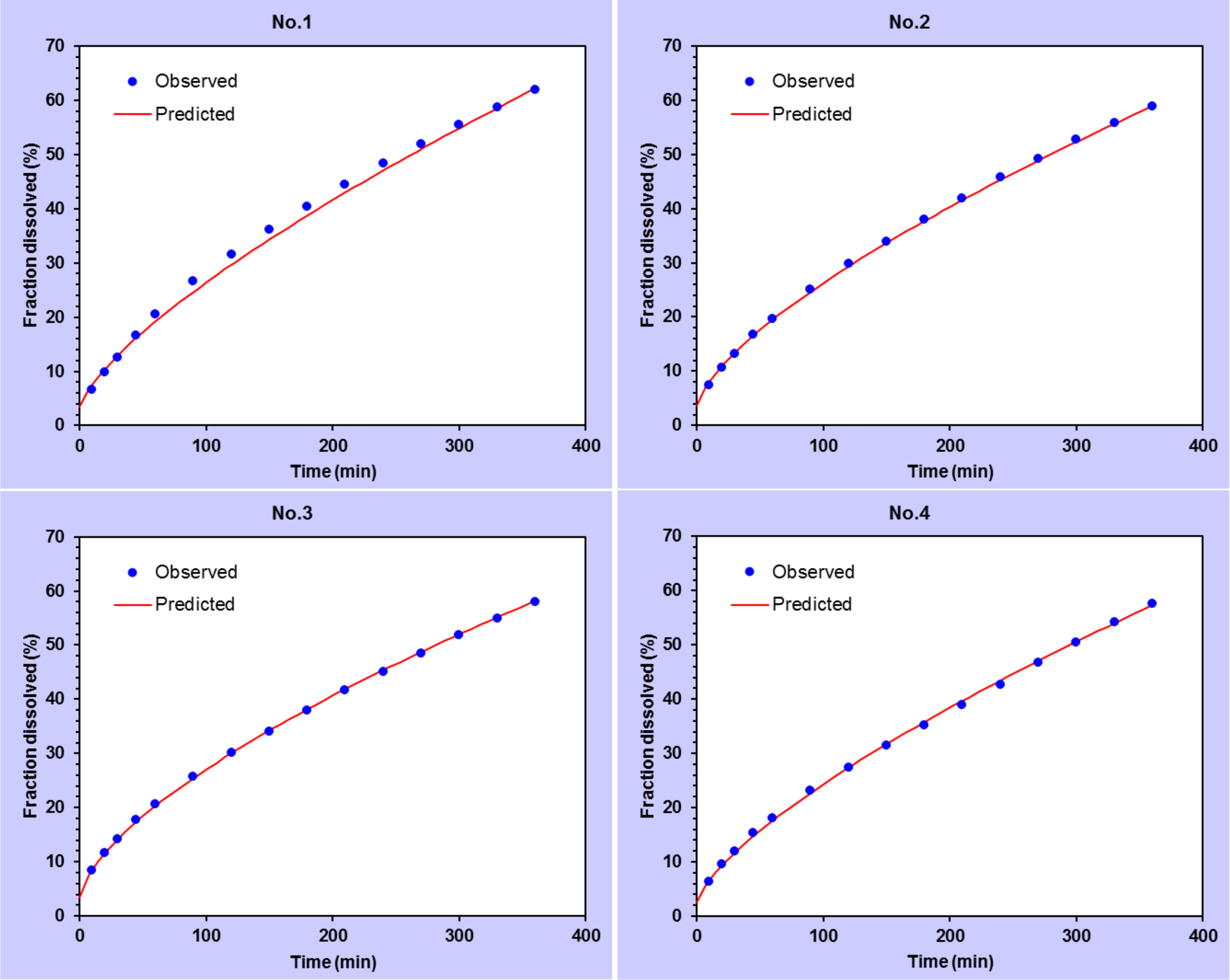

Supplementary materials – Model fitting summary – Lactochem® Fine Powder (modelling up to app. 60 % of carvedilol released)

Model: **Hixson–Crowell**

Model equation: $F = 100 \cdot [1 - (1 - k_{HC} \cdot t)^3]$

Fitted model parameters per tested tablet (N = 4) with statistics – mean, standard deviation (SD), and relative standard deviation expressed in % (RSD%) (output from DDSolver):

Parameter	No.1	No.2	No.3	No.4	Mean	SD	RSD(%)
k_{HC}	0.001	0.001	0.001	0.001	0.001	0.000	5.349

Number of dissolution data points (N), degrees of freedom (df), and selected goodness of fit criteria – Pearson correlation coefficient (R), coefficient of determination (R^2), adjusted coefficient of determination ($R^2_{adjusted}$), and residual sum of squares (RSS) (manual calculation in MS Excel):

Parameter	No.1	No.2	No.3	No.4
N	15	15	15	15
df	14	14	14	14
R	0.997815657	0.998633036	0.998171663	0.998861302
R^2	0.995636086	0.99726794	0.996346669	0.9977239
$R^2_{adjusted}$	0.995636086	0.99726794	0.996346669	0.9977239
RSS	255.1719124	286.1723844	389.7275173	199.3178164

Graphical abstract of model fit presented as mean ± 1 SD of the fraction % of released carvedilol:

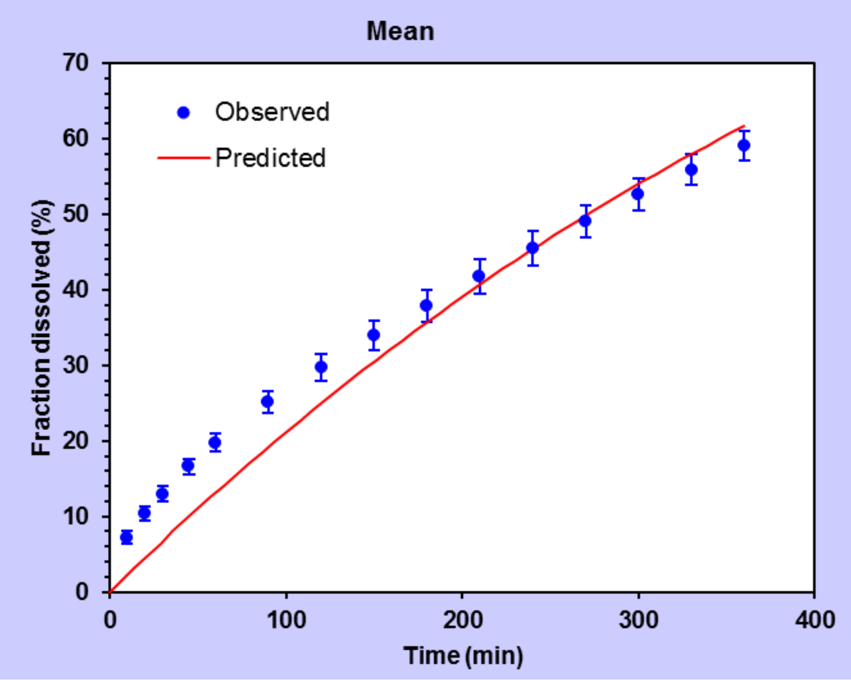

Supplementary materials – Model fitting summary – Lactochem® Fine Powder (modelling up to app. 60 % of carvedilol released)

Graphical abstract of model fit presented as the fraction % of released carvedilol per tested tablet:

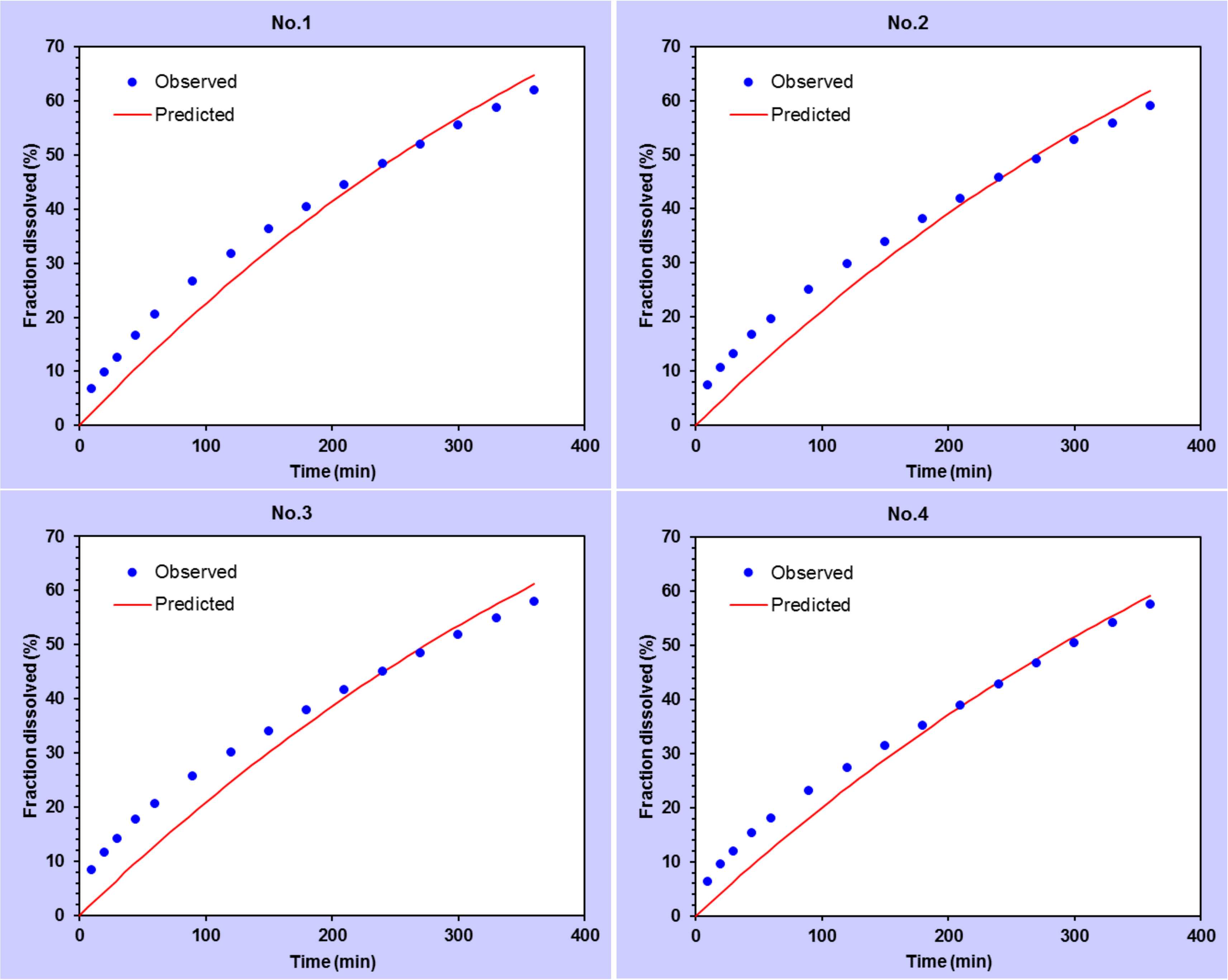

Supplementary materials – Model fitting summary – Lactochem® Fine Powder (modelling up to app. 60 % of carvedilol released)

Model: **Hixson–Crowell with T_{lag}**

Model equation: $F = 100 \cdot \left\{1 - \left[1 - k_{HC} \cdot \left(t - T_{lag}\right)\right]^3\right\}$

Fitted model parameters per tested tablet (N = 4) with statistics – mean, standard deviation (SD), and relative standard deviation expressed in % (RSD%) (output from DDSolver):

Parameter	No.1	No.2	No.3	No.4	Mean	SD	RSD(%)
k_{HC}	0.001	0.001	0.001	0.001	0.001	0.000	6.293
T_{lag}	-38.387	-44.296	-54.318	-37.065	-43.516	7.857	-18.056

Number of dissolution data points (N), degrees of freedom (df), and selected goodness of fit criteria – Pearson correlation coefficient (R), coefficient of determination (R^2), adjusted coefficient of determination ($R^2_{adjusted}$), and residual sum of squares (RSS) (manual calculation in MS Excel):

Parameter	No.1	No.2	No.3	No.4
N	15	15	15	15
df	13	13	13	13
R	0.997205986	0.998164108	0.997566405	0.998754756
R^2	0.994419779	0.996331586	0.995138733	0.997511062
$R^2_{adjusted}$	0.993990532	0.996049401	0.994764789	0.997319606
RSS	27.9210269	15.6823371	18.86470215	9.985072429

Graphical abstract of model fit presented as mean ± 1 SD of the fraction % of released carvedilol:

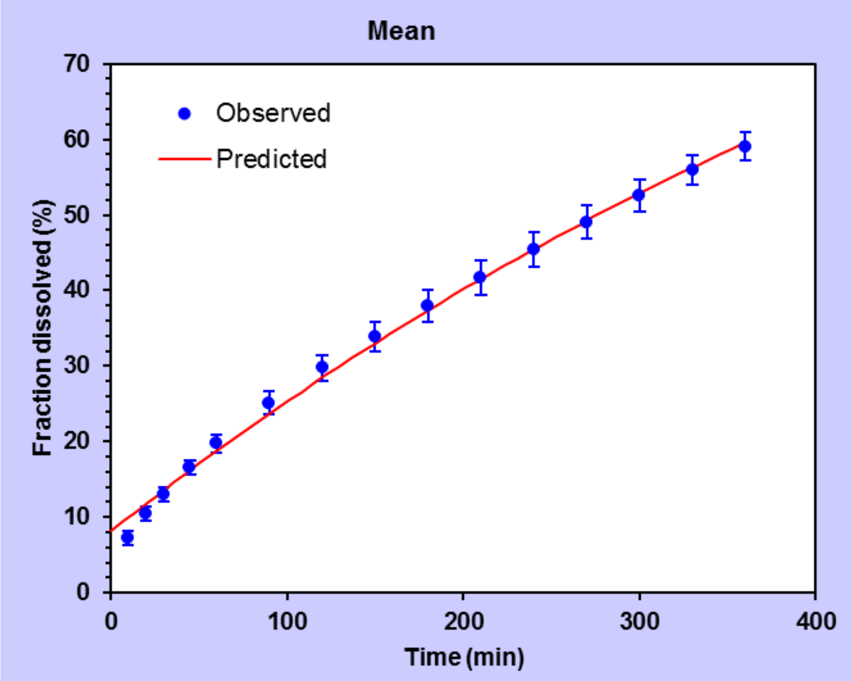

Supplementary materials – Model fitting summary – Lactochem® Fine Powder (modelling up to app. 60 % of carvedilol released)

Graphical abstract of model fit presented as the fraction % of released carvedilol per tested tablet:

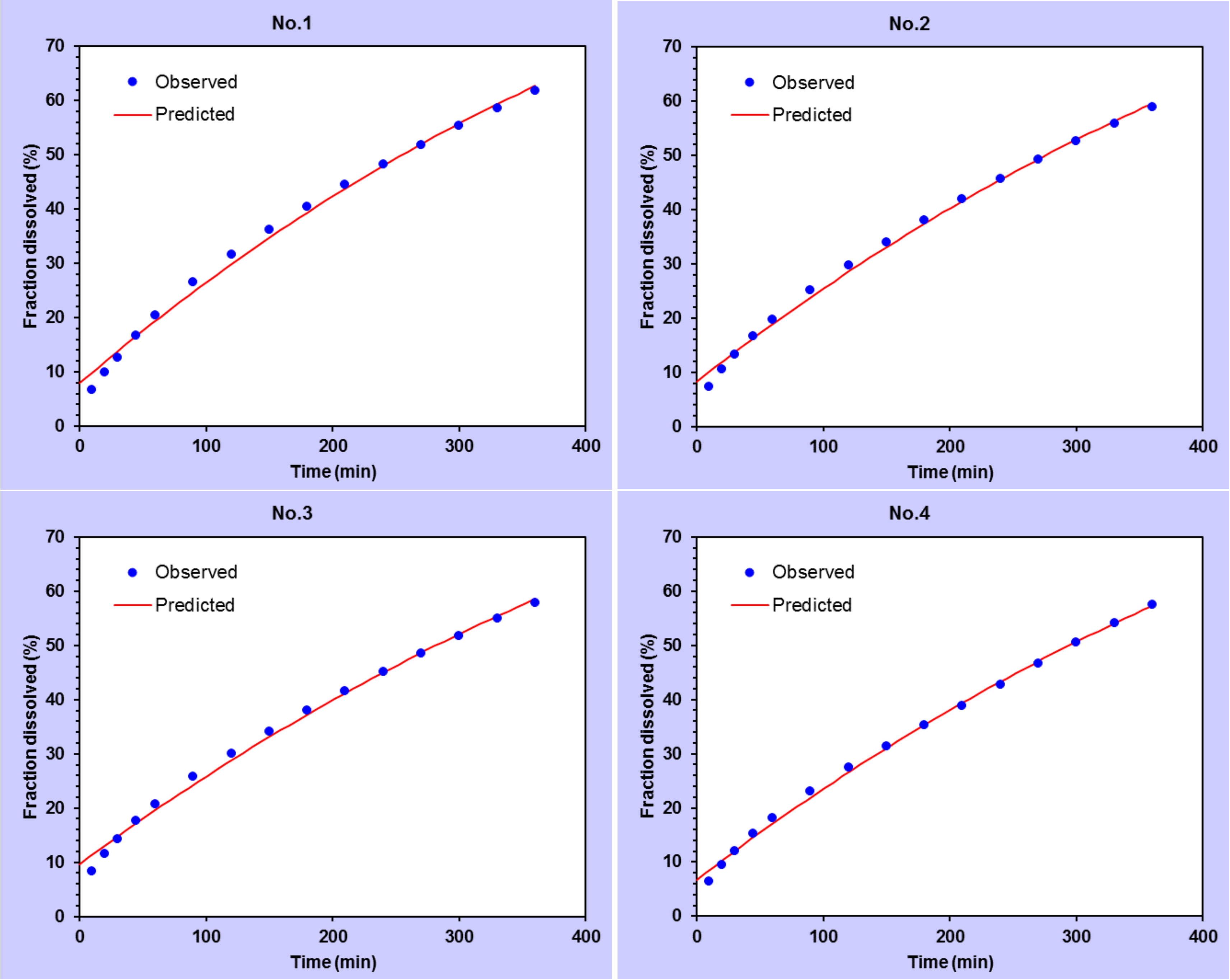

Supplementary materials – Model fitting summary – Lactochem® Fine Powder (modelling up to app. 60 % of carvedilol released)

Model: **Hopfenberg**

Model equation: $F = 100 \cdot [1 - (1 - k_{HB} \cdot t)^n]$

Fitted model parameters per tested tablet (N = 4) with statistics – mean, standard deviation (SD), and relative standard deviation expressed in % (RSD%) (output from DDSolver):

Parameter	No.1	No.2	No.3	No.4	Mean	SD	RSD(%)
k_{HB}	0.001	0.001	0.001	0.001	0.001	0.000	5.349
n	3.000	3.000	3.000	3.000	3.000	0.000	0.000

Number of dissolution data points (N), degrees of freedom (df), and selected goodness of fit criteria – Pearson correlation coefficient (R), coefficient of determination (R^2), adjusted coefficient of determination ($R^2_{adjusted}$), and residual sum of squares (RSS) (manual calculation in MS Excel):

Parameter	No.1	No.2	No.3	No.4
N	15	15	15	15
df	13	13	13	13
R	0.997815657	0.998633036	0.998171663	0.998861302
R^2	0.995636086	0.99726794	0.996346669	0.9977239
$R^2_{adjusted}$	0.995300401	0.997057782	0.996065644	0.997548816
RSS	255.1719124	286.1723844	389.7275173	199.3178164

Graphical abstract of model fit presented as mean ± 1 SD of the fraction % of released carvedilol:

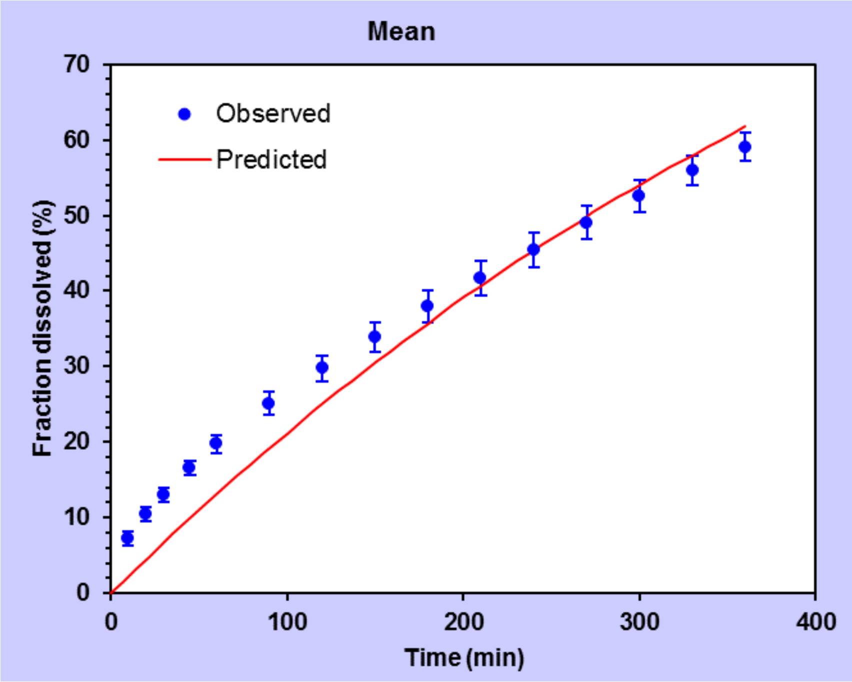

Supplementary materials – Model fitting summary – Lactochem® Fine Powder (modelling up to app. 60 % of carvedilol released)

Graphical abstract of model fit presented as the fraction % of released carvedilol per tested tablet:

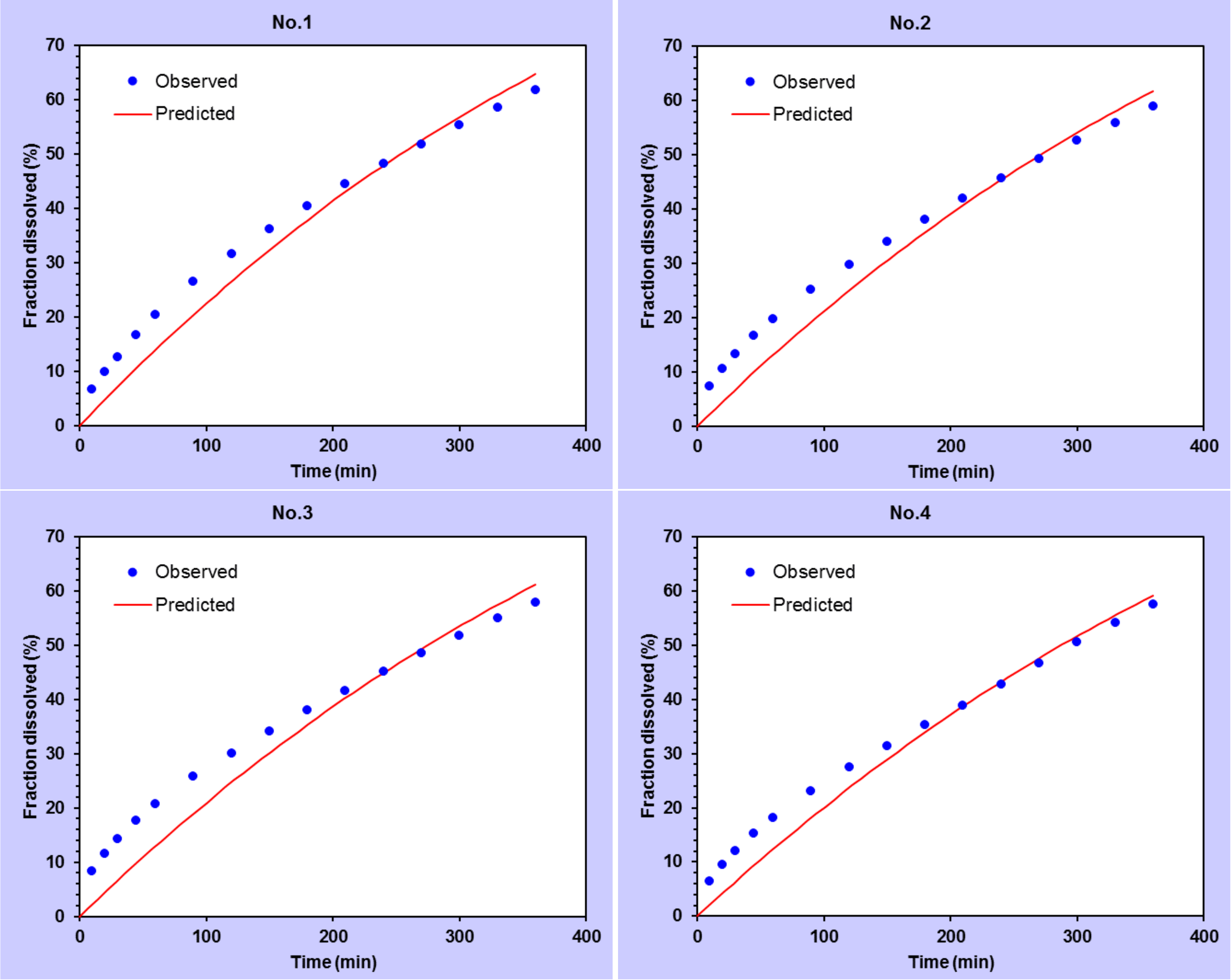

Supplementary materials – Model fitting summary – Lactochem® Fine Powder (modelling up to app. 60 % of carvedilol released)

Model: **Hopfenberg with T_{lag}**

Model equation: $F = 100 \cdot \{1 - [1 - k_{HB} \cdot (t - T_{lag})]^n\}$

Fitted model parameters per tested tablet (N = 4) with statistics – mean, standard deviation (SD), and relative standard deviation expressed in % (RSD%) (output from DDSolver):

Parameter	No.1	No.2	No.3	No.4	Mean	SD	RSD(%)
k_{HB}	0.001	0.001	0.001	0.001	0.001	0.000	11.365
n	3.000	3.000	3.627	3.000	3.157	0.313	9.924
T_{lag}	-38.387	-44.296	-46.550	-37.065	-41.575	4.570	-10.993

Number of dissolution data points (N), degrees of freedom (df), and selected goodness of fit criteria – Pearson correlation coefficient (R), coefficient of determination (R^2), adjusted coefficient of determination ($R^2_{adjusted}$), and residual sum of squares (RSS) (manual calculation in MS Excel):

Parameter	No.1	No.2	No.3	No.4
N	15	15	15	15
df	12	12	12	12
R	0.997205986	0.998164108	0.997916099	0.998754756
R^2	0.994419779	0.996331586	0.99583654	0.997511062
$R^2_{adjusted}$	0.993489743	0.995720184	0.99514263	0.997096239
RSS	27.9210269	15.6823371	17.15742438	9.985072429

Graphical abstract of model fit presented as mean ± 1 SD of the fraction % of released carvedilol:

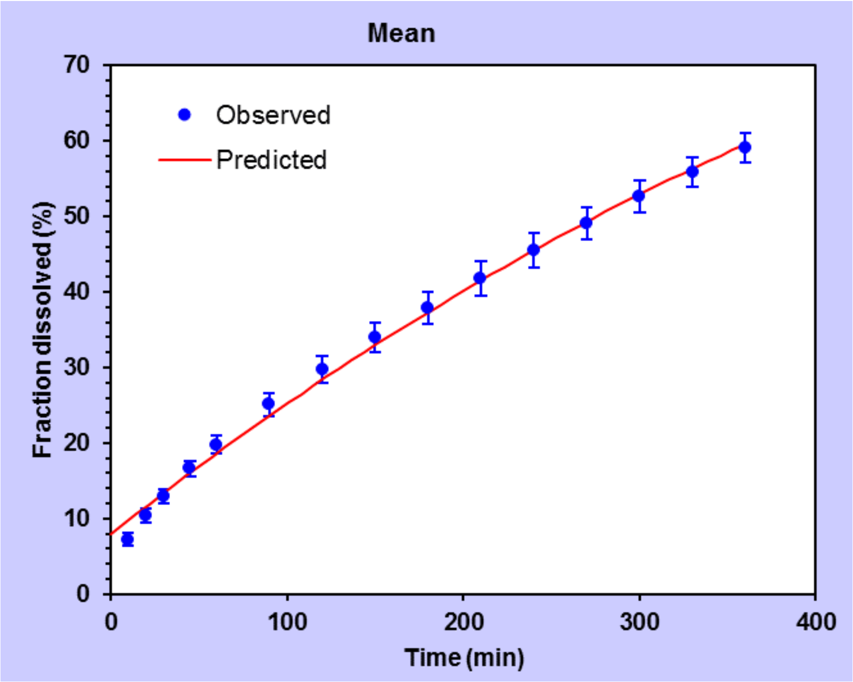

Supplementary materials – Model fitting summary – Lactochem® Fine Powder (modelling up to app. 60 % of carvedilol released)

Graphical abstract of model fit presented as the fraction % of released carvedilol per tested tablet:

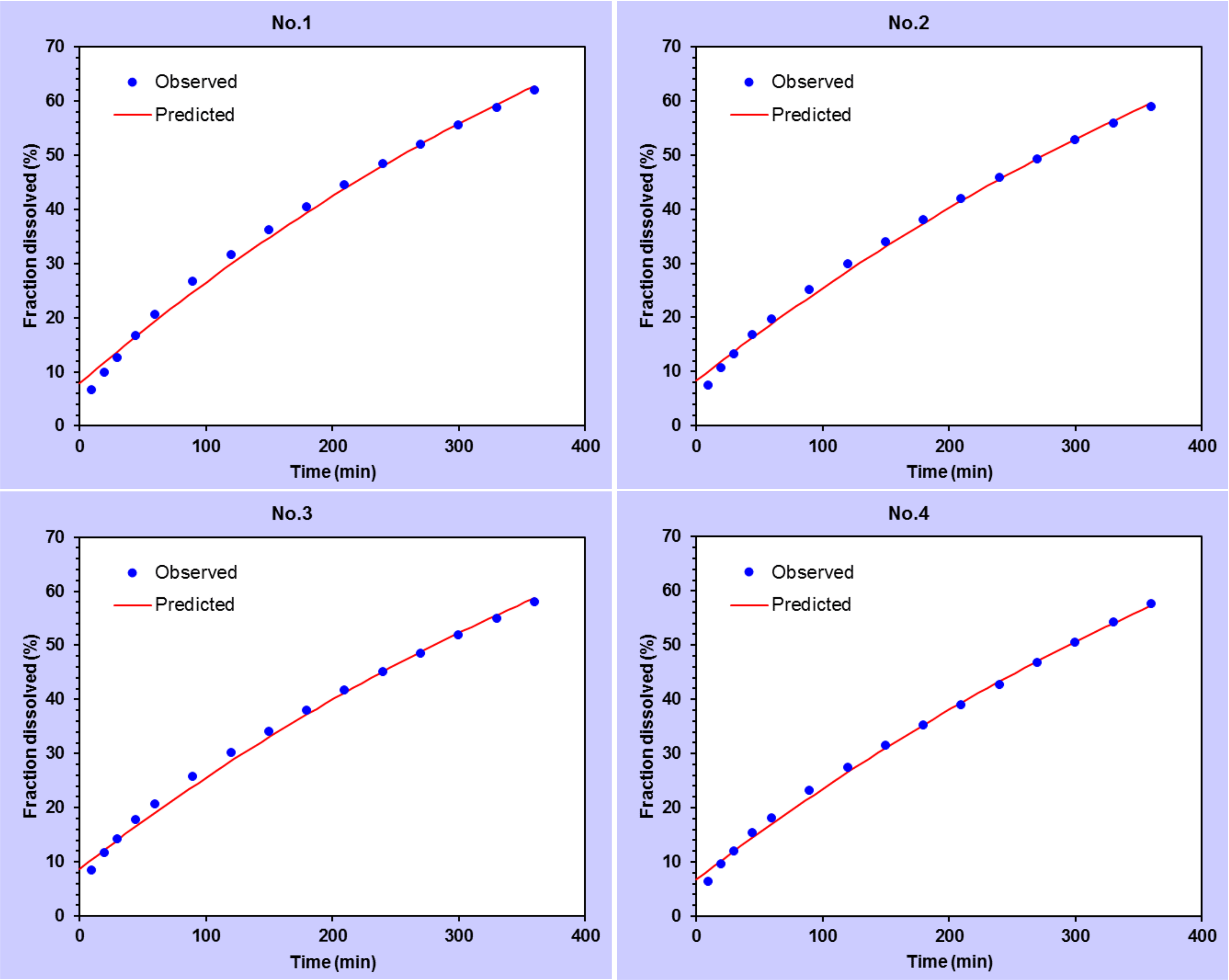

Supplementary materials – Model fitting summary – Lactochem® Fine Powder (modelling up to app. 60 % of carvedilol released)

Model: **Baker–Lonsdale**

Model equation: $\frac{3}{2} \cdot \left[1 - \left(1 - \frac{F}{100}\right)^{\frac{2}{3}}\right] - \frac{F}{100} = k_{BL} \cdot t$

Fitted model parameters per tested tablet (N = 4) with statistics – mean, standard deviation (SD), and relative standard deviation expressed in % (RSD%) (output from DDSolver):

Parameter	No.1	No.2	No.3	No.4	Mean	SD	RSD(%)
k_{BL}	0.0002	0.0002	0.0002	0.0002	0.0002	0.0000	9.5766

Number of dissolution data points (N), degrees of freedom (df), and selected goodness of fit criteria – Pearson correlation coefficient (R), coefficient of determination (R^2), adjusted coefficient of determination ($R^2_{adjusted}$), and residual sum of squares (RSS) (manual calculation in MS Excel):

Parameter	No.1	No.2	No.3	No.4
N	15	15	15	15
df	14	14	14	14
R	0.996731673	0.995213732	0.995749696	0.992082227
R^2	0.993474028	0.990450373	0.991517457	0.984227144
$R^2_{adjusted}$	0.993474028	0.990450373	0.991517457	0.984227144
RSS	238.3275423	182.5184734	141.5824886	222.0182232

Graphical abstract of model fit presented as mean ± 1 SD of the fraction % of released carvedilol:

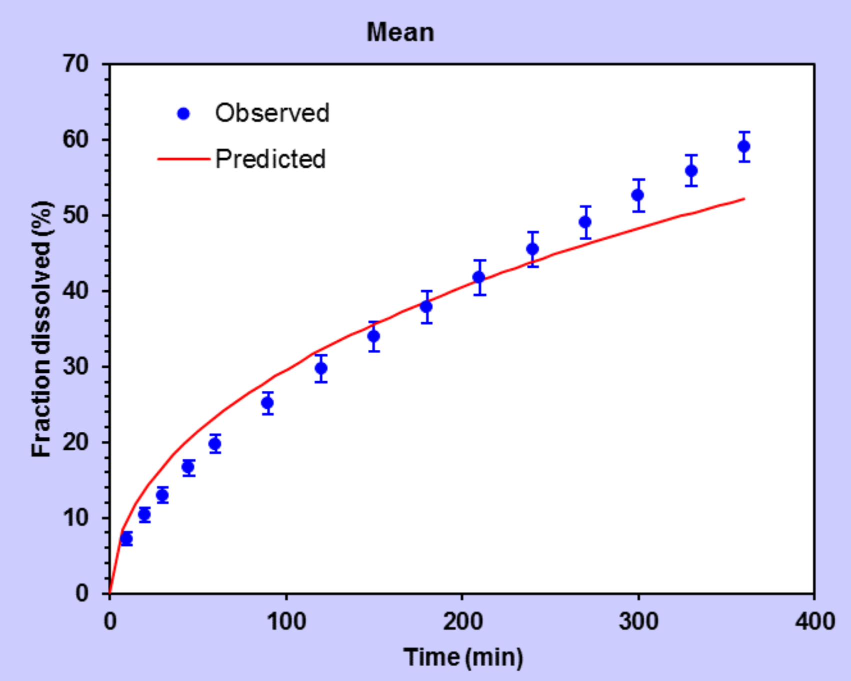

Supplementary materials – Model fitting summary – Lactochem® Fine Powder (modelling up to app. 60 % of carvedilol released)

Graphical abstract of model fit presented as the fraction % of released carvedilol per tested tablet:

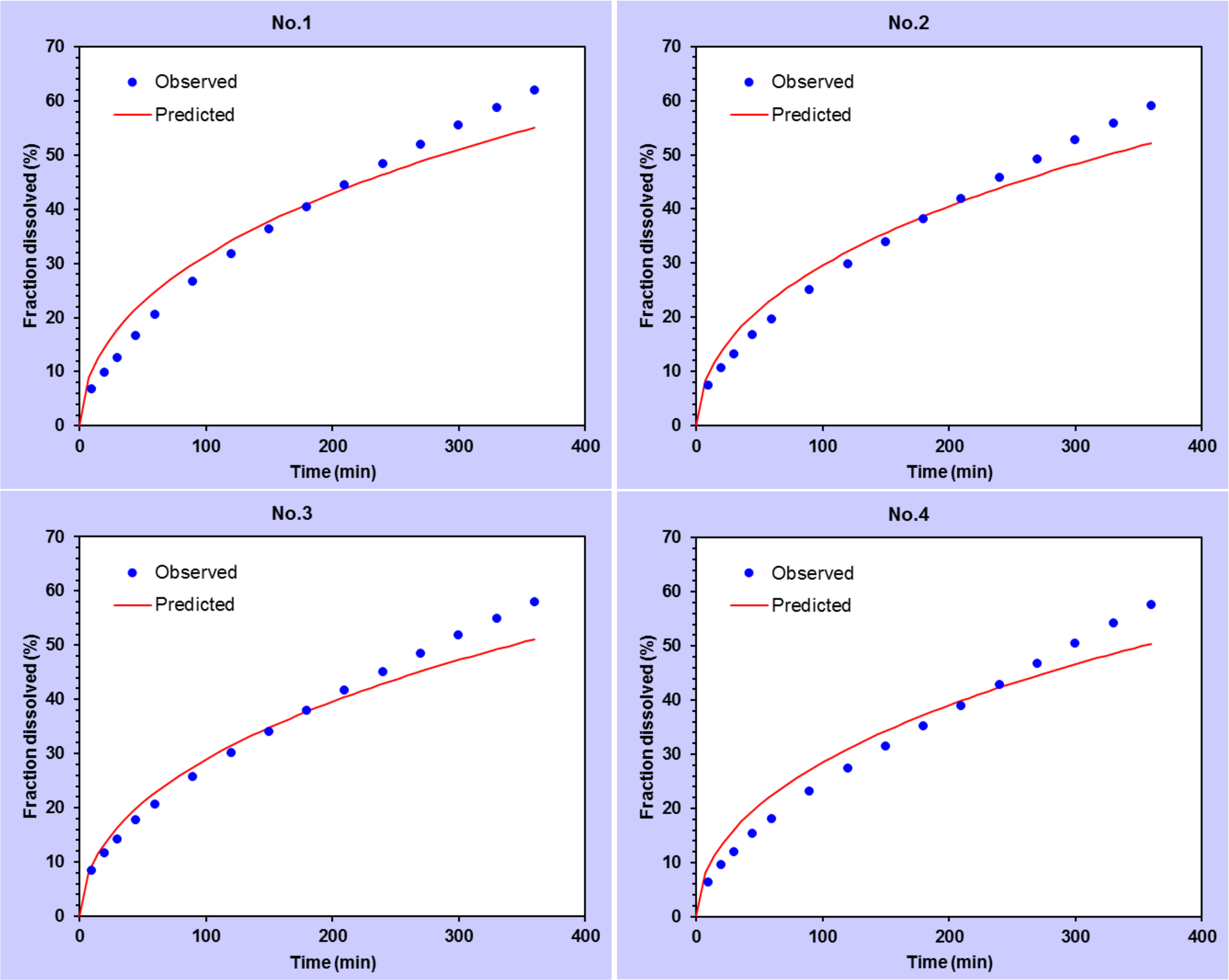

Supplementary materials – Model fitting summary – Lactochem® Fine Powder (modelling up to app. 60 % of carvedilol released)

Model: **Baker–Lonsdale with T_{lag}**

Model equation: $\frac{3}{2} \cdot \left[1 - \left(1 - \frac{F}{100}\right)^{\frac{2}{3}}\right] - \frac{F}{100} = k_{BL} \cdot (t - T_{lag})$

Fitted model parameters per tested tablet (N = 4) with statistics – mean, standard deviation (SD), and relative standard deviation expressed in % (RSD%) (output from DDSolver):

Parameter	No.1	No.2	No.3	No.4	Mean	SD	RSD(%)
k_{BL}	0.0003	0.0002	0.0002	0.0002	0.0002	0.0000	9.5766
T_{lag}	29.1661	28.1143	23.9285	32.9288	28.5344	3.7014	12.9717

Number of dissolution data points (N), degrees of freedom (df), and selected goodness of fit criteria – Pearson correlation coefficient (R), coefficient of determination (R^2), adjusted coefficient of determination ($R^2_{adjusted}$), and residual sum of squares (RSS) (manual calculation in MS Excel):

Parameter	No.1	No.2	No.3	No.4
N	15	15	15	15
df	13	13	13	13
R	0.986623912	0.983834089	0.98379091	0.977577041
R^2	0.973426743	0.967929514	0.967844555	0.955656871
$R^2_{adjusted}$	0.971382646	0.965462554	0.965371059	0.952245861
RSS	264.0378655	278.5751749	269.7903042	352.1096431

Graphical abstract of model fit presented as mean ± 1 SD of the fraction % of released carvedilol:

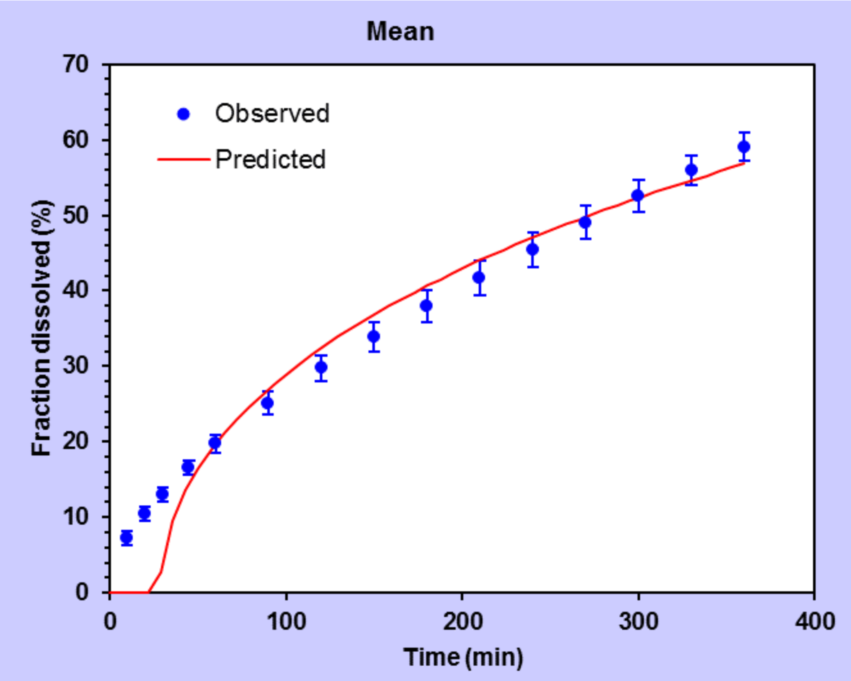

Supplementary materials – Model fitting summary – Lactochem® Fine Powder (modelling up to app. 60 % of carvedilol released)

Graphical abstract of model fit presented as the fraction % of released carvedilol per tested tablet:

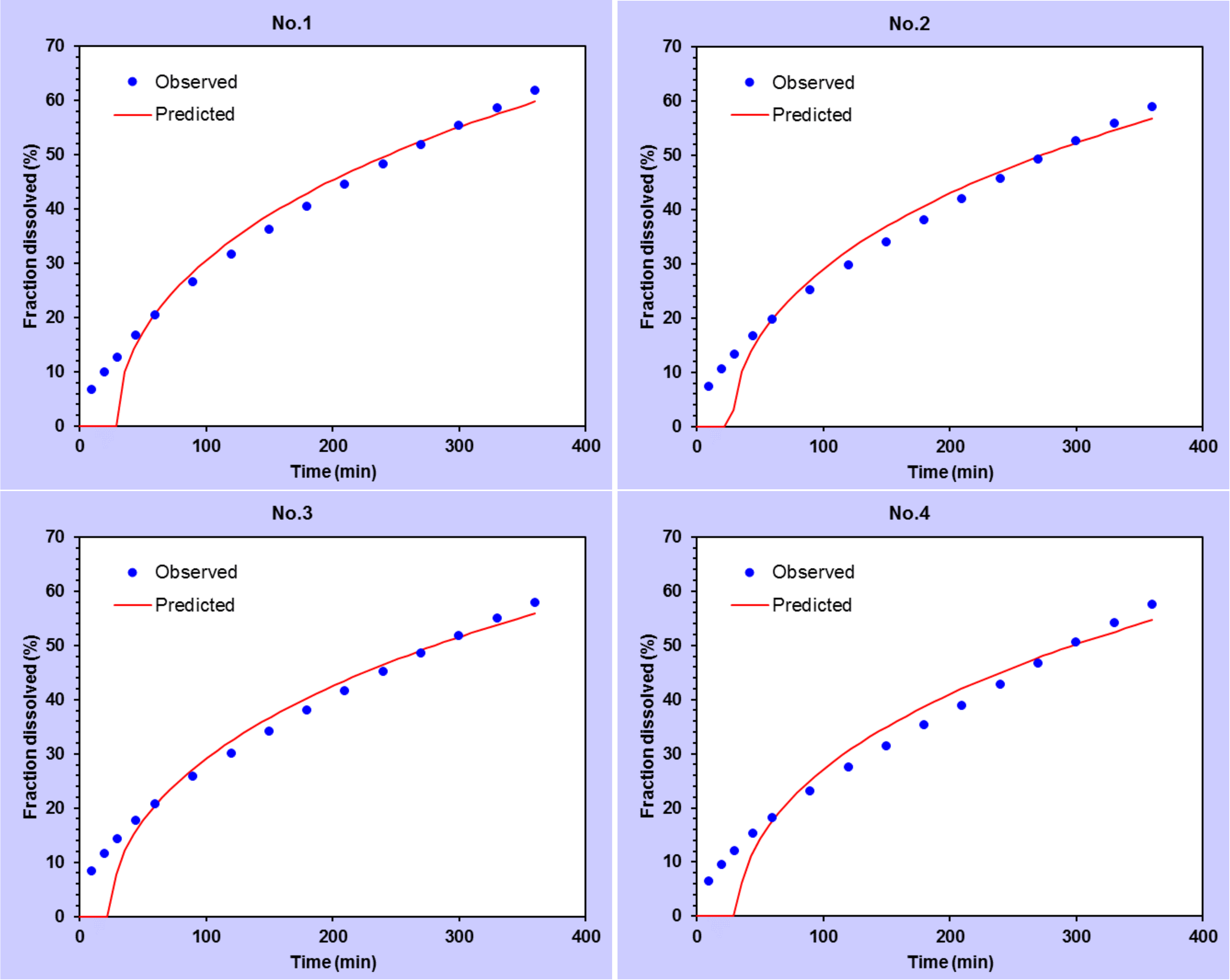

Supplementary materials – Model fitting summary – Lactochem® Fine Powder (modelling up to app. 60 % of carvedilol released)

Model: **Makoid–Banakar**

Model equation: $F = k_{MB} \cdot t^n \cdot e^{-k \cdot t}$

Fitted model parameters per tested tablet (N = 4) with statistics – mean, standard deviation (SD), and relative standard deviation expressed in % (RSD%) (output from DDSolver):

Parameter	No.1	No.2	No.3	No.4	Mean	SD	RSD(%)
k_{MB}	1.5046	2.0581	2.6169	1.7561	1.9839	0.4788	24.1353
n	0.6357	0.5469	0.4973	0.5606	0.5601	0.0572	10.2200
k	0.0001	-0.0004	-0.0005	-0.0005	-0.0003	0.0003	-80.1428

Number of dissolution data points (N), degrees of freedom (df), and selected goodness of fit criteria – Pearson correlation coefficient (R), coefficient of determination (R^2), adjusted coefficient of determination ($R^2_{adjusted}$), and residual sum of squares (RSS) (manual calculation in MS Excel):

Parameter	No.1	No.2	No.3	No.4
N	15	15	15	15
df	12	12	12	12
R	0.999923004	0.999917536	0.999950587	0.999943096
R^2	0.999846015	0.999835079	0.999901176	0.999886195
$R^2_{adjusted}$	0.99982035	0.999807592	0.999884706	0.999867228
RSS	0.754730184	0.697710787	0.380242454	0.458871263

Graphical abstract of model fit presented as mean ± 1 SD of the fraction % of released carvedilol:

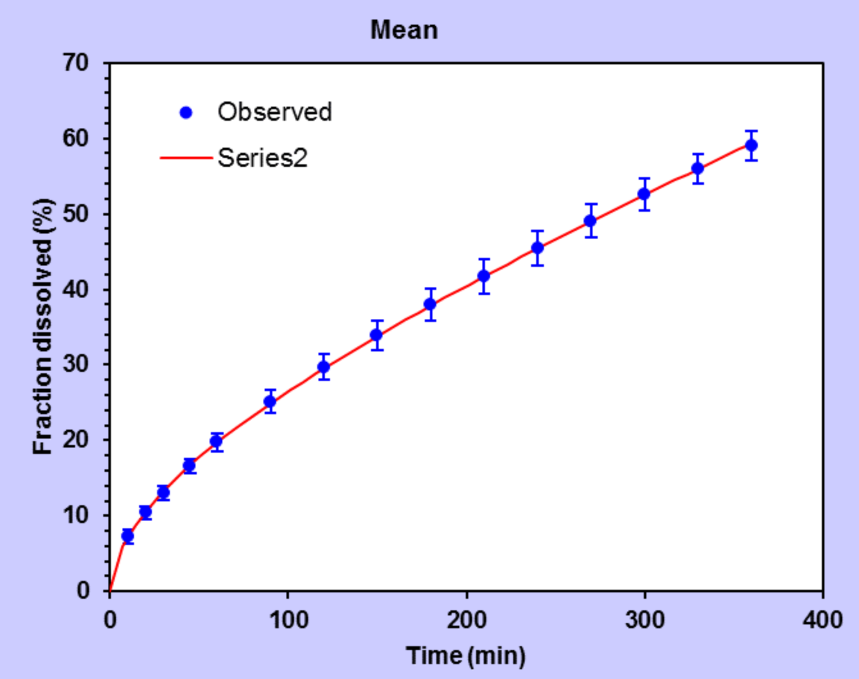

Supplementary materials – Model fitting summary – Lactochem® Fine Powder (modelling up to app. 60 % of carvedilol released)

Graphical abstract of model fit presented as the fraction % of released carvedilol per tested tablet:

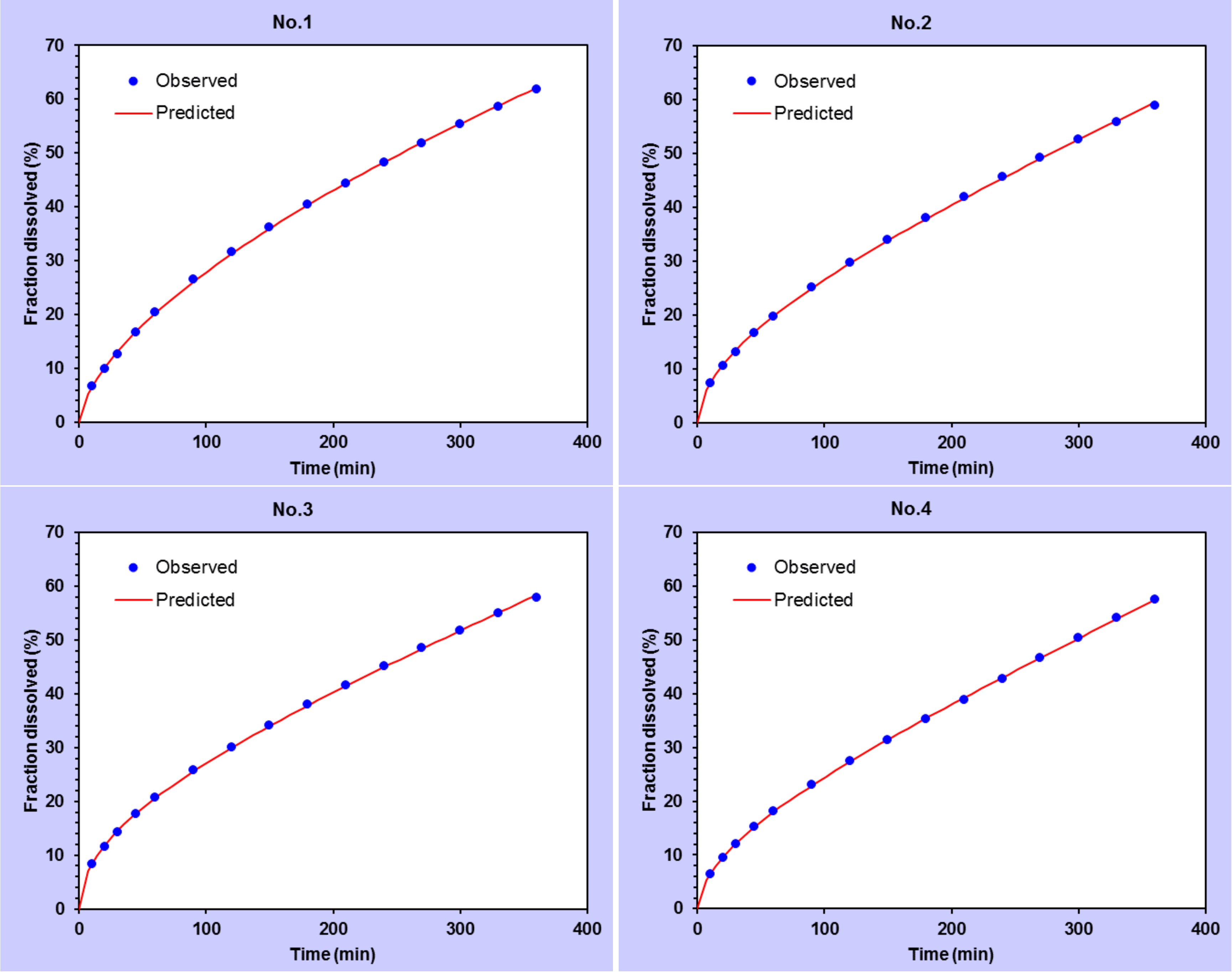

Supplementary materials – Model fitting summary – Lactochem® Fine Powder (modelling up to app. 60 % of carvedilol released)

Model: **Makoid–Banakar with T_{lag}**

Model equation: $F = k_{MB} \cdot (t - T_{lag})^n \cdot e^{-k \cdot (t - T_{lag})}$

Fitted model parameters per tested tablet (N = 4) with statistics – mean, standard deviation (SD), and relative standard deviation expressed in % (RSD%) (output from DDSolver):

Parameter	No.1	No.2	No.3	No.4	Mean	SD	RSD(%)
k_{MB}	2.4545	3.1370	3.8412	2.6945	3.0318	0.6091	20.0920
n	0.5182	0.4463	0.4058	0.4589	0.4573	0.0465	10.1677
k	-0.0006	-0.0010	-0.0010	-0.0011	-0.0009	0.0002	-24.1501
T_{lag}	4.0000	4.0000	4.0000	4.0000	4.0000	0.0000	0.0000

Number of dissolution data points (N), degrees of freedom (df), and selected goodness of fit criteria – Pearson correlation coefficient (R), coefficient of determination (R^2), adjusted coefficient of determination ($R^2_{adjusted}$), and residual sum of squares (RSS) (manual calculation in MS Excel):

Parameter	No.1	No.2	No.3	No.4
N	15	15	15	15
df	11	11	11	11
R	0.999208839	0.999195333	0.999361269	0.999779464
R^2	0.998418304	0.998391313	0.998722946	0.999558976
$R^2_{adjusted}$	0.997986933	0.99795258	0.998374658	0.999438697
RSS	7.892163885	6.919160504	4.986598833	1.83451949

Graphical abstract of model fit presented as mean ± 1 SD of the fraction % of released carvedilol:

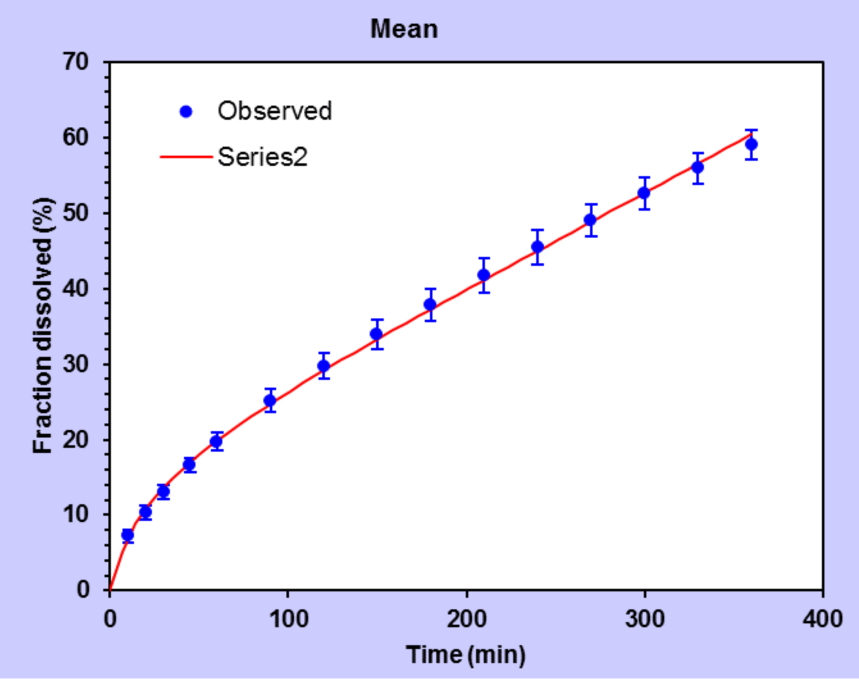

Supplementary materials – Model fitting summary – Lactochem® Fine Powder (modelling up to app. 60 % of carvedilol released)

Graphical abstract of model fit presented as the fraction % of released carvedilol per tested tablet:

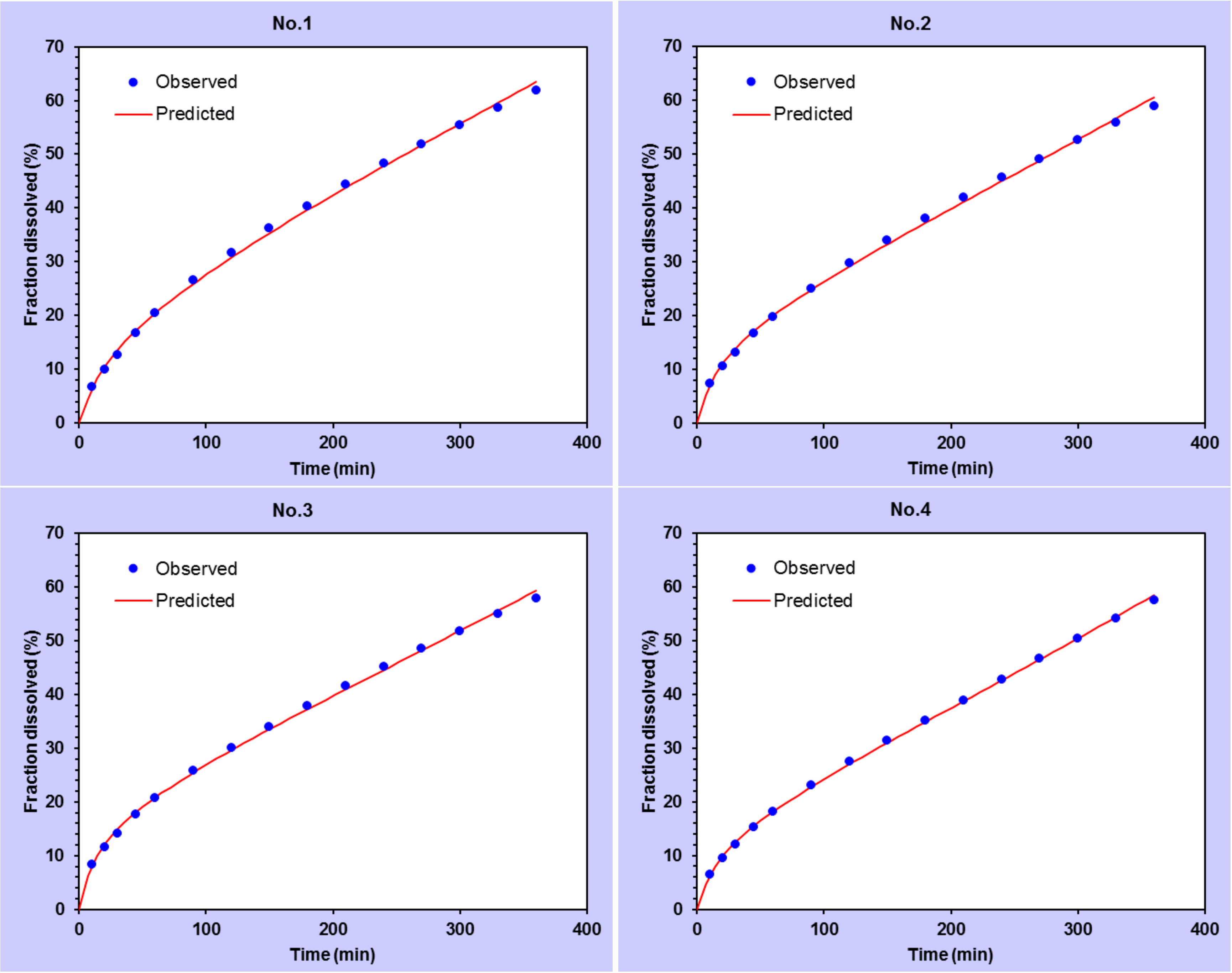

Supplementary materials – Model fitting summary – Lactochem® Fine Powder (modelling up to app. 60 % of carvedilol released)

Model: **Peppas–Sahlin_1**

Model equation: $F = k_1 \cdot t^m + k_2 \cdot t^{2m}$

Fitted model parameters per tested tablet (N = 4) with statistics – mean, standard deviation (SD), and relative standard deviation expressed in % (RSD%) (output from DDSolver):

Parameter	No.1	No.2	No.3	No.4	Mean	SD	RSD(%)
k_1	2.253	2.268	2.581	1.829	2.233	0.309	13.832
k_2	0.154	0.136	0.107	0.156	0.138	0.023	16.628
m	0.450	0.450	0.450	0.450	0.450	0.000	0.000

Number of dissolution data points (N), degrees of freedom (df), and selected goodness of fit criteria – Pearson correlation coefficient (R), coefficient of determination (R^2), adjusted coefficient of determination ($R^2_{adjusted}$), and residual sum of squares (RSS) (manual calculation in MS Excel):

Parameter	No.1	No.2	No.3	No.4
N	15	15	15	15
df	12	12	12	12
R	0.999396703	0.999975714	0.999963133	0.999797043
R^2	0.998793771	0.999951429	0.999926267	0.999594128
$R^2_{adjusted}$	0.998592733	0.999943334	0.999913978	0.999526482
RSS	6.490412151	0.214895848	0.303928386	1.662089886

Graphical abstract of model fit presented as mean ± 1 SD of the fraction % of released carvedilol:

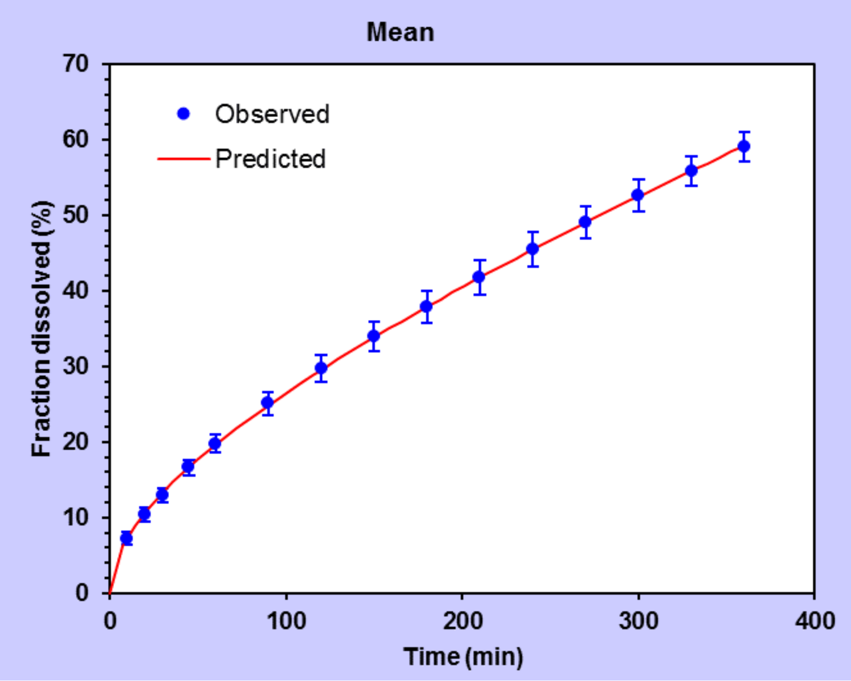

Supplementary materials – Model fitting summary – Lactochem® Fine Powder (modelling up to app. 60 % of carvedilol released)

Graphical abstract of model fit presented as the fraction % of released carvedilol per tested tablet:

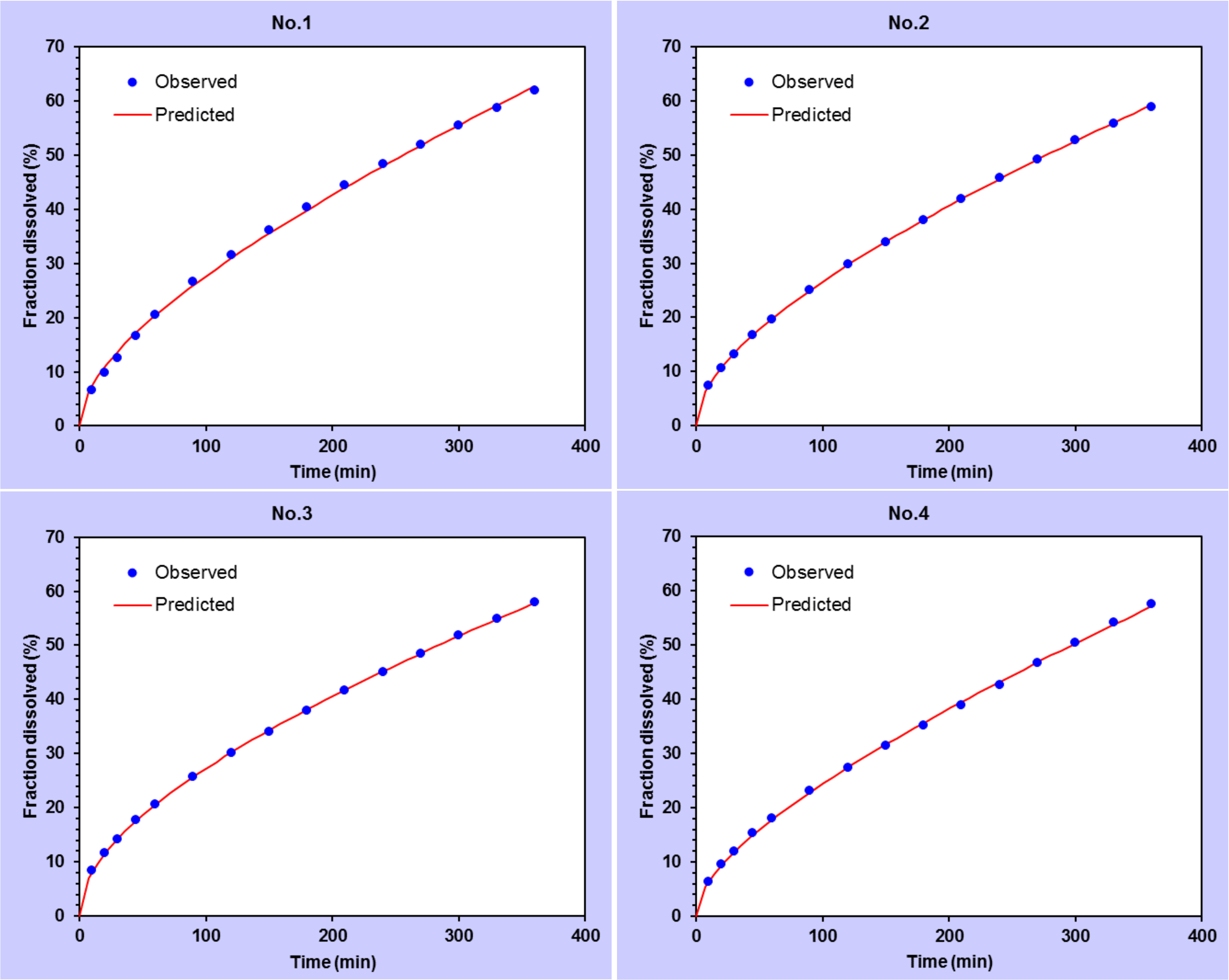

Supplementary materials – Model fitting summary – Lactochem® Fine Powder (modelling up to app. 60 % of carvedilol released)

Model: **Peppas–Sahlin_1 with T_{lag}**

Model equation: $F = k_1 \cdot (t - T_{lag})^m + k_2 \cdot (t - T_{lag})^{2m}$

Fitted model parameters per tested tablet (N = 4) with statistics – mean, standard deviation (SD), and relative standard deviation expressed in % (RSD%) (output from DDSolver):

Parameter	No.1	No.2	No.3	No.4	Mean	SD	RSD(%)
k_1	2.527	2.522	2.836	2.062	2.487	0.319	12.822
k_2	0.136	0.118	0.089	0.141	0.121	0.023	19.273
m	0.450	0.450	0.450	0.450	0.450	0.000	0.000
T_{lag}	4.000	4.000	4.000	4.000	4.000	0.000	0.000

Number of dissolution data points (N), degrees of freedom (df), and selected goodness of fit criteria – Pearson correlation coefficient (R), coefficient of determination (R^2), adjusted coefficient of determination ($R^2_{adjusted}$), and residual sum of squares (RSS) (manual calculation in MS Excel):

Parameter	No.1	No.2	No.3	No.4
N	15	15	15	15
df	11	11	11	11
R	0.999745178	0.999815047	0.999538457	0.999503933
R^2	0.999490421	0.999630128	0.999077128	0.999008113
$R^2_{adjusted}$	0.999351445	0.999529253	0.998825435	0.998737598
RSS	2.552156893	1.786405953	4.234984671	4.502120029

Graphical abstract of model fit presented as mean ± 1 SD of the fraction % of released carvedilol:

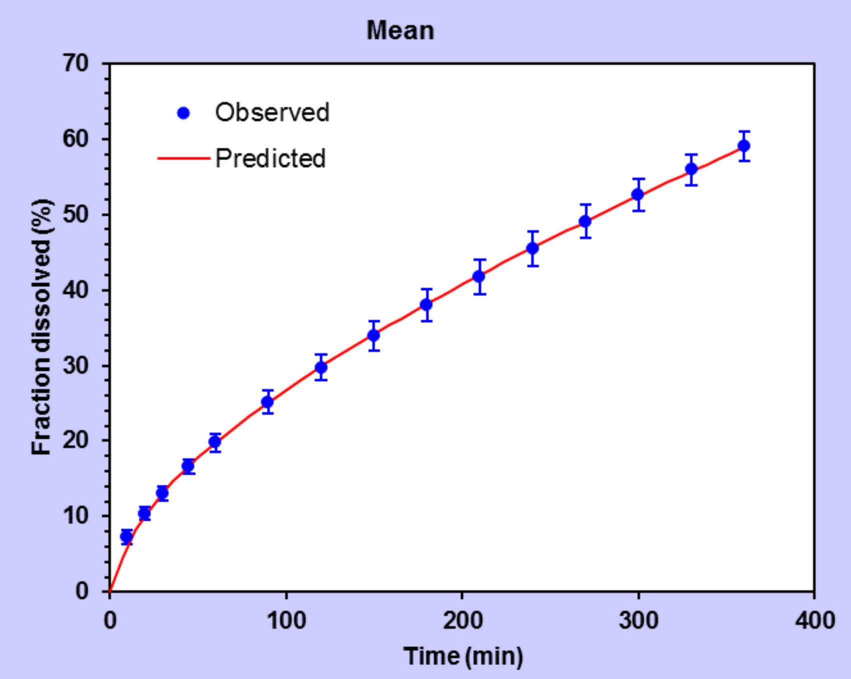

Supplementary materials – Model fitting summary – Lactochem® Fine Powder (modelling up to app. 60 % of carvedilol released)

Graphical abstract of model fit presented as the fraction % of released carvedilol per tested tablet:

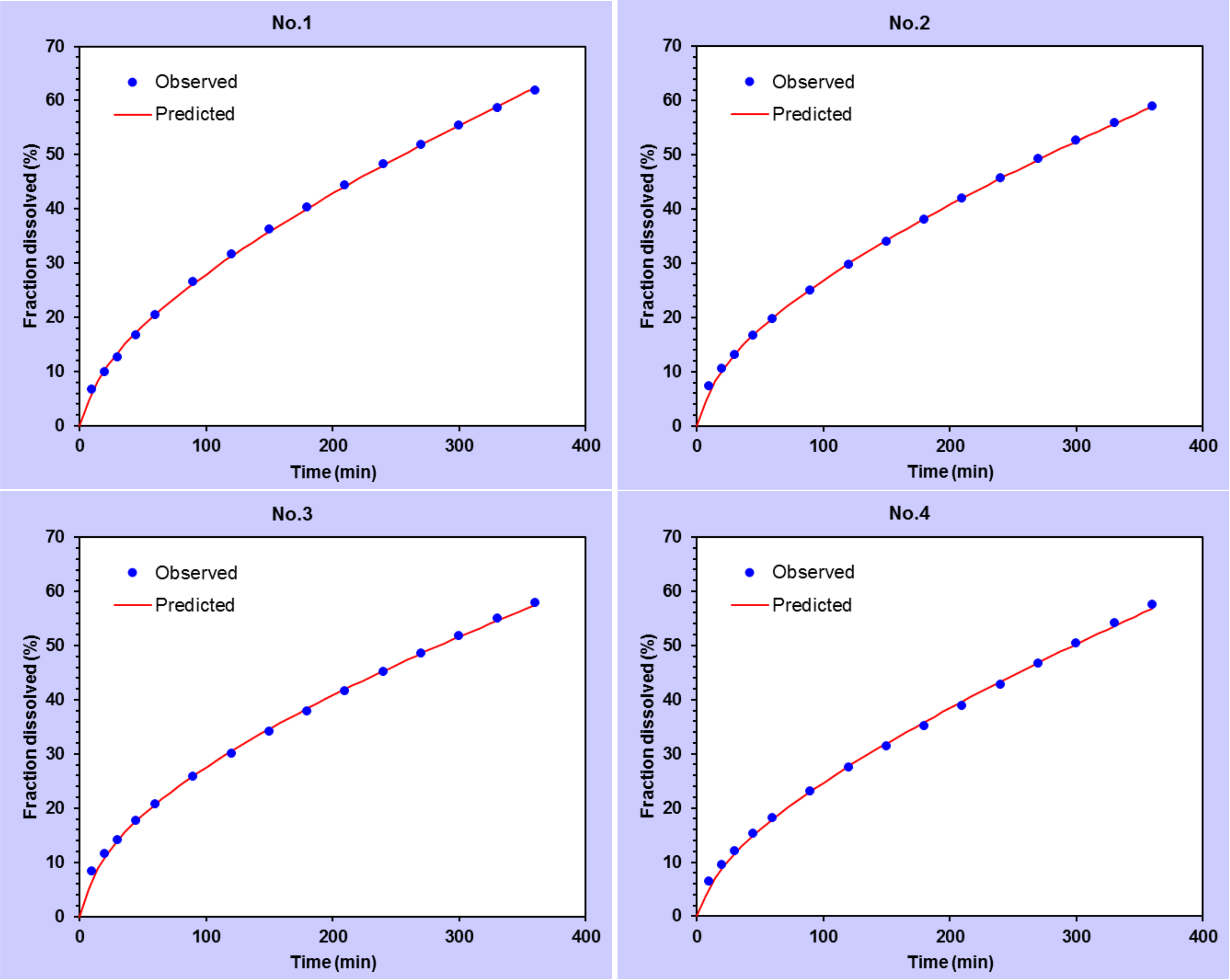

Supplementary materials – Model fitting summary – Lactochem® Fine Powder (modelling up to app. 60 % of carvedilol released)

Model: **Peppas–Sahlin_2**

Model equation: $F = k_1 \cdot t^{0.5} + k_2 \cdot t$

Fitted model parameters per tested tablet (N = 4) with statistics – mean, standard deviation (SD), and relative standard deviation expressed in % (RSD%) (output from DDSolver):

Parameter	No.1	No.2	No.3	No.4	Mean	SD	RSD(%)
k_1	2.184	2.158	2.382	1.801	2.131	0.242	11.345
k_2	0.059	0.051	0.035	0.064	0.052	0.013	24.414

Number of dissolution data points (N), degrees of freedom (df), and selected goodness of fit criteria – Pearson correlation coefficient (R), coefficient of determination (R^2), adjusted coefficient of determination ($R^2_{adjusted}$), and residual sum of squares (RSS) (manual calculation in MS Excel):

Parameter	No.1	No.2	No.3	No.4
N	15	15	15	15
df	13	13	13	13
R	0.99947768	0.999983529	0.999914531	0.999815208
R^2	0.998955632	0.999967059	0.99982907	0.999630449
$R^2_{adjusted}$	0.998875296	0.999964525	0.999815922	0.999602022
RSS	5.863584921	0.139785647	0.768692073	1.543304712

Graphical abstract of model fit presented as mean ± 1 SD of the fraction % of released carvedilol:

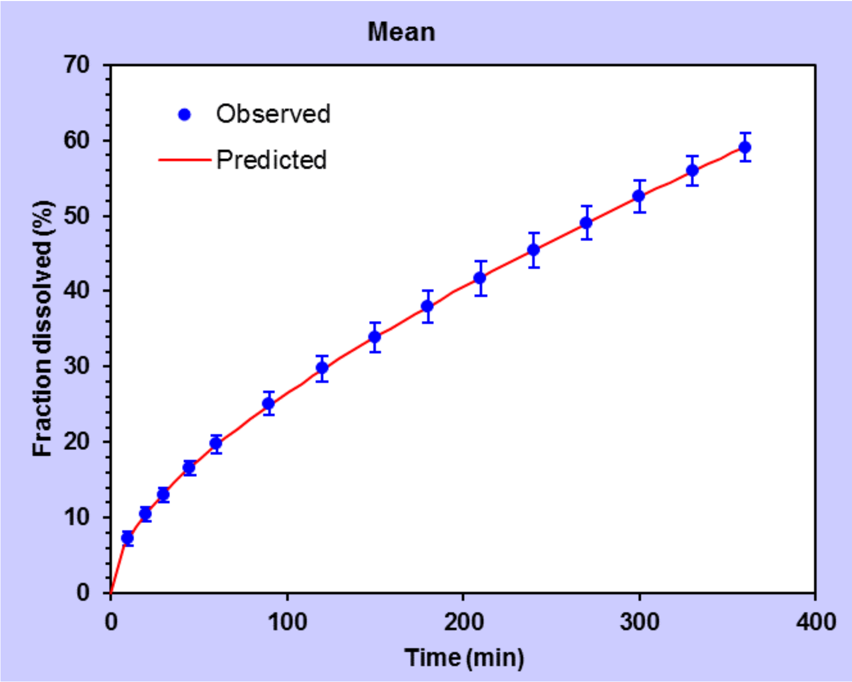

Supplementary materials – Model fitting summary – Lactochem® Fine Powder (modelling up to app. 60 % of carvedilol released)

Graphical abstract of model fit presented as the fraction % of released carvedilol per tested tablet:

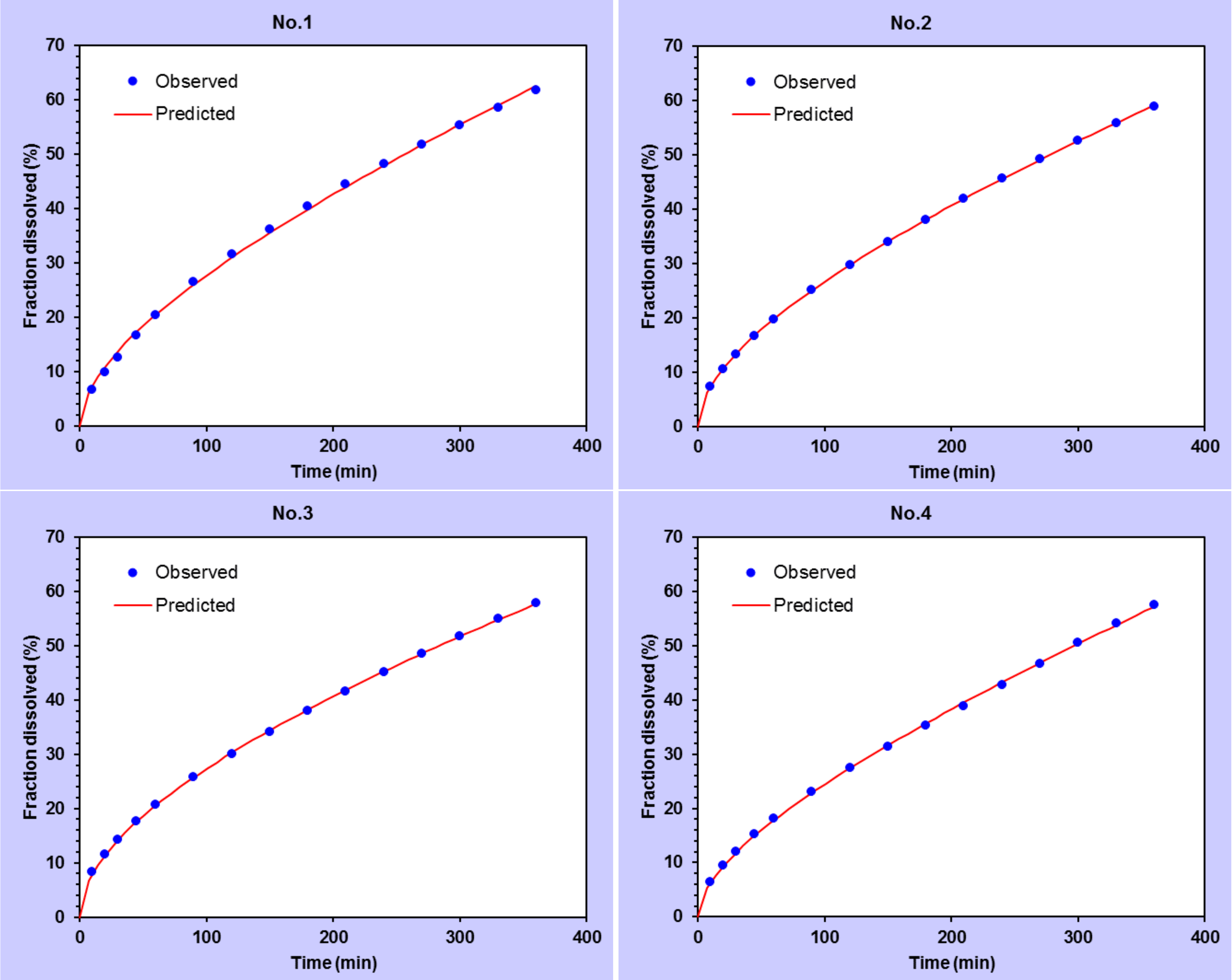

Supplementary materials – Model fitting summary – Lactochem® Fine Powder (modelling up to app. 60 % of carvedilol released)

Model: **Peppas–Sahlin_2 with T_{lag}**

Model equation: $F = k_1 \cdot (t - T_{lag})^{0.5} + k_2 \cdot (t - T_{lag})$

Fitted model parameters per tested tablet (N = 4) with statistics – mean, standard deviation (SD), and relative standard deviation expressed in % (RSD%) (output from DDSolver):

Parameter	No.1	No.2	No.3	No.4	Mean	SD	RSD(%)
k_1	2.388	2.221	2.569	1.973	2.288	0.254	11.083
k_2	0.048	0.049	0.025	0.055	0.044	0.013	29.903
T_{lag}	4.000	1.697	4.000	4.000	3.424	1.152	33.628

Number of dissolution data points (N), degrees of freedom (df), and selected goodness of fit criteria – Pearson correlation coefficient (R), coefficient of determination (R^2), adjusted coefficient of determination ($R^2_{adjusted}$), and residual sum of squares (RSS) (manual calculation in MS Excel):

Parameter	No.1	No.2	No.3	No.4
N	15	15	15	15
df	12	12	12	12
R	0.99978177	0.999965646	0.999326885	0.999475964
R^2	0.999563587	0.999931294	0.998654224	0.998952203
$R^2_{adjusted}$	0.999490852	0.999919843	0.998429927	0.99877757
RSS	2.159489052	2.263969495	6.648118887	5.060424664

Graphical abstract of model fit presented as mean ± 1 SD of the fraction % of released carvedilol:

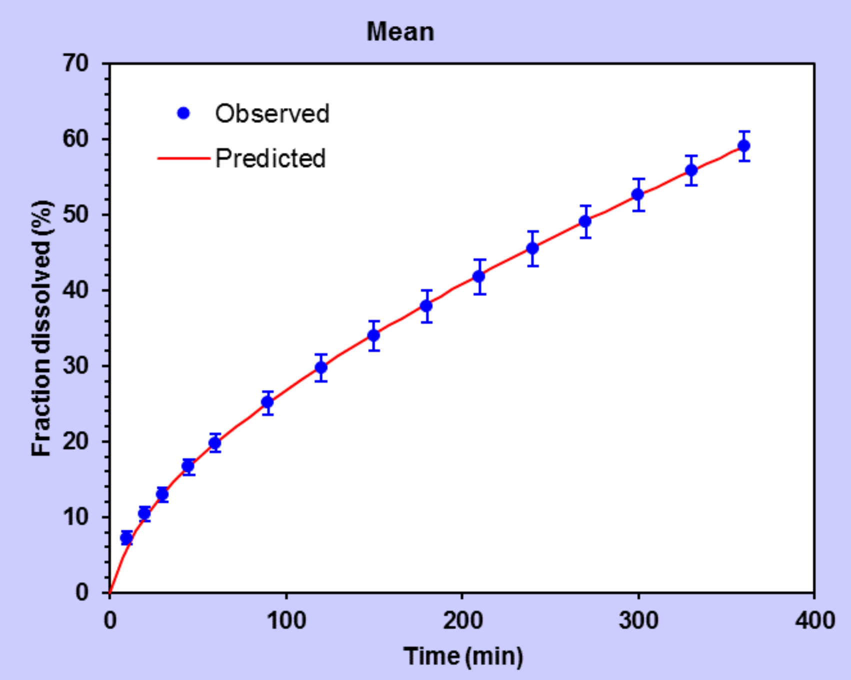

Supplementary materials – Model fitting summary – Lactochem® Fine Powder (modelling up to app. 60 % of carvedilol released)

Graphical abstract of model fit presented as the fraction % of released carvedilol per tested tablet:

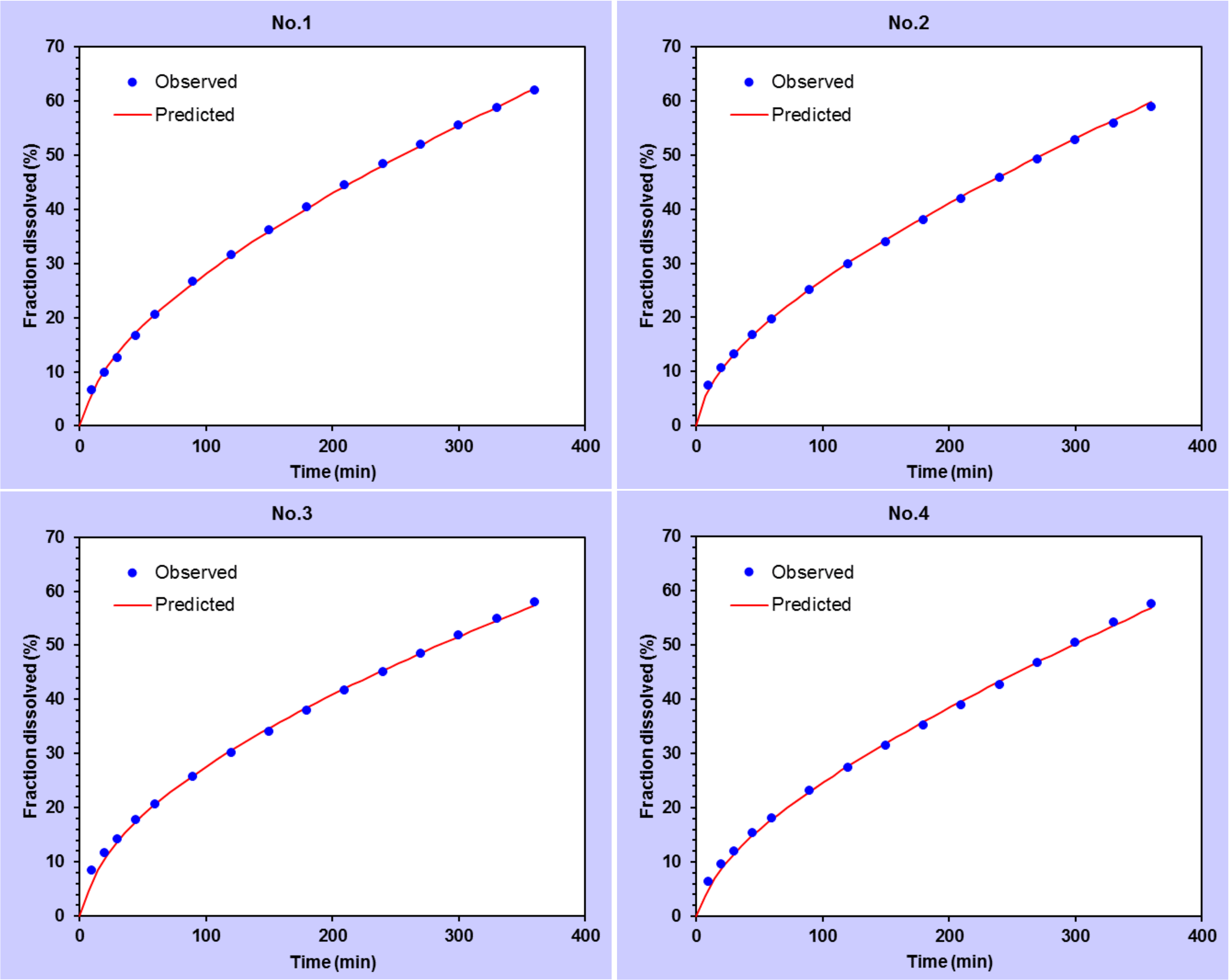

Supplementary materials – Model fitting summary – Lactochem® Fine Powder (modelling up to app. 60 % of carvedilol released)

Model: **Quadratic**

Model equation: $F = 100 \cdot (k_1 \cdot t^2 + k_2 \cdot t)$

Fitted model parameters per tested tablet (N = 4) with statistics – mean, standard deviation (SD), and relative standard deviation expressed in % (RSD%) (output from DDSolver):

Parameter	No.1	No.2	No.3	No.4	Mean	SD	RSD(%)
k_1	-0.000004	-0.000004	-0.000004	-0.000003	-0.000004	0.000000	-12.673385
k_2	0.003050	0.002892	0.002956	0.002598	0.002874	0.000195	6.785228

Number of dissolution data points (N), degrees of freedom (df), and selected goodness of fit criteria – Pearson correlation coefficient (R), coefficient of determination (R^2), adjusted coefficient of determination ($R^2_{adjusted}$), and residual sum of squares (RSS) (manual calculation in MS Excel):

Parameter	No.1	No.2	No.3	No.4
N	15	15	15	15
df	13	13	13	13
R	0.995957288	0.994391519	0.991885631	0.994265856
R^2	0.99193092	0.988814493	0.983837105	0.988564591
$R^2_{adjusted}$	0.991310222	0.98795407	0.982593805	0.987684945
RSS	103.6736396	135.8364981	189.2012859	117.2872329

Graphical abstract of model fit presented as mean ± 1 SD of the fraction % of released carvedilol:

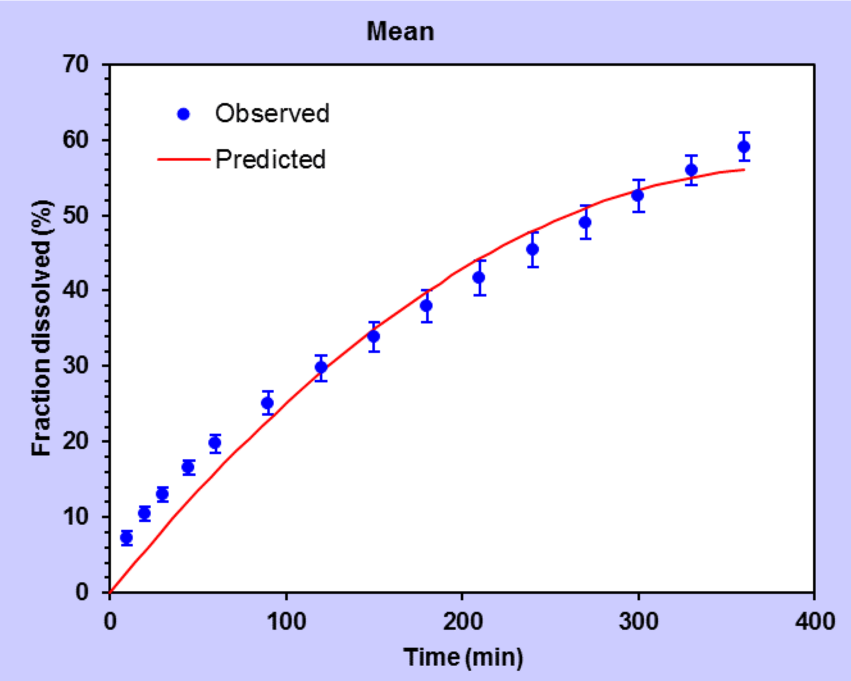

Supplementary materials – Model fitting summary – Lactochem® Fine Powder (modelling up to app. 60 % of carvedilol released)

Graphical abstract of model fit presented as the fraction % of released carvedilol per tested tablet:

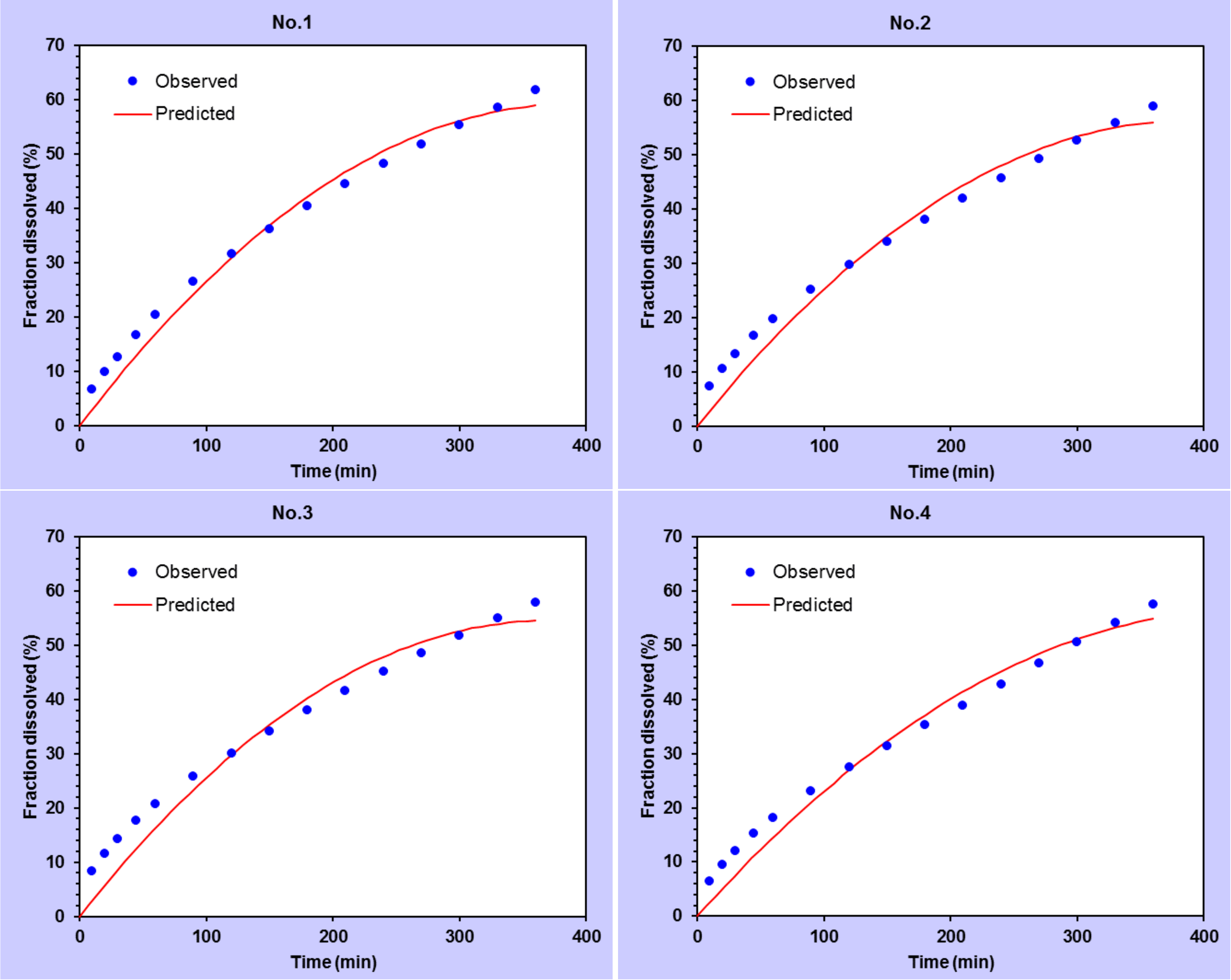

Supplementary materials – Model fitting summary – Lactochem® Fine Powder (modelling up to app. 60 % of carvedilol released)

Model: **Quadratic with T_{lag}**

Model equation: $F = 100 \cdot \left[k_1 \cdot (t - T_{lag})^2 + k_2 \cdot (t - T_{lag})\right]$

Fitted model parameters per tested tablet (N = 4) with statistics – mean, standard deviation (SD), and relative standard deviation expressed in % (RSD%) (output from DDSolver):

Parameter	No.1	No.2	No.3	No.4	Mean	SD	RSD(%)
k_1	-0.000004	-0.000004	-0.000004	-0.000003	-0.000004	0.000000	-12.294225
k_2	0.003146	0.002978	0.003039	0.002679	0.002960	0.000200	6.763616
T_{lag}	4.000000	4.000000	4.000000	4.000000	4.000000	0.000000	0.000000

Number of dissolution data points (N), degrees of freedom (df), and selected goodness of fit criteria – Pearson correlation coefficient (R), coefficient of determination (R^2), adjusted coefficient of determination ($R^2_{adjusted}$), and residual sum of squares (RSS) (manual calculation in MS Excel):

Parameter	No.1	No.2	No.3	No.4
N	15	15	15	15
df	12	12	12	12
R	0.995083186	0.993385305	0.99077744	0.993289177
R^2	0.990190547	0.986814365	0.981639935	0.986623388
$R^2_{adjusted}$	0.988555639	0.984616759	0.978579924	0.984393953
RSS	147.7537473	183.9940631	246.7393387	156.8556182

Graphical abstract of model fit presented as mean ± 1 SD of the fraction % of released carvedilol:

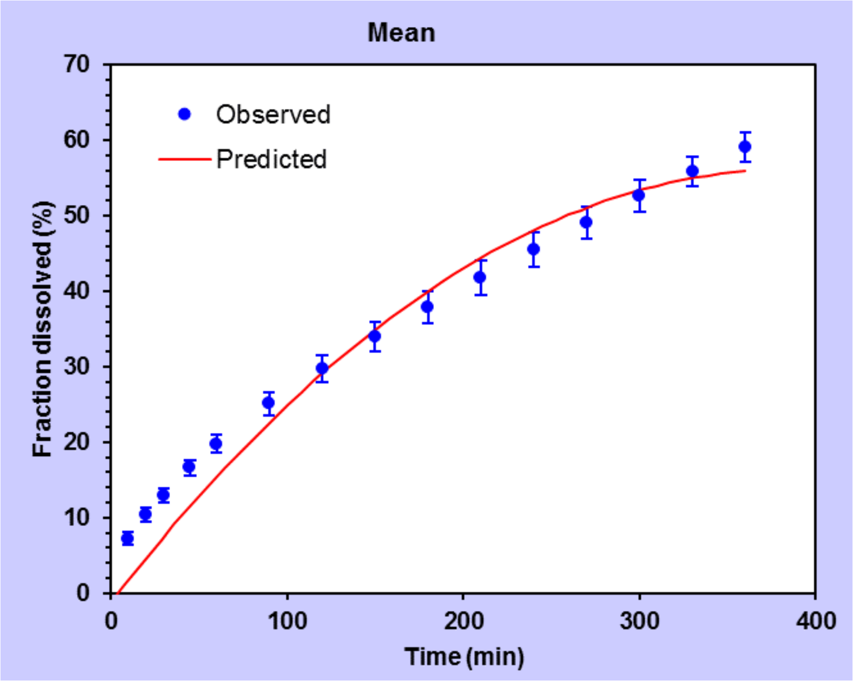

Supplementary materials – Model fitting summary – Lactochem® Fine Powder (modelling up to app. 60 % of carvedilol released)

Graphical abstract of model fit presented as the fraction % of released carvedilol per tested tablet:

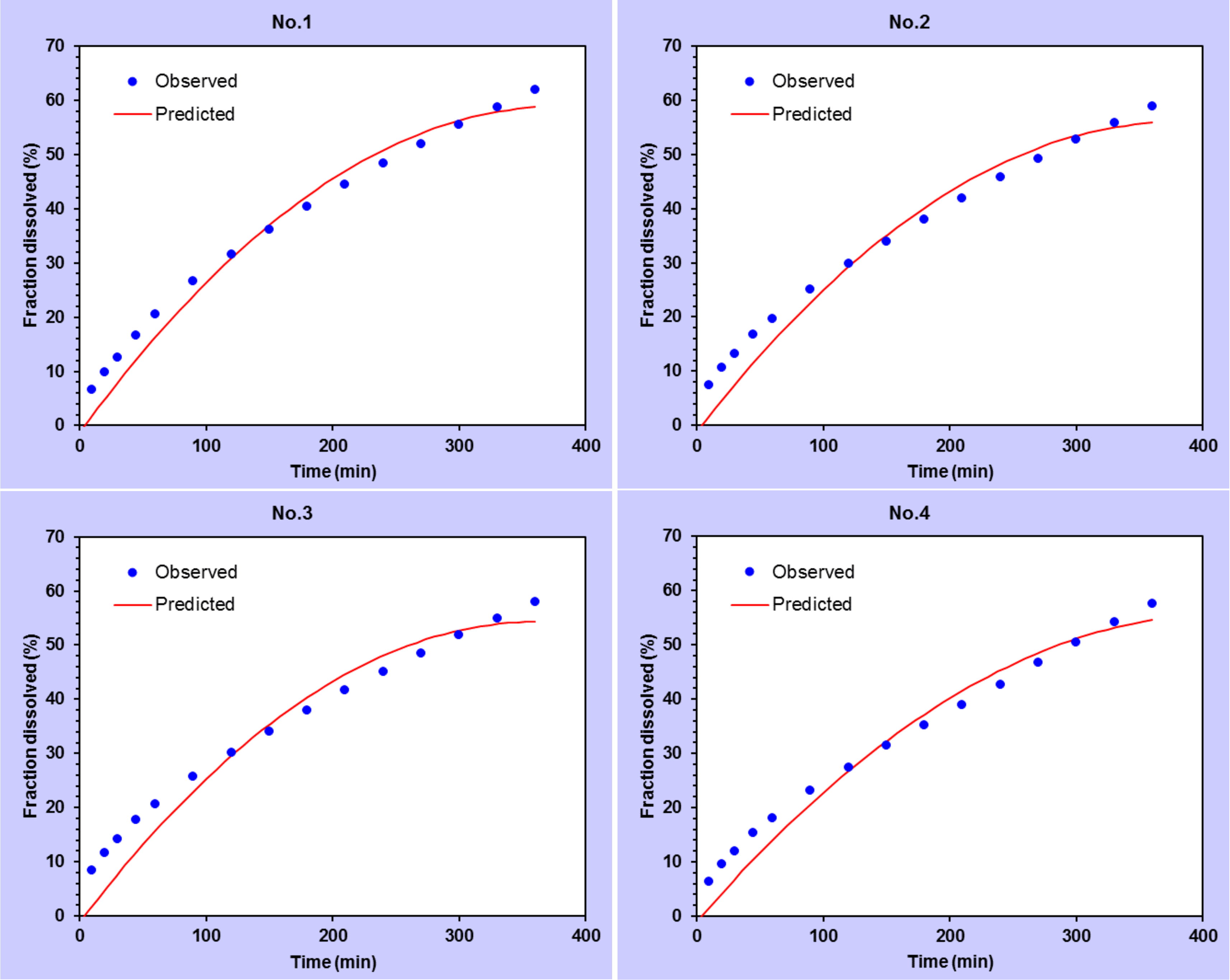

Supplementary materials – Model fitting summary – Lactochem® Fine Powder (modelling up to app. 60 % of carvedilol released)

Model: **Weibull_1**

Model equation: $F = 100 \cdot \left[1 - e^{-\frac{(t-T_i)^{\beta}}{\alpha}}\right]$

Fitted model parameters per tested tablet (N = 4) with statistics – mean, standard deviation (SD), and relative standard deviation expressed in % (RSD%) (output from DDSolver):

Parameter	No.1	No.2	No.3	No.4	Mean	SD	RSD(%)
α	64.819	49.916	40.398	59.457	53.648	10.771	20.077
β	0.696	0.622	0.581	0.641	0.635	0.048	7.491
T_i	4.768	4.000	4.000	4.000	4.192	0.384	9.162

Number of dissolution data points (N), degrees of freedom (df), and selected goodness of fit criteria – Pearson correlation coefficient (R), coefficient of determination (R^2), adjusted coefficient of determination ($R^2_{adjusted}$), and residual sum of squares (RSS) (manual calculation in MS Excel):

Parameter	No.1	No.2	No.3	No.4
N	15	15	15	15
df	12	12	12	12
R	0.996464133	0.99345422	0.992369877	0.991321079
R^2	0.992940768	0.986951287	0.984797973	0.982717482
$R^2_{adjusted}$	0.991764229	0.984776502	0.982264302	0.979837062
RSS	47.79651015	75.97341661	77.22297397	93.57177895

Graphical abstract of model fit presented as mean ± 1 SD of the fraction % of released carvedilol:

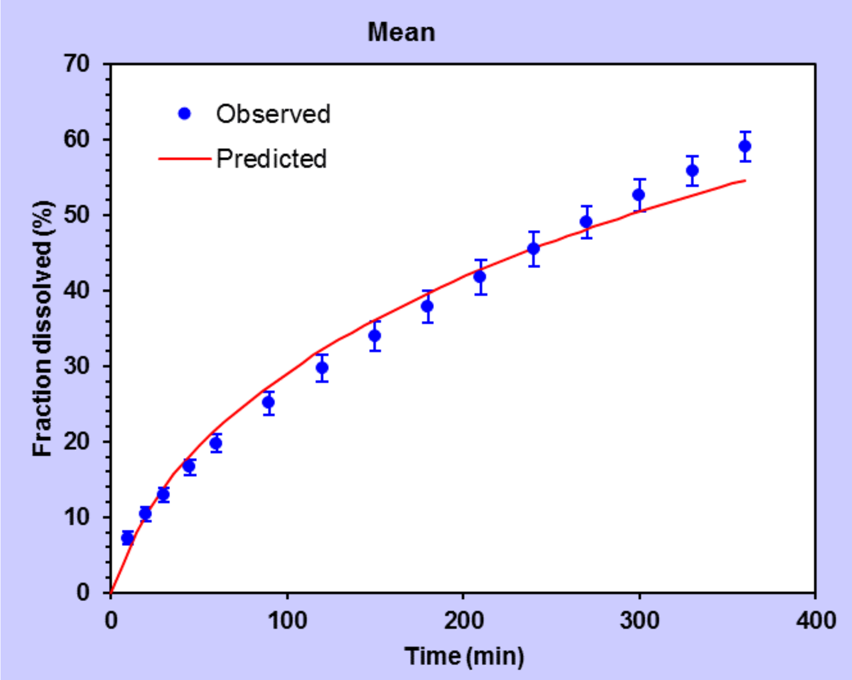

Supplementary materials – Model fitting summary – Lactochem® Fine Powder (modelling up to app. 60 % of carvedilol released)

Graphical abstract of model fit presented as the fraction % of released carvedilol per tested tablet:

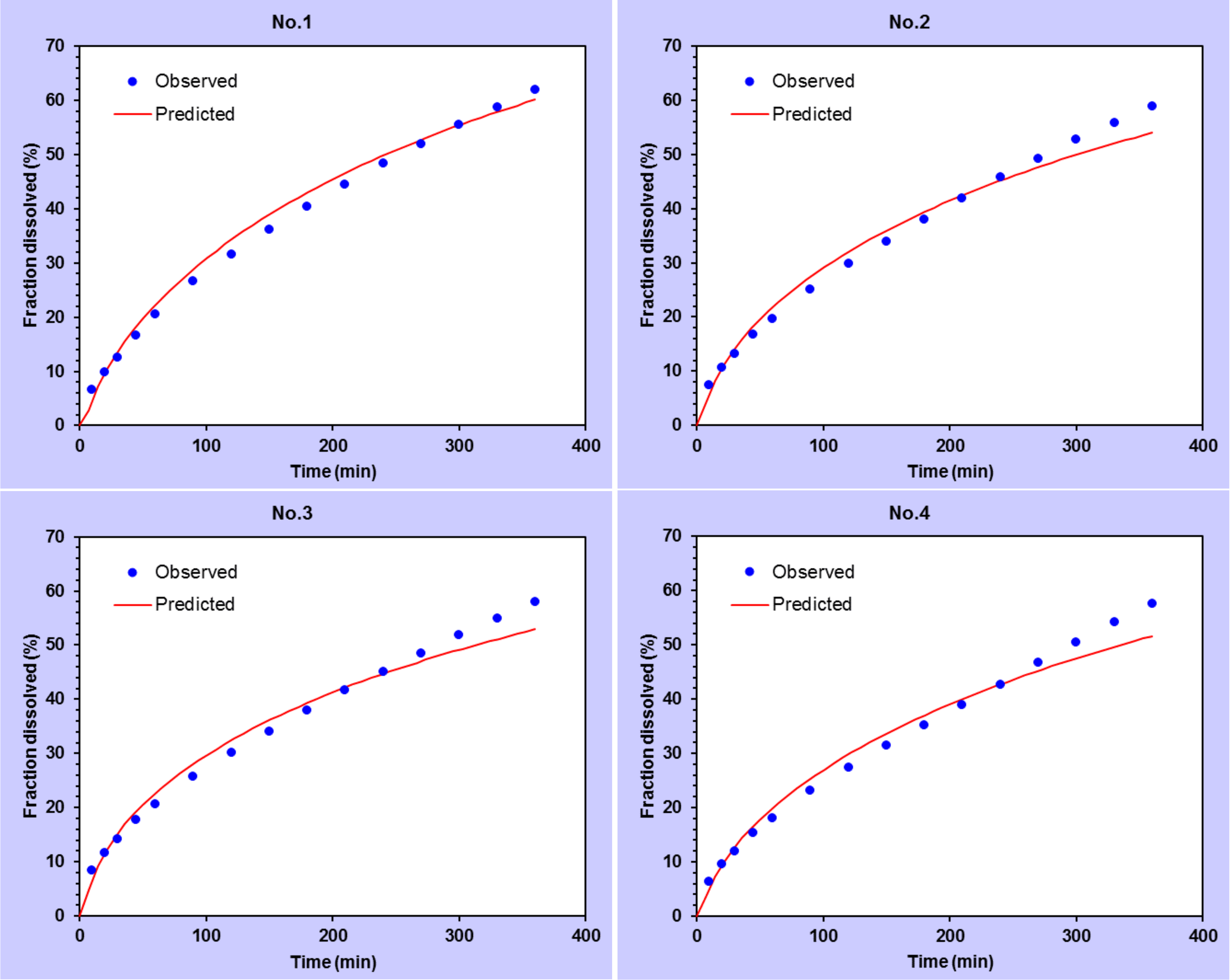

Supplementary materials – Model fitting summary – Lactochem® Fine Powder (modelling up to app. 60 % of carvedilol released)

Model: **Weibull_2**

Model equation: $F = 100 \cdot \left(1 - e^{-\frac{t^{\beta}}{\alpha}}\right)$

Fitted model parameters per tested tablet (N = 4) with statistics – mean, standard deviation (SD), and relative standard deviation expressed in % (RSD%) (output from DDSolver):

Parameter	No.1	No.2	No.3	No.4	Mean	SD	RSD(%)
α	89.306	81.878	57.192	87.066	78.860	14.777	18.738
β	0.744	0.721	0.645	0.711	0.705	0.042	6.015

Number of dissolution data points (N), degrees of freedom (df), and selected goodness of fit criteria – Pearson correlation coefficient (R), coefficient of determination (R^2), adjusted coefficient of determination ($R^2_{adjusted}$), and residual sum of squares (RSS) (manual calculation in MS Excel):

Parameter	No.1	No.2	No.3	No.4
N	15	15	15	15
df	13	13	13	13
R	0.998567987	0.997004759	0.995839211	0.994752433
R^2	0.997138025	0.994018489	0.991695733	0.989532404
$R^2_{adjusted}$	0.996917873	0.993558373	0.991056944	0.988727204
RSS	18.13127941	31.76242731	39.42612121	51.96245335

Graphical abstract of model fit presented as mean ± 1 SD of the fraction % of released carvedilol:

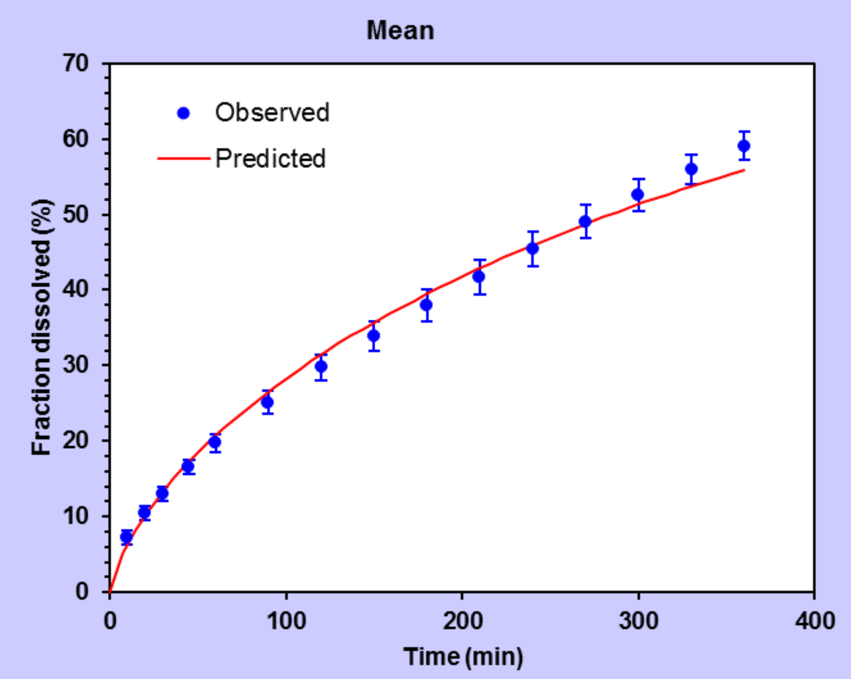

Supplementary materials – Model fitting summary – Lactochem® Fine Powder (modelling up to app. 60 % of carvedilol released)

Graphical abstract of model fit presented as the fraction % of released carvedilol per tested tablet:

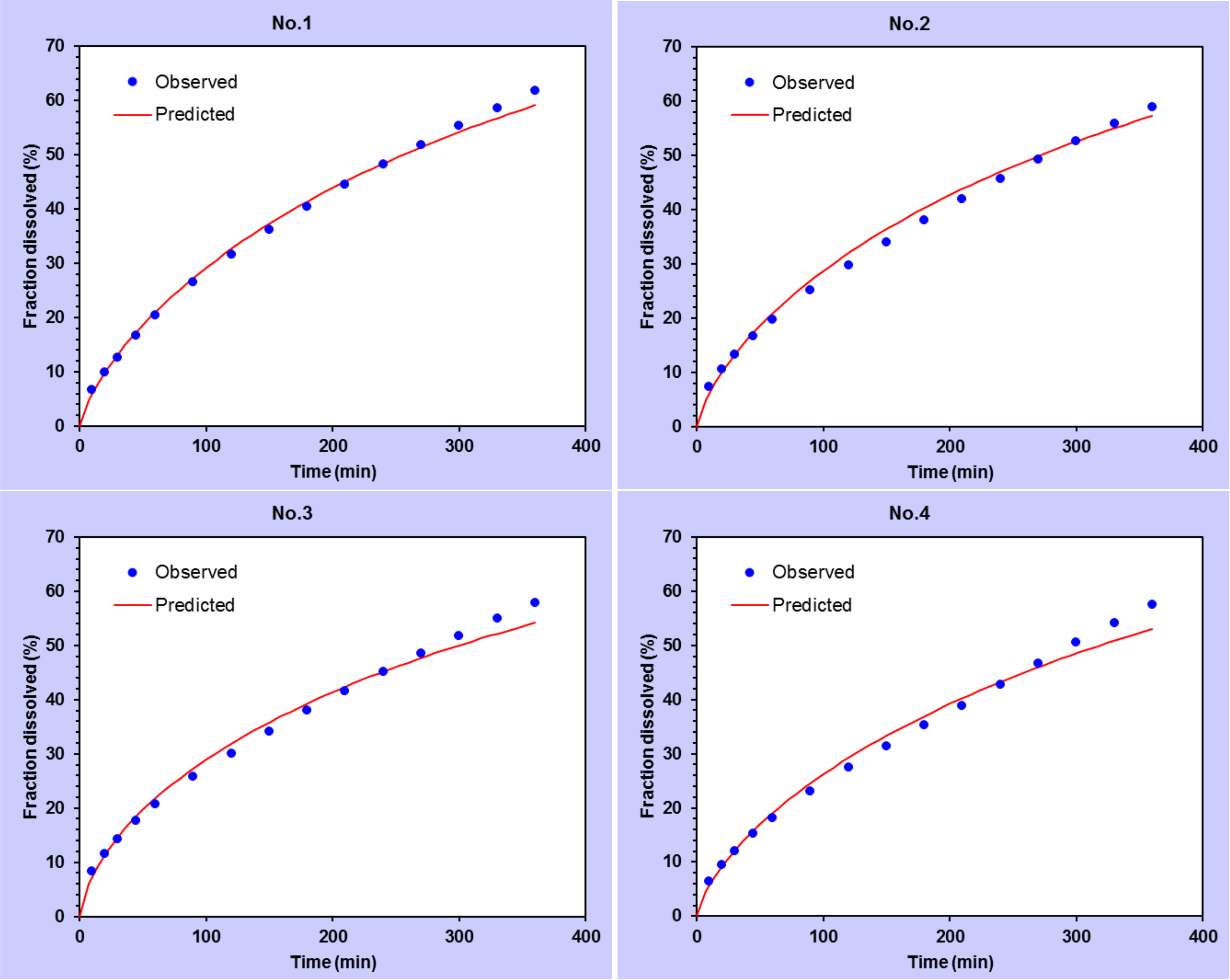

Supplementary materials – Model fitting summary – Lactochem® Fine Powder (modelling up to app. 60 % of carvedilol released)

Model: **Weibull_3**

Model equation: $F = F_{max} \cdot \left(1 - e^{-\frac{t^\beta}{\alpha}}\right)$

Fitted model parameters per tested tablet (N = 4) with statistics – mean, standard deviation (SD), and relative standard deviation expressed in % (RSD%) (output from DDSolver):

Parameter	No.1	No.2	No.3	No.4	Mean	SD	RSD(%)
α	122.056	94.483	56.629	85.375	89.636	26.970	30.089
β	0.909	0.847	0.803	0.864	0.856	0.044	5.130
F_{max}	67.829	66.803	60.836	60.457	63.981	3.876	6.059

Number of dissolution data points (N), degrees of freedom (df), and selected goodness of fit criteria – Pearson correlation coefficient (R), coefficient of determination (R^2), adjusted coefficient of determination ($R^2_{adjusted}$), and residual sum of squares (RSS) (manual calculation in MS Excel):

Parameter	No.1	No.2	No.3	No.4
N	15	15	15	15
df	12	12	12	12
R	0.995442402	0.993228852	0.984926842	0.98363698
R^2	0.990905575	0.986503552	0.970080883	0.967541709
$R^2_{adjusted}$	0.989389838	0.984254144	0.965094364	0.962131994
RSS	87.88958202	100.4328872	122.4241004	140.2068478

Graphical abstract of model fit presented as mean ± 1 SD of the fraction % of released carvedilol:

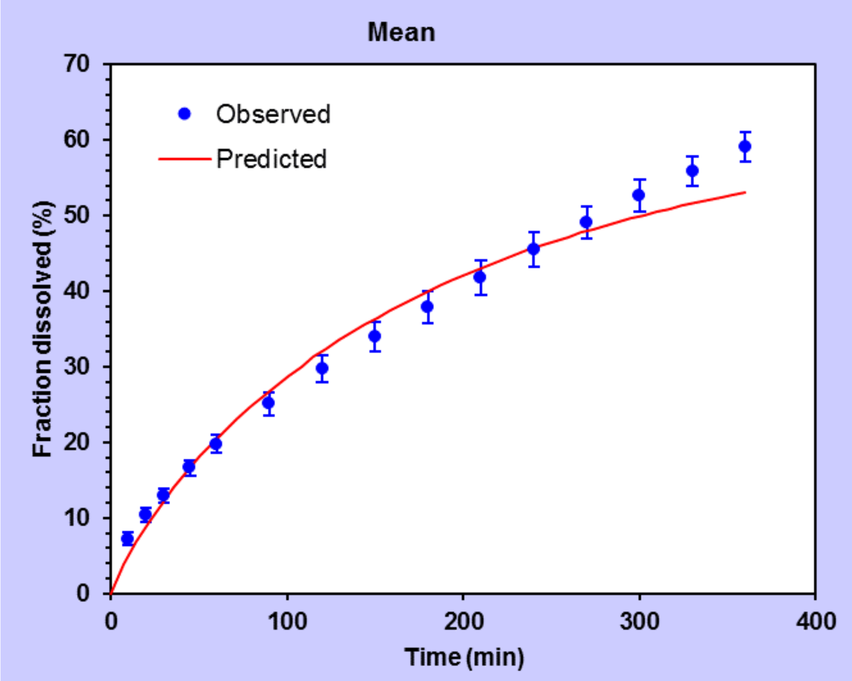

Supplementary materials – Model fitting summary – Lactochem® Fine Powder (modelling up to app. 60 % of carvedilol released)

Graphical abstract of model fit presented as the fraction % of released carvedilol per tested tablet:

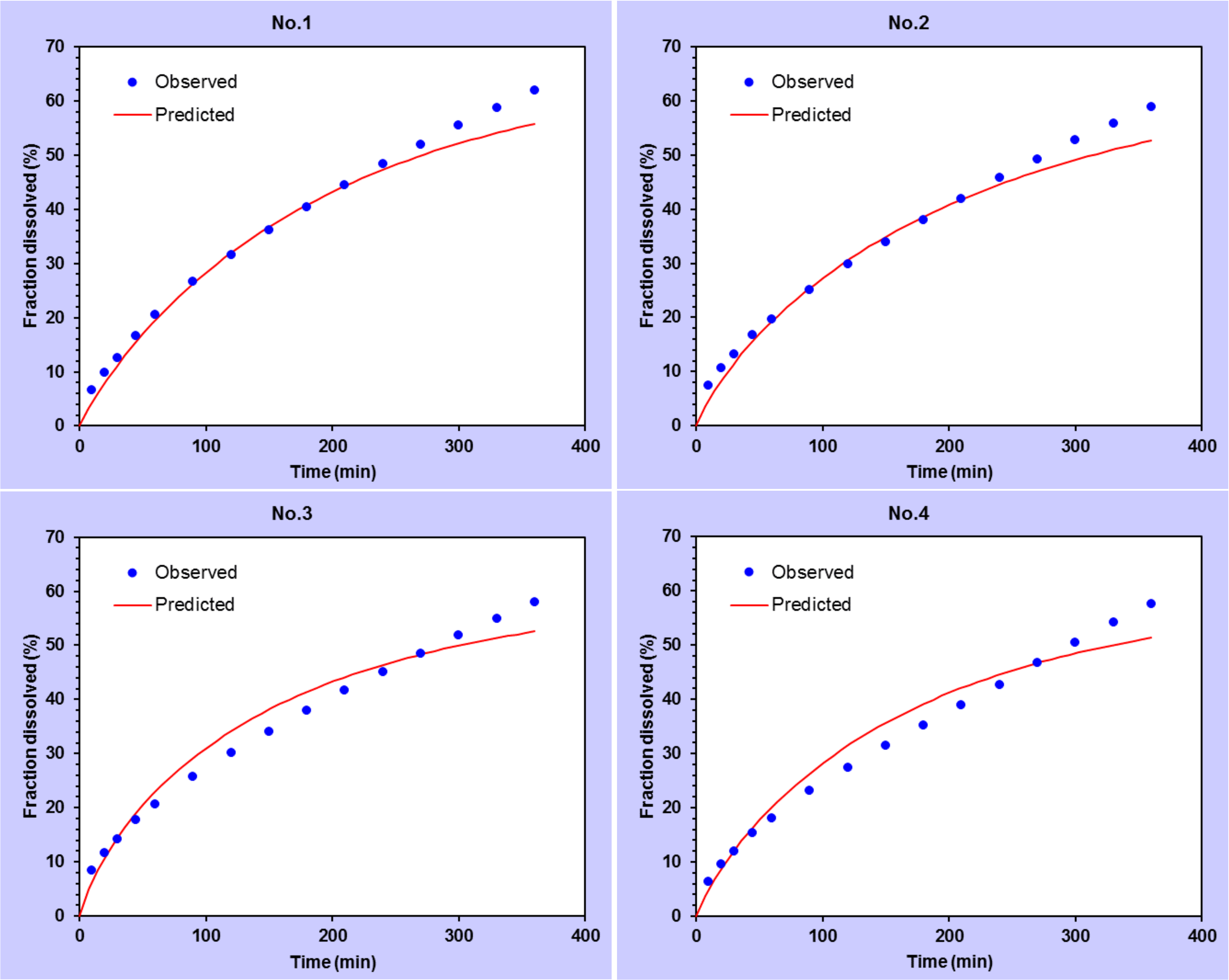

Supplementary materials – Model fitting summary – Lactochem® Fine Powder (modelling up to app. 60 % of carvedilol released)

Model: **Weibull_4**

Model equation: $F = F_{max} \cdot \left[1 - e^{-\frac{(t-T_i)^{\beta}}{\alpha}}\right]$

Fitted model parameters per tested tablet (N = 4) with statistics – mean, standard deviation (SD), and relative standard deviation expressed in % (RSD%) (output from DDSolver):

Parameter	No.1	No.2	No.3	No.4	Mean	SD	RSD(%)
α	57.311	45.728	36.275	52.989	48.076	9.205	19.147
β	0.805	0.760	0.720	0.776	0.765	0.035	4.602
T_i	6.000	6.000	6.000	6.000	6.000	0.000	0.000
F_{max}	64.947	61.883	60.836	60.457	62.031	2.036	3.282

Number of dissolution data points (N), degrees of freedom (df), and selected goodness of fit criteria – Pearson correlation coefficient (R), coefficient of determination (R^2), adjusted coefficient of determination ($R^2_{adjusted}$), and residual sum of squares (RSS) (manual calculation in MS Excel):

Parameter	No.1	No.2	No.3	No.4
N	15	15	15	15
df	11	11	11	11
R	0.986473555	0.980852671	0.978673343	0.978128577
R^2	0.973130074	0.962071962	0.957801512	0.956735513
$R^2_{adjusted}$	0.965801912	0.951727952	0.946292834	0.944936107
RSS	134.9745993	163.4686981	163.8782375	179.5545913

Graphical abstract of model fit presented as mean ± 1 SD of the fraction % of released carvedilol:

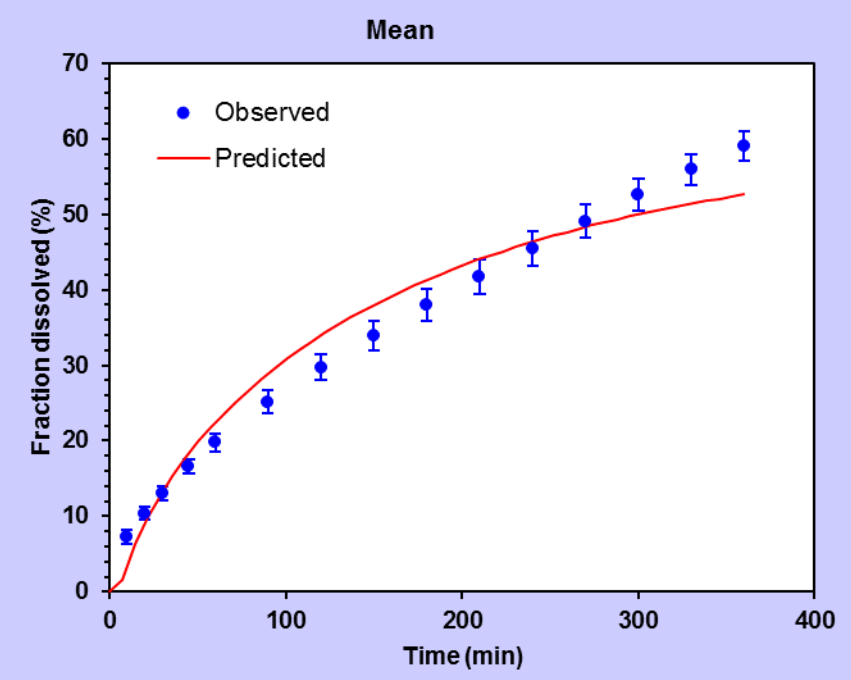

Supplementary materials – Model fitting summary – Lactochem® Fine Powder (modelling up to app. 60 % of carvedilol released)

Graphical abstract of model fit presented as the fraction % of released carvedilol per tested tablet:

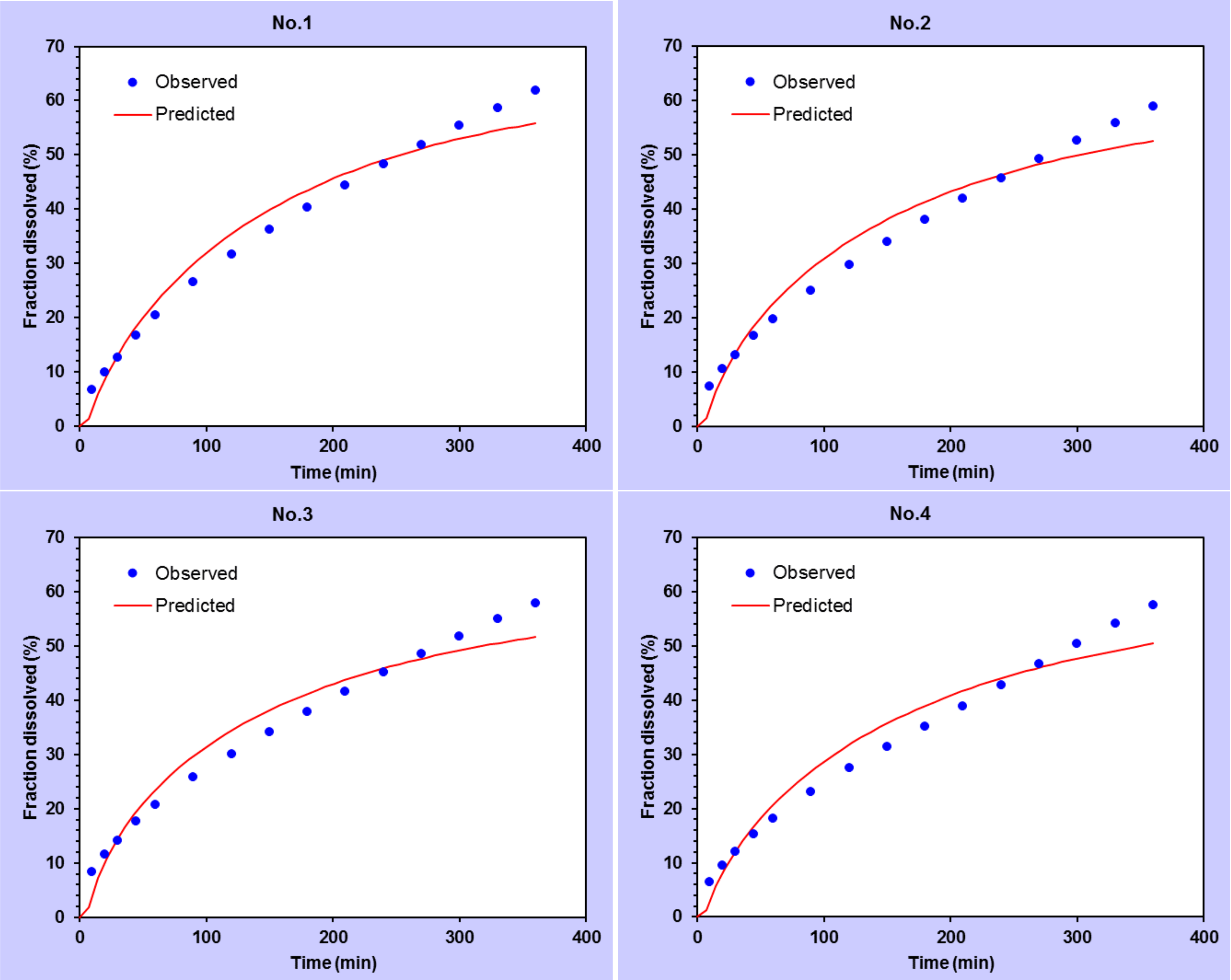

Supplementary materials – Model fitting summary – Lactochem® Fine Powder (modelling up to app. 60 % of carvedilol released)

Model: **Logistic_1**

Model equation: $F = 100 \cdot \frac{e^{\alpha+\beta \cdot \log(t)}}{1+e^{\alpha+\beta \cdot \log(t)}}$

Fitted model parameters per tested tablet (N = 4) with statistics – mean, standard deviation (SD), and relative standard deviation expressed in % (RSD%) (output from DDSolver):

Parameter	No.1	No.2	No.3	No.4	Mean	SD	RSD(%)
α	-5.262	-4.612	-4.352	-4.783	-4.752	0.383	-8.058
β	2.214	1.867	1.750	1.898	1.932	0.198	10.263

Number of dissolution data points (N), degrees of freedom (df), and selected goodness of fit criteria – Pearson correlation coefficient (R), coefficient of determination (R^2), adjusted coefficient of determination ($R^2_{adjusted}$), and residual sum of squares (RSS) (manual calculation in MS Excel):

Parameter	No.1	No.2	No.3	No.4
N	15	15	15	15
df	13	13	13	13
R	0.995859395	0.99205453	0.991210497	0.989701256
R^2	0.991735935	0.98417219	0.982498249	0.979508577
$R^2_{adjusted}$	0.991100238	0.982954666	0.981151961	0.977932313
RSS	49.38655513	77.43272083	76.47333458	94.98258025

Graphical abstract of model fit presented as mean ± 1 SD of the fraction % of released carvedilol:

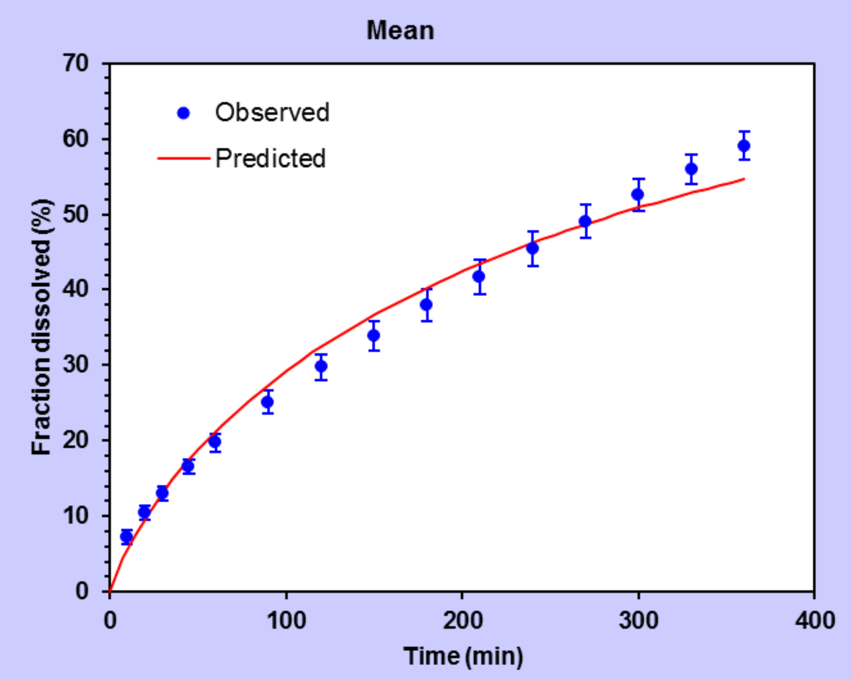

Supplementary materials – Model fitting summary – Lactochem® Fine Powder (modelling up to app. 60 % of carvedilol released)

Graphical abstract of model fit presented as the fraction % of released carvedilol per tested tablet:

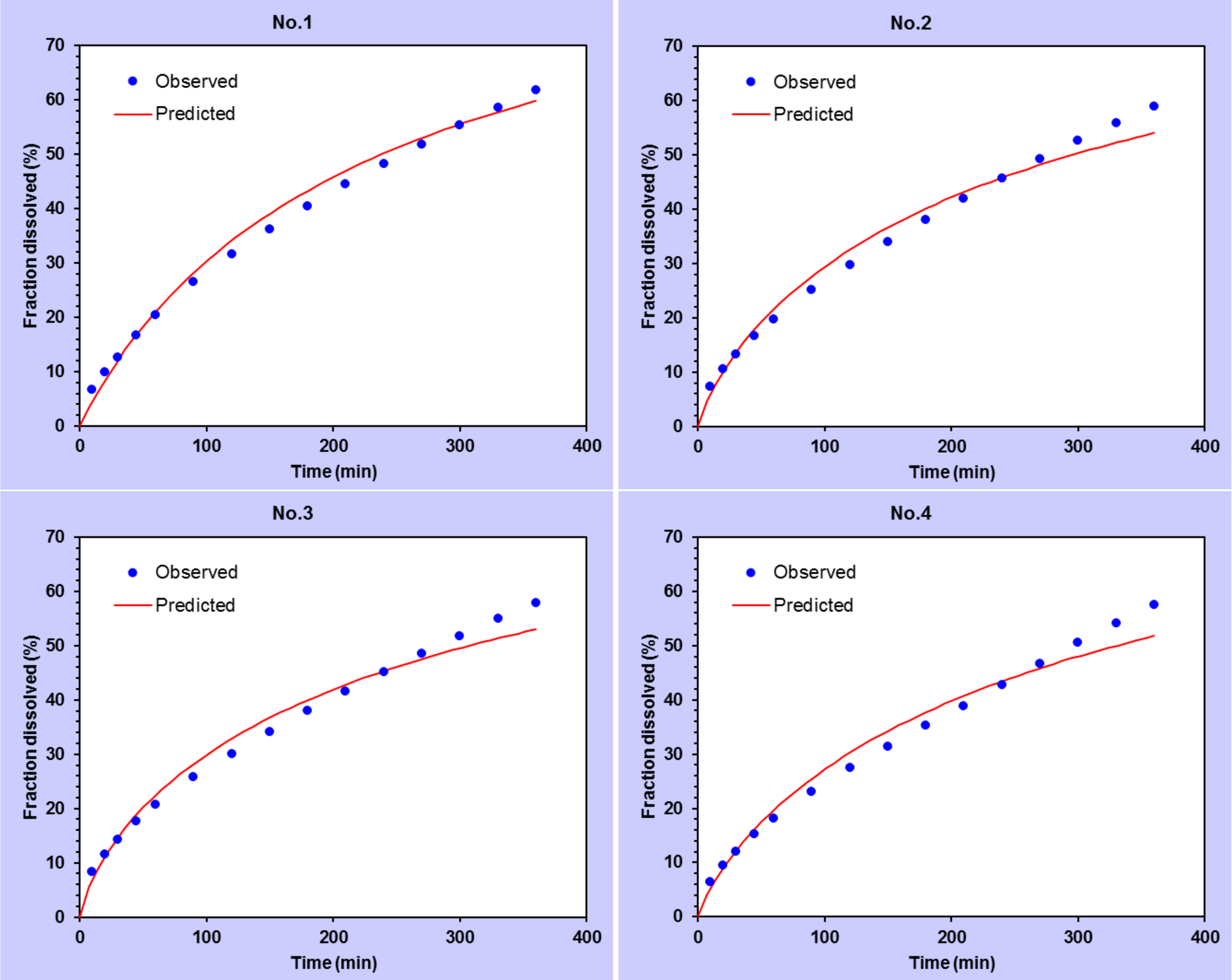

Supplementary materials – Model fitting summary – Lactochem® Fine Powder (modelling up to app. 60 % of carvedilol released)

Model: **Logistic_2**

Model equation: $F = F_{max} \cdot \frac{e^{\alpha+\beta\cdot\log(t)}}{1+e^{\alpha+\beta\cdot\log(t)}}$

Fitted model parameters per tested tablet (N = 4) with statistics – mean, standard deviation (SD), and relative standard deviation expressed in % (RSD%) (output from DDSolver):

Parameter	No.1	No.2	No.3	No.4	Mean	SD	RSD(%)
α	-5.761	-5.482	-5.203	-5.601	-5.512	0.235	-4.270
β	2.958	2.829	2.721	2.835	2.836	0.097	3.413
F_{max}	64.947	61.883	60.836	60.457	62.031	2.036	3.282

Number of dissolution data points (N), degrees of freedom (df), and selected goodness of fit criteria – Pearson correlation coefficient (R), coefficient of determination (R^2), adjusted coefficient of determination ($R^2_{adjusted}$), and residual sum of squares (RSS) (manual calculation in MS Excel):

Parameter	No.1	No.2	No.3	No.4
N	15	15	15	15
df	12	12	12	12
R	0.974215423	0.967613661	0.965698861	0.964372338
R^2	0.949095691	0.936276197	0.932574291	0.930014006
$R^2_{adjusted}$	0.94061164	0.925655564	0.921336672	0.918349674
RSS	285.2055855	304.0663743	291.4723667	319.0855494

Graphical abstract of model fit presented as mean ± 1 SD of the fraction % of released carvedilol:

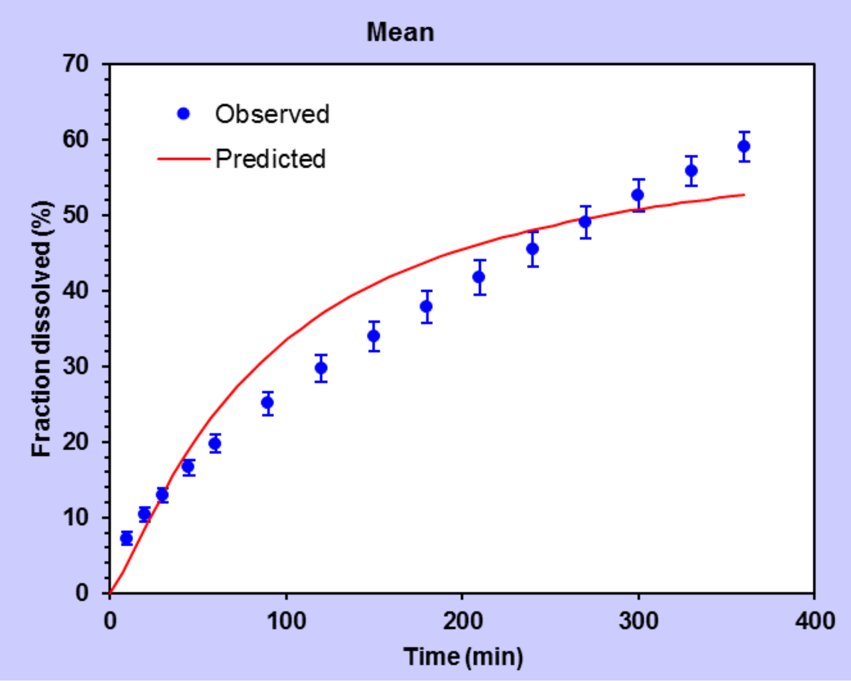

Supplementary materials – Model fitting summary – Lactochem® Fine Powder (modelling up to app. 60 % of carvedilol released)

Graphical abstract of model fit presented as the fraction % of released carvedilol per tested tablet:

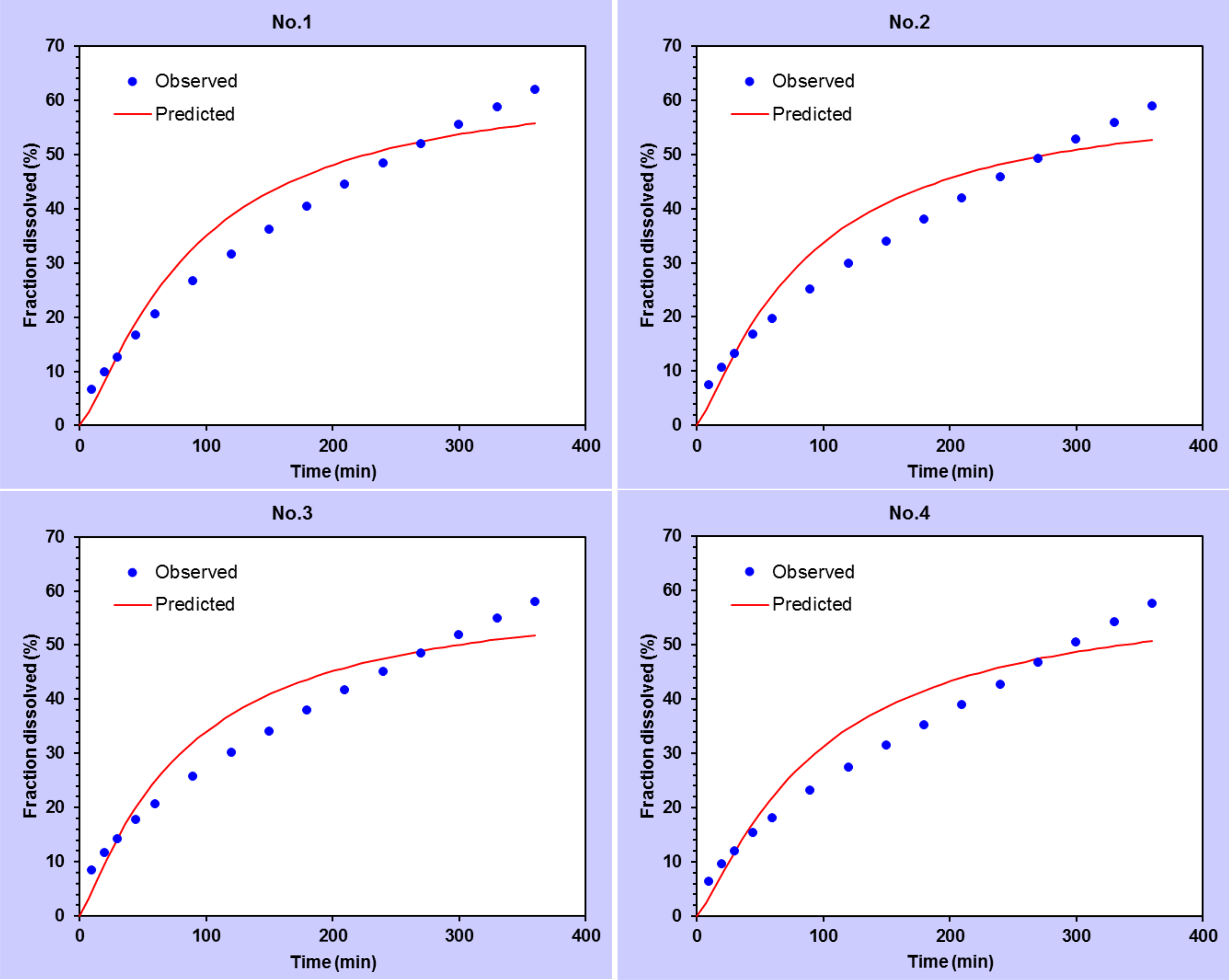

Supplementary materials – Model fitting summary – Lactochem® Fine Powder (modelling up to app. 60 % of carvedilol released)

Model: **Logistic_3**

Model equation: $F = F_{max} \cdot \frac{1}{1+e^{-k\cdot(t-\gamma)}}$

Fitted model parameters per tested tablet (N = 4) with statistics – mean, standard deviation (SD), and relative standard deviation expressed in % (RSD%) (output from DDSolver):

Parameter	No.1	No.2	No.3	No.4	Mean	SD	RSD(%)
k	0.013	0.012	0.012	0.012	0.012	0.000	2.746
γ	142.929	140.774	134.952	149.756	142.103	6.114	4.303
F_{max}	64.947	61.883	60.836	60.457	62.031	2.036	3.282

Number of dissolution data points (N), degrees of freedom (df), and selected goodness of fit criteria – Pearson correlation coefficient (R), coefficient of determination (R^2), adjusted coefficient of determination ($R^2_{adjusted}$), and residual sum of squares (RSS) (manual calculation in MS Excel):

Parameter	No.1	No.2	No.3	No.4
N	15	15	15	15
df	12	12	12	12
R	0.992027745	0.993941794	0.993778746	0.992954158
R^2	0.984119047	0.987920291	0.987596196	0.98595796
$R^2_{adjusted}$	0.981472221	0.985907006	0.985528896	0.98361762
RSS	83.17517188	54.91865293	51.1598361	63.21481884

Graphical abstract of model fit presented as mean ± 1 SD of the fraction % of released carvedilol:

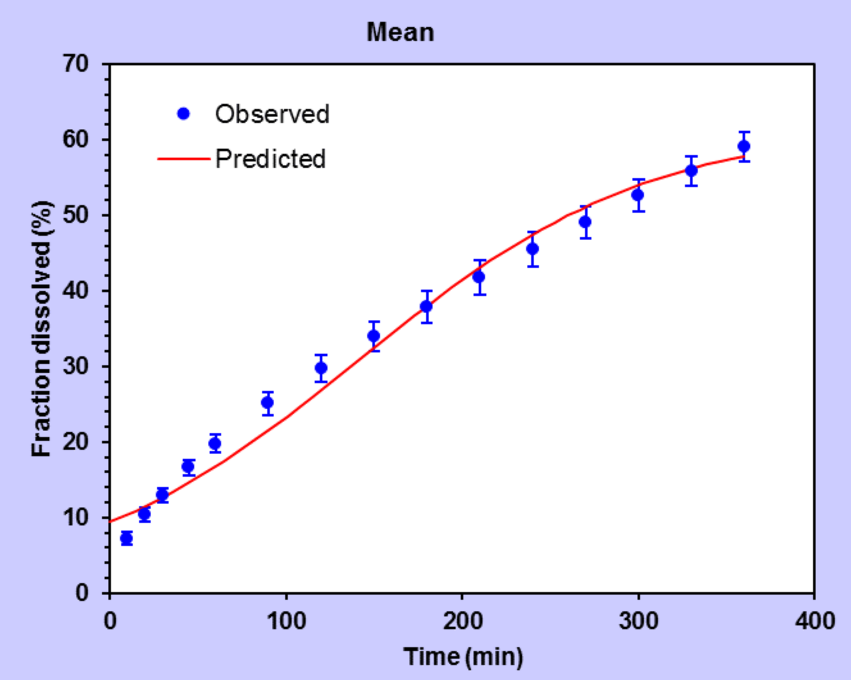

Supplementary materials – Model fitting summary – Lactochem® Fine Powder (modelling up to app. 60 % of carvedilol released)

Graphical abstract of model fit presented as the fraction % of released carvedilol per tested tablet:

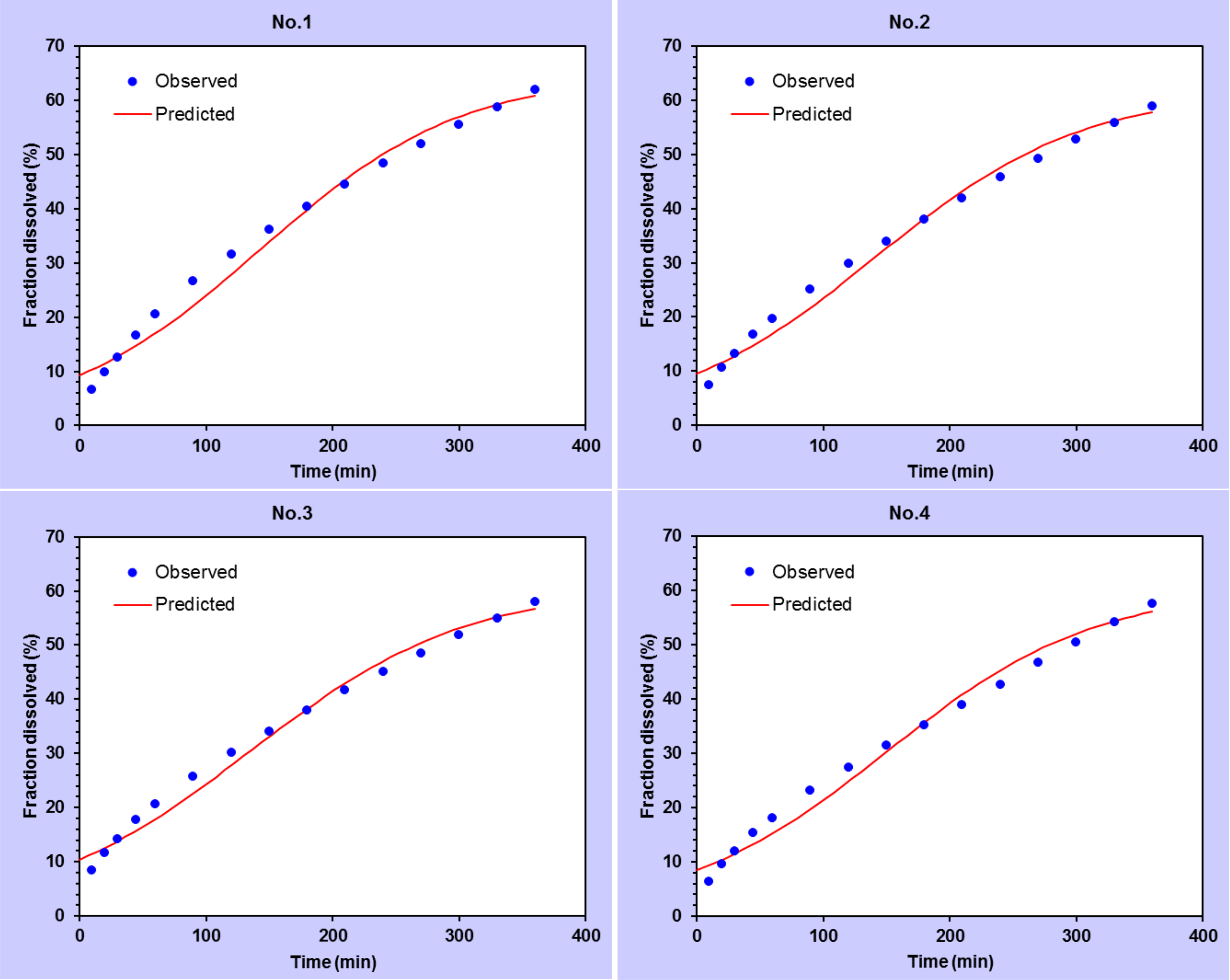

Supplementary materials – Model fitting summary – Lactochem® Fine Powder (modelling up to app. 60 % of carvedilol released)

Model: **Gompertz_1**

Model equation: $F = 100 \cdot e^{-\alpha \cdot e^{-\beta \cdot \log(t)}}$

Fitted model parameters per tested tablet (N = 4) with statistics – mean, standard deviation (SD), and relative standard deviation expressed in % (RSD%) (output from DDSolver):

Parameter	No.1	No.2	No.3	No.4	Mean	SD	RSD(%)
α	12.516	9.064	8.056	9.377	9.753	1.926	19.750
β	1.177	1.032	0.976	1.018	1.051	0.087	8.305

Number of dissolution data points (N), degrees of freedom (df), and selected goodness of fit criteria – Pearson correlation coefficient (R), coefficient of determination (R^2), adjusted coefficient of determination ($R^2_{adjusted}$), and residual sum of squares (RSS) (manual calculation in MS Excel):

Parameter	No.1	No.2	No.3	No.4
N	15	15	15	15
df	13	13	13	13
R	0.986014828	0.977768434	0.97733231	0.974186345
R^2	0.972225242	0.956031111	0.955178444	0.949039034
$R^2_{adjusted}$	0.970088722	0.952648889	0.951730632	0.94511896
RSS	184.3306596	194.9755178	179.0187889	217.2810949

Graphical abstract of model fit presented as mean ± 1 SD of the fraction % of released carvedilol:

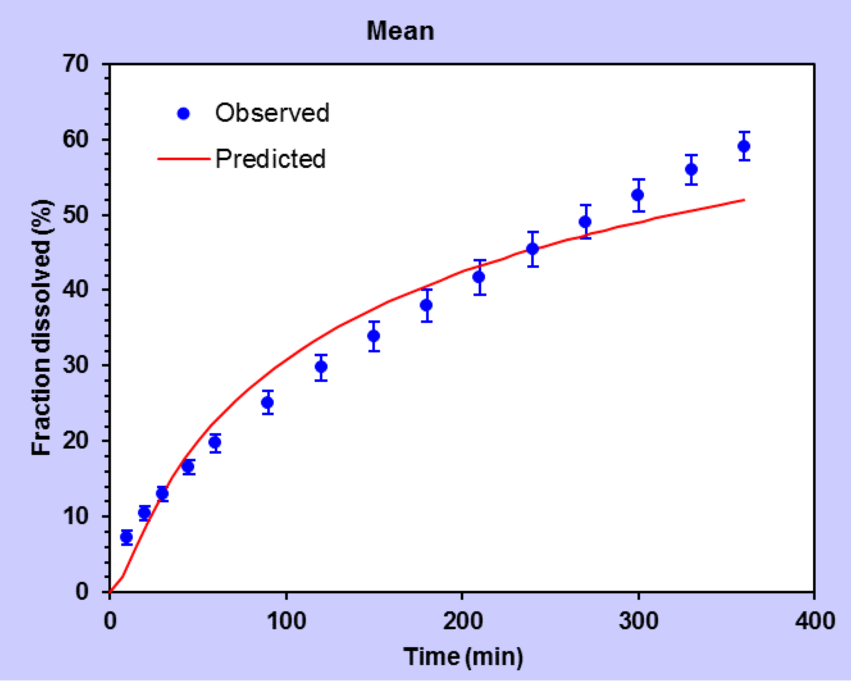

Supplementary materials – Model fitting summary – Lactochem® Fine Powder (modelling up to app. 60 % of carvedilol released)

Graphical abstract of model fit presented as the fraction % of released carvedilol per tested tablet:

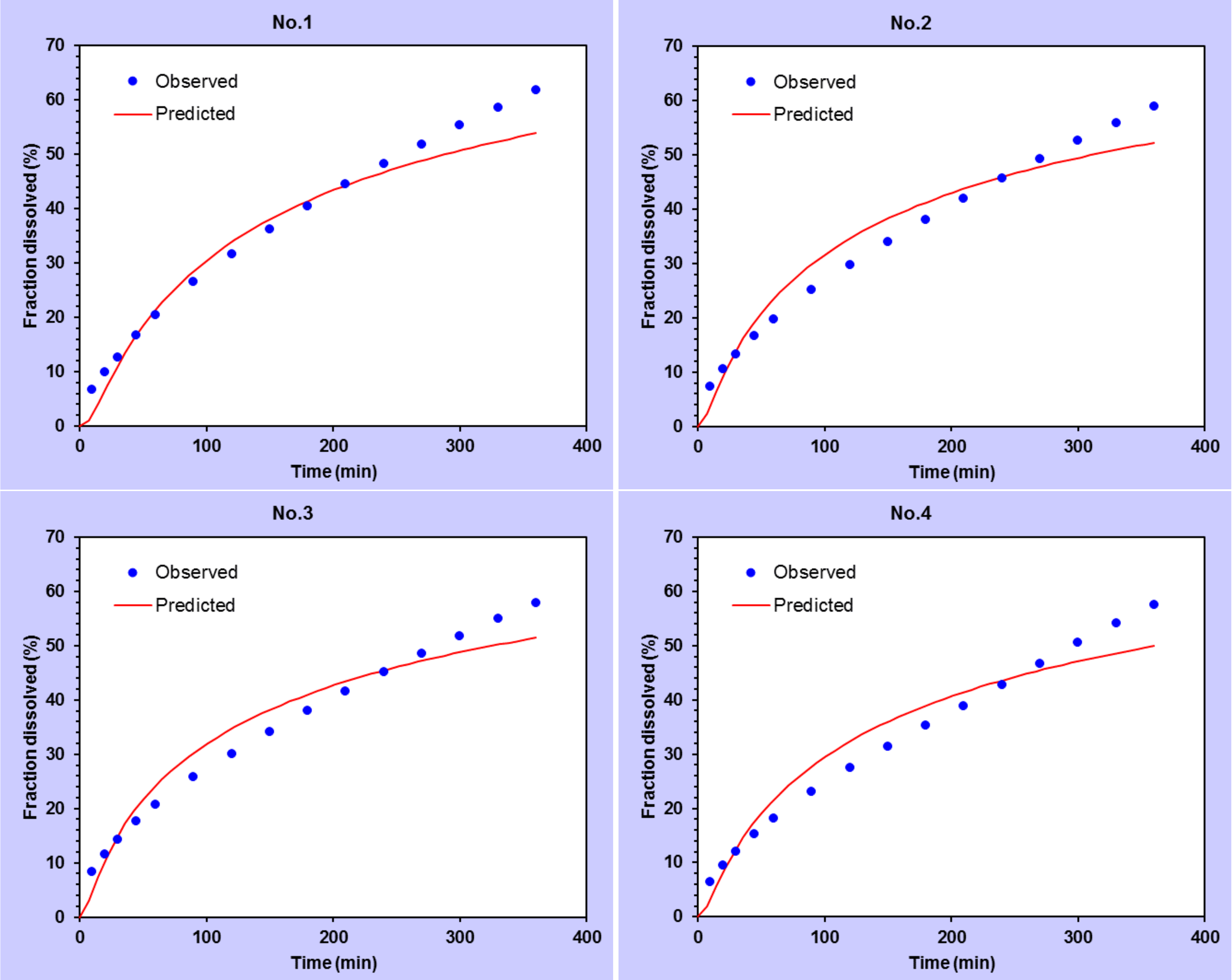

Supplementary materials – Model fitting summary – Lactochem® Fine Powder (modelling up to app. 60 % of carvedilol released)

Model: **Gompertz_2**

Model equation: $F = F_{max} \cdot e^{-\alpha \cdot e^{-\beta \cdot \log(t)}}$

Fitted model parameters per tested tablet (N = 4) with statistics – mean, standard deviation (SD), and relative standard deviation expressed in % (RSD%) (output from DDSolver):

Parameter	No.1	No.2	No.3	No.4	Mean	SD	RSD(%)
α	44.545	47.438	41.622	47.739	45.336	2.864	6.318
β	1.940	2.043	1.996	2.008	1.997	0.043	2.150
F_{max}	72.690	61.883	60.836	60.457	63.966	5.847	9.140

Number of dissolution data points (N), degrees of freedom (df), and selected goodness of fit criteria – Pearson correlation coefficient (R), coefficient of determination (R^2), adjusted coefficient of determination ($R^2_{adjusted}$), and residual sum of squares (RSS) (manual calculation in MS Excel):

Parameter	No.1	No.2	No.3	No.4
N	15	15	15	15
df	12	12	12	12
R	0.982686672	0.971126729	0.969591711	0.967543528
R^2	0.965673095	0.943087125	0.940108085	0.936140478
$R^2_{adjusted}$	0.959951945	0.933601645	0.9301261	0.925497225
RSS	345.7855799	482.3172215	492.3371566	485.4903324

Graphical abstract of model fit presented as mean ± 1 SD of the fraction % of released carvedilol:

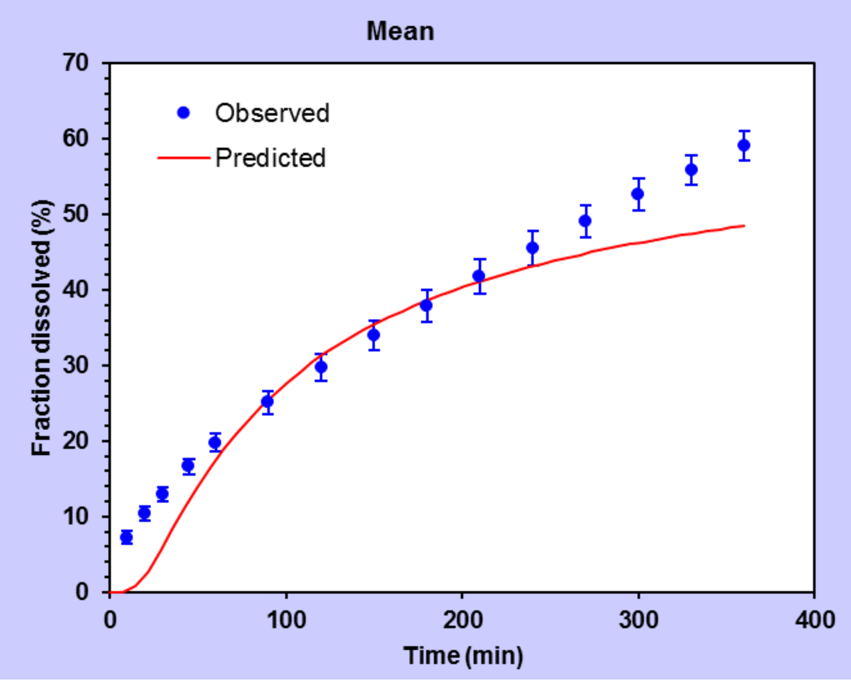

Supplementary materials – Model fitting summary – Lactochem® Fine Powder (modelling up to app. 60 % of carvedilol released)

Graphical abstract of model fit presented as the fraction % of released carvedilol per tested tablet:

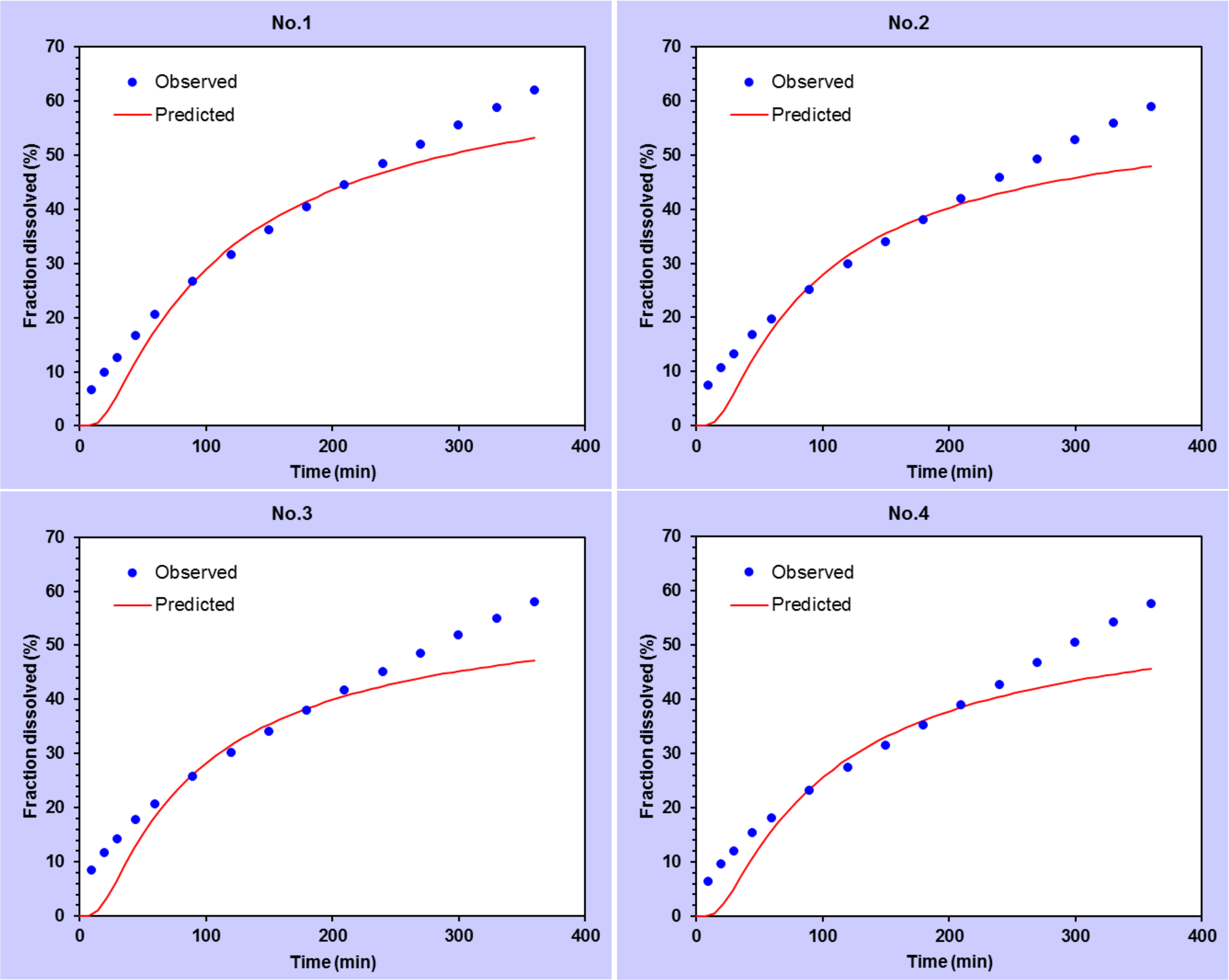

Supplementary materials – Model fitting summary – Lactochem® Fine Powder (modelling up to app. 60 % of carvedilol released)

Model: **Gompertz_3**

Model equation: $F = F_{max} \cdot e^{-e^{-k\cdot(t-\gamma)}}$

Fitted model parameters per tested tablet (N = 4) with statistics – mean, standard deviation (SD), and relative standard deviation expressed in % (RSD%) (output from DDSolver):

Parameter	No.1	No.2	No.3	No.4	Mean	SD	RSD(%)
k	0.009	0.009	0.009	0.009	0.009	0.000	1.902
γ	88.836	86.815	81.263	94.473	87.847	5.456	6.210
F_{max}	64.947	61.883	60.836	60.457	62.031	2.036	3.282

Number of dissolution data points (N), degrees of freedom (df), and selected goodness of fit criteria – Pearson correlation coefficient (R), coefficient of determination (R^2), adjusted coefficient of determination ($R^2_{adjusted}$), and residual sum of squares (RSS) (manual calculation in MS Excel):

Parameter	No.1	No.2	No.3	No.4
N	15	15	15	15
df	12	12	12	12
R	0.995689252	0.995349036	0.994950837	0.993054167
R^2	0.991397087	0.990719704	0.989927167	0.986156578
$R^2_{adjusted}$	0.989963269	0.989172988	0.988248362	0.983849341
RSS	52.67171194	52.1291587	51.58925416	74.90685751

Graphical abstract of model fit presented as mean ± 1 SD of the fraction % of released carvedilol:

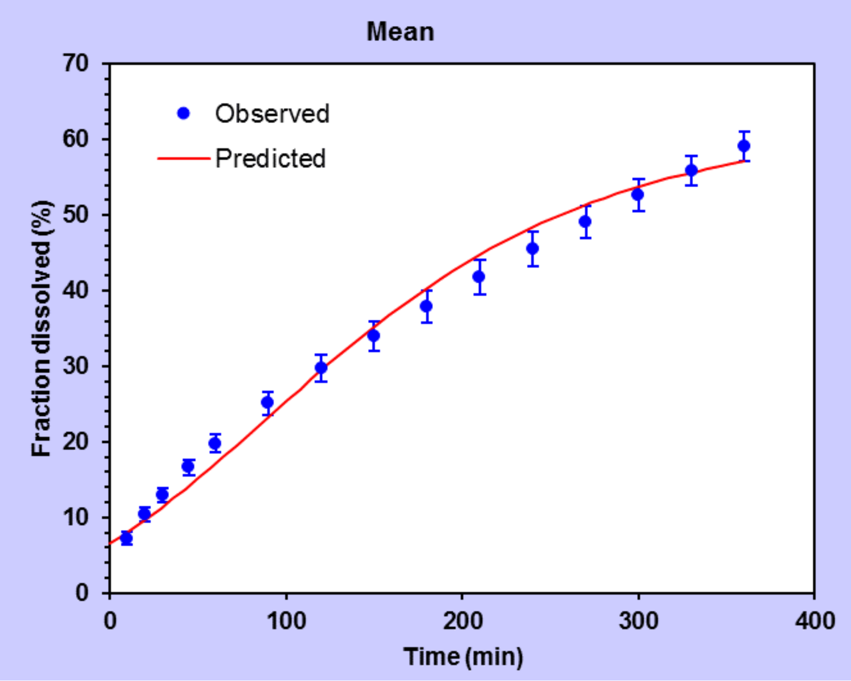

Supplementary materials – Model fitting summary – Lactochem® Fine Powder (modelling up to app. 60 % of carvedilol released)

Graphical abstract of model fit presented as the fraction % of released carvedilol per tested tablet:

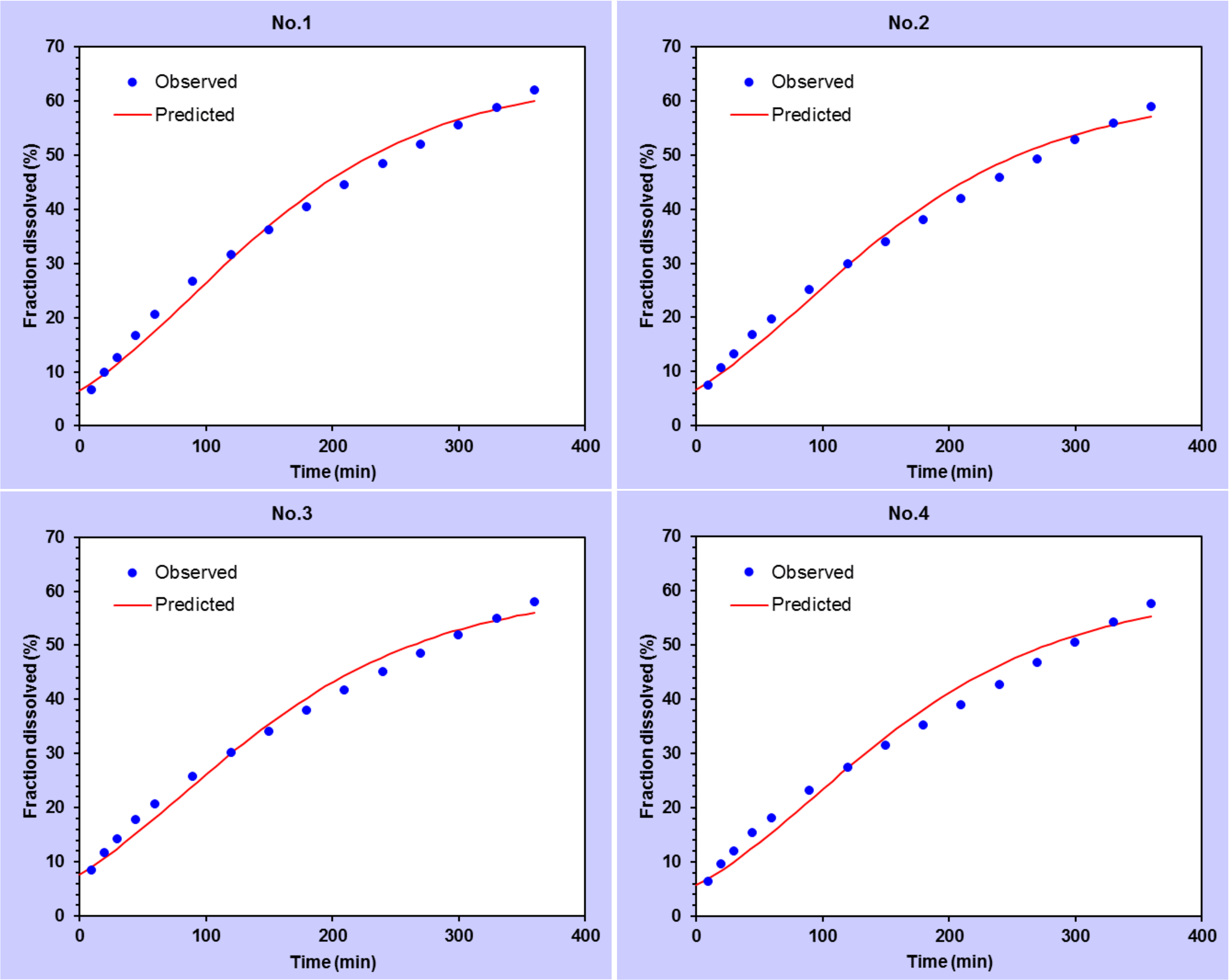

Supplementary materials – Model fitting summary – Lactochem® Fine Powder (modelling up to app. 60 % of carvedilol released)

Model: **Gompertz_4**

Model equation: $F = F_{max} \cdot e^{-\beta \cdot e^{-k \cdot t}}$

Fitted model parameters per tested tablet (N = 4) with statistics – mean, standard deviation (SD), and relative standard deviation expressed in % (RSD%) (output from DDSolver):

Parameter	No.1	No.2	No.3	No.4	Mean	SD	RSD(%)
k	0.009	0.009	0.009	0.009	0.009	0.000	1.902
β	2.303	2.219	2.075	2.359	2.239	0.123	5.514
F_{max}	64.947	61.883	60.836	60.457	62.031	2.036	3.282

Number of dissolution data points (N), degrees of freedom (df), and selected goodness of fit criteria – Pearson correlation coefficient (R), coefficient of determination (R^2), adjusted coefficient of determination ($R^2_{adjusted}$), and residual sum of squares (RSS) (manual calculation in MS Excel):

Parameter	No.1	No.2	No.3	No.4
N	15	15	15	15
df	12	12	12	12
R	0.995689252	0.995349036	0.994950837	0.993054167
R^2	0.991397087	0.990719704	0.989927167	0.986156578
$R^2_{adjusted}$	0.989963269	0.989172988	0.988248362	0.983849341
RSS	52.67171194	52.1291587	51.58925416	74.90685751

Graphical abstract of model fit presented as mean ± 1 SD of the fraction % of released carvedilol:

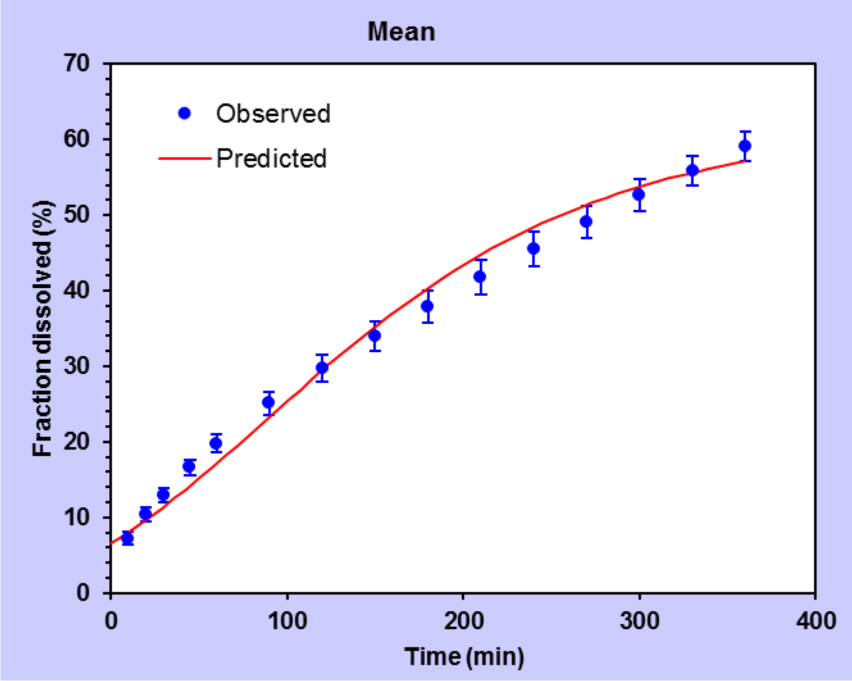

Supplementary materials – Model fitting summary – Lactochem® Fine Powder (modelling up to app. 60 % of carvedilol released)

Graphical abstract of model fit presented as the fraction % of released carvedilol per tested tablet:

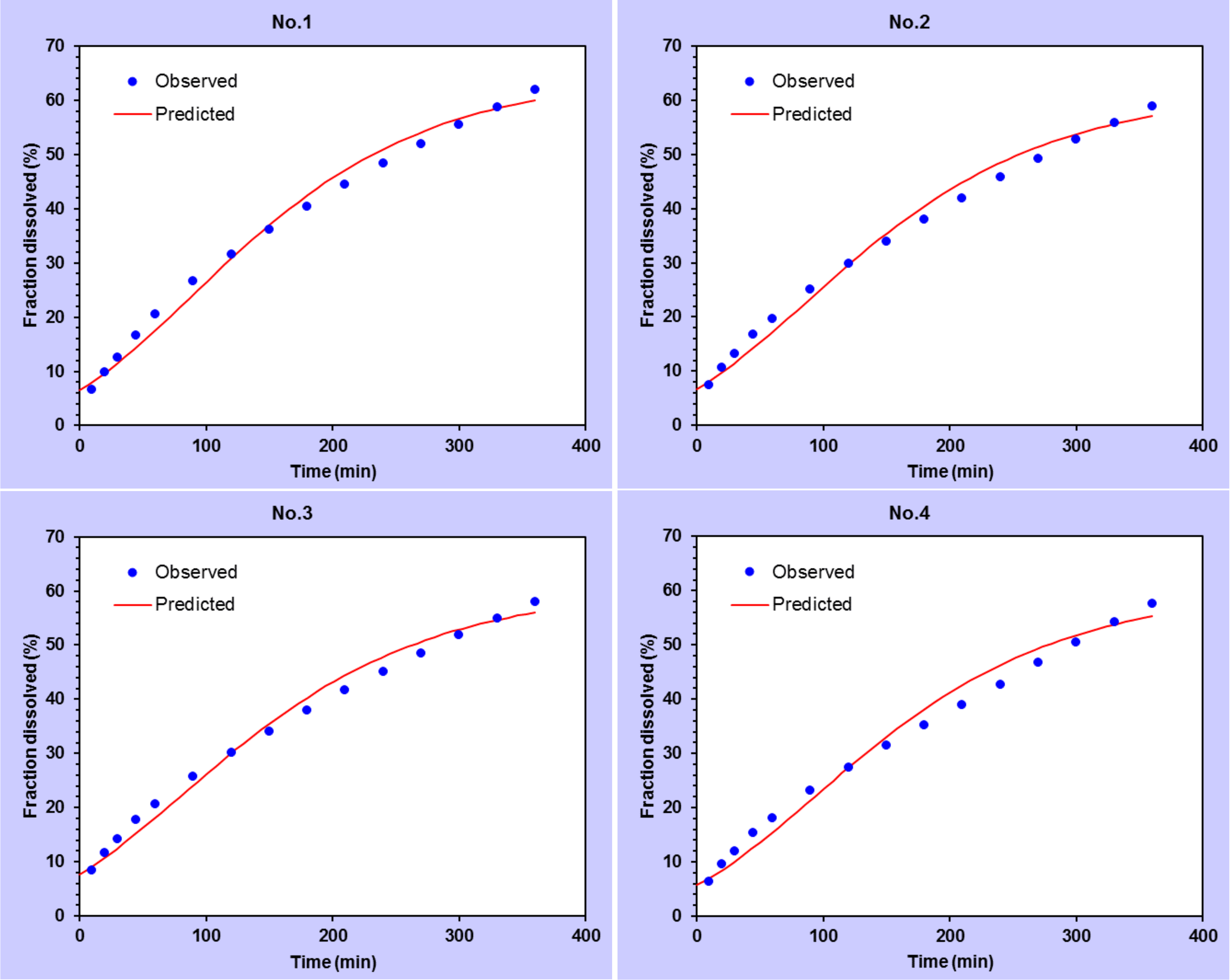

Supplementary materials – Model fitting summary – Lactochem® Fine Powder (modelling up to app. 60 % of carvedilol released)

Model: **Probit_1**

Model equation: $F = 100 \cdot \phi[\alpha + \beta \cdot \log(t)]$

Fitted model parameters per tested tablet (N = 4) with statistics – mean, standard deviation (SD), and relative standard deviation expressed in % (RSD%) (output from DDSolver):

Parameter	No.1	No.2	No.3	No.4	Mean	SD	RSD(%)
α	-2.847	-2.703	-2.564	-2.780	-2.724	0.121	-4.459
β	1.180	1.090	1.027	1.097	1.099	0.063	5.694

Number of dissolution data points (N), degrees of freedom (df), and selected goodness of fit criteria – Pearson correlation coefficient (R), coefficient of determination (R^2), adjusted coefficient of determination ($R^2_{adjusted}$), and residual sum of squares (RSS) (manual calculation in MS Excel):

Parameter	No.1	No.2	No.3	No.4
N	15	15	15	15
df	13	13	13	13
R	0.991770058	0.988326837	0.98759605	0.985339744
R^2	0.983607849	0.976789937	0.975345957	0.970894411
$R^2_{adjusted}$	0.982346914	0.975004548	0.973449493	0.968655519
RSS	91.96697547	110.4693241	104.9478875	132.2624357

Graphical abstract of model fit presented as mean ± 1 SD of the fraction % of released carvedilol:

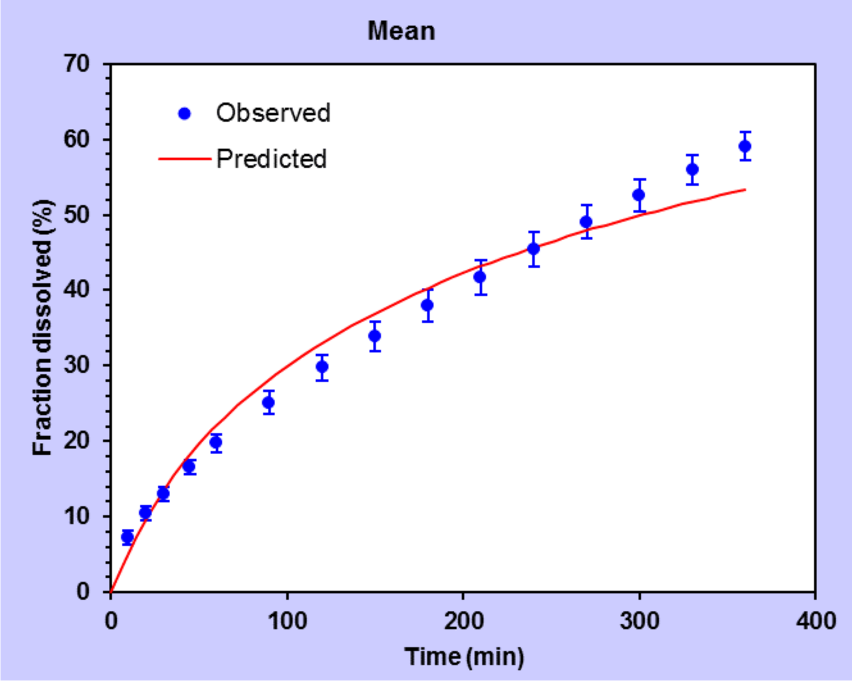

Supplementary materials – Model fitting summary – Lactochem® Fine Powder (modelling up to app. 60 % of carvedilol released)

Graphical abstract of model fit presented as the fraction % of released carvedilol per tested tablet:

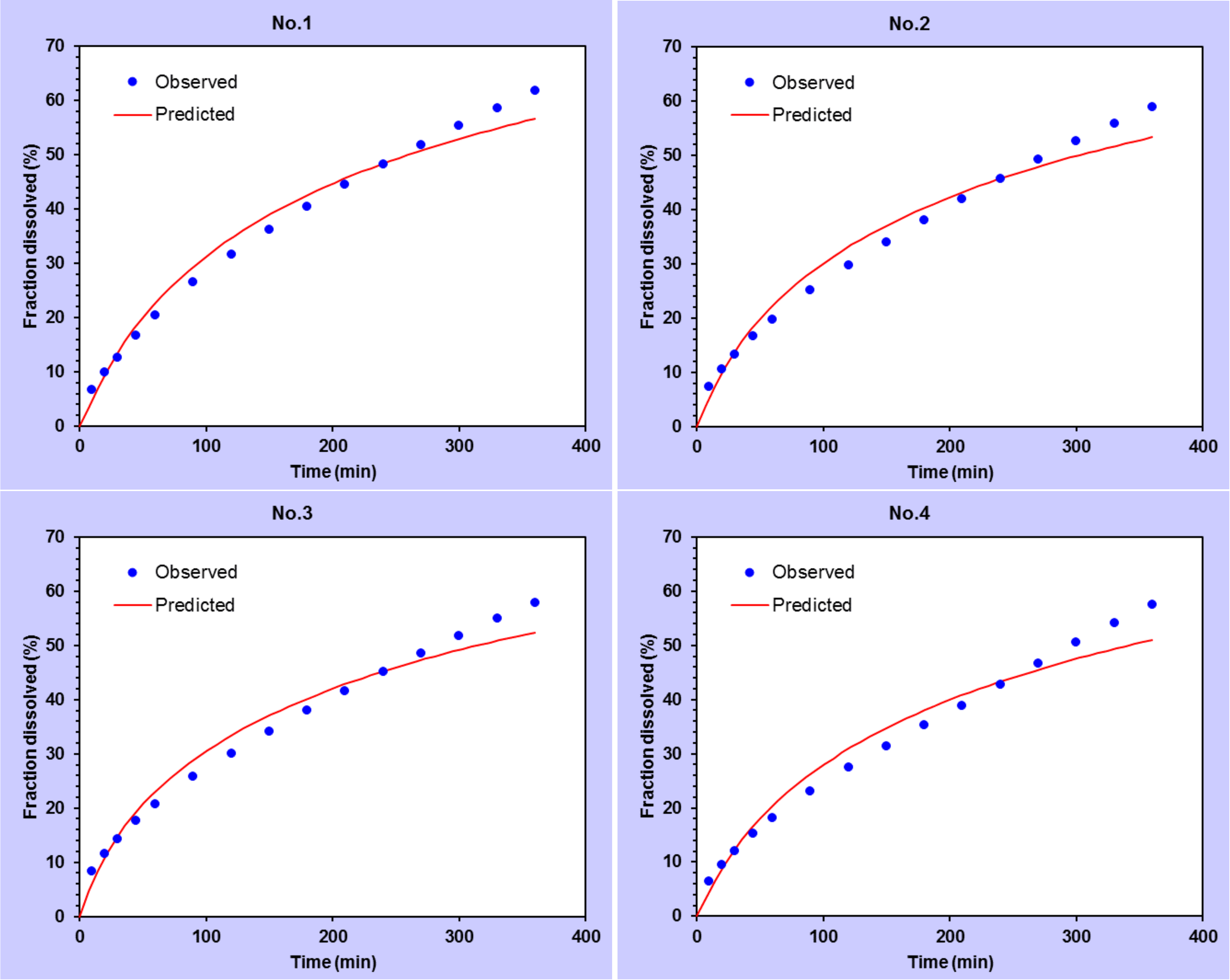

Supplementary materials – Model fitting summary – Lactochem® Fine Powder (modelling up to app. 60 % of carvedilol released)

Model: **Probit_2**

Model equation: $F = F_{max} \cdot \phi[\alpha + \beta \cdot \log(t)]$

Fitted model parameters per tested tablet (N = 4) with statistics – mean, standard deviation (SD), and relative standard deviation expressed in % (RSD%) (output from DDSolver):

Parameter	No.1	No.2	No.3	No.4	Mean	SD	RSD(%)
α	-3.932	-3.253	-3.097	-3.317	-3.400	0.367	-10.794
β	1.855	1.675	1.615	1.675	1.705	0.104	6.111
F_{max}	70.718	61.883	60.836	60.457	63.473	4.867	7.668

Number of dissolution data points (N), degrees of freedom (df), and selected goodness of fit criteria – Pearson correlation coefficient (R), coefficient of determination (R^2), adjusted coefficient of determination ($R^2_{adjusted}$), and residual sum of squares (RSS) (manual calculation in MS Excel):

Parameter	No.1	No.2	No.3	No.4
N	15	15	15	15
df	12	12	12	12
R	0.989344811	0.968395154	0.966855118	0.964719284
R^2	0.978803154	0.937789175	0.934808819	0.930683297
$R^2_{adjusted}$	0.975270347	0.927420704	0.923943622	0.919130514
RSS	162.1780811	284.1888757	269.6453713	302.8460573

Graphical abstract of model fit presented as mean ± 1 SD of the fraction % of released carvedilol:

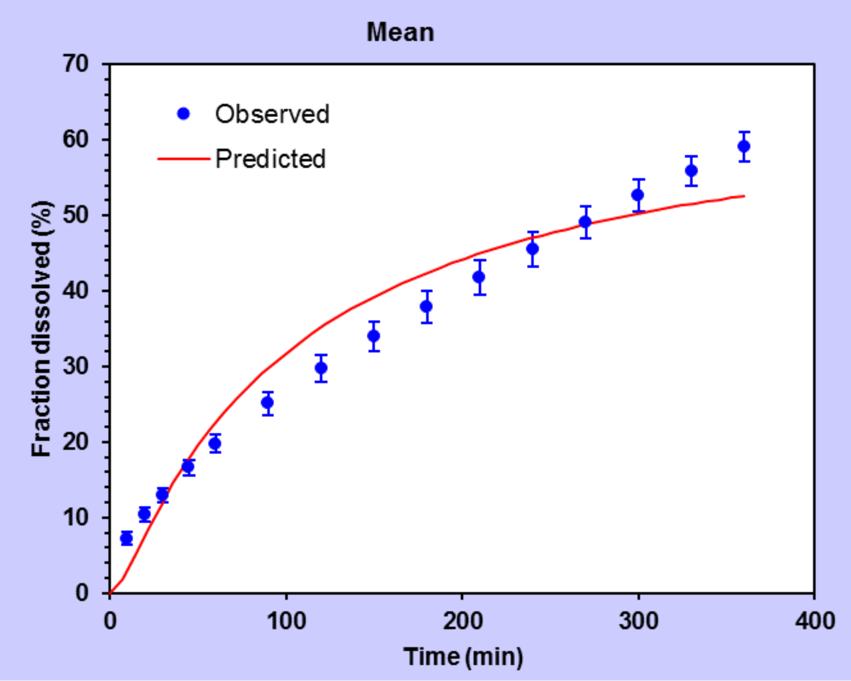

Supplementary materials – Model fitting summary – Lactochem® Fine Powder (modelling up to app. 60 % of carvedilol released)

Graphical abstract of model fit presented as the fraction % of released carvedilol per tested tablet:

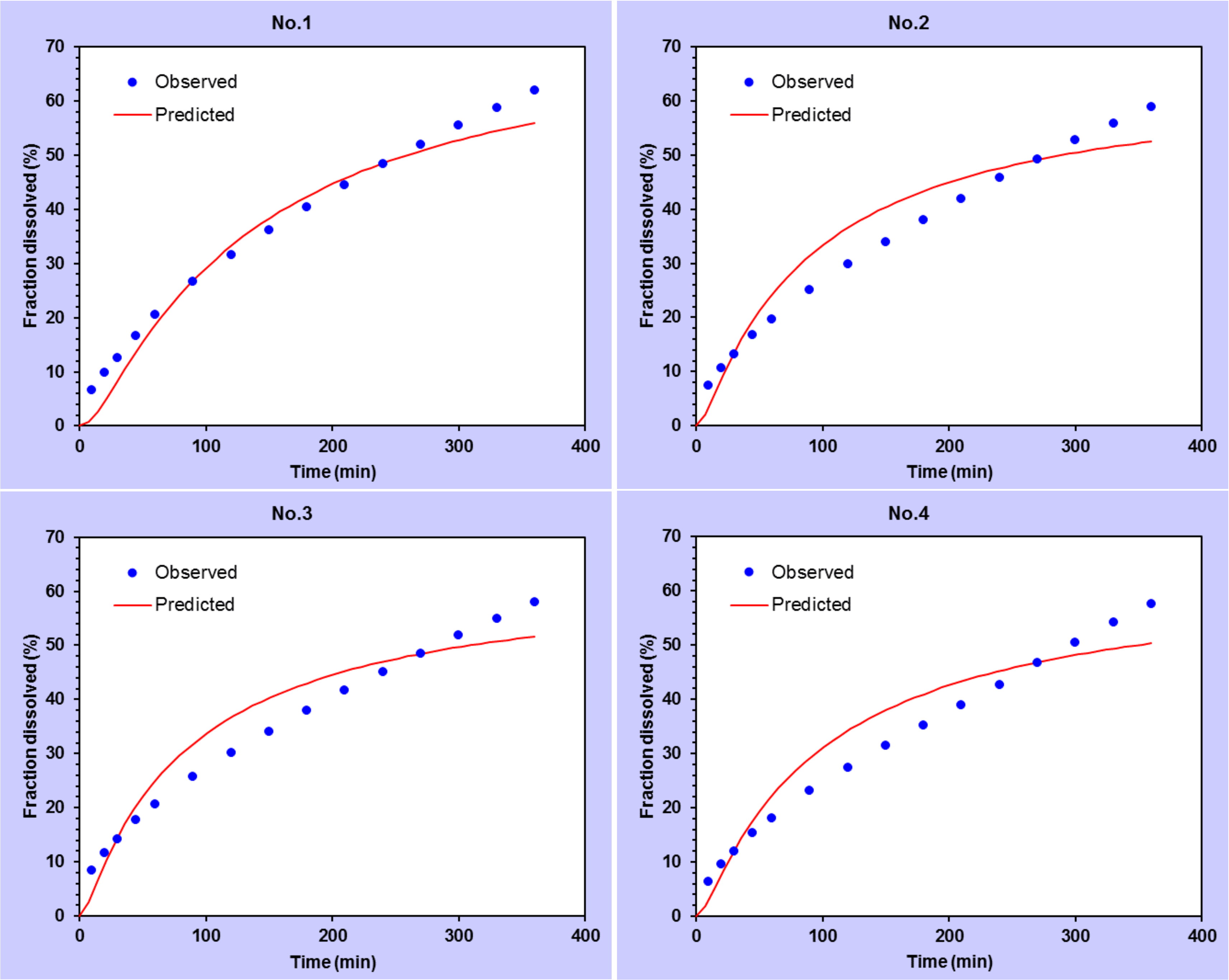

Supplement: Supplementary file 1 [file pharmaceutics-16-00498-s001.zip › Supplementary materials_Model fitting summary_Lactochem® Fine Powder.pdf]
