# Supplementary material for: Comparative Fitting of Mathematical Models to Carvedilol Release Profiles Obtained from Hypromellose Matrix Tablets
Source: Pharmaceutics. 2024 Apr 4;16(4):498. doi: 10.3390/pharmaceutics16040498 (PMC11053526; doi:10.3390/pharmaceutics16040498)

Supplementary materials – Model fitting summary – Parteck® M 100 (modelling the entire relevant dissolution data)

Model: **Zero–order**

Model equation: $F = k_0 \cdot t$

Fitted model parameters per tested tablet (N = 4) with statistics – mean, standard deviation (SD), and relative standard deviation expressed in % (RSD%) (output from DDSolver):

Parameter	No.1	No.2	No.3	No.4	Mean	SD	RSD(%)
k_0	0.582	0.544	0.546	0.591	0.566	0.024	4.279

Number of dissolution data points (N), degrees of freedom (df), and selected goodness of fit criteria – Pearson correlation coefficient (R), coefficient of determination (R^2), adjusted coefficient of determination ($R^2_{adjusted}$), and residual sum of squares (RSS) (manual calculation in MS Excel):

Parameter	No.1	No.2	No.3	No.4
N	11	11	11	11
df	10	10	10	10
R	0.844170926	0.916244095	0.91972513	0.787764747
R^2	0.712624552	0.839503242	0.845894315	0.620573297
$R^2_{adjusted}$	0.712624552	0.839503242	0.845894315	0.620573297
RSS	7799.283989	3881.028157	3677.914928	10713.83634

Graphical abstract of model fit presented as mean ± 1 SD of the fraction % of released carvedilol:

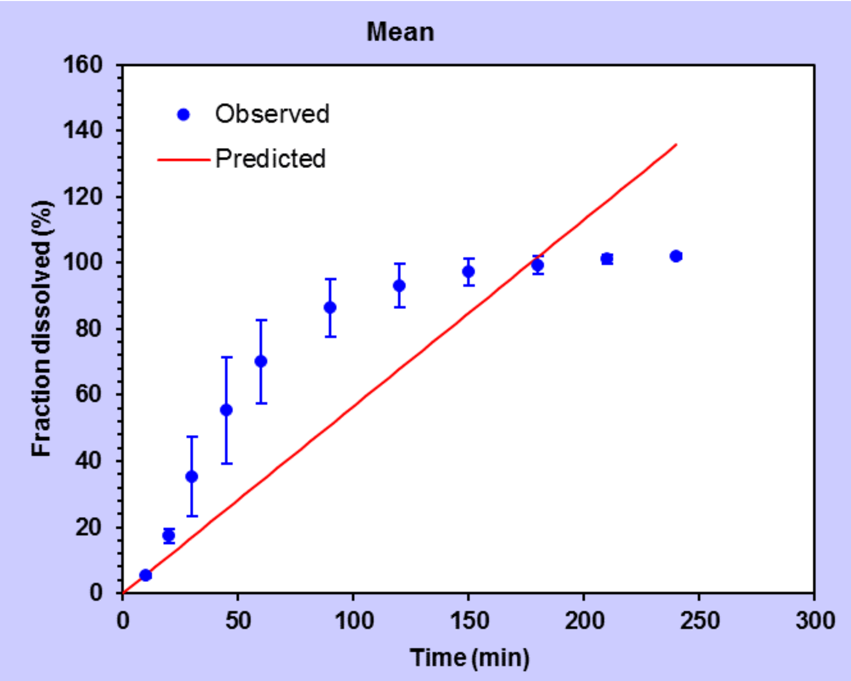

Supplementary materials – Model fitting summary – Parteck® M 100 (modelling the entire relevant dissolution data)

Graphical abstract of model fit presented as the fraction % of released carvedilol per tested tablet:

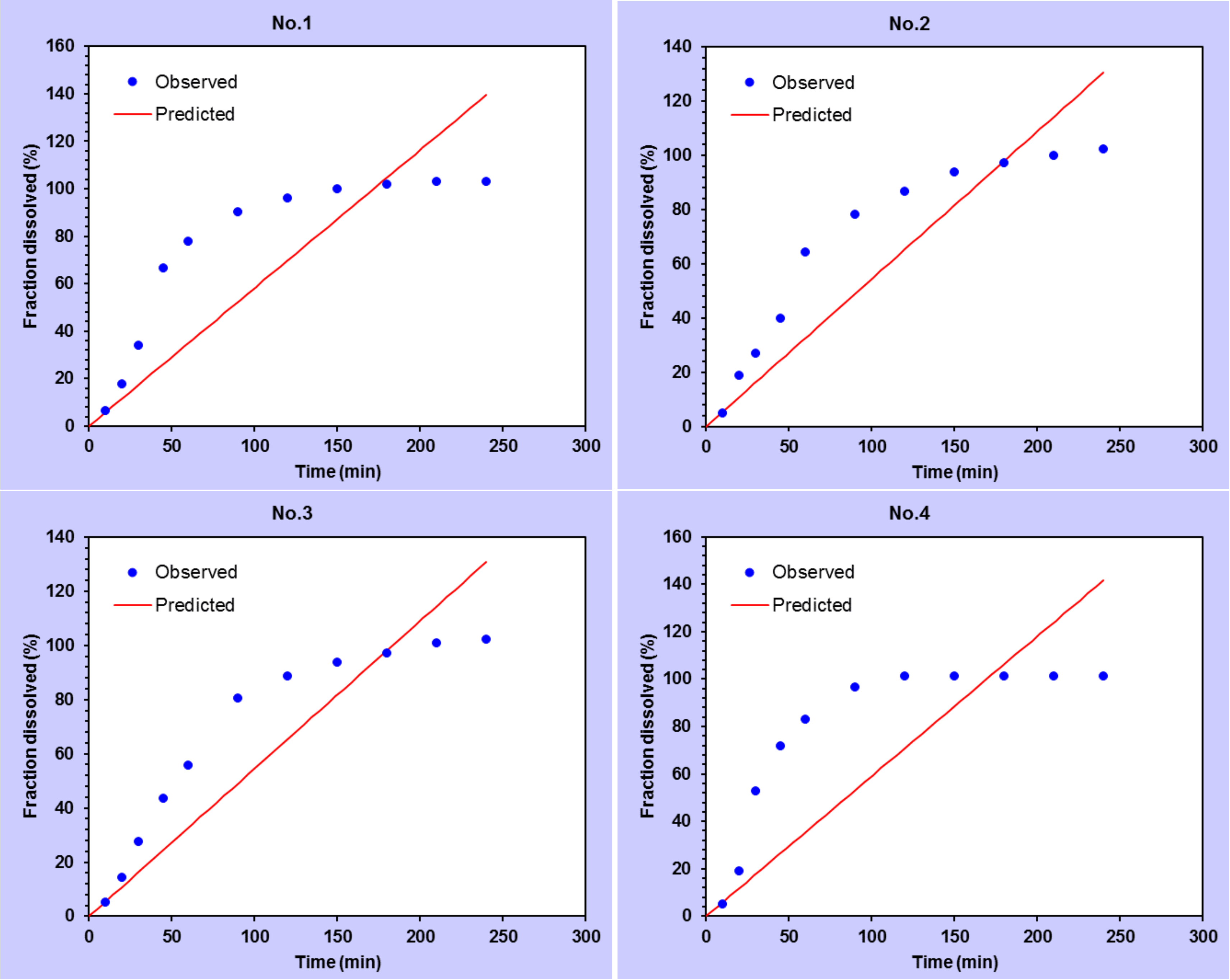

Supplementary materials – Model fitting summary – Parteck® M 100 (modelling the entire relevant dissolution data)

Model: **Zero–order with T_{lag}**

Model equation: $F = k_0 \cdot (t - T_{lag})$

Fitted model parameters per tested tablet (N = 4) with statistics – mean, standard deviation (SD), and relative standard deviation expressed in % (RSD%) (output from DDSolver):

Parameter	No.1	No.2	No.3	No.4	Mean	SD	RSD(%)
k_0	0.382	0.410	0.418	0.346	0.389	0.033	8.409
T_{lag}	-84.459	-52.811	-49.013	-114.116	-75.100	30.480	-40.586

Number of dissolution data points (N), degrees of freedom (df), and selected goodness of fit criteria – Pearson correlation coefficient (R), coefficient of determination (R^2), adjusted coefficient of determination ($R^2_{adjusted}$), and residual sum of squares (RSS) (manual calculation in MS Excel):

Parameter	No.1	No.2	No.3	No.4
N	11	11	11	11
df	9	9	9	9
R	0.844170926	0.916244095	0.91972513	0.787764747
R^2	0.712624552	0.839503242	0.845894315	0.620573297
$R^2_{adjusted}$	0.680693947	0.821670269	0.828771461	0.578414774
RSS	3813.028909	2084.136936	2067.034148	4743.220481

Graphical abstract of model fit presented as mean ± 1 SD of the fraction % of released carvedilol:

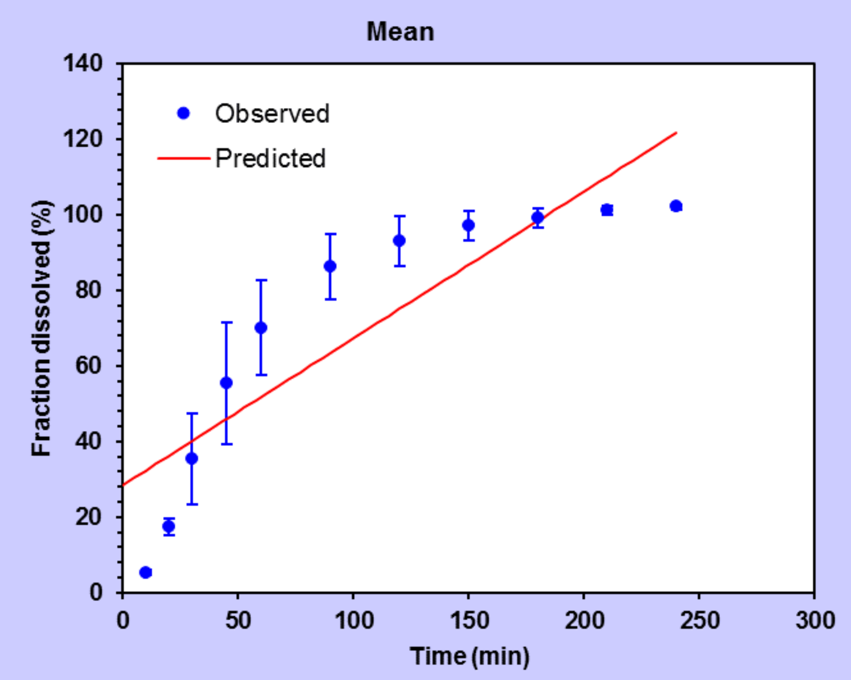

Supplementary materials – Model fitting summary – Parteck® M 100 (modelling the entire relevant dissolution data)

Graphical abstract of model fit presented as the fraction % of released carvedilol per tested tablet:

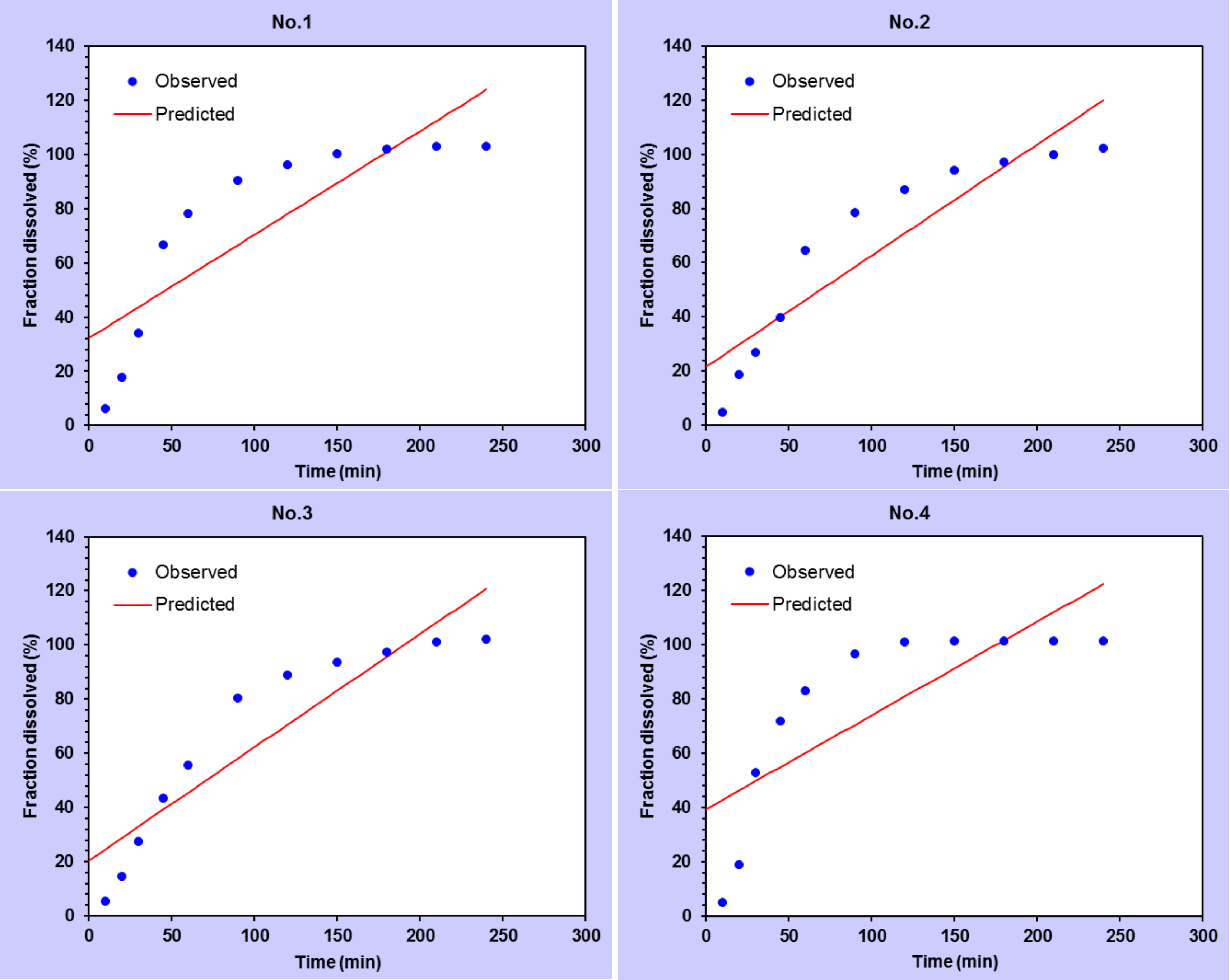

Supplementary materials – Model fitting summary – Parteck® M 100 (modelling the entire relevant dissolution data)

Model: **Zero–order with F_0**

Model equation: $F = F_0 + k_0 \cdot t$

Fitted model parameters per tested tablet (N = 4) with statistics – mean, standard deviation (SD), and relative standard deviation expressed in % (RSD%) (output from DDSolver):

Parameter	No.1	No.2	No.3	No.4	Mean	SD	RSD(%)
k_0	0.382	0.410	0.418	0.346	0.389	0.033	8.409
F_0	32.250	21.653	20.501	39.469	28.468	9.042	31.760

Number of dissolution data points (N), degrees of freedom (df), and selected goodness of fit criteria – Pearson correlation coefficient (R), coefficient of determination (R^2), adjusted coefficient of determination ($R^2_{adjusted}$), and residual sum of squares (RSS) (manual calculation in MS Excel):

Parameter	No.1	No.2	No.3	No.4
N	11	11	11	11
df	9	9	9	9
R	0.844170926	0.916244095	0.91972513	0.787764747
R^2	0.712624552	0.839503242	0.845894315	0.620573297
$R^2_{adjusted}$	0.680693947	0.821670269	0.828771461	0.578414774
RSS	3813.028909	2084.136936	2067.034148	4743.220481

Graphical abstract of model fit presented as mean ± 1 SD of the fraction % of released carvedilol:

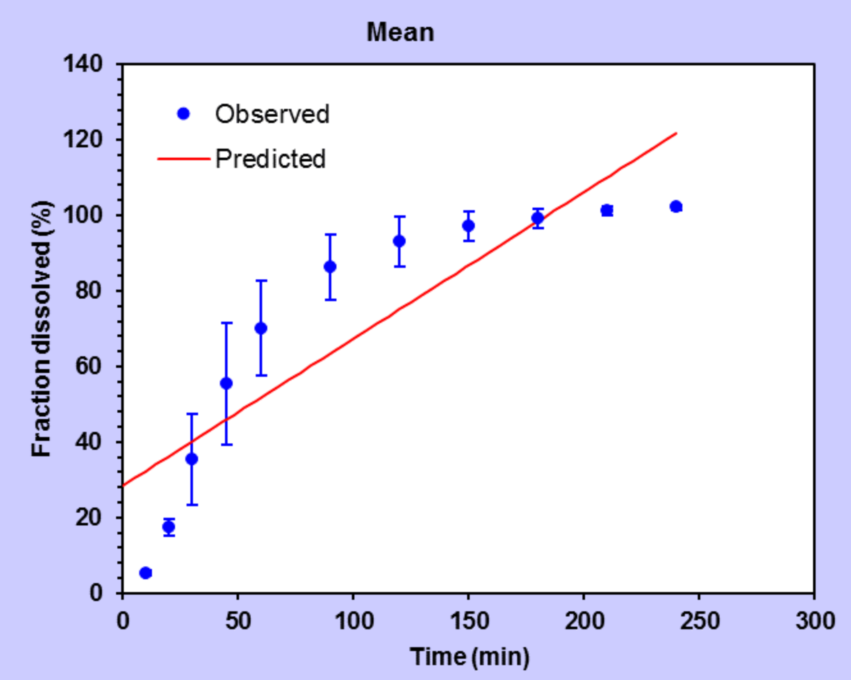

Supplementary materials – Model fitting summary – Parteck® M 100 (modelling the entire relevant dissolution data)

Graphical abstract of model fit presented as the fraction % of released carvedilol per tested tablet:

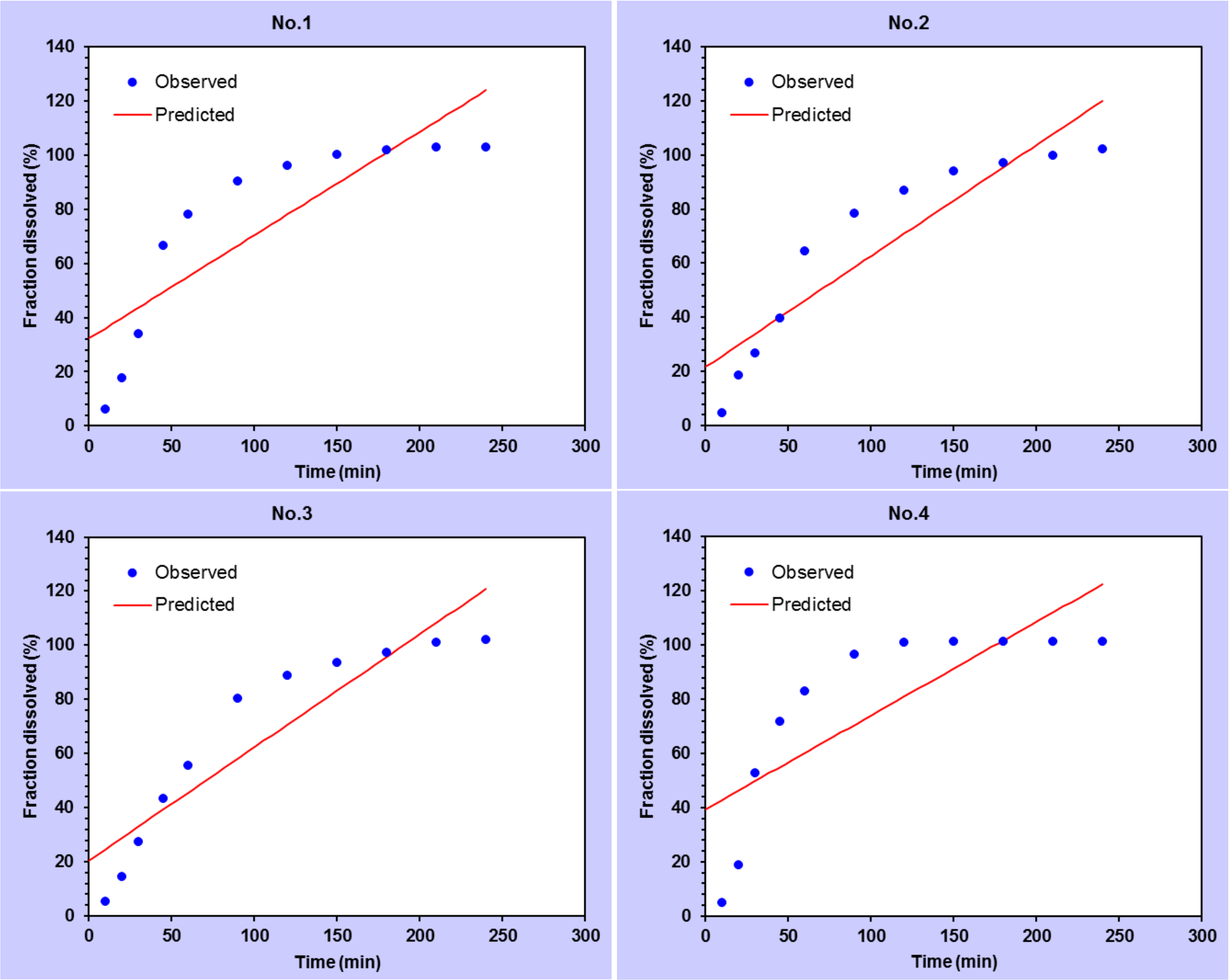

Supplementary materials – Model fitting summary – Parteck® M 100 (modelling the entire relevant dissolution data)

Model: **First–order**

Model equation: $F = 100 \cdot (1 - e^{-k_1 \cdot t})$

Fitted model parameters per tested tablet (N = 4) with statistics – mean, standard deviation (SD), and relative standard deviation expressed in % (RSD%) (output from DDSolver):

Parameter	No.1	No.2	No.3	No.4	Mean	SD	RSD(%)
k_1	0.036	0.022	0.014	0.033	0.026	0.010	39.449

Number of dissolution data points (N), degrees of freedom (df), and selected goodness of fit criteria – Pearson correlation coefficient (R), coefficient of determination (R^2), adjusted coefficient of determination ($R^2_{adjusted}$), and residual sum of squares (RSS) (manual calculation in MS Excel):

Parameter	No.1	No.2	No.3	No.4
N	11	11	11	11
df	10	10	10	10
R	0.981073735	0.98944465	0.997275789	0.994283432
R^2	0.962505674	0.979000715	0.994559	0.988599543
$R^2_{adjusted}$	0.962505674	0.979000715	0.994559	0.988599543
RSS	3203.246685	1686.405326	495.8863577	1502.61604

Graphical abstract of model fit presented as mean ± 1 SD of the fraction % of released carvedilol:

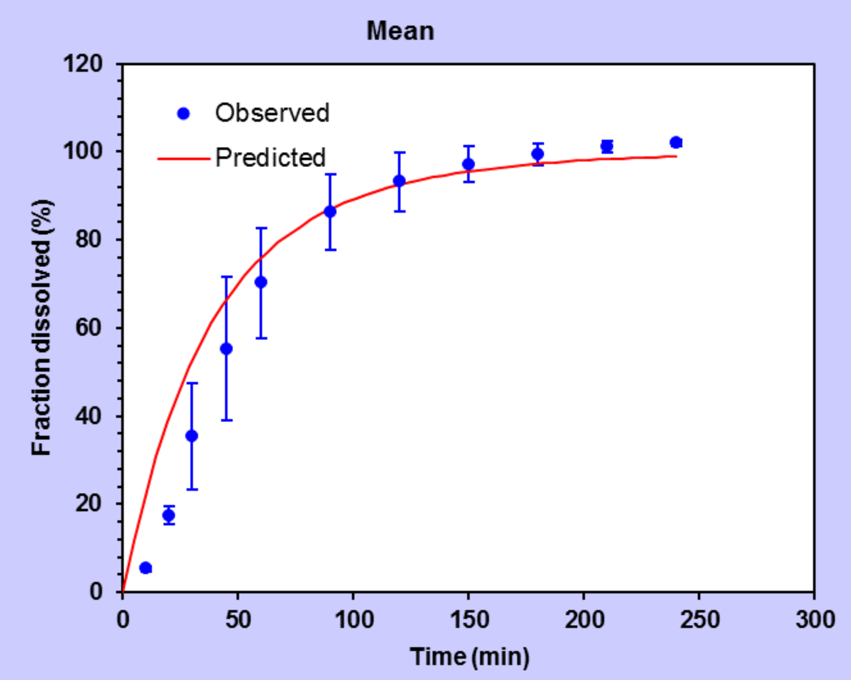

Supplementary materials – Model fitting summary – Parteck® M 100 (modelling the entire relevant dissolution data)

Graphical abstract of model fit presented as the fraction % of released carvedilol per tested tablet:

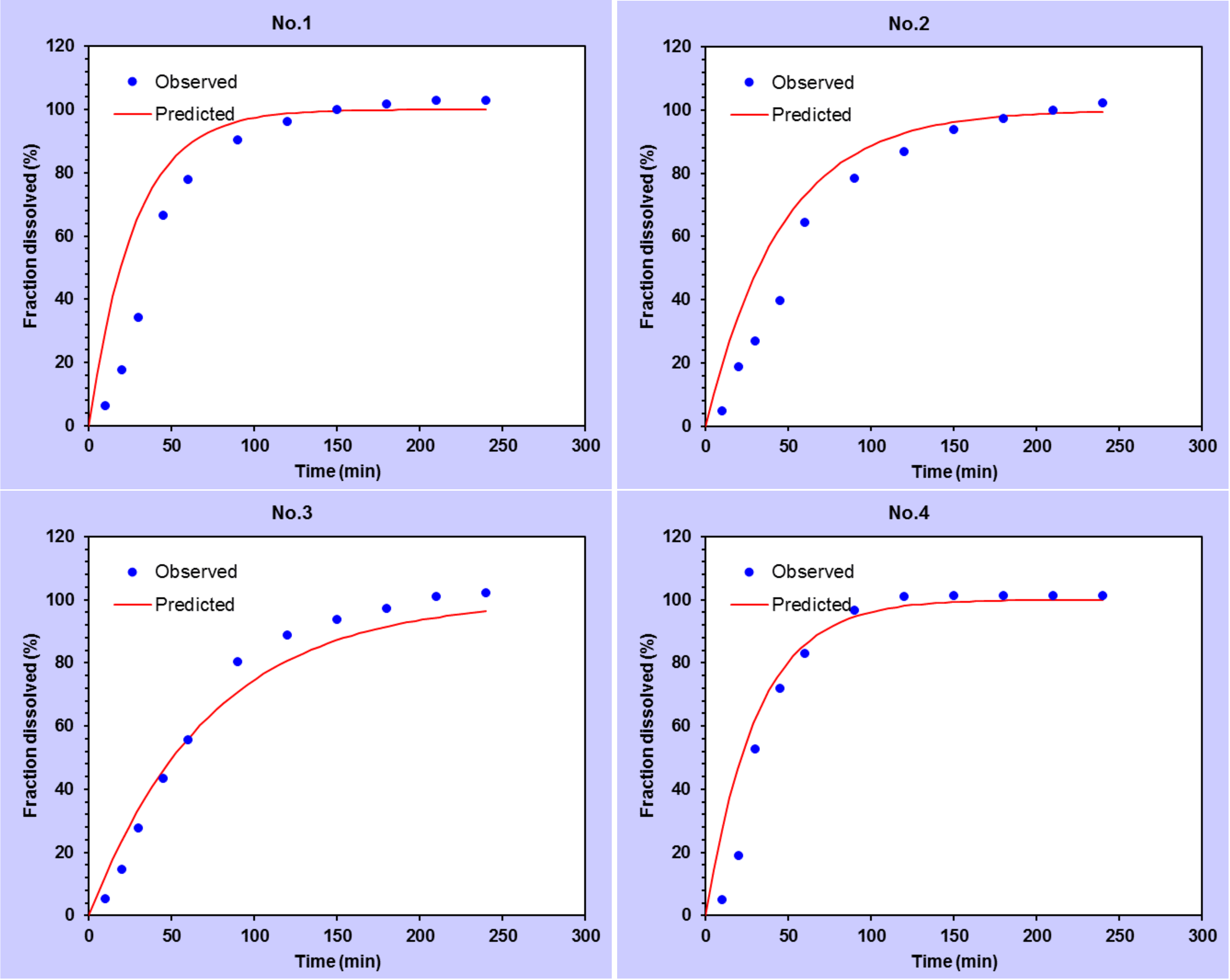

Supplementary materials – Model fitting summary – Parteck® M 100 (modelling the entire relevant dissolution data)

Model: **First–order with T_{lag}**

Model equation: $F = 100 \cdot \left[1 - e^{-k_1 \cdot (t - T_{lag})}\right]$

Fitted model parameters per tested tablet (N = 4) with statistics – mean, standard deviation (SD), and relative standard deviation expressed in % (RSD%) (output from DDSolver):

Parameter	No.1	No.2	No.3	No.4	Mean	SD	RSD(%)
k_1	/	/	/	/	/	/	/
T_{lag}	/	/	/	/	/	/	/

Number of dissolution data points (N), degrees of freedom (df), and selected goodness of fit criteria – Pearson correlation coefficient (R), coefficient of determination (R^2), adjusted coefficient of determination ($R^2_{adjusted}$), and residual sum of squares (RSS) (manual calculation in MS Excel):

Parameter	No.1	No.2	No.3	No.4
N	/	/	/	/
df	/	/	/	/
R	/	/	/	/
R^2	/	/	/	/
$R^2_{adjusted}$	/	/	/	/
RSS	/	/	/	/

Graphical abstract of model fit presented as mean ± 1 SD of the fraction % of released carvedilol: /

Graphical abstract of model fit presented as the fraction % of released carvedilol per tested tablet: /

Note: the model could not be fitted

Supplementary materials – Model fitting summary – Parteck® M 100 (modelling the entire relevant dissolution data)

Model: **First–order with F_{max}**

Model equation: $F = F_{max} \cdot (1 - e^{-k_1 \cdot t})$

Fitted model parameters per tested tablet (N = 4) with statistics – mean, standard deviation (SD), and relative standard deviation expressed in % (RSD%) (output from DDSolver):

Parameter	No.1	No.2	No.3	No.4	Mean	SD	RSD(%)
k_1	0.018	0.013	0.014	0.025	0.018	0.006	31.589
F_{max}	107.967	107.226	107.226	106.340	107.190	0.665	0.621

Number of dissolution data points (N), degrees of freedom (df), and selected goodness of fit criteria – Pearson correlation coefficient (R), coefficient of determination (R^2), adjusted coefficient of determination ($R^2_{adjusted}$), and residual sum of squares (RSS) (manual calculation in MS Excel):

Parameter	No.1	No.2	No.3	No.4
N	11	11	11	11
df	9	9	9	9
R	0.986883568	0.994698778	0.997171299	0.98837407
R^2	0.973939177	0.989425659	0.994350599	0.976883302
$R^2_{adjusted}$	0.97104353	0.988250732	0.993722888	0.97431478
RSS	621.1775627	302.6549703	348.5802512	966.6047277

Graphical abstract of model fit presented as mean ± 1 SD of the fraction % of released carvedilol:

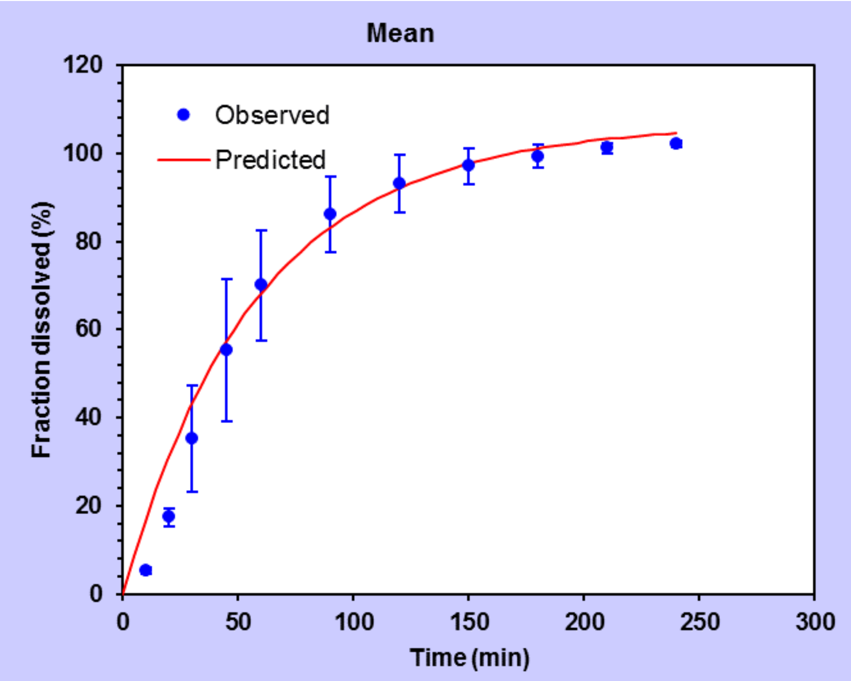

Supplementary materials – Model fitting summary – Parteck® M 100 (modelling the entire relevant dissolution data)

Graphical abstract of model fit presented as the fraction % of released carvedilol per tested tablet:

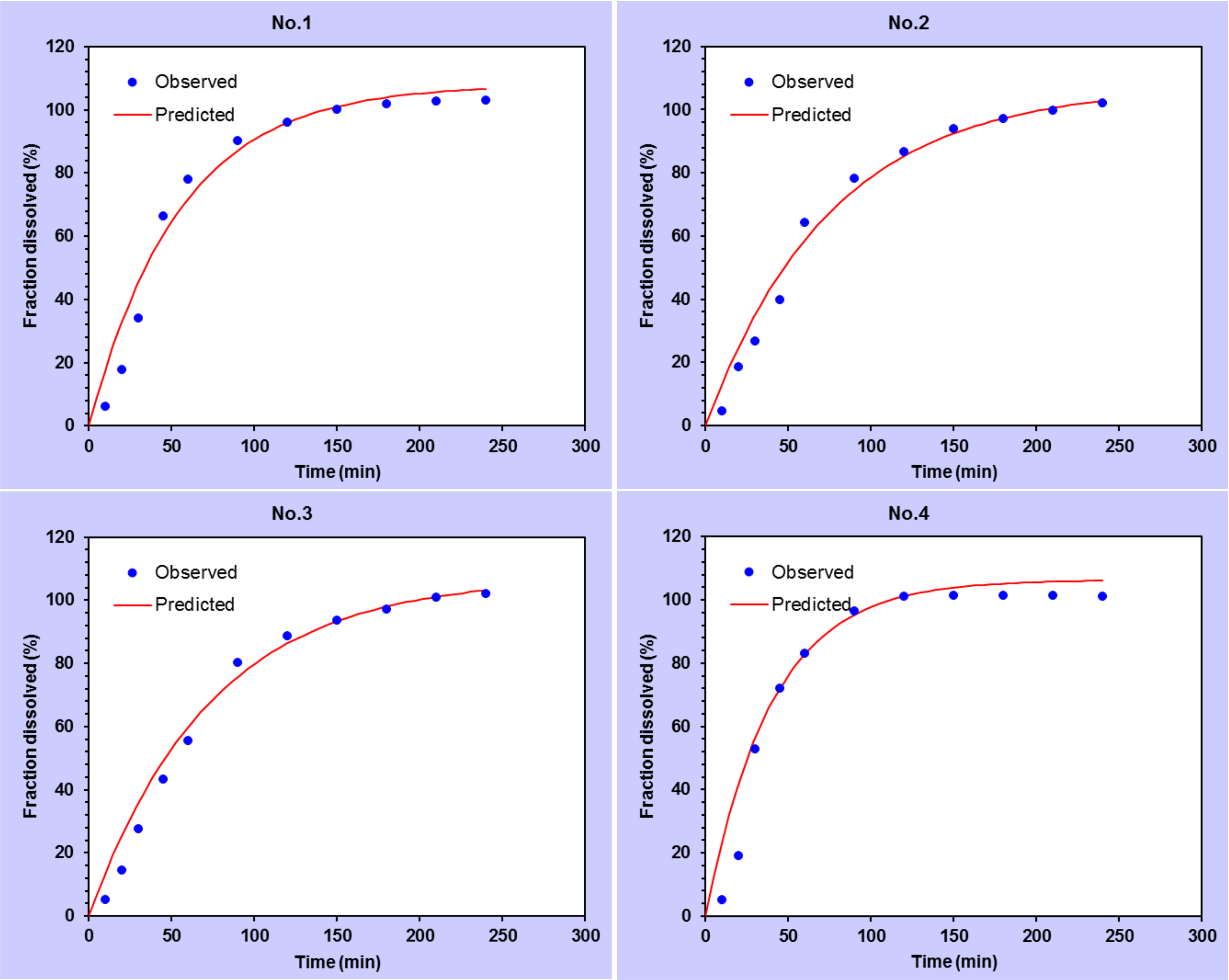

Supplementary materials – Model fitting summary – Parteck® M 100 (modelling the entire relevant dissolution data)

Model: **First–order with T_{lag} and F_{max}**

Model equation: $F = F_{max} \cdot \left[1 - e^{-k_1 \cdot (t - T_{lag})}\right]$

Fitted model parameters per tested tablet (N = 4) with statistics – mean, standard deviation (SD), and relative standard deviation expressed in % (RSD%) (output from DDSolver):

Parameter	No.1	No.2	No.3	No.4	Mean	SD	RSD(%)
k_1	0.014	0.013	0.014	0.014	0.014	0.000	2.295
T_{lag}	-15.064	2.432	5.103	-34.286	-10.454	18.233	-174.411
F_{max}	107.967	107.226	107.226	106.340	107.190	0.665	0.621

Number of dissolution data points (N), degrees of freedom (df), and selected goodness of fit criteria – Pearson correlation coefficient (R), coefficient of determination (R^2), adjusted coefficient of determination ($R^2_{adjusted}$), and residual sum of squares (RSS) (manual calculation in MS Excel):

Parameter	No.1	No.2	No.3	No.4
N	11	11	11	11
df	8	8	8	8
R	0.972530454	0.994650932	0.997140213	0.943624176
R^2	0.945815483	0.989330477	0.994288604	0.890426585
$R^2_{adjusted}$	0.932269354	0.986663096	0.992860755	0.863033232
RSS	1678.384387	201.9124788	110.853815	3557.734501

Graphical abstract of model fit presented as mean ± 1 SD of the fraction % of released carvedilol:

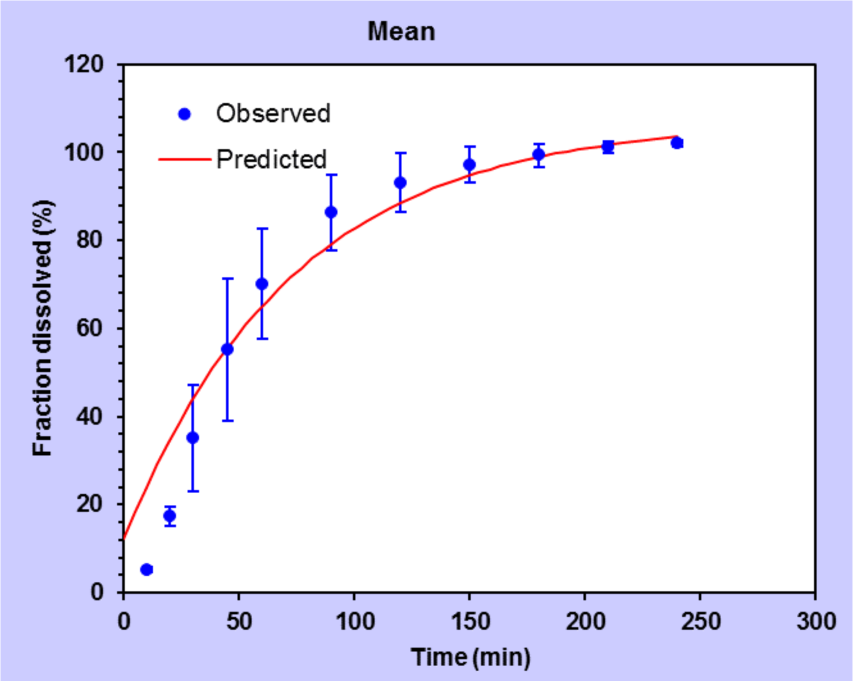

Supplementary materials – Model fitting summary – Parteck® M 100 (modelling the entire relevant dissolution data)

Graphical abstract of model fit presented as the fraction % of released carvedilol per tested tablet:

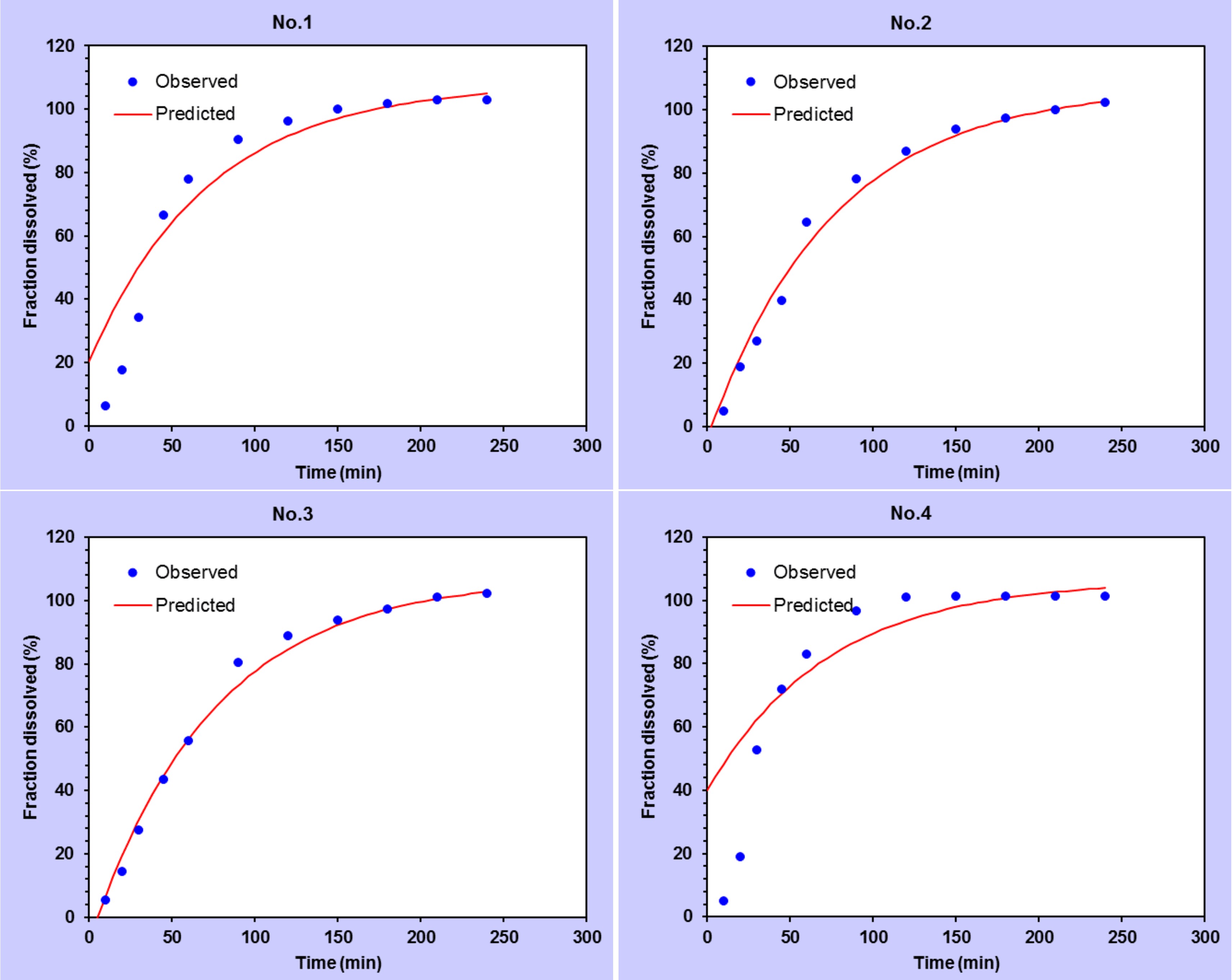

Supplementary materials – Model fitting summary – Parteck® M 100 (modelling the entire relevant dissolution data)

Model: **Higuchi**

Model equation: $F = k_H \cdot t^{0.5}$

Fitted model parameters per tested tablet (N = 4) with statistics – mean, standard deviation (SD), and relative standard deviation expressed in % (RSD%) (output from DDSolver):

Parameter	No.1	No.2	No.3	No.4	Mean	SD	RSD(%)
k_H	7.718	7.082	7.081	7.937	7.454	0.440	5.897

Number of dissolution data points (N), degrees of freedom (df), and selected goodness of fit criteria – Pearson correlation coefficient (R), coefficient of determination (R^2), adjusted coefficient of determination ($R^2_{adjusted}$), and residual sum of squares (RSS) (manual calculation in MS Excel):

Parameter	No.1	No.2	No.3	No.4
N	11	11	11	11
df	10	10	10	10
R	0.920210592	0.968408017	0.970886699	0.878428977
R^2	0.846787534	0.937814087	0.942620983	0.771637468
$R^2_{adjusted}$	0.846787534	0.937814087	0.942620983	0.771637468
RSS	2043.236043	1102.589675	1148.569787	2925.754528

Graphical abstract of model fit presented as mean ± 1 SD of the fraction % of released carvedilol:

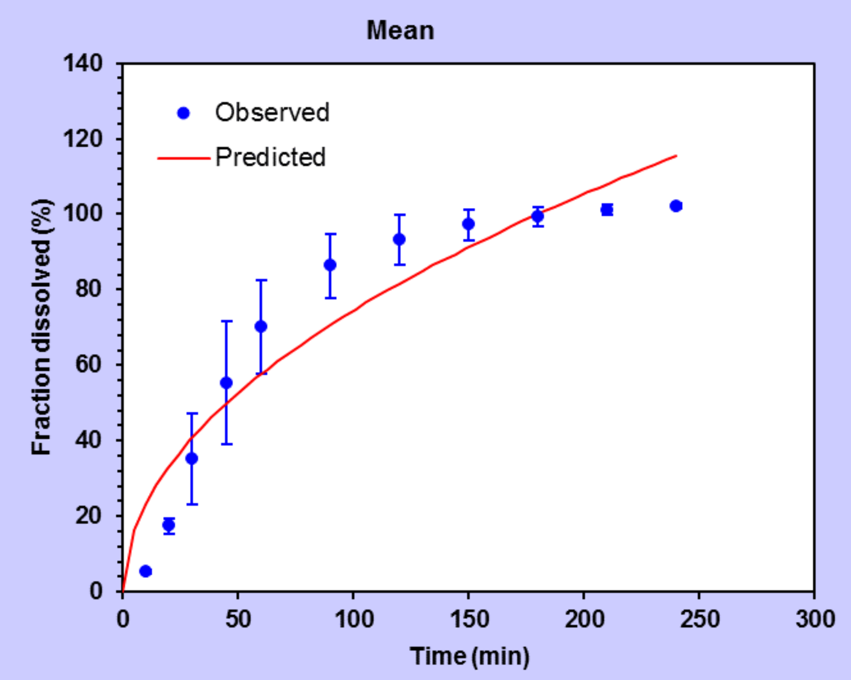

Supplementary materials – Model fitting summary – Parteck® M 100 (modelling the entire relevant dissolution data)

Graphical abstract of model fit presented as the fraction % of released carvedilol per tested tablet:

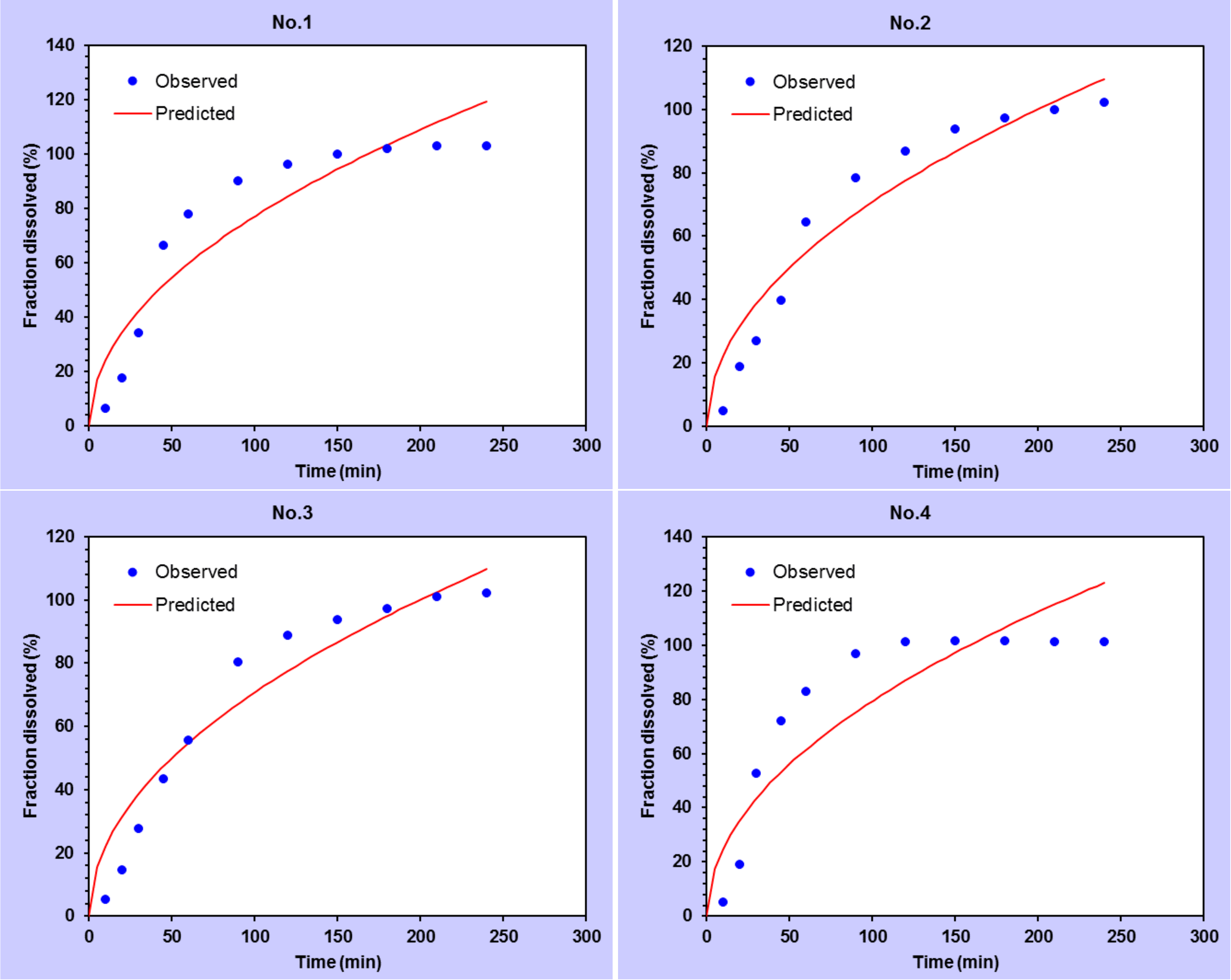

Supplementary materials – Model fitting summary – Parteck® M 100 (modelling the entire relevant dissolution data)

Model: **Higuchi with T_{lag}**

Model equation: $F = k_H \cdot (t - T_{lag})^{0.5}$

Fitted model parameters per tested tablet (N = 4) with statistics – mean, standard deviation (SD), and relative standard deviation expressed in % (RSD%) (output from DDSolver):

Parameter	No.1	No.2	No.3	No.4	Mean	SD	RSD(%)
k_H	6.942	7.038	7.102	6.619	6.925	0.215	3.101
T_{lag}	-28.650	-3.352	-1.443	-52.053	-21.375	23.918	-111.898

Number of dissolution data points (N), degrees of freedom (df), and selected goodness of fit criteria – Pearson correlation coefficient (R), coefficient of determination (R^2), adjusted coefficient of determination ($R^2_{adjusted}$), and residual sum of squares (RSS) (manual calculation in MS Excel):

Parameter	No.1	No.2	No.3	No.4
N	11	11	11	11
df	9	9	9	9
R	0.898302188	0.96629071	0.970008544	0.839290634
R^2	0.80694682	0.933717736	0.940916575	0.704408769
$R^2_{adjusted}$	0.785496467	0.92635304	0.93435175	0.671565299
RSS	3420.679843	1317.520934	1251.628249	4616.226525

Graphical abstract of model fit presented as mean ± 1 SD of the fraction % of released carvedilol:

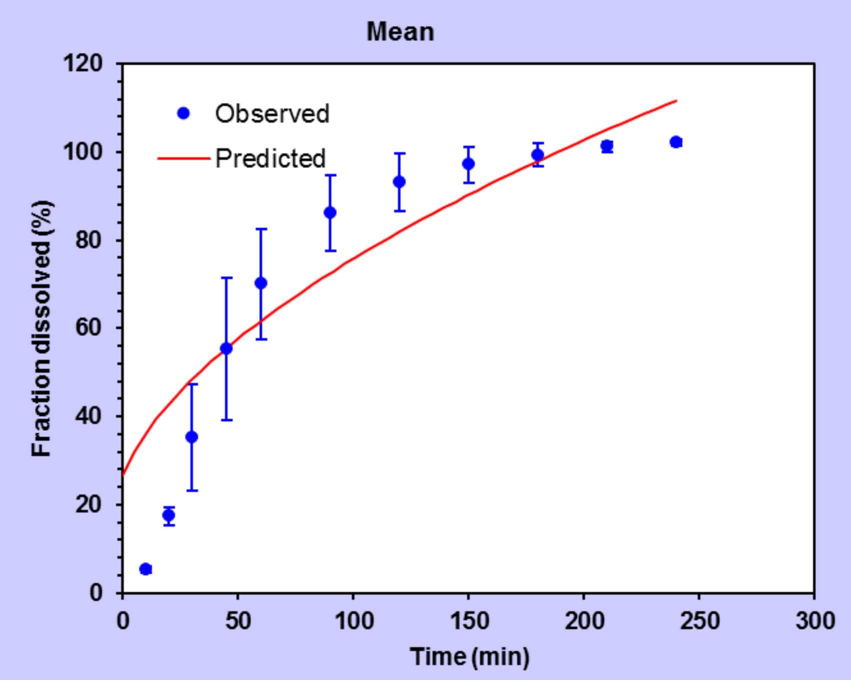

Supplementary materials – Model fitting summary – Parteck® M 100 (modelling the entire relevant dissolution data)

Graphical abstract of model fit presented as the fraction % of released carvedilol per tested tablet:

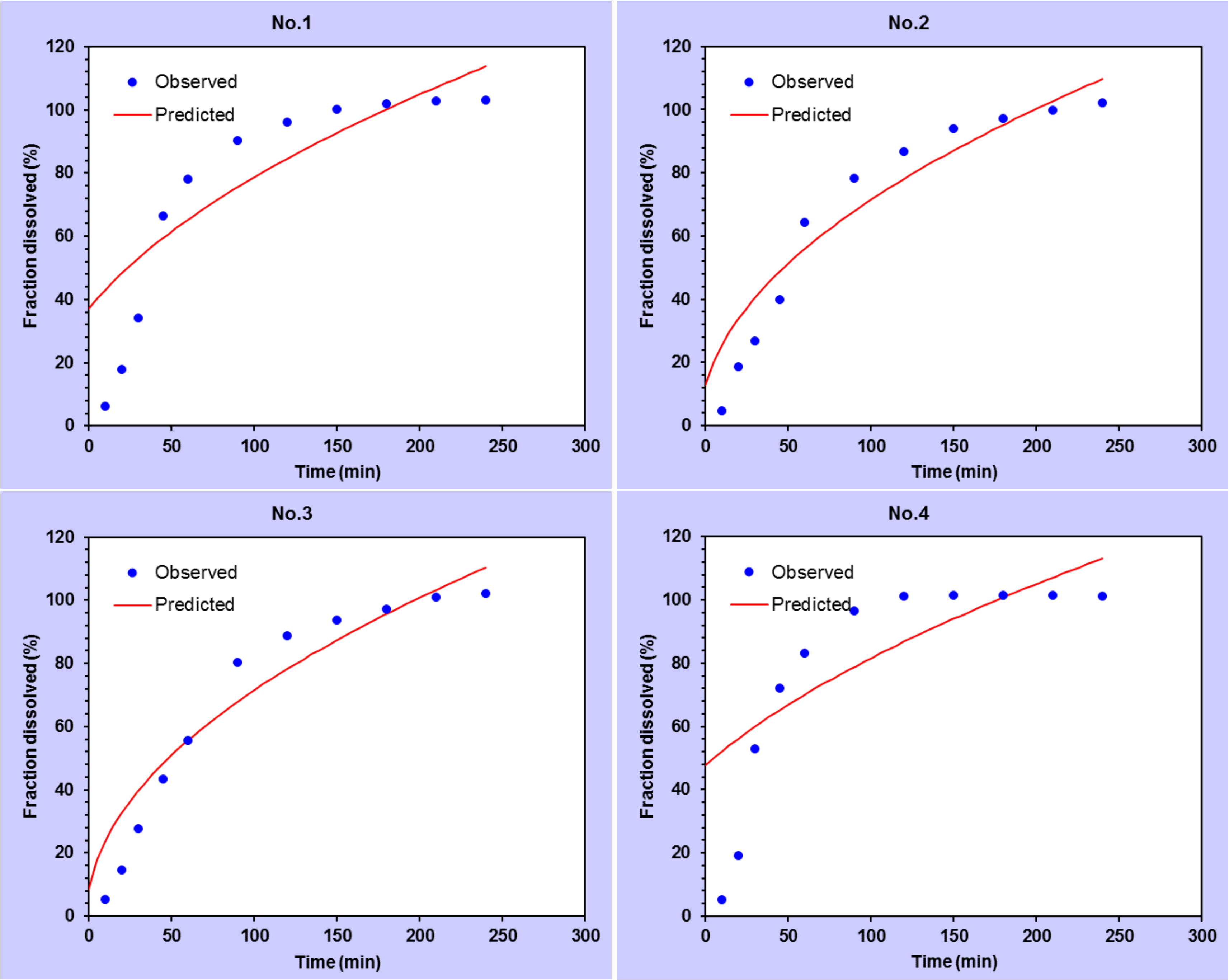

Supplementary materials – Model fitting summary – Parteck® M 100 (modelling the entire relevant dissolution data)

Model: **Higuchi with F_0**

Model equation: $F = F_0 + k_H \cdot t^{0.5}$

Fitted model parameters per tested tablet (N = 4) with statistics – mean, standard deviation (SD), and relative standard deviation expressed in % (RSD%) (output from DDSolver):

Parameter	No.1	No.2	No.3	No.4	Mean	SD	RSD(%)
k_H	7.939	8.265	8.422	7.356	7.996	0.471	5.895
F_0	-2.468	-13.184	-14.940	6.467	-6.031	9.990	-165.637

Number of dissolution data points (N), degrees of freedom (df), and selected goodness of fit criteria – Pearson correlation coefficient (R), coefficient of determination (R^2), adjusted coefficient of determination ($R^2_{adjusted}$), and residual sum of squares (RSS) (manual calculation in MS Excel):

Parameter	No.1	No.2	No.3	No.4
N	11	11	11	11
df	9	9	9	9
R	0.920210592	0.968408017	0.970886699	0.878428977
R^2	0.846787534	0.937814087	0.942620983	0.771637468
$R^2_{adjusted}$	0.829763927	0.930904542	0.936245536	0.746263853
RSS	2032.893087	807.517604	769.6301916	2854.764386

Graphical abstract of model fit presented as mean ± 1 SD of the fraction % of released carvedilol:

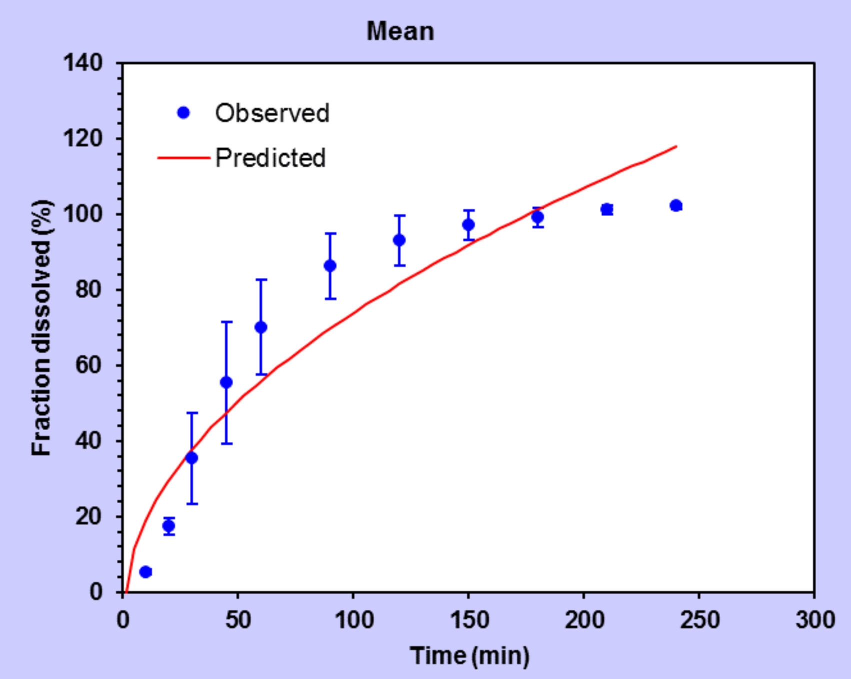

Supplementary materials – Model fitting summary – Parteck® M 100 (modelling the entire relevant dissolution data)

Graphical abstract of model fit presented as the fraction % of released carvedilol per tested tablet:

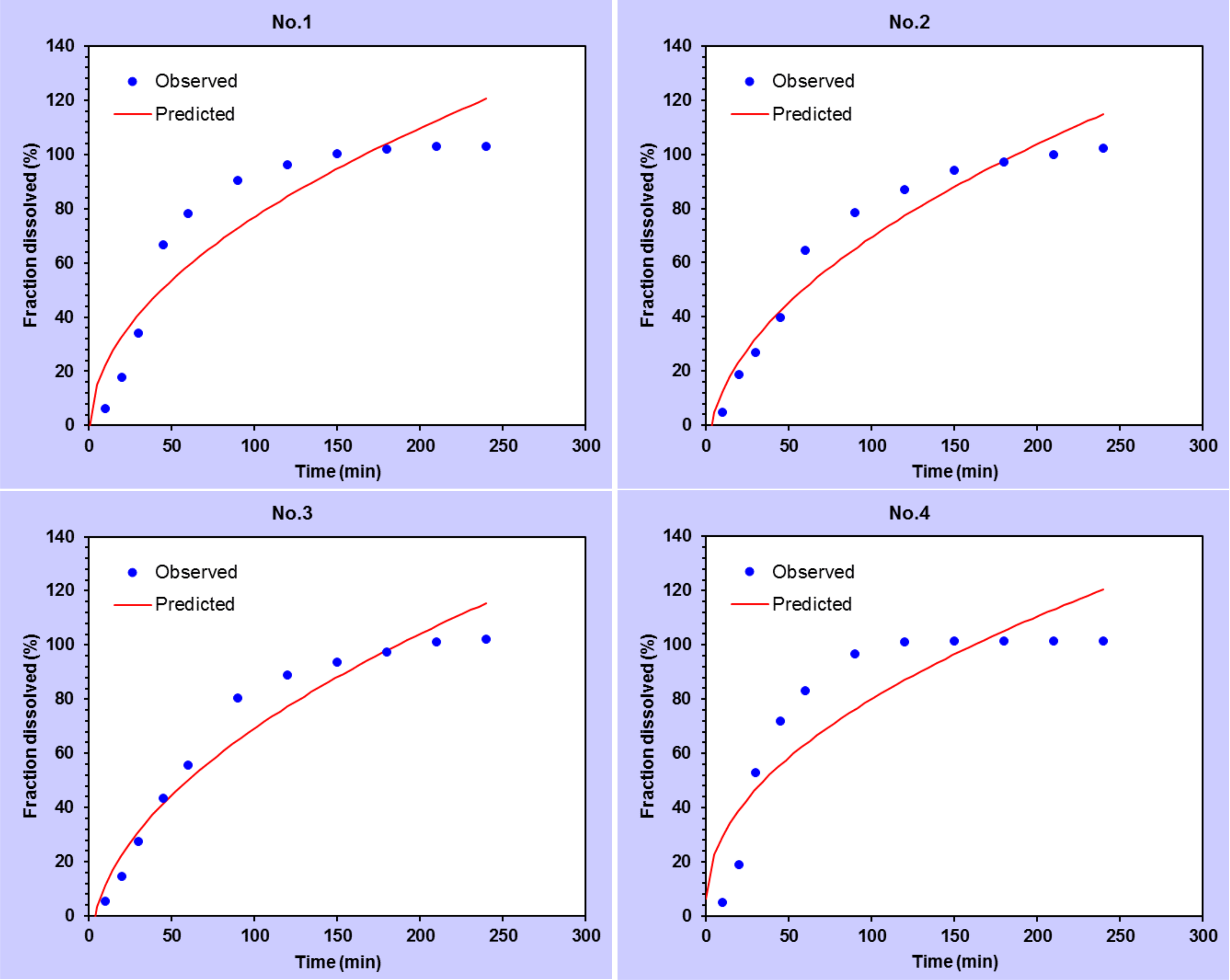

Supplementary materials – Model fitting summary – Parteck® M 100 (modelling the entire relevant dissolution data)

Model: **Korsmeyer–Peppas**

Model equation: $F = k_{KP} \cdot t^n$

Fitted model parameters per tested tablet (N = 4) with statistics – mean, standard deviation (SD), and relative standard deviation expressed in % (RSD%) (output from DDSolver):

Parameter	No.1	No.2	No.3	No.4	Mean	SD	RSD(%)
k_{KP}	0.849	1.302	0.736	0.513	0.850	0.332	39.062
n	1.028	0.817	1.002	0.000	0.712	0.484	67.957

Number of dissolution data points (N), degrees of freedom (df), and selected goodness of fit criteria – Pearson correlation coefficient (R), coefficient of determination (R^2), adjusted coefficient of determination ($R^2_{adjusted}$), and residual sum of squares (RSS) (manual calculation in MS Excel):

Parameter	No.1	No.2	No.3	No.4
N	11	11	11	11
df	9	9	9	9
R	0.839852799	0.937383581	0.919523754	0.697060585
R^2	0.705352724	0.878687979	0.845523935	0.485893459
$R^2_{adjusted}$	0.672614138	0.865208865	0.828359928	0.42877051
RSS	38011.15087	2559.929114	10962.24383	74827.43264

Graphical abstract of model fit presented as mean ± 1 SD of the fraction % of released carvedilol: / (no charts were produced by DDSolver)

Graphical abstract of model fit presented as the fraction % of released carvedilol per tested tablet: / (no charts were produced by DDSolver)

Supplementary materials – Model fitting summary – Parteck® M 100 (modelling the entire relevant dissolution data)

Model: **Korsmeyer–Peppas with T_{lag}**

Model equation: $F = k_{KP} \cdot \left(t - T_{lag}\right)^n$

Fitted model parameters per tested tablet (N = 4) with statistics – mean, standard deviation (SD), and relative standard deviation expressed in % (RSD%) (output from DDSolver):

Parameter	No.1	No.2	No.3	No.4	Mean	SD	RSD(%)
k_{KP}	2.596	1.769	1.694	2.729	2.197	0.541	24.629
n	0.740	0.798	0.806	0.740	0.771	0.036	4.673
T_{lag}	4.000	4.000	4.000	4.000	4.000	0.000	0.000

Number of dissolution data points (N), degrees of freedom (df), and selected goodness of fit criteria – Pearson correlation coefficient (R), coefficient of determination (R^2), adjusted coefficient of determination ($R^2_{adjusted}$), and residual sum of squares (RSS) (manual calculation in MS Excel):

Parameter	No.1	No.2	No.3	No.4
N	11	11	11	11
df	8	8	8	8
R	0.887800998	0.940998654	0.943270187	0.83887505
R^2	0.788190611	0.885478467	0.889758646	0.703711349
$R^2_{adjusted}$	0.735238264	0.856848084	0.862198307	0.629639187
RSS	5253.276507	2893.532689	2735.61609	7946.766068

Graphical abstract of model fit presented as mean ± 1 SD of the fraction % of released carvedilol:

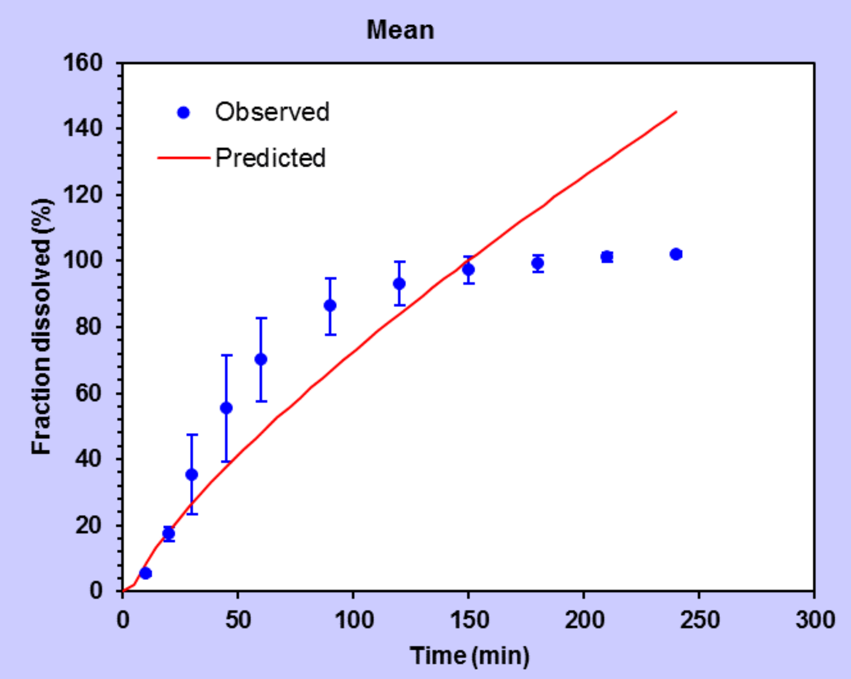

Supplementary materials – Model fitting summary – Parteck® M 100 (modelling the entire relevant dissolution data)

Graphical abstract of model fit presented as the fraction % of released carvedilol per tested tablet:

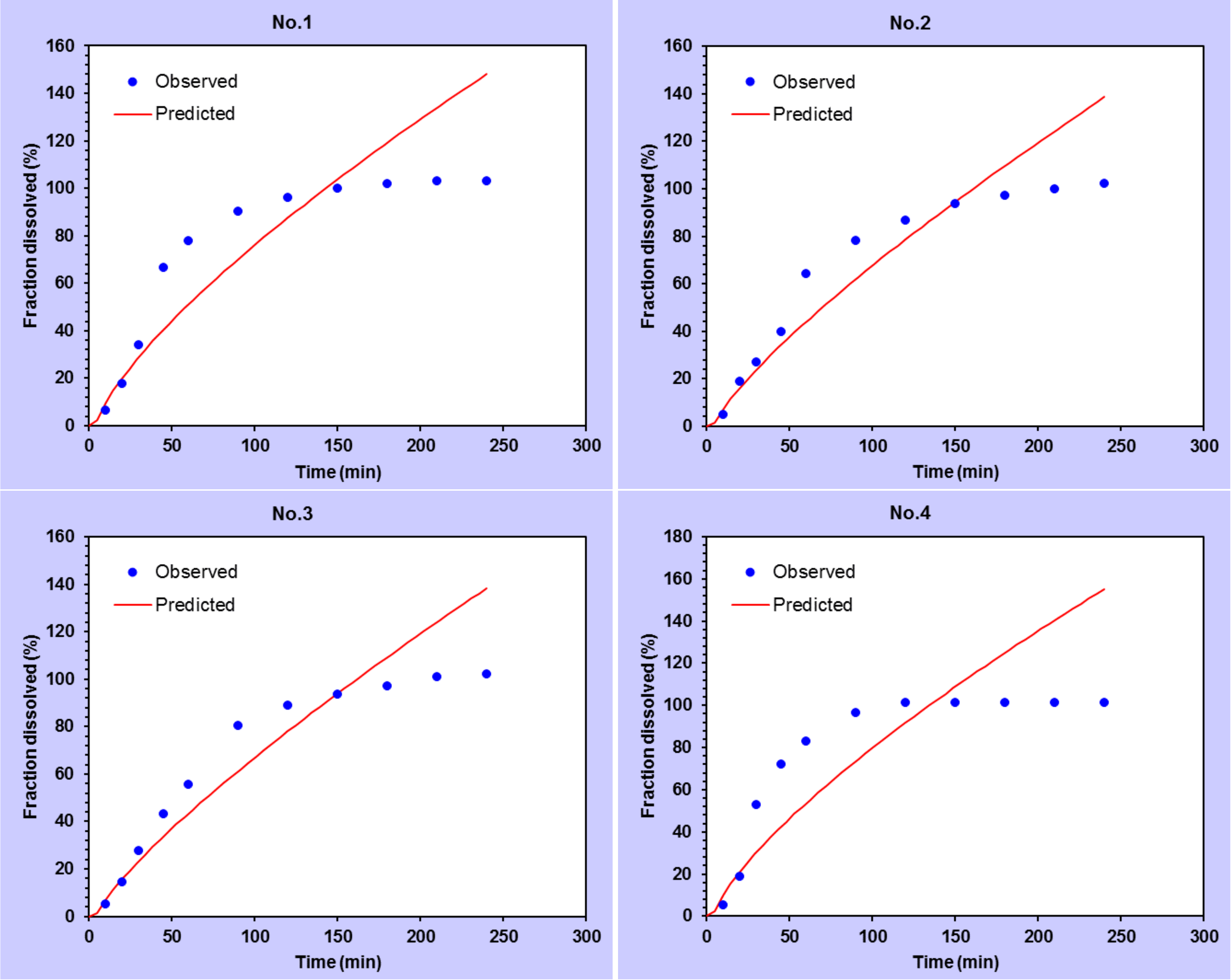

Supplementary materials – Model fitting summary – Parteck® M 100 (modelling the entire relevant dissolution data)

Model: **Korsmeyer–Peppas with F_0**

Model equation: $F = F_0 + k_{KP} \cdot t^n$

Fitted model parameters per tested tablet (N = 4) with statistics – mean, standard deviation (SD), and relative standard deviation expressed in % (RSD%) (output from DDSolver):

Parameter	No.1	No.2	No.3	No.4	Mean	SD	RSD(%)
k_{KP}	0.998	0.662	0.605	1.122	0.847	0.253	29.820
n	0.925	0.989	1.006	0.911	0.958	0.047	4.883
F_0	2.479	1.880	2.080	2.000	2.110	0.260	12.320

Number of dissolution data points (N), degrees of freedom (df), and selected goodness of fit criteria – Pearson correlation coefficient (R), coefficient of determination (R^2), adjusted coefficient of determination ($R^2_{adjusted}$), and residual sum of squares (RSS) (manual calculation in MS Excel):

Parameter	No.1	No.2	No.3	No.4
N	11	11	11	11
df	8	8	8	8
R	0.855977939	0.917501987	0.919035869	0.803713072
R^2	0.732698232	0.841809897	0.844626928	0.645954702
$R^2_{adjusted}$	0.66587279	0.802262371	0.805783661	0.557443378
RSS	8164.977474	5092.267238	4984.089785	11371.94075

Graphical abstract of model fit presented as mean ± 1 SD of the fraction % of released carvedilol:

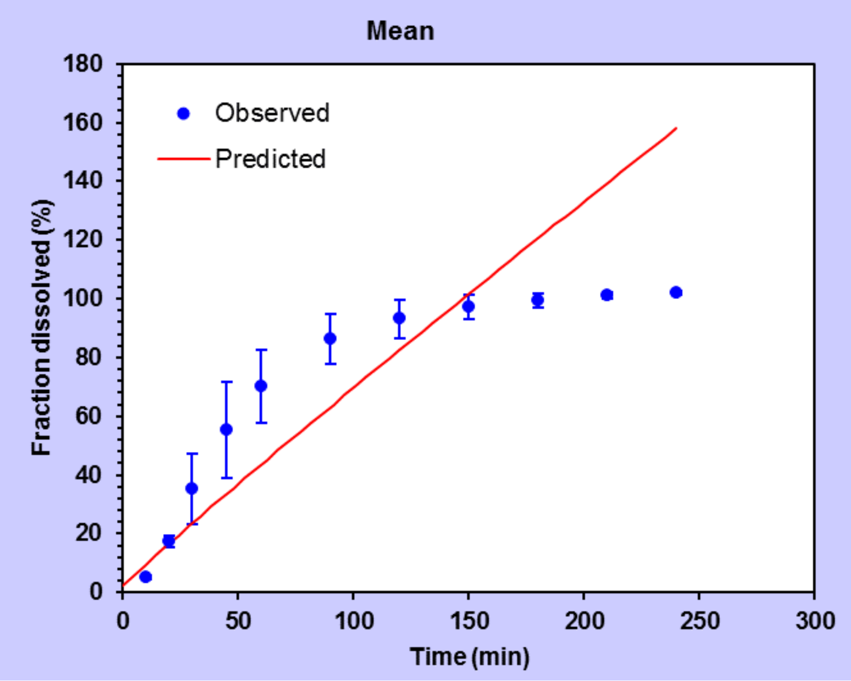

Supplementary materials – Model fitting summary – Parteck® M 100 (modelling the entire relevant dissolution data)

Graphical abstract of model fit presented as the fraction % of released carvedilol per tested tablet:

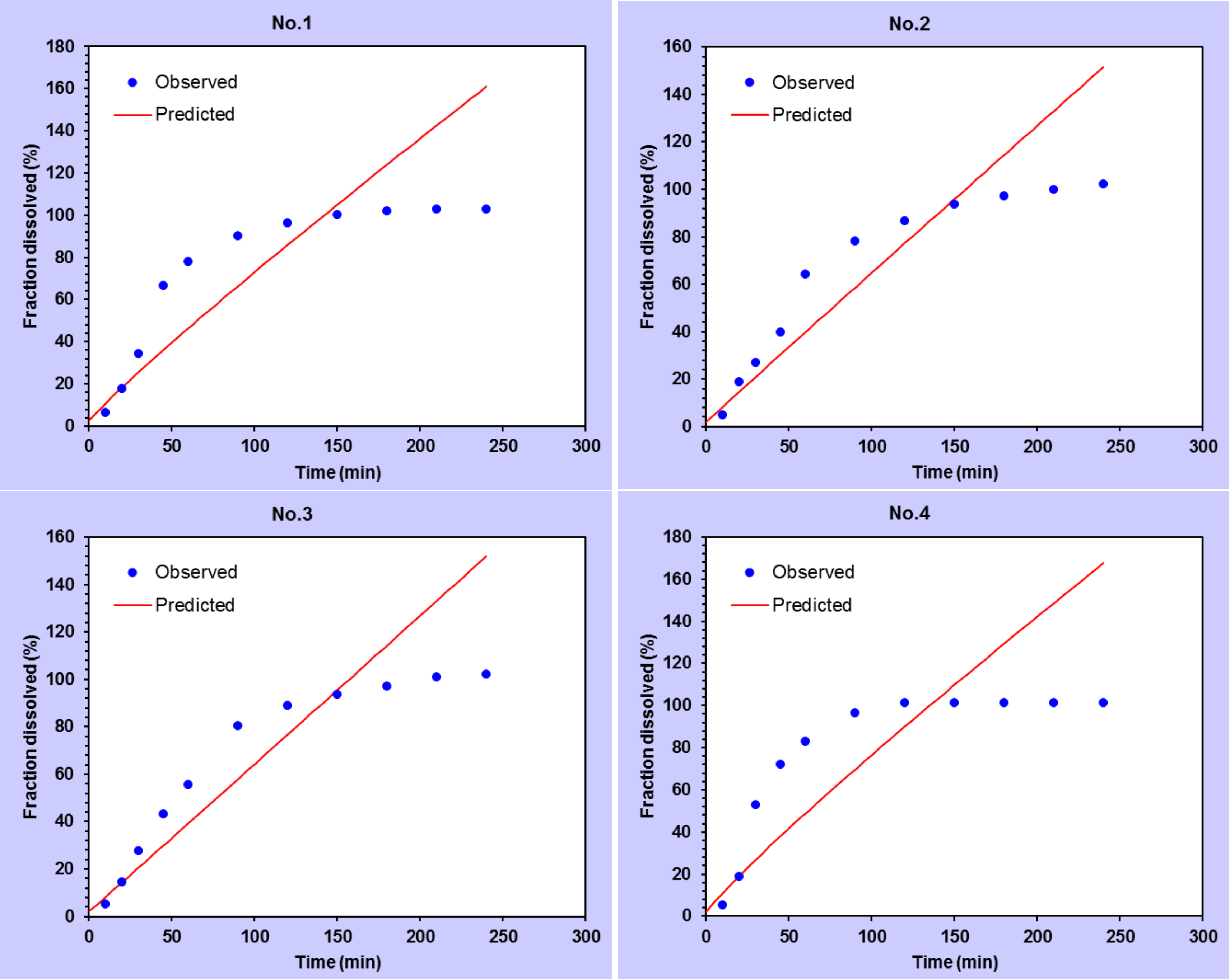

Supplementary materials – Model fitting summary – Parteck® M 100 (modelling the entire relevant dissolution data)

Model: **Hixson–Crowell**

Model equation: $F = 100 \cdot [1 - (1 - k_{HC} \cdot t)^3]$

Fitted model parameters per tested tablet (N = 4) with statistics – mean, standard deviation (SD), and relative standard deviation expressed in % (RSD%) (output from DDSolver):

Parameter	No.1	No.2	No.3	No.4	Mean	SD	RSD(%)
k_{HC}	0.006	0.004	0.004	0.006	0.005	0.001	24.342

Number of dissolution data points (N), degrees of freedom (df), and selected goodness of fit criteria – Pearson correlation coefficient (R), coefficient of determination (R^2), adjusted coefficient of determination ($R^2_{adjusted}$), and residual sum of squares (RSS) (manual calculation in MS Excel):

Parameter	No.1	No.2	No.3	No.4
N	11	11	11	11
df	10	10	10	10
R	0.990319905	0.995006712	0.99756823	0.971953852
R^2	0.980733515	0.990038358	0.995142374	0.944694291
$R^2_{adjusted}$	0.980733515	0.990038358	0.995142374	0.944694291
RSS	499.0948319	202.5351833	179.8738095	763.4176245

Graphical abstract of model fit presented as mean ± 1 SD of the fraction % of released carvedilol:

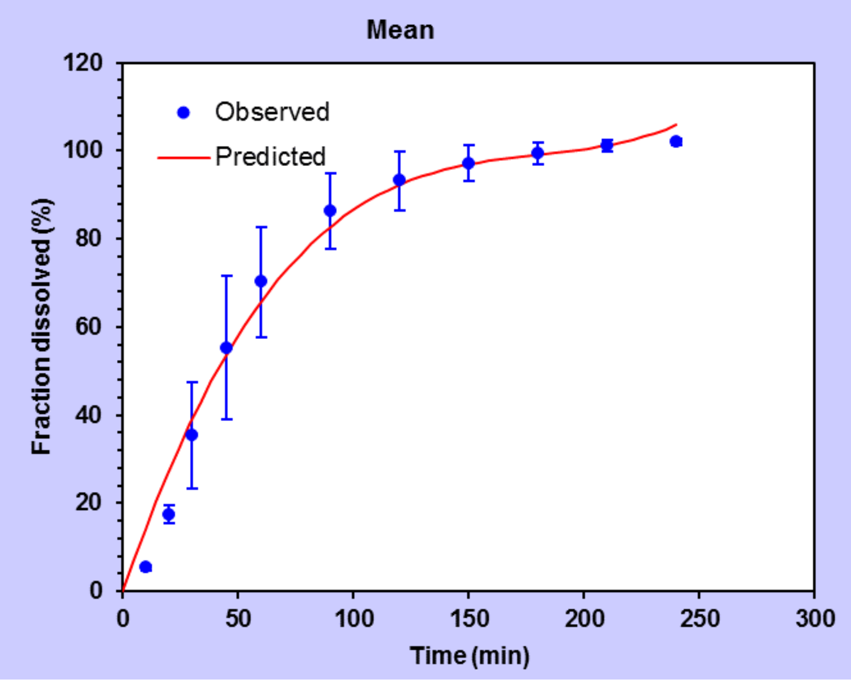

Supplementary materials – Model fitting summary – Parteck® M 100 (modelling the entire relevant dissolution data)

Graphical abstract of model fit presented as the fraction % of released carvedilol per tested tablet:

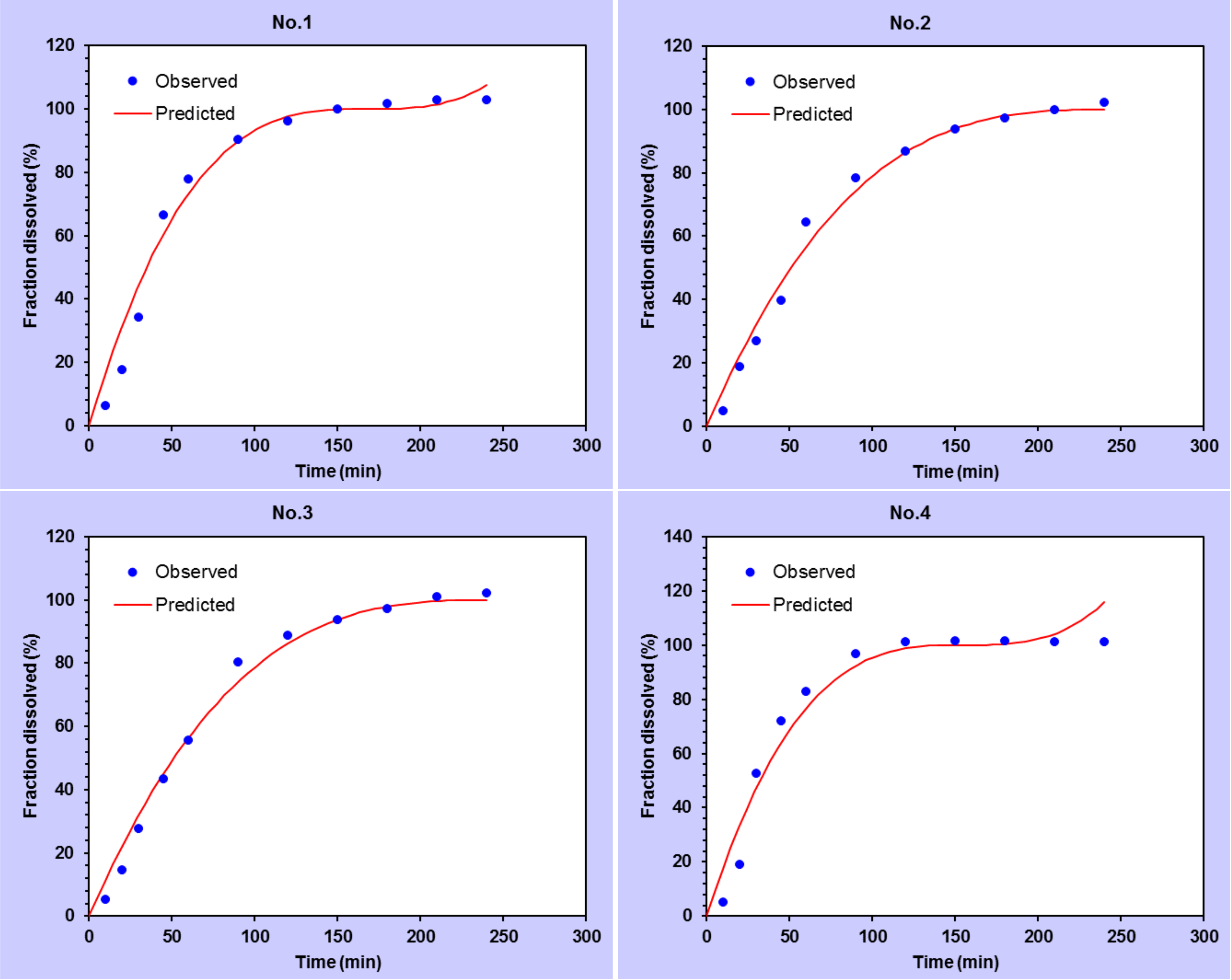

Supplementary materials – Model fitting summary – Parteck® M 100 (modelling the entire relevant dissolution data)

Model: **Hixson–Crowell with T_{lag}**

Model equation: $F = 100 \cdot \left\{1 - \left[1 - k_{HC} \cdot \left(t - T_{lag}\right)\right]^3\right\}$

Fitted model parameters per tested tablet (N = 4) with statistics – mean, standard deviation (SD), and relative standard deviation expressed in % (RSD%) (output from DDSolver):

Parameter	No.1	No.2	No.3	No.4	Mean	SD	RSD(%)
k_{HC}	0.006	0.004	0.004	0.004	0.005	0.001	22.422
T_{lag}	7.230	3.260	4.396	10.812	6.425	3.368	52.422

Number of dissolution data points (N), degrees of freedom (df), and selected goodness of fit criteria – Pearson correlation coefficient (R), coefficient of determination (R^2), adjusted coefficient of determination ($R^2_{adjusted}$), and residual sum of squares (RSS) (manual calculation in MS Excel):

Parameter	No.1	No.2	No.3	No.4
N	11	11	11	11
df	9	9	9	9
R	0.990279716	0.995052277	0.997690114	0.937207433
R^2	0.980653916	0.990129035	0.995385563	0.878357772
$R^2_{adjusted}$	0.978504351	0.989032261	0.994872848	0.864841969
RSS	266.2993067	145.4303078	87.47054743	4428.054698

Graphical abstract of model fit presented as mean ± 1 SD of the fraction % of released carvedilol:

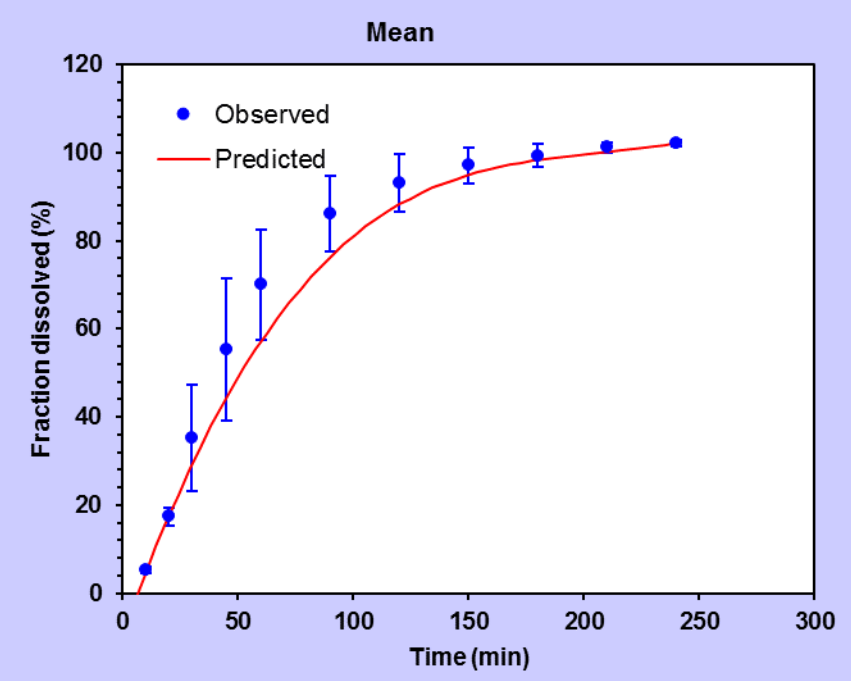

Supplementary materials – Model fitting summary – Parteck® M 100 (modelling the entire relevant dissolution data)

Graphical abstract of model fit presented as the fraction % of released carvedilol per tested tablet:

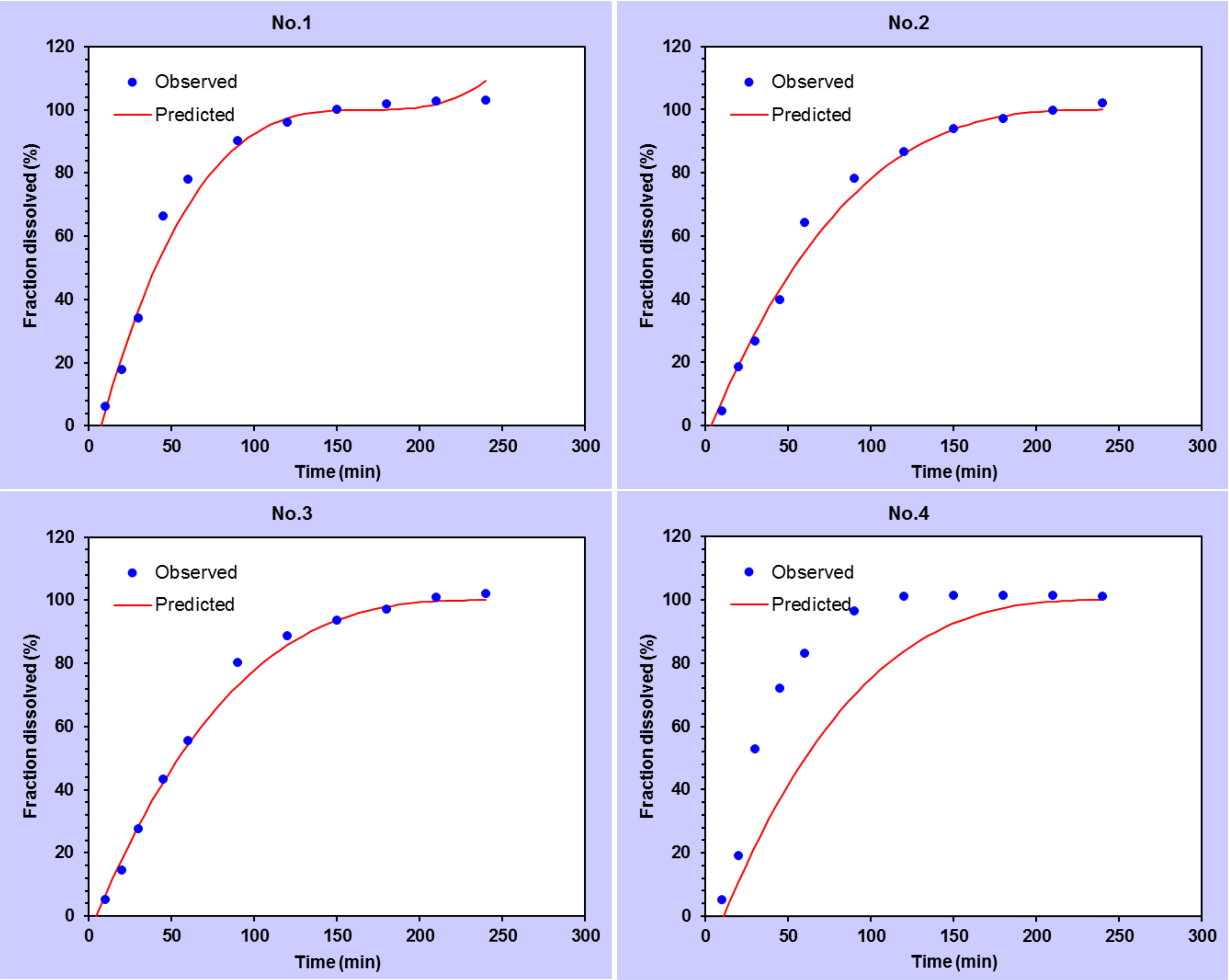

Supplementary materials – Model fitting summary – Parteck® M 100 (modelling the entire relevant dissolution data)

Model: **Hopfenberg**

Model equation: $F = 100 \cdot [1 - (1 - k_{HB} \cdot t)^n]$

Fitted model parameters per tested tablet (N = 4) with statistics – mean, standard deviation (SD), and relative standard deviation expressed in % (RSD%) (output from DDSolver):

Parameter	No.1	No.2	No.3	No.4	Mean	SD	RSD(%)
k_{HB}	0.007	0.004	0.004	0.009	0.006	0.003	41.922
n	2.000	3.000	3.000	2.000	2.500	0.577	23.094

Number of dissolution data points (N), degrees of freedom (df), and selected goodness of fit criteria – Pearson correlation coefficient (R), coefficient of determination (R^2), adjusted coefficient of determination ($R^2_{adjusted}$), and residual sum of squares (RSS) (manual calculation in MS Excel):

Parameter	No.1	No.2	No.3	No.4
N	11	11	11	11
df	9	9	9	9
R	0.984796682	0.995006712	0.99756823	0.989910609
R^2	0.969824505	0.990038358	0.995142374	0.979923014
$R^2_{adjusted}$	0.966471672	0.988931509	0.994602637	0.977692238
RSS	489.5287706	202.5351833	179.8738095	450.3043061

Graphical abstract of model fit presented as mean ± 1 SD of the fraction % of released carvedilol:

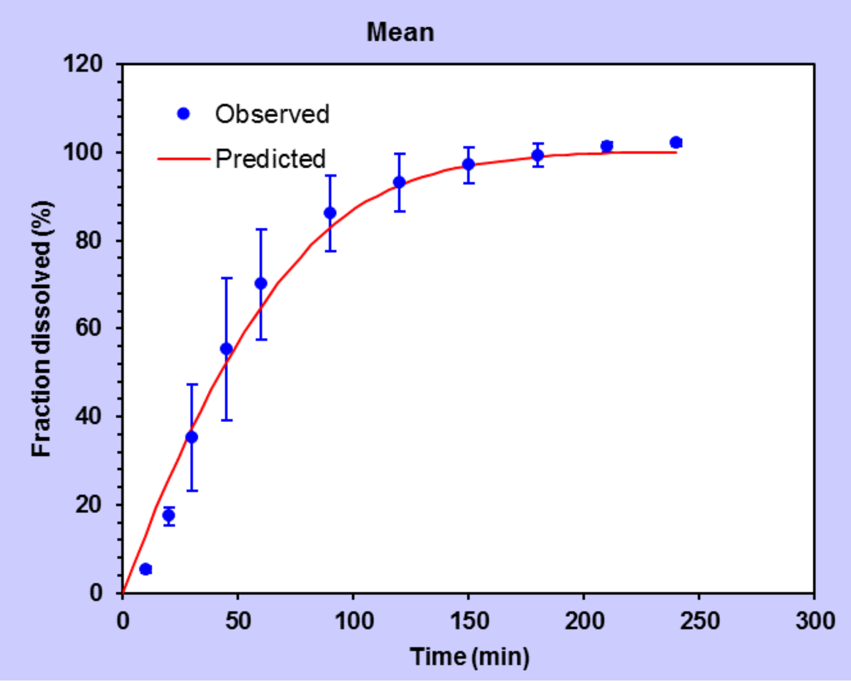

Supplementary materials – Model fitting summary – Parteck® M 100 (modelling the entire relevant dissolution data)

Graphical abstract of model fit presented as the fraction % of released carvedilol per tested tablet:

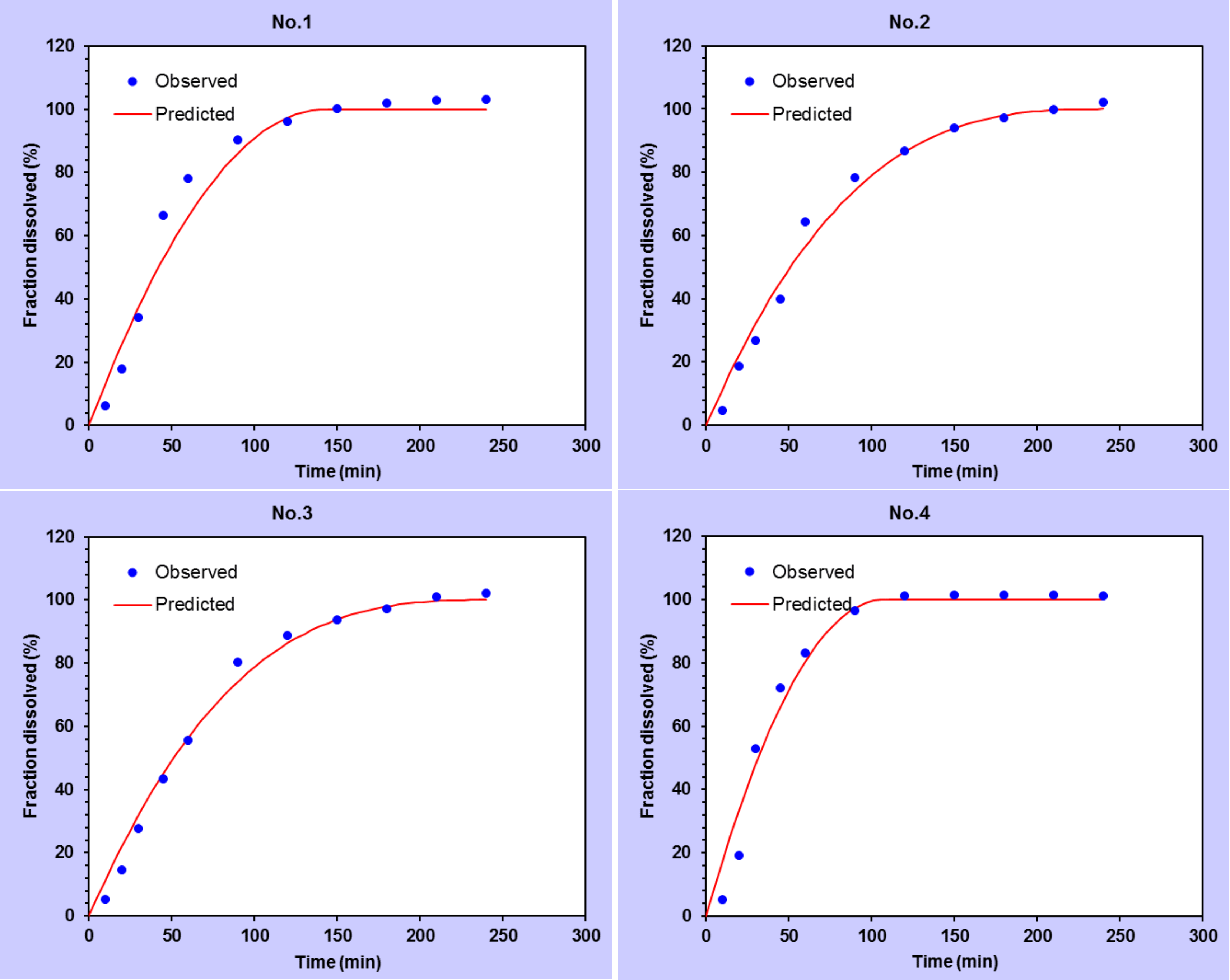

Supplementary materials – Model fitting summary – Parteck® M 100 (modelling the entire relevant dissolution data)

Model: **Hopfenberg with T_{lag}**

Model equation: $F = 100 \cdot \{1 - [1 - k_{HB} \cdot (t - T_{lag})]^n\}$

Fitted model parameters per tested tablet (N = 4) with statistics – mean, standard deviation (SD), and relative standard deviation expressed in % (RSD%) (output from DDSolver):

Parameter	No.1	No.2	No.3	No.4	Mean	SD	RSD(%)
k_{HB}	0.006	0.004	0.004	0.008	0.006	0.002	35.349
n	3.000	3.000	3.000	3.000	3.000	0.000	0.000
T_{lag}	7.230	3.260	4.396	7.208	5.524	2.012	36.424

Number of dissolution data points (N), degrees of freedom (df), and selected goodness of fit criteria – Pearson correlation coefficient (R), coefficient of determination (R^2), adjusted coefficient of determination ($R^2_{adjusted}$), and residual sum of squares (RSS) (manual calculation in MS Excel):

Parameter	No.1	No.2	No.3	No.4
N	11	11	11	11
df	8	8	8	8
R	0.992607861	0.995052277	0.997690114	0.99477478
R^2	0.985270365	0.990129035	0.995385563	0.989576864
$R^2_{adjusted}$	0.981587957	0.987661294	0.994231954	0.98697108
RSS	240.7317305	145.4303078	87.47054743	156.1583124

Graphical abstract of model fit presented as mean ± 1 SD of the fraction % of released carvedilol:

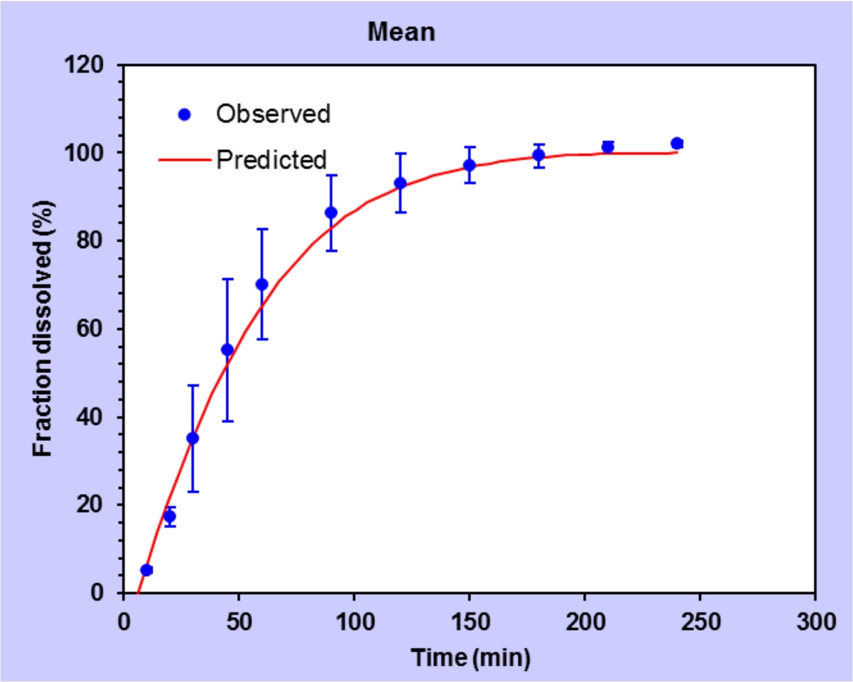

Supplementary materials – Model fitting summary – Parteck® M 100 (modelling the entire relevant dissolution data)

Graphical abstract of model fit presented as the fraction % of released carvedilol per tested tablet:

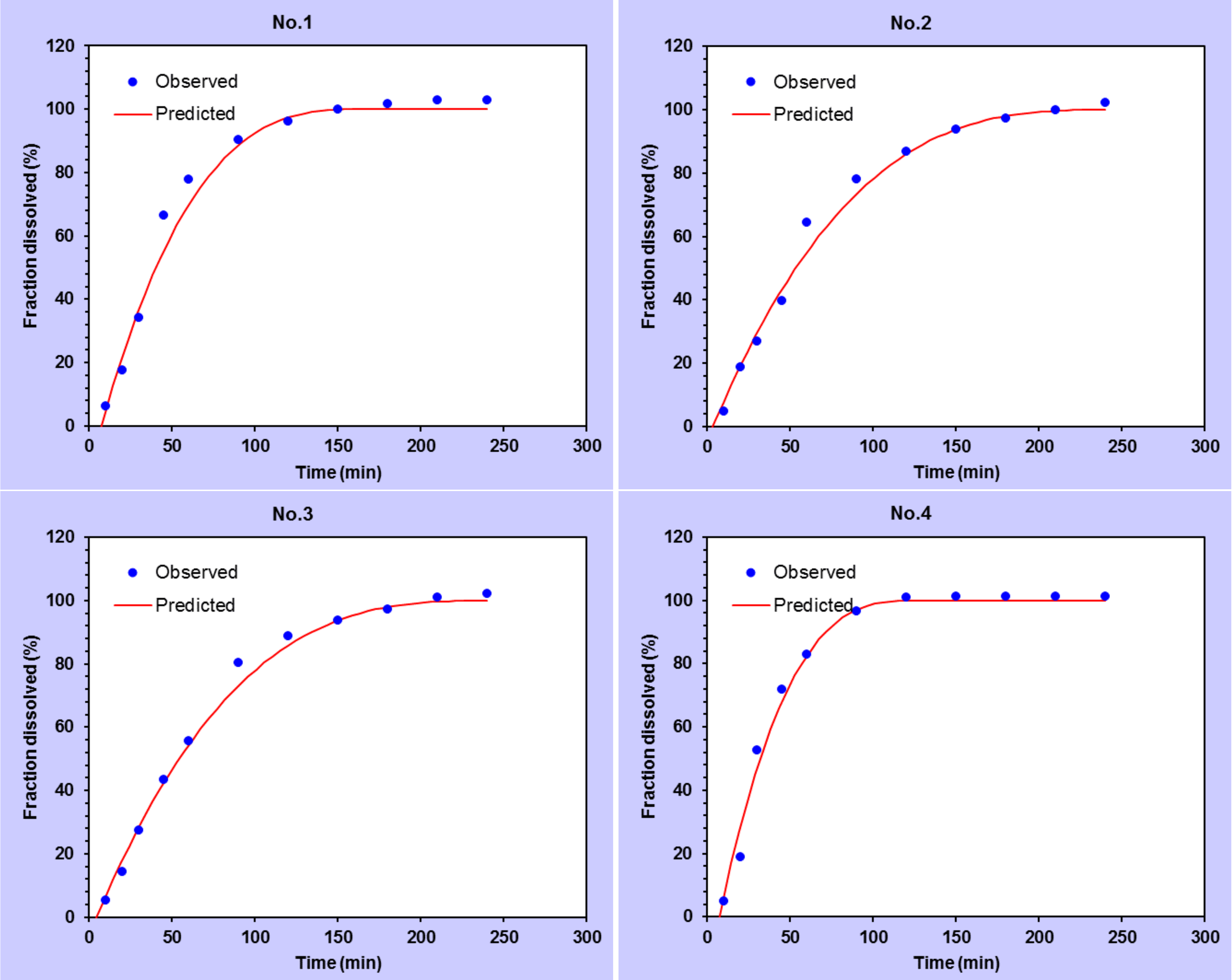

Supplementary materials – Model fitting summary – Parteck® M 100 (modelling the entire relevant dissolution data)

Model: **Baker–Lonsdale**

Model equation: $\frac{3}{2}\cdot\left[1-\left(1-\frac{F}{100}\right)^{\frac{2}{3}}\right]-\frac{F}{100}=k_{BL}\cdot t$

Fitted model parameters per tested tablet (N = 4) with statistics – mean, standard deviation (SD), and relative standard deviation expressed in % (RSD%) (output from DDSolver):

Parameter	No.1	No.2	No.3	No.4	Mean	SD	RSD(%)
k_{BL}	0.004	0.002	0.002	0.005	0.003	0.001	33.988

Number of dissolution data points (N), degrees of freedom (df), and selected goodness of fit criteria – Pearson correlation coefficient (R), coefficient of determination (R^2), adjusted coefficient of determination ($R^2_{adjusted}$), and residual sum of squares (RSS) (manual calculation in MS Excel):

Parameter	No.1	No.2	No.3	No.4
N	11	11	11	11
df	10	10	10	10
R	-0.57377779	-0.361528835	-0.364767838	-0.632480283
R^2	0.329220952	0.130703098	0.133055575	0.400031308
$R^2_{adjusted}$	0.329220952	0.130703098	0.133055575	0.400031308
RSS	640741.8567	255362.4721	257010.7541	987467.7904

Graphical abstract of model fit presented as mean ± 1 SD of the fraction % of released carvedilol:

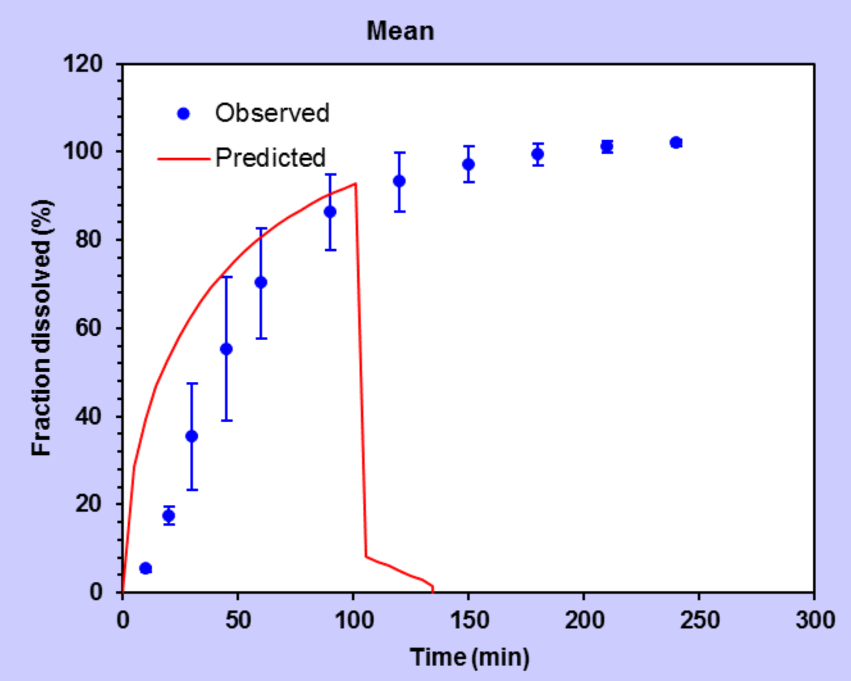

Supplementary materials – Model fitting summary – Parteck® M 100 (modelling the entire relevant dissolution data)

Graphical abstract of model fit presented as the fraction % of released carvedilol per tested tablet:

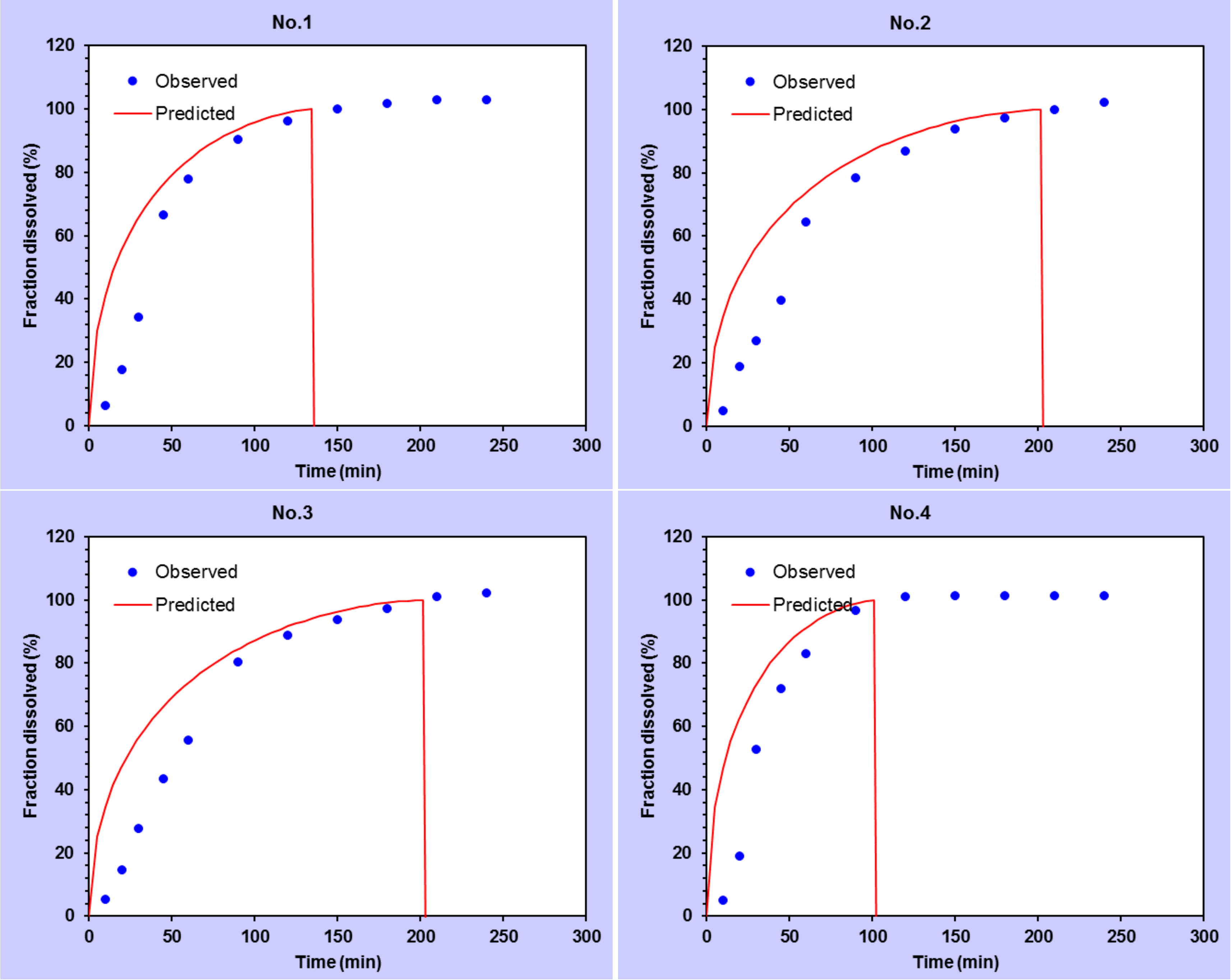

Supplementary materials – Model fitting summary – Parteck® M 100 (modelling the entire relevant dissolution data)

Model: **Baker–Lonsdale with T_{lag}**

Model equation: $\frac{3}{2}\cdot\left[1-\left(1-\frac{F}{100}\right)^{\frac{2}{3}}\right]-\frac{F}{100}=k_{BL}\cdot\left(t-T_{lag}\right)$

Fitted model parameters per tested tablet (N = 4) with statistics – mean, standard deviation (SD), and relative standard deviation expressed in % (RSD%) (output from DDSolver):

Parameter	No.1	No.2	No.3	No.4	Mean	SD	RSD(%)
k_{BL}	0.004	0.000	0.000	0.005	0.002	0.003	117.887
T_{lag}	23.542	35.080	35.523	23.150	29.324	6.907	23.553

Number of dissolution data points (N), degrees of freedom (df), and selected goodness of fit criteria – Pearson correlation coefficient (R), coefficient of determination (R^2), adjusted coefficient of determination ($R^2_{adjusted}$), and residual sum of squares (RSS) (manual calculation in MS Excel):

Parameter	No.1	No.2	No.3	No.4
N	11	11	11	11
df	9	9	9	9
R	-0.346419295	0.855670565	0.854506531	-0.423995847
R^2	0.120006328	0.732172116	0.730181412	0.179772478
$R^2_{adjusted}$	0.022229253	0.702413463	0.700201569	0.088636087
RSS	458748.5775	58956.34116	58983.20331	749459.7805

Graphical abstract of model fit presented as mean ± 1 SD of the fraction % of released carvedilol: / (no charts were produced by DDSolver)

Graphical abstract of model fit presented as the fraction % of released carvedilol per tested tablet: / (no charts were produced by DDSolver)

Supplementary materials – Model fitting summary – Parteck® M 100 (modelling the entire relevant dissolution data)

Model: **Makoid–Banakar**

Model equation: $F = k_{MB} \cdot t^n \cdot e^{-k \cdot t}$

Fitted model parameters per tested tablet (N = 4) with statistics – mean, standard deviation (SD), and relative standard deviation expressed in % (RSD%) (output from DDSolver):

Parameter	No.1	No.2	No.3	No.4	Mean	SD	RSD(%)
k_{MB}	0.176	0.168	0.174	0.110	0.157	0.031	19.980
n	1.631	1.562	1.539	1.818	1.638	0.127	7.742
k	0.011	0.009	0.009	0.014	0.011	0.002	21.114

Number of dissolution data points (N), degrees of freedom (df), and selected goodness of fit criteria – Pearson correlation coefficient (R), coefficient of determination (R^2), adjusted coefficient of determination ($R^2_{adjusted}$), and residual sum of squares (RSS) (manual calculation in MS Excel):

Parameter	No.1	No.2	No.3	No.4
N	11	11	11	11
df	8	8	8	8
R	0.968250909	0.986617397	0.992649539	0.941094525
R^2	0.937509823	0.973413888	0.985353107	0.885658906
$R^2_{adjusted}$	0.921887279	0.96676736	0.981691383	0.857073632
RSS	1001.219525	382.3614794	215.0214652	2149.558911

Graphical abstract of model fit presented as mean ± 1 SD of the fraction % of released carvedilol:

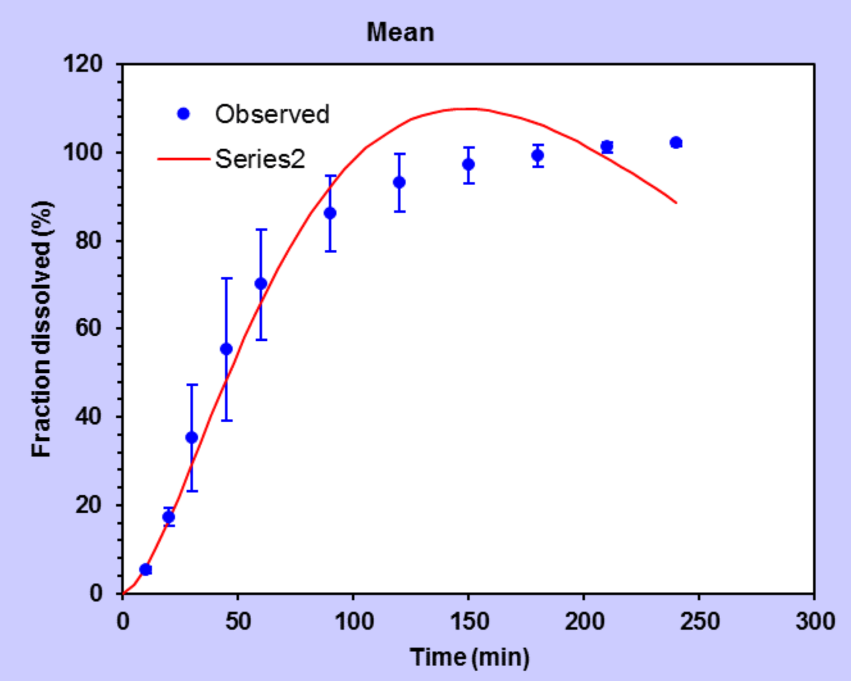

Supplementary materials – Model fitting summary – Parteck® M 100 (modelling the entire relevant dissolution data)

Graphical abstract of model fit presented as the fraction % of released carvedilol per tested tablet:

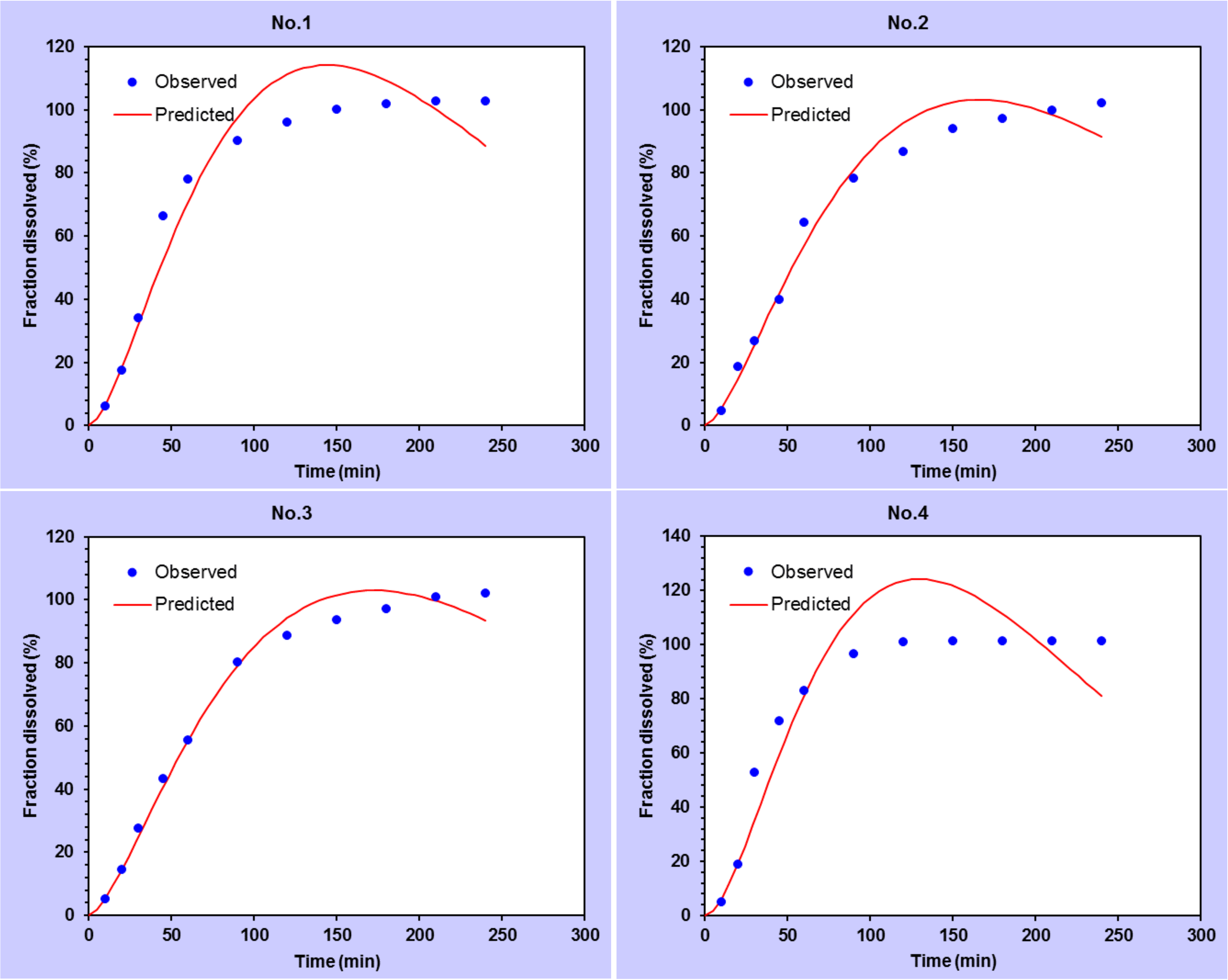

Supplementary materials – Model fitting summary – Parteck® M 100 (modelling the entire relevant dissolution data)

Model: **Makoid–Banakar with T_{lag}**

Model equation: $F = k_{MB} \cdot (t - T_{lag})^n \cdot e^{-k \cdot (t - T_{lag})}$

Fitted model parameters per tested tablet (N = 4) with statistics – mean, standard deviation (SD), and relative standard deviation expressed in % (RSD%) (output from DDSolver):

Parameter	No.1	No.2	No.3	No.4	Mean	SD	RSD(%)
k_{MB}	0.642	0.577	0.601	0.447	0.567	0.084	14.898
n	1.291	1.240	1.214	1.453	1.299	0.107	8.241
k	0.009	0.007	0.007	0.011	0.008	0.002	25.983
T_{lag}	4.000	4.000	4.000	4.000	4.000	0.000	0.000

Number of dissolution data points (N), degrees of freedom (df), and selected goodness of fit criteria – Pearson correlation coefficient (R), coefficient of determination (R^2), adjusted coefficient of determination ($R^2_{adjusted}$), and residual sum of squares (RSS) (manual calculation in MS Excel):

Parameter	No.1	No.2	No.3	No.4
N	11	11	11	11
df	7	7	7	7
R	0.979776456	0.992856098	0.997065247	0.958025482
R^2	0.959961904	0.985763231	0.994139106	0.917812824
$R^2_{adjusted}$	0.94280272	0.979661759	0.991627295	0.882589749
RSS	587.9200786	192.4948854	80.73514771	1395.416474

Graphical abstract of model fit presented as mean ± 1 SD of the fraction % of released carvedilol:

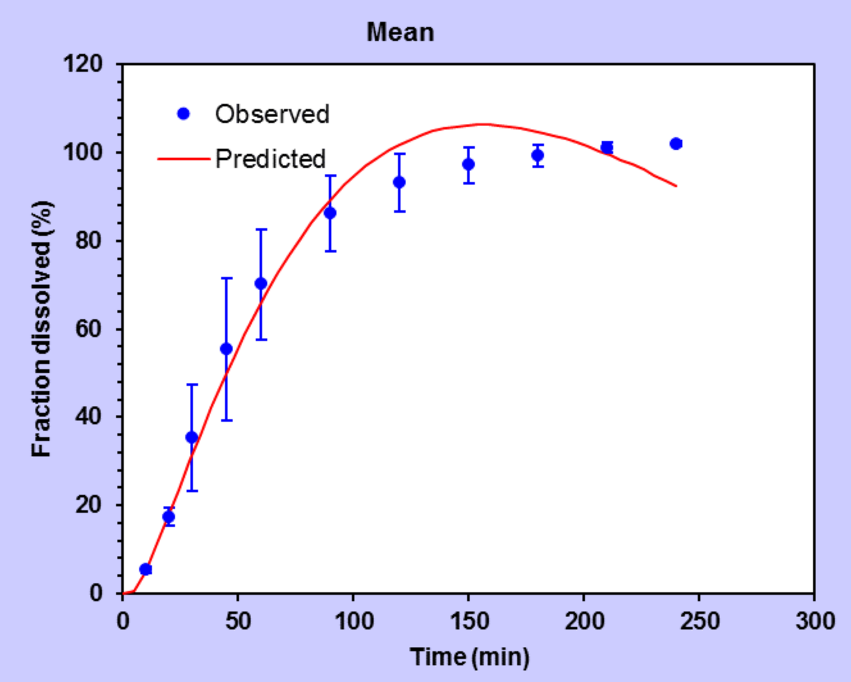

Supplementary materials – Model fitting summary – Parteck® M 100 (modelling the entire relevant dissolution data)

Graphical abstract of model fit presented as the fraction % of released carvedilol per tested tablet:

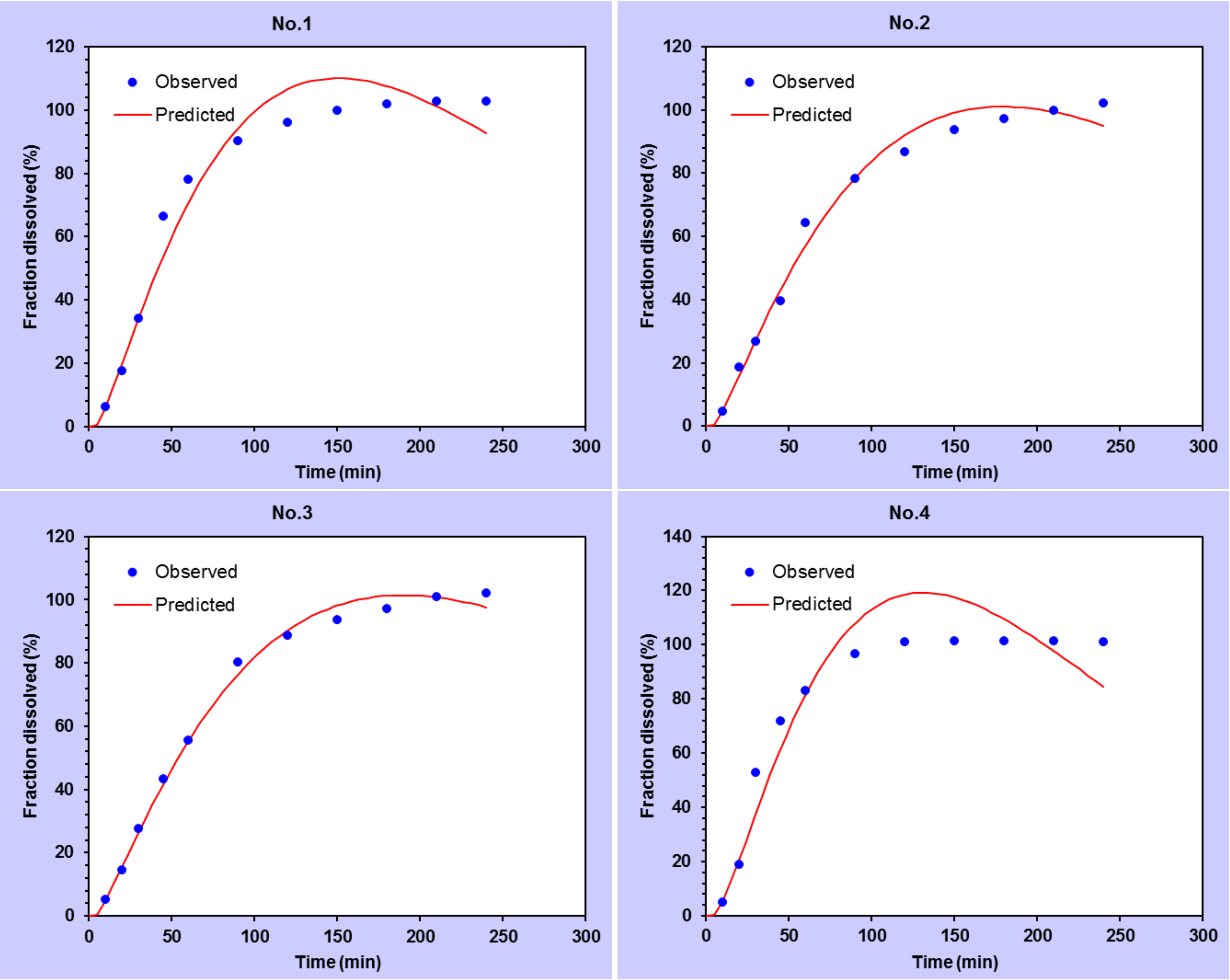

Supplementary materials – Model fitting summary – Parteck® M 100 (modelling the entire relevant dissolution data)

Model: **Peppas–Sahlin_1**

Model equation: $F = k_1 \cdot t^m + k_2 \cdot t^{2m}$

Fitted model parameters per tested tablet (N = 4) with statistics – mean, standard deviation (SD), and relative standard deviation expressed in % (RSD%) (output from DDSolver):

Parameter	No.1	No.2	No.3	No.4	Mean	SD	RSD(%)
k_1	10.518	6.770	6.410	12.995	9.173	3.153	34.370
k_2	-0.069	0.241	0.279	-0.300	0.038	0.274	725.497
m	0.450	0.450	0.450	0.450	0.450	0.000	0.000

Number of dissolution data points (N), degrees of freedom (df), and selected goodness of fit criteria – Pearson correlation coefficient (R), coefficient of determination (R^2), adjusted coefficient of determination ($R^2_{adjusted}$), and residual sum of squares (RSS) (manual calculation in MS Excel):

Parameter	No.1	No.2	No.3	No.4
N	11	11	11	11
df	8	8	8	8
R	0.93285847	0.959681417	0.960497298	0.920659247
R^2	0.870224924	0.920988423	0.92255506	0.847613449
$R^2_{adjusted}$	0.837781155	0.901235528	0.903193825	0.809516812
RSS	1997.067616	1132.531831	1144.592068	2338.958778

Graphical abstract of model fit presented as mean ± 1 SD of the fraction % of released carvedilol:

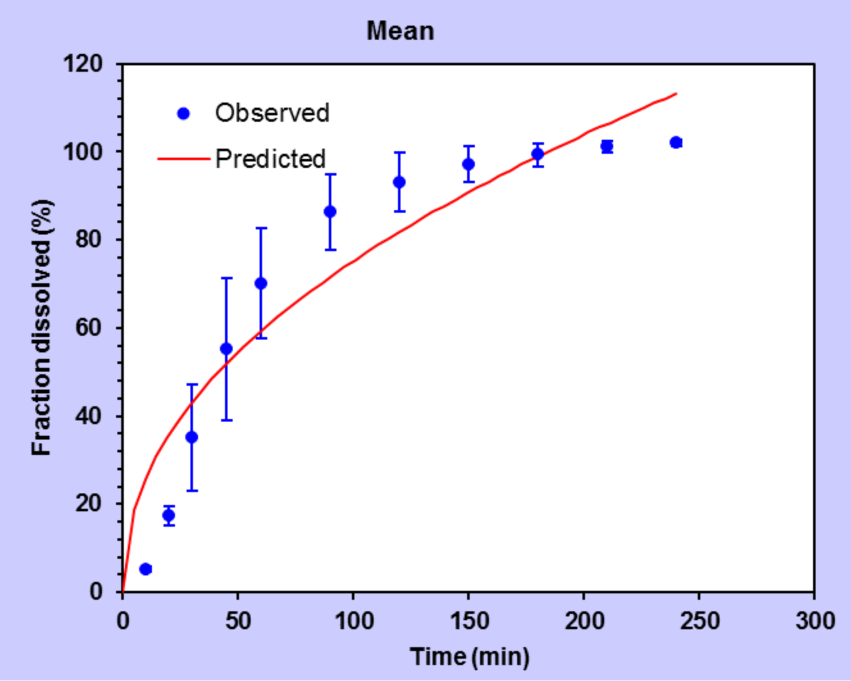

Supplementary materials – Model fitting summary – Parteck® M 100 (modelling the entire relevant dissolution data)

Graphical abstract of model fit presented as the fraction % of released carvedilol per tested tablet:

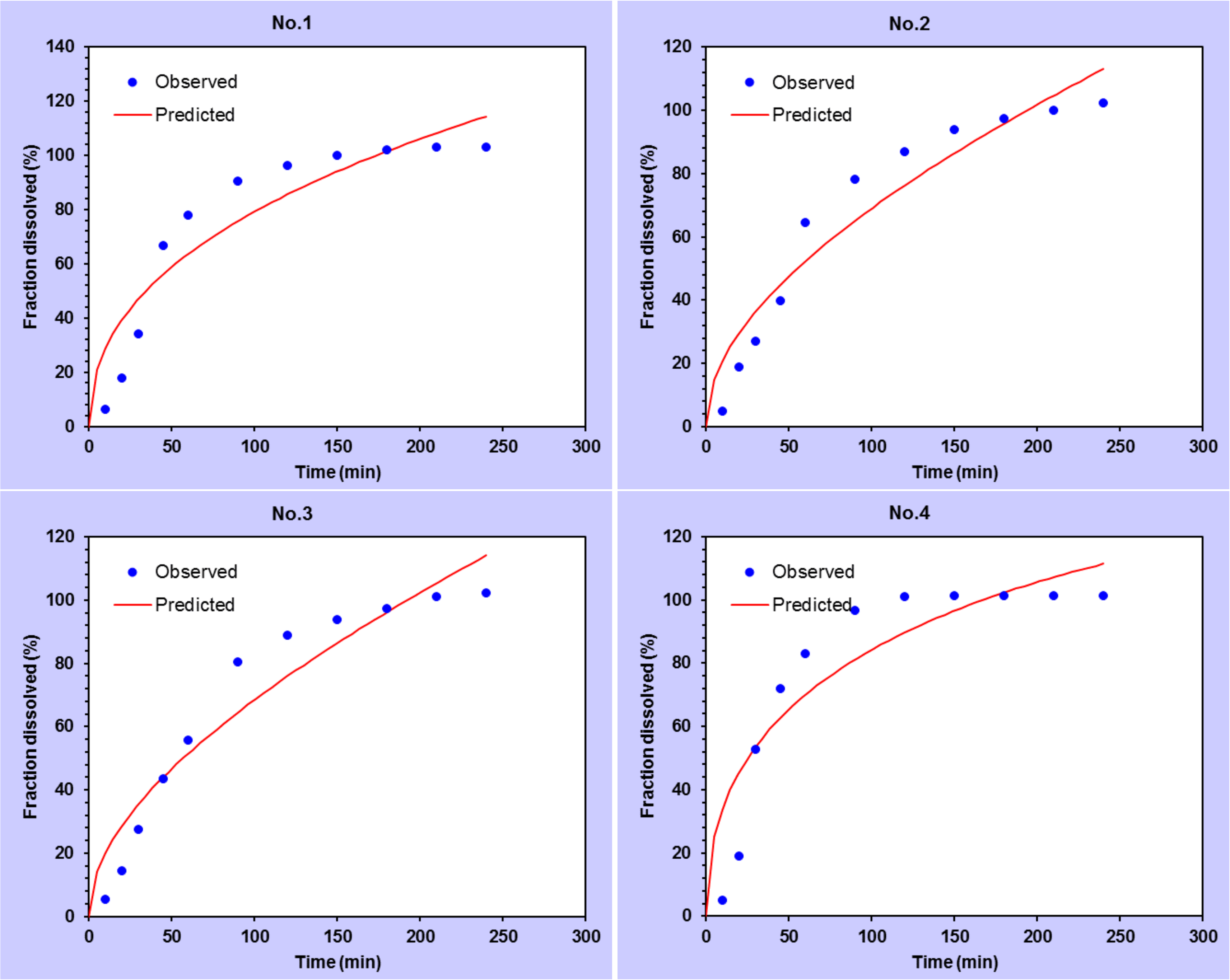

Supplementary materials – Model fitting summary – Parteck® M 100 (modelling the entire relevant dissolution data)

Model: **Peppas–Sahlin_1 with T_{lag}**

Model equation: $F = k_1 \cdot (t - T_{lag})^m + k_2 \cdot (t - T_{lag})^{2m}$

Fitted model parameters per tested tablet (N = 4) with statistics – mean, standard deviation (SD), and relative standard deviation expressed in % (RSD%) (output from DDSolver):

Parameter	No.1	No.2	No.3	No.4	Mean	SD	RSD(%)
k_1	11.813	7.848	7.481	14.402	10.386	3.319	31.954
k_2	-0.185	0.148	0.187	-0.430	-0.070	0.292	-418.340
m	0.450	0.450	0.450	0.450	0.450	0.000	0.000
T_{lag}	6.000	6.000	6.000	6.000	6.000	0.000	0.000

Number of dissolution data points (N), degrees of freedom (df), and selected goodness of fit criteria – Pearson correlation coefficient (R), coefficient of determination (R^2), adjusted coefficient of determination ($R^2_{adjusted}$), and residual sum of squares (RSS) (manual calculation in MS Excel):

Parameter	No.1	No.2	No.3	No.4
N	11	11	11	11
df	7	7	7	7
R	0.951666032	0.969607147	0.969874719	0.947258217
R^2	0.905668236	0.940138019	0.94065697	0.897298129
$R^2_{adjusted}$	0.865240337	0.914482884	0.915224243	0.853283042
RSS	1442.609916	841.0695867	860.3109981	1597.482971

Graphical abstract of model fit presented as mean ± 1 SD of the fraction % of released carvedilol:

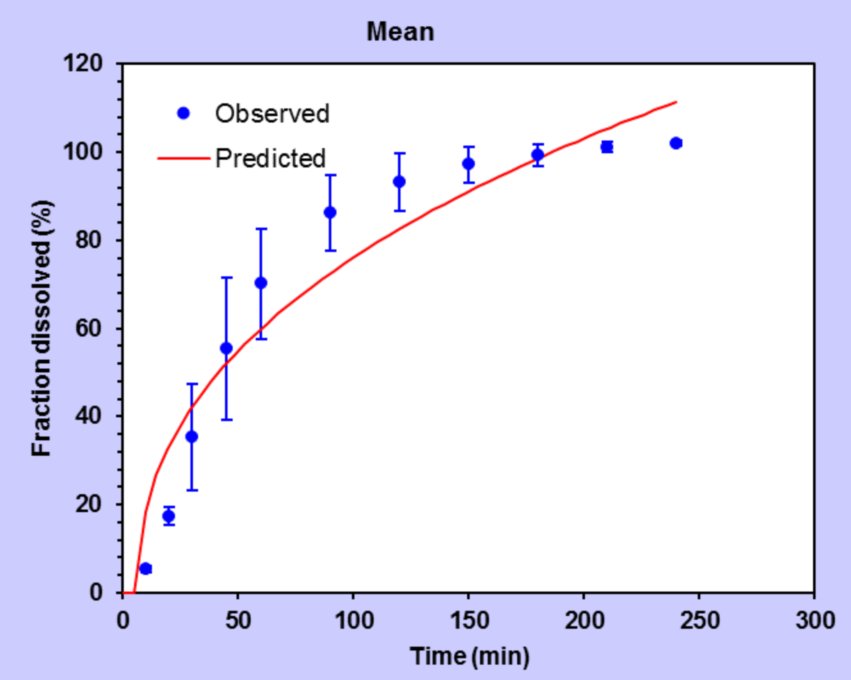

Supplementary materials – Model fitting summary – Parteck® M 100 (modelling the entire relevant dissolution data)

Graphical abstract of model fit presented as the fraction % of released carvedilol per tested tablet:

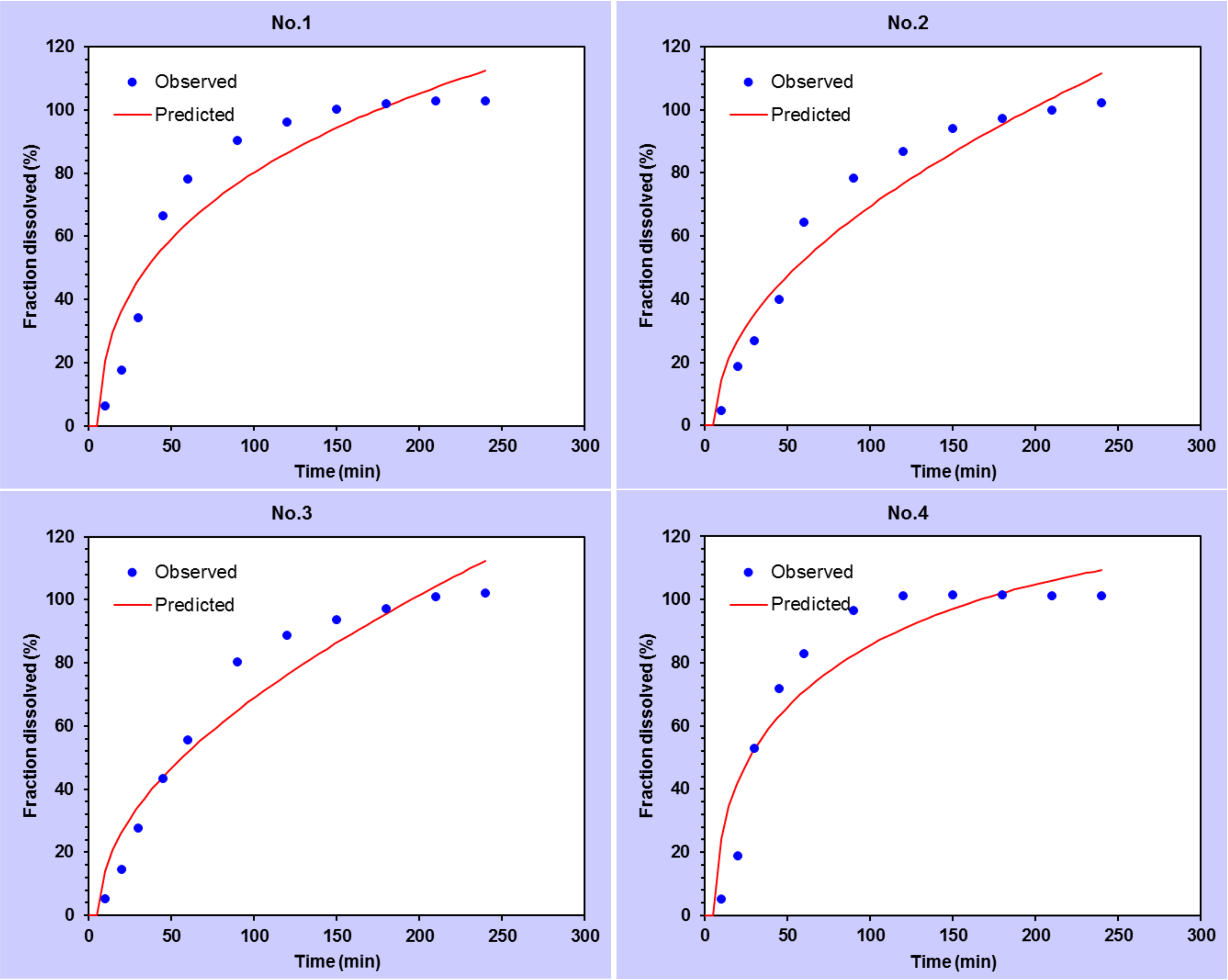

Supplementary materials – Model fitting summary – Parteck® M 100 (modelling the entire relevant dissolution data)

Model: **Peppas–Sahlin_2**

Model equation: $F = k_1 \cdot t^{0.5} + k_2 \cdot t$

Fitted model parameters per tested tablet (N = 4) with statistics – mean, standard deviation (SD), and relative standard deviation expressed in % (RSD%) (output from DDSolver):

Parameter	No.1	No.2	No.3	No.4	Mean	SD	RSD(%)
k_1	9.479	6.492	6.230	11.393	8.398	2.481	29.545
k_2	-0.143	0.048	0.069	-0.280	-0.077	0.166	-216.714

Number of dissolution data points (N), degrees of freedom (df), and selected goodness of fit criteria – Pearson correlation coefficient (R), coefficient of determination (R^2), adjusted coefficient of determination ($R^2_{adjusted}$), and residual sum of squares (RSS) (manual calculation in MS Excel):

Parameter	No.1	No.2	No.3	No.4
N	11	11	11	11
df	9	9	9	9
R	0.943084632	0.963509436	0.963996635	0.93599271
R^2	0.889408624	0.928350433	0.929289512	0.876082354
$R^2_{adjusted}$	0.877120693	0.92038937	0.921432791	0.862313727
RSS	1824.320182	1078.04133	1097.393704	2082.957091

Graphical abstract of model fit presented as mean ± 1 SD of the fraction % of released carvedilol:

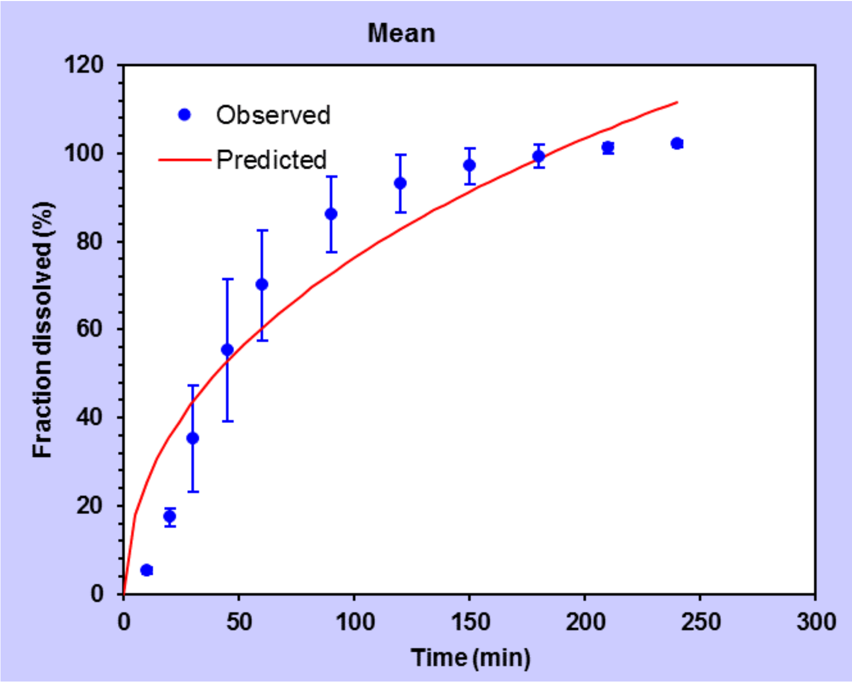

Supplementary materials – Model fitting summary – Parteck® M 100 (modelling the entire relevant dissolution data)

Graphical abstract of model fit presented as the fraction % of released carvedilol per tested tablet:

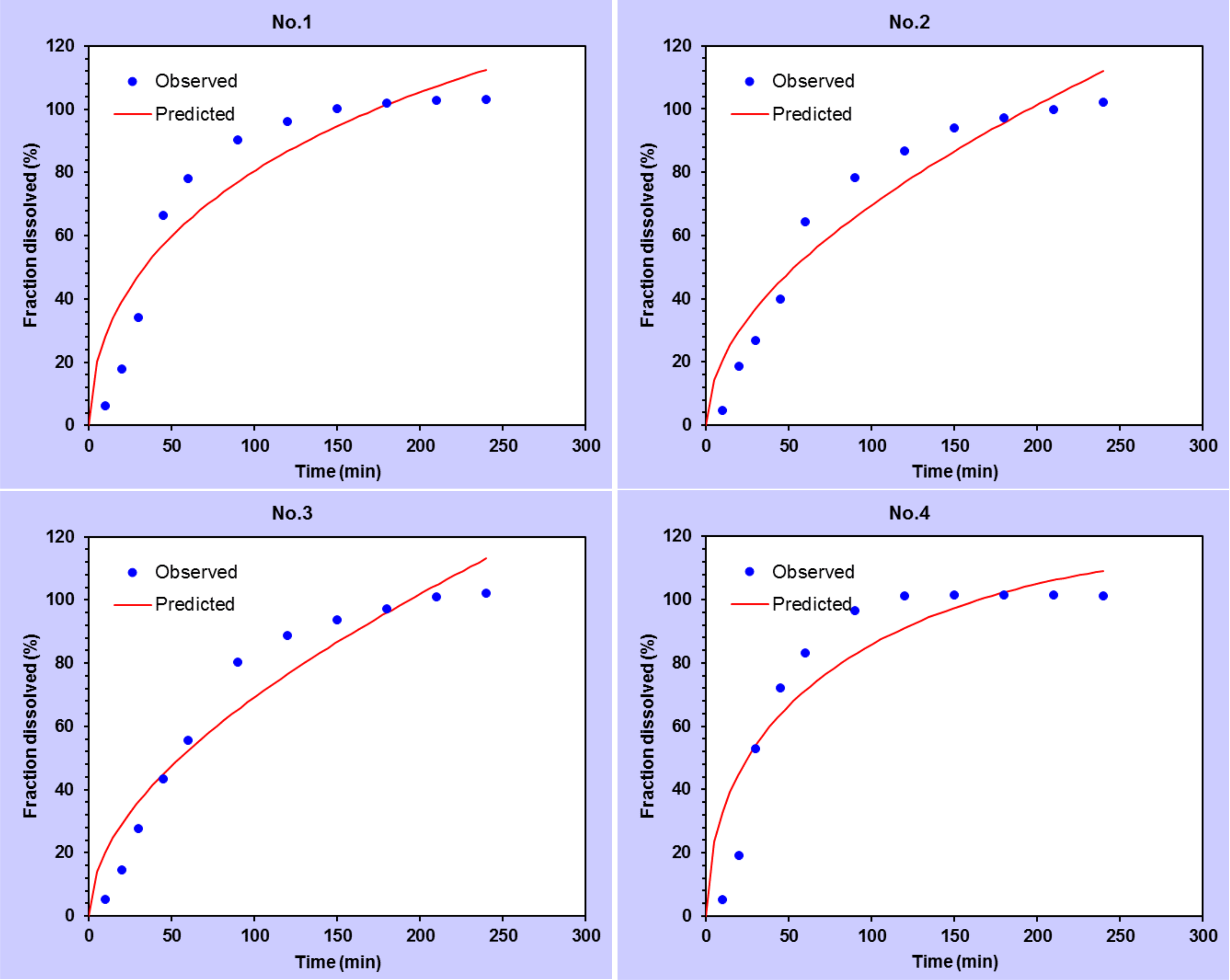

Supplementary materials – Model fitting summary – Parteck® M 100 (modelling the entire relevant dissolution data)

Model: **Peppas–Sahlin_2 with T_{lag}**

Model equation: $F = k_1 \cdot (t - T_{lag})^{0.5} + k_2 \cdot (t - T_{lag})$

Fitted model parameters per tested tablet (N = 4) with statistics – mean, standard deviation (SD), and relative standard deviation expressed in % (RSD%) (output from DDSolver):

Parameter	No.1	No.2	No.3	No.4	Mean	SD	RSD(%)
k_1	10.503	7.342	7.074	12.505	9.356	2.614	27.937
k_2	-0.214	-0.009	0.013	-0.360	-0.142	0.177	-124.690
T_{lag}	6.000	6.000	6.000	6.000	6.000	0.000	0.000

Number of dissolution data points (N), degrees of freedom (df), and selected goodness of fit criteria – Pearson correlation coefficient (R), coefficient of determination (R^2), adjusted coefficient of determination ($R^2_{adjusted}$), and residual sum of squares (RSS) (manual calculation in MS Excel):

Parameter	No.1	No.2	No.3	No.4
N	11	11	11	11
df	8	8	8	8
R	0.961136775	0.973760354	0.973818794	0.959962799
R^2	0.923783901	0.948209226	0.948323044	0.921528575
$R^2_{adjusted}$	0.904729876	0.935261533	0.935403805	0.901910718
RSS	1236.098339	757.1733946	781.0179267	1317.352972

Graphical abstract of model fit presented as mean ± 1 SD of the fraction % of released carvedilol:

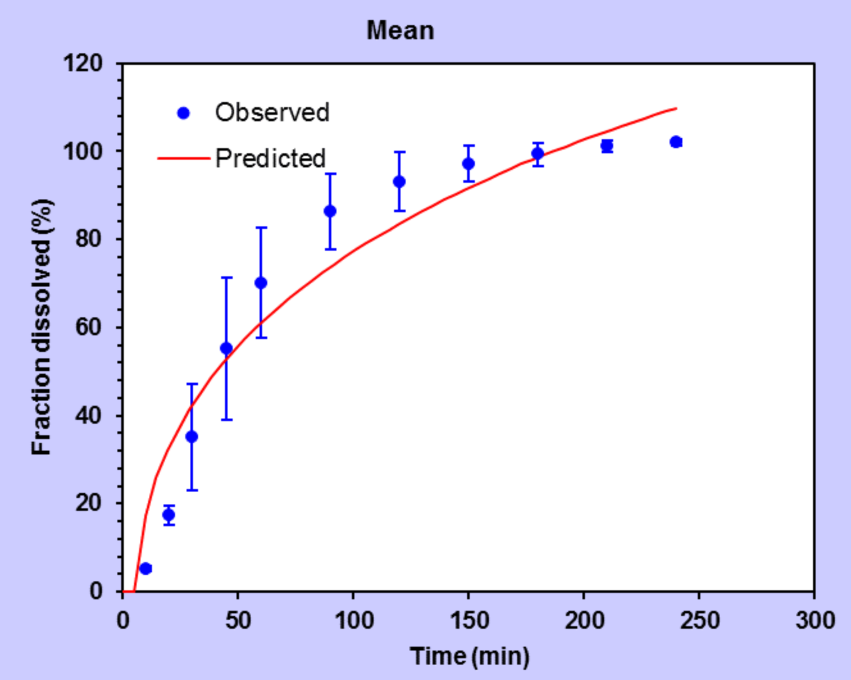

Supplementary materials – Model fitting summary – Parteck® M 100 (modelling the entire relevant dissolution data)

Graphical abstract of model fit presented as the fraction % of released carvedilol per tested tablet:

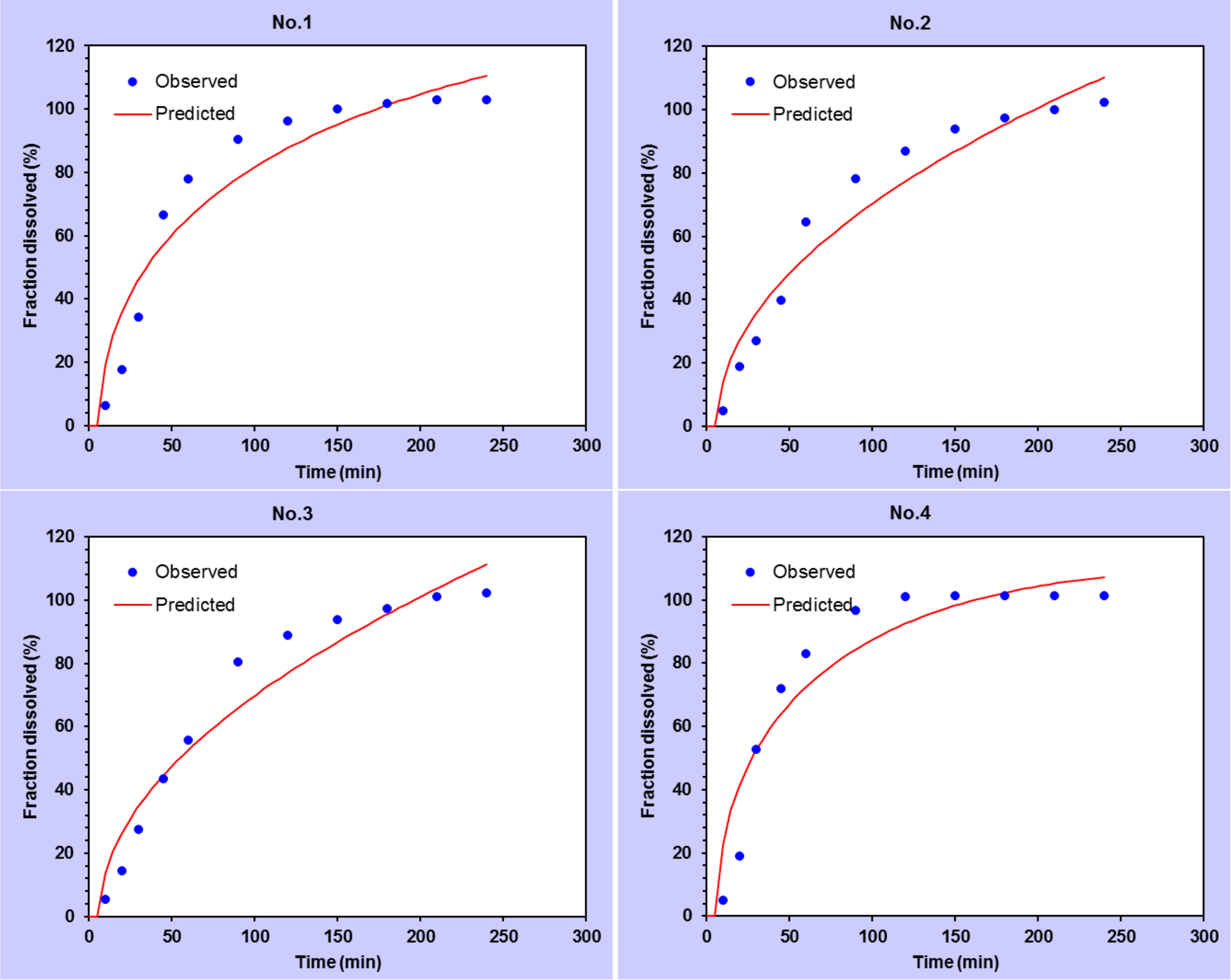

Supplementary materials – Model fitting summary – Parteck® M 100 (modelling the entire relevant dissolution data)

Model: **Quadratic**

Model equation: $F = 100 \cdot (k_1 \cdot t^2 + k_2 \cdot t)$

Fitted model parameters per tested tablet (N = 4) with statistics – mean, standard deviation (SD), and relative standard deviation expressed in % (RSD%) (output from DDSolver):

Parameter	No.1	No.2	No.3	No.4	Mean	SD	RSD(%)
k_1	-0.00004	-0.00003	-0.00003	-0.00004	-0.00003	0.00001	-24.56254
k_2	0.01290	0.01056	0.01047	0.01412	0.01201	0.00180	14.99051

Number of dissolution data points (N), degrees of freedom (df), and selected goodness of fit criteria – Pearson correlation coefficient (R), coefficient of determination (R^2), adjusted coefficient of determination ($R^2_{adjusted}$), and residual sum of squares (RSS) (manual calculation in MS Excel):

Parameter	No.1	No.2	No.3	No.4
N	11	11	11	11
df	9	9	9	9
R	0.968386436	0.990320135	0.993635307	0.948240893
R^2	0.937772289	0.980733969	0.987311123	0.899160791
$R^2_{adjusted}$	0.930858099	0.978593299	0.985901248	0.887956434
RSS	836.5499518	253.5922973	183.777736	1353.394511

Graphical abstract of model fit presented as mean ± 1 SD of the fraction % of released carvedilol:

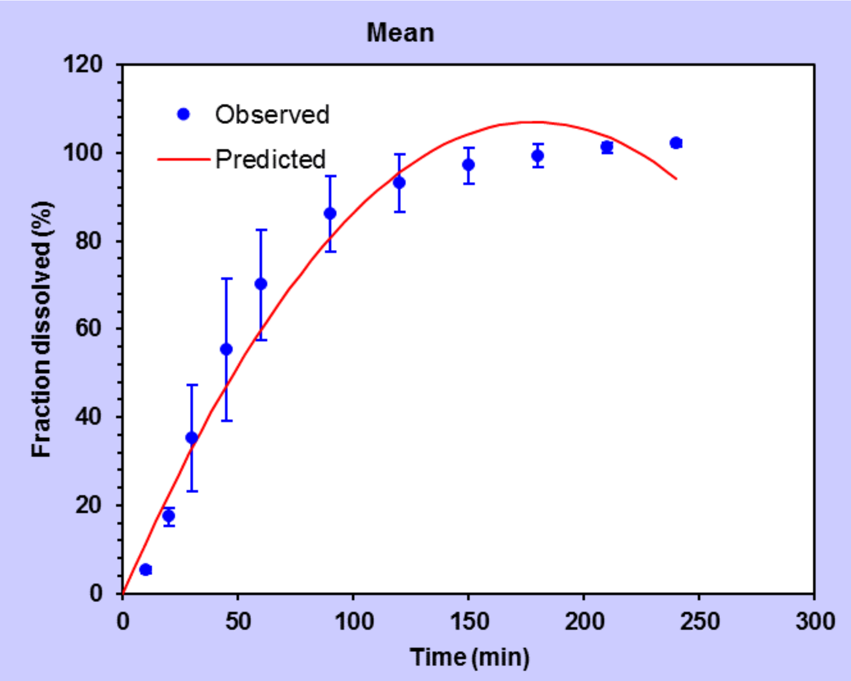

Supplementary materials – Model fitting summary – Parteck® M 100 (modelling the entire relevant dissolution data)

Graphical abstract of model fit presented as the fraction % of released carvedilol per tested tablet:

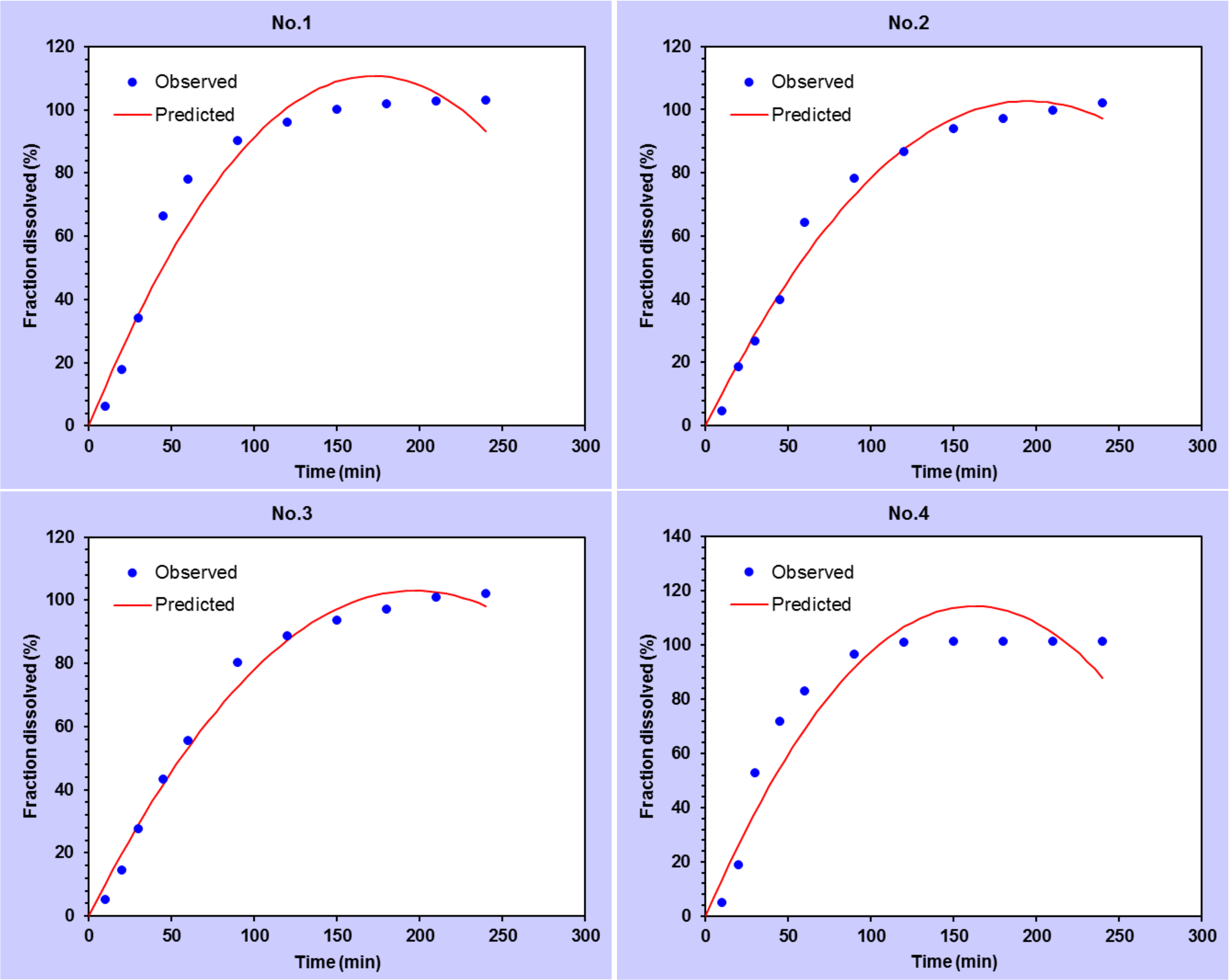

Supplementary materials – Model fitting summary – Parteck® M 100 (modelling the entire relevant dissolution data)

Model: **Quadratic with T_{lag}**

Model equation: $F = 100 \cdot \left[k_1 \cdot (t - T_{lag})^2 + k_2 \cdot (t - T_{lag})\right]$

Fitted model parameters per tested tablet (N = 4) with statistics – mean, standard deviation (SD), and relative standard deviation expressed in % (RSD%) (output from DDSolver):

Parameter	No.1	No.2	No.3	No.4	Mean	SD	RSD(%)
k_1	-0.00004	-0.00003	-0.00003	-0.00005	-0.00004	0.00001	-23.37894
k_2	0.01353	0.01112	0.01105	0.01476	0.01261	0.00184	14.56342
T_{lag}	4.00000	4.00000	4.00000	4.00000	4.00000	0.00000	0.00000

Number of dissolution data points (N), degrees of freedom (df), and selected goodness of fit criteria – Pearson correlation coefficient (R), coefficient of determination (R^2), adjusted coefficient of determination ($R^2_{adjusted}$), and residual sum of squares (RSS) (manual calculation in MS Excel):

Parameter	No.1	No.2	No.3	No.4
N	11	11	11	11
df	8	8	8	8
R	0.968033494	0.990254329	0.993764087	0.947867802
R^2	0.937088846	0.980603636	0.987567061	0.89845337
$R^2_{adjusted}$	0.921361058	0.975754545	0.984458827	0.873066712
RSS	942.8407519	264.8232387	169.4497182	1579.499658

Graphical abstract of model fit presented as mean ± 1 SD of the fraction % of released carvedilol:

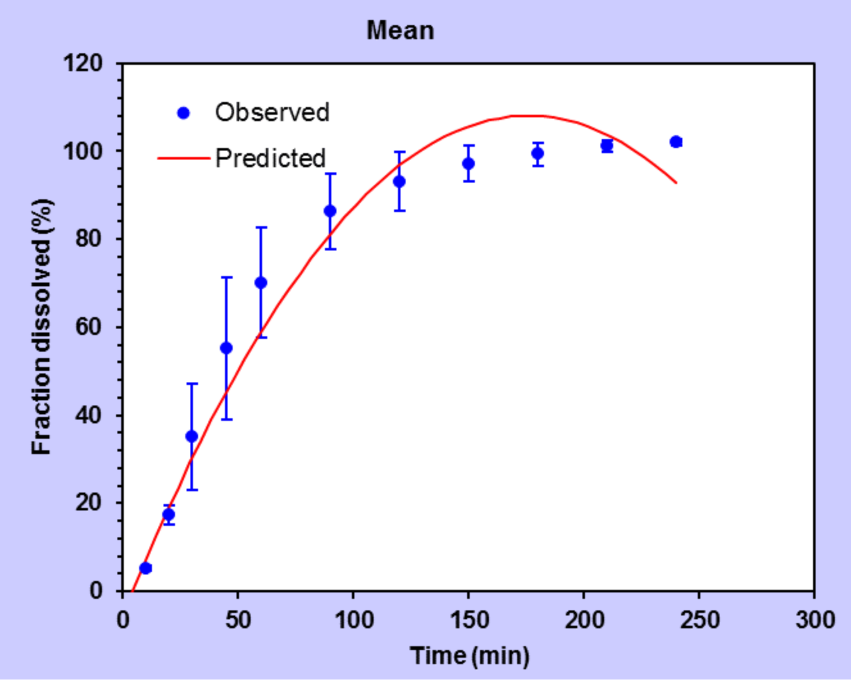

Supplementary materials – Model fitting summary – Parteck® M 100 (modelling the entire relevant dissolution data)

Graphical abstract of model fit presented as the fraction % of released carvedilol per tested tablet:

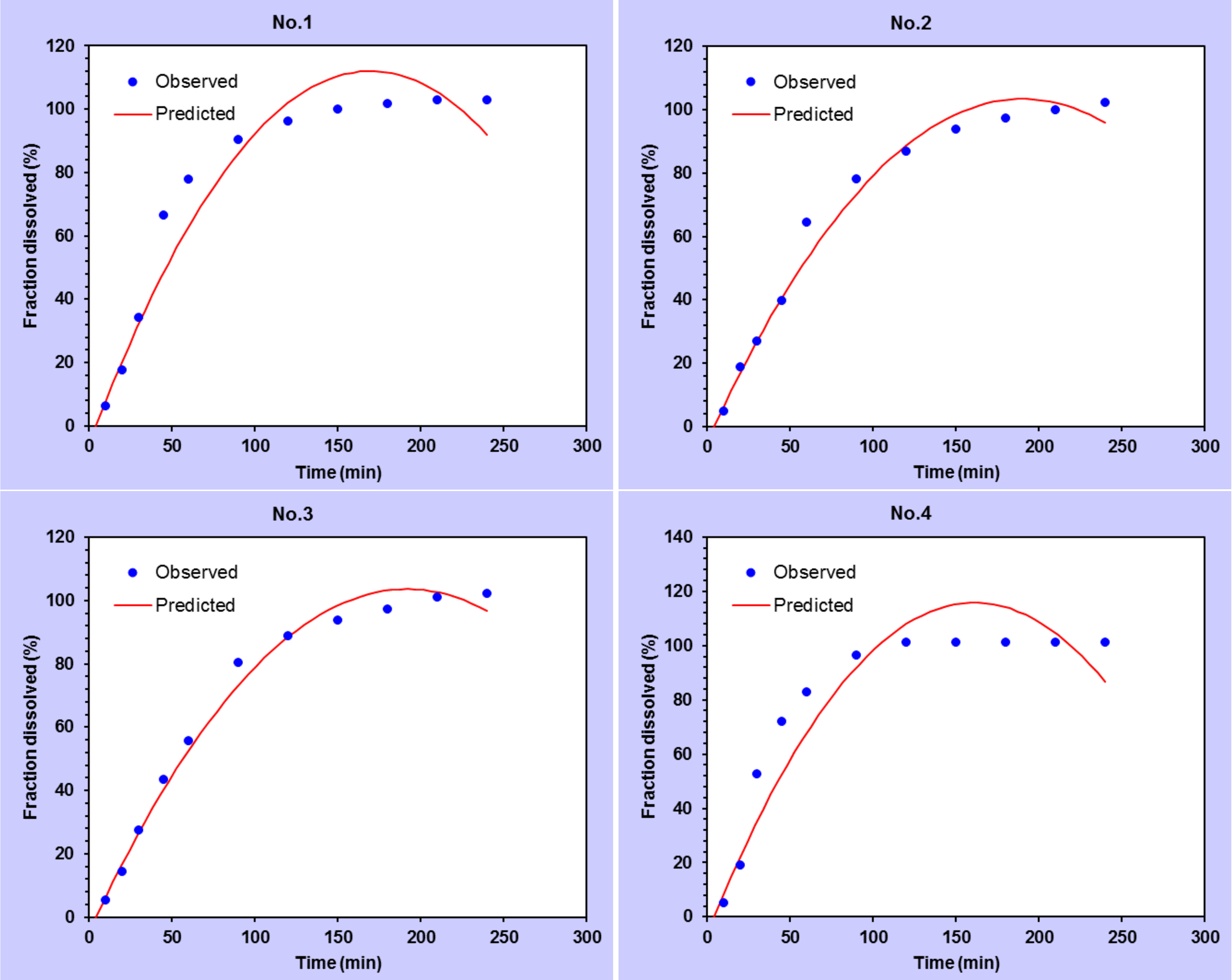

Supplementary materials – Model fitting summary – Parteck® M 100 (modelling the entire relevant dissolution data)

Model: **Weibull_1**

Model equation: $F = 100 \cdot \left[1 - e^{-\frac{(t-T_i)^{\beta}}{\alpha}}\right]$

Fitted model parameters per tested tablet (N = 4) with statistics – mean, standard deviation (SD), and relative standard deviation expressed in % (RSD%) (output from DDSolver):

Parameter	No.1	No.2	No.3	No.4	Mean	SD	RSD(%)
α	240.634	203.834	193.457	332.641	242.642	63.322	26.097
β	1.450	1.291	1.266	1.600	1.402	0.155	11.082
T_i	4.000	4.000	4.000	4.000	4.000	0.000	0.000

Number of dissolution data points (N), degrees of freedom (df), and selected goodness of fit criteria – Pearson correlation coefficient (R), coefficient of determination (R^2), adjusted coefficient of determination ($R^2_{adjusted}$), and residual sum of squares (RSS) (manual calculation in MS Excel):

Parameter	No.1	No.2	No.3	No.4
N	11	11	11	11
df	8	8	8	8
R	0.996382973	0.99688794	0.999193216	0.994920145
R^2	0.99277903	0.993785565	0.998387083	0.989866094
$R^2_{adjusted}$	0.990973787	0.992231956	0.997983854	0.987332618
RSS	103.6000464	80.91550503	30.01186539	142.5977942

Graphical abstract of model fit presented as mean ± 1 SD of the fraction % of released carvedilol:

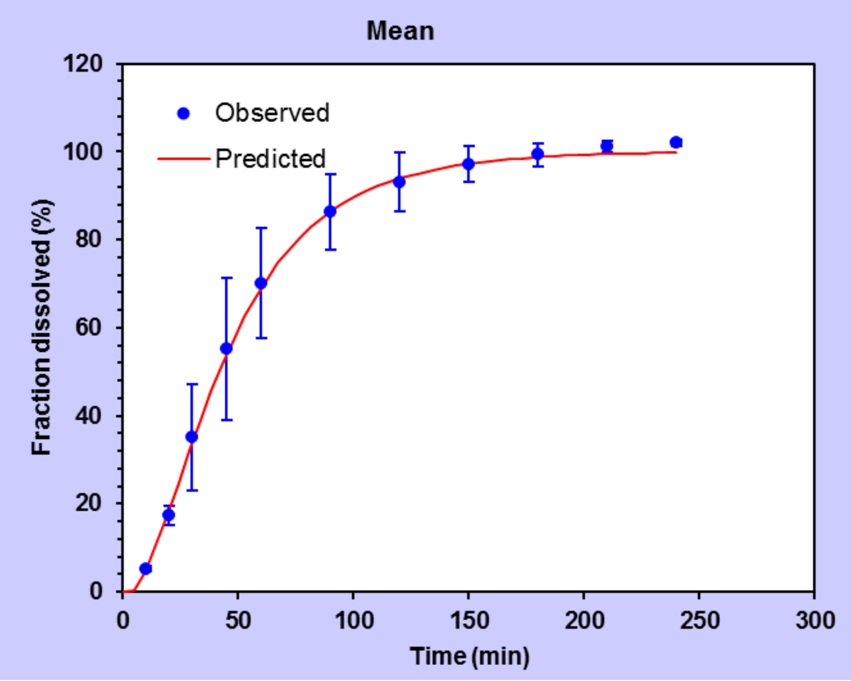

Supplementary materials – Model fitting summary – Parteck® M 100 (modelling the entire relevant dissolution data)

Graphical abstract of model fit presented as the fraction % of released carvedilol per tested tablet:

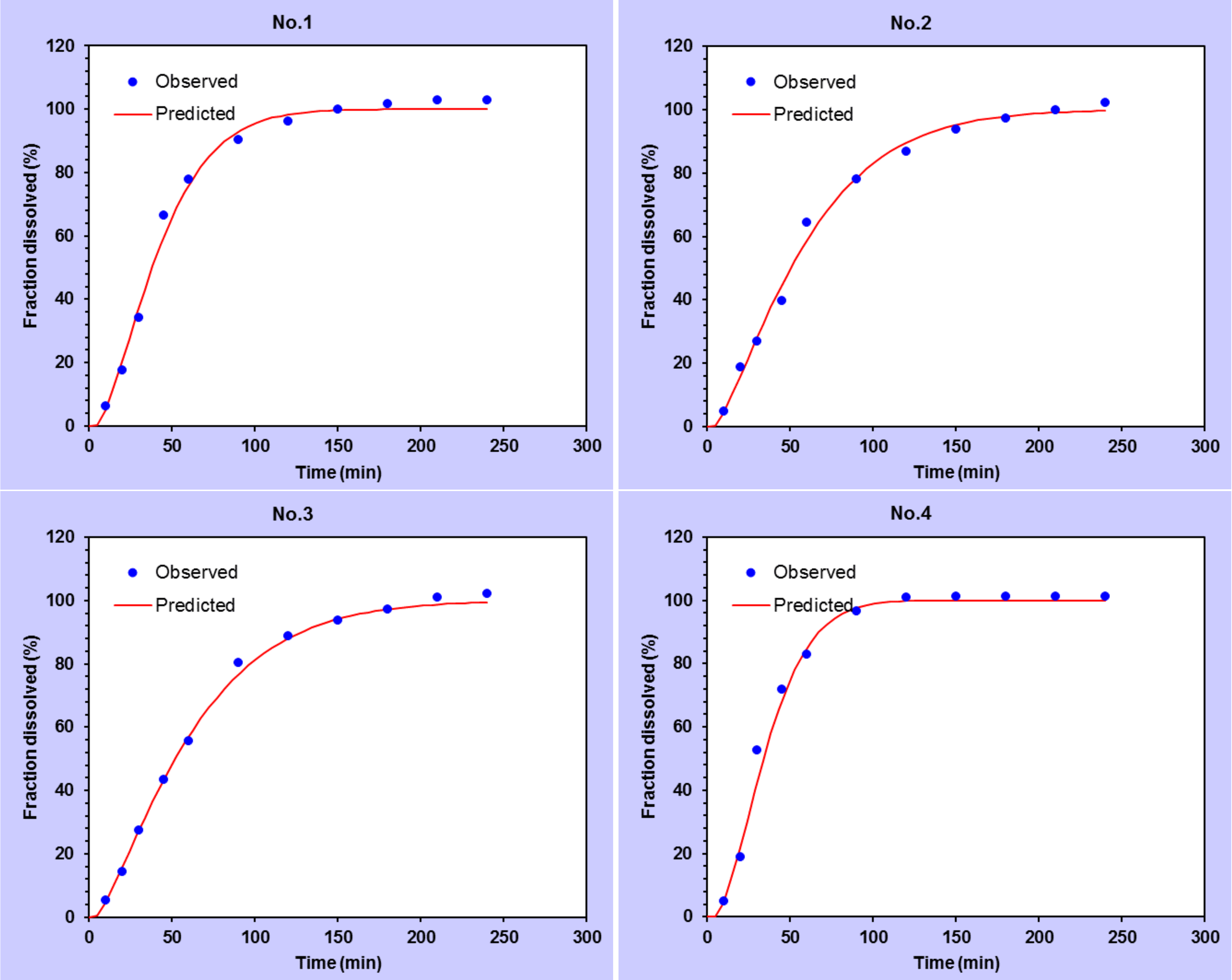

Supplementary materials – Model fitting summary – Parteck® M 100 (modelling the entire relevant dissolution data)

Model: **Weibull_2**

Model equation: $F = 100 \cdot \left(1 - e^{-\frac{t^\beta}{\alpha}}\right)$

Fitted model parameters per tested tablet (N = 4) with statistics – mean, standard deviation (SD), and relative standard deviation expressed in % (RSD%) (output from DDSolver):

Parameter	No.1	No.2	No.3	No.4	Mean	SD	RSD(%)
α	730.632	485.817	476.968	1376.175	767.398	422.532	55.060
β	1.684	1.463	1.450	1.922	1.630	0.222	13.631

Number of dissolution data points (N), degrees of freedom (df), and selected goodness of fit criteria – Pearson correlation coefficient (R), coefficient of determination (R^2), adjusted coefficient of determination ($R^2_{adjusted}$), and residual sum of squares (RSS) (manual calculation in MS Excel):

Parameter	No.1	No.2	No.3	No.4
N	11	11	11	11
df	9	9	9	9
R	0.994981262	0.996298949	0.998838555	0.992455516
R^2	0.989987713	0.992611596	0.997678458	0.984967952
$R^2_{adjusted}$	0.988875236	0.991790662	0.997420509	0.983297724
RSS	150.2003194	103.7626309	40.89962489	222.6854988

Graphical abstract of model fit presented as mean ± 1 SD of the fraction % of released carvedilol:

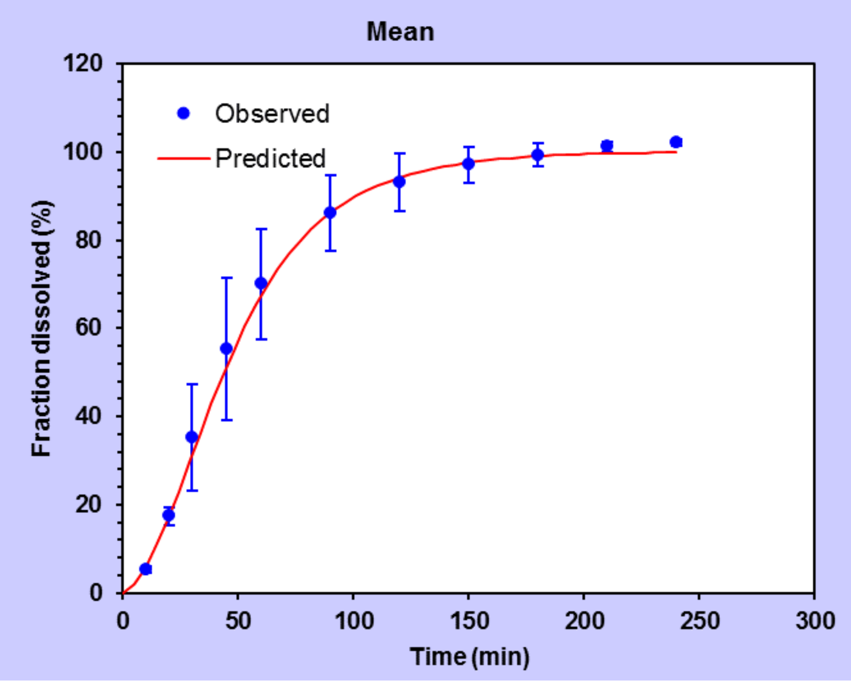

Supplementary materials – Model fitting summary – Parteck® M 100 (modelling the entire relevant dissolution data)

Graphical abstract of model fit presented as the fraction % of released carvedilol per tested tablet:

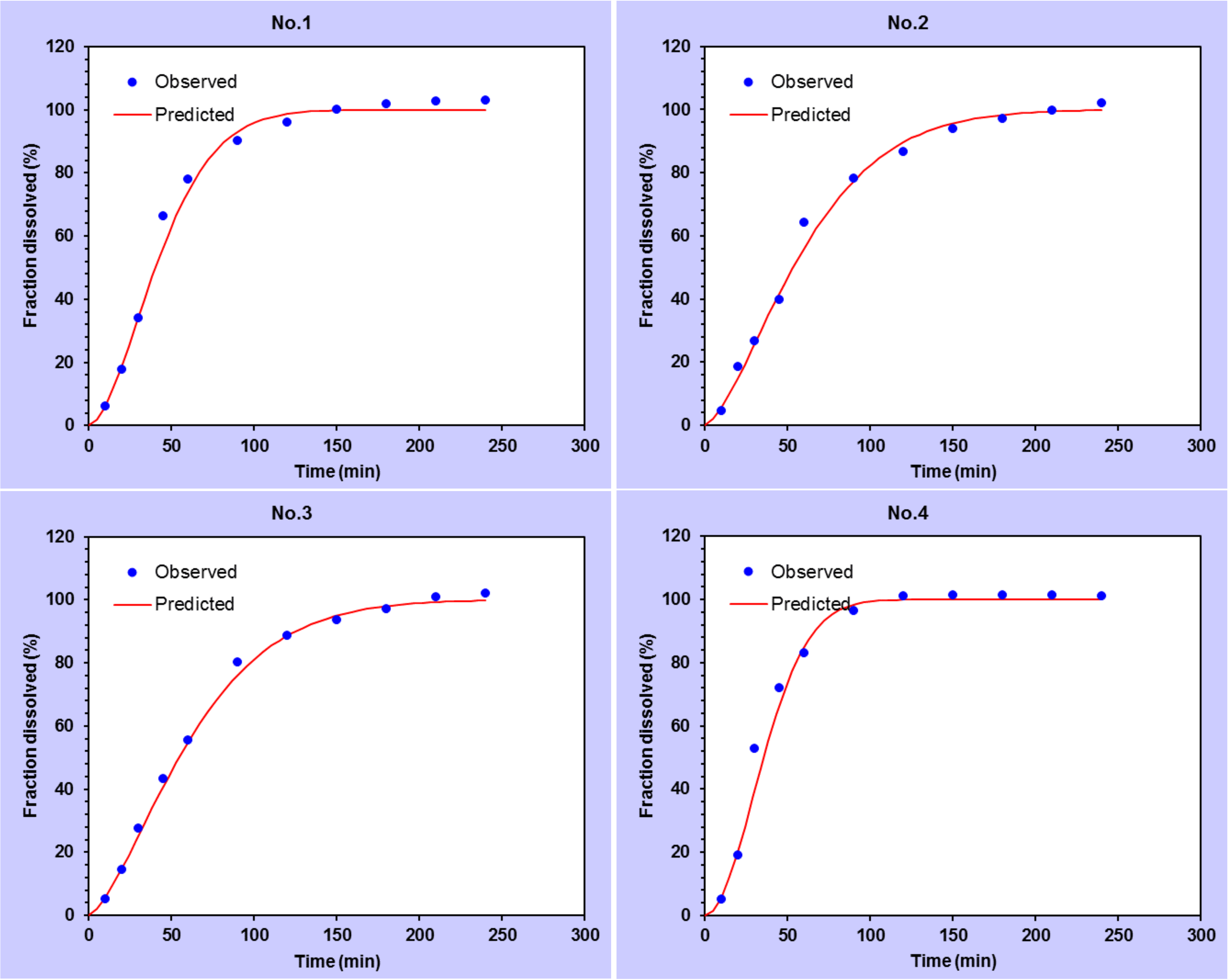

Supplementary materials – Model fitting summary – Parteck® M 100 (modelling the entire relevant dissolution data)

Model: **Weibull_3**

Model equation: $F = F_{max} \cdot \left(1 - e^{-\frac{t^{\beta}}{\alpha}}\right)$

Fitted model parameters per tested tablet (N = 4) with statistics – mean, standard deviation (SD), and relative standard deviation expressed in % (RSD%) (output from DDSolver):

Parameter	No.1	No.2	No.3	No.4	Mean	SD	RSD(%)
α	244.541	281.911	356.275	216.052	274.695	60.706	22.099
β	1.343	1.370	1.416	1.348	1.369	0.033	2.439
F_{max}	102.718	98.513	102.283	101.171	101.171	1.888	1.866

Number of dissolution data points (N), degrees of freedom (df), and selected goodness of fit criteria – Pearson correlation coefficient (R), coefficient of determination (R^2), adjusted coefficient of determination ($R^2_{adjusted}$), and residual sum of squares (RSS) (manual calculation in MS Excel):

Parameter	No.1	No.2	No.3	No.4
N	11	11	11	11
df	8	8	8	8
R	0.985647619	0.996408193	0.998690274	0.97539044
R^2	0.971501229	0.992829286	0.997382264	0.95138651
$R^2_{adjusted}$	0.964376536	0.991036608	0.99672783	0.939233138
RSS	475.819184	129.988601	150.0536823	871.6532626

Graphical abstract of model fit presented as mean ± 1 SD of the fraction % of released carvedilol:

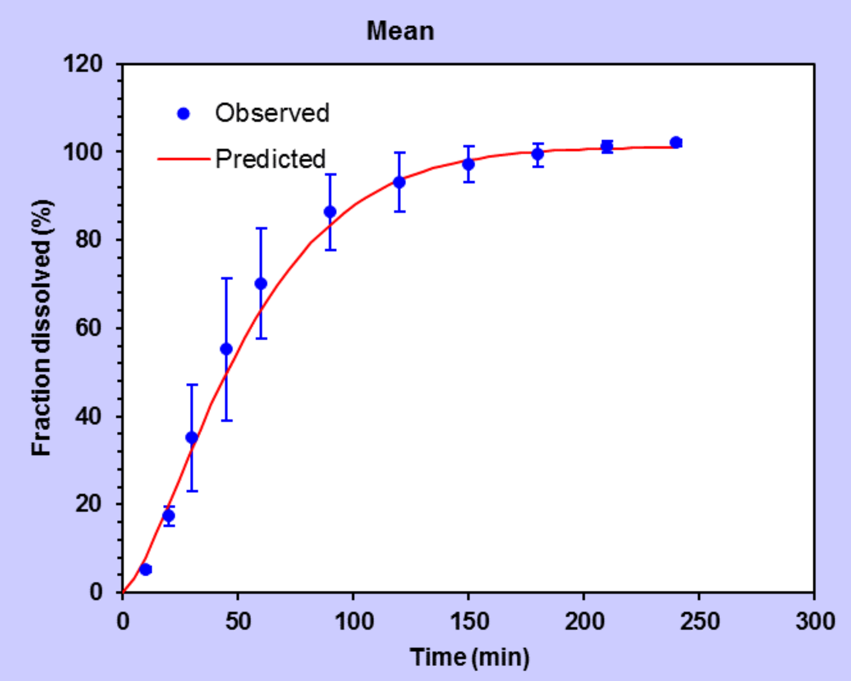

Supplementary materials – Model fitting summary – Parteck® M 100 (modelling the entire relevant dissolution data)

Graphical abstract of model fit presented as the fraction % of released carvedilol per tested tablet:

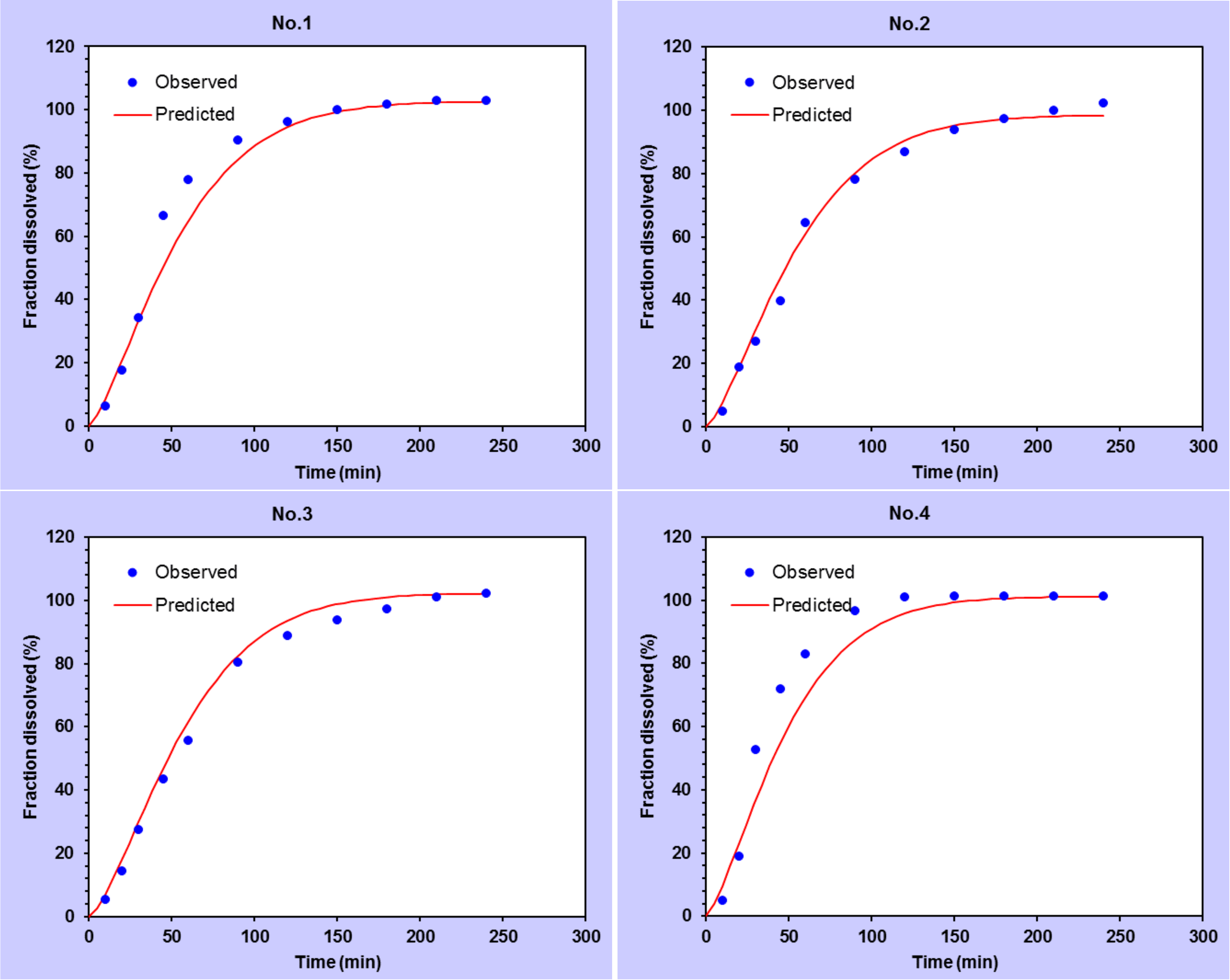

Supplementary materials – Model fitting summary – Parteck® M 100 (modelling the entire relevant dissolution data)

Model: **Weibull_4**

Model equation: $F = F_{max} \cdot \left[1 - e^{-\frac{(t-T_i)^{\beta}}{\alpha}}\right]$

Fitted model parameters per tested tablet (N = 4) with statistics – mean, standard deviation (SD), and relative standard deviation expressed in % (RSD%) (output from DDSolver):

Parameter	No.1	No.2	No.3	No.4	Mean	SD	RSD(%)
α	103.996	113.739	120.677	87.263	106.419	14.488	13.614
β	1.269	1.172	1.178	1.117	1.184	0.063	5.328
T_i	4.813	5.448	5.444	4.000	4.926	0.686	13.920
F_{max}	92.626	97.566	99.760	106.340	99.073	5.690	5.743

Number of dissolution data points (N), degrees of freedom (df), and selected goodness of fit criteria – Pearson correlation coefficient (R), coefficient of determination (R^2), adjusted coefficient of determination ($R^2_{adjusted}$), and residual sum of squares (RSS) (manual calculation in MS Excel):

Parameter	No.1	No.2	No.3	No.4
N	11	11	11	11
df	7	7	7	7
R	0.995516921	0.996247197	0.998358737	0.969170757
R^2	0.991053939	0.992508477	0.996720168	0.939291955
$R^2_{adjusted}$	0.987219913	0.989297824	0.995314526	0.913274222
RSS	545.8668787	126.2133898	68.04892026	947.2572963

Graphical abstract of model fit presented as mean ± 1 SD of the fraction % of released carvedilol:

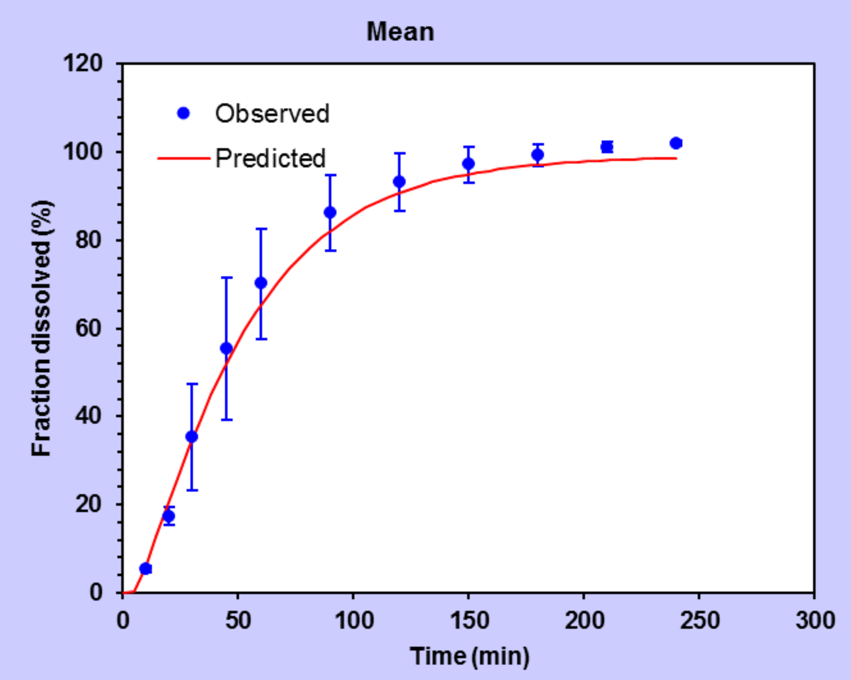

Supplementary materials – Model fitting summary – Parteck® M 100 (modelling the entire relevant dissolution data)

Graphical abstract of model fit presented as the fraction % of released carvedilol per tested tablet:

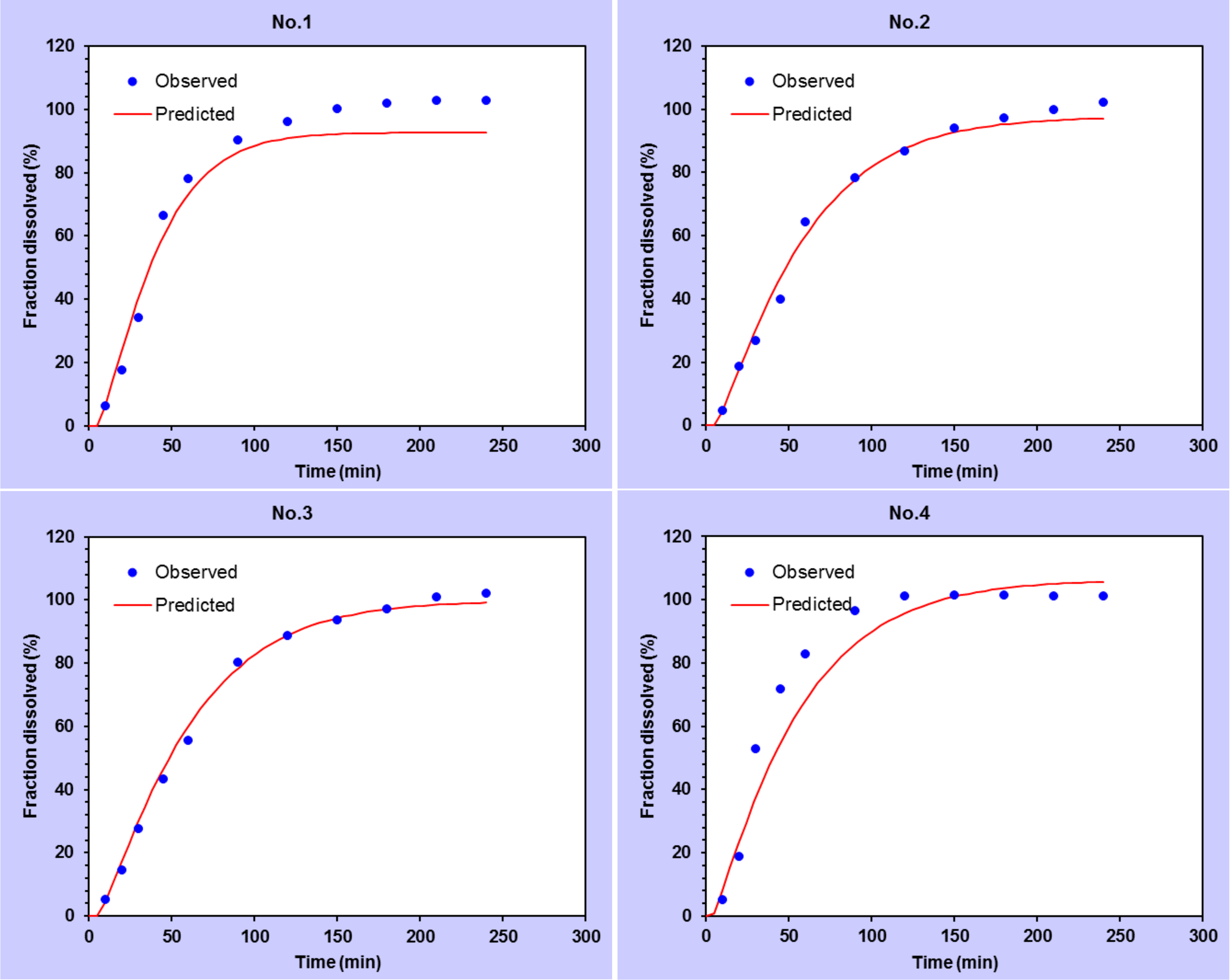

Supplementary materials – Model fitting summary – Parteck® M 100 (modelling the entire relevant dissolution data)

Model: **Logistic_1**

Model equation: $F = 100 \cdot \frac{e^{\alpha+\beta\cdot\log(t)}}{1+e^{\alpha+\beta\cdot\log(t)}}$

Fitted model parameters per tested tablet (N = 4) with statistics – mean, standard deviation (SD), and relative standard deviation expressed in % (RSD%) (output from DDSolver):

Parameter	No.1	No.2	No.3	No.4	Mean	SD	RSD(%)
α	-11.074	-9.388	-8.293	-9.551	-9.577	1.144	-11.946
β	7.352	5.781	5.015	6.421	6.142	0.990	16.124

Number of dissolution data points (N), degrees of freedom (df), and selected goodness of fit criteria – Pearson correlation coefficient (R), coefficient of determination (R^2), adjusted coefficient of determination ($R^2_{adjusted}$), and residual sum of squares (RSS) (manual calculation in MS Excel):

Parameter	No.1	No.2	No.3	No.4
N	11	11	11	11
df	9	9	9	9
R	0.988352094	0.986668793	0.99362726	0.997045224
R^2	0.976839861	0.973515307	0.987295132	0.994099179
$R^2_{adjusted}$	0.974266512	0.970572563	0.985883479	0.993443532
RSS	366.5495394	439.3089709	187.5529047	84.81605343

Graphical abstract of model fit presented as mean ± 1 SD of the fraction % of released carvedilol:

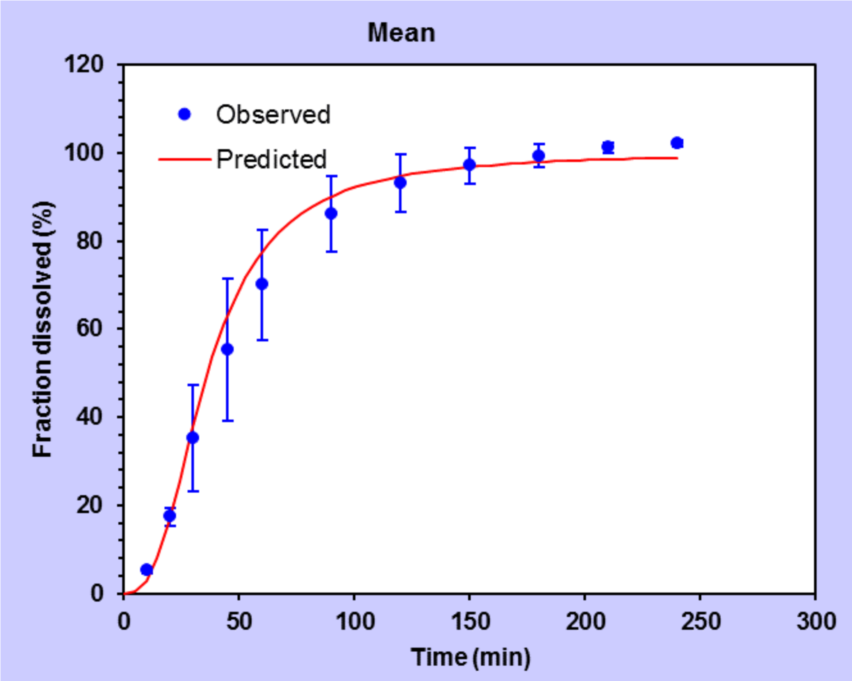

Supplementary materials – Model fitting summary – Parteck® M 100 (modelling the entire relevant dissolution data)

Graphical abstract of model fit presented as the fraction % of released carvedilol per tested tablet:

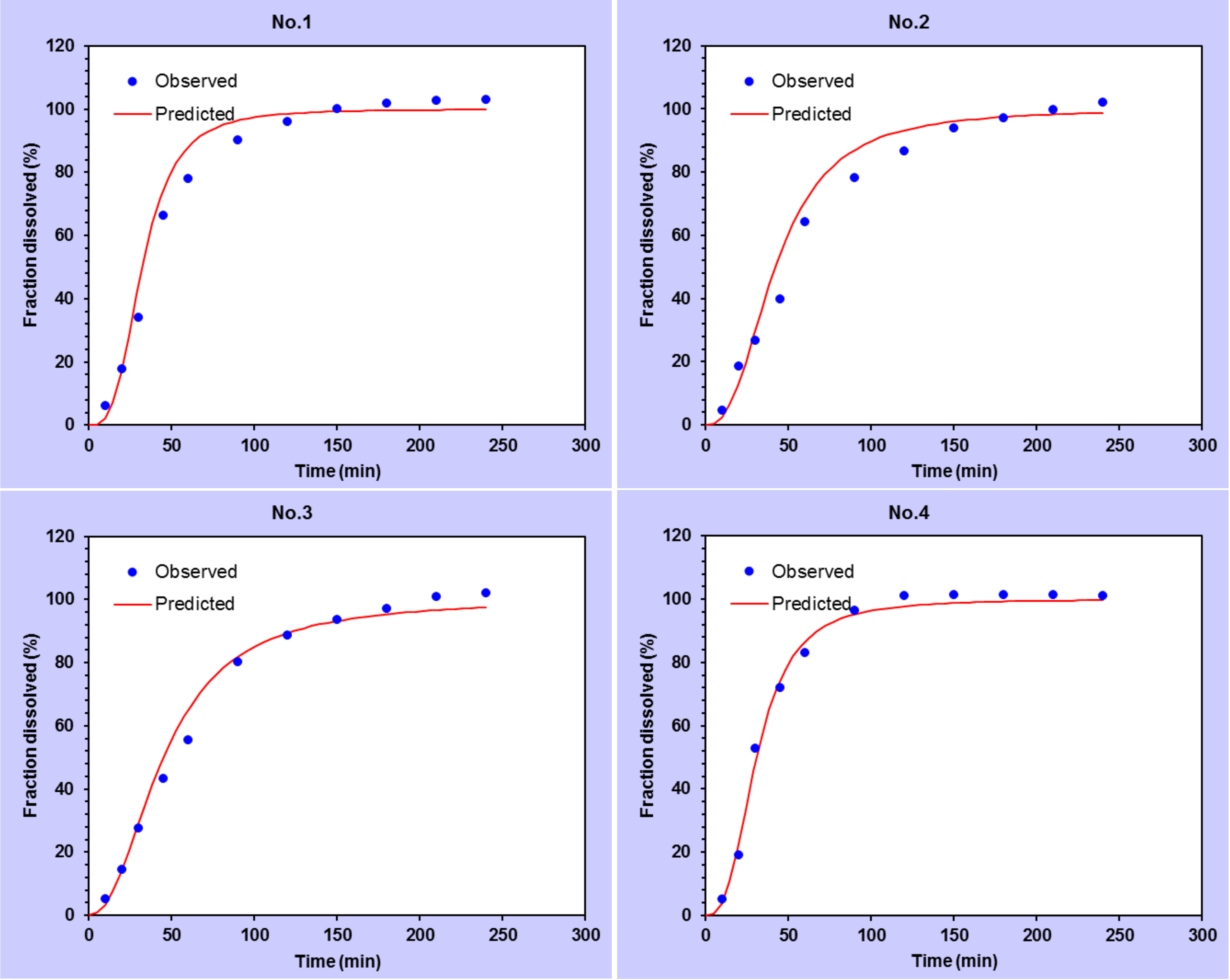

Supplementary materials – Model fitting summary – Parteck® M 100 (modelling the entire relevant dissolution data)

Model: **Logistic_2**

Model equation: $F = F_{max} \cdot \frac{e^{\alpha+\beta \cdot \log(t)}}{1+e^{\alpha+\beta \cdot \log(t)}}$

Fitted model parameters per tested tablet (N = 4) with statistics – mean, standard deviation (SD), and relative standard deviation expressed in % (RSD%) (output from DDSolver):

Parameter	No.1	No.2	No.3	No.4	Mean	SD	RSD(%)
α	-7.116	-7.346	-7.500	-7.024	-7.246	0.216	-2.984
β	4.393	4.277	4.362	4.508	4.385	0.095	2.176
F_{max}	107.967	107.226	107.226	106.340	107.190	0.665	0.621

Number of dissolution data points (N), degrees of freedom (df), and selected goodness of fit criteria – Pearson correlation coefficient (R), coefficient of determination (R^2), adjusted coefficient of determination ($R^2_{adjusted}$), and residual sum of squares (RSS) (manual calculation in MS Excel):

Parameter	No.1	No.2	No.3	No.4
N	11	11	11	11
df	8	8	8	8
R	0.995032735	0.997201251	0.998805367	0.99031268
R^2	0.990090144	0.994410335	0.997612162	0.980719204
$R^2_{adjusted}$	0.98761268	0.993012919	0.997015202	0.975899005
RSS	142.7824664	73.53508444	35.99632186	274.3096418

Graphical abstract of model fit presented as mean ± 1 SD of the fraction % of released carvedilol:

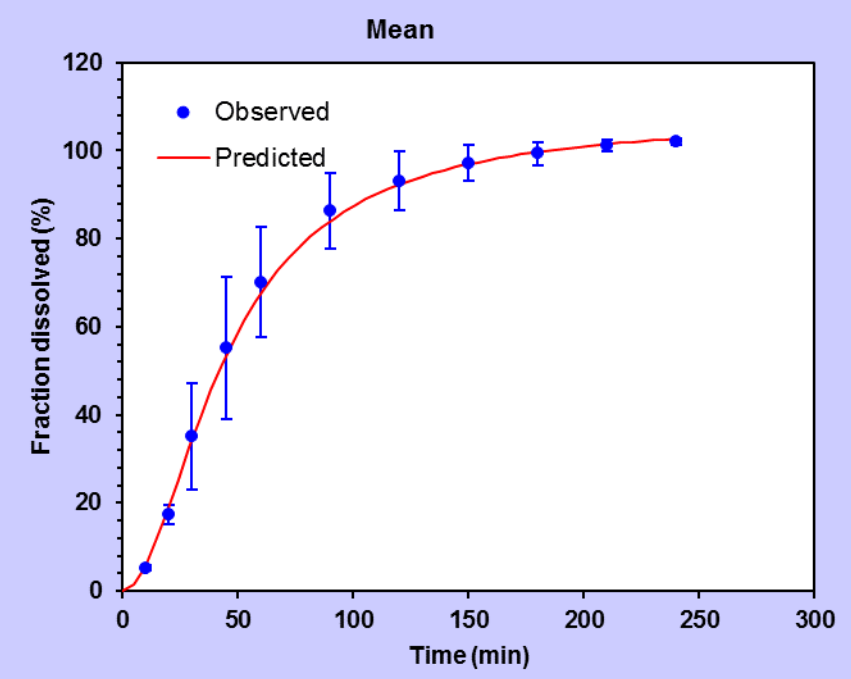

Supplementary materials – Model fitting summary – Parteck® M 100 (modelling the entire relevant dissolution data)

Graphical abstract of model fit presented as the fraction % of released carvedilol per tested tablet:

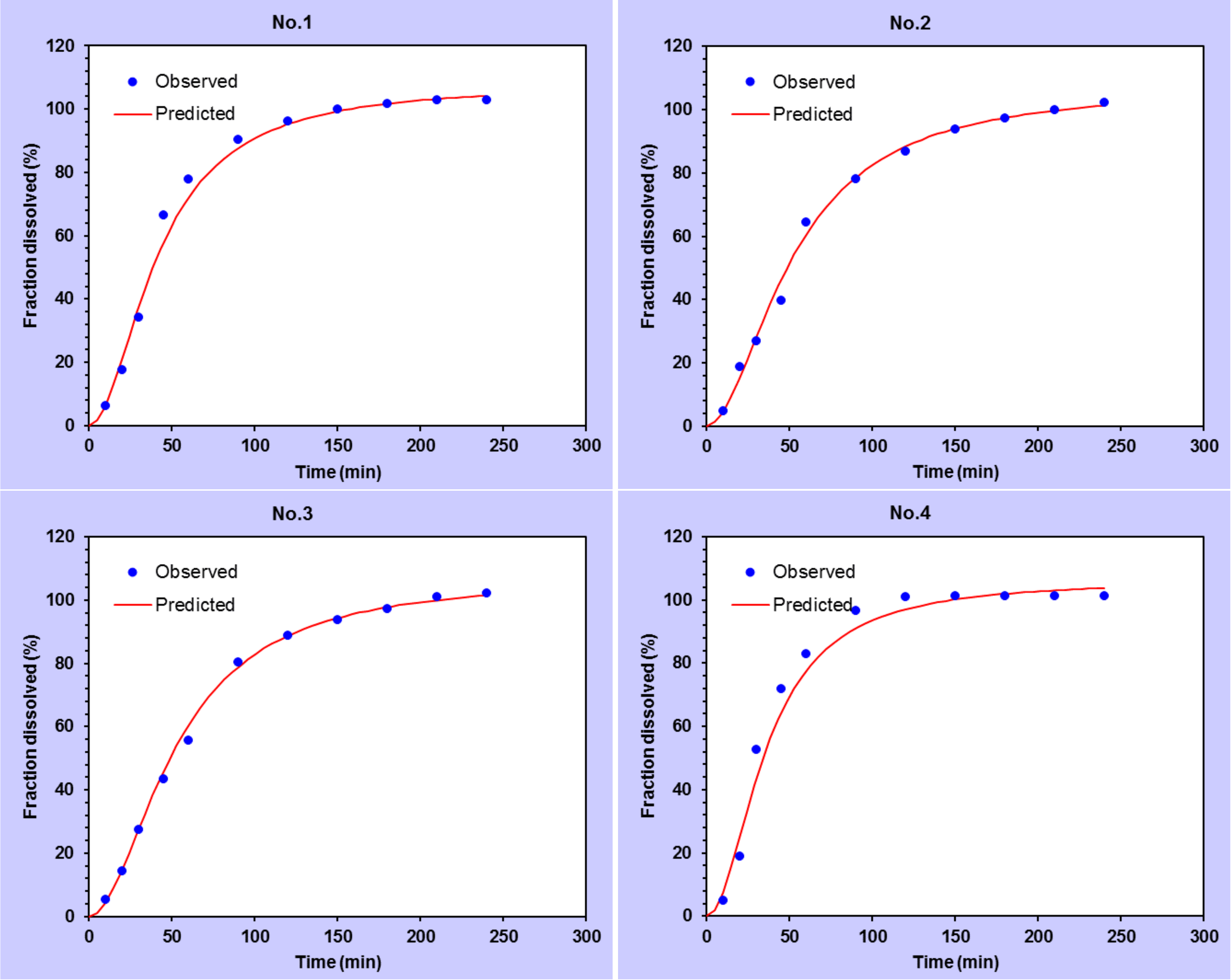

Supplementary materials – Model fitting summary – Parteck® M 100 (modelling the entire relevant dissolution data)

Model: **Logistic_3**

Model equation: $F = F_{max} \cdot \frac{1}{1+e^{-k\cdot(t-\gamma)}}$

Fitted model parameters per tested tablet (N = 4) with statistics – mean, standard deviation (SD), and relative standard deviation expressed in % (RSD%) (output from DDSolver):

Parameter	No.1	No.2	No.3	No.4	Mean	SD	RSD(%)
k	0.033	0.023	0.023	0.032	0.028	0.006	20.815
γ	59.042	79.383	79.894	43.518	65.459	17.558	26.822
F_{max}	107.967	107.226	107.226	106.340	107.190	0.665	0.621

Number of dissolution data points (N), degrees of freedom (df), and selected goodness of fit criteria – Pearson correlation coefficient (R), coefficient of determination (R^2), adjusted coefficient of determination ($R^2_{adjusted}$), and residual sum of squares (RSS) (manual calculation in MS Excel):

Parameter	No.1	No.2	No.3	No.4
N	11	11	11	11
df	8	8	8	8
R	0.946618235	0.95866361	0.9637745	0.93777321
R^2	0.896086083	0.919035918	0.928861286	0.879418593
$R^2_{adjusted}$	0.870107604	0.898794897	0.911076608	0.849273241
RSS	1513.283586	1196.365114	1096.577538	1538.151114

Graphical abstract of model fit presented as mean ± 1 SD of the fraction % of released carvedilol:

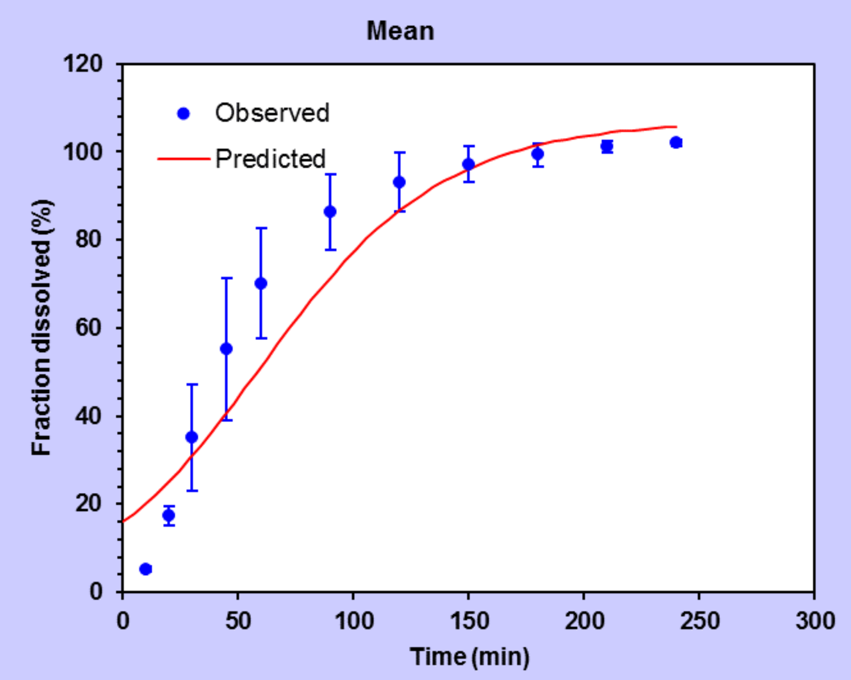

Supplementary materials – Model fitting summary – Parteck® M 100 (modelling the entire relevant dissolution data)

Graphical abstract of model fit presented as the fraction % of released carvedilol per tested tablet:

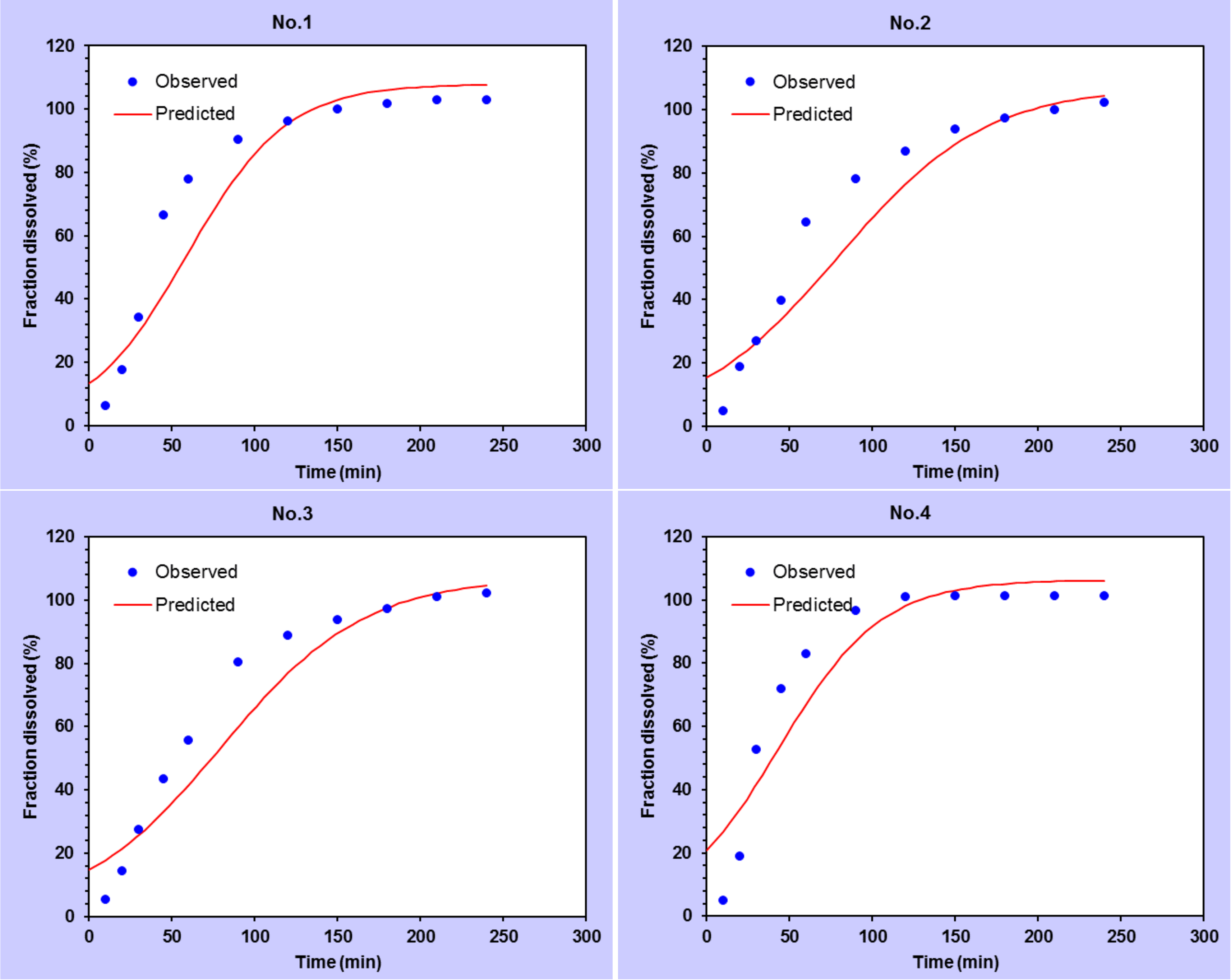

Supplementary materials – Model fitting summary – Parteck® M 100 (modelling the entire relevant dissolution data)

Model: **Gompertz_1**

Model equation: $F = 100 \cdot e^{-\alpha \cdot e^{-\beta \cdot \log(t)}}$

Fitted model parameters per tested tablet (N = 4) with statistics – mean, standard deviation (SD), and relative standard deviation expressed in % (RSD%) (output from DDSolver):

Parameter	No.1	No.2	No.3	No.4	Mean	SD	RSD(%)
α	5706.656	1113.915	318.478	613.280	1938.082	2533.747	130.735
β	5.939	4.491	3.625	4.477	4.633	0.960	20.728

Number of dissolution data points (N), degrees of freedom (df), and selected goodness of fit criteria – Pearson correlation coefficient (R), coefficient of determination (R^2), adjusted coefficient of determination ($R^2_{adjusted}$), and residual sum of squares (RSS) (manual calculation in MS Excel):

Parameter	No.1	No.2	No.3	No.4
N	11	11	11	11
df	9	9	9	9
R	0.988346328	0.984464893	0.993795981	0.998373608
R^2	0.976828463	0.969171126	0.987630452	0.996749862
$R^2_{adjusted}$	0.974253848	0.965745696	0.986256057	0.996388736
RSS	353.8143533	490.3936317	361.4670457	248.3606275

Graphical abstract of model fit presented as mean ± 1 SD of the fraction % of released carvedilol:

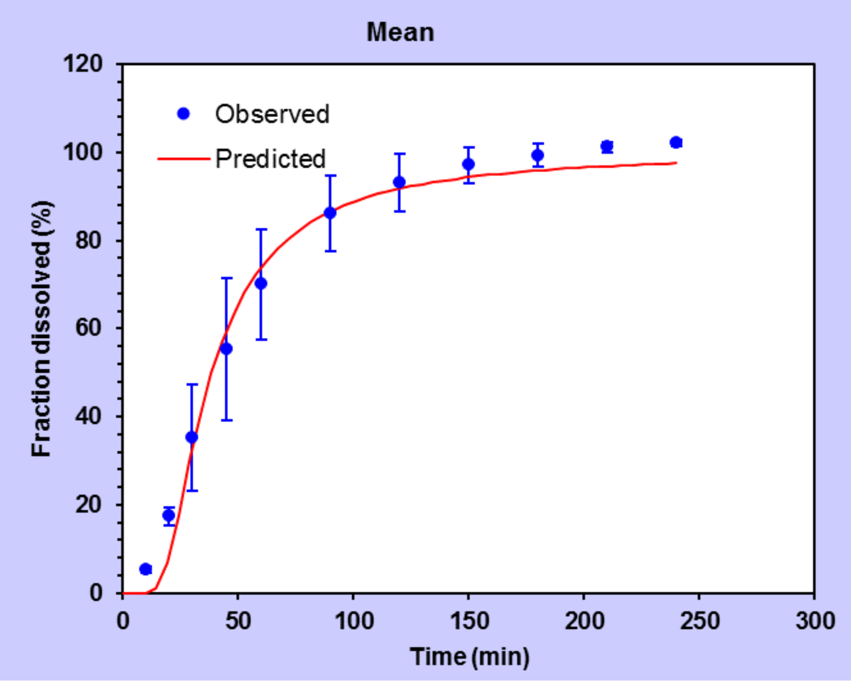

Supplementary materials – Model fitting summary – Parteck® M 100 (modelling the entire relevant dissolution data)

Graphical abstract of model fit presented as the fraction % of released carvedilol per tested tablet:

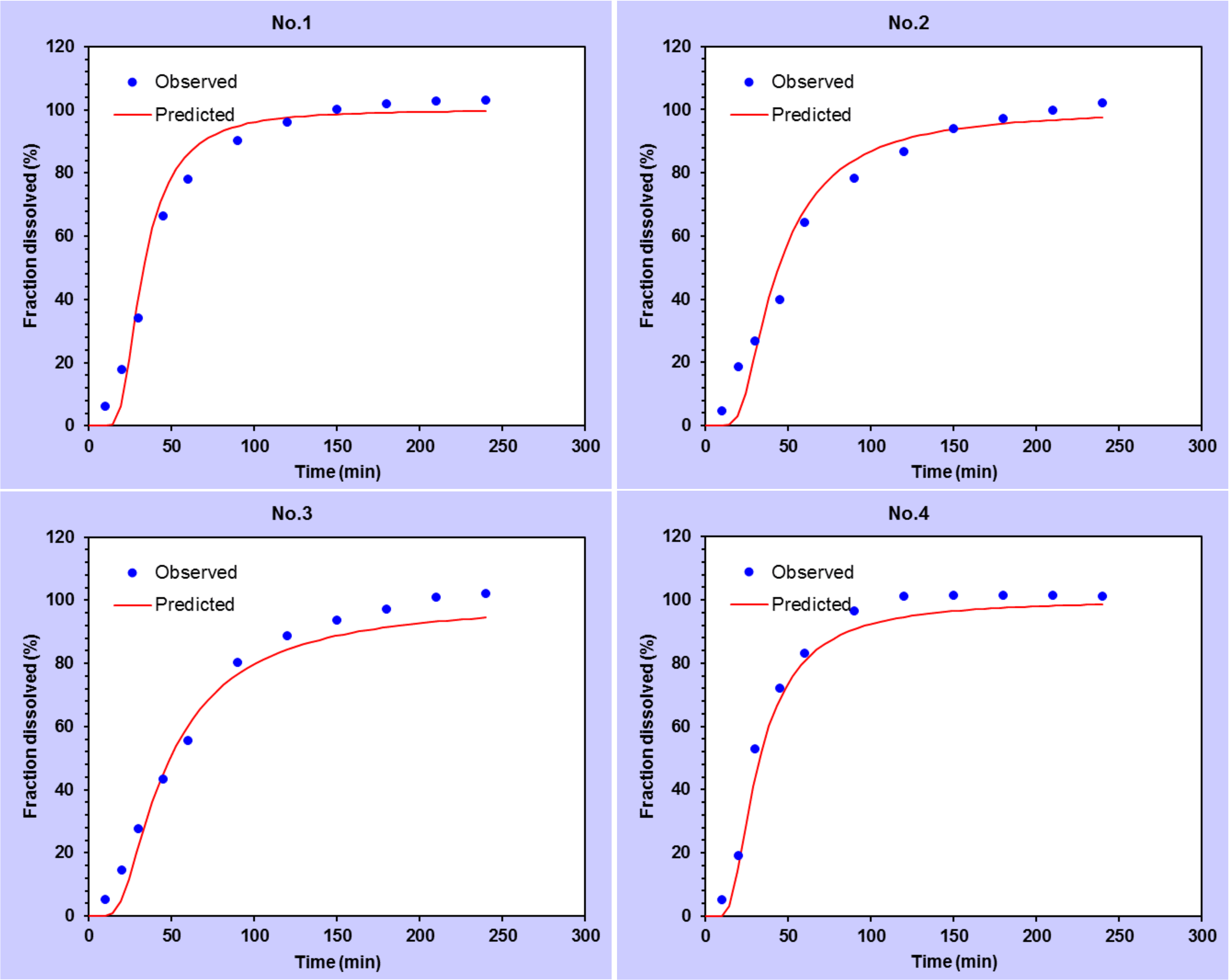

Supplementary materials – Model fitting summary – Parteck® M 100 (modelling the entire relevant dissolution data)

Model: **Gompertz_2**

Model equation: $F = F_{max} \cdot e^{-\alpha \cdot e^{-\beta \cdot \log(t)}}$

Fitted model parameters per tested tablet (N = 4) with statistics – mean, standard deviation (SD), and relative standard deviation expressed in % (RSD%) (output from DDSolver):

Parameter	No.1	No.2	No.3	No.4	Mean	SD	RSD(%)
α	106.294	101.361	172.076	132.486	128.054	32.371	25.279
β	3.272	3.049	3.121	3.400	3.211	0.157	4.884
F_{max}	107.967	107.226	107.226	108.258	107.669	0.525	0.488

Number of dissolution data points (N), degrees of freedom (df), and selected goodness of fit criteria – Pearson correlation coefficient (R), coefficient of determination (R^2), adjusted coefficient of determination ($R^2_{adjusted}$), and residual sum of squares (RSS) (manual calculation in MS Excel):

Parameter	No.1	No.2	No.3	No.4
N	11	11	11	11
df	8	8	8	8
R	0.993164574	0.986782872	0.998161192	0.994901104
R^2	0.986375872	0.973740436	0.996325764	0.989828207
$R^2_{adjusted}$	0.982969839	0.967175545	0.995407206	0.987285259
RSS	211.2410958	397.4647638	391.4473485	177.822142

Graphical abstract of model fit presented as mean ± 1 SD of the fraction % of released carvedilol:

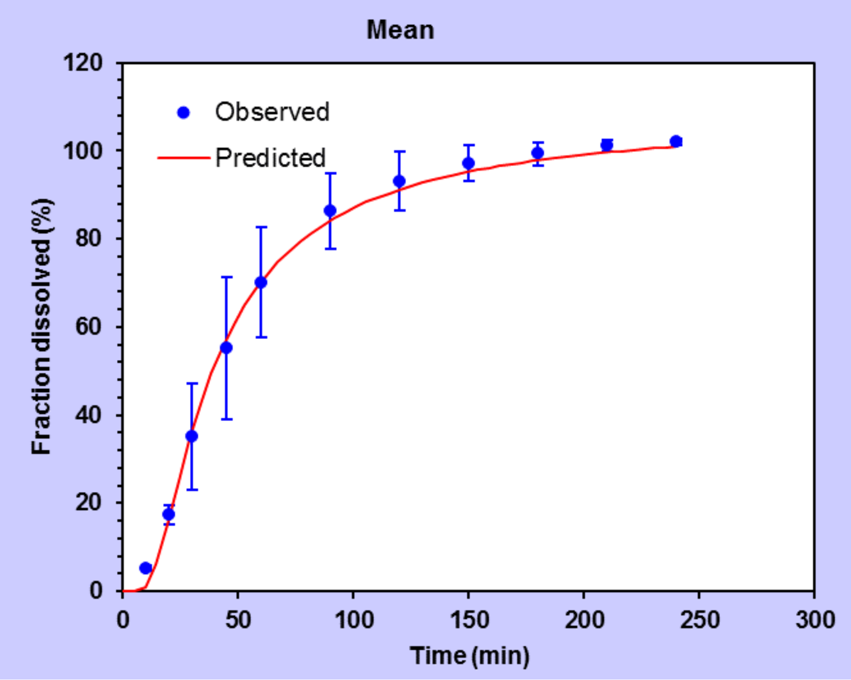

Supplementary materials – Model fitting summary – Parteck® M 100 (modelling the entire relevant dissolution data)

Graphical abstract of model fit presented as the fraction % of released carvedilol per tested tablet:

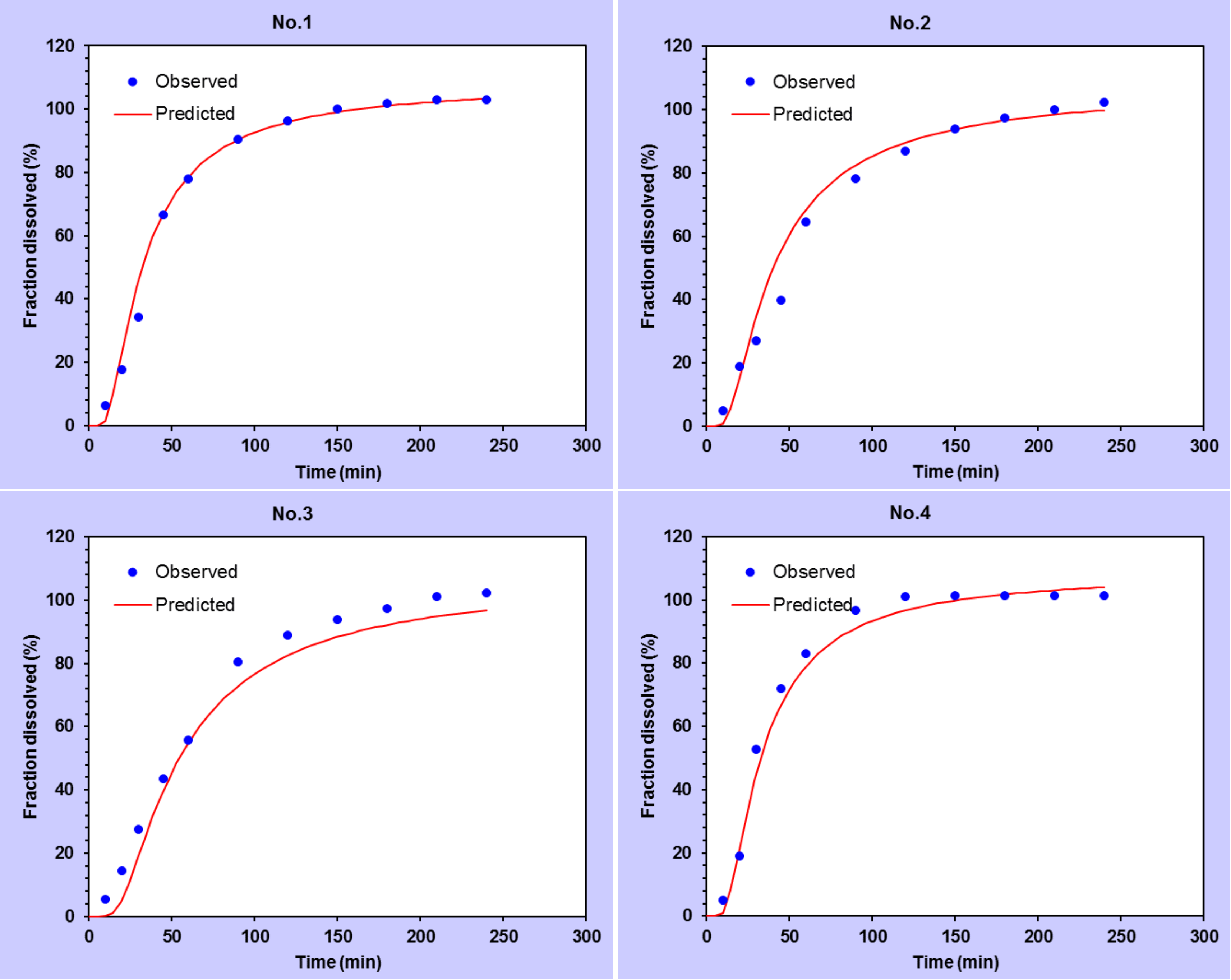

Supplementary materials – Model fitting summary – Parteck® M 100 (modelling the entire relevant dissolution data)

Model: **Gompertz_3**

Model equation: $F = F_{max} \cdot e^{-e^{-k\cdot(t-\gamma)}}$

Fitted model parameters per tested tablet (N = 4) with statistics – mean, standard deviation (SD), and relative standard deviation expressed in % (RSD%) (output from DDSolver):

Parameter	No.1	No.2	No.3	No.4	Mean	SD	RSD(%)
k	0.026	0.026	0.026	0.017	0.024	0.005	19.013
γ	24.648	44.524	45.558	9.845	31.144	17.153	55.076
F_{max}	107.967	107.226	107.226	106.340	107.190	0.665	0.621

Number of dissolution data points (N), degrees of freedom (df), and selected goodness of fit criteria – Pearson correlation coefficient (R), coefficient of determination (R^2), adjusted coefficient of determination ($R^2_{adjusted}$), and residual sum of squares (RSS) (manual calculation in MS Excel):

Parameter	No.1	No.2	No.3	No.4
N	11	11	11	11
df	8	8	8	8
R	0.982421124	0.992843147	0.996218211	0.925072482
R^2	0.965151264	0.985737515	0.992450723	0.855759097
$R^2_{adjusted}$	0.95643908	0.982171894	0.990563404	0.819698871
RSS	934.9236466	299.2026241	208.7532079	2524.547517

Graphical abstract of model fit presented as mean ± 1 SD of the fraction % of released carvedilol:

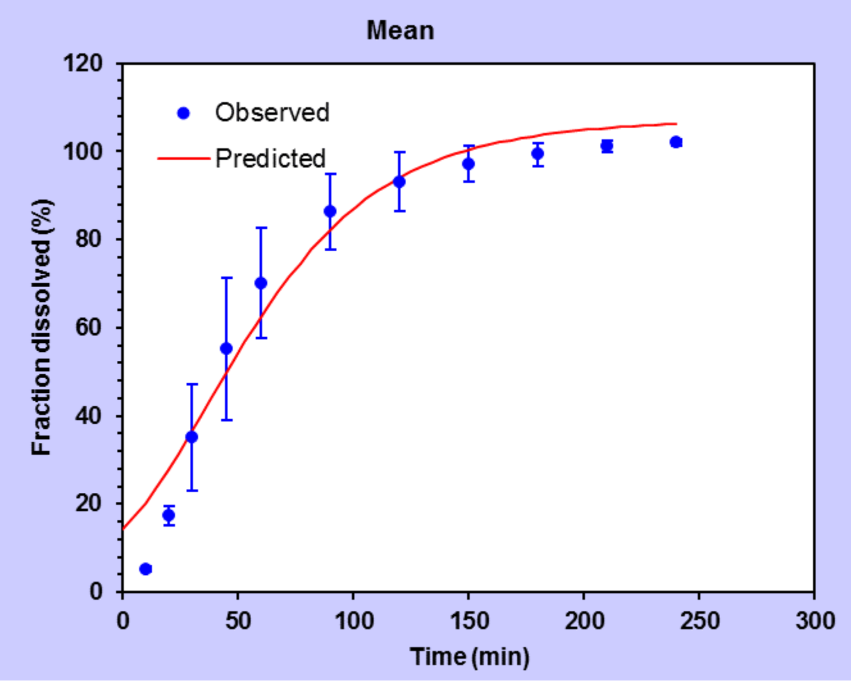

Supplementary materials – Model fitting summary – Parteck® M 100 (modelling the entire relevant dissolution data)

Graphical abstract of model fit presented as the fraction % of released carvedilol per tested tablet:

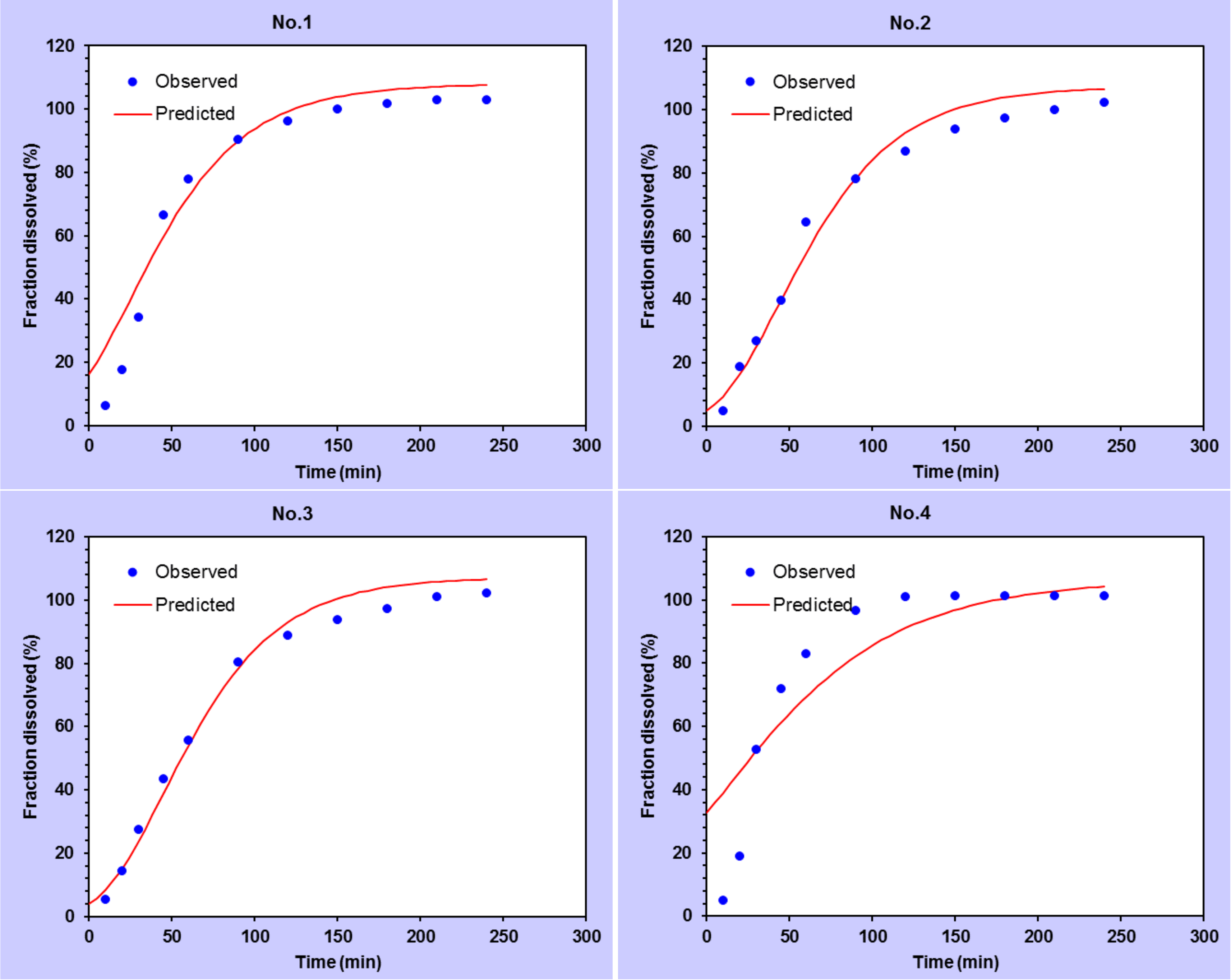

Supplementary materials – Model fitting summary – Parteck® M 100 (modelling the entire relevant dissolution data)

Model: **Gompertz_4**

Model equation: $F = F_{max} \cdot e^{-\beta \cdot e^{-k \cdot t}}$

Fitted model parameters per tested tablet (N = 4) with statistics – mean, standard deviation (SD), and relative standard deviation expressed in % (RSD%) (output from DDSolver):

Parameter	No.1	No.2	No.3	No.4	Mean	SD	RSD(%)
k	0.017	0.017	0.017	0.017	0.017	0.000	1.505
β	1.534	2.134	2.218	1.118	1.751	0.521	29.731
F_{max}	107.967	107.226	107.226	106.340	107.190	0.665	0.621

Number of dissolution data points (N), degrees of freedom (df), and selected goodness of fit criteria – Pearson correlation coefficient (R), coefficient of determination (R^2), adjusted coefficient of determination ($R^2_{adjusted}$), and residual sum of squares (RSS) (manual calculation in MS Excel):

Parameter	No.1	No.2	No.3	No.4
N	11	11	11	11
df	8	8	8	8
R	0.95253123	0.980723873	0.985154489	0.927321037
R^2	0.907315745	0.961819316	0.970529366	0.859924305
$R^2_{adjusted}$	0.884144681	0.952274145	0.963161708	0.824905381
RSS	1604.269949	566.7276792	471.1180266	2673.991965

Graphical abstract of model fit presented as mean ± 1 SD of the fraction % of released carvedilol:

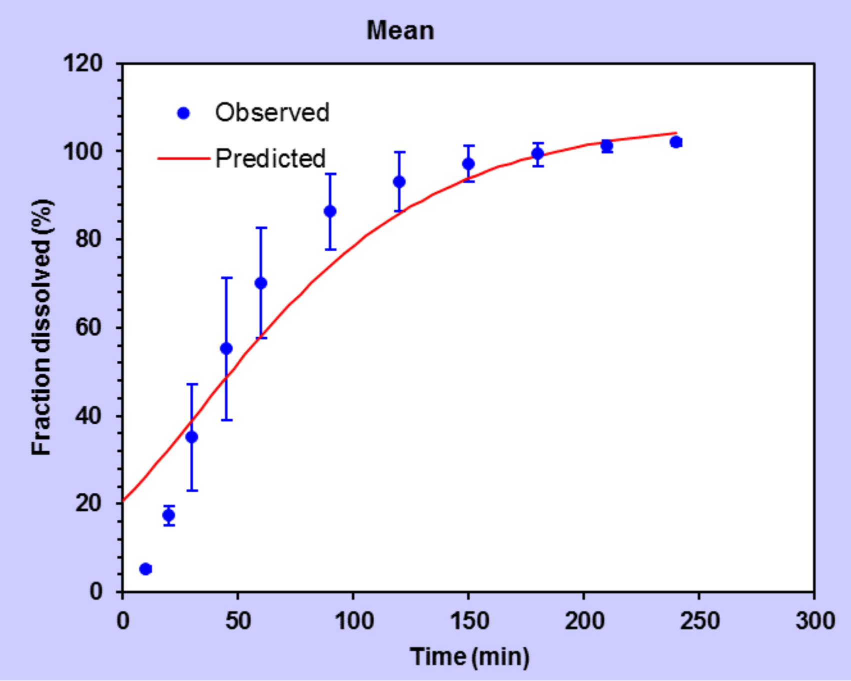

Supplementary materials – Model fitting summary – Parteck® M 100 (modelling the entire relevant dissolution data)

Graphical abstract of model fit presented as the fraction % of released carvedilol per tested tablet:

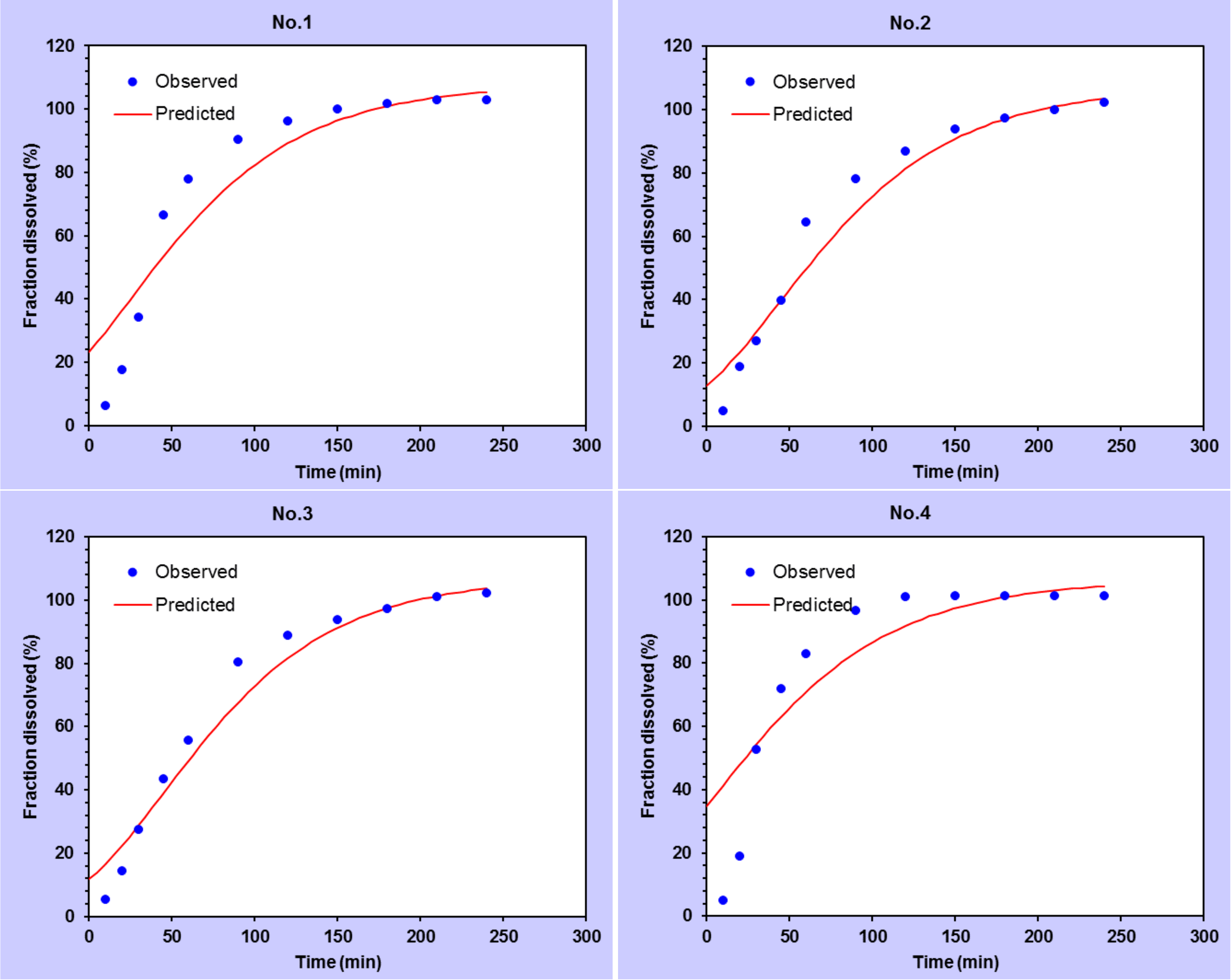

Supplementary materials – Model fitting summary – Parteck® M 100 (modelling the entire relevant dissolution data)

Model: **Probit_1**

Model equation: $F = 100 \cdot \phi[\alpha + \beta \cdot \log(t)]$

Fitted model parameters per tested tablet (N = 4) with statistics – mean, standard deviation (SD), and relative standard deviation expressed in % (RSD%) (output from DDSolver):

Parameter	No.1	No.2	No.3	No.4	Mean	SD	RSD(%)
α	-5.682	-5.827	-5.409	-5.388	-5.576	0.214	-3.838
β	3.735	3.376	3.143	3.623	3.469	0.264	7.620

Number of dissolution data points (N), degrees of freedom (df), and selected goodness of fit criteria – Pearson correlation coefficient (R), coefficient of determination (R^2), adjusted coefficient of determination ($R^2_{adjusted}$), and residual sum of squares (RSS) (manual calculation in MS Excel):

Parameter	No.1	No.2	No.3	No.4
N	11	11	11	11
df	9	9	9	9
R	0.993705915	0.996422124	0.998673663	0.997258982
R^2	0.987451446	0.99285705	0.997349085	0.994525477
$R^2_{adjusted}$	0.986057162	0.992063389	0.997054539	0.993917197
RSS	189.1521469	254.6220619	126.6516744	79.74848309

Graphical abstract of model fit presented as mean ± 1 SD of the fraction % of released carvedilol:

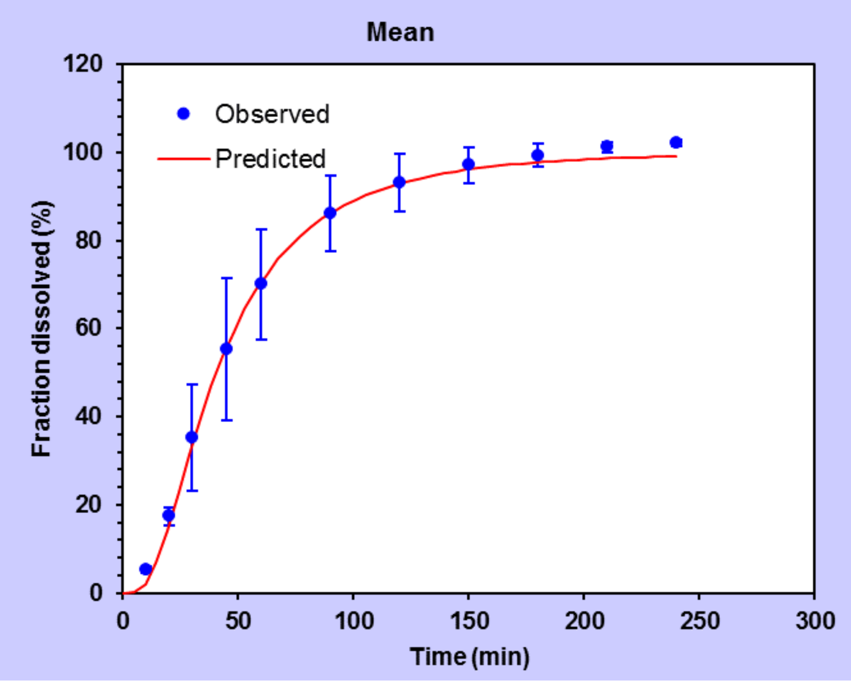

Supplementary materials – Model fitting summary – Parteck® M 100 (modelling the entire relevant dissolution data)

Graphical abstract of model fit presented as the fraction % of released carvedilol per tested tablet:

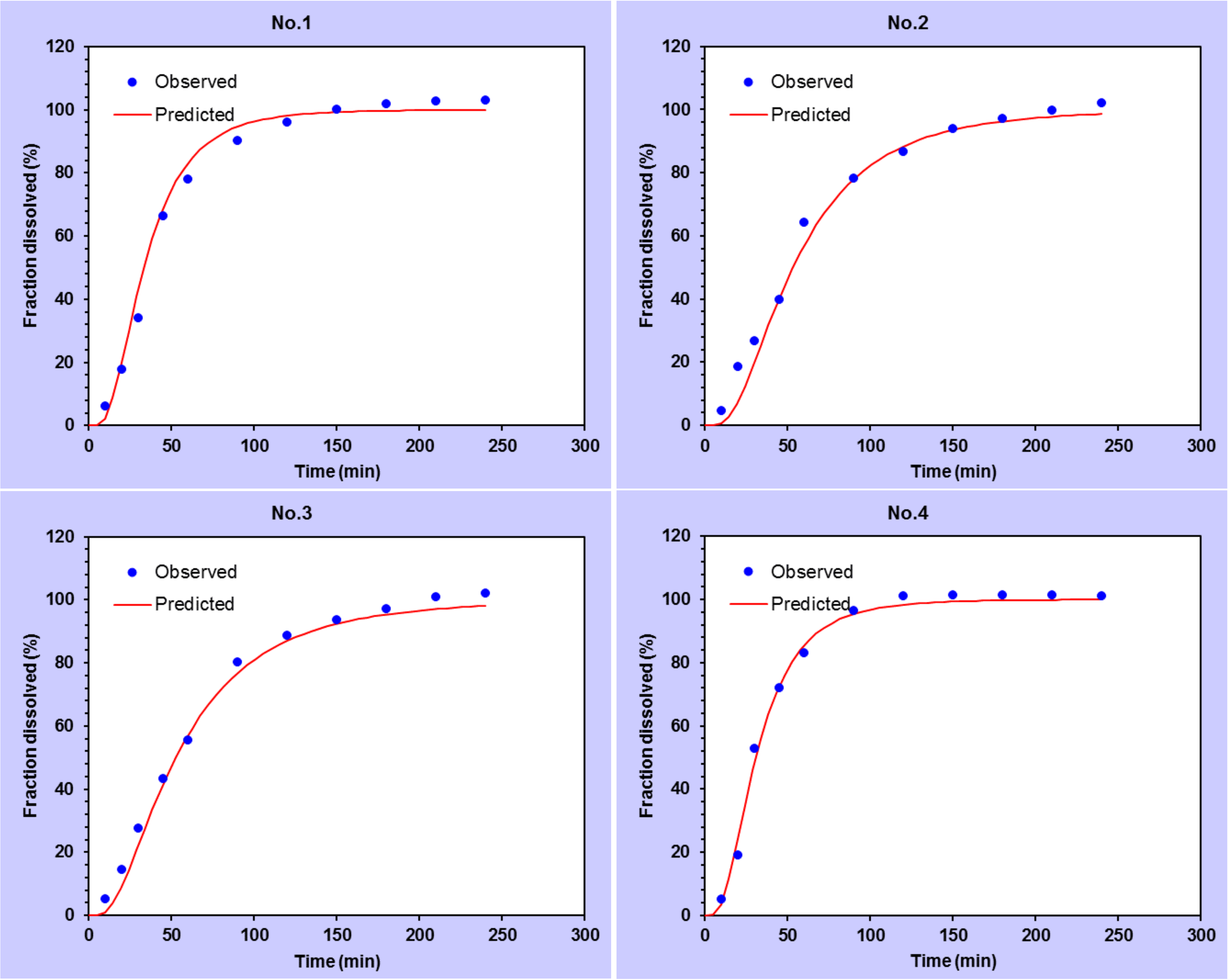

Supplementary materials – Model fitting summary – Parteck® M 100 (modelling the entire relevant dissolution data)

Model: **Probit_2**

Model equation: $F = F_{max} \cdot \phi[\alpha + \beta \cdot \log(t)]$

Fitted model parameters per tested tablet (N = 4) with statistics – mean, standard deviation (SD), and relative standard deviation expressed in % (RSD%) (output from DDSolver):

Parameter	No.1	No.2	No.3	No.4	Mean	SD	RSD(%)
α	-4.356	-4.206	-4.296	-4.547	-4.351	0.144	-3.316
β	2.776	2.452	2.499	2.887	2.653	0.211	7.961
F_{max}	102.009	107.226	107.226	106.709	105.793	2.534	2.396

Number of dissolution data points (N), degrees of freedom (df), and selected goodness of fit criteria – Pearson correlation coefficient (R), coefficient of determination (R^2), adjusted coefficient of determination ($R^2_{adjusted}$), and residual sum of squares (RSS) (manual calculation in MS Excel):

Parameter	No.1	No.2	No.3	No.4
N	11	11	11	11
df	8	8	8	8
R	0.996102206	0.996696151	0.998426408	0.988626775
R^2	0.992219604	0.993403217	0.996855291	0.9773829
$R^2_{adjusted}$	0.990274506	0.991754022	0.996069114	0.971728625
RSS	170.3661353	87.66335463	48.4829172	324.0377005

Graphical abstract of model fit presented as mean ± 1 SD of the fraction % of released carvedilol:

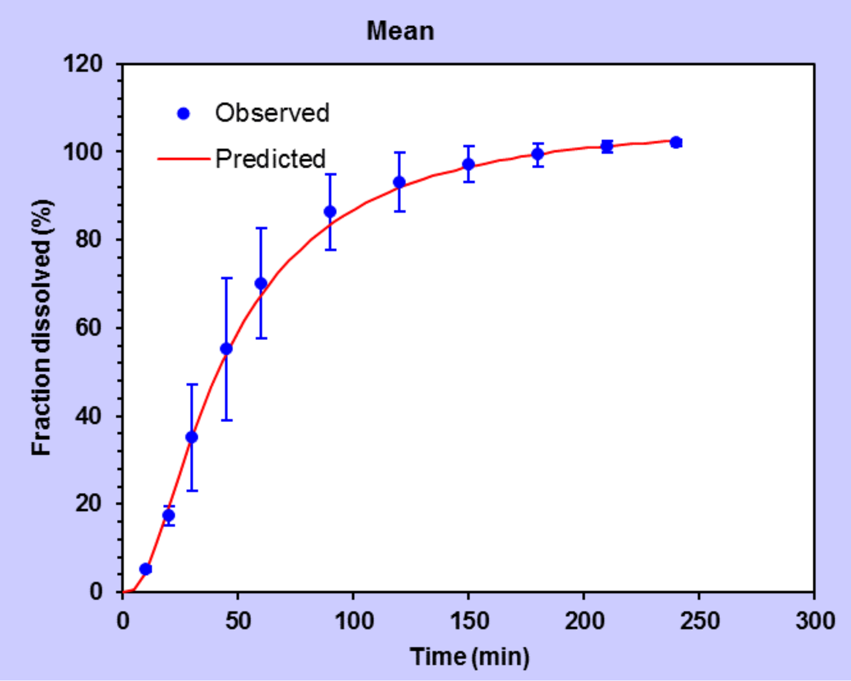

Supplementary materials – Model fitting summary – Parteck® M 100 (modelling the entire relevant dissolution data)

Graphical abstract of model fit presented as the fraction % of released carvedilol per tested tablet:

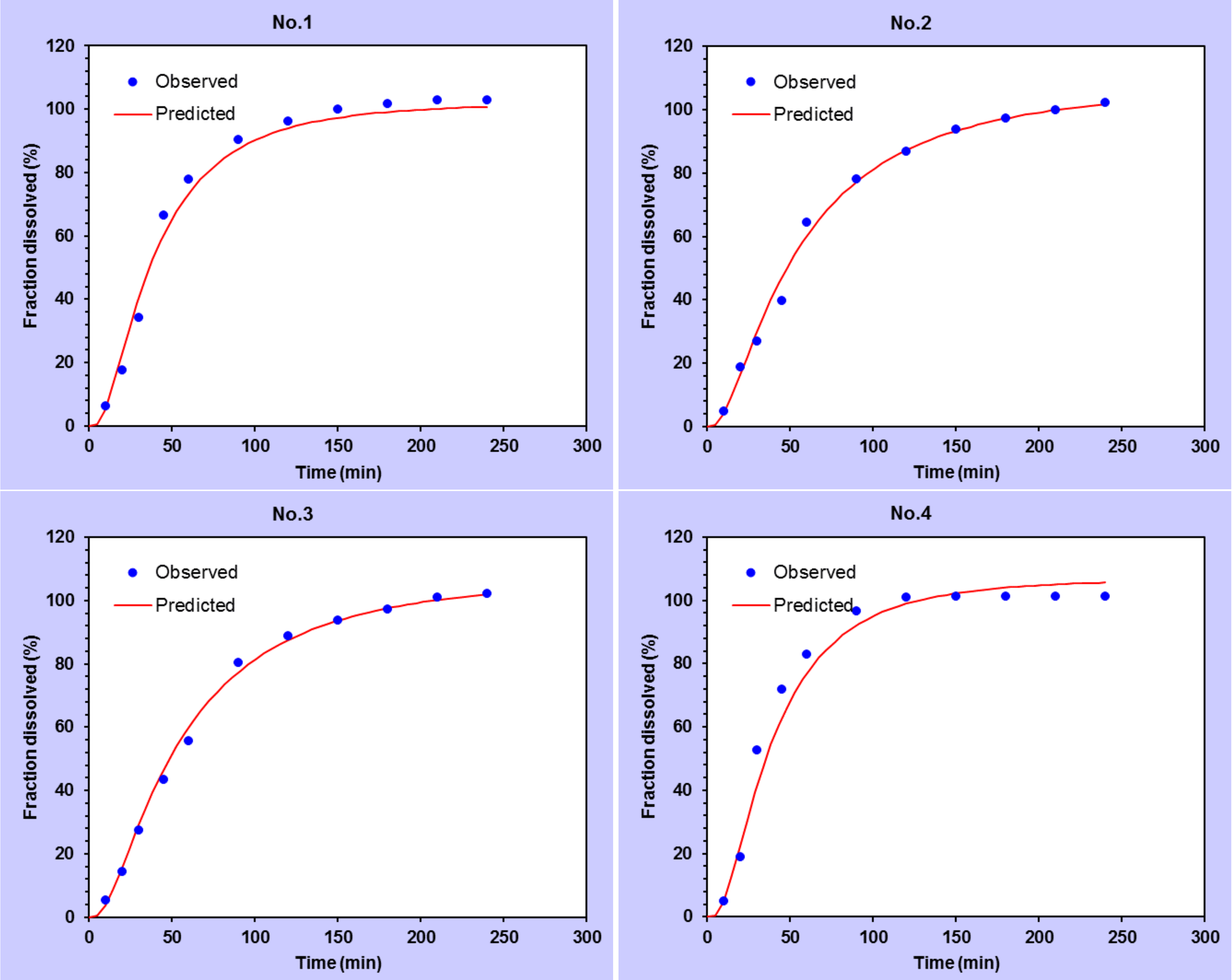

Supplementary materials – Model fitting summary – Parteck® M 100 (modelling up to app. 60 % of carvedilol released)

Model: **Zero–order**

Model equation: $F = k_0 \cdot t$

Fitted model parameters per tested tablet (N = 4) with statistics – mean, standard deviation (SD), and relative standard deviation expressed in % (RSD%) (output from DDSolver):

Parameter	No.1	No.2	No.3	No.4	Mean	SD	RSD(%)
k_0	1.292	0.878	0.909	1.530	1.152	0.314	27.294

Number of dissolution data points (N), degrees of freedom (df), and selected goodness of fit criteria – Pearson correlation coefficient (R), coefficient of determination (R^2), adjusted coefficient of determination ($R^2_{adjusted}$), and residual sum of squares (RSS) (manual calculation in MS Excel):

Parameter	No.1	No.2	No.3	No.4
N	4	4	4	4
df	3	3	3	3
R	0.990677239	0.992042875	0.998448029	0.98106963
R^2	0.981441392	0.984149067	0.996898467	0.962497618
$R^2_{adjusted}$	0.981441392	0.984149067	0.996898467	0.962497618
RSS	203.8826469	17.94862361	35.09966412	297.8034846

Graphical abstract of model fit presented as mean ± 1 SD of the fraction % of released carvedilol:

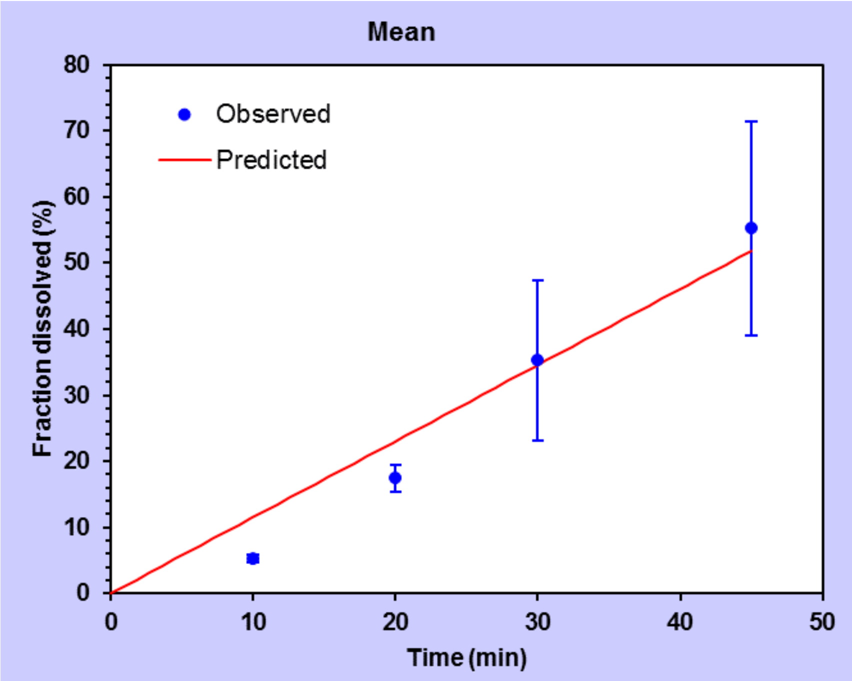

Supplementary materials – Model fitting summary – Parteck® M 100 (modelling up to app. 60 % of carvedilol released)

Graphical abstract of model fit presented as the fraction % of released carvedilol per tested tablet:

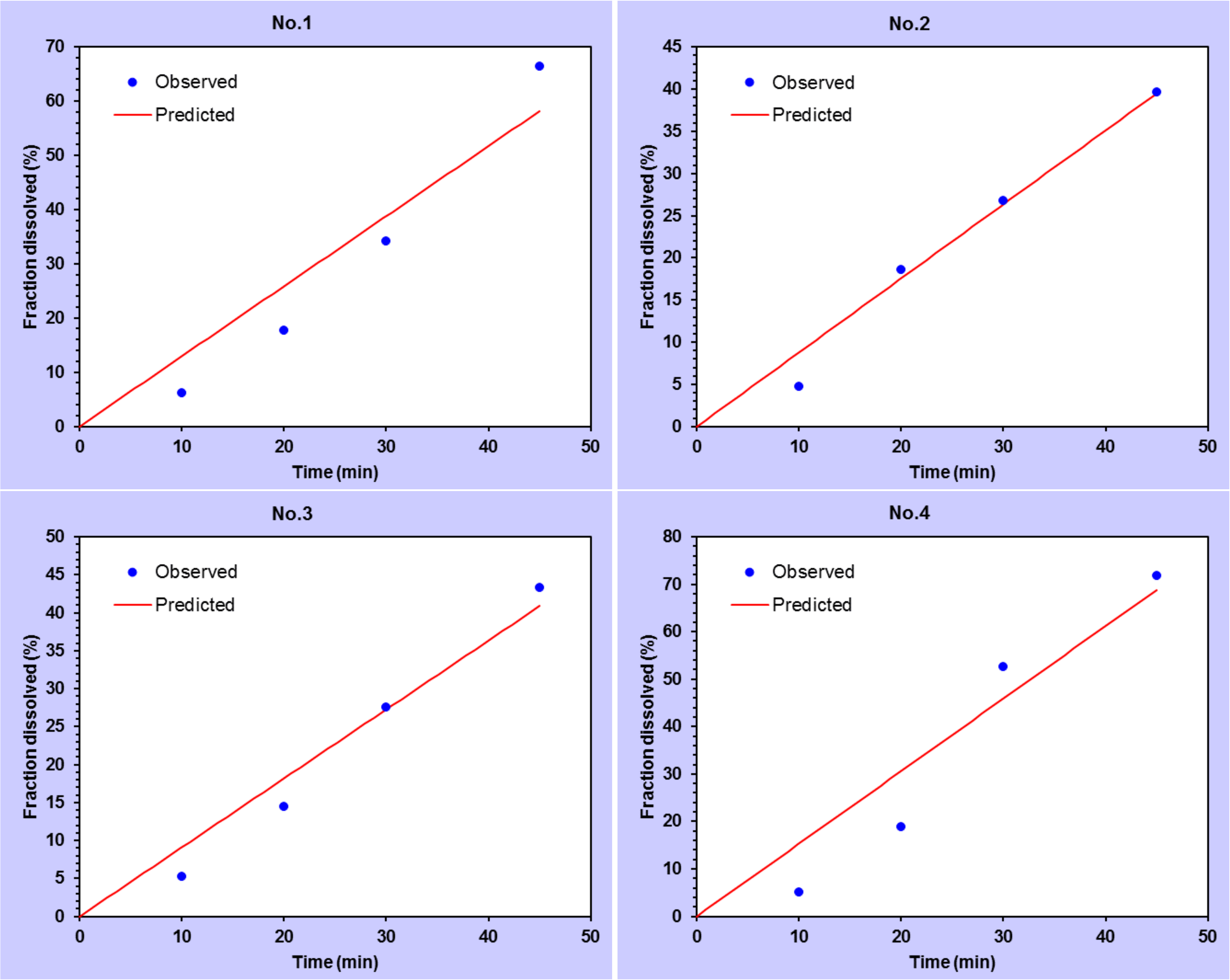

Supplementary materials – Model fitting summary – Parteck® M 100 (modelling up to app. 60 % of carvedilol released)

Model: **Zero–order with T_{lag}**

Model equation: $F = k_0 \cdot (t - T_{lag})$

Fitted model parameters per tested tablet (N = 4) with statistics – mean, standard deviation (SD), and relative standard deviation expressed in % (RSD%) (output from DDSolver):

Parameter	No.1	No.2	No.3	No.4	Mean	SD	RSD(%)
k_0	1.739	0.975	1.106	2.011	1.458	0.497	34.117
T_{lag}	8.377	3.226	5.835	7.805	6.311	2.327	36.874

Number of dissolution data points (N), degrees of freedom (df), and selected goodness of fit criteria – Pearson correlation coefficient (R), coefficient of determination (R^2), adjusted coefficient of determination ($R^2_{adjusted}$), and residual sum of squares (RSS) (manual calculation in MS Excel):

Parameter	No.1	No.2	No.3	No.4
N	4	4	4	4
df	2	2	2	2
R	0.990677239	0.992042875	0.998448029	0.98106963
R^2	0.981441392	0.984149067	0.996898467	0.962497618
$R^2_{adjusted}$	0.972162088	0.9762236	0.995347701	0.943746427
RSS	38.22456767	10.22989545	2.547077157	105.3852605

Graphical abstract of model fit presented as mean ± 1 SD of the fraction % of released carvedilol:

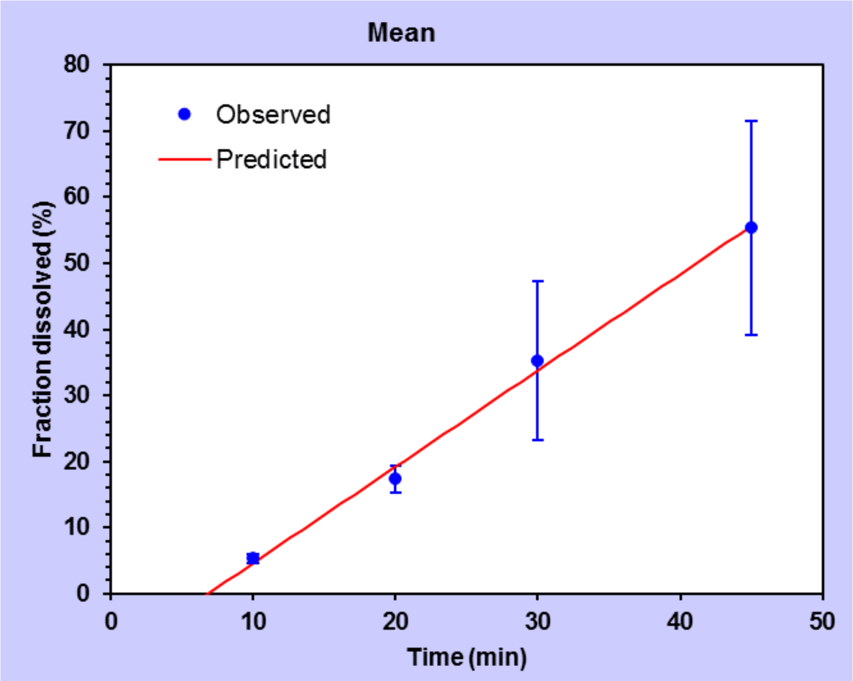

Supplementary materials – Model fitting summary – Parteck® M 100 (modelling up to app. 60 % of carvedilol released)

Graphical abstract of model fit presented as the fraction % of released carvedilol per tested tablet:

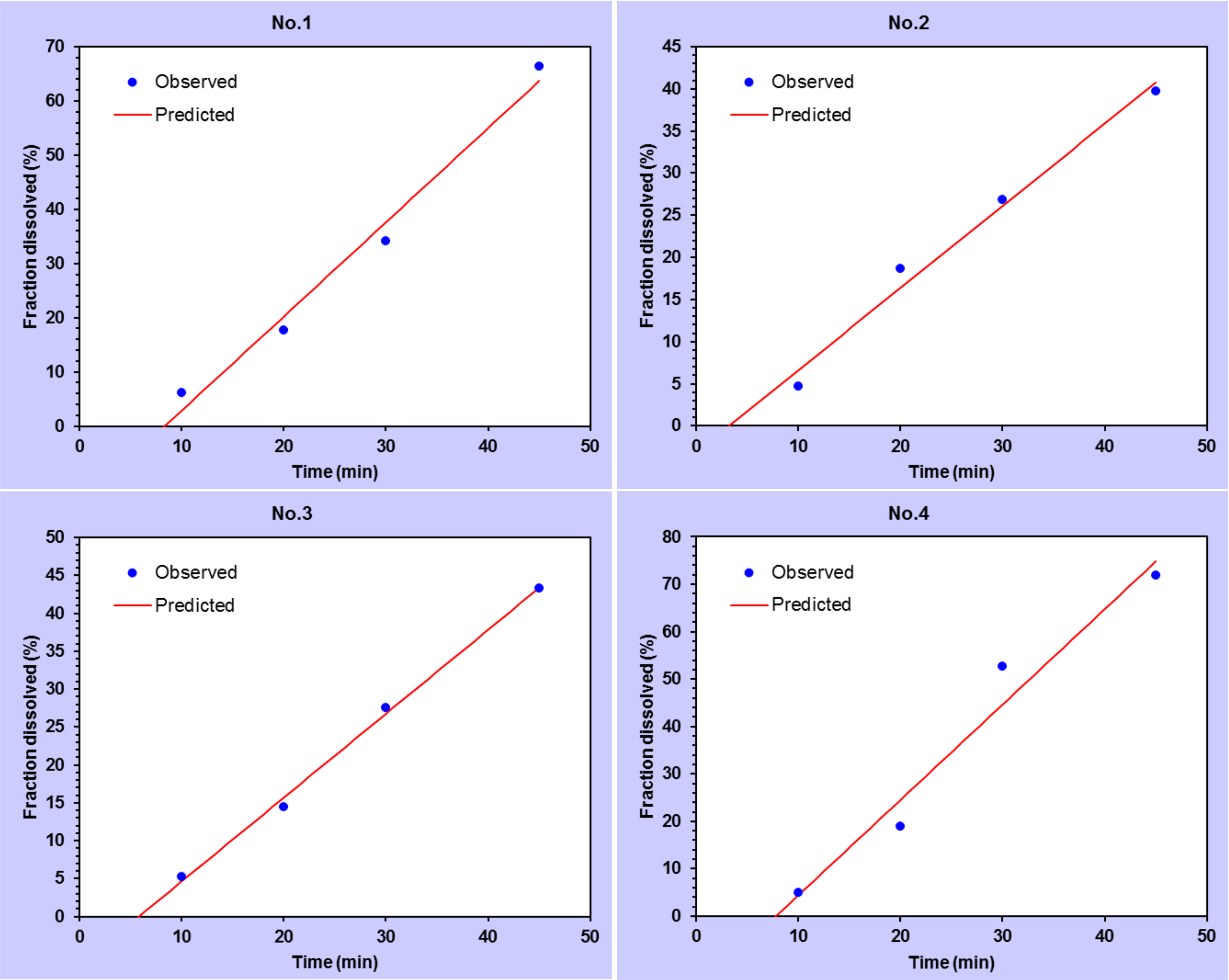

Supplementary materials – Model fitting summary – Parteck® M 100 (modelling up to app. 60 % of carvedilol released)

Model: **Zero–order with F_0**

Model equation: $F = F_0 + k_0 \cdot t$

Fitted model parameters per tested tablet (N = 4) with statistics – mean, standard deviation (SD), and relative standard deviation expressed in % (RSD%) (output from DDSolver):

Parameter	No.1	No.2	No.3	No.4	Mean	SD	RSD(%)
k_0	1.739	0.975	1.106	2.011	1.458	0.497	34.117
F_0	-14.564	-3.144	-6.456	-15.696	-9.965	6.133	-61.545

Number of dissolution data points (N), degrees of freedom (df), and selected goodness of fit criteria – Pearson correlation coefficient (R), coefficient of determination (R^2), adjusted coefficient of determination ($R^2_{adjusted}$), and residual sum of squares (RSS) (manual calculation in MS Excel):

Parameter	No.1	No.2	No.3	No.4
N	4	4	4	4
df	2	2	2	2
R	0.990677239	0.992042875	0.998448029	0.98106963
R^2	0.981441392	0.984149067	0.996898467	0.962497618
$R^2_{adjusted}$	0.972162088	0.9762236	0.995347701	0.943746427
RSS	38.22456767	10.22989545	2.547077157	105.3852605

Graphical abstract of model fit presented as mean ± 1 SD of the fraction % of released carvedilol:

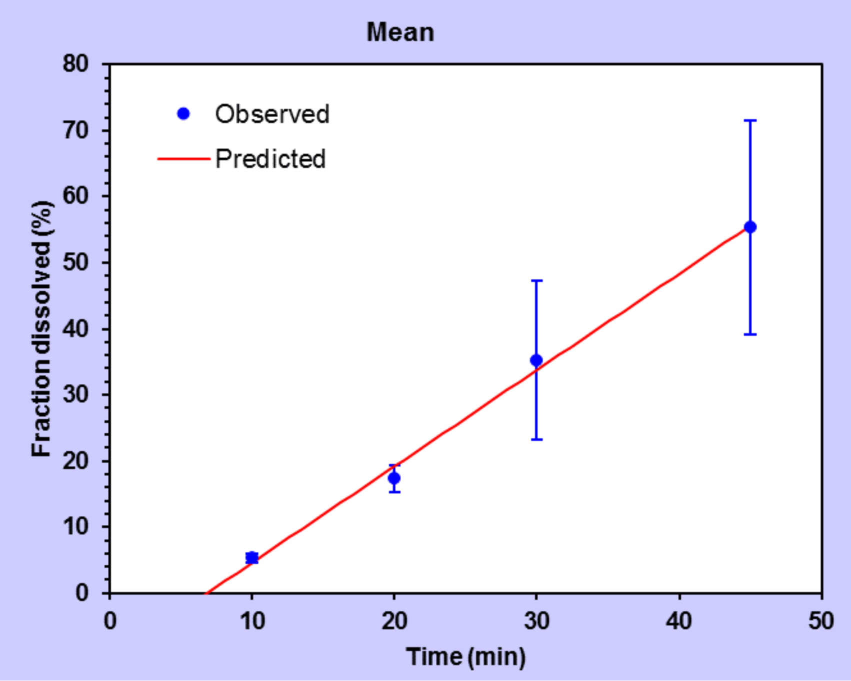

Supplementary materials – Model fitting summary – Parteck® M 100 (modelling up to app. 60 % of carvedilol released)

Graphical abstract of model fit presented as the fraction % of released carvedilol per tested tablet:

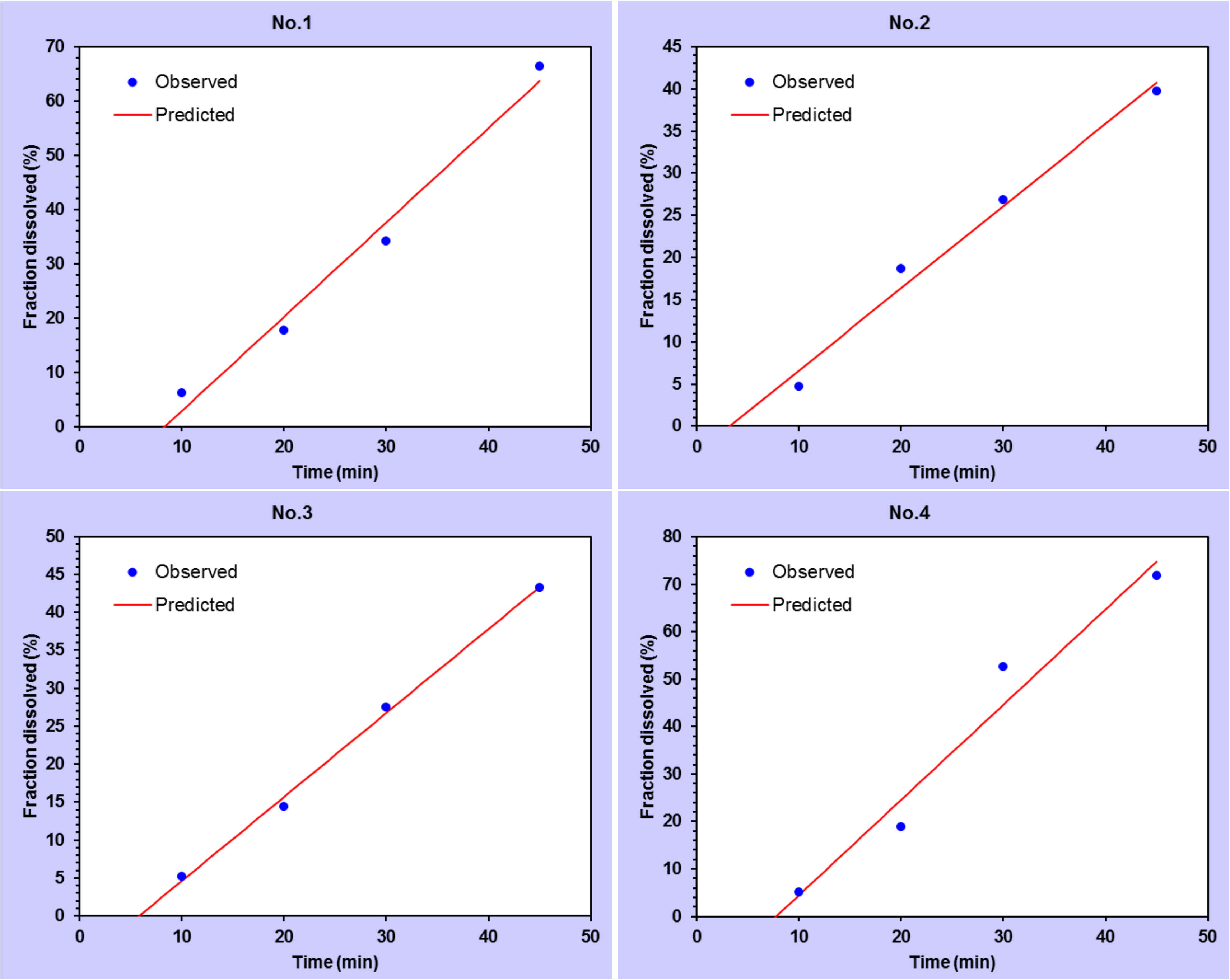

Supplementary materials – Model fitting summary – Parteck® M 100 (modelling up to app. 60 % of carvedilol released)

Model: **First–order**

Model equation: $F = 100 \cdot (1 - e^{-k_1 \cdot t})$

Fitted model parameters per tested tablet (N = 4) with statistics – mean, standard deviation (SD), and relative standard deviation expressed in % (RSD%) (output from DDSolver):

Parameter	No.1	No.2	No.3	No.4	Mean	SD	RSD(%)
k_1	0.014	0.011	0.011	0.025	0.015	0.006	41.990

Number of dissolution data points (N), degrees of freedom (df), and selected goodness of fit criteria – Pearson correlation coefficient (R), coefficient of determination (R^2), adjusted coefficient of determination ($R^2_{adjusted}$), and residual sum of squares (RSS) (manual calculation in MS Excel):

Parameter	No.1	No.2	No.3	No.4
N	4	4	4	4
df	3	3	3	3
R	0.977092749	0.996784682	0.995793402	0.980212627
R^2	0.95471024	0.993579702	0.9916045	0.960816794
$R^2_{adjusted}$	0.95471024	0.993579702	0.9916045	0.960816794
RSS	455.5216217	32.79822271	77.75772378	703.3389858

Graphical abstract of model fit presented as mean ± 1 SD of the fraction % of released carvedilol:

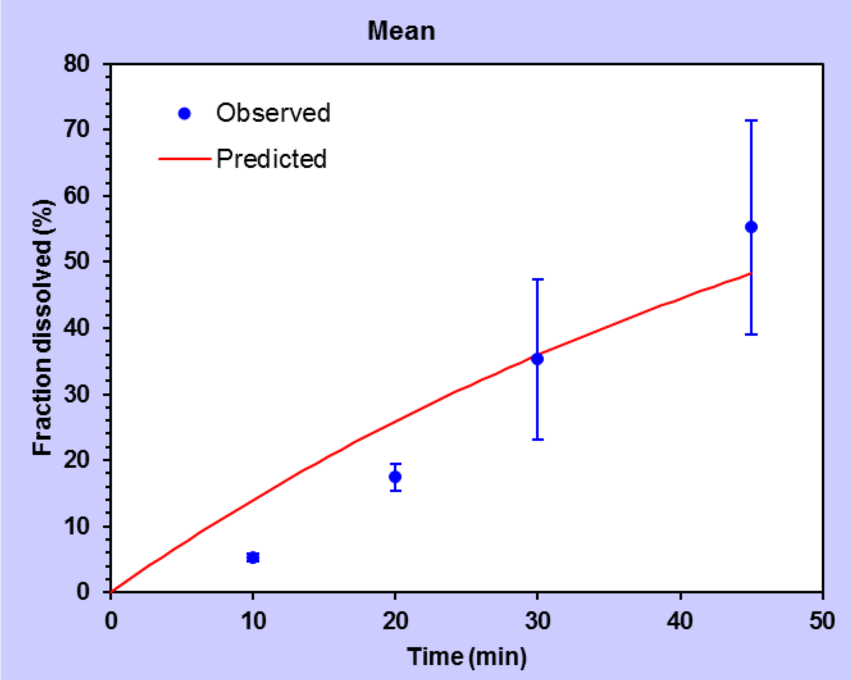

Supplementary materials – Model fitting summary – Parteck® M 100 (modelling up to app. 60 % of carvedilol released)

Graphical abstract of model fit presented as the fraction % of released carvedilol per tested tablet:

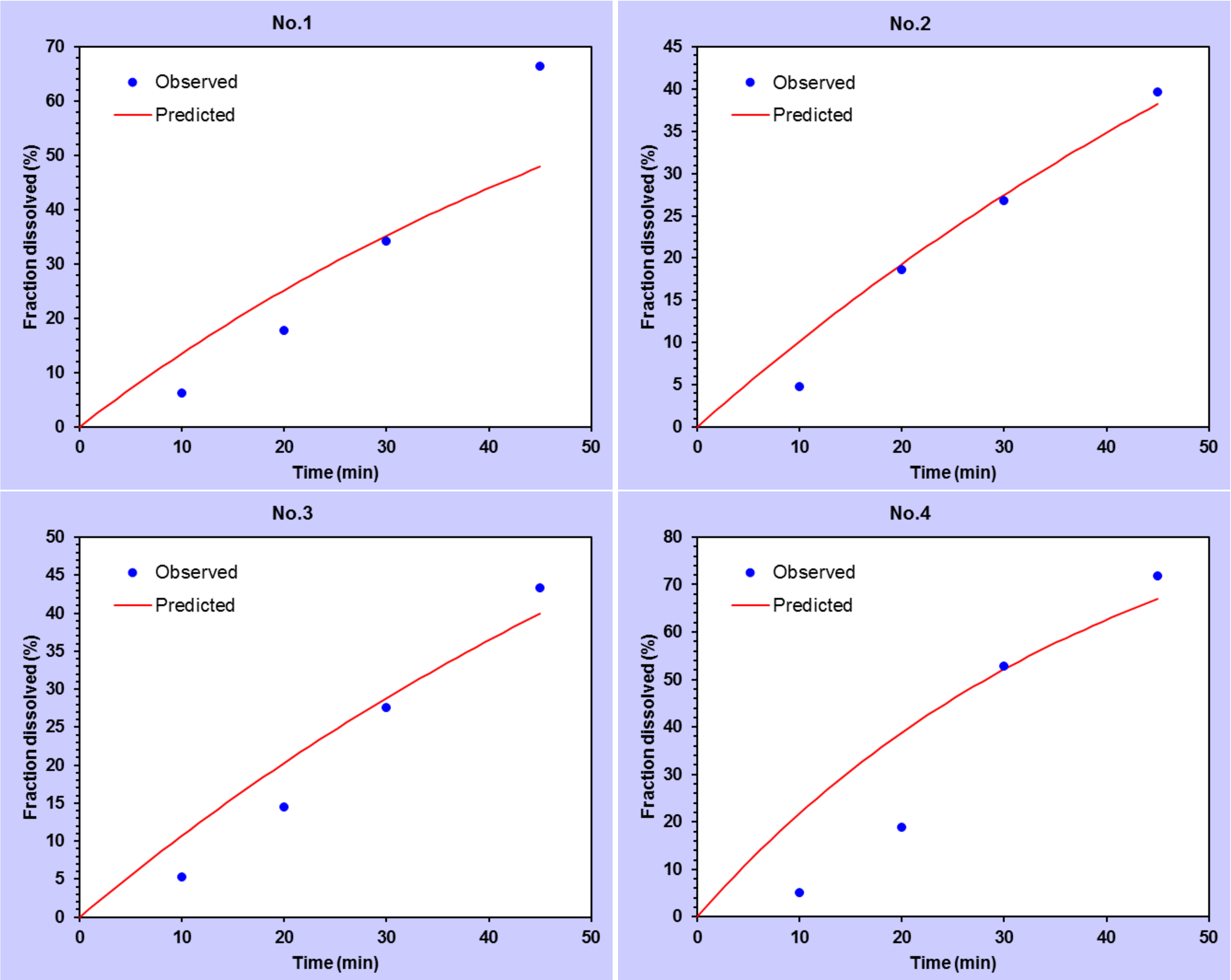

Supplementary materials – Model fitting summary – Parteck® M 100 (modelling up to app. 60 % of carvedilol released)

Model: **First–order with T_{lag}**

Model equation: $F = 100 \cdot \left[1 - e^{-k_1 \cdot (t - T_{lag})}\right]$

Fitted model parameters per tested tablet (N = 4) with statistics – mean, standard deviation (SD), and relative standard deviation expressed in % (RSD%) (output from DDSolver):

Parameter	No.1	No.2	No.3	No.4	Mean	SD	RSD(%)
k_1	0.030	0.013	0.015	0.037	0.023	0.011	48.793
T_{lag}	11.326	5.373	7.899	10.669	8.817	2.734	31.011

Number of dissolution data points (N), degrees of freedom (df), and selected goodness of fit criteria – Pearson correlation coefficient (R), coefficient of determination (R^2), adjusted coefficient of determination ($R^2_{adjusted}$), and residual sum of squares (RSS) (manual calculation in MS Excel):

Parameter	No.1	No.2	No.3	No.4
N	4	4	4	4
df	2	2	2	2
R	0.95699179	0.99734993	0.994132837	0.972999607
R^2	0.915833286	0.994706883	0.988300098	0.946728235
$R^2_{adjusted}$	0.873749929	0.992060325	0.982450147	0.920092353
RSS	210.6512667	3.462698421	9.97304072	159.6728842

Graphical abstract of model fit presented as mean ± 1 SD of the fraction % of released carvedilol:

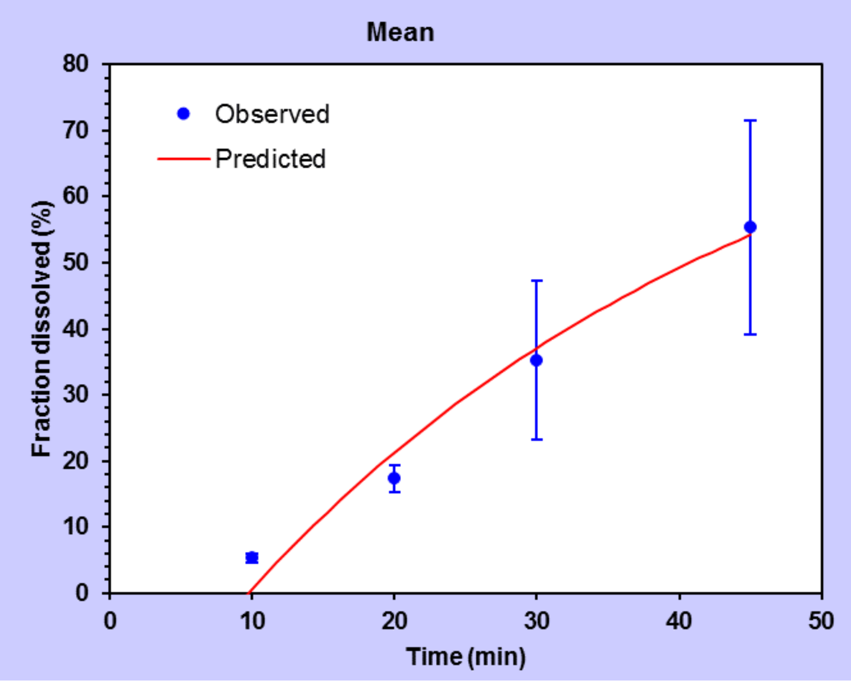

Supplementary materials – Model fitting summary – Parteck® M 100 (modelling up to app. 60 % of carvedilol released)

Graphical abstract of model fit presented as the fraction % of released carvedilol per tested tablet:

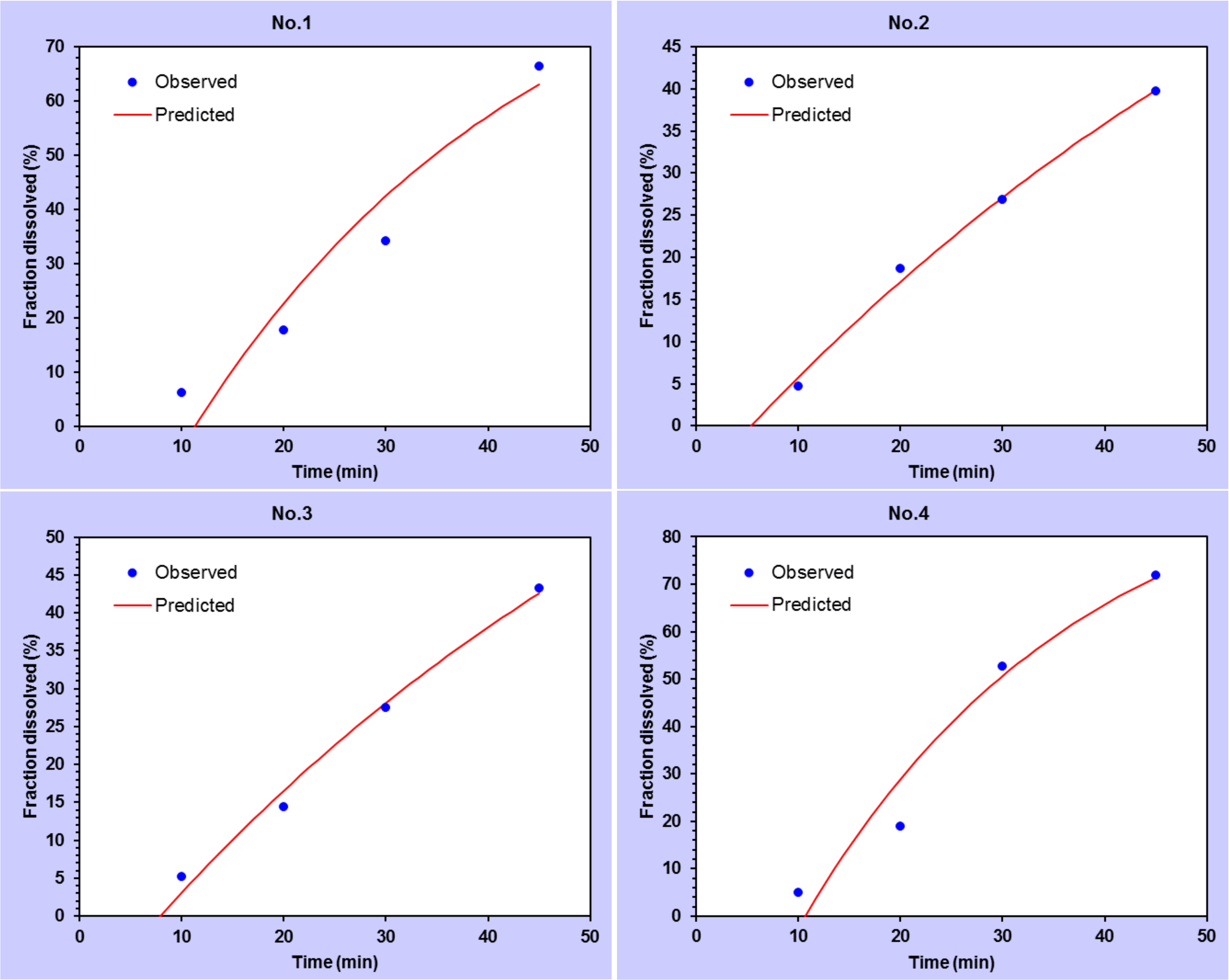

Supplementary materials – Model fitting summary – Parteck® M 100 (modelling up to app. 60 % of carvedilol released)

Model: **First–order with F_{max}**

Model equation: $F = F_{max} \cdot (1 - e^{-k_1 \cdot t})$

Fitted model parameters per tested tablet (N = 4) with statistics – mean, standard deviation (SD), and relative standard deviation expressed in % (RSD%) (output from DDSolver):

Parameter	No.1	No.2	No.3	No.4	Mean	SD	RSD(%)
k_1	0.072	0.053	0.051	0.052	0.057	0.010	17.455
F_{max}	34.876	41.656	45.433	75.416	49.345	17.921	36.317

Number of dissolution data points (N), degrees of freedom (df), and selected goodness of fit criteria – Pearson correlation coefficient (R), coefficient of determination (R^2), adjusted coefficient of determination ($R^2_{adjusted}$), and residual sum of squares (RSS) (manual calculation in MS Excel):

Parameter	No.1	No.2	No.3	No.4
N	4	4	4	4
df	2	2	2	2
R	0.881903708	0.986675392	0.959527298	0.957816312
R^2	0.777754151	0.973528329	0.920692636	0.917412087
$R^2_{adjusted}$	0.666631226	0.960292493	0.881038954	0.876118131
RSS	1311.952513	270.8623396	448.3013749	1630.236368

Graphical abstract of model fit presented as mean ± 1 SD of the fraction % of released carvedilol:

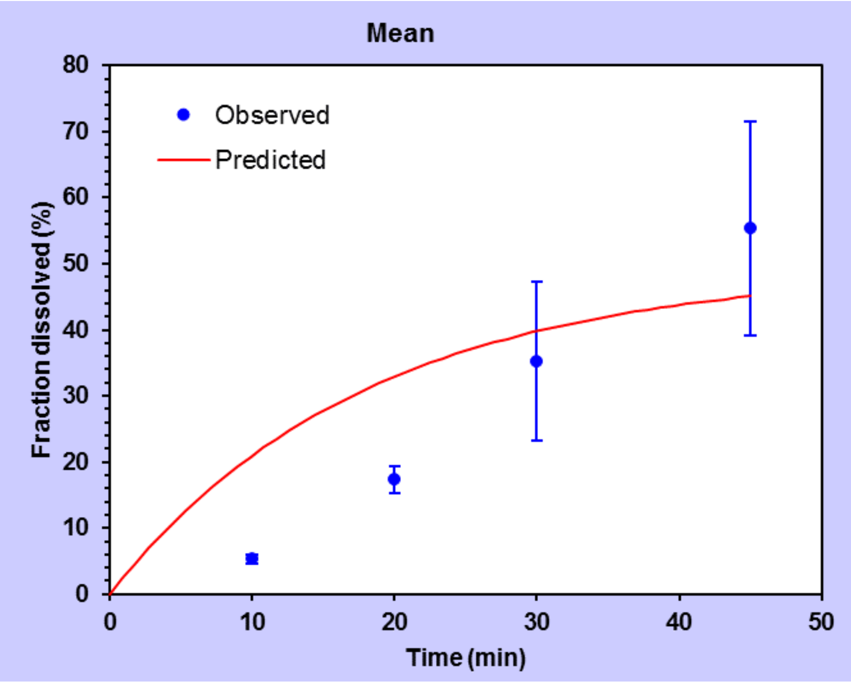

Supplementary materials – Model fitting summary – Parteck® M 100 (modelling up to app. 60 % of carvedilol released)

Graphical abstract of model fit presented as the fraction % of released carvedilol per tested tablet:

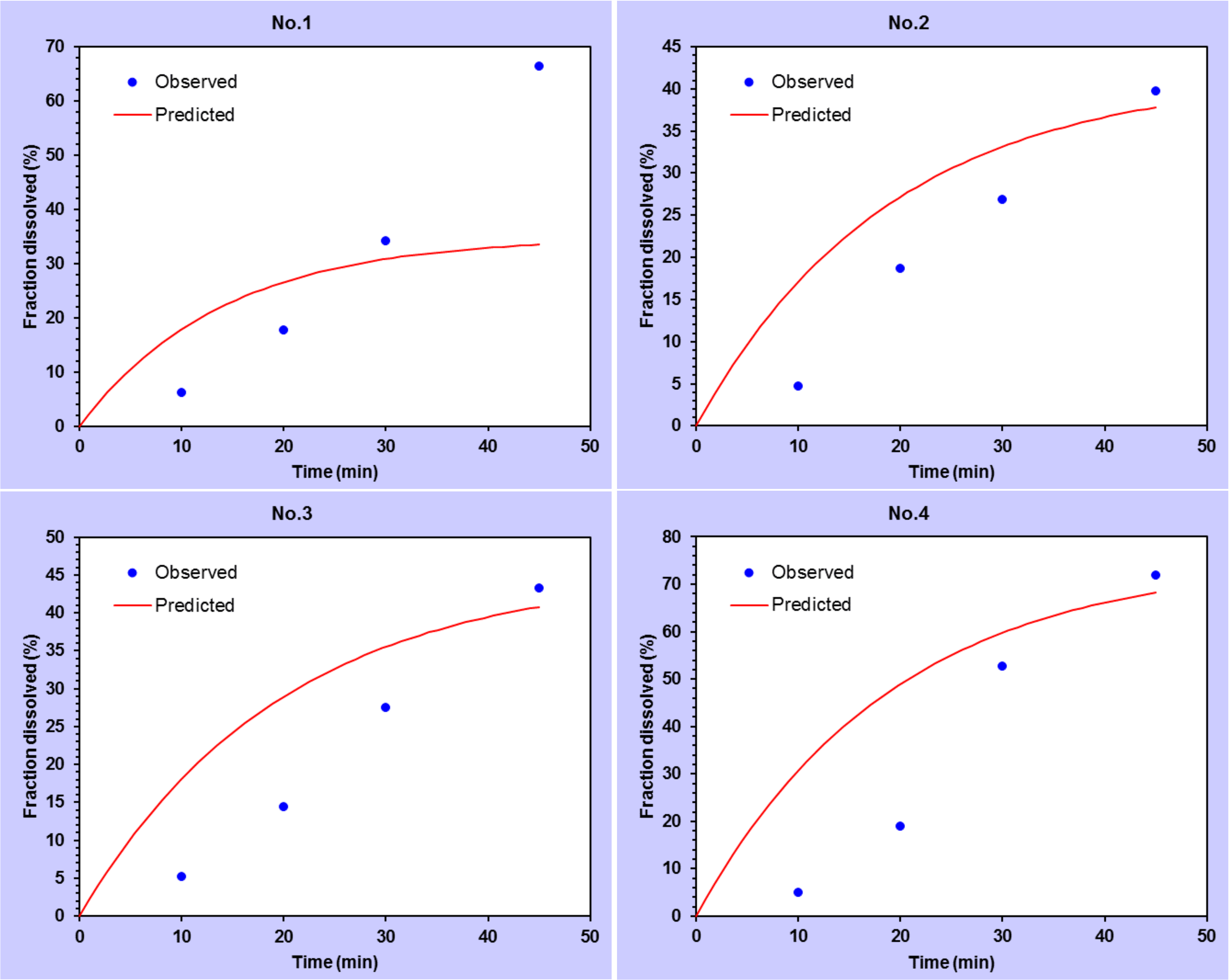

Supplementary materials – Model fitting summary – Parteck® M 100 (modelling up to app. 60 % of carvedilol released)

Model: **First–order with T_{lag} and F_{max}**

Model equation: $F = F_{max} \cdot \left[1 - e^{-k_1 \cdot (t - T_{lag})}\right]$

Fitted model parameters per tested tablet (N = 4) with statistics – mean, standard deviation (SD), and relative standard deviation expressed in % (RSD%) (output from DDSolver):

Parameter	No.1	No.2	No.3	No.4	Mean	SD	RSD(%)
k_1	0.063	0.083	0.126	0.132	0.101	0.033	33.054
T_{lag}	8.209	11.783	10.781	10.950	10.431	1.545	14.810
F_{max}	63.940	41.656	30.289	50.278	46.541	14.197	30.505

Number of dissolution data points (N), degrees of freedom (df), and selected goodness of fit criteria – Pearson correlation coefficient (R), coefficient of determination (R^2), adjusted coefficient of determination ($R^2_{adjusted}$), and residual sum of squares (RSS) (manual calculation in MS Excel):

Parameter	No.1	No.2	No.3	No.4
N	4	4	4	4
df	1	1	1	1
R	0.898323522	0.960796521	0.849315447	0.849766072
R^2	0.806985149	0.923129955	0.721336728	0.722102378
$R^2_{adjusted}$	0.420955448	0.769389866	0.164010183	0.166307134
RSS	519.8075046	164.1512727	289.8803083	927.3253615

Graphical abstract of model fit presented as mean ± 1 SD of the fraction % of released carvedilol:

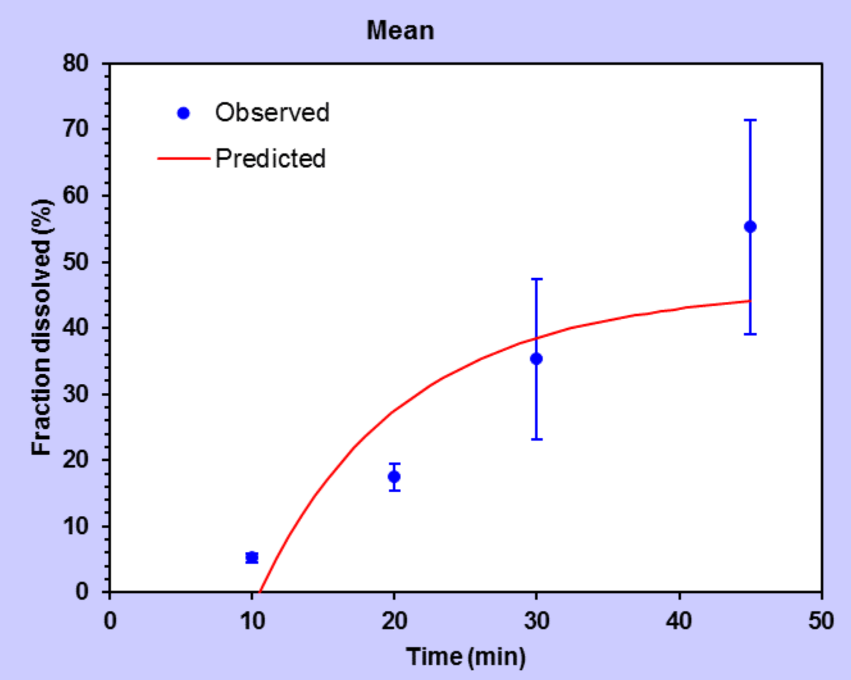

Supplementary materials – Model fitting summary – Parteck® M 100 (modelling up to app. 60 % of carvedilol released)

Graphical abstract of model fit presented as the fraction % of released carvedilol per tested tablet:

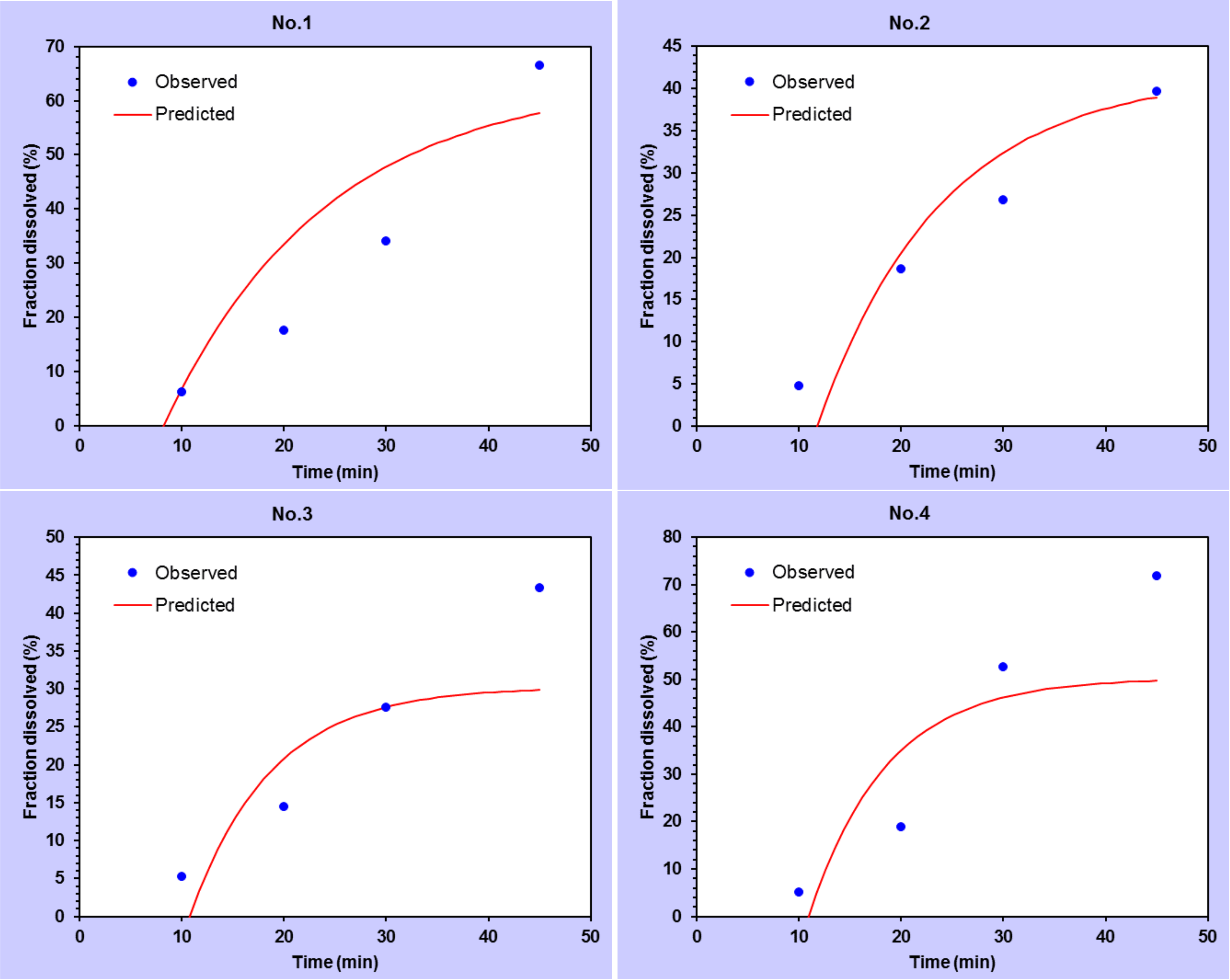

Supplementary materials – Model fitting summary – Parteck® M 100 (modelling up to app. 60 % of carvedilol released)

Model: **Higuchi**

Model equation: $F = k_H \cdot t^{0.5}$

Fitted model parameters per tested tablet (N = 4) with statistics – mean, standard deviation (SD), and relative standard deviation expressed in % (RSD%) (output from DDSolver):

Parameter	No.1	No.2	No.3	No.4	Mean	SD	RSD(%)
k_H	6.958	4.865	4.968	8.291	6.271	1.656	26.407

Number of dissolution data points (N), degrees of freedom (df), and selected goodness of fit criteria – Pearson correlation coefficient (R), coefficient of determination (R^2), adjusted coefficient of determination ($R^2_{adjusted}$), and residual sum of squares (RSS) (manual calculation in MS Excel):

Parameter	No.1	No.2	No.3	No.4
N	4	4	4	4
df	3	3	3	3
R	0.969323867	0.998927578	0.990020335	0.979598118
R^2	0.939588759	0.997856306	0.980140264	0.959612472
$R^2_{adjusted}$	0.939588759	0.997856306	0.980140264	0.959612472
RSS	839.2538825	173.7156484	270.6160646	1096.313657

Graphical abstract of model fit presented as mean ± 1 SD of the fraction % of released carvedilol:

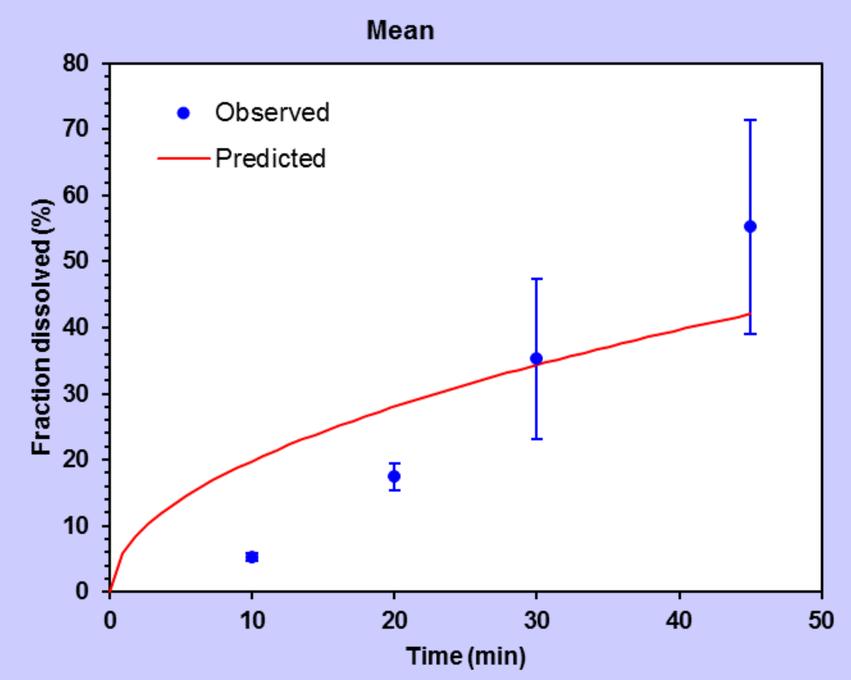

Supplementary materials – Model fitting summary – Parteck® M 100 (modelling up to app. 60 % of carvedilol released)

Graphical abstract of model fit presented as the fraction % of released carvedilol per tested tablet:

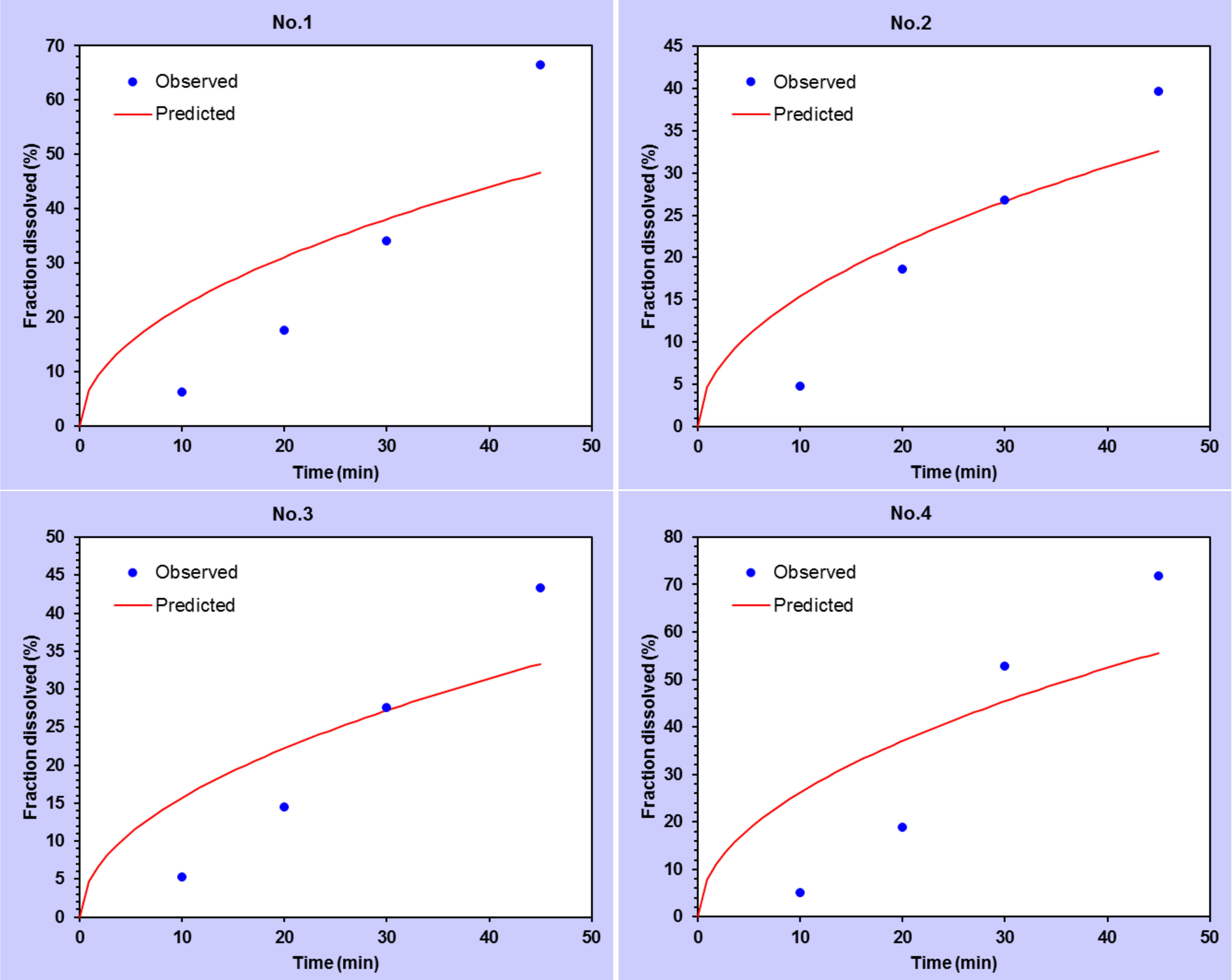

Supplementary materials – Model fitting summary – Parteck® M 100 (modelling up to app. 60 % of carvedilol released)

Model: **Higuchi with T_{lag}**

Model equation: $F = k_H \cdot (t - T_{lag})^{0.5}$

Fitted model parameters per tested tablet (N = 4) with statistics – mean, standard deviation (SD), and relative standard deviation expressed in % (RSD%) (output from DDSolver):

Parameter	No.1	No.2	No.3	No.4	Mean	SD	RSD(%)
k_H	12.649	6.662	8.278	12.500	10.022	3.021	30.140
T_{lag}	17.264	11.271	15.477	12.947	14.240	2.656	18.651

Number of dissolution data points (N), degrees of freedom (df), and selected goodness of fit criteria – Pearson correlation coefficient (R), coefficient of determination (R^2), adjusted coefficient of determination ($R^2_{adjusted}$), and residual sum of squares (RSS) (manual calculation in MS Excel):

Parameter	No.1	No.2	No.3	No.4
N	4	4	4	4
df	2	2	2	2
R	0.970995146	0.988303648	0.983776715	0.960567619
R^2	0.942831573	0.976744101	0.967816624	0.922690151
$R^2_{adjusted}$	0.914247359	0.965116152	0.951724937	0.884035226
RSS	171.951458	28.40960057	56.7053078	231.8218077

Graphical abstract of model fit presented as mean ± 1 SD of the fraction % of released carvedilol:

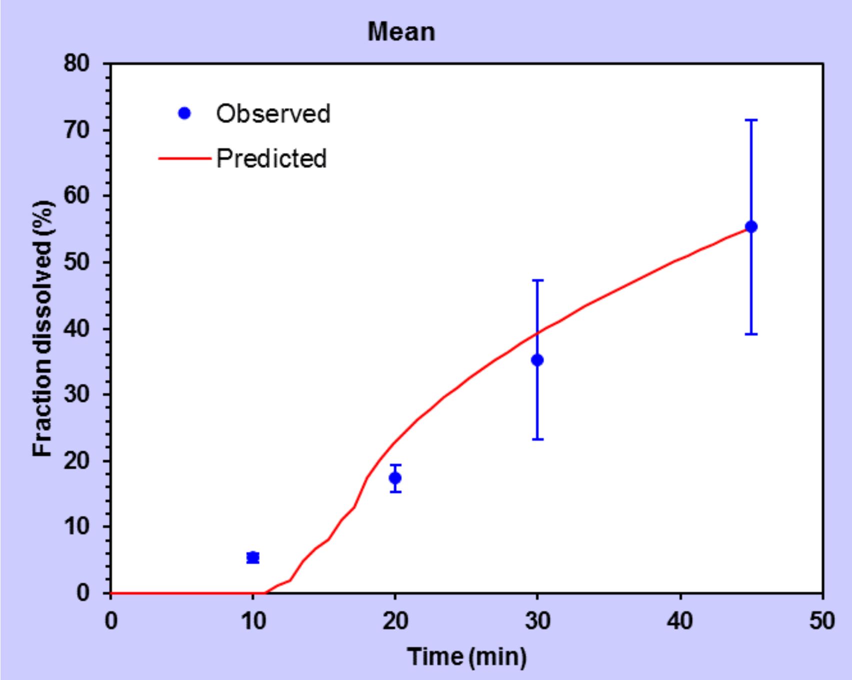

Supplementary materials – Model fitting summary – Parteck® M 100 (modelling up to app. 60 % of carvedilol released)

Graphical abstract of model fit presented as the fraction % of released carvedilol per tested tablet:

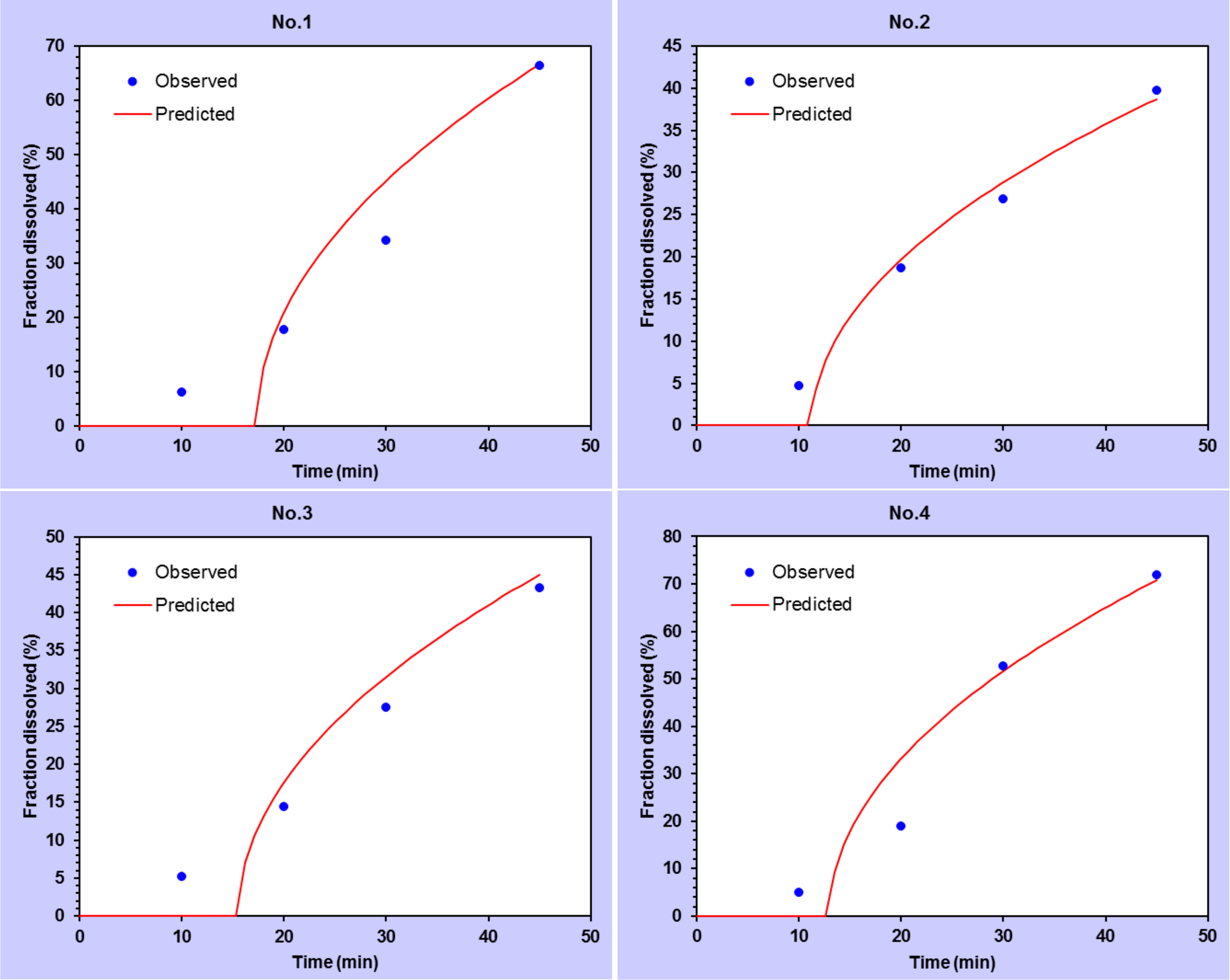

Supplementary materials – Model fitting summary – Parteck® M 100 (modelling up to app. 60 % of carvedilol released)

Model: **Higuchi with F_0**

Model equation: $F = F_0 + k_H \cdot t^{0.5}$

Fitted model parameters per tested tablet (N = 4) with statistics – mean, standard deviation (SD), and relative standard deviation expressed in % (RSD%) (output from DDSolver):

Parameter	No.1	No.2	No.3	No.4	Mean	SD	RSD(%)
k_H	16.878	9.736	10.885	19.923	14.356	4.856	33.827
F_0	-52.556	-25.805	-31.347	-61.625	-42.833	17.025	-39.747

Number of dissolution data points (N), degrees of freedom (df), and selected goodness of fit criteria – Pearson correlation coefficient (R), coefficient of determination (R^2), adjusted coefficient of determination ($R^2_{adjusted}$), and residual sum of squares (RSS) (manual calculation in MS Excel):

Parameter	No.1	No.2	No.3	No.4
N	4	4	4	4
df	2	2	2	2
R	0.969323867	0.998927578	0.990020335	0.979598118
R^2	0.939588759	0.997856306	0.980140264	0.959612472
$R^2_{adjusted}$	0.909383138	0.996784459	0.970210395	0.939418708
RSS	124.4270888	1.383499816	16.30944473	113.4927959

Graphical abstract of model fit presented as mean ± 1 SD of the fraction % of released carvedilol:

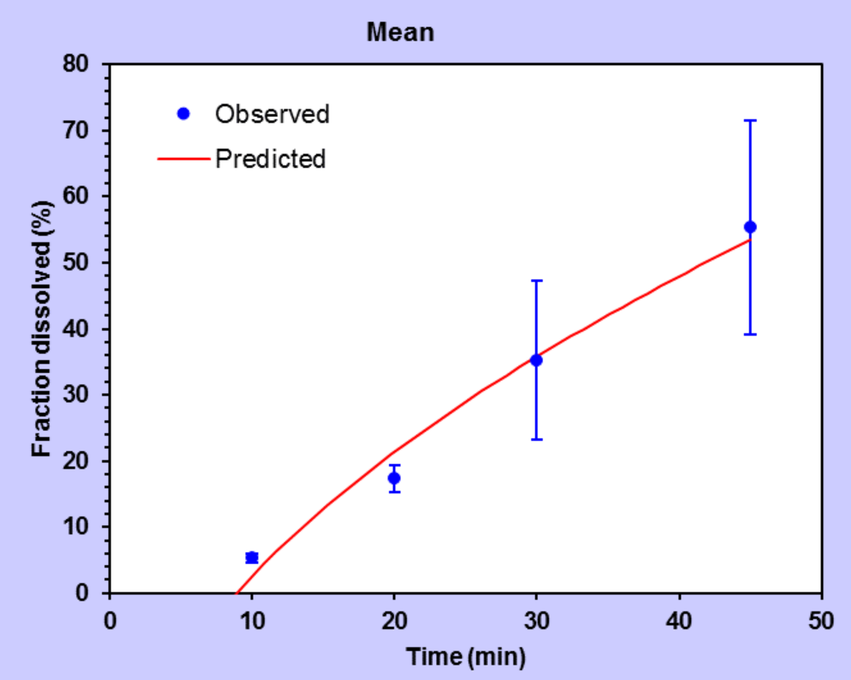

Supplementary materials – Model fitting summary – Parteck® M 100 (modelling up to app. 60 % of carvedilol released)

Graphical abstract of model fit presented as the fraction % of released carvedilol per tested tablet:

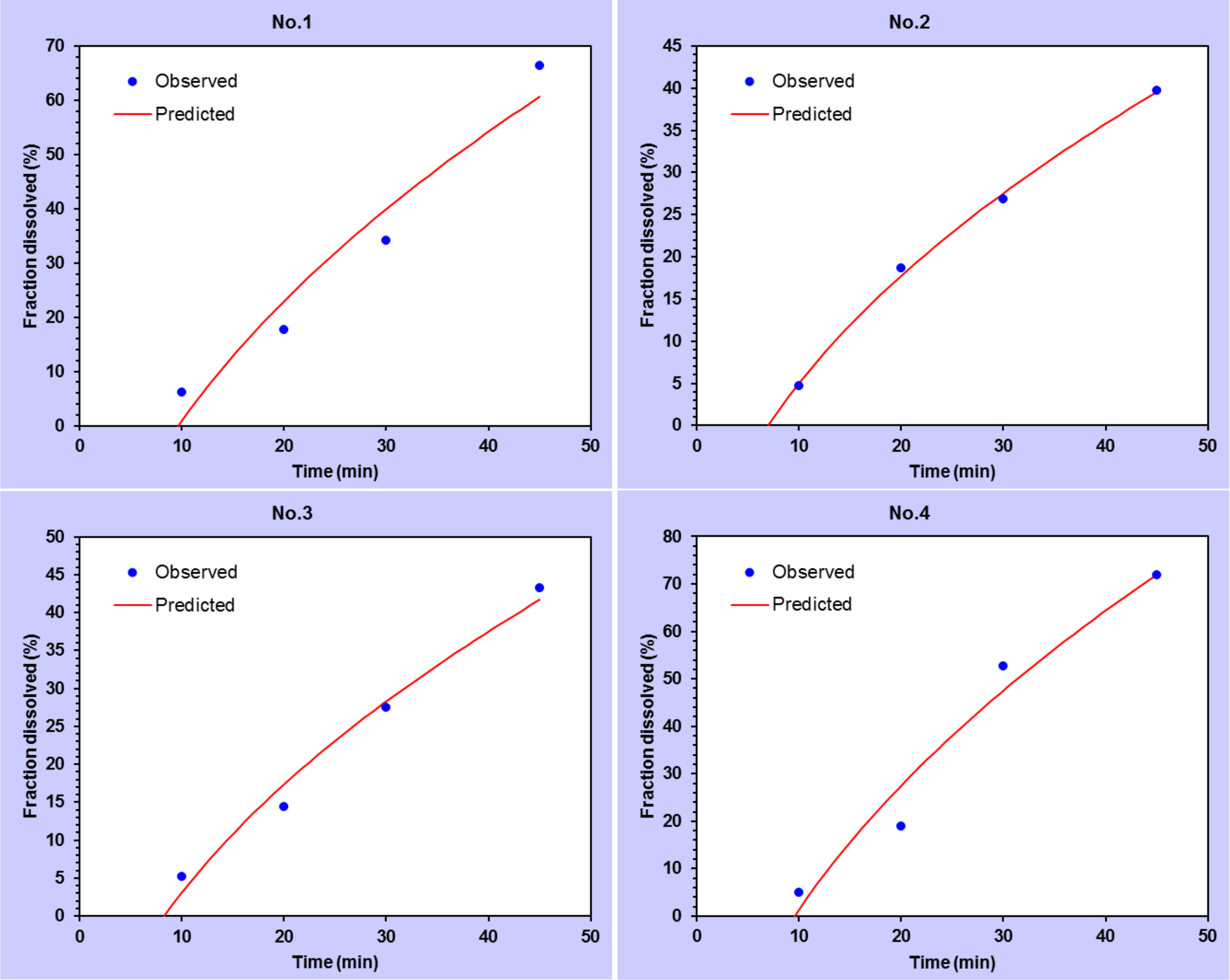

Supplementary materials – Model fitting summary – Parteck® M 100 (modelling up to app. 60 % of carvedilol released)

Model: **Korsmeyer–Peppas**

Model equation: $F = k_{KP} \cdot t^n$

Fitted model parameters per tested tablet (N = 4) with statistics – mean, standard deviation (SD), and relative standard deviation expressed in % (RSD%) (output from DDSolver):

Parameter	No.1	No.2	No.3	No.4	Mean	SD	RSD(%)
k_{KP}	0.162	0.279	0.197	0.075	0.178	0.085	47.553
n	1.576	1.329	1.431	1.852	1.547	0.227	14.698

Number of dissolution data points (N), degrees of freedom (df), and selected goodness of fit criteria – Pearson correlation coefficient (R), coefficient of determination (R^2), adjusted coefficient of determination ($R^2_{adjusted}$), and residual sum of squares (RSS) (manual calculation in MS Excel):

Parameter	No.1	No.2	No.3	No.4
N	4	4	4	4
df	2	2	2	2
R	0.999790777	0.981984029	0.995840642	0.956854158
R^2	0.999581598	0.964292634	0.991698584	0.91556988
$R^2_{adjusted}$	0.999372397	0.946438951	0.987547876	0.873354819
RSS	2.15535453	34.12603681	9.531776819	349.4983917

Graphical abstract of model fit presented as mean ± 1 SD of the fraction % of released carvedilol:

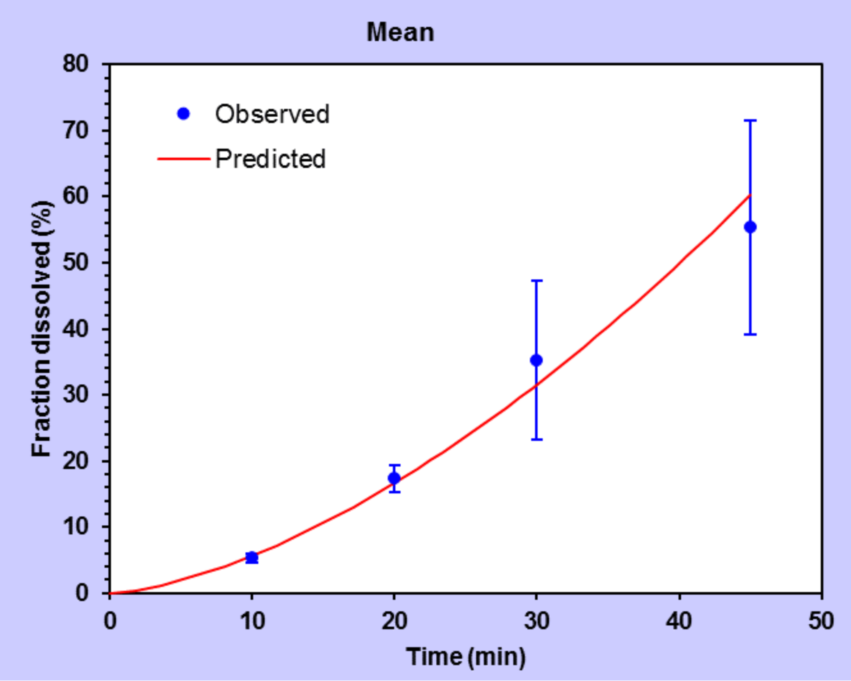

Supplementary materials – Model fitting summary – Parteck® M 100 (modelling up to app. 60 % of carvedilol released)

Graphical abstract of model fit presented as the fraction % of released carvedilol per tested tablet:

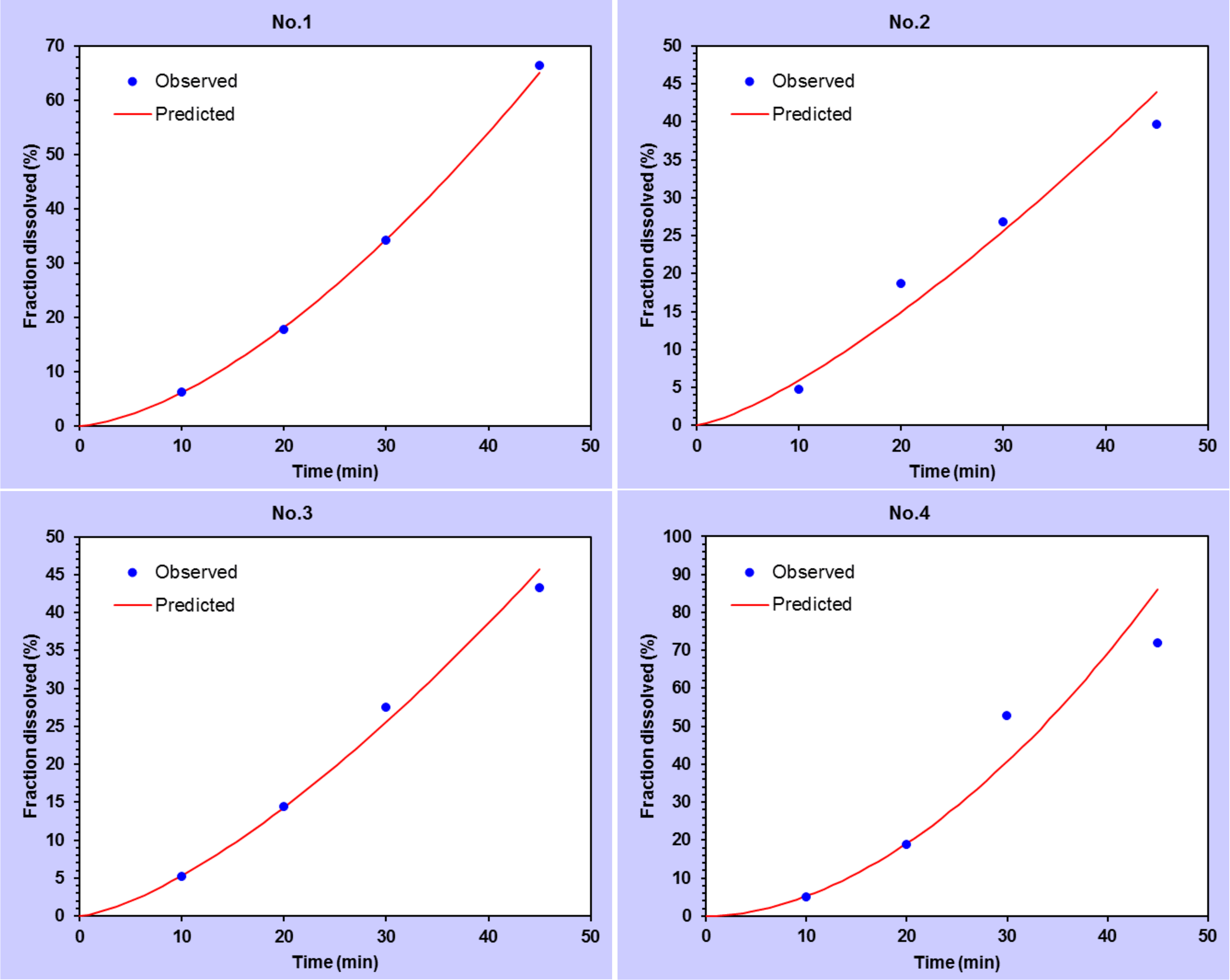

Supplementary materials – Model fitting summary – Parteck® M 100 (modelling up to app. 60 % of carvedilol released)

Model: **Korsmeyer–Peppas with T_{lag}**

Model equation: $F = k_{KP} \cdot \left(t - T_{lag}\right)^n$

Fitted model parameters per tested tablet (N = 4) with statistics – mean, standard deviation (SD), and relative standard deviation expressed in % (RSD%) (output from DDSolver):

Parameter	No.1	No.2	No.3	No.4	Mean	SD	RSD(%)
k_{KP}	0.754	0.700	0.693	0.376	0.631	0.172	27.262
n	1.216	1.115	1.115	1.448	1.223	0.157	12.819
T_{lag}	4.706	4.000	4.000	4.000	4.177	0.353	8.457

Number of dissolution data points (N), degrees of freedom (df), and selected goodness of fit criteria – Pearson correlation coefficient (R), coefficient of determination (R^2), adjusted coefficient of determination ($R^2_{adjusted}$), and residual sum of squares (RSS) (manual calculation in MS Excel):

Parameter	No.1	No.2	No.3	No.4
N	4	4	4	4
df	1	1	1	1
R	0.996850322	0.98815632	0.998520357	0.968913803
R^2	0.993710565	0.976452913	0.997042902	0.938793957
$R^2_{adjusted}$	0.981131696	0.92935874	0.991128707	0.816381872
RSS	28.84686377	29.33732134	2.454046592	205.5773

Graphical abstract of model fit presented as mean ± 1 SD of the fraction % of released carvedilol:

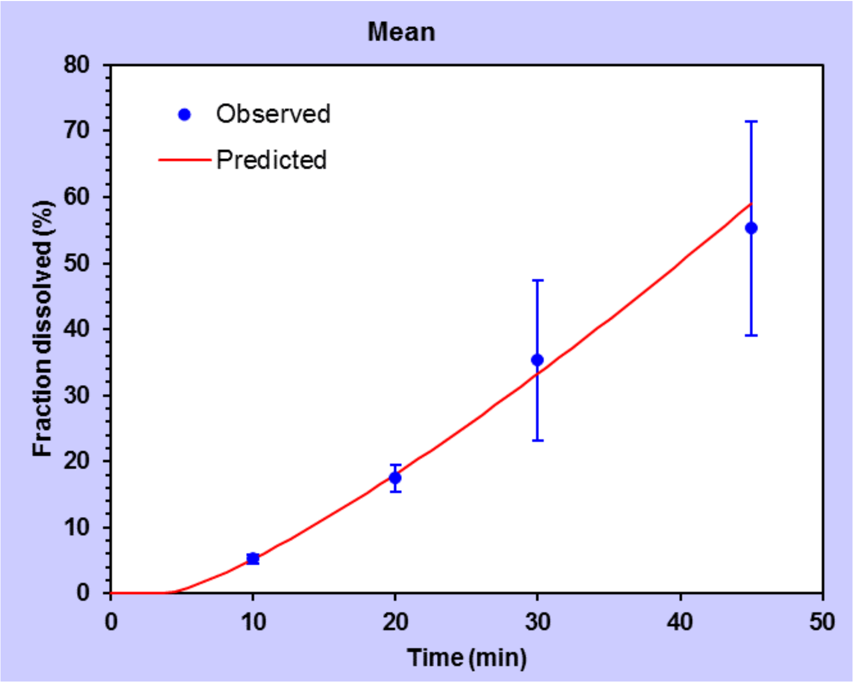

Supplementary materials – Model fitting summary – Parteck® M 100 (modelling up to app. 60 % of carvedilol released)

Graphical abstract of model fit presented as the fraction % of released carvedilol per tested tablet:

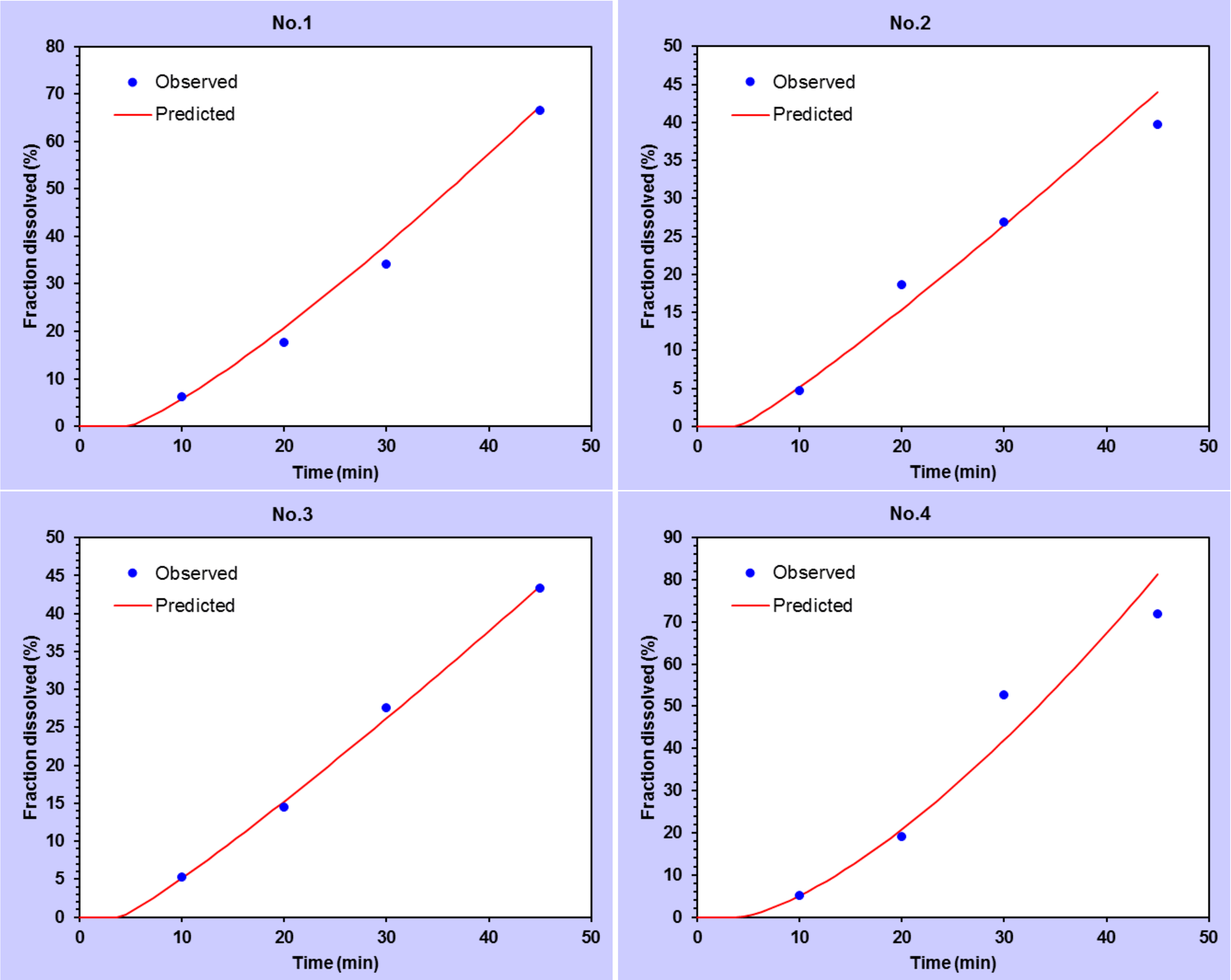

Supplementary materials – Model fitting summary – Parteck® M 100 (modelling up to app. 60 % of carvedilol released)

Model: **Korsmeyer–Peppas with F_0**

Model equation: $F = F_0 + k_{KP} \cdot t^n$

Fitted model parameters per tested tablet (N = 4) with statistics – mean, standard deviation (SD), and relative standard deviation expressed in % (RSD%) (output from DDSolver):

Parameter	No.1	No.2	No.3	No.4	Mean	SD	RSD(%)
k_{KP}	0.047	0.085	0.061	0.023	0.054	0.026	48.365
n	1.890	1.655	1.743	2.181	1.867	0.230	12.339
F_0	3.080	2.115	2.080	2.000	2.319	0.510	22.001

Number of dissolution data points (N), degrees of freedom (df), and selected goodness of fit criteria – Pearson correlation coefficient (R), coefficient of determination (R^2), adjusted coefficient of determination ($R^2_{adjusted}$), and residual sum of squares (RSS) (manual calculation in MS Excel):

Parameter	No.1	No.2	No.3	No.4
N	4	4	4	4
df	1	1	1	1
R	0.999366679	0.969055951	0.98965669	0.942439236
R^2	0.998733759	0.939069435	0.979420364	0.888191713
$R^2_{adjusted}$	0.996201278	0.817208306	0.938261091	0.664575139
RSS	4.578958502	95.80197104	34.27353906	611.3614423

Graphical abstract of model fit presented as mean ± 1 SD of the fraction % of released carvedilol:

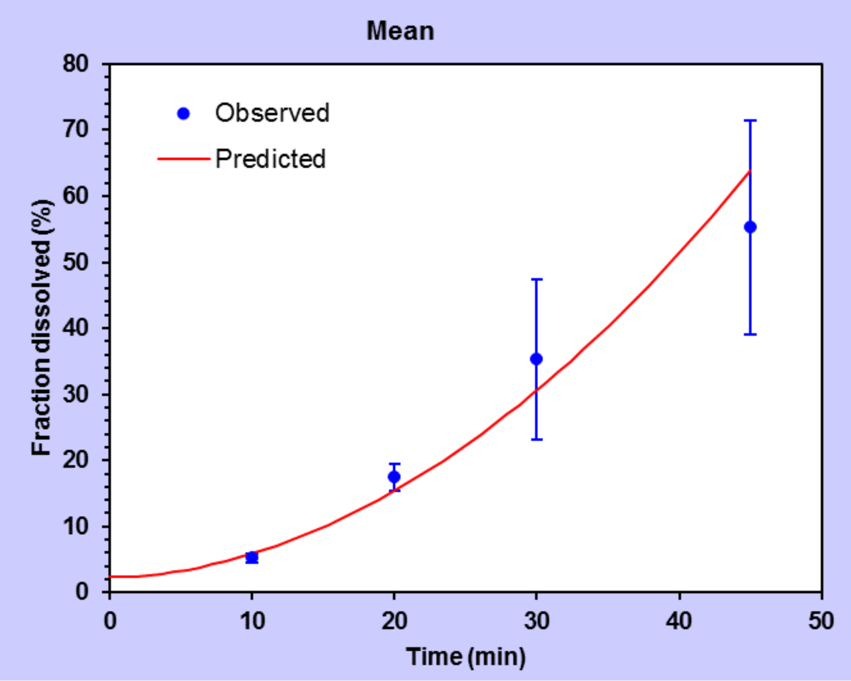

Supplementary materials – Model fitting summary – Parteck® M 100 (modelling up to app. 60 % of carvedilol released)

Graphical abstract of model fit presented as the fraction % of released carvedilol per tested tablet:

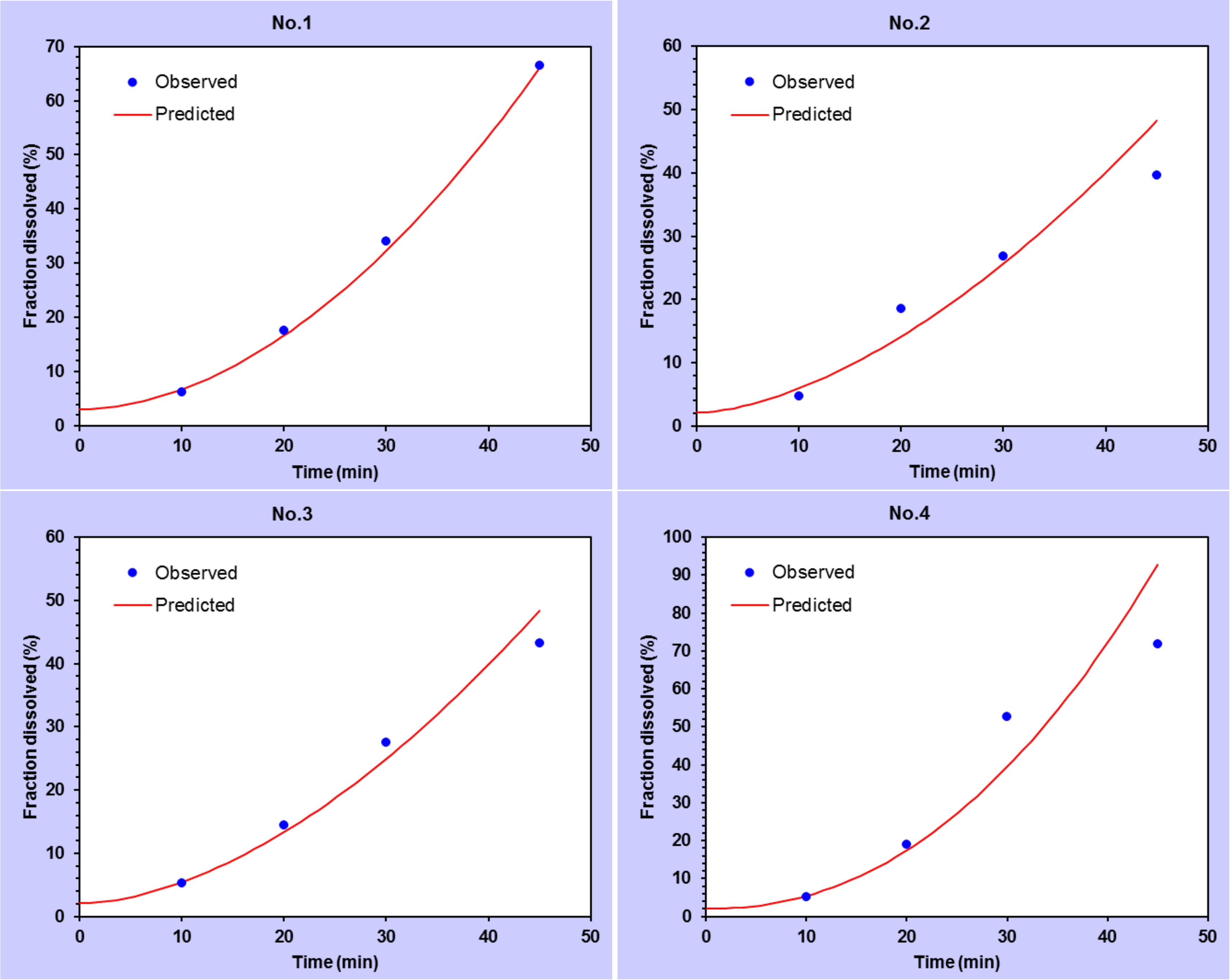

Supplementary materials – Model fitting summary – Parteck® M 100 (modelling up to app. 60 % of carvedilol released)

Model: **Hixson–Crowell**

Model equation: $F = 100 \cdot [1 - (1 - k_{HC} \cdot t)^3]$

Fitted model parameters per tested tablet (N = 4) with statistics – mean, standard deviation (SD), and relative standard deviation expressed in % (RSD%) (output from DDSolver):

Parameter	No.1	No.2	No.3	No.4	Mean	SD	RSD(%)
k_{HC}	0.006	0.003	0.003	0.007	0.005	0.002	35.578

Number of dissolution data points (N), degrees of freedom (df), and selected goodness of fit criteria – Pearson correlation coefficient (R), coefficient of determination (R^2), adjusted coefficient of determination ($R^2_{adjusted}$), and residual sum of squares (RSS) (manual calculation in MS Excel):

Parameter	No.1	No.2	No.3	No.4
N	4	4	4	4
df	3	3	3	3
R	0.978634506	0.99560511	0.997075835	0.98289067
R^2	0.957725497	0.991229535	0.99416022	0.96607407
$R^2_{adjusted}$	0.957725497	0.991229535	0.99416022	0.96607407
RSS	380.6785382	26.01939552	61.14765616	523.4121165

Graphical abstract of model fit presented as mean ± 1 SD of the fraction % of released carvedilol:

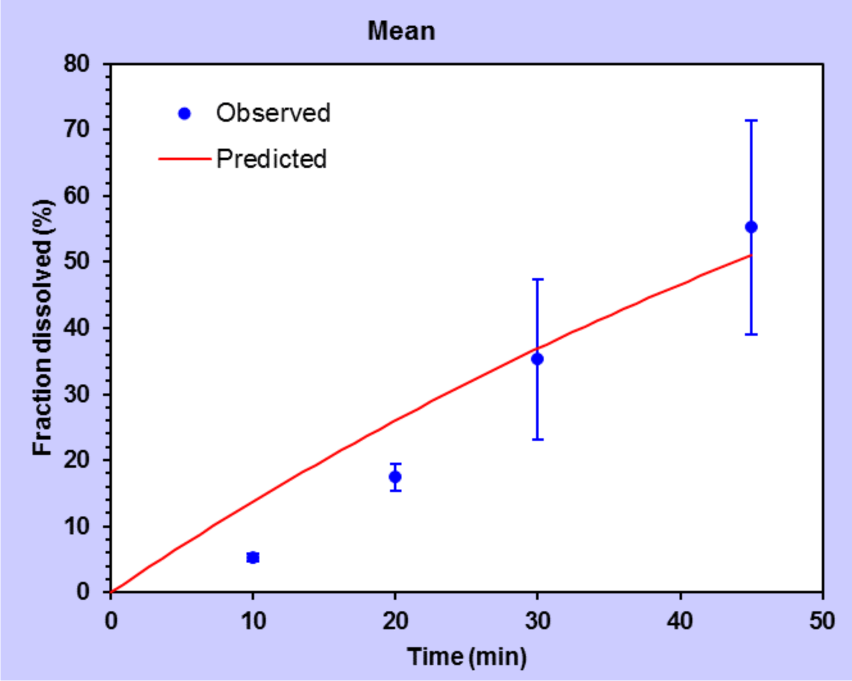

Supplementary materials – Model fitting summary – Parteck® M 100 (modelling up to app. 60 % of carvedilol released)

Graphical abstract of model fit presented as the fraction % of released carvedilol per tested tablet:

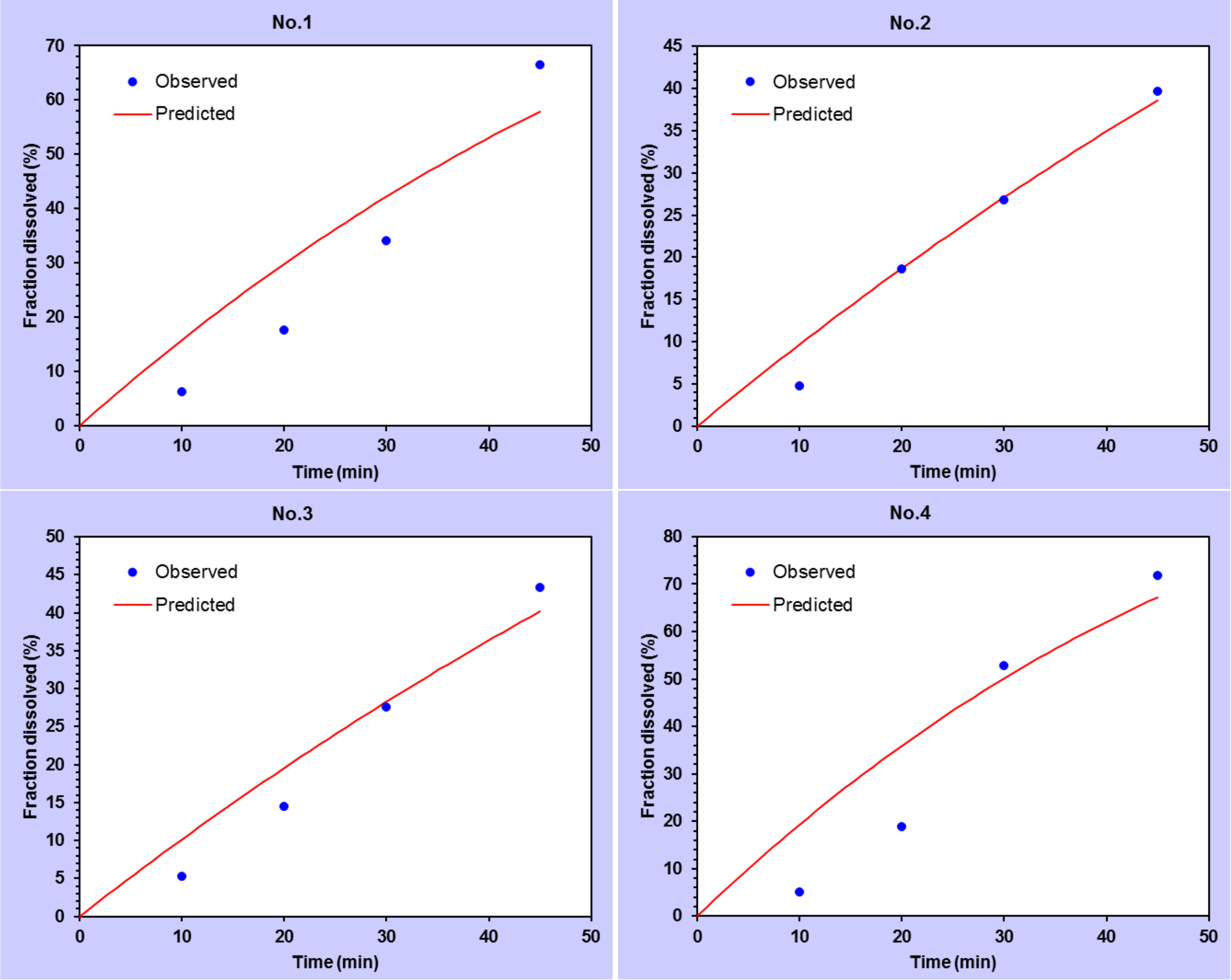

Supplementary materials – Model fitting summary – Parteck® M 100 (modelling up to app. 60 % of carvedilol released)

Model: **Hixson–Crowell with T_{lag}**

Model equation: $F = 100 \cdot \left\{1 - \left[1 - k_{HC} \cdot \left(t - T_{lag}\right)\right]^3\right\}$

Fitted model parameters per tested tablet (N = 4) with statistics – mean, standard deviation (SD), and relative standard deviation expressed in % (RSD%) (output from DDSolver):

Parameter	No.1	No.2	No.3	No.4	Mean	SD	RSD(%)
k_{HC}	0.008	0.004	0.004	0.010	0.007	0.003	43.545
T_{lag}	10.415	4.683	7.241	9.771	8.027	2.617	32.600

Number of dissolution data points (N), degrees of freedom (df), and selected goodness of fit criteria – Pearson correlation coefficient (R), coefficient of determination (R^2), adjusted coefficient of determination ($R^2_{adjusted}$), and residual sum of squares (RSS) (manual calculation in MS Excel):

Parameter	No.1	No.2	No.3	No.4
N	4	4	4	4
df	2	2	2	2
R	0.971764373	0.996045698	0.996382649	0.981198659
R^2	0.944325996	0.992107032	0.992778383	0.962750808
$R^2_{adjusted}$	0.916488993	0.988160548	0.989167574	0.944126212
RSS	125.0897973	5.144005344	6.008490979	105.3144133

Graphical abstract of model fit presented as mean ± 1 SD of the fraction % of released carvedilol:

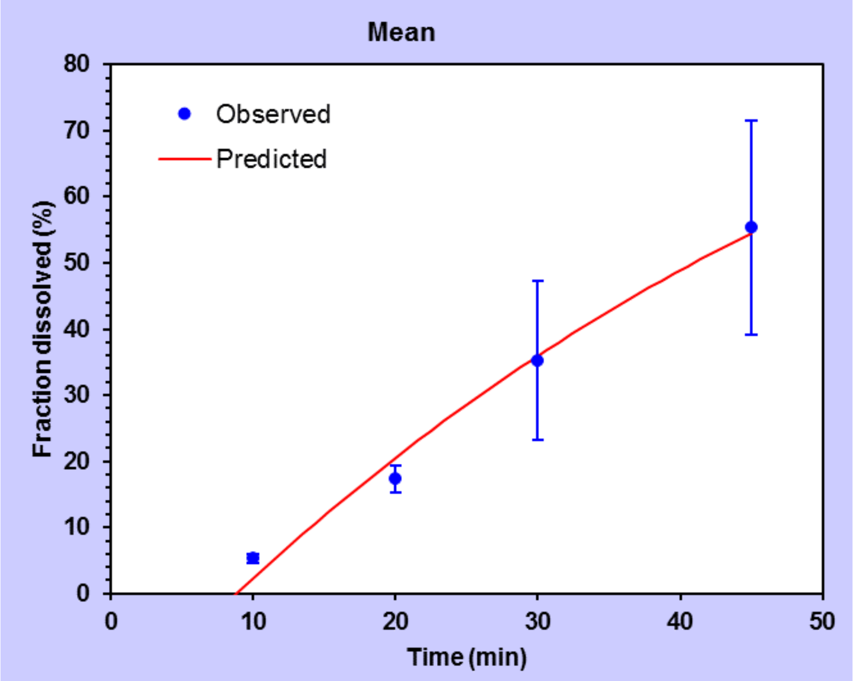

Supplementary materials – Model fitting summary – Parteck® M 100 (modelling up to app. 60 % of carvedilol released)

Graphical abstract of model fit presented as the fraction % of released carvedilol per tested tablet:

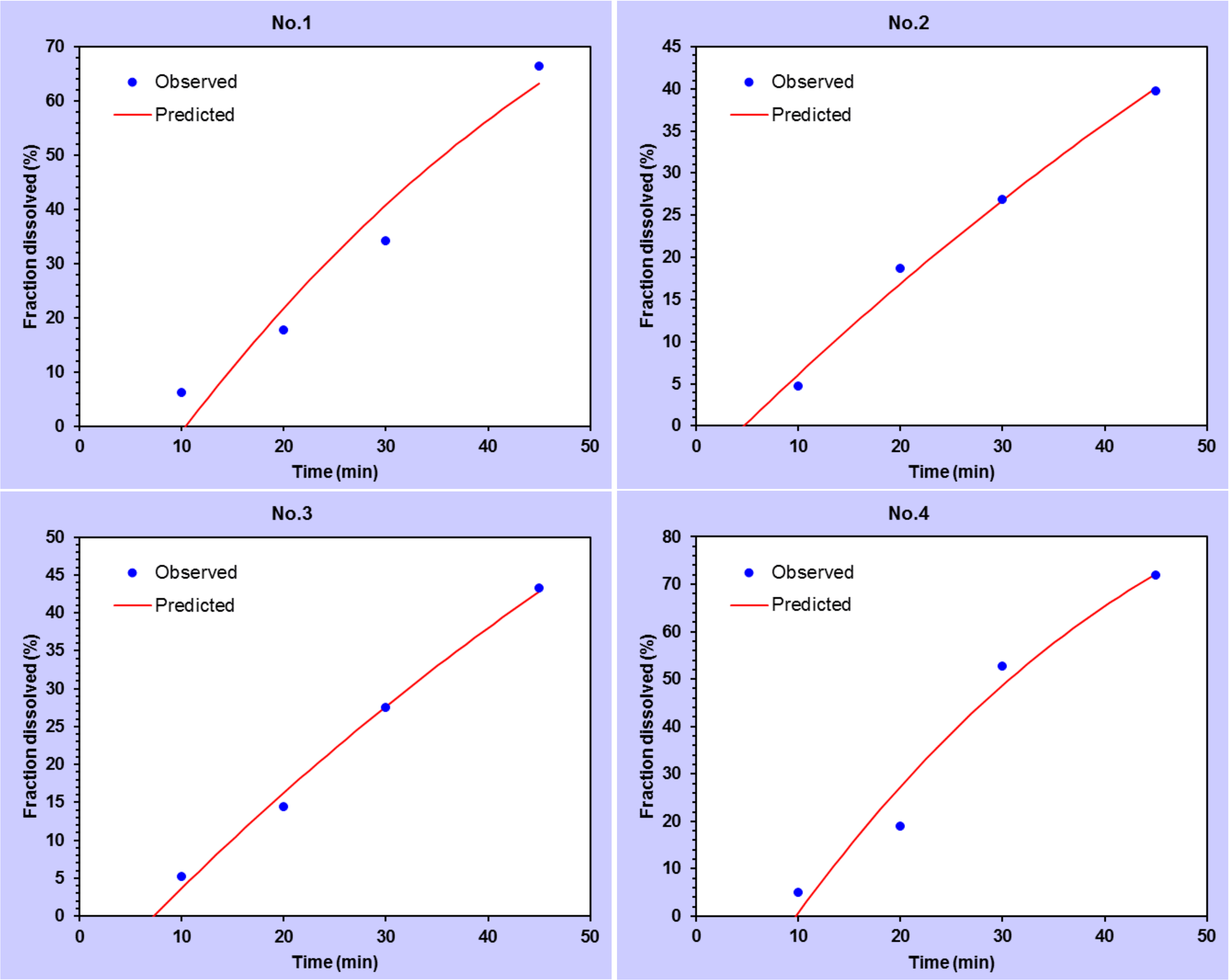

Supplementary materials – Model fitting summary – Parteck® M 100 (modelling up to app. 60 % of carvedilol released)

Model: **Hopfenberg**

Model equation: $F = 100 \cdot [1 - (1 - k_{HB} \cdot t)^n]$

Fitted model parameters per tested tablet (N = 4) with statistics – mean, standard deviation (SD), and relative standard deviation expressed in % (RSD%) (output from DDSolver):

Parameter	No.1	No.2	No.3	No.4	Mean	SD	RSD(%)
k_{HB}	0.013	0.009	0.009	0.015	0.012	0.003	27.294
n	1.000	1.000	1.000	1.000	1.000	0.000	0.000

Number of dissolution data points (N), degrees of freedom (df), and selected goodness of fit criteria – Pearson correlation coefficient (R), coefficient of determination (R^2), adjusted coefficient of determination ($R^2_{adjusted}$), and residual sum of squares (RSS) (manual calculation in MS Excel):

Parameter	No.1	No.2	No.3	No.4
N	4	4	4	4
df	2	2	2	2
R	0.990677239	0.992042875	0.998448029	0.98106963
R^2	0.981441392	0.984149067	0.996898467	0.962497618
$R^2_{adjusted}$	0.972162088	0.9762236	0.995347701	0.943746427
RSS	203.8826469	17.94862361	35.09966412	297.8034846

Graphical abstract of model fit presented as mean ± 1 SD of the fraction % of released carvedilol:

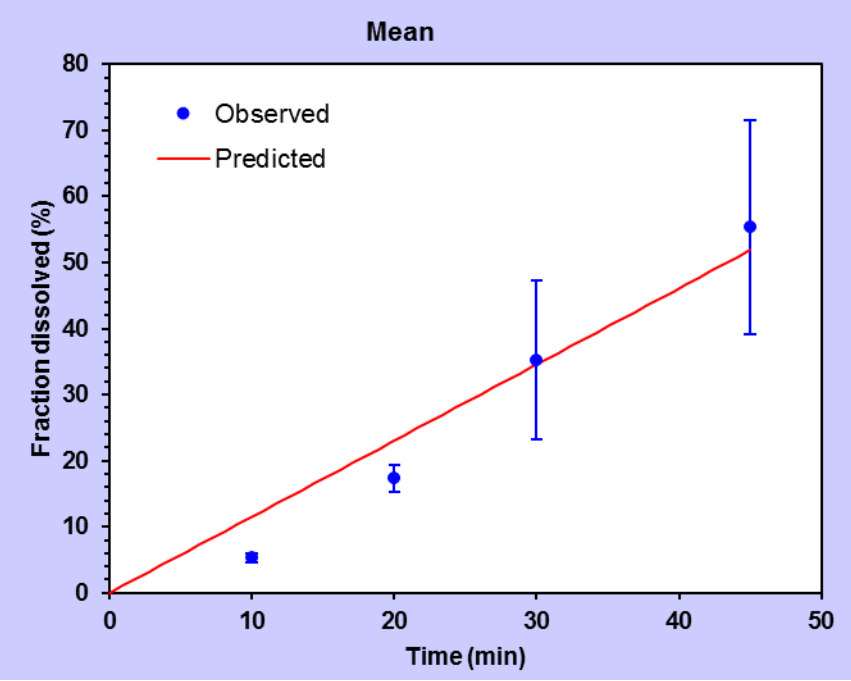

Supplementary materials – Model fitting summary – Parteck® M 100 (modelling up to app. 60 % of carvedilol released)

Graphical abstract of model fit presented as the fraction % of released carvedilol per tested tablet:

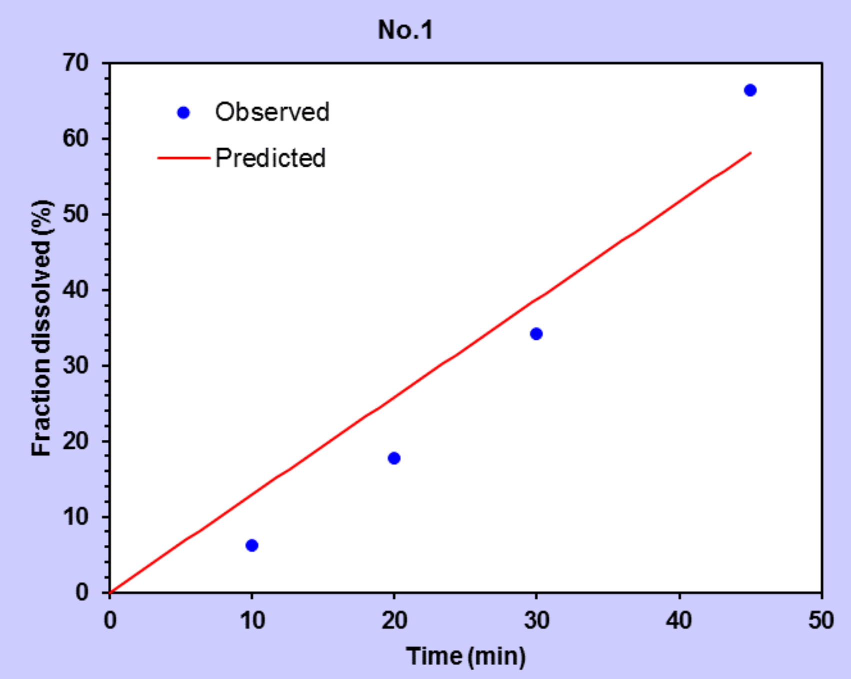

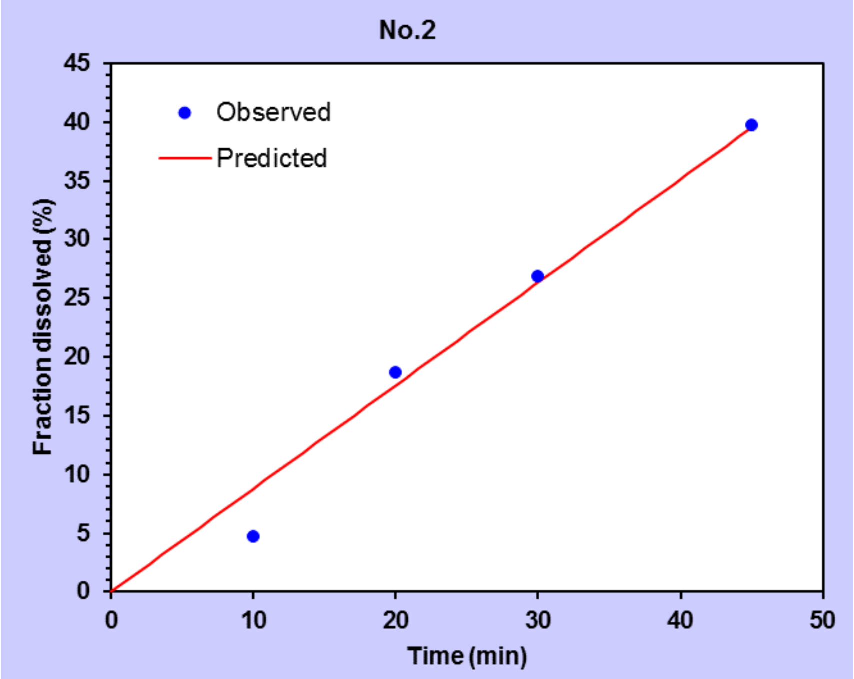

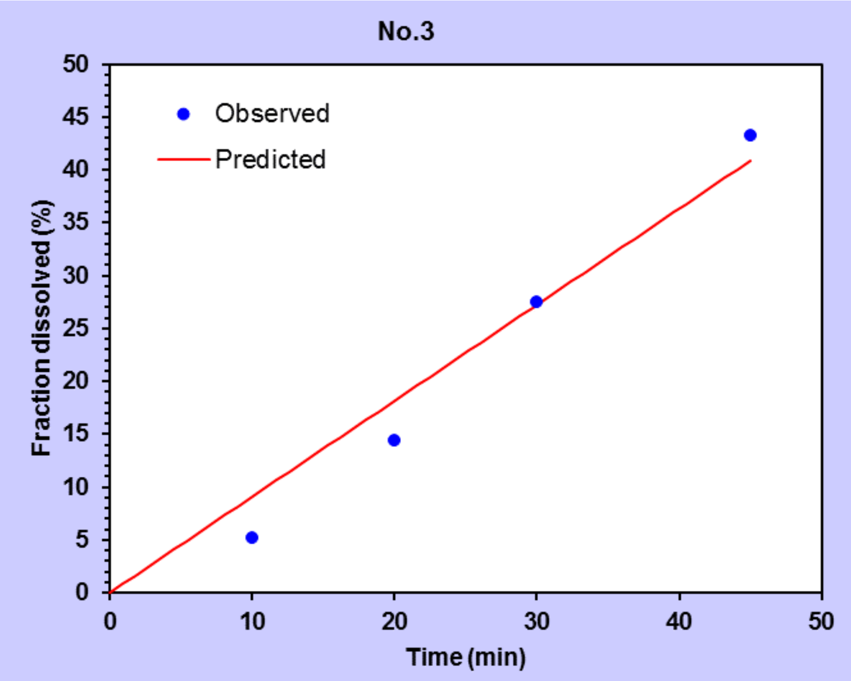

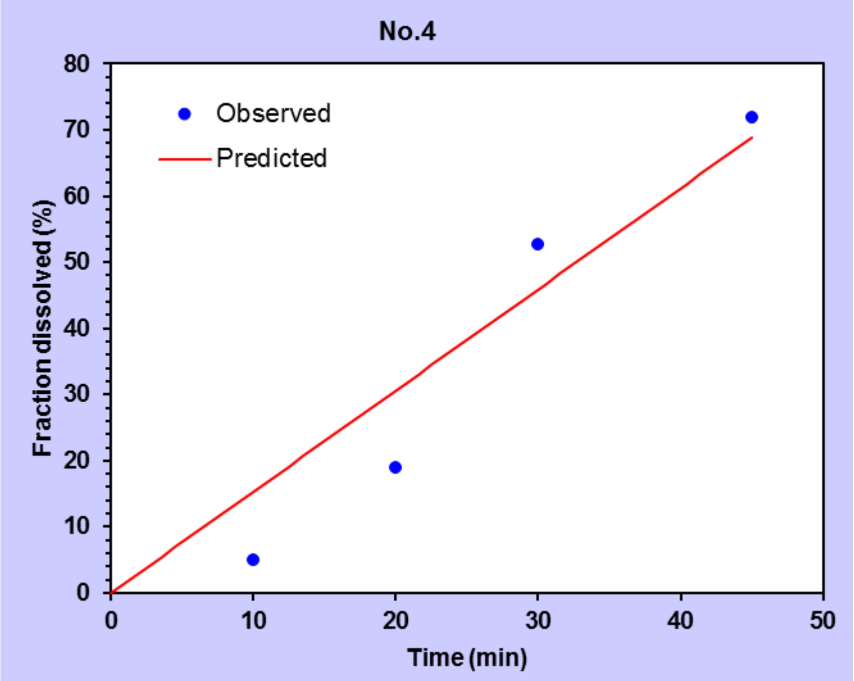

Supplementary materials – Model fitting summary – Parteck® M 100 (modelling up to app. 60 % of carvedilol released)

Model: **Hopfenberg with T_{lag}**

Model equation: $F = 100 \cdot \{1 - [1 - k_{HB} \cdot (t - T_{lag})]^n\}$

Fitted model parameters per tested tablet (N = 4) with statistics – mean, standard deviation (SD), and relative standard deviation expressed in % (RSD%) (output from DDSolver):

Parameter	No.1	No.2	No.3	No.4	Mean	SD	RSD(%)
k_{HB}	0.017	0.004	0.011	0.013	0.011	0.006	49.539
n	1.000	3.000	1.000	2.000	1.750	0.957	54.710
T_{lag}	8.377	4.683	5.835	9.299	7.048	2.152	30.537

Number of dissolution data points (N), degrees of freedom (df), and selected goodness of fit criteria – Pearson correlation coefficient (R), coefficient of determination (R^2), adjusted coefficient of determination ($R^2_{adjusted}$), and residual sum of squares (RSS) (manual calculation in MS Excel):

Parameter	No.1	No.2	No.3	No.4
N	4	4	4	4
df	1	1	1	1
R	0.990677239	0.996045698	0.998448029	0.983116224
R^2	0.981441392	0.992107032	0.996898467	0.96651751
$R^2_{adjusted}$	0.944324176	0.976321097	0.990695401	0.899552529
RSS	38.22456767	5.144005344	2.547077157	94.13211963

Graphical abstract of model fit presented as mean ± 1 SD of the fraction % of released carvedilol:

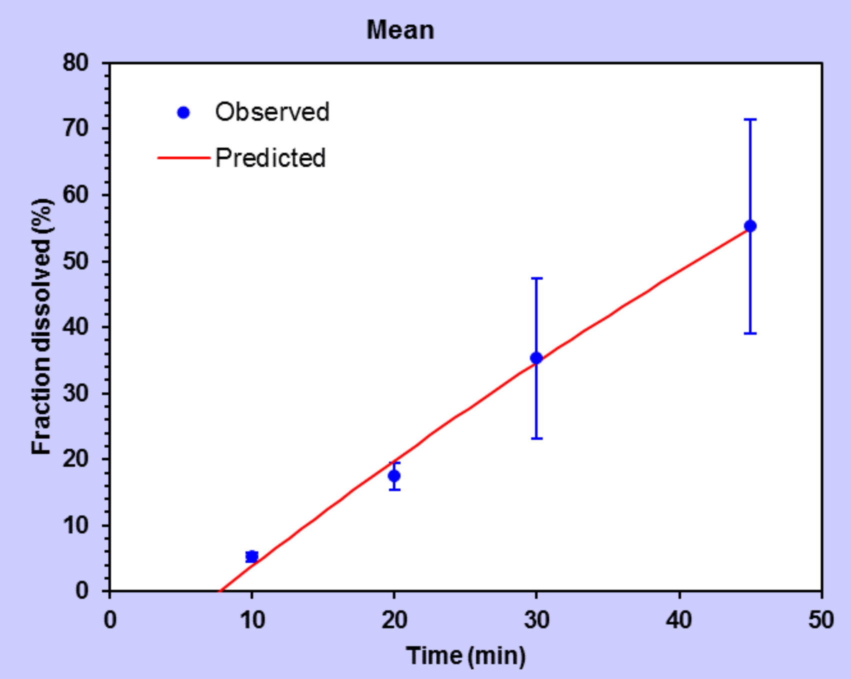

Supplementary materials – Model fitting summary – Parteck® M 100 (modelling up to app. 60 % of carvedilol released)

Graphical abstract of model fit presented as the fraction % of released carvedilol per tested tablet:

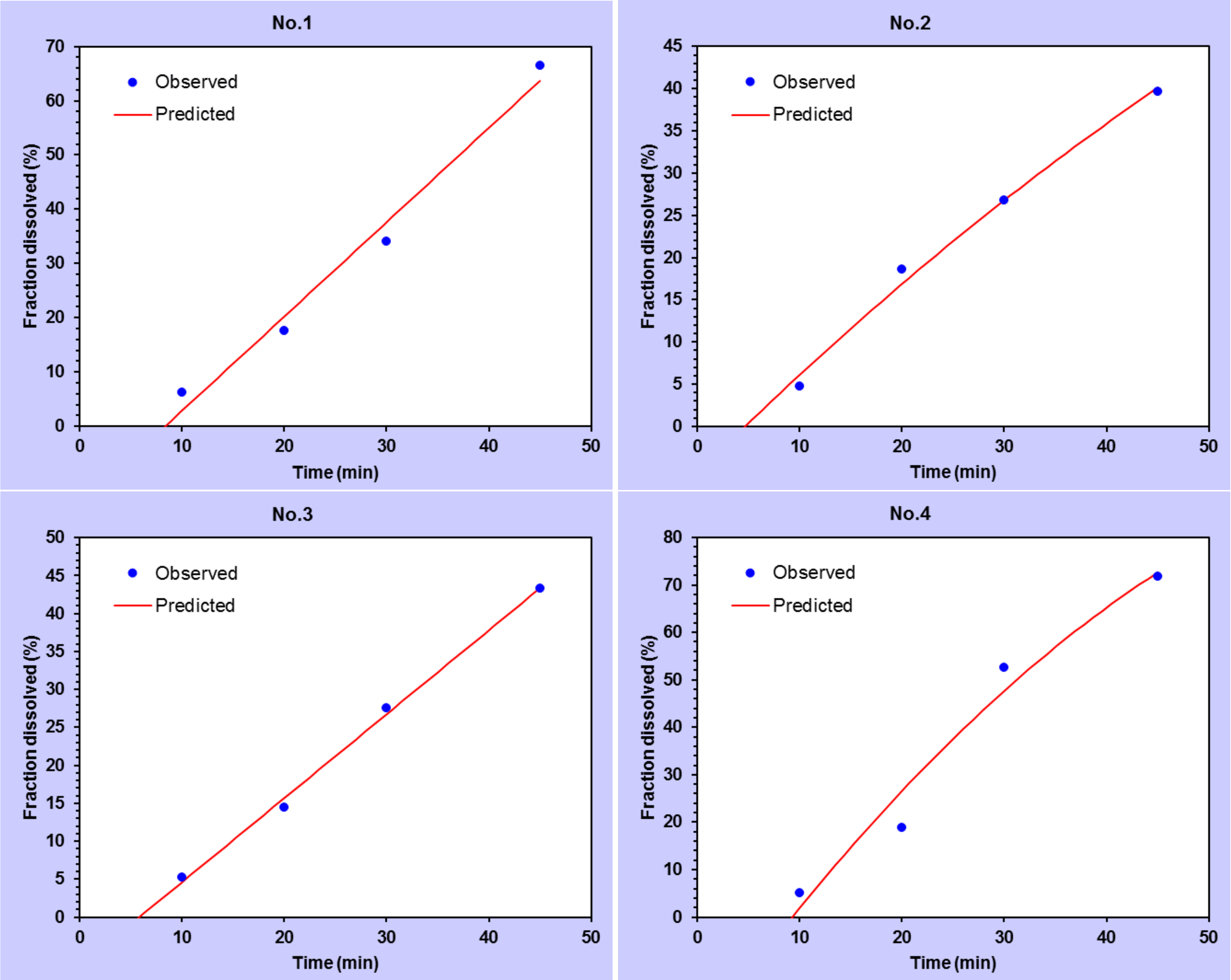

Supplementary materials – Model fitting summary – Parteck® M 100 (modelling up to app. 60 % of carvedilol released)

Model: **Baker–Lonsdale**

Model equation: $\frac{3}{2}\cdot\left[1-\left(1-\frac{F}{100}\right)^{\frac{2}{3}}\right]-\frac{F}{100}=k_{BL}\cdot t$

Fitted model parameters per tested tablet (N = 4) with statistics – mean, standard deviation (SD), and relative standard deviation expressed in % (RSD%) (output from DDSolver):

Parameter	No.1	No.2	No.3	No.4	Mean	SD	RSD(%)
k_{BL}	0.003	0.001	0.001	0.004	0.002	0.002	66.938

Number of dissolution data points (N), degrees of freedom (df), and selected goodness of fit criteria – Pearson correlation coefficient (R), coefficient of determination (R^2), adjusted coefficient of determination ($R^2_{adjusted}$), and residual sum of squares (RSS) (manual calculation in MS Excel):

Parameter	No.1	No.2	No.3	No.4
N	4	4	4	4
df	3	3	3	3
R	0.954739081	0.998796419	0.986281341	0.972962416
R^2	0.911526712	0.997594288	0.972750884	0.946655862
$R^2_{adjusted}$	0.911526712	0.997594288	0.972750884	0.946655862
RSS	3217.217427	573.8364084	957.8149564	3482.793945

Graphical abstract of model fit presented as mean ± 1 SD of the fraction % of released carvedilol:

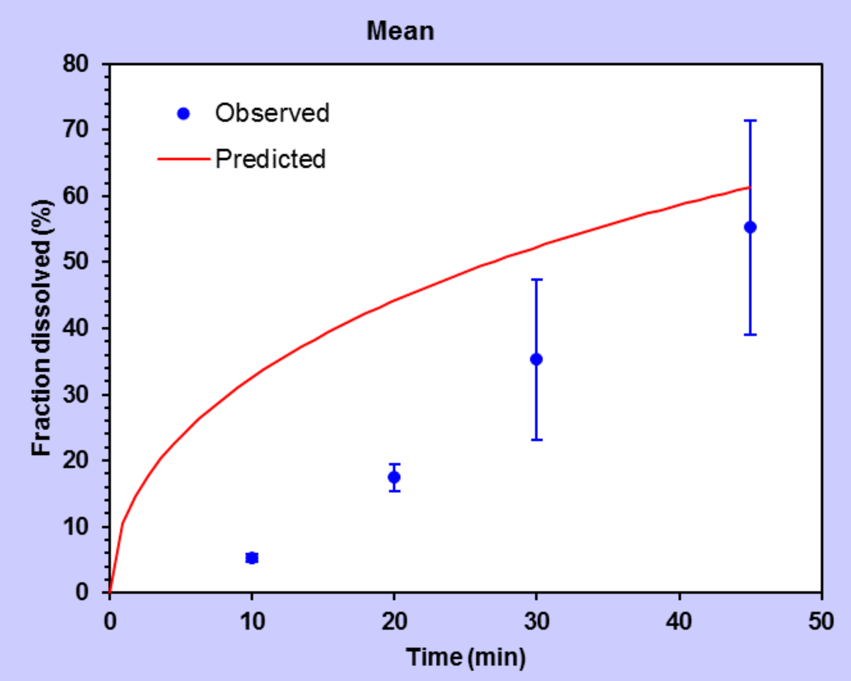

Supplementary materials – Model fitting summary – Parteck® M 100 (modelling up to app. 60 % of carvedilol released)

Graphical abstract of model fit presented as the fraction % of released carvedilol per tested tablet:

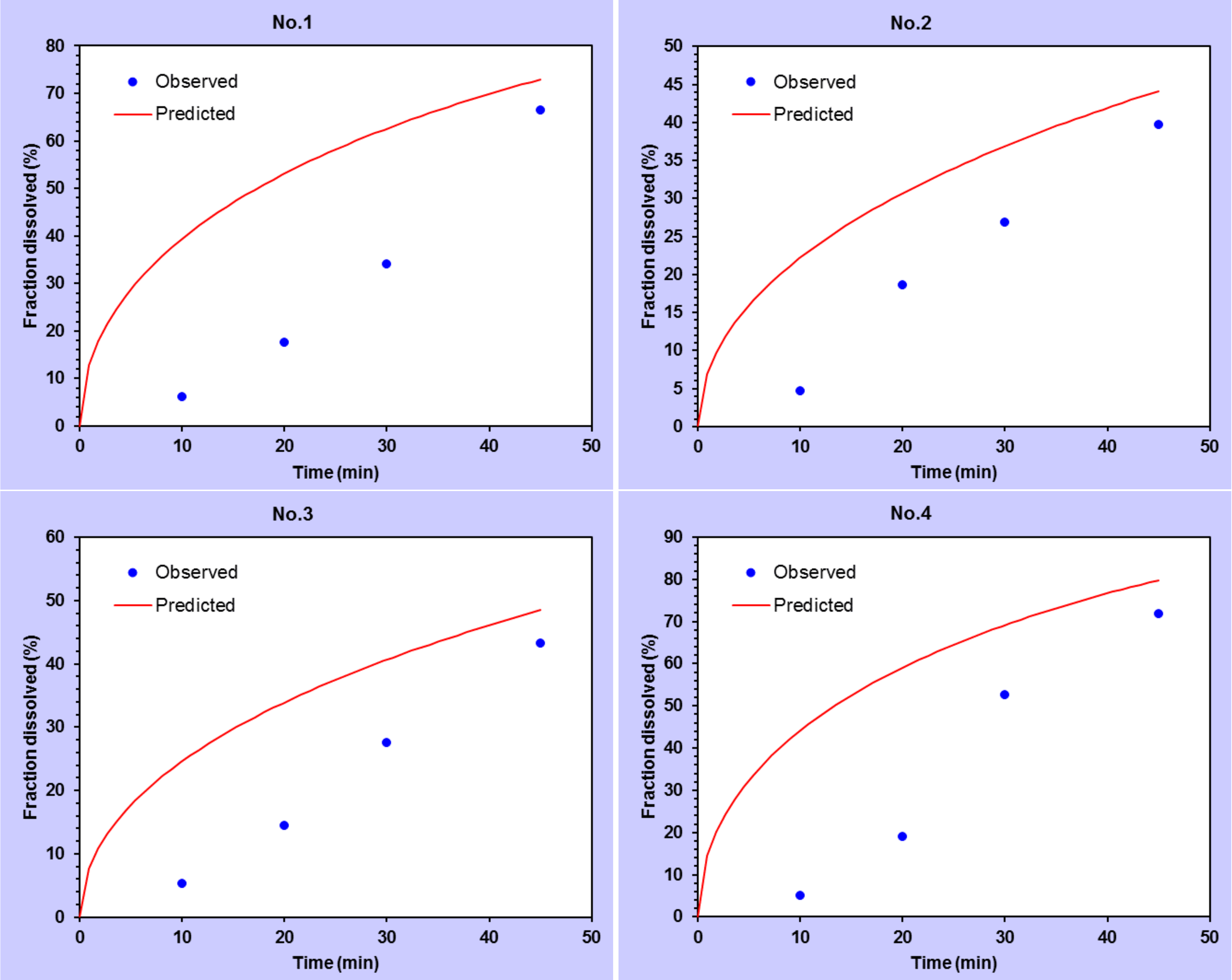

Supplementary materials – Model fitting summary – Parteck® M 100 (modelling up to app. 60 % of carvedilol released)

Model: **Baker–Lonsdale with T_{lag}**

Model equation: $\frac{3}{2} \cdot \left[1 - \left(1 - \frac{F}{100}\right)^{\frac{2}{3}}\right] - \frac{F}{100} = k_{BL} \cdot (t - T_{lag})$

Fitted model parameters per tested tablet (N = 4) with statistics – mean, standard deviation (SD), and relative standard deviation expressed in % (RSD%) (output from DDSolver):

Parameter	No.1	No.2	No.3	No.4	Mean	SD	RSD(%)
k_{BL}	0.003	0.001	0.001	0.004	0.002	0.002	64.454
T_{lag}	15.198	11.862	16.910	13.736	14.427	2.146	14.874

Number of dissolution data points (N), degrees of freedom (df), and selected goodness of fit criteria – Pearson correlation coefficient (R), coefficient of determination (R^2), adjusted coefficient of determination ($R^2_{adjusted}$), and residual sum of squares (RSS) (manual calculation in MS Excel):

Parameter	No.1	No.2	No.3	No.4
N	4	4	4	4
df	2	2	2	2
R	0.932766802	0.985198682	0.987338401	0.949115096
R^2	0.870053907	0.970616443	0.974837118	0.900819465
$R^2_{adjusted}$	0.80508086	0.955924664	0.962255677	0.851229197
RSS	327.615693	32.13054803	33.96383439	318.6660969

Graphical abstract of model fit presented as mean ± 1 SD of the fraction % of released carvedilol:

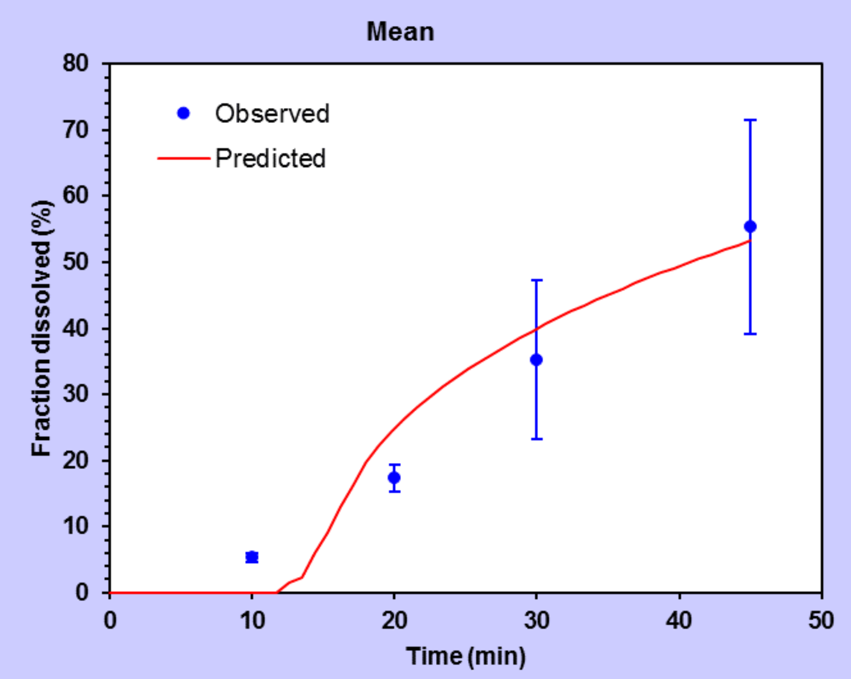

Supplementary materials – Model fitting summary – Parteck® M 100 (modelling up to app. 60 % of carvedilol released)

Graphical abstract of model fit presented as the fraction % of released carvedilol per tested tablet:

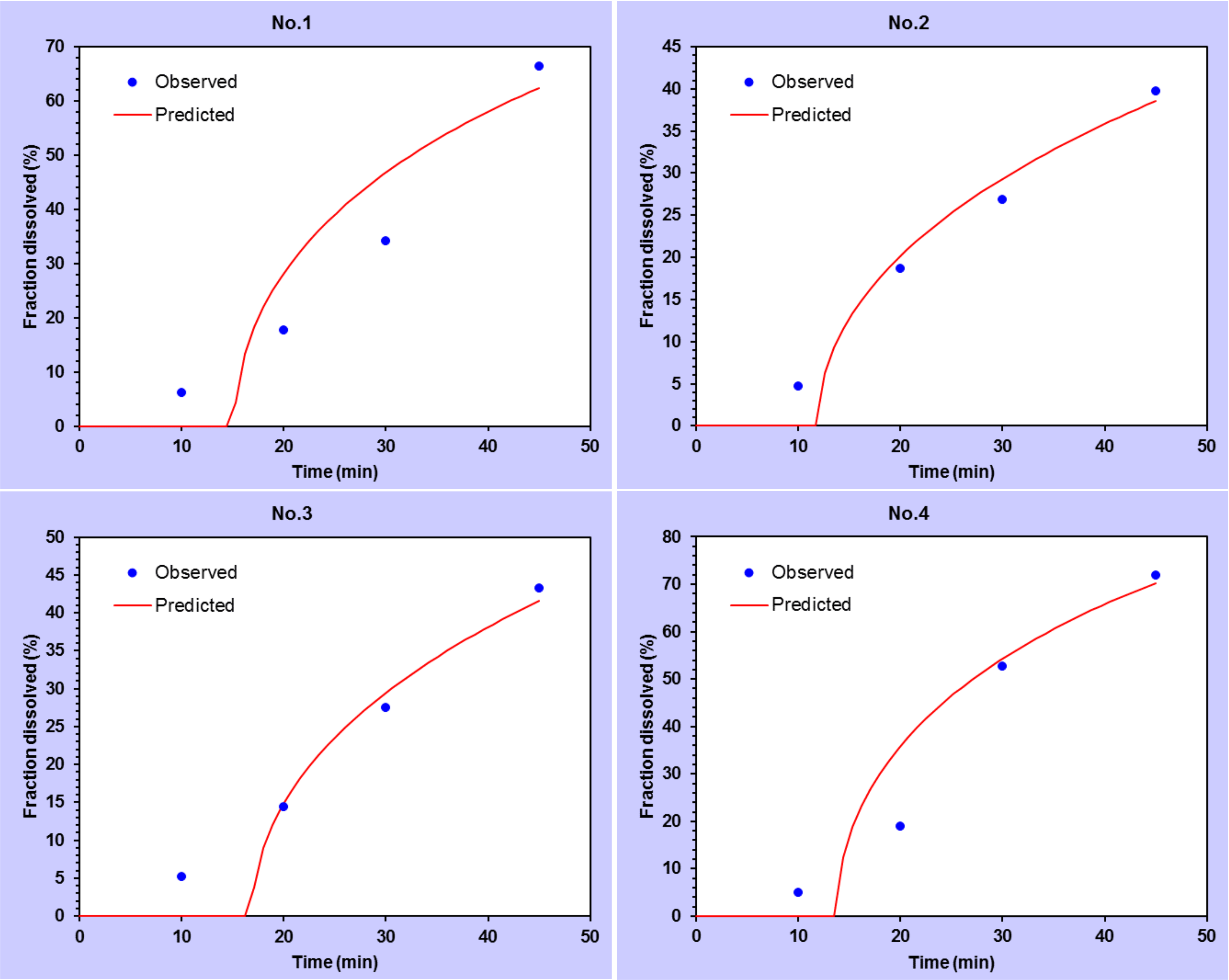

Supplementary materials – Model fitting summary – Parteck® M 100 (modelling up to app. 60 % of carvedilol released)

Model: **Makoid–Banakar**

Model equation: $F = k_{MB} \cdot t^n \cdot e^{-k \cdot t}$

Fitted model parameters per tested tablet (N = 4) with statistics – mean, standard deviation (SD), and relative standard deviation expressed in % (RSD%) (output from DDSolver):

Parameter	No.1	No.2	No.3	No.4	Mean	SD	RSD(%)
k_{MB}	0.220	0.021	0.112	0.013	0.091	0.097	105.781
n	1.420	2.581	1.717	2.751	2.117	0.649	30.649
k	-0.007	0.051	0.013	0.040	0.024	0.026	108.747

Number of dissolution data points (N), degrees of freedom (df), and selected goodness of fit criteria – Pearson correlation coefficient (R), coefficient of determination (R^2), adjusted coefficient of determination ($R^2_{adjusted}$), and residual sum of squares (RSS) (manual calculation in MS Excel):

Parameter	No.1	No.2	No.3	No.4
N	4	4	4	4
df	1	1	1	1
R	0.99996799	0.992783668	0.998934441	0.985241641
R^2	0.999935981	0.985619411	0.997870018	0.970701092
$R^2_{adjusted}$	0.999807944	0.956858234	0.993610055	0.912103275
RSS	0.139872697	9.326341582	1.770885631	83.37626782

Graphical abstract of model fit presented as mean ± 1 SD of the fraction % of released carvedilol:

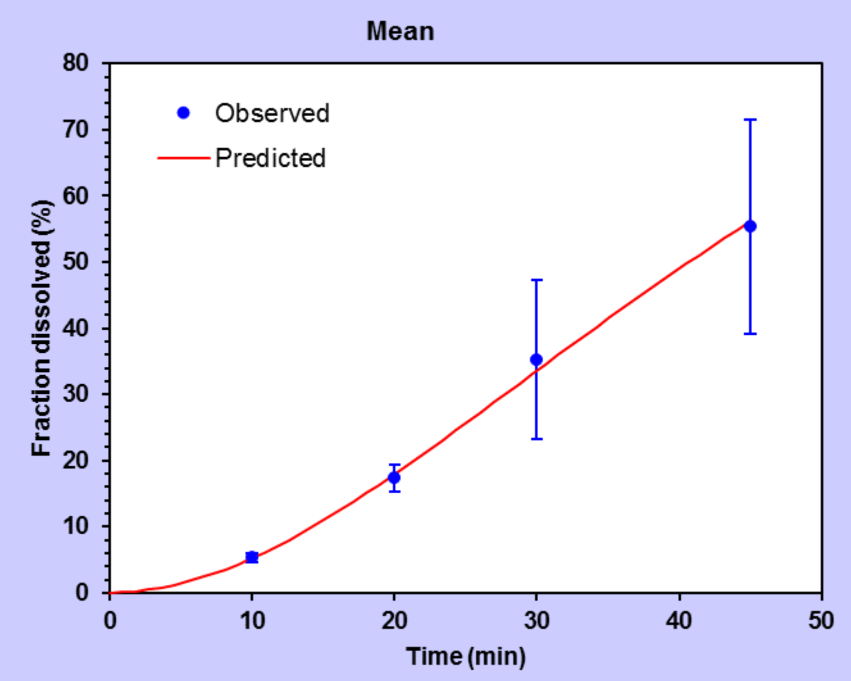

Supplementary materials – Model fitting summary – Parteck® M 100 (modelling up to app. 60 % of carvedilol released)

Graphical abstract of model fit presented as the fraction % of released carvedilol per tested tablet:

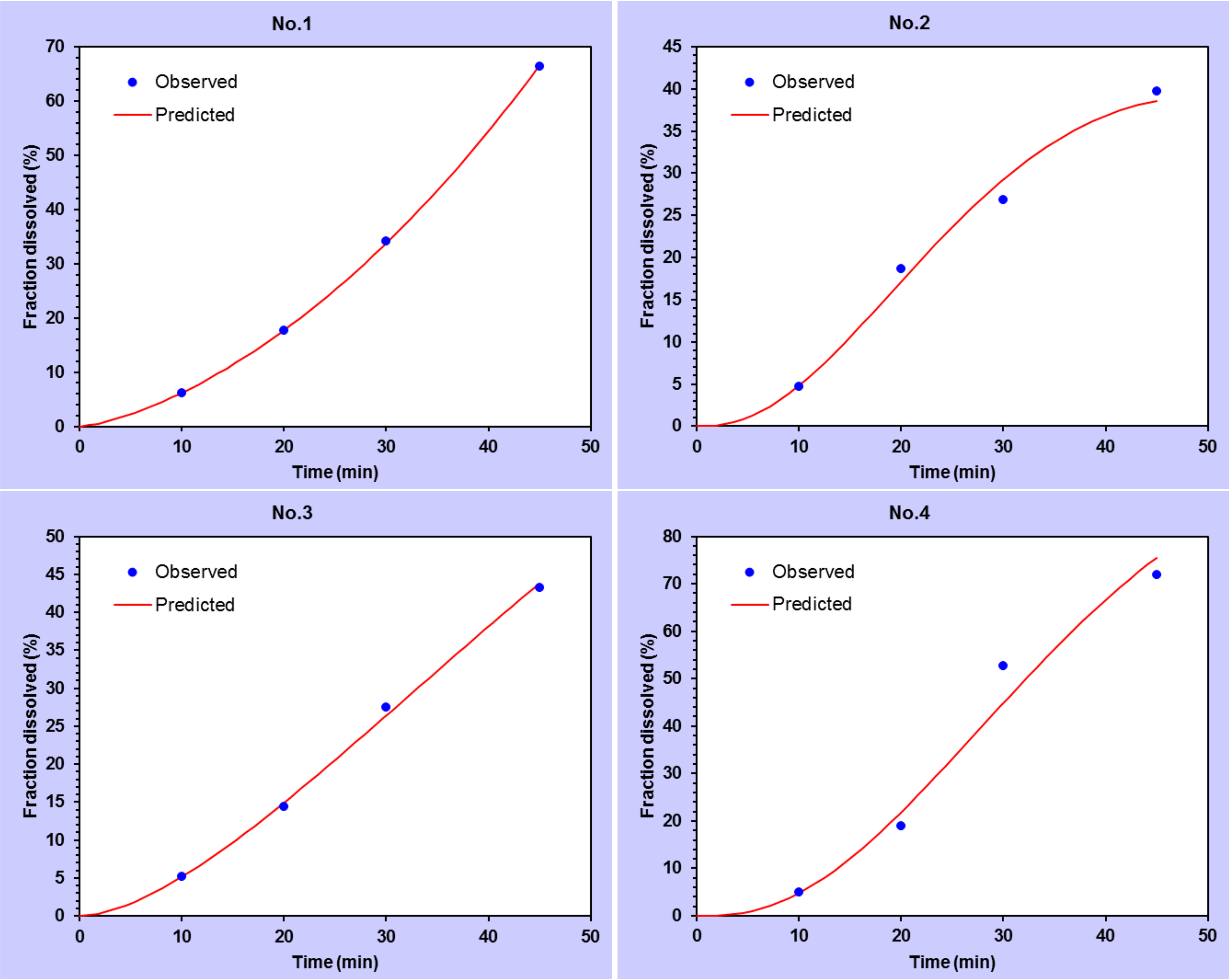

Supplementary materials – Model fitting summary – Parteck® M 100 (modelling up to app. 60 % of carvedilol released)

Model: **Makoid–Banakar with T_{lag}**

Model equation: $F = k_{MB} \cdot (t - T_{lag})^n \cdot e^{-k \cdot (t - T_{lag})}$

Fitted model parameters per tested tablet (N = 4) with statistics – mean, standard deviation (SD), and relative standard deviation expressed in % (RSD%) (output from DDSolver):

Parameter	No.1	No.2	No.3	No.4	Mean	SD	RSD(%)
k_{MB}	/	/	/	/	/	/	/
n	/	/	/	/	/	/	/
k	/	/	/	/	/	/	/
T_{lag}	/	/	/	/	/	/	/

Number of dissolution data points (N), degrees of freedom (df), and selected goodness of fit criteria – Pearson correlation coefficient (R), coefficient of determination (R^2), adjusted coefficient of determination ($R^2_{adjusted}$), and residual sum of squares (RSS) (manual calculation in MS Excel):

Parameter	No.1	No.2	No.3	No.4
N	/	/	/	/
df	/	/	/	/
R	/	/	/	/
R^2	/	/	/	/
$R^2_{adjusted}$	/	/	/	/
RSS	/	/	/	/

Graphical abstract of model fit presented as mean ± 1 SD of the fraction % of released carvedilol: /

Graphical abstract of model fit presented as the fraction % of released carvedilol per tested tablet: /

Note: model could not be fitted to experimental dissolution data due too few data points being available for fitting

Supplementary materials – Model fitting summary – Parteck® M 100 (modelling up to app. 60 % of carvedilol released)

Model: **Peppas–Sahlin_1**

Model equation: $F = k_1 \cdot t^m + k_2 \cdot t^{2m}$

Fitted model parameters per tested tablet (N = 4) with statistics – mean, standard deviation (SD), and relative standard deviation expressed in % (RSD%) (output from DDSolver):

Parameter	No.1	No.2	No.3	No.4	Mean	SD	RSD(%)
k_1	-9.831	-2.619	-4.655	-10.228	-6.833	3.787	-55.417
k_2	3.864	1.792	2.253	4.286	3.049	1.212	39.760
m	0.450	0.450	0.450	0.450	0.450	0.000	0.000

Number of dissolution data points (N), degrees of freedom (df), and selected goodness of fit criteria – Pearson correlation coefficient (R), coefficient of determination (R^2), adjusted coefficient of determination ($R^2_{adjusted}$), and residual sum of squares (RSS) (manual calculation in MS Excel):

Parameter	No.1	No.2	No.3	No.4
N	4	4	4	4
df	1	1	1	1
R	0.99343431	0.992031982	0.998671888	0.980393194
R^2	0.986911728	0.984127454	0.99734554	0.961170816
$R^2_{adjusted}$	0.960735184	0.952382362	0.992036619	0.883512447
RSS	27.15316167	10.34283746	2.180509096	109.1871898

Graphical abstract of model fit presented as mean ± 1 SD of the fraction % of released carvedilol:

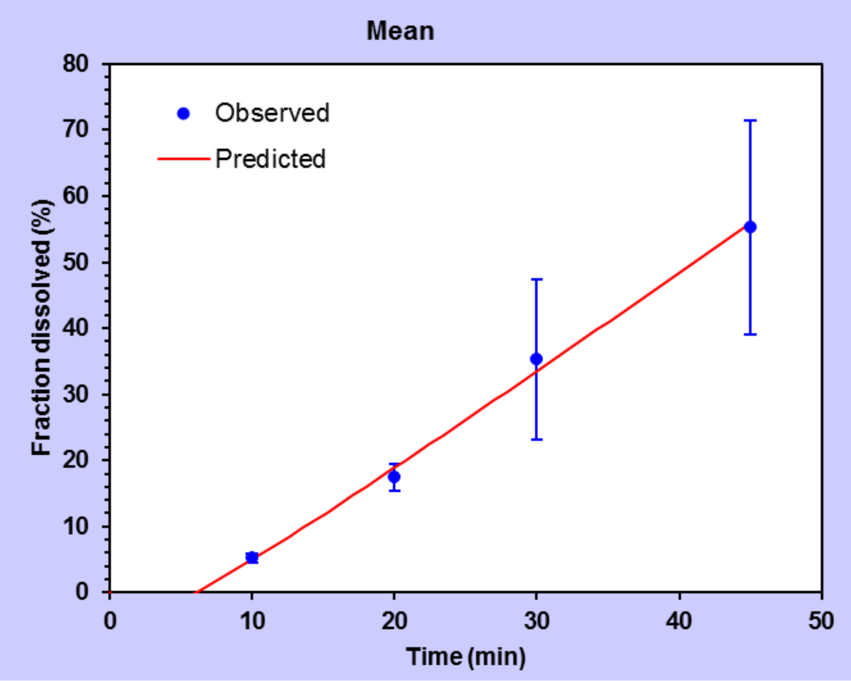

Supplementary materials – Model fitting summary – Parteck® M 100 (modelling up to app. 60 % of carvedilol released)

Graphical abstract of model fit presented as the fraction % of released carvedilol per tested tablet:

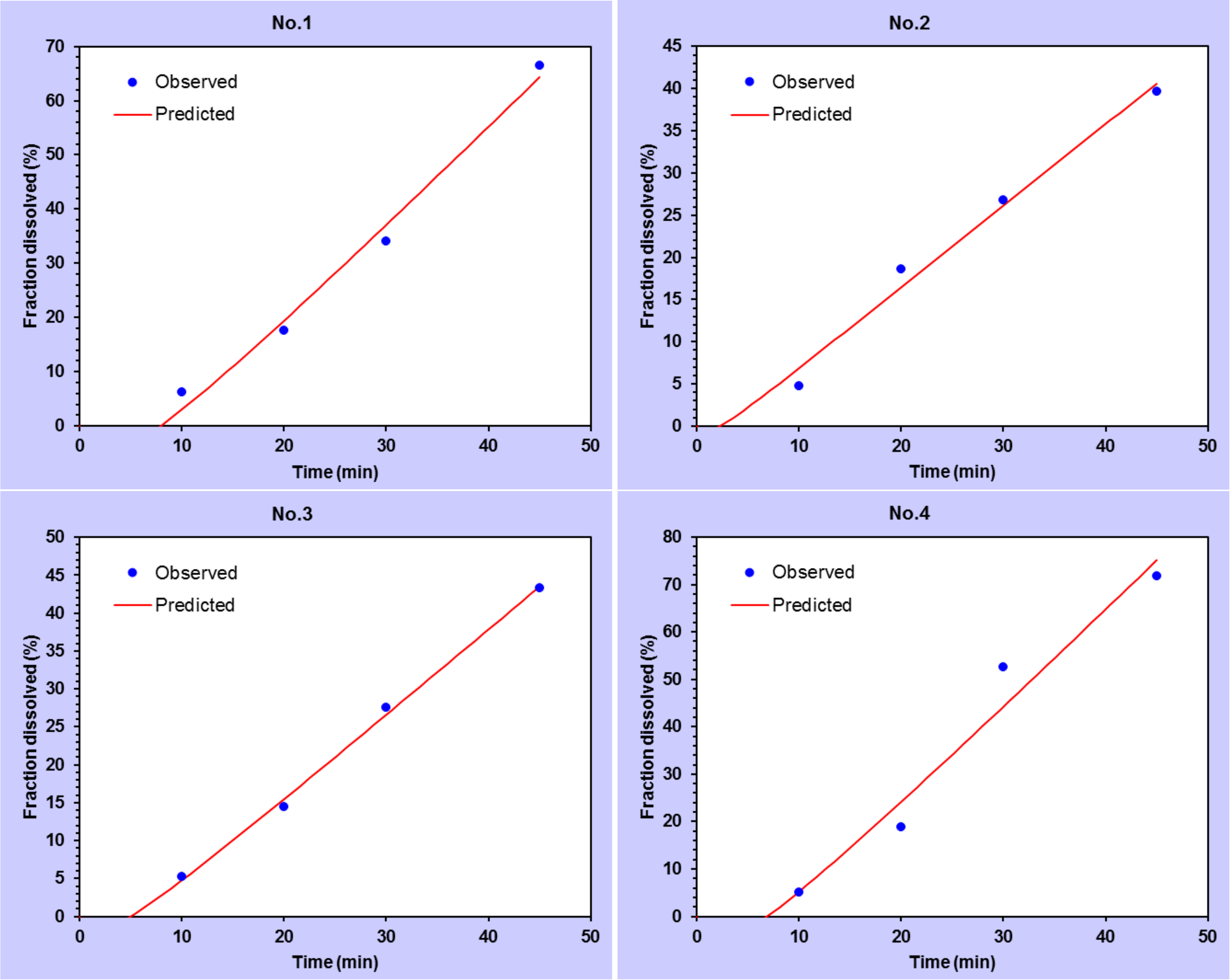

Supplementary materials – Model fitting summary – Parteck® M 100 (modelling up to app. 60 % of carvedilol released)

Model: **Peppas–Sahlin_1 with T_{lag}**

Model equation: $F = k_1 \cdot (t - T_{lag})^m + k_2 \cdot (t - T_{lag})^{2m}$

Fitted model parameters per tested tablet (N = 4) with statistics – mean, standard deviation (SD), and relative standard deviation expressed in % (RSD%) (output from DDSolver):

Parameter	No.1	No.2	No.3	No.4	Mean	SD	RSD(%)
k_1	/	/	/	/	/	/	/
k_2	/	/	/	/	/	/	/
m	/	/	/	/	/	/	/
T_{lag}	/	/	/	/	/	/	/

Number of dissolution data points (N), degrees of freedom (df), and selected goodness of fit criteria – Pearson correlation coefficient (R), coefficient of determination (R^2), adjusted coefficient of determination ($R^2_{adjusted}$), and residual sum of squares (RSS) (manual calculation in MS Excel):

Parameter	No.1	No.2	No.3	No.4
N	/	/	/	/
df	/	/	/	/
R	/	/	/	/
R^2	/	/	/	/
$R^2_{adjusted}$	/	/	/	/
RSS	/	/	/	/

Graphical abstract of model fit presented as mean ± 1 SD of the fraction % of released carvedilol: /

Graphical abstract of model fit presented as the fraction % of released carvedilol per tested tablet: /

Note: model could not be fitted to experimental dissolution data due too few data points being available for fitting

Supplementary materials – Model fitting summary – Parteck® M 100 (modelling up to app. 60 % of carvedilol released)

Model: **Peppas–Sahlin_2**

Model equation: $F = k_1 \cdot t^{0.5} + k_2 \cdot t$

Fitted model parameters per tested tablet (N = 4) with statistics – mean, standard deviation (SD), and relative standard deviation expressed in % (RSD%) (output from DDSolver):

Parameter	No.1	No.2	No.3	No.4	Mean	SD	RSD(%)
k_1	-6.554	-1.124	-2.759	-6.466	-4.226	2.721	-64.396
k_2	2.416	1.071	1.382	2.639	1.877	0.767	40.879

Number of dissolution data points (N), degrees of freedom (df), and selected goodness of fit criteria – Pearson correlation coefficient (R), coefficient of determination (R^2), adjusted coefficient of determination ($R^2_{adjusted}$), and residual sum of squares (RSS) (manual calculation in MS Excel):

Parameter	No.1	No.2	No.3	No.4
N	4	4	4	4
df	2	2	2	2
R	0.995489295	0.990408398	0.998600578	0.978885442
R^2	0.990998936	0.980908795	0.997203115	0.958216708
$R^2_{adjusted}$	0.986498404	0.971363193	0.995804673	0.937325062
RSS	18.74016636	12.5056245	2.297719032	117.6185222

Graphical abstract of model fit presented as mean ± 1 SD of the fraction % of released carvedilol:

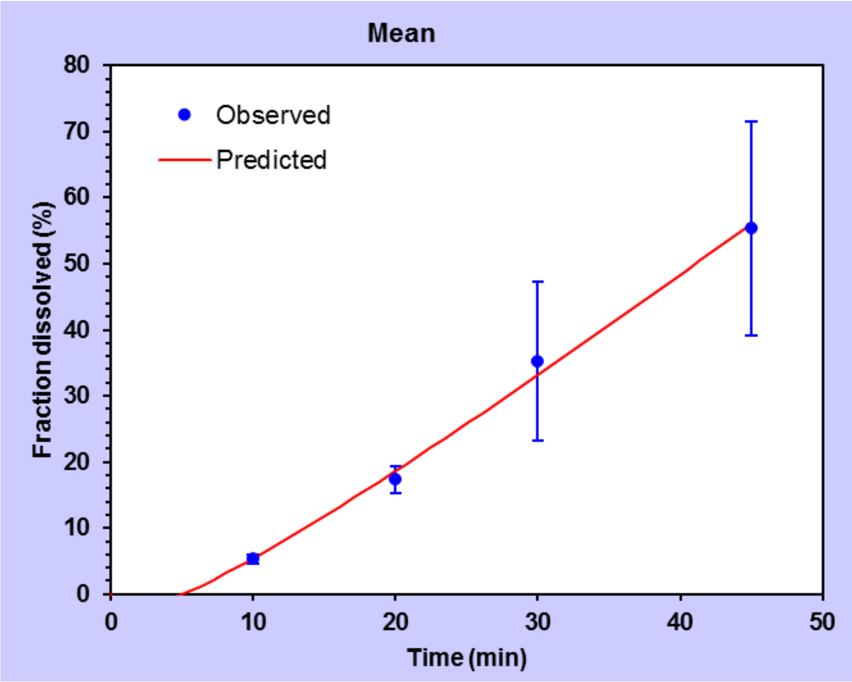

Supplementary materials – Model fitting summary – Parteck® M 100 (modelling up to app. 60 % of carvedilol released)

Graphical abstract of model fit presented as the fraction % of released carvedilol per tested tablet:

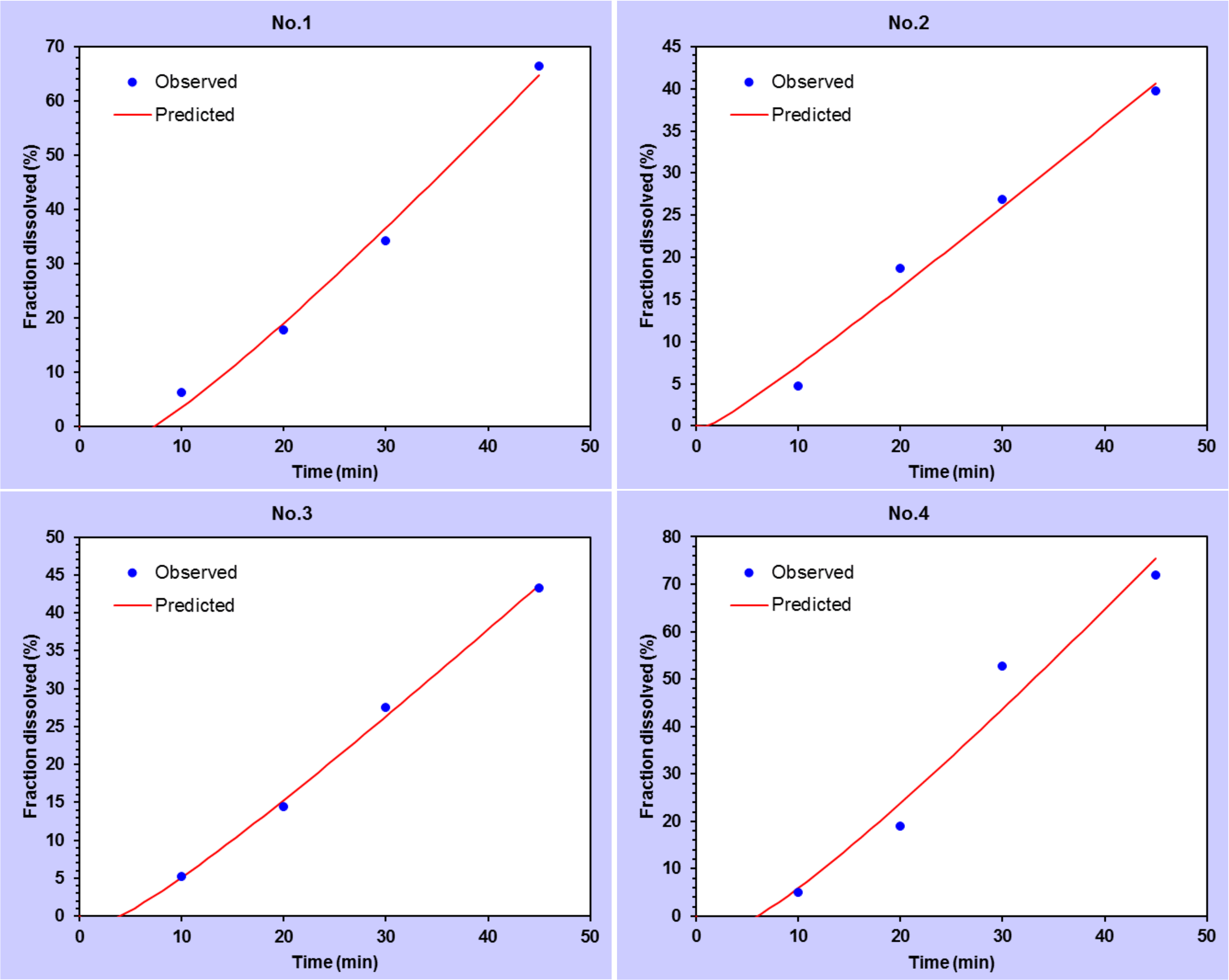

Supplementary materials – Model fitting summary – Parteck® M 100 (modelling up to app. 60 % of carvedilol released)

Model: **Peppas–Sahlin_2 with T_{lag}**

Model equation: $F = k_1 \cdot (t - T_{lag})^{0.5} + k_2 \cdot (t - T_{lag})$

Fitted model parameters per tested tablet (N = 4) with statistics – mean, standard deviation (SD), and relative standard deviation expressed in % (RSD%) (output from DDSolver):

Parameter	No.1	No.2	No.3	No.4	Mean	SD	RSD(%)
k_1	-4.216	0.646	-1.027	-3.476	-2.018	2.239	-110.929
k_2	2.237	0.884	1.222	2.376	1.680	0.739	43.979
T_{lag}	4.000	4.000	4.000	4.000	4.000	0.000	0.000

Number of dissolution data points (N), degrees of freedom (df), and selected goodness of fit criteria – Pearson correlation coefficient (R), coefficient of determination (R^2), adjusted coefficient of determination ($R^2_{adjusted}$), and residual sum of squares (RSS) (manual calculation in MS Excel):

Parameter	No.1	No.2	No.3	No.4
N	4	4	4	4
df	1	1	1	1
R	0.995015169	0.993239503	0.998649724	0.979734218
R^2	0.990055187	0.98652471	0.997301272	0.959879138
$R^2_{adjusted}$	0.97016556	0.95957413	0.991903817	0.879637415
RSS	21.03827161	9.026927845	2.217567983	113.080349

Graphical abstract of model fit presented as mean ± 1 SD of the fraction % of released carvedilol:

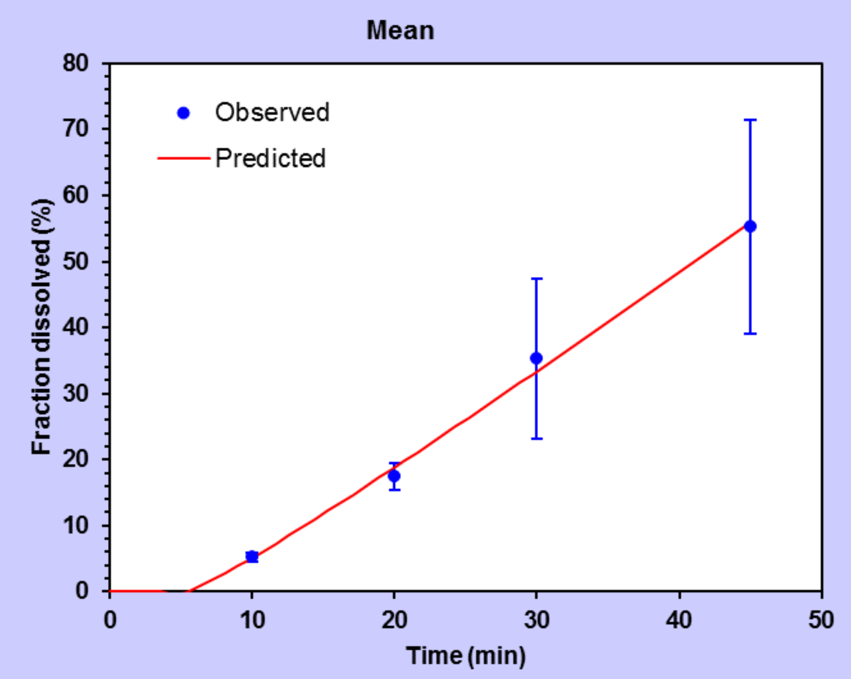

Supplementary materials – Model fitting summary – Parteck® M 100 (modelling up to app. 60 % of carvedilol released)

Graphical abstract of model fit presented as the fraction % of released carvedilol per tested tablet:

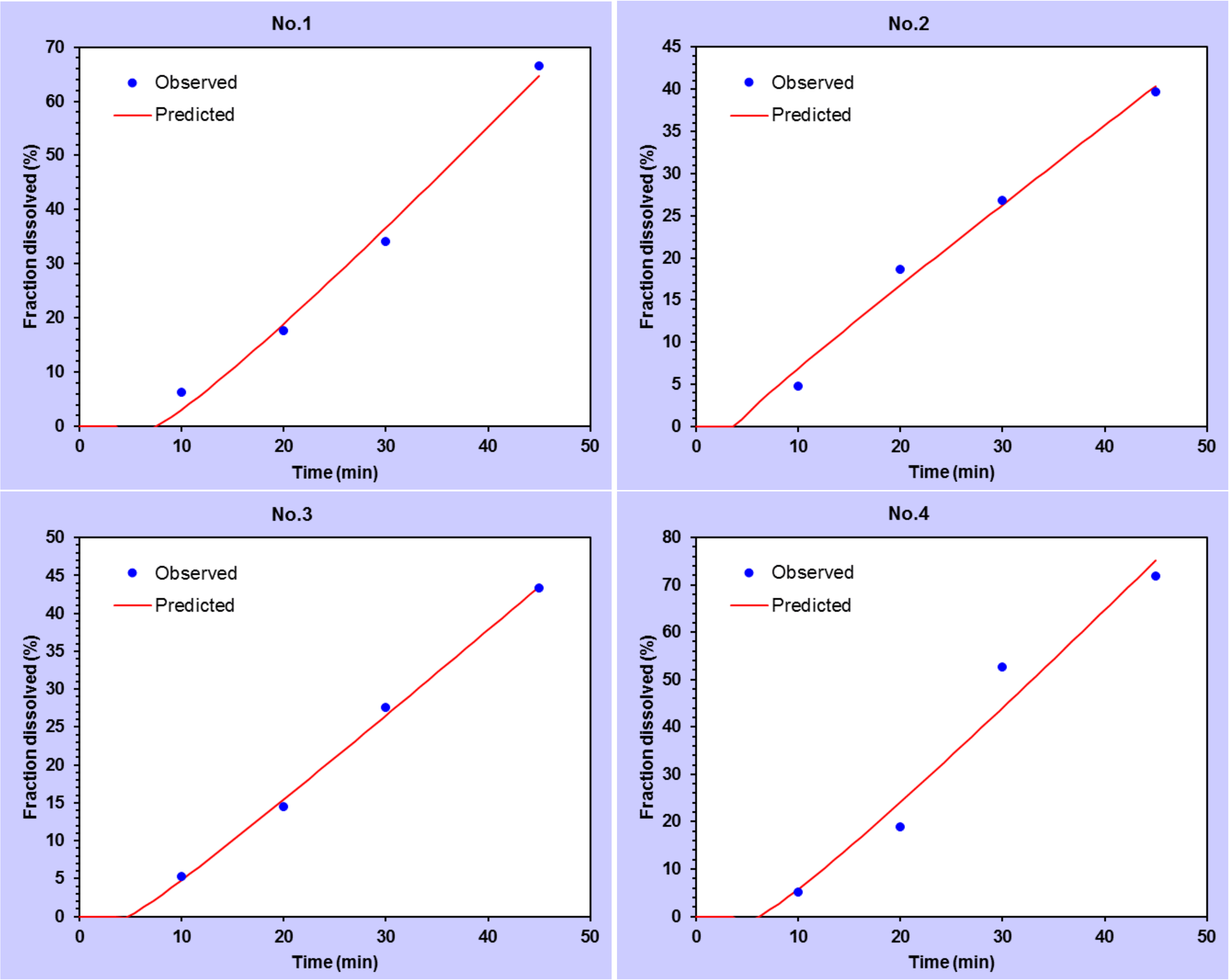

Supplementary materials – Model fitting summary – Parteck® M 100 (modelling up to app. 60 % of carvedilol released)

Model: **Quadratic**

Model equation: $F = 100 \cdot (k_1 \cdot t^2 + k_2 \cdot t)$

Fitted model parameters per tested tablet (N = 4) with statistics – mean, standard deviation (SD), and relative standard deviation expressed in % (RSD%) (output from DDSolver):

Parameter	No.1	No.2	No.3	No.4	Mean	SD	RSD(%)
k_1	0.00024	0.00002	0.00009	0.00018	0.00013	0.00010	73.83612
k_2	0.00412	0.00805	0.00586	0.00873	0.00669	0.00210	31.45159

Number of dissolution data points (N), degrees of freedom (df), and selected goodness of fit criteria – Pearson correlation coefficient (R), coefficient of determination (R^2), adjusted coefficient of determination ($R^2_{adjusted}$), and residual sum of squares (RSS) (manual calculation in MS Excel):

Parameter	No.1	No.2	No.3	No.4
N	4	4	4	4
df	2	2	2	2
R	0.999942287	0.989250973	0.995731731	0.969093525
R^2	0.999884578	0.978617488	0.991481679	0.939142261
$R^2_{adjusted}$	0.999826866	0.967926232	0.987222519	0.908713391
RSS	0.260219252	16.53874863	7.819731913	184.2402209

Graphical abstract of model fit presented as mean ± 1 SD of the fraction % of released carvedilol:

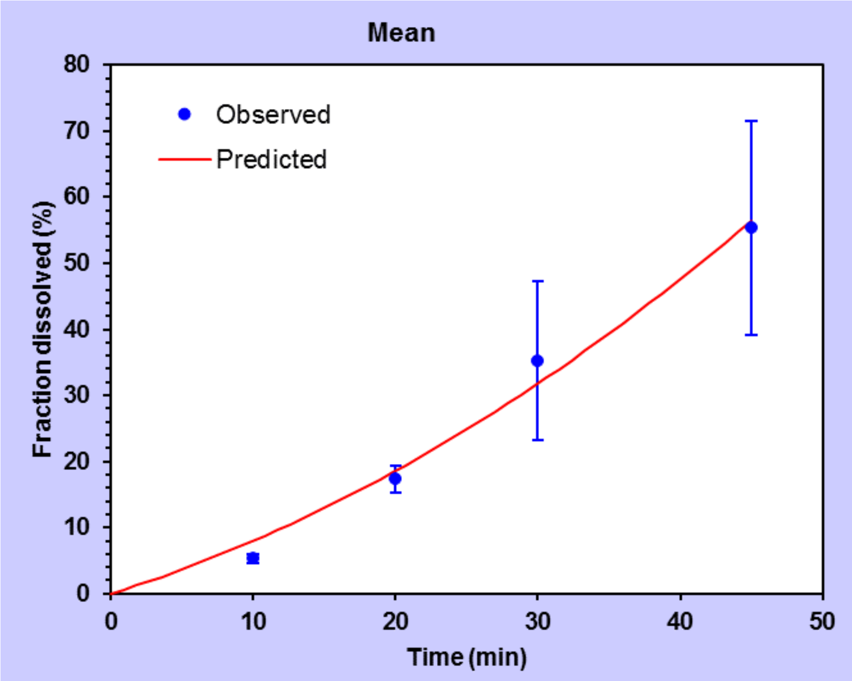

Supplementary materials – Model fitting summary – Parteck® M 100 (modelling up to app. 60 % of carvedilol released)

Graphical abstract of model fit presented as the fraction % of released carvedilol per tested tablet:

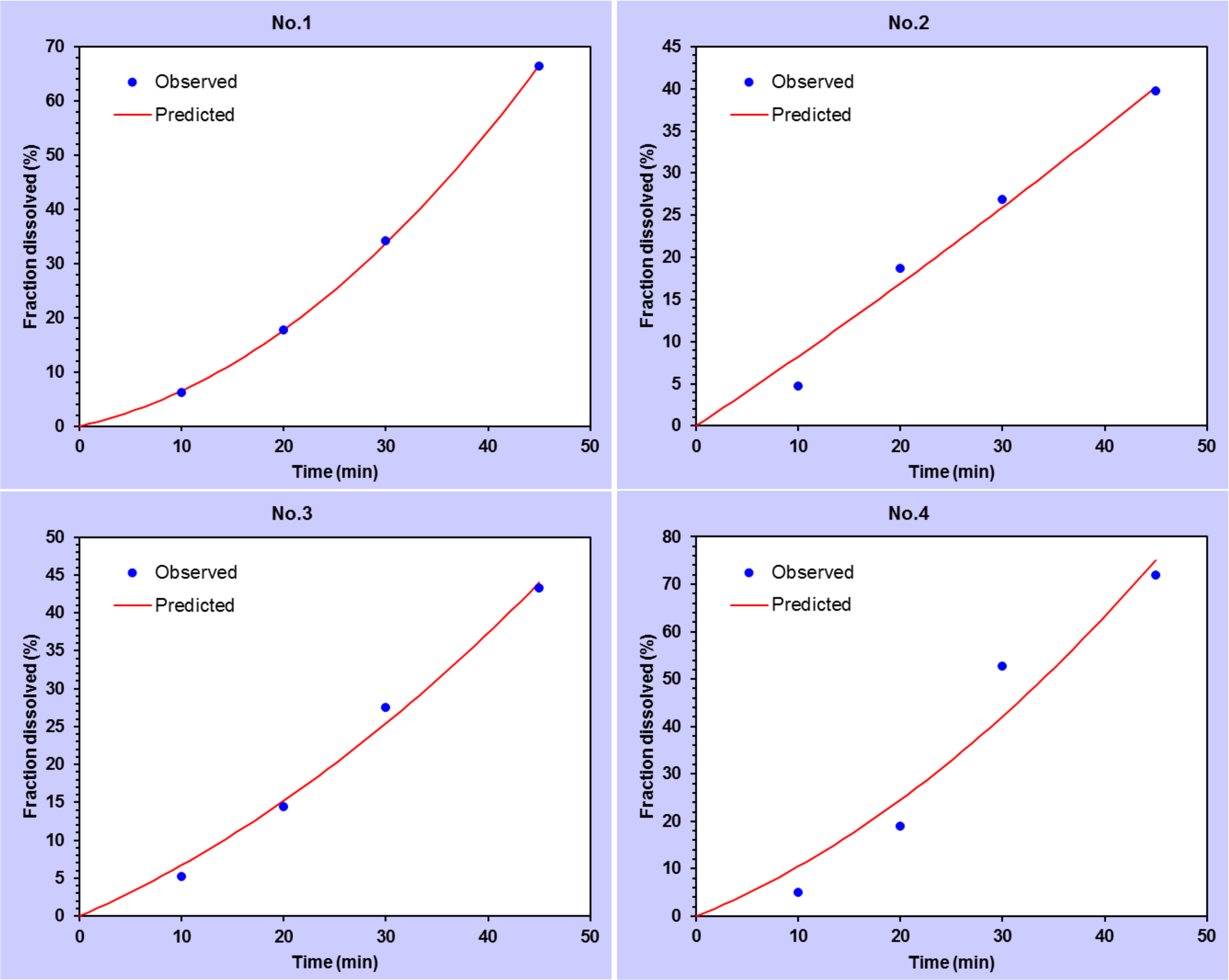

Supplementary materials – Model fitting summary – Parteck® M 100 (modelling up to app. 60 % of carvedilol released)

Model: **Quadratic with T_{lag}**

Model equation: $F = 100 \cdot \left[k_1 \cdot \left(t - T_{lag}\right)^2 + k_2 \cdot \left(t - T_{lag}\right)\right]$

Fitted model parameters per tested tablet (N = 4) with statistics – mean, standard deviation (SD), and relative standard deviation expressed in % (RSD%) (output from DDSolver):

Parameter	No.1	No.2	No.3	No.4	Mean	SD	RSD(%)
k_1	0.00020	-0.00005	0.00004	0.00009	0.00007	0.00010	146.10546
k_2	0.00792	0.01155	0.00906	0.01453	0.01077	0.00293	27.23402
T_{lag}	4.00000	4.00000	4.00000	4.00000	4.00000	0.00000	0.00000

Number of dissolution data points (N), degrees of freedom (df), and selected goodness of fit criteria – Pearson correlation coefficient (R), coefficient of determination (R^2), adjusted coefficient of determination ($R^2_{adjusted}$), and residual sum of squares (RSS) (manual calculation in MS Excel):

Parameter	No.1	No.2	No.3	No.4
N	4	4	4	4
df	1	1	1	1
R	0.999883506	0.996374747	0.998184739	0.976421677
R^2	0.999767026	0.992762637	0.996372774	0.953399292
$R^2_{adjusted}$	0.999301078	0.97828791	0.989118323	0.860197877
RSS	0.603175624	5.934985892	3.206665981	146.2917574

Graphical abstract of model fit presented as mean ± 1 SD of the fraction % of released carvedilol:

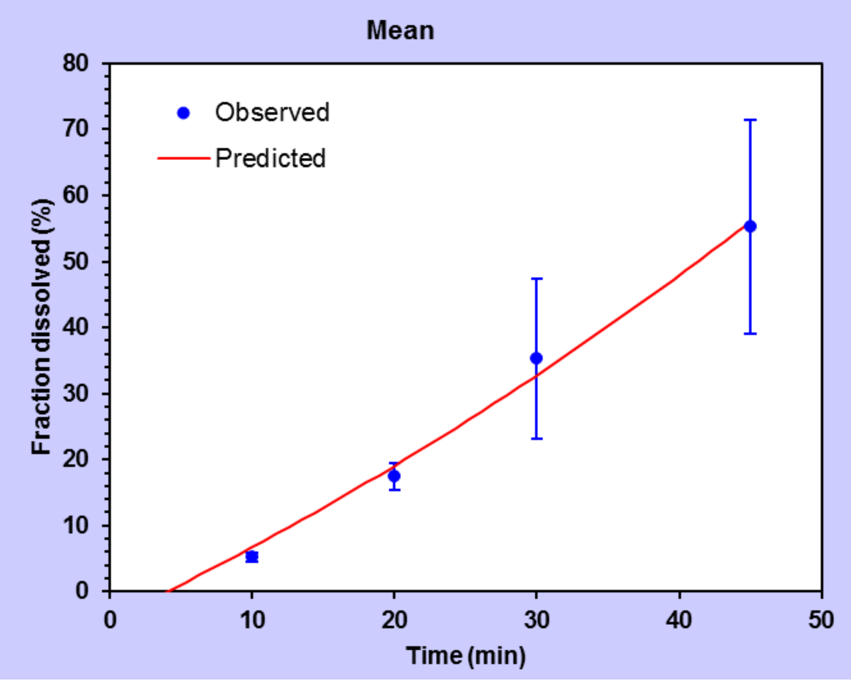

Supplementary materials – Model fitting summary – Parteck® M 100 (modelling up to app. 60 % of carvedilol released)

Graphical abstract of model fit presented as the fraction % of released carvedilol per tested tablet:

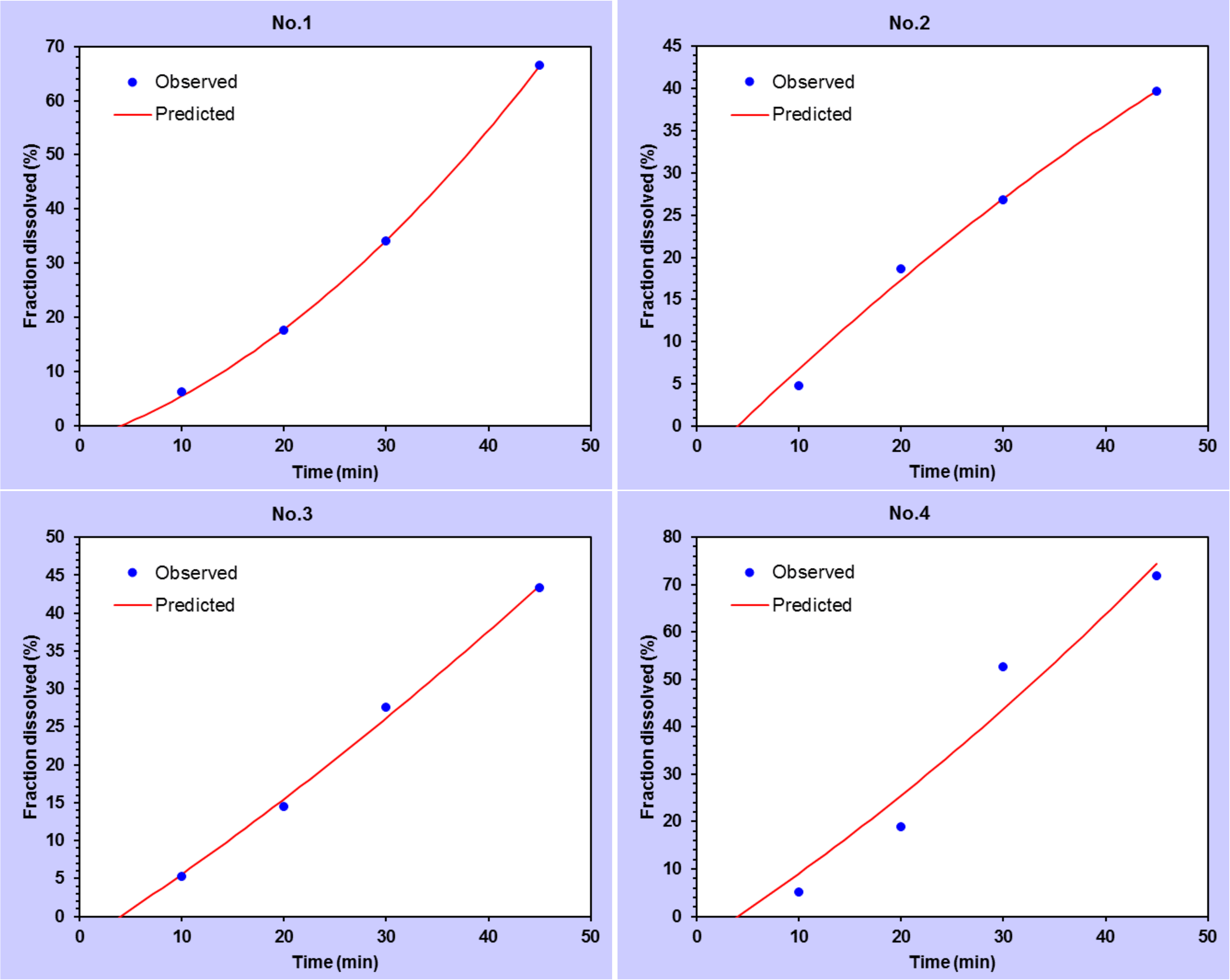

Supplementary materials – Model fitting summary – Parteck® M 100 (modelling up to app. 60 % of carvedilol released)

Model: **Weibull_1**

Model equation: $F = 100 \cdot \left[1 - e^{-\frac{(t-T_i)^{\beta}}{\alpha}}\right]$

Fitted model parameters per tested tablet (N = 4) with statistics – mean, standard deviation (SD), and relative standard deviation expressed in % (RSD%) (output from DDSolver):

Parameter	No.1	No.2	No.3	No.4	Mean	SD	RSD(%)
α	233.075	171.373	178.848	454.625	259.480	132.970	51.245
β	1.441	1.223	1.235	1.724	1.406	0.234	16.675
T_i	4.000	4.000	4.000	4.000	4.000	0.000	0.000

Number of dissolution data points (N), degrees of freedom (df), and selected goodness of fit criteria – Pearson correlation coefficient (R), coefficient of determination (R^2), adjusted coefficient of determination ($R^2_{adjusted}$), and residual sum of squares (RSS) (manual calculation in MS Excel):

Parameter	No.1	No.2	No.3	No.4
N	4	4	4	4
df	1	1	1	1
R	0.988527942	0.993236899	0.99861517	0.987264507
R^2	0.977187492	0.986519538	0.997232258	0.974691207
$R^2_{adjusted}$	0.931562475	0.959558613	0.991696774	0.92407362
RSS	69.51399868	13.7567728	3.340985797	72.79952341

Graphical abstract of model fit presented as mean ± 1 SD of the fraction % of released carvedilol:

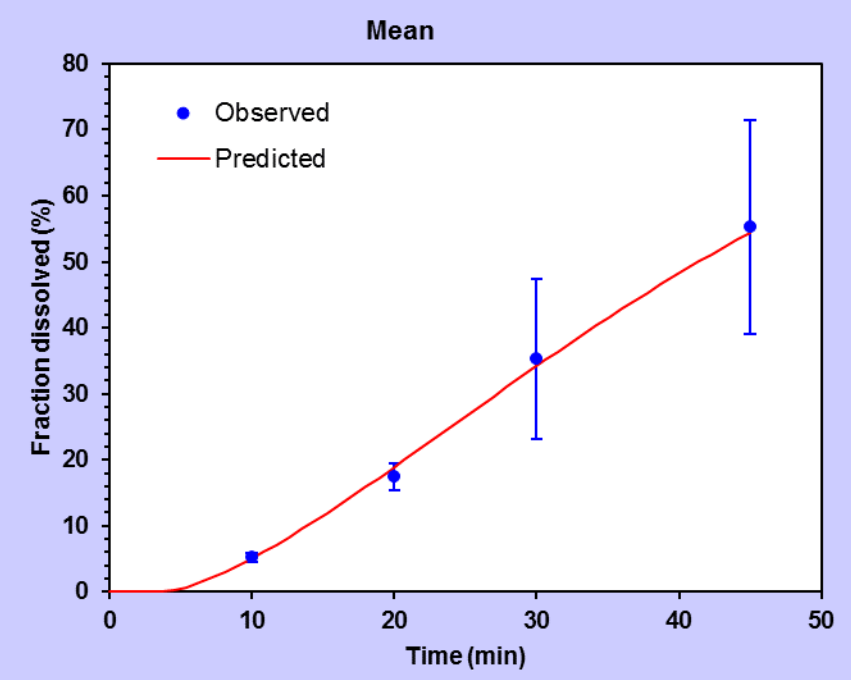

Supplementary materials – Model fitting summary – Parteck® M 100 (modelling up to app. 60 % of carvedilol released)

Graphical abstract of model fit presented as the fraction % of released carvedilol per tested tablet:

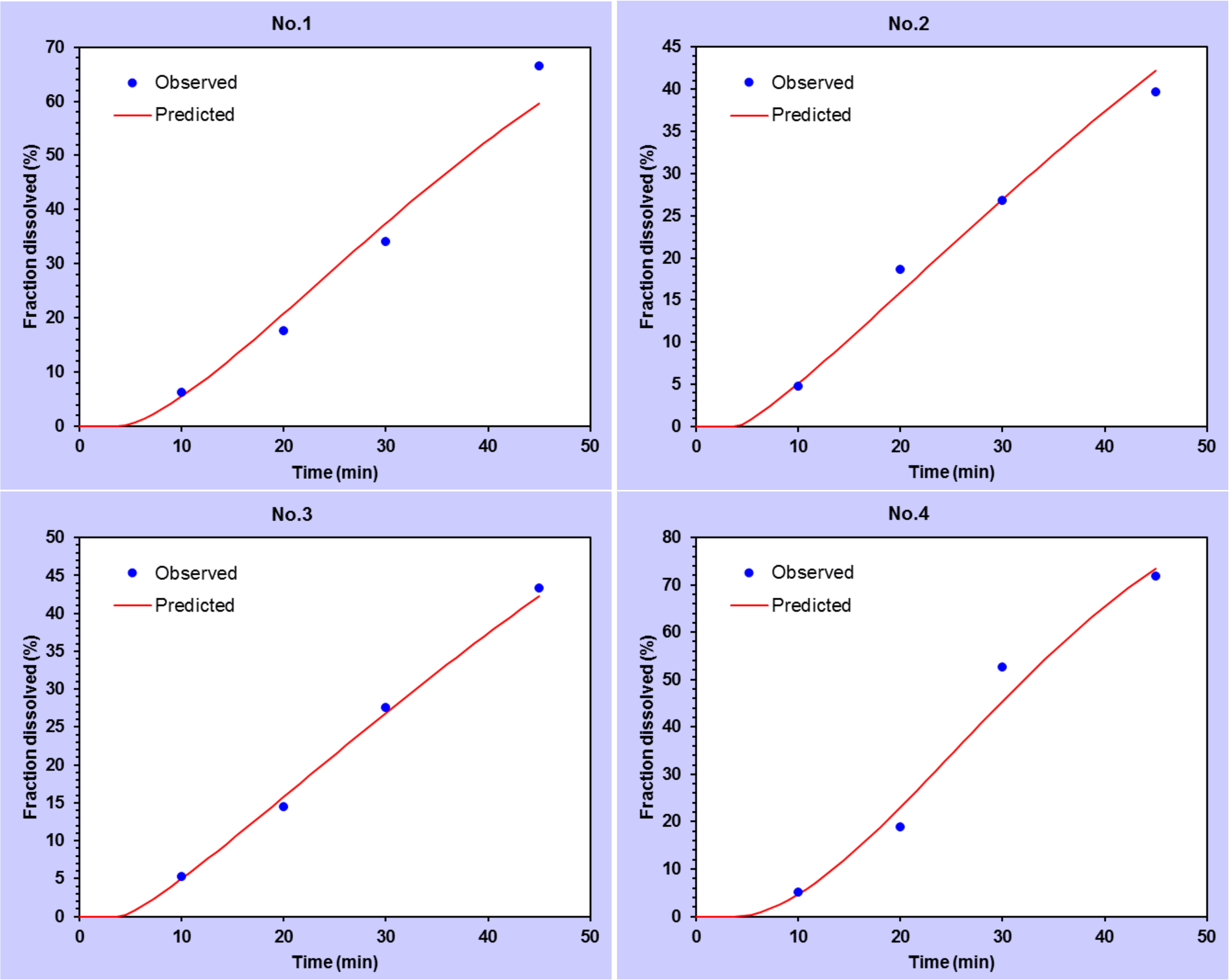

Supplementary materials – Model fitting summary – Parteck® M 100 (modelling up to app. 60 % of carvedilol released)

Model: **Weibull_2**

Model equation: $F = 100 \cdot \left(1 - e^{-\frac{t^{\beta}}{\alpha}}\right)$

Fitted model parameters per tested tablet (N = 4) with statistics – mean, standard deviation (SD), and relative standard deviation expressed in % (RSD%) (output from DDSolver):

Parameter	No.1	No.2	No.3	No.4	Mean	SD	RSD(%)
α	1465.735	656.047	727.389	3202.360	1512.883	1184.303	78.281
β	1.929	1.558	1.588	2.215	1.822	0.311	17.063

Number of dissolution data points (N), degrees of freedom (df), and selected goodness of fit criteria – Pearson correlation coefficient (R), coefficient of determination (R^2), adjusted coefficient of determination ($R^2_{adjusted}$), and residual sum of squares (RSS) (manual calculation in MS Excel):

Parameter	No.1	No.2	No.3	No.4
N	4	4	4	4
df	2	2	2	2
R	0.995453434	0.986180972	0.99871492	0.983405271
R^2	0.990927539	0.97255291	0.997431491	0.967085926
$R^2_{adjusted}$	0.986391309	0.958829365	0.996147237	0.950628889
RSS	24.0558527	29.58733027	2.215867819	95.15379579

Graphical abstract of model fit presented as mean ± 1 SD of the fraction % of released carvedilol:

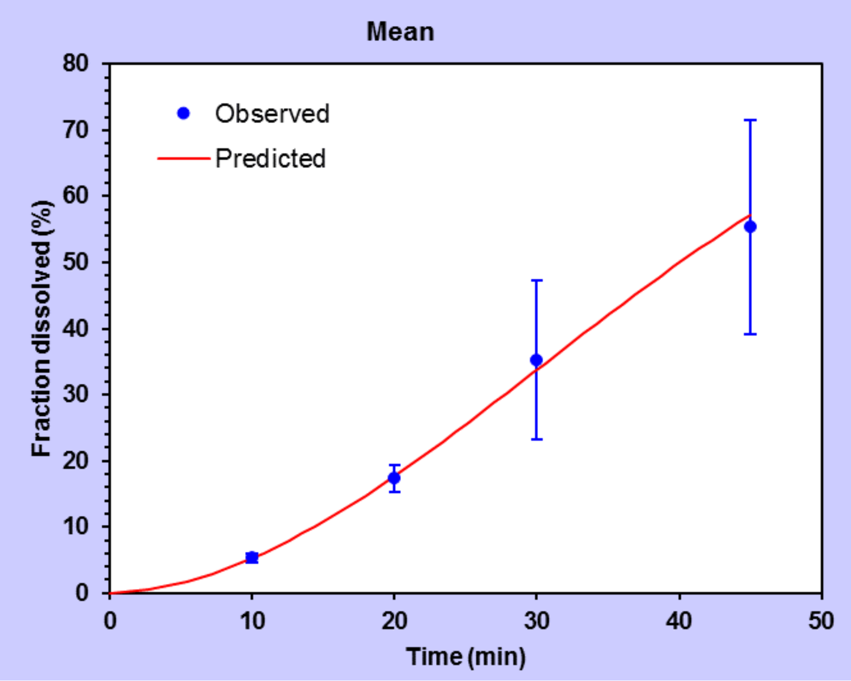

Supplementary materials – Model fitting summary – Parteck® M 100 (modelling up to app. 60 % of carvedilol released)

Graphical abstract of model fit presented as the fraction % of released carvedilol per tested tablet:

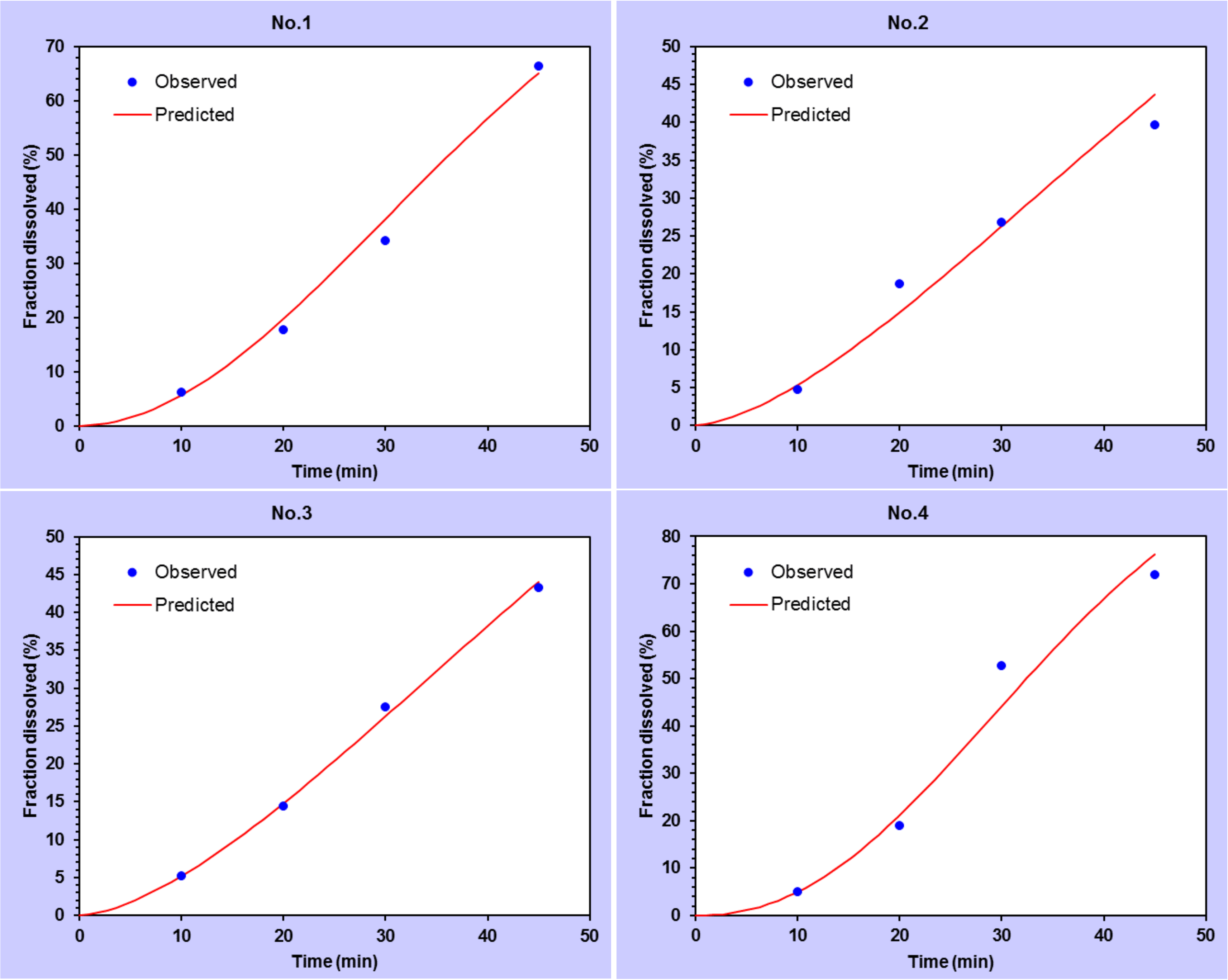

Supplementary materials – Model fitting summary – Parteck® M 100 (modelling up to app. 60 % of carvedilol released)

Model: **Weibull_3**

Model equation: $F = F_{max} \cdot \left(1 - e^{-\frac{t^{\beta}}{\alpha}}\right)$

Fitted model parameters per tested tablet (N = 4) with statistics – mean, standard deviation (SD), and relative standard deviation expressed in % (RSD%) (output from DDSolver):

Parameter	No.1	No.2	No.3	No.4	Mean	SD	RSD(%)
α	2748.342	1013.531	1580.759	6051.388	2848.505	2254.093	79.132
β	2.159	2.093	2.119	2.575	2.237	0.227	10.159
F_{max}	78.350	41.656	47.217	75.416	60.660	18.908	31.171

Number of dissolution data points (N), degrees of freedom (df), and selected goodness of fit criteria – Pearson correlation coefficient (R), coefficient of determination (R^2), adjusted coefficient of determination ($R^2_{adjusted}$), and residual sum of squares (RSS) (manual calculation in MS Excel):

Parameter	No.1	No.2	No.3	No.4
N	4	4	4	4
df	1	1	1	1
R	0.995468957	0.993037349	0.998418714	0.994772989
R^2	0.990958444	0.986123177	0.996839928	0.9895733
$R^2_{adjusted}$	0.972875333	0.958369532	0.990519783	0.968719899
RSS	76.29232632	9.518906464	7.79477157	32.92876764

Graphical abstract of model fit presented as mean ± 1 SD of the fraction % of released carvedilol:

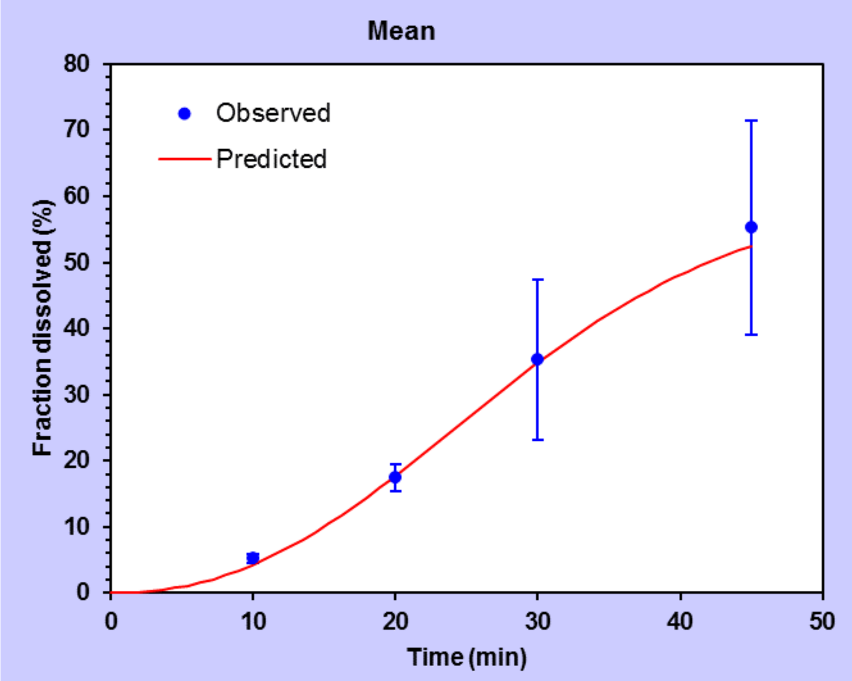

Supplementary materials – Model fitting summary – Parteck® M 100 (modelling up to app. 60 % of carvedilol released)

Graphical abstract of model fit presented as the fraction % of released carvedilol per tested tablet:

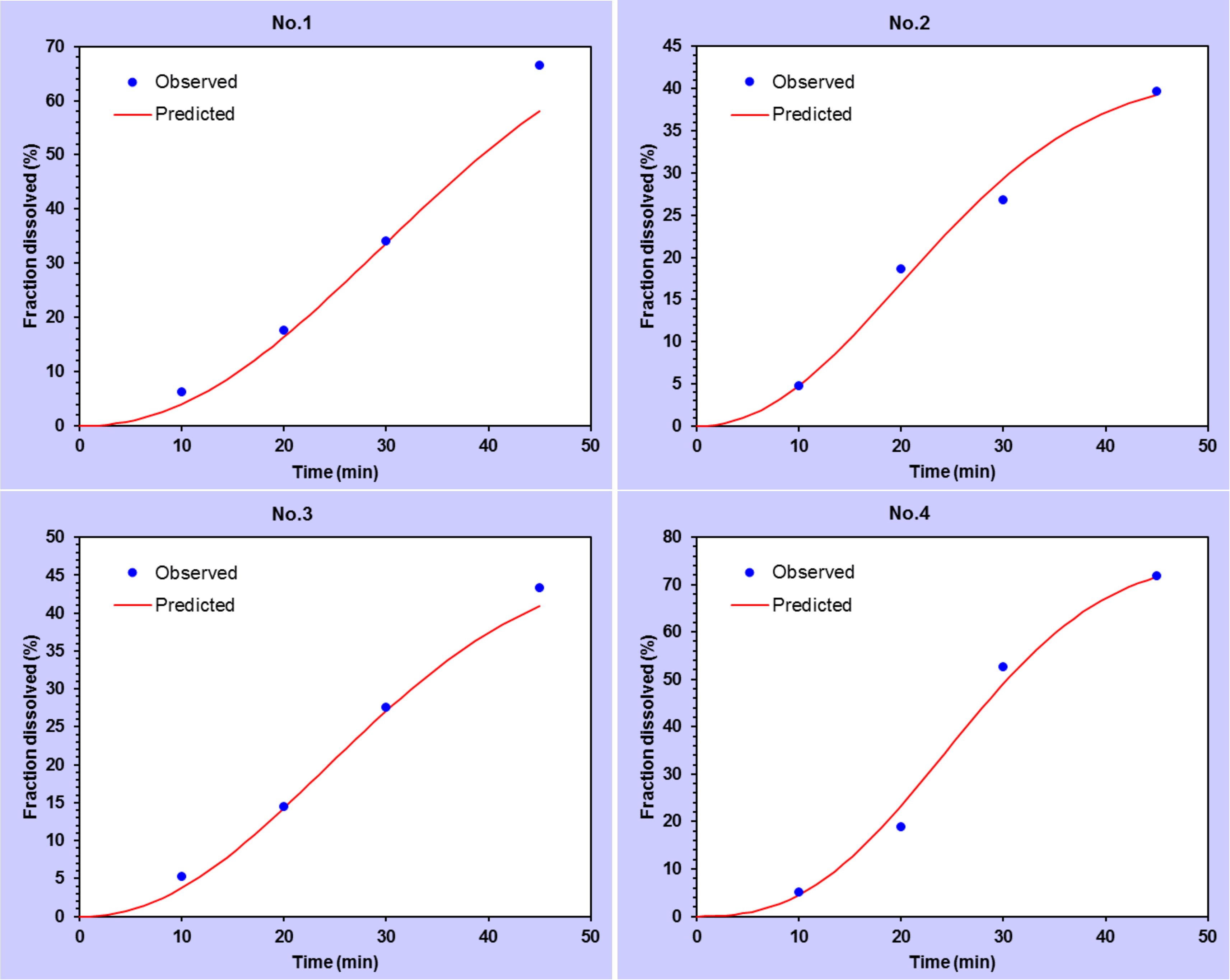

Supplementary materials – Model fitting summary – Parteck® M 100 (modelling up to app. 60 % of carvedilol released)

Model: **Weibull_4**

Model equation: $F = F_{max} \cdot \left[1 - e^{-\frac{(t-T_i)^\beta}{\alpha}}\right]$

Fitted model parameters per tested tablet (N = 4) with statistics – mean, standard deviation (SD), and relative standard deviation expressed in % (RSD%) (output from DDSolver):

Parameter	No.1	No.2	No.3	No.4	Mean	SD	RSD(%)
α	/	/	/	/	/	/	/
β	/	/	/	/	/	/	/
T_i	/	/	/	/	/	/	/
F_{max}	/	/	/	/	/	/	/

Number of dissolution data points (N), degrees of freedom (df), and selected goodness of fit criteria – Pearson correlation coefficient (R), coefficient of determination (R^2), adjusted coefficient of determination ($R^2_{adjusted}$), and residual sum of squares (RSS) (manual calculation in MS Excel):

Parameter	No.1	No.2	No.3	No.4
N	/	/	/	/
df	/	/	/	/
R	/	/	/	/
R^2	/	/	/	/
$R^2_{adjusted}$	/	/	/	/
RSS	/	/	/	/

Graphical abstract of model fit presented as mean ± 1 SD of the fraction % of released carvedilol: /

Graphical abstract of model fit presented as the fraction % of released carvedilol per tested tablet: /

Note: model could not be fitted to experimental dissolution data due too few data points being available for fitting

Supplementary materials – Model fitting summary – Parteck® M 100 (modelling up to app. 60 % of carvedilol released)

Model: **Logistic_1**

Model equation: $F = 100 \cdot \frac{e^{\alpha+\beta\cdot\log(t)}}{1+e^{\alpha+\beta\cdot\log(t)}}$

Fitted model parameters per tested tablet (N = 4) with statistics – mean, standard deviation (SD), and relative standard deviation expressed in % (RSD%) (output from DDSolver):

Parameter	No.1	No.2	No.3	No.4	Mean	SD	RSD(%)
α	-7.960	-6.828	-6.992	-9.157	-7.734	1.072	-13.860
β	5.082	3.940	4.058	6.125	4.801	1.021	21.257

Number of dissolution data points (N), degrees of freedom (df), and selected goodness of fit criteria – Pearson correlation coefficient (R), coefficient of determination (R^2), adjusted coefficient of determination ($R^2_{adjusted}$), and residual sum of squares (RSS) (manual calculation in MS Excel):

Parameter	No.1	No.2	No.3	No.4
N	4	4	4	4
df	2	2	2	2
R	0.987253088	0.990917037	0.999384584	0.991574495
R^2	0.974668659	0.981916575	0.998769547	0.98321998
$R^2_{adjusted}$	0.962002988	0.972874862	0.99815432	0.97482997
RSS	63.75695455	16.85341409	1.207784915	49.69236952

Graphical abstract of model fit presented as mean ± 1 SD of the fraction % of released carvedilol:

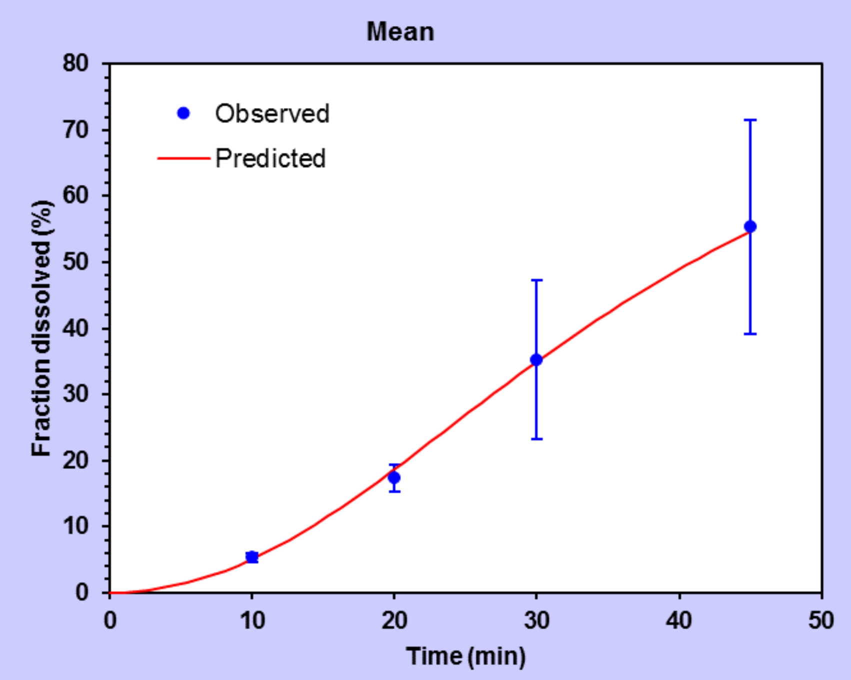

Supplementary materials – Model fitting summary – Parteck® M 100 (modelling up to app. 60 % of carvedilol released)

Graphical abstract of model fit presented as the fraction % of released carvedilol per tested tablet:

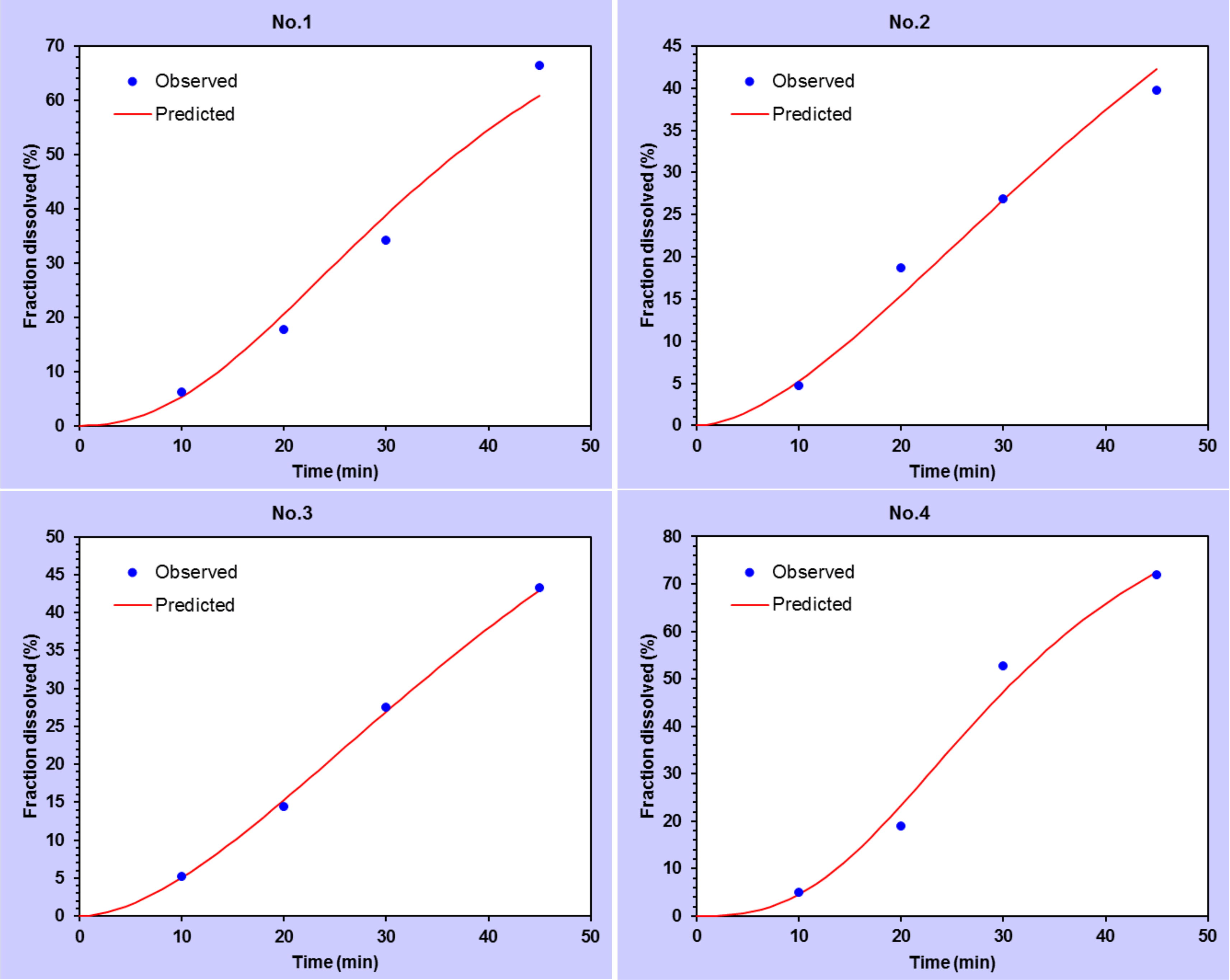

Supplementary materials – Model fitting summary – Parteck® M 100 (modelling up to app. 60 % of carvedilol released)

Model: **Logistic_2**

Model equation: $F = F_{max} \cdot \frac{e^{\alpha+\beta\cdot\log(t)}}{1+e^{\alpha+\beta\cdot\log(t)}}$

Fitted model parameters per tested tablet (N = 4) with statistics – mean, standard deviation (SD), and relative standard deviation expressed in % (RSD%) (output from DDSolver):

Parameter	No.1	No.2	No.3	No.4	Mean	SD	RSD(%)
α	-11.745	-9.622	-10.283	-11.608	-10.814	1.033	-9.551
β	8.388	7.327	6.947	8.566	7.807	0.793	10.152
F_{max}	56.820	41.656	53.645	75.416	56.885	13.974	24.566

Number of dissolution data points (N), degrees of freedom (df), and selected goodness of fit criteria – Pearson correlation coefficient (R), coefficient of determination (R^2), adjusted coefficient of determination ($R^2_{adjusted}$), and residual sum of squares (RSS) (manual calculation in MS Excel):

Parameter	No.1	No.2	No.3	No.4
N	4	4	4	4
df	1	1	1	1
R	0.959728115	0.981101094	0.99901303	0.983043963
R^2	0.921078055	0.962559356	0.998027034	0.966375434
$R^2_{adjusted}$	0.763234166	0.887678069	0.994081103	0.899126301
RSS	274.8538366	31.28954162	22.07663998	120.4721166

Graphical abstract of model fit presented as mean ± 1 SD of the fraction % of released carvedilol:

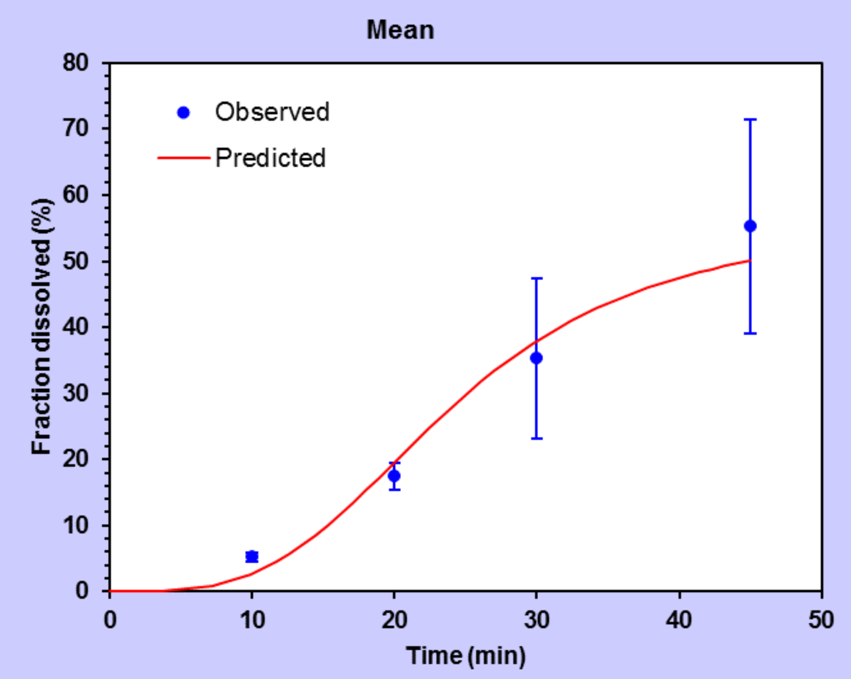

Supplementary materials – Model fitting summary – Parteck® M 100 (modelling up to app. 60 % of carvedilol released)

Graphical abstract of model fit presented as the fraction % of released carvedilol per tested tablet:

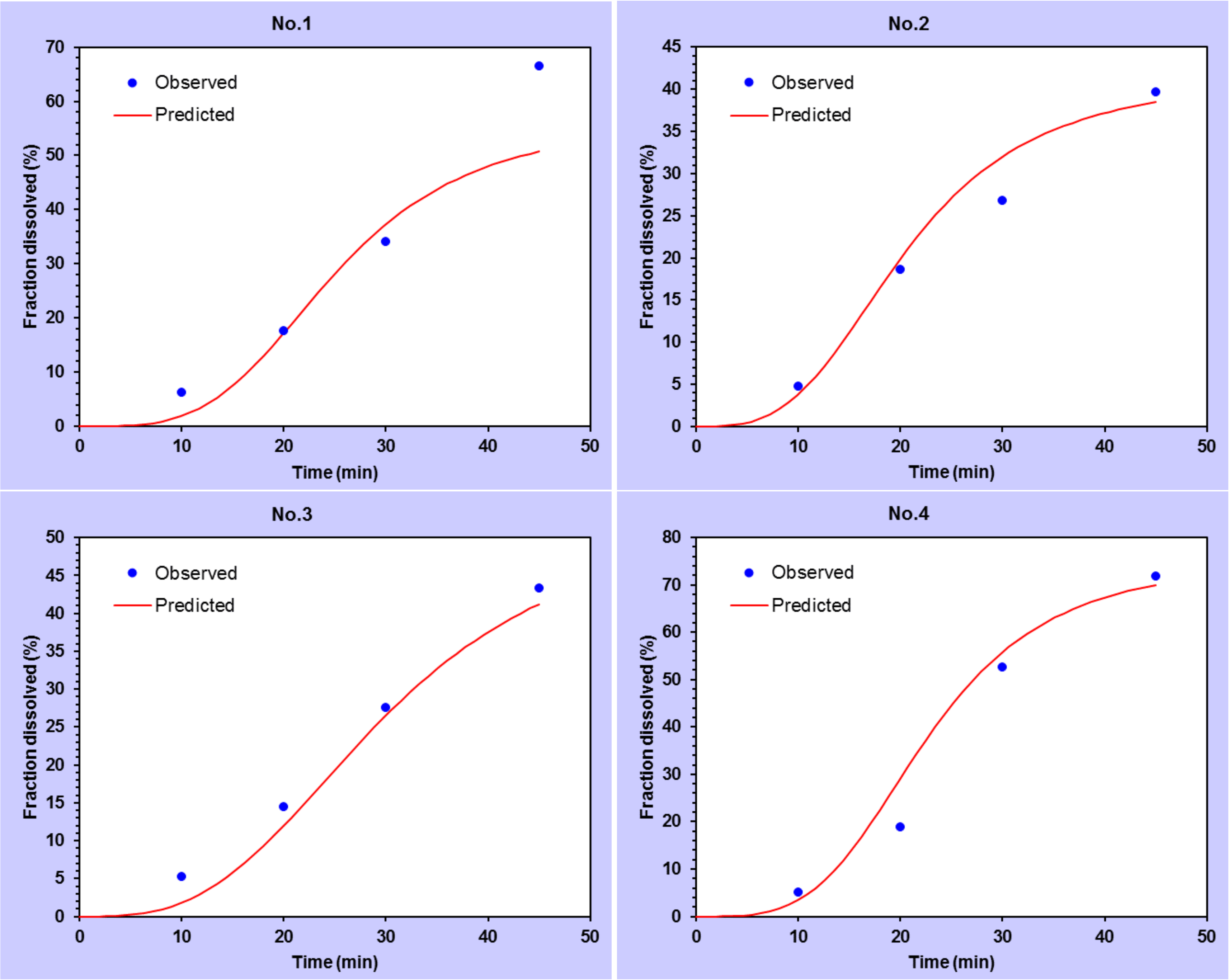

Supplementary materials – Model fitting summary – Parteck® M 100 (modelling up to app. 60 % of carvedilol released)

Model: **Logistic_3**

Model equation: $F = F_{max} \cdot \frac{1}{1+e^{-k\cdot(t-\gamma)}}$

Fitted model parameters per tested tablet (N = 4) with statistics – mean, standard deviation (SD), and relative standard deviation expressed in % (RSD%) (output from DDSolver):

Parameter	No.1	No.2	No.3	No.4	Mean	SD	RSD(%)
k	0.150	0.139	0.100	0.215	0.151	0.048	31.663
γ	27.022	23.907	29.084	24.342	26.089	2.426	9.298
F_{max}	69.752	41.656	50.727	69.985	58.030	14.163	24.406

Number of dissolution data points (N), degrees of freedom (df), and selected goodness of fit criteria – Pearson correlation coefficient (R), coefficient of determination (R^2), adjusted coefficient of determination ($R^2_{adjusted}$), and residual sum of squares (RSS) (manual calculation in MS Excel):

Parameter	No.1	No.2	No.3	No.4
N	4	4	4	4
df	1	1	1	1
R	0.985089924	0.987778194	0.99961043	0.997888384
R^2	0.970402159	0.975705761	0.999221012	0.995781227
$R^2_{adjusted}$	0.911206477	0.927117283	0.997663035	0.987343681
RSS	74.69199421	16.92500408	4.062691709	13.35452018

Graphical abstract of model fit presented as mean ± 1 SD of the fraction % of released carvedilol:

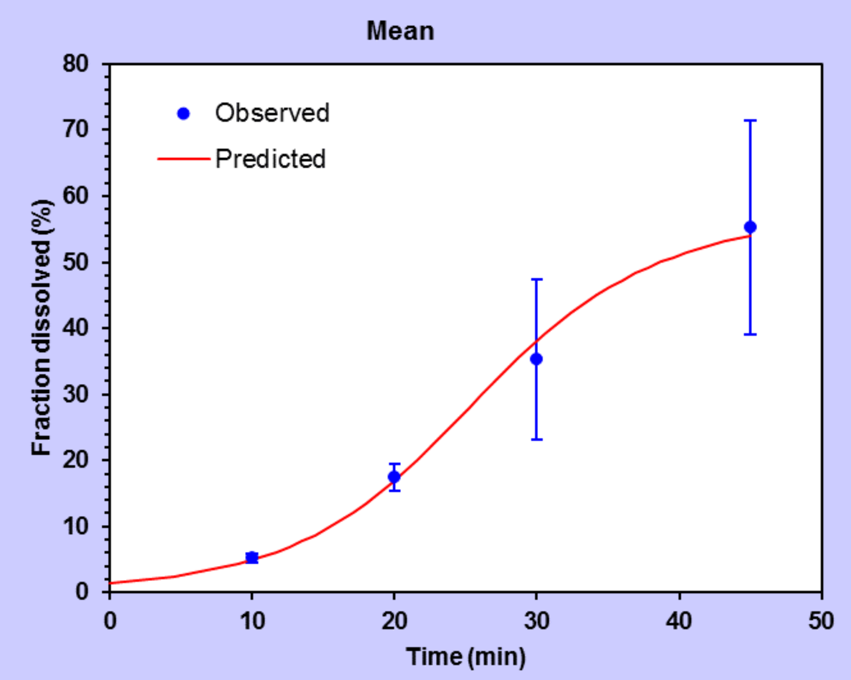

Supplementary materials – Model fitting summary – Parteck® M 100 (modelling up to app. 60 % of carvedilol released)

Graphical abstract of model fit presented as the fraction % of released carvedilol per tested tablet:

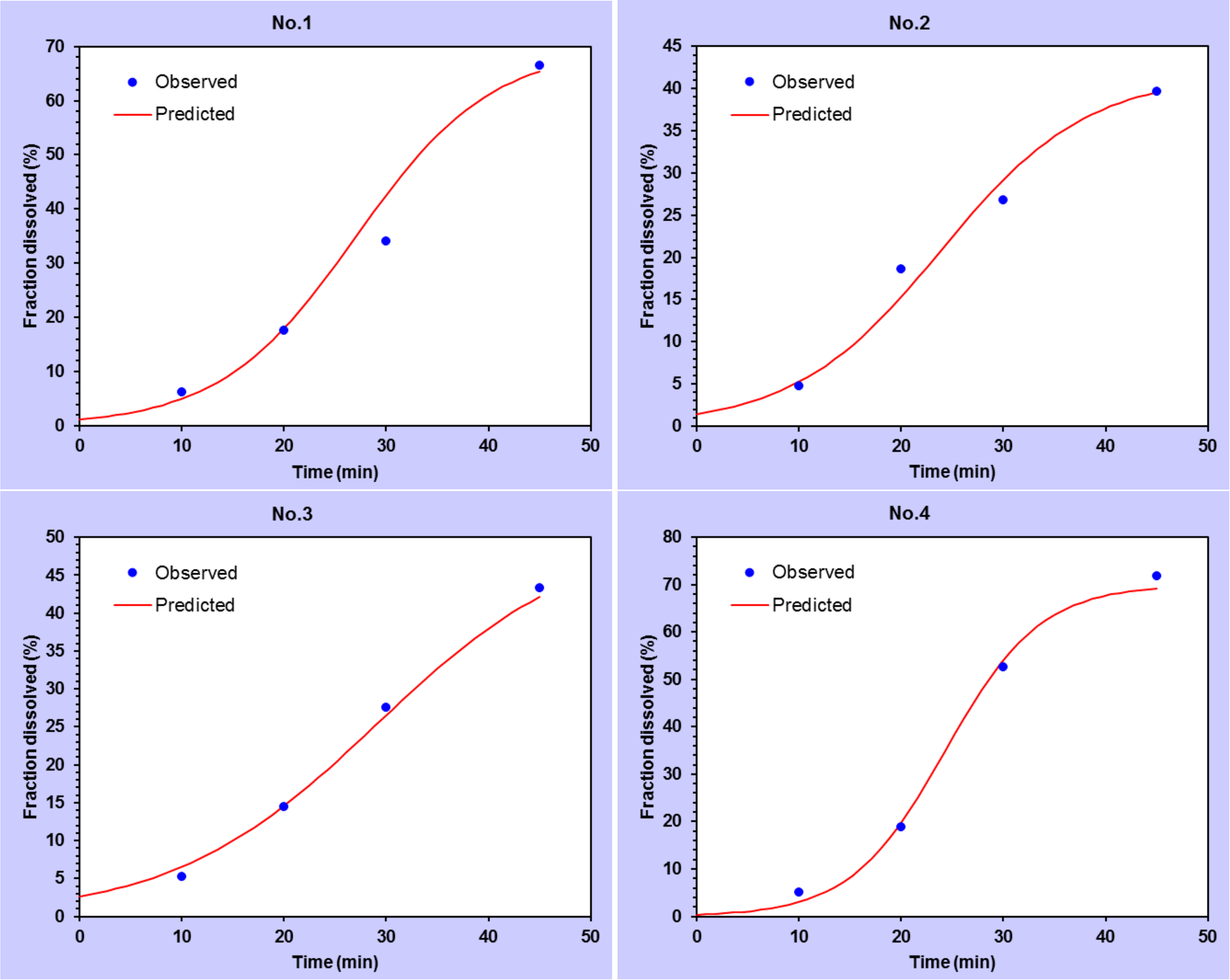

Supplementary materials – Model fitting summary – Parteck® M 100 (modelling up to app. 60 % of carvedilol released)

Model: **Gompertz_1**

Model equation: $F = 100 \cdot e^{-\alpha \cdot e^{-\beta \cdot \log(t)}}$

Fitted model parameters per tested tablet (N = 4) with statistics – mean, standard deviation (SD), and relative standard deviation expressed in % (RSD%) (output from DDSolver):

Parameter	No.1	No.2	No.3	No.4	Mean	SD	RSD(%)
α	54.393	18.319	21.450	108.757	50.730	41.985	82.763
β	2.804	1.804	1.922	3.443	2.493	0.775	31.069

Number of dissolution data points (N), degrees of freedom (df), and selected goodness of fit criteria – Pearson correlation coefficient (R), coefficient of determination (R^2), adjusted coefficient of determination ($R^2_{adjusted}$), and residual sum of squares (RSS) (manual calculation in MS Excel):

Parameter	No.1	No.2	No.3	No.4
N	4	4	4	4
df	2	2	2	2
R	0.960673011	0.997763487	0.991780884	0.980825723
R^2	0.922892634	0.995531976	0.983629322	0.962019099
$R^2_{adjusted}$	0.884338951	0.993297964	0.975443982	0.943028648
RSS	170.6782889	2.949312601	15.39697811	117.4494893

Graphical abstract of model fit presented as mean ± 1 SD of the fraction % of released carvedilol:

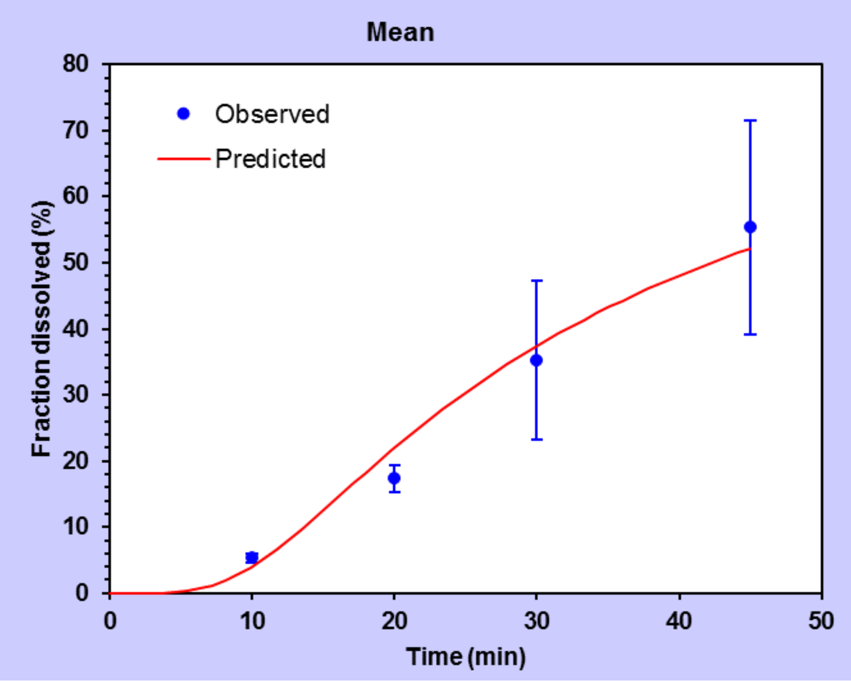

Supplementary materials – Model fitting summary – Parteck® M 100 (modelling up to app. 60 % of carvedilol released)

Graphical abstract of model fit presented as the fraction % of released carvedilol per tested tablet:

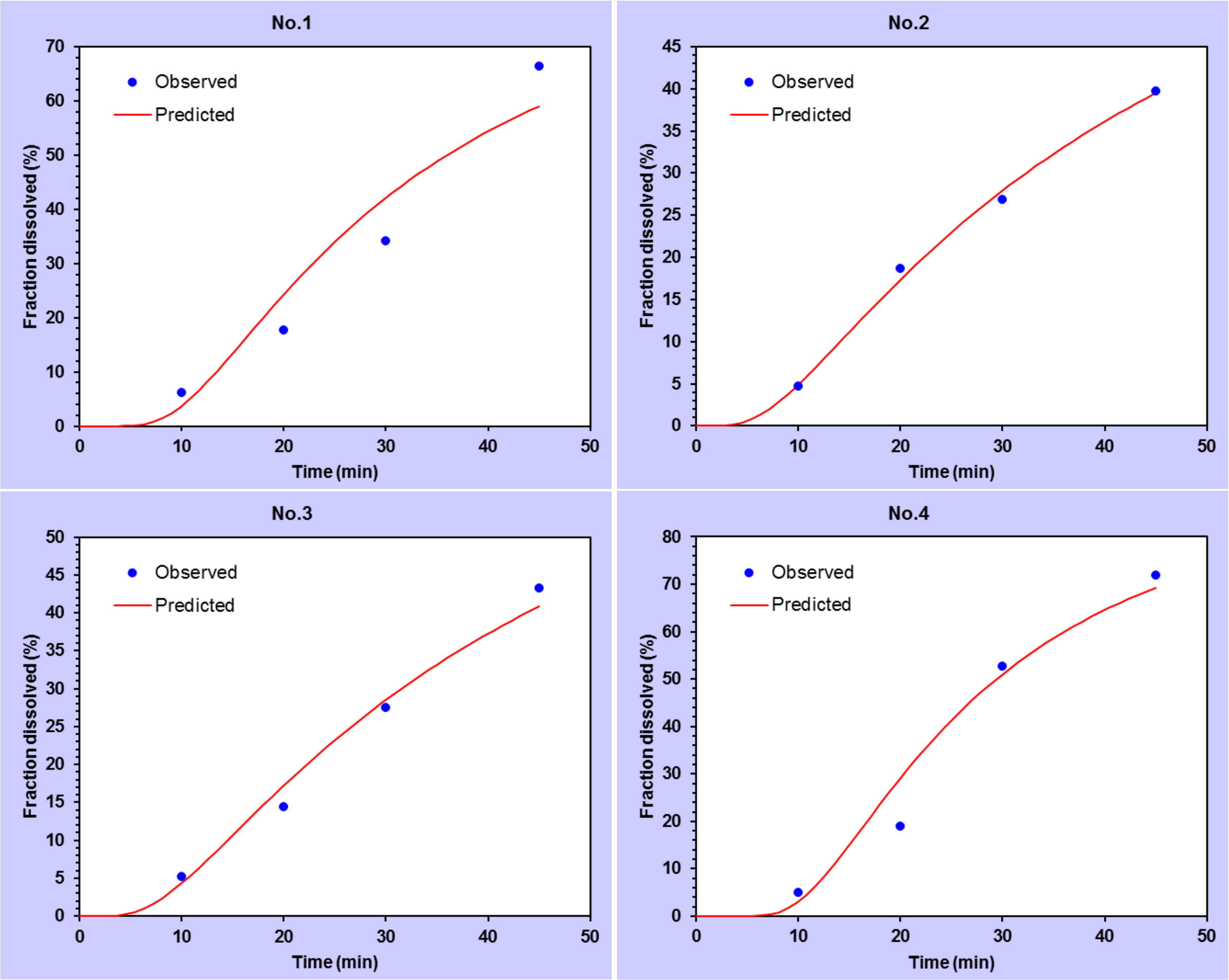

Supplementary materials – Model fitting summary – Parteck® M 100 (modelling up to app. 60 % of carvedilol released)

Model: **Gompertz_2**

Model equation: $F = F_{max} \cdot e^{-\alpha \cdot e^{-\beta \cdot \log(t)}}$

Fitted model parameters per tested tablet (N = 4) with statistics – mean, standard deviation (SD), and relative standard deviation expressed in % (RSD%) (output from DDSolver):

Parameter	No.1	No.2	No.3	No.4	Mean	SD	RSD(%)
α	1416.931	1010.574	1154.109	1987.809	1392.356	431.156	30.966
β	5.441	5.399	5.409	5.391	5.410	0.022	0.405
F_{max}	69.752	41.656	45.433	93.223	62.516	23.960	38.326

Number of dissolution data points (N), degrees of freedom (df), and selected goodness of fit criteria – Pearson correlation coefficient (R), coefficient of determination (R^2), adjusted coefficient of determination ($R^2_{adjusted}$), and residual sum of squares (RSS) (manual calculation in MS Excel):

Parameter	No.1	No.2	No.3	No.4
N	4	4	4	4
df	1	1	1	1
R	0.947509707	0.982724425	0.968924103	0.997859367
R^2	0.897774644	0.965747295	0.938813918	0.995723316
$R^2_{adjusted}$	0.693323932	0.897241885	0.816441754	0.987169949
RSS	212.7621632	38.46651007	56.88300452	71.5493603

Graphical abstract of model fit presented as mean ± 1 SD of the fraction % of released carvedilol:

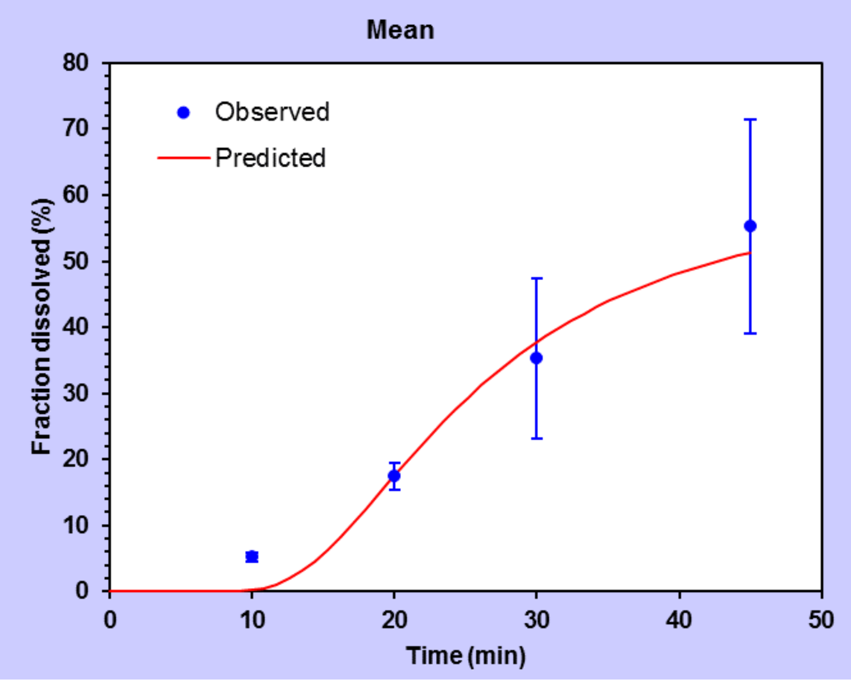

Supplementary materials – Model fitting summary – Parteck® M 100 (modelling up to app. 60 % of carvedilol released)

Graphical abstract of model fit presented as the fraction % of released carvedilol per tested tablet:

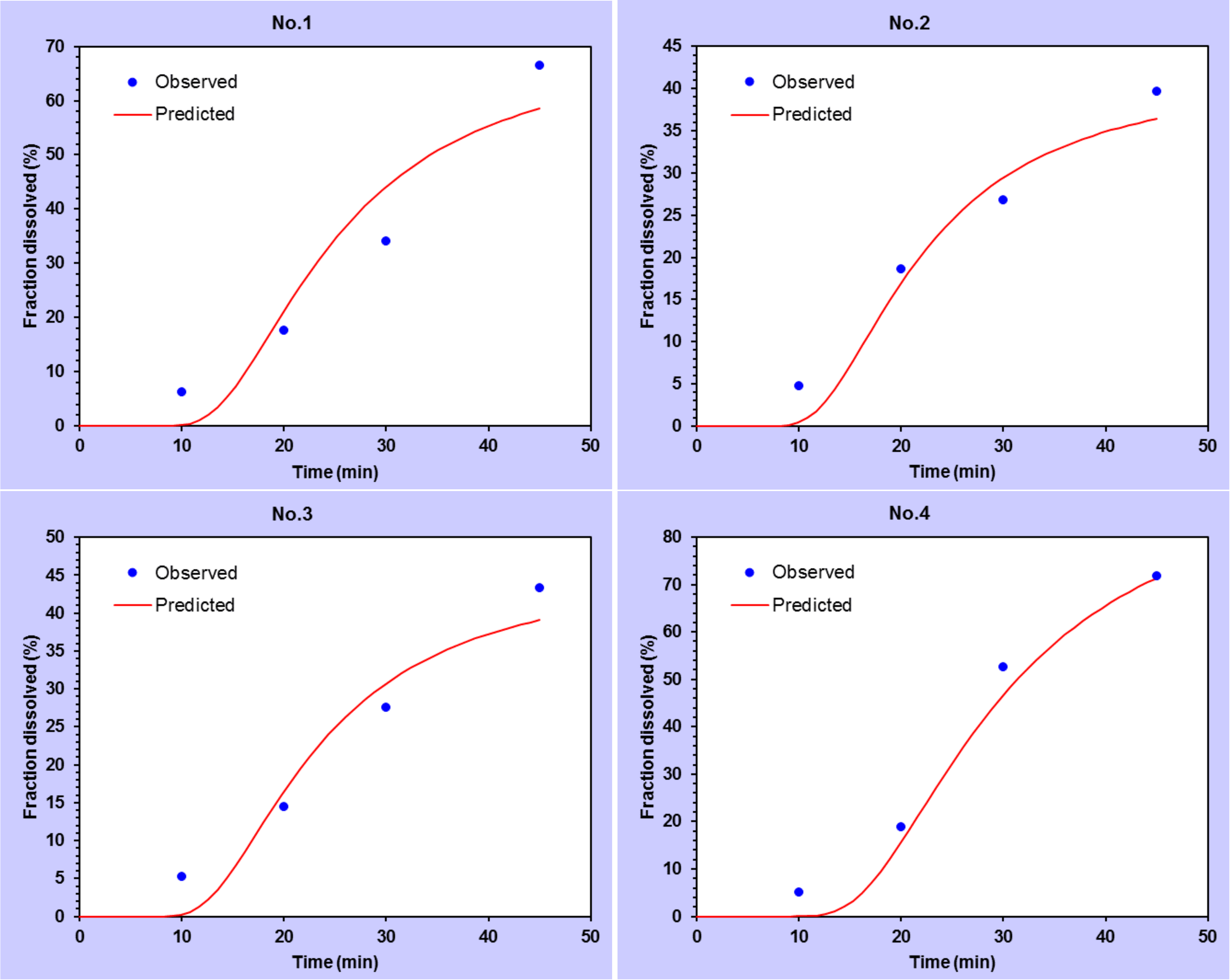

Supplementary materials – Model fitting summary – Parteck® M 100 (modelling up to app. 60 % of carvedilol released)

Model: **Gompertz_3**

Model equation: $F = F_{max} \cdot e^{-e^{-k\cdot(t-\gamma)}}$

Fitted model parameters per tested tablet (N = 4) with statistics – mean, standard deviation (SD), and relative standard deviation expressed in % (RSD%) (output from DDSolver):

Parameter	No.1	No.2	No.3	No.4	Mean	SD	RSD(%)
k	0.111	0.106	0.109	0.138	0.116	0.015	12.895
γ	21.410	18.548	19.817	23.481	20.814	2.129	10.229
F_{max}	69.752	41.656	45.433	75.678	58.130	17.084	29.390

Number of dissolution data points (N), degrees of freedom (df), and selected goodness of fit criteria – Pearson correlation coefficient (R), coefficient of determination (R^2), adjusted coefficient of determination ($R^2_{adjusted}$), and residual sum of squares (RSS) (manual calculation in MS Excel):

Parameter	No.1	No.2	No.3	No.4
N	4	4	4	4
df	1	1	1	1
R	0.960025876	0.988074675	0.981056594	0.999917869
R^2	0.921649683	0.976291563	0.962472041	0.999835745
$R^2_{adjusted}$	0.76494905	0.92887469	0.887416124	0.999507234
RSS	215.4564082	20.02671509	41.37981685	43.94808868

Graphical abstract of model fit presented as mean ± 1 SD of the fraction % of released carvedilol:

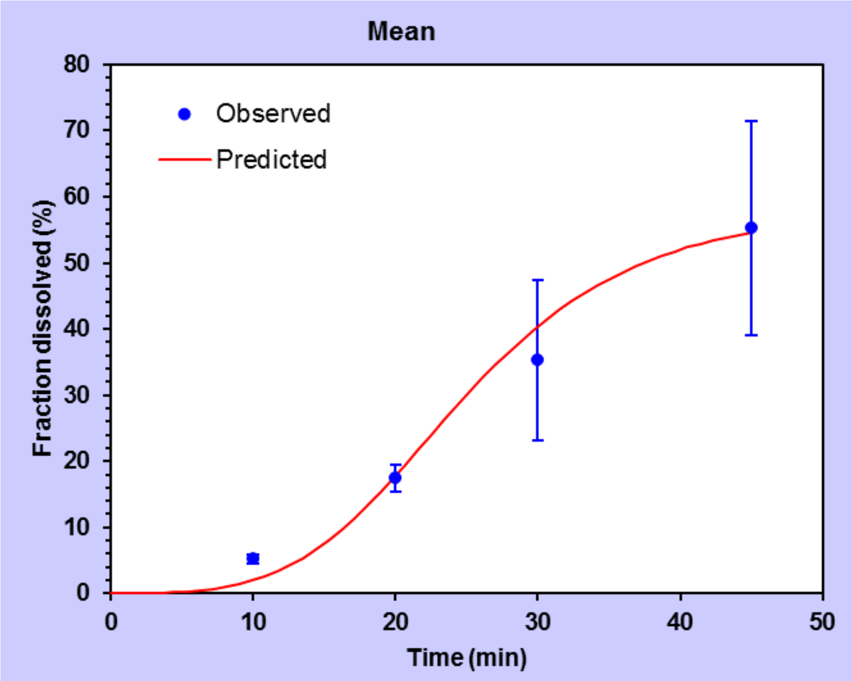

Supplementary materials – Model fitting summary – Parteck® M 100 (modelling up to app. 60 % of carvedilol released)

Graphical abstract of model fit presented as the fraction % of released carvedilol per tested tablet:

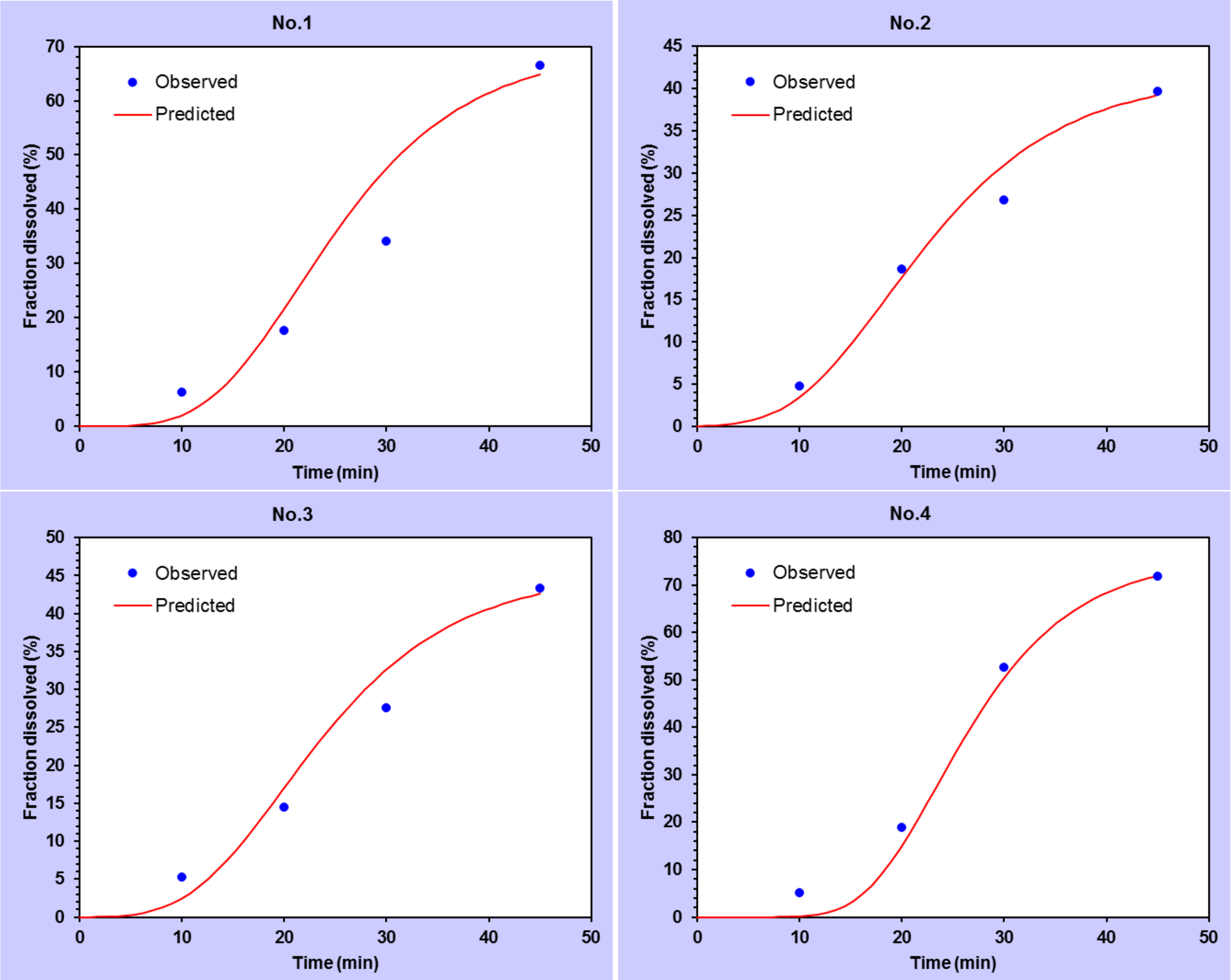

Supplementary materials – Model fitting summary – Parteck® M 100 (modelling up to app. 60 % of carvedilol released)

Model: **Gompertz_4**

Model equation: $F = F_{max} \cdot e^{-\beta \cdot e^{-k \cdot t}}$

Fitted model parameters per tested tablet (N = 4) with statistics – mean, standard deviation (SD), and relative standard deviation expressed in % (RSD%) (output from DDSolver):

Parameter	No.1	No.2	No.3	No.4	Mean	SD	RSD(%)
k	0.093	0.106	0.109	0.118	0.106	0.010	9.471
β	13.541	7.171	12.915	11.134	11.190	2.867	25.620
F_{max}	79.924	41.656	45.433	75.416	60.608	19.848	32.749

Number of dissolution data points (N), degrees of freedom (df), and selected goodness of fit criteria – Pearson correlation coefficient (R), coefficient of determination (R^2), adjusted coefficient of determination ($R^2_{adjusted}$), and residual sum of squares (RSS) (manual calculation in MS Excel):

Parameter	No.1	No.2	No.3	No.4
N	4	4	4	4
df	1	1	1	1
R	0.994550683	0.988074675	0.996035921	0.9903008
R^2	0.98913106	0.976291563	0.992087555	0.980695675
$R^2_{adjusted}$	0.967393181	0.92887469	0.976262666	0.942087024
RSS	96.43087153	20.02671509	41.17781907	63.45247706

Graphical abstract of model fit presented as mean ± 1 SD of the fraction % of released carvedilol:

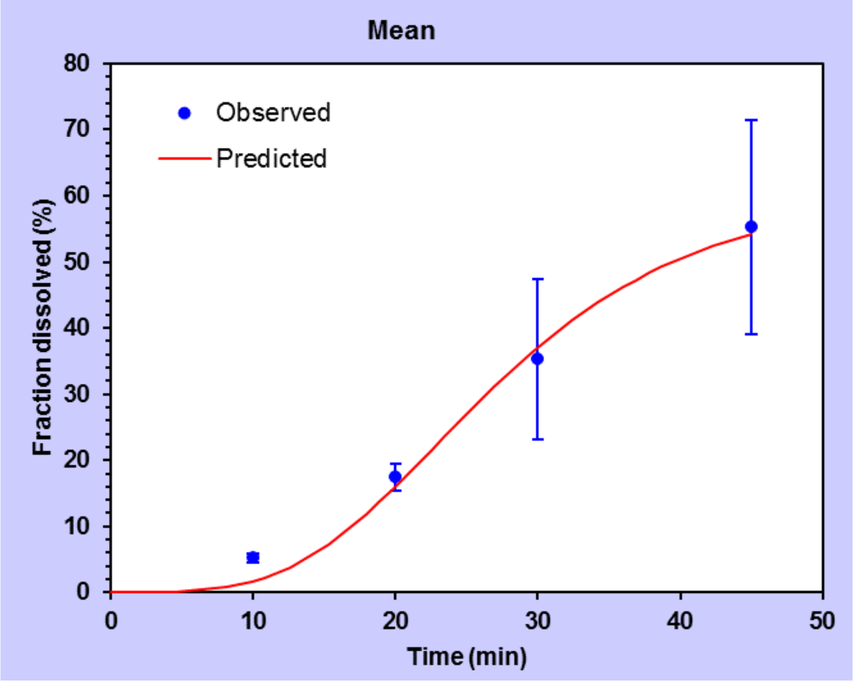

Supplementary materials – Model fitting summary – Parteck® M 100 (modelling up to app. 60 % of carvedilol released)

Graphical abstract of model fit presented as the fraction % of released carvedilol per tested tablet:

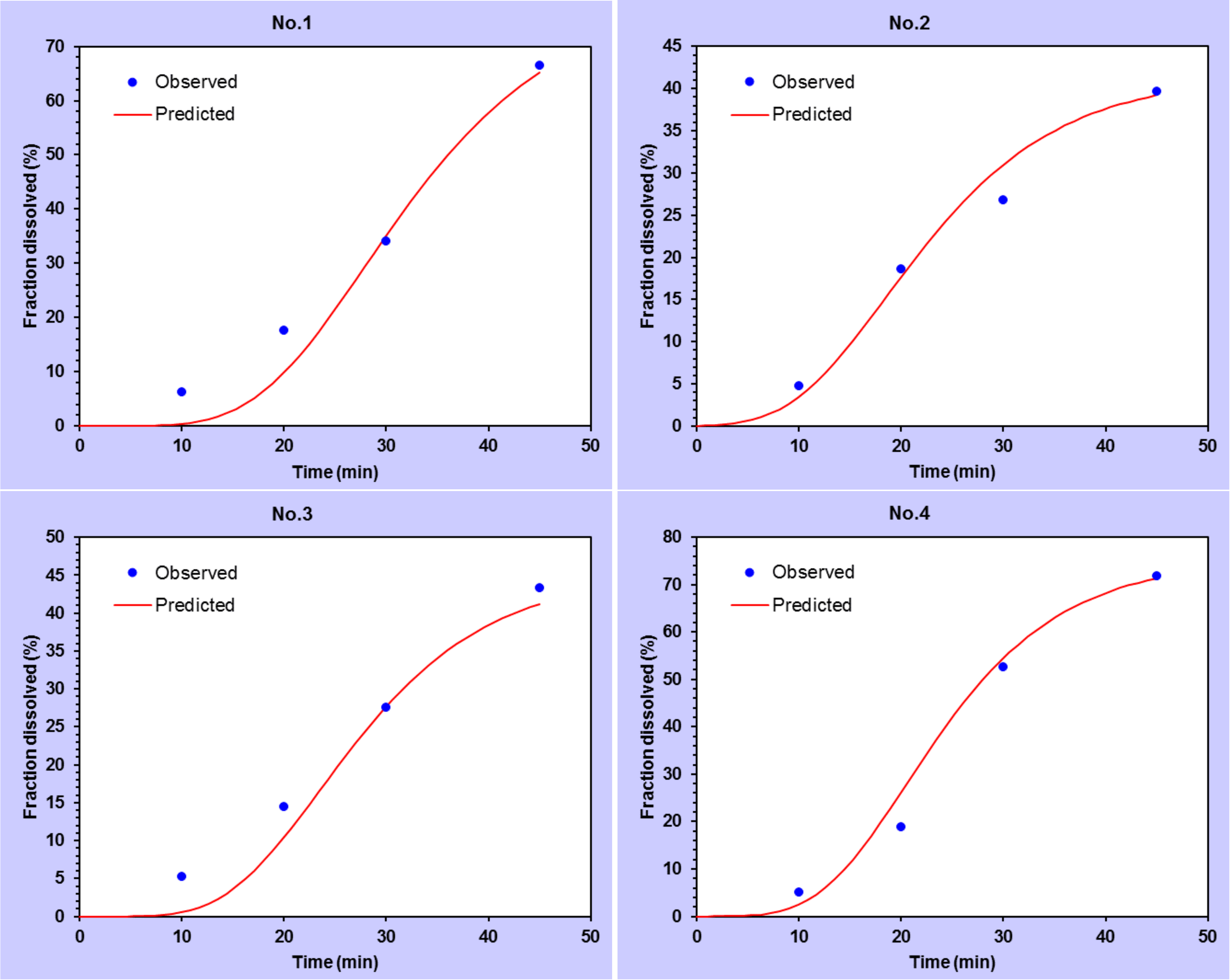

Supplementary materials – Model fitting summary – Parteck® M 100 (modelling up to app. 60 % of carvedilol released)

Model: **Probit_1**

Model equation: $F = 100 \cdot \phi[\alpha + \beta \cdot \log(t)]$

Fitted model parameters per tested tablet (N = 4) with statistics – mean, standard deviation (SD), and relative standard deviation expressed in % (RSD%) (output from DDSolver):

Parameter	No.1	No.2	No.3	No.4	Mean	SD	RSD(%)
α	-4.579	-3.772	-3.902	-5.239	-4.373	0.677	-15.481
β	2.920	2.143	2.237	3.512	2.703	0.641	23.701

Number of dissolution data points (N), degrees of freedom (df), and selected goodness of fit criteria – Pearson correlation coefficient (R), coefficient of determination (R^2), adjusted coefficient of determination ($R^2_{adjusted}$), and residual sum of squares (RSS) (manual calculation in MS Excel):

Parameter	No.1	No.2	No.3	No.4
N	4	4	4	4
df	2	2	2	2
R	0.980669795	0.995337355	0.997796509	0.989792094
R^2	0.961713247	0.99069645	0.995597873	0.97968839
$R^2_{adjusted}$	0.942569871	0.986044674	0.99339681	0.969532585
RSS	92.73215816	7.453864873	4.743992579	62.14861951

Graphical abstract of model fit presented as mean ± 1 SD of the fraction % of released carvedilol:

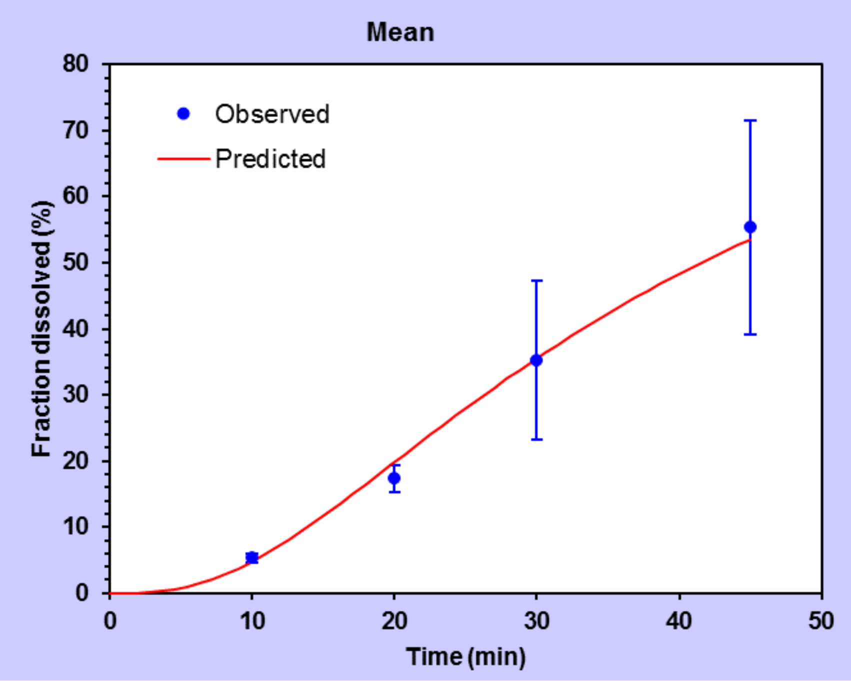

Supplementary materials – Model fitting summary – Parteck® M 100 (modelling up to app. 60 % of carvedilol released)

Graphical abstract of model fit presented as the fraction % of released carvedilol per tested tablet:

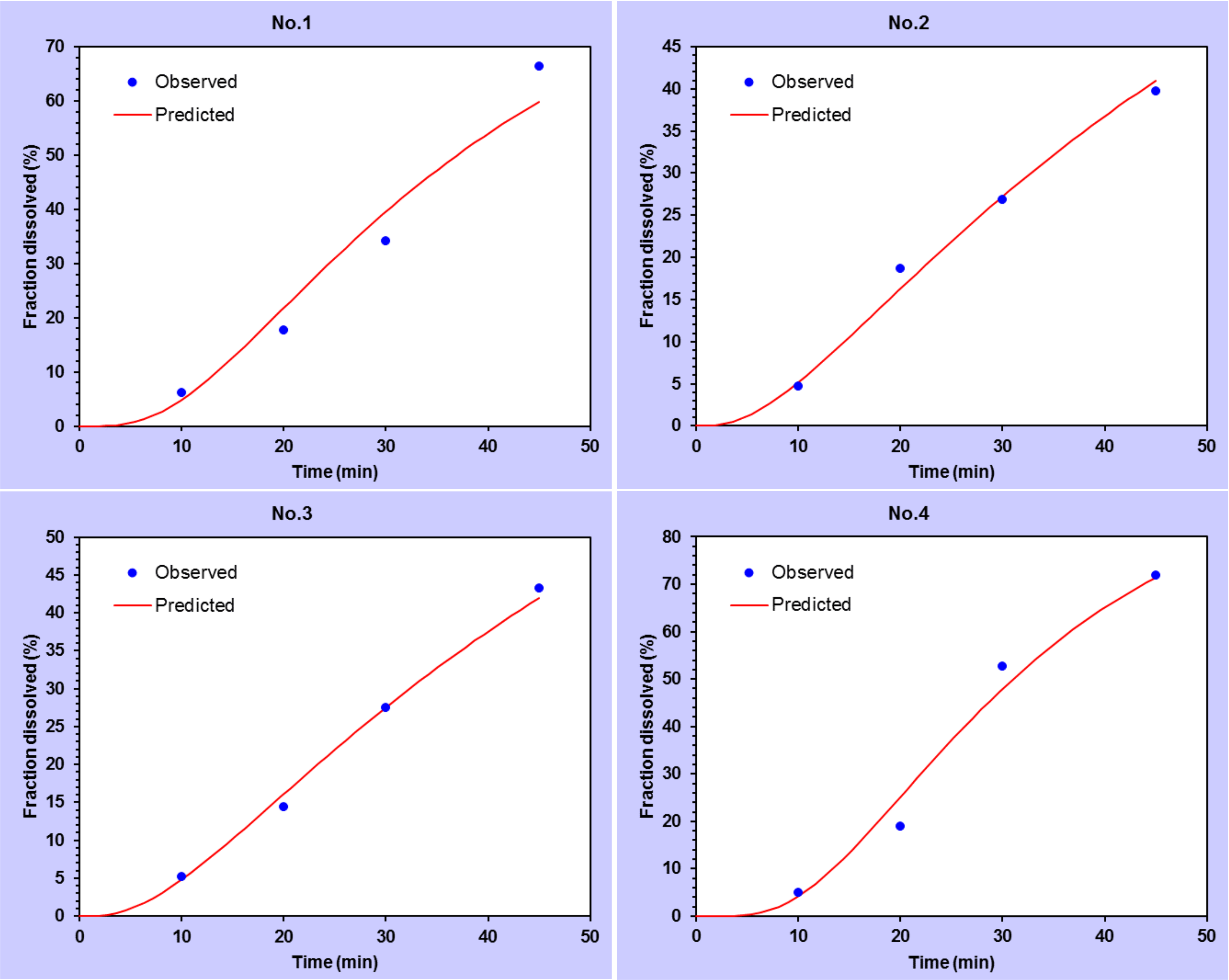

Supplementary materials – Model fitting summary – Parteck® M 100 (modelling up to app. 60 % of carvedilol released)

Model: **Probit_2**

Model equation: $F = F_{max} \cdot \phi[\alpha + \beta \cdot \log(t)]$

Fitted model parameters per tested tablet (N = 4) with statistics – mean, standard deviation (SD), and relative standard deviation expressed in % (RSD%) (output from DDSolver):

Parameter	No.1	No.2	No.3	No.4	Mean	SD	RSD(%)
α	-5.984	-5.532	-6.547	-6.597	-6.165	0.505	-8.193
β	4.338	4.201	4.490	4.860	4.472	0.284	6.357
F_{max}	69.752	41.656	49.285	75.416	59.027	16.128	27.322

Number of dissolution data points (N), degrees of freedom (df), and selected goodness of fit criteria – Pearson correlation coefficient (R), coefficient of determination (R^2), adjusted coefficient of determination ($R^2_{adjusted}$), and residual sum of squares (RSS) (manual calculation in MS Excel):

Parameter	No.1	No.2	No.3	No.4
N	4	4	4	4
df	1	1	1	1
R	0.949042257	0.984883542	0.997313391	0.981081839
R^2	0.900681206	0.969995592	0.994633999	0.962521574
$R^2_{adjusted}$	0.702043618	0.909986775	0.983901997	0.887564723
RSS	244.9229201	23.44170764	37.10260963	124.8721994

Graphical abstract of model fit presented as mean ± 1 SD of the fraction % of released carvedilol:

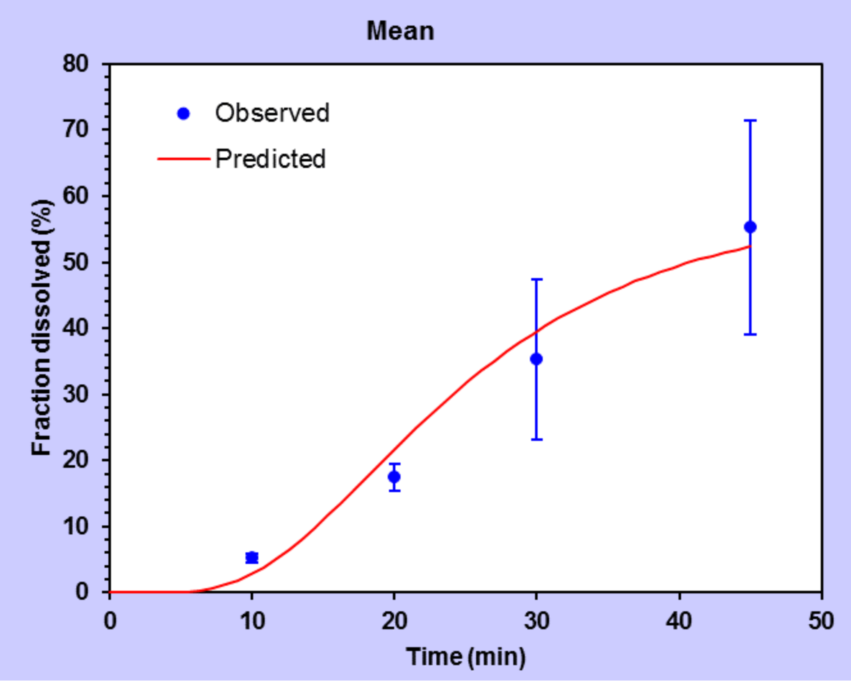

Supplementary materials – Model fitting summary – Parteck® M 100 (modelling up to app. 60 % of carvedilol released)

Graphical abstract of model fit presented as the fraction % of released carvedilol per tested tablet:

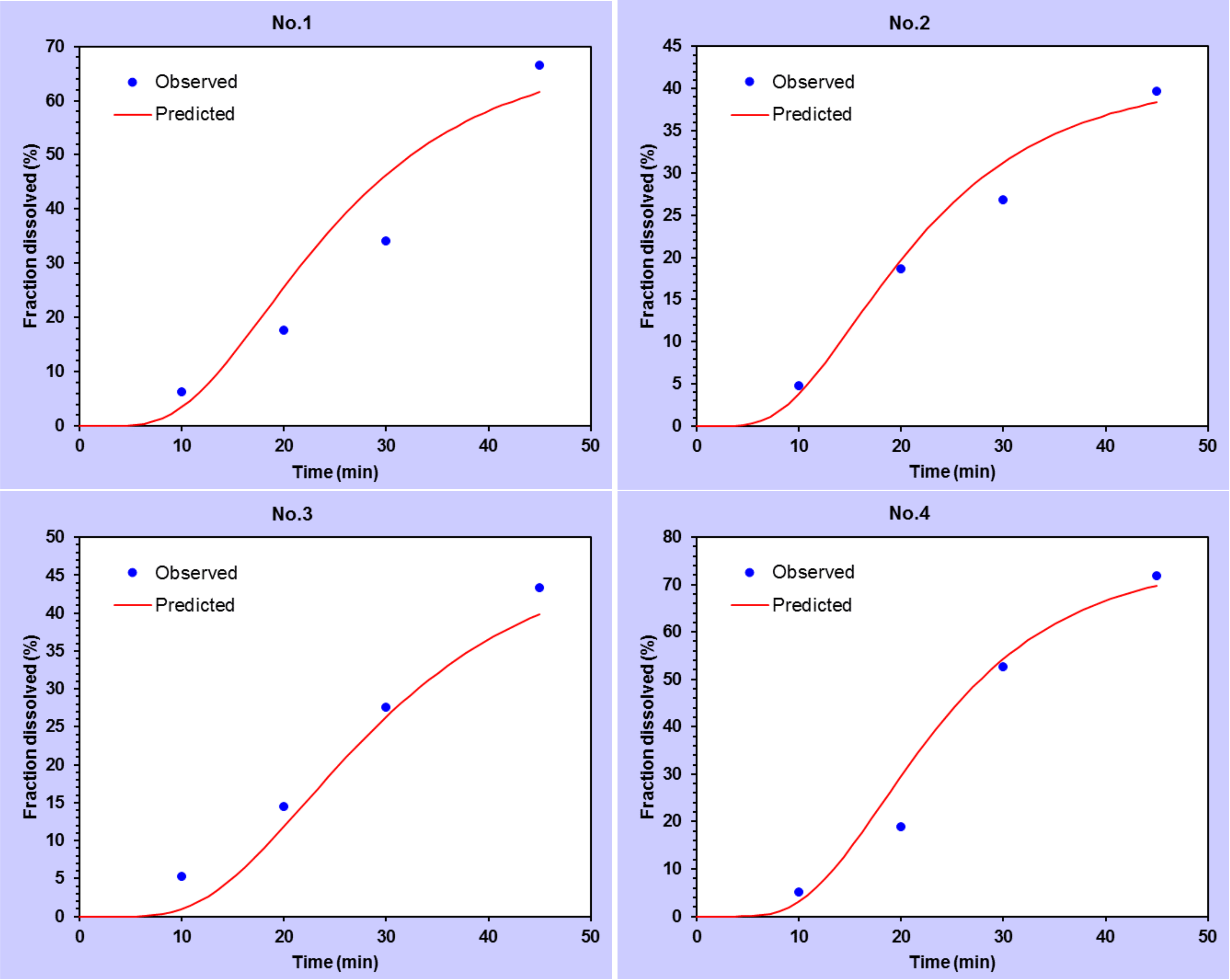

Supplementary materials – Model fitting summary – Parteck® M 100 (modelling up to app. 70 % of carvedilol released)

Model: **Zero–order**

Model equation: $F = k_0 \cdot t$

Fitted model parameters per tested tablet (N = 4) with statistics – mean, standard deviation (SD), and relative standard deviation expressed in % (RSD%) (output from DDSolver):

Parameter	No.1	No.2	No.3	No.4	Mean	SD	RSD(%)
k_0	1.295	0.977	0.917	1.454	1.161	0.256	22.077

Number of dissolution data points (N), degrees of freedom (df), and selected goodness of fit criteria – Pearson correlation coefficient (R), coefficient of determination (R^2), adjusted coefficient of determination ($R^2_{adjusted}$), and residual sum of squares (RSS) (manual calculation in MS Excel):

Parameter	No.1	No.2	No.3	No.4
N	5	5	5	5
df	4	4	4	4
R	0.988678076	0.990876008	0.997320012	0.969389722
R^2	0.977484337	0.981835263	0.994647207	0.939716433
$R^2_{adjusted}$	0.977484337	0.981835263	0.994647207	0.939716433
RSS	203.9475095	82.98476008	35.62450654	336.4929592

Graphical abstract of model fit presented as mean ± 1 SD of the fraction % of released carvedilol:

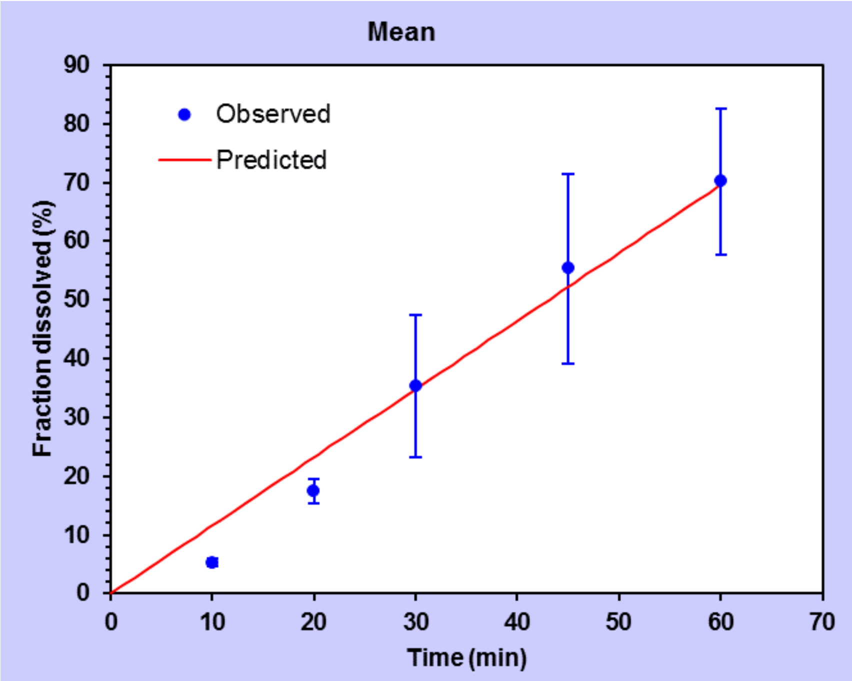

Supplementary materials – Model fitting summary – Parteck® M 100 (modelling up to app. 70 % of carvedilol released)

Graphical abstract of model fit presented as the fraction % of released carvedilol per tested tablet:

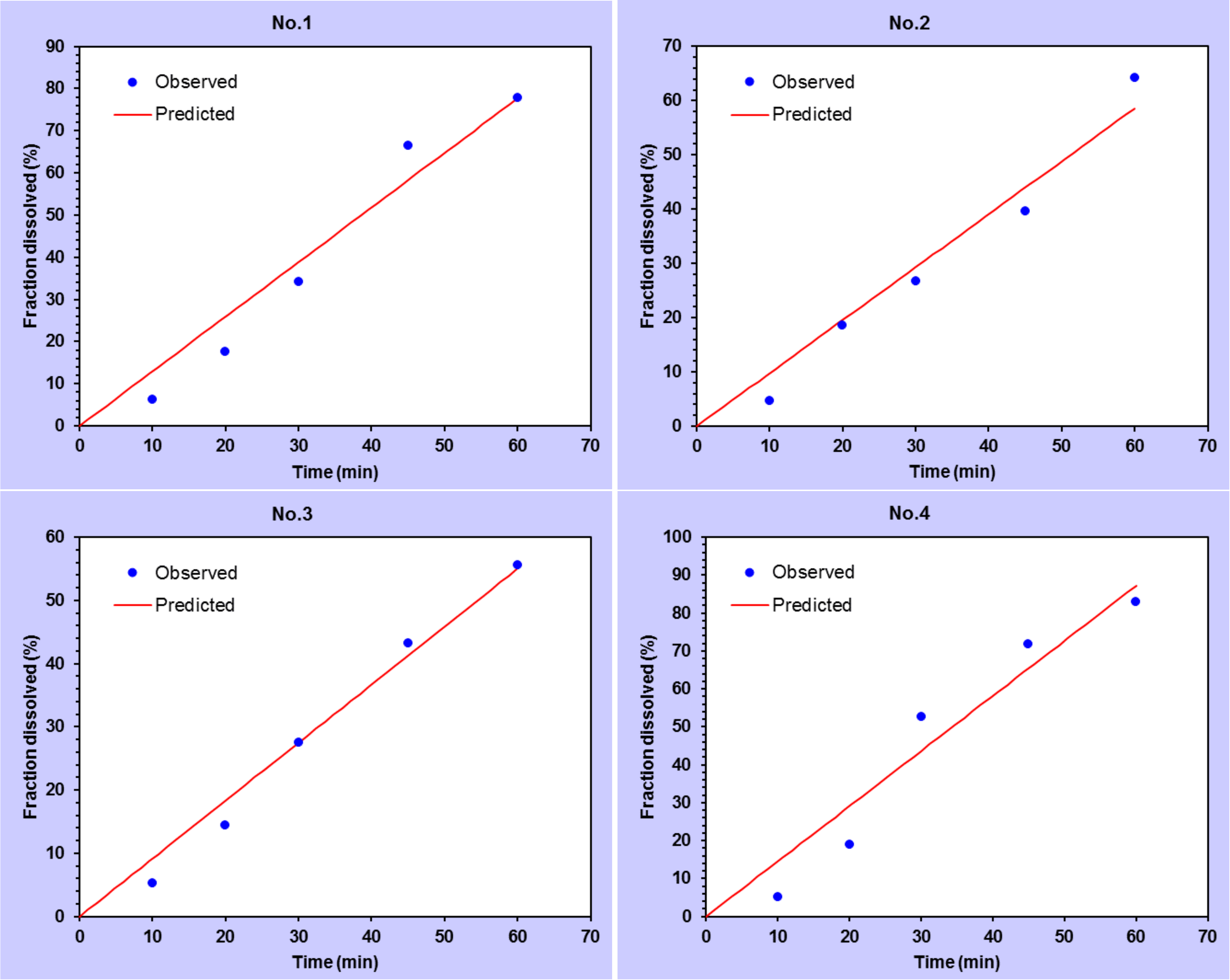

Supplementary materials – Model fitting summary – Parteck® M 100 (modelling up to app. 70 % of carvedilol released)

Model: **Zero–order with T_{lag}**

Model equation: $F = k_0 \cdot (t - T_{lag})$

Fitted model parameters per tested tablet (N = 4) with statistics – mean, standard deviation (SD), and relative standard deviation expressed in % (RSD%) (output from DDSolver):

Parameter	No.1	No.2	No.3	No.4	Mean	SD	RSD(%)
k_0	1.536	1.127	1.032	1.634	1.332	0.297	22.311
T_{lag}	6.672	5.668	4.713	4.688	5.435	0.942	17.336

Number of dissolution data points (N), degrees of freedom (df), and selected goodness of fit criteria – Pearson correlation coefficient (R), coefficient of determination (R^2), adjusted coefficient of determination ($R^2_{adjusted}$), and residual sum of squares (RSS) (manual calculation in MS Excel):

Parameter	No.1	No.2	No.3	No.4
N	5	5	5	5
df	3	3	3	3
R	0.988678076	0.990876008	0.997320012	0.969389722
R^2	0.977484337	0.981835263	0.994647207	0.939716433
$R^2_{adjusted}$	0.969979116	0.975780351	0.992862943	0.919621911
RSS	85.85572368	37.1162236	9.04808791	270.5212831

Graphical abstract of model fit presented as mean ± 1 SD of the fraction % of released carvedilol:

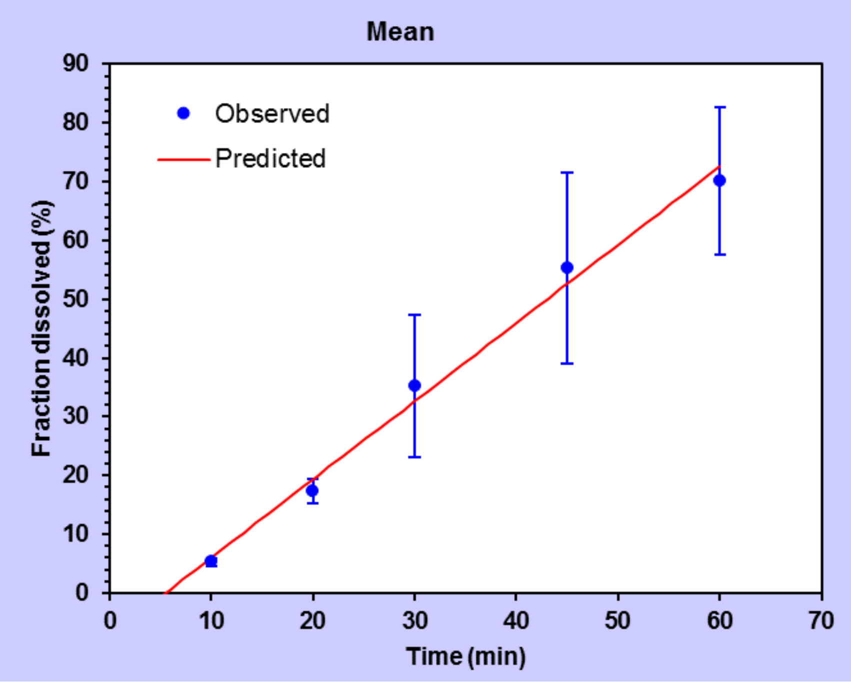

Supplementary materials – Model fitting summary – Parteck® M 100 (modelling up to app. 70 % of carvedilol released)

Graphical abstract of model fit presented as the fraction % of released carvedilol per tested tablet:

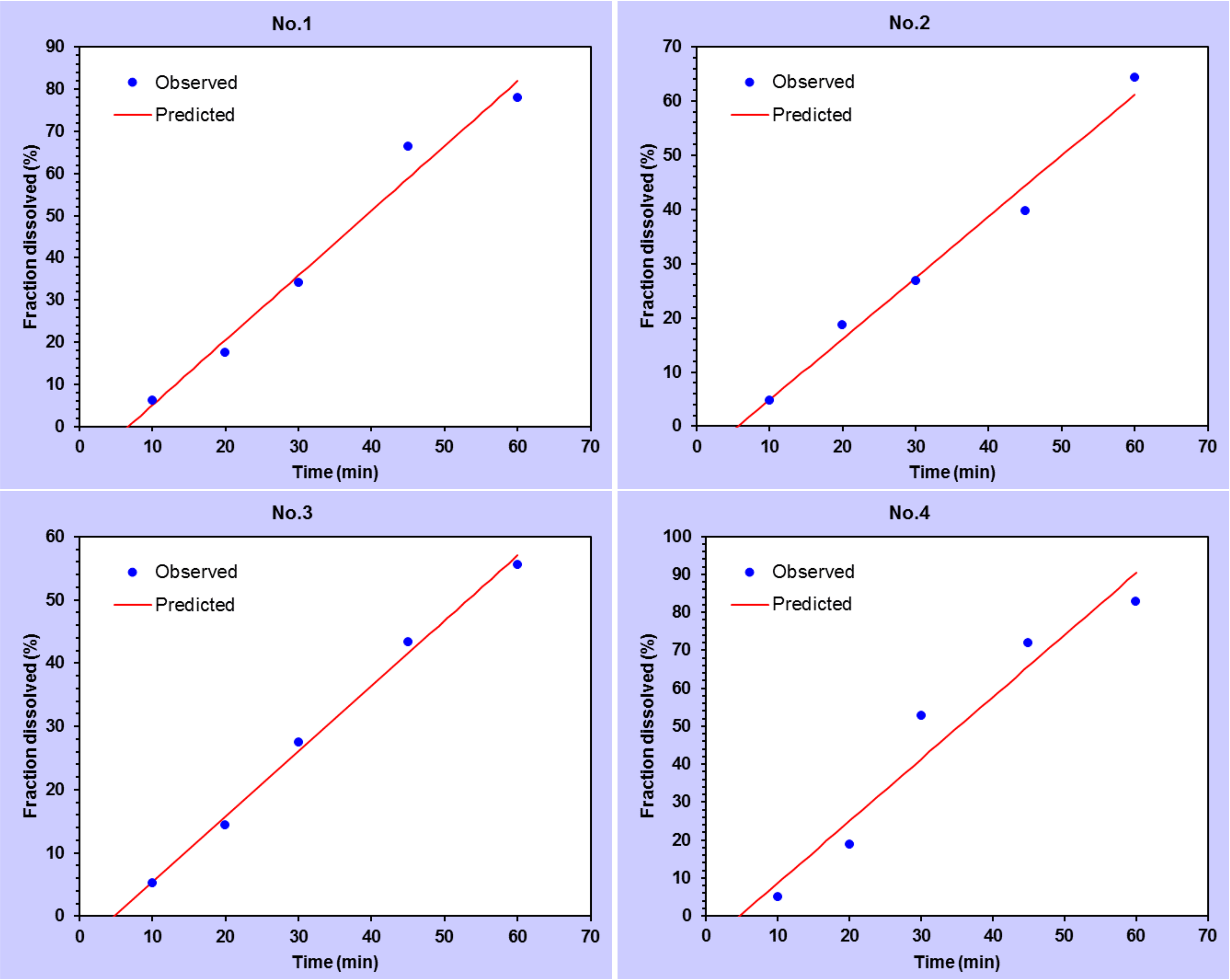

Supplementary materials – Model fitting summary – Parteck® M 100 (modelling up to app. 70 % of carvedilol released)

Model: **Zero–order with F_0**

Model equation: $F = F_0 + k_0 \cdot t$

Fitted model parameters per tested tablet (N = 4) with statistics – mean, standard deviation (SD), and relative standard deviation expressed in % (RSD%) (output from DDSolver):

Parameter	No.1	No.2	No.3	No.4	Mean	SD	RSD(%)
k_0	1.536	1.127	1.032	1.634	1.332	0.297	22.311
F_0	-10.248	-6.387	-4.861	-7.659	-7.289	2.280	-31.284

Number of dissolution data points (N), degrees of freedom (df), and selected goodness of fit criteria – Pearson correlation coefficient (R), coefficient of determination (R^2), adjusted coefficient of determination ($R^2_{adjusted}$), and residual sum of squares (RSS) (manual calculation in MS Excel):

Parameter	No.1	No.2	No.3	No.4
N	5	5	5	5
df	3	3	3	3
R	0.988678076	0.990876008	0.997320012	0.969389722
R^2	0.977484337	0.981835263	0.994647207	0.939716433
$R^2_{adjusted}$	0.969979116	0.975780351	0.992862943	0.919621911
RSS	85.85572368	37.1162236	9.04808791	270.5212831

Graphical abstract of model fit presented as mean ± 1 SD of the fraction % of released carvedilol:

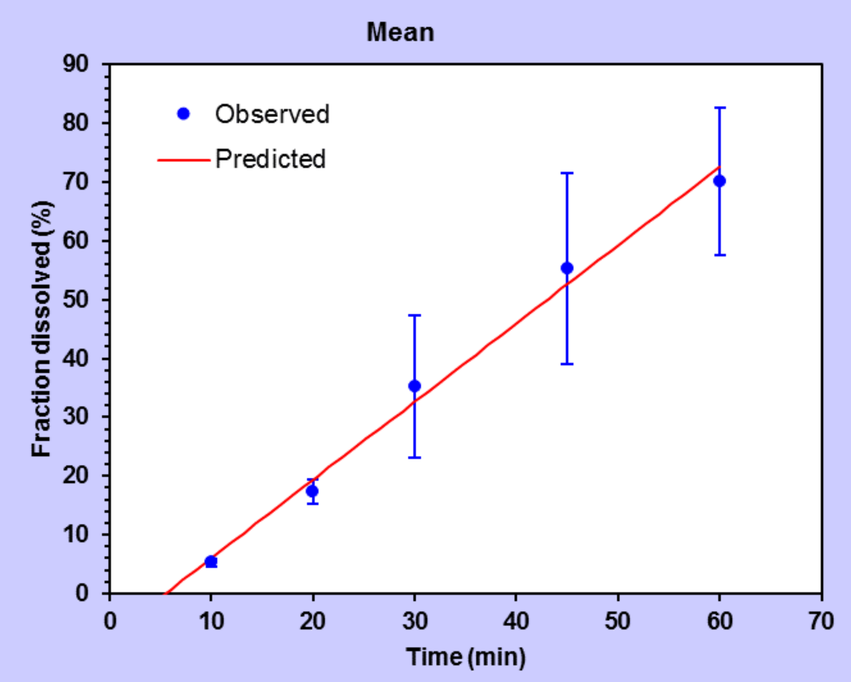

Supplementary materials – Model fitting summary – Parteck® M 100 (modelling up to app. 70 % of carvedilol released)

Graphical abstract of model fit presented as the fraction % of released carvedilol per tested tablet:

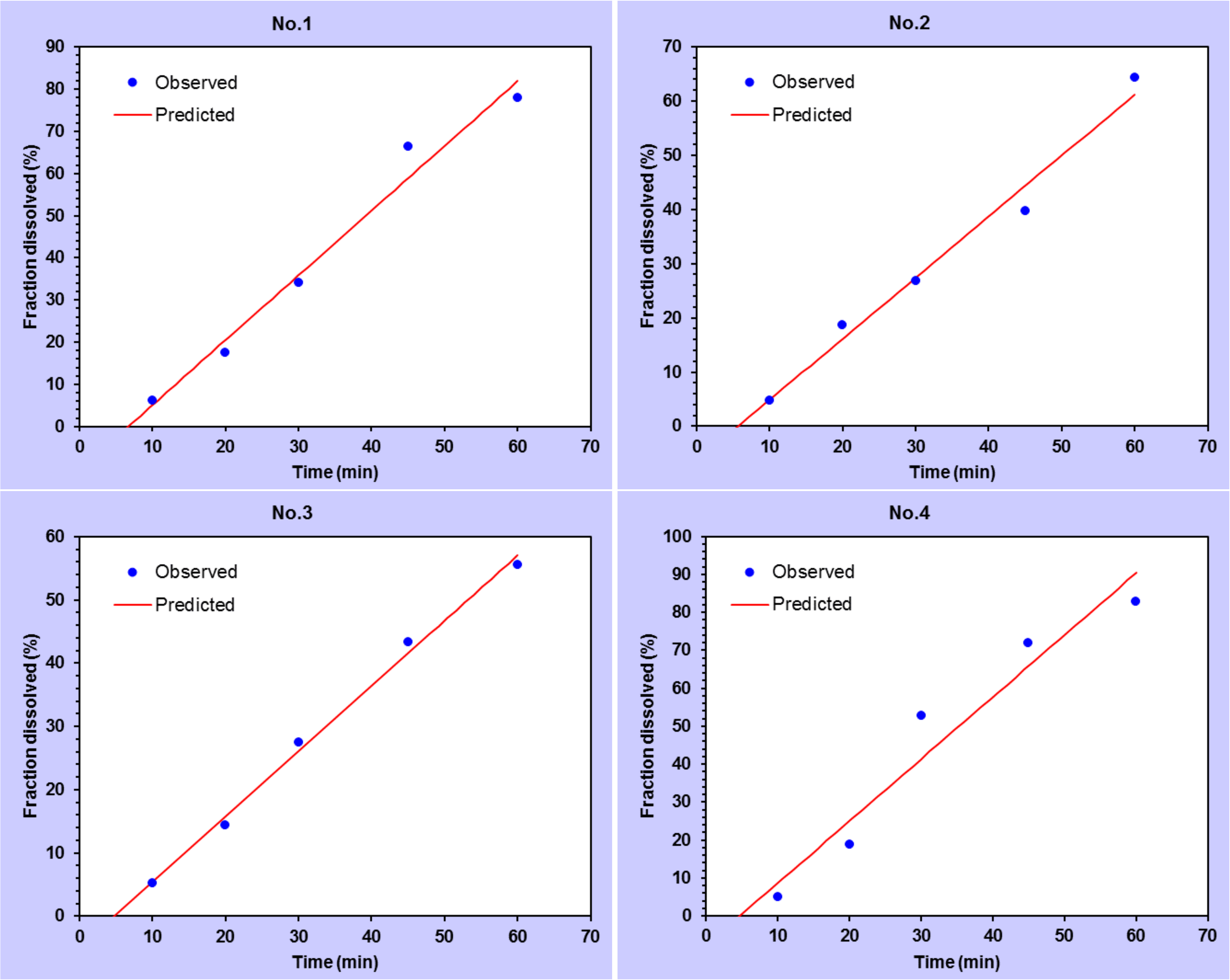

Supplementary materials – Model fitting summary – Parteck® M 100 (modelling up to app. 70 % of carvedilol released)

Model: **First–order**

Model equation: $F = 100 \cdot (1 - e^{-k_1 \cdot t})$

Fitted model parameters per tested tablet (N = 4) with statistics – mean, standard deviation (SD), and relative standard deviation expressed in % (RSD%) (output from DDSolver):

Parameter	No.1	No.2	No.3	No.4	Mean	SD	RSD(%)
k_1	0.017	0.014	0.012	0.020	0.016	0.003	21.668

Number of dissolution data points (N), degrees of freedom (df), and selected goodness of fit criteria – Pearson correlation coefficient (R), coefficient of determination (R^2), adjusted coefficient of determination ($R^2_{adjusted}$), and residual sum of squares (RSS) (manual calculation in MS Excel):

Parameter	No.1	No.2	No.3	No.4
N	5	5	5	5
df	4	4	4	4
R	0.986286415	0.979368818	0.99758327	0.986121555
R^2	0.972760891	0.959163281	0.995172381	0.972435721
$R^2_{adjusted}$	0.972760891	0.959163281	0.995172381	0.972435721
RSS	626.0831966	265.5420452	122.9696196	736.5925019

Graphical abstract of model fit presented as mean ± 1 SD of the fraction % of released carvedilol:

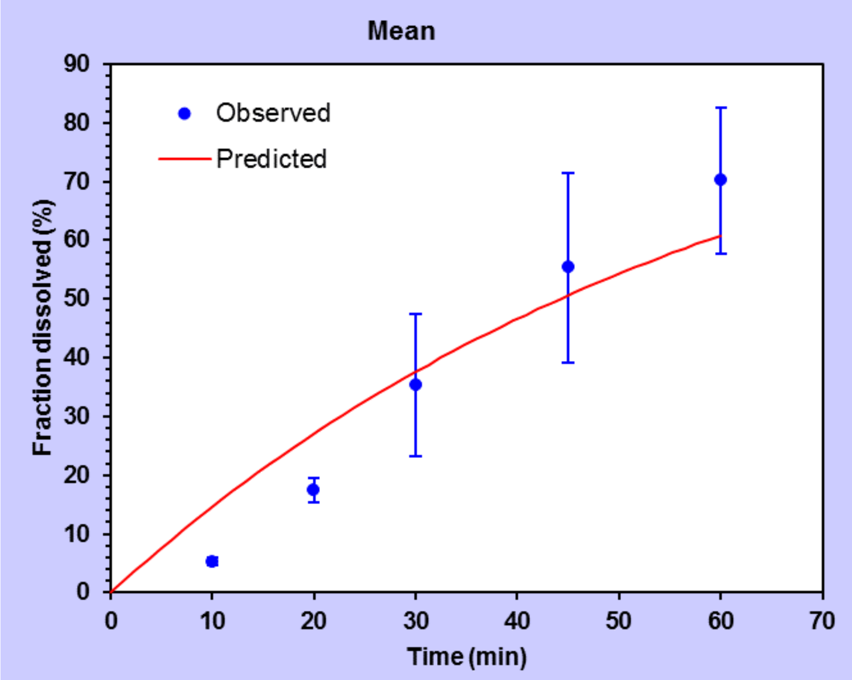

Supplementary materials – Model fitting summary – Parteck® M 100 (modelling up to app. 70 % of carvedilol released)

Graphical abstract of model fit presented as the fraction % of released carvedilol per tested tablet:

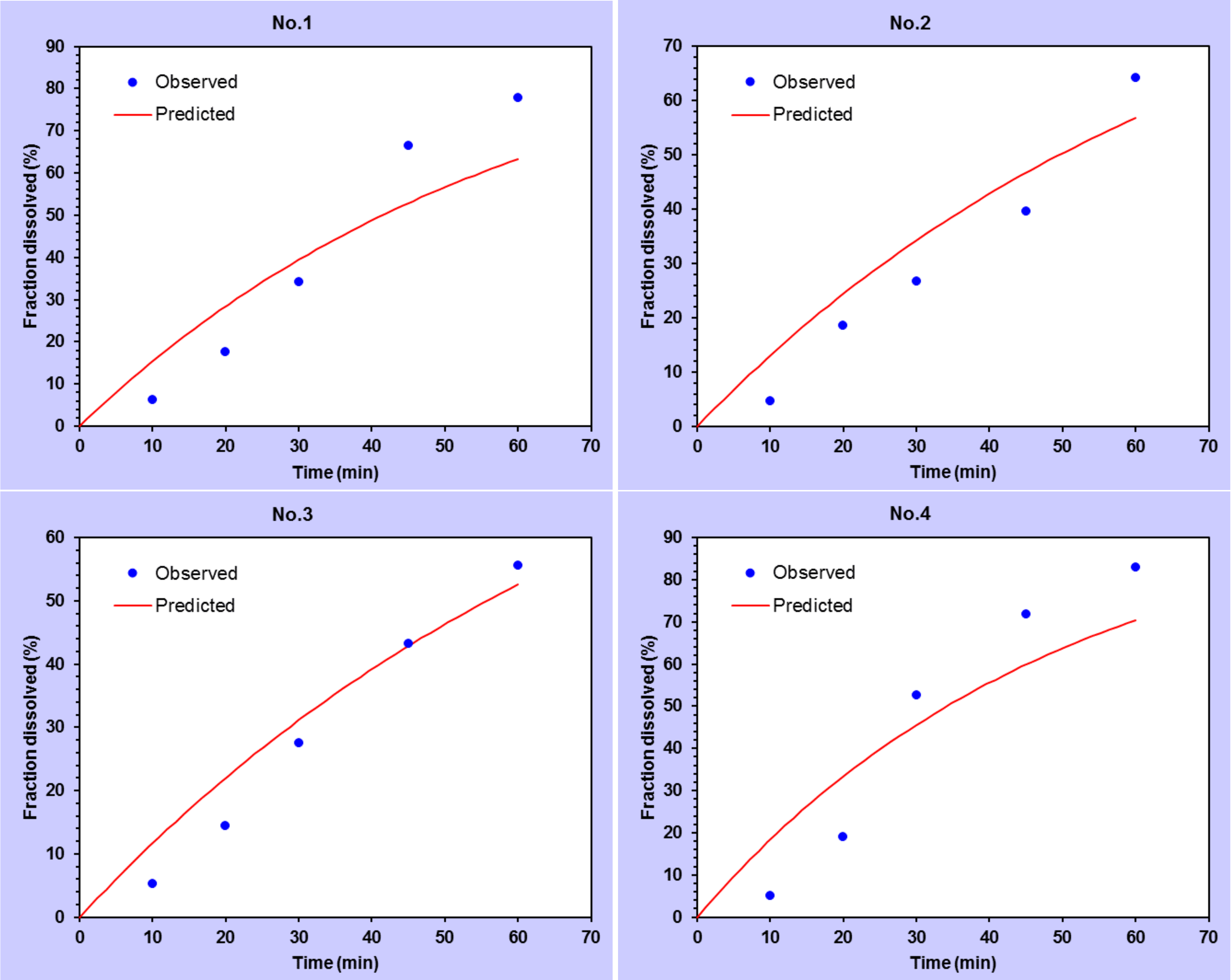

Supplementary materials – Model fitting summary – Parteck® M 100 (modelling up to app. 70 % of carvedilol released)

Model: **First–order with T_{lag}**

Model equation: $F = 100 \cdot \left[1 - e^{-k_1 \cdot (t - T_{lag})}\right]$

Fitted model parameters per tested tablet (N = 4) with statistics – mean, standard deviation (SD), and relative standard deviation expressed in % (RSD%) (output from DDSolver):

Parameter	No.1	No.2	No.3	No.4	Mean	SD	RSD(%)
k_1	0.031	0.018	0.015	0.036	0.025	0.010	38.794
T_{lag}	11.711	10.213	8.373	10.488	10.196	1.379	13.525

Number of dissolution data points (N), degrees of freedom (df), and selected goodness of fit criteria – Pearson correlation coefficient (R), coefficient of determination (R^2), adjusted coefficient of determination ($R^2_{adjusted}$), and residual sum of squares (RSS) (manual calculation in MS Excel):

Parameter	No.1	No.2	No.3	No.4
N	5	5	5	5
df	3	3	3	3
R	0.972155761	0.974037229	0.996357806	0.983283843
R^2	0.945086823	0.948748523	0.992728878	0.966847117
$R^2_{adjusted}$	0.92678243	0.931664697	0.990305171	0.955796156
RSS	244.7860694	120.6303005	12.9175153	152.4459312

Graphical abstract of model fit presented as mean ± 1 SD of the fraction % of released carvedilol:

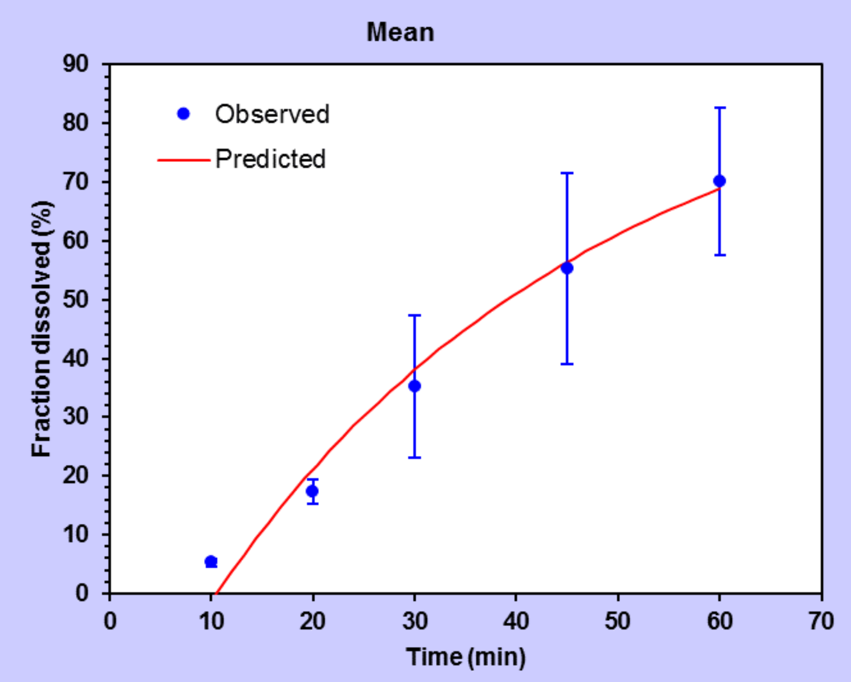

Supplementary materials – Model fitting summary – Parteck® M 100 (modelling up to app. 70 % of carvedilol released)

Graphical abstract of model fit presented as the fraction % of released carvedilol per tested tablet:

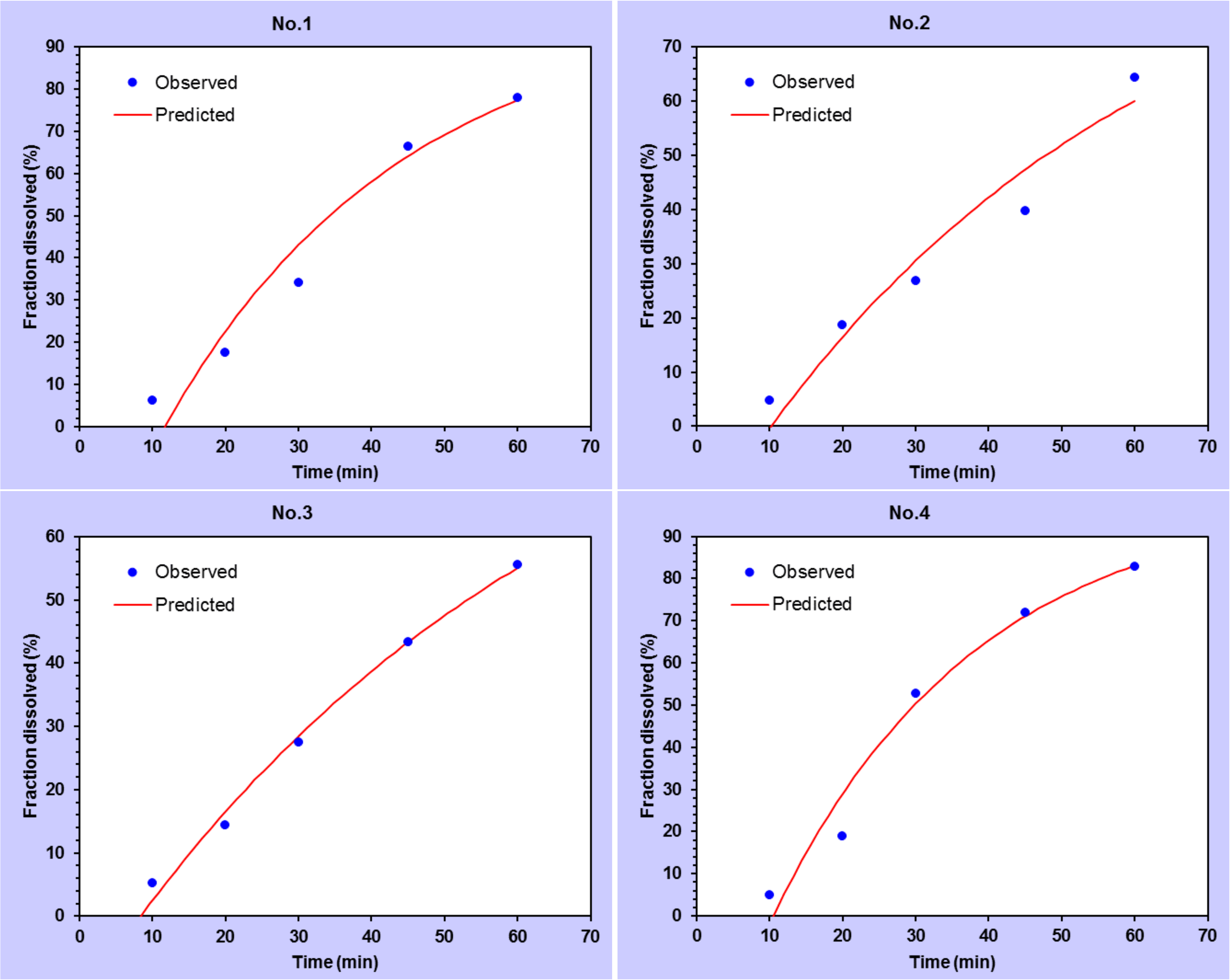

Supplementary materials – Model fitting summary – Parteck® M 100 (modelling up to app. 70 % of carvedilol released)

Model: **First–order with F_{max}**

Model equation: $F = F_{max} \cdot (1 - e^{-k_1 \cdot t})$

Fitted model parameters per tested tablet (N = 4) with statistics – mean, standard deviation (SD), and relative standard deviation expressed in % (RSD%) (output from DDSolver):

Parameter	No.1	No.2	No.3	No.4	Mean	SD	RSD(%)
k_1	0.040	0.035	0.038	0.042	0.039	0.003	7.670
F_{max}	81.786	67.452	58.326	87.029	73.648	13.146	17.849

Number of dissolution data points (N), degrees of freedom (df), and selected goodness of fit criteria – Pearson correlation coefficient (R), coefficient of determination (R^2), adjusted coefficient of determination ($R^2_{adjusted}$), and residual sum of squares (RSS) (manual calculation in MS Excel):

Parameter	No.1	No.2	No.3	No.4
N	5	5	5	5
df	3	3	3	3
R	0.958314353	0.948411641	0.973008752	0.979033645
R^2	0.918366399	0.89948464	0.946746031	0.958506878
$R^2_{adjusted}$	0.891155199	0.86597952	0.928994708	0.944675838
RSS	1715.514078	965.6539764	646.7092757	1651.429651

Graphical abstract of model fit presented as mean ± 1 SD of the fraction % of released carvedilol:

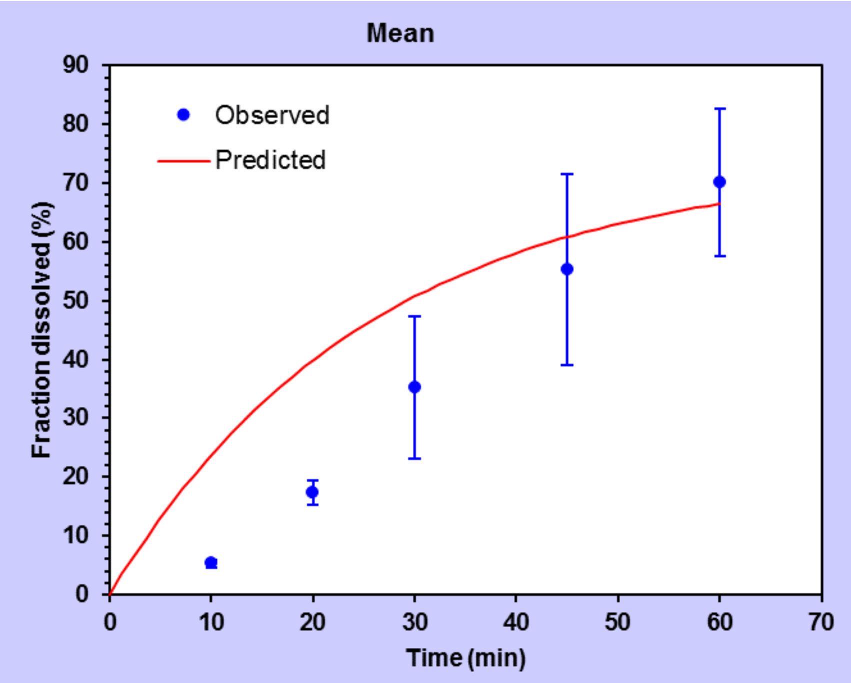

Supplementary materials – Model fitting summary – Parteck® M 100 (modelling up to app. 70 % of carvedilol released)

Graphical abstract of model fit presented as the fraction % of released carvedilol per tested tablet:

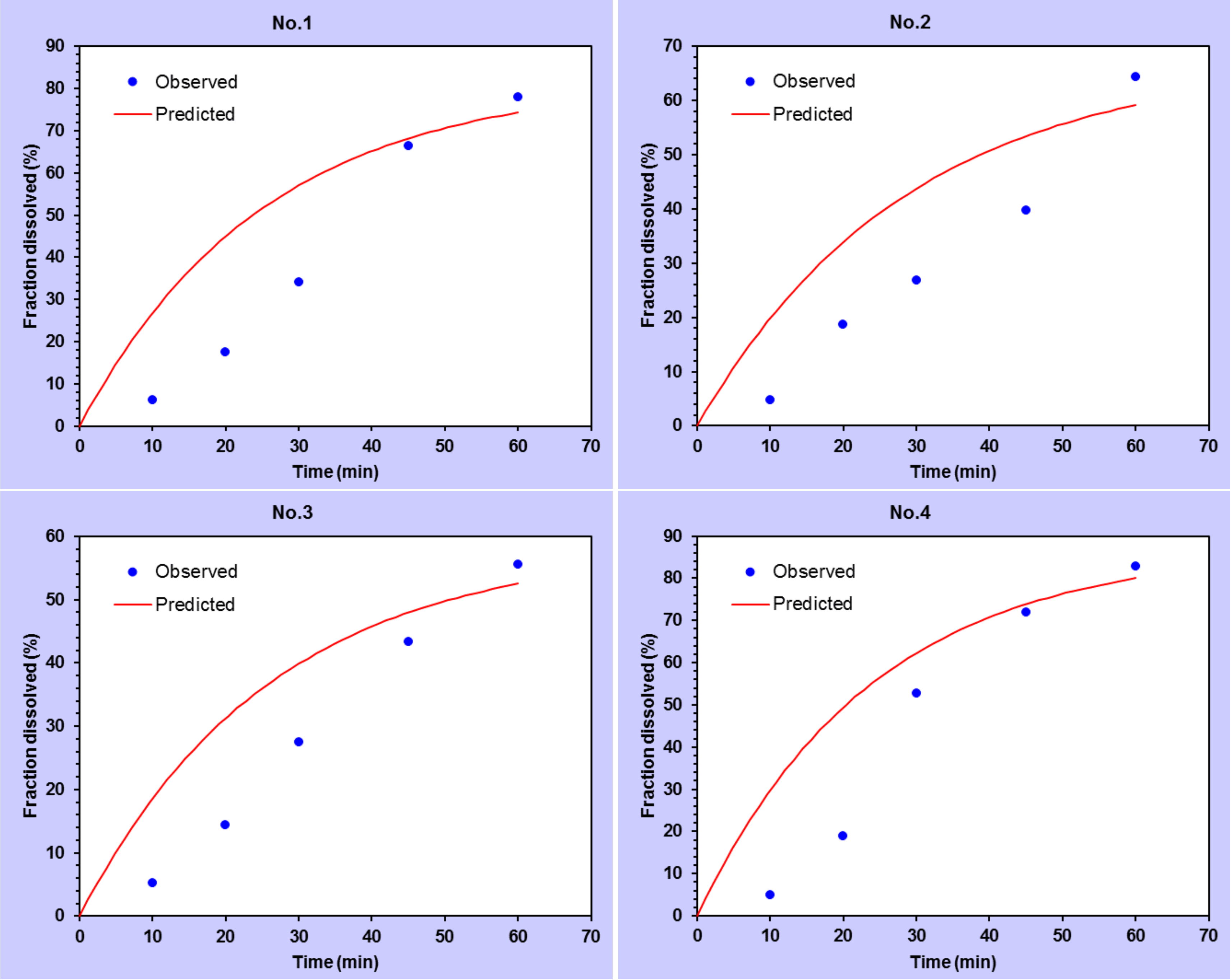

Supplementary materials – Model fitting summary – Parteck® M 100 (modelling up to app. 70 % of carvedilol released)

Model: **First–order with T_{lag} and F_{max}**

Model equation: $F = F_{max} \cdot \left[1 - e^{-k_1 \cdot (t - T_{lag})}\right]$

Fitted model parameters per tested tablet (N = 4) with statistics – mean, standard deviation (SD), and relative standard deviation expressed in % (RSD%) (output from DDSolver):

Parameter	No.1	No.2	No.3	No.4	Mean	SD	RSD(%)
k_1	0.091	0.081	0.057	0.061	0.073	0.016	22.235
T_{lag}	12.153	12.612	14.143	13.137	13.011	0.855	6.572
F_{max}	54.524	44.968	58.326	87.029	61.212	18.106	29.578

Number of dissolution data points (N), degrees of freedom (df), and selected goodness of fit criteria – Pearson correlation coefficient (R), coefficient of determination (R^2), adjusted coefficient of determination ($R^2_{adjusted}$), and residual sum of squares (RSS) (manual calculation in MS Excel):

Parameter	No.1	No.2	No.3	No.4
N	5	5	5	5
df	2	2	2	2
R	0.852529901	0.856416182	0.940043777	0.957655543
R^2	0.726807233	0.733448678	0.883682303	0.917104138
$R^2_{adjusted}$	0.453614465	0.466897355	0.767364605	0.834208277
RSS	1315.448542	703.0773432	523.0479445	672.0827797

Graphical abstract of model fit presented as mean ± 1 SD of the fraction % of released carvedilol:

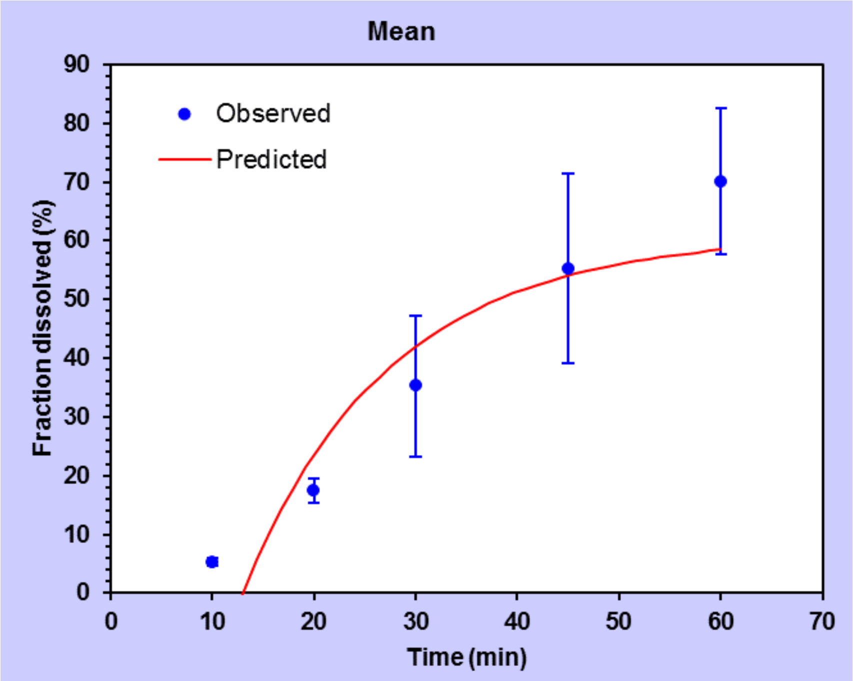

Supplementary materials – Model fitting summary – Parteck® M 100 (modelling up to app. 70 % of carvedilol released)

Graphical abstract of model fit presented as the fraction % of released carvedilol per tested tablet:

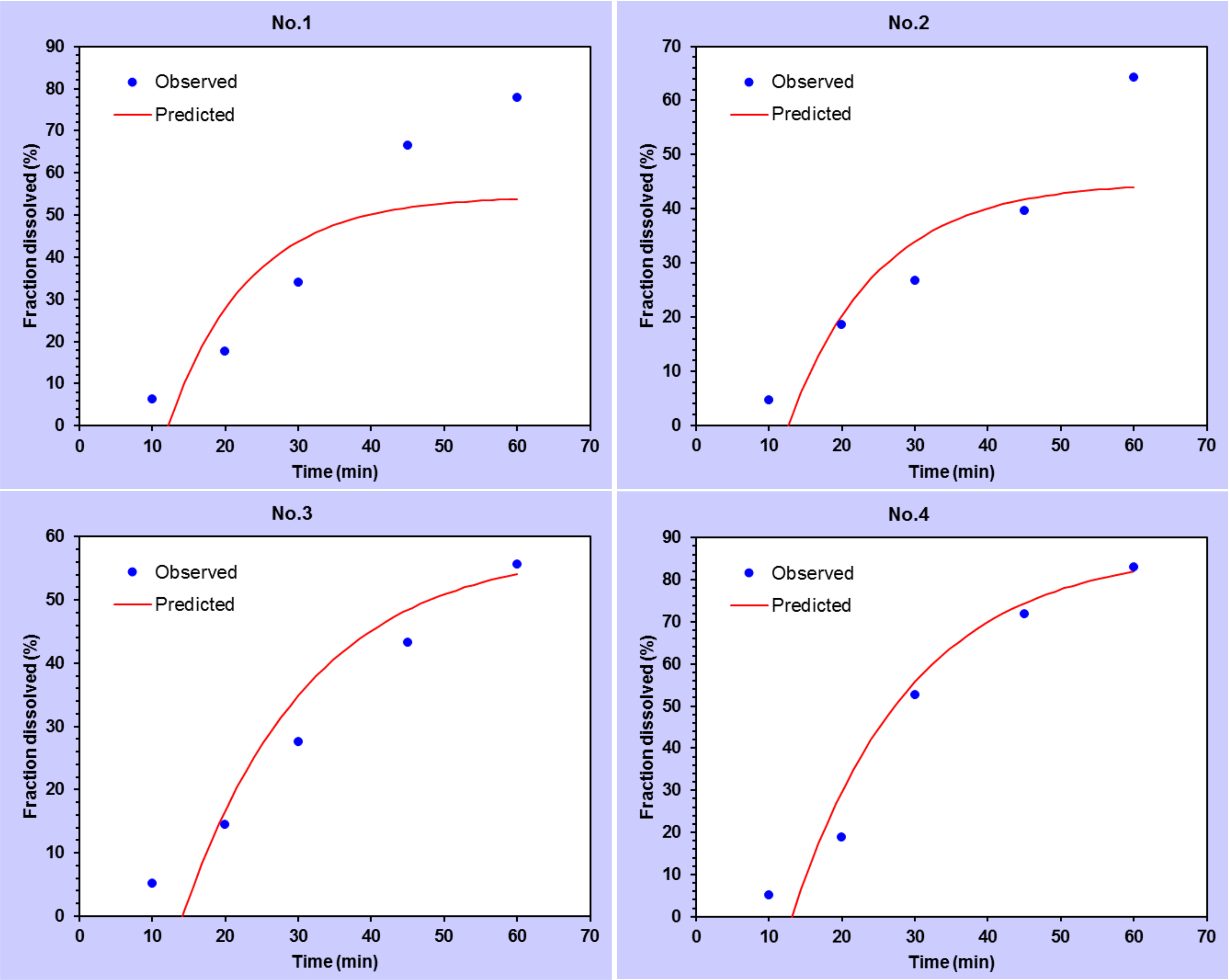

Supplementary materials – Model fitting summary – Parteck® M 100 (modelling up to app. 70 % of carvedilol released)

Model: **Higuchi**

Model equation: $F = k_H \cdot t^{0.5}$

Fitted model parameters per tested tablet (N = 4) with statistics – mean, standard deviation (SD), and relative standard deviation expressed in % (RSD%) (output from DDSolver):

Parameter	No.1	No.2	No.3	No.4	Mean	SD	RSD(%)
k_H	8.084	6.112	5.769	9.167	7.283	1.618	22.217

Number of dissolution data points (N), degrees of freedom (df), and selected goodness of fit criteria – Pearson correlation coefficient (R), coefficient of determination (R^2), adjusted coefficient of determination ($R^2_{adjusted}$), and residual sum of squares (RSS) (manual calculation in MS Excel):

Parameter	No.1	No.2	No.3	No.4
N	5	5	5	5
df	4	4	4	4
R	0.983544524	0.977562832	0.994338016	0.982725585
R^2	0.967359831	0.95562909	0.988708089	0.965749576
$R^2_{adjusted}$	0.967359831	0.95562909	0.988708089	0.965749576
RSS	1205.745355	622.394583	455.9717276	1317.955277

Graphical abstract of model fit presented as mean ± 1 SD of the fraction % of released carvedilol:

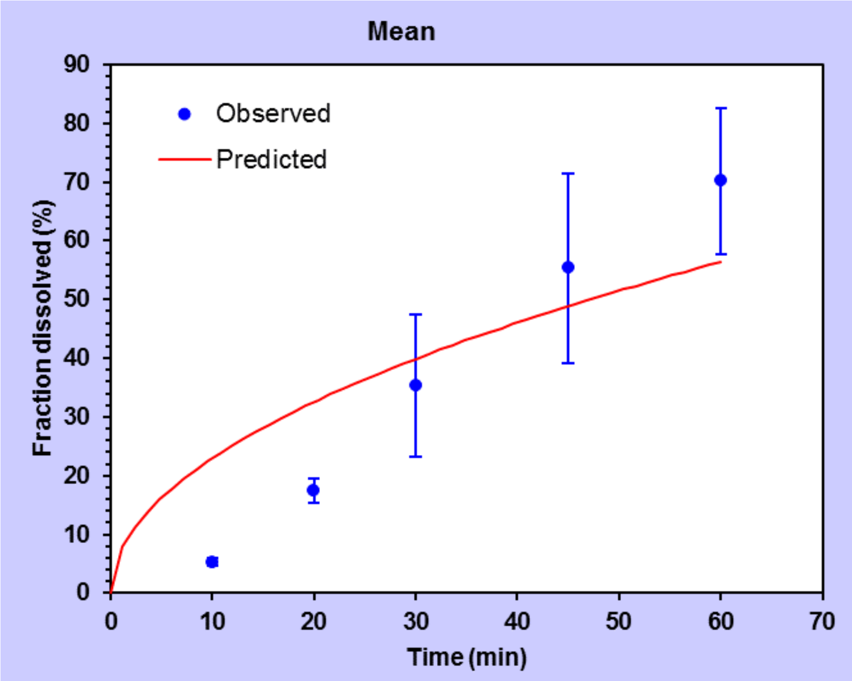

Supplementary materials – Model fitting summary – Parteck® M 100 (modelling up to app. 70 % of carvedilol released)

Graphical abstract of model fit presented as the fraction % of released carvedilol per tested tablet:

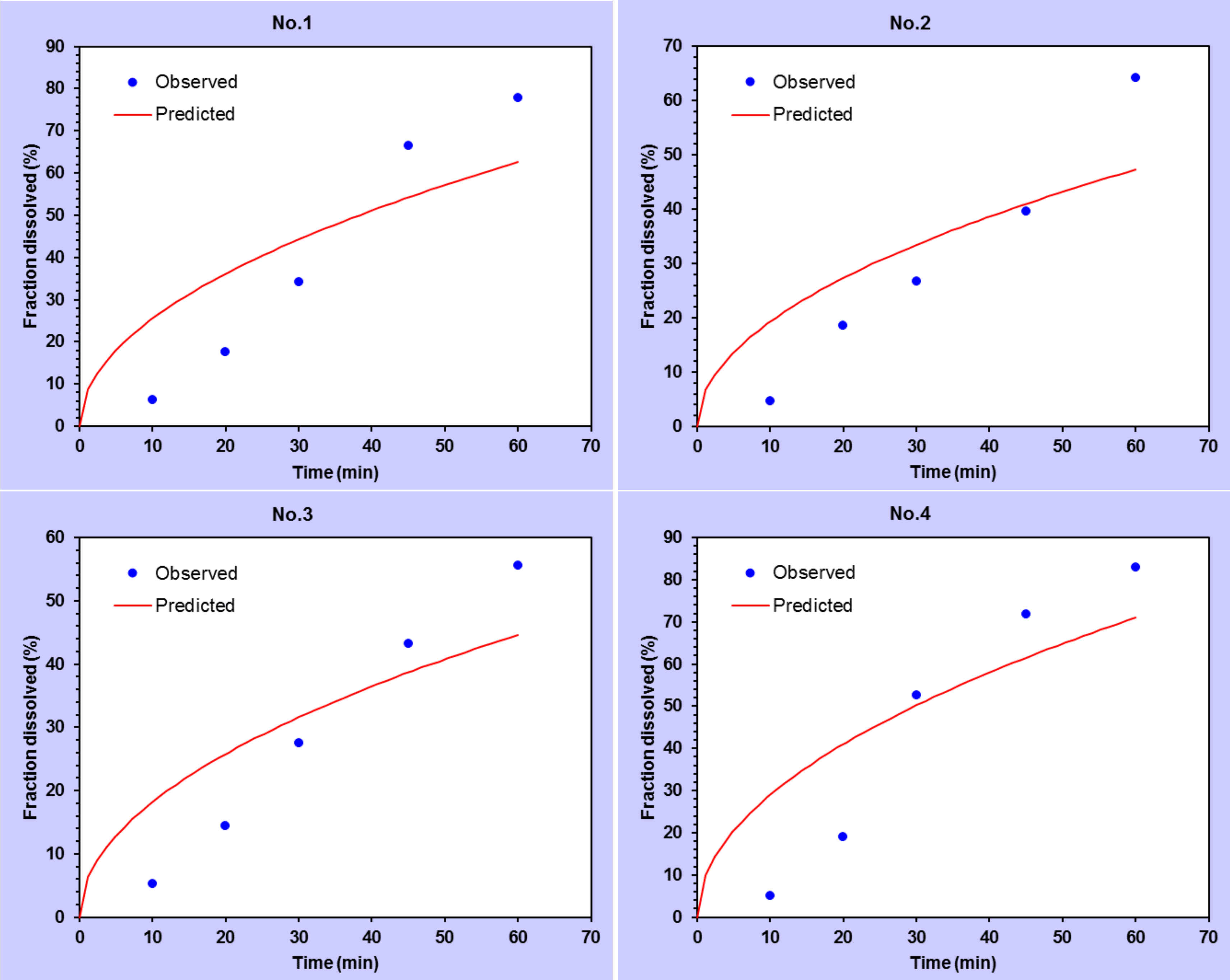

Supplementary materials – Model fitting summary – Parteck® M 100 (modelling up to app. 70 % of carvedilol released)

Model: **Higuchi with T_{lag}**

Model equation: $F = k_H \cdot (t - T_{lag})^{0.5}$

Fitted model parameters per tested tablet (N = 4) with statistics – mean, standard deviation (SD), and relative standard deviation expressed in % (RSD%) (output from DDSolver):

Parameter	No.1	No.2	No.3	No.4	Mean	SD	RSD(%)
k_H	12.920	8.829	8.212	12.166	10.532	2.356	22.371
T_{lag}	17.597	15.588	18.470	12.483	16.034	2.658	16.575

Number of dissolution data points (N), degrees of freedom (df), and selected goodness of fit criteria – Pearson correlation coefficient (R), coefficient of determination (R^2), adjusted coefficient of determination ($R^2_{adjusted}$), and residual sum of squares (RSS) (manual calculation in MS Excel):

Parameter	No.1	No.2	No.3	No.4
N	5	5	5	5
df	3	3	3	3
R	0.98517838	0.96276079	0.996165747	0.973303956
R^2	0.970576441	0.926908338	0.992346196	0.94732059
$R^2_{adjusted}$	0.960768587	0.90254445	0.989794928	0.929760787
RSS	215.203683	163.9483197	52.99197189	244.2324553

Graphical abstract of model fit presented as mean ± 1 SD of the fraction % of released carvedilol:

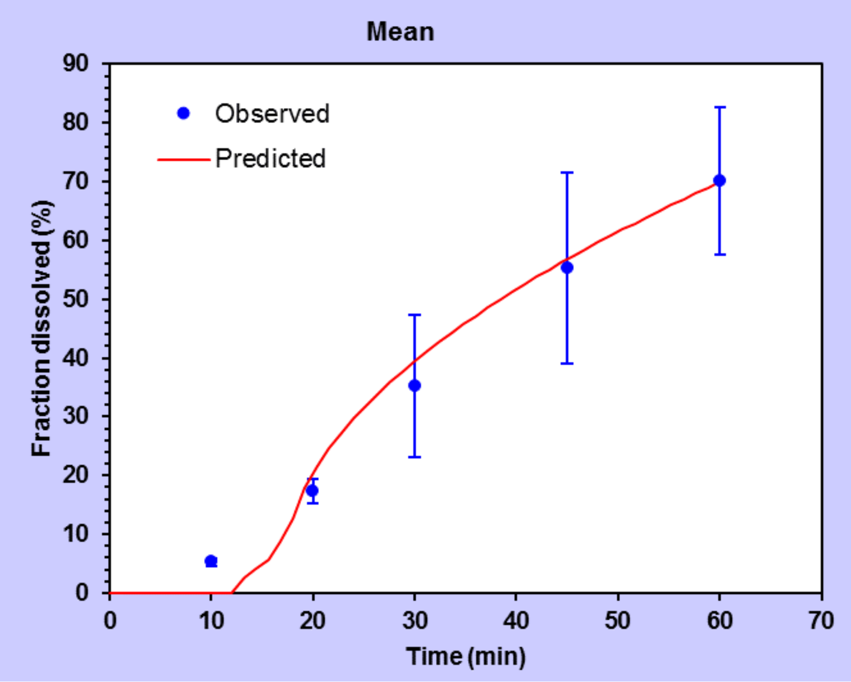

Supplementary materials – Model fitting summary – Parteck® M 100 (modelling up to app. 70 % of carvedilol released)

Graphical abstract of model fit presented as the fraction % of released carvedilol per tested tablet:

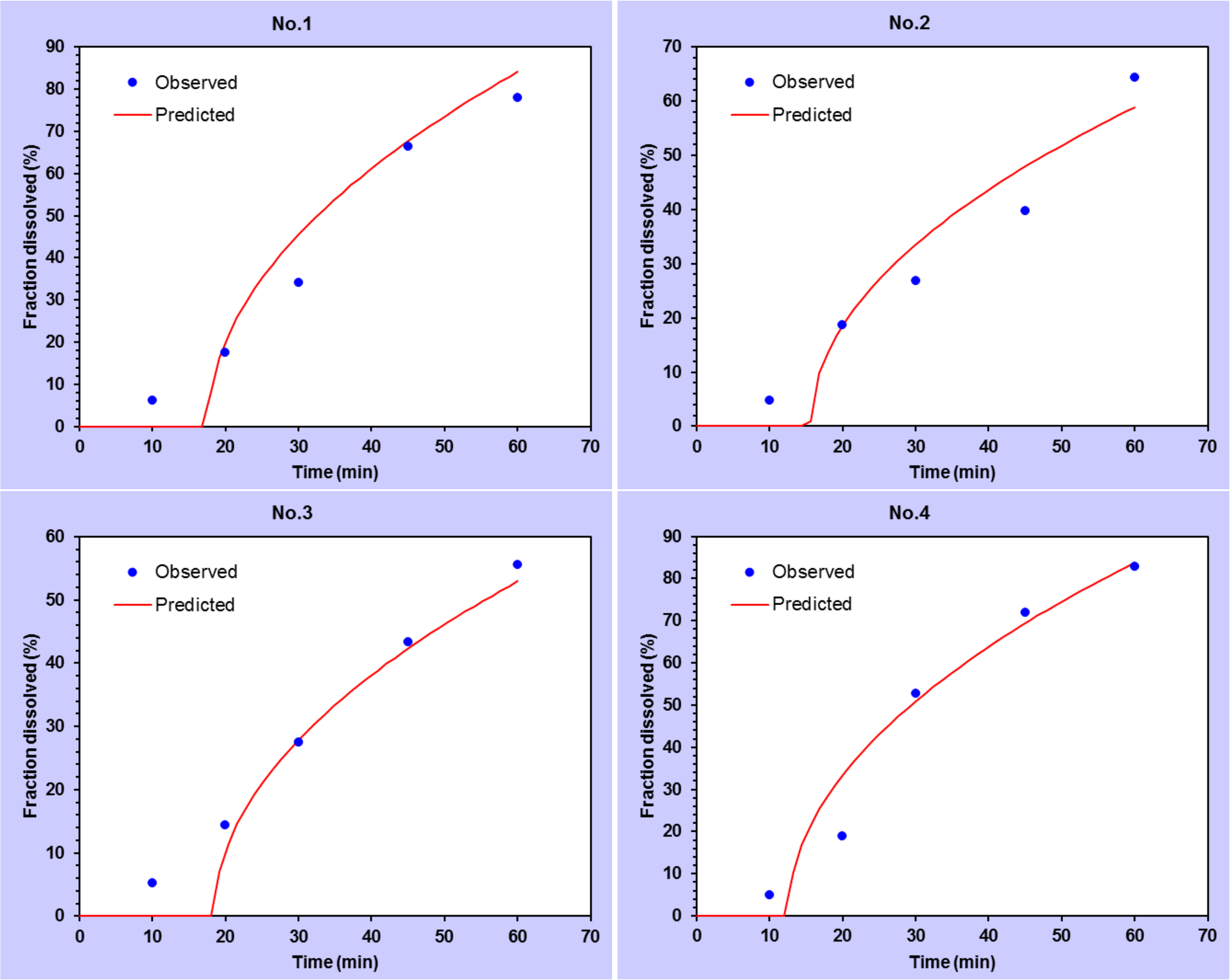

Supplementary materials – Model fitting summary – Parteck® M 100 (modelling up to app. 70 % of carvedilol released)

Model: **Higuchi with F_0**

Model equation: $F = F_0 + k_H \cdot t^{0.5}$

Fitted model parameters per tested tablet (N = 4) with statistics – mean, standard deviation (SD), and relative standard deviation expressed in % (RSD%) (output from DDSolver):

Parameter	No.1	No.2	No.3	No.4	Mean	SD	RSD(%)
k_H	16.828	12.244	11.327	18.241	14.660	3.390	23.121
F_0	-52.340	-36.704	-33.270	-54.311	-44.156	10.711	-24.256

Number of dissolution data points (N), degrees of freedom (df), and selected goodness of fit criteria – Pearson correlation coefficient (R), coefficient of determination (R^2), adjusted coefficient of determination ($R^2_{adjusted}$), and residual sum of squares (RSS) (manual calculation in MS Excel):

Parameter	No.1	No.2	No.3	No.4
N	5	5	5	5
df	3	3	3	3
R	0.983544524	0.977562832	0.994338016	0.982725585
R^2	0.967359831	0.95562909	0.988708089	0.965749576
$R^2_{adjusted}$	0.956479775	0.940838787	0.984944119	0.954332767
RSS	124.4620404	90.66361096	19.08726958	153.6980851

Graphical abstract of model fit presented as mean ± 1 SD of the fraction % of released carvedilol:

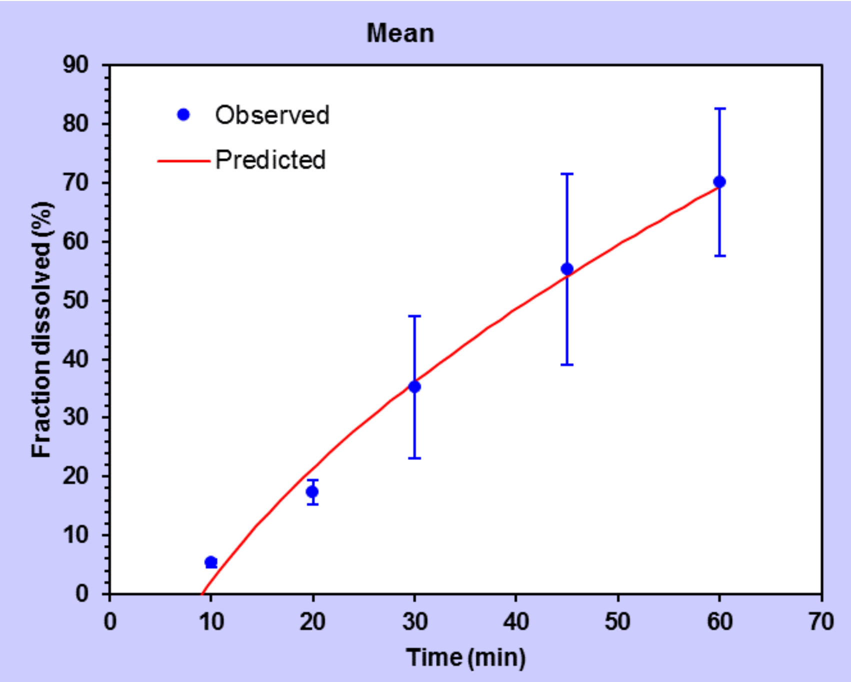

Supplementary materials – Model fitting summary – Parteck® M 100 (modelling up to app. 70 % of carvedilol released)

Graphical abstract of model fit presented as the fraction % of released carvedilol per tested tablet:

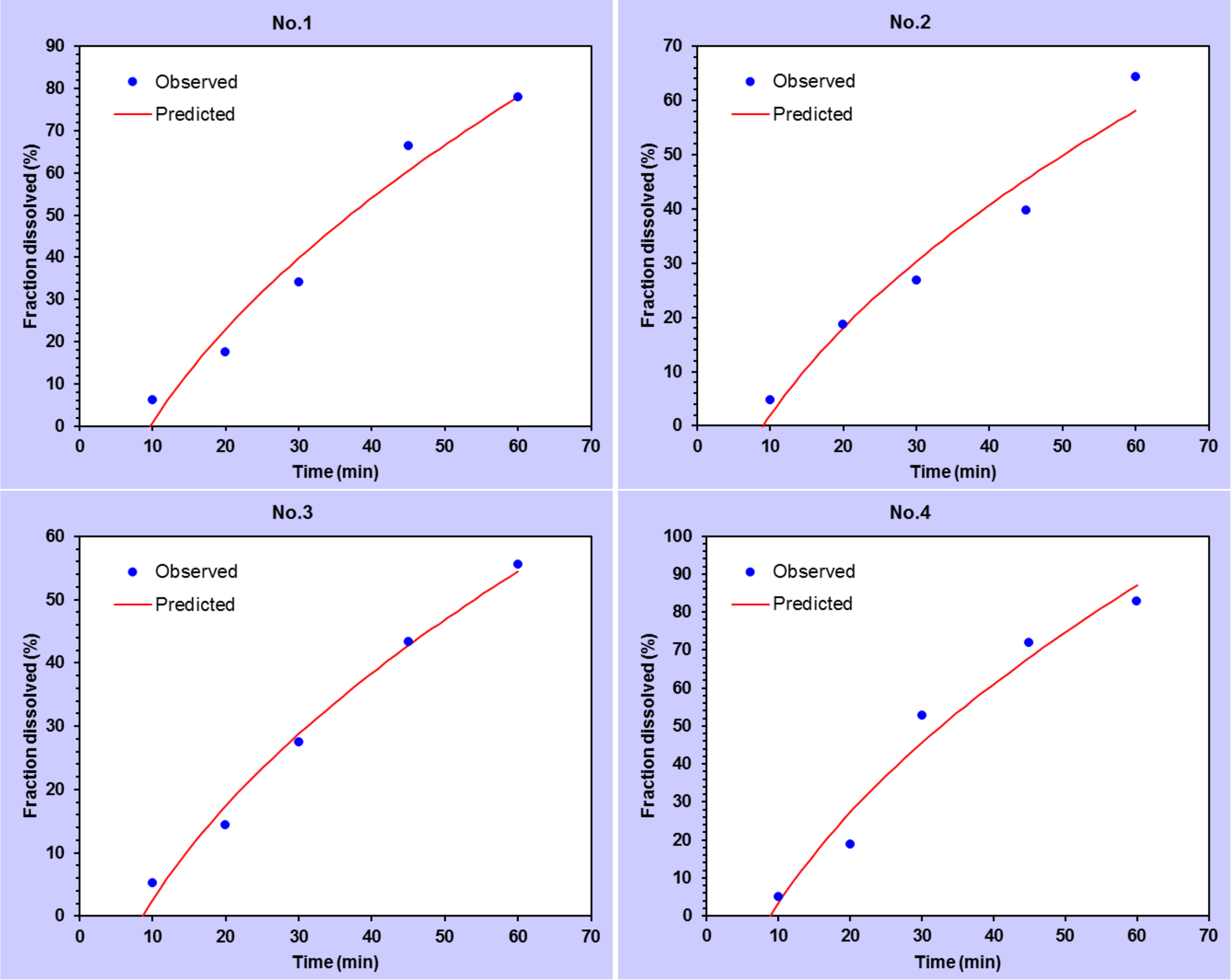

Supplementary materials – Model fitting summary – Parteck® M 100 (modelling up to app. 70 % of carvedilol released)

Model: **Korsmeyer–Peppas**

Model equation: $F = k_{KP} \cdot t^n$

Fitted model parameters per tested tablet (N = 4) with statistics – mean, standard deviation (SD), and relative standard deviation expressed in % (RSD%) (output from DDSolver):

Parameter	No.1	No.2	No.3	No.4	Mean	SD	RSD(%)
k_{KP}	0.218	0.224	0.250	0.188	0.220	0.026	11.594
n	1.469	1.390	1.346	1.527	1.433	0.081	5.628

Number of dissolution data points (N), degrees of freedom (df), and selected goodness of fit criteria – Pearson correlation coefficient (R), coefficient of determination (R^2), adjusted coefficient of determination ($R^2_{adjusted}$), and residual sum of squares (RSS) (manual calculation in MS Excel):

Parameter	No.1	No.2	No.3	No.4
N	5	5	5	5
df	3	3	3	3
R	0.979297925	0.992409035	0.990756751	0.941409554
R^2	0.959024427	0.984875692	0.981598941	0.886251948
$R^2_{adjusted}$	0.945365902	0.979834257	0.975465254	0.848335931
RSS	195.8114109	48.93203883	51.64126569	651.5930938

Graphical abstract of model fit presented as mean ± 1 SD of the fraction % of released carvedilol:

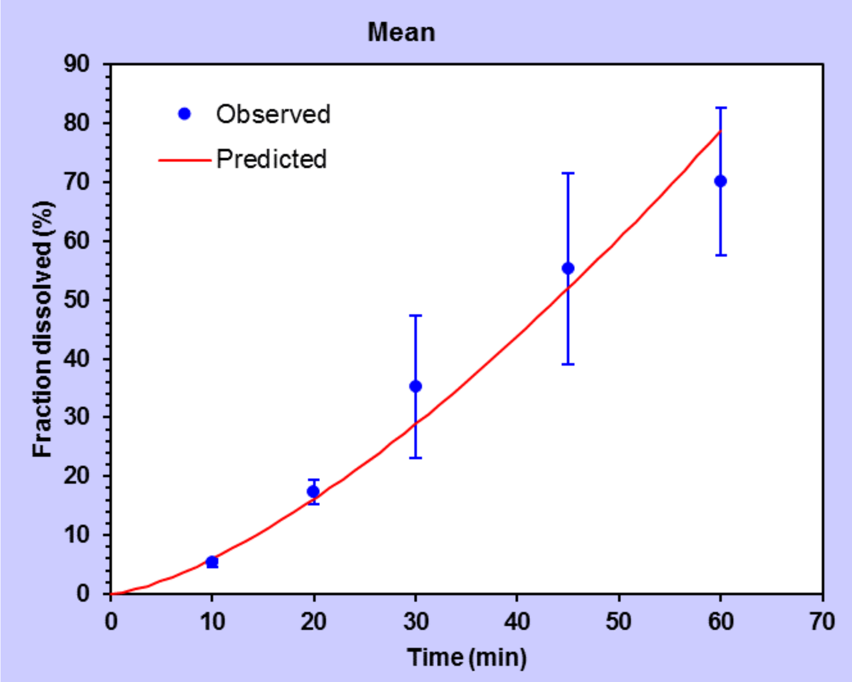

Supplementary materials – Model fitting summary – Parteck® M 100 (modelling up to app. 70 % of carvedilol released)

Graphical abstract of model fit presented as the fraction % of released carvedilol per tested tablet:

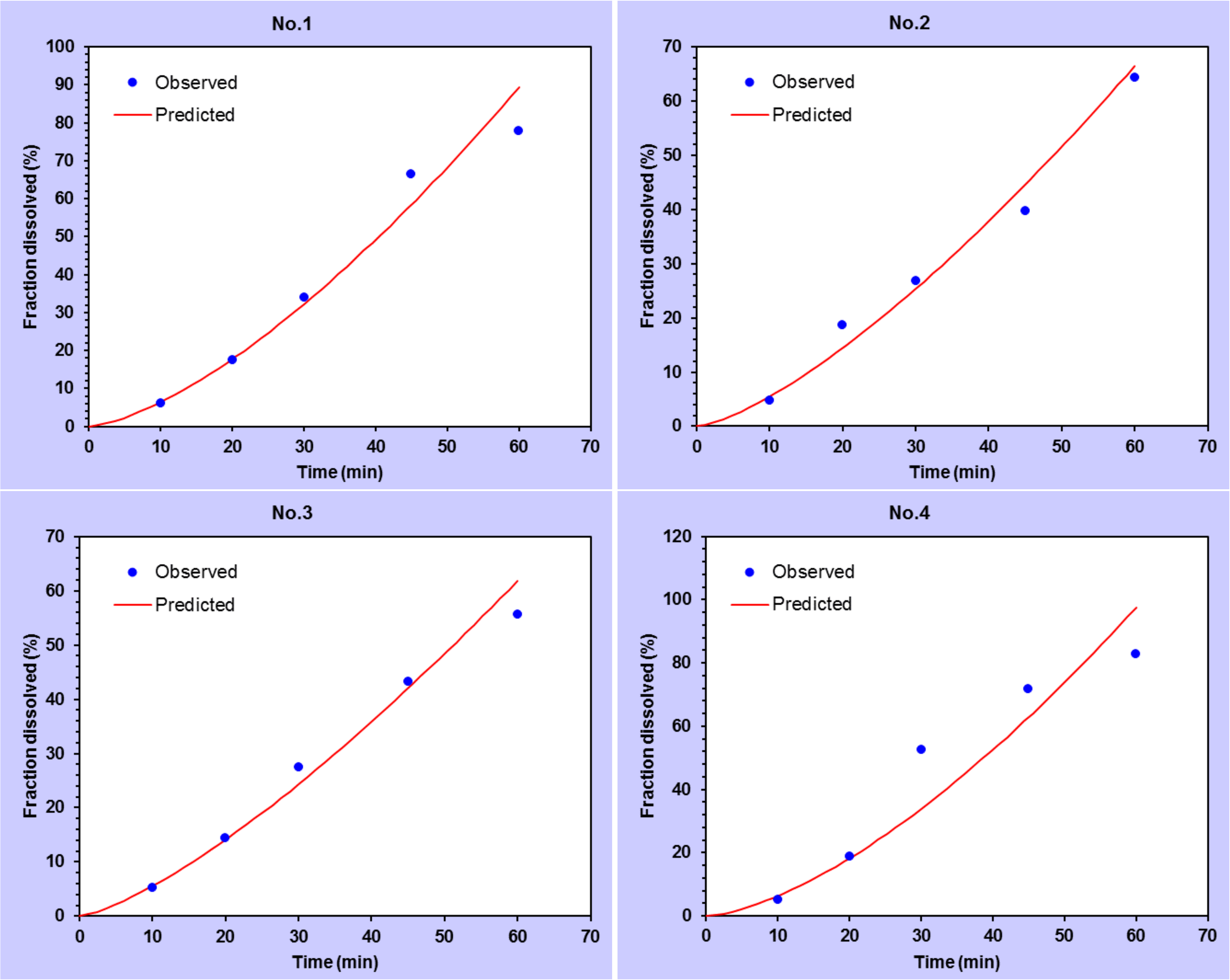

Supplementary materials – Model fitting summary – Parteck® M 100 (modelling up to app. 70 % of carvedilol released)

Model: **Korsmeyer–Peppas with T_{lag}**

Model equation: $F = k_{KP} \cdot (t - T_{lag})^n$

Fitted model parameters per tested tablet (N = 4) with statistics – mean, standard deviation (SD), and relative standard deviation expressed in % (RSD%) (output from DDSolver):

Parameter	No.1	No.2	No.3	No.4	Mean	SD	RSD(%)
k_{KP}	0.726	0.685	0.748	0.514	0.668	0.106	15.885
n	1.181	1.124	1.084	1.320	1.177	0.103	8.745
T_{lag}	4.000	4.000	4.000	6.000	4.500	1.000	22.222

Number of dissolution data points (N), degrees of freedom (df), and selected goodness of fit criteria – Pearson correlation coefficient (R), coefficient of determination (R^2), adjusted coefficient of determination ($R^2_{adjusted}$), and residual sum of squares (RSS) (manual calculation in MS Excel):

Parameter	No.1	No.2	No.3	No.4
N	5	5	5	5
df	2	2	2	2
R	0.985974801	0.992113296	0.996079043	0.948766088
R^2	0.972146308	0.984288792	0.99217346	0.900157089
$R^2_{adjusted}$	0.944292615	0.968577585	0.984346921	0.800314179
RSS	108.8398176	34.64644968	16.14324588	674.5851908

Graphical abstract of model fit presented as mean ± 1 SD of the fraction % of released carvedilol:

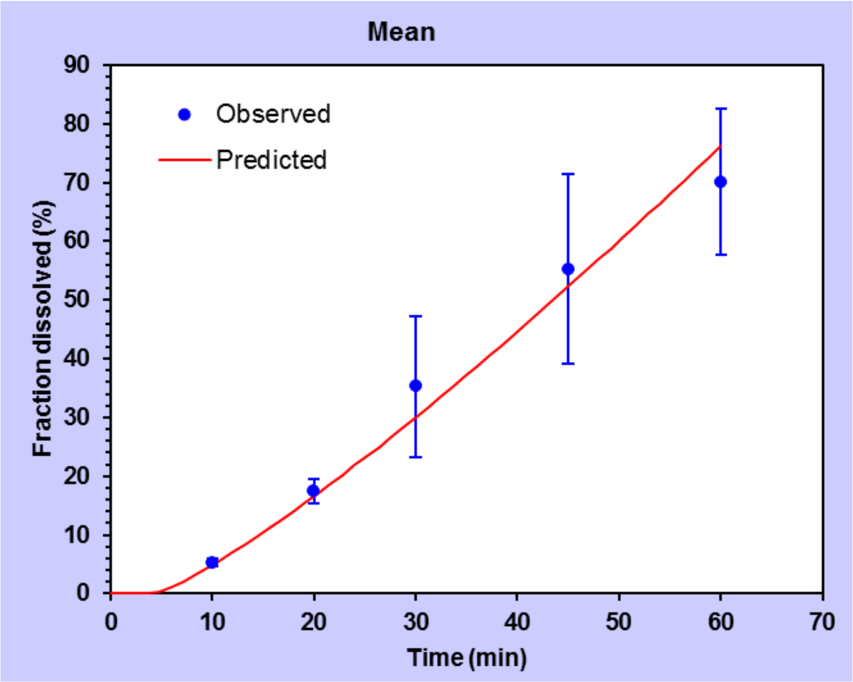

Supplementary materials – Model fitting summary – Parteck® M 100 (modelling up to app. 70 % of carvedilol released)

Graphical abstract of model fit presented as the fraction % of released carvedilol per tested tablet:

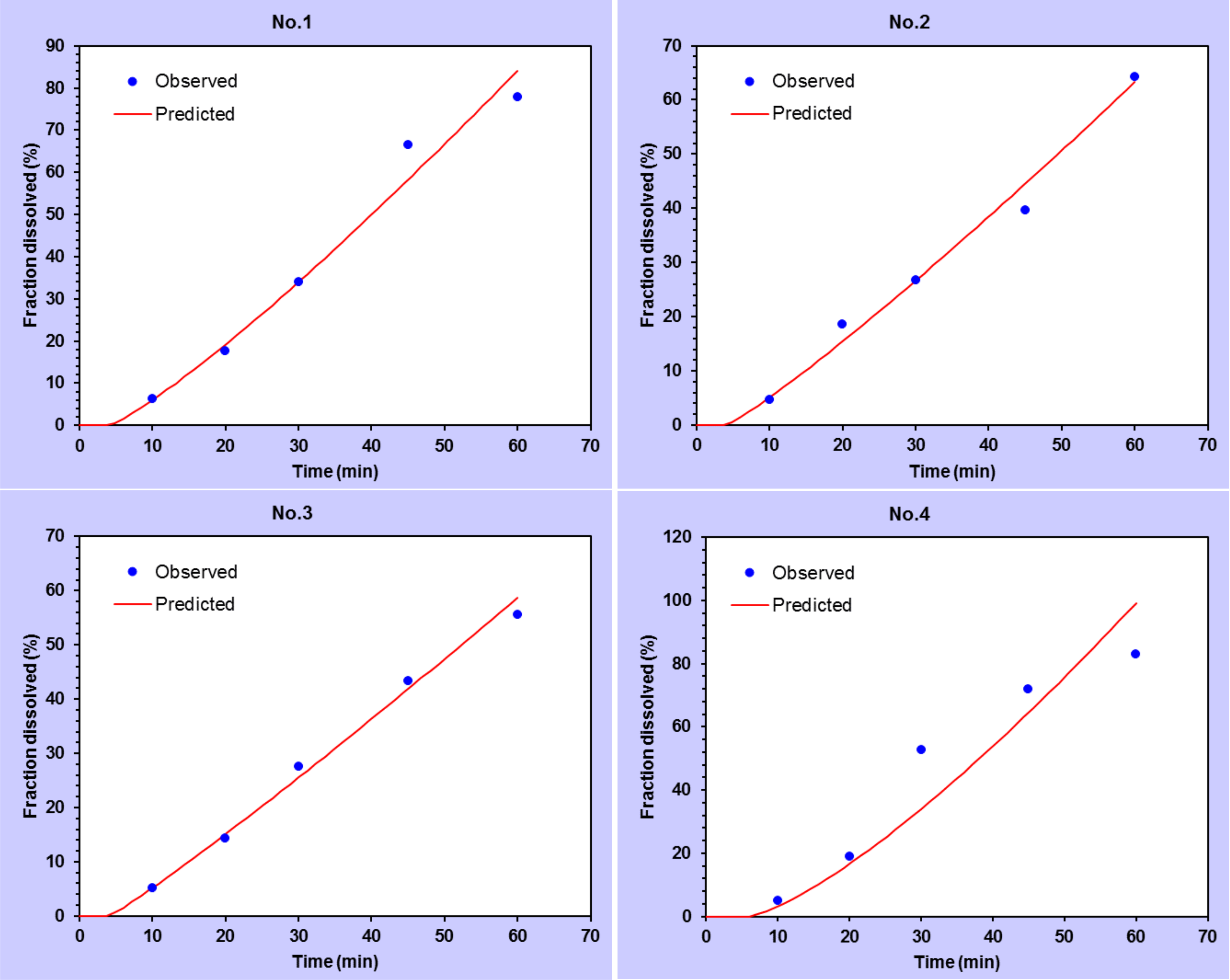

Supplementary materials – Model fitting summary – Parteck® M 100 (modelling up to app. 70 % of carvedilol released)

Model: **Korsmeyer–Peppas with F_0**

Model equation: $F = F_0 + k_{KP} \cdot t^n$

Fitted model parameters per tested tablet (N = 4) with statistics – mean, standard deviation (SD), and relative standard deviation expressed in % (RSD%) (output from DDSolver):

Parameter	No.1	No.2	No.3	No.4	Mean	SD	RSD(%)
k_{KP}	0.078	0.083	0.090	0.051	0.075	0.017	22.790
n	1.731	1.643	1.603	1.891	1.717	0.128	7.430
F_0	2.479	1.880	2.080	2.000	2.110	0.260	12.320

Number of dissolution data points (N), degrees of freedom (df), and selected goodness of fit criteria – Pearson correlation coefficient (R), coefficient of determination (R^2), adjusted coefficient of determination ($R^2_{adjusted}$), and residual sum of squares (RSS) (manual calculation in MS Excel):

Parameter	No.1	No.2	No.3	No.4
N	5	5	5	5
df	2	2	2	2
R	0.969996205	0.990412056	0.982677013	0.917886388
R^2	0.940892638	0.980916041	0.965654111	0.842515422
$R^2_{adjusted}$	0.881785275	0.961832082	0.931308222	0.685030843
RSS	379.3160535	114.5076405	131.1733502	1679.48628

Graphical abstract of model fit presented as mean ± 1 SD of the fraction % of released carvedilol:

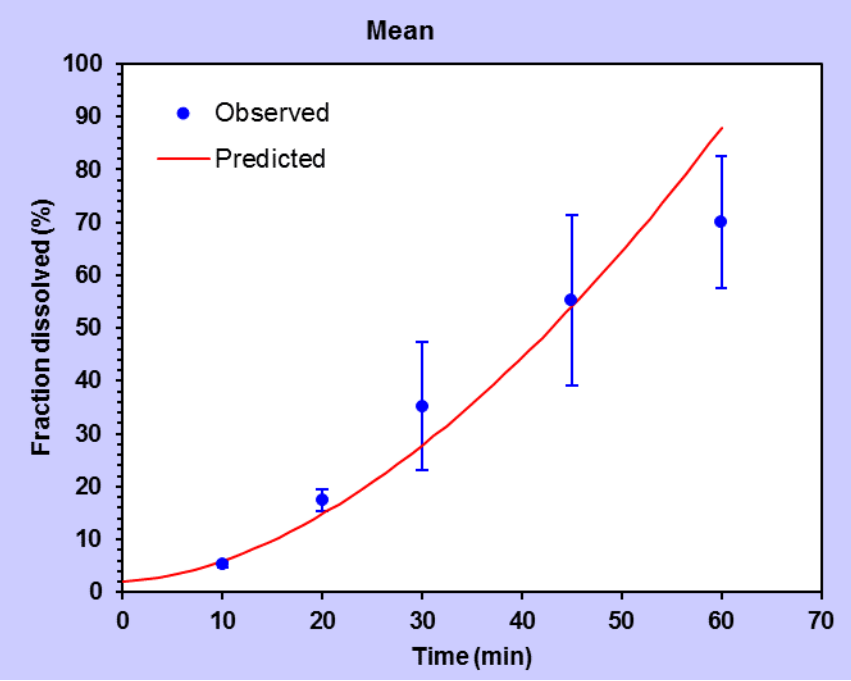

Supplementary materials – Model fitting summary – Parteck® M 100 (modelling up to app. 70 % of carvedilol released)

Graphical abstract of model fit presented as the fraction % of released carvedilol per tested tablet:

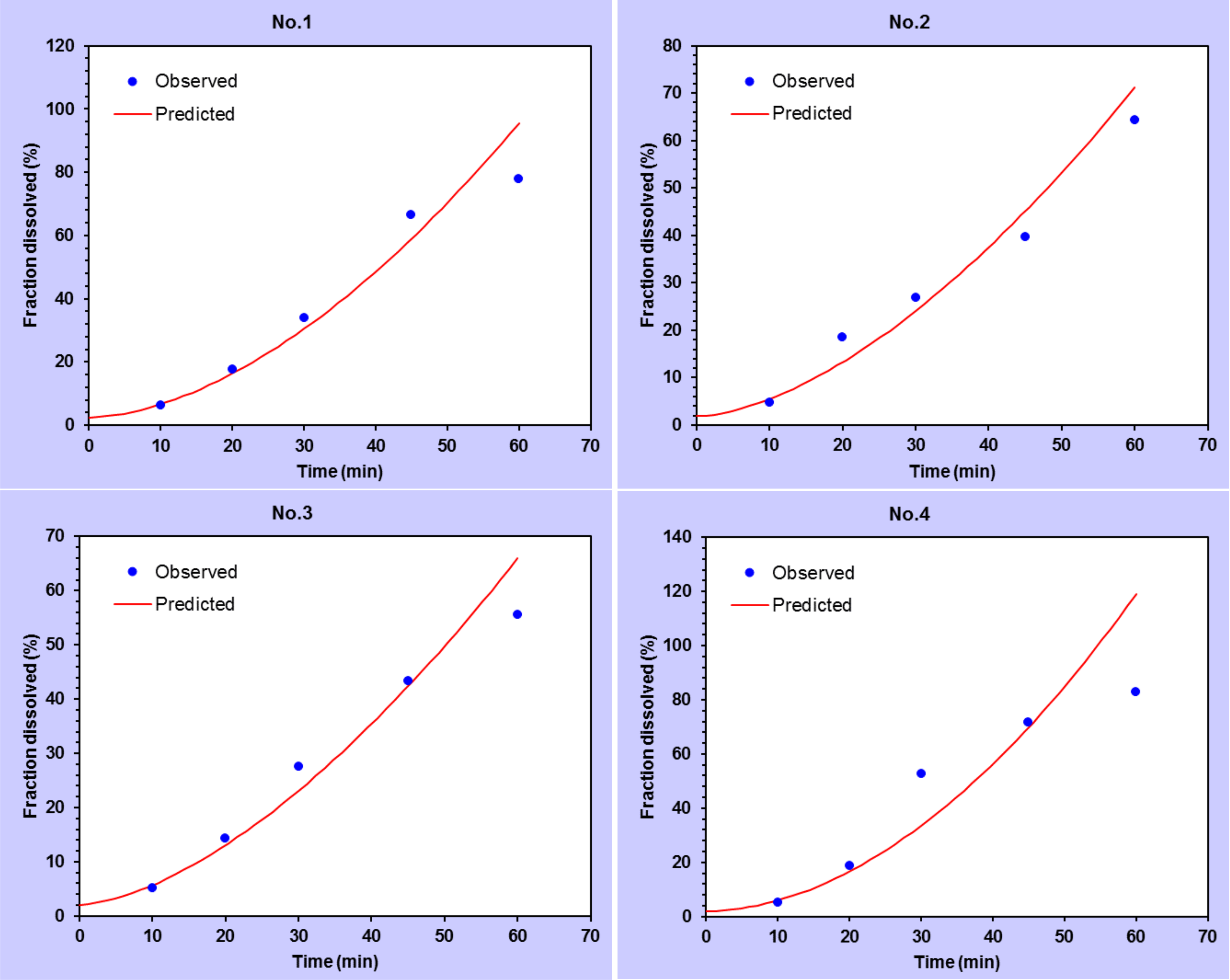

Supplementary materials – Model fitting summary – Parteck® M 100 (modelling up to app. 70 % of carvedilol released)

Model: **Hixson–Crowell**

Model equation: $F = 100 \cdot [1 - (1 - k_{HC} \cdot t)^3]$

Fitted model parameters per tested tablet (N = 4) with statistics – mean, standard deviation (SD), and relative standard deviation expressed in % (RSD%) (output from DDSolver):

Parameter	No.1	No.2	No.3	No.4	Mean	SD	RSD(%)
k_{HC}	0.006	0.004	0.004	0.007	0.005	0.002	30.966

Number of dissolution data points (N), degrees of freedom (df), and selected goodness of fit criteria – Pearson correlation coefficient (R), coefficient of determination (R^2), adjusted coefficient of determination ($R^2_{adjusted}$), and residual sum of squares (RSS) (manual calculation in MS Excel):

Parameter	No.1	No.2	No.3	No.4
N	5	5	5	5
df	4	4	4	4
R	0.987597439	0.983775948	0.998472928	0.98629874
R^2	0.975348702	0.967815115	0.996948189	0.972785204
$R^2_{adjusted}$	0.975348702	0.967815115	0.996948189	0.972785204
RSS	498.6837859	188.2018202	83.64882464	568.6470202

Graphical abstract of model fit presented as mean ± 1 SD of the fraction % of released carvedilol:

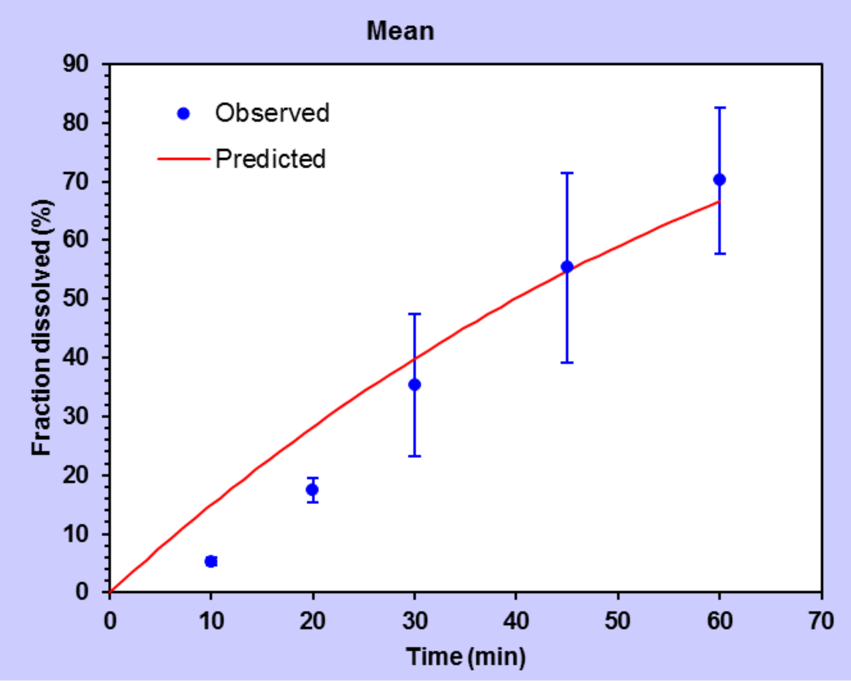

Supplementary materials – Model fitting summary – Parteck® M 100 (modelling up to app. 70 % of carvedilol released)

Graphical abstract of model fit presented as the fraction % of released carvedilol per tested tablet:

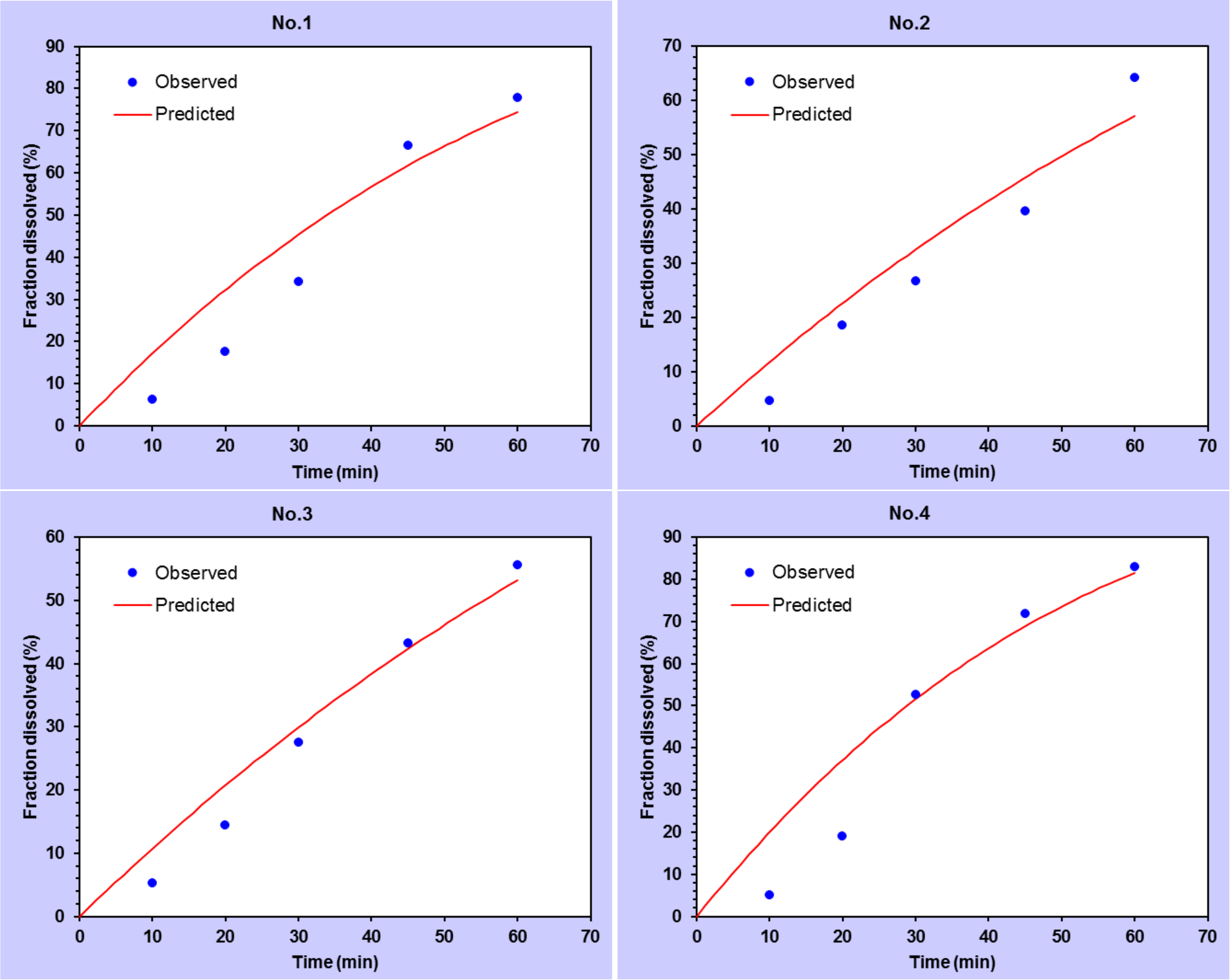

Supplementary materials – Model fitting summary – Parteck® M 100 (modelling up to app. 70 % of carvedilol released)

Model: **Hixson–Crowell with T_{lag}**

Model equation: $F = 100 \cdot \left\{1 - \left[1 - k_{HC} \cdot \left(t - T_{lag}\right)\right]^3\right\}$

Fitted model parameters per tested tablet (N = 4) with statistics – mean, standard deviation (SD), and relative standard deviation expressed in % (RSD%) (output from DDSolver):

Parameter	No.1	No.2	No.3	No.4	Mean	SD	RSD(%)
k_{HC}	0.008	0.005	0.004	0.009	0.007	0.002	32.629
T_{lag}	10.174	8.788	7.218	8.674	8.714	1.208	13.861

Number of dissolution data points (N), degrees of freedom (df), and selected goodness of fit criteria – Pearson correlation coefficient (R), coefficient of determination (R^2), adjusted coefficient of determination ($R^2_{adjusted}$), and residual sum of squares (RSS) (manual calculation in MS Excel):

Parameter	No.1	No.2	No.3	No.4
N	5	5	5	5
df	3	3	3	3
R	0.984920218	0.981362946	0.998240196	0.98743961
R^2	0.970067836	0.963073231	0.996483488	0.975036983
$R^2_{adjusted}$	0.960090448	0.950764308	0.995311318	0.966715978
RSS	117.8071062	79.75319164	5.977435996	114.6576271

Graphical abstract of model fit presented as mean ± 1 SD of the fraction % of released carvedilol:

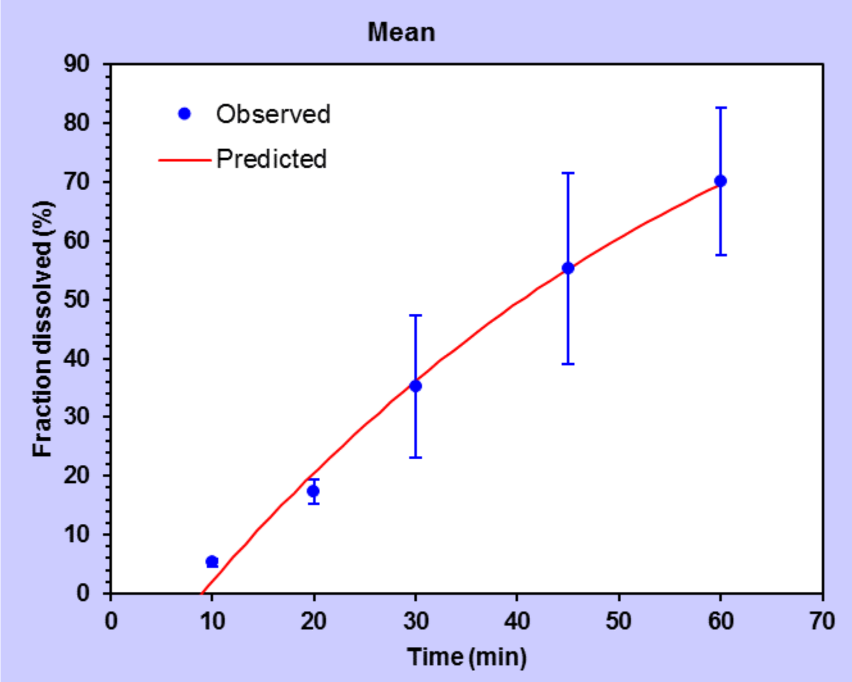

Supplementary materials – Model fitting summary – Parteck® M 100 (modelling up to app. 70 % of carvedilol released)

Graphical abstract of model fit presented as the fraction % of released carvedilol per tested tablet:

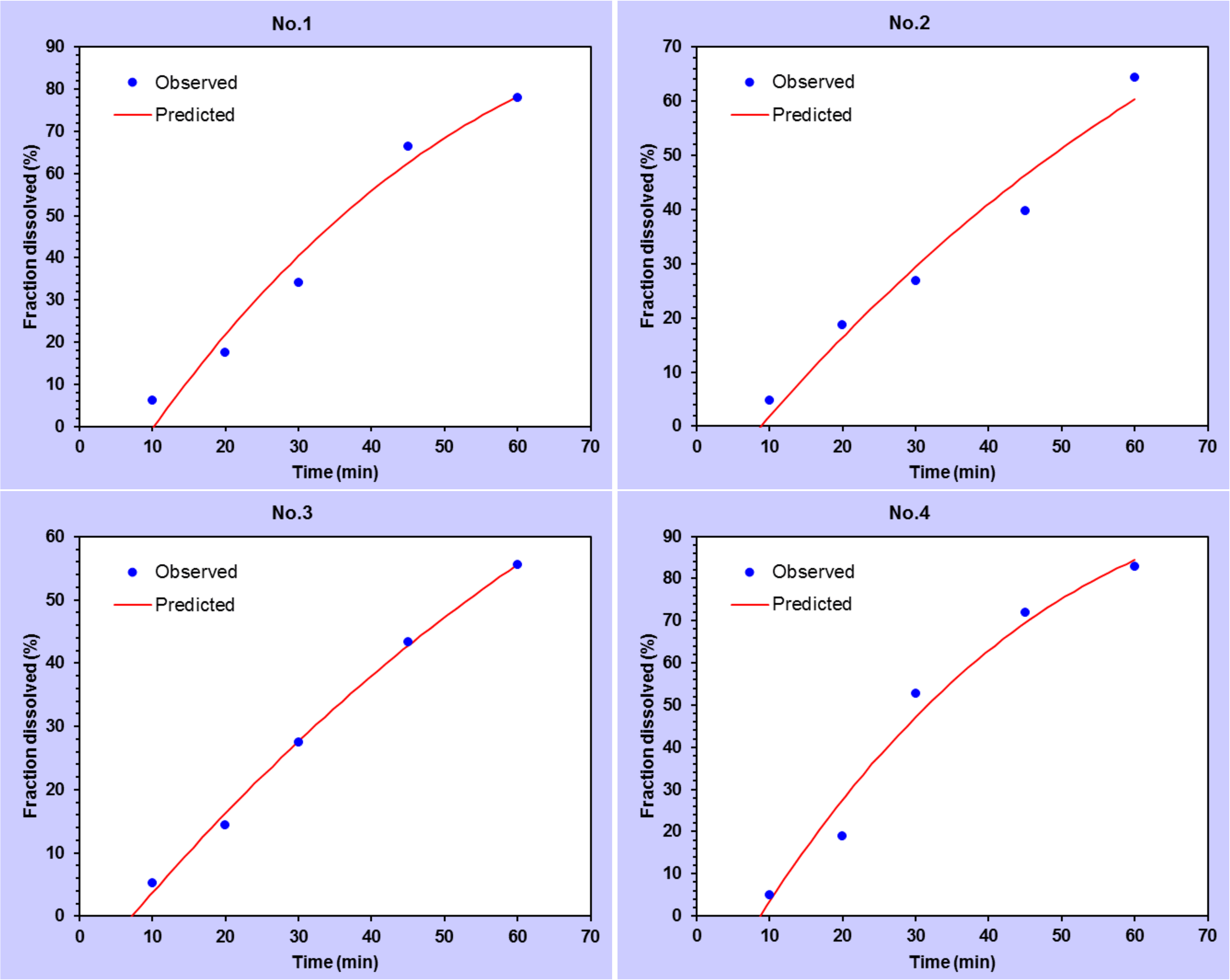

Supplementary materials – Model fitting summary – Parteck® M 100 (modelling up to app. 70 % of carvedilol released)

Model: **Hopfenberg**

Model equation: $F = 100 \cdot [1 - (1 - k_{HB} \cdot t)^n]$

Fitted model parameters per tested tablet (N = 4) with statistics – mean, standard deviation (SD), and relative standard deviation expressed in % (RSD%) (output from DDSolver):

Parameter	No.1	No.2	No.3	No.4	Mean	SD	RSD(%)
k_{HB}	0.013	0.010	0.009	0.015	0.012	0.003	22.077
n	1.000	1.000	1.000	1.000	1.000	0.000	0.000

Number of dissolution data points (N), degrees of freedom (df), and selected goodness of fit criteria – Pearson correlation coefficient (R), coefficient of determination (R^2), adjusted coefficient of determination ($R^2_{adjusted}$), and residual sum of squares (RSS) (manual calculation in MS Excel):

Parameter	No.1	No.2	No.3	No.4
N	5	5	5	5
df	3	3	3	3
R	0.988678076	0.990876008	0.997320012	0.969389722
R^2	0.977484337	0.981835263	0.994647207	0.939716433
$R^2_{adjusted}$	0.969979116	0.975780351	0.992862943	0.919621911
RSS	203.9475095	82.98476008	35.62450654	336.4929592

Graphical abstract of model fit presented as mean ± 1 SD of the fraction % of released carvedilol:

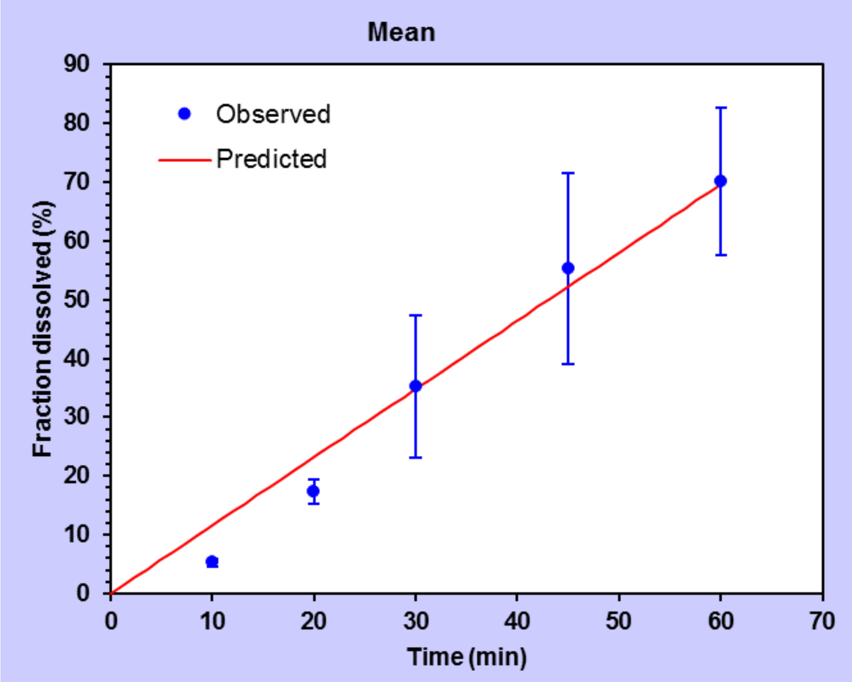

Supplementary materials – Model fitting summary – Parteck® M 100 (modelling up to app. 70 % of carvedilol released)

Graphical abstract of model fit presented as the fraction % of released carvedilol per tested tablet:

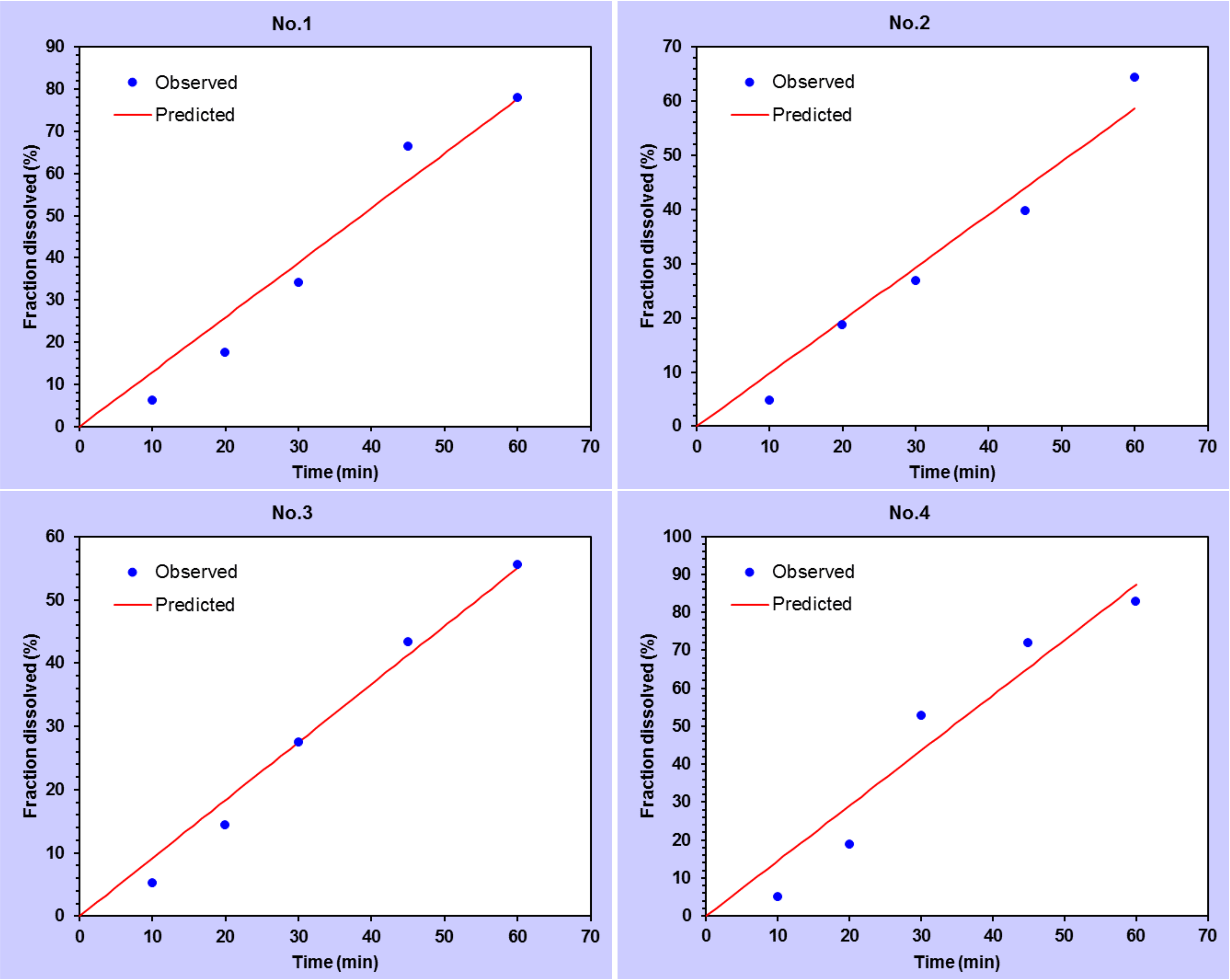

Supplementary materials – Model fitting summary – Parteck® M 100 (modelling up to app. 70 % of carvedilol released)

Model: **Hopfenberg with T_{lag}**

Model equation: $F = 100 \cdot \{1 - [1 - k_{HB} \cdot (t - T_{lag})]^n\}$

Fitted model parameters per tested tablet (N = 4) with statistics – mean, standard deviation (SD), and relative standard deviation expressed in % (RSD%) (output from DDSolver):

Parameter	No.1	No.2	No.3	No.4	Mean	SD	RSD(%)
k_{HB}	0.015	0.011	0.006	0.009	0.010	0.004	36.629
n	1.000	1.000	2.000	3.000	1.750	0.957	54.710
T_{lag}	6.672	5.668	6.616	8.674	6.908	1.265	18.308

Number of dissolution data points (N), degrees of freedom (df), and selected goodness of fit criteria – Pearson correlation coefficient (R), coefficient of determination (R^2), adjusted coefficient of determination ($R^2_{adjusted}$), and residual sum of squares (RSS) (manual calculation in MS Excel):

Parameter	No.1	No.2	No.3	No.4
N	5	5	5	5
df	2	2	2	2
R	0.988678076	0.990876008	0.998578567	0.98743961
R^2	0.977484337	0.981835263	0.997159154	0.975036983
$R^2_{adjusted}$	0.954968674	0.963670527	0.994318307	0.950073966
RSS	85.85572368	37.1162236	4.802185717	114.6576271

Graphical abstract of model fit presented as mean ± 1 SD of the fraction % of released carvedilol:

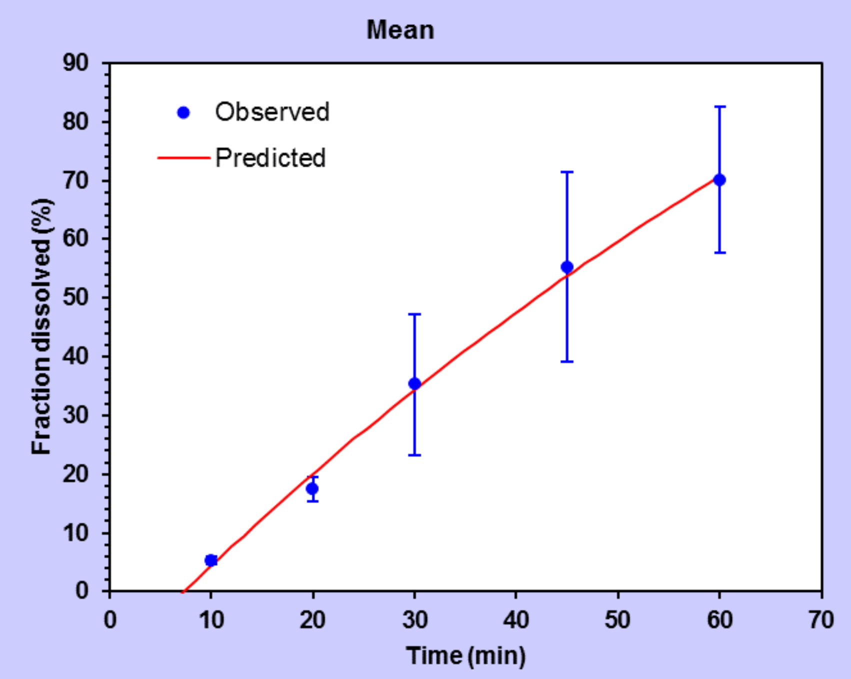

Supplementary materials – Model fitting summary – Parteck® M 100 (modelling up to app. 70 % of carvedilol released)

Graphical abstract of model fit presented as the fraction % of released carvedilol per tested tablet:

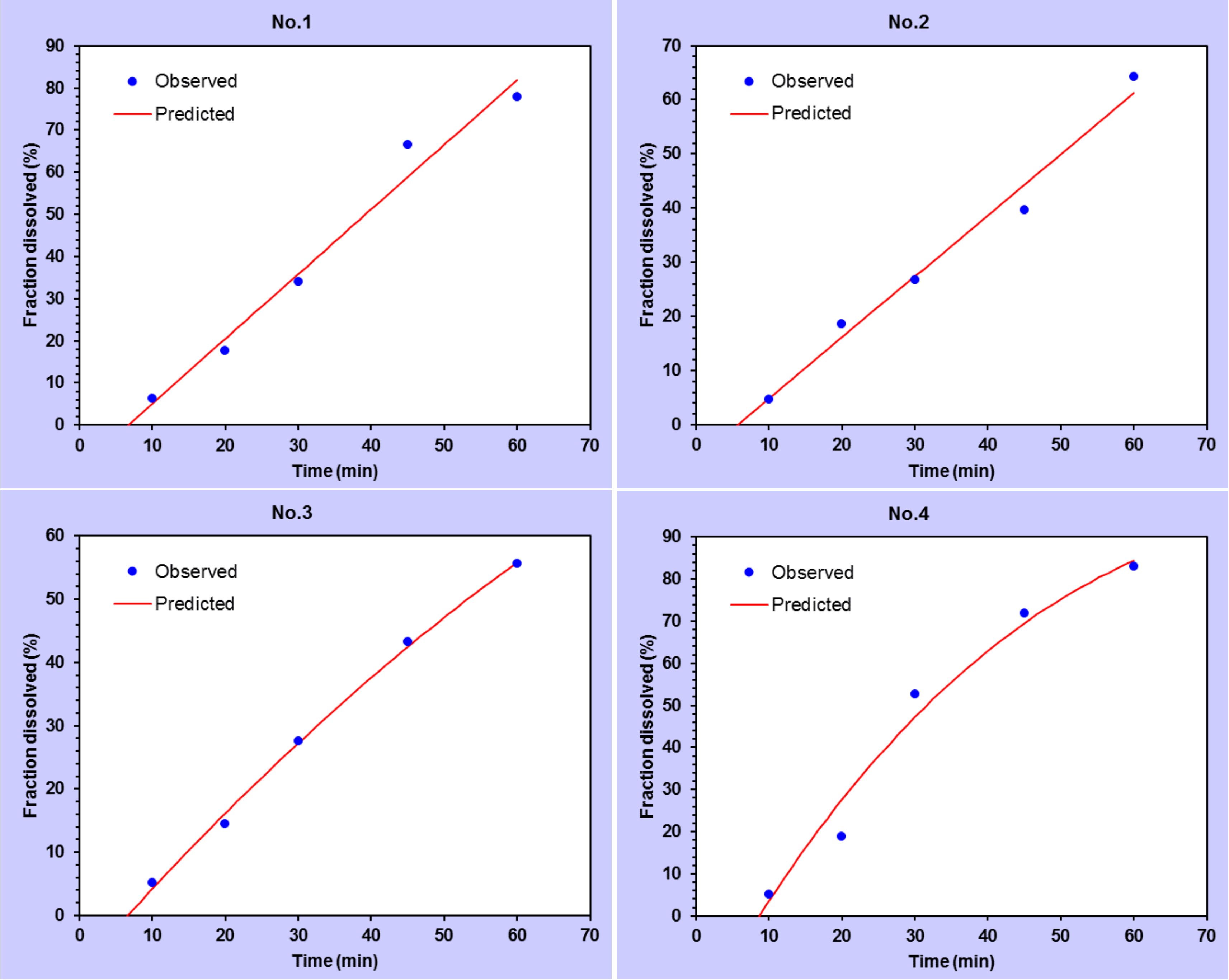

Supplementary materials – Model fitting summary – Parteck® M 100 (modelling up to app. 70 % of carvedilol released)

Model: **Baker–Lonsdale**

Model equation: $\frac{3}{2}\cdot\left[1-\left(1-\frac{F}{100}\right)^{\frac{2}{3}}\right]-\frac{F}{100}=k_{BL}\cdot t$

Fitted model parameters per tested tablet (N = 4) with statistics – mean, standard deviation (SD), and relative standard deviation expressed in % (RSD%) (output from DDSolver):

Parameter	No.1	No.2	No.3	No.4	Mean	SD	RSD(%)
k_{BL}	0.004	0.002	0.001	0.004	0.003	0.001	49.631

Number of dissolution data points (N), degrees of freedom (df), and selected goodness of fit criteria – Pearson correlation coefficient (R), coefficient of determination (R^2), adjusted coefficient of determination ($R^2_{adjusted}$), and residual sum of squares (RSS) (manual calculation in MS Excel):

Parameter	No.1	No.2	No.3	No.4
N	5	5	5	5
df	4	4	4	4
R	0.972088221	0.968452205	0.990229695	0.982724453
R^2	0.94495551	0.937899673	0.98055485	0.96574735
$R^2_{adjusted}$	0.94495551	0.937899673	0.98055485	0.96574735
RSS	3997.989582	2278.932273	1482.981896	3873.860951

Graphical abstract of model fit presented as mean ± 1 SD of the fraction % of released carvedilol:

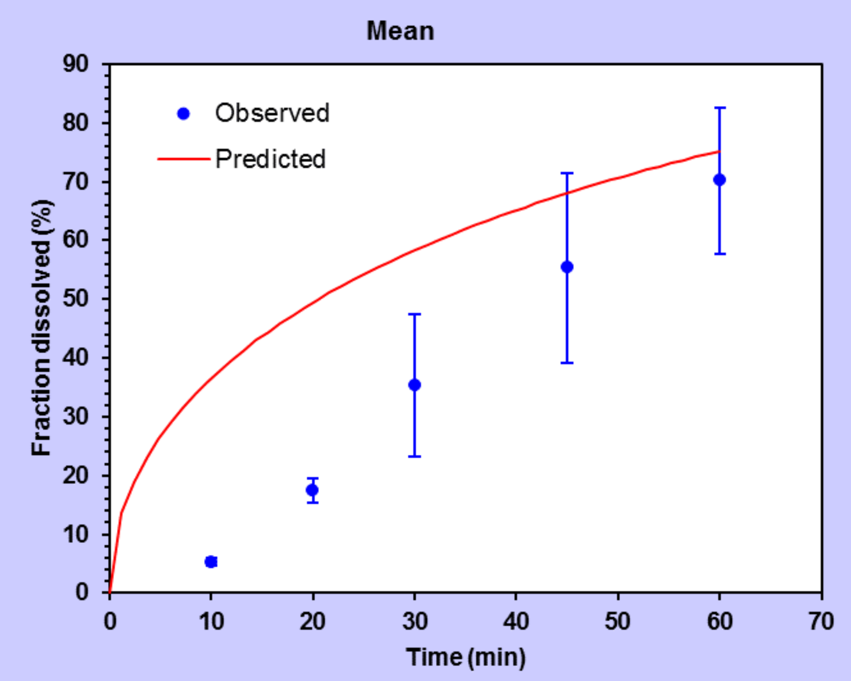

Supplementary materials – Model fitting summary – Parteck® M 100 (modelling up to app. 70 % of carvedilol released)

Graphical abstract of model fit presented as the fraction % of released carvedilol per tested tablet:

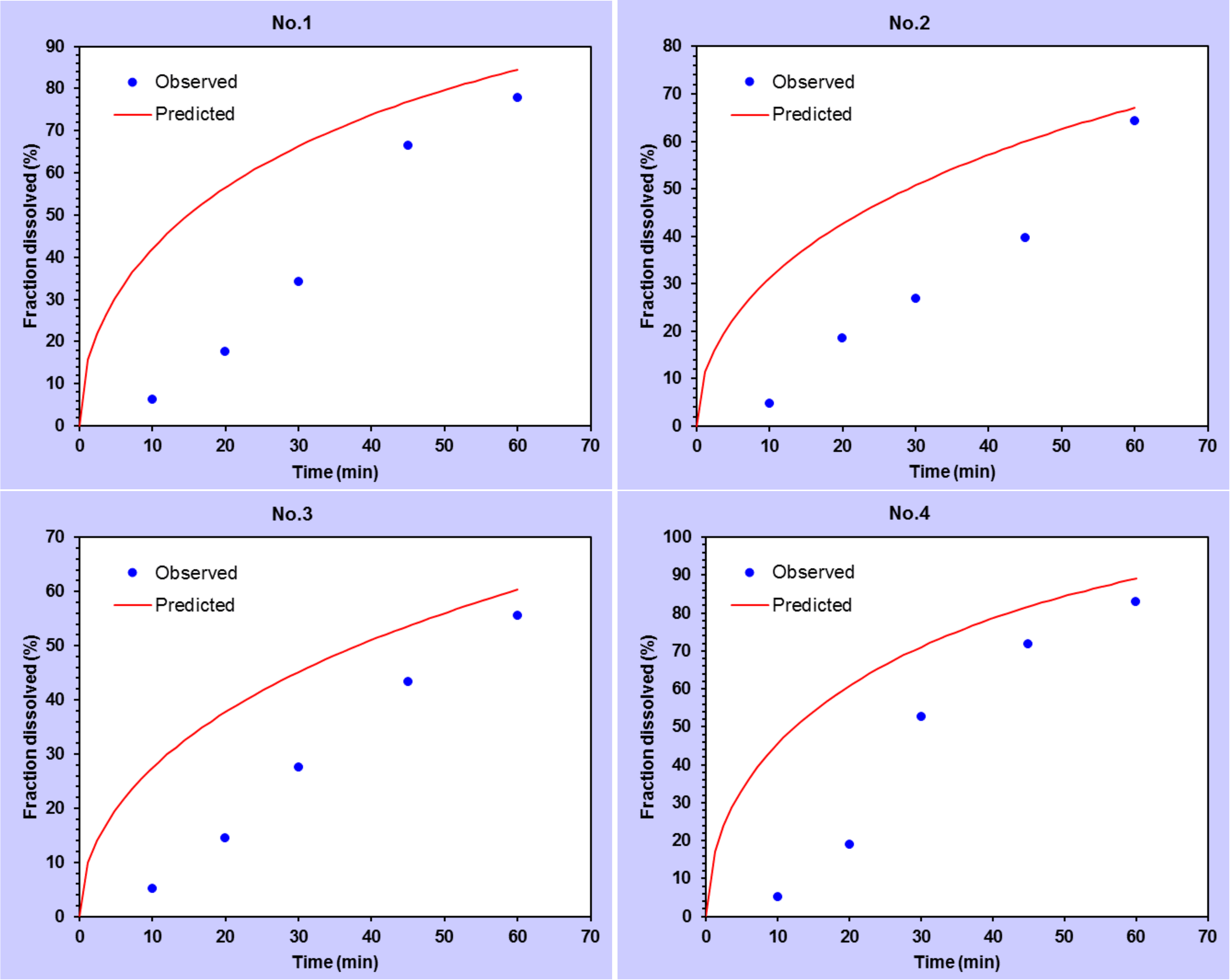

Supplementary materials – Model fitting summary – Parteck® M 100 (modelling up to app. 70 % of carvedilol released)

Model: **Baker–Lonsdale with T_{lag}**

Model equation: $\frac{3}{2}\cdot\left[1-\left(1-\frac{F}{100}\right)^{\frac{2}{3}}\right]-\frac{F}{100}=k_{BL}\cdot\left(t-T_{lag}\right)$

Fitted model parameters per tested tablet (N = 4) with statistics – mean, standard deviation (SD), and relative standard deviation expressed in % (RSD%) (output from DDSolver):

Parameter	No.1	No.2	No.3	No.4	Mean	SD	RSD(%)
k_{BL}	0.004	0.002	0.001	0.006	0.003	0.002	59.367
T_{lag}	16.052	16.762	15.174	16.046	16.008	0.650	4.060

Number of dissolution data points (N), degrees of freedom (df), and selected goodness of fit criteria – Pearson correlation coefficient (R), coefficient of determination (R^2), adjusted coefficient of determination ($R^2_{adjusted}$), and residual sum of squares (RSS) (manual calculation in MS Excel):

Parameter	No.1	No.2	No.3	No.4
N	5	5	5	5
df	3	3	3	3
R	0.959720971	0.953042777	0.976044411	0.980586445
R^2	0.921064342	0.908290535	0.952662693	0.961549776
$R^2_{adjusted}$	0.894752456	0.877720714	0.93688359	0.948733035
RSS	353.9945888	221.2062401	90.42120255	288.9279123

Graphical abstract of model fit presented as mean ± 1 SD of the fraction % of released carvedilol:

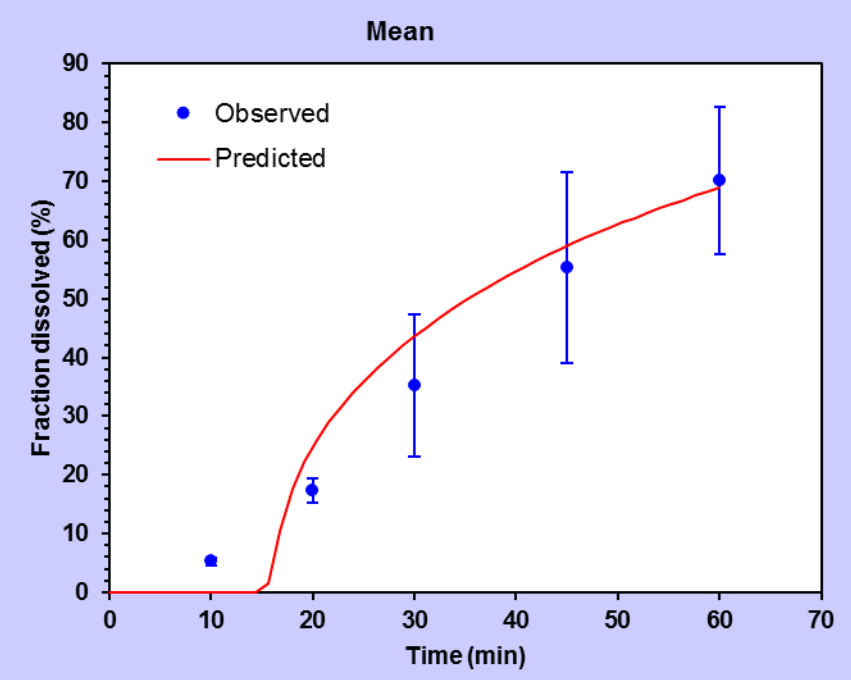

Supplementary materials – Model fitting summary – Parteck® M 100 (modelling up to app. 70 % of carvedilol released)

Graphical abstract of model fit presented as the fraction % of released carvedilol per tested tablet:

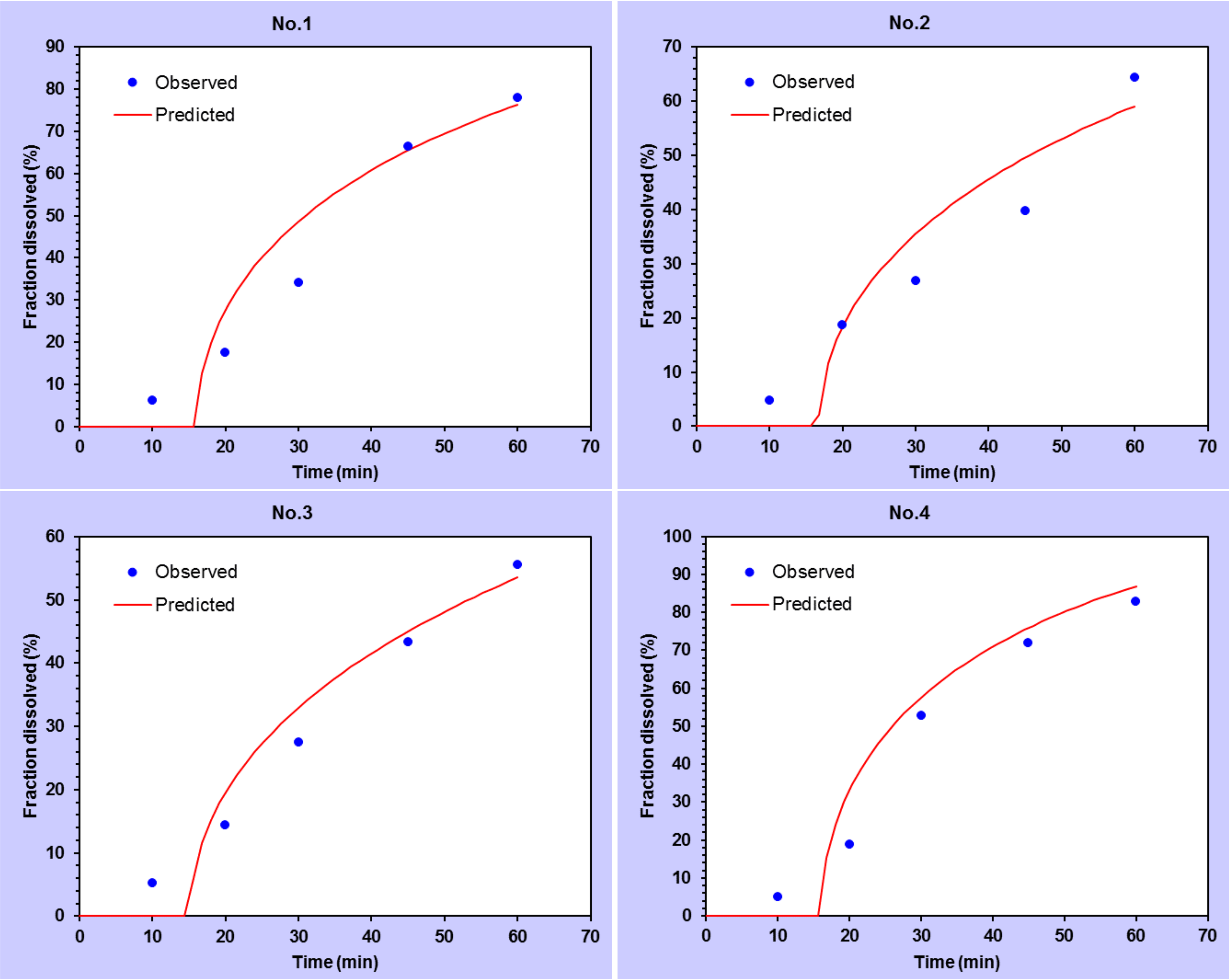

Supplementary materials – Model fitting summary – Parteck® M 100 (modelling up to app. 70 % of carvedilol released)

Model: **Makoid–Banakar**

Model equation: $F = k_{MB} \cdot t^n \cdot e^{-k \cdot t}$

Fitted model parameters per tested tablet (N = 4) with statistics – mean, standard deviation (SD), and relative standard deviation expressed in % (RSD%) (output from DDSolver):

Parameter	No.1	No.2	No.3	No.4	Mean	SD	RSD(%)
k_{MB}	0.101	0.068	0.097	0.013	0.070	0.041	58.094
n	1.833	1.957	1.795	2.851	2.109	0.499	23.676
k	0.013	0.021	0.016	0.049	0.025	0.016	65.384

Number of dissolution data points (N), degrees of freedom (df), and selected goodness of fit criteria – Pearson correlation coefficient (R), coefficient of determination (R^2), adjusted coefficient of determination ($R^2_{adjusted}$), and residual sum of squares (RSS) (manual calculation in MS Excel):

Parameter	No.1	No.2	No.3	No.4
N	5	5	5	5
df	2	2	2	2
R	0.990675544	0.981183314	0.999427475	0.993965896
R^2	0.981438033	0.962720696	0.998855278	0.987968203
$R^2_{adjusted}$	0.962876066	0.925441391	0.997710556	0.975936406
RSS	71.33816053	76.62865576	1.957158107	71.019587

Graphical abstract of model fit presented as mean ± 1 SD of the fraction % of released carvedilol:

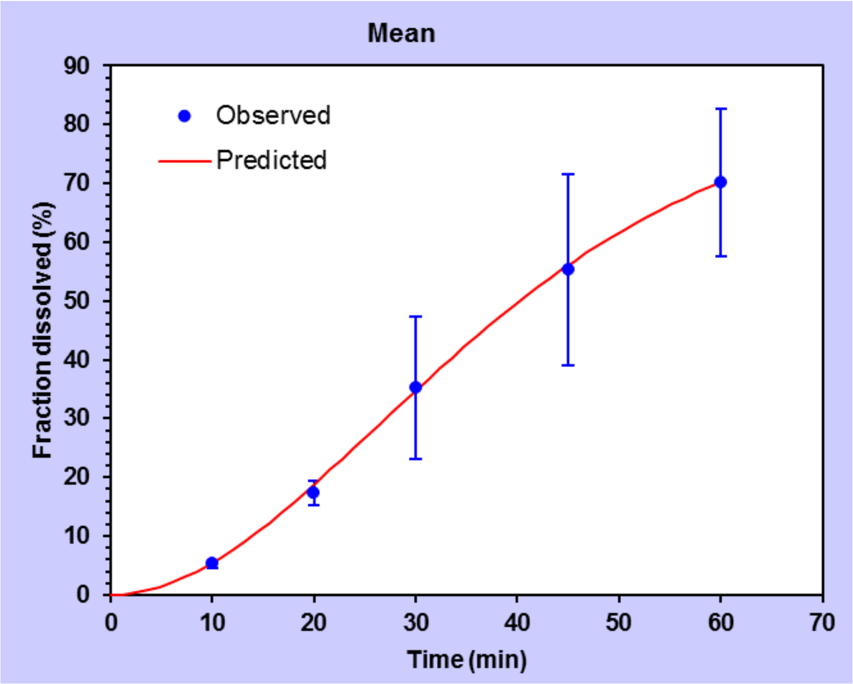

Supplementary materials – Model fitting summary – Parteck® M 100 (modelling up to app. 70 % of carvedilol released)

Graphical abstract of model fit presented as the fraction % of released carvedilol per tested tablet:

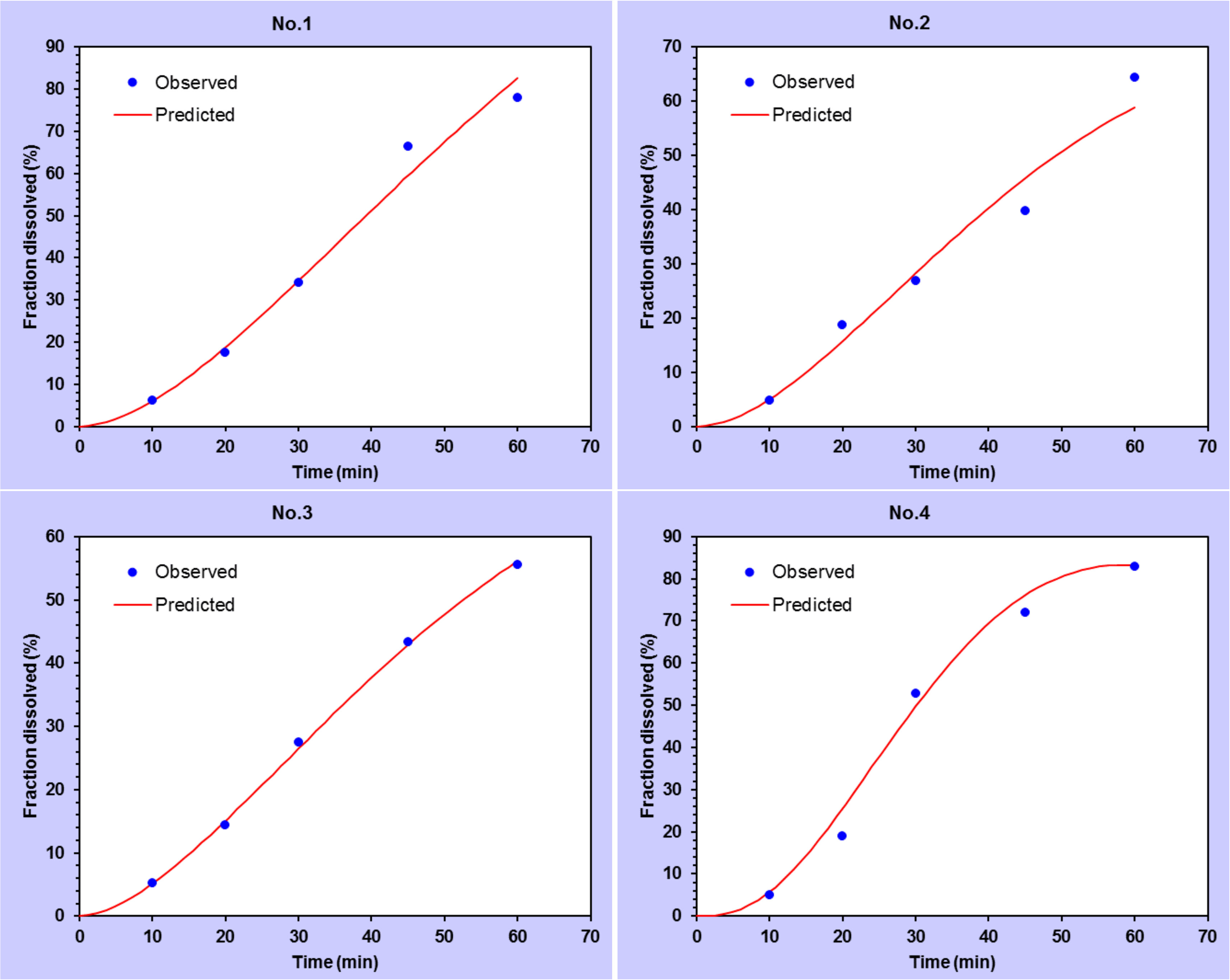

Supplementary materials – Model fitting summary – Parteck® M 100 (modelling up to app. 70 % of carvedilol released)

Model: **Makoid–Banakar with T_{lag}**

Model equation: $F = k_{MB} \cdot (t - T_{lag})^n \cdot e^{-k \cdot (t - T_{lag})}$

Fitted model parameters per tested tablet (N = 4) with statistics – mean, standard deviation (SD), and relative standard deviation expressed in % (RSD%) (output from DDSolver):

Parameter	No.1	No.2	No.3	No.4	Mean	SD	RSD(%)
k_{MB}	0.716	0.494	0.640	0.196	0.512	0.230	44.927
n	1.189	1.309	1.172	1.867	1.384	0.327	23.662
k	0.001	0.009	0.004	0.025	0.010	0.011	113.928
T_{lag}	4.000	4.000	4.000	4.000	4.000	0.000	0.000

Number of dissolution data points (N), degrees of freedom (df), and selected goodness of fit criteria – Pearson correlation coefficient (R), coefficient of determination (R^2), adjusted coefficient of determination ($R^2_{adjusted}$), and residual sum of squares (RSS) (manual calculation in MS Excel):

Parameter	No.1	No.2	No.3	No.4
N	5	5	5	5
df	1	1	1	1
R	0.986548895	0.985799628	0.9981646	0.988698822
R^2	0.973278722	0.971800907	0.996332569	0.97752536
$R^2_{adjusted}$	0.893114888	0.887203627	0.985330275	0.91010144
RSS	103.4116524	58.52012013	6.34090167	102.2133715

Graphical abstract of model fit presented as mean ± 1 SD of the fraction % of released carvedilol:

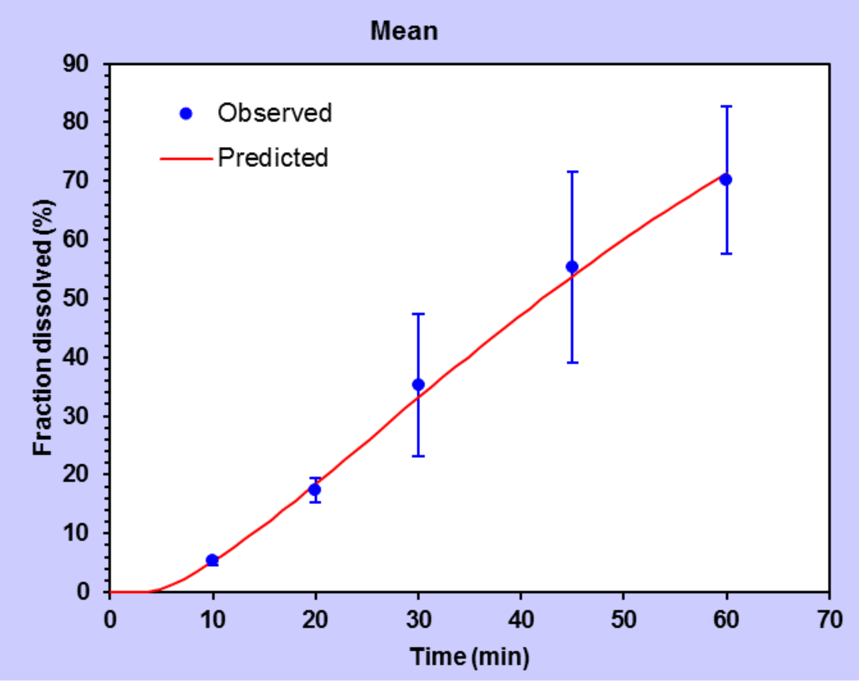

Supplementary materials – Model fitting summary – Parteck® M 100 (modelling up to app. 70 % of carvedilol released)

Graphical abstract of model fit presented as the fraction % of released carvedilol per tested tablet:

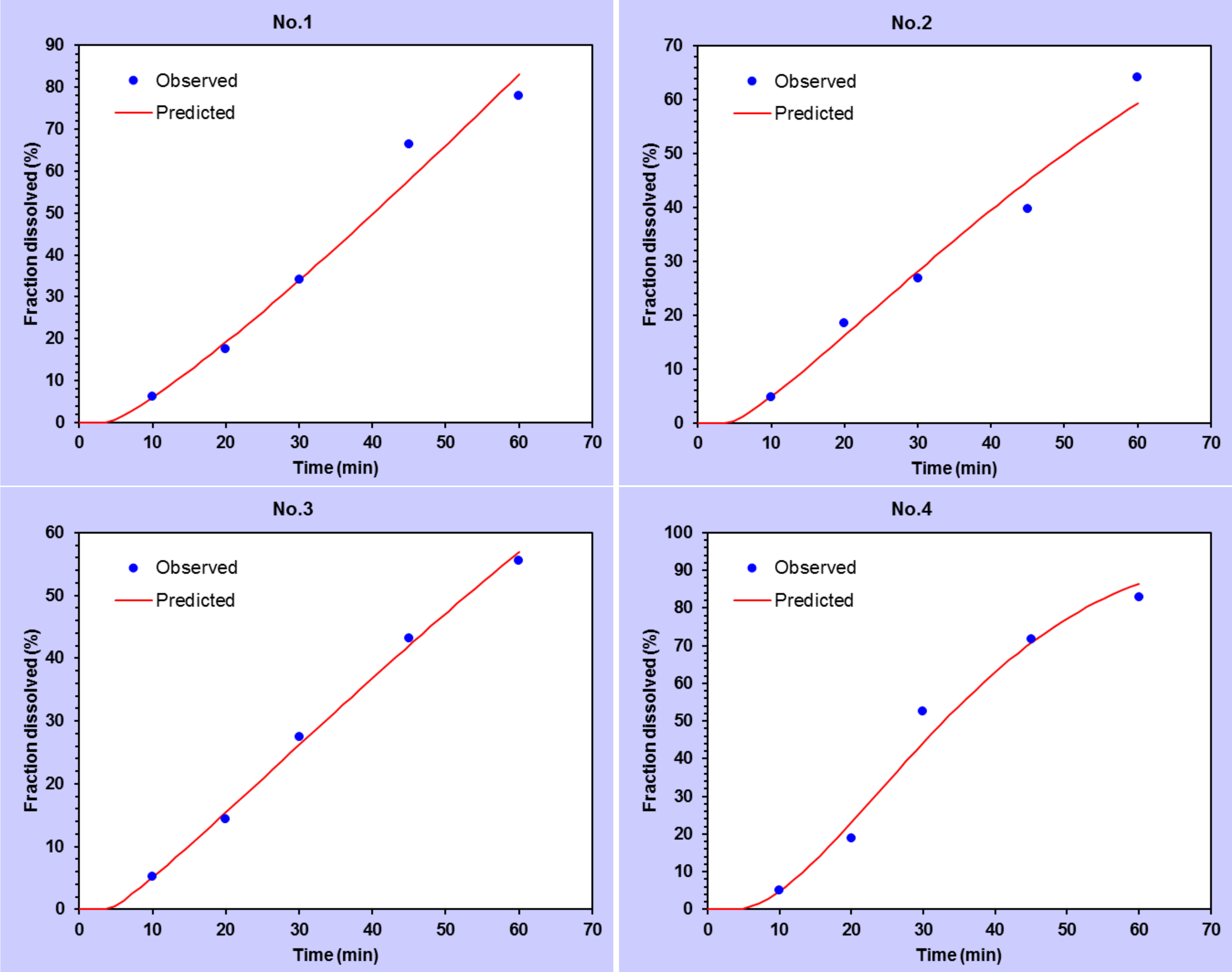

Supplementary materials – Model fitting summary – Parteck® M 100 (modelling up to app. 70 % of carvedilol released)

Model: **Peppas–Sahlin_1**

Model equation: $F = k_1 \cdot t^m + k_2 \cdot t^{2m}$

Fitted model parameters per tested tablet (N = 4) with statistics – mean, standard deviation (SD), and relative standard deviation expressed in % (RSD%) (output from DDSolver):

Parameter	No.1	No.2	No.3	No.4	Mean	SD	RSD(%)
k_1	-6.821	-4.638	-3.615	-5.214	-5.072	1.341	-26.435
k_2	3.141	2.276	2.003	3.082	2.626	0.573	21.807
m	0.450	0.450	0.450	0.450	0.450	0.000	0.000

Number of dissolution data points (N), degrees of freedom (df), and selected goodness of fit criteria – Pearson correlation coefficient (R), coefficient of determination (R^2), adjusted coefficient of determination ($R^2_{adjusted}$), and residual sum of squares (RSS) (manual calculation in MS Excel):

Parameter	No.1	No.2	No.3	No.4
N	5	5	5	5
df	2	2	2	2
R	0.988665045	0.990956658	0.997283104	0.969386064
R^2	0.977458571	0.981995098	0.99457359	0.93970934
$R^2_{adjusted}$	0.954917142	0.963990197	0.989147179	0.879418681
RSS	85.9975232	36.82231083	9.215378287	272.468254

Graphical abstract of model fit presented as mean ± 1 SD of the fraction % of released carvedilol:

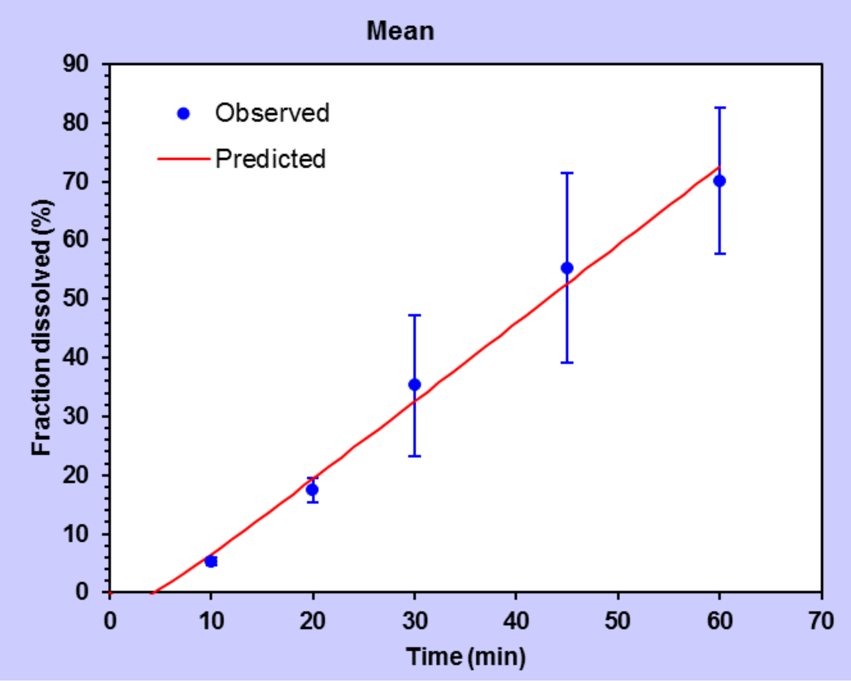

Supplementary materials – Model fitting summary – Parteck® M 100 (modelling up to app. 70 % of carvedilol released)

Graphical abstract of model fit presented as the fraction % of released carvedilol per tested tablet:

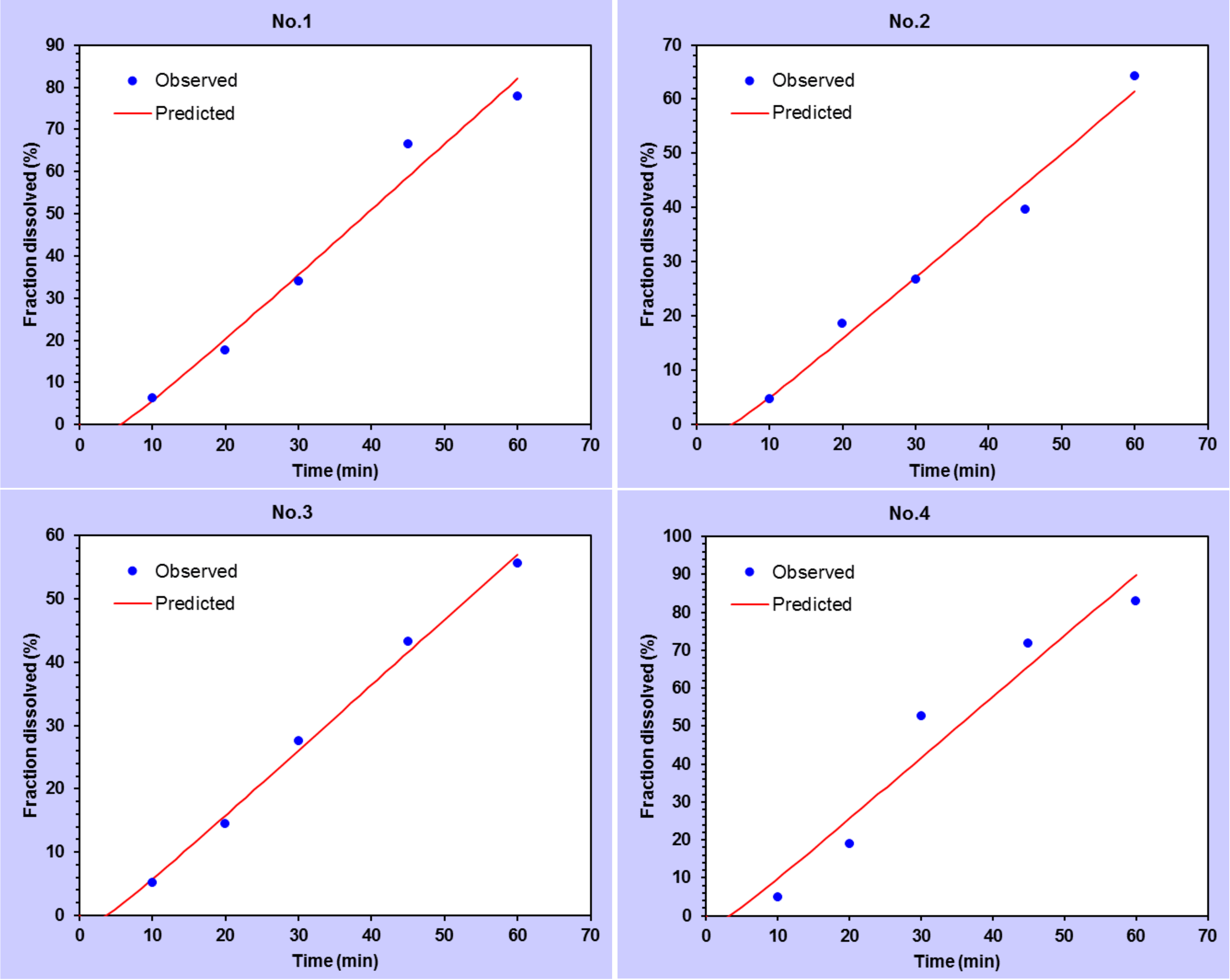

Supplementary materials – Model fitting summary – Parteck® M 100 (modelling up to app. 70 % of carvedilol released)

Model: **Peppas–Sahlin_1 with T_{lag}**

Model equation: $F = k_1 \cdot (t - T_{lag})^m + k_2 \cdot (t - T_{lag})^{2m}$

Fitted model parameters per tested tablet (N = 4) with statistics – mean, standard deviation (SD), and relative standard deviation expressed in % (RSD%) (output from DDSolver):

Parameter	No.1	No.2	No.3	No.4	Mean	SD	RSD(%)
k_1	-4.041	-2.561	-1.624	-1.895	-2.530	1.081	-42.729
k_2	2.845	2.053	1.781	2.697	2.344	0.509	21.722
m	0.450	0.450	0.450	0.450	0.450	0.000	0.000
T_{lag}	4.000	4.000	4.000	4.000	4.000	0.000	0.000

Number of dissolution data points (N), degrees of freedom (df), and selected goodness of fit criteria – Pearson correlation coefficient (R), coefficient of determination (R^2), adjusted coefficient of determination ($R^2_{adjusted}$), and residual sum of squares (RSS) (manual calculation in MS Excel):

Parameter	No.1	No.2	No.3	No.4
N	5	5	5	5
df	1	1	1	1
R	0.989032474	0.990362756	0.997819679	0.972184866
R^2	0.978185235	0.980818389	0.995644113	0.945143414
$R^2_{adjusted}$	0.912740939	0.923273558	0.982576451	0.780573654
RSS	83.20009582	39.30376615	7.393152336	249.391185

Graphical abstract of model fit presented as mean ± 1 SD of the fraction % of released carvedilol:

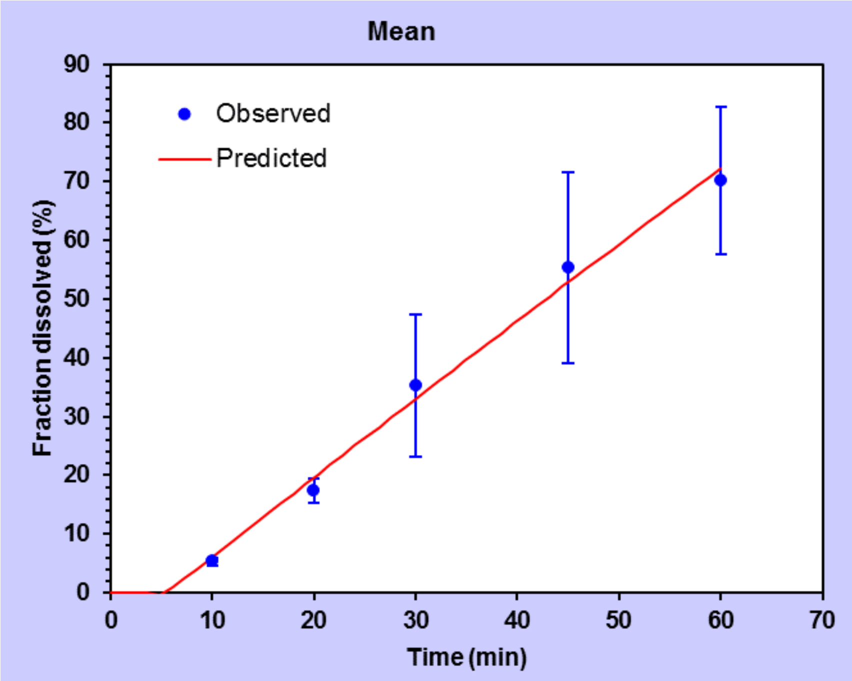

Supplementary materials – Model fitting summary – Parteck® M 100 (modelling up to app. 70 % of carvedilol released)

Graphical abstract of model fit presented as the fraction % of released carvedilol per tested tablet:

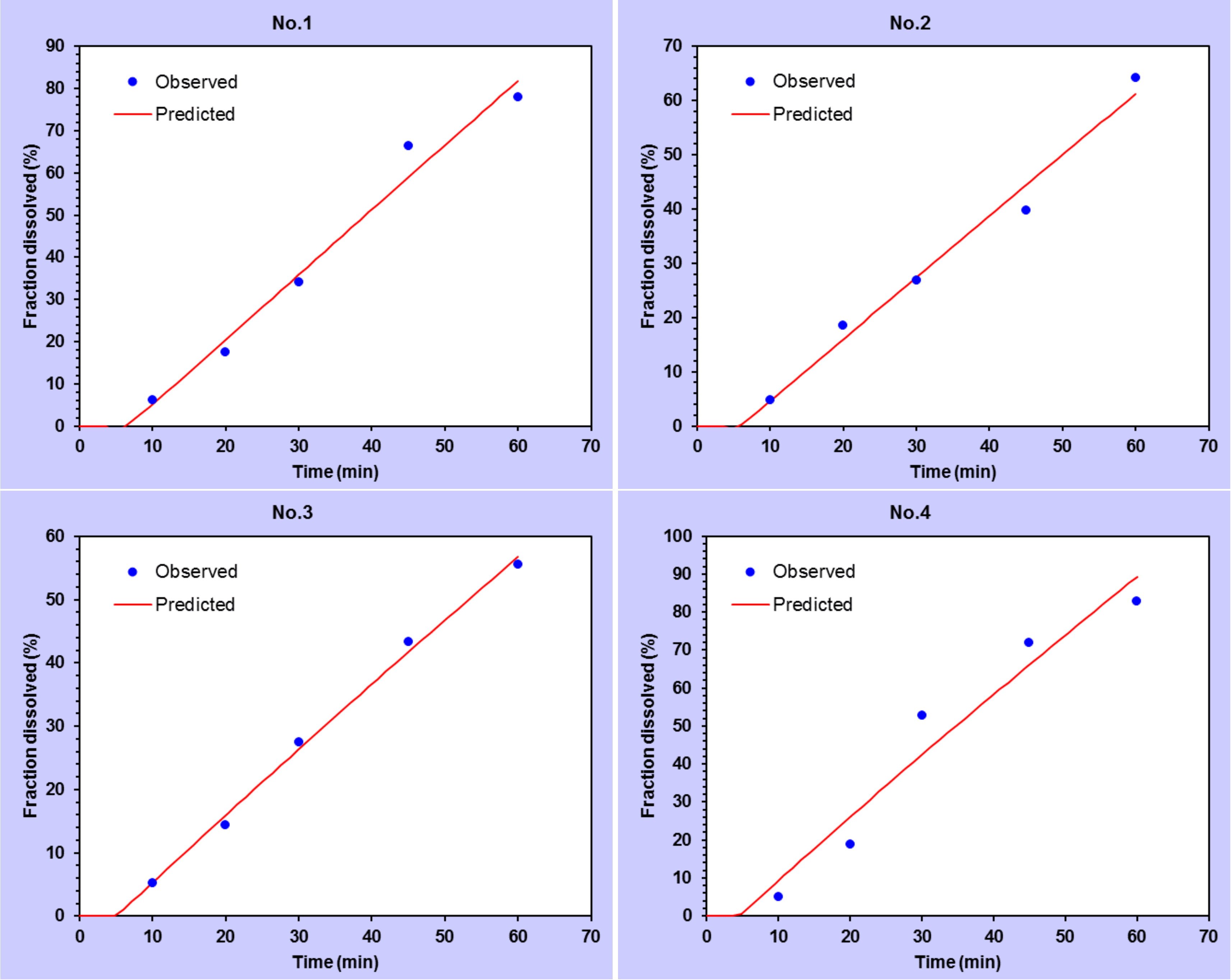

Supplementary materials – Model fitting summary – Parteck® M 100 (modelling up to app. 70 % of carvedilol released)

Model: **Peppas–Sahlin_2**

Model equation: $F = k_1 \cdot t^{0.5} + k_2 \cdot t$

Fitted model parameters per tested tablet (N = 4) with statistics – mean, standard deviation (SD), and relative standard deviation expressed in % (RSD%) (output from DDSolver):

Parameter	No.1	No.2	No.3	No.4	Mean	SD	RSD(%)
k_1	-3.847	-2.568	-1.766	-2.256	-2.609	0.889	-34.063
k_2	1.871	1.361	1.182	1.792	1.552	0.333	21.465

Number of dissolution data points (N), degrees of freedom (df), and selected goodness of fit criteria – Pearson correlation coefficient (R), coefficient of determination (R^2), adjusted coefficient of determination ($R^2_{adjusted}$), and residual sum of squares (RSS) (manual calculation in MS Excel):

Parameter	No.1	No.2	No.3	No.4
N	5	5	5	5
df	3	3	3	3
R	0.987684457	0.991700034	0.996377312	0.966569936
R^2	0.975520587	0.983468958	0.992767749	0.934257442
$R^2_{adjusted}$	0.967360783	0.977958611	0.990356998	0.912343256
RSS	93.52754714	33.79392669	12.35228741	298.5299208

Graphical abstract of model fit presented as mean ± 1 SD of the fraction % of released carvedilol:

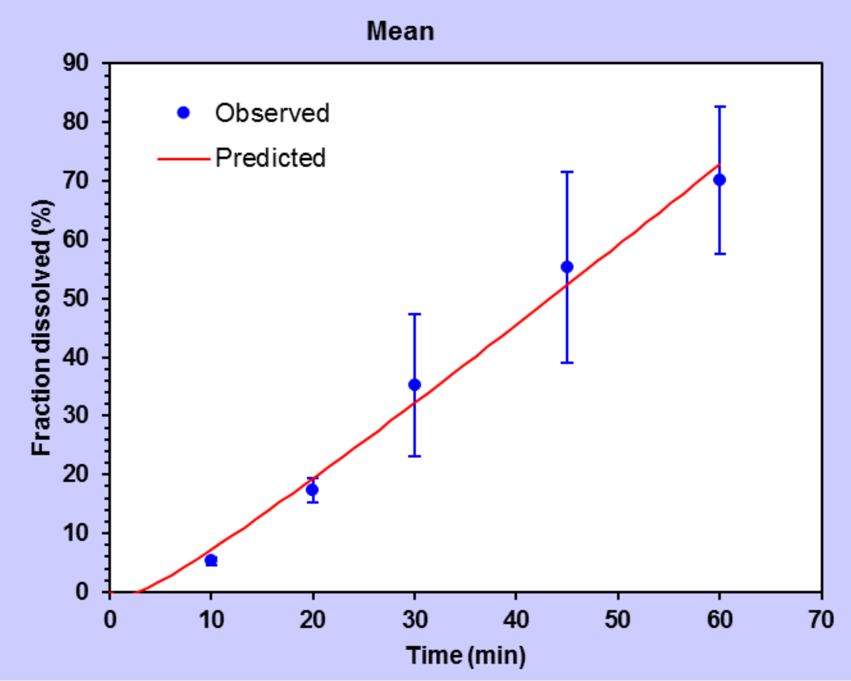

Supplementary materials – Model fitting summary – Parteck® M 100 (modelling up to app. 70 % of carvedilol released)

Graphical abstract of model fit presented as the fraction % of released carvedilol per tested tablet:

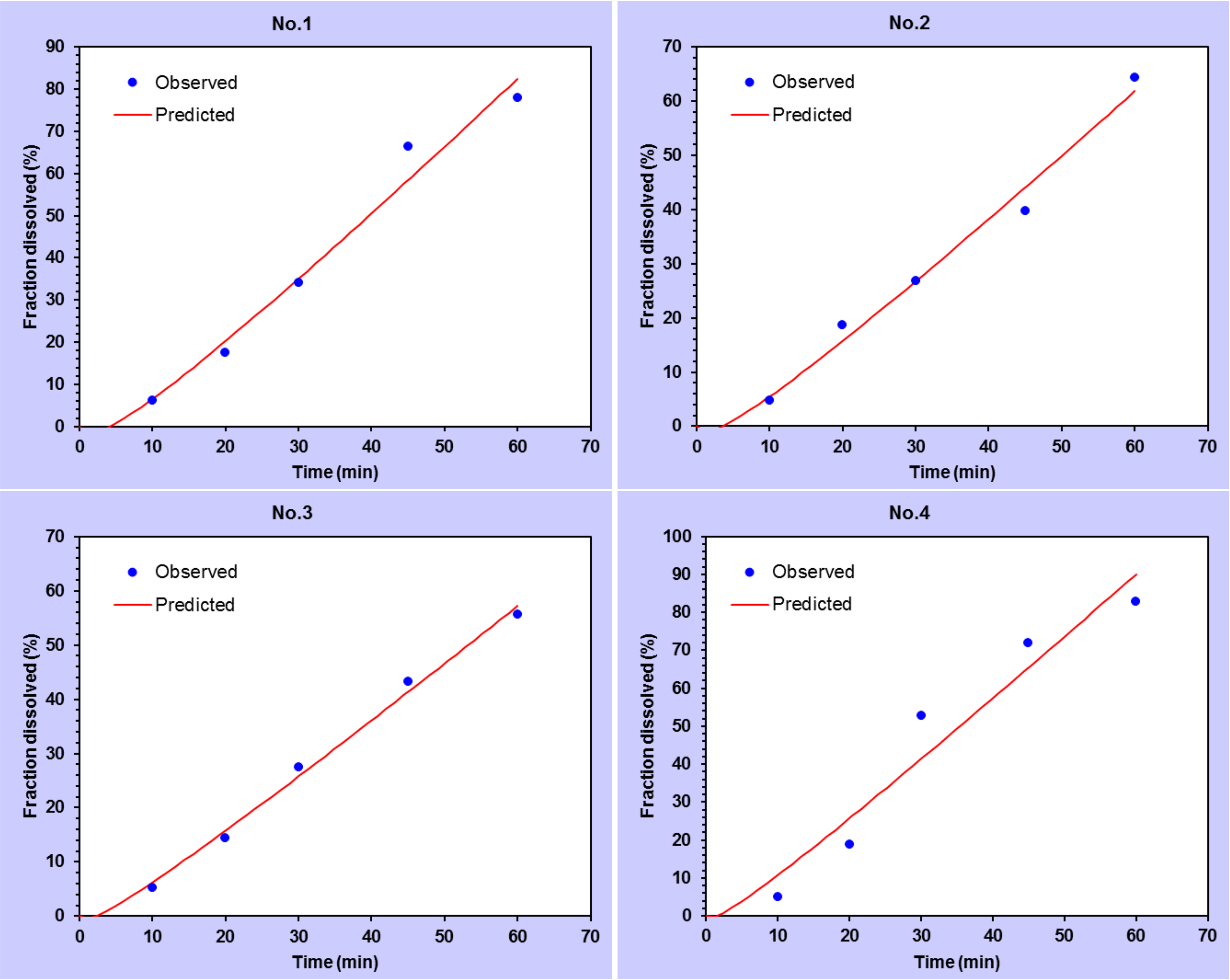

Supplementary materials – Model fitting summary – Parteck® M 100 (modelling up to app. 70 % of carvedilol released)

Model: **Peppas–Sahlin_2 with T_{lag}**

Model equation: $F = k_1 \cdot (t - T_{lag})^{0.5} + k_2 \cdot (t - T_{lag})$

Fitted model parameters per tested tablet (N = 4) with statistics – mean, standard deviation (SD), and relative standard deviation expressed in % (RSD%) (output from DDSolver):

Parameter	No.1	No.2	No.3	No.4	Mean	SD	RSD(%)
k_1	-1.664	-0.947	-0.190	0.416	-0.596	0.904	-151.698
k_2	1.690	1.226	1.042	1.541	1.375	0.294	21.396
T_{lag}	4.000	4.000	4.000	4.000	4.000	0.000	0.000

Number of dissolution data points (N), degrees of freedom (df), and selected goodness of fit criteria – Pearson correlation coefficient (R), coefficient of determination (R^2), adjusted coefficient of determination ($R^2_{adjusted}$), and residual sum of squares (RSS) (manual calculation in MS Excel):

Parameter	No.1	No.2	No.3	No.4
N	5	5	5	5
df	2	2	2	2
R	0.988291906	0.991295757	0.997216247	0.970017606
R^2	0.976720891	0.982667278	0.994440244	0.940934155
$R^2_{adjusted}$	0.953441781	0.965334556	0.988880489	0.88186831
RSS	88.94221227	35.48742906	9.526653527	271.0012733

Graphical abstract of model fit presented as mean ± 1 SD of the fraction % of released carvedilol:

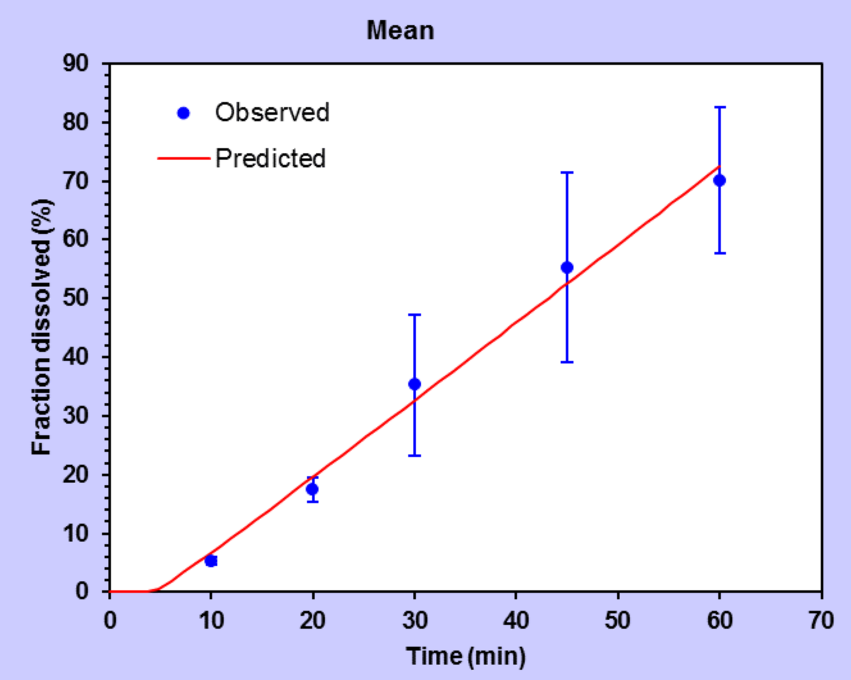

Supplementary materials – Model fitting summary – Parteck® M 100 (modelling up to app. 70 % of carvedilol released)

Graphical abstract of model fit presented as the fraction % of released carvedilol per tested tablet:

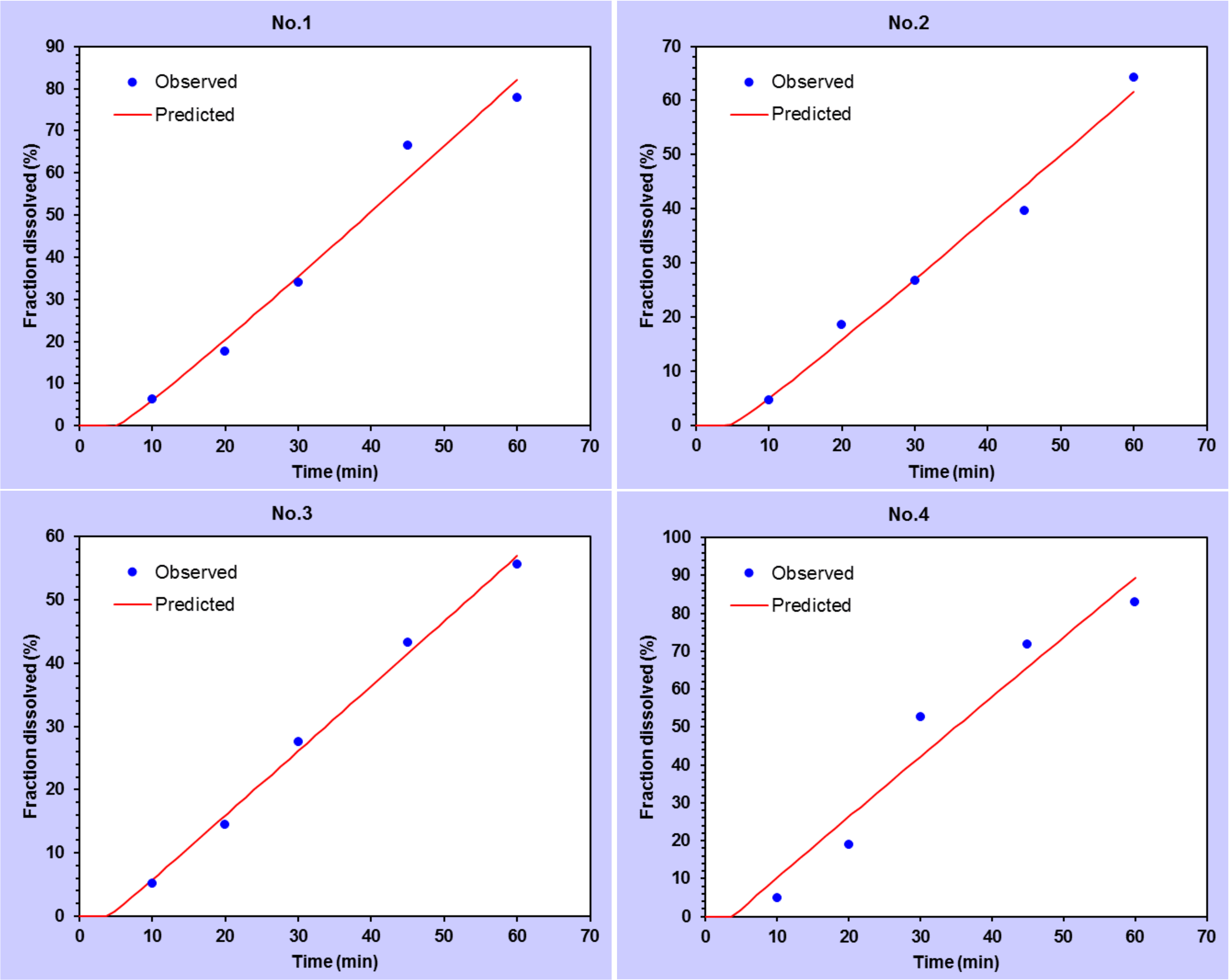

Supplementary materials – Model fitting summary – Parteck® M 100 (modelling up to app. 70 % of carvedilol released)

Model: **Quadratic**

Model equation: $F = 100 \cdot (k_1 \cdot t^2 + k_2 \cdot t)$

Fitted model parameters per tested tablet (N = 4) with statistics – mean, standard deviation (SD), and relative standard deviation expressed in % (RSD%) (output from DDSolver):

Parameter	No.1	No.2	No.3	No.4	Mean	SD	RSD(%)
k1	0.000069	0.000066	0.000030	0.000004	0.000042	0.000031	74.191001
k2	0.009586	0.006548	0.007711	0.014366	0.009553	0.003444	36.056209

Number of dissolution data points (N), degrees of freedom (df), and selected goodness of fit criteria – Pearson correlation coefficient (R), coefficient of determination (R^2), adjusted coefficient of determination ($R^2_{adjusted}$), and residual sum of squares (RSS) (manual calculation in MS Excel):

Parameter	No.1	No.2	No.3	No.4
N	5	5	5	5
df	3	3	3	3
R	0.98290649	0.993317072	0.994133736	0.968709606
R^2	0.966105167	0.986678805	0.988301884	0.9383983
$R^2_{adjusted}$	0.95480689	0.982238407	0.984402512	0.917864401
RSS	143.0719176	27.30960936	24.1293404	336.3349343

Graphical abstract of model fit presented as mean ± 1 SD of the fraction % of released carvedilol:

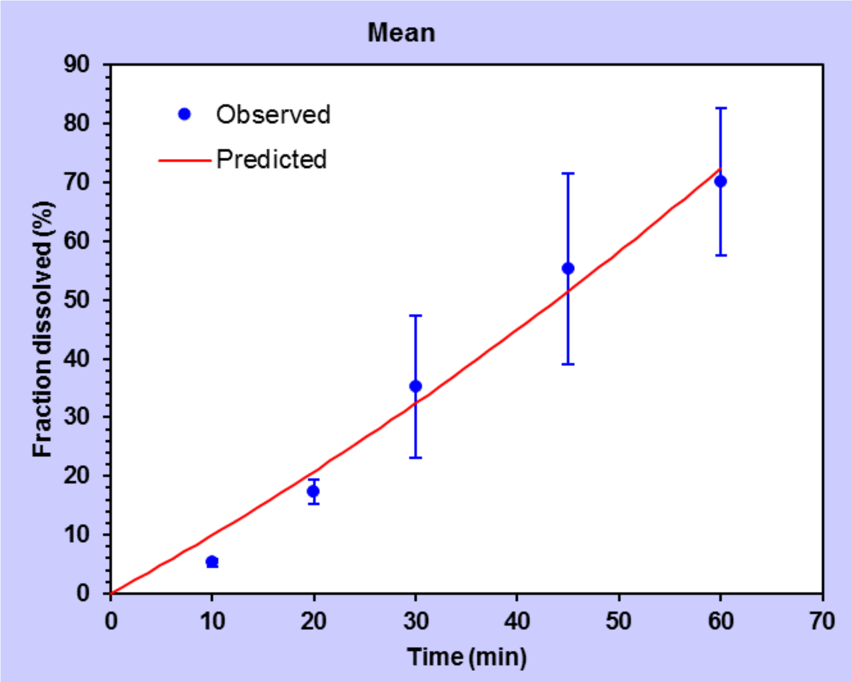

Supplementary materials – Model fitting summary – Parteck® M 100 (modelling up to app. 70 % of carvedilol released)

Graphical abstract of model fit presented as the fraction % of released carvedilol per tested tablet:

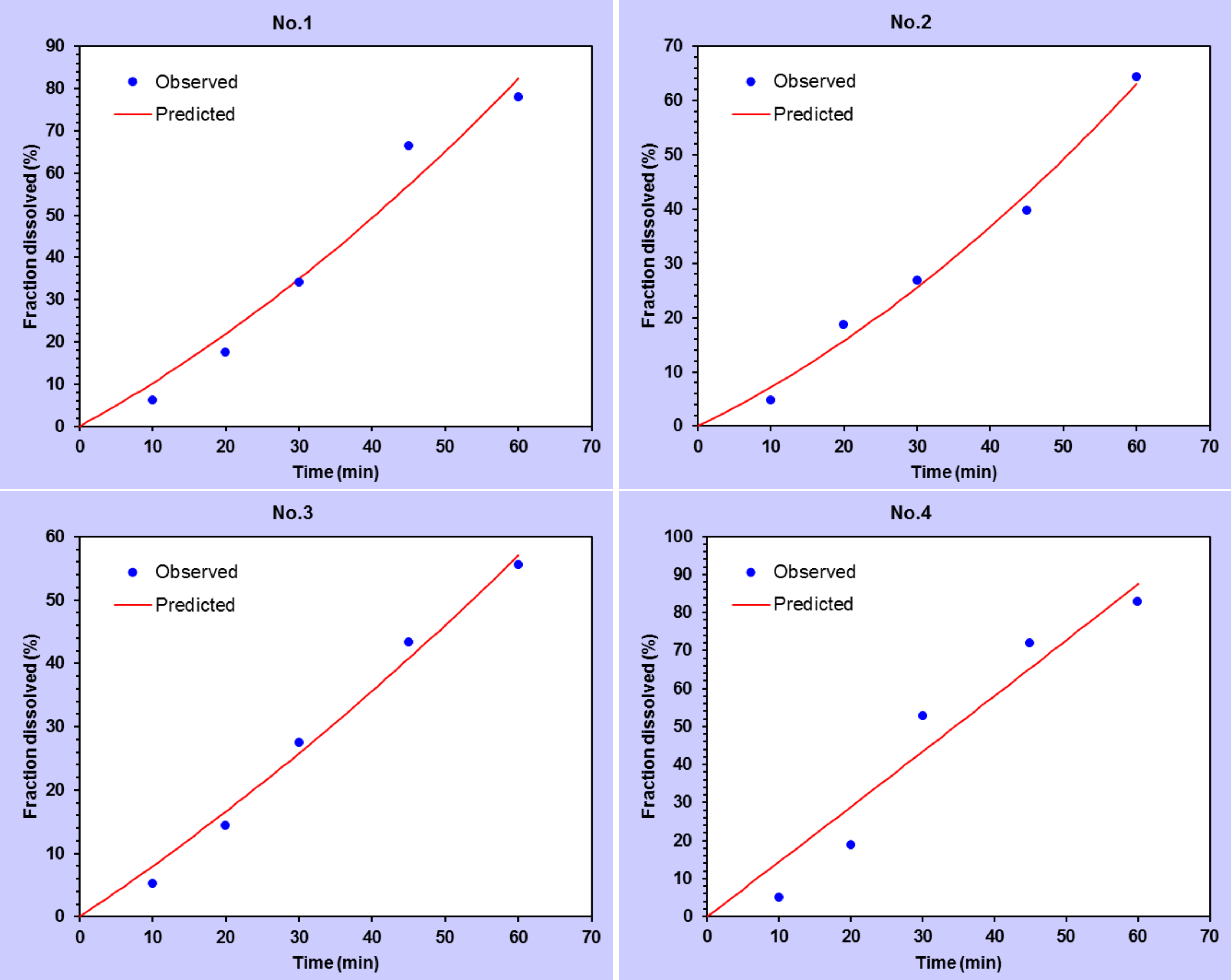

Supplementary materials – Model fitting summary – Parteck® M 100 (modelling up to app. 70 % of carvedilol released)

Model: **Quadratic with T_{lag}**

Model equation: $F = 100 \cdot \left[k_1 \cdot (t - T_{lag})^2 + k_2 \cdot (t - T_{lag})\right]$

Fitted model parameters per tested tablet (N = 4) with statistics – mean, standard deviation (SD), and relative standard deviation expressed in % (RSD%) (output from DDSolver):

Parameter	No.1	No.2	No.3	No.4	Mean	SD	RSD(%)
k_1	0.000021	0.000039	-0.000006	-0.000071	-0.000004	0.000048	-1105.910905
k_2	0.013374	0.009024	0.010400	0.019314	0.013028	0.004567	35.053515
T_{lag}	4.000000	4.000000	4.000000	6.000000	4.500000	1.000000	22.222222

Number of dissolution data points (N), degrees of freedom (df), and selected goodness of fit criteria – Pearson correlation coefficient (R), coefficient of determination (R^2), adjusted coefficient of determination ($R^2_{adjusted}$), and residual sum of squares (RSS) (manual calculation in MS Excel):

Parameter	No.1	No.2	No.3	No.4
N	5	5	5	5
df	2	2	2	2
R	0.987452791	0.993150953	0.997708074	0.980245394
R^2	0.975063015	0.986348816	0.995421402	0.960881032
$R^2_{adjusted}$	0.95012603	0.972697632	0.990842804	0.921762065
RSS	104.9125532	28.38989695	9.474835688	213.7772149

Graphical abstract of model fit presented as mean ± 1 SD of the fraction % of released carvedilol:

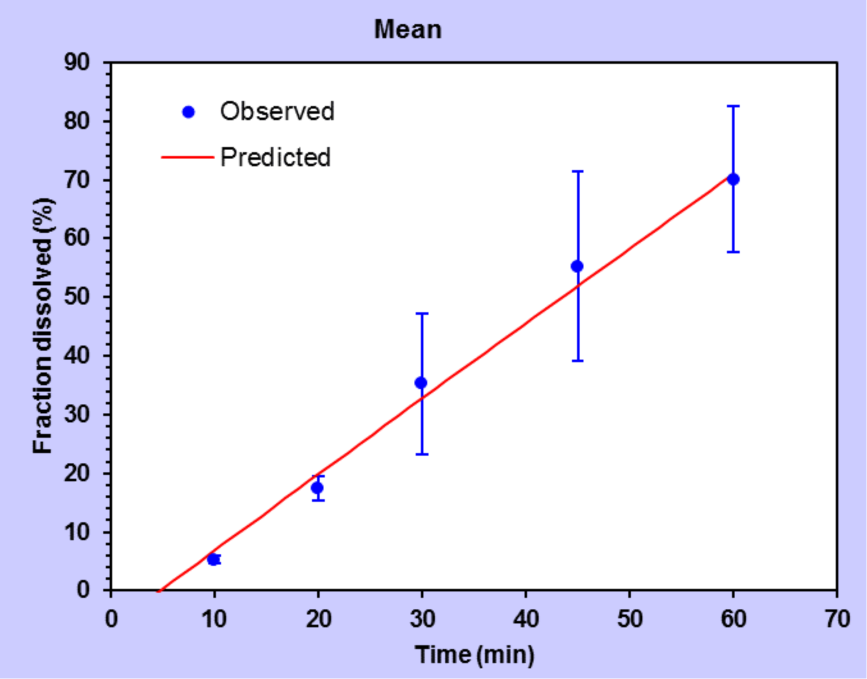

Supplementary materials – Model fitting summary – Parteck® M 100 (modelling up to app. 70 % of carvedilol released)

Graphical abstract of model fit presented as the fraction % of released carvedilol per tested tablet:

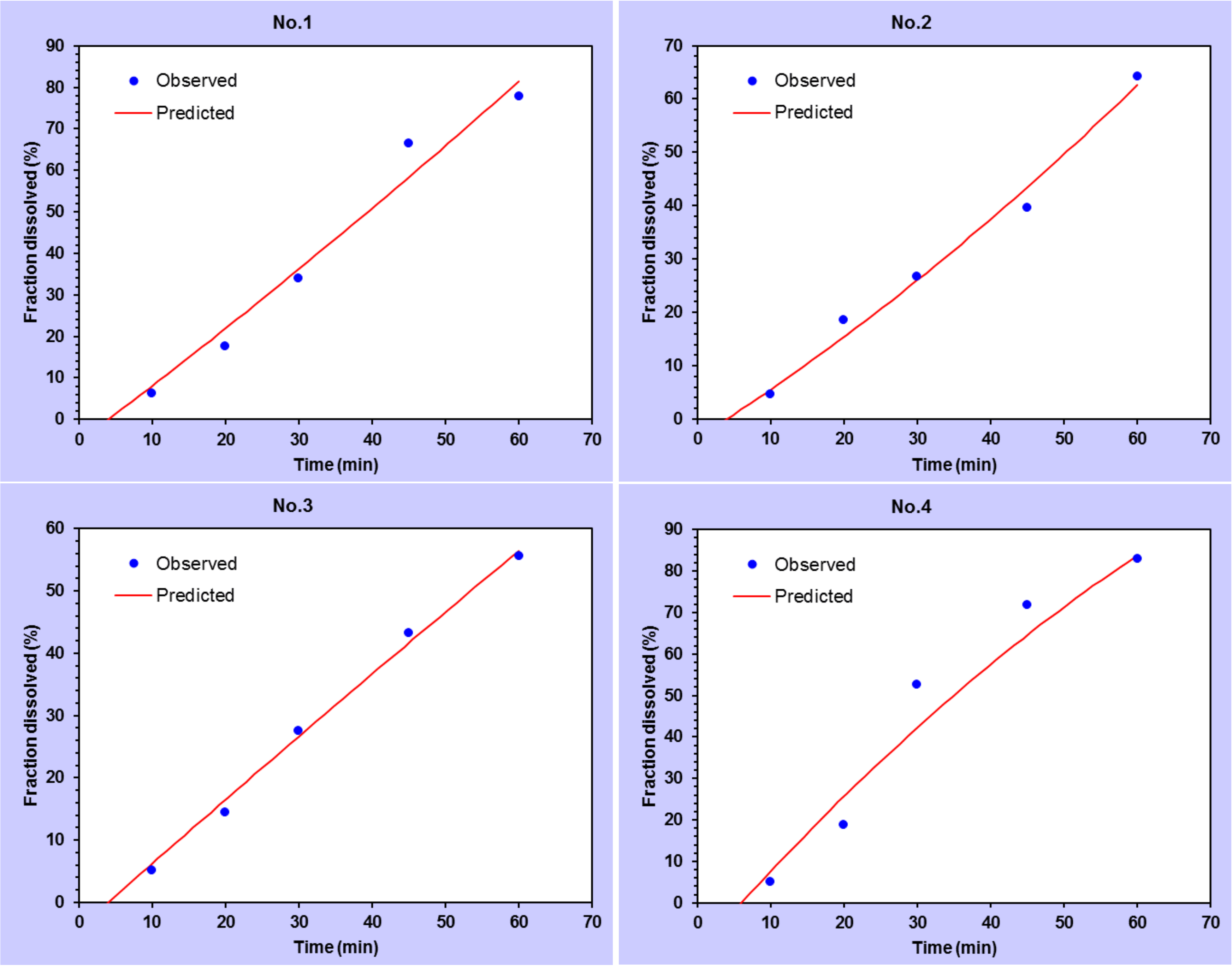

Supplementary materials – Model fitting summary – Parteck® M 100 (modelling up to app. 70 % of carvedilol released)

Model: **Weibull_1**

Model equation: $F = 100 \cdot \left[1 - e^{-\frac{(t-T_i)^\beta}{\alpha}}\right]$

Fitted model parameters per tested tablet (N = 4) with statistics – mean, standard deviation (SD), and relative standard deviation expressed in % (RSD%) (output from DDSolver):

Parameter	No.1	No.2	No.3	No.4	Mean	SD	RSD(%)
α	243.688	204.988	179.336	378.657	251.667	88.696	35.243
β	1.459	1.297	1.237	1.649	1.410	0.185	13.108
T_i	4.000	4.000	4.000	4.000	4.000	0.000	0.000

Number of dissolution data points (N), degrees of freedom (df), and selected goodness of fit criteria – Pearson correlation coefficient (R), coefficient of determination (R^2), adjusted coefficient of determination ($R^2_{adjusted}$), and residual sum of squares (RSS) (manual calculation in MS Excel):

Parameter	No.1	No.2	No.3	No.4
N	5	5	5	5
df	2	2	2	2
R	0.993342849	0.98485955	0.999176337	0.987258851
R^2	0.986730015	0.969948333	0.998353352	0.974680038
$R^2_{adjusted}$	0.97346003	0.939896665	0.996706704	0.949360077
RSS	63.7518661	61.93762844	3.249912021	116.7968265

Graphical abstract of model fit presented as mean ± 1 SD of the fraction % of released carvedilol:

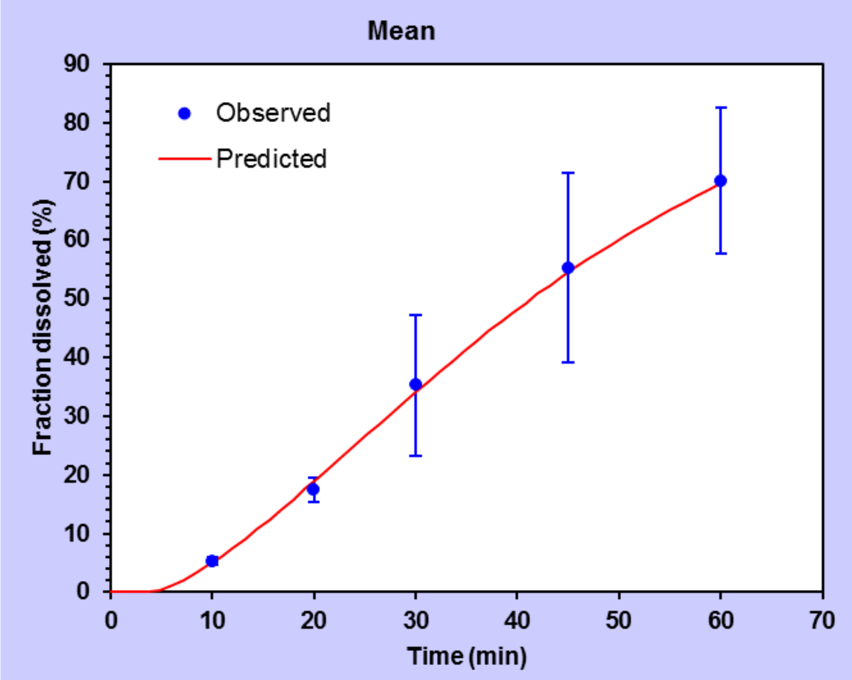

Supplementary materials – Model fitting summary – Parteck® M 100 (modelling up to app. 70 % of carvedilol released)

Graphical abstract of model fit presented as the fraction % of released carvedilol per tested tablet:

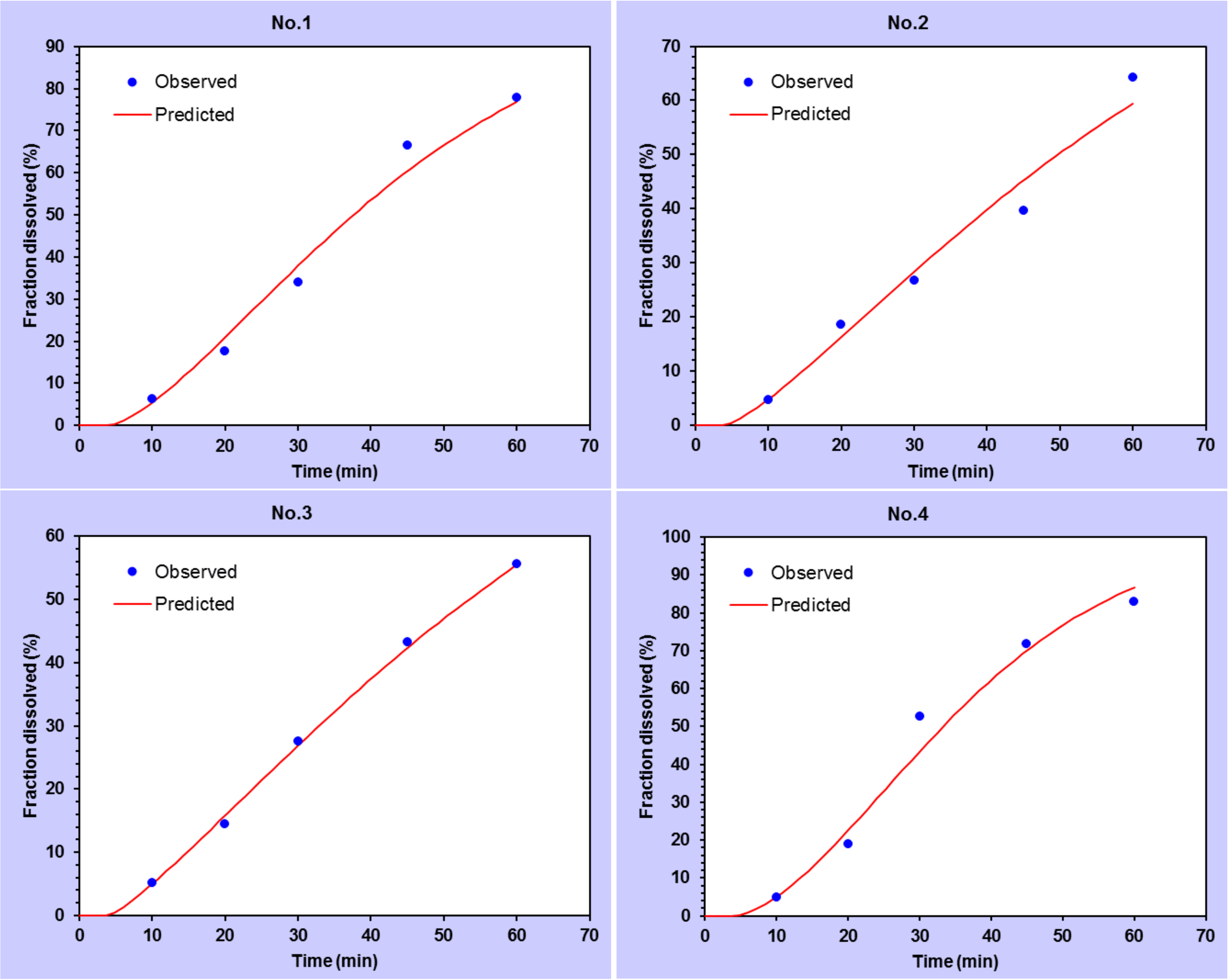

Supplementary materials – Model fitting summary – Parteck® M 100 (modelling up to app. 70 % of carvedilol released)

Model: **Weibull_2**

Model equation: $F = 100 \cdot \left(1 - e^{-\frac{t^{\beta}}{\alpha}}\right)$

Fitted model parameters per tested tablet (N = 4) with statistics – mean, standard deviation (SD), and relative standard deviation expressed in % (RSD%) (output from DDSolver):

Parameter	No.1	No.2	No.3	No.4	Mean	SD	RSD(%)
α	1112.052	761.531	635.404	2002.840	1127.957	617.130	54.712
β	1.825	1.611	1.540	2.048	1.756	0.229	13.049

Number of dissolution data points (N), degrees of freedom (df), and selected goodness of fit criteria – Pearson correlation coefficient (R), coefficient of determination (R^2), adjusted coefficient of determination ($R^2_{adjusted}$), and residual sum of squares (RSS) (manual calculation in MS Excel):

Parameter	No.1	No.2	No.3	No.4
N	5	5	5	5
df	3	3	3	3
R	0.994921079	0.987665746	0.997806791	0.981961125
R^2	0.989867954	0.975483625	0.995618391	0.96424765
$R^2_{adjusted}$	0.986490605	0.9673115	0.994157855	0.9523302
RSS	40.68781252	51.17639331	8.983788792	174.873194

Graphical abstract of model fit presented as mean ± 1 SD of the fraction % of released carvedilol:

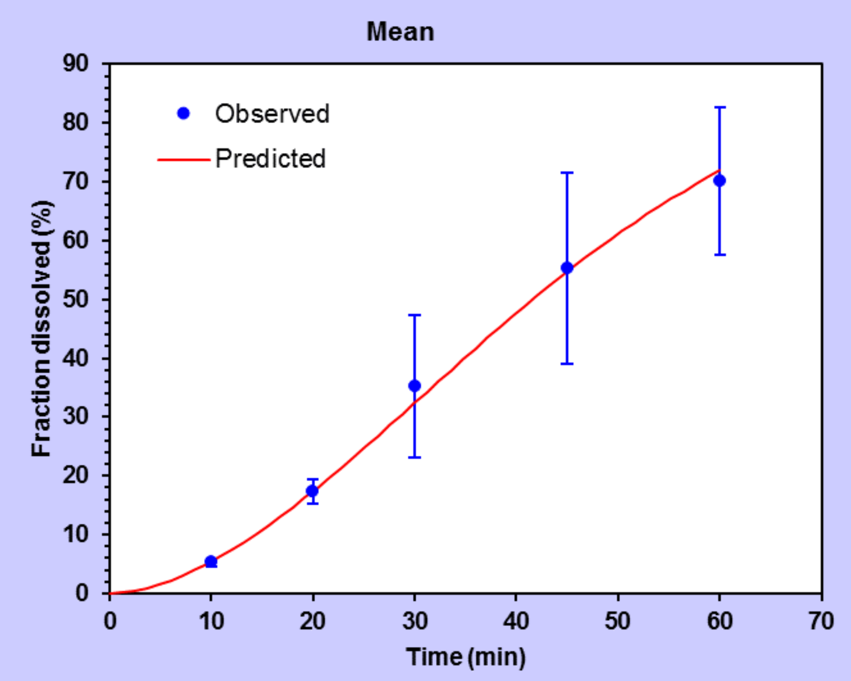

Supplementary materials – Model fitting summary – Parteck® M 100 (modelling up to app. 70 % of carvedilol released)

Graphical abstract of model fit presented as the fraction % of released carvedilol per tested tablet:

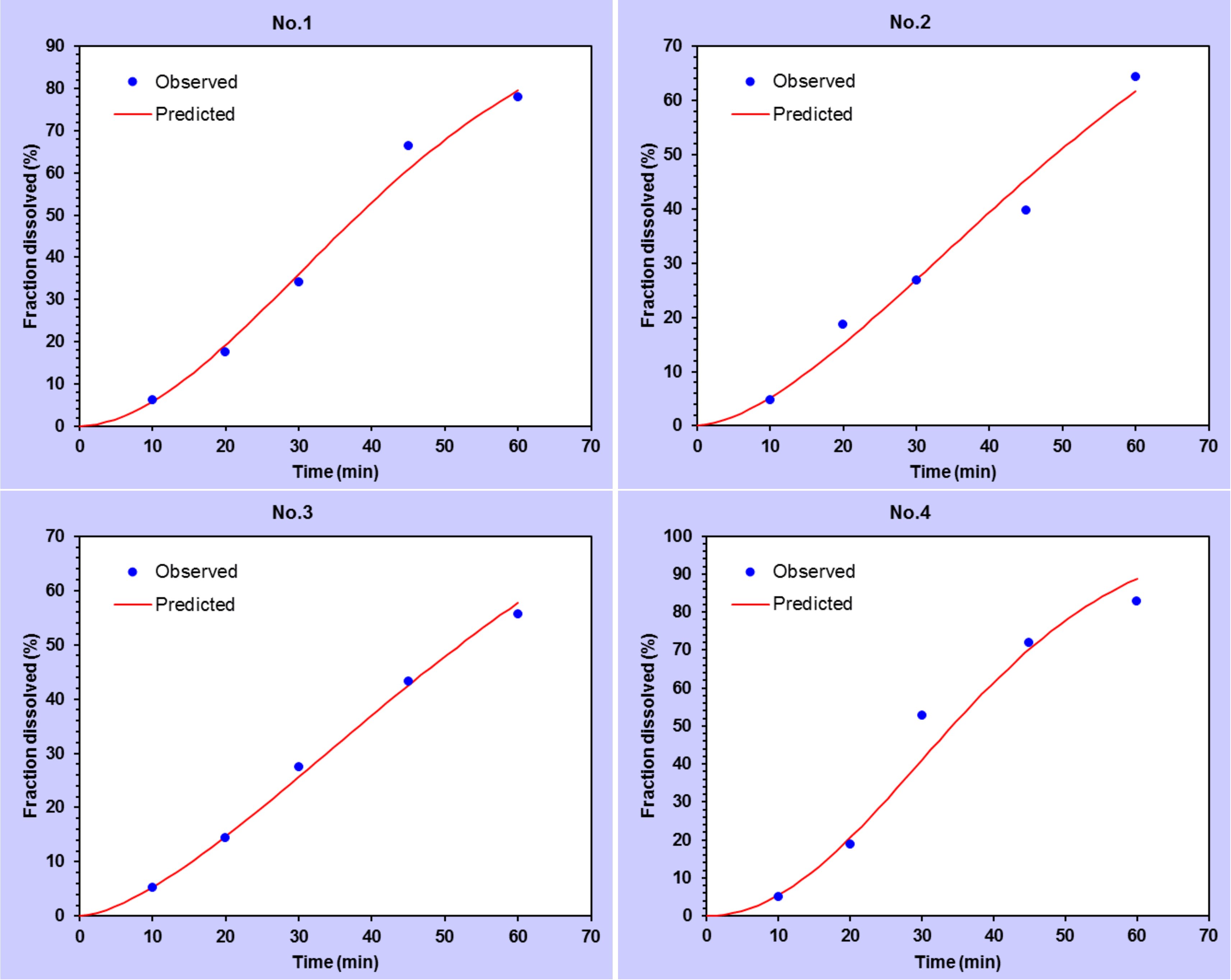

Supplementary materials – Model fitting summary – Parteck® M 100 (modelling up to app. 70 % of carvedilol released)

Model: **Weibull_3**

Model equation: $F = F_{max} \cdot \left(1 - e^{-\frac{t^{\beta}}{\alpha}}\right)$

Fitted model parameters per tested tablet (N = 4) with statistics – mean, standard deviation (SD), and relative standard deviation expressed in % (RSD%) (output from DDSolver):

Parameter	No.1	No.2	No.3	No.4	Mean	SD	RSD(%)
α	2289.365	1133.984	1282.040	2965.633	1917.756	866.905	45.204
β	2.096	1.911	1.937	2.249	2.048	0.157	7.667
F_{max}	84.484	67.452	60.250	87.029	74.804	13.026	17.413

Number of dissolution data points (N), degrees of freedom (df), and selected goodness of fit criteria – Pearson correlation coefficient (R), coefficient of determination (R^2), adjusted coefficient of determination ($R^2_{adjusted}$), and residual sum of squares (RSS) (manual calculation in MS Excel):

Parameter	No.1	No.2	No.3	No.4
N	5	5	5	5
df	2	2	2	2
R	0.997035183	0.973715022	0.999501394	0.991390337
R^2	0.994079157	0.948120945	0.999003037	0.982854801
$R^2_{adjusted}$	0.988158314	0.89624189	0.998006074	0.965709602
RSS	38.44513607	113.2592058	9.282646453	80.81517349

Graphical abstract of model fit presented as mean ± 1 SD of the fraction % of released carvedilol:

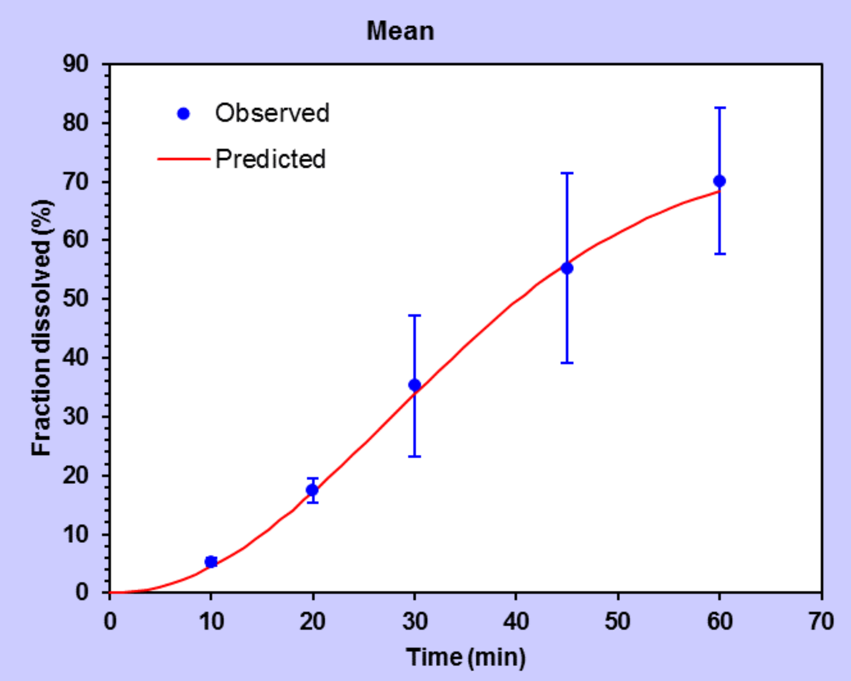

Supplementary materials – Model fitting summary – Parteck® M 100 (modelling up to app. 70 % of carvedilol released)

Graphical abstract of model fit presented as the fraction % of released carvedilol per tested tablet:

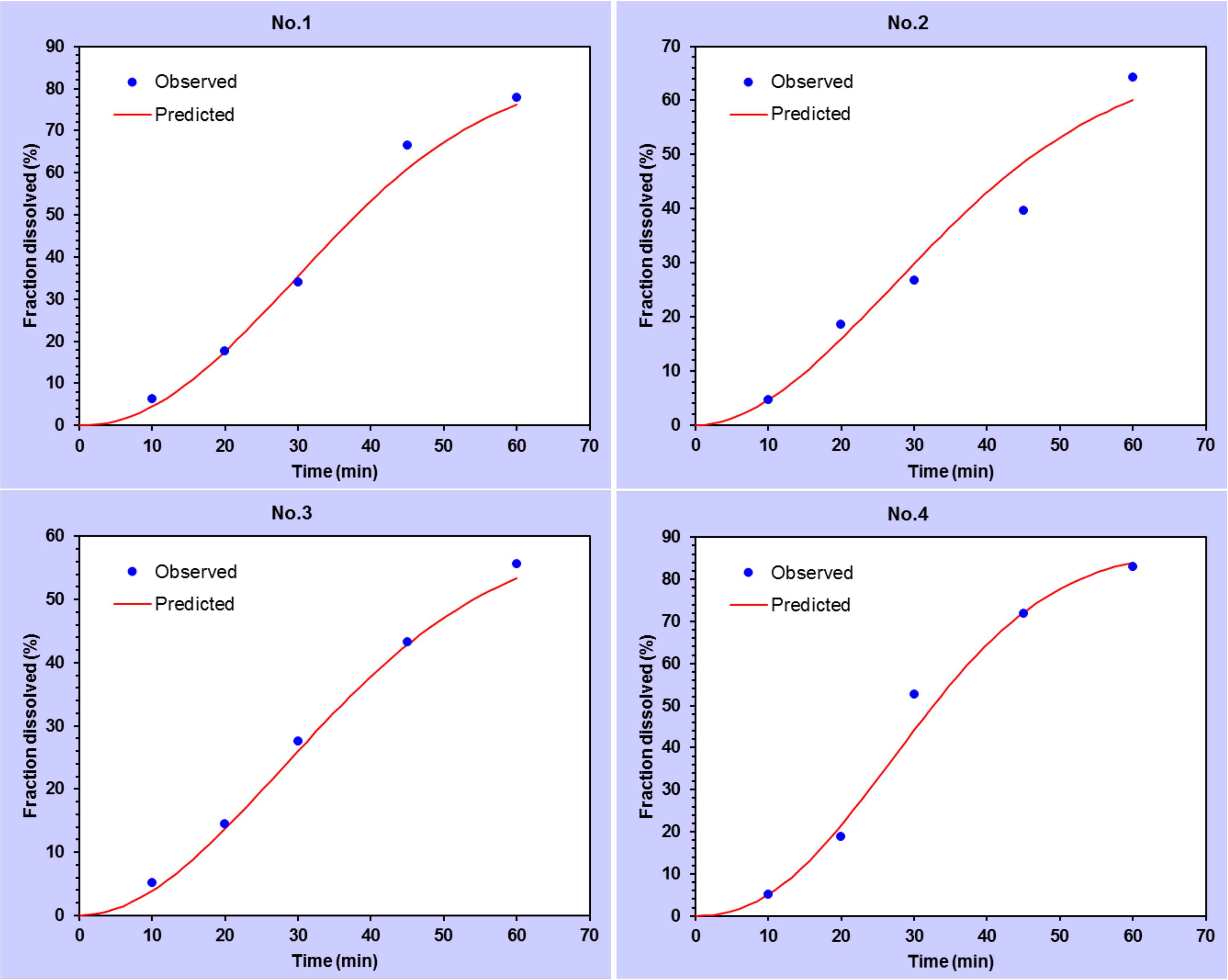

Supplementary materials – Model fitting summary – Parteck® M 100 (modelling up to app. 70 % of carvedilol released)

Model: **Weibull_4**

Model equation: $F = F_{max} \cdot \left[1 - e^{-\frac{(t-T_i)^\beta}{\alpha}}\right]$

Fitted model parameters per tested tablet (N = 4) with statistics – mean, standard deviation (SD), and relative standard deviation expressed in % (RSD%) (output from DDSolver):

Parameter	No.1	No.2	No.3	No.4	Mean	SD	RSD(%)
α	332.932	231.710	196.710	467.040	307.098	121.268	39.488
β	1.625	1.528	1.526	1.805	1.621	0.131	8.097
T_i	4.591	6.000	6.000	4.000	5.148	1.013	19.683
F_{max}	92.395	67.452	58.326	87.029	76.301	16.078	21.072

Number of dissolution data points (N), degrees of freedom (df), and selected goodness of fit criteria – Pearson correlation coefficient (R), coefficient of determination (R^2), adjusted coefficient of determination ($R^2_{adjusted}$), and residual sum of squares (RSS) (manual calculation in MS Excel):

Parameter	No.1	No.2	No.3	No.4
N	5	5	5	5
df	1	1	1	1
R	0.993940127	0.972487253	0.996075211	0.993493809
R^2	0.987916975	0.945731457	0.992165826	0.987029948
$R^2_{adjusted}$	0.951667901	0.782925829	0.968663302	0.948119791
RSS	64.332571	115.892641	19.90310525	60.60095548

Graphical abstract of model fit presented as mean ± 1 SD of the fraction % of released carvedilol:

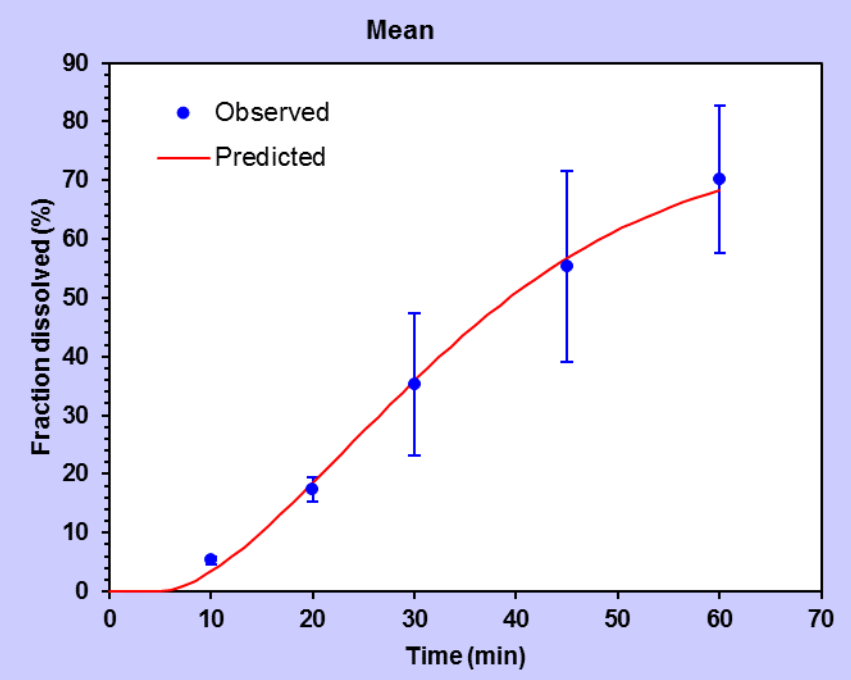

Supplementary materials – Model fitting summary – Parteck® M 100 (modelling up to app. 70 % of carvedilol released)

Graphical abstract of model fit presented as the fraction % of released carvedilol per tested tablet:

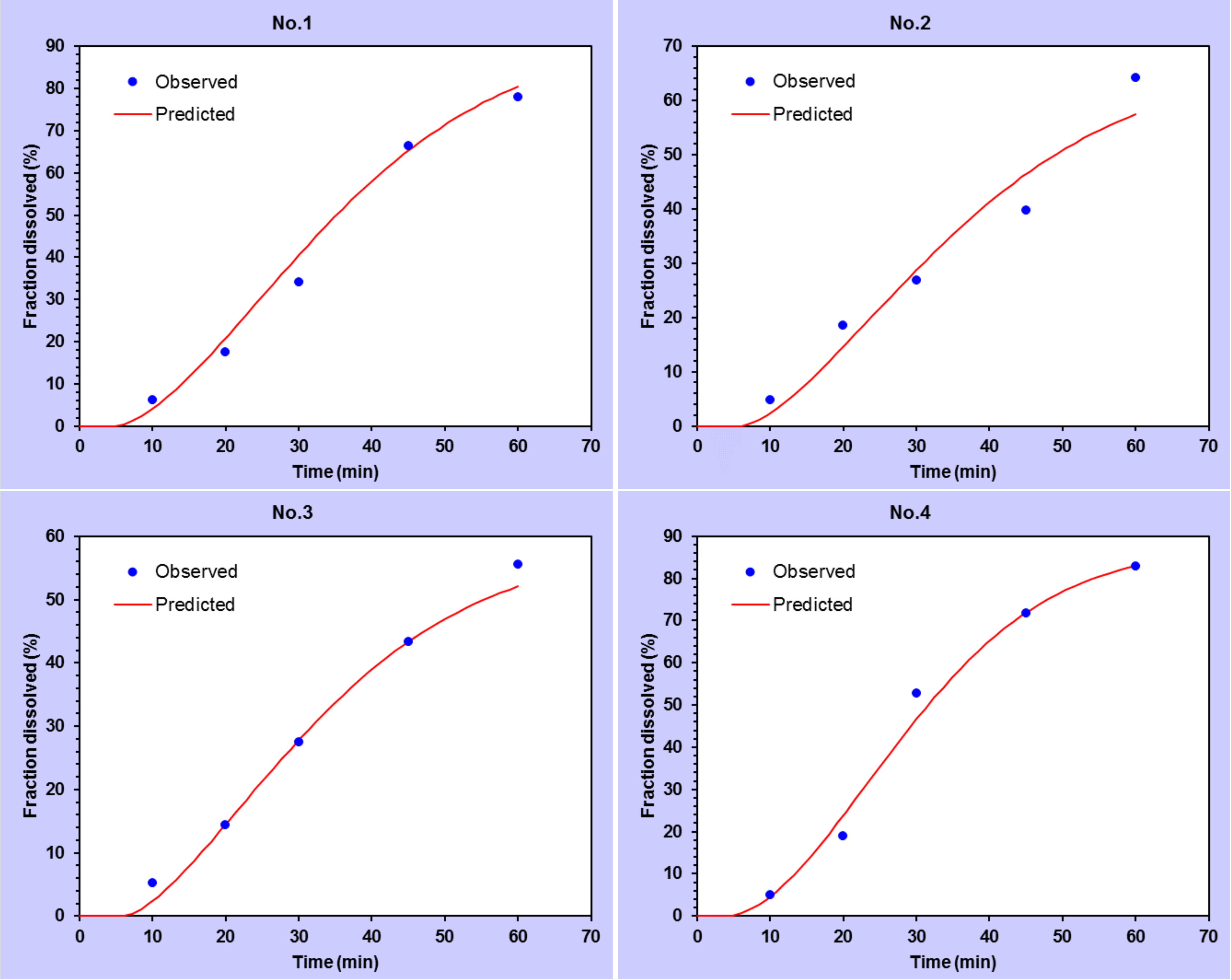

Supplementary materials – Model fitting summary – Parteck® M 100 (modelling up to app. 70 % of carvedilol released)

Model: **Logistic_1**

Model equation: $F = 100 \cdot \frac{e^{\alpha+\beta \cdot \log(t)}}{1+e^{\alpha+\beta \cdot \log(t)}}$

Fitted model parameters per tested tablet (N = 4) with statistics – mean, standard deviation (SD), and relative standard deviation expressed in % (RSD%) (output from DDSolver):

Parameter	No.1	No.2	No.3	No.4	Mean	SD	RSD(%)
α	-8.161	-7.275	-6.991	-8.985	-7.853	0.904	-11.516
β	5.246	4.307	4.057	5.984	4.898	0.887	18.097

Number of dissolution data points (N), degrees of freedom (df), and selected goodness of fit criteria – Pearson correlation coefficient (R), coefficient of determination (R^2), adjusted coefficient of determination ($R^2_{adjusted}$), and residual sum of squares (RSS) (manual calculation in MS Excel):

Parameter	No.1	No.2	No.3	No.4
N	5	5	5	5
df	3	3	3	3
R	0.99307375	0.981345715	0.999664331	0.993528126
R^2	0.986195474	0.963039413	0.999328774	0.987098137
$R^2_{adjusted}$	0.981593965	0.950719217	0.999105032	0.982797516
RSS	62.82276386	75.71453256	1.216789915	59.43135761

Graphical abstract of model fit presented as mean ± 1 SD of the fraction % of released carvedilol:

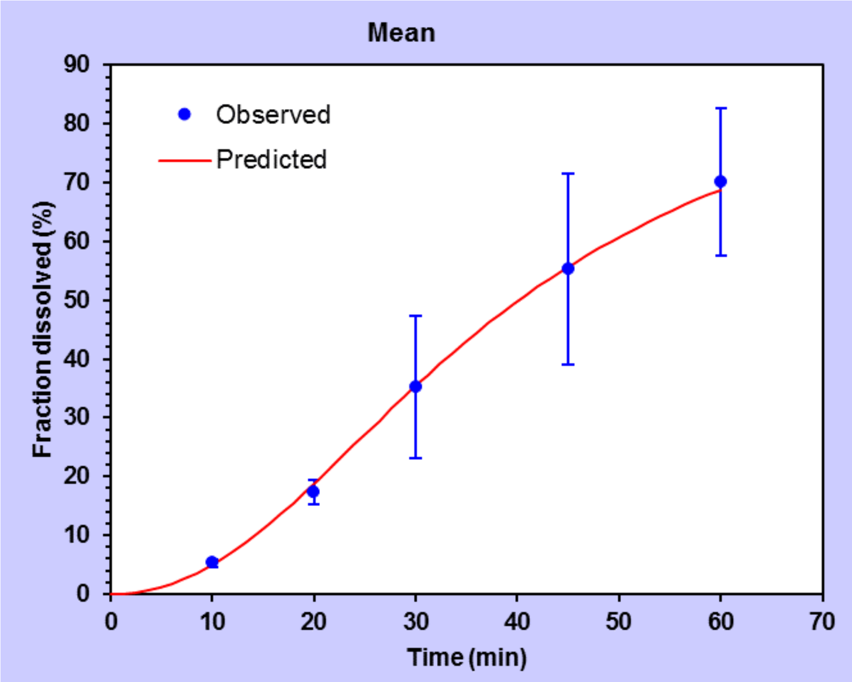

Supplementary materials – Model fitting summary – Parteck® M 100 (modelling up to app. 70 % of carvedilol released)

Graphical abstract of model fit presented as the fraction % of released carvedilol per tested tablet:

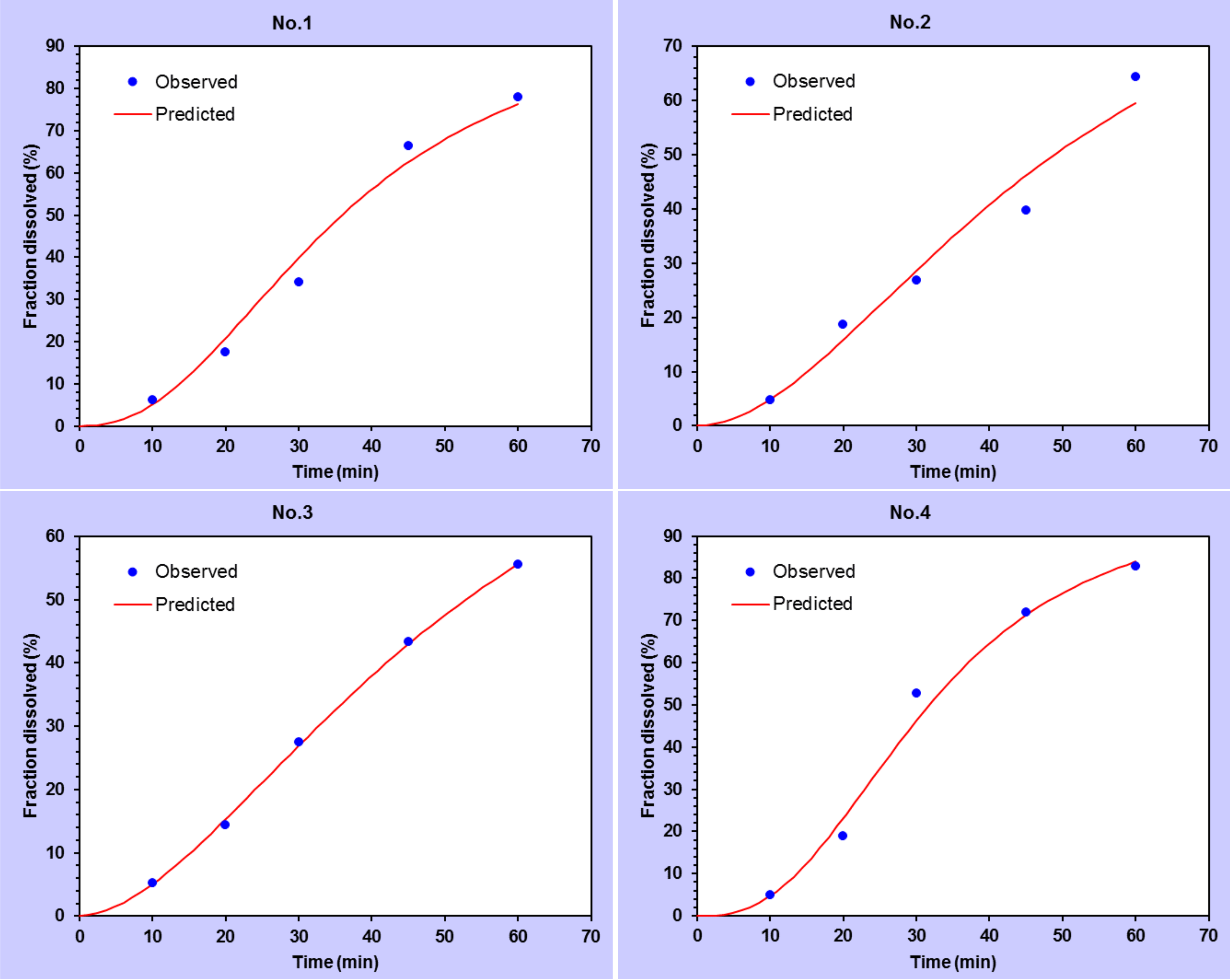

Supplementary materials – Model fitting summary – Parteck® M 100 (modelling up to app. 70 % of carvedilol released)

Model: **Logistic_2**

Model equation: $F = F_{max} \cdot \frac{e^{\alpha+\beta\cdot\log(t)}}{1+e^{\alpha+\beta\cdot\log(t)}}$

Fitted model parameters per tested tablet (N = 4) with statistics – mean, standard deviation (SD), and relative standard deviation expressed in % (RSD%) (output from DDSolver):

Parameter	No.1	No.2	No.3	No.4	Mean	SD	RSD(%)
α	-11.470	-9.180	-10.621	-10.489	-10.440	0.946	-9.058
β	7.374	6.280	6.856	7.398	6.977	0.528	7.561
F_{max}	88.720	67.452	63.271	87.029	76.618	13.128	17.134

Number of dissolution data points (N), degrees of freedom (df), and selected goodness of fit criteria – Pearson correlation coefficient (R), coefficient of determination (R^2), adjusted coefficient of determination ($R^2_{adjusted}$), and residual sum of squares (RSS) (manual calculation in MS Excel):

Parameter	No.1	No.2	No.3	No.4
N	5	5	5	5
df	2	2	2	2
R	0.998303339	0.9505465	0.998549293	0.994863651
R^2	0.996609557	0.903538649	0.997100691	0.989753684
$R^2_{adjusted}$	0.993219113	0.807077298	0.994201382	0.979507367
RSS	119.4173513	248.0390298	58.54684138	56.77823391

Graphical abstract of model fit presented as mean ± 1 SD of the fraction % of released carvedilol:

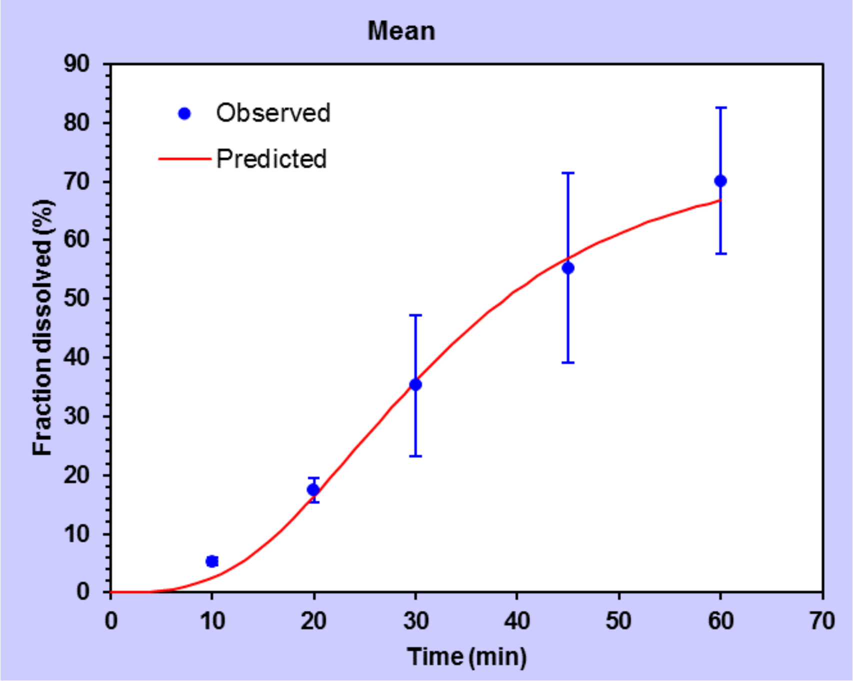

Supplementary materials – Model fitting summary – Parteck® M 100 (modelling up to app. 70 % of carvedilol released)

Graphical abstract of model fit presented as the fraction % of released carvedilol per tested tablet:

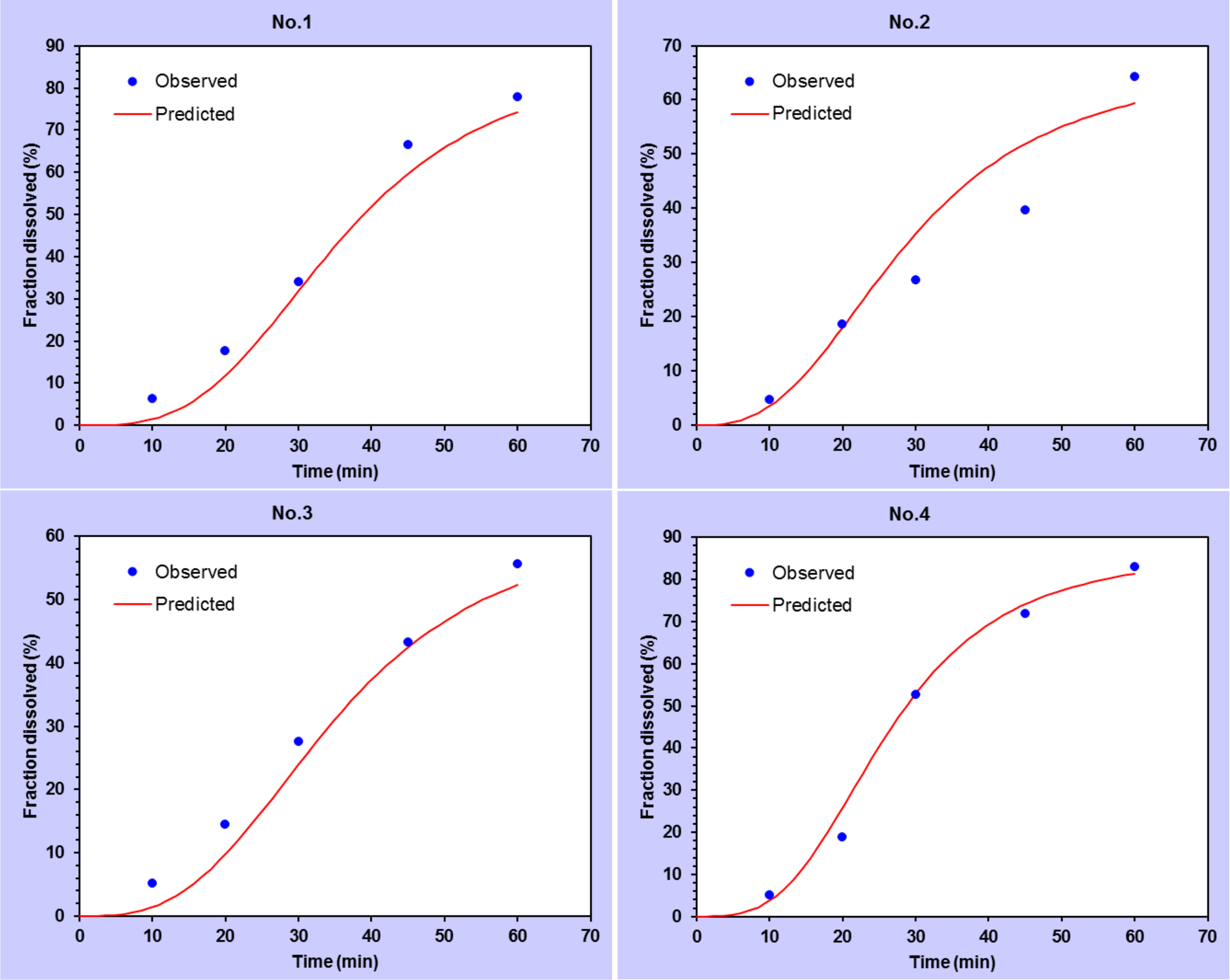

Supplementary materials – Model fitting summary – Parteck® M 100 (modelling up to app. 70 % of carvedilol released)

Model: **Logistic_3**

Model equation: $F = F_{max} \cdot \frac{1}{1+e^{-k\cdot(t-\gamma)}}$

Fitted model parameters per tested tablet (N = 4) with statistics – mean, standard deviation (SD), and relative standard deviation expressed in % (RSD%) (output from DDSolver):

Parameter	No.1	No.2	No.3	No.4	Mean	SD	RSD(%)
k	0.110	0.100	0.102	0.113	0.107	0.006	5.805
γ	32.403	34.242	32.031	31.423	32.525	1.214	3.732
F_{max}	81.786	67.452	58.326	87.029	73.648	13.146	17.849

Number of dissolution data points (N), degrees of freedom (df), and selected goodness of fit criteria – Pearson correlation coefficient (R), coefficient of determination (R^2), adjusted coefficient of determination ($R^2_{adjusted}$), and residual sum of squares (RSS) (manual calculation in MS Excel):

Parameter	No.1	No.2	No.3	No.4
N	5	5	5	5
df	2	2	2	2
R	0.99947248	0.970053077	0.997047089	0.983745589
R^2	0.998945239	0.941002972	0.994102898	0.967755384
$R^2_{adjusted}$	0.997890477	0.882005944	0.988205795	0.935510767
RSS	4.06108087	147.8324826	11.64703687	165.108798

Graphical abstract of model fit presented as mean ± 1 SD of the fraction % of released carvedilol:

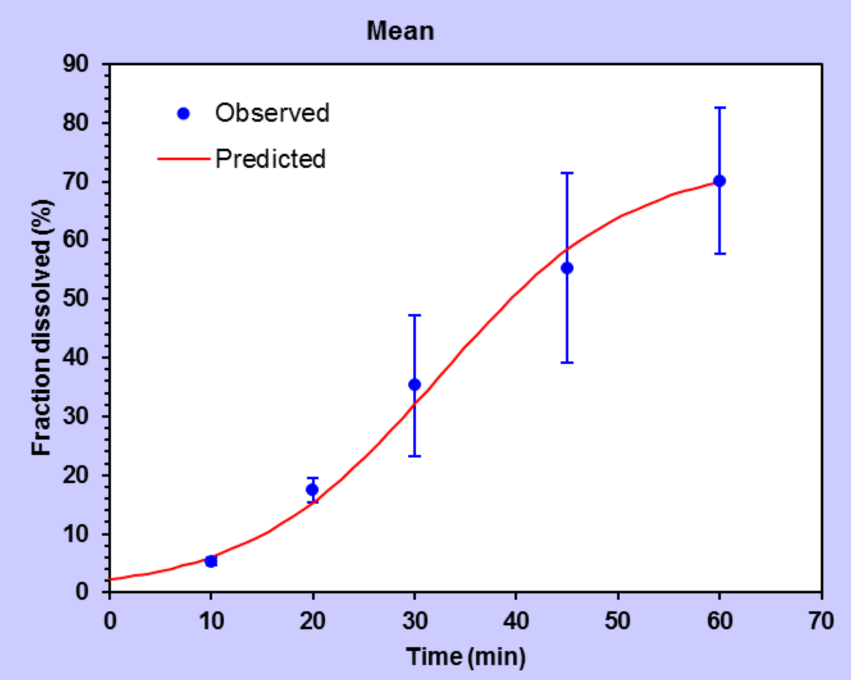

Supplementary materials – Model fitting summary – Parteck® M 100 (modelling up to app. 70 % of carvedilol released)

Graphical abstract of model fit presented as the fraction % of released carvedilol per tested tablet:

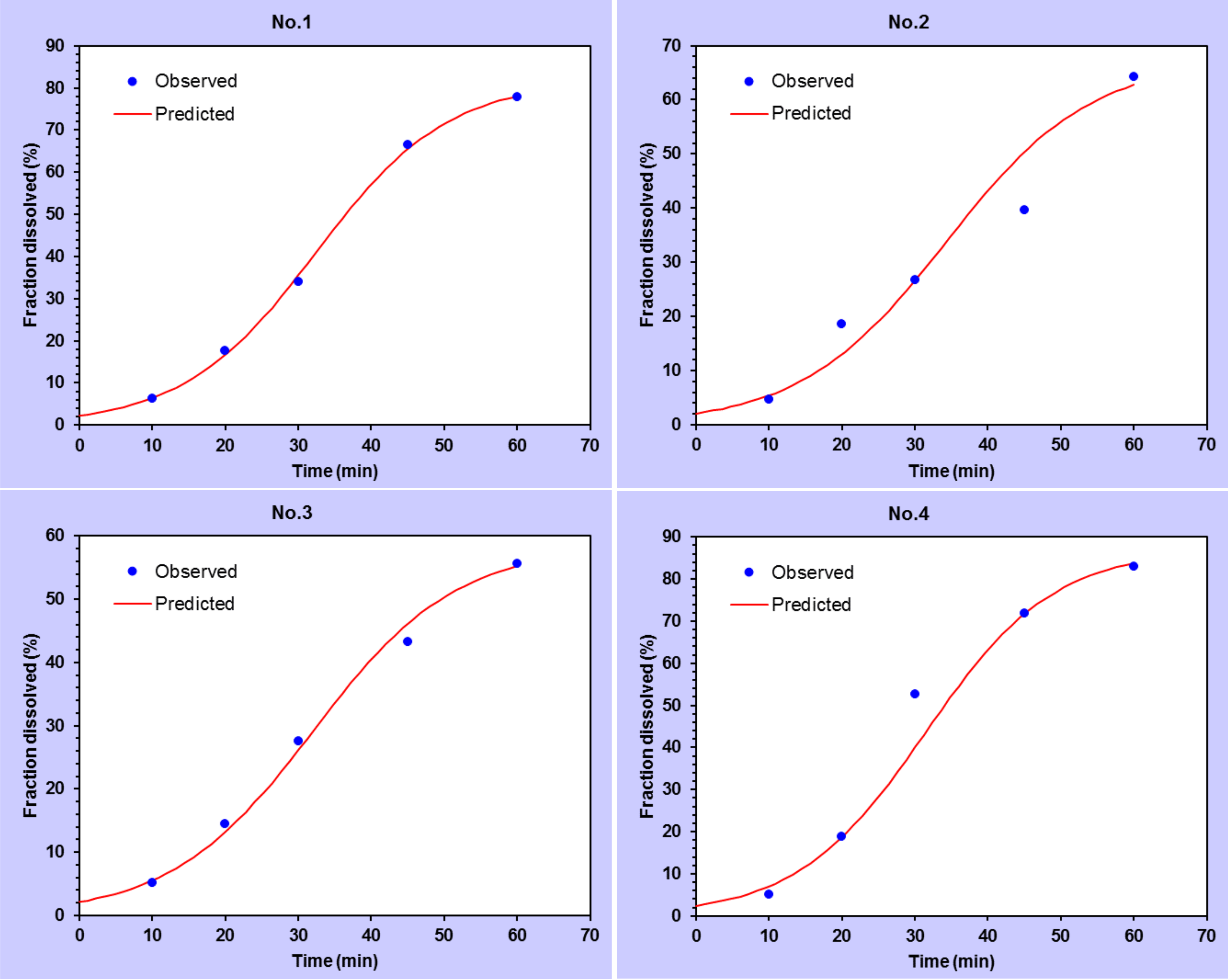

Supplementary materials – Model fitting summary – Parteck® M 100 (modelling up to app. 70 % of carvedilol released)

Model: **Gompertz_1**

Model equation: $F = 100 \cdot e^{-\alpha \cdot e^{-\beta \cdot \log(t)}}$

Fitted model parameters per tested tablet (N = 4) with statistics – mean, standard deviation (SD), and relative standard deviation expressed in % (RSD%) (output from DDSolver):

Parameter	No.1	No.2	No.3	No.4	Mean	SD	RSD(%)
α	84.056	32.243	26.115	184.641	81.764	73.344	89.702
β	3.161	2.267	2.083	3.716	2.807	0.767	27.337

Number of dissolution data points (N), degrees of freedom (df), and selected goodness of fit criteria – Pearson correlation coefficient (R), coefficient of determination (R^2), adjusted coefficient of determination ($R^2_{adjusted}$), and residual sum of squares (RSS) (manual calculation in MS Excel):

Parameter	No.1	No.2	No.3	No.4
N	5	5	5	5
df	3	3	3	3
R	0.973107545	0.965410723	0.992979677	0.993873423
R^2	0.946938294	0.932017865	0.986008639	0.98778438
$R^2_{adjusted}$	0.929251059	0.909357153	0.981344853	0.983712507
RSS	224.5886524	142.6545421	27.29098741	114.1653727

Graphical abstract of model fit presented as mean ± 1 SD of the fraction % of released carvedilol:

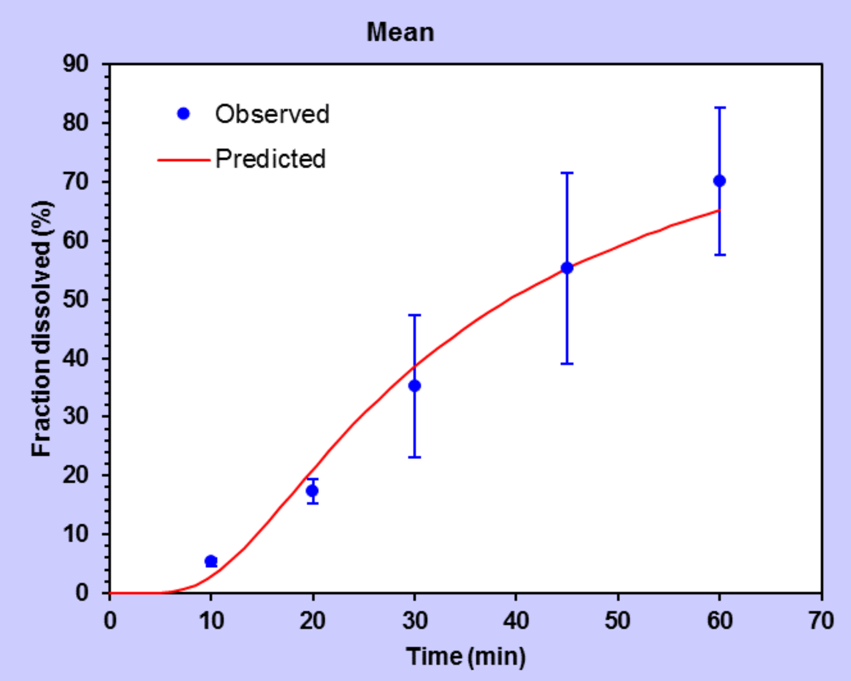

Supplementary materials – Model fitting summary – Parteck® M 100 (modelling up to app. 70 % of carvedilol released)

Graphical abstract of model fit presented as the fraction % of released carvedilol per tested tablet:

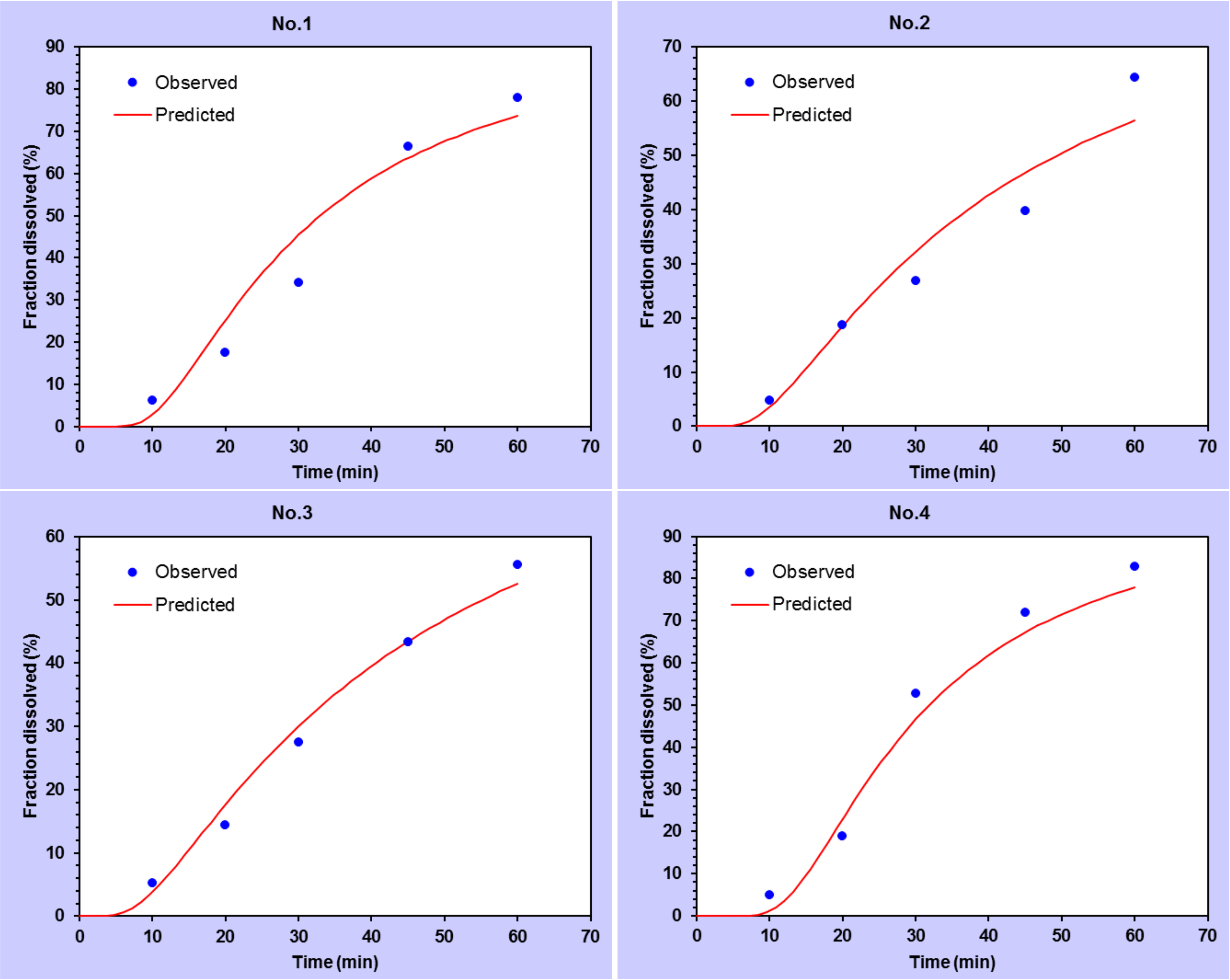

Supplementary materials – Model fitting summary – Parteck® M 100 (modelling up to app. 70 % of carvedilol released)

Model: **Gompertz_2**

Model equation: $F = F_{max} \cdot e^{-\alpha \cdot e^{-\beta \cdot \log(t)}}$

Fitted model parameters per tested tablet (N = 4) with statistics – mean, standard deviation (SD), and relative standard deviation expressed in % (RSD%) (output from DDSolver):

Parameter	No.1	No.2	No.3	No.4	Mean	SD	RSD(%)
α	893.741	470.291	584.091	876.633	706.189	211.961	30.015
β	4.896	4.333	4.594	4.792	4.654	0.248	5.323
F_{max}	81.786	67.452	58.326	101.039	77.151	18.624	24.140

Number of dissolution data points (N), degrees of freedom (df), and selected goodness of fit criteria – Pearson correlation coefficient (R), coefficient of determination (R^2), adjusted coefficient of determination ($R^2_{adjusted}$), and residual sum of squares (RSS) (manual calculation in MS Excel):

Parameter	No.1	No.2	No.3	No.4
N	5	5	5	5
df	2	2	2	2
R	0.97755213	0.950881472	0.984211332	0.997945043
R^2	0.955608168	0.904175574	0.968671945	0.995894308
$R^2_{adjusted}$	0.911216335	0.808351149	0.93734389	0.991788616
RSS	185.8012315	218.3668852	71.32394979	51.1497567

Graphical abstract of model fit presented as mean ± 1 SD of the fraction % of released carvedilol:

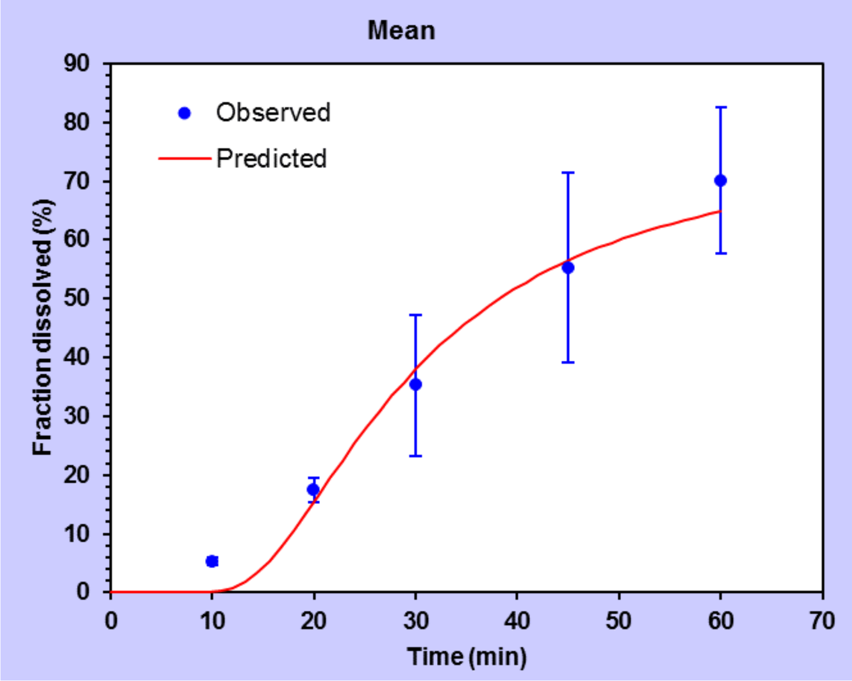

Supplementary materials – Model fitting summary – Parteck® M 100 (modelling up to app. 70 % of carvedilol released)

Graphical abstract of model fit presented as the fraction % of released carvedilol per tested tablet:

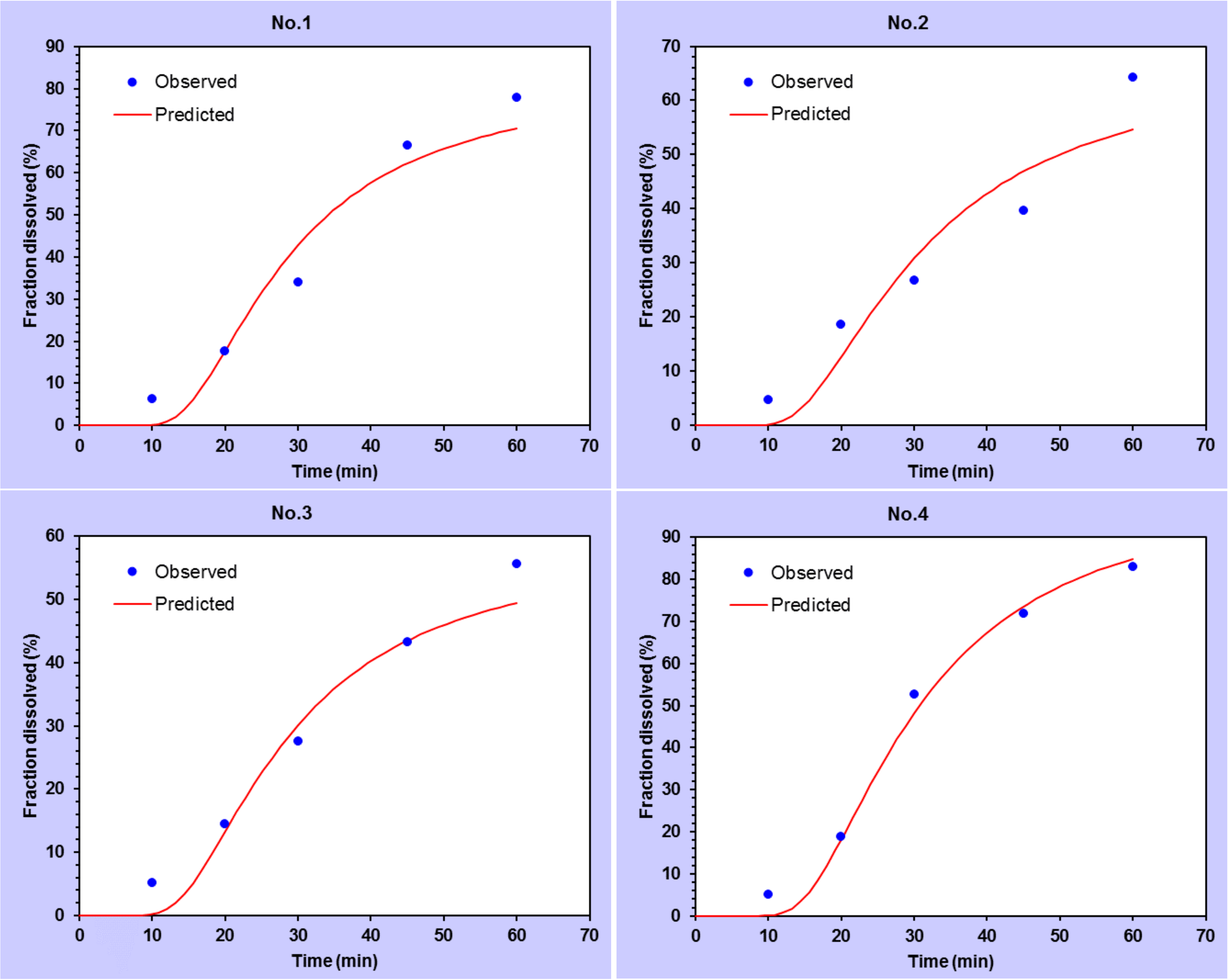

Supplementary materials – Model fitting summary – Parteck® M 100 (modelling up to app. 70 % of carvedilol released)

Model: **Gompertz_3**

Model equation: $F = F_{max} \cdot e^{-e^{-k \cdot (t-\gamma)}}$

Fitted model parameters per tested tablet (N = 4) with statistics – mean, standard deviation (SD), and relative standard deviation expressed in % (RSD%) (output from DDSolver):

Parameter	No.1	No.2	No.3	No.4	Mean	SD	RSD(%)
k	0.081	0.073	0.076	0.082	0.078	0.004	5.407
γ	24.698	26.115	24.314	23.477	24.651	1.101	4.467
F_{max}	81.786	67.452	58.326	87.029	73.648	13.146	17.849

Number of dissolution data points (N), degrees of freedom (df), and selected goodness of fit criteria – Pearson correlation coefficient (R), coefficient of determination (R^2), adjusted coefficient of determination ($R^2_{adjusted}$), and residual sum of squares (RSS) (manual calculation in MS Excel):

Parameter	No.1	No.2	No.3	No.4
N	5	5	5	5
df	2	2	2	2
R	0.990306383	0.960884556	0.993403781	0.995788247
R^2	0.980706733	0.92329913	0.986851072	0.991594234
$R^2_{adjusted}$	0.961413466	0.84659826	0.973702144	0.983188467
RSS	85.50691173	213.5720306	31.37524101	38.23219794

Graphical abstract of model fit presented as mean ± 1 SD of the fraction % of released carvedilol:

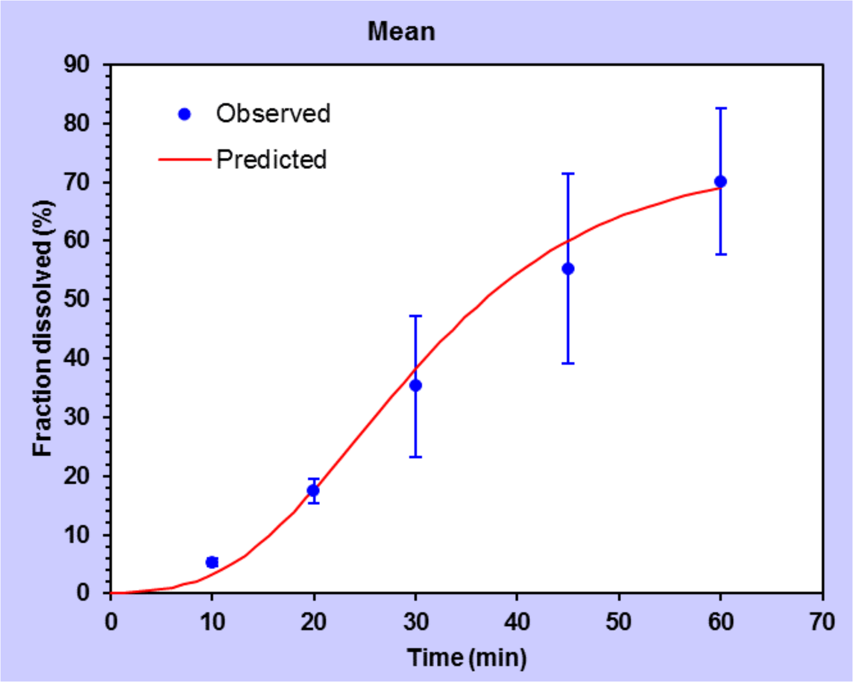

Supplementary materials – Model fitting summary – Parteck® M 100 (modelling up to app. 70 % of carvedilol released)

Graphical abstract of model fit presented as the fraction % of released carvedilol per tested tablet:

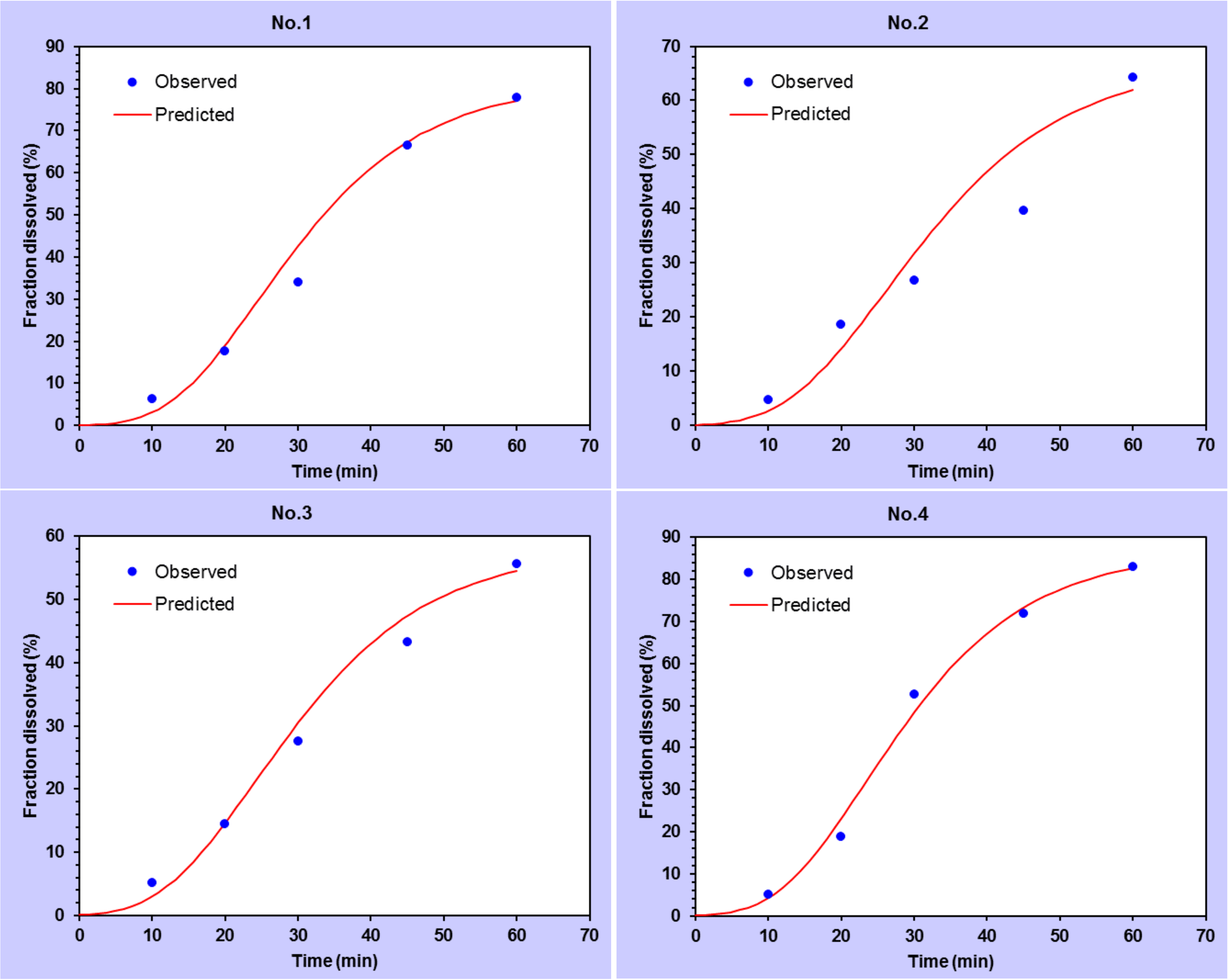

Supplementary materials – Model fitting summary – Parteck® M 100 (modelling up to app. 70 % of carvedilol released)

Model: **Gompertz_4**

Model equation: $F = F_{max} \cdot e^{-\beta \cdot e^{-k \cdot t}}$

Fitted model parameters per tested tablet (N = 4) with statistics – mean, standard deviation (SD), and relative standard deviation expressed in % (RSD%) (output from DDSolver):

Parameter	No.1	No.2	No.3	No.4	Mean	SD	RSD(%)
k	0.081	0.073	0.076	0.082	0.078	0.004	5.407
β	7.324	6.663	6.325	6.793	6.776	0.415	6.122
F_{max}	81.786	67.452	58.326	87.029	73.648	13.146	17.849

Number of dissolution data points (N), degrees of freedom (df), and selected goodness of fit criteria – Pearson correlation coefficient (R), coefficient of determination (R^2), adjusted coefficient of determination ($R^2_{adjusted}$), and residual sum of squares (RSS) (manual calculation in MS Excel):

Parameter	No.1	No.2	No.3	No.4
N	5	5	5	5
df	2	2	2	2
R	0.990306383	0.960884556	0.993403781	0.995788247
R^2	0.980706733	0.92329913	0.986851072	0.991594234
$R^2_{adjusted}$	0.961413466	0.84659826	0.973702144	0.983188467
RSS	85.50691173	213.5720306	31.37524101	38.23219794

Graphical abstract of model fit presented as mean ± 1 SD of the fraction % of released carvedilol:

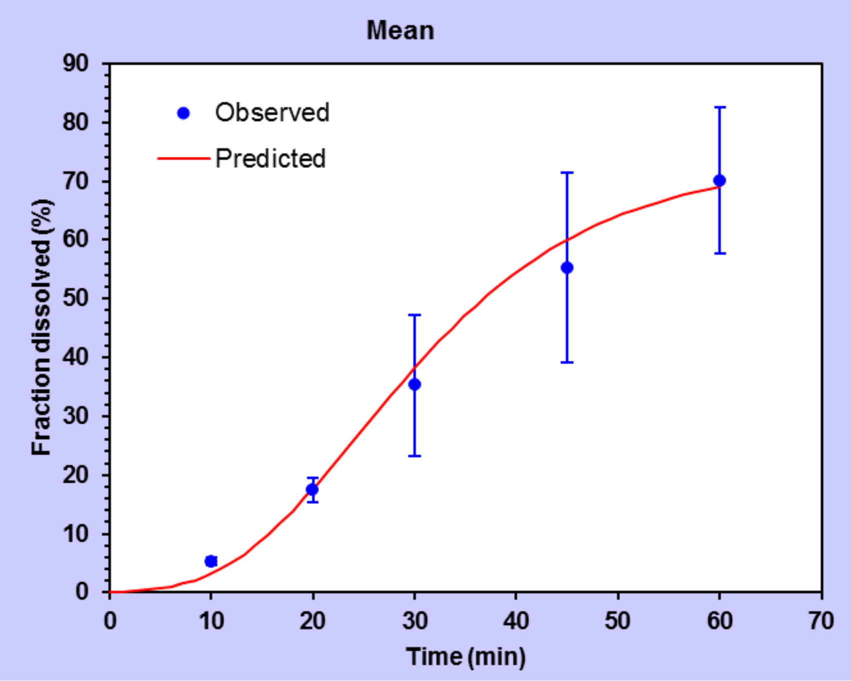

Supplementary materials – Model fitting summary – Parteck® M 100 (modelling up to app. 70 % of carvedilol released)

Graphical abstract of model fit presented as the fraction % of released carvedilol per tested tablet:

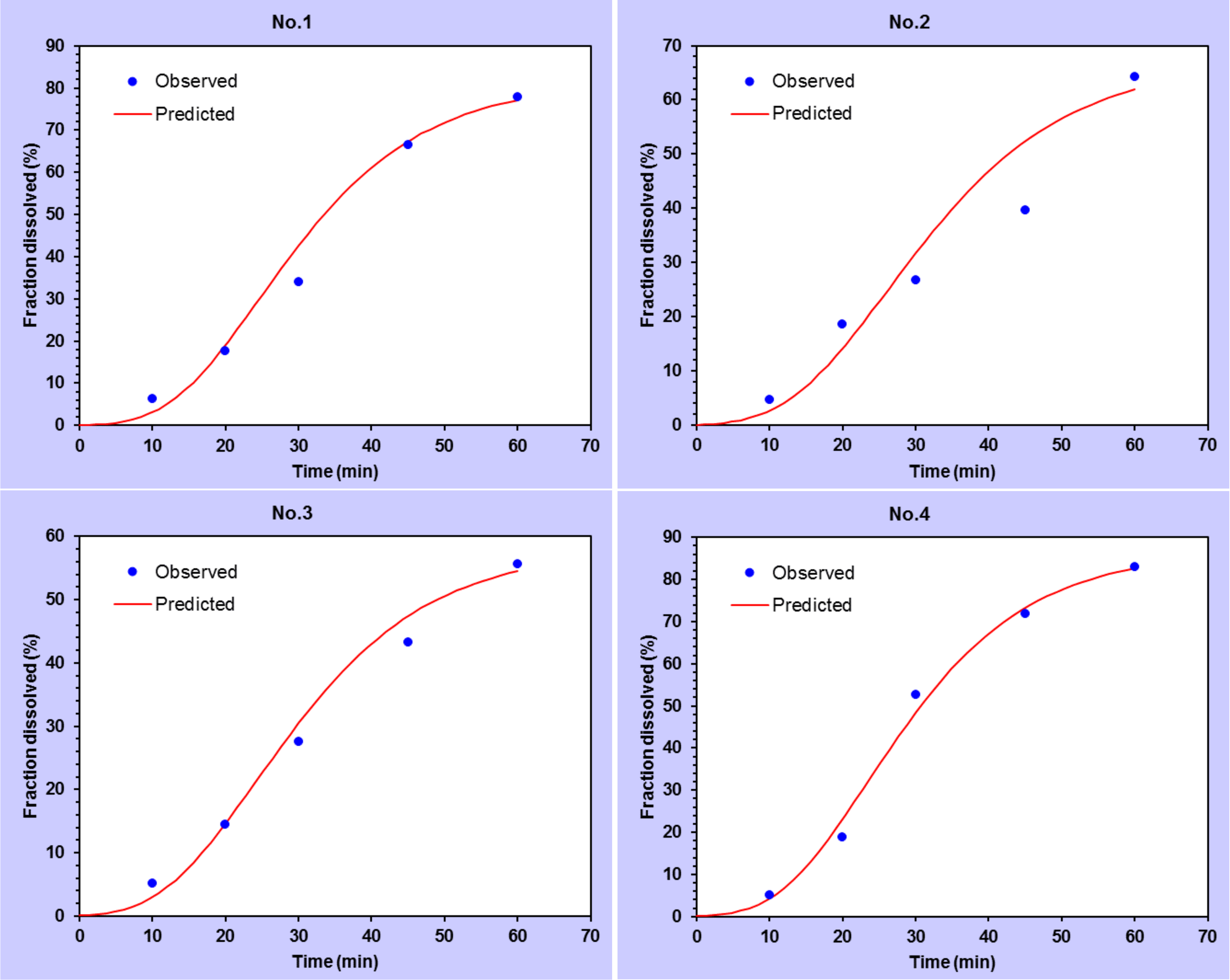

Supplementary materials – Model fitting summary – Parteck® M 100 (modelling up to app. 70 % of carvedilol released)

Model: **Probit_1**

Model equation: $F = 100 \cdot \phi[\alpha + \beta \cdot \log(t)]$

Fitted model parameters per tested tablet (N = 4) with statistics – mean, standard deviation (SD), and relative standard deviation expressed in % (RSD%) (output from DDSolver):

Parameter	No.1	No.2	No.3	No.4	Mean	SD	RSD(%)
α	-4.749	-4.130	-4.199	-5.178	-4.564	0.494	-10.828
β	3.060	2.436	2.447	3.462	2.851	0.500	17.548

Number of dissolution data points (N), degrees of freedom (df), and selected goodness of fit criteria – Pearson correlation coefficient (R), coefficient of determination (R^2), adjusted coefficient of determination ($R^2_{adjusted}$), and residual sum of squares (RSS) (manual calculation in MS Excel):

Parameter	No.1	No.2	No.3	No.4
N	5	5	5	5
df	3	3	3	3
R	0.989428714	0.978129076	0.999311862	0.992809416
R^2	0.97896918	0.95673649	0.998624198	0.985670537
$R^2_{adjusted}$	0.971958906	0.942315319	0.998165598	0.980894049
RSS	93.91684659	89.92240985	3.630299347	67.10877382

Graphical abstract of model fit presented as mean ± 1 SD of the fraction % of released carvedilol:

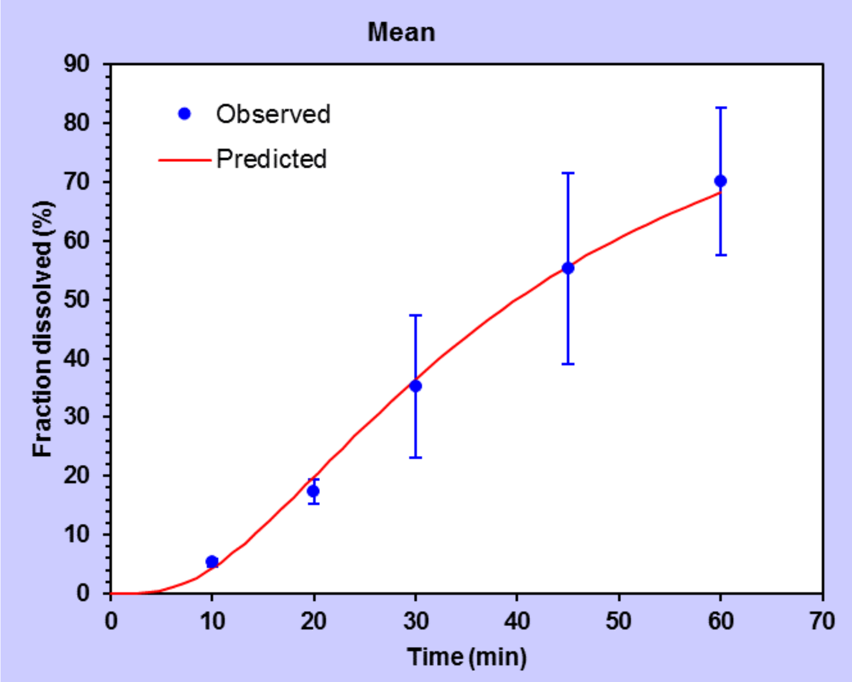

Supplementary materials – Model fitting summary – Parteck® M 100 (modelling up to app. 70 % of carvedilol released)

Graphical abstract of model fit presented as the fraction % of released carvedilol per tested tablet:

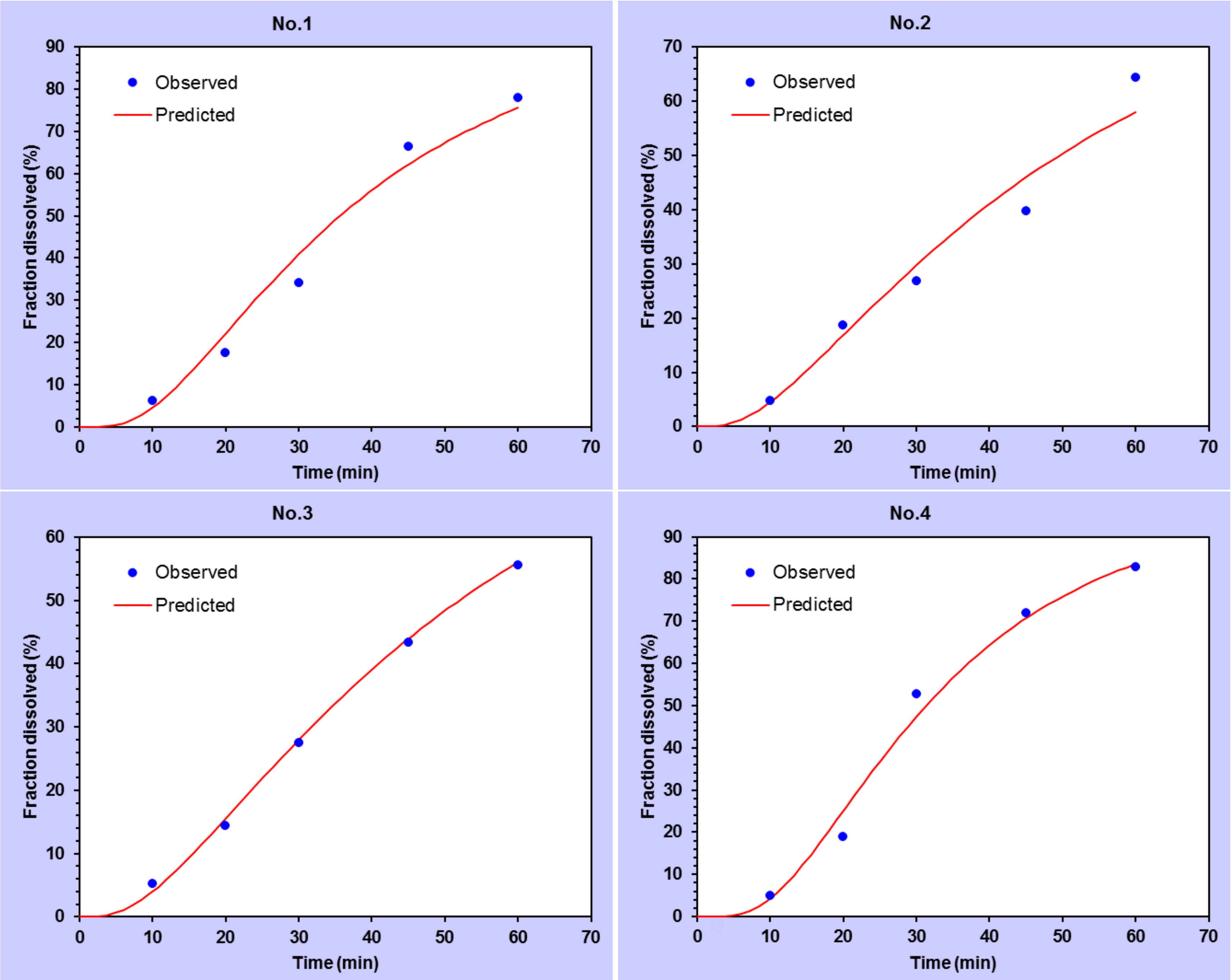

Supplementary materials – Model fitting summary – Parteck® M 100 (modelling up to app. 70 % of carvedilol released)

Model: **Probit_2**

Model equation: $F = F_{max} \cdot \phi[\alpha + \beta \cdot \log(t)]$

Fitted model parameters per tested tablet (N = 4) with statistics – mean, standard deviation (SD), and relative standard deviation expressed in % (RSD%) (output from DDSolver):

Parameter	No.1	No.2	No.3	No.4	Mean	SD	RSD(%)
α	-6.546	-6.053	-6.127	-6.485	-6.302	0.249	-3.948
β	4.196	3.800	3.945	4.536	4.119	0.322	7.828
F_{max}	91.342	73.171	63.271	89.877	79.415	13.556	17.070

Number of dissolution data points (N), degrees of freedom (df), and selected goodness of fit criteria – Pearson correlation coefficient (R), coefficient of determination (R^2), adjusted coefficient of determination ($R^2_{adjusted}$), and residual sum of squares (RSS) (manual calculation in MS Excel):

Parameter	No.1	No.2	No.3	No.4
N	5	5	5	5
df	2	2	2	2
R	0.997174526	0.974717221	0.999051875	0.995165261
R^2	0.994357034	0.95007366	0.998104649	0.990353897
$R^2_{adjusted}$	0.988714069	0.90014732	0.996209299	0.980707794
RSS	109.0692517	186.8912666	68.49736618	66.7119249

Graphical abstract of model fit presented as mean ± 1 SD of the fraction % of released carvedilol:

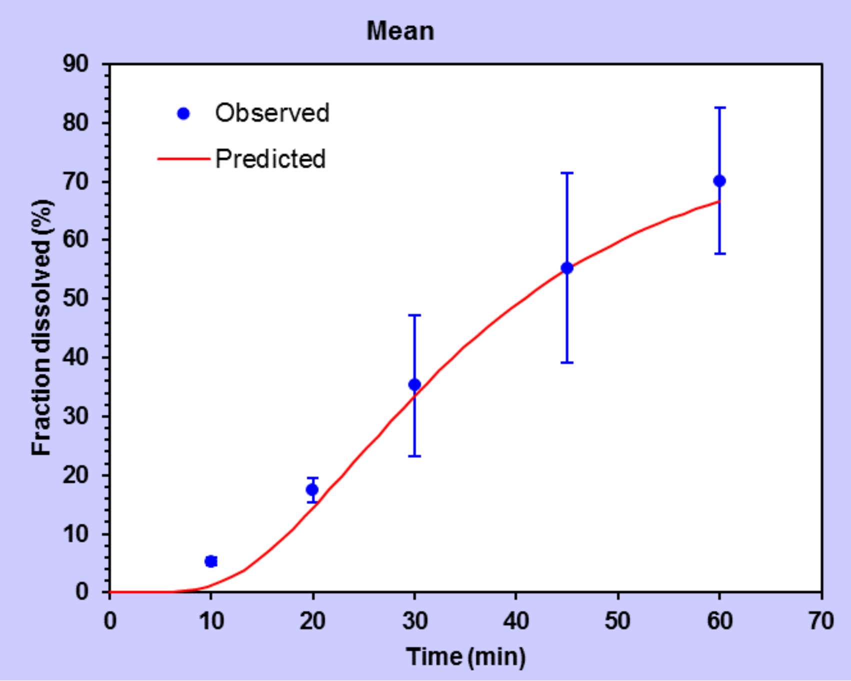

Supplementary materials – Model fitting summary – Parteck® M 100 (modelling up to app. 70 % of carvedilol released)

Graphical abstract of model fit presented as the fraction % of released carvedilol per tested tablet:

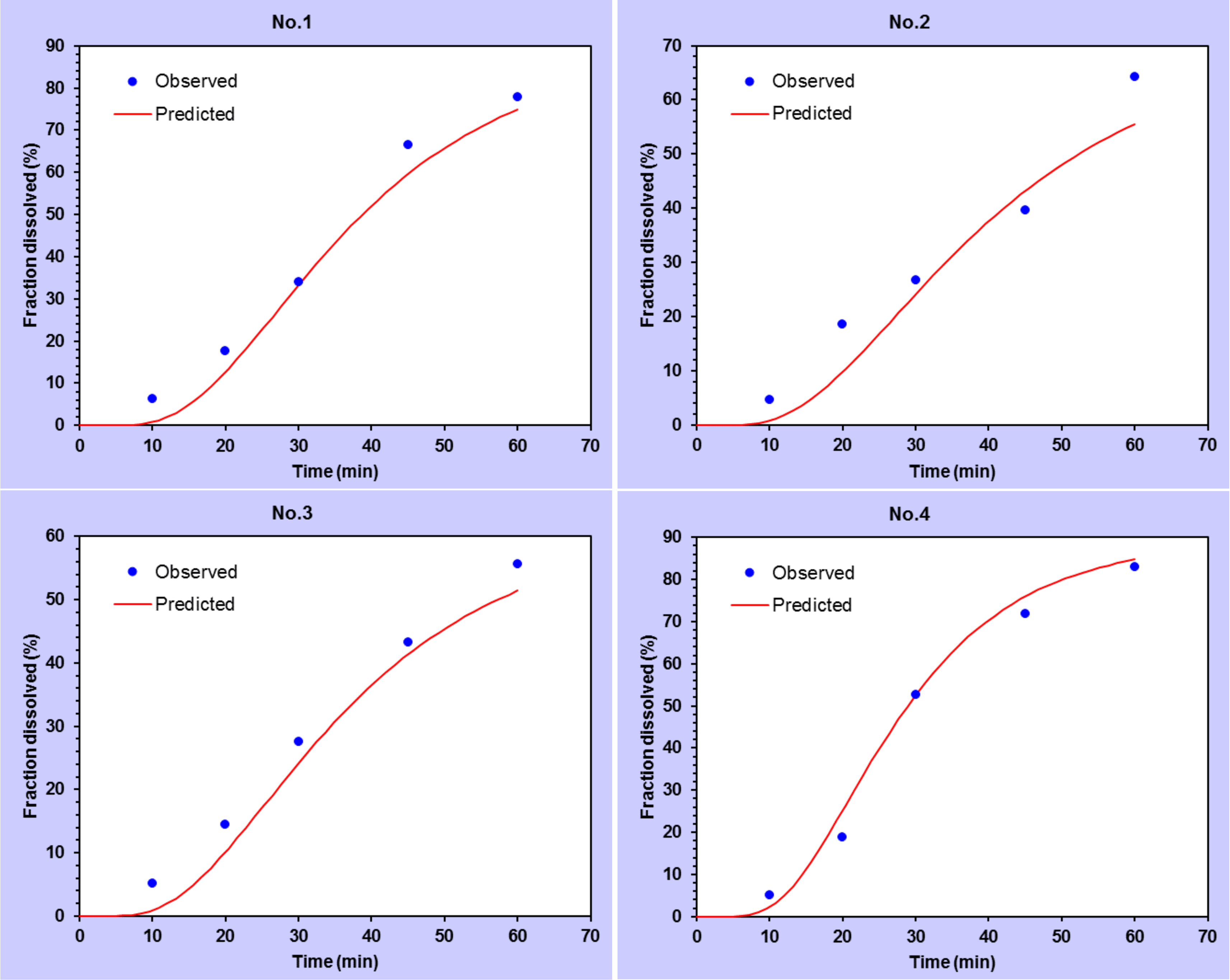

Supplement: Supplementary file 1 [file pharmaceutics-16-00498-s001.zip › Supplementary materials_Model fitting summary_Parteck® M 100.pdf]
