# Supplementary material for: Comparative Fitting of Mathematical Models to Carvedilol Release Profiles Obtained from Hypromellose Matrix Tablets
Source: Pharmaceutics. 2024 Apr 4;16(4):498. doi: 10.3390/pharmaceutics16040498 (PMC11053526; doi:10.3390/pharmaceutics16040498)

Supplementary materials – Model fitting summary – Parteck® M 200 (modelling the entire relevant dissolution data)

Model: **Zero–order**

Model equation: $F = k_0 \cdot t$

Fitted model parameters per tested tablet (N = 4) with statistics – mean, standard deviation (SD), and relative standard deviation expressed in % (RSD%) (output from DDSolver):

Parameter	No.1	No.2	No.3	No.4	Mean	SD	RSD(%)
k_0	0.562	0.658	0.620	0.531	0.593	0.057	9.594

Number of dissolution data points (N), degrees of freedom (df), and selected goodness of fit criteria – Pearson correlation coefficient (R), coefficient of determination (R^2), adjusted coefficient of determination ($R^2_{adjusted}$), and residual sum of squares (RSS) (manual calculation in MS Excel):

Parameter	No.1	No.2	No.3	No.4
N	10	10	10	10
df	9	9	9	9
R	0.956206748	0.803297391	0.910871659	0.956787606
R^2	0.914331346	0.645286698	0.829687179	0.915442522
$R^2_{adjusted}$	0.914331346	0.645286698	0.829687179	0.915442522
RSS	1496.664665	8494.941769	3513.552033	1604.023067

Graphical abstract of model fit presented as mean ± 1 SD of the fraction % of released carvedilol:

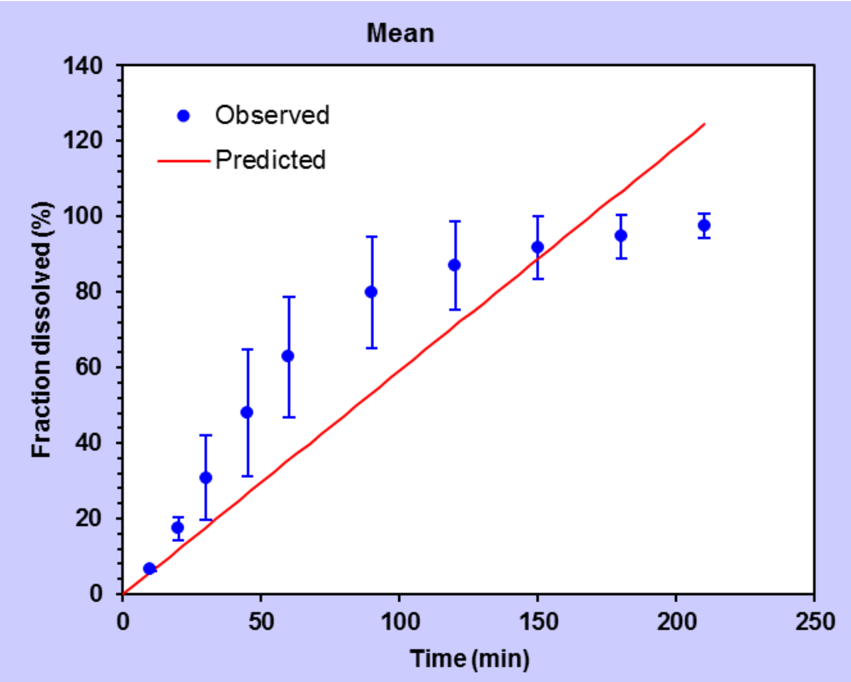

Supplementary materials – Model fitting summary – Parteck® M 200 (modelling the entire relevant dissolution data)

Graphical abstract of model fit presented as the fraction % of released carvedilol per tested tablet:

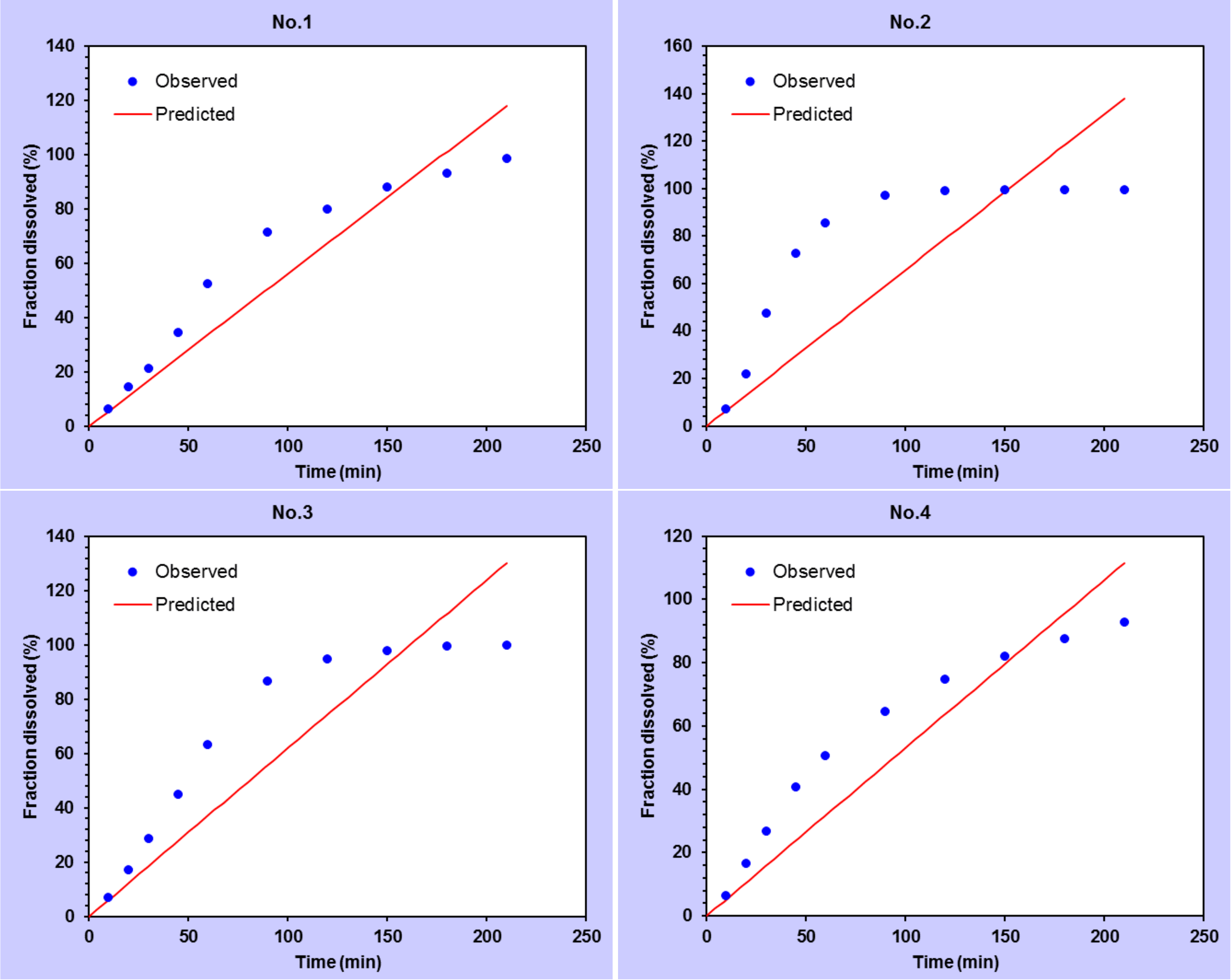

Supplementary materials – Model fitting summary – Parteck® M 200 (modelling the entire relevant dissolution data)

Model: **Zero–order with T_{lag}**

Model equation: $F = k_0 \cdot (t - T_{lag})$

Fitted model parameters per tested tablet (N = 4) with statistics – mean, standard deviation (SD), and relative standard deviation expressed in % (RSD%) (output from DDSolver):

Parameter	No.1	No.2	No.3	No.4	Mean	SD	RSD(%)
k_0	0.471	0.401	0.476	0.418	0.442	0.038	8.591
T_{lag}	-26.969	-90.093	-42.521	-38.031	-49.404	27.902	-56.478

Number of dissolution data points (N), degrees of freedom (df), and selected goodness of fit criteria – Pearson correlation coefficient (R), coefficient of determination (R^2), adjusted coefficient of determination ($R^2_{adjusted}$), and residual sum of squares (RSS) (manual calculation in MS Excel):

Parameter	No.1	No.2	No.3	No.4
N	10	10	10	10
df	8	8	8	8
R	0.956206748	0.803297391	0.910871659	0.956787606
R^2	0.914331346	0.645286698	0.829687179	0.915442522
$R^2_{adjusted}$	0.903622764	0.600947535	0.808398076	0.904872838
RSS	933.080873	3953.235524	2084.814399	723.072665

Graphical abstract of model fit presented as mean ± 1 SD of the fraction % of released carvedilol:

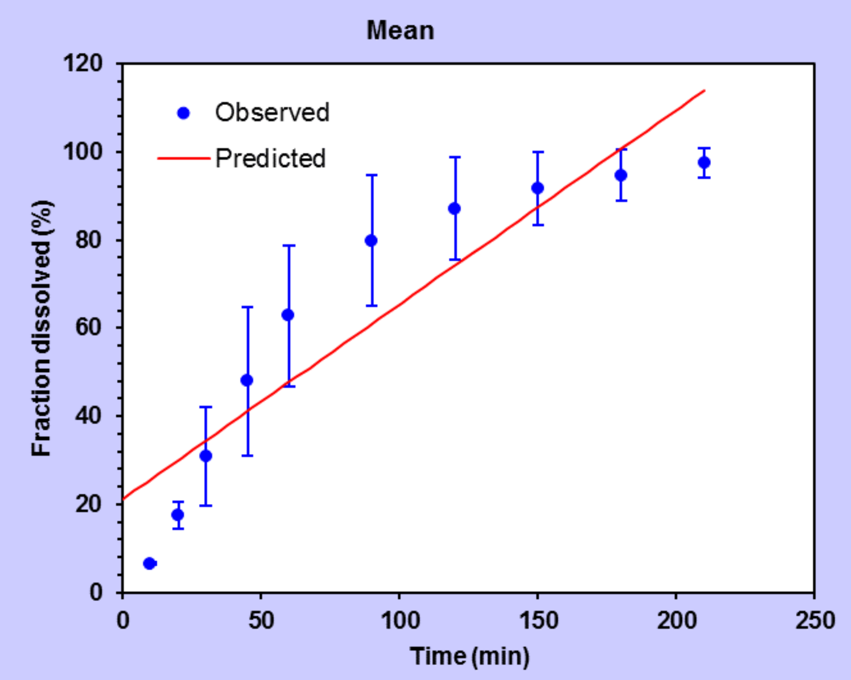

Supplementary materials – Model fitting summary – Parteck® M 200 (modelling the entire relevant dissolution data)

Graphical abstract of model fit presented as the fraction % of released carvedilol per tested tablet:

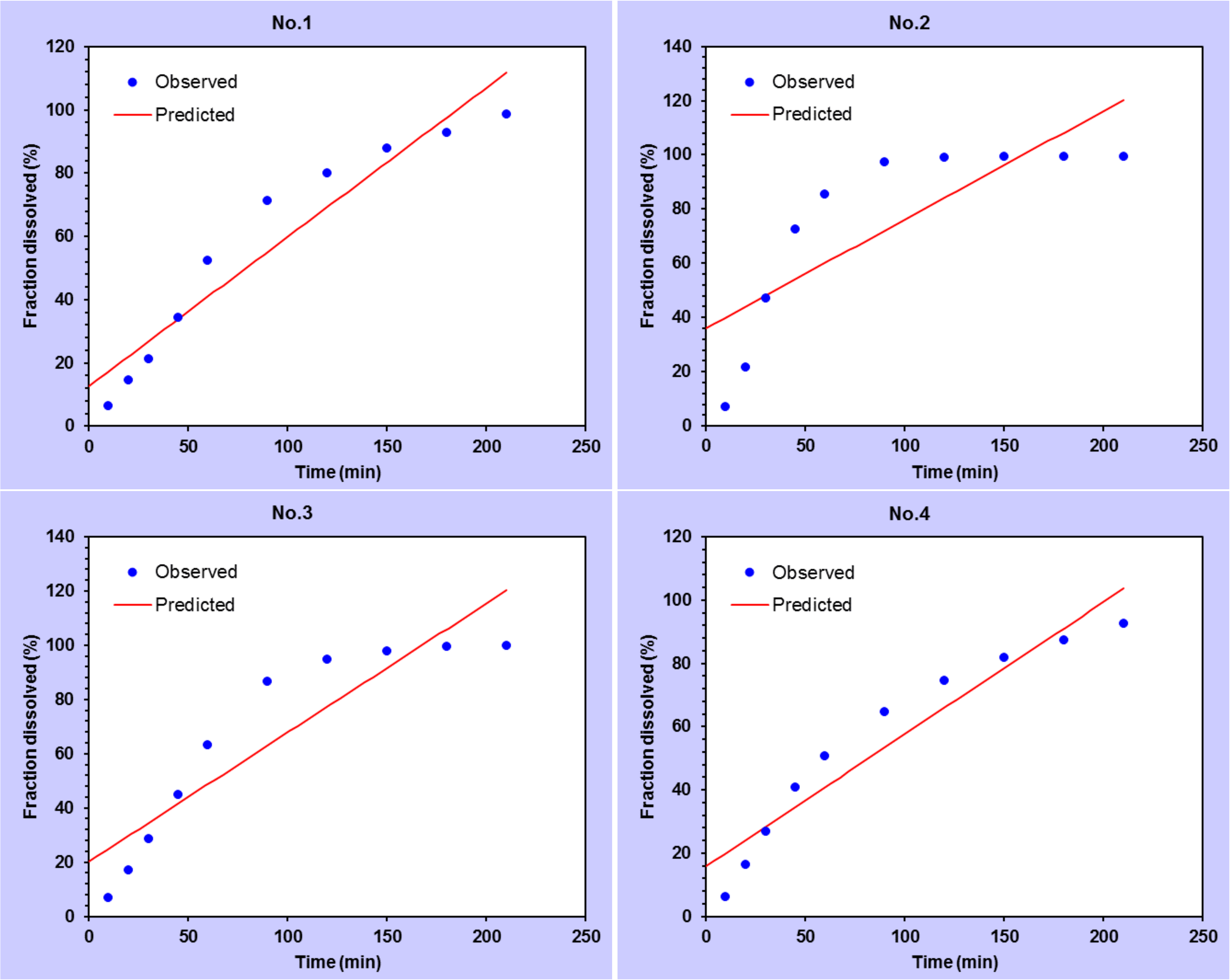

Supplementary materials – Model fitting summary – Parteck® M 200 (modelling the entire relevant dissolution data)

Model: **Zero–order with F_0**

Model equation: $F = F_0 + k_0 \cdot t$

Fitted model parameters per tested tablet (N = 4) with statistics – mean, standard deviation (SD), and relative standard deviation expressed in % (RSD%) (output from DDSolver):

Parameter	No.1	No.2	No.3	No.4	Mean	SD	RSD(%)
k_0	0.471	0.401	0.476	0.418	0.442	0.038	8.591
F_0	12.715	36.095	20.245	15.897	21.238	10.375	48.848

Number of dissolution data points (N), degrees of freedom (df), and selected goodness of fit criteria – Pearson correlation coefficient (R), coefficient of determination (R^2), adjusted coefficient of determination ($R^2_{adjusted}$), and residual sum of squares (RSS) (manual calculation in MS Excel):

Parameter	No.1	No.2	No.3	No.4
N	10	10	10	10
df	8	8	8	8
R	0.956206748	0.803297391	0.910871659	0.956787606
R^2	0.914331346	0.645286698	0.829687179	0.915442522
$R^2_{adjusted}$	0.903622764	0.600947535	0.808398076	0.904872838
RSS	933.080873	3953.235524	2084.814399	723.072665

Graphical abstract of model fit presented as mean ± 1 SD of the fraction % of released carvedilol:

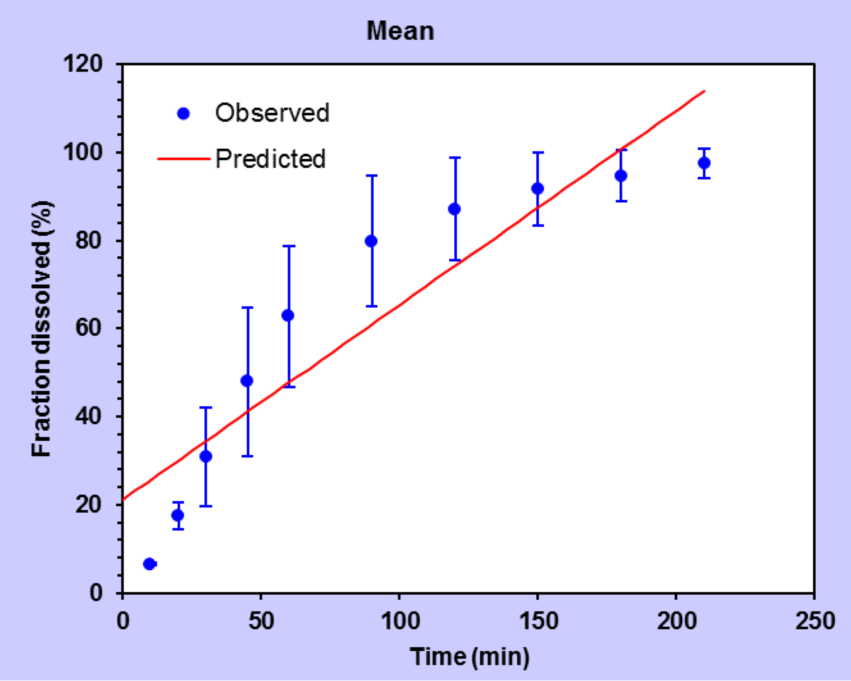

Supplementary materials – Model fitting summary – Parteck® M 200 (modelling the entire relevant dissolution data)

Graphical abstract of model fit presented as the fraction % of released carvedilol per tested tablet:

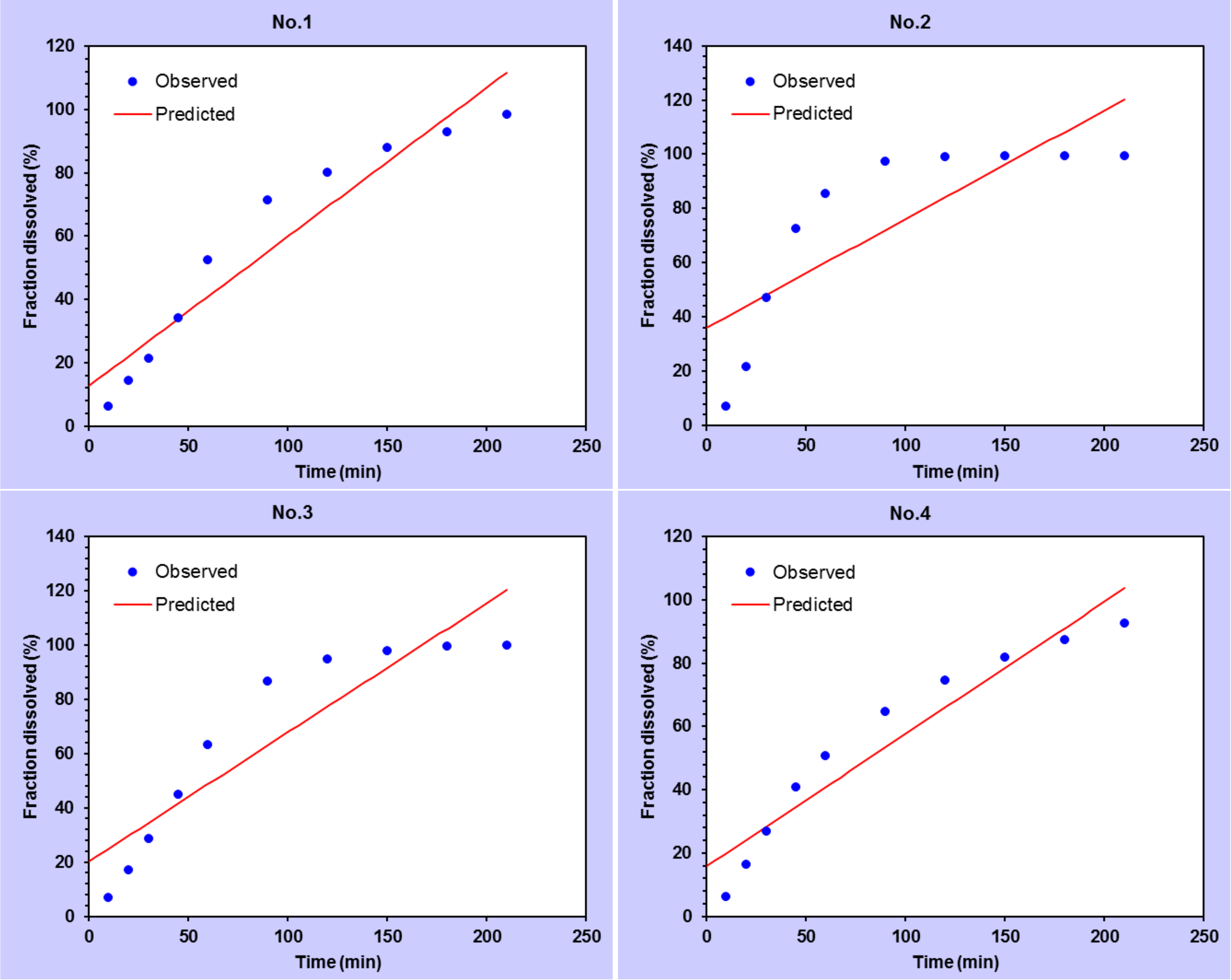

Supplementary materials – Model fitting summary – Parteck® M 200 (modelling the entire relevant dissolution data)

Model: **First–order**

Model equation: $F = 100 \cdot (1 - e^{-k_1 \cdot t})$

Fitted model parameters per tested tablet (N = 4) with statistics – mean, standard deviation (SD), and relative standard deviation expressed in % (RSD%) (output from DDSolver):

Parameter	No.1	No.2	No.3	No.4	Mean	SD	RSD(%)
k_1	0.012	0.022	0.028	0.012	0.018	0.008	42.563

Number of dissolution data points (N), degrees of freedom (df), and selected goodness of fit criteria – Pearson correlation coefficient (R), coefficient of determination (R^2), adjusted coefficient of determination ($R^2_{adjusted}$), and residual sum of squares (RSS) (manual calculation in MS Excel):

Parameter	No.1	No.2	No.3	No.4
N	10	10	10	10
df	9	9	9	9
R	0.996892555	0.979919817	0.97850979	0.999336074
R^2	0.993794765	0.960242849	0.957481409	0.998672589
$R^2_{adjusted}$	0.993794765	0.960242849	0.957481409	0.998672589
RSS	334.981699	785.9111871	2771.082573	59.97595004

Graphical abstract of model fit presented as mean ± 1 SD of the fraction % of released carvedilol:

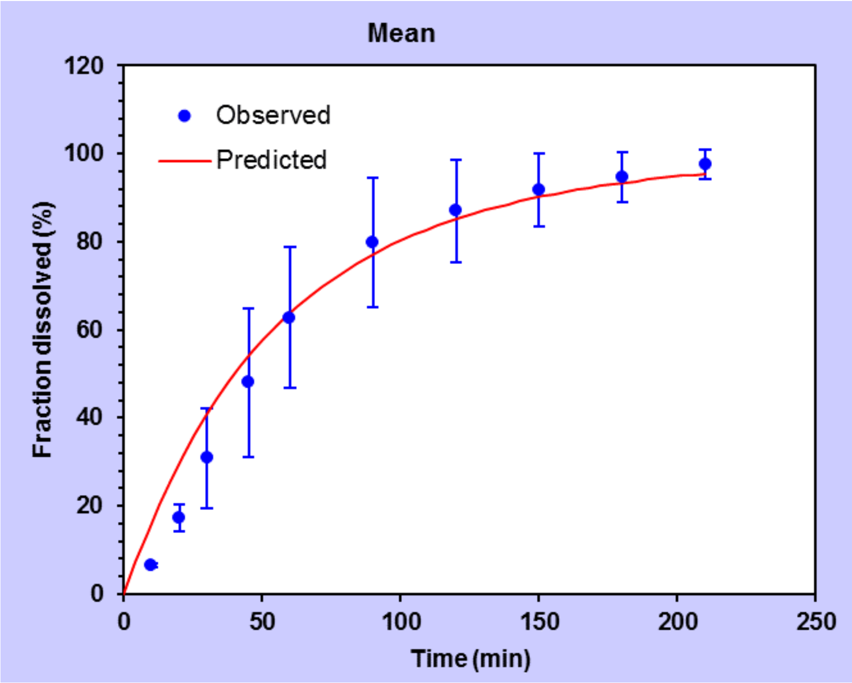

Supplementary materials – Model fitting summary – Parteck® M 200 (modelling the entire relevant dissolution data)

Graphical abstract of model fit presented as the fraction % of released carvedilol per tested tablet:

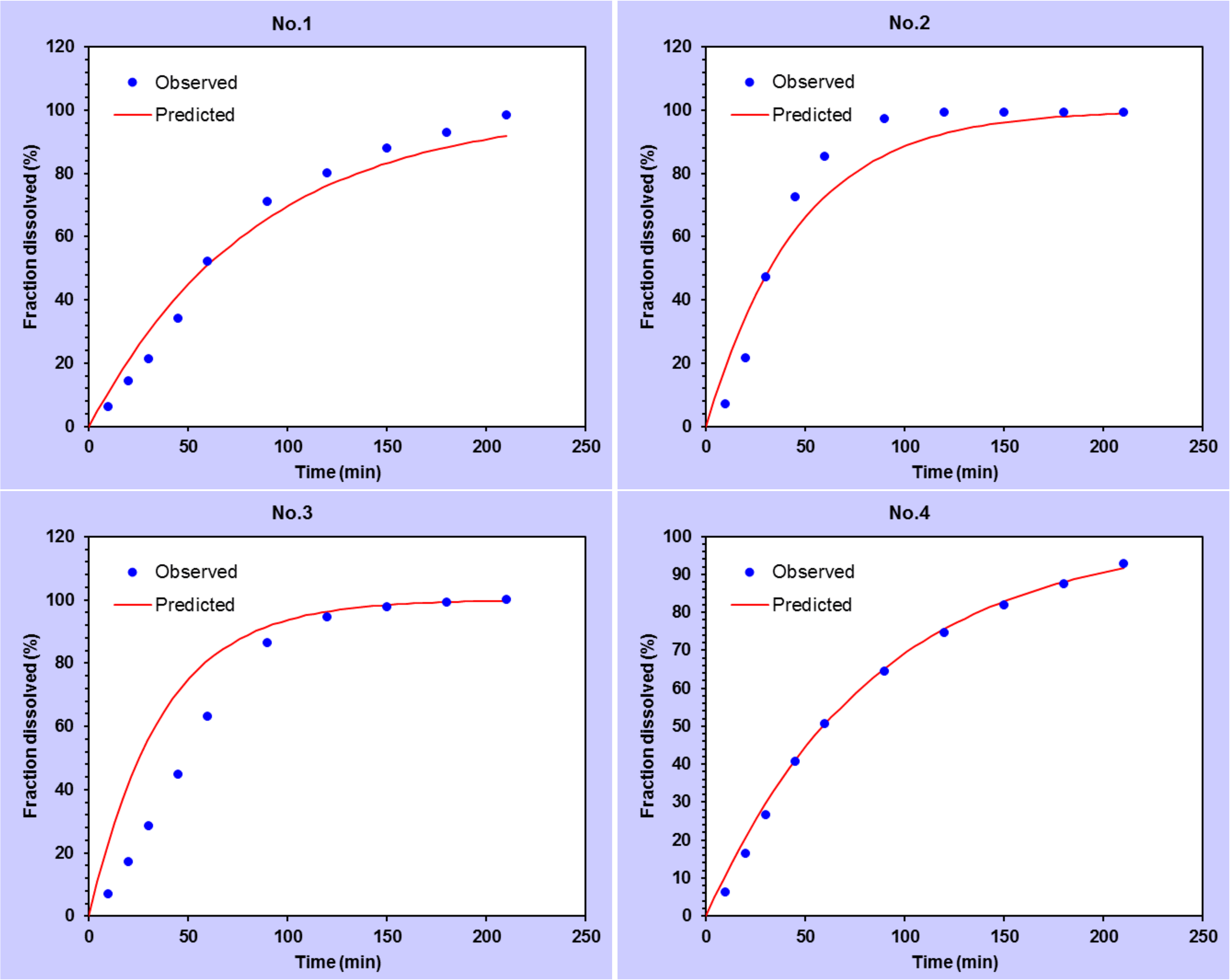

Supplementary materials – Model fitting summary – Parteck® M 200 (modelling the entire relevant dissolution data)

Model: **First–order with T_{lag}**

Model equation: $F = 100 \cdot \left[1 - e^{-k_1 \cdot (t - T_{lag})}\right]$

Fitted model parameters per tested tablet (N = 4) with statistics – mean, standard deviation (SD), and relative standard deviation expressed in % (RSD%) (output from DDSolver):

Parameter	No.1	No.2	No.3	No.4	Mean	SD	RSD(%)
k_1	0.016	0.028	0.033	0.012	0.022	0.010	43.419
T_{lag}	13.836	-6.179	21.696	4.850	8.551	11.992	140.242

Number of dissolution data points (N), degrees of freedom (df), and selected goodness of fit criteria – Pearson correlation coefficient (R), coefficient of determination (R^2), adjusted coefficient of determination ($R^2_{adjusted}$), and residual sum of squares (RSS) (manual calculation in MS Excel):

Parameter	No.1	No.2	No.3	No.4
N	10	10	10	10
df	8	8	8	8
R	0.9928419	0.991473912	0.963230736	0.999455206
R^2	0.985735038	0.983020518	0.927813451	0.998910708
$R^2_{adjusted}$	0.983951918	0.980898083	0.918790132	0.998774547
RSS	223.0581156	2036.489679	3528.566403	10.24378842

Graphical abstract of model fit presented as mean ± 1 SD of the fraction % of released carvedilol:

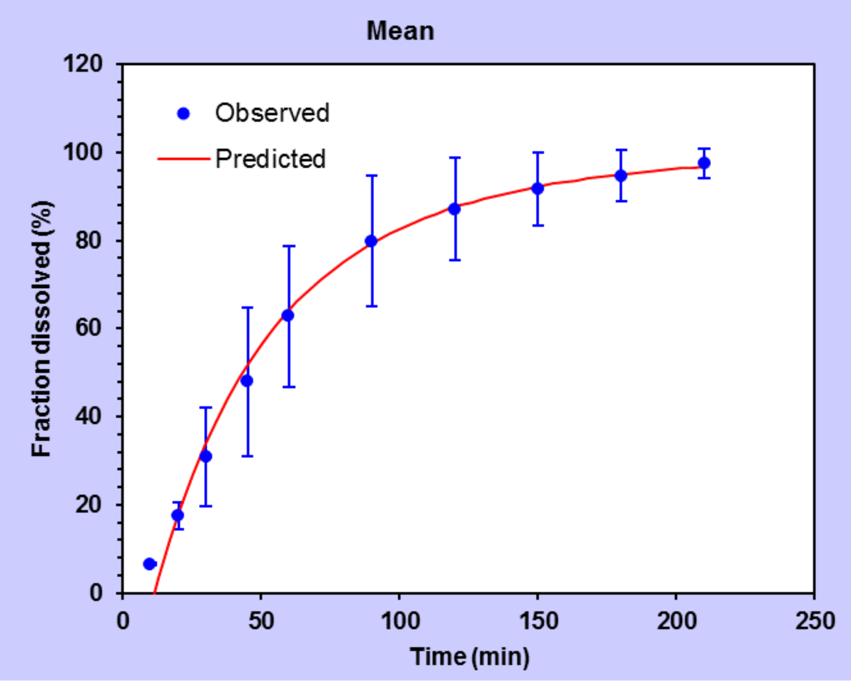

Supplementary materials – Model fitting summary – Parteck® M 200 (modelling the entire relevant dissolution data)

Graphical abstract of model fit presented as the fraction % of released carvedilol per tested tablet:

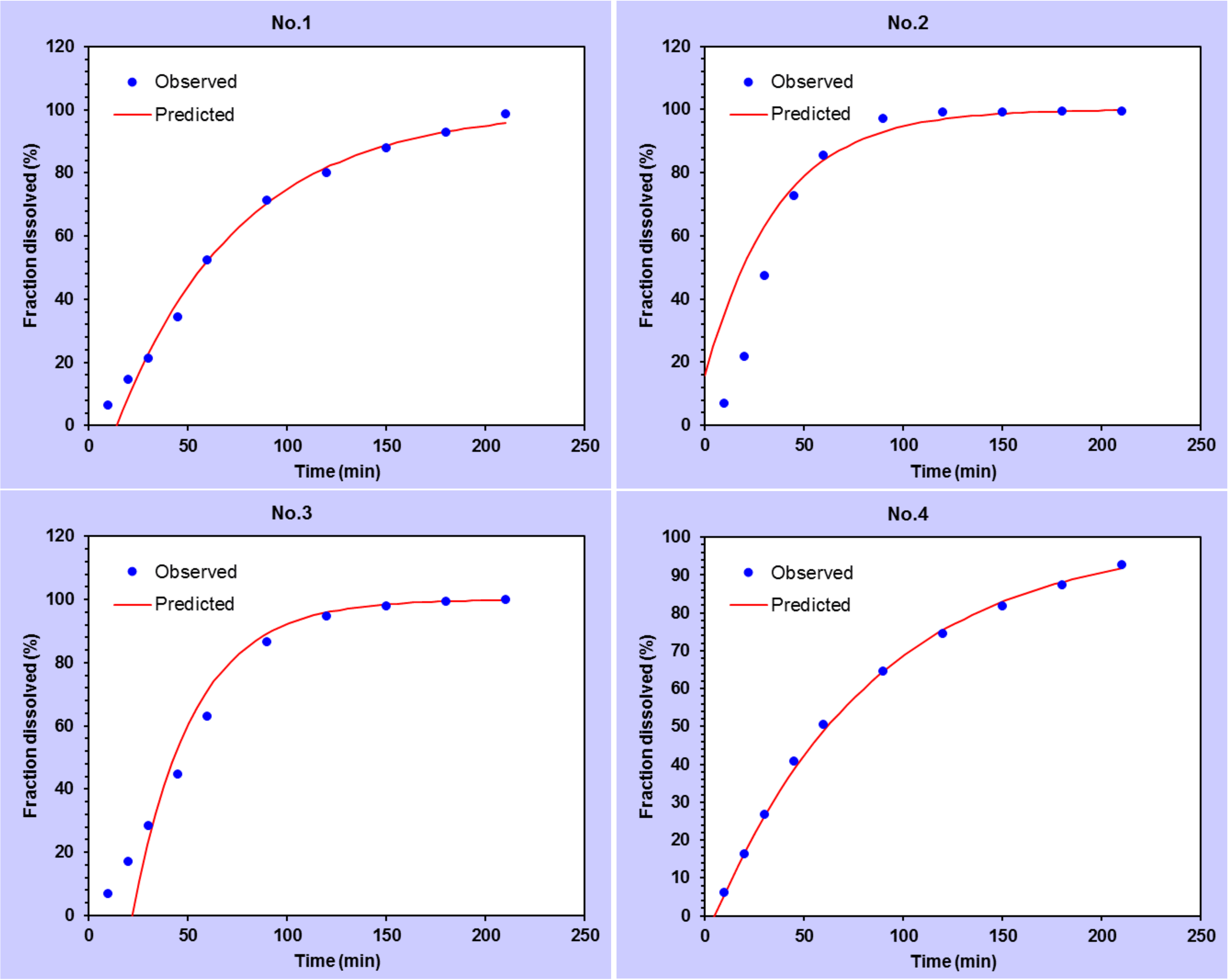

Supplementary materials – Model fitting summary – Parteck® M 200 (modelling the entire relevant dissolution data)

Model: **First–order with F_{max}**

Model equation: $F = F_{max} \cdot (1 - e^{-k_1 \cdot t})$

Fitted model parameters per tested tablet (N = 4) with statistics – mean, standard deviation (SD), and relative standard deviation expressed in % (RSD%) (output from DDSolver):

Parameter	No.1	No.2	No.3	No.4	Mean	SD	RSD(%)
k_1	0.013	0.019	0.016	0.013	0.015	0.003	18.174
F_{max}	103.362	104.225	104.895	97.334	102.454	3.471	3.388

Number of dissolution data points (N), degrees of freedom (df), and selected goodness of fit criteria – Pearson correlation coefficient (R), coefficient of determination (R^2), adjusted coefficient of determination ($R^2_{adjusted}$), and residual sum of squares (RSS) (manual calculation in MS Excel):

Parameter	No.1	No.2	No.3	No.4
N	10	10	10	10
df	8	8	8	8
R	0.996420416	0.970286449	0.994401257	0.999462085
R^2	0.992853644	0.941455794	0.98883386	0.998924459
$R^2_{adjusted}$	0.99196035	0.934137768	0.987438093	0.998790017
RSS	458.7601147	820.2563248	544.2060584	124.8557806

Graphical abstract of model fit presented as mean ± 1 SD of the fraction % of released carvedilol:

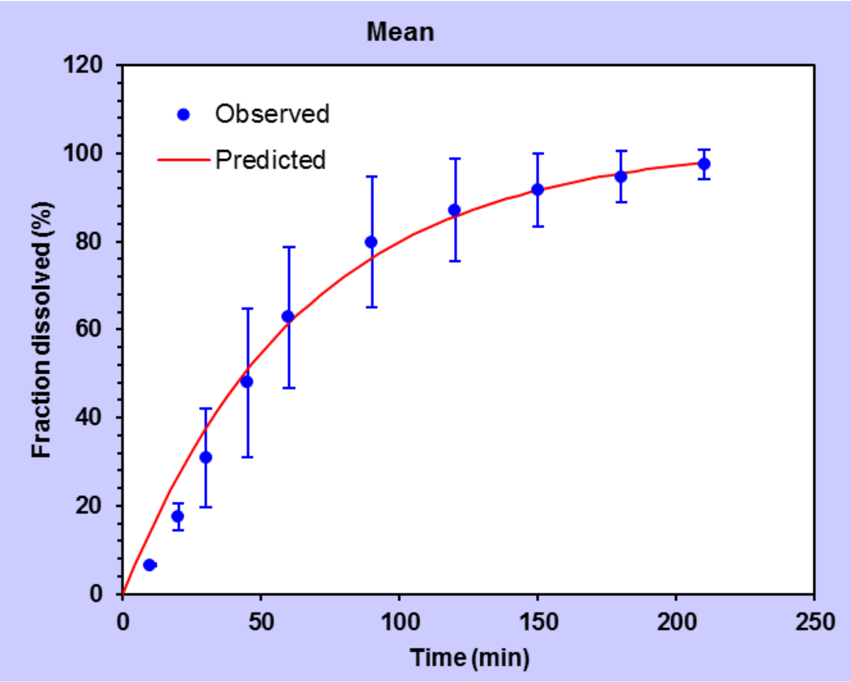

Supplementary materials – Model fitting summary – Parteck® M 200 (modelling the entire relevant dissolution data)

Graphical abstract of model fit presented as the fraction % of released carvedilol per tested tablet:

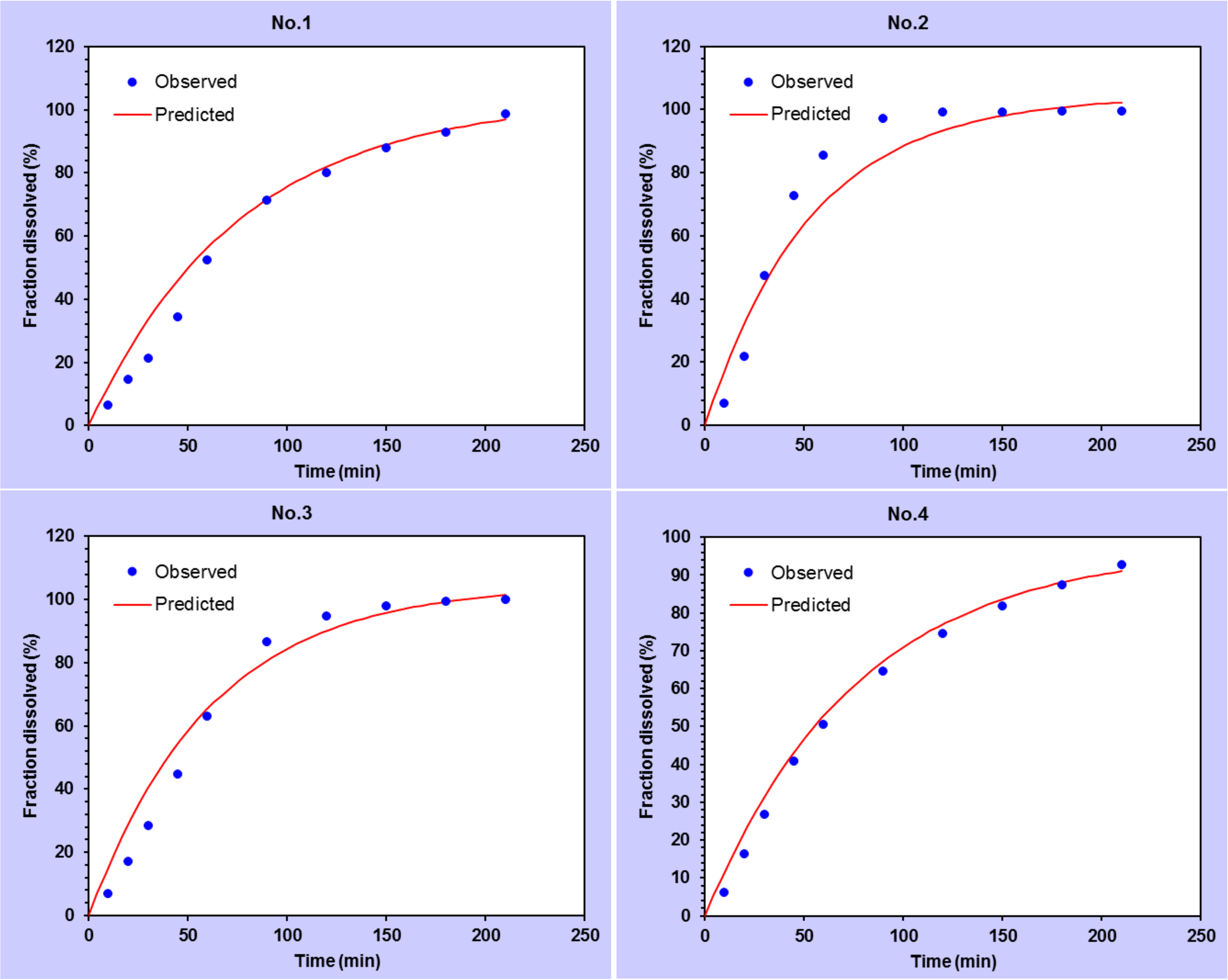

Supplementary materials – Model fitting summary – Parteck® M 200 (modelling the entire relevant dissolution data)

Model: **First–order with T_{lag} and F_{max}**

Model equation: $F = F_{max} \cdot \left[1 - e^{-k_1 \cdot (t - T_{lag})}\right]$

Fitted model parameters per tested tablet (N = 4) with statistics – mean, standard deviation (SD), and relative standard deviation expressed in % (RSD%) (output from DDSolver):

Parameter	No.1	No.2	No.3	No.4	Mean	SD	RSD(%)
k_1	0.014	0.016	0.017	0.014	0.015	0.001	8.787
T_{lag}	12.006	-24.304	4.752	8.499	0.238	16.627	6977.525
F_{max}	103.362	104.225	104.895	97.334	102.454	3.471	3.388

Number of dissolution data points (N), degrees of freedom (df), and selected goodness of fit criteria – Pearson correlation coefficient (R), coefficient of determination (R^2), adjusted coefficient of determination ($R^2_{adjusted}$), and residual sum of squares (RSS) (manual calculation in MS Excel):

Parameter	No.1	No.2	No.3	No.4
N	10	10	10	10
df	7	7	7	7
R	0.99528693	0.9574915	0.994522931	0.999177839
R^2	0.990596073	0.916789973	0.98907586	0.998356354
$R^2_{adjusted}$	0.987909237	0.893015679	0.985954677	0.99788674
RSS	133.788939	2753.437411	230.4057437	41.30283806

Graphical abstract of model fit presented as mean ± 1 SD of the fraction % of released carvedilol:

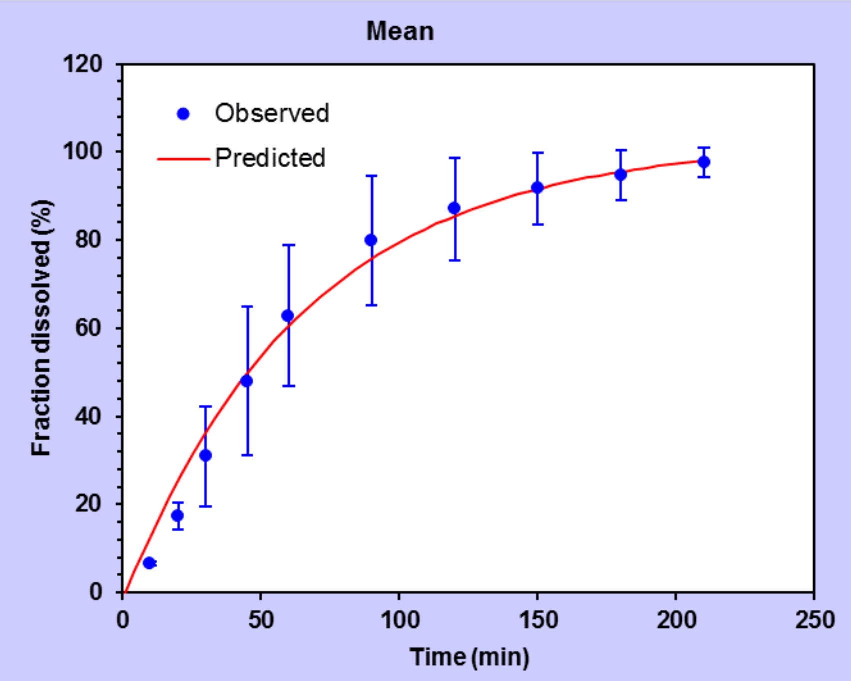

Supplementary materials – Model fitting summary – Parteck® M 200 (modelling the entire relevant dissolution data)

Graphical abstract of model fit presented as the fraction % of released carvedilol per tested tablet:

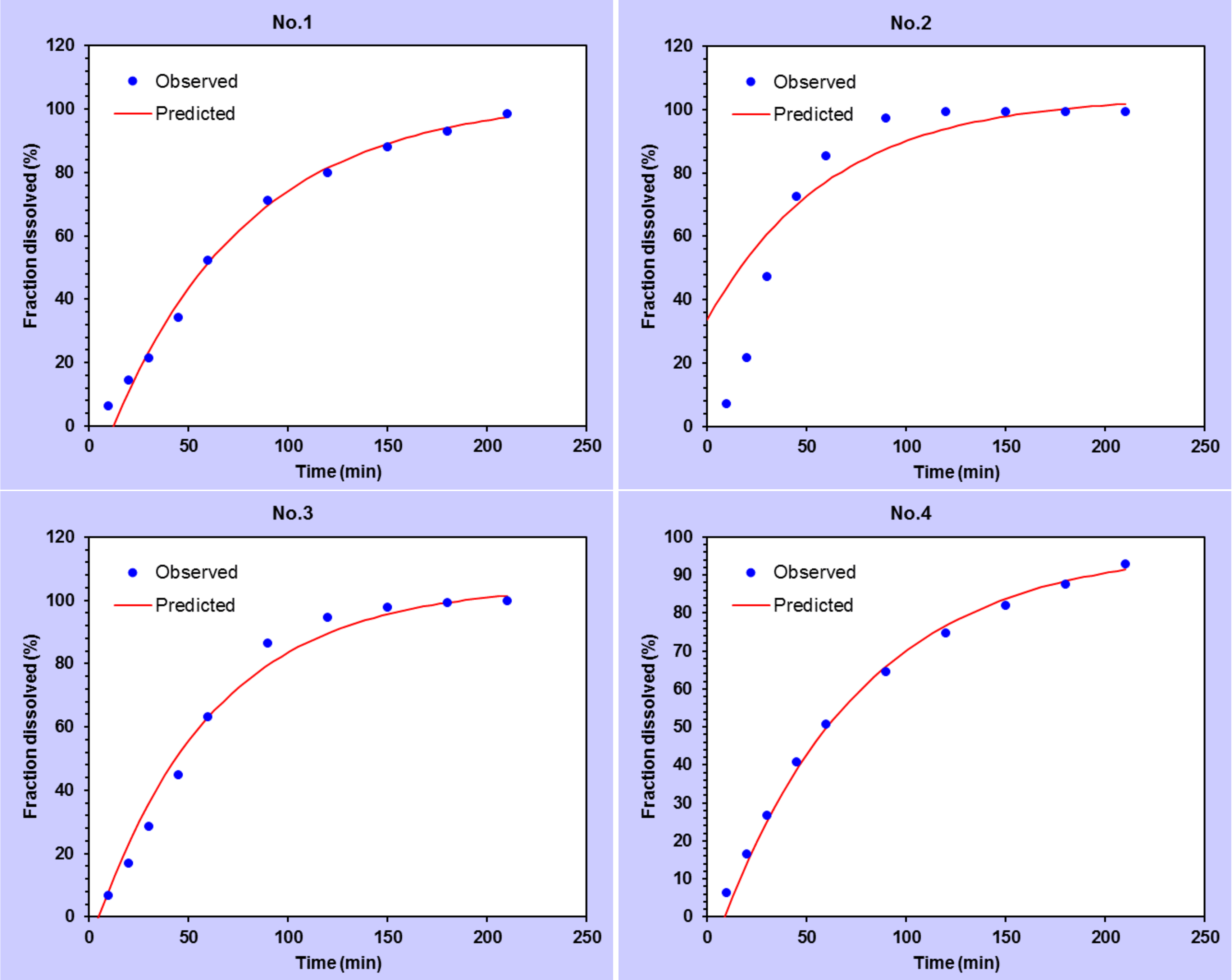

Supplementary materials – Model fitting summary – Parteck® M 200 (modelling the entire relevant dissolution data)

Model: **Higuchi**

Model equation: $F = k_H \cdot t^{0.5}$

Fitted model parameters per tested tablet (N = 4) with statistics – mean, standard deviation (SD), and relative standard deviation expressed in % (RSD%) (output from DDSolver):

Parameter	No.1	No.2	No.3	No.4	Mean	SD	RSD(%)
k_H	6.704	8.215	7.515	6.393	7.207	0.822	11.403

Number of dissolution data points (N), degrees of freedom (df), and selected goodness of fit criteria – Pearson correlation coefficient (R), coefficient of determination (R^2), adjusted coefficient of determination ($R^2_{adjusted}$), and residual sum of squares (RSS) (manual calculation in MS Excel):

Parameter	No.1	No.2	No.3	No.4
N	10	10	10	10
df	9	9	9	9
R	0.987646181	0.889514017	0.963200744	0.991540927
R^2	0.975444979	0.791235186	0.927755672	0.98315341
$R^2_{adjusted}$	0.975444979	0.791235186	0.927755672	0.98315341
RSS	968.1355529	2334.033585	1281.530235	470.823856

Graphical abstract of model fit presented as mean ± 1 SD of the fraction % of released carvedilol:

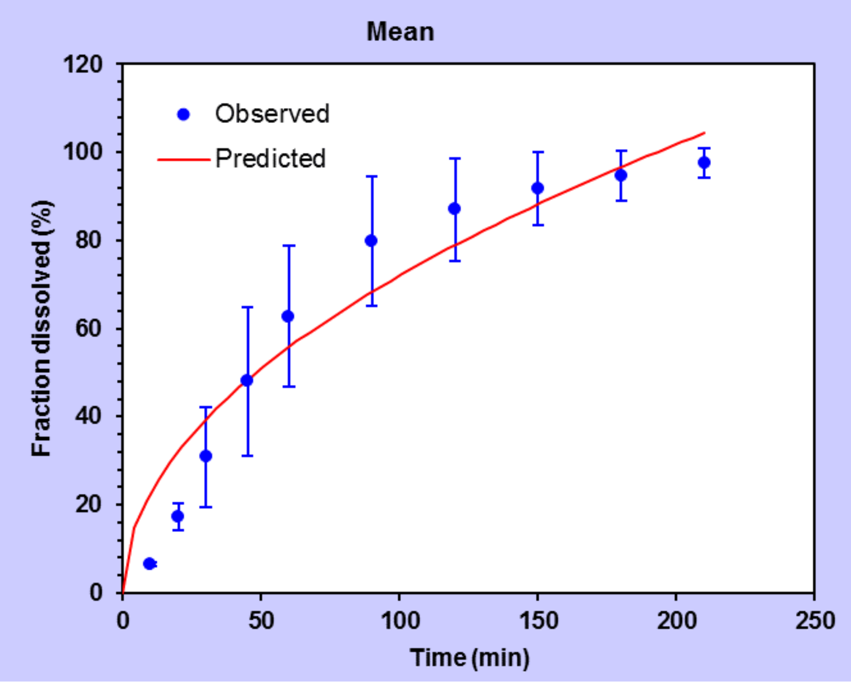

Supplementary materials – Model fitting summary – Parteck® M 200 (modelling the entire relevant dissolution data)

Graphical abstract of model fit presented as the fraction % of released carvedilol per tested tablet:

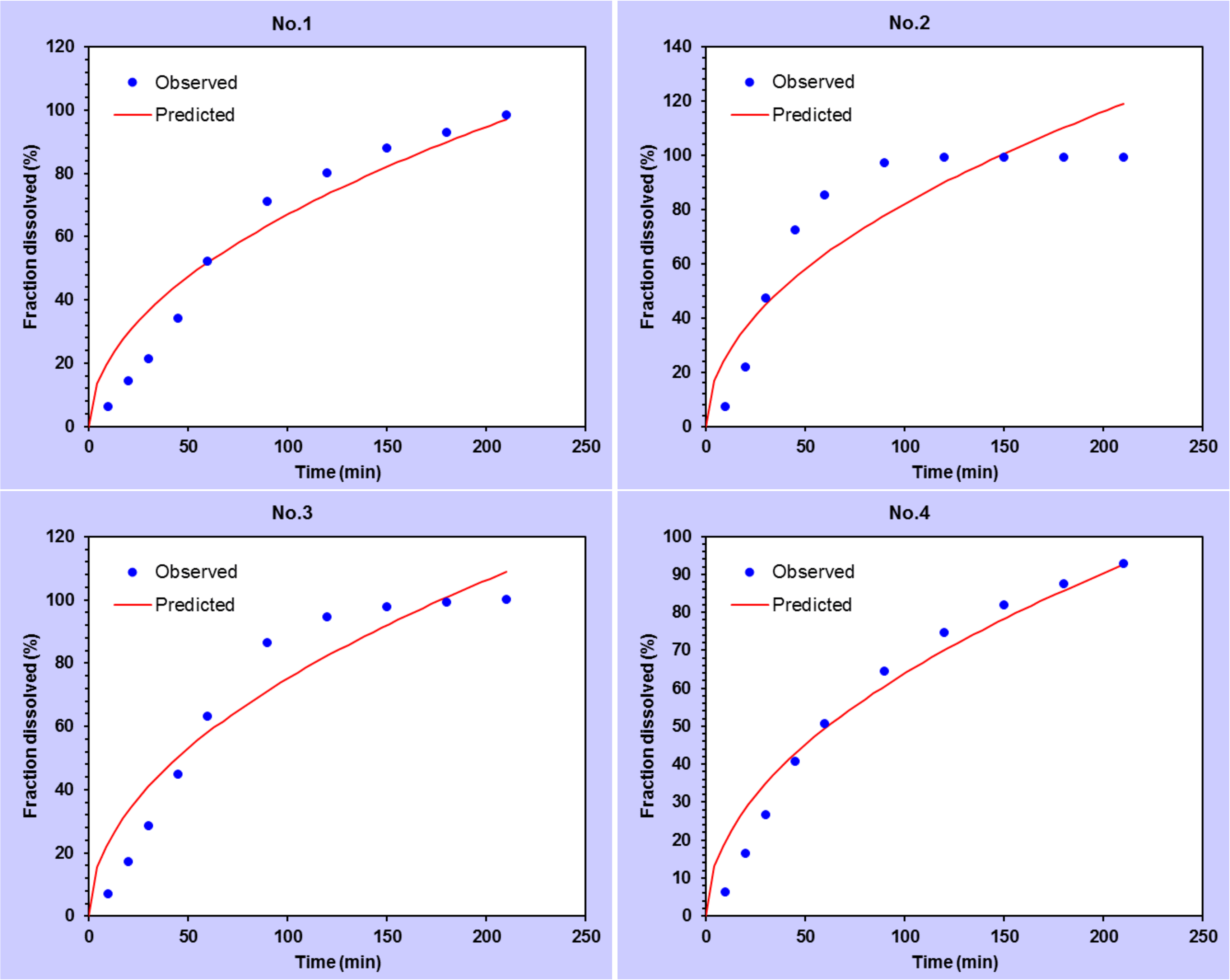

Supplementary materials – Model fitting summary – Parteck® M 200 (modelling the entire relevant dissolution data)

Model: **Higuchi with T_{lag}**

Model equation: $F = k_H \cdot (t - T_{lag})^{0.5}$

Fitted model parameters per tested tablet (N = 4) with statistics – mean, standard deviation (SD), and relative standard deviation expressed in % (RSD%) (output from DDSolver):

Parameter	No.1	No.2	No.3	No.4	Mean	SD	RSD(%)
k_H	7.246	7.059	7.568	6.704	7.144	0.361	5.053
T_{lag}	16.993	-37.107	-0.955	10.880	-2.547	24.215	-950.662

Number of dissolution data points (N), degrees of freedom (df), and selected goodness of fit criteria – Pearson correlation coefficient (R), coefficient of determination (R^2), adjusted coefficient of determination ($R^2_{adjusted}$), and residual sum of squares (RSS) (manual calculation in MS Excel):

Parameter	No.1	No.2	No.3	No.4
N	10	10	10	10
df	8	8	8	8
R	0.990377339	0.857579409	0.962578579	0.993728942
R^2	0.980847273	0.735442442	0.926557521	0.987497209
$R^2_{adjusted}$	0.978453182	0.702372748	0.917377211	0.98593436
RSS	256.1749804	3748.852086	1356.224317	130.5645348

Graphical abstract of model fit presented as mean ± 1 SD of the fraction % of released carvedilol:

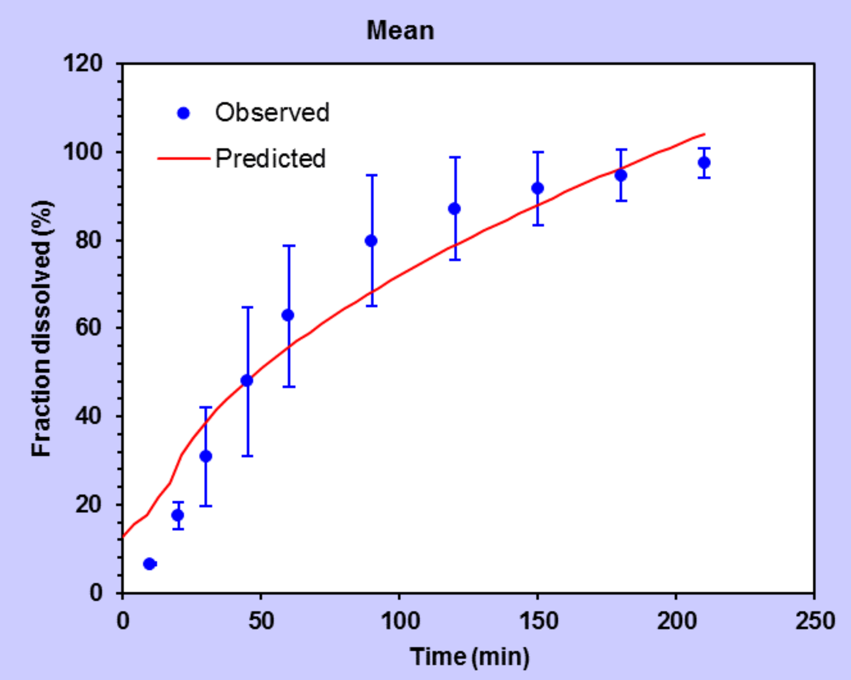

Supplementary materials – Model fitting summary – Parteck® M 200 (modelling the entire relevant dissolution data)

Graphical abstract of model fit presented as the fraction % of released carvedilol per tested tablet:

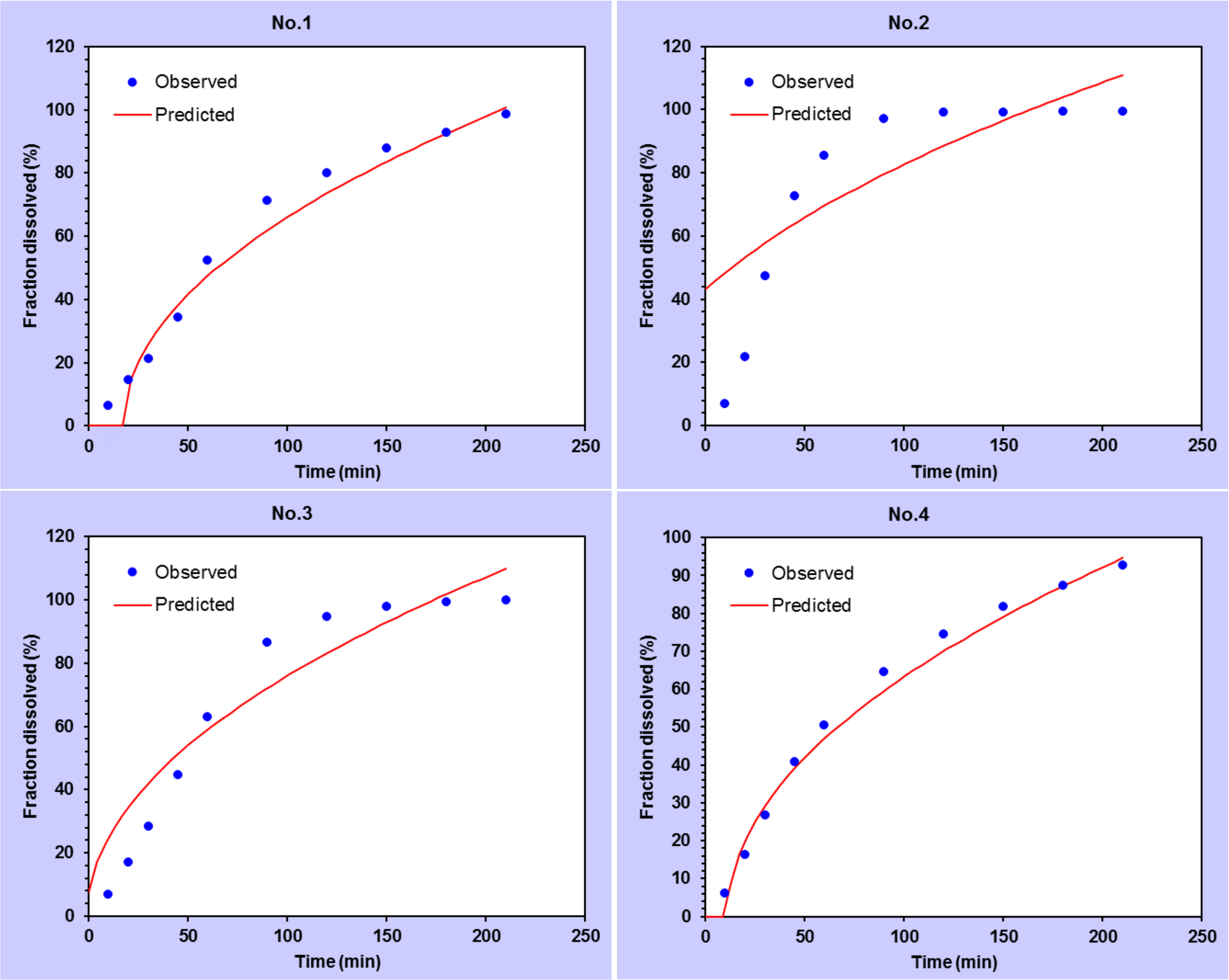

Supplementary materials – Model fitting summary – Parteck® M 200 (modelling the entire relevant dissolution data)

Model: **Higuchi with F_0**

Model equation: $F = F_0 + k_H \cdot t^{0.5}$

Fitted model parameters per tested tablet (N = 4) with statistics – mean, standard deviation (SD), and relative standard deviation expressed in % (RSD%) (output from DDSolver):

Parameter	No.1	No.2	No.3	No.4	Mean	SD	RSD(%)
k_H	8.783	8.001	9.080	7.813	8.419	0.609	7.230
F_0	-21.575	2.214	-16.243	-14.733	-12.584	10.293	-81.788

Number of dissolution data points (N), degrees of freedom (df), and selected goodness of fit criteria – Pearson correlation coefficient (R), coefficient of determination (R^2), adjusted coefficient of determination ($R^2_{adjusted}$), and residual sum of squares (RSS) (manual calculation in MS Excel):

Parameter	No.1	No.2	No.3	No.4
N	10	10	10	10
df	8	8	8	8
R	0.987646181	0.889514017	0.963200744	0.991540927
R^2	0.975444979	0.791235186	0.927755672	0.98315341
$R^2_{adjusted}$	0.972375601	0.765139585	0.918725131	0.981047586
RSS	267.4469554	2326.657815	884.3492444	144.0595132

Graphical abstract of model fit presented as mean ± 1 SD of the fraction % of released carvedilol:

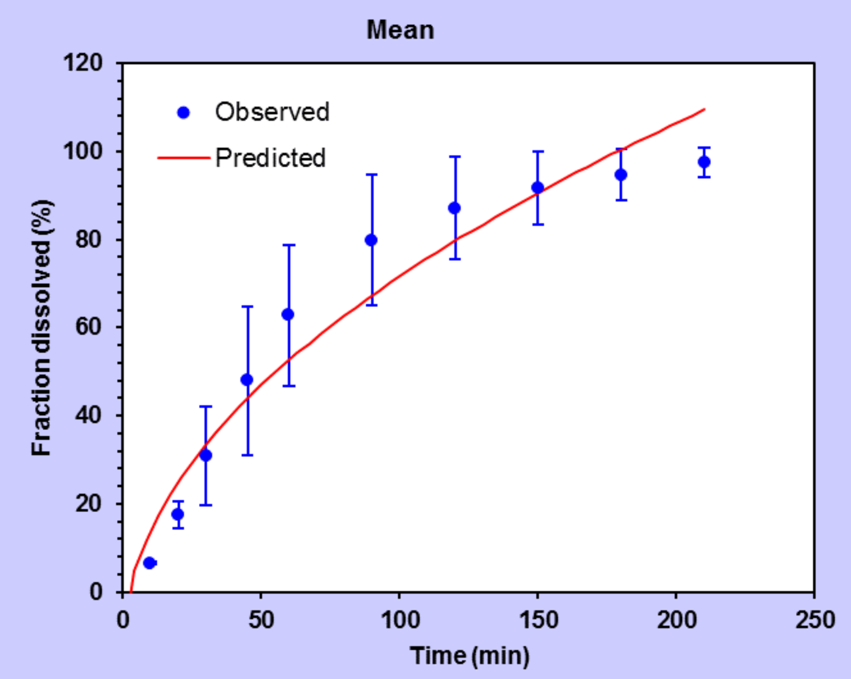

Supplementary materials – Model fitting summary – Parteck® M 200 (modelling the entire relevant dissolution data)

Graphical abstract of model fit presented as the fraction % of released carvedilol per tested tablet:

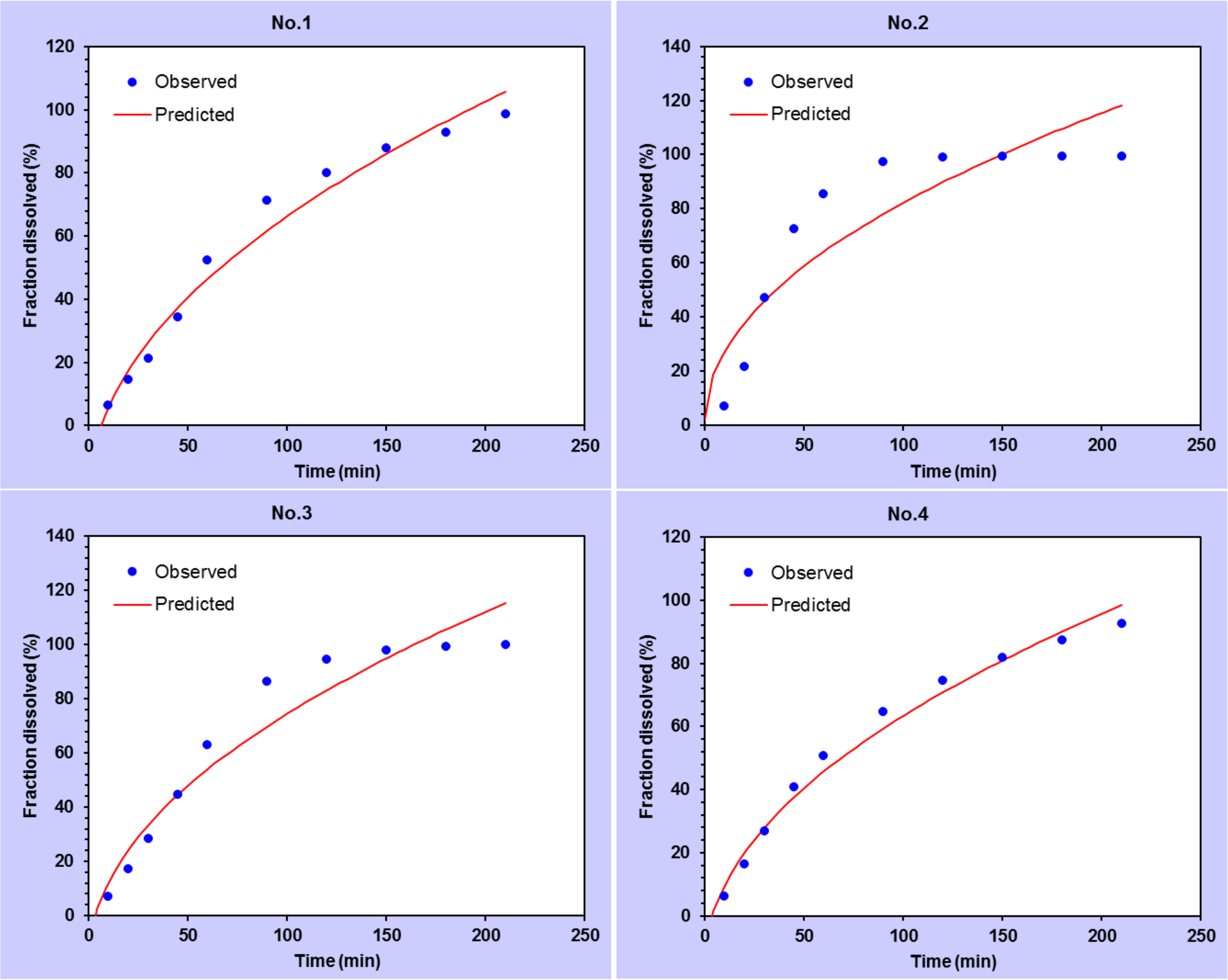

Supplementary materials – Model fitting summary – Parteck® M 200 (modelling the entire relevant dissolution data)

Model: **Korsmeyer–Peppas**

Model equation: $F = k_{KP} \cdot t^n$

Fitted model parameters per tested tablet (N = 4) with statistics – mean, standard deviation (SD), and relative standard deviation expressed in % (RSD%) (output from DDSolver):

Parameter	No.1	No.2	No.3	No.4	Mean	SD	RSD(%)
k_{KP}	1.269	2.180	1.713	1.286	1.612	0.430	26.699
n	0.856	0.791	0.824	0.842	0.828	0.028	3.406

Number of dissolution data points (N), degrees of freedom (df), and selected goodness of fit criteria – Pearson correlation coefficient (R), coefficient of determination (R^2), adjusted coefficient of determination ($R^2_{adjusted}$), and residual sum of squares (RSS) (manual calculation in MS Excel):

Parameter	No.1	No.2	No.3	No.4
N	10	10	10	10
df	8	8	8	8
R	0.96764012	0.839742309	0.931526834	0.969897727
R^2	0.936327403	0.705167146	0.867742242	0.9407016
$R^2_{adjusted}$	0.928368328	0.668313039	0.851210022	0.9332893
RSS	1150.665647	6275.762064	2825.314686	1066.608863

Graphical abstract of model fit presented as mean ± 1 SD of the fraction % of released carvedilol:

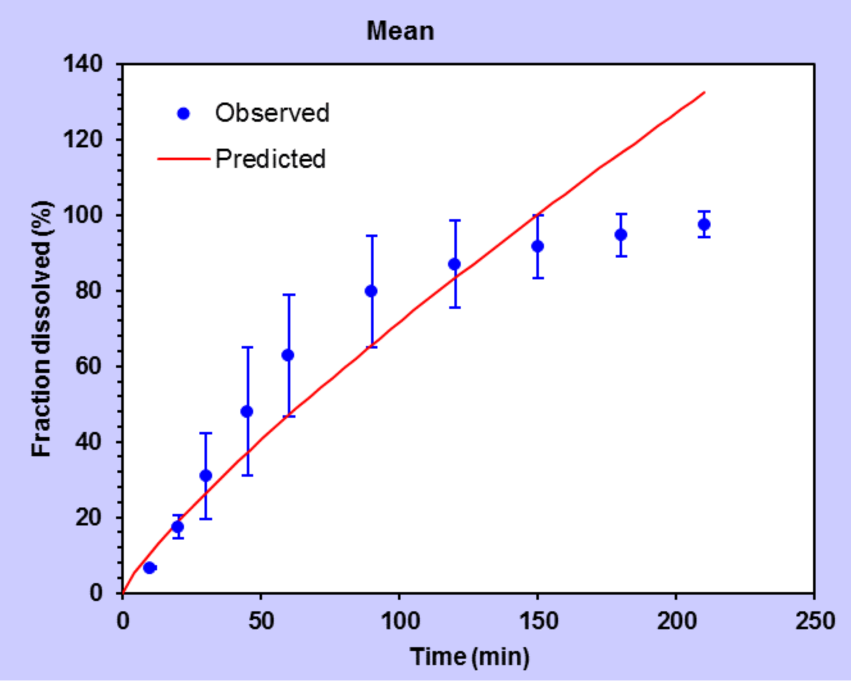

Supplementary materials – Model fitting summary – Parteck® M 200 (modelling the entire relevant dissolution data)

Graphical abstract of model fit presented as the fraction % of released carvedilol per tested tablet:

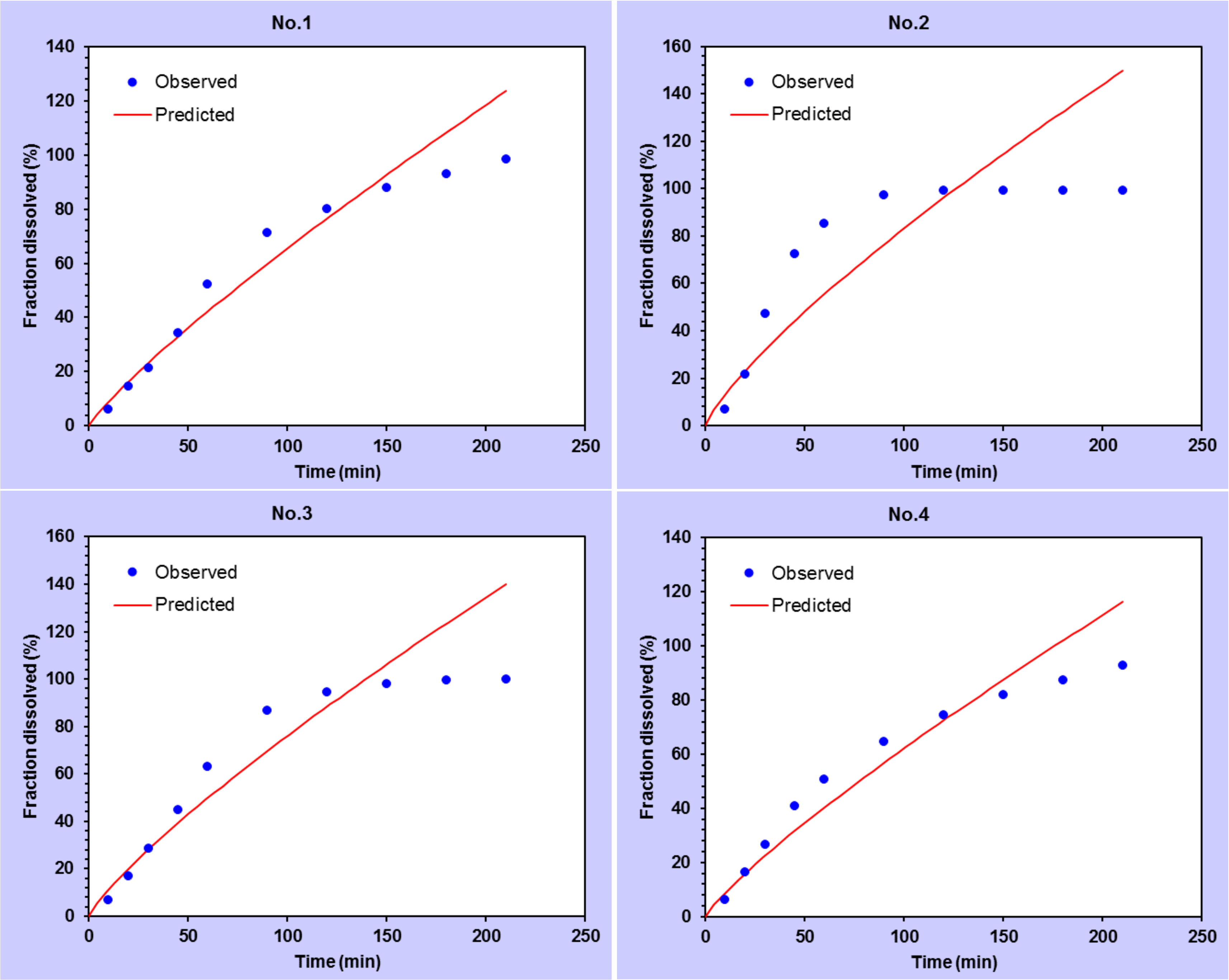

Supplementary materials – Model fitting summary – Parteck® M 200 (modelling the entire relevant dissolution data)

Model: **Korsmeyer–Peppas with T_{lag}**

Model equation: $F = k_{KP} \cdot \left(t - T_{lag}\right)^n$

Fitted model parameters per tested tablet (N = 4) with statistics – mean, standard deviation (SD), and relative standard deviation expressed in % (RSD%) (output from DDSolver):

Parameter	No.1	No.2	No.3	No.4	Mean	SD	RSD(%)
k_{KP}	1.597	3.259	2.079	2.334	2.317	0.698	30.129
n	0.810	0.714	0.783	0.696	0.751	0.054	7.240
T_{lag}	4.000	4.000	4.000	4.875	4.219	0.438	10.370

Number of dissolution data points (N), degrees of freedom (df), and selected goodness of fit criteria – Pearson correlation coefficient (R), coefficient of determination (R^2), adjusted coefficient of determination ($R^2_{adjusted}$), and residual sum of squares (RSS) (manual calculation in MS Excel):

Parameter	No.1	No.2	No.3	No.4
N	10	10	10	10
df	7	7	7	7
R	0.971944302	0.857379701	0.937772213	0.982609358
R^2	0.944675726	0.735099951	0.879416723	0.96552115
$R^2_{adjusted}$	0.928868791	0.659414223	0.844964359	0.95567005
RSS	891.0583931	5494.326904	2315.578127	644.7433034

Graphical abstract of model fit presented as mean ± 1 SD of the fraction % of released carvedilol:

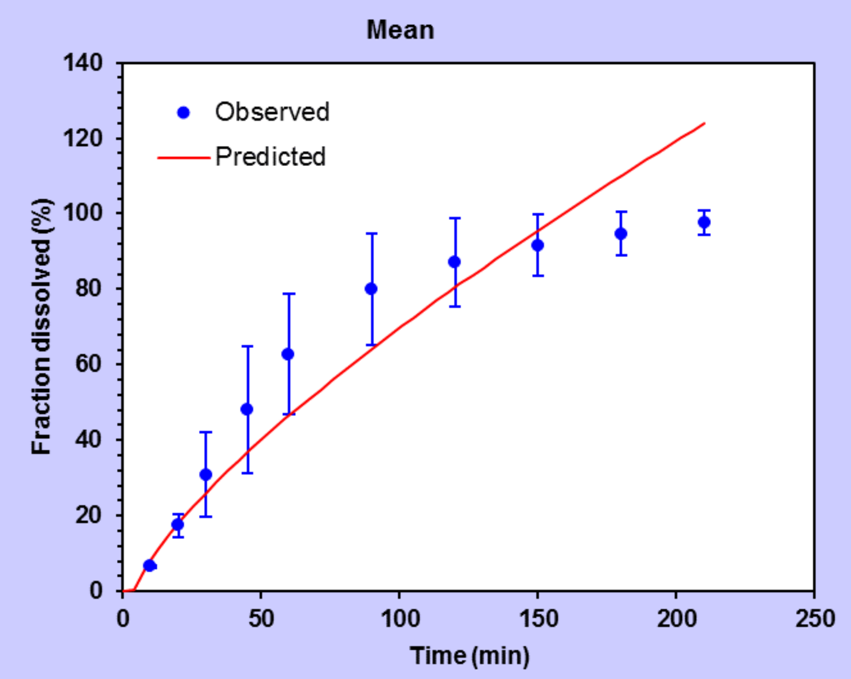

Supplementary materials – Model fitting summary – Parteck® M 200 (modelling the entire relevant dissolution data)

Graphical abstract of model fit presented as the fraction % of released carvedilol per tested tablet:

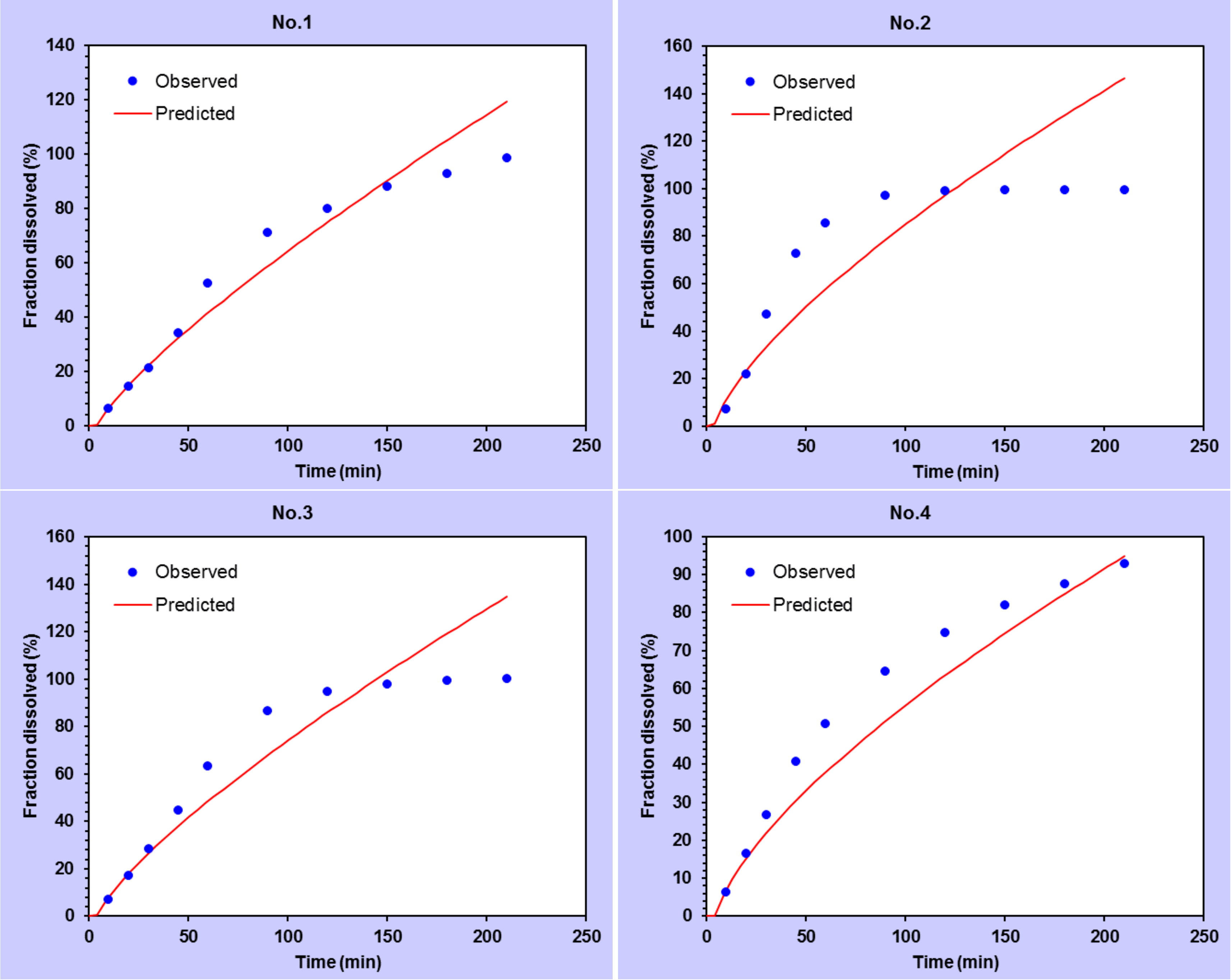

Supplementary materials – Model fitting summary – Parteck® M 200 (modelling the entire relevant dissolution data)

Model: **Korsmeyer–Peppas with F_0**

Model equation: $F = F_0 + k_{KP} \cdot t^n$

Fitted model parameters per tested tablet (N = 4) with statistics – mean, standard deviation (SD), and relative standard deviation expressed in % (RSD%) (output from DDSolver):

Parameter	No.1	No.2	No.3	No.4	Mean	SD	RSD(%)
k_{KP}	0.502	1.243	0.694	0.707	0.787	0.319	40.498
n	1.039	0.904	1.000	0.962	0.976	0.058	5.909
F_0	2.480	2.800	2.720	2.480	2.620	0.165	6.298

Number of dissolution data points (N), degrees of freedom (df), and selected goodness of fit criteria – Pearson correlation coefficient (R), coefficient of determination (R^2), adjusted coefficient of determination ($R^2_{adjusted}$), and residual sum of squares (RSS) (manual calculation in MS Excel):

Parameter	No.1	No.2	No.3	No.4
N	10	10	10	10
df	7	7	7	7
R	0.952864558	0.819965549	0.910829832	0.960108214
R^2	0.907950865	0.672343501	0.829610984	0.921807783
$R^2_{adjusted}$	0.881651113	0.578727359	0.780928407	0.89946715
RSS	2108.105777	8302.842633	4300.355002	1743.002616

Graphical abstract of model fit presented as mean ± 1 SD of the fraction % of released carvedilol:

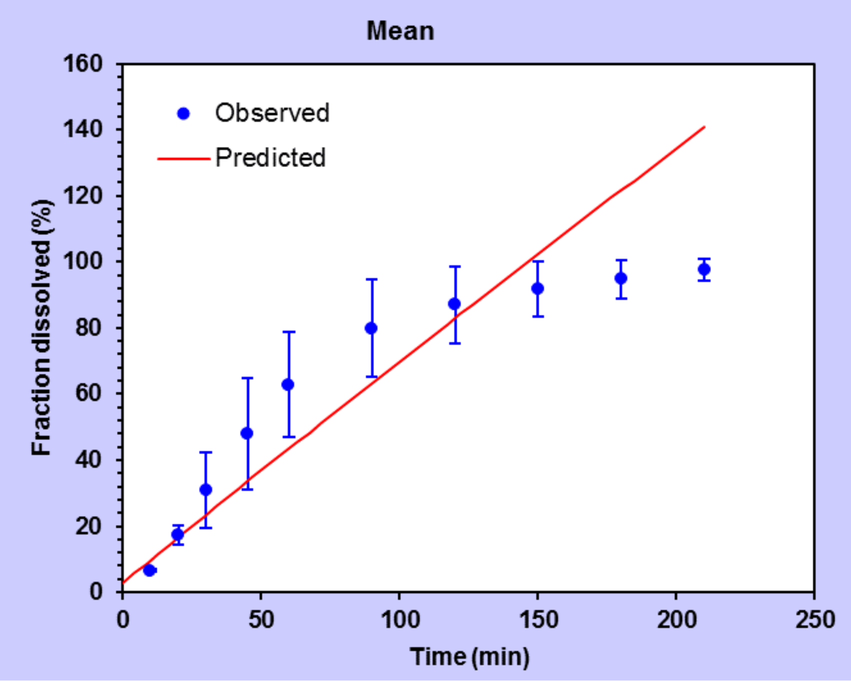

Supplementary materials – Model fitting summary – Parteck® M 200 (modelling the entire relevant dissolution data)

Graphical abstract of model fit presented as the fraction % of released carvedilol per tested tablet:

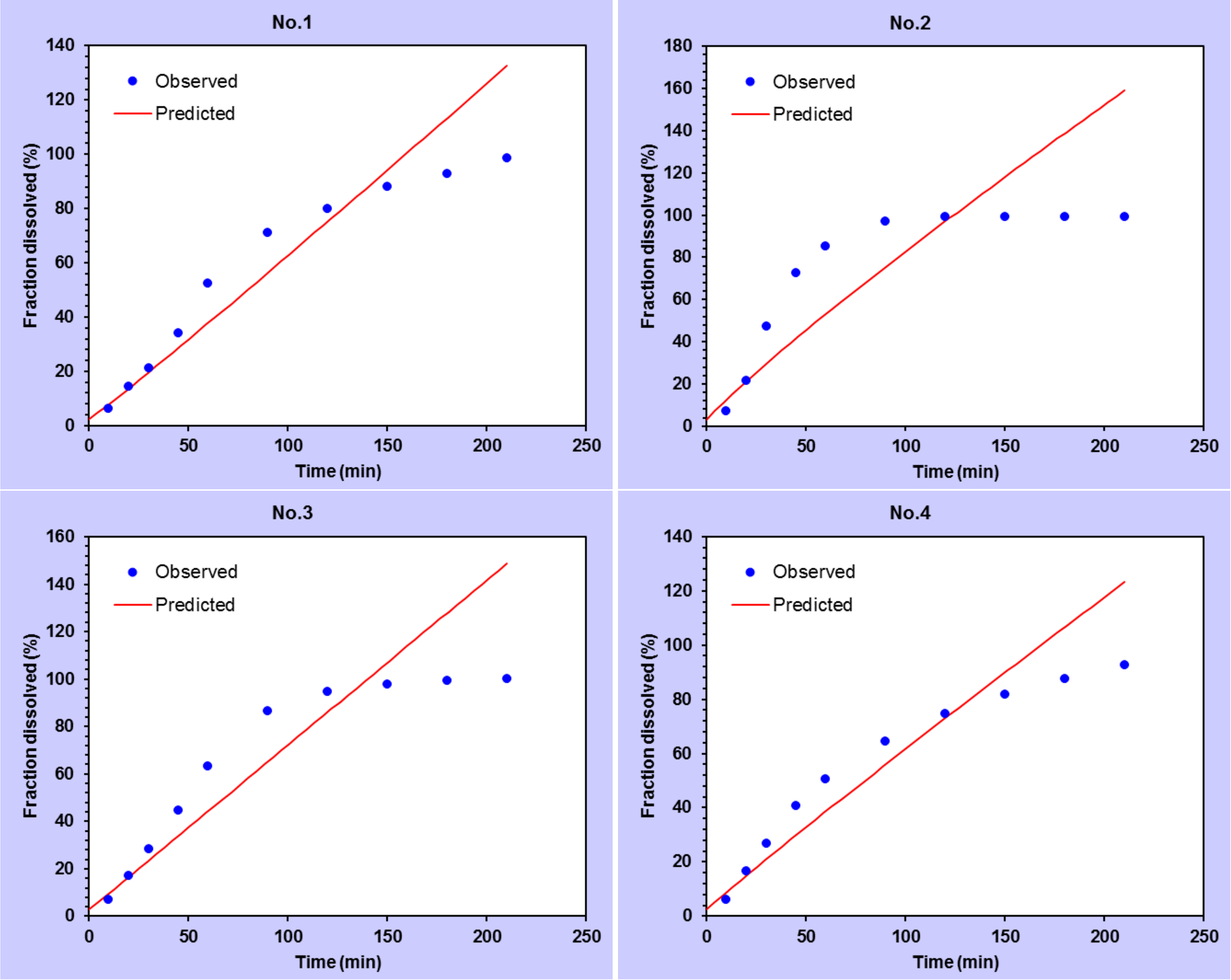

Supplementary materials – Model fitting summary – Parteck® M 200 (modelling the entire relevant dissolution data)

Model: **Hixson–Crowell**

Model equation: $F = 100 \cdot [1 - (1 - k_{HC} \cdot t)^3]$

Fitted model parameters per tested tablet (N = 4) with statistics – mean, standard deviation (SD), and relative standard deviation expressed in % (RSD%) (output from DDSolver):

Parameter	No.1	No.2	No.3	No.4	Mean	SD	RSD(%)
k_{HC}	0.003	0.008	0.005	0.003	0.005	0.002	44.686

Number of dissolution data points (N), degrees of freedom (df), and selected goodness of fit criteria – Pearson correlation coefficient (R), coefficient of determination (R^2), adjusted coefficient of determination ($R^2_{adjusted}$), and residual sum of squares (RSS) (manual calculation in MS Excel):

Parameter	No.1	No.2	No.3	No.4
N	10	10	10	10
df	9	9	9	9
R	0.996751203	0.975537144	0.996415492	0.994088759
R^2	0.993512961	0.951672719	0.992843833	0.988212461
$R^2_{adjusted}$	0.993512961	0.951672719	0.992843833	0.988212461
RSS	136.2595047	945.1894022	251.4258936	141.0506801

Graphical abstract of model fit presented as mean ± 1 SD of the fraction % of released carvedilol:

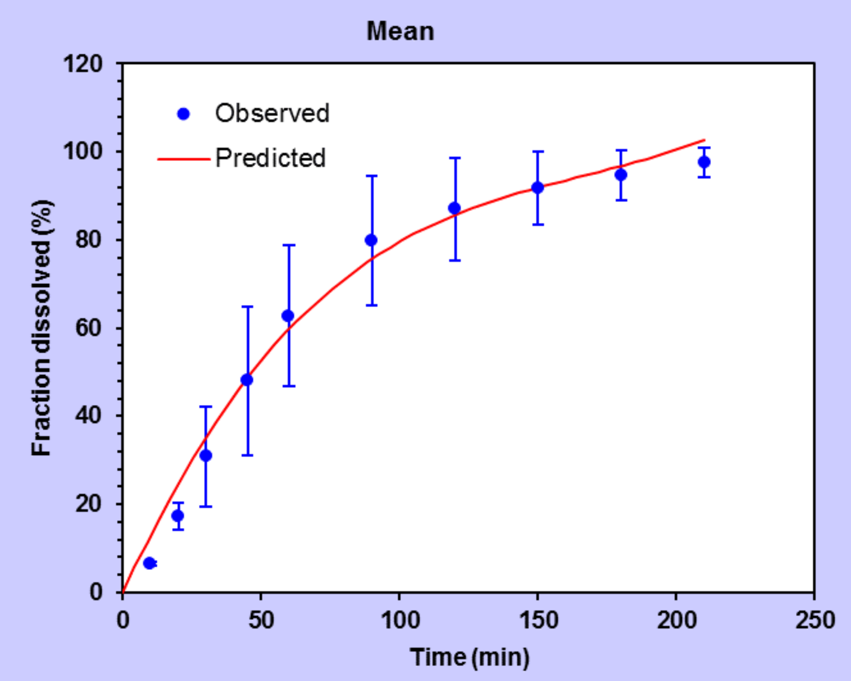

Supplementary materials – Model fitting summary – Parteck® M 200 (modelling the entire relevant dissolution data)

Graphical abstract of model fit presented as the fraction % of released carvedilol per tested tablet:

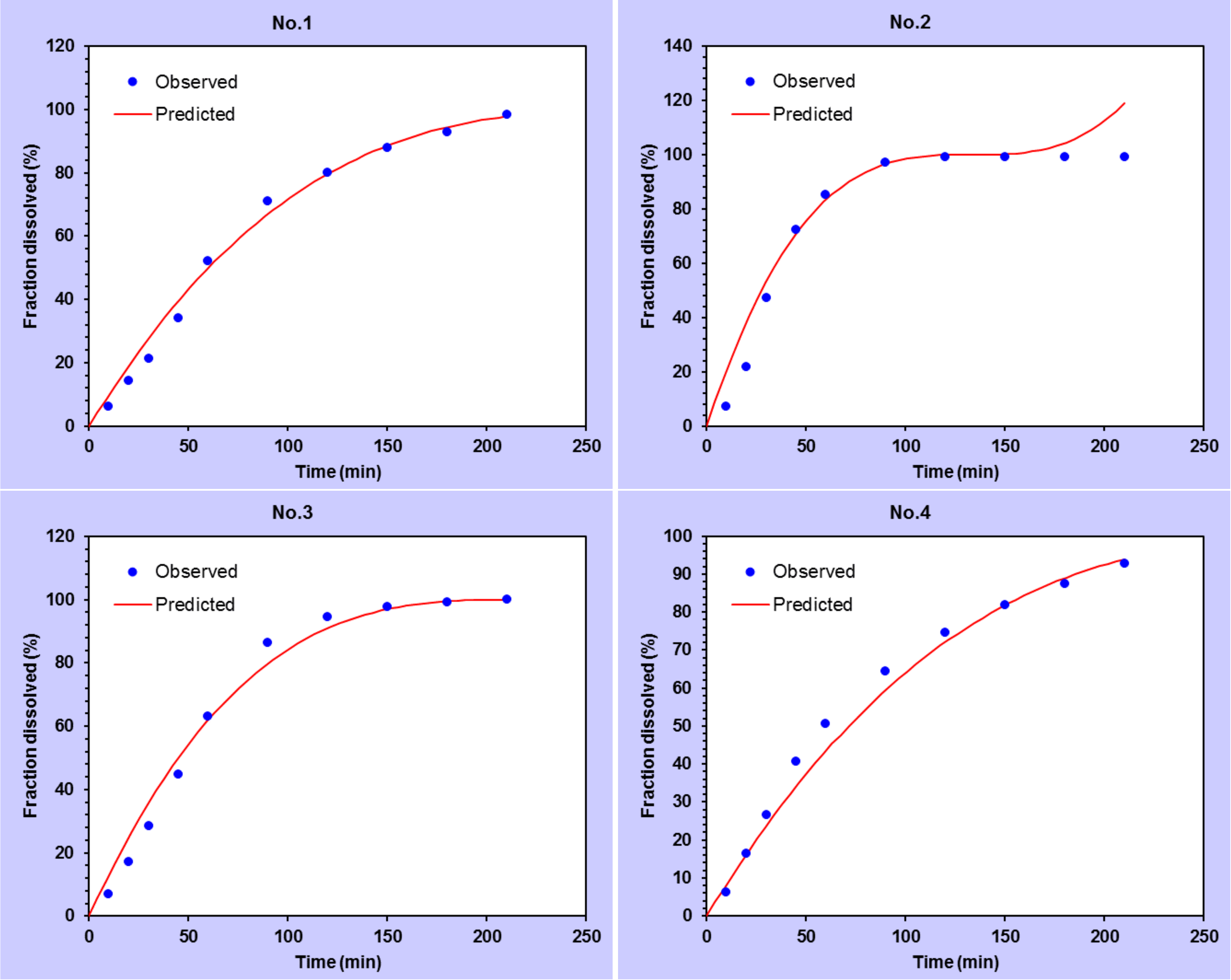

Supplementary materials – Model fitting summary – Parteck® M 200 (modelling the entire relevant dissolution data)

Model: **Hixson–Crowell with T_{lag}**

Model equation: $F = 100 \cdot \left\{1 - \left[1 - k_{HC} \cdot \left(t - T_{lag}\right)\right]^3\right\}$

Fitted model parameters per tested tablet (N = 4) with statistics – mean, standard deviation (SD), and relative standard deviation expressed in % (RSD%) (output from DDSolver):

Parameter	No.1	No.2	No.3	No.4	Mean	SD	RSD(%)
k_{HC}	0.004	0.004	0.005	0.003	0.004	0.001	21.650
T_{lag}	4.178	-30.301	1.544	-7.928	-8.127	15.670	-192.821

Number of dissolution data points (N), degrees of freedom (df), and selected goodness of fit criteria – Pearson correlation coefficient (R), coefficient of determination (R^2), adjusted coefficient of determination ($R^2_{adjusted}$), and residual sum of squares (RSS) (manual calculation in MS Excel):

Parameter	No.1	No.2	No.3	No.4
N	10	10	10	10
df	8	8	8	8
R	0.996922063	0.95477556	0.996420902	0.99331212
R^2	0.9938536	0.91159637	0.992854615	0.986668967
$R^2_{adjusted}$	0.9930853	0.900545917	0.991961441	0.985002588
RSS	70.38540993	2396.73536	188.1164153	137.0869717

Graphical abstract of model fit presented as mean ± 1 SD of the fraction % of released carvedilol:

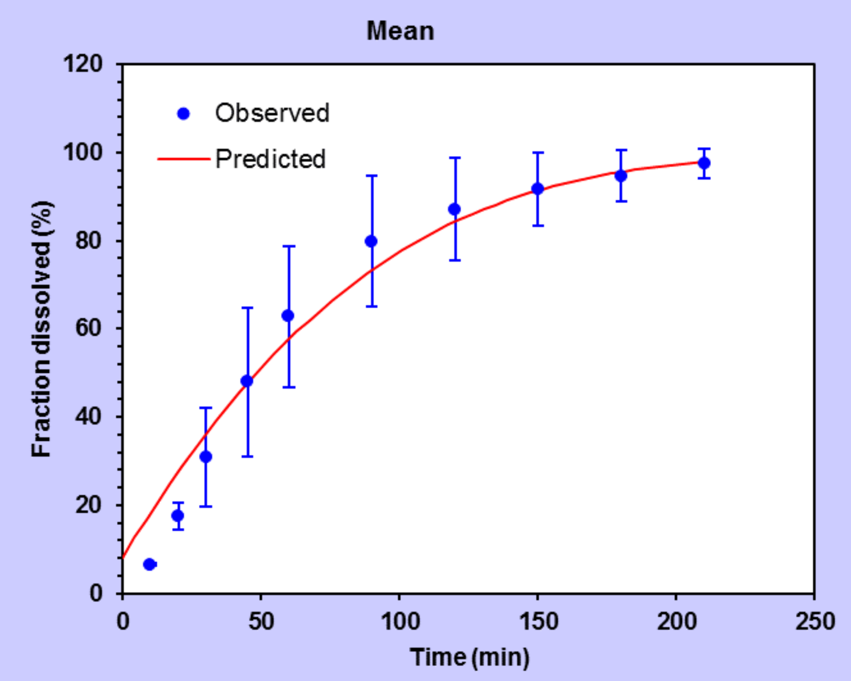

Supplementary materials – Model fitting summary – Parteck® M 200 (modelling the entire relevant dissolution data)

Graphical abstract of model fit presented as the fraction % of released carvedilol per tested tablet:

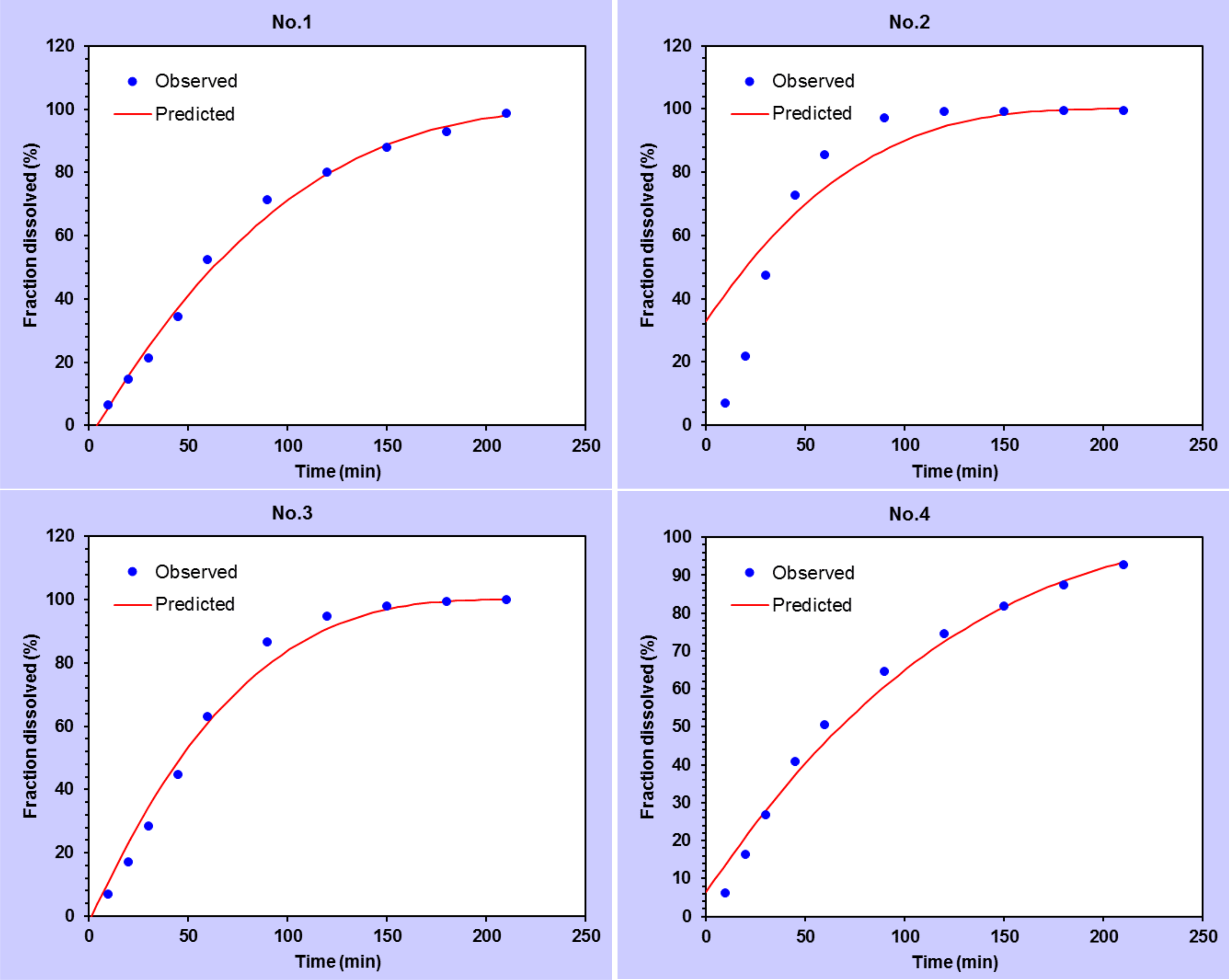

Supplementary materials – Model fitting summary – Parteck® M 200 (modelling the entire relevant dissolution data)

Model: **Hopfenberg**

Model equation: $F = 100 \cdot [1 - (1 - k_{HB} \cdot t)^n]$

Fitted model parameters per tested tablet (N = 4) with statistics – mean, standard deviation (SD), and relative standard deviation expressed in % (RSD%) (output from DDSolver):

Parameter	No.1	No.2	No.3	No.4	Mean	SD	RSD(%)
k_{HB}	0.003	0.008	0.005	0.003	0.005	0.002	44.686
n	3.000	3.000	3.000	3.000	3.000	0.000	0.000

Number of dissolution data points (N), degrees of freedom (df), and selected goodness of fit criteria – Pearson correlation coefficient (R), coefficient of determination (R^2), adjusted coefficient of determination ($R^2_{adjusted}$), and residual sum of squares (RSS) (manual calculation in MS Excel):

Parameter	No.1	No.2	No.3	No.4
N	10	10	10	10
df	8	8	8	8
R	0.996751203	0.995095738	0.996415492	0.994088759
R^2	0.993512961	0.990215528	0.992843833	0.988212461
$R^2_{adjusted}$	0.992702081	0.988992469	0.991949312	0.986739019
RSS	136.2595047	529.1732966	251.4258936	141.0506801

Graphical abstract of model fit presented as mean ± 1 SD of the fraction % of released carvedilol:

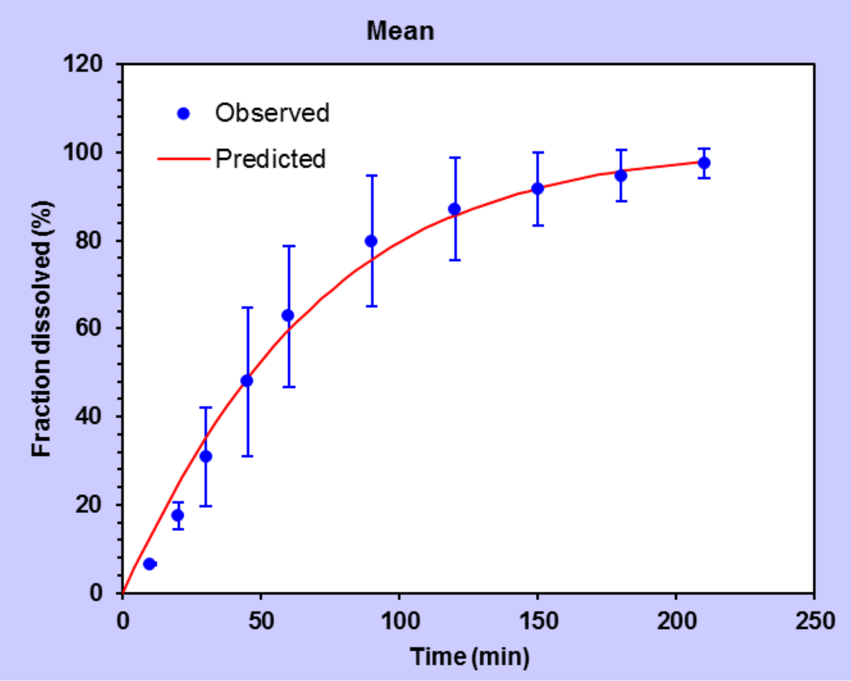

Supplementary materials – Model fitting summary – Parteck® M 200 (modelling the entire relevant dissolution data)

Graphical abstract of model fit presented as the fraction % of released carvedilol per tested tablet:

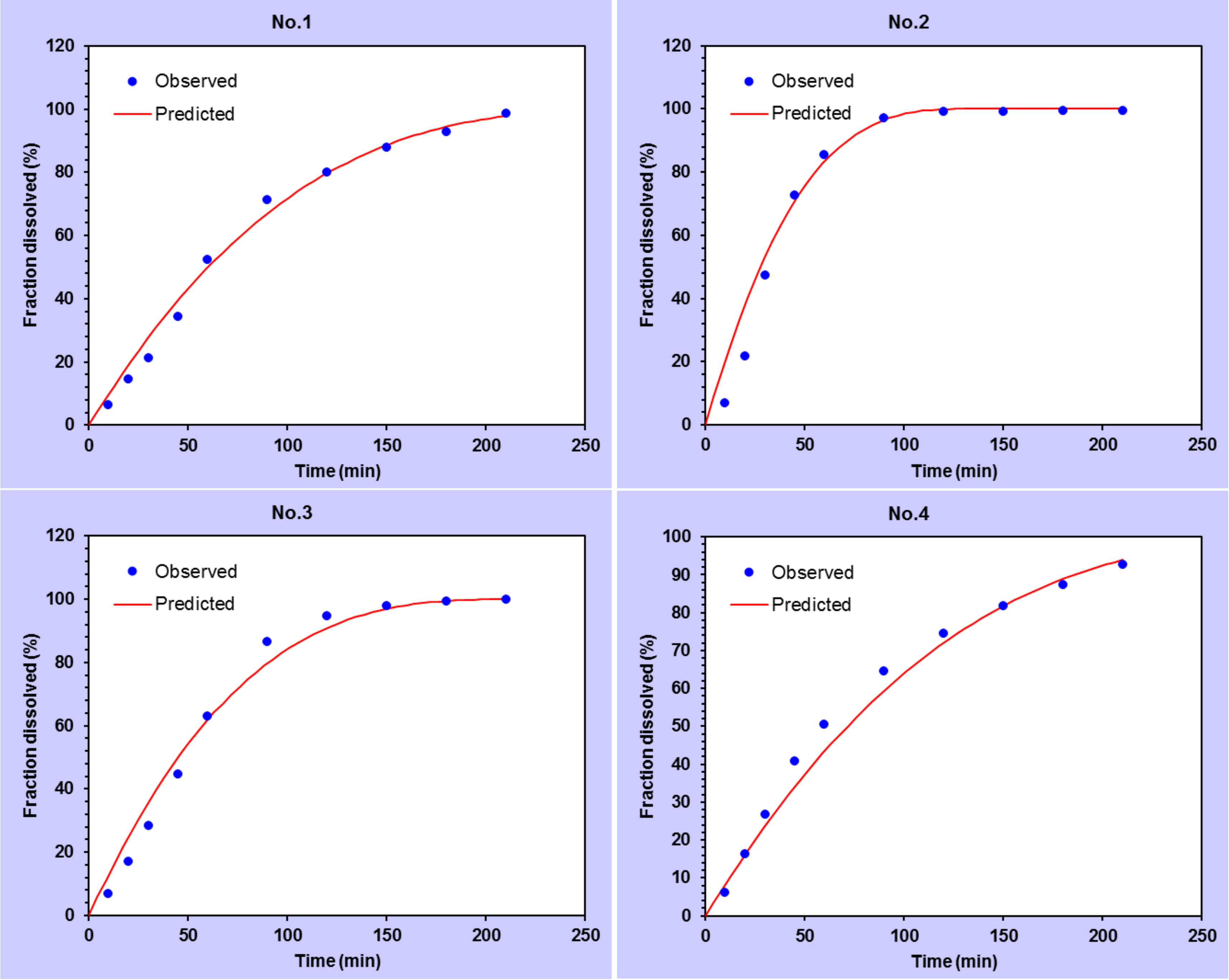

Supplementary materials – Model fitting summary – Parteck® M 200 (modelling the entire relevant dissolution data)

Model: **Hopfenberg with T_{lag}**

Model equation: $F = 100 \cdot \{1 - [1 - k_{HB} \cdot (t - T_{lag})]^n\}$

Fitted model parameters per tested tablet (N = 4) with statistics – mean, standard deviation (SD), and relative standard deviation expressed in % (RSD%) (output from DDSolver):

Parameter	No.1	No.2	No.3	No.4	Mean	SD	RSD(%)
k_{HB}	0.004	0.004	0.005	0.003	0.004	0.001	21.650
n	3.000	3.000	3.000	3.000	3.000	0.000	0.000
T_{lag}	4.178	-30.301	1.544	-7.928	-8.127	15.670	-192.821

Number of dissolution data points (N), degrees of freedom (df), and selected goodness of fit criteria – Pearson correlation coefficient (R), coefficient of determination (R^2), adjusted coefficient of determination ($R^2_{adjusted}$), and residual sum of squares (RSS) (manual calculation in MS Excel):

Parameter	No.1	No.2	No.3	No.4
N	10	10	10	10
df	7	7	7	7
R	0.996922063	0.95477556	0.996420902	0.99331212
R^2	0.9938536	0.91159637	0.992854615	0.986668967
$R^2_{adjusted}$	0.992097486	0.886338191	0.990813076	0.9828601
RSS	70.38540993	2396.73536	188.1164153	137.0869717

Graphical abstract of model fit presented as mean ± 1 SD of the fraction % of released carvedilol:

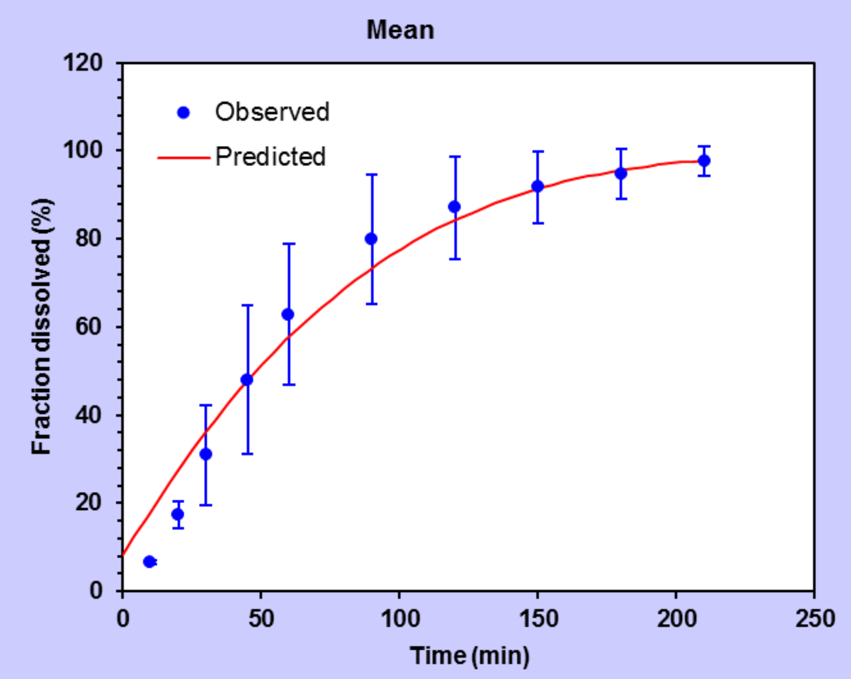

Supplementary materials – Model fitting summary – Parteck® M 200 (modelling the entire relevant dissolution data)

Graphical abstract of model fit presented as the fraction % of released carvedilol per tested tablet:

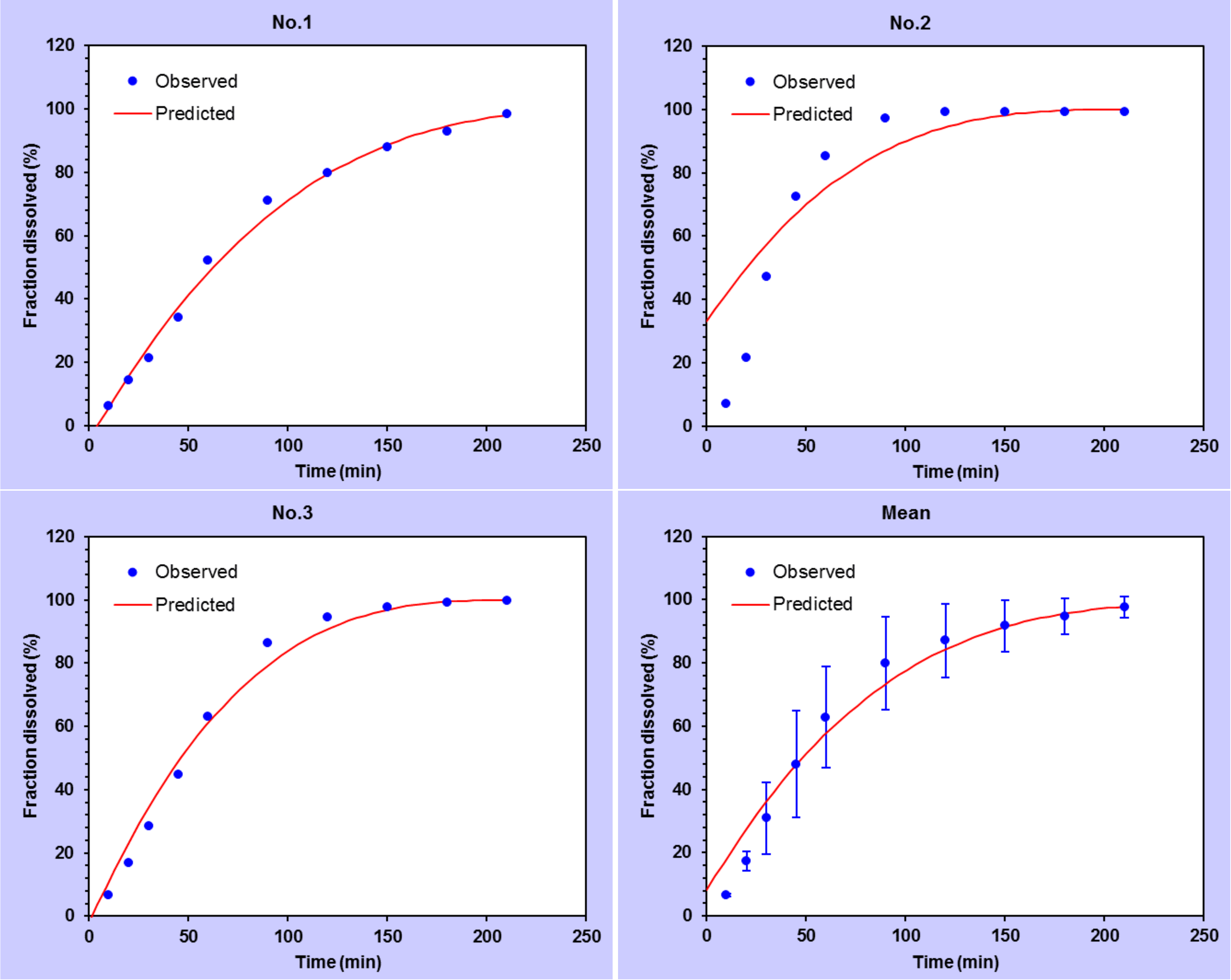

Supplementary materials – Model fitting summary – Parteck® M 200 (modelling the entire relevant dissolution data)

Model: **Baker–Lonsdale**

Model equation: $\frac{3}{2}\cdot\left[1-\left(1-\frac{F}{100}\right)^{\frac{2}{3}}\right]-\frac{F}{100}=k_{BL}\cdot t$

Fitted model parameters per tested tablet (N = 4) with statistics – mean, standard deviation (SD), and relative standard deviation expressed in % (RSD%) (output from DDSolver):

Parameter	No.1	No.2	No.3	No.4	Mean	SD	RSD(%)
k_{BL}	0.00104767	0.00000000	0.00000000	0.00156574	0.000653353	0.000783513	119.9217974

Number of dissolution data points (N), degrees of freedom (df), and selected goodness of fit criteria – Pearson correlation coefficient (R), coefficient of determination (R^2), adjusted coefficient of determination ($R^2_{adjusted}$), and residual sum of squares (RSS) (manual calculation in MS Excel):

Parameter	No.1	No.2	No.3	No.4
N	10	10	10	10
df	9	9	9	9
R	0.99297465	/	0.21871712	0.999144005
R^2	0.985998655	/	0.047837179	0.998288742
$R^2_{adjusted}$	0.985998655	/	0.047837179	0.998288742
RSS	1822.641156	63989.00308	52880.76778	1894.180479

Graphical abstract of model fit presented as mean ± 1 SD of the fraction % of released carvedilol: / (no charts were produced by DDSolver)

Graphical abstract of model fit presented as the fraction % of released carvedilol per tested tablet: / (no charts were produced by DDSolver)

Supplementary materials – Model fitting summary – Parteck® M 200 (modelling the entire relevant dissolution data)

Model: **Baker–Lonsdale with T_{lag}**

Model equation: $\frac{3}{2} \cdot \left[1 - \left(1 - \frac{F}{100}\right)^{\frac{2}{3}}\right] - \frac{F}{100} = k_{BL} \cdot (t - T_{lag})$

Fitted model parameters per tested tablet (N = 4) with statistics – mean, standard deviation (SD), and relative standard deviation expressed in % (RSD%) (output from DDSolver):

Parameter	No.1	No.2	No.3	No.4	Mean	SD	RSD(%)
k_{BL}	0.00209535	0.00000000	0.00000000	0.00156574	0.00091527	0.00107875	117.86163453
T_{lag}	24.37535542	-19.25347139	28.53718362	19.66086232	13.32998249	22.02287247	165.21306375

Number of dissolution data points (N), degrees of freedom (df), and selected goodness of fit criteria – Pearson correlation coefficient (R), coefficient of determination (R^2), adjusted coefficient of determination ($R^2_{adjusted}$), and residual sum of squares (RSS) (manual calculation in MS Excel):

Parameter	No.1	No.2	No.3	No.4
N	10	10	10	10
df	8	8	8	8
R	0.984870637	/	0.741841296	0.993483912
R^2	0.969970172	/	0.550328509	0.987010283
$R^2_{adjusted}$	0.972973154	/	0.595295658	0.988309254
RSS	405.9133914	63989.00308	52883.65173	178.969492

Graphical abstract of model fit presented as mean ± 1 SD of the fraction % of released carvedilol: / (no charts were produced by DDSolver)

Graphical abstract of model fit presented as the fraction % of released carvedilol per tested tablet: / (no charts were produced by DDSolver)

Supplementary materials – Model fitting summary – Parteck® M 200 (modelling the entire relevant dissolution data)

Model: **Makoid–Banakar**

Model equation: $F = k_{MB} \cdot t^n \cdot e^{-k \cdot t}$

Fitted model parameters per tested tablet (N = 4) with statistics – mean, standard deviation (SD), and relative standard deviation expressed in % (RSD%) (output from DDSolver):

Parameter	No.1	No.2	No.3	No.4	Mean	SD	RSD(%)
k_{MB}	0.299	0.173	0.244	0.335	0.263	0.071	26.807
n	1.333	1.716	1.483	1.334	1.466	0.181	12.321
k	0.006	0.014	0.009	0.008	0.009	0.003	36.585

Number of dissolution data points (N), degrees of freedom (df), and selected goodness of fit criteria – Pearson correlation coefficient (R), coefficient of determination (R^2), adjusted coefficient of determination ($R^2_{adjusted}$), and residual sum of squares (RSS) (manual calculation in MS Excel):

Parameter	No.1	No.2	No.3	No.4
N	10	10	10	10
df	7	7	7	7
R	0.997273318	0.96604191	0.997749167	0.992946819
R^2	0.994554071	0.933236971	0.9955034	0.985943386
$R^2_{adjusted}$	0.992998091	0.91416182	0.994218657	0.98192721
RSS	59.55818697	965.2759727	55.93342511	126.1121234

Graphical abstract of model fit presented as mean ± 1 SD of the fraction % of released carvedilol:

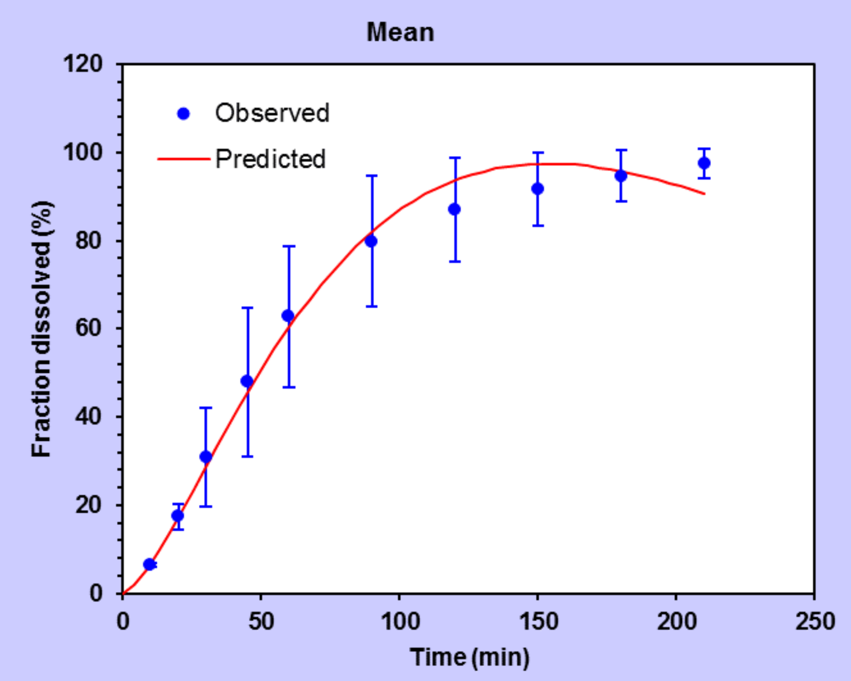

Supplementary materials – Model fitting summary – Parteck® M 200 (modelling the entire relevant dissolution data)

Graphical abstract of model fit presented as the fraction % of released carvedilol per tested tablet:

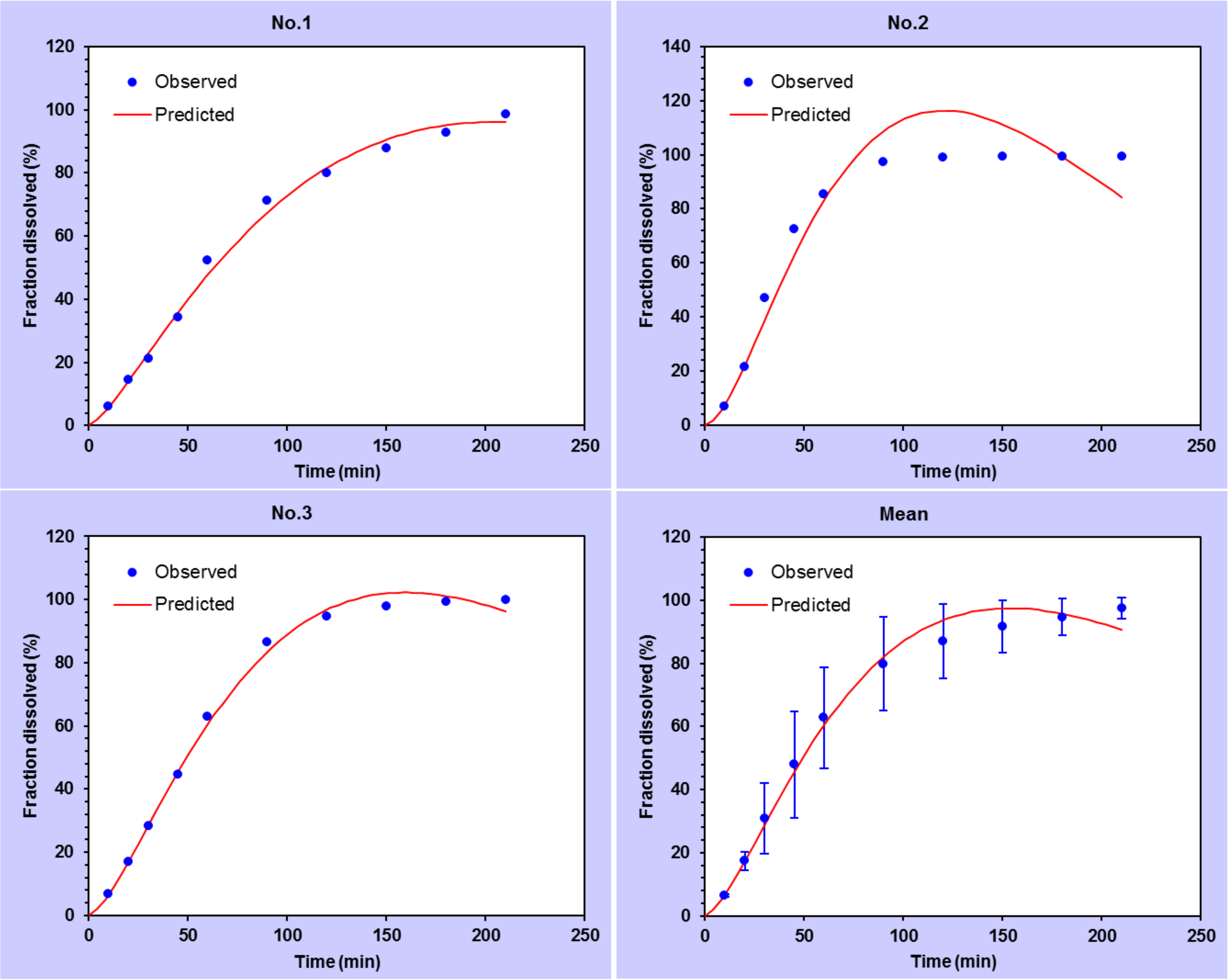

Supplementary materials – Model fitting summary – Parteck® M 200 (modelling the entire relevant dissolution data)

Model: **Makoid–Banakar with T_{lag}**

Model equation: $F = k_{MB} \cdot (t - T_{lag})^n \cdot e^{-k \cdot (t - T_{lag})}$

Fitted model parameters per tested tablet (N = 4) with statistics – mean, standard deviation (SD), and relative standard deviation expressed in % (RSD%) (output from DDSolver):

Parameter	No.1	No.2	No.3	No.4	Mean	SD	RSD(%)
k_{MB}	0.934	0.695	0.851	1.008	0.872	0.134	15.382
n	1.028	1.342	1.146	1.040	1.139	0.145	12.764
k	0.004	0.011	0.006	0.005	0.007	0.003	47.753
T_{lag}	4.000	4.000	4.000	4.000	4.000	0.000	0.000

Number of dissolution data points (N), degrees of freedom (df), and selected goodness of fit criteria – Pearson correlation coefficient (R), coefficient of determination (R^2), adjusted coefficient of determination ($R^2_{adjusted}$), and residual sum of squares (RSS) (manual calculation in MS Excel):

Parameter	No.1	No.2	No.3	No.4
N	10	10	10	10
df	6	6	6	6
R	0.995891725	0.978741184	0.997188741	0.997594403
R^2	0.991800328	0.957934305	0.994385385	0.995194593
$R^2_{adjusted}$	0.987700492	0.936901457	0.991578077	0.992791889
RSS	89.9763625	548.57273	69.82442217	41.98206799

Graphical abstract of model fit presented as mean ± 1 SD of the fraction % of released carvedilol:

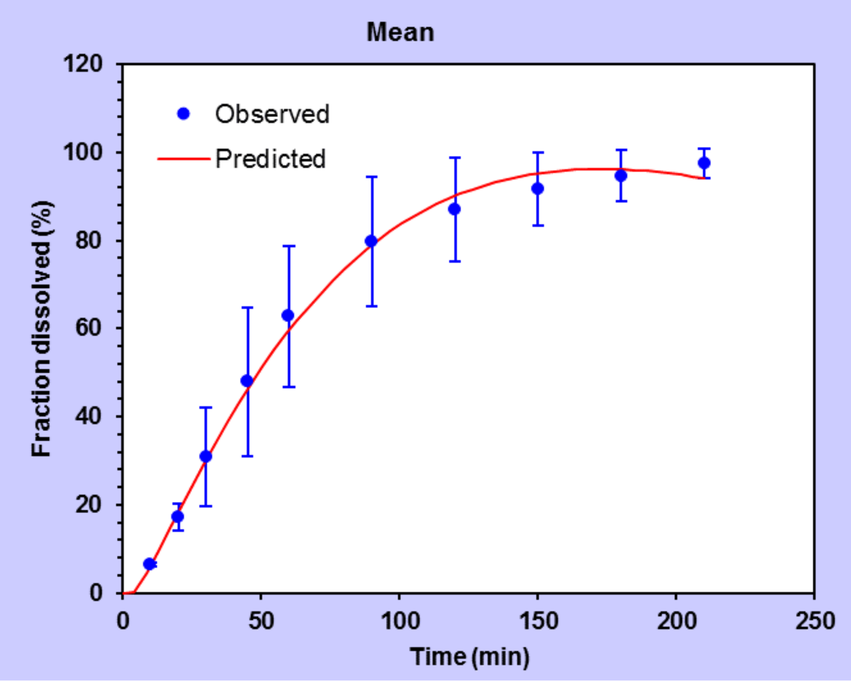

Supplementary materials – Model fitting summary – Parteck® M 200 (modelling the entire relevant dissolution data)

Graphical abstract of model fit presented as the fraction % of released carvedilol per tested tablet:

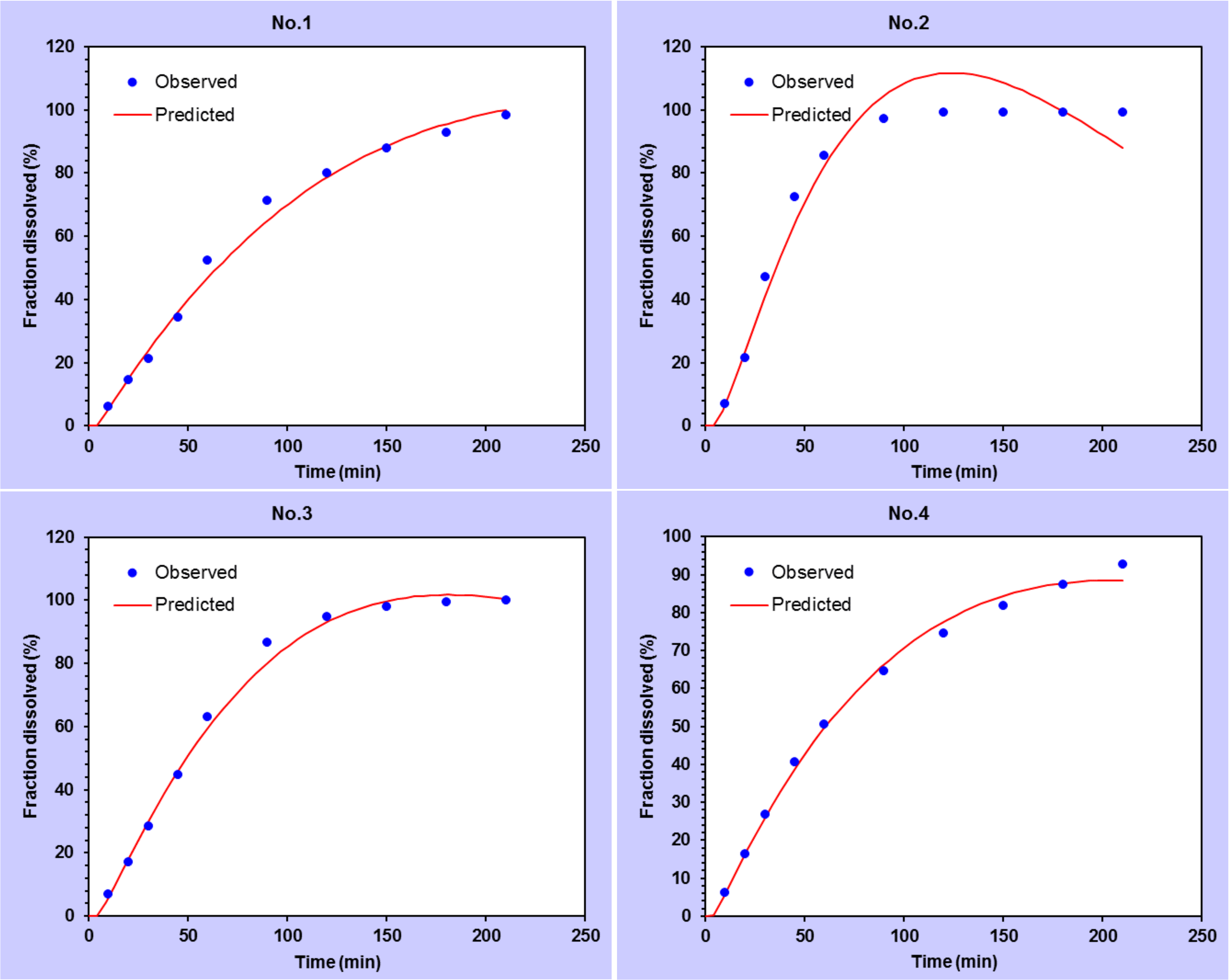

Supplementary materials – Model fitting summary – Parteck® M 200 (modelling the entire relevant dissolution data)

Model: **Peppas–Sahlin_1**

Model equation: $F = k_1 \cdot t^m + k_2 \cdot t^{2m}$

Fitted model parameters per tested tablet (N = 4) with statistics – mean, standard deviation (SD), and relative standard deviation expressed in % (RSD%) (output from DDSolver):

Parameter	No.1	No.2	No.3	No.4	Mean	SD	RSD(%)
k_1	3.730	12.327	6.574	4.801	6.858	3.830	55.846
k_2	0.537	-0.213	0.333	0.372	0.257	0.326	126.657
m	0.450	0.450	0.450	0.450	0.450	0.000	0.000

Number of dissolution data points (N), degrees of freedom (df), and selected goodness of fit criteria – Pearson correlation coefficient (R), coefficient of determination (R^2), adjusted coefficient of determination ($R^2_{adjusted}$), and residual sum of squares (RSS) (manual calculation in MS Excel):

Parameter	No.1	No.2	No.3	No.4
N	10	10	10	10
df	7	7	7	7
R	0.975117056	0.917821755	0.951349672	0.98175584
R^2	0.950853274	0.842396774	0.905066198	0.96384453
$R^2_{adjusted}$	0.936811352	0.797367281	0.877942255	0.953514396
RSS	566.8124667	2081.033765	1259.12658	337.7639571

Graphical abstract of model fit presented as mean ± 1 SD of the fraction % of released carvedilol:

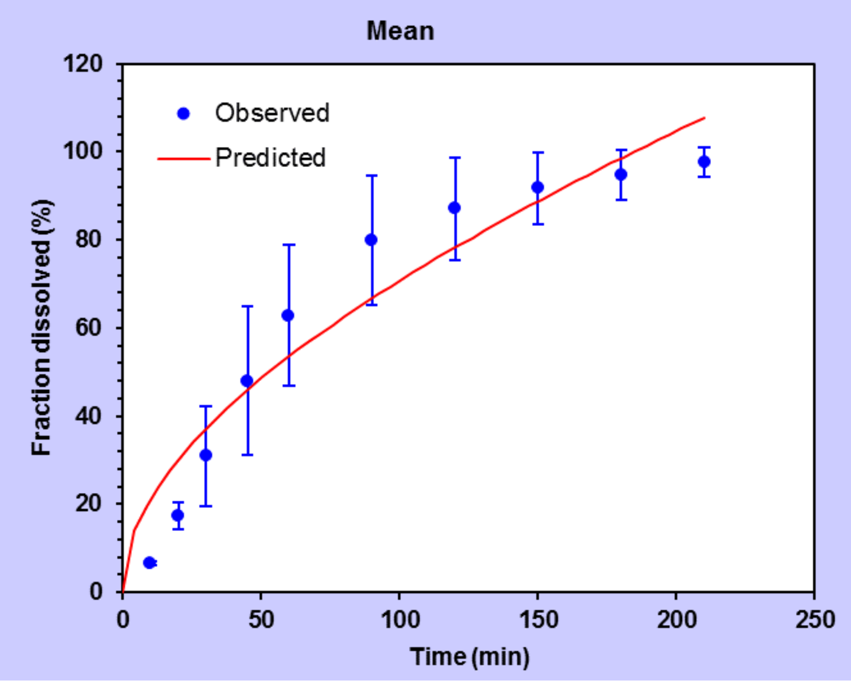

Supplementary materials – Model fitting summary – Parteck® M 200 (modelling the entire relevant dissolution data)

Graphical abstract of model fit presented as the fraction % of released carvedilol per tested tablet:

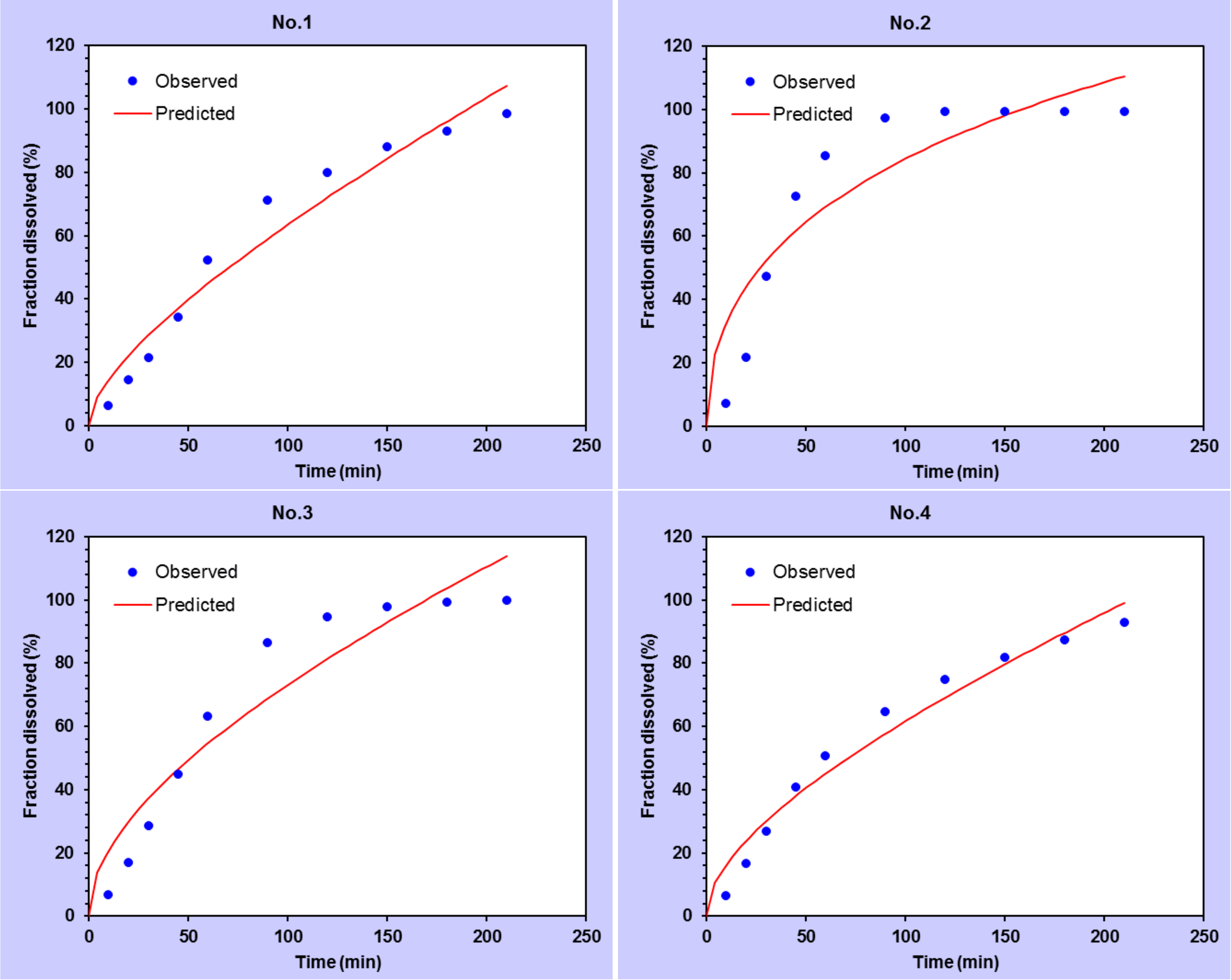

Supplementary materials – Model fitting summary – Parteck® M 200 (modelling the entire relevant dissolution data)

Model: **Peppas–Sahlin_1 with T_{lag}**

Model equation: $F = k_1 \cdot (t - T_{lag})^m + k_2 \cdot (t - T_{lag})^{2m}$

Fitted model parameters per tested tablet (N = 4) with statistics – mean, standard deviation (SD), and relative standard deviation expressed in % (RSD%) (output from DDSolver):

Parameter	No.1	No.2	No.3	No.4	Mean	SD	RSD(%)
k_1	4.740	13.866	7.810	5.791	8.052	4.080	50.671
k_2	0.450	-0.362	0.222	0.285	0.149	0.354	237.534
m	0.450	0.450	0.450	0.450	0.450	0.000	0.000
T_{lag}	6.000	6.000	6.000	6.000	6.000	0.000	0.000

Number of dissolution data points (N), degrees of freedom (df), and selected goodness of fit criteria – Pearson correlation coefficient (R), coefficient of determination (R^2), adjusted coefficient of determination ($R^2_{adjusted}$), and residual sum of squares (RSS) (manual calculation in MS Excel):

Parameter	No.1	No.2	No.3	No.4
N	10	10	10	10
df	6	6	6	6
R	0.980136789	0.943941275	0.961560348	0.988232465
R^2	0.960668126	0.89102513	0.924598302	0.976603404
$R^2_{adjusted}$	0.941002188	0.836537695	0.886897453	0.964905106
RSS	443.9925136	1446.135234	981.0317651	212.8889798

Graphical abstract of model fit presented as mean ± 1 SD of the fraction % of released carvedilol:

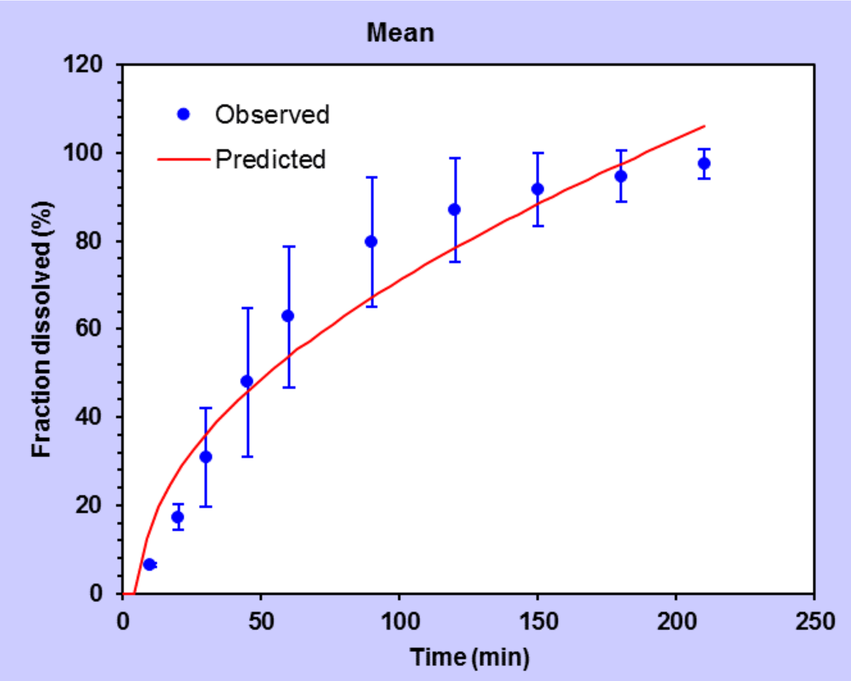

Supplementary materials – Model fitting summary – Parteck® M 200 (modelling the entire relevant dissolution data)

Graphical abstract of model fit presented as the fraction % of released carvedilol per tested tablet:

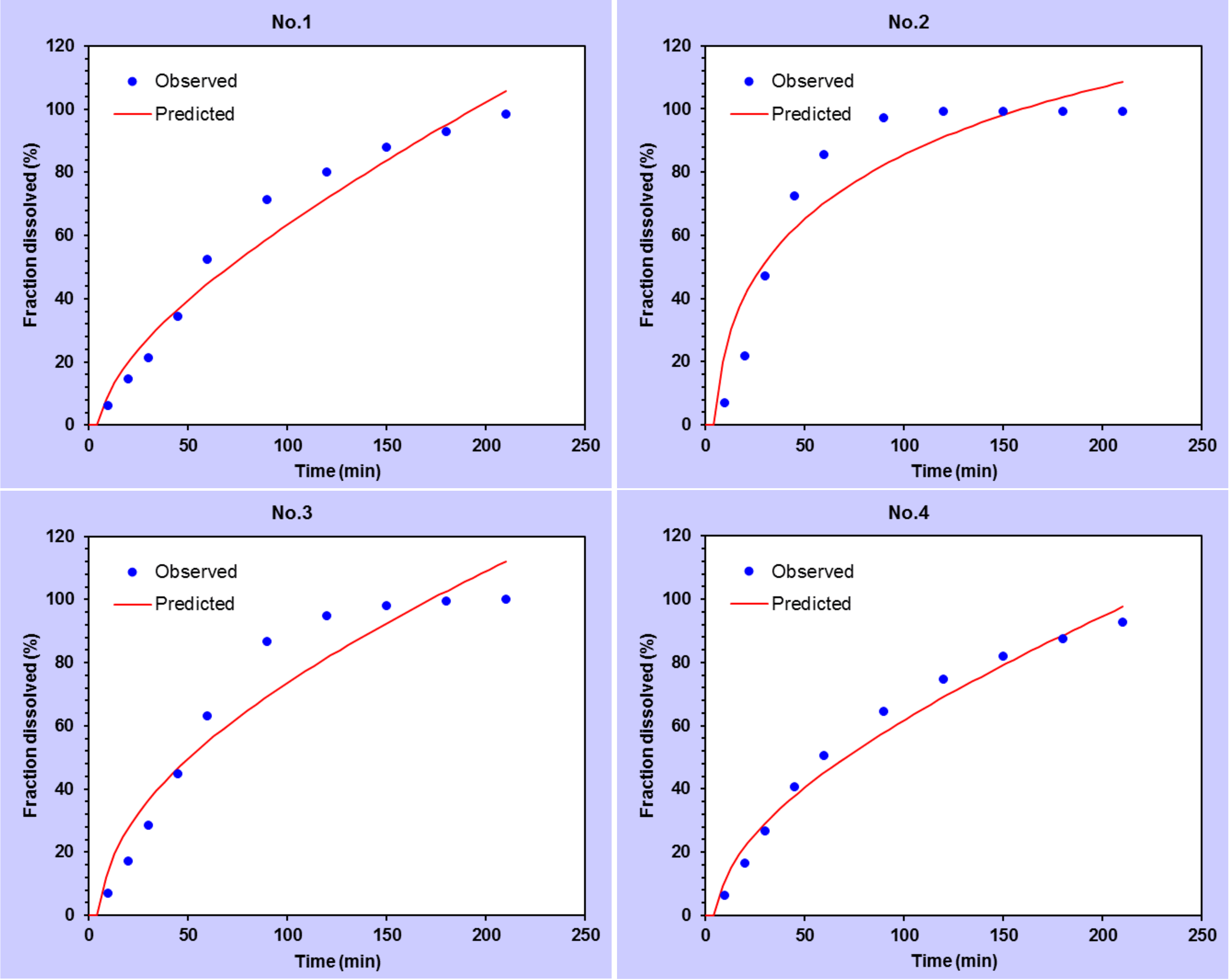

Supplementary materials – Model fitting summary – Parteck® M 200 (modelling the entire relevant dissolution data)

Model: **Peppas–Sahlin_2**

Model equation: $F = k_1 \cdot t^{0.5} + k_2 \cdot t$

Fitted model parameters per tested tablet (N = 4) with statistics – mean, standard deviation (SD), and relative standard deviation expressed in % (RSD%) (output from DDSolver):

Parameter	No.1	No.2	No.3	No.4	Mean	SD	RSD(%)
k_1	4.105	11.001	6.480	4.831	6.604	3.095	46.862
k_2	0.226	-0.242	0.090	0.136	0.052	0.205	390.230

Number of dissolution data points (N), degrees of freedom (df), and selected goodness of fit criteria – Pearson correlation coefficient (R), coefficient of determination (R^2), adjusted coefficient of determination ($R^2_{adjusted}$), and residual sum of squares (RSS) (manual calculation in MS Excel):

Parameter	No.1	No.2	No.3	No.4
N	10	10	10	10
df	8	8	8	8
R	0.975461683	0.931957046	0.954896446	0.982974953
R^2	0.951525496	0.868543936	0.911827222	0.966239758
$R^2_{adjusted}$	0.945466183	0.852111928	0.900805625	0.962019728
RSS	575.8218169	1882.940355	1219.280422	329.0102025

Graphical abstract of model fit presented as mean ± 1 SD of the fraction % of released carvedilol:

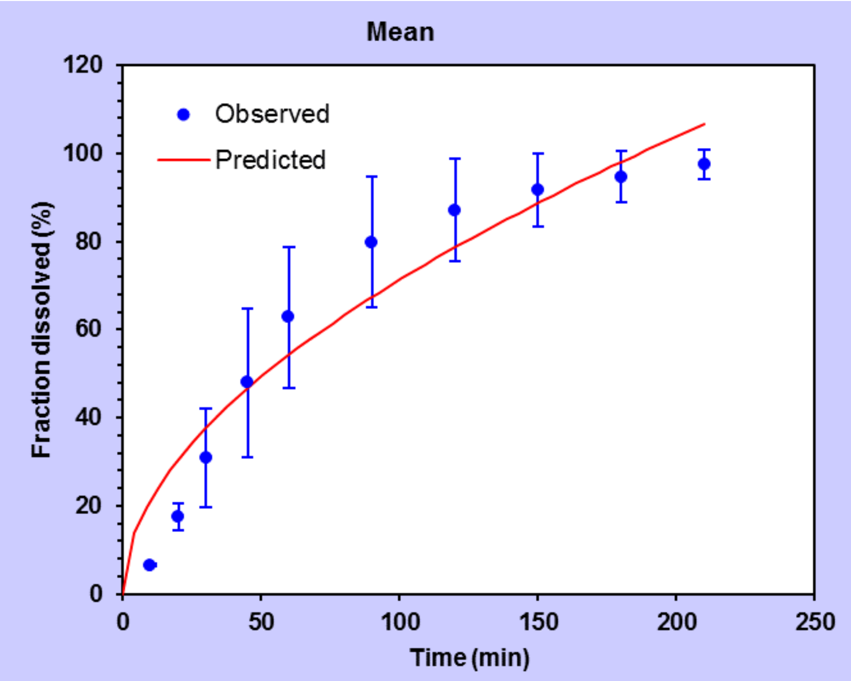

Supplementary materials – Model fitting summary – Parteck® M 200 (modelling the entire relevant dissolution data)

Graphical abstract of model fit presented as the fraction % of released carvedilol per tested tablet:

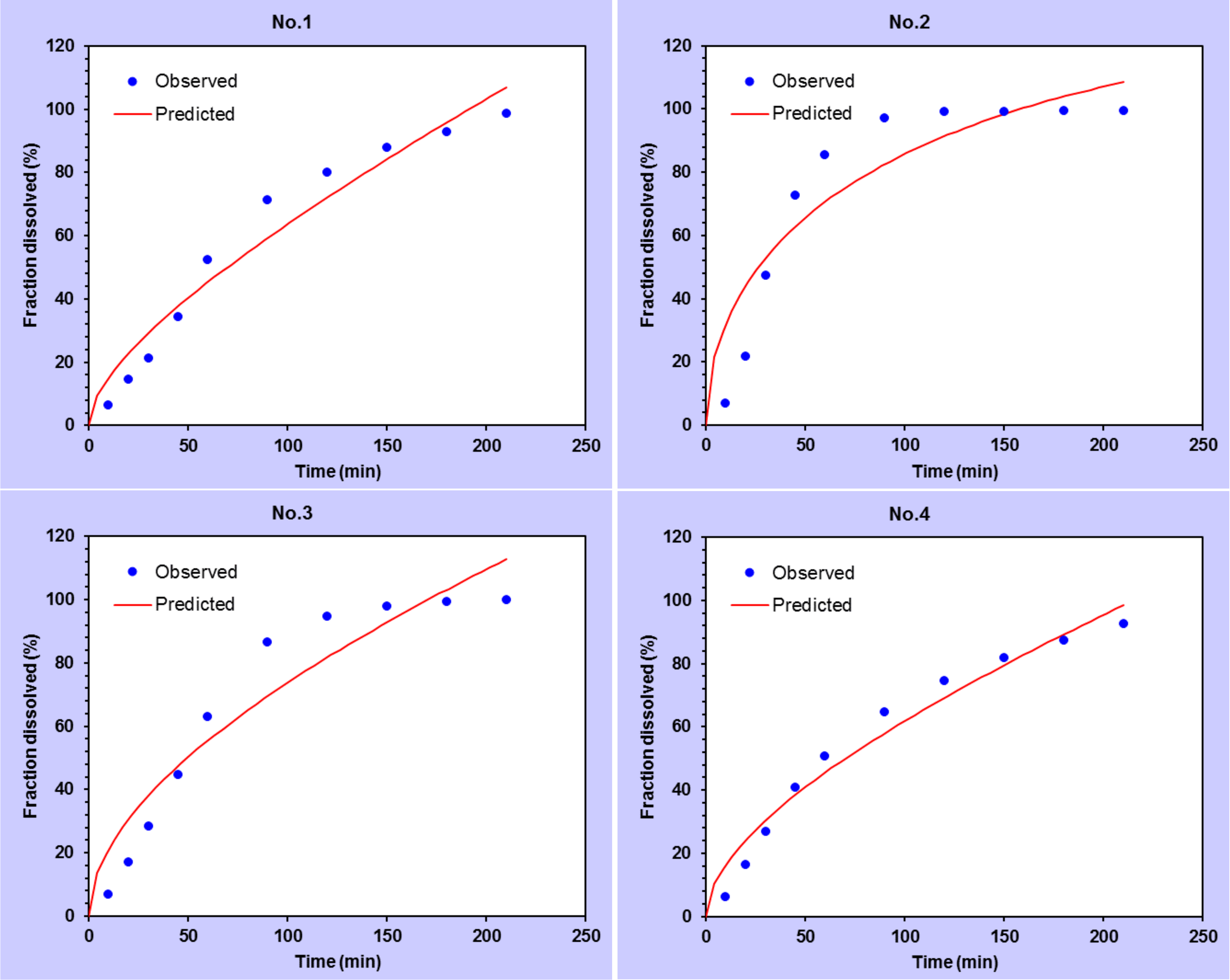

Supplementary materials – Model fitting summary – Parteck® M 200 (modelling the entire relevant dissolution data)

Model: **Peppas–Sahlin_2 with T_{lag}**

Model equation: $F = k_1 \cdot (t - T_{lag})^{0.5} + k_2 \cdot (t - T_{lag})$

Fitted model parameters per tested tablet (N = 4) with statistics – mean, standard deviation (SD), and relative standard deviation expressed in % (RSD%) (output from DDSolver):

Parameter	No.1	No.2	No.3	No.4	Mean	SD	RSD(%)
k_1	4.903	12.233	7.466	5.610	7.553	3.302	43.714
k_2	0.173	-0.335	0.021	0.083	-0.015	0.223	-1520.196
T_{lag}	6.000	6.000	6.000	6.000	6.000	0.000	0.000

Number of dissolution data points (N), degrees of freedom (df), and selected goodness of fit criteria – Pearson correlation coefficient (R), coefficient of determination (R^2), adjusted coefficient of determination ($R^2_{adjusted}$), and residual sum of squares (RSS) (manual calculation in MS Excel):

Parameter	No.1	No.2	No.3	No.4
N	10	10	10	10
df	7	7	7	7
R	0.981063935	0.956487218	0.965775079	0.989793647
R^2	0.962486444	0.914867797	0.932721503	0.979691463
$R^2_{adjusted}$	0.951768285	0.890544311	0.913499075	0.973889024
RSS	431.9367887	1213.008572	905.4097538	190.4066965

Graphical abstract of model fit presented as mean ± 1 SD of the fraction % of released carvedilol:

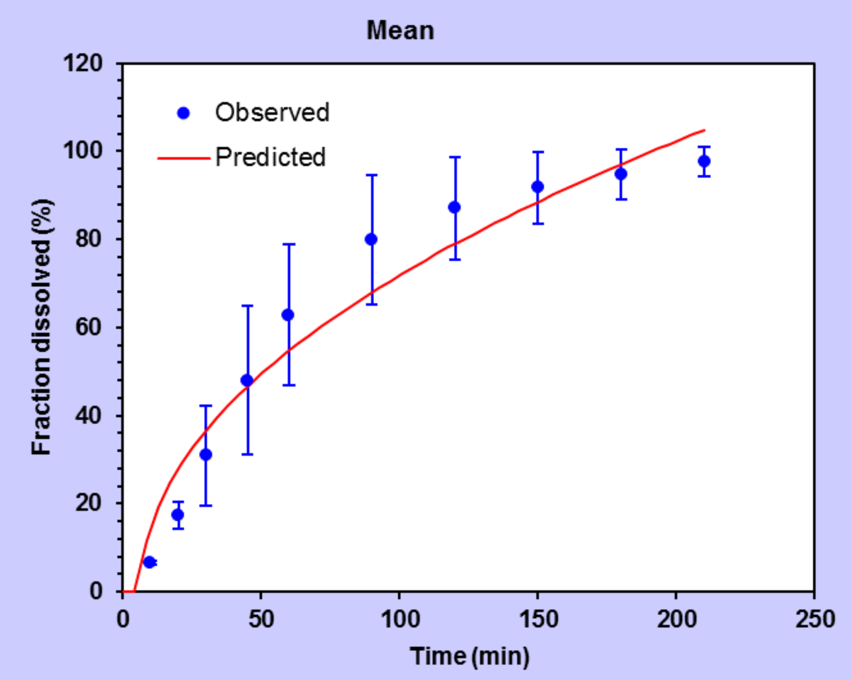

Supplementary materials – Model fitting summary – Parteck® M 200 (modelling the entire relevant dissolution data)

Graphical abstract of model fit presented as the fraction % of released carvedilol per tested tablet:

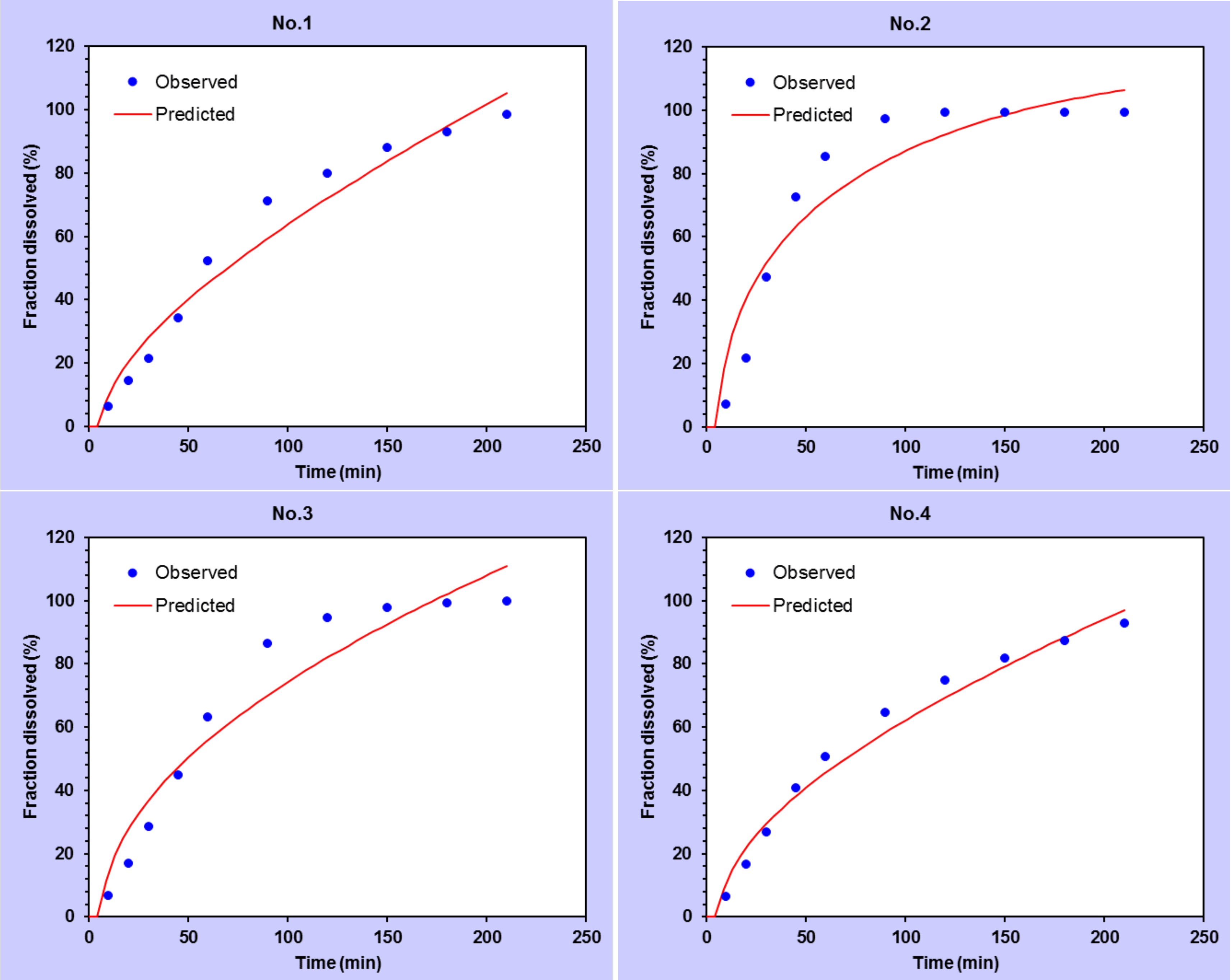

Supplementary materials – Model fitting summary – Parteck® M 200 (modelling the entire relevant dissolution data)

Model: **Quadratic**

Model equation: $F = 100 \cdot (k_1 \cdot t^2 + k_2 \cdot t)$

Fitted model parameters per tested tablet (N = 4) with statistics – mean, standard deviation (SD), and relative standard deviation expressed in % (RSD%) (output from DDSolver):

Parameter	No.1	No.2	No.3	No.4	Mean	SD	RSD(%)
k_1	-0.00002	-0.00005	-0.00004	-0.00002	-0.00003	0.00001	-41.99025
k_2	0.00935	0.01534	0.01200	0.00924	0.01148	0.00287	24.99580

Number of dissolution data points (N), degrees of freedom (df), and selected goodness of fit criteria – Pearson correlation coefficient (R), coefficient of determination (R^2), adjusted coefficient of determination ($R^2_{adjusted}$), and residual sum of squares (RSS) (manual calculation in MS Excel):

Parameter	No.1	No.2	No.3	No.4
N	10	10	10	10
df	8	8	8	8
R	0.995915807	0.961312609	0.994927856	0.995892011
R^2	0.991848294	0.924121933	0.989881439	0.991800897
$R^2_{adjusted}$	0.990829331	0.914637174	0.988616619	0.99077601
RSS	111.1999188	858.0709608	169.0007784	70.65799147

Graphical abstract of model fit presented as mean ± 1 SD of the fraction % of released carvedilol:

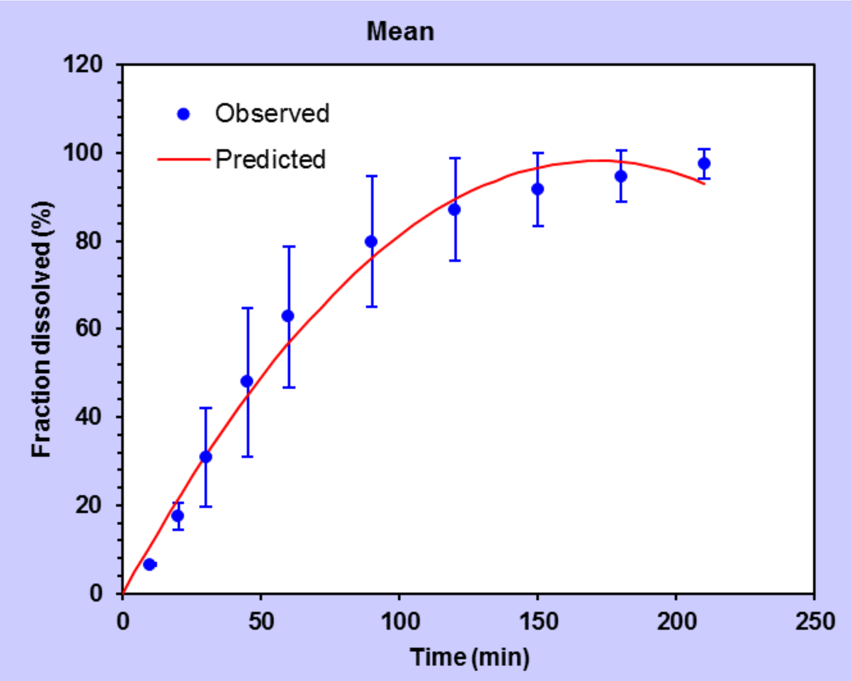

Supplementary materials – Model fitting summary – Parteck® M 200 (modelling the entire relevant dissolution data)

Graphical abstract of model fit presented as the fraction % of released carvedilol per tested tablet:

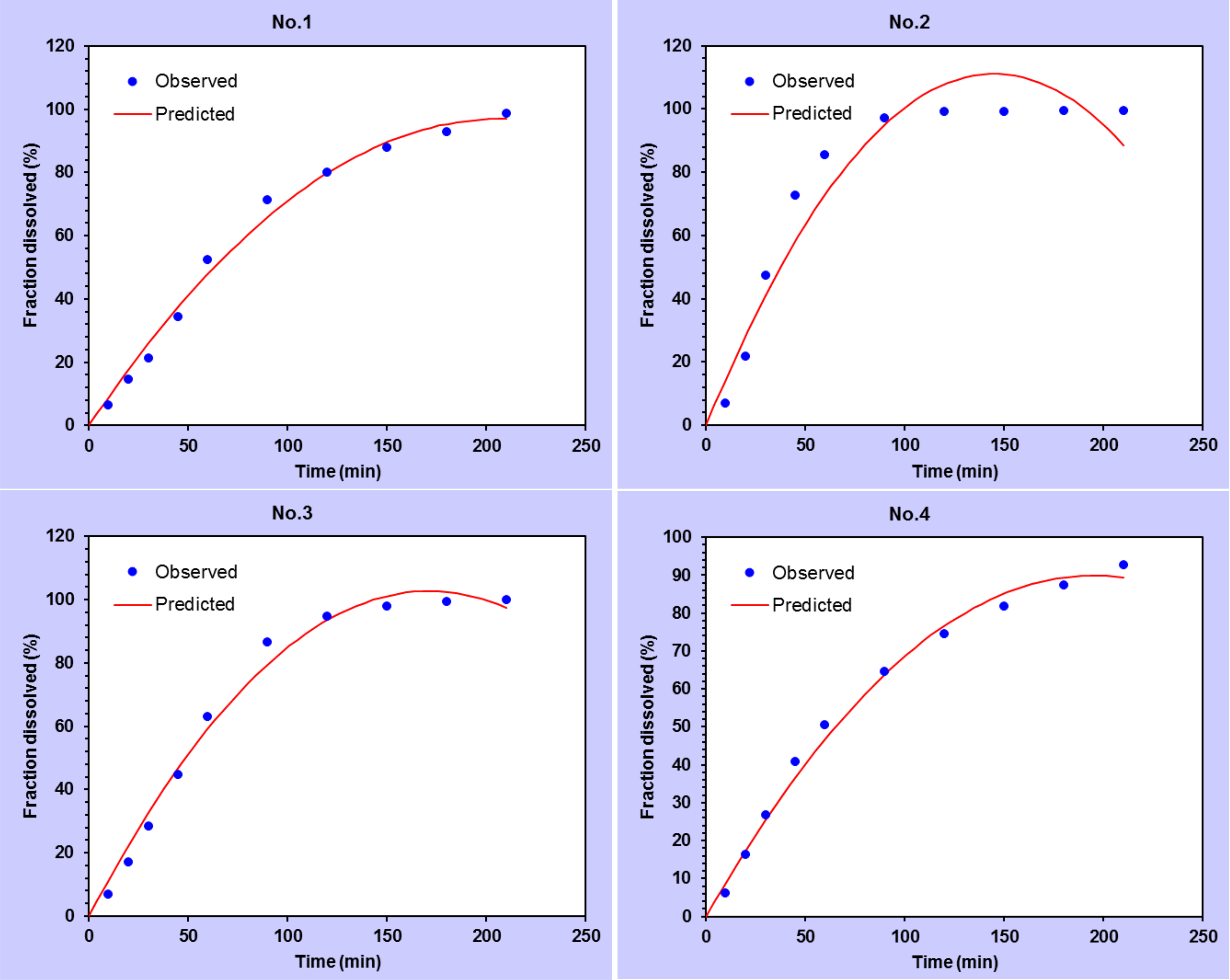

Supplementary materials – Model fitting summary – Parteck® M 200 (modelling the entire relevant dissolution data)

Model: **Quadratic with T_{lag}**

Model equation: $F = 100 \cdot \left[k_1 \cdot (t - T_{lag})^2 + k_2 \cdot (t - T_{lag})\right]$

Fitted model parameters per tested tablet (N = 4) with statistics – mean, standard deviation (SD), and relative standard deviation expressed in % (RSD%) (output from DDSolver):

Parameter	No.1	No.2	No.3	No.4	Mean	SD	RSD(%)
k_1	-0.00003	-0.00006	-0.00004	-0.00003	-0.00004	0.00001	-39.84732
k_2	0.01000	0.01618	0.01278	0.00982	0.01220	0.00298	24.46418
T_{lag}	4.00000	4.00000	4.00000	4.00000	4.00000	0.00000	0.00000

Number of dissolution data points (N), degrees of freedom (df), and selected goodness of fit criteria – Pearson correlation coefficient (R), coefficient of determination (R^2), adjusted coefficient of determination ($R^2_{adjusted}$), and residual sum of squares (RSS) (manual calculation in MS Excel):

Parameter	No.1	No.2	No.3	No.4
N	10	10	10	10
df	7	7	7	7
R	0.996443379	0.960943441	0.995496994	0.995404472
R^2	0.992899408	0.923412296	0.991014266	0.990830062
$R^2_{adjusted}$	0.990870667	0.901530095	0.988446913	0.98821008
RSS	77.78395147	986.8483487	111.2400984	104.2802409

Graphical abstract of model fit presented as mean ± 1 SD of the fraction % of released carvedilol:

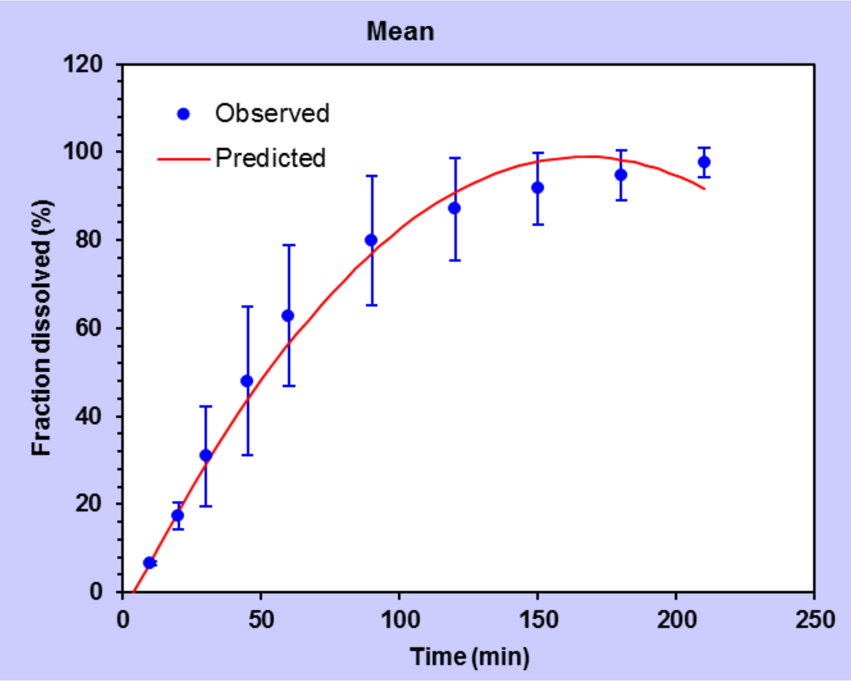

Supplementary materials – Model fitting summary – Parteck® M 200 (modelling the entire relevant dissolution data)

Graphical abstract of model fit presented as the fraction % of released carvedilol per tested tablet:

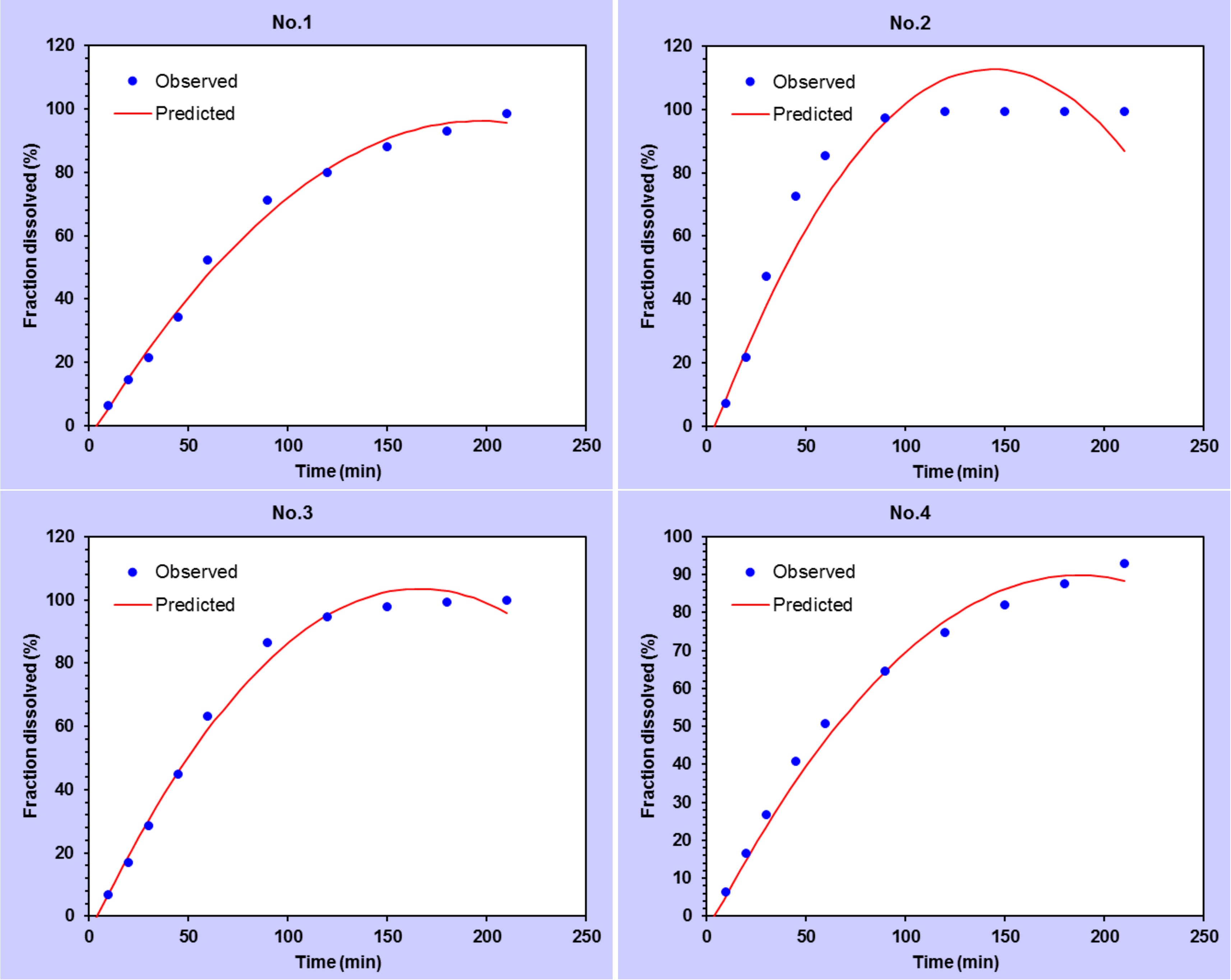

Supplementary materials – Model fitting summary – Parteck® M 200 (modelling the entire relevant dissolution data)

Model: **Weibull_1**

Model equation: $F = 100 \cdot \left[1 - e^{-\frac{(t-T_i)^\beta}{\alpha}}\right]$

Fitted model parameters per tested tablet (N = 4) with statistics – mean, standard deviation (SD), and relative standard deviation expressed in % (RSD%) (output from DDSolver):

Parameter	No.1	No.2	No.3	No.4	Mean	SD	RSD(%)
α	158.947	106.498	193.546	94.650	138.410	46.170	33.357
β	1.175	1.259	1.325	1.029	1.197	0.128	10.655
T_i	6.000	4.000	6.000	4.000	5.000	1.155	23.094

Number of dissolution data points (N), degrees of freedom (df), and selected goodness of fit criteria – Pearson correlation coefficient (R), coefficient of determination (R^2), adjusted coefficient of determination ($R^2_{adjusted}$), and residual sum of squares (RSS) (manual calculation in MS Excel):

Parameter	No.1	No.2	No.3	No.4
N	10	10	10	10
df	7	7	7	7
R	0.998002159	0.99251302	0.998512385	0.999255446
R^2	0.996008309	0.985082095	0.997026982	0.998511447
$R^2_{adjusted}$	0.994867825	0.980819837	0.996177548	0.998086146
RSS	46.5786949	212.179213	38.11009584	14.5398211

Graphical abstract of model fit presented as mean ± 1 SD of the fraction % of released carvedilol:

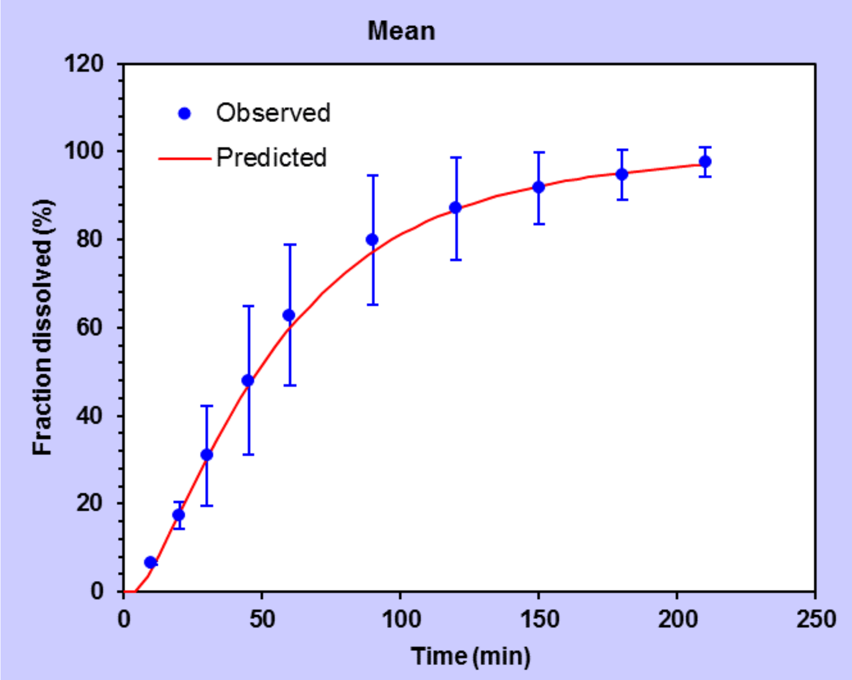

Supplementary materials – Model fitting summary – Parteck® M 200 (modelling the entire relevant dissolution data)

Graphical abstract of model fit presented as the fraction % of released carvedilol per tested tablet:

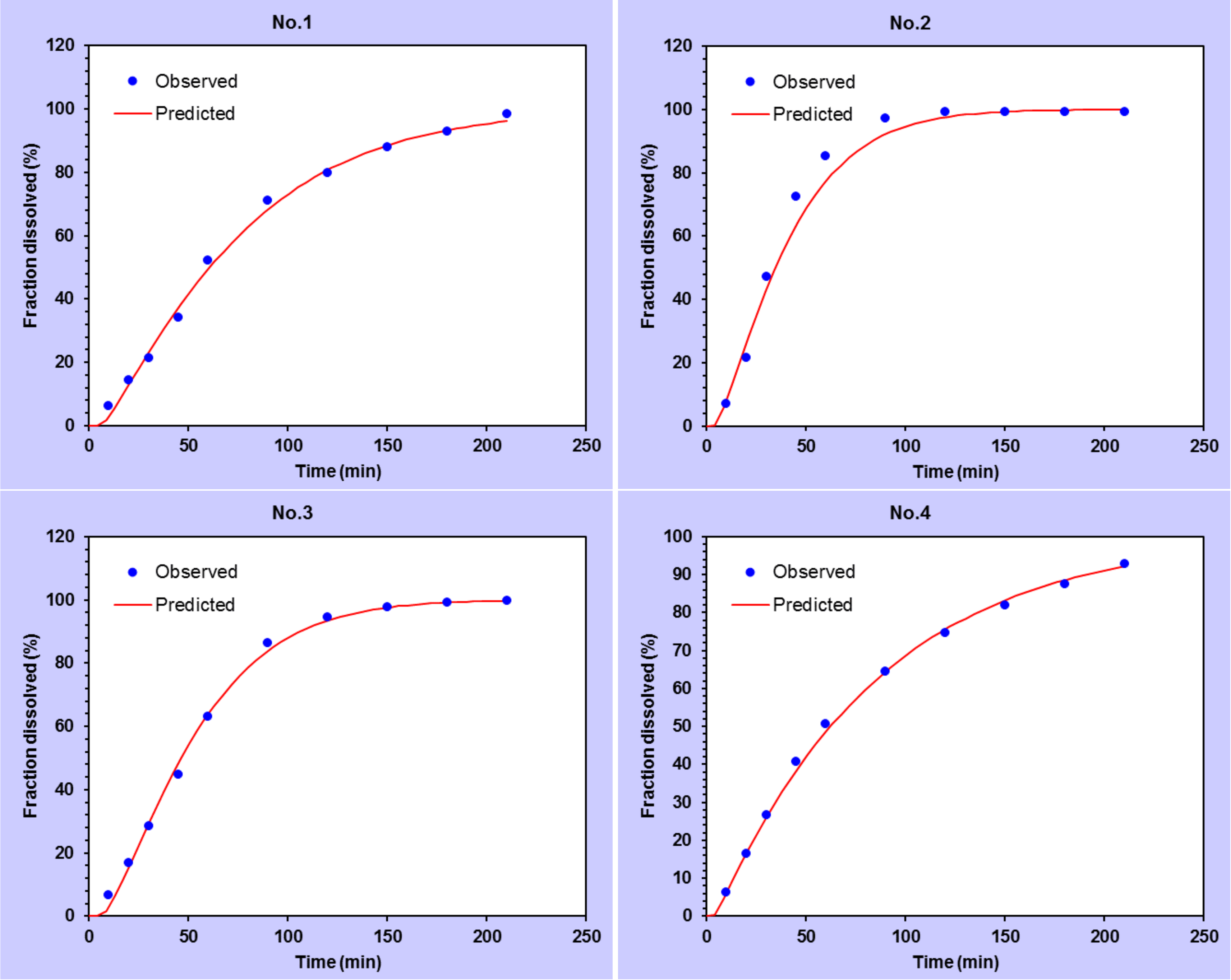

Supplementary materials – Model fitting summary – Parteck® M 200 (modelling the entire relevant dissolution data)

Model: **Weibull_2**

Model equation: $F = 100 \cdot \left(1 - e^{-\frac{t^{\beta}}{\alpha}}\right)$

Fitted model parameters per tested tablet (N = 4) with statistics – mean, standard deviation (SD), and relative standard deviation expressed in % (RSD%) (output from DDSolver):

Parameter	No.1	No.2	No.3	No.4	Mean	SD	RSD(%)
α	359.589	282.217	485.441	187.667	328.729	125.927	38.307
β	1.339	1.594	1.509	1.165	1.402	0.190	13.557

Number of dissolution data points (N), degrees of freedom (df), and selected goodness of fit criteria – Pearson correlation coefficient (R), coefficient of determination (R^2), adjusted coefficient of determination ($R^2_{adjusted}$), and residual sum of squares (RSS) (manual calculation in MS Excel):

Parameter	No.1	No.2	No.3	No.4
N	10	10	10	10
df	8	8	8	8
R	0.998140232	0.997416736	0.999416975	0.997581143
R^2	0.996283923	0.994840145	0.998834289	0.995168136
$R^2_{adjusted}$	0.995819413	0.994195163	0.998688575	0.994564153
RSS	40.63304277	330.4732432	15.65645872	53.73779479

Graphical abstract of model fit presented as mean ± 1 SD of the fraction % of released carvedilol:

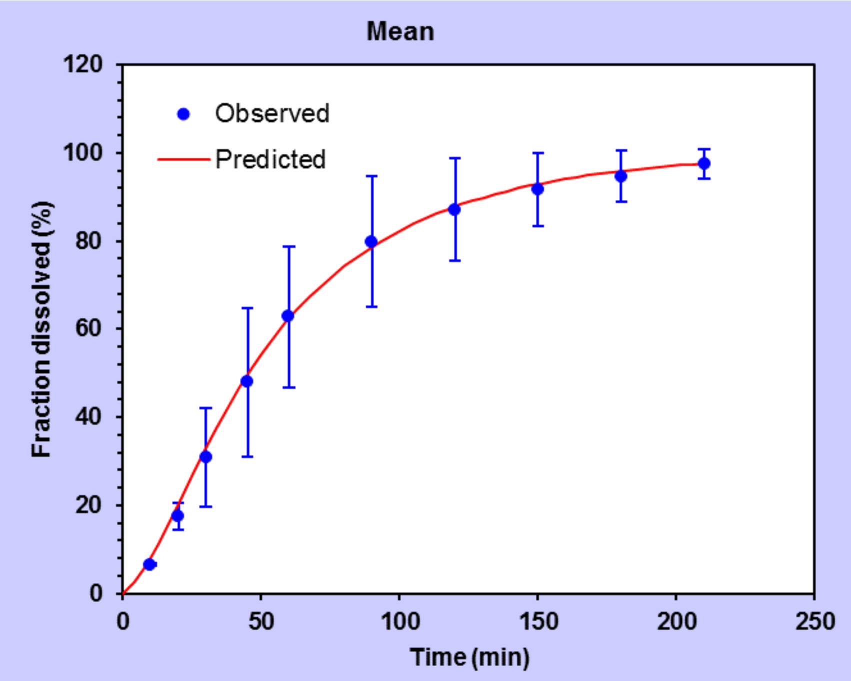

Supplementary materials – Model fitting summary – Parteck® M 200 (modelling the entire relevant dissolution data)

Graphical abstract of model fit presented as the fraction % of released carvedilol per tested tablet:

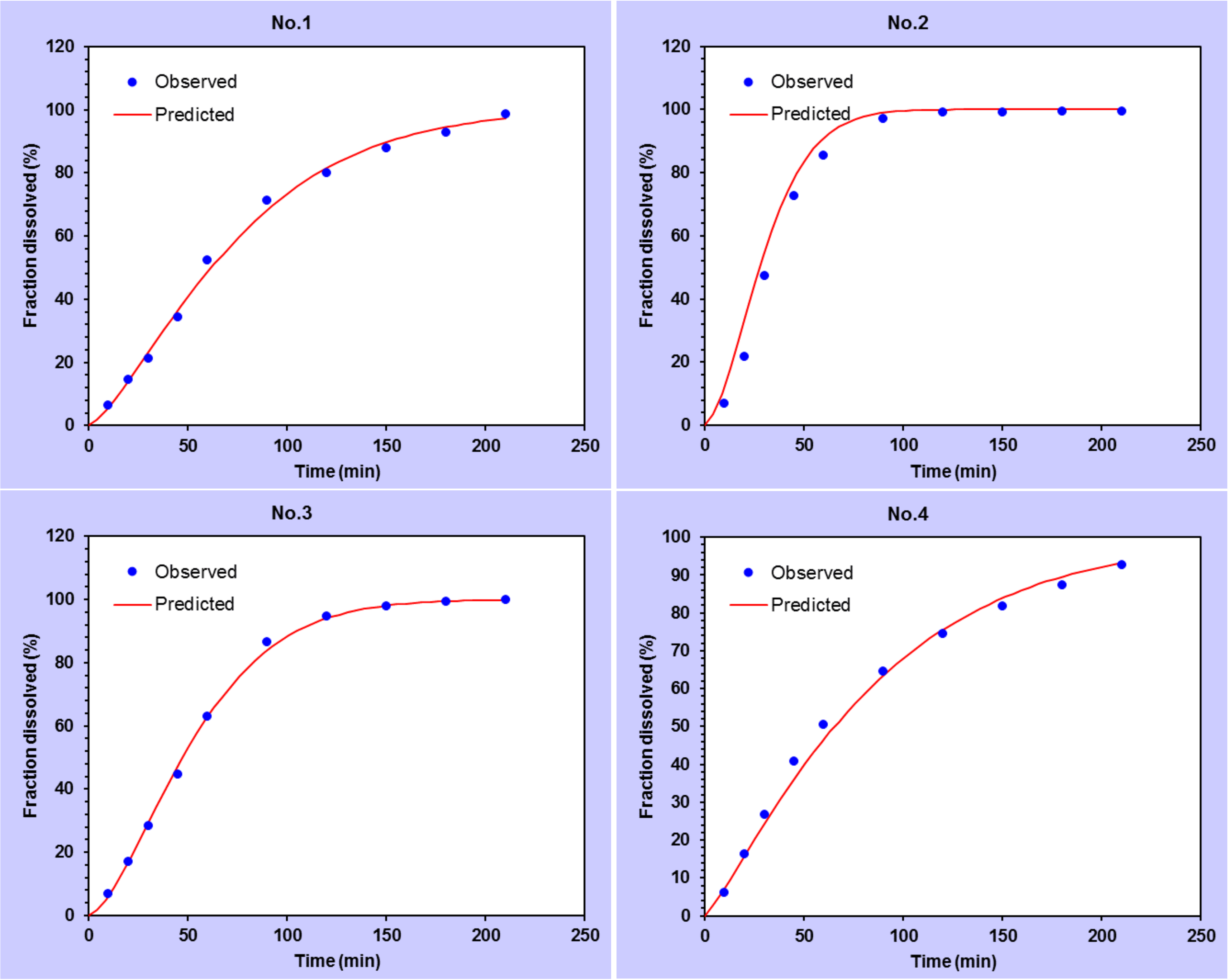

Supplementary materials – Model fitting summary – Parteck® M 200 (modelling the entire relevant dissolution data)

Model: **Weibull_3**

Model equation: $F = F_{max} \cdot \left(1 - e^{-\frac{t^{\beta}}{\alpha}}\right)$

Fitted model parameters per tested tablet (N = 4) with statistics – mean, standard deviation (SD), and relative standard deviation expressed in % (RSD%) (output from DDSolver):

Parameter	No.1	No.2	No.3	No.4	Mean	SD	RSD(%)
α	308.115	190.451	271.536	200.576	242.669	56.613	23.329
β	1.280	1.349	1.312	1.195	1.284	0.066	5.101
F_{max}	103.362	99.159	104.895	97.334	101.187	3.533	3.491

Number of dissolution data points (N), degrees of freedom (df), and selected goodness of fit criteria – Pearson correlation coefficient (R), coefficient of determination (R^2), adjusted coefficient of determination ($R^2_{adjusted}$), and residual sum of squares (RSS) (manual calculation in MS Excel):

Parameter	No.1	No.2	No.3	No.4
N	10	10	10	10
df	7	7	7	7
R	0.997371697	0.984097311	0.994721276	0.997990069
R^2	0.994750302	0.968447517	0.989470417	0.995984178
$R^2_{adjusted}$	0.993250388	0.959432522	0.986461965	0.9948368
RSS	58.35806161	521.8700072	146.9263616	43.64426876

Graphical abstract of model fit presented as mean ± 1 SD of the fraction % of released carvedilol:

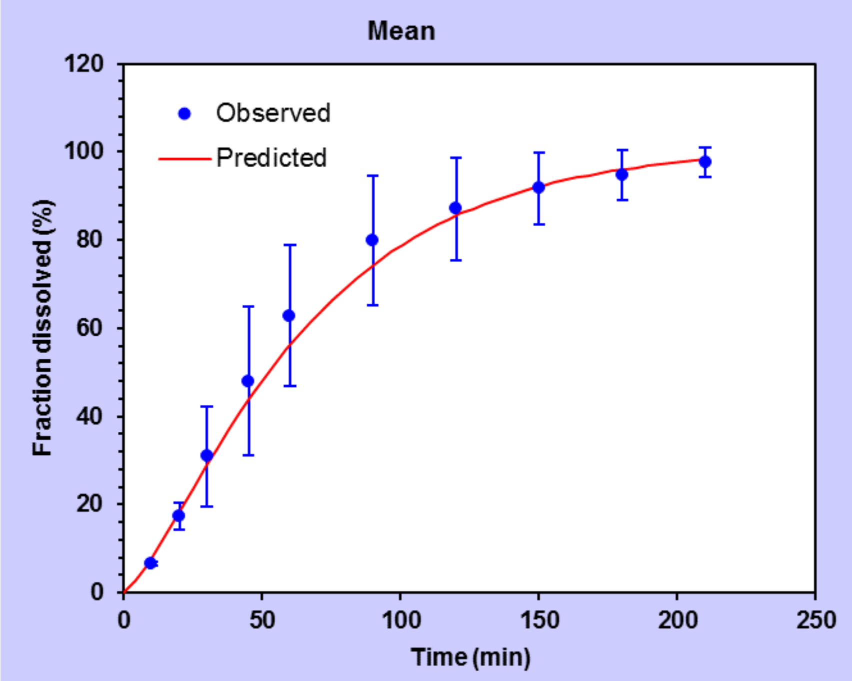

Supplementary materials – Model fitting summary – Parteck® M 200 (modelling the entire relevant dissolution data)

Graphical abstract of model fit presented as the fraction % of released carvedilol per tested tablet:

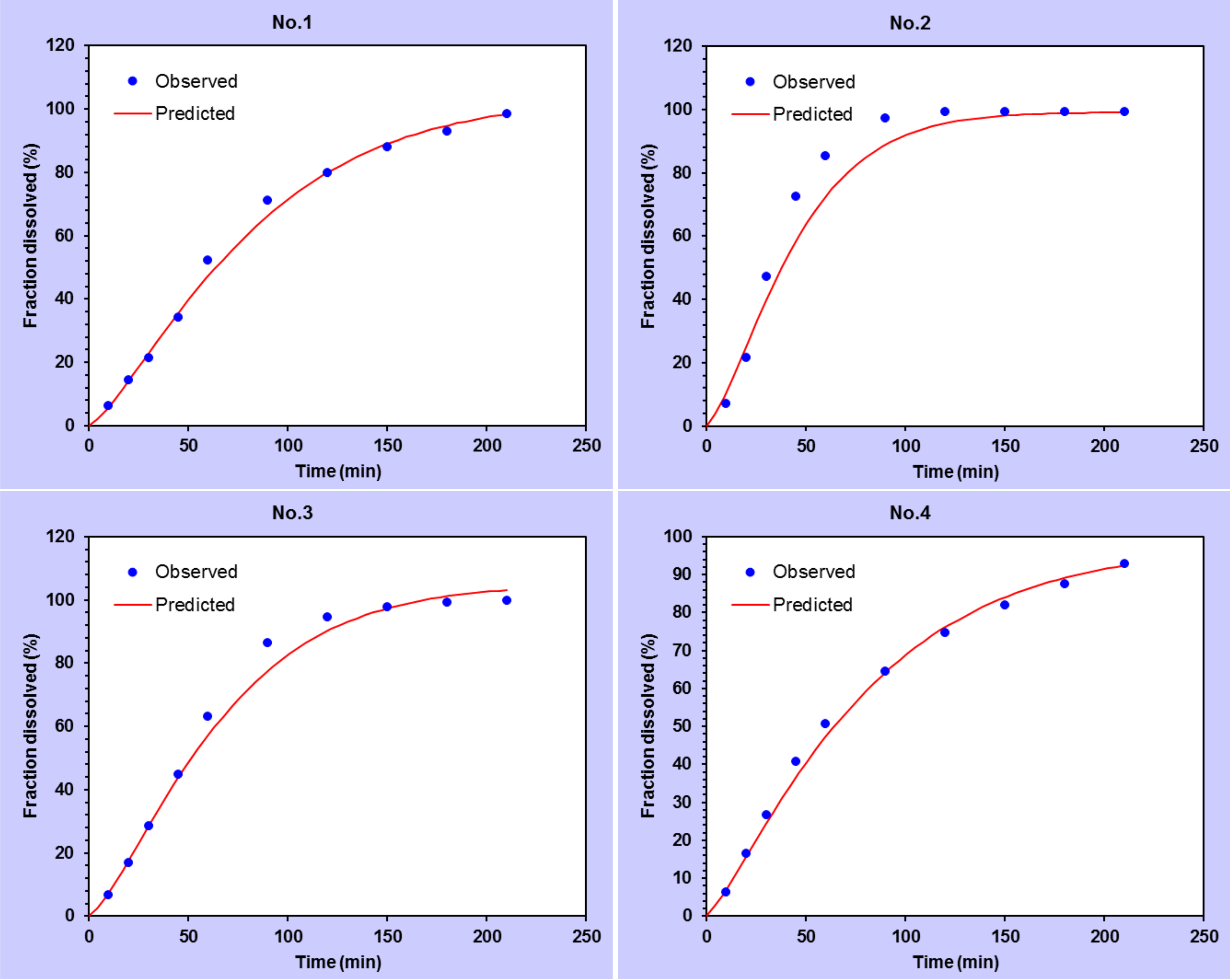

Supplementary materials – Model fitting summary – Parteck® M 200 (modelling the entire relevant dissolution data)

Model: **Weibull_4**

Model equation: $F = F_{max} \cdot \left[1 - e^{-\frac{(t-T_i)^{\beta}}{\alpha}}\right]$

Fitted model parameters per tested tablet (N = 4) with statistics – mean, standard deviation (SD), and relative standard deviation expressed in % (RSD%) (output from DDSolver):

Parameter	No.1	No.2	No.3	No.4	Mean	SD	RSD(%)
α	142.168	73.499	120.633	98.993	108.823	29.415	27.030
β	1.125	1.105	1.185	1.055	1.118	0.054	4.813
T_i	4.000	4.000	5.101	4.000	4.275	0.550	12.873
F_{max}	103.362	104.225	100.688	97.334	101.402	3.102	3.060

Number of dissolution data points (N), degrees of freedom (df), and selected goodness of fit criteria – Pearson correlation coefficient (R), coefficient of determination (R^2), adjusted coefficient of determination ($R^2_{adjusted}$), and residual sum of squares (RSS) (manual calculation in MS Excel):

Parameter	No.1	No.2	No.3	No.4
N	10	10	10	10
df	6	6	6	6
R	0.997743236	0.977910806	0.997444077	0.999286231
R^2	0.995491565	0.956309544	0.994894688	0.998572972
$R^2_{adjusted}$	0.993237348	0.934464316	0.992342032	0.997859457
RSS	53.47774649	572.784468	87.25204945	13.32642036

Graphical abstract of model fit presented as mean ± 1 SD of the fraction % of released carvedilol:

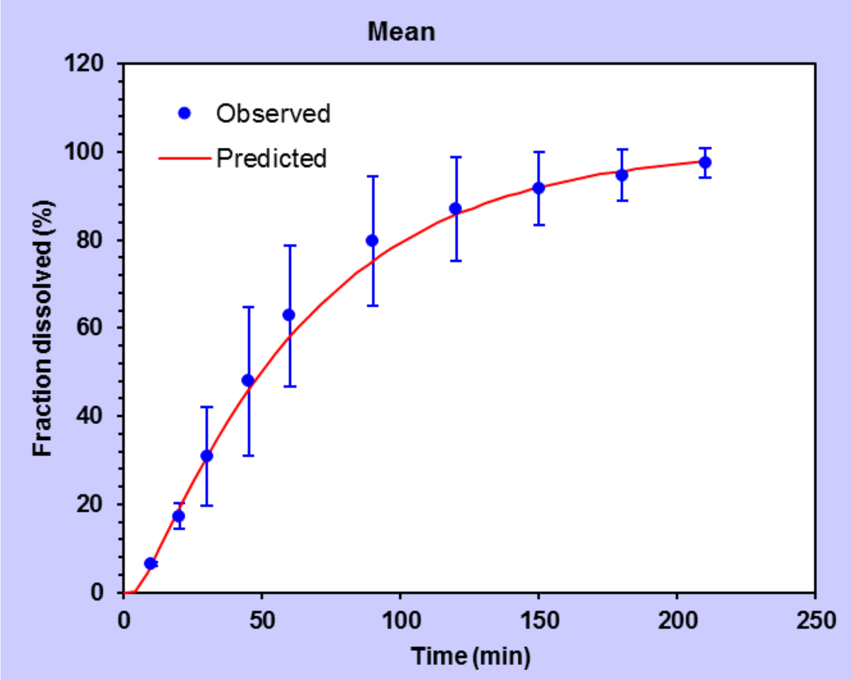

Supplementary materials – Model fitting summary – Parteck® M 200 (modelling the entire relevant dissolution data)

Graphical abstract of model fit presented as the fraction % of released carvedilol per tested tablet:

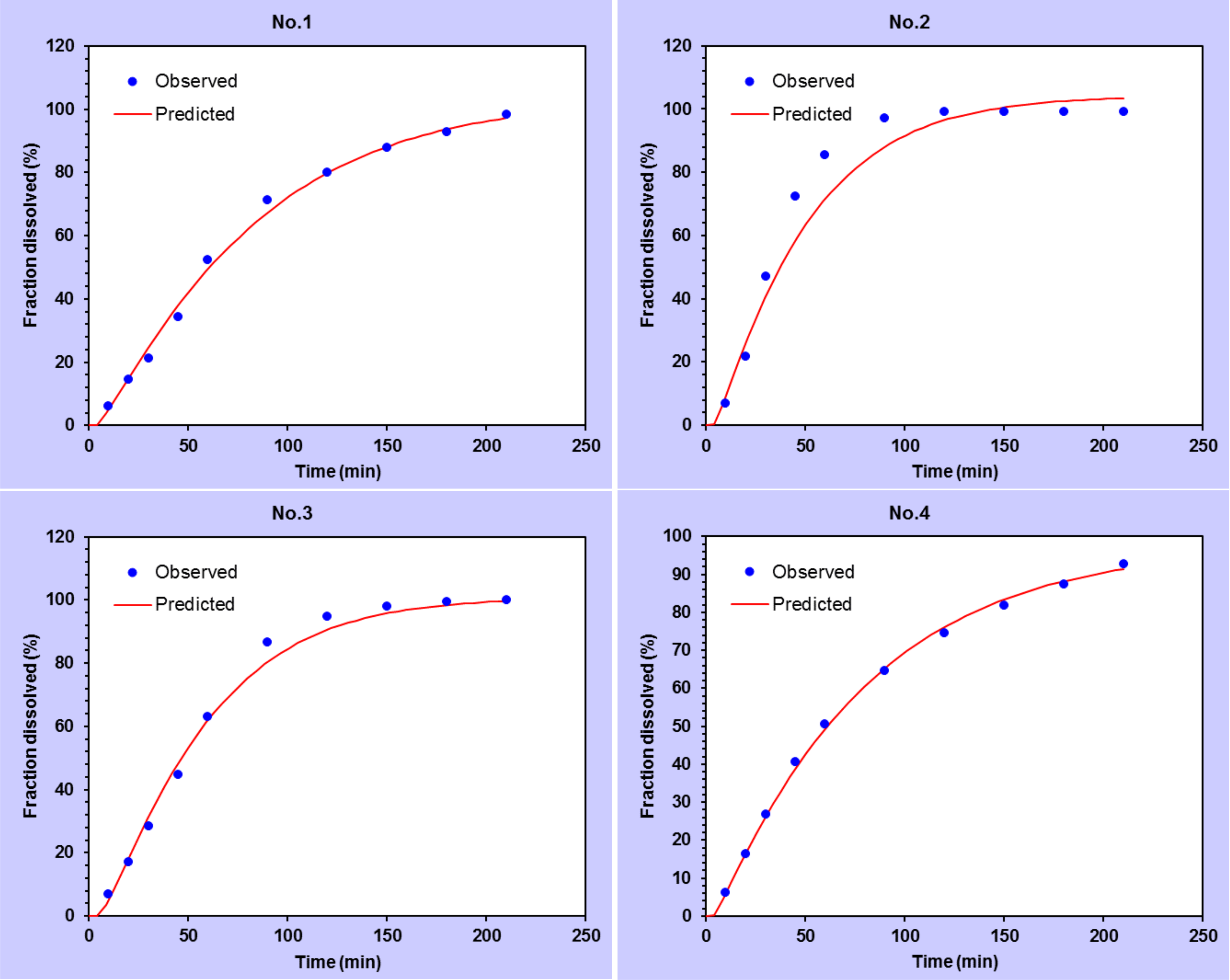

Supplementary materials – Model fitting summary – Parteck® M 200 (modelling the entire relevant dissolution data)

Model: **Logistic_1**

Model equation: $F = 100 \cdot \frac{e^{\alpha+\beta \cdot \log(t)}}{1+e^{\alpha+\beta \cdot \log(t)}}$

Fitted model parameters per tested tablet (N = 4) with statistics – mean, standard deviation (SD), and relative standard deviation expressed in % (RSD%) (output from DDSolver):

Parameter	No.1	No.2	No.3	No.4	Mean	SD	RSD(%)
α	-9.236	-9.248	-13.044	-6.569	-9.524	2.663	-27.964
β	5.142	6.336	7.497	3.758	5.683	1.604	28.218

Number of dissolution data points (N), degrees of freedom (df), and selected goodness of fit criteria – Pearson correlation coefficient (R), coefficient of determination (R^2), adjusted coefficient of determination ($R^2_{adjusted}$), and residual sum of squares (RSS) (manual calculation in MS Excel):

Parameter	No.1	No.2	No.3	No.4
N	10	10	10	10
df	8	8	8	8
R	0.998452393	0.996831142	0.997269216	0.997803888
R^2	0.996907182	0.993672325	0.994545889	0.995612598
$R^2_{adjusted}$	0.996520579	0.992881365	0.993864126	0.995064173
RSS	150.1692028	96.80961817	645.7532659	42.92673477

Graphical abstract of model fit presented as mean ± 1 SD of the fraction % of released carvedilol:

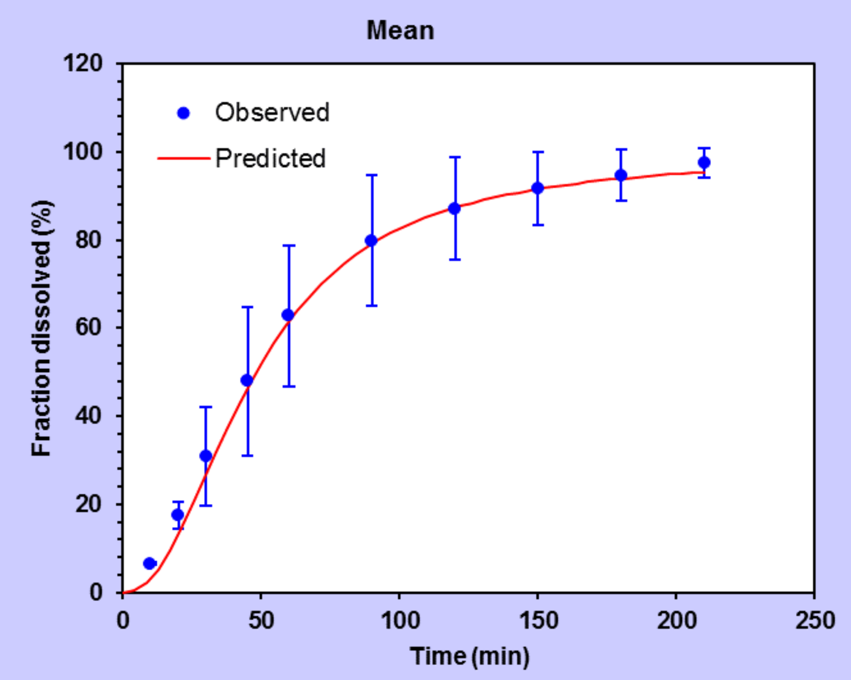

Supplementary materials – Model fitting summary – Parteck® M 200 (modelling the entire relevant dissolution data)

Graphical abstract of model fit presented as the fraction % of released carvedilol per tested tablet:

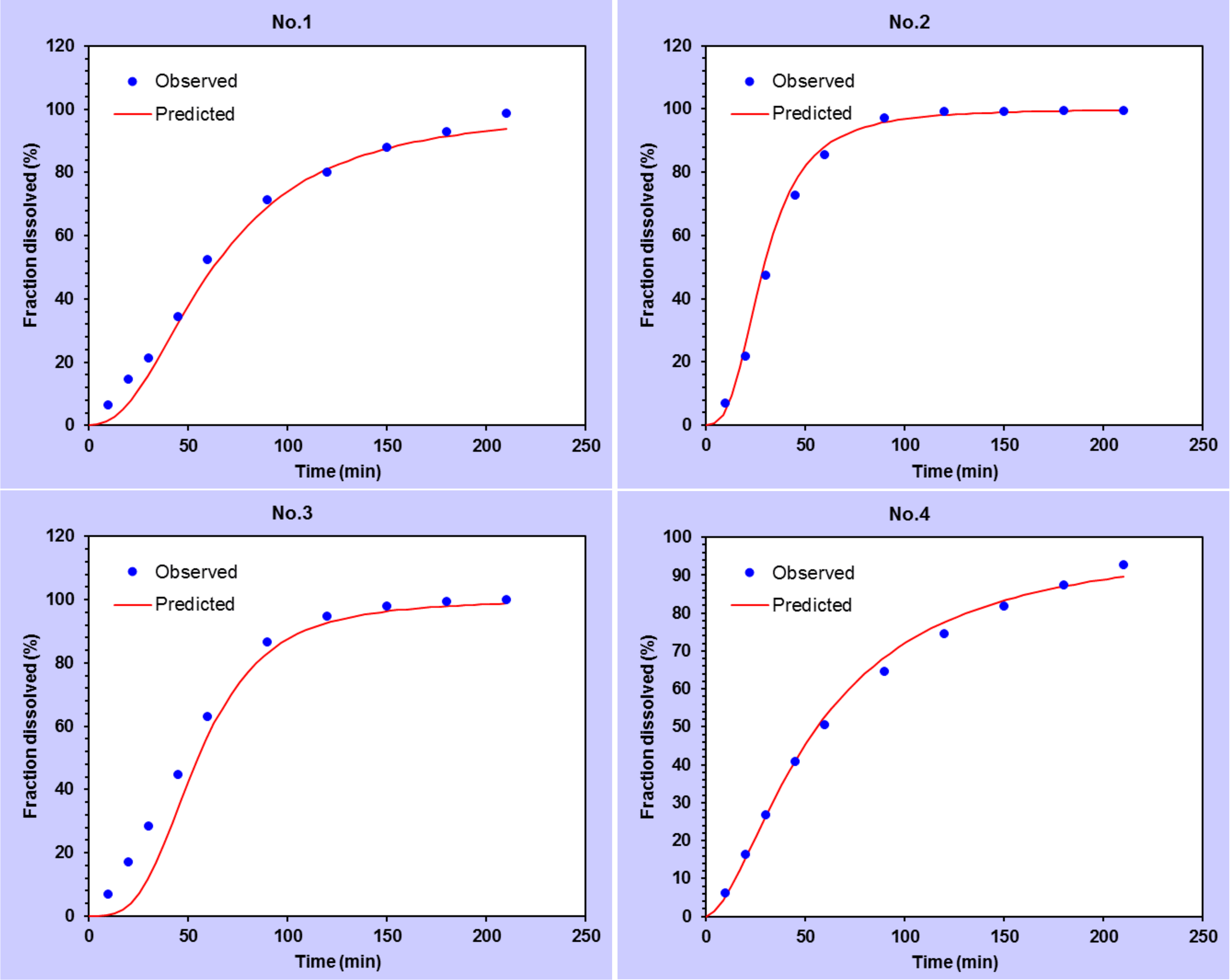

Supplementary materials – Model fitting summary – Parteck® M 200 (modelling the entire relevant dissolution data)

Model: **Logistic_2**

Model equation: $F = F_{max} \cdot \frac{e^{\alpha+\beta\cdot\log(t)}}{1+e^{\alpha+\beta\cdot\log(t)}}$

Fitted model parameters per tested tablet (N = 4) with statistics – mean, standard deviation (SD), and relative standard deviation expressed in % (RSD%) (output from DDSolver):

Parameter	No.1	No.2	No.3	No.4	Mean	SD	RSD(%)
α	-7.353	-6.972	-7.606	-6.848	-7.195	0.348	-4.841
β	4.215	4.581	4.620	3.982	4.349	0.305	7.021
F_{max}	103.362	104.225	104.895	97.334	102.454	3.471	3.388

Number of dissolution data points (N), degrees of freedom (df), and selected goodness of fit criteria – Pearson correlation coefficient (R), coefficient of determination (R^2), adjusted coefficient of determination ($R^2_{adjusted}$), and residual sum of squares (RSS) (manual calculation in MS Excel):

Parameter	No.1	No.2	No.3	No.4
N	10	10	10	10
df	7	7	7	7
R	0.995760459	0.99458278	0.995463533	0.996258739
R^2	0.991538892	0.989194907	0.990947647	0.992531476
$R^2_{adjusted}$	0.989121433	0.986107737	0.98836126	0.990397611
RSS	115.6510454	145.6426856	131.3194591	78.62850267

Graphical abstract of model fit presented as mean ± 1 SD of the fraction % of released carvedilol:

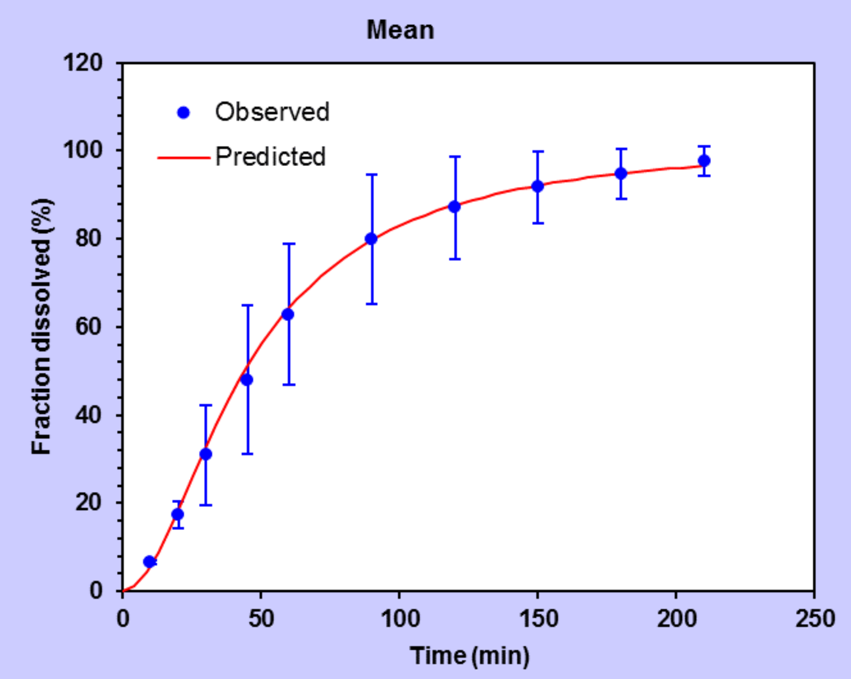

Supplementary materials – Model fitting summary – Parteck® M 200 (modelling the entire relevant dissolution data)

Graphical abstract of model fit presented as the fraction % of released carvedilol per tested tablet:

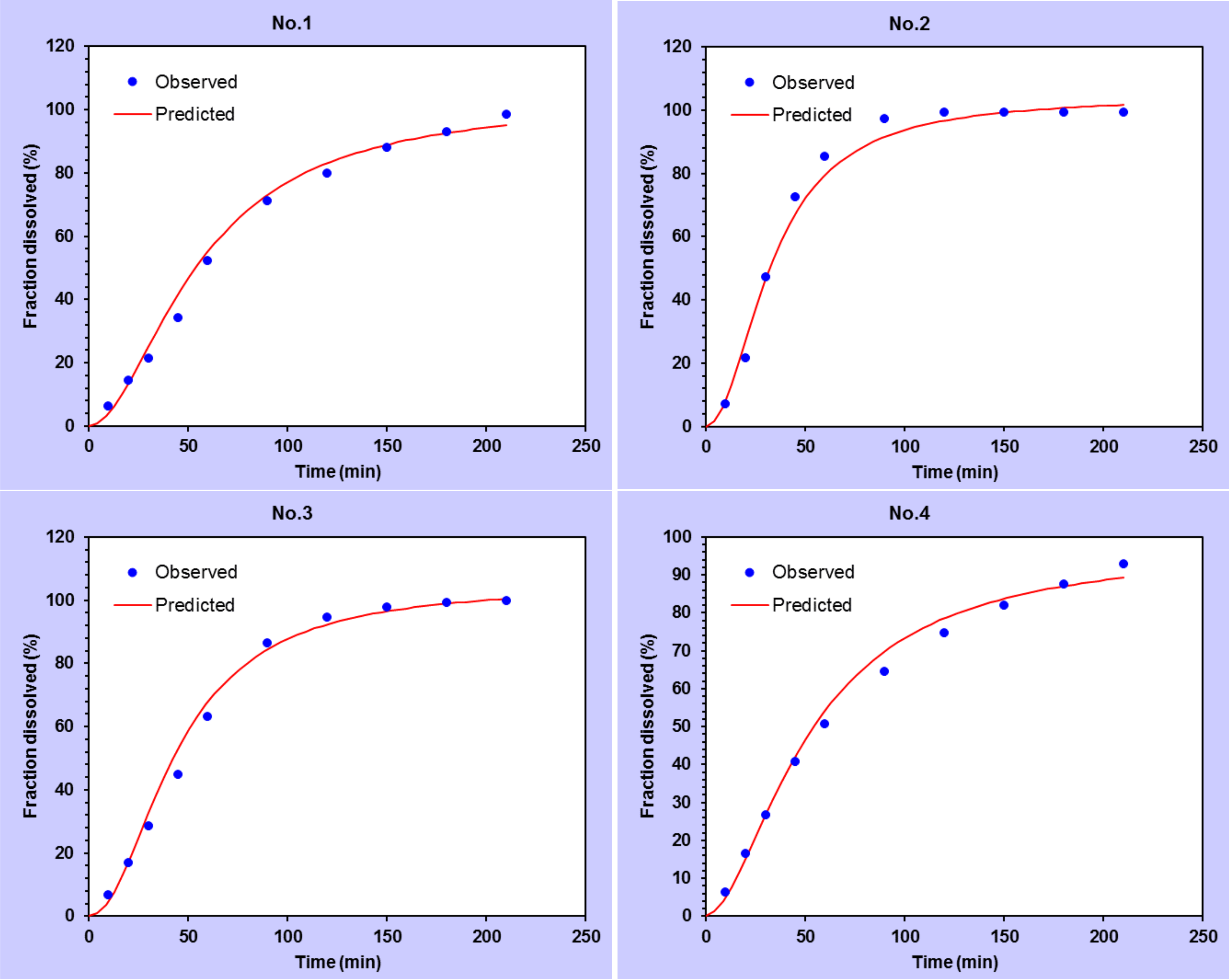

Supplementary materials – Model fitting summary – Parteck® M 200 (modelling the entire relevant dissolution data)

Model: **Logistic_3**

Model equation: $F = F_{max} \cdot \frac{1}{1+e^{-k\cdot(t-\gamma)}}$

Fitted model parameters per tested tablet (N = 4) with statistics – mean, standard deviation (SD), and relative standard deviation expressed in % (RSD%) (output from DDSolver):

Parameter	No.1	No.2	No.3	No.4	Mean	SD	RSD(%)
k	0.026	0.037	0.041	0.024	0.032	0.008	25.896
γ	82.474	40.184	65.540	78.358	66.639	19.054	28.592
F_{max}	103.362	104.225	104.895	97.334	102.454	3.471	3.388

Number of dissolution data points (N), degrees of freedom (df), and selected goodness of fit criteria – Pearson correlation coefficient (R), coefficient of determination (R^2), adjusted coefficient of determination ($R^2_{adjusted}$), and residual sum of squares (RSS) (manual calculation in MS Excel):

Parameter	No.1	No.2	No.3	No.4
N	10	10	10	10
df	7	7	7	7
R	0.976529117	0.952569592	0.981642688	0.974926619
R^2	0.953609116	0.907388828	0.963622367	0.950481913
$R^2_{adjusted}$	0.940354578	0.880928493	0.953228757	0.936333888
RSS	572.1083866	1080.056805	689.8975419	478.2378581

Graphical abstract of model fit presented as mean ± 1 SD of the fraction % of released carvedilol:

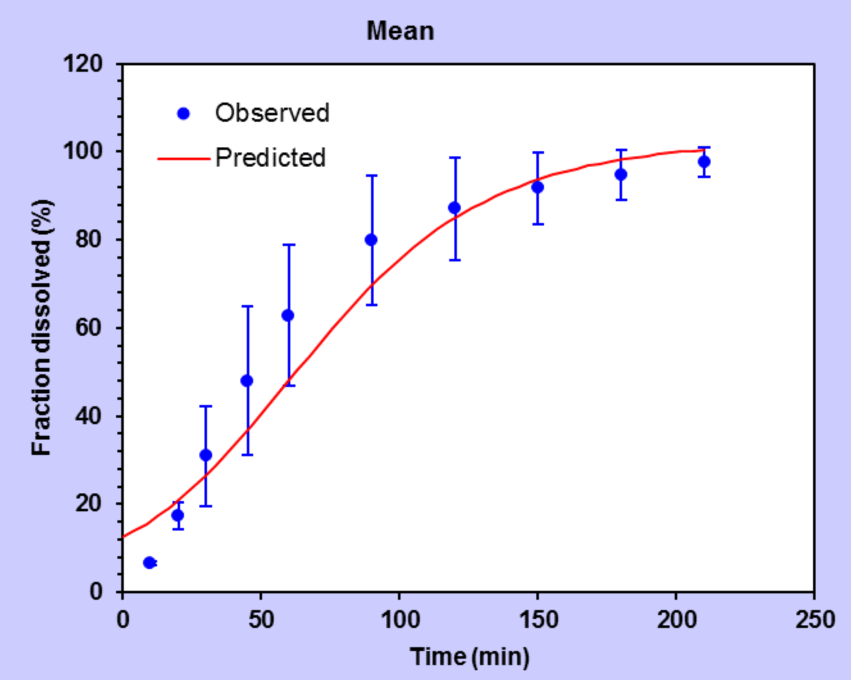

Supplementary materials – Model fitting summary – Parteck® M 200 (modelling the entire relevant dissolution data)

Graphical abstract of model fit presented as the fraction % of released carvedilol per tested tablet:

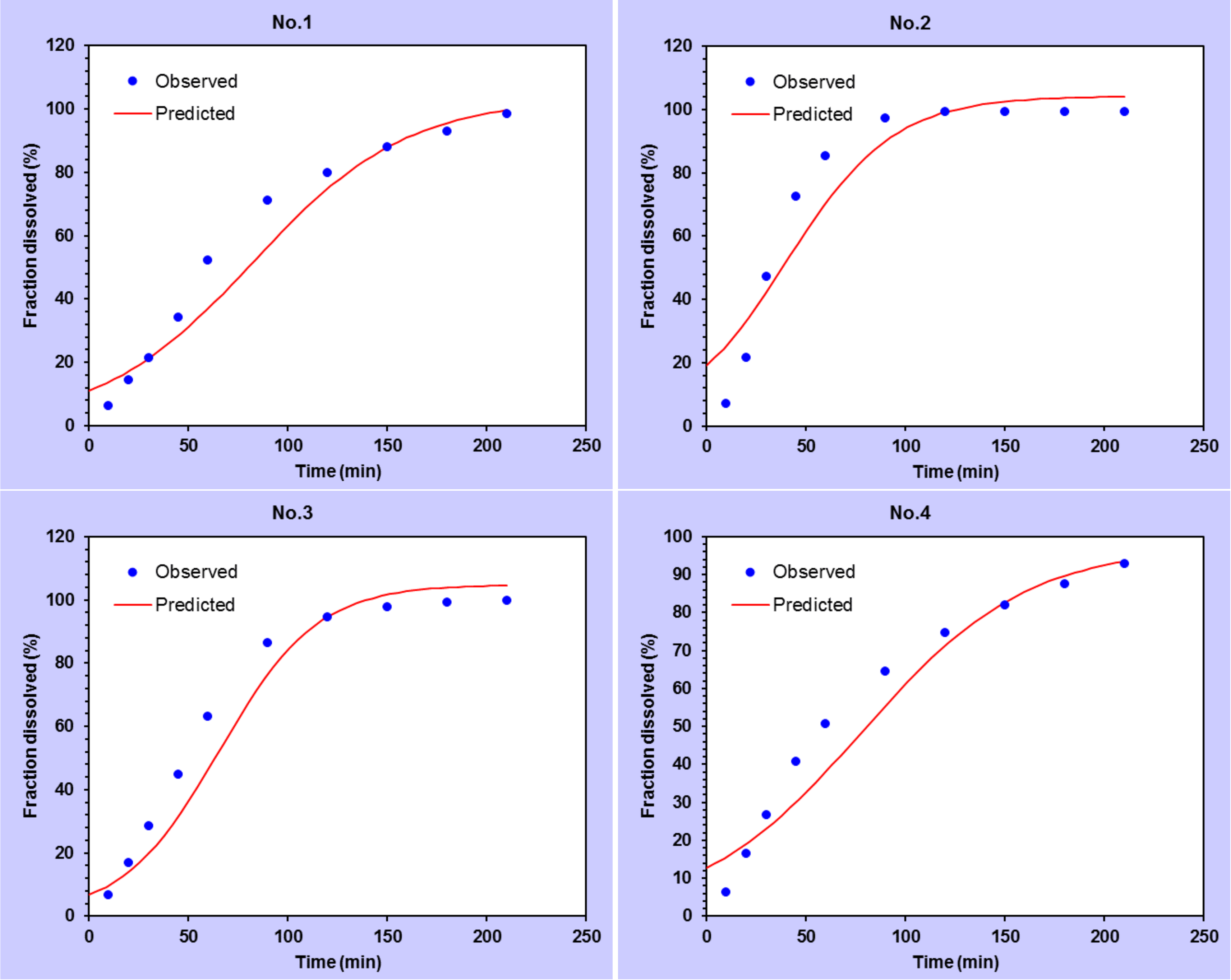

Supplementary materials – Model fitting summary – Parteck® M 200 (modelling the entire relevant dissolution data)

Model: **Gompertz_1**

Model equation: $F = 100 \cdot e^{-\alpha \cdot e^{-\beta \cdot \log(t)}}$

Fitted model parameters per tested tablet (N = 4) with statistics – mean, standard deviation (SD), and relative standard deviation expressed in % (RSD%) (output from DDSolver):

Parameter	No.1	No.2	No.3	No.4	Mean	SD	RSD(%)
α	286.572	1654.791	3947.194	54.020	1485.644	1786.538	120.253
β	3.453	5.265	5.470	2.596	4.196	1.400	33.358

Number of dissolution data points (N), degrees of freedom (df), and selected goodness of fit criteria – Pearson correlation coefficient (R), coefficient of determination (R^2), adjusted coefficient of determination ($R^2_{adjusted}$), and residual sum of squares (RSS) (manual calculation in MS Excel):

Parameter	No.1	No.2	No.3	No.4
N	10	10	10	10
df	8	8	8	8
R	0.992159086	0.997043731	0.973421271	0.98802625
R^2	0.984379652	0.994096201	0.947548971	0.976195871
$R^2_{adjusted}$	0.982427108	0.993358226	0.940992592	0.973220355
RSS	260.1207876	100.7908104	820.6628835	243.2274305

Graphical abstract of model fit presented as mean ± 1 SD of the fraction % of released carvedilol:

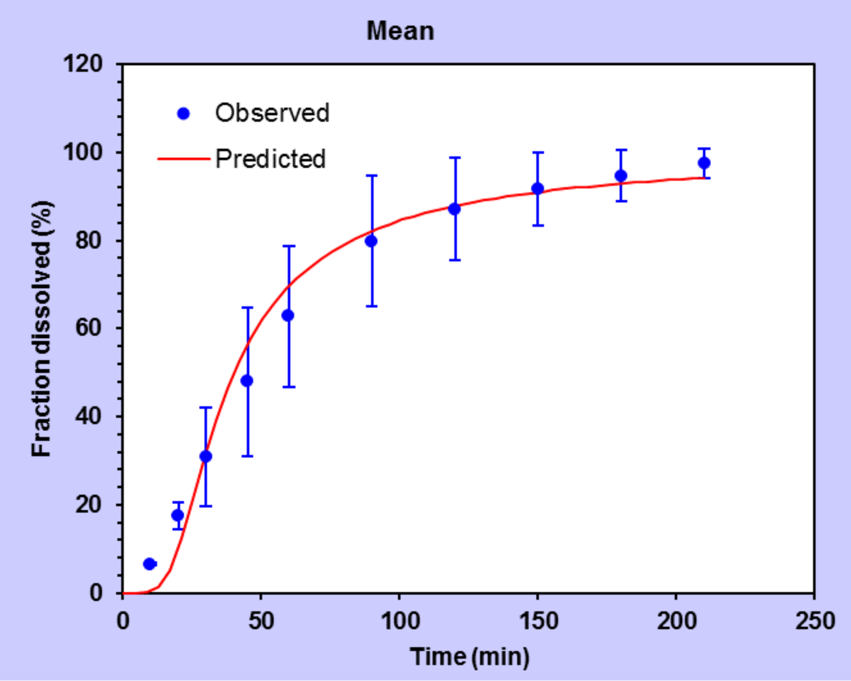

Supplementary materials – Model fitting summary – Parteck® M 200 (modelling the entire relevant dissolution data)

Graphical abstract of model fit presented as the fraction % of released carvedilol per tested tablet:

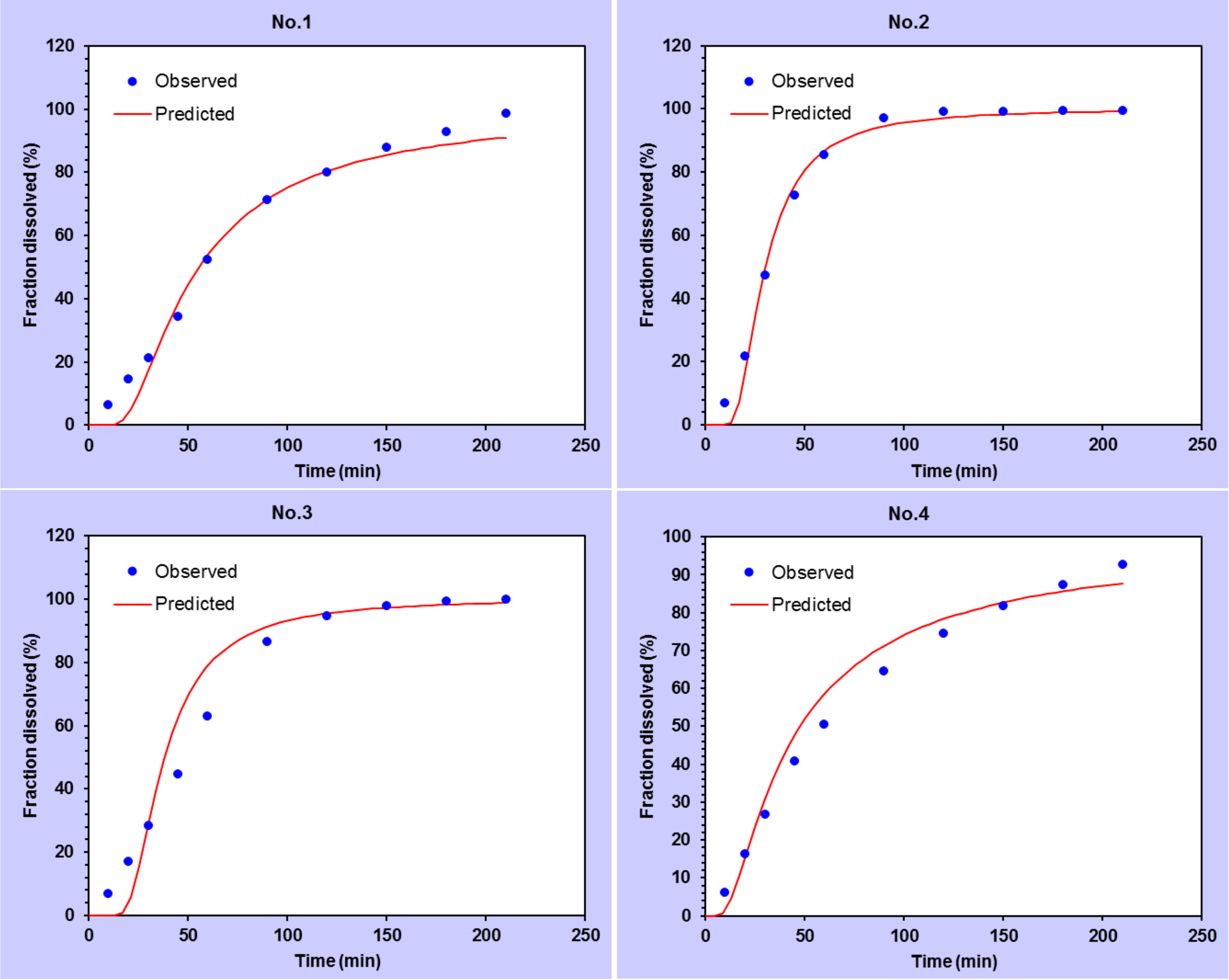

Supplementary materials – Model fitting summary – Parteck® M 200 (modelling the entire relevant dissolution data)

Model: **Gompertz_2**

Model equation: $F = F_{max} \cdot e^{-\alpha \cdot e^{-\beta \cdot \log(t)}}$

Fitted model parameters per tested tablet (N = 4) with statistics – mean, standard deviation (SD), and relative standard deviation expressed in % (RSD%) (output from DDSolver):

Parameter	No.1	No.2	No.3	No.4	Mean	SD	RSD(%)
α	143.696	109.577	223.989	73.019	137.570	64.437	46.839
β	2.953	3.504	3.419	2.824	3.175	0.337	10.600
F_{max}	103.362	104.225	104.895	97.334	102.454	3.471	3.388

Number of dissolution data points (N), degrees of freedom (df), and selected goodness of fit criteria – Pearson correlation coefficient (R), coefficient of determination (R^2), adjusted coefficient of determination ($R^2_{adjusted}$), and residual sum of squares (RSS) (manual calculation in MS Excel):

Parameter	No.1	No.2	No.3	No.4
N	10	10	10	10
df	7	7	7	7
R	0.996349065	0.990415299	0.994991312	0.984652645
R^2	0.992711459	0.980922465	0.99000771	0.969540831
$R^2_{adjusted}$	0.990629018	0.975471741	0.98715277	0.960838211
RSS	382.9075518	255.8846919	324.089477	327.7086192

Graphical abstract of model fit presented as mean ± 1 SD of the fraction % of released carvedilol:

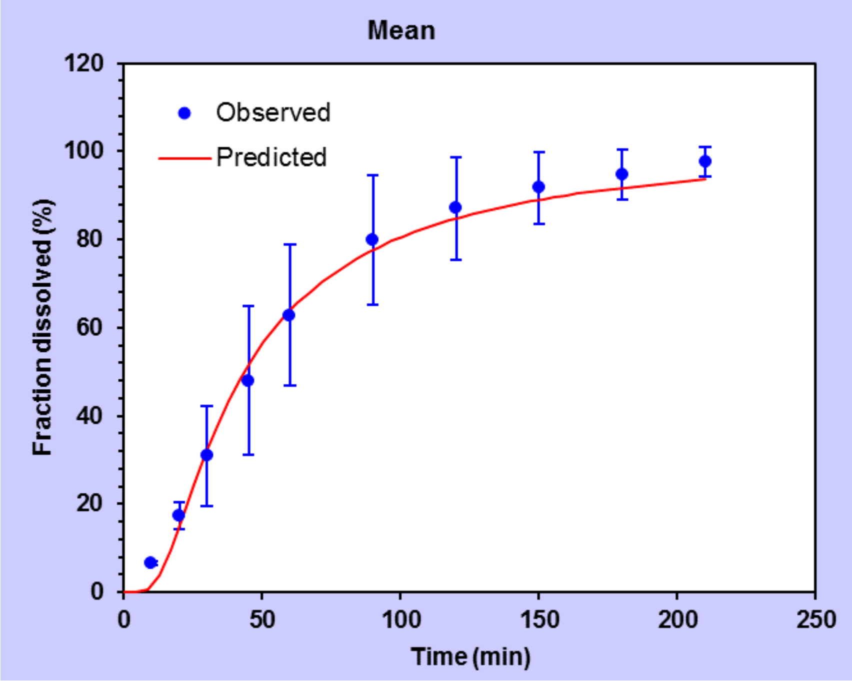

Supplementary materials – Model fitting summary – Parteck® M 200 (modelling the entire relevant dissolution data)

Graphical abstract of model fit presented as the fraction % of released carvedilol per tested tablet:

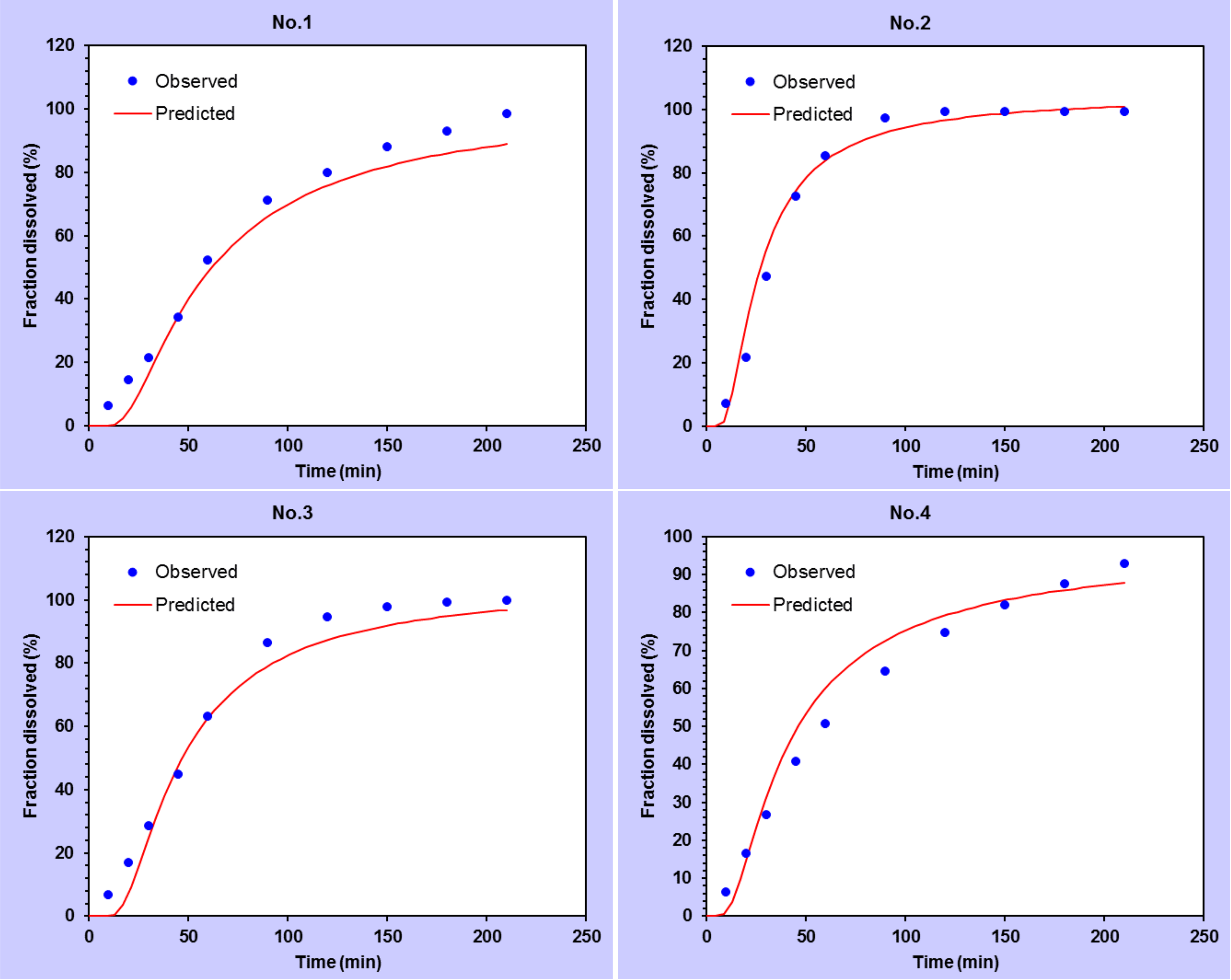

Supplementary materials – Model fitting summary – Parteck® M 200 (modelling the entire relevant dissolution data)

Model: **Gompertz_3**

Model equation: $F = F_{max} \cdot e^{-e^{-k\cdot(t-\gamma)}}$

Fitted model parameters per tested tablet (N = 4) with statistics – mean, standard deviation (SD), and relative standard deviation expressed in % (RSD%) (output from DDSolver):

Parameter	No.1	No.2	No.3	No.4	Mean	SD	RSD(%)
k	0.019	0.020	0.031	0.026	0.024	0.006	23.439
γ	51.974	14.766	36.958	36.701	35.100	15.321	43.650
F_{max}	103.362	104.225	100.397	88.096	99.020	7.465	7.539

Number of dissolution data points (N), degrees of freedom (df), and selected goodness of fit criteria – Pearson correlation coefficient (R), coefficient of determination (R^2), adjusted coefficient of determination ($R^2_{adjusted}$), and residual sum of squares (RSS) (manual calculation in MS Excel):

Parameter	No.1	No.2	No.3	No.4
N	10	10	10	10
df	7	7	7	7
R	0.992850591	0.937397062	0.999213332	0.995244918
R^2	0.985752296	0.878713252	0.998427283	0.990512446
$R^2_{adjusted}$	0.981681523	0.844059896	0.997977935	0.987801717
RSS	165.8356821	1883.462438	34.98673916	108.3655148

Graphical abstract of model fit presented as mean ± 1 SD of the fraction % of released carvedilol:

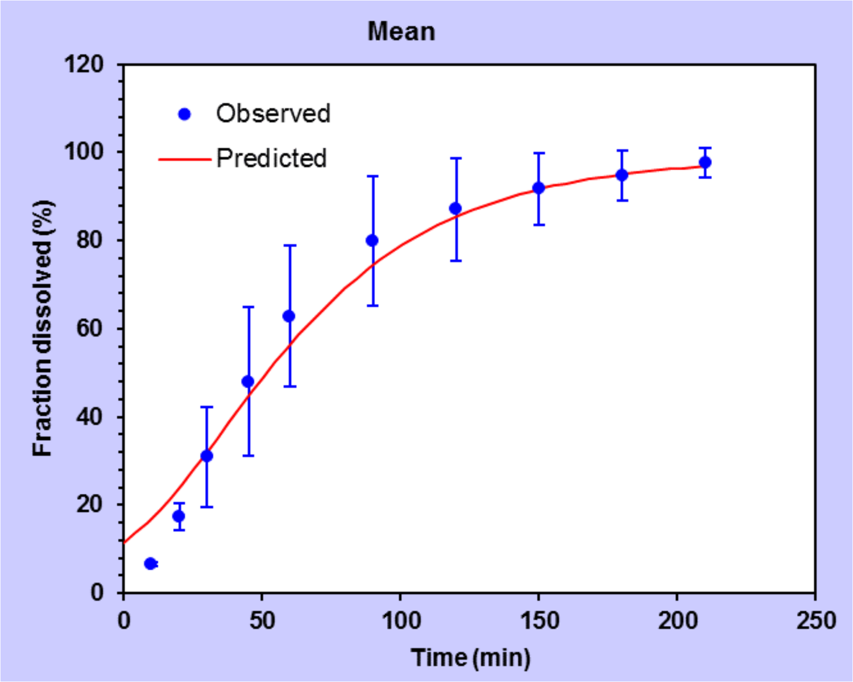

Supplementary materials – Model fitting summary – Parteck® M 200 (modelling the entire relevant dissolution data)

Graphical abstract of model fit presented as the fraction % of released carvedilol per tested tablet:

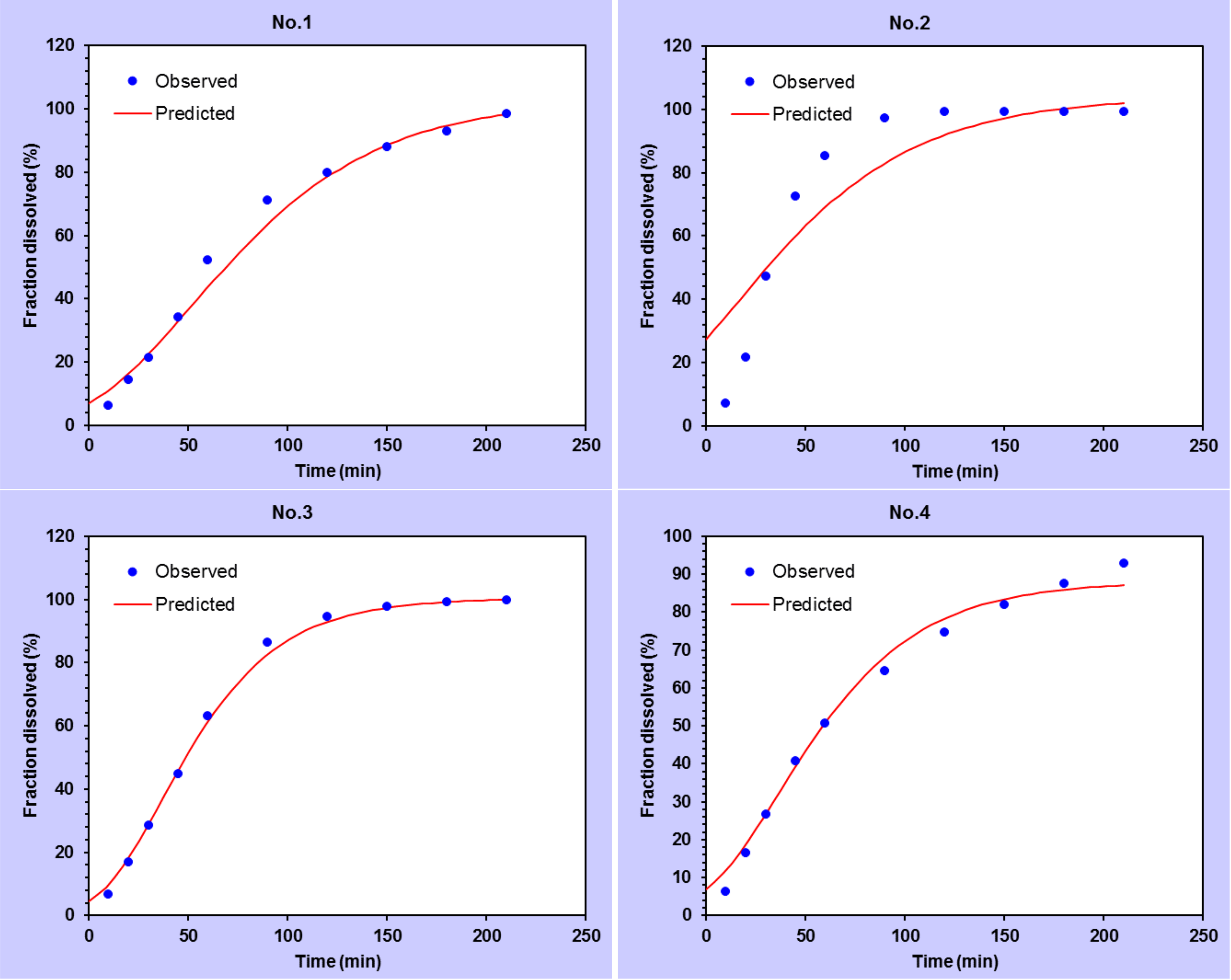

Supplementary materials – Model fitting summary – Parteck® M 200 (modelling the entire relevant dissolution data)

Model: **Gompertz_4**

Model equation: $F = F_{max} \cdot e^{-\beta \cdot e^{-k \cdot t}}$

Fitted model parameters per tested tablet (N = 4) with statistics – mean, standard deviation (SD), and relative standard deviation expressed in % (RSD%) (output from DDSolver):

Parameter	No.1	No.2	No.3	No.4	Mean	SD	RSD(%)
k	0.019	0.020	0.023	0.018	0.020	0.002	11.244
β	2.690	1.214	2.784	2.386	2.269	0.723	31.881
F_{max}	103.362	104.225	107.080	97.334	103.000	4.098	3.979

Number of dissolution data points (N), degrees of freedom (df), and selected goodness of fit criteria – Pearson correlation coefficient (R), coefficient of determination (R^2), adjusted coefficient of determination ($R^2_{adjusted}$), and residual sum of squares (RSS) (manual calculation in MS Excel):

Parameter	No.1	No.2	No.3	No.4
N	10	10	10	10
df	7	7	7	7
R	0.992850591	0.94133046	0.988557504	0.990382218
R^2	0.985752296	0.886103036	0.977245939	0.980856937
$R^2_{adjusted}$	0.981681523	0.853561046	0.970744778	0.975387491
RSS	165.8356821	2090.72315	299.0411903	170.0933462

Graphical abstract of model fit presented as mean ± 1 SD of the fraction % of released carvedilol:

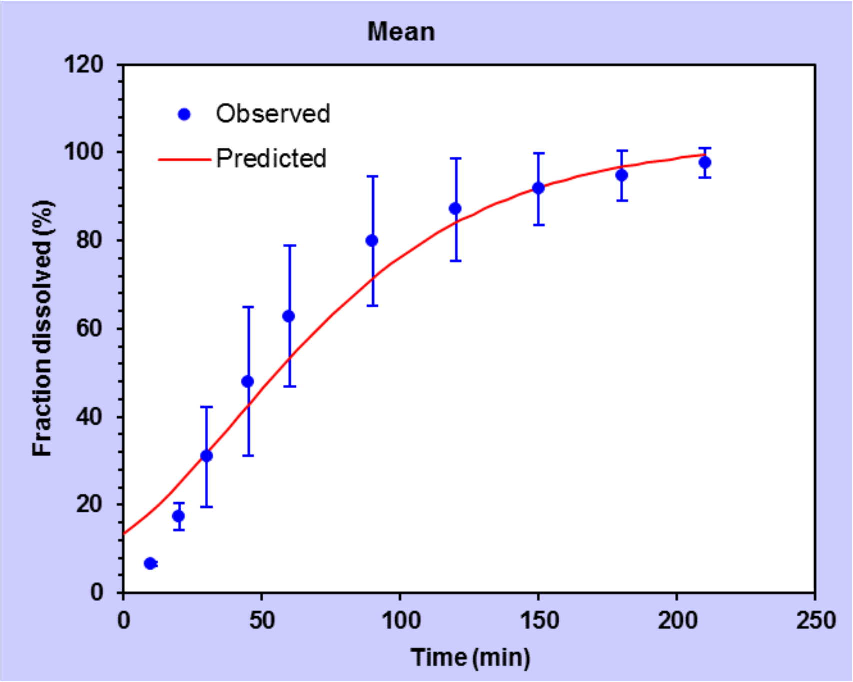

Supplementary materials – Model fitting summary – Parteck® M 200 (modelling the entire relevant dissolution data)

Graphical abstract of model fit presented as the fraction % of released carvedilol per tested tablet:

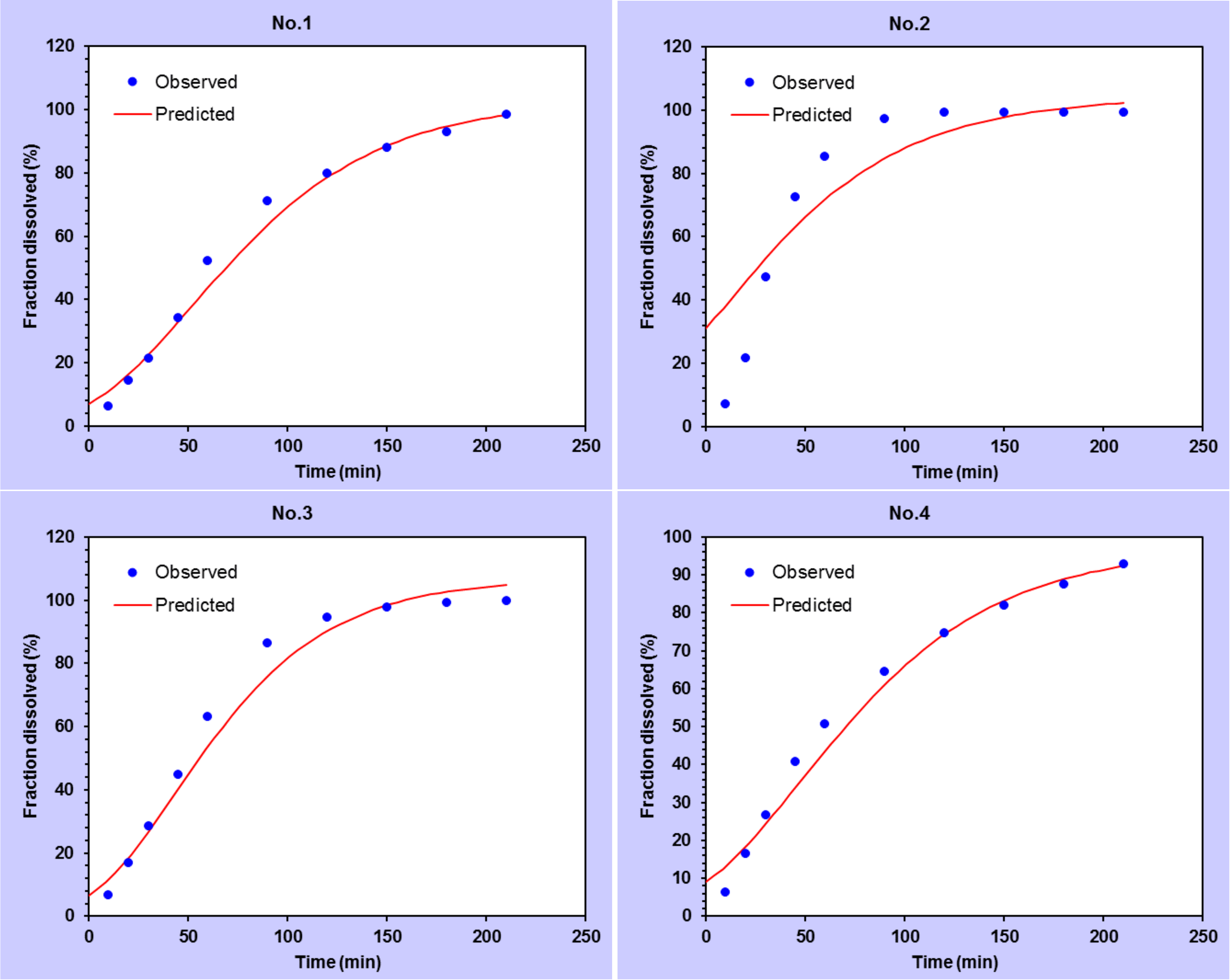

Supplementary materials – Model fitting summary – Parteck® M 200 (modelling the entire relevant dissolution data)

Model: **Probit_1**

Model equation: $F = 100 \cdot \phi[\alpha + \beta \cdot \log(t)]$

Fitted model parameters per tested tablet (N = 4) with statistics – mean, standard deviation (SD), and relative standard deviation expressed in % (RSD%) (output from DDSolver):

Parameter	No.1	No.2	No.3	No.4	Mean	SD	RSD(%)
α	-5.244	-5.223	-5.442	-3.834	-4.936	0.741	-15.010
β	2.909	3.564	3.438	2.196	3.027	0.622	20.565

Number of dissolution data points (N), degrees of freedom (df), and selected goodness of fit criteria – Pearson correlation coefficient (R), coefficient of determination (R^2), adjusted coefficient of determination ($R^2_{adjusted}$), and residual sum of squares (RSS) (manual calculation in MS Excel):

Parameter	No.1	No.2	No.3	No.4
N	10	10	10	10
df	8	8	8	8
R	0.99867427	0.997526399	0.986049059	0.998251062
R^2	0.997350297	0.995058917	0.972292746	0.996505182
$R^2_{adjusted}$	0.997019084	0.994441282	0.96882934	0.99606833
RSS	174.0697127	74.38525576	449.6580058	33.74147437

Graphical abstract of model fit presented as mean ± 1 SD of the fraction % of released carvedilol:

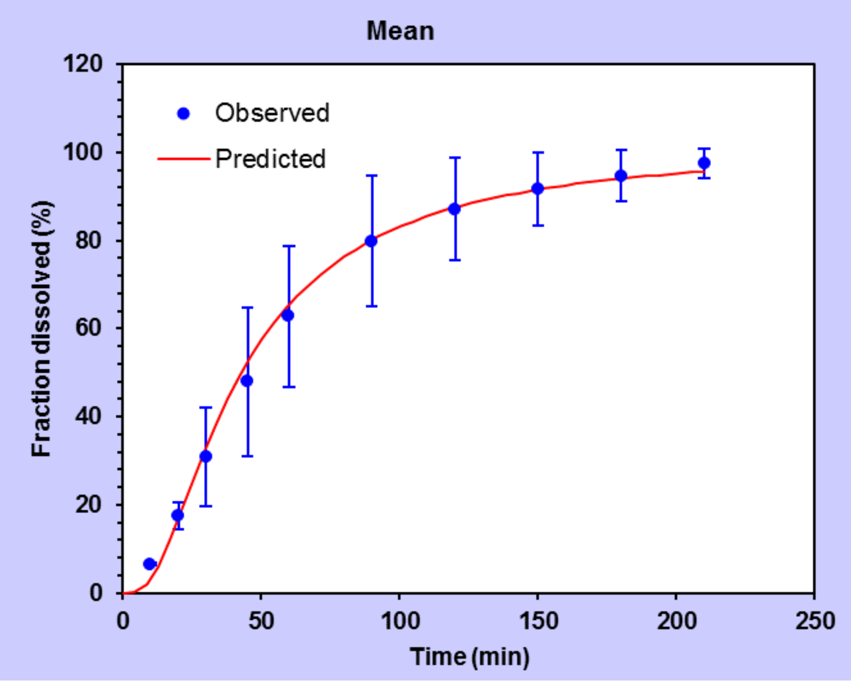

Supplementary materials – Model fitting summary – Parteck® M 200 (modelling the entire relevant dissolution data)

Graphical abstract of model fit presented as the fraction % of released carvedilol per tested tablet:

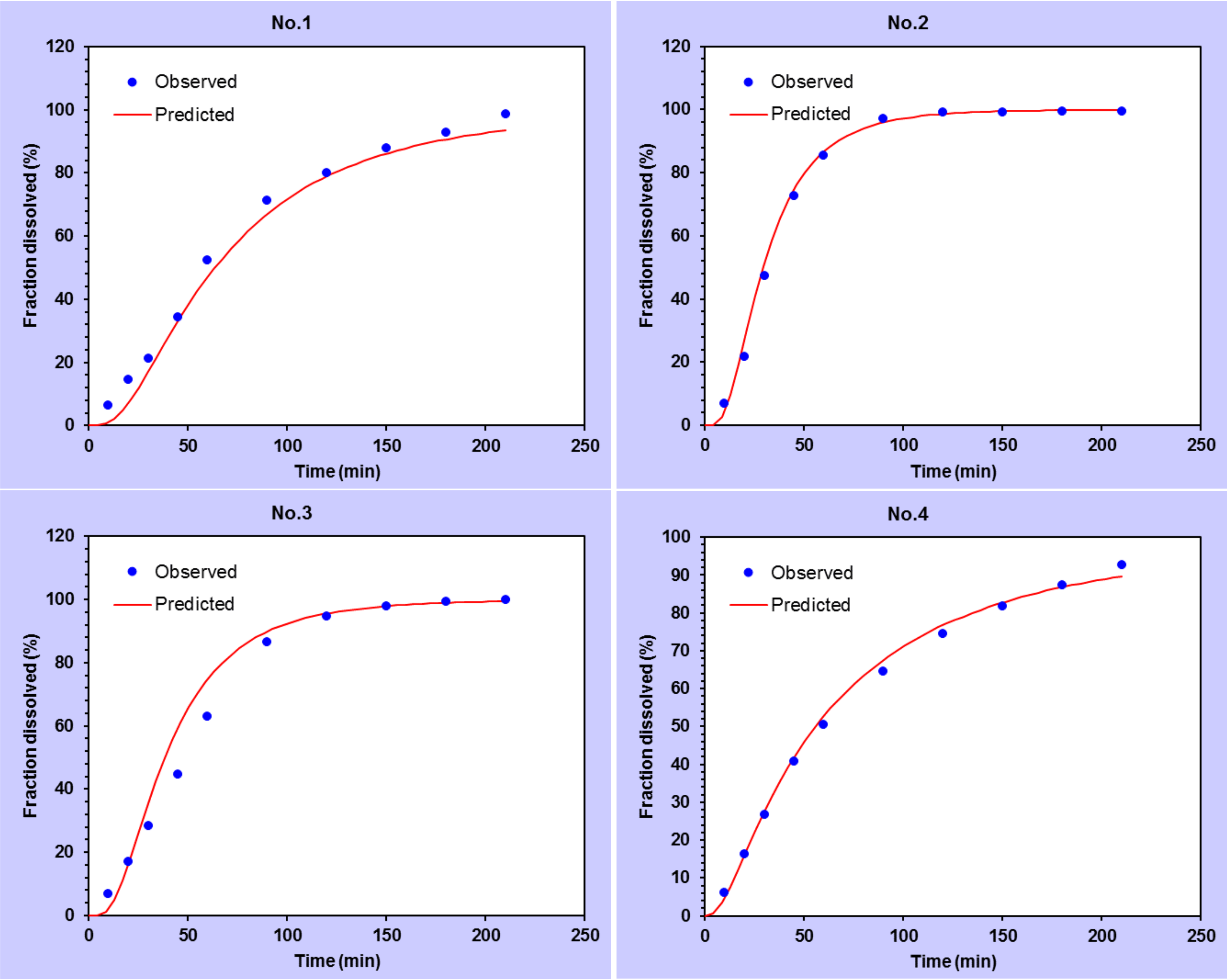

Supplementary materials – Model fitting summary – Parteck® M 200 (modelling the entire relevant dissolution data)

Model: **Probit_2**

Model equation: $F = F_{max} \cdot \phi[\alpha + \beta \cdot \log(t)]$

Fitted model parameters per tested tablet (N = 4) with statistics – mean, standard deviation (SD), and relative standard deviation expressed in % (RSD%) (output from DDSolver):

Parameter	No.1	No.2	No.3	No.4	Mean	SD	RSD(%)
α	-4.619	-3.942	-4.363	-3.971	-4.224	0.326	-7.718
β	2.617	2.587	2.644	2.309	2.539	0.156	6.126
F_{max}	99.521	104.225	104.895	97.334	101.494	3.662	3.608

Number of dissolution data points (N), degrees of freedom (df), and selected goodness of fit criteria – Pearson correlation coefficient (R), coefficient of determination (R^2), adjusted coefficient of determination ($R^2_{adjusted}$), and residual sum of squares (RSS) (manual calculation in MS Excel):

Parameter	No.1	No.2	No.3	No.4
N	10	10	10	10
df	7	7	7	7
R	0.996857908	0.99087294	0.994968409	0.997115229
R^2	0.993725688	0.981829184	0.989962134	0.994238779
$R^2_{adjusted}$	0.991933028	0.976637522	0.987094172	0.992592716
RSS	106.8005669	236.4485143	142.6267027	58.24616172

Graphical abstract of model fit presented as mean ± 1 SD of the fraction % of released carvedilol:

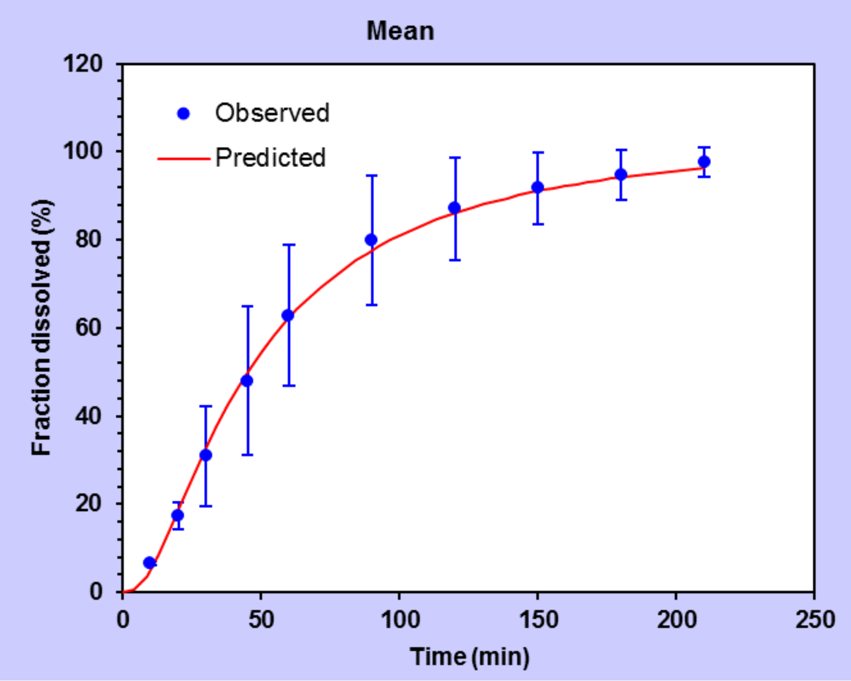

Supplementary materials – Model fitting summary – Parteck® M 200 (modelling the entire relevant dissolution data)

Graphical abstract of model fit presented as the fraction % of released carvedilol per tested tablet:

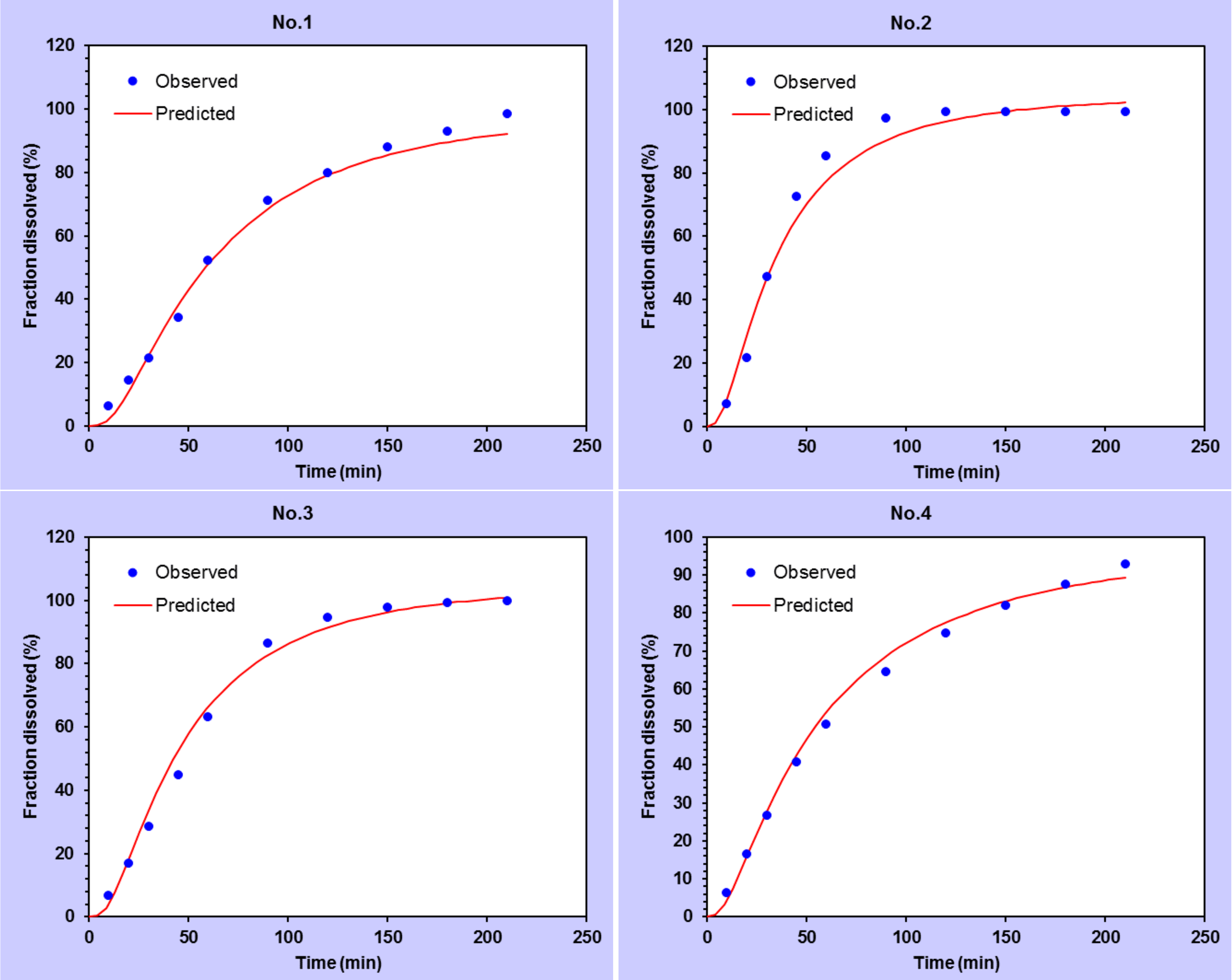

Supplementary materials – Model fitting summary – Parteck® M 200 (modelling up to app. 60 % of carvedilol released)

Model: **Zero–order**

Model equation: $F = k_0 \cdot t$

Fitted model parameters per tested tablet (N = 4) with statistics – mean, standard deviation (SD), and relative standard deviation expressed in % (RSD%) (output from DDSolver):

Parameter	No.1	No.2	No.3	No.4	Mean	SD	RSD(%)
k_0	0.806	1.466	1.005	0.862	1.035	0.300	28.967

Number of dissolution data points (N), degrees of freedom (df), and selected goodness of fit criteria – Pearson correlation coefficient (R), coefficient of determination (R^2), adjusted coefficient of determination ($R^2_{adjusted}$), and residual sum of squares (RSS) (manual calculation in MS Excel):

Parameter	No.1	No.2	No.3	No.4
N	5	5	5	5
df	4	4	4	4
R	0.994112033	0.986599376	0.999547112	0.996231412
R^2	0.988258735	0.973378328	0.999094429	0.992477026
$R^2_{adjusted}$	0.988258735	0.973378328	0.999094429	0.992477026
RSS	35.00443994	177.7017298	31.34964413	12.16225017

Graphical abstract of model fit presented as mean ± 1 SD of the fraction % of released carvedilol:

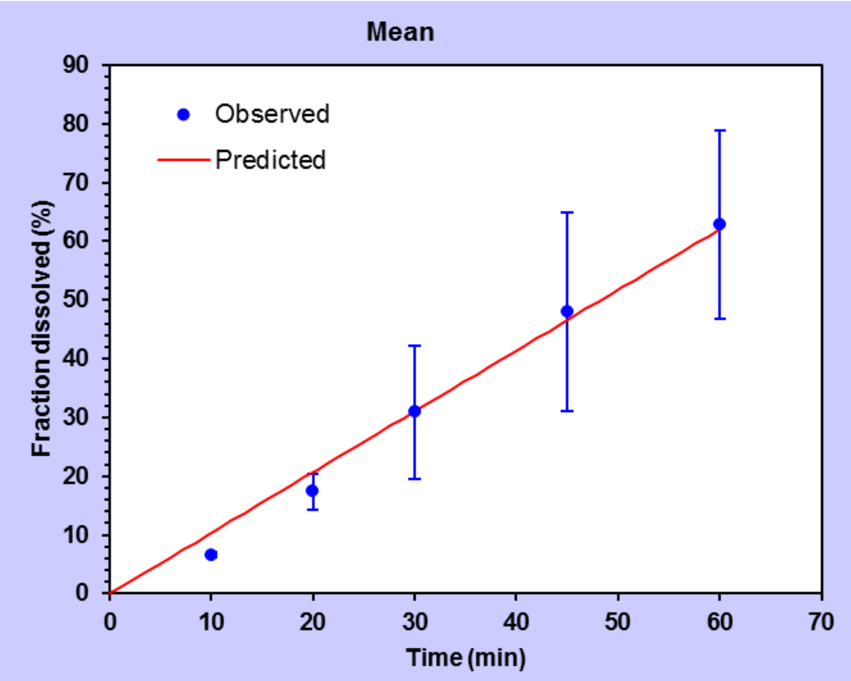

Supplementary materials – Model fitting summary – Parteck® M 200 (modelling up to app. 60 % of carvedilol released)

Graphical abstract of model fit presented as the fraction % of released carvedilol per tested tablet:

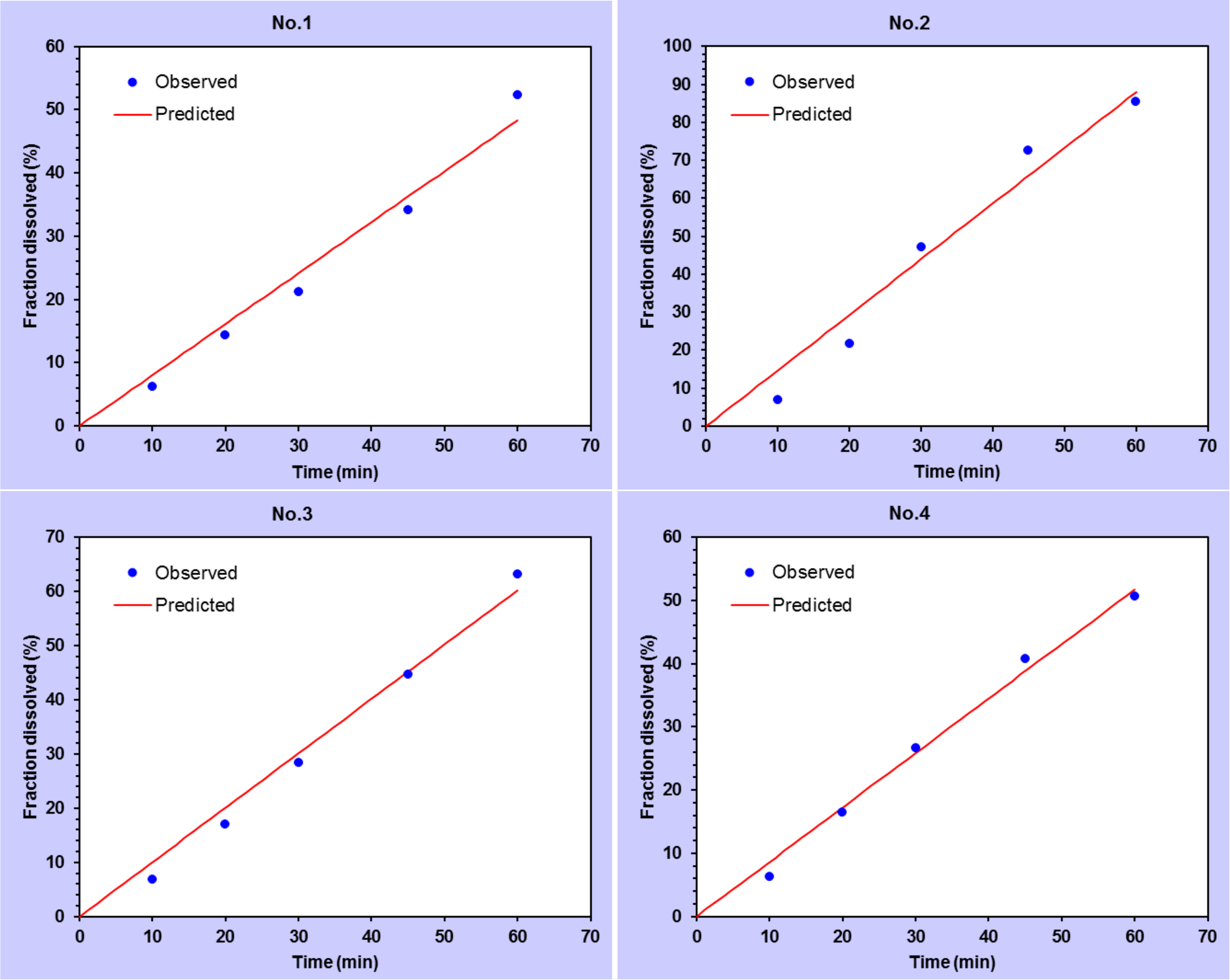

Supplementary materials – Model fitting summary – Parteck® M 200 (modelling up to app. 60 % of carvedilol released)

Model: **Zero–order with T_{lag}**

Model equation: $F = k_0 \cdot (t - T_{lag})$

Fitted model parameters per tested tablet (N = 4) with statistics – mean, standard deviation (SD), and relative standard deviation expressed in % (RSD%) (output from DDSolver):

Parameter	No.1	No.2	No.3	No.4	Mean	SD	RSD(%)
k_0	0.904	1.640	1.125	0.897	1.141	0.349	30.554
T_{lag}	4.625	4.509	4.556	1.671	3.840	1.447	37.678

Number of dissolution data points (N), degrees of freedom (df), and selected goodness of fit criteria – Pearson correlation coefficient (R), coefficient of determination (R^2), adjusted coefficient of determination ($R^2_{adjusted}$), and residual sum of squares (RSS) (manual calculation in MS Excel):

Parameter	No.1	No.2	No.3	No.4
N	5	5	5	5
df	3	3	3	3
R	0.994112033	0.986599376	0.999547112	0.996231412
R^2	0.988258735	0.973378328	0.999094429	0.992477026
$R^2_{adjusted}$	0.98434498	0.964504438	0.998792572	0.989969369
RSS	15.34127725	116.2174301	1.8125	9.635782265

Graphical abstract of model fit presented as mean ± 1 SD of the fraction % of released carvedilol:

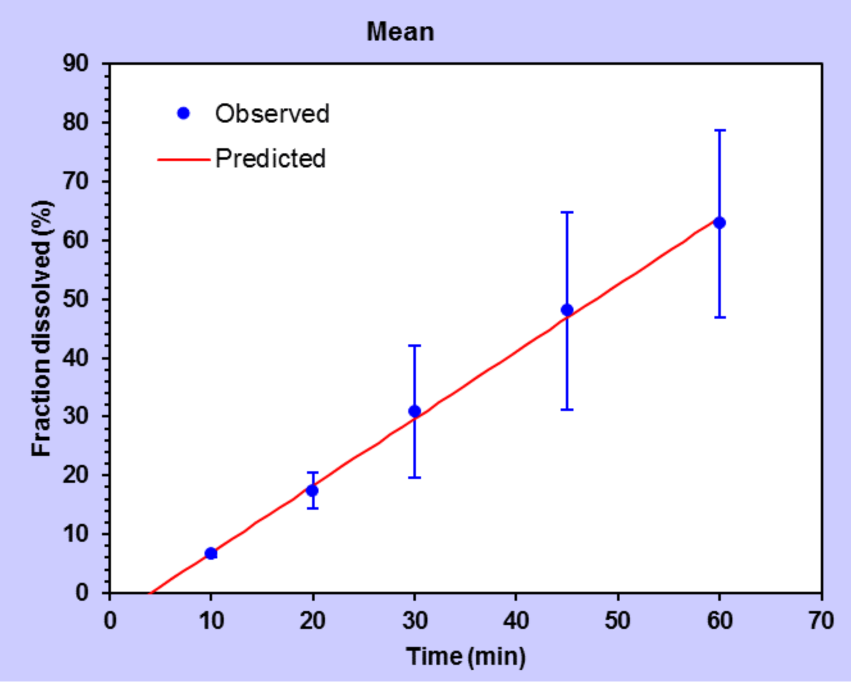

Supplementary materials – Model fitting summary – Parteck® M 200 (modelling up to app. 60 % of carvedilol released)

Graphical abstract of model fit presented as the fraction % of released carvedilol per tested tablet:

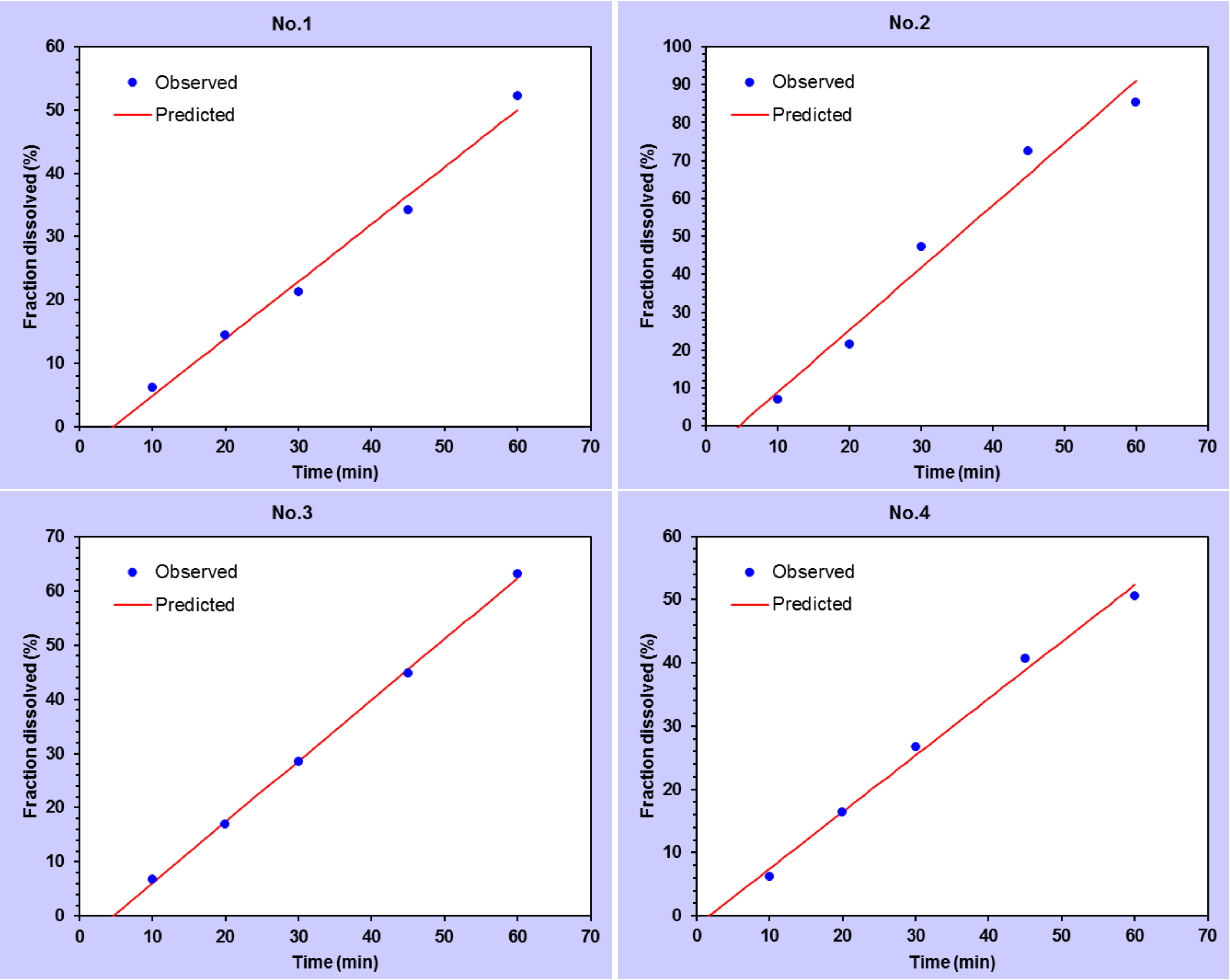

Supplementary materials – Model fitting summary – Parteck® M 200 (modelling up to app. 60 % of carvedilol released)

Model: **Zero–order with F_0**

Model equation: $F = F_0 + k_0 \cdot t$

Fitted model parameters per tested tablet (N = 4) with statistics – mean, standard deviation (SD), and relative standard deviation expressed in % (RSD%) (output from DDSolver):

Parameter	No.1	No.2	No.3	No.4	Mean	SD	RSD(%)
k_0	0.904	1.640	1.125	0.897	1.141	0.349	30.554
F_0	-4.182	-7.394	-5.125	-1.499	-4.550	2.440	-53.634

Number of dissolution data points (N), degrees of freedom (df), and selected goodness of fit criteria – Pearson correlation coefficient (R), coefficient of determination (R^2), adjusted coefficient of determination ($R^2_{adjusted}$), and residual sum of squares (RSS) (manual calculation in MS Excel):

Parameter	No.1	No.2	No.3	No.4
N	5	5	5	5
df	3	3	3	3
R	0.994112033	0.986599376	0.999547112	0.996231412
R^2	0.988258735	0.973378328	0.999094429	0.992477026
$R^2_{adjusted}$	0.98434498	0.964504438	0.998792572	0.989969369
RSS	15.34127725	116.2174301	1.8125	9.635782265

Graphical abstract of model fit presented as mean ± 1 SD of the fraction % of released carvedilol:

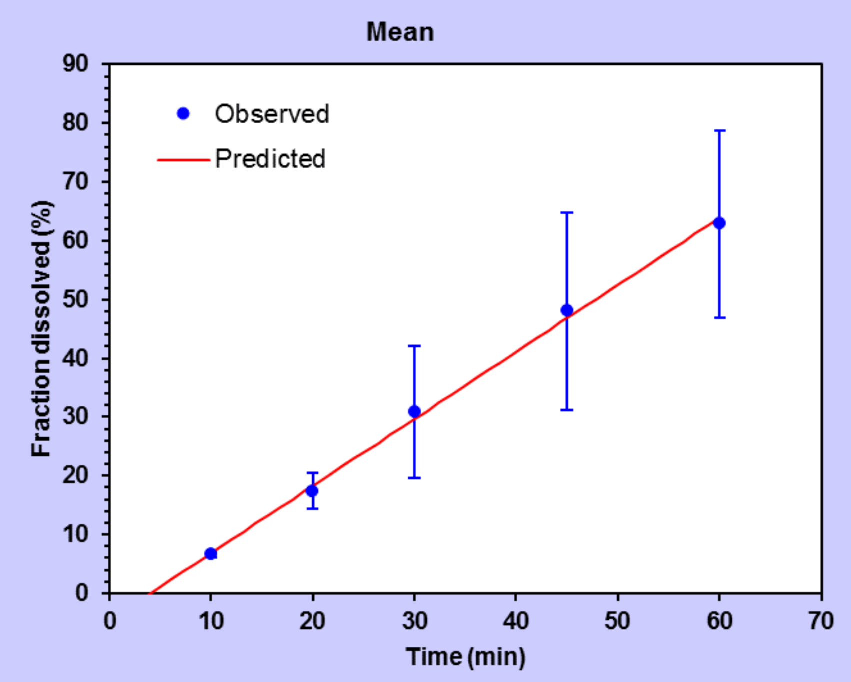

Supplementary materials – Model fitting summary – Parteck® M 200 (modelling up to app. 60 % of carvedilol released)

Graphical abstract of model fit presented as the fraction % of released carvedilol per tested tablet:

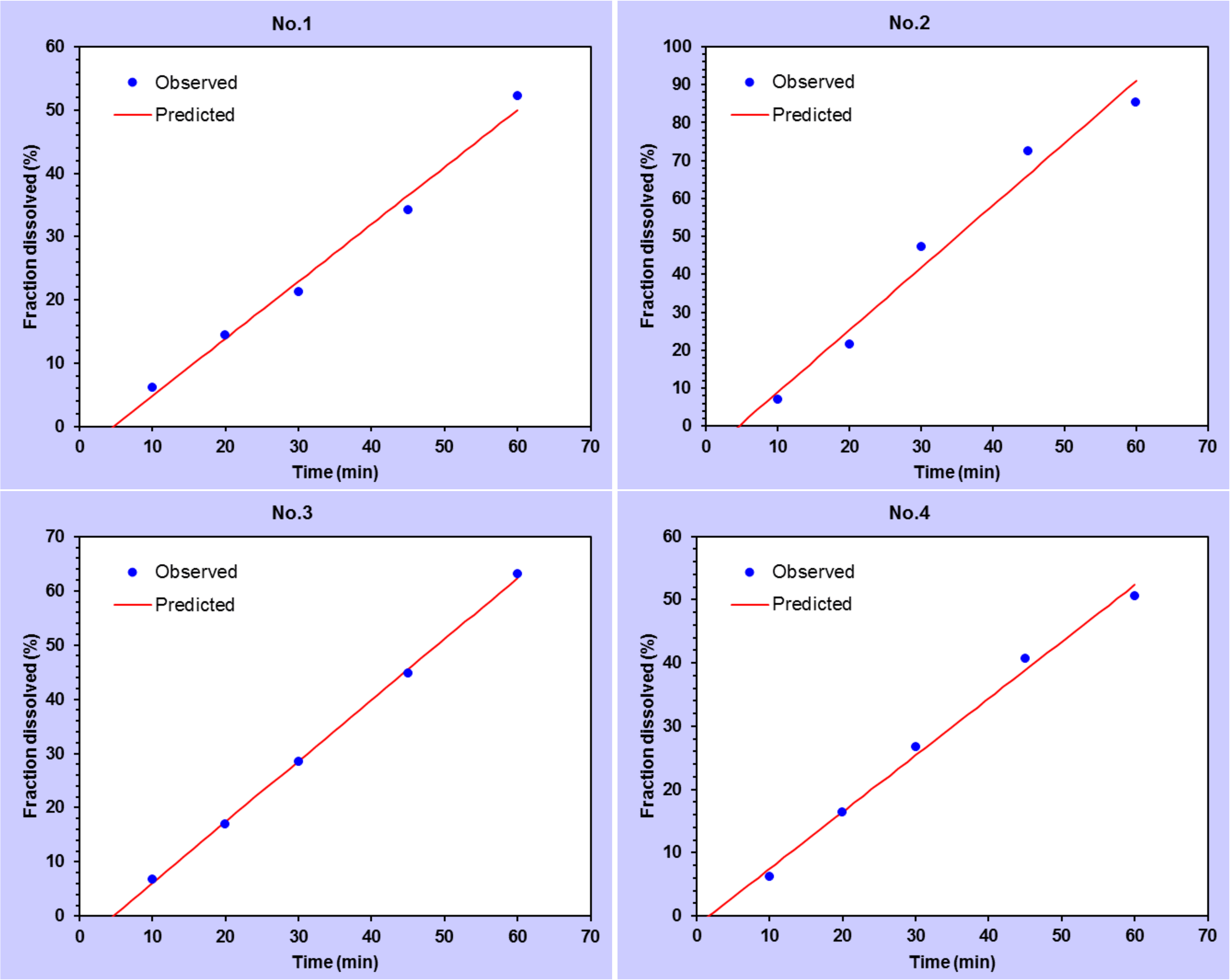

Supplementary materials – Model fitting summary – Parteck® M 200 (modelling up to app. 60 % of carvedilol released)

Model: **First–order**

Model equation: $F = 100 \cdot (1 - e^{-k_1 \cdot t})$

Fitted model parameters per tested tablet (N = 4) with statistics – mean, standard deviation (SD), and relative standard deviation expressed in % (RSD%) (output from DDSolver):

Parameter	No.1	No.2	No.3	No.4	Mean	SD	RSD(%)
k_1	0.011	0.021	0.014	0.011	0.014	0.005	33.703

Number of dissolution data points (N), degrees of freedom (df), and selected goodness of fit criteria – Pearson correlation coefficient (R), coefficient of determination (R^2), adjusted coefficient of determination ($R^2_{adjusted}$), and residual sum of squares (RSS) (manual calculation in MS Excel):

Parameter	No.1	No.2	No.3	No.4
N	5	5	5	5
df	4	4	4	4
R	0.983814867	0.993466939	0.991374457	0.99952684
R^2	0.967891693	0.98697656	0.982823315	0.999053903
$R^2_{adjusted}$	0.967891693	0.98697656	0.982823315	0.999053903
RSS	113.0294406	616.2169959	187.5825097	41.24124754

Graphical abstract of model fit presented as mean ± 1 SD of the fraction % of released carvedilol:

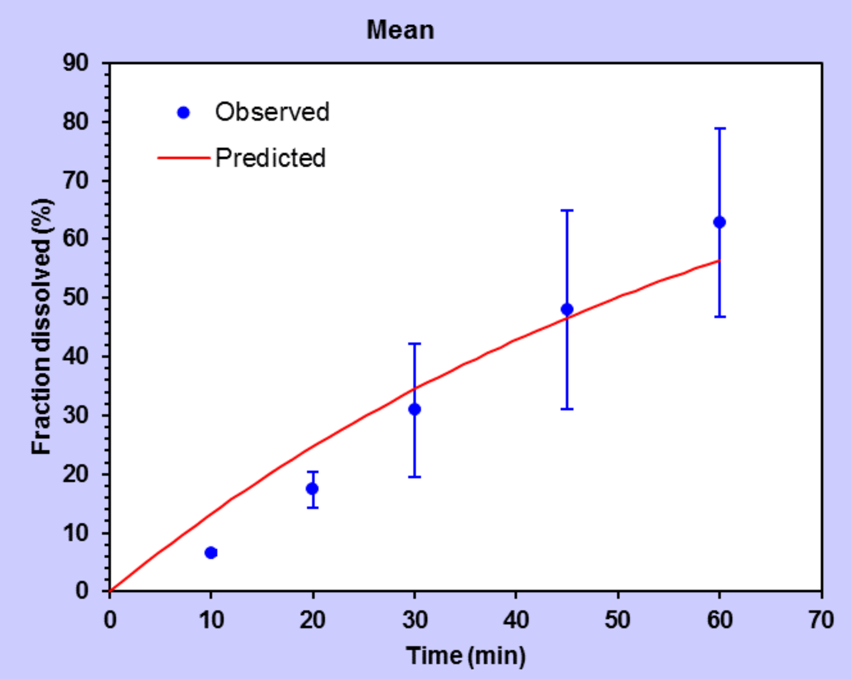

Supplementary materials – Model fitting summary – Parteck® M 200 (modelling up to app. 60 % of carvedilol released)

Graphical abstract of model fit presented as the fraction % of released carvedilol per tested tablet:

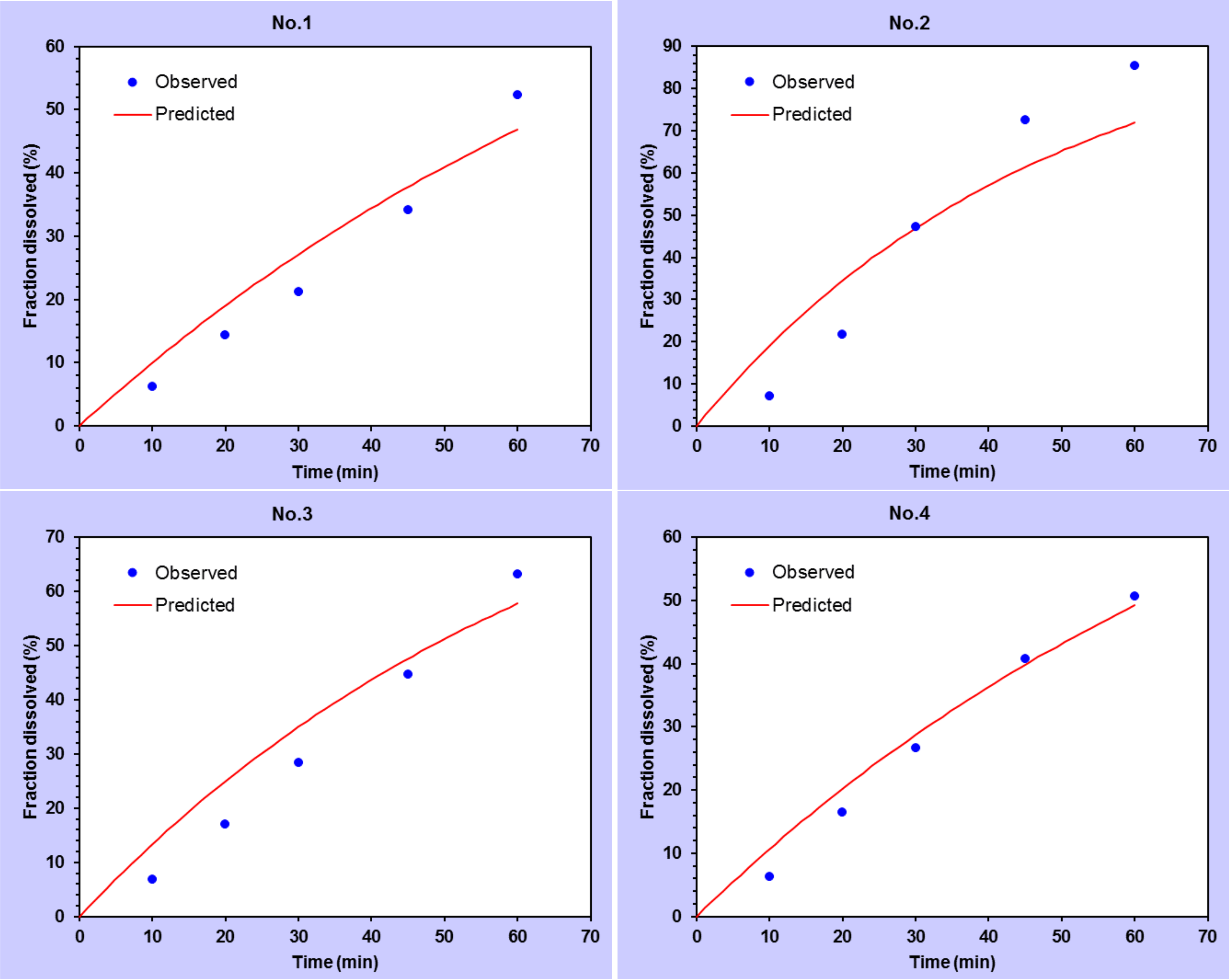

Supplementary materials – Model fitting summary – Parteck® M 200 (modelling up to app. 60 % of carvedilol released)

Model: **First–order with T_{lag}**

Model equation: $F = 100 \cdot \left[1 - e^{-k_1 \cdot (t - T_{lag})}\right]$

Fitted model parameters per tested tablet (N = 4) with statistics – mean, standard deviation (SD), and relative standard deviation expressed in % (RSD%) (output from DDSolver):

Parameter	No.1	No.2	No.3	No.4	Mean	SD	RSD(%)
k_1	0.013	0.034	0.018	0.013	0.020	0.010	49.643
T_{lag}	8.436	8.461	9.229	5.630	7.939	1.583	19.937

Number of dissolution data points (N), degrees of freedom (df), and selected goodness of fit criteria – Pearson correlation coefficient (R), coefficient of determination (R^2), adjusted coefficient of determination ($R^2_{adjusted}$), and residual sum of squares (RSS) (manual calculation in MS Excel):

Parameter	No.1	No.2	No.3	No.4
N	5	5	5	5
df	3	3	3	3
R	0.980457291	0.986092318	0.987302612	0.999424334
R^2	0.961296499	0.972378059	0.974766447	0.998849
$R^2_{adjusted}$	0.948395332	0.963170745	0.966355263	0.998465334
RSS	54.79263762	145.5842089	58.69007629	1.484221425

Graphical abstract of model fit presented as mean ± 1 SD of the fraction % of released carvedilol:

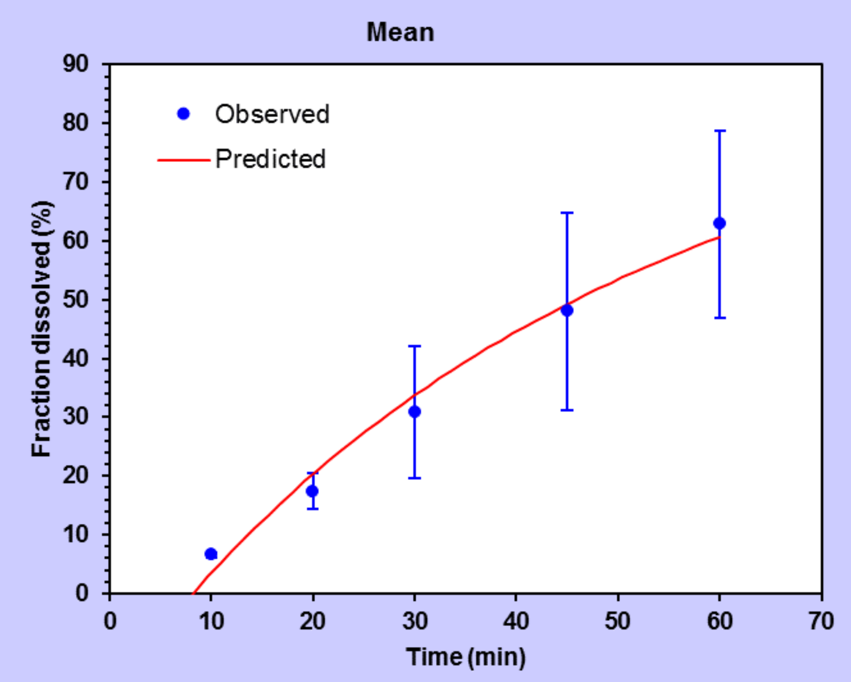

Supplementary materials – Model fitting summary – Parteck® M 200 (modelling up to app. 60 % of carvedilol released)

Graphical abstract of model fit presented as the fraction % of released carvedilol per tested tablet:

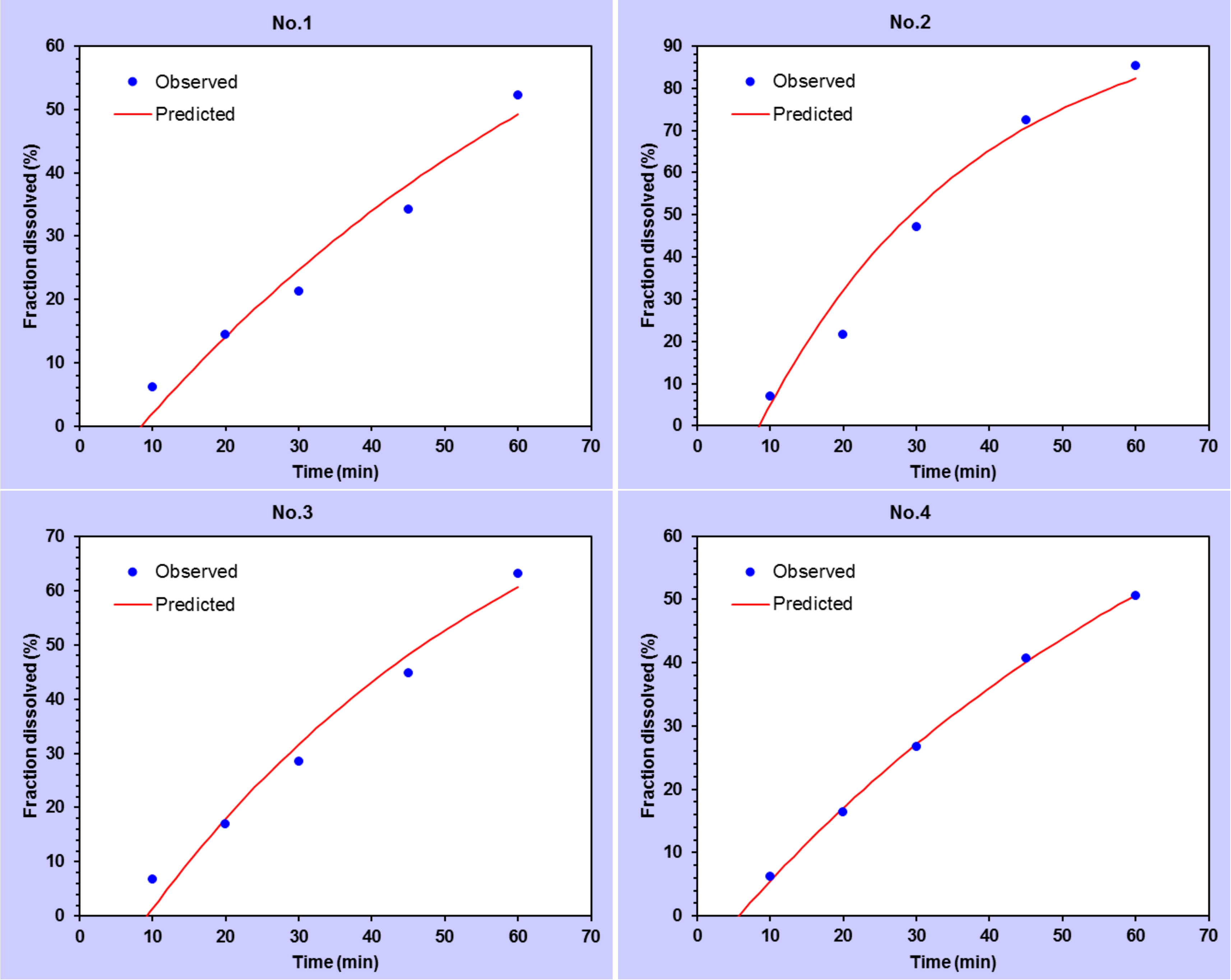

Supplementary materials – Model fitting summary – Parteck® M 200 (modelling up to app. 60 % of carvedilol released)

Model: **First–order with F_{max}**

Model equation: $F = F_{max} \cdot (1 - e^{-k_1 \cdot t})$

Fitted model parameters per tested tablet (N = 4) with statistics – mean, standard deviation (SD), and relative standard deviation expressed in % (RSD%) (output from DDSolver):

Parameter	No.1	No.2	No.3	No.4	Mean	SD	RSD(%)
k_1	0.035	0.041	0.037	0.040	0.038	0.002	6.521
F_{max}	54.890	89.628	66.255	53.081	65.963	16.820	25.499

Number of dissolution data points (N), degrees of freedom (df), and selected goodness of fit criteria – Pearson correlation coefficient (R), coefficient of determination (R^2), adjusted coefficient of determination ($R^2_{adjusted}$), and residual sum of squares (RSS) (manual calculation in MS Excel):

Parameter	No.1	No.2	No.3	No.4
N	5	5	5	5
df	3	3	3	3
R	0.941142084	0.97870043	0.960274086	0.979720883
R^2	0.885748421	0.957854531	0.922126321	0.959853009
$R^2_{adjusted}$	0.847664562	0.943806041	0.896168428	0.946470678
RSS	606.0777319	1604.750875	827.2464334	404.1753176

Graphical abstract of model fit presented as mean ± 1 SD of the fraction % of released carvedilol:

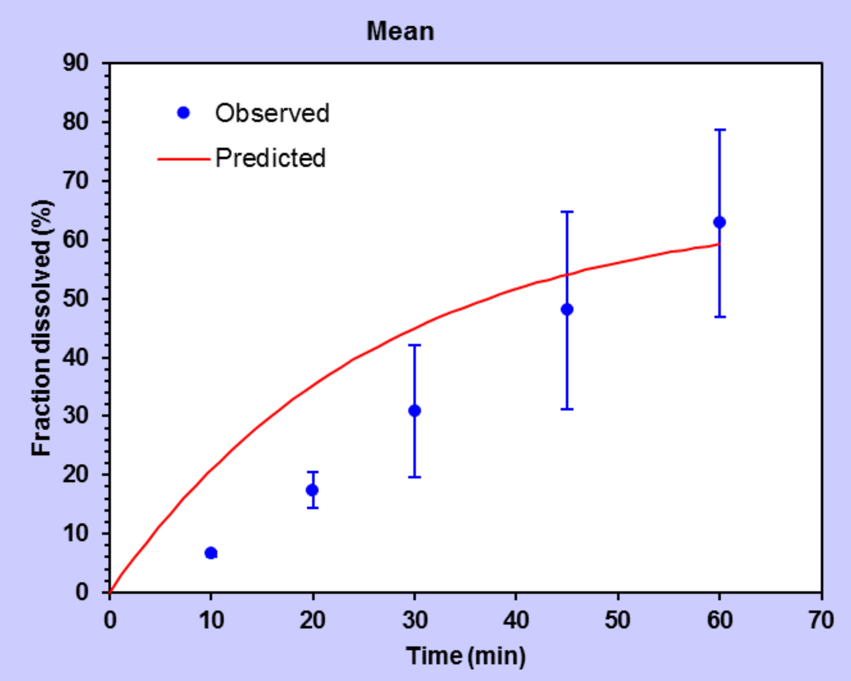

Supplementary materials – Model fitting summary – Parteck® M 200 (modelling up to app. 60 % of carvedilol released)

Graphical abstract of model fit presented as the fraction % of released carvedilol per tested tablet:

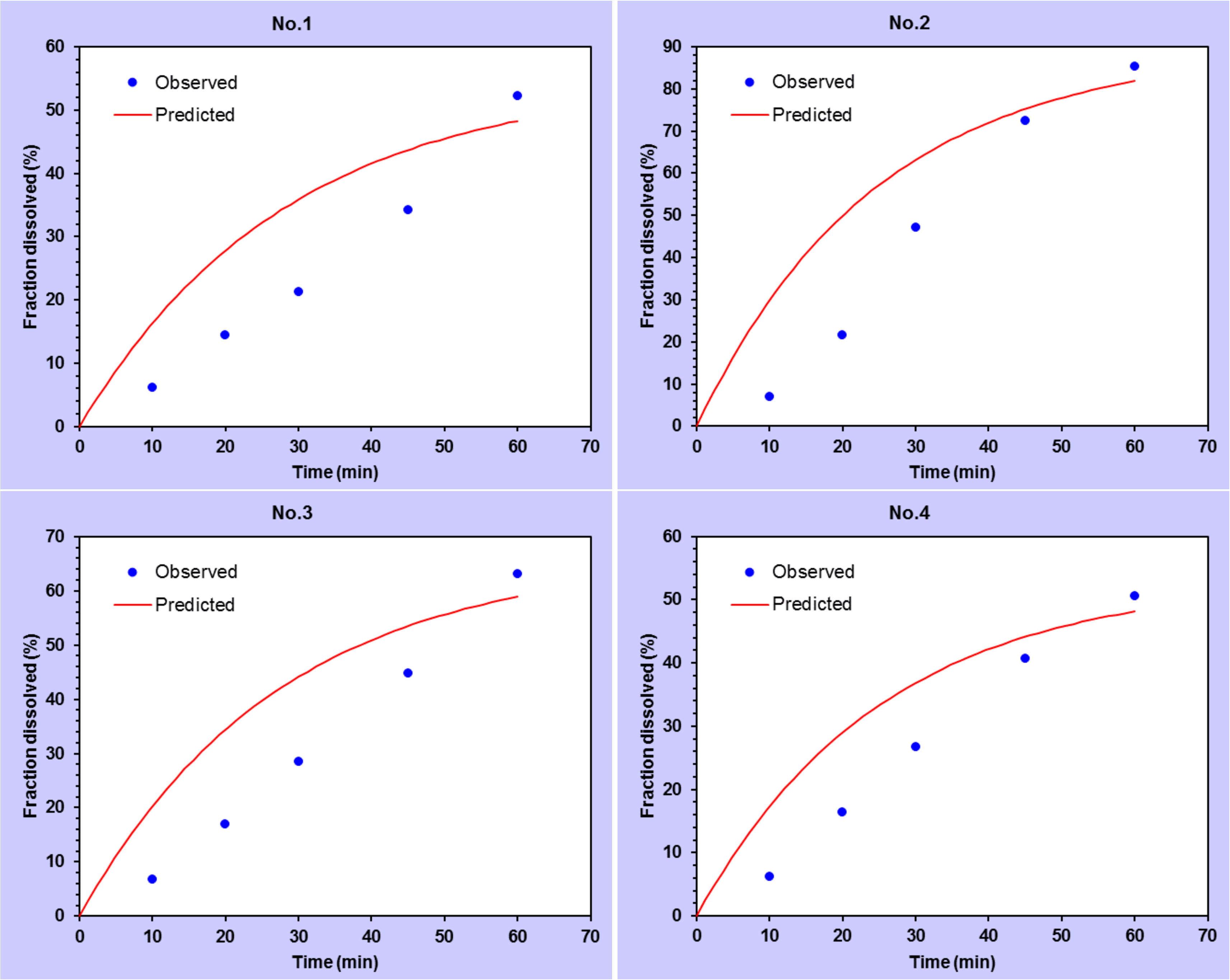

Supplementary materials – Model fitting summary – Parteck® M 200 (modelling up to app. 60 % of carvedilol released)

Model: **First–order with T_{lag} and F_{max}**

Model equation: $F = F_{max} \cdot \left[1 - e^{-k_1 \cdot (t - T_{lag})}\right]$

Fitted model parameters per tested tablet (N = 4) with statistics – mean, standard deviation (SD), and relative standard deviation expressed in % (RSD%) (output from DDSolver):

Parameter	No.1	No.2	No.3	No.4	Mean	SD	RSD(%)
k_1	0.082	0.060	0.084	0.057	0.071	0.014	20.148
T_{lag}	12.763	13.565	12.485	12.996	12.952	0.459	3.543
F_{max}	34.052	89.628	41.103	53.081	54.466	24.723	45.391

Number of dissolution data points (N), degrees of freedom (df), and selected goodness of fit criteria – Pearson correlation coefficient (R), coefficient of determination (R^2), adjusted coefficient of determination ($R^2_{adjusted}$), and residual sum of squares (RSS) (manual calculation in MS Excel):

Parameter	No.1	No.2	No.3	No.4
N	5	5	5	5
df	2	2	2	2
R	0.834498029	0.950949011	0.862084654	0.953408818
R^2	0.696386961	0.904304021	0.743189951	0.908988374
$R^2_{adjusted}$	0.392773922	0.808608043	0.486379902	0.817976749
RSS	606.9536671	939.6243766	839.0571975	313.6375068

Graphical abstract of model fit presented as mean ± 1 SD of the fraction % of released carvedilol:

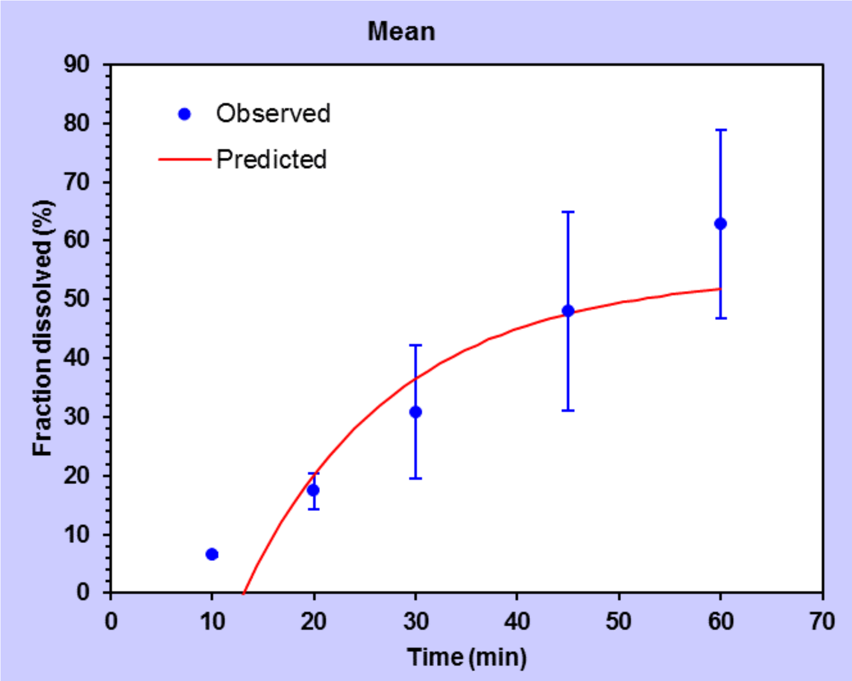

Supplementary materials – Model fitting summary – Parteck® M 200 (modelling up to app. 60 % of carvedilol released)

Graphical abstract of model fit presented as the fraction % of released carvedilol per tested tablet:

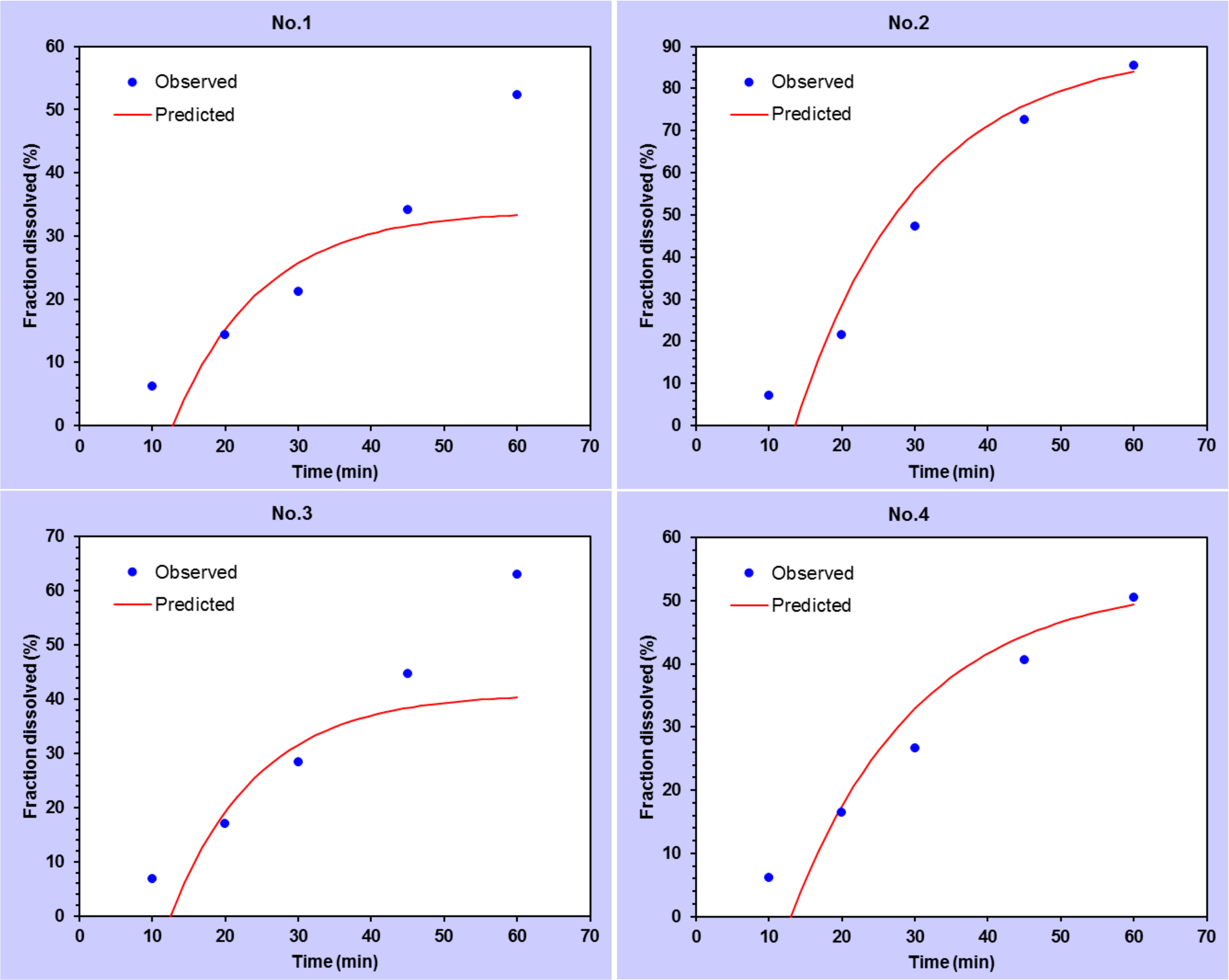

Supplementary materials – Model fitting summary – Parteck® M 200 (modelling up to app. 60 % of carvedilol released)

Model: **Higuchi**

Model equation: $F = k_H \cdot t^{0.5}$

Fitted model parameters per tested tablet (N = 4) with statistics – mean, standard deviation (SD), and relative standard deviation expressed in % (RSD%) (output from DDSolver):

Parameter	No.1	No.2	No.3	No.4	Mean	SD	RSD(%)
k_H	5.057	9.240	6.313	5.476	6.521	1.886	28.920

Number of dissolution data points (N), degrees of freedom (df), and selected goodness of fit criteria – Pearson correlation coefficient (R), coefficient of determination (R^2), adjusted coefficient of determination ($R^2_{adjusted}$), and residual sum of squares (RSS) (manual calculation in MS Excel):

Parameter	No.1	No.2	No.3	No.4
N	5	5	5	5
df	4	4	4	4
R	0.975446307	0.992086628	0.988494312	0.997800719
R^2	0.951495497	0.984235878	0.977121005	0.995606274
$R^2_{adjusted}$	0.951495497	0.984235878	0.977121005	0.995606274
RSS	377.5063472	1194.700499	544.7716249	281.7517717

Graphical abstract of model fit presented as mean ± 1 SD of the fraction % of released carvedilol:

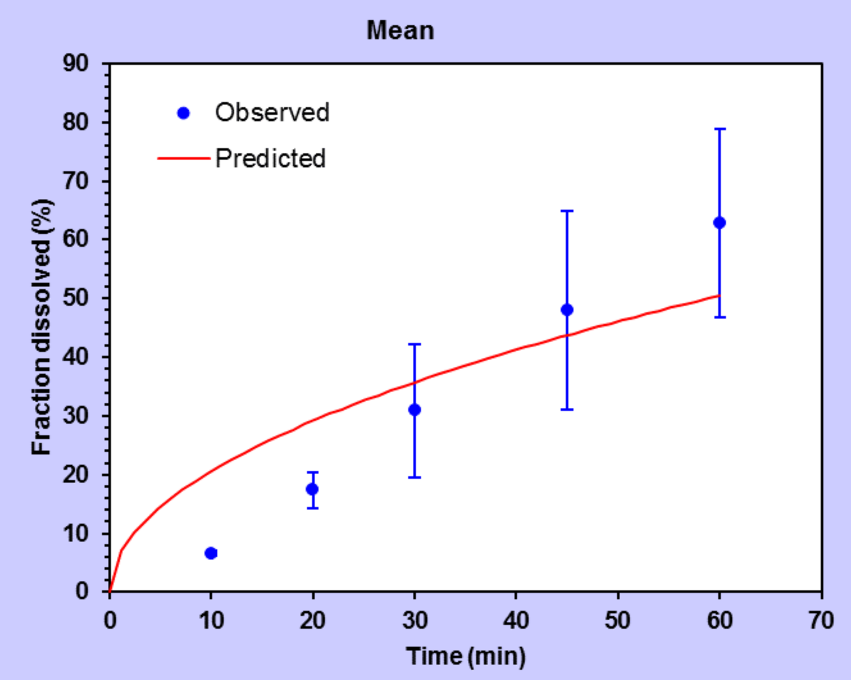

Supplementary materials – Model fitting summary – Parteck® M 200 (modelling up to app. 60 % of carvedilol released)

Graphical abstract of model fit presented as the fraction % of released carvedilol per tested tablet:

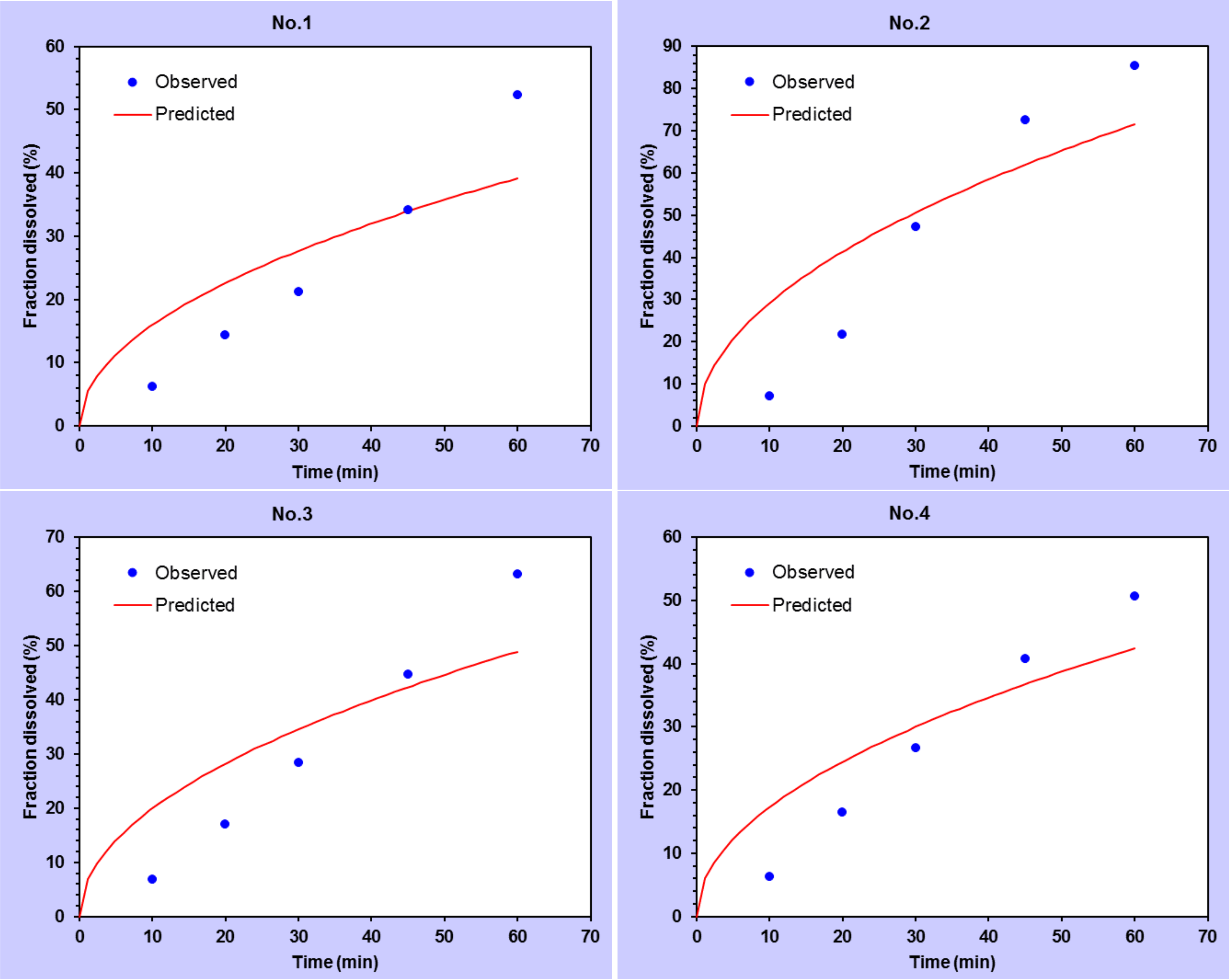

Supplementary materials – Model fitting summary – Parteck® M 200 (modelling up to app. 60 % of carvedilol released)

Model: **Higuchi with T_{lag}**

Model equation: $F = k_H \cdot (t - T_{lag})^{0.5}$

Fitted model parameters per tested tablet (N = 4) with statistics – mean, standard deviation (SD), and relative standard deviation expressed in % (RSD%) (output from DDSolver):

Parameter	No.1	No.2	No.3	No.4	Mean	SD	RSD(%)
k_H	7.243	12.865	8.868	7.444	9.105	2.609	28.654
T_{lag}	15.475	17.329	14.887	16.770	16.115	1.128	7.002

Number of dissolution data points (N), degrees of freedom (df), and selected goodness of fit criteria – Pearson correlation coefficient (R), coefficient of determination (R^2), adjusted coefficient of determination ($R^2_{adjusted}$), and residual sum of squares (RSS) (manual calculation in MS Excel):

Parameter	No.1	No.2	No.3	No.4
N	5	5	5	5
df	3	3	3	3
R	0.957782679	0.997037138	0.972875391	0.995621459
R^2	0.91734766	0.994083055	0.946486526	0.991262089
$R^2_{adjusted}$	0.889796879	0.992110739	0.928648701	0.988349452
RSS	122.7882668	76.05587792	120.6576991	51.49664842

Graphical abstract of model fit presented as mean ± 1 SD of the fraction % of released carvedilol:

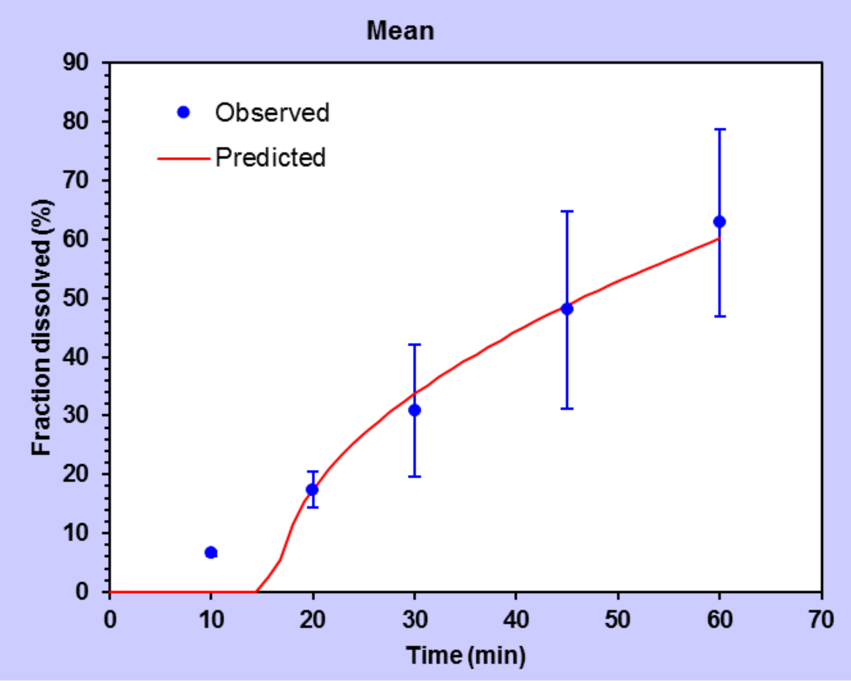

Supplementary materials – Model fitting summary – Parteck® M 200 (modelling up to app. 60 % of carvedilol released)

Graphical abstract of model fit presented as the fraction % of released carvedilol per tested tablet:

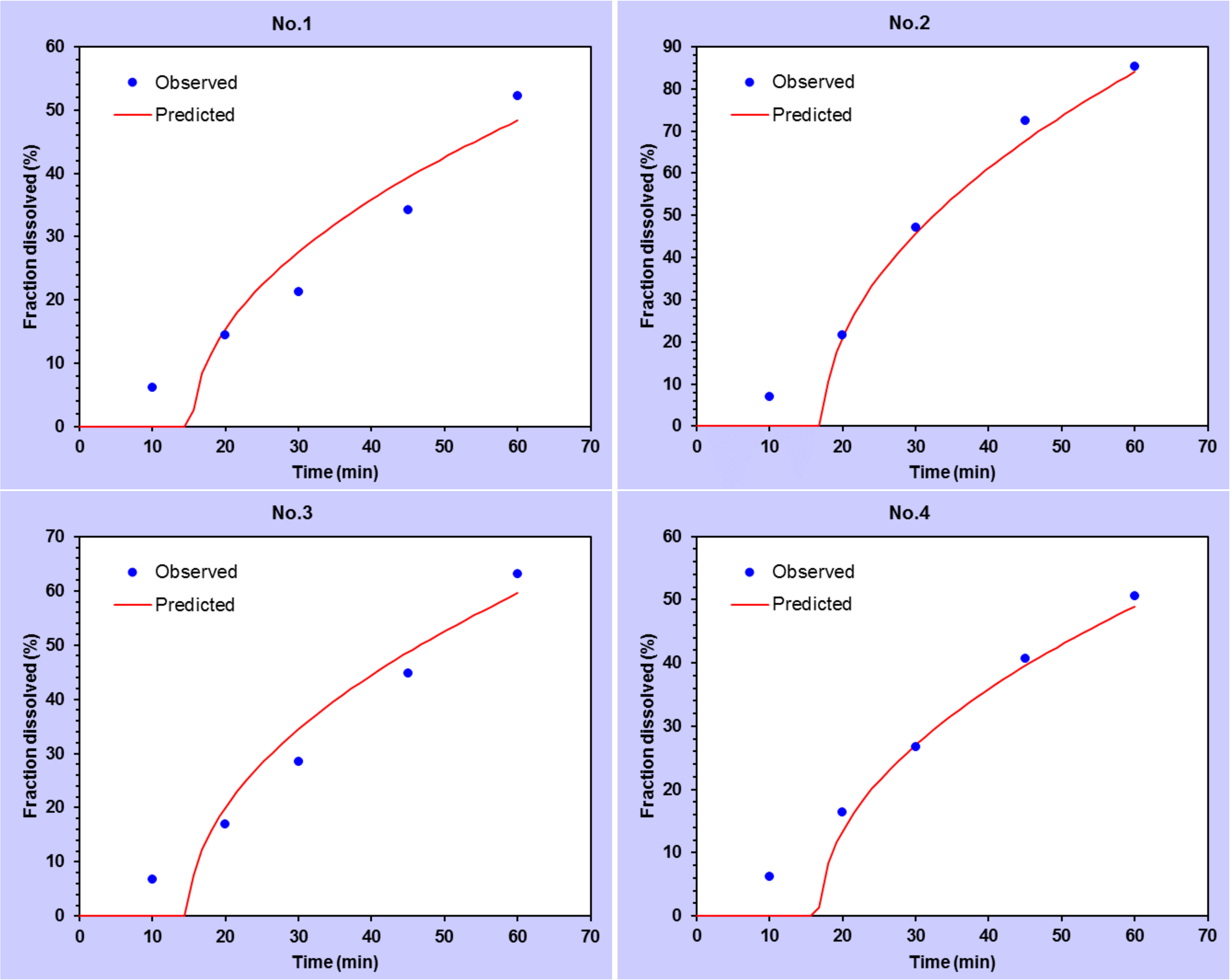

Supplementary materials – Model fitting summary – Parteck® M 200 (modelling up to app. 60 % of carvedilol released)

Model: **Higuchi with F_0**

Model equation: $F = F_0 + k_H \cdot t^{0.5}$

Fitted model parameters per tested tablet (N = 4) with statistics – mean, standard deviation (SD), and relative standard deviation expressed in % (RSD%) (output from DDSolver):

Parameter	No.1	No.2	No.3	No.4	Mean	SD	RSD(%)
k_H	9.770	18.162	12.253	9.895	12.520	3.931	31.400
F_0	-28.211	-53.409	-35.555	-26.449	-35.906	12.317	-34.302

Number of dissolution data points (N), degrees of freedom (df), and selected goodness of fit criteria – Pearson correlation coefficient (R), coefficient of determination (R^2), adjusted coefficient of determination ($R^2_{adjusted}$), and residual sum of squares (RSS) (manual calculation in MS Excel):

Parameter	No.1	No.2	No.3	No.4
N	5	5	5	5
df	3	3	3	3
R	0.975446307	0.992086628	0.988494312	0.997800719
R^2	0.951495497	0.984235878	0.977121005	0.995606274
$R^2_{adjusted}$	0.935327329	0.978981171	0.969494674	0.994141699
RSS	63.37656168	68.81858254	45.79230791	5.627692938

Graphical abstract of model fit presented as mean ± 1 SD of the fraction % of released carvedilol:

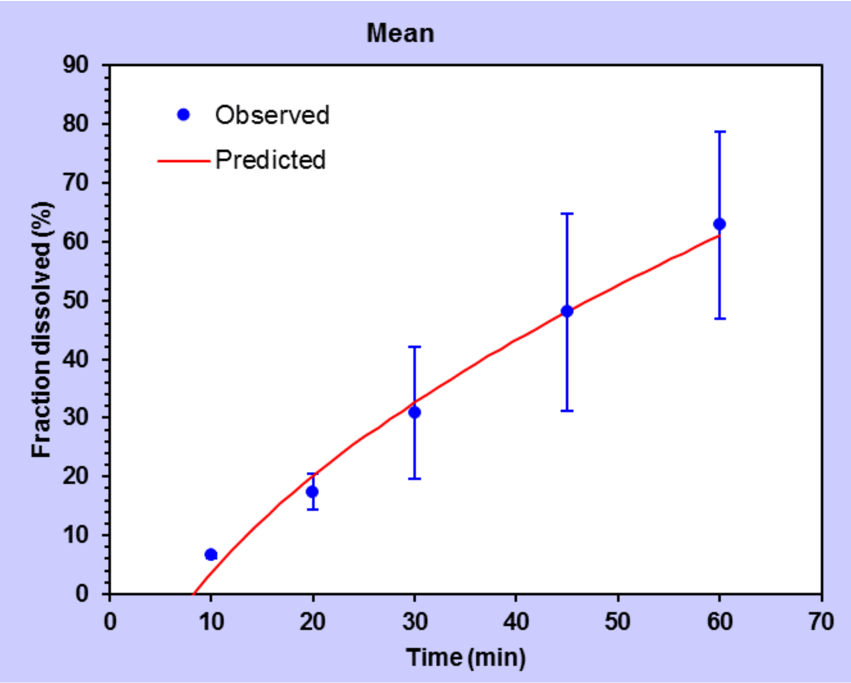

Supplementary materials – Model fitting summary – Parteck® M 200 (modelling up to app. 60 % of carvedilol released)

Graphical abstract of model fit presented as the fraction % of released carvedilol per tested tablet:

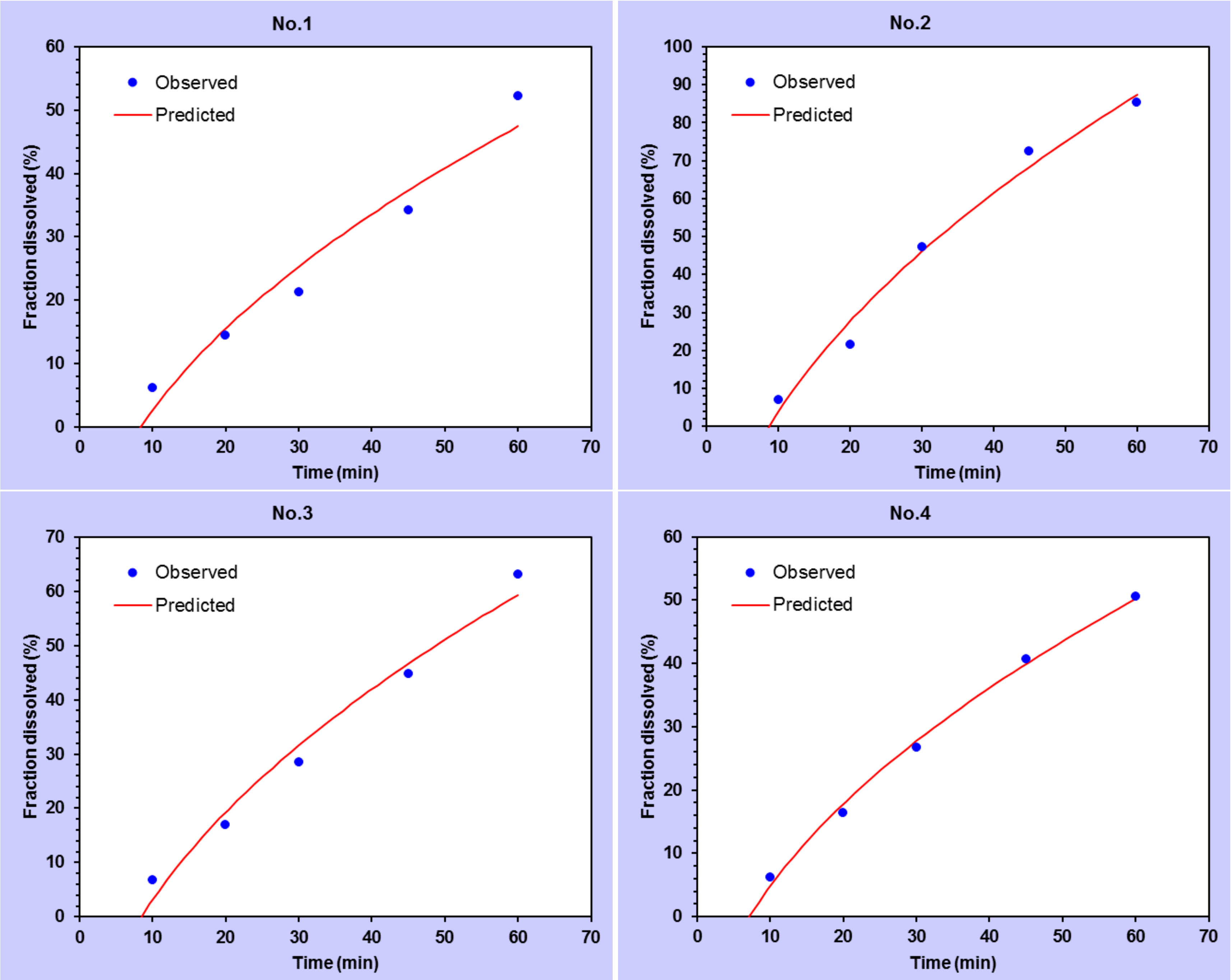

Supplementary materials – Model fitting summary – Parteck® M 200 (modelling up to app. 60 % of carvedilol released)

Model: **Korsmeyer–Peppas**

Model equation: $F = k_{KP} \cdot t^n$

Fitted model parameters per tested tablet (N = 4) with statistics – mean, standard deviation (SD), and relative standard deviation expressed in % (RSD%) (output from DDSolver):

Parameter	No.1	No.2	No.3	No.4	Mean	SD	RSD(%)
k_{KP}	0.425	0.347	0.403	0.595	0.442	0.107	24.121
n	1.164	1.342	1.240	1.108	1.213	0.102	8.371

Number of dissolution data points (N), degrees of freedom (df), and selected goodness of fit criteria – Pearson correlation coefficient (R), coefficient of determination (R^2), adjusted coefficient of determination ($R^2_{adjusted}$), and residual sum of squares (RSS) (manual calculation in MS Excel):

Parameter	No.1	No.2	No.3	No.4
N	5	5	5	5
df	3	3	3	3
R	0.996928091	0.97447017	0.999516593	0.993956831
R^2	0.993865619	0.949592112	0.99903342	0.987950182
$R^2_{adjusted}$	0.991820825	0.932789483	0.998711227	0.983933576
RSS	9.606323936	425.6143327	3.924719285	26.63270072

Graphical abstract of model fit presented as mean ± 1 SD of the fraction % of released carvedilol:

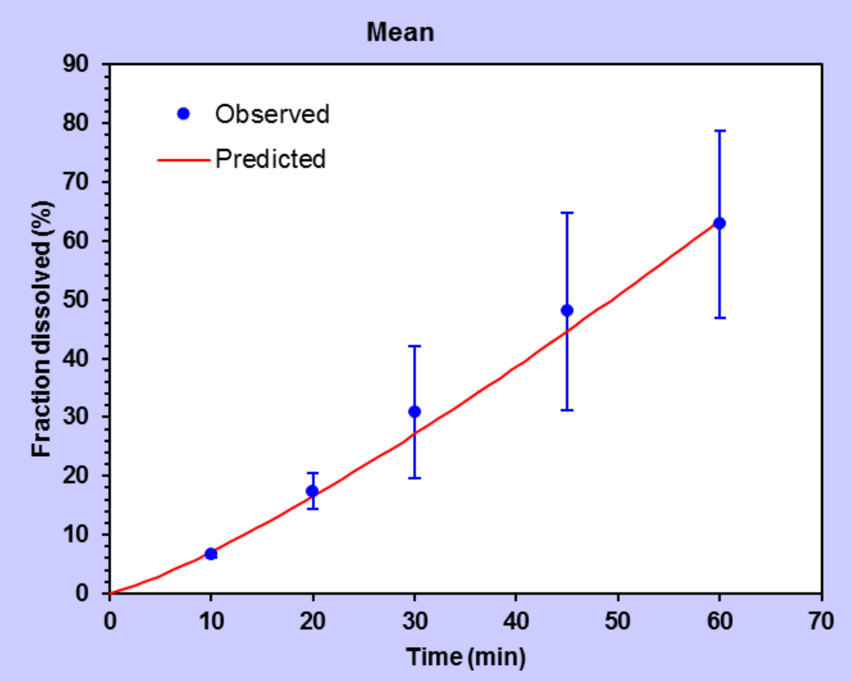

Supplementary materials – Model fitting summary – Parteck® M 200 (modelling up to app. 60 % of carvedilol released)

Graphical abstract of model fit presented as the fraction % of released carvedilol per tested tablet:

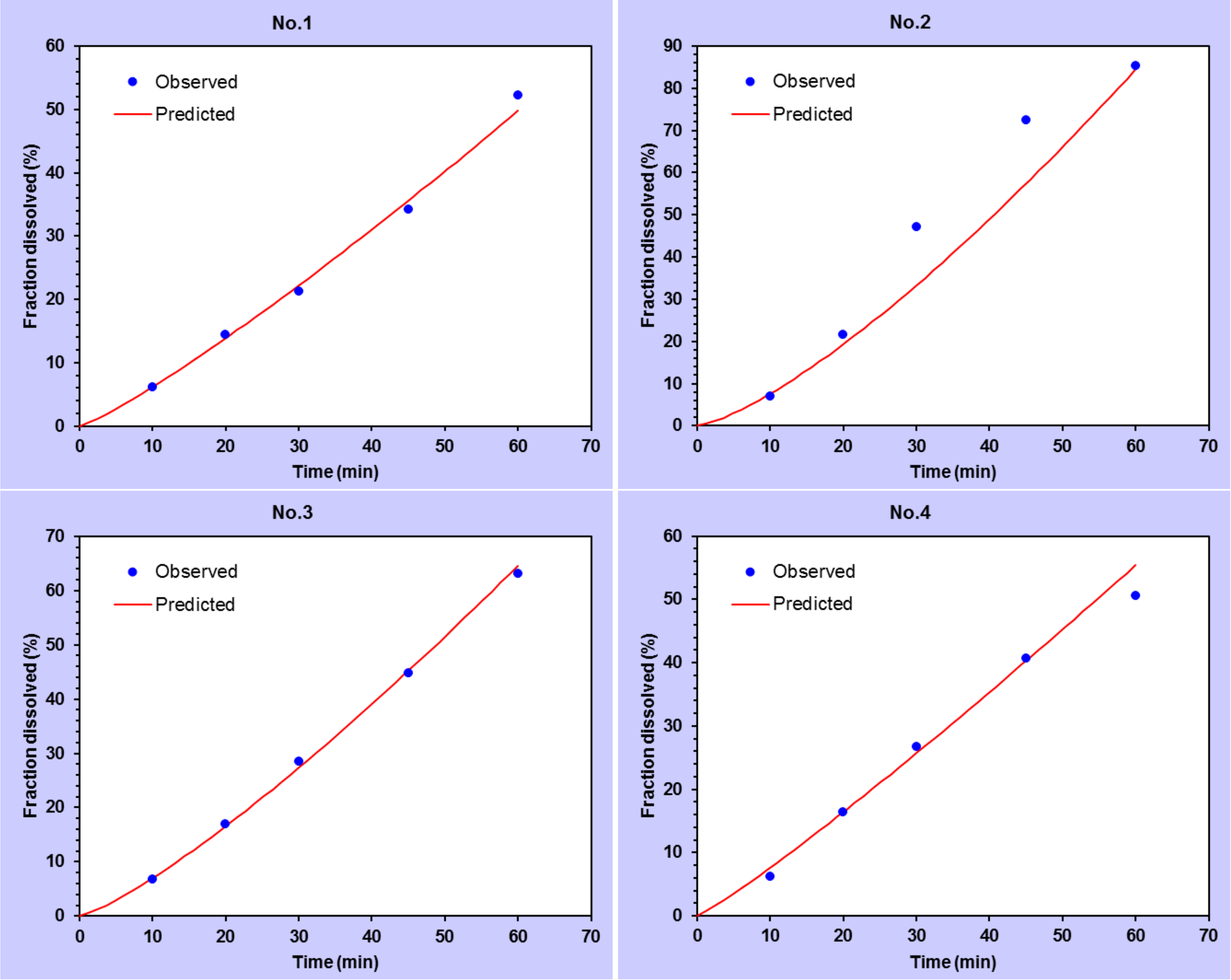

Supplementary materials – Model fitting summary – Parteck® M 200 (modelling up to app. 60 % of carvedilol released)

Model: **Korsmeyer–Peppas with T_{lag}**

Model equation: $F = k_{KP} \cdot (t - T_{lag})^n$

Fitted model parameters per tested tablet (N = 4) with statistics – mean, standard deviation (SD), and relative standard deviation expressed in % (RSD%) (output from DDSolver):

Parameter	No.1	No.2	No.3	No.4	Mean	SD	RSD(%)
k_{KP}	1.121	0.899	1.114	1.153	1.072	0.117	10.872
n	0.953	1.165	0.996	0.953	1.017	0.101	9.961
T_{lag}	4.939	4.000	4.000	4.000	4.235	0.470	11.092

Number of dissolution data points (N), degrees of freedom (df), and selected goodness of fit criteria – Pearson correlation coefficient (R), coefficient of determination (R^2), adjusted coefficient of determination ($R^2_{adjusted}$), and residual sum of squares (RSS) (manual calculation in MS Excel):

Parameter	No.1	No.2	No.3	No.4
N	5	5	5	5
df	2	2	2	2
R	0.992762481	0.98046372	0.99951744	0.997167016
R^2	0.985577343	0.961309107	0.999035114	0.994342058
$R^2_{adjusted}$	0.971154686	0.922618213	0.998070227	0.988684115
RSS	23.59855114	230.2236762	3.395711028	9.914316448

Graphical abstract of model fit presented as mean ± 1 SD of the fraction % of released carvedilol:

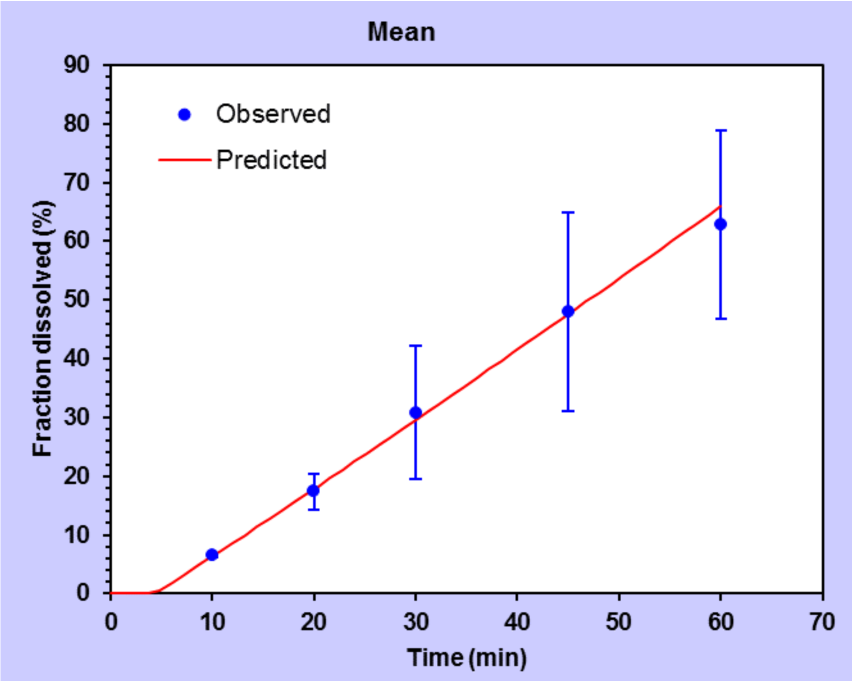

Supplementary materials – Model fitting summary – Parteck® M 200 (modelling up to app. 60 % of carvedilol released)

Graphical abstract of model fit presented as the fraction % of released carvedilol per tested tablet:

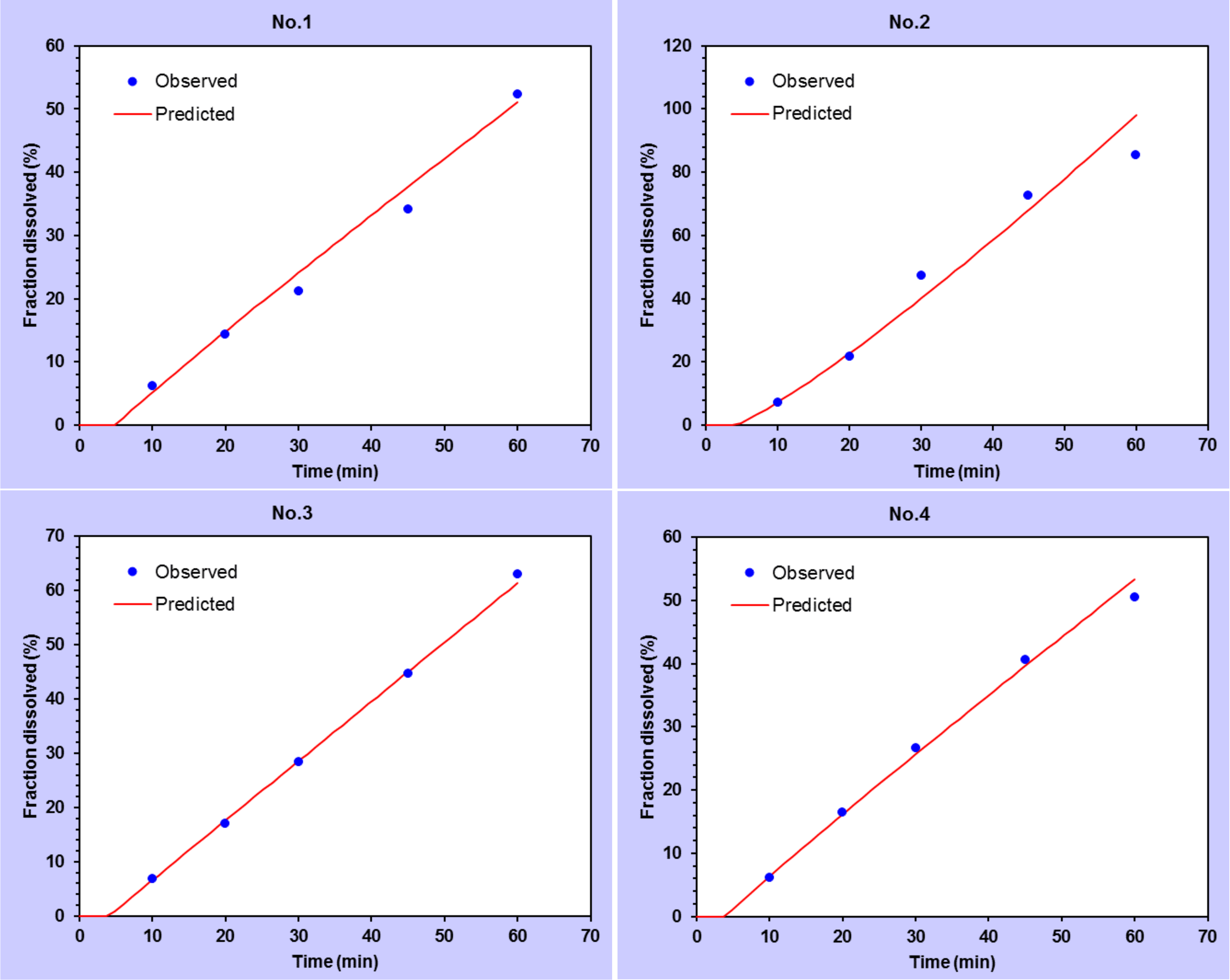

Supplementary materials – Model fitting summary – Parteck® M 200 (modelling up to app. 60 % of carvedilol released)

Model: **Korsmeyer–Peppas with F_0**

Model equation: $F = F_0 + k_{KP} \cdot t^n$

Fitted model parameters per tested tablet (N = 4) with statistics – mean, standard deviation (SD), and relative standard deviation expressed in % (RSD%) (output from DDSolver):

Parameter	No.1	No.2	No.3	No.4	Mean	SD	RSD(%)
k_{KP}	0.153	0.101	0.132	0.176	0.141	0.032	22.725
n	1.414	1.703	1.491	1.359	1.492	0.151	10.114
F_0	2.480	2.800	3.527	3.049	2.964	0.442	14.901

Number of dissolution data points (N), degrees of freedom (df), and selected goodness of fit criteria – Pearson correlation coefficient (R), coefficient of determination (R^2), adjusted coefficient of determination ($R^2_{adjusted}$), and residual sum of squares (RSS) (manual calculation in MS Excel):

Parameter	No.1	No.2	No.3	No.4
N	5	5	5	5
df	2	2	2	2
R	0.998664198	0.956982566	0.996652102	0.986583678
R^2	0.997330181	0.915815632	0.993315412	0.973347354
$R^2_{adjusted}$	0.994660361	0.831631265	0.986630824	0.946694708
RSS	4.399161462	792.1902879	27.53262811	86.14972281

Graphical abstract of model fit presented as mean ± 1 SD of the fraction % of released carvedilol:

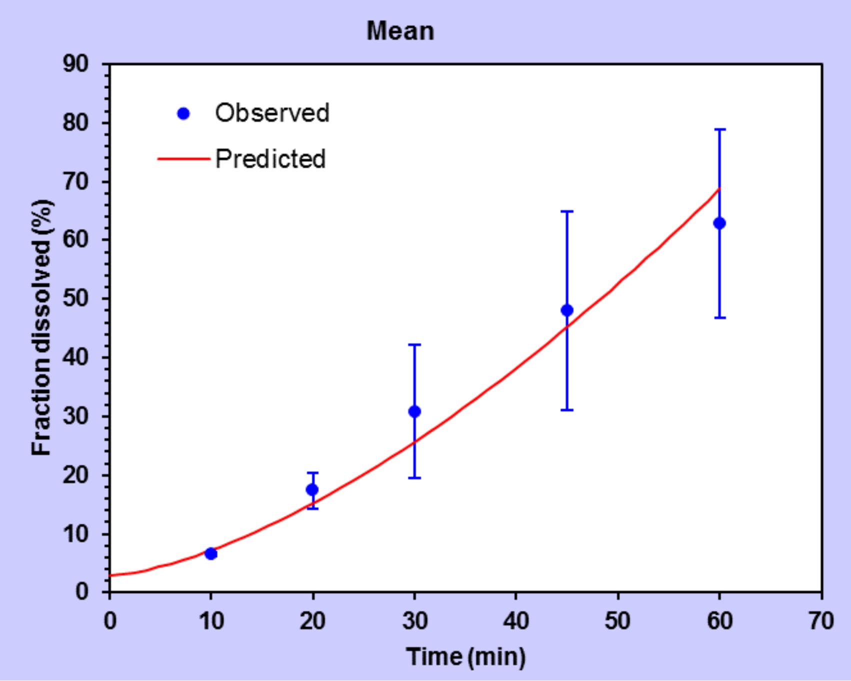

Supplementary materials – Model fitting summary – Parteck® M 200 (modelling up to app. 60 % of carvedilol released)

Graphical abstract of model fit presented as the fraction % of released carvedilol per tested tablet:

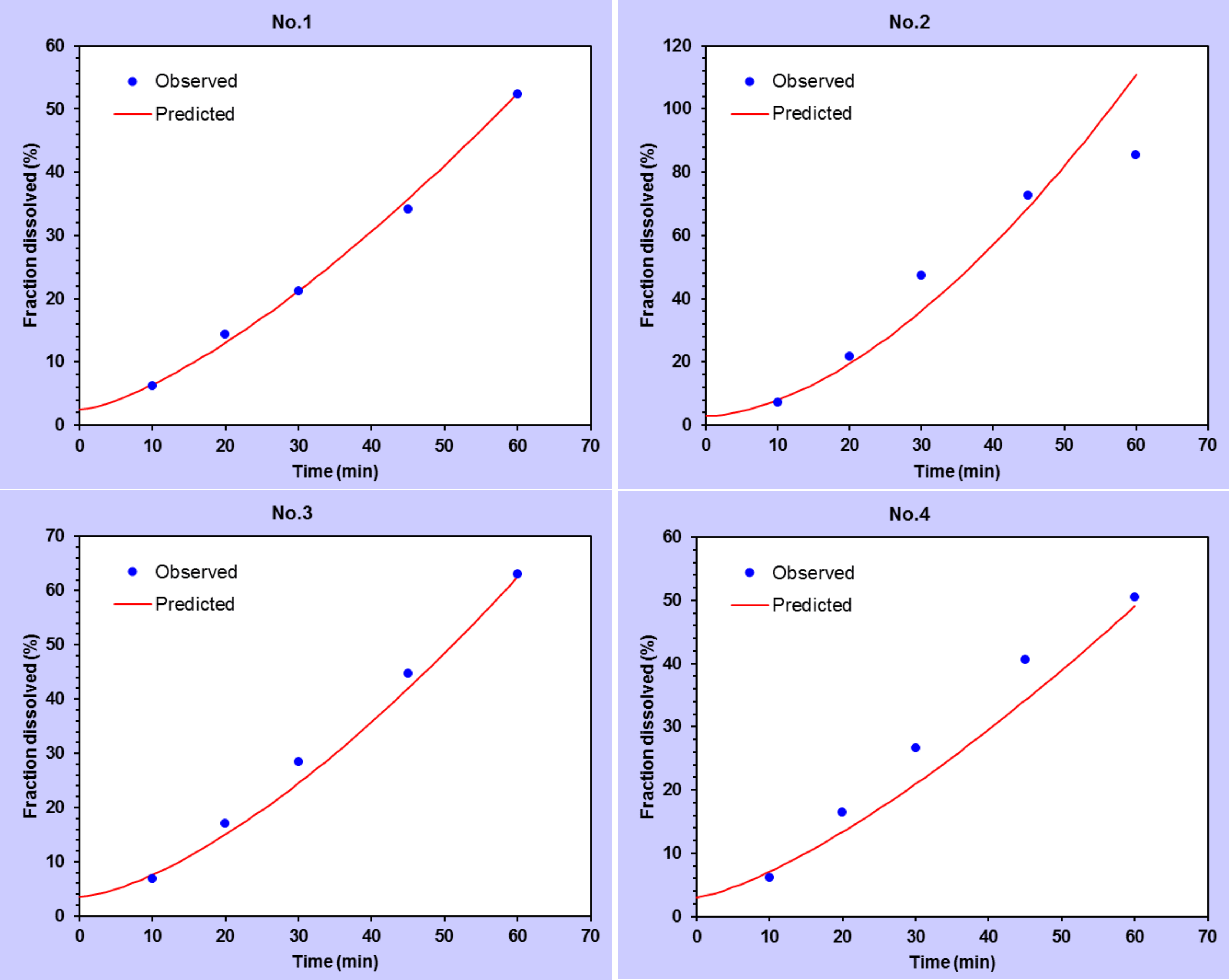

Supplementary materials – Model fitting summary – Parteck® M 200 (modelling up to app. 60 % of carvedilol released)

Model: **Hixson–Crowell**

Model equation: $F = 100 \cdot [1 - (1 - k_{HC} \cdot t)^3]$

Fitted model parameters per tested tablet (N = 4) with statistics – mean, standard deviation (SD), and relative standard deviation expressed in % (RSD%) (output from DDSolver):

Parameter	No.1	No.2	No.3	No.4	Mean	SD	RSD(%)
k_{HC}	0.003	0.007	0.004	0.003	0.005	0.002	42.200

Number of dissolution data points (N), degrees of freedom (df), and selected goodness of fit criteria – Pearson correlation coefficient (R), coefficient of determination (R^2), adjusted coefficient of determination ($R^2_{adjusted}$), and residual sum of squares (RSS) (manual calculation in MS Excel):

Parameter	No.1	No.2	No.3	No.4
N	5	5	5	5
df	4	4	4	4
R	0.987637738	0.994402057	0.995057664	0.999266836
R^2	0.975428302	0.98883545	0.990139755	0.99853421
$R^2_{adjusted}$	0.975428302	0.98883545	0.990139755	0.99853421
RSS	81.65841402	492.6261593	119.0076903	24.7119394

Graphical abstract of model fit presented as mean ± 1 SD of the fraction % of released carvedilol:

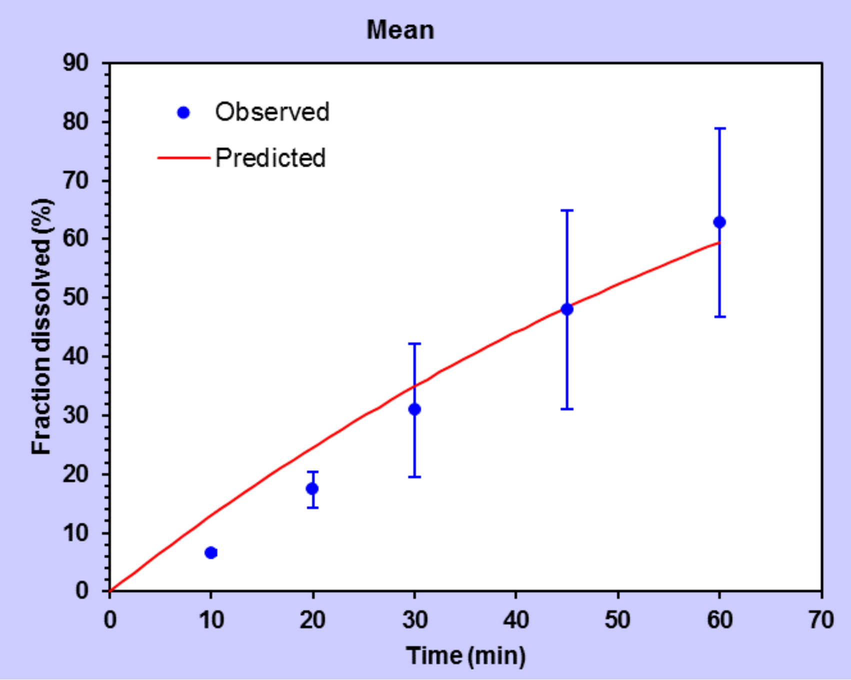

Supplementary materials – Model fitting summary – Parteck® M 200 (modelling up to app. 60 % of carvedilol released)

Graphical abstract of model fit presented as the fraction % of released carvedilol per tested tablet:

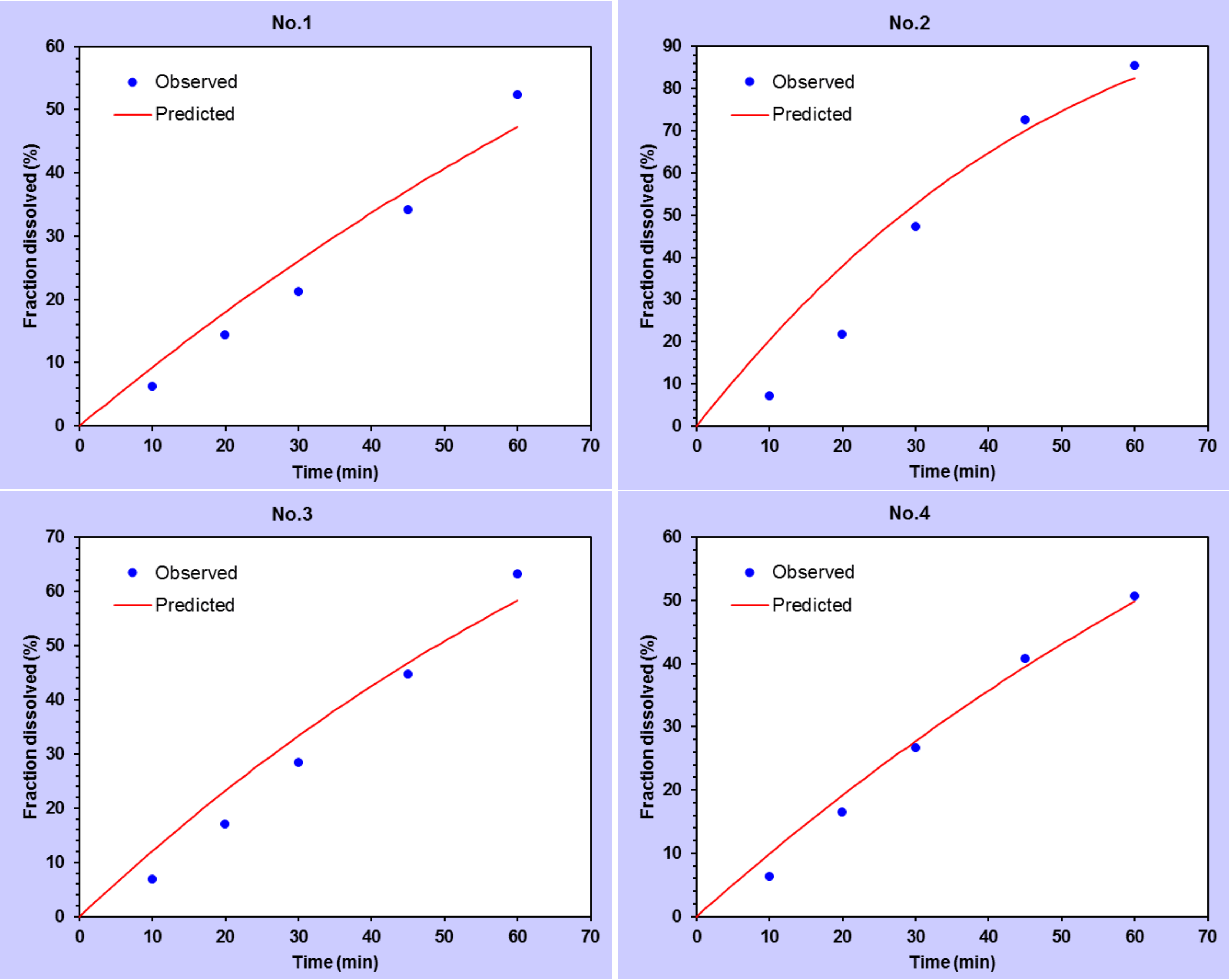

Supplementary materials – Model fitting summary – Parteck® M 200 (modelling up to app. 60 % of carvedilol released)

Model: **Hixson–Crowell with T_{lag}**

Model equation: $F = 100 \cdot \left\{1 - \left[1 - k_{HC} \cdot \left(t - T_{lag}\right)\right]^3\right\}$

Fitted model parameters per tested tablet (N = 4) with statistics – mean, standard deviation (SD), and relative standard deviation expressed in % (RSD%) (output from DDSolver):

Parameter	No.1	No.2	No.3	No.4	Mean	SD	RSD(%)
k_{HC}	0.004	0.009	0.005	0.004	0.006	0.003	47.321
T_{lag}	7.236	9.215	7.774	4.382	7.152	2.027	28.338

Number of dissolution data points (N), degrees of freedom (df), and selected goodness of fit criteria – Pearson correlation coefficient (R), coefficient of determination (R^2), adjusted coefficient of determination ($R^2_{adjusted}$), and residual sum of squares (RSS) (manual calculation in MS Excel):

Parameter	No.1	No.2	No.3	No.4
N	5	5	5	5
df	3	3	3	3
R	0.986054101	0.993196722	0.99346624	0.999405298
R^2	0.97230269	0.986439729	0.98697517	0.99881095
$R^2_{adjusted}$	0.963070253	0.981919639	0.98263356	0.998414601
RSS	37.48623777	60.36005987	27.88367181	1.543278572

Graphical abstract of model fit presented as mean ± 1 SD of the fraction % of released carvedilol:

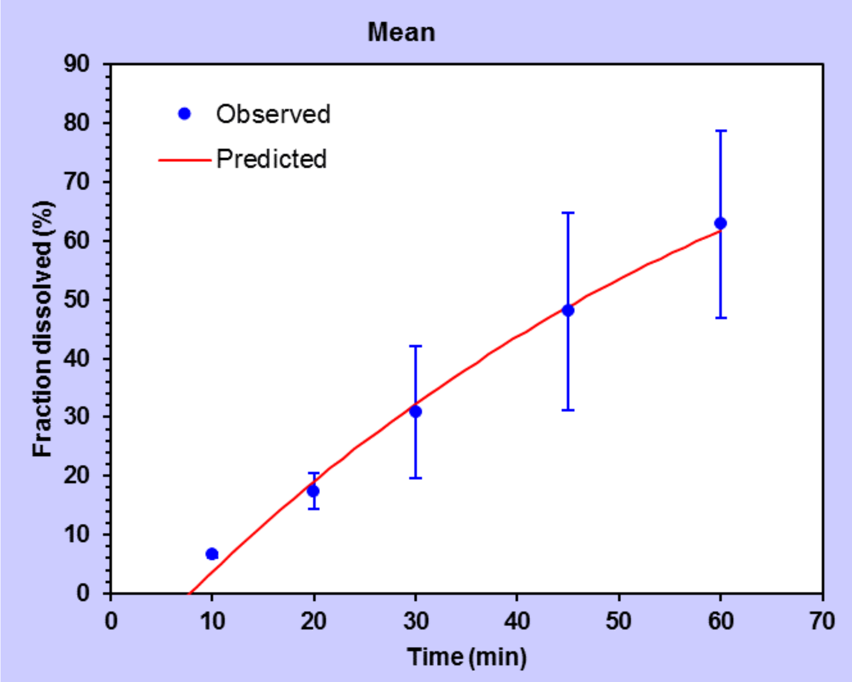

Supplementary materials – Model fitting summary – Parteck® M 200 (modelling up to app. 60 % of carvedilol released)

Graphical abstract of model fit presented as the fraction % of released carvedilol per tested tablet:

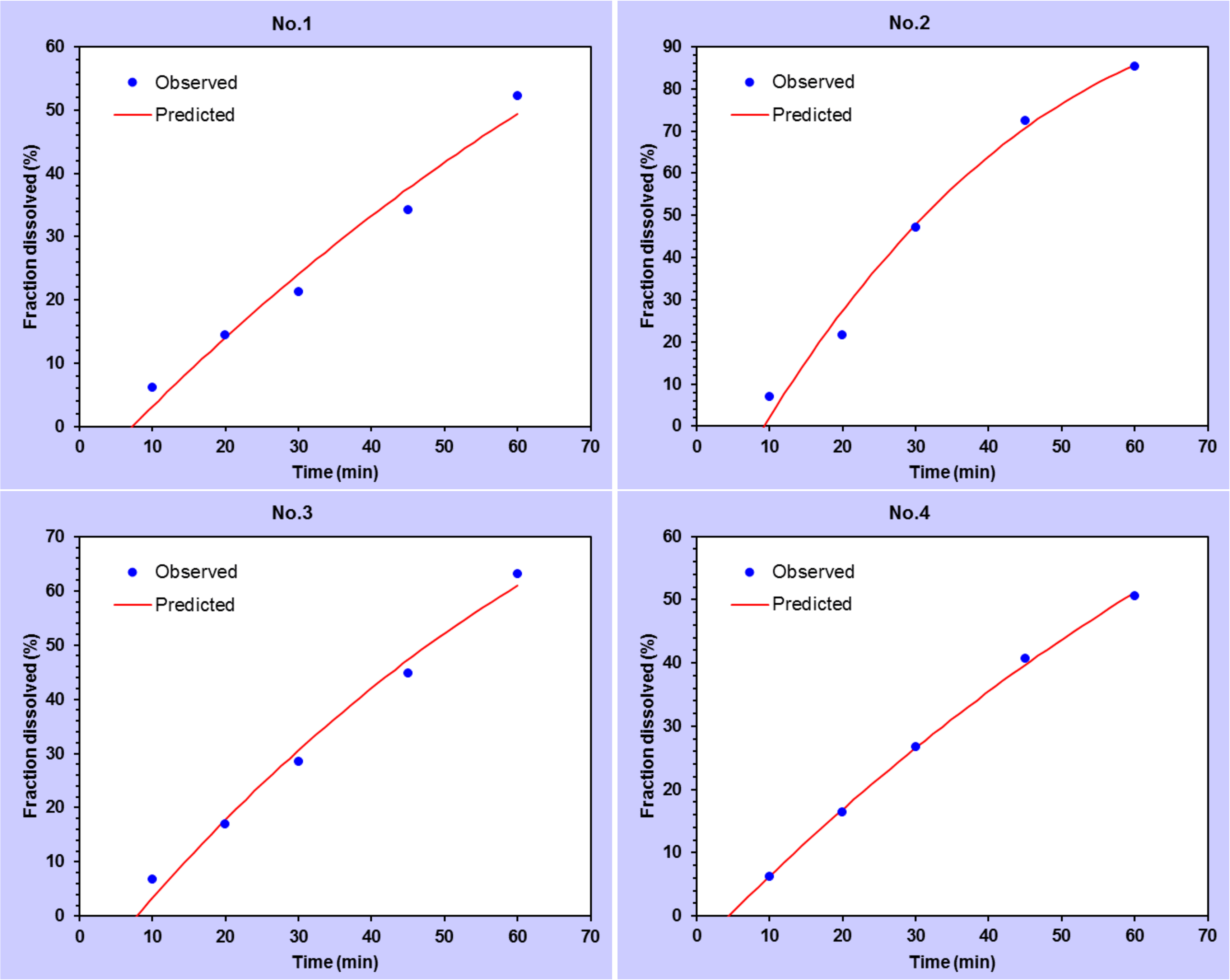

Supplementary materials – Model fitting summary – Parteck® M 200 (modelling up to app. 60 % of carvedilol released)

Model: **Hopfenberg**

Model equation: $F = 100 \cdot [1 - (1 - k_{HB} \cdot t)^n]$

Fitted model parameters per tested tablet (N = 4) with statistics – mean, standard deviation (SD), and relative standard deviation expressed in % (RSD%) (output from DDSolver):

Parameter	No.1	No.2	No.3	No.4	Mean	SD	RSD(%)
k_{HB}	0.008	0.015	0.010	0.009	0.010	0.003	28.967
n	1.000	1.000	1.000	1.000	1.000	0.000	0.000

Number of dissolution data points (N), degrees of freedom (df), and selected goodness of fit criteria – Pearson correlation coefficient (R), coefficient of determination (R^2), adjusted coefficient of determination ($R^2_{adjusted}$), and residual sum of squares (RSS) (manual calculation in MS Excel):

Parameter	No.1	No.2	No.3	No.4
N	5	5	5	5
df	3	3	3	3
R	0.994112033	0.986599376	0.999547112	0.996231412
R^2	0.988258735	0.973378328	0.999094429	0.992477026
$R^2_{adjusted}$	0.98434498	0.964504438	0.998792572	0.989969369
RSS	35.00443994	177.7017298	31.34964413	12.16225017

Graphical abstract of model fit presented as mean ± 1 SD of the fraction % of released carvedilol:

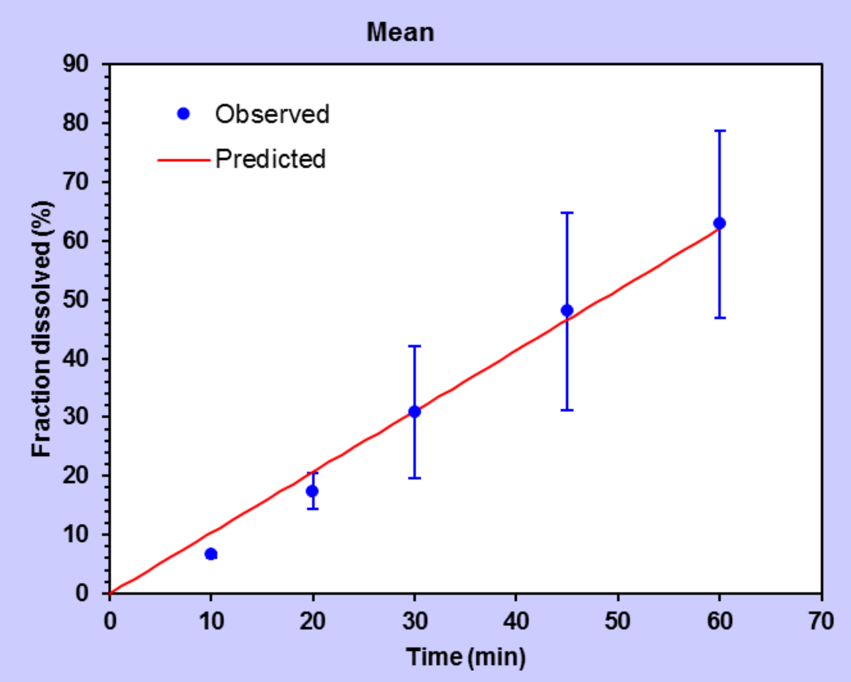

Supplementary materials – Model fitting summary – Parteck® M 200 (modelling up to app. 60 % of carvedilol released)

Graphical abstract of model fit presented as the fraction % of released carvedilol per tested tablet:

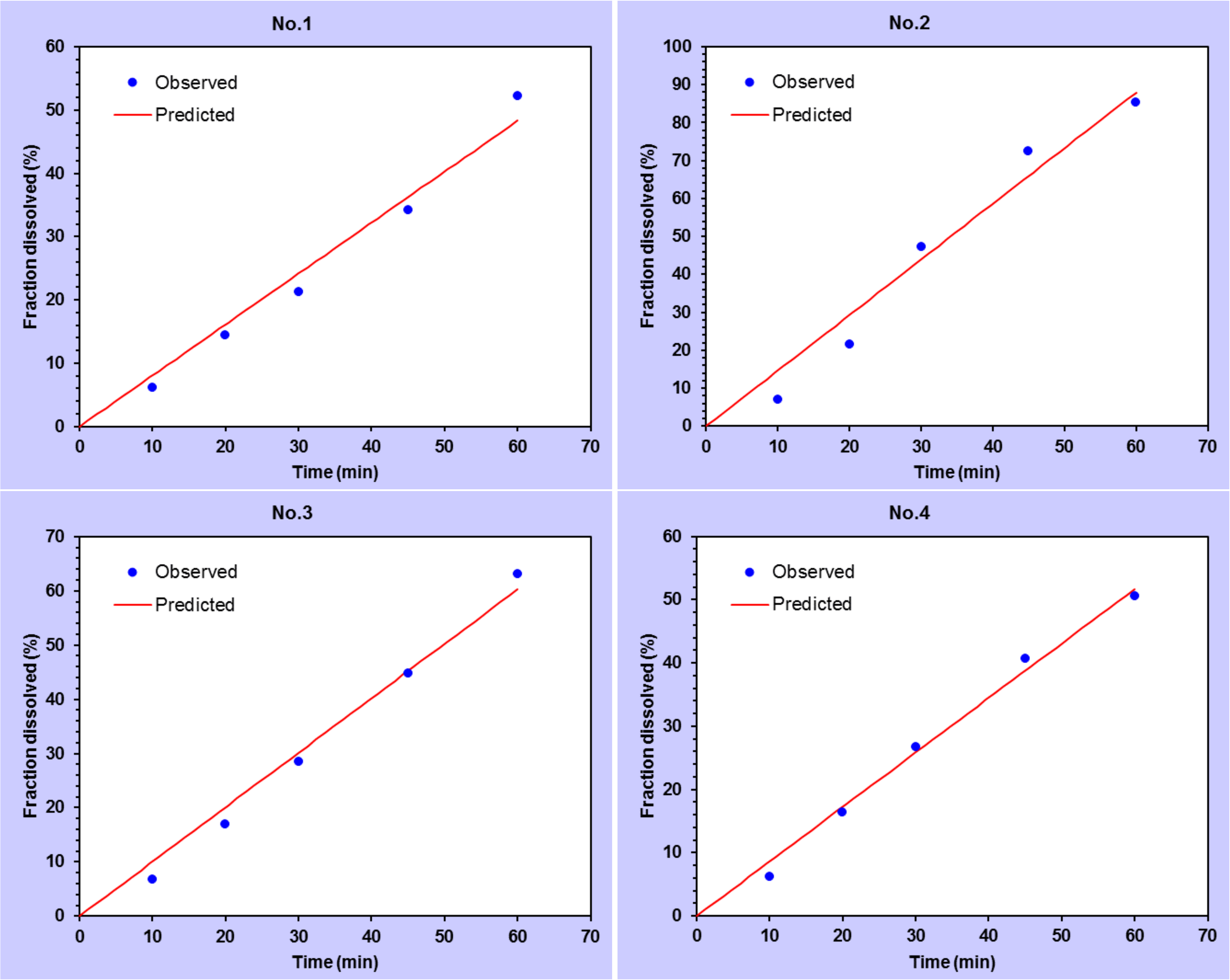

Supplementary materials – Model fitting summary – Parteck® M 200 (modelling up to app. 60 % of carvedilol released)

Model: **Hopfenberg with T_{lag}**

Model equation: $F = 100 \cdot \{1 - [1 - k_{HB} \cdot (t - T_{lag})]^n\}$

Fitted model parameters per tested tablet (N = 4) with statistics – mean, standard deviation (SD), and relative standard deviation expressed in % (RSD%) (output from DDSolver):

Parameter	No.1	No.2	No.3	No.4	Mean	SD	RSD(%)
k_{HB}	0.009	0.012	0.011	0.004	0.009	0.004	41.249
n	1.000	2.000	1.000	3.000	1.750	0.957	54.710
T_{lag}	4.625	8.104	4.556	4.382	5.417	1.795	33.128

Number of dissolution data points (N), degrees of freedom (df), and selected goodness of fit criteria – Pearson correlation coefficient (R), coefficient of determination (R^2), adjusted coefficient of determination ($R^2_{adjusted}$), and residual sum of squares (RSS) (manual calculation in MS Excel):

Parameter	No.1	No.2	No.3	No.4
N	5	5	5	5
df	2	2	2	2
R	0.994112033	0.994988115	0.999547112	0.999405298
R^2	0.988258735	0.990001348	0.999094429	0.99881095
$R^2_{adjusted}$	0.976517469	0.980002696	0.998188858	0.997621901
RSS	15.34127725	43.76893618	1.8125	1.543278572

Graphical abstract of model fit presented as mean ± 1 SD of the fraction % of released carvedilol:

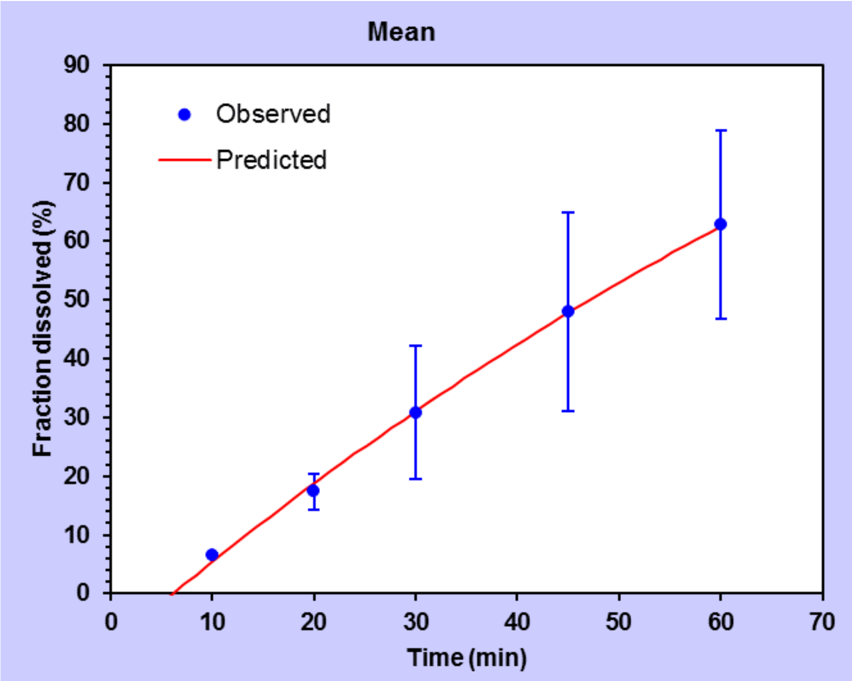

Supplementary materials – Model fitting summary – Parteck® M 200 (modelling up to app. 60 % of carvedilol released)

Graphical abstract of model fit presented as the fraction % of released carvedilol per tested tablet:

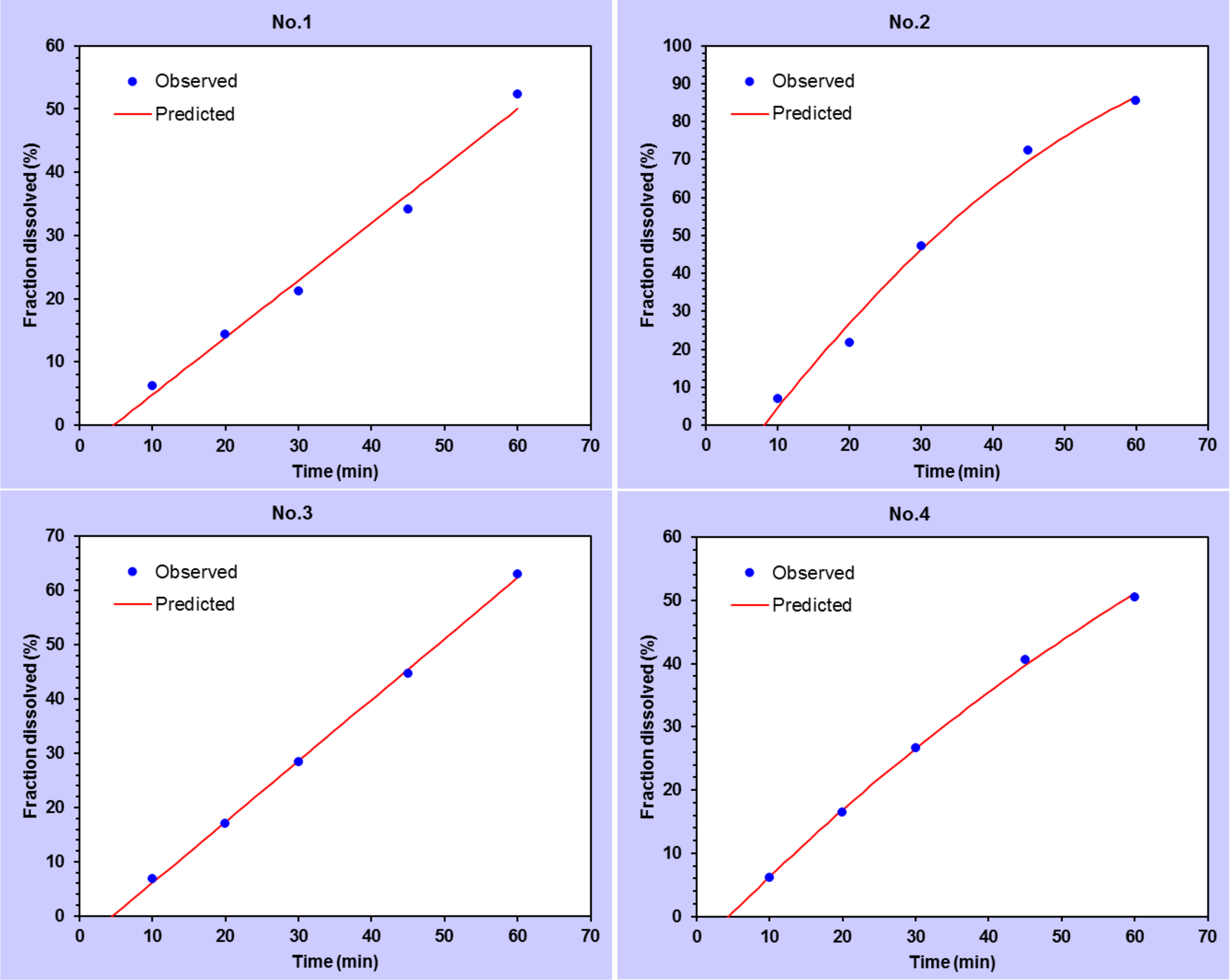

Supplementary materials – Model fitting summary – Parteck® M 200 (modelling up to app. 60 % of carvedilol released)

Model: **Baker–Lonsdale**

Model equation: $\frac{3}{2} \cdot \left[1 - \left(1 - \frac{F}{100}\right)^{\frac{2}{3}}\right] - \frac{F}{100} = k_{BL} \cdot t$

Fitted model parameters per tested tablet (N = 4) with statistics – mean, standard deviation (SD), and relative standard deviation expressed in % (RSD%) (output from DDSolver):

Parameter	No.1	No.2	No.3	No.4	Mean	SD	RSD(%)
k_{BL}	0.001	0.005	0.002	0.001	0.002	0.002	77.111

Number of dissolution data points (N), degrees of freedom (df), and selected goodness of fit criteria – Pearson correlation coefficient (R), coefficient of determination (R^2), adjusted coefficient of determination ($R^2_{adjusted}$), and residual sum of squares (RSS) (manual calculation in MS Excel):

Parameter	No.1	No.2	No.3	No.4
N	5	5	5	5
df	4	4	4	4
R	0.967765773	0.984901254	0.980241687	0.995580516
R^2	0.936570591	0.970030481	0.960873764	0.991180563
$R^2_{adjusted}$	0.936570591	0.970030481	0.960873764	0.991180563
RSS	1367.706881	4161.126234	2000.667471	938.97462

Graphical abstract of model fit presented as mean ± 1 SD of the fraction % of released carvedilol:

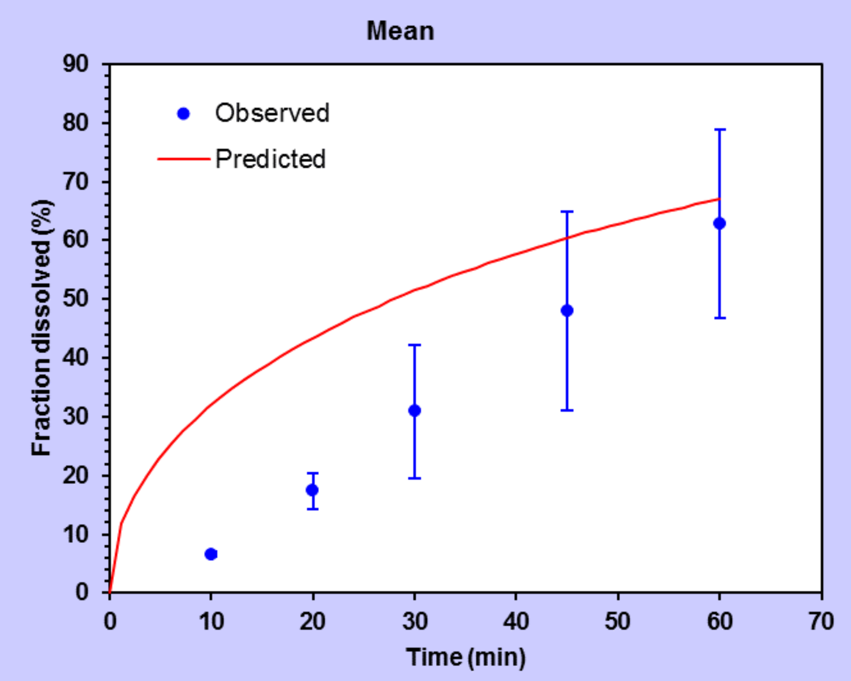

Supplementary materials – Model fitting summary – Parteck® M 200 (modelling up to app. 60 % of carvedilol released)

Graphical abstract of model fit presented as the fraction % of released carvedilol per tested tablet:

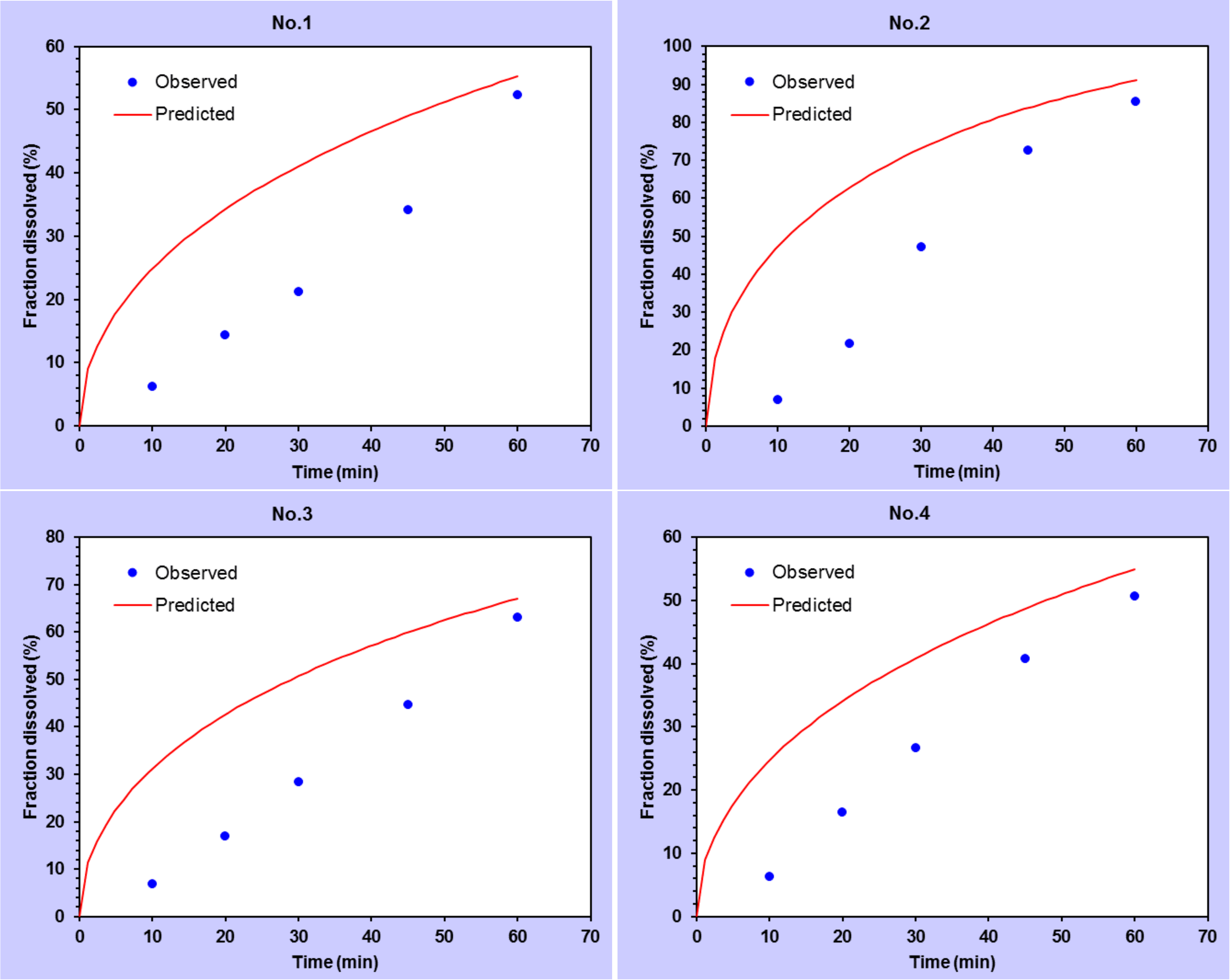

Supplementary materials – Model fitting summary – Parteck® M 200 (modelling up to app. 60 % of carvedilol released)

Model: **Baker–Lonsdale with T_{lag}**

Model equation: $\frac{3}{2}\cdot\left[1-\left(1-\frac{F}{100}\right)^{\frac{2}{3}}\right]-\frac{F}{100}=k_{BL}\cdot(t-T_{lag})$

Fitted model parameters per tested tablet (N = 4) with statistics – mean, standard deviation (SD), and relative standard deviation expressed in % (RSD%) (output from DDSolver):

Parameter	No.1	No.2	No.3	No.4	Mean	SD	RSD(%)
k_{BL}	0.001	0.006	0.002	0.001	0.003	0.002	88.868
T_{lag}	16.356	17.149	16.038	17.396	16.735	0.642	3.836

Number of dissolution data points (N), degrees of freedom (df), and selected goodness of fit criteria – Pearson correlation coefficient (R), coefficient of determination (R^2), adjusted coefficient of determination ($R^2_{adjusted}$), and residual sum of squares (RSS) (manual calculation in MS Excel):

Parameter	No.1	No.2	No.3	No.4
N	5	5	5	5
df	3	3	3	3
R	0.950596918	0.984493564	0.965365309	0.992662374
R^2	0.903634501	0.969227578	0.93193018	0.985378589
$R^2_{adjusted}$	0.871512668	0.958970104	0.90924024	0.980504786
RSS	148.7875569	254.7565508	161.3035746	51.99281426

Graphical abstract of model fit presented as mean ± 1 SD of the fraction % of released carvedilol:

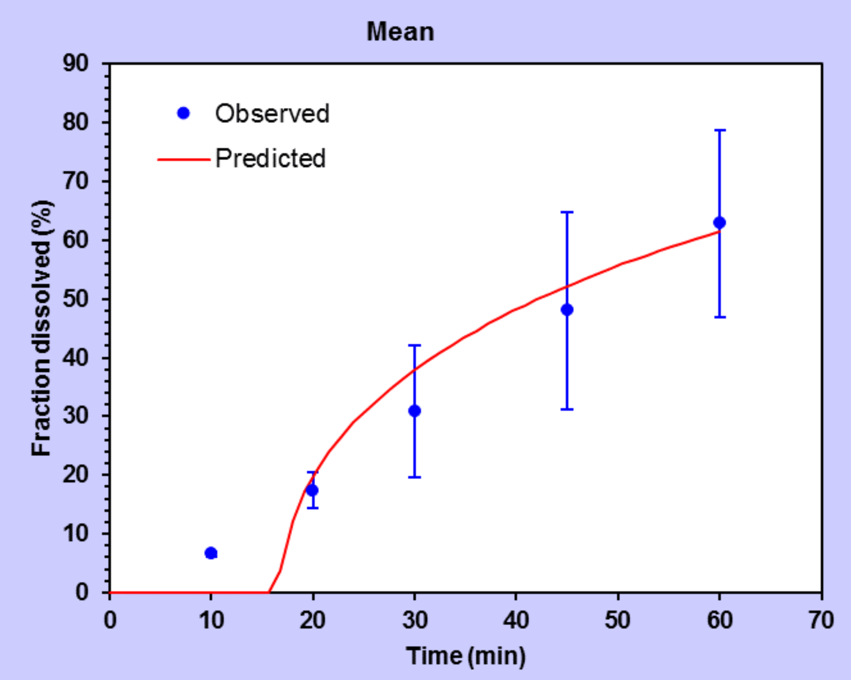

Supplementary materials – Model fitting summary – Parteck® M 200 (modelling up to app. 60 % of carvedilol released)

Graphical abstract of model fit presented as the fraction % of released carvedilol per tested tablet:

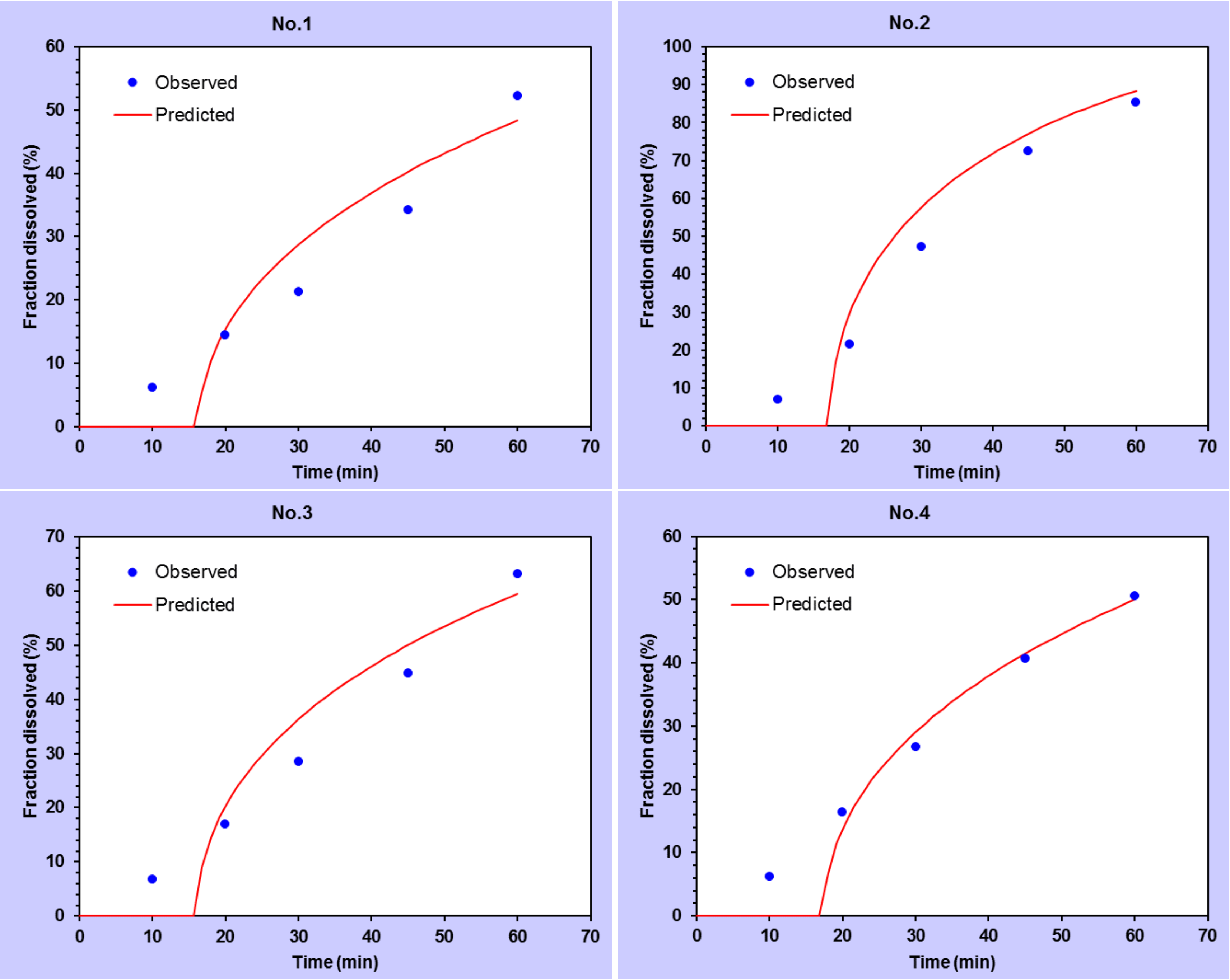

Supplementary materials – Model fitting summary – Parteck® M 200 (modelling up to app. 60 % of carvedilol released)

Model: **Makoid–Banakar**

Model equation: $F = k_{MB} \cdot t^n \cdot e^{-k \cdot t}$

Fitted model parameters per tested tablet (N = 4) with statistics – mean, standard deviation (SD), and relative standard deviation expressed in % (RSD%) (output from DDSolver):

Parameter	No.1	No.2	No.3	No.4	Mean	SD	RSD(%)
k_{MB}	0.542	0.055	0.303	0.169	0.267	0.209	78.296
n	1.048	2.215	1.370	1.636	1.567	0.494	31.542
k	-0.004	0.028	0.005	0.017	0.011	0.014	125.145

Number of dissolution data points (N), degrees of freedom (df), and selected goodness of fit criteria – Pearson correlation coefficient (R), coefficient of determination (R^2), adjusted coefficient of determination ($R^2_{adjusted}$), and residual sum of squares (RSS) (manual calculation in MS Excel):

Parameter	No.1	No.2	No.3	No.4
N	5	5	5	5
df	2	2	2	2
R	0.998539774	0.997422186	0.999797479	0.999985512
R^2	0.99708168	0.994851017	0.999594998	0.999971025
$R^2_{adjusted}$	0.99416336	0.989702034	0.999189997	0.99994205
RSS	3.976805735	22.6182928	0.964851583	0.037156577

Graphical abstract of model fit presented as mean ± 1 SD of the fraction % of released carvedilol:

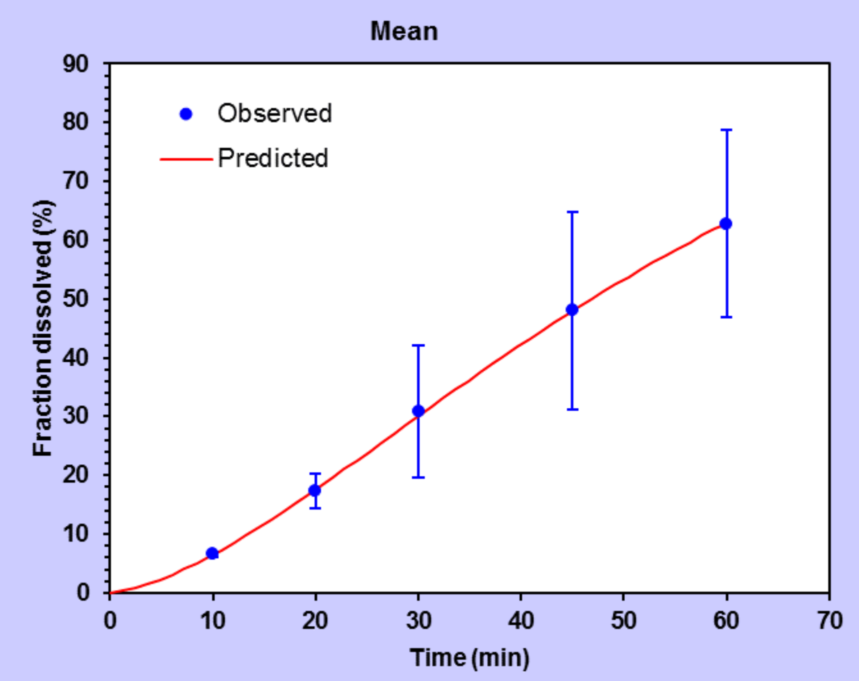

Supplementary materials – Model fitting summary – Parteck® M 200 (modelling up to app. 60 % of carvedilol released)

Graphical abstract of model fit presented as the fraction % of released carvedilol per tested tablet:

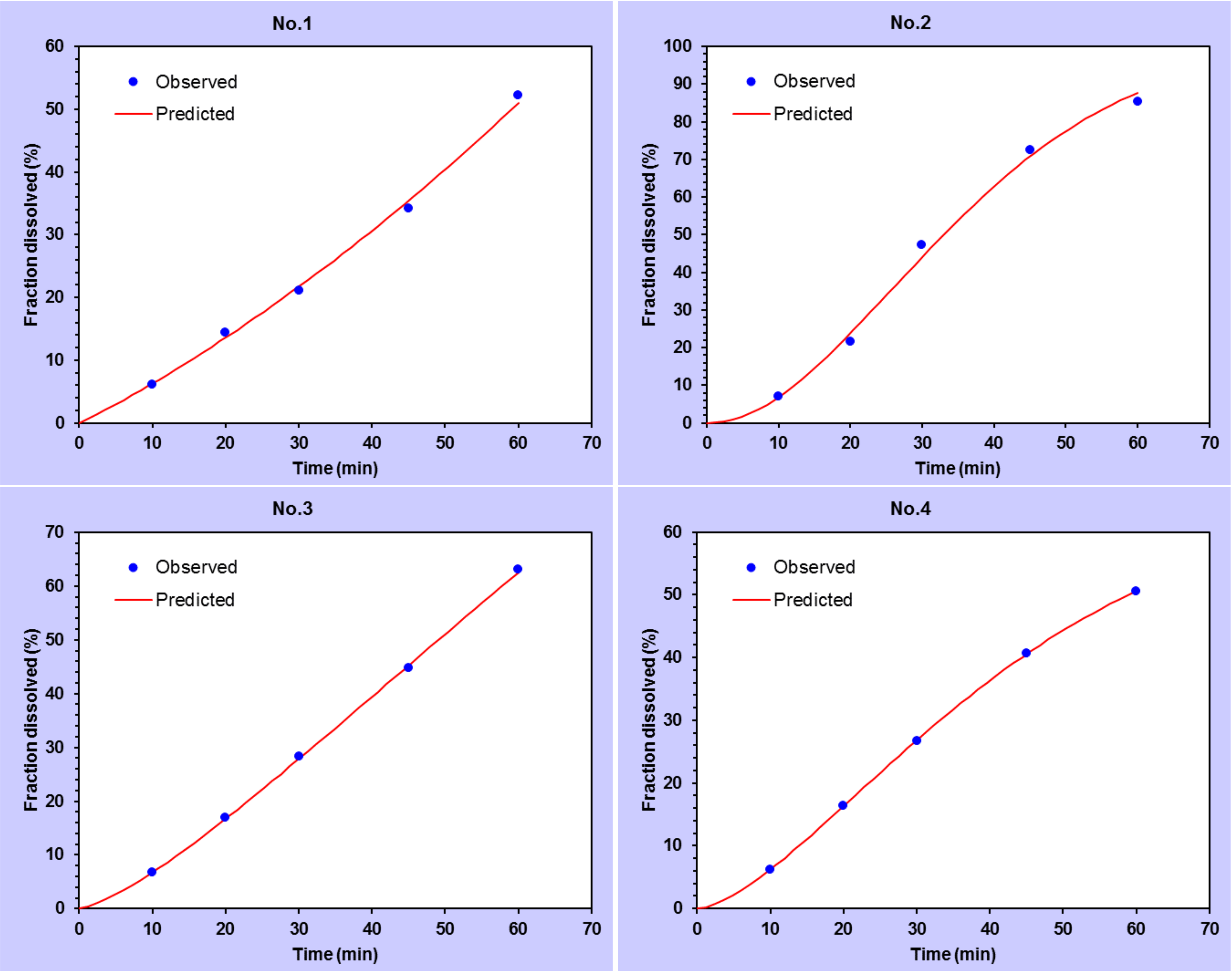

Supplementary materials – Model fitting summary – Parteck® M 200 (modelling up to app. 60 % of carvedilol released)

Model: **Makoid–Banakar with T_{lag}**

Model equation: $F = k_{MB} \cdot (t - T_{lag})^n \cdot e^{-k\cdot(t-T_{lag})}$

Fitted model parameters per tested tablet (N = 4) with statistics – mean, standard deviation (SD), and relative standard deviation expressed in % (RSD%) (output from DDSolver):

Parameter	No.1	No.2	No.3	No.4	Mean	SD	RSD(%)
k_{MB}	1.690	0.553	1.288	0.931	1.115	0.486	43.610
n	0.695	1.441	0.914	1.073	1.031	0.314	30.486
k	-0.011	0.013	-0.004	0.006	0.001	0.010	1168.545
T_{lag}	4.000	4.000	4.000	4.000	4.000	0.000	0.000

Number of dissolution data points (N), degrees of freedom (df), and selected goodness of fit criteria – Pearson correlation coefficient (R), coefficient of determination (R^2), adjusted coefficient of determination ($R^2_{adjusted}$), and residual sum of squares (RSS) (manual calculation in MS Excel):

Parameter	No.1	No.2	No.3	No.4
N	5	5	5	5
df	1	1	1	1
R	0.999230551	0.99429236	0.99992424	0.999536878
R^2	0.998461693	0.988617297	0.999848486	0.999073969
$R^2_{adjusted}$	0.993846774	0.954469189	0.999393946	0.996295878
RSS	2.134250844	50.27396016	0.306711217	1.190840994

Graphical abstract of model fit presented as mean ± 1 SD of the fraction % of released carvedilol:

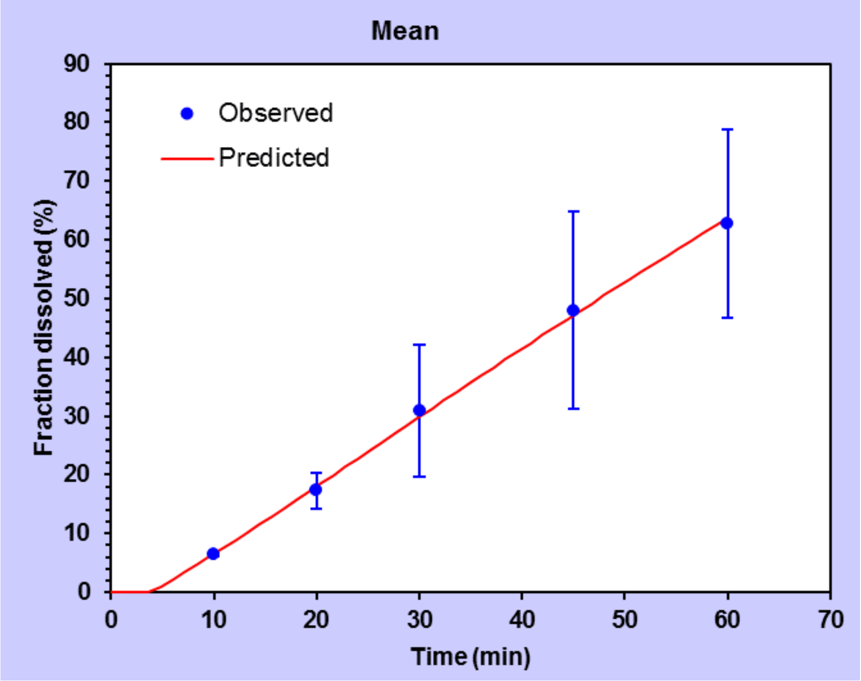

Supplementary materials – Model fitting summary – Parteck® M 200 (modelling up to app. 60 % of carvedilol released)

Graphical abstract of model fit presented as the fraction % of released carvedilol per tested tablet:

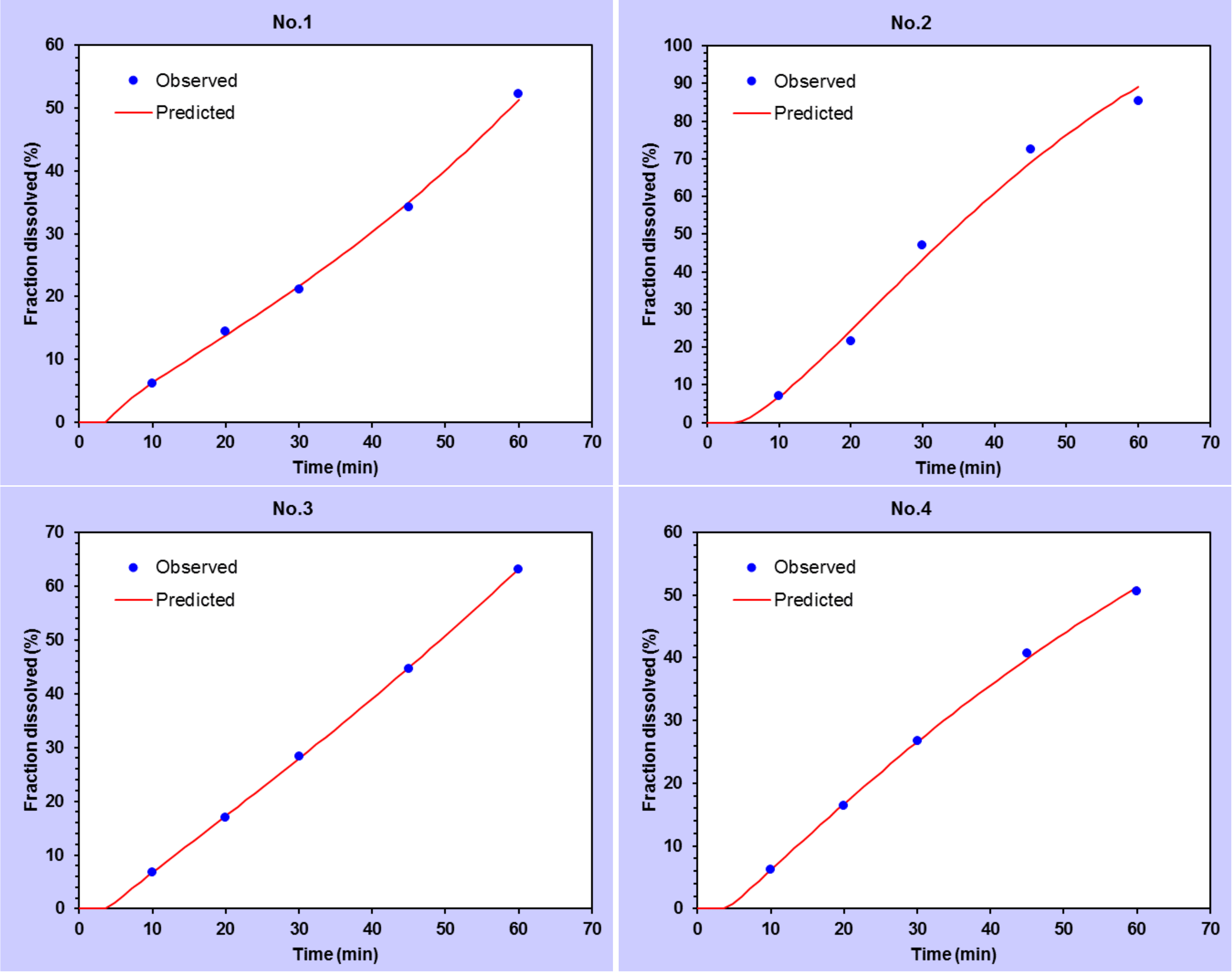

Supplementary materials – Model fitting summary – Parteck® M 200 (modelling up to app. 60 % of carvedilol released)

Model: **Peppas–Sahlin_1**

Model equation: $F = k_1 \cdot t^m + k_2 \cdot t^{2m}$

Fitted model parameters per tested tablet (N = 4) with statistics – mean, standard deviation (SD), and relative standard deviation expressed in % (RSD%) (output from DDSolver):

Parameter	No.1	No.2	No.3	No.4	Mean	SD	RSD(%)
k_1	-3.386	-5.344	-4.023	-1.826	-3.645	1.461	-40.097
k_2	1.798	3.124	2.205	1.596	2.181	0.678	31.082
m	0.450	0.450	0.450	0.450	0.450	0.000	0.000

Number of dissolution data points (N), degrees of freedom (df), and selected goodness of fit criteria – Pearson correlation coefficient (R), coefficient of determination (R^2), adjusted coefficient of determination ($R^2_{adjusted}$), and residual sum of squares (RSS) (manual calculation in MS Excel):

Parameter	No.1	No.2	No.3	No.4
N	5	5	5	5
df	2	2	2	2
R	0.994438713	0.986611288	0.99963141	0.996853216
R^2	0.988908355	0.973401835	0.999262956	0.993716333
$R^2_{adjusted}$	0.97781671	0.946803669	0.998525912	0.987432667
RSS	14.61760086	116.8591628	1.489201524	8.147675563

Graphical abstract of model fit presented as mean ± 1 SD of the fraction % of released carvedilol:

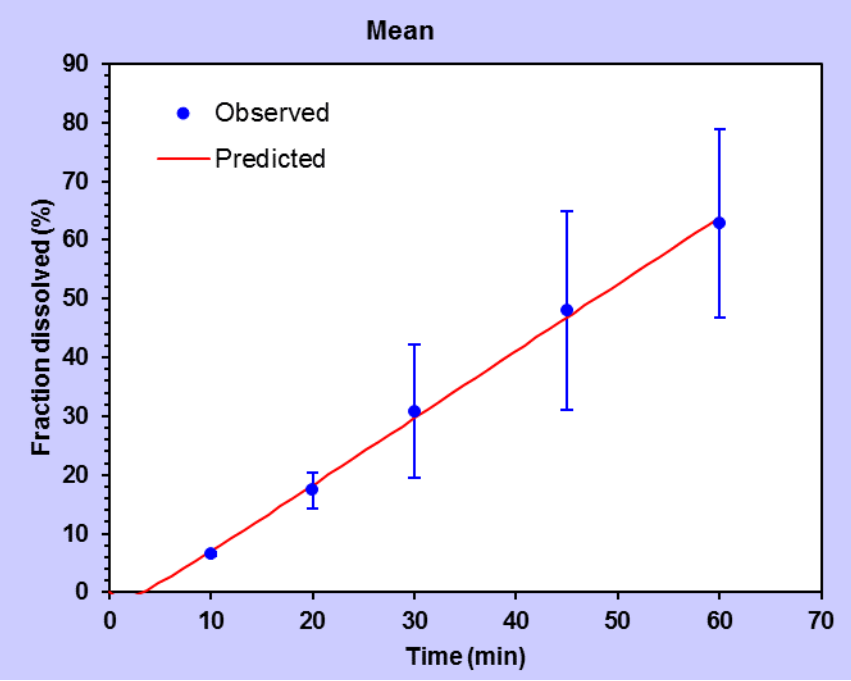

Supplementary materials – Model fitting summary – Parteck® M 200 (modelling up to app. 60 % of carvedilol released)

Graphical abstract of model fit presented as the fraction % of released carvedilol per tested tablet:

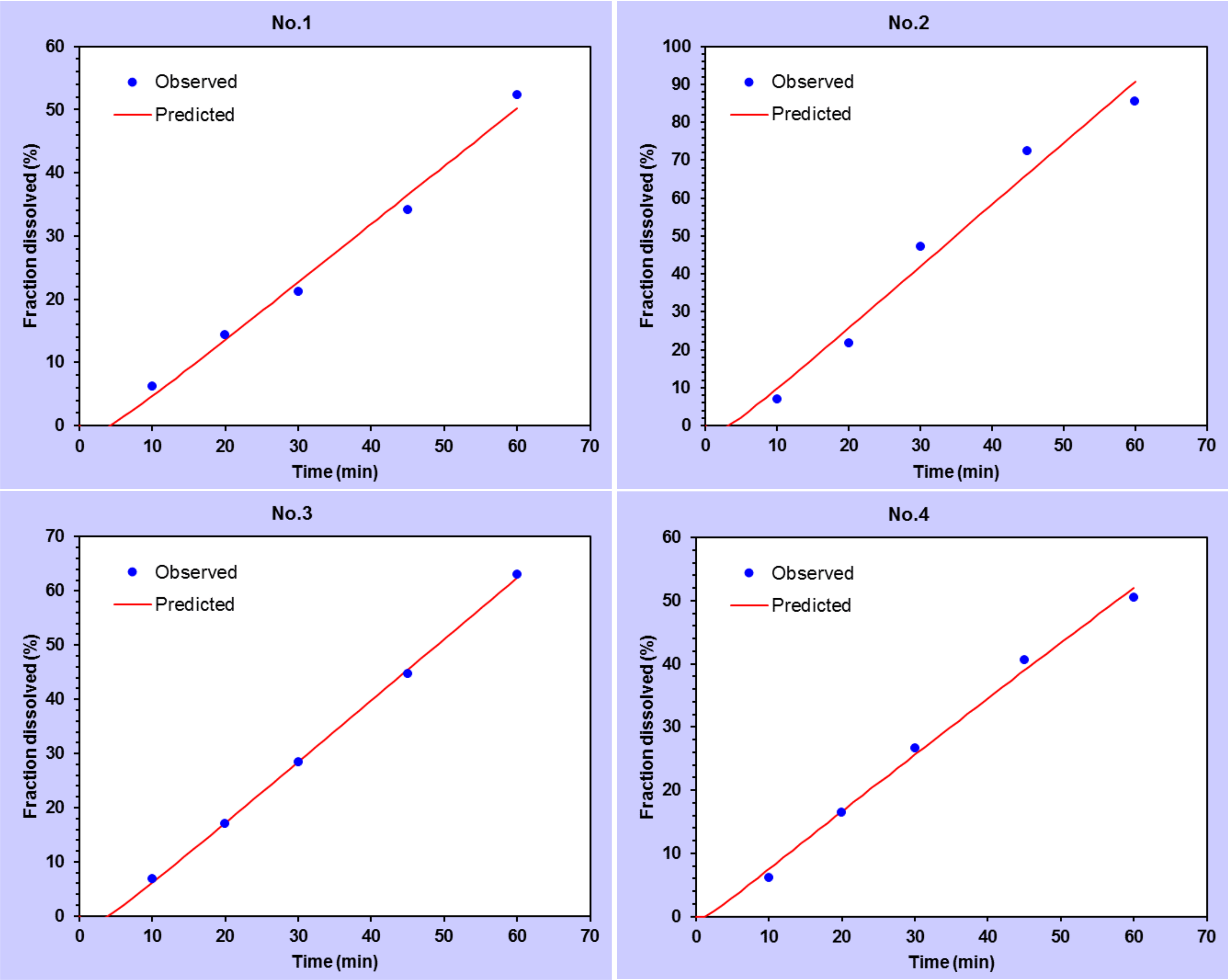

Supplementary materials – Model fitting summary – Parteck® M 200 (modelling up to app. 60 % of carvedilol released)

Model: **Peppas–Sahlin_1 with T_{lag}**

Model equation: $F = k_1 \cdot (t - T_{lag})^m + k_2 \cdot (t - T_{lag})^{2m}$

Fitted model parameters per tested tablet (N = 4) with statistics – mean, standard deviation (SD), and relative standard deviation expressed in % (RSD%) (output from DDSolver):

Parameter	No.1	No.2	No.3	No.4	Mean	SD	RSD(%)
k_1	-1.719	-2.082	-1.894	0.067	-1.407	0.994	-70.629
k_2	1.618	2.751	1.971	1.372	1.928	0.601	31.182
m	0.450	0.450	0.450	0.450	0.450	0.000	0.000
T_{lag}	4.000	4.000	4.000	4.000	4.000	0.000	0.000

Number of dissolution data points (N), degrees of freedom (df), and selected goodness of fit criteria – Pearson correlation coefficient (R), coefficient of determination (R^2), adjusted coefficient of determination ($R^2_{adjusted}$), and residual sum of squares (RSS) (manual calculation in MS Excel):

Parameter	No.1	No.2	No.3	No.4
N	5	5	5	5
df	1	1	1	1
R	0.993188305	0.988271293	0.999193964	0.998032327
R^2	0.986423008	0.976680149	0.998388577	0.996068525
$R^2_{adjusted}$	0.945692033	0.906720596	0.993554308	0.9842741
RSS	18.10685214	102.8676637	3.315005251	5.131475257

Graphical abstract of model fit presented as mean ± 1 SD of the fraction % of released carvedilol:

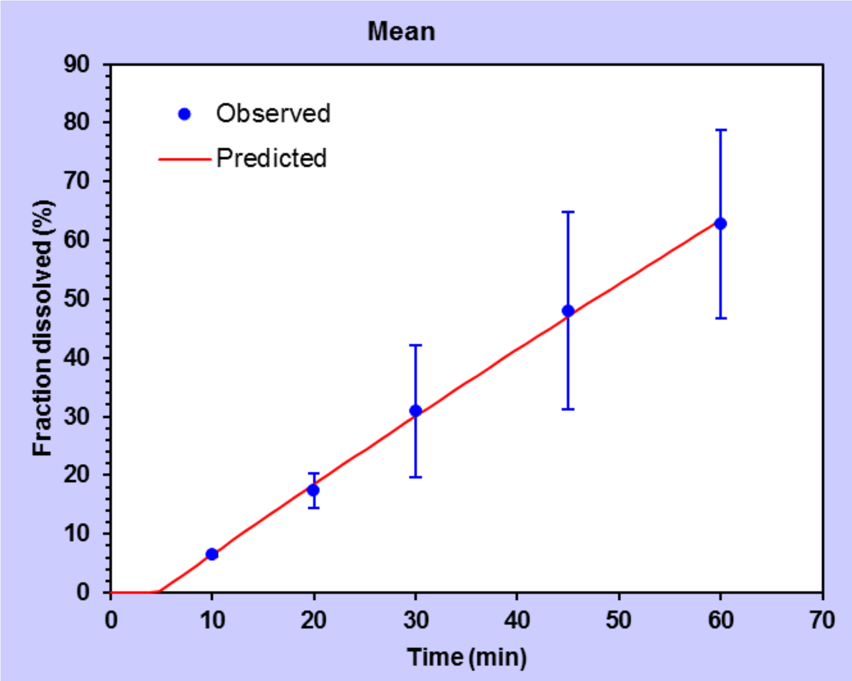

Supplementary materials – Model fitting summary – Parteck® M 200 (modelling up to app. 60 % of carvedilol released)

Graphical abstract of model fit presented as the fraction % of released carvedilol per tested tablet:

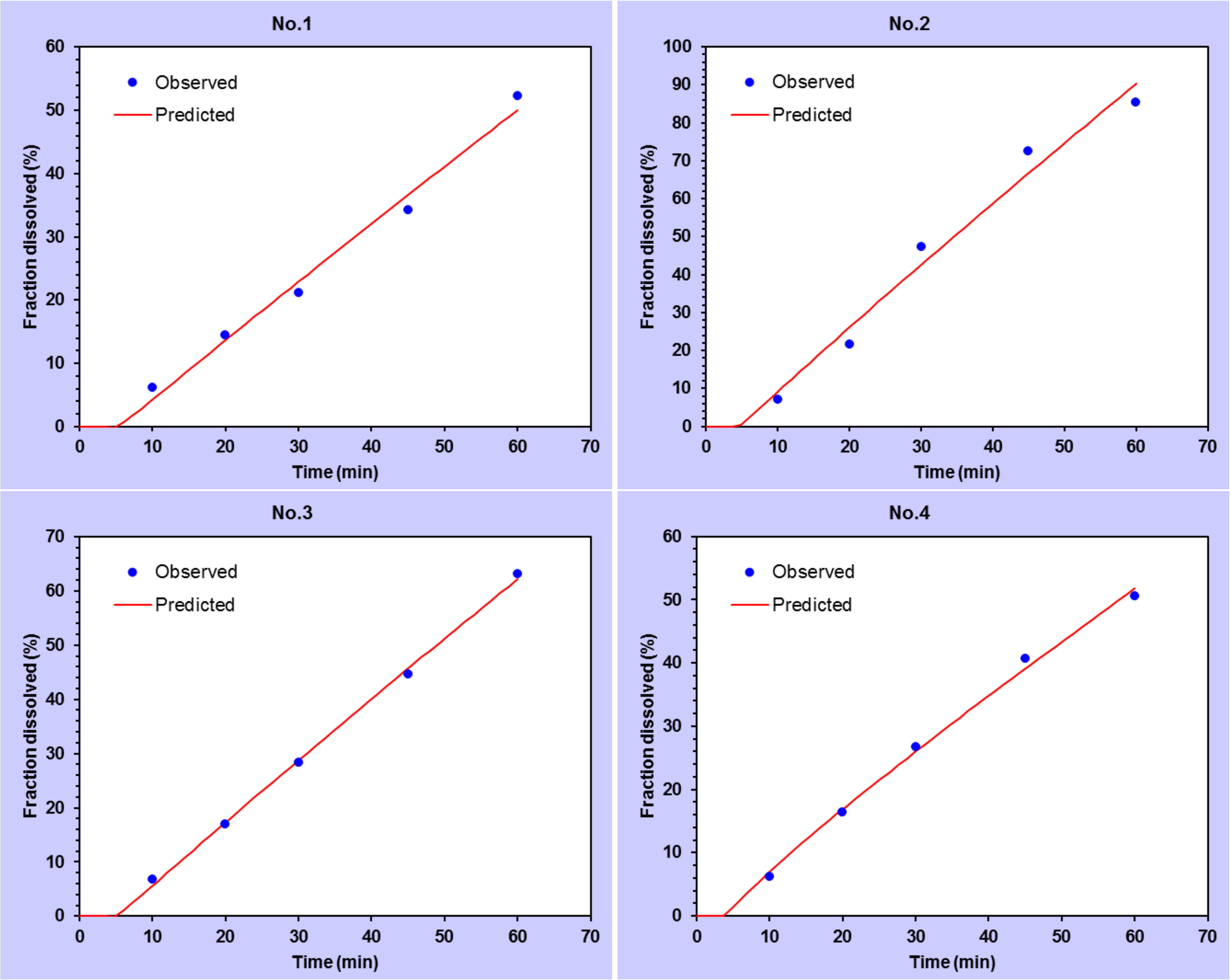

Supplementary materials – Model fitting summary – Parteck® M 200 (modelling up to app. 60 % of carvedilol released)

Model: **Peppas–Sahlin_2**

Model equation: $F = k_1 \cdot t^{0.5} + k_2 \cdot t$

Fitted model parameters per tested tablet (N = 4) with statistics – mean, standard deviation (SD), and relative standard deviation expressed in % (RSD%) (output from DDSolver):

Parameter	No.1	No.2	No.3	No.4	Mean	SD	RSD(%)
k_1	-1.792	-2.410	-2.033	-0.412	-1.662	0.871	-52.413
k_2	1.074	1.827	1.309	0.924	1.284	0.396	30.828

Number of dissolution data points (N), degrees of freedom (df), and selected goodness of fit criteria – Pearson correlation coefficient (R), coefficient of determination (R^2), adjusted coefficient of determination ($R^2_{adjusted}$), and residual sum of squares (RSS) (manual calculation in MS Excel):

Parameter	No.1	No.2	No.3	No.4
N	5	5	5	5
df	3	3	3	3
R	0.995811319	0.984666078	0.999872024	0.995823124
R^2	0.991640184	0.969567285	0.999744065	0.991663694
$R^2_{adjusted}$	0.988853579	0.959423046	0.999658753	0.988884925
RSS	11.0444223	134.3657809	0.513686876	10.89433299

Graphical abstract of model fit presented as mean ± 1 SD of the fraction % of released carvedilol:

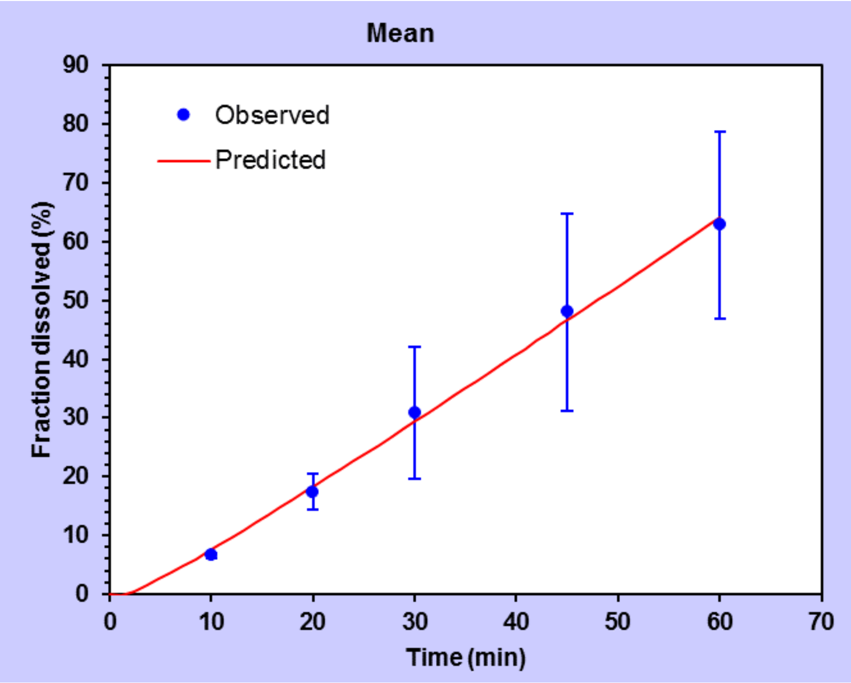

Supplementary materials – Model fitting summary – Parteck® M 200 (modelling up to app. 60 % of carvedilol released)

Graphical abstract of model fit presented as the fraction % of released carvedilol per tested tablet:

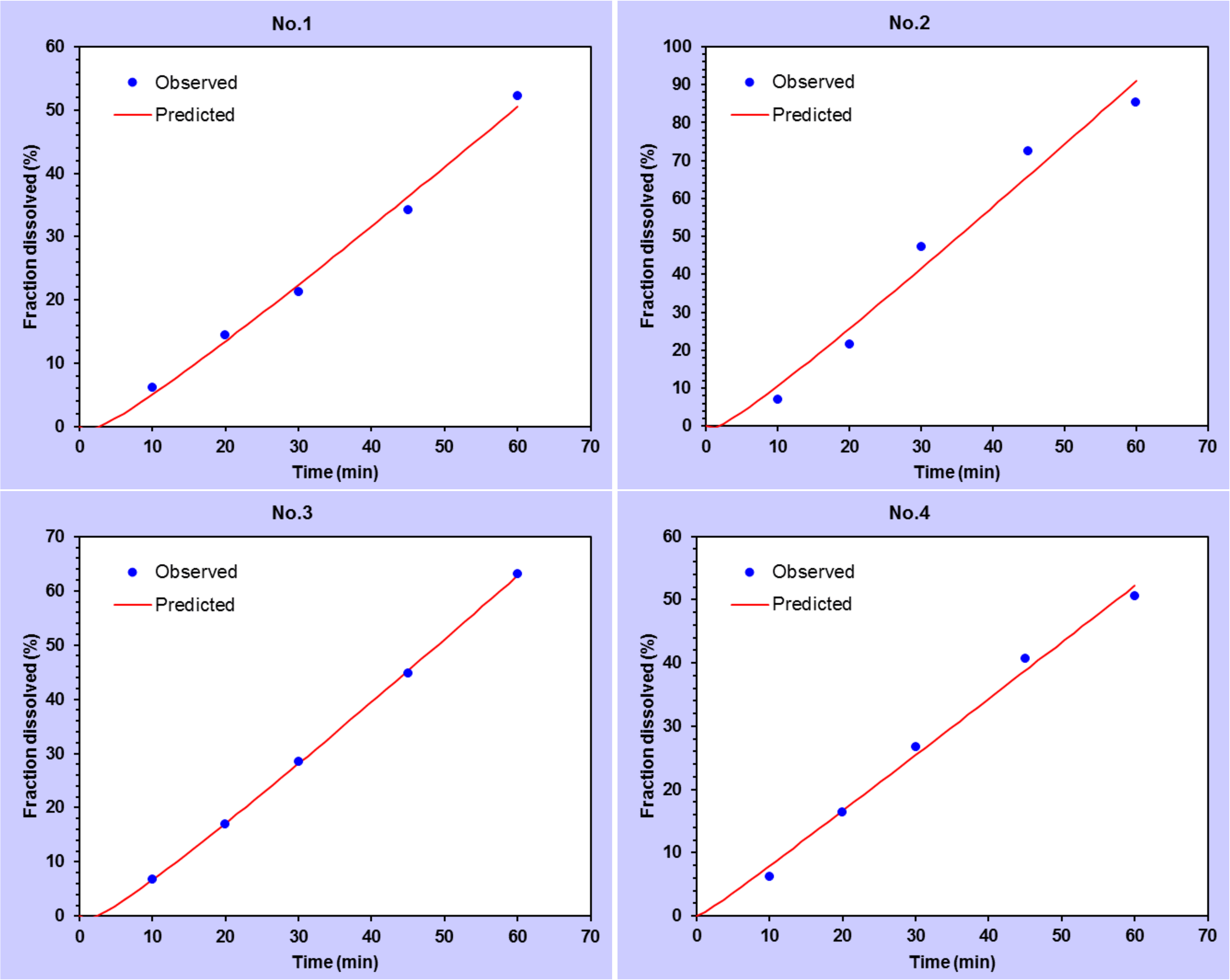

Supplementary materials – Model fitting summary – Parteck® M 200 (modelling up to app. 60 % of carvedilol released)

Model: **Peppas–Sahlin_2 with T_{lag}**

Model equation: $F = k_1 \cdot (t - T_{lag})^{0.5} + k_2 \cdot (t - T_{lag})$

Fitted model parameters per tested tablet (N = 4) with statistics – mean, standard deviation (SD), and relative standard deviation expressed in % (RSD%) (output from DDSolver):

Parameter	No.1	No.2	No.3	No.4	Mean	SD	RSD(%)
k_1	-0.495	0.197	-0.362	1.105	0.111	0.727	653.207
k_2	0.966	1.589	1.165	0.779	1.125	0.347	30.870
T_{lag}	4.000	4.000	4.000	4.000	4.000	0.000	0.000

Number of dissolution data points (N), degrees of freedom (df), and selected goodness of fit criteria – Pearson correlation coefficient (R), coefficient of determination (R^2), adjusted coefficient of determination ($R^2_{adjusted}$), and residual sum of squares (RSS) (manual calculation in MS Excel):

Parameter	No.1	No.2	No.3	No.4
N	5	5	5	5
df	2	2	2	2
R	0.994766771	0.986782358	0.999659875	0.997425454
R^2	0.989560929	0.973739422	0.999319865	0.994857536
$R^2_{adjusted}$	0.979121857	0.947478844	0.99863973	0.989715072
RSS	14.01667965	116.8898949	1.405805933	6.815920224

Graphical abstract of model fit presented as mean ± 1 SD of the fraction % of released carvedilol:

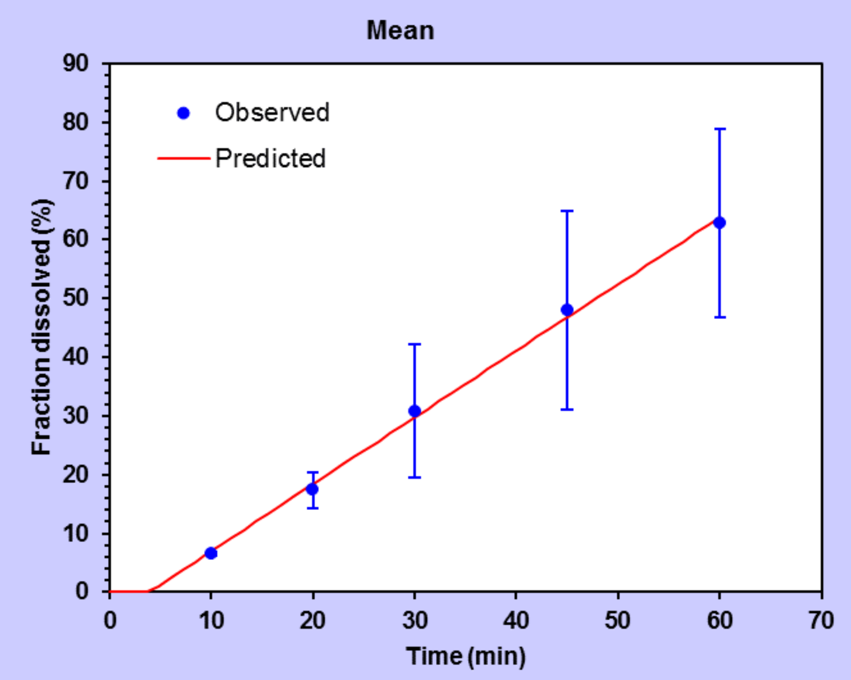

Supplementary materials – Model fitting summary – Parteck® M 200 (modelling up to app. 60 % of carvedilol released)

Graphical abstract of model fit presented as the fraction % of released carvedilol per tested tablet:

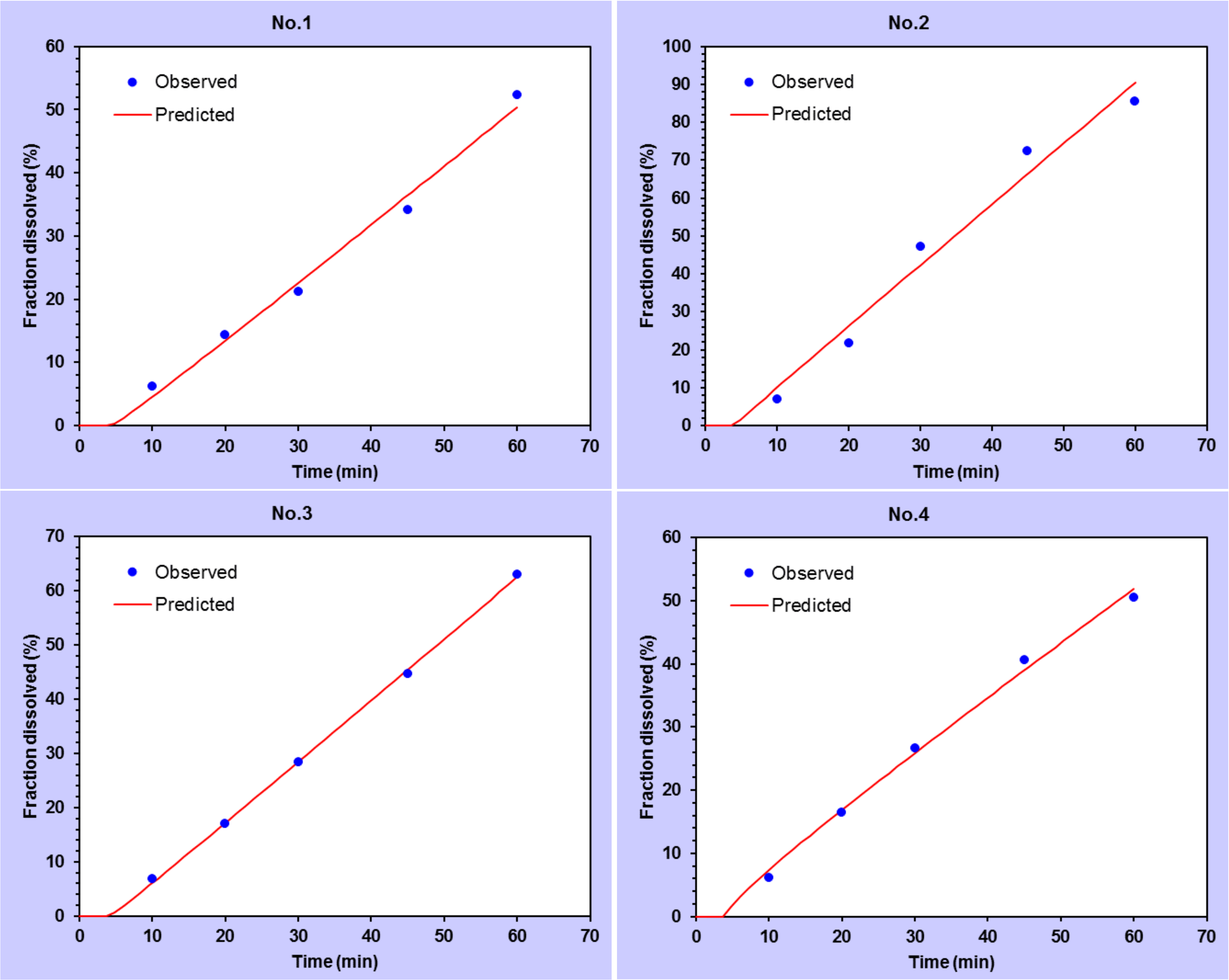

Supplementary materials – Model fitting summary – Parteck® M 200 (modelling up to app. 60 % of carvedilol released)

Model: **Quadratic**

Model equation: $F = 100 \cdot (k_1 \cdot t^2 + k_2 \cdot t)$

Fitted model parameters per tested tablet (N = 4) with statistics – mean, standard deviation (SD), and relative standard deviation expressed in % (RSD%) (output from DDSolver):

Parameter	No.1	No.2	No.3	No.4	Mean	SD	RSD(%)
k_1	0.000050	0.000021	0.000047	-0.000001	0.000029	0.000024	81.795727
k_2	0.005624	0.013614	0.007757	0.008674	0.008917	0.003382	37.923900

Number of dissolution data points (N), degrees of freedom (df), and selected goodness of fit criteria – Pearson correlation coefficient (R), coefficient of determination (R^2), adjusted coefficient of determination ($R^2_{adjusted}$), and residual sum of squares (RSS) (manual calculation in MS Excel):

Parameter	No.1	No.2	No.3	No.4
N	5	5	5	5
df	3	3	3	3
R	0.998833879	0.983745817	0.999353354	0.996388302
R^2	0.997669117	0.967755832	0.998707125	0.992789648
$R^2_{adjusted}$	0.996892156	0.957007775	0.998276167	0.990386198
RSS	3.186571095	171.7918319	3.203911812	12.14549136

Graphical abstract of model fit presented as mean ± 1 SD of the fraction % of released carvedilol:

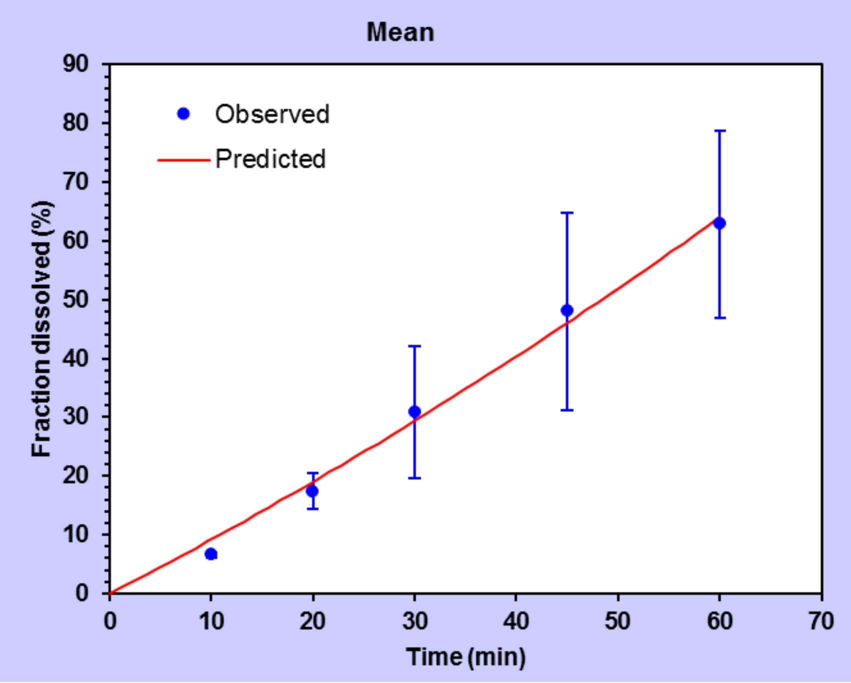

Supplementary materials – Model fitting summary – Parteck® M 200 (modelling up to app. 60 % of carvedilol released)

Graphical abstract of model fit presented as the fraction % of released carvedilol per tested tablet:

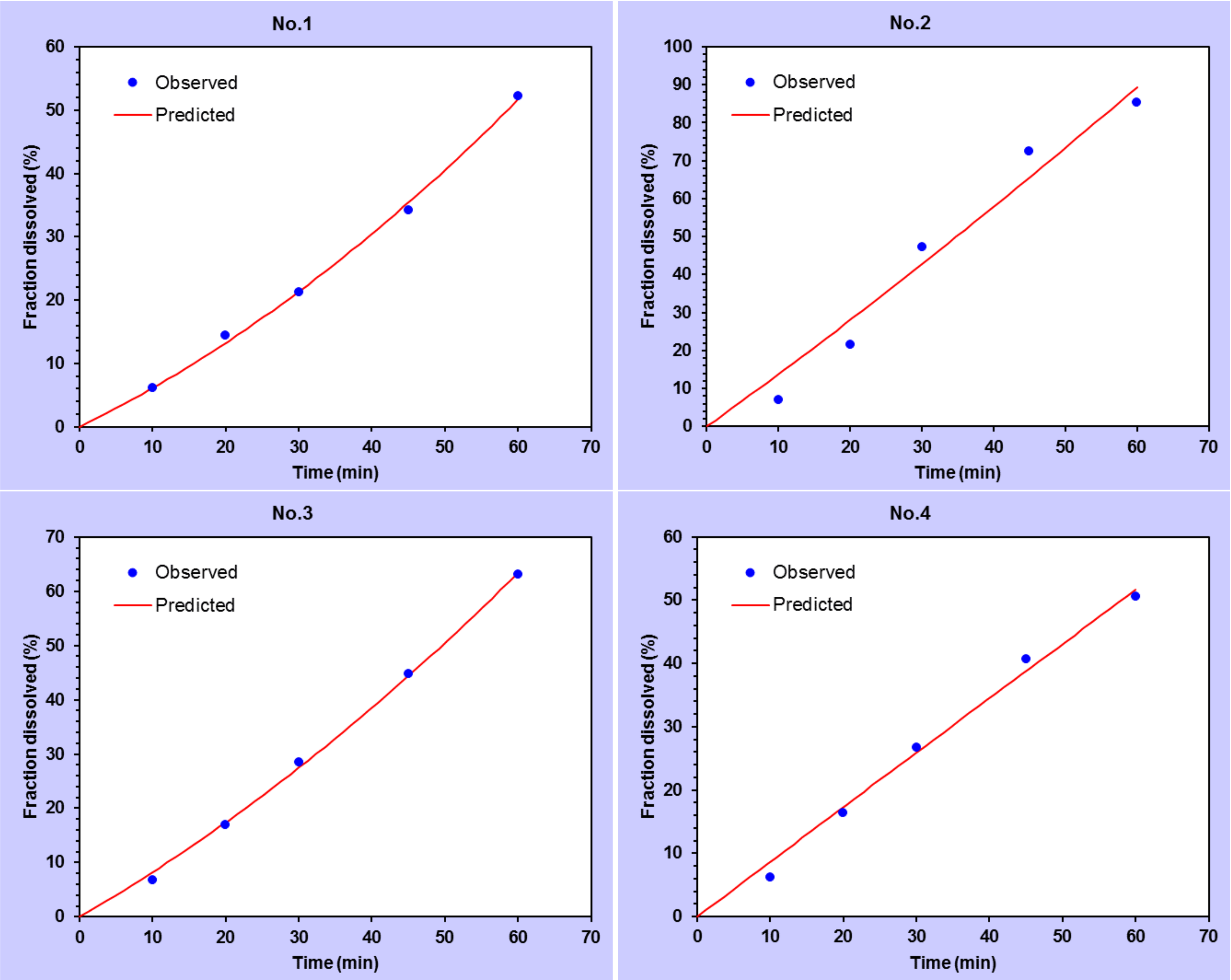

Supplementary materials – Model fitting summary – Parteck® M 200 (modelling up to app. 60 % of carvedilol released)

Model: **Quadratic with T_{lag}**

Model equation: $F = 100 \cdot \left[k_1 \cdot \left(t - T_{lag}\right)^2 + k_2 \cdot \left(t - T_{lag}\right)\right]$

Fitted model parameters per tested tablet (N = 4) with statistics – mean, standard deviation (SD), and relative standard deviation expressed in % (RSD%) (output from DDSolver):

Parameter	No.1	No.2	No.3	No.4	Mean	SD	RSD(%)
k_1	0.000028	-0.000045	0.000014	-0.000039	-0.000011	0.000037	-348.346551
k_2	0.007606	0.018281	0.010457	0.011288	0.011908	0.004532	38.058600
T_{lag}	4.000000	4.000000	4.000000	4.000000	4.000000	0.000000	0.000000

Number of dissolution data points (N), degrees of freedom (df), and selected goodness of fit criteria – Pearson correlation coefficient (R), coefficient of determination (R^2), adjusted coefficient of determination ($R^2_{adjusted}$), and residual sum of squares (RSS) (manual calculation in MS Excel):

Parameter	No.1	No.2	No.3	No.4
N	5	5	5	5
df	2	2	2	2
R	0.997475718	0.991183802	0.999875392	0.999459492
R^2	0.994957808	0.982445329	0.999750799	0.998919275
$R^2_{adjusted}$	0.989915615	0.964890658	0.999501598	0.99783855
RSS	8.577967397	98.32480129	0.600526448	1.714536467

Graphical abstract of model fit presented as mean ± 1 SD of the fraction % of released carvedilol:

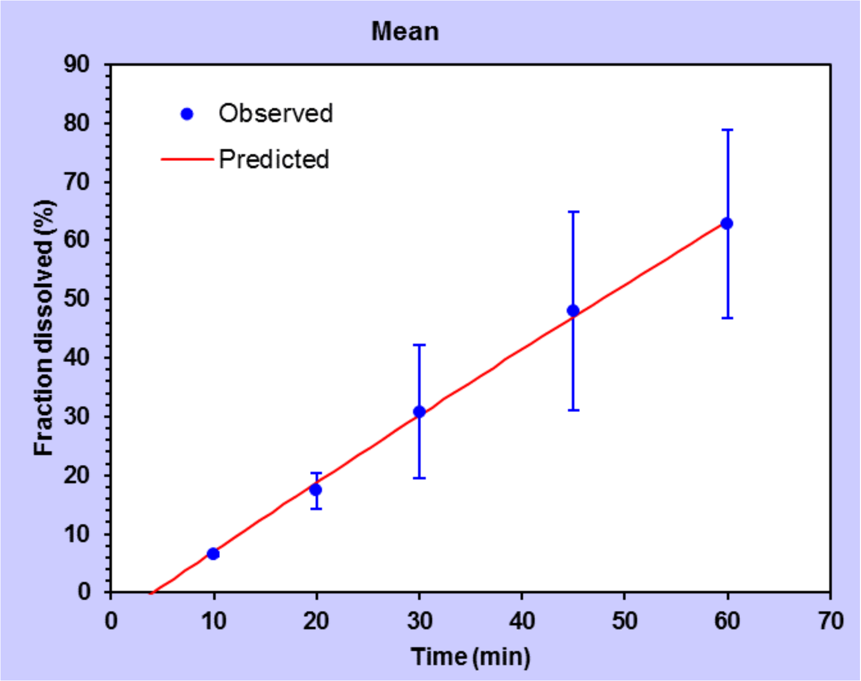

Supplementary materials – Model fitting summary – Parteck® M 200 (modelling up to app. 60 % of carvedilol released)

Graphical abstract of model fit presented as the fraction % of released carvedilol per tested tablet:

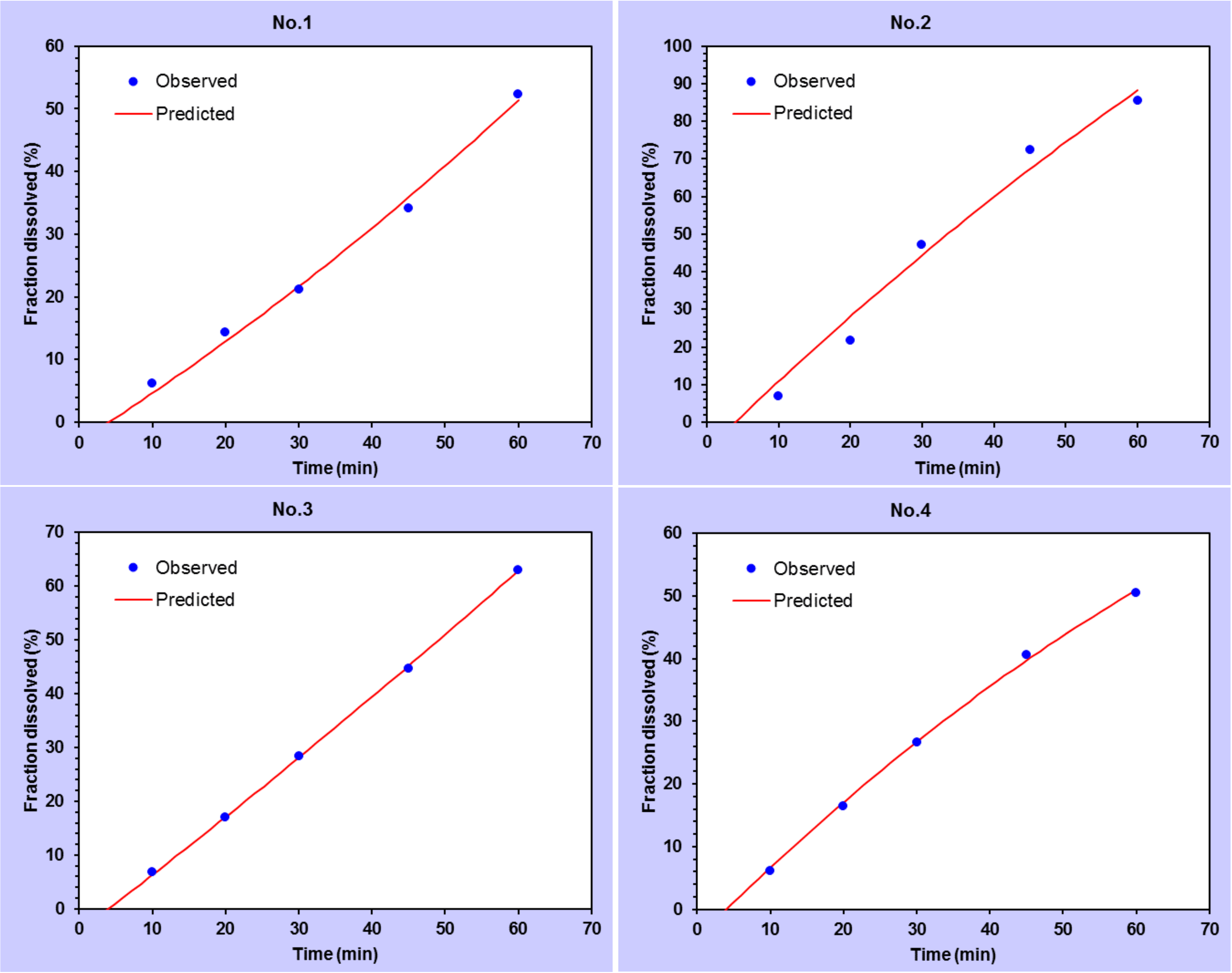

Supplementary materials – Model fitting summary – Parteck® M 200 (modelling up to app. 60 % of carvedilol released)

Model: **Weibull_1**

Model equation: $F = 100 \cdot \left[1 - e^{-\frac{(t-T_i)^{\beta}}{\alpha}}\right]$

Fitted model parameters per tested tablet (N = 4) with statistics – mean, standard deviation (SD), and relative standard deviation expressed in % (RSD%) (output from DDSolver):

Parameter	No.1	No.2	No.3	No.4	Mean	SD	RSD(%)
α	113.981	243.222	125.337	109.979	148.130	63.728	43.022
β	1.058	1.556	1.169	1.084	1.217	0.231	18.987
T_i	4.000	4.733	4.000	4.000	4.183	0.366	8.757

Number of dissolution data points (N), degrees of freedom (df), and selected goodness of fit criteria – Pearson correlation coefficient (R), coefficient of determination (R^2), adjusted coefficient of determination ($R^2_{adjusted}$), and residual sum of squares (RSS) (manual calculation in MS Excel):

Parameter	No.1	No.2	No.3	No.4
N	5	5	5	5
df	2	2	2	2
R	0.98553305	0.998198265	0.995080372	0.999622596
R^2	0.971275392	0.996399776	0.990184947	0.999245335
$R^2_{adjusted}$	0.942550784	0.992799552	0.980369893	0.99849067
RSS	49.11727208	20.34297245	26.77853881	0.973650435

Graphical abstract of model fit presented as mean ± 1 SD of the fraction % of released carvedilol:

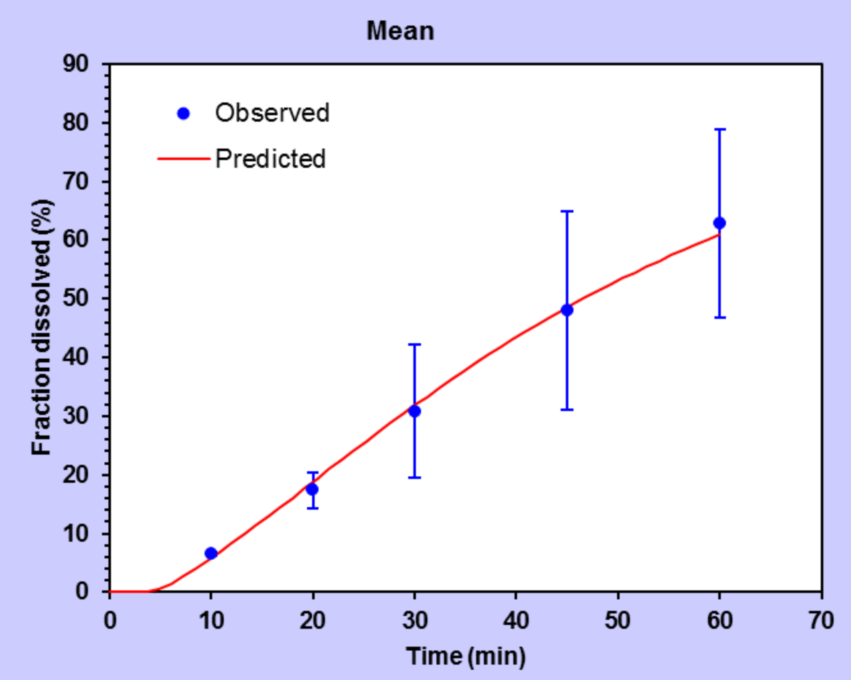

Supplementary materials – Model fitting summary – Parteck® M 200 (modelling up to app. 60 % of carvedilol released)

Graphical abstract of model fit presented as the fraction % of released carvedilol per tested tablet:

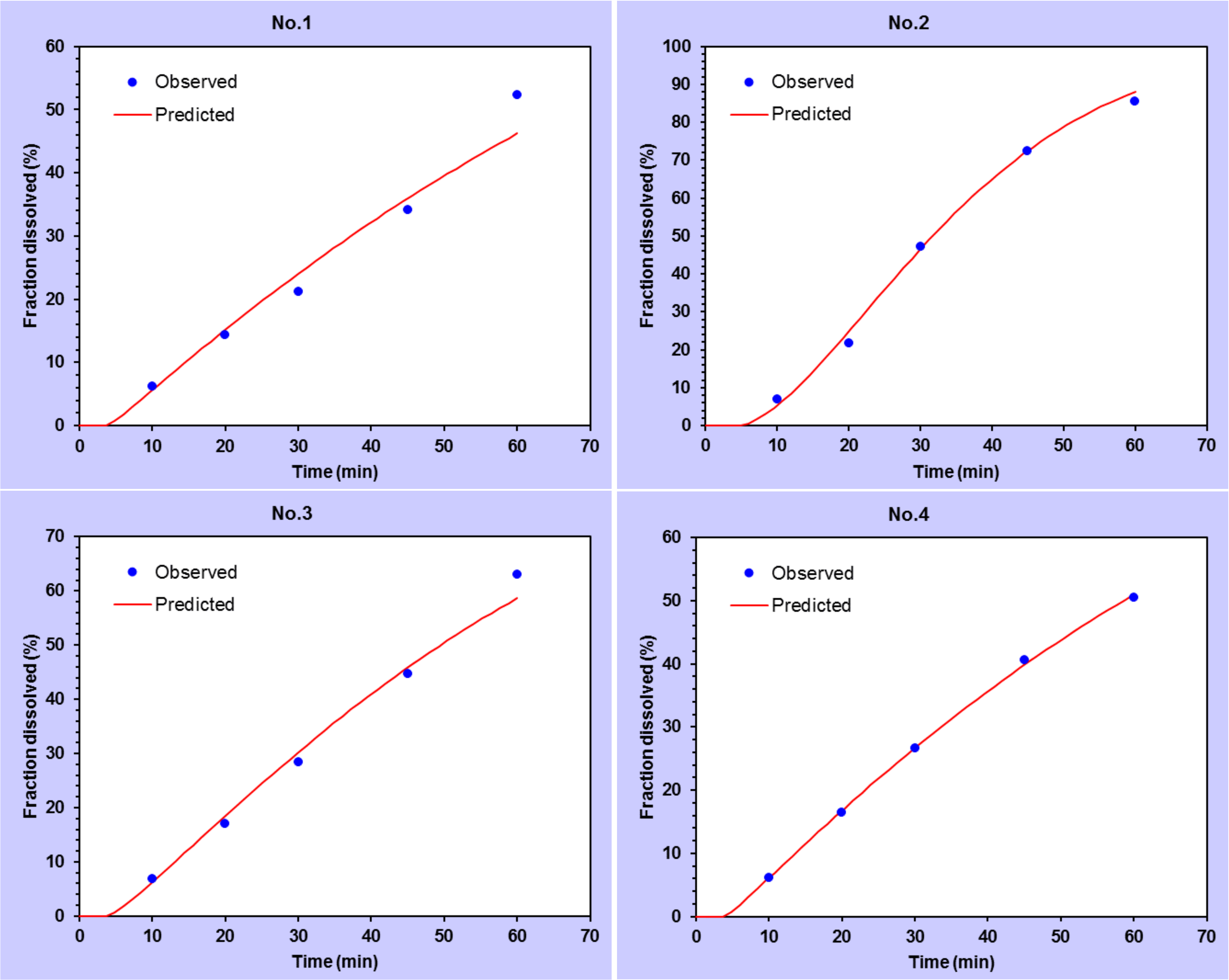

Supplementary materials – Model fitting summary – Parteck® M 200 (modelling up to app. 60 % of carvedilol released)

Model: **Weibull_2**

Model equation: $F = 100 \cdot \left(1 - e^{-\frac{t^{\beta}}{\alpha}}\right)$

Fitted model parameters per tested tablet (N = 4) with statistics – mean, standard deviation (SD), and relative standard deviation expressed in % (RSD%) (output from DDSolver):

Parameter	No.1	No.2	No.3	No.4	Mean	SD	RSD(%)
α	344.225	1037.255	421.491	330.840	533.453	338.236	63.405
β	1.325	1.876	1.462	1.347	1.502	0.256	17.053

Number of dissolution data points (N), degrees of freedom (df), and selected goodness of fit criteria – Pearson correlation coefficient (R), coefficient of determination (R^2), adjusted coefficient of determination ($R^2_{adjusted}$), and residual sum of squares (RSS) (manual calculation in MS Excel):

Parameter	No.1	No.2	No.3	No.4
N	5	5	5	5
df	3	3	3	3
R	0.992246344	0.997010362	0.998521173	0.997853384
R^2	0.984552806	0.994029662	0.997044534	0.995711377
$R^2_{adjusted}$	0.979403742	0.992039549	0.996059378	0.994281836
RSS	24.27590466	26.77437723	6.801435459	7.580131817

Graphical abstract of model fit presented as mean ± 1 SD of the fraction % of released carvedilol:

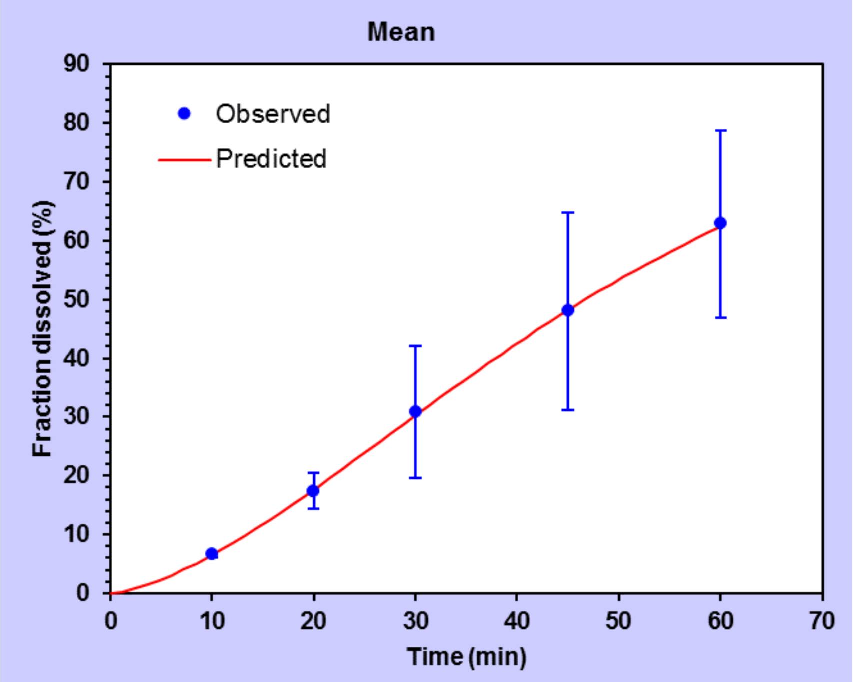

Supplementary materials – Model fitting summary – Parteck® M 200 (modelling up to app. 60 % of carvedilol released)

Graphical abstract of model fit presented as the fraction % of released carvedilol per tested tablet:

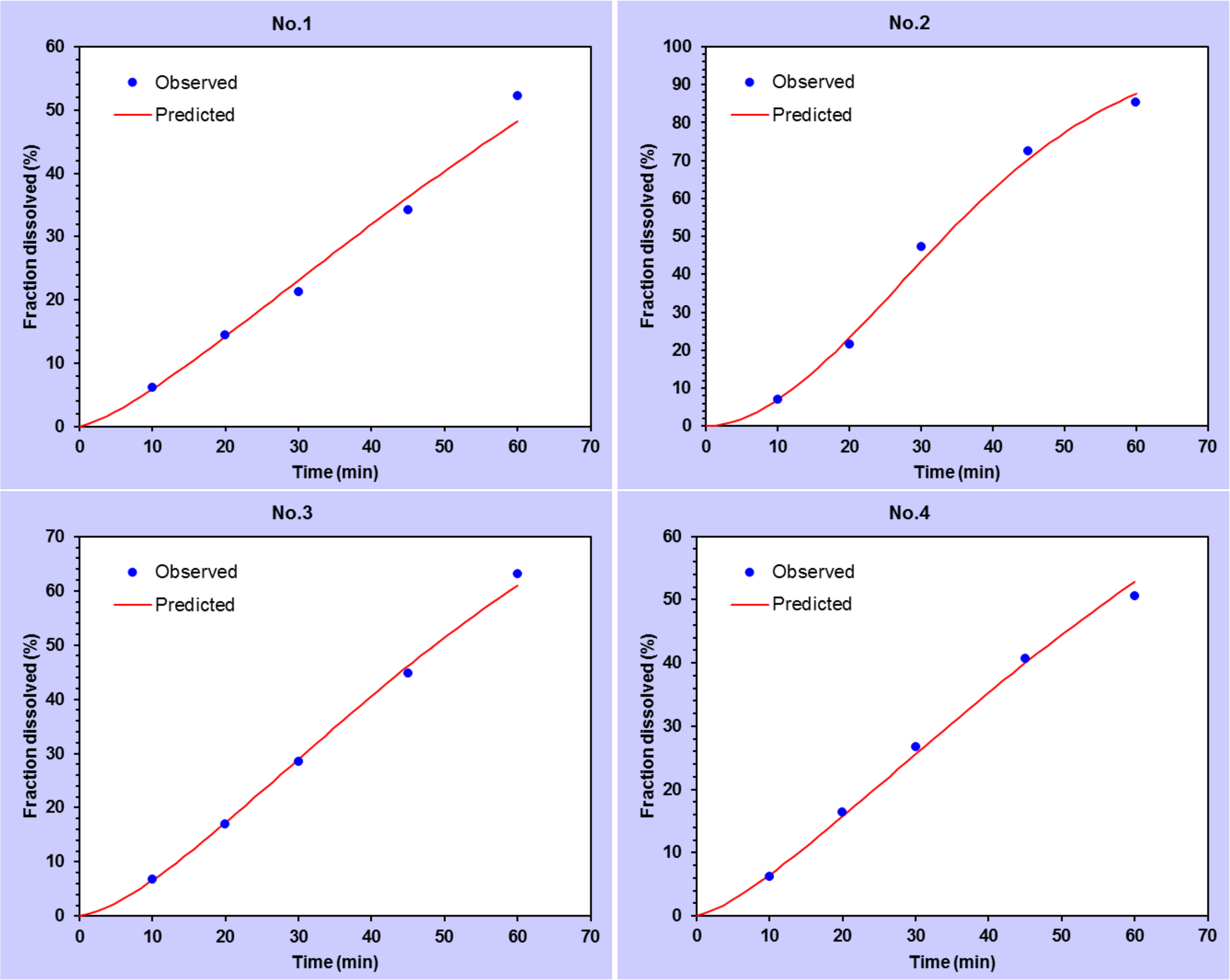

Supplementary materials – Model fitting summary – Parteck® M 200 (modelling up to app. 60 % of carvedilol released)

Model: **Weibull_3**

Model equation: $F = F_{max} \cdot \left(1 - e^{-\frac{t^{\beta}}{\alpha}}\right)$

Fitted model parameters per tested tablet (N = 4) with statistics – mean, standard deviation (SD), and relative standard deviation expressed in % (RSD%) (output from DDSolver):

Parameter	No.1	No.2	No.3	No.4	Mean	SD	RSD(%)
α	479.659	1453.444	732.454	484.591	787.537	459.359	58.329
β	1.682	2.044	1.738	1.745	1.802	0.164	9.083
F_{max}	54.890	89.628	70.949	53.081	67.137	17.010	25.336

Number of dissolution data points (N), degrees of freedom (df), and selected goodness of fit criteria – Pearson correlation coefficient (R), coefficient of determination (R^2), adjusted coefficient of determination ($R^2_{adjusted}$), and residual sum of squares (RSS) (manual calculation in MS Excel):

Parameter	No.1	No.2	No.3	No.4
N	5	5	5	5
df	2	2	2	2
R	0.975157198	0.999132476	0.995473452	0.996972346
R^2	0.95093156	0.998265705	0.990967393	0.993953859
$R^2_{adjusted}$	0.90186312	0.99653141	0.981934787	0.987907718
RSS	69.51634687	8.34031703	33.72036932	8.77690238

Graphical abstract of model fit presented as mean ± 1 SD of the fraction % of released carvedilol:

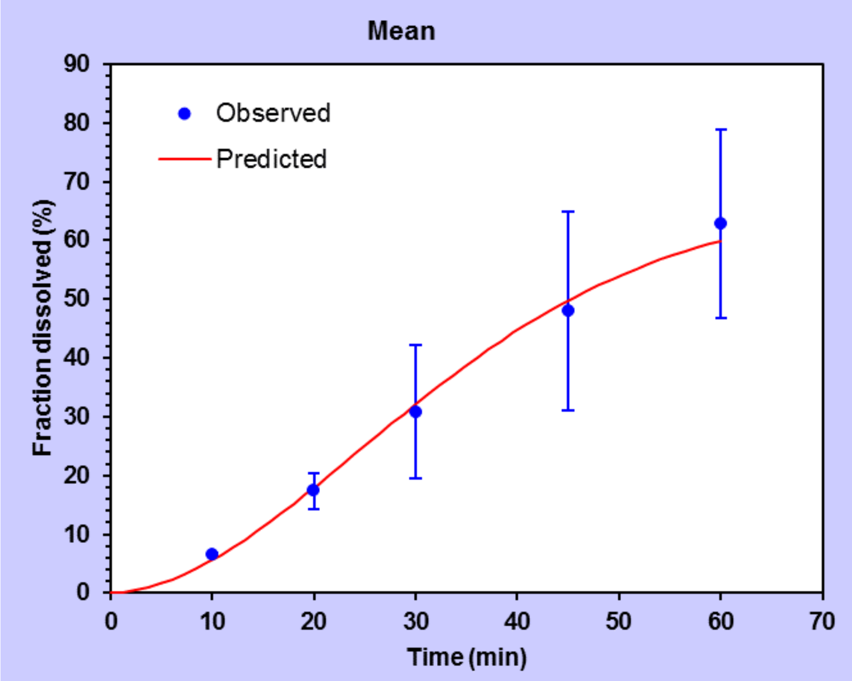

Supplementary materials – Model fitting summary – Parteck® M 200 (modelling up to app. 60 % of carvedilol released)

Graphical abstract of model fit presented as the fraction % of released carvedilol per tested tablet:

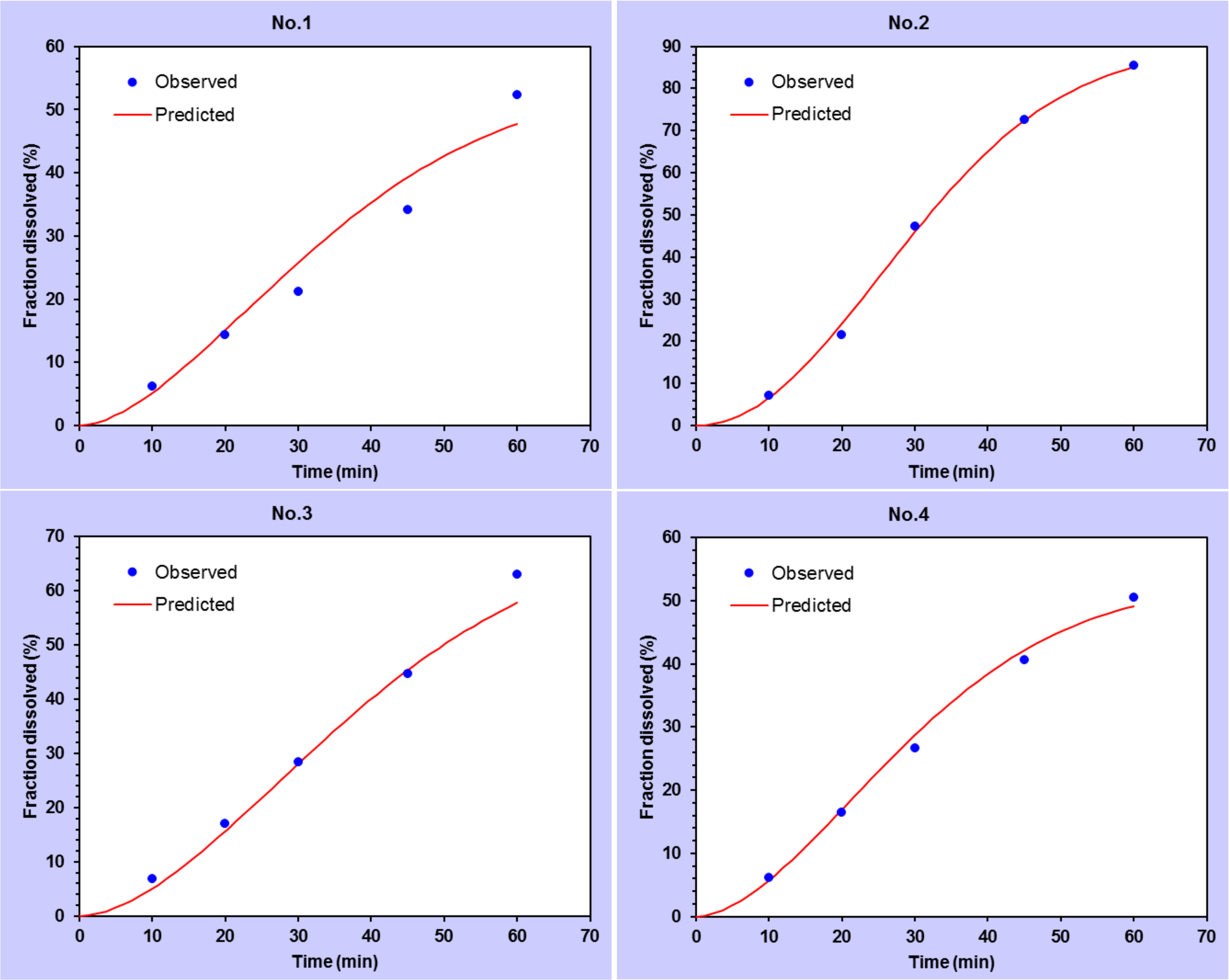

Supplementary materials – Model fitting summary – Parteck® M 200 (modelling up to app. 60 % of carvedilol released)

Model: **Weibull_4**

Model equation: $F = F_{max} \cdot \left[1 - e^{-\frac{(t-T_i)^{\beta}}{\alpha}}\right]$

Fitted model parameters per tested tablet (N = 4) with statistics – mean, standard deviation (SD), and relative standard deviation expressed in % (RSD%) (output from DDSolver):

Parameter	No.1	No.2	No.3	No.4	Mean	SD	RSD(%)
α	114.377	265.685	142.553	112.560	158.794	72.572	45.702
β	1.334	1.635	1.414	1.392	1.444	0.132	9.126
T_i	6.000	4.000	6.000	6.000	5.500	1.000	18.182
F_{max}	54.890	89.628	66.255	53.081	65.963	16.820	25.499

Number of dissolution data points (N), degrees of freedom (df), and selected goodness of fit criteria – Pearson correlation coefficient (R), coefficient of determination (R^2), adjusted coefficient of determination ($R^2_{adjusted}$), and residual sum of squares (RSS) (manual calculation in MS Excel):

Parameter	No.1	No.2	No.3	No.4
N	5	5	5	5
df	1	1	1	1
R	0.969726997	0.997415181	0.985615755	0.995028097
R^2	0.940370449	0.994837043	0.971438416	0.990080913
$R^2_{adjusted}$	0.761481795	0.979348173	0.885753665	0.960323652
RSS	79.86807148	28.75817497	61.70898694	19.03746271

Graphical abstract of model fit presented as mean ± 1 SD of the fraction % of released carvedilol:

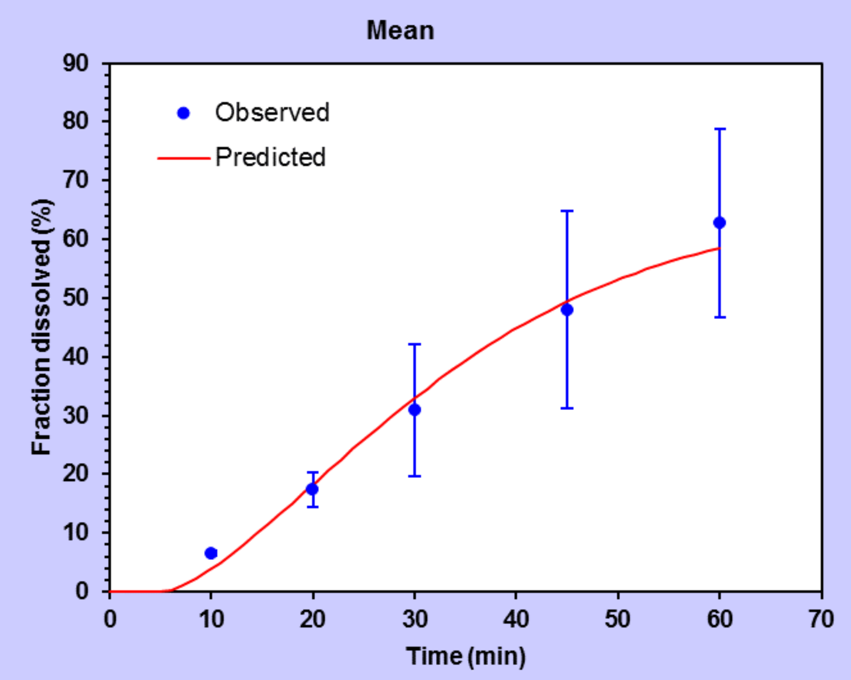

Supplementary materials – Model fitting summary – Parteck® M 200 (modelling up to app. 60 % of carvedilol released)

Graphical abstract of model fit presented as the fraction % of released carvedilol per tested tablet:

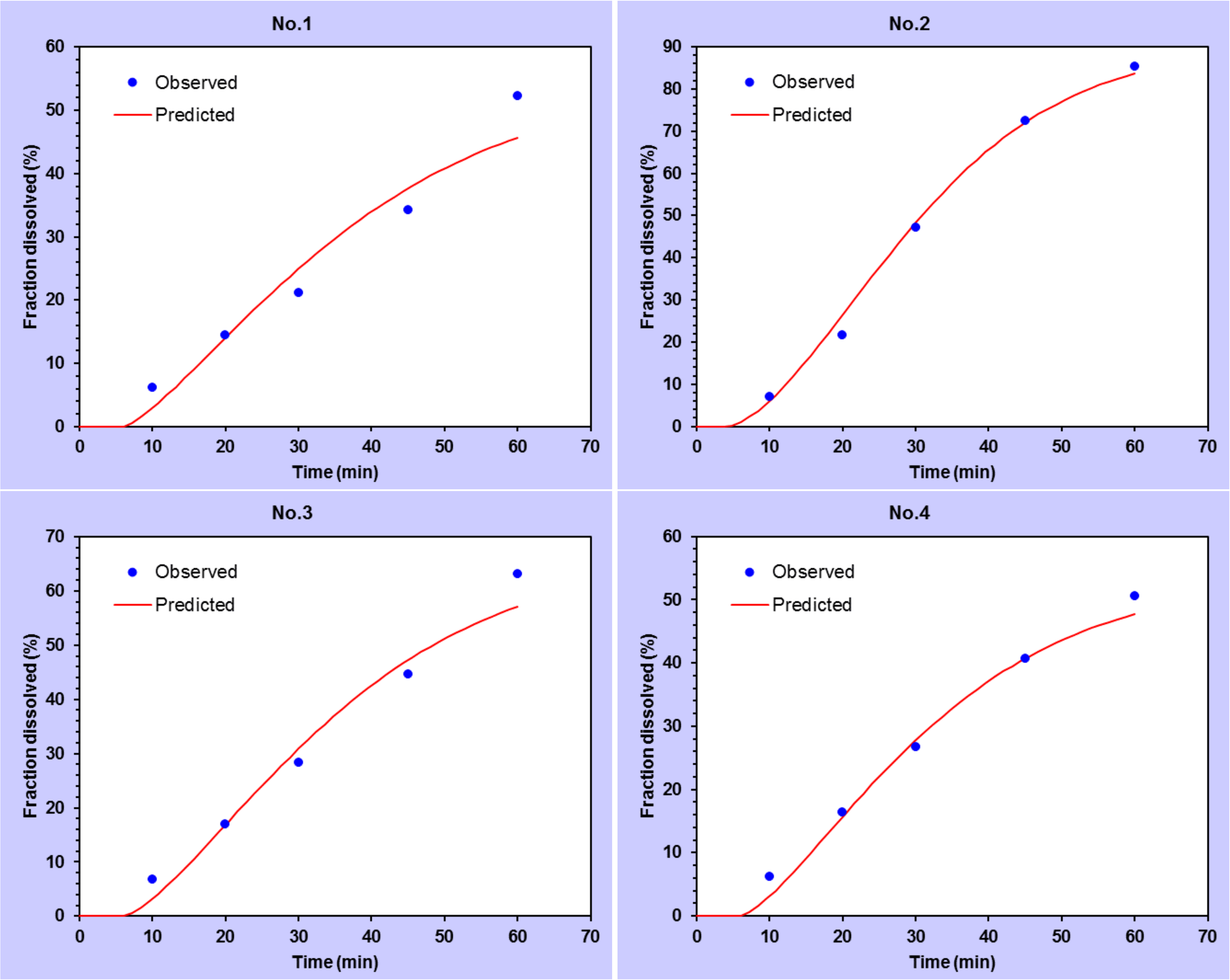

Supplementary materials – Model fitting summary – Parteck® M 200 (modelling up to app. 60 % of carvedilol released)

Model: **Logistic_1**

Model equation: $F = 100 \cdot \frac{e^{\alpha+\beta \cdot \log(t)}}{1+e^{\alpha+\beta \cdot \log(t)}}$

Fitted model parameters per tested tablet (N = 4) with statistics – mean, standard deviation (SD), and relative standard deviation expressed in % (RSD%) (output from DDSolver):

Parameter	No.1	No.2	No.3	No.4	Mean	SD	RSD(%)
α	-6.277	-9.082	-6.677	-6.239	-7.069	1.357	-19.193
β	3.469	6.130	3.965	3.534	4.274	1.256	29.391

Number of dissolution data points (N), degrees of freedom (df), and selected goodness of fit criteria – Pearson correlation coefficient (R), coefficient of determination (R^2), adjusted coefficient of determination ($R^2_{adjusted}$), and residual sum of squares (RSS) (manual calculation in MS Excel):

Parameter	No.1	No.2	No.3	No.4
N	5	5	5	5
df	3	3	3	3
R	0.986711555	0.998350449	0.994277684	0.999792355
R^2	0.973599694	0.996703619	0.988588112	0.999584752
$R^2_{adjusted}$	0.964799591	0.995604826	0.98478415	0.999446336
RSS	39.73303913	22.3170166	26.03829718	0.594187002

Graphical abstract of model fit presented as mean ± 1 SD of the fraction % of released carvedilol:

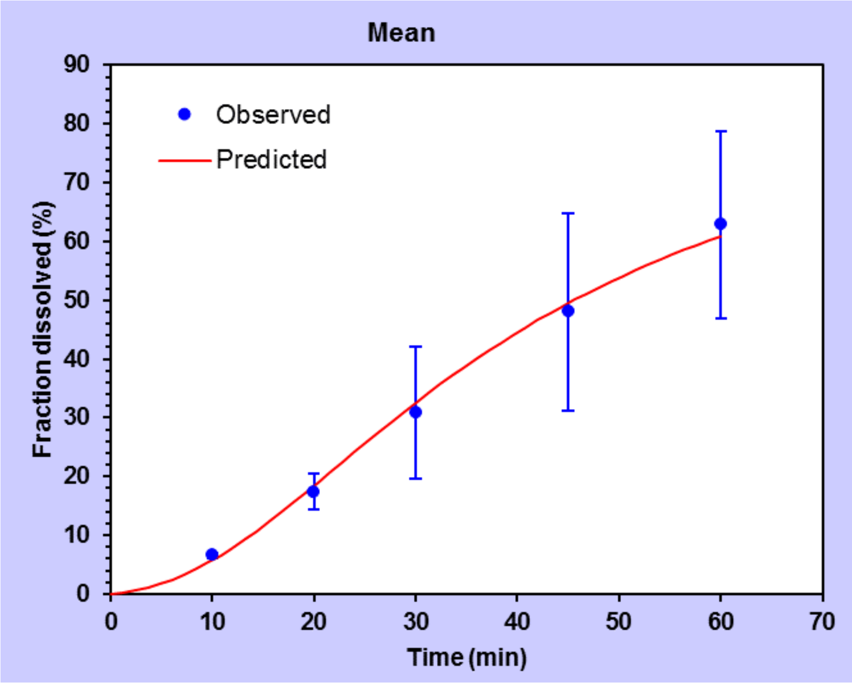

Supplementary materials – Model fitting summary – Parteck® M 200 (modelling up to app. 60 % of carvedilol released)

Graphical abstract of model fit presented as the fraction % of released carvedilol per tested tablet:

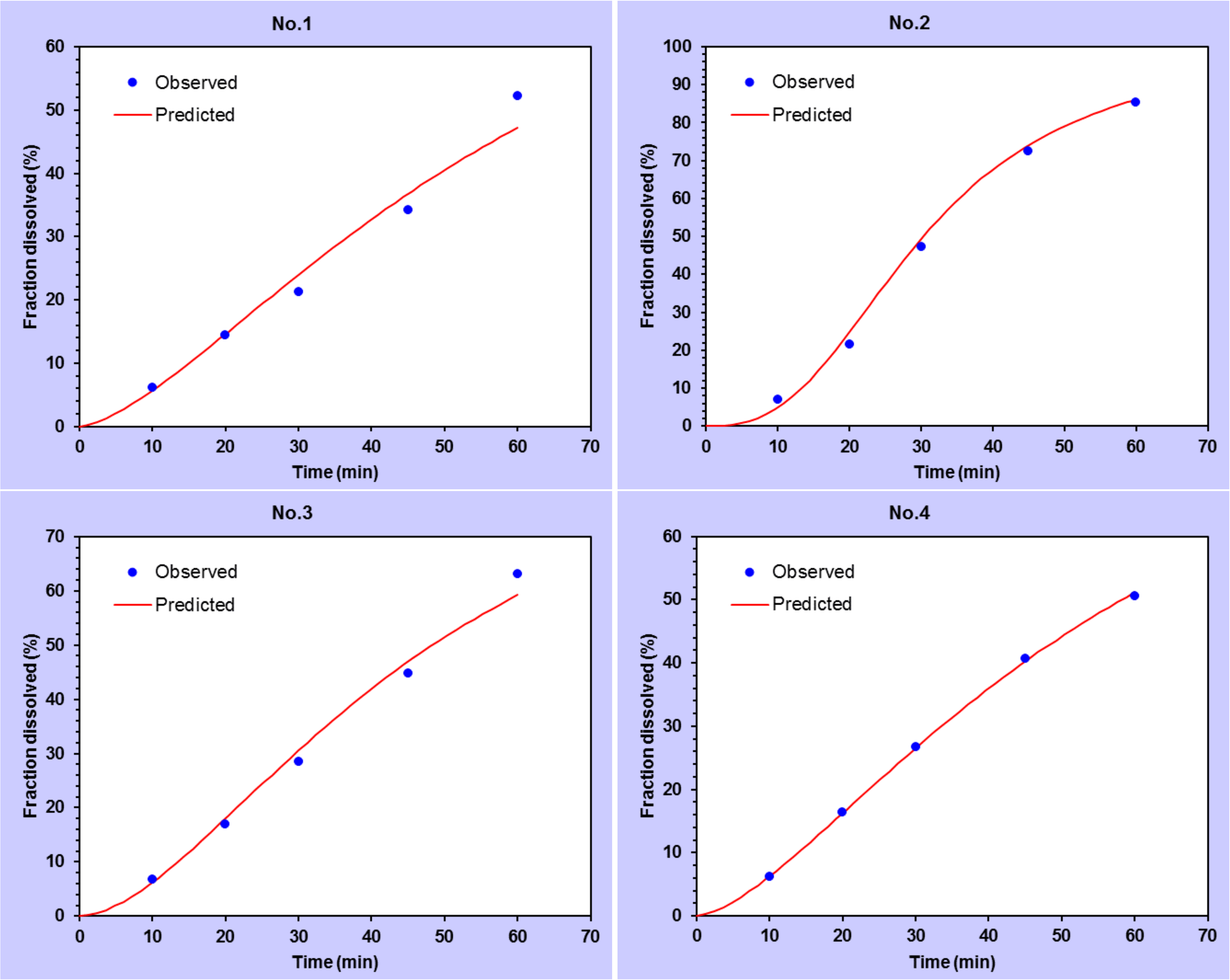

Supplementary materials – Model fitting summary – Parteck® M 200 (modelling up to app. 60 % of carvedilol released)

Model: **Logistic_2**

Model equation: $F = F_{max} \cdot \frac{e^{\alpha+\beta\cdot\log(t)}}{1+e^{\alpha+\beta\cdot\log(t)}}$

Fitted model parameters per tested tablet (N = 4) with statistics – mean, standard deviation (SD), and relative standard deviation expressed in % (RSD%) (output from DDSolver):

Parameter	No.1	No.2	No.3	No.4	Mean	SD	RSD(%)
α	-8.313	-9.713	-10.014	-9.776	-9.454	0.772	-8.165
β	5.757	6.865	6.426	6.445	6.373	0.458	7.190
F_{max}	54.890	89.628	71.872	57.581	68.493	15.940	23.273

Number of dissolution data points (N), degrees of freedom (df), and selected goodness of fit criteria – Pearson correlation coefficient (R), coefficient of determination (R^2), adjusted coefficient of determination ($R^2_{adjusted}$), and residual sum of squares (RSS) (manual calculation in MS Excel):

Parameter	No.1	No.2	No.3	No.4
N	5	5	5	5
df	2	2	2	2
R	0.946056687	0.990394705	0.99127534	0.997615919
R^2	0.895023254	0.980881673	0.9826268	0.995237523
$R^2_{adjusted}$	0.790046509	0.961763345	0.9652536	0.990475045
RSS	172.7942583	109.9991122	87.82448069	48.3760464

Graphical abstract of model fit presented as mean ± 1 SD of the fraction % of released carvedilol:

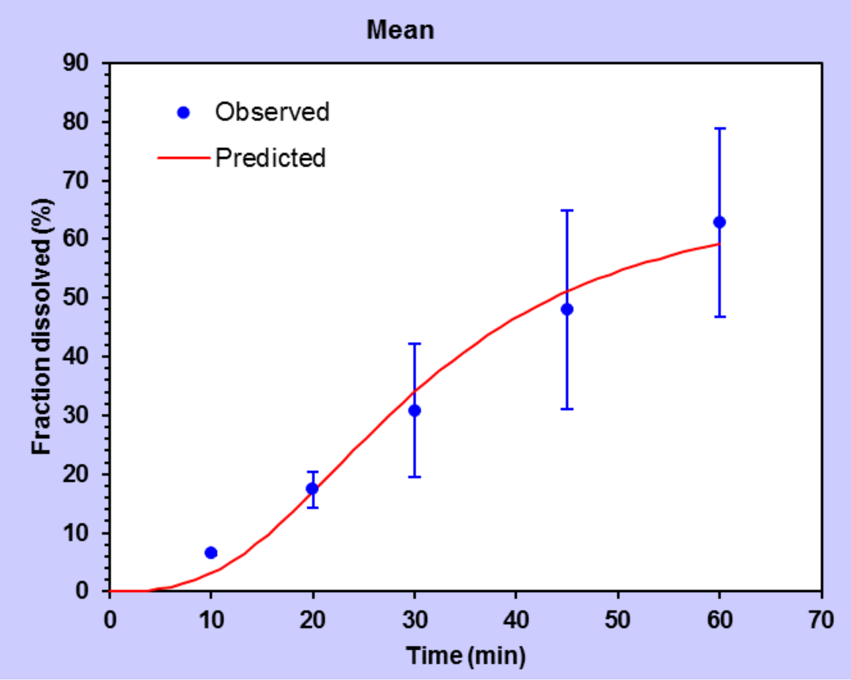

Supplementary materials – Model fitting summary – Parteck® M 200 (modelling up to app. 60 % of carvedilol released)

Graphical abstract of model fit presented as the fraction % of released carvedilol per tested tablet:

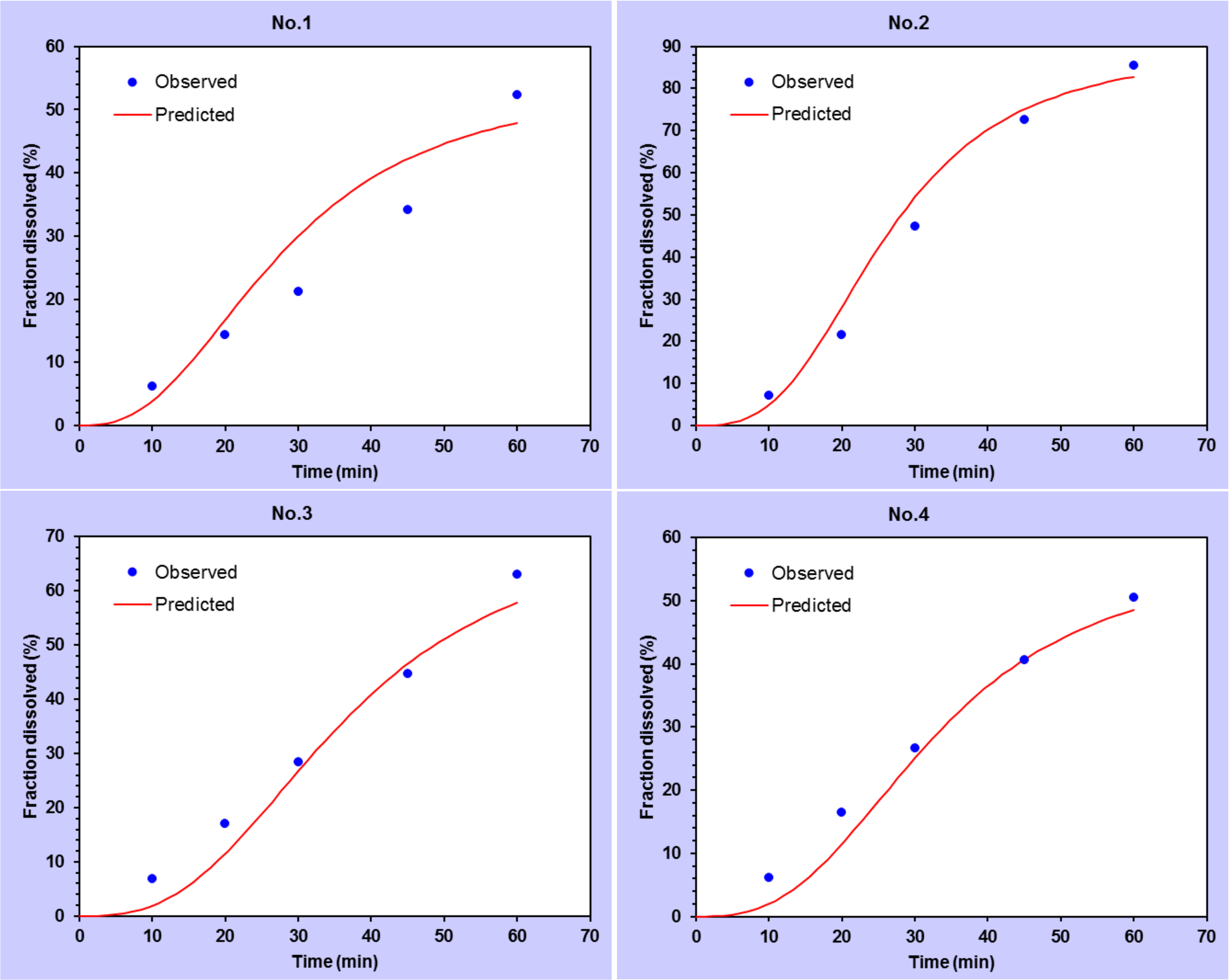

Supplementary materials – Model fitting summary – Parteck® M 200 (modelling up to app. 60 % of carvedilol released)

Model: **Logistic_3**

Model equation: $F = F_{max} \cdot \frac{1}{1+e^{-k\cdot(t-\gamma)}}$

Fitted model parameters per tested tablet (N = 4) with statistics – mean, standard deviation (SD), and relative standard deviation expressed in % (RSD%) (output from DDSolver):

Parameter	No.1	No.2	No.3	No.4	Mean	SD	RSD(%)
k	0.094	0.107	0.068	0.096	0.092	0.017	18.160
γ	33.130	31.273	37.615	30.164	33.045	3.283	9.935
F_{max}	54.890	89.628	73.975	53.081	67.893	17.300	25.482

Number of dissolution data points (N), degrees of freedom (df), and selected goodness of fit criteria – Pearson correlation coefficient (R), coefficient of determination (R^2), adjusted coefficient of determination ($R^2_{adjusted}$), and residual sum of squares (RSS) (manual calculation in MS Excel):

Parameter	No.1	No.2	No.3	No.4
N	5	5	5	5
df	2	2	2	2
R	0.980683704	0.996724529	0.997645485	0.9971144
R^2	0.961740528	0.993459786	0.995296514	0.994237126
$R^2_{adjusted}$	0.923481055	0.986919572	0.990593028	0.988474252
RSS	63.5325911	32.48236027	17.0655556	8.624441191

Graphical abstract of model fit presented as mean ± 1 SD of the fraction % of released carvedilol:

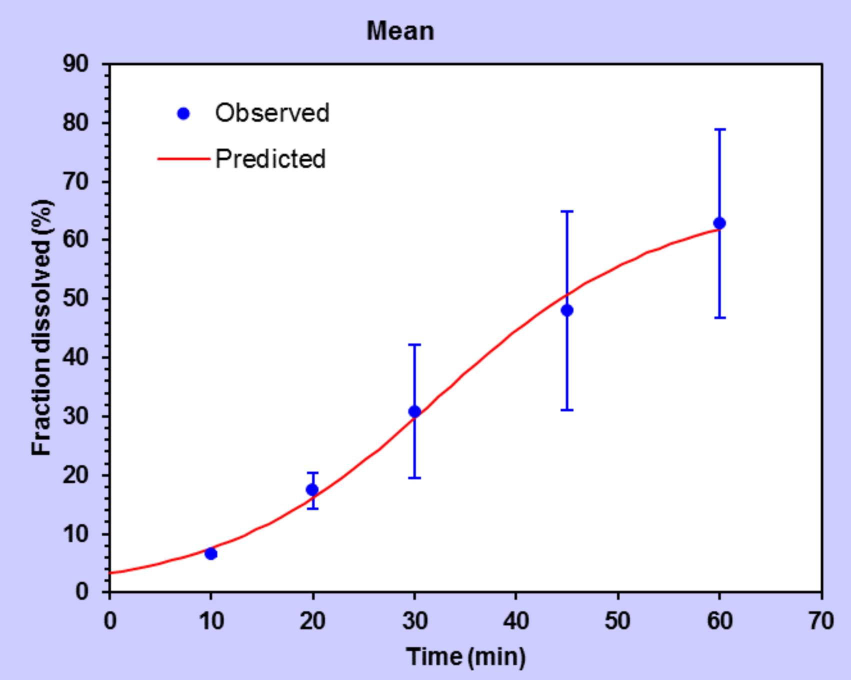

Supplementary materials – Model fitting summary – Parteck® M 200 (modelling up to app. 60 % of carvedilol released)

Graphical abstract of model fit presented as the fraction % of released carvedilol per tested tablet:

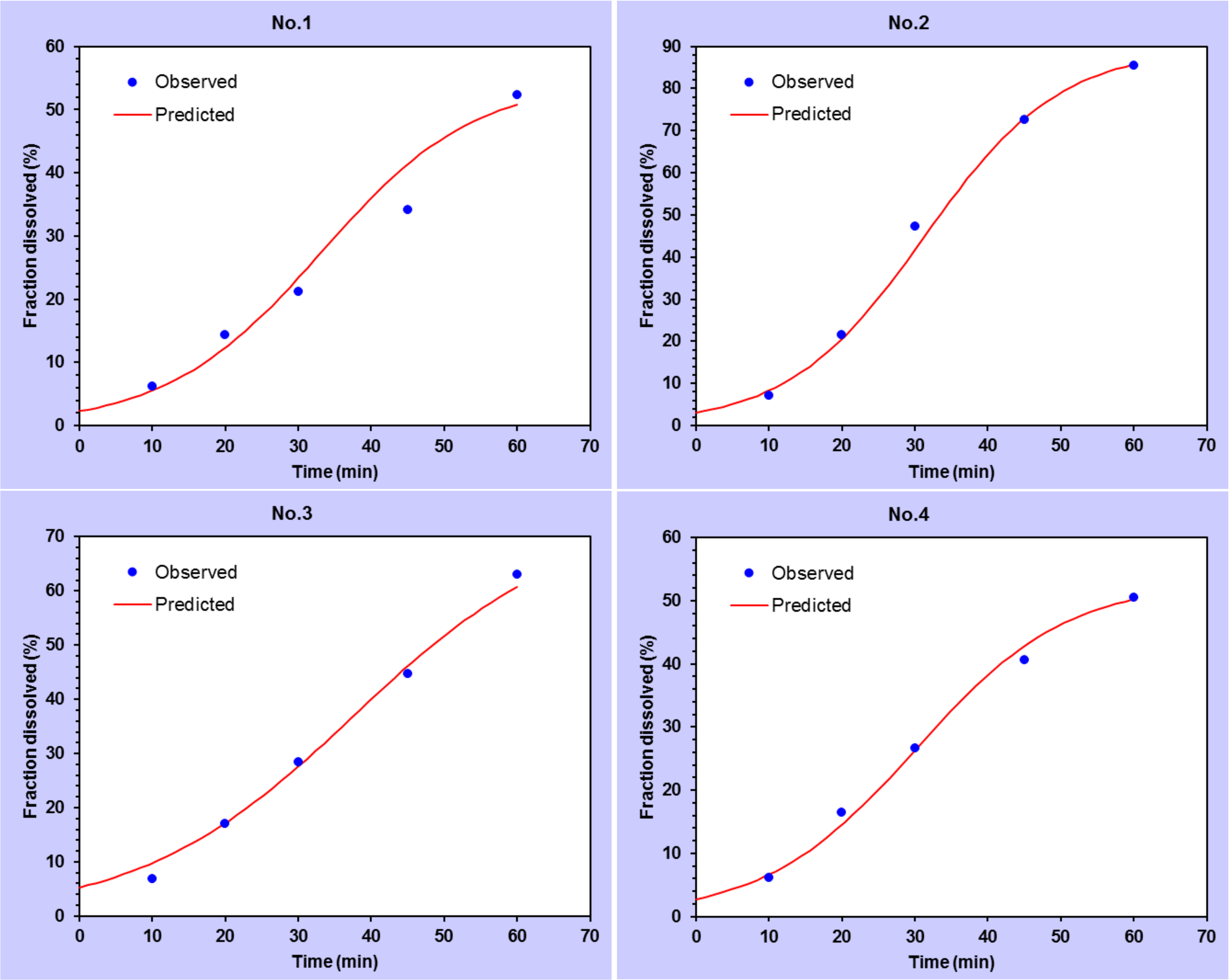

Supplementary materials – Model fitting summary – Parteck® M 200 (modelling up to app. 60 % of carvedilol released)

Model: **Gompertz_1**

Model equation: $F = 100 \cdot e^{-\alpha \cdot e^{-\beta \cdot \log(t)}}$

Fitted model parameters per tested tablet (N = 4) with statistics – mean, standard deviation (SD), and relative standard deviation expressed in % (RSD%) (output from DDSolver):

Parameter	No.1	No.2	No.3	No.4	Mean	SD	RSD(%)
α	18.127	131.291	27.255	17.973	48.661	55.257	113.554
β	1.765	3.640	2.181	1.809	2.349	0.881	37.508

Number of dissolution data points (N), degrees of freedom (df), and selected goodness of fit criteria – Pearson correlation coefficient (R), coefficient of determination (R^2), adjusted coefficient of determination ($R^2_{adjusted}$), and residual sum of squares (RSS) (manual calculation in MS Excel):

Parameter	No.1	No.2	No.3	No.4
N	5	5	5	5
df	3	3	3	3
R	0.967918913	0.98138225	0.978361396	0.99567233
R^2	0.936867023	0.963111121	0.95719102	0.991363388
$R^2_{adjusted}$	0.915822697	0.950814829	0.942921361	0.988484517
RSS	87.85285536	182.3351704	91.2871854	12.27404336

Graphical abstract of model fit presented as mean ± 1 SD of the fraction % of released carvedilol:

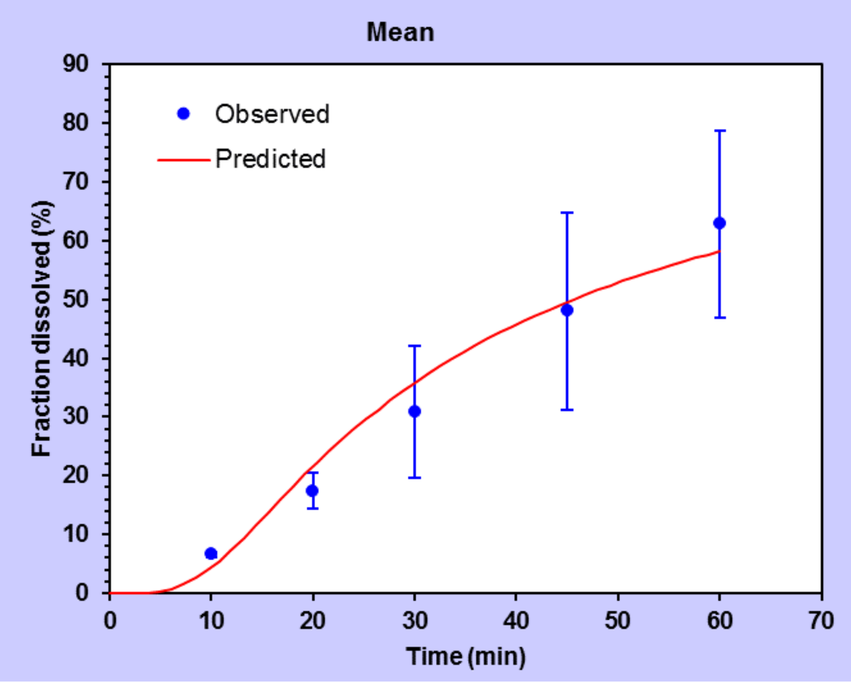

Supplementary materials – Model fitting summary – Parteck® M 200 (modelling up to app. 60 % of carvedilol released)

Graphical abstract of model fit presented as the fraction % of released carvedilol per tested tablet:

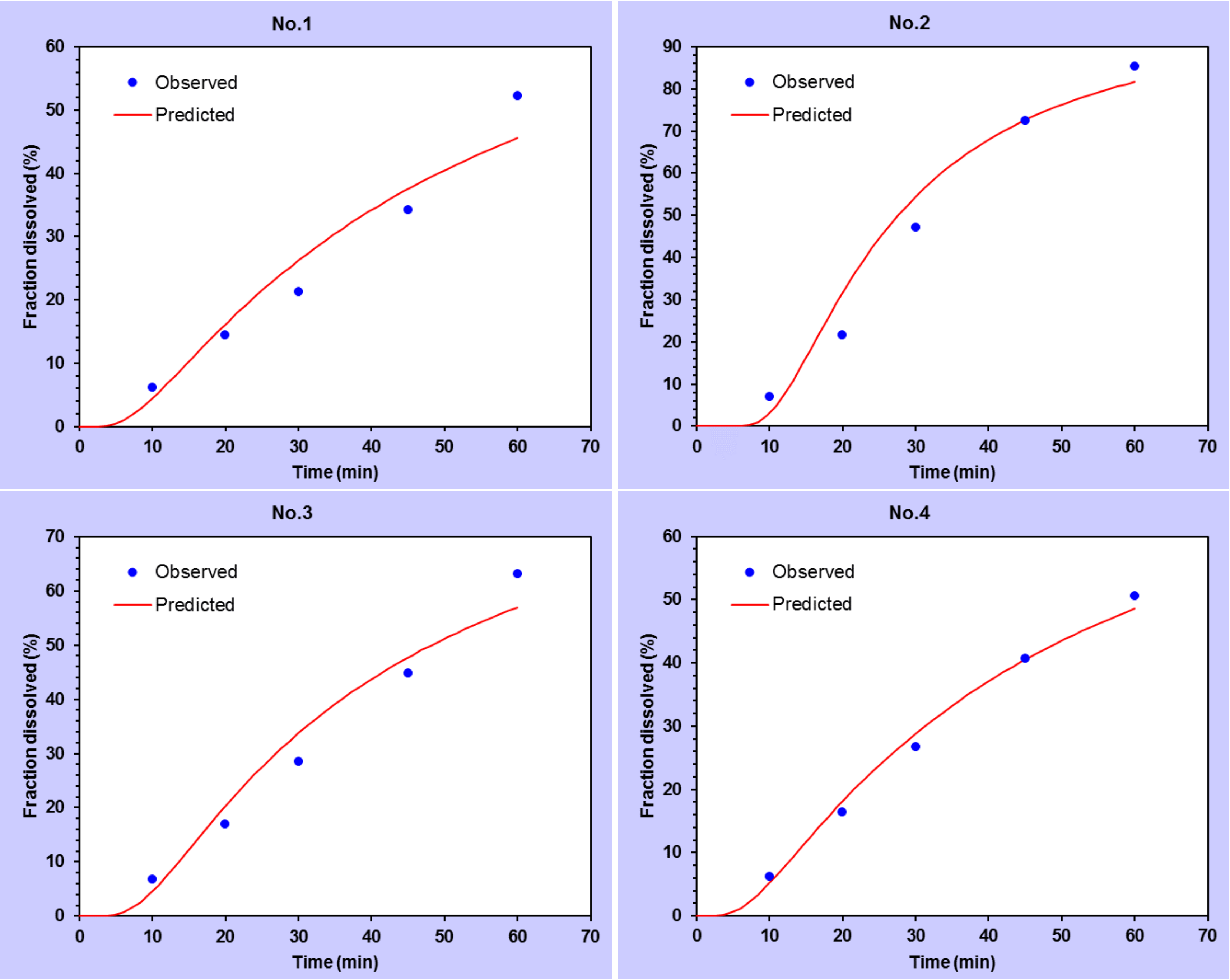

Supplementary materials – Model fitting summary – Parteck® M 200 (modelling up to app. 60 % of carvedilol released)

Model: **Gompertz_2**

Model equation: $F = F_{max} \cdot e^{-\alpha \cdot e^{-\beta \cdot \log(t)}}$

Fitted model parameters per tested tablet (N = 4) with statistics – mean, standard deviation (SD), and relative standard deviation expressed in % (RSD%) (output from DDSolver):

Parameter	No.1	No.2	No.3	No.4	Mean	SD	RSD(%)
α	355.213	644.271	355.370	436.731	447.896	136.429	30.460
β	4.173	4.570	4.027	4.461	4.308	0.251	5.827
F_{max}	54.890	102.354	73.936	53.081	71.065	22.893	32.214

Number of dissolution data points (N), degrees of freedom (df), and selected goodness of fit criteria – Pearson correlation coefficient (R), coefficient of determination (R^2), adjusted coefficient of determination ($R^2_{adjusted}$), and residual sum of squares (RSS) (manual calculation in MS Excel):

Parameter	No.1	No.2	No.3	No.4
N	5	5	5	5
df	2	2	2	2
R	0.949343705	0.998370285	0.982569566	0.983094663
R^2	0.90125347	0.996743226	0.965442951	0.966475117
$R^2_{adjusted}$	0.80250694	0.993486451	0.930885902	0.932950233
RSS	146.6557665	56.04207997	131.9050993	71.79973271

Graphical abstract of model fit presented as mean ± 1 SD of the fraction % of released carvedilol:

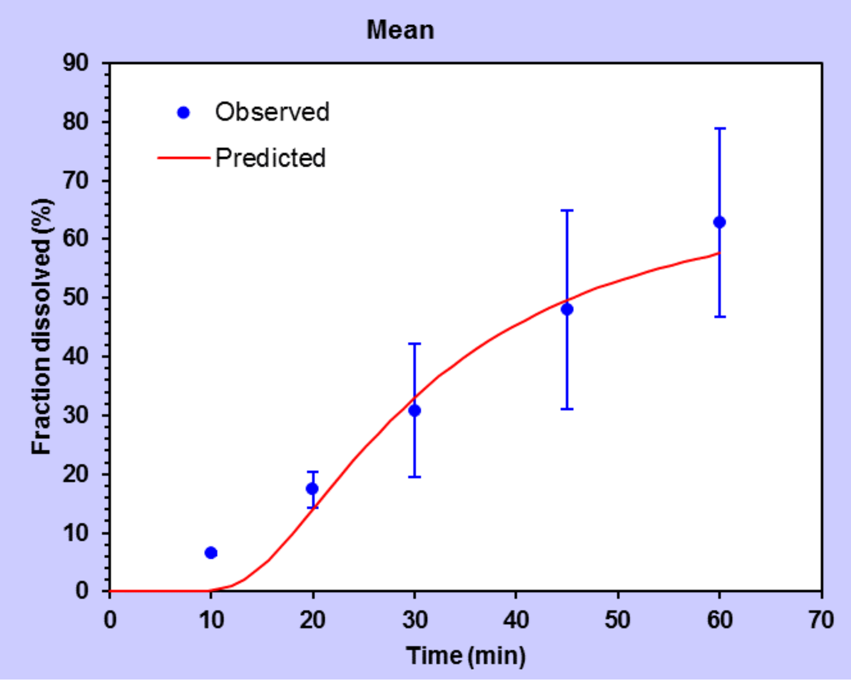

Supplementary materials – Model fitting summary – Parteck® M 200 (modelling up to app. 60 % of carvedilol released)

Graphical abstract of model fit presented as the fraction % of released carvedilol per tested tablet:

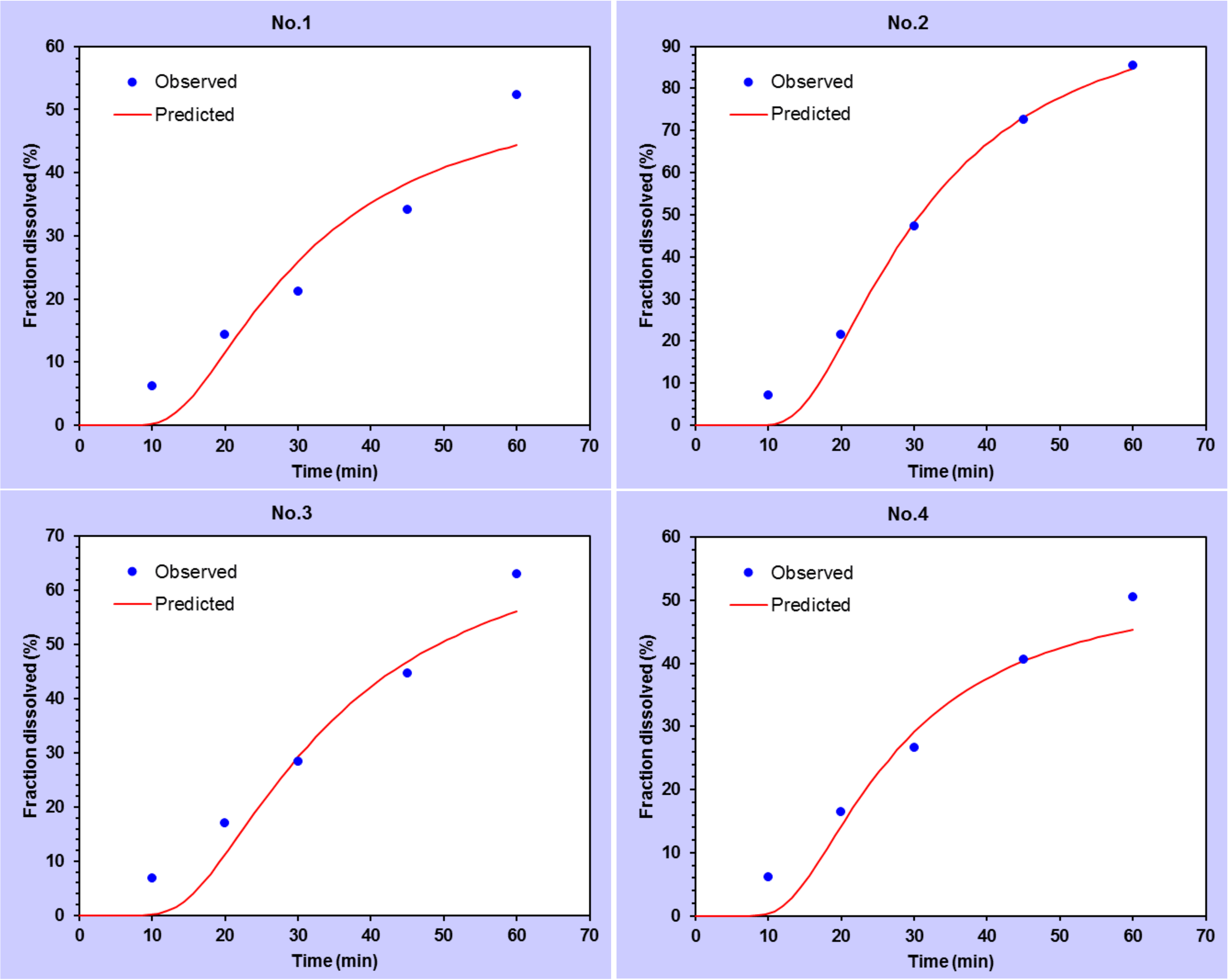

Supplementary materials – Model fitting summary – Parteck® M 200 (modelling up to app. 60 % of carvedilol released)

Model: **Gompertz_3**

Model equation: $F = F_{max} \cdot e^{-e^{-k \cdot (t - \gamma)}}$

Fitted model parameters per tested tablet (N = 4) with statistics – mean, standard deviation (SD), and relative standard deviation expressed in % (RSD%) (output from DDSolver):

Parameter	No.1	No.2	No.3	No.4	Mean	SD	RSD(%)
k	0.071	0.067	0.073	0.065	0.069	0.003	4.960
γ	25.257	24.147	24.800	24.539	24.686	0.466	1.887
F_{max}	54.890	91.407	66.255	52.596	66.287	17.780	26.822

Number of dissolution data points (N), degrees of freedom (df), and selected goodness of fit criteria – Pearson correlation coefficient (R), coefficient of determination (R^2), adjusted coefficient of determination ($R^2_{adjusted}$), and residual sum of squares (RSS) (manual calculation in MS Excel):

Parameter	No.1	No.2	No.3	No.4
N	5	5	5	5
df	2	2	2	2
R	0.965212282	0.999211519	0.981546619	0.997706618
R^2	0.93163475	0.99842366	0.963433766	0.995418495
$R^2_{adjusted}$	0.8632695	0.99684732	0.926867532	0.990836989
RSS	125.0759712	12.35821204	103.6006534	21.00344198

Graphical abstract of model fit presented as mean ± 1 SD of the fraction % of released carvedilol:

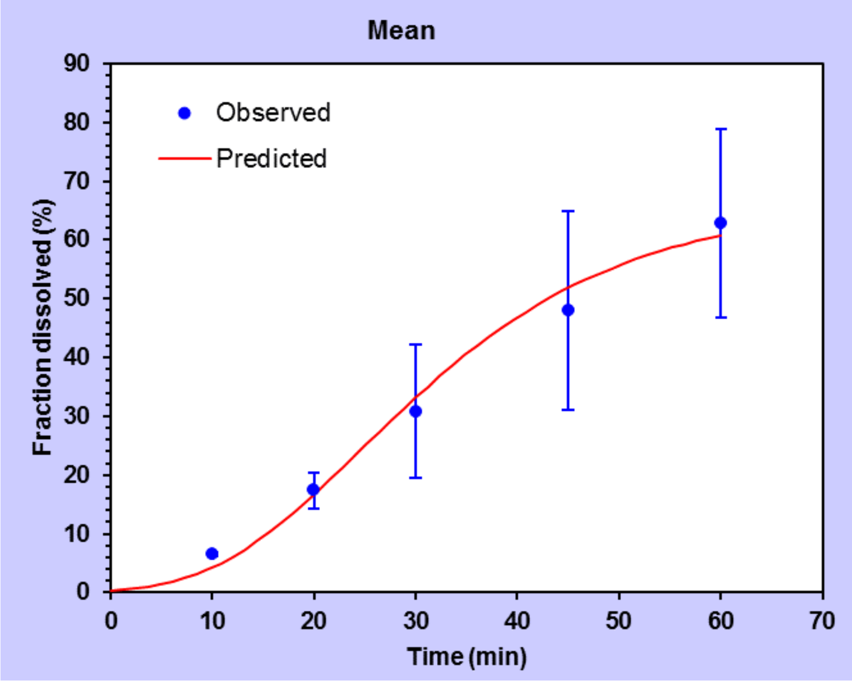

Supplementary materials – Model fitting summary – Parteck® M 200 (modelling up to app. 60 % of carvedilol released)

Graphical abstract of model fit presented as the fraction % of released carvedilol per tested tablet:

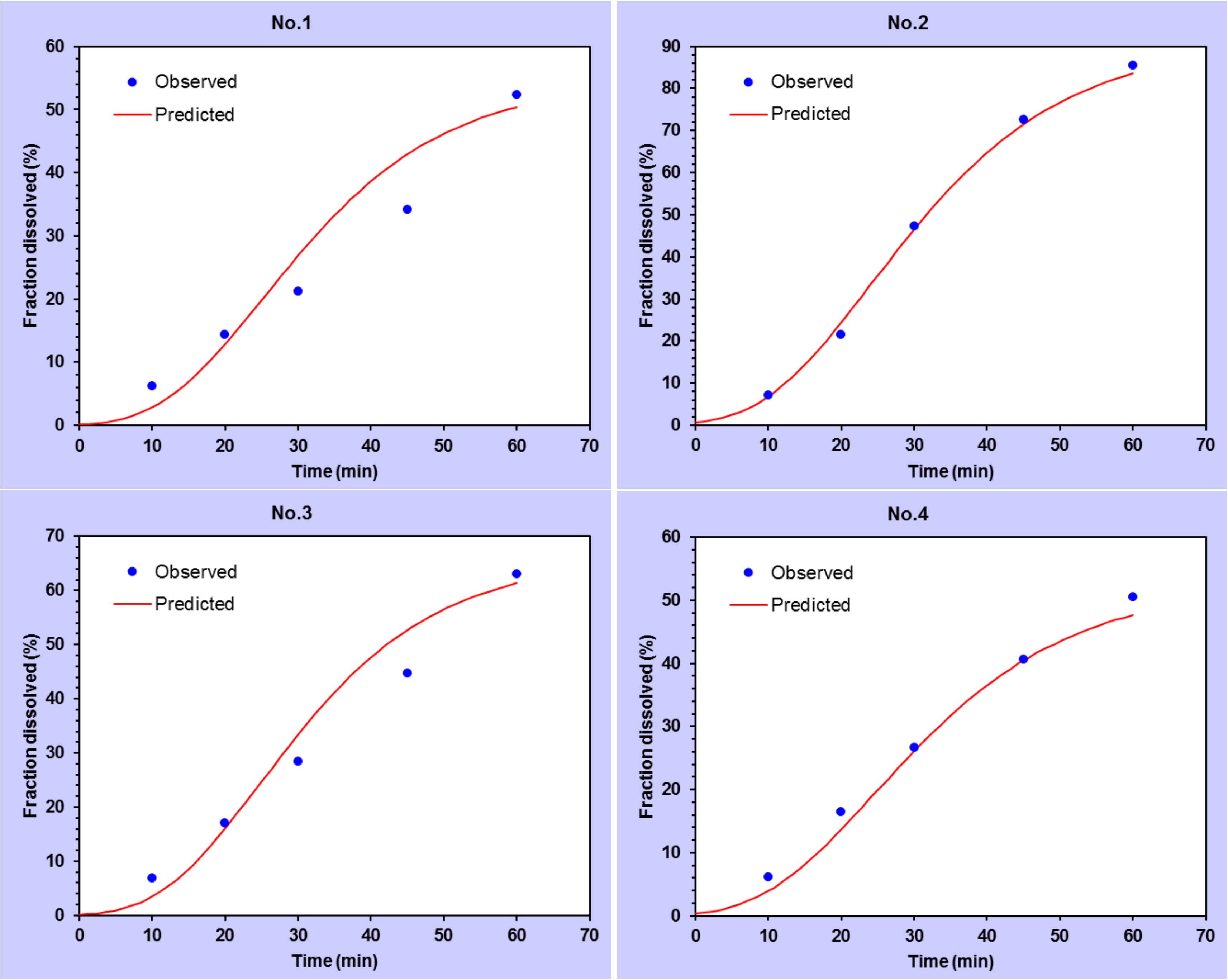

Supplementary materials – Model fitting summary – Parteck® M 200 (modelling up to app. 60 % of carvedilol released)

Model: **Gompertz_4**

Model equation: $F = F_{max} \cdot e^{-\beta \cdot e^{-k \cdot t}}$

Fitted model parameters per tested tablet (N = 4) with statistics – mean, standard deviation (SD), and relative standard deviation expressed in % (RSD%) (output from DDSolver):

Parameter	No.1	No.2	No.3	No.4	Mean	SD	RSD(%)
k	0.071	0.079	0.073	0.073	0.074	0.003	4.712
β	6.000	6.451	6.096	5.279	5.956	0.492	8.252
F_{max}	54.890	89.628	66.255	53.081	65.963	16.820	25.499

Number of dissolution data points (N), degrees of freedom (df), and selected goodness of fit criteria – Pearson correlation coefficient (R), coefficient of determination (R^2), adjusted coefficient of determination ($R^2_{adjusted}$), and residual sum of squares (RSS) (manual calculation in MS Excel):

Parameter	No.1	No.2	No.3	No.4
N	5	5	5	5
df	2	2	2	2
R	0.965212282	0.998354493	0.981546619	0.993733724
R^2	0.93163475	0.996711693	0.963433766	0.987506714
$R^2_{adjusted}$	0.8632695	0.993423386	0.926867532	0.975013428
RSS	125.0759712	18.00482656	103.6006534	22.81153068

Graphical abstract of model fit presented as mean ± 1 SD of the fraction % of released carvedilol:

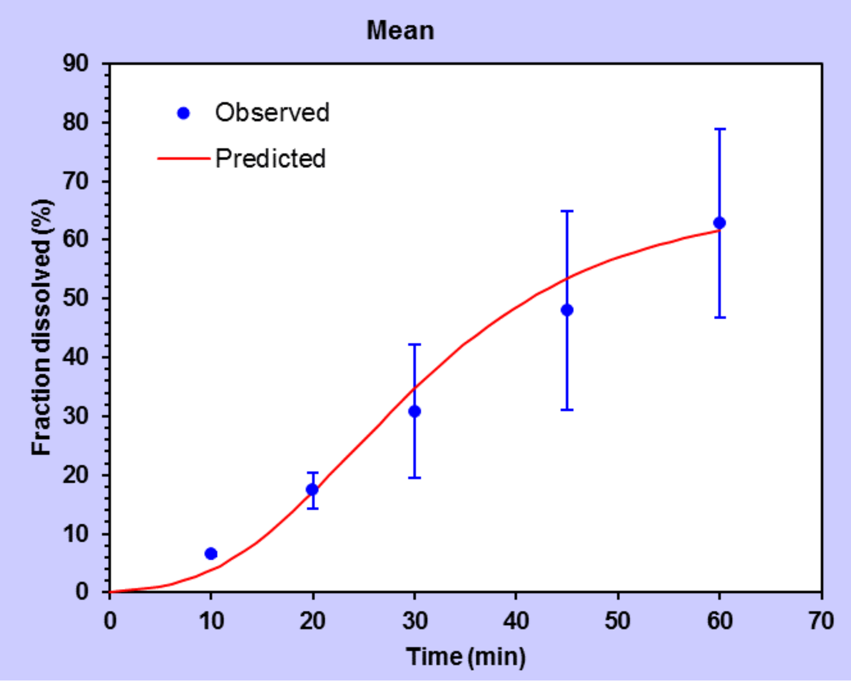

Supplementary materials – Model fitting summary – Parteck® M 200 (modelling up to app. 60 % of carvedilol released)

Graphical abstract of model fit presented as the fraction % of released carvedilol per tested tablet:

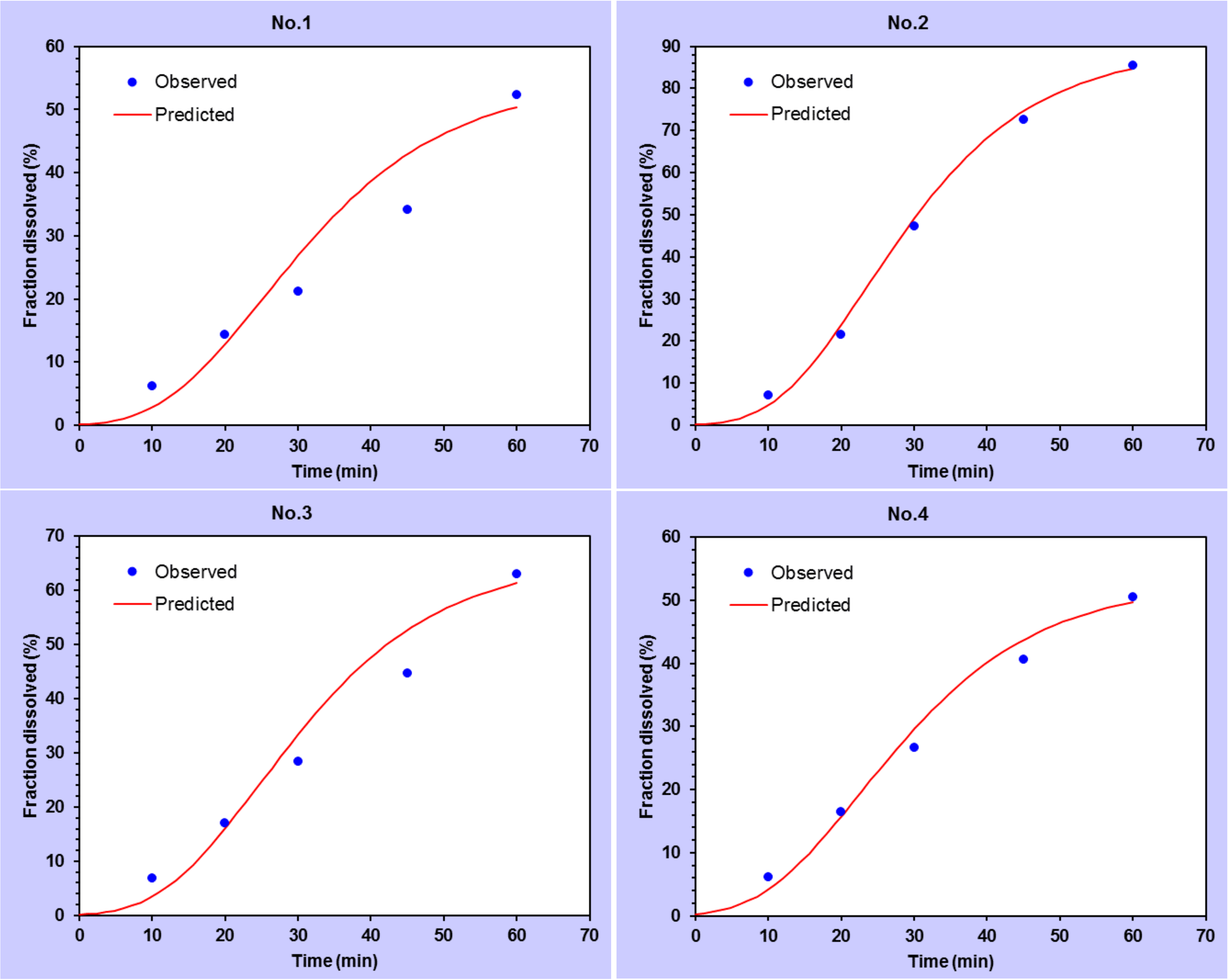

Supplementary materials – Model fitting summary – Parteck® M 200 (modelling up to app. 60 % of carvedilol released)

Model: **Probit_1**

Model equation: $F = 100 \cdot \phi[\alpha + \beta \cdot \log(t)]$

Fitted model parameters per tested tablet (N = 4) with statistics – mean, standard deviation (SD), and relative standard deviation expressed in % (RSD%) (output from DDSolver):

Parameter	No.1	No.2	No.3	No.4	Mean	SD	RSD(%)
α	-3.581	-5.380	-4.181	-3.565	-4.177	0.852	-20.393
β	1.963	3.559	2.510	2.006	2.510	0.742	29.576

Number of dissolution data points (N), degrees of freedom (df), and selected goodness of fit criteria – Pearson correlation coefficient (R), coefficient of determination (R^2), adjusted coefficient of determination ($R^2_{adjusted}$), and residual sum of squares (RSS) (manual calculation in MS Excel):

Parameter	No.1	No.2	No.3	No.4
N	5	5	5	5
df	3	3	3	3
R	0.980932251	0.998510711	0.991902723	0.999582907
R^2	0.962228082	0.99702364	0.983871012	0.999165987
$R^2_{adjusted}$	0.949637443	0.99603152	0.978494683	0.998887983
RSS	56.02655309	34.65272645	37.55871766	1.314153739

Graphical abstract of model fit presented as mean ± 1 SD of the fraction % of released carvedilol:

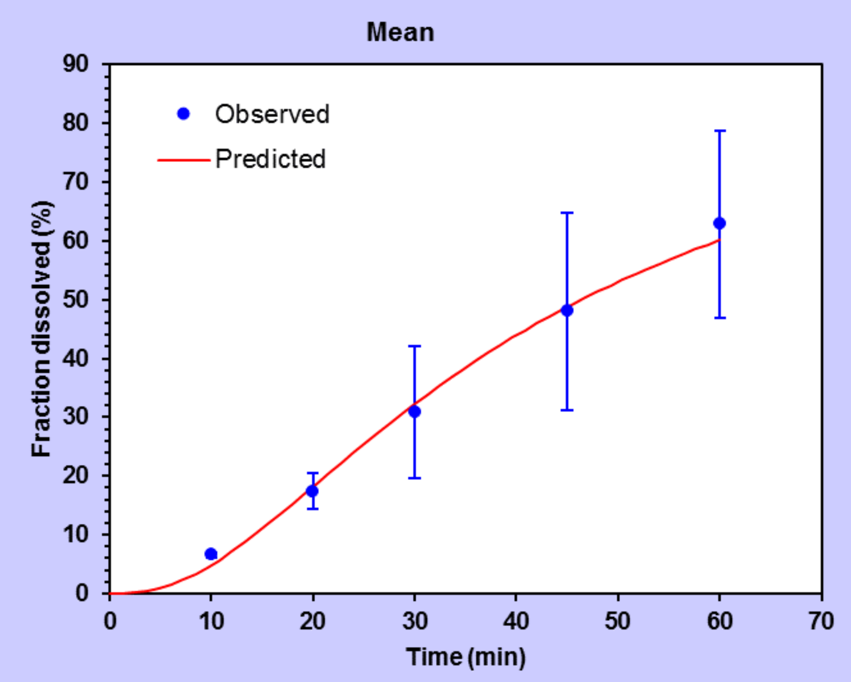

Supplementary materials – Model fitting summary – Parteck® M 200 (modelling up to app. 60 % of carvedilol released)

Graphical abstract of model fit presented as the fraction % of released carvedilol per tested tablet:

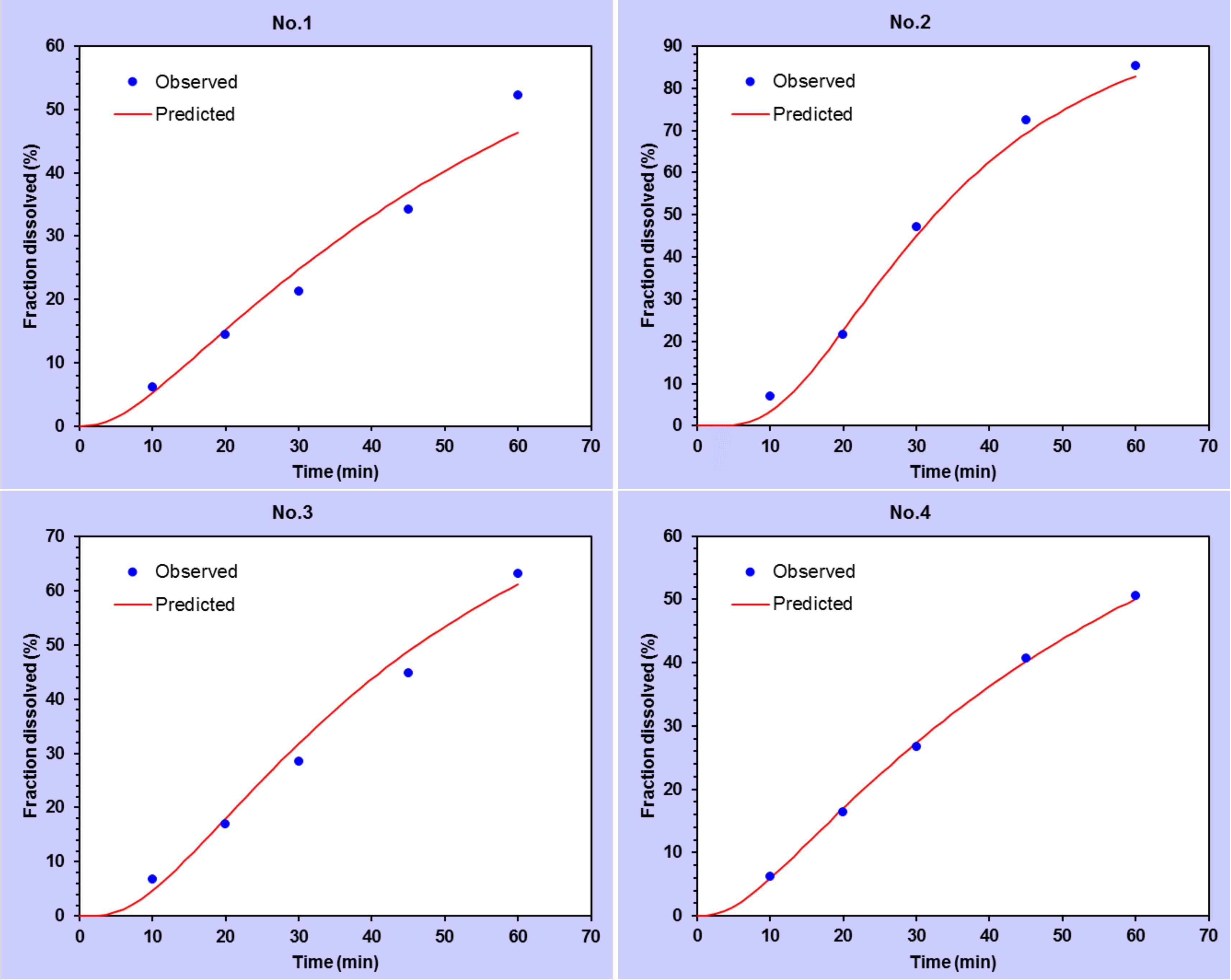

Supplementary materials – Model fitting summary – Parteck® M 200 (modelling up to app. 60 % of carvedilol released)

Model: **Probit_2**

Model equation: $F = F_{max} \cdot \phi[\alpha + \beta \cdot \log(t)]$

Fitted model parameters per tested tablet (N = 4) with statistics – mean, standard deviation (SD), and relative standard deviation expressed in % (RSD%) (output from DDSolver):

Parameter	No.1	No.2	No.3	No.4	Mean	SD	RSD(%)
α	-4.817	-6.135	-5.795	-4.913	-5.415	0.651	-12.023
β	3.319	4.238	3.704	3.506	3.692	0.397	10.743
F_{max}	54.890	85.615	71.872	53.081	66.364	15.374	23.165

Number of dissolution data points (N), degrees of freedom (df), and selected goodness of fit criteria – Pearson correlation coefficient (R), coefficient of determination (R^2), adjusted coefficient of determination ($R^2_{adjusted}$), and residual sum of squares (RSS) (manual calculation in MS Excel):

Parameter	No.1	No.2	No.3	No.4
N	5	5	5	5
df	2	2	2	2
R	0.948872882	0.995853616	0.991364562	0.982415552
R^2	0.900359746	0.991724424	0.982803694	0.965140317
$R^2_{adjusted}$	0.800719492	0.983448848	0.965607388	0.930280634
RSS	151.7946552	76.9249871	103.6770325	52.93984075

Graphical abstract of model fit presented as mean ± 1 SD of the fraction % of released carvedilol:

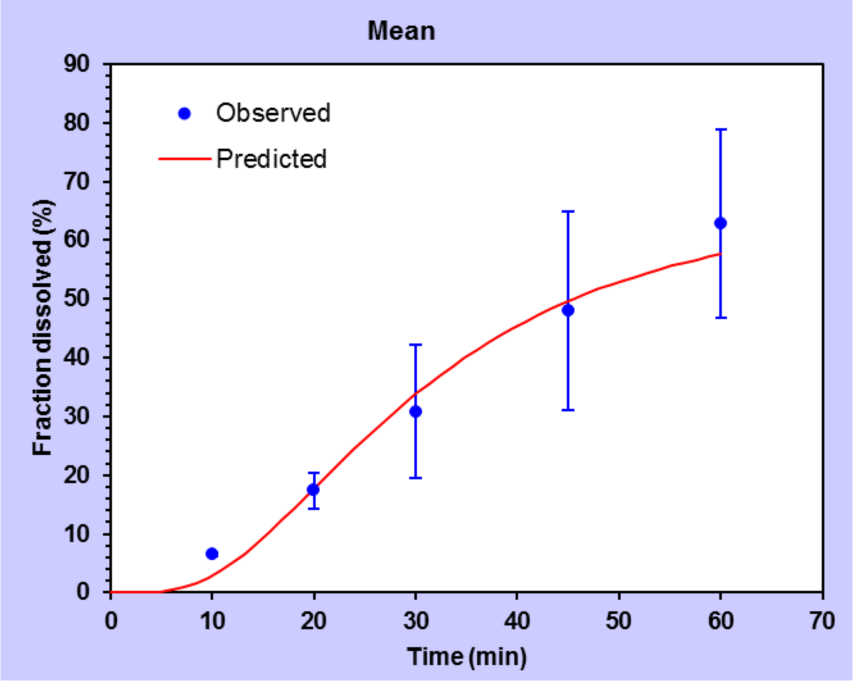

Supplementary materials – Model fitting summary – Parteck® M 200 (modelling up to app. 60 % of carvedilol released)

Graphical abstract of model fit presented as the fraction % of released carvedilol per tested tablet:

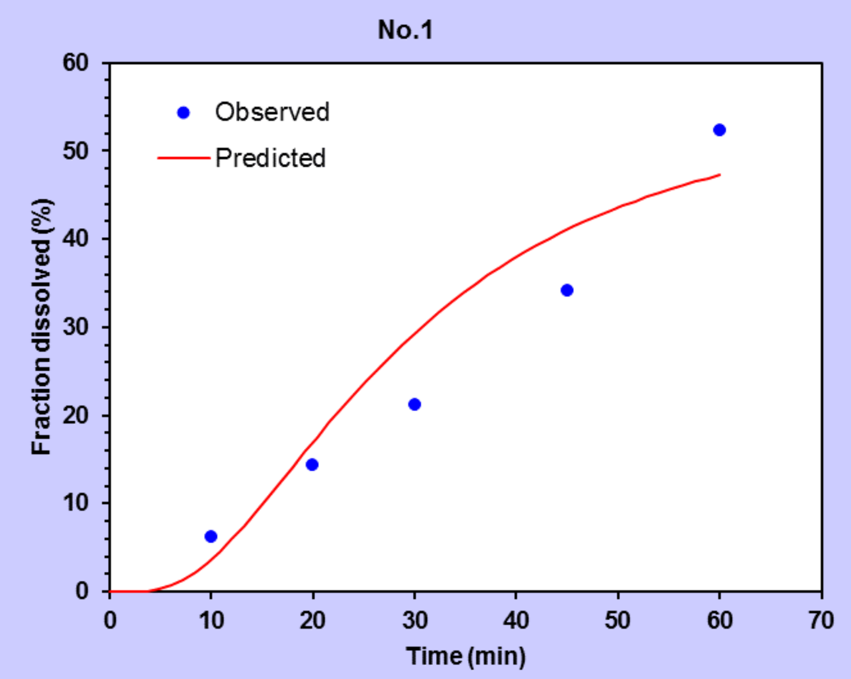

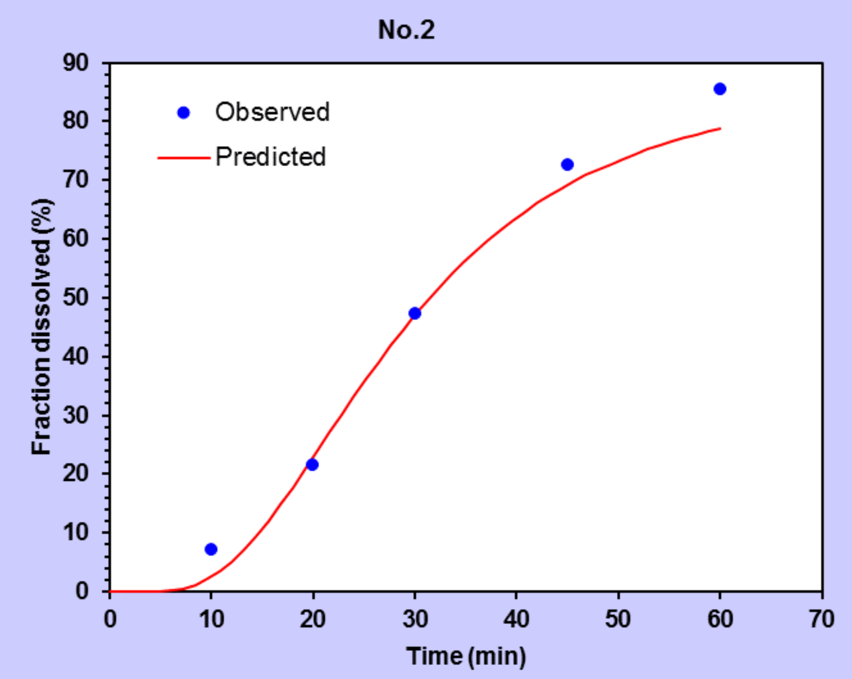

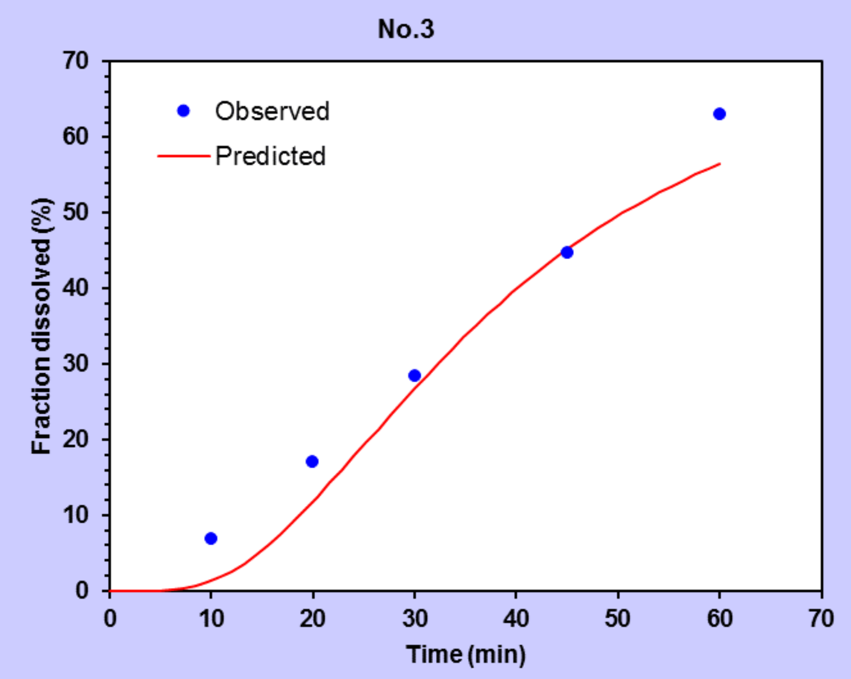

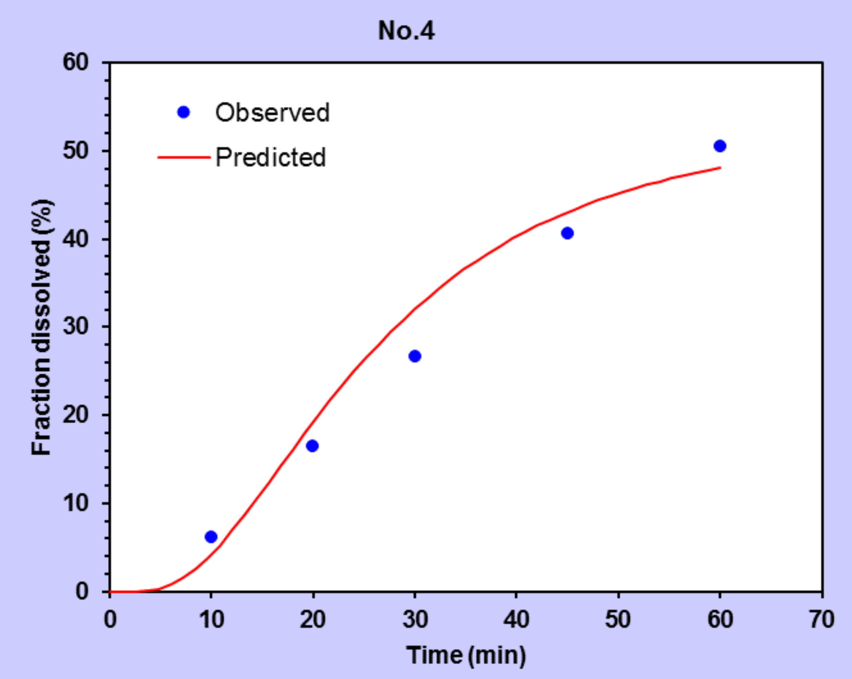

Supplement: Supplementary file 1 [file pharmaceutics-16-00498-s001.zip › Supplementary materials_Model fitting summary_Parteck® M 200.pdf]
