# Supplementary material for: Comparative Fitting of Mathematical Models to Carvedilol Release Profiles Obtained from Hypromellose Matrix Tablets
Source: Pharmaceutics. 2024 Apr 4;16(4):498. doi: 10.3390/pharmaceutics16040498 (PMC11053526; doi:10.3390/pharmaceutics16040498)

Supplementary materials – Model fitting summary – Pearlitol® 160C (modelling the entire relevant dissolution data)

Model: **Zero–order**

Model equation: $F = k_0 \cdot t$

Fitted model parameters per tested tablet (N = 4) with statistics – mean, standard deviation (SD), and relative standard deviation expressed in % (RSD%) (output from DDSolver):

Parameter	No.1	No.2	No.3	No.4	Mean	SD	RSD(%)
k_0	0.316	0.336	0.366	0.297	0.329	0.030	8.984

Number of dissolution data points (N), degrees of freedom (df), and selected goodness of fit criteria – Pearson correlation coefficient (R), coefficient of determination (R^2), adjusted coefficient of determination ($R^2_{adjusted}$), and residual sum of squares (RSS) (manual calculation in MS Excel):

Parameter	No.1	No.2	No.3	No.4
N	16	16	16	16
df	15	15	15	15
R	0.960482951	0.932306095	0.840690874	0.973689911
R^2	0.9225275	0.869194654	0.706761145	0.948072042
$R^2_{adjusted}$	0.9225275	0.869194654	0.706761145	0.948072042
RSS	3504.916332	5862.728513	12719.4646	2664.95986

Graphical abstract of model fit presented as mean ± 1 SD of the fraction % of released carvedilol:

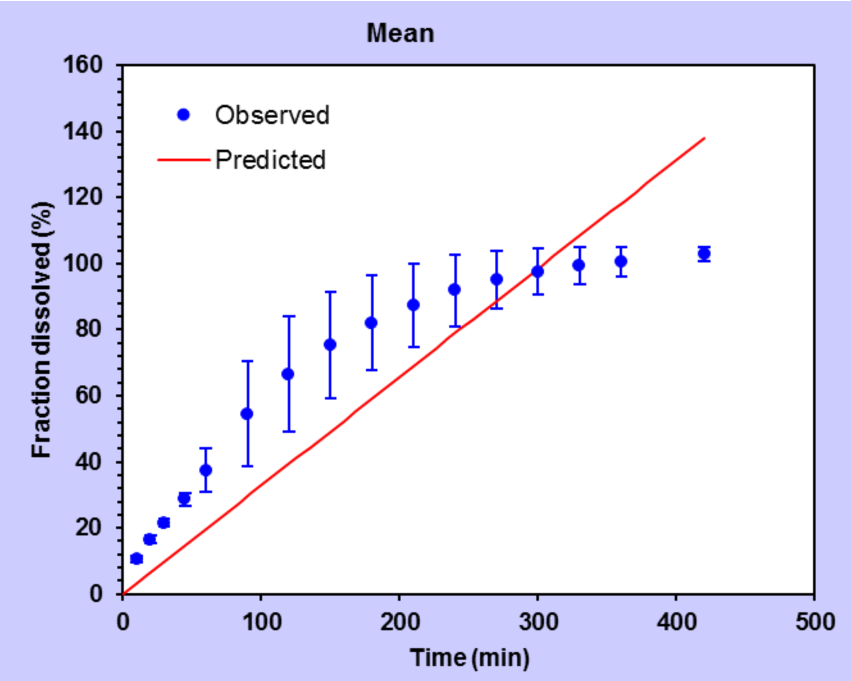

Supplementary materials – Model fitting summary – Pearlitol® 160C (modelling the entire relevant dissolution data)

Graphical abstract of model fit presented as the fraction % of released carvedilol per tested tablet:

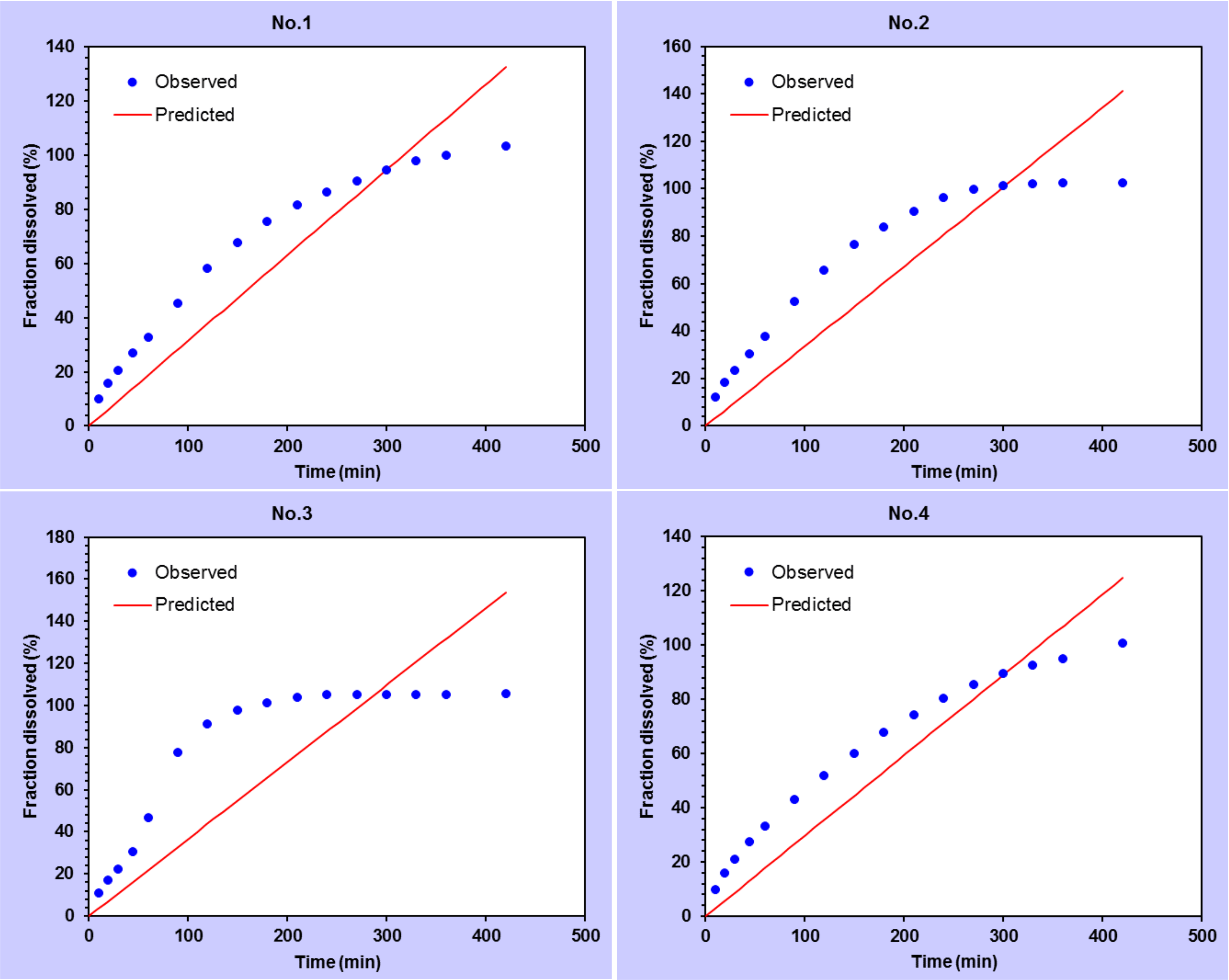

Supplementary materials – Model fitting summary – Pearlitol® 160C (modelling the entire relevant dissolution data)

Model: **Zero–order with T_{lag}**

Model equation: $F = k_0 \cdot (t - T_{lag})$

Fitted model parameters per tested tablet (N = 4) with statistics – mean, standard deviation (SD), and relative standard deviation expressed in % (RSD%) (output from DDSolver):

Parameter	No.1	No.2	No.3	No.4	Mean	SD	RSD(%)
k_0	0.241	0.241	0.237	0.228	0.237	0.006	2.662
T_{lag}	-83.916	-105.846	-146.832	-82.322	-104.729	30.051	-28.694

Number of dissolution data points (N), degrees of freedom (df), and selected goodness of fit criteria – Pearson correlation coefficient (R), coefficient of determination (R^2), adjusted coefficient of determination ($R^2_{adjusted}$), and residual sum of squares (RSS) (manual calculation in MS Excel):

Parameter	No.1	No.2	No.3	No.4
N	16	16	16	16
df	14	14	14	14
R	0.960482951	0.932306095	0.840690874	0.973689911
R^2	0.9225275	0.869194654	0.706761145	0.948072042
$R^2_{adjusted}$	0.91699375	0.859851415	0.685815512	0.944362902
RSS	1271.618459	2290.826723	6090.740917	742.0255641

Graphical abstract of model fit presented as mean ± 1 SD of the fraction % of released carvedilol:

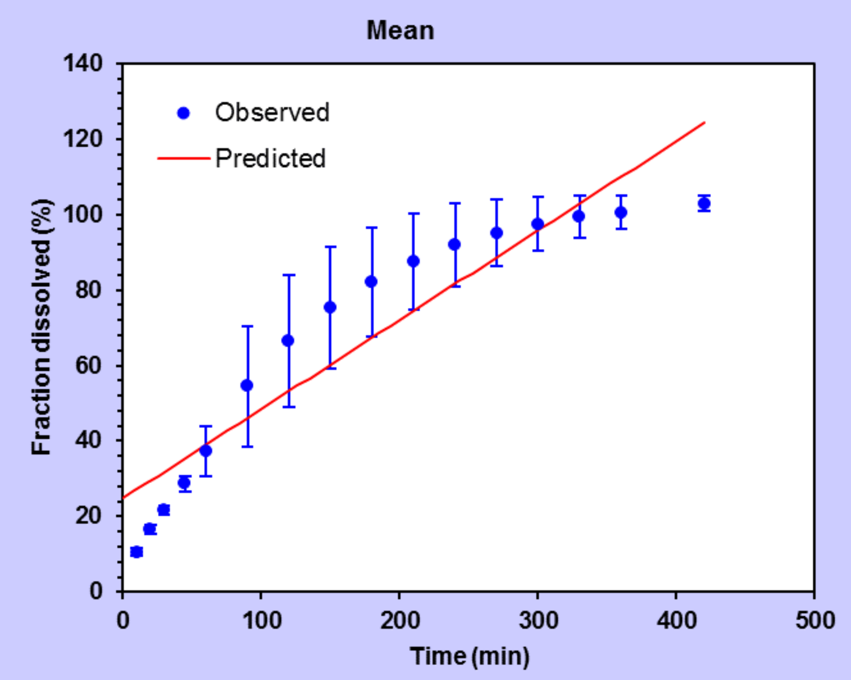

Supplementary materials – Model fitting summary – Pearlitol® 160C (modelling the entire relevant dissolution data)

Graphical abstract of model fit presented as the fraction % of released carvedilol per tested tablet:

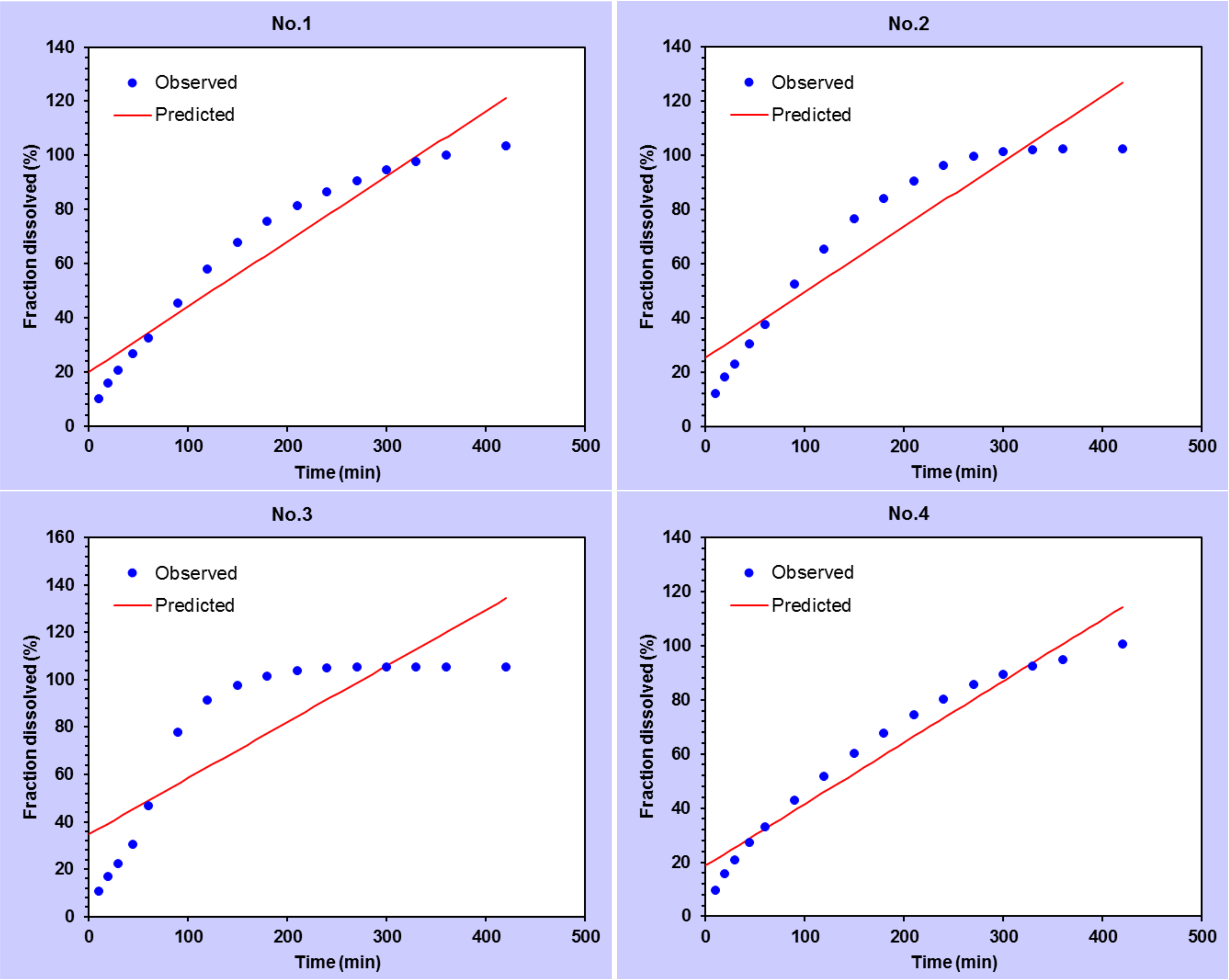

Supplementary materials – Model fitting summary – Pearlitol® 160C (modelling the entire relevant dissolution data)

Model: **Zero–order with F_0**

Model equation: $F = F_0 + k_0 \cdot t$

Fitted model parameters per tested tablet (N = 4) with statistics – mean, standard deviation (SD), and relative standard deviation expressed in % (RSD%) (output from DDSolver):

Parameter	No.1	No.2	No.3	No.4	Mean	SD	RSD(%)
k_0	0.241	0.241	0.237	0.228	0.237	0.006	2.662
F_0	20.189	25.533	34.783	18.734	24.810	7.263	29.274

Number of dissolution data points (N), degrees of freedom (df), and selected goodness of fit criteria – Pearson correlation coefficient (R), coefficient of determination (R^2), adjusted coefficient of determination ($R^2_{adjusted}$), and residual sum of squares (RSS) (manual calculation in MS Excel):

Parameter	No.1	No.2	No.3	No.4
N	16	16	16	16
df	14	14	14	14
R	0.960482951	0.932306095	0.840690874	0.973689911
R^2	0.9225275	0.869194654	0.706761145	0.948072042
$R^2_{adjusted}$	0.91699375	0.859851415	0.685815512	0.944362902
RSS	1271.618459	2290.826723	6090.740917	742.0255641

Graphical abstract of model fit presented as mean ± 1 SD of the fraction % of released carvedilol:

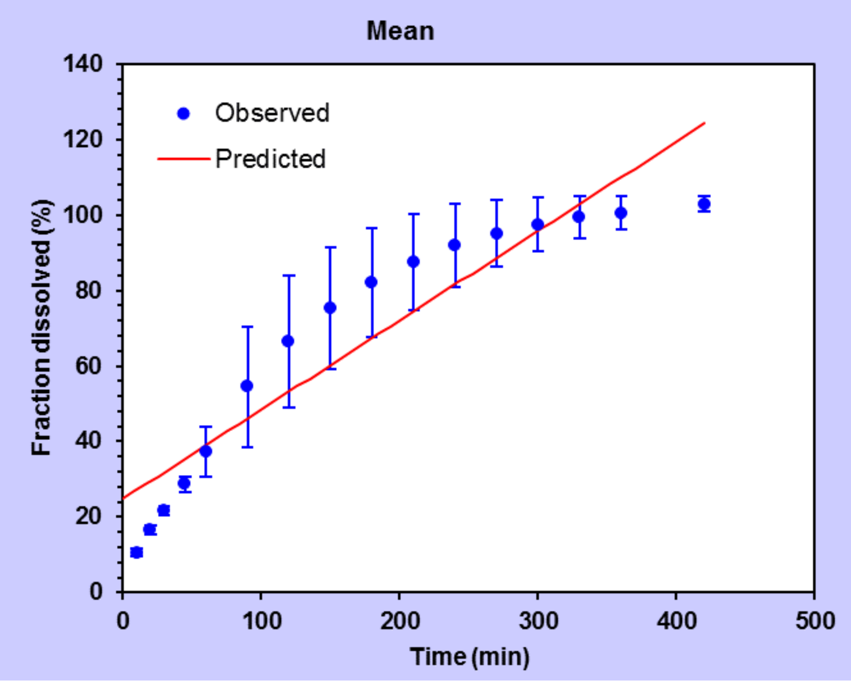

Supplementary materials – Model fitting summary – Pearlitol® 160C (modelling the entire relevant dissolution data)

Graphical abstract of model fit presented as the fraction % of released carvedilol per tested tablet:

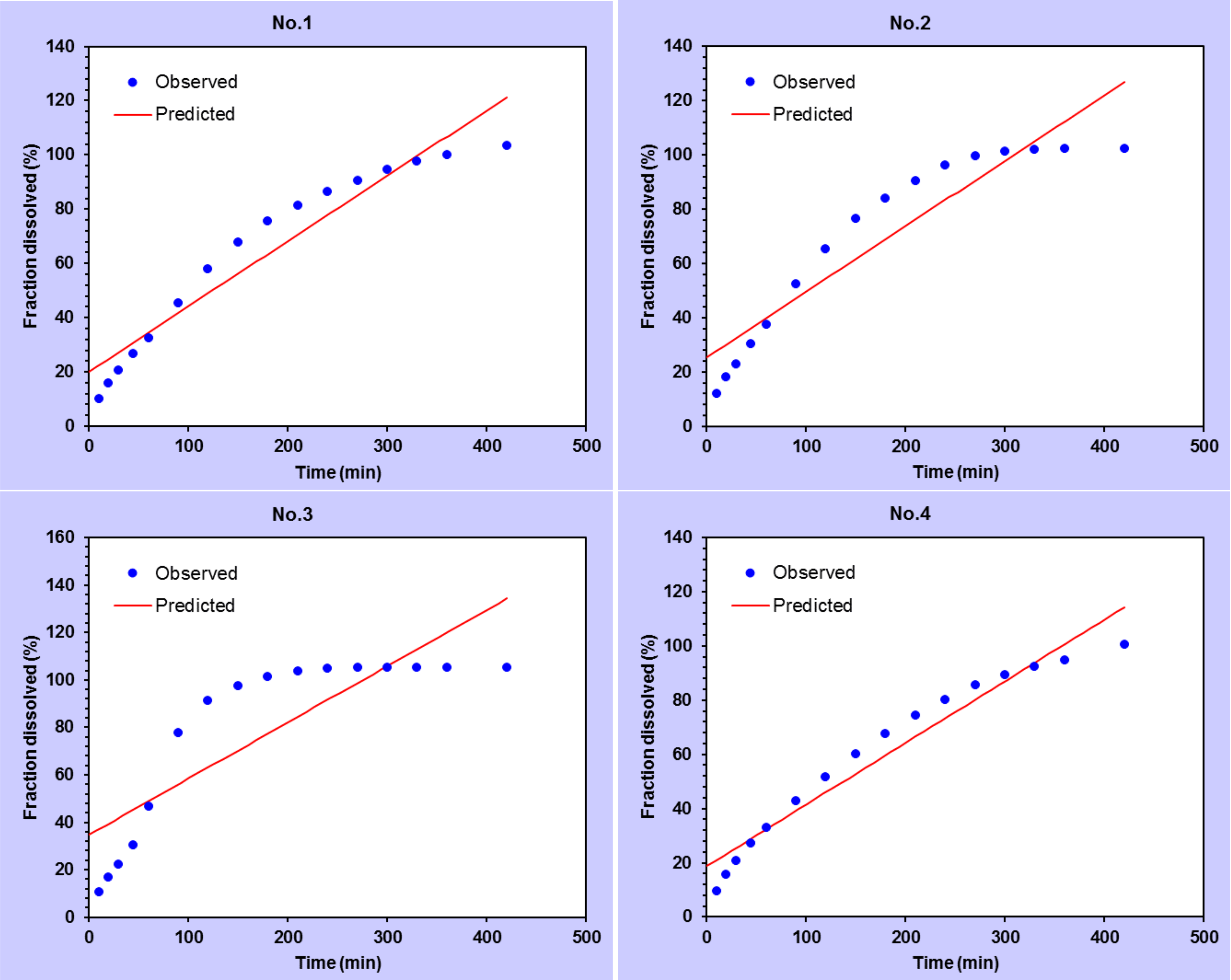

Supplementary materials – Model fitting summary – Pearlitol® 160C (modelling the entire relevant dissolution data)

Model: **First–order**

Model equation: $F = 100 \cdot (1 - e^{-k_1 \cdot t})$

Fitted model parameters per tested tablet (N = 4) with statistics – mean, standard deviation (SD), and relative standard deviation expressed in % (RSD%) (output from DDSolver):

Parameter	No.1	No.2	No.3	No.4	Mean	SD	RSD(%)
k_1	0.006	0.010	0.020	0.006	0.011	0.007	62.067

Number of dissolution data points (N), degrees of freedom (df), and selected goodness of fit criteria – Pearson correlation coefficient (R), coefficient of determination (R^2), adjusted coefficient of determination ($R^2_{adjusted}$), and residual sum of squares (RSS) (manual calculation in MS Excel):

Parameter	No.1	No.2	No.3	No.4
N	16	16	16	16
df	15	15	15	15
R	0.999418683	0.992056166	0.975616161	0.996525624
R^2	0.998837703	0.984175436	0.951826894	0.993063319
$R^2_{adjusted}$	0.998837703	0.984175436	0.951826894	0.993063319
RSS	1488.402062	400.2966415	2467.794129	172.1205026

Graphical abstract of model fit presented as mean ± 1 SD of the fraction % of released carvedilol:

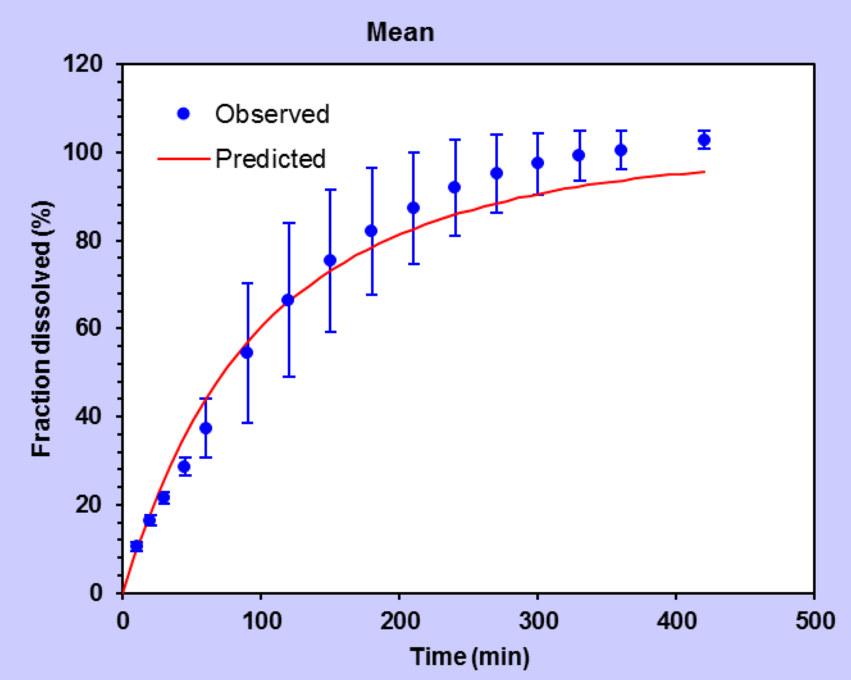

Supplementary materials – Model fitting summary – Pearlitol® 160C (modelling the entire relevant dissolution data)

Graphical abstract of model fit presented as the fraction % of released carvedilol per tested tablet:

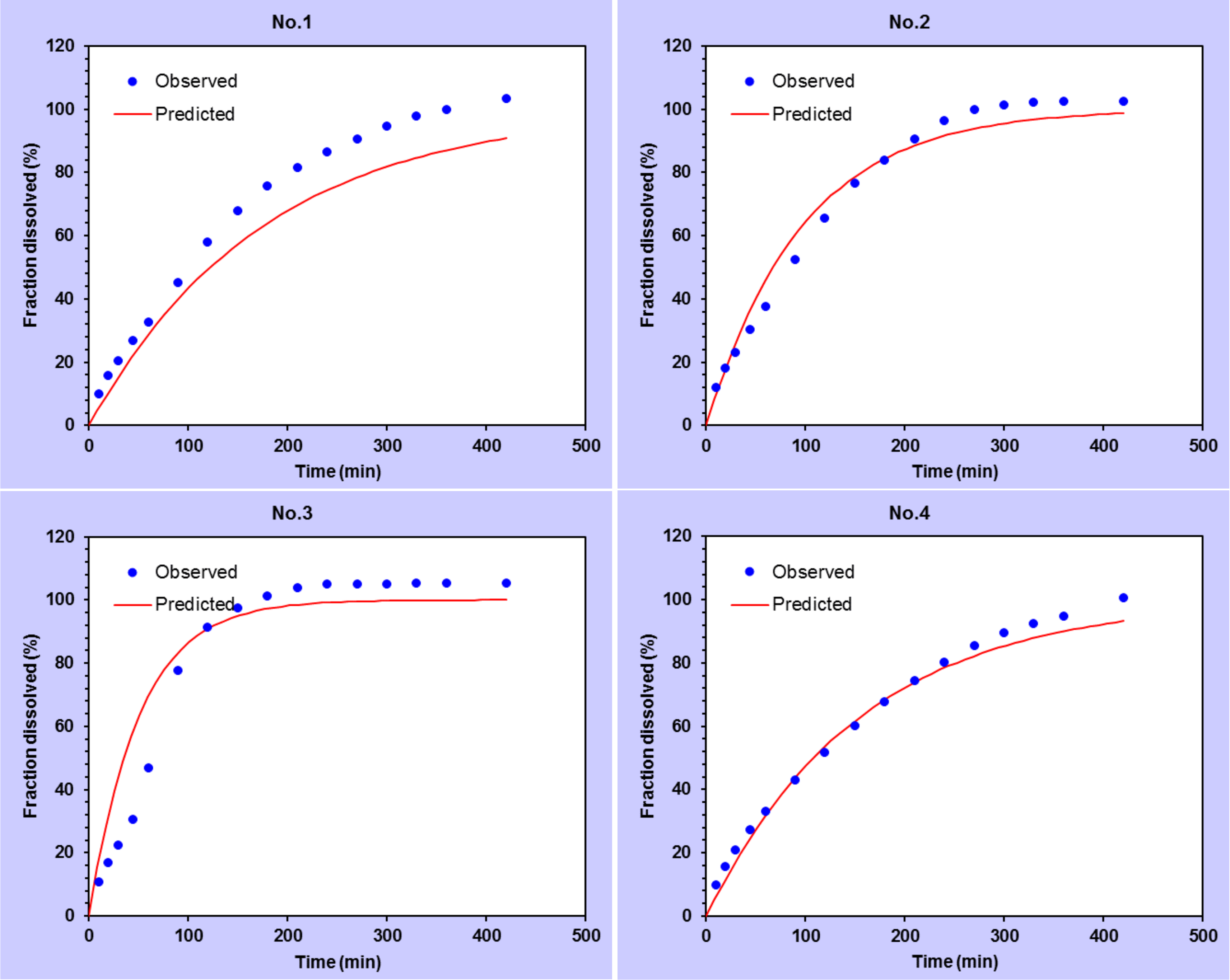

Supplementary materials – Model fitting summary – Pearlitol® 160C (modelling the entire relevant dissolution data)

Model: **First–order with T_{lag}**

Model equation: $F = 100 \cdot \left[1 - e^{-k_1 \cdot (t - T_{lag})}\right]$

Fitted model parameters per tested tablet (N = 4) with statistics – mean, standard deviation (SD), and relative standard deviation expressed in % (RSD%) (output from DDSolver):

Parameter	No.1	No.2	No.3	No.4	Mean	SD	RSD(%)
k_1	/	/	/	/	/	/	/
T_{lag}	/	/	/	/	/	/	/

Number of dissolution data points (N), degrees of freedom (df), and selected goodness of fit criteria – Pearson correlation coefficient (R), coefficient of determination (R^2), adjusted coefficient of determination ($R^2_{adjusted}$), and residual sum of squares (RSS) (manual calculation in MS Excel):

Parameter	No.1	No.2	No.3	No.4
N	/	/	/	/
df	/	/	/	/
R	/	/	/	/
R^2	/	/	/	/
$R^2_{adjusted}$	/	/	/	/
RSS	/	/	/	/

Graphical abstract of model fit presented as mean ± 1 SD of the fraction % of released carvedilol: /

Graphical abstract of model fit presented as the fraction % of released carvedilol per tested tablet: /

Note: the model could not be fitted

Supplementary materials – Model fitting summary – Pearlitol® 160C (modelling the entire relevant dissolution data)

Model: **First–order with F_{max}**

Model equation: $F = F_{max} \cdot (1 - e^{-k_1 \cdot t})$

Fitted model parameters per tested tablet (N = 4) with statistics – mean, standard deviation (SD), and relative standard deviation expressed in % (RSD%) (output from DDSolver):

Parameter	No.1	No.2	No.3	No.4	Mean	SD	RSD(%)
k_1	0.005	0.009	0.013	0.006	0.008	0.004	42.350
F_{max}	117.778	107.520	110.565	105.525	110.347	5.370	4.866

Number of dissolution data points (N), degrees of freedom (df), and selected goodness of fit criteria – Pearson correlation coefficient (R), coefficient of determination (R^2), adjusted coefficient of determination ($R^2_{adjusted}$), and residual sum of squares (RSS) (manual calculation in MS Excel):

Parameter	No.1	No.2	No.3	No.4
N	16	16	16	16
df	14	14	14	14
R	0.999401795	0.995982055	0.989642938	0.997376615
R^2	0.998803948	0.991980253	0.979393145	0.994760112
$R^2_{adjusted}$	0.998718516	0.991407414	0.977921227	0.994385834
RSS	53.98131776	197.644451	977.9701956	86.26367792

Graphical abstract of model fit presented as mean ± 1 SD of the fraction % of released carvedilol:

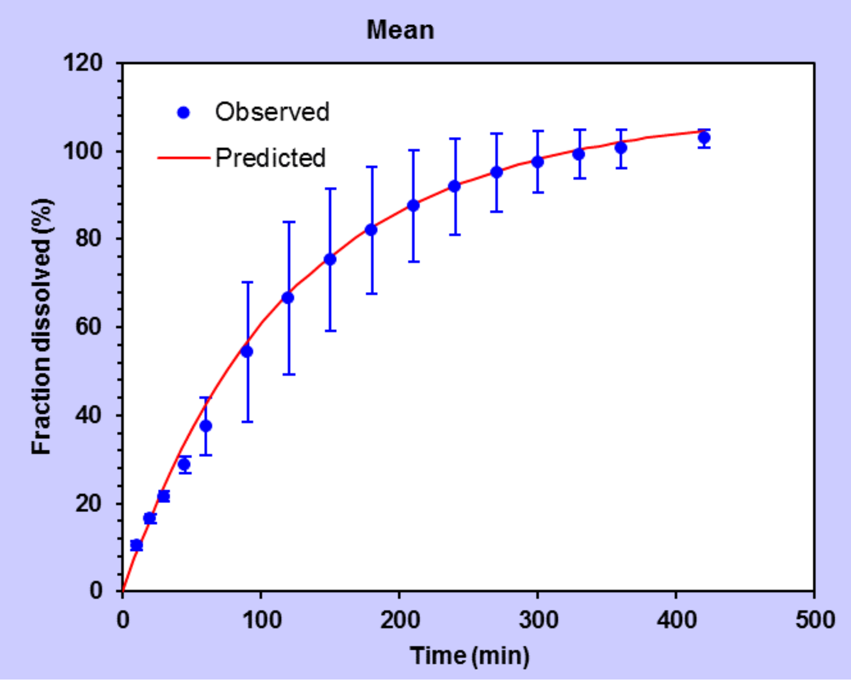

Supplementary materials – Model fitting summary – Pearlitol® 160C (modelling the entire relevant dissolution data)

Graphical abstract of model fit presented as the fraction % of released carvedilol per tested tablet:

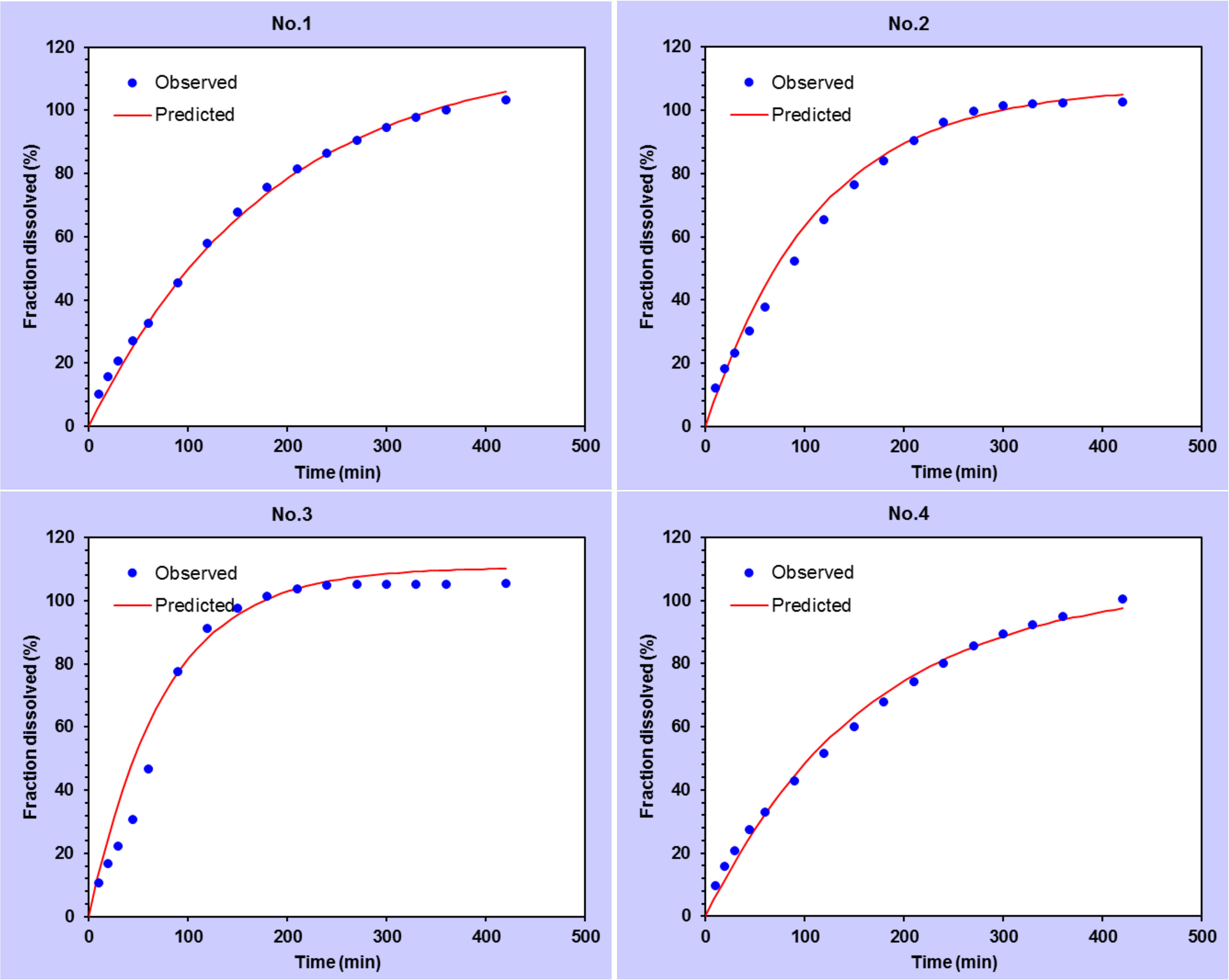

Supplementary materials – Model fitting summary – Pearlitol® 160C (modelling the entire relevant dissolution data)

Model: **First–order with T_{lag} and F_{max}**

Model equation: $F = F_{max} \cdot \left[1 - e^{-k_1 \cdot (t - T_{lag})}\right]$

Fitted model parameters per tested tablet (N = 4) with statistics – mean, standard deviation (SD), and relative standard deviation expressed in % (RSD%) (output from DDSolver):

Parameter	No.1	No.2	No.3	No.4	Mean	SD	RSD(%)
k_1	0.007	0.007	0.009	0.007	0.007	0.001	11.780
T_{lag}	7.218	-1.631	-40.272	8.896	-6.448	23.018	-357.000
F_{max}	108.360	114.240	110.565	105.525	109.673	3.678	3.354

Number of dissolution data points (N), degrees of freedom (df), and selected goodness of fit criteria – Pearson correlation coefficient (R), coefficient of determination (R^2), adjusted coefficient of determination ($R^2_{adjusted}$), and residual sum of squares (RSS) (manual calculation in MS Excel):

Parameter	No.1	No.2	No.3	No.4
N	16	16	16	16
df	13	13	13	13
R	0.997544586	0.997348566	0.97684632	0.995857752
R^2	0.995095202	0.994704163	0.954228733	0.991732662
$R^2_{adjusted}$	0.994340618	0.993889419	0.947187	0.990460764
RSS	144.9458756	100.0146092	3610.659304	287.8359929

Graphical abstract of model fit presented as mean ± 1 SD of the fraction % of released carvedilol:

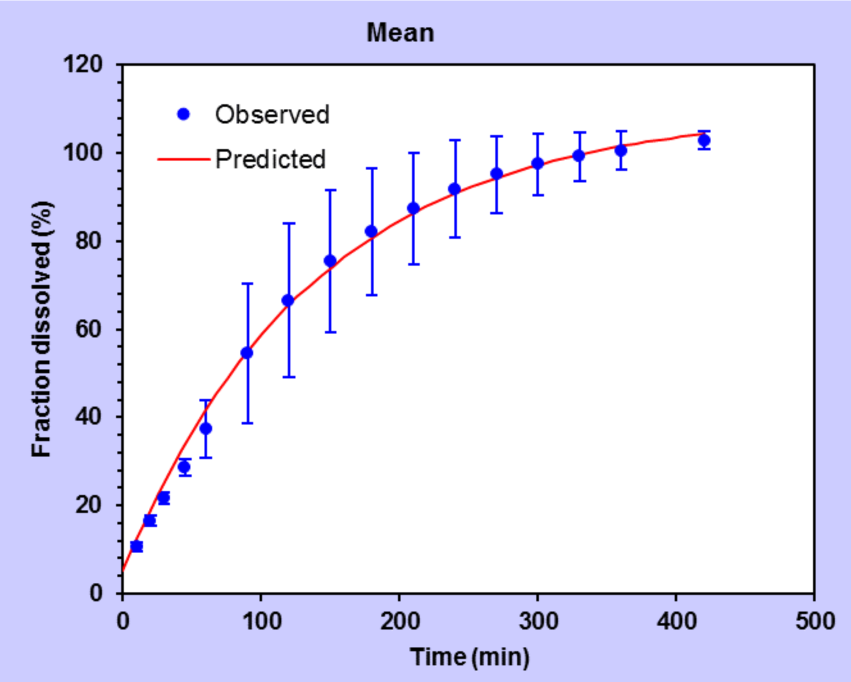

Supplementary materials – Model fitting summary – Pearlitol® 160C (modelling the entire relevant dissolution data)

Graphical abstract of model fit presented as the fraction % of released carvedilol per tested tablet:

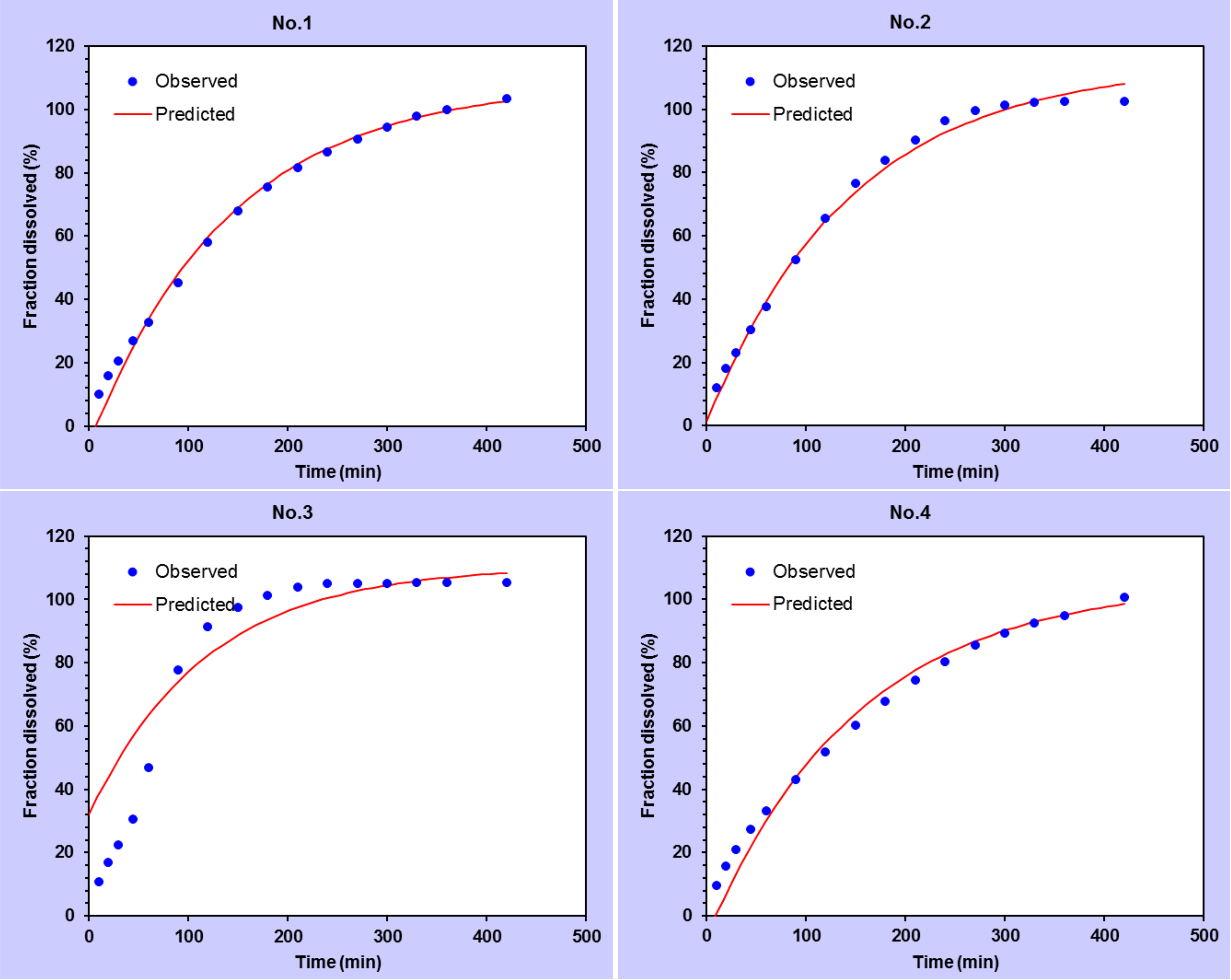

Supplementary materials – Model fitting summary – Pearlitol® 160C (modelling the entire relevant dissolution data)

Model: **Higuchi**

Model equation: $F = k_H \cdot t^{0.5}$

Fitted model parameters per tested tablet (N = 4) with statistics – mean, standard deviation (SD), and relative standard deviation expressed in % (RSD%) (output from DDSolver):

Parameter	No.1	No.2	No.3	No.4	Mean	SD	RSD(%)
k_H	5.282	5.675	6.284	4.963	5.551	0.569	10.249

Number of dissolution data points (N), degrees of freedom (df), and selected goodness of fit criteria – Pearson correlation coefficient (R), coefficient of determination (R^2), adjusted coefficient of determination ($R^2_{adjusted}$), and residual sum of squares (RSS) (manual calculation in MS Excel):

Parameter	No.1	No.2	No.3	No.4
N	16	16	16	16
df	15	15	15	15
R	0.992960581	0.97973519	0.922542459	0.997831582
R^2	0.985970716	0.959881042	0.851084588	0.995667865
$R^2_{adjusted}$	0.985970716	0.959881042	0.851084588	0.995667865
RSS	472.454034	784.1674441	3095.536835	267.5723835

Graphical abstract of model fit presented as mean ± 1 SD of the fraction % of released carvedilol:

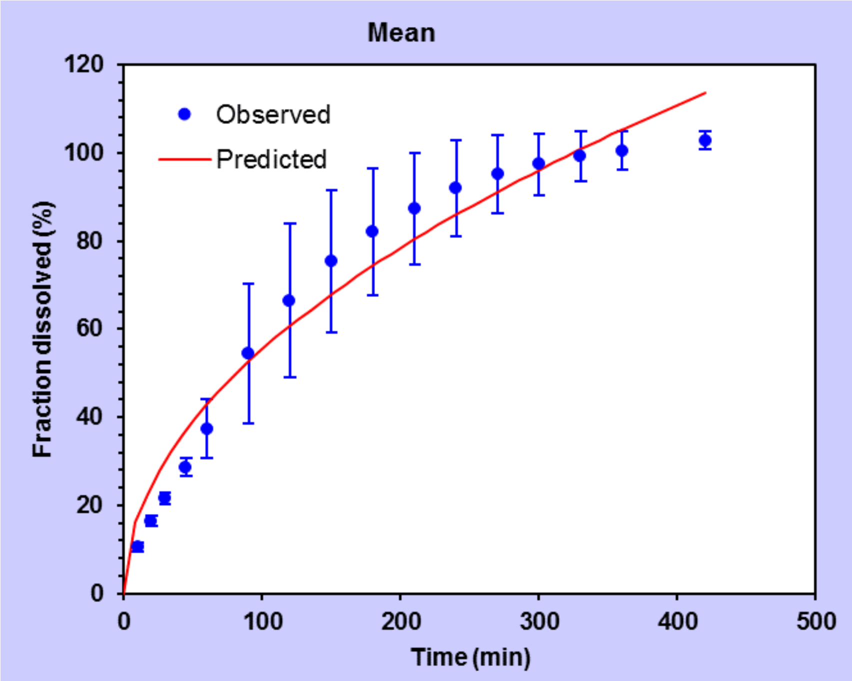

Supplementary materials – Model fitting summary – Pearlitol® 160C (modelling the entire relevant dissolution data)

Graphical abstract of model fit presented as the fraction % of released carvedilol per tested tablet:

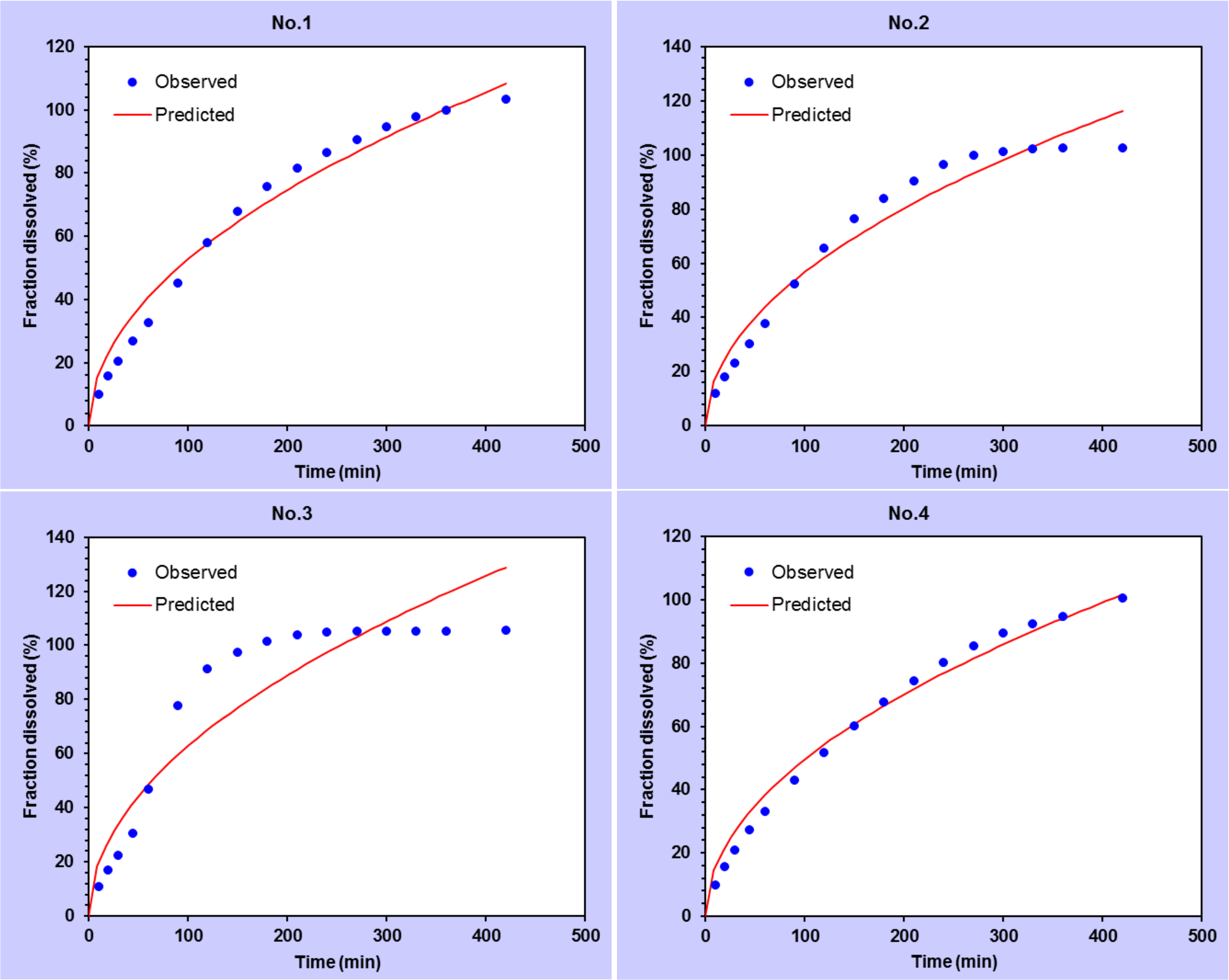

Supplementary materials – Model fitting summary – Pearlitol® 160C (modelling the entire relevant dissolution data)

Model: **Higuchi with T_{lag}**

Model equation: $F = k_H \cdot (t - T_{lag})^{0.5}$

Fitted model parameters per tested tablet (N = 4) with statistics – mean, standard deviation (SD), and relative standard deviation expressed in % (RSD%) (output from DDSolver):

Parameter	No.1	No.2	No.3	No.4	Mean	SD	RSD(%)
k_H	5.387	5.536	5.557	5.136	5.404	0.194	3.590
T_{lag}	8.824	-10.627	-55.620	11.124	-11.575	30.942	-267.321

Number of dissolution data points (N), degrees of freedom (df), and selected goodness of fit criteria – Pearson correlation coefficient (R), coefficient of determination (R^2), adjusted coefficient of determination ($R^2_{adjusted}$), and residual sum of squares (RSS) (manual calculation in MS Excel):

Parameter	No.1	No.2	No.3	No.4
N	16	16	16	16
df	14	14	14	14
R	0.992457172	0.977061482	0.897863764	0.994670664
R^2	0.984971238	0.95464914	0.806159339	0.98936973
$R^2_{adjusted}$	0.983897754	0.951409793	0.792313578	0.988610425
RSS	260.3044466	1170.885344	5448.080523	158.5768926

Graphical abstract of model fit presented as mean ± 1 SD of the fraction % of released carvedilol:

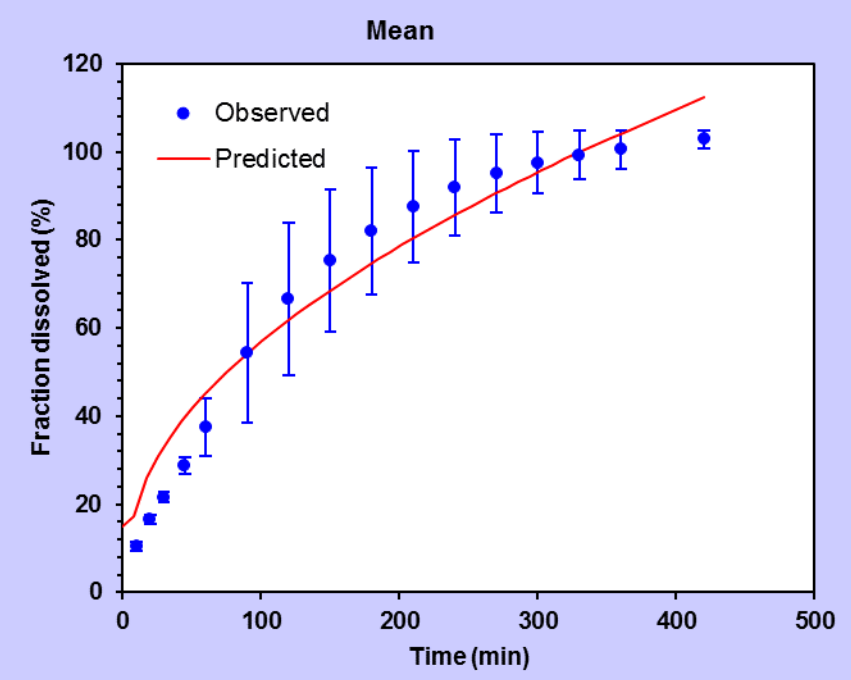

Supplementary materials – Model fitting summary – Pearlitol® 160C (modelling the entire relevant dissolution data)

Graphical abstract of model fit presented as the fraction % of released carvedilol per tested tablet:

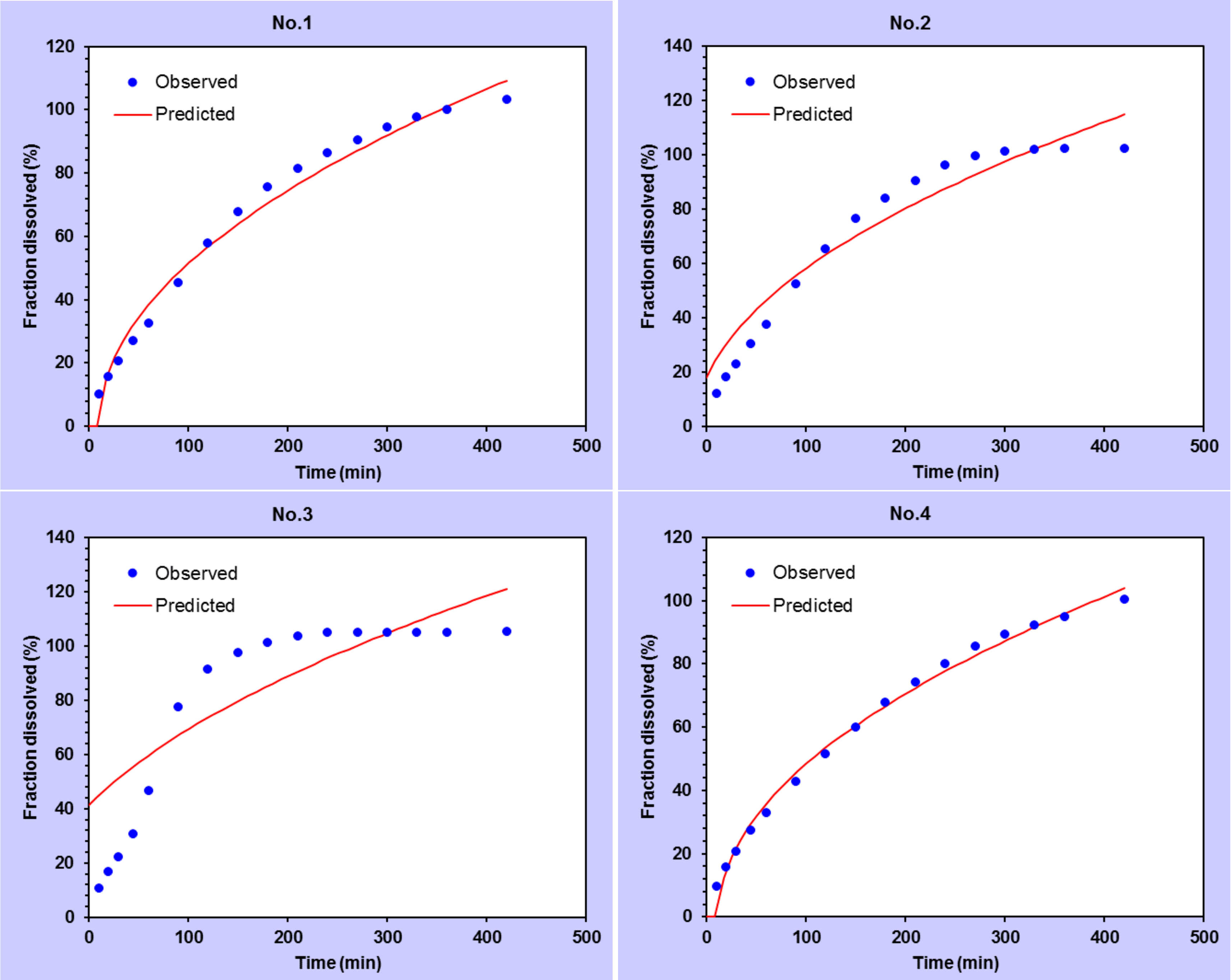

Supplementary materials – Model fitting summary – Pearlitol® 160C (modelling the entire relevant dissolution data)

Model: **Higuchi with F_0**

Model equation: $F = F_0 + k_H \cdot t^{0.5}$

Fitted model parameters per tested tablet (N = 4) with statistics – mean, standard deviation (SD), and relative standard deviation expressed in % (RSD%) (output from DDSolver):

Parameter	No.1	No.2	No.3	No.4	Mean	SD	RSD(%)
k_H	5.948	6.062	6.216	5.577	5.951	0.272	4.579
F_0	-9.685	-5.620	0.980	-8.925	-5.813	4.860	-83.607

Number of dissolution data points (N), degrees of freedom (df), and selected goodness of fit criteria – Pearson correlation coefficient (R), coefficient of determination (R^2), adjusted coefficient of determination ($R^2_{adjusted}$), and residual sum of squares (RSS) (manual calculation in MS Excel):

Parameter	No.1	No.2	No.3	No.4
N	16	16	16	16
df	14	14	14	14
R	0.992960581	0.97973519	0.922542459	0.997831582
R^2	0.985970716	0.959881042	0.851084588	0.995667865
$R^2_{adjusted}$	0.984968624	0.957015402	0.840447773	0.995358427
RSS	230.2739293	702.6133434	3093.059375	61.90412297

Graphical abstract of model fit presented as mean ± 1 SD of the fraction % of released carvedilol:

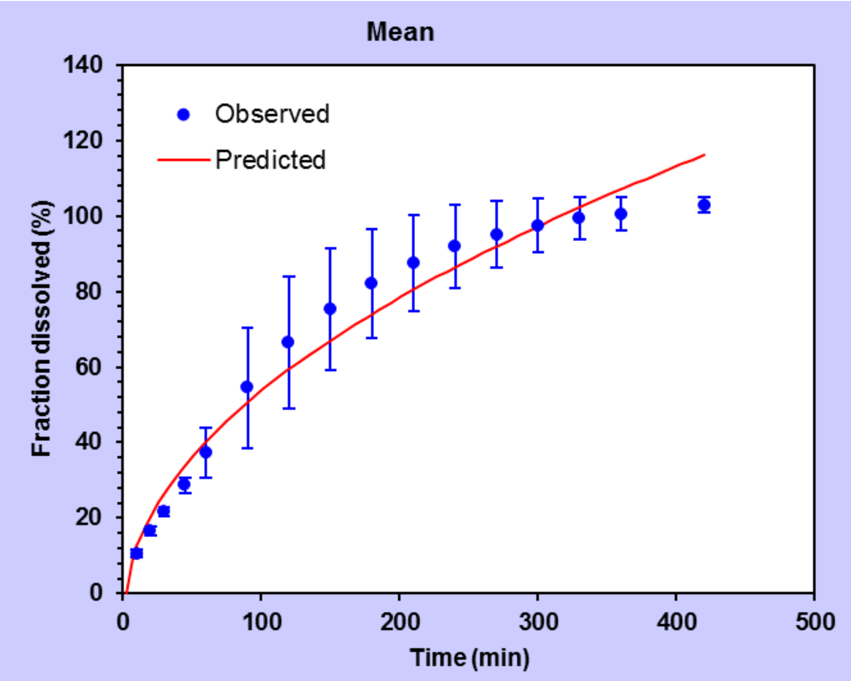

Supplementary materials – Model fitting summary – Pearlitol® 160C (modelling the entire relevant dissolution data)

Graphical abstract of model fit presented as the fraction % of released carvedilol per tested tablet:

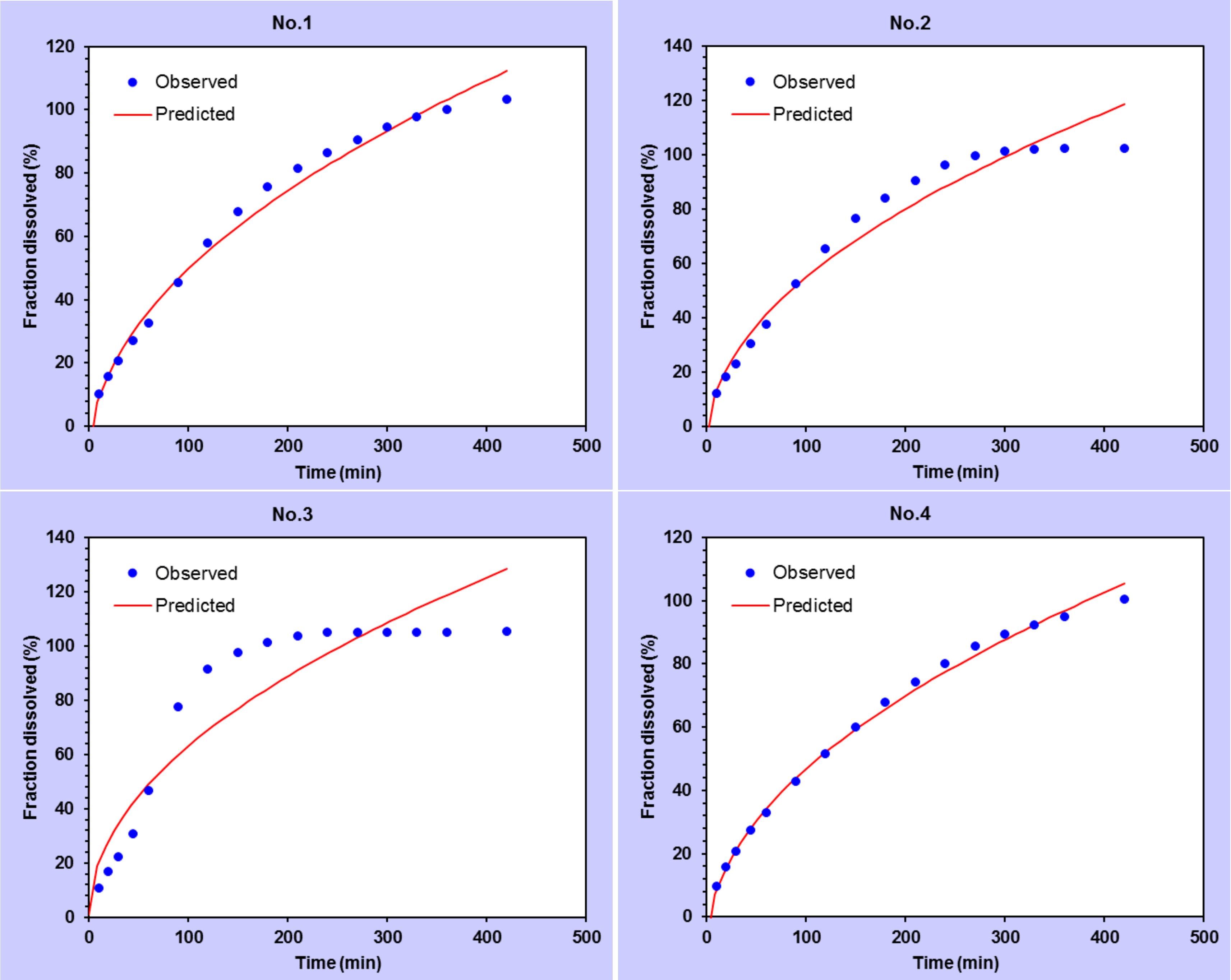

Supplementary materials – Model fitting summary – Pearlitol® 160C (modelling the entire relevant dissolution data)

Model: **Korsmeyer–Peppas**

Model equation: $F = k_{KP} \cdot t^n$

Fitted model parameters per tested tablet (N = 4) with statistics – mean, standard deviation (SD), and relative standard deviation expressed in % (RSD%) (output from DDSolver):

Parameter	No.1	No.2	No.3	No.4	Mean	SD	RSD(%)
k_{KP}	2.162	3.052	1.366	2.280	2.215	0.690	31.156
n	0.669	0.583	0.555	0.646	0.613	0.053	8.716

Number of dissolution data points (N), degrees of freedom (df), and selected goodness of fit criteria – Pearson correlation coefficient (R), coefficient of determination (R^2), adjusted coefficient of determination ($R^2_{adjusted}$), and residual sum of squares (RSS) (manual calculation in MS Excel):

Parameter	No.1	No.2	No.3	No.4
N	16	16	16	16
df	14	14	14	14
R	0.985877388	0.974297724	0.914738507	0.994427835
R^2	0.971954223	0.949256055	0.836746537	0.988886719
$R^2_{adjusted}$	0.969950954	0.945631488	0.825085575	0.988092914
RSS	700.4346019	3014.592954	59089.94388	260.7088044

Graphical abstract of model fit presented as mean ± 1 SD of the fraction % of released carvedilol:

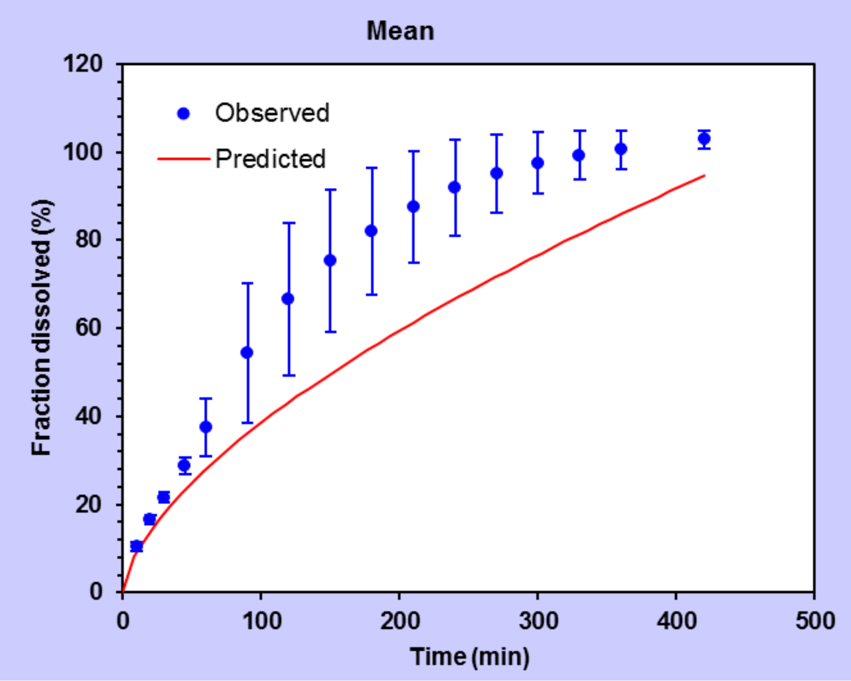

Supplementary materials – Model fitting summary – Pearlitol® 160C (modelling the entire relevant dissolution data)

Graphical abstract of model fit presented as the fraction % of released carvedilol per tested tablet:

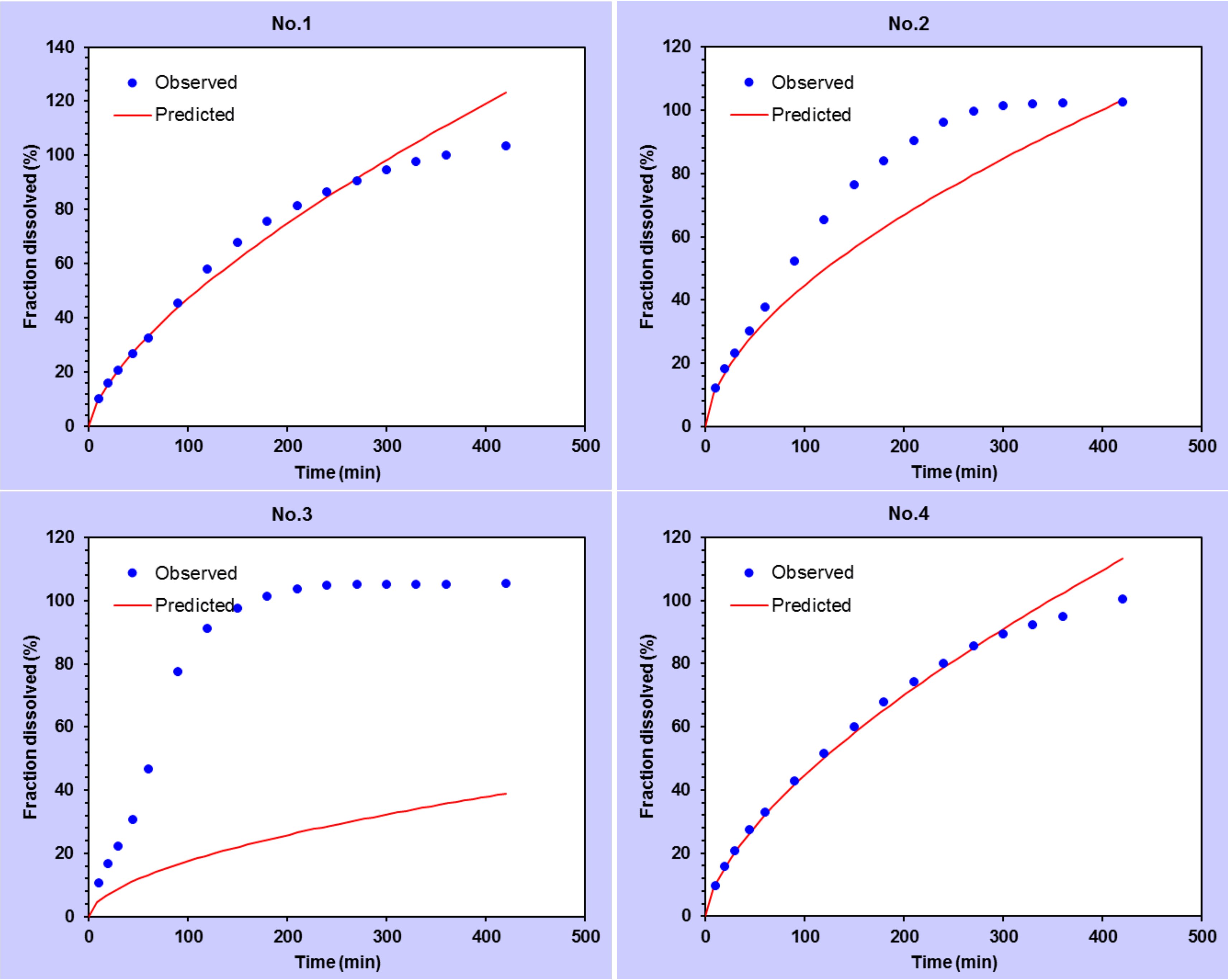

Supplementary materials – Model fitting summary – Pearlitol® 160C (modelling the entire relevant dissolution data)

Model: **Korsmeyer–Peppas with T_{lag}**

Model equation: $F = k_{KP} \cdot (t - T_{lag})^n$

Fitted model parameters per tested tablet (N = 4) with statistics – mean, standard deviation (SD), and relative standard deviation expressed in % (RSD%) (output from DDSolver):

Parameter	No.1	No.2	No.3	No.4	Mean	SD	RSD(%)
k_{KP}	3.146	3.990	3.711	3.232	3.520	0.400	11.373
n	0.598	0.569	0.605	0.581	0.588	0.016	2.796
T_{lag}	4.000	4.000	4.000	4.000	4.000	0.000	0.000

Number of dissolution data points (N), degrees of freedom (df), and selected goodness of fit criteria – Pearson correlation coefficient (R), coefficient of determination (R^2), adjusted coefficient of determination ($R^2_{adjusted}$), and residual sum of squares (RSS) (manual calculation in MS Excel):

Parameter	No.1	No.2	No.3	No.4
N	16	16	16	16
df	13	13	13	13
R	0.990121196	0.976512512	0.910050789	0.996773777
R^2	0.980339982	0.953576687	0.828192438	0.993557963
$R^2_{adjusted}$	0.977315364	0.946434639	0.801760505	0.99256688
RSS	343.9946871	887.5912246	4547.031909	95.93292848

Graphical abstract of model fit presented as mean ± 1 SD of the fraction % of released carvedilol:

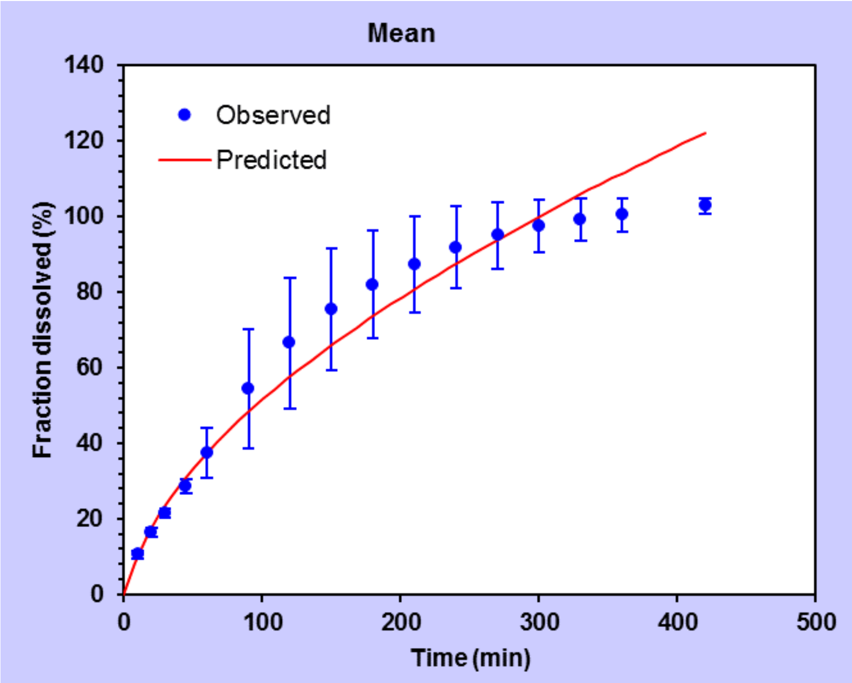

Supplementary materials – Model fitting summary – Pearlitol® 160C (modelling the entire relevant dissolution data)

Graphical abstract of model fit presented as the fraction % of released carvedilol per tested tablet:

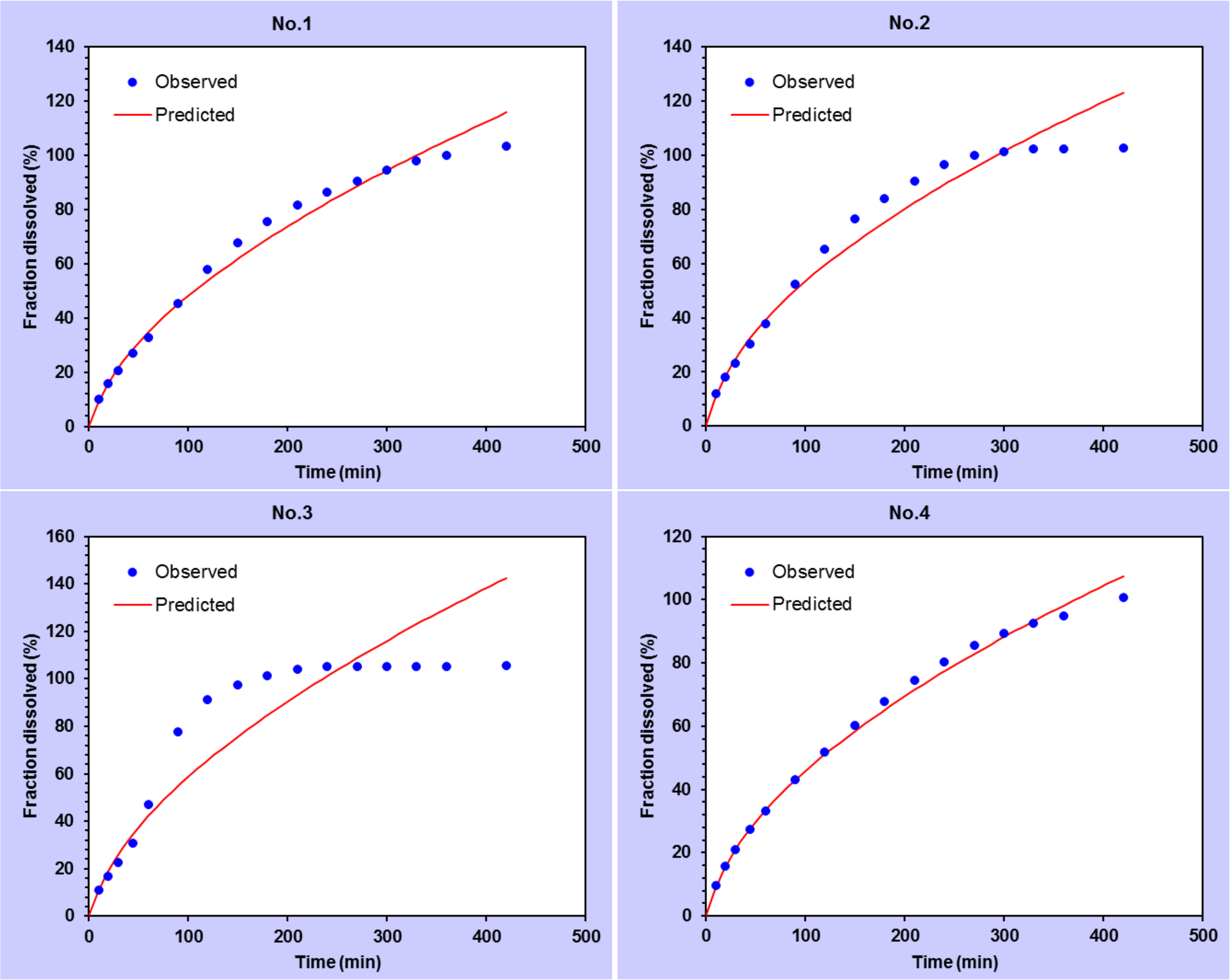

Supplementary materials – Model fitting summary – Pearlitol® 160C (modelling the entire relevant dissolution data)

Model: **Korsmeyer–Peppas with F_0**

Model equation: $F = F_0 + k_{KP} \cdot t^n$

Fitted model parameters per tested tablet (N = 4) with statistics – mean, standard deviation (SD), and relative standard deviation expressed in % (RSD%) (output from DDSolver):

Parameter	No.1	No.2	No.3	No.4	Mean	SD	RSD(%)
k_{KP}	1.227	1.570	1.495	1.294	1.397	0.162	11.613
n	0.761	0.729	0.764	0.739	0.748	0.017	2.235
F_0	4.000	4.760	4.240	3.840	4.210	0.402	9.545

Number of dissolution data points (N), degrees of freedom (df), and selected goodness of fit criteria – Pearson correlation coefficient (R), coefficient of determination (R^2), adjusted coefficient of determination ($R^2_{adjusted}$), and residual sum of squares (RSS) (manual calculation in MS Excel):

Parameter	No.1	No.2	No.3	No.4
N	16	16	16	16
df	13	13	13	13
R	0.980109814	0.961907781	0.881706865	0.990466424
R^2	0.960615248	0.925266579	0.777406996	0.981023738
$R^2_{adjusted}$	0.954556056	0.91376913	0.743161918	0.978104313
RSS	963.2479228	1823.442238	7131.431734	451.4557093

Graphical abstract of model fit presented as mean ± 1 SD of the fraction % of released carvedilol:

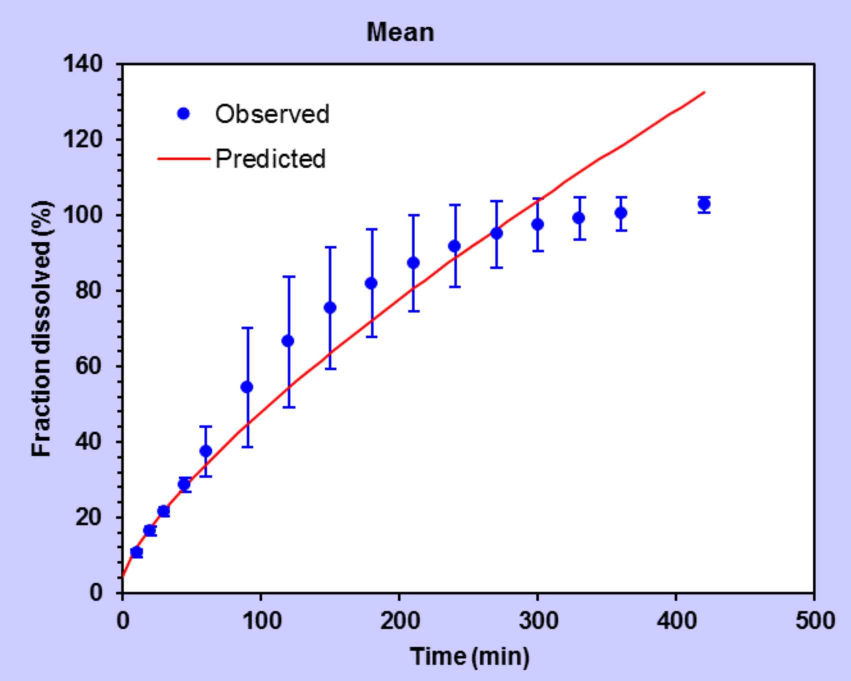

Supplementary materials – Model fitting summary – Pearlitol® 160C (modelling the entire relevant dissolution data)

Graphical abstract of model fit presented as the fraction % of released carvedilol per tested tablet:

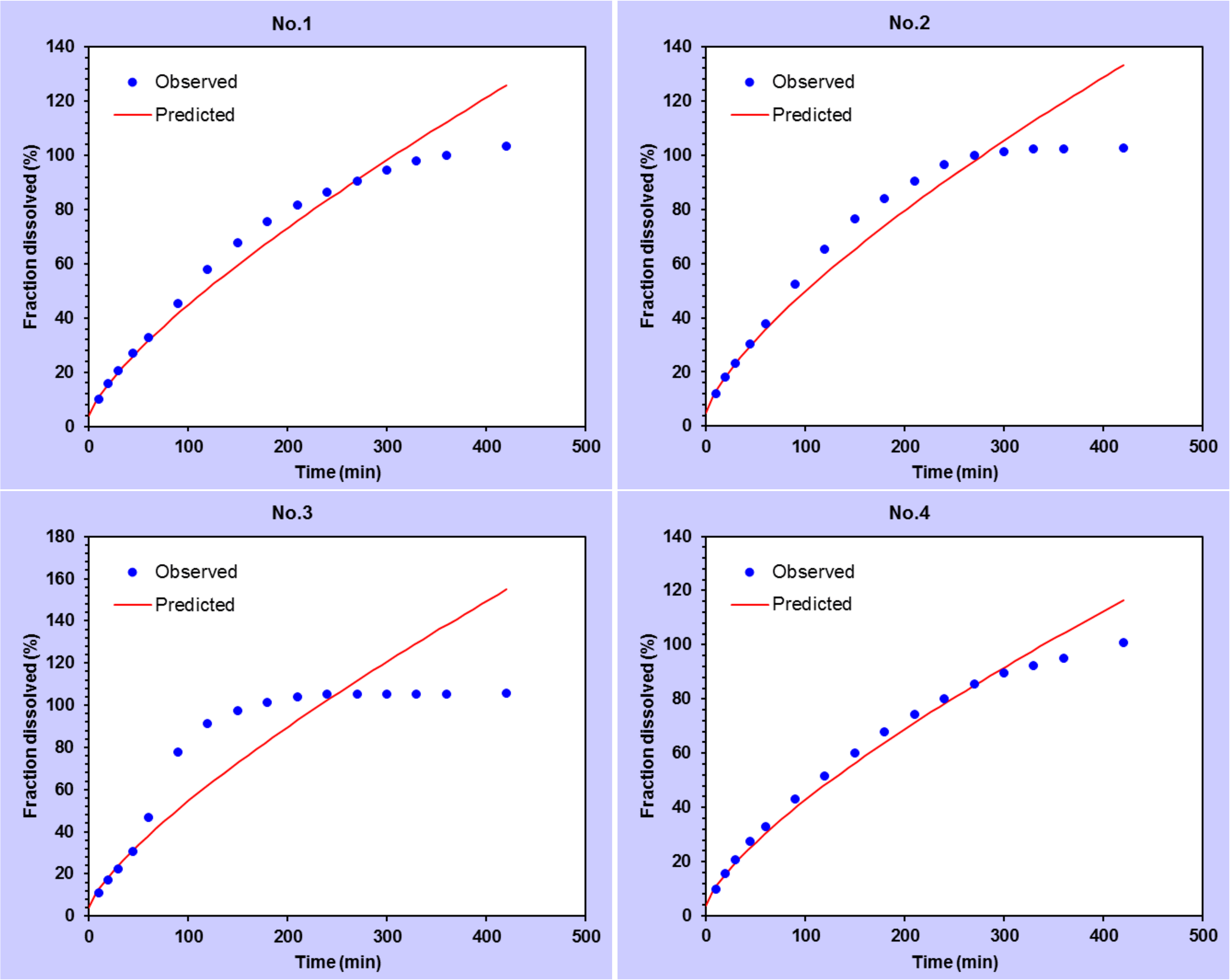

Supplementary materials – Model fitting summary – Pearlitol® 160C (modelling the entire relevant dissolution data)

Model: **Hixson–Crowell**

Model equation: $F = 100 \cdot [1 - (1 - k_{HC} \cdot t)^3]$

Fitted model parameters per tested tablet (N = 4) with statistics – mean, standard deviation (SD), and relative standard deviation expressed in % (RSD%) (output from DDSolver):

Parameter	No.1	No.2	No.3	No.4	Mean	SD	RSD(%)
k_{HC}	0.002	0.002	0.003	0.002	0.002	0.001	28.127

Number of dissolution data points (N), degrees of freedom (df), and selected goodness of fit criteria – Pearson correlation coefficient (R), coefficient of determination (R^2), adjusted coefficient of determination ($R^2_{adjusted}$), and residual sum of squares (RSS) (manual calculation in MS Excel):

Parameter	No.1	No.2	No.3	No.4
N	16	16	16	16
df	15	15	15	15
R	0.999178649	0.999232938	0.991142606	0.999181895
R^2	0.998357972	0.998466464	0.982363666	0.99836446
$R^2_{adjusted}$	0.998357972	0.998466464	0.982363666	0.99836446
RSS	67.59238689	129.4793385	769.7149428	144.6805114

Graphical abstract of model fit presented as mean ± 1 SD of the fraction % of released carvedilol:

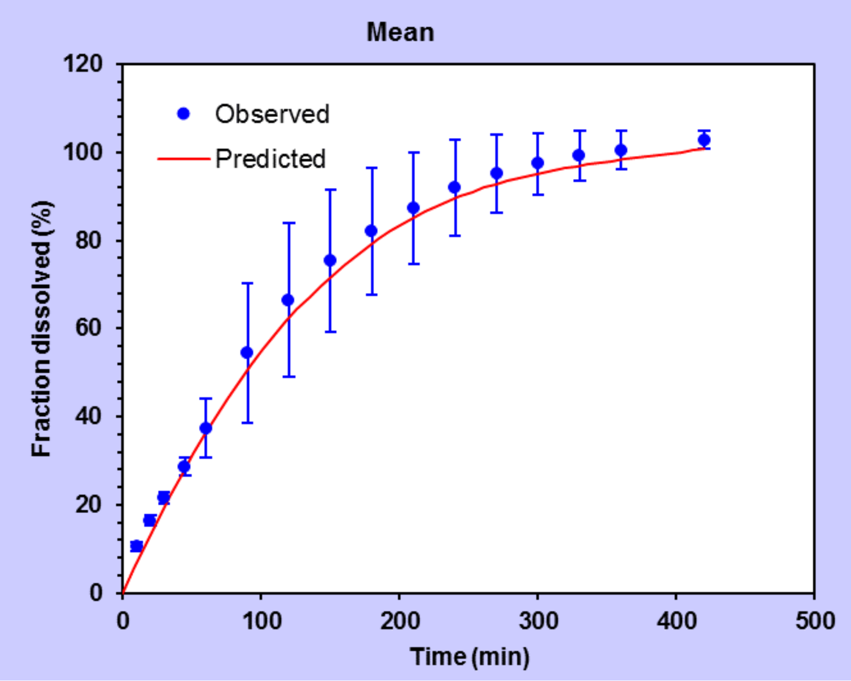

Supplementary materials – Model fitting summary – Pearlitol® 160C (modelling the entire relevant dissolution data)

Graphical abstract of model fit presented as the fraction % of released carvedilol per tested tablet:

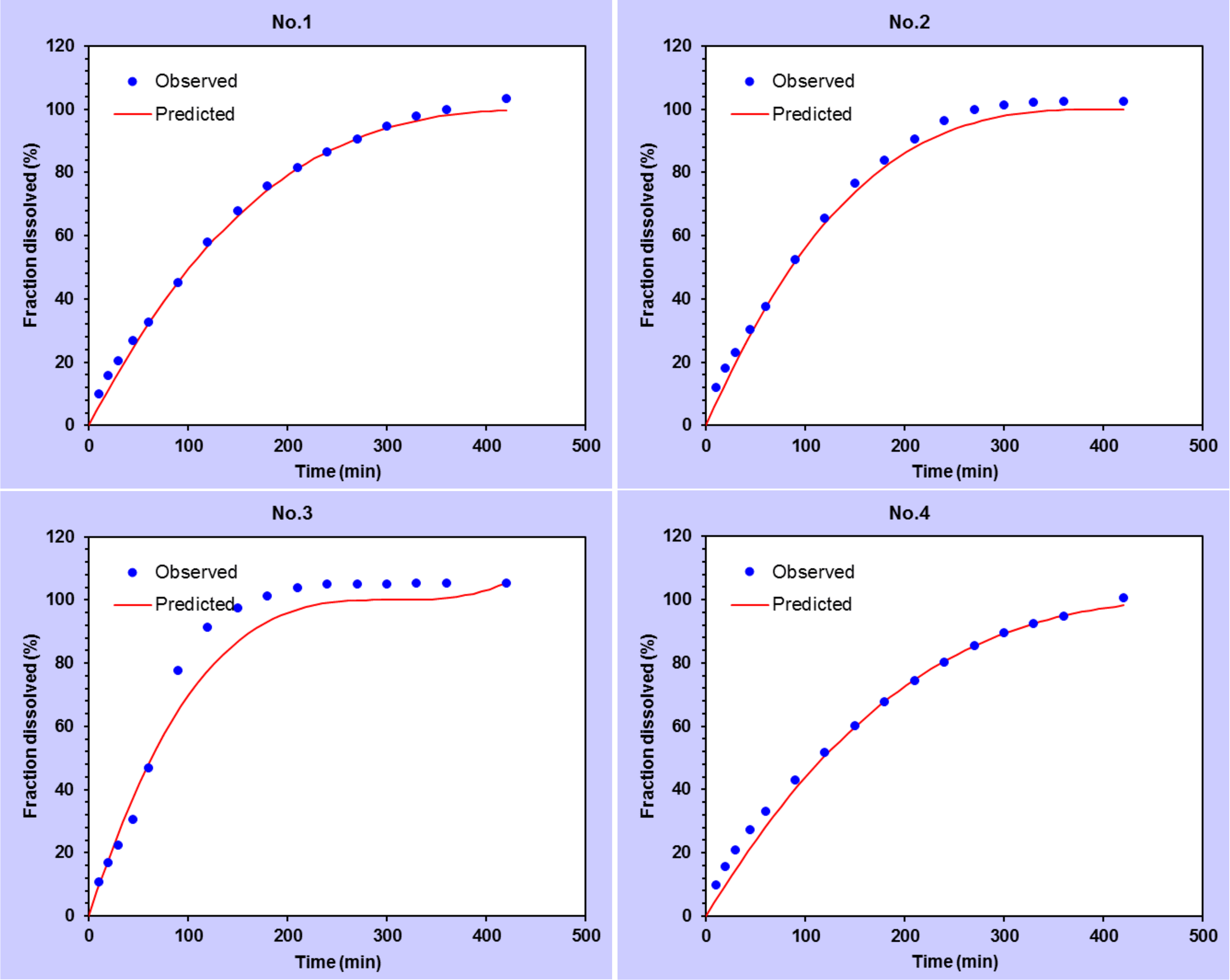

Supplementary materials – Model fitting summary – Pearlitol® 160C (modelling the entire relevant dissolution data)

Model: **Hixson–Crowell with T_{lag}**

Model equation: $F = 100 \cdot \left\{1 - \left[1 - k_{HC} \cdot \left(t - T_{lag}\right)\right]^3\right\}$

Fitted model parameters per tested tablet (N = 4) with statistics – mean, standard deviation (SD), and relative standard deviation expressed in % (RSD%) (output from DDSolver):

Parameter	No.1	No.2	No.3	No.4	Mean	SD	RSD(%)
k_{HC}	0.002	0.003	0.003	0.002	0.002	0.000	21.567
T_{lag}	3.982	6.280	18.777	-11.404	4.409	12.385	280.918

Number of dissolution data points (N), degrees of freedom (df), and selected goodness of fit criteria – Pearson correlation coefficient (R), coefficient of determination (R^2), adjusted coefficient of determination ($R^2_{adjusted}$), and residual sum of squares (RSS) (manual calculation in MS Excel):

Parameter	No.1	No.2	No.3	No.4
N	16	16	16	16
df	14	14	14	14
R	0.997862178	0.995534322	0.973679337	0.999416466
R^2	0.995728927	0.991088586	0.948051452	0.998833273
$R^2_{adjusted}$	0.995423851	0.990452057	0.944340842	0.998749936
RSS	128.8632146	211.0817362	5423.477865	17.2663834

Graphical abstract of model fit presented as mean ± 1 SD of the fraction % of released carvedilol:

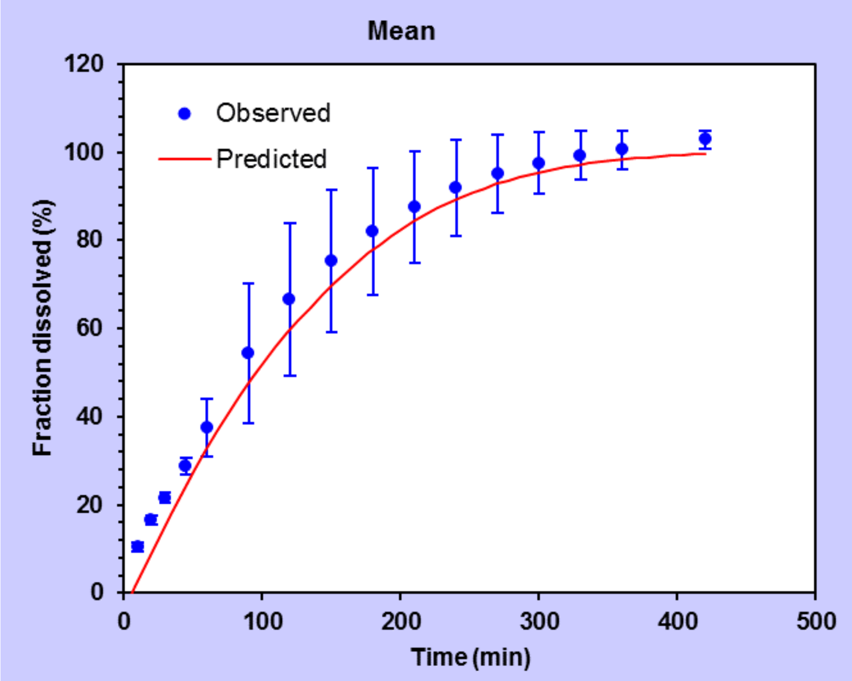

Supplementary materials – Model fitting summary – Pearlitol® 160C (modelling the entire relevant dissolution data)

Graphical abstract of model fit presented as the fraction % of released carvedilol per tested tablet:

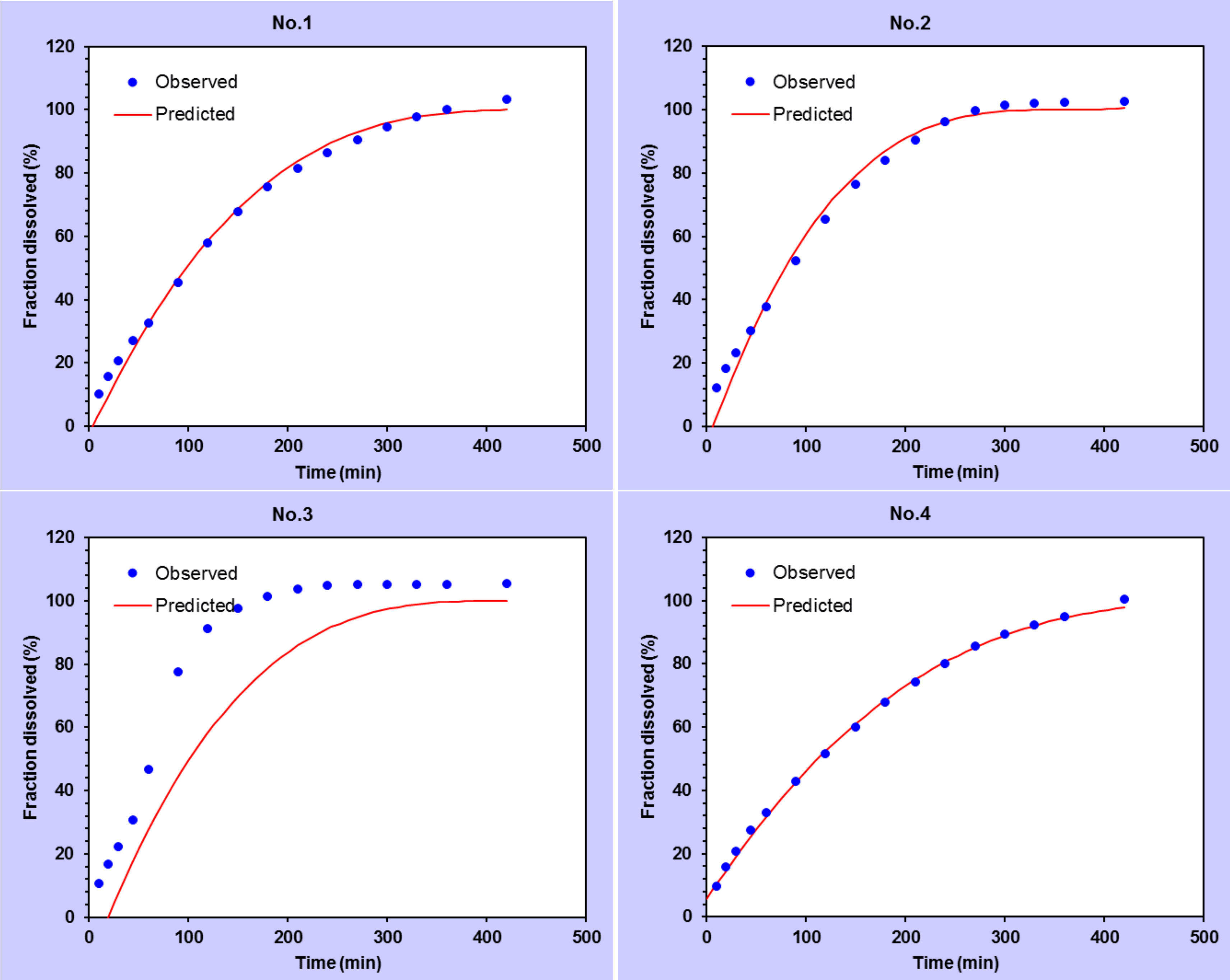

Supplementary materials – Model fitting summary – Pearlitol® 160C (modelling the entire relevant dissolution data)

Model: **Hopfenberg**

Model equation: $F = 100 \cdot [1 - (1 - k_{HB} \cdot t)^n]$

Fitted model parameters per tested tablet (N = 4) with statistics – mean, standard deviation (SD), and relative standard deviation expressed in % (RSD%) (output from DDSolver):

Parameter	No.1	No.2	No.3	No.4	Mean	SD	RSD(%)
k_{HB}	0.002	0.003	0.007	0.002	0.004	0.003	68.929
n	3.000	2.000	1.000	3.000	2.250	0.957	42.552

Number of dissolution data points (N), degrees of freedom (df), and selected goodness of fit criteria – Pearson correlation coefficient (R), coefficient of determination (R^2), adjusted coefficient of determination ($R^2_{adjusted}$), and residual sum of squares (RSS) (manual calculation in MS Excel):

Parameter	No.1	No.2	No.3	No.4
N	16	16	16	16
df	14	14	14	14
R	0.998046967	0.999071247	0.996029719	0.999181895
R^2	0.996097748	0.998143357	0.992075201	0.99836446
$R^2_{adjusted}$	0.995819016	0.99801074	0.991509144	0.998247636
RSS	80.20864351	90.31177946	368.5227983	144.6805114

Graphical abstract of model fit presented as mean ± 1 SD of the fraction % of released carvedilol:

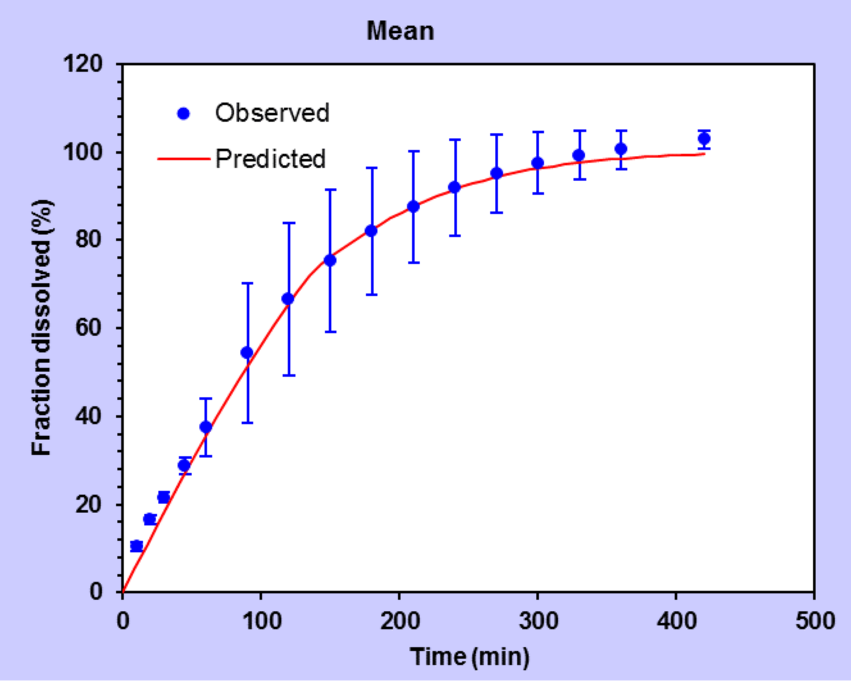

Supplementary materials – Model fitting summary – Pearlitol® 160C (modelling the entire relevant dissolution data)

Graphical abstract of model fit presented as the fraction % of released carvedilol per tested tablet:

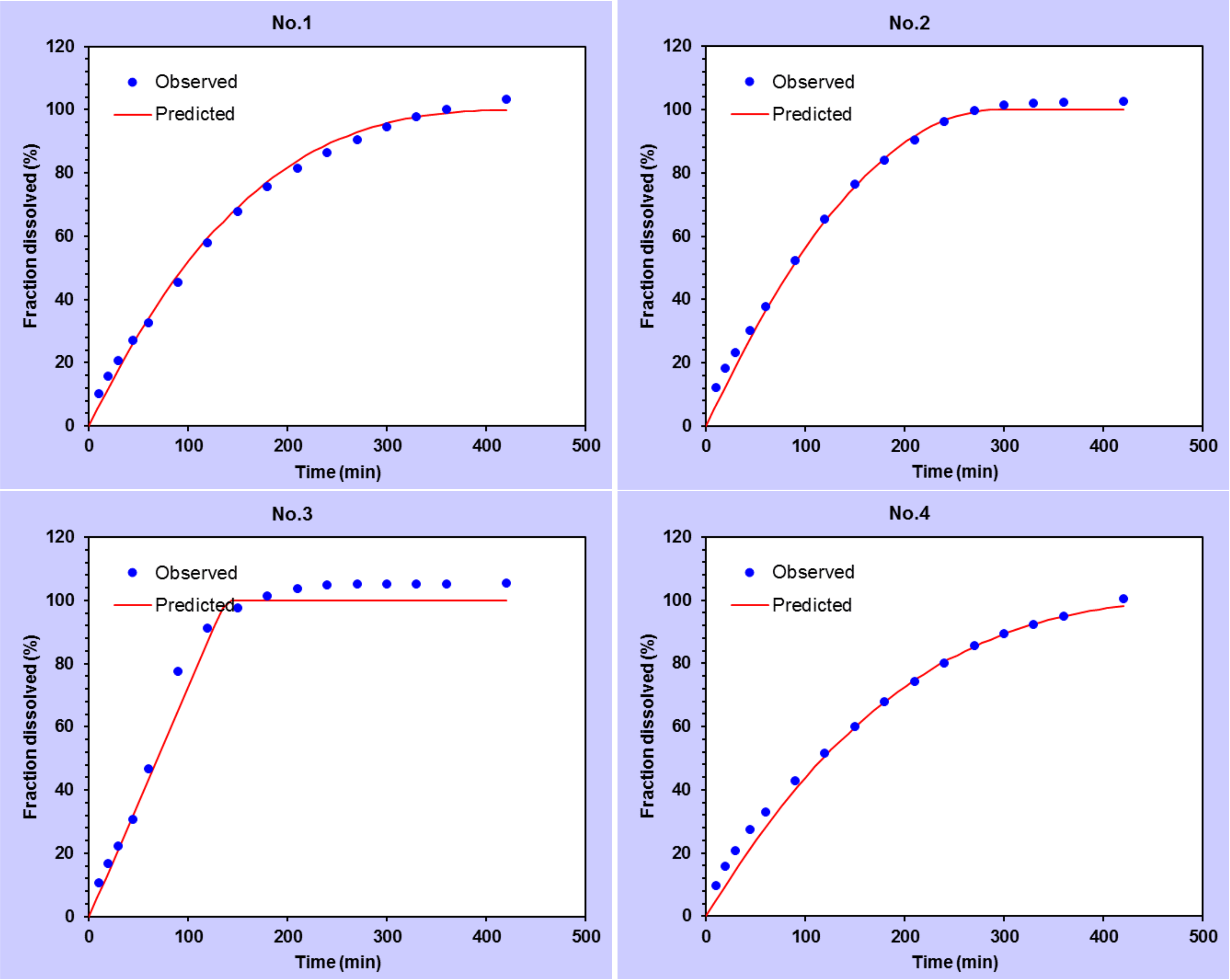

Supplementary materials – Model fitting summary – Pearlitol® 160C (modelling the entire relevant dissolution data)

Model: **Hopfenberg with T_{lag}**

Model equation: $F = 100 \cdot \{1 - [1 - k_{HB} \cdot (t - T_{lag})]^n\}$

Fitted model parameters per tested tablet (N = 4) with statistics – mean, standard deviation (SD), and relative standard deviation expressed in % (RSD%) (output from DDSolver):

Parameter	No.1	No.2	No.3	No.4	Mean	SD	RSD(%)
k_{HB}	0.003	0.003	0.007	0.002	0.004	0.002	63.576
n	2.000	2.000	1.000	3.000	2.000	0.816	40.825
T_{lag}	-13.780	-5.888	-9.123	-11.404	-10.049	3.363	-33.466

Number of dissolution data points (N), degrees of freedom (df), and selected goodness of fit criteria – Pearson correlation coefficient (R), coefficient of determination (R^2), adjusted coefficient of determination ($R^2_{adjusted}$), and residual sum of squares (RSS) (manual calculation in MS Excel):

Parameter	No.1	No.2	No.3	No.4
N	16	16	16	16
df	13	13	13	13
R	0.999143396	0.999306632	0.9961272	0.999416466
R^2	0.998287525	0.998613745	0.992269399	0.998833273
$R^2_{adjusted}$	0.998024068	0.998400476	0.991080075	0.998653777
RSS	31.72632073	28.18514256	357.6679848	17.2663834

Graphical abstract of model fit presented as mean ± 1 SD of the fraction % of released carvedilol:

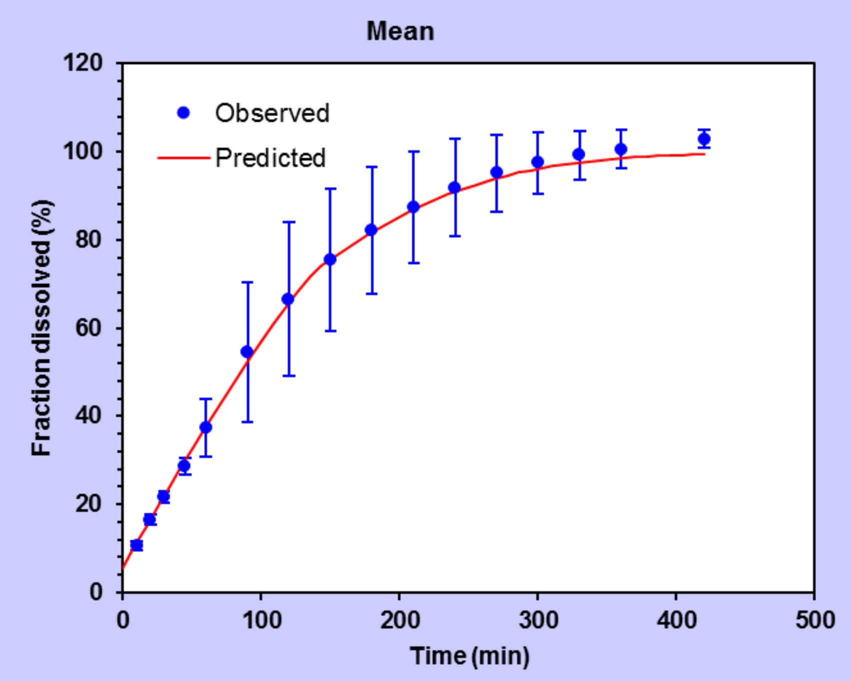

Supplementary materials – Model fitting summary – Pearlitol® 160C (modelling the entire relevant dissolution data)

Graphical abstract of model fit presented as the fraction % of released carvedilol per tested tablet:

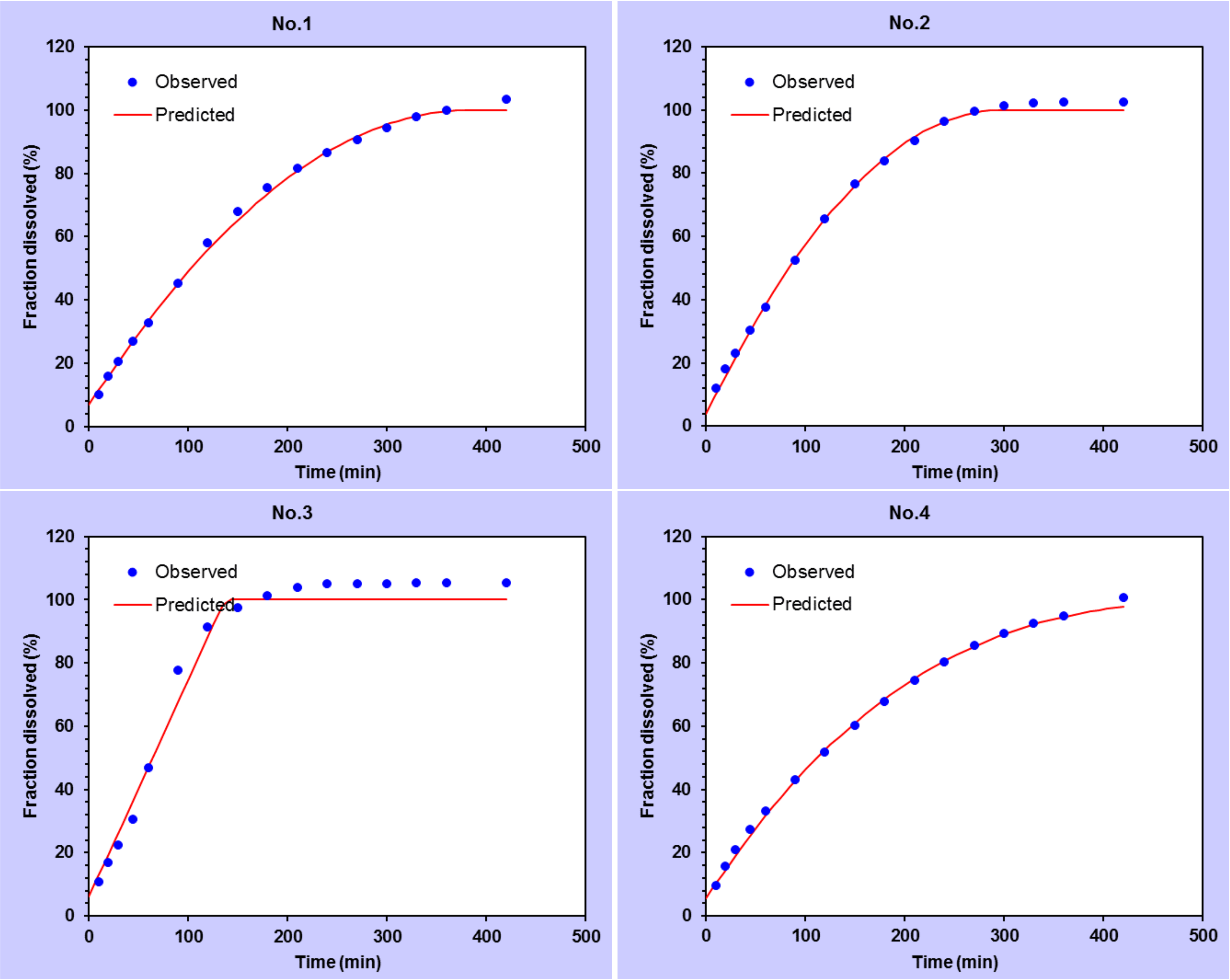

Supplementary materials – Model fitting summary – Pearlitol® 160C (modelling the entire relevant dissolution data)

Model: **Baker–Lonsdale**

Model equation: $\frac{3}{2}\cdot\left[1-\left(1-\frac{F}{100}\right)^{\frac{2}{3}}\right]-\frac{F}{100}=k_{BL}\cdot t$

Fitted model parameters per tested tablet (N = 4) with statistics – mean, standard deviation (SD), and relative standard deviation expressed in % (RSD%) (output from DDSolver):

Parameter	No.1	No.2	No.3	No.4	Mean	SD	RSD(%)
k_{BL}	0.0000	0.0017	0.0030	0.0005	0.0013	0.0013	102.7666

Number of dissolution data points (N), degrees of freedom (df), and selected goodness of fit criteria – Pearson correlation coefficient (R), coefficient of determination (R^2), adjusted coefficient of determination ($R^2_{adjusted}$), and residual sum of squares (RSS) (manual calculation in MS Excel):

Parameter	No.1	No.2	No.3	No.4
N	16	16	16	16
df	15	15	15	15
R	-0.15532633	-0.474968019	-0.712536371	0.997041282
R^2	0.024126269	0.225594619	0.507708081	0.994091318
$R^2_{adjusted}$	0.024126269	0.225594619	0.507708081	0.994091318
RSS	79430.88519	548106.3954	1565638.814	1830.218296

Graphical abstract of model fit presented as mean ± 1 SD of the fraction % of released carvedilol:

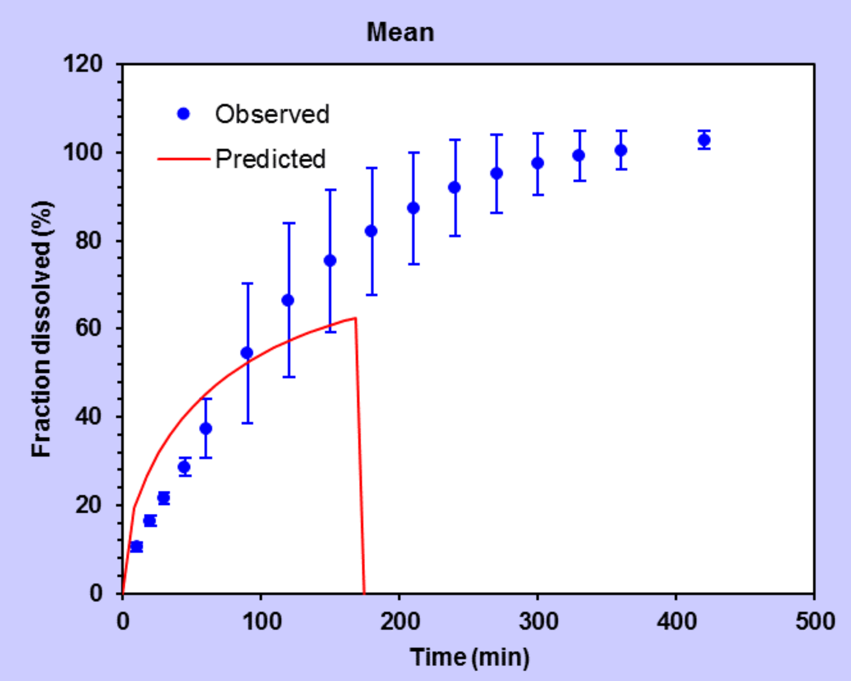

Supplementary materials – Model fitting summary – Pearlitol® 160C (modelling the entire relevant dissolution data)

Graphical abstract of model fit presented as the fraction % of released carvedilol per tested tablet:

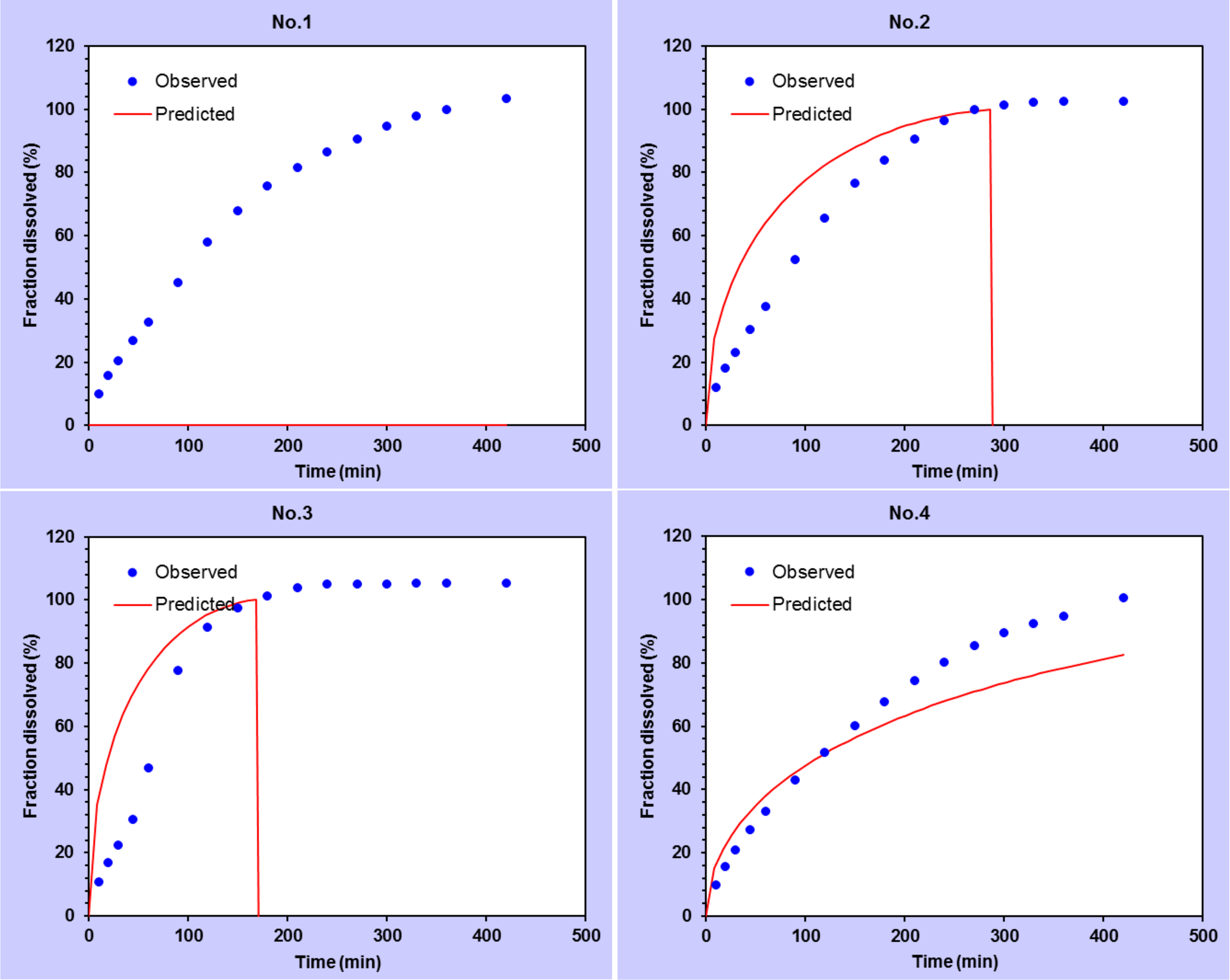

Supplementary materials – Model fitting summary – Pearlitol® 160C (modelling the entire relevant dissolution data)

Model: **Baker–Lonsdale with T_{lag}**

Model equation: $\frac{3}{2}\cdot\left[1-\left(1-\frac{F}{100}\right)^{\frac{2}{3}}\right]-\frac{F}{100}=k_{BL}\cdot\left(t-T_{lag}\right)$

Fitted model parameters per tested tablet (N = 4) with statistics – mean, standard deviation (SD), and relative standard deviation expressed in % (RSD%) (output from DDSolver):

Parameter	No.1	No.2	No.3	No.4	Mean	SD	RSD(%)
k_{BL}	0.001	0.000	0.003	0.001	0.001	0.001	93.700
T_{lag}	39.599	59.820	39.027	36.309	43.689	10.849	24.833

Number of dissolution data points (N), degrees of freedom (df), and selected goodness of fit criteria – Pearson correlation coefficient (R), coefficient of determination (R^2), adjusted coefficient of determination ($R^2_{adjusted}$), and residual sum of squares (RSS) (manual calculation in MS Excel):

Parameter	No.1	No.2	No.3	No.4
N	16	16	16	16
df	14	14	14	14
R	0.982801366	0.828914239	-0.554900659	0.980556987
R^2	0.965898525	0.687098815	0.307914742	0.961492004
$R^2_{adjusted}$	0.963462705	0.664748731	0.258480081	0.958741433
RSS	1041.127268	91974.2902	1256193.652	993.1392671

Graphical abstract of model fit presented as mean ± 1 SD of the fraction % of released carvedilol:

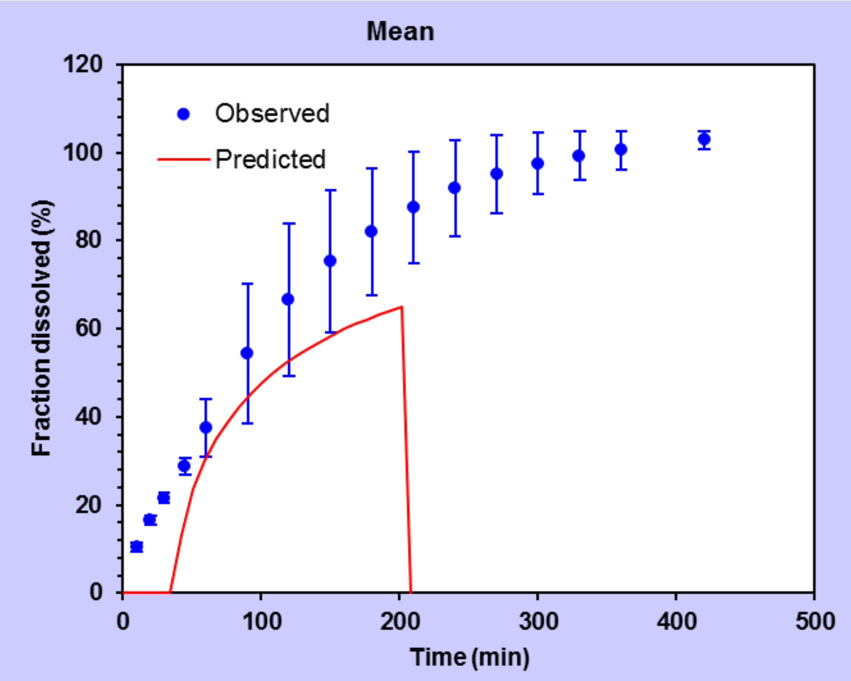

Supplementary materials – Model fitting summary – Pearlitol® 160C (modelling the entire relevant dissolution data)

Graphical abstract of model fit presented as the fraction % of released carvedilol per tested tablet:

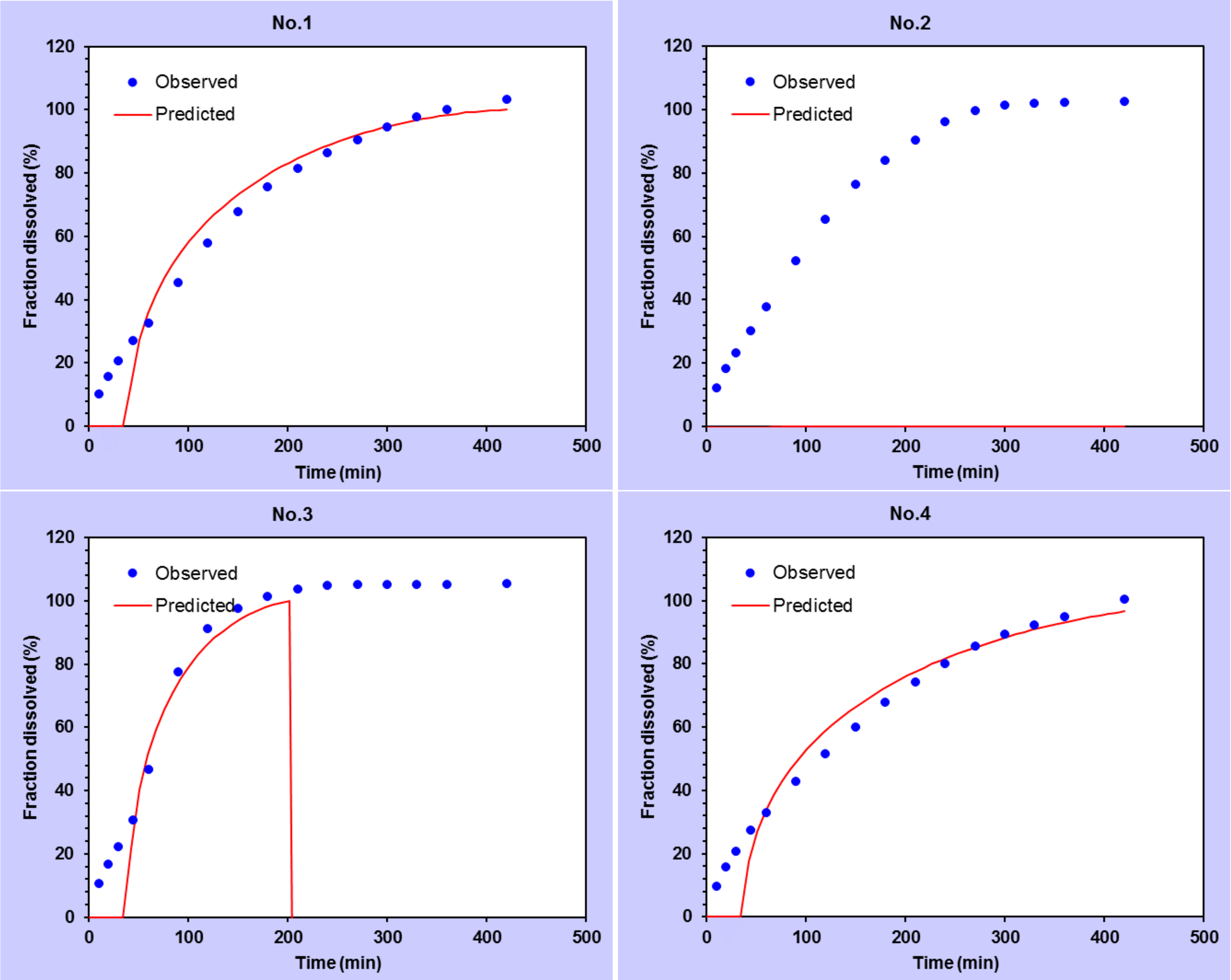

Supplementary materials – Model fitting summary – Pearlitol® 160C (modelling the entire relevant dissolution data)

Model: **Makoid–Banakar**

Model equation: $F = k_{MB} \cdot t^n \cdot e^{-k \cdot t}$

Fitted model parameters per tested tablet (N = 4) with statistics – mean, standard deviation (SD), and relative standard deviation expressed in % (RSD%) (output from DDSolver):

Parameter	No.1	No.2	No.3	No.4	Mean	SD	RSD(%)
k_{MB}	1.609	1.798	0.847	1.822	1.519	0.458	30.134
n	0.768	0.782	1.038	0.721	0.827	0.143	17.259
k	0.001	0.001	0.004	0.001	0.002	0.001	73.412

Number of dissolution data points (N), degrees of freedom (df), and selected goodness of fit criteria – Pearson correlation coefficient (R), coefficient of determination (R^2), adjusted coefficient of determination ($R^2_{adjusted}$), and residual sum of squares (RSS) (manual calculation in MS Excel):

Parameter	No.1	No.2	No.3	No.4
N	16	16	16	16
df	13	13	13	13
R	0.997767052	0.995599764	0.988834503	0.999468389
R^2	0.99553909	0.99121889	0.977793674	0.998937061
$R^2_{adjusted}$	0.994852796	0.98986795	0.974377316	0.998773532
RSS	73.48677867	155.8158972	465.8716611	15.20408728

Graphical abstract of model fit presented as mean ± 1 SD of the fraction % of released carvedilol:

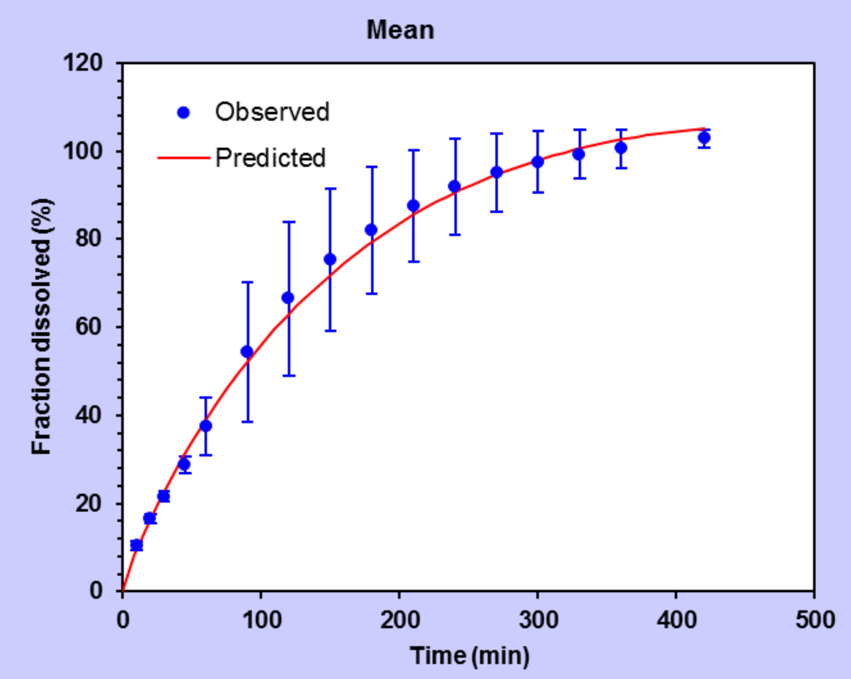

Supplementary materials – Model fitting summary – Pearlitol® 160C (modelling the entire relevant dissolution data)

Graphical abstract of model fit presented as the fraction % of released carvedilol per tested tablet:

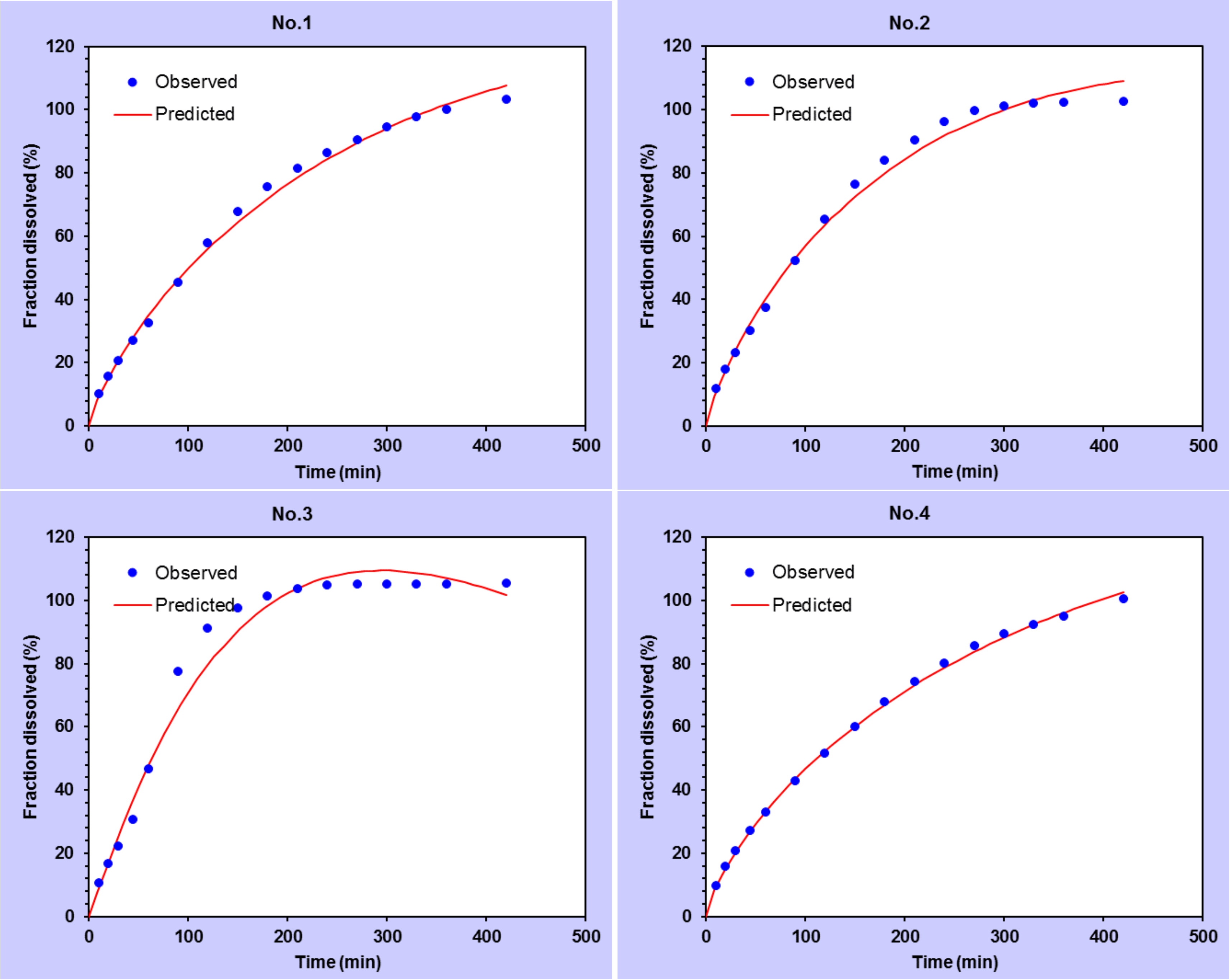

Supplementary materials – Model fitting summary – Pearlitol® 160C (modelling the entire relevant dissolution data)

Model: **Makoid–Banakar with T_{lag}**

Model equation: $F = k_{MB} \cdot (t - T_{lag})^n \cdot e^{-k \cdot (t - T_{lag})}$

Fitted model parameters per tested tablet (N = 4) with statistics – mean, standard deviation (SD), and relative standard deviation expressed in % (RSD%) (output from DDSolver):

Parameter	No.1	No.2	No.3	No.4	Mean	SD	RSD(%)
k_{MB}	2.865	3.251	1.851	3.098	2.767	0.630	22.784
n	0.631	0.641	0.849	0.596	0.679	0.115	16.938
k	0.000	0.001	0.003	0.000	0.001	0.001	115.413
T_{lag}	4.000	4.000	4.000	4.000	4.000	0.000	0.000

Number of dissolution data points (N), degrees of freedom (df), and selected goodness of fit criteria – Pearson correlation coefficient (R), coefficient of determination (R^2), adjusted coefficient of determination ($R^2_{adjusted}$), and residual sum of squares (RSS) (manual calculation in MS Excel):

Parameter	No.1	No.2	No.3	No.4
N	16	16	16	16
df	12	12	12	12
R	0.994208451	0.990239462	0.984884114	0.997805512
R^2	0.988450444	0.980574192	0.969996718	0.995615839
$R^2_{adjusted}$	0.985563055	0.975717739	0.962495898	0.994519799
RSS	190.8496185	342.341089	638.7816783	62.93117204

Graphical abstract of model fit presented as mean ± 1 SD of the fraction % of released carvedilol:

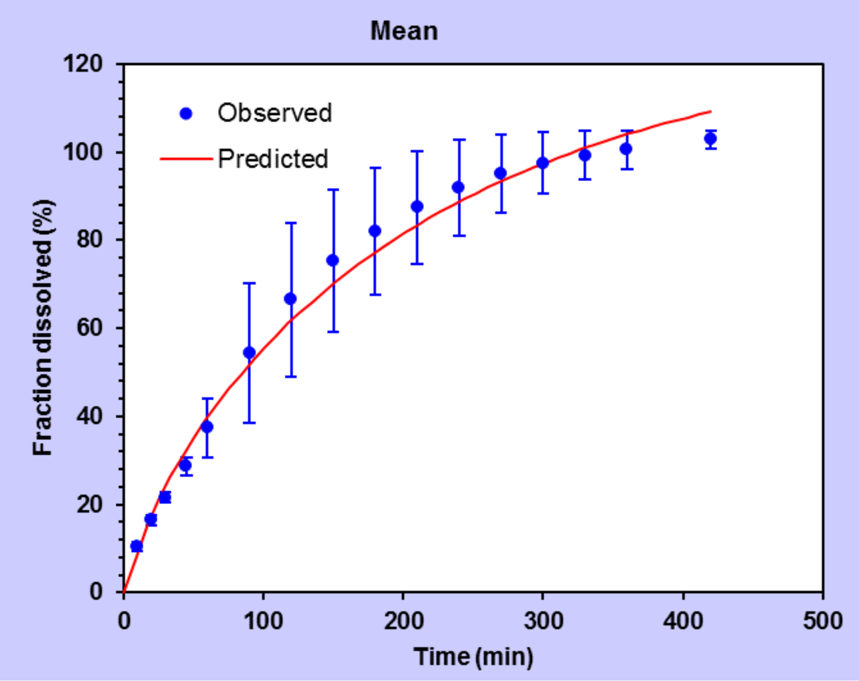

Supplementary materials – Model fitting summary – Pearlitol® 160C (modelling the entire relevant dissolution data)

Graphical abstract of model fit presented as the fraction % of released carvedilol per tested tablet:

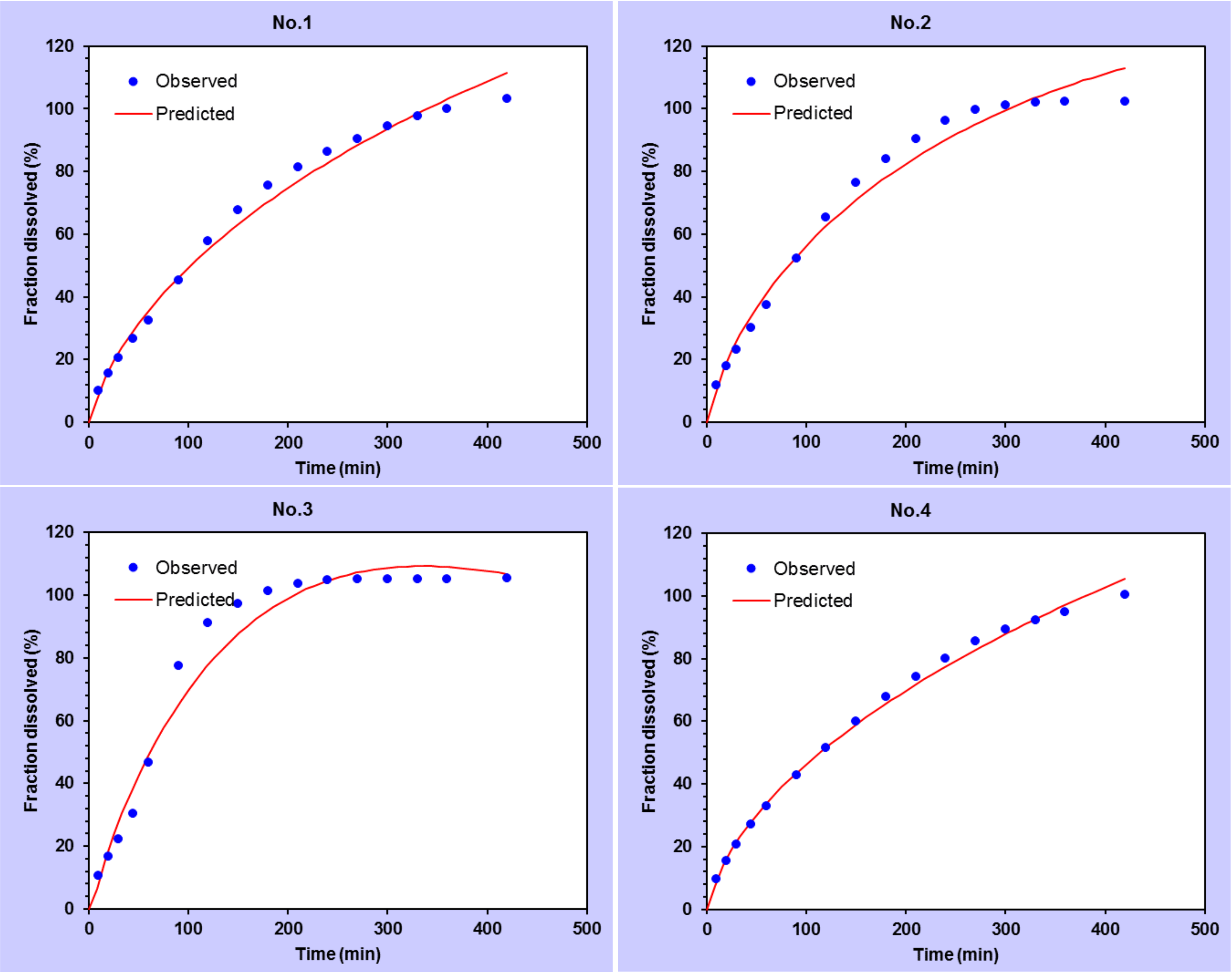

Supplementary materials – Model fitting summary – Pearlitol® 160C (modelling the entire relevant dissolution data)

Model: **Peppas–Sahlin_1**

Model equation: $F = k_1 \cdot t^m + k_2 \cdot t^{2m}$

Fitted model parameters per tested tablet (N = 4) with statistics – mean, standard deviation (SD), and relative standard deviation expressed in % (RSD%) (output from DDSolver):

Parameter	No.1	No.2	No.3	No.4	Mean	SD	RSD(%)
k_1	4.898	6.482	9.550	4.390	6.330	2.324	36.712
k_2	0.170	0.081	-0.108	0.177	0.080	0.133	166.477
m	0.450	0.450	0.450	0.450	0.450	0.000	0.000

Number of dissolution data points (N), degrees of freedom (df), and selected goodness of fit criteria – Pearson correlation coefficient (R), coefficient of determination (R^2), adjusted coefficient of determination ($R^2_{adjusted}$), and residual sum of squares (RSS) (manual calculation in MS Excel):

Parameter	No.1	No.2	No.3	No.4
N	16	16	16	16
df	13	13	13	13
R	0.988191921	0.977602827	0.942884338	0.994663148
R^2	0.976523274	0.955707287	0.889030874	0.989354777
$R^2_{adjusted}$	0.97291147	0.948893023	0.871958701	0.987717051
RSS	426.0444575	871.3319355	2779.744668	168.79479

Graphical abstract of model fit presented as mean ± 1 SD of the fraction % of released carvedilol:

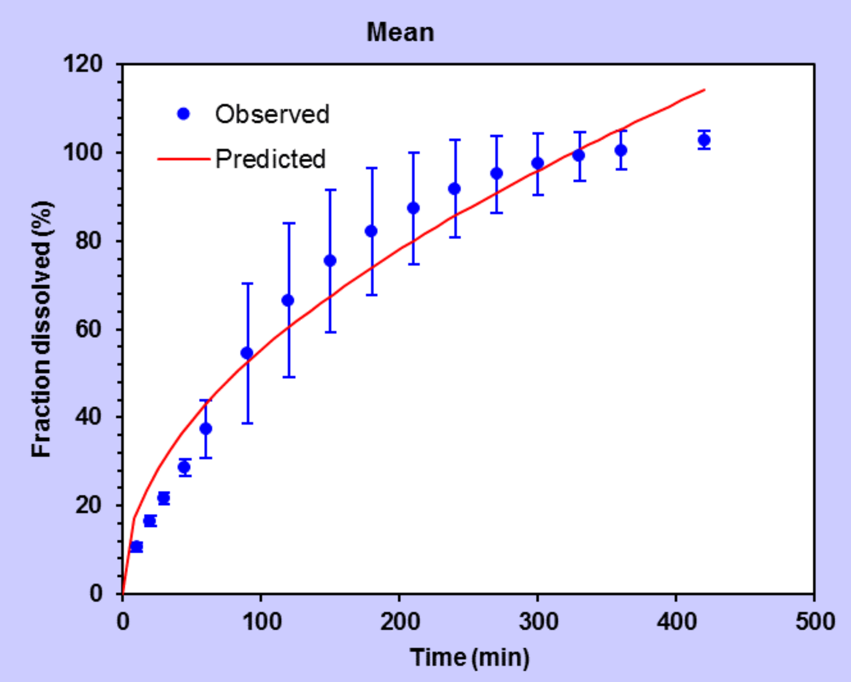

Supplementary materials – Model fitting summary – Pearlitol® 160C (modelling the entire relevant dissolution data)

Graphical abstract of model fit presented as the fraction % of released carvedilol per tested tablet:

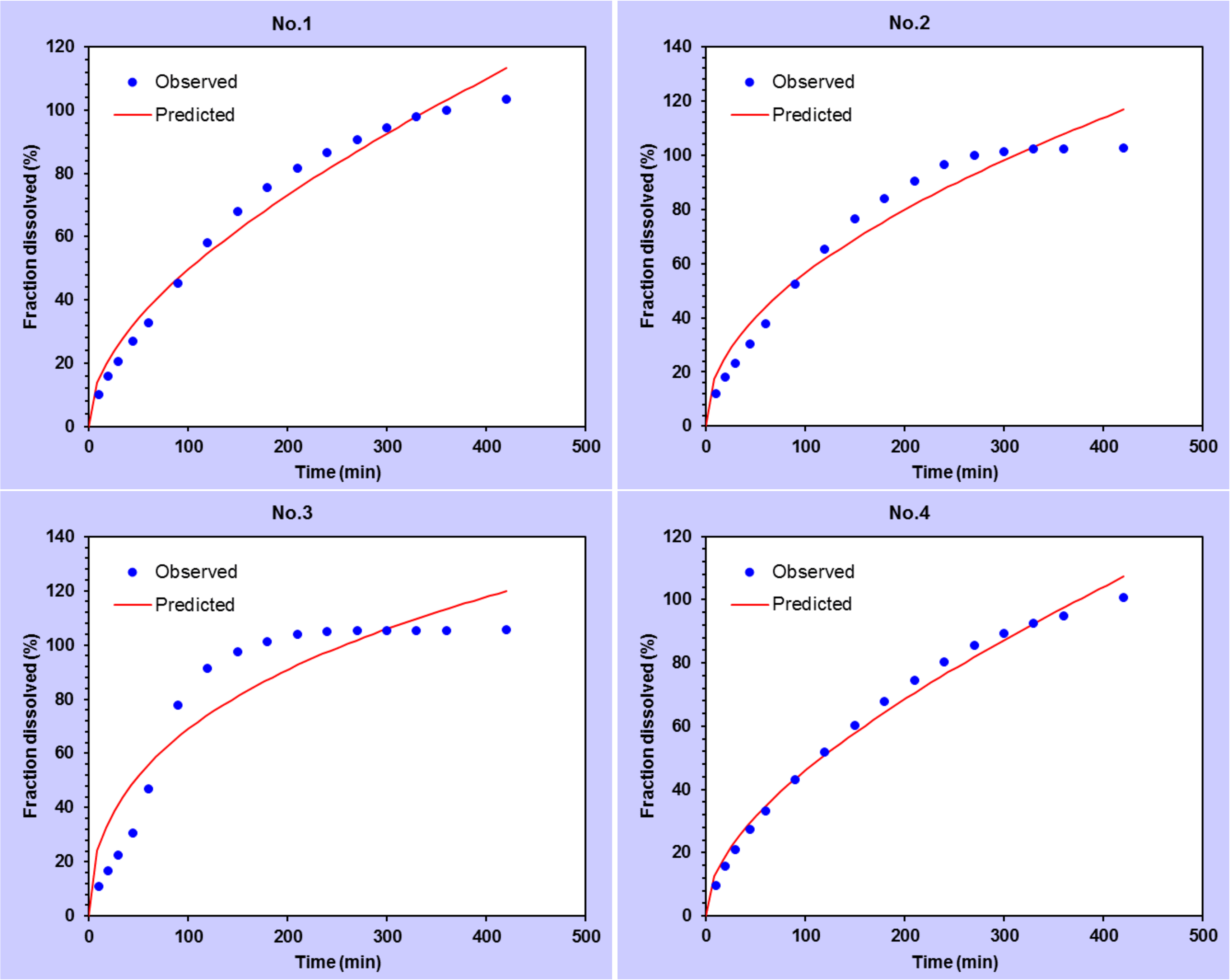

Supplementary materials – Model fitting summary – Pearlitol® 160C (modelling the entire relevant dissolution data)

Model: **Peppas–Sahlin_1 with T_{lag}**

Model equation: $F = k_1 \cdot (t - T_{lag})^m + k_2 \cdot (t - T_{lag})^{2m}$

Fitted model parameters per tested tablet (N = 4) with statistics – mean, standard deviation (SD), and relative standard deviation expressed in % (RSD%) (output from DDSolver):

Parameter	No.1	No.2	No.3	No.4	Mean	SD	RSD(%)
k_1	5.375	7.030	10.264	4.822	6.873	2.448	35.615
k_2	0.137	0.043	-0.160	0.148	0.042	0.143	340.864
m	0.450	0.450	0.450	0.450	0.450	0.000	0.000
T_{lag}	6.000	6.000	6.000	6.000	6.000	0.000	0.000

Number of dissolution data points (N), degrees of freedom (df), and selected goodness of fit criteria – Pearson correlation coefficient (R), coefficient of determination (R^2), adjusted coefficient of determination ($R^2_{adjusted}$), and residual sum of squares (RSS) (manual calculation in MS Excel):

Parameter	No.1	No.2	No.3	No.4
N	16	16	16	16
df	12	12	12	12
R	0.990483296	0.981280179	0.952594793	0.99617428
R^2	0.98105716	0.96291079	0.907436839	0.992363196
$R^2_{adjusted}$	0.976321451	0.953638487	0.884296048	0.990453995
RSS	324.0322154	689.9246993	2241.670971	112.7028364

Graphical abstract of model fit presented as mean ± 1 SD of the fraction % of released carvedilol:

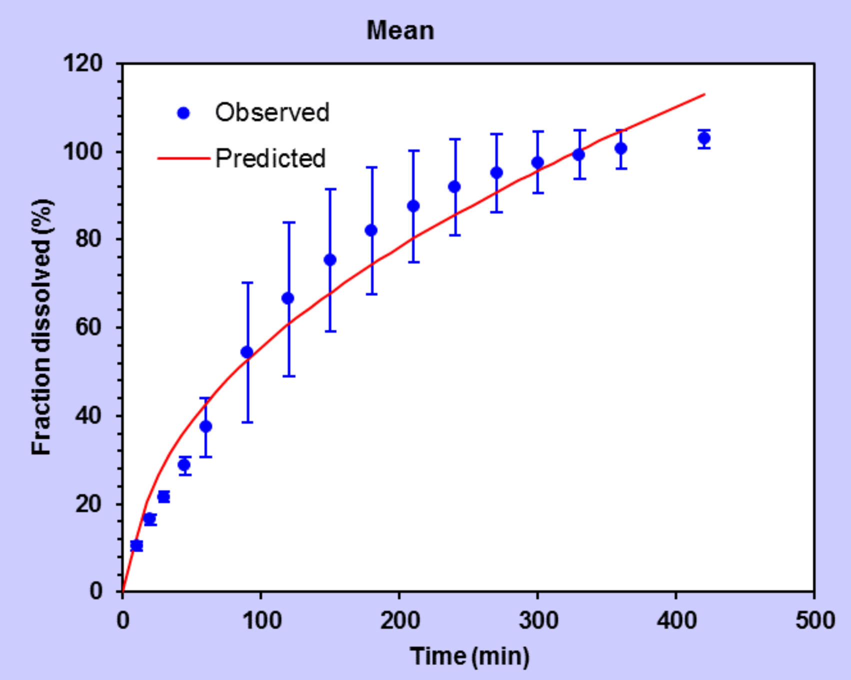

Supplementary materials – Model fitting summary – Pearlitol® 160C (modelling the entire relevant dissolution data)

Graphical abstract of model fit presented as the fraction % of released carvedilol per tested tablet:

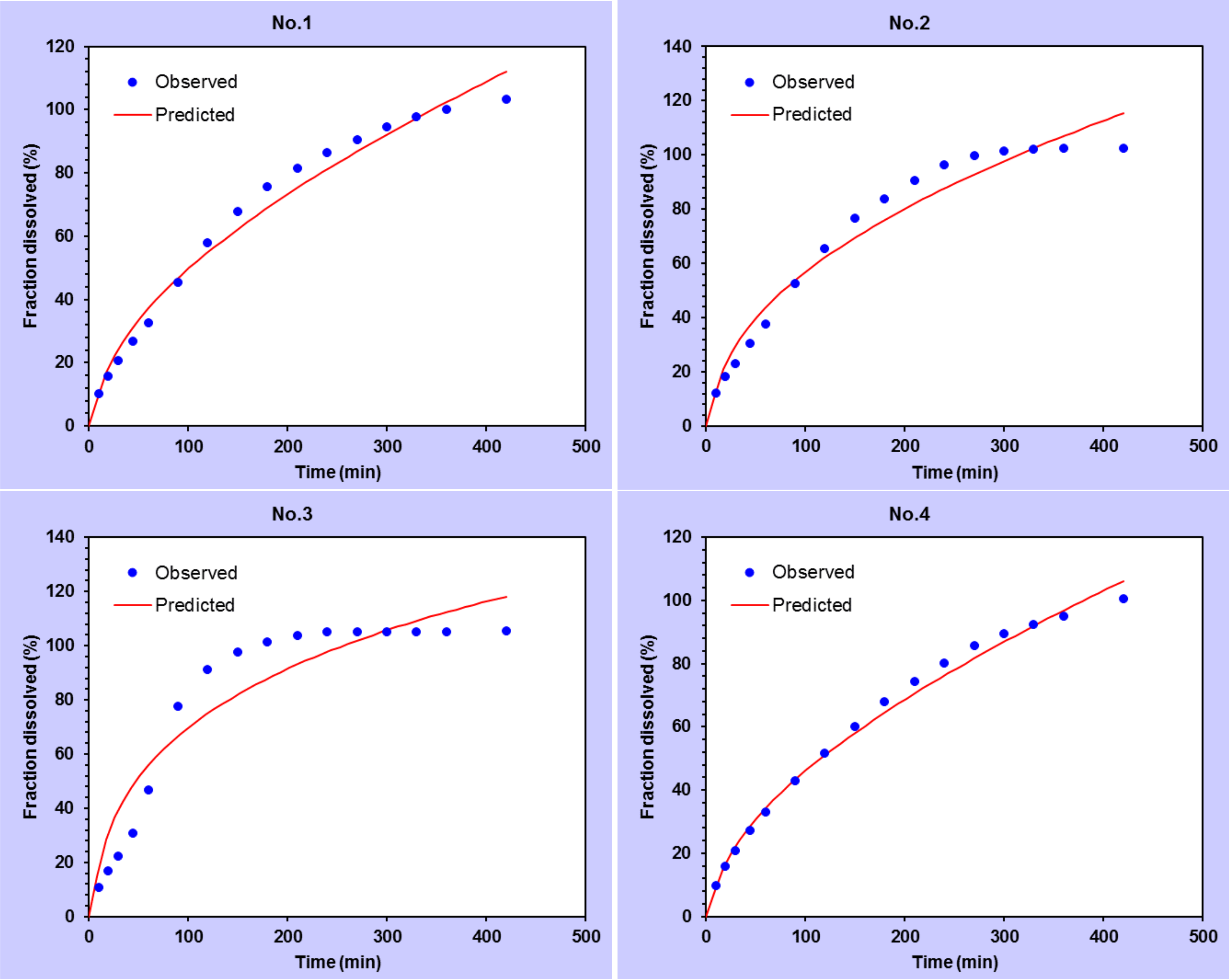

Supplementary materials – Model fitting summary – Pearlitol® 160C (modelling the entire relevant dissolution data)

Model: **Peppas–Sahlin_2**

Model equation: $F = k_1 \cdot t^{0.5} + k_2 \cdot t$

Fitted model parameters per tested tablet (N = 4) with statistics – mean, standard deviation (SD), and relative standard deviation expressed in % (RSD%) (output from DDSolver):

Parameter	No.1	No.2	No.3	No.4	Mean	SD	RSD(%)
k_1	4.572	5.843	8.304	4.108	5.707	1.880	32.951
k_2	0.044	-0.011	-0.126	0.053	-0.010	0.083	-847.520

Number of dissolution data points (N), degrees of freedom (df), and selected goodness of fit criteria – Pearson correlation coefficient (R), coefficient of determination (R^2), adjusted coefficient of determination ($R^2_{adjusted}$), and residual sum of squares (RSS) (manual calculation in MS Excel):

Parameter	No.1	No.2	No.3	No.4
N	16	16	16	16
df	14	14	14	14
R	0.989596272	0.981030557	0.953911885	0.995383239
R^2	0.979300781	0.962420953	0.909947884	0.990787793
$R^2_{adjusted}$	0.977822265	0.959736736	0.90351559	0.990129778
RSS	393.3436567	779.7262543	2454.192111	152.7813392

Graphical abstract of model fit presented as mean ± 1 SD of the fraction % of released carvedilol:

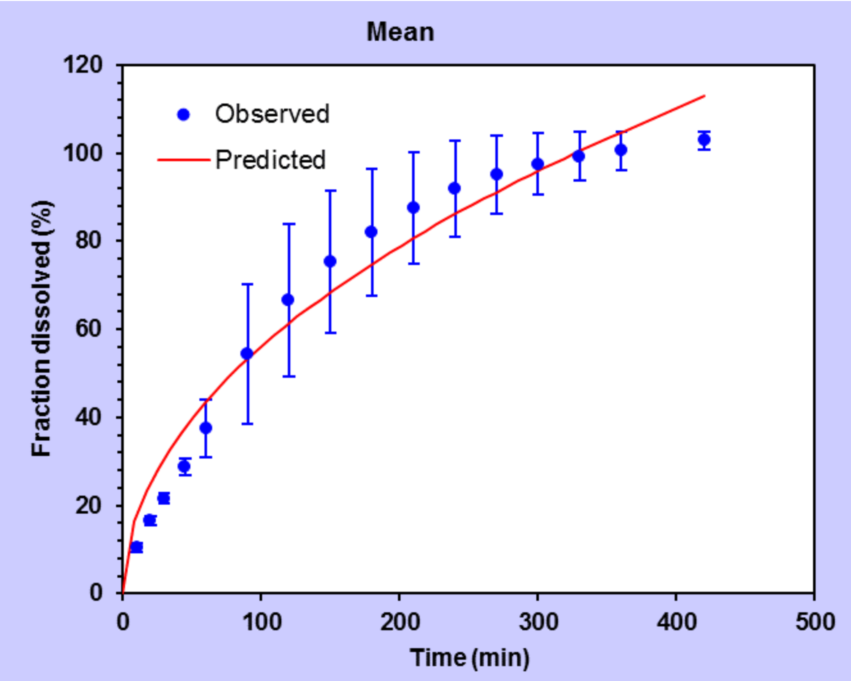

Supplementary materials – Model fitting summary – Pearlitol® 160C (modelling the entire relevant dissolution data)

Graphical abstract of model fit presented as the fraction % of released carvedilol per tested tablet:

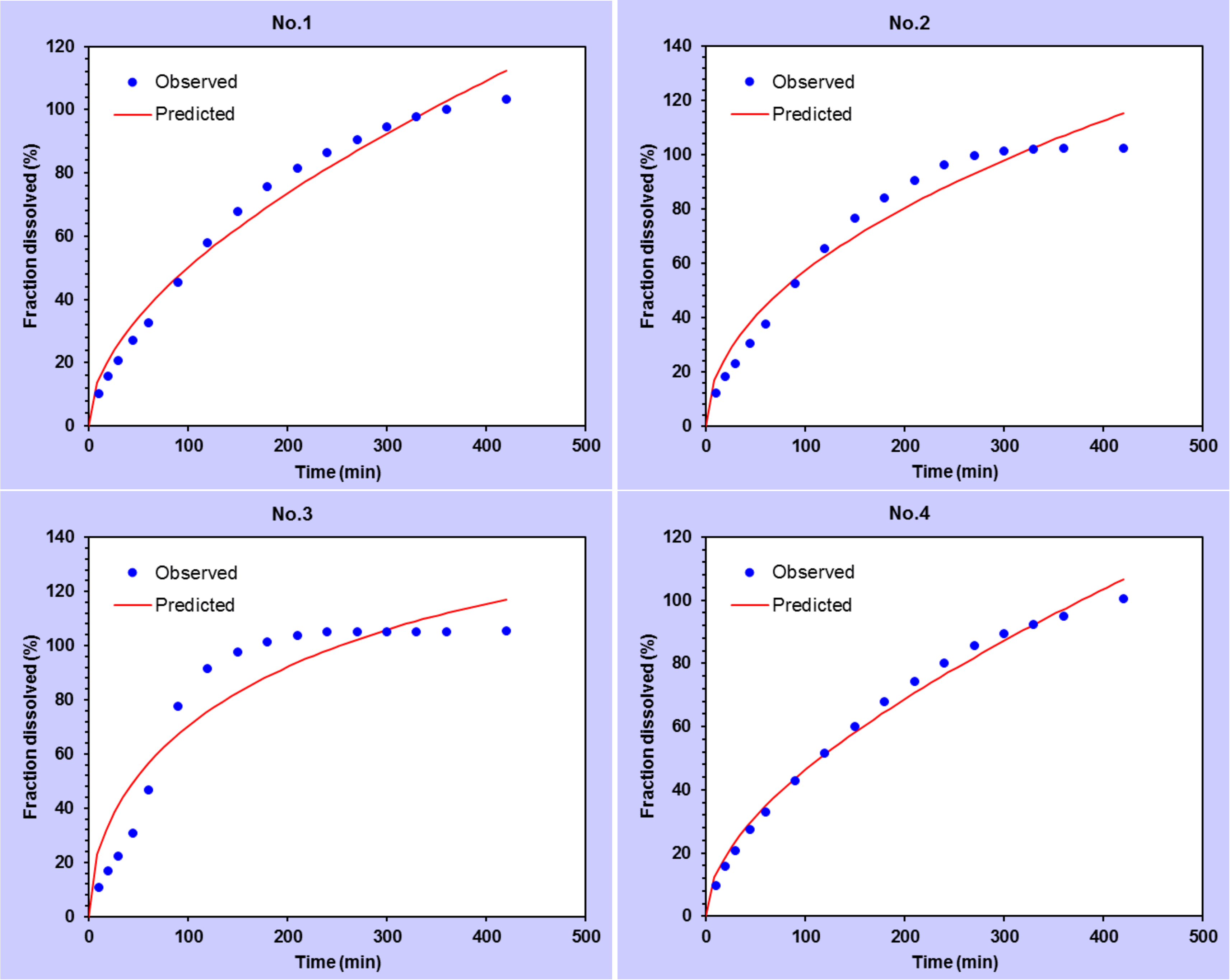

Supplementary materials – Model fitting summary – Pearlitol® 160C (modelling the entire relevant dissolution data)

Model: **Peppas–Sahlin_2 with T_{lag}**

Model equation: $F = k_1 \cdot (t - T_{lag})^{0.5} + k_2 \cdot (t - T_{lag})$

Fitted model parameters per tested tablet (N = 4) with statistics – mean, standard deviation (SD), and relative standard deviation expressed in % (RSD%) (output from DDSolver):

Parameter	No.1	No.2	No.3	No.4	Mean	SD	RSD(%)
k_1	4.927	6.252	8.841	4.428	6.112	1.976	32.324
k_2	0.026	-0.032	-0.156	0.037	-0.031	0.089	-285.790
T_{lag}	6.000	6.000	6.000	6.000	6.000	0.000	0.000

Number of dissolution data points (N), degrees of freedom (df), and selected goodness of fit criteria – Pearson correlation coefficient (R), coefficient of determination (R^2), adjusted coefficient of determination ($R^2_{adjusted}$), and residual sum of squares (RSS) (manual calculation in MS Excel):

Parameter	No.1	No.2	No.3	No.4
N	16	16	16	16
df	13	13	13	13
R	0.991747002	0.984315766	0.962255379	0.996749115
R^2	0.983562115	0.968877528	0.925935415	0.993508799
$R^2_{adjusted}$	0.98103321	0.964089455	0.914540863	0.992510153
RSS	286.0345445	592.579233	1899.100298	96.48295786

Graphical abstract of model fit presented as mean ± 1 SD of the fraction % of released carvedilol:

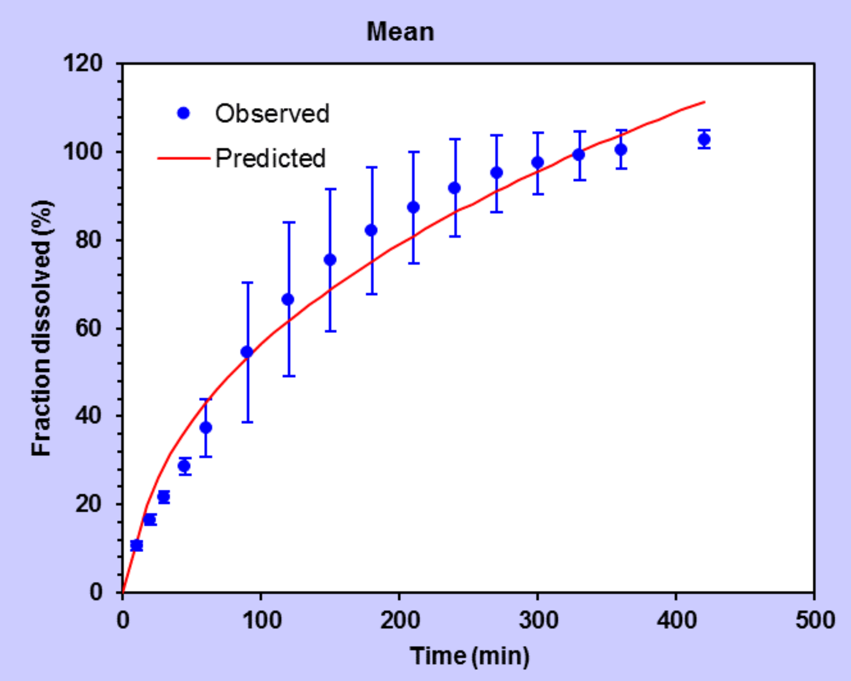

Supplementary materials – Model fitting summary – Pearlitol® 160C (modelling the entire relevant dissolution data)

Graphical abstract of model fit presented as the fraction % of released carvedilol per tested tablet:

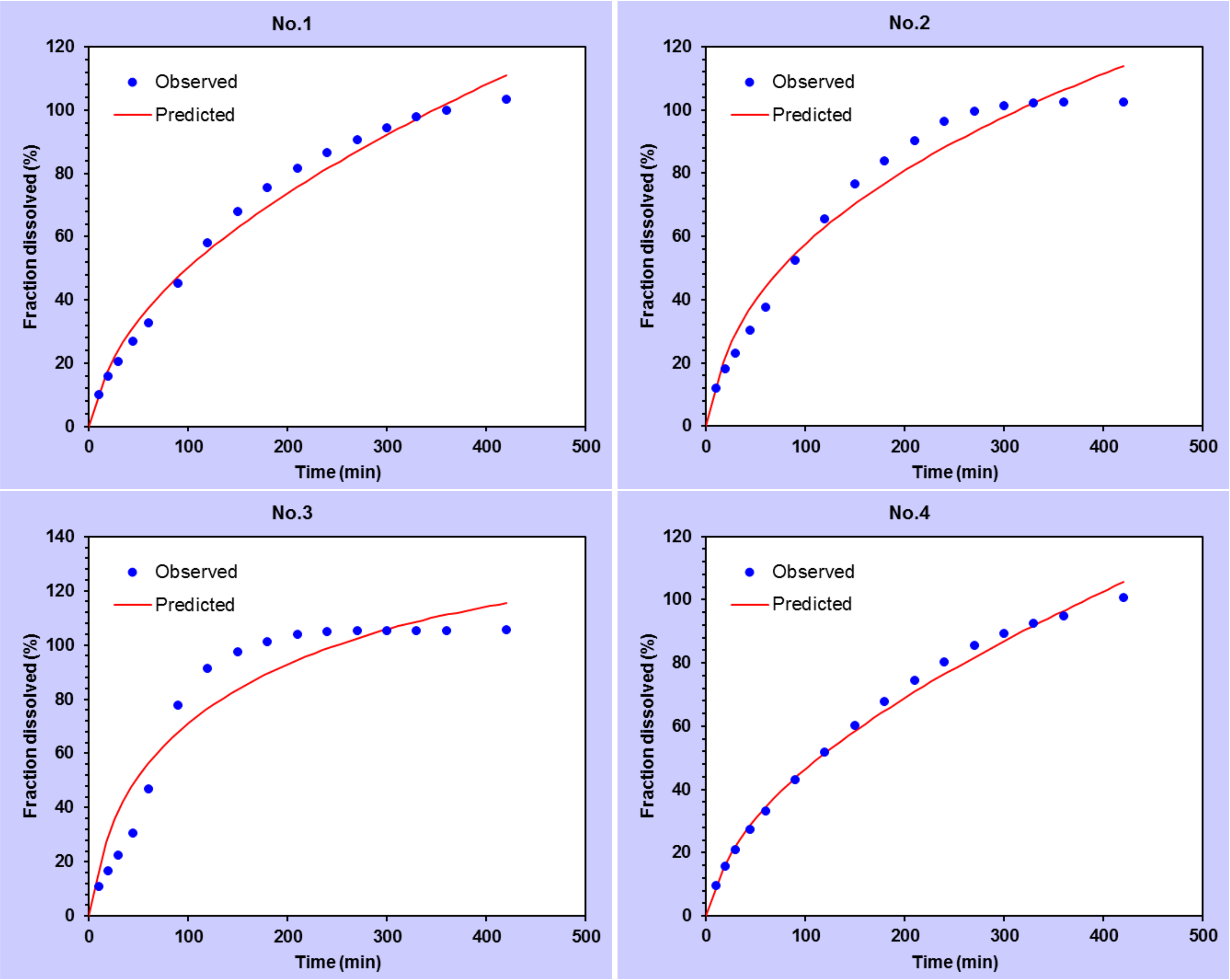

Supplementary materials – Model fitting summary – Pearlitol® 160C (modelling the entire relevant dissolution data)

Model: **Quadratic**

Model equation: $F = 100 \cdot (k_1 \cdot t^2 + k_2 \cdot t)$

Fitted model parameters per tested tablet (N = 4) with statistics – mean, standard deviation (SD), and relative standard deviation expressed in % (RSD%) (output from DDSolver):

Parameter	No.1	No.2	No.3	No.4	Mean	SD	RSD(%)
k_1	-0.000008	-0.000010	-0.000014	-0.000006	-0.000009	0.000003	-35.446083
k_2	0.005515	0.006433	0.008045	0.004986	0.006245	0.001341	21.474412

Number of dissolution data points (N), degrees of freedom (df), and selected goodness of fit criteria – Pearson correlation coefficient (R), coefficient of determination (R^2), adjusted coefficient of determination ($R^2_{adjusted}$), and residual sum of squares (RSS) (manual calculation in MS Excel):

Parameter	No.1	No.2	No.3	No.4
N	16	16	16	16
df	14	14	14	14
R	0.998259875	0.998351899	0.975290442	0.997666793
R^2	0.996522779	0.996706515	0.951191446	0.99533903
$R^2_{adjusted}$	0.996274406	0.996471266	0.94770512	0.995006104
RSS	154.6520253	180.7274381	1146.055324	221.8521594

Graphical abstract of model fit presented as mean ± 1 SD of the fraction % of released carvedilol:

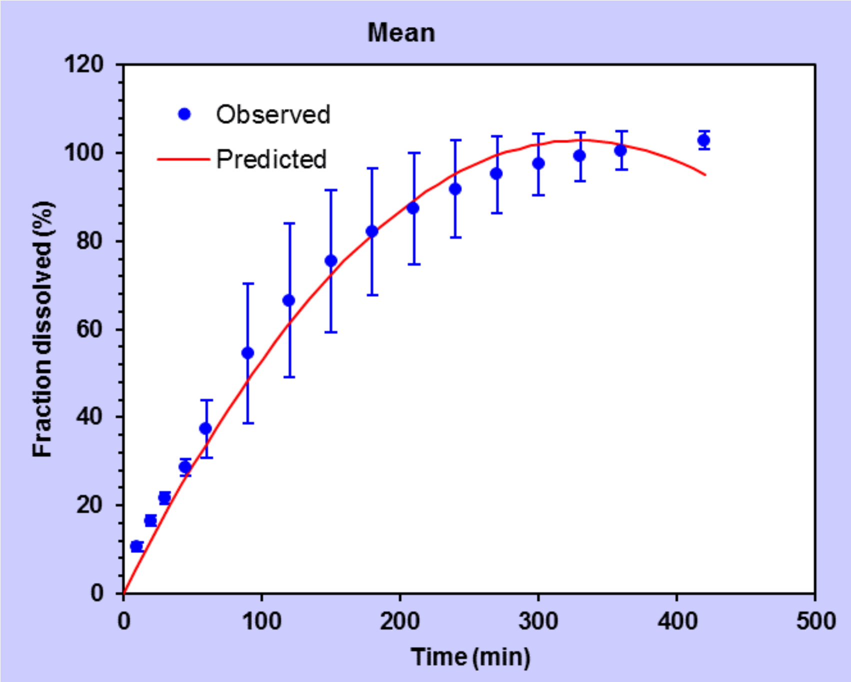

Supplementary materials – Model fitting summary – Pearlitol® 160C (modelling the entire relevant dissolution data)

Graphical abstract of model fit presented as the fraction % of released carvedilol per tested tablet:

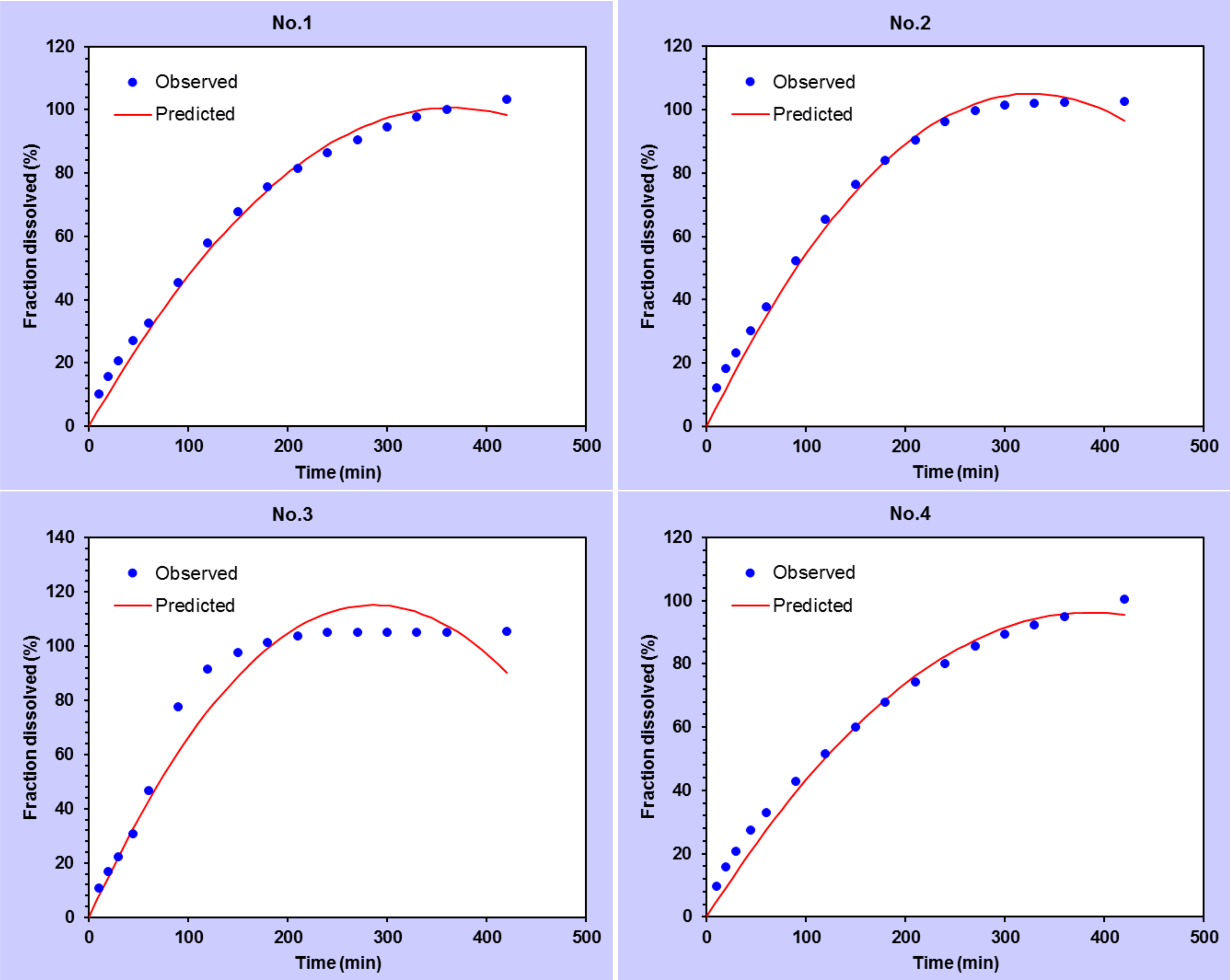

Supplementary materials – Model fitting summary – Pearlitol® 160C (modelling the entire relevant dissolution data)

Model: **Quadratic with T_{lag}**

Model equation: $F = 100 \cdot \left[k_1 \cdot (t - T_{lag})^2 + k_2 \cdot (t - T_{lag})\right]$

Fitted model parameters per tested tablet (N = 4) with statistics – mean, standard deviation (SD), and relative standard deviation expressed in % (RSD%) (output from DDSolver):

Parameter	No.1	No.2	No.3	No.4	Mean	SD	RSD(%)
k_1	-0.000008	-0.000010	-0.000015	-0.000007	-0.000010	0.000003	-34.906635
k_2	0.005663	0.006599	0.008243	0.005118	0.006405	0.001369	21.370853
T_{lag}	4.000000	4.000000	4.000000	4.000000	4.000000	0.000000	0.000000

Number of dissolution data points (N), degrees of freedom (df), and selected goodness of fit criteria – Pearson correlation coefficient (R), coefficient of determination (R^2), adjusted coefficient of determination ($R^2_{adjusted}$), and residual sum of squares (RSS) (manual calculation in MS Excel):

Parameter	No.1	No.2	No.3	No.4
N	16	16	16	16
df	13	13	13	13
R	0.997805086	0.997975515	0.975121138	0.997066527
R^2	0.995614989	0.995955129	0.950861234	0.994141658
$R^2_{adjusted}$	0.994940372	0.995332841	0.943301424	0.993240375
RSS	257.5556003	312.3402816	1315.770787	335.7363676

Graphical abstract of model fit presented as mean ± 1 SD of the fraction % of released carvedilol:

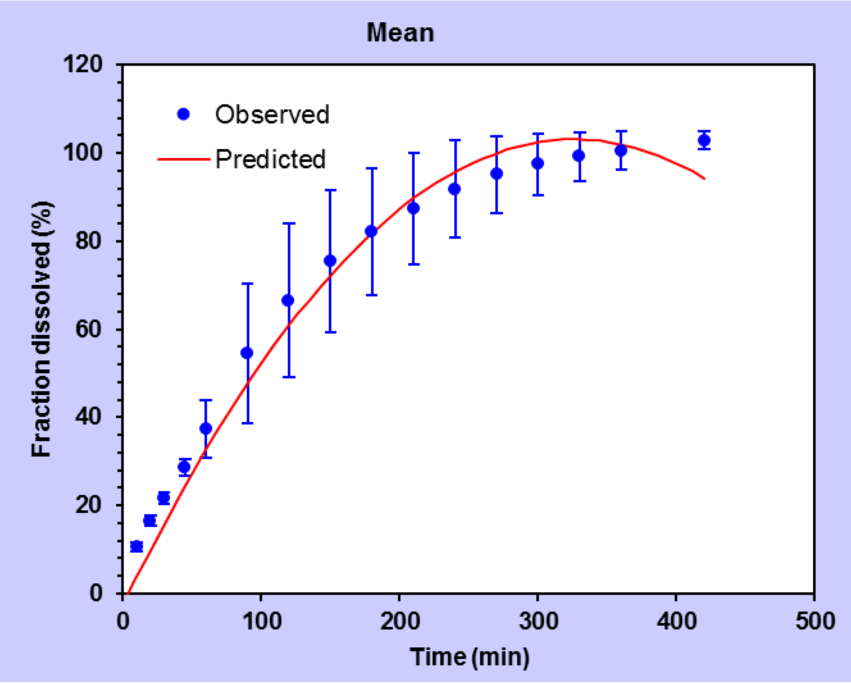

Supplementary materials – Model fitting summary – Pearlitol® 160C (modelling the entire relevant dissolution data)

Graphical abstract of model fit presented as the fraction % of released carvedilol per tested tablet:

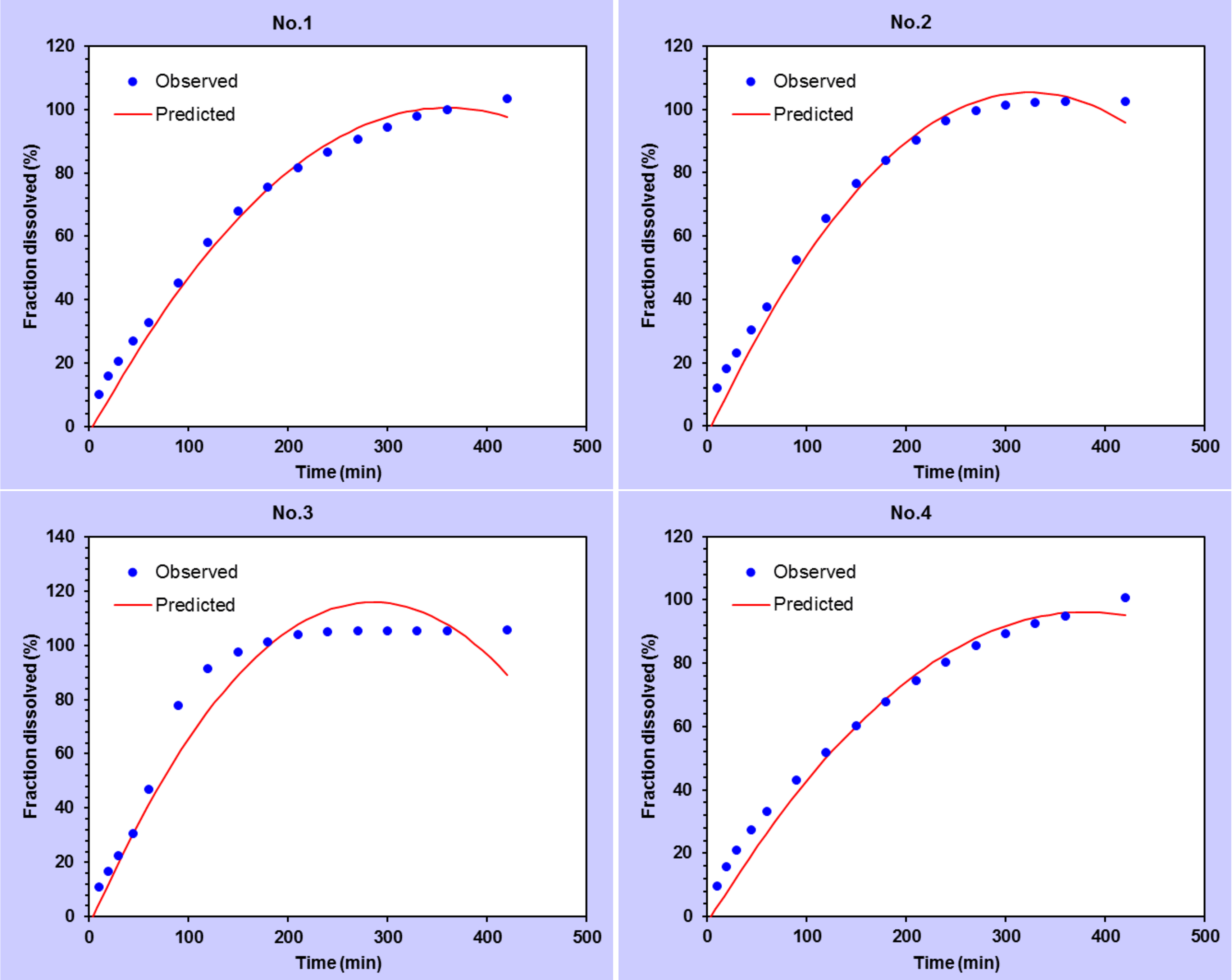

Supplementary materials – Model fitting summary – Pearlitol® 160C (modelling the entire relevant dissolution data)

Model: **Weibull_1**

Model equation: $F = 100 \cdot \left[1 - e^{-\frac{(t-T_i)^{\beta}}{\alpha}}\right]$

Fitted model parameters per tested tablet (N = 4) with statistics – mean, standard deviation (SD), and relative standard deviation expressed in % (RSD%) (output from DDSolver):

Parameter	No.1	No.2	No.3	No.4	Mean	SD	RSD(%)
α	90.511	73.296	112.246	62.933	84.746	21.574	25.458
β	0.964	0.959	1.129	0.844	0.974	0.117	12.056
T_i	6.000	6.000	6.000	6.000	6.000	0.000	0.000

Number of dissolution data points (N), degrees of freedom (df), and selected goodness of fit criteria – Pearson correlation coefficient (R), coefficient of determination (R^2), adjusted coefficient of determination ($R^2_{adjusted}$), and residual sum of squares (RSS) (manual calculation in MS Excel):

Parameter	No.1	No.2	No.3	No.4
N	16	16	16	16
df	13	13	13	13
R	0.988831428	0.987653053	0.990627752	0.990280691
R^2	0.977787594	0.975458554	0.981343343	0.980655846
$R^2_{adjusted}$	0.9743703	0.971682947	0.978473088	0.977679823
RSS	398.3175424	460.6179428	657.1958835	300.2113238

Graphical abstract of model fit presented as mean ± 1 SD of the fraction % of released carvedilol:

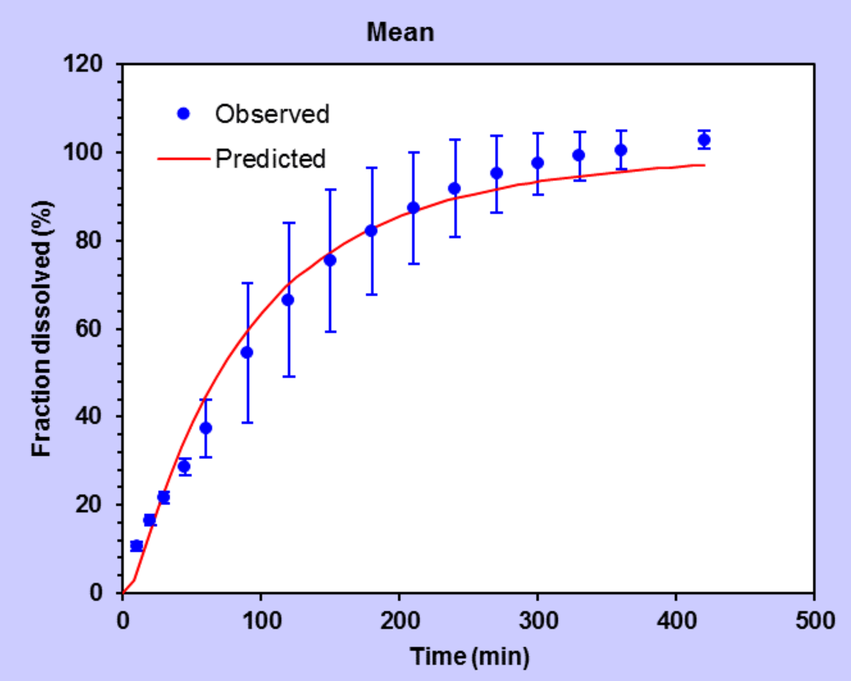

Supplementary materials – Model fitting summary – Pearlitol® 160C (modelling the entire relevant dissolution data)

Graphical abstract of model fit presented as the fraction % of released carvedilol per tested tablet:

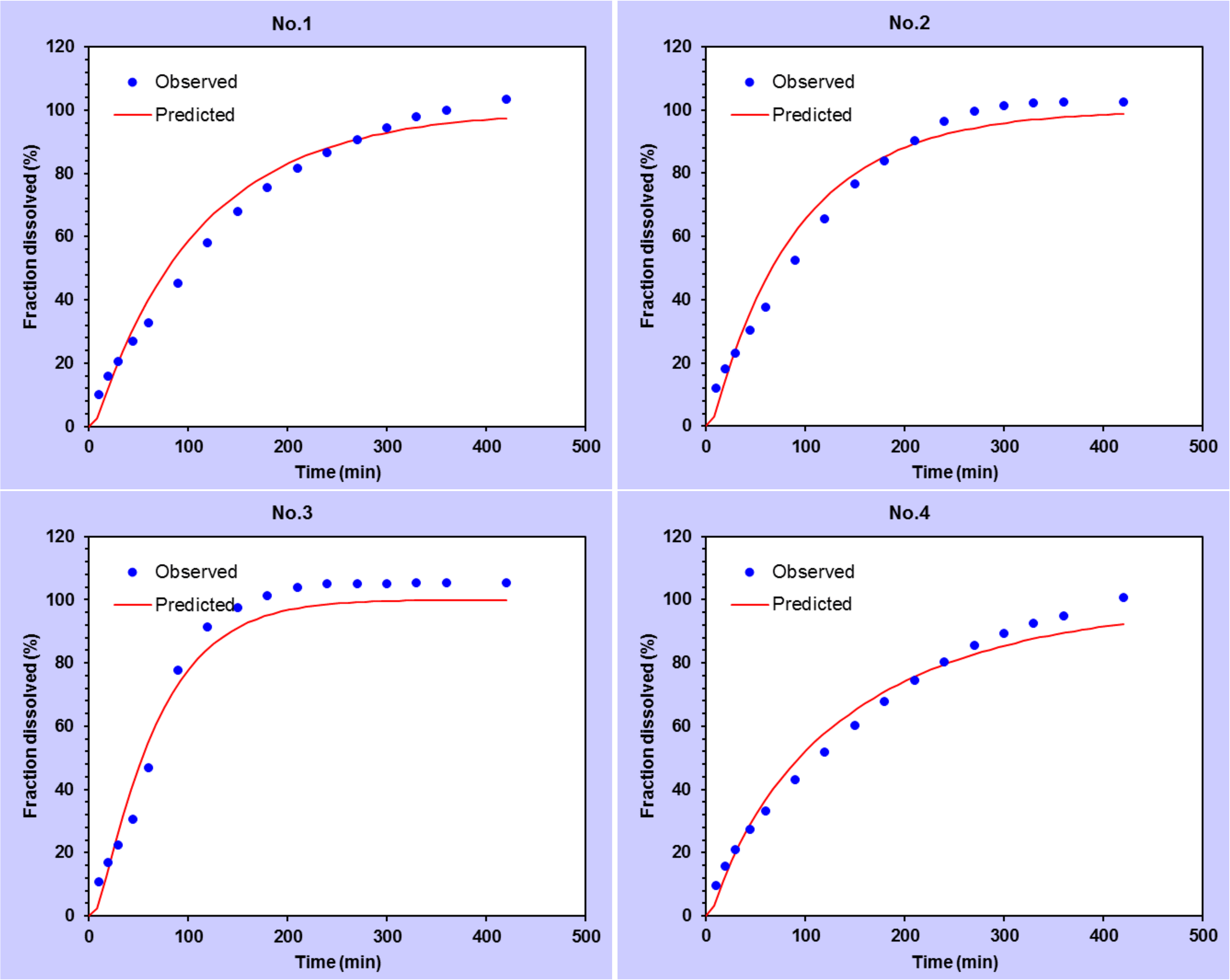

Supplementary materials – Model fitting summary – Pearlitol® 160C (modelling the entire relevant dissolution data)

Model: **Weibull_2**

Model equation: $F = 100 \cdot \left(1 - e^{-\frac{t^{\beta}}{\alpha}}\right)$

Fitted model parameters per tested tablet (N = 4) with statistics – mean, standard deviation (SD), and relative standard deviation expressed in % (RSD%) (output from DDSolver):

Parameter	No.1	No.2	No.3	No.4	Mean	SD	RSD(%)
α	214.285	184.174	288.303	104.601	197.841	76.014	38.422
β	1.091	1.103	1.332	0.938	1.116	0.162	14.545

Number of dissolution data points (N), degrees of freedom (df), and selected goodness of fit criteria – Pearson correlation coefficient (R), coefficient of determination (R^2), adjusted coefficient of determination ($R^2_{adjusted}$), and residual sum of squares (RSS) (manual calculation in MS Excel):

Parameter	No.1	No.2	No.3	No.4
N	16	16	16	16
df	14	14	14	14
R	0.997638278	0.997008738	0.993200964	0.993810507
R^2	0.995282134	0.994026424	0.986448156	0.987659325
$R^2_{adjusted}$	0.994945143	0.99359974	0.985480167	0.986777848
RSS	181.1099418	286.7598963	505.7659834	199.1470507

Graphical abstract of model fit presented as mean ± 1 SD of the fraction % of released carvedilol:

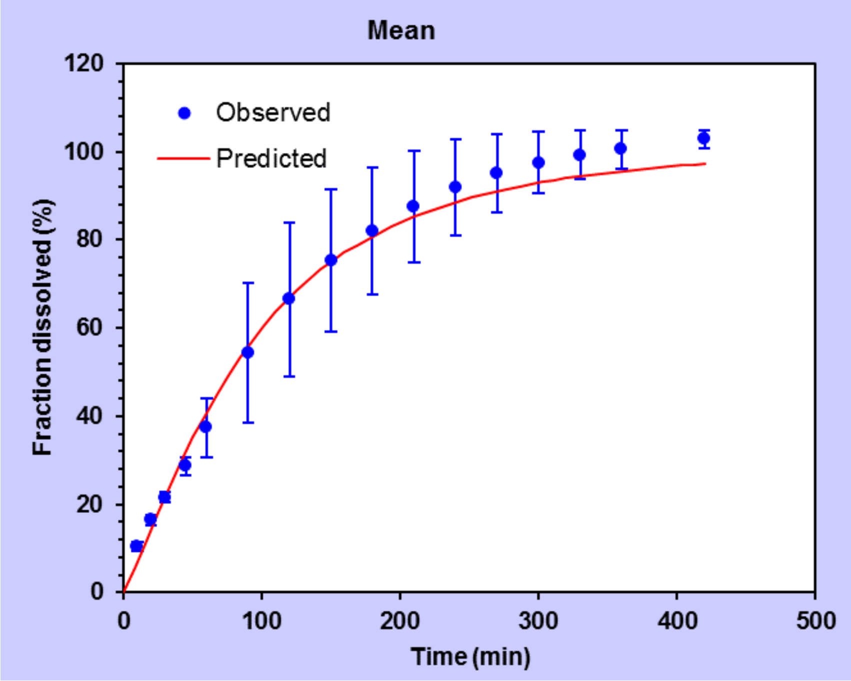

Supplementary materials – Model fitting summary – Pearlitol® 160C (modelling the entire relevant dissolution data)

Graphical abstract of model fit presented as the fraction % of released carvedilol per tested tablet:

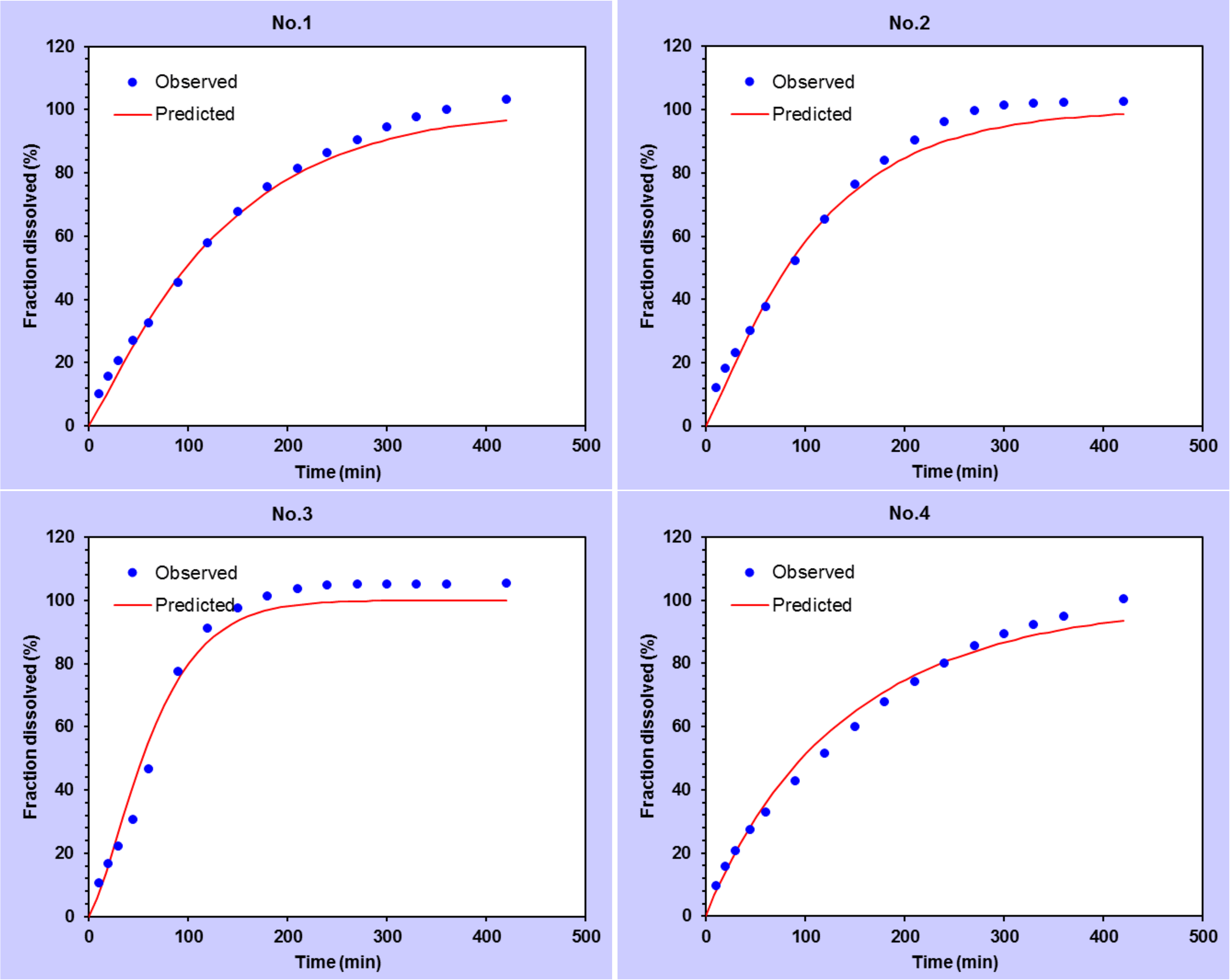

Supplementary materials – Model fitting summary – Pearlitol® 160C (modelling the entire relevant dissolution data)

Model: **Weibull_3**

Model equation: $F = F_{max} \cdot \left(1 - e^{-\frac{t^{\beta}}{\alpha}}\right)$

Fitted model parameters per tested tablet (N = 4) with statistics – mean, standard deviation (SD), and relative standard deviation expressed in % (RSD%) (output from DDSolver):

Parameter	No.1	No.2	No.3	No.4	Mean	SD	RSD(%)
α	117.639	123.647	123.581	117.904	120.693	3.375	2.797
β	0.955	1.006	1.050	0.898	0.977	0.065	6.699
F_{max}	108.360	109.719	110.565	119.827	112.118	5.219	4.655

Number of dissolution data points (N), degrees of freedom (df), and selected goodness of fit criteria – Pearson correlation coefficient (R), coefficient of determination (R^2), adjusted coefficient of determination ($R^2_{adjusted}$), and residual sum of squares (RSS) (manual calculation in MS Excel):

Parameter	No.1	No.2	No.3	No.4
N	16	16	16	16
df	13	13	13	13
R	0.997820229	0.997076621	0.985853486	0.998972816
R^2	0.995645209	0.994161788	0.971907096	0.997946687
$R^2_{adjusted}$	0.994975241	0.993263602	0.96758511	0.997630792
RSS	87.3512658	135.6170602	640.6303729	99.65888567

Graphical abstract of model fit presented as mean ± 1 SD of the fraction % of released carvedilol:

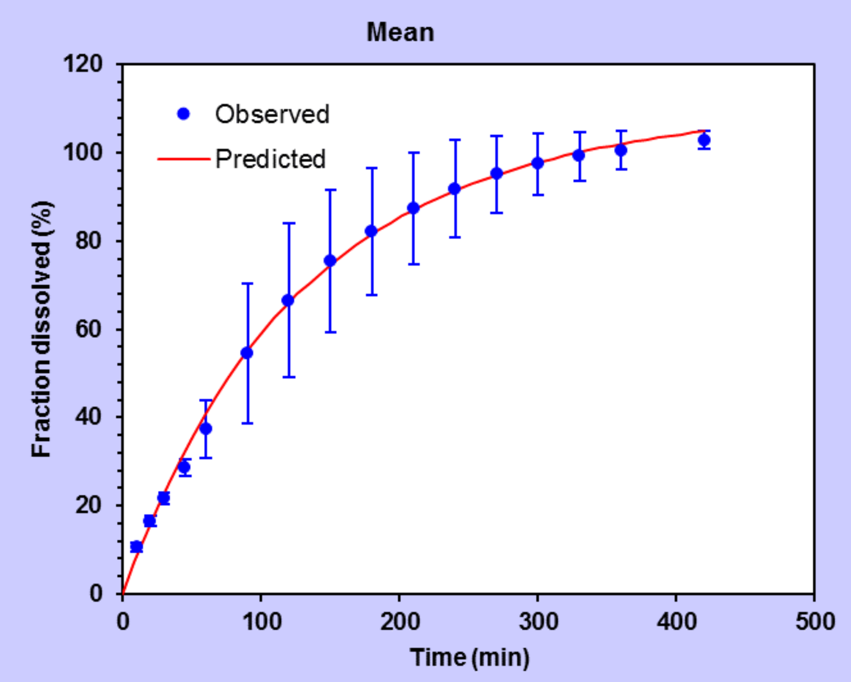

Supplementary materials – Model fitting summary – Pearlitol® 160C (modelling the entire relevant dissolution data)

Graphical abstract of model fit presented as the fraction % of released carvedilol per tested tablet:

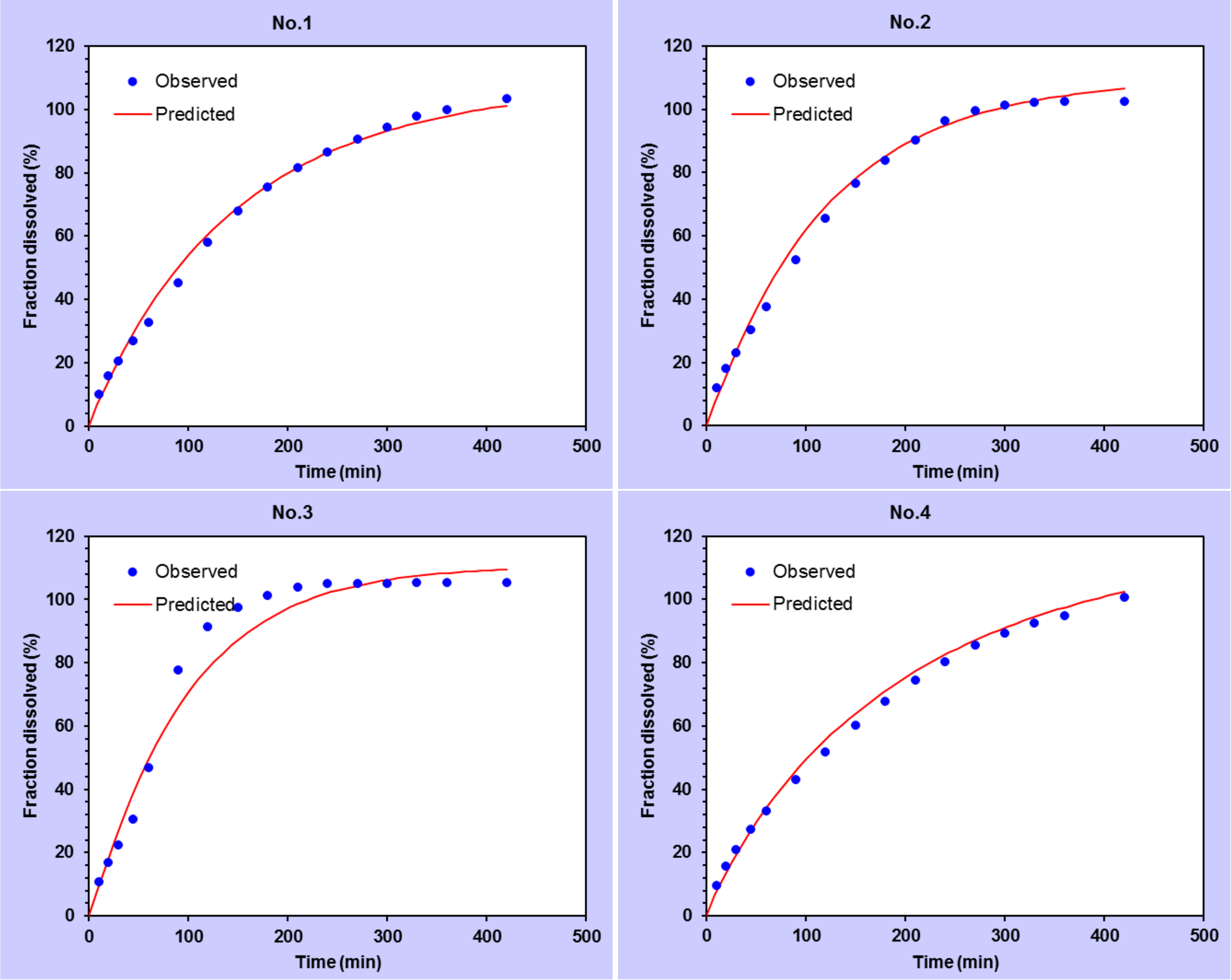

Supplementary materials – Model fitting summary – Pearlitol® 160C (modelling the entire relevant dissolution data)

Model: **Weibull_4**

Model equation: $F = F_{max} \cdot \left[1 - e^{-\frac{(t-T_i)^\beta}{\alpha}}\right]$

Fitted model parameters per tested tablet (N = 4) with statistics – mean, standard deviation (SD), and relative standard deviation expressed in % (RSD%) (output from DDSolver):

Parameter	No.1	No.2	No.3	No.4	Mean	SD	RSD(%)
α	77.762	63.095	72.710	63.516	69.271	7.192	10.383
β	0.862	0.883	0.954	0.826	0.882	0.054	6.132
T_i	4.676	6.000	6.000	6.000	5.669	0.662	11.677
F_{max}	115.786	107.520	110.565	105.525	109.849	4.468	4.067

Number of dissolution data points (N), degrees of freedom (df), and selected goodness of fit criteria – Pearson correlation coefficient (R), coefficient of determination (R^2), adjusted coefficient of determination ($R^2_{adjusted}$), and residual sum of squares (RSS) (manual calculation in MS Excel):

Parameter	No.1	No.2	No.3	No.4
N	16	16	16	16
df	12	12	12	12
R	0.996882138	0.993289773	0.986179596	0.99298088
R^2	0.993773997	0.986624574	0.972550195	0.986011029
$R^2_{adjusted}$	0.992217496	0.983280717	0.965687744	0.982513786
RSS	167.0925939	244.7102489	623.7791914	207.9203567

Graphical abstract of model fit presented as mean ± 1 SD of the fraction % of released carvedilol:

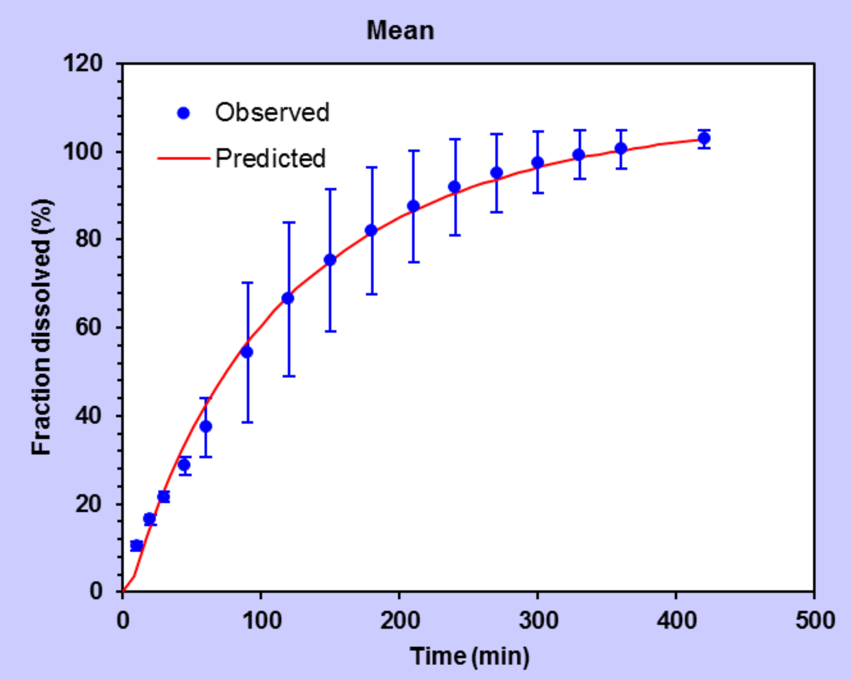

Supplementary materials – Model fitting summary – Pearlitol® 160C (modelling the entire relevant dissolution data)

Graphical abstract of model fit presented as the fraction % of released carvedilol per tested tablet:

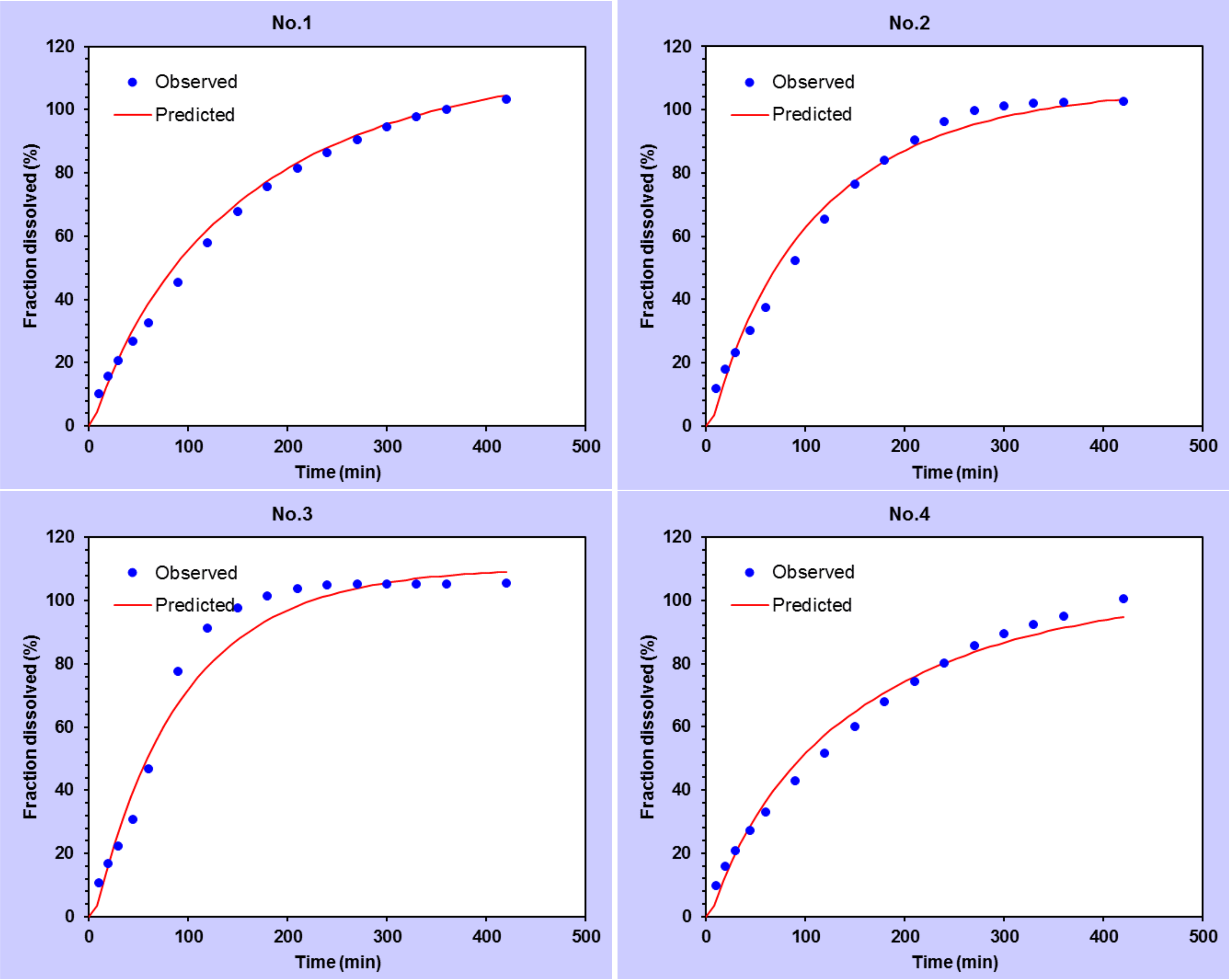

Supplementary materials – Model fitting summary – Pearlitol® 160C (modelling the entire relevant dissolution data)

Model: **Logistic_1**

Model equation: $F = 100 \cdot \frac{e^{\alpha+\beta \cdot \log(t)}}{1+e^{\alpha+\beta \cdot \log(t)}}$

Fitted model parameters per tested tablet (N = 4) with statistics – mean, standard deviation (SD), and relative standard deviation expressed in % (RSD%) (output from DDSolver):

Parameter	No.1	No.2	No.3	No.4	Mean	SD	RSD(%)
α	-9.653	-9.157	-9.745	-6.012	-8.642	1.772	-20.506
β	4.814	4.753	5.334	3.158	4.514	0.941	20.850

Number of dissolution data points (N), degrees of freedom (df), and selected goodness of fit criteria – Pearson correlation coefficient (R), coefficient of determination (R^2), adjusted coefficient of determination ($R^2_{adjusted}$), and residual sum of squares (RSS) (manual calculation in MS Excel):

Parameter	No.1	No.2	No.3	No.4
N	16	16	16	16
df	14	14	14	14
R	0.992826813	0.992687234	0.997287702	0.976444085
R^2	0.985705081	0.985427944	0.994582761	0.953443051
$R^2_{adjusted}$	0.984684015	0.984387083	0.994195815	0.950117555
RSS	759.8621777	872.0744949	1294.788717	716.1773457

Graphical abstract of model fit presented as mean ± 1 SD of the fraction % of released carvedilol:

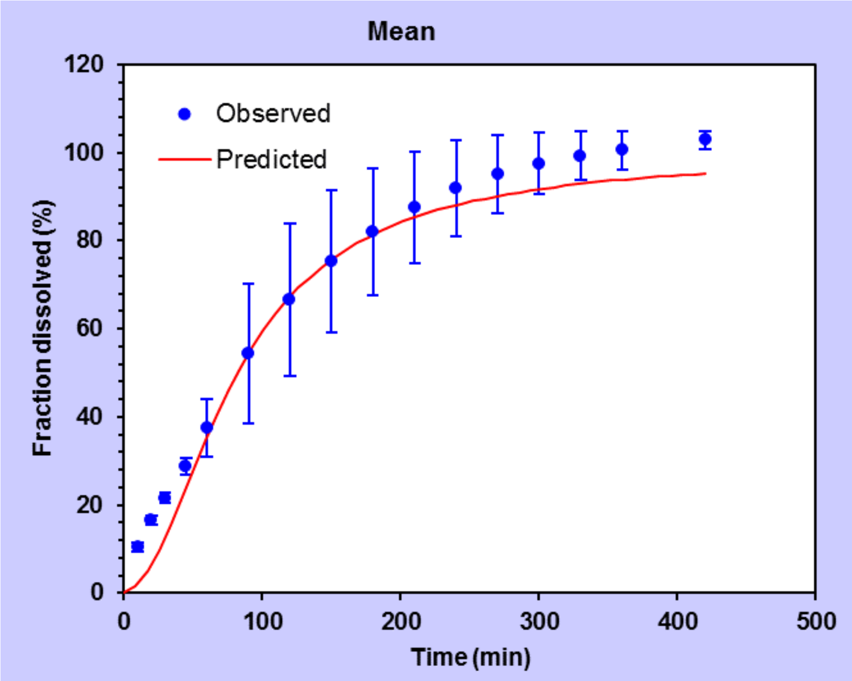

Supplementary materials – Model fitting summary – Pearlitol® 160C (modelling the entire relevant dissolution data)

Graphical abstract of model fit presented as the fraction % of released carvedilol per tested tablet:

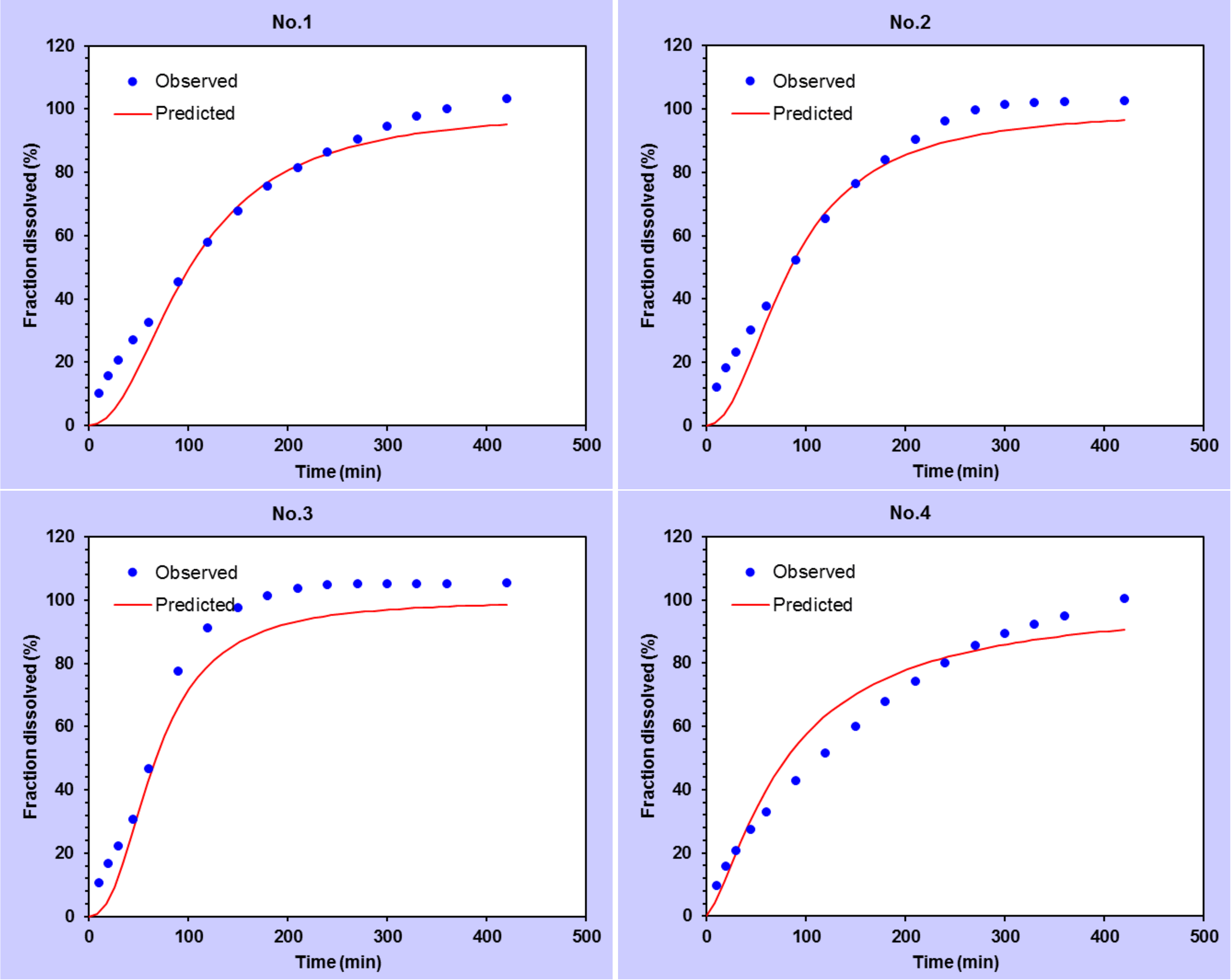

Supplementary materials – Model fitting summary – Pearlitol® 160C (modelling the entire relevant dissolution data)

Model: **Logistic_2**

Model equation: $F = F_{max} \cdot \frac{e^{\alpha+\beta\cdot\log(t)}}{1+e^{\alpha+\beta\cdot\log(t)}}$

Fitted model parameters per tested tablet (N = 4) with statistics – mean, standard deviation (SD), and relative standard deviation expressed in % (RSD%) (output from DDSolver):

Parameter	No.1	No.2	No.3	No.4	Mean	SD	RSD(%)
α	-7.202	-6.450	-7.856	-6.887	-7.099	0.592	-8.334
β	3.461	3.553	4.183	3.257	3.613	0.399	11.045
F_{max}	112.950	107.520	115.989	109.995	111.613	3.666	3.284

Number of dissolution data points (N), degrees of freedom (df), and selected goodness of fit criteria – Pearson correlation coefficient (R), coefficient of determination (R^2), adjusted coefficient of determination ($R^2_{adjusted}$), and residual sum of squares (RSS) (manual calculation in MS Excel):

Parameter	No.1	No.2	No.3	No.4
N	16	16	16	16
df	13	13	13	13
R	0.998104874	0.981391117	0.992090919	0.995446986
R^2	0.99621334	0.963128525	0.984244392	0.990914701
$R^2_{adjusted}$	0.995630777	0.95745599	0.981820453	0.989516963
RSS	376.4314875	757.586251	506.4969304	456.1077999

Graphical abstract of model fit presented as mean ± 1 SD of the fraction % of released carvedilol:

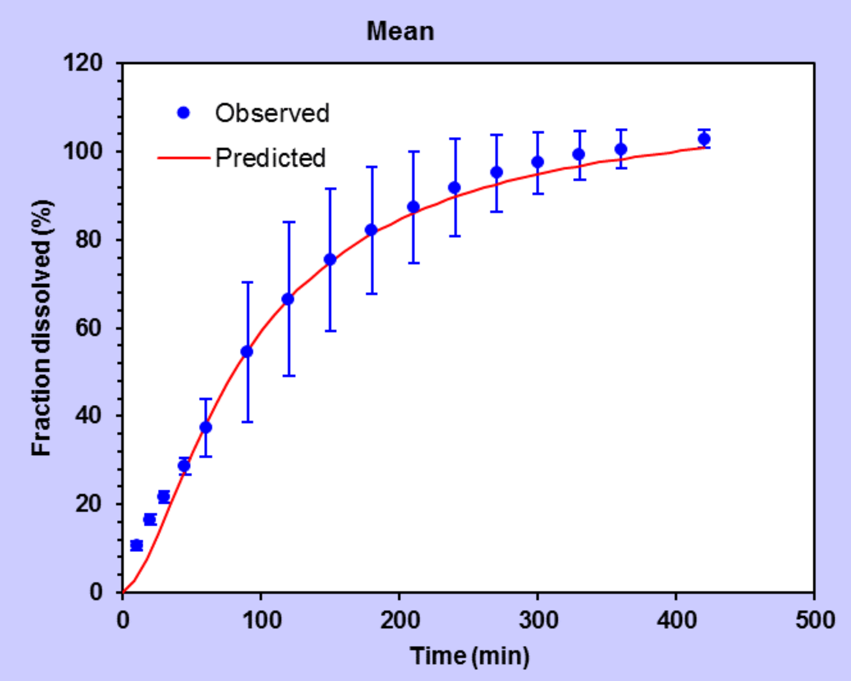

Supplementary materials – Model fitting summary – Pearlitol® 160C (modelling the entire relevant dissolution data)

Graphical abstract of model fit presented as the fraction % of released carvedilol per tested tablet:

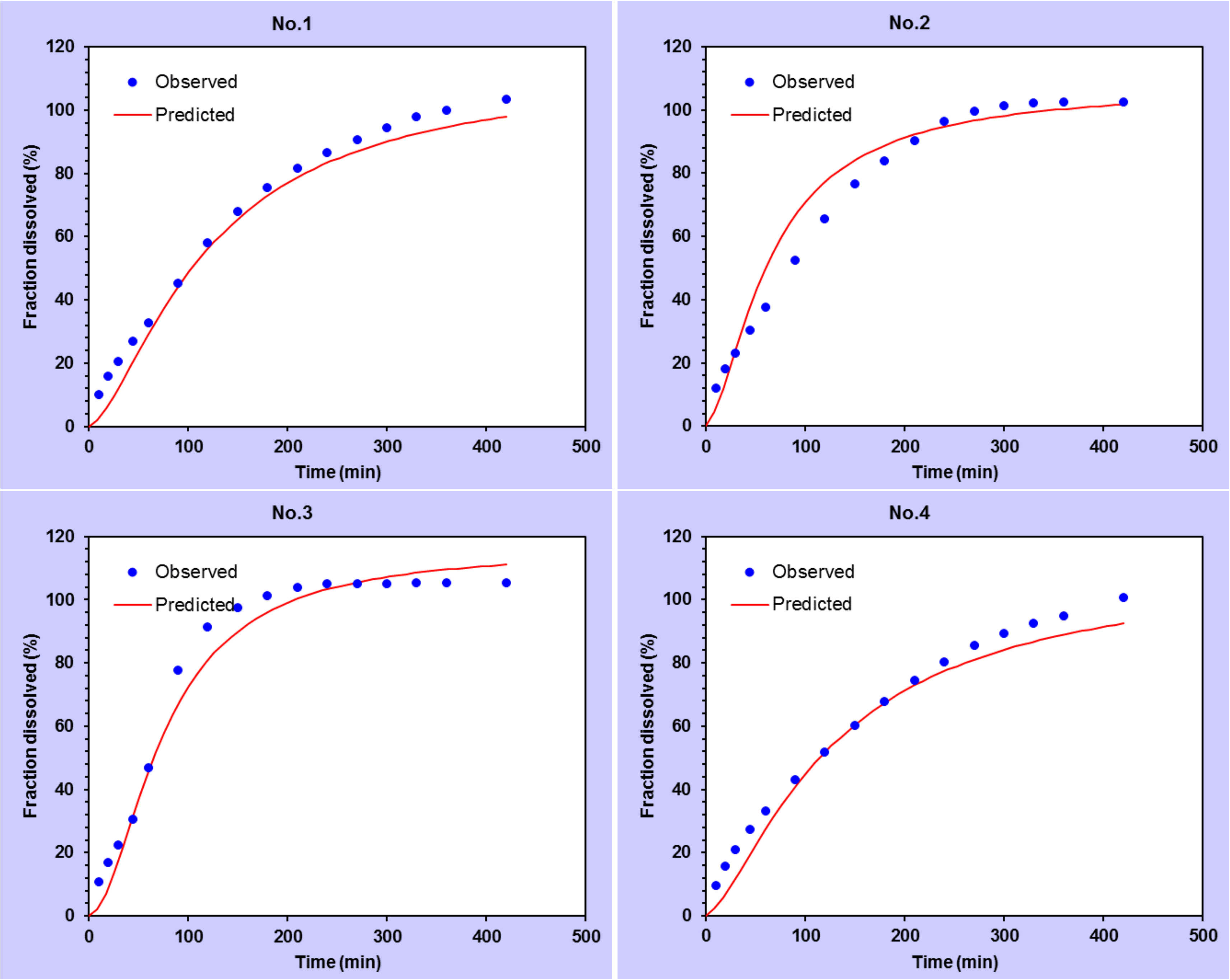

Supplementary materials – Model fitting summary – Pearlitol® 160C (modelling the entire relevant dissolution data)

Model: **Logistic_3**

Model equation: $F = F_{max} \cdot \frac{1}{1+e^{-k\cdot(t-\gamma)}}$

Fitted model parameters per tested tablet (N = 4) with statistics – mean, standard deviation (SD), and relative standard deviation expressed in % (RSD%) (output from DDSolver):

Parameter	No.1	No.2	No.3	No.4	Mean	SD	RSD(%)
k	0.017	0.017	0.020	0.011	0.016	0.003	21.238
γ	108.039	95.007	79.106	147.693	107.461	29.315	27.279
F_{max}	97.407	108.198	110.565	105.525	105.424	5.727	5.433

Number of dissolution data points (N), degrees of freedom (df), and selected goodness of fit criteria – Pearson correlation coefficient (R), coefficient of determination (R^2), adjusted coefficient of determination ($R^2_{adjusted}$), and residual sum of squares (RSS) (manual calculation in MS Excel):

Parameter	No.1	No.2	No.3	No.4
N	16	16	16	16
df	13	13	13	13
R	0.996265336	0.99786075	0.976194751	0.989993783
R^2	0.99254462	0.995726076	0.952956192	0.980087691
$R^2_{adjusted}$	0.991397639	0.995068549	0.945718683	0.977024259
RSS	150.633358	271.9576873	1038.179494	310.0373558

Graphical abstract of model fit presented as mean ± 1 SD of the fraction % of released carvedilol:

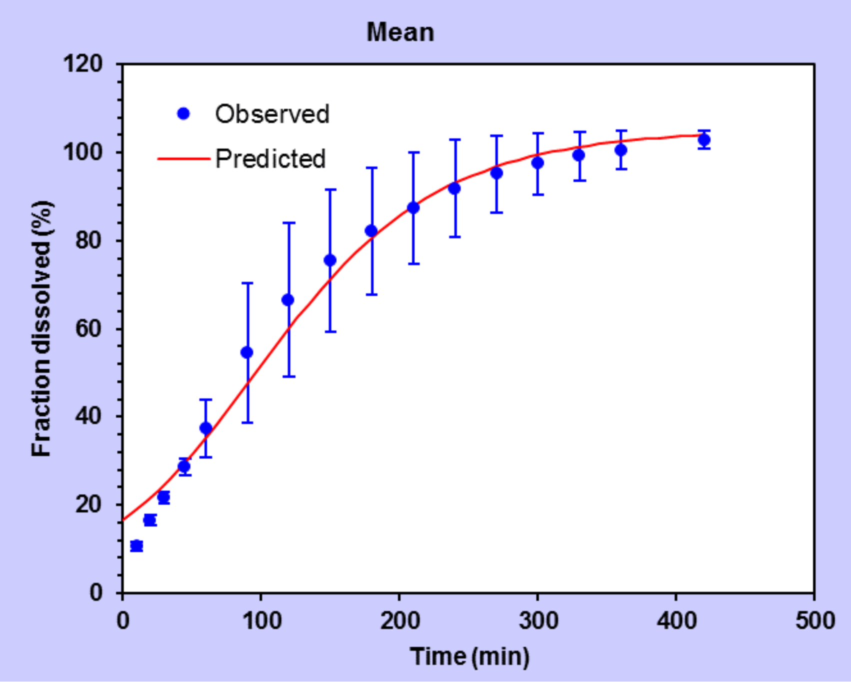

Supplementary materials – Model fitting summary – Pearlitol® 160C (modelling the entire relevant dissolution data)

Graphical abstract of model fit presented as the fraction % of released carvedilol per tested tablet:

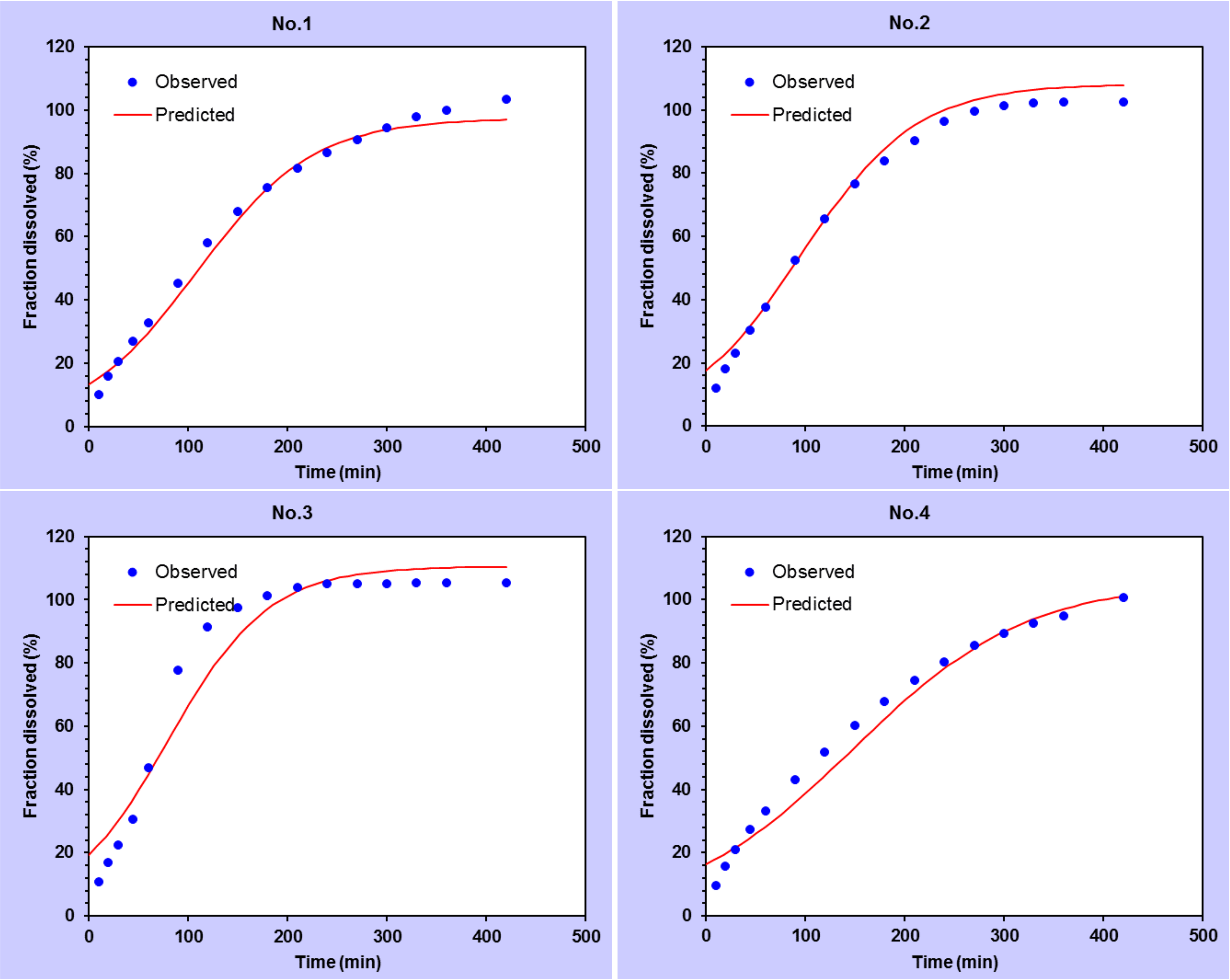

Supplementary materials – Model fitting summary – Pearlitol® 160C (modelling the entire relevant dissolution data)

Model: **Gompertz_1**

Model equation: $F = 100 \cdot e^{-\alpha \cdot e^{-\beta \cdot \log(t)}}$

Fitted model parameters per tested tablet (N = 4) with statistics – mean, standard deviation (SD), and relative standard deviation expressed in % (RSD%) (output from DDSolver):

Parameter	No.1	No.2	No.3	No.4	Mean	SD	RSD(%)
α	370.457	267.361	279.834	68.492	246.536	127.277	51.626
β	3.383	3.321	3.541	2.287	3.133	0.572	18.250

Number of dissolution data points (N), degrees of freedom (df), and selected goodness of fit criteria – Pearson correlation coefficient (R), coefficient of determination (R^2), adjusted coefficient of determination ($R^2_{adjusted}$), and residual sum of squares (RSS) (manual calculation in MS Excel):

Parameter	No.1	No.2	No.3	No.4
N	16	16	16	16
df	14	14	14	14
R	0.965396557	0.966572719	0.977671483	0.979188572
R^2	0.931990512	0.934262821	0.955841529	0.958810259
$R^2_{adjusted}$	0.927132692	0.929567308	0.952687352	0.955868135
RSS	1338.070734	1275.605235	1477.413752	1229.677998

Graphical abstract of model fit presented as mean ± 1 SD of the fraction % of released carvedilol:

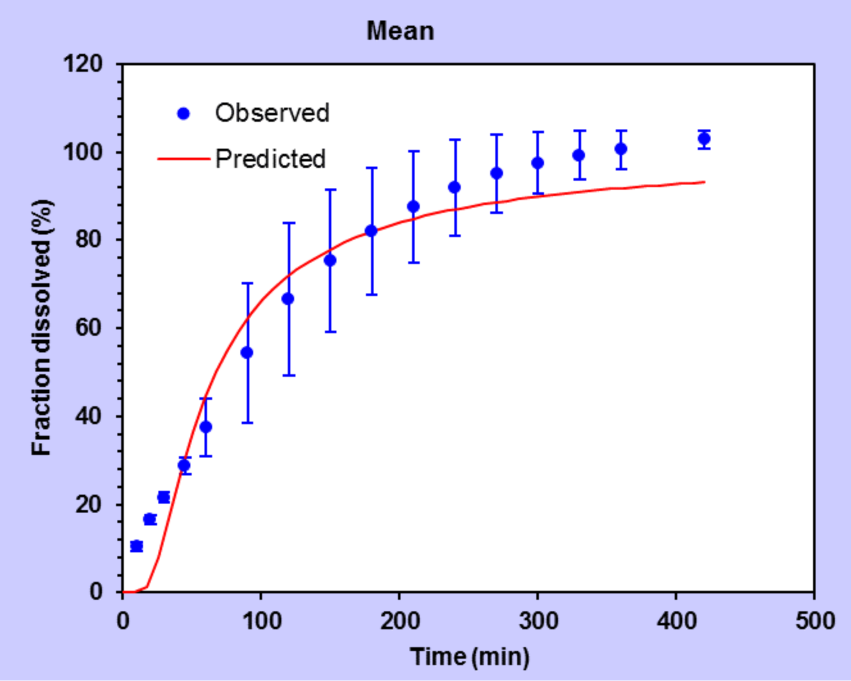

Supplementary materials – Model fitting summary – Pearlitol® 160C (modelling the entire relevant dissolution data)

Graphical abstract of model fit presented as the fraction % of released carvedilol per tested tablet:

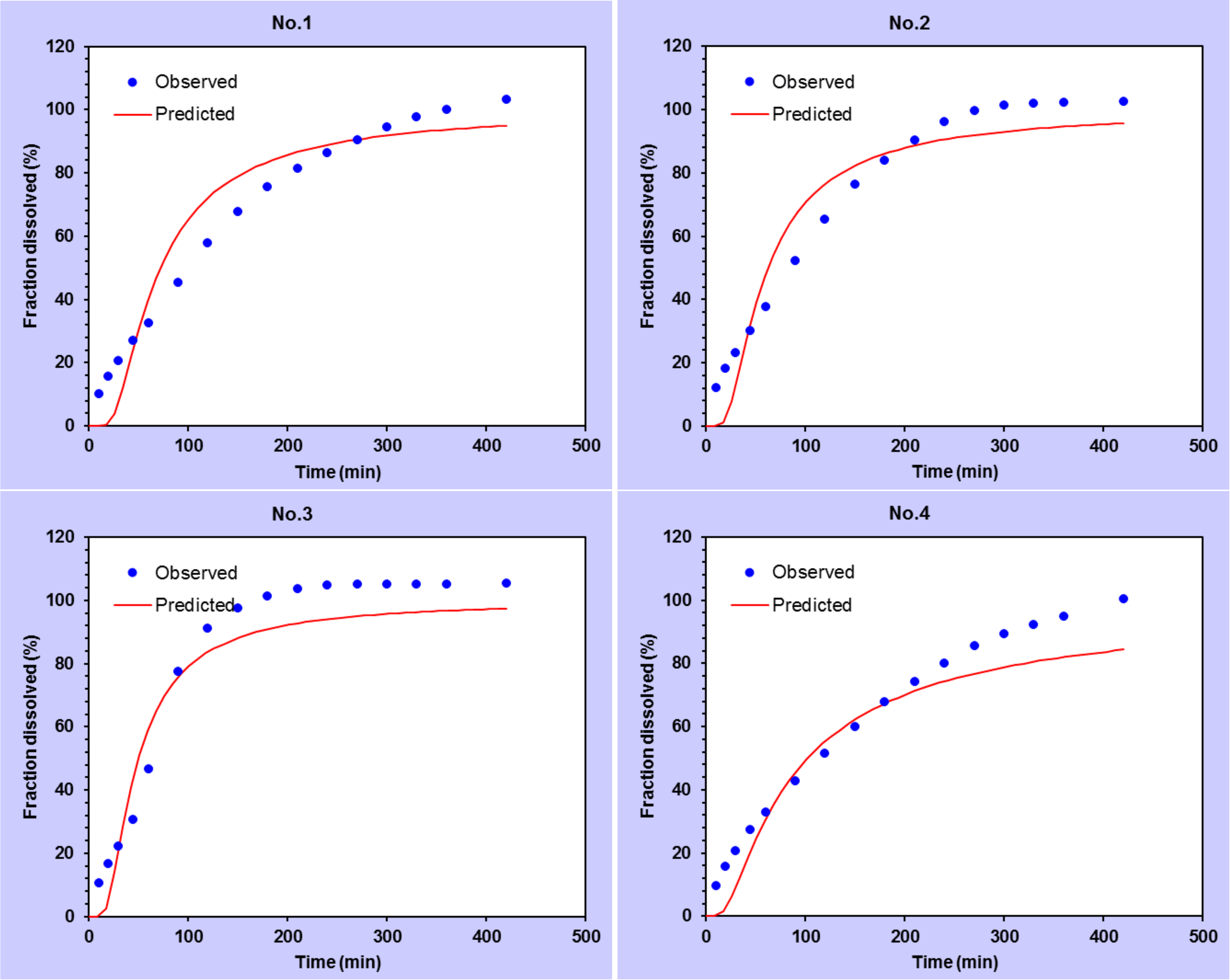

Supplementary materials – Model fitting summary – Pearlitol® 160C (modelling the entire relevant dissolution data)

Model: **Gompertz_2**

Model equation: $F = F_{max} \cdot e^{-\alpha \cdot e^{-\beta \cdot \log(t)}}$

Fitted model parameters per tested tablet (N = 4) with statistics – mean, standard deviation (SD), and relative standard deviation expressed in % (RSD%) (output from DDSolver):

Parameter	No.1	No.2	No.3	No.4	Mean	SD	RSD(%)
α	76.813	120.025	150.723	60.770	102.083	40.960	40.124
β	2.335	2.722	3.016	2.169	2.560	0.382	14.922
F_{max}	108.360	107.520	110.565	105.525	107.993	2.087	1.932

Number of dissolution data points (N), degrees of freedom (df), and selected goodness of fit criteria – Pearson correlation coefficient (R), coefficient of determination (R^2), adjusted coefficient of determination ($R^2_{adjusted}$), and residual sum of squares (RSS) (manual calculation in MS Excel):

Parameter	No.1	No.2	No.3	No.4
N	16	16	16	16
df	13	13	13	13
R	0.987811999	0.983769651	0.988307802	0.984166856
R^2	0.975772545	0.967802727	0.976752312	0.968584401
$R^2_{adjusted}$	0.972045244	0.9628493	0.973175745	0.963751232
RSS	977.4410088	875.3861636	733.5445066	1141.141266

Graphical abstract of model fit presented as mean ± 1 SD of the fraction % of released carvedilol:

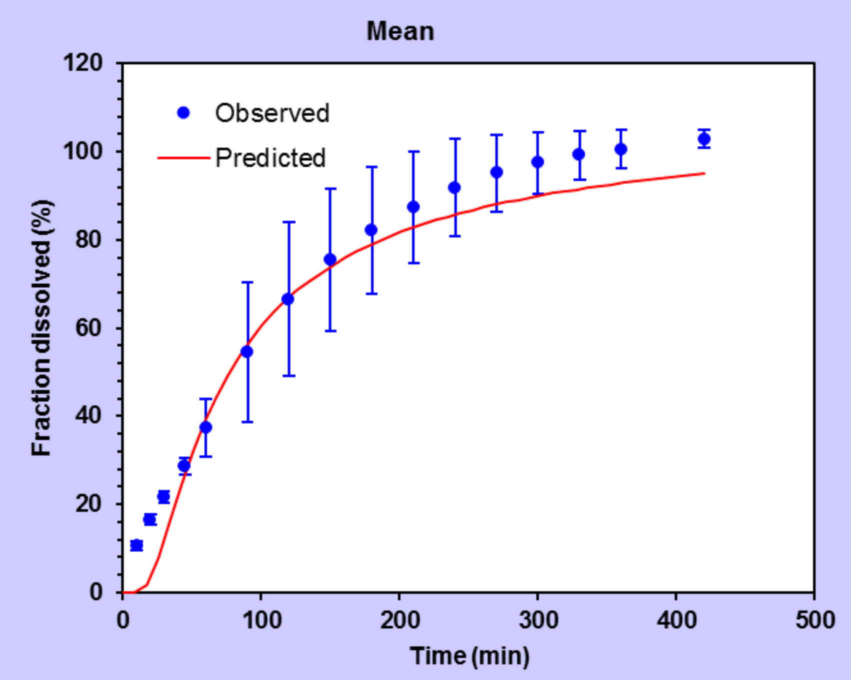

Supplementary materials – Model fitting summary – Pearlitol® 160C (modelling the entire relevant dissolution data)

Graphical abstract of model fit presented as the fraction % of released carvedilol per tested tablet:

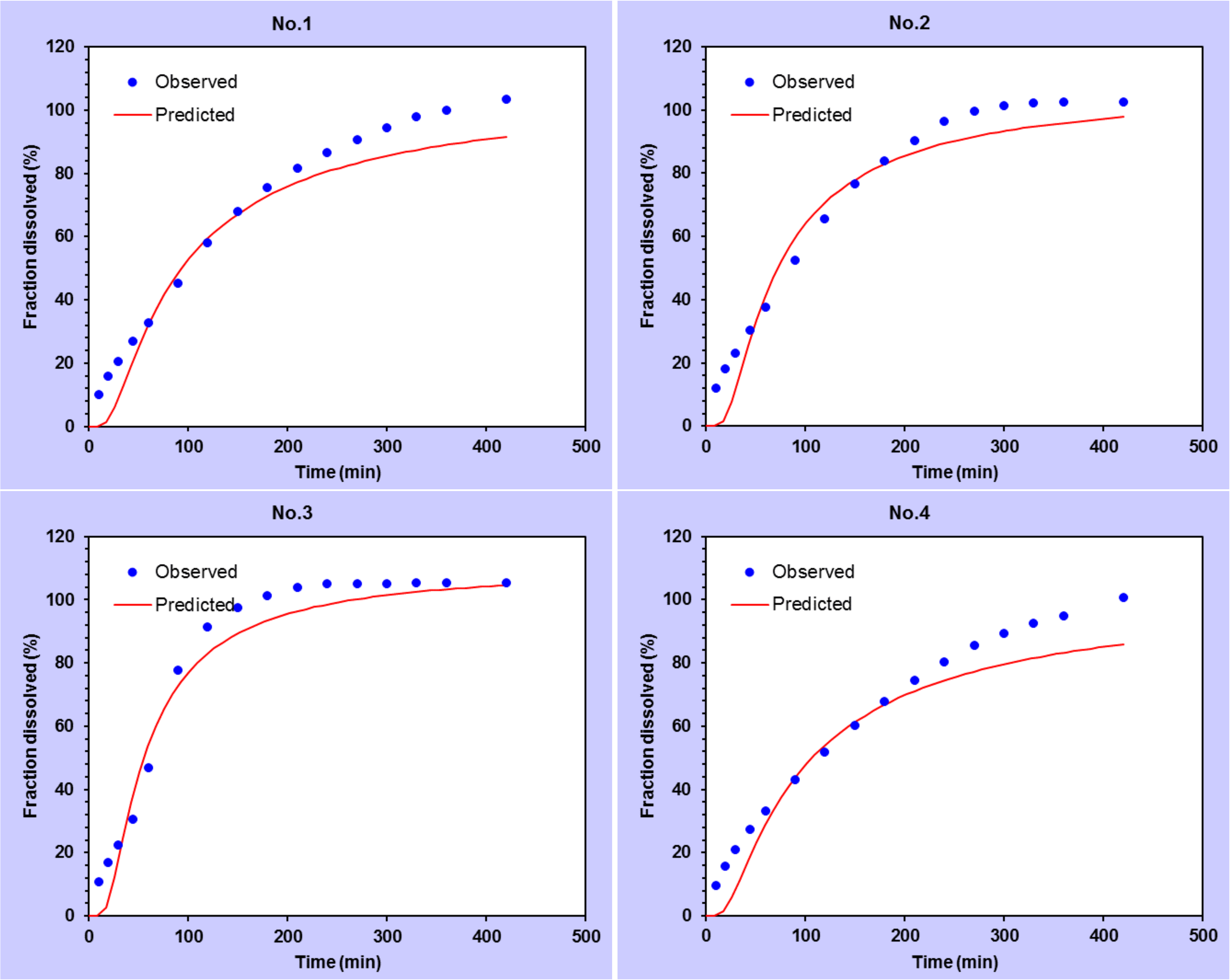

Supplementary materials – Model fitting summary – Pearlitol® 160C (modelling the entire relevant dissolution data)

Model: **Gompertz_3**

Model equation: $F = F_{max} \cdot e^{-e^{-k\cdot(t-\gamma)}}$

Fitted model parameters per tested tablet (N = 4) with statistics – mean, standard deviation (SD), and relative standard deviation expressed in % (RSD%) (output from DDSolver):

Parameter	No.1	No.2	No.3	No.4	Mean	SD	RSD(%)
k	0.011	0.010	0.010	0.009	0.010	0.001	11.035
γ	71.597	59.831	35.904	88.394	63.931	22.057	34.501
F_{max}	101.755	107.520	110.565	105.525	106.341	3.694	3.474

Number of dissolution data points (N), degrees of freedom (df), and selected goodness of fit criteria – Pearson correlation coefficient (R), coefficient of determination (R^2), adjusted coefficient of determination ($R^2_{adjusted}$), and residual sum of squares (RSS) (manual calculation in MS Excel):

Parameter	No.1	No.2	No.3	No.4
N	16	16	16	16
df	13	13	13	13
R	0.999095464	0.997190996	0.962852908	0.997577936
R^2	0.998191747	0.994389882	0.927085722	0.995161739
$R^2_{adjusted}$	0.997913554	0.993526787	0.915868141	0.994417391
RSS	37.36380843	201.3931222	2247.772033	71.01440606

Graphical abstract of model fit presented as mean ± 1 SD of the fraction % of released carvedilol:

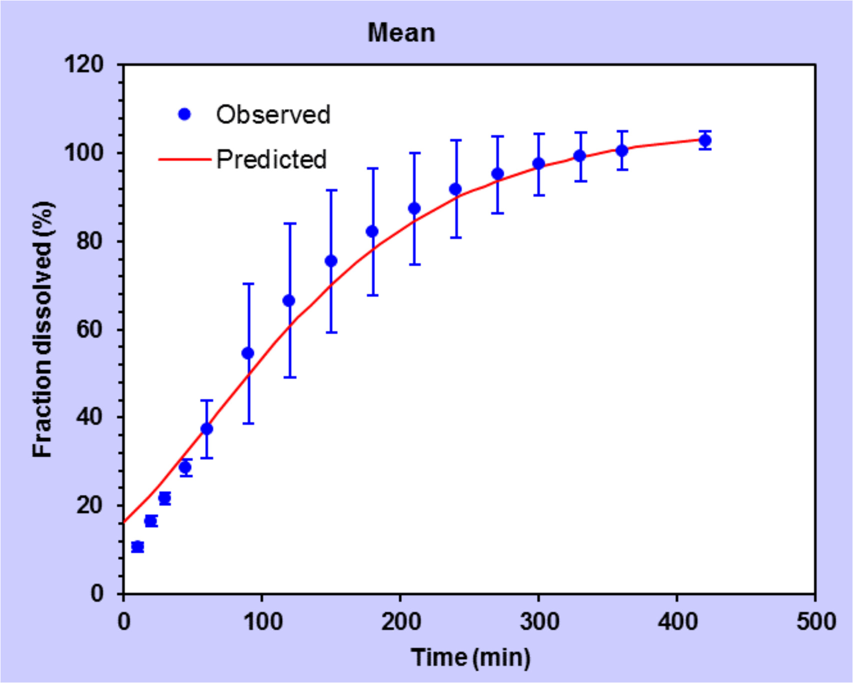

Supplementary materials – Model fitting summary – Pearlitol® 160C (modelling the entire relevant dissolution data)

Graphical abstract of model fit presented as the fraction % of released carvedilol per tested tablet:

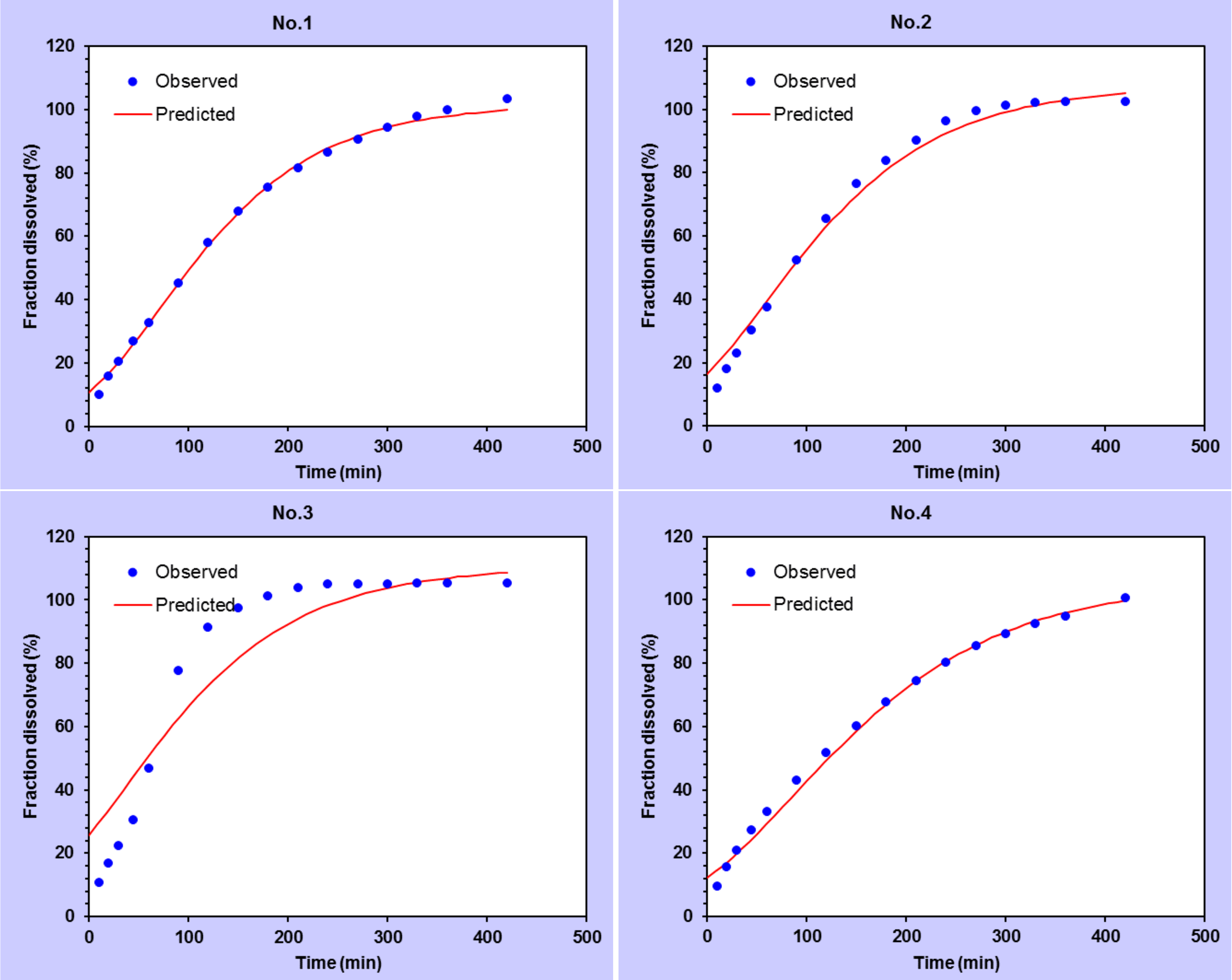

Supplementary materials – Model fitting summary – Pearlitol® 160C (modelling the entire relevant dissolution data)

Model: **Gompertz_4**

Model equation: $F = F_{max} \cdot e^{-\beta \cdot e^{-k \cdot t}}$

Fitted model parameters per tested tablet (N = 4) with statistics – mean, standard deviation (SD), and relative standard deviation expressed in % (RSD%) (output from DDSolver):

Parameter	No.1	No.2	No.3	No.4	Mean	SD	RSD(%)
k	0.009	0.010	0.010	0.009	0.010	0.001	9.491
β	2.125	1.872	1.285	2.150	1.858	0.402	21.638
F_{max}	108.360	107.520	110.565	105.525	107.993	2.087	1.932

Number of dissolution data points (N), degrees of freedom (df), and selected goodness of fit criteria – Pearson correlation coefficient (R), coefficient of determination (R^2), adjusted coefficient of determination ($R^2_{adjusted}$), and residual sum of squares (RSS) (manual calculation in MS Excel):

Parameter	No.1	No.2	No.3	No.4
N	16	16	16	16
df	13	13	13	13
R	0.997329636	0.997190996	0.966675439	0.997577936
R^2	0.994666402	0.994389882	0.934461404	0.995161739
$R^2_{adjusted}$	0.993845849	0.993526787	0.924378543	0.994417391
RSS	96.94685017	201.3931222	2667.261543	71.01440606

Graphical abstract of model fit presented as mean ± 1 SD of the fraction % of released carvedilol:

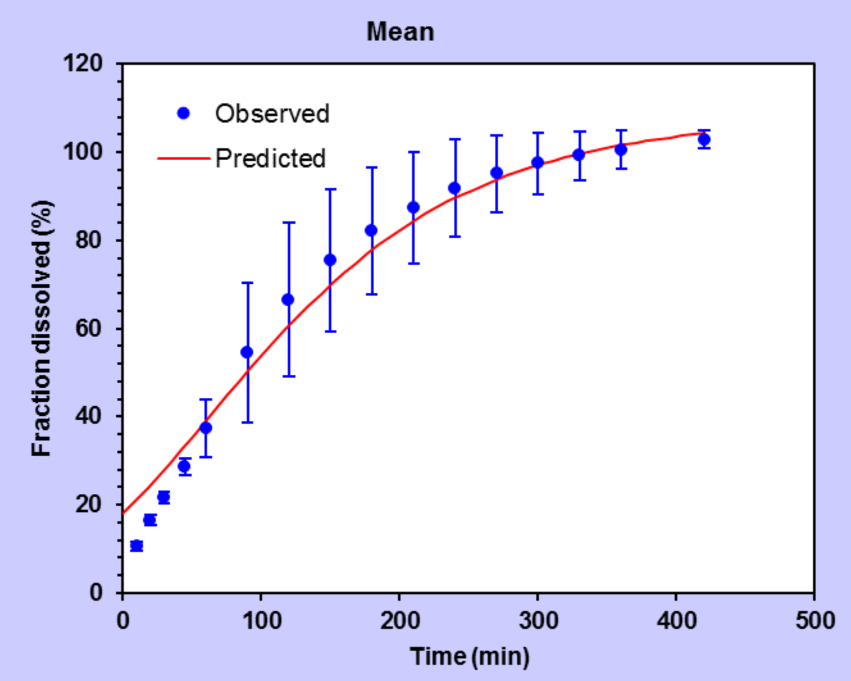

Supplementary materials – Model fitting summary – Pearlitol® 160C (modelling the entire relevant dissolution data)

Graphical abstract of model fit presented as the fraction % of released carvedilol per tested tablet:

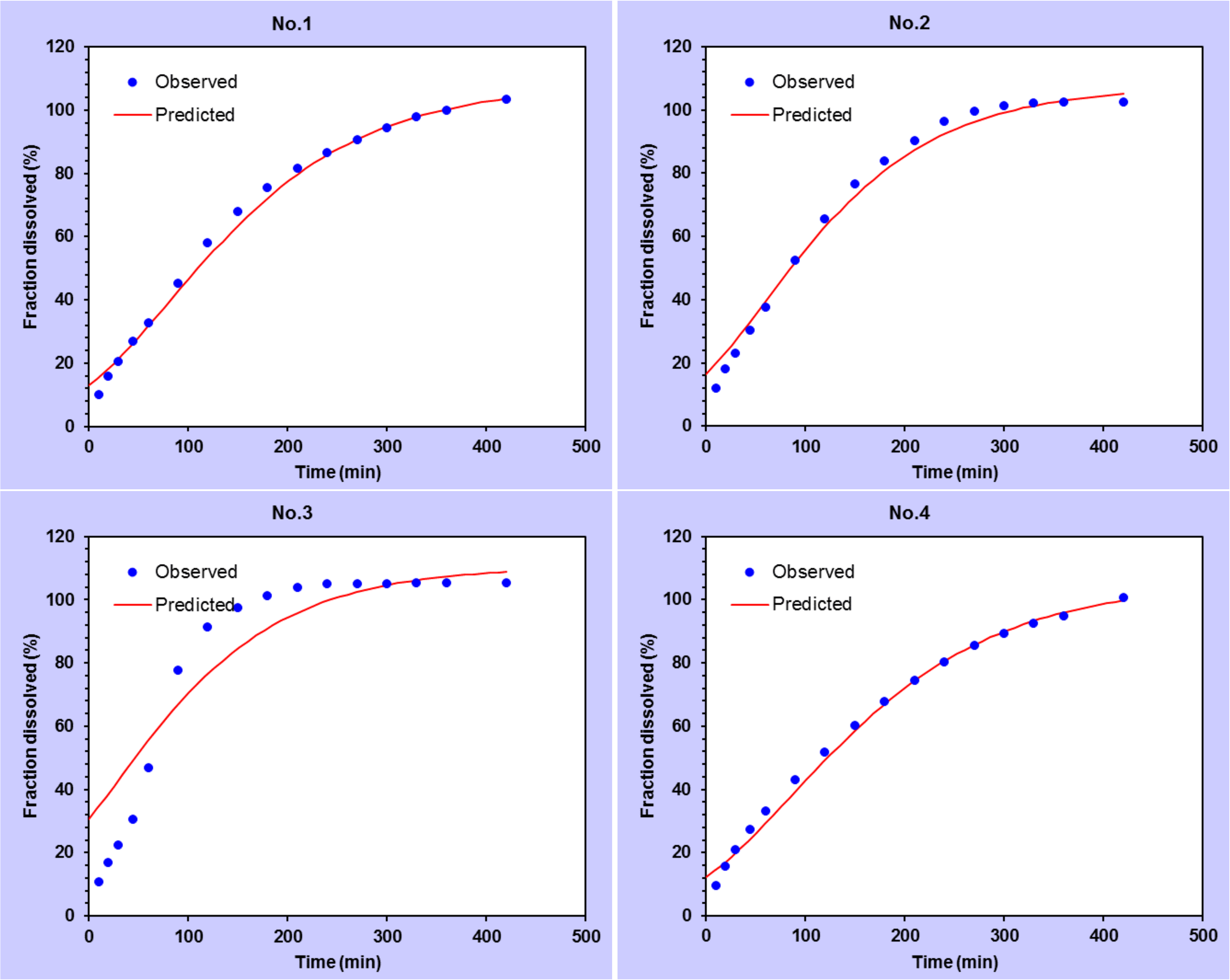

Supplementary materials – Model fitting summary – Pearlitol® 160C (modelling the entire relevant dissolution data)

Model: **Probit_1**

Model equation: $F = 100 \cdot \phi[\alpha + \beta \cdot \log(t)]$

Fitted model parameters per tested tablet (N = 4) with statistics – mean, standard deviation (SD), and relative standard deviation expressed in % (RSD%) (output from DDSolver):

Parameter	No.1	No.2	No.3	No.4	Mean	SD	RSD(%)
α	-5.315	-5.136	-4.491	-3.542	-4.621	0.802	-17.350
β	2.623	2.640	2.713	1.857	2.458	0.403	16.392

Number of dissolution data points (N), degrees of freedom (df), and selected goodness of fit criteria – Pearson correlation coefficient (R), coefficient of determination (R^2), adjusted coefficient of determination ($R^2_{adjusted}$), and residual sum of squares (RSS) (manual calculation in MS Excel):

Parameter	No.1	No.2	No.3	No.4
N	16	16	16	16
df	14	14	14	14
R	0.995733811	0.995244023	0.978233437	0.97814547
R^2	0.991485821	0.990510664	0.956940658	0.95676856
$R^2_{adjusted}$	0.990877666	0.989832855	0.953864991	0.9536806
RSS	901.2298085	1047.366737	1150.946155	658.0637623

Graphical abstract of model fit presented as mean ± 1 SD of the fraction % of released carvedilol:

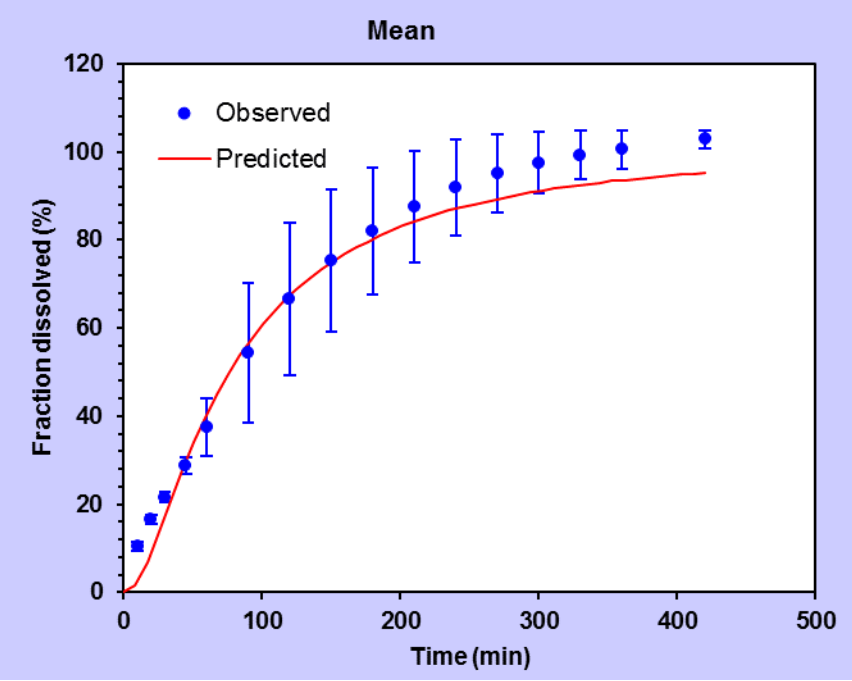

Supplementary materials – Model fitting summary – Pearlitol® 160C (modelling the entire relevant dissolution data)

Graphical abstract of model fit presented as the fraction % of released carvedilol per tested tablet:

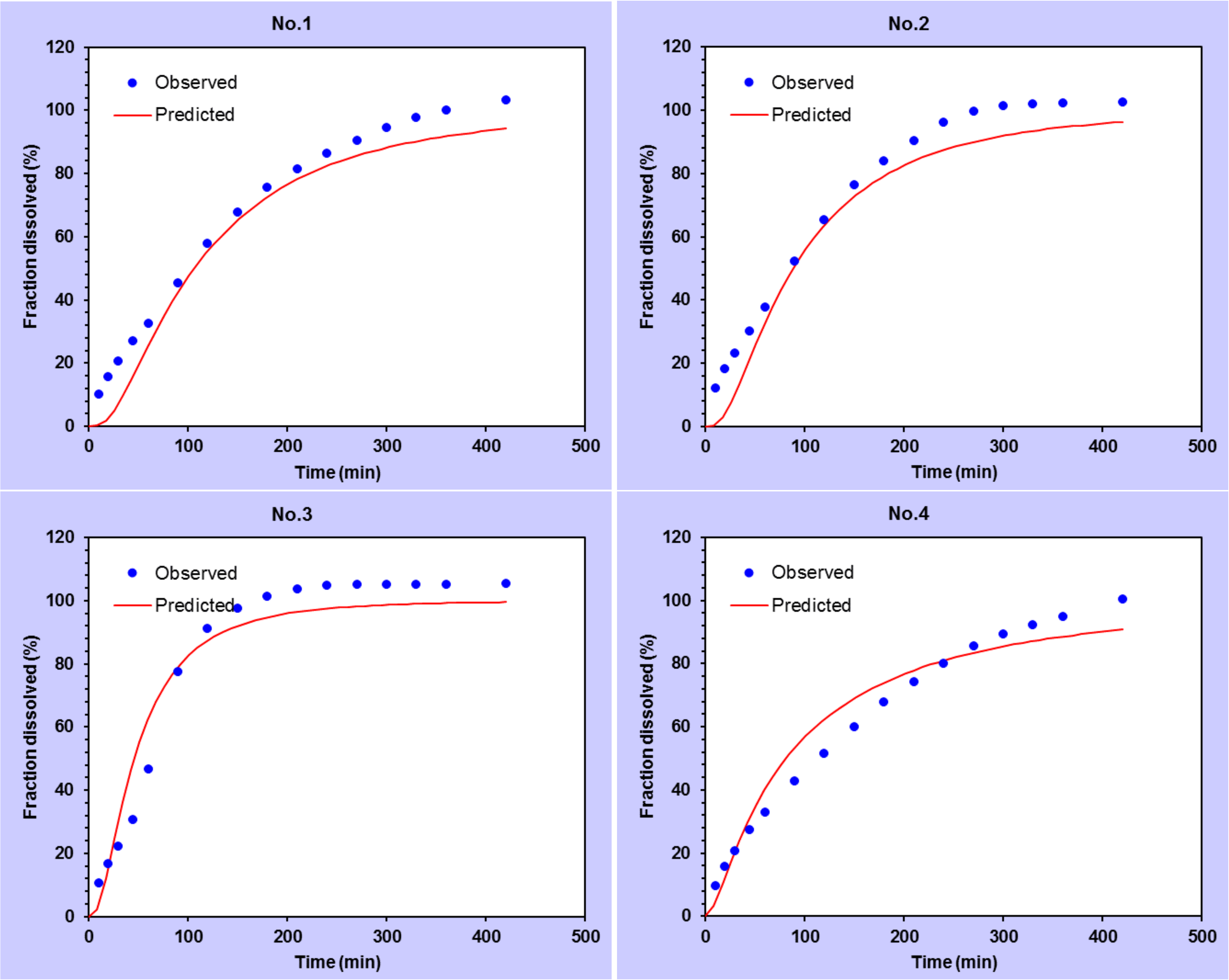

Supplementary materials – Model fitting summary – Pearlitol® 160C (modelling the entire relevant dissolution data)

Model: **Probit_2**

Model equation: $F = F_{max} \cdot \phi[\alpha + \beta \cdot \log(t)]$

Fitted model parameters per tested tablet (N = 4) with statistics – mean, standard deviation (SD), and relative standard deviation expressed in % (RSD%) (output from DDSolver):

Parameter	No.1	No.2	No.3	No.4	Mean	SD	RSD(%)
α	-4.195	-4.134	-3.881	-4.025	-4.059	0.138	-3.393
β	2.010	1.948	2.231	1.898	2.022	0.147	7.251
F_{max}	117.988	130.231	110.565	114.901	118.421	8.441	7.128

Number of dissolution data points (N), degrees of freedom (df), and selected goodness of fit criteria – Pearson correlation coefficient (R), coefficient of determination (R^2), adjusted coefficient of determination ($R^2_{adjusted}$), and residual sum of squares (RSS) (manual calculation in MS Excel):

Parameter	No.1	No.2	No.3	No.4
N	16	16	16	16
df	13	13	13	13
R	0.997583823	0.996728961	0.986487354	0.995054975
R^2	0.995173484	0.993468623	0.973157299	0.990134403
$R^2_{adjusted}$	0.994430943	0.992463795	0.969027652	0.988616618
RSS	250.6525326	614.0041957	634.5803512	317.462508

Graphical abstract of model fit presented as mean ± 1 SD of the fraction % of released carvedilol:

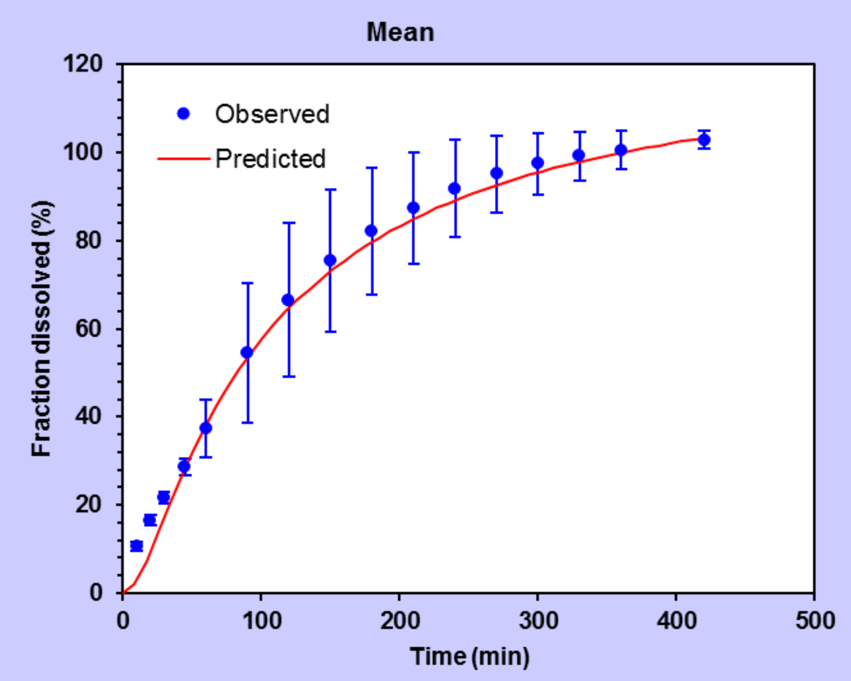

Supplementary materials – Model fitting summary – Pearlitol® 160C (modelling the entire relevant dissolution data)

Graphical abstract of model fit presented as the fraction % of released carvedilol per tested tablet:

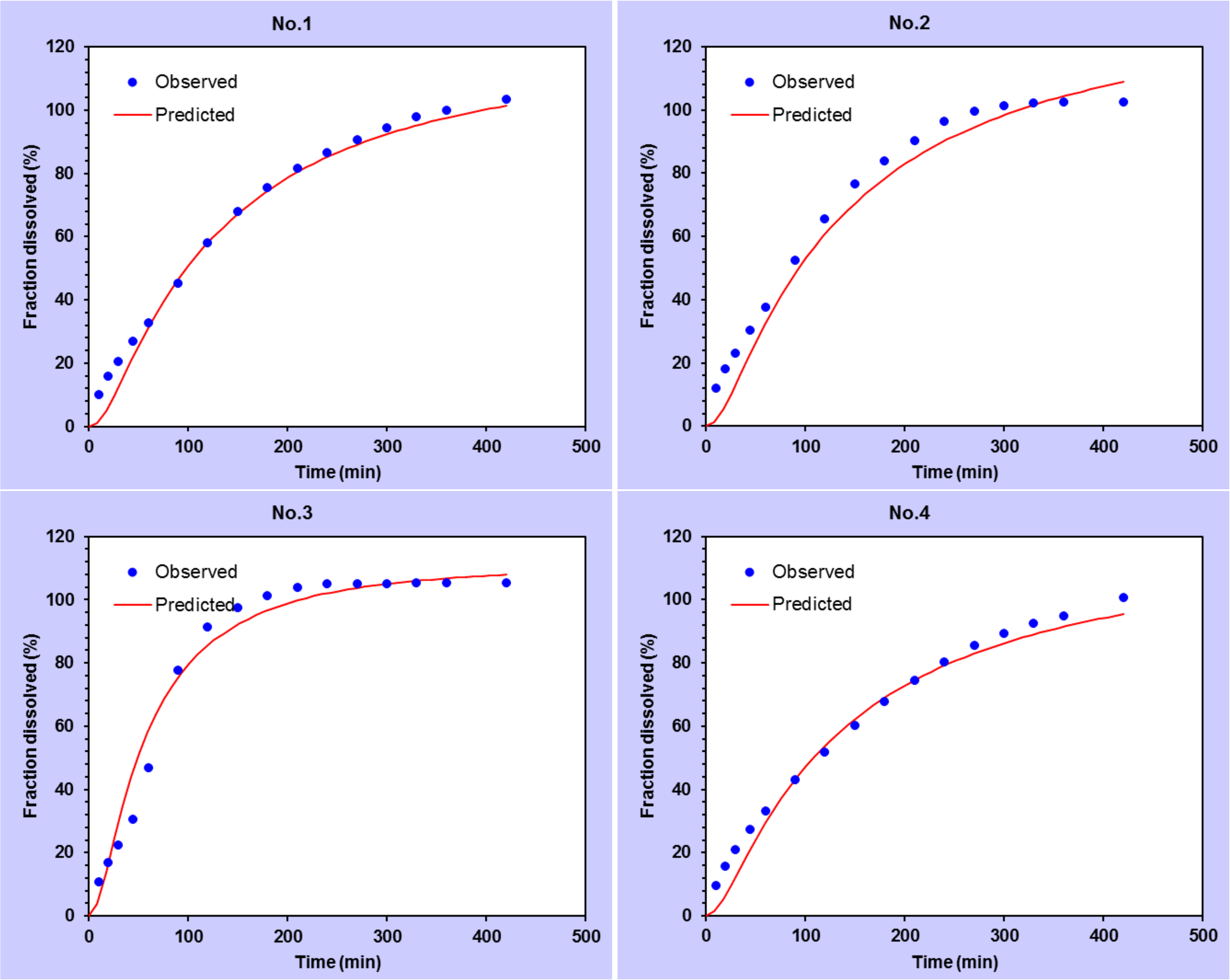

Supplementary materials – Model fitting summary – Pearlitol® 160C (modelling up to app. 60 % of carvedilol released)

Model: **Zero–order**

Model equation: $F = k_0 \cdot t$

Fitted model parameters per tested tablet (N = 4) with statistics – mean, standard deviation (SD), and relative standard deviation expressed in % (RSD%) (output from DDSolver):

Parameter	No.1	No.2	No.3	No.4	Mean	SD	RSD(%)
k_0	0.546	0.627	0.810	0.534	0.629	0.128	20.269

Number of dissolution data points (N), degrees of freedom (df), and selected goodness of fit criteria – Pearson correlation coefficient (R), coefficient of determination (R^2), adjusted coefficient of determination ($R^2_{adjusted}$), and residual sum of squares (RSS) (manual calculation in MS Excel):

Parameter	No.1	No.2	No.3	No.4
N	6	6	6	6
df	5	5	5	5
R	0.998467839	0.999576794	0.988857294	0.993461697
R^2	0.996938026	0.999153767	0.977838748	0.986966144
$R^2_{adjusted}$	0.996938026	0.999153767	0.977838748	0.986966144
RSS	80.99667769	100.0759504	71.08130579	102.7121653

Graphical abstract of model fit presented as mean ± 1 SD of the fraction % of released carvedilol:

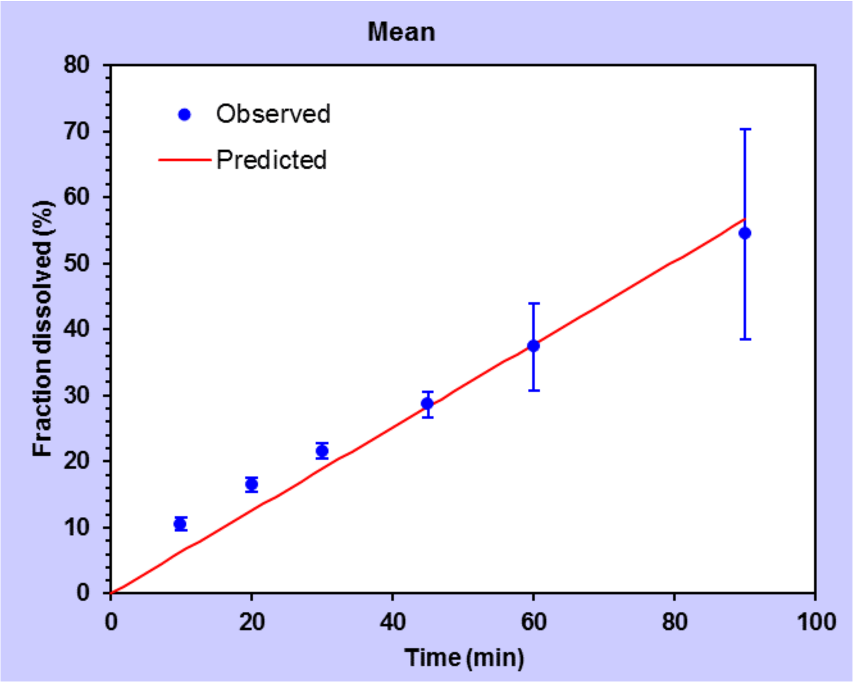

Supplementary materials – Model fitting summary – Pearlitol® 160C (modelling up to app. 60 % of carvedilol released)

Graphical abstract of model fit presented as the fraction % of released carvedilol per tested tablet:

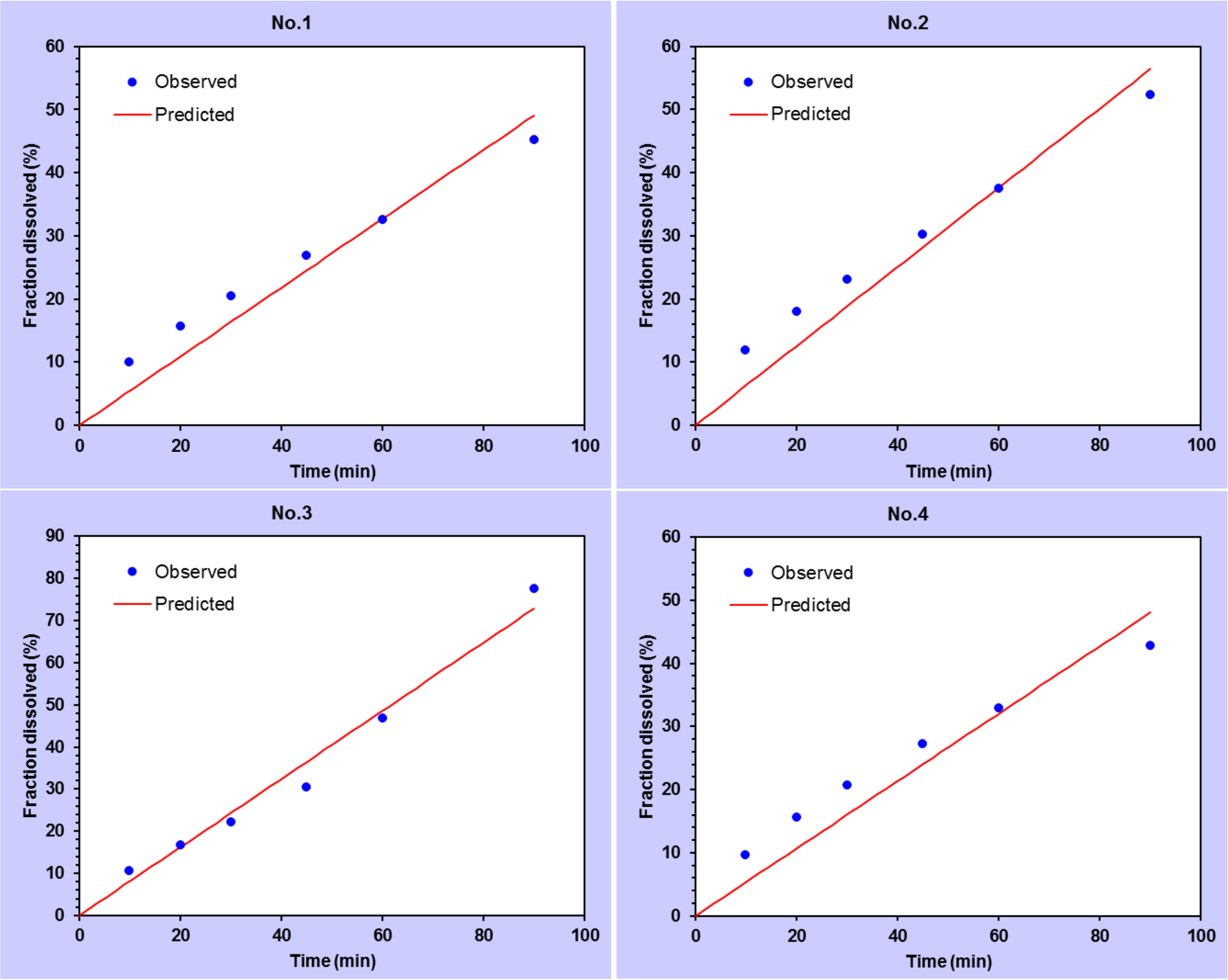

Supplementary materials – Model fitting summary – Pearlitol® 160C (modelling up to app. 60 % of carvedilol released)

Model: **Zero–order with T_{lag}**

Model equation: $F = k_0 \cdot (t - T_{lag})$

Fitted model parameters per tested tablet (N = 4) with statistics – mean, standard deviation (SD), and relative standard deviation expressed in % (RSD%) (output from DDSolver):

Parameter	No.1	No.2	No.3	No.4	Mean	SD	RSD(%)
k_0	0.431	0.498	0.834	0.409	0.543	0.198	36.388
T_{lag}	-15.765	-15.322	1.707	-18.087	-11.867	9.130	-76.936

Number of dissolution data points (N), degrees of freedom (df), and selected goodness of fit criteria – Pearson correlation coefficient (R), coefficient of determination (R^2), adjusted coefficient of determination ($R^2_{adjusted}$), and residual sum of squares (RSS) (manual calculation in MS Excel):

Parameter	No.1	No.2	No.3	No.4
N	6	6	6	6
df	4	4	4	4
R	0.998467839	0.999576794	0.988857294	0.993461697
R^2	0.996938026	0.999153767	0.977838748	0.986966144
$R^2_{adjusted}$	0.996172532	0.998942209	0.972298435	0.98370768
RSS	2.447094266	0.901904762	67.63333333	9.486822157

Graphical abstract of model fit presented as mean ± 1 SD of the fraction % of released carvedilol:

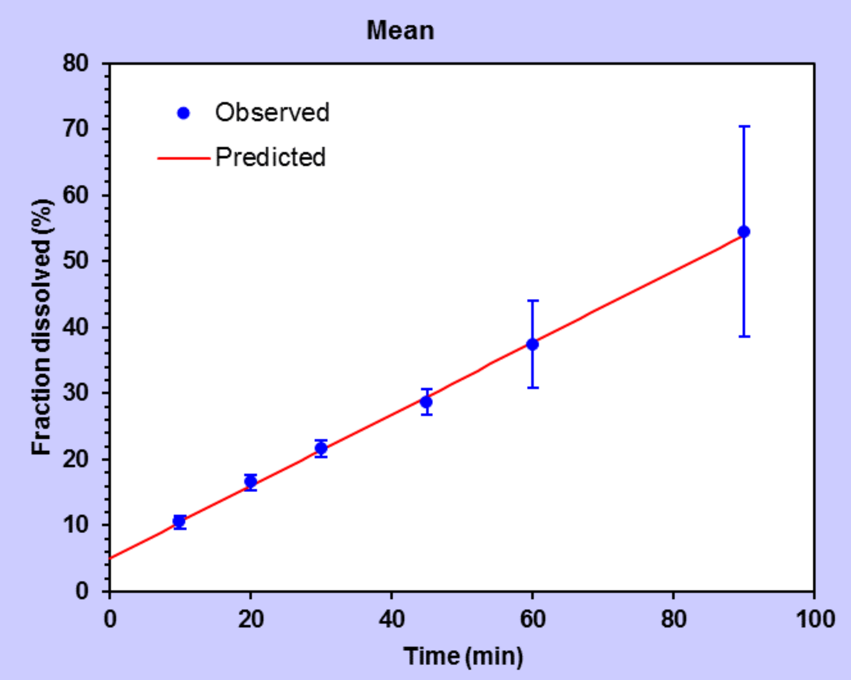

Supplementary materials – Model fitting summary – Pearlitol® 160C (modelling up to app. 60 % of carvedilol released)

Graphical abstract of model fit presented as the fraction % of released carvedilol per tested tablet:

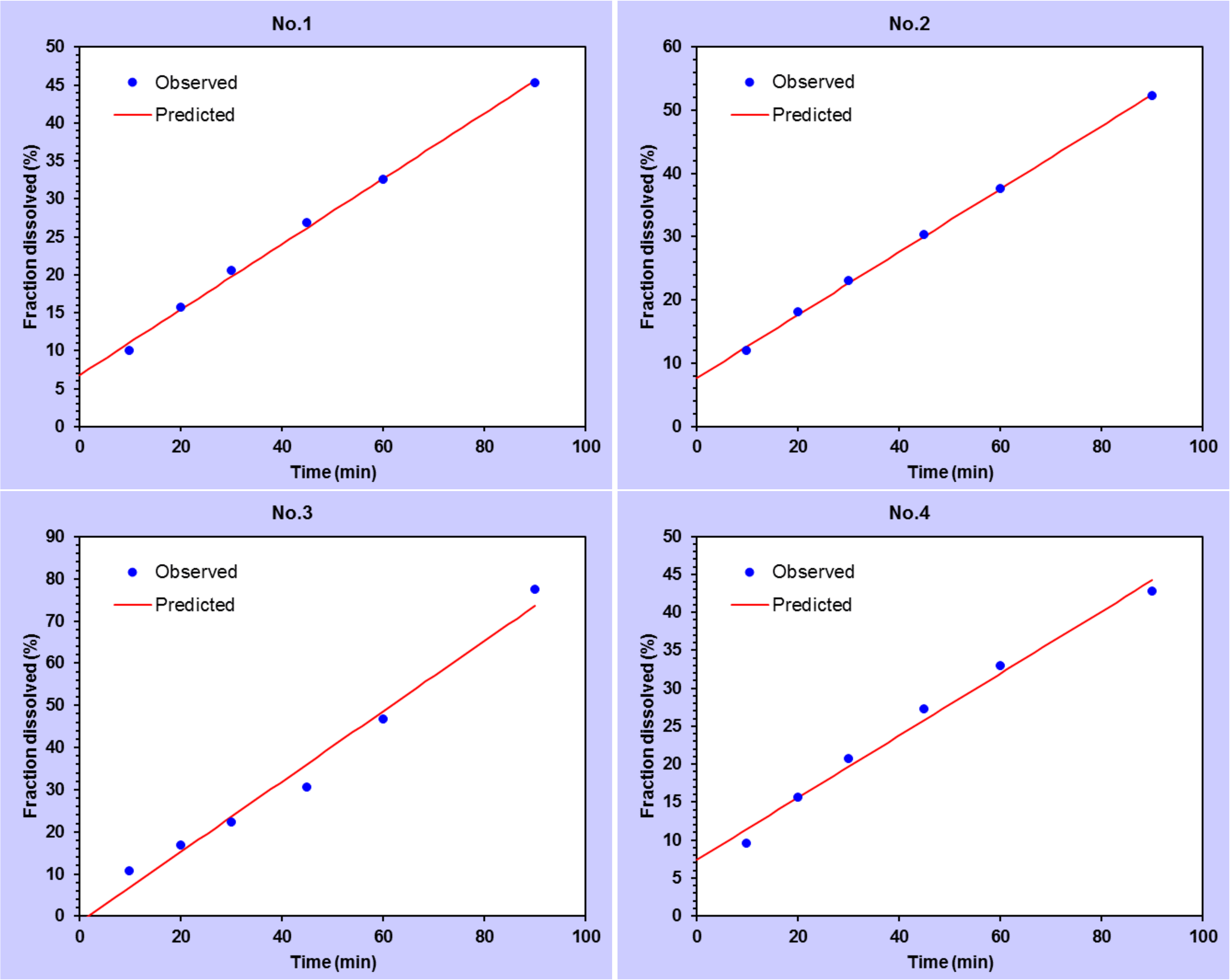

Supplementary materials – Model fitting summary – Pearlitol® 160C (modelling up to app. 60 % of carvedilol released)

Model: **Zero–order with F_0**

Model equation: $F = F_0 + k_0 \cdot t$

Fitted model parameters per tested tablet (N = 4) with statistics – mean, standard deviation (SD), and relative standard deviation expressed in % (RSD%) (output from DDSolver):

Parameter	No.1	No.2	No.3	No.4	Mean	SD	RSD(%)
k_0	0.431	0.498	0.834	0.409	0.543	0.198	36.388
F_0	6.796	7.636	-1.424	7.403	5.103	4.366	85.550

Number of dissolution data points (N), degrees of freedom (df), and selected goodness of fit criteria – Pearson correlation coefficient (R), coefficient of determination (R^2), adjusted coefficient of determination ($R^2_{adjusted}$), and residual sum of squares (RSS) (manual calculation in MS Excel):

Parameter	No.1	No.2	No.3	No.4
N	6	6	6	6
df	4	4	4	4
R	0.998467839	0.999576794	0.988857294	0.993461697
R^2	0.996938026	0.999153767	0.977838748	0.986966144
$R^2_{adjusted}$	0.996172532	0.998942209	0.972298435	0.98370768
RSS	2.447094266	0.901904762	67.63333333	9.486822157

Graphical abstract of model fit presented as mean ± 1 SD of the fraction % of released carvedilol:

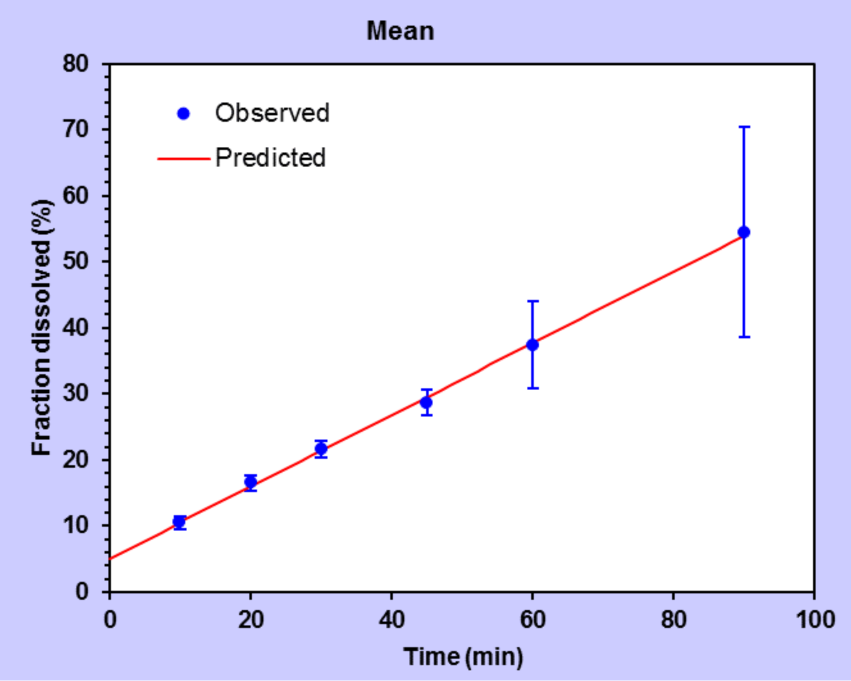

Supplementary materials – Model fitting summary – Pearlitol® 160C (modelling up to app. 60 % of carvedilol released)

Graphical abstract of model fit presented as the fraction % of released carvedilol per tested tablet:

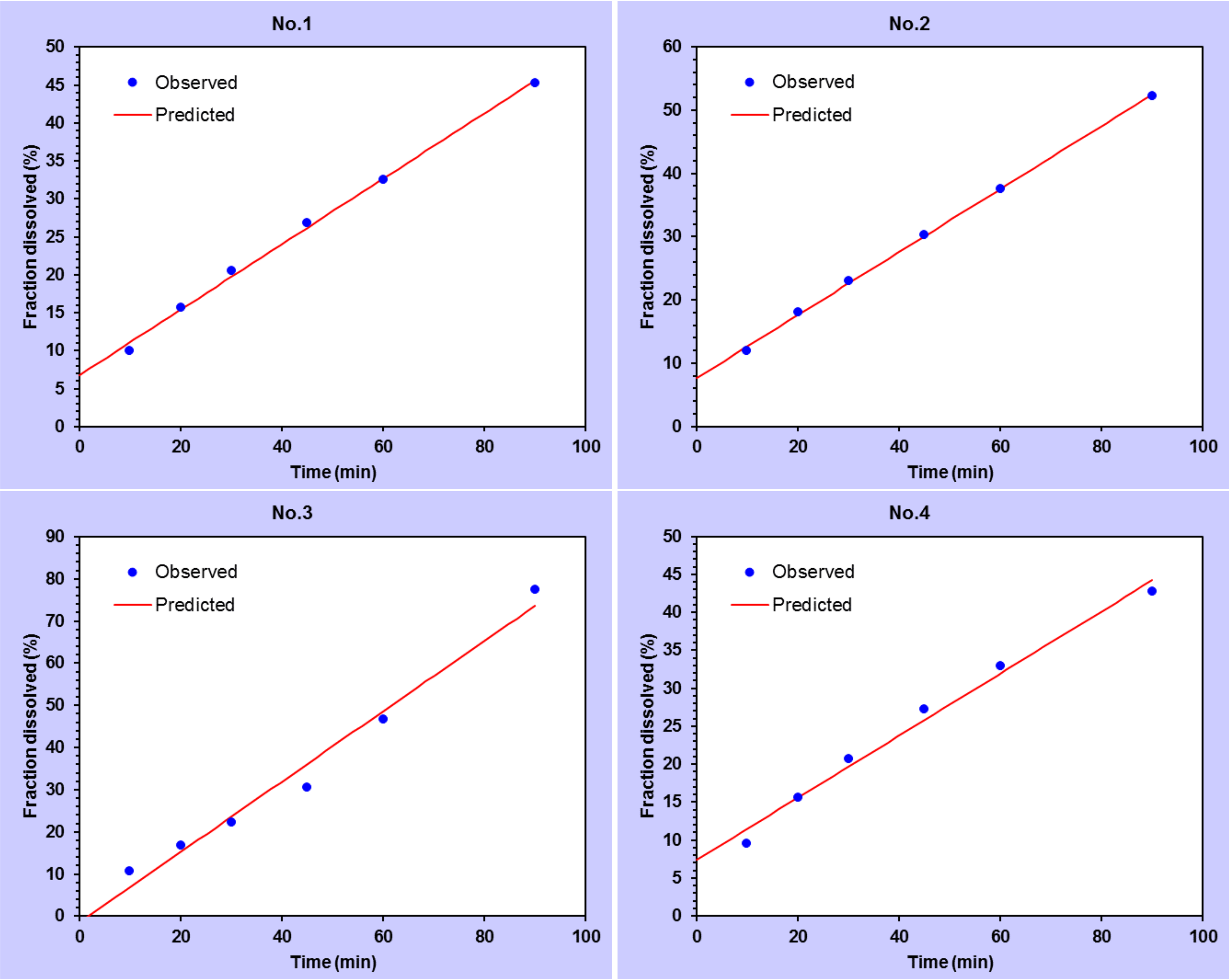

Supplementary materials – Model fitting summary – Pearlitol® 160C (modelling up to app. 60 % of carvedilol released)

Model: **First–order**

Model equation: $F = 100 \cdot (1 - e^{-k_1 \cdot t})$

Fitted model parameters per tested tablet (N = 4) with statistics – mean, standard deviation (SD), and relative standard deviation expressed in % (RSD%) (output from DDSolver):

Parameter	No.1	No.2	No.3	No.4	Mean	SD	RSD(%)
k_1	0.007	0.008	0.010	0.007	0.008	0.002	19.567

Number of dissolution data points (N), degrees of freedom (df), and selected goodness of fit criteria – Pearson correlation coefficient (R), coefficient of determination (R^2), adjusted coefficient of determination ($R^2_{adjusted}$), and residual sum of squares (RSS) (manual calculation in MS Excel):

Parameter	No.1	No.2	No.3	No.4
N	6	6	6	6
df	5	5	5	5
R	0.998555417	0.9965378	0.964827893	0.999282637
R^2	0.997112922	0.993087587	0.930892863	0.998565788
$R^2_{adjusted}$	0.997112922	0.993087587	0.930892863	0.998565788
RSS	26.09991409	28.16041562	386.6874178	34.48093889

Graphical abstract of model fit presented as mean ± 1 SD of the fraction % of released carvedilol:

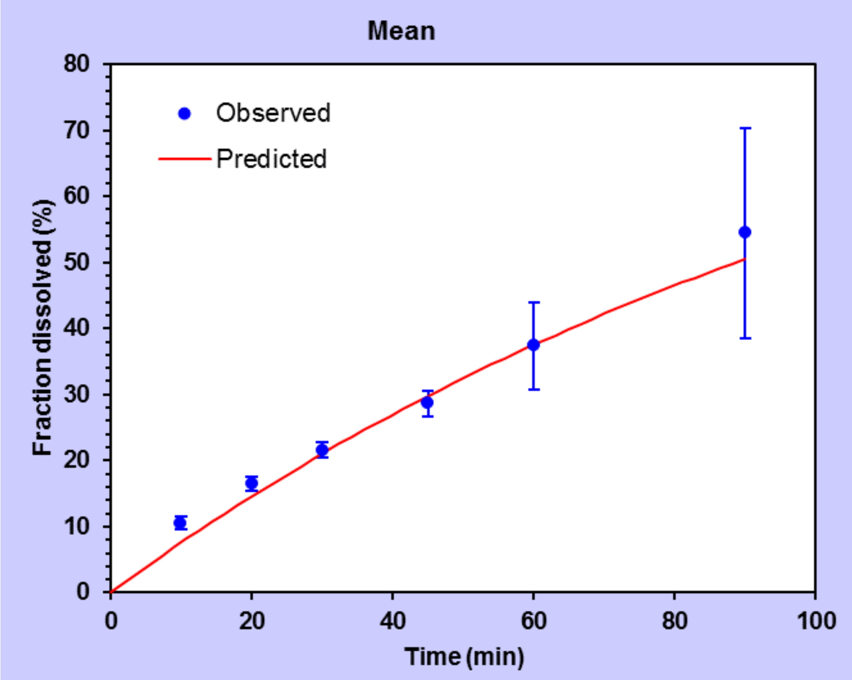

Supplementary materials – Model fitting summary – Pearlitol® 160C (modelling up to app. 60 % of carvedilol released)

Graphical abstract of model fit presented as the fraction % of released carvedilol per tested tablet:

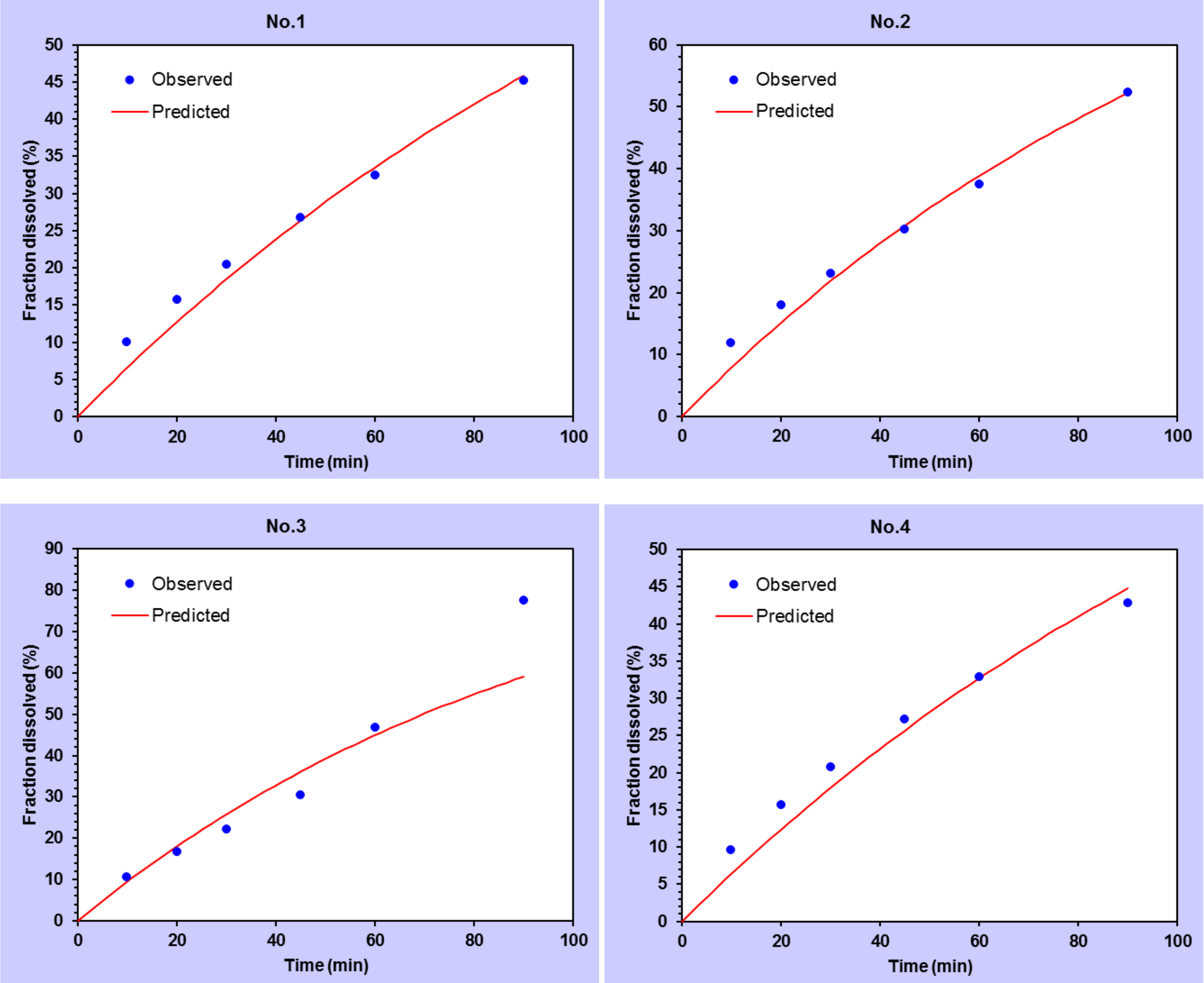

Supplementary materials – Model fitting summary – Pearlitol® 160C (modelling up to app. 60 % of carvedilol released)

Model: **First–order with T_{lag}**

Model equation: $F = 100 \cdot \left[1 - e^{-k_1 \cdot (t - T_{lag})}\right]$

Fitted model parameters per tested tablet (N = 4) with statistics – mean, standard deviation (SD), and relative standard deviation expressed in % (RSD%) (output from DDSolver):

Parameter	No.1	No.2	No.3	No.4	Mean	SD	RSD(%)
k_1	0.006	0.008	0.017	0.006	0.009	0.005	57.927
T_{lag}	-7.123	-5.005	12.374	-9.739	-2.373	10.021	-422.204

Number of dissolution data points (N), degrees of freedom (df), and selected goodness of fit criteria – Pearson correlation coefficient (R), coefficient of determination (R^2), adjusted coefficient of determination ($R^2_{adjusted}$), and residual sum of squares (RSS) (manual calculation in MS Excel):

Parameter	No.1	No.2	No.3	No.4
N	6	6	6	6
df	4	4	4	4
R	0.998858547	0.997113501	0.941599074	0.99884434
R^2	0.997718398	0.994235333	0.886608816	0.997690015
$R^2_{adjusted}$	0.997147997	0.992794167	0.85826102	0.997112519
RSS	1.868954421	6.591706242	474.8643843	1.738489936

Graphical abstract of model fit presented as mean ± 1 SD of the fraction % of released carvedilol:

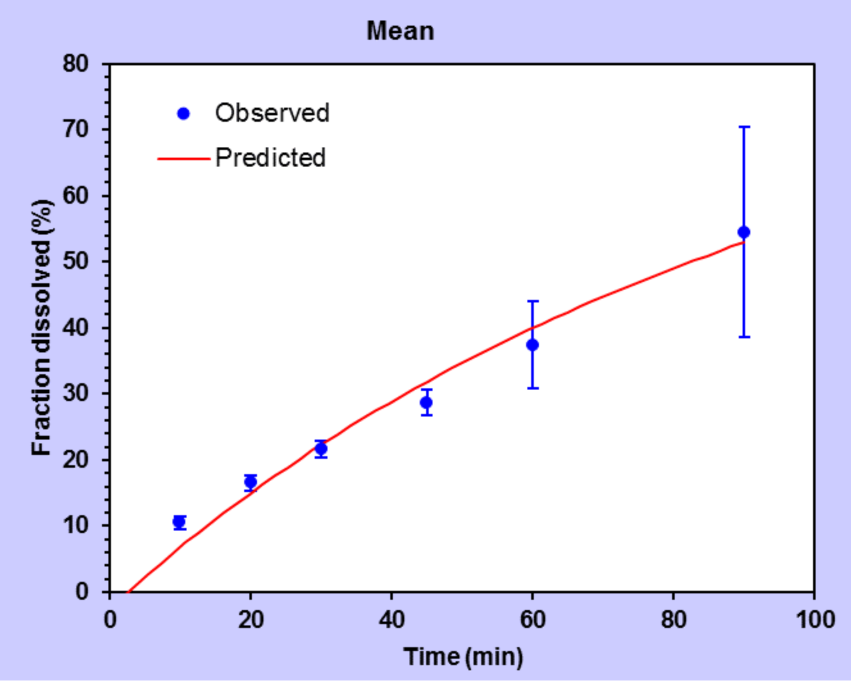

Supplementary materials – Model fitting summary – Pearlitol® 160C (modelling up to app. 60 % of carvedilol released)

Graphical abstract of model fit presented as the fraction % of released carvedilol per tested tablet:

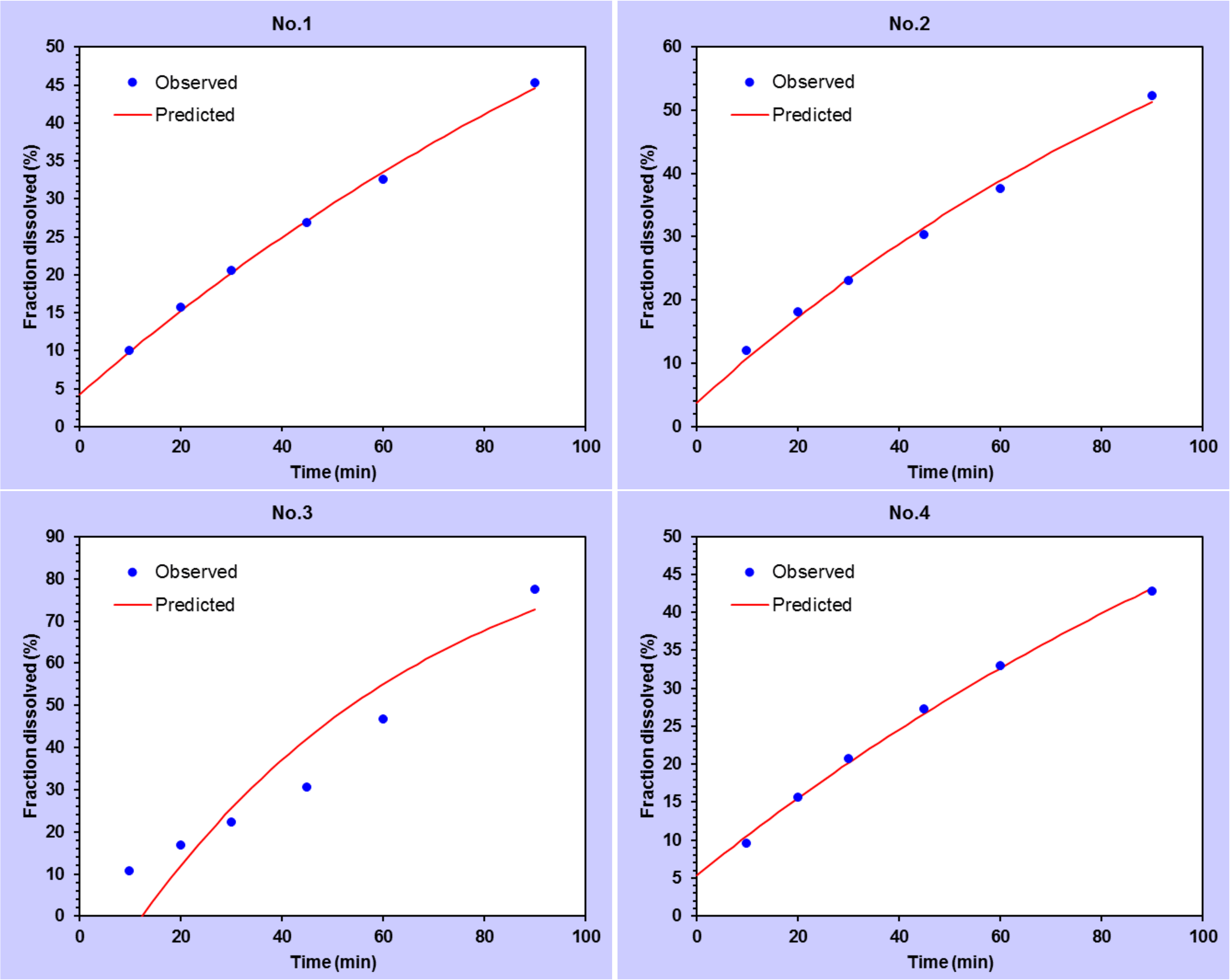

Supplementary materials – Model fitting summary – Pearlitol® 160C (modelling up to app. 60 % of carvedilol released)

Model: **First–order with F_{max}**

Model equation: $F = F_{max} \cdot (1 - e^{-k_1 \cdot t})$

Fitted model parameters per tested tablet (N = 4) with statistics – mean, standard deviation (SD), and relative standard deviation expressed in % (RSD%) (output from DDSolver):

Parameter	No.1	No.2	No.3	No.4	Mean	SD	RSD(%)
k_1	0.028	0.028	0.024	0.028	0.027	0.002	7.315
F_{max}	38.561	44.618	81.375	44.940	52.374	19.556	37.339

Number of dissolution data points (N), degrees of freedom (df), and selected goodness of fit criteria – Pearson correlation coefficient (R), coefficient of determination (R^2), adjusted coefficient of determination ($R^2_{adjusted}$), and residual sum of squares (RSS) (manual calculation in MS Excel):

Parameter	No.1	No.2	No.3	No.4
N	6	6	6	6
df	4	4	4	4
R	0.963725257	0.957433492	0.913284241	0.979428168
R^2	0.928766371	0.916678891	0.834088106	0.959279537
$R^2_{adjusted}$	0.910957964	0.895848614	0.792610132	0.949099421
RSS	100.5860394	140.7844471	1428.625481	80.31569058

Graphical abstract of model fit presented as mean ± 1 SD of the fraction % of released carvedilol:

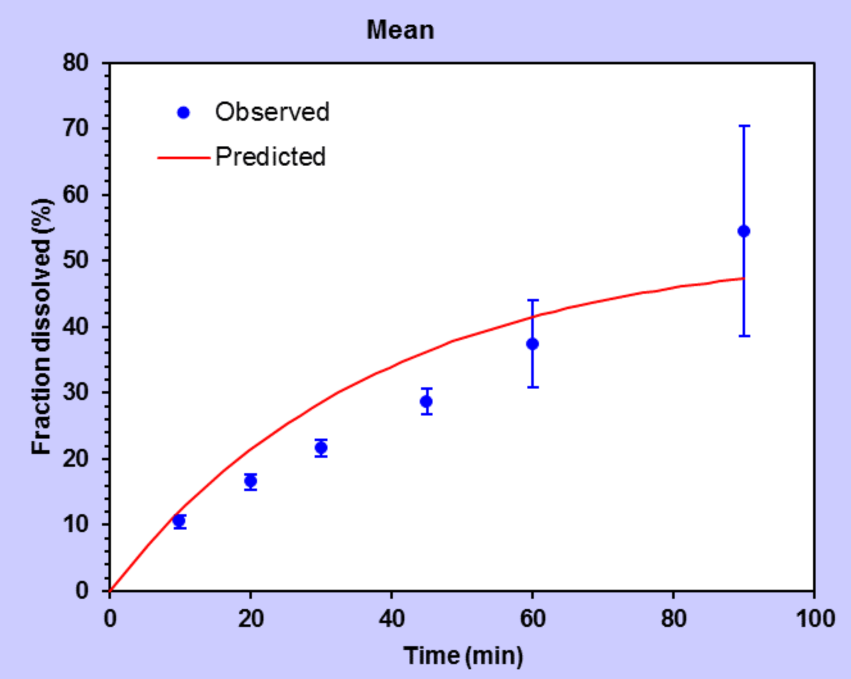

Supplementary materials – Model fitting summary – Pearlitol® 160C (modelling up to app. 60 % of carvedilol released)

Graphical abstract of model fit presented as the fraction % of released carvedilol per tested tablet:

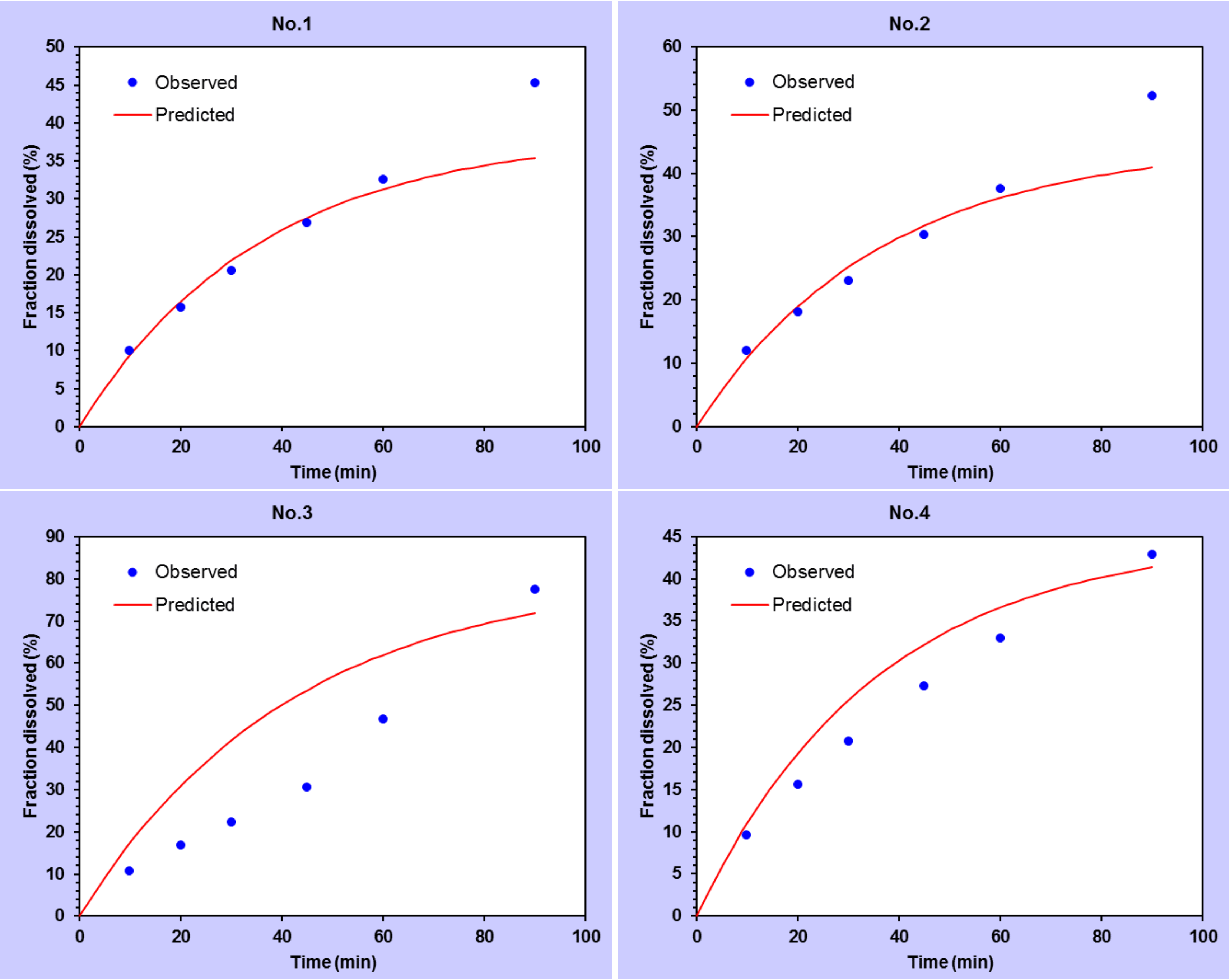

Supplementary materials – Model fitting summary – Pearlitol® 160C (modelling up to app. 60 % of carvedilol released)

Model: **First–order with T_{lag} and F_{max}**

Model equation: $F = F_{max} \cdot \left[1 - e^{-k_1 \cdot (t - T_{lag})}\right]$

Fitted model parameters per tested tablet (N = 4) with statistics – mean, standard deviation (SD), and relative standard deviation expressed in % (RSD%) (output from DDSolver):

Parameter	No.1	No.2	No.3	No.4	Mean	SD	RSD(%)
k_1	0.033	0.033	0.063	0.051	0.045	0.014	31.949
T_{lag}	11.367	11.652	5.977	6.711	8.927	2.999	33.599
F_{max}	47.460	54.915	40.688	37.450	45.128	7.744	17.159

Number of dissolution data points (N), degrees of freedom (df), and selected goodness of fit criteria – Pearson correlation coefficient (R), coefficient of determination (R^2), adjusted coefficient of determination ($R^2_{adjusted}$), and residual sum of squares (RSS) (manual calculation in MS Excel):

Parameter	No.1	No.2	No.3	No.4
N	6	6	6	6
df	3	3	3	3
R	0.948854747	0.940718203	0.755695066	0.92455129
R^2	0.900325331	0.884950737	0.571075033	0.854795087
$R^2_{adjusted}$	0.833875552	0.808251228	0.285125055	0.757991812
RSS	225.9157455	339.8344833	1613.064515	112.6088117

Graphical abstract of model fit presented as mean ± 1 SD of the fraction % of released carvedilol:

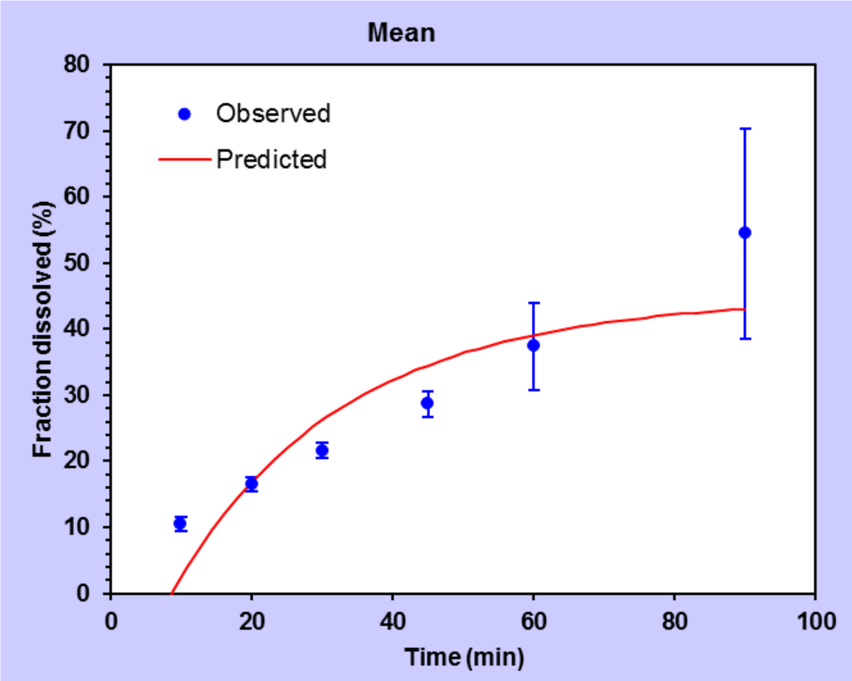

Supplementary materials – Model fitting summary – Pearlitol® 160C (modelling up to app. 60 % of carvedilol released)

Graphical abstract of model fit presented as the fraction % of released carvedilol per tested tablet:

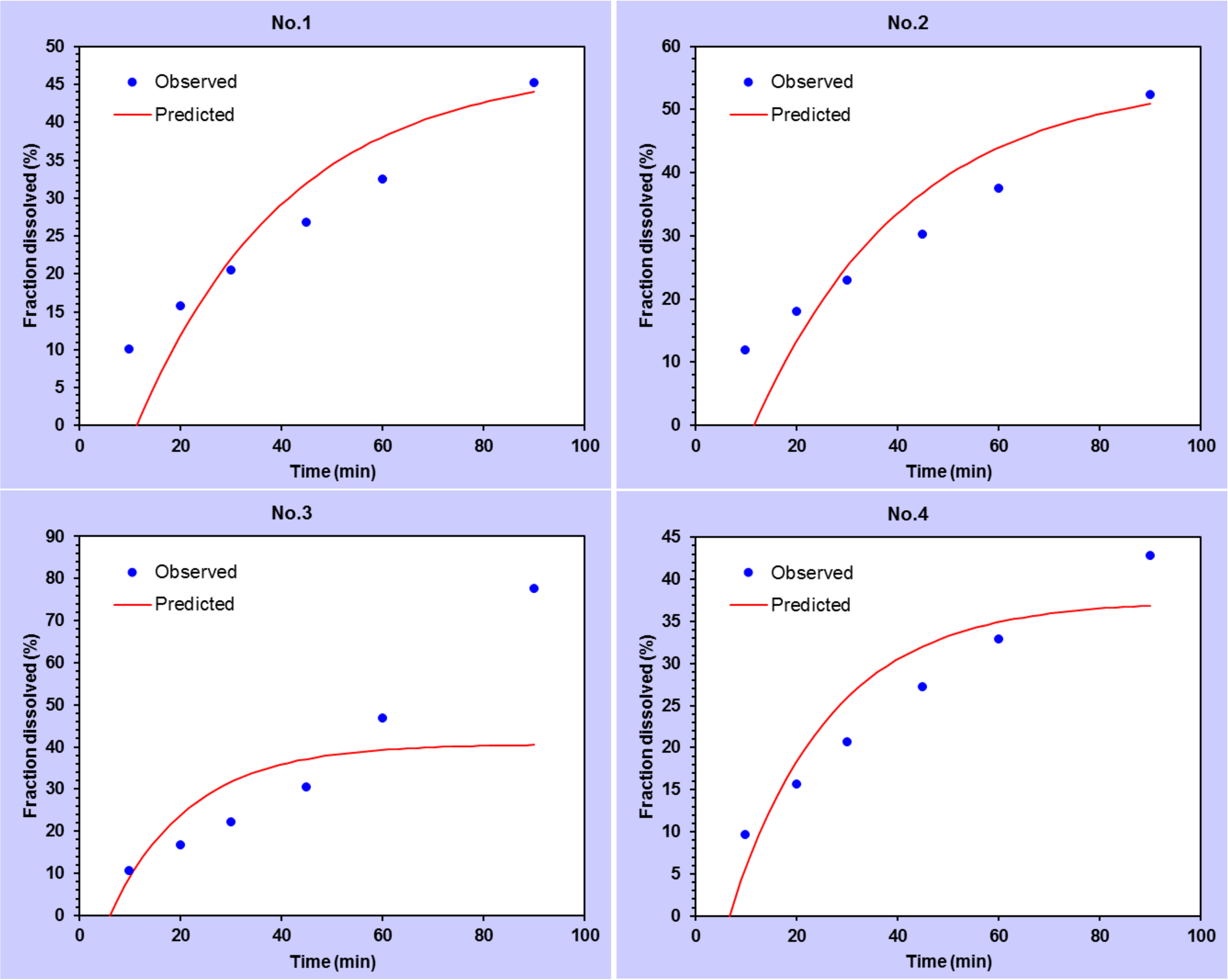

Supplementary materials – Model fitting summary – Pearlitol® 160C (modelling up to app. 60 % of carvedilol released)

Model: **Higuchi**

Model equation: $F = k_H \cdot t^{0.5}$

Fitted model parameters per tested tablet (N = 4) with statistics – mean, standard deviation (SD), and relative standard deviation expressed in % (RSD%) (output from DDSolver):

Parameter	No.1	No.2	No.3	No.4	Mean	SD	RSD(%)
k_H	4.214	4.837	6.005	4.144	4.800	0.862	17.953

Number of dissolution data points (N), degrees of freedom (df), and selected goodness of fit criteria – Pearson correlation coefficient (R), coefficient of determination (R^2), adjusted coefficient of determination ($R^2_{adjusted}$), and residual sum of squares (RSS) (manual calculation in MS Excel):

Parameter	No.1	No.2	No.3	No.4
N	6	6	6	6
df	5	5	5	5
R	0.994132543	0.991281185	0.95634241	0.999184303
R^2	0.988299512	0.982638387	0.914590805	0.998369272
$R^2_{adjusted}$	0.988299512	0.982638387	0.914590805	0.998369272
RSS	57.06802323	83.09403704	805.0849838	38.02737798

Graphical abstract of model fit presented as mean ± 1 SD of the fraction % of released carvedilol:

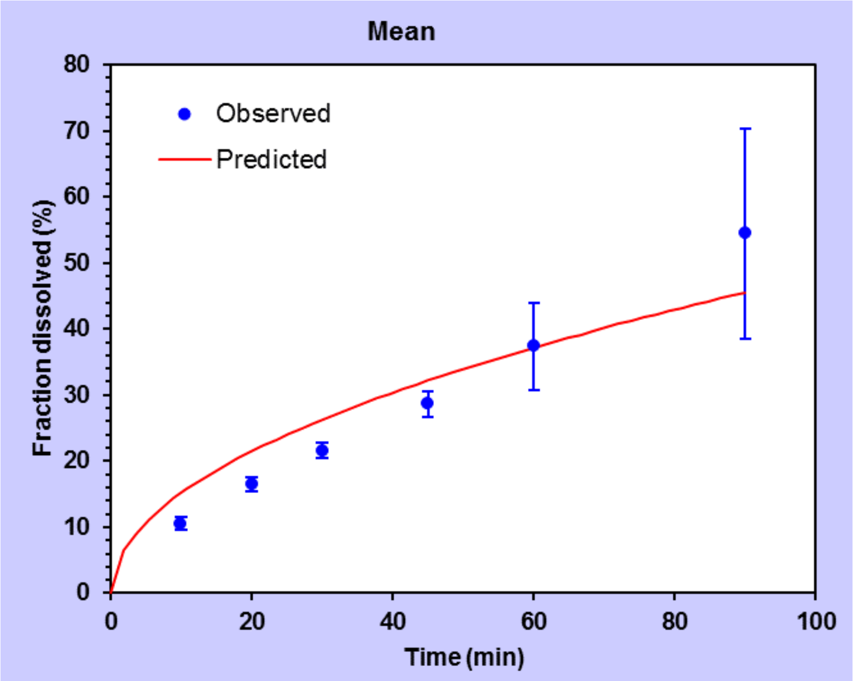

Supplementary materials – Model fitting summary – Pearlitol® 160C (modelling up to app. 60 % of carvedilol released)

Graphical abstract of model fit presented as the fraction % of released carvedilol per tested tablet:

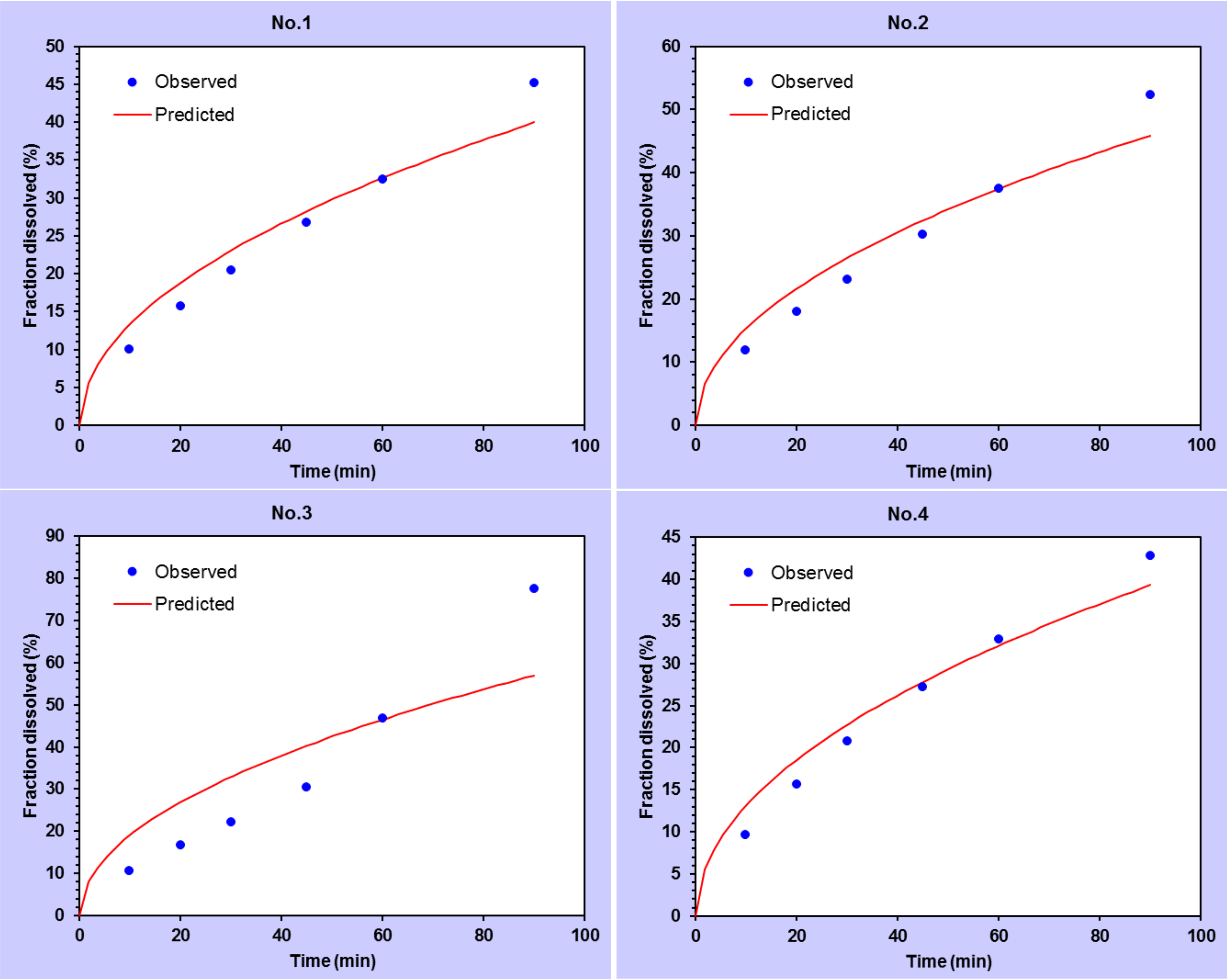

Supplementary materials – Model fitting summary – Pearlitol® 160C (modelling up to app. 60 % of carvedilol released)

Model: **Higuchi with T_{lag}**

Model equation: $F = k_H \cdot (t - T_{lag})^{0.5}$

Fitted model parameters per tested tablet (N = 4) with statistics – mean, standard deviation (SD), and relative standard deviation expressed in % (RSD%) (output from DDSolver):

Parameter	No.1	No.2	No.3	No.4	Mean	SD	RSD(%)
k_H	4.908	5.680	8.499	4.682	5.942	1.757	29.574
T_{lag}	10.780	11.252	19.423	8.906	12.590	4.667	37.065

Number of dissolution data points (N), degrees of freedom (df), and selected goodness of fit criteria – Pearson correlation coefficient (R), coefficient of determination (R^2), adjusted coefficient of determination ($R^2_{adjusted}$), and residual sum of squares (RSS) (manual calculation in MS Excel):

Parameter	No.1	No.2	No.3	No.4
N	6	6	6	6
df	4	4	4	4
R	0.966824624	0.961875795	0.934323147	0.990126175
R^2	0.934749854	0.925205044	0.872959742	0.980349843
$R^2_{adjusted}$	0.934749854	0.925205044	0.872959742	0.980349843
RSS	111.3521864	161.6515523	495.2821071	24.34601802

Graphical abstract of model fit presented as mean ± 1 SD of the fraction % of released carvedilol:

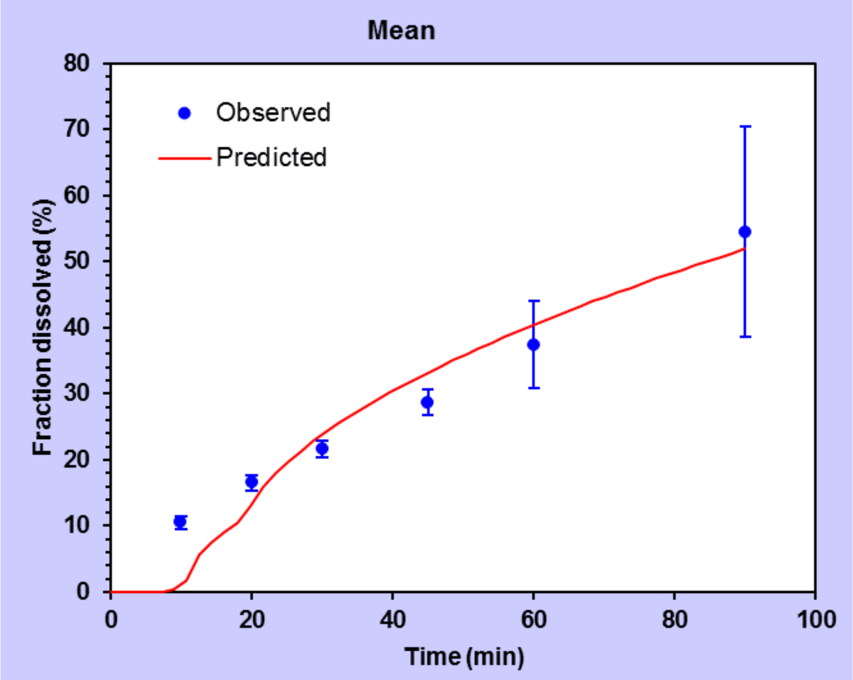

Supplementary materials – Model fitting summary – Pearlitol® 160C (modelling up to app. 60 % of carvedilol released)

Graphical abstract of model fit presented as the fraction % of released carvedilol per tested tablet:

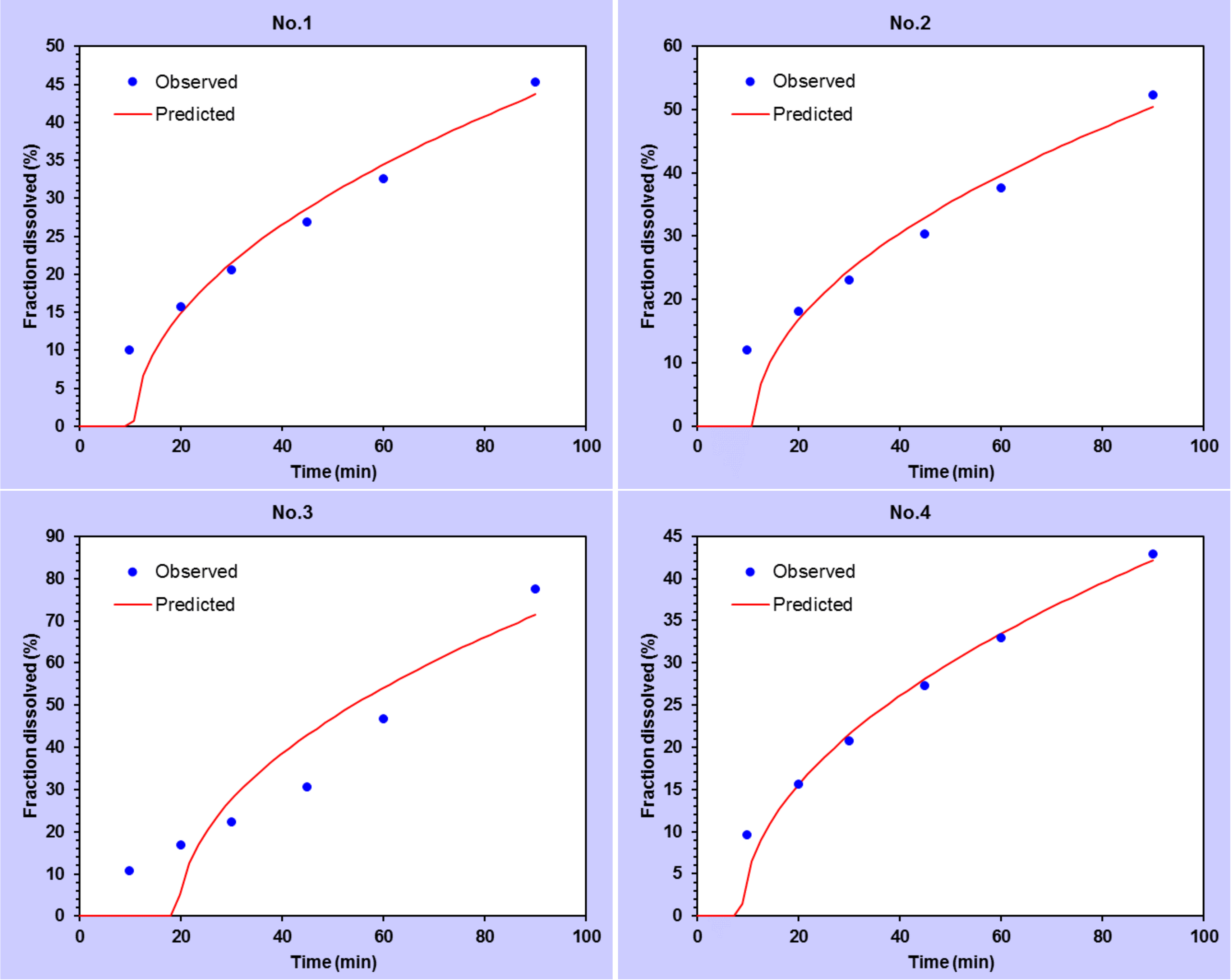

Supplementary materials – Model fitting summary – Pearlitol® 160C (modelling up to app. 60 % of carvedilol released)

Model: **Higuchi with F_0**

Model equation: $F = F_0 + k_H \cdot t^{0.5}$

Fitted model parameters per tested tablet (N = 4) with statistics – mean, standard deviation (SD), and relative standard deviation expressed in % (RSD%) (output from DDSolver):

Parameter	No.1	No.2	No.3	No.4	Mean	SD	RSD(%)
k_H	5.492	6.324	10.325	5.268	6.852	2.359	34.428
F_0	-8.801	-10.239	-29.727	-7.733	-14.125	10.452	-73.996

Number of dissolution data points (N), degrees of freedom (df), and selected goodness of fit criteria – Pearson correlation coefficient (R), coefficient of determination (R^2), adjusted coefficient of determination ($R^2_{adjusted}$), and residual sum of squares (RSS) (manual calculation in MS Excel):

Parameter	No.1	No.2	No.3	No.4
N	6	6	6	6
df	4	4	4	4
R	0.994132543	0.991281185	0.95634241	0.999184303
R^2	0.988299512	0.982638387	0.914590805	0.998369272
$R^2_{adjusted}$	0.988299512	0.982638387	0.914590805	0.998369272
RSS	9.350893397	18.50380441	260.6580453	1.186941688

Graphical abstract of model fit presented as mean ± 1 SD of the fraction % of released carvedilol:

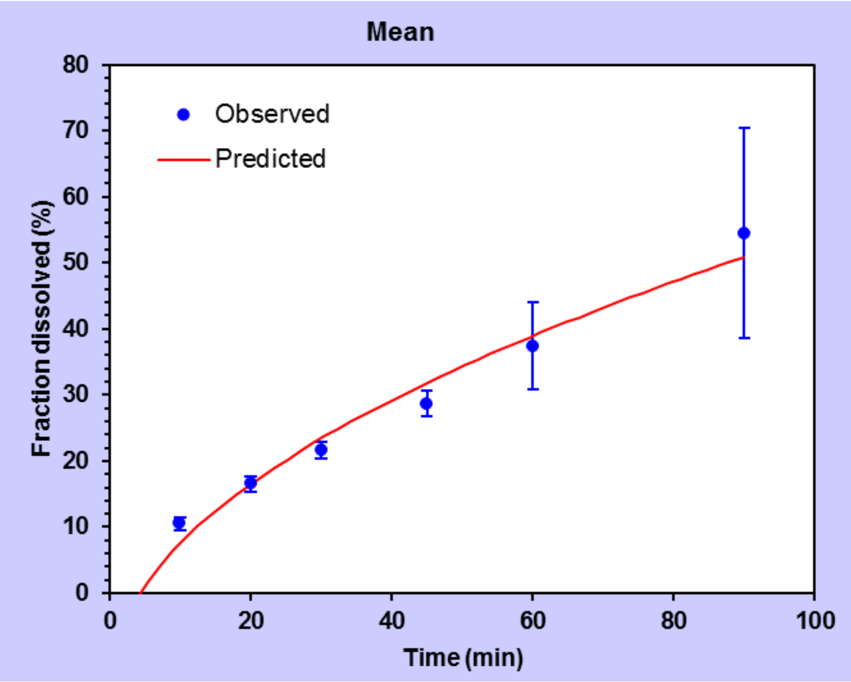

Supplementary materials – Model fitting summary – Pearlitol® 160C (modelling up to app. 60 % of carvedilol released)

Graphical abstract of model fit presented as the fraction % of released carvedilol per tested tablet:

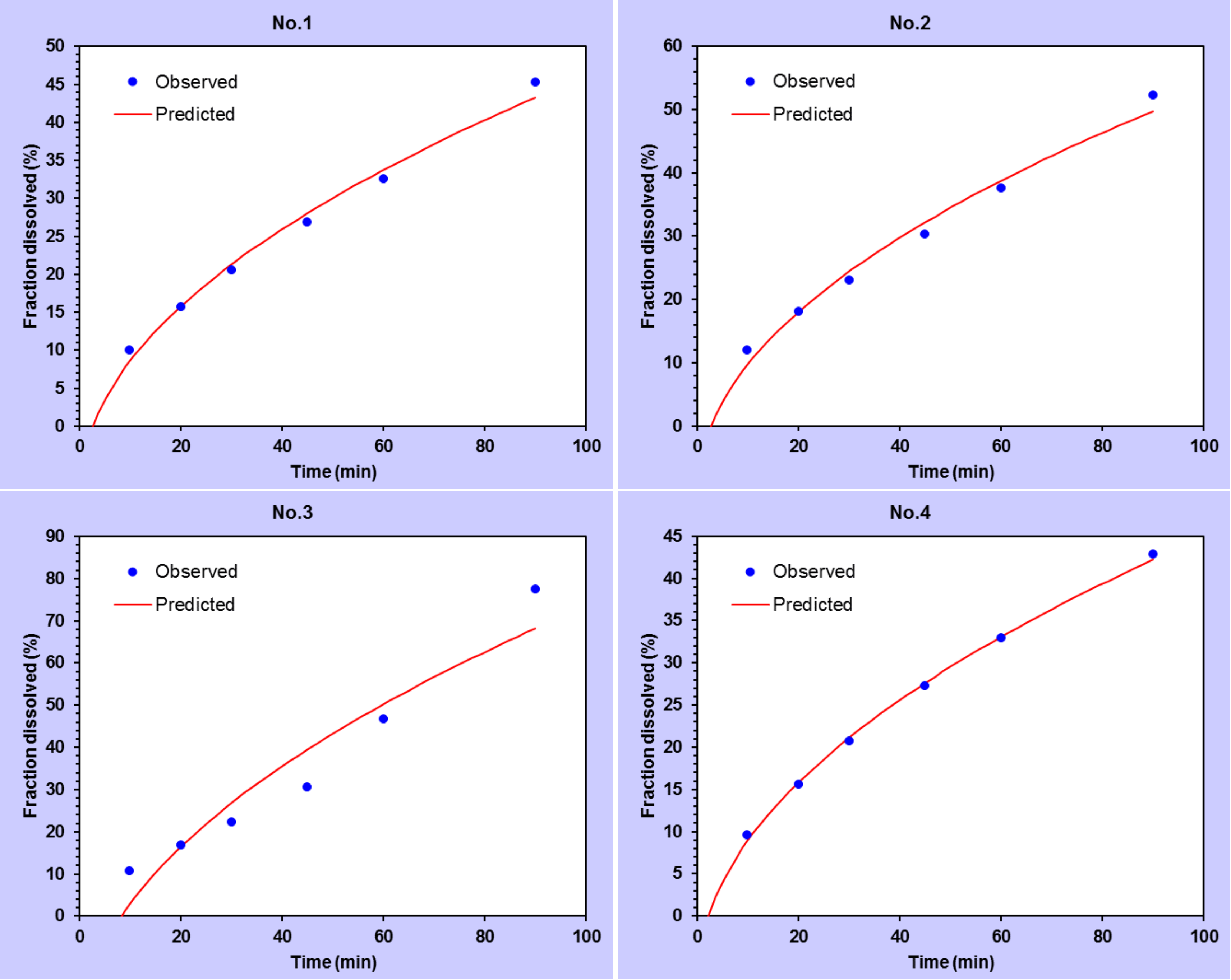

Supplementary materials – Model fitting summary – Pearlitol® 160C (modelling up to app. 60 % of carvedilol released)

Model: **Korsmeyer–Peppas**

Model equation: $F = k_{KP} \cdot t^n$

Fitted model parameters per tested tablet (N = 4) with statistics – mean, standard deviation (SD), and relative standard deviation expressed in % (RSD%) (output from DDSolver):

Parameter	No.1	No.2	No.3	No.4	Mean	SD	RSD(%)
k_{KP}	2.064	2.466	1.204	2.202	1.984	0.546	27.525
n	0.678	0.667	0.890	0.659	0.723	0.111	15.365

Number of dissolution data points (N), degrees of freedom (df), and selected goodness of fit criteria – Pearson correlation coefficient (R), coefficient of determination (R^2), adjusted coefficient of determination ($R^2_{adjusted}$), and residual sum of squares (RSS) (manual calculation in MS Excel):

Parameter	No.1	No.2	No.3	No.4
N	6	6	6	6
df	4	4	4	4
R	0.998421437	0.996794054	0.983884017	0.999931076
R^2	0.996845365	0.993598387	0.968027759	0.999862157
$R^2_{adjusted}$	0.996845365	0.993598387	0.968027759	0.999862157
RSS	3.130989483	9.357621523	168.1295529	0.347765177

Graphical abstract of model fit presented as mean ± 1 SD of the fraction % of released carvedilol:

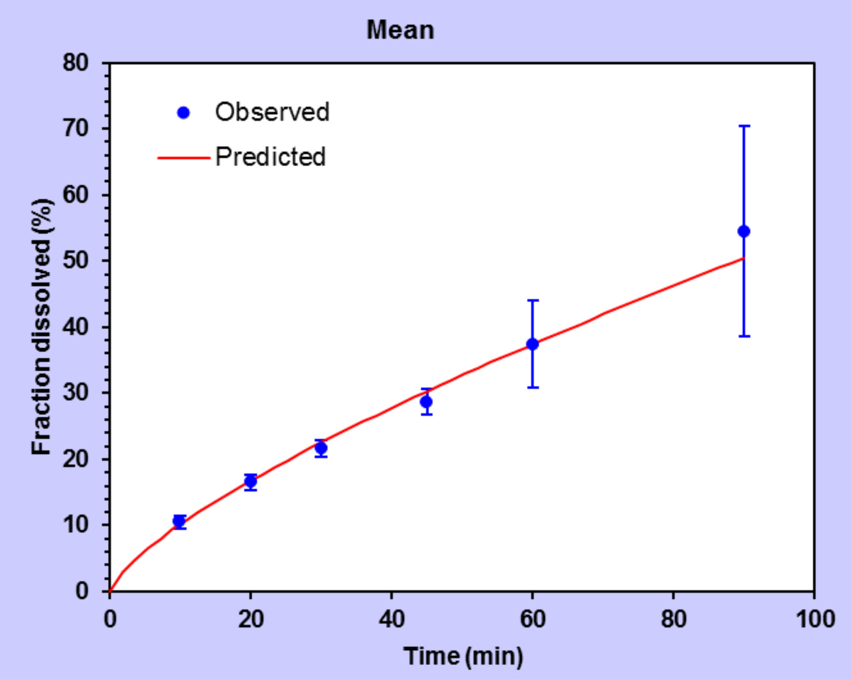

Supplementary materials – Model fitting summary – Pearlitol® 160C (modelling up to app. 60 % of carvedilol released)

Graphical abstract of model fit presented as the fraction % of released carvedilol per tested tablet:

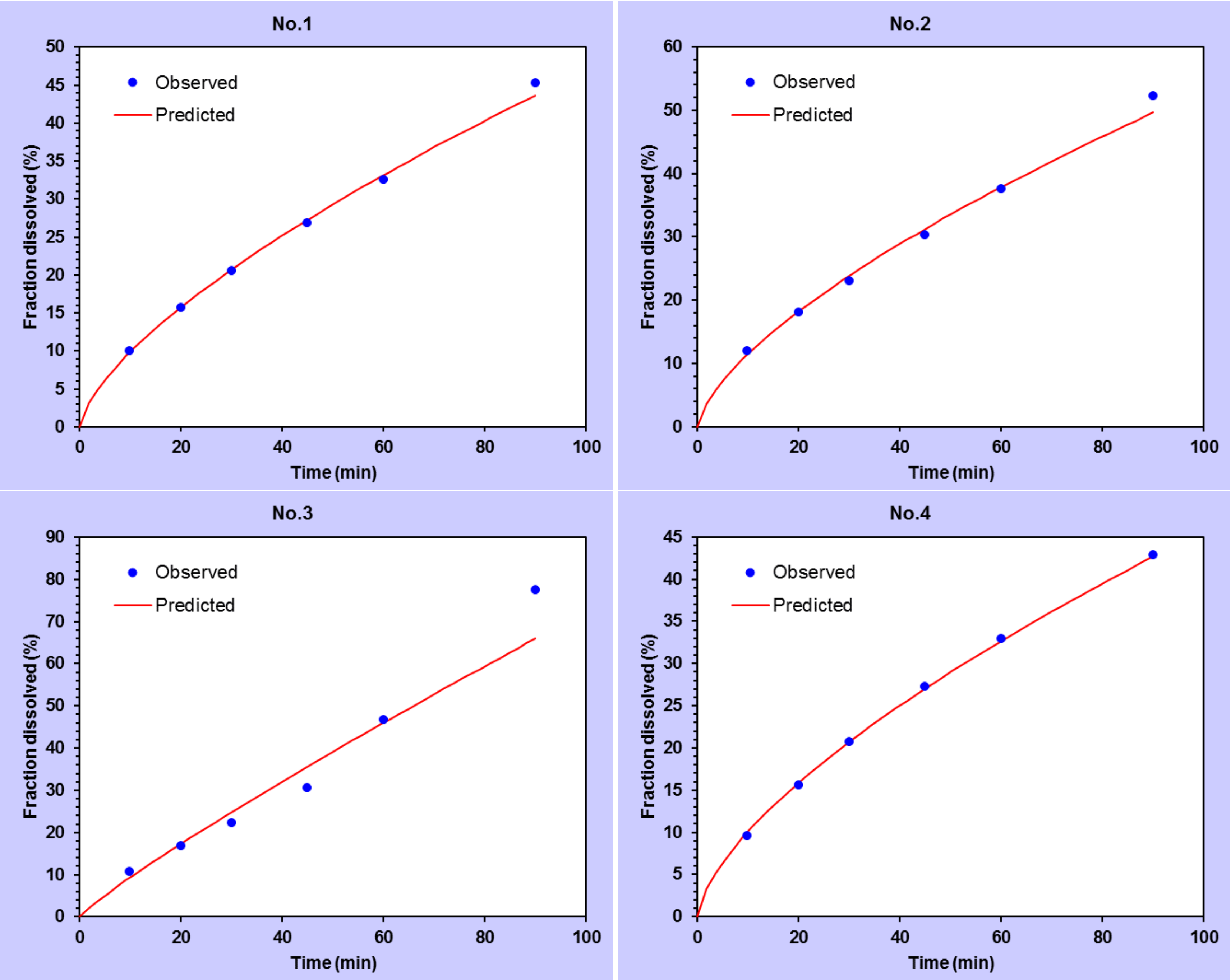

Supplementary materials – Model fitting summary – Pearlitol® 160C (modelling up to app. 60 % of carvedilol released)

Model: **Korsmeyer–Peppas with T_{lag}**

Model equation: $F = k_{KP} \cdot \left(t - T_{lag}\right)^{n}$

Fitted model parameters per tested tablet (N = 4) with statistics – mean, standard deviation (SD), and relative standard deviation expressed in % (RSD%) (output from DDSolver):

Parameter	No.1	No.2	No.3	No.4	Mean	SD	RSD(%)
k_{KP}	3.463	4.120	2.424	3.379	3.347	0.699	20.877
n	0.560	0.549	0.728	0.564	0.600	0.085	14.239
T_{lag}	4.000	4.000	4.000	4.000	4.000	0.000	0.000

Number of dissolution data points (N), degrees of freedom (df), and selected goodness of fit criteria – Pearson correlation coefficient (R), coefficient of determination (R^2), adjusted coefficient of determination ($R^2_{adjusted}$), and residual sum of squares (RSS) (manual calculation in MS Excel):

Parameter	No.1	No.2	No.3	No.4
N	6	6	6	6
df	3	3	3	3
R	0.9939147	0.990432928	0.971744968	0.999079208
R^2	0.987866431	0.980957385	0.944288283	0.998159263
$R^2_{adjusted}$	0.979777384	0.968262309	0.907147138	0.996932105
RSS	13.44617633	28.6290632	290.3758607	2.061159847

Graphical abstract of model fit presented as mean ± 1 SD of the fraction % of released carvedilol:

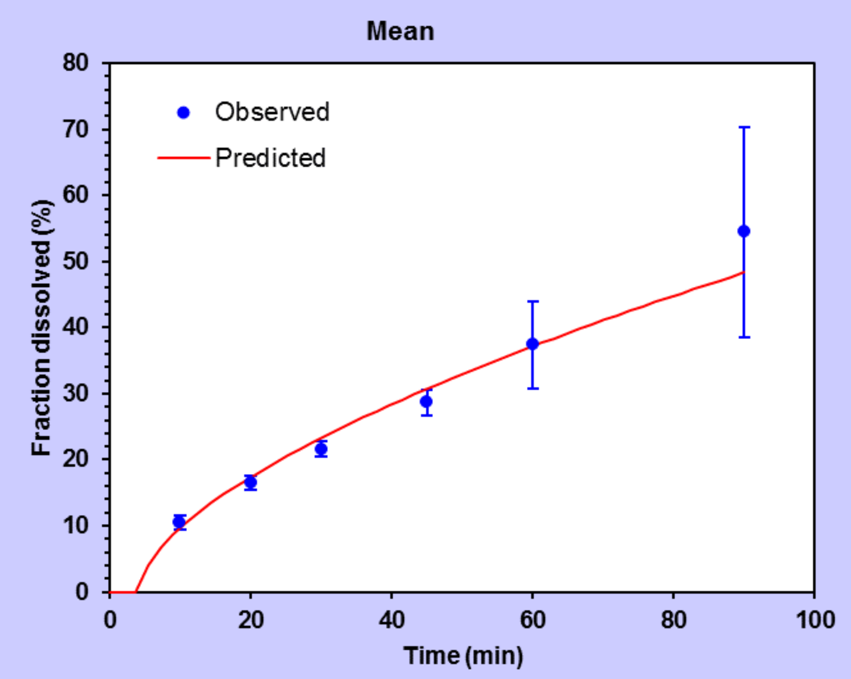

Supplementary materials – Model fitting summary – Pearlitol® 160C (modelling up to app. 60 % of carvedilol released)

Graphical abstract of model fit presented as the fraction % of released carvedilol per tested tablet:

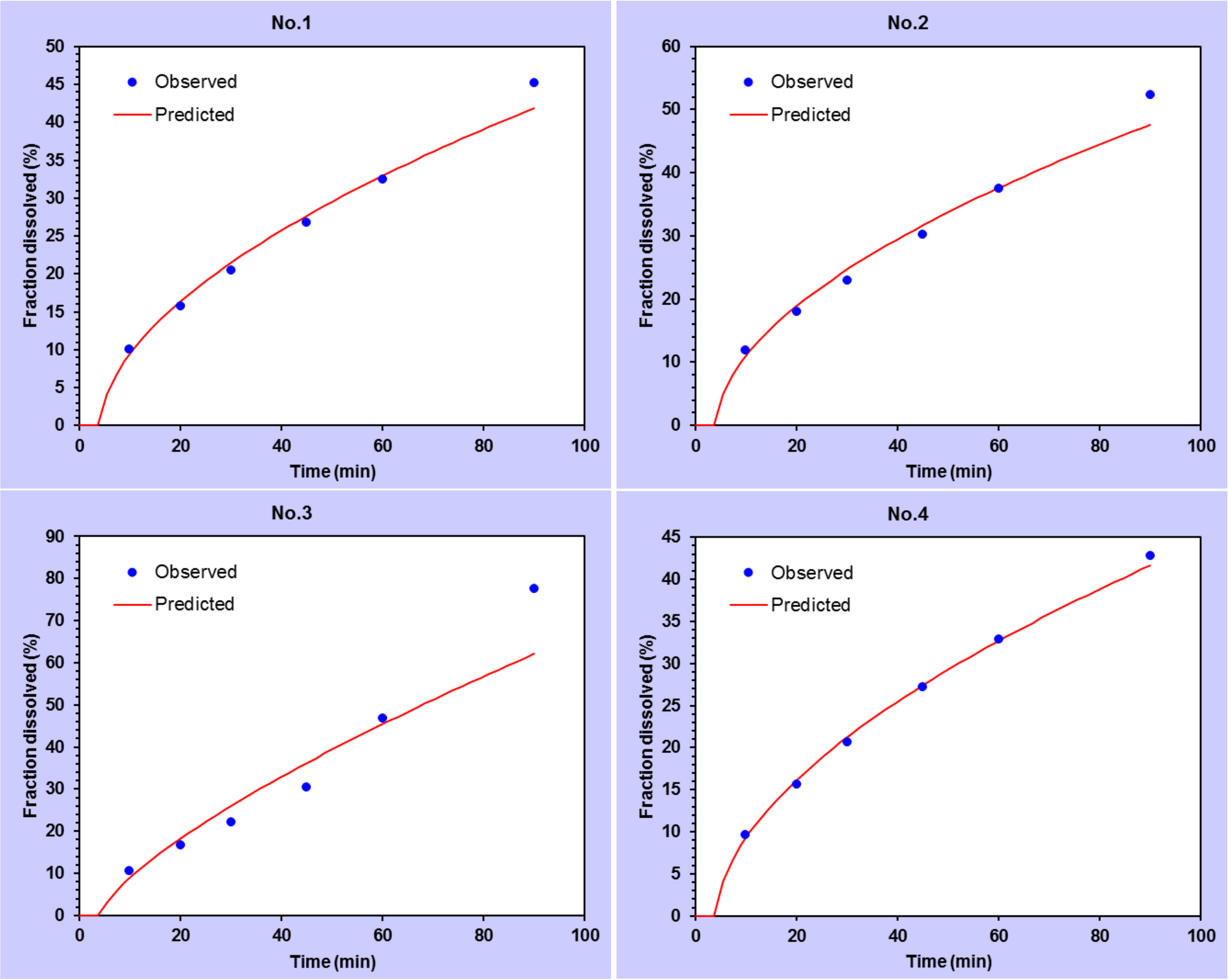

Supplementary materials – Model fitting summary – Pearlitol® 160C (modelling up to app. 60 % of carvedilol released)

Model: **Korsmeyer–Peppas with F_0**

Model equation: $F = F_0 + k_{KP} \cdot t^n$

Fitted model parameters per tested tablet (N = 4) with statistics – mean, standard deviation (SD), and relative standard deviation expressed in % (RSD%) (output from DDSolver):

Parameter	No.1	No.2	No.3	No.4	Mean	SD	RSD(%)
k_{KP}	0.848	1.006	0.471	0.955	0.820	0.242	29.519
n	0.864	0.853	1.095	0.832	0.911	0.123	13.519
F_0	4.000	4.760	4.240	4.181	4.295	0.326	7.595

Number of dissolution data points (N), degrees of freedom (df), and selected goodness of fit criteria – Pearson correlation coefficient (R), coefficient of determination (R^2), adjusted coefficient of determination ($R^2_{adjusted}$), and residual sum of squares (RSS) (manual calculation in MS Excel):

Parameter	No.1	No.2	No.3	No.4
N	6	6	6	6
df	3	3	3	3
R	0.999548177	0.999589132	0.992232036	0.997900966
R^2	0.999096557	0.999178432	0.984524414	0.995806338
$R^2_{adjusted}$	0.998494262	0.99863072	0.974207357	0.993010563
RSS	0.970473776	1.001756758	91.6574384	4.259610905

Graphical abstract of model fit presented as mean ± 1 SD of the fraction % of released carvedilol:

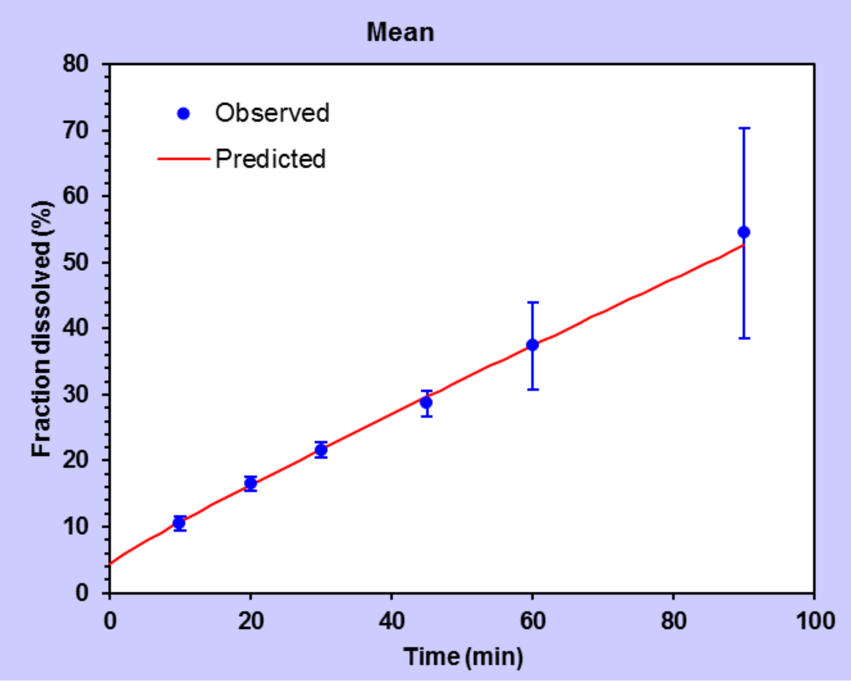

Supplementary materials – Model fitting summary – Pearlitol® 160C (modelling up to app. 60 % of carvedilol released)

Graphical abstract of model fit presented as the fraction % of released carvedilol per tested tablet:

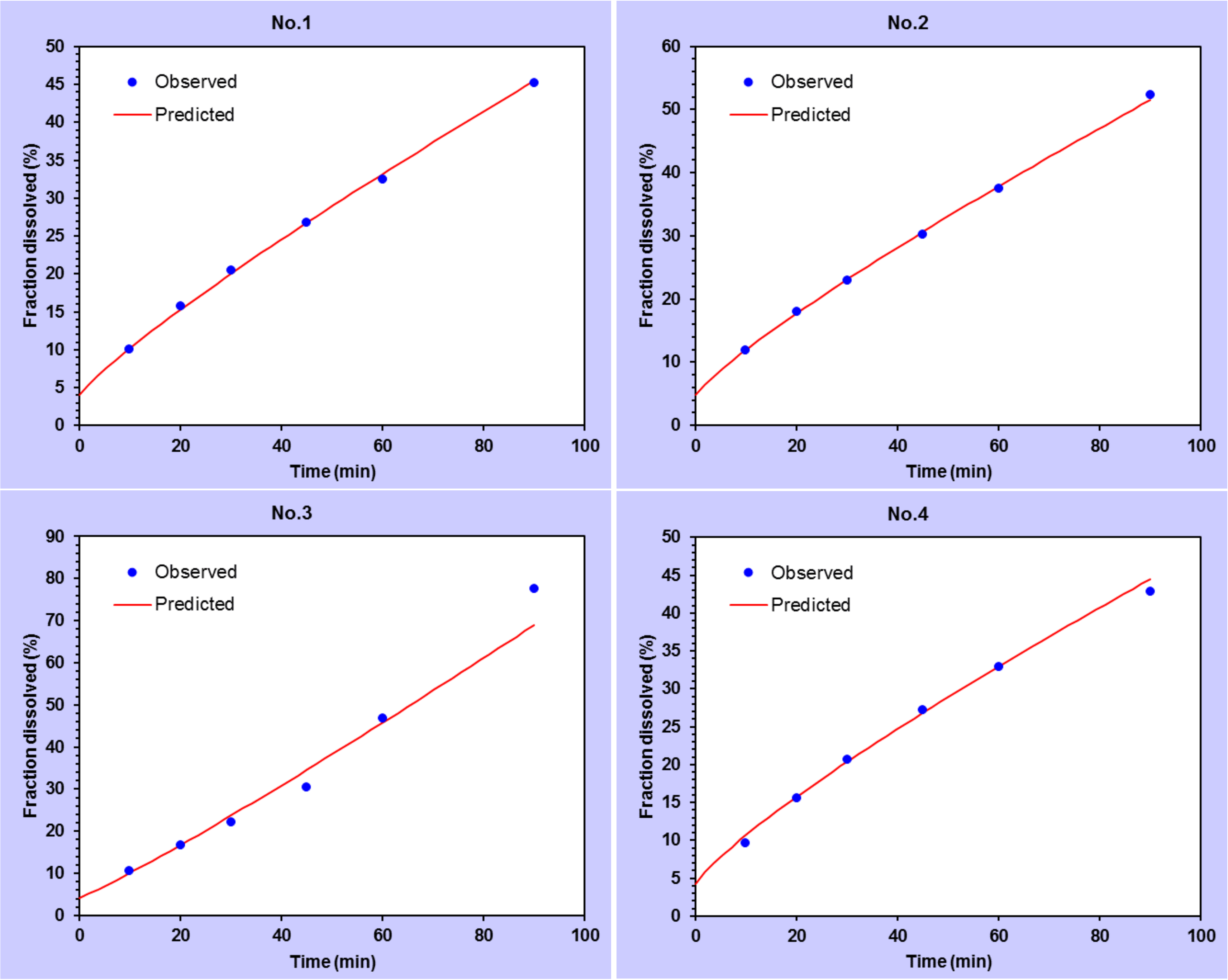

Supplementary materials – Model fitting summary – Pearlitol® 160C (modelling up to app. 60 % of carvedilol released)

Model: **Hixson–Crowell**

Model equation: $F = 100 \cdot [1 - (1 - k_{HC} \cdot t)^3]$

Fitted model parameters per tested tablet (N = 4) with statistics – mean, standard deviation (SD), and relative standard deviation expressed in % (RSD%) (output from DDSolver):

Parameter	No.1	No.2	No.3	No.4	Mean	SD	RSD(%)
k_{HC}	0.002	0.002	0.004	0.002	0.003	0.001	29.486

Number of dissolution data points (N), degrees of freedom (df), and selected goodness of fit criteria – Pearson correlation coefficient (R), coefficient of determination (R^2), adjusted coefficient of determination ($R^2_{adjusted}$), and residual sum of squares (RSS) (manual calculation in MS Excel):

Parameter	No.1	No.2	No.3	No.4
N	6	6	6	6
df	5	5	5	5
R	0.999200505	0.998464949	0.967711217	0.998135171
R^2	0.99840165	0.996932255	0.936465	0.996273819
$R^2_{adjusted}$	0.99840165	0.996932255	0.936465	0.996273819
RSS	38.99291095	42.55432505	291.1366191	51.69854073

Graphical abstract of model fit presented as mean ± 1 SD of the fraction % of released carvedilol:

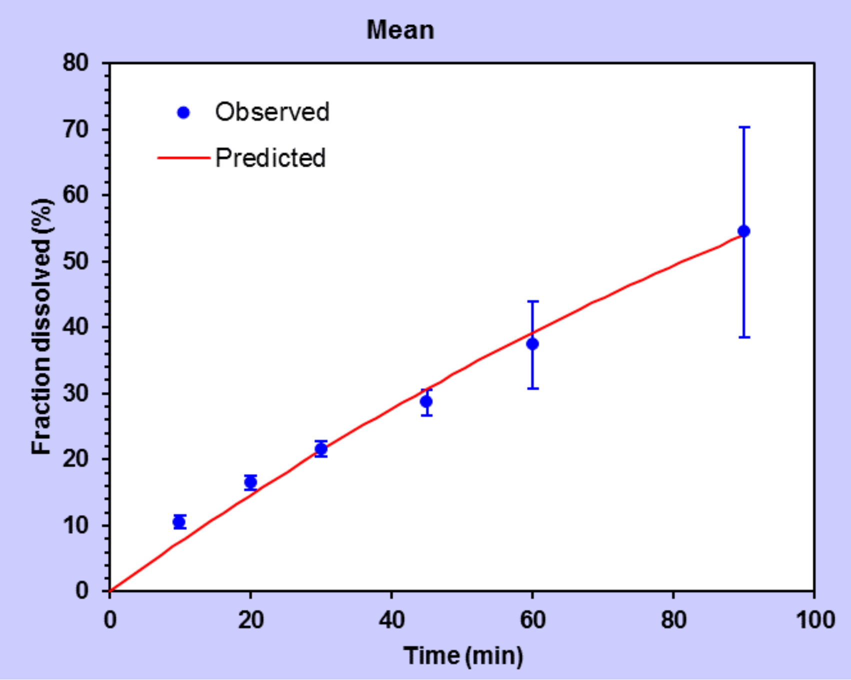

Supplementary materials – Model fitting summary – Pearlitol® 160C (modelling up to app. 60 % of carvedilol released)

Graphical abstract of model fit presented as the fraction % of released carvedilol per tested tablet:

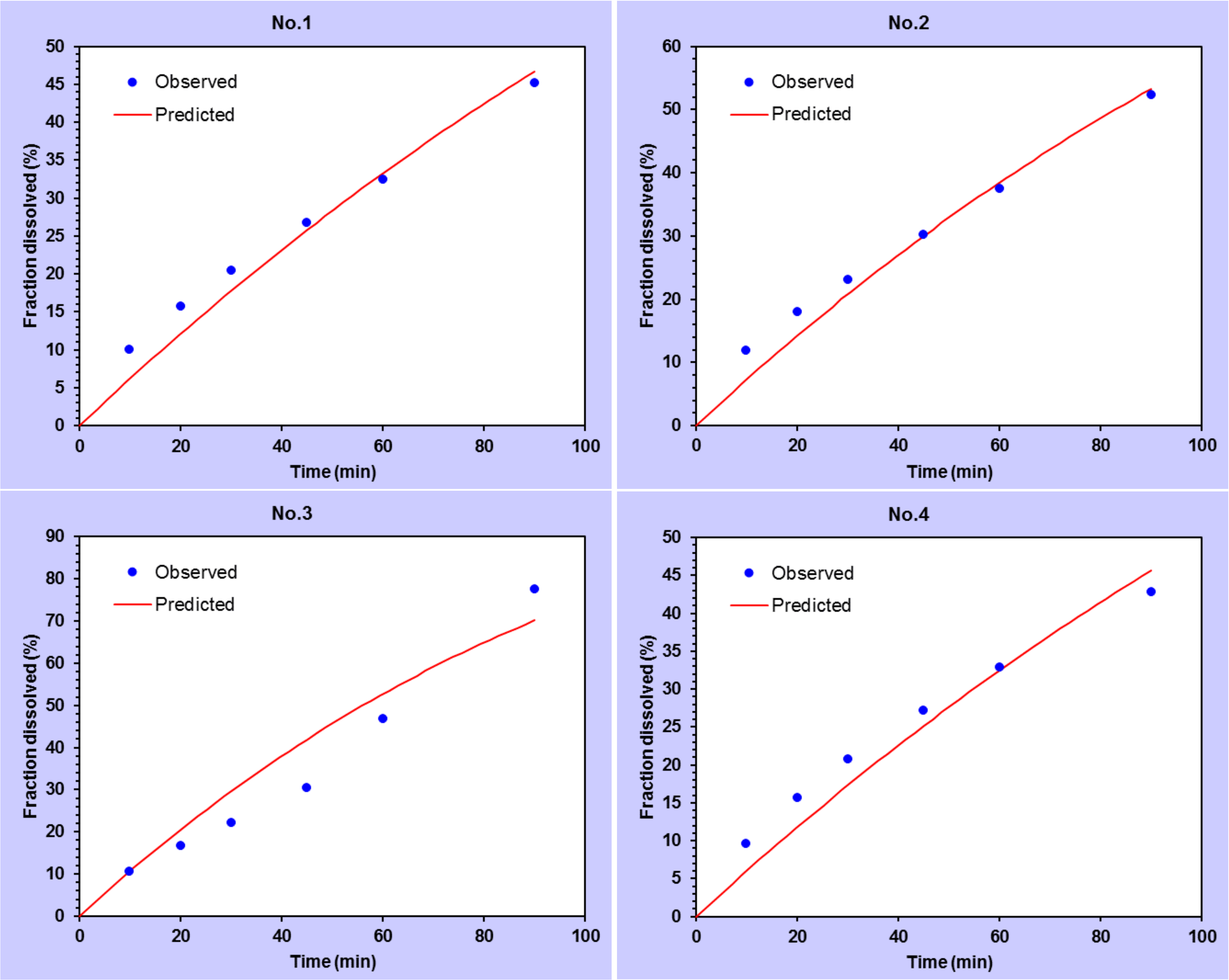

Supplementary materials – Model fitting summary – Pearlitol® 160C (modelling up to app. 60 % of carvedilol released)

Model: **Hixson–Crowell with T_{lag}**

Model equation: $F = 100 \cdot \left\{1 - \left[1 - k_{HC} \cdot \left(t - T_{lag}\right)\right]^3\right\}$

Fitted model parameters per tested tablet (N = 4) with statistics – mean, standard deviation (SD), and relative standard deviation expressed in % (RSD%) (output from DDSolver):

Parameter	No.1	No.2	No.3	No.4	Mean	SD	RSD(%)
k_{HC}	0.002	0.002	0.004	0.002	0.003	0.001	49.754
T_{lag}	-9.832	-8.196	9.176	-12.363	-5.304	9.804	-184.848

Number of dissolution data points (N), degrees of freedom (df), and selected goodness of fit criteria – Pearson correlation coefficient (R), coefficient of determination (R^2), adjusted coefficient of determination ($R^2_{adjusted}$), and residual sum of squares (RSS) (manual calculation in MS Excel):

Parameter	No.1	No.2	No.3	No.4
N	6	6	6	6
df	4	4	4	4
R	0.99928952	0.998839442	0.963009818	0.997557963
R^2	0.998579545	0.99768023	0.927387909	0.995121889
$R^2_{adjusted}$	0.998224431	0.997100287	0.909234886	0.993902361
RSS	1.136777938	2.541184518	255.5925436	3.610096003

Graphical abstract of model fit presented as mean ± 1 SD of the fraction % of released carvedilol:

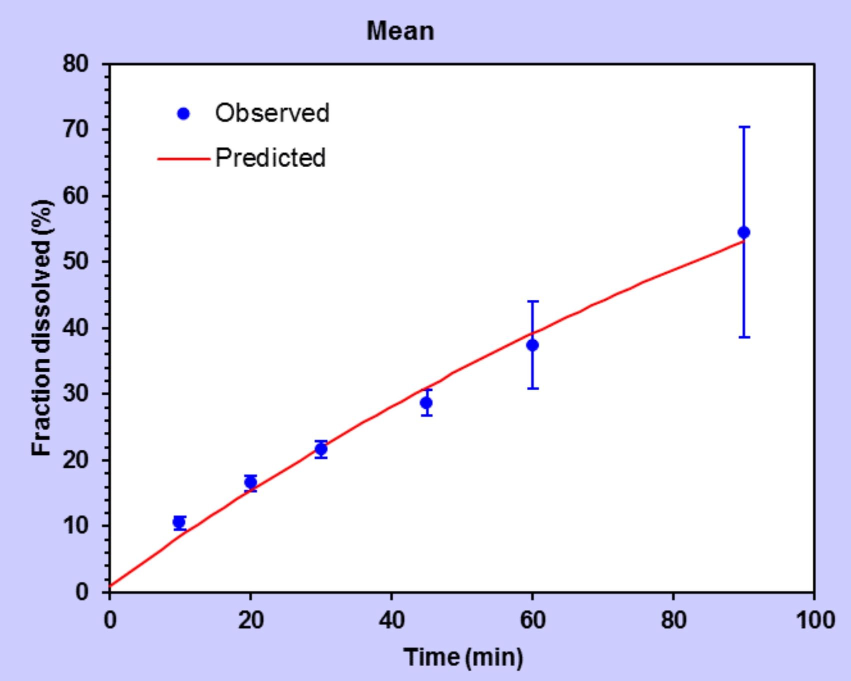

Supplementary materials – Model fitting summary – Pearlitol® 160C (modelling up to app. 60 % of carvedilol released)

Graphical abstract of model fit presented as the fraction % of released carvedilol per tested tablet:

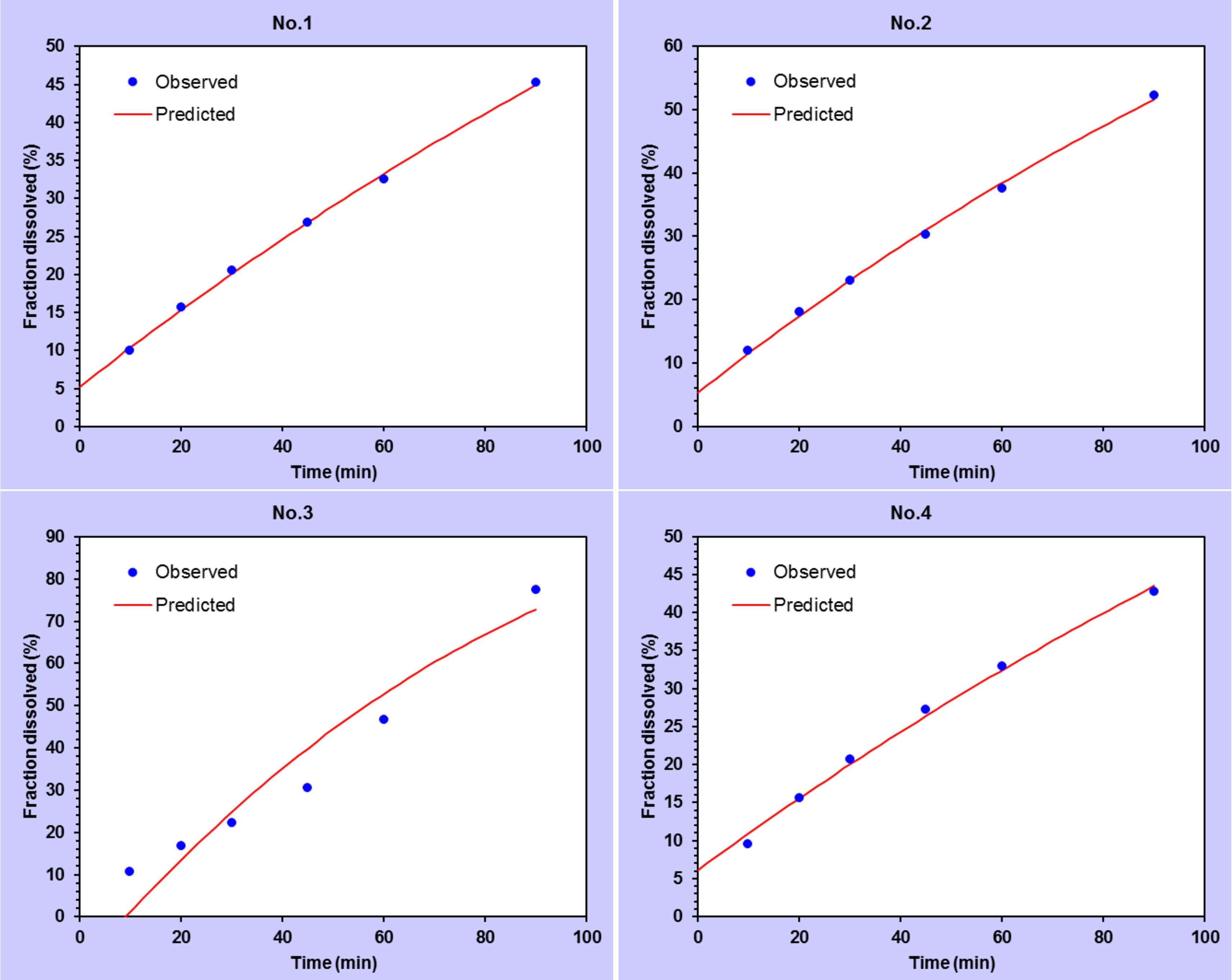

Supplementary materials – Model fitting summary – Pearlitol® 160C (modelling up to app. 60 % of carvedilol released)

Model: **Hopfenberg**

Model equation: $F = 100 \cdot [1 - (1 - k_{HB} \cdot t)^n]$

Fitted model parameters per tested tablet (N = 4) with statistics – mean, standard deviation (SD), and relative standard deviation expressed in % (RSD%) (output from DDSolver):

Parameter	No.1	No.2	No.3	No.4	Mean	SD	RSD(%)
k_{HB}	0.002	0.002	0.008	0.002	0.004	0.003	80.078
n	3.000	3.000	1.000	3.000	2.500	1.000	40.000

Number of dissolution data points (N), degrees of freedom (df), and selected goodness of fit criteria – Pearson correlation coefficient (R), coefficient of determination (R^2), adjusted coefficient of determination ($R^2_{adjusted}$), and residual sum of squares (RSS) (manual calculation in MS Excel):

Parameter	No.1	No.2	No.3	No.4
N	6	6	6	6
df	4	4	4	4
R	0.999200505	0.998464949	0.988857294	0.998135171
R^2	0.99840165	0.996932255	0.977838748	0.996273819
$R^2_{adjusted}$	0.998002063	0.996165318	0.972298435	0.995342274
RSS	38.99291095	42.55432505	71.08130579	51.69854073

Graphical abstract of model fit presented as mean ± 1 SD of the fraction % of released carvedilol:

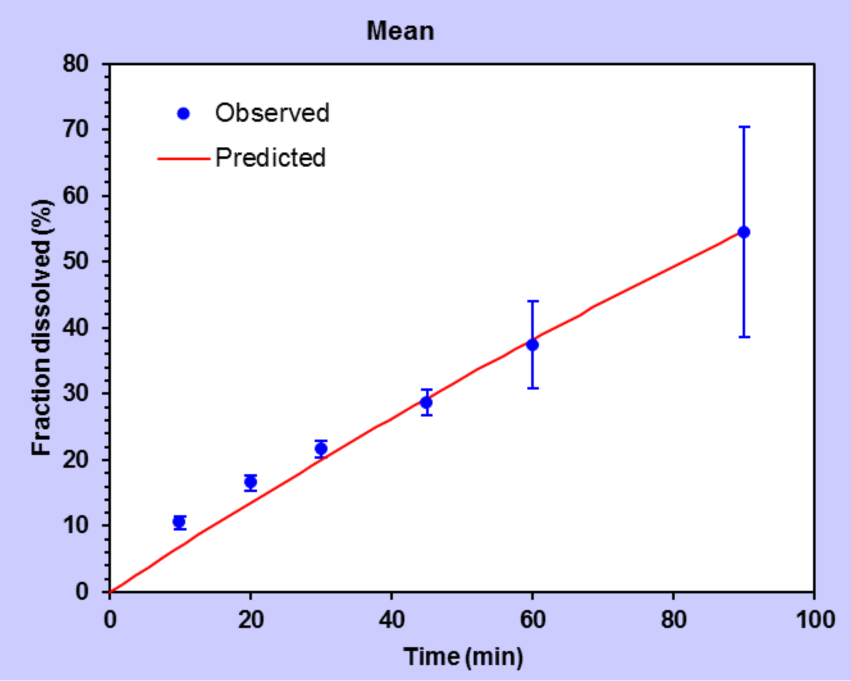

Supplementary materials – Model fitting summary – Pearlitol® 160C (modelling up to app. 60 % of carvedilol released)

Graphical abstract of model fit presented as the fraction % of released carvedilol per tested tablet:

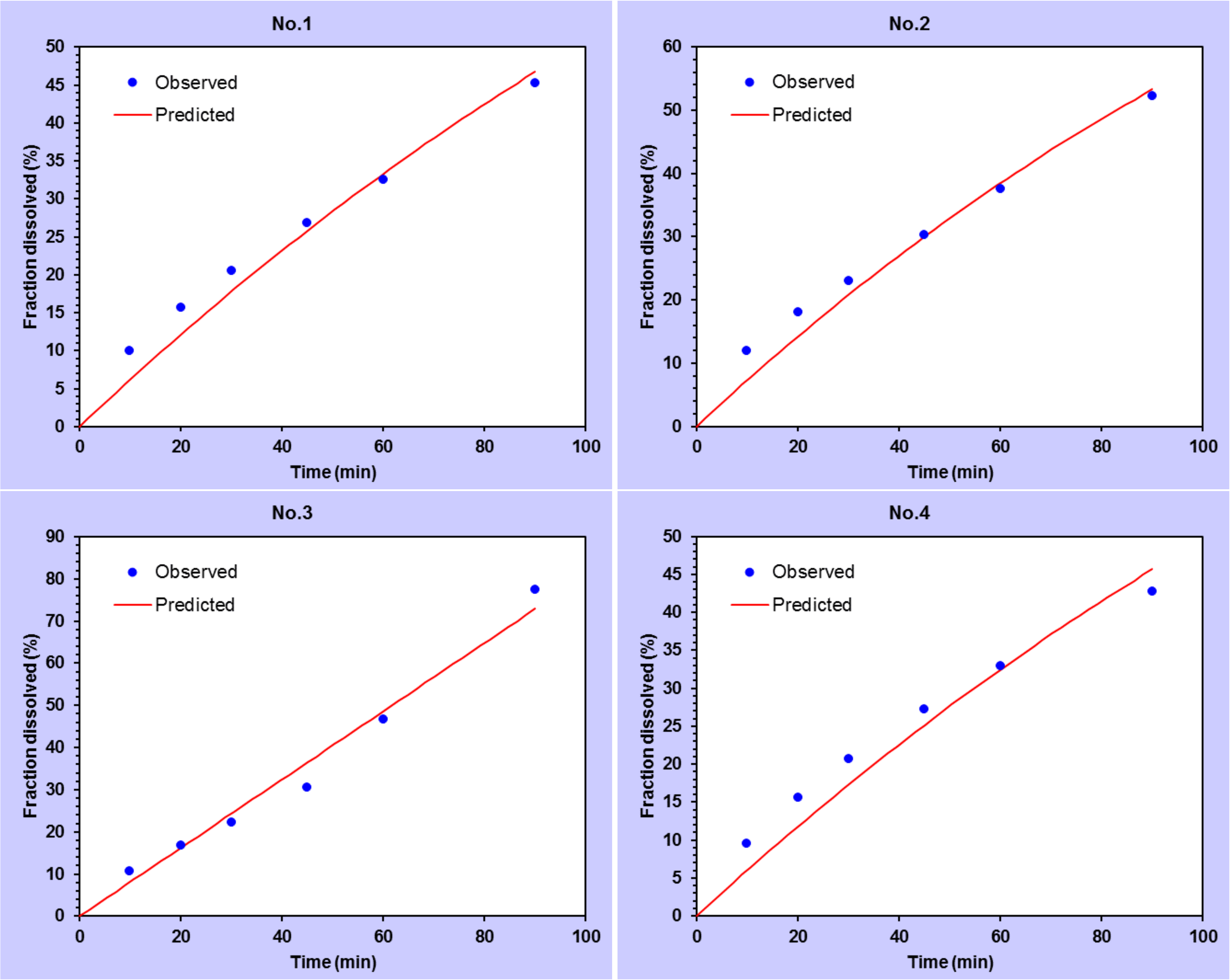

Supplementary materials – Model fitting summary – Pearlitol® 160C (modelling up to app. 60 % of carvedilol released)

Model: **Hopfenberg with T_{lag}**

Model equation: $F = 100 \cdot \{1 - [1 - k_{HB} \cdot (t - T_{lag})]^n\}$

Fitted model parameters per tested tablet (N = 4) with statistics – mean, standard deviation (SD), and relative standard deviation expressed in % (RSD%) (output from DDSolver):

Parameter	No.1	No.2	No.3	No.4	Mean	SD	RSD(%)
k_{HB}	0.003	0.005	0.008	0.001	0.004	0.003	69.943
n	2.000	1.000	1.000	3.477	1.869	1.171	62.646
T_{lag}	-11.249	-15.322	1.707	-10.963	-8.957	7.383	-82.423

Number of dissolution data points (N), degrees of freedom (df), and selected goodness of fit criteria – Pearson correlation coefficient (R), coefficient of determination (R^2), adjusted coefficient of determination ($R^2_{adjusted}$), and residual sum of squares (RSS) (manual calculation in MS Excel):

Parameter	No.1	No.2	No.3	No.4
N	6	6	6	6
df	3	3	3	3
R	0.999293435	0.999576794	0.988857294	0.997791993
R^2	0.998587369	0.999153767	0.977838748	0.995588861
$R^2_{adjusted}$	0.997645615	0.998589612	0.96306458	0.992648101
RSS	1.129037949	0.901904762	67.63333333	3.509663481

Graphical abstract of model fit presented as mean ± 1 SD of the fraction % of released carvedilol:

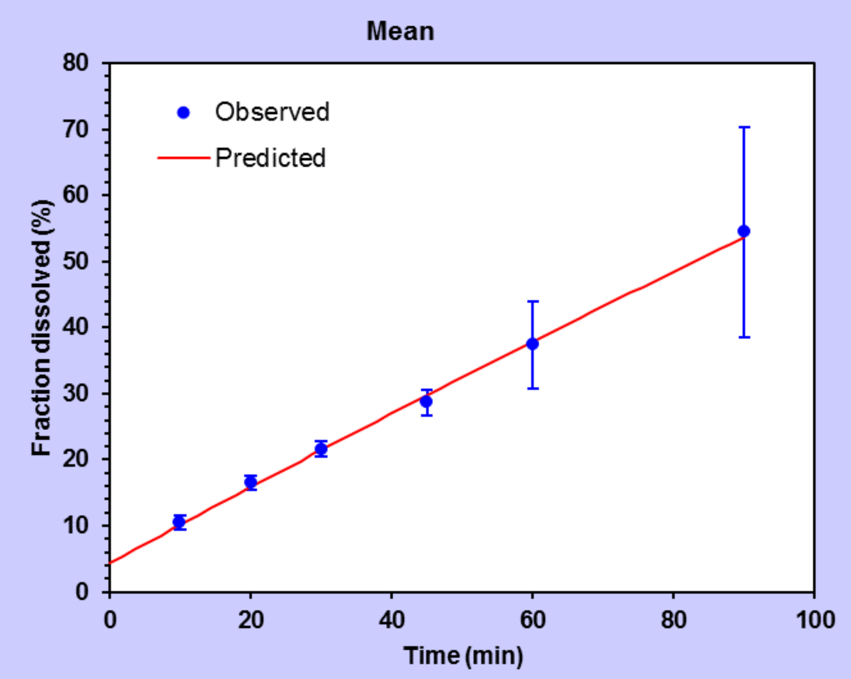

Supplementary materials – Model fitting summary – Pearlitol® 160C (modelling up to app. 60 % of carvedilol released)

Graphical abstract of model fit presented as the fraction % of released carvedilol per tested tablet:

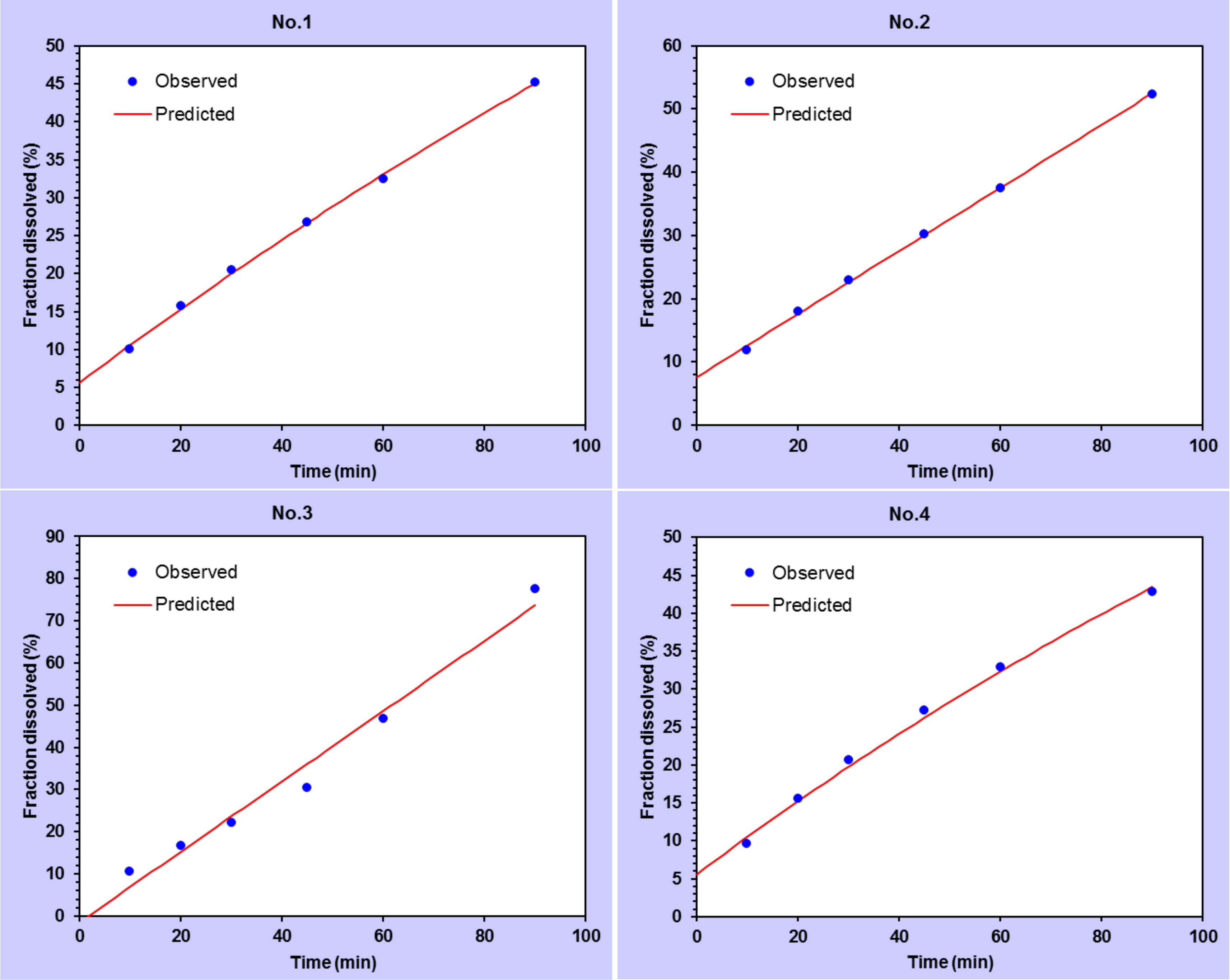

Supplementary materials – Model fitting summary – Pearlitol® 160C (modelling up to app. 60 % of carvedilol released)

Model: **Baker–Lonsdale**

Model equation: $\frac{3}{2}\cdot\left[1-\left(1-\frac{F}{100}\right)^{\frac{2}{3}}\right]-\frac{F}{100}=k_{BL}\cdot t$

Fitted model parameters per tested tablet (N = 4) with statistics – mean, standard deviation (SD), and relative standard deviation expressed in % (RSD%) (output from DDSolver):

Parameter	No.1	No.2	No.3	No.4	Mean	SD	RSD(%)
k_{BL}	0.0003	0.0004	0.0020	0.0002	0.0007	0.0009	121.0763

Number of dissolution data points (N), degrees of freedom (df), and selected goodness of fit criteria – Pearson correlation coefficient (R), coefficient of determination (R^2), adjusted coefficient of determination ($R^2_{adjusted}$), and residual sum of squares (RSS) (manual calculation in MS Excel):

Parameter	No.1	No.2	No.3	No.4
N	6	6	6	6
df	5	5	5	5
R	0.991860438	0.987834037	0.93151073	0.998272153
R^2	0.983787128	0.975816085	0.867712239	0.996547292
$R^2_{adjusted}$	0.983787128	0.975816085	0.867712239	0.996547292
RSS	147.8774645	184.2226006	3523.085735	158.5387813

Graphical abstract of model fit presented as mean ± 1 SD of the fraction % of released carvedilol:

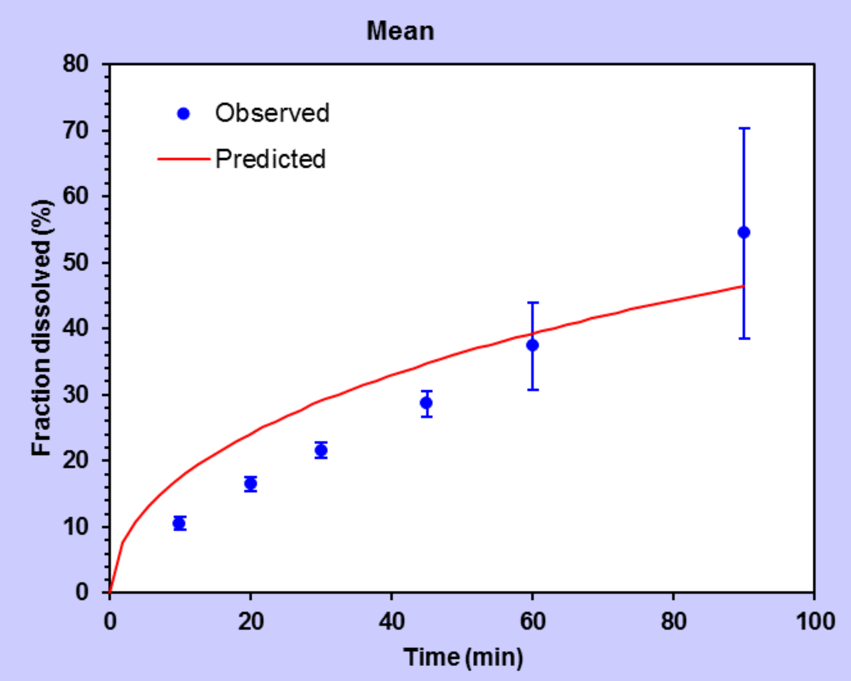

Supplementary materials – Model fitting summary – Pearlitol® 160C (modelling up to app. 60 % of carvedilol released)

Graphical abstract of model fit presented as the fraction % of released carvedilol per tested tablet:

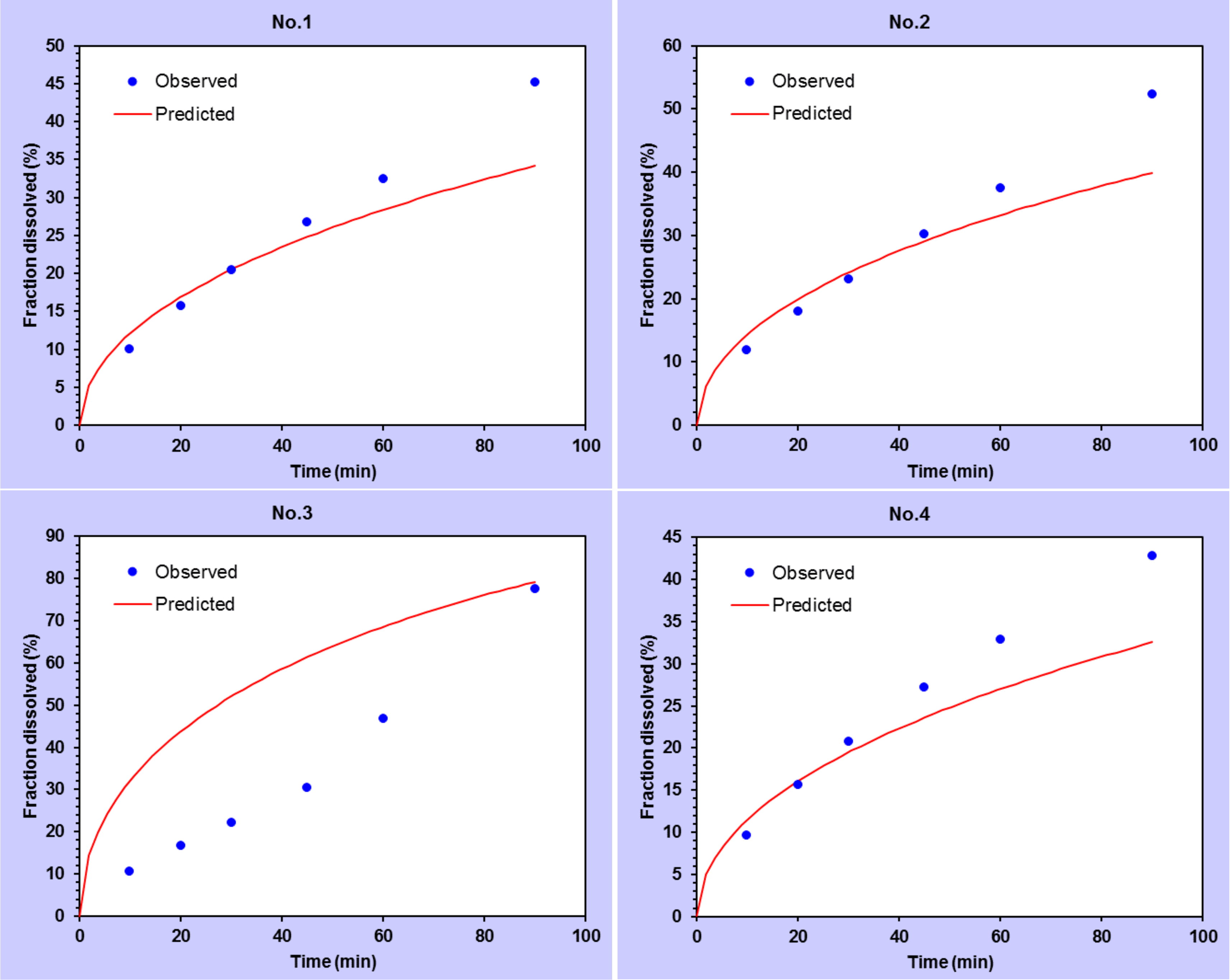

Supplementary materials – Model fitting summary – Pearlitol® 160C (modelling up to app. 60 % of carvedilol released)

Model: **Baker–Lonsdale with T_{lag}**

Model equation: $\frac{3}{2} \cdot \left[1 - \left(1 - \frac{F}{100}\right)^{\frac{2}{3}}\right] - \frac{F}{100} = k_{BL} \cdot (t - T_{lag})$

Fitted model parameters per tested tablet (N = 4) with statistics – mean, standard deviation (SD), and relative standard deviation expressed in % (RSD%) (output from DDSolver):

Parameter	No.1	No.2	No.3	No.4	Mean	SD	RSD(%)
k_{BL}	0.0005	0.0007	0.0020	0.0005	0.0009	0.0007	78.8486
T_{lag}	12.8218	13.6778	21.7554	10.9011	14.7890	4.7871	32.3696

Number of dissolution data points (N), degrees of freedom (df), and selected goodness of fit criteria – Pearson correlation coefficient (R), coefficient of determination (R^2), adjusted coefficient of determination ($R^2_{adjusted}$), and residual sum of squares (RSS) (manual calculation in MS Excel):

Parameter	No.1	No.2	No.3	No.4
N	6	6	6	6
df	4	4	4	4
R	0.963891334	0.957842998	0.903464615	0.971201755
R^2	0.929086504	0.917463209	0.816248311	0.943232849
$R^2_{adjusted}$	0.91135813	0.896829011	0.770310389	0.929041062
RSS	118.7480728	176.5704133	853.0426986	97.31226928

Graphical abstract of model fit presented as mean ± 1 SD of the fraction % of released carvedilol:

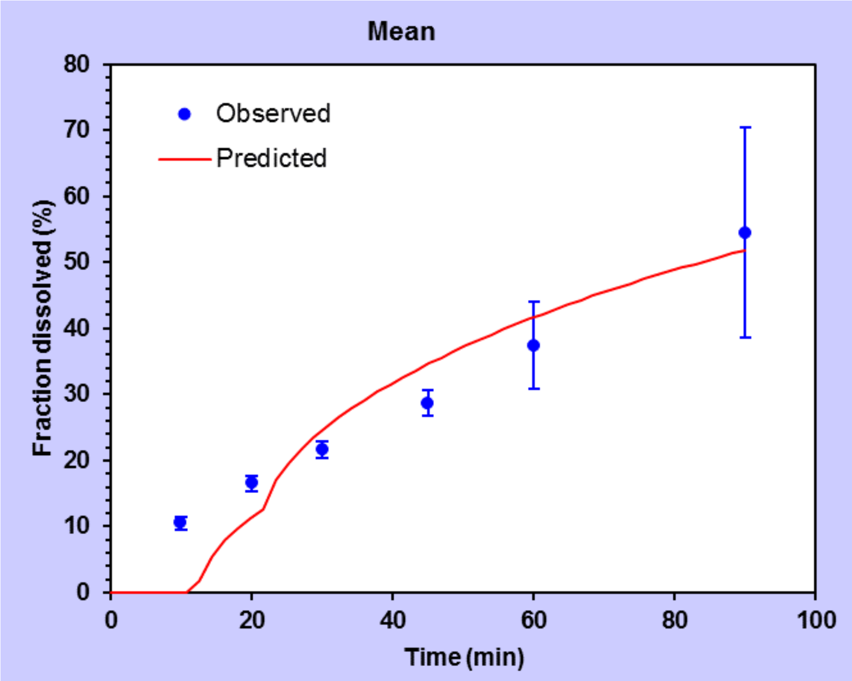

Supplementary materials – Model fitting summary – Pearlitol® 160C (modelling up to app. 60 % of carvedilol released)

Graphical abstract of model fit presented as the fraction % of released carvedilol per tested tablet:

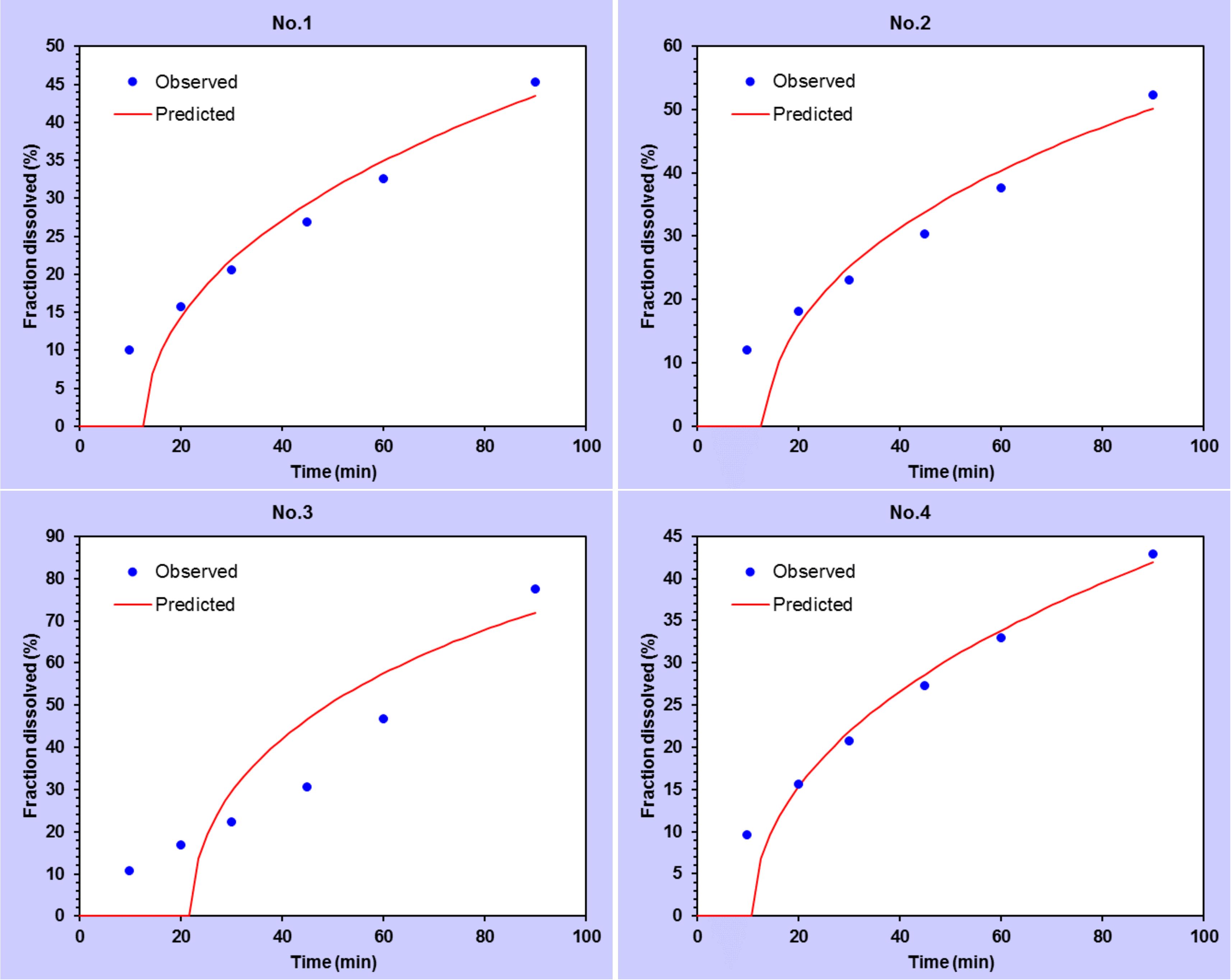

Supplementary materials – Model fitting summary – Pearlitol® 160C (modelling up to app. 60 % of carvedilol released)

Model: **Makoid–Banakar**

Model equation: $F = k_{MB} \cdot t^n \cdot e^{-k \cdot t}$

Fitted model parameters per tested tablet (N = 4) with statistics – mean, standard deviation (SD), and relative standard deviation expressed in % (RSD%) (output from DDSolver):

Parameter	No.1	No.2	No.3	No.4	Mean	SD	RSD(%)
k_{MB}	2.453	3.405	3.481	1.869	2.802	0.778	27.781
n	0.603	0.527	0.429	0.715	0.569	0.121	21.262
k	-0.002	-0.004	-0.013	0.001	-0.005	0.006	-132.496

Number of dissolution data points (N), degrees of freedom (df), and selected goodness of fit criteria – Pearson correlation coefficient (R), coefficient of determination (R^2), adjusted coefficient of determination ($R^2_{adjusted}$), and residual sum of squares (RSS) (manual calculation in MS Excel):

Parameter	No.1	No.2	No.3	No.4
N	6	6	6	6
df	3	3	3	3
R	0.999783374	0.99998548	0.998515481	0.999996715
R^2	0.999566795	0.99997096	0.997033167	0.99999343
$R^2_{adjusted}$	0.999277992	0.9999516	0.995055278	0.999989049
RSS	0.348994265	0.031116411	9.115785753	0.004784606

Graphical abstract of model fit presented as mean ± 1 SD of the fraction % of released carvedilol:

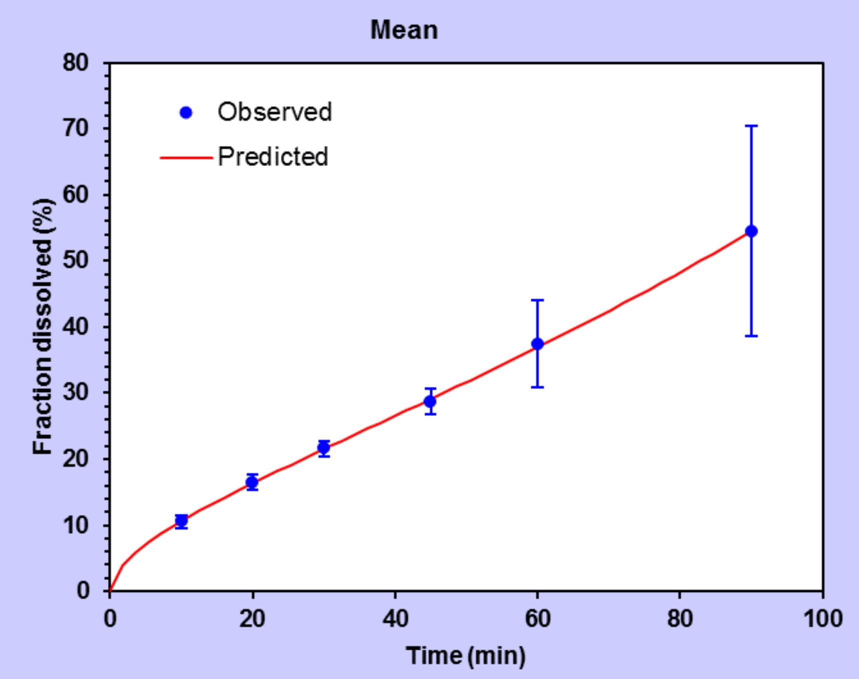

Supplementary materials – Model fitting summary – Pearlitol® 160C (modelling up to app. 60 % of carvedilol released)

Graphical abstract of model fit presented as the fraction % of released carvedilol per tested tablet:

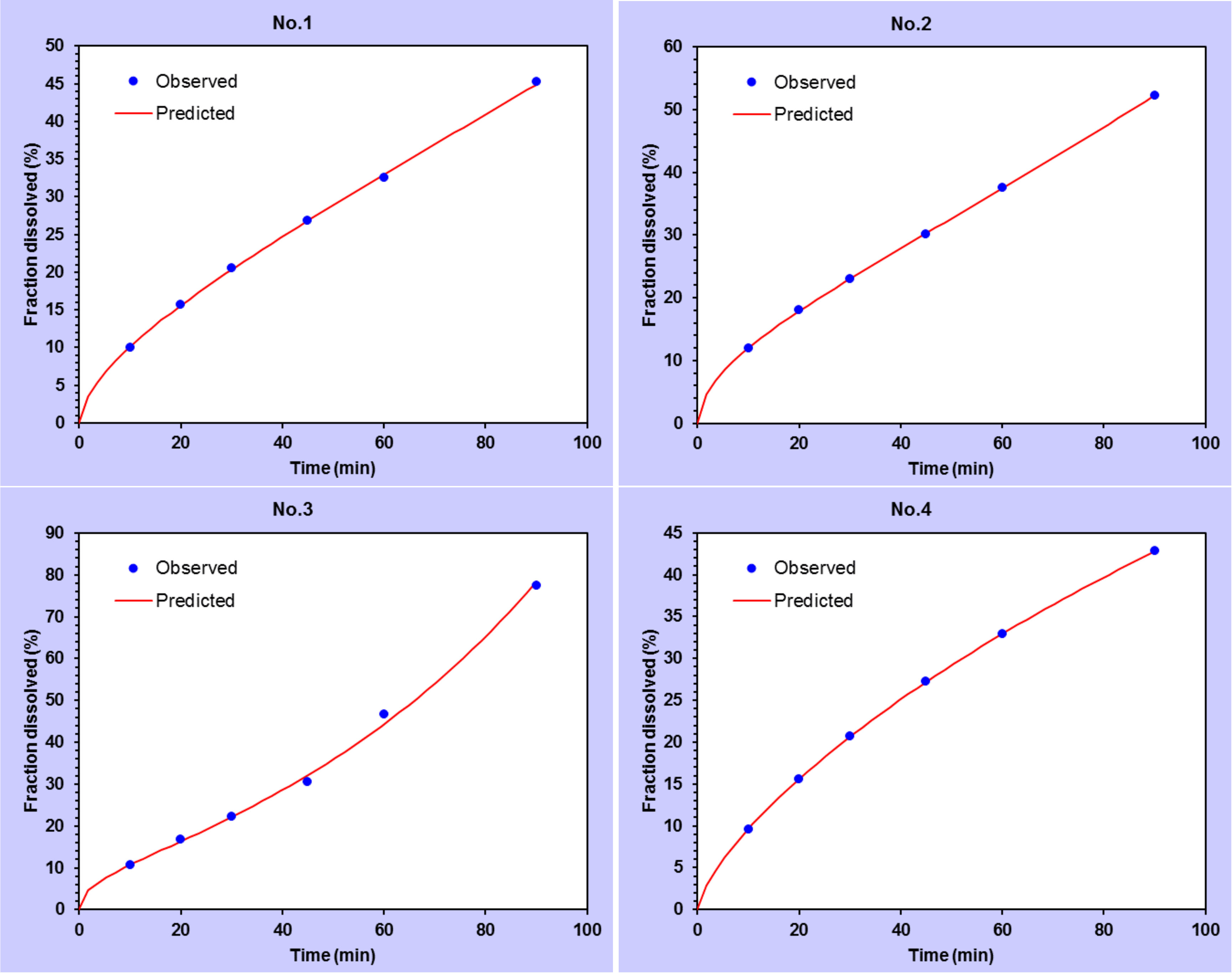

Supplementary materials – Model fitting summary – Pearlitol® 160C (modelling up to app. 60 % of carvedilol released)

Model: **Makoid–Banakar with T_{lag}**

Model equation: $F = k_{MB} \cdot (t - T_{lag})^n \cdot e^{-k \cdot (t - T_{lag})}$

Fitted model parameters per tested tablet (N = 4) with statistics – mean, standard deviation (SD), and relative standard deviation expressed in % (RSD%) (output from DDSolver):

Parameter	No.1	No.2	No.3	No.4	Mean	SD	RSD(%)
k_{MB}	4.538	5.888	5.650	3.834	4.978	0.963	19.344
n	0.423	0.369	0.301	0.500	0.398	0.084	21.170
k	-0.005	-0.006	-0.015	-0.002	-0.007	0.006	-78.071
T_{lag}	4.000	4.000	4.000	4.000	4.000	0.000	0.000

Number of dissolution data points (N), degrees of freedom (df), and selected goodness of fit criteria – Pearson correlation coefficient (R), coefficient of determination (R^2), adjusted coefficient of determination ($R^2_{adjusted}$), and residual sum of squares (RSS) (manual calculation in MS Excel):

Parameter	No.1	No.2	No.3	No.4
N	6	6	6	6
df	2	2	2	2
R	0.999948303	0.999867289	0.998268903	0.999714169
R^2	0.99989661	0.999734596	0.996540803	0.99942842
$R^2_{adjusted}$	0.999741524	0.99933649	0.991352008	0.99857105
RSS	0.08284297	0.29049539	10.79196745	0.419699371

Graphical abstract of model fit presented as mean ± 1 SD of the fraction % of released carvedilol:

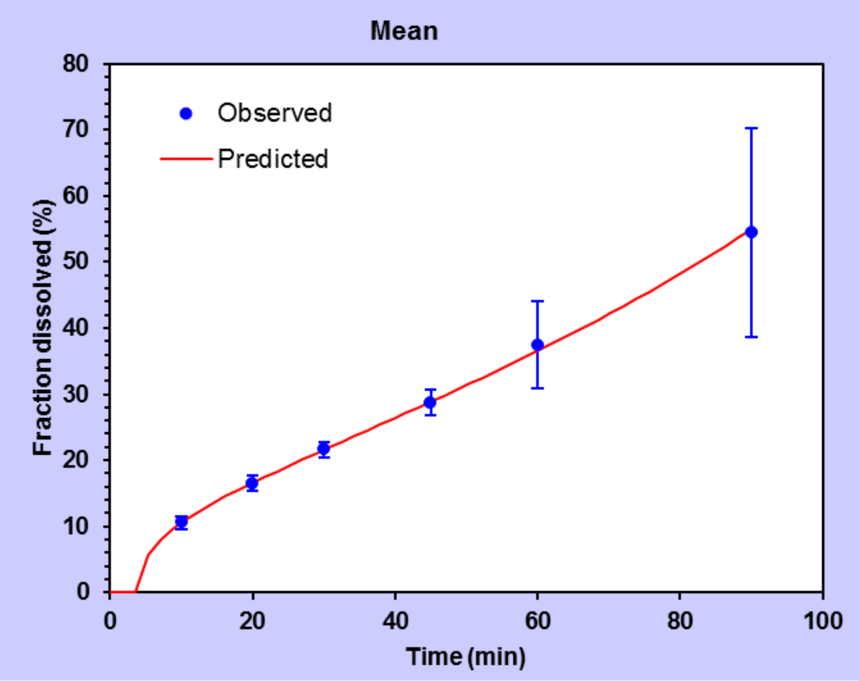

Supplementary materials – Model fitting summary – Pearlitol® 160C (modelling up to app. 60 % of carvedilol released)

Graphical abstract of model fit presented as the fraction % of released carvedilol per tested tablet:

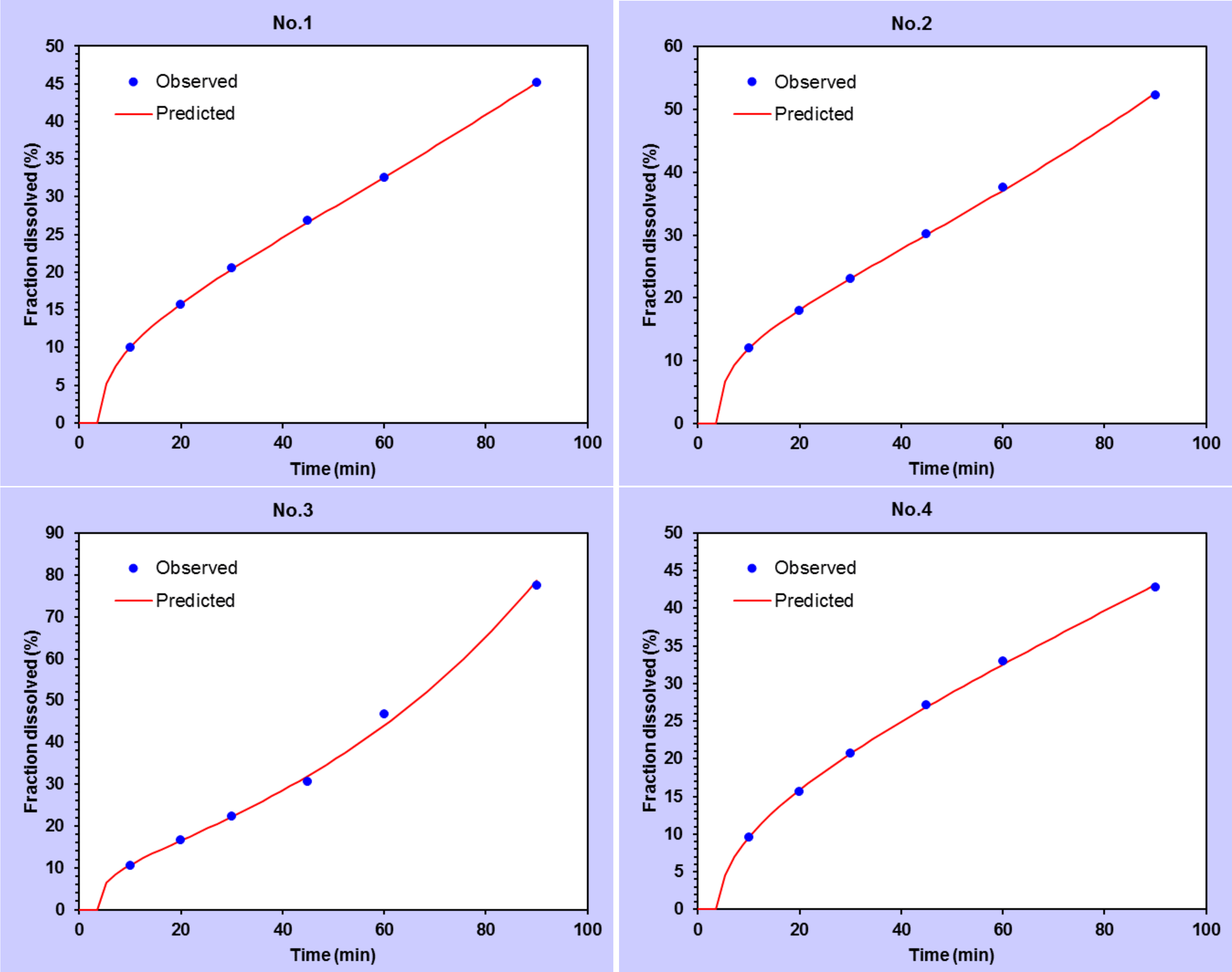

Supplementary materials – Model fitting summary – Pearlitol® 160C (modelling up to app. 60 % of carvedilol released)

Model: **Peppas–Sahlin_1**

Model equation: $F = k_1 \cdot t^m + k_2 \cdot t^{2m}$

Fitted model parameters per tested tablet (N = 4) with statistics – mean, standard deviation (SD), and relative standard deviation expressed in % (RSD%) (output from DDSolver):

Parameter	No.1	No.2	No.3	No.4	Mean	SD	RSD(%)
k_1	2.053	2.241	-2.393	2.420	1.080	2.320	214.856
k_2	0.509	0.603	1.604	0.434	0.787	0.549	69.699
m	0.450	0.450	0.450	0.450	0.450	0.000	0.000

Number of dissolution data points (N), degrees of freedom (df), and selected goodness of fit criteria – Pearson correlation coefficient (R), coefficient of determination (R^2), adjusted coefficient of determination ($R^2_{adjusted}$), and residual sum of squares (RSS) (manual calculation in MS Excel):

Parameter	No.1	No.2	No.3	No.4
N	6	6	6	6
df	3	3	3	3
R	0.99944254	0.998901089	0.987926894	0.999330966
R^2	0.998885392	0.997803385	0.975999549	0.99866238
$R^2_{adjusted}$	0.998142319	0.996338975	0.959999248	0.997770633
RSS	0.904426972	2.432075953	74.84494428	1.023201916

Graphical abstract of model fit presented as mean ± 1 SD of the fraction % of released carvedilol:

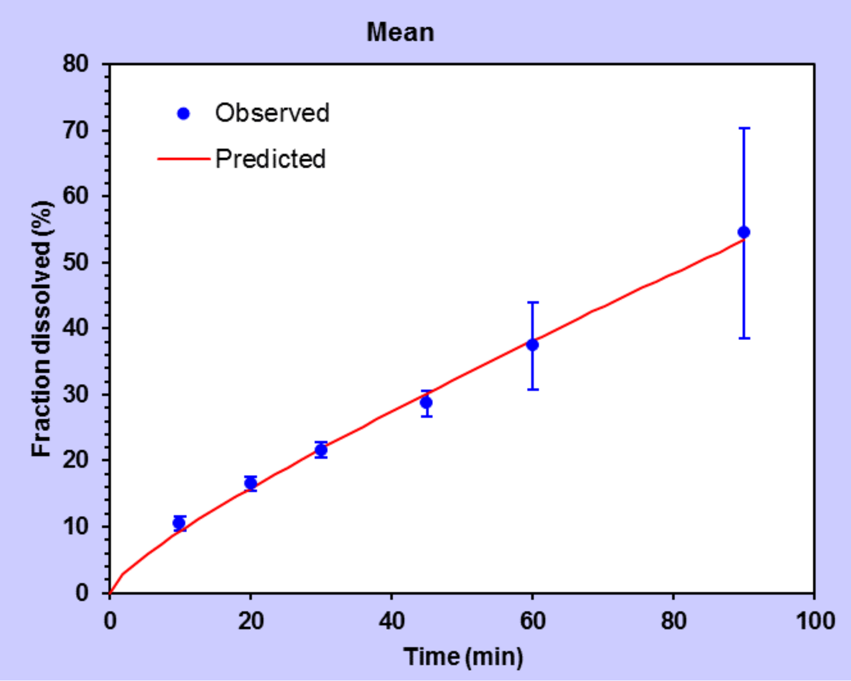

Supplementary materials – Model fitting summary – Pearlitol® 160C (modelling up to app. 60 % of carvedilol released)

Graphical abstract of model fit presented as the fraction % of released carvedilol per tested tablet:

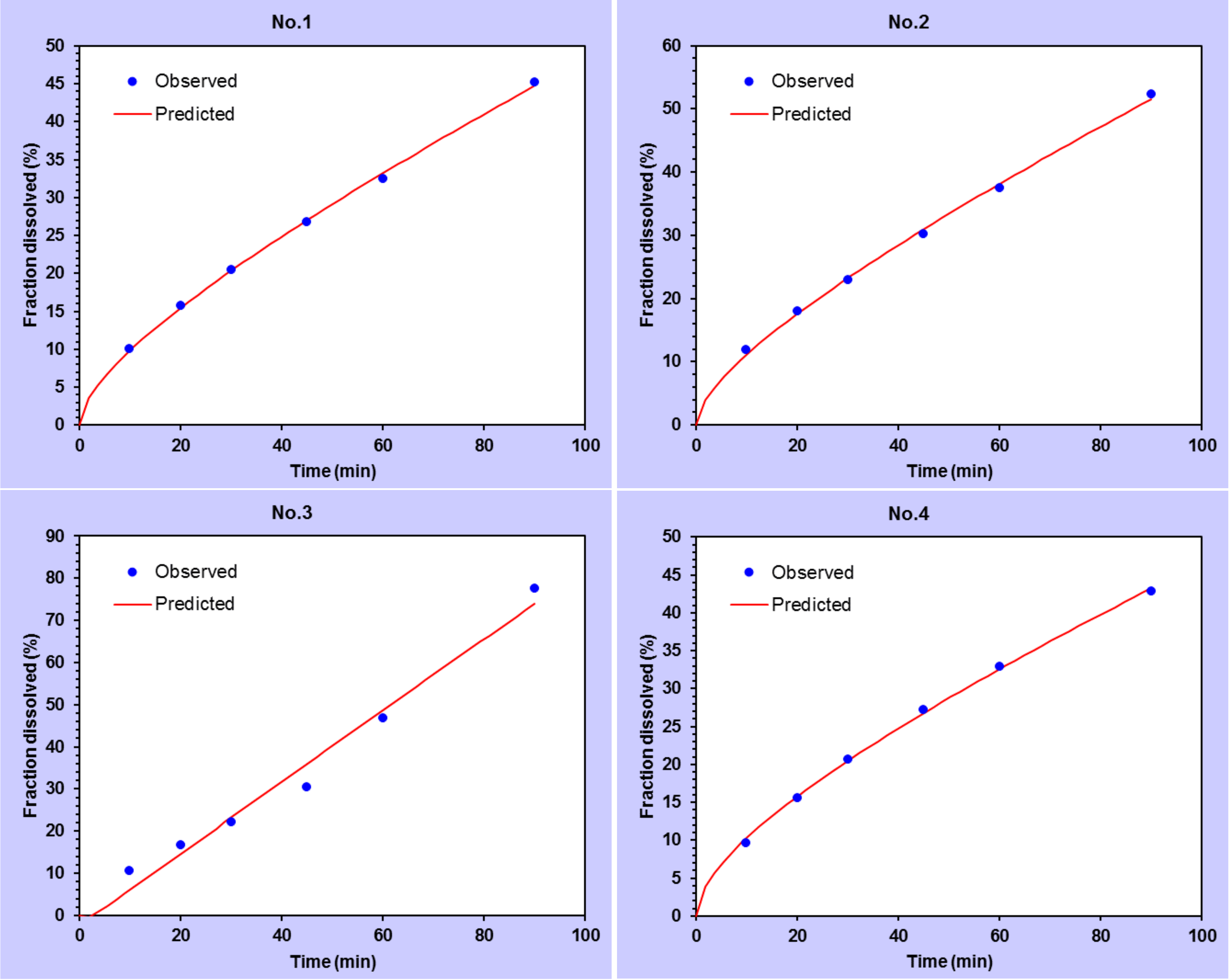

Supplementary materials – Model fitting summary – Pearlitol® 160C (modelling up to app. 60 % of carvedilol released)

Model: **Peppas–Sahlin_1 with T_{lag}**

Model equation: $F = k_1 \cdot (t - T_{lag})^m + k_2 \cdot (t - T_{lag})^{2m}$

Fitted model parameters per tested tablet (N = 4) with statistics – mean, standard deviation (SD), and relative standard deviation expressed in % (RSD%) (output from DDSolver):

Parameter	No.1	No.2	No.3	No.4	Mean	SD	RSD(%)
k_1	3.081	3.408	-1.029	3.453	2.228	2.178	97.752
k_2	0.390	0.469	1.476	0.312	0.662	0.547	82.606
m	0.450	0.450	0.450	0.450	0.450	0.000	0.000
T_{lag}	4.000	4.000	4.000	4.000	4.000	0.000	0.000

Number of dissolution data points (N), degrees of freedom (df), and selected goodness of fit criteria – Pearson correlation coefficient (R), coefficient of determination (R^2), adjusted coefficient of determination ($R^2_{adjusted}$), and residual sum of squares (RSS) (manual calculation in MS Excel):

Parameter	No.1	No.2	No.3	No.4
N	6	6	6	6
df	2	2	2	2
R	0.998023673	0.996630727	0.985725865	0.999896579
R^2	0.996051252	0.993272806	0.97165548	0.999793169
$R^2_{adjusted}$	0.990128129	0.983182016	0.929138701	0.999482922
RSS	3.406493891	7.805531653	90.17226406	0.155864262

Graphical abstract of model fit presented as mean ± 1 SD of the fraction % of released carvedilol:

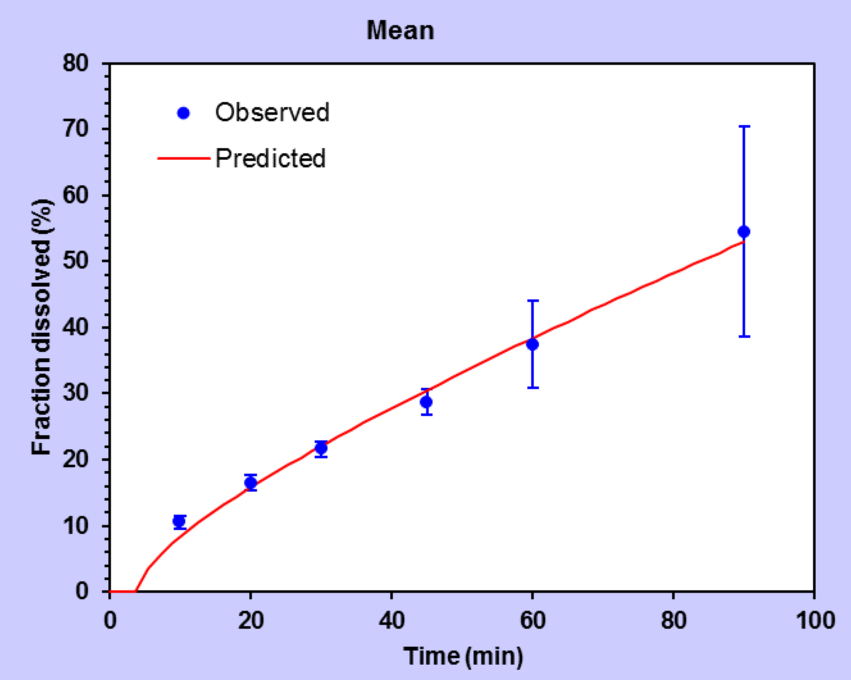

Supplementary materials – Model fitting summary – Pearlitol® 160C (modelling up to app. 60 % of carvedilol released)

Graphical abstract of model fit presented as the fraction % of released carvedilol per tested tablet:

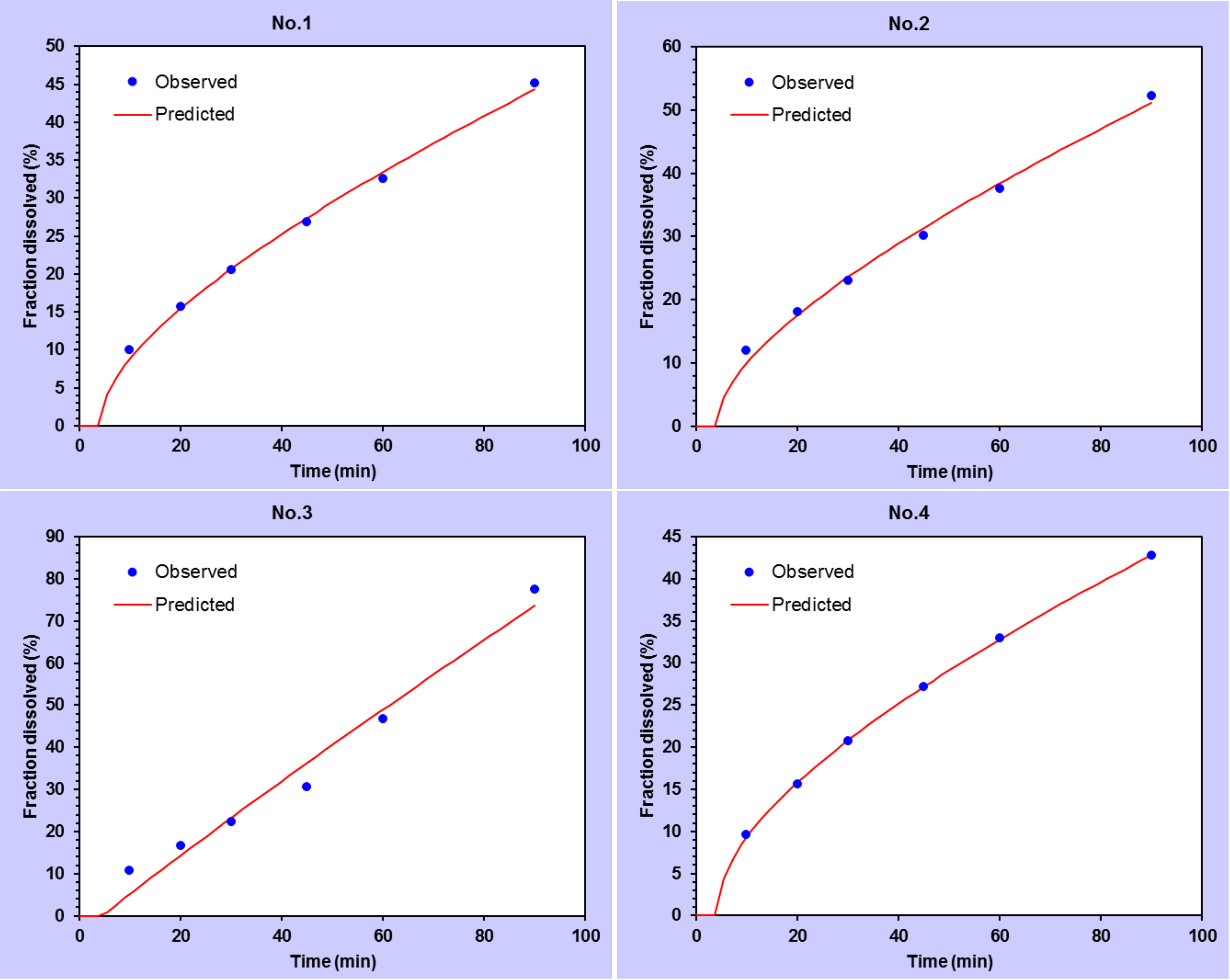

Supplementary materials – Model fitting summary – Pearlitol® 160C (modelling up to app. 60 % of carvedilol released)

Model: **Peppas–Sahlin_2**

Model equation: $F = k_1 \cdot t^{0.5} + k_2 \cdot t$

Fitted model parameters per tested tablet (N = 4) with statistics – mean, standard deviation (SD), and relative standard deviation expressed in % (RSD%) (output from DDSolver):

Parameter	No.1	No.2	No.3	No.4	Mean	SD	RSD(%)
k_1	2.324	2.569	-0.867	2.615	1.660	1.690	101.791
k_2	0.253	0.303	0.920	0.205	0.420	0.335	79.843

Number of dissolution data points (N), degrees of freedom (df), and selected goodness of fit criteria – Pearson correlation coefficient (R), coefficient of determination (R^2), adjusted coefficient of determination ($R^2_{adjusted}$), and residual sum of squares (RSS) (manual calculation in MS Excel):

Parameter	No.1	No.2	No.3	No.4
N	6	6	6	6
df	4	4	4	4
R	0.999539765	0.999112895	0.99043758	0.999284243
R^2	0.999079742	0.998226578	0.9809666	0.998568998
$R^2_{adjusted}$	0.998849678	0.997783222	0.97620825	0.998211247
RSS	0.749303838	1.999451575	59.90177032	1.120910114

Graphical abstract of model fit presented as mean ± 1 SD of the fraction % of released carvedilol:

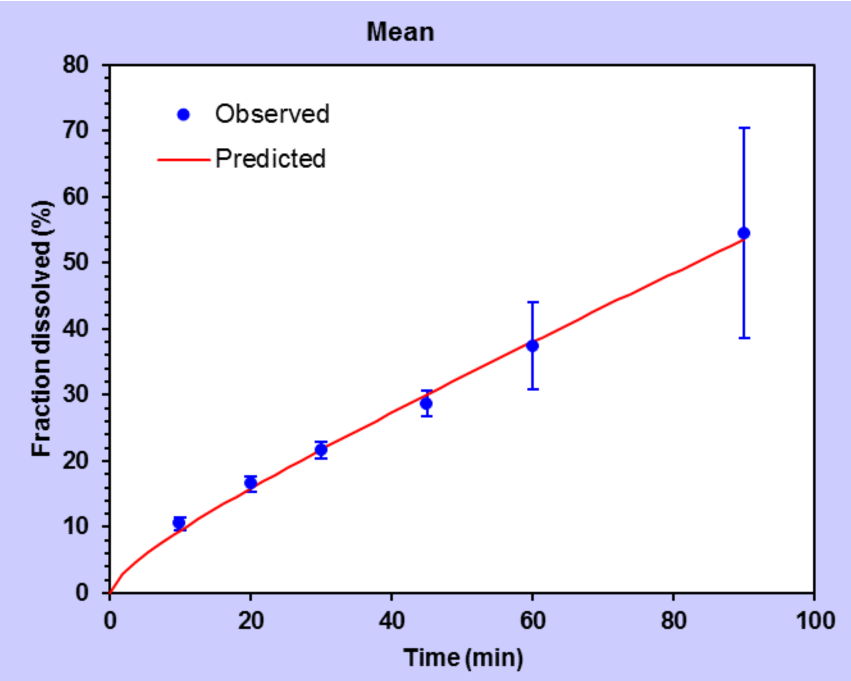

Supplementary materials – Model fitting summary – Pearlitol® 160C (modelling up to app. 60 % of carvedilol released)

Graphical abstract of model fit presented as the fraction % of released carvedilol per tested tablet:

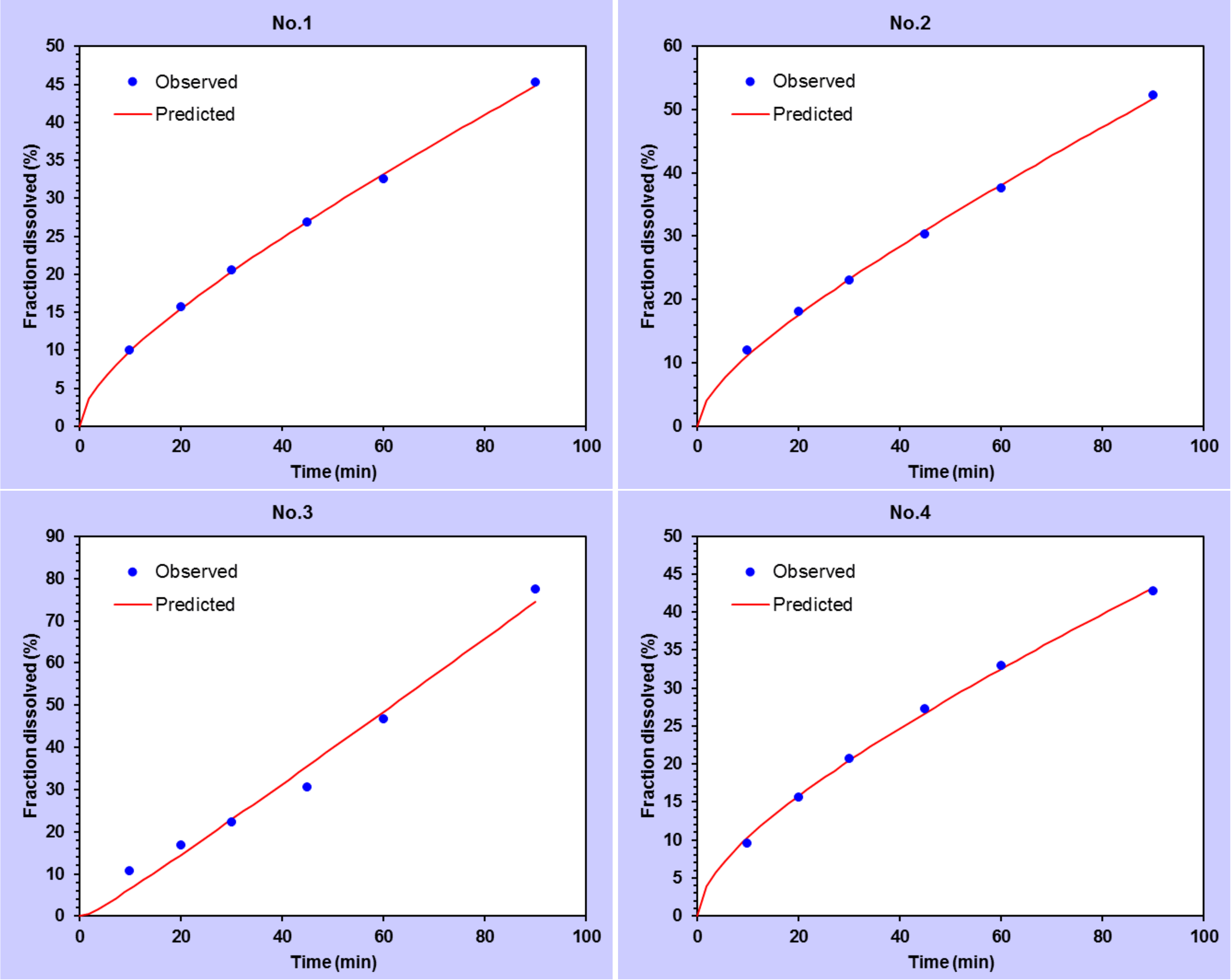

Supplementary materials – Model fitting summary – Pearlitol® 160C (modelling up to app. 60 % of carvedilol released)

Model: **Peppas–Sahlin_2 with T_{lag}**

Model equation: $F = k_1 \cdot (t - T_{lag})^{0.5} + k_2 \cdot (t - T_{lag})$

Fitted model parameters per tested tablet (N = 4) with statistics – mean, standard deviation (SD), and relative standard deviation expressed in % (RSD%) (output from DDSolver):

Parameter	No.1	No.2	No.3	No.4	Mean	SD	RSD(%)
k_1	3.145	3.498	0.174	3.294	2.528	1.576	62.331
k_2	0.177	0.219	0.845	0.143	0.346	0.334	96.626
T_{lag}	4.000	4.000	4.000	3.300	3.825	0.350	9.155

Number of dissolution data points (N), degrees of freedom (df), and selected goodness of fit criteria – Pearson correlation coefficient (R), coefficient of determination (R^2), adjusted coefficient of determination ($R^2_{adjusted}$), and residual sum of squares (RSS) (manual calculation in MS Excel):

Parameter	No.1	No.2	No.3	No.4
N	6	6	6	6
df	3	3	3	3
R	0.998022056	0.996706335	0.988446275	0.999938918
R^2	0.996048024	0.993423519	0.977026039	0.999877839
$R^2_{adjusted}$	0.993413373	0.989039198	0.961710065	0.999796399
RSS	3.536458533	7.928315225	74.34574797	0.177989509

Graphical abstract of model fit presented as mean ± 1 SD of the fraction % of released carvedilol:

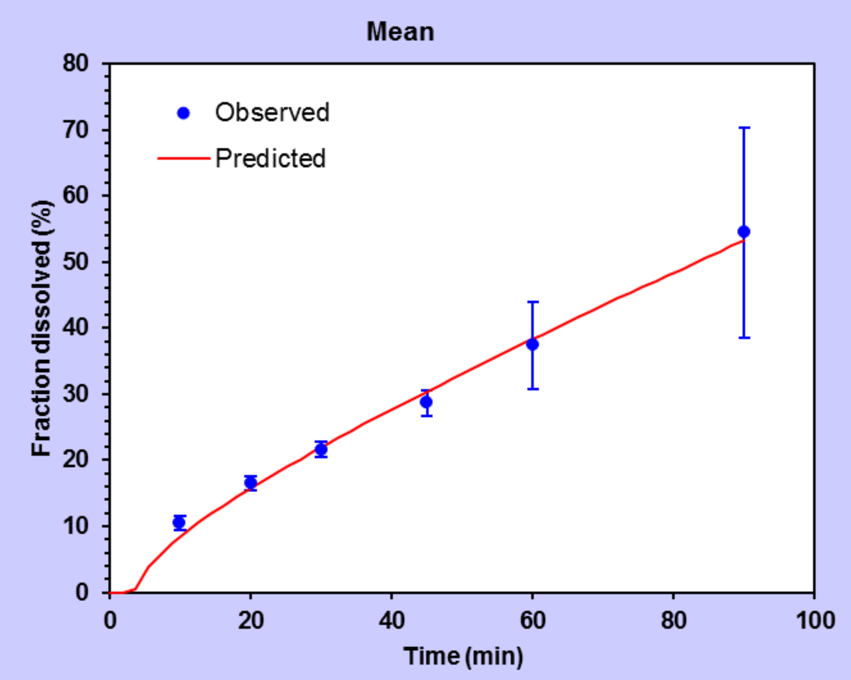

Supplementary materials – Model fitting summary – Pearlitol® 160C (modelling up to app. 60 % of carvedilol released)

Graphical abstract of model fit presented as the fraction % of released carvedilol per tested tablet:

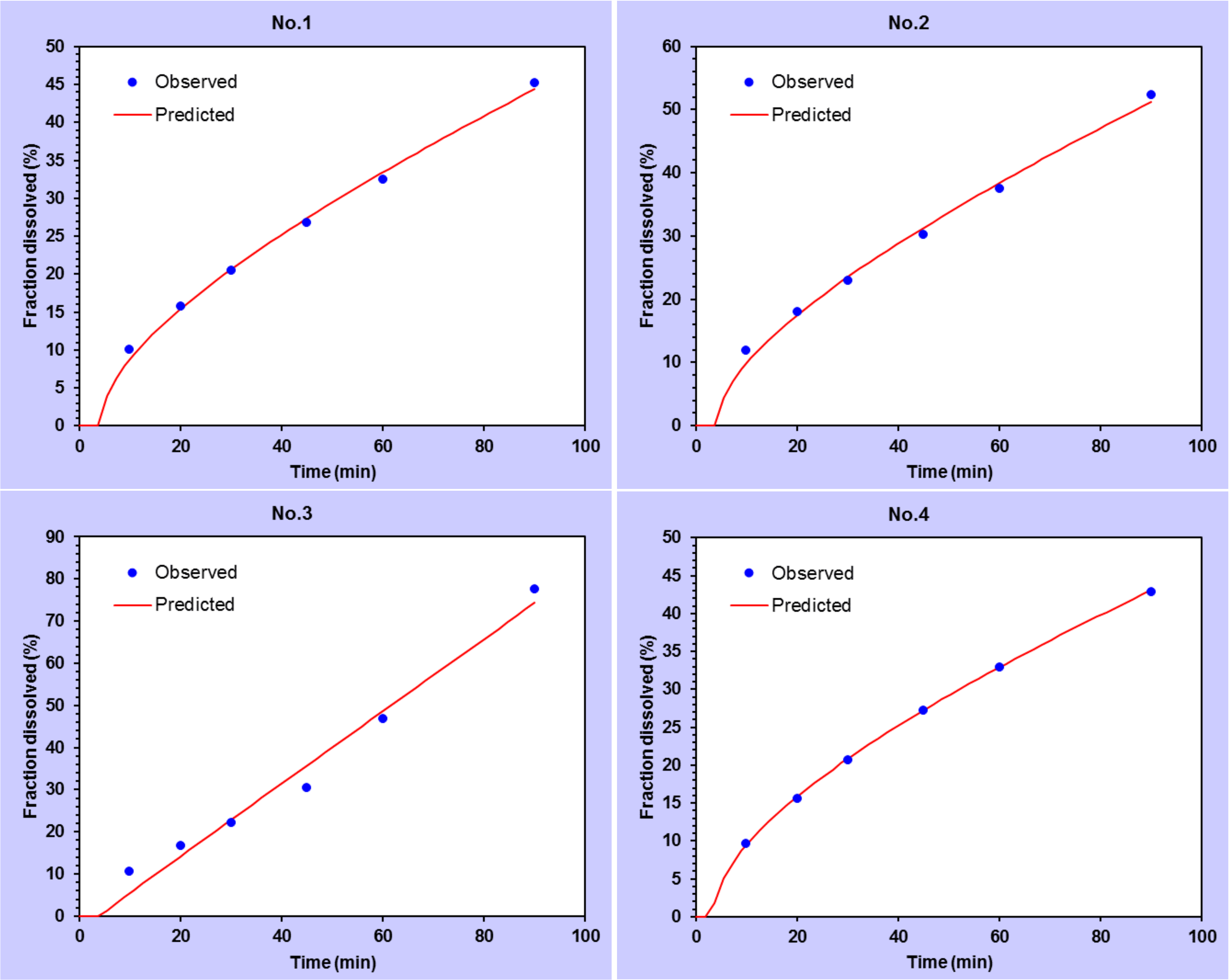

Supplementary materials – Model fitting summary – Pearlitol® 160C (modelling up to app. 60 % of carvedilol released)

Model: **Quadratic**

Model equation: $F = 100 \cdot (k_1 \cdot t^2 + k_2 \cdot t)$

Fitted model parameters per tested tablet (N = 4) with statistics – mean, standard deviation (SD), and relative standard deviation expressed in % (RSD%) (output from DDSolver):

Parameter	No.1	No.2	No.3	No.4	Mean	SD	RSD(%)
k_1	-0.00003	-0.00003	0.00002	-0.00003	-0.00002	0.00003	-147.45979
k_2	0.00748	0.00844	0.00656	0.00781	0.00757	0.00078	10.34783

Number of dissolution data points (N), degrees of freedom (df), and selected goodness of fit criteria – Pearson correlation coefficient (R), coefficient of determination (R^2), adjusted coefficient of determination ($R^2_{adjusted}$), and residual sum of squares (RSS) (manual calculation in MS Excel):

Parameter	No.1	No.2	No.3	No.4
N	6	6	6	6
df	4	4	4	4
R	0.9934198	0.992562518	0.99558255	0.996419372
R^2	0.986882899	0.985180352	0.991184615	0.992851565
$R^2_{adjusted}$	0.983603623	0.981475439	0.988980768	0.991064456
RSS	18.40170494	28.15193971	34.94804979	9.958796044

Graphical abstract of model fit presented as mean ± 1 SD of the fraction % of released carvedilol:

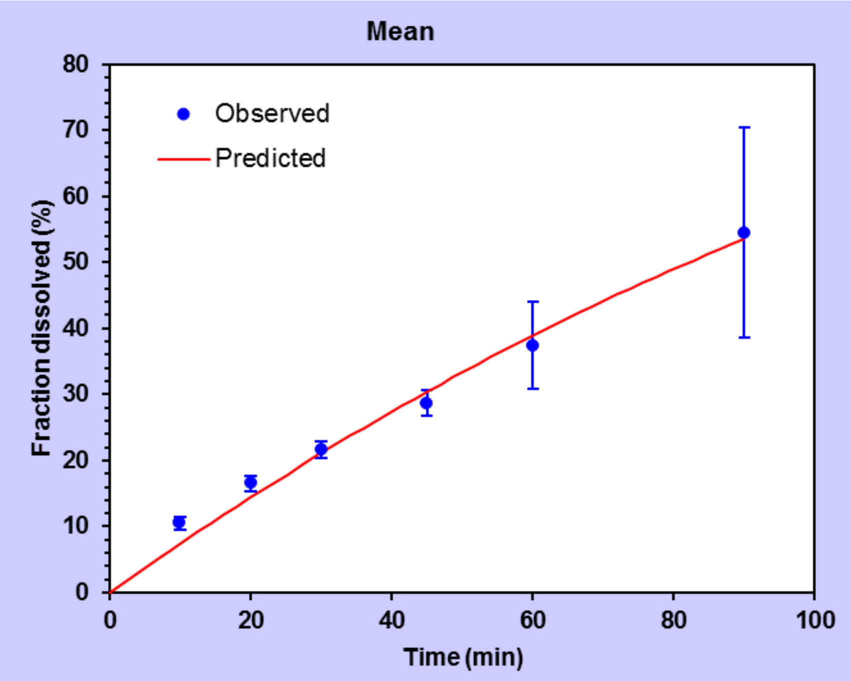

Supplementary materials – Model fitting summary – Pearlitol® 160C (modelling up to app. 60 % of carvedilol released)

Graphical abstract of model fit presented as the fraction % of released carvedilol per tested tablet:

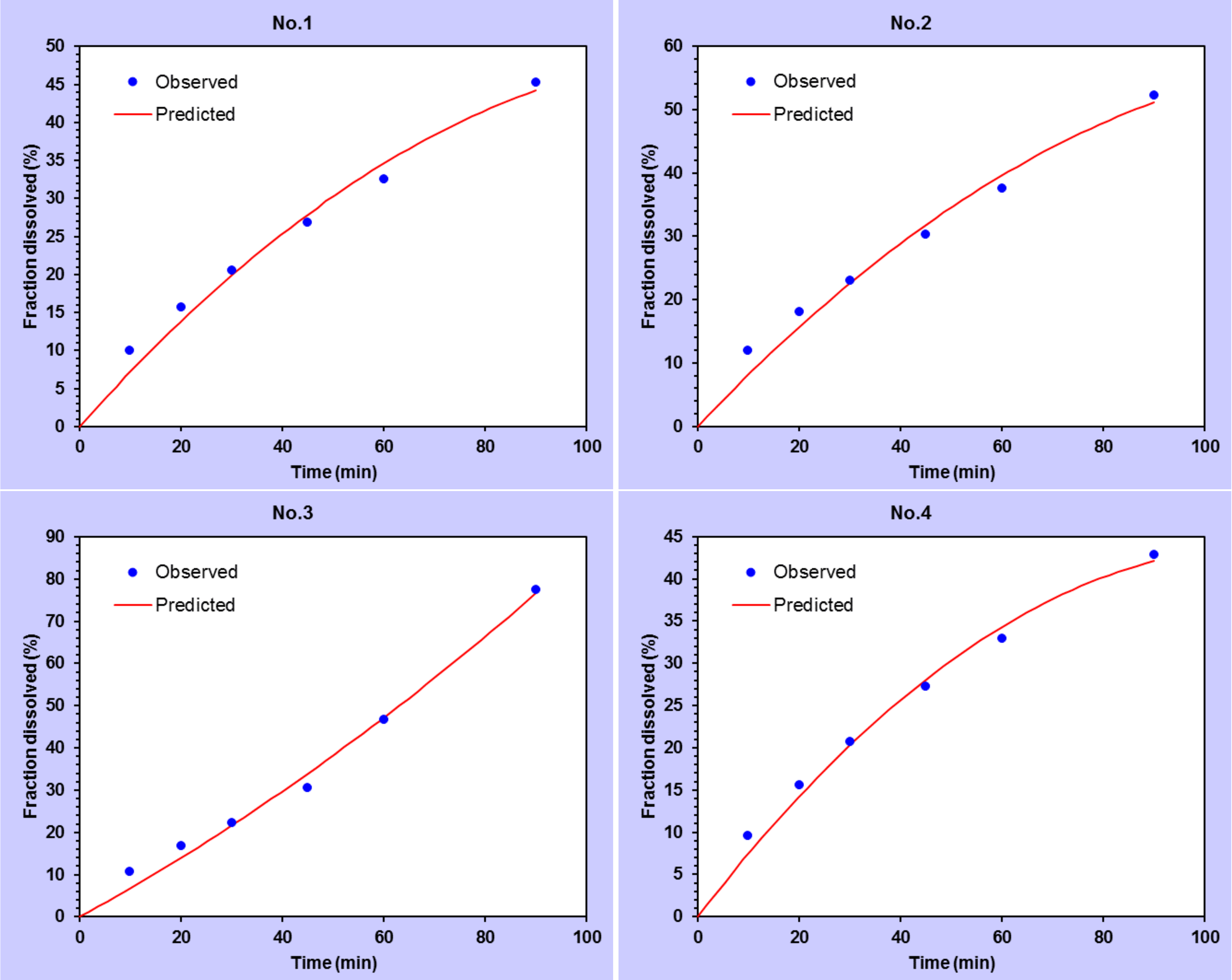

Supplementary materials – Model fitting summary – Pearlitol® 160C (modelling up to app. 60 % of carvedilol released)

Model: **Quadratic with T_{lag}**

Model equation: $F = 100 \cdot \left[k_1 \cdot (t - T_{lag})^2 + k_2 \cdot (t - T_{lag})\right]$

Fitted model parameters per tested tablet (N = 4) with statistics – mean, standard deviation (SD), and relative standard deviation expressed in % (RSD%) (output from DDSolver):

Parameter	No.1	No.2	No.3	No.4	Mean	SD	RSD(%)
k_1	-0.00004	-0.00004	0.00001	-0.00005	-0.00003	0.00003	-94.60471
k_2	0.00853	0.00963	0.00785	0.00889	0.00873	0.00074	8.49517
T_{lag}	4.00000	4.00000	4.00000	4.00000	4.00000	0.00000	0.00000

Number of dissolution data points (N), degrees of freedom (df), and selected goodness of fit criteria – Pearson correlation coefficient (R), coefficient of determination (R^2), adjusted coefficient of determination ($R^2_{adjusted}$), and residual sum of squares (RSS) (manual calculation in MS Excel):

Parameter	No.1	No.2	No.3	No.4
N	6	6	6	6
df	3	3	3	3
R	0.987168708	0.986028869	0.992966851	0.990878711
R^2	0.974502058	0.97225293	0.985983167	0.981840621
$R^2_{adjusted}$	0.95750343	0.953754883	0.976638612	0.969734368
RSS	47.30596134	68.29482557	65.82289787	34.54535383

Graphical abstract of model fit presented as mean ± 1 SD of the fraction % of released carvedilol:

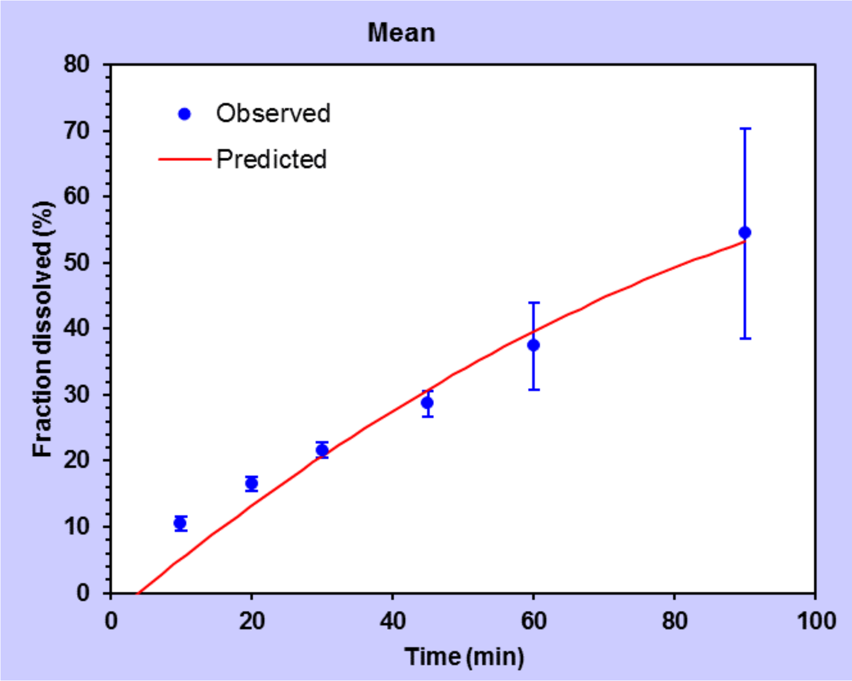

Supplementary materials – Model fitting summary – Pearlitol® 160C (modelling up to app. 60 % of carvedilol released)

Graphical abstract of model fit presented as the fraction % of released carvedilol per tested tablet:

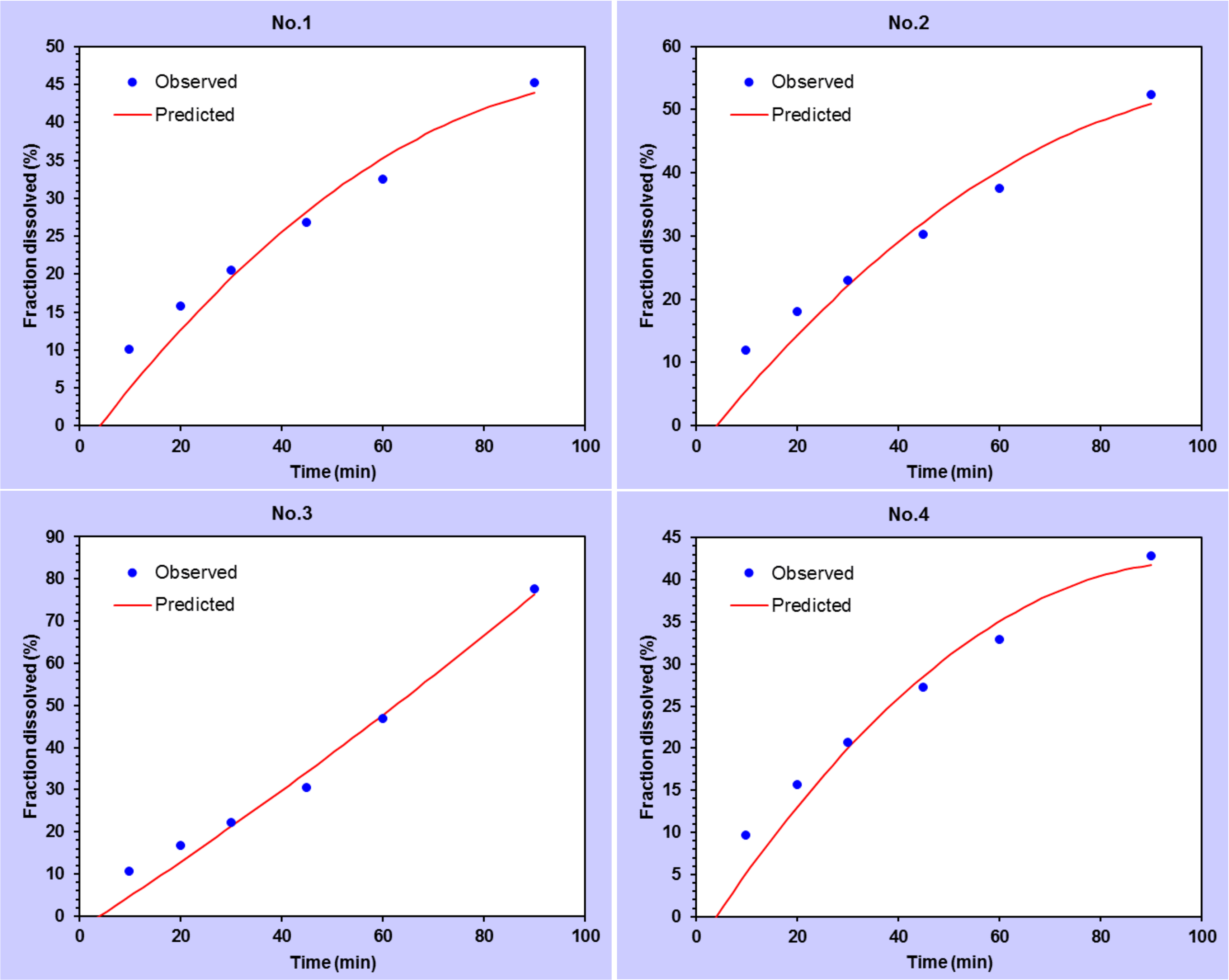

Supplementary materials – Model fitting summary – Pearlitol® 160C (modelling up to app. 60 % of carvedilol released)

Model: **Weibull_1**

Model equation: $F = 100 \cdot \left[1 - e^{-\frac{(t-T_i)^\beta}{\alpha}}\right]$

Fitted model parameters per tested tablet (N = 4) with statistics – mean, standard deviation (SD), and relative standard deviation expressed in % (RSD%) (output from DDSolver):

Parameter	No.1	No.2	No.3	No.4	Mean	SD	RSD(%)
α	32.702	28.316	61.103	33.234	38.839	15.006	38.636
β	0.641	0.647	0.916	0.642	0.711	0.136	19.155
T_i	4.000	4.000	4.000	4.000	4.000	0.000	0.000

Number of dissolution data points (N), degrees of freedom (df), and selected goodness of fit criteria – Pearson correlation coefficient (R), coefficient of determination (R^2), adjusted coefficient of determination ($R^2_{adjusted}$), and residual sum of squares (RSS) (manual calculation in MS Excel):

Parameter	No.1	No.2	No.3	No.4
N	6	6	6	6
df	3	3	3	3
R	0.989212965	0.983520225	0.953504224	0.996897223
R^2	0.97854229	0.967312034	0.909170306	0.993804074
$R^2_{adjusted}$	0.964237149	0.945520056	0.848617176	0.989673457
RSS	21.44480824	42.96155438	350.7713595	5.970173609

Graphical abstract of model fit presented as mean ± 1 SD of the fraction % of released carvedilol:

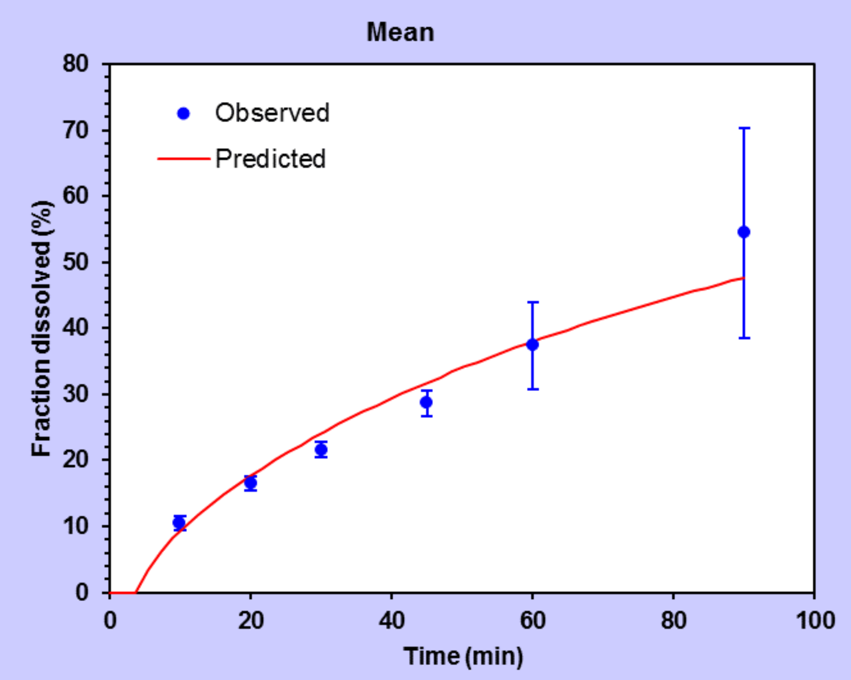

Supplementary materials – Model fitting summary – Pearlitol® 160C (modelling up to app. 60 % of carvedilol released)

Graphical abstract of model fit presented as the fraction % of released carvedilol per tested tablet:

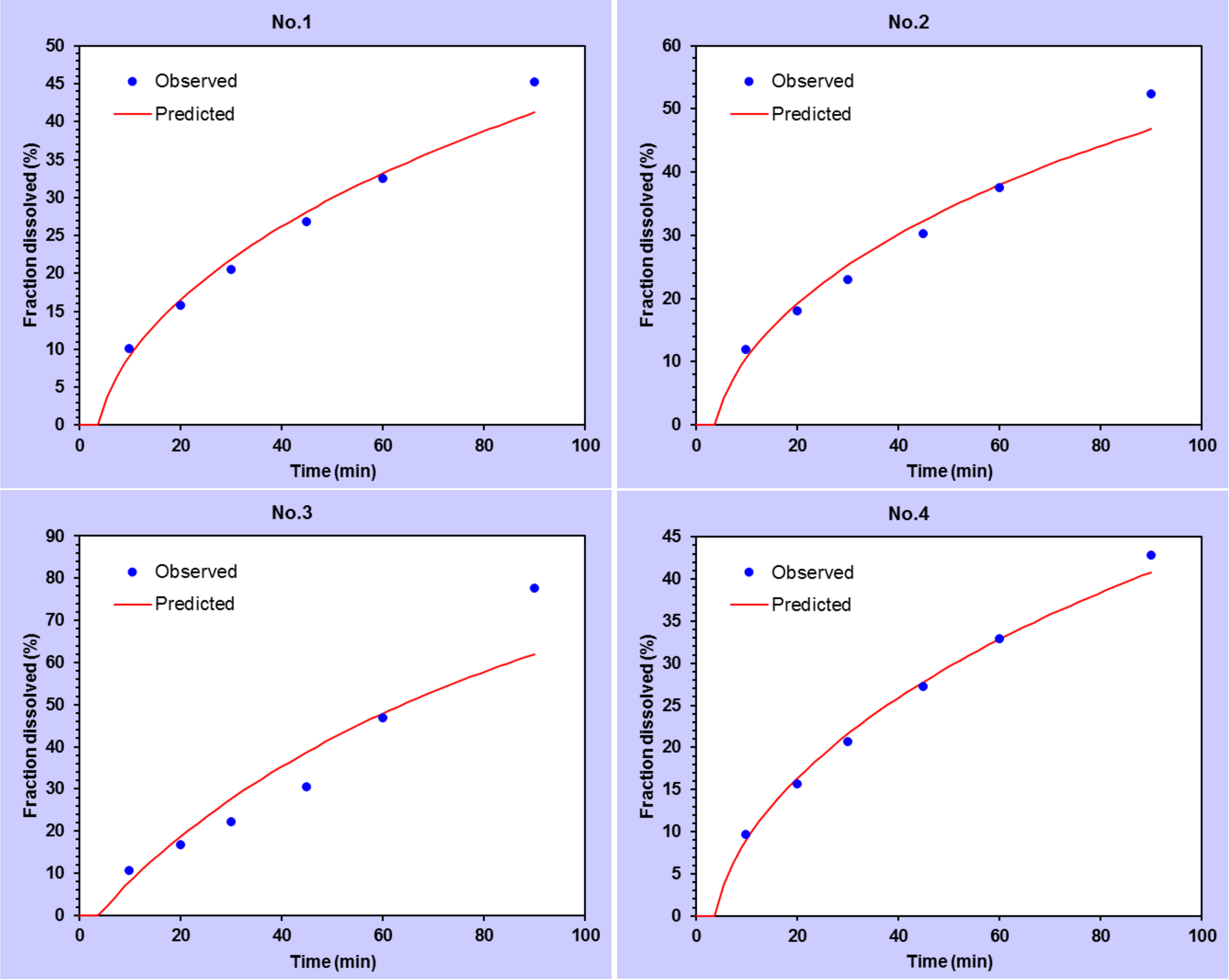

Supplementary materials – Model fitting summary – Pearlitol® 160C (modelling up to app. 60 % of carvedilol released)

Model: **Weibull_2**

Model equation: $F = 100 \cdot \left(1 - e^{-\frac{t^{\beta}}{\alpha}}\right)$

Fitted model parameters per tested tablet (N = 4) with statistics – mean, standard deviation (SD), and relative standard deviation expressed in % (RSD%) (output from DDSolver):

Parameter	No.1	No.2	No.3	No.4	Mean	SD	RSD(%)
α	59.530	52.247	150.348	60.039	80.541	46.674	57.950
β	0.778	0.788	1.125	0.777	0.867	0.172	19.824

Number of dissolution data points (N), degrees of freedom (df), and selected goodness of fit criteria – Pearson correlation coefficient (R), coefficient of determination (R^2), adjusted coefficient of determination ($R^2_{adjusted}$), and residual sum of squares (RSS) (manual calculation in MS Excel):

Parameter	No.1	No.2	No.3	No.4
N	6	6	6	6
df	4	4	4	4
R	0.99558766	0.992015346	0.967663496	0.999777041
R^2	0.991194789	0.984094446	0.936372642	0.999554131
$R^2_{adjusted}$	0.988993486	0.980118058	0.920465802	0.999442664
RSS	8.378261707	20.46870044	241.3690842	0.403493621

Graphical abstract of model fit presented as mean ± 1 SD of the fraction % of released carvedilol:

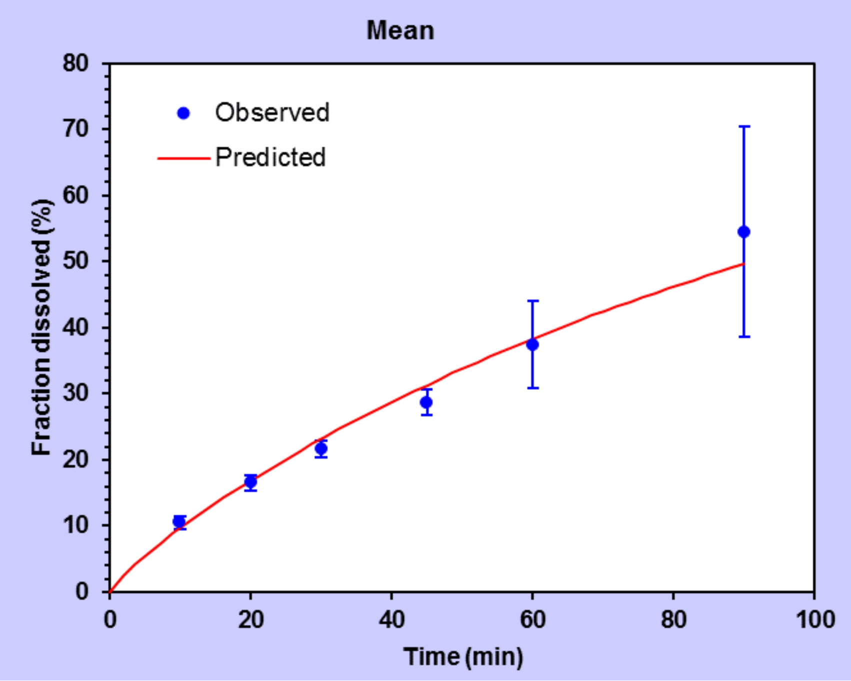

Supplementary materials – Model fitting summary – Pearlitol® 160C (modelling up to app. 60 % of carvedilol released)

Graphical abstract of model fit presented as the fraction % of released carvedilol per tested tablet:

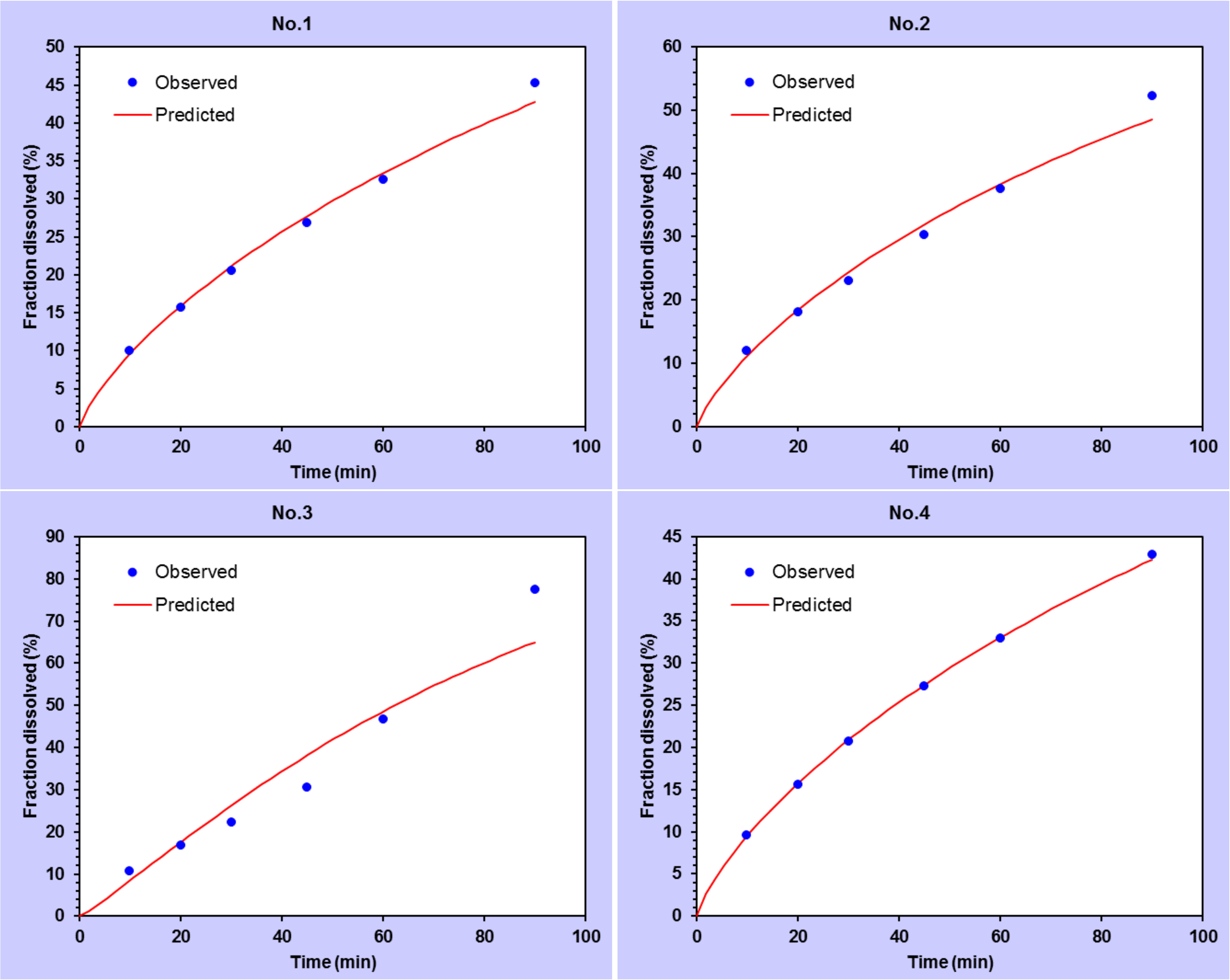

Supplementary materials – Model fitting summary – Pearlitol® 160C (modelling up to app. 60 % of carvedilol released)

Model: **Weibull_3**

Model equation: $F = F_{max} \cdot \left(1 - e^{-\frac{t^{\beta}}{\alpha}}\right)$

Fitted model parameters per tested tablet (N = 4) with statistics – mean, standard deviation (SD), and relative standard deviation expressed in % (RSD%) (output from DDSolver):

Parameter	No.1	No.2	No.3	No.4	Mean	SD	RSD(%)
α	81.029	78.110	197.982	61.309	104.607	62.853	60.085
β	1.087	1.074	1.296	1.102	1.140	0.105	9.197
F_{max}	50.234	58.125	81.375	44.940	58.668	16.078	27.405

Number of dissolution data points (N), degrees of freedom (df), and selected goodness of fit criteria – Pearson correlation coefficient (R), coefficient of determination (R^2), adjusted coefficient of determination ($R^2_{adjusted}$), and residual sum of squares (RSS) (manual calculation in MS Excel):

Parameter	No.1	No.2	No.3	No.4
N	6	6	6	6
df	3	3	3	3
R	0.98799362	0.984769063	0.955365174	0.987536995
R^2	0.976131393	0.969770108	0.912722616	0.975229317
$R^2_{adjusted}$	0.960218989	0.949616847	0.854537694	0.958715528
RSS	35.10453784	53.45390897	290.6935939	19.67158208

Graphical abstract of model fit presented as mean ± 1 SD of the fraction % of released carvedilol:

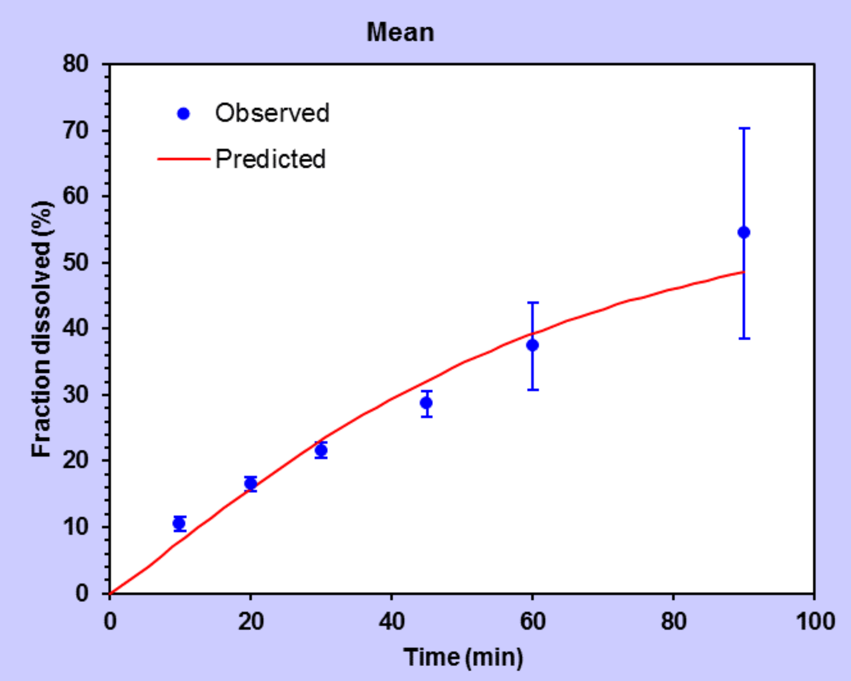

Supplementary materials – Model fitting summary – Pearlitol® 160C (modelling up to app. 60 % of carvedilol released)

Graphical abstract of model fit presented as the fraction % of released carvedilol per tested tablet:

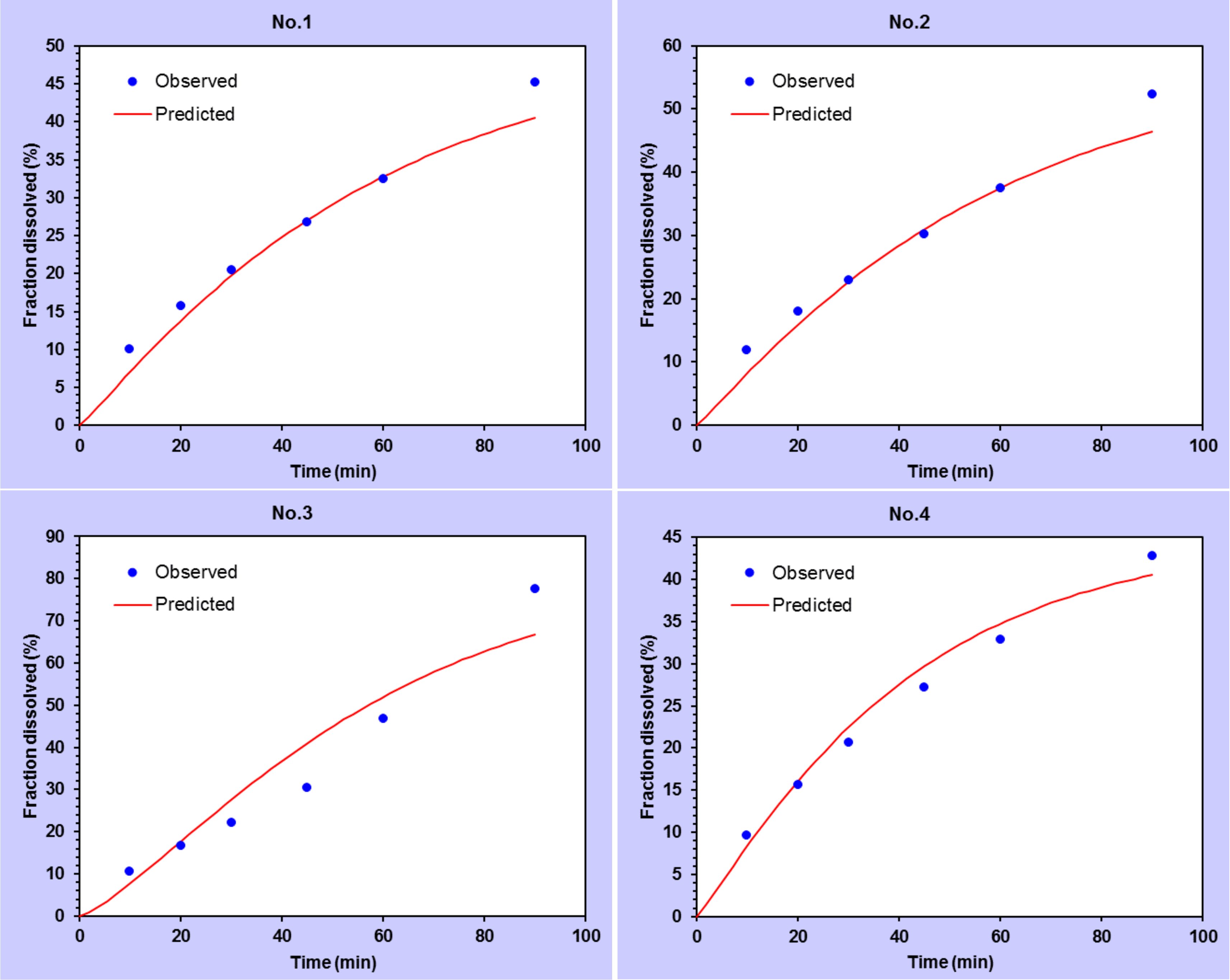

Supplementary materials – Model fitting summary – Pearlitol® 160C (modelling up to app. 60 % of carvedilol released)

Model: **Weibull_4**

Model equation: $F = F_{max} \cdot \left[1 - e^{-\frac{(t-T_i)^{\beta}}{\alpha}}\right]$

Fitted model parameters per tested tablet (N = 4) with statistics – mean, standard deviation (SD), and relative standard deviation expressed in % (RSD%) (output from DDSolver):

Parameter	No.1	No.2	No.3	No.4	Mean	SD	RSD(%)
α	26.226	25.413	69.141	28.689	37.367	21.228	56.810
β	0.887	0.874	1.051	0.853	0.916	0.091	9.904
T_i	4.000	4.000	6.000	4.454	4.614	0.949	20.565
F_{max}	47.460	54.915	81.375	54.518	59.567	14.937	25.075

Number of dissolution data points (N), degrees of freedom (df), and selected goodness of fit criteria – Pearson correlation coefficient (R), coefficient of determination (R^2), adjusted coefficient of determination ($R^2_{adjusted}$), and residual sum of squares (RSS) (manual calculation in MS Excel):

Parameter	No.1	No.2	No.3	No.4
N	6	6	6	6
df	2	2	2	2
R	0.967967894	0.962132025	0.942110767	0.989499159
R^2	0.936961844	0.925698034	0.887572697	0.979108586
$R^2_{adjusted}$	0.842404611	0.814245084	0.718931741	0.947771464
RSS	53.95327772	84.45956802	355.4392219	31.11316481

Graphical abstract of model fit presented as mean ± 1 SD of the fraction % of released carvedilol:

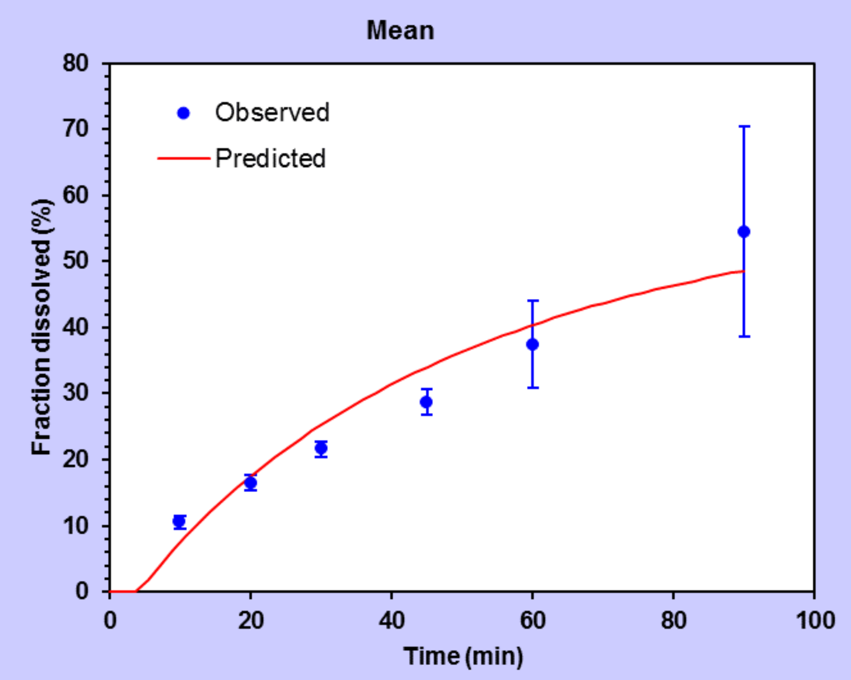

Supplementary materials – Model fitting summary – Pearlitol® 160C (modelling up to app. 60 % of carvedilol released)

Graphical abstract of model fit presented as the fraction % of released carvedilol per tested tablet:

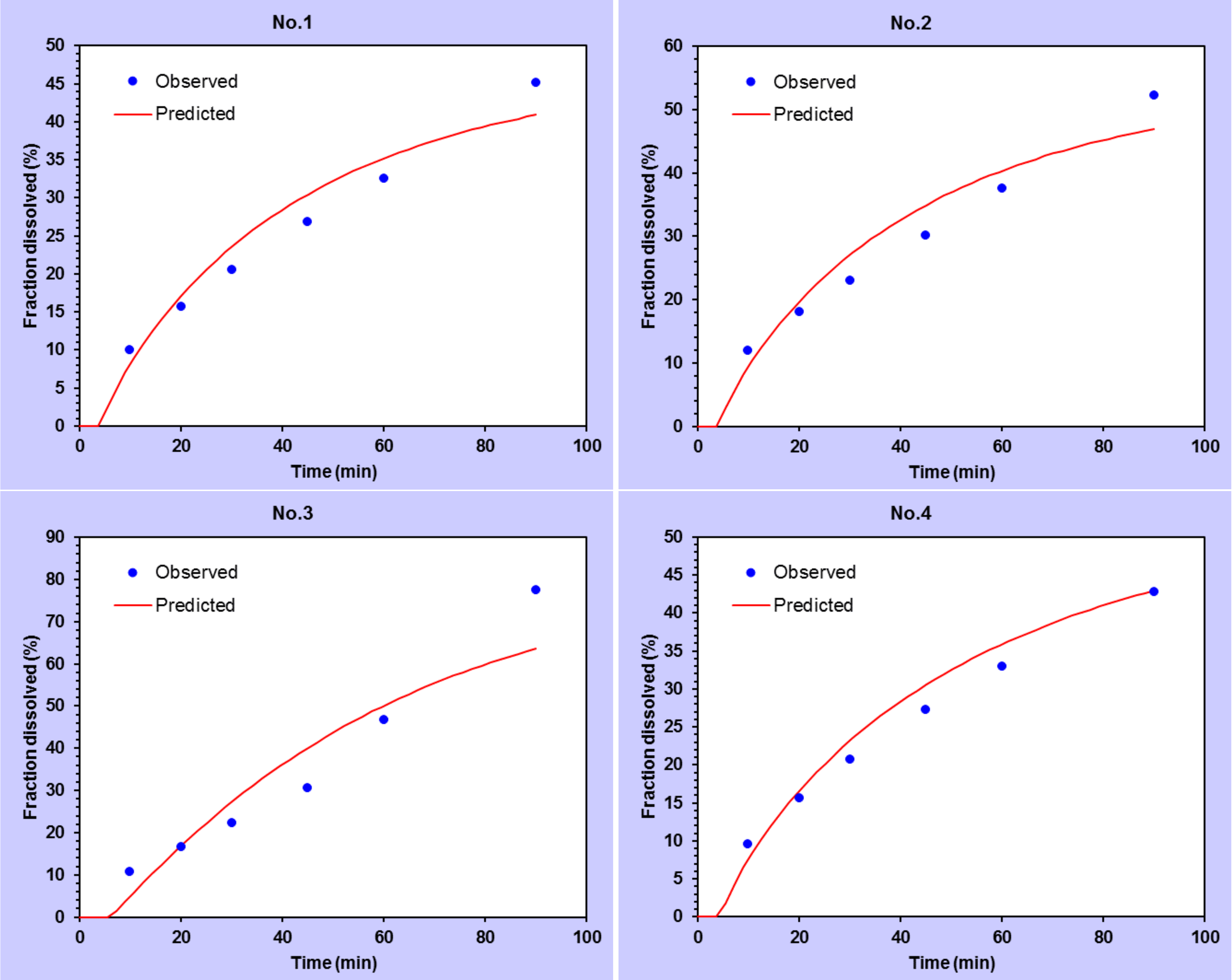

Supplementary materials – Model fitting summary – Pearlitol® 160C (modelling up to app. 60 % of carvedilol released)

Model: **Logistic_1**

Model equation: $F = 100 \cdot \frac{e^{\alpha+\beta\cdot\log(t)}}{1+e^{\alpha+\beta\cdot\log(t)}}$

Fitted model parameters per tested tablet (N = 4) with statistics – mean, standard deviation (SD), and relative standard deviation expressed in % (RSD%) (output from DDSolver):

Parameter	No.1	No.2	No.3	No.4	Mean	SD	RSD(%)
α	-4.648	-4.256	-5.789	-4.315	-4.752	0.712	-14.988
β	2.261	2.134	3.276	2.033	2.426	0.574	23.670

Number of dissolution data points (N), degrees of freedom (df), and selected goodness of fit criteria – Pearson correlation coefficient (R), coefficient of determination (R^2), adjusted coefficient of determination ($R^2_{adjusted}$), and residual sum of squares (RSS) (manual calculation in MS Excel):

Parameter	No.1	No.2	No.3	No.4
N	6	6	6	6
df	4	4	4	4
R	0.993370822	0.986472715	0.949771015	0.998534209
R^2	0.98678559	0.973128417	0.902064981	0.997070566
$R^2_{adjusted}$	0.983481988	0.966410521	0.877581226	0.996338208
RSS	12.99178083	32.02509918	326.4125341	2.49428913

Graphical abstract of model fit presented as mean ± 1 SD of the fraction % of released carvedilol:

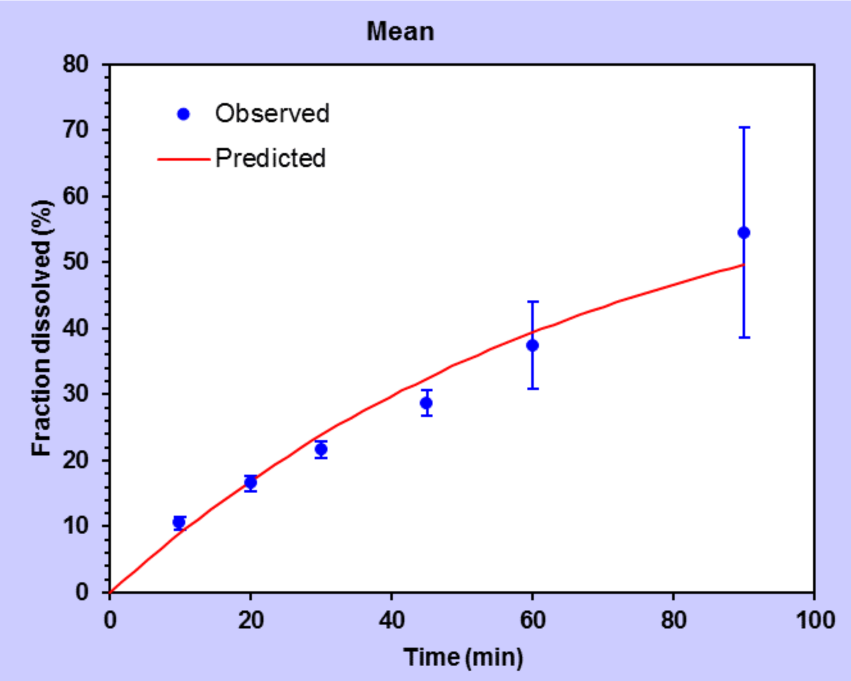

Supplementary materials – Model fitting summary – Pearlitol® 160C (modelling up to app. 60 % of carvedilol released)

Graphical abstract of model fit presented as the fraction % of released carvedilol per tested tablet:

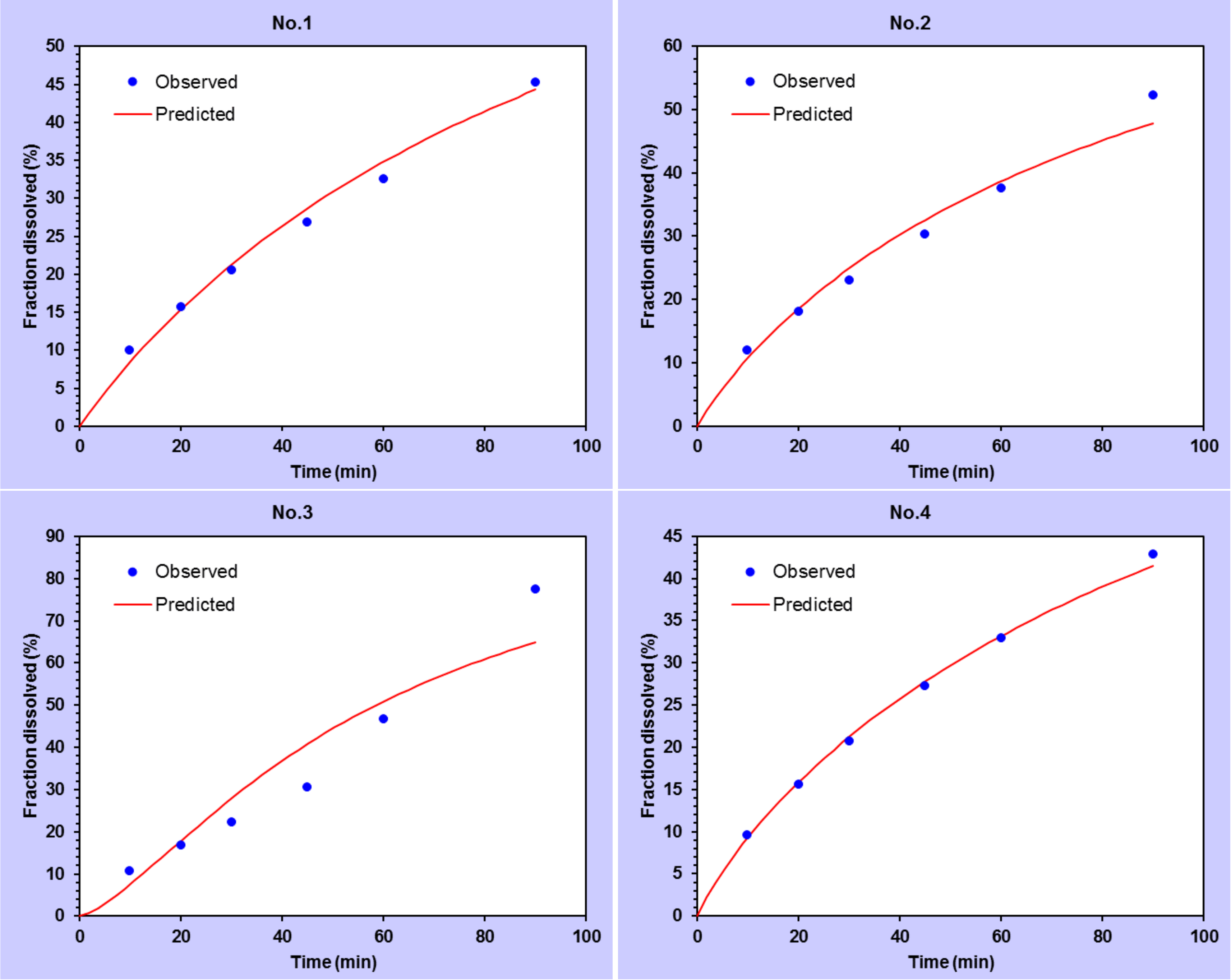

Supplementary materials – Model fitting summary – Pearlitol® 160C (modelling up to app. 60 % of carvedilol released)

Model: **Logistic_2**

Model equation: $F = F_{max} \cdot \frac{e^{\alpha+\beta\cdot\log(t)}}{1+e^{\alpha+\beta\cdot\log(t)}}$

Fitted model parameters per tested tablet (N = 4) with statistics – mean, standard deviation (SD), and relative standard deviation expressed in % (RSD%) (output from DDSolver):

Parameter	No.1	No.2	No.3	No.4	Mean	SD	RSD(%)
α	-5.832	-5.791	-7.059	-5.853	-6.134	0.617	-10.062
β	4.008	3.969	4.463	4.087	4.132	0.226	5.476
F_{max}	47.460	54.915	81.375	44.940	57.173	16.682	29.178

Number of dissolution data points (N), degrees of freedom (df), and selected goodness of fit criteria – Pearson correlation coefficient (R), coefficient of determination (R^2), adjusted coefficient of determination ($R^2_{adjusted}$), and residual sum of squares (RSS) (manual calculation in MS Excel):

Parameter	No.1	No.2	No.3	No.4
N	6	6	6	6
df	3	3	3	3
R	0.950260365	0.943316881	0.917599057	0.964519182
R^2	0.902994762	0.889846738	0.841988029	0.930297253
$R^2_{adjusted}$	0.838324603	0.816411229	0.736646715	0.883828755
RSS	99.36562658	148.9513902	586.5319374	65.23769001

Graphical abstract of model fit presented as mean ± 1 SD of the fraction % of released carvedilol:

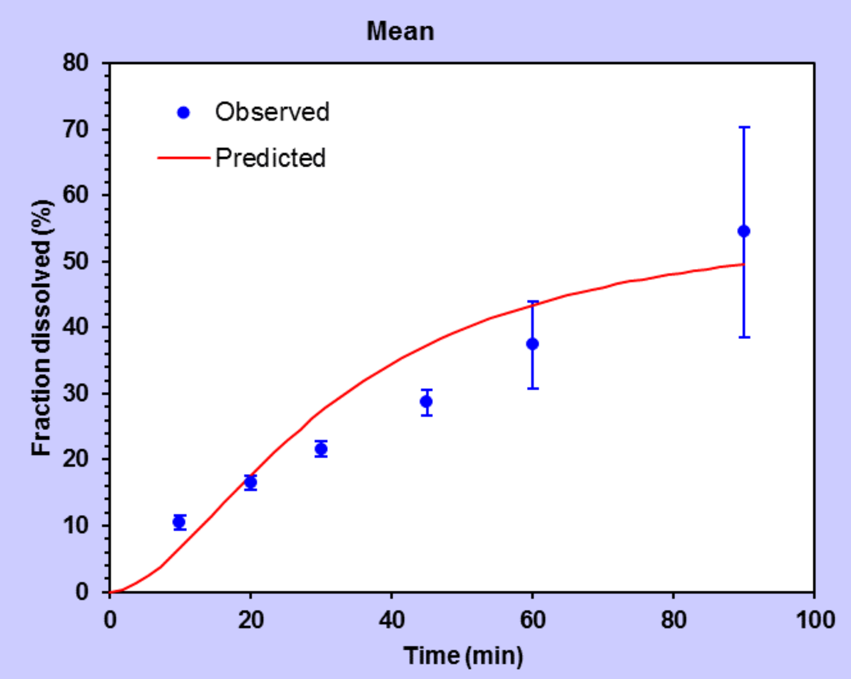

Supplementary materials – Model fitting summary – Pearlitol® 160C (modelling up to app. 60 % of carvedilol released)

Graphical abstract of model fit presented as the fraction % of released carvedilol per tested tablet:

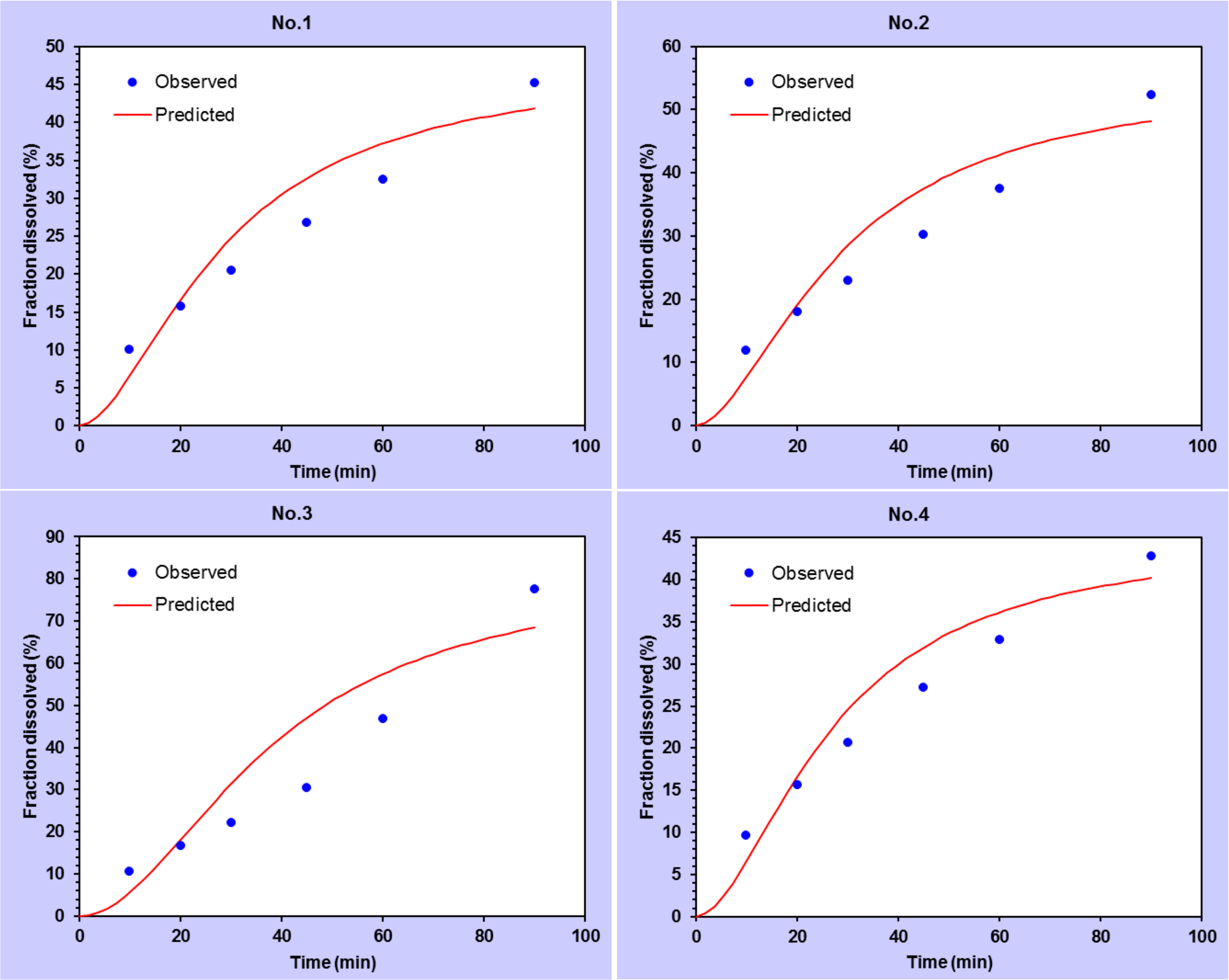

Supplementary materials – Model fitting summary – Pearlitol® 160C (modelling up to app. 60 % of carvedilol released)

Model: **Logistic_3**

Model equation: $F = F_{max} \cdot \frac{1}{1+e^{-k\cdot(t-\gamma)}}$

Fitted model parameters per tested tablet (N = 4) with statistics – mean, standard deviation (SD), and relative standard deviation expressed in % (RSD%) (output from DDSolver):

Parameter	No.1	No.2	No.3	No.4	Mean	SD	RSD(%)
k	0.032	0.032	0.058	0.051	0.044	0.013	30.784
γ	45.793	46.186	46.637	34.897	43.378	5.665	13.060
F_{max}	52.404	60.635	81.375	44.940	59.839	15.724	26.277

Number of dissolution data points (N), degrees of freedom (df), and selected goodness of fit criteria – Pearson correlation coefficient (R), coefficient of determination (R^2), adjusted coefficient of determination ($R^2_{adjusted}$), and residual sum of squares (RSS) (manual calculation in MS Excel):

Parameter	No.1	No.2	No.3	No.4
N	6	6	6	6
df	3	3	3	3
R	0.997177388	0.998410284	0.97835424	0.994871956
R^2	0.994362744	0.996823096	0.957177019	0.98977021
$R^2_{adjusted}$	0.990604573	0.99470516	0.928628365	0.98295035
RSS	17.06716097	19.87743562	166.1348711	9.180801203

Graphical abstract of model fit presented as mean ± 1 SD of the fraction % of released carvedilol:

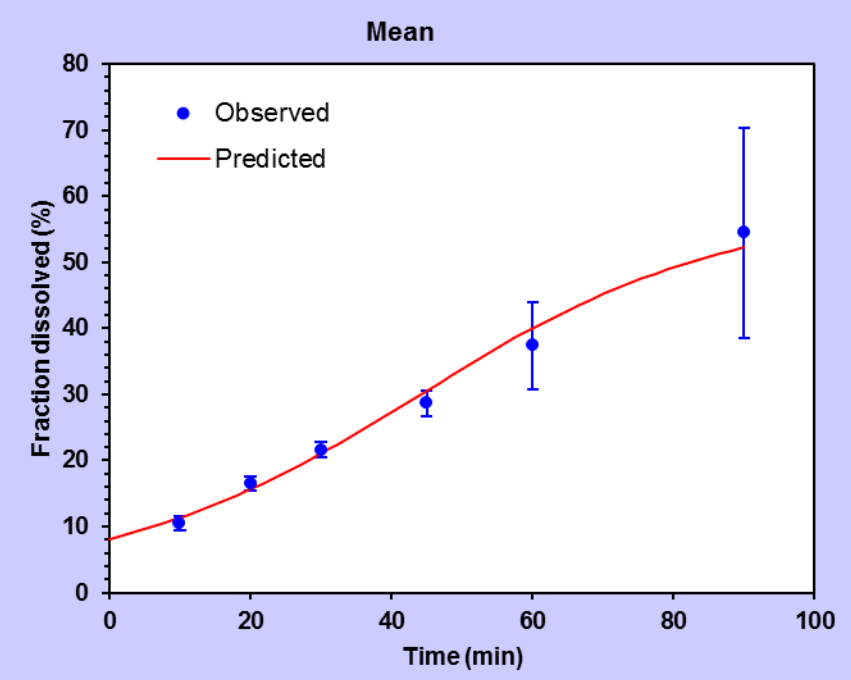

Supplementary materials – Model fitting summary – Pearlitol® 160C (modelling up to app. 60 % of carvedilol released)

Graphical abstract of model fit presented as the fraction % of released carvedilol per tested tablet:

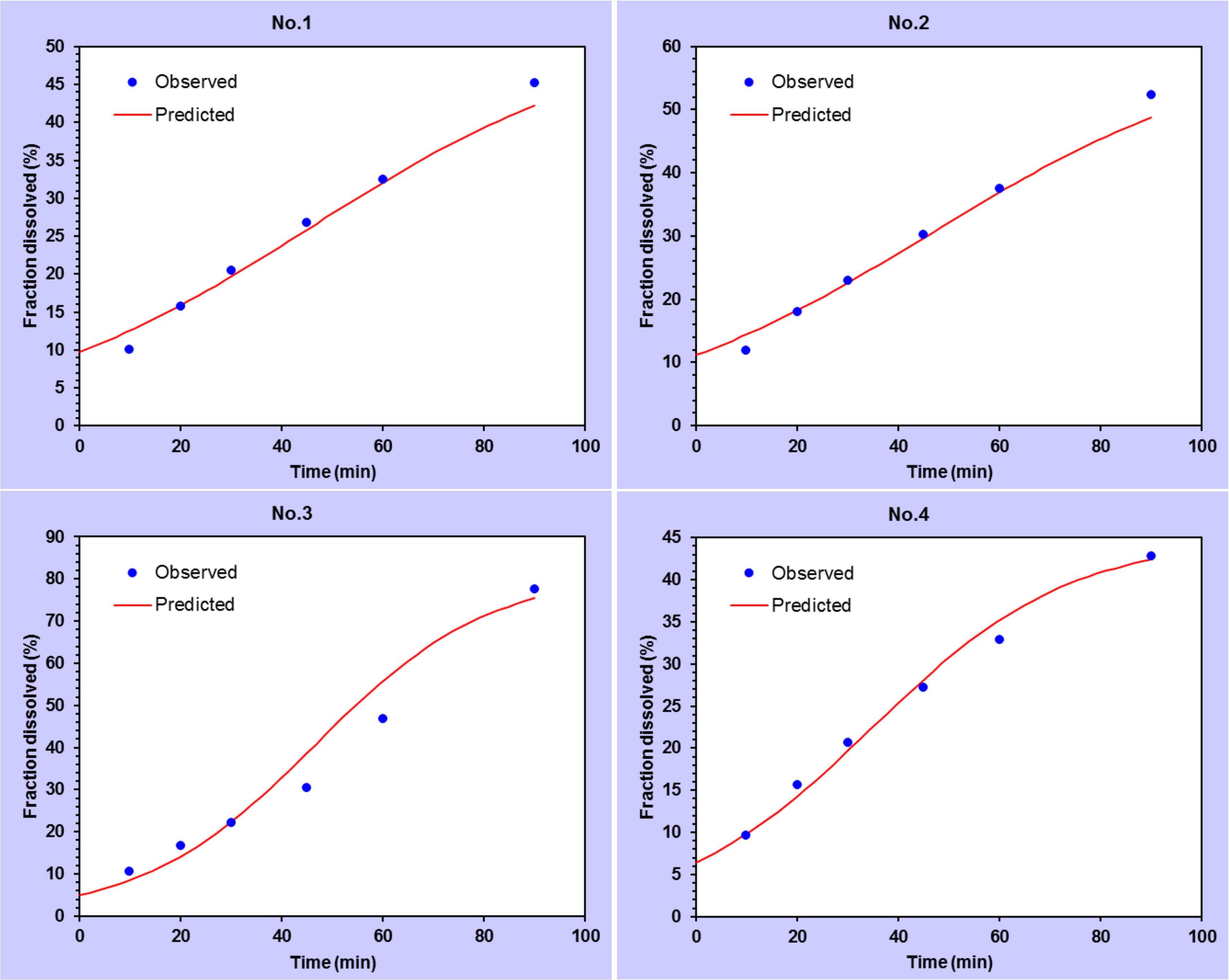

Supplementary materials – Model fitting summary – Pearlitol® 160C (modelling up to app. 60 % of carvedilol released)

Model: **Gompertz_1**

Model equation: $F = 100 \cdot e^{-\alpha \cdot e^{-\beta \cdot \log(t)}}$

Fitted model parameters per tested tablet (N = 4) with statistics – mean, standard deviation (SD), and relative standard deviation expressed in % (RSD%) (output from DDSolver):

Parameter	No.1	No.2	No.3	No.4	Mean	SD	RSD(%)
α	7.285	7.814	23.798	7.088	11.496	8.207	71.388
β	1.074	1.193	2.039	1.050	1.339	0.471	35.177

Number of dissolution data points (N), degrees of freedom (df), and selected goodness of fit criteria – Pearson correlation coefficient (R), coefficient of determination (R^2), adjusted coefficient of determination ($R^2_{adjusted}$), and residual sum of squares (RSS) (manual calculation in MS Excel):

Parameter	No.1	No.2	No.3	No.4
N	6	6	6	6
df	4	4	4	4
R	0.97969586	0.97073759	0.914659535	0.991050009
R^2	0.959803978	0.942331468	0.836602064	0.98218012
$R^2_{adjusted}$	0.949754972	0.927914335	0.79575258	0.97772515
RSS	33.35569527	63.00332136	521.3886595	13.61321906

Graphical abstract of model fit presented as mean ± 1 SD of the fraction % of released carvedilol:

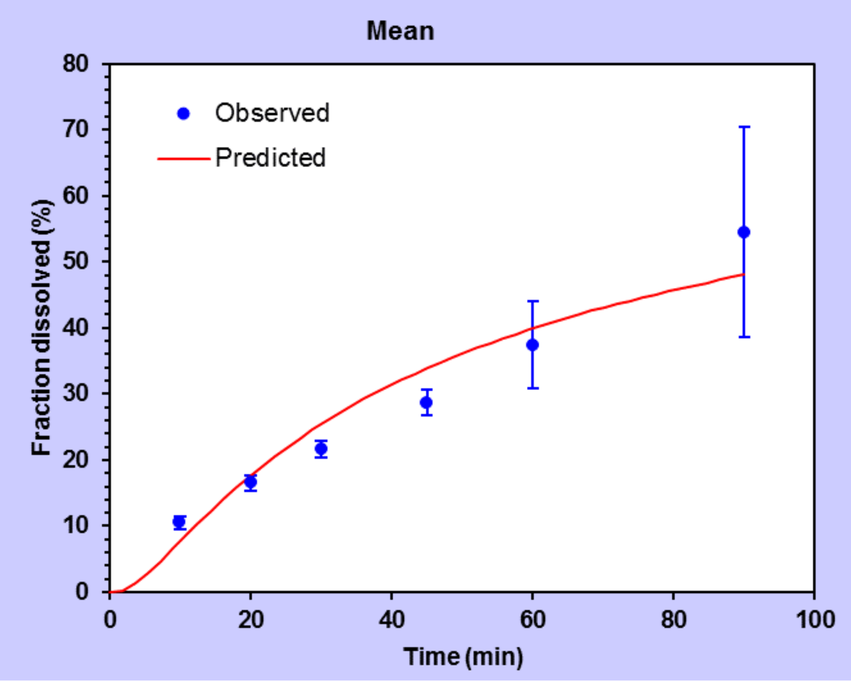

Supplementary materials – Model fitting summary – Pearlitol® 160C (modelling up to app. 60 % of carvedilol released)

Graphical abstract of model fit presented as the fraction % of released carvedilol per tested tablet:

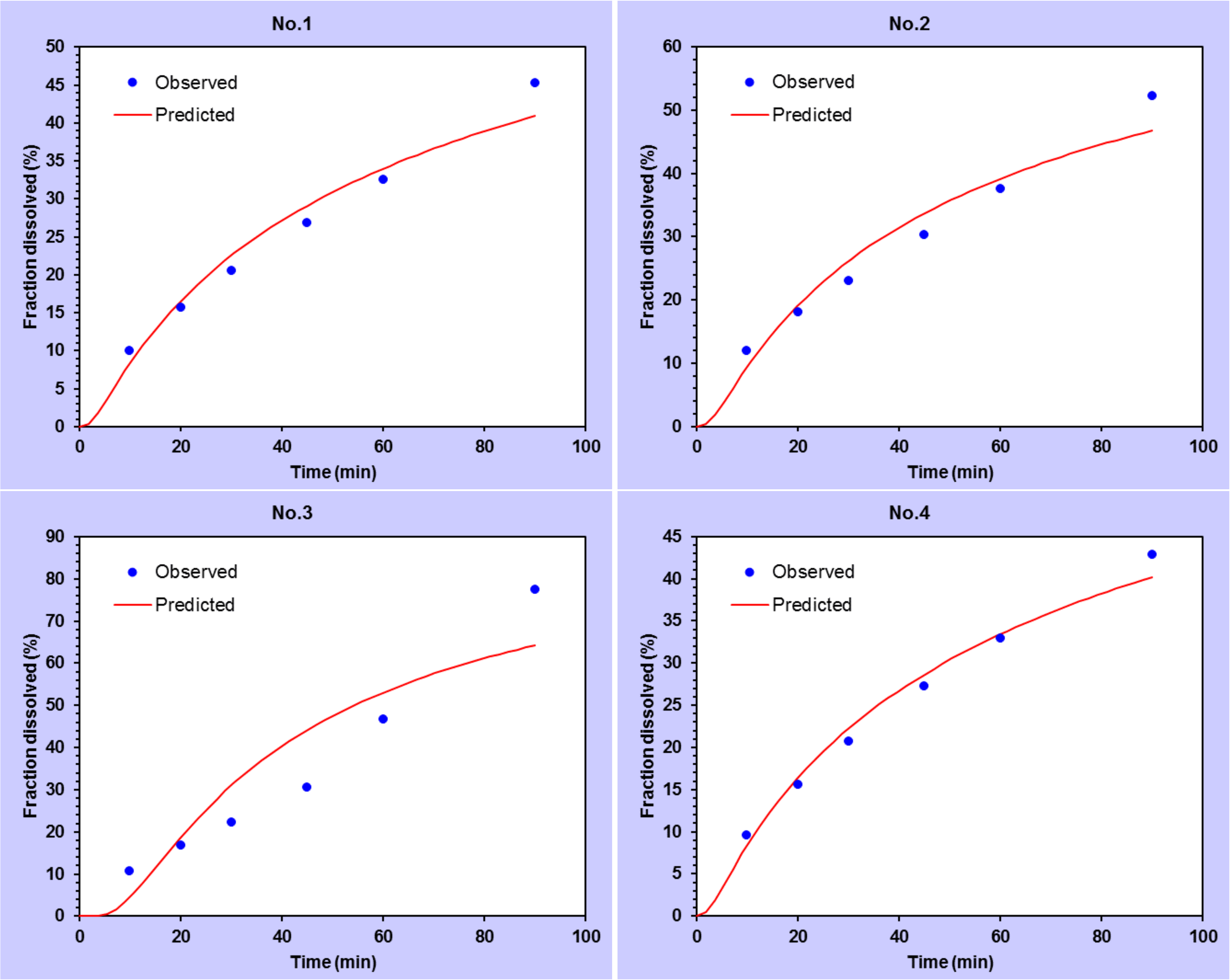

Supplementary materials – Model fitting summary – Pearlitol® 160C (modelling up to app. 60 % of carvedilol released)

Model: **Gompertz_2**

Model equation: $F = F_{max} \cdot e^{-\alpha \cdot e^{-\beta \cdot \log(t)}}$

Fitted model parameters per tested tablet (N = 4) with statistics – mean, standard deviation (SD), and relative standard deviation expressed in % (RSD%) (output from DDSolver):

Parameter	No.1	No.2	No.3	No.4	Mean	SD	RSD(%)
α	88.033	86.158	151.007	61.269	96.617	38.257	39.597
β	3.123	3.100	3.259	3.201	3.171	0.073	2.304
F_{max}	47.460	54.915	81.375	44.940	57.173	16.682	29.178

Number of dissolution data points (N), degrees of freedom (df), and selected goodness of fit criteria – Pearson correlation coefficient (R), coefficient of determination (R^2), adjusted coefficient of determination ($R^2_{adjusted}$), and residual sum of squares (RSS) (manual calculation in MS Excel):

Parameter	No.1	No.2	No.3	No.4
N	6	6	6	6
df	3	3	3	3
R	0.955524697	0.9490442	0.922651958	0.939049568
R^2	0.913027447	0.900684893	0.851286635	0.88181409
$R^2_{adjusted}$	0.855045745	0.834474822	0.752144392	0.803023484
RSS	153.2180592	216.6551654	509.4646879	122.5185542

Graphical abstract of model fit presented as mean ± 1 SD of the fraction % of released carvedilol:

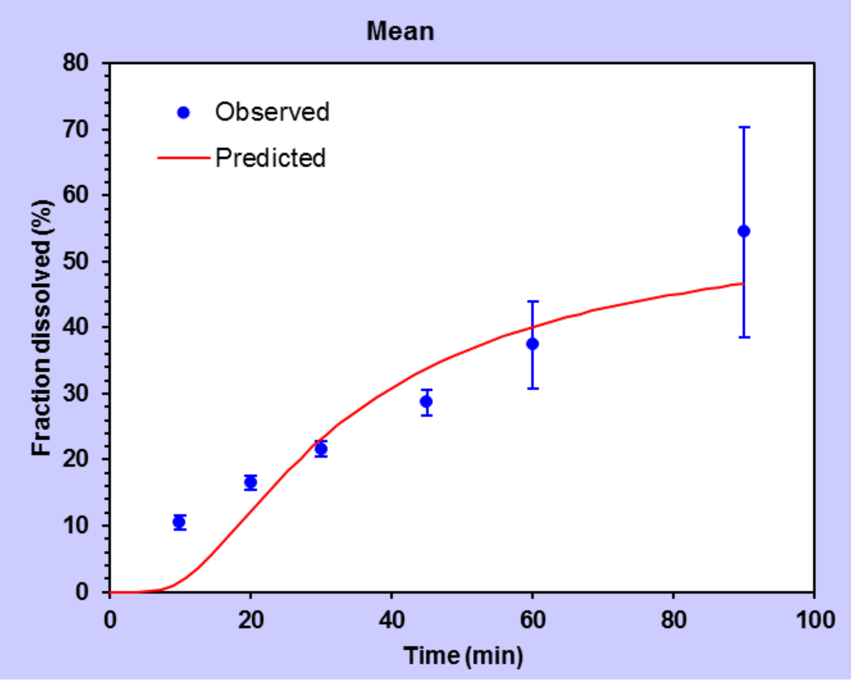

Supplementary materials – Model fitting summary – Pearlitol® 160C (modelling up to app. 60 % of carvedilol released)

Graphical abstract of model fit presented as the fraction % of released carvedilol per tested tablet:

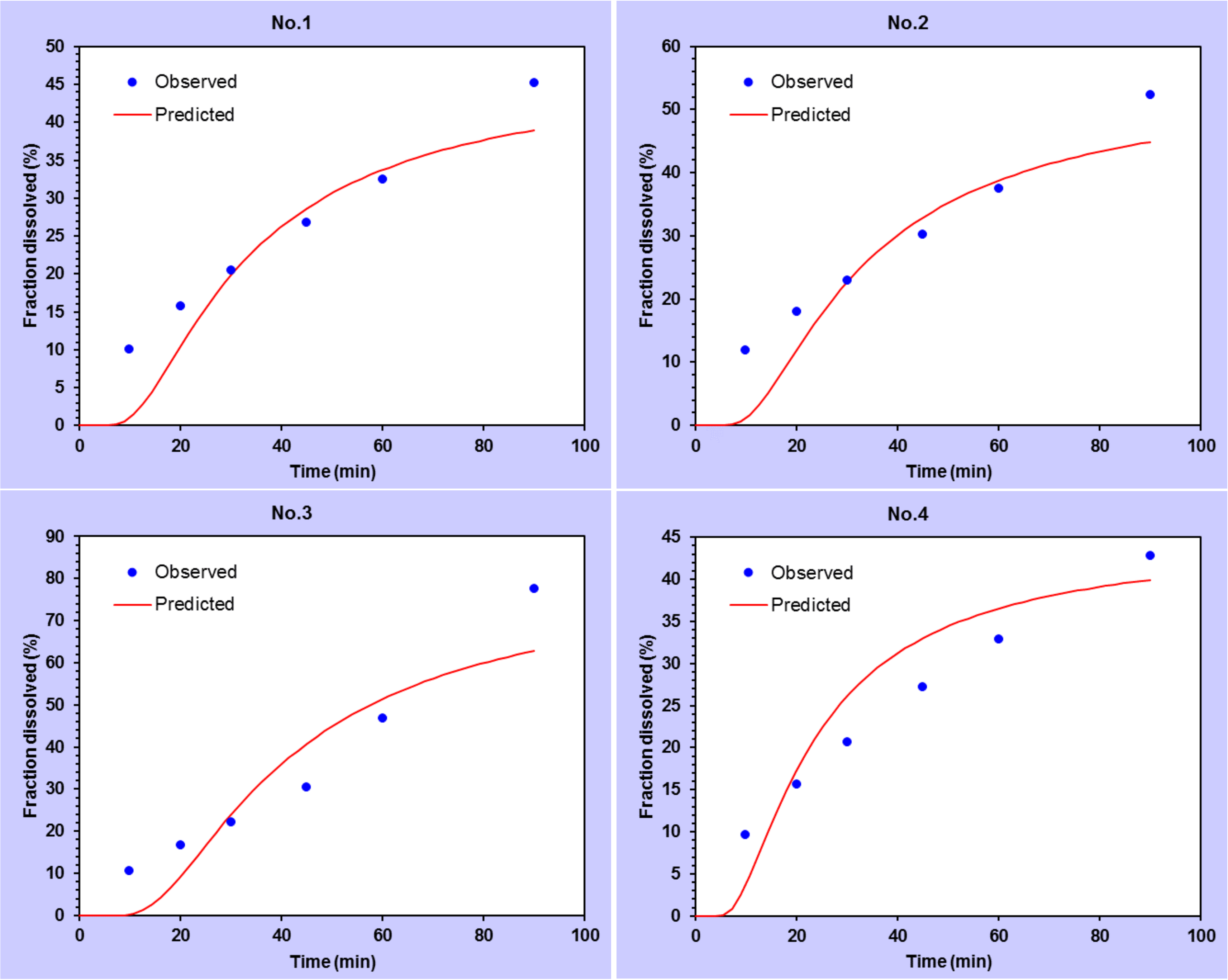

Supplementary materials – Model fitting summary – Pearlitol® 160C (modelling up to app. 60 % of carvedilol released)

Model: **Gompertz_3**

Model equation: $F = F_{max} \cdot e^{-e^{-k\cdot(t-\gamma)}}$

Fitted model parameters per tested tablet (N = 4) with statistics – mean, standard deviation (SD), and relative standard deviation expressed in % (RSD%) (output from DDSolver):

Parameter	No.1	No.2	No.3	No.4	Mean	SD	RSD(%)
k	0.041	0.041	0.044	0.028	0.039	0.007	18.433
γ	25.553	25.854	34.273	26.215	27.974	4.208	15.044
F_{max}	47.460	54.915	81.375	46.578	57.582	16.297	28.302

Number of dissolution data points (N), degrees of freedom (df), and selected goodness of fit criteria – Pearson correlation coefficient (R), coefficient of determination (R^2), adjusted coefficient of determination ($R^2_{adjusted}$), and residual sum of squares (RSS) (manual calculation in MS Excel):

Parameter	No.1	No.2	No.3	No.4
N	6	6	6	6
df	3	3	3	3
R	0.980760735	0.977734032	0.952344119	0.998435799
R^2	0.96189162	0.955963837	0.906959322	0.996874044
$R^2_{adjusted}$	0.936486034	0.926606395	0.844932203	0.994790073
RSS	48.59414428	74.32008636	400.954978	19.12851788

Graphical abstract of model fit presented as mean ± 1 SD of the fraction % of released carvedilol:

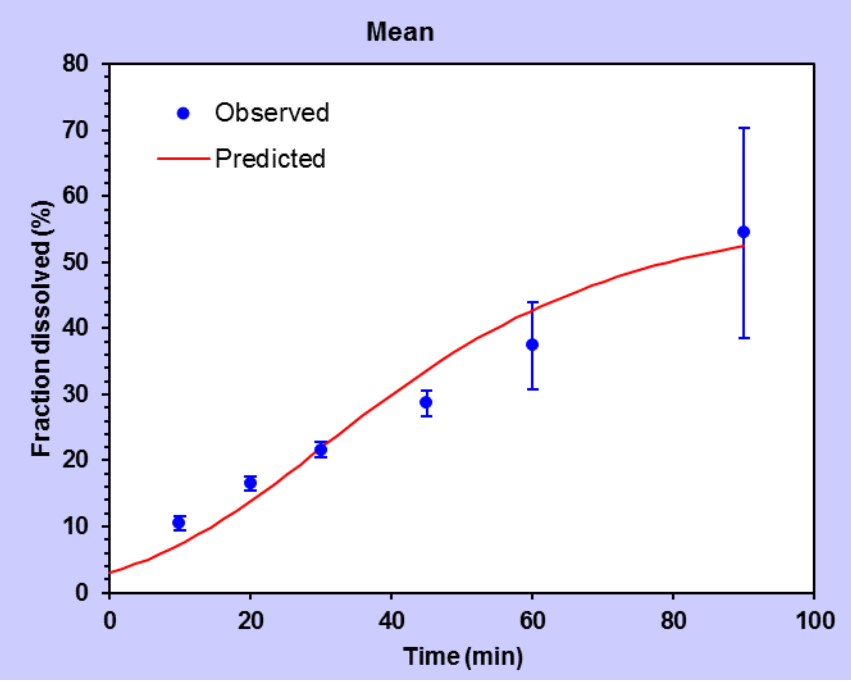

Supplementary materials – Model fitting summary – Pearlitol® 160C (modelling up to app. 60 % of carvedilol released)

Graphical abstract of model fit presented as the fraction % of released carvedilol per tested tablet:

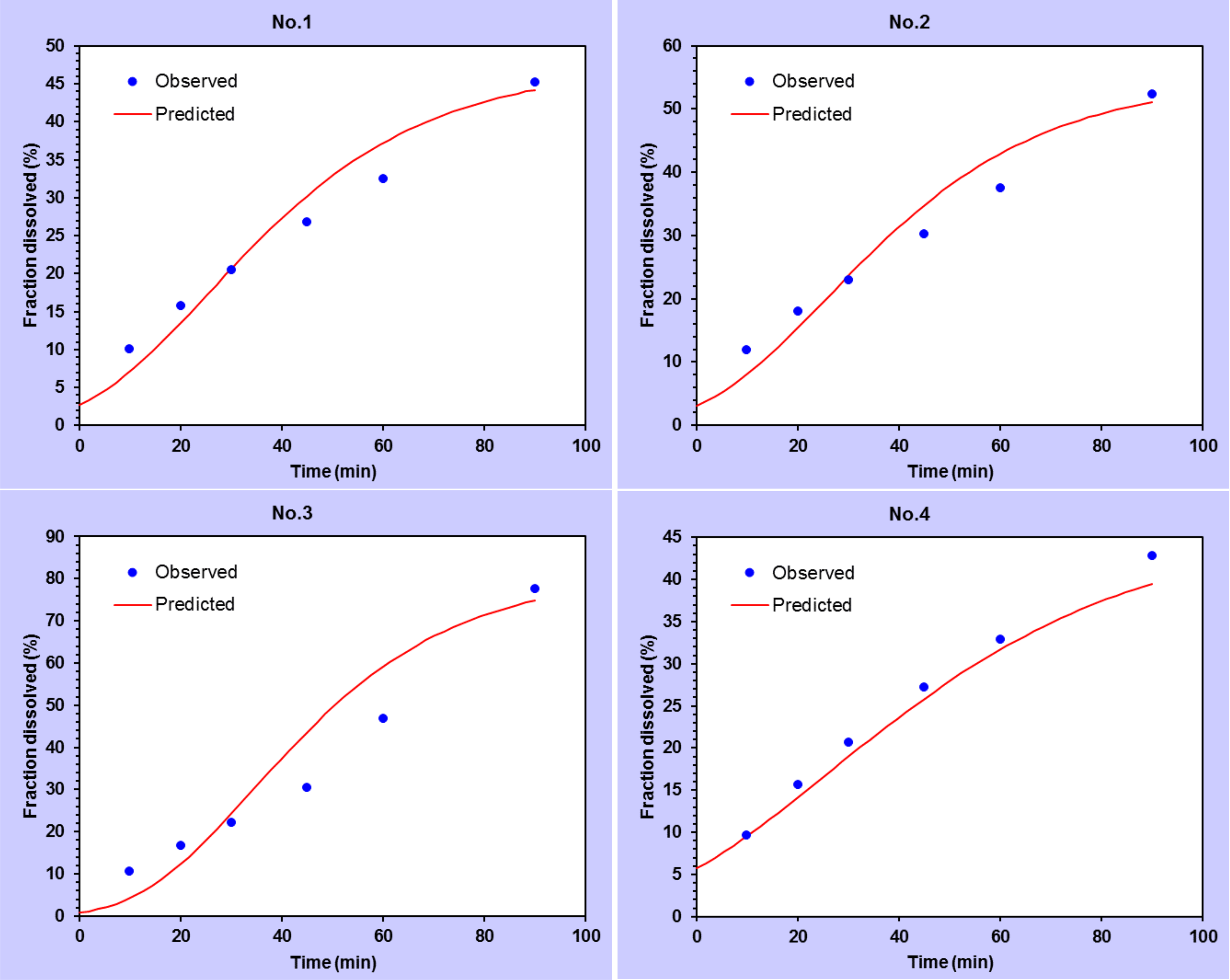

Supplementary materials – Model fitting summary – Pearlitol® 160C (modelling up to app. 60 % of carvedilol released)

Model: **Gompertz_4**

Model equation: $F = F_{max} \cdot e^{-\beta \cdot e^{-k \cdot t}}$

Fitted model parameters per tested tablet (N = 4) with statistics – mean, standard deviation (SD), and relative standard deviation expressed in % (RSD%) (output from DDSolver):

Parameter	No.1	No.2	No.3	No.4	Mean	SD	RSD(%)
k	0.041	0.041	0.044	0.041	0.042	0.002	3.853
β	2.860	2.892	6.881	2.689	3.831	2.035	53.136
F_{max}	47.460	54.915	81.375	44.940	57.173	16.682	29.178

Number of dissolution data points (N), degrees of freedom (df), and selected goodness of fit criteria – Pearson correlation coefficient (R), coefficient of determination (R^2), adjusted coefficient of determination ($R^2_{adjusted}$), and residual sum of squares (RSS) (manual calculation in MS Excel):

Parameter	No.1	No.2	No.3	No.4
N	6	6	6	6
df	3	3	3	3
R	0.980760735	0.977734032	0.978170499	0.990521222
R^2	0.96189162	0.955963837	0.956817526	0.981132292
$R^2_{adjusted}$	0.936486034	0.926606395	0.928029209	0.968553819
RSS	48.59414428	74.32008636	363.3004139	22.50702054

Graphical abstract of model fit presented as mean ± 1 SD of the fraction % of released carvedilol:

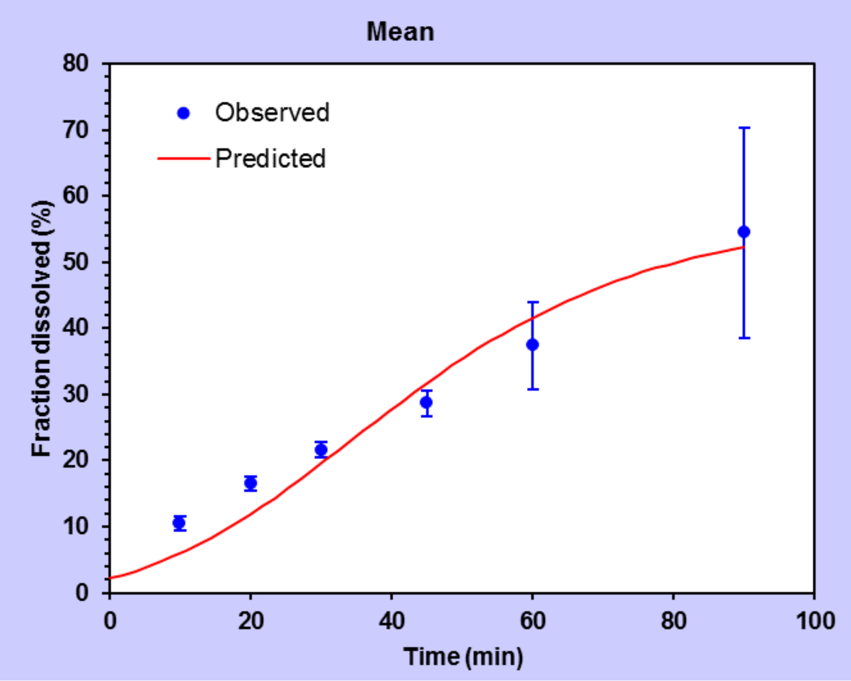

Supplementary materials – Model fitting summary – Pearlitol® 160C (modelling up to app. 60 % of carvedilol released)

Graphical abstract of model fit presented as the fraction % of released carvedilol per tested tablet:

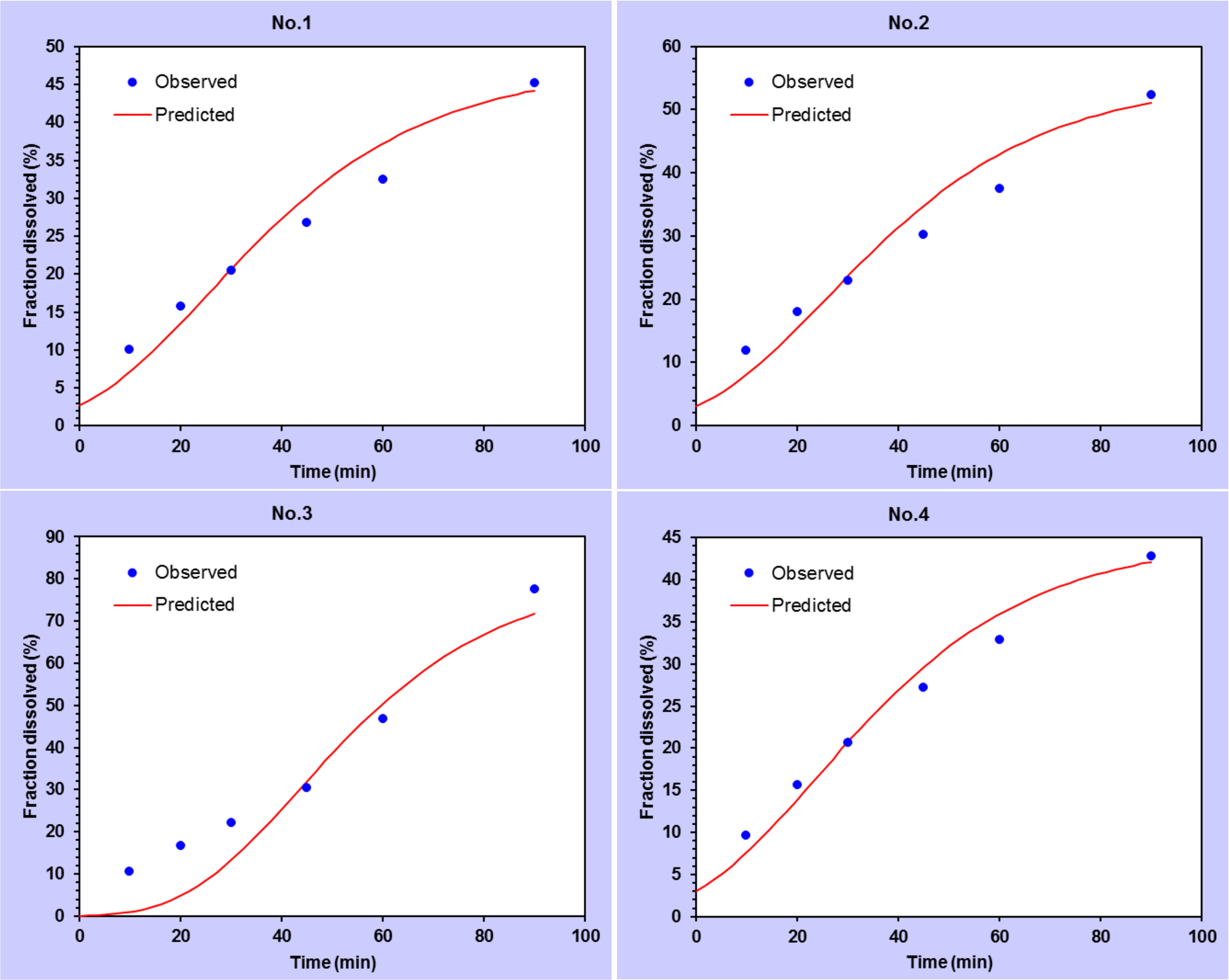

Supplementary materials – Model fitting summary – Pearlitol® 160C (modelling up to app. 60 % of carvedilol released)

Model: **Probit_1**

Model equation: $F = 100 \cdot \phi[\alpha + \beta \cdot \log(t)]$

Fitted model parameters per tested tablet (N = 4) with statistics – mean, standard deviation (SD), and relative standard deviation expressed in % (RSD%) (output from DDSolver):

Parameter	No.1	No.2	No.3	No.4	Mean	SD	RSD(%)
α	-2.525	-2.524	-3.439	-2.512	-2.750	0.459	-16.705
β	1.184	1.258	1.944	1.169	1.389	0.373	26.826

Number of dissolution data points (N), degrees of freedom (df), and selected goodness of fit criteria – Pearson correlation coefficient (R), coefficient of determination (R^2), adjusted coefficient of determination ($R^2_{adjusted}$), and residual sum of squares (RSS) (manual calculation in MS Excel):

Parameter	No.1	No.2	No.3	No.4
N	6	6	6	6
df	4	4	4	4
R	0.988318394	0.982127046	0.942581458	0.996530191
R^2	0.976773247	0.964573534	0.888459805	0.993072422
$R^2_{adjusted}$	0.970966559	0.955716918	0.860574756	0.991340527
RSS	20.41438251	40.96331805	365.9649642	5.680208882

Graphical abstract of model fit presented as mean ± 1 SD of the fraction % of released carvedilol:

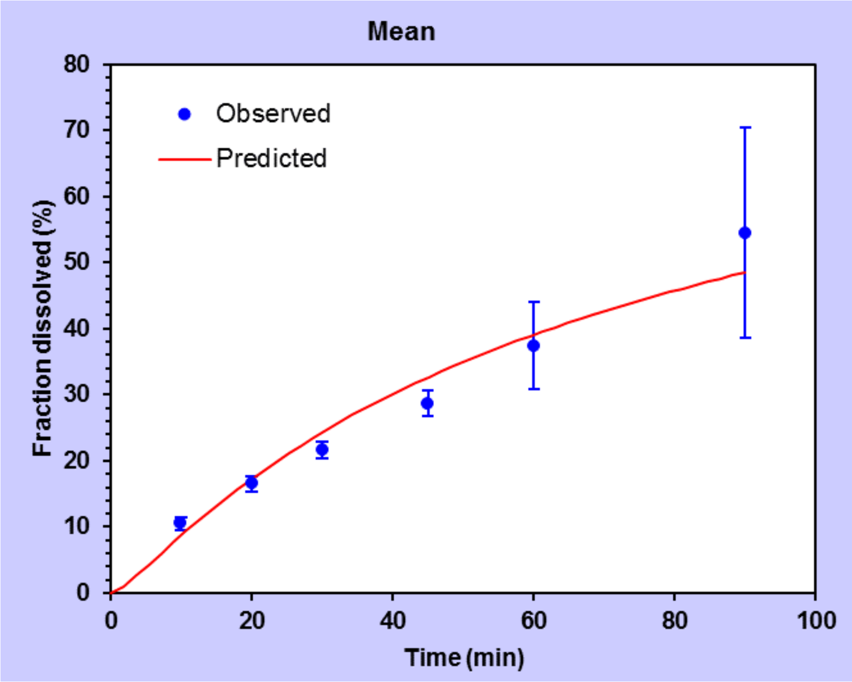

Supplementary materials – Model fitting summary – Pearlitol® 160C (modelling up to app. 60 % of carvedilol released)

Graphical abstract of model fit presented as the fraction % of released carvedilol per tested tablet:

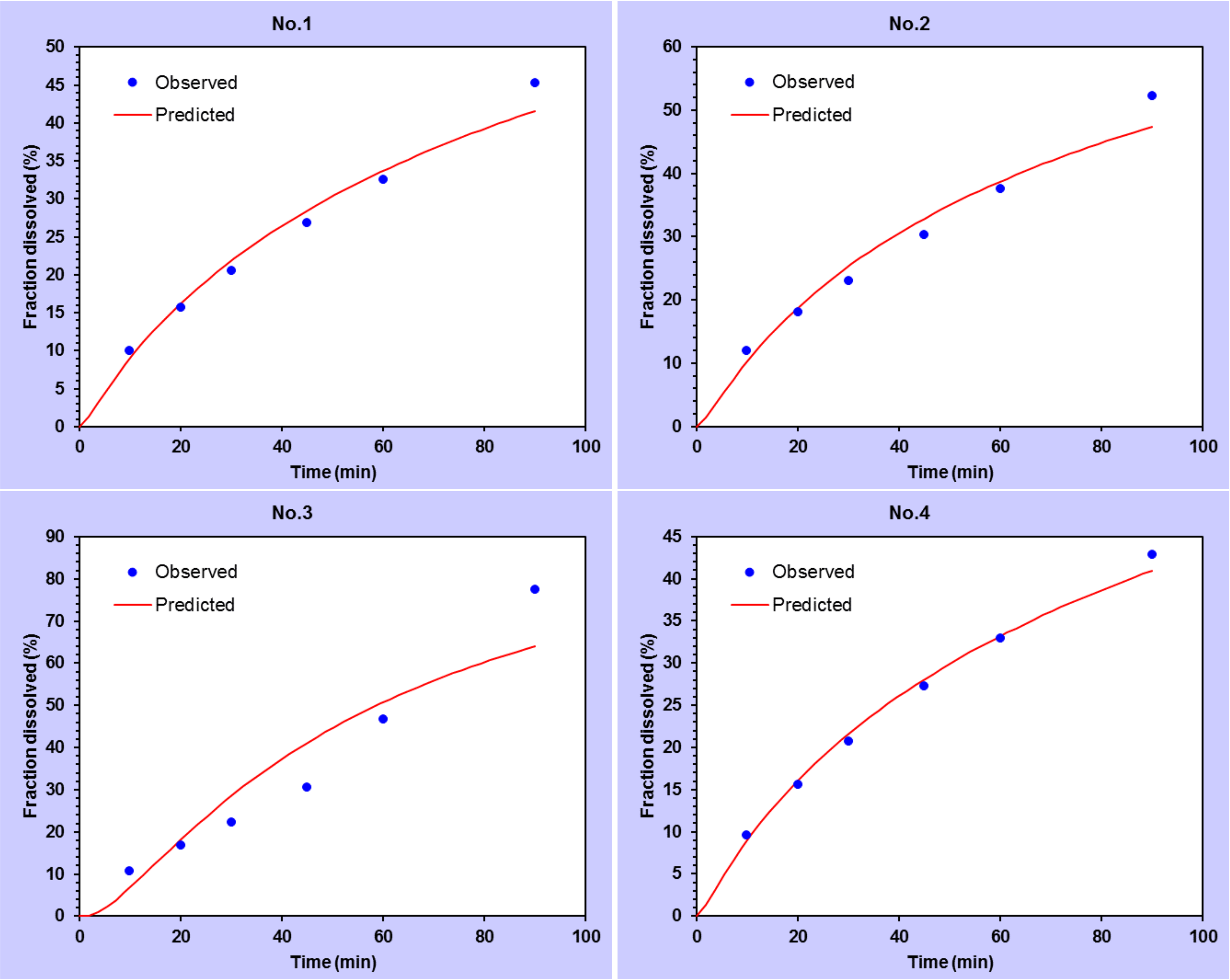

Supplementary materials – Model fitting summary – Pearlitol® 160C (modelling up to app. 60 % of carvedilol released)

Model: **Probit_2**

Model equation: $F = F_{max} \cdot \phi[\alpha + \beta \cdot \log(t)]$

Fitted model parameters per tested tablet (N = 4) with statistics – mean, standard deviation (SD), and relative standard deviation expressed in % (RSD%) (output from DDSolver):

Parameter	No.1	No.2	No.3	No.4	Mean	SD	RSD(%)
α	-3.419	-3.395	-4.775	-3.431	-3.755	0.680	-18.115
β	2.336	2.314	2.718	2.385	2.438	0.189	7.749
F_{max}	47.460	54.915	89.143	44.940	59.115	20.462	34.615

Number of dissolution data points (N), degrees of freedom (df), and selected goodness of fit criteria – Pearson correlation coefficient (R), coefficient of determination (R^2), adjusted coefficient of determination ($R^2_{adjusted}$), and residual sum of squares (RSS) (manual calculation in MS Excel):

Parameter	No.1	No.2	No.3	No.4
N	6	6	6	6
df	3	3	3	3
R	0.955035557	0.948284008	0.961906662	0.968878937
R^2	0.912092916	0.89924256	0.925264426	0.938726395
$R^2_{adjusted}$	0.853488193	0.832070934	0.875440711	0.897877326
RSS	82.67476277	125.3364891	367.0327658	52.78415965

Graphical abstract of model fit presented as mean ± 1 SD of the fraction % of released carvedilol:

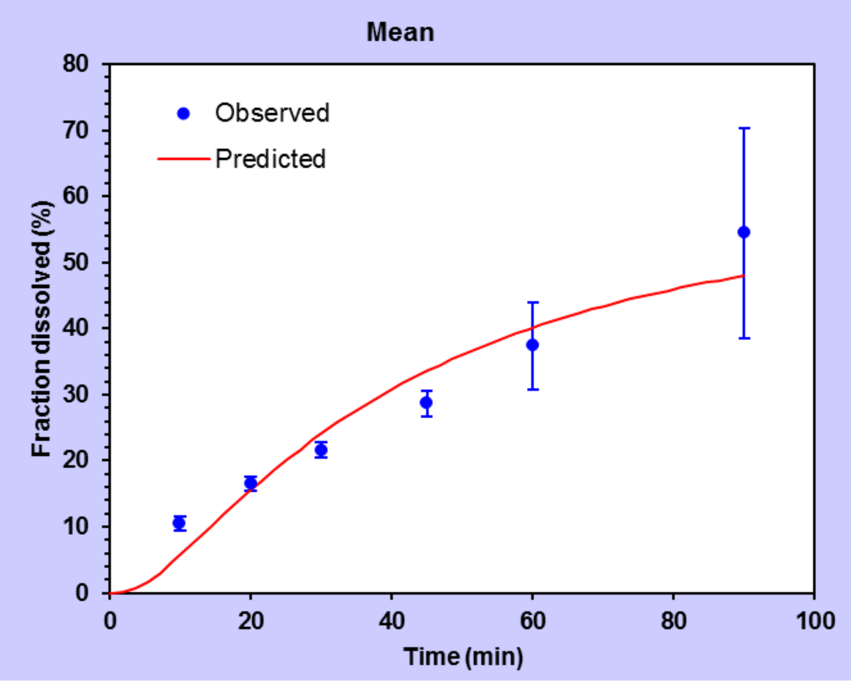

Supplementary materials – Model fitting summary – Pearlitol® 160C (modelling up to app. 60 % of carvedilol released)

Graphical abstract of model fit presented as the fraction % of released carvedilol per tested table:

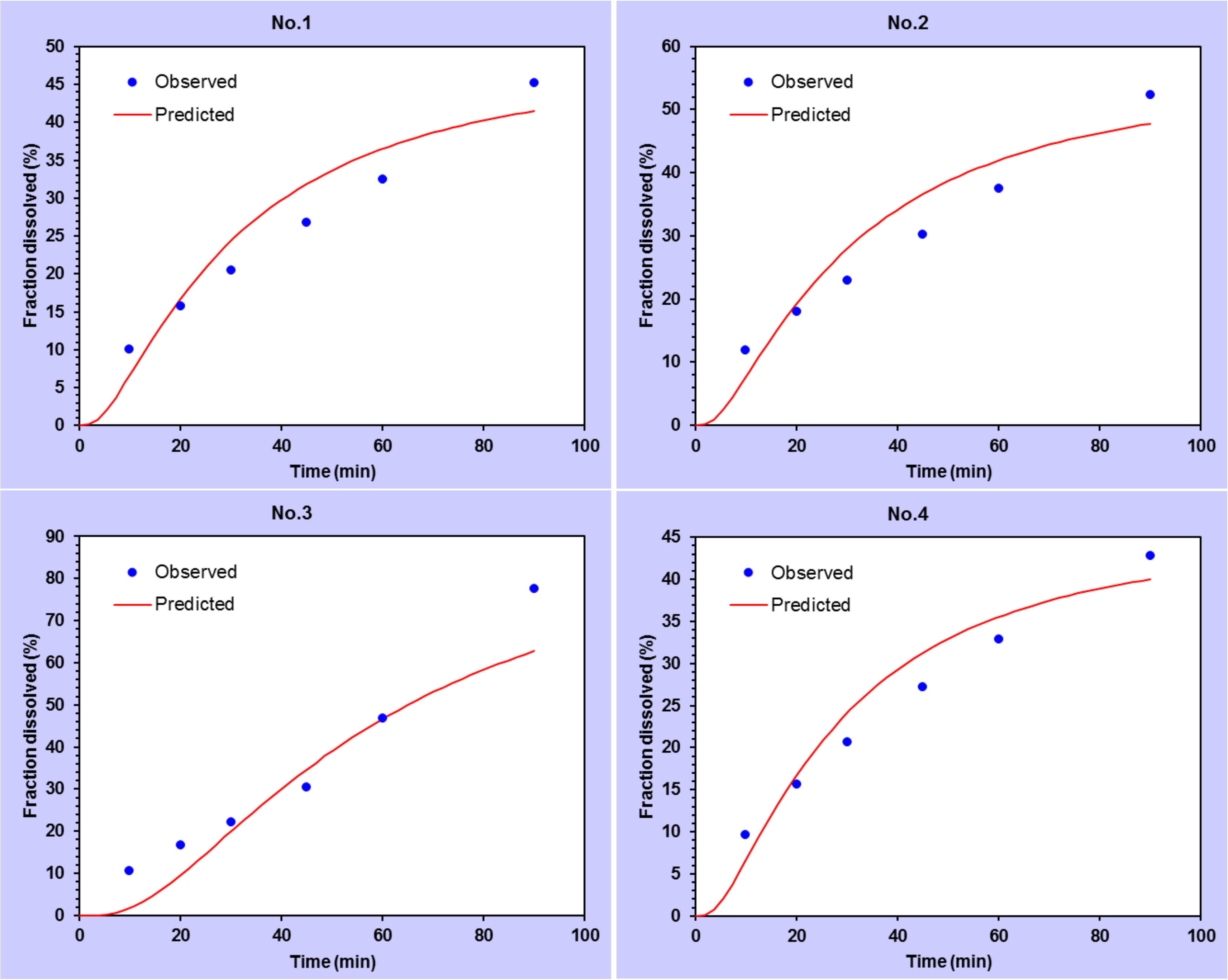

Supplement: Supplementary file 1 [file pharmaceutics-16-00498-s001.zip › Supplementary materials_Model fitting summary_Pearlitol® 160 C.pdf]
