# Supplementary material for: Comparative Fitting of Mathematical Models to Carvedilol Release Profiles Obtained from Hypromellose Matrix Tablets
Source: Pharmaceutics. 2024 Apr 4;16(4):498. doi: 10.3390/pharmaceutics16040498 (PMC11053526; doi:10.3390/pharmaceutics16040498)

Supplementary materials – Model fitting summary – Polyglykol® 4000 P (modelling the entire relevant dissolution data)

Model: **Zero–order**

Model equation: $F = k_0 \cdot t$

Fitted model parameters per tested tablet (N = 4) with statistics – mean, standard deviation (SD), and relative standard deviation expressed in % (RSD%) (output from DDSolver):

Parameter	No.1	No.2	No.3	No.4	Mean	SD	RSD(%)
k_0	0.921	1.062	0.894	1.021	0.975	0.080	8.181

Number of dissolution data points (N), degrees of freedom (df), and selected goodness of fit criteria – Pearson correlation coefficient (R), coefficient of determination (R^2), adjusted coefficient of determination ($R^2_{adjusted}$), and residual sum of squares (RSS) (manual calculation in MS Excel):

Parameter	No.1	No.2	No.3	No.4
N	7	7	7	7
df	6	6	6	6
R	0.94996594	0.90686639	0.94628882	0.92982708
R^2	0.90243529	0.82240664	0.89546253	0.8645784
$R^2_{adjusted}$	0.90243529	0.82240664	0.89546253	0.8645784
RSS	1522.255883	2771.174875	1475.504905	2035.49856

Graphical abstract of model fit presented as mean ± 1 SD of the fraction % of released carvedilol:

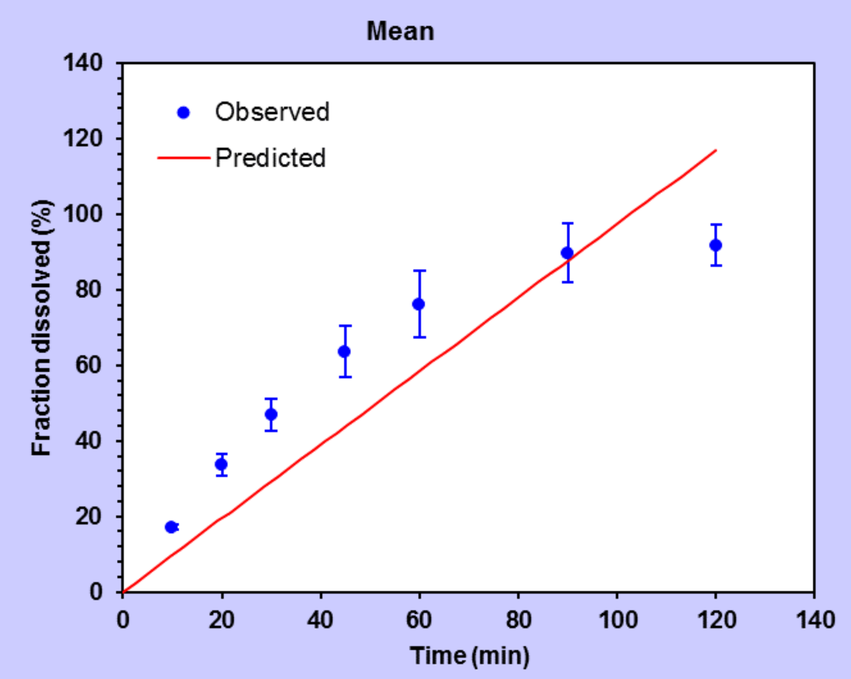

Supplementary materials – Model fitting summary – Polyglykol® 4000 P (modelling the entire relevant dissolution data)

Graphical abstract of model fit presented as the fraction % of released carvedilol per tested tablet:

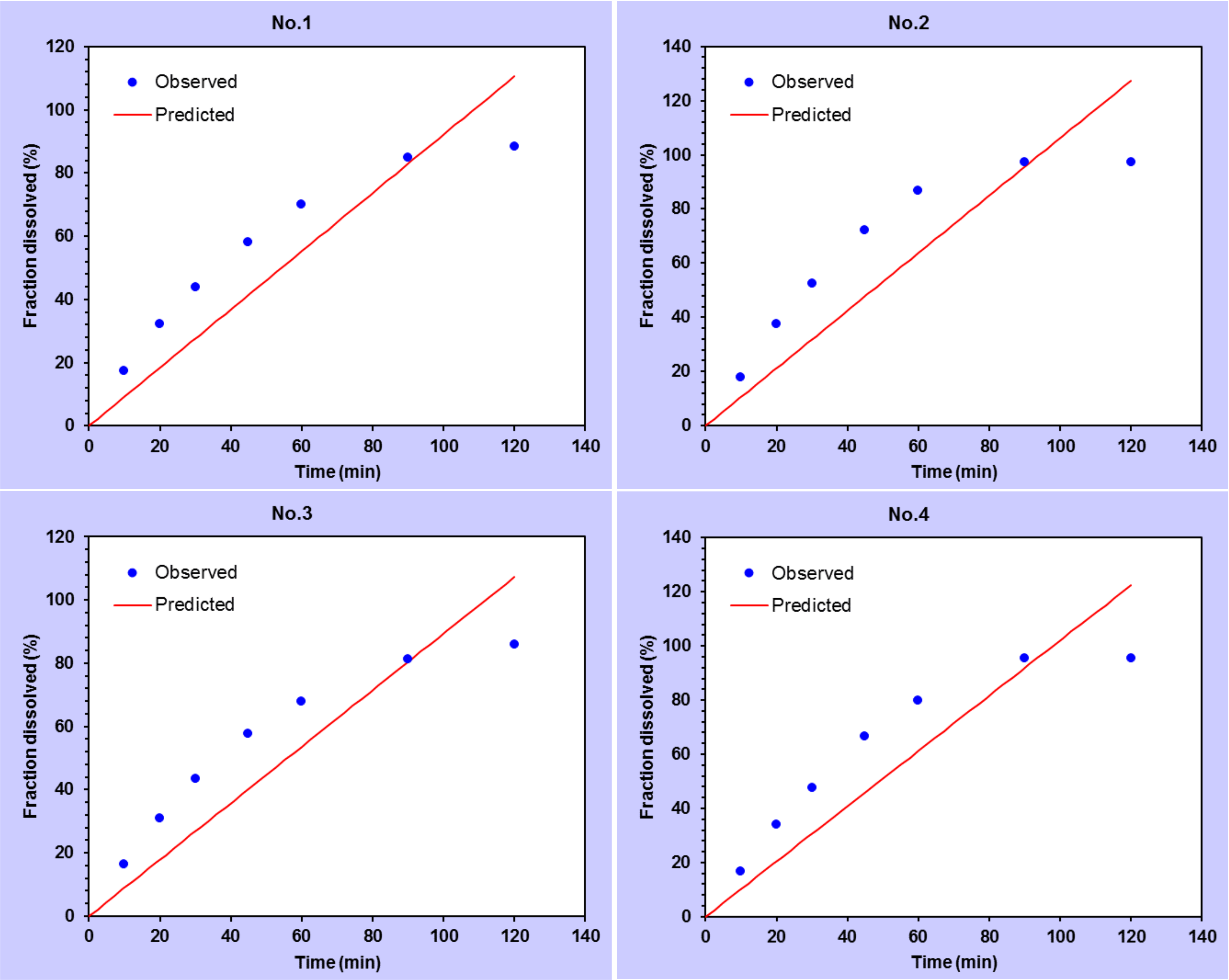

Supplementary materials – Model fitting summary – Polyglykol® 4000 P (modelling the entire relevant dissolution data)

Model: **Zero–order with T_{lag}**

Model equation: $F = k_0 \cdot (t - T_{lag})$

Fitted model parameters per tested tablet (N = 4) with statistics – mean, standard deviation (SD), and relative standard deviation expressed in % (RSD%) (output from DDSolver):

Parameter	No.1	No.2	No.3	No.4	Mean	SD	RSD(%)
k_0	0.639	0.706	0.619	0.718	0.670	0.049	7.309
T_{lag}	-34.787	-39.650	-35.095	-33.253	-35.696	2.756	-7.721

Number of dissolution data points (N), degrees of freedom (df), and selected goodness of fit criteria – Pearson correlation coefficient (R), coefficient of determination (R^2), adjusted coefficient of determination ($R^2_{adjusted}$), and residual sum of squares (RSS) (manual calculation in MS Excel):

Parameter	No.1	No.2	No.3	No.4
N	7	7	7	7
df	5	5	5	5
R	0.94996594	0.90686639	0.94628882	0.92982708
R^2	0.90243529	0.82240664	0.89546253	0.8645784
$R^2_{adjusted}$	0.88292235	0.78688797	0.87455503	0.83749407
RSS	416.6244943	1016.554428	421.4053064	761.2891389

Graphical abstract of model fit presented as mean ± 1 SD of the fraction % of released carvedilol:

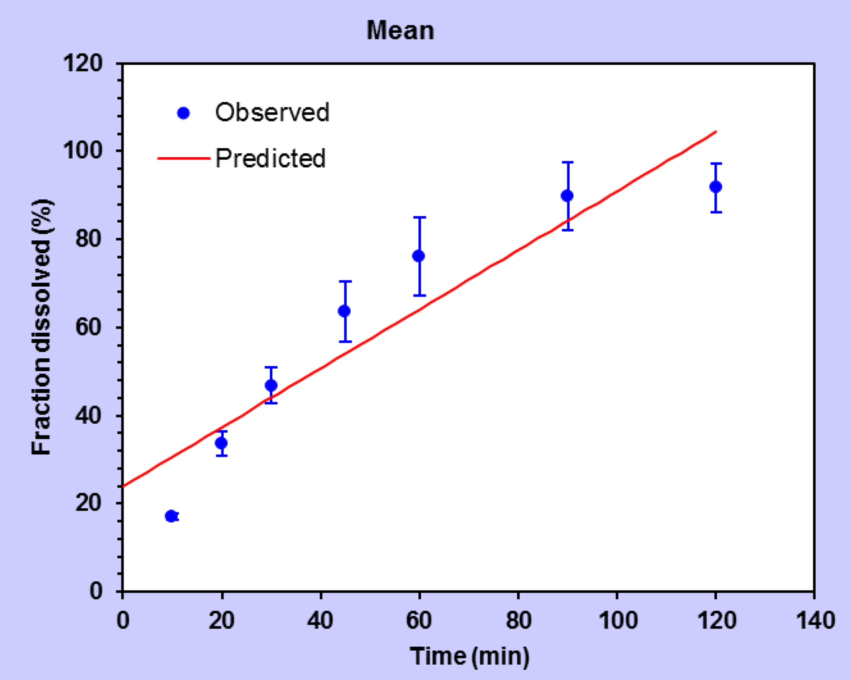

Supplementary materials – Model fitting summary – Polyglykol® 4000 P (modelling the entire relevant dissolution data)

Graphical abstract of model fit presented as the fraction % of released carvedilol per tested tablet:

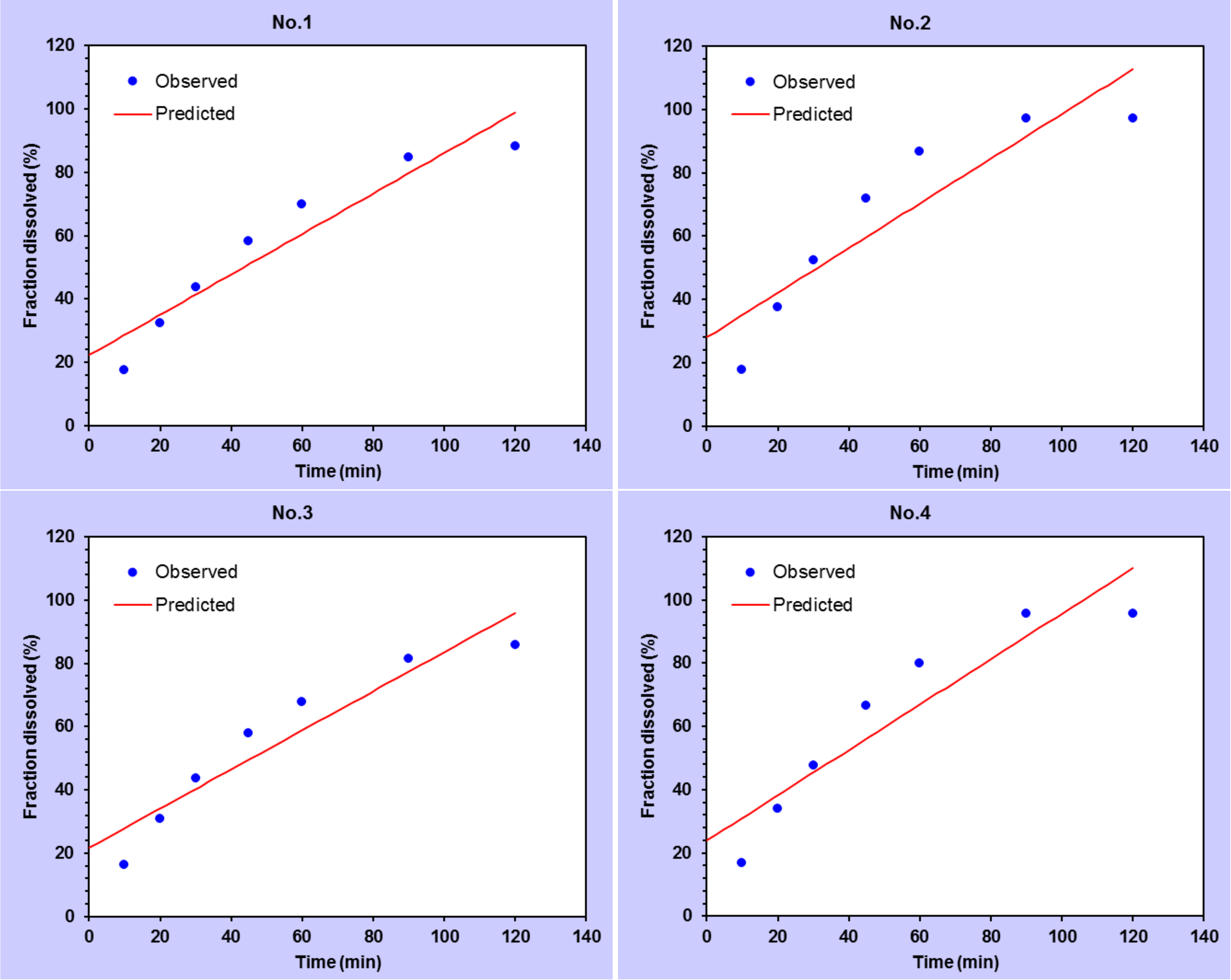

Supplementary materials – Model fitting summary – Polyglykol® 4000 P (modelling the entire relevant dissolution data)

Model: **Zero–order with F_0**

Model equation: $F = F_0 + k_0 \cdot t$

Fitted model parameters per tested tablet (N = 4) with statistics – mean, standard deviation (SD), and relative standard deviation expressed in % (RSD%) (output from DDSolver):

Parameter	No.1	No.2	No.3	No.4	Mean	SD	RSD(%)
k_0	0.639	0.706	0.619	0.718	0.670	0.049	7.309
F_0	22.231	28.006	21.707	23.866	23.953	2.854	11.917

Number of dissolution data points (N), degrees of freedom (df), and selected goodness of fit criteria – Pearson correlation coefficient (R), coefficient of determination (R^2), adjusted coefficient of determination ($R^2_{adjusted}$), and residual sum of squares (RSS) (manual calculation in MS Excel):

Parameter	No.1	No.2	No.3	No.4
N	7	7	7	7
df	5	5	5	5
R	0.94996594	0.90686639	0.94628882	0.92982708
R^2	0.90243529	0.82240664	0.89546253	0.8645784
$R^2_{adjusted}$	0.88292235	0.78688797	0.87455503	0.83749407
RSS	416.6244943	1016.554428	421.4053064	761.2891389

Graphical abstract of model fit presented as mean ± 1 SD of the fraction % of released carvedilol:

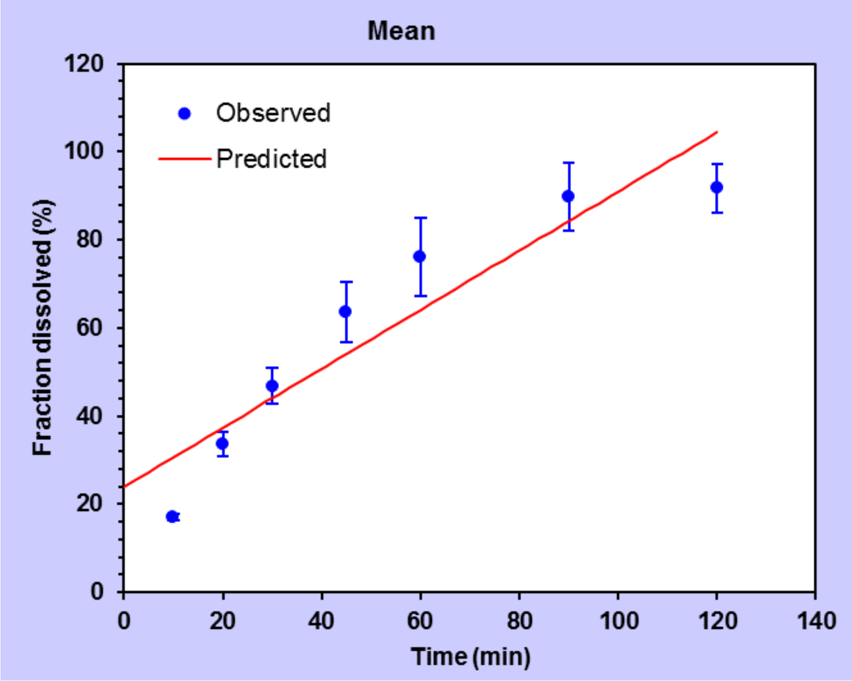

Supplementary materials – Model fitting summary – Polyglykol® 4000 P (modelling the entire relevant dissolution data)

Graphical abstract of model fit presented as the fraction % of released carvedilol per tested tablet:

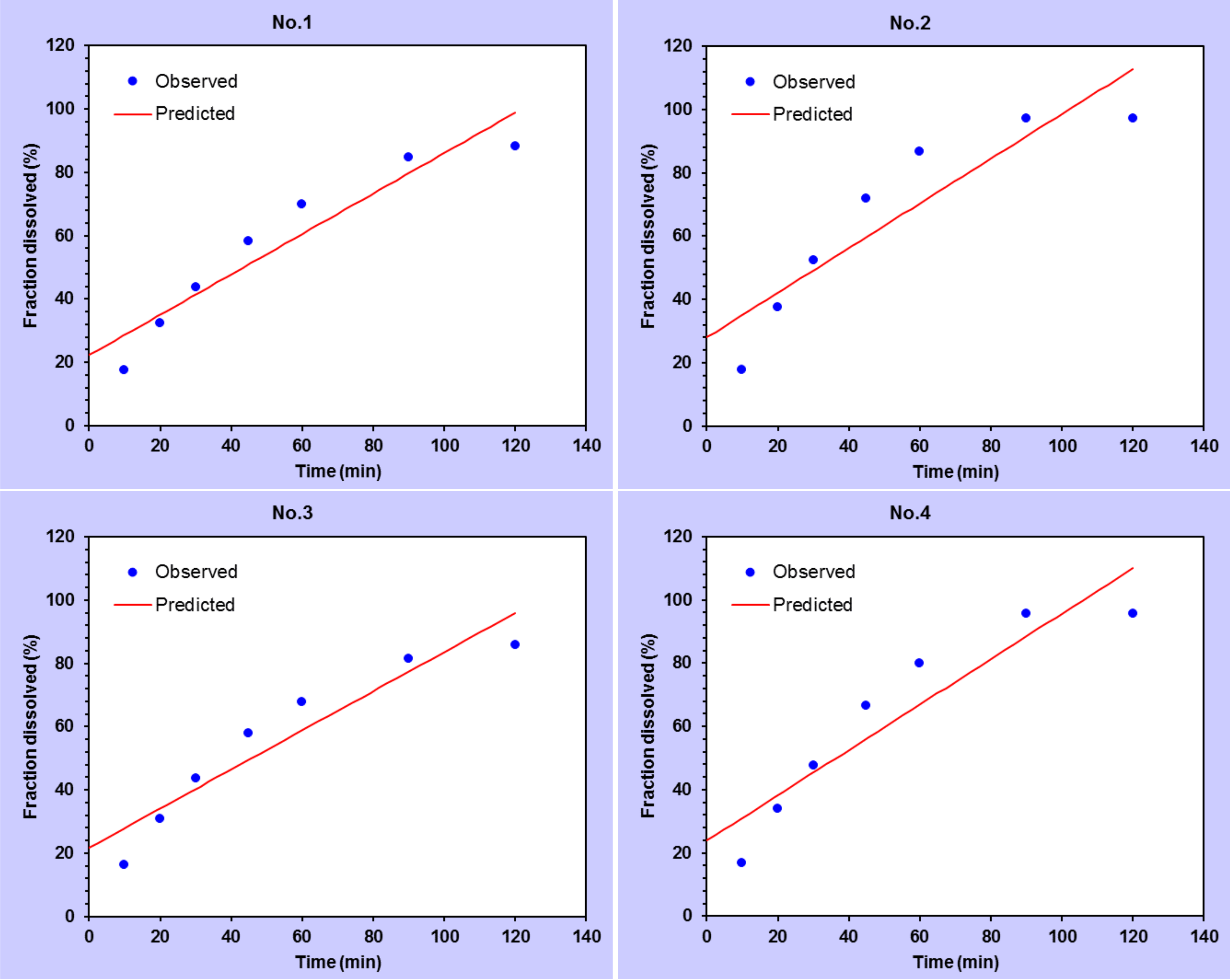

Supplementary materials – Model fitting summary – Polyglykol® 4000 P (modelling the entire relevant dissolution data)

Model: **First–order**

Model equation: $F = 100 \cdot (1 - e^{-k_1 \cdot t})$

Fitted model parameters per tested tablet (N = 4) with statistics – mean, standard deviation (SD), and relative standard deviation expressed in % (RSD%) (output from DDSolver):

Parameter	No.1	No.2	No.3	No.4	Mean	SD	RSD(%)
k_1	0.019	0.024	0.018	0.021	0.021	0.003	14.288

Number of dissolution data points (N), degrees of freedom (df), and selected goodness of fit criteria – Pearson correlation coefficient (R), coefficient of determination (R^2), adjusted coefficient of determination ($R^2_{adjusted}$), and residual sum of squares (RSS) (manual calculation in MS Excel):

Parameter	No.1	No.2	No.3	No.4
N	7	7	7	7
df	6	6	6	6
R	0.99871498	0.99513246	0.99773628	0.99600325
R^2	0.99743162	0.9902886	0.99547768	0.99202248
$R^2_{adjusted}$	0.99743162	0.9902886	0.99547768	0.99202248
RSS	12.93315149	214.0901621	32.04741032	225.7070708

Graphical abstract of model fit presented as mean ± 1 SD of the fraction % of released carvedilol:

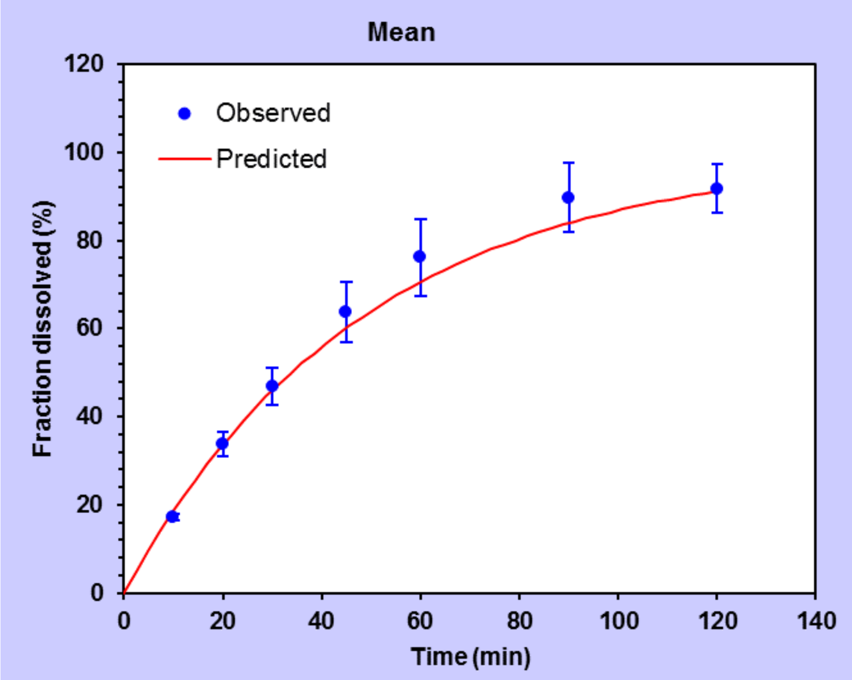

Supplementary materials – Model fitting summary – Polyglykol® 4000 P (modelling the entire relevant dissolution data)

Graphical abstract of model fit presented as the fraction % of released carvedilol per tested tablet:

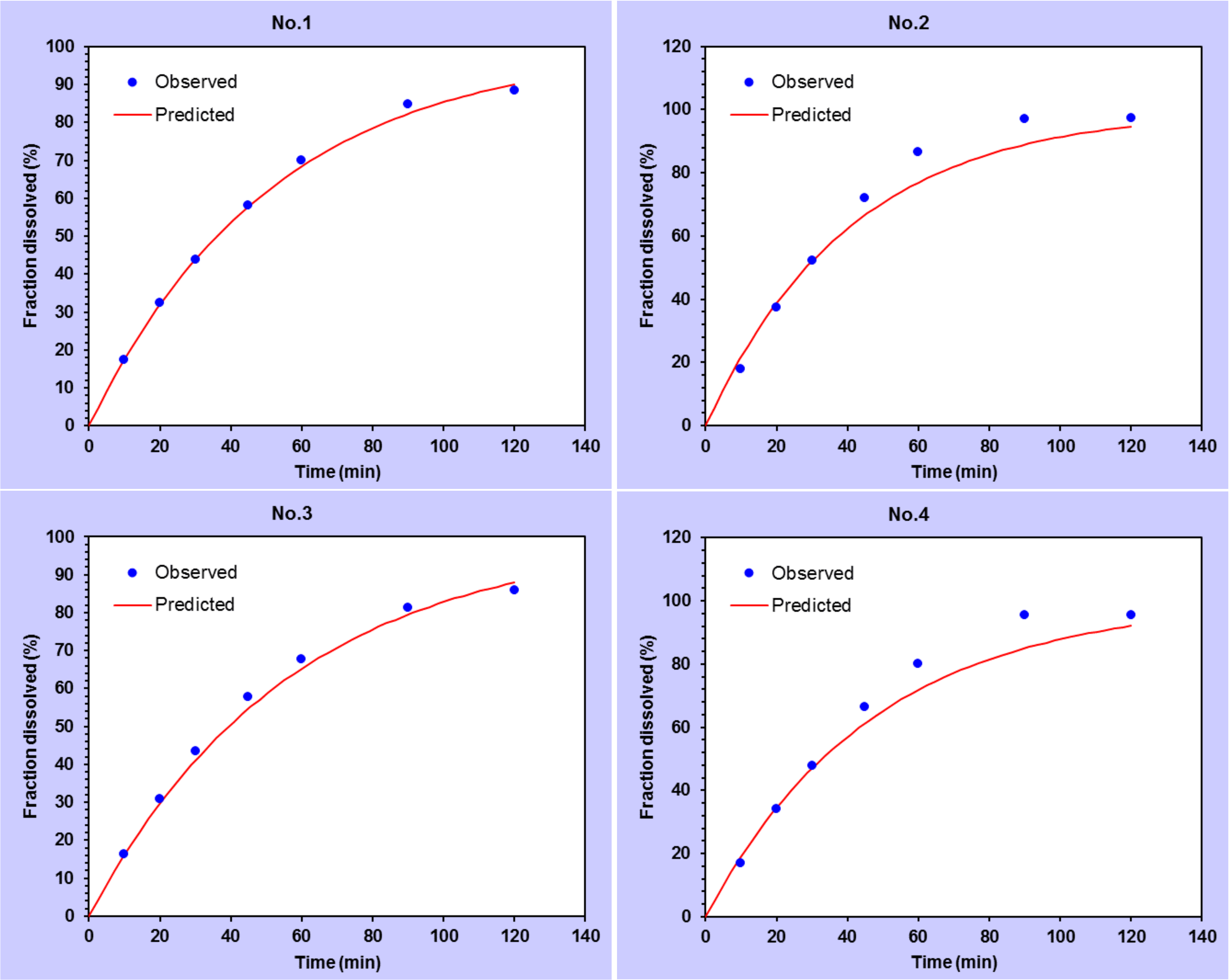

Supplementary materials – Model fitting summary – Polyglykol® 4000 P (modelling the entire relevant dissolution data)

Model: **First–order with T_{lag}**

Model equation: $F = 100 \cdot \left[1 - e^{-k_1 \cdot (t - T_{lag})}\right]$

Fitted model parameters per tested tablet (N = 4) with statistics – mean, standard deviation (SD), and relative standard deviation expressed in % (RSD%) (output from DDSolver):

Parameter	No.1	No.2	No.3	No.4	Mean	SD	RSD(%)
k_1	0.019	0.035	0.017	0.030	0.025	0.009	34.826
T_{lag}	-1.912	4.878	-4.212	5.234	0.997	4.782	479.638

Number of dissolution data points (N), degrees of freedom (df), and selected goodness of fit criteria – Pearson correlation coefficient (R), coefficient of determination (R^2), adjusted coefficient of determination ($R^2_{adjusted}$), and residual sum of squares (RSS) (manual calculation in MS Excel):

Parameter	No.1	No.2	No.3	No.4
N	7	7	7	7
df	5	5	5	5
R	0.99854662	0.9950847	0.99685797	0.99465013
R^2	0.99709535	0.99019355	0.99372582	0.98932887
$R^2_{adjusted}$	0.99651442	0.98823226	0.99247098	0.98719465
RSS	21.06275654	66.92370966	42.73106285	64.31407156

Graphical abstract of model fit presented as mean ± 1 SD of the fraction % of released carvedilol:

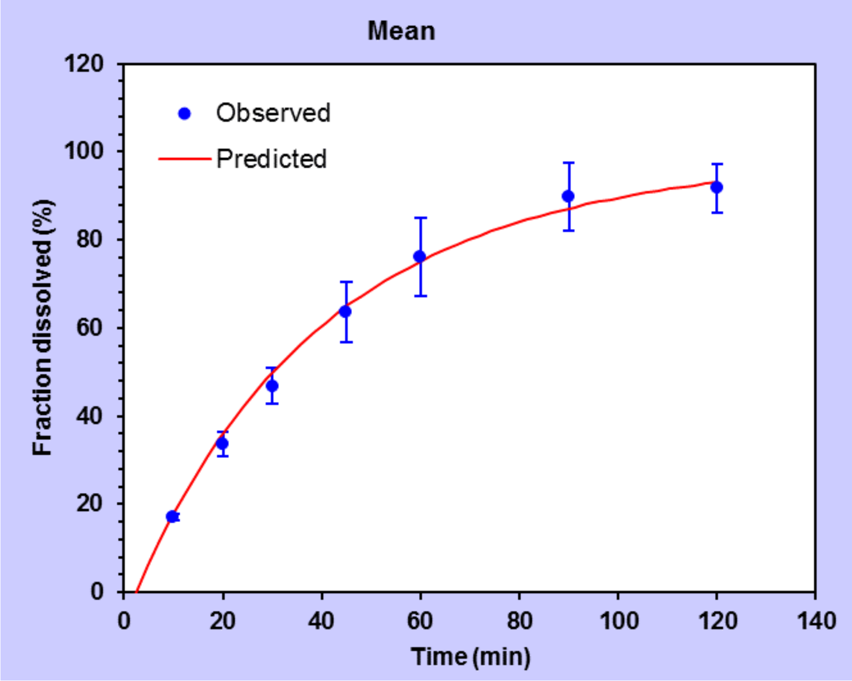

Supplementary materials – Model fitting summary – Polyglykol® 4000 P (modelling the entire relevant dissolution data)

Graphical abstract of model fit presented as the fraction % of released carvedilol per tested tablet:

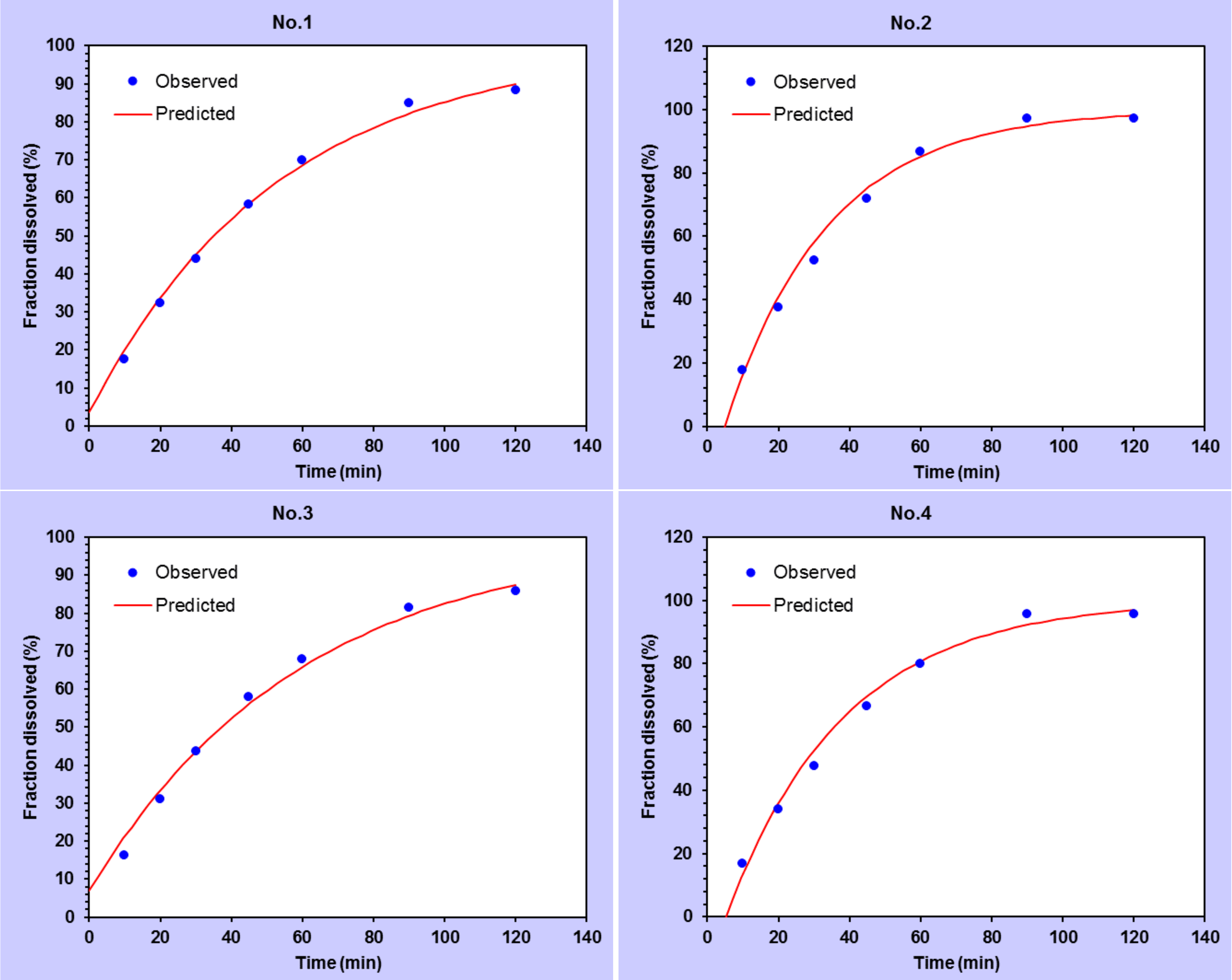

Supplementary materials – Model fitting summary – Polyglykol® 4000 P (modelling the entire relevant dissolution data)

Model: **First–order with F_{max}**

Model equation: $F = F_{max} \cdot (1 - e^{-k_1 \cdot t})$

Fitted model parameters per tested tablet (N = 4) with statistics – mean, standard deviation (SD), and relative standard deviation expressed in % (RSD%) (output from DDSolver):

Parameter	No.1	No.2	No.3	No.4	Mean	SD	RSD(%)
k_1	0.023	0.028	0.025	0.027	0.026	0.003	10.264
F_{max}	97.211	102.081	90.238	100.380	97.477	5.231	5.367

Number of dissolution data points (N), degrees of freedom (df), and selected goodness of fit criteria – Pearson correlation coefficient (R), coefficient of determination (R^2), adjusted coefficient of determination ($R^2_{adjusted}$), and residual sum of squares (RSS) (manual calculation in MS Excel):

Parameter	No.1	No.2	No.3	No.4
N	7	7	7	7
df	5	5	5	5
R	0.99873842	0.99700187	0.99920039	0.99637215
R^2	0.99747843	0.99401273	0.99840142	0.99275747
$R^2_{adjusted}$	0.99697412	0.99281528	0.9980817	0.99130896
RSS	53.40860818	162.9710886	60.34346953	238.2130464

Graphical abstract of model fit presented as mean ± 1 SD of the fraction % of released carvedilol:

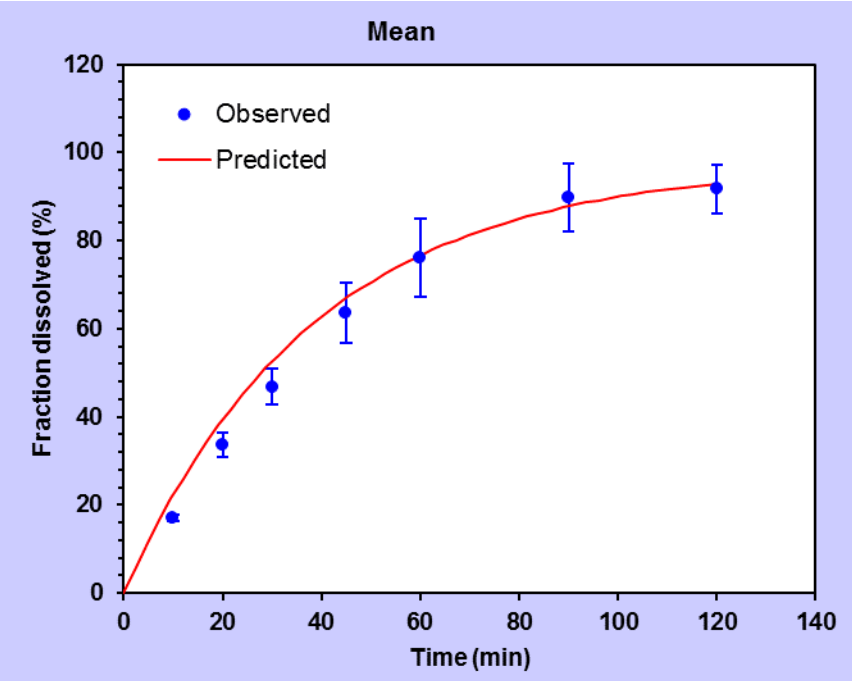

Supplementary materials – Model fitting summary – Polyglykol® 4000 P (modelling the entire relevant dissolution data)

Graphical abstract of model fit presented as the fraction % of released carvedilol per tested tablet:

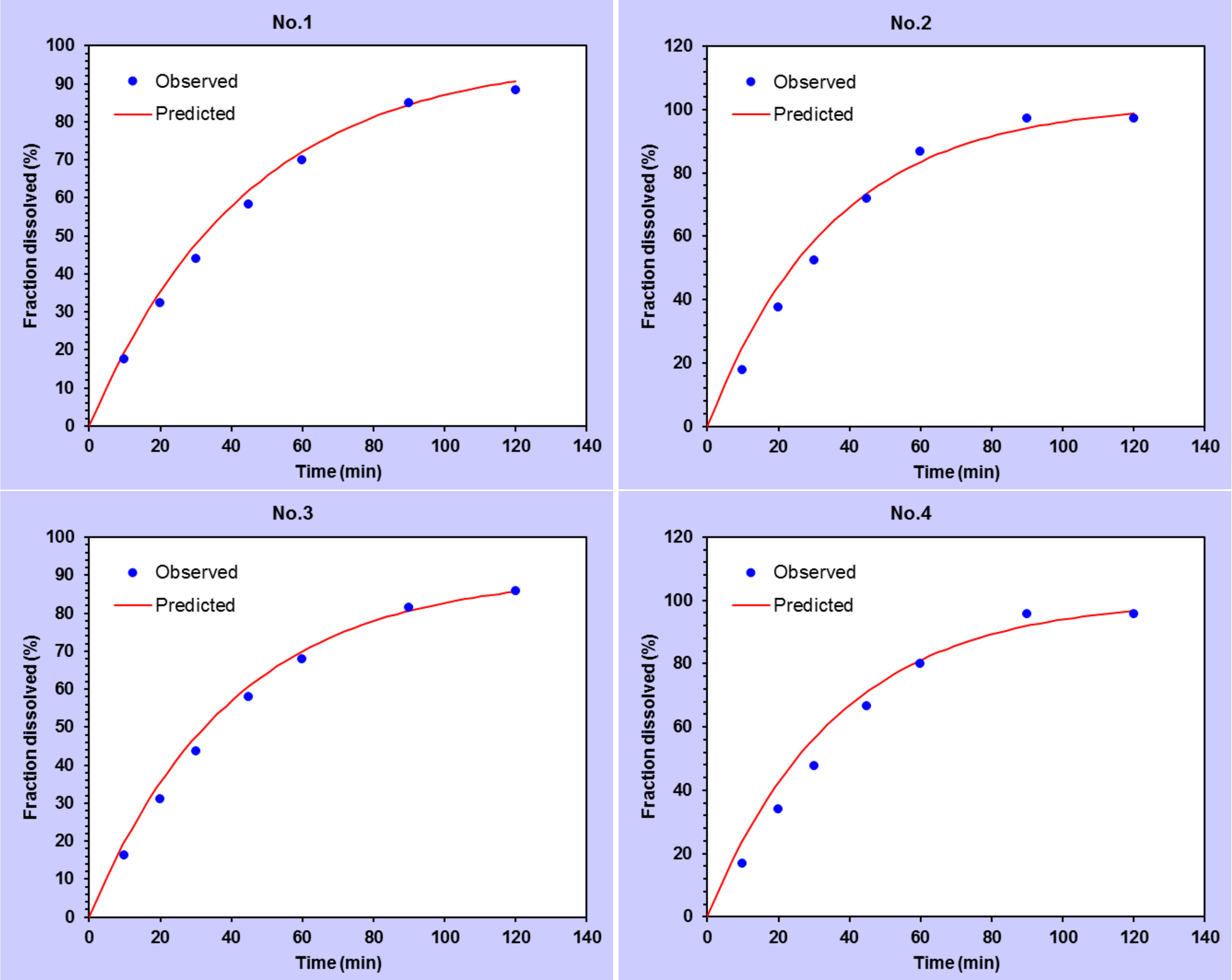

Supplementary materials – Model fitting summary – Polyglykol® 4000 P (modelling the entire relevant dissolution data)

Model: **First–order with T_{lag} and F_{max}**

Model equation: $F = F_{max} \cdot \left[1 - e^{-k_1 \cdot (t - T_{lag})}\right]$

Fitted model parameters per tested tablet (N = 4) with statistics – mean, standard deviation (SD), and relative standard deviation expressed in % (RSD%) (output from DDSolver):

Parameter	No.1	No.2	No.3	No.4	Mean	SD	RSD(%)
k_1	0.027	0.029	0.026	0.029	0.028	0.001	5.236
T_{lag}	4.853	2.307	4.432	4.756	4.087	1.200	29.368
F_{max}	92.772	102.081	90.238	100.380	96.368	5.752	5.969

Number of dissolution data points (N), degrees of freedom (df), and selected goodness of fit criteria – Pearson correlation coefficient (R), coefficient of determination (R^2), adjusted coefficient of determination ($R^2_{adjusted}$), and residual sum of squares (RSS) (manual calculation in MS Excel):

Parameter	No.1	No.2	No.3	No.4
N	7	7	7	7
df	4	4	4	4
R	0.99593789	0.99705605	0.99841594	0.99530625
R^2	0.99189228	0.99412078	0.99683439	0.99063454
$R^2_{adjusted}$	0.98783842	0.99118116	0.99525158	0.98595181
RSS	49.8545276	60.87995882	21.36787081	57.05452361

Graphical abstract of model fit presented as mean ± 1 SD of the fraction % of released carvedilol:

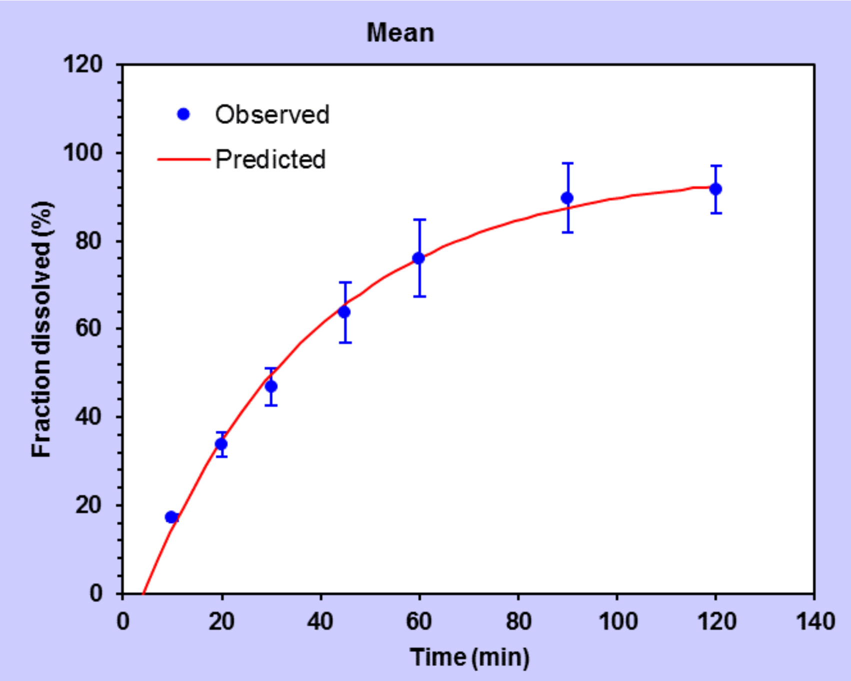

Supplementary materials – Model fitting summary – Polyglykol® 4000 P (modelling the entire relevant dissolution data)

Graphical abstract of model fit presented as the fraction % of released carvedilol per tested tablet:

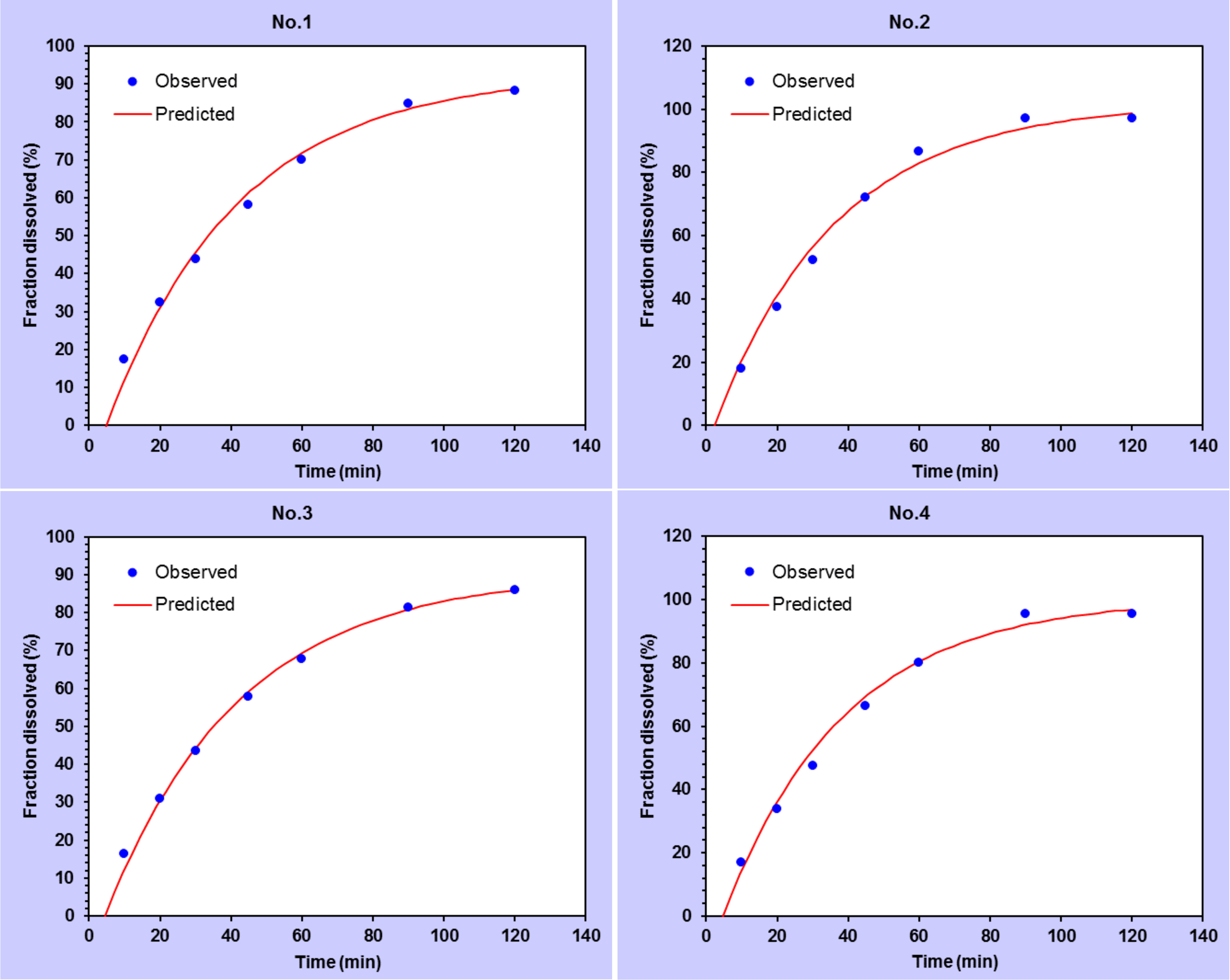

Supplementary materials – Model fitting summary – Polyglykol® 4000 P (modelling the entire relevant dissolution data)

Model: **Higuchi**

Model equation: $F = k_H \cdot t^{0.5}$

Fitted model parameters per tested tablet (N = 4) with statistics – mean, standard deviation (SD), and relative standard deviation expressed in % (RSD%) (output from DDSolver):

Parameter	No.1	No.2	No.3	No.4	Mean	SD	RSD(%)
k_H	8.392	9.741	8.149	9.296	8.894	0.750	8.429

Number of dissolution data points (N), degrees of freedom (df), and selected goodness of fit criteria – Pearson correlation coefficient (R), coefficient of determination (R^2), adjusted coefficient of determination ($R^2_{adjusted}$), and residual sum of squares (RSS) (manual calculation in MS Excel):

Parameter	No.1	No.2	No.3	No.4
N	7	7	7	7
df	6	6	6	6
R	0.98645414	0.96117081	0.98527691	0.9746161
R^2	0.97309177	0.92384933	0.9707706	0.94987654
$R^2_{adjusted}$	0.97309177	0.92384933	0.9707706	0.94987654
RSS	182.3574857	488.9364256	180.7085218	400.1168768

Graphical abstract of model fit presented as mean ± 1 SD of the fraction % of released carvedilol:

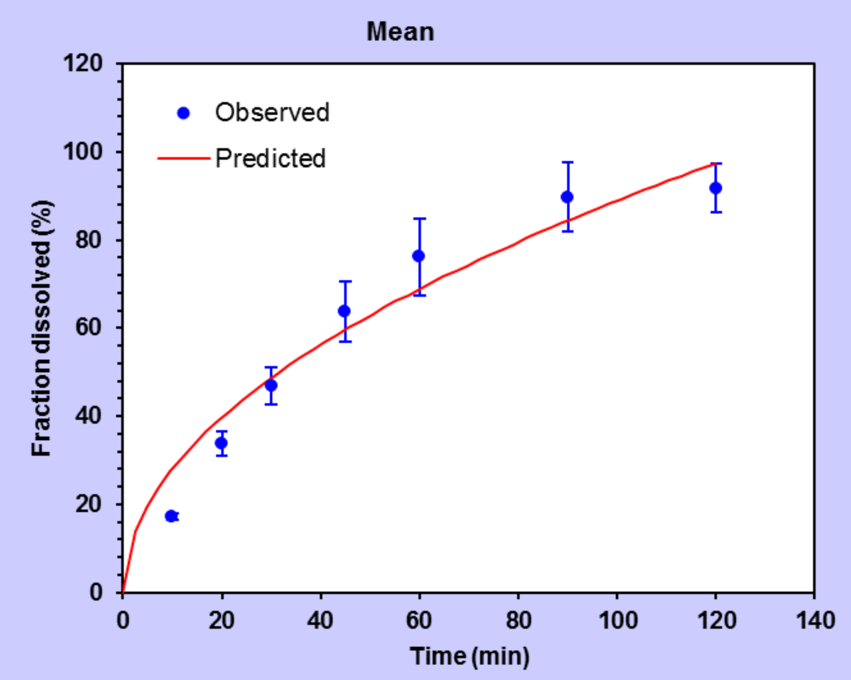

Supplementary materials – Model fitting summary – Polyglykol® 4000 P (modelling the entire relevant dissolution data)

Graphical abstract of model fit presented as the fraction % of released carvedilol per tested tablet:

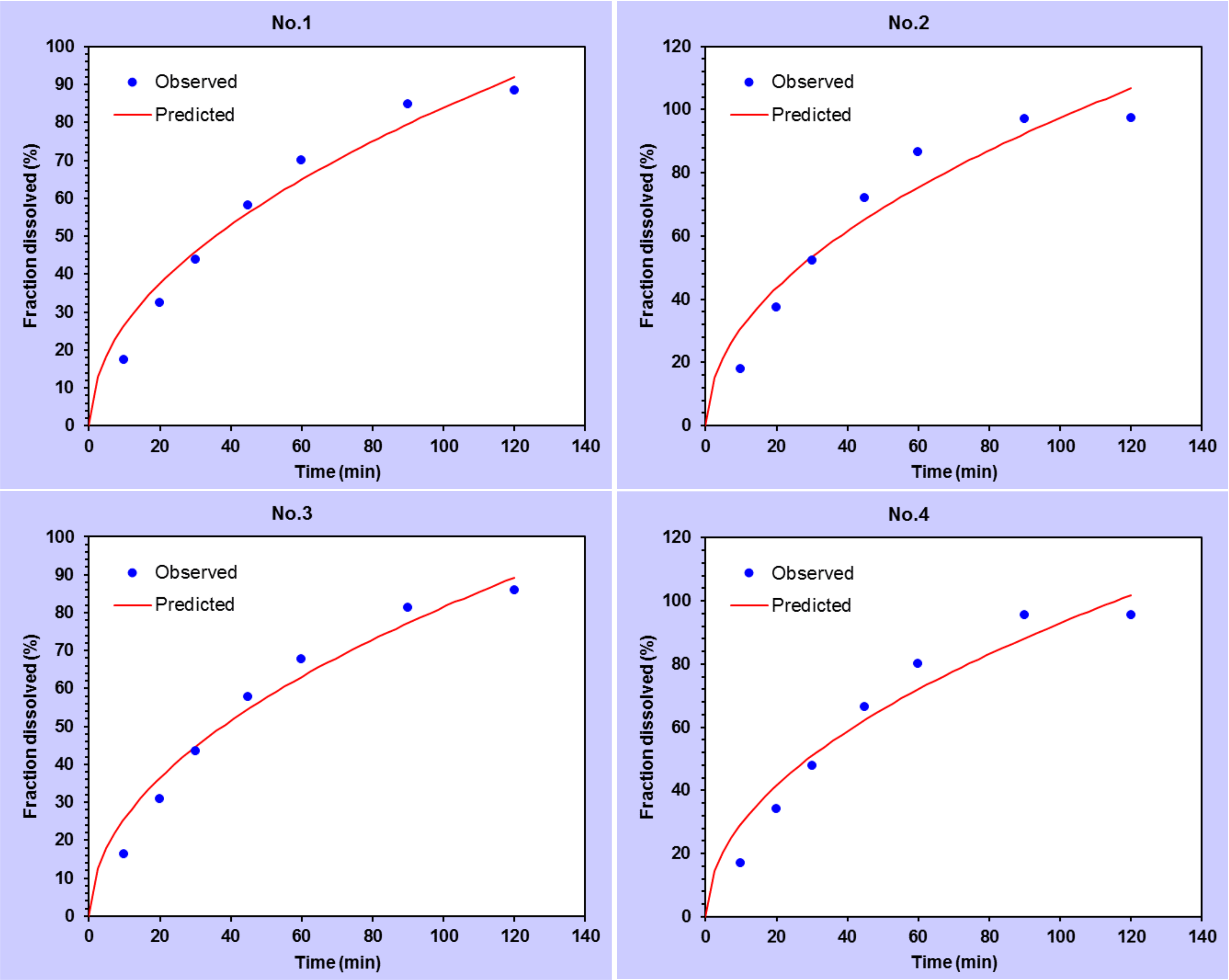

Supplementary materials – Model fitting summary – Polyglykol® 4000 P (modelling the entire relevant dissolution data)

Model: **Higuchi with T_{lag}**

Model equation: $F = k_H \cdot (t - T_{lag})^{0.5}$

Fitted model parameters per tested tablet (N = 4) with statistics – mean, standard deviation (SD), and relative standard deviation expressed in % (RSD%) (output from DDSolver):

Parameter	No.1	No.2	No.3	No.4	Mean	SD	RSD(%)
k_H	8.550	9.490	8.263	9.422	8.931	0.618	6.918
T_{lag}	2.418	-3.646	1.629	1.175	0.394	2.742	696.260

Number of dissolution data points (N), degrees of freedom (df), and selected goodness of fit criteria – Pearson correlation coefficient (R), coefficient of determination (R^2), adjusted coefficient of determination ($R^2_{adjusted}$), and residual sum of squares (RSS) (manual calculation in MS Excel):

Parameter	No.1	No.2	No.3	No.4
N	7	7	7	7
df	5	5	5	5
R	0.9880031	0.95740775	0.98647949	0.97560991
R^2	0.97615012	0.91662961	0.97314178	0.9518147
$R^2_{adjusted}$	0.97138015	0.89995553	0.96777014	0.94217764
RSS	124.9505714	629.5024469	141.1383728	353.7988029

Graphical abstract of model fit presented as mean ± 1 SD of the fraction % of released carvedilol:

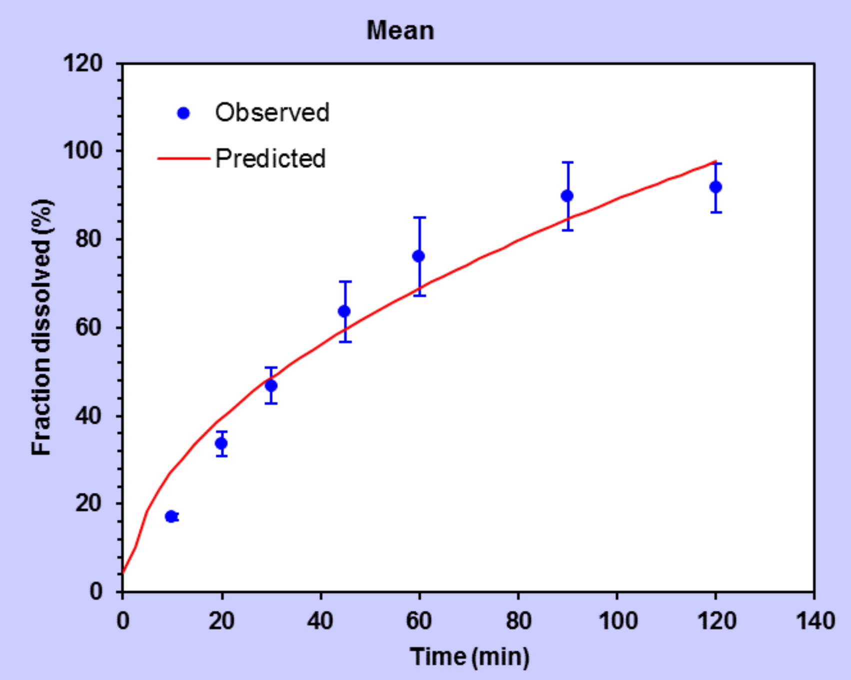

Supplementary materials – Model fitting summary – Polyglykol® 4000 P (modelling the entire relevant dissolution data)

Graphical abstract of model fit presented as the fraction % of released carvedilol per tested tablet:

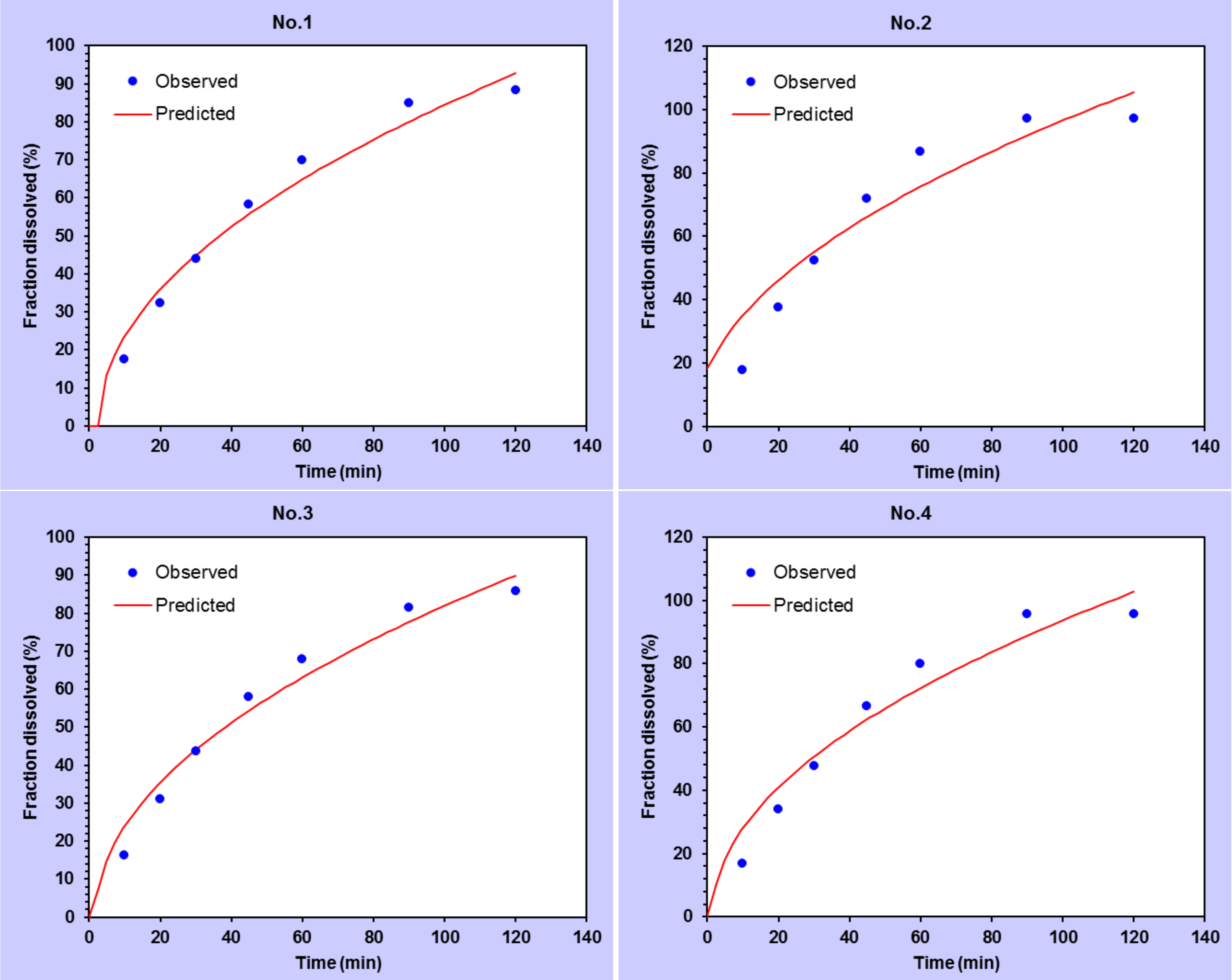

Supplementary materials – Model fitting summary – Polyglykol® 4000 P (modelling the entire relevant dissolution data)

Model: **Higuchi with F_0**

Model equation: $F = F_0 + k_H \cdot t^{0.5}$

Fitted model parameters per tested tablet (N = 4) with statistics – mean, standard deviation (SD), and relative standard deviation expressed in % (RSD%) (output from DDSolver):

Parameter	No.1	No.2	No.3	No.4	Mean	SD	RSD(%)
k_H	9.529	10.750	9.248	10.802	10.082	0.810	8.031
F_0	-8.886	-7.880	-8.580	-11.770	-9.279	1.713	-18.464

Number of dissolution data points (N), degrees of freedom (df), and selected goodness of fit criteria – Pearson correlation coefficient (R), coefficient of determination (R^2), adjusted coefficient of determination ($R^2_{adjusted}$), and residual sum of squares (RSS) (manual calculation in MS Excel):

Parameter	No.1	No.2	No.3	No.4
N	7	7	7	7
df	5	5	5	5
R	0.98645414	0.96117081	0.98527691	0.9746161
R^2	0.97309177	0.92384933	0.9707706	0.94987654
$R^2_{adjusted}$	0.96771013	0.90861919	0.96492472	0.93985184
RSS	114.9045278	435.8907698	117.8278465	281.7751971

Graphical abstract of model fit presented as mean ± 1 SD of the fraction % of released carvedilol:

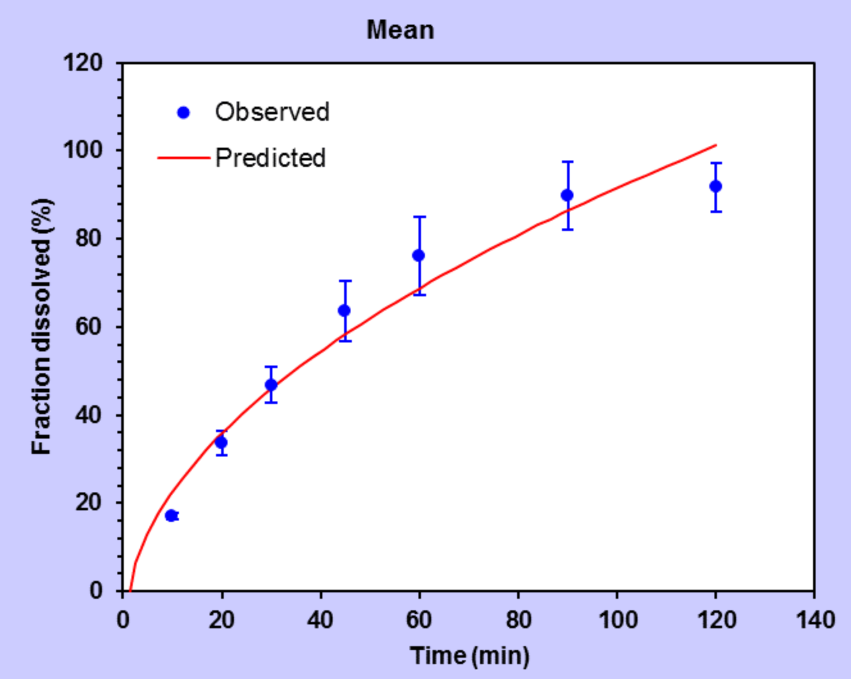

Supplementary materials – Model fitting summary – Polyglykol® 4000 P (modelling the entire relevant dissolution data)

Graphical abstract of model fit presented as the fraction % of released carvedilol per tested tablet:

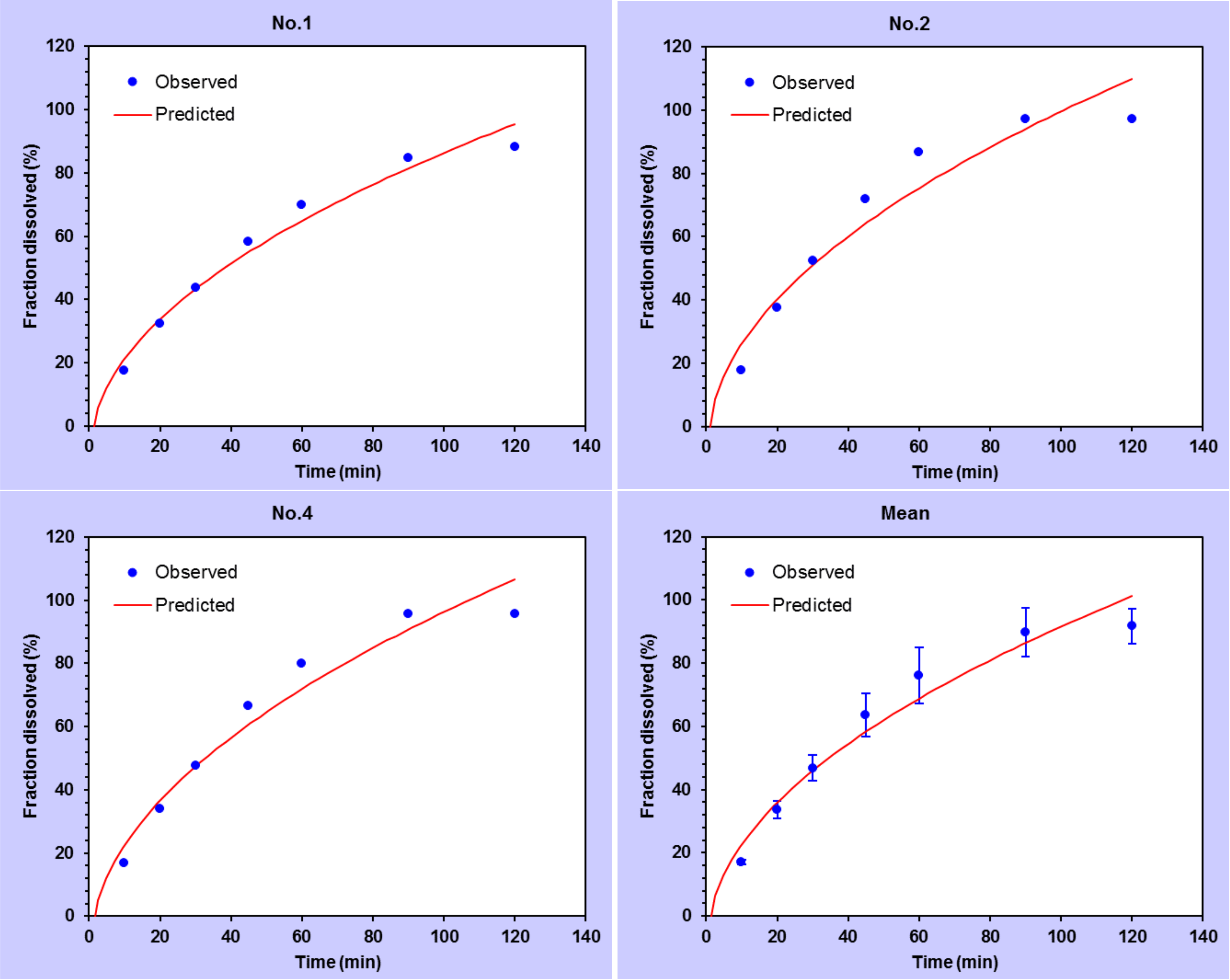

Supplementary materials – Model fitting summary – Polyglykol® 4000 P (modelling the entire relevant dissolution data)

Model: **Korsmeyer–Peppas**

Model equation: $F = k_{KP} \cdot t^n$

Fitted model parameters per tested tablet (N = 4) with statistics – mean, standard deviation (SD), and relative standard deviation expressed in % (RSD%) (output from DDSolver):

Parameter	No.1	No.2	No.3	No.4	Mean	SD	RSD(%)
k_{KP}	5.309	4.432	3.962	3.819	4.381	0.672	15.342
n	0.619	0.693	0.676	0.716	0.676	0.041	6.087

Number of dissolution data points (N), degrees of freedom (df), and selected goodness of fit criteria – Pearson correlation coefficient (R), coefficient of determination (R^2), adjusted coefficient of determination ($R^2_{adjusted}$), and residual sum of squares (RSS) (manual calculation in MS Excel):

Parameter	No.1	No.2	No.3	No.4
N	7	7	7	7
df	5	5	5	5
R	0.97979259	0.94240308	0.97396034	0.95779929
R^2	0.95999352	0.88812357	0.94859875	0.91737948
$R^2_{adjusted}$	0.95199223	0.86574829	0.9383185	0.90085538
RSS	254.6276525	912.6031293	300.9964857	654.8312095

Graphical abstract of model fit presented as mean ± 1 SD of the fraction % of released carvedilol:

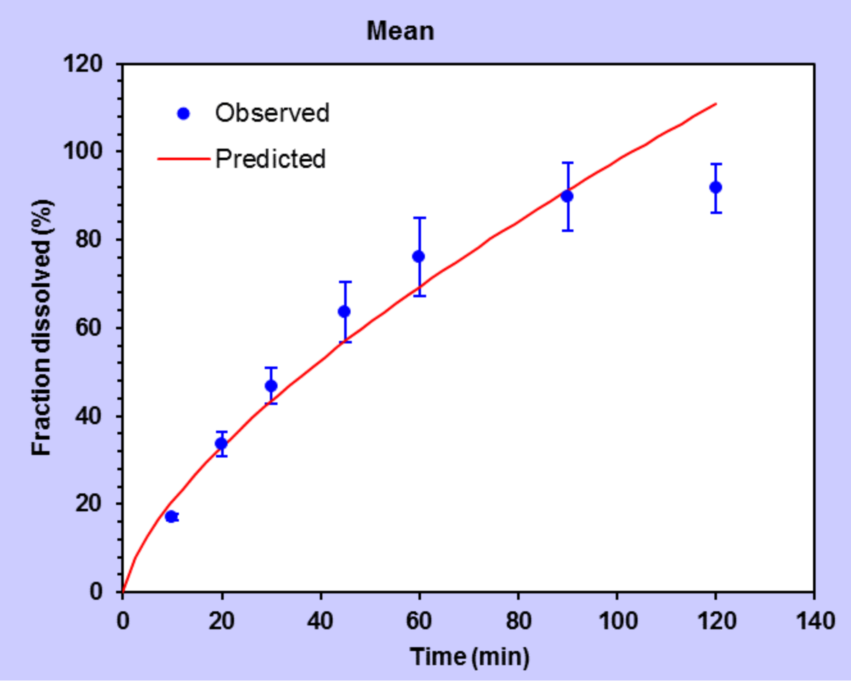

Supplementary materials – Model fitting summary – Polyglykol® 4000 P (modelling the entire relevant dissolution data)

Graphical abstract of model fit presented as the fraction % of released carvedilol per tested tablet:

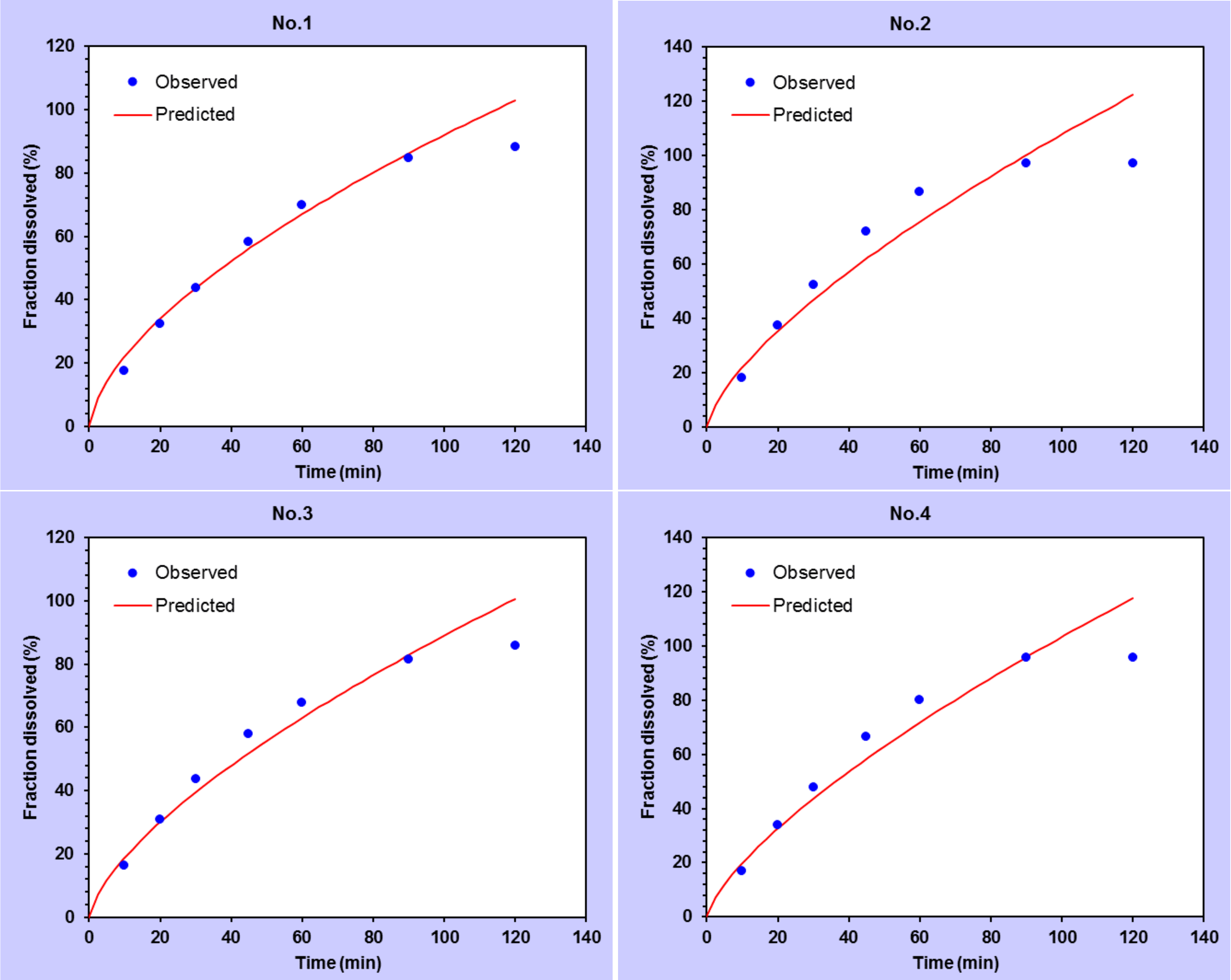

Supplementary materials – Model fitting summary – Polyglykol® 4000 P (modelling the entire relevant dissolution data)

Model: **Korsmeyer–Peppas with T_{lag}**

Model equation: $F = k_{KP} \cdot (t - T_{lag})^n$

Fitted model parameters per tested tablet (N = 4) with statistics – mean, standard deviation (SD), and relative standard deviation expressed in % (RSD%) (output from DDSolver):

Parameter	No.1	No.2	No.3	No.4	Mean	SD	RSD(%)
k_{KP}	6.701	6.991	6.233	6.159	6.521	0.395	6.055
n	0.567	0.595	0.578	0.613	0.588	0.020	3.453
T_{lag}	4.000	4.000	4.000	4.000	4.000	0.000	0.000

Number of dissolution data points (N), degrees of freedom (df), and selected goodness of fit criteria – Pearson correlation coefficient (R), coefficient of determination (R^2), adjusted coefficient of determination ($R^2_{adjusted}$), and residual sum of squares (RSS) (manual calculation in MS Excel):

Parameter	No.1	No.2	No.3	No.4
N	7	7	7	7
df	4	4	4	4
R	0.98565877	0.95696017	0.98376012	0.96982169
R^2	0.97152321	0.91577276	0.96778398	0.94055411
$R^2_{adjusted}$	0.95728482	0.87365914	0.95167597	0.91083116
RSS	151.2396129	644.3571303	173.7334126	432.0570118

Graphical abstract of model fit presented as mean ± 1 SD of the fraction % of released carvedilol:

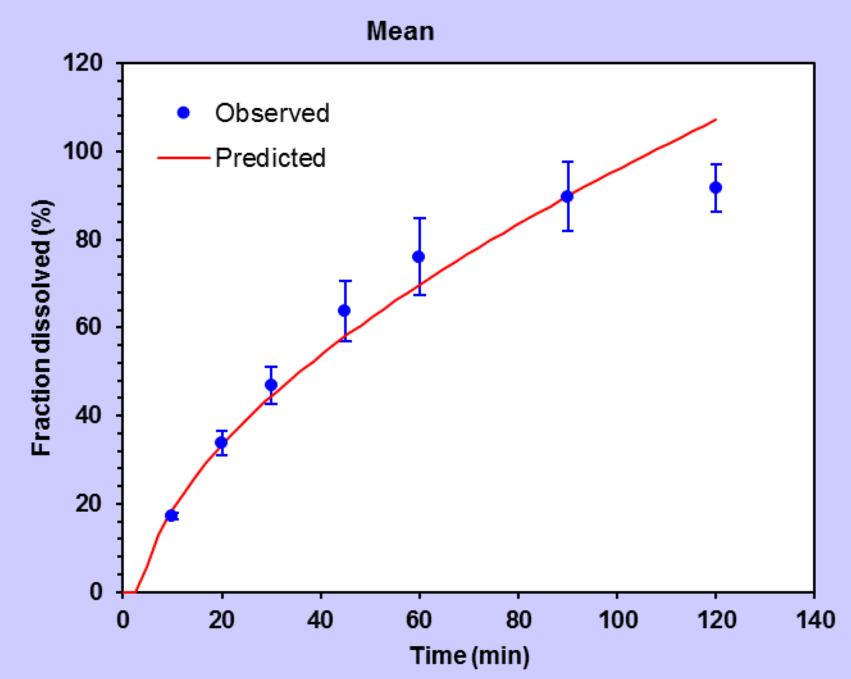

Supplementary materials – Model fitting summary – Polyglykol® 4000 P (modelling the entire relevant dissolution data)

Graphical abstract of model fit presented as the fraction % of released carvedilol per tested tablet:

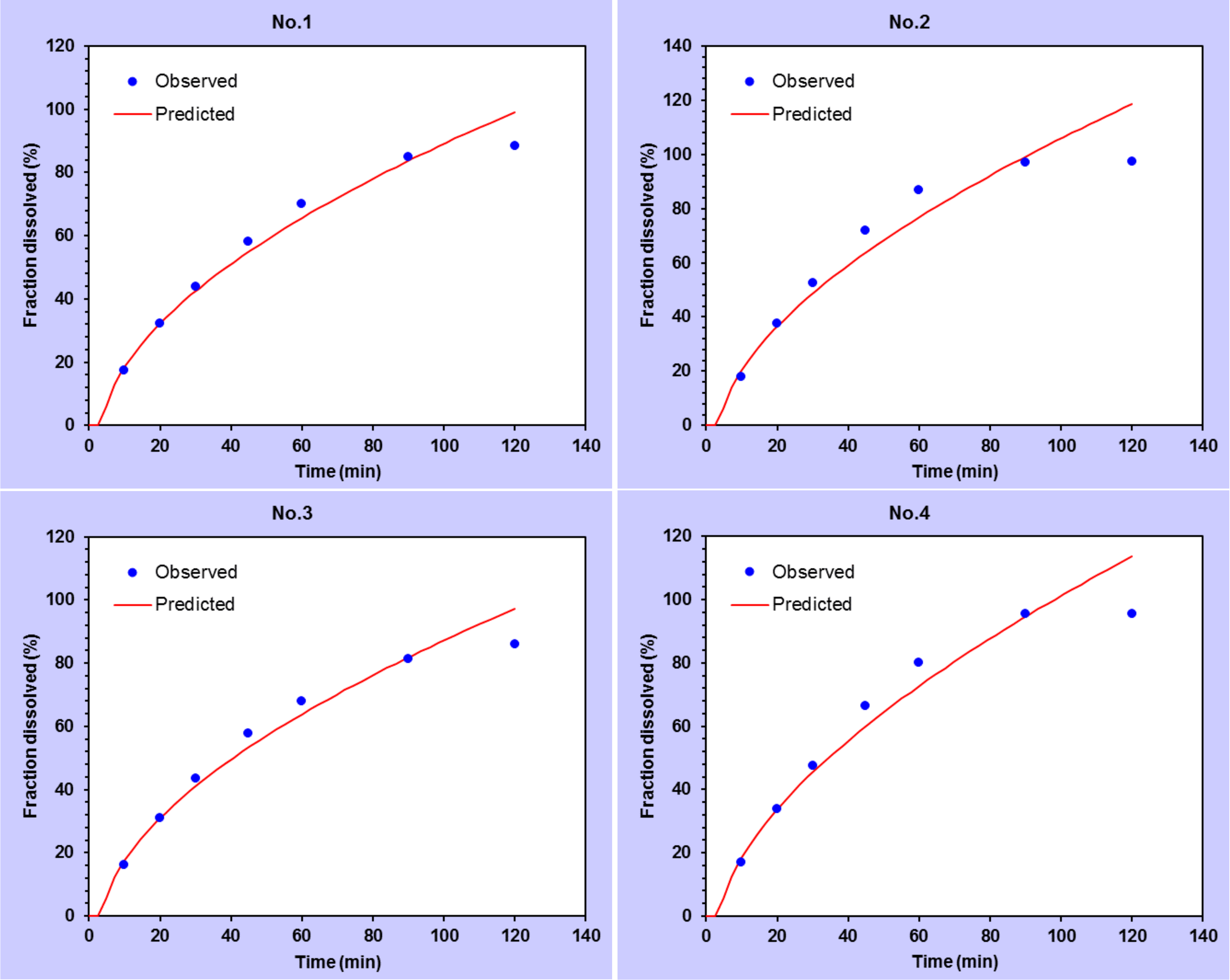

Supplementary materials – Model fitting summary – Polyglykol® 4000 P (modelling the entire relevant dissolution data)

Model: **Korsmeyer–Peppas with F_0**

Model equation: $F = F_0 + k_{KP} \cdot t^n$

Fitted model parameters per tested tablet (N = 4) with statistics – mean, standard deviation (SD), and relative standard deviation expressed in % (RSD%) (output from DDSolver):

Parameter	No.1	No.2	No.3	No.4	Mean	SD	RSD(%)
k_{KP}	1.964	2.078	1.828	1.767	1.909	0.139	7.297
n	0.823	0.851	0.835	0.877	0.846	0.023	2.765
F_0	6.998	7.159	6.519	6.758	6.859	0.280	4.081

Number of dissolution data points (N), degrees of freedom (df), and selected goodness of fit criteria – Pearson correlation coefficient (R), coefficient of determination (R^2), adjusted coefficient of determination ($R^2_{adjusted}$), and residual sum of squares (RSS) (manual calculation in MS Excel):

Parameter	No.1	No.2	No.3	No.4
N	7	7	7	7
df	4	4	4	4
R	0.96520057	0.92476665	0.96125405	0.94256915
R^2	0.93161215	0.85519335	0.92400935	0.8884366
$R^2_{adjusted}$	0.89741822	0.78279003	0.88601403	0.8326549
RSS	493.1862325	1429.774736	546.6682024	1079.624103

Graphical abstract of model fit presented as mean ± 1 SD of the fraction % of released carvedilol:

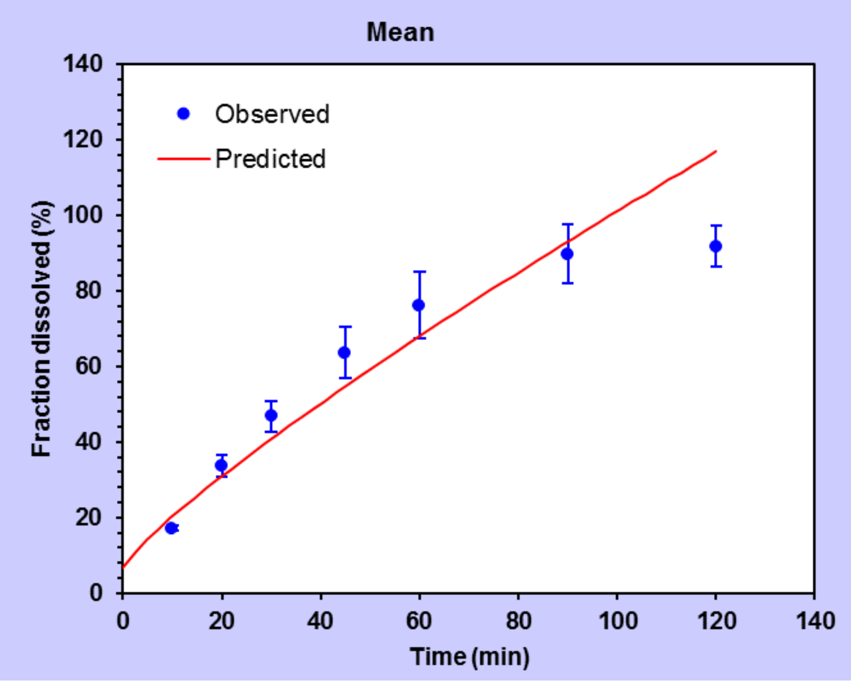

Supplementary materials – Model fitting summary – Polyglykol® 4000 P (modelling the entire relevant dissolution data)

Graphical abstract of model fit presented as the fraction % of released carvedilol per tested tablet:

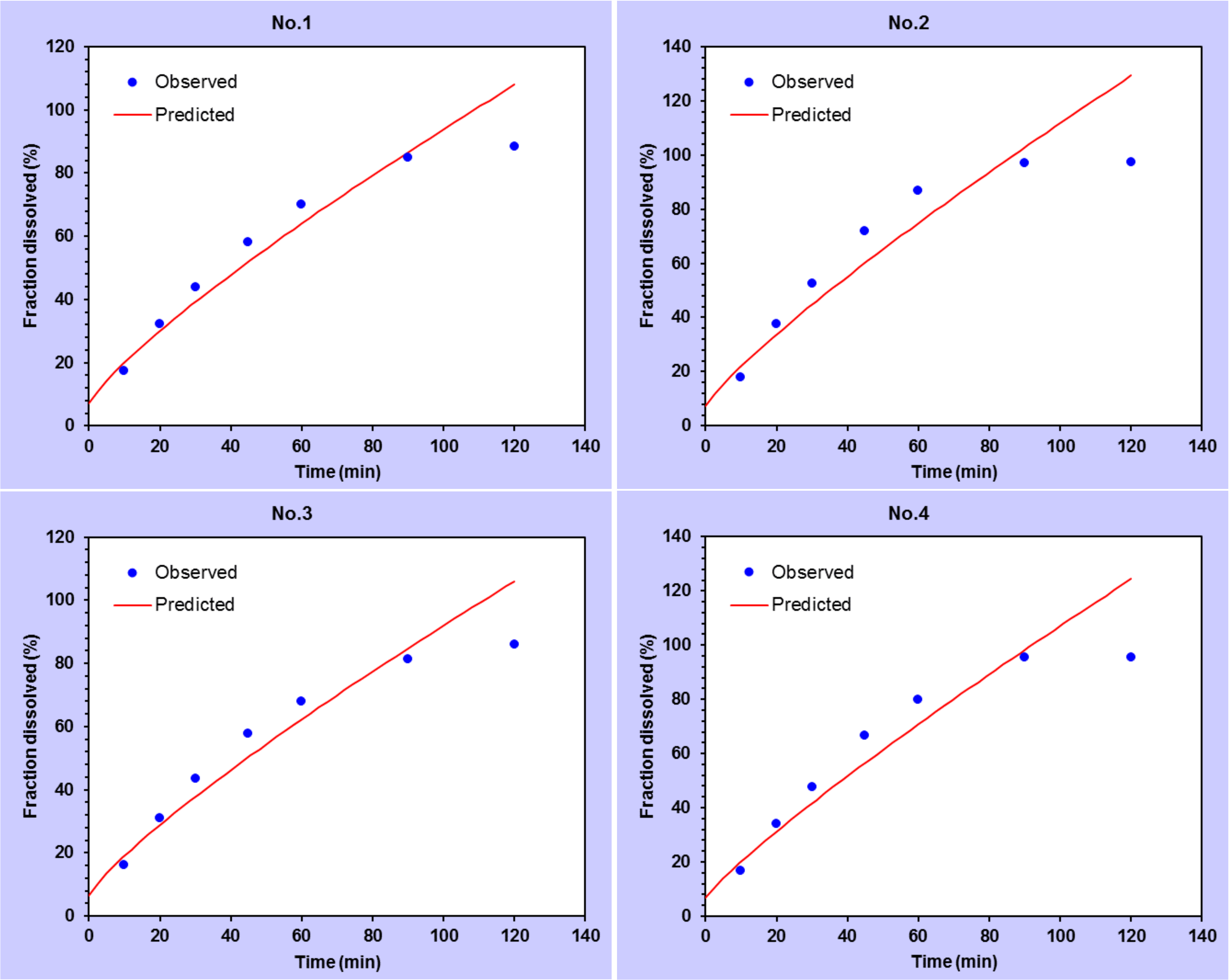

Supplementary materials – Model fitting summary – Polyglykol® 4000 P (modelling the entire relevant dissolution data)

Model: **Hixson–Crowell**

Model equation: $F = 100 \cdot [1 - (1 - k_{HC} \cdot t)^3]$

Fitted model parameters per tested tablet (N = 4) with statistics – mean, standard deviation (SD), and relative standard deviation expressed in % (RSD%) (output from DDSolver):

Parameter	No.1	No.2	No.3	No.4	Mean	SD	RSD(%)
k_{HC}	0.006	0.007	0.005	0.006	0.006	0.001	16.261

Number of dissolution data points (N), degrees of freedom (df), and selected goodness of fit criteria – Pearson correlation coefficient (R), coefficient of determination (R^2), adjusted coefficient of determination ($R^2_{adjusted}$), and residual sum of squares (RSS) (manual calculation in MS Excel):

Parameter	No.1	No.2	No.3	No.4
N	7	7	7	7
df	6	6	6	6
R	0.99913574	0.99424698	0.99044931	0.99597292
R^2	0.99827223	0.98852705	0.98098984	0.99196206
$R^2_{adjusted}$	0.99827223	0.98852705	0.98098984	0.99196206
RSS	154.2664448	108.7215969	240.9814647	67.14263798

Graphical abstract of model fit presented as mean ± 1 SD of the fraction % of released carvedilol:

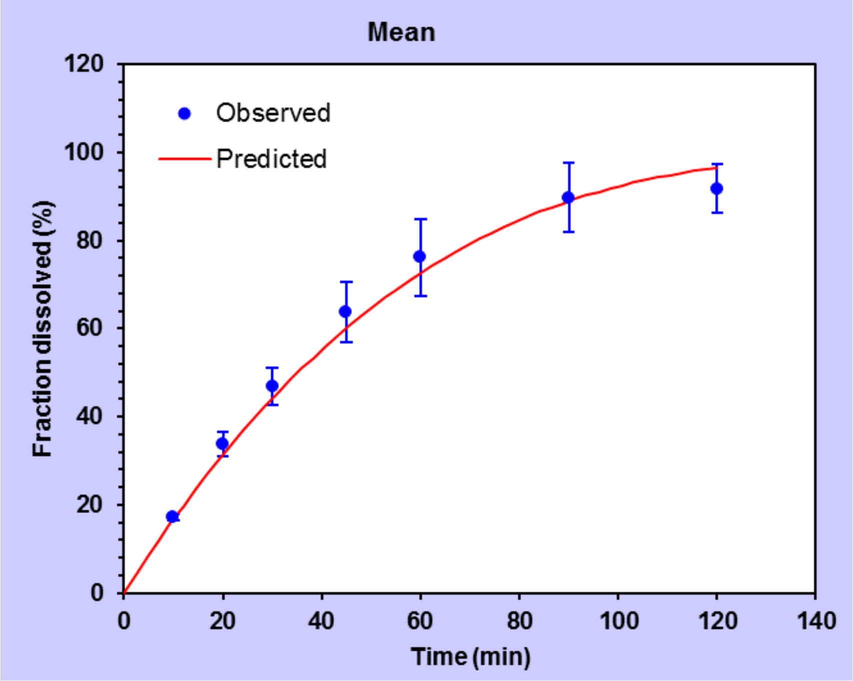

Supplementary materials – Model fitting summary – Polyglykol® 4000 P (modelling the entire relevant dissolution data)

Graphical abstract of model fit presented as the fraction % of released carvedilol per tested tablet:

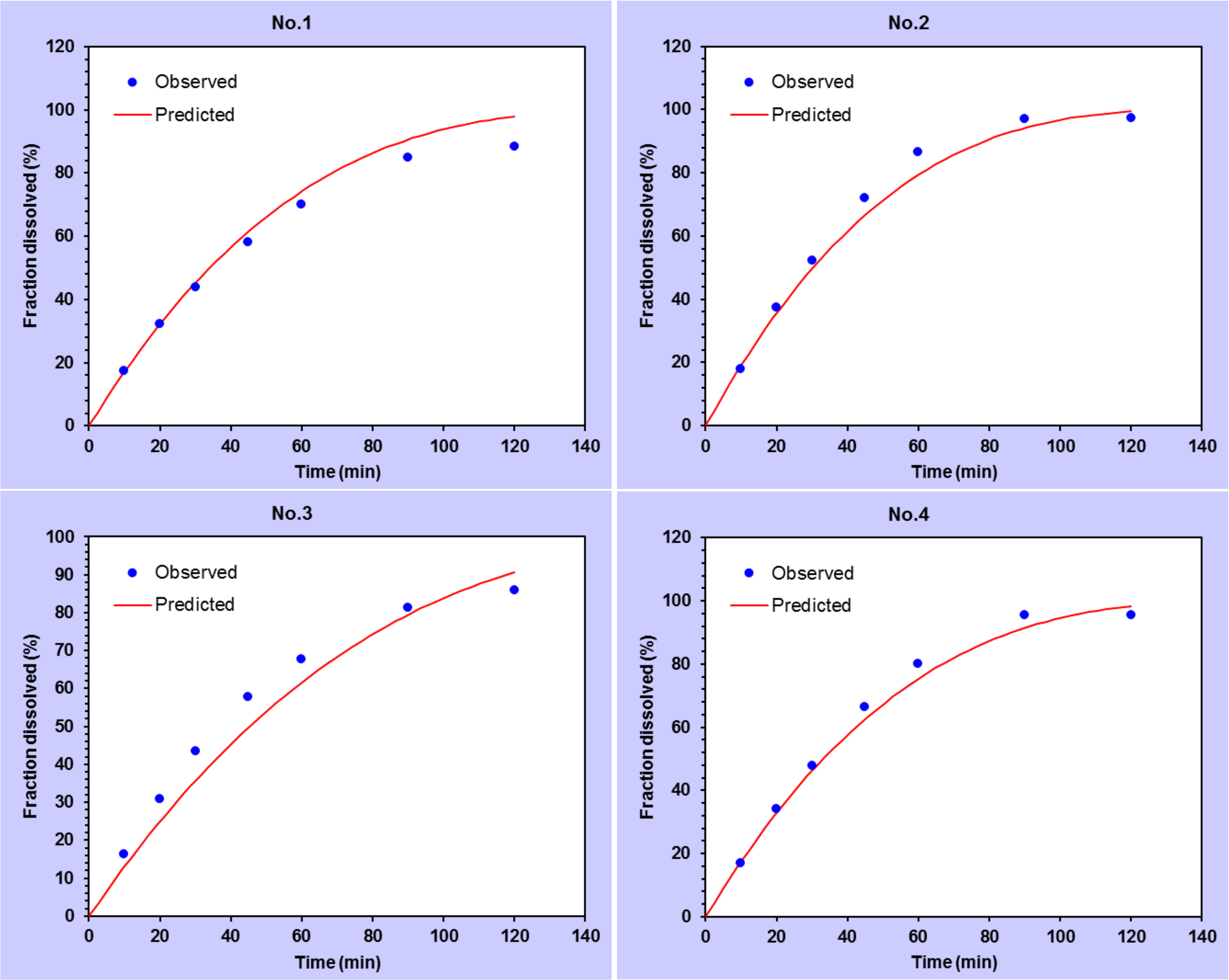

Supplementary materials – Model fitting summary – Polyglykol® 4000 P (modelling the entire relevant dissolution data)

Model: **Hixson–Crowell with T_{lag}**

Model equation: $F = 100 \cdot \left\{1 - \left[1 - k_{HC} \cdot \left(t - T_{lag}\right)\right]^3\right\}$

Fitted model parameters per tested tablet (N = 4) with statistics – mean, standard deviation (SD), and relative standard deviation expressed in % (RSD%) (output from DDSolver):

Parameter	No.1	No.2	No.3	No.4	Mean	SD	RSD(%)
k_{HC}	0.004	0.006	0.004	0.006	0.005	0.001	22.820
T_{lag}	-11.118	-7.100	-13.000	-5.205	-9.106	3.580	-39.317

Number of dissolution data points (N), degrees of freedom (df), and selected goodness of fit criteria – Pearson correlation coefficient (R), coefficient of determination (R^2), adjusted coefficient of determination ($R^2_{adjusted}$), and residual sum of squares (RSS) (manual calculation in MS Excel):

Parameter	No.1	No.2	No.3	No.4
N	7	7	7	7
df	5	5	5	5
R	0.99142304	0.99226043	0.98698212	0.99485323
R^2	0.98291965	0.98458076	0.9741337	0.98973296
$R^2_{adjusted}$	0.97950357	0.98149692	0.96896044	0.98767955
RSS	97.99751215	211.602729	131.0215144	118.7706838

Graphical abstract of model fit presented as mean ± 1 SD of the fraction % of released carvedilol:

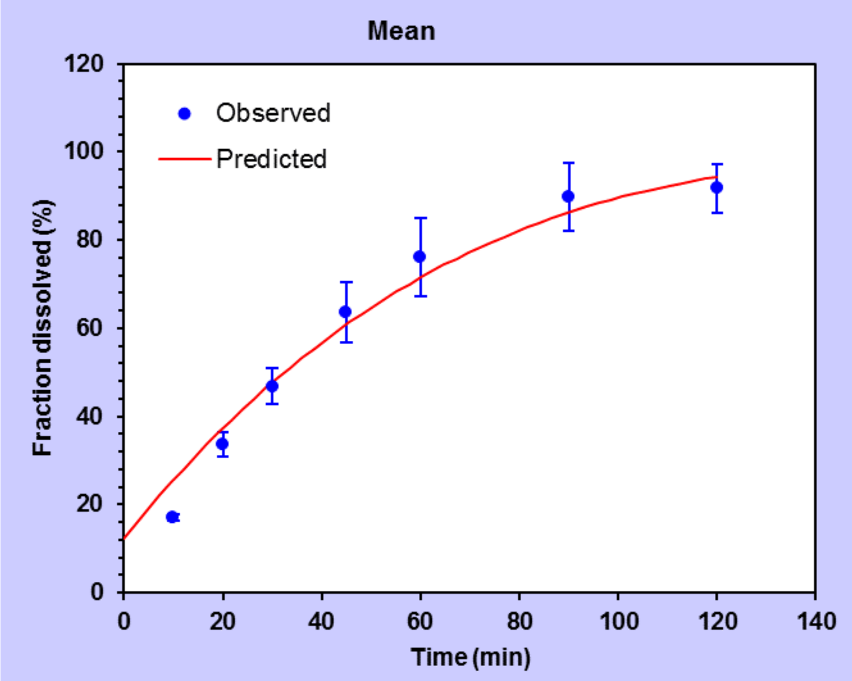

Supplementary materials – Model fitting summary – Polyglykol® 4000 P (modelling the entire relevant dissolution data)

Graphical abstract of model fit presented as the fraction % of released carvedilol per tested tablet:

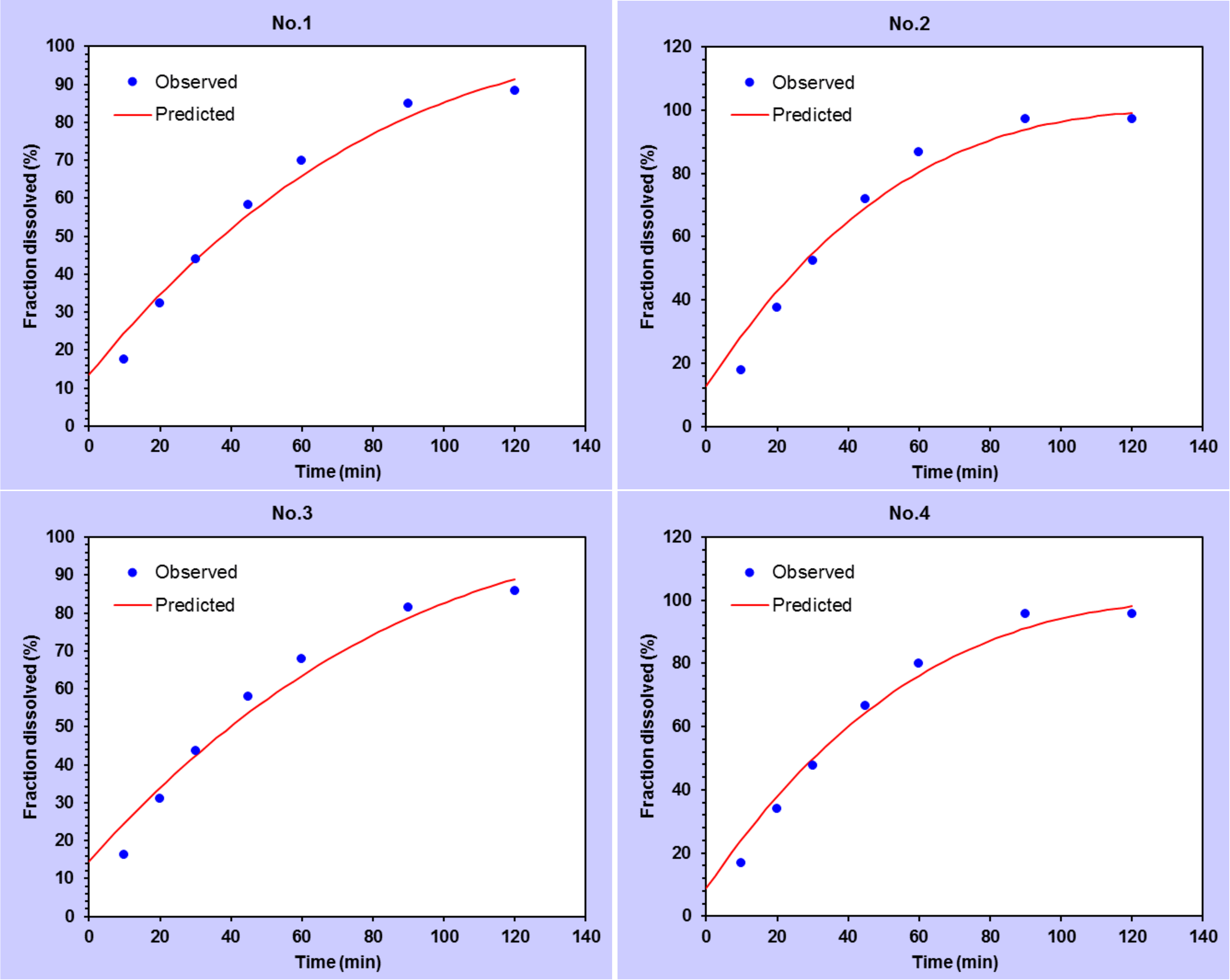

Supplementary materials – Model fitting summary – Polyglykol® 4000 P (modelling the entire relevant dissolution data)

Model: **Hopfenberg**

Model equation: $F = 100 \cdot [1 - (1 - k_{HB} \cdot t)^n]$

Fitted model parameters per tested tablet (N = 4) with statistics – mean, standard deviation (SD), and relative standard deviation expressed in % (RSD%) (output from DDSolver):

Parameter	No.1	No.2	No.3	No.4	Mean	SD	RSD(%)
k_{HB}	0.005	0.007	0.005	0.007	0.006	0.001	24.075
n	3.000	3.533	3.000	2.906	3.110	0.286	9.186

Number of dissolution data points (N), degrees of freedom (df), and selected goodness of fit criteria – Pearson correlation coefficient (R), coefficient of determination (R^2), adjusted coefficient of determination ($R^2_{adjusted}$), and residual sum of squares (RSS) (manual calculation in MS Excel):

Parameter	No.1	No.2	No.3	No.4
N	7	7	7	7
df	5	5	5	5
R	0.99398736	0.99862713	0.99044931	0.99906746
R^2	0.98801087	0.99725614	0.98098984	0.9981358
$R^2_{adjusted}$	0.98561305	0.99670736	0.97718781	0.99776295
RSS	185.6526718	54.50388678	240.9814647	62.29729579

Graphical abstract of model fit presented as mean ± 1 SD of the fraction % of released carvedilol:

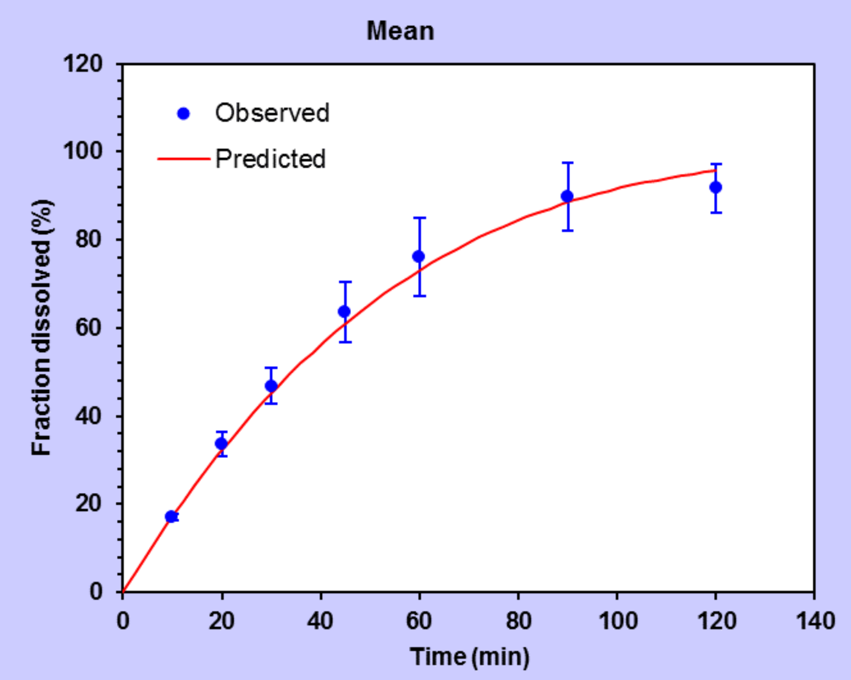

Supplementary materials – Model fitting summary – Polyglykol® 4000 P (modelling the entire relevant dissolution data)

Graphical abstract of model fit presented as the fraction % of released carvedilol per tested tablet:

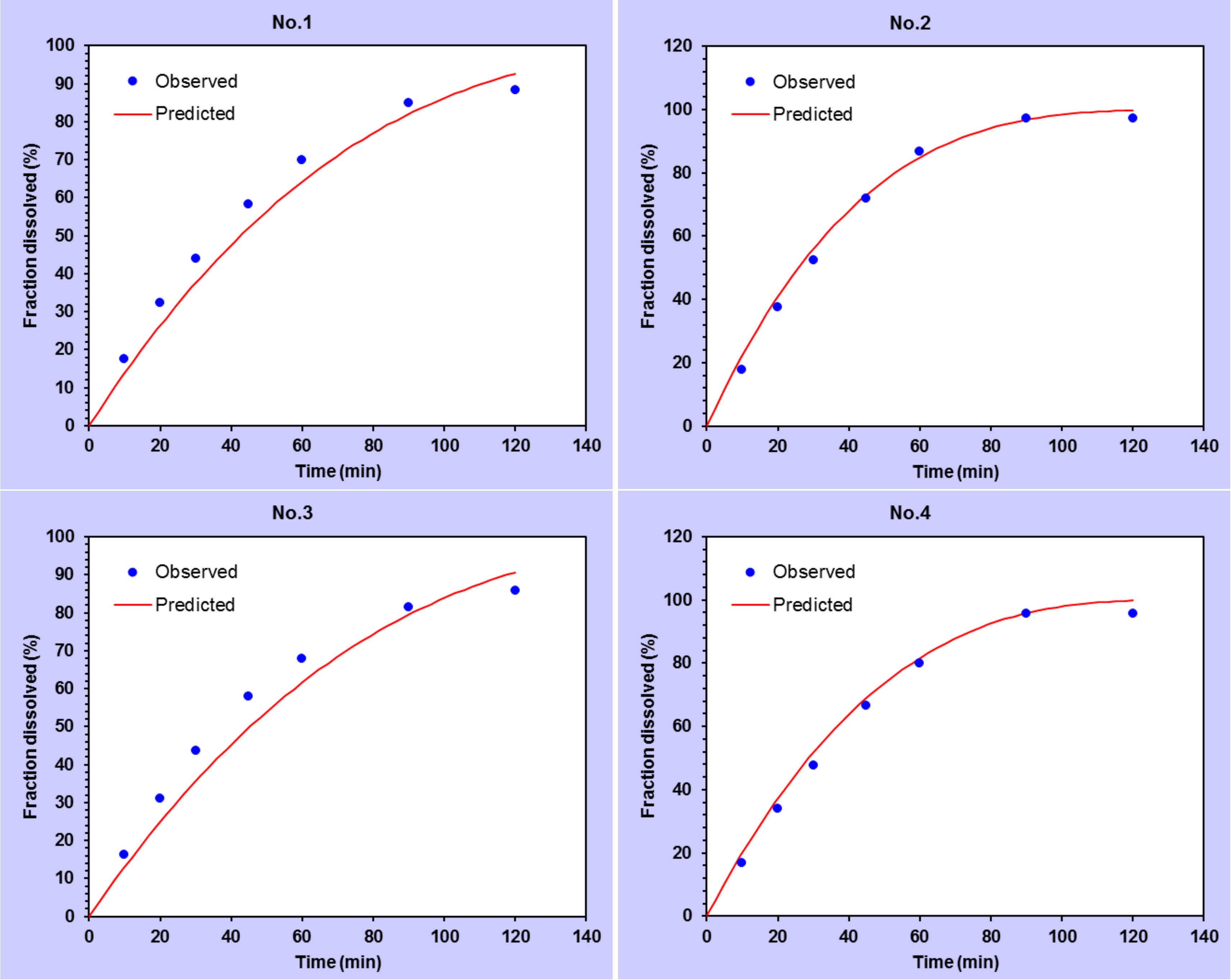

Supplementary materials – Model fitting summary – Polyglykol® 4000 P (modelling the entire relevant dissolution data)

Model: **Hopfenberg with T_{lag}**

Model equation: $F = 100 \cdot \{1 - [1 - k_{HB} \cdot (t - T_{lag})]^n\}$

Fitted model parameters per tested tablet (N = 4) with statistics – mean, standard deviation (SD), and relative standard deviation expressed in % (RSD%) (output from DDSolver):

Parameter	No.1	No.2	No.3	No.4	Mean	SD	RSD(%)
k_{HB}	0.004	0.006	0.004	0.006	0.005	0.001	22.820
n	3.000	3.000	3.000	3.000	3.000	0.000	0.000
T_{lag}	-11.118	-7.100	-13.000	-5.205	-9.106	3.580	-39.317

Number of dissolution data points (N), degrees of freedom (df), and selected goodness of fit criteria – Pearson correlation coefficient (R), coefficient of determination (R^2), adjusted coefficient of determination ($R^2_{adjusted}$), and residual sum of squares (RSS) (manual calculation in MS Excel):

Parameter	No.1	No.2	No.3	No.4
N	7	7	7	7
df	4	4	4	4
R	0.99142304	0.99226043	0.98698212	0.99485323
R^2	0.98291965	0.98458076	0.9741337	0.98973296
$R^2_{adjusted}$	0.97437947	0.97687114	0.96120055	0.98459943
RSS	97.99751215	211.602729	131.0215144	118.7706838

Graphical abstract of model fit presented as mean ± 1 SD of the fraction % of released carvedilol:

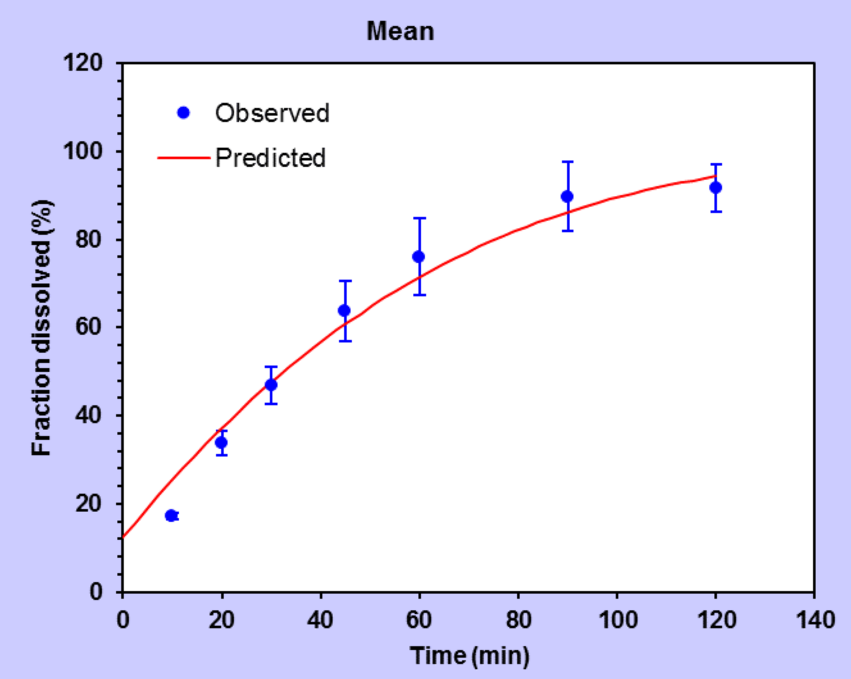

Supplementary materials – Model fitting summary – Polyglykol® 4000 P (modelling the entire relevant dissolution data)

Graphical abstract of model fit presented as the fraction % of released carvedilol per tested tablet:

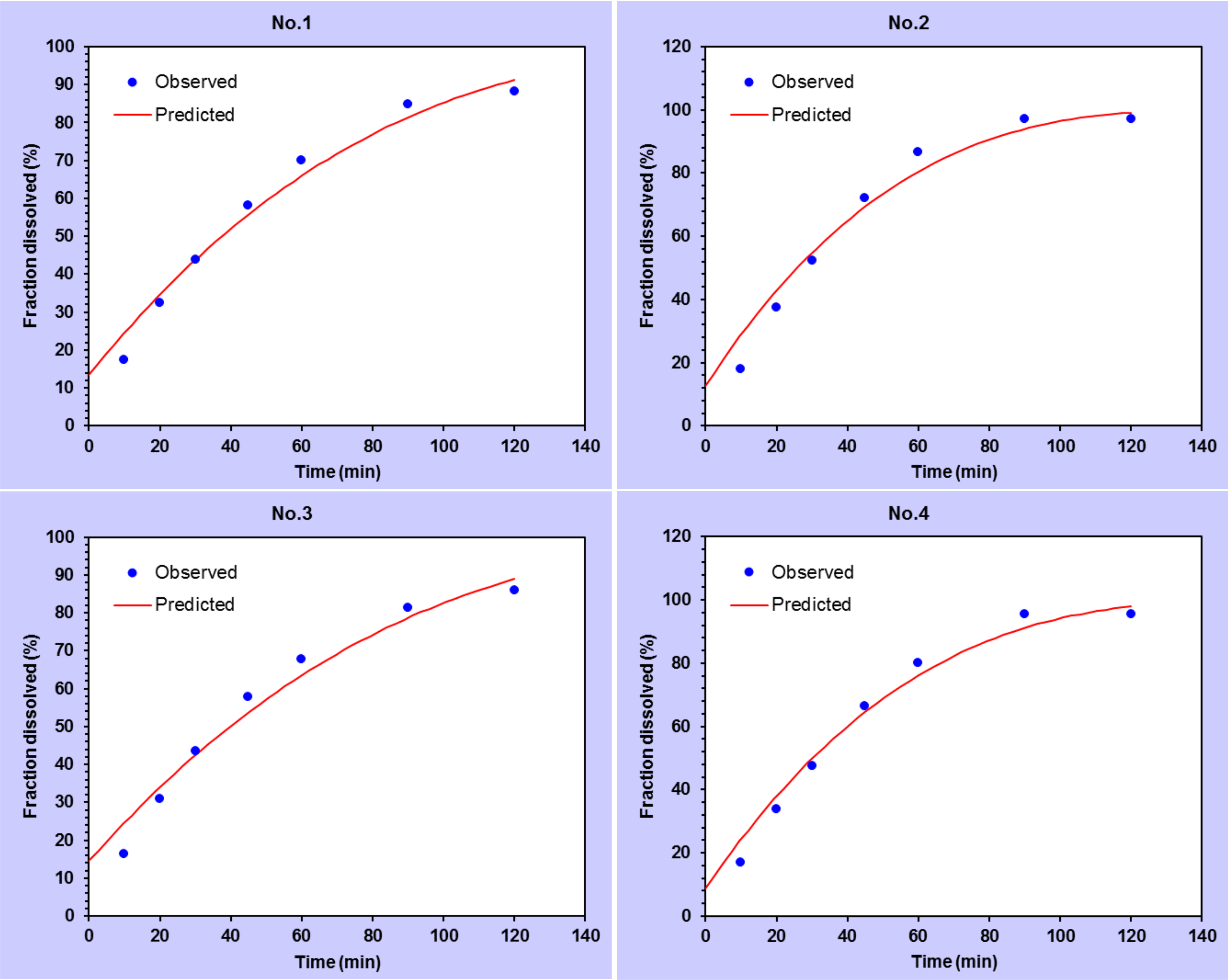

Supplementary materials – Model fitting summary – Polyglykol® 4000 P (modelling the entire relevant dissolution data)

Model: **Baker–Lonsdale**

Model equation: $\frac{3}{2}\cdot\left[1-\left(1-\frac{F}{100}\right)^{\frac{2}{3}}\right]-\frac{F}{100}=k_{BL}\cdot t$

Fitted model parameters per tested tablet (N = 4) with statistics – mean, standard deviation (SD), and relative standard deviation expressed in % (RSD%) (output from DDSolver):

Parameter	No.1	No.2	No.3	No.4	Mean	SD	RSD(%)
k_{BL}	0.001	0.002	0.002	0.002	0.002	0.000	19.006

Number of dissolution data points (N), degrees of freedom (df), and selected goodness of fit criteria – Pearson correlation coefficient (R), coefficient of determination (R^2), adjusted coefficient of determination ($R^2_{adjusted}$), and residual sum of squares (RSS) (manual calculation in MS Excel):

Parameter	No.1	No.2	No.3	No.4
N	7	7	7	7
df	6	6	6	6
R	0.99409254	0.98198328	0.9954758	0.98877954
R^2	0.98821999	0.96429117	0.99097207	0.97768498
$R^2_{adjusted}$	0.98821999	0.96429117	0.99097207	0.97768498
RSS	836.0164481	1131.367023	362.7579291	982.9878161

Graphical abstract of model fit presented as mean ± 1 SD of the fraction % of released carvedilol:

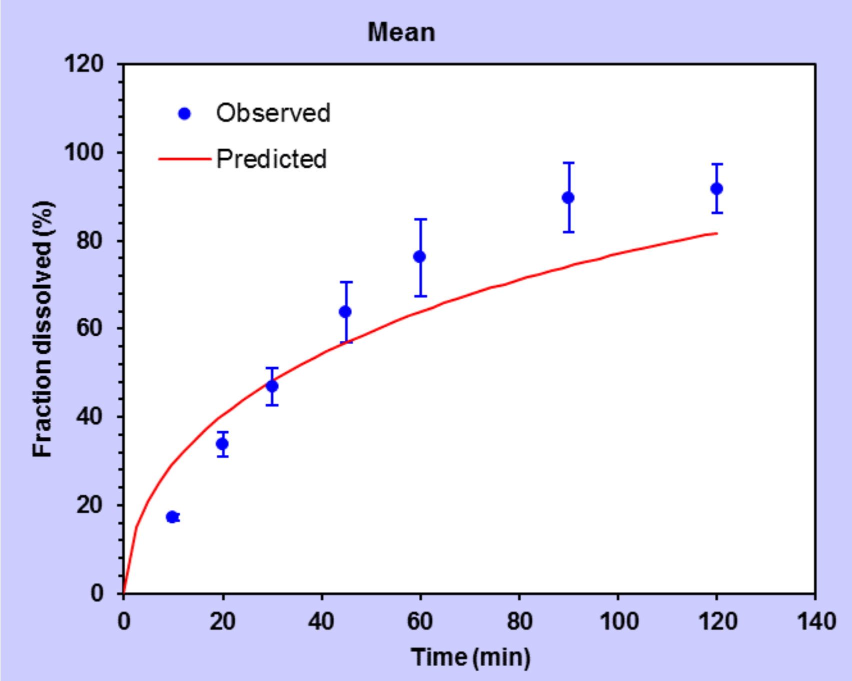

Supplementary materials – Model fitting summary – Polyglykol® 4000 P (modelling the entire relevant dissolution data)

Graphical abstract of model fit presented as the fraction % of released carvedilol per tested tablet:

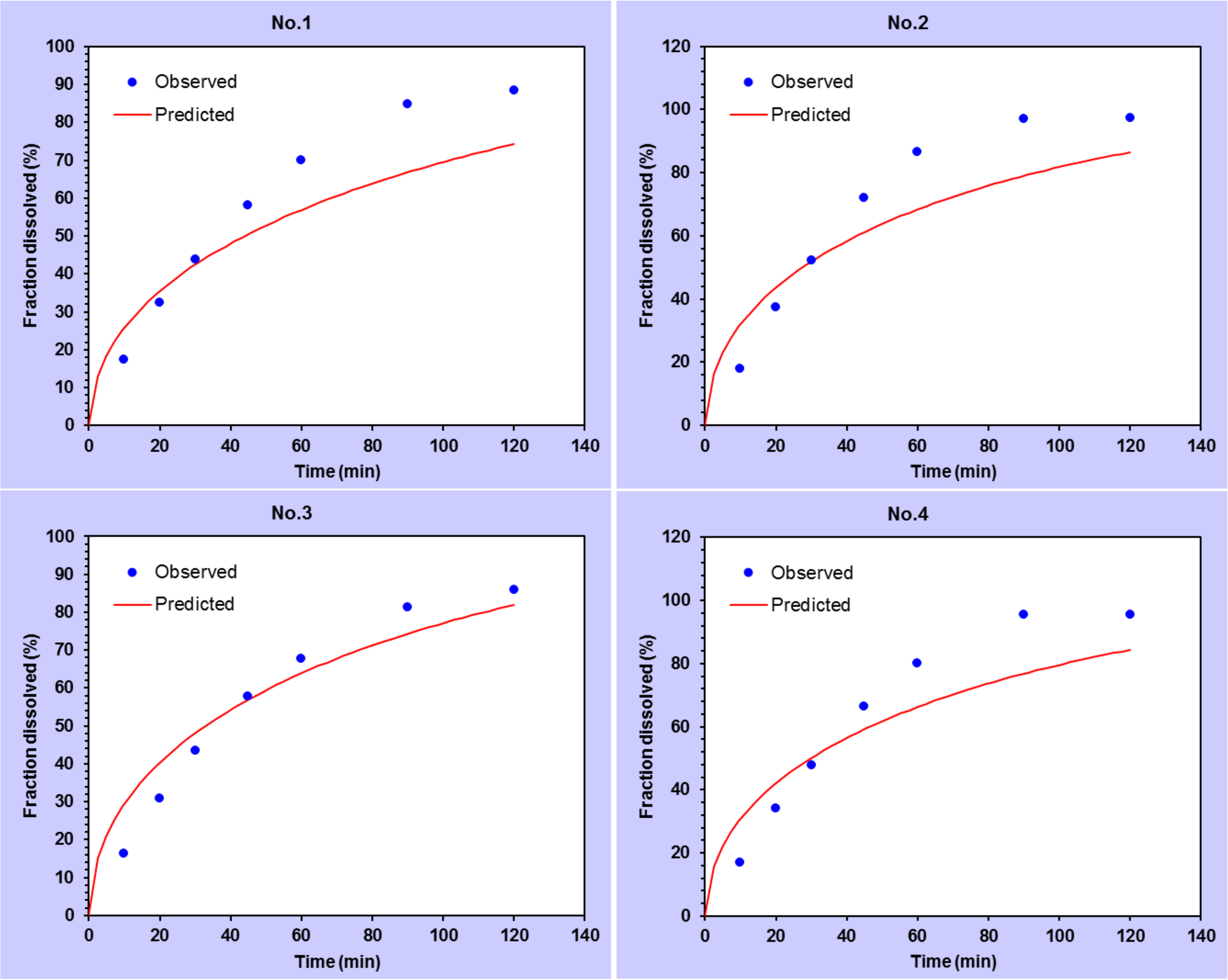

Supplementary materials – Model fitting summary – Polyglykol® 4000 P (modelling the entire relevant dissolution data)

Model: **Baker–Lonsdale with T_{lag}**

Model equation: $\frac{3}{2}\cdot\left[1-\left(1-\frac{F}{100}\right)^{\frac{2}{3}}\right]-\frac{F}{100}=k_{BL}\cdot(t-T_{lag})$

Fitted model parameters per tested tablet (N = 4) with statistics – mean, standard deviation (SD), and relative standard deviation expressed in % (RSD%) (output from DDSolver):

Parameter	No.1	No.2	No.3	No.4	Mean	SD	RSD(%)
k_{BL}	0.003	0.004	0.002	0.004	0.003	0.001	27.899
T_{lag}	10.353	8.800	9.395	11.323	9.968	1.107	11.108

Number of dissolution data points (N), degrees of freedom (df), and selected goodness of fit criteria – Pearson correlation coefficient (R), coefficient of determination (R^2), adjusted coefficient of determination ($R^2_{adjusted}$), and residual sum of squares (RSS) (manual calculation in MS Excel):

Parameter	No.1	No.2	No.3	No.4
N	7	7	7	7
df	5	5	5	5
R	0.97667805	0.98789711	0.99105372	0.9729279
R^2	0.95390001	0.9759407	0.98218748	0.9465887
$R^2_{adjusted}$	0.94468001	0.97112884	0.97862498	0.93590644
RSS	343.9686405	162.3161597	84.16109892	400.7324259

Graphical abstract of model fit presented as mean ± 1 SD of the fraction % of released carvedilol:

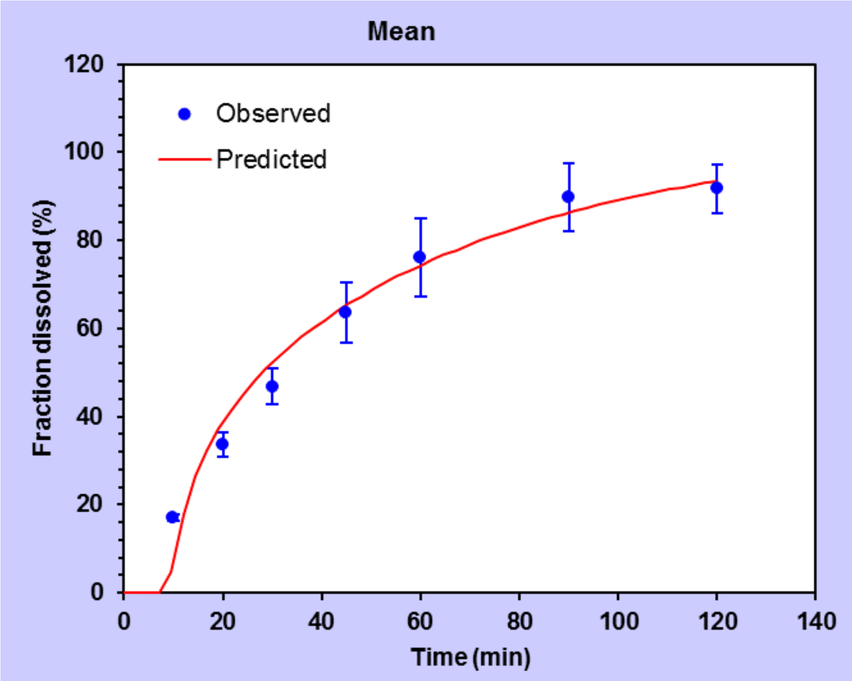

Supplementary materials – Model fitting summary – Polyglykol® 4000 P (modelling the entire relevant dissolution data)

Graphical abstract of model fit presented as the fraction % of released carvedilol per tested tablet:

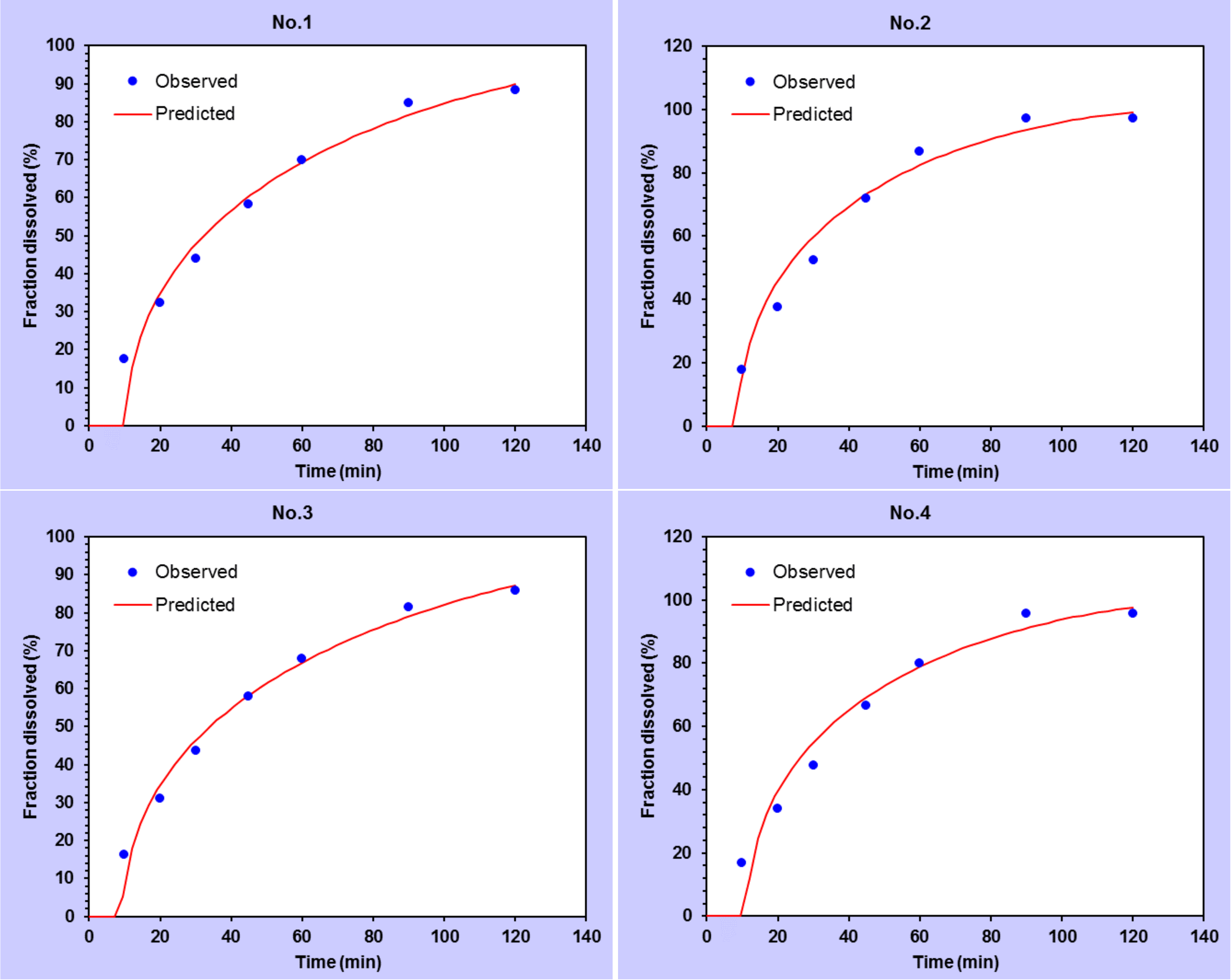

Supplementary materials – Model fitting summary – Polyglykol® 4000 P (modelling the entire relevant dissolution data)

Model: **Makoid–Banakar**

Model equation: $F = k_{MB} \cdot t^n \cdot e^{-k \cdot t}$

Fitted model parameters per tested tablet (N = 4) with statistics – mean, standard deviation (SD), and relative standard deviation expressed in % (RSD%) (output from DDSolver):

Parameter	No.1	No.2	No.3	No.4	Mean	SD	RSD(%)
k_{MB}	2.007	1.274	1.659	1.328	1.567	0.339	21.612
n	0.973	1.201	1.031	1.147	1.088	0.105	9.623
k	0.007	0.012	0.008	0.010	0.009	0.002	21.829

Number of dissolution data points (N), degrees of freedom (df), and selected goodness of fit criteria – Pearson correlation coefficient (R), coefficient of determination (R^2), adjusted coefficient of determination ($R^2_{adjusted}$), and residual sum of squares (RSS) (manual calculation in MS Excel):

Parameter	No.1	No.2	No.3	No.4
N	7	7	7	7
df	4	4	4	4
R	0.99964803	0.99975165	0.99984826	0.99954149
R^2	0.99929618	0.99950336	0.99969654	0.99908318
$R^2_{adjusted}$	0.99894427	0.99925503	0.99954481	0.99862477
RSS	3.021353848	2.844418549	1.244425464	5.264080946

Graphical abstract of model fit presented as mean ± 1 SD of the fraction % of released carvedilol:

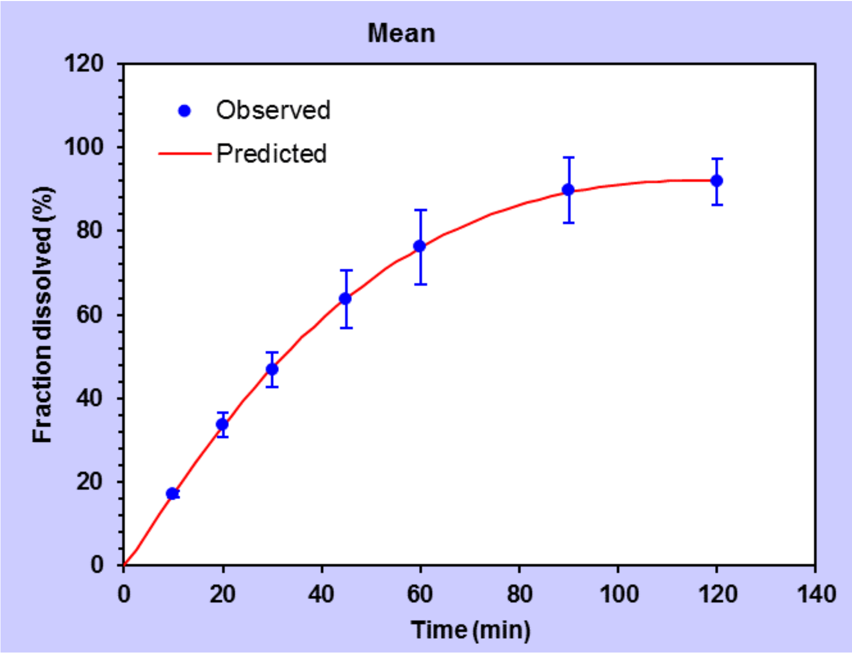

Supplementary materials – Model fitting summary – Polyglykol® 4000 P (modelling the entire relevant dissolution data)

Graphical abstract of model fit presented as the fraction % of released carvedilol per tested tablet:

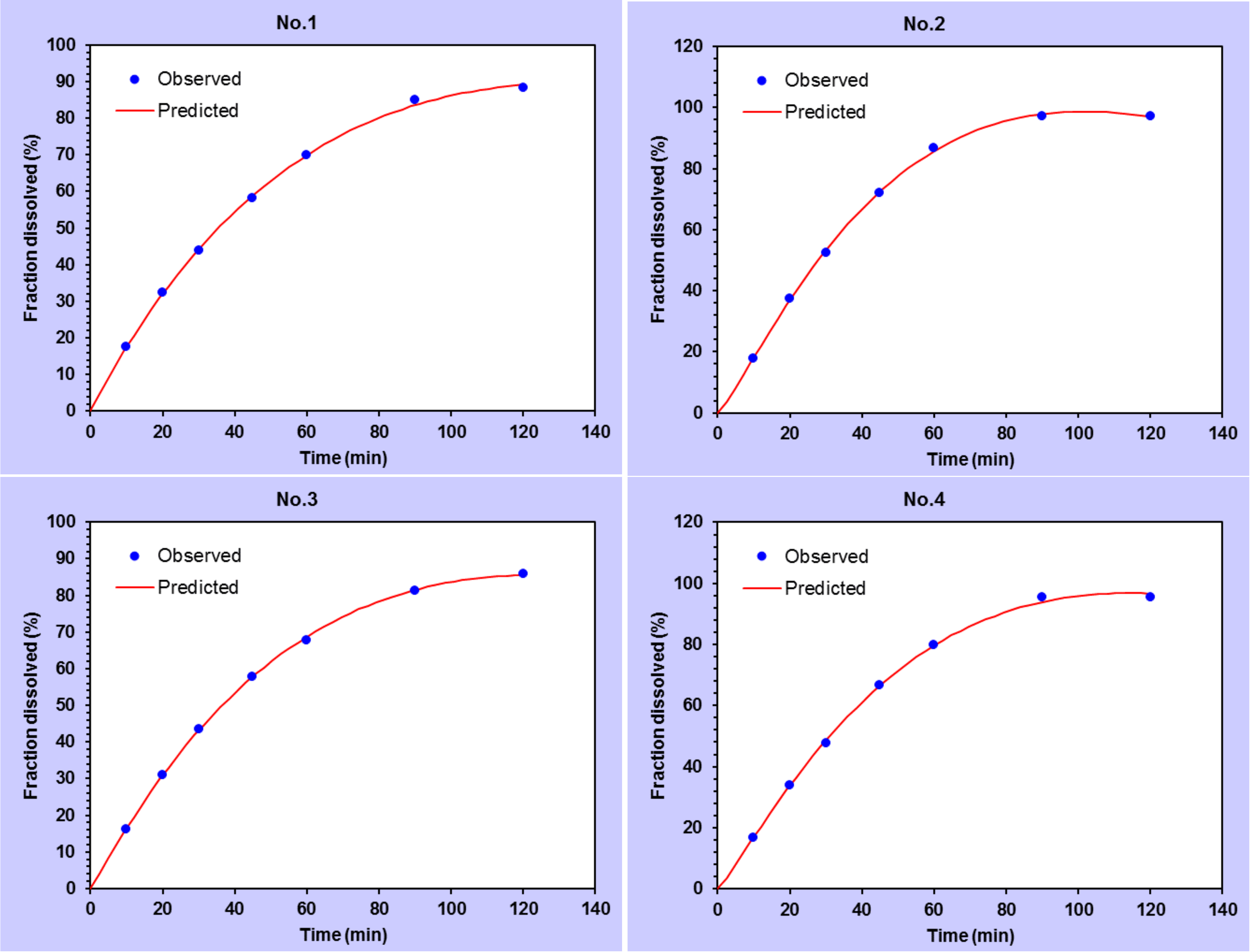

Supplementary materials – Model fitting summary – Polyglykol® 4000 P (modelling the entire relevant dissolution data)

Model: **Makoid–Banakar with T_{lag}**

Model equation: $F = k_{MB} \cdot \left(t - T_{lag}\right)^{n} \cdot e^{-k \cdot \left(t - T_{lag}\right)}$

Fitted model parameters per tested tablet (N = 4) with statistics – mean, standard deviation (SD), and relative standard deviation expressed in % (RSD%) (output from DDSolver):

Parameter	No.1	No.2	No.3	No.4	Mean	SD	RSD(%)
k_{MB}	4.981	3.866	4.325	3.866	4.260	0.528	12.388
n	0.705	0.872	0.748	0.830	0.789	0.076	9.604
k	0.004	0.008	0.005	0.006	0.006	0.002	29.920
T_{lag}	4.000	4.000	4.000	4.000	4.000	0.000	0.000

Number of dissolution data points (N), degrees of freedom (df), and selected goodness of fit criteria – Pearson correlation coefficient (R), coefficient of determination (R^2), adjusted coefficient of determination ($R^2_{adjusted}$), and residual sum of squares (RSS) (manual calculation in MS Excel):

Parameter	No.1	No.2	No.3	No.4
N	7	7	7	7
df	3	3	3	3
R	0.99825855	0.99851995	0.99951403	0.9973914
R^2	0.99652013	0.99704209	0.99902829	0.9947896
$R^2_{adjusted}$	0.99304026	0.99408418	0.99805657	0.9895792
RSS	14.96894027	17.49591265	3.960742309	29.8671924

Graphical abstract of model fit presented as mean ± 1 SD of the fraction % of released carvedilol:

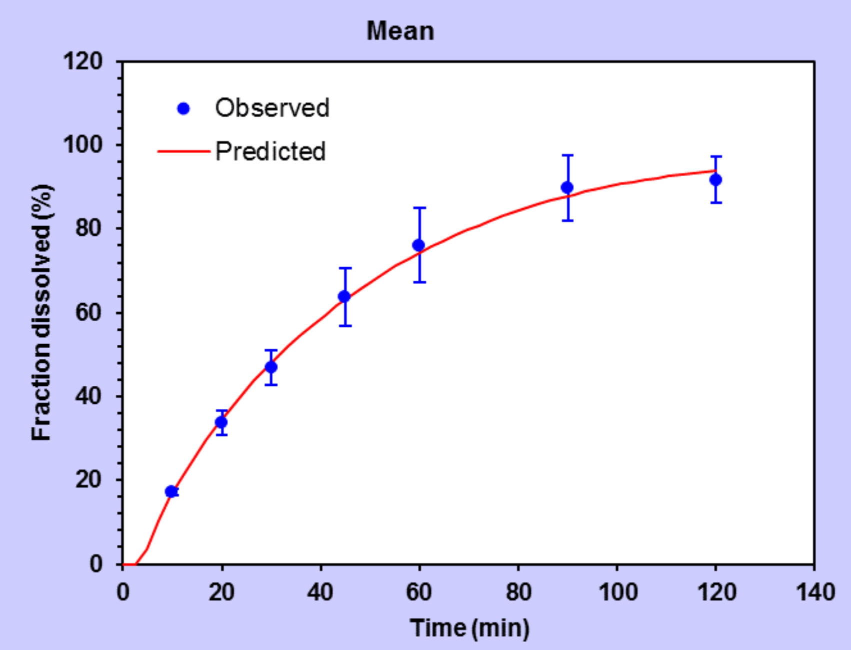

Supplementary materials – Model fitting summary – Polyglykol® 4000 P (modelling the entire relevant dissolution data)

Graphical abstract of model fit presented as the fraction % of released carvedilol per tested tablet:

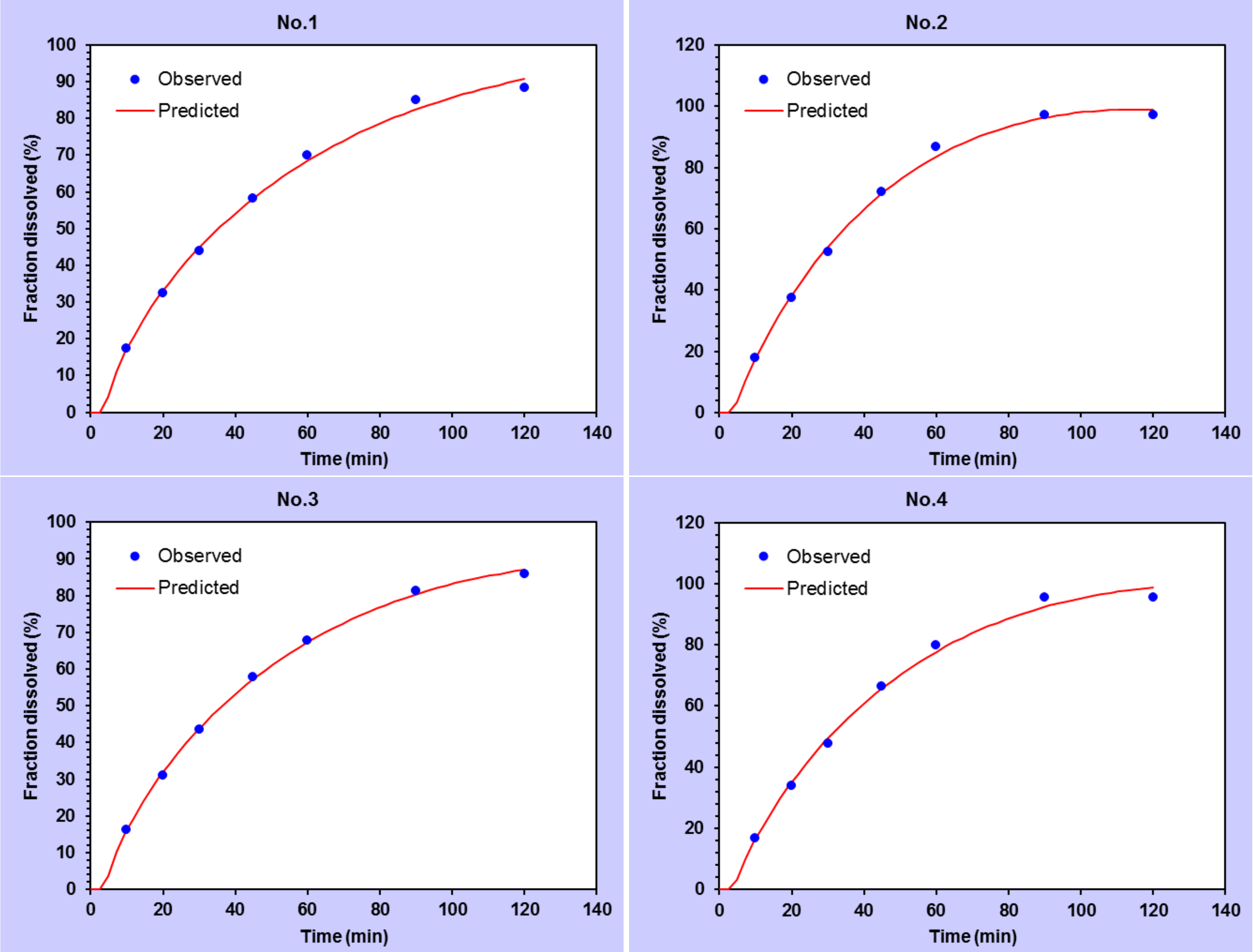

Supplementary materials – Model fitting summary – Polyglykol® 4000 P (modelling the entire relevant dissolution data)

Model: **Peppas–Sahlin_1**

Model equation: $F = k_1 \cdot t^m + k_2 \cdot t^{2m}$

Fitted model parameters per tested tablet (N = 4) with statistics – mean, standard deviation (SD), and relative standard deviation expressed in % (RSD%) (output from DDSolver):

Parameter	No.1	No.2	No.3	No.4	Mean	SD	RSD(%)
k_1	7.795	10.292	7.665	8.542	8.573	1.209	14.100
k_2	0.373	0.252	0.349	0.427	0.350	0.073	20.950
m	0.450	0.450	0.450	0.450	0.450	0.000	0.000

Number of dissolution data points (N), degrees of freedom (df), and selected goodness of fit criteria – Pearson correlation coefficient (R), coefficient of determination (R^2), adjusted coefficient of determination ($R^2_{adjusted}$), and residual sum of squares (RSS) (manual calculation in MS Excel):

Parameter	No.1	No.2	No.3	No.4
N	7	7	7	7
df	4	4	4	4
R	0.98054845	0.95696949	0.97916004	0.96648512
R^2	0.96147526	0.9157906	0.95875438	0.93409348
$R^2_{adjusted}$	0.94221289	0.8736859	0.93813157	0.90114022
RSS	176.4482221	523.8308721	179.5722025	396.4754568

Graphical abstract of model fit presented as mean ± 1 SD of the fraction % of released carvedilol:

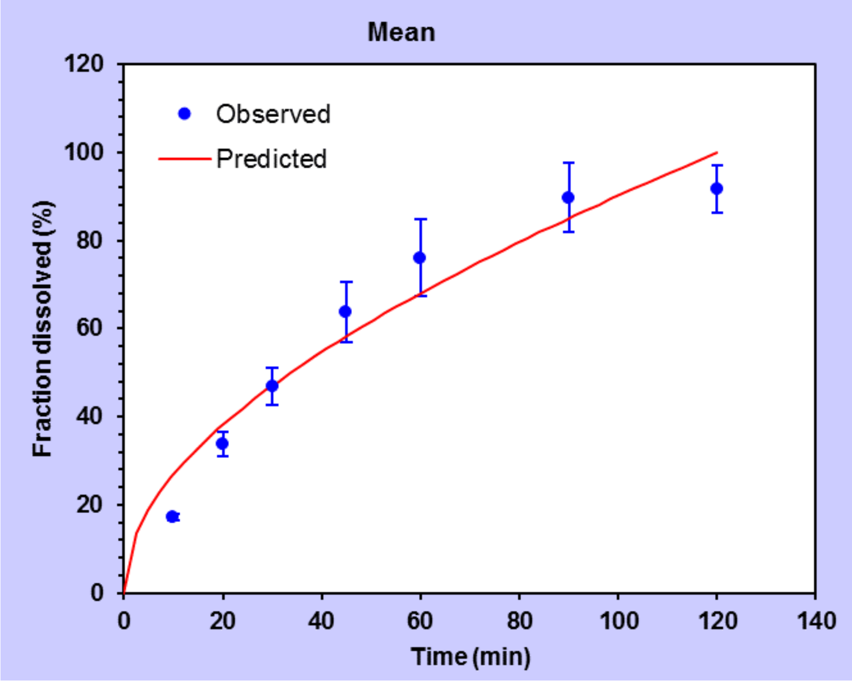

Supplementary materials – Model fitting summary – Polyglykol® 4000 P (modelling the entire relevant dissolution data)

Graphical abstract of model fit presented as the fraction % of released carvedilol per tested tablet:

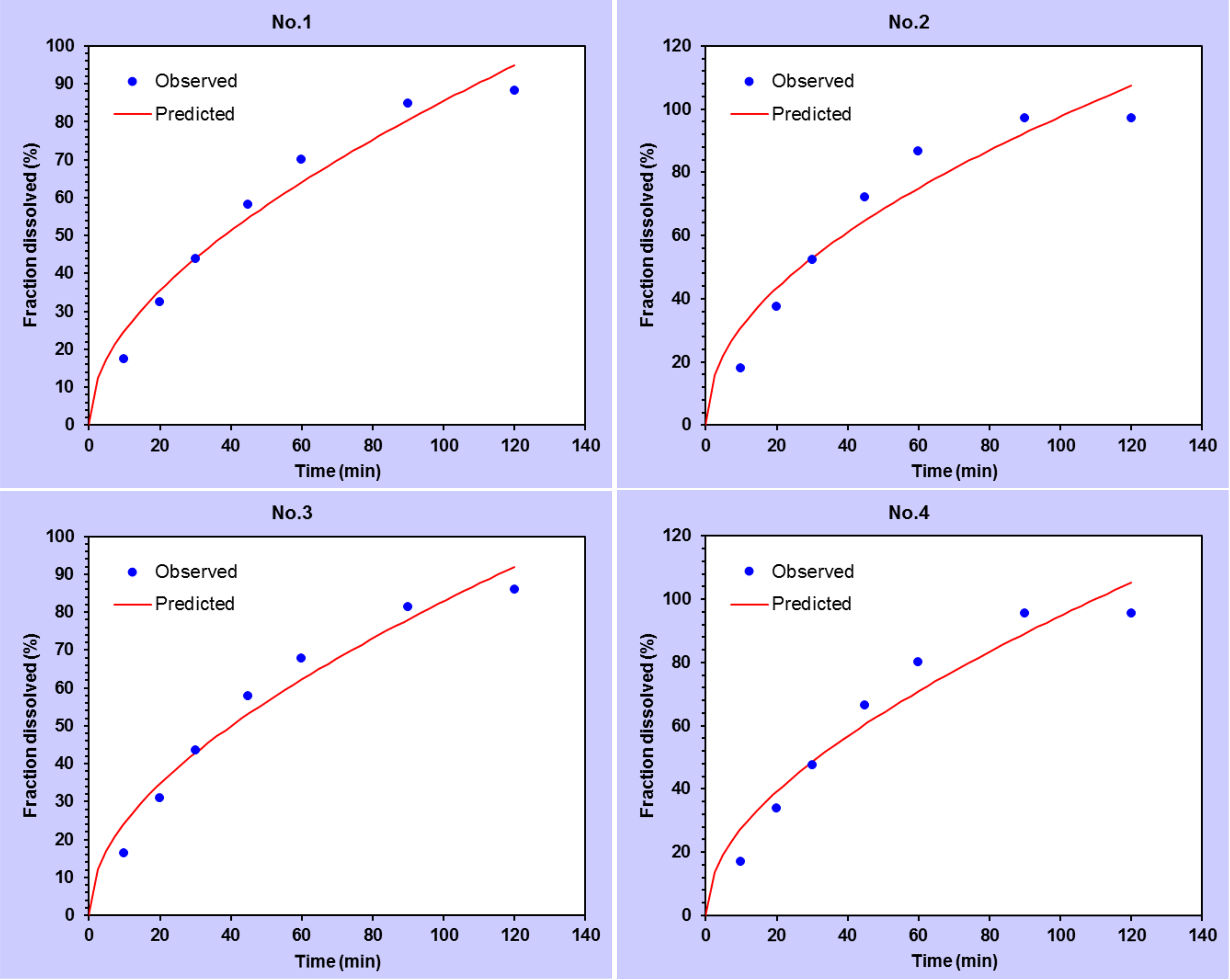

Supplementary materials – Model fitting summary – Polyglykol® 4000 P (modelling the entire relevant dissolution data)

Model: **Peppas–Sahlin_1 with T_{lag}**

Model equation: $F = k_1 \cdot (t - T_{lag})^m + k_2 \cdot (t - T_{lag})^{2m}$

Fitted model parameters per tested tablet (N = 4) with statistics – mean, standard deviation (SD), and relative standard deviation expressed in % (RSD%) (output from DDSolver):

Parameter	No.1	No.2	No.3	No.4	Mean	SD	RSD(%)
k_1	9.630	12.519	9.462	10.625	10.559	1.404	13.294
k_2	0.167	-0.004	0.146	0.192	0.125	0.089	70.608
m	0.450	0.450	0.450	0.450	0.450	0.000	0.000
T_{lag}	6.000	6.000	6.000	6.000	6.000	0.000	0.000

Number of dissolution data points (N), degrees of freedom (df), and selected goodness of fit criteria – Pearson correlation coefficient (R), coefficient of determination (R^2), adjusted coefficient of determination ($R^2_{adjusted}$), and residual sum of squares (RSS) (manual calculation in MS Excel):

Parameter	No.1	No.2	No.3	No.4
N	7	7	7	7
df	3	3	3	3
R	0.98959971	0.97370953	0.98950338	0.97885124
R^2	0.97930758	0.94811025	0.97911693	0.95814976
$R^2_{adjusted}$	0.95861516	0.89622049	0.95823386	0.91629951
RSS	100.0282566	317.8039794	94.96242478	250.8356982

Graphical abstract of model fit presented as mean ± 1 SD of the fraction % of released carvedilol:

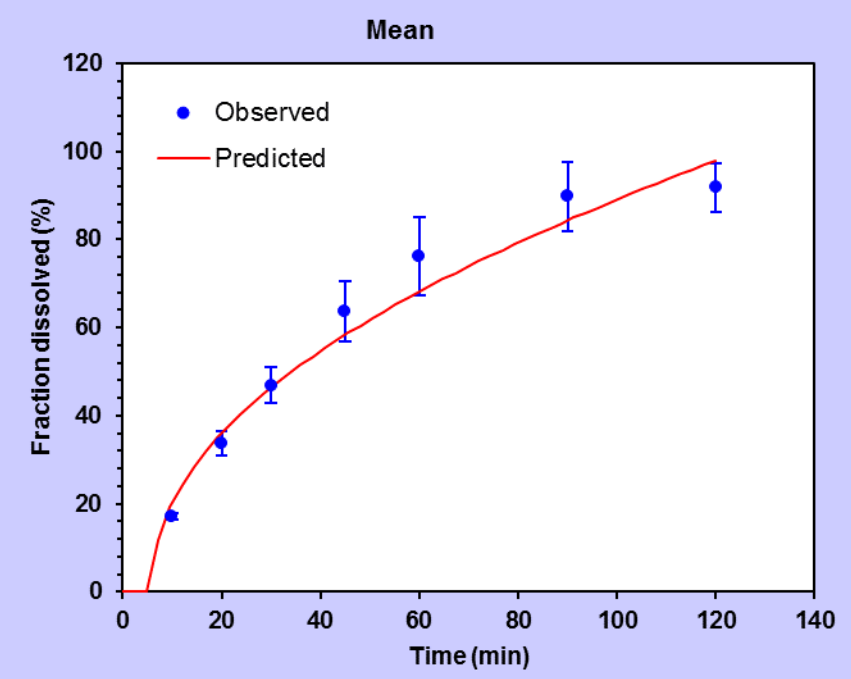

Supplementary materials – Model fitting summary – Polyglykol® 4000 P (modelling the entire relevant dissolution data)

Graphical abstract of model fit presented as the fraction % of released carvedilol per tested tablet:

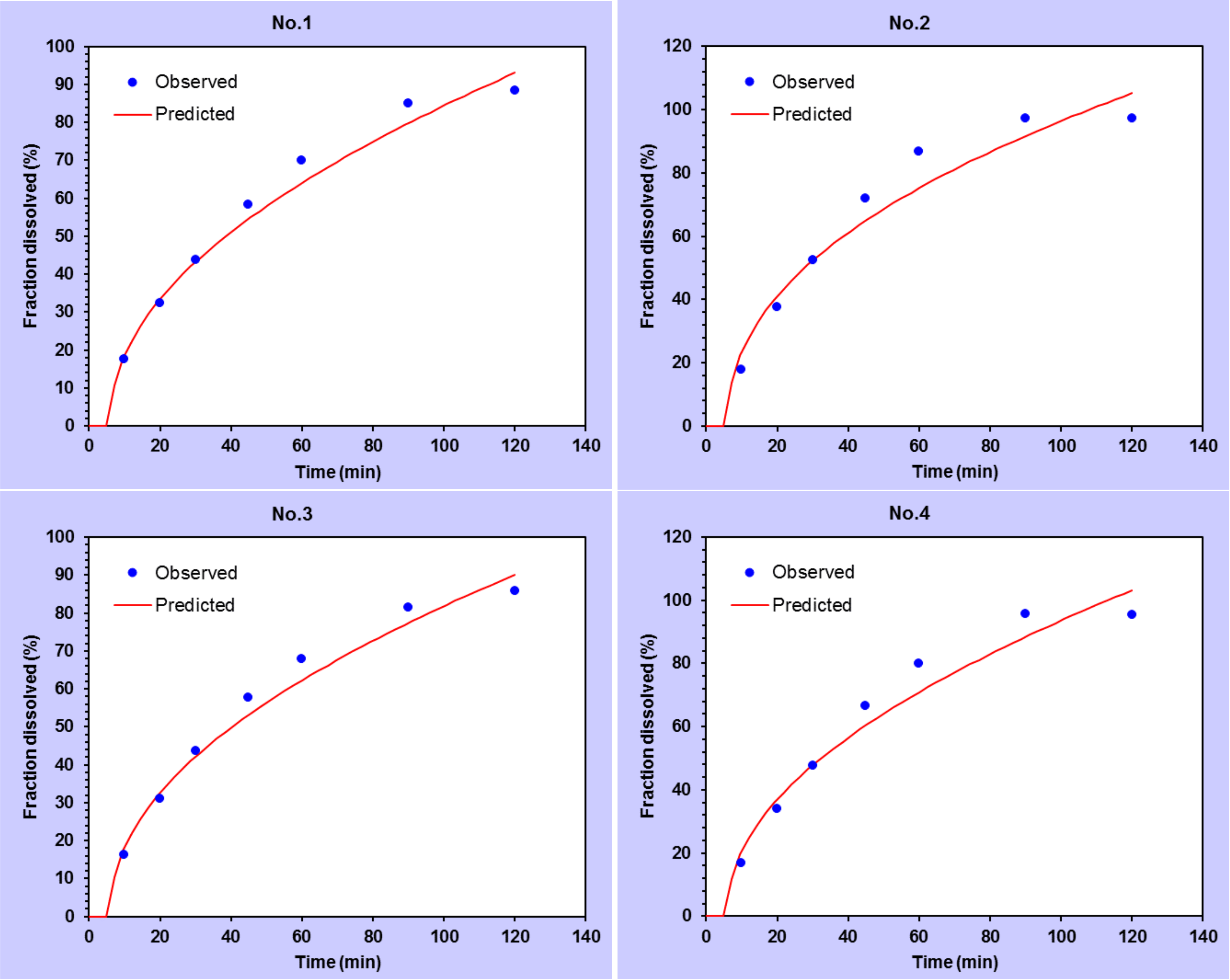

Supplementary materials – Model fitting summary – Polyglykol® 4000 P (modelling the entire relevant dissolution data)

Model: **Peppas–Sahlin_2**

Model equation: $F = k_1 \cdot t^{0.5} + k_2 \cdot t$

Fitted model parameters per tested tablet (N = 4) with statistics – mean, standard deviation (SD), and relative standard deviation expressed in % (RSD%) (output from DDSolver):

Parameter	No.1	No.2	No.3	No.4	Mean	SD	RSD(%)
k_1	7.560	9.803	7.422	8.355	8.285	1.092	13.185
k_2	0.097	-0.007	0.085	0.110	0.071	0.053	74.845

Number of dissolution data points (N), degrees of freedom (df), and selected goodness of fit criteria – Pearson correlation coefficient (R), coefficient of determination (R^2), adjusted coefficient of determination ($R^2_{adjusted}$), and residual sum of squares (RSS) (manual calculation in MS Excel):

Parameter	No.1	No.2	No.3	No.4
N	7	7	7	7
df	5	5	5	5
R	0.98246517	0.96161717	0.98133369	0.96920327
R^2	0.9652378	0.92470759	0.96301581	0.93935499
$R^2_{adjusted}$	0.95828536	0.90964911	0.95561897	0.92722598
RSS	164.8356246	488.8385055	167.300702	377.6901925

Graphical abstract of model fit presented as mean ± 1 SD of the fraction % of released carvedilol:

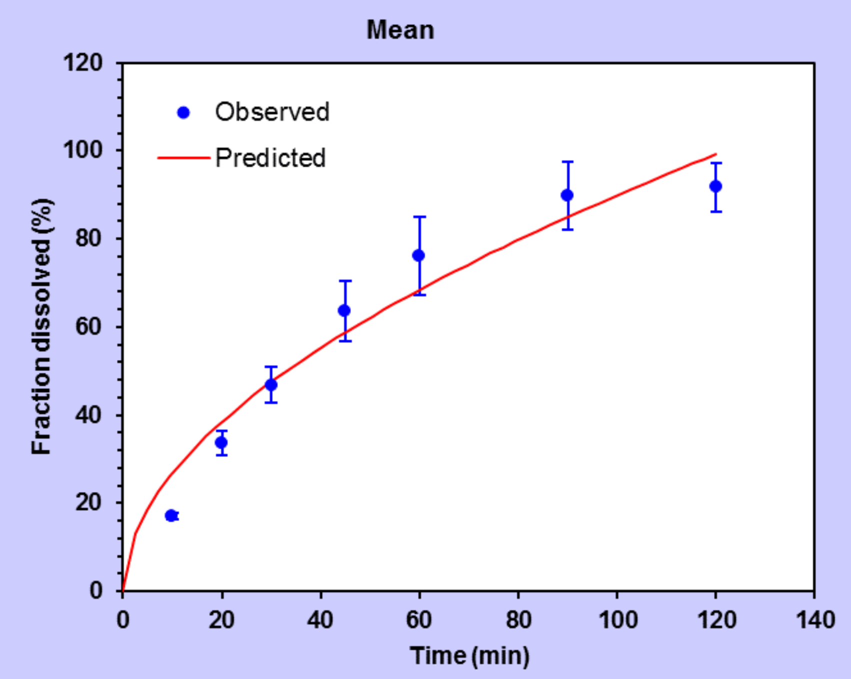

Supplementary materials – Model fitting summary – Polyglykol® 4000 P (modelling the entire relevant dissolution data)

Graphical abstract of model fit presented as the fraction % of released carvedilol per tested tablet:

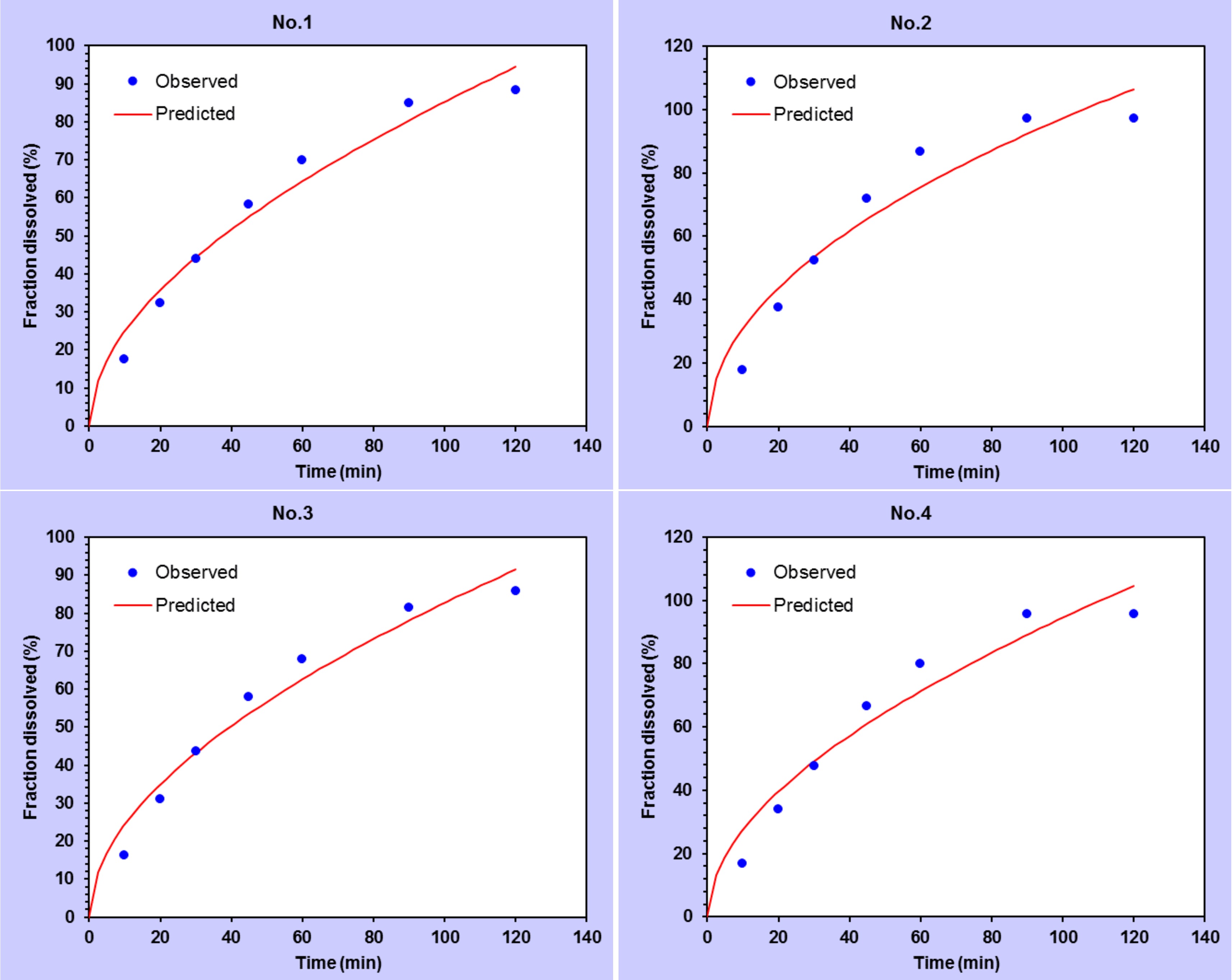

Supplementary materials – Model fitting summary – Polyglykol® 4000 P (modelling the entire relevant dissolution data)

Model: **Peppas–Sahlin_2 with T_{lag}**

Model equation: $F = k_1 \cdot (t - T_{lag})^{0.5} + k_2 \cdot (t - T_{lag})$

Fitted model parameters per tested tablet (N = 4) with statistics – mean, standard deviation (SD), and relative standard deviation expressed in % (RSD%) (output from DDSolver):

Parameter	No.1	No.2	No.3	No.4	Mean	SD	RSD(%)
k_1	9.044	11.621	8.878	10.048	9.898	1.260	12.729
k_2	-0.036	-0.175	-0.046	-0.043	-0.075	0.067	-88.718
T_{lag}	6.000	6.000	6.000	6.000	6.000	0.000	0.000

Number of dissolution data points (N), degrees of freedom (df), and selected goodness of fit criteria – Pearson correlation coefficient (R), coefficient of determination (R^2), adjusted coefficient of determination ($R^2_{adjusted}$), and residual sum of squares (RSS) (manual calculation in MS Excel):

Parameter	No.1	No.2	No.3	No.4
N	7	7	7	7
df	4	4	4	4
R	0.99148506	0.9785126	0.99168858	0.9819506
R^2	0.98304262	0.95748691	0.98344624	0.96422698
$R^2_{adjusted}$	0.97456393	0.93623036	0.97516936	0.94634047
RSS	83.93058979	266.0873313	77.40606245	217.6241959

Graphical abstract of model fit presented as mean ± 1 SD of the fraction % of released carvedilol:

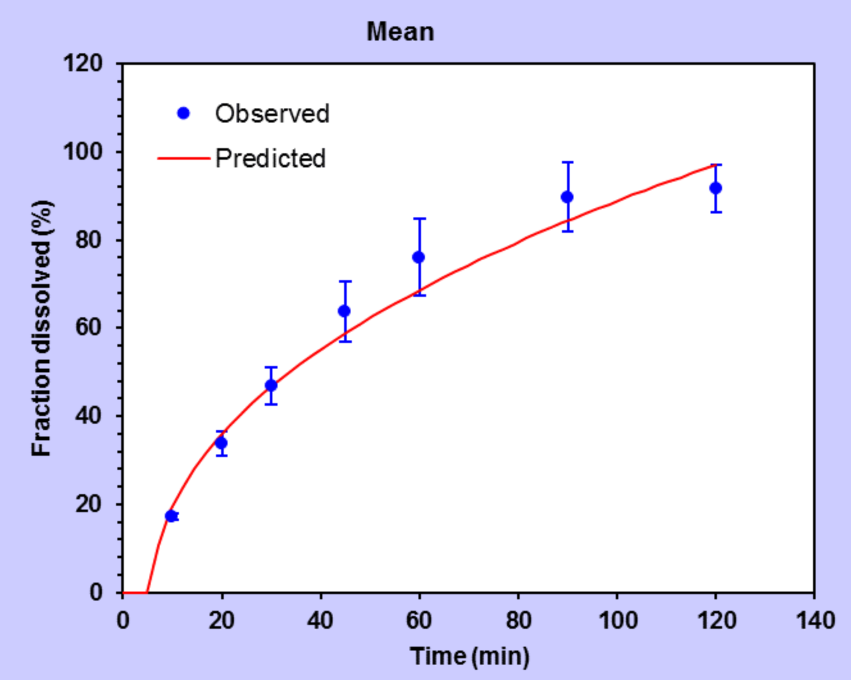

Supplementary materials – Model fitting summary – Polyglykol® 4000 P (modelling the entire relevant dissolution data)

Graphical abstract of model fit presented as the fraction % of released carvedilol per tested tablet:

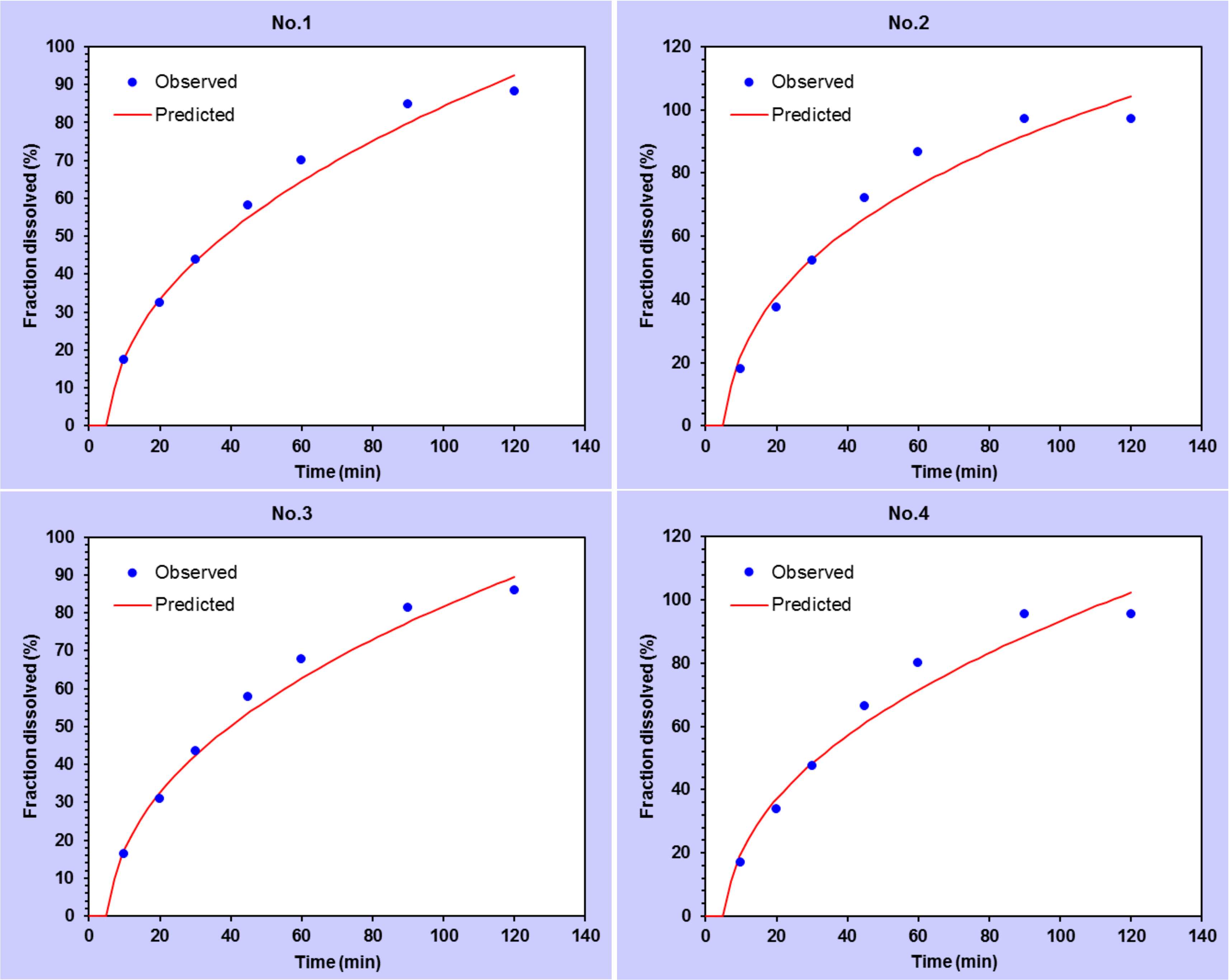

Supplementary materials – Model fitting summary – Polyglykol® 4000 P (modelling the entire relevant dissolution data)

Model: **Quadratic**

Model equation: $F = 100 \cdot (k_1 \cdot t^2 + k_2 \cdot t)$

Fitted model parameters per tested tablet (N = 4) with statistics – mean, standard deviation (SD), and relative standard deviation expressed in % (RSD%) (output from DDSolver):

Parameter	No.1	No.2	No.3	No.4	Mean	SD	RSD(%)
k_1	0.000	0.000	0.000	0.000	0.000	0.000	-15.304
k_2	0.017	0.020	0.016	0.019	0.018	0.002	11.336

Number of dissolution data points (N), degrees of freedom (df), and selected goodness of fit criteria – Pearson correlation coefficient (R), coefficient of determination (R^2), adjusted coefficient of determination ($R^2_{adjusted}$), and residual sum of squares (RSS) (manual calculation in MS Excel):

Parameter	No.1	No.2	No.3	No.4
N	7	7	7	7
df	5	5	5	5
R	0.99919823	0.99865152	0.99846792	0.9999133
R^2	0.99839709	0.99730487	0.99693819	0.99982661
$R^2_{adjusted}$	0.99807651	0.99676584	0.99632583	0.99979193
RSS	15.58501334	15.46207405	19.17008147	1.286434083

Graphical abstract of model fit presented as mean ± 1 SD of the fraction % of released carvedilol:

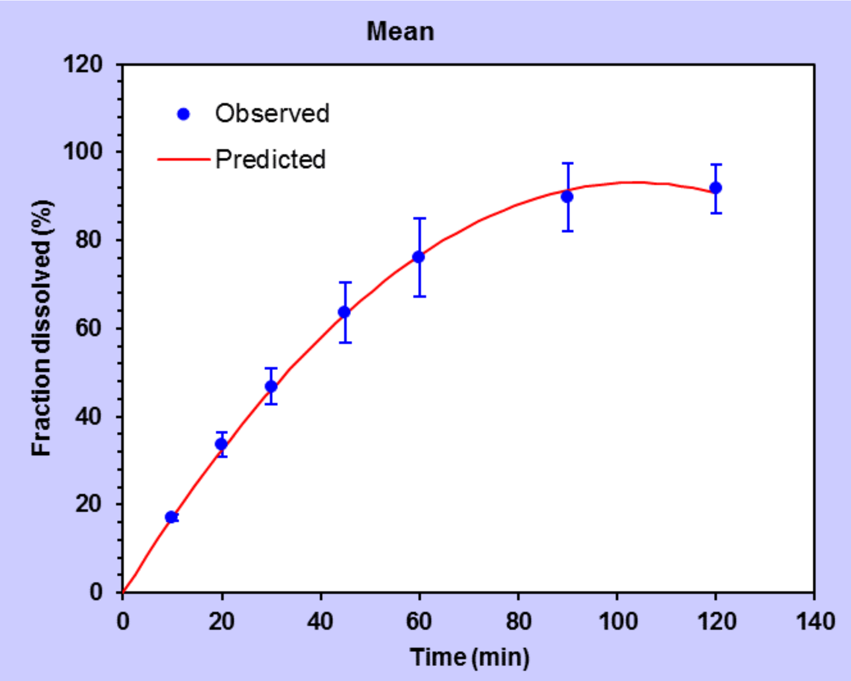

Supplementary materials – Model fitting summary – Polyglykol® 4000 P (modelling the entire relevant dissolution data)

Graphical abstract of model fit presented as the fraction % of released carvedilol per tested tablet:

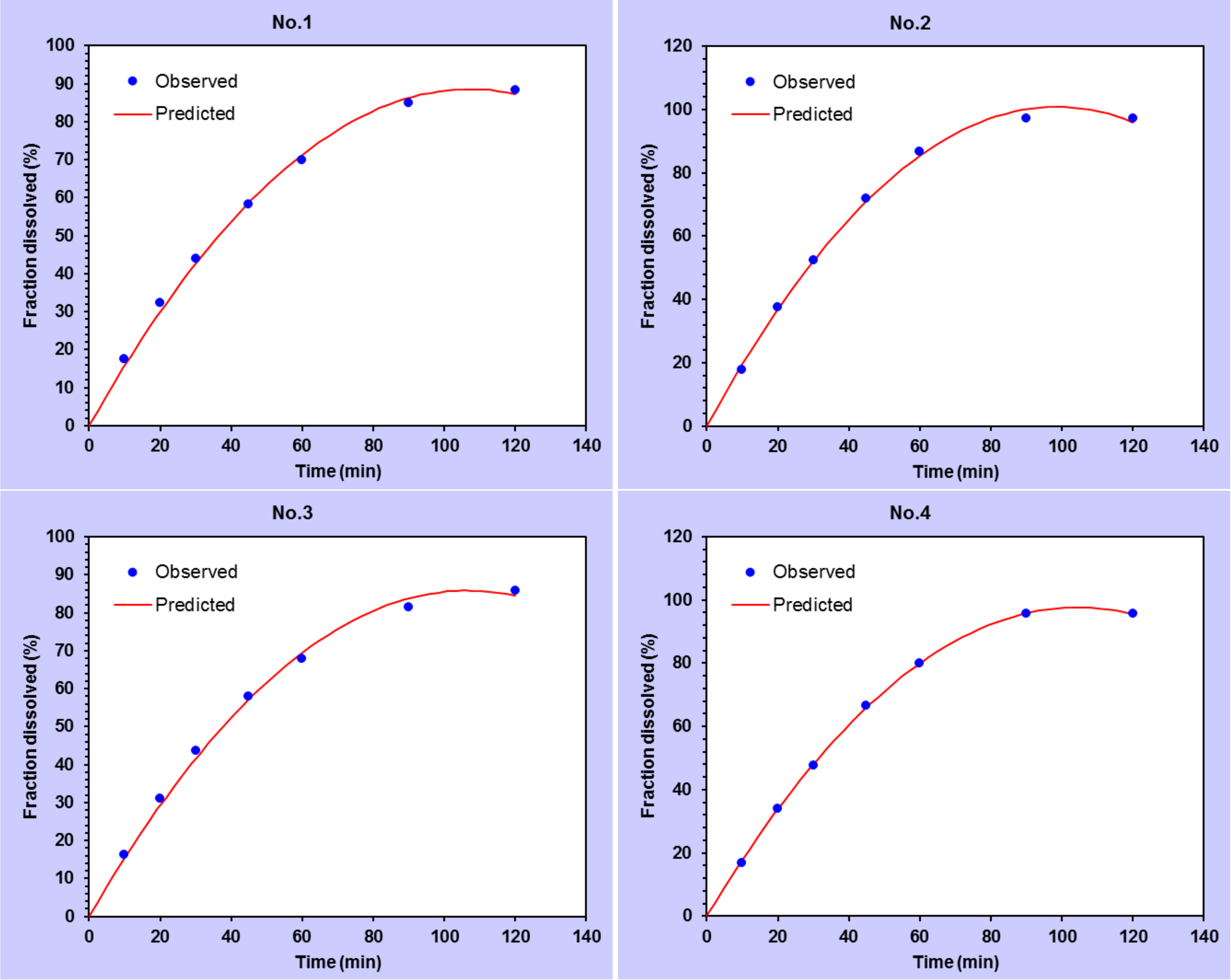

Supplementary materials – Model fitting summary – Polyglykol® 4000 P (modelling the entire relevant dissolution data)

Model: **Quadratic with T_{lag}**

Model equation: $F = 100 \cdot \left[k_1 \cdot \left(t - T_{lag}\right)^2 + k_2 \cdot \left(t - T_{lag}\right)\right]$

Fitted model parameters per tested tablet (N = 4) with statistics – mean, standard deviation (SD), and relative standard deviation expressed in % (RSD%) (output from DDSolver):

Parameter	No.1	No.2	No.3	No.4	Mean	SD	RSD(%)
k_1	0.000	0.000	0.000	0.000	0.000	0.000	-14.927
k_2	0.018	0.023	0.018	0.021	0.020	0.002	11.371
T_{lag}	4.000	4.000	4.000	4.000	4.000	0.000	0.000

Number of dissolution data points (N), degrees of freedom (df), and selected goodness of fit criteria – Pearson correlation coefficient (R), coefficient of determination (R^2), adjusted coefficient of determination ($R^2_{adjusted}$), and residual sum of squares (RSS) (manual calculation in MS Excel):

Parameter	No.1	No.2	No.3	No.4
N	7	7	7	7
df	4	4	4	4
R	0.9966888	0.99761179	0.99609086	0.99897711
R^2	0.99338856	0.99522929	0.99219699	0.99795526
$R^2_{adjusted}$	0.99008284	0.99284393	0.98829549	0.99693289
RSS	109.2627254	80.97919471	103.8705594	48.68910285

Graphical abstract of model fit presented as mean ± 1 SD of the fraction % of released carvedilol:

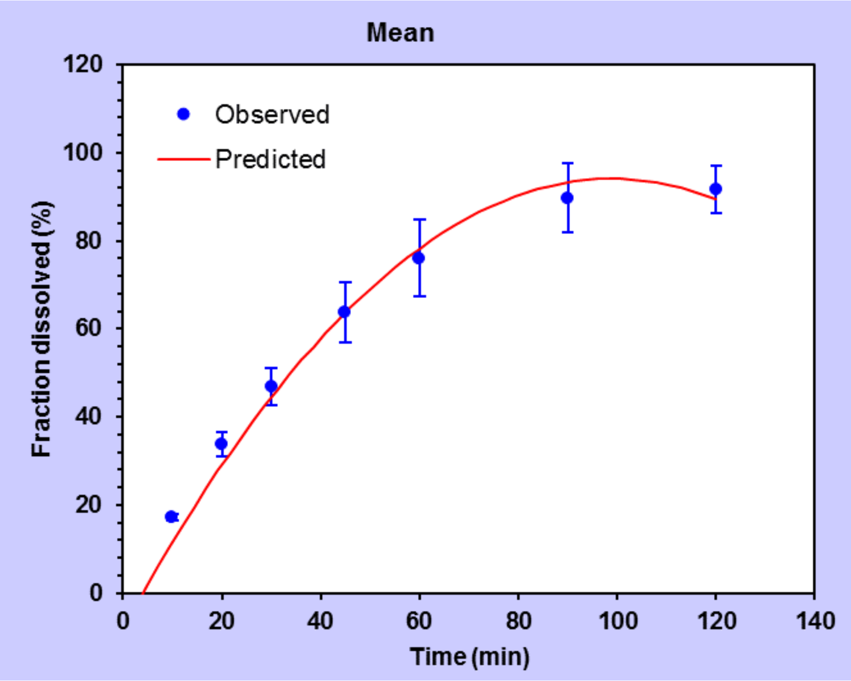

Supplementary materials – Model fitting summary – Polyglykol® 4000 P (modelling the entire relevant dissolution data)

Graphical abstract of model fit presented as the fraction % of released carvedilol per tested tablet:

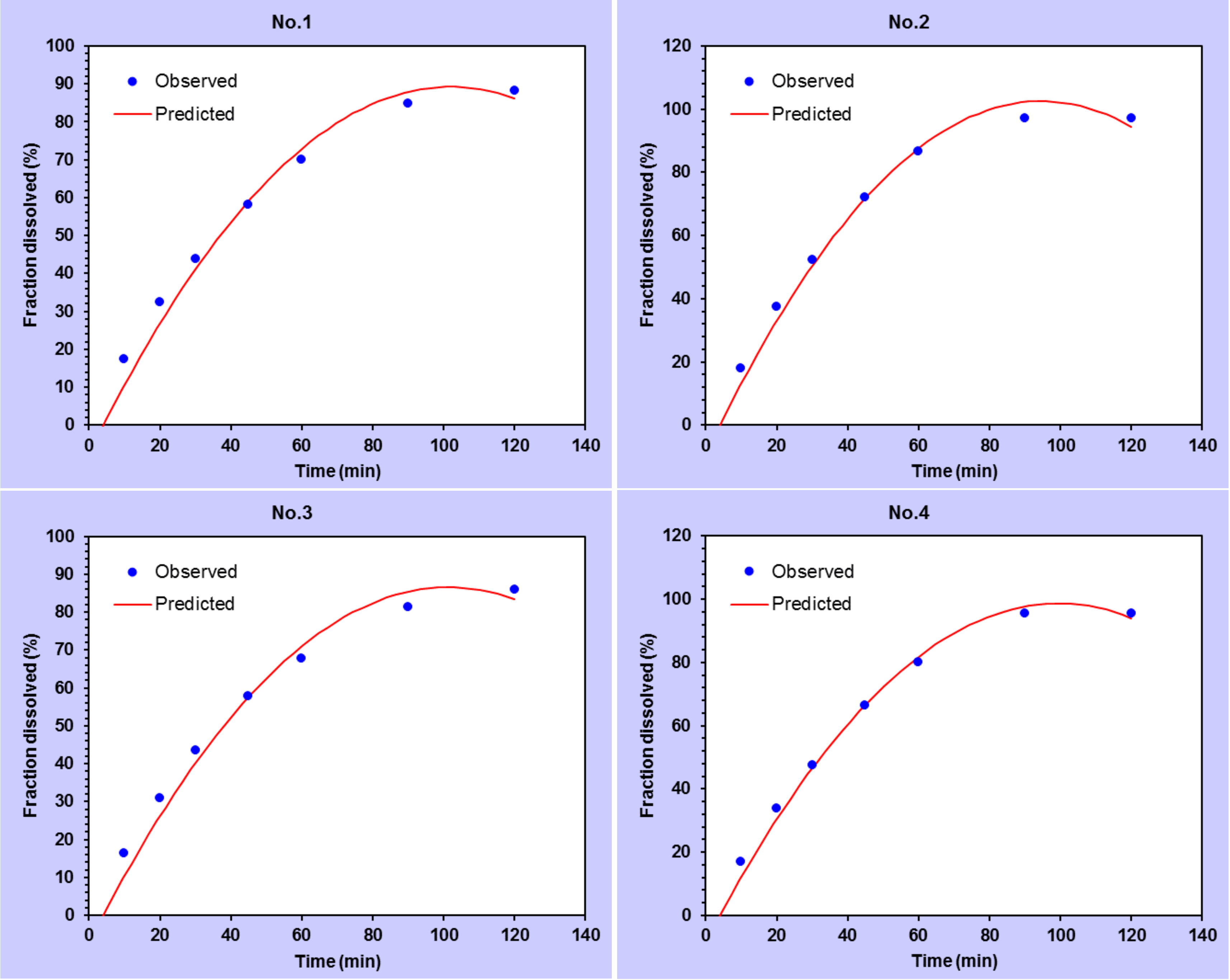

Supplementary materials – Model fitting summary – Polyglykol® 4000 P (modelling the entire relevant dissolution data)

Model: **Weibull_1**

Model equation: $F = 100 \cdot \left[1 - e^{-\frac{(t-T_i)^{\beta}}{\alpha}}\right]$

Fitted model parameters per tested tablet (N = 4) with statistics – mean, standard deviation (SD), and relative standard deviation expressed in % (RSD%) (output from DDSolver):

Parameter	No.1	No.2	No.3	No.4	Mean	SD	RSD(%)
α	25.433	36.213	25.956	37.483	31.271	6.464	20.670
β	0.847	1.047	0.835	1.018	0.937	0.111	11.881
T_i	4.000	4.000	4.000	4.000	4.000	0.000	0.000

Number of dissolution data points (N), degrees of freedom (df), and selected goodness of fit criteria – Pearson correlation coefficient (R), coefficient of determination (R^2), adjusted coefficient of determination ($R^2_{adjusted}$), and residual sum of squares (RSS) (manual calculation in MS Excel):

Parameter	No.1	No.2	No.3	No.4
N	7	7	7	7
df	4	4	4	4
R	0.99772176	0.99669611	0.99911315	0.99601876
R^2	0.9954487	0.99340313	0.99822708	0.99205337
$R^2_{adjusted}$	0.99317305	0.99010469	0.99734062	0.98808006
RSS	20.87473466	40.91422603	7.487739992	48.72985024

Graphical abstract of model fit presented as mean ± 1 SD of the fraction % of released carvedilol:

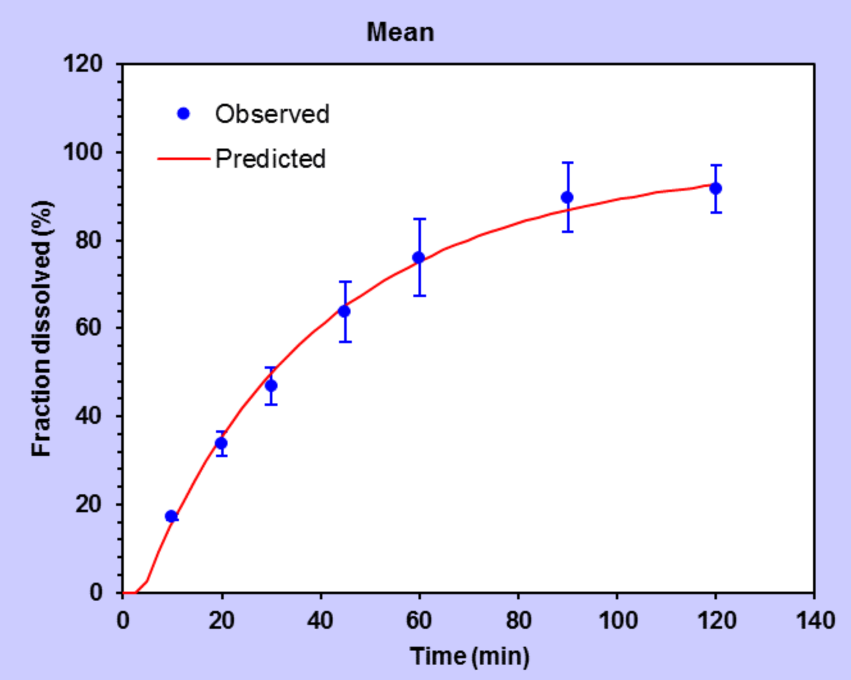

Supplementary materials – Model fitting summary – Polyglykol® 4000 P (modelling the entire relevant dissolution data)

Graphical abstract of model fit presented as the fraction % of released carvedilol per tested tablet:

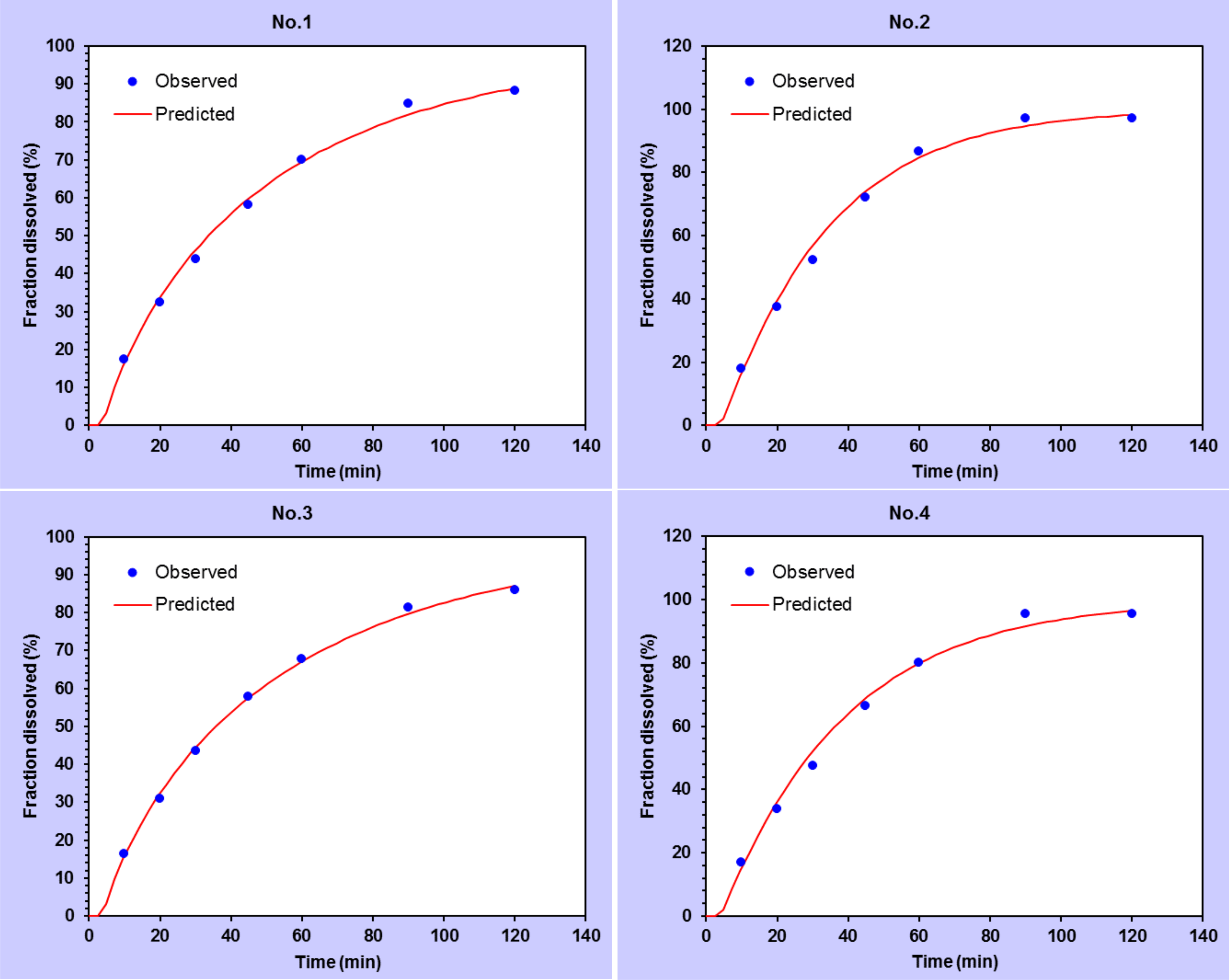

Supplementary materials – Model fitting summary – Polyglykol® 4000 P (modelling the entire relevant dissolution data)

Model: **Weibull_2**

Model equation: $F = 100 \cdot \left(1 - e^{-\frac{t^{\beta}}{\alpha}}\right)$

Fitted model parameters per tested tablet (N = 4) with statistics – mean, standard deviation (SD), and relative standard deviation expressed in % (RSD%) (output from DDSolver):

Parameter	No.1	No.2	No.3	No.4	Mean	SD	RSD(%)
α	51.333	86.219	51.475	87.621	69.162	20.513	29.660
β	1.000	1.237	0.984	1.203	1.106	0.132	11.965

Number of dissolution data points (N), degrees of freedom (df), and selected goodness of fit criteria – Pearson correlation coefficient (R), coefficient of determination (R^2), adjusted coefficient of determination ($R^2_{adjusted}$), and residual sum of squares (RSS) (manual calculation in MS Excel):

Parameter	No.1	No.2	No.3	No.4
N	7	7	7	7
df	5	5	5	5
R	0.9987963	0.99870627	0.998315	0.99841655
R^2	0.99759405	0.99741421	0.99663283	0.99683561
$R^2_{adjusted}$	0.99711286	0.99689705	0.9959594	0.99620273
RSS	10.40982892	16.48034333	14.21956937	18.60066066

Graphical abstract of model fit presented as mean ± 1 SD of the fraction % of released carvedilol:

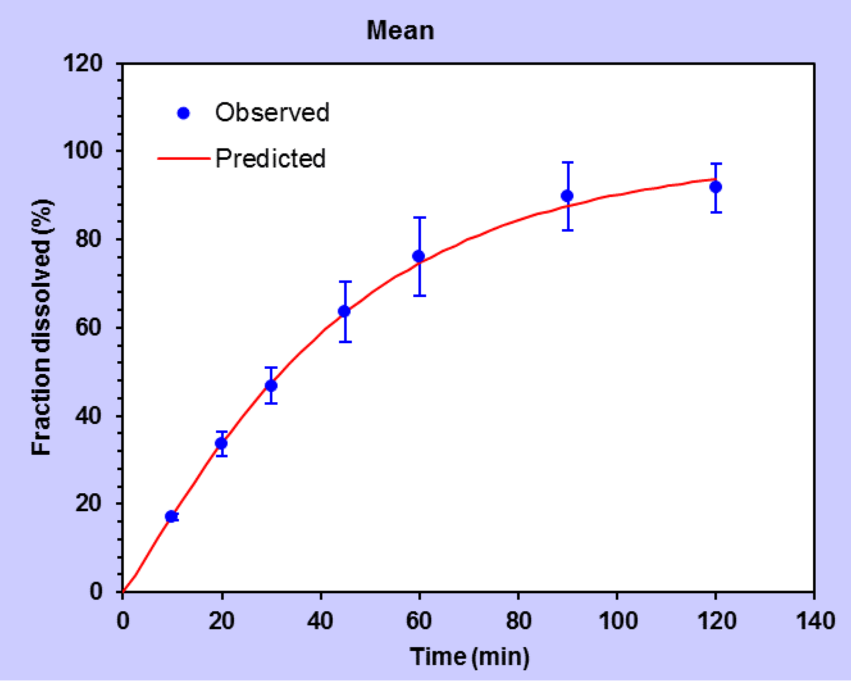

Supplementary materials – Model fitting summary – Polyglykol® 4000 P (modelling the entire relevant dissolution data)

Graphical abstract of model fit presented as the fraction % of released carvedilol per tested tablet:

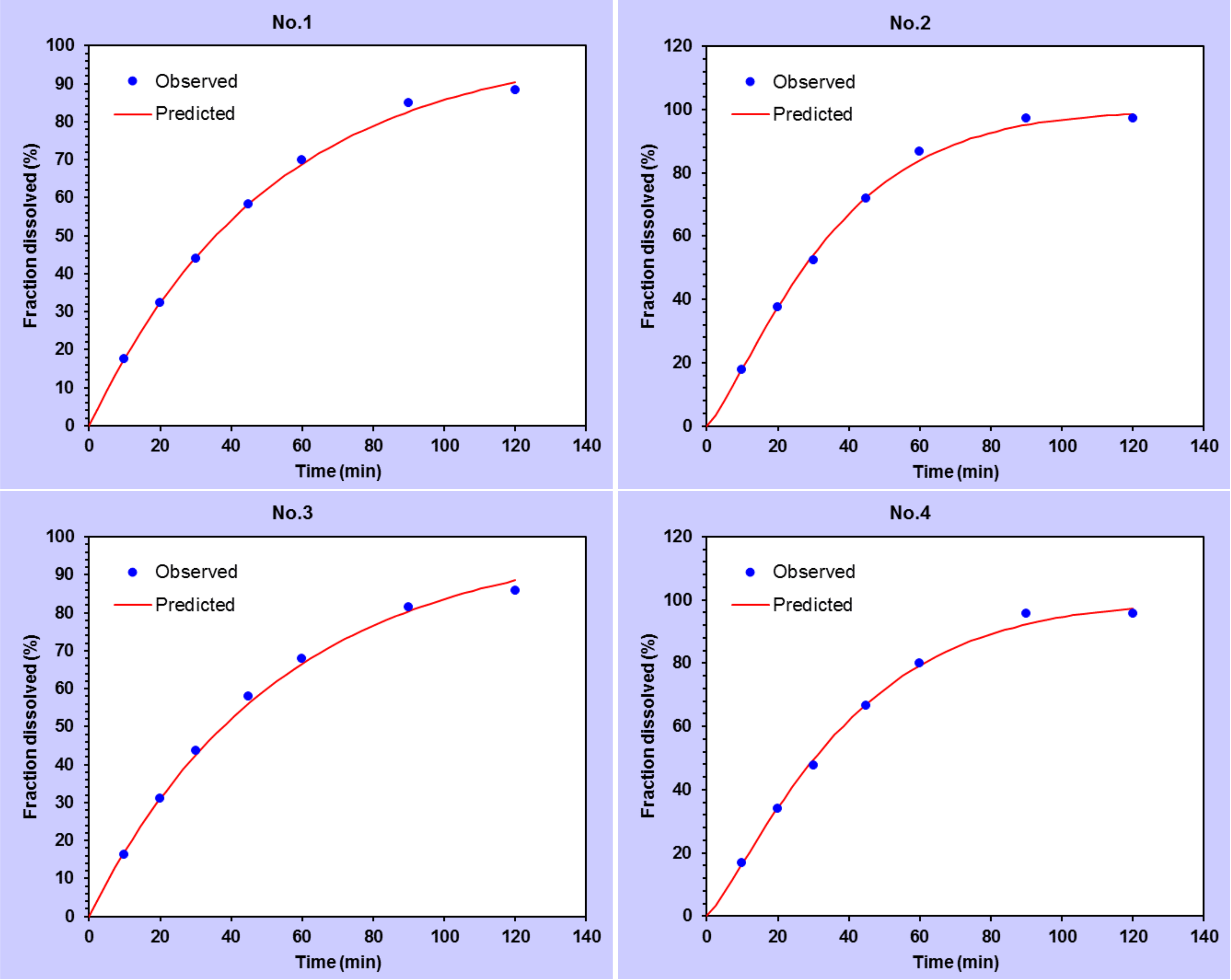

Supplementary materials – Model fitting summary – Polyglykol® 4000 P (modelling the entire relevant dissolution data)

Model: **Weibull_3**

Model equation: $F = F_{max} \cdot \left(1 - e^{-\frac{t^{\beta}}{\alpha}}\right)$

Fitted model parameters per tested tablet (N = 4) with statistics – mean, standard deviation (SD), and relative standard deviation expressed in % (RSD%) (output from DDSolver):

Parameter	No.1	No.2	No.3	No.4	Mean	SD	RSD(%)
α	62.978	74.374	64.874	85.659	71.971	10.398	14.448
β	1.100	1.203	1.106	1.194	1.151	0.055	4.803
F_{max}	92.772	99.466	90.238	100.380	95.714	4.983	5.206

Number of dissolution data points (N), degrees of freedom (df), and selected goodness of fit criteria – Pearson correlation coefficient (R), coefficient of determination (R^2), adjusted coefficient of determination ($R^2_{adjusted}$), and residual sum of squares (RSS) (manual calculation in MS Excel):

Parameter	No.1	No.2	No.3	No.4
N	7	7	7	7
df	4	4	4	4
R	0.99877655	0.99854124	0.99982603	0.99838016
R^2	0.99755459	0.9970846	0.99965209	0.99676295
$R^2_{adjusted}$	0.99633188	0.9956269	0.99947813	0.99514443
RSS	10.91963598	25.97322134	1.437828185	19.0380459

Graphical abstract of model fit presented as mean ± 1 SD of the fraction % of released carvedilol:

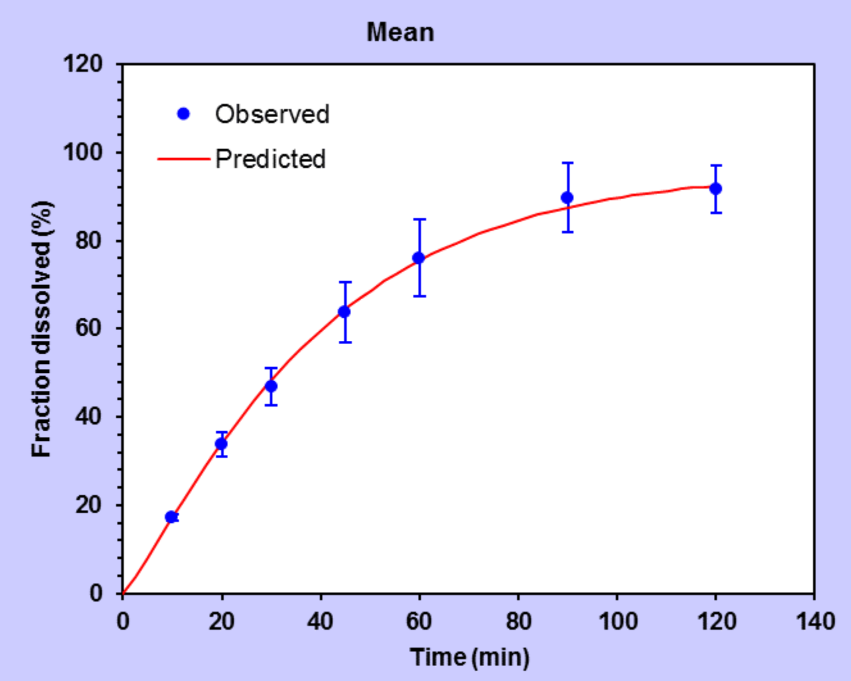

Supplementary materials – Model fitting summary – Polyglykol® 4000 P (modelling the entire relevant dissolution data)

Graphical abstract of model fit presented as the fraction % of released carvedilol per tested tablet:

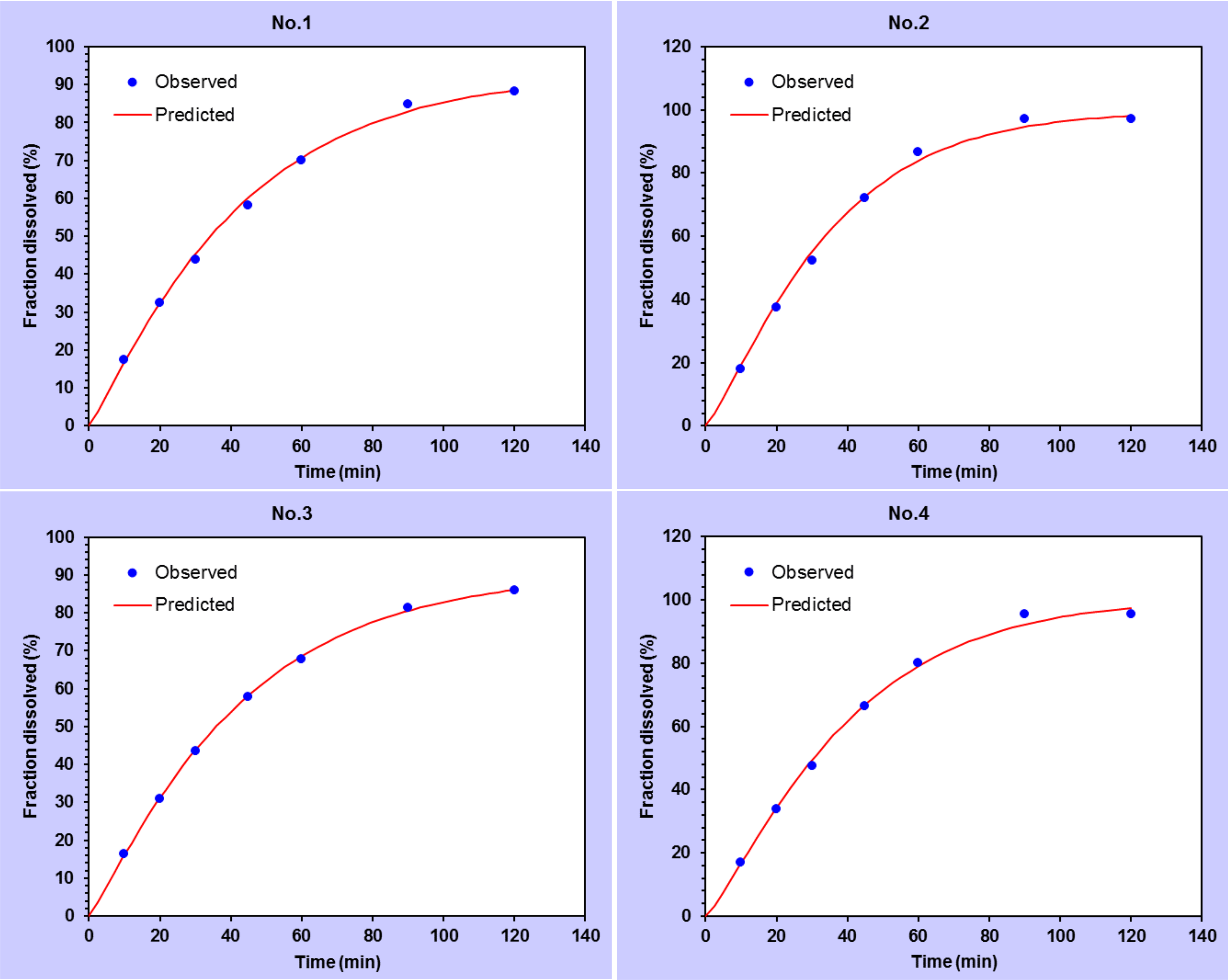

Supplementary materials – Model fitting summary – Polyglykol® 4000 P (modelling the entire relevant dissolution data)

Model: **Weibull_4**

Model equation: $F = F_{max} \cdot \left[1 - e^{-\frac{(t-T_i)^\beta}{\alpha}}\right]$

Fitted model parameters per tested tablet (N = 4) with statistics – mean, standard deviation (SD), and relative standard deviation expressed in % (RSD%) (output from DDSolver):

Parameter	No.1	No.2	No.3	No.4	Mean	SD	RSD(%)
α	31.528	32.620	29.694	36.918	32.690	3.067	9.381
β	0.920	0.997	0.935	1.010	0.965	0.045	4.638
T_i	4.681	4.000	4.000	4.000	4.170	0.341	8.169
F_{max}	99.611	102.081	90.238	100.380	98.077	5.327	5.432

Number of dissolution data points (N), degrees of freedom (df), and selected goodness of fit criteria – Pearson correlation coefficient (R), coefficient of determination (R^2), adjusted coefficient of determination ($R^2_{adjusted}$), and residual sum of squares (RSS) (manual calculation in MS Excel):

Parameter	No.1	No.2	No.3	No.4
N	7	7	7	7
df	3	3	3	3
R	0.99773225	0.99696353	0.998026	0.99615509
R^2	0.99546963	0.99393628	0.9960559	0.99232496
$R^2_{adjusted}$	0.99093927	0.98787256	0.99211181	0.98464993
RSS	40.15541614	37.14725314	17.70268894	46.87255752

Graphical abstract of model fit presented as mean ± 1 SD of the fraction % of released carvedilol:

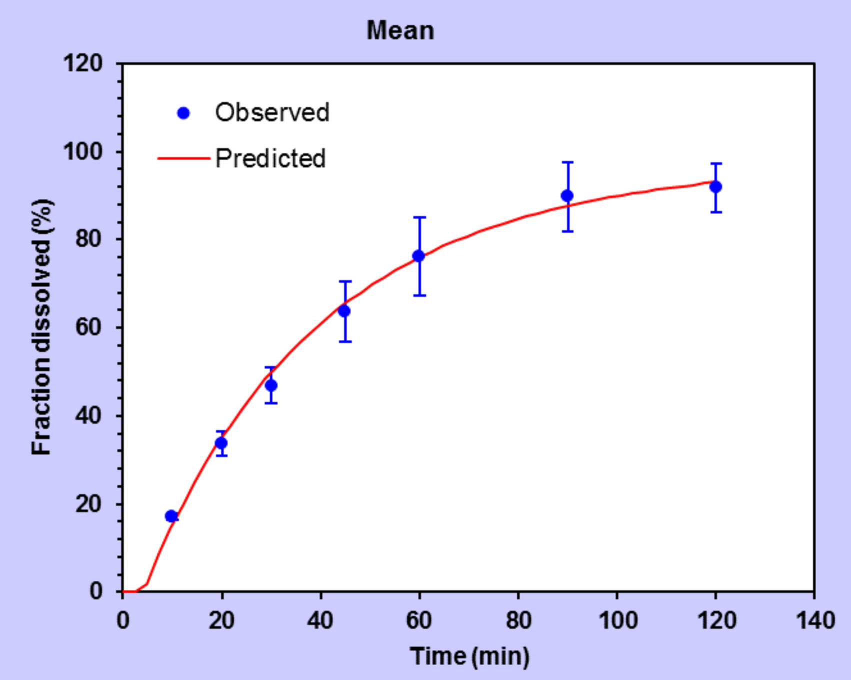

Supplementary materials – Model fitting summary – Polyglykol® 4000 P (modelling the entire relevant dissolution data)

Graphical abstract of model fit presented as the fraction % of released carvedilol per tested tablet:

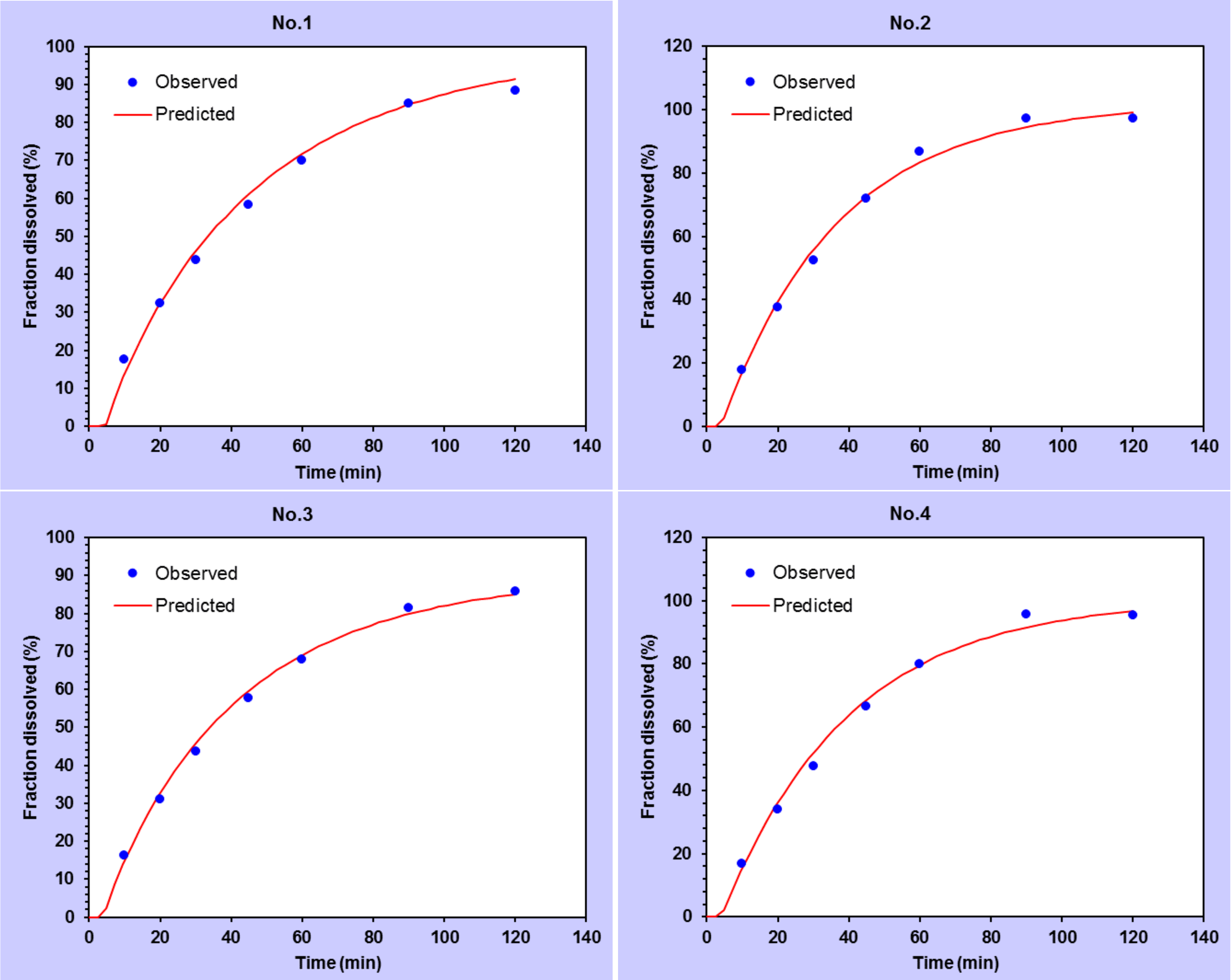

Supplementary materials – Model fitting summary – Polyglykol® 4000 P (modelling the entire relevant dissolution data)

Model: **Logistic_1**

Model equation: $F = 100 \cdot \frac{e^{\alpha+\beta\cdot\log(t)}}{1+e^{\alpha+\beta\cdot\log(t)}}$

Fitted model parameters per tested tablet (N = 4) with statistics – mean, standard deviation (SD), and relative standard deviation expressed in % (RSD%) (output from DDSolver):

Parameter	No.1	No.2	No.3	No.4	Mean	SD	RSD(%)
α	-5.146	-7.036	-4.998	-6.633	-5.953	1.033	-17.346
β	3.417	5.090	3.259	4.651	4.104	0.905	22.049

Number of dissolution data points (N), degrees of freedom (df), and selected goodness of fit criteria – Pearson correlation coefficient (R), coefficient of determination (R^2), adjusted coefficient of determination ($R^2_{adjusted}$), and residual sum of squares (RSS) (manual calculation in MS Excel):

Parameter	No.1	No.2	No.3	No.4
N	7	7	7	7
df	5	5	5	5
R	0.99476019	0.98612112	0.99845992	0.98662223
R^2	0.98954784	0.97243487	0.9969222	0.97342343
$R^2_{adjusted}$	0.9874574	0.96692184	0.99630664	0.96810811
RSS	47.5473455	192.7843784	13.15734813	179.4458697

Graphical abstract of model fit presented as mean ± 1 SD of the fraction % of released carvedilol:

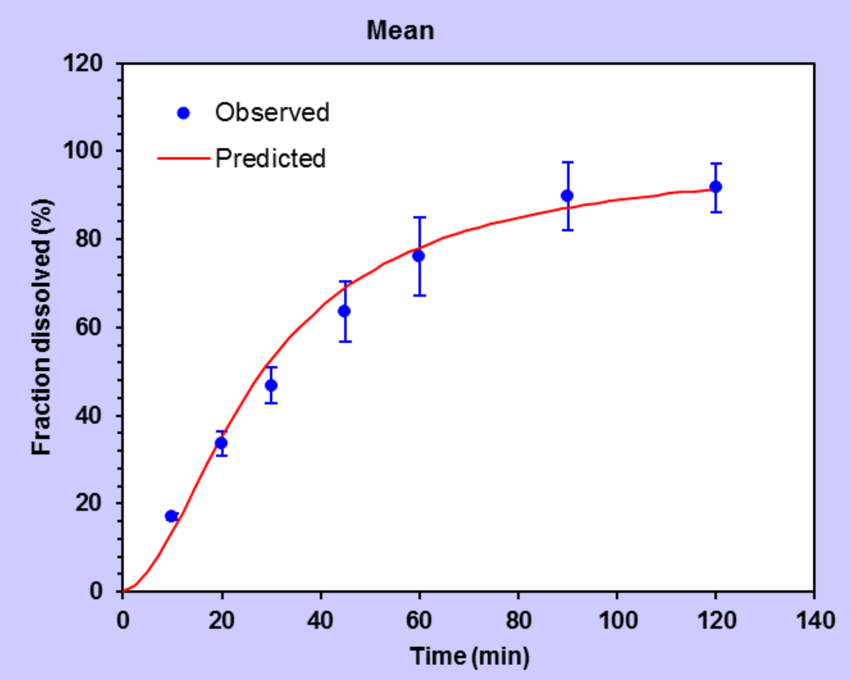

Supplementary materials – Model fitting summary – Polyglykol® 4000 P (modelling the entire relevant dissolution data)

Graphical abstract of model fit presented as the fraction % of released carvedilol per tested tablet:

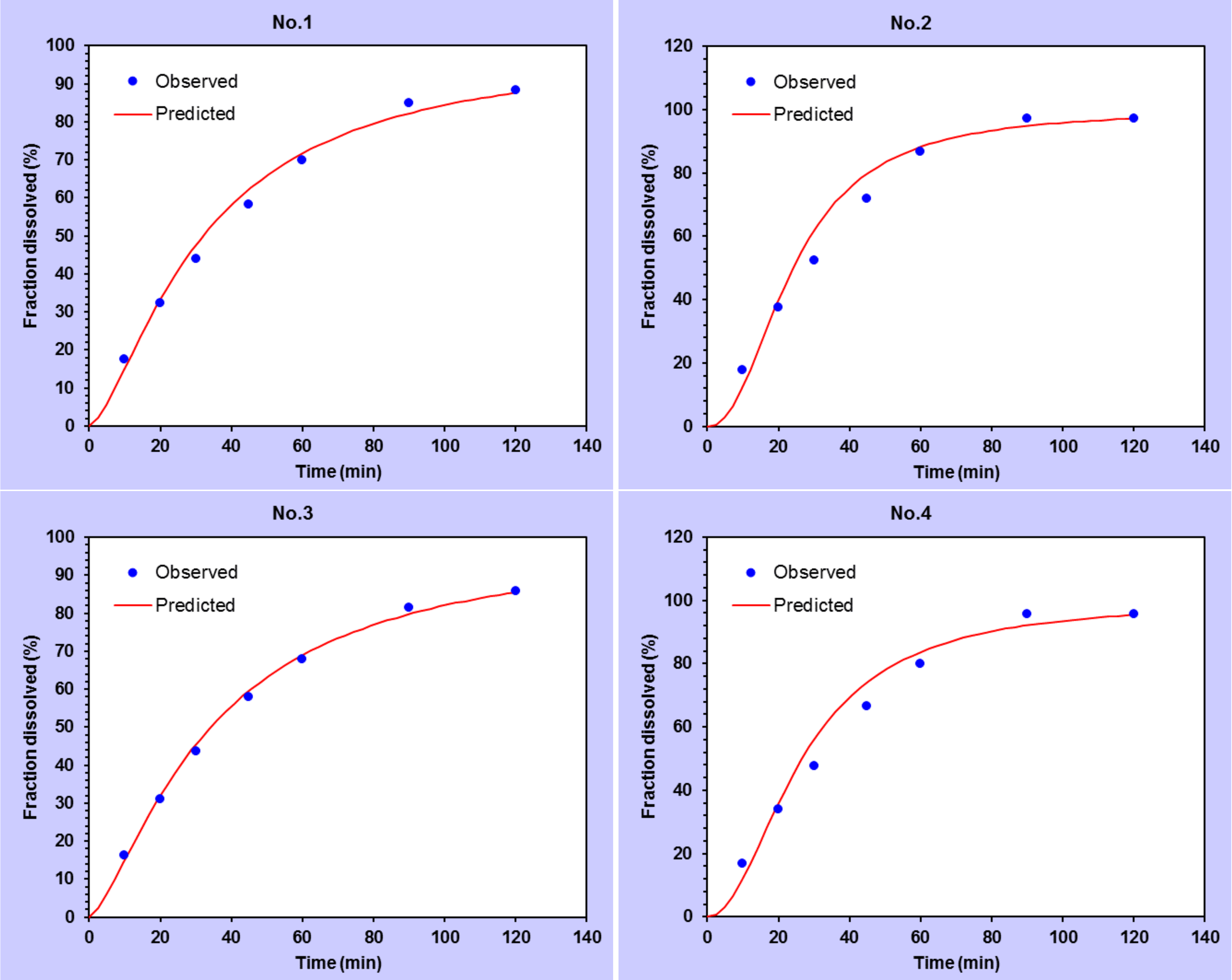

Supplementary materials – Model fitting summary – Polyglykol® 4000 P (modelling the entire relevant dissolution data)

Model: **Logistic_2**

Model equation: $F = F_{max} \cdot \frac{e^{\alpha+\beta \cdot \log(t)}}{1+e^{\alpha+\beta \cdot \log(t)}}$

Fitted model parameters per tested tablet (N = 4) with statistics – mean, standard deviation (SD), and relative standard deviation expressed in % (RSD%) (output from DDSolver):

Parameter	No.1	No.2	No.3	No.4	Mean	SD	RSD(%)
α	-5.992	-6.387	-5.973	-6.539	-6.223	0.284	-4.570
β	4.160	4.559	4.135	4.574	4.357	0.242	5.557
F_{max}	92.772	102.081	90.238	100.380	96.368	5.752	5.969

Number of dissolution data points (N), degrees of freedom (df), and selected goodness of fit criteria – Pearson correlation coefficient (R), coefficient of determination (R^2), adjusted coefficient of determination ($R^2_{adjusted}$), and residual sum of squares (RSS) (manual calculation in MS Excel):

Parameter	No.1	No.2	No.3	No.4
N	7	7	7	7
df	4	4	4	4
R	0.98599216	0.99132822	0.99068625	0.98751652
R^2	0.97218053	0.98273164	0.98145924	0.97518888
$R^2_{adjusted}$	0.9582708	0.97409746	0.97218886	0.96278331
RSS	141.9866591	111.193107	90.84512332	165.4037007

Graphical abstract of model fit presented as mean ± 1 SD of the fraction % of released carvedilol:

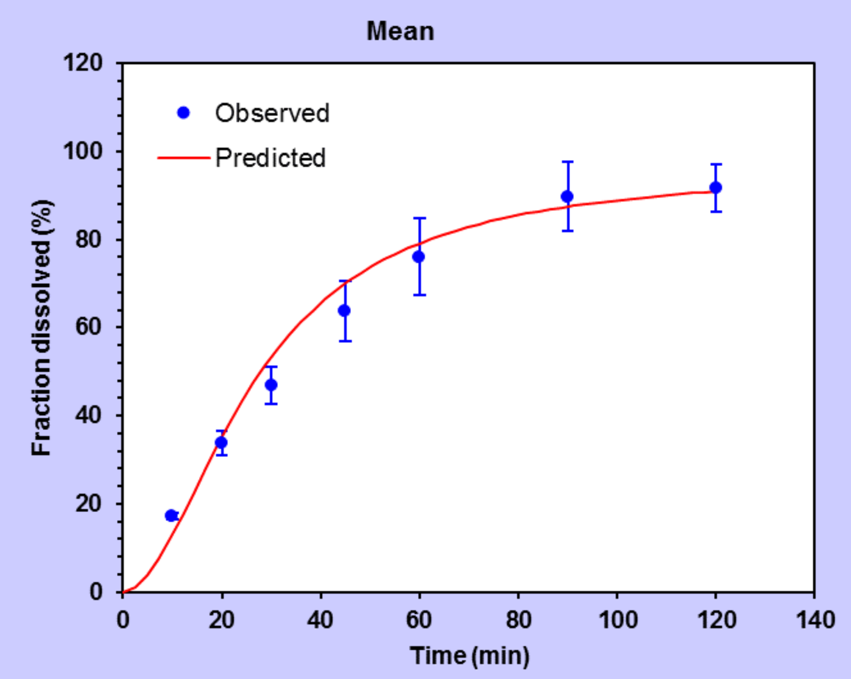

Supplementary materials – Model fitting summary – Polyglykol® 4000 P (modelling the entire relevant dissolution data)

Graphical abstract of model fit presented as the fraction % of released carvedilol per tested tablet:

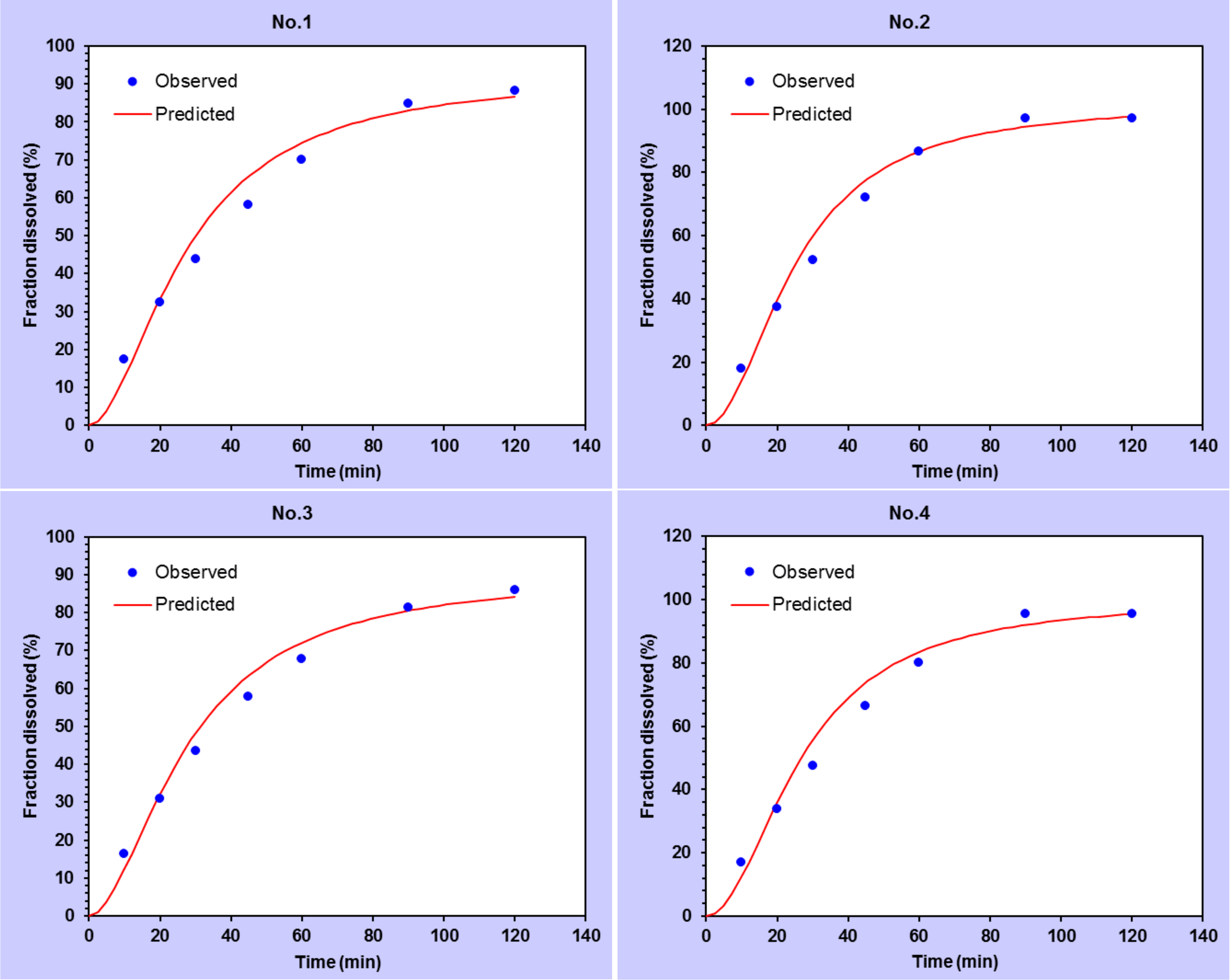

Supplementary materials – Model fitting summary – Polyglykol® 4000 P (modelling the entire relevant dissolution data)

Model: **Logistic_3**

Model equation: $F = F_{max} \cdot \frac{1}{1+e^{-k\cdot(t-\gamma)}}$

Fitted model parameters per tested tablet (N = 4) with statistics – mean, standard deviation (SD), and relative standard deviation expressed in % (RSD%) (output from DDSolver):

Parameter	No.1	No.2	No.3	No.4	Mean	SD	RSD(%)
k	0.040	0.074	0.039	0.064	0.054	0.018	32.251
γ	36.228	29.108	36.598	34.744	34.170	3.468	10.149
F_{max}	92.772	94.992	90.238	100.380	94.595	4.318	4.565

Number of dissolution data points (N), degrees of freedom (df), and selected goodness of fit criteria – Pearson correlation coefficient (R), coefficient of determination (R^2), adjusted coefficient of determination ($R^2_{adjusted}$), and residual sum of squares (RSS) (manual calculation in MS Excel):

Parameter	No.1	No.2	No.3	No.4
N	7	7	7	7
df	4	4	4	4
R	0.9915285	0.99730034	0.98777098	0.99616227
R^2	0.98312878	0.99460796	0.9756915	0.99233927
$R^2_{adjusted}$	0.97469316	0.99191194	0.96353726	0.9885089
RSS	84.84792023	57.14625719	111.1307864	99.97515369

Graphical abstract of model fit presented as mean ± 1 SD of the fraction % of released carvedilol:

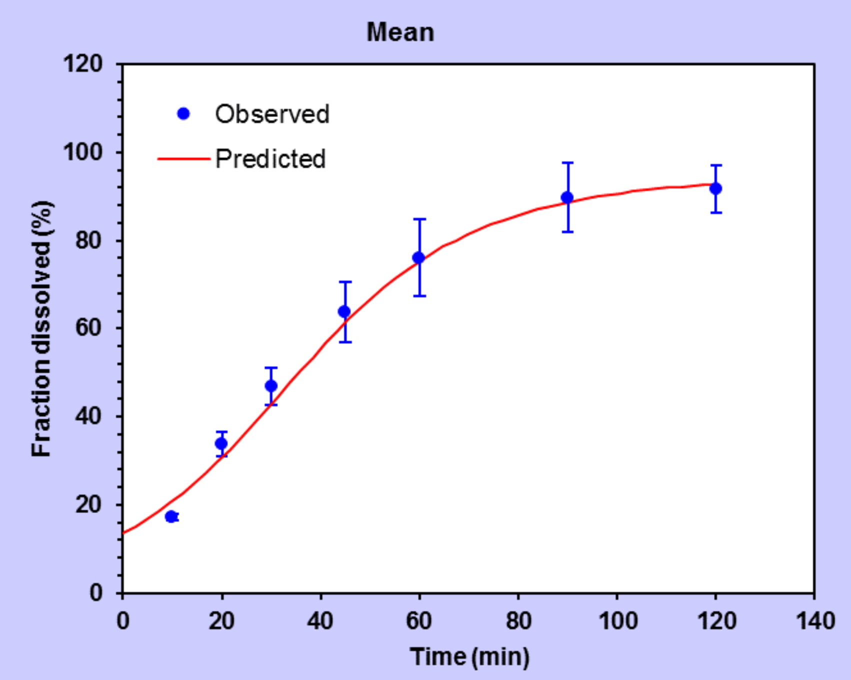

Supplementary materials – Model fitting summary – Polyglykol® 4000 P (modelling the entire relevant dissolution data)

Graphical abstract of model fit presented as the fraction % of released carvedilol per tested tablet:

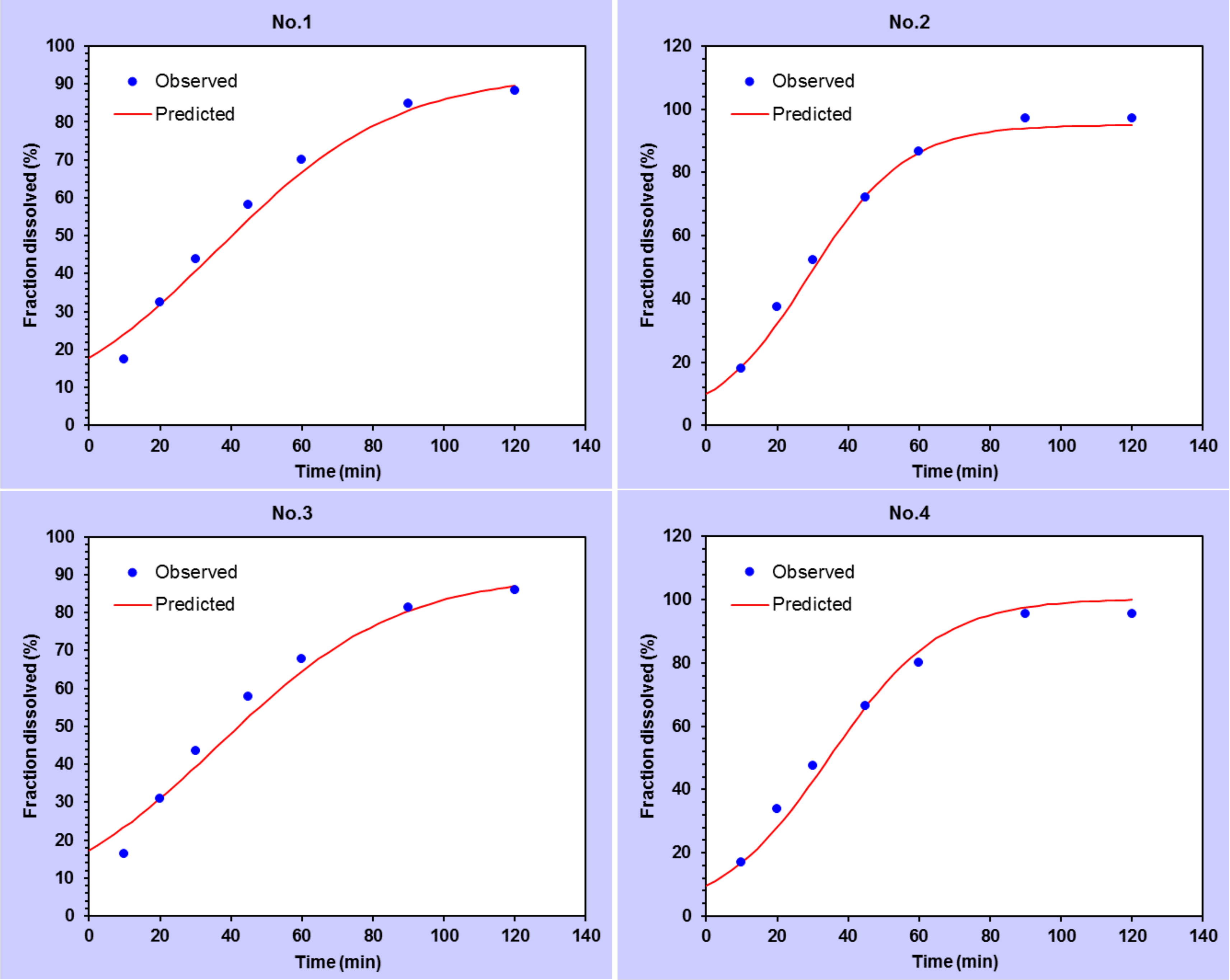

Supplementary materials – Model fitting summary – Polyglykol® 4000 P (modelling the entire relevant dissolution data)

Model: **Gompertz_1**

Model equation: $F = 100 \cdot e^{-\alpha \cdot e^{-\beta \cdot \log(t)}}$

Fitted model parameters per tested tablet (N = 4) with statistics – mean, standard deviation (SD), and relative standard deviation expressed in % (RSD%) (output from DDSolver):

Parameter	No.1	No.2	No.3	No.4	Mean	SD	RSD(%)
α	28.927	296.956	23.635	180.847	132.591	131.608	99.259
β	2.545	4.190	2.365	3.716	3.204	0.889	27.755

Number of dissolution data points (N), degrees of freedom (df), and selected goodness of fit criteria – Pearson correlation coefficient (R), coefficient of determination (R^2), adjusted coefficient of determination ($R^2_{adjusted}$), and residual sum of squares (RSS) (manual calculation in MS Excel):

Parameter	No.1	No.2	No.3	No.4
N	7	7	7	7
df	5	5	5	5
R	0.98115892	0.98861424	0.98883257	0.98915292
R^2	0.96267283	0.97735812	0.97778984	0.97842349
$R^2_{adjusted}$	0.9552074	0.97282974	0.97334781	0.97410819
RSS	176.5839914	419.7744585	96.50681507	421.7050307

Graphical abstract of model fit presented as mean ± 1 SD of the fraction % of released carvedilol:

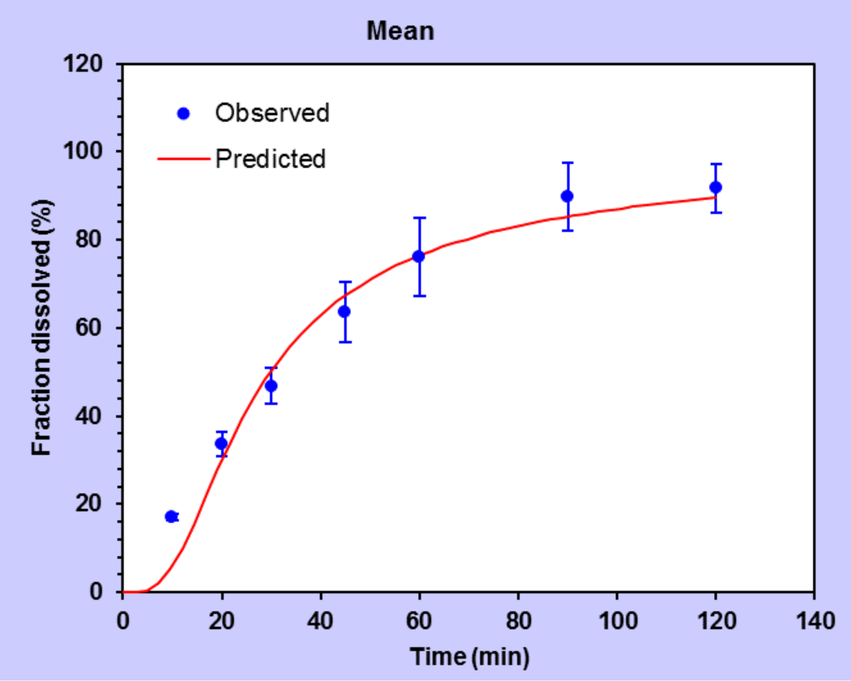

Supplementary materials – Model fitting summary – Polyglykol® 4000 P (modelling the entire relevant dissolution data)

Graphical abstract of model fit presented as the fraction % of released carvedilol per tested tablet:

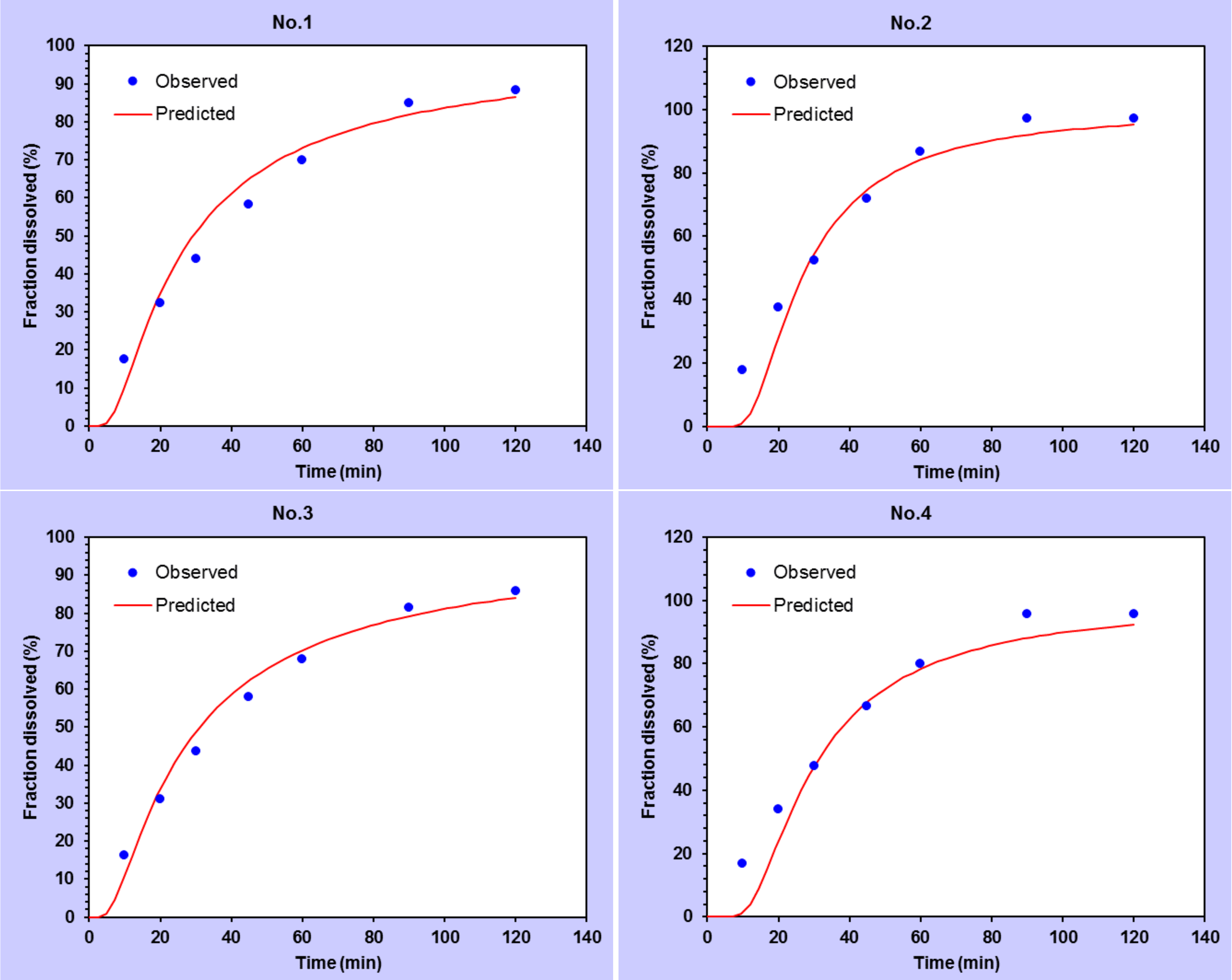

Supplementary materials – Model fitting summary – Polyglykol® 4000 P (modelling the entire relevant dissolution data)

Model: **Gompertz_2**

Model equation: $F = F_{max} \cdot e^{-\alpha \cdot e^{-\beta \cdot \log(t)}}$

Fitted model parameters per tested tablet (N = 4) with statistics – mean, standard deviation (SD), and relative standard deviation expressed in % (RSD%) (output from DDSolver):

Parameter	No.1	No.2	No.3	No.4	Mean	SD	RSD(%)
α	71.303	101.830	67.728	164.223	101.271	44.671	44.110
β	3.297	3.656	3.253	3.638	3.461	0.216	6.228
F_{max}	92.772	102.081	90.238	100.380	96.368	5.752	5.969

Number of dissolution data points (N), degrees of freedom (df), and selected goodness of fit criteria – Pearson correlation coefficient (R), coefficient of determination (R^2), adjusted coefficient of determination ($R^2_{adjusted}$), and residual sum of squares (RSS) (manual calculation in MS Excel):

Parameter	No.1	No.2	No.3	No.4
N	7	7	7	7
df	4	4	4	4
R	0.96634948	0.97373395	0.97348006	0.98991262
R^2	0.93383132	0.9481578	0.94766343	0.97992699
$R^2_{adjusted}$	0.90074698	0.9222367	0.92149515	0.96989049
RSS	365.1952778	358.9893716	274.0220338	426.4891097

Graphical abstract of model fit presented as mean ± 1 SD of the fraction % of released carvedilol:

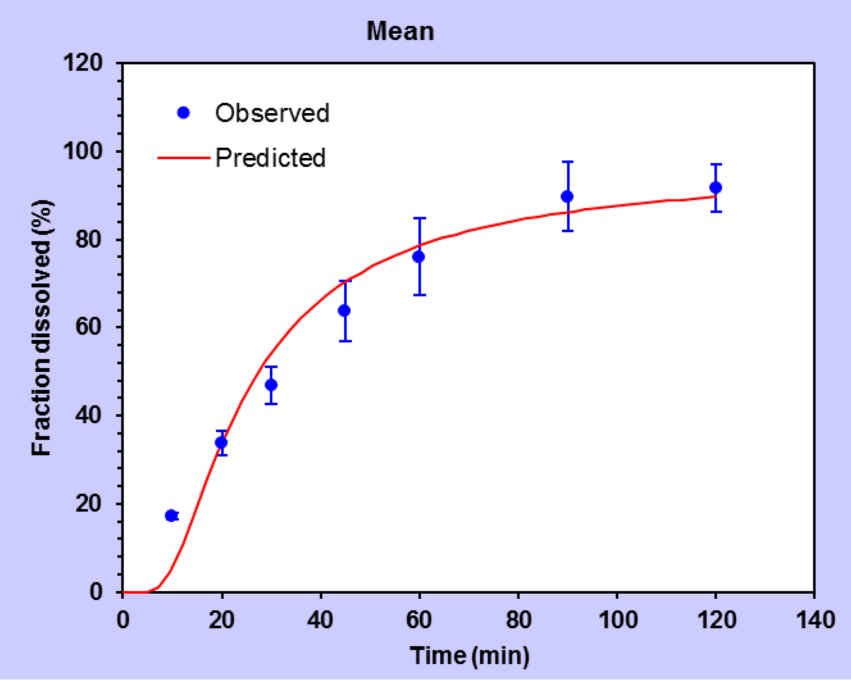

Supplementary materials – Model fitting summary – Polyglykol® 4000 P (modelling the entire relevant dissolution data)

Graphical abstract of model fit presented as the fraction % of released carvedilol per tested tablet:

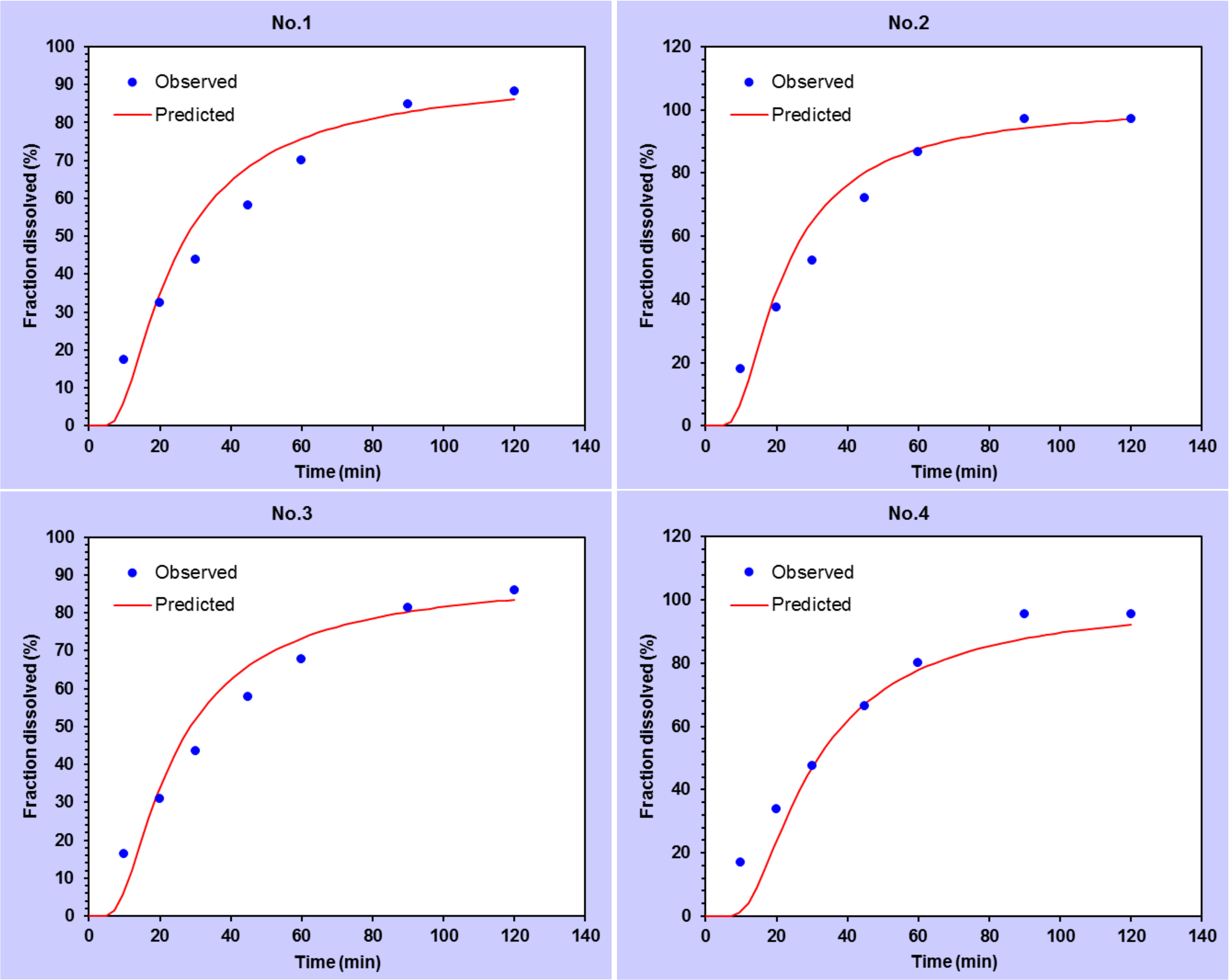

Supplementary materials – Model fitting summary – Polyglykol® 4000 P (modelling the entire relevant dissolution data)

Model: **Gompertz_3**

Model equation: $F = F_{max} \cdot e^{-e^{-k\cdot(t-\gamma)}}$

Fitted model parameters per tested tablet (N = 4) with statistics – mean, standard deviation (SD), and relative standard deviation expressed in % (RSD%) (output from DDSolver):

Parameter	No.1	No.2	No.3	No.4	Mean	SD	RSD(%)
k	0.033	0.035	0.039	0.046	0.038	0.006	15.586
γ	22.021	17.582	22.837	22.206	21.162	2.412	11.398
F_{max}	92.772	102.081	87.222	99.858	95.483	6.789	7.110

Number of dissolution data points (N), degrees of freedom (df), and selected goodness of fit criteria – Pearson correlation coefficient (R), coefficient of determination (R^2), adjusted coefficient of determination ($R^2_{adjusted}$), and residual sum of squares (RSS) (manual calculation in MS Excel):

Parameter	No.1	No.2	No.3	No.4
N	7	7	7	7
df	4	4	4	4
R	0.99839484	0.99309599	0.99868149	0.99847531
R^2	0.99679226	0.98623965	0.99736472	0.99695294
$R^2_{adjusted}$	0.99518839	0.97935947	0.99604708	0.99542941
RSS	18.19661011	160.1592558	15.65173939	44.84062033

Graphical abstract of model fit presented as mean ± 1 SD of the fraction % of released carvedilol:

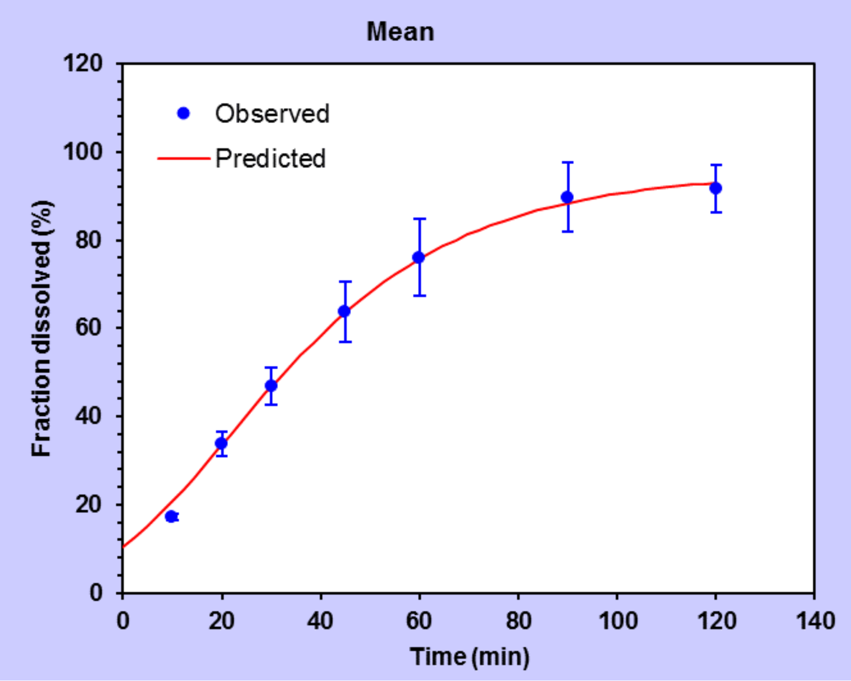

Supplementary materials – Model fitting summary – Polyglykol® 4000 P (modelling the entire relevant dissolution data)

Graphical abstract of model fit presented as the fraction % of released carvedilol per tested tablet:

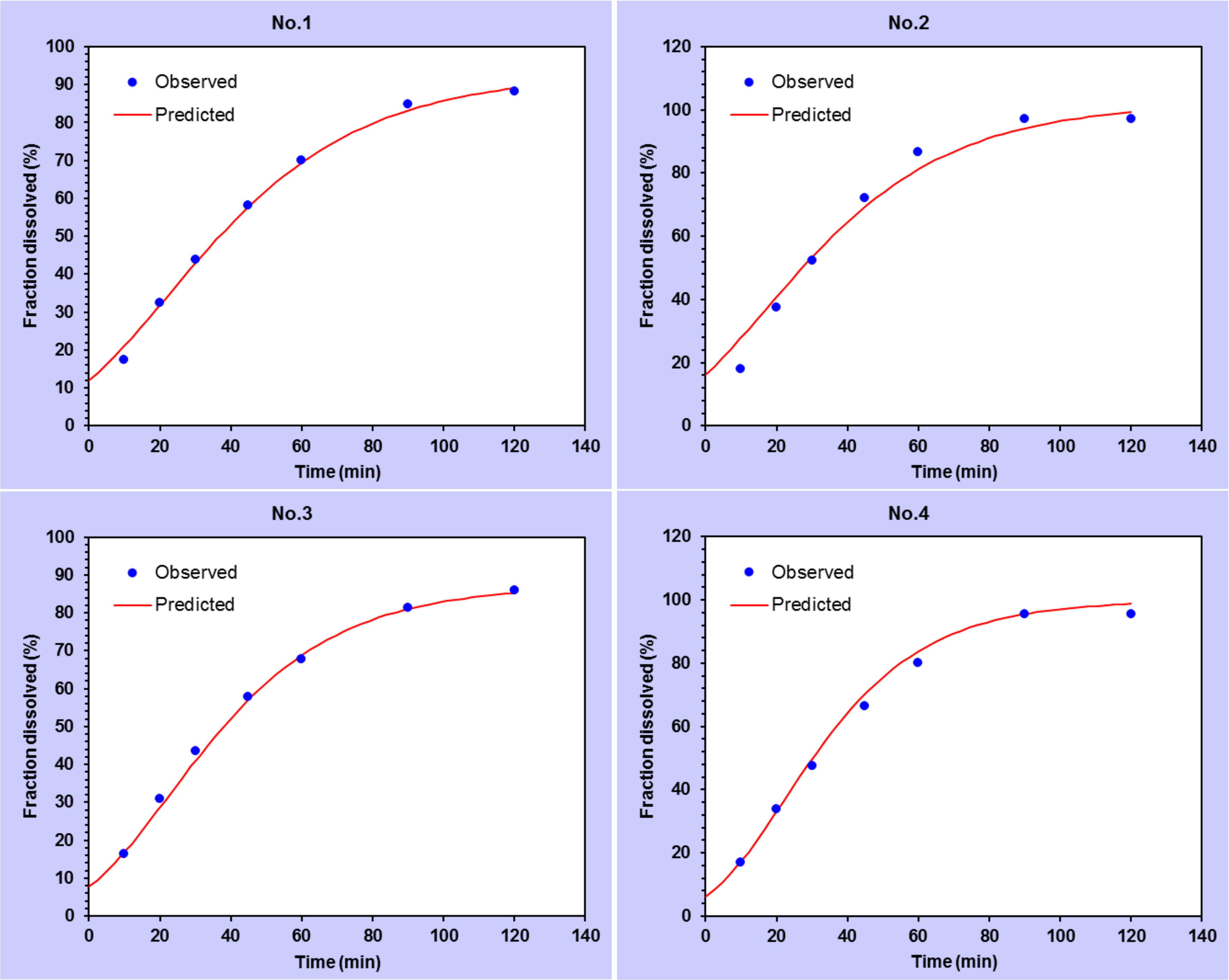

Supplementary materials – Model fitting summary – Polyglykol® 4000 P (modelling the entire relevant dissolution data)

Model: **Gompertz_4**

Model equation: $F = F_{max} \cdot e^{-\beta \cdot e^{-k \cdot t}}$

Fitted model parameters per tested tablet (N = 4) with statistics – mean, standard deviation (SD), and relative standard deviation expressed in % (RSD%) (output from DDSolver):

Parameter	No.1	No.2	No.3	No.4	Mean	SD	RSD(%)
k	0.039	0.037	0.039	0.039	0.038	0.001	1.647
β	2.291	2.038	2.232	2.494	2.264	0.188	8.291
F_{max}	90.705	106.157	87.695	99.480	96.009	8.412	8.762

Number of dissolution data points (N), degrees of freedom (df), and selected goodness of fit criteria – Pearson correlation coefficient (R), coefficient of determination (R^2), adjusted coefficient of determination ($R^2_{adjusted}$), and residual sum of squares (RSS) (manual calculation in MS Excel):

Parameter	No.1	No.2	No.3	No.4
N	7	7	7	7
df	4	4	4	4
R	0.99875154	0.99509071	0.99896042	0.99798337
R^2	0.99750465	0.99020552	0.99792192	0.99597081
$R^2_{adjusted}$	0.99625697	0.98530828	0.99688289	0.99395622
RSS	15.98203644	130.7212287	17.40530718	34.00352393

Graphical abstract of model fit presented as mean ± 1 SD of the fraction % of released carvedilol:

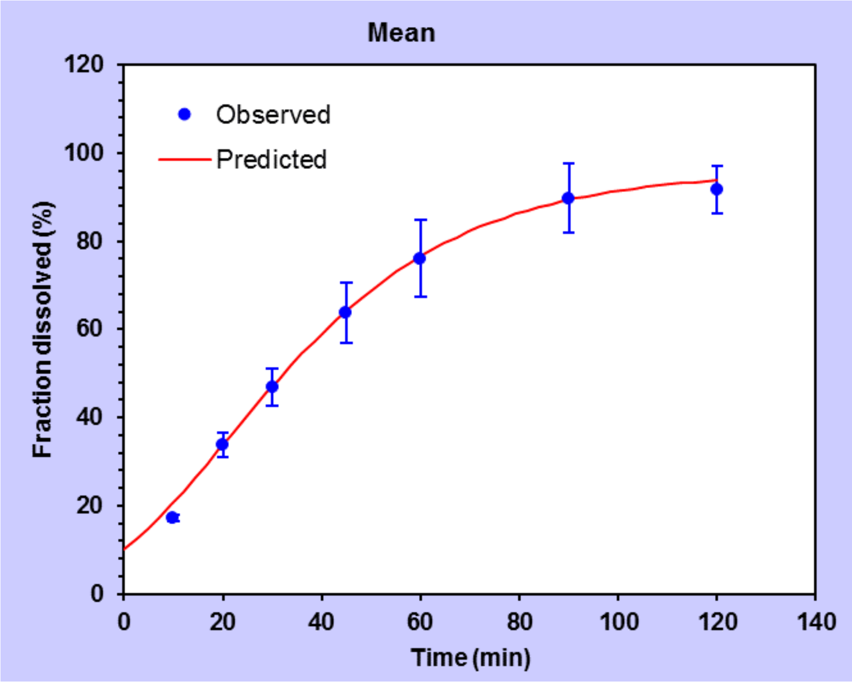

Supplementary materials – Model fitting summary – Polyglykol® 4000 P (modelling the entire relevant dissolution data)

Graphical abstract of model fit presented as the fraction % of released carvedilol per tested tablet:

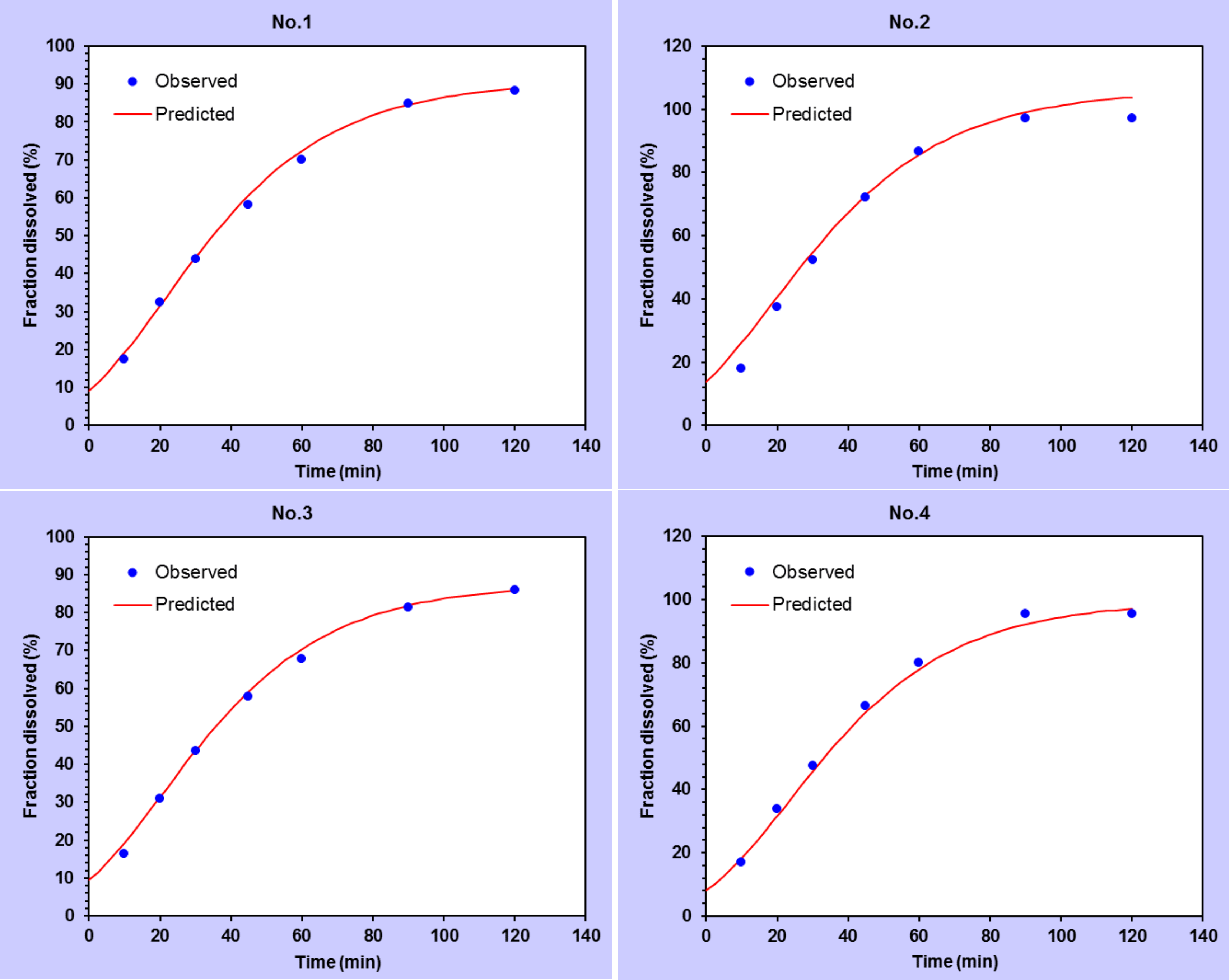

Supplementary materials – Model fitting summary – Polyglykol® 4000 P (modelling the entire relevant dissolution data)

Model: **Probit_1**

Model equation: $F = 100 \cdot \phi[\alpha + \beta \cdot \log(t)]$

Fitted model parameters per tested tablet (N = 4) with statistics – mean, standard deviation (SD), and relative standard deviation expressed in % (RSD%) (output from DDSolver):

Parameter	No.1	No.2	No.3	No.4	Mean	SD	RSD(%)
α	-3.090	-3.960	-3.013	-3.819	-3.470	0.488	-14.072
β	2.049	2.851	1.964	2.666	2.383	0.442	18.557

Number of dissolution data points (N), degrees of freedom (df), and selected goodness of fit criteria – Pearson correlation coefficient (R), coefficient of determination (R^2), adjusted coefficient of determination ($R^2_{adjusted}$), and residual sum of squares (RSS) (manual calculation in MS Excel):

Parameter	No.1	No.2	No.3	No.4
N	7	7	7	7
df	5	5	5	5
R	0.99511072	0.99061767	0.99833169	0.9898836
R^2	0.99024535	0.98132338	0.99666617	0.97986955
$R^2_{adjusted}$	0.98829442	0.97758805	0.9959994	0.97584346
RSS	43.34385185	120.294852	13.99307454	126.8532355

Graphical abstract of model fit presented as mean ± 1 SD of the fraction % of released carvedilol:

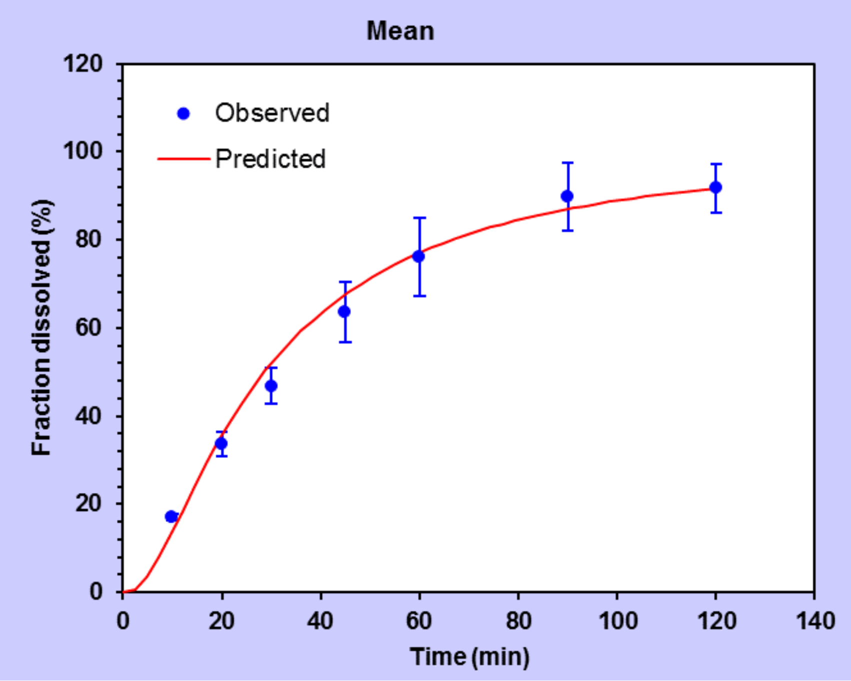

Supplementary materials – Model fitting summary – Polyglykol® 4000 P (modelling the entire relevant dissolution data)

Graphical abstract of model fit presented as the fraction % of released carvedilol per tested tablet:

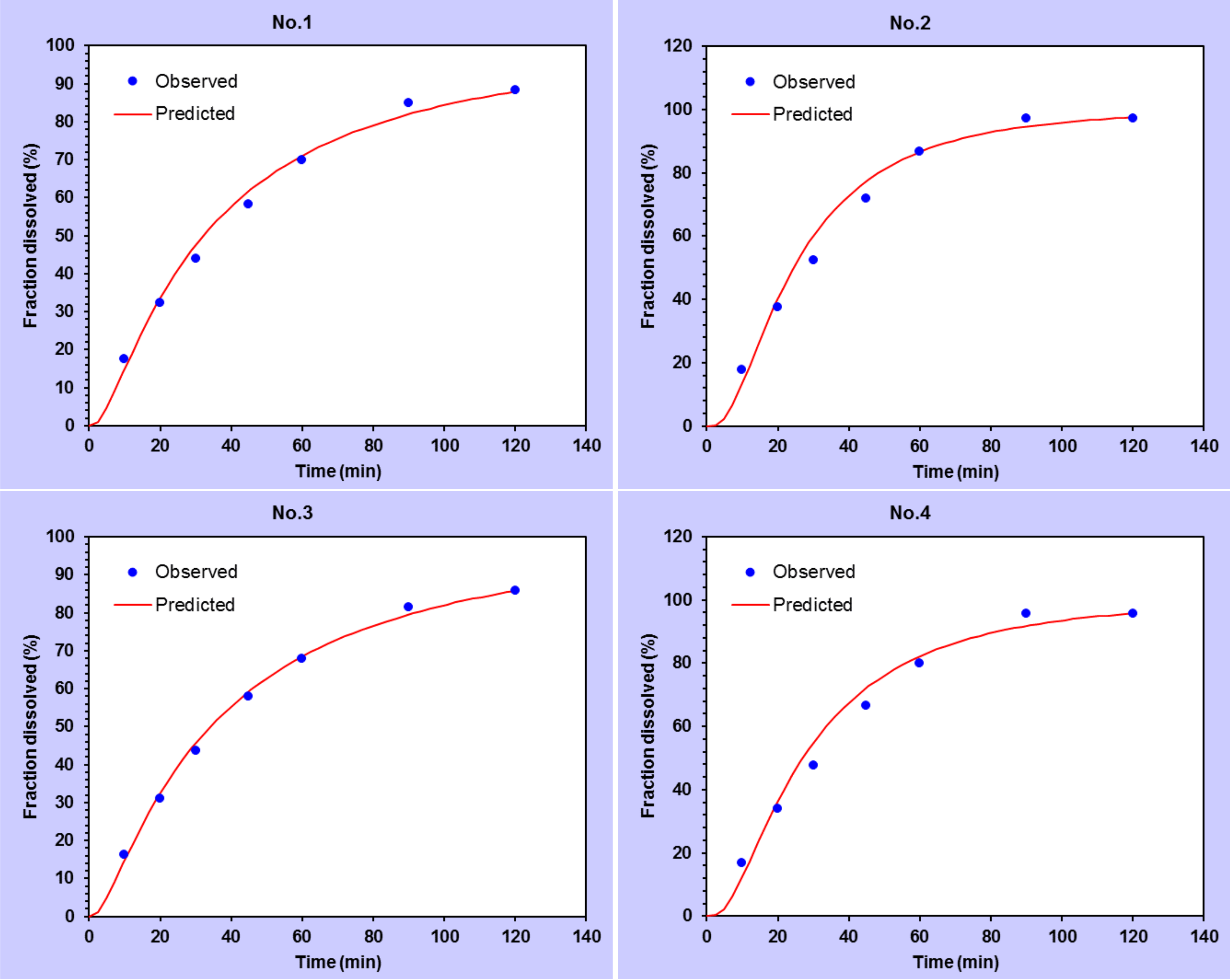

Supplementary materials – Model fitting summary – Polyglykol® 4000 P (modelling the entire relevant dissolution data)

Model: **Probit_2**

Model equation: $F = F_{max} \cdot \phi[\alpha + \beta \cdot \log(t)]$

Fitted model parameters per tested tablet (N = 4) with statistics – mean, standard deviation (SD), and relative standard deviation expressed in % (RSD%) (output from DDSolver):

Parameter	No.1	No.2	No.3	No.4	Mean	SD	RSD(%)
α	-3.494	-3.683	-3.488	-3.778	-3.611	0.144	-3.979
β	2.417	2.619	2.407	2.632	2.519	0.123	4.899
F_{max}	92.772	102.081	90.238	100.380	96.368	5.752	5.969

Number of dissolution data points (N), degrees of freedom (df), and selected goodness of fit criteria – Pearson correlation coefficient (R), coefficient of determination (R^2), adjusted coefficient of determination ($R^2_{adjusted}$), and residual sum of squares (RSS) (manual calculation in MS Excel):

Parameter	No.1	No.2	No.3	No.4
N	7	7	7	7
df	4	4	4	4
R	0.98916322	0.99349916	0.9931023	0.99042878
R^2	0.97844387	0.98704059	0.98625218	0.98094917
$R^2_{adjusted}$	0.96766581	0.98056088	0.97937828	0.97142376
RSS	103.5140641	78.80523749	63.04493903	118.9129208

Graphical abstract of model fit presented as mean ± 1 SD of the fraction % of released carvedilol:

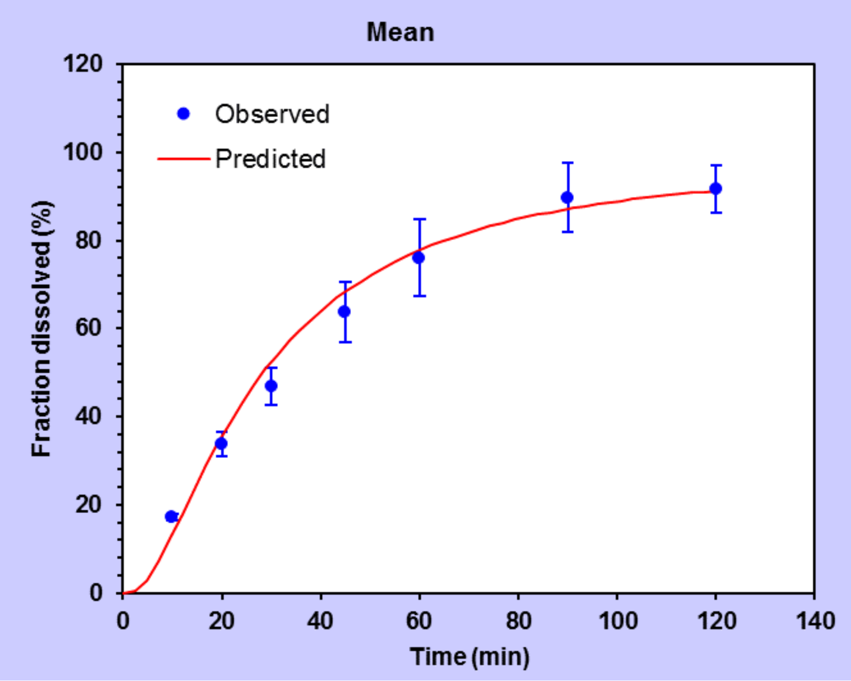

Supplementary materials – Model fitting summary – Polyglykol® 4000 P (modelling the entire relevant dissolution data)

Graphical abstract of model fit presented as the fraction % of released carvedilol per tested tablet:

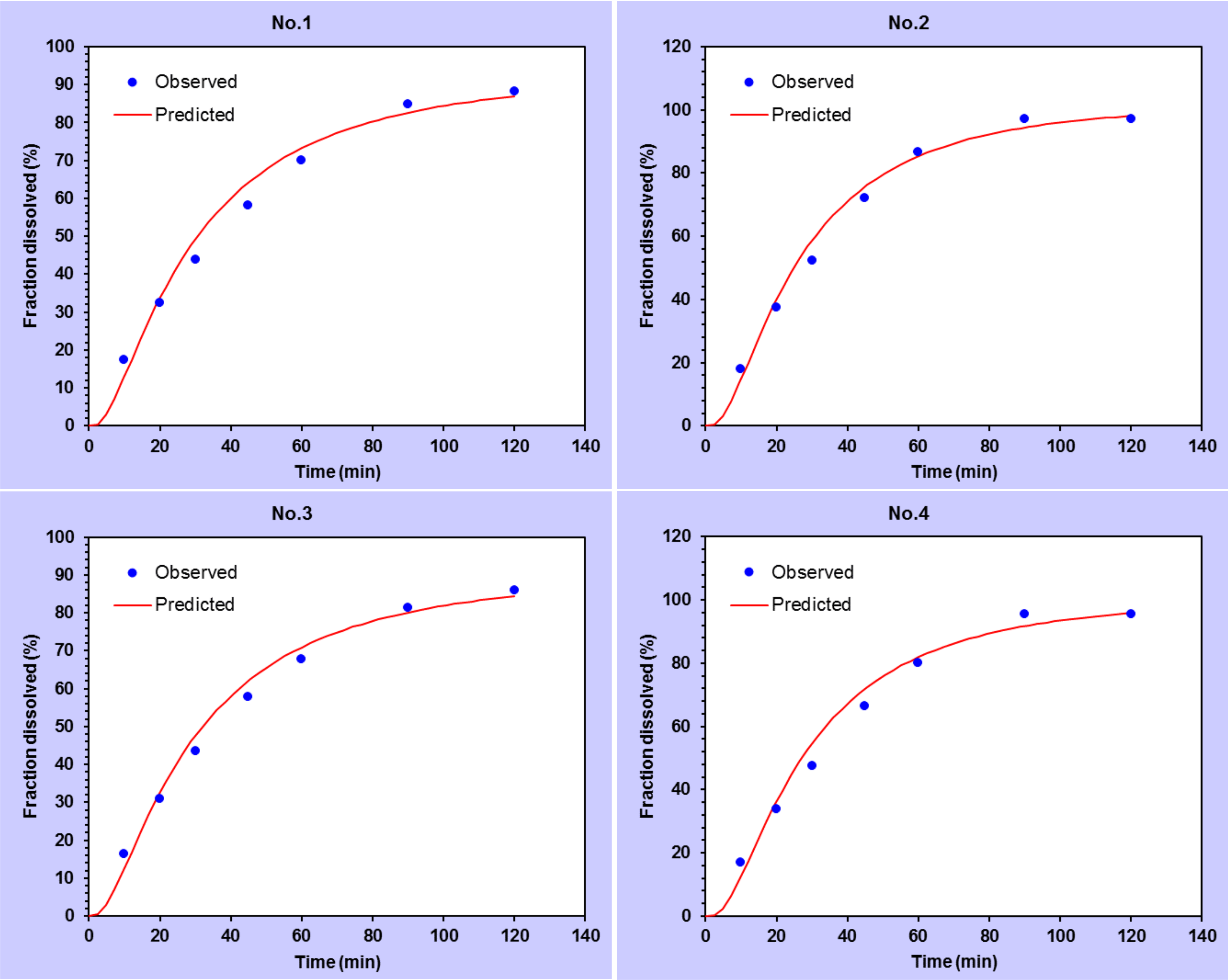

Supplementary materials – Model fitting summary – Polyglykol® 4000 P (modelling up to app. 60 % of carvedilol released)

Model: **Zero–order**

Model equation: $F = k_0 \cdot t$

Fitted model parameters per tested tablet (N = 4) with statistics – mean, standard deviation (SD), and relative standard deviation expressed in % (RSD%) (output from DDSolver):

Parameter	No.1	No.2	No.3	No.4	Mean	SD	RSD(%)
k_0	1.390	1.677	1.370	1.539	1.494	0.143	9.587

Number of dissolution data points (N), degrees of freedom (df), and selected goodness of fit criteria – Pearson correlation coefficient (R), coefficient of determination (R^2), adjusted coefficient of determination ($R^2_{adjusted}$), and residual sum of squares (RSS) (manual calculation in MS Excel):

Parameter	No.1	No.2	No.3	No.4
N	4	4	4	4
df	3	3	3	3
R	0.994433851	0.995366715	0.994519061	0.997333934
R^2	0.988898683	0.990754898	0.989068162	0.994674975
$R^2_{adjusted}$	0.988898683	0.990754898	0.989068162	0.994674975
RSS	57.24180379	32.7900895	40.15252458	22.19781378

Graphical abstract of model fit presented as mean ± 1 SD of the fraction % of released carvedilol:

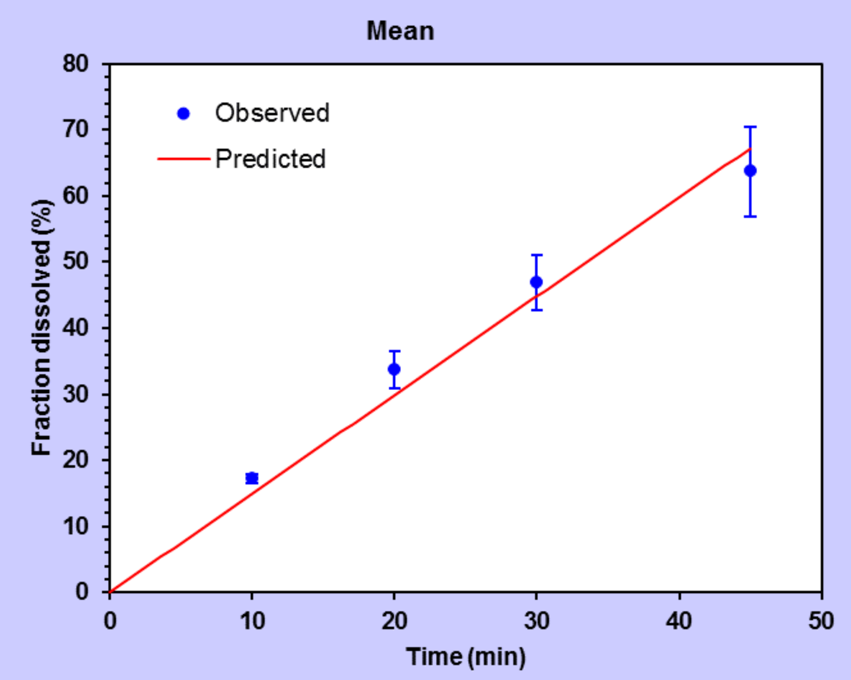

Supplementary materials – Model fitting summary – Polyglykol® 4000 P (modelling up to app. 60 % of carvedilol released)

Graphical abstract of model fit presented as the fraction % of released carvedilol per tested tablet:

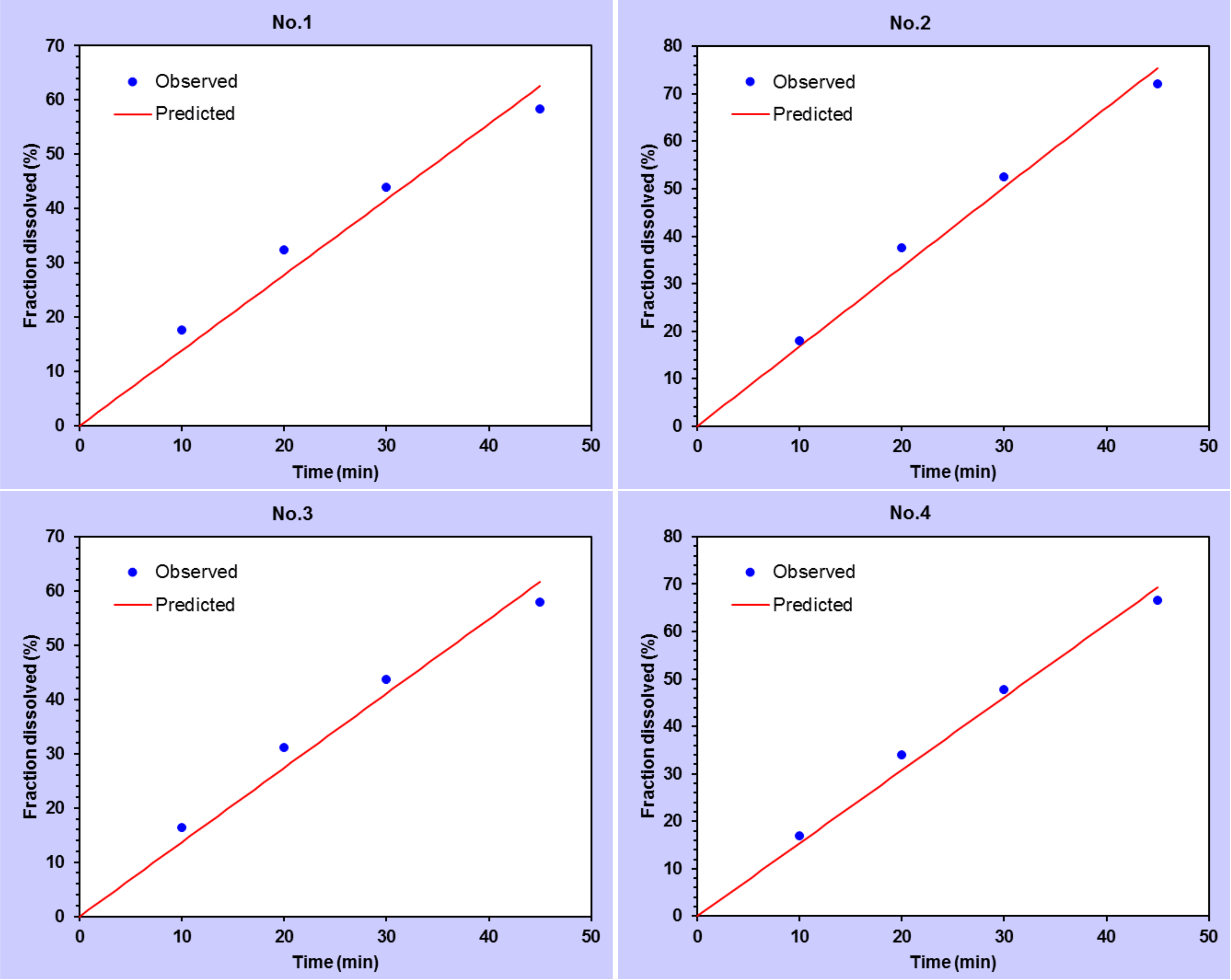

Supplementary materials – Model fitting summary – Polyglykol® 4000 P (modelling up to app. 60 % of carvedilol released)

Model: **Zero–order with T_{lag}**

Model equation: $F = k_0 \cdot (t - T_{lag})$

Fitted model parameters per tested tablet (N = 4) with statistics – mean, standard deviation (SD), and relative standard deviation expressed in % (RSD%) (output from DDSolver):

Parameter	No.1	No.2	No.3	No.4	Mean	SD	RSD(%)
k_0	1.151	1.529	1.181	1.404	1.316	0.181	13.762
T_{lag}	-6.760	-3.159	-5.234	-3.134	-4.572	1.760	-38.491

Number of dissolution data points (N), degrees of freedom (df), and selected goodness of fit criteria – Pearson correlation coefficient (R), coefficient of determination (R^2), adjusted coefficient of determination ($R^2_{adjusted}$), and residual sum of squares (RSS) (manual calculation in MS Excel):

Parameter	No.1	No.2	No.3	No.4
N	4	4	4	4
df	2	2	2	2
R	0.994433851	0.995366715	0.994519061	0.997333934
R^2	0.988898683	0.990754898	0.989068162	0.994674975
$R^2_{adjusted}$	0.983348025	0.986132346	0.983602243	0.992012463
RSS	9.94704164	14.5802187	10.3087406	7.06198836

Graphical abstract of model fit presented as mean ± 1 SD of the fraction % of released carvedilol:

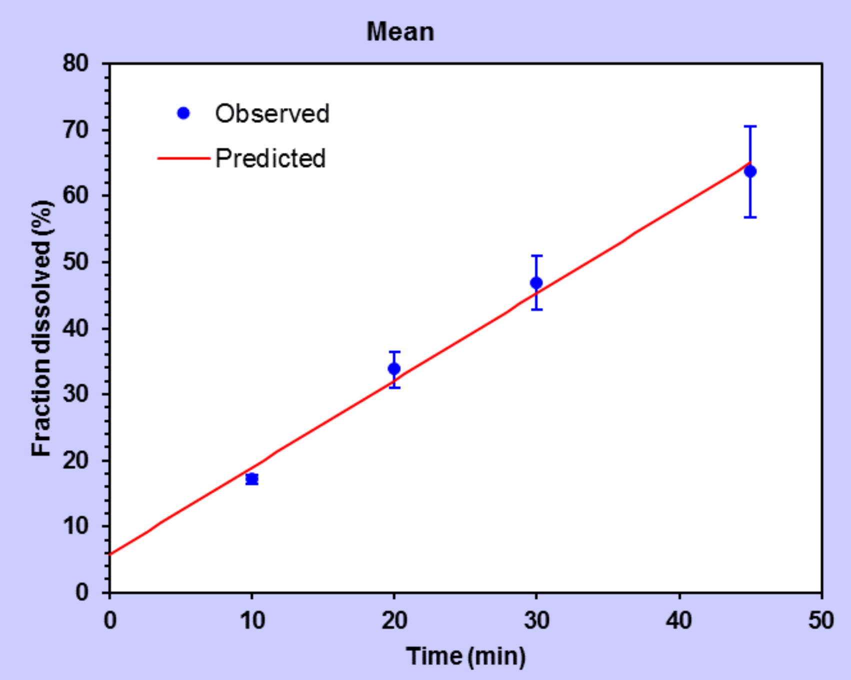

Supplementary materials – Model fitting summary – Polyglykol® 4000 P (modelling up to app. 60 % of carvedilol released)

Graphical abstract of model fit presented as the fraction % of released carvedilol per tested tablet:

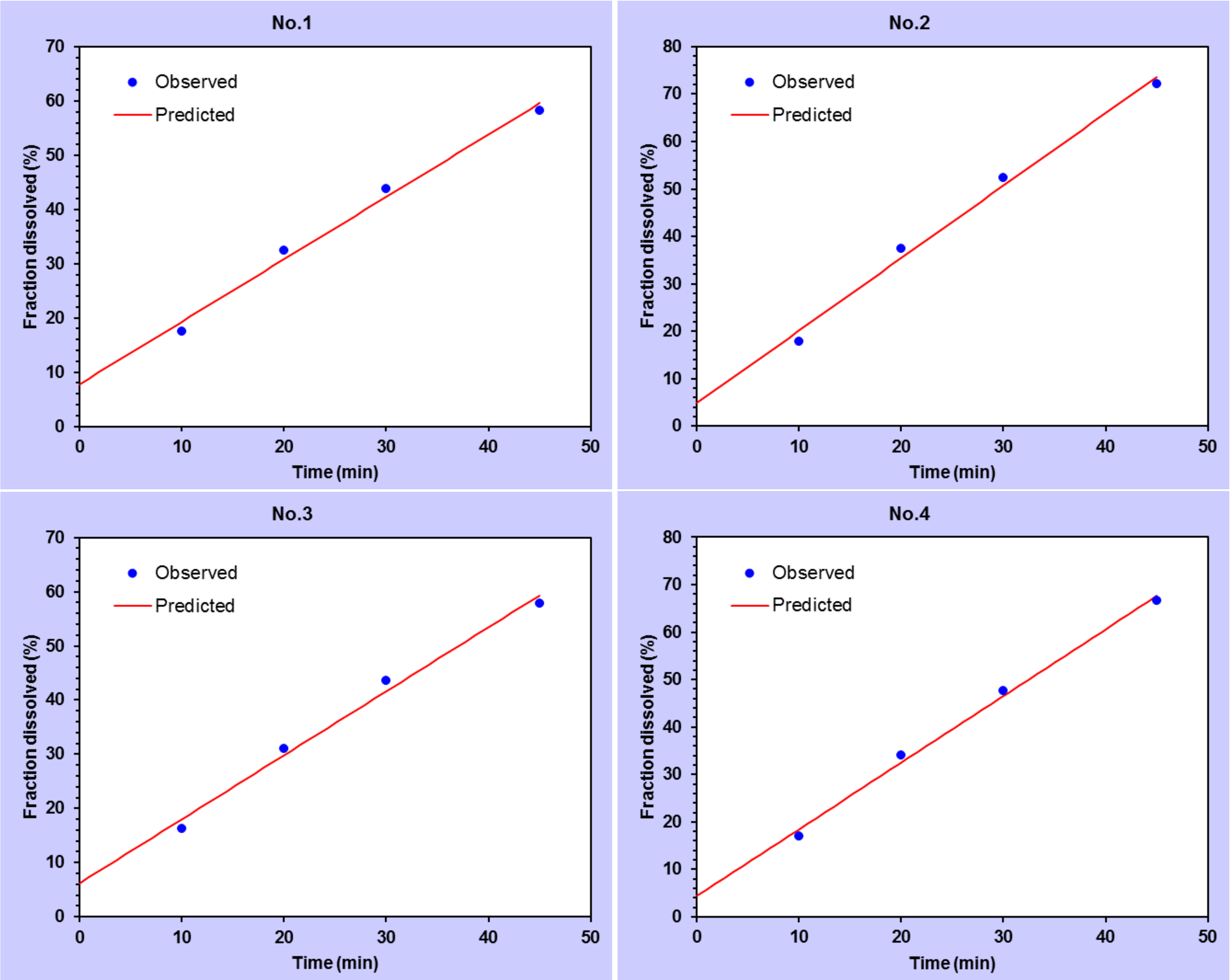

Supplementary materials – Model fitting summary – Polyglykol® 4000 P (modelling up to app. 60 % of carvedilol released)

Model: **Zero–order with F_0**

Model equation: $F = F_0 + k_0 \cdot t$

Fitted model parameters per tested tablet (N = 4) with statistics – mean, standard deviation (SD), and relative standard deviation expressed in % (RSD%) (output from DDSolver):

Parameter	No.1	No.2	No.3	No.4	Mean	SD	RSD(%)
k_0	1.151	1.529	1.181	1.404	1.316	0.181	13.762
F_0	7.782	4.829	6.182	4.402	5.799	1.524	26.287

Number of dissolution data points (N), degrees of freedom (df), and selected goodness of fit criteria – Pearson correlation coefficient (R), coefficient of determination (R^2), adjusted coefficient of determination ($R^2_{adjusted}$), and residual sum of squares (RSS) (manual calculation in MS Excel):

Parameter	No.1	No.2	No.3	No.4
N	4	4	4	4
df	2	2	2	2
R	0.99443385	0.99536672	0.99451906	0.99733393
R^2	0.98889868	0.9907549	0.98906816	0.99467498
$R^2_{adjusted}$	0.98334802	0.98613235	0.98360224	0.99201246
RSS	9.947041642	14.58021867	10.30874057	7.061988363

Graphical abstract of model fit presented as mean ± 1 SD of the fraction % of released carvedilol:

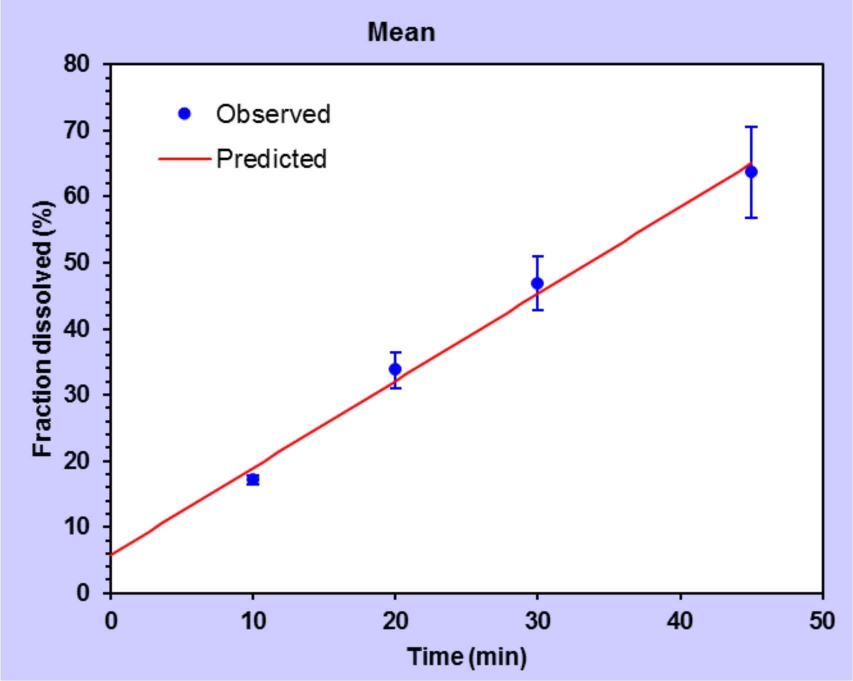

Supplementary materials – Model fitting summary – Polyglykol® 4000 P (modelling up to app. 60 % of carvedilol released)

Graphical abstract of model fit presented as the fraction % of released carvedilol per tested tablet:

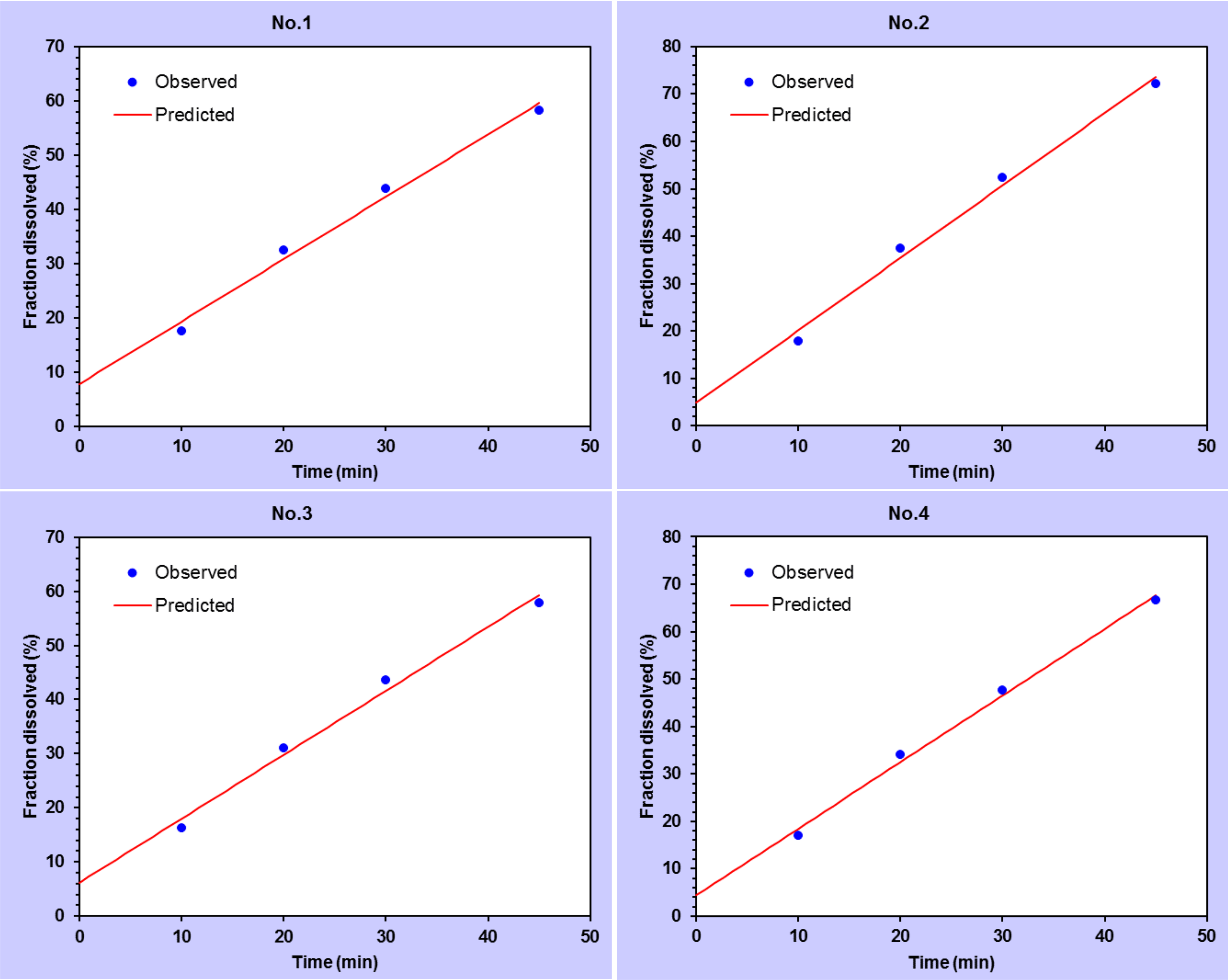

Supplementary materials – Model fitting summary – Polyglykol® 4000 P (modelling up to app. 60 % of carvedilol released)

Model: **First–order**

Model equation: $F = 100 \cdot (1 - e^{-k_1 \cdot t})$

Fitted model parameters per tested tablet (N = 4) with statistics – mean, standard deviation (SD), and relative standard deviation expressed in % (RSD%) (output from DDSolver):

Parameter	No.1	No.2	No.3	No.4	Mean	SD	RSD(%)
k_1	0.019	0.027	0.019	0.023	0.022	0.004	16.056

Number of dissolution data points (N), degrees of freedom (df), and selected goodness of fit criteria – Pearson correlation coefficient (R), coefficient of determination (R^2), adjusted coefficient of determination ($R^2_{adjusted}$), and residual sum of squares (RSS) (manual calculation in MS Excel):

Parameter	No.1	No.2	No.3	No.4
N	4	4	4	4
df	3	3	3	3
R	0.999928373	0.998764748	0.999969747	0.998558194
R^2	0.99985675	0.997531022	0.999939495	0.997118467
$R^2_{adjusted}$	0.99985675	0.997531022	0.999939495	0.997118467
RSS	0.12903759	55.25881	1.67087454	30.8504844

Graphical abstract of model fit presented as mean ± 1 SD of the fraction % of released carvedilol:

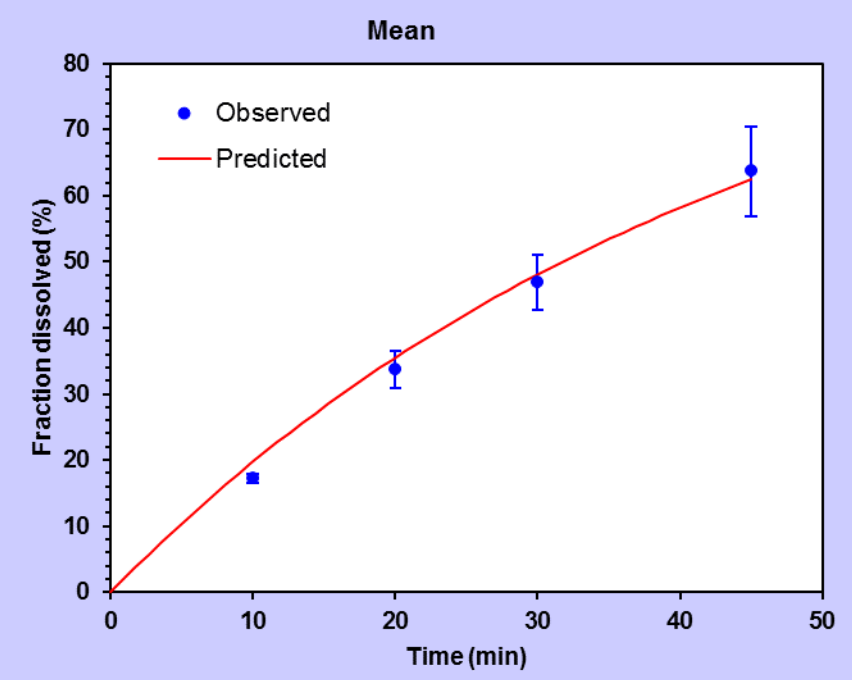

Supplementary materials – Model fitting summary – Polyglykol® 4000 P (modelling up to app. 60 % of carvedilol released)

Graphical abstract of model fit presented as the fraction % of released carvedilol per tested tablet:

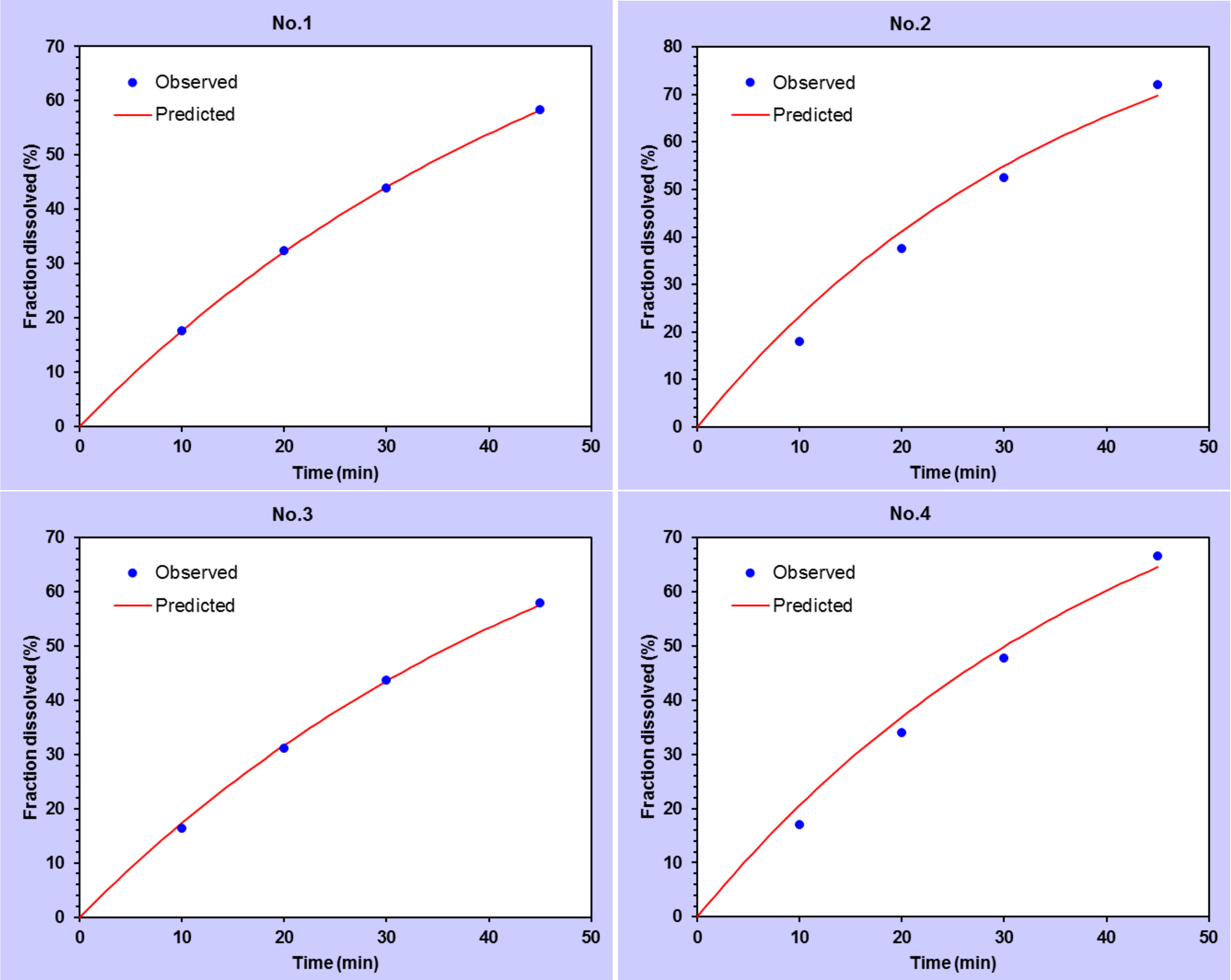

Supplementary materials – Model fitting summary – Polyglykol® 4000 P (modelling up to app. 60 % of carvedilol released)

Model: **First–order with T_{lag}**

Model equation: $F = 100 \cdot \left[1 - e^{-k_1 \cdot (t - T_{lag})}\right]$

Fitted model parameters per tested tablet (N = 4) with statistics – mean, standard deviation (SD), and relative standard deviation expressed in % (RSD%) (output from DDSolver):

Parameter	No.1	No.2	No.3	No.4	Mean	SD	RSD(%)
k_1	0.019	0.031	0.020	0.026	0.024	0.005	22.773
T_{lag}	0.021	4.409	0.990	3.675	2.274	2.101	92.423

Number of dissolution data points (N), degrees of freedom (df), and selected goodness of fit criteria – Pearson correlation coefficient (R), coefficient of determination (R^2), adjusted coefficient of determination ($R^2_{adjusted}$), and residual sum of squares (RSS) (manual calculation in MS Excel):

Parameter	No.1	No.2	No.3	No.4
N	4	4	4	4
df	2	2	2	2
R	0.999928464	0.997538452	0.999970705	0.997656541
R^2	0.999856932	0.995082963	0.999941411	0.995318574
$R^2_{adjusted}$	0.999785399	0.992624444	0.999912117	0.992977862
RSS	0.12819199	9.681411341	0.055265156	7.288628186

Graphical abstract of model fit presented as mean ± 1 SD of the fraction % of released carvedilol:

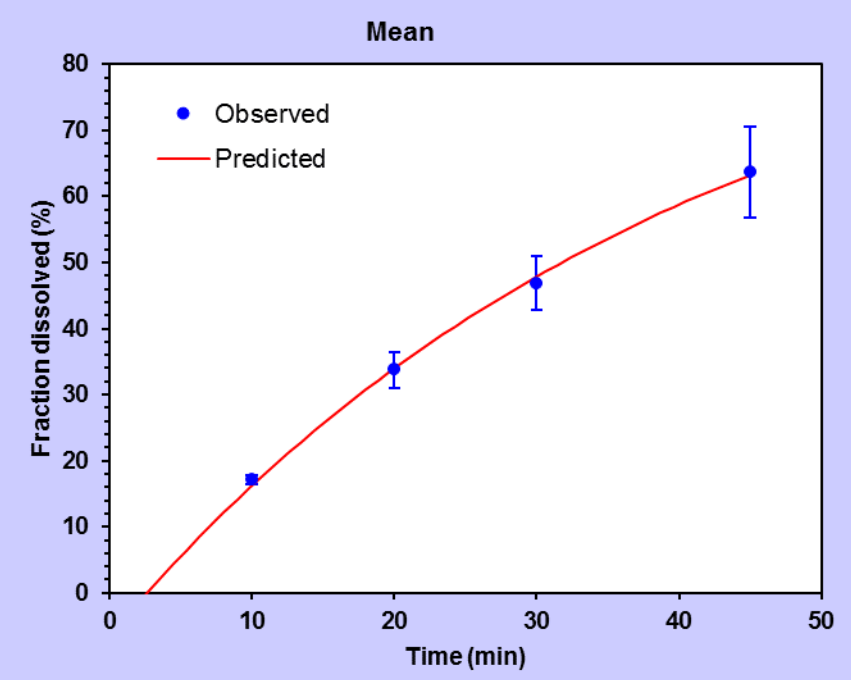

Supplementary materials – Model fitting summary – Polyglykol® 4000 P (modelling up to app. 60 % of carvedilol released)

Graphical abstract of model fit presented as the fraction % of released carvedilol per tested tablet:

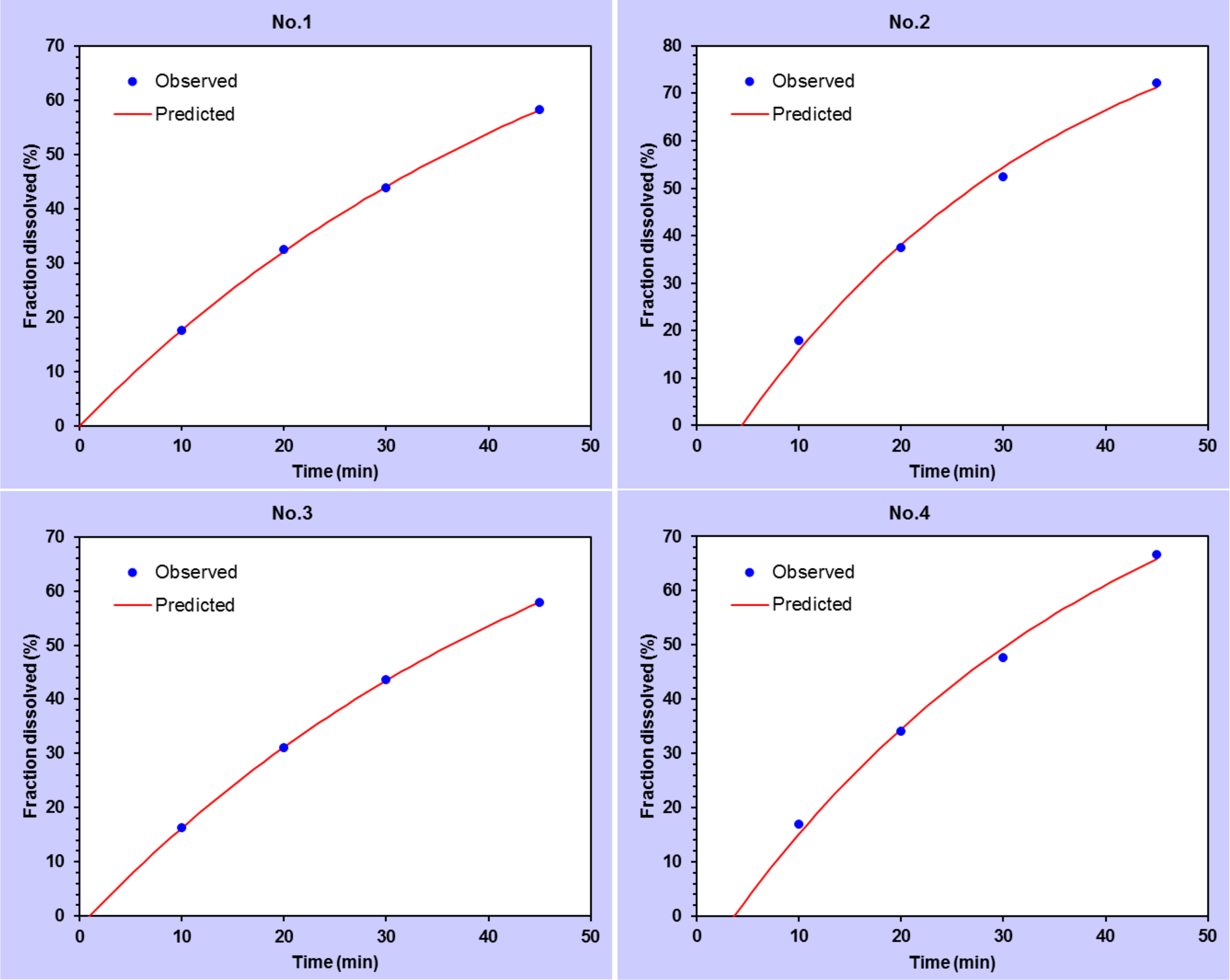

Supplementary materials – Model fitting summary – Polyglykol® 4000 P (modelling up to app. 60 % of carvedilol released)

Model: **First–order with F_{max}**

Model equation: $F = F_{max} \cdot (1 - e^{-k_1 \cdot t})$

Fitted model parameters per tested tablet (N = 4) with statistics – mean, standard deviation (SD), and relative standard deviation expressed in % (RSD%) (output from DDSolver):

Parameter	No.1	No.2	No.3	No.4	Mean	SD	RSD(%)
k_1	0.058	0.057	0.058	0.056	0.057	0.001	1.469
F_{max}	49.685	61.468	49.362	56.759	54.318	5.862	10.793

Number of dissolution data points (N), degrees of freedom (df), and selected goodness of fit criteria – Pearson correlation coefficient (R), coefficient of determination (R^2), adjusted coefficient of determination ($R^2_{adjusted}$), and residual sum of squares (RSS) (manual calculation in MS Excel):

Parameter	No.1	No.2	No.3	No.4
N	4	4	4	4
df	2	2	2	2
R	0.98169135	0.980987632	0.981441978	0.976566226
R^2	0.963717906	0.962336734	0.963228356	0.953681593
$R^2_{adjusted}$	0.945576859	0.943505101	0.944842533	0.93052239
RSS	179.1060427	334.5105506	193.3042771	281.9075162

Graphical abstract of model fit presented as mean ± 1 SD of the fraction % of released carvedilol:

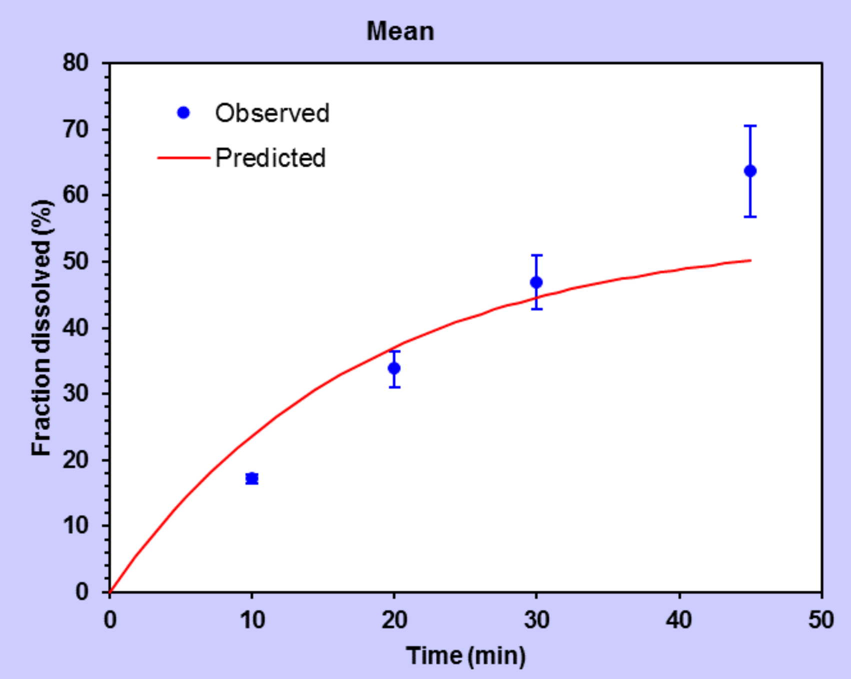

Supplementary materials – Model fitting summary – Polyglykol® 4000 P (modelling up to app. 60 % of carvedilol released)

Graphical abstract of model fit presented as the fraction % of released carvedilol per tested tablet:

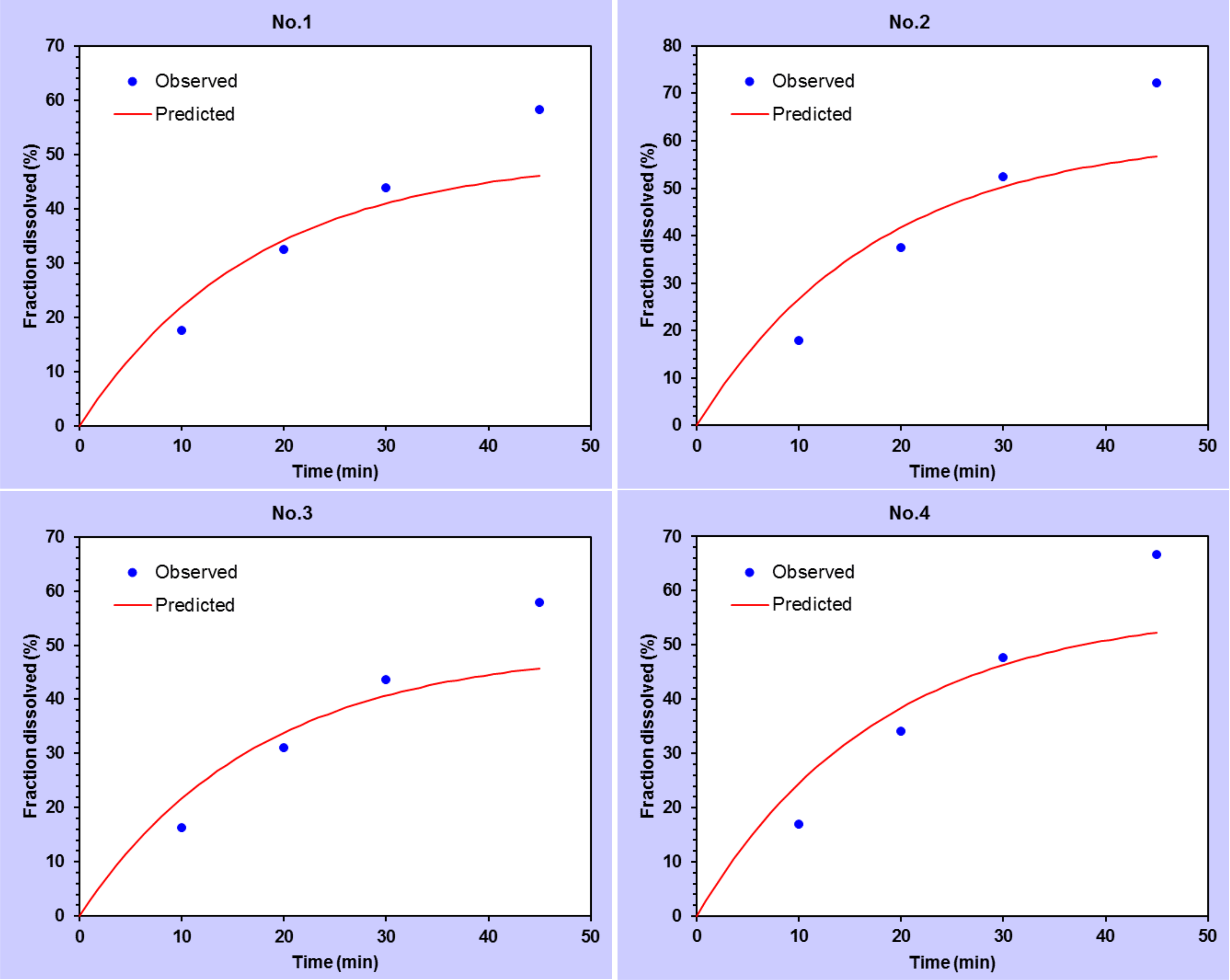

Supplementary materials – Model fitting summary – Polyglykol® 4000 P (modelling up to app. 60 % of carvedilol released)

Model: **First–order with T_{lag} and F_{max}**

Model equation: $F = F_{max} \cdot \left[1 - e^{-k_1 \cdot (t - T_{lag})}\right]$

Fitted model parameters per tested tablet (N = 4) with statistics – mean, standard deviation (SD), and relative standard deviation expressed in % (RSD%) (output from DDSolver):

Parameter	No.1	No.2	No.3	No.4	Mean	SD	RSD(%)
k_1	0.116	0.119	0.117	0.118	0.117	0.001	1.043
T_{lag}	5.845	6.579	6.130	6.645	6.300	0.380	6.031
F_{max}	50.959	63.044	50.627	58.214	55.711	6.013	10.793

Number of dissolution data points (N), degrees of freedom (df), and selected goodness of fit criteria – Pearson correlation coefficient (R), coefficient of determination (R^2), adjusted coefficient of determination ($R^2_{adjusted}$), and residual sum of squares (RSS) (manual calculation in MS Excel):

Parameter	No.1	No.2	No.3	No.4
N	4	4	4	4
df	1	1	1	1
R	0.922087836	0.915784808	0.918546194	0.906505099
R^2	0.850245978	0.838661814	0.843727111	0.821751494
$R^2_{adjusted}$	0.550737933	0.515985443	0.531181334	0.465254482
RSS	156.4017719	310.1345572	174.5118791	281.4294413

Graphical abstract of model fit presented as mean ± 1 SD of the fraction % of released carvedilol:

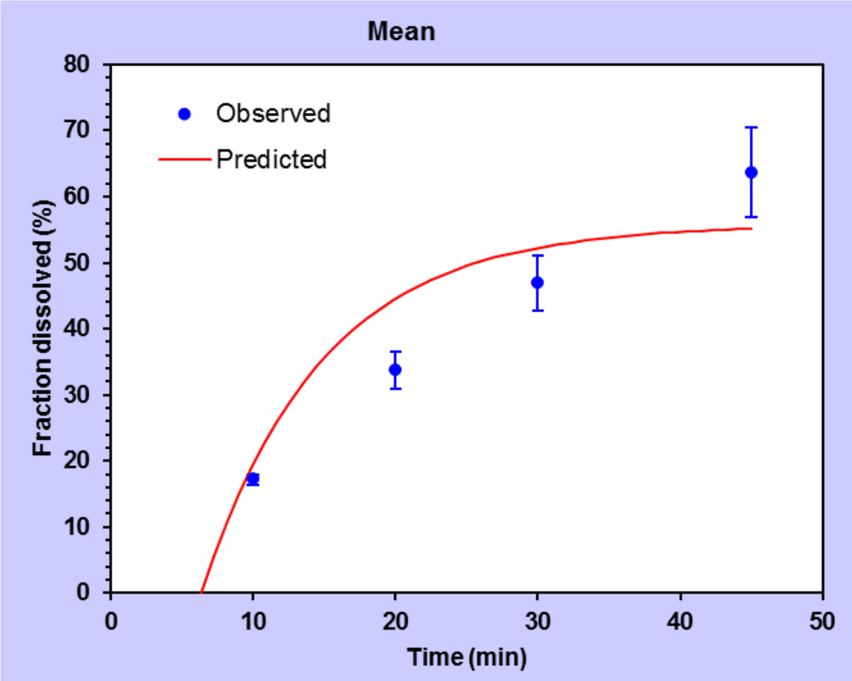

Supplementary materials – Model fitting summary – Polyglykol® 4000 P (modelling up to app. 60 % of carvedilol released)

Graphical abstract of model fit presented as the fraction % of released carvedilol per tested tablet:

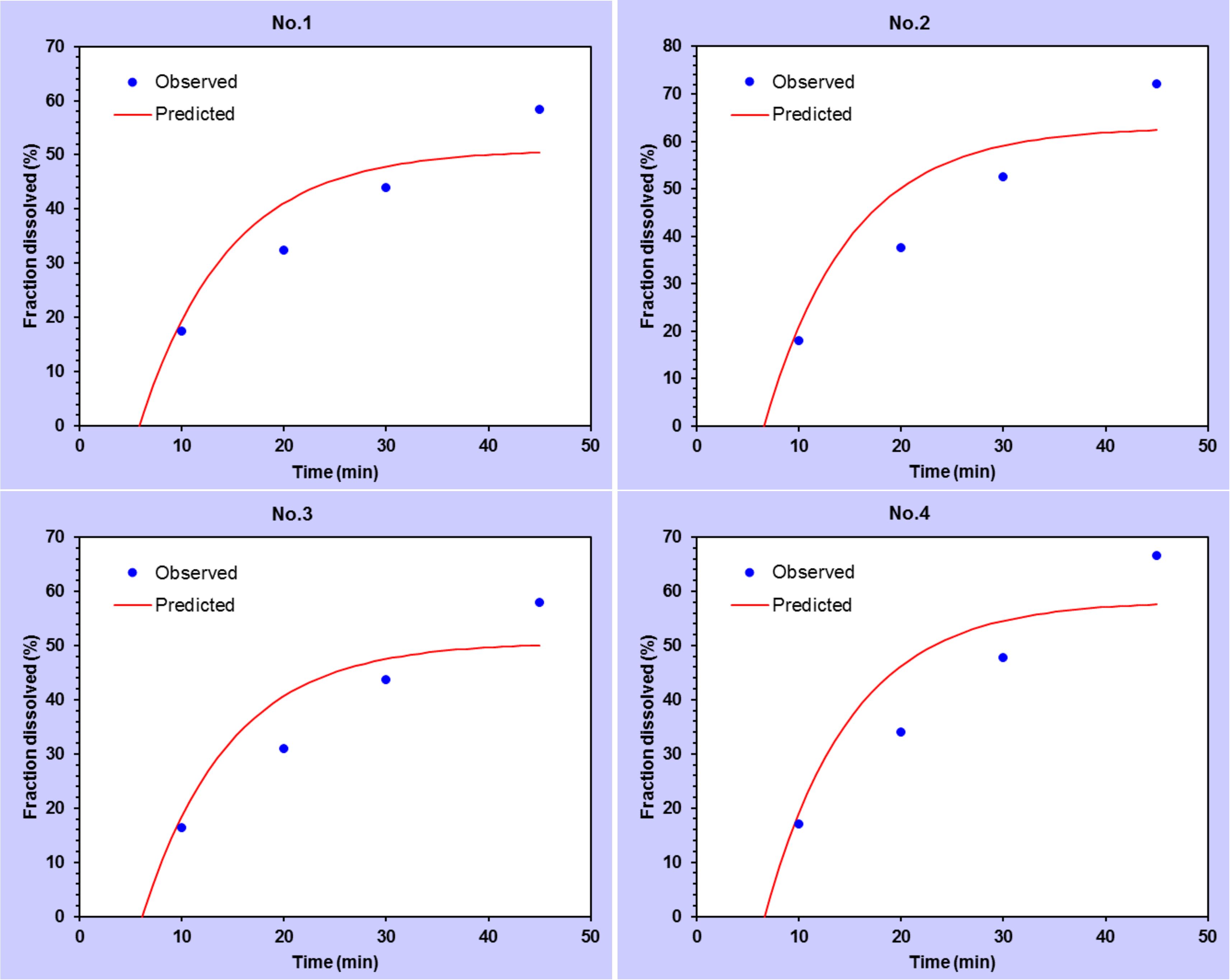

Supplementary materials – Model fitting summary – Polyglykol® 4000 P (modelling up to app. 60 % of carvedilol released)

Model: **Higuchi**

Model equation: $F = k_H \cdot t^{0.5}$

Fitted model parameters per tested tablet (N = 4) with statistics – mean, standard deviation (SD), and relative standard deviation expressed in % (RSD%) (output from DDSolver):

Parameter	No.1	No.2	No.3	No.4	Mean	SD	RSD(%)
k_H	7.915	9.471	7.781	8.693	8.465	0.782	9.238

Number of dissolution data points (N), degrees of freedom (df), and selected goodness of fit criteria – Pearson correlation coefficient (R), coefficient of determination (R^2), adjusted coefficient of determination ($R^2_{adjusted}$), and residual sum of squares (RSS) (manual calculation in MS Excel):

Parameter	No.1	No.2	No.3	No.4
N	4	4	4	4
df	3	3	3	3
R	0.999978083	0.999837803	0.999804211	0.999193413
R^2	0.999956167	0.999675632	0.999608461	0.998387476
$R^2_{adjusted}$	0.999956167	0.999675632	0.999608461	0.998387476
RSS	92.5517869	241.739054	116.518351	203.664947

Graphical abstract of model fit presented as mean ± 1 SD of the fraction % of released carvedilol:

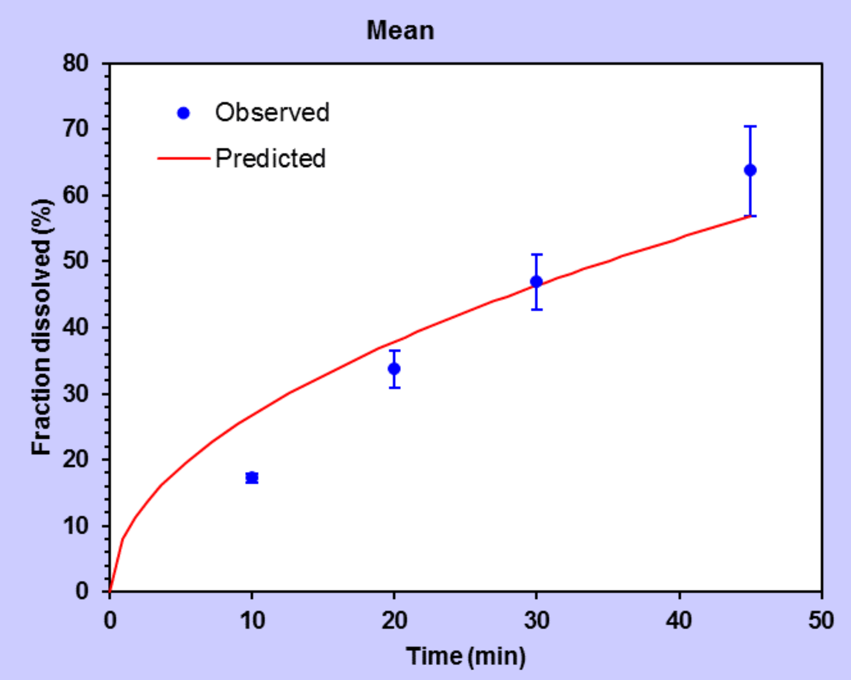

Supplementary materials – Model fitting summary – Polyglykol® 4000 P (modelling up to app. 60 % of carvedilol released)

Graphical abstract of model fit presented as the fraction % of released carvedilol per tested tablet:

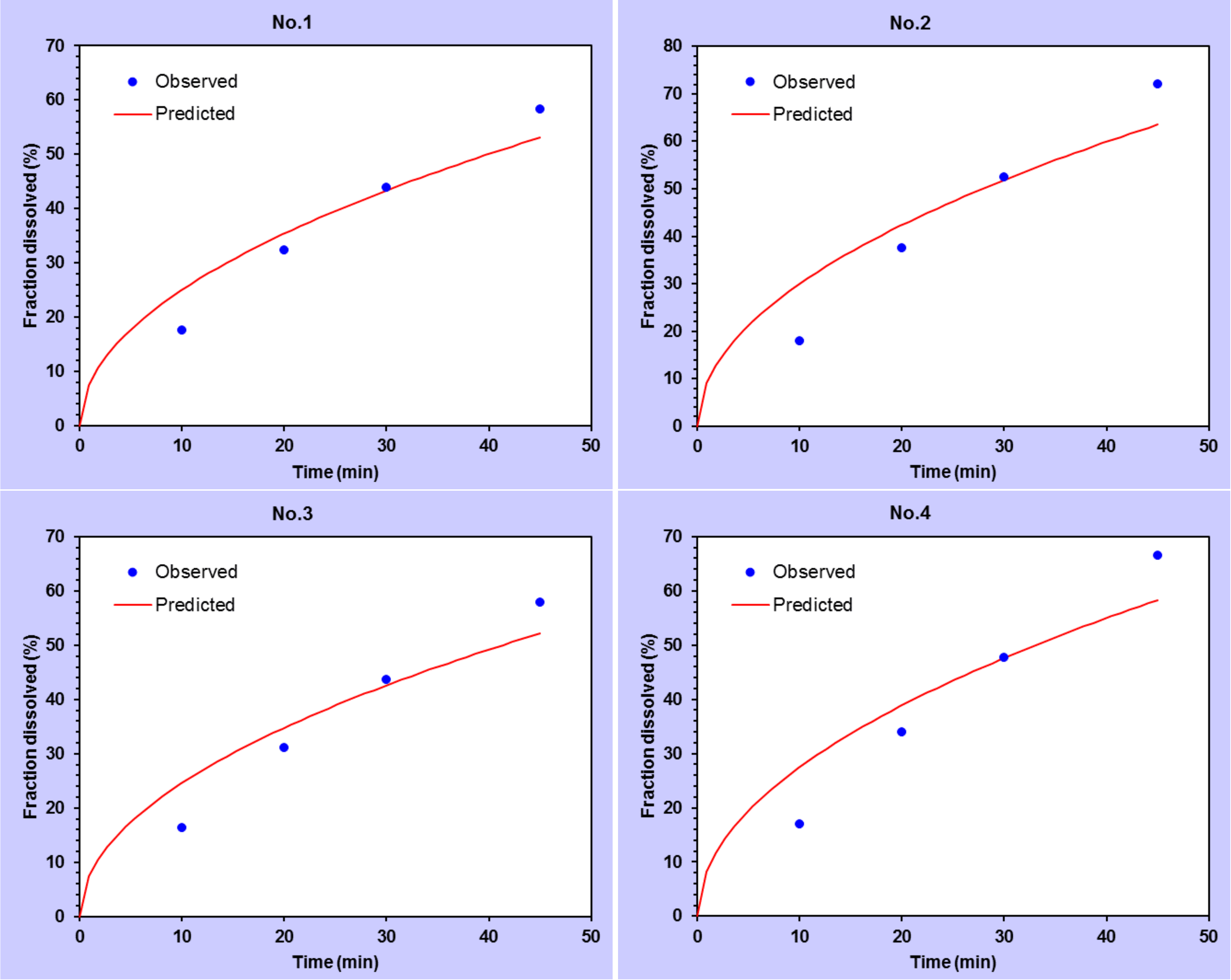

Supplementary materials – Model fitting summary – Polyglykol® 4000 P (modelling up to app. 60 % of carvedilol released)

Model: **Higuchi with T_{lag}**

Model equation: $F = k_H \cdot \left(t - T_{lag}\right)^{0.5}$

Fitted model parameters per tested tablet (N = 4) with statistics – mean, standard deviation (SD), and relative standard deviation expressed in % (RSD%) (output from DDSolver):

Parameter	No.1	No.2	No.3	No.4	Mean	SD	RSD(%)
k_H	9.415	11.682	9.438	11.042	10.394	1.148	11.040
T_{lag}	7.436	7.353	8.084	7.896	7.693	0.354	4.601

Number of dissolution data points (N), degrees of freedom (df), and selected goodness of fit criteria – Pearson correlation coefficient (R), coefficient of determination (R^2), adjusted coefficient of determination ($R^2_{adjusted}$), and residual sum of squares (RSS) (manual calculation in MS Excel):

Parameter	No.1	No.2	No.3	No.4
N	4	4	4	4
df	2	2	2	2
R	0.996983636	0.996397125	0.995137891	0.992954144
R^2	0.99397637	0.992807231	0.990299422	0.985957933
$R^2_{adjusted}$	0.990964555	0.989210846	0.985449132	0.978936899
RSS	7.851331331	28.19395061	13.61645093	38.9577782

Graphical abstract of model fit presented as mean ± 1 SD of the fraction % of released carvedilol:

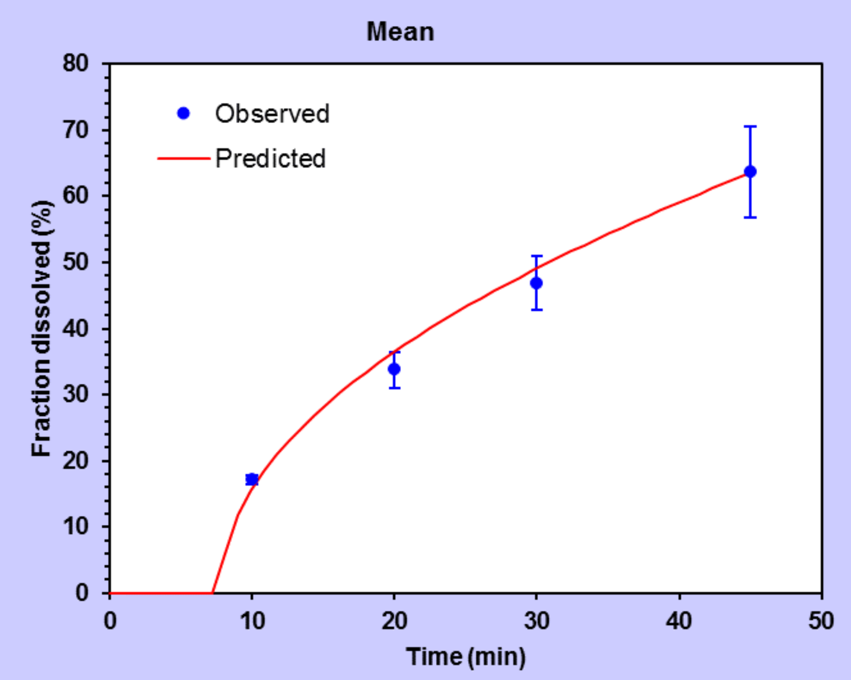

Supplementary materials – Model fitting summary – Polyglykol® 4000 P (modelling up to app. 60 % of carvedilol released)

Graphical abstract of model fit presented as the fraction % of released carvedilol per tested tablet:

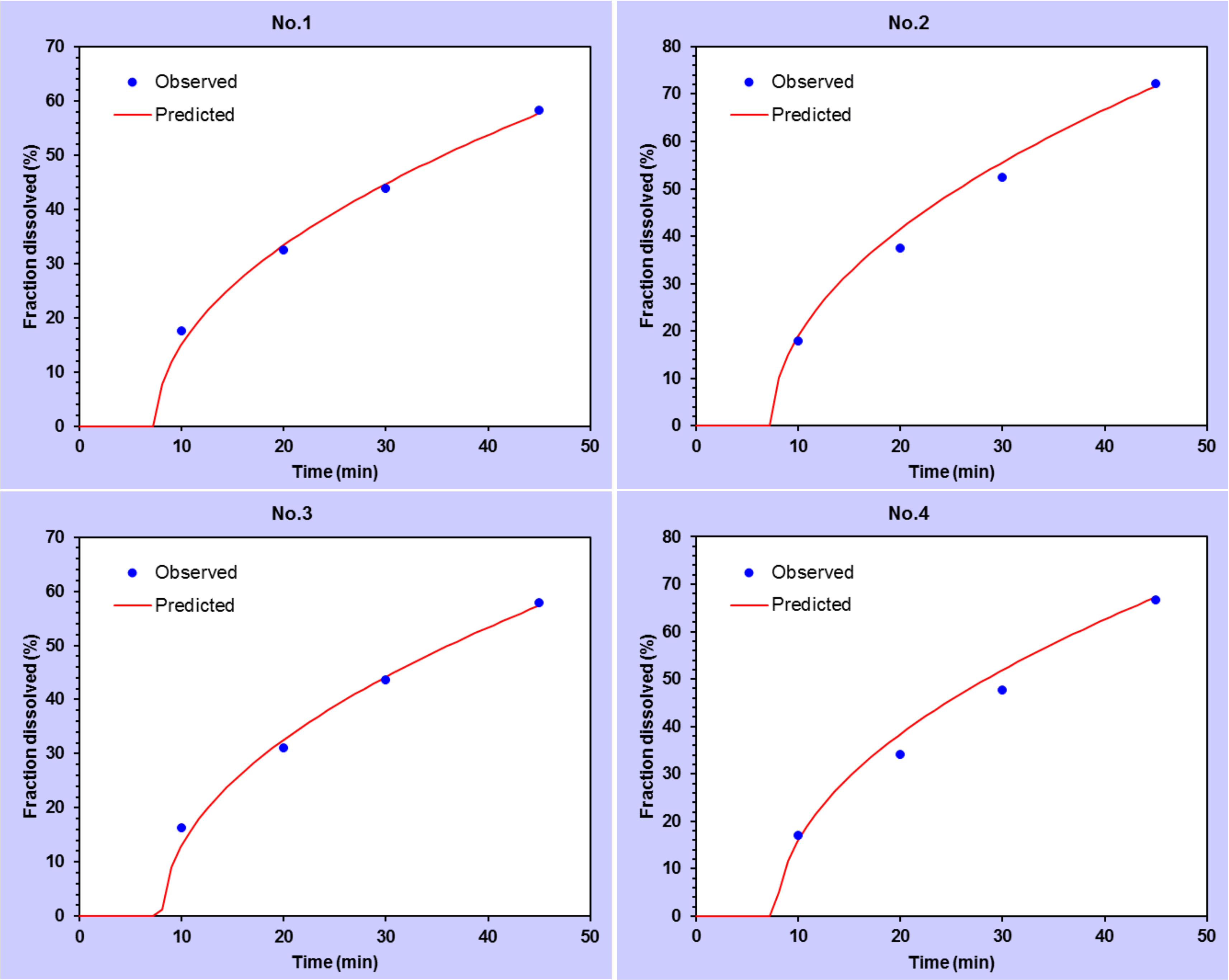

Supplementary materials – Model fitting summary – Polyglykol® 4000 P (modelling up to app. 60 % of carvedilol released)

Model: **Higuchi with F_0**

Model equation: $F = F_0 + k_H \cdot t^{0.5}$

Fitted model parameters per tested tablet (N = 4) with statistics – mean, standard deviation (SD), and relative standard deviation expressed in % (RSD%) (output from DDSolver):

Parameter	No.1	No.2	No.3	No.4	Mean	SD	RSD(%)
k_H	11.484	15.234	11.779	13.961	13.115	1.793	13.674
F_0	-18.907	-30.530	-21.185	-27.905	-24.632	5.482	-22.257

Number of dissolution data points (N), degrees of freedom (df), and selected goodness of fit criteria – Pearson correlation coefficient (R), coefficient of determination (R^2), adjusted coefficient of determination ($R^2_{adjusted}$), and residual sum of squares (RSS) (manual calculation in MS Excel):

Parameter	No.1	No.2	No.3	No.4
N	4	4	4	4
df	2	2	2	2
R	0.999978083	0.999837803	0.999804211	0.999193413
R^2	0.999956167	0.999675632	0.999608461	0.998387476
$R^2_{adjusted}$	0.999934251	0.999513447	0.999412691	0.997581214
RSS	0.039275285	0.511553241	0.369221917	2.138511016

Graphical abstract of model fit presented as mean ± 1 SD of the fraction % of released carvedilol:

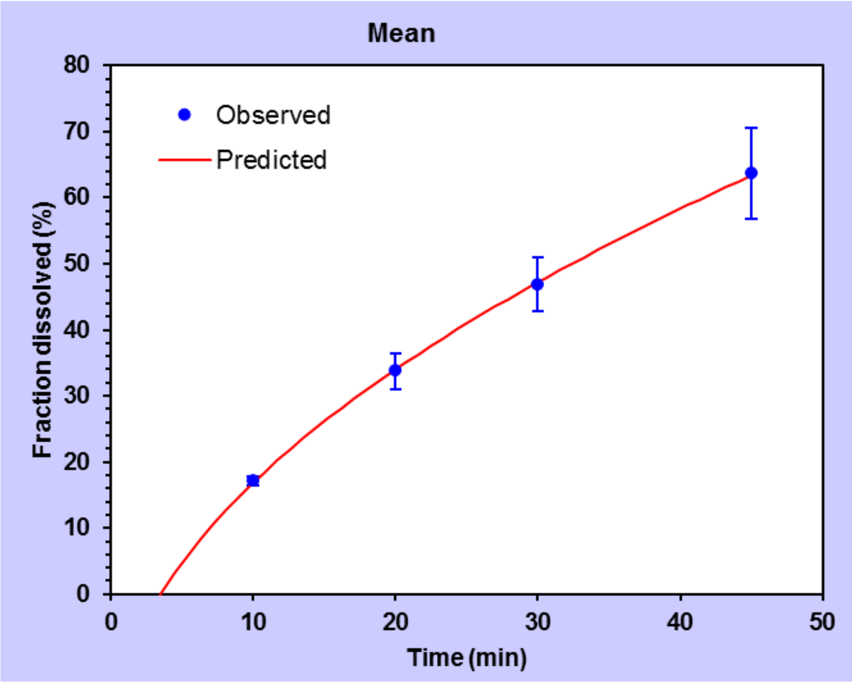

Supplementary materials – Model fitting summary – Polyglykol® 4000 P (modelling up to app. 60 % of carvedilol released)

Graphical abstract of model fit presented as the fraction % of released carvedilol per tested tablet:

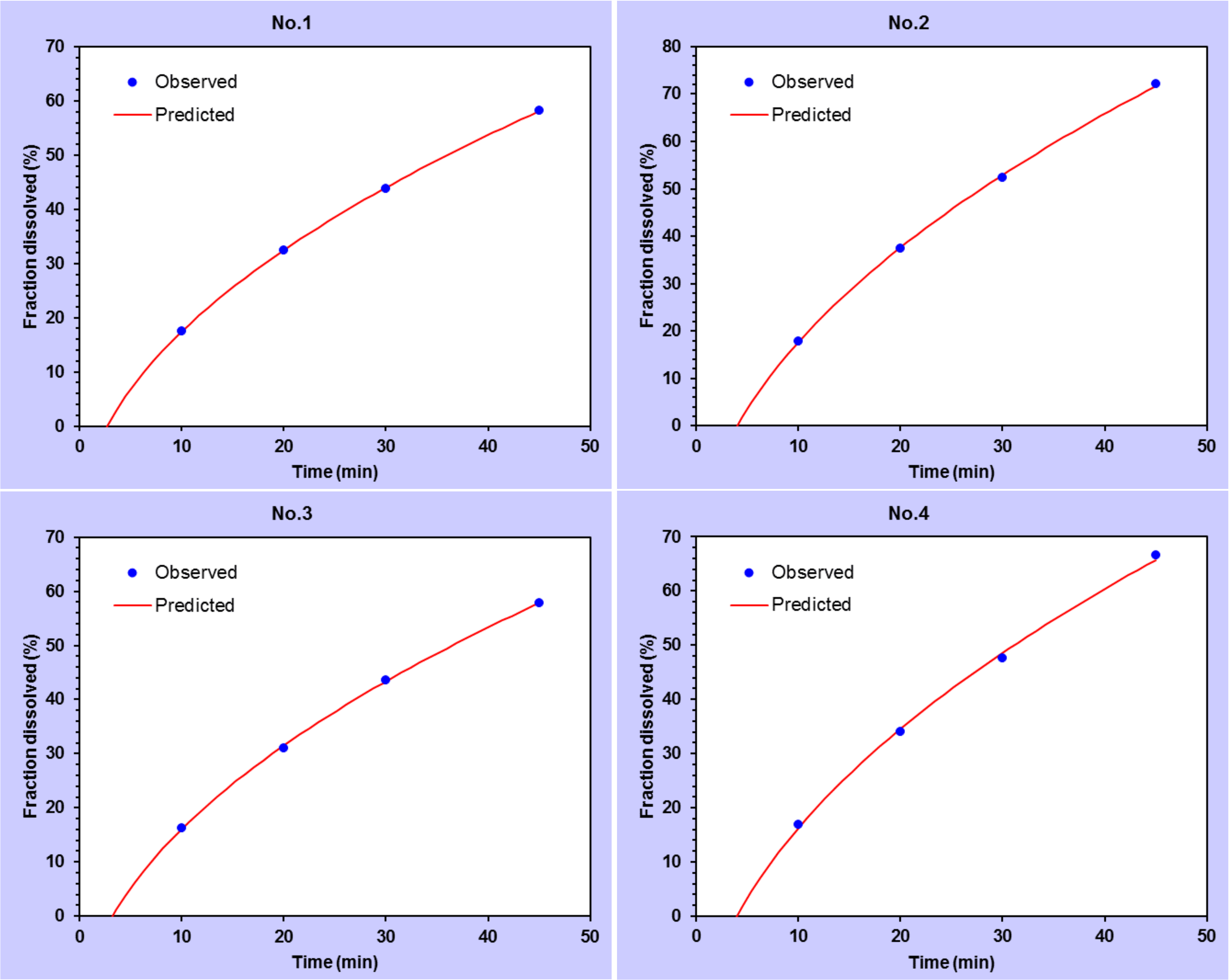

Supplementary materials – Model fitting summary – Polyglykol® 4000 P (modelling up to app. 60 % of carvedilol released)

Model: **Korsmeyer–Peppas**

Model equation: $F = k_{KP} \cdot t^n$

Fitted model parameters per tested tablet (N = 4) with statistics – mean, standard deviation (SD), and relative standard deviation expressed in % (RSD%) (output from DDSolver):

Parameter	No.1	No.2	No.3	No.4	Mean	SD	RSD(%)
k_{KP}	2.825	2.559	2.362	2.478	2.556	0.197	7.694
n	0.802	0.876	0.849	0.861	0.847	0.032	3.769

Number of dissolution data points (N), degrees of freedom (df), and selected goodness of fit criteria – Pearson correlation coefficient (R), coefficient of determination (R^2), adjusted coefficient of determination ($R^2_{adjusted}$), and residual sum of squares (RSS) (manual calculation in MS Excel):

Parameter	No.1	No.2	No.3	No.4
N	4	4	4	4
df	2	2	2	2
R	0.998066107	0.997582553	0.997394533	0.999067146
R^2	0.996135953	0.995170951	0.994795854	0.998135163
$R^2_{adjusted}$	0.99420393	0.992756426	0.992193781	0.997202744
RSS	4.51034337	10.65225892	6.322304264	5.48936042

Graphical abstract of model fit presented as mean ± 1 SD of the fraction % of released carvedilol:

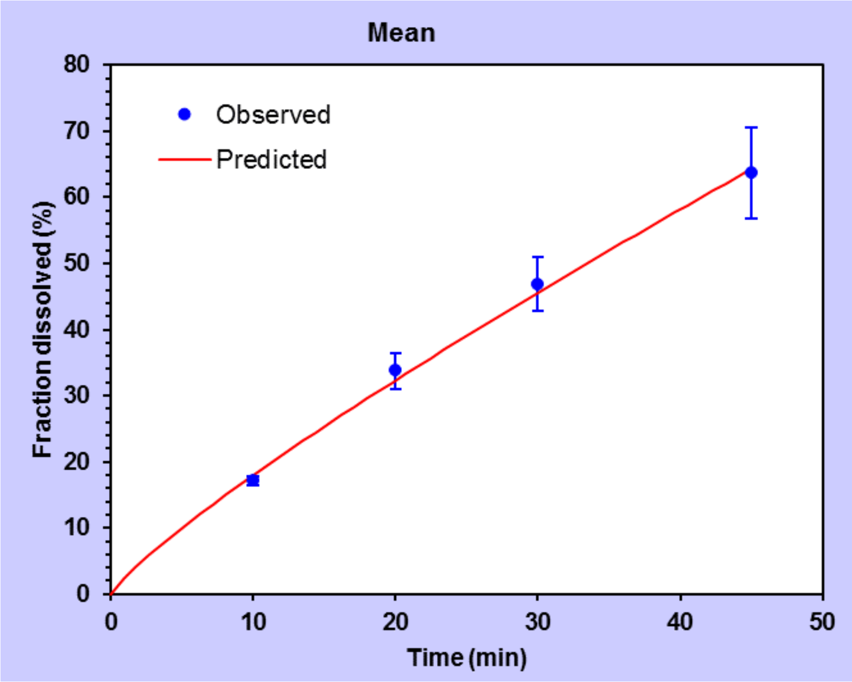

Supplementary materials – Model fitting summary – Polyglykol® 4000 P (modelling up to app. 60 % of carvedilol released)

Graphical abstract of model fit presented as the fraction % of released carvedilol per tested tablet:

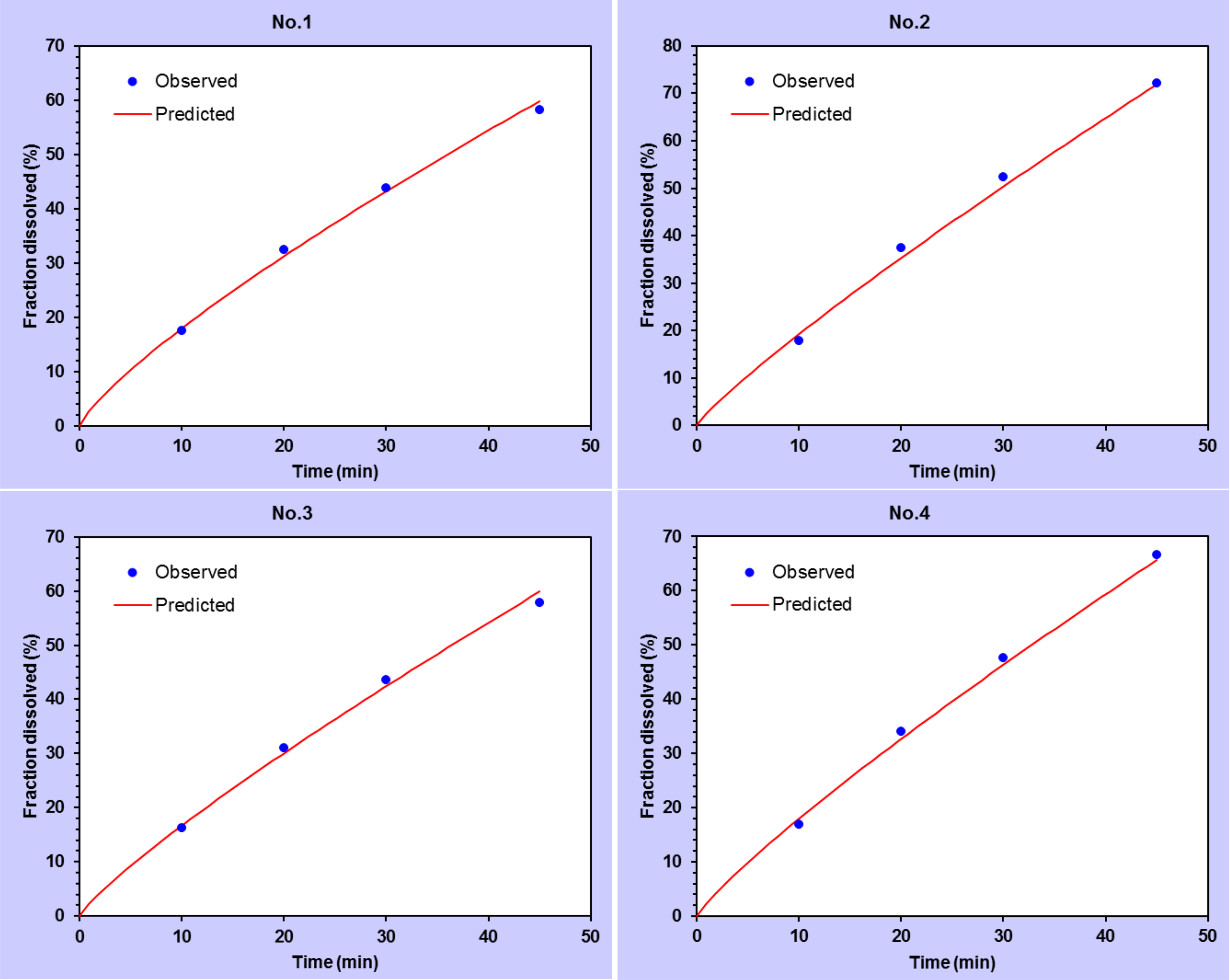

Supplementary materials – Model fitting summary – Polyglykol® 4000 P (modelling up to app. 60 % of carvedilol released)

Model: **Korsmeyer–Peppas with T_{lag}**

Model equation: $F = k_{KP} \cdot \left(t - T_{lag}\right)^{n}$

Fitted model parameters per tested tablet (N = 4) with statistics – mean, standard deviation (SD), and relative standard deviation expressed in % (RSD%) (output from DDSolver):

Parameter	No.1	No.2	No.3	No.4	Mean	SD	RSD(%)
k_{KP}	5.704	5.407	4.970	4.718	5.200	0.440	8.469
n	0.626	0.697	0.663	0.712	0.674	0.038	5.676
T_{lag}	4.000	4.223	4.000	4.000	4.056	0.111	2.743

Number of dissolution data points (N), degrees of freedom (df), and selected goodness of fit criteria – Pearson correlation coefficient (R), coefficient of determination (R^2), adjusted coefficient of determination ($R^2_{adjusted}$), and residual sum of squares (RSS) (manual calculation in MS Excel):

Parameter	No.1	No.2	No.3	No.4
N	4	4	4	4
df	1	1	1	1
R	0.999997223	0.999903725	0.999764643	0.999953165
R^2	0.999994446	0.999807458	0.999529342	0.999906331
$R^2_{adjusted}$	0.999983337	0.999422375	0.998588025	0.999718994
RSS	0.006218314	0.606128472	0.453451467	0.133907469

Graphical abstract of model fit presented as mean ± 1 SD of the fraction % of released carvedilol:

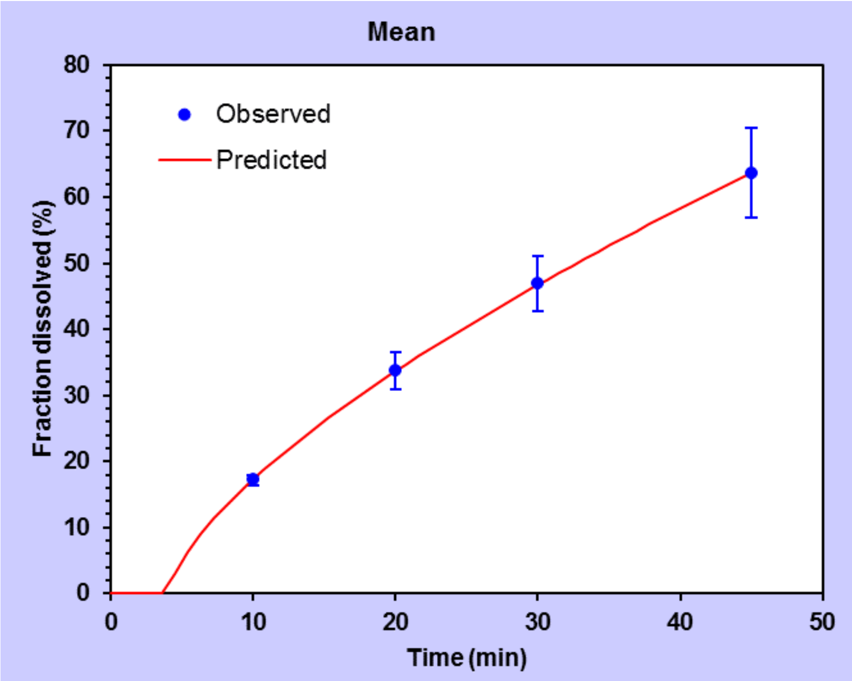

Supplementary materials – Model fitting summary – Polyglykol® 4000 P (modelling up to app. 60 % of carvedilol released)

Graphical abstract of model fit presented as the fraction % of released carvedilol per tested tablet:

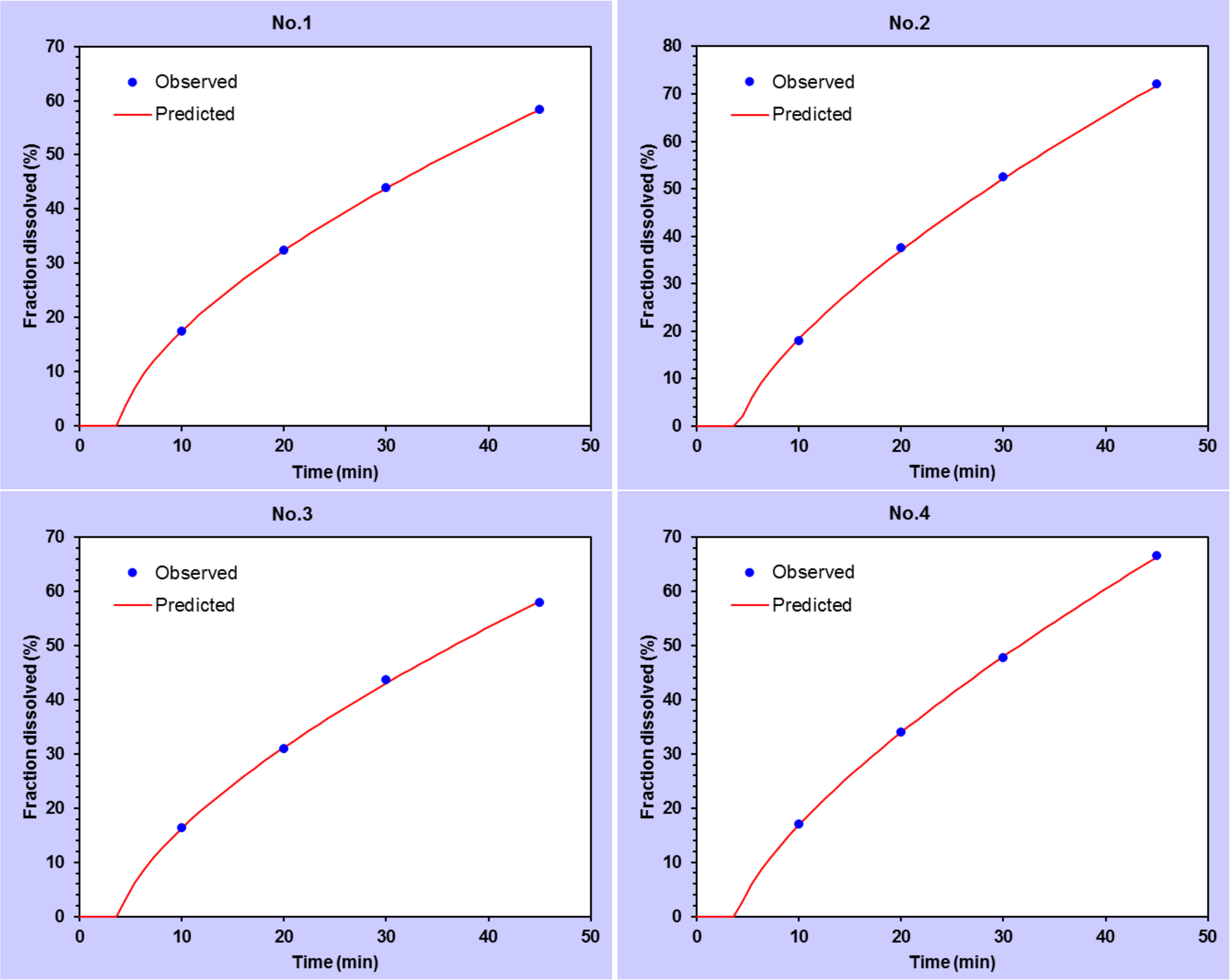

Supplementary materials – Model fitting summary – Polyglykol® 4000 P (modelling up to app. 60 % of carvedilol released)

Model: **Korsmeyer–Peppas with F_0**

Model equation: $F = F_0 + k_{KP} \cdot t^n$

Fitted model parameters per tested tablet (N = 4) with statistics – mean, standard deviation (SD), and relative standard deviation expressed in % (RSD%) (output from DDSolver):

Parameter	No.1	No.2	No.3	No.4	Mean	SD	RSD(%)
k_{KP}	0.979	0.731	0.800	0.776	0.821	0.109	13.238
n	1.044	1.201	1.113	1.134	1.123	0.065	5.764
F_0	9.349	7.159	6.519	8.266	7.823	1.247	15.944

Number of dissolution data points (N), degrees of freedom (df), and selected goodness of fit criteria – Pearson correlation coefficient (R), coefficient of determination (R^2), adjusted coefficient of determination ($R^2_{adjusted}$), and residual sum of squares (RSS) (manual calculation in MS Excel):

Parameter	No.1	No.2	No.3	No.4
N	4	4	4	4
df	1	1	1	1
R	0.993392831	0.990426553	0.991719742	0.994878559
R^2	0.986829316	0.980944758	0.983508048	0.989783347
$R^2_{adjusted}$	0.960487949	0.942834273	0.950524143	0.969350041
RSS	18.14285711	51.49362783	24.0096656	17.38281377

Graphical abstract of model fit presented as mean ± 1 SD of the fraction % of released carvedilol:

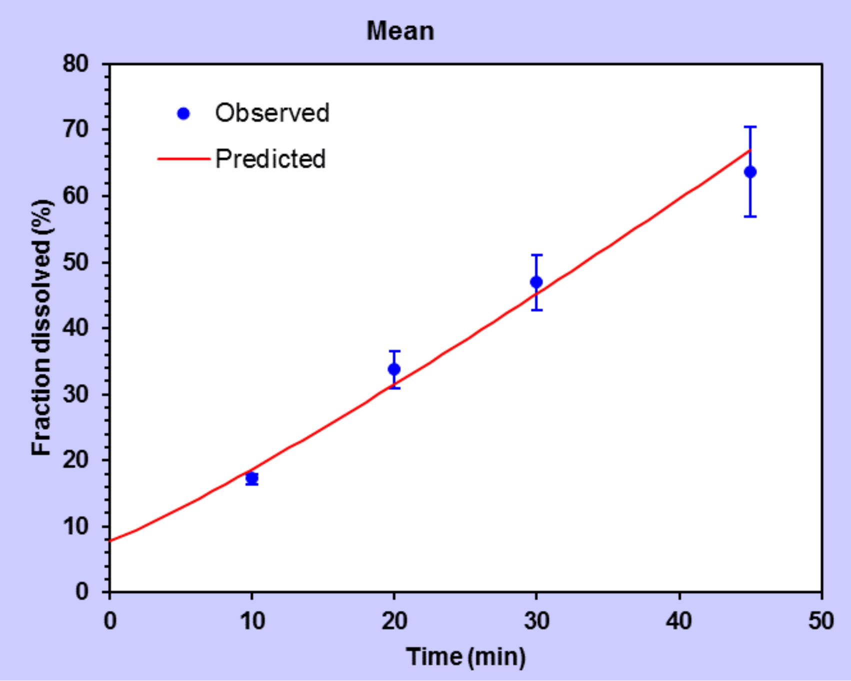

Supplementary materials – Model fitting summary – Polyglykol® 4000 P (modelling up to app. 60 % of carvedilol released)

Graphical abstract of model fit presented as the fraction % of released carvedilol per tested tablet:

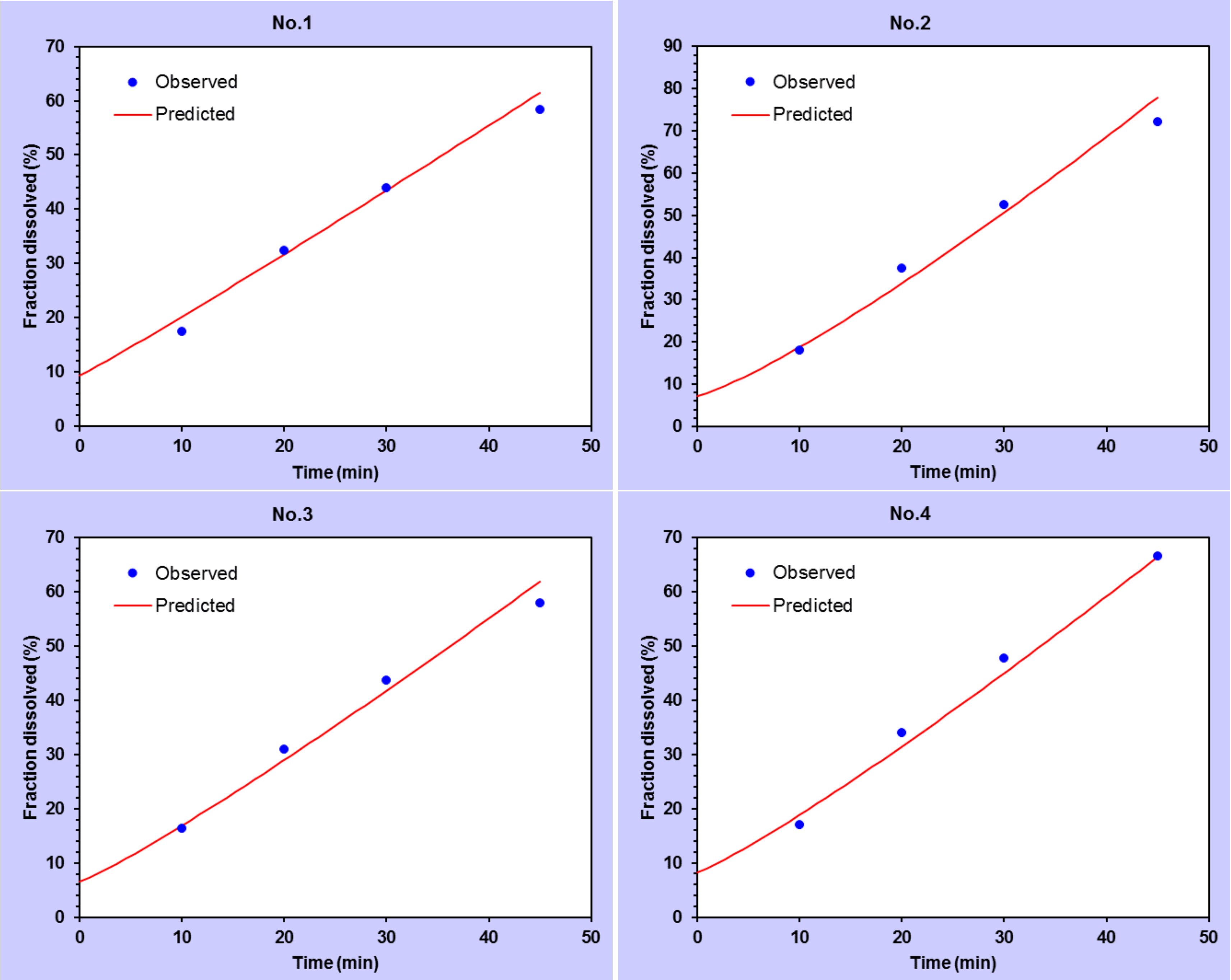

Supplementary materials – Model fitting summary – Polyglykol® 4000 P (modelling up to app. 60 % of carvedilol released)

Model: **Hixson–Crowell**

Model equation: $F = 100 \cdot [1 - (1 - k_{HC} \cdot t)^3]$

Fitted model parameters per tested tablet (N = 4) with statistics – mean, standard deviation (SD), and relative standard deviation expressed in % (RSD%) (output from DDSolver):

Parameter	No.1	No.2	No.3	No.4	Mean	SD	RSD(%)
k_{HC}	0.006	0.007	0.006	0.007	0.006	0.001	13.549

Number of dissolution data points (N), degrees of freedom (df), and selected goodness of fit criteria – Pearson correlation coefficient (R), coefficient of determination (R^2), adjusted coefficient of determination ($R^2_{adjusted}$), and residual sum of squares (RSS) (manual calculation in MS Excel):

Parameter	No.1	No.2	No.3	No.4
N	4	4	4	4
df	3	3	3	3
R	0.999396108	0.999783384	0.999448608	0.999731051
R^2	0.998792581	0.999566814	0.99889752	0.999462174
$R^2_{adjusted}$	0.998792581	0.999566814	0.99889752	0.999462174
RSS	5.766724332	12.36988729	1.811659657	5.704148101

Graphical abstract of model fit presented as mean ± 1 SD of the fraction % of released carvedilol:

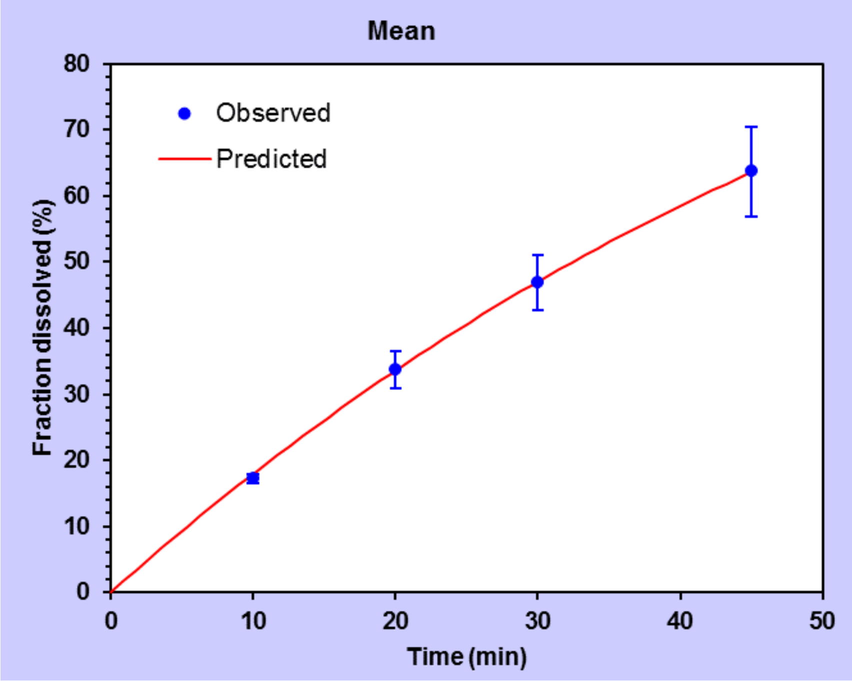

Supplementary materials – Model fitting summary – Polyglykol® 4000 P (modelling up to app. 60 % of carvedilol released)

Graphical abstract of model fit presented as the fraction % of released carvedilol per tested tablet:

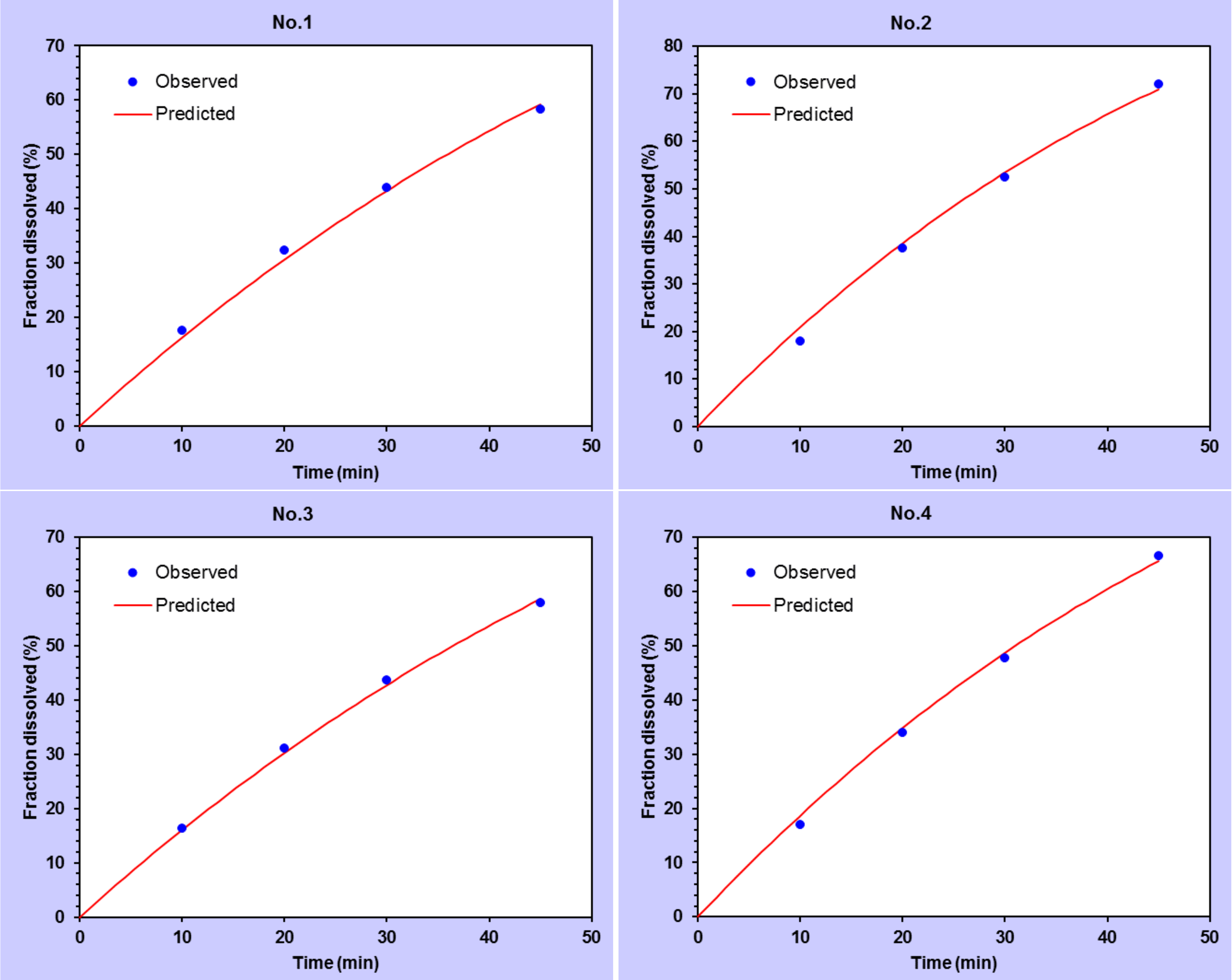

Supplementary materials – Model fitting summary – Polyglykol® 4000 P (modelling up to app. 60 % of carvedilol released)

Model: **Hixson–Crowell with T_{lag}**

Model equation: $F = 100 \cdot \left\{1 - \left[1 - k_{HC} \cdot \left(t - T_{lag}\right)\right]^3\right\}$

Fitted model parameters per tested tablet (N = 4) with statistics – mean, standard deviation (SD), and relative standard deviation expressed in % (RSD%) (output from DDSolver):

Parameter	No.1	No.2	No.3	No.4	Mean	SD	RSD(%)
k_{HC}	0.005	0.008	0.006	0.007	0.006	0.001	19.451
T_{lag}	-2.030	2.169	-0.899	1.637	0.219	2.010	916.838

Number of dissolution data points (N), degrees of freedom (df), and selected goodness of fit criteria – Pearson correlation coefficient (R), coefficient of determination (R^2), adjusted coefficient of determination ($R^2_{adjusted}$), and residual sum of squares (RSS) (manual calculation in MS Excel):

Parameter	No.1	No.2	No.3	No.4
N	4	4	4	4
df	2	2	2	2
R	0.99925735	0.9997194	0.99938609	0.9996537
R^2	0.99851525	0.99943887	0.99877256	0.99930751
$R^2_{adjusted}$	0.99777288	0.99915831	0.99815884	0.99896127
RSS	1.376751406	0.921193328	1.207509682	0.963775216

Graphical abstract of model fit presented as mean ± 1 SD of the fraction % of released carvedilol:

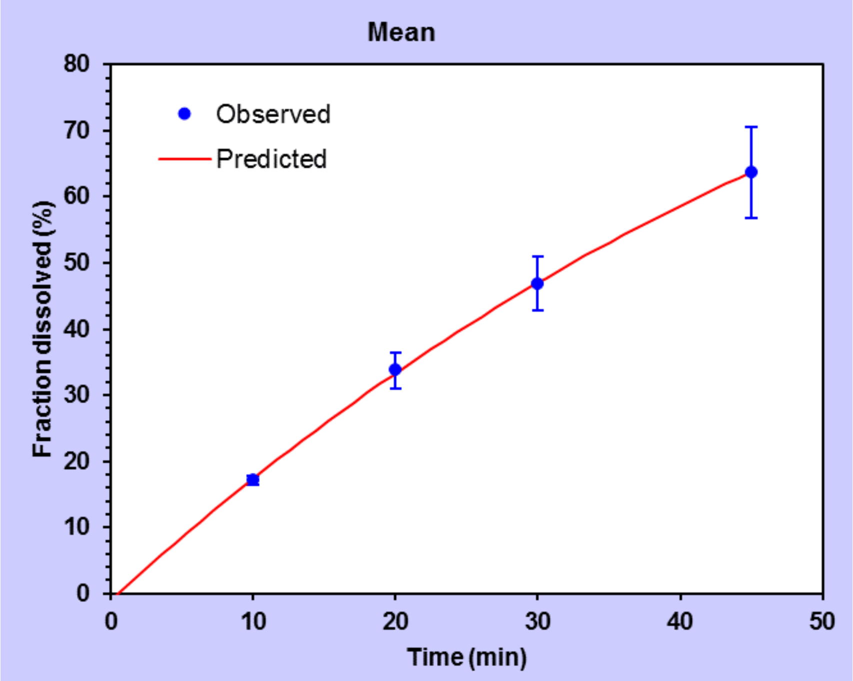

Supplementary materials – Model fitting summary – Polyglykol® 4000 P (modelling up to app. 60 % of carvedilol released)

Graphical abstract of model fit presented as the fraction % of released carvedilol per tested tablet:

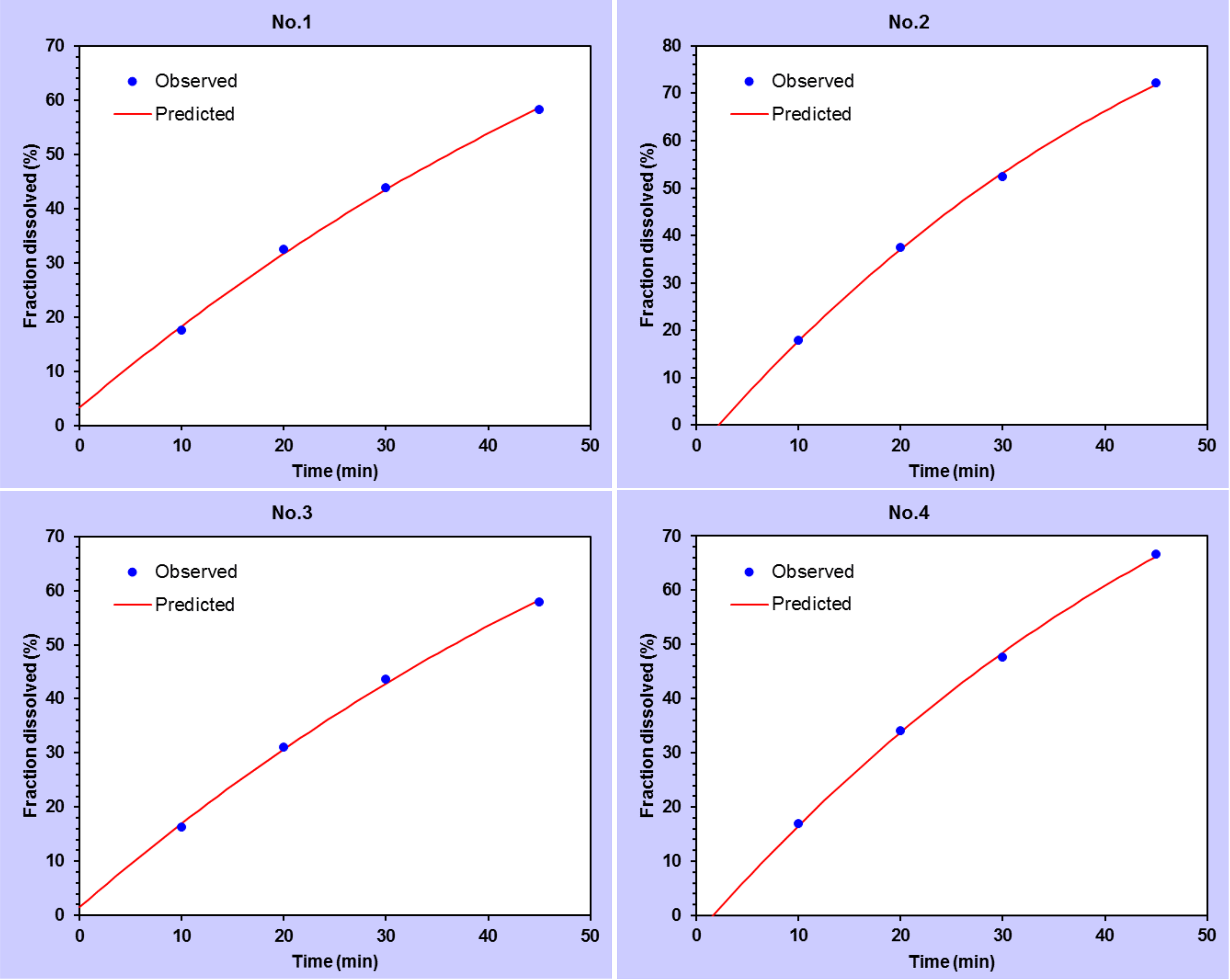

Supplementary materials – Model fitting summary – Polyglykol® 4000 P (modelling up to app. 60 % of carvedilol released)

Model: **Hopfenberg**

Model equation: $F = 100 \cdot [1 - (1 - k_{HB} \cdot t)^n]$

Fitted model parameters per tested tablet (N = 4) with statistics – mean, standard deviation (SD), and relative standard deviation expressed in % (RSD%) (output from DDSolver):

Parameter	No.1	No.2	No.3	No.4	Mean	SD	RSD(%)
k_{HB}	0.005	0.010	0.006	0.009	0.008	0.003	36.516
n	3.721	2.000	3.000	2.000	2.680	0.839	31.293

Number of dissolution data points (N), degrees of freedom (df), and selected goodness of fit criteria – Pearson correlation coefficient (R), coefficient of determination (R^2), adjusted coefficient of determination ($R^2_{adjusted}$), and residual sum of squares (RSS) (manual calculation in MS Excel):

Parameter	No.1	No.2	No.3	No.4
N	4	4	4	4
df	2	2	2	2
R	0.999584459	0.999642544	0.999448608	0.999829185
R^2	0.99916909	0.999285216	0.99889752	0.9996584
$R^2_{adjusted}$	0.998753635	0.998927825	0.998346279	0.9994876
RSS	3.633558808	3.586880287	1.811659657	1.025987972

Graphical abstract of model fit presented as mean ± 1 SD of the fraction % of released carvedilol:

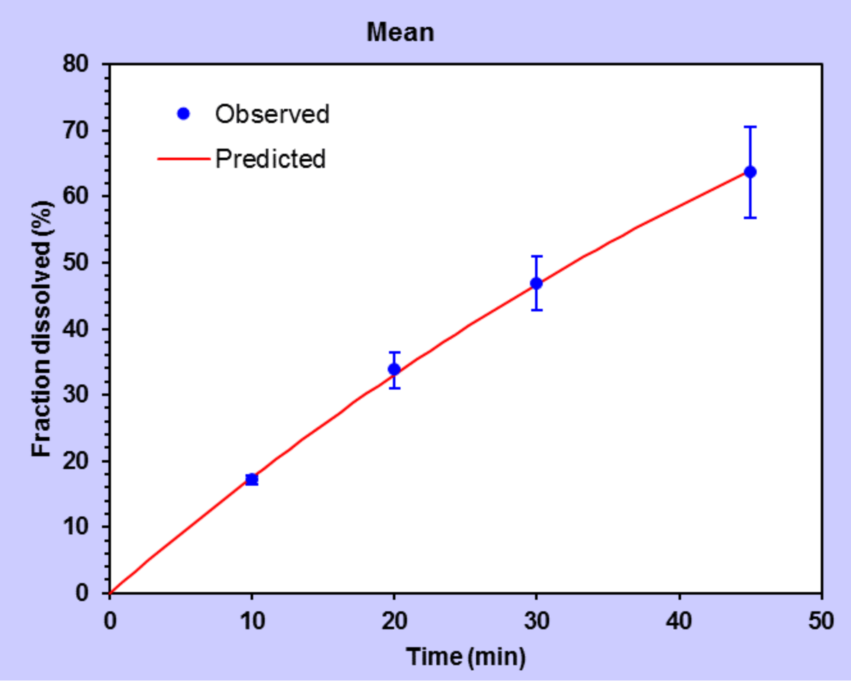

Supplementary materials – Model fitting summary – Polyglykol® 4000 P (modelling up to app. 60 % of carvedilol released)

Graphical abstract of model fit presented as the fraction % of released carvedilol per tested tablet:

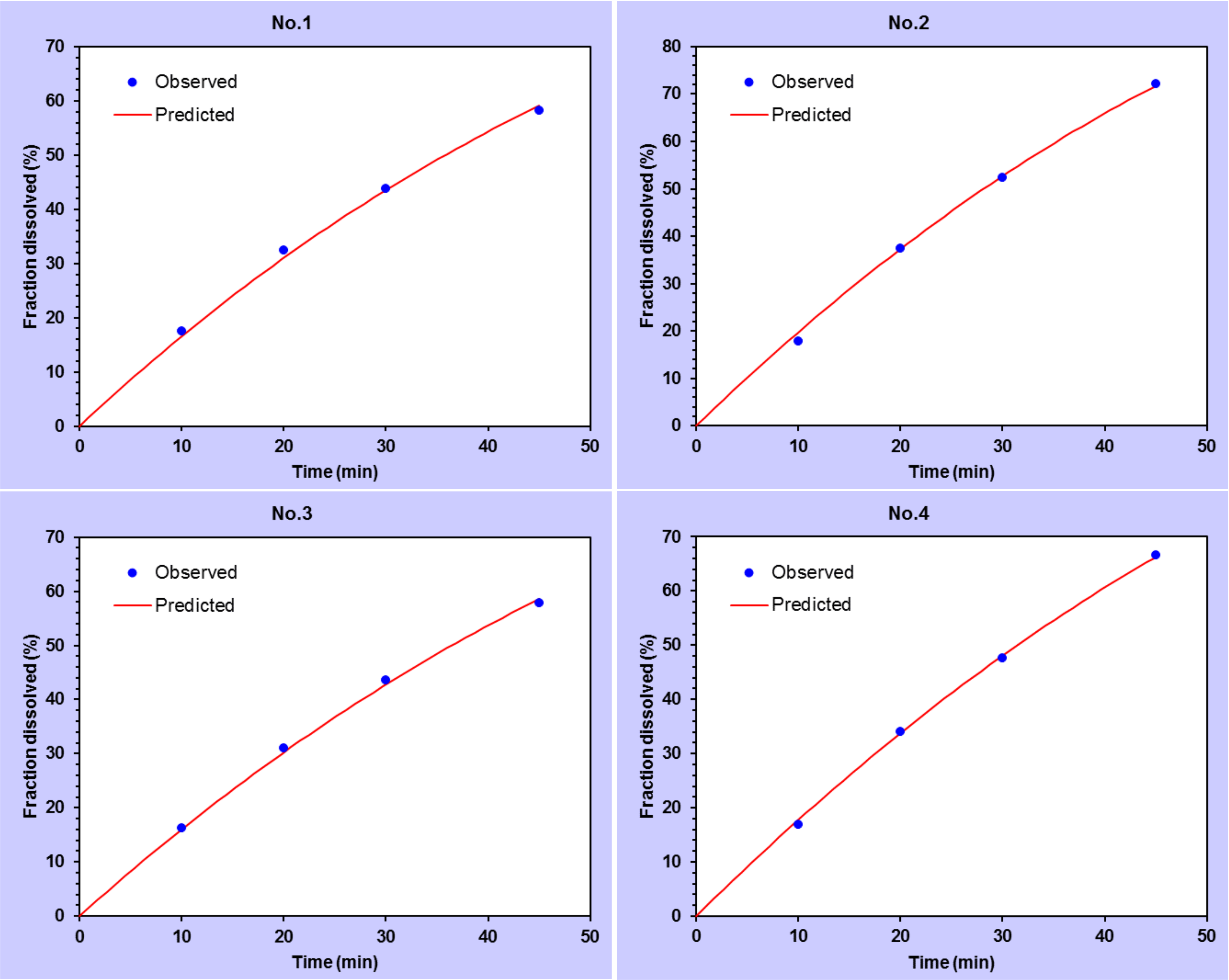

Supplementary materials – Model fitting summary – Polyglykol® 4000 P (modelling up to app. 60 % of carvedilol released)

Model: **Hopfenberg with T_{lag}**

Model equation: $F = 100 \cdot \{1 - [1 - k_{HB} \cdot (t - T_{lag})]^n\}$

Fitted model parameters per tested tablet (N = 4) with statistics – mean, standard deviation (SD), and relative standard deviation expressed in % (RSD%) (output from DDSolver):

Parameter	No.1	No.2	No.3	No.4	Mean	SD	RSD(%)
k_{HB}	0.005	0.008	0.006	0.009	0.007	0.002	28.007
n	3.000	3.000	3.000	2.000	2.750	0.500	18.182
T_{lag}	-2.030	2.169	-0.899	0.533	-0.057	1.817	-3204.003

Number of dissolution data points (N), degrees of freedom (df), and selected goodness of fit criteria – Pearson correlation coefficient (R), coefficient of determination (R^2), adjusted coefficient of determination ($R^2_{adjusted}$), and residual sum of squares (RSS) (manual calculation in MS Excel):

Parameter	No.1	No.2	No.3	No.4
N	4	4	4	4
df	1	1	1	1
R	0.999257352	0.999719397	0.999386092	0.999832389
R^2	0.998515255	0.999438873	0.998772561	0.999664807
$R^2_{adjusted}$	0.995545765	0.99831662	0.996317682	0.99899442
RSS	1.376751406	0.921193328	1.207509682	0.444676485

Graphical abstract of model fit presented as mean ± 1 SD of the fraction % of released carvedilol:

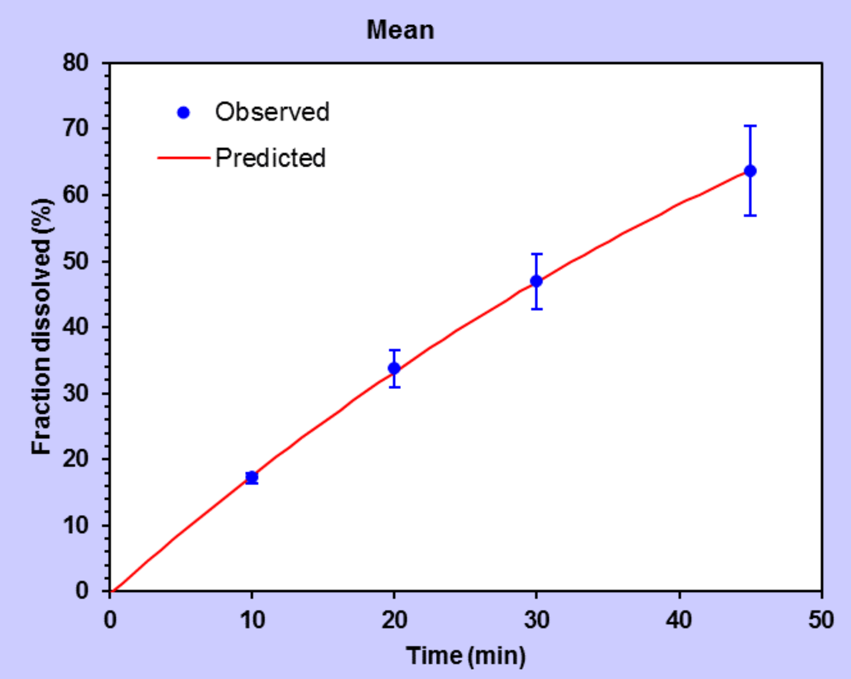

Supplementary materials – Model fitting summary – Polyglykol® 4000 P (modelling up to app. 60 % of carvedilol released)

Graphical abstract of model fit presented as the fraction % of released carvedilol per tested tablet:

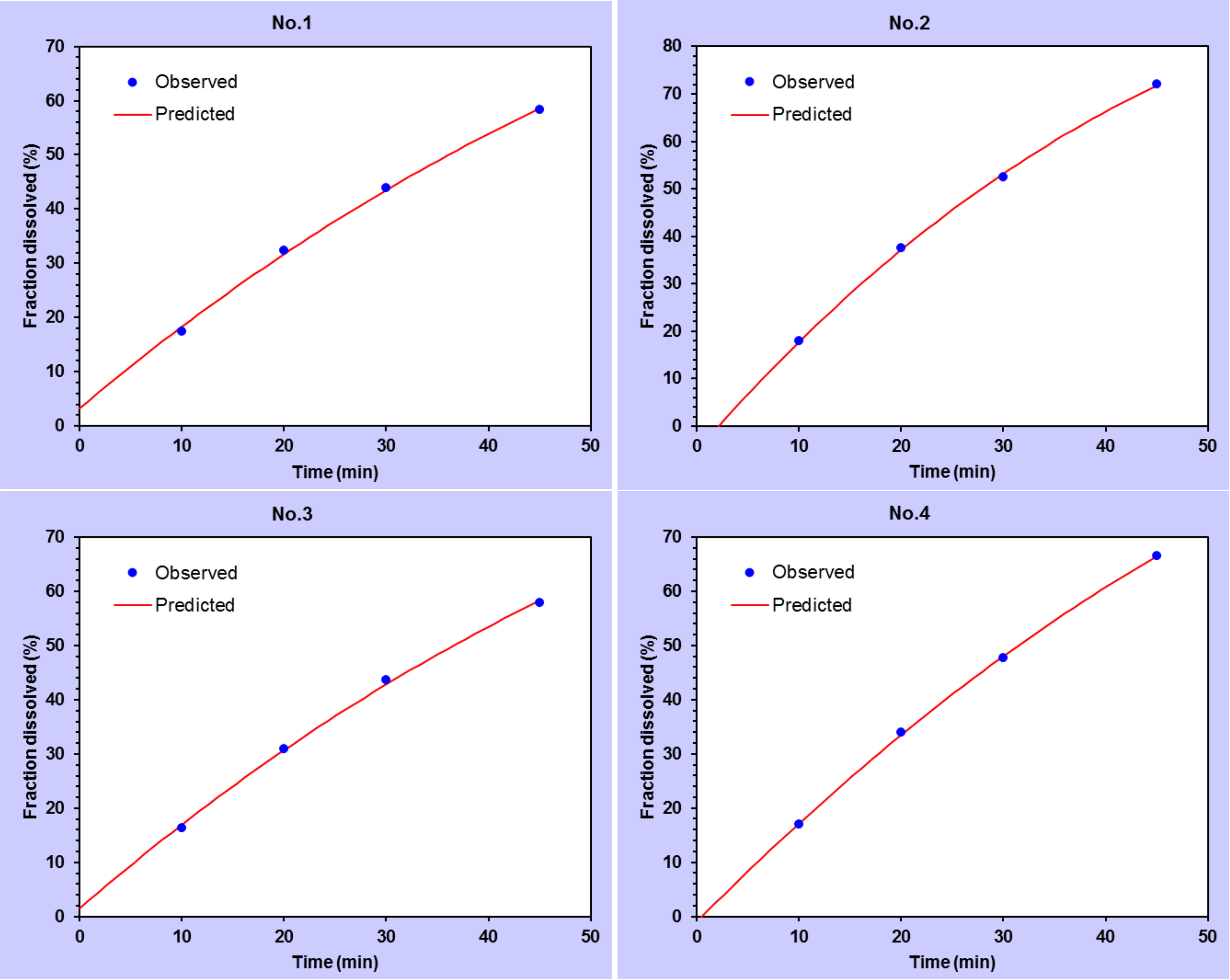

Supplementary materials – Model fitting summary – Polyglykol® 4000 P (modelling up to app. 60 % of carvedilol released)

Model: **Baker–Lonsdale**

Model equation: $\frac{3}{2}\cdot\left[1-\left(1-\frac{F}{100}\right)^{\frac{2}{3}}\right]-\frac{F}{100}=k_{BL}\cdot t$

Fitted model parameters per tested tablet (N = 4) with statistics – mean, standard deviation (SD), and relative standard deviation expressed in % (RSD%) (output from DDSolver):

Parameter	No.1	No.2	No.3	No.4	Mean	SD	RSD(%)
k_{BL}	0.002	0.004	0.002	0.003	0.003	0.001	29.299

Number of dissolution data points (N), degrees of freedom (df), and selected goodness of fit criteria – Pearson correlation coefficient (R), coefficient of determination (R^2), adjusted coefficient of determination ($R^2_{adjusted}$), and residual sum of squares (RSS) (manual calculation in MS Excel):

Parameter	No.1	No.2	No.3	No.4
N	4	4	4	4
df	3	3	3	3
R	0.998932608	0.996885936	0.998671902	0.995773747
R^2	0.997866356	0.99378157	0.997345568	0.991565354
$R^2_{adjusted}$	0.997866356	0.99378157	0.997345568	0.991565354
RSS	498.9323315	1255.749789	580.5152763	1040.864068

Graphical abstract of model fit presented as mean ± 1 SD of the fraction % of released carvedilol:

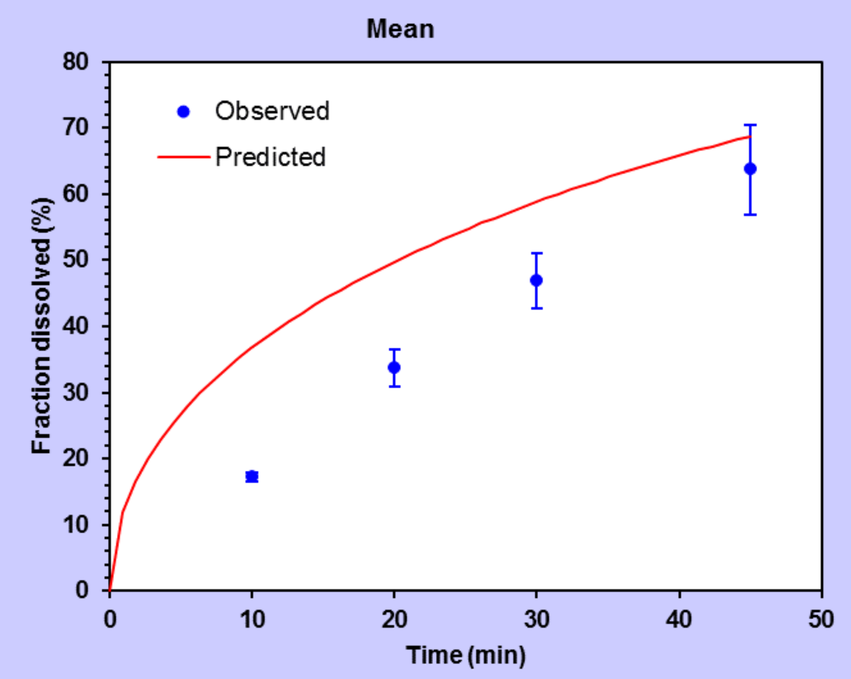

Supplementary materials – Model fitting summary – Polyglykol® 4000 P (modelling up to app. 60 % of carvedilol released)

Graphical abstract of model fit presented as the fraction % of released carvedilol per tested tablet:

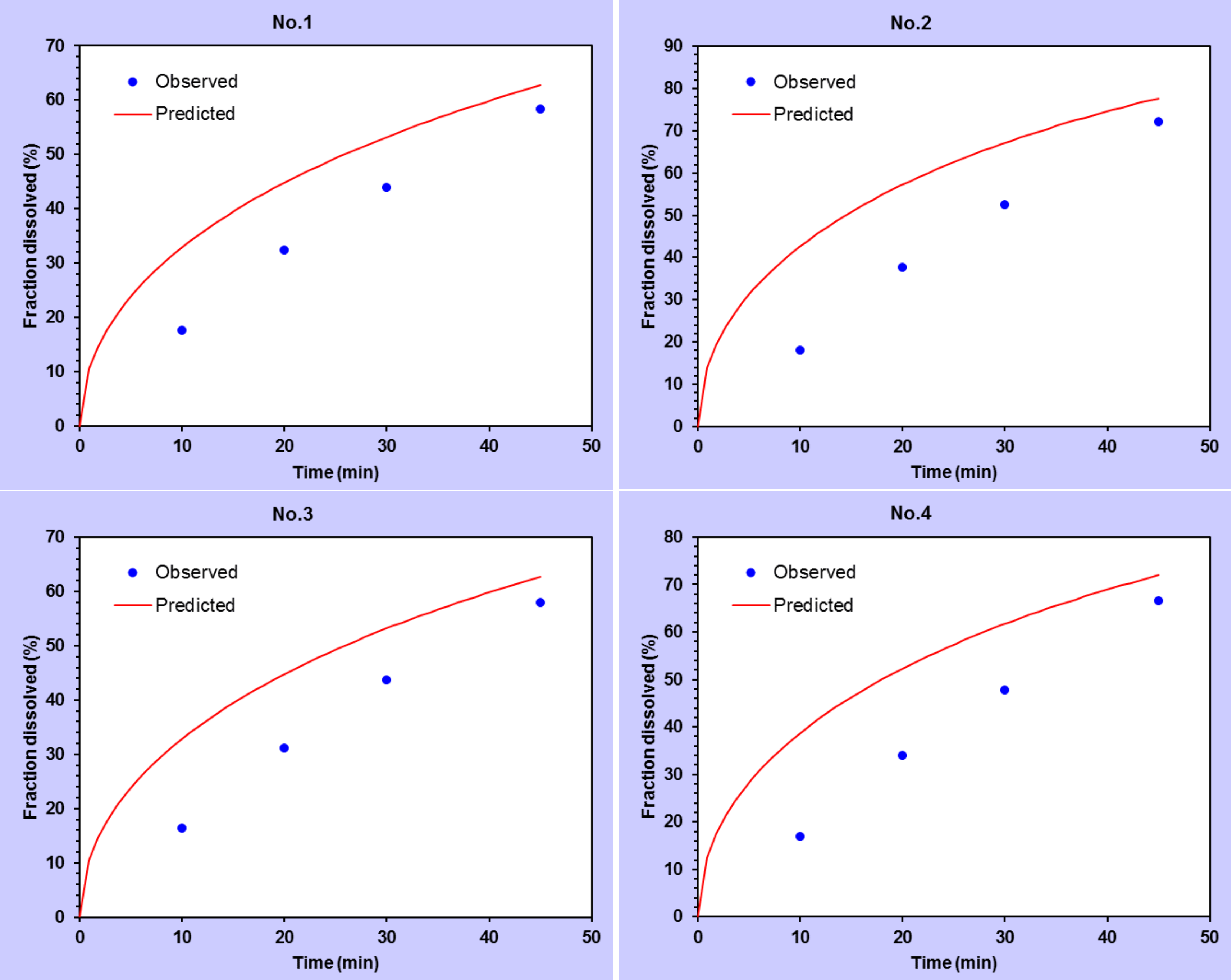

Supplementary materials – Model fitting summary – Polyglykol® 4000 P (modelling up to app. 60 % of carvedilol released)

Model: **Baker–Lonsdale with T_{lag}**

Model equation: $\frac{3}{2}\cdot\left[1-\left(1-\frac{F}{100}\right)^{\frac{2}{3}}\right]-\frac{F}{100}=k_{BL}\cdot\left(t-T_{lag}\right)$

Fitted model parameters per tested tablet (N = 4) with statistics – mean, standard deviation (SD), and relative standard deviation expressed in % (RSD%) (output from DDSolver):

Parameter	No.1	No.2	No.3	No.4	Mean	SD	RSD(%)
k_{BL}	0.002	0.004	0.002	0.003	0.003	0.001	29.299
T_{lag}	9.084	10.910	9.593	10.847	10.108	0.914	9.039

Number of dissolution data points (N), degrees of freedom (df), and selected goodness of fit criteria – Pearson correlation coefficient (R), coefficient of determination (R^2), adjusted coefficient of determination ($R^2_{adjusted}$), and residual sum of squares (RSS) (manual calculation in MS Excel):

Parameter	No.1	No.2	No.3	No.4
N	4	4	4	4
df	2	2	2	2
R	0.985699444	0.965756921	0.979687184	0.961939553
R^2	0.971603394	0.932686431	0.959786979	0.925327704
$R^2_{adjusted}$	0.957405092	0.899029646	0.939680468	0.887991556
RSS	55.52531374	349.1085181	94.12806904	312.4548916

Graphical abstract of model fit presented as mean ± 1 SD of the fraction % of released carvedilol:

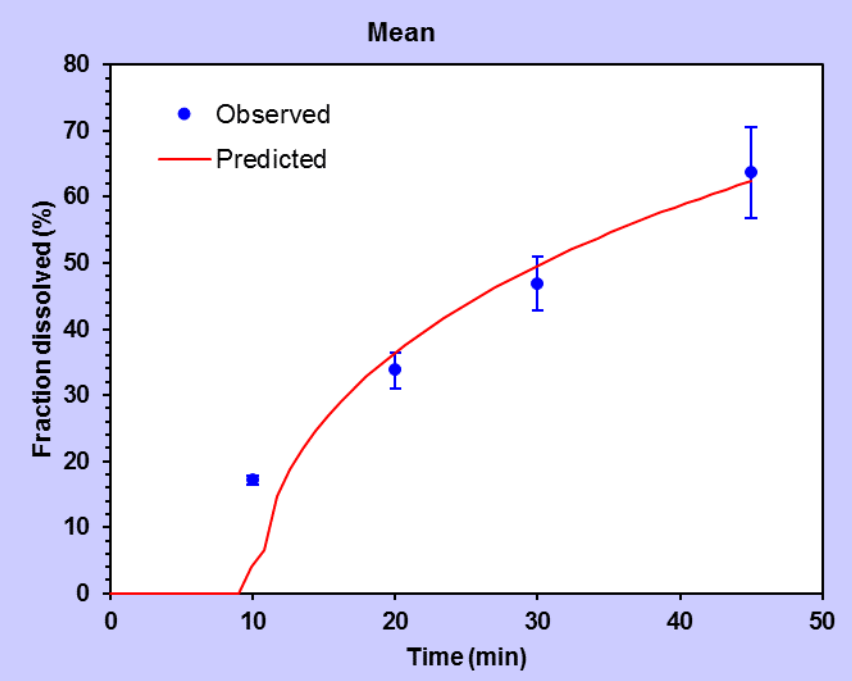

Supplementary materials – Model fitting summary – Polyglykol® 4000 P (modelling up to app. 60 % of carvedilol released)

Graphical abstract of model fit presented as the fraction % of released carvedilol per tested tablet:

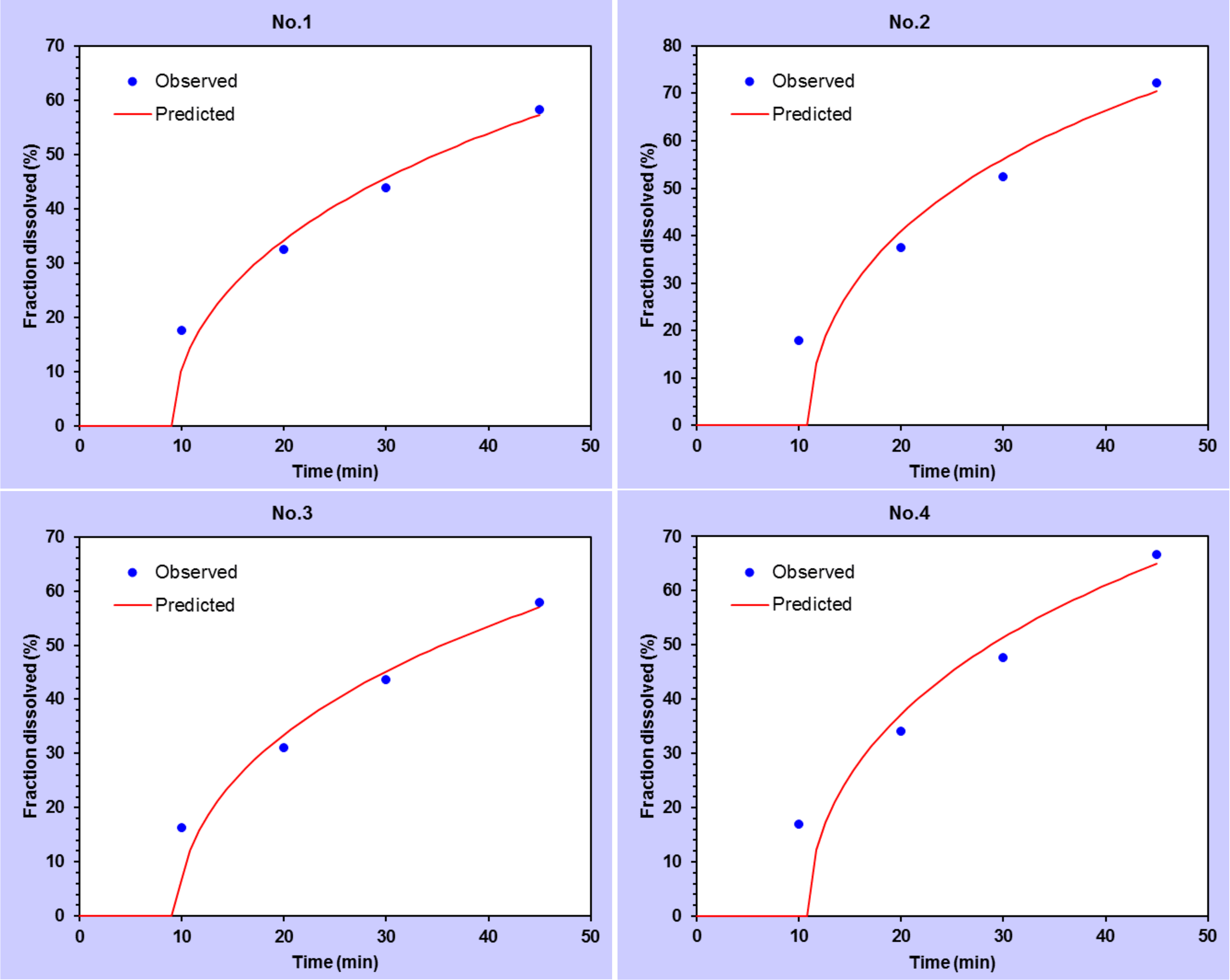

Supplementary materials – Model fitting summary – Polyglykol® 4000 P (modelling up to app. 60 % of carvedilol released)

Model: **Makoid–Banakar**

Model equation: $F = k_{MB} \cdot t^n \cdot e^{-k \cdot t}$

Fitted model parameters per tested tablet (N = 4) with statistics – mean, standard deviation (SD), and relative standard deviation expressed in % (RSD%) (output from DDSolver):

Parameter	No.1	No.2	No.3	No.4	Mean	SD	RSD(%)
k_{MB}	1.895	1.195	1.516	1.410	1.504	0.293	19.483
n	1.005	1.236	1.074	1.119	1.109	0.097	8.758
k	0.009	0.014	0.010	0.009	0.010	0.002	20.787

Number of dissolution data points (N), degrees of freedom (df), and selected goodness of fit criteria – Pearson correlation coefficient (R), coefficient of determination (R^2), adjusted coefficient of determination ($R^2_{adjusted}$), and residual sum of squares (RSS) (manual calculation in MS Excel):

Parameter	No.1	No.2	No.3	No.4
N	4	4	4	4
df	1	1	1	1
R	0.99989836	0.99962681	0.999990578	0.99977721
R^2	0.99979673	0.999253759	0.999981155	0.99955447
$R^2_{adjusted}$	0.999390191	0.997761276	0.999943466	0.998663411
RSS	0.182137255	1.176898333	0.017771827	0.591026048

Graphical abstract of model fit presented as mean ± 1 SD of the fraction % of released carvedilol:

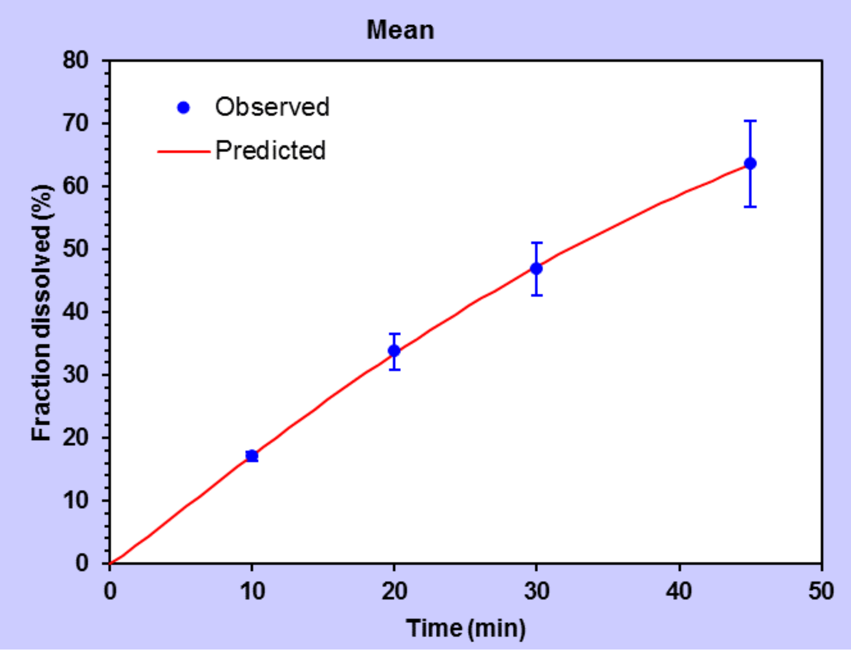

Supplementary materials – Model fitting summary – Polyglykol® 4000 P (modelling up to app. 60 % of carvedilol released)

Graphical abstract of model fit presented as the fraction % of released carvedilol per tested tablet:

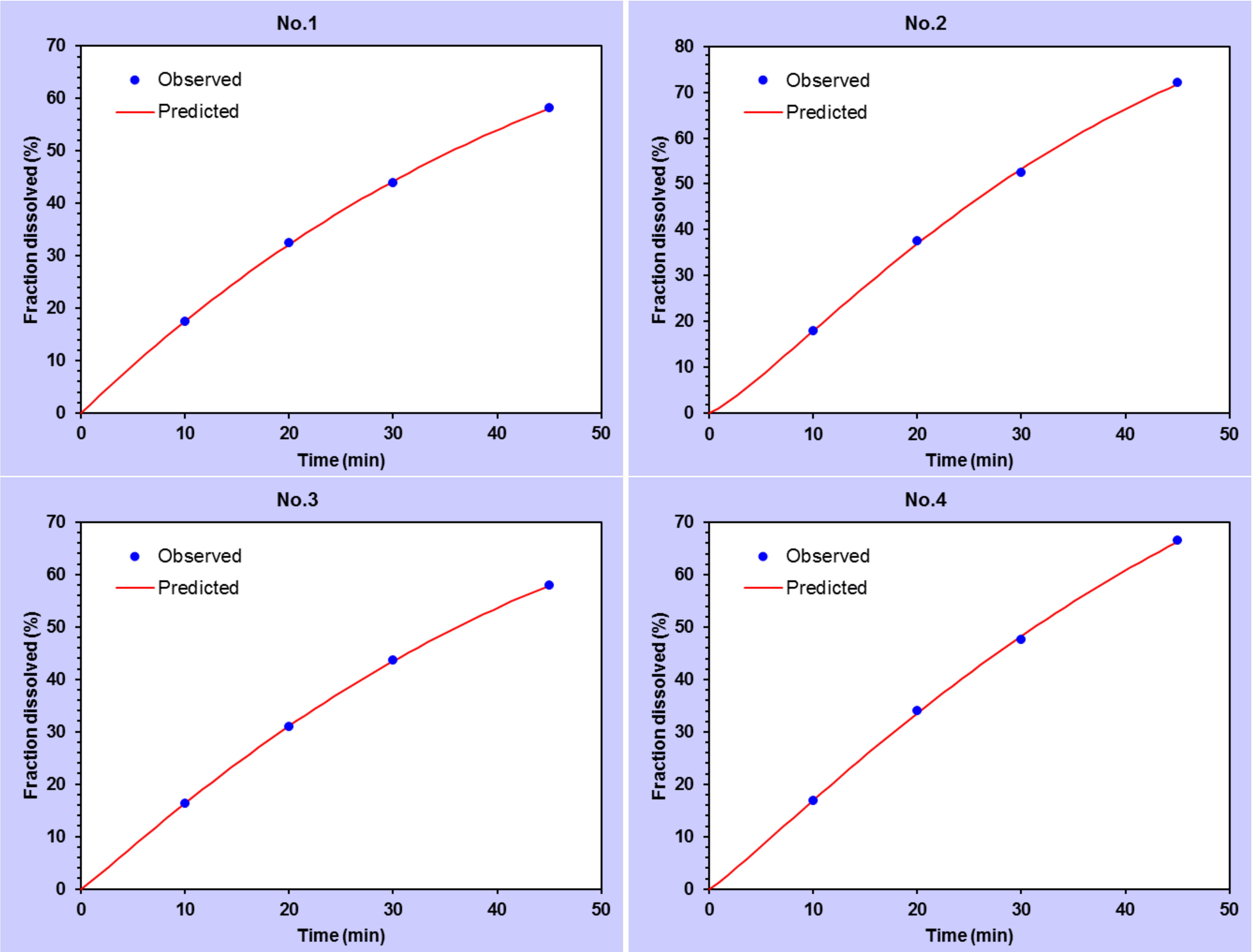

Supplementary materials – Model fitting summary – Polyglykol® 4000 P (modelling up to app. 60 % of carvedilol released)

Model: **Makoid–Banakar with T_{lag}**

Model equation: $F = k_{MB} \cdot (t - T_{lag})^n \cdot e^{-k\cdot(t-T_{lag})}$

Fitted model parameters per tested tablet (N = 4) with statistics – mean, standard deviation (SD), and relative standard deviation expressed in % (RSD%) (output from DDSolver):

Parameter	No.1	No.2	No.3	No.4	Mean	SD	RSD(%)
k_{MB}	/	/	/	/	/	/	/
n	/	/	/	/	/	/	/
k	/	/	/	/	/	/	/
T_{lag}	/	/	/	/	/	/	/

Number of dissolution data points (N), degrees of freedom (df), and selected goodness of fit criteria – Pearson correlation coefficient (R), coefficient of determination (R^2), adjusted coefficient of determination ($R^2_{adjusted}$), and residual sum of squares (RSS) (manual calculation in MS Excel):

Parameter	No.1	No.2	No.3	No.4
N	/	/	/	/
df	/	/	/	/
R	/	/	/	/
R^2	/	/	/	/
$R^2_{adjusted}$	/	/	/	/
RSS	/	/	/	/

Graphical abstract of model fit presented as mean ± 1 SD of the fraction % of released carvedilol: /

Graphical abstract of model fit presented as the fraction % of released carvedilol per tested tablet: /

Note: model could not be fitted to experimental dissolution data due too few data points being available for fitting

Supplementary materials – Model fitting summary – Polyglykol® 4000 P (modelling up to app. 60 % of carvedilol released)

Model: **Peppas–Sahlin_1**

Model equation: $F = k_1 \cdot t^m + k_2 \cdot t^{2m}$

Fitted model parameters per tested tablet (N = 4) with statistics – mean, standard deviation (SD), and relative standard deviation expressed in % (RSD%) (output from DDSolver):

Parameter	No.1	No.2	No.3	No.4	Mean	SD	RSD(%)
k_1	2.570	0.665	1.739	0.540	1.378	0.960	69.625
k_2	1.455	2.256	1.598	2.086	1.849	0.383	20.713
m	0.450	0.450	0.450	0.450	0.450	0.000	0.000

Number of dissolution data points (N), degrees of freedom (df), and selected goodness of fit criteria – Pearson correlation coefficient (R), coefficient of determination (R^2), adjusted coefficient of determination ($R^2_{adjusted}$), and residual sum of squares (RSS) (manual calculation in MS Excel):

Parameter	No.1	No.2	No.3	No.4
N	4	4	4	4
df	1	1	1	1
R	0.997812195	0.997457436	0.99741797	0.998818765
R^2	0.995629177	0.994921337	0.994842607	0.997638925
$R^2_{adjusted}$	0.986887532	0.98476401	0.98452782	0.992916775
RSS	3.994632049	8.139002724	4.947333986	3.180821955

Graphical abstract of model fit presented as mean ± 1 SD of the fraction % of released carvedilol:

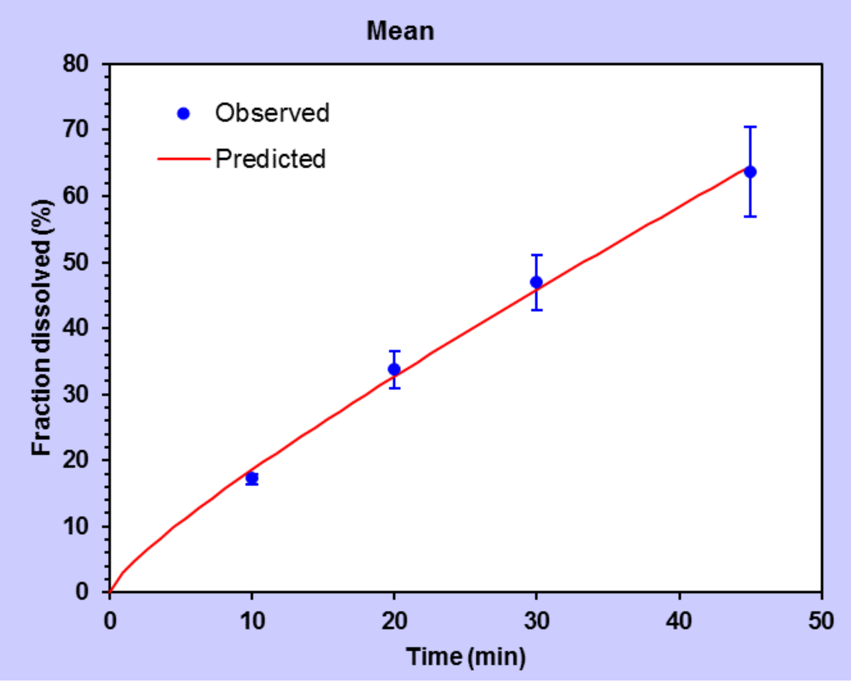

Supplementary materials – Model fitting summary – Polyglykol® 4000 P (modelling up to app. 60 % of carvedilol released)

Graphical abstract of model fit presented as the fraction % of released carvedilol per tested tablet:

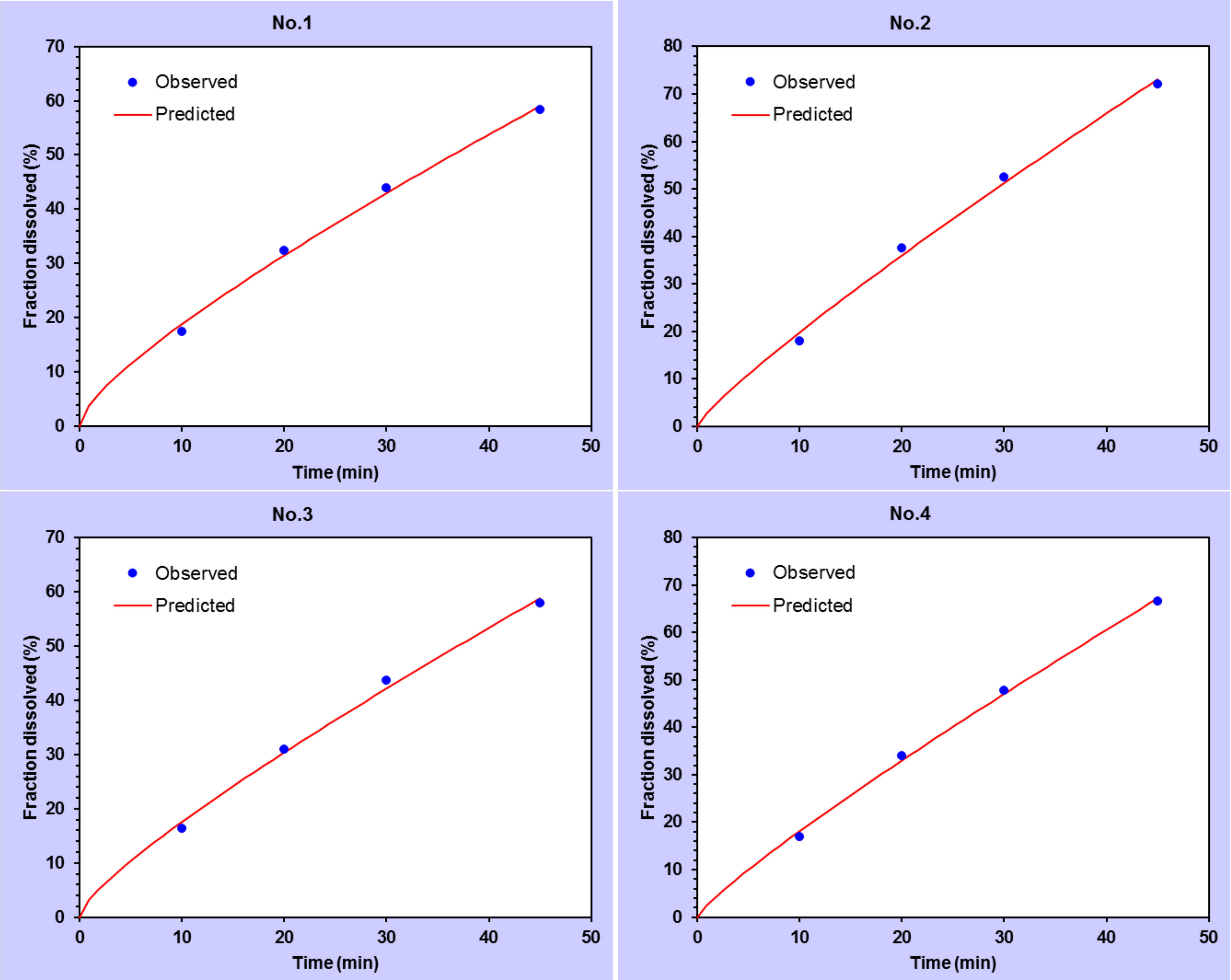

Supplementary materials – Model fitting summary – Polyglykol® 4000 P (modelling up to app. 60 % of carvedilol released)

Model: **Peppas–Sahlin_1 with T_{lag}**

Model equation: $F = k_1 \cdot (t - T_{lag})^m + k_2 \cdot (t - T_{lag})^{2m}$

Fitted model parameters per tested tablet (N = 4) with statistics – mean, standard deviation (SD), and relative standard deviation expressed in % (RSD%) (output from DDSolver):

Parameter	No.1	No.2	No.3	No.4	Mean	SD	RSD(%)
k_1	/	/	/	/	/	/	/
k_2	/	/	/	/	/	/	/
m	/	/	/	/	/	/	/
T_{lag}	/	/	/	/	/	/	/

Number of dissolution data points (N), degrees of freedom (df), and selected goodness of fit criteria – Pearson correlation coefficient (R), coefficient of determination (R^2), adjusted coefficient of determination ($R^2_{adjusted}$), and residual sum of squares (RSS) (manual calculation in MS Excel):

Parameter	No.1	No.2	No.3	No.4
N	/	/	/	/
df	/	/	/	/
R	/	/	/	/
R^2	/	/	/	/
$R^2_{adjusted}$	/	/	/	/
RSS	/	/	/	/

Graphical abstract of model fit presented as mean ± 1 SD of the fraction % of released carvedilol: /

Graphical abstract of model fit presented as the fraction % of released carvedilol per tested tablet: /

Note: model could not be fitted to experimental dissolution data due too few data points being available for fitting

Supplementary materials – Model fitting summary – Polyglykol® 4000 P (modelling up to app. 60 % of carvedilol released)

Model: **Peppas–Sahlin_2**

Model equation: $F = k_1 \cdot t^{0.5} + k_2 \cdot t$

Fitted model parameters per tested tablet (N = 4) with statistics – mean, standard deviation (SD), and relative standard deviation expressed in % (RSD%) (output from DDSolver):

Parameter	No.1	No.2	No.3	No.4	Mean	SD	RSD(%)
k_1	3.493	2.292	2.819	2.034	2.660	0.645	24.235
k_2	0.791	1.284	0.887	1.191	1.038	0.236	22.765

Number of dissolution data points (N), degrees of freedom (df), and selected goodness of fit criteria – Pearson correlation coefficient (R), coefficient of determination (R^2), adjusted coefficient of determination ($R^2_{adjusted}$), and residual sum of squares (RSS) (manual calculation in MS Excel):

Parameter	No.1	No.2	No.3	No.4
N	4	4	4	4
df	2	2	2	2
R	0.997474429	0.99685618	0.996949265	0.998390921
R^2	0.994955236	0.993722243	0.993907836	0.996784432
$R^2_{adjusted}$	0.992432854	0.990583364	0.990861754	0.995176648
RSS	4.657149664	10.1435329	5.896981187	4.367410878

Graphical abstract of model fit presented as mean ± 1 SD of the fraction % of released carvedilol:

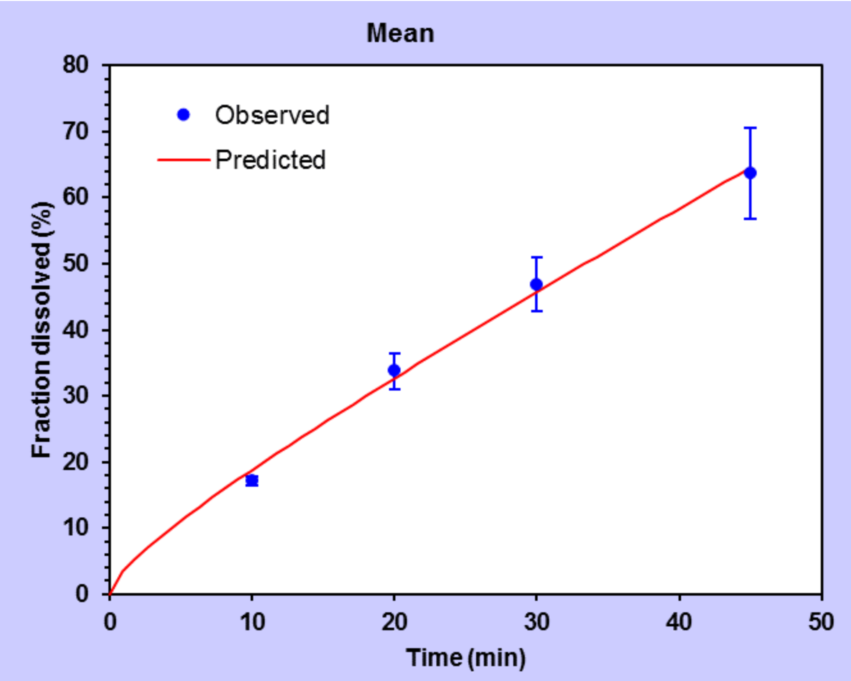

Supplementary materials – Model fitting summary – Polyglykol® 4000 P (modelling up to app. 60 % of carvedilol released)

Graphical abstract of model fit presented as the fraction % of released carvedilol per tested tablet:

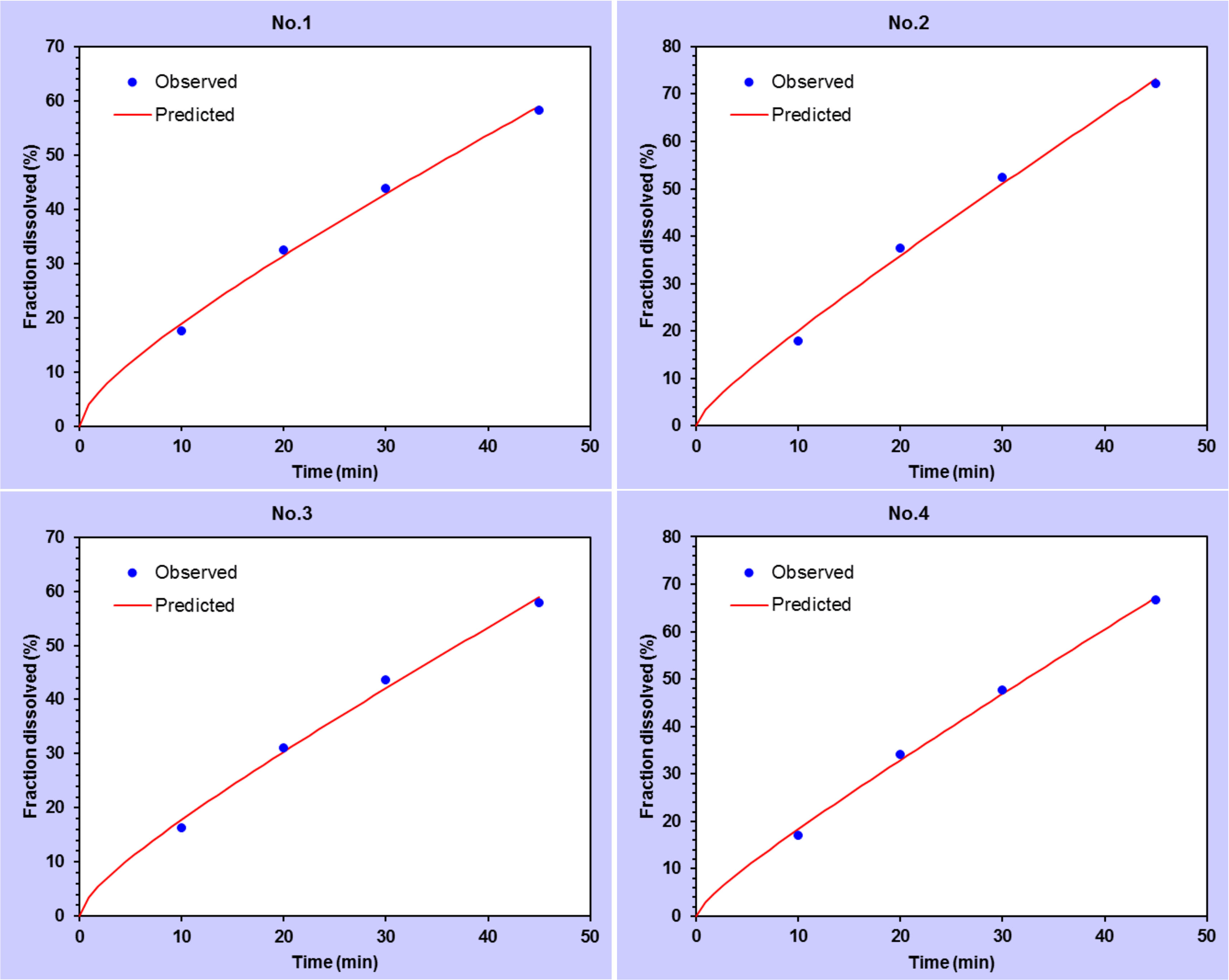

Supplementary materials – Model fitting summary – Polyglykol® 4000 P (modelling up to app. 60 % of carvedilol released)

Model: **Peppas–Sahlin_2 with T_{lag}**

Model equation: $F = k_1 \cdot (t - T_{lag})^{0.5} + k_2 \cdot (t - T_{lag})$

Fitted model parameters per tested tablet (N = 4) with statistics – mean, standard deviation (SD), and relative standard deviation expressed in % (RSD%) (output from DDSolver):

Parameter	No.1	No.2	No.3	No.4	Mean	SD	RSD(%)
k_1	6.215	5.589	5.507	5.038	5.587	0.484	8.665
k_2	0.456	0.897	0.562	0.840	0.689	0.213	30.975
T_{lag}	4.000	4.000	4.000	4.000	4.000	0.000	0.000

Number of dissolution data points (N), degrees of freedom (df), and selected goodness of fit criteria – Pearson correlation coefficient (R), coefficient of determination (R^2), adjusted coefficient of determination ($R^2_{adjusted}$), and residual sum of squares (RSS) (manual calculation in MS Excel):

Parameter	No.1	No.2	No.3	No.4
N	4	4	4	4
df	1	1	1	1
R	0.999774802	0.999235766	0.999340222	0.999844534
R^2	0.999549655	0.998472115	0.998680879	0.999689093
$R^2_{adjusted}$	0.998648965	0.995416346	0.996042637	0.999067278
RSS	0.434948631	2.561760681	1.303431594	0.435926745

Graphical abstract of model fit presented as mean ± 1 SD of the fraction % of released carvedilol:

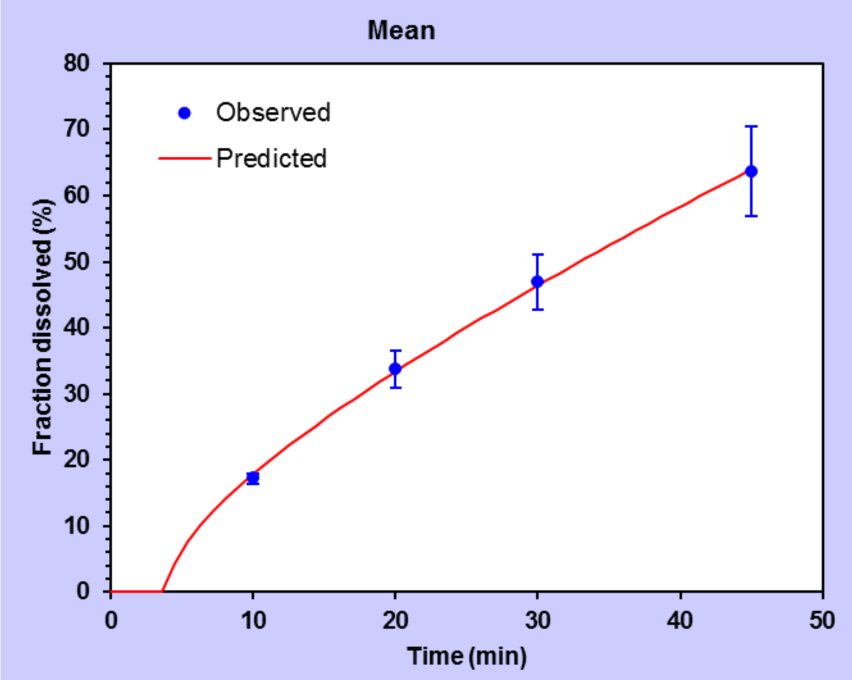

Supplementary materials – Model fitting summary – Polyglykol® 4000 P (modelling up to app. 60 % of carvedilol released)

Graphical abstract of model fit presented as the fraction % of released carvedilol per tested tablet:

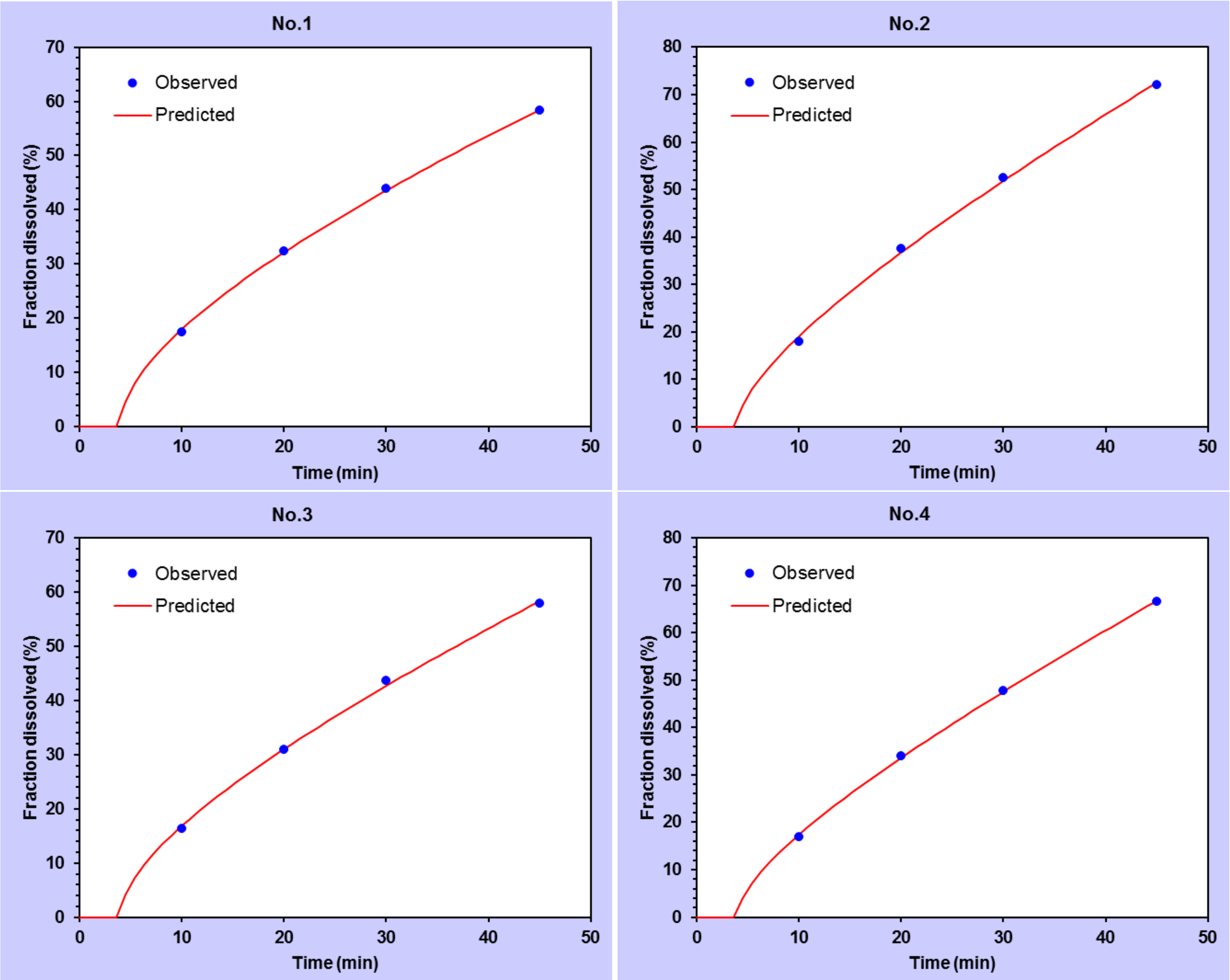

Supplementary materials – Model fitting summary – Polyglykol® 4000 P (modelling up to app. 60 % of carvedilol released)

Model: **Quadratic**

Model equation: $F = 100 \cdot (k_1 \cdot t^2 + k_2 \cdot t)$

Fitted model parameters per tested tablet (N = 4) with statistics – mean, standard deviation (SD), and relative standard deviation expressed in % (RSD%) (output from DDSolver):

Parameter	No.1	No.2	No.3	No.4	Mean	SD	RSD(%)
k_1	0.000	0.000	0.000	0.000	0.000	0.000	-20.590
k_2	0.019	0.020	0.018	0.018	0.019	0.001	5.818

Number of dissolution data points (N), degrees of freedom (df), and selected goodness of fit criteria – Pearson correlation coefficient (R), coefficient of determination (R^2), adjusted coefficient of determination ($R^2_{adjusted}$), and residual sum of squares (RSS) (manual calculation in MS Excel):

Parameter	No.1	No.2	No.3	No.4
N	4	4	4	4
df	2	2	2	2
R	0.999803421	0.999343635	0.999952366	0.999766497
R^2	0.99960688	0.9986877	0.999904734	0.999533048
$R^2_{adjusted}$	0.99941032	0.99803155	0.999857101	0.999299572
RSS	0.39991455	2.539652019	0.122507746	0.682598722

Graphical abstract of model fit presented as mean ± 1 SD of the fraction % of released carvedilol:

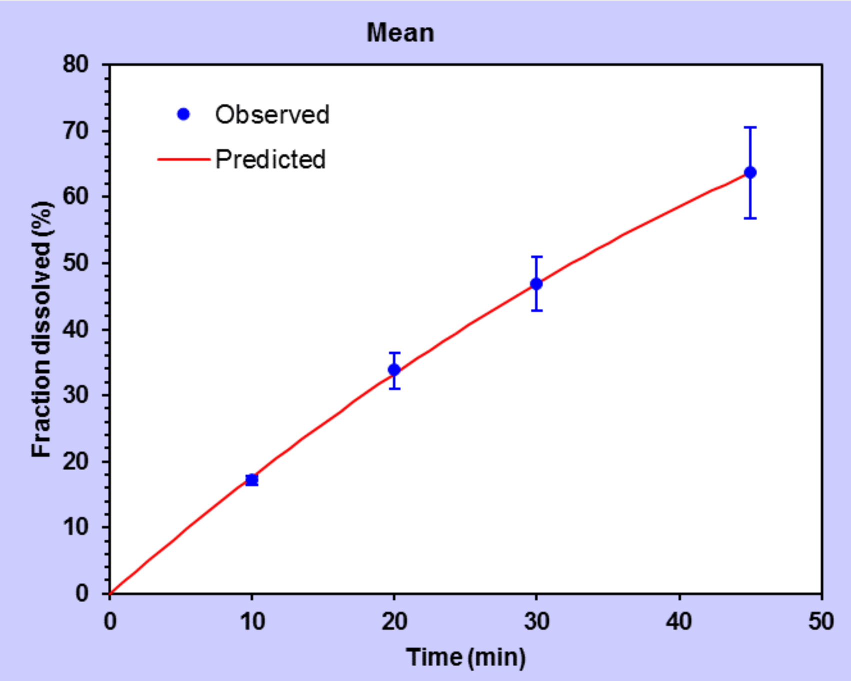

Supplementary materials – Model fitting summary – Polyglykol® 4000 P (modelling up to app. 60 % of carvedilol released)

Graphical abstract of model fit presented as the fraction % of released carvedilol per tested tablet:

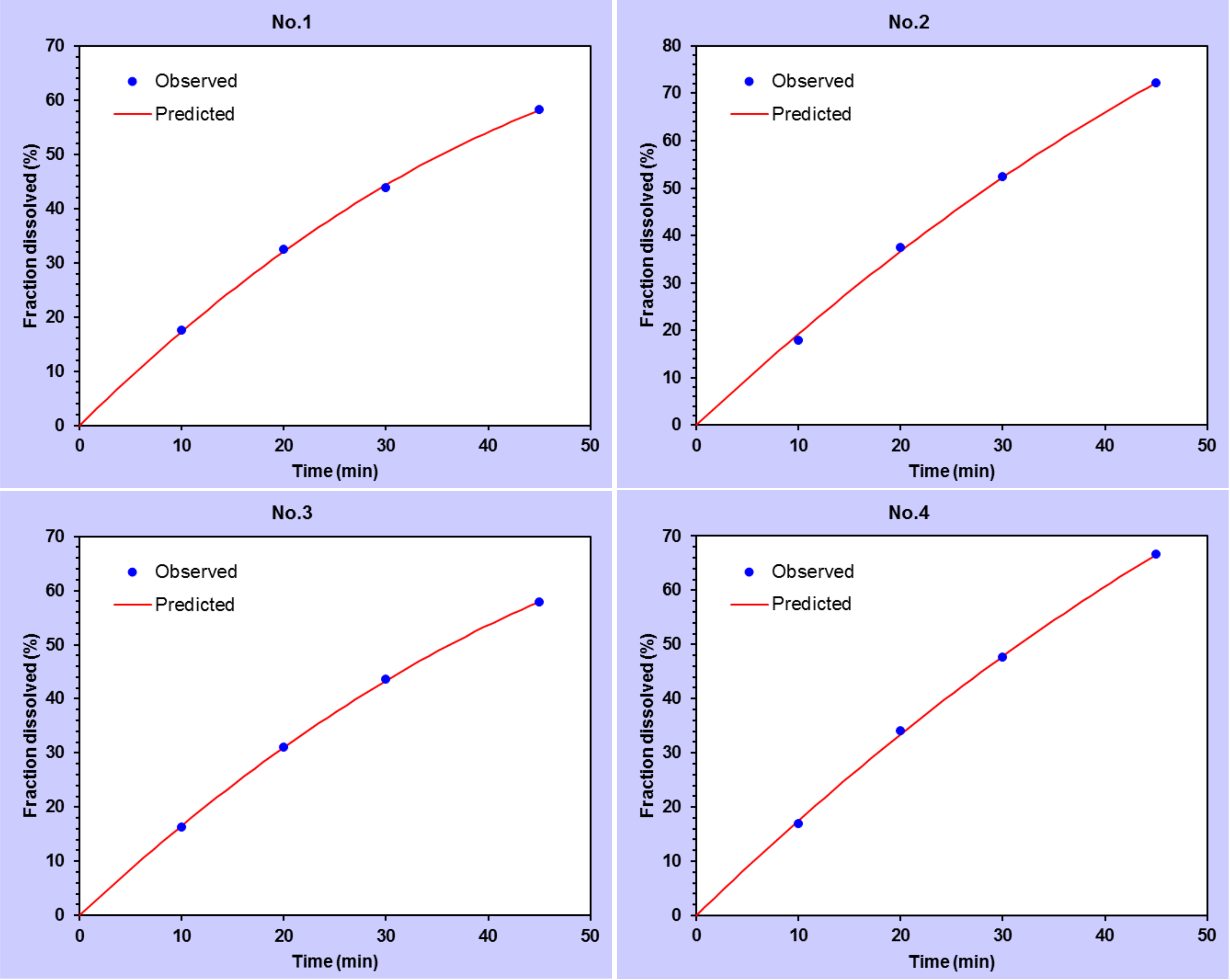

Supplementary materials – Model fitting summary – Polyglykol® 4000 P (modelling up to app. 60 % of carvedilol released)

Model: **Quadratic with T_{lag}**

Model equation: $F = 100 \cdot \left[k_1 \cdot (t - T_{lag})^2 + k_2 \cdot (t - T_{lag})\right]$

Fitted model parameters per tested tablet (N = 4) with statistics – mean, standard deviation (SD), and relative standard deviation expressed in % (RSD%) (output from DDSolver):

Parameter	No.1	No.2	No.3	No.4	Mean	SD	RSD(%)
k_1	0.000	0.000	0.000	0.000	0.000	0.000	-8.556
k_2	0.024	0.027	0.023	0.024	0.025	0.002	6.427
T_{lag}	4.000	4.000	4.000	4.000	4.000	0.000	0.000

Number of dissolution data points (N), degrees of freedom (df), and selected goodness of fit criteria – Pearson correlation coefficient (R), coefficient of determination (R^2), adjusted coefficient of determination ($R^2_{adjusted}$), and residual sum of squares (RSS) (manual calculation in MS Excel):

Parameter	No.1	No.2	No.3	No.4
N	4	4	4	4
df	1	1	1	1
R	0.99423826	0.997999528	0.996479006	0.997116359
R^2	0.988509717	0.996003058	0.99297041	0.994241033
$R^2_{adjusted}$	0.96552915	0.988009174	0.978911231	0.9827231
RSS	20.67182333	10.96403941	12.93835643	13.4830513

Graphical abstract of model fit presented as mean ± 1 SD of the fraction % of released carvedilol:

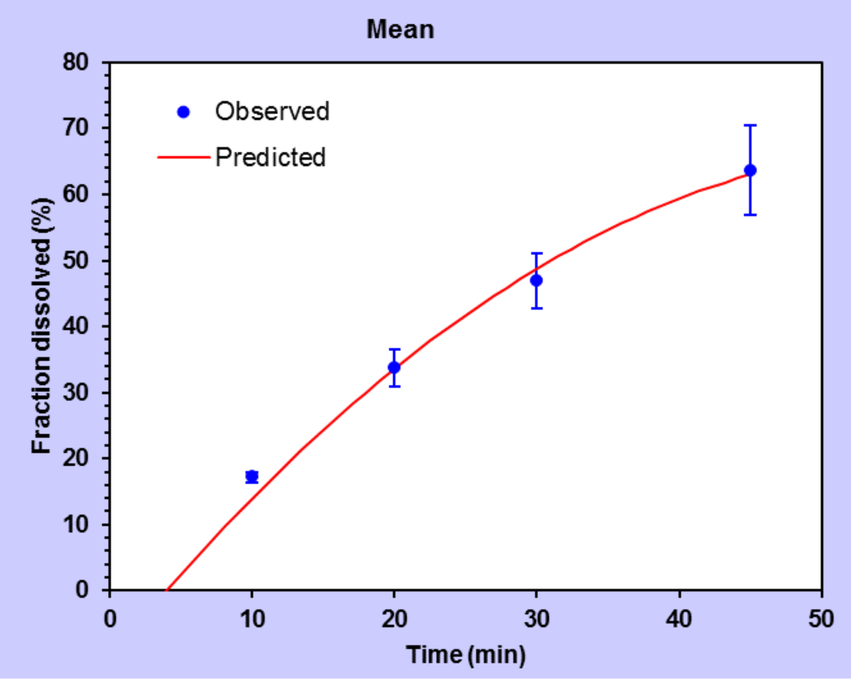

Supplementary materials – Model fitting summary – Polyglykol® 4000 P (modelling up to app. 60 % of carvedilol released)

Graphical abstract of model fit presented as the fraction % of released carvedilol per tested tablet:

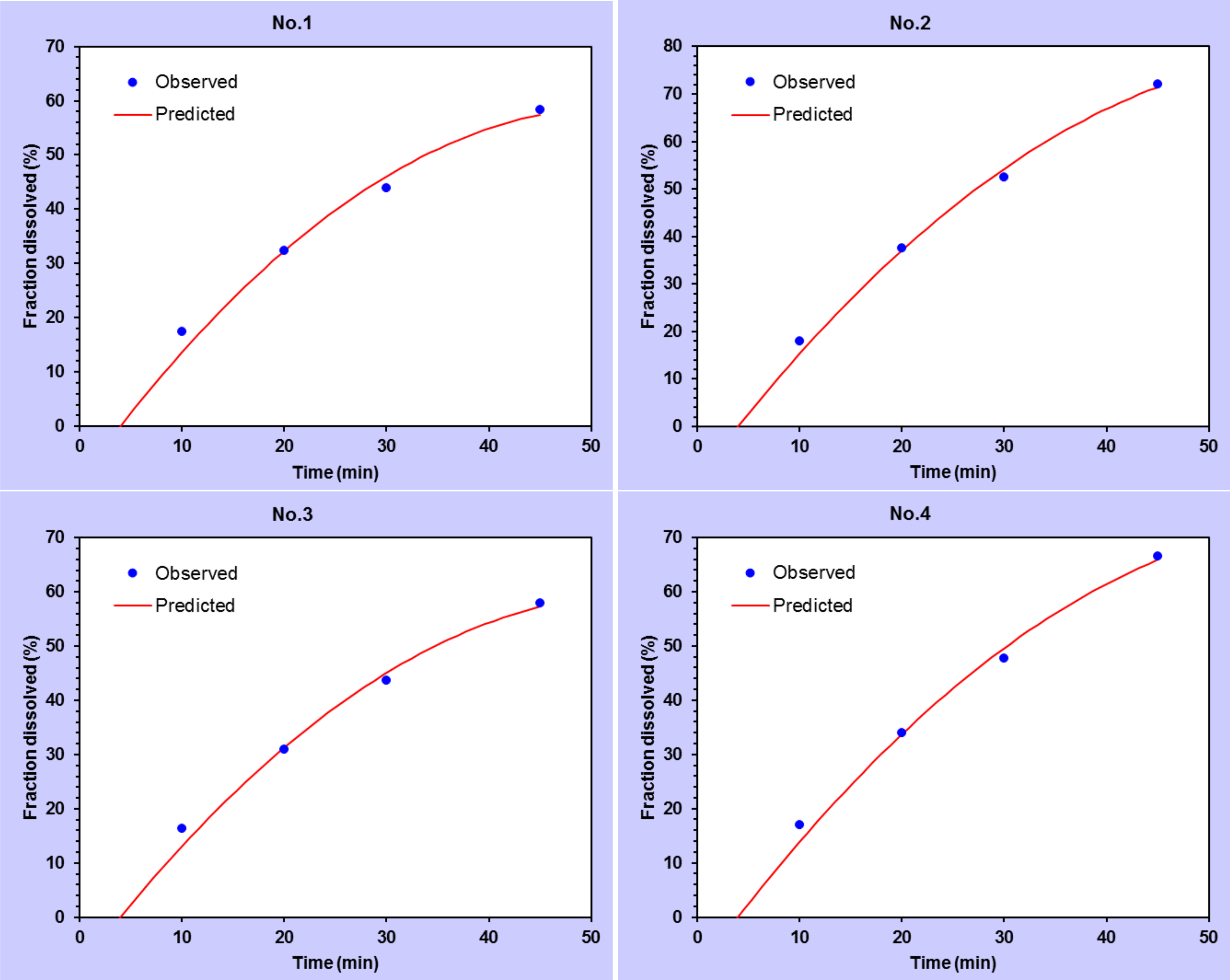

Supplementary materials – Model fitting summary – Polyglykol® 4000 P (modelling up to app. 60 % of carvedilol released)

Model: **Weibull_1**

Model equation: $F = 100 \cdot \left[1 - e^{-\frac{(t-T_i)^{\beta}}{\alpha}}\right]$

Fitted model parameters per tested tablet (N = 4) with statistics – mean, standard deviation (SD), and relative standard deviation expressed in % (RSD%) (output from DDSolver):

Parameter	No.1	No.2	No.3	No.4	Mean	SD	RSD(%)
α	21.520	29.083	25.001	28.654	26.065	3.540	13.582
β	0.781	0.958	0.819	0.911	0.867	0.081	9.385
T_i	4.000	4.000	4.000	4.000	4.000	0.000	0.000

Number of dissolution data points (N), degrees of freedom (df), and selected goodness of fit criteria – Pearson correlation coefficient (R), coefficient of determination (R^2), adjusted coefficient of determination ($R^2_{adjusted}$), and residual sum of squares (RSS) (manual calculation in MS Excel):

Parameter	No.1	No.2	No.3	No.4
N	4	4	4	4
df	1	1	1	1
R	0.998445382	0.997404448	0.998727443	0.996547199
R^2	0.99689318	0.994815632	0.997456506	0.99310632
$R^2_{adjusted}$	0.99067954	0.984446896	0.992369517	0.97931896
RSS	3.082443079	8.786383817	2.739281693	10.06960059

Graphical abstract of model fit presented as mean ± 1 SD of the fraction % of released carvedilol:

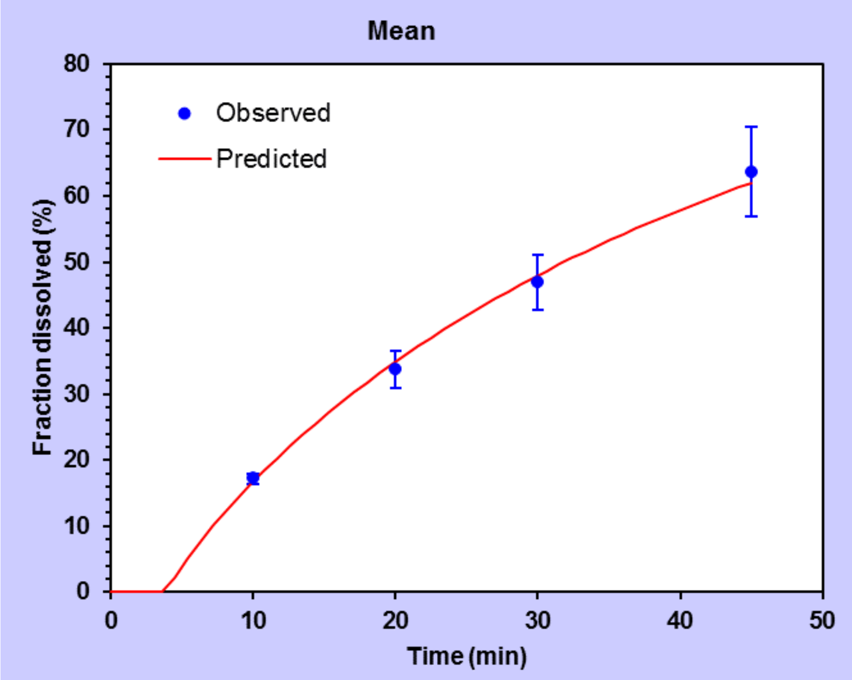

Supplementary materials – Model fitting summary – Polyglykol® 4000 P (modelling up to app. 60 % of carvedilol released)

Graphical abstract of model fit presented as the fraction % of released carvedilol per tested tablet:

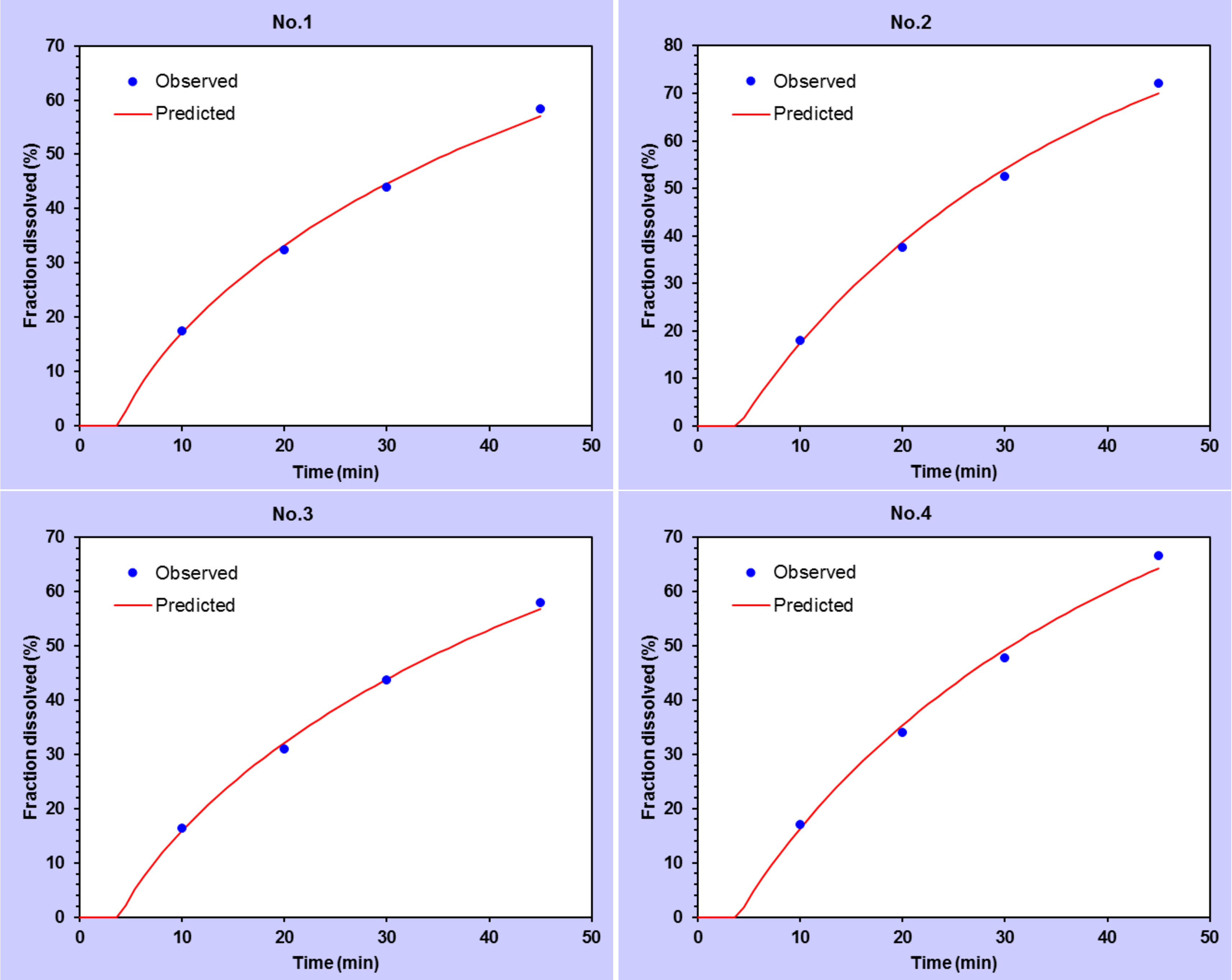

Supplementary materials – Model fitting summary – Polyglykol® 4000 P (modelling up to app. 60 % of carvedilol released)

Model: **Weibull_2**

Model equation: $F = 100 \cdot \left(1 - e^{-\frac{t^{\beta}}{\alpha}}\right)$

Fitted model parameters per tested tablet (N = 4) with statistics – mean, standard deviation (SD), and relative standard deviation expressed in % (RSD%) (output from DDSolver):

Parameter	No.1	No.2	No.3	No.4	Mean	SD	RSD(%)
α	52.179	86.392	63.353	80.945	70.717	15.792	22.332
β	1.003	1.231	1.053	1.173	1.115	0.105	9.429

Number of dissolution data points (N), degrees of freedom (df), and selected goodness of fit criteria – Pearson correlation coefficient (R), coefficient of determination (R^2), adjusted coefficient of determination ($R^2_{adjusted}$), and residual sum of squares (RSS) (manual calculation in MS Excel):

Parameter	No.1	No.2	No.3	No.4
N	4	4	4	4
df	2	2	2	2
R	0.999926451	0.999550582	0.999932515	0.999433286
R^2	0.999852907	0.999101366	0.999865034	0.998866894
$R^2_{adjusted}$	0.99977936	0.998652049	0.99979755	0.998300341
RSS	0.135324458	1.420500106	0.135744788	1.553332303

Graphical abstract of model fit presented as mean ± 1 SD of the fraction % of released carvedilol:

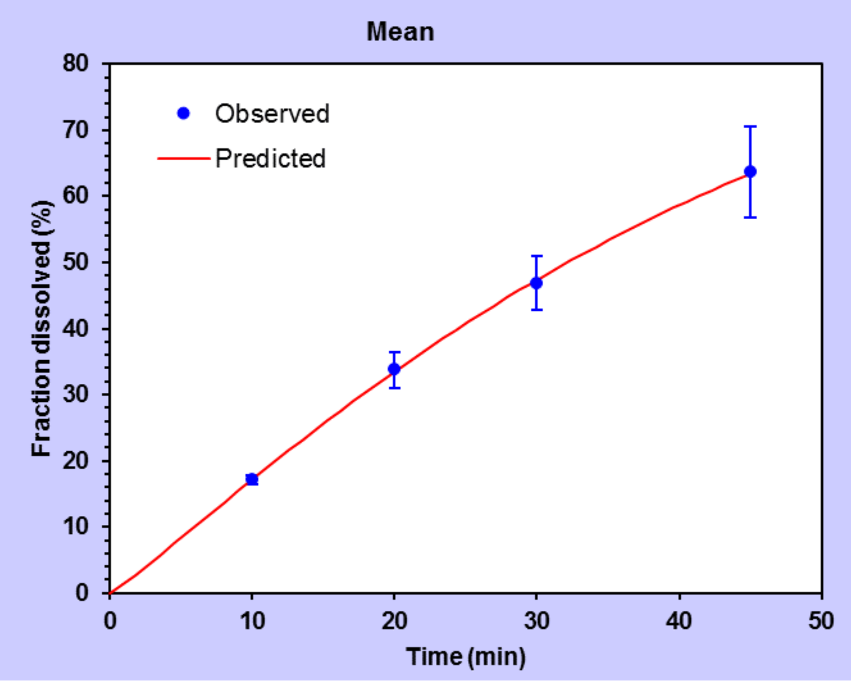

Supplementary materials – Model fitting summary – Polyglykol® 4000 P (modelling up to app. 60 % of carvedilol released)

Graphical abstract of model fit presented as the fraction % of released carvedilol per tested tablet:

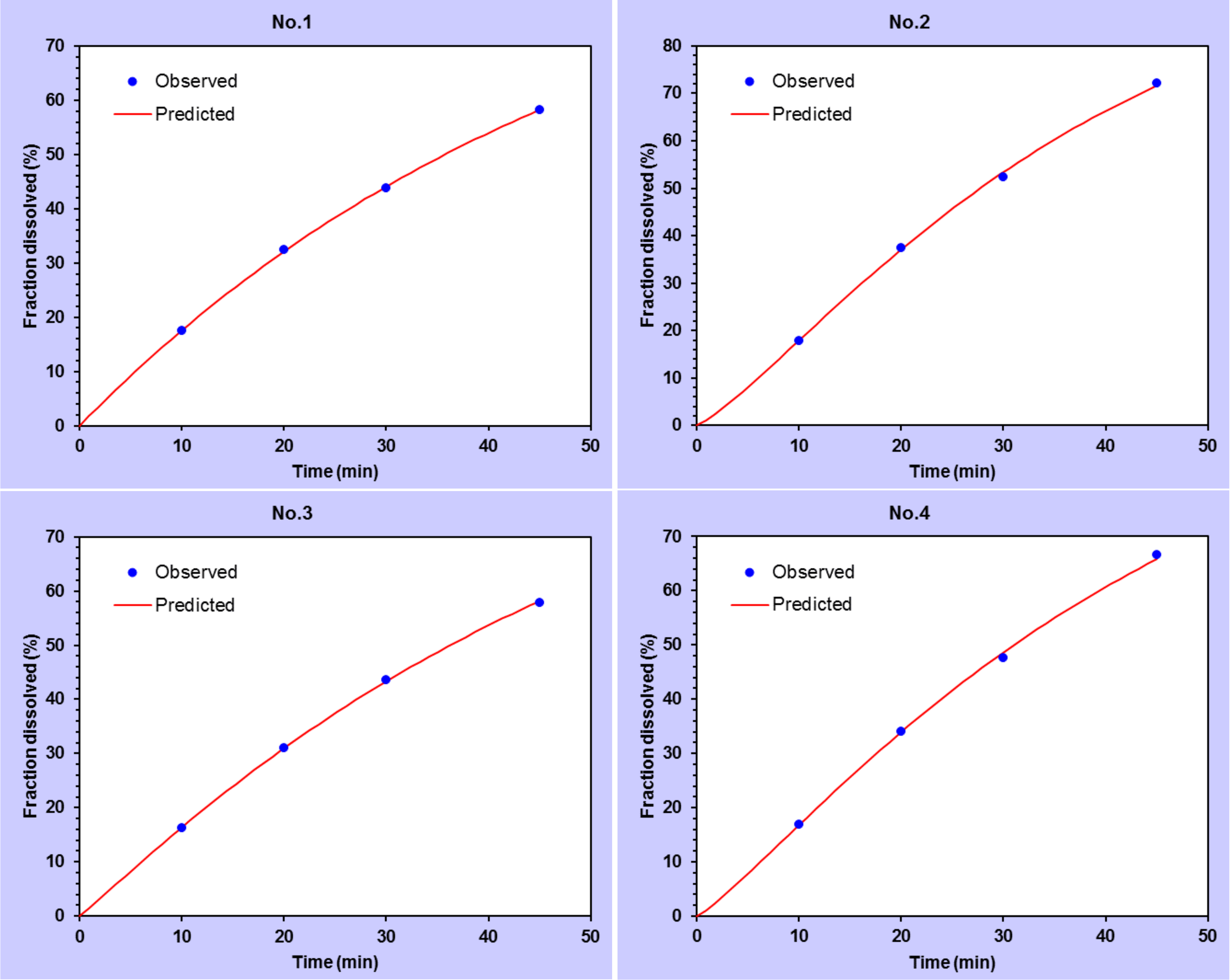

Supplementary materials – Model fitting summary – Polyglykol® 4000 P (modelling up to app. 60 % of carvedilol released)

Model: **Weibull_3**

Model equation: $F = F_{max} \cdot \left(1 - e^{-\frac{t^{\beta}}{\alpha}}\right)$

Fitted model parameters per tested tablet (N = 4) with statistics – mean, standard deviation (SD), and relative standard deviation expressed in % (RSD%) (output from DDSolver):

Parameter	No.1	No.2	No.3	No.4	Mean	SD	RSD(%)
α	85.707	147.721	105.733	158.153	124.328	34.296	27.585
β	1.422	1.565	1.478	1.483	1.487	0.059	3.959
F_{max}	61.151	75.652	60.753	76.306	68.466	8.682	12.680

Number of dissolution data points (N), degrees of freedom (df), and selected goodness of fit criteria – Pearson correlation coefficient (R), coefficient of determination (R^2), adjusted coefficient of determination ($R^2_{adjusted}$), and residual sum of squares (RSS) (manual calculation in MS Excel):

Parameter	No.1	No.2	No.3	No.4
N	4	4	4	4
df	1	1	1	1
R	0.990726017	0.991973304	0.991827384	0.997572514
R^2	0.981538041	0.984011037	0.98372156	0.995150922
$R^2_{adjusted}$	0.944614124	0.952033111	0.951164681	0.985452765
RSS	18.34089932	28.00820723	17.00094387	26.73230762

Graphical abstract of model fit presented as mean ± 1 SD of the fraction % of released carvedilol:

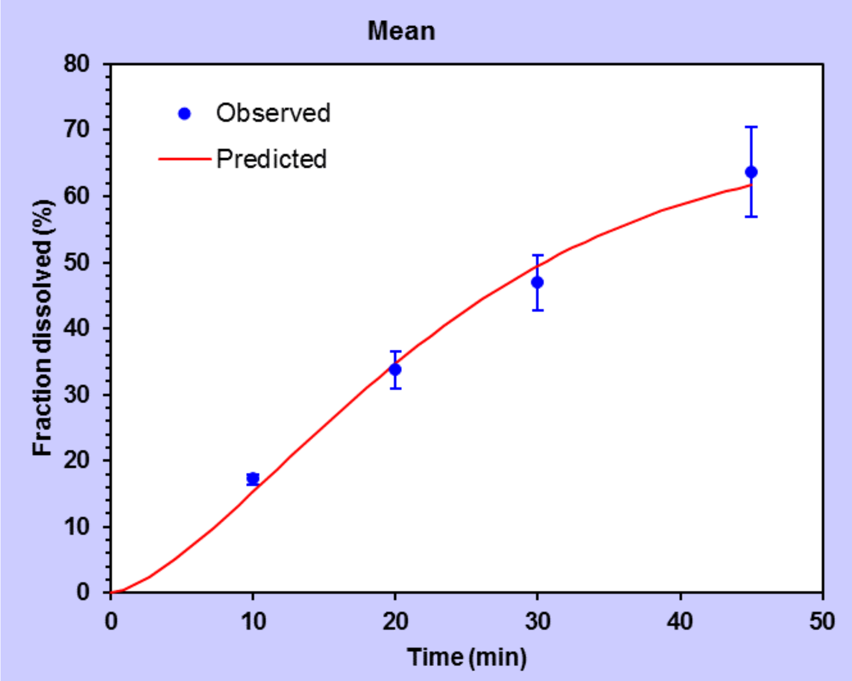

Supplementary materials – Model fitting summary – Polyglykol® 4000 P (modelling up to app. 60 % of carvedilol released)

Graphical abstract of model fit presented as the fraction % of released carvedilol per tested tablet:

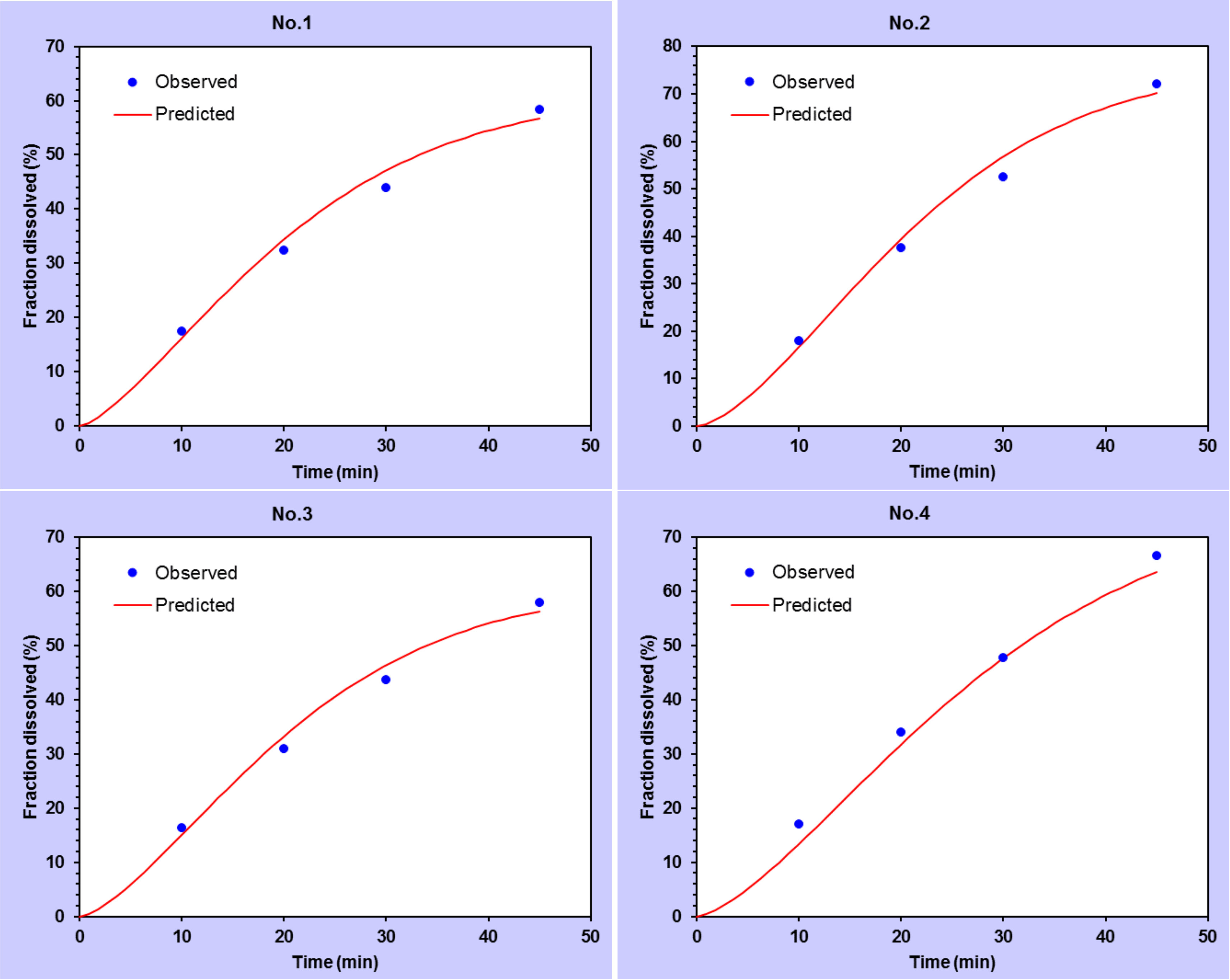

Supplementary materials – Model fitting summary – Polyglykol® 4000 P (modelling up to app. 60 % of carvedilol released)

Model: **Weibull_4**

Model equation: $F = F_{max} \cdot \left[1 - e^{-\frac{(t-T_i)^{\beta}}{\alpha}}\right]$

Fitted model parameters per tested tablet (N = 4) with statistics – mean, standard deviation (SD), and relative standard deviation expressed in % (RSD%) (output from DDSolver):

Parameter	No.1	No.2	No.3	No.4	Mean	SD	RSD(%)
α	/	/	/	/	/	/	/
β	/	/	/	/	/	/	/
T_i	/	/	/	/	/	/	/
F_{max}	/	/	/	/	/	/	/

Number of dissolution data points (N), degrees of freedom (df), and selected goodness of fit criteria – Pearson correlation coefficient (R), coefficient of determination (R^2), adjusted coefficient of determination ($R^2_{adjusted}$), and residual sum of squares (RSS) (manual calculation in MS Excel):

Parameter	No.1	No.2	No.3	No.4
N	/	/	/	/
df	/	/	/	/
R	/	/	/	/
R^2	/	/	/	/
$R^2_{adjusted}$	/	/	/	/
RSS	/	/	/	/

Graphical abstract of model fit presented as mean ± 1 SD of the fraction % of released carvedilol: /

Graphical abstract of model fit presented as the fraction % of released carvedilol per tested tablet: /

Note: model could not be fitted to experimental dissolution data due too few data points being available for fitting

Supplementary materials – Model fitting summary – Polyglykol® 4000 P (modelling up to app. 60 % of carvedilol released)

Model: **Logistic_1**

Model equation: $F = 100 \cdot \frac{e^{\alpha+\beta \cdot \log(t)}}{1+e^{\alpha+\beta \cdot \log(t)}}$

Fitted model parameters per tested tablet (N = 4) with statistics – mean, standard deviation (SD), and relative standard deviation expressed in % (RSD%) (output from DDSolver):

Parameter	No.1	No.2	No.3	No.4	Mean	SD	RSD(%)
α	-4.433	-5.296	-4.642	-5.080	-4.863	0.395	-8.119
β	2.860	3.717	2.981	3.435	3.248	0.399	12.278

Number of dissolution data points (N), degrees of freedom (df), and selected goodness of fit criteria – Pearson correlation coefficient (R), coefficient of determination (R^2), adjusted coefficient of determination ($R^2_{adjusted}$), and residual sum of squares (RSS) (manual calculation in MS Excel):

Parameter	No.1	No.2	No.3	No.4
N	4	4	4	4
df	2	2	2	2
R	0.9988762	0.99617984	0.9992332	0.99609382
R^2	0.99775366	0.99237427	0.99846699	0.99220291
$R^2_{adjusted}$	0.99663048	0.98856141	0.99770048	0.98830436
RSS	2.085841122	12.32117555	1.539235865	10.67780156

Graphical abstract of model fit presented as mean ± 1 SD of the fraction % of released carvedilol:

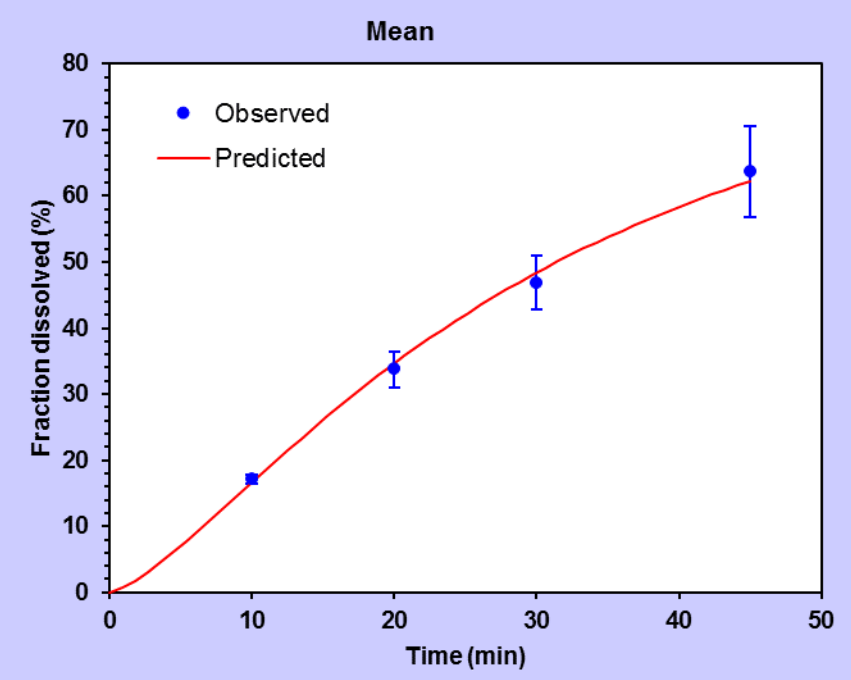

Supplementary materials – Model fitting summary – Polyglykol® 4000 P (modelling up to app. 60 % of carvedilol released)

Graphical abstract of model fit presented as the fraction % of released carvedilol per tested tablet:

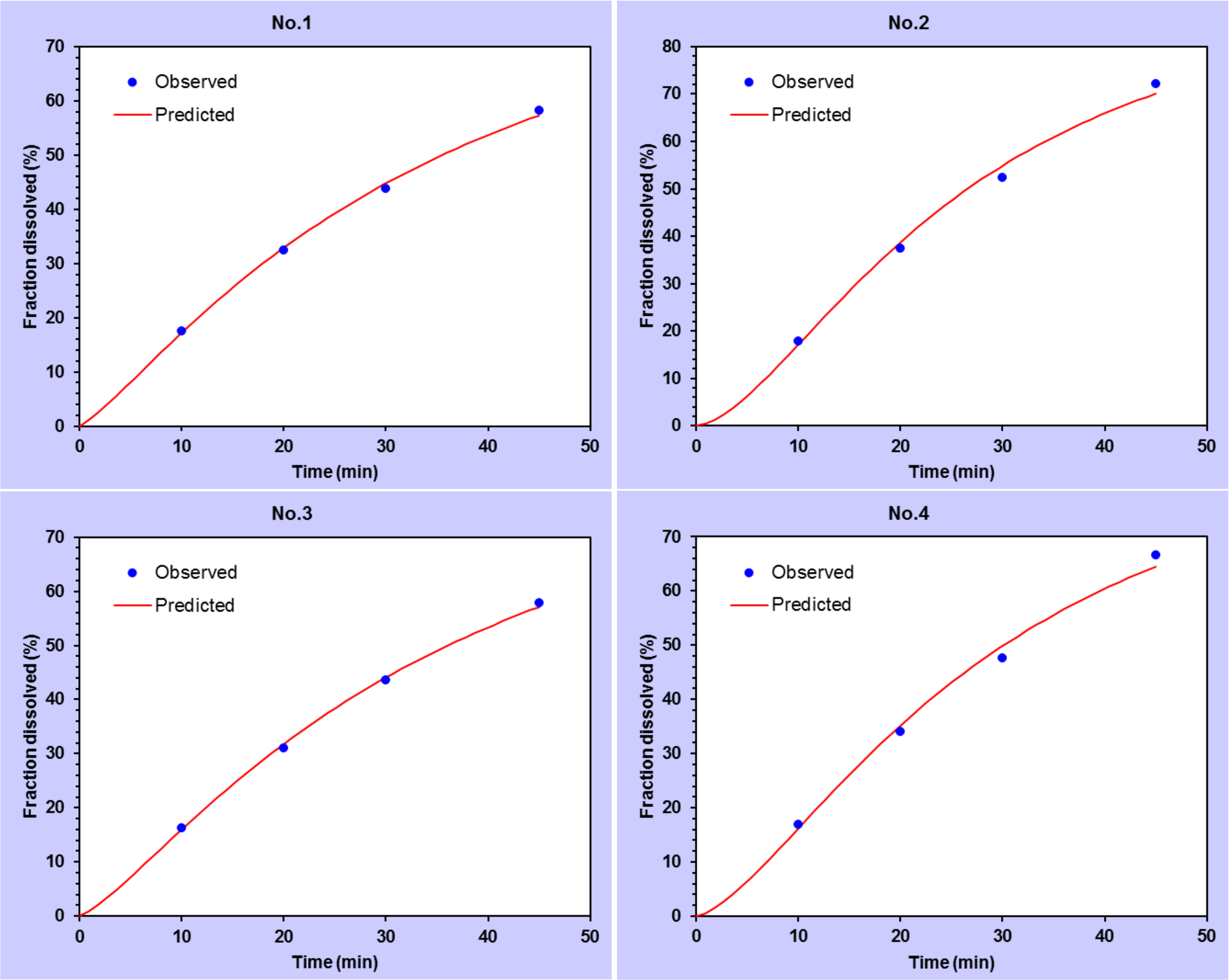

Supplementary materials – Model fitting summary – Polyglykol® 4000 P (modelling up to app. 60 % of carvedilol released)

Model: **Logistic_2**

Model equation: $F = F_{max} \cdot \frac{e^{\alpha+\beta \cdot \log(t)}}{1+e^{\alpha+\beta \cdot \log(t)}}$

Fitted model parameters per tested tablet (N = 4) with statistics – mean, standard deviation (SD), and relative standard deviation expressed in % (RSD%) (output from DDSolver):

Parameter	No.1	No.2	No.3	No.4	Mean	SD	RSD(%)
α	-6.897	-7.524	-7.151	-8.608	-7.545	0.754	-9.994
β	5.656	6.023	5.812	6.338	5.957	0.295	4.954
F_{max}	61.151	75.652	60.753	76.064	68.405	8.609	12.586

Number of dissolution data points (N), degrees of freedom (df), and selected goodness of fit criteria – Pearson correlation coefficient (R), coefficient of determination (R^2), adjusted coefficient of determination ($R^2_{adjusted}$), and residual sum of squares (RSS) (manual calculation in MS Excel):

Parameter	No.1	No.2	No.3	No.4
N	4	4	4	4
df	1	1	1	1
R	0.966964978	0.969304135	0.967880903	0.98847823
R^2	0.935021269	0.939550505	0.936793441	0.977089212
$R^2_{adjusted}$	0.805063807	0.818651516	0.810380324	0.931267636
RSS	77.16302327	125.1879455	77.86687522	120.0264313

Graphical abstract of model fit presented as mean ± 1 SD of the fraction % of released carvedilol:

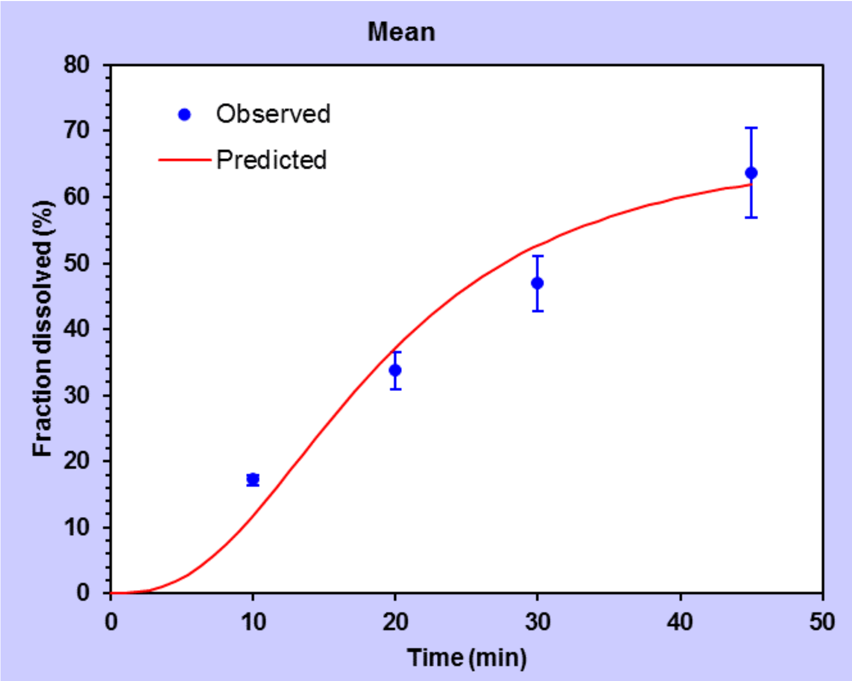

Supplementary materials – Model fitting summary – Polyglykol® 4000 P (modelling up to app. 60 % of carvedilol released)

Graphical abstract of model fit presented as the fraction % of released carvedilol per tested tablet:

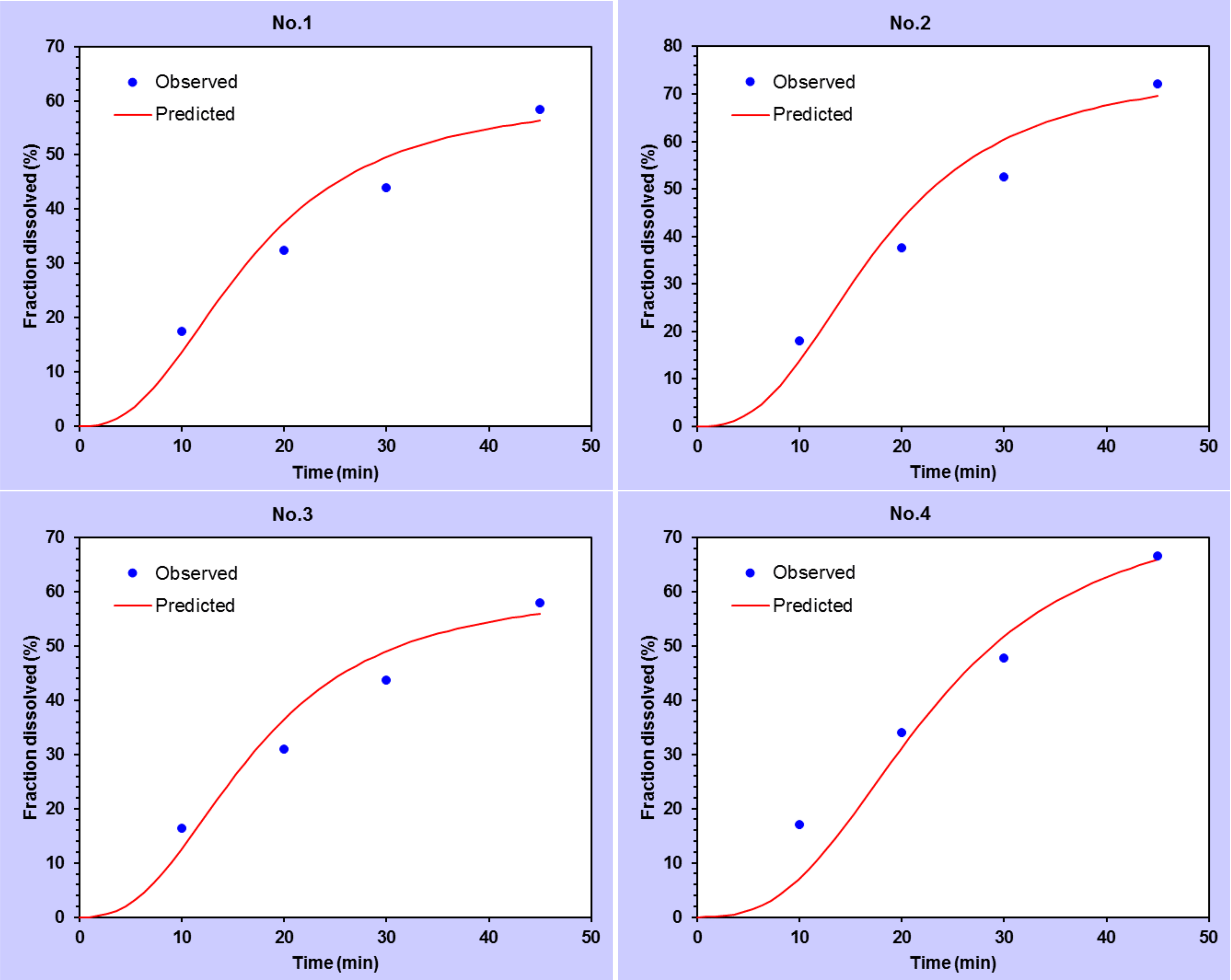

Supplementary materials – Model fitting summary – Polyglykol® 4000 P (modelling up to app. 60 % of carvedilol released)

Model: **Logistic_3**

Model equation: $F = F_{max} \cdot \frac{1}{1+e^{-k\cdot(t-\gamma)}}$

Fitted model parameters per tested tablet (N = 4) with statistics – mean, standard deviation (SD), and relative standard deviation expressed in % (RSD%) (output from DDSolver):

Parameter	No.1	No.2	No.3	No.4	Mean	SD	RSD(%)
k	0.110	0.088	0.113	0.117	0.107	0.013	12.039
γ	19.154	21.505	19.705	20.751	20.279	1.052	5.188
F_{max}	61.151	80.707	60.753	69.857	68.117	9.386	13.779

Number of dissolution data points (N), degrees of freedom (df), and selected goodness of fit criteria – Pearson correlation coefficient (R), coefficient of determination (R^2), adjusted coefficient of determination ($R^2_{adjusted}$), and residual sum of squares (RSS) (manual calculation in MS Excel):

Parameter	No.1	No.2	No.3	No.4
N	4	4	4	4
df	1	1	1	1
R	0.995122815	0.998151959	0.996373744	0.993263304
R^2	0.990269417	0.996307333	0.992760638	0.986571991
$R^2_{adjusted}$	0.97080825	0.988921998	0.978281913	0.959715972
RSS	11.24213455	18.90484722	8.874563789	22.40713803

Graphical abstract of model fit presented as mean ± 1 SD of the fraction % of released carvedilol:

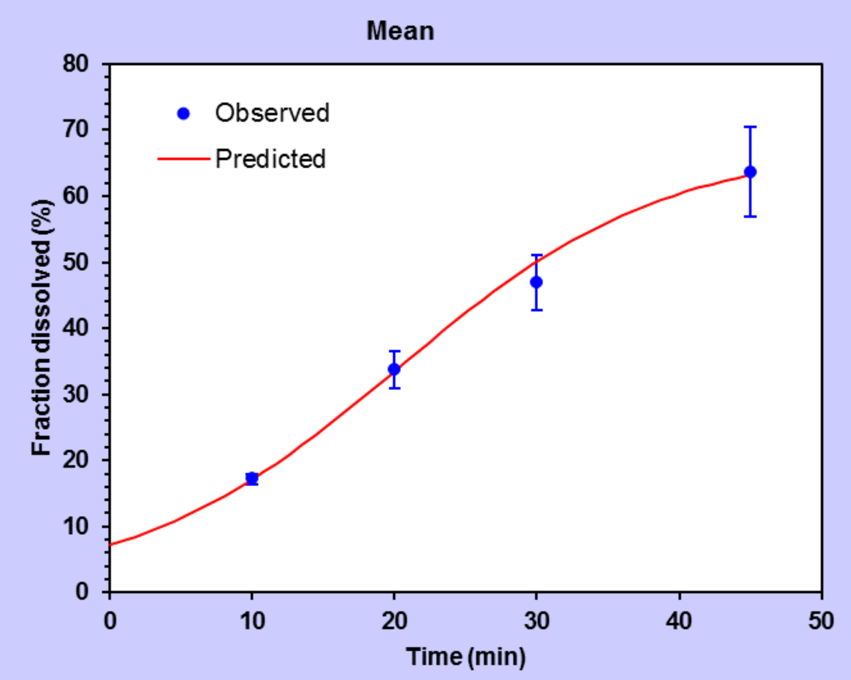

Supplementary materials – Model fitting summary – Polyglykol® 4000 P (modelling up to app. 60 % of carvedilol released)

Graphical abstract of model fit presented as the fraction % of released carvedilol per tested tablet:

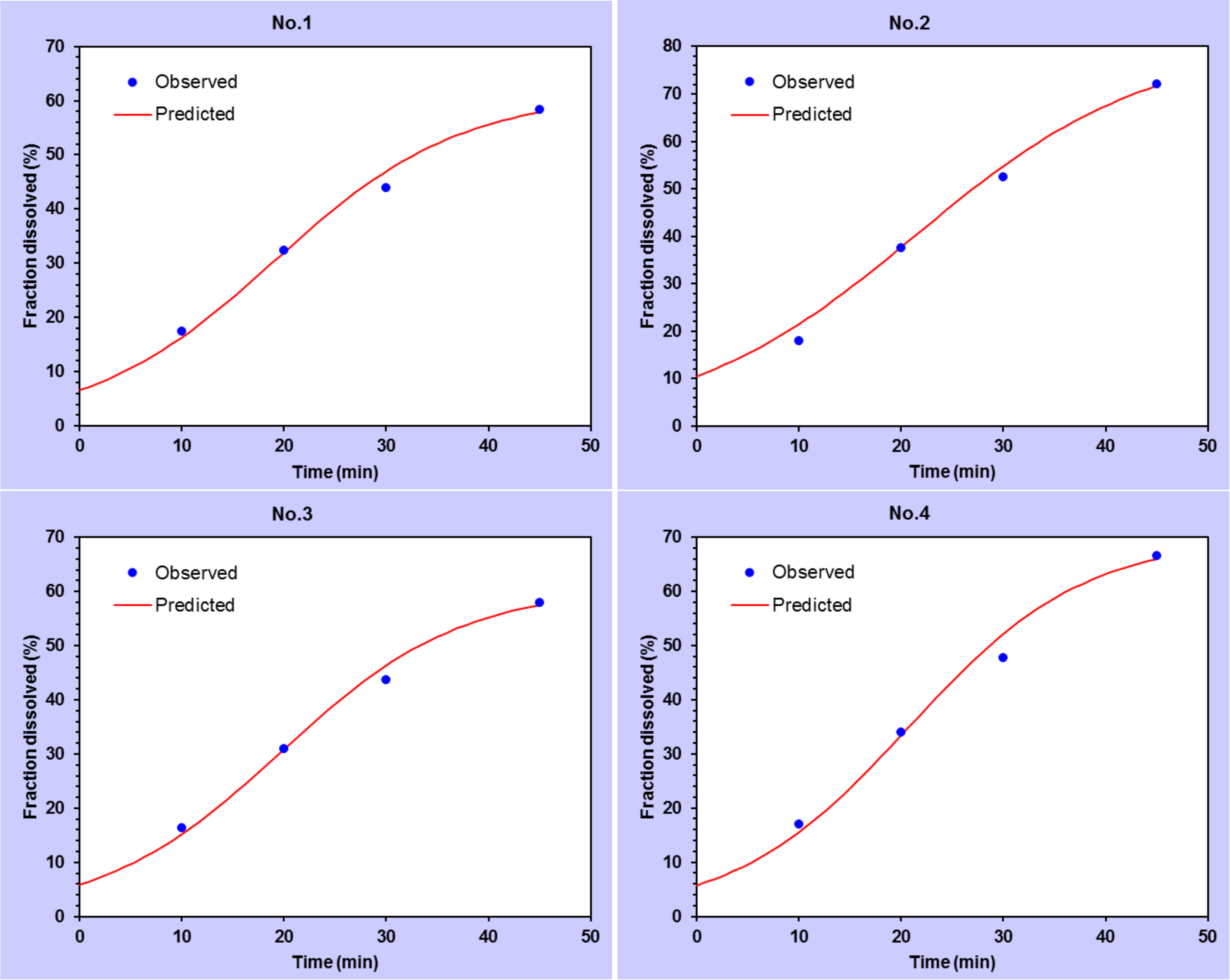

Supplementary materials – Model fitting summary – Polyglykol® 4000 P (modelling up to app. 60 % of carvedilol released)

Model: **Gompertz_1**

Model equation: $F = 100 \cdot e^{-\alpha \cdot e^{-\beta \cdot \log(t)}}$

Fitted model parameters per tested tablet (N = 4) with statistics – mean, standard deviation (SD), and relative standard deviation expressed in % (RSD%) (output from DDSolver):

Parameter	No.1	No.2	No.3	No.4	Mean	SD	RSD(%)
α	10.604	22.082	11.652	17.217	15.389	5.323	34.588
β	1.763	2.468	1.815	2.196	2.061	0.333	16.178

Number of dissolution data points (N), degrees of freedom (df), and selected goodness of fit criteria – Pearson correlation coefficient (R), coefficient of determination (R^2), adjusted coefficient of determination ($R^2_{adjusted}$), and residual sum of squares (RSS) (manual calculation in MS Excel):

Parameter	No.1	No.2	No.3	No.4
N	4	4	4	4
df	2	2	2	2
R	0.993109768	0.986363135	0.993314576	0.986214633
R^2	0.986267011	0.972912234	0.986673847	0.972619302
$R^2_{adjusted}$	0.979400517	0.959368352	0.98001077	0.958928953
RSS	12.37852389	43.88822717	12.66780382	36.81805372

Graphical abstract of model fit presented as mean ± 1 SD of the fraction % of released carvedilol:

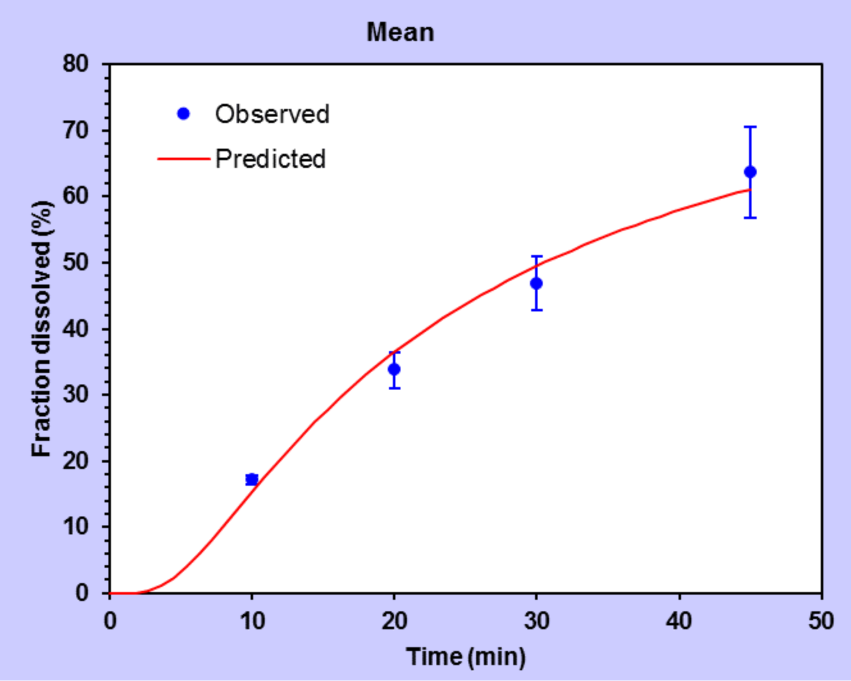

Supplementary materials – Model fitting summary – Polyglykol® 4000 P (modelling up to app. 60 % of carvedilol released)

Graphical abstract of model fit presented as the fraction % of released carvedilol per tested tablet:

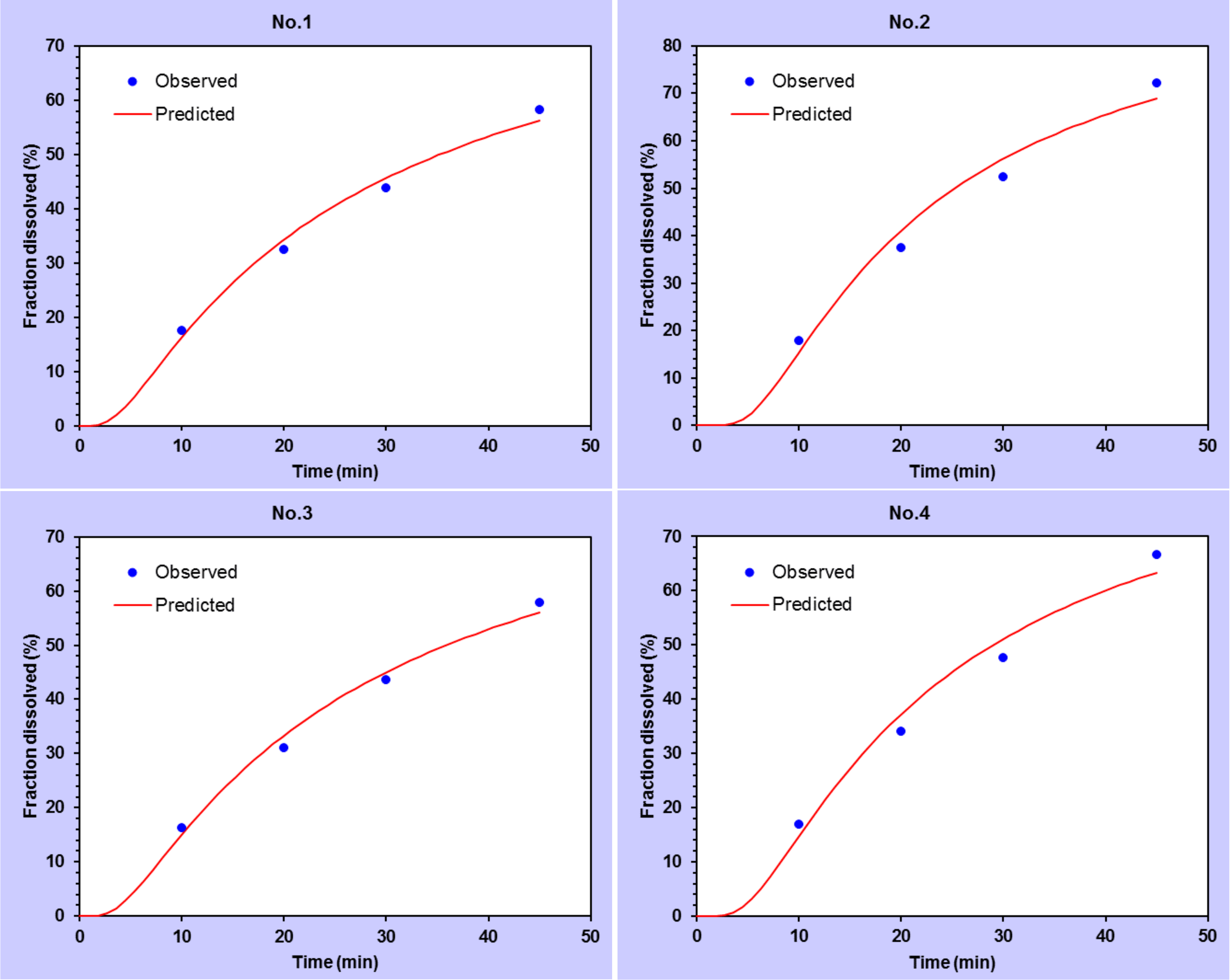

Supplementary materials – Model fitting summary – Polyglykol® 4000 P (modelling up to app. 60 % of carvedilol released)

Model: **Gompertz_2**

Model equation: $F = F_{max} \cdot e^{-\alpha \cdot e^{-\beta \cdot \log(t)}}$

Fitted model parameters per tested tablet (N = 4) with statistics – mean, standard deviation (SD), and relative standard deviation expressed in % (RSD%) (output from DDSolver):

Parameter	No.1	No.2	No.3	No.4	Mean	SD	RSD(%)
α	181.548	255.852	211.041	369.336	254.444	82.462	32.409
β	4.632	4.821	4.723	4.784	4.740	0.083	1.747
F_{max}	61.151	75.652	60.753	69.857	66.853	7.215	10.793

Number of dissolution data points (N), degrees of freedom (df), and selected goodness of fit criteria – Pearson correlation coefficient (R), coefficient of determination (R^2), adjusted coefficient of determination ($R^2_{adjusted}$), and residual sum of squares (RSS) (manual calculation in MS Excel):

Parameter	No.1	No.2	No.3	No.4
N	4	4	4	4
df	1	1	1	1
R	0.945911096	0.946864495	0.945413215	0.966365524
R^2	0.894747801	0.896552373	0.893806148	0.933862326
$R^2_{adjusted}$	0.684243403	0.689657118	0.681418443	0.801586979
RSS	146.4126978	244.5601739	151.4812816	229.7610751

Graphical abstract of model fit presented as mean ± 1 SD of the fraction % of released carvedilol:

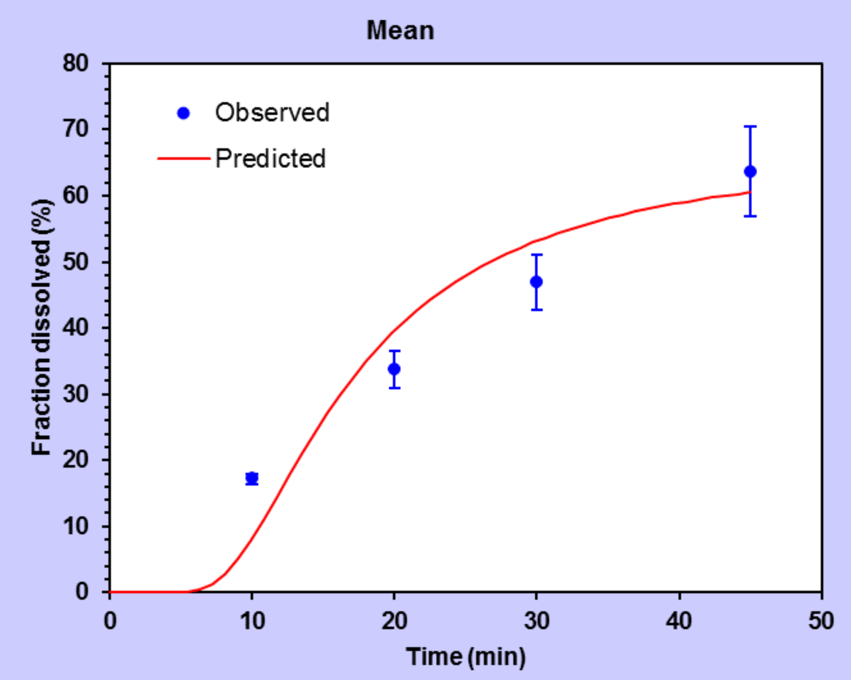

Supplementary materials – Model fitting summary – Polyglykol® 4000 P (modelling up to app. 60 % of carvedilol released)

Graphical abstract of model fit presented as the fraction % of released carvedilol per tested tablet:

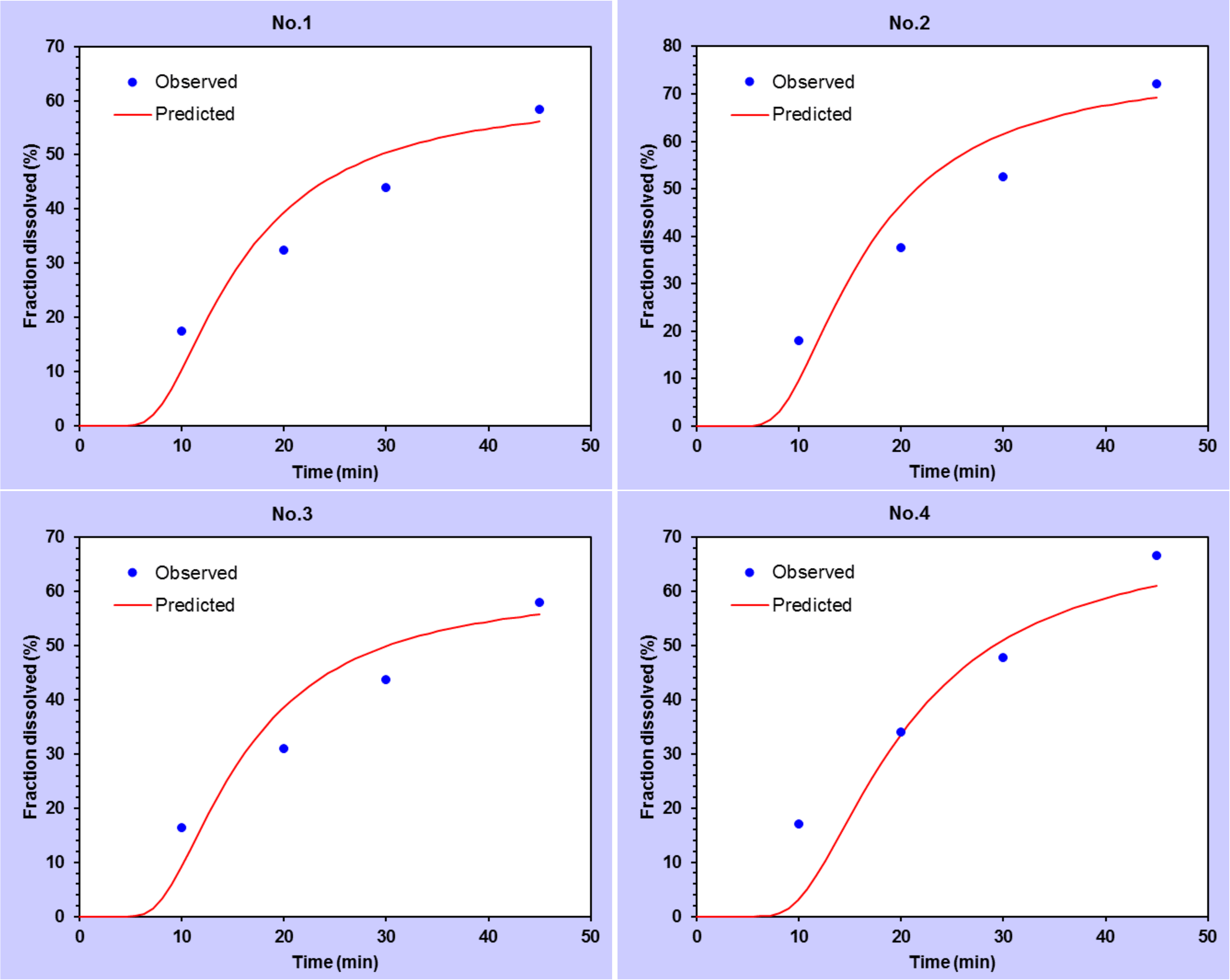

Supplementary materials – Model fitting summary – Polyglykol® 4000 P (modelling up to app. 60 % of carvedilol released)

Model: **Gompertz_3**

Model equation: $F = F_{max} \cdot e^{-e^{-k\cdot(t-\gamma)}}$

Fitted model parameters per tested tablet (N = 4) with statistics – mean, standard deviation (SD), and relative standard deviation expressed in % (RSD%) (output from DDSolver):

Parameter	No.1	No.2	No.3	No.4	Mean	SD	RSD(%)
k	0.092	0.065	0.066	0.096	0.080	0.016	20.553
γ	14.435	15.894	15.298	15.887	15.379	0.688	4.474
F_{max}	61.151	83.124	64.425	69.857	69.639	9.680	13.901

Number of dissolution data points (N), degrees of freedom (df), and selected goodness of fit criteria – Pearson correlation coefficient (R), coefficient of determination (R^2), adjusted coefficient of determination ($R^2_{adjusted}$), and residual sum of squares (RSS) (manual calculation in MS Excel):

Parameter	No.1	No.2	No.3	No.4
N	4	4	4	4
df	1	1	1	1
R	0.987552481	0.997672045	0.998461492	0.98386912
R^2	0.975259903	0.99534951	0.996925352	0.967998445
$R^2_{adjusted}$	0.92577971	0.98604853	0.990776055	0.903995335
RSS	35.68220765	14.72313469	4.371325018	65.00294176

Graphical abstract of model fit presented as mean ± 1 SD of the fraction % of released carvedilol:

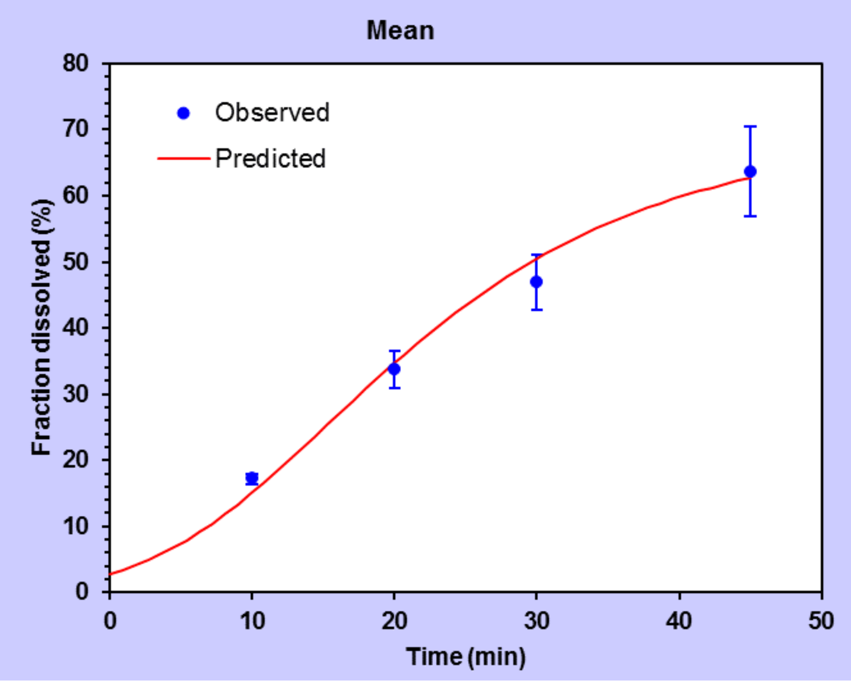

Supplementary materials – Model fitting summary – Polyglykol® 4000 P (modelling up to app. 60 % of carvedilol released)

Graphical abstract of model fit presented as the fraction % of released carvedilol per tested tablet:

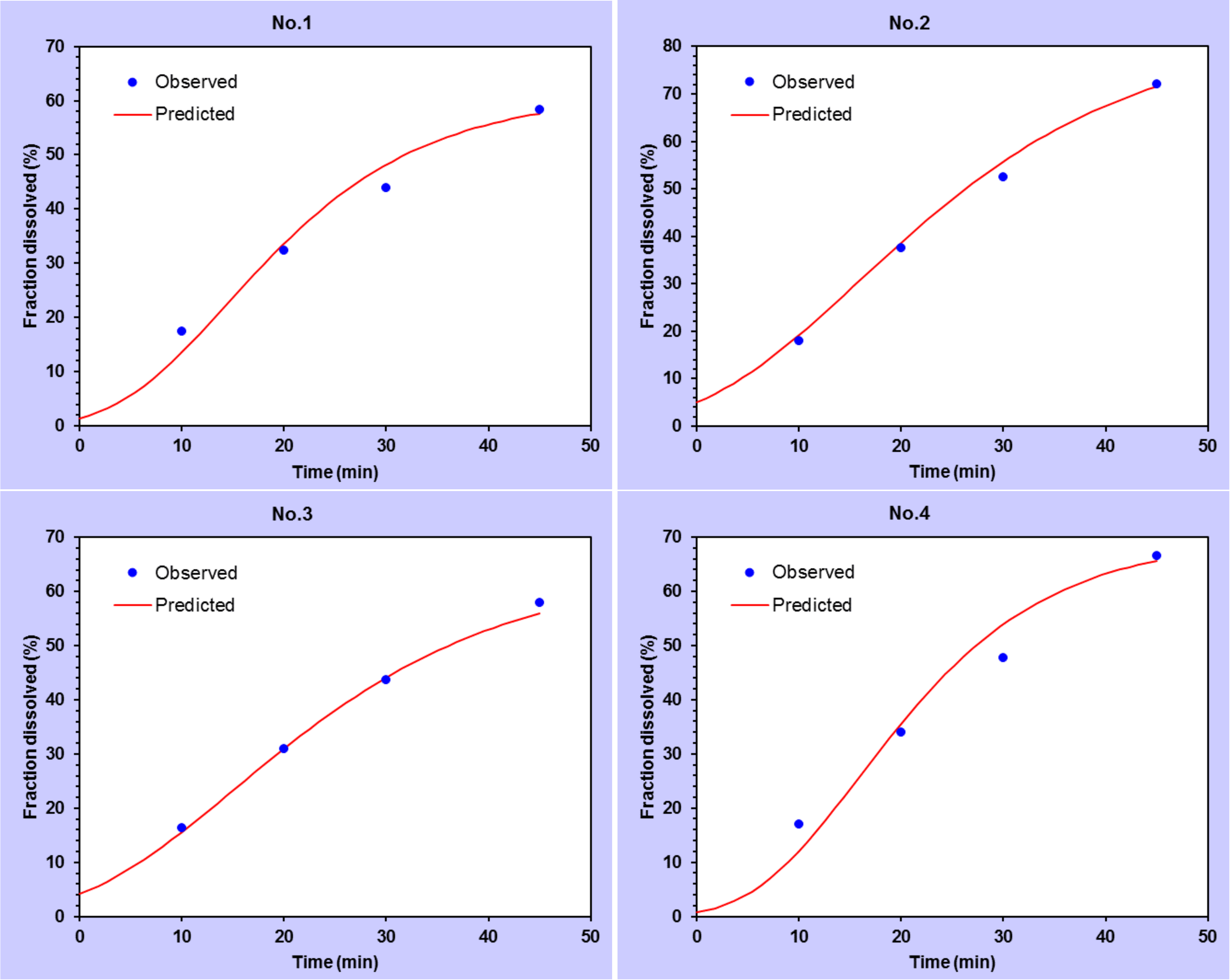

Supplementary materials – Model fitting summary – Polyglykol® 4000 P (modelling up to app. 60 % of carvedilol released)

Model: **Gompertz_4**

Model equation: $F = F_{max} \cdot e^{-\beta \cdot e^{-k \cdot t}}$

Fitted model parameters per tested tablet (N = 4) with statistics – mean, standard deviation (SD), and relative standard deviation expressed in % (RSD%) (output from DDSolver):

Parameter	No.1	No.2	No.3	No.4	Mean	SD	RSD(%)
k	0.092	0.096	0.094	0.096	0.094	0.002	1.849
β	3.778	4.547	4.064	4.561	4.237	0.384	9.056
F_{max}	61.151	75.652	60.753	69.857	66.853	7.215	10.793

Number of dissolution data points (N), degrees of freedom (df), and selected goodness of fit criteria – Pearson correlation coefficient (R), coefficient of determination (R^2), adjusted coefficient of determination ($R^2_{adjusted}$), and residual sum of squares (RSS) (manual calculation in MS Excel):

Parameter	No.1	No.2	No.3	No.4
N	4	4	4	4
df	1	1	1	1
R	0.987552481	0.987109479	0.988274288	0.98386912
R^2	0.975259903	0.974385124	0.976686069	0.967998445
$R^2_{adjusted}$	0.92577971	0.923155372	0.930058206	0.903995335
RSS	35.68220765	62.27800959	34.65197301	65.00294176

Graphical abstract of model fit presented as mean ± 1 SD of the fraction % of released carvedilol:

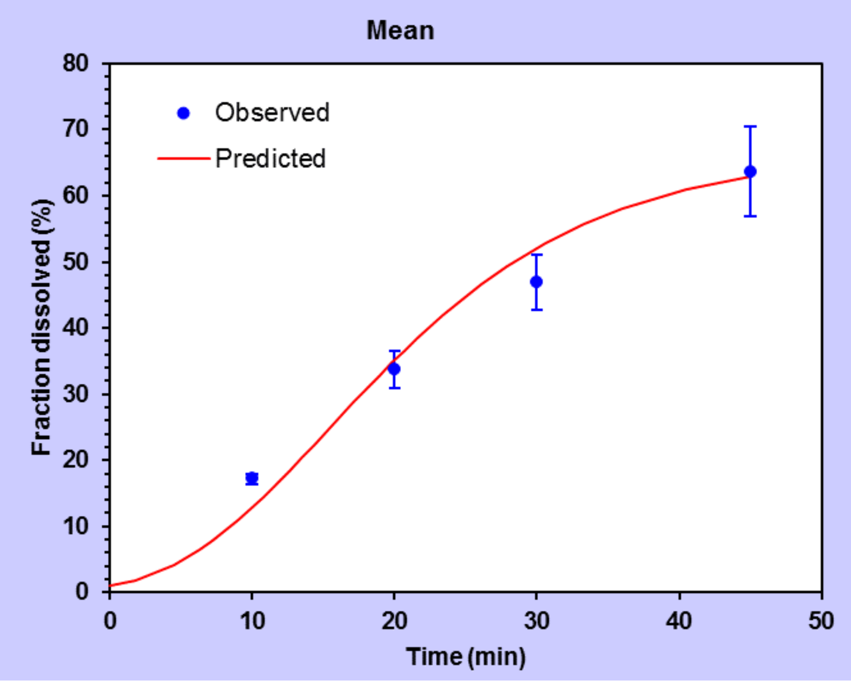

Supplementary materials – Model fitting summary – Polyglykol® 4000 P (modelling up to app. 60 % of carvedilol released)

Graphical abstract of model fit presented as the fraction % of released carvedilol per tested tablet:

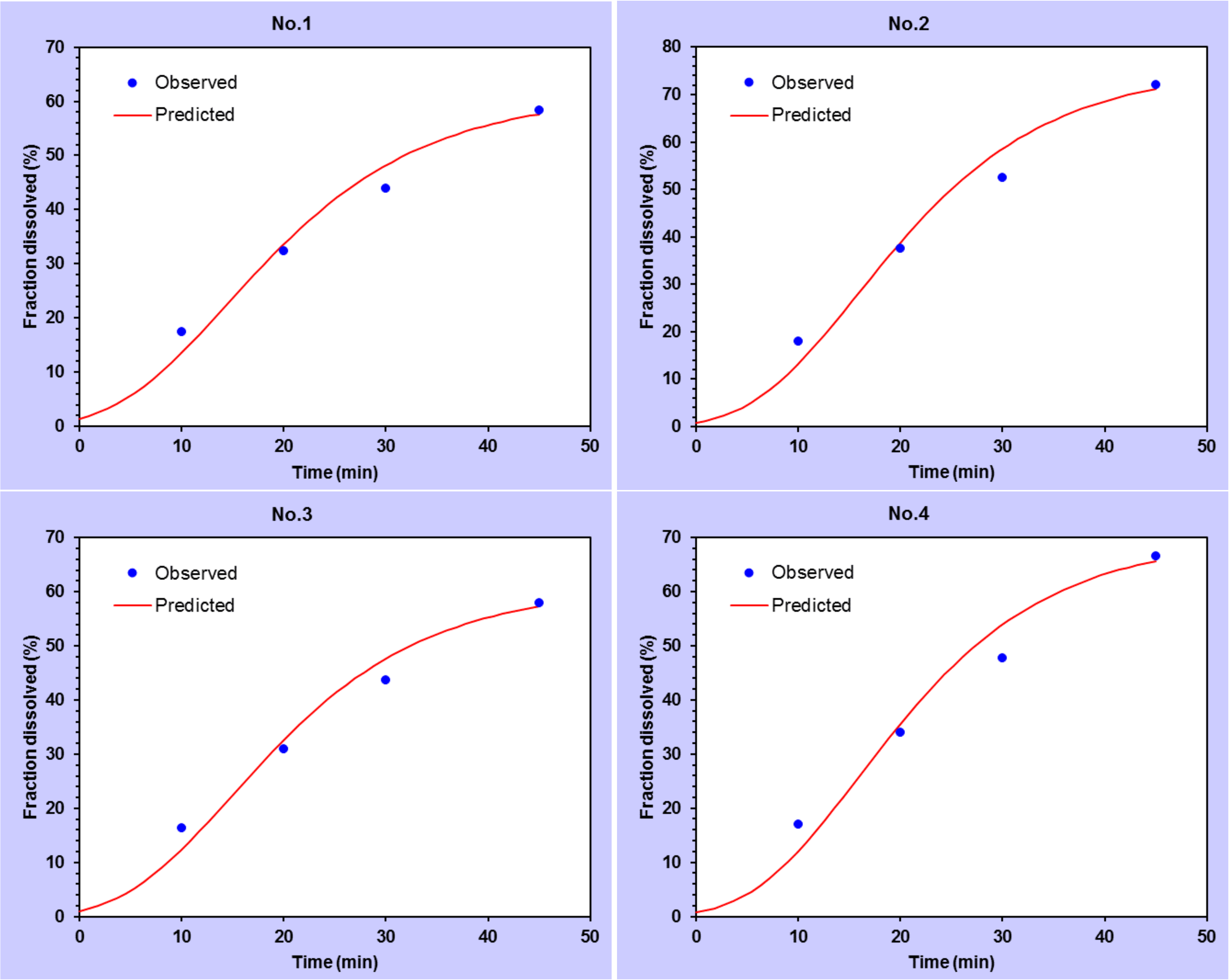

Supplementary materials – Model fitting summary – Polyglykol® 4000 P (modelling up to app. 60 % of carvedilol released)

Model: **Probit_1**

Model equation: $F = 100 \cdot \phi[\alpha + \beta \cdot \log(t)]$

Fitted model parameters per tested tablet (N = 4) with statistics – mean, standard deviation (SD), and relative standard deviation expressed in % (RSD%) (output from DDSolver):

Parameter	No.1	No.2	No.3	No.4	Mean	SD	RSD(%)
α	-2.688	-3.222	-2.805	-3.083	-2.950	0.246	-8.330
β	1.734	2.263	1.800	2.086	1.971	0.248	12.573

Number of dissolution data points (N), degrees of freedom (df), and selected goodness of fit criteria – Pearson correlation coefficient (R), coefficient of determination (R^2), adjusted coefficient of determination ($R^2_{adjusted}$), and residual sum of squares (RSS) (manual calculation in MS Excel):

Parameter	No.1	No.2	No.3	No.4
N	4	4	4	4
df	2	2	2	2
R	0.99807948	0.995494969	0.998361036	0.994920322
R^2	0.996162648	0.991010232	0.996724759	0.989866447
$R^2_{adjusted}$	0.994243972	0.986515349	0.995087139	0.984799671
RSS	3.537919288	14.44580783	3.224508828	13.78361595

Graphical abstract of model fit presented as mean ± 1 SD of the fraction % of released carvedilol:

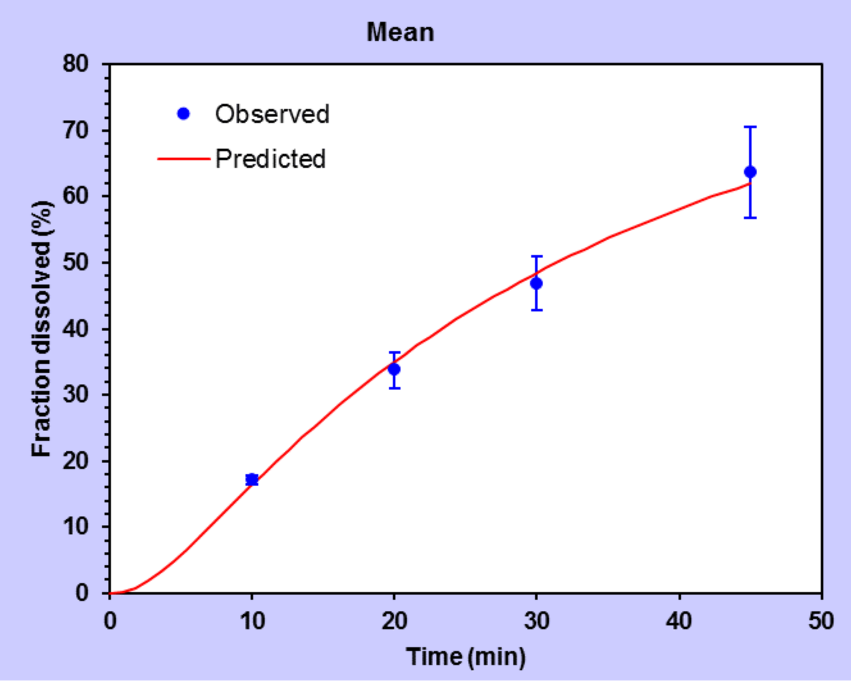

Supplementary materials – Model fitting summary – Polyglykol® 4000 P (modelling up to app. 60 % of carvedilol released)

Graphical abstract of model fit presented as the fraction % of released carvedilol per tested tablet:

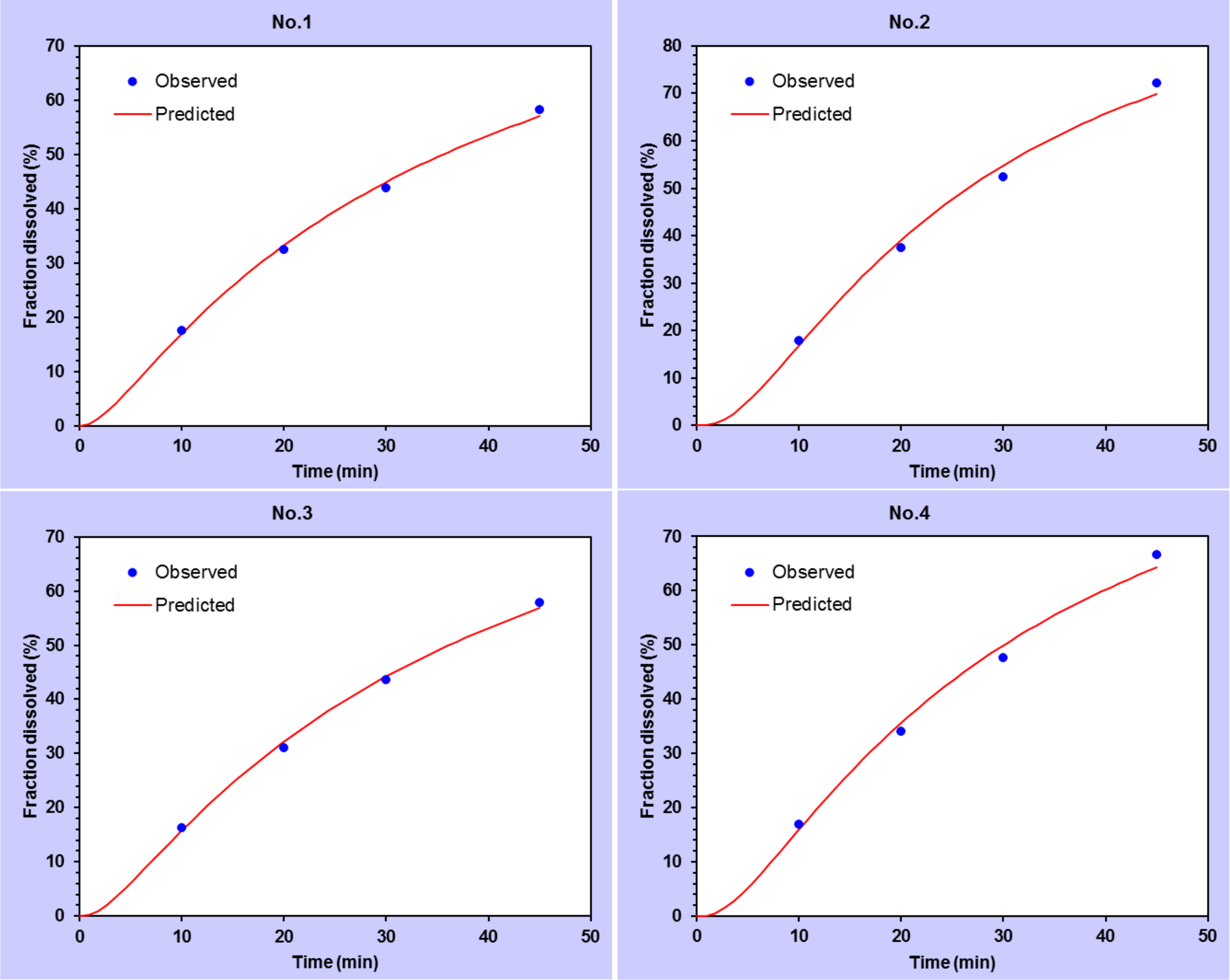

Supplementary materials – Model fitting summary – Polyglykol® 4000 P (modelling up to app. 60 % of carvedilol released)

Model: **Probit_2**

Model equation: $F = F_{max} \cdot \phi[\alpha + \beta \cdot \log(t)]$

Fitted model parameters per tested tablet (N = 4) with statistics – mean, standard deviation (SD), and relative standard deviation expressed in % (RSD%) (output from DDSolver):

Parameter	No.1	No.2	No.3	No.4	Mean	SD	RSD(%)
α	-3.991	-4.365	-4.144	-4.326	-4.207	0.173	-4.110
β	3.262	3.480	3.356	3.445	3.386	0.098	2.886
F_{max}	61.151	75.652	60.753	69.857	66.853	7.215	10.793

Number of dissolution data points (N), degrees of freedom (df), and selected goodness of fit criteria – Pearson correlation coefficient (R), coefficient of determination (R^2), adjusted coefficient of determination ($R^2_{adjusted}$), and residual sum of squares (RSS) (manual calculation in MS Excel):

Parameter	No.1	No.2	No.3	No.4
N	4	4	4	4
df	1	1	1	1
R	0.974160244	0.975573258	0.974752982	0.970896625
R^2	0.948988181	0.951743183	0.950143376	0.942640257
$R^2_{adjusted}$	0.846964542	0.855229548	0.850430129	0.827920772
RSS	56.14764294	92.8058353	57.08641401	92.40013639

Graphical abstract of model fit presented as mean ± 1 SD of the fraction % of released carvedilol:

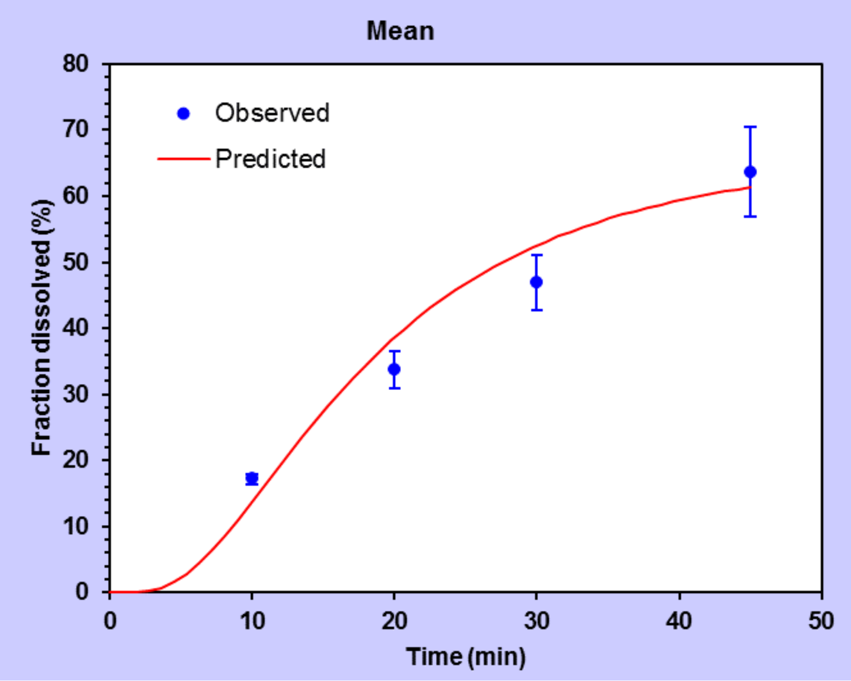

Supplementary materials – Model fitting summary – Polyglykol® 4000 P (modelling up to app. 60 % of carvedilol released)

Graphical abstract of model fit presented as the fraction % of released carvedilol per tested tablet:

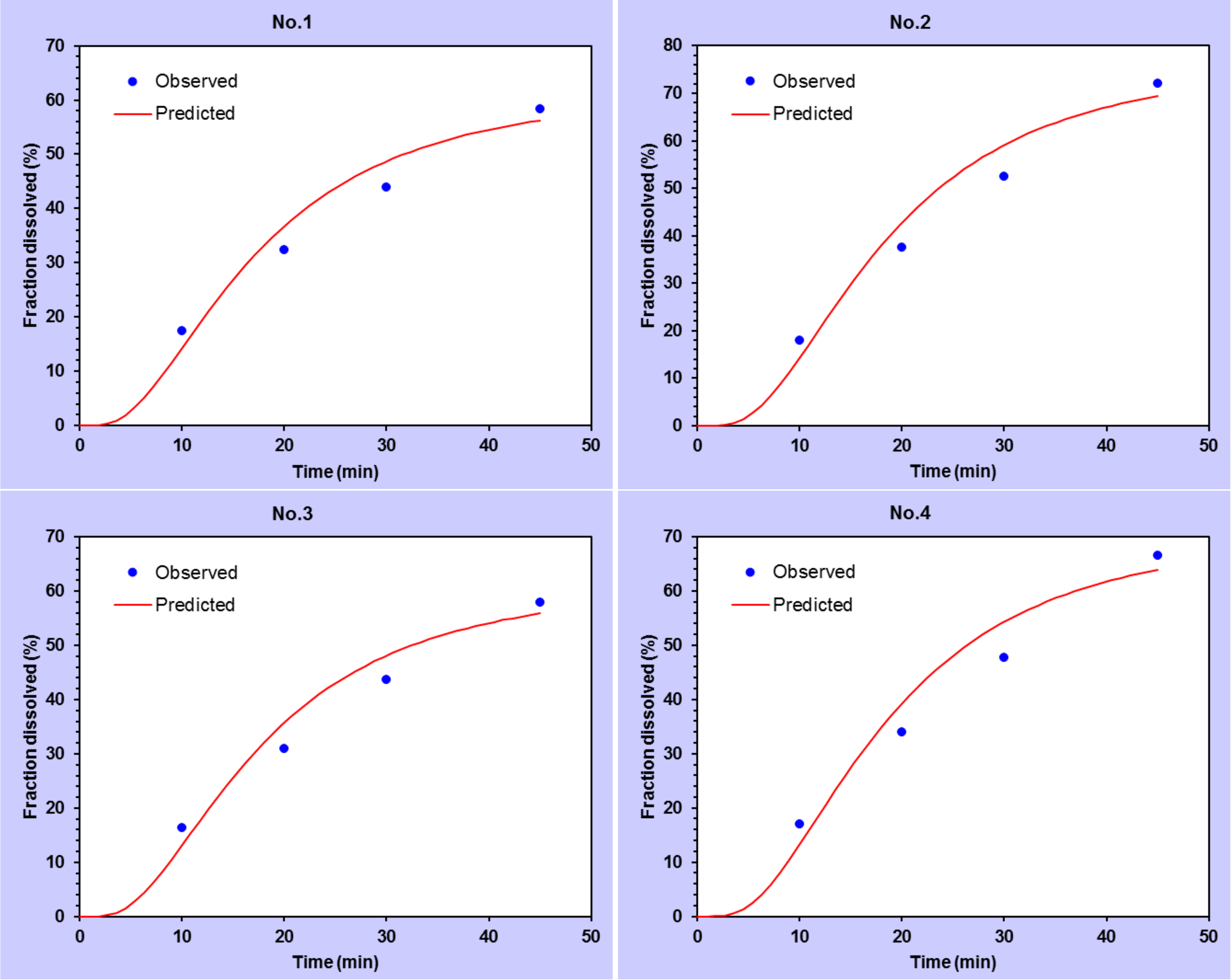

Supplementary materials – Model fitting summary – Polyglykol® 4000 P (modelling up to app. 75 % of carvedilol released)

Model: **Zero–order**

Model equation: $F = k_0 \cdot t$

Fitted model parameters per tested tablet (N = 4) with statistics – mean, standard deviation (SD), and relative standard deviation expressed in % (RSD%) (output from DDSolver):

Parameter	No.1	No.2	No.3	No.4	Mean	SD	RSD(%)
k_0	1.275	1.558	1.248	1.434	1.379	0.145	10.517

Number of dissolution data points (N), degrees of freedom (df), and selected goodness of fit criteria – Pearson correlation coefficient (R), coefficient of determination (R^2), adjusted coefficient of determination ($R^2_{adjusted}$), and residual sum of squares (RSS) (manual calculation in MS Excel):

Parameter	No.1	No.2	No.3	No.4
N	5	5	5	5
df	4	4	4	4
R	0.992450148	0.992021905	0.988432255	0.993528537
R^2	0.984957296	0.98410746	0.976998323	0.987098953
$R^2_{adjusted}$	0.984957296	0.98410746	0.976998323	0.987098953
RSS	144.4677471	126.646043	141.1265692	96.88175988

Graphical abstract of model fit presented as mean ± 1 SD of the fraction % of released carvedilol:

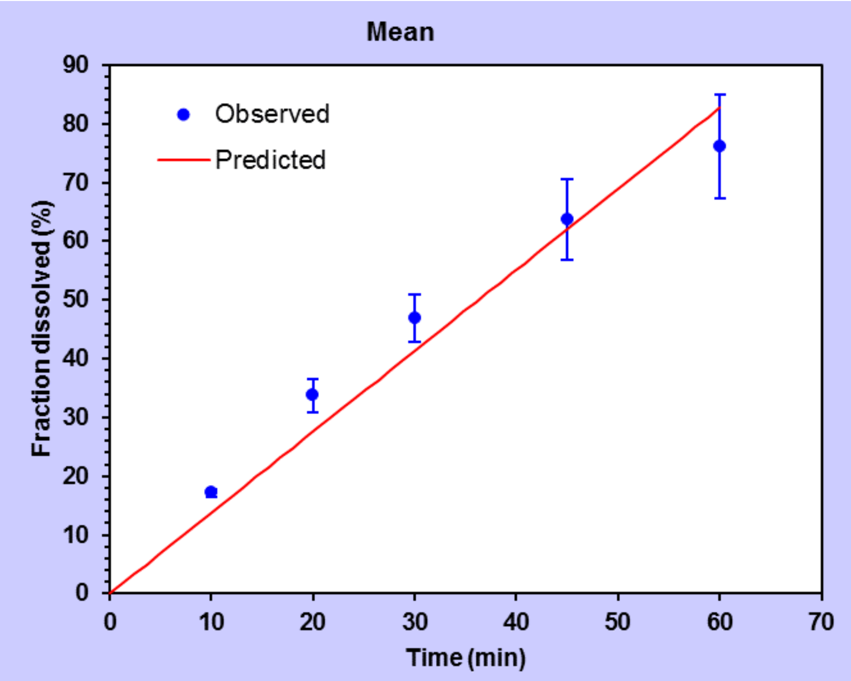

Supplementary materials – Model fitting summary – Polyglykol® 4000 P (modelling up to app. 75 % of carvedilol released)

Graphical abstract of model fit presented as the fraction % of released carvedilol per tested tablet:

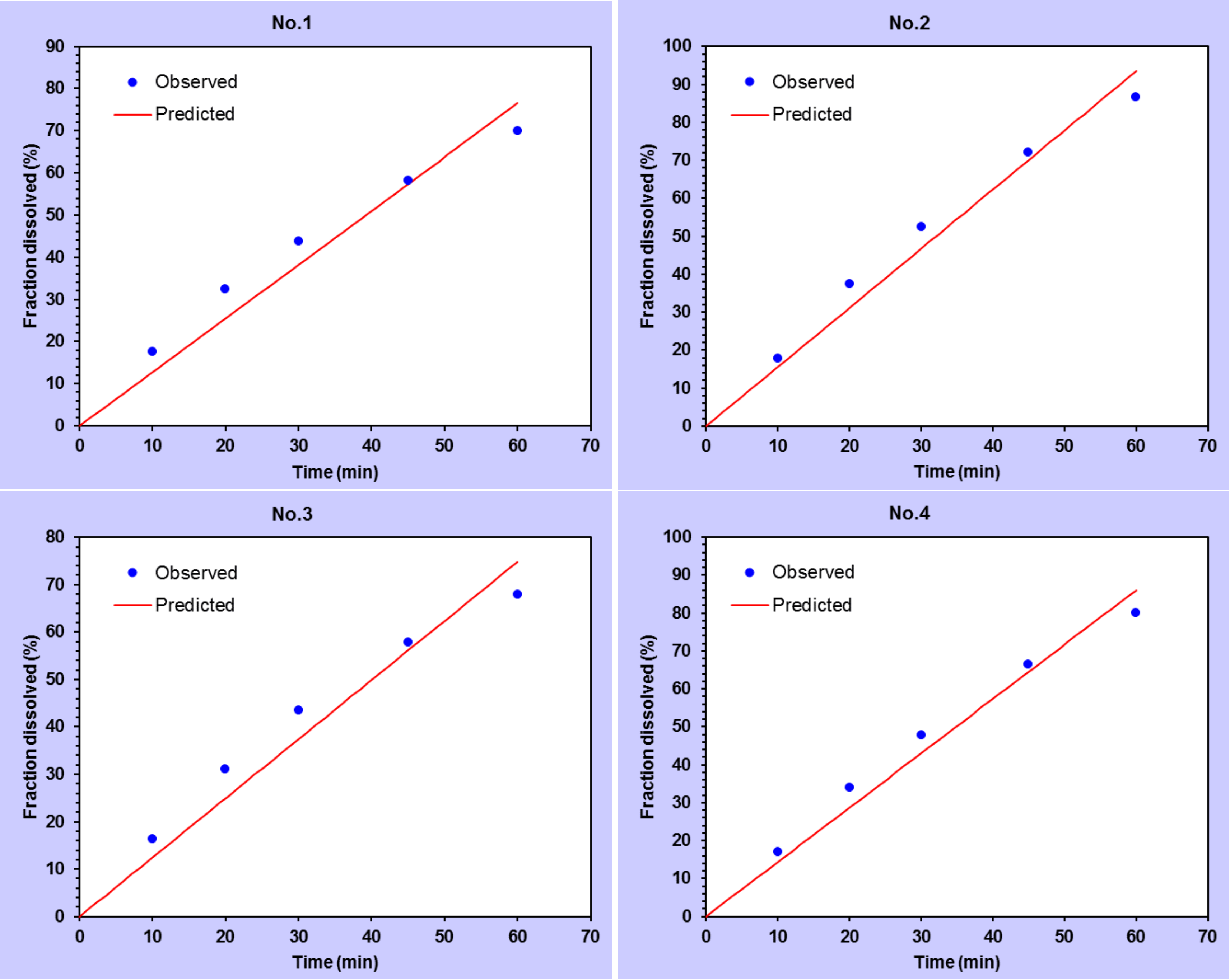

Supplementary materials – Model fitting summary – Polyglykol® 4000 P (modelling up to app. 75 % of carvedilol released)

Model: **Zero–order with T_{lag}**

Model equation: $F = k_0 \cdot (t - T_{lag})$

Fitted model parameters per tested tablet (N = 4) with statistics – mean, standard deviation (SD), and relative standard deviation expressed in % (RSD%) (output from DDSolver):

Parameter	No.1	No.2	No.3	No.4	Mean	SD	RSD(%)
k_0	1.034	1.361	1.024	1.256	1.169	0.167	14.287
T_{lag}	-9.933	-6.175	-9.310	-6.020	-7.860	2.051	-26.100

Number of dissolution data points (N), degrees of freedom (df), and selected goodness of fit criteria – Pearson correlation coefficient (R), coefficient of determination (R^2), adjusted coefficient of determination ($R^2_{adjusted}$), and residual sum of squares (RSS) (manual calculation in MS Excel):

Parameter	No.1	No.2	No.3	No.4
N	5	5	5	5
df	3	3	3	3
R	0.992450148	0.992021905	0.988432255	0.993528537
R^2	0.984957296	0.98410746	0.976998323	0.987098953
$R^2_{adjusted}$	0.979943061	0.978809947	0.969331098	0.982798604
RSS	25.80561044	47.24291157	38.98317295	32.58205128

Graphical abstract of model fit presented as mean ± 1 SD of the fraction % of released carvedilol:

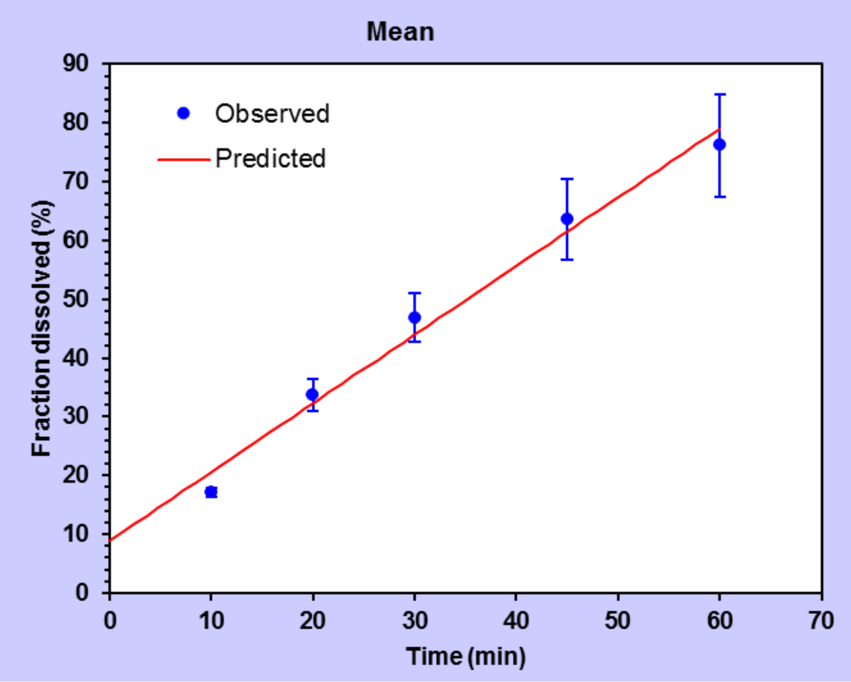

Supplementary materials – Model fitting summary – Polyglykol® 4000 P (modelling up to app. 75 % of carvedilol released)

Graphical abstract of model fit presented as the fraction % of released carvedilol per tested tablet:

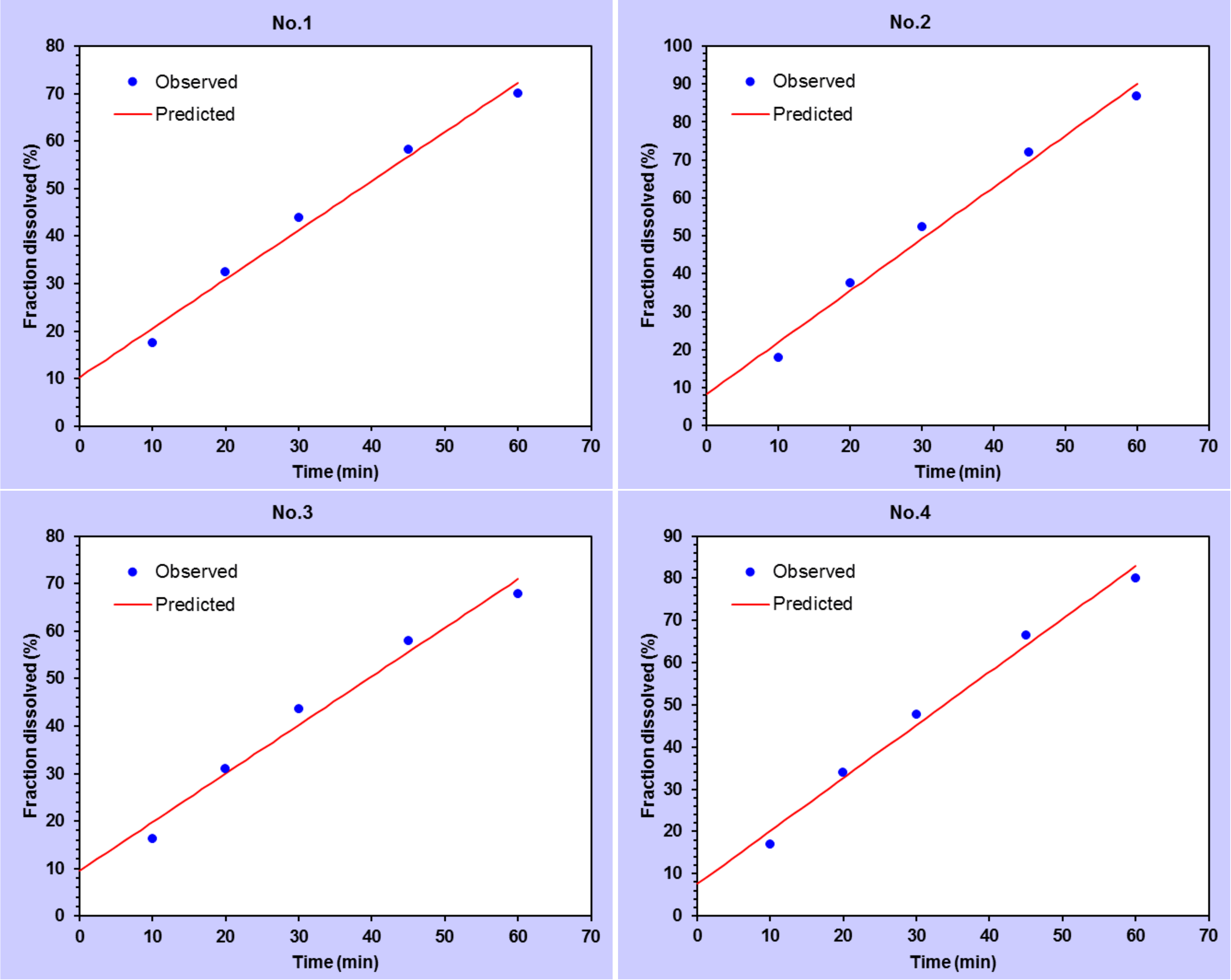

Supplementary materials – Model fitting summary – Polyglykol® 4000 P (modelling up to app. 75 % of carvedilol released)

Model: **Zero–order with F_0**

Model equation: $F = F_0 + k_0 \cdot t$

Fitted model parameters per tested tablet (N = 4) with statistics – mean, standard deviation (SD), and relative standard deviation expressed in % (RSD%) (output from DDSolver):

Parameter	No.1	No.2	No.3	No.4	Mean	SD	RSD(%)
k_0	1.034	1.361	1.024	1.256	1.169	0.167	14.287
F_0	10.272	8.403	9.530	7.562	8.942	1.199	13.408

Number of dissolution data points (N), degrees of freedom (df), and selected goodness of fit criteria – Pearson correlation coefficient (R), coefficient of determination (R^2), adjusted coefficient of determination ($R^2_{adjusted}$), and residual sum of squares (RSS) (manual calculation in MS Excel):

Parameter	No.1	No.2	No.3	No.4
N	5	5	5	5
df	3	3	3	3
R	0.992450148	0.992021905	0.988432255	0.993528537
R^2	0.984957296	0.98410746	0.976998323	0.987098953
$R^2_{adjusted}$	0.979943061	0.978809947	0.969331098	0.982798604
RSS	25.80561044	47.24291157	38.98317295	32.58205128

Graphical abstract of model fit presented as mean ± 1 SD of the fraction % of released carvedilol:

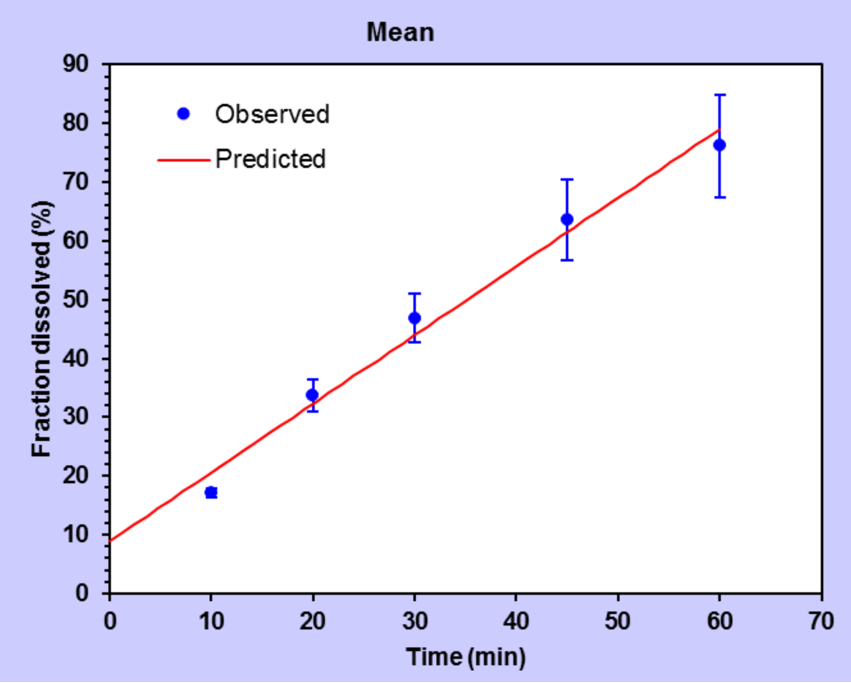

Supplementary materials – Model fitting summary – Polyglykol® 4000 P (modelling up to app. 75 % of carvedilol released)

Graphical abstract of model fit presented as the fraction % of released carvedilol per tested tablet:

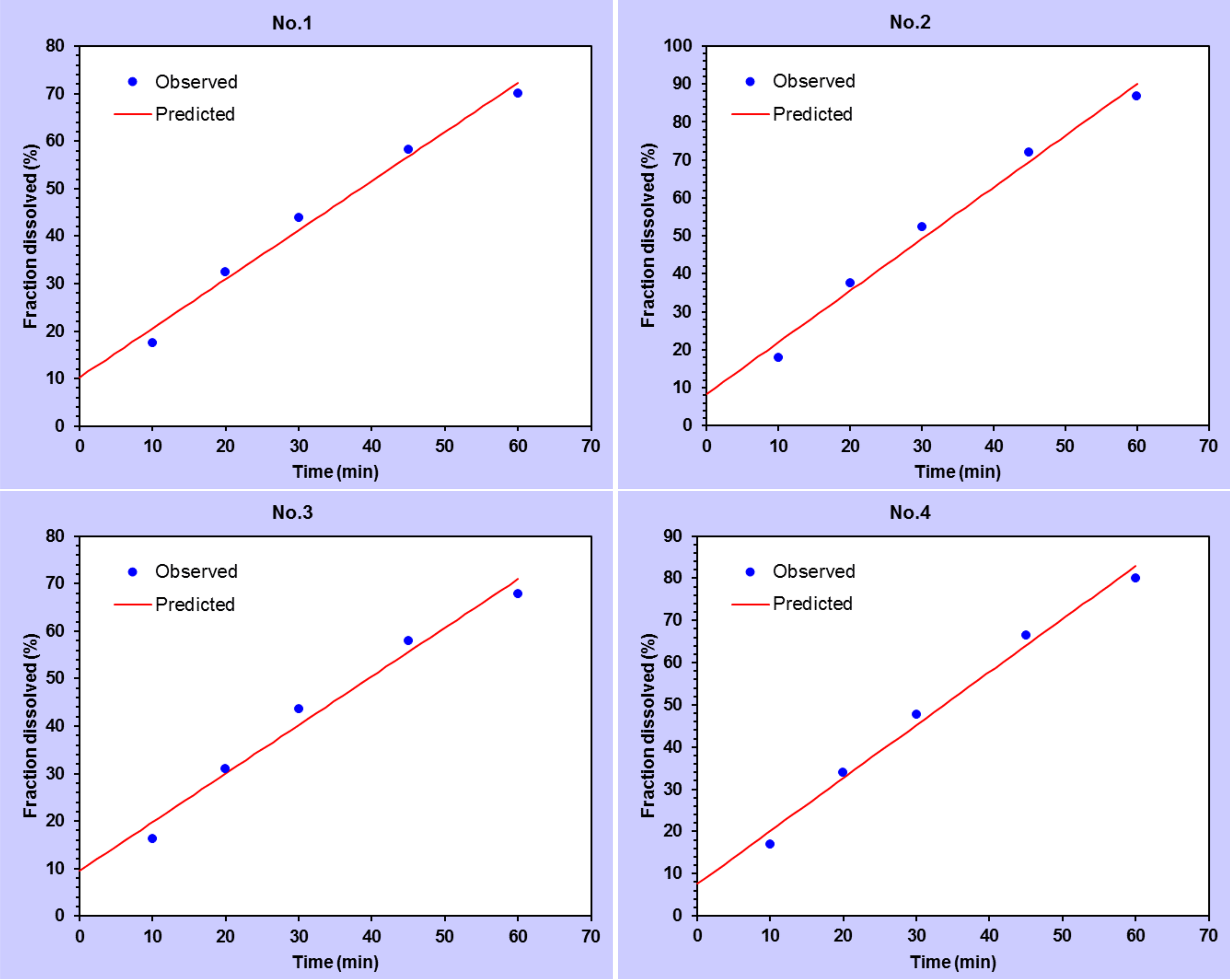

Supplementary materials – Model fitting summary – Polyglykol® 4000 P (modelling up to app. 75 % of carvedilol released)

Model: **First–order**

Model equation: $F = 100 \cdot (1 - e^{-k_1 \cdot t})$

Fitted model parameters per tested tablet (N = 4) with statistics – mean, standard deviation (SD), and relative standard deviation expressed in % (RSD%) (output from DDSolver):

Parameter	No.1	No.2	No.3	No.4	Mean	SD	RSD(%)
k_1	0.020	0.026	0.019	0.022	0.022	0.003	15.388

Number of dissolution data points (N), degrees of freedom (df), and selected goodness of fit criteria – Pearson correlation coefficient (R), coefficient of determination (R^2), adjusted coefficient of determination ($R^2_{adjusted}$), and residual sum of squares (RSS) (manual calculation in MS Excel):

Parameter	No.1	No.2	No.3	No.4
N	5	5	5	5
df	4	4	4	4
R	0.999721226	0.997972452	0.99984369	0.998886176
R^2	0.999442529	0.995949015	0.999687404	0.997773593
$R^2_{adjusted}$	0.999442529	0.995949015	0.999687404	0.997773593
RSS	1.611596051	104.785229	1.561323515	73.56644014

Graphical abstract of model fit presented as mean ± 1 SD of the fraction % of released carvedilol:

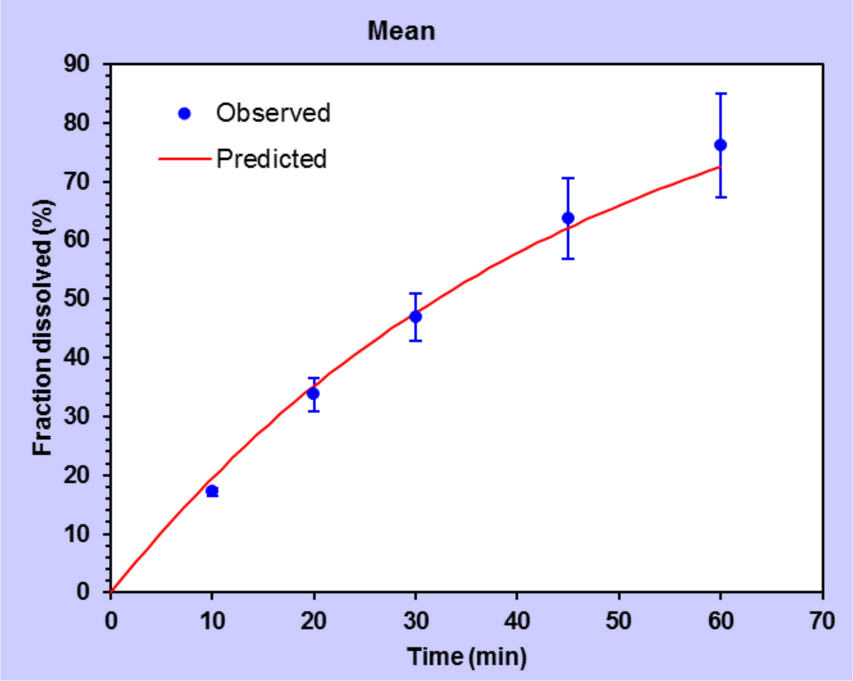

Supplementary materials – Model fitting summary – Polyglykol® 4000 P (modelling up to app. 75 % of carvedilol released)

Graphical abstract of model fit presented as the fraction % of released carvedilol per tested tablet:

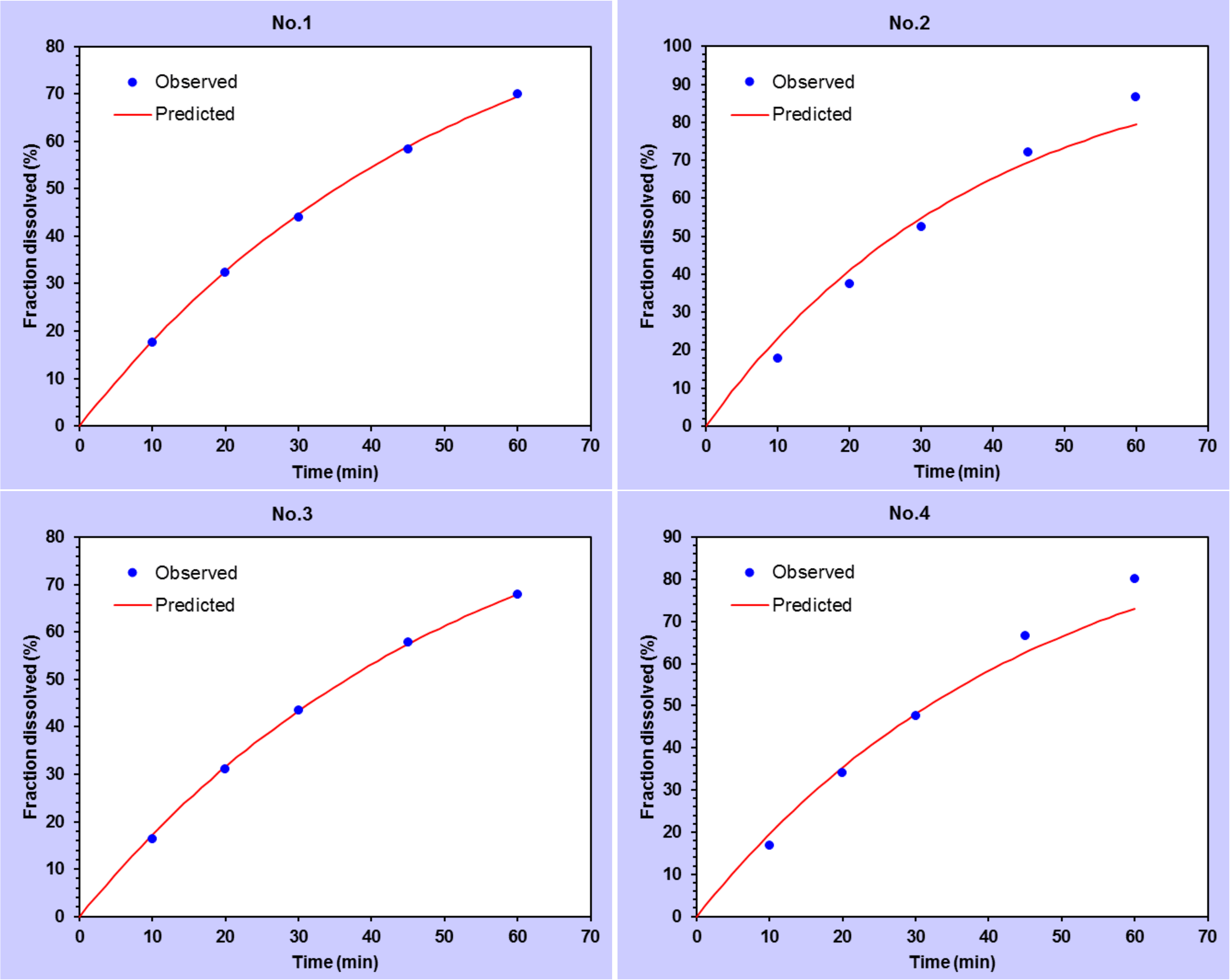

Supplementary materials – Model fitting summary – Polyglykol® 4000 P (modelling up to app. 75 % of carvedilol released)

Model: **First–order with T_{lag}**

Model equation: $F = 100 \cdot \left[1 - e^{-k_1 \cdot (t - T_{lag})}\right]$

Fitted model parameters per tested tablet (N = 4) with statistics – mean, standard deviation (SD), and relative standard deviation expressed in % (RSD%) (output from DDSolver):

Parameter	No.1	No.2	No.3	No.4	Mean	SD	RSD(%)
k_1	0.020	0.036	0.019	0.028	0.026	0.008	30.477
T_{lag}	0.764	6.902	0.536	5.238	3.360	3.204	95.344

Number of dissolution data points (N), degrees of freedom (df), and selected goodness of fit criteria – Pearson correlation coefficient (R), coefficient of determination (R^2), adjusted coefficient of determination ($R^2_{adjusted}$), and residual sum of squares (RSS) (manual calculation in MS Excel):

Parameter	No.1	No.2	No.3	No.4
N	5	5	5	5
df	3	3	3	3
R	0.999671068	0.991891922	0.999867643	0.99577825
R^2	0.999342244	0.983849584	0.999735303	0.991574322
$R^2_{adjusted}$	0.999122992	0.978466112	0.999647071	0.988765763
RSS	1.319406475	79.80464129	0.515424497	28.97034007

Graphical abstract of model fit presented as mean ± 1 SD of the fraction % of released carvedilol:

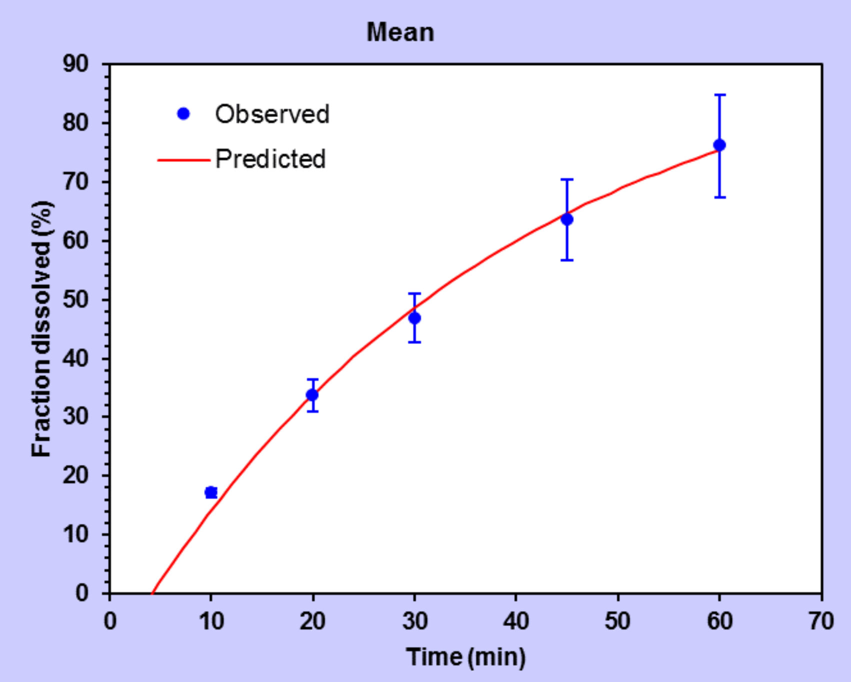

Supplementary materials – Model fitting summary – Polyglykol® 4000 P (modelling up to app. 75 % of carvedilol released)

Graphical abstract of model fit presented as the fraction % of released carvedilol per tested tablet:

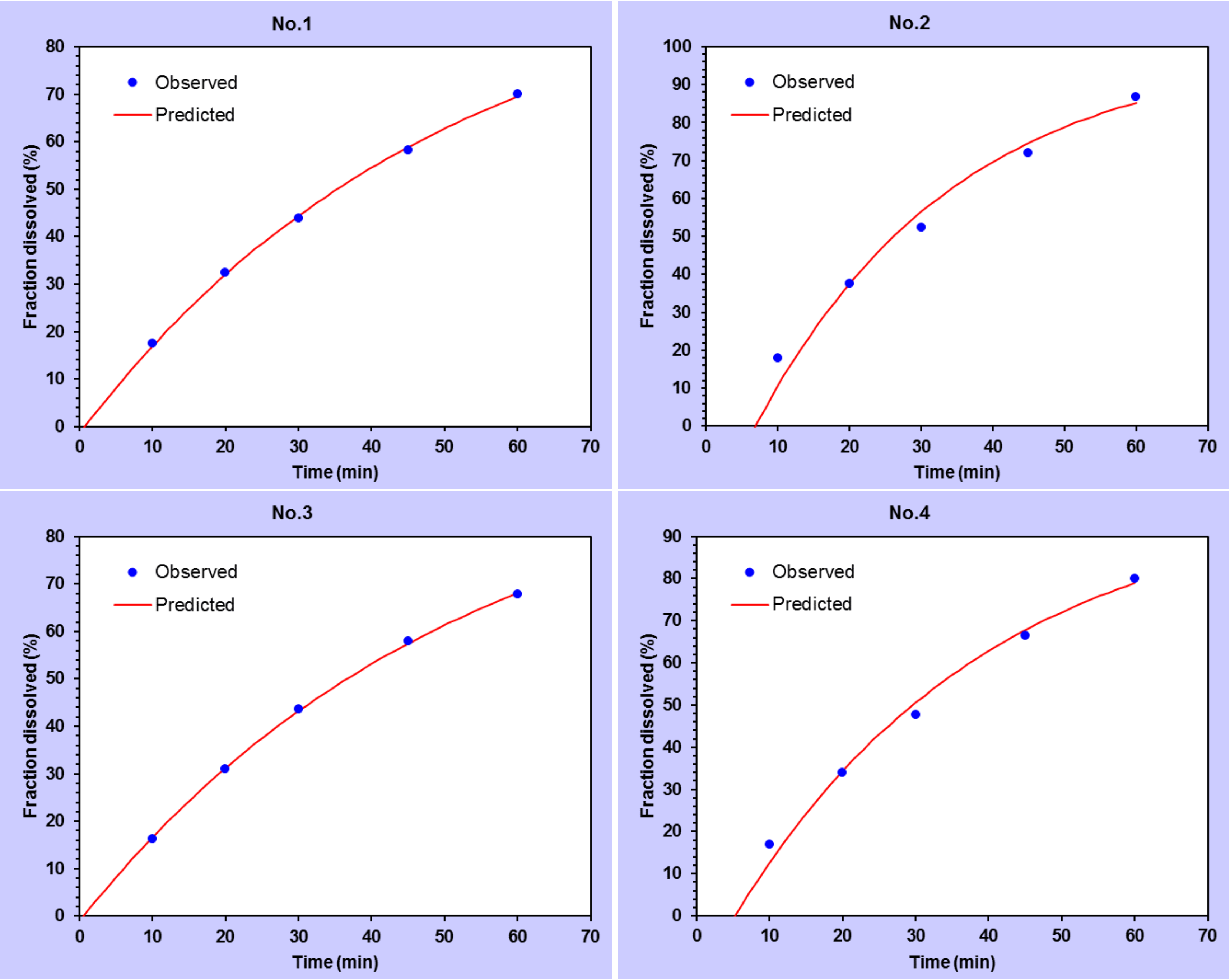

Supplementary materials – Model fitting summary – Polyglykol® 4000 P (modelling up to app. 75 % of carvedilol released)

Model: **First–order with F_{max}**

Model equation: $F = F_{max} \cdot (1 - e^{-k_1 \cdot t})$

Fitted model parameters per tested tablet (N = 4) with statistics – mean, standard deviation (SD), and relative standard deviation expressed in % (RSD%) (output from DDSolver):

Parameter	No.1	No.2	No.3	No.4	Mean	SD	RSD(%)
k_1	0.042	0.043	0.043	0.043	0.043	0.000	0.916
F_{max}	73.503	73.983	71.229	68.240	71.739	2.623	3.657

Number of dissolution data points (N), degrees of freedom (df), and selected goodness of fit criteria – Pearson correlation coefficient (R), coefficient of determination (R^2), adjusted coefficient of determination ($R^2_{adjusted}$), and residual sum of squares (RSS) (manual calculation in MS Excel):

Parameter	No.1	No.2	No.3	No.4
N	5	5	5	5
df	3	3	3	3
R	0.986000526	0.985507531	0.98931585	0.982937227
R^2	0.972197038	0.971225094	0.97874585	0.966165593
$R^2_{adjusted}$	0.962929383	0.961633459	0.971661134	0.954887457
RSS	247.5918016	505.8651599	246.504191	434.0784926

Graphical abstract of model fit presented as mean ± 1 SD of the fraction % of released carvedilol:

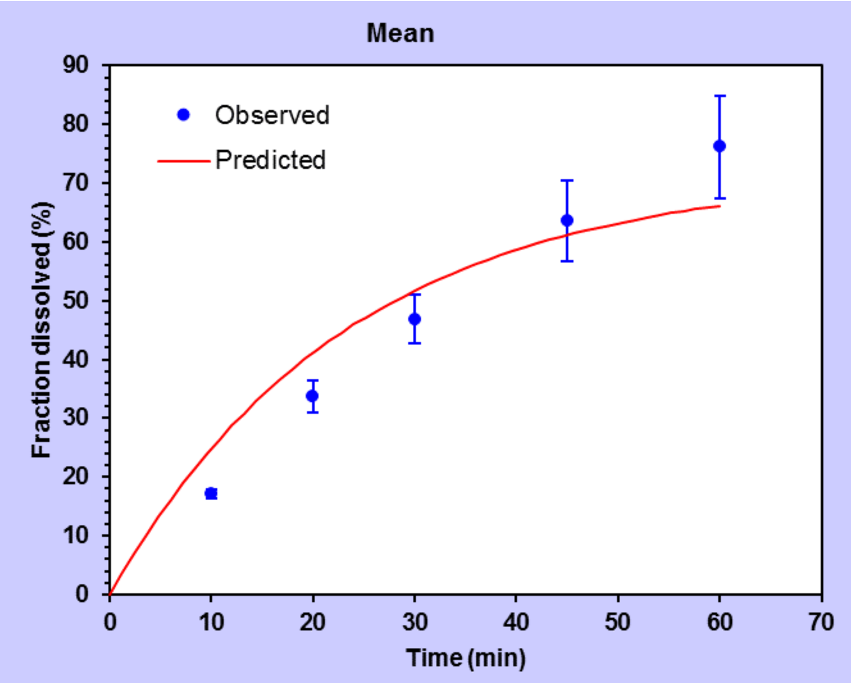

Supplementary materials – Model fitting summary – Polyglykol® 4000 P (modelling up to app. 75 % of carvedilol released)

Graphical abstract of model fit presented as the fraction % of released carvedilol per tested tablet:

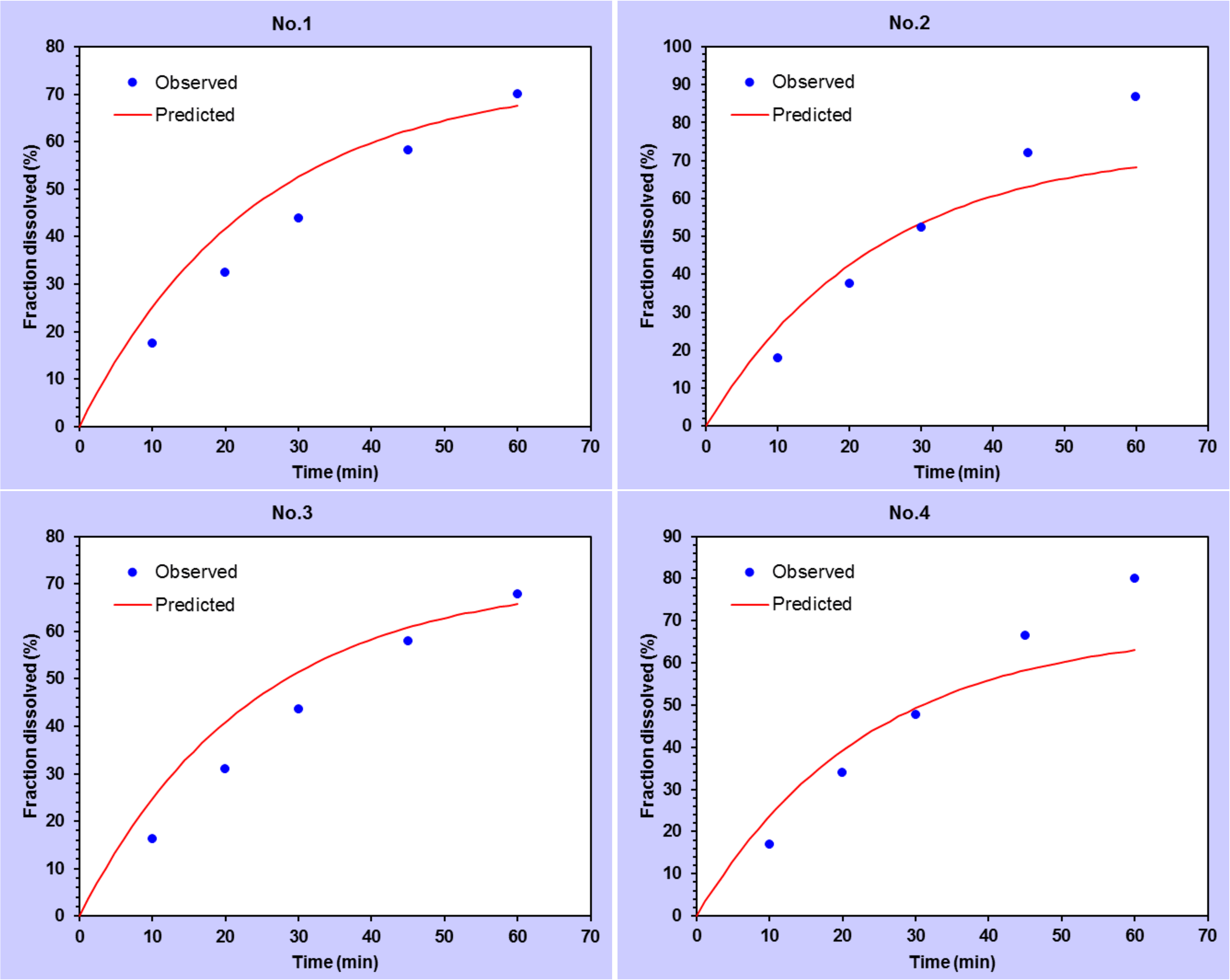

Supplementary materials – Model fitting summary – Polyglykol® 4000 P (modelling up to app. 75 % of carvedilol released)

Model: **First–order with T_{lag} and F_{max}**

Model equation: $F = F_{max} \cdot \left[1 - e^{-k_1 \cdot (t - T_{lag})}\right]$

Fitted model parameters per tested tablet (N = 4) with statistics – mean, standard deviation (SD), and relative standard deviation expressed in % (RSD%) (output from DDSolver):

Parameter	No.1	No.2	No.3	No.4	Mean	SD	RSD(%)
k_1	0.080	0.082	0.054	0.082	0.075	0.014	18.154
T_{lag}	6.107	6.860	9.147	6.931	7.261	1.311	18.059
F_{max}	61.252	75.880	71.229	69.990	69.588	6.108	8.777

Number of dissolution data points (N), degrees of freedom (df), and selected goodness of fit criteria – Pearson correlation coefficient (R), coefficient of determination (R^2), adjusted coefficient of determination ($R^2_{adjusted}$), and residual sum of squares (RSS) (manual calculation in MS Excel):

Parameter	No.1	No.2	No.3	No.4
N	5	5	5	5
df	2	2	2	2
R	0.932373695	0.929828424	0.976621961	0.923602303
R^2	0.869320706	0.864580898	0.953790454	0.853041214
$R^2_{adjusted}$	0.738641413	0.729161796	0.907580909	0.706082429
RSS	239.9818859	446.6554563	205.7924893	407.03778

Graphical abstract of model fit presented as mean ± 1 SD of the fraction % of released carvedilol:

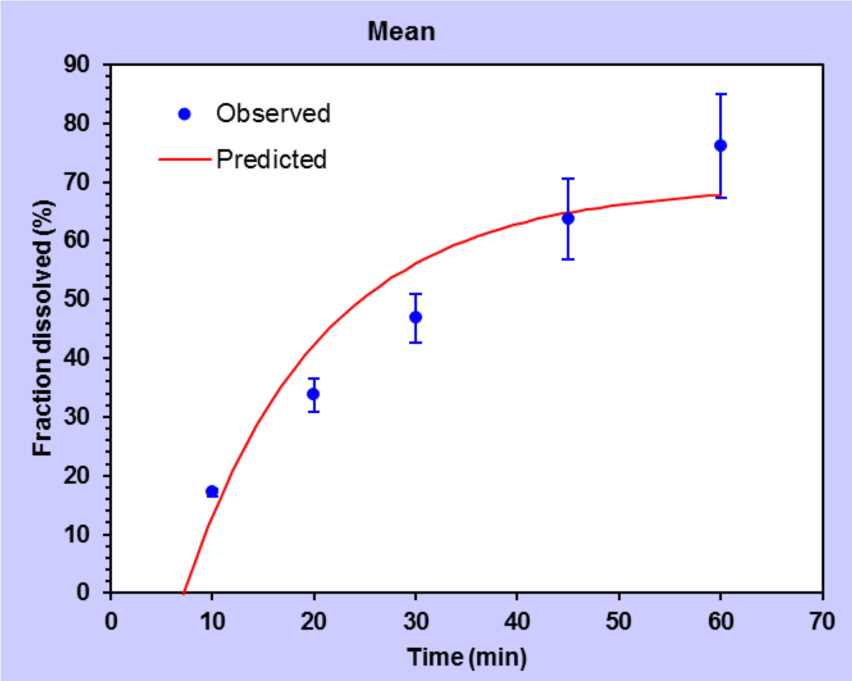

Supplementary materials – Model fitting summary – Polyglykol® 4000 P (modelling up to app. 75 % of carvedilol released)

Graphical abstract of model fit presented as the fraction % of released carvedilol per tested tablet:

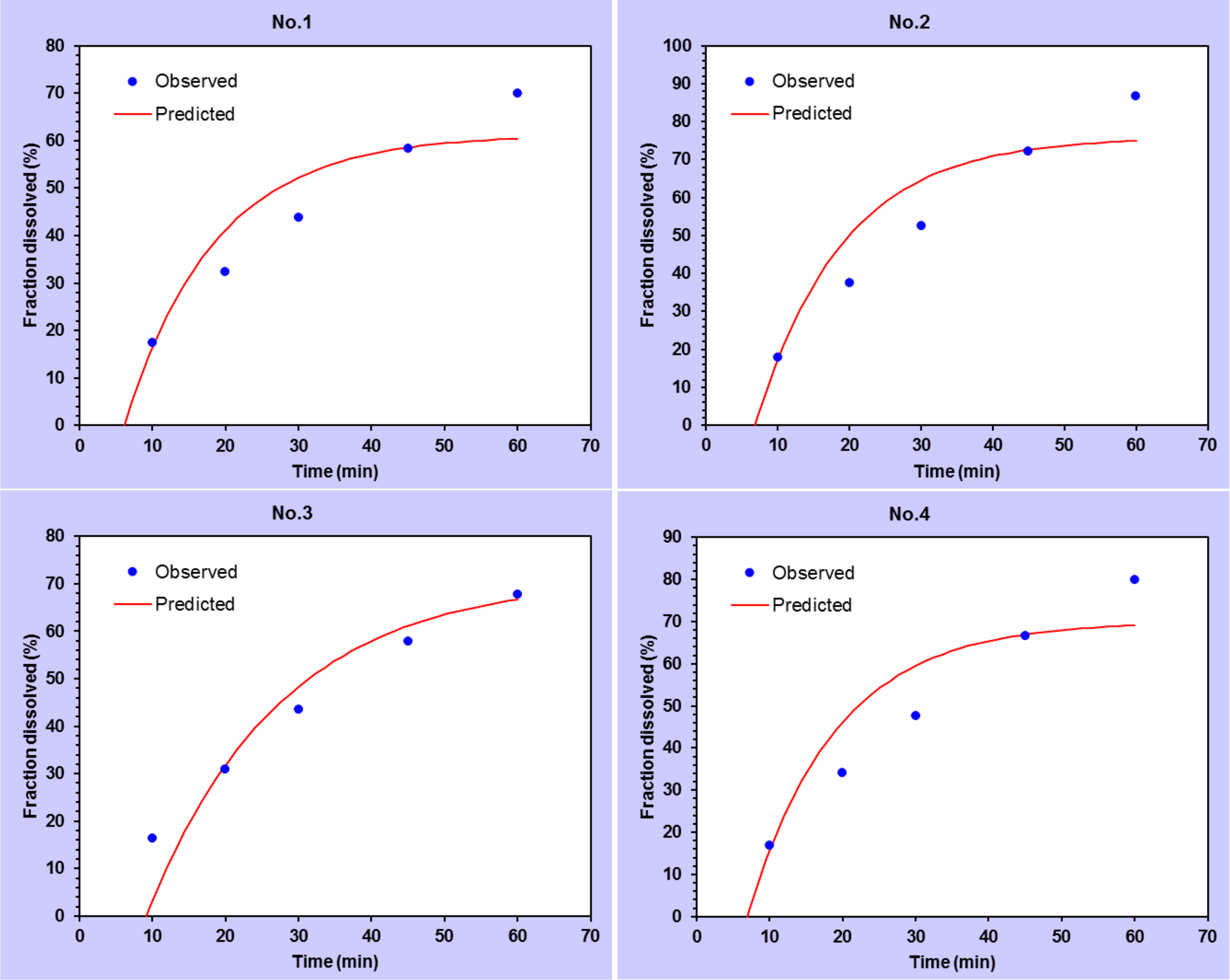

Supplementary materials – Model fitting summary – Polyglykol® 4000 P (modelling up to app. 75 % of carvedilol released)

Model: **Higuchi**

Model equation: $F = k_H \cdot t^{0.5}$

Fitted model parameters per tested tablet (N = 4) with statistics – mean, standard deviation (SD), and relative standard deviation expressed in % (RSD%) (output from DDSolver):

Parameter	No.1	No.2	No.3	No.4	Mean	SD	RSD(%)
k_H	8.323	10.098	8.136	9.287	8.961	0.910	10.160

Number of dissolution data points (N), degrees of freedom (df), and selected goodness of fit criteria – Pearson correlation coefficient (R), coefficient of determination (R^2), adjusted coefficient of determination ($R^2_{adjusted}$), and residual sum of squares (RSS) (manual calculation in MS Excel):

Parameter	No.1	No.2	No.3	No.4
N	5	5	5	5
df	4	4	4	4
R	0.999988278	0.999874362	0.999283418	0.999571569
R^2	0.999976555	0.999748741	0.998567349	0.999143321
$R^2_{adjusted}$	0.999976555	0.999748741	0.998567349	0.999143321
RSS	140.606346	355.2900274	152.9745925	305.5104222

Graphical abstract of model fit presented as mean ± 1 SD of the fraction % of released carvedilol:

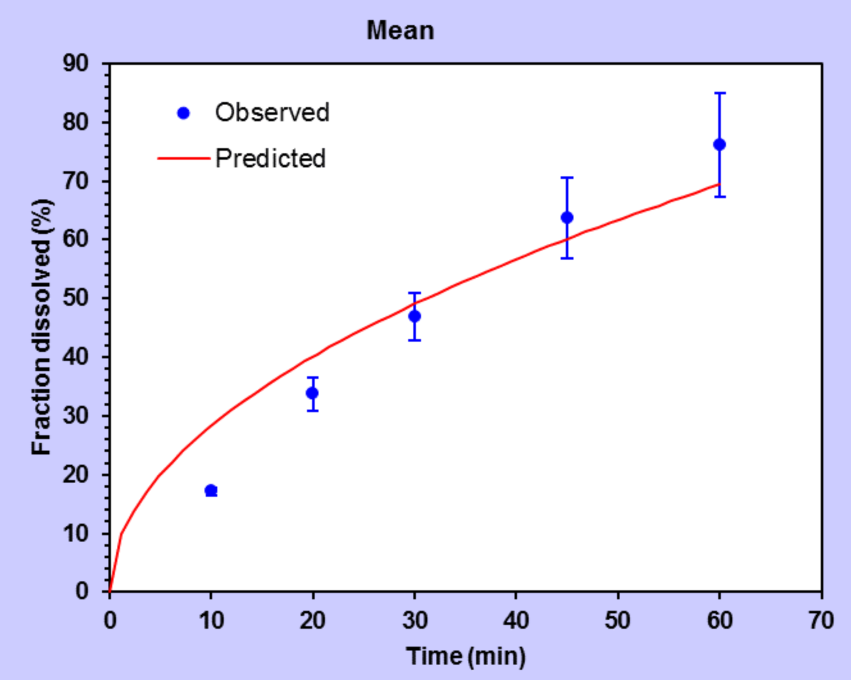

Supplementary materials – Model fitting summary – Polyglykol® 4000 P (modelling up to app. 75 % of carvedilol released)

Graphical abstract of model fit presented as the fraction % of released carvedilol per tested tablet:

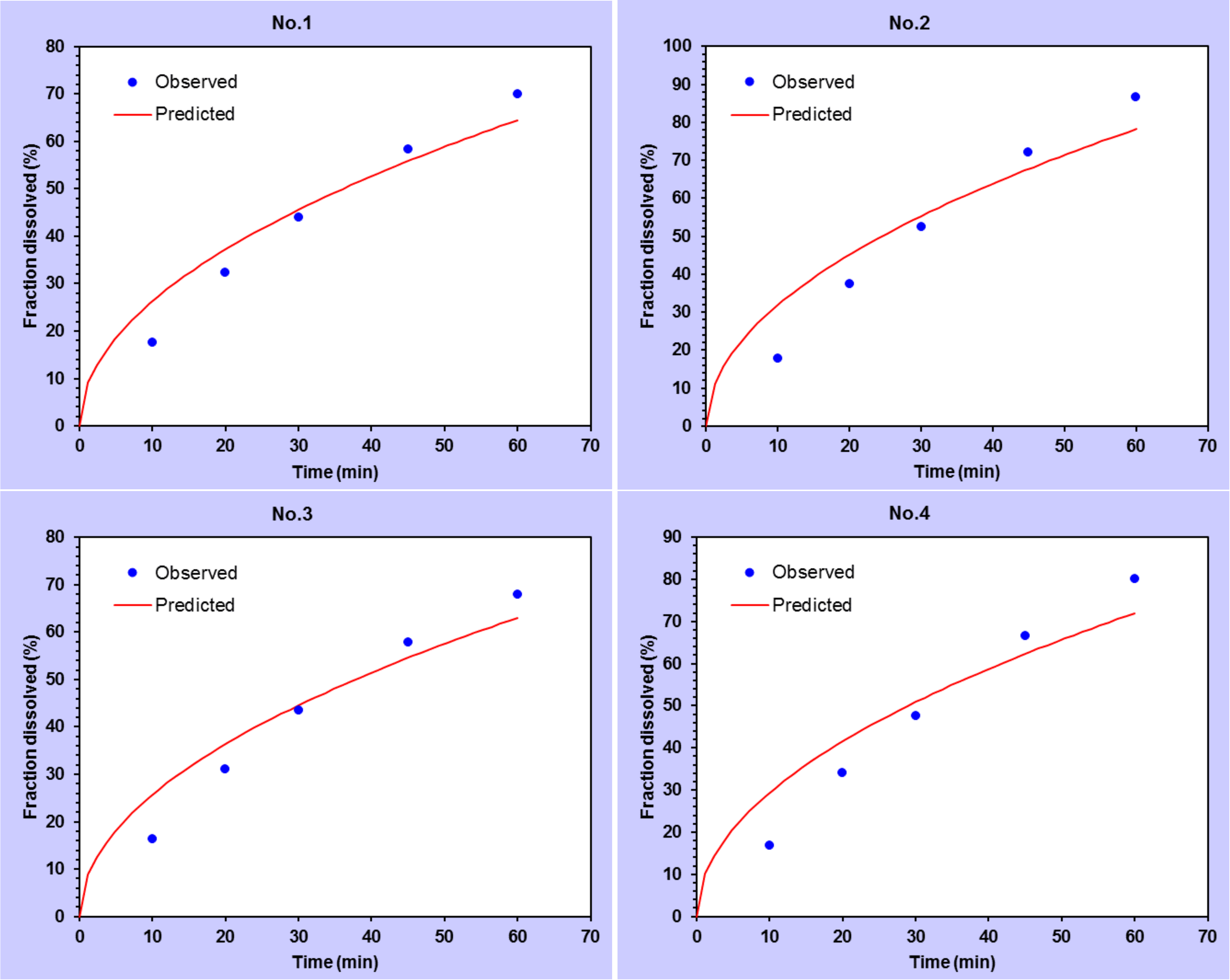

Supplementary materials – Model fitting summary – Polyglykol® 4000 P (modelling up to app. 75 % of carvedilol released)

Model: **Higuchi with T_{lag}**

Model equation: $F = k_H \cdot (t - T_{lag})^{0.5}$

Fitted model parameters per tested tablet (N = 4) with statistics – mean, standard deviation (SD), and relative standard deviation expressed in % (RSD%) (output from DDSolver):

Parameter	No.1	No.2	No.3	No.4	Mean	SD	RSD(%)
k_H	9.330	12.104	9.418	11.180	10.508	1.363	12.972
T_{lag}	5.661	9.546	8.026	9.737	8.243	1.884	22.854

Number of dissolution data points (N), degrees of freedom (df), and selected goodness of fit criteria – Pearson correlation coefficient (R), coefficient of determination (R^2), adjusted coefficient of determination ($R^2_{adjusted}$), and residual sum of squares (RSS) (manual calculation in MS Excel):

Parameter	No.1	No.2	No.3	No.4
N	5	5	5	5
df	3	3	3	3
R	0.998871195	0.99082523	0.997147466	0.986385177
R^2	0.997743665	0.981734637	0.994303068	0.972955718
$R^2_{adjusted}$	0.996991553	0.975646183	0.992404091	0.963940957
RSS	18.69261143	103.7759724	12.63037561	135.6336933

Graphical abstract of model fit presented as mean ± 1 SD of the fraction % of released carvedilol:

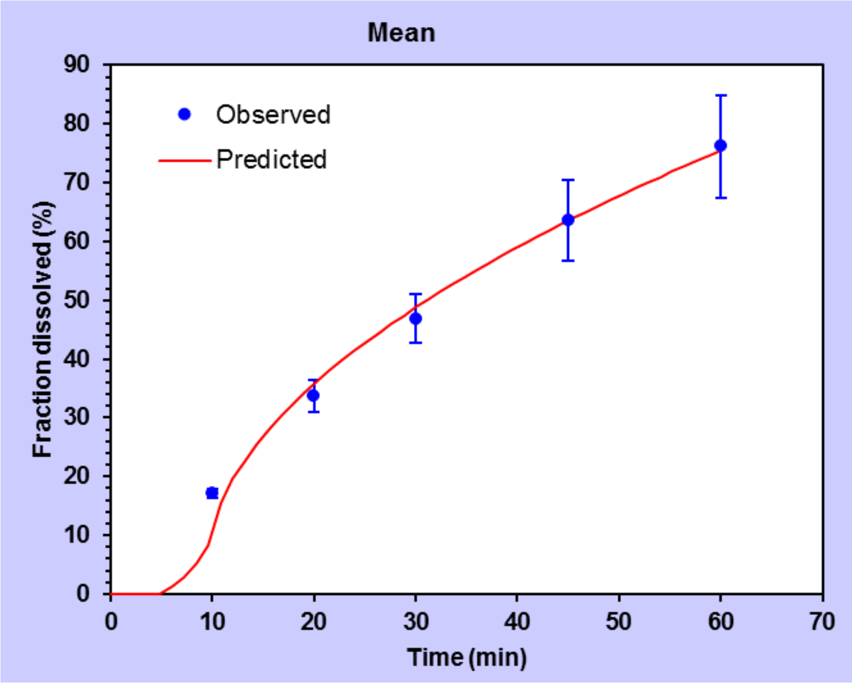

Supplementary materials – Model fitting summary – Polyglykol® 4000 P (modelling up to app. 75 % of carvedilol released)

Graphical abstract of model fit presented as the fraction % of released carvedilol per tested tablet:

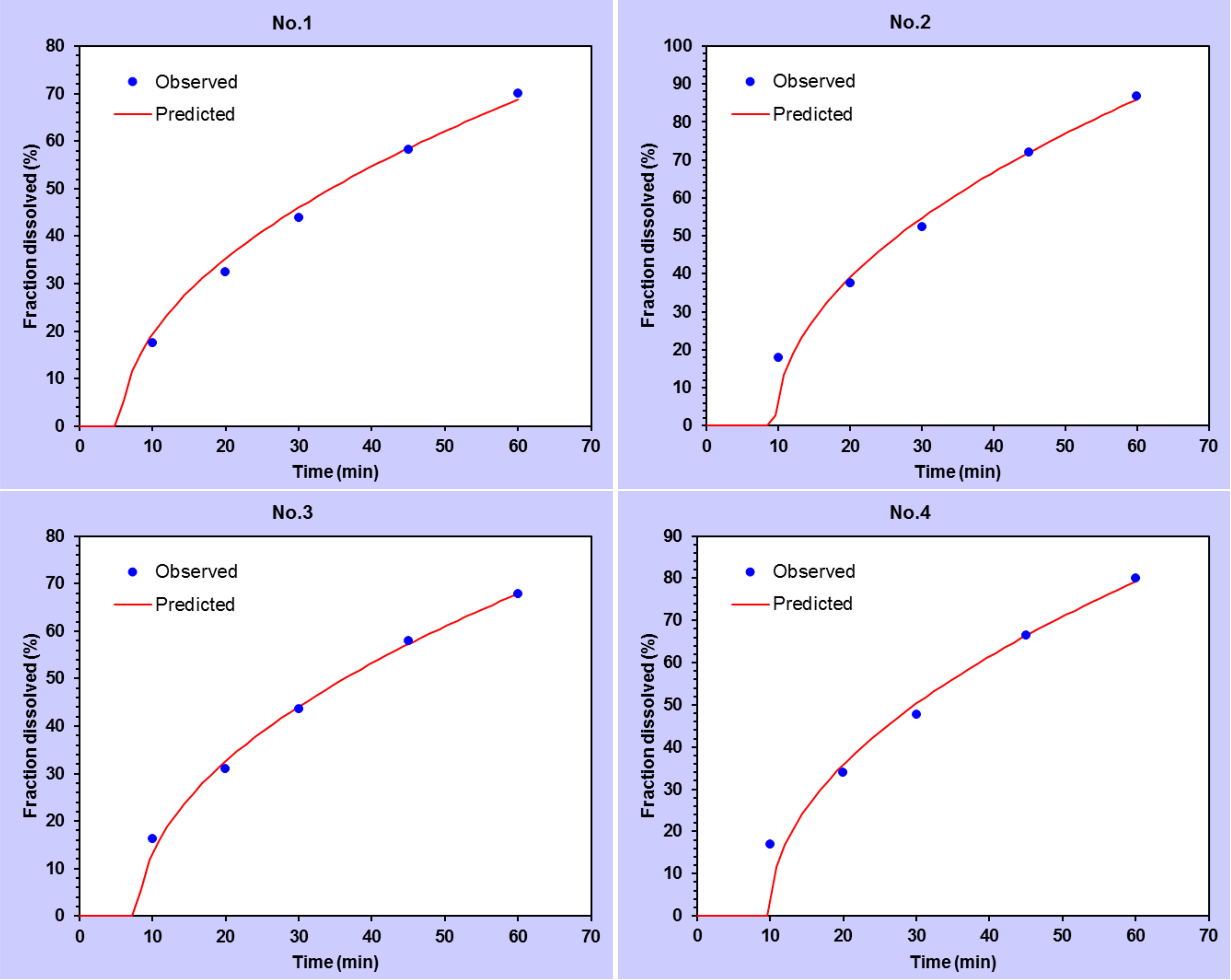

Supplementary materials – Model fitting summary – Polyglykol® 4000 P (modelling up to app. 75 % of carvedilol released)

Model: **Higuchi with F_0**

Model equation: $F = F_0 + k_H \cdot t^{0.5}$

Fitted model parameters per tested tablet (N = 4) with statistics – mean, standard deviation (SD), and relative standard deviation expressed in % (RSD%) (output from DDSolver):

Parameter	No.1	No.2	No.3	No.4	Mean	SD	RSD(%)
k_H	11.476	15.105	11.399	13.919	12.975	1.840	14.183
F_0	-18.871	-29.971	-19.530	-27.723	-24.024	5.651	-23.521

Number of dissolution data points (N), degrees of freedom (df), and selected goodness of fit criteria – Pearson correlation coefficient (R), coefficient of determination (R^2), adjusted coefficient of determination ($R^2_{adjusted}$), and residual sum of squares (RSS) (manual calculation in MS Excel):

Parameter	No.1	No.2	No.3	No.4
N	5	5	5	5
df	3	3	3	3
R	0.999988278	0.999874362	0.999283418	0.999571569
R^2	0.999976555	0.999748741	0.998567349	0.999143321
$R^2_{adjusted}$	0.99996874	0.999664987	0.998089798	0.998857761
RSS	0.040219411	0.746905558	2.42805347	2.163573049

Graphical abstract of model fit presented as mean ± 1 SD of the fraction % of released carvedilol:

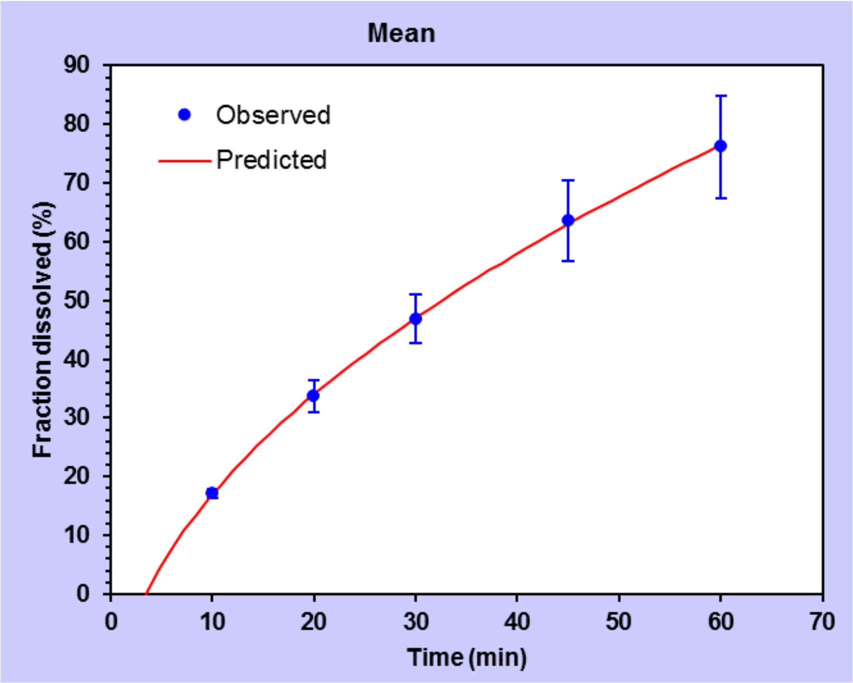

Supplementary materials – Model fitting summary – Polyglykol® 4000 P (modelling up to app. 75 % of carvedilol released)

Graphical abstract of model fit presented as the fraction % of released carvedilol per tested tablet:

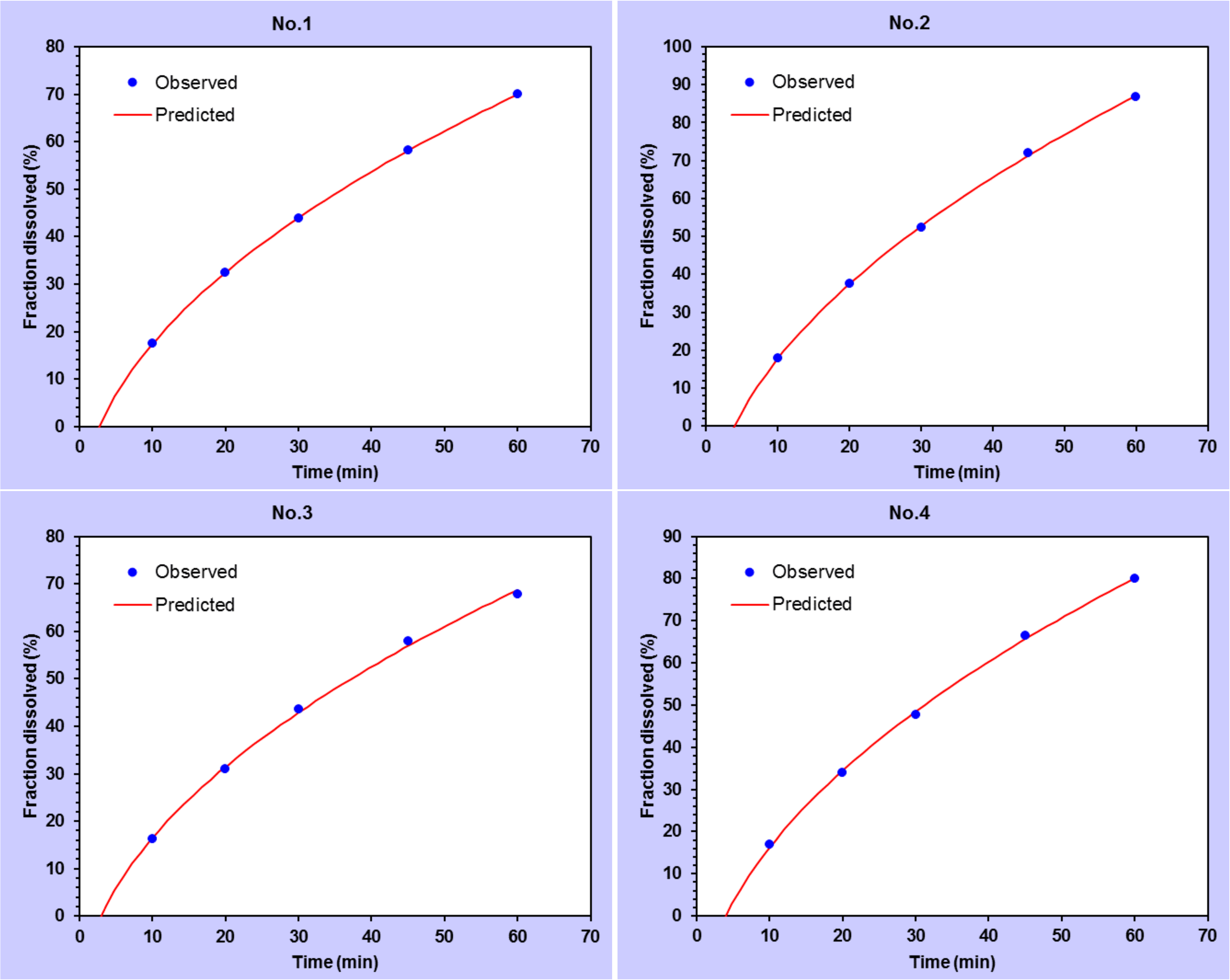

Supplementary materials – Model fitting summary – Polyglykol® 4000 P (modelling up to app. 75 % of carvedilol released)

Model: **Korsmeyer–Peppas**

Model equation: $F = k_{KP} \cdot t^n$

Fitted model parameters per tested tablet (N = 4) with statistics – mean, standard deviation (SD), and relative standard deviation expressed in % (RSD%) (output from DDSolver):

Parameter	No.1	No.2	No.3	No.4	Mean	SD	RSD(%)
k_{KP}	3.064	2.503	2.691	2.779	2.759	0.233	8.461
n	0.773	0.881	0.803	0.822	0.820	0.045	5.536

Number of dissolution data points (N), degrees of freedom (df), and selected goodness of fit criteria – Pearson correlation coefficient (R), coefficient of determination (R^2), adjusted coefficient of determination ($R^2_{adjusted}$), and residual sum of squares (RSS) (manual calculation in MS Excel):

Parameter	No.1	No.2	No.3	No.4
N	5	5	5	5
df	3	3	3	3
R	0.997695428	0.995205548	0.994468572	0.997395494
R^2	0.995396167	0.990434083	0.988967742	0.994797771
$R^2_{adjusted}$	0.993861556	0.987245444	0.985290322	0.993063695
RSS	11.01826533	42.46081323	25.05374009	17.63178719

Graphical abstract of model fit presented as mean ± 1 SD of the fraction % of released carvedilol:

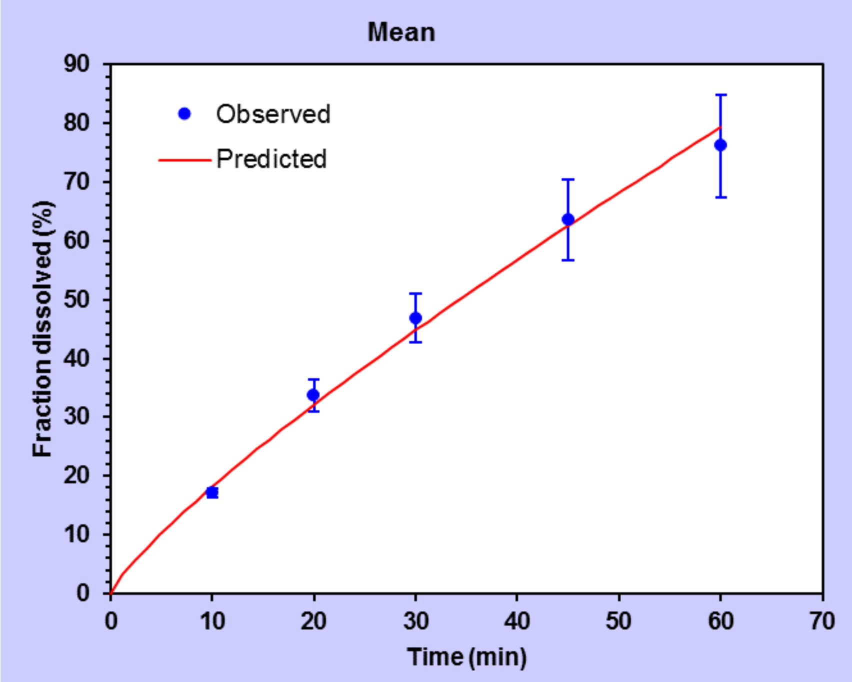

Supplementary materials – Model fitting summary – Polyglykol® 4000 P (modelling up to app. 75 % of carvedilol released)

Graphical abstract of model fit presented as the fraction % of released carvedilol per tested tablet:

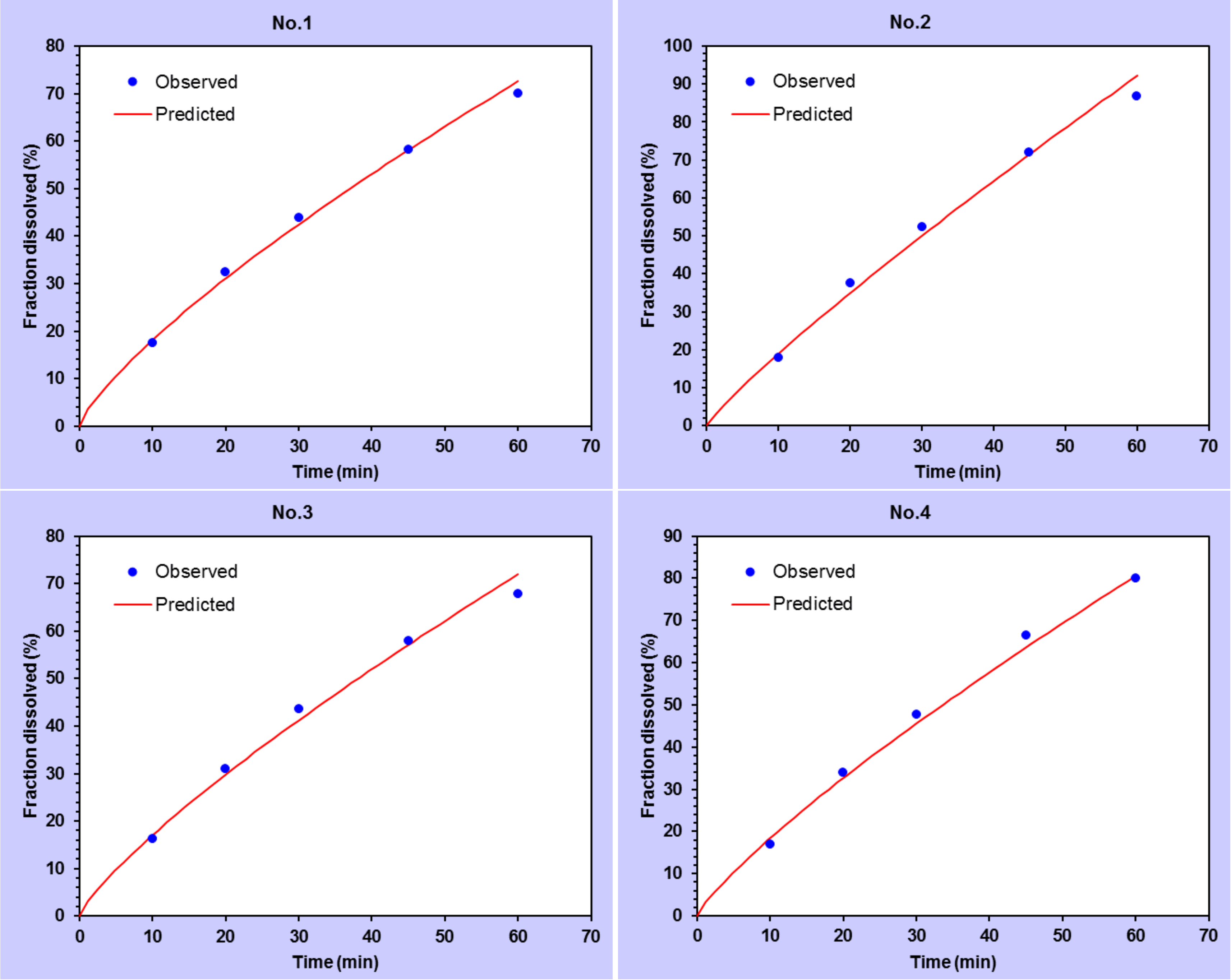

Supplementary materials – Model fitting summary – Polyglykol® 4000 P (modelling up to app. 75 % of carvedilol released)

Model: **Korsmeyer–Peppas with T_{lag}**

Model equation: $F = k_{KP} \cdot \left(t - T_{lag}\right)^n$

Fitted model parameters per tested tablet (N = 4) with statistics – mean, standard deviation (SD), and relative standard deviation expressed in % (RSD%) (output from DDSolver):

Parameter	No.1	No.2	No.3	No.4	Mean	SD	RSD(%)
k_{KP}	5.754	5.113	5.166	4.836	5.217	0.386	7.404
n	0.622	0.710	0.647	0.702	0.670	0.042	6.336
T_{lag}	4.000	4.000	4.000	4.000	4.000	0.000	0.000

Number of dissolution data points (N), degrees of freedom (df), and selected goodness of fit criteria – Pearson correlation coefficient (R), coefficient of determination (R^2), adjusted coefficient of determination ($R^2_{adjusted}$), and residual sum of squares (RSS) (manual calculation in MS Excel):

Parameter	No.1	No.2	No.3	No.4
N	5	5	5	5
df	2	2	2	2
R	0.999929746	0.999073817	0.998545998	0.999401505
R^2	0.999859497	0.998148491	0.99709411	0.998803367
$R^2_{adjusted}$	0.999718995	0.996296982	0.994188221	0.997606735
RSS	0.284816003	7.453551557	5.68197708	3.348436012

Graphical abstract of model fit presented as mean ± 1 SD of the fraction % of released carvedilol:

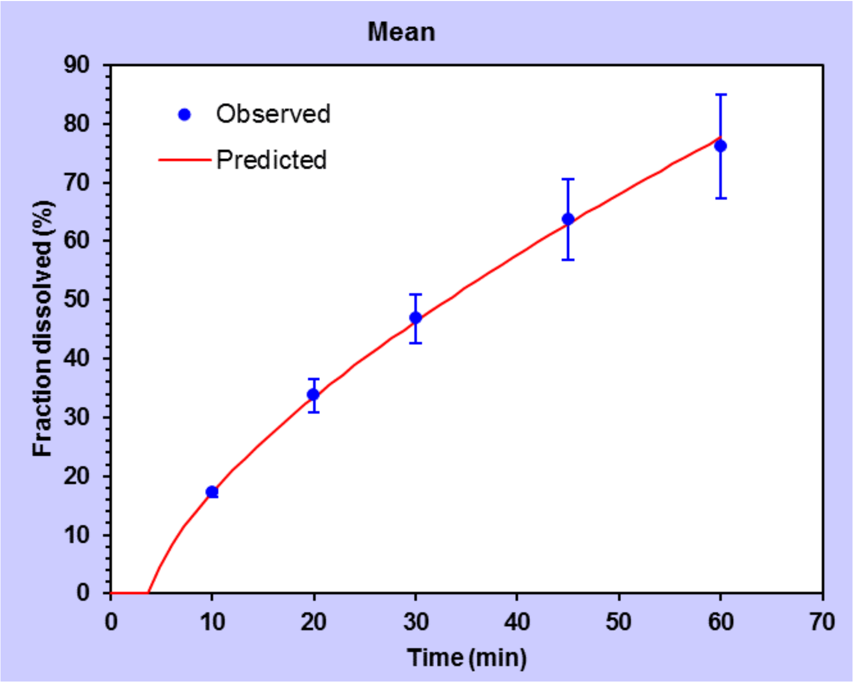

Supplementary materials – Model fitting summary – Polyglykol® 4000 P (modelling up to app. 75 % of carvedilol released)

Graphical abstract of model fit presented as the fraction % of released carvedilol per tested tablet:

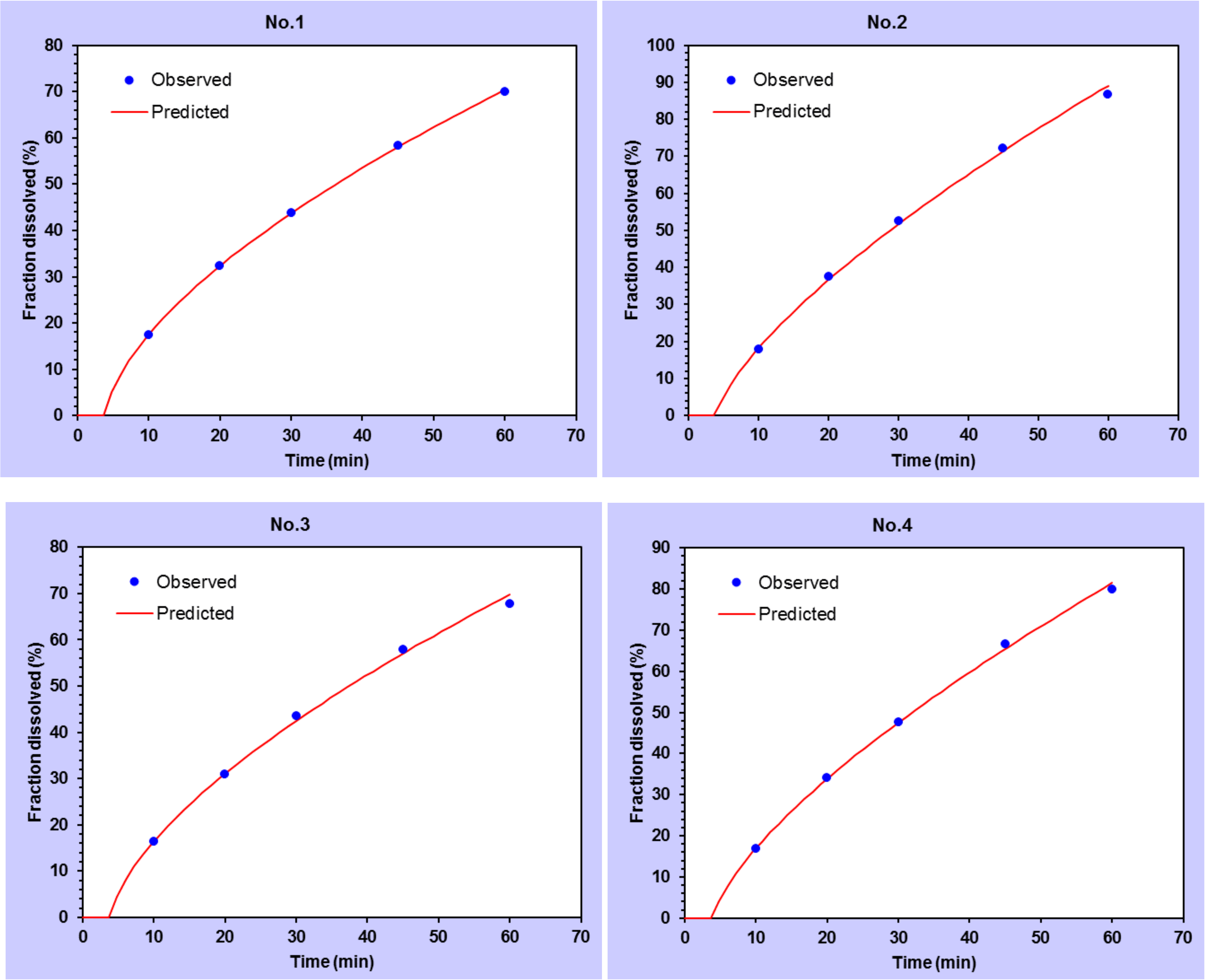

Supplementary materials – Model fitting summary – Polyglykol® 4000 P (modelling up to app. 75 % of carvedilol released)

Model: **Korsmeyer–Peppas with F_0**

Model equation: $F = F_0 + k_{KP} \cdot t^n$

Fitted model parameters per tested tablet (N = 4) with statistics – mean, standard deviation (SD), and relative standard deviation expressed in % (RSD%) (output from DDSolver):

Parameter	No.1	No.2	No.3	No.4	Mean	SD	RSD(%)
k_{KP}	1.161	0.939	1.016	0.889	1.001	0.119	11.860
n	0.995	1.112	1.028	1.103	1.060	0.057	5.374
F_0	6.998	7.159	6.519	6.758	6.859	0.280	4.081

Number of dissolution data points (N), degrees of freedom (df), and selected goodness of fit criteria – Pearson correlation coefficient (R), coefficient of determination (R^2), adjusted coefficient of determination ($R^2_{adjusted}$), and residual sum of squares (RSS) (manual calculation in MS Excel):

Parameter	No.1	No.2	No.3	No.4
N	5	5	5	5
df	2	2	2	2
R	0.992581245	0.988413279	0.98742013	0.990567006
R^2	0.985217528	0.97696081	0.974998512	0.981222992
$R^2_{adjusted}$	0.970435055	0.953921619	0.949997024	0.962445985
RSS	42.73148974	126.2553938	69.12001994	84.04434068

Graphical abstract of model fit presented as mean ± 1 SD of the fraction % of released carvedilol:

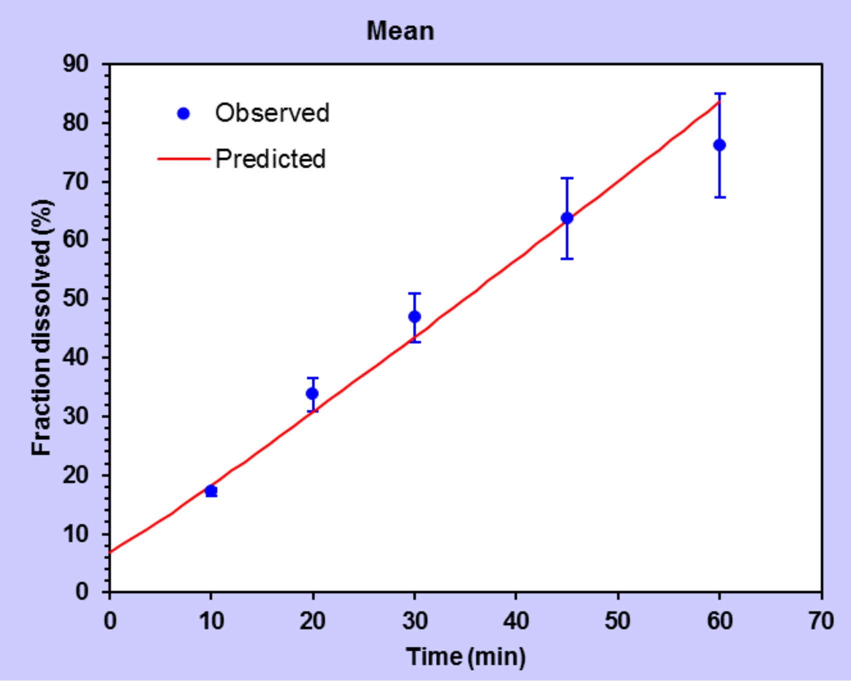

Supplementary materials – Model fitting summary – Polyglykol® 4000 P (modelling up to app. 75 % of carvedilol released)

Graphical abstract of model fit presented as the fraction % of released carvedilol per tested tablet:

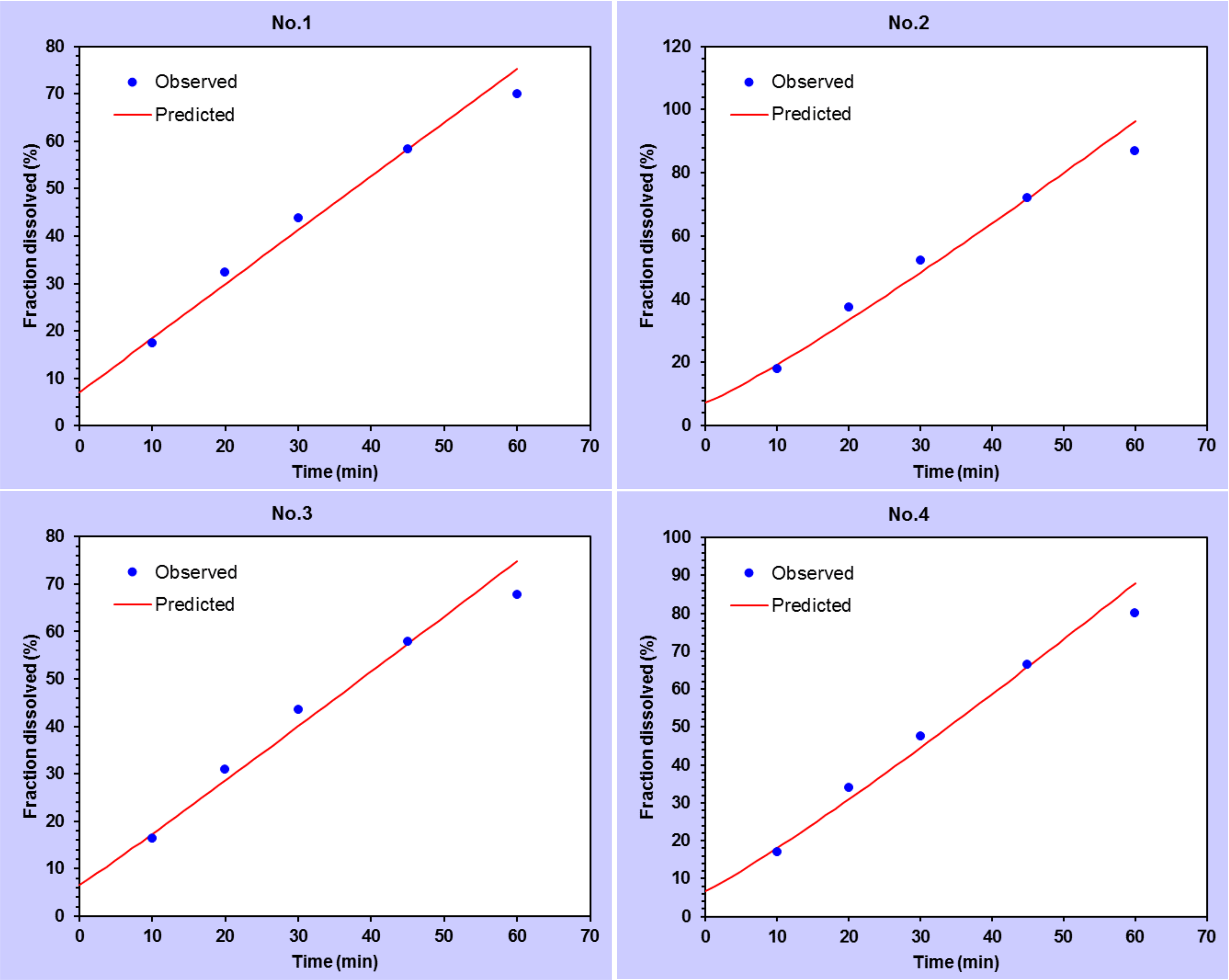

Supplementary materials – Model fitting summary – Polyglykol® 4000 P (modelling up to app. 75 % of carvedilol released)

Model: **Hixson–Crowell**

Model equation: $F = 100 \cdot [1 - (1 - k_{HC} \cdot t)^3]$

Fitted model parameters per tested tablet (N = 4) with statistics – mean, standard deviation (SD), and relative standard deviation expressed in % (RSD%) (output from DDSolver):

Parameter	No.1	No.2	No.3	No.4	Mean	SD	RSD(%)
k_{HC}	0.006	0.008	0.005	0.007	0.006	0.001	17.349

Number of dissolution data points (N), degrees of freedom (df), and selected goodness of fit criteria – Pearson correlation coefficient (R), coefficient of determination (R^2), adjusted coefficient of determination ($R^2_{adjusted}$), and residual sum of squares (RSS) (manual calculation in MS Excel):

Parameter	No.1	No.2	No.3	No.4
N	5	5	5	5
df	4	4	4	4
R	0.999593163	0.999510092	0.998453278	0.999797791
R^2	0.999186492	0.999020423	0.996908949	0.999595622
$R^2_{adjusted}$	0.999186492	0.999020423	0.996908949	0.999595622
RSS	10.19937729	32.71874609	11.76585467	10.55347872

Graphical abstract of model fit presented as mean ± 1 SD of the fraction % of released carvedilol:

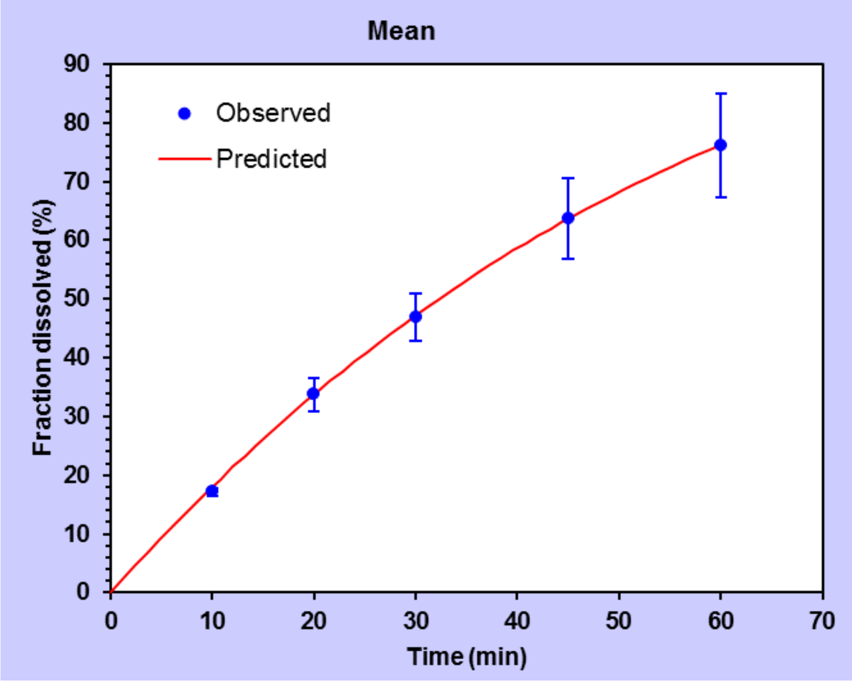

Supplementary materials – Model fitting summary – Polyglykol® 4000 P (modelling up to app. 75 % of carvedilol released)

Graphical abstract of model fit presented as the fraction % of released carvedilol per tested tablet:

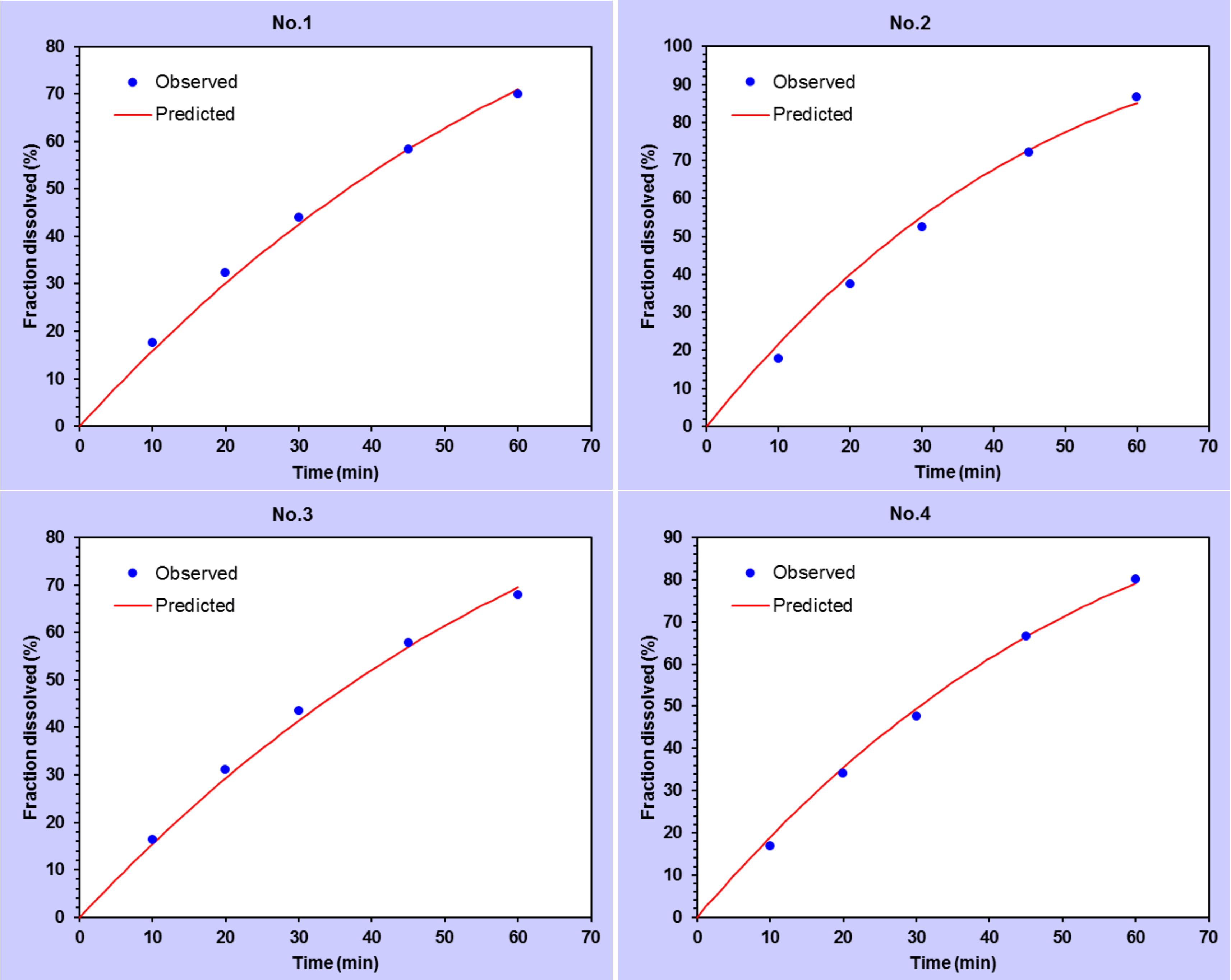

Supplementary materials – Model fitting summary – Polyglykol® 4000 P (modelling up to app. 75 % of carvedilol released)

Model: **Hixson–Crowell with T_{lag}**

Model equation: $F = 100 \cdot \left\{1 - \left[1 - k_{HC} \cdot \left(t - T_{lag}\right)\right]^3\right\}$

Fitted model parameters per tested tablet (N = 4) with statistics – mean, standard deviation (SD), and relative standard deviation expressed in % (RSD%) (output from DDSolver):

Parameter	No.1	No.2	No.3	No.4	Mean	SD	RSD(%)
k_{HC}	0.005	0.008	0.005	0.007	0.007	0.002	24.141
T_{lag}	-2.406	3.143	-2.401	1.952	0.072	2.899	4021.235

Number of dissolution data points (N), degrees of freedom (df), and selected goodness of fit criteria – Pearson correlation coefficient (R), coefficient of determination (R^2), adjusted coefficient of determination ($R^2_{adjusted}$), and residual sum of squares (RSS) (manual calculation in MS Excel):

Parameter	No.1	No.2	No.3	No.4
N	5	5	5	5
df	3	3	3	3
R	0.999473223	0.999167166	0.998173195	0.999704228
R^2	0.998946724	0.998335026	0.996349726	0.999408544
$R^2_{adjusted}$	0.998595631	0.997780035	0.995132969	0.999211392
RSS	1.891329378	6.179527842	6.661482122	1.642219755

Graphical abstract of model fit presented as mean ± 1 SD of the fraction % of released carvedilol:

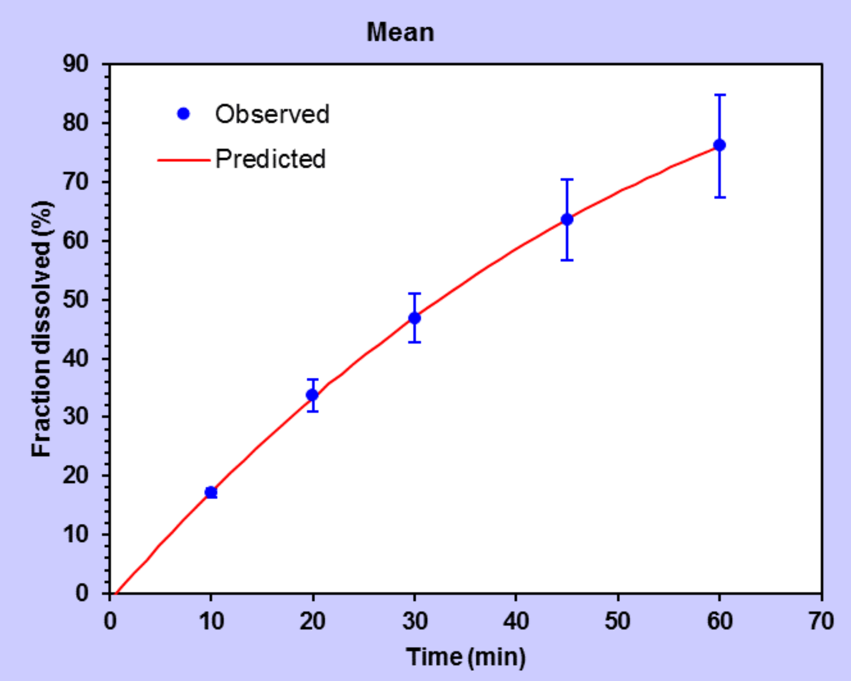

Supplementary materials – Model fitting summary – Polyglykol® 4000 P (modelling up to app. 75 % of carvedilol released)

Graphical abstract of model fit presented as the fraction % of released carvedilol per tested tablet:

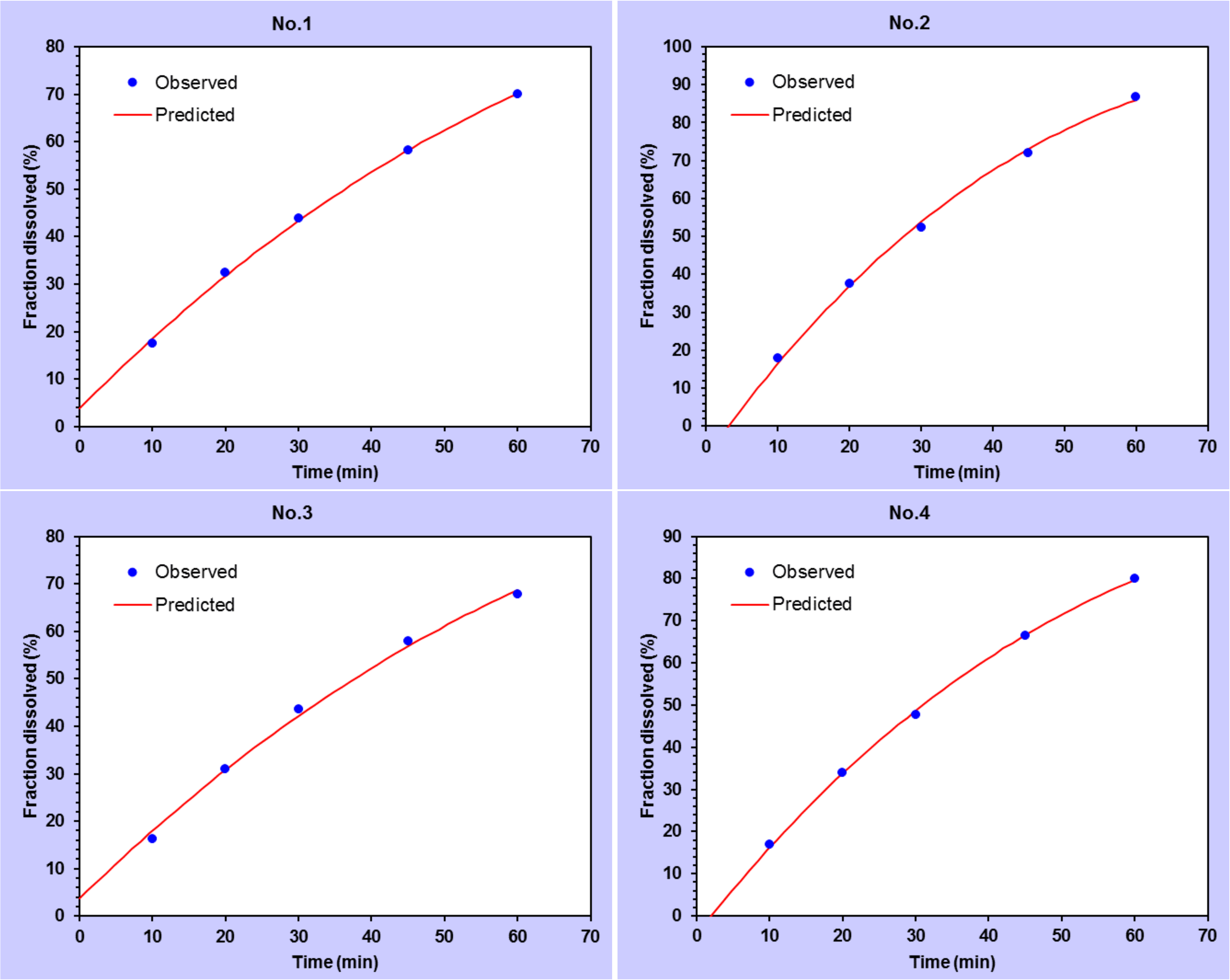

Supplementary materials – Model fitting summary – Polyglykol® 4000 P (modelling up to app. 75 % of carvedilol released)

Model: **Hopfenberg**

Model equation: $F = 100 \cdot [1 - (1 - k_{HB} \cdot t)^n]$

Fitted model parameters per tested tablet (N = 4) with statistics – mean, standard deviation (SD), and relative standard deviation expressed in % (RSD%) (output from DDSolver):

Parameter	No.1	No.2	No.3	No.4	Mean	SD	RSD(%)
k_{HB}	0.006	0.011	0.005	0.009	0.008	0.003	33.208
n	3.000	2.000	3.000	2.000	2.500	0.577	23.094

Number of dissolution data points (N), degrees of freedom (df), and selected goodness of fit criteria – Pearson correlation coefficient (R), coefficient of determination (R^2), adjusted coefficient of determination ($R^2_{adjusted}$), and residual sum of squares (RSS) (manual calculation in MS Excel):

Parameter	No.1	No.2	No.3	No.4
N	5	5	5	5
df	3	3	3	3
R	0.999593163	0.999818371	0.998453278	0.999809494
R^2	0.999186492	0.999636775	0.996908949	0.999619024
$R^2_{adjusted}$	0.998915322	0.9995157	0.995878599	0.999492031
RSS	10.19937729	4.694463175	11.76585467	1.133638815

Graphical abstract of model fit presented as mean ± 1 SD of the fraction % of released carvedilol:

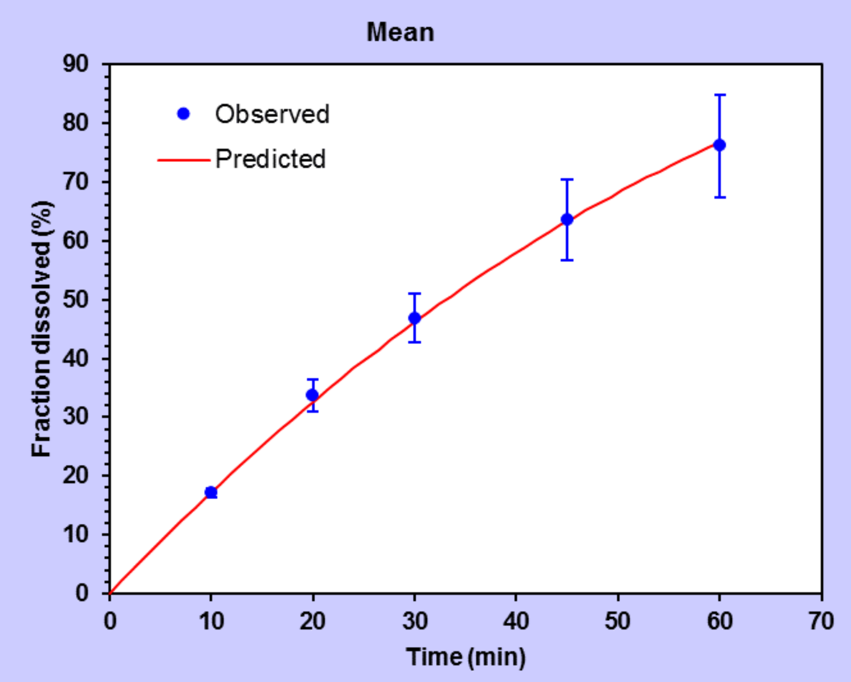

Supplementary materials – Model fitting summary – Polyglykol® 4000 P (modelling up to app. 75 % of carvedilol released)

Graphical abstract of model fit presented as the fraction % of released carvedilol per tested tablet:

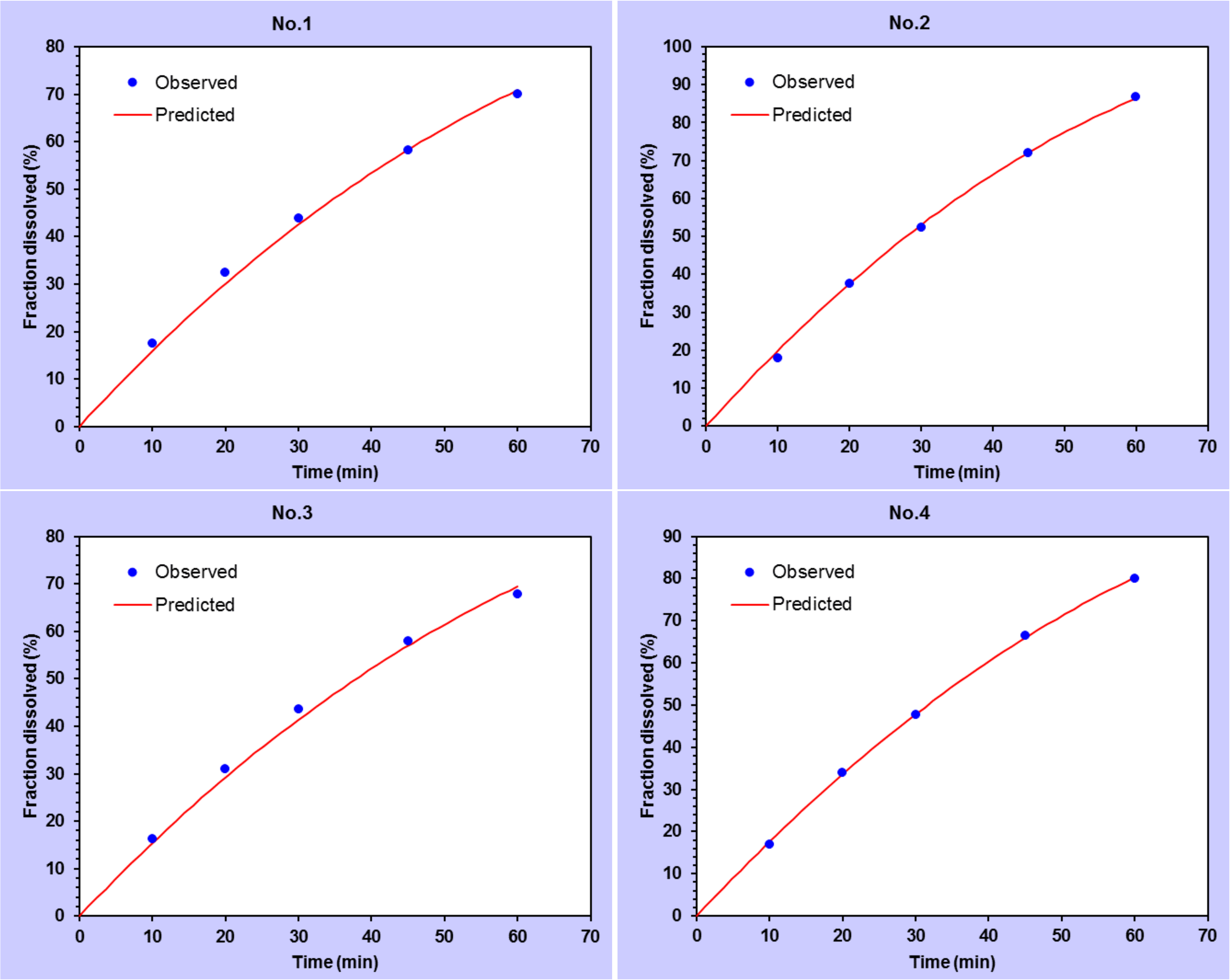

Supplementary materials – Model fitting summary – Polyglykol® 4000 P (modelling up to app. 75 % of carvedilol released)

Model: **Hopfenberg with T_{lag}**

Model equation: $F = 100 \cdot \{1 - [1 - k_{HB} \cdot (t - T_{lag})]^n\}$

Fitted model parameters per tested tablet (N = 4) with statistics – mean, standard deviation (SD), and relative standard deviation expressed in % (RSD%) (output from DDSolver):

Parameter	No.1	No.2	No.3	No.4	Mean	SD	RSD(%)
k_{HB}	0.005	0.011	0.005	0.009	0.008	0.003	37.058
n	3.000	2.000	3.000	2.000	2.500	0.577	23.094
T_{lag}	-2.406	1.038	-2.401	0.137	-0.908	1.765	-194.445

Number of dissolution data points (N), degrees of freedom (df), and selected goodness of fit criteria – Pearson correlation coefficient (R), coefficient of determination (R^2), adjusted coefficient of determination ($R^2_{adjusted}$), and residual sum of squares (RSS) (manual calculation in MS Excel):

Parameter	No.1	No.2	No.3	No.4
N	5	5	5	5
df	2	2	2	2
R	0.999473223	0.9998285	0.998173195	0.999813451
R^2	0.998946724	0.99965703	0.996349726	0.999626937
$R^2_{adjusted}$	0.997893447	0.999314061	0.992699453	0.999253874
RSS	1.891329378	1.019976002	6.661482122	0.988111221

Graphical abstract of model fit presented as mean ± 1 SD of the fraction % of released carvedilol:

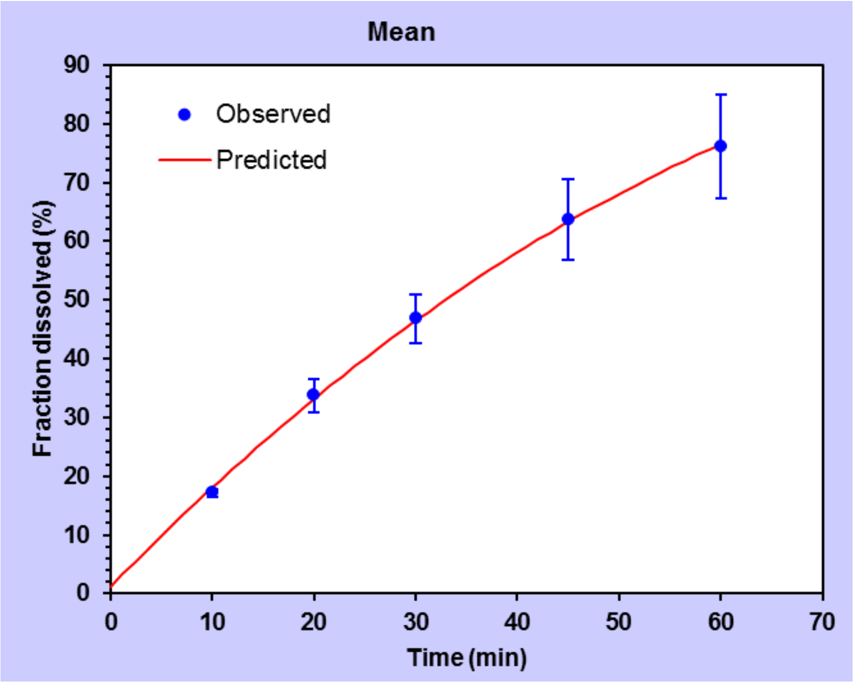

Supplementary materials – Model fitting summary – Polyglykol® 4000 P (modelling up to app. 75 % of carvedilol released)

Graphical abstract of model fit presented as the fraction % of released carvedilol per tested tablet:

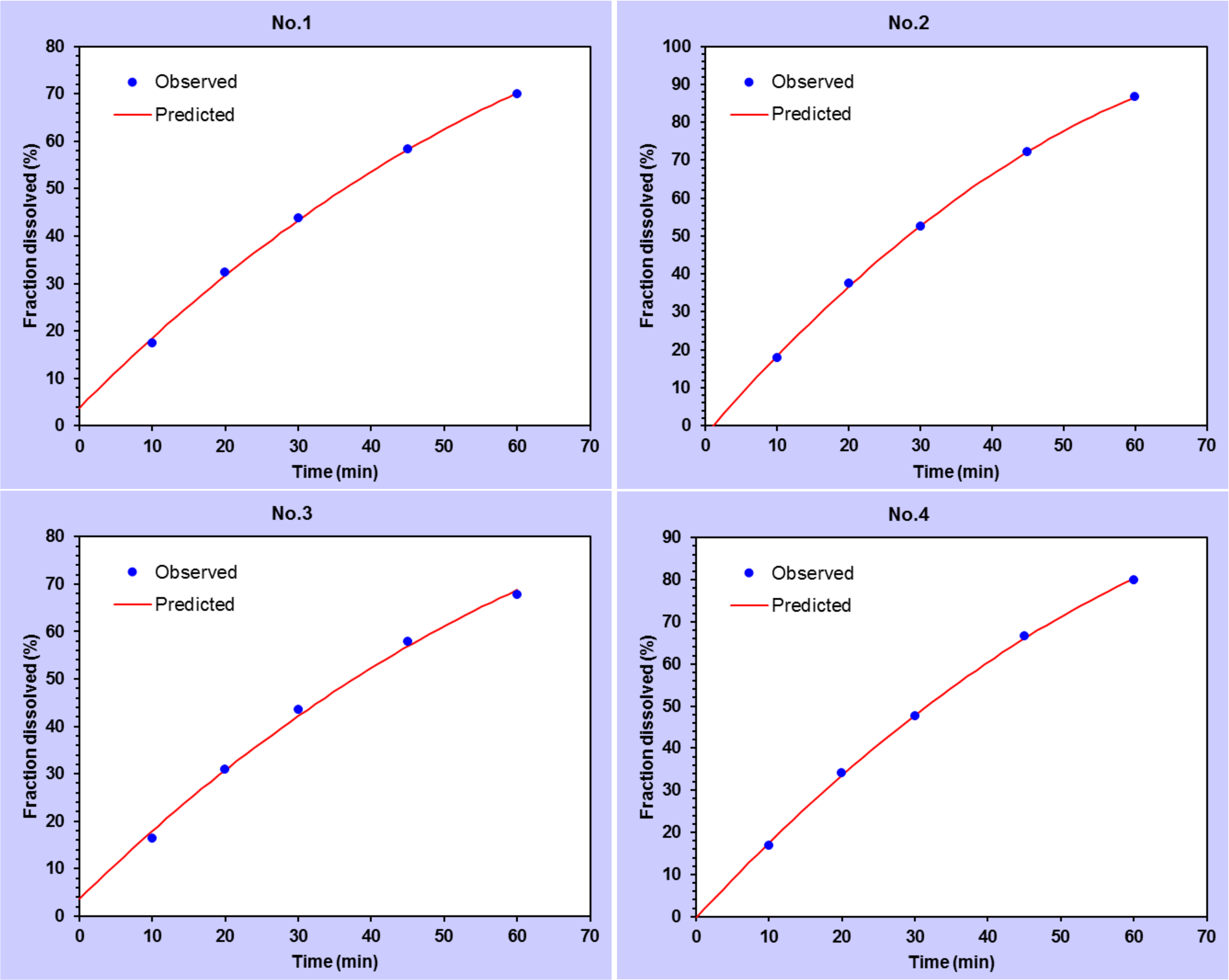

Supplementary materials – Model fitting summary – Polyglykol® 4000 P (modelling up to app. 75 % of carvedilol released)

Model: **Baker–Lonsdale**

Model equation: $\frac{3}{2}\cdot\left[1-\left(1-\frac{F}{100}\right)^{\frac{2}{3}}\right]-\frac{F}{100}=k_{BL}\cdot t$

Fitted model parameters per tested tablet (N = 4) with statistics – mean, standard deviation (SD), and relative standard deviation expressed in % (RSD%) (output from DDSolver):

Parameter	No.1	No.2	No.3	No.4	Mean	SD	RSD(%)
k_{BL}	0.002	0.005	0.002	0.004	0.003	0.001	34.835

Number of dissolution data points (N), degrees of freedom (df), and selected goodness of fit criteria – Pearson correlation coefficient (R), coefficient of determination (R^2), adjusted coefficient of determination ($R^2_{adjusted}$), and residual sum of squares (RSS) (manual calculation in MS Excel):

Parameter	No.1	No.2	No.3	No.4
N	5	5	5	5
df	4	4	4	4
R	0.99820443	0.994553253	0.999137416	0.995168698
R^2	0.996412084	0.989136173	0.998275577	0.990360737
$R^2_{adjusted}$	0.996412084	0.989136173	0.998275577	0.990360737
RSS	782.6888042	2040.722299	744.4491384	1609.087292

Graphical abstract of model fit presented as mean ± 1 SD of the fraction % of released carvedilol:

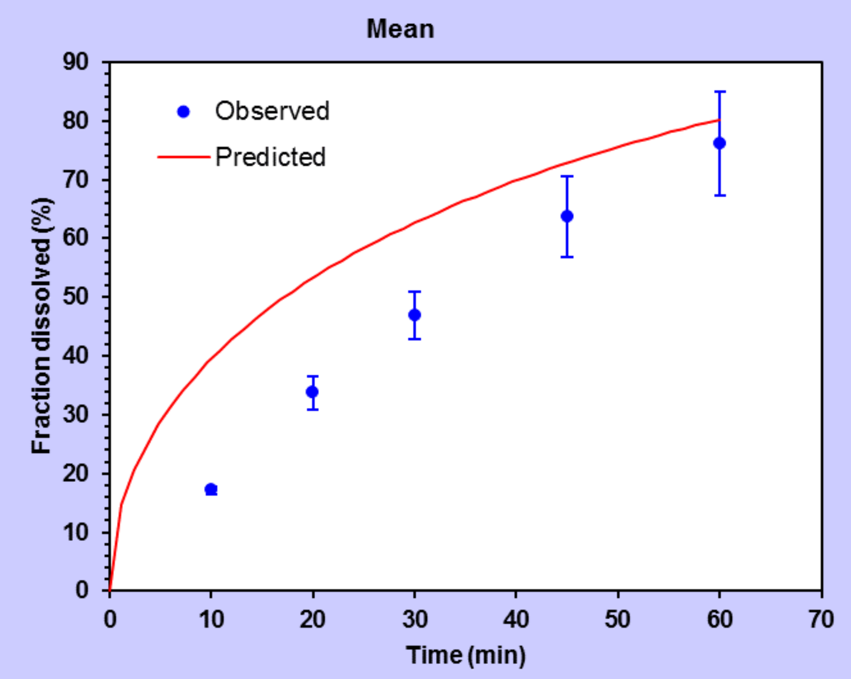

Supplementary materials – Model fitting summary – Polyglykol® 4000 P (modelling up to app. 75 % of carvedilol released)

Graphical abstract of model fit presented as the fraction % of released carvedilol per tested tablet:

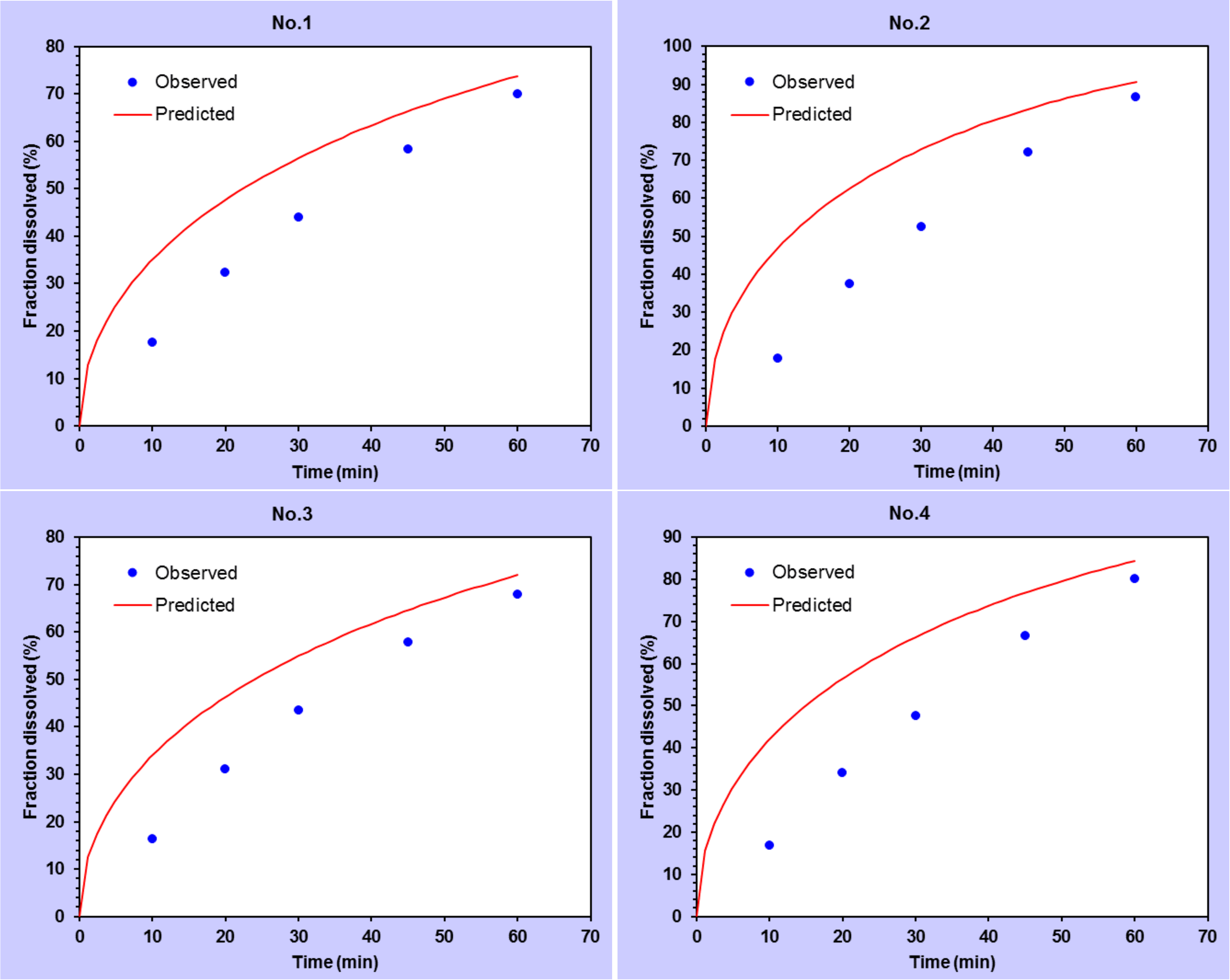

Supplementary materials – Model fitting summary – Polyglykol® 4000 P (modelling up to app. 75 % of carvedilol released)

Model: **Baker–Lonsdale with T_{lag}**

Model equation: $\frac{3}{2}\cdot\left[1-\left(1-\frac{F}{100}\right)^{\frac{2}{3}}\right]-\frac{F}{100}=k_{BL}\cdot\left(t-T_{lag}\right)$

Fitted model parameters per tested tablet (N = 4) with statistics – mean, standard deviation (SD), and relative standard deviation expressed in % (RSD%) (output from DDSolver):

Parameter	No.1	No.2	No.3	No.4	Mean	SD	RSD(%)
k_{BL}	0.002	0.005	0.002	0.004	0.003	0.001	34.835
T_{lag}	10.720	12.967	10.480	12.603	11.693	1.274	10.895

Number of dissolution data points (N), degrees of freedom (df), and selected goodness of fit criteria – Pearson correlation coefficient (R), coefficient of determination (R^2), adjusted coefficient of determination ($R^2_{adjusted}$), and residual sum of squares (RSS) (manual calculation in MS Excel):

Parameter	No.1	No.2	No.3	No.4
N	5	5	5	5
df	3	3	3	3
R	0.968498647	0.970691011	0.971566622	0.96911859
R^2	0.93798963	0.942241038	0.9439417	0.939190841
$R^2_{adjusted}$	0.917319506	0.922988051	0.9252556	0.918921121
RSS	321.5896963	378.3072538	276.814223	331.0788736

Graphical abstract of model fit presented as mean ± 1 SD of the fraction % of released carvedilol:

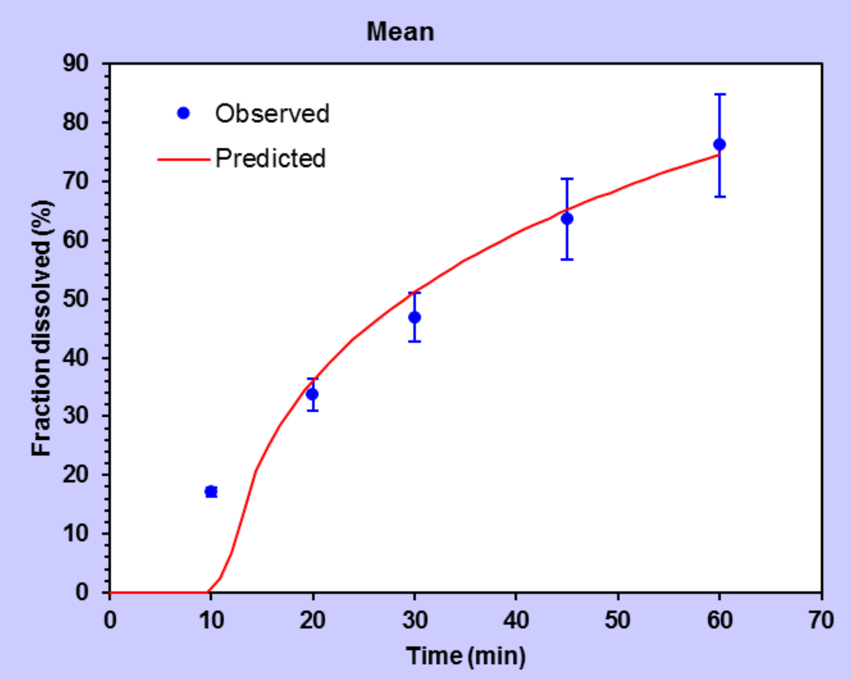

Supplementary materials – Model fitting summary – Polyglykol® 4000 P (modelling up to app. 75 % of carvedilol released)

Graphical abstract of model fit presented as the fraction % of released carvedilol per tested tablet:

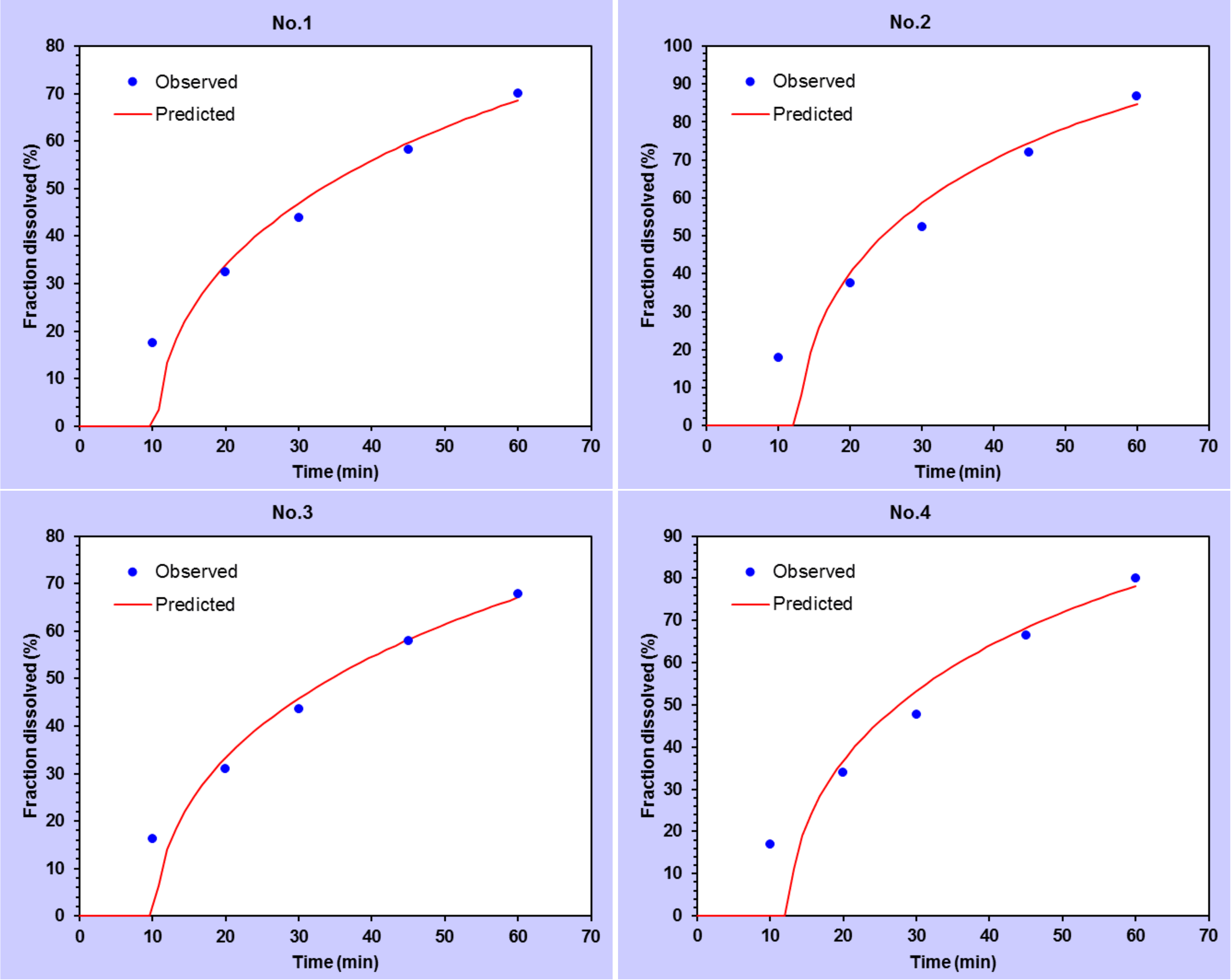

Supplementary materials – Model fitting summary – Polyglykol® 4000 P (modelling up to app. 75 % of carvedilol released)

Model: **Makoid–Banakar**

Model equation: $F = k_{MB} \cdot t^n \cdot e^{-k \cdot t}$

Fitted model parameters per tested tablet (N = 4) with statistics – mean, standard deviation (SD), and relative standard deviation expressed in % (RSD%) (output from DDSolver):

Parameter	No.1	No.2	No.3	No.4	Mean	SD	RSD(%)
k_{MB}	2.029	1.299	1.510	1.420	1.565	0.322	20.553
n	0.968	1.191	1.076	1.116	1.088	0.093	8.538
k	0.007	0.011	0.010	0.009	0.009	0.002	19.055

Number of dissolution data points (N), degrees of freedom (df), and selected goodness of fit criteria – Pearson correlation coefficient (R), coefficient of determination (R^2), adjusted coefficient of determination ($R^2_{adjusted}$), and residual sum of squares (RSS) (manual calculation in MS Excel):

Parameter	No.1	No.2	No.3	No.4
N	5	5	5	5
df	2	2	2	2
R	0.999842915	0.99971273	0.999995082	0.999888334
R^2	0.999685854	0.999425543	0.999990165	0.999776681
$R^2_{adjusted}$	0.999371708	0.998851087	0.99998033	0.999553363
RSS	0.53895922	1.708074207	0.016670839	0.564102516

Graphical abstract of model fit presented as mean ± 1 SD of the fraction % of released carvedilol:

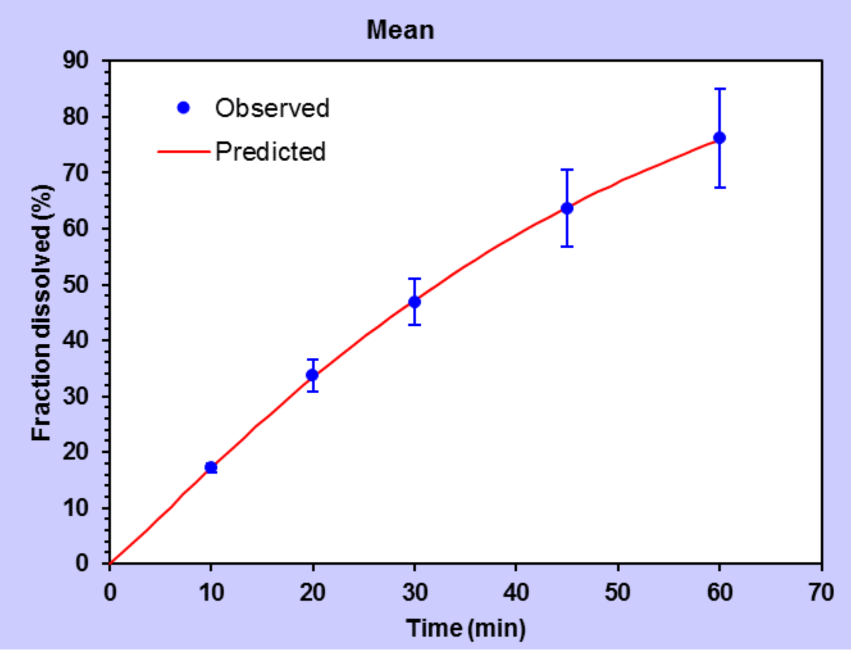

Supplementary materials – Model fitting summary – Polyglykol® 4000 P (modelling up to app. 75 % of carvedilol released)

Graphical abstract of model fit presented as the fraction % of released carvedilol per tested tablet:

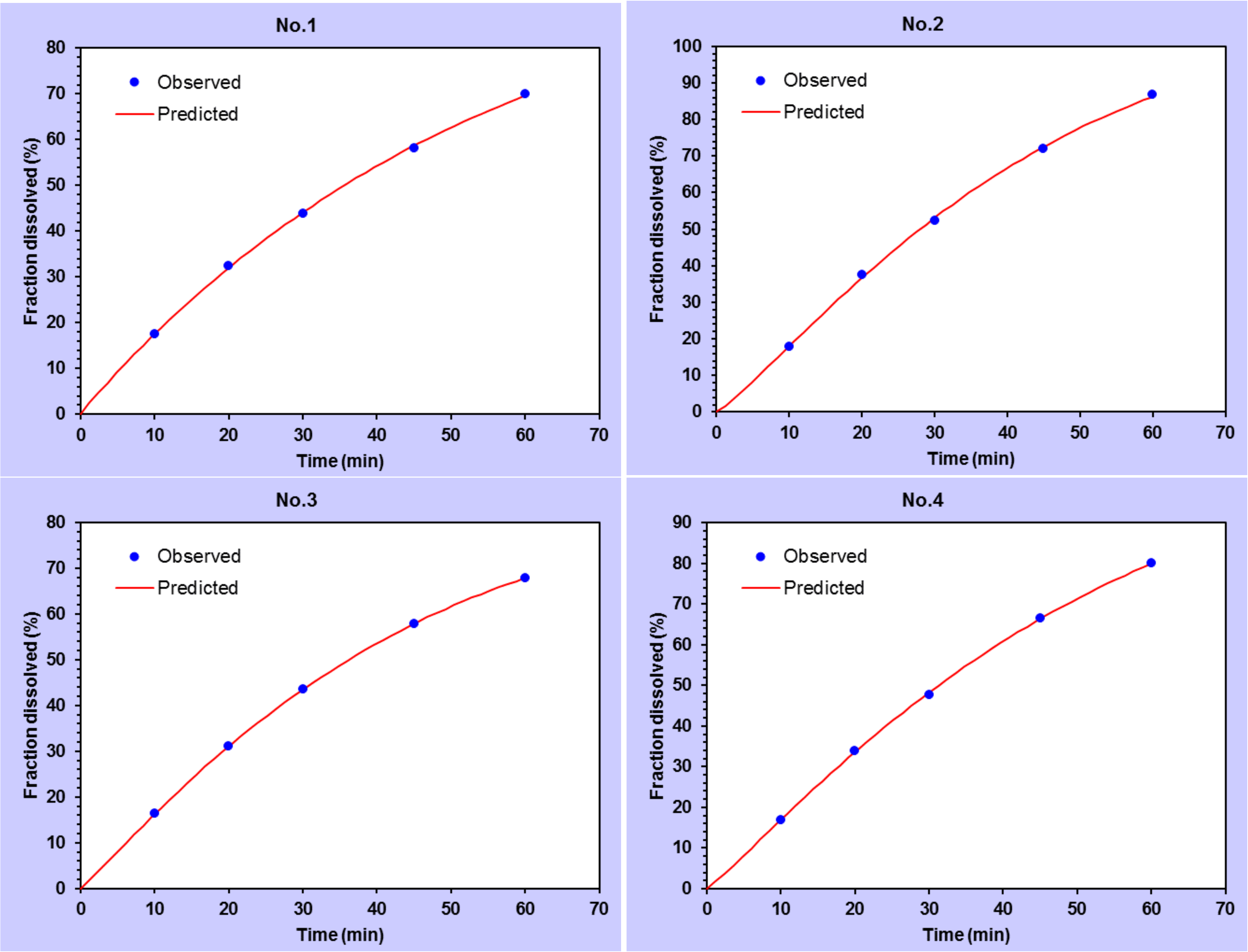

Supplementary materials – Model fitting summary – Polyglykol® 4000 P (modelling up to app. 75 % of carvedilol released)

Model: **Makoid–Banakar with T_{lag}**

Model equation: $F = k_{MB} \cdot (t - T_{lag})^n \cdot e^{-k \cdot (t - T_{lag})}$

Fitted model parameters per tested tablet (N = 4) with statistics – mean, standard deviation (SD), and relative standard deviation expressed in % (RSD%) (output from DDSolver):

Parameter	No.1	No.2	No.3	No.4	Mean	SD	RSD(%)
k_{MB}	5.610	4.488	4.656	4.577	4.833	0.523	10.818
n	0.637	0.784	0.706	0.733	0.715	0.061	8.576
k	0.001	0.003	0.003	0.001	0.002	0.001	60.110
T_{lag}	4.000	4.000	4.000	4.000	4.000	0.000	0.000

Number of dissolution data points (N), degrees of freedom (df), and selected goodness of fit criteria – Pearson correlation coefficient (R), coefficient of determination (R^2), adjusted coefficient of determination ($R^2_{adjusted}$), and residual sum of squares (RSS) (manual calculation in MS Excel):

Parameter	No.1	No.2	No.3	No.4
N	5	5	5	5
df	1	1	1	1
R	0.999987123	0.99993579	0.999647588	0.999712384
R^2	0.999974245	0.999871585	0.999295299	0.99942485
$R^2_{adjusted}$	0.999896981	0.99948634	0.997181197	0.9976994
RSS	0.044205303	0.381751316	1.194801345	1.453280274

Graphical abstract of model fit presented as mean ± 1 SD of the fraction % of released carvedilol:

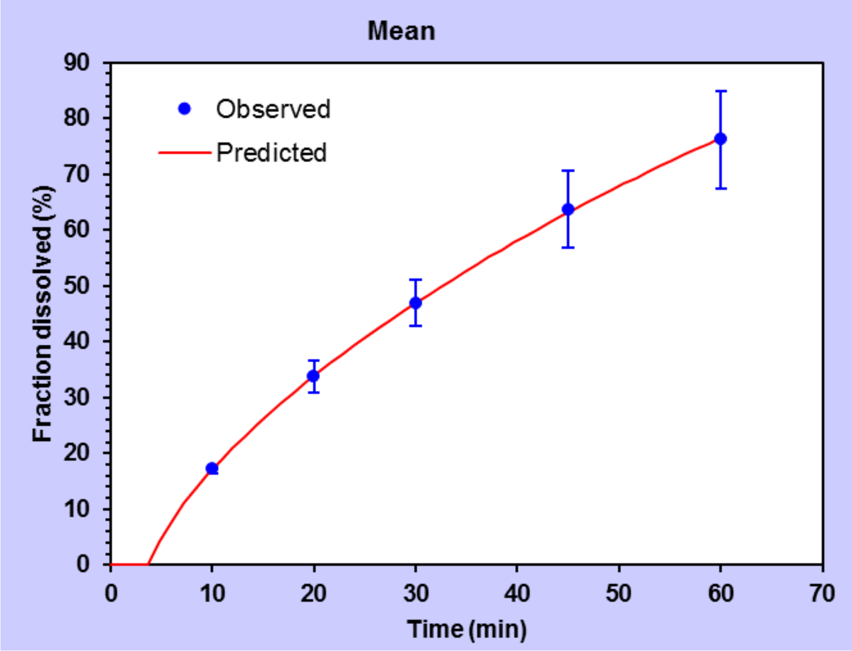

Supplementary materials – Model fitting summary – Polyglykol® 4000 P (modelling up to app. 75 % of carvedilol released)

Graphical abstract of model fit presented as the fraction % of released carvedilol per tested tablet:

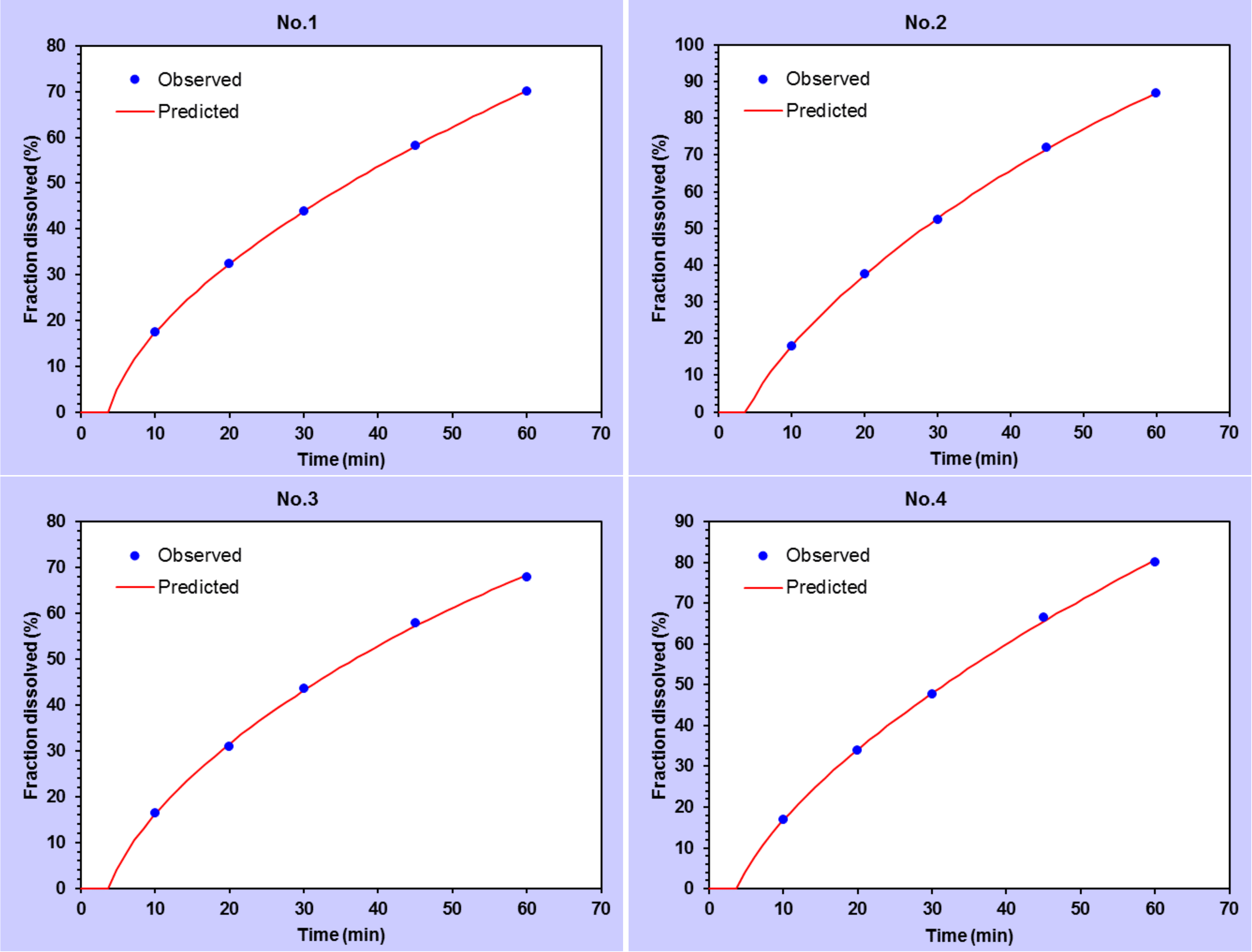

Supplementary materials – Model fitting summary – Polyglykol® 4000 P (modelling up to app. 75 % of carvedilol released)

Model: **Peppas–Sahlin_1**

Model equation: $F = k_1 \cdot t^m + k_2 \cdot t^{2m}$

Fitted model parameters per tested tablet (N = 4) with statistics – mean, standard deviation (SD), and relative standard deviation expressed in % (RSD%) (output from DDSolver):

Parameter	No.1	No.2	No.3	No.4	Mean	SD	RSD(%)
k_1	3.470	2.248	3.207	1.943	2.717	0.736	27.097
k_2	1.239	1.876	1.246	1.749	1.528	0.333	21.813
m	0.450	0.450	0.450	0.450	0.450	0.000	0.000

Number of dissolution data points (N), degrees of freedom (df), and selected goodness of fit criteria – Pearson correlation coefficient (R), coefficient of determination (R^2), adjusted coefficient of determination ($R^2_{adjusted}$), and residual sum of squares (RSS) (manual calculation in MS Excel):

Parameter	No.1	No.2	No.3	No.4
N	5	5	5	5
df	2	2	2	2
R	0.997375202	0.995982691	0.994545117	0.996902324
R^2	0.994757295	0.991981522	0.989119991	0.993814244
$R^2_{adjusted}$	0.989514589	0.983963043	0.978239981	0.987628487
RSS	9.259133978	24.42162626	18.93108825	15.97242896

Graphical abstract of model fit presented as mean ± 1 SD of the fraction % of released carvedilol:

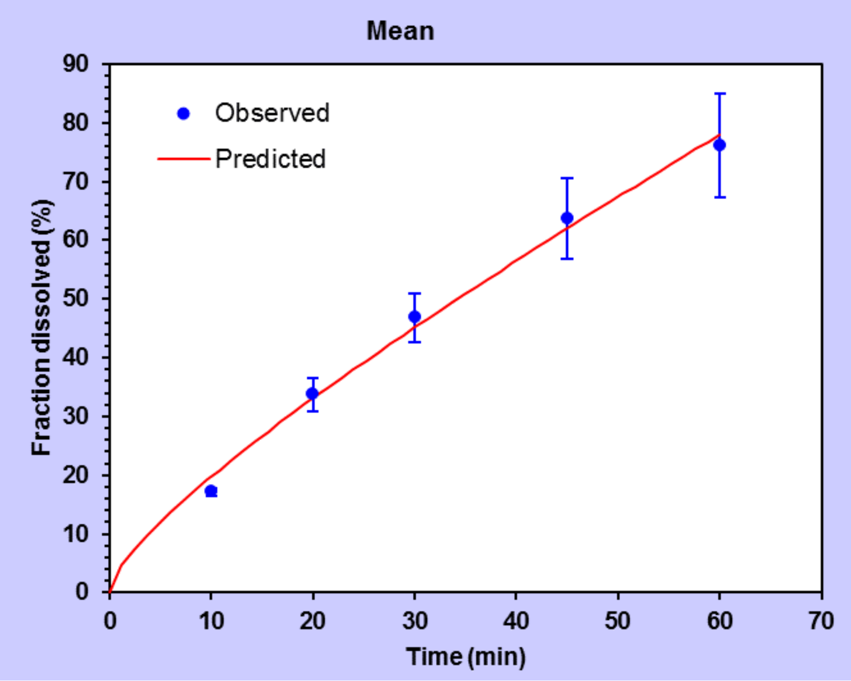

Supplementary materials – Model fitting summary – Polyglykol® 4000 P (modelling up to app. 75 % of carvedilol released)

Graphical abstract of model fit presented as the fraction % of released carvedilol per tested tablet:

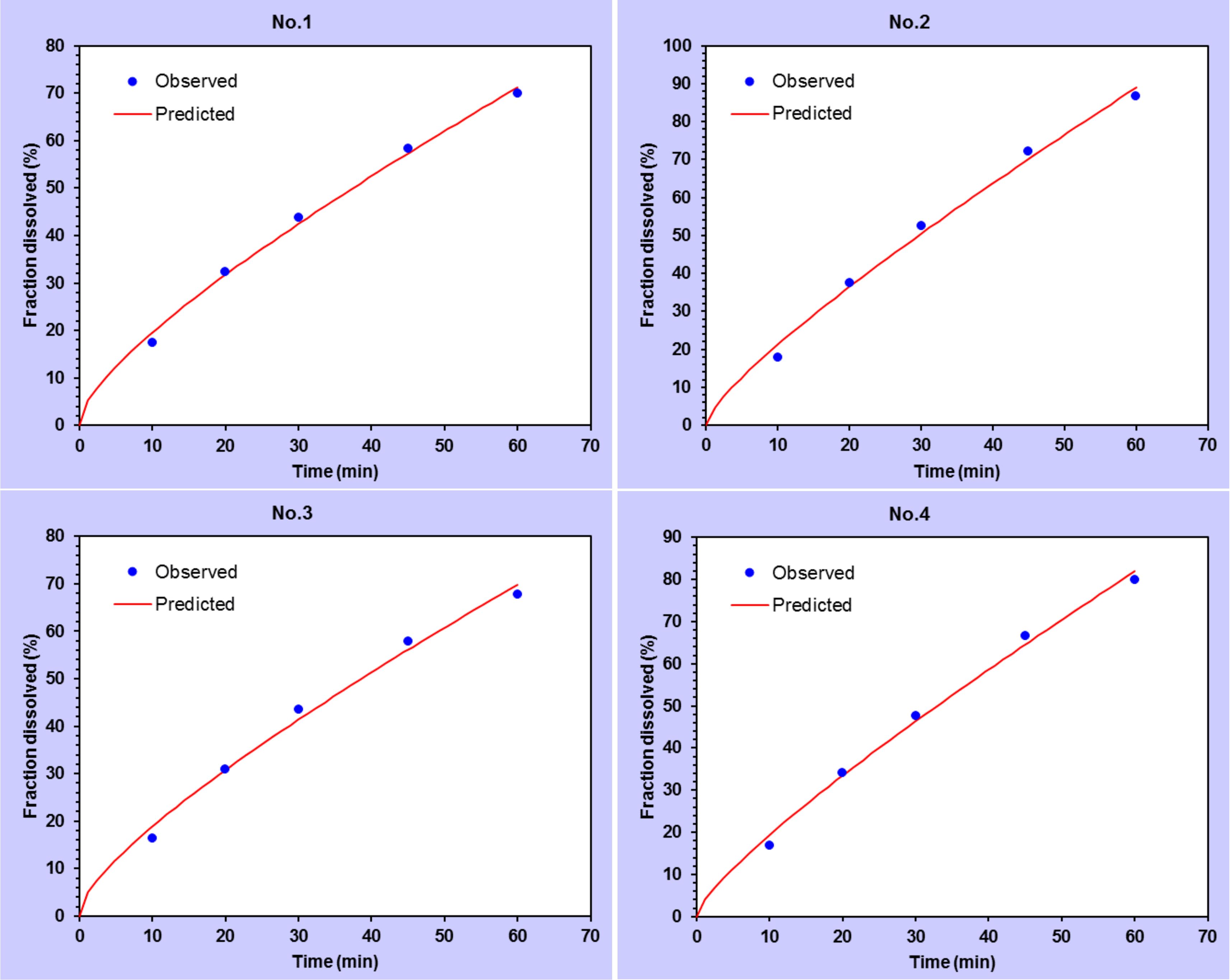

Supplementary materials – Model fitting summary – Polyglykol® 4000 P (modelling up to app. 75 % of carvedilol released)

Model: **Peppas–Sahlin_1 with T_{lag}**

Model equation: $F = k_1 \cdot (t - T_{lag})^m + k_2 \cdot (t - T_{lag})^{2m}$

Fitted model parameters per tested tablet (N = 4) with statistics – mean, standard deviation (SD), and relative standard deviation expressed in % (RSD%) (output from DDSolver):

Parameter	No.1	No.2	No.3	No.4	Mean	SD	RSD(%)
k_1	6.271	5.693	5.969	5.095	5.757	0.500	8.689
k_2	0.861	1.425	0.874	1.338	1.124	0.299	26.586
m	0.450	0.450	0.450	0.450	0.450	0.000	0.000
T_{lag}	4.000	4.000	4.000	4.000	4.000	0.000	0.000

Number of dissolution data points (N), degrees of freedom (df), and selected goodness of fit criteria – Pearson correlation coefficient (R), coefficient of determination (R^2), adjusted coefficient of determination ($R^2_{adjusted}$), and residual sum of squares (RSS) (manual calculation in MS Excel):

Parameter	No.1	No.2	No.3	No.4
N	5	5	5	5
df	1	1	1	1
R	0.99952968	0.998493142	0.997760899	0.998867106
R^2	0.999059581	0.996988554	0.995526811	0.997735496
$R^2_{adjusted}$	0.996238322	0.987954215	0.982107244	0.990941985
RSS	1.714698593	9.412753033	7.945355562	5.927463371

Graphical abstract of model fit presented as mean ± 1 SD of the fraction % of released carvedilol:

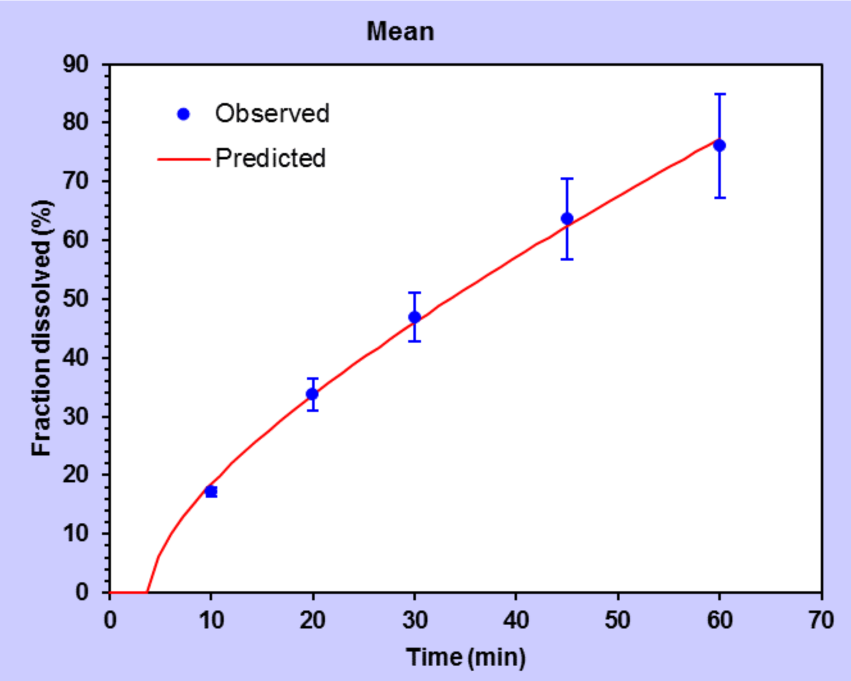

Supplementary materials – Model fitting summary – Polyglykol® 4000 P (modelling up to app. 75 % of carvedilol released)

Graphical abstract of model fit presented as the fraction % of released carvedilol per tested tablet:

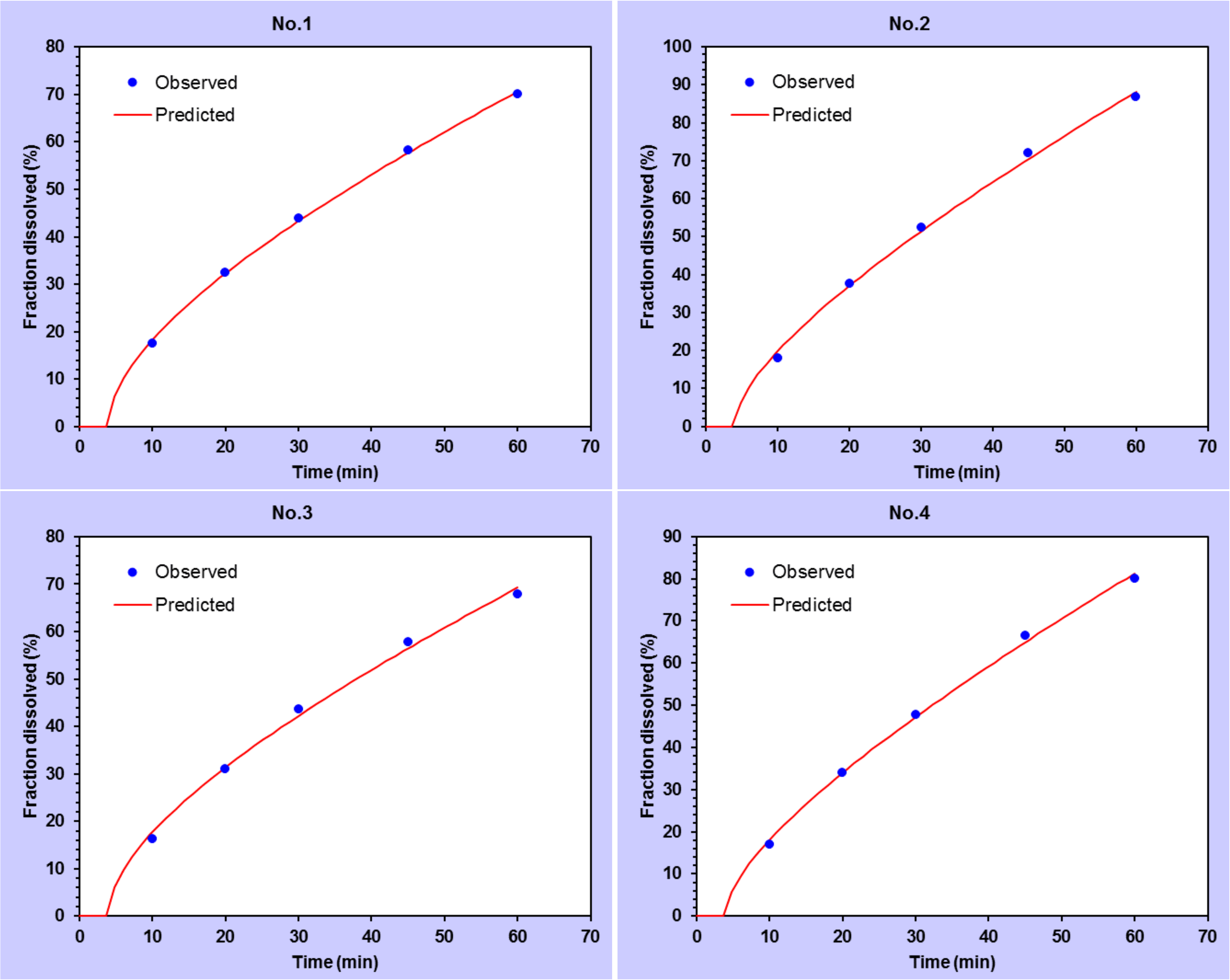

Supplementary materials – Model fitting summary – Polyglykol® 4000 P (modelling up to app. 75 % of carvedilol released)

Model: **Peppas–Sahlin_2**

Model equation: $F = k_1 \cdot t^{0.5} + k_2 \cdot t$

Fitted model parameters per tested tablet (N = 4) with statistics – mean, standard deviation (SD), and relative standard deviation expressed in % (RSD%) (output from DDSolver):

Parameter	No.1	No.2	No.3	No.4	Mean	SD	RSD(%)
k_1	4.240	3.629	4.020	3.231	3.780	0.445	11.761
k_2	0.640	1.015	0.646	0.950	0.813	0.198	24.319

Number of dissolution data points (N), degrees of freedom (df), and selected goodness of fit criteria – Pearson correlation coefficient (R), coefficient of determination (R^2), adjusted coefficient of determination ($R^2_{adjusted}$), and residual sum of squares (RSS) (manual calculation in MS Excel):

Parameter	No.1	No.2	No.3	No.4
N	5	5	5	5
df	3	3	3	3
R	0.997105953	0.995385112	0.99414202	0.996356997
R^2	0.994220282	0.990791522	0.988318357	0.992727265
$R^2_{adjusted}$	0.992293709	0.987722029	0.984424476	0.99030302
RSS	10.35510833	28.39035574	20.60347295	18.99834832

Graphical abstract of model fit presented as mean ± 1 SD of the fraction % of released carvedilol:

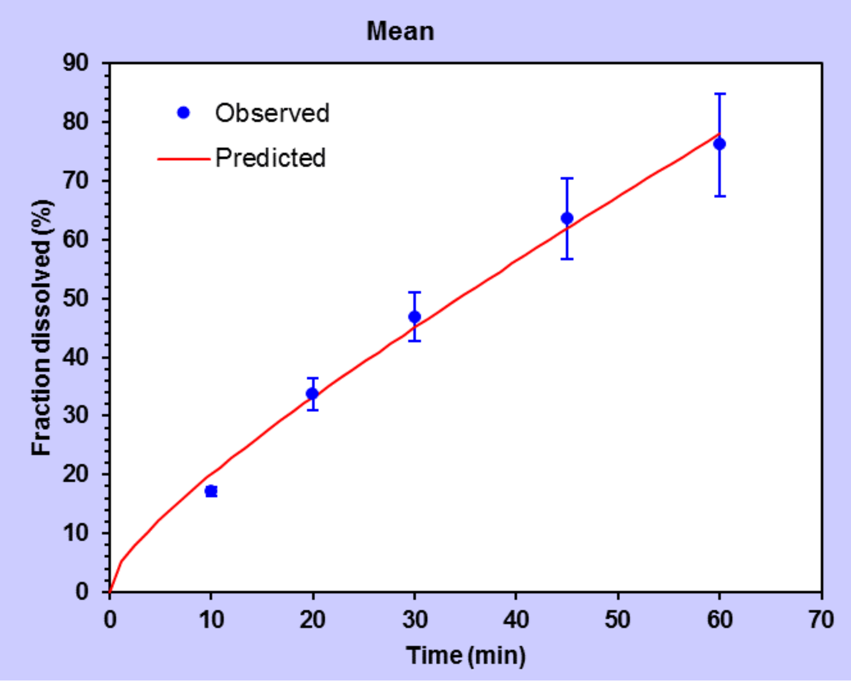

Supplementary materials – Model fitting summary – Polyglykol® 4000 P (modelling up to app. 75 % of carvedilol released)

Graphical abstract of model fit presented as the fraction % of released carvedilol per tested tablet:

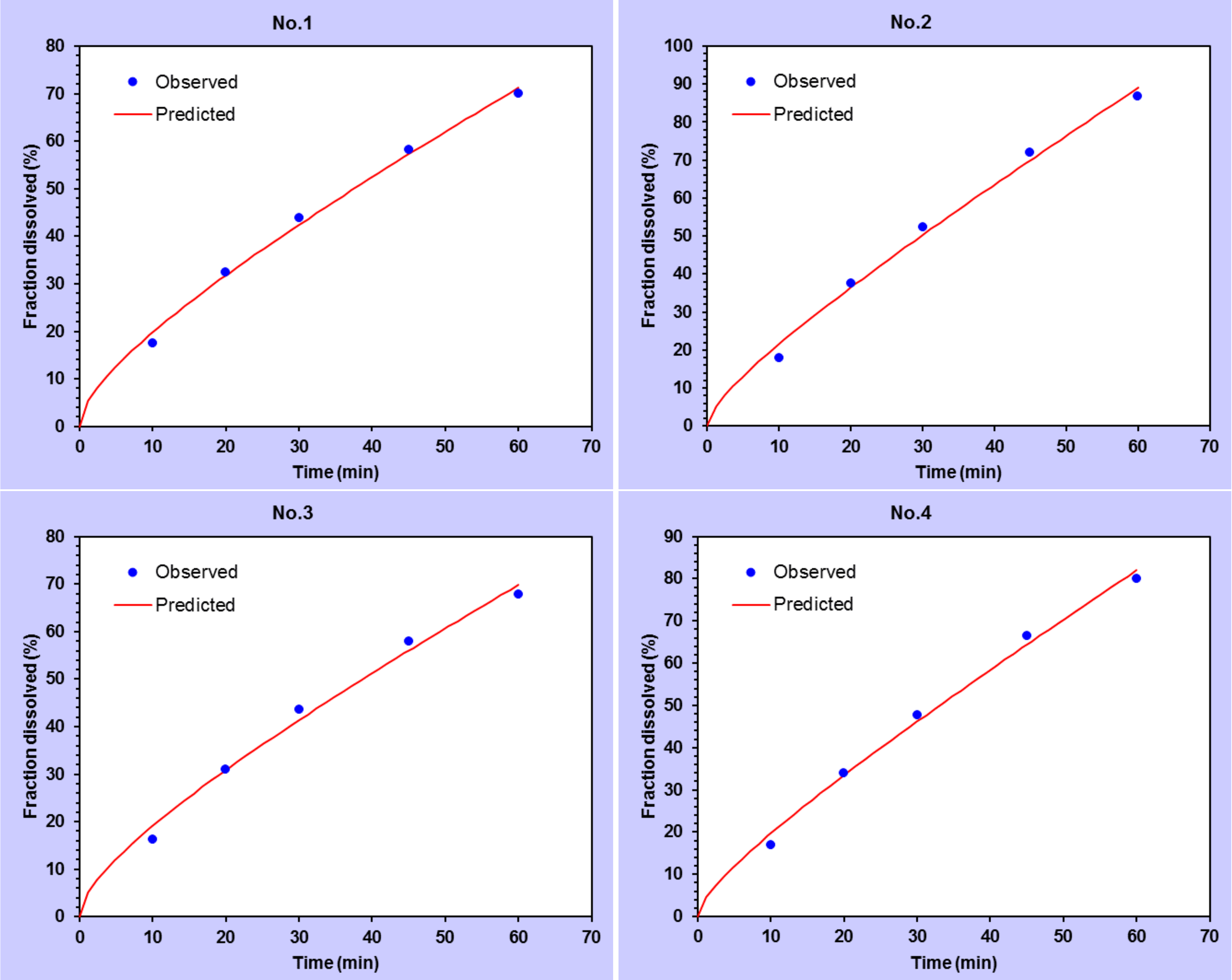

Supplementary materials – Model fitting summary – Polyglykol® 4000 P (modelling up to app. 75 % of carvedilol released)

Model: **Peppas–Sahlin_2 with T_{lag}**

Model equation: $F = k_1 \cdot (t - T_{lag})^{0.5} + k_2 \cdot (t - T_{lag})$

Fitted model parameters per tested tablet (N = 4) with statistics – mean, standard deviation (SD), and relative standard deviation expressed in % (RSD%) (output from DDSolver):

Parameter	No.1	No.2	No.3	No.4	Mean	SD	RSD(%)
k_1	6.541	6.445	6.647	5.804	6.359	0.379	5.962
k_2	0.386	0.713	0.340	0.676	0.529	0.193	36.506
T_{lag}	4.000	4.000	4.718	4.000	4.180	0.359	8.594

Number of dissolution data points (N), degrees of freedom (df), and selected goodness of fit criteria – Pearson correlation coefficient (R), coefficient of determination (R^2), adjusted coefficient of determination ($R^2_{adjusted}$), and residual sum of squares (RSS) (manual calculation in MS Excel):

Parameter	No.1	No.2	No.3	No.4
N	5	5	5	5
df	2	2	2	2
R	0.999589506	0.998405558	0.99842983	0.998770193
R^2	0.99917918	0.996813658	0.996862126	0.997541899
$R^2_{adjusted}$	0.998358361	0.993627317	0.993724253	0.995083797
RSS	1.538943143	10.20661798	7.172176206	6.560134487

Graphical abstract of model fit presented as mean ± 1 SD of the fraction % of released carvedilol:

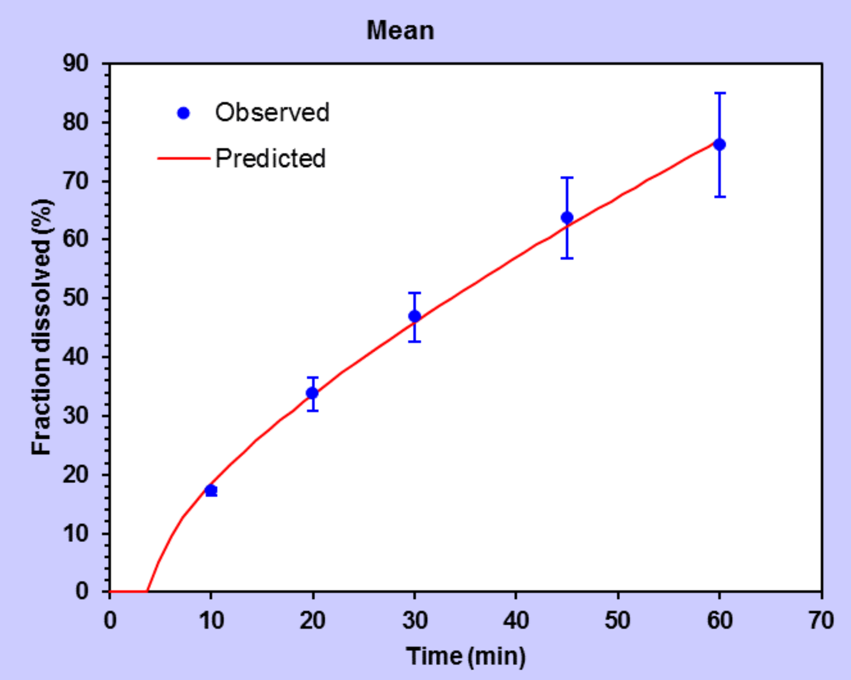

Supplementary materials – Model fitting summary – Polyglykol® 4000 P (modelling up to app. 75 % of carvedilol released)

Graphical abstract of model fit presented as the fraction % of released carvedilol per tested tablet:

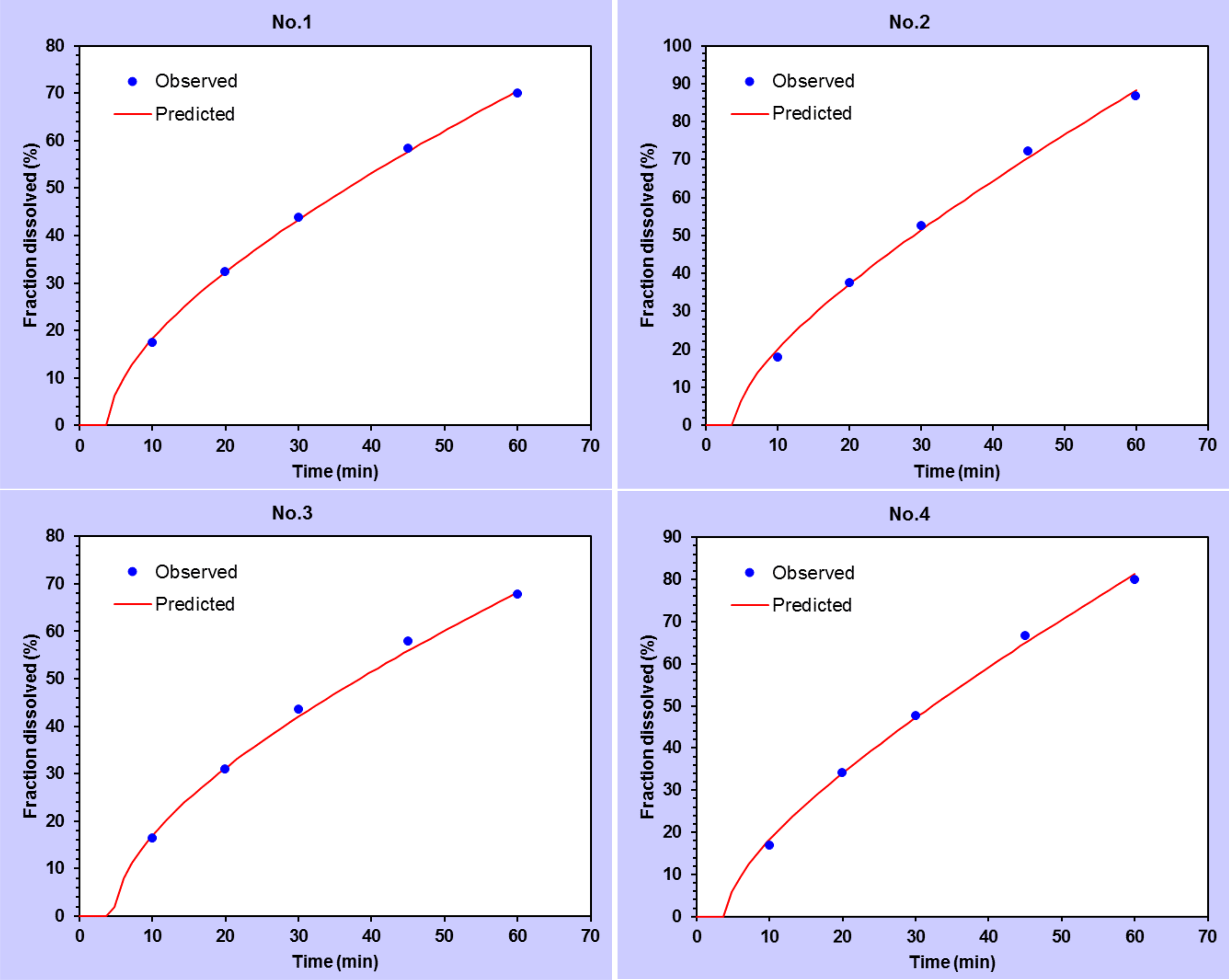

Supplementary materials – Model fitting summary – Polyglykol® 4000 P (modelling up to app. 75 % of carvedilol released)

Model: **Quadratic**

Model equation: $F = 100 \cdot (k_1 \cdot t^2 + k_2 \cdot t)$

Fitted model parameters per tested tablet (N = 4) with statistics – mean, standard deviation (SD), and relative standard deviation expressed in % (RSD%) (output from DDSolver):

Parameter	No.1	No.2	No.3	No.4	Mean	SD	RSD(%)
k_1	0.000	0.000	0.000	0.000	0.000	0.000	-8.910
k_2	0.018	0.020	0.018	0.019	0.019	0.001	6.709

Number of dissolution data points (N), degrees of freedom (df), and selected goodness of fit criteria – Pearson correlation coefficient (R), coefficient of determination (R^2), adjusted coefficient of determination ($R^2_{adjusted}$), and residual sum of squares (RSS) (manual calculation in MS Excel):

Parameter	No.1	No.2	No.3	No.4
N	5	5	5	5
df	3	3	3	3
R	0.999462541	0.999635184	0.999970492	0.999821315
R^2	0.998925371	0.999270501	0.999940985	0.999642663
$R^2_{adjusted}$	0.998567161	0.999027335	0.999921313	0.99952355
RSS	2.416608928	2.782077049	0.122771072	1.126173682

Graphical abstract of model fit presented as mean ± 1 SD of the fraction % of released carvedilol:

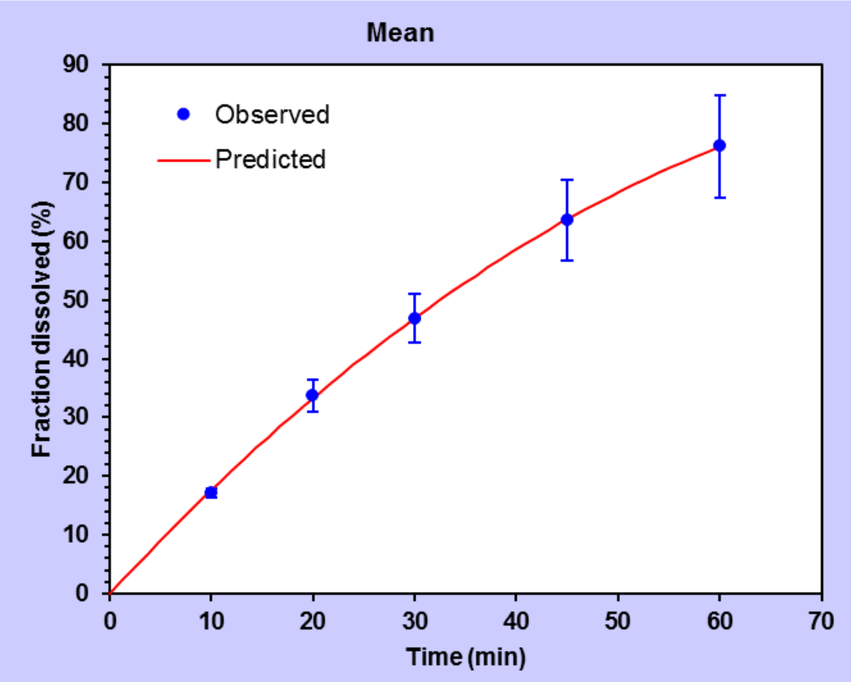

Supplementary materials – Model fitting summary – Polyglykol® 4000 P (modelling up to app. 75 % of carvedilol released)

Graphical abstract of model fit presented as the fraction % of released carvedilol per tested tablet:

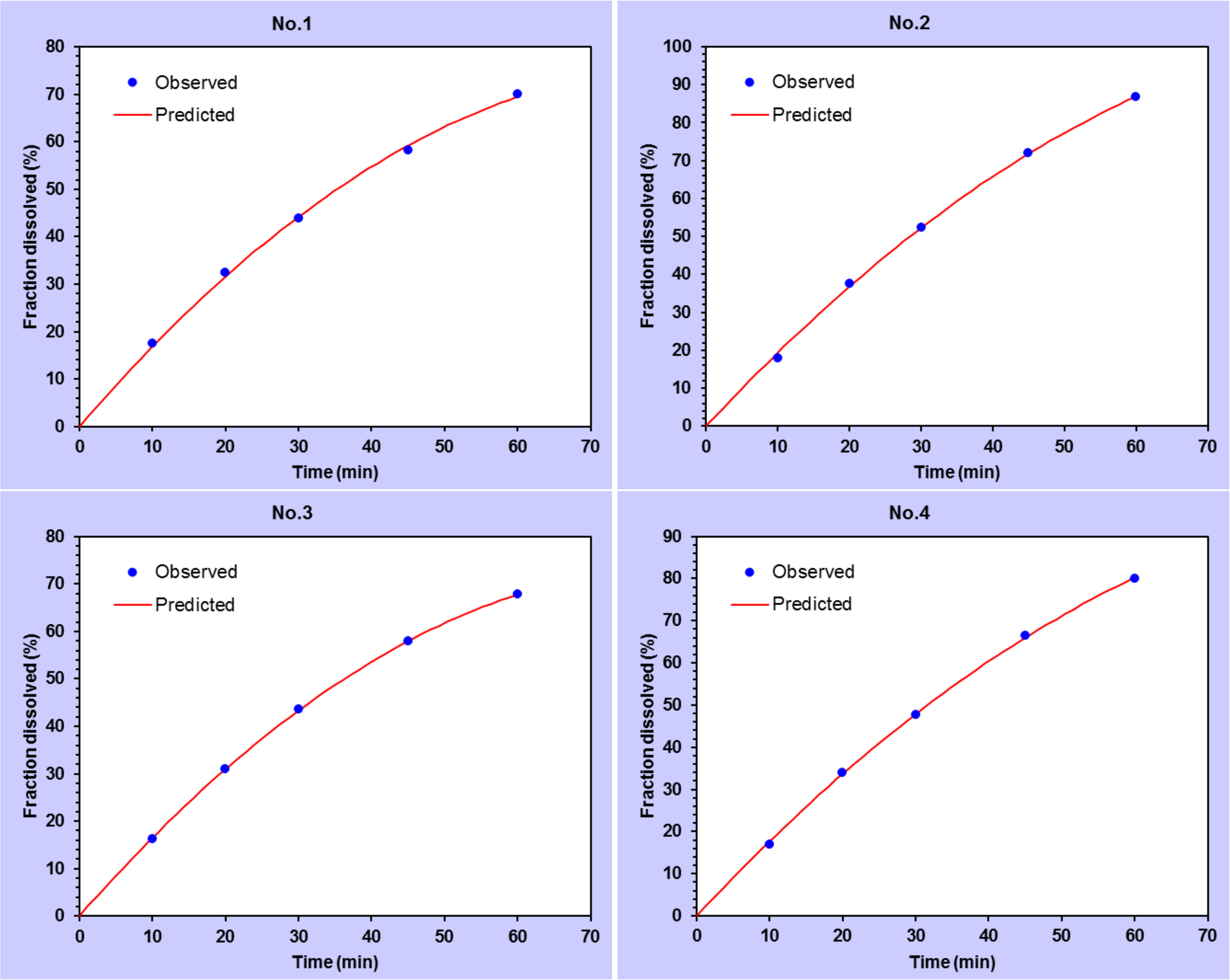

Supplementary materials – Model fitting summary – Polyglykol® 4000 P (modelling up to app. 75 % of carvedilol released)

Model: **Quadratic with T_{lag}**

Model equation: $F = 100 \cdot \left[k_1 \cdot (t - T_{lag})^2 + k_2 \cdot (t - T_{lag})\right]$

Fitted model parameters per tested tablet (N = 4) with statistics – mean, standard deviation (SD), and relative standard deviation expressed in % (RSD%) (output from DDSolver):

Parameter	No.1	No.2	No.3	No.4	Mean	SD	RSD(%)
k_1	0.000	0.000	0.000	0.000	0.000	0.000	-4.846
k_2	0.022	0.025	0.022	0.023	0.023	0.002	7.299
T_{lag}	4.000	4.000	4.000	4.000	4.000	0.000	0.000

Number of dissolution data points (N), degrees of freedom (df), and selected goodness of fit criteria – Pearson correlation coefficient (R), coefficient of determination (R^2), adjusted coefficient of determination ($R^2_{adjusted}$), and residual sum of squares (RSS) (manual calculation in MS Excel):

Parameter	No.1	No.2	No.3	No.4
N	5	5	5	5
df	2	2	2	2
R	0.994830687	0.99833654	0.997244433	0.998107663
R^2	0.989688095	0.996675848	0.99449646	0.996218908
$R^2_{adjusted}$	0.97937619	0.993351695	0.98899292	0.992437815
RSS	38.50399174	19.40339842	21.07707886	19.05186677

Graphical abstract of model fit presented as mean ± 1 SD of the fraction % of released carvedilol:

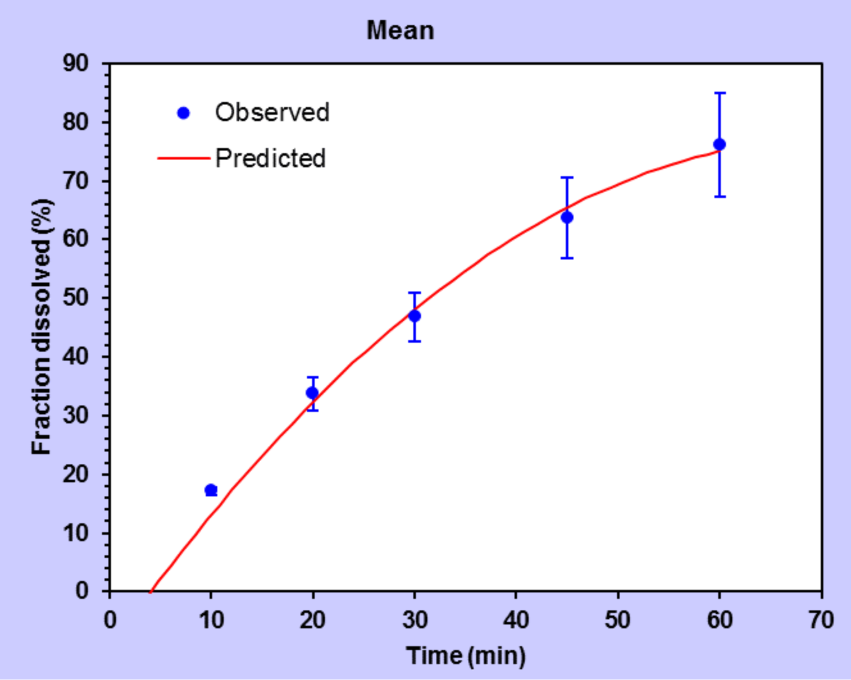

Supplementary materials – Model fitting summary – Polyglykol® 4000 P (modelling up to app. 75 % of carvedilol released)

Graphical abstract of model fit presented as the fraction % of released carvedilol per tested tablet:

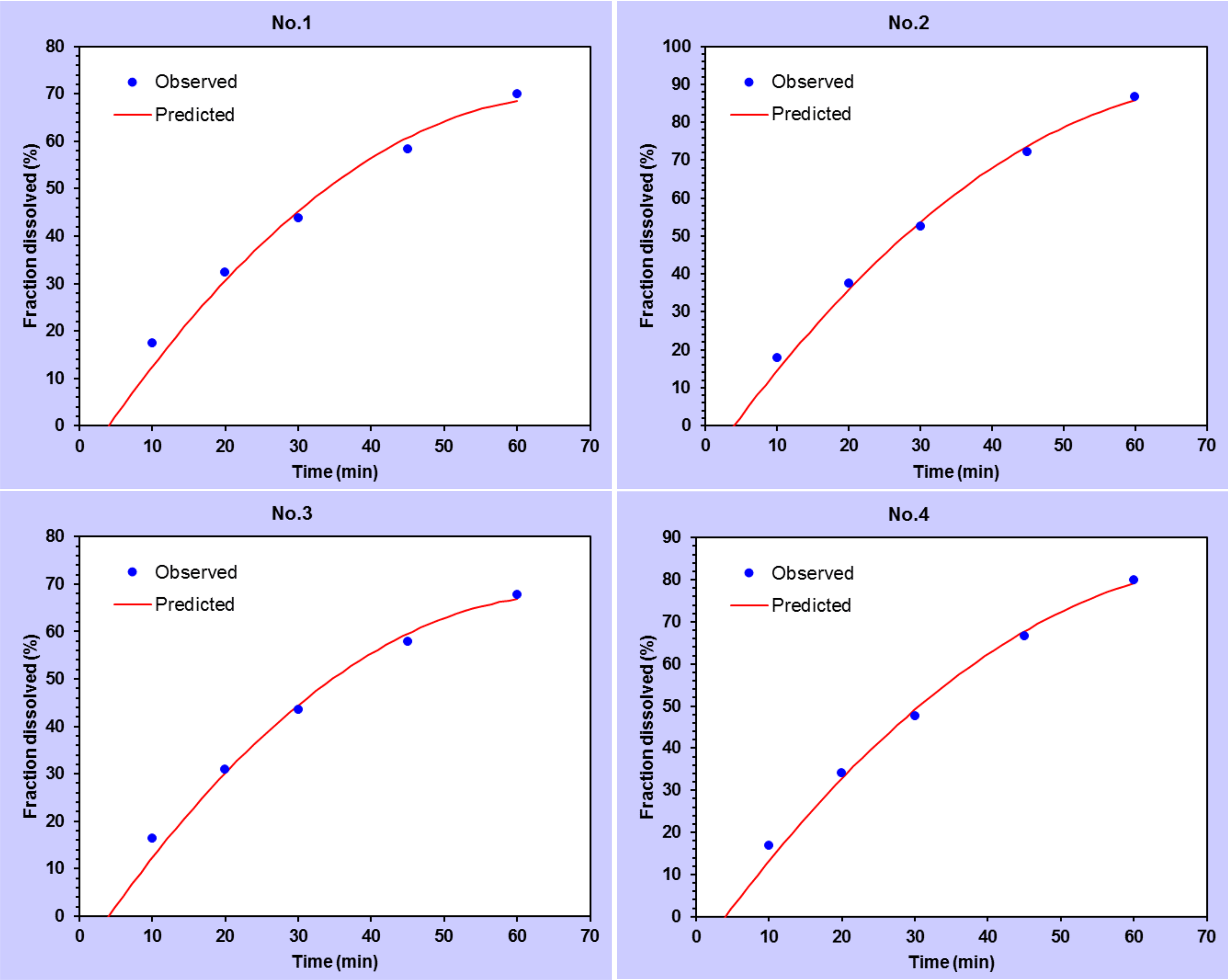

Supplementary materials – Model fitting summary – Polyglykol® 4000 P (modelling up to app. 75 % of carvedilol released)

Model: **Weibull_1**

Model equation: $F = 100 \cdot \left[1 - e^{-\frac{(t-T_i)^{\beta}}{\alpha}}\right]$

Fitted model parameters per tested tablet (N = 4) with statistics – mean, standard deviation (SD), and relative standard deviation expressed in % (RSD%) (output from DDSolver):

Parameter	No.1	No.2	No.3	No.4	Mean	SD	RSD(%)
α	23.349	34.065	25.854	32.245	28.878	5.098	17.652
β	0.814	1.022	0.833	0.960	0.907	0.100	11.051
T_i	4.000	4.000	4.000	4.000	4.000	0.000	0.000

Number of dissolution data points (N), degrees of freedom (df), and selected goodness of fit criteria – Pearson correlation coefficient (R), coefficient of determination (R^2), adjusted coefficient of determination ($R^2_{adjusted}$), and residual sum of squares (RSS) (manual calculation in MS Excel):

Parameter	No.1	No.2	No.3	No.4
N	5	5	5	5
df	2	2	2	2
R	0.997534964	0.995366555	0.999158722	0.996128544
R^2	0.995076004	0.990754578	0.998318152	0.992272075
$R^2_{adjusted}$	0.990152008	0.981509156	0.996636305	0.984544151
RSS	9.400287574	29.33537953	3.321972772	21.57849234

Graphical abstract of model fit presented as mean ± 1 SD of the fraction % of released carvedilol:

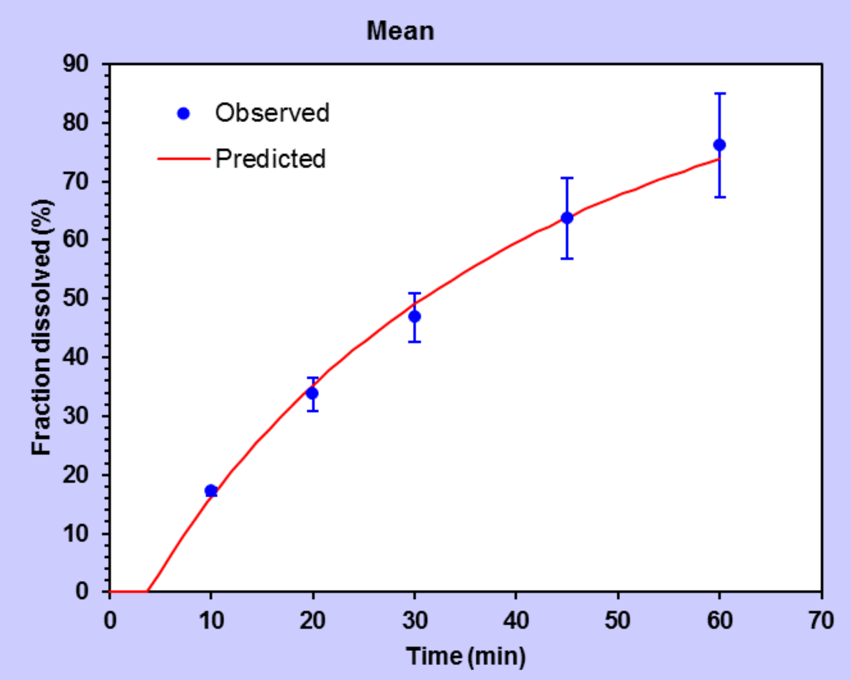

Supplementary materials – Model fitting summary – Polyglykol® 4000 P (modelling up to app. 75 % of carvedilol released)

Graphical abstract of model fit presented as the fraction % of released carvedilol per tested tablet:

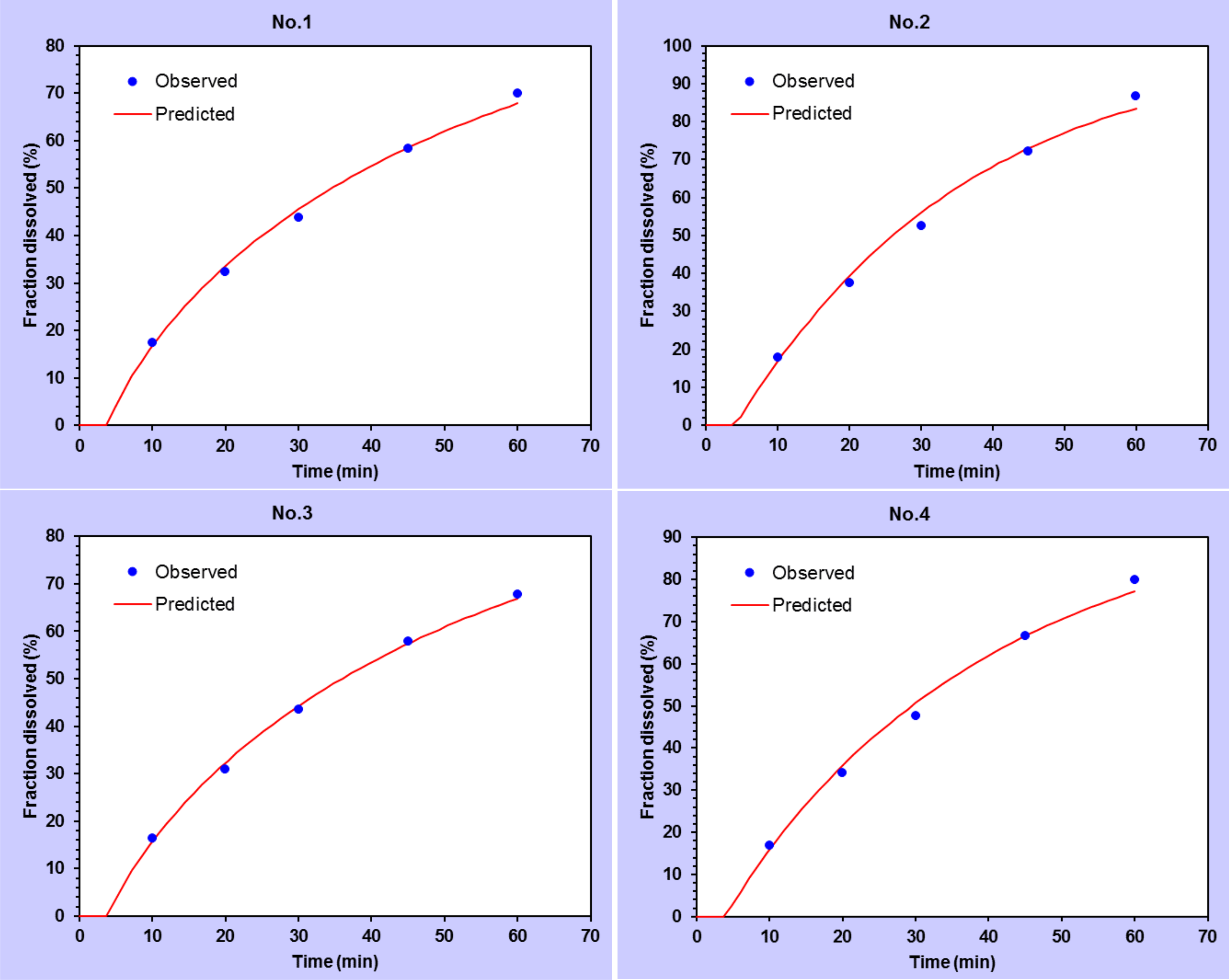

Supplementary materials – Model fitting summary – Polyglykol® 4000 P (modelling up to app. 75 % of carvedilol released)

Model: **Weibull_2**

Model equation: $F = 100 \cdot \left(1 - e^{-\frac{t^{\beta}}{\alpha}}\right)$

Fitted model parameters per tested tablet (N = 4) with statistics – mean, standard deviation (SD), and relative standard deviation expressed in % (RSD%) (output from DDSolver):

Parameter	No.1	No.2	No.3	No.4	Mean	SD	RSD(%)
α	54.075	98.477	60.786	87.226	75.141	21.138	28.131
β	1.016	1.278	1.039	1.199	1.133	0.127	11.170

Number of dissolution data points (N), degrees of freedom (df), and selected goodness of fit criteria – Pearson correlation coefficient (R), coefficient of determination (R^2), adjusted coefficient of determination ($R^2_{adjusted}$), and residual sum of squares (RSS) (manual calculation in MS Excel):

Parameter	No.1	No.2	No.3	No.4
N	5	5	5	5
df	3	3	3	3
R	0.999761579	0.998667144	0.999757268	0.999301062
R^2	0.999523215	0.997336064	0.999514596	0.998602613
$R^2_{adjusted}$	0.999364287	0.996448085	0.999352794	0.998136818
RSS	0.829955593	8.146177054	0.888470151	3.725407771

Graphical abstract of model fit presented as mean ± 1 SD of the fraction % of released carvedilol:

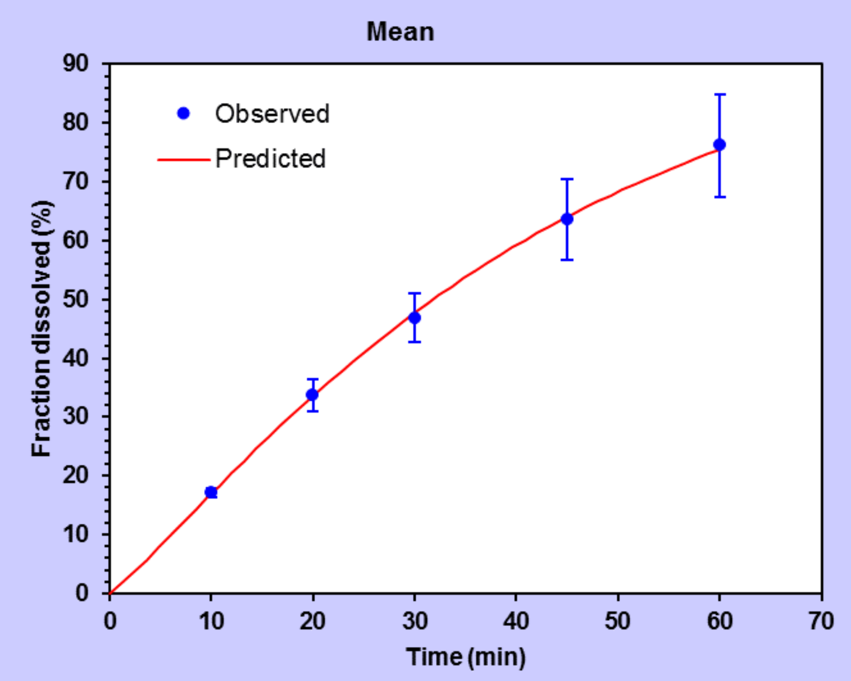

Supplementary materials – Model fitting summary – Polyglykol® 4000 P (modelling up to app. 75 % of carvedilol released)

Graphical abstract of model fit presented as the fraction % of released carvedilol per tested tablet:

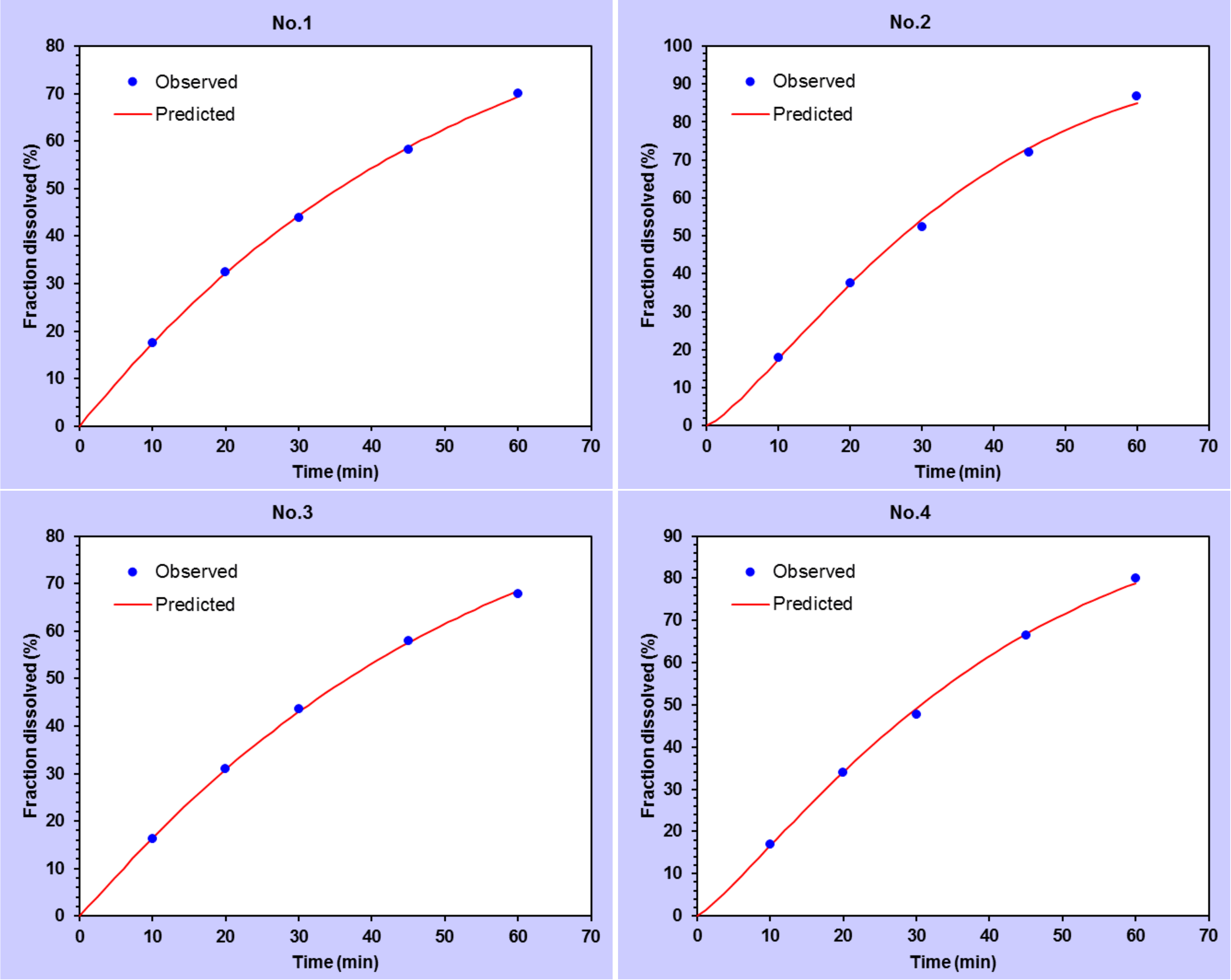

Supplementary materials – Model fitting summary – Polyglykol® 4000 P (modelling up to app. 75 % of carvedilol released)

Model: **Weibull_3**

Model equation: $F = F_{max} \cdot \left(1 - e^{-\frac{t^{\beta}}{\alpha}}\right)$

Fitted model parameters per tested tablet (N = 4) with statistics – mean, standard deviation (SD), and relative standard deviation expressed in % (RSD%) (output from DDSolver):

Parameter	No.1	No.2	No.3	No.4	Mean	SD	RSD(%)
α	80.408	130.203	91.986	126.409	107.252	24.815	23.137
β	1.299	1.422	1.342	1.412	1.368	0.059	4.278
F_{max}	73.503	91.056	71.229	83.988	79.944	9.260	11.584

Number of dissolution data points (N), degrees of freedom (df), and selected goodness of fit criteria – Pearson correlation coefficient (R), coefficient of determination (R^2), adjusted coefficient of determination ($R^2_{adjusted}$), and residual sum of squares (RSS) (manual calculation in MS Excel):

Parameter	No.1	No.2	No.3	No.4
N	5	5	5	5
df	2	2	2	2
R	0.993057853	0.995144154	0.99602558	0.99412195
R^2	0.986163899	0.990311887	0.992066957	0.988278452
$R^2_{adjusted}$	0.972327797	0.980623775	0.984133913	0.976556904
RSS	26.07514038	31.77752912	14.85494532	32.587886

Graphical abstract of model fit presented as mean ± 1 SD of the fraction % of released carvedilol:

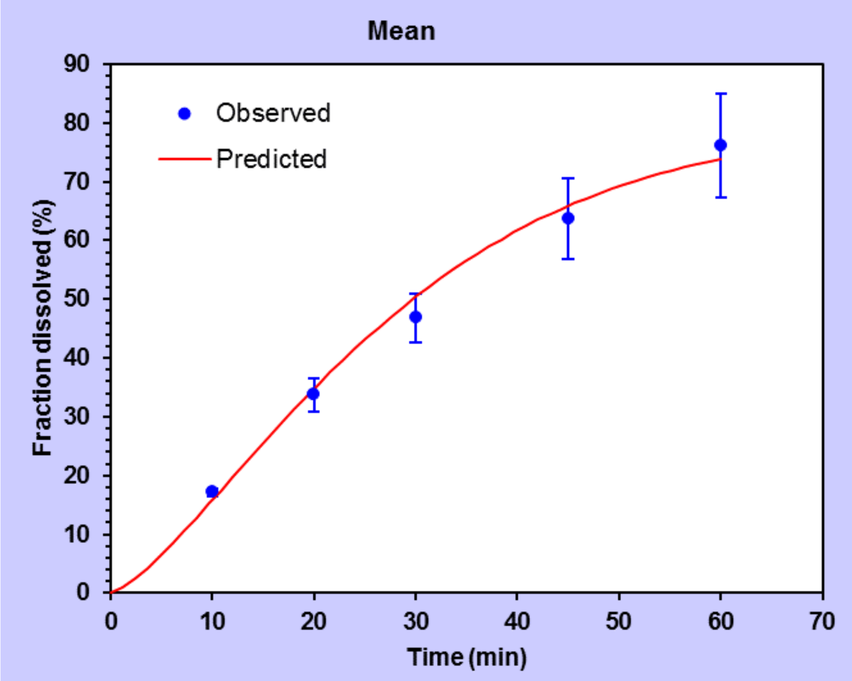

Supplementary materials – Model fitting summary – Polyglykol® 4000 P (modelling up to app. 75 % of carvedilol released)

Graphical abstract of model fit presented as the fraction % of released carvedilol per tested tablet:

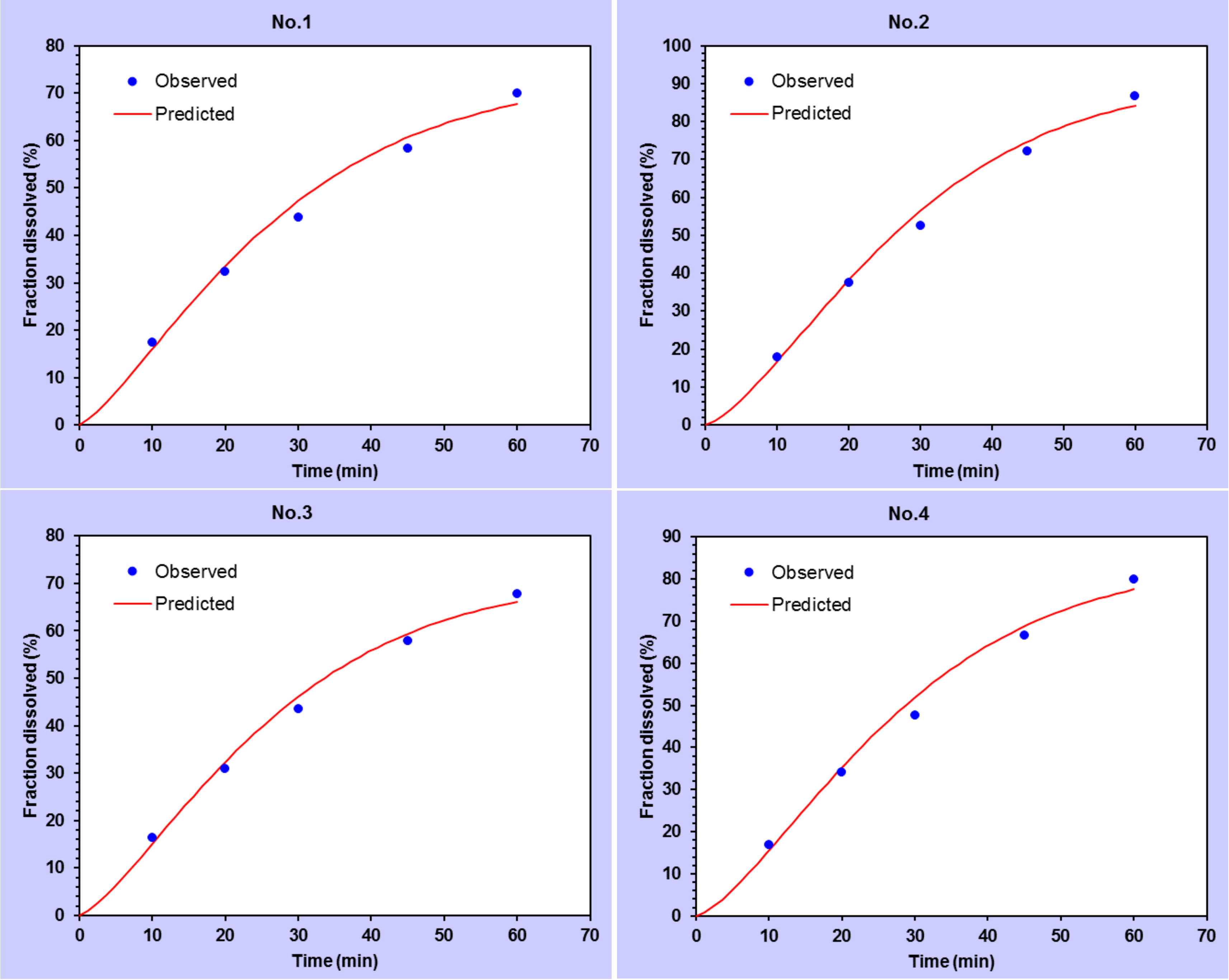

Supplementary materials – Model fitting summary – Polyglykol® 4000 P (modelling up to app. 75 % of carvedilol released)

Model: **Weibull_4**

Model equation: $F = F_{max} \cdot \left[1 - e^{-\frac{(t-T_i)^{\beta}}{\alpha}}\right]$

Fitted model parameters per tested tablet (N = 4) with statistics – mean, standard deviation (SD), and relative standard deviation expressed in % (RSD%) (output from DDSolver):

Parameter	No.1	No.2	No.3	No.4	Mean	SD	RSD(%)
α	26.898	43.201	29.791	38.512	34.600	7.566	21.866
β	1.034	1.095	1.069	1.124	1.081	0.039	3.567
T_i	4.000	4.609	4.000	4.000	4.152	0.305	7.334
F_{max}	73.503	103.374	71.229	83.988	83.023	14.661	17.659

Number of dissolution data points (N), degrees of freedom (df), and selected goodness of fit criteria – Pearson correlation coefficient (R), coefficient of determination (R^2), adjusted coefficient of determination ($R^2_{adjusted}$), and residual sum of squares (RSS) (manual calculation in MS Excel):

Parameter	No.1	No.2	No.3	No.4
N	5	5	5	5
df	1	1	1	1
R	0.986733243	0.995991682	0.990453548	0.988118788
R^2	0.973642494	0.99199943	0.980998232	0.976378738
$R^2_{adjusted}$	0.894569974	0.967997721	0.923992927	0.905514953
RSS	49.14580164	52.58293108	35.1468422	65.31178067

Graphical abstract of model fit presented as mean ± 1 SD of the fraction % of released carvedilol:

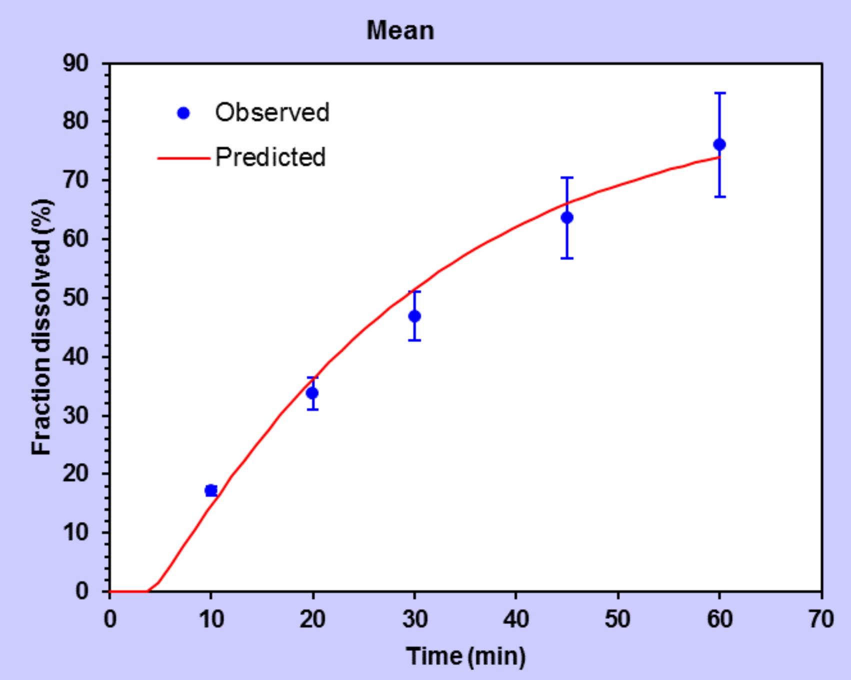

Supplementary materials – Model fitting summary – Polyglykol® 4000 P (modelling up to app. 75 % of carvedilol released)

Graphical abstract of model fit presented as the fraction % of released carvedilol per tested tablet:

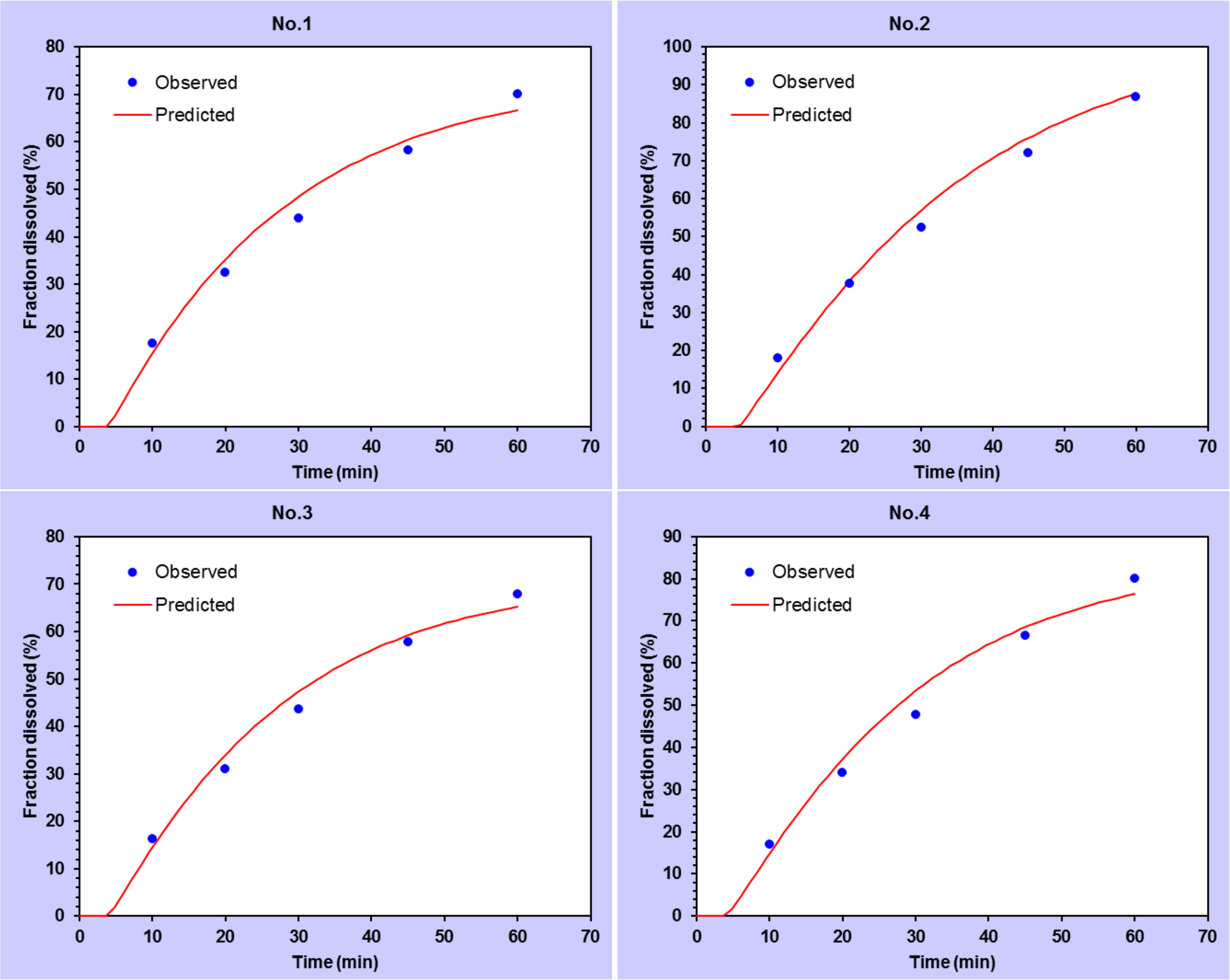

Supplementary materials – Model fitting summary – Polyglykol® 4000 P (modelling up to app. 75 % of carvedilol released)

Model: **Logistic_1**

Model equation: $F = 100 \cdot \frac{e^{\alpha+\beta \cdot \log(t)}}{1+e^{\alpha+\beta \cdot \log(t)}}$

Fitted model parameters per tested tablet (N = 4) with statistics – mean, standard deviation (SD), and relative standard deviation expressed in % (RSD%) (output from DDSolver):

Parameter	No.1	No.2	No.3	No.4	Mean	SD	RSD(%)
α	-4.648	-5.912	-4.739	-5.472	-5.193	0.605	-11.652
β	3.036	4.223	3.060	3.757	3.519	0.576	16.373

Number of dissolution data points (N), degrees of freedom (df), and selected goodness of fit criteria – Pearson correlation coefficient (R), coefficient of determination (R^2), adjusted coefficient of determination ($R^2_{adjusted}$), and residual sum of squares (RSS) (manual calculation in MS Excel):

Parameter	No.1	No.2	No.3	No.4
N	5	5	5	5
df	3	3	3	3
R	0.996936381	0.990379863	0.999145965	0.993467709
R^2	0.993882147	0.980852274	0.998292659	0.986978089
$R^2_{adjusted}$	0.991842863	0.974469698	0.997723545	0.982637451
RSS	10.82656837	61.01847447	3.074708224	34.3246097

Graphical abstract of model fit presented as mean ± 1 SD of the fraction % of released carvedilol:

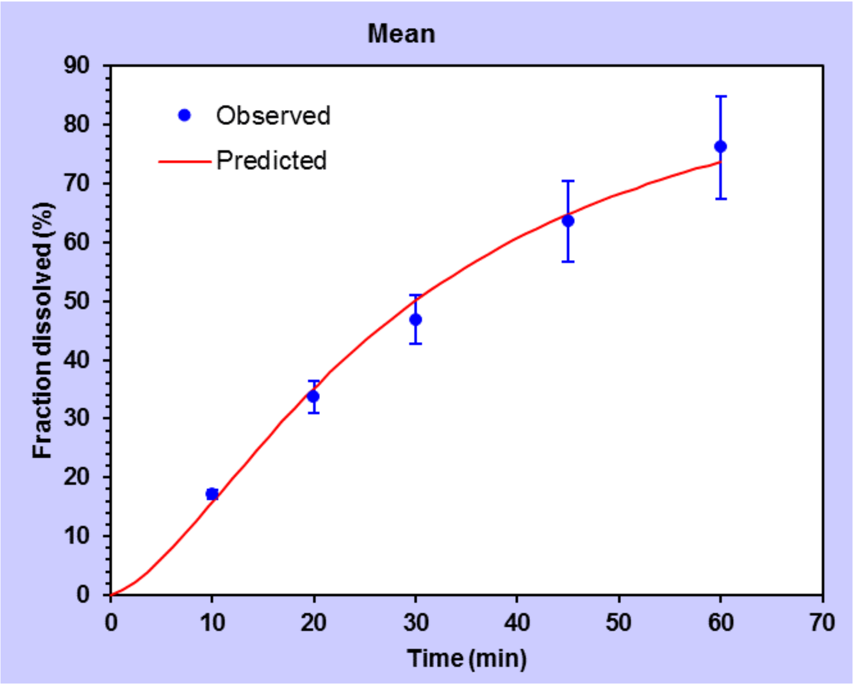

Supplementary materials – Model fitting summary – Polyglykol® 4000 P (modelling up to app. 75 % of carvedilol released)

Graphical abstract of model fit presented as the fraction % of released carvedilol per tested tablet:

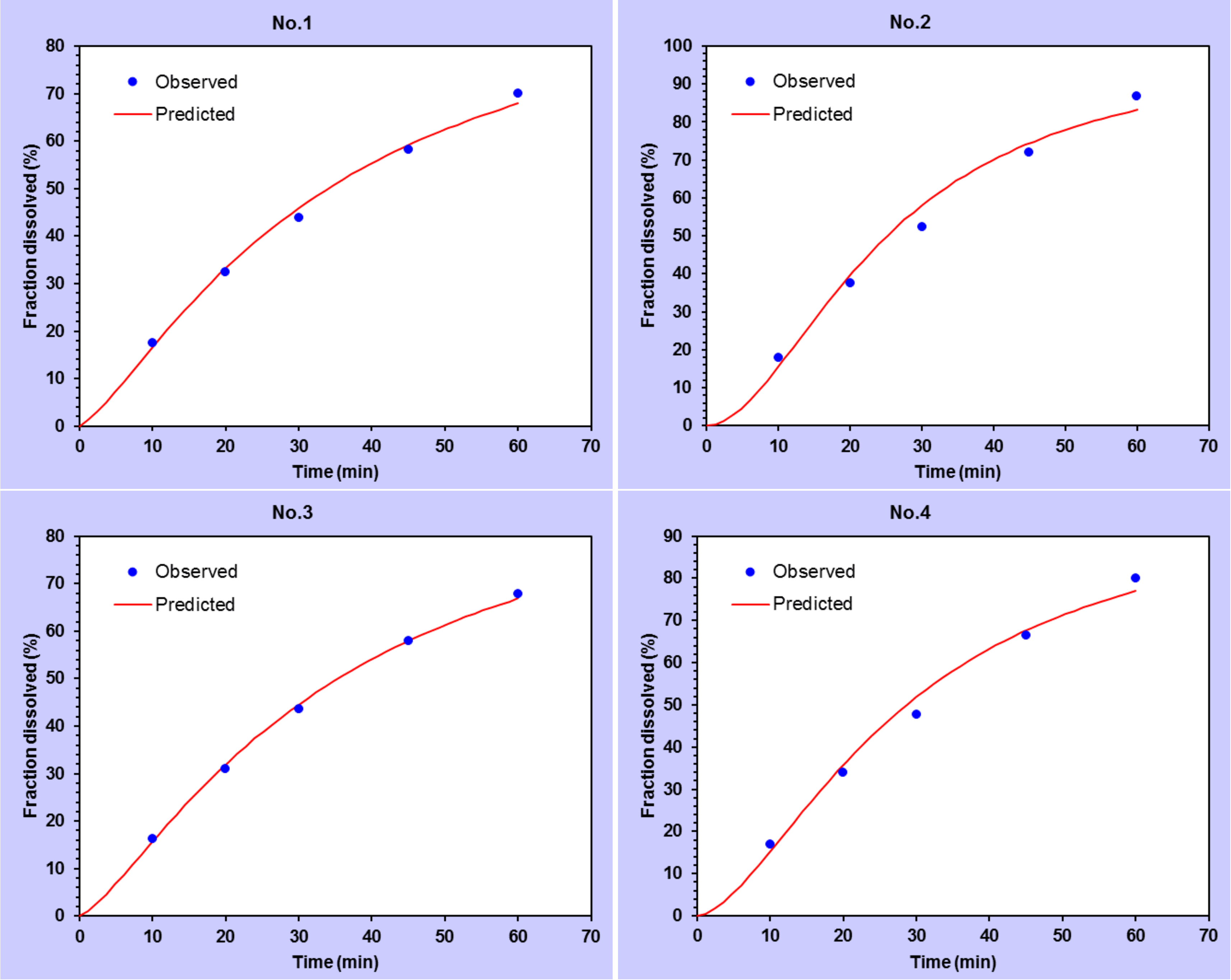

Supplementary materials – Model fitting summary – Polyglykol® 4000 P (modelling up to app. 75 % of carvedilol released)

Model: **Logistic_2**

Model equation: $F = F_{max} \cdot \frac{e^{\alpha+\beta\cdot\log(t)}}{1+e^{\alpha+\beta\cdot\log(t)}}$

Fitted model parameters per tested tablet (N = 4) with statistics – mean, standard deviation (SD), and relative standard deviation expressed in % (RSD%) (output from DDSolver):

Parameter	No.1	No.2	No.3	No.4	Mean	SD	RSD(%)
α	-6.473	-7.028	-6.661	-8.081	-7.061	0.718	-10.171
β	4.951	5.272	5.097	5.579	5.224	0.270	5.171
F_{max}	73.503	91.056	71.229	91.451	81.810	10.945	13.379

Number of dissolution data points (N), degrees of freedom (df), and selected goodness of fit criteria – Pearson correlation coefficient (R), coefficient of determination (R^2), adjusted coefficient of determination ($R^2_{adjusted}$), and residual sum of squares (RSS) (manual calculation in MS Excel):

Parameter	No.1	No.2	No.3	No.4
N	5	5	5	5
df	2	2	2	2
R	0.973385492	0.977436152	0.978254177	0.993565606
R^2	0.947479315	0.95538143	0.956981235	0.987172613
$R^2_{adjusted}$	0.894958631	0.910762861	0.913962469	0.974345226
RSS	113.8864161	166.8155277	90.91694855	148.3344272

Graphical abstract of model fit presented as mean ± 1 SD of the fraction % of released carvedilol:

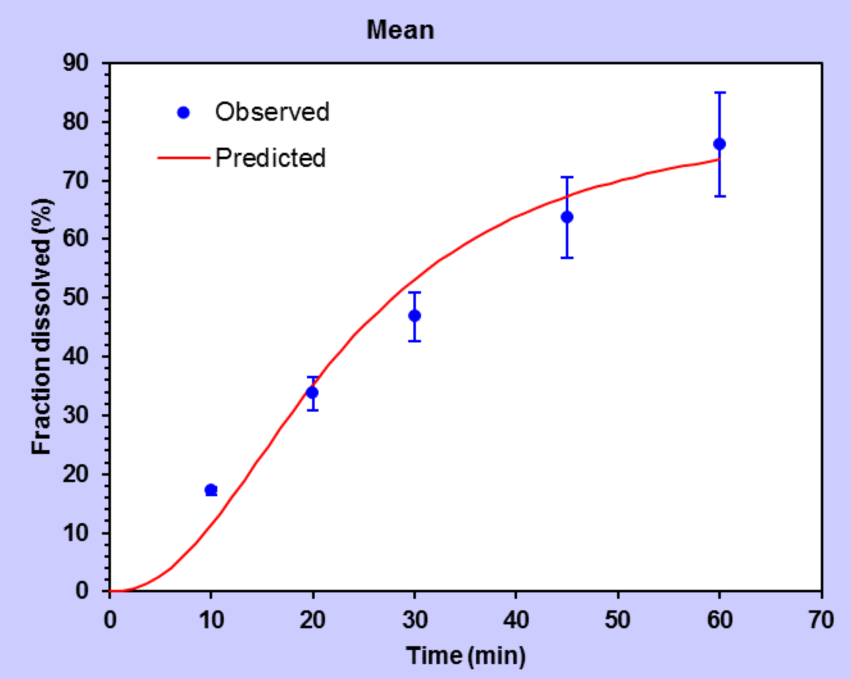

Supplementary materials – Model fitting summary – Polyglykol® 4000 P (modelling up to app. 75 % of carvedilol released)

Graphical abstract of model fit presented as the fraction % of released carvedilol per tested tablet:

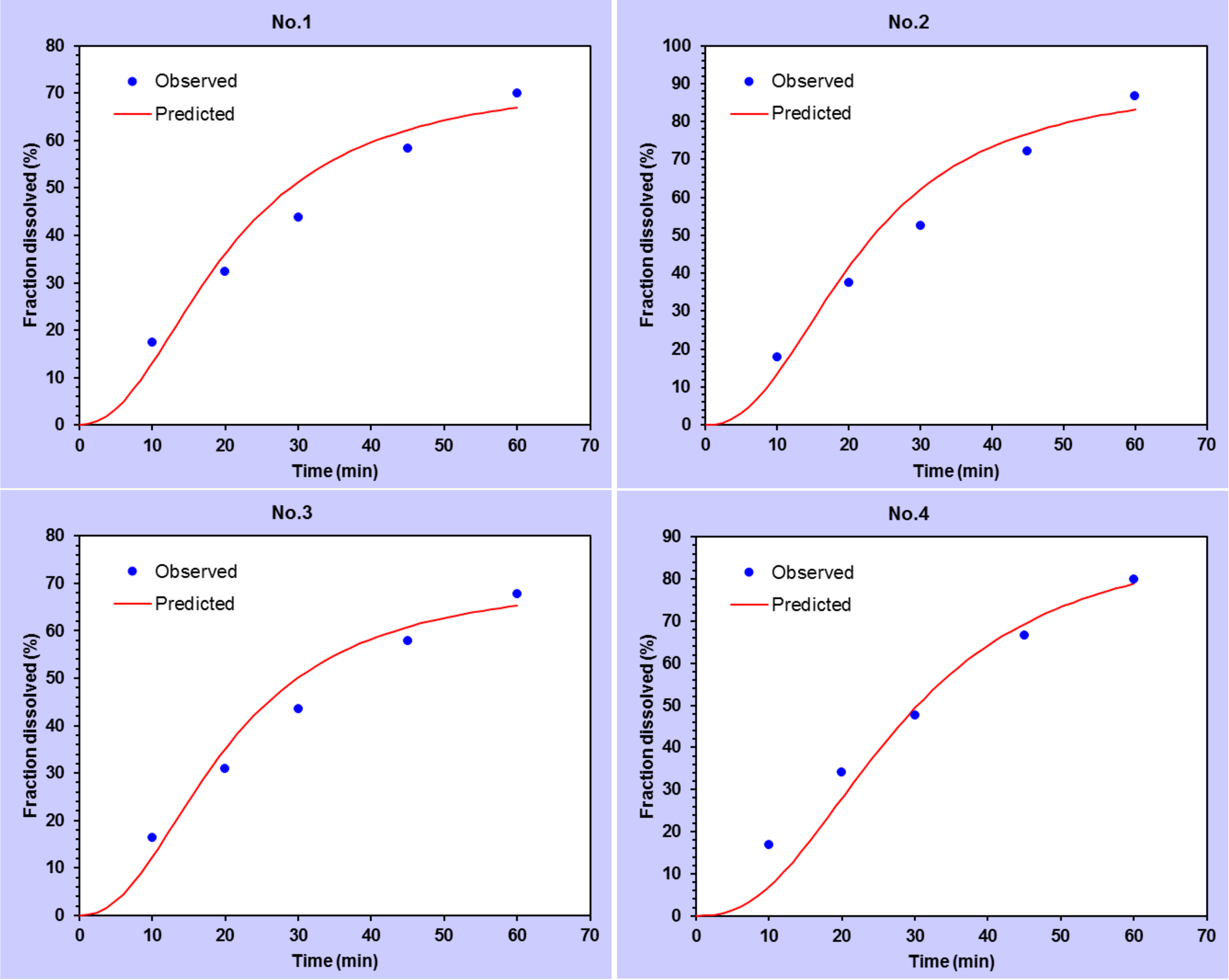

Supplementary materials – Model fitting summary – Polyglykol® 4000 P (modelling up to app. 75 % of carvedilol released)

Model: **Logistic_3**

Model equation: $F = F_{max} \cdot \frac{1}{1+e^{-k\cdot(t-\gamma)}}$

Fitted model parameters per tested tablet (N = 4) with statistics – mean, standard deviation (SD), and relative standard deviation expressed in % (RSD%) (output from DDSolver):

Parameter	No.1	No.2	No.3	No.4	Mean	SD	RSD(%)
k	0.080	0.066	0.081	0.069	0.074	0.007	10.033
γ	24.638	28.054	24.538	28.282	26.378	2.069	7.845
F_{max}	73.503	96.711	71.229	88.530	82.493	12.197	14.785

Number of dissolution data points (N), degrees of freedom (df), and selected goodness of fit criteria – Pearson correlation coefficient (R), coefficient of determination (R^2), adjusted coefficient of determination ($R^2_{adjusted}$), and residual sum of squares (RSS) (manual calculation in MS Excel):

Parameter	No.1	No.2	No.3	No.4
N	5	5	5	5
df	2	2	2	2
R	0.996146611	0.996561795	0.997913599	0.997649487
R^2	0.99230807	0.993135411	0.995831551	0.9953045
$R^2_{adjusted}$	0.98461614	0.986270823	0.991663102	0.990608999
RSS	15.94195819	25.49563479	7.95113399	12.35188928

Graphical abstract of model fit presented as mean ± 1 SD of the fraction % of released carvedilol:

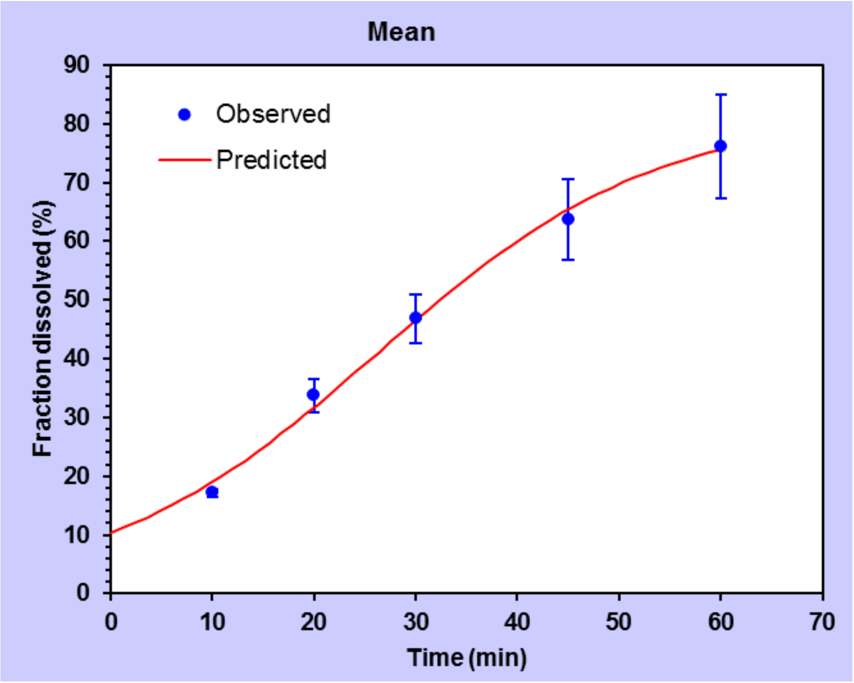

Supplementary materials – Model fitting summary – Polyglykol® 4000 P (modelling up to app. 75 % of carvedilol released)

Graphical abstract of model fit presented as the fraction % of released carvedilol per tested tablet:

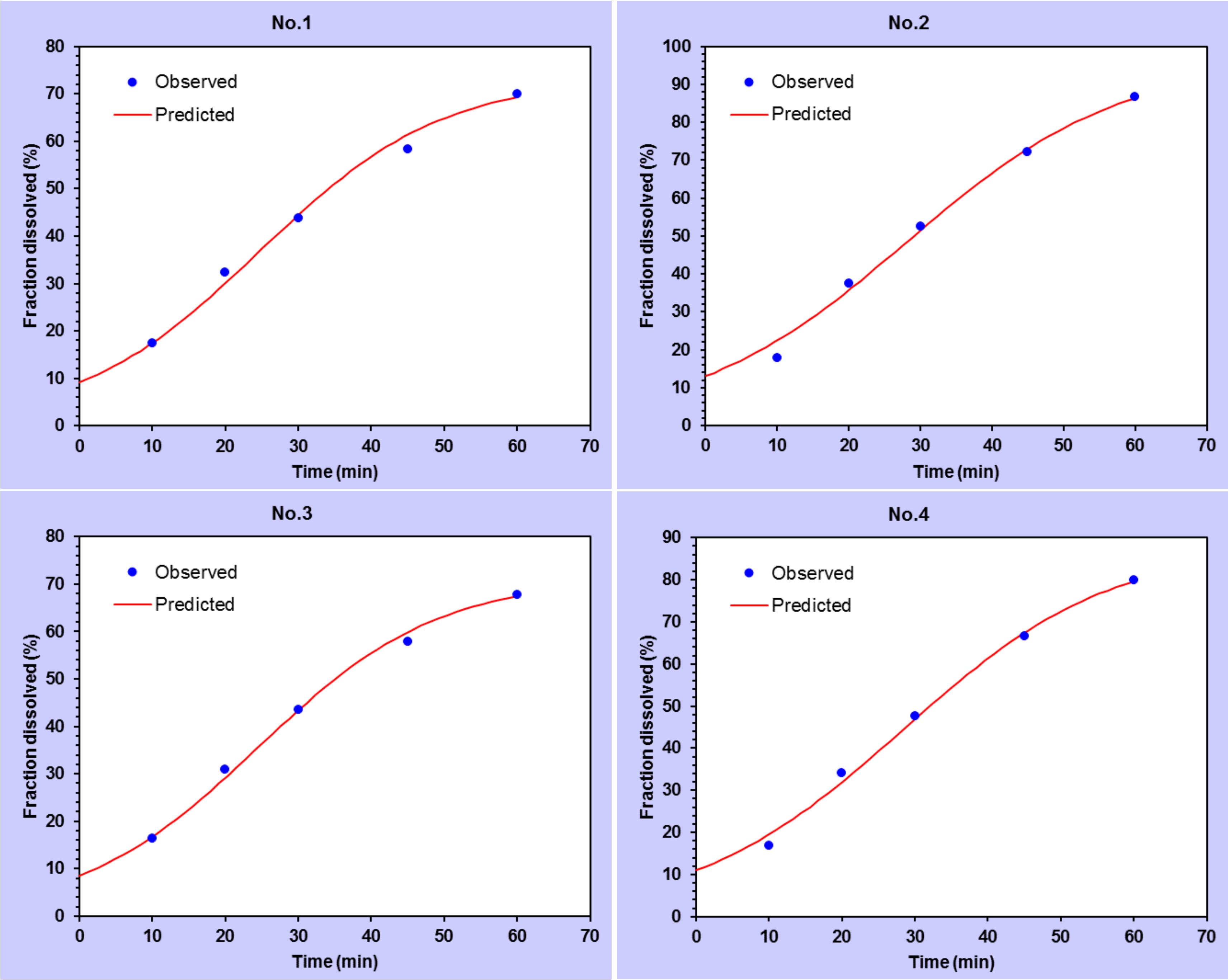

Supplementary materials – Model fitting summary – Polyglykol® 4000 P (modelling up to app. 75 % of carvedilol released)

Model: **Gompertz_1**

Model equation: $F = 100 \cdot e^{-\alpha \cdot e^{-\beta \cdot \log(t)}}$

Fitted model parameters per tested tablet (N = 4) with statistics – mean, standard deviation (SD), and relative standard deviation expressed in % (RSD%) (output from DDSolver):

Parameter	No.1	No.2	No.3	No.4	Mean	SD	RSD(%)
α	14.048	45.228	14.118	27.879	25.318	14.781	58.380
β	1.994	3.056	1.972	2.592	2.404	0.521	21.688

Number of dissolution data points (N), degrees of freedom (df), and selected goodness of fit criteria – Pearson correlation coefficient (R), coefficient of determination (R^2), adjusted coefficient of determination ($R^2_{adjusted}$), and residual sum of squares (RSS) (manual calculation in MS Excel):

Parameter	No.1	No.2	No.3	No.4
N	5	5	5	5
df	3	3	3	3
R	0.987840245	0.974483492	0.992128776	0.979539993
R^2	0.975828349	0.949618076	0.984319507	0.959498597
$R^2_{adjusted}$	0.967771132	0.932824101	0.979092676	0.945998129
RSS	42.15816089	167.3221627	26.94693372	107.502879

Graphical abstract of model fit presented as mean ± 1 SD of the fraction % of released carvedilol:

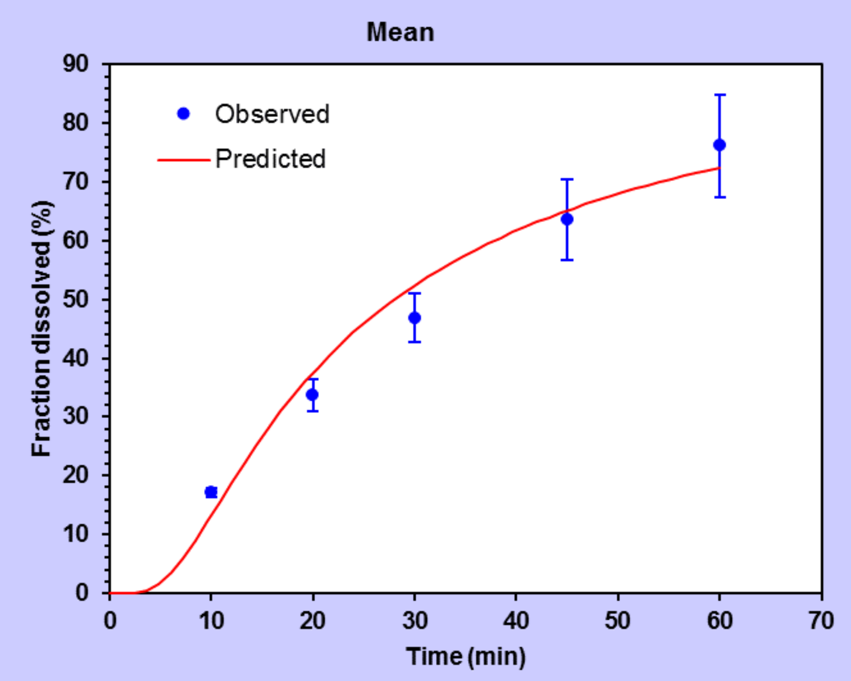

Supplementary materials – Model fitting summary – Polyglykol® 4000 P (modelling up to app. 75 % of carvedilol released)

Graphical abstract of model fit presented as the fraction % of released carvedilol per tested tablet:

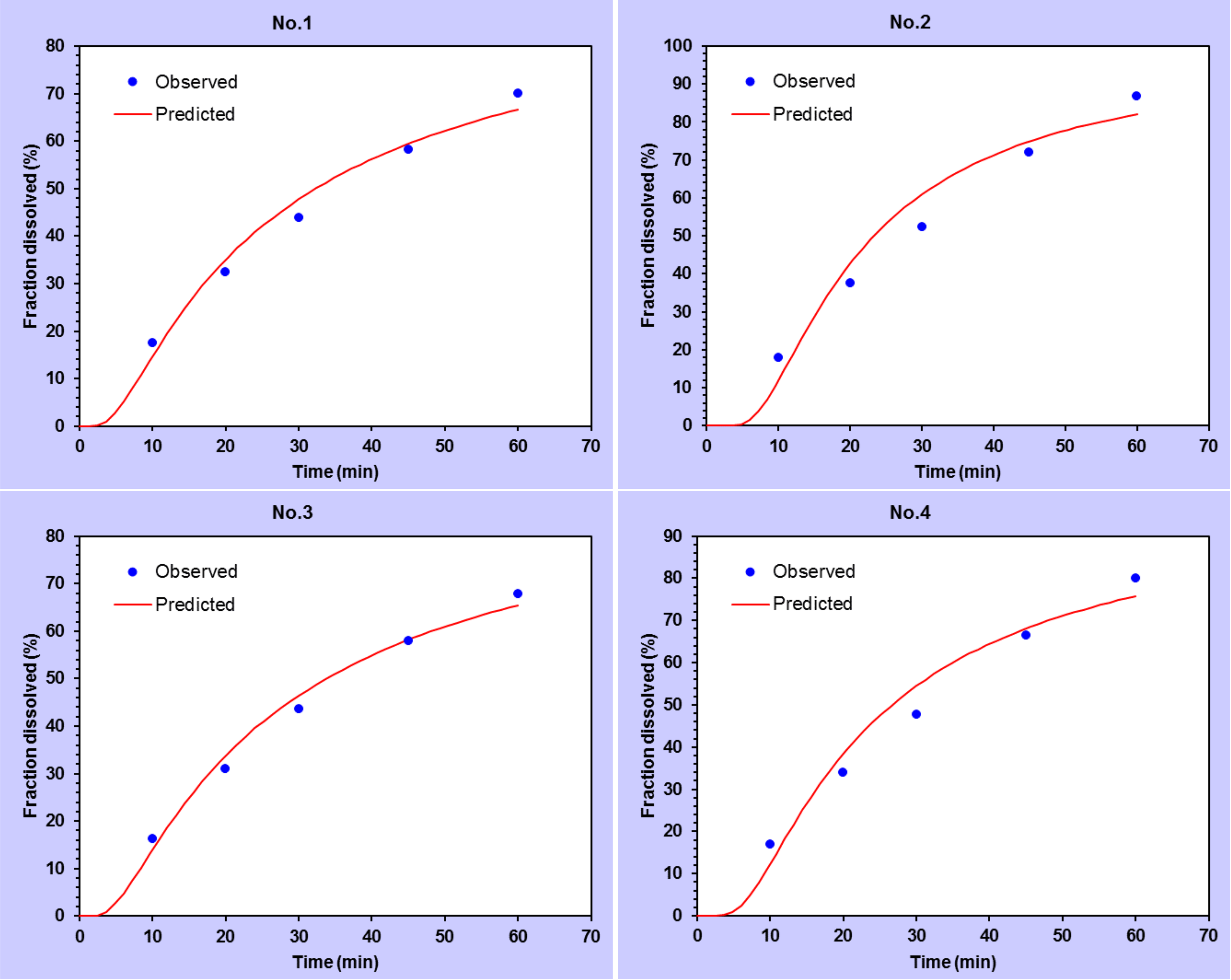

Supplementary materials – Model fitting summary – Polyglykol® 4000 P (modelling up to app. 75 % of carvedilol released)

Model: **Gompertz_2**

Model equation: $F = F_{max} \cdot e^{-\alpha \cdot e^{-\beta \cdot \log(t)}}$

Fitted model parameters per tested tablet (N = 4) with statistics – mean, standard deviation (SD), and relative standard deviation expressed in % (RSD%) (output from DDSolver):

Parameter	No.1	No.2	No.3	No.4	Mean	SD	RSD(%)
α	110.367	223.086	125.609	221.183	170.061	60.455	35.549
β	3.953	4.121	4.059	4.111	4.061	0.077	1.900
F_{max}	73.503	91.056	71.229	83.988	79.944	9.260	11.584

Number of dissolution data points (N), degrees of freedom (df), and selected goodness of fit criteria – Pearson correlation coefficient (R), coefficient of determination (R^2), adjusted coefficient of determination ($R^2_{adjusted}$), and residual sum of squares (RSS) (manual calculation in MS Excel):

Parameter	No.1	No.2	No.3	No.4
N	5	5	5	5
df	2	2	2	2
R	0.950869218	0.980280113	0.956014417	0.977701765
R^2	0.90415227	0.9609491	0.913963565	0.955900741
$R^2_{adjusted}$	0.80830454	0.921898201	0.827927129	0.911801481
RSS	232.5169941	341.9760192	201.5758704	301.7782715

Graphical abstract of model fit presented as mean ± 1 SD of the fraction % of released carvedilol:

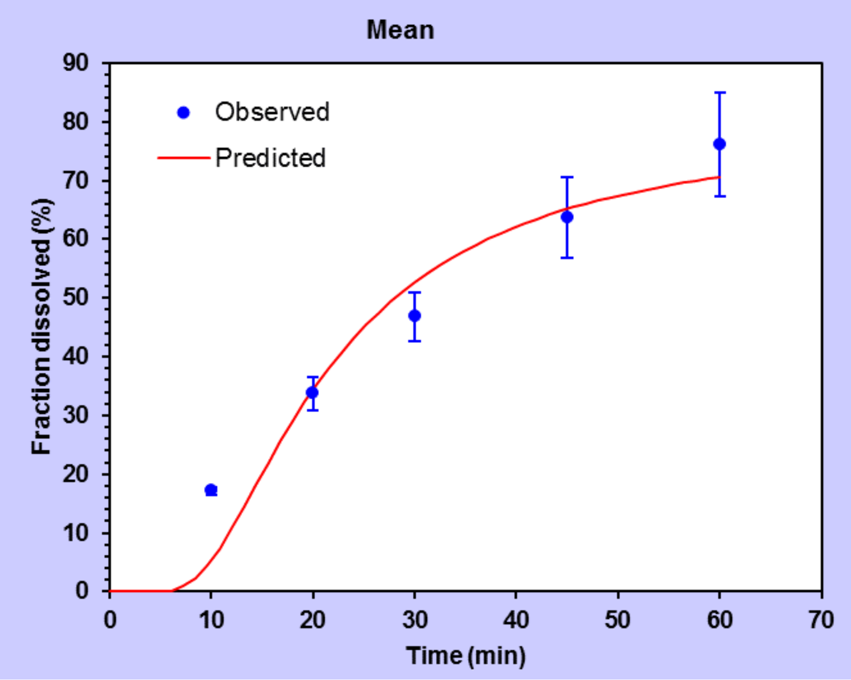

Supplementary materials – Model fitting summary – Polyglykol® 4000 P (modelling up to app. 75 % of carvedilol released)

Graphical abstract of model fit presented as the fraction % of released carvedilol per tested tablet:

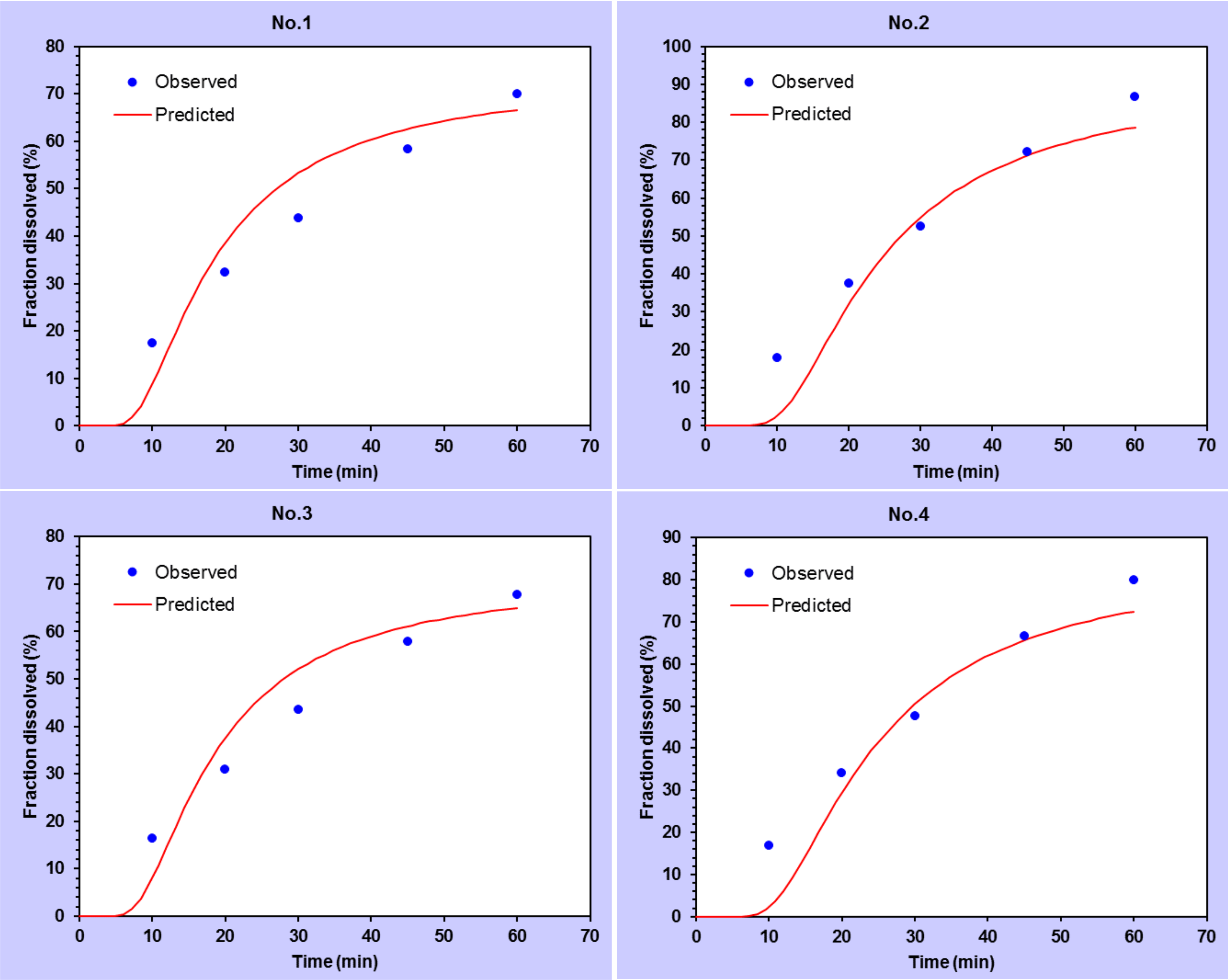

Supplementary materials – Model fitting summary – Polyglykol® 4000 P (modelling up to app. 75 % of carvedilol released)

Model: **Gompertz_3**

Model equation: $F = F_{max} \cdot e^{-e^{-k\cdot(t-\gamma)}}$

Fitted model parameters per tested tablet (N = 4) with statistics – mean, standard deviation (SD), and relative standard deviation expressed in % (RSD%) (output from DDSolver):

Parameter	No.1	No.2	No.3	No.4	Mean	SD	RSD(%)
k	0.065	0.068	0.066	0.053	0.063	0.007	10.967
γ	17.699	19.106	17.634	18.496	18.233	0.701	3.846
F_{max}	73.503	91.056	71.229	82.141	79.482	9.035	11.367

Number of dissolution data points (N), degrees of freedom (df), and selected goodness of fit criteria – Pearson correlation coefficient (R), coefficient of determination (R^2), adjusted coefficient of determination ($R^2_{adjusted}$), and residual sum of squares (RSS) (manual calculation in MS Excel):

Parameter	No.1	No.2	No.3	No.4
N	5	5	5	5
df	2	2	2	2
R	0.9930998	0.993996965	0.996213694	0.997900208
R^2	0.986247213	0.988029966	0.992441724	0.995804825
$R^2_{adjusted}$	0.972494426	0.976059932	0.984883447	0.99160965
RSS	37.99578444	54.77172082	20.93171624	50.86732266

Graphical abstract of model fit presented as mean ± 1 SD of the fraction % of released carvedilol:

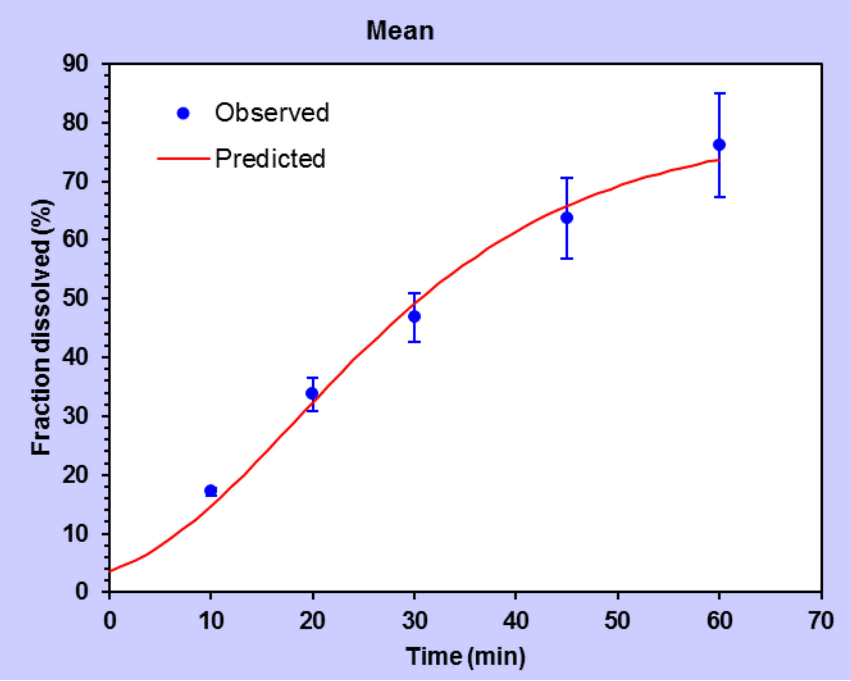

Supplementary materials – Model fitting summary – Polyglykol® 4000 P (modelling up to app. 75 % of carvedilol released)

Graphical abstract of model fit presented as the fraction % of released carvedilol per tested tablet:

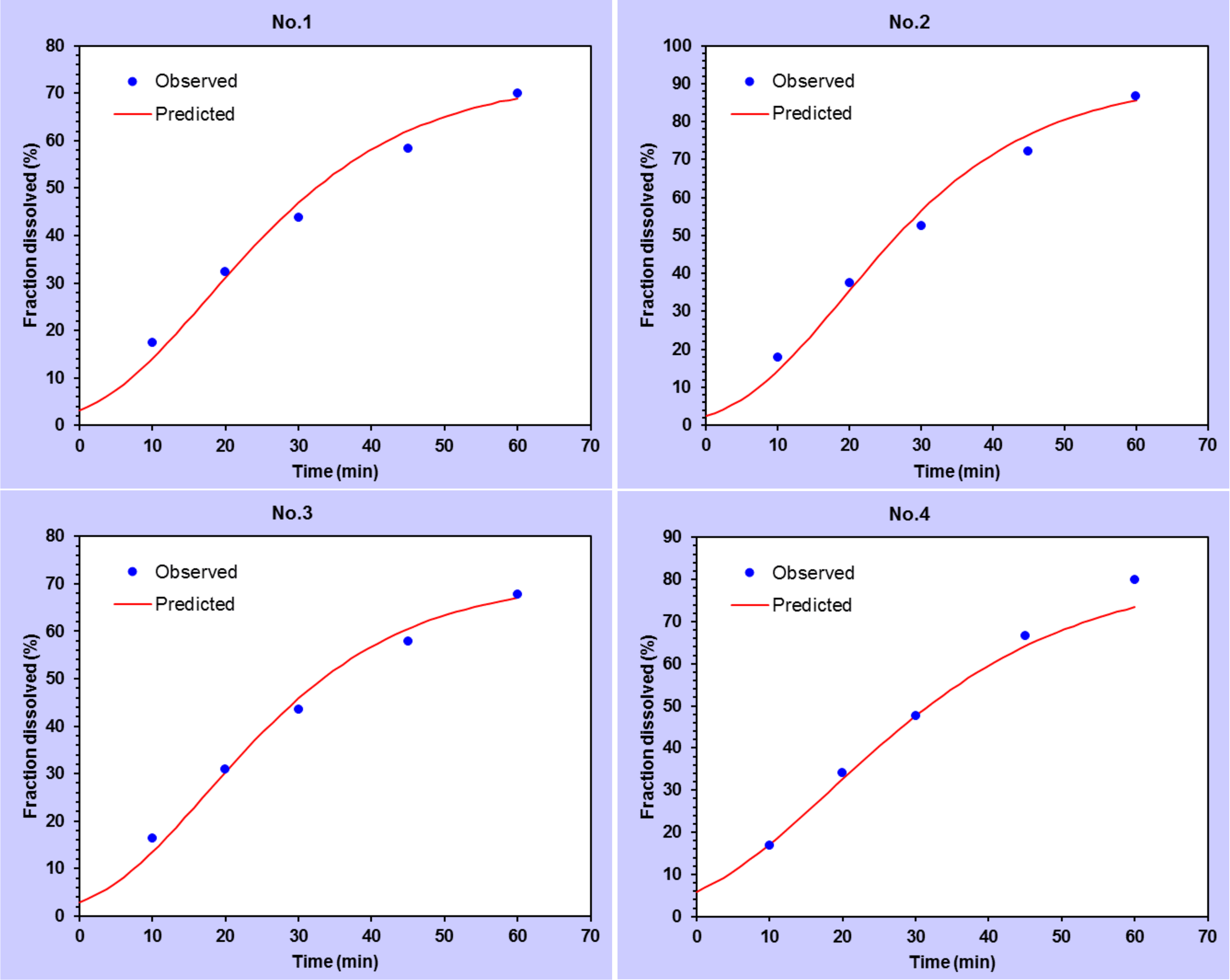

Supplementary materials – Model fitting summary – Polyglykol® 4000 P (modelling up to app. 75 % of carvedilol released)

Model: **Gompertz_4**

Model equation: $F = F_{max} \cdot e^{-\beta \cdot e^{-k \cdot t}}$

Fitted model parameters per tested tablet (N = 4) with statistics – mean, standard deviation (SD), and relative standard deviation expressed in % (RSD%) (output from DDSolver):

Parameter	No.1	No.2	No.3	No.4	Mean	SD	RSD(%)
k	0.065	0.068	0.066	0.068	0.067	0.001	1.853
β	3.162	3.640	3.221	3.661	3.421	0.266	7.772
F_{max}	73.503	91.056	71.229	83.988	79.944	9.260	11.584

Number of dissolution data points (N), degrees of freedom (df), and selected goodness of fit criteria – Pearson correlation coefficient (R), coefficient of determination (R^2), adjusted coefficient of determination ($R^2_{adjusted}$), and residual sum of squares (RSS) (manual calculation in MS Excel):

Parameter	No.1	No.2	No.3	No.4
N	5	5	5	5
df	2	2	2	2
R	0.9930998	0.993996965	0.996213694	0.993217231
R^2	0.986247213	0.988029966	0.992441724	0.986480468
$R^2_{adjusted}$	0.972494426	0.976059932	0.984883447	0.972960937
RSS	37.99578444	54.77172082	20.93171624	52.11166267

Graphical abstract of model fit presented as mean ± 1 SD of the fraction % of released carvedilol:

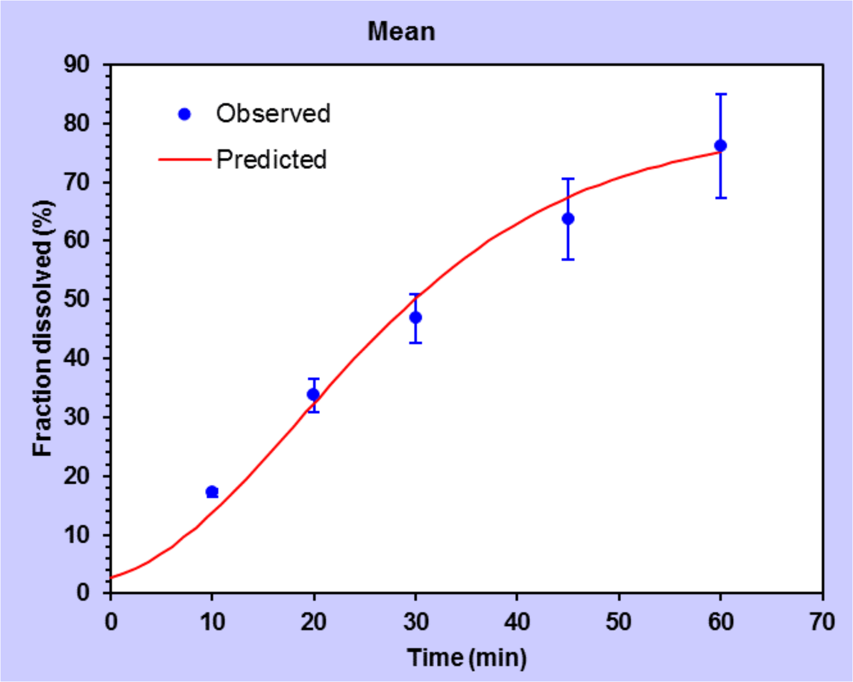

Supplementary materials – Model fitting summary – Polyglykol® 4000 P (modelling up to app. 75 % of carvedilol released)

Graphical abstract of model fit presented as the fraction % of released carvedilol per tested tablet:

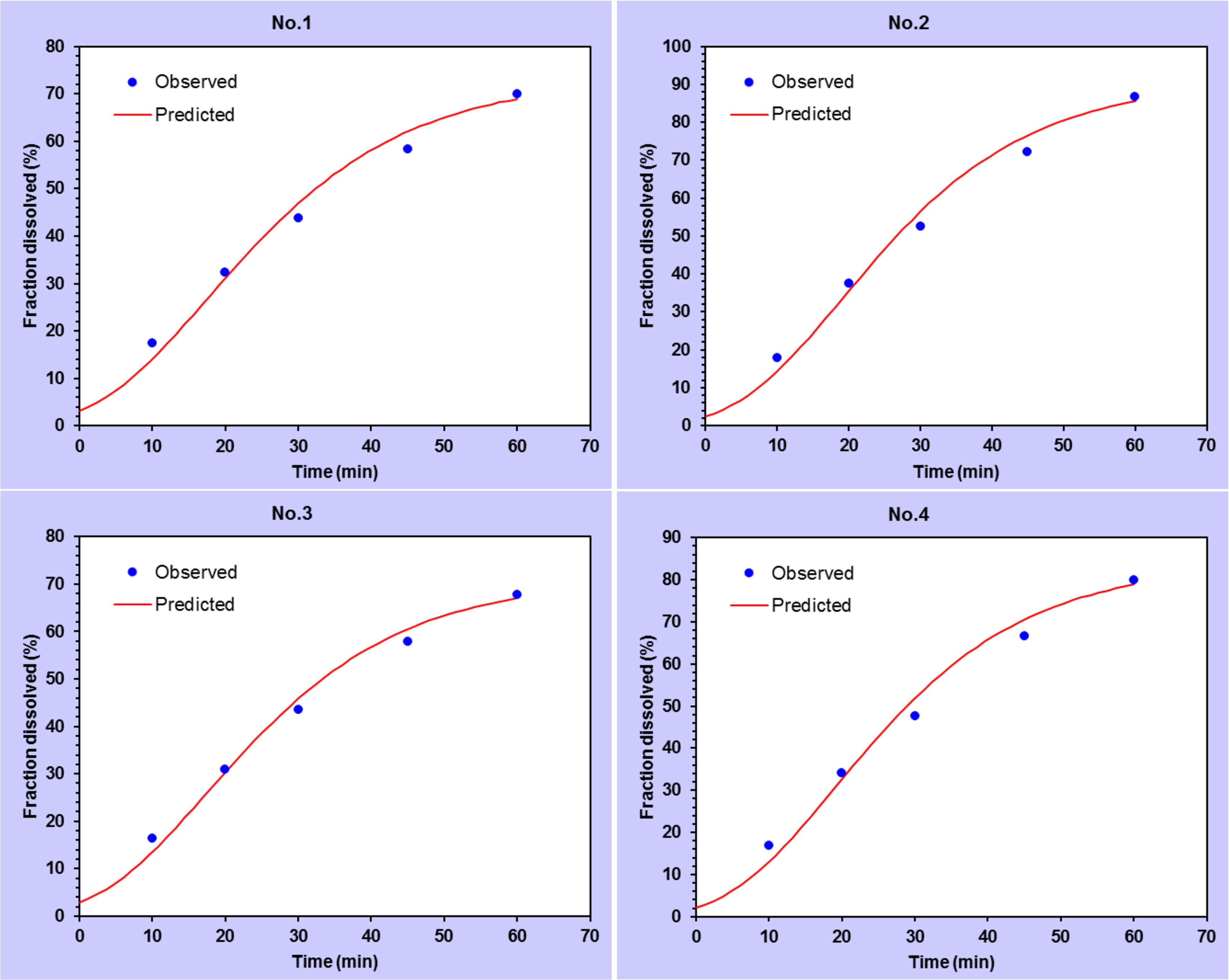

Supplementary materials – Model fitting summary – Polyglykol® 4000 P (modelling up to app. 75 % of carvedilol released)

Model: **Probit_1**

Model equation: $F = 100 \cdot \phi[\alpha + \beta \cdot \log(t)]$

Fitted model parameters per tested tablet (N = 4) with statistics – mean, standard deviation (SD), and relative standard deviation expressed in % (RSD%) (output from DDSolver):

Parameter	No.1	No.2	No.3	No.4	Mean	SD	RSD(%)
α	-2.831	-3.562	-2.878	-3.319	-3.148	0.353	-11.215
β	1.851	2.542	1.860	2.279	2.133	0.338	15.852

Number of dissolution data points (N), degrees of freedom (df), and selected goodness of fit criteria – Pearson correlation coefficient (R), coefficient of determination (R^2), adjusted coefficient of determination ($R^2_{adjusted}$), and residual sum of squares (RSS) (manual calculation in MS Excel):

Parameter	No.1	No.2	No.3	No.4
N	5	5	5	5
df	3	3	3	3
R	0.996087683	0.990820939	0.99842141	0.99282656
R^2	0.992190672	0.981726134	0.996845312	0.985704579
$R^2_{adjusted}$	0.989587562	0.975634845	0.995793749	0.980939438
RSS	13.73970636	56.87074892	5.588809365	37.23878647

Graphical abstract of model fit presented as mean ± 1 SD of the fraction % of released carvedilol:

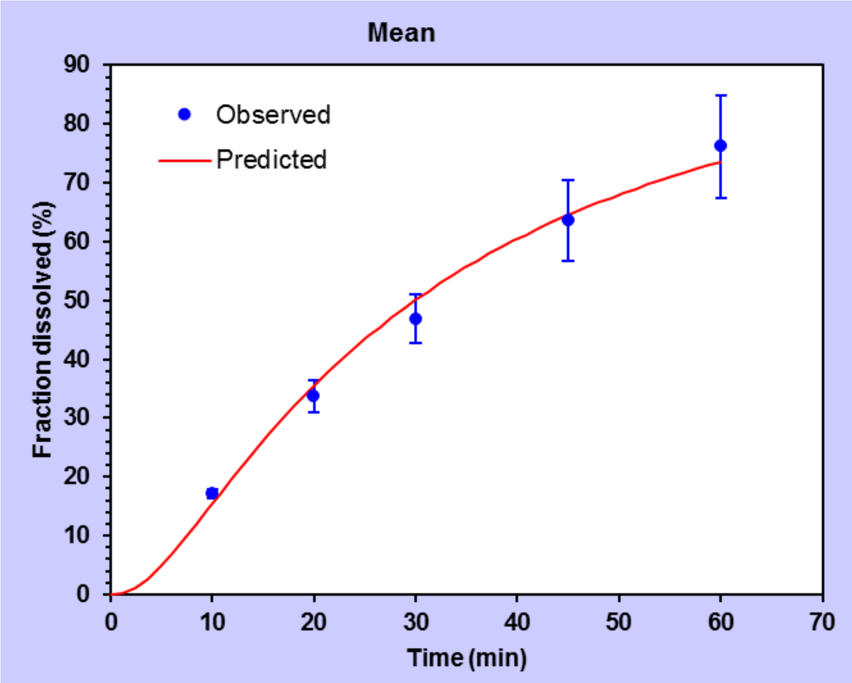

Supplementary materials – Model fitting summary – Polyglykol® 4000 P (modelling up to app. 75 % of carvedilol released)

Graphical abstract of model fit presented as the fraction % of released carvedilol per tested tablet:

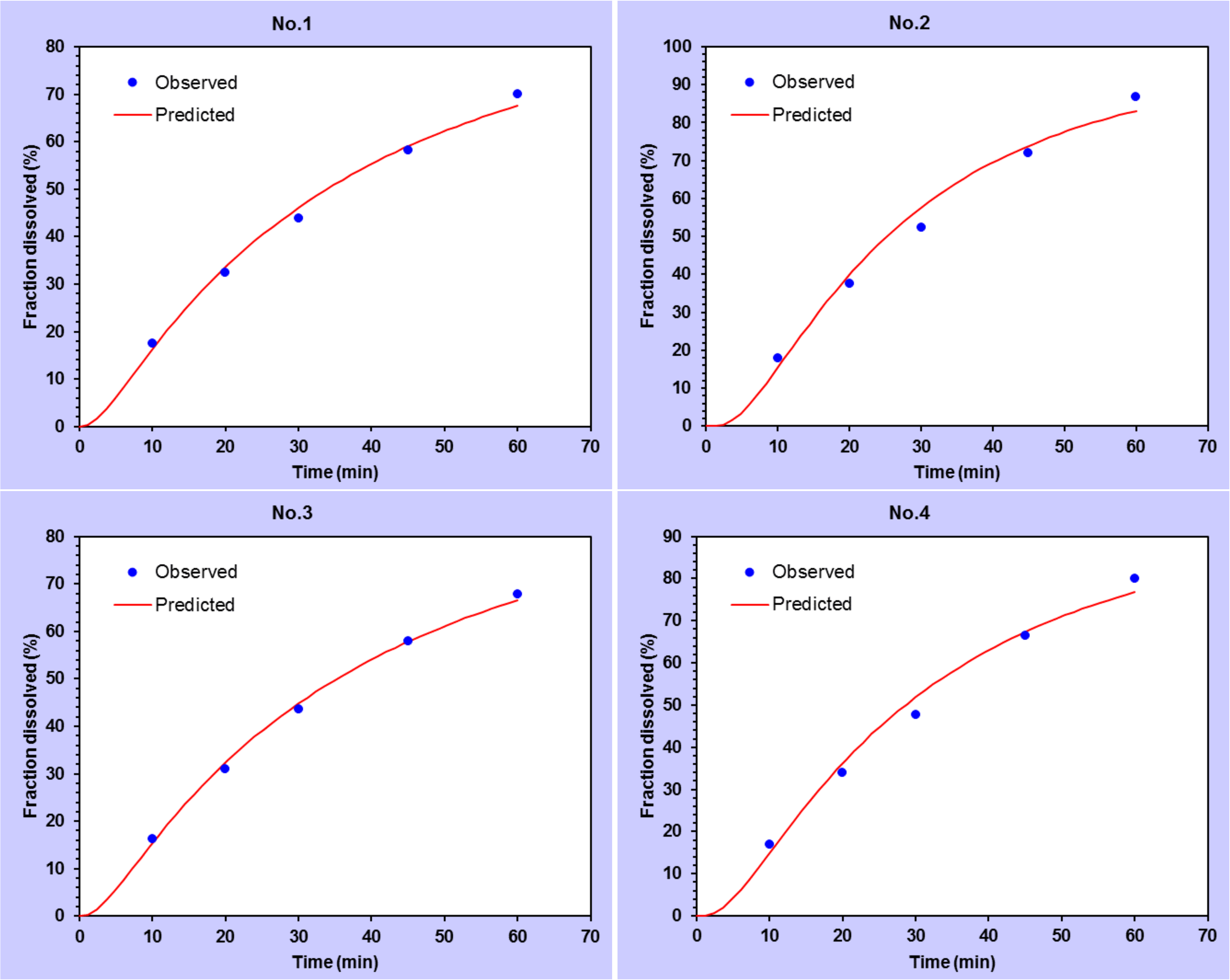

Supplementary materials – Model fitting summary – Polyglykol® 4000 P (modelling up to app. 75 % of carvedilol released)

Model: **Probit_2**

Model equation: $F = F_{max} \cdot \phi[\alpha + \beta \cdot \log(t)]$

Fitted model parameters per tested tablet (N = 4) with statistics – mean, standard deviation (SD), and relative standard deviation expressed in % (RSD%) (output from DDSolver):

Parameter	No.1	No.2	No.3	No.4	Mean	SD	RSD(%)
α	-3.781	-4.106	-3.890	-4.091	-3.967	0.158	-3.992
β	2.881	3.068	2.966	3.056	2.993	0.087	2.917
F_{max}	73.503	91.056	71.229	83.988	79.944	9.260	11.584

Number of dissolution data points (N), degrees of freedom (df), and selected goodness of fit criteria – Pearson correlation coefficient (R), coefficient of determination (R^2), adjusted coefficient of determination ($R^2_{adjusted}$), and residual sum of squares (RSS) (manual calculation in MS Excel):

Parameter	No.1	No.2	No.3	No.4
N	5	5	5	5
df	2	2	2	2
R	0.978205205	0.981387549	0.982544257	0.978718641
R^2	0.956885422	0.963121521	0.965393217	0.957890177
$R^2_{adjusted}$	0.913770845	0.926243042	0.930786433	0.915780355
RSS	87.34567662	128.862487	68.55568871	124.0676448

Graphical abstract of model fit presented as mean ± 1 SD of the fraction % of released carvedilol:

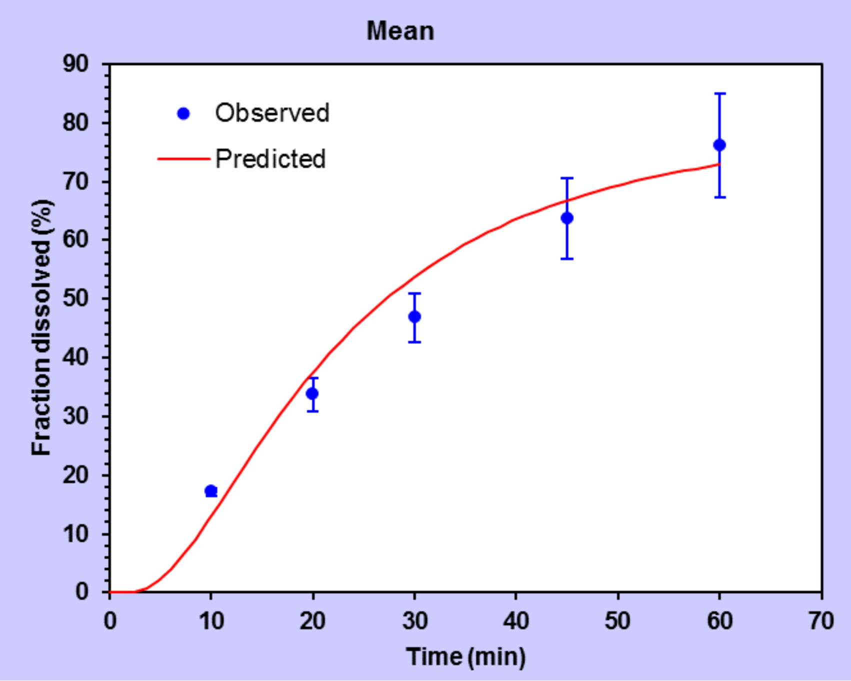

Supplementary materials – Model fitting summary – Polyglykol® 4000 P (modelling up to app. 75 % of carvedilol released)

Graphical abstract of model fit presented as the fraction % of released carvedilol per tested tablet:

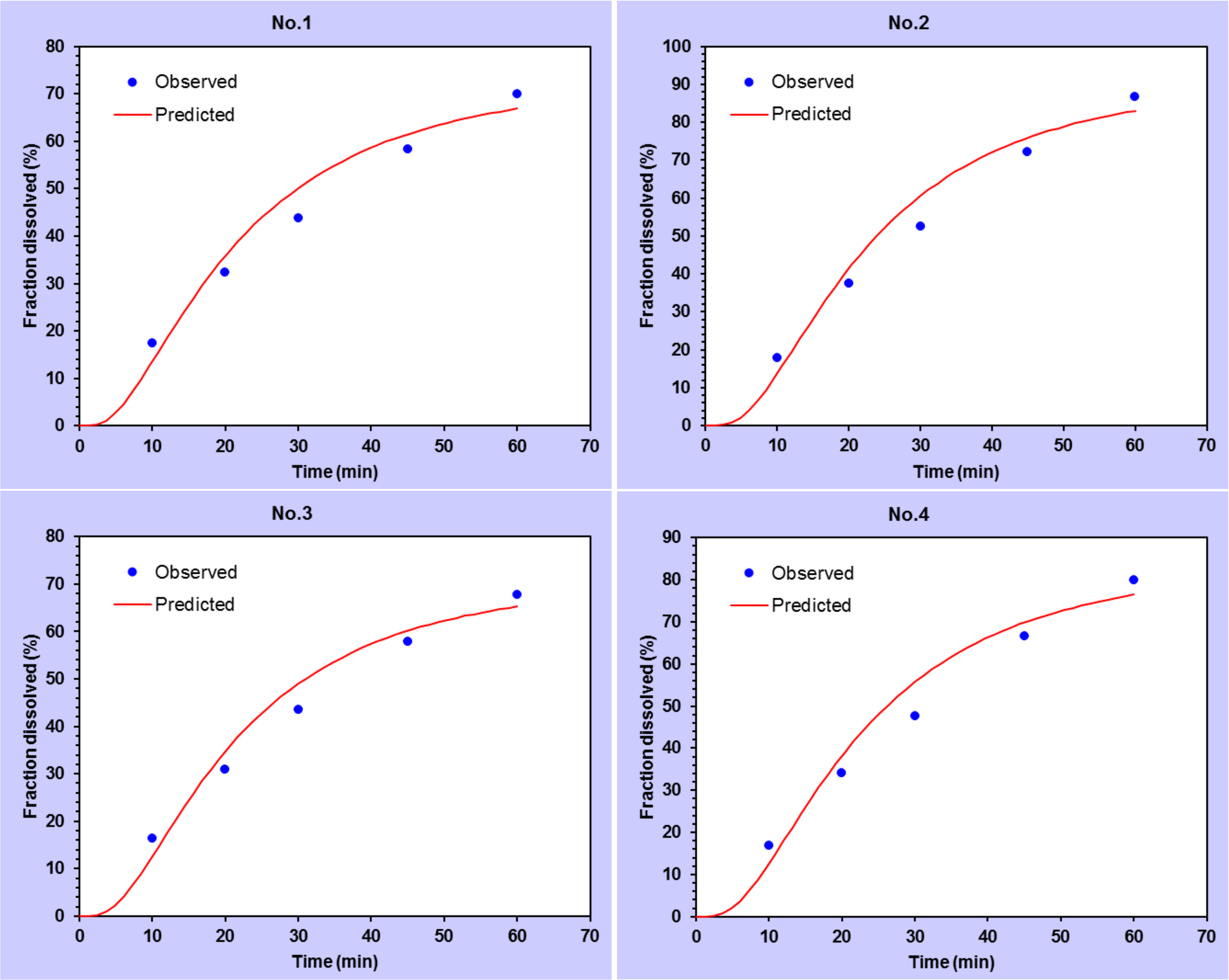

Supplement: Supplementary file 1 [file pharmaceutics-16-00498-s001.zip › Supplementary materials_Model fitting summary_Polyglykol® 4000 P.pdf]
