# Supplementary material for: Comparative Fitting of Mathematical Models to Carvedilol Release Profiles Obtained from Hypromellose Matrix Tablets
Source: Pharmaceutics. 2024 Apr 4;16(4):498. doi: 10.3390/pharmaceutics16040498 (PMC11053526; doi:10.3390/pharmaceutics16040498)

Supplementary materials – Model fitting summary – Polyglykol® 8000 P (modelling the entire relevant dissolution data)

Model: **Zero–order**

Model equation: $F = k_0 \cdot t$

Fitted model parameters per tested tablet (N = 4) with statistics – mean, standard deviation (SD), and relative standard deviation expressed in % (RSD%) (output from DDSolver):

Parameter	No.1	No.2	No.3	No.4	Mean	SD	RSD(%)
k_0	0.996	1.003	1.009	1.015	1.006	0.008	0.786

Number of dissolution data points (N), degrees of freedom (df), and selected goodness of fit criteria – Pearson correlation coefficient (R), coefficient of determination (R^2), adjusted coefficient of determination ($R^2_{adjusted}$), and residual sum of squares (RSS) (manual calculation in MS Excel):

Parameter	No.1	No.2	No.3	No.4
N	7	7	7	7
df	6	6	6	6
R	0.92498906	0.93789075	0.95081171	0.9306074
R^2	0.85560475	0.87963907	0.90404292	0.86603013
$R^2_{adjusted}$	0.85560475	0.87963907	0.90404292	0.86603013
RSS	1800.29475	1535.55595	1234.74872	1858.02436

Graphical abstract of model fit presented as mean ± 1 SD of the fraction % of released carvedilol:

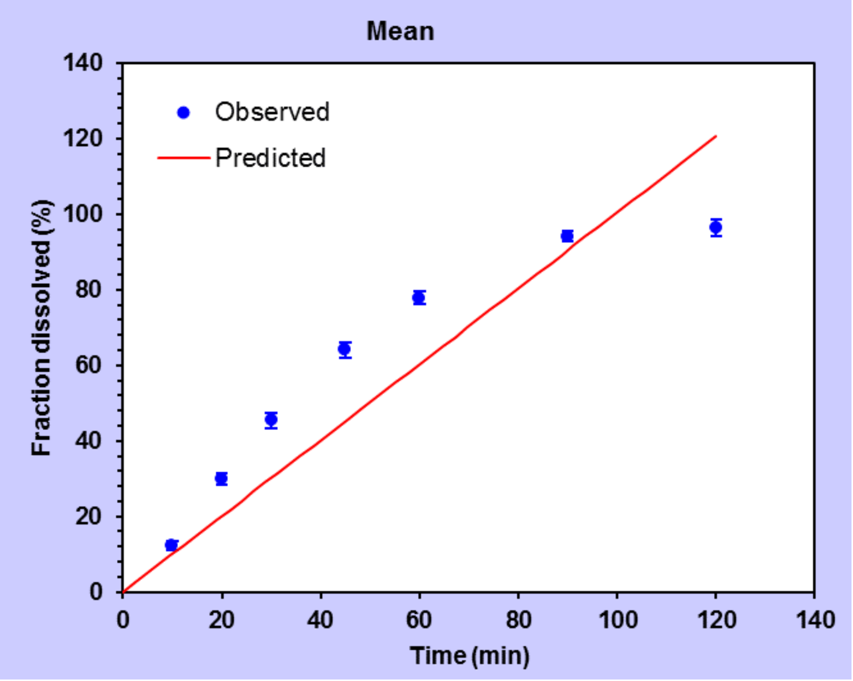

Supplementary materials – Model fitting summary – Polyglykol® 8000 P (modelling the entire relevant dissolution data)

Graphical abstract of model fit presented as the fraction % of released carvedilol per tested tablet:

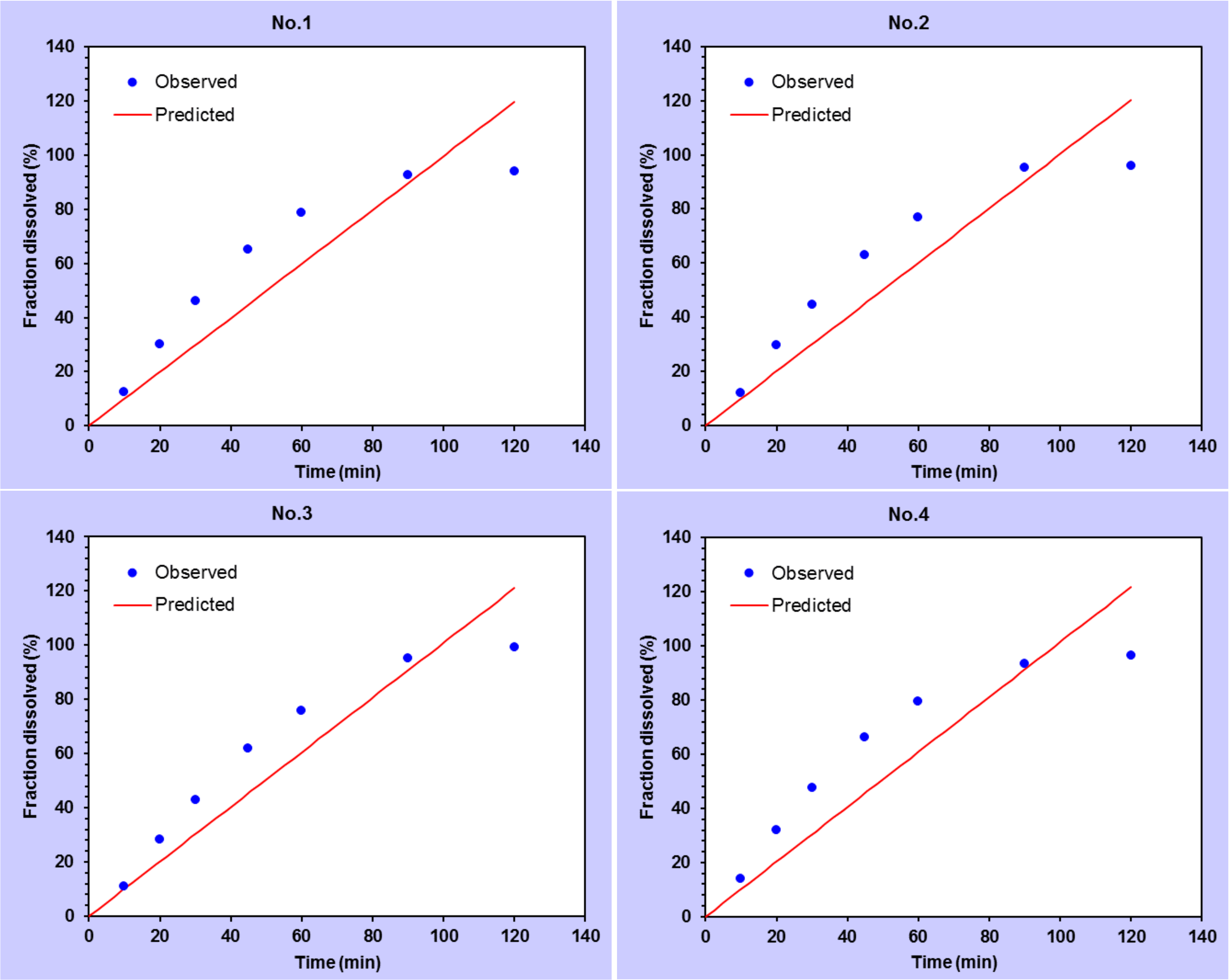

Supplementary materials – Model fitting summary – Polyglykol® 8000 P (modelling the entire relevant dissolution data)

Model: **Zero–order with T_{lag}**

Model equation: $F = k_0 \cdot (t - T_{lag})$

Fitted model parameters per tested tablet (N = 4) with statistics – mean, standard deviation (SD), and relative standard deviation expressed in % (RSD%) (output from DDSolver):

Parameter	No.1	No.2	No.3	No.4	Mean	SD	RSD(%)
k_0	0.736	0.766	0.803	0.738	0.761	0.031	4.101
T_{lag}	-27.805	-24.360	-20.229	-29.571	-25.491	4.122	-16.170

Number of dissolution data points (N), degrees of freedom (df), and selected goodness of fit criteria – Pearson correlation coefficient (R), coefficient of determination (R^2), adjusted coefficient of determination ($R^2_{adjusted}$), and residual sum of squares (RSS) (manual calculation in MS Excel):

Parameter	No.1	No.2	No.3	No.4
N	7	7	7	7
df	5	5	5	5
R	0.92498906	0.93789075	0.95081171	0.9306074
R^2	0.85560475	0.87963907	0.90404292	0.86603013
$R^2_{adjusted}$	0.82672571	0.85556688	0.8848515	0.83923616
RSS	862.984811	757.094737	645.101171	793.970524

Graphical abstract of model fit presented as mean ± 1 SD of the fraction % of released carvedilol:

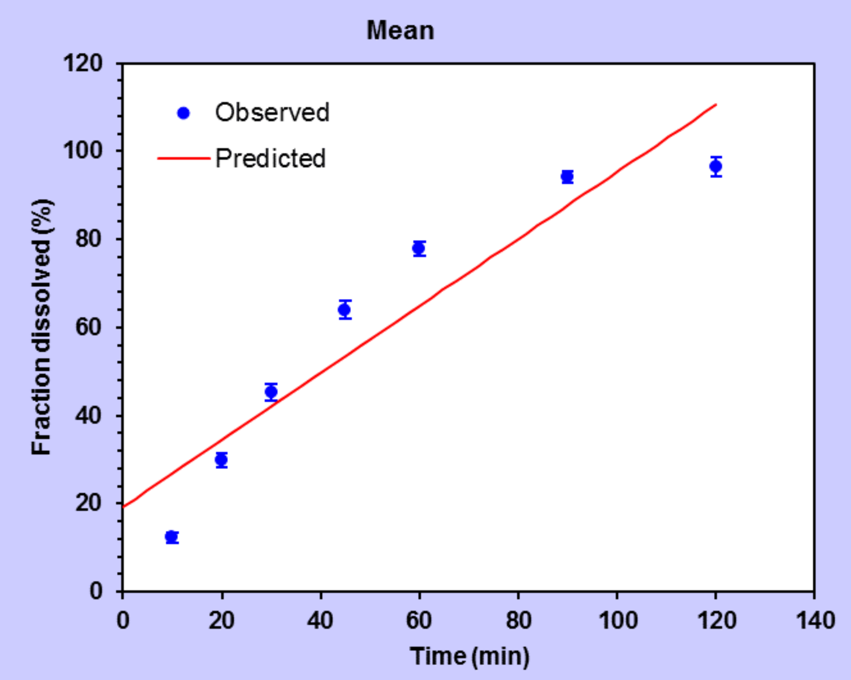

Supplementary materials – Model fitting summary – Polyglykol® 8000 P (modelling the entire relevant dissolution data)

Graphical abstract of model fit presented as the fraction % of released carvedilol per tested tablet:

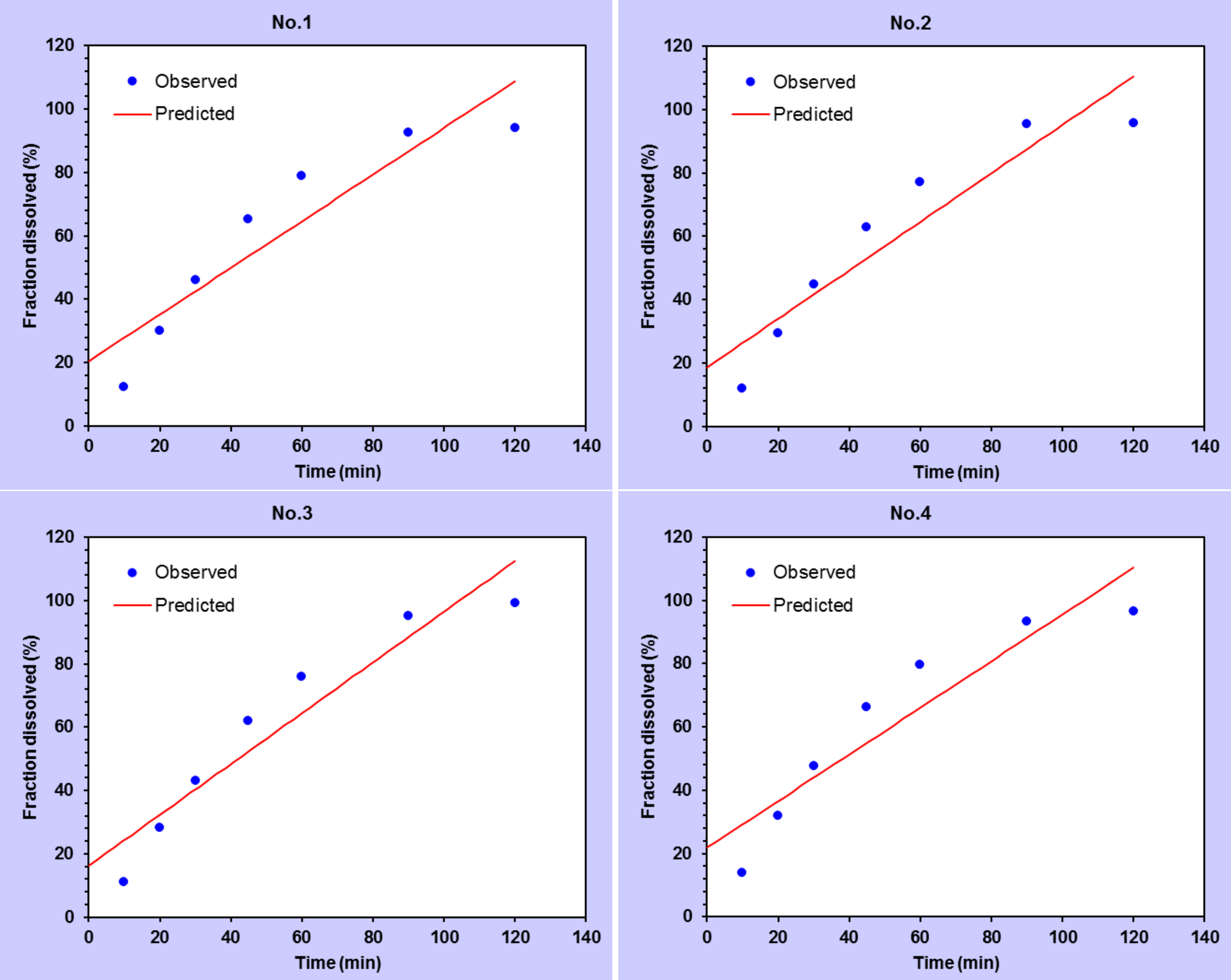

Supplementary materials – Model fitting summary – Polyglykol® 8000 P (modelling the entire relevant dissolution data)

Model: **Zero–order with F_0**

Model equation: $F = F_0 + k_0 \cdot t$

Fitted model parameters per tested tablet (N = 4) with statistics – mean, standard deviation (SD), and relative standard deviation expressed in % (RSD%) (output from DDSolver):

Parameter	No.1	No.2	No.3	No.4	Mean	SD	RSD(%)
k_0	0.736	0.766	0.803	0.738	0.761	0.031	4.101
F_0	20.469	18.654	16.235	21.809	19.292	2.413	12.510

Number of dissolution data points (N), degrees of freedom (df), and selected goodness of fit criteria – Pearson correlation coefficient (R), coefficient of determination (R^2), adjusted coefficient of determination ($R^2_{adjusted}$), and residual sum of squares (RSS) (manual calculation in MS Excel):

Parameter	No.1	No.2	No.3	No.4
N	7	7	7	7
df	5	5	5	5
R	0.92498906	0.93789075	0.95081171	0.9306074
R^2	0.85560475	0.87963907	0.90404292	0.86603013
$R^2_{adjusted}$	0.82672571	0.85556688	0.8848515	0.83923616
RSS	862.984811	757.094737	645.101171	793.970524

Graphical abstract of model fit presented as mean ± 1 SD of the fraction % of released carvedilol:

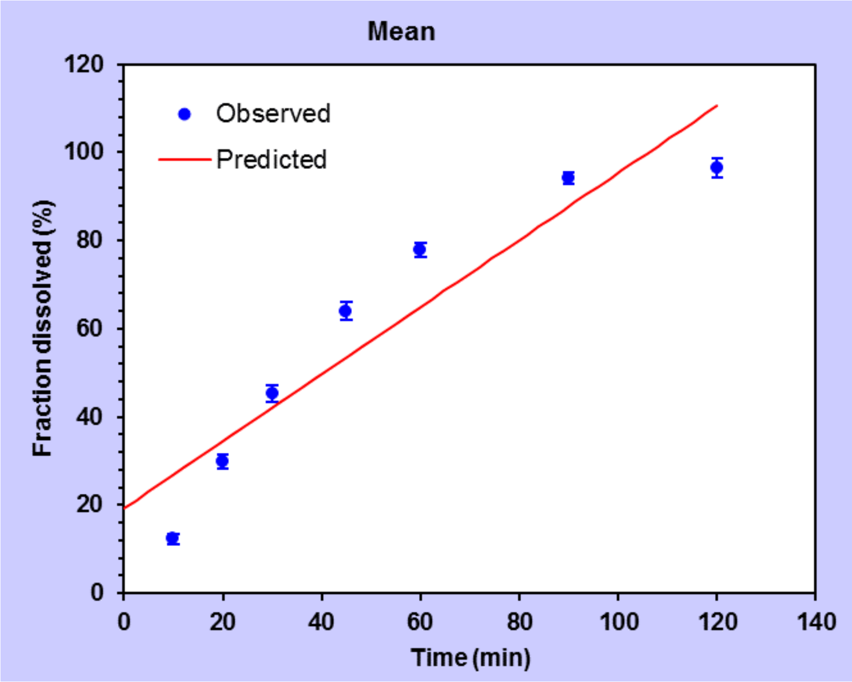

Supplementary materials – Model fitting summary – Polyglykol® 8000 P (modelling the entire relevant dissolution data)

Graphical abstract of model fit presented as the fraction % of released carvedilol per tested tablet:

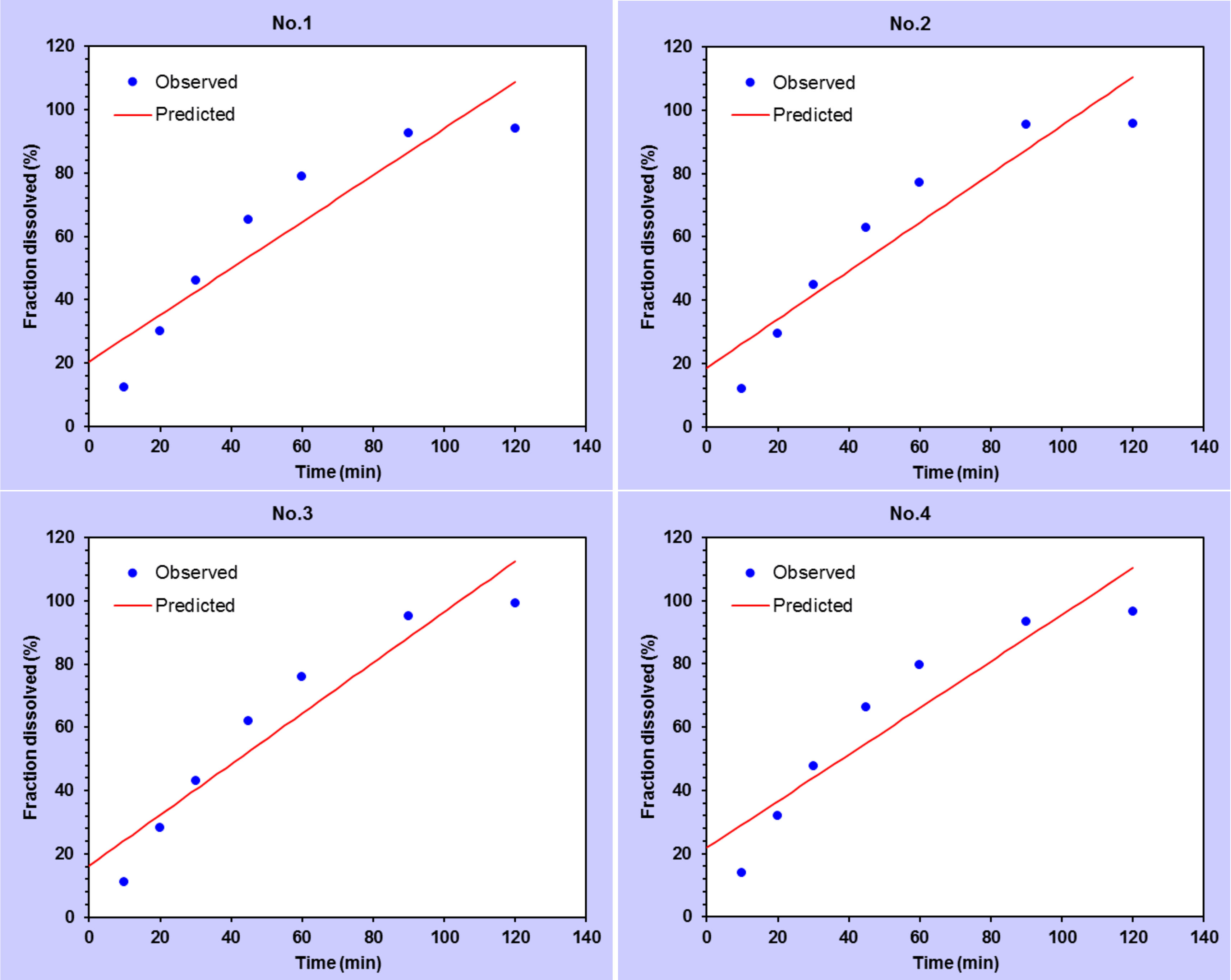

Supplementary materials – Model fitting summary – Polyglykol® 8000 P (modelling the entire relevant dissolution data)

Model: **First–order**

Model equation: $F = 100 \cdot (1 - e^{-k_1 \cdot t})$

Fitted model parameters per tested tablet (N = 4) with statistics – mean, standard deviation (SD), and relative standard deviation expressed in % (RSD%) (output from DDSolver):

Parameter	No.1	No.2	No.3	No.4	Mean	SD	RSD(%)
k_1	0.022	0.021	0.034	0.021	0.024	0.007	26.576

Number of dissolution data points (N), degrees of freedom (df), and selected goodness of fit criteria – Pearson correlation coefficient (R), coefficient of determination (R^2), adjusted coefficient of determination ($R^2_{adjusted}$), and residual sum of squares (RSS) (manual calculation in MS Excel):

Parameter	No.1	No.2	No.3	No.4
N	7	7	7	7
df	6	6	6	6
R	0.99673594	0.99711664	0.98456719	0.99727942
R^2	0.99348254	0.9942416	0.96937255	0.99456624
$R^2_{adjusted}$	0.99348254	0.9942416	0.96937255	0.99456624
RSS	170.165929	236.932529	1625.37094	223.801412

Graphical abstract of model fit presented as mean ± 1 SD of the fraction % of released carvedilol:

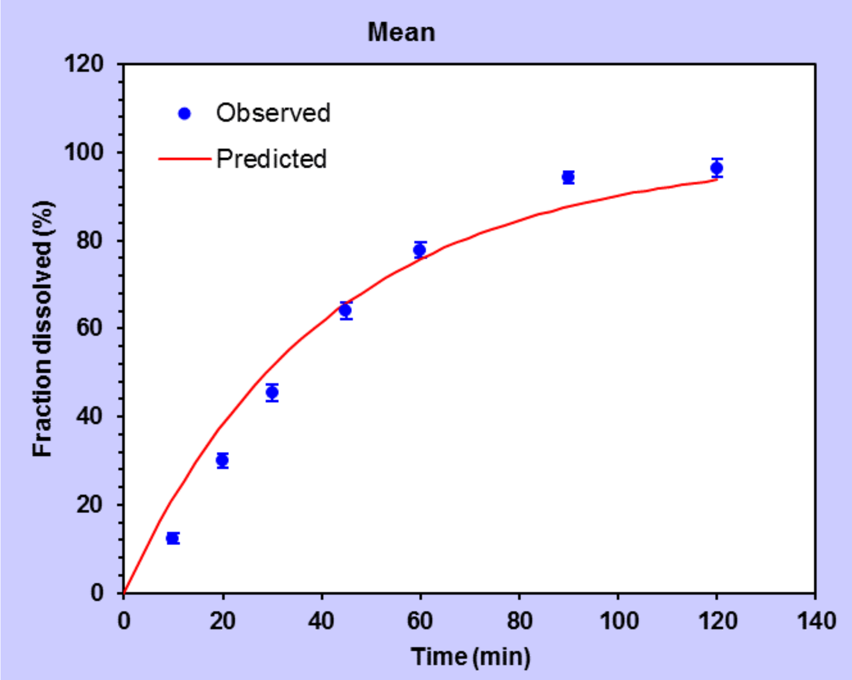

Supplementary materials – Model fitting summary – Polyglykol® 8000 P (modelling the entire relevant dissolution data)

Graphical abstract of model fit presented as the fraction % of released carvedilol per tested tablet:

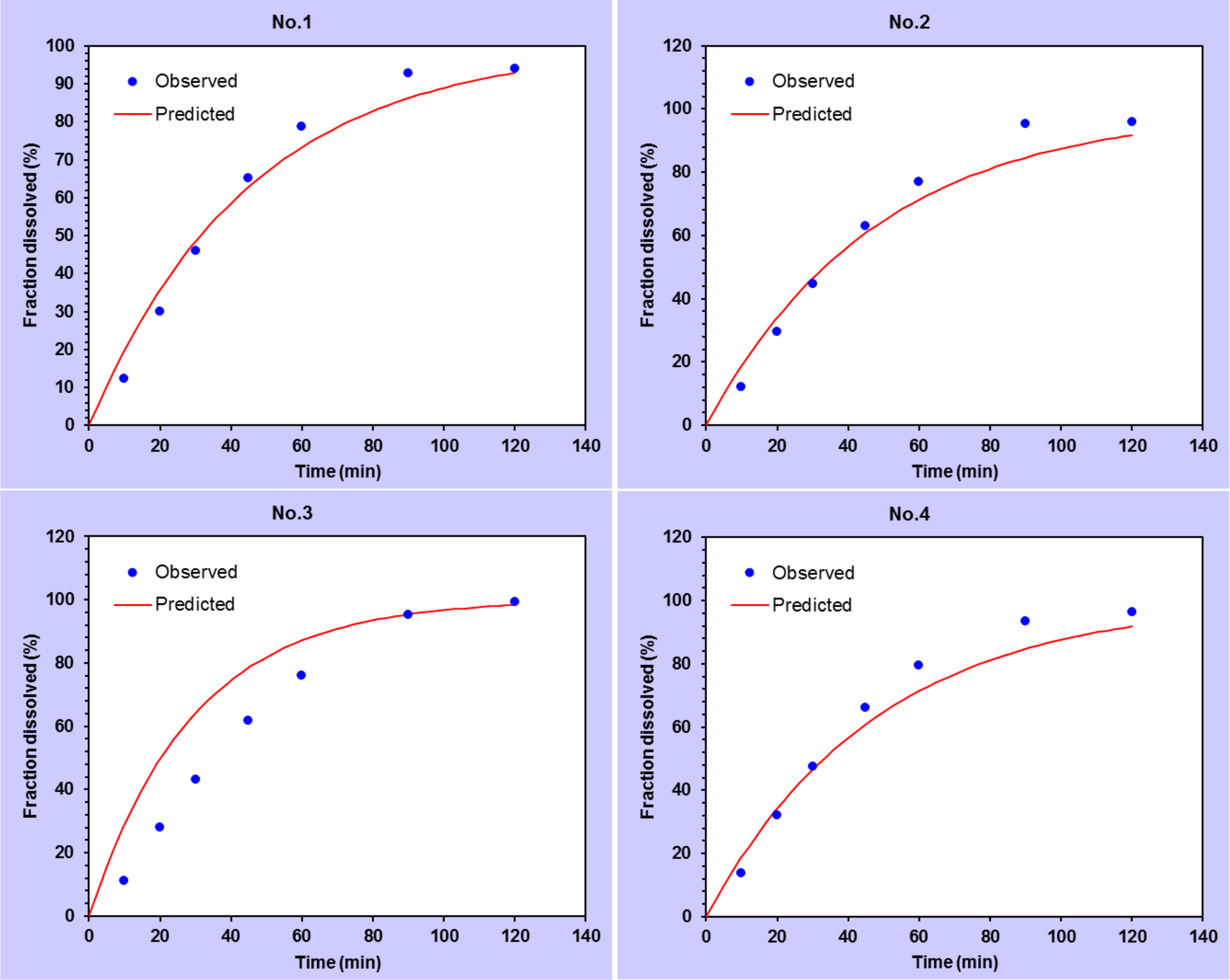

Supplementary materials – Model fitting summary – Polyglykol® 8000 P (modelling the entire relevant dissolution data)

Model: **First–order with T_{lag}**

Model equation: $F = 100 \cdot \left[1 - e^{-k_1 \cdot (t - T_{lag})}\right]$

Fitted model parameters per tested tablet (N = 4) with statistics – mean, standard deviation (SD), and relative standard deviation expressed in % (RSD%) (output from DDSolver):

Parameter	No.1	No.2	No.3	No.4	Mean	SD	RSD(%)
k_1	0.027	0.031	0.043	0.031	0.033	0.007	21.215
T_{lag}	4.621	8.598	15.948	7.158	9.081	4.864	53.561

Number of dissolution data points (N), degrees of freedom (df), and selected goodness of fit criteria – Pearson correlation coefficient (R), coefficient of determination (R^2), adjusted coefficient of determination ($R^2_{adjusted}$), and residual sum of squares (RSS) (manual calculation in MS Excel):

Parameter	No.1	No.2	No.3	No.4
N	7	7	7	7
df	5	5	5	5
R	0.99804594	0.99236587	0.966166	0.99688797
R^2	0.9960957	0.98479002	0.93347674	0.99378563
$R^2_{adjusted}$	0.99531484	0.98174802	0.92017208	0.99254276
RSS	39.1290055	119.839152	1935.31837	46.4739142

Graphical abstract of model fit presented as mean ± 1 SD of the fraction % of released carvedilol:

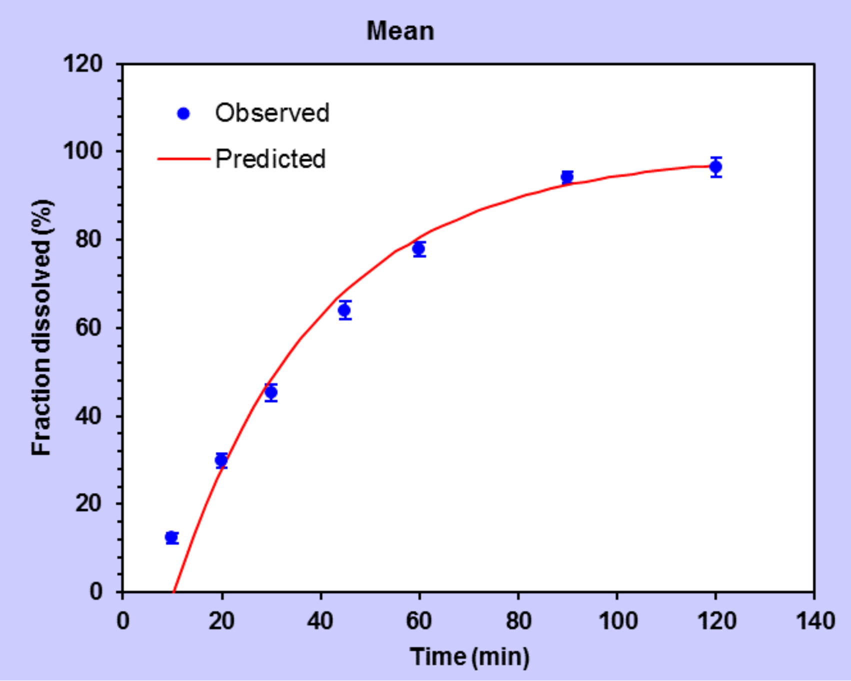

Supplementary materials – Model fitting summary – Polyglykol® 8000 P (modelling the entire relevant dissolution data)

Graphical abstract of model fit presented as the fraction % of released carvedilol per tested tablet:

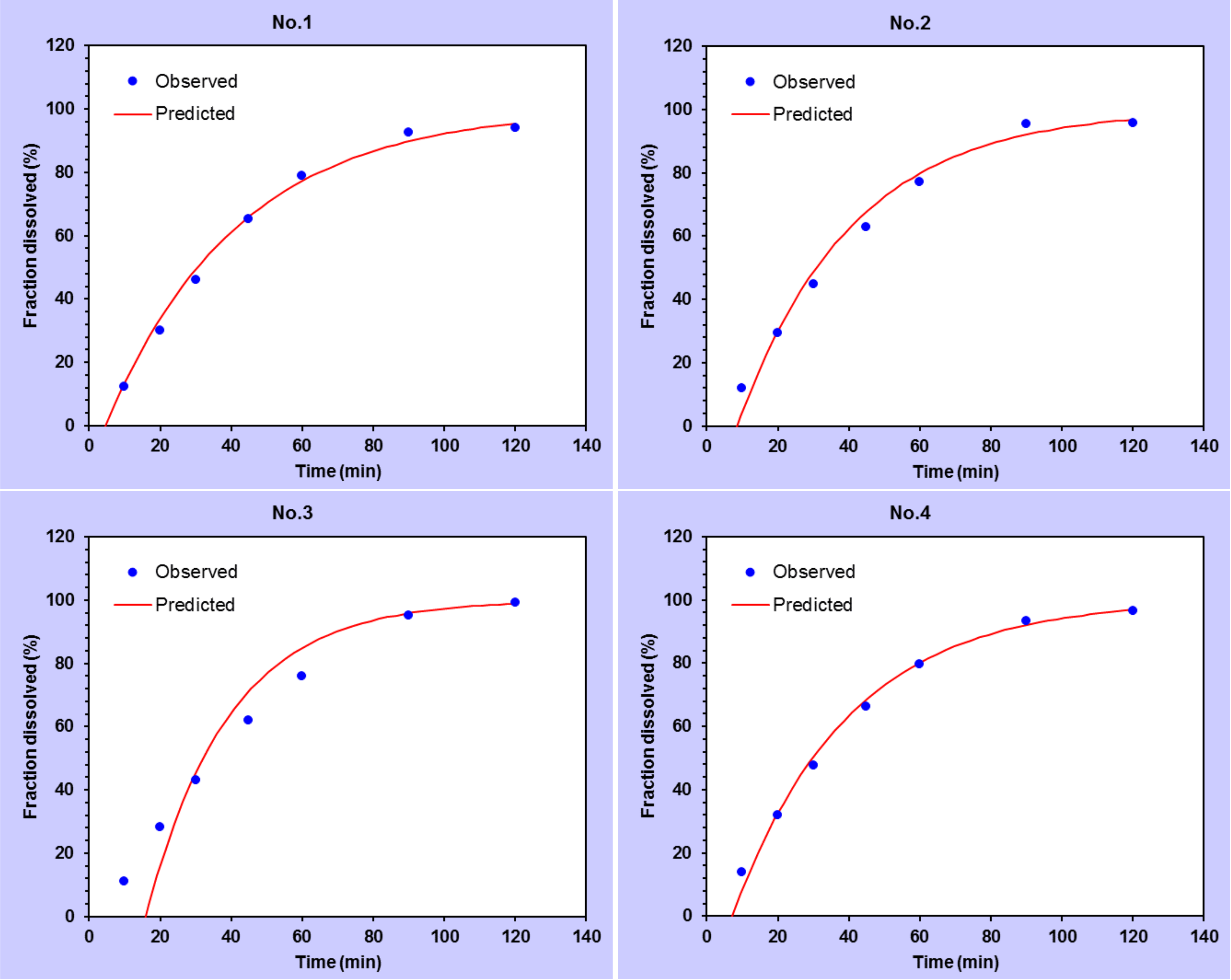

Supplementary materials – Model fitting summary – Polyglykol® 8000 P (modelling the entire relevant dissolution data)

Model: **First–order with F_{max}**

Model equation: $F = F_{max} \cdot (1 - e^{-k_1 \cdot t})$

Fitted model parameters per tested tablet (N = 4) with statistics – mean, standard deviation (SD), and relative standard deviation expressed in % (RSD%) (output from DDSolver):

Parameter	No.1	No.2	No.3	No.4	Mean	SD	RSD(%)
k_1	0.027	0.027	0.025	0.026	0.026	0.001	3.643
F_{max}	98.832	100.718	104.185	101.347	101.270	2.218	2.190

Number of dissolution data points (N), degrees of freedom (df), and selected goodness of fit criteria – Pearson correlation coefficient (R), coefficient of determination (R^2), adjusted coefficient of determination ($R^2_{adjusted}$), and residual sum of squares (RSS) (manual calculation in MS Excel):

Parameter	No.1	No.2	No.3	No.4
N	7	7	7	7
df	5	5	5	5
R	0.99805483	0.99645201	0.99721626	0.99896739
R^2	0.99611344	0.9929166	0.99444026	0.99793584
$R^2_{adjusted}$	0.99533613	0.99149992	0.99332831	0.99752301
RSS	328.783265	468.849836	509.735572	230.411777

Graphical abstract of model fit presented as mean ± 1 SD of the fraction % of released carvedilol:

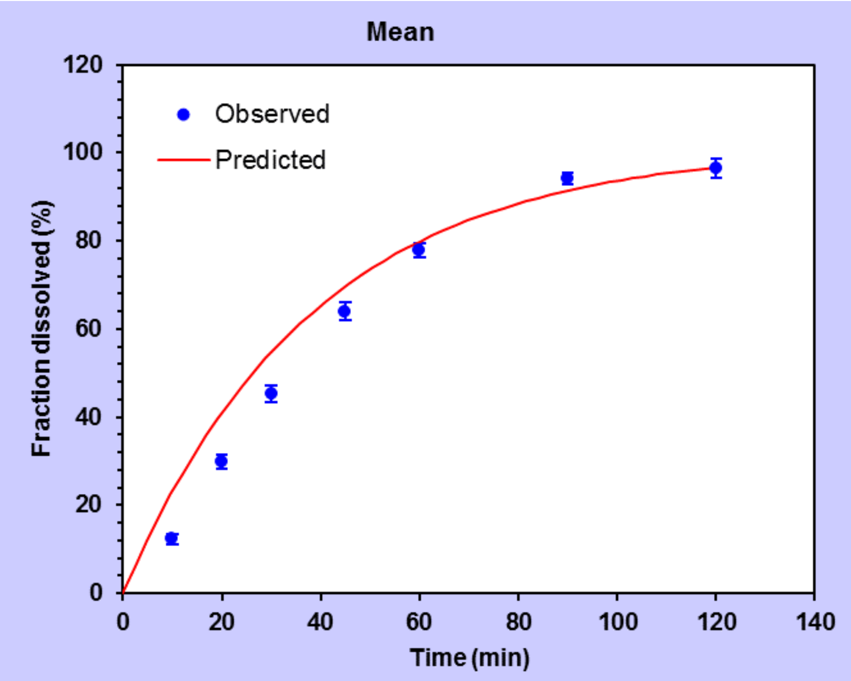

Supplementary materials – Model fitting summary – Polyglykol® 8000 P (modelling the entire relevant dissolution data)

Graphical abstract of model fit presented as the fraction % of released carvedilol per tested tablet:

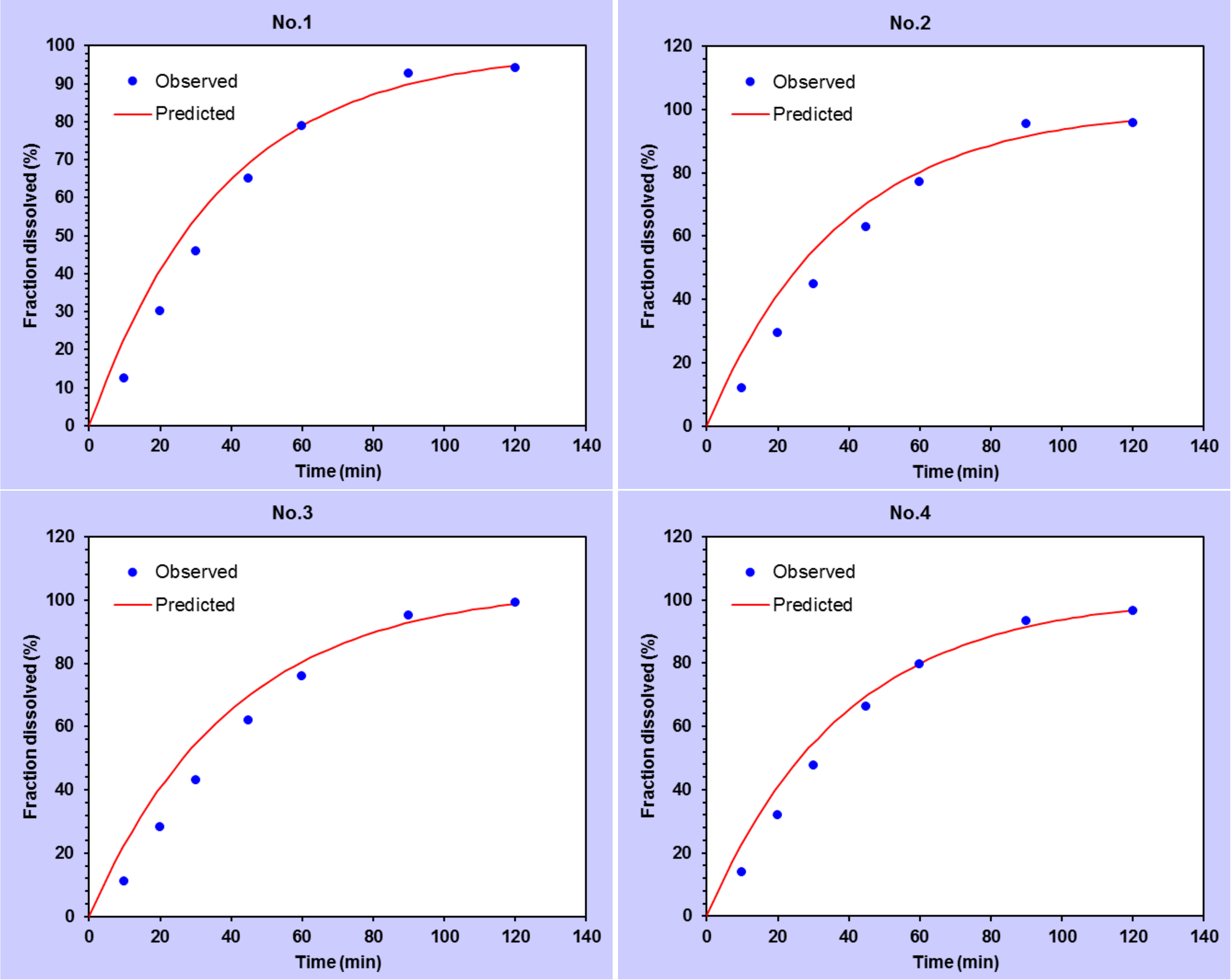

Supplementary materials – Model fitting summary – Polyglykol® 8000 P (modelling the entire relevant dissolution data)

Model: **First–order with T_{lag} and F_{max}**

Model equation: $F = F_{max} \cdot \left[1 - e^{-k_1 \cdot (t - T_{lag})}\right]$

Fitted model parameters per tested tablet (N = 4) with statistics – mean, standard deviation (SD), and relative standard deviation expressed in % (RSD%) (output from DDSolver):

Parameter	No.1	No.2	No.3	No.4	Mean	SD	RSD(%)
k_1	0.029	0.030	0.028	0.028	0.029	0.001	2.889
T_{lag}	5.866	7.800	9.356	5.433	7.114	1.815	25.509
F_{max}	98.832	100.718	104.185	101.347	101.270	2.218	2.190

Number of dissolution data points (N), degrees of freedom (df), and selected goodness of fit criteria – Pearson correlation coefficient (R), coefficient of determination (R^2), adjusted coefficient of determination ($R^2_{adjusted}$), and residual sum of squares (RSS) (manual calculation in MS Excel):

Parameter	No.1	No.2	No.3	No.4
N	7	7	7	7
df	4	4	4	4
R	0.99746683	0.99410955	0.99405146	0.99851312
R^2	0.99494007	0.98825379	0.9881383	0.99702845
$R^2_{adjusted}$	0.99241011	0.98238069	0.98220745	0.99554268
RSS	35.1934475	83.7859404	122.105377	18.2982668

Graphical abstract of model fit presented as mean ± 1 SD of the fraction % of released carvedilol:

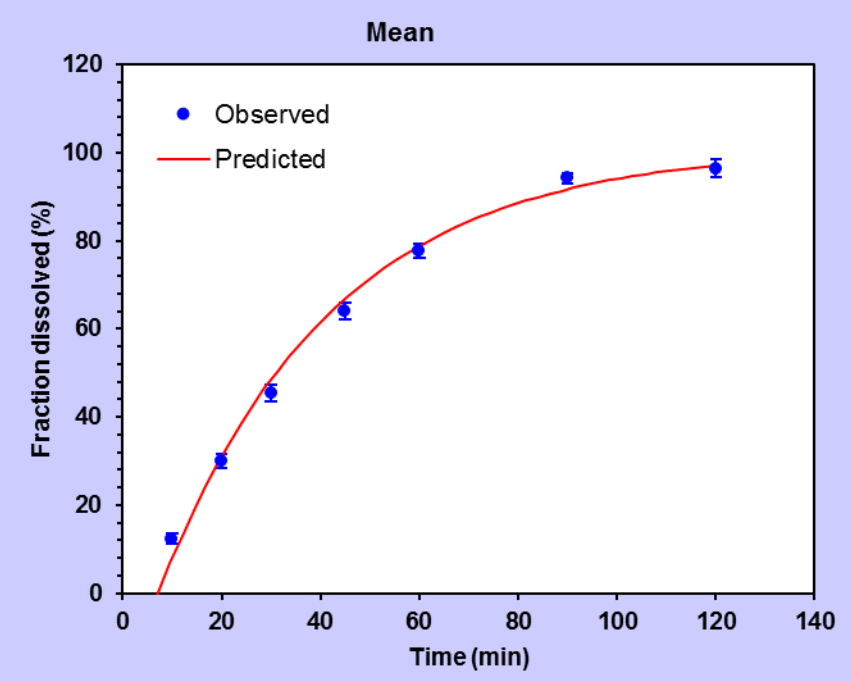

Supplementary materials – Model fitting summary – Polyglykol® 8000 P (modelling the entire relevant dissolution data)

Graphical abstract of model fit presented as the fraction % of released carvedilol per tested tablet:

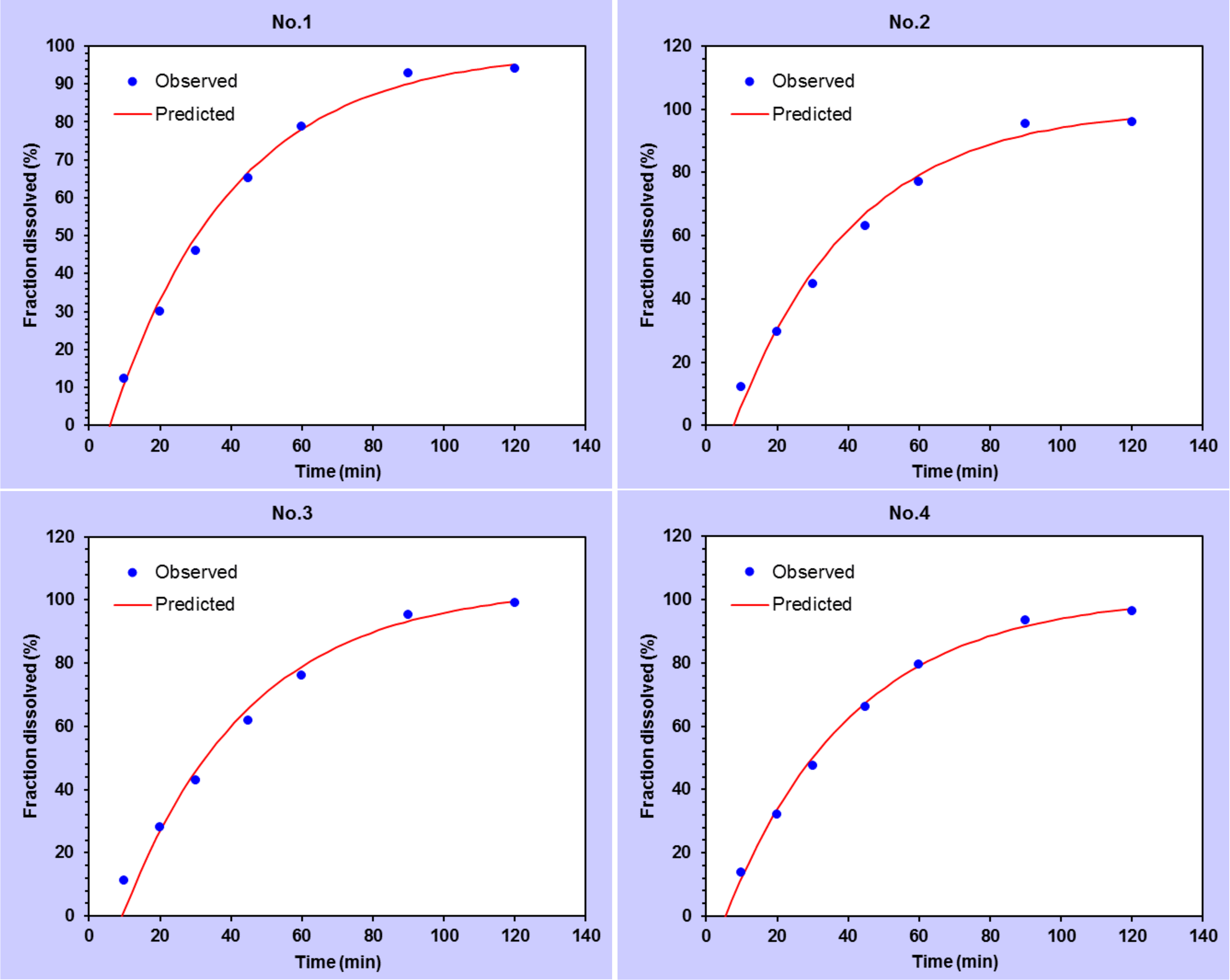

Supplementary materials – Model fitting summary – Polyglykol® 8000 P (modelling the entire relevant dissolution data)

Model: **Higuchi**

Model equation: $F = k_H \cdot t^{0.5}$

Fitted model parameters per tested tablet (N = 4) with statistics – mean, standard deviation (SD), and relative standard deviation expressed in % (RSD%) (output from DDSolver):

Parameter	No.1	No.2	No.3	No.4	Mean	SD	RSD(%)
k_H	9.025	9.041	9.042	9.206	9.078	0.085	0.939

Number of dissolution data points (N), degrees of freedom (df), and selected goodness of fit criteria – Pearson correlation coefficient (R), coefficient of determination (R^2), adjusted coefficient of determination ($R^2_{adjusted}$), and residual sum of squares (RSS) (manual calculation in MS Excel):

Parameter	No.1	No.2	No.3	No.4
N	7	7	7	7
df	6	6	6	6
R	0.97241168	0.97931158	0.98648545	0.97617007
R^2	0.94558448	0.95905116	0.97315355	0.95290801
$R^2_{adjusted}$	0.94558448	0.95905116	0.97315355	0.95290801
RSS	552.401362	568.104131	623.486808	467.935873

Graphical abstract of model fit presented as mean ± 1 SD of the fraction % of released carvedilol:

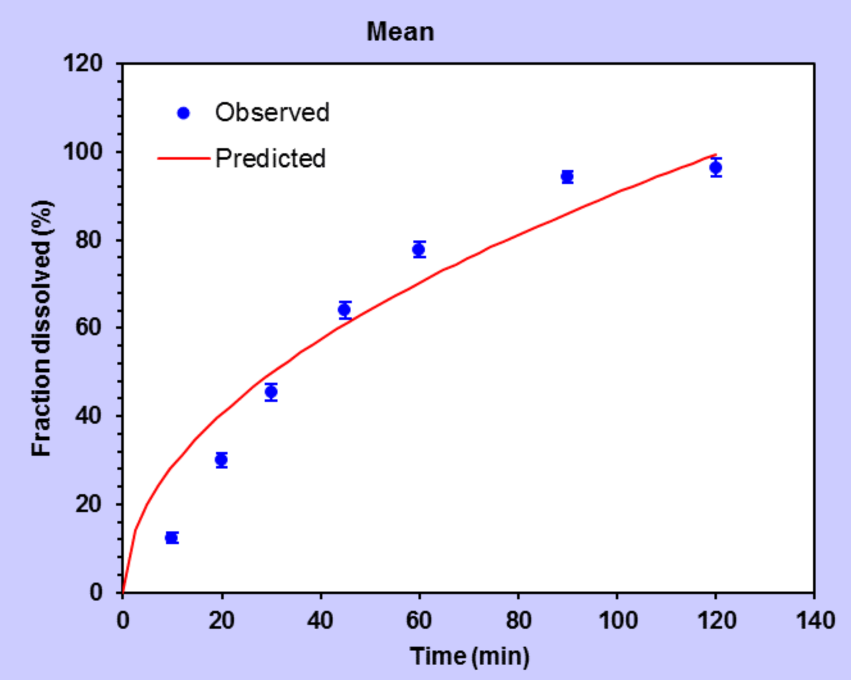

Supplementary materials – Model fitting summary – Polyglykol® 8000 P (modelling the entire relevant dissolution data)

Graphical abstract of model fit presented as the fraction % of released carvedilol per tested tablet:

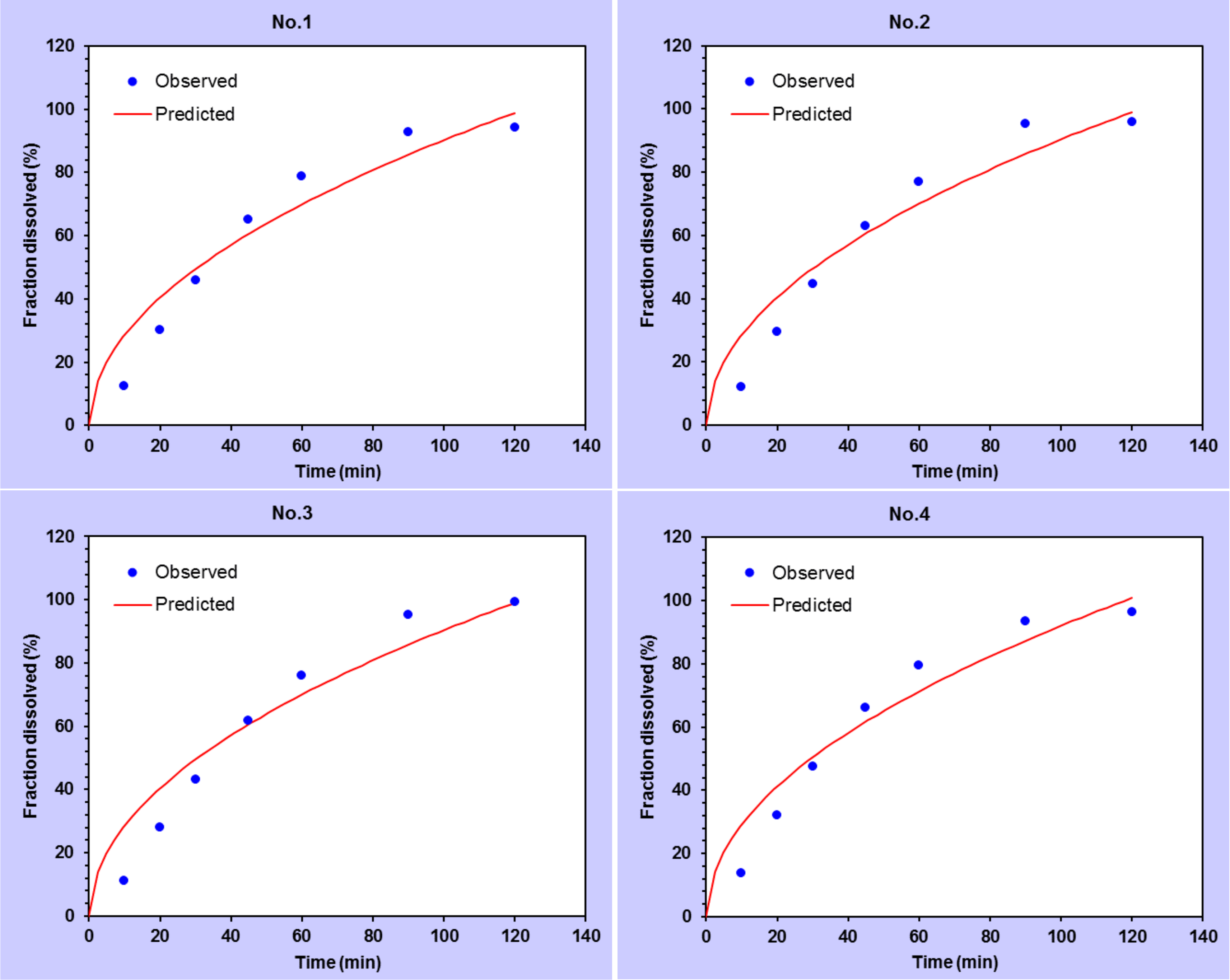

Supplementary materials – Model fitting summary – Polyglykol® 8000 P (modelling the entire relevant dissolution data)

Model: **Higuchi with T_{lag}**

Model equation: $F = k_H \cdot (t - T_{lag})^{0.5}$

Fitted model parameters per tested tablet (N = 4) with statistics – mean, standard deviation (SD), and relative standard deviation expressed in % (RSD%) (output from DDSolver):

Parameter	No.1	No.2	No.3	No.4	Mean	SD	RSD(%)
k_H	9.315	9.567	9.835	9.458	9.544	0.220	2.307
T_{lag}	3.553	7.267	7.377	3.107	5.326	2.313	43.419

Number of dissolution data points (N), degrees of freedom (df), and selected goodness of fit criteria – Pearson correlation coefficient (R), coefficient of determination (R^2), adjusted coefficient of determination ($R^2_{adjusted}$), and residual sum of squares (RSS) (manual calculation in MS Excel):

Parameter	No.1	No.2	No.3	No.4
N	7	7	7	7
df	5	5	5	5
R	0.97597149	0.98528282	0.9907275	0.97912919
R^2	0.95252034	0.97078223	0.98154097	0.95869397
$R^2_{adjusted}$	0.94302441	0.96493868	0.97784917	0.95043276
RSS	373.375188	211.490652	166.827112	322.548102

Graphical abstract of model fit presented as mean ± 1 SD of the fraction % of released carvedilol:

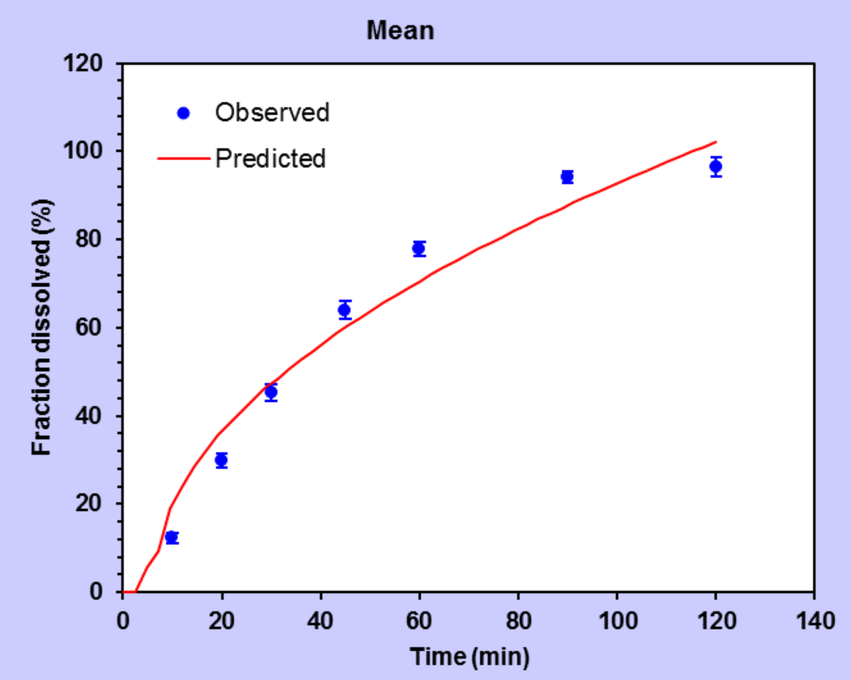

Supplementary materials – Model fitting summary – Polyglykol® 8000 P (modelling the entire relevant dissolution data)

Graphical abstract of model fit presented as the fraction % of released carvedilol per tested tablet:

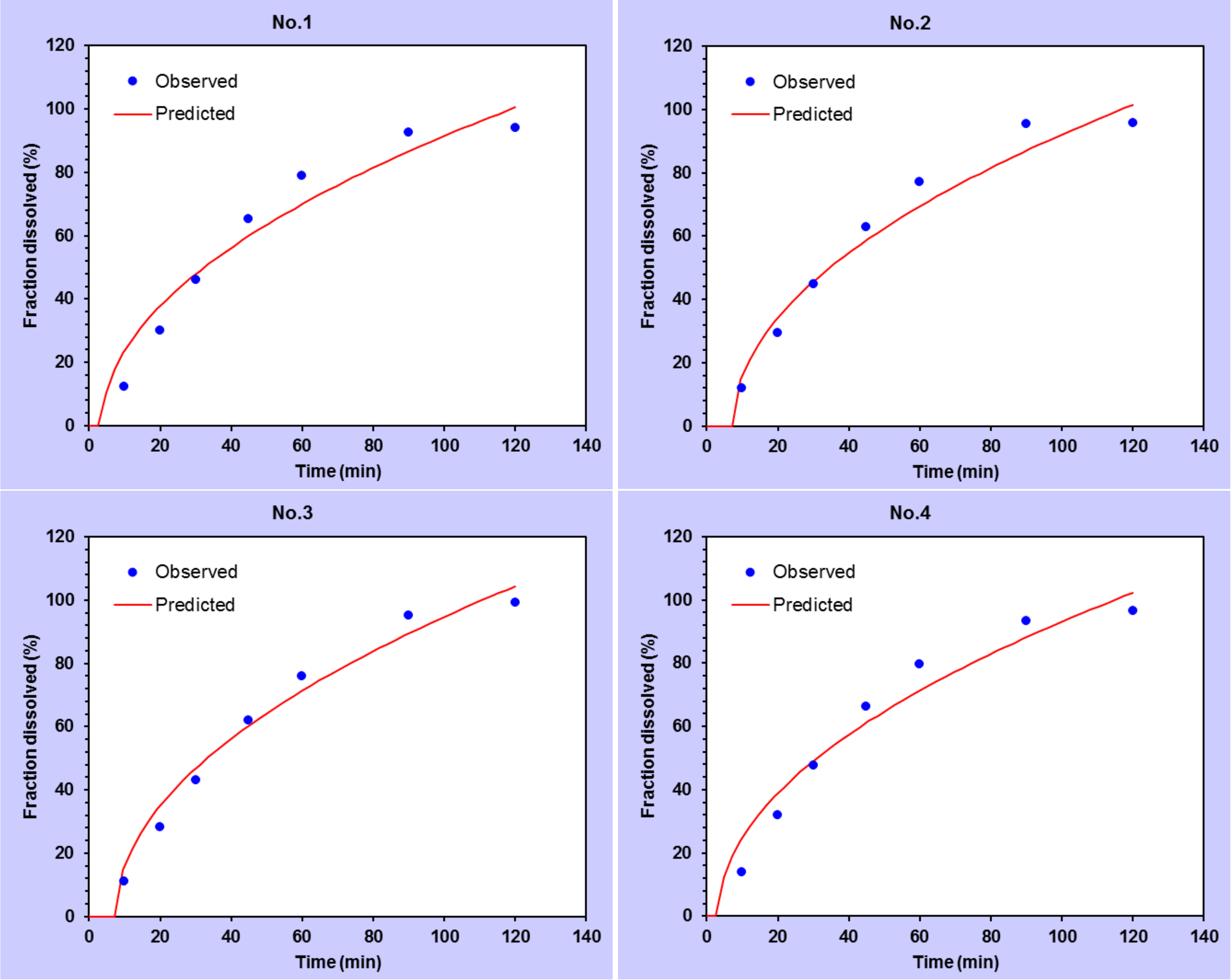

Supplementary materials – Model fitting summary – Polyglykol® 8000 P (modelling the entire relevant dissolution data)

Model: **Higuchi with F_0**

Model equation: $F = F_0 + k_H \cdot t^{0.5}$

Fitted model parameters per tested tablet (N = 4) with statistics – mean, standard deviation (SD), and relative standard deviation expressed in % (RSD%) (output from DDSolver):

Parameter	No.1	No.2	No.3	No.4	Mean	SD	RSD(%)
k_H	11.113	11.482	11.957	11.109	11.415	0.401	3.515
F_0	-16.308	-19.067	-22.773	-14.869	-18.254	3.480	-19.063

Number of dissolution data points (N), degrees of freedom (df), and selected goodness of fit criteria – Pearson correlation coefficient (R), coefficient of determination (R^2), adjusted coefficient of determination ($R^2_{adjusted}$), and residual sum of squares (RSS) (manual calculation in MS Excel):

Parameter	No.1	No.2	No.3	No.4
N	7	7	7	7
df	5	5	5	5
R	0.97241168	0.97931158	0.98648545	0.97617007
R^2	0.94558448	0.95905116	0.97315355	0.95290801
$R^2_{adjusted}$	0.93470138	0.9508614	0.96778426	0.94348962
RSS	325.216852	257.576512	180.48357	279.089992

Graphical abstract of model fit presented as mean ± 1 SD of the fraction % of released carvedilol:

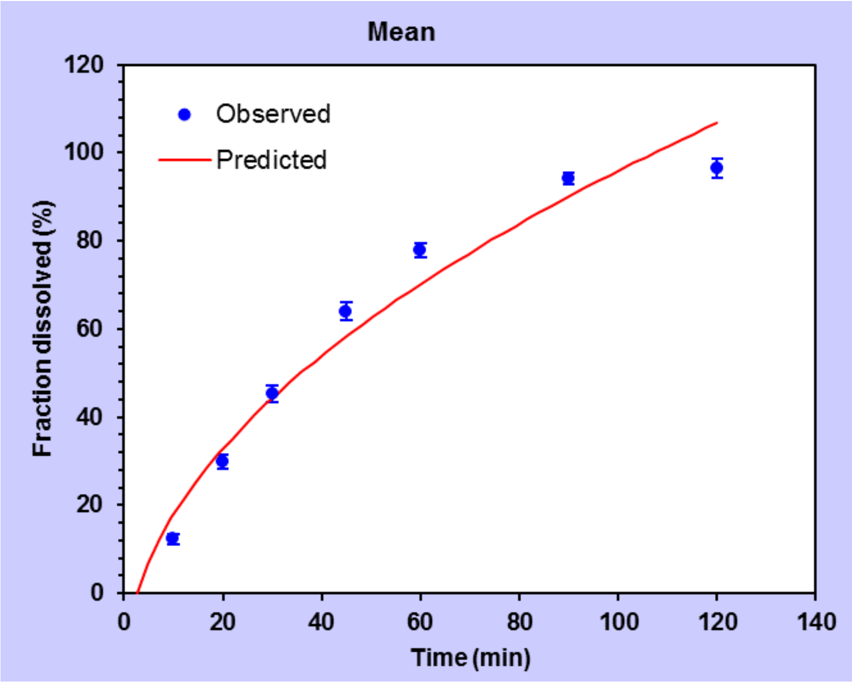

Supplementary materials – Model fitting summary – Polyglykol® 8000 P (modelling the entire relevant dissolution data)

Graphical abstract of model fit presented as the fraction % of released carvedilol per tested tablet:

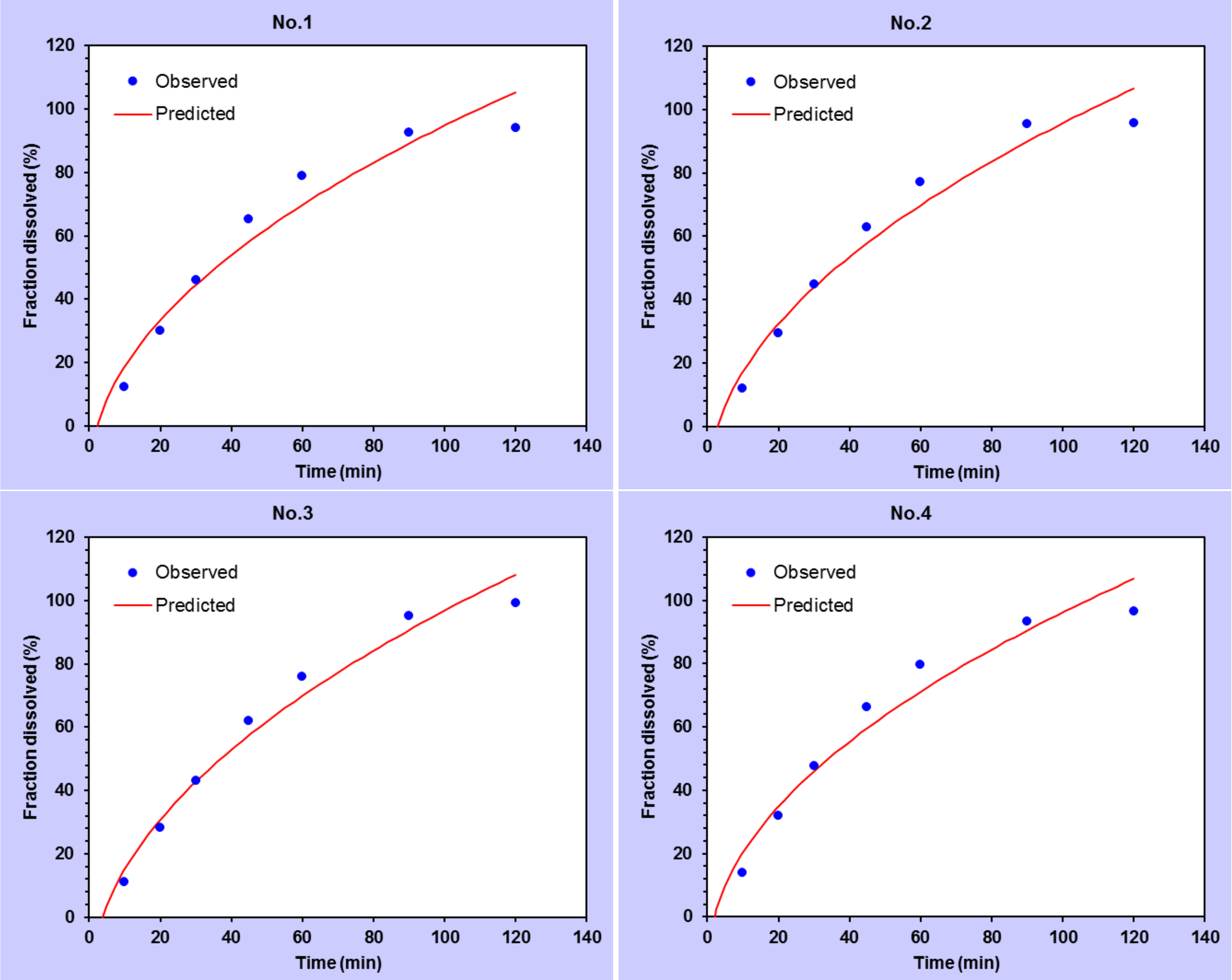

Supplementary materials – Model fitting summary – Polyglykol® 8000 P (modelling the entire relevant dissolution data)

Model: **Korsmeyer–Peppas**

Model equation: $F = k_{KP} \cdot t^n$

Fitted model parameters per tested tablet (N = 4) with statistics – mean, standard deviation (SD), and relative standard deviation expressed in % (RSD%) (output from DDSolver):

Parameter	No.1	No.2	No.3	No.4	Mean	SD	RSD(%)
k_{KP}	2.387	2.204	1.834	2.858	2.321	0.425	18.332
n	0.822	0.840	0.883	0.784	0.832	0.041	4.981

Number of dissolution data points (N), degrees of freedom (df), and selected goodness of fit criteria – Pearson correlation coefficient (R), coefficient of determination (R^2), adjusted coefficient of determination ($R^2_{adjusted}$), and residual sum of squares (RSS) (manual calculation in MS Excel):

Parameter	No.1	No.2	No.3	No.4
N	7	7	7	7
df	5	5	5	5
R	0.94396848	0.95323171	0.96091513	0.95262651
R^2	0.89107649	0.90865068	0.92335789	0.90749727
$R^2_{adjusted}$	0.86929179	0.89038082	0.90802946	0.88899672
RSS	1069.41627	950.109287	910.55497	880.890766

Graphical abstract of model fit presented as mean ± 1 SD of the fraction % of released carvedilol:

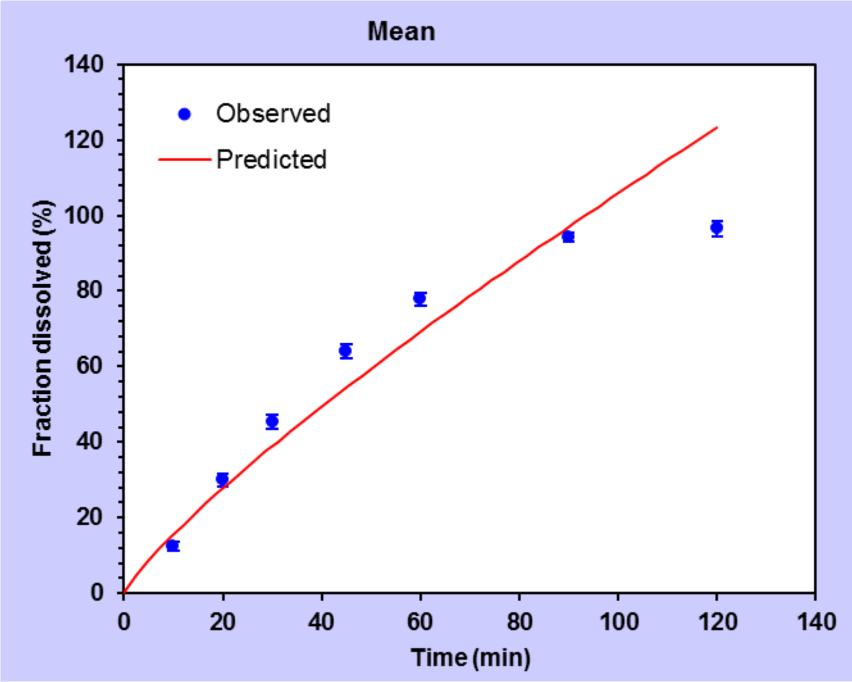

Supplementary materials – Model fitting summary – Polyglykol® 8000 P (modelling the entire relevant dissolution data)

Graphical abstract of model fit presented as the fraction % of released carvedilol per tested tablet:

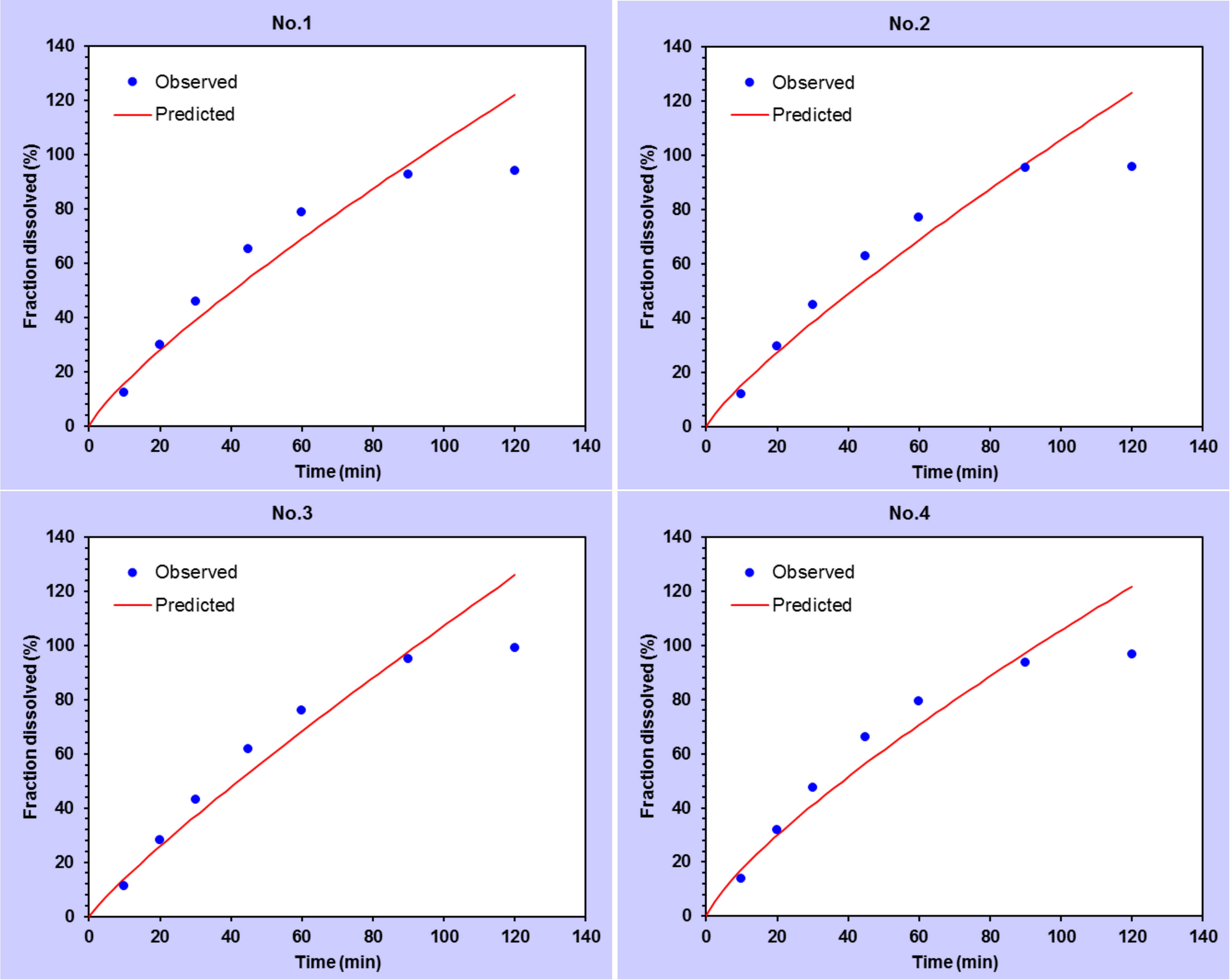

Supplementary materials – Model fitting summary – Polyglykol® 8000 P (modelling the entire relevant dissolution data)

Model: **Korsmeyer–Peppas with T_{lag}**

Model equation: $F = k_{KP} \cdot (t - T_{lag})^n$

Fitted model parameters per tested tablet (N = 4) with statistics – mean, standard deviation (SD), and relative standard deviation expressed in % (RSD%) (output from DDSolver):

Parameter	No.1	No.2	No.3	No.4	Mean	SD	RSD(%)
k_{KP}	4.094	3.840	3.295	4.790	4.005	0.621	15.499
n	0.707	0.722	0.758	0.673	0.715	0.035	4.918
T_{lag}	4.000	4.000	4.000	4.000	4.000	0.000	0.000

Number of dissolution data points (N), degrees of freedom (df), and selected goodness of fit criteria – Pearson correlation coefficient (R), coefficient of determination (R^2), adjusted coefficient of determination ($R^2_{adjusted}$), and residual sum of squares (RSS) (manual calculation in MS Excel):

Parameter	No.1	No.2	No.3	No.4
N	7	7	7	7
df	4	4	4	4
R	0.95835777	0.96614569	0.97277815	0.96597169
R^2	0.91844962	0.93343749	0.94629733	0.93310131
$R^2_{adjusted}$	0.87767444	0.90015623	0.919446	0.89965197
RSS	741.810072	633.847902	579.701837	591.739756

Graphical abstract of model fit presented as mean ± 1 SD of the fraction % of released carvedilol:

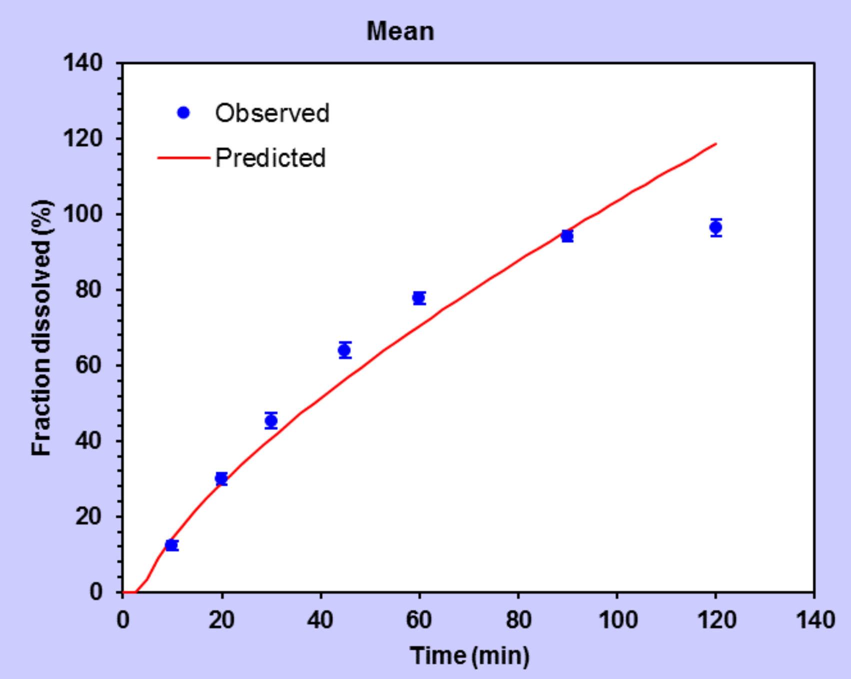

Supplementary materials – Model fitting summary – Polyglykol® 8000 P (modelling the entire relevant dissolution data)

Graphical abstract of model fit presented as the fraction % of released carvedilol per tested tablet:

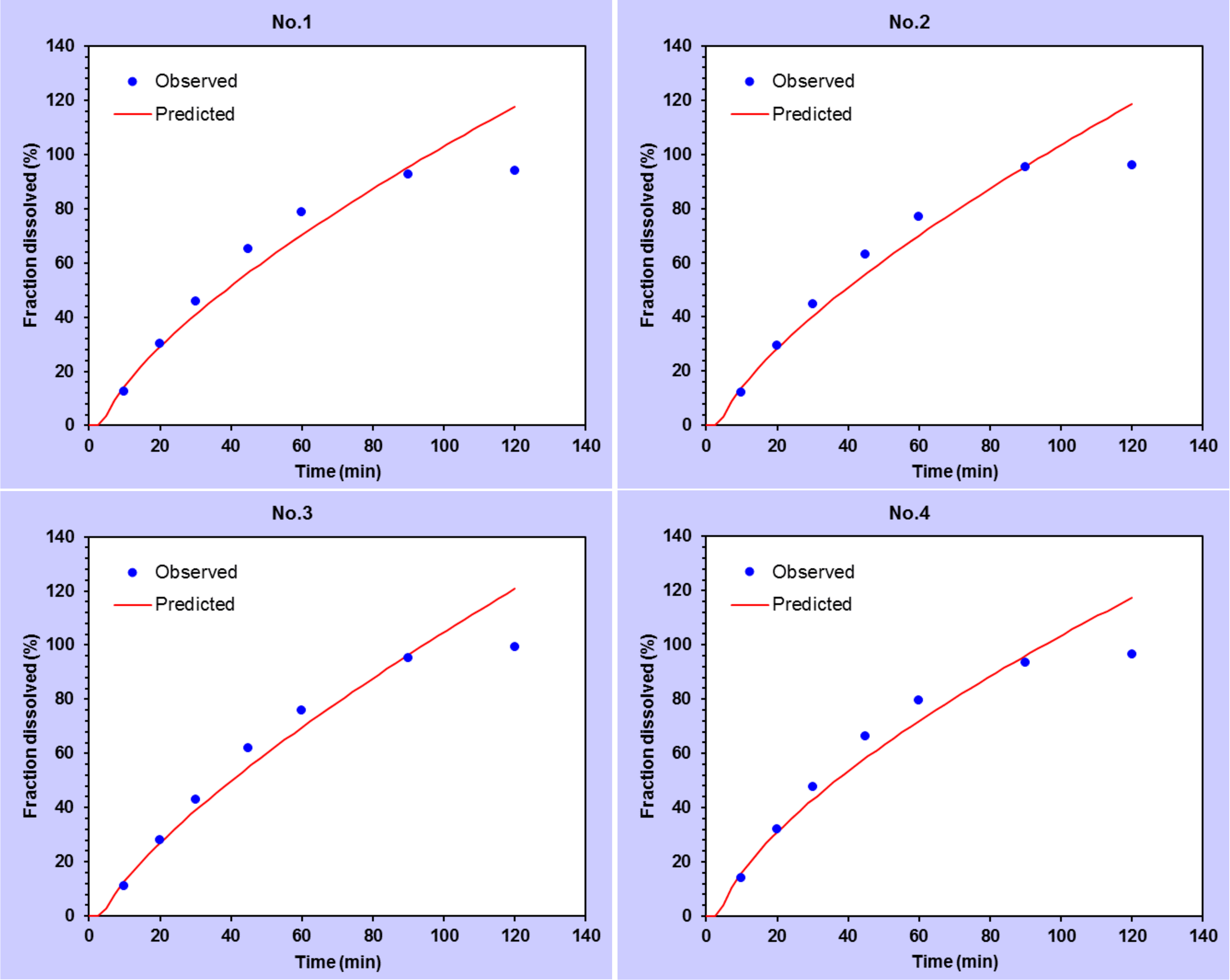

Supplementary materials – Model fitting summary – Polyglykol® 8000 P (modelling the entire relevant dissolution data)

Model: **Korsmeyer–Peppas with F_0**

Model equation: $F = F_0 + k_{KP} \cdot t^n$

Fitted model parameters per tested tablet (N = 4) with statistics – mean, standard deviation (SD), and relative standard deviation expressed in % (RSD%) (output from DDSolver):

Parameter	No.1	No.2	No.3	No.4	Mean	SD	RSD(%)
k_{KP}	1.125	1.036	0.862	1.342	1.091	0.199	18.271
n	0.985	1.004	1.048	0.945	0.995	0.043	4.293
F_0	4.959	4.839	4.439	5.559	4.949	0.464	9.366

Number of dissolution data points (N), degrees of freedom (df), and selected goodness of fit criteria – Pearson correlation coefficient (R), coefficient of determination (R^2), adjusted coefficient of determination ($R^2_{adjusted}$), and residual sum of squares (RSS) (manual calculation in MS Excel):

Parameter	No.1	No.2	No.3	No.4
N	7	7	7	7
df	4	4	4	4
R	0.9266936	0.93749753	0.94642805	0.93640712
R^2	0.85876103	0.87890162	0.89572605	0.87685829
$R^2_{adjusted}$	0.78814155	0.81835243	0.84358907	0.81528744
RSS	1725.22299	1580.44372	1572.58946	1456.28391

Graphical abstract of model fit presented as mean ± 1 SD of the fraction % of released carvedilol:

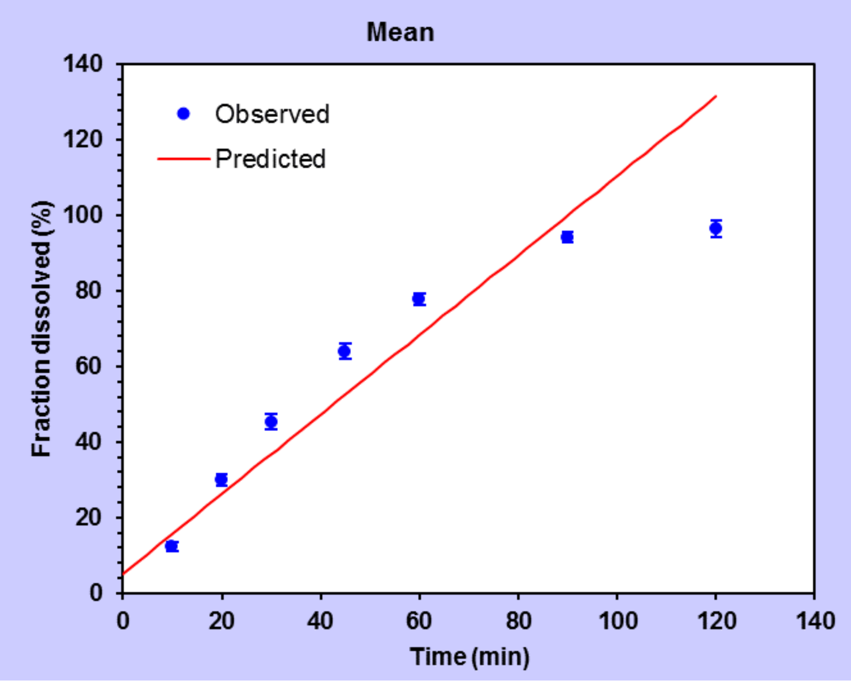

Supplementary materials – Model fitting summary – Polyglykol® 8000 P (modelling the entire relevant dissolution data)

Graphical abstract of model fit presented as the fraction % of released carvedilol per tested tablet:

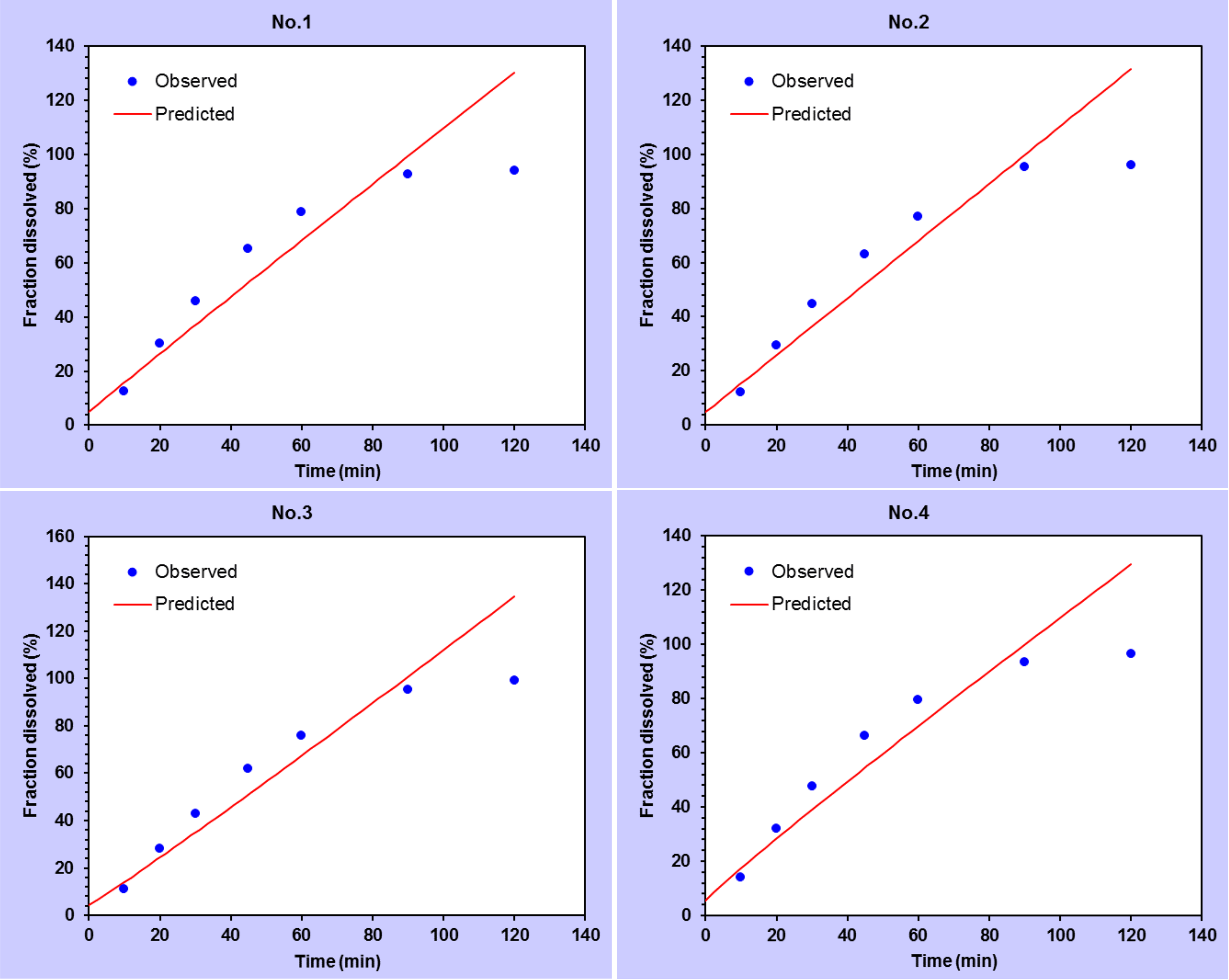

Supplementary materials – Model fitting summary – Polyglykol® 8000 P (modelling the entire relevant dissolution data)

Model: **Hixson–Crowell**

Model equation: $F = 100 \cdot [1 - (1 - k_{HC} \cdot t)^3]$

Fitted model parameters per tested tablet (N = 4) with statistics – mean, standard deviation (SD), and relative standard deviation expressed in % (RSD%) (output from DDSolver):

Parameter	No.1	No.2	No.3	No.4	Mean	SD	RSD(%)
k_{HC}	0.006	0.006	0.006	0.006	0.006	0.000	3.052

Number of dissolution data points (N), degrees of freedom (df), and selected goodness of fit criteria – Pearson correlation coefficient (R), coefficient of determination (R^2), adjusted coefficient of determination ($R^2_{adjusted}$), and residual sum of squares (RSS) (manual calculation in MS Excel):

Parameter	No.1	No.2	No.3	No.4
N	7	7	7	7
df	6	6	6	6
R	0.99236759	0.99713369	0.99864323	0.99614519
R^2	0.98479343	0.99427559	0.9972883	0.99230524
$R^2_{adjusted}$	0.98479343	0.99427559	0.9972883	0.99230524
RSS	115.493066	66.6498342	94.5332225	62.1054443

Graphical abstract of model fit presented as mean ± 1 SD of the fraction % of released carvedilol:

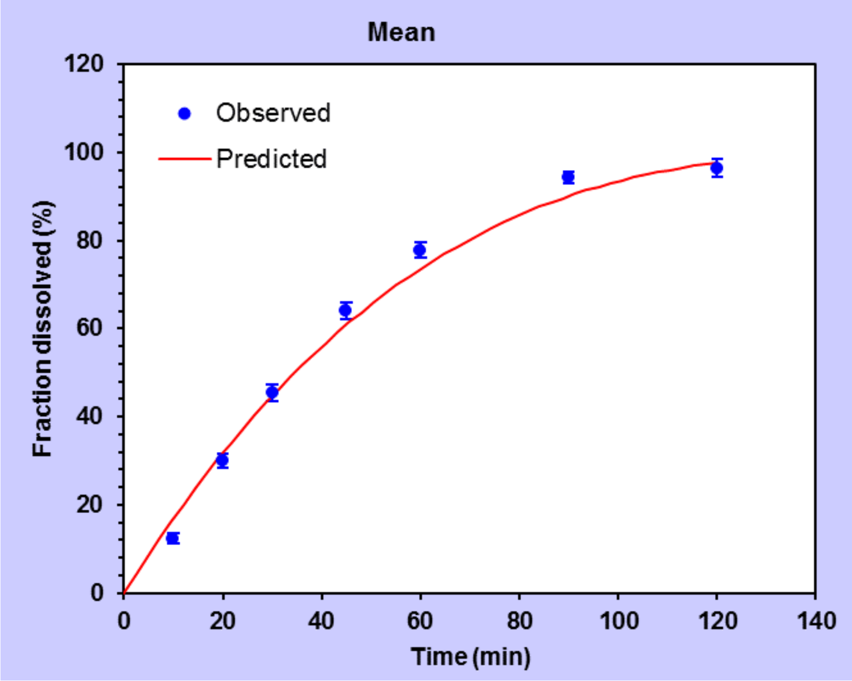

Supplementary materials – Model fitting summary – Polyglykol® 8000 P (modelling the entire relevant dissolution data)

Graphical abstract of model fit presented as the fraction % of released carvedilol per tested tablet:

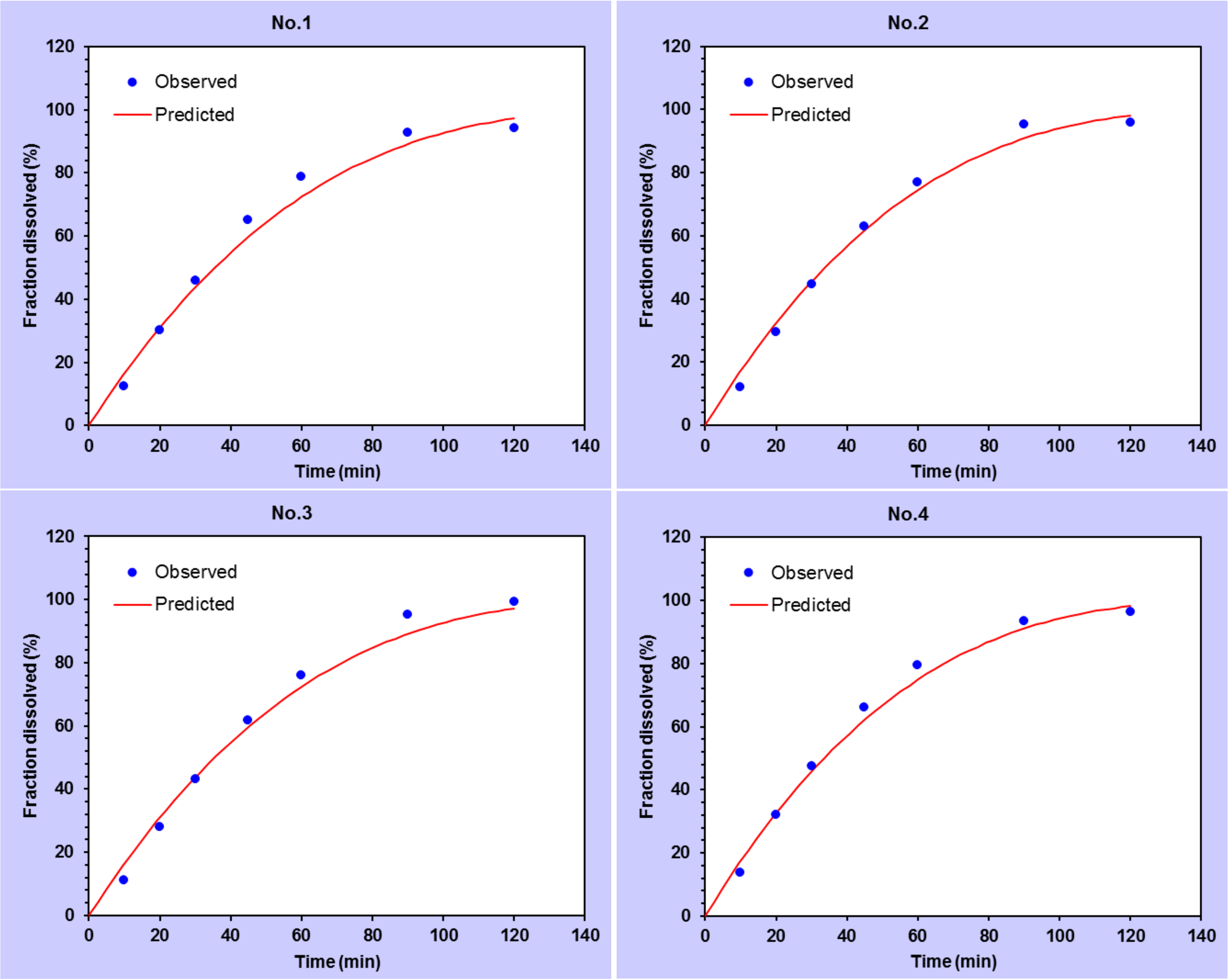

Supplementary materials – Model fitting summary – Polyglykol® 8000 P (modelling the entire relevant dissolution data)

Model: **Hixson–Crowell with T_{lag}**

Model equation: $F = 100 \cdot \left\{1 - \left[1 - k_{HC} \cdot \left(t - T_{lag}\right)\right]^3\right\}$

Fitted model parameters per tested tablet (N = 4) with statistics – mean, standard deviation (SD), and relative standard deviation expressed in % (RSD%) (output from DDSolver):

Parameter	No.1	No.2	No.3	No.4	Mean	SD	RSD(%)
k_{HC}	0.005	0.006	0.007	0.006	0.006	0.001	9.637
T_{lag}	-4.524	-0.571	4.373	-3.150	-0.968	3.920	-404.941

Number of dissolution data points (N), degrees of freedom (df), and selected goodness of fit criteria – Pearson correlation coefficient (R), coefficient of determination (R^2), adjusted coefficient of determination ($R^2_{adjusted}$), and residual sum of squares (RSS) (manual calculation in MS Excel):

Parameter	No.1	No.2	No.3	No.4
N	7	7	7	7
df	5	5	5	5
R	0.99098858	0.9970395	0.99960187	0.99542846
R^2	0.98205837	0.99408776	0.9992039	0.99087782
$R^2_{adjusted}$	0.97847004	0.99290532	0.99904468	0.98905338
RSS	189.576743	79.0793133	7.31561059	106.402104

Graphical abstract of model fit presented as mean ± 1 SD of the fraction % of released carvedilol:

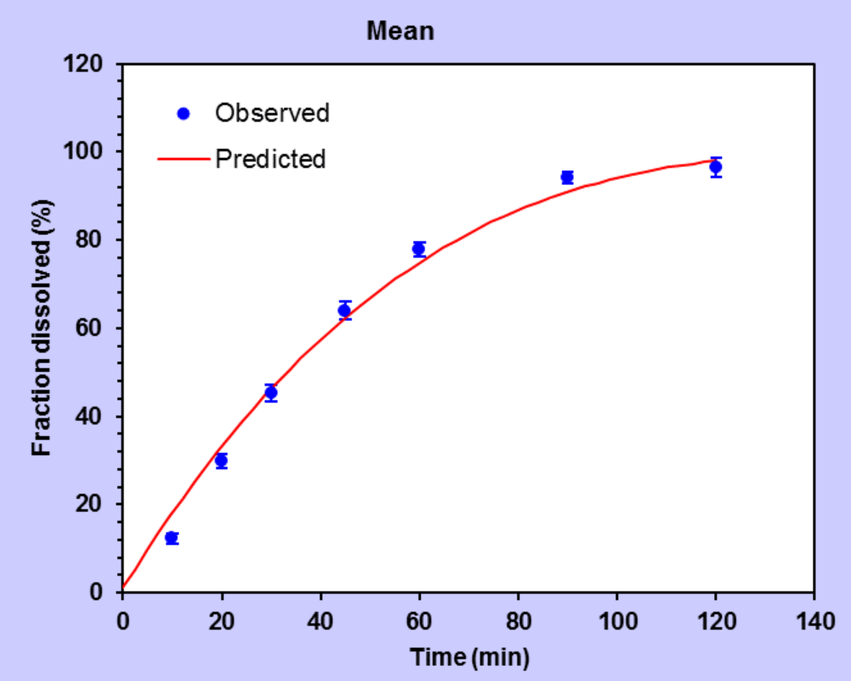

Supplementary materials – Model fitting summary – Polyglykol® 8000 P (modelling the entire relevant dissolution data)

Graphical abstract of model fit presented as the fraction % of released carvedilol per tested tablet:

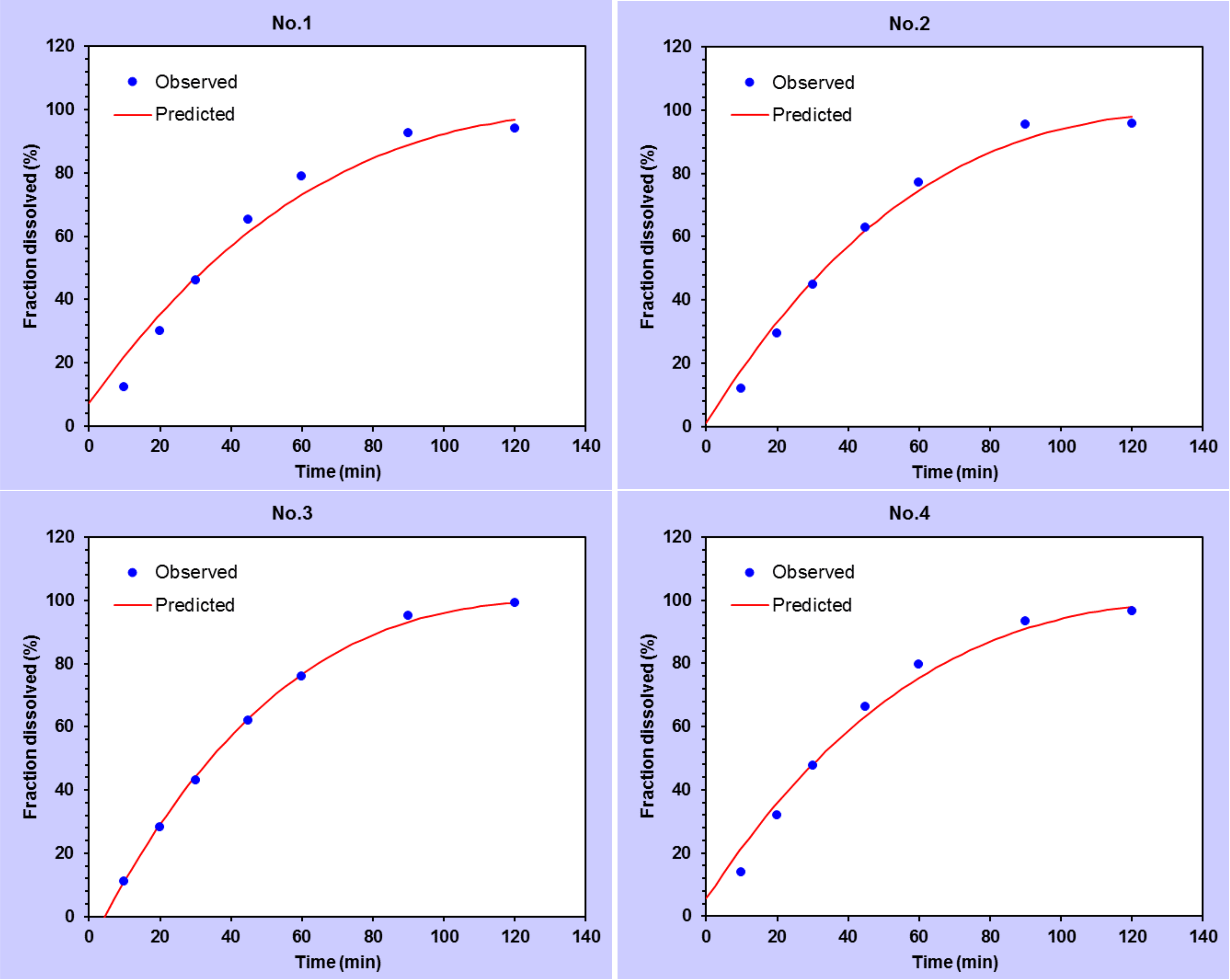

Supplementary materials – Model fitting summary – Polyglykol® 8000 P (modelling the entire relevant dissolution data)

Model: **Hopfenberg**

Model equation: $F = 100 \cdot [1 - (1 - k_{HB} \cdot t)^n]$

Fitted model parameters per tested tablet (N = 4) with statistics – mean, standard deviation (SD), and relative standard deviation expressed in % (RSD%) (output from DDSolver):

Parameter	No.1	No.2	No.3	No.4	Mean	SD	RSD(%)
k_{HB}	0.007	0.006	0.009	0.006	0.007	0.002	22.107
n	2.906	3.000	1.609	3.000	2.629	0.681	25.909

Number of dissolution data points (N), degrees of freedom (df), and selected goodness of fit criteria – Pearson correlation coefficient (R), coefficient of determination (R^2), adjusted coefficient of determination ($R^2_{adjusted}$), and residual sum of squares (RSS) (manual calculation in MS Excel):

Parameter	No.1	No.2	No.3	No.4
N	7	7	7	7
df	5	5	5	5
R	0.99776249	0.99713369	0.99816671	0.99614519
R^2	0.99552998	0.99427559	0.99633679	0.99230524
$R^2_{adjusted}$	0.99463598	0.99313071	0.99560415	0.99076629
RSS	105.426917	66.6498342	27.9414886	62.1054443

Graphical abstract of model fit presented as mean ± 1 SD of the fraction % of released carvedilol:

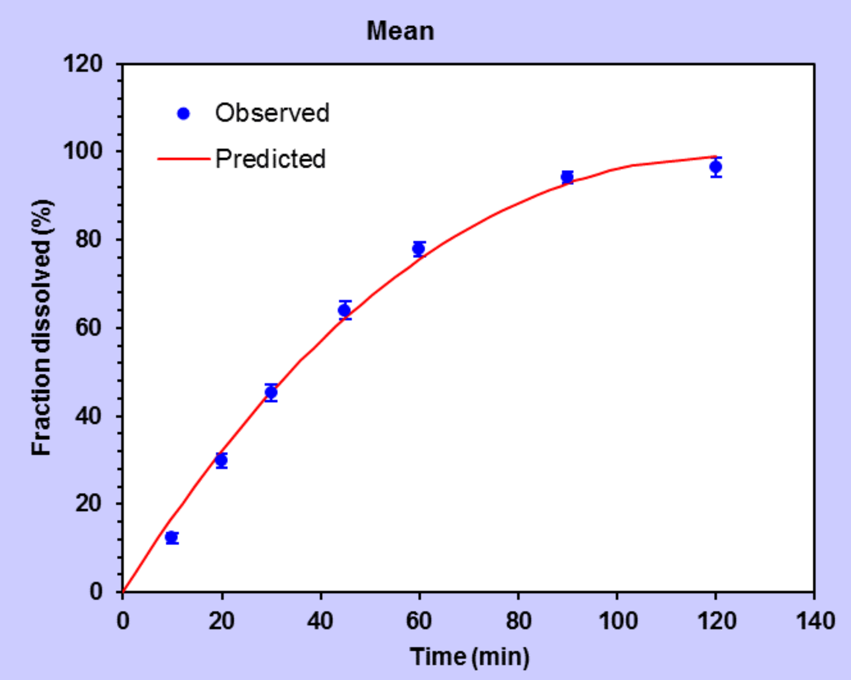

Supplementary materials – Model fitting summary – Polyglykol® 8000 P (modelling the entire relevant dissolution data)

Graphical abstract of model fit presented as the fraction % of released carvedilol per tested tablet:

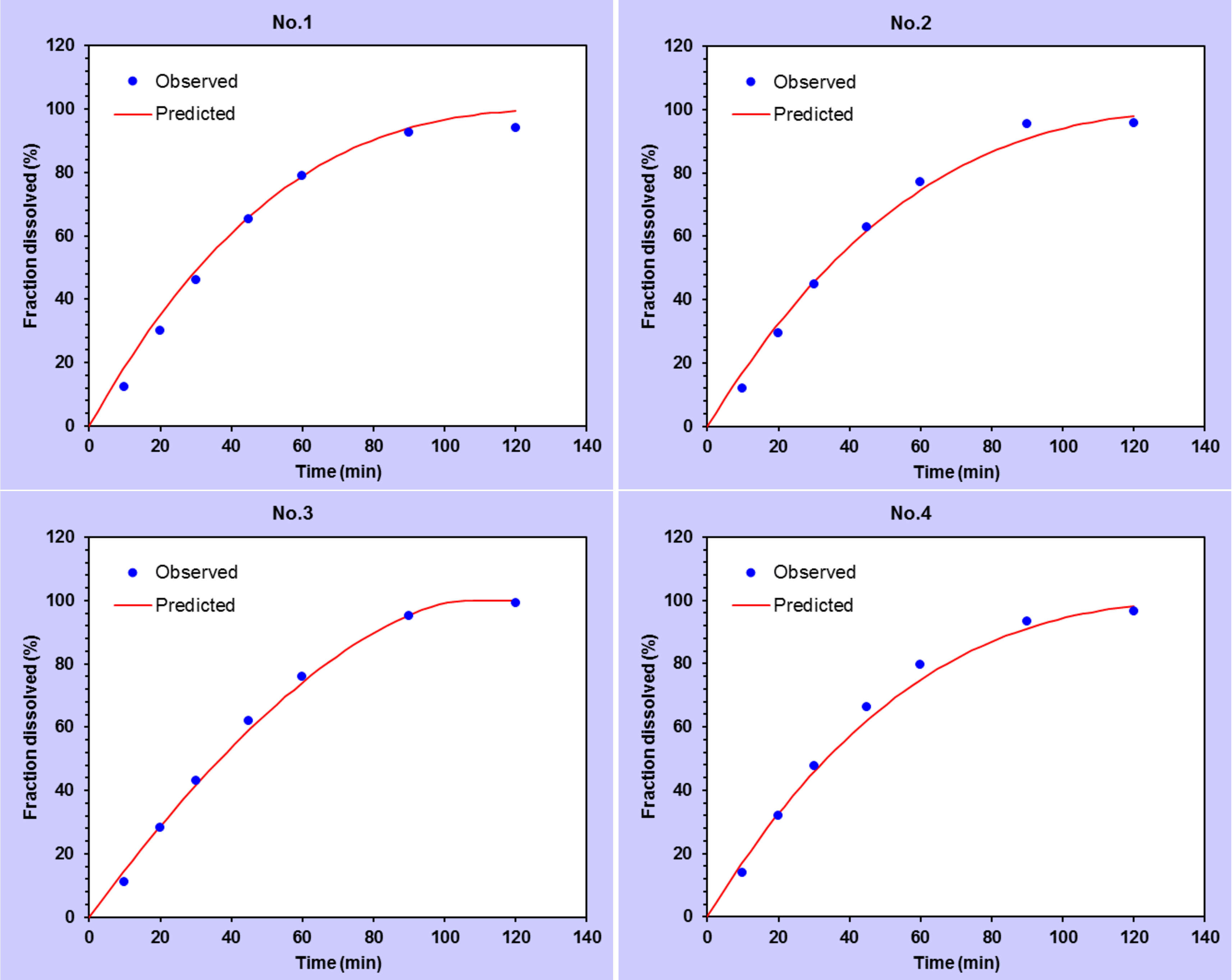

Supplementary materials – Model fitting summary – Polyglykol® 8000 P (modelling the entire relevant dissolution data)

Model: **Hopfenberg with T_{lag}**

Model equation: $F = 100 \cdot \{1 - [1 - k_{HB} \cdot (t - T_{lag})]^n\}$

Fitted model parameters per tested tablet (N = 4) with statistics – mean, standard deviation (SD), and relative standard deviation expressed in % (RSD%) (output from DDSolver):

Parameter	No.1	No.2	No.3	No.4	Mean	SD	RSD(%)
k_{HB}	0.005	0.006	0.007	0.006	0.006	0.001	11.330
n	3.000	3.000	3.000	3.000	3.000	0.000	0.000
T_{lag}	-4.524	-0.571	5.402	-3.150	-0.711	4.392	-617.920

Number of dissolution data points (N), degrees of freedom (df), and selected goodness of fit criteria – Pearson correlation coefficient (R), coefficient of determination (R^2), adjusted coefficient of determination ($R^2_{adjusted}$), and residual sum of squares (RSS) (manual calculation in MS Excel):

Parameter	No.1	No.2	No.3	No.4
N	7	7	7	7
df	4	4	4	4
R	0.99098858	0.9970395	0.99940388	0.99542846
R^2	0.98205837	0.99408776	0.99880812	0.99087782
$R^2_{adjusted}$	0.97308755	0.99113165	0.99821218	0.98631672
RSS	189.576743	79.0793133	8.44251152	106.402104

Graphical abstract of model fit presented as mean ± 1 SD of the fraction % of released carvedilol:

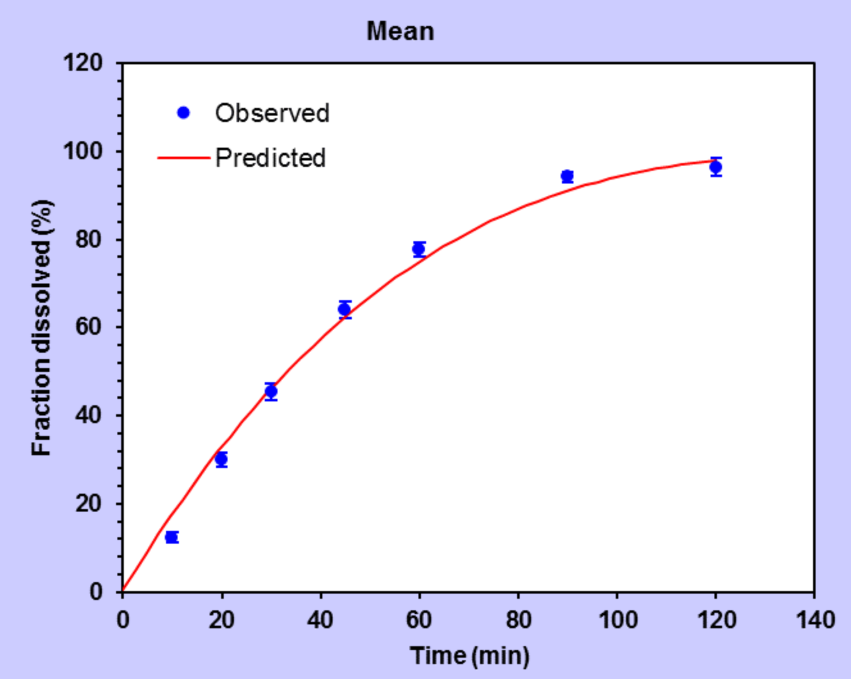

Supplementary materials – Model fitting summary – Polyglykol® 8000 P (modelling the entire relevant dissolution data)

Graphical abstract of model fit presented as the fraction % of released carvedilol per tested tablet:

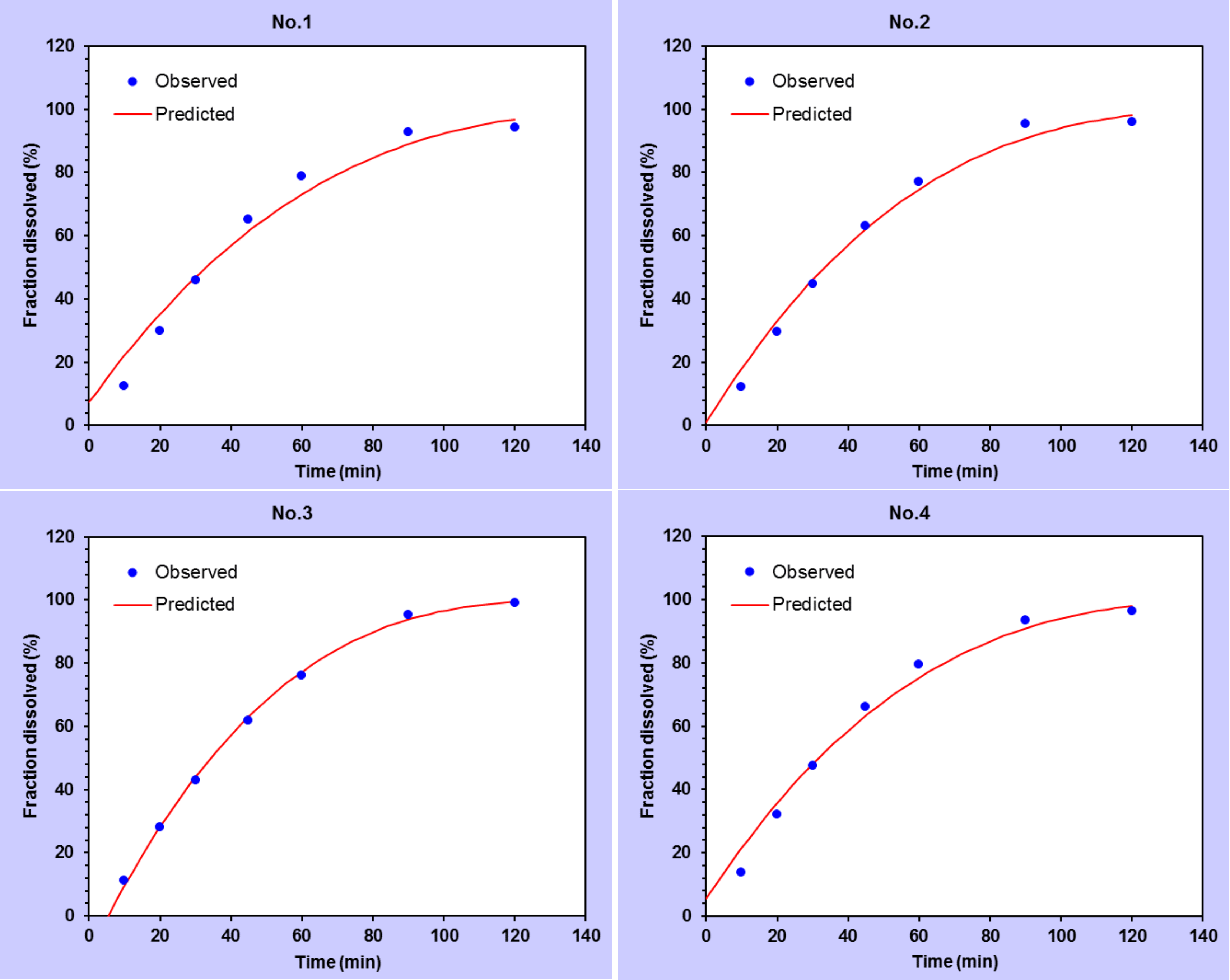

Supplementary materials – Model fitting summary – Polyglykol® 8000 P (modelling the entire relevant dissolution data)

Model: **Baker–Lonsdale**

Model equation: $\frac{3}{2}\cdot\left[1-\left(1-\frac{F}{100}\right)^{\frac{2}{3}}\right]-\frac{F}{100}=k_{BL}\cdot t$

Fitted model parameters per tested tablet (N = 4) with statistics – mean, standard deviation (SD), and relative standard deviation expressed in % (RSD%) (output from DDSolver):

Parameter	No.1	No.2	No.3	No.4	Mean	SD	RSD(%)
k_{BL}	0.002	0.002	0.004	0.002	0.002	0.001	52.539

Number of dissolution data points (N), degrees of freedom (df), and selected goodness of fit criteria – Pearson correlation coefficient (R), coefficient of determination (R^2), adjusted coefficient of determination ($R^2_{adjusted}$), and residual sum of squares (RSS) (manual calculation in MS Excel):

Parameter	No.1	No.2	No.3	No.4
N	7	7	7	7
df	6	6	6	6
R	0.98722794	0.99168611	-0.4004347	0.99075441
R^2	0.974619	0.98344135	0.16034794	0.9815943
$R^2_{adjusted}$	0.974619	0.98344135	0.16034794	0.9815943
RSS	1163.41078	1116.24157	121992.371	1035.52442

Graphical abstract of model fit presented as mean ± 1 SD of the fraction % of released carvedilol:

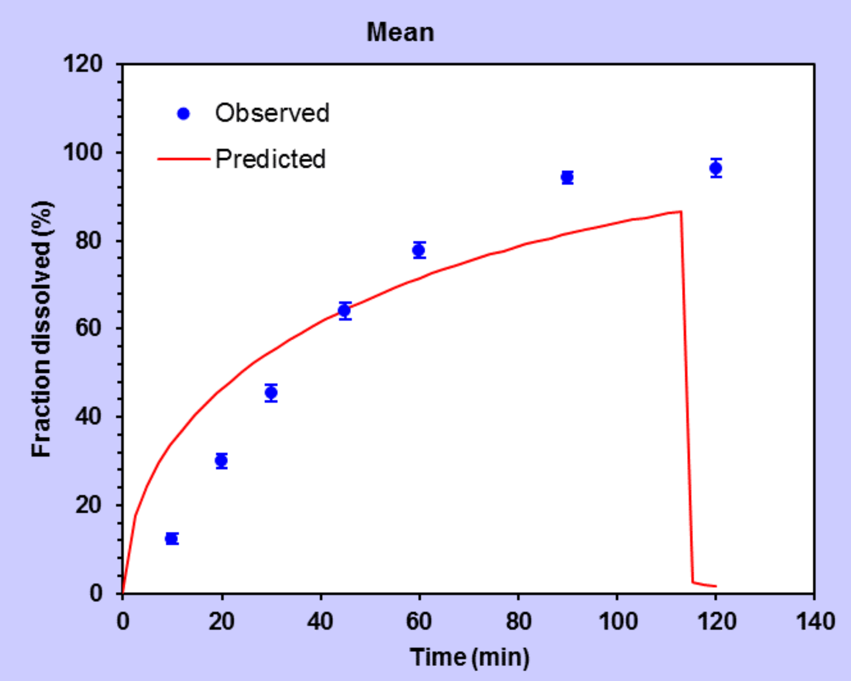

Supplementary materials – Model fitting summary – Polyglykol® 8000 P (modelling the entire relevant dissolution data)

Graphical abstract of model fit presented as the fraction % of released carvedilol per tested tablet:

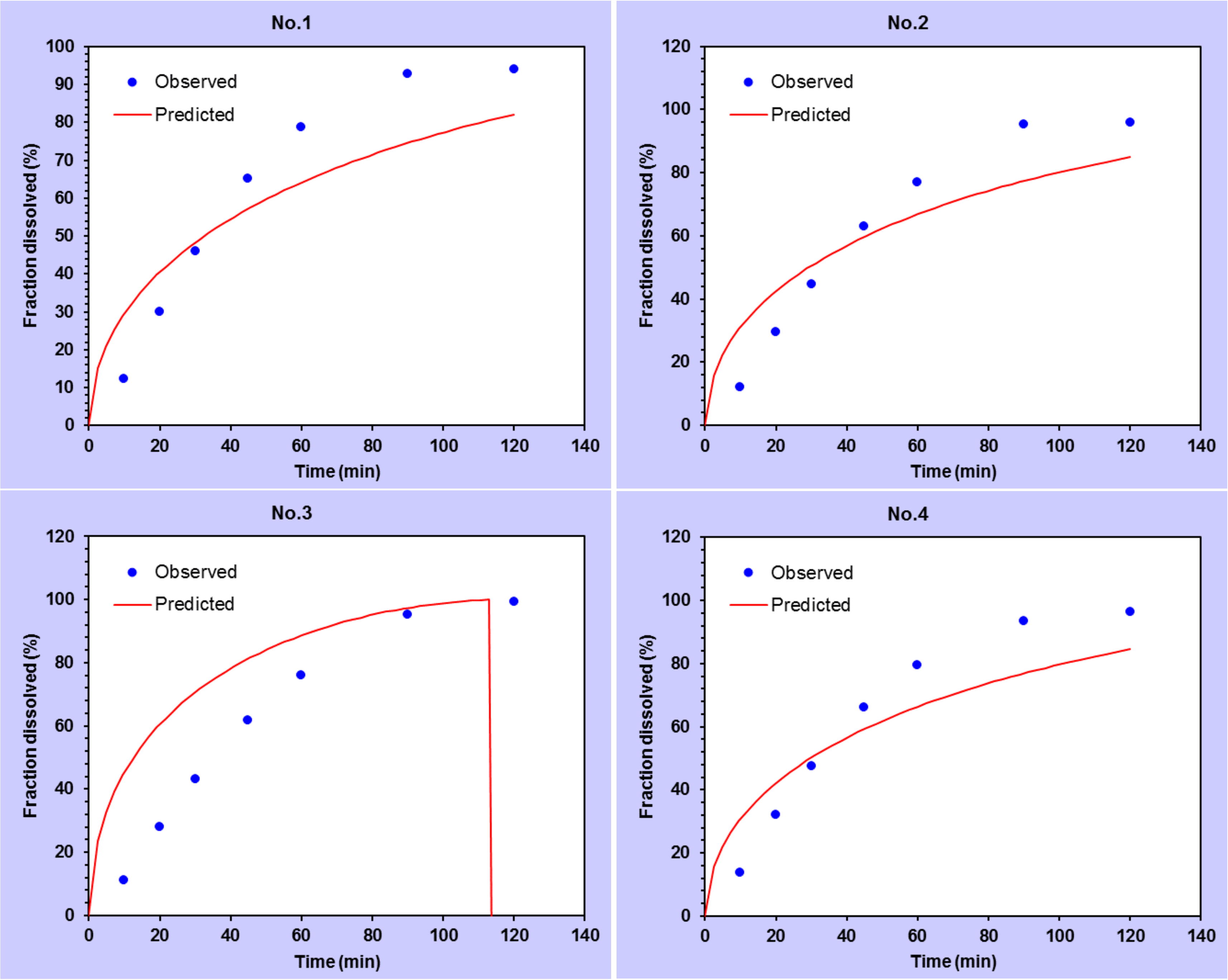

Supplementary materials – Model fitting summary – Polyglykol® 8000 P (modelling the entire relevant dissolution data)

Model: **Baker–Lonsdale with T_{lag}**

Model equation: $\frac{3}{2}\cdot\left[1-\left(1-\frac{F}{100}\right)^{\frac{2}{3}}\right]-\frac{F}{100}=k_{BL}\cdot\left(t-T_{lag}\right)$

Fitted model parameters per tested tablet (N = 4) with statistics – mean, standard deviation (SD), and relative standard deviation expressed in % (RSD%) (output from DDSolver):

Parameter	No.1	No.2	No.3	No.4	Mean	SD	RSD(%)
k_{BL}	0.003	0.004	0.004	0.004	0.004	0.000	10.932
T_{lag}	11.555	14.048	17.387	12.487	13.869	2.561	18.464

Number of dissolution data points (N), degrees of freedom (df), and selected goodness of fit criteria – Pearson correlation coefficient (R), coefficient of determination (R^2), adjusted coefficient of determination ($R^2_{adjusted}$), and residual sum of squares (RSS) (manual calculation in MS Excel):

Parameter	No.1	No.2	No.3	No.4
N	7	7	7	7
df	5	5	5	5
R	0.97898416	0.98243831	0.98717356	0.98224121
R^2	0.95840999	0.96518503	0.97451164	0.96479779
$R^2_{adjusted}$	0.95009198	0.95822204	0.96941397	0.95775735
RSS	280.834599	259.749752	249.033406	268.55782

Graphical abstract of model fit presented as mean ± 1 SD of the fraction % of released carvedilol:

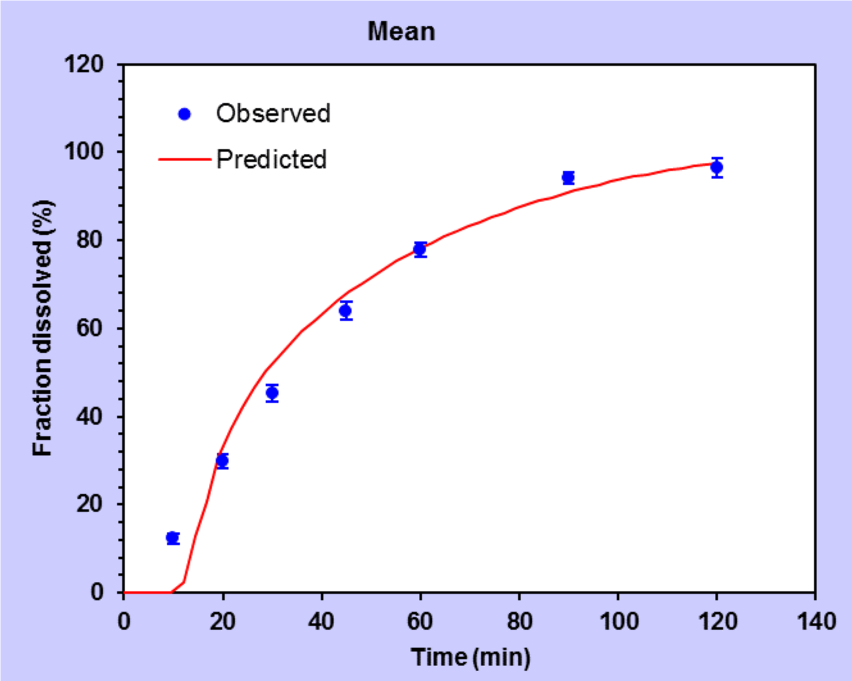

Supplementary materials – Model fitting summary – Polyglykol® 8000 P (modelling the entire relevant dissolution data)

Graphical abstract of model fit presented as the fraction % of released carvedilol per tested tablet:

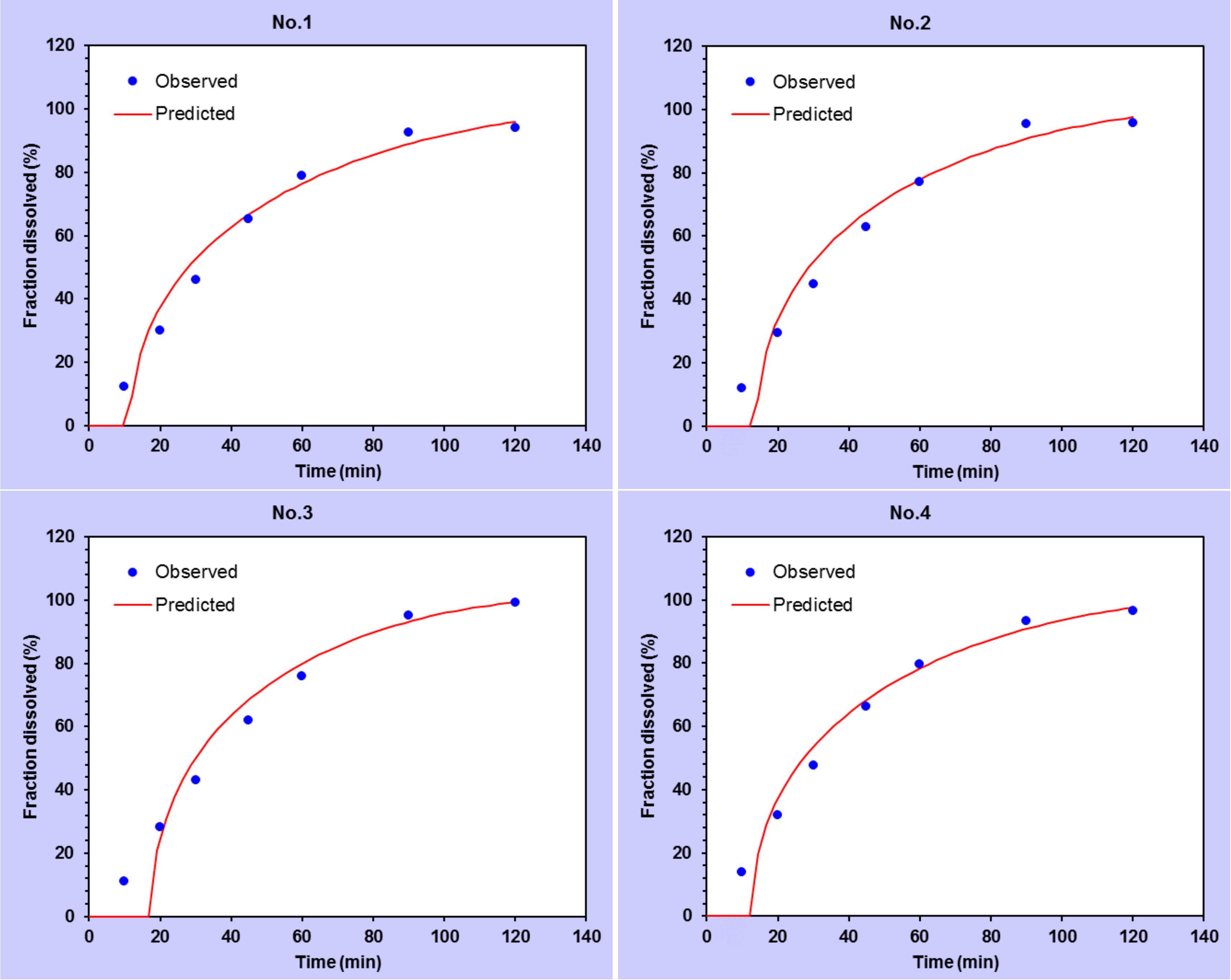

Supplementary materials – Model fitting summary – Polyglykol® 8000 P (modelling the entire relevant dissolution data)

Model: **Makoid–Banakar**

Model equation: $F = k_{MB} \cdot t^n \cdot e^{-k \cdot t}$

Fitted model parameters per tested tablet (N = 4) with statistics – mean, standard deviation (SD), and relative standard deviation expressed in % (RSD%) (output from DDSolver):

Parameter	No.1	No.2	No.3	No.4	Mean	SD	RSD(%)
k_{MB}	0.550	0.564	0.479	0.762	0.589	0.121	20.641
n	1.421	1.397	1.431	1.323	1.393	0.049	3.514
k	0.014	0.013	0.013	0.013	0.013	0.001	4.724

Number of dissolution data points (N), degrees of freedom (df), and selected goodness of fit criteria – Pearson correlation coefficient (R), coefficient of determination (R^2), adjusted coefficient of determination ($R^2_{adjusted}$), and residual sum of squares (RSS) (manual calculation in MS Excel):

Parameter	No.1	No.2	No.3	No.4
N	7	7	7	7
df	4	4	4	4
R	0.99960083	0.99939761	0.99938556	0.99950939
R^2	0.99920182	0.99879558	0.99877149	0.99901901
$R^2_{adjusted}$	0.99880273	0.99819337	0.99815724	0.99852852
RSS	5.11097173	7.68073762	8.49816166	6.15241302

Graphical abstract of model fit presented as mean ± 1 SD of the fraction % of released carvedilol:

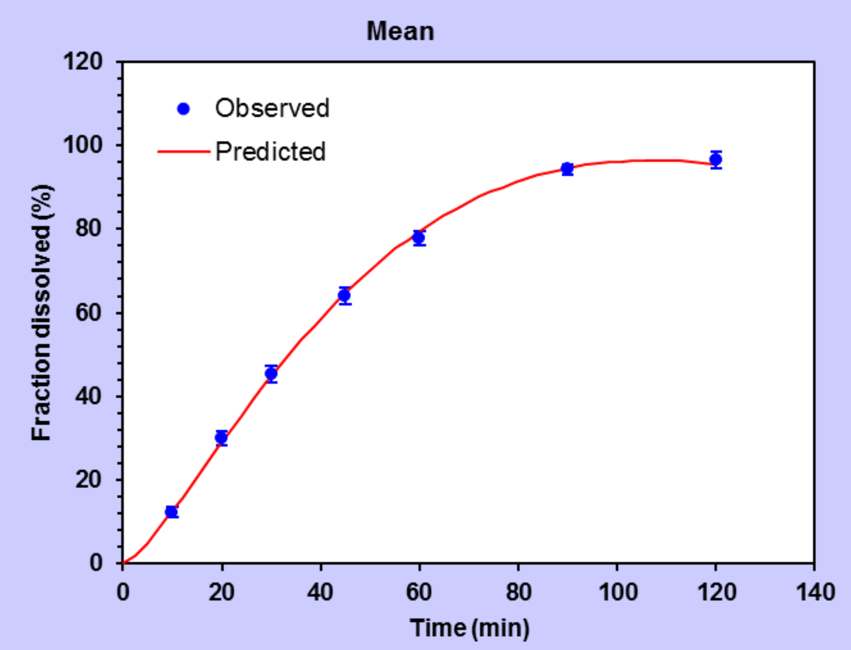

Supplementary materials – Model fitting summary – Polyglykol® 8000 P (modelling the entire relevant dissolution data)

Graphical abstract of model fit presented as the fraction % of released carvedilol per tested tablet:

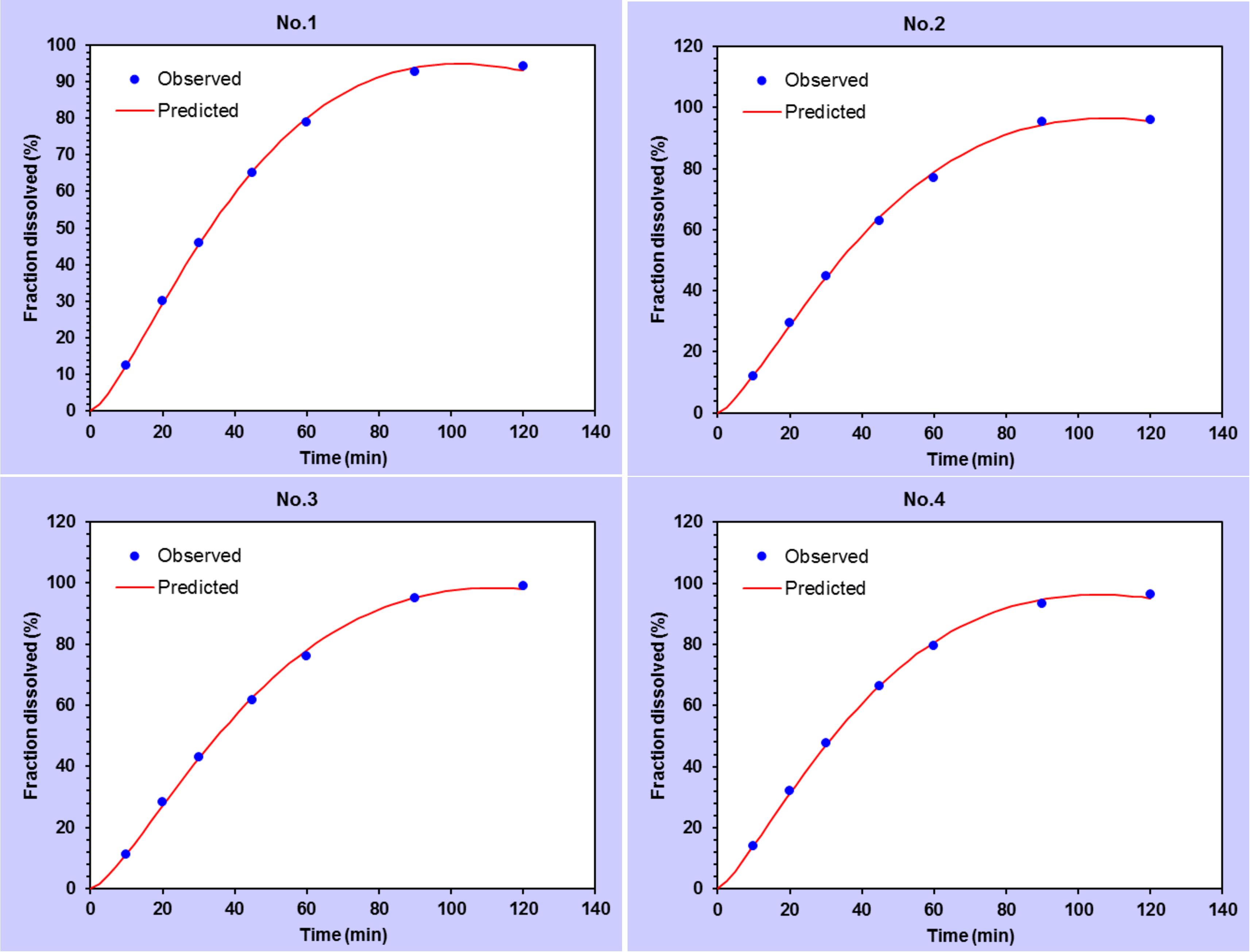

Supplementary materials – Model fitting summary – Polyglykol® 8000 P (modelling the entire relevant dissolution data)

Model: **Makoid–Banakar with T_{lag}**

Model equation: $F = k_{MB} \cdot (t - T_{lag})^n \cdot e^{-k \cdot (t - T_{lag})}$

Fitted model parameters per tested tablet (N = 4) with statistics – mean, standard deviation (SD), and relative standard deviation expressed in % (RSD%) (output from DDSolver):

Parameter	No.1	No.2	No.3	No.4	Mean	SD	RSD(%)
k_{MB}	2.033	2.042	1.791	2.577	2.111	0.332	15.722
n	1.033	1.016	1.042	0.962	1.013	0.036	3.534
k	0.009	0.008	0.008	0.008	0.008	0.001	6.404
T_{lag}	4.000	4.000	4.000	4.000	4.000	0.000	0.000

Number of dissolution data points (N), degrees of freedom (df), and selected goodness of fit criteria – Pearson correlation coefficient (R), coefficient of determination (R^2), adjusted coefficient of determination ($R^2_{adjusted}$), and residual sum of squares (RSS) (manual calculation in MS Excel):

Parameter	No.1	No.2	No.3	No.4
N	7	7	7	7
df	3	3	3	3
R	0.99970364	0.99904994	0.99965256	0.99980667
R^2	0.99940737	0.99810078	0.99930523	0.99961338
$R^2_{adjusted}$	0.99881473	0.99620156	0.99861046	0.99922677
RSS	3.68179928	12.1385535	4.72461696	2.36166484

Graphical abstract of model fit presented as mean ± 1 SD of the fraction % of released carvedilol:

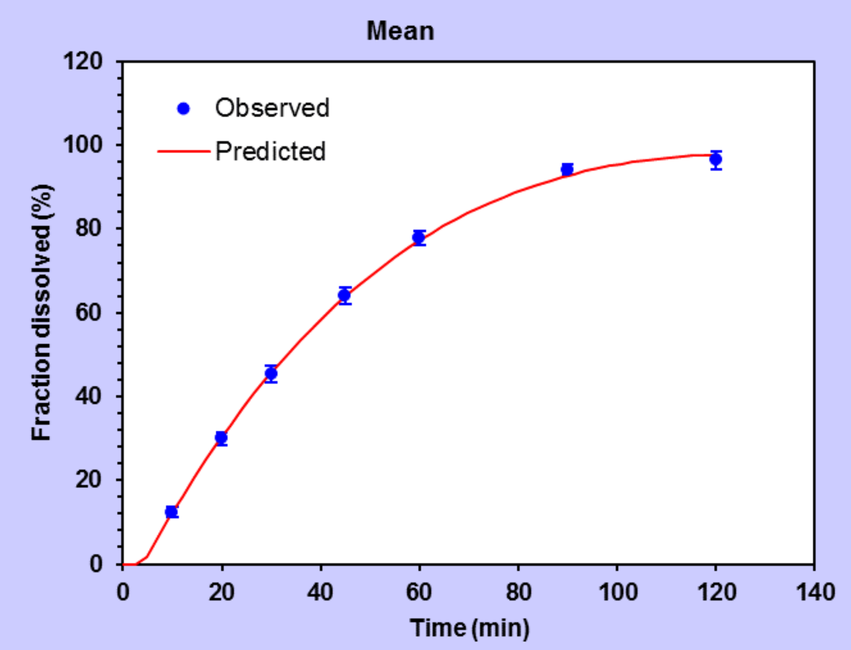

Supplementary materials – Model fitting summary – Polyglykol® 8000 P (modelling the entire relevant dissolution data)

Graphical abstract of model fit presented as the fraction % of released carvedilol per tested tablet:

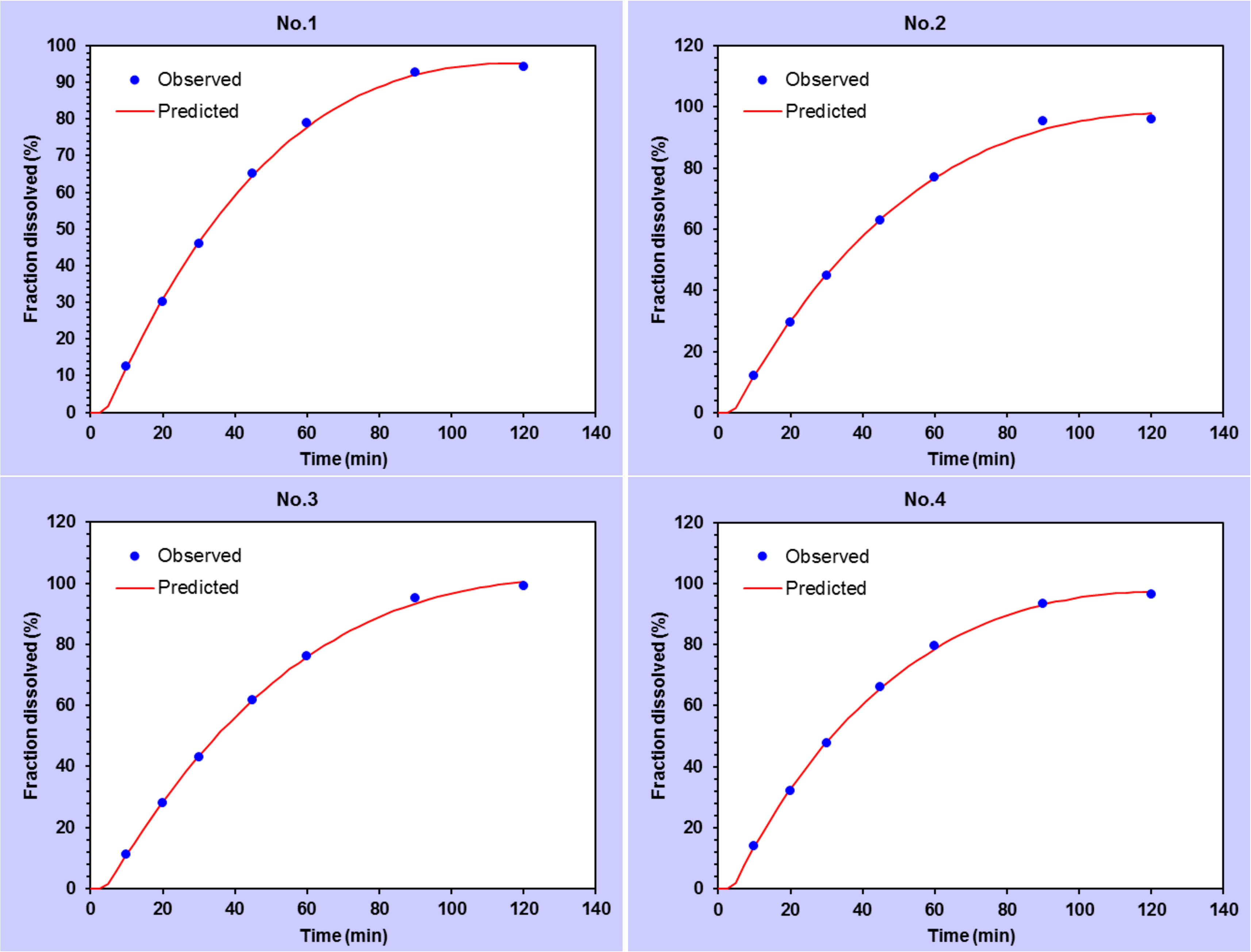

Supplementary materials – Model fitting summary – Polyglykol® 8000 P (modelling the entire relevant dissolution data)

Model: **Peppas–Sahlin_1**

Model equation: $F = k_1 \cdot t^m + k_2 \cdot t^{2m}$

Fitted model parameters per tested tablet (N = 4) with statistics – mean, standard deviation (SD), and relative standard deviation expressed in % (RSD%) (output from DDSolver):

Parameter	No.1	No.2	No.3	No.4	Mean	SD	RSD(%)
k_1	7.359	6.537	5.491	7.804	6.798	1.017	14.960
k_2	0.551	0.674	0.826	0.518	0.642	0.140	21.764
m	0.450	0.450	0.450	0.450	0.450	0.000	0.000

Number of dissolution data points (N), degrees of freedom (df), and selected goodness of fit criteria – Pearson correlation coefficient (R), coefficient of determination (R^2), adjusted coefficient of determination ($R^2_{adjusted}$), and residual sum of squares (RSS) (manual calculation in MS Excel):

Parameter	No.1	No.2	No.3	No.4
N	7	7	7	7
df	4	4	4	4
R	0.95992232	0.96601558	0.97263448	0.96539381
R^2	0.92145085	0.9331861	0.94601783	0.93198521
$R^2_{adjusted}$	0.88217628	0.89977916	0.91902674	0.89797782
RSS	501.438849	445.349404	382.816048	432.089399

Graphical abstract of model fit presented as mean ± 1 SD of the fraction % of released carvedilol:

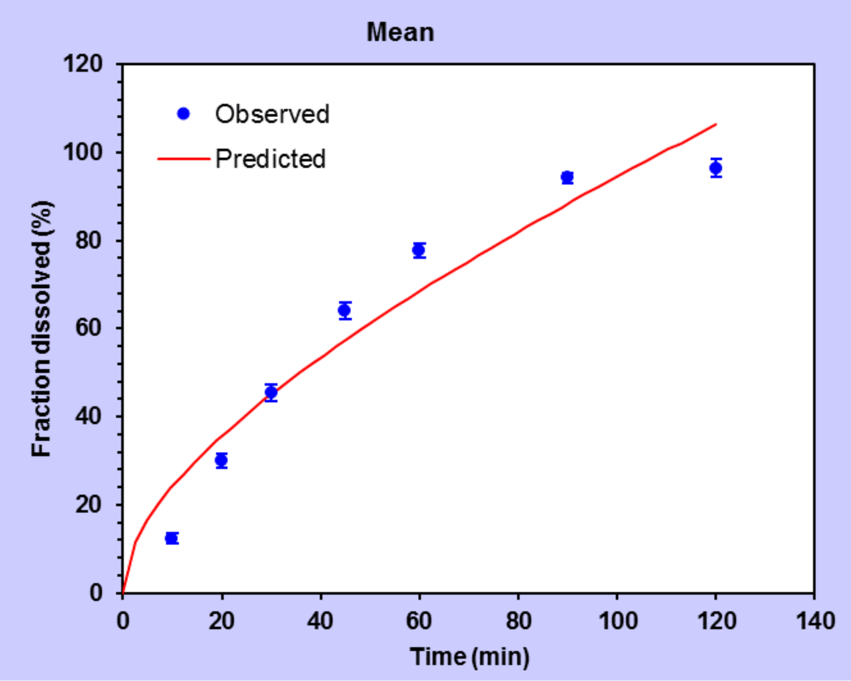

Supplementary materials – Model fitting summary – Polyglykol® 8000 P (modelling the entire relevant dissolution data)

Graphical abstract of model fit presented as the fraction % of released carvedilol per tested tablet:

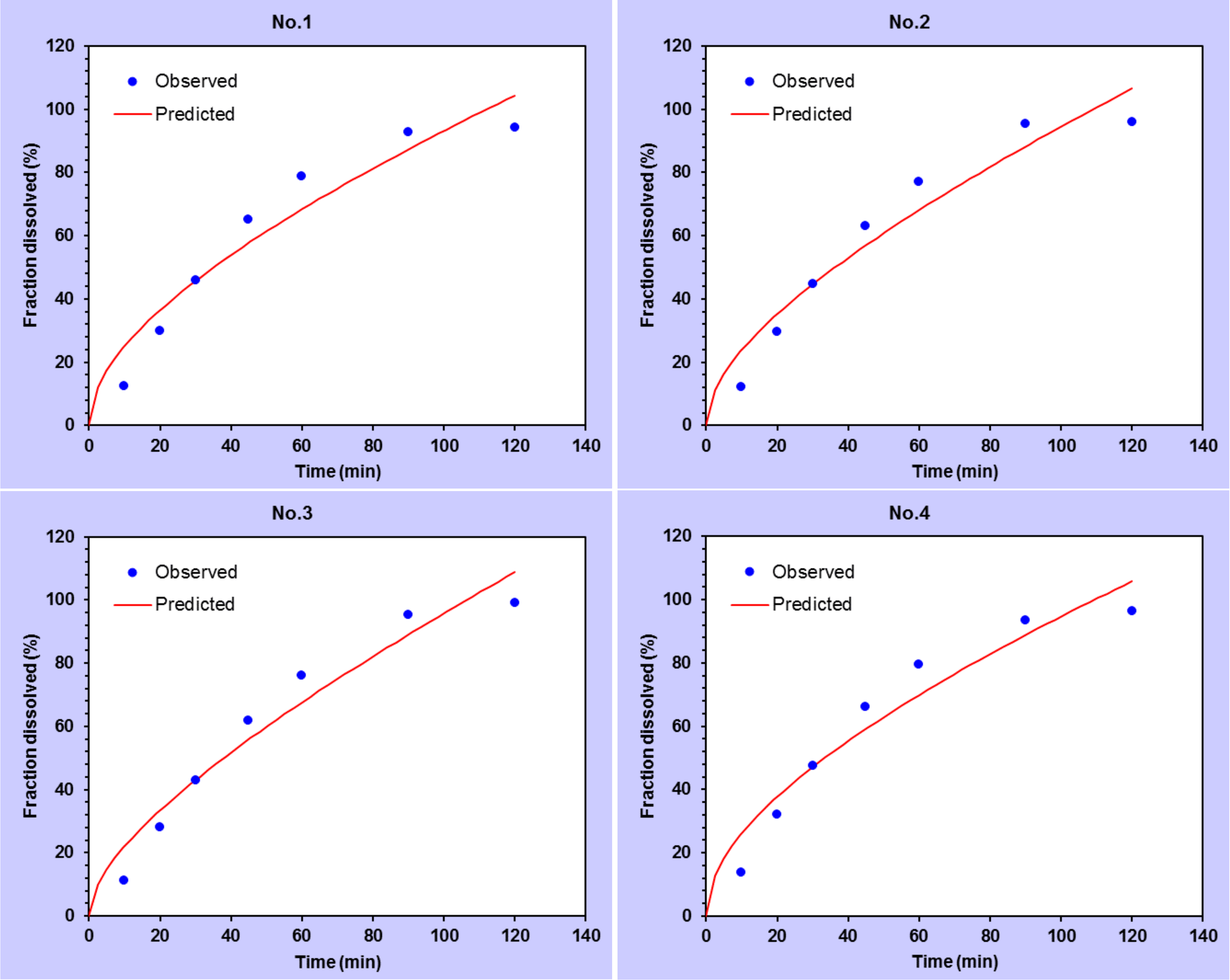

Supplementary materials – Model fitting summary – Polyglykol® 8000 P (modelling the entire relevant dissolution data)

Model: **Peppas–Sahlin_1 with T_{lag}**

Model equation: $F = k_1 \cdot (t - T_{lag})^m + k_2 \cdot (t - T_{lag})^{2m}$

Fitted model parameters per tested tablet (N = 4) with statistics – mean, standard deviation (SD), and relative standard deviation expressed in % (RSD%) (output from DDSolver):

Parameter	No.1	No.2	No.3	No.4	Mean	SD	RSD(%)
k_1	k1	9.422	8.576	7.509	9.892	8.850	1.046
k_2	k2	0.321	0.449	0.608	0.285	0.416	0.147
m	m	0.450	0.450	0.450	0.450	0.450	0.000
T_{lag}	Tlag	6.000	6.000	6.000	6.000	6.000	0.000

Number of dissolution data points (N), degrees of freedom (df), and selected goodness of fit criteria – Pearson correlation coefficient (R), coefficient of determination (R^2), adjusted coefficient of determination ($R^2_{adjusted}$), and residual sum of squares (RSS) (manual calculation in MS Excel):

Parameter	No.1	No.2	No.3	No.4
N	7	7	7	7
df	3	3	3	3
R	0.97377392	0.97738415	0.98194987	0.97863989
R^2	0.94823566	0.95527978	0.96422555	0.95773604
$R^2_{adjusted}$	0.89647131	0.91055957	0.92845109	0.91547207
RSS	328.872327	298.435266	256.816083	268.17704

Graphical abstract of model fit presented as mean ± 1 SD of the fraction % of released carvedilol:

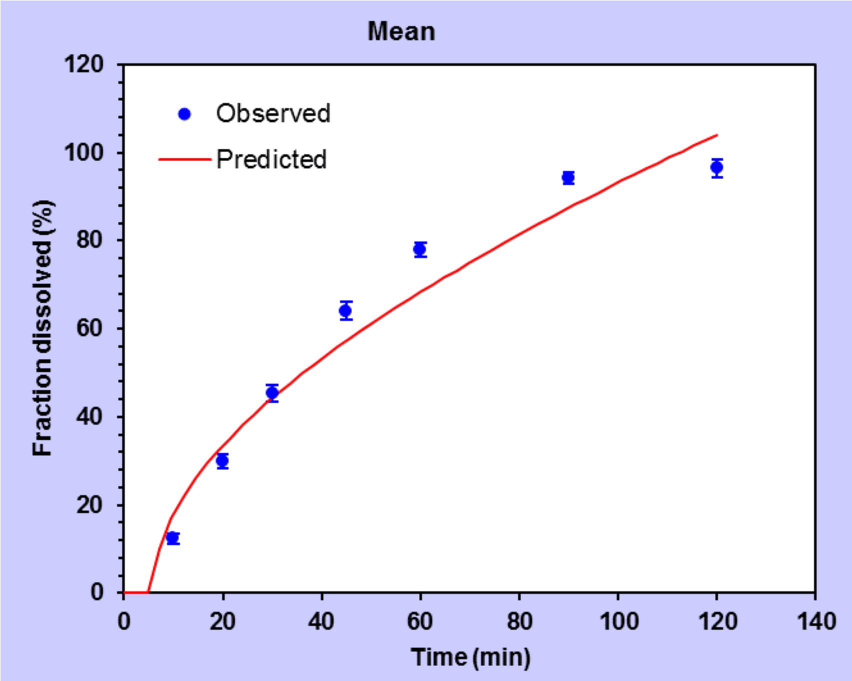

Supplementary materials – Model fitting summary – Polyglykol® 8000 P (modelling the entire relevant dissolution data)

Graphical abstract of model fit presented as the fraction % of released carvedilol per tested tablet:

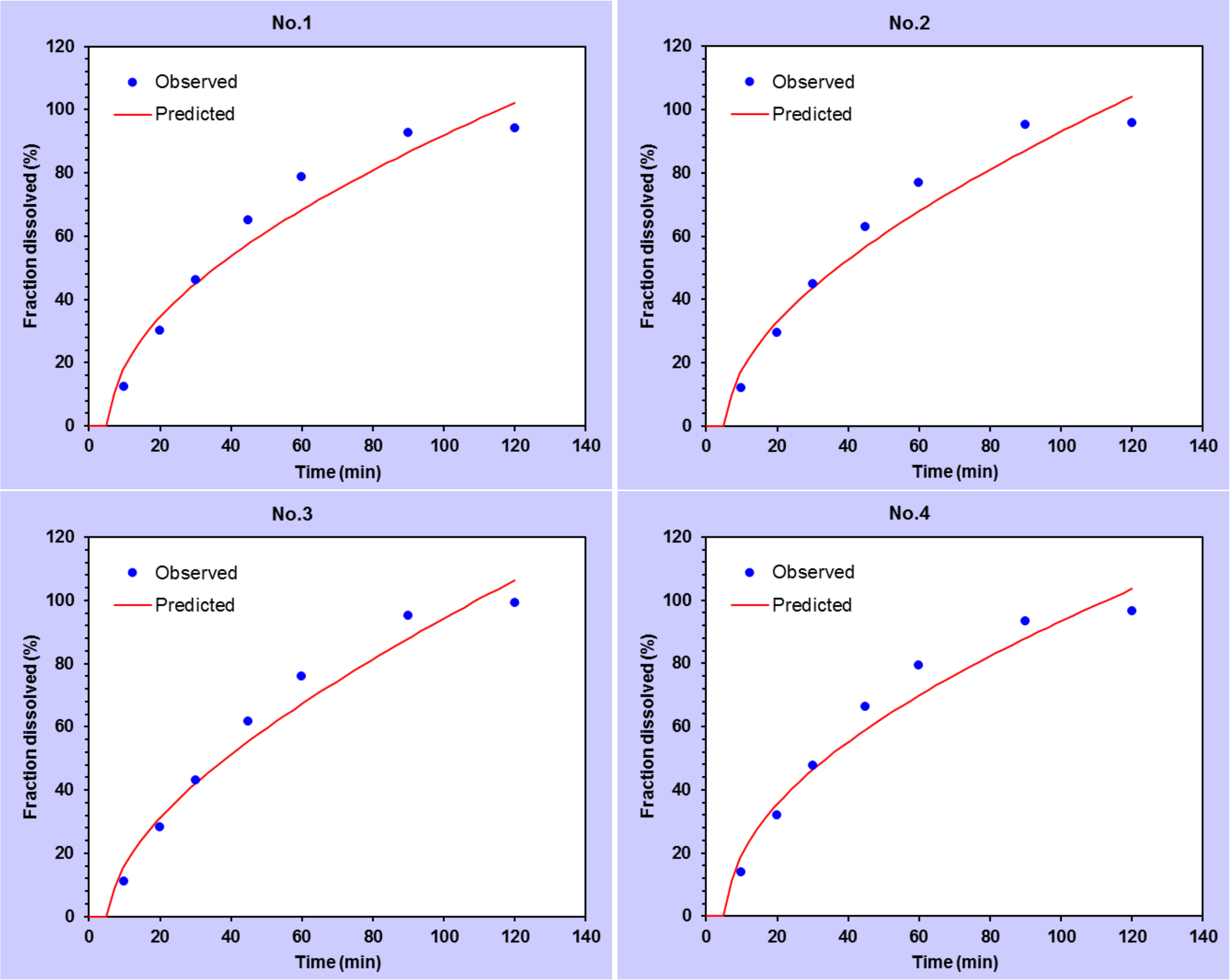

Supplementary materials – Model fitting summary – Polyglykol® 8000 P (modelling the entire relevant dissolution data)

Model: **Peppas–Sahlin_2**

Model equation: $F = k_1 \cdot t^{0.5} + k_2 \cdot t$

Fitted model parameters per tested tablet (N = 4) with statistics – mean, standard deviation (SD), and relative standard deviation expressed in % (RSD%) (output from DDSolver):

Parameter	No.1	No.2	No.3	No.4	Mean	SD	RSD(%)
k_1	7.433	6.792	5.979	7.789	6.999	0.795	11.359
k_2	0.185	0.262	0.357	0.165	0.242	0.087	35.897

Number of dissolution data points (N), degrees of freedom (df), and selected goodness of fit criteria – Pearson correlation coefficient (R), coefficient of determination (R^2), adjusted coefficient of determination ($R^2_{adjusted}$), and residual sum of squares (RSS) (manual calculation in MS Excel):

Parameter	No.1	No.2	No.3	No.4
N	7	7	7	7
df	5	5	5	5
R	0.96234008	0.96744324	0.97317909	0.96780104
R^2	0.92609844	0.93594642	0.94707755	0.93663885
$R^2_{adjusted}$	0.91131812	0.9231357	0.93649306	0.92396662
RSS	488.140702	439.910965	385.716211	417.084534

Graphical abstract of model fit presented as mean ± 1 SD of the fraction % of released carvedilol:

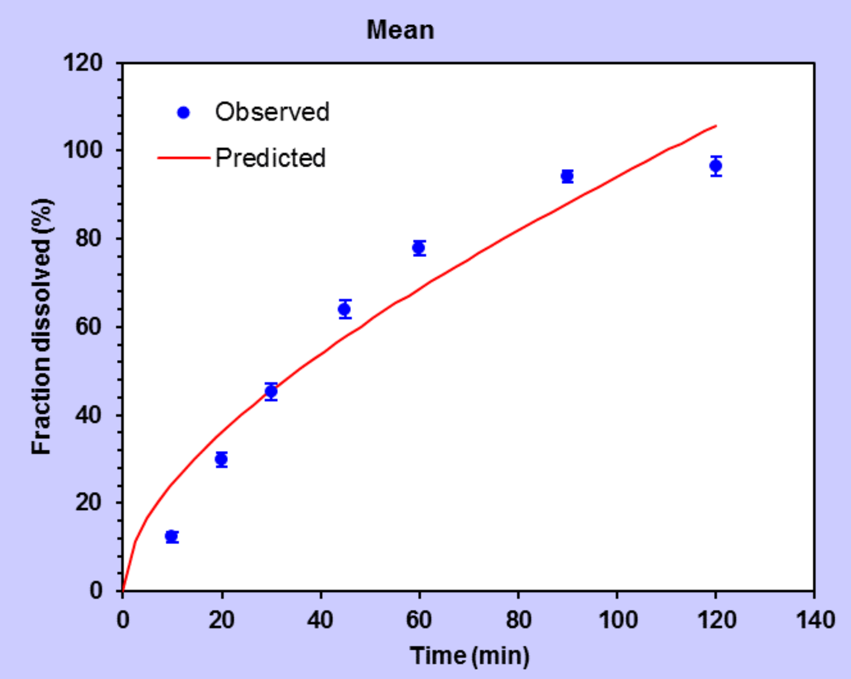

Supplementary materials – Model fitting summary – Polyglykol® 8000 P (modelling the entire relevant dissolution data)

Graphical abstract of model fit presented as the fraction % of released carvedilol per tested tablet:

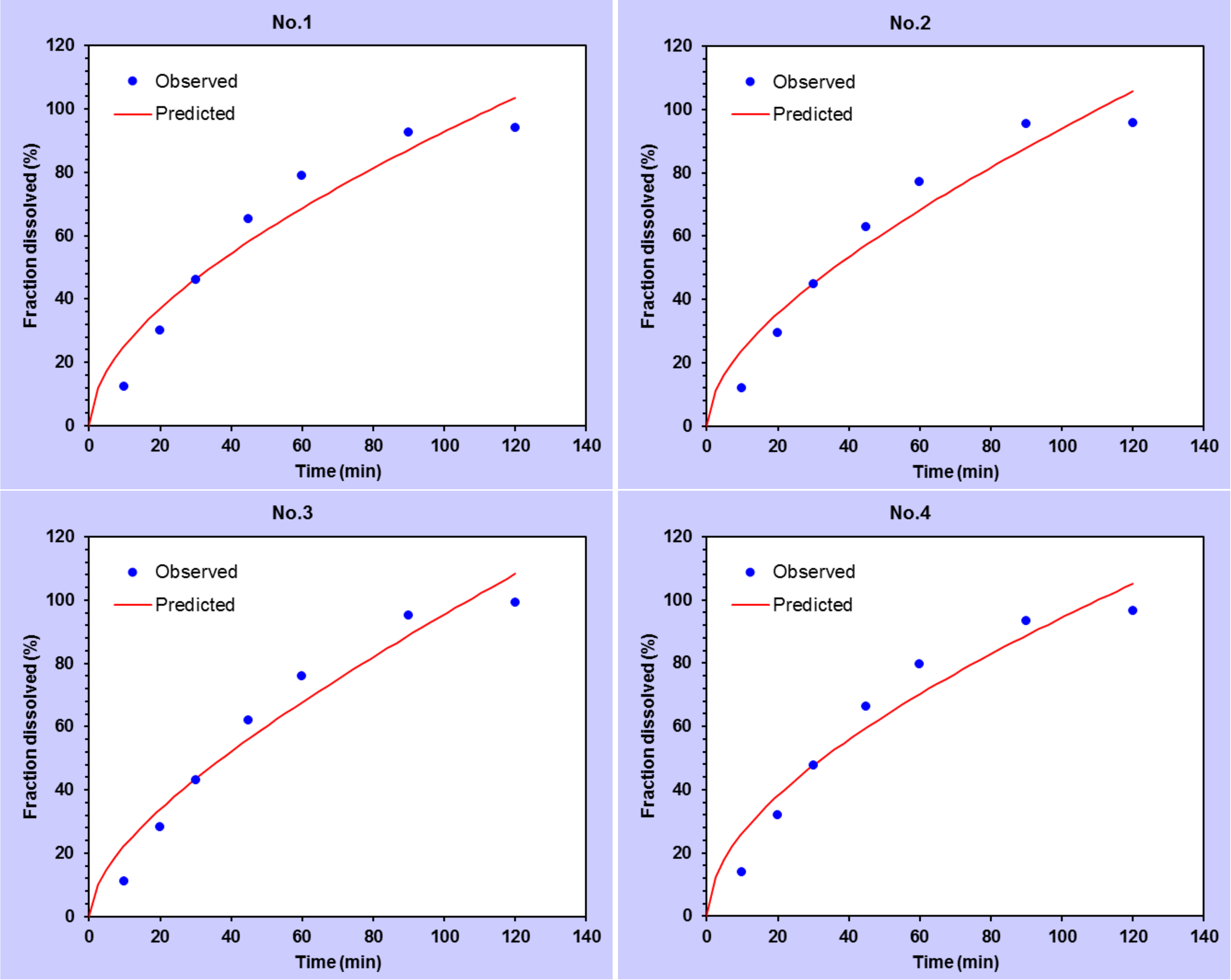

Supplementary materials – Model fitting summary – Polyglykol® 8000 P (modelling the entire relevant dissolution data)

Model: **Peppas–Sahlin_2 with T_{lag}**

Model equation: $F = k_1 \cdot (t - T_{lag})^{0.5} + k_2 \cdot (t - T_{lag})$

Fitted model parameters per tested tablet (N = 4) with statistics – mean, standard deviation (SD), and relative standard deviation expressed in % (RSD%) (output from DDSolver):

Parameter	No.1	No.2	No.3	No.4	Mean	SD	RSD(%)
k_1	k1	9.112	8.446	7.610	9.486	8.663	0.824
k_2	k2	0.036	0.117	0.216	0.013	0.095	0.092
T_{lag}	Tlag	6.000	6.000	6.000	6.000	6.000	0.000

Number of dissolution data points (N), degrees of freedom (df), and selected goodness of fit criteria – Pearson correlation coefficient (R), coefficient of determination (R^2), adjusted coefficient of determination ($R^2_{adjusted}$), and residual sum of squares (RSS) (manual calculation in MS Excel):

Parameter	No.1	No.2	No.3	No.4
N	7	7	7	7
df	4	4	4	4
R	0.97712779	0.97973734	0.98343288	0.98175182
R^2	0.95477871	0.95988525	0.96714022	0.96383663
$R^2_{adjusted}$	0.93216807	0.93982787	0.95071034	0.94575494
RSS	293.629056	272.188678	239.243581	234.352359

Graphical abstract of model fit presented as mean ± 1 SD of the fraction % of released carvedilol:

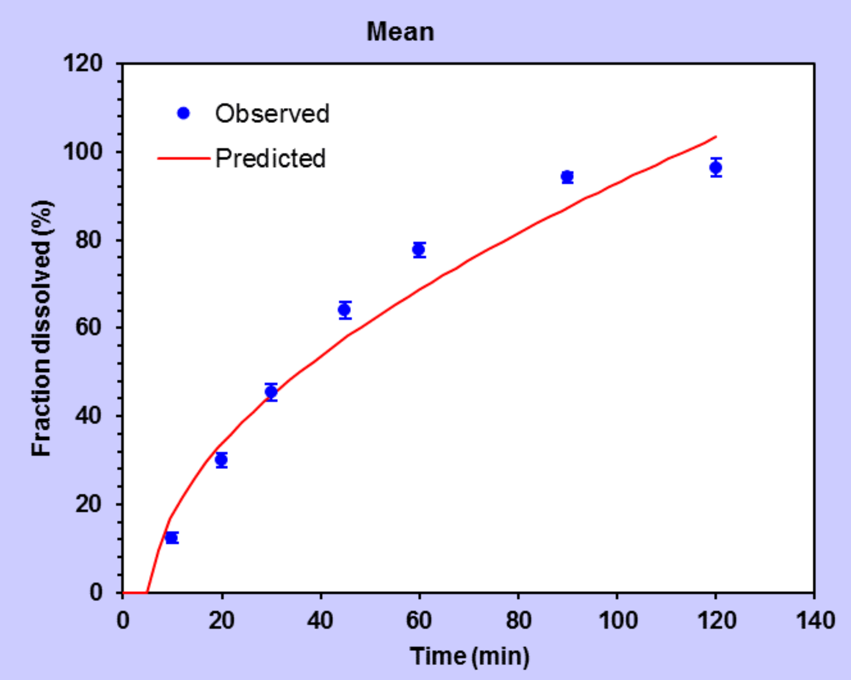

Supplementary materials – Model fitting summary – Polyglykol® 8000 P (modelling the entire relevant dissolution data)

Graphical abstract of model fit presented as the fraction % of released carvedilol per tested tablet:

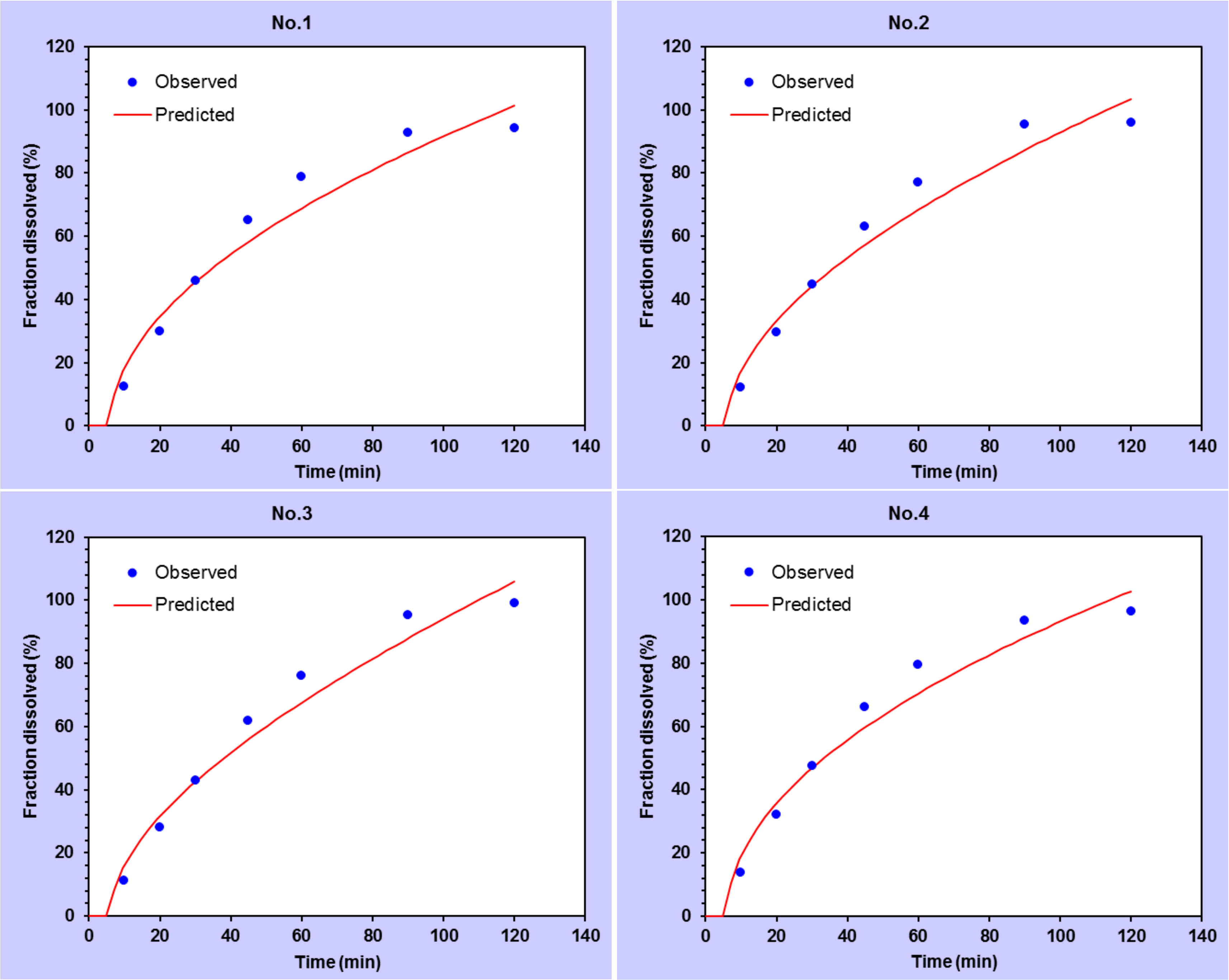

Supplementary materials – Model fitting summary – Polyglykol® 8000 P (modelling the entire relevant dissolution data)

Model: **Quadratic**

Model equation: $F = 100 \cdot (k_1 \cdot t^2 + k_2 \cdot t)$

Fitted model parameters per tested tablet (N = 4) with statistics – mean, standard deviation (SD), and relative standard deviation expressed in % (RSD%) (output from DDSolver):

Parameter	No.1	No.2	No.3	No.4	Mean	SD	RSD(%)
k_1	0.000	0.000	0.000	0.000	0.000	0.000	-9.437
k_2	0.018	0.017	0.017	0.018	0.017	0.001	4.003

Number of dissolution data points (N), degrees of freedom (df), and selected goodness of fit criteria – Pearson correlation coefficient (R), coefficient of determination (R^2), adjusted coefficient of determination ($R^2_{adjusted}$), and residual sum of squares (RSS) (manual calculation in MS Excel):

Parameter	No.1	No.2	No.3	No.4
N	7	7	7	7
df	5	5	5	5
R	0.9987031	0.9990634	0.99891497	0.99884054
R^2	0.99740788	0.99812767	0.99783111	0.99768242
$R^2_{adjusted}$	0.99688945	0.99775321	0.99739733	0.9972189
RSS	32.4901114	29.3155456	34.1781646	19.092189

Graphical abstract of model fit presented as mean ± 1 SD of the fraction % of released carvedilol:

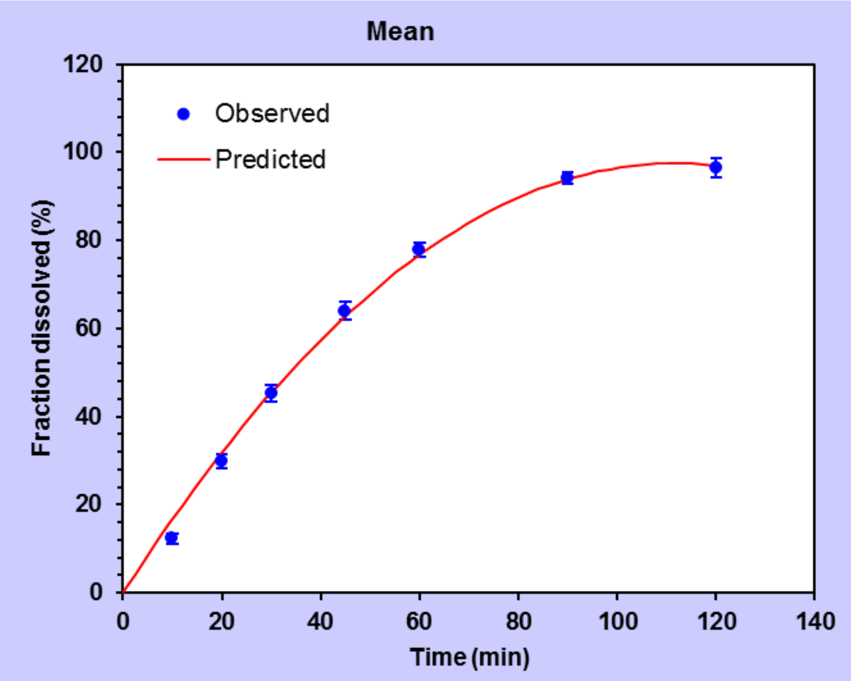

Supplementary materials – Model fitting summary – Polyglykol® 8000 P (modelling the entire relevant dissolution data)

Graphical abstract of model fit presented as the fraction % of released carvedilol per tested tablet:

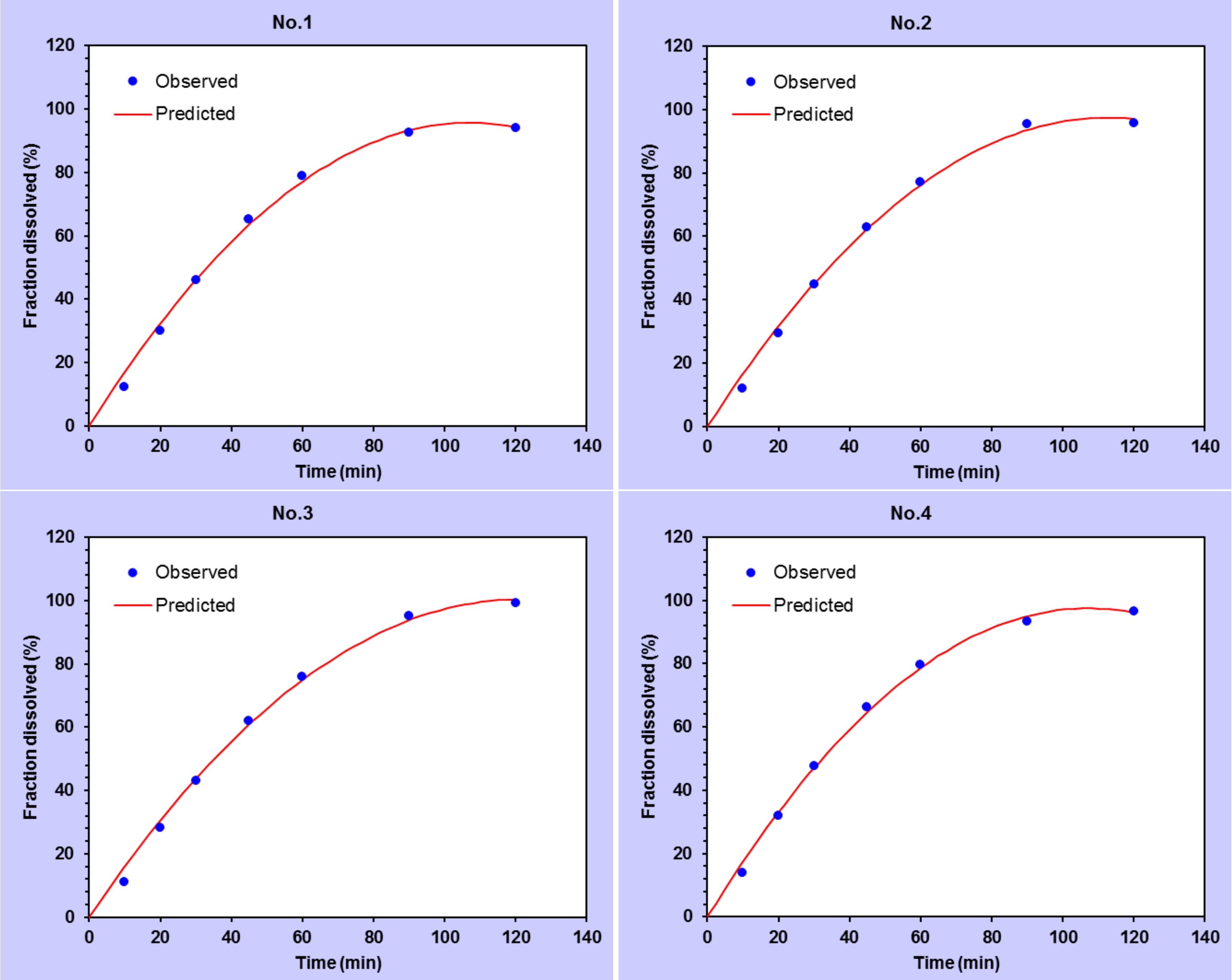

Supplementary materials – Model fitting summary – Polyglykol® 8000 P (modelling the entire relevant dissolution data)

Model: **Quadratic with T_{lag}**

Model equation: $F = 100 \cdot \left[k_1 \cdot \left(t - T_{lag}\right)^2 + k_2 \cdot \left(t - T_{lag}\right)\right]$

Fitted model parameters per tested tablet (N = 4) with statistics – mean, standard deviation (SD), and relative standard deviation expressed in % (RSD%) (output from DDSolver):

Parameter	No.1	No.2	No.3	No.4	Mean	SD	RSD(%)
k_1	0.000	0.000	0.000	0.000	0.000	0.000	-8.181
k_2	0.020	0.019	0.019	0.020	0.019	0.001	3.711
T_{lag}	4.000	4.000	4.000	4.000	4.000	0.000	0.000

Number of dissolution data points (N), degrees of freedom (df), and selected goodness of fit criteria – Pearson correlation coefficient (R), coefficient of determination (R^2), adjusted coefficient of determination ($R^2_{adjusted}$), and residual sum of squares (RSS) (manual calculation in MS Excel):

Parameter	No.1	No.2	No.3	No.4
N	7	7	7	7
df	4	4	4	4
R	0.9993367	0.99981864	0.99990201	0.99865456
R^2	0.99867383	0.99963731	0.99980403	0.99731094
$R^2_{adjusted}$	0.99801075	0.99945596	0.99970604	0.9959664
RSS	11.6476979	4.62486716	2.04853985	31.8330691

Graphical abstract of model fit presented as mean ± 1 SD of the fraction % of released carvedilol:

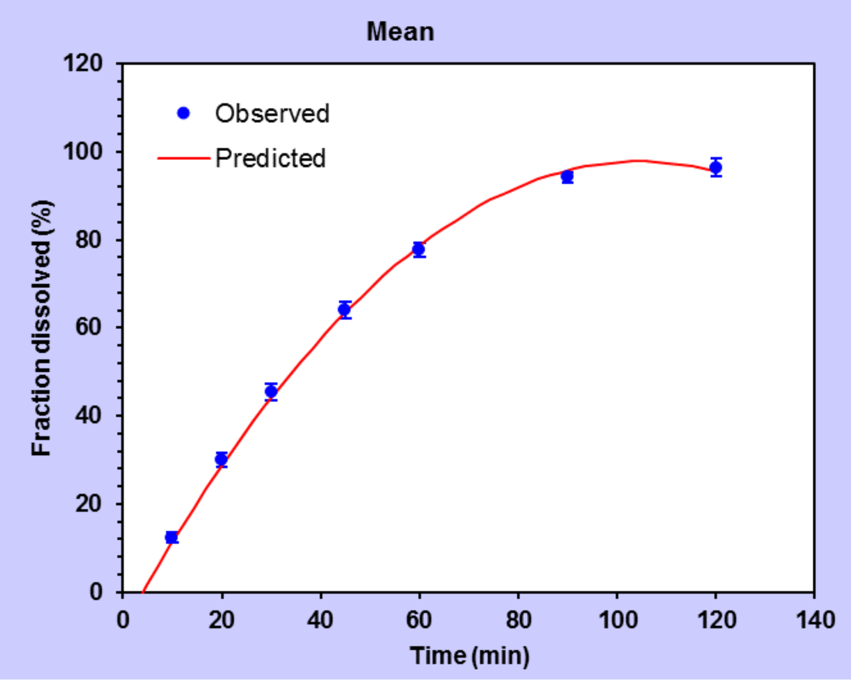

Supplementary materials – Model fitting summary – Polyglykol® 8000 P (modelling the entire relevant dissolution data)

Graphical abstract of model fit presented as the fraction % of released carvedilol per tested tablet:

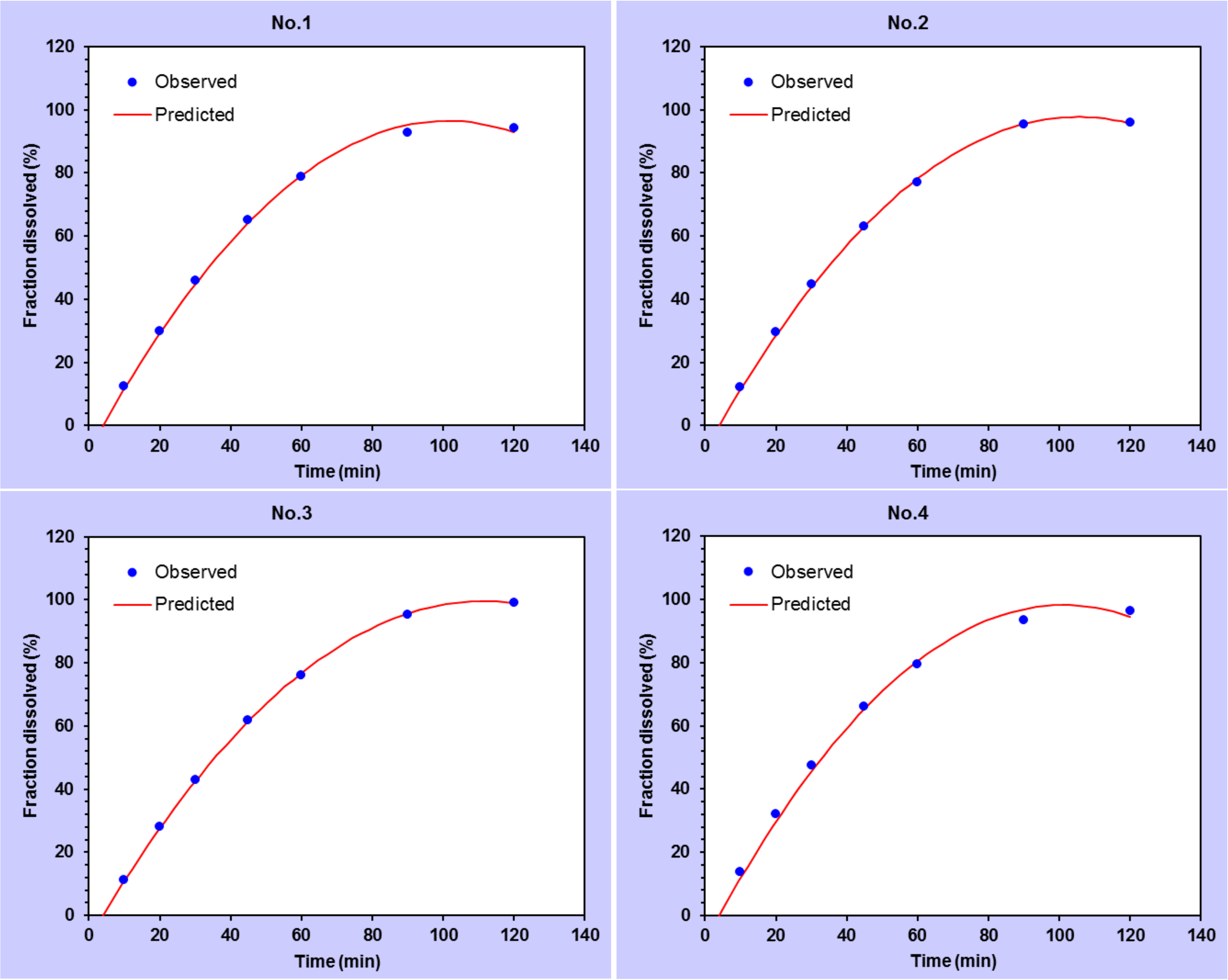

Supplementary materials – Model fitting summary – Polyglykol® 8000 P (modelling the entire relevant dissolution data)

Model: **Weibull_1**

Model equation: $F = 100 \cdot \left[1 - e^{-\frac{(t-T_i)^{\beta}}{\alpha}}\right]$

Fitted model parameters per tested tablet (N = 4) with statistics – mean, standard deviation (SD), and relative standard deviation expressed in % (RSD%) (output from DDSolver):

Parameter	No.1	No.2	No.3	No.4	Mean	SD	RSD(%)
α	53.715	63.731	92.341	49.214	64.751	19.369	29.913
β	1.084	1.136	1.246	1.080	1.136	0.078	6.820
T_i	4.000	4.000	6.000	4.000	4.500	1.000	22.222

Number of dissolution data points (N), degrees of freedom (df), and selected goodness of fit criteria – Pearson correlation coefficient (R), coefficient of determination (R^2), adjusted coefficient of determination ($R^2_{adjusted}$), and residual sum of squares (RSS) (manual calculation in MS Excel):

Parameter	No.1	No.2	No.3	No.4
N	7	7	7	7
df	4	4	4	4
R	0.99869945	0.99781302	0.99710857	0.99918306
R^2	0.99740058	0.99563082	0.99422549	0.99836679
$R^2_{adjusted}$	0.99610087	0.99344623	0.99133824	0.99755019
RSS	16.8564783	29.8423201	57.9392431	10.946526

Graphical abstract of model fit presented as mean ± 1 SD of the fraction % of released carvedilol:

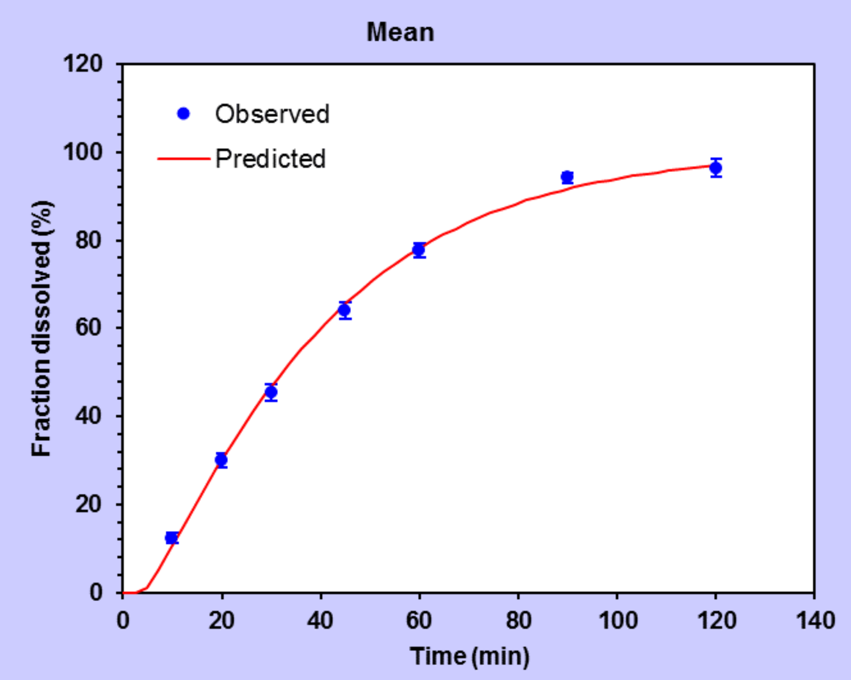

Supplementary materials – Model fitting summary – Polyglykol® 8000 P (modelling the entire relevant dissolution data)

Graphical abstract of model fit presented as the fraction % of released carvedilol per tested tablet:

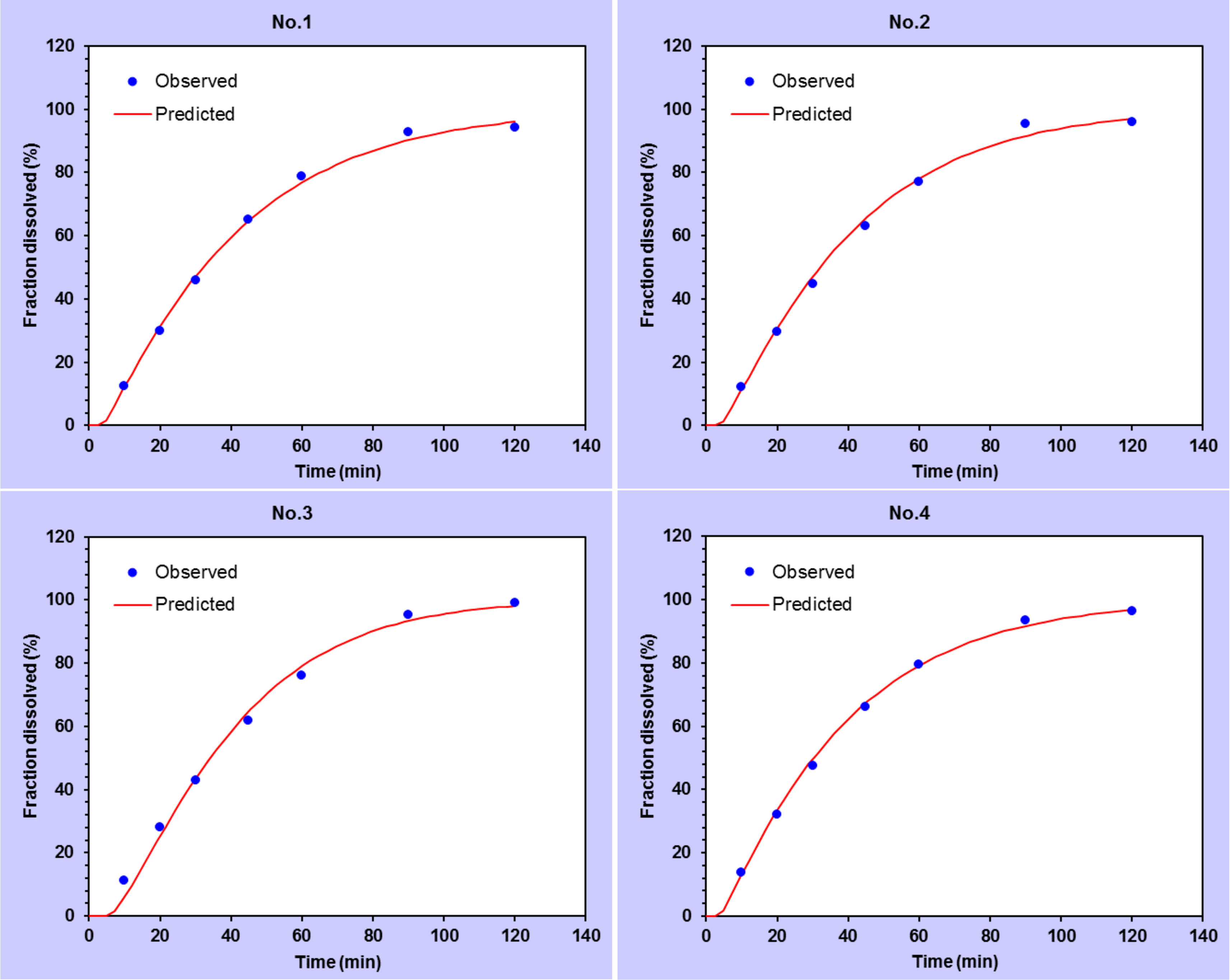

Supplementary materials – Model fitting summary – Polyglykol® 8000 P (modelling the entire relevant dissolution data)

Model: **Weibull_2**

Model equation: $F = 100 \cdot \left(1 - e^{-\frac{t^{\beta}}{\alpha}}\right)$

Fitted model parameters per tested tablet (N = 4) with statistics – mean, standard deviation (SD), and relative standard deviation expressed in % (RSD%) (output from DDSolver):

Parameter	No.1	No.2	No.3	No.4	Mean	SD	RSD(%)
α	147.011	162.869	263.717	119.871	173.367	62.796	36.221
β	1.332	1.340	1.476	1.274	1.355	0.086	6.325

Number of dissolution data points (N), degrees of freedom (df), and selected goodness of fit criteria – Pearson correlation coefficient (R), coefficient of determination (R^2), adjusted coefficient of determination ($R^2_{adjusted}$), and residual sum of squares (RSS) (manual calculation in MS Excel):

Parameter	No.1	No.2	No.3	No.4
N	7	7	7	7
df	5	5	5	5
R	0.9993763	0.99888738	0.99844023	0.99966096
R^2	0.99875299	0.997776	0.9968829	0.99932203
$R^2_{adjusted}$	0.99850359	0.9973312	0.99625948	0.99918643
RSS	20.3852738	14.2634796	25.7095718	4.88550596

Graphical abstract of model fit presented as mean ± 1 SD of the fraction % of released carvedilol:

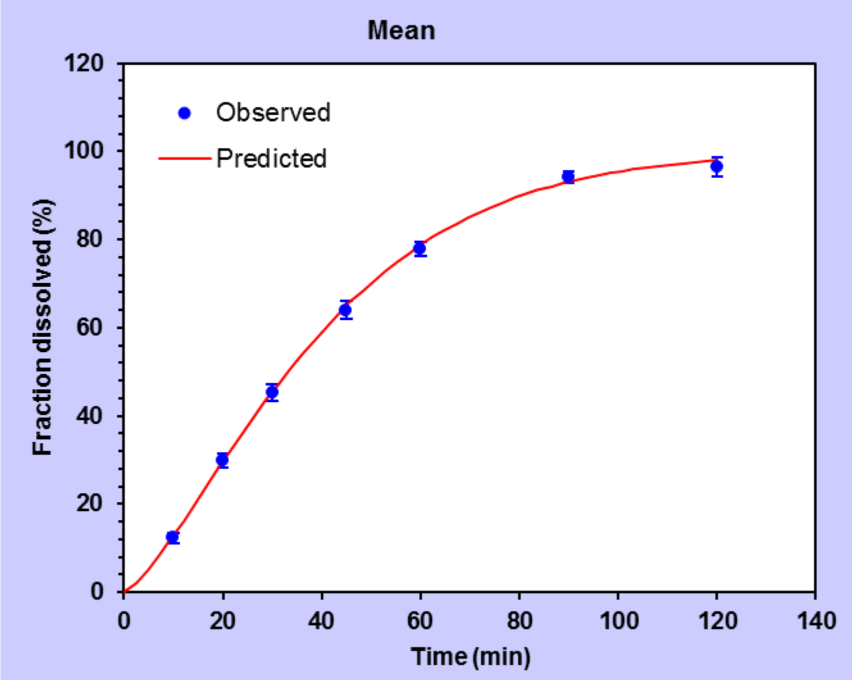

Supplementary materials – Model fitting summary – Polyglykol® 8000 P (modelling the entire relevant dissolution data)

Graphical abstract of model fit presented as the fraction % of released carvedilol per tested tablet:

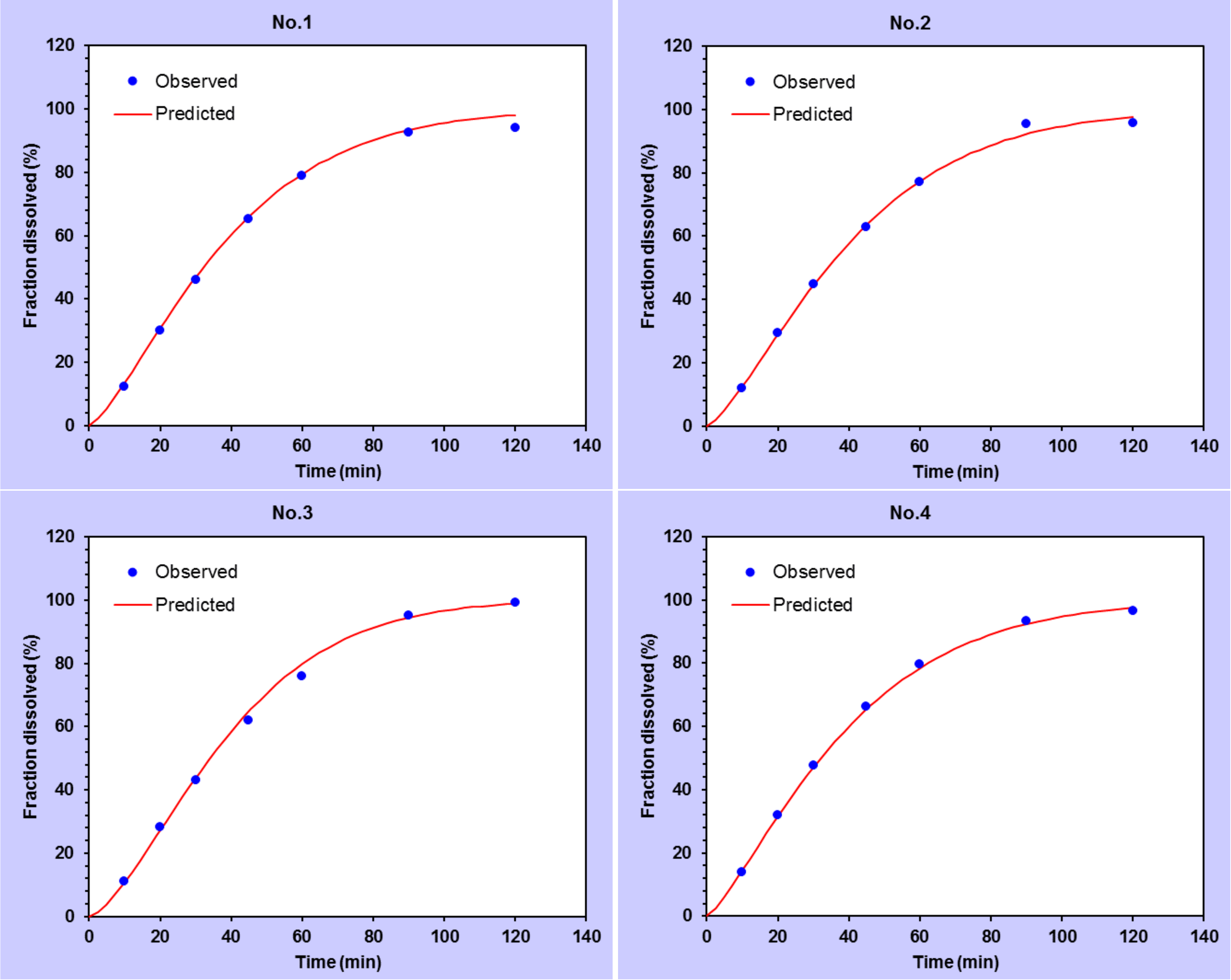

Supplementary materials – Model fitting summary – Polyglykol® 8000 P (modelling the entire relevant dissolution data)

Model: **Weibull_3**

Model equation: $F = F_{max} \cdot \left(1 - e^{-\frac{t^{\beta}}{\alpha}}\right)$

Fitted model parameters per tested tablet (N = 4) with statistics – mean, standard deviation (SD), and relative standard deviation expressed in % (RSD%) (output from DDSolver):

Parameter	No.1	No.2	No.3	No.4	Mean	SD	RSD(%)
α	137.392	156.011	184.081	111.302	147.197	30.674	20.839
β	1.300	1.323	1.341	1.243	1.302	0.042	3.262
F_{max}	98.832	100.718	104.185	101.347	101.270	2.218	2.190

Number of dissolution data points (N), degrees of freedom (df), and selected goodness of fit criteria – Pearson correlation coefficient (R), coefficient of determination (R^2), adjusted coefficient of determination ($R^2_{adjusted}$), and residual sum of squares (RSS) (manual calculation in MS Excel):

Parameter	No.1	No.2	No.3	No.4
N	7	7	7	7
df	4	4	4	4
R	0.99867265	0.99878028	0.9995065	0.99921819
R^2	0.99734706	0.99756204	0.99901325	0.99843699
$R^2_{adjusted}$	0.99602059	0.99634306	0.99851987	0.99765548
RSS	18.6477881	15.8913147	7.12613744	10.8763954

Graphical abstract of model fit presented as mean ± 1 SD of the fraction % of released carvedilol:

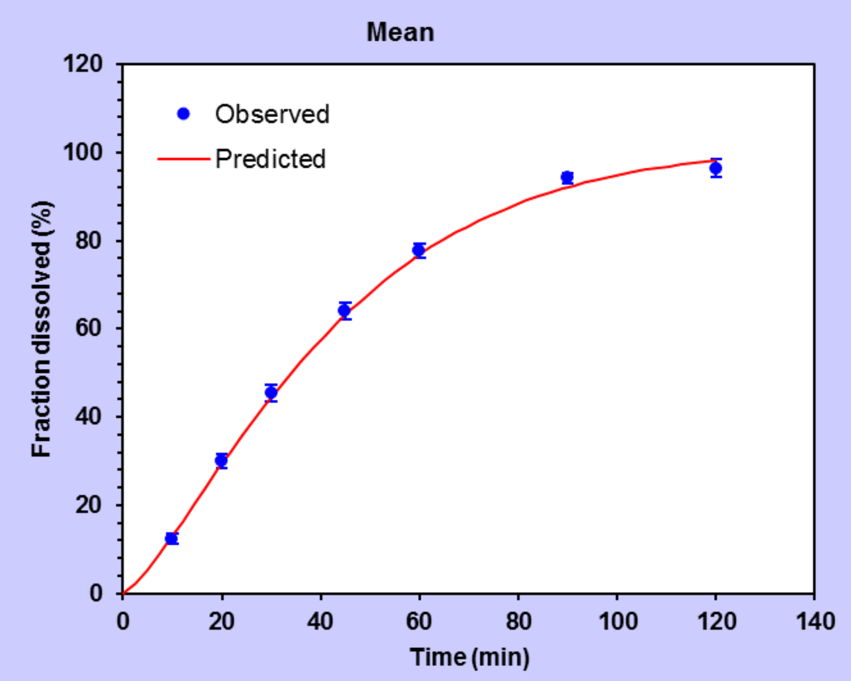

Supplementary materials – Model fitting summary – Polyglykol® 8000 P (modelling the entire relevant dissolution data)

Graphical abstract of model fit presented as the fraction % of released carvedilol per tested tablet:

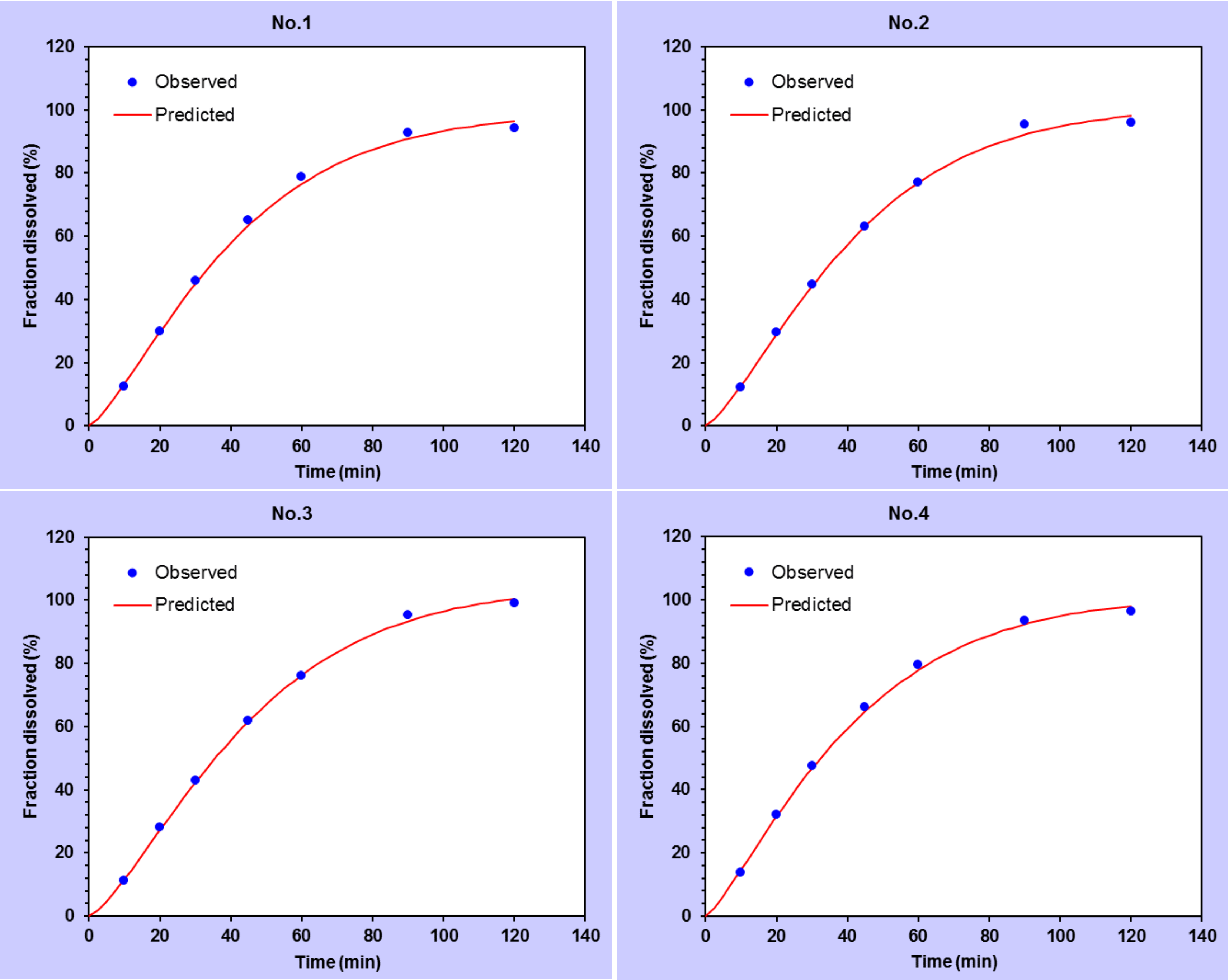

Supplementary materials – Model fitting summary – Polyglykol® 8000 P (modelling the entire relevant dissolution data)

Model: **Weibull_4**

Model equation: $F = F_{max} \cdot \left[1 - e^{-\frac{(t-T_i)^{\beta}}{\alpha}}\right]$

Fitted model parameters per tested tablet (N = 4) with statistics – mean, standard deviation (SD), and relative standard deviation expressed in % (RSD%) (output from DDSolver):

Parameter	No.1	No.2	No.3	No.4	Mean	SD	RSD(%)
α	55.730	61.915	72.105	46.841	59.148	10.626	17.964
β	1.104	1.121	1.137	1.055	1.104	0.036	3.219
T_i	4.000	4.000	4.000	4.000	4.000	0.000	0.000
F_{max}	98.832	100.718	104.185	101.347	101.270	2.218	2.190

Number of dissolution data points (N), degrees of freedom (df), and selected goodness of fit criteria – Pearson correlation coefficient (R), coefficient of determination (R^2), adjusted coefficient of determination ($R^2_{adjusted}$), and residual sum of squares (RSS) (manual calculation in MS Excel):

Parameter	No.1	No.2	No.3	No.4
N	7	7	7	7
df	3	3	3	3
R	0.9988939	0.99798591	0.9989595	0.99927609
R^2	0.99778902	0.99597587	0.99792008	0.9985527
$R^2_{adjusted}$	0.99557804	0.99195175	0.99584017	0.9971054
RSS	14.5504556	27.1335302	15.4860315	9.54746465

Graphical abstract of model fit presented as mean ± 1 SD of the fraction % of released carvedilol:

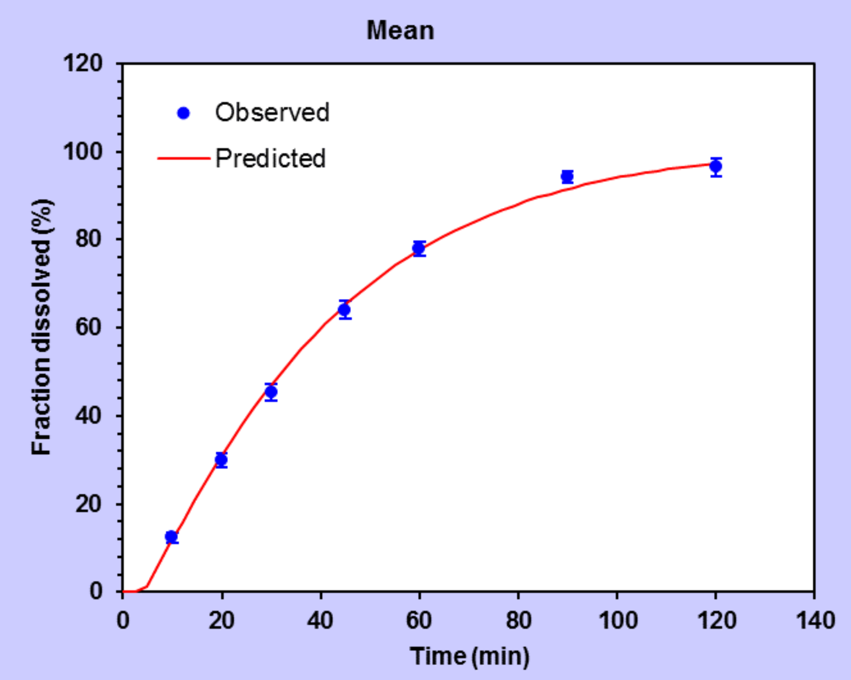

Supplementary materials – Model fitting summary – Polyglykol® 8000 P (modelling the entire relevant dissolution data)

Graphical abstract of model fit presented as the fraction % of released carvedilol per tested tablet:

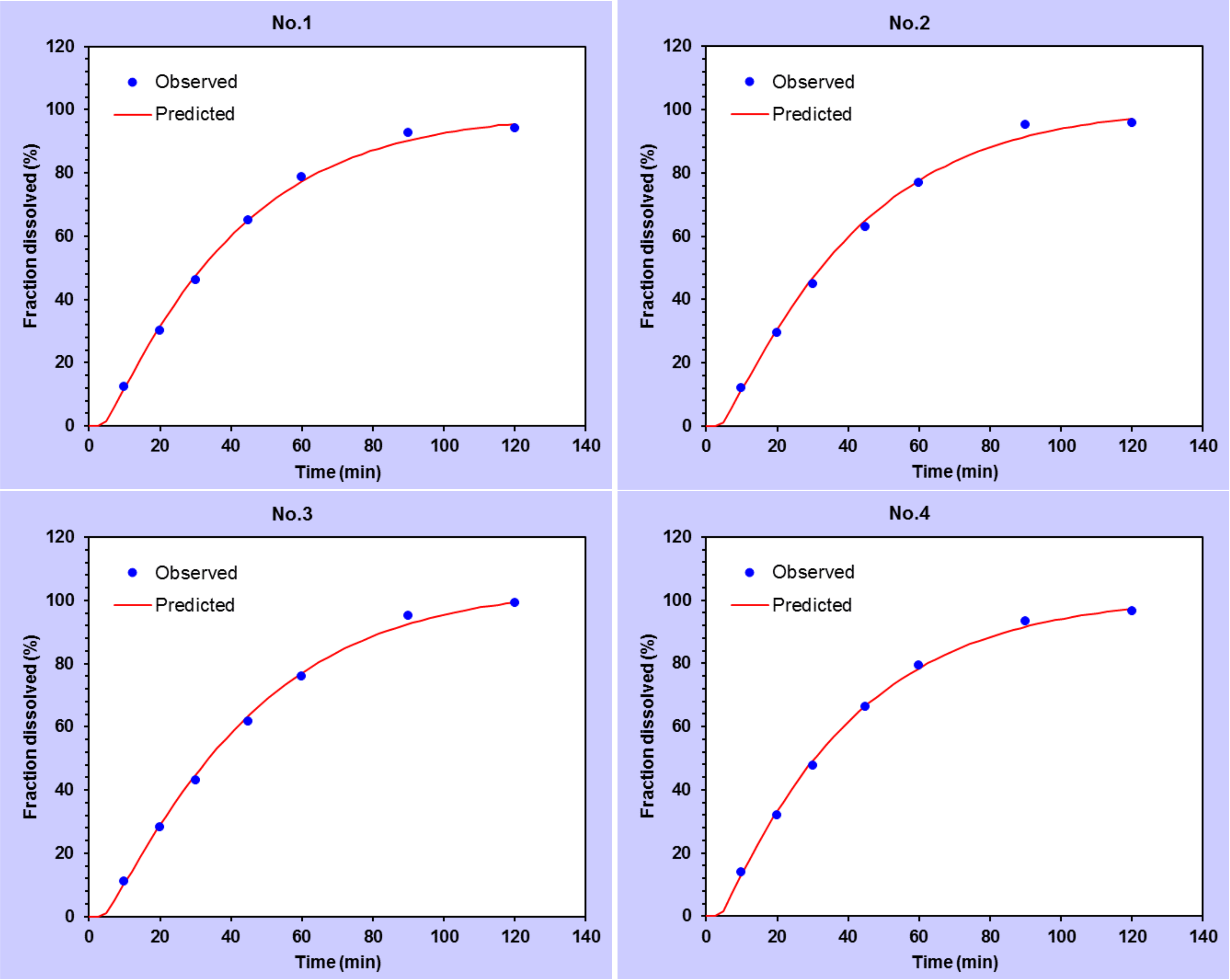

Supplementary materials – Model fitting summary – Polyglykol® 8000 P (modelling the entire relevant dissolution data)

Model: **Logistic_1**

Model equation: $F = 100 \cdot \frac{e^{\alpha+\beta \cdot \log(t)}}{1+e^{\alpha+\beta \cdot \log(t)}}$

Fitted model parameters per tested tablet (N = 4) with statistics – mean, standard deviation (SD), and relative standard deviation expressed in % (RSD%) (output from DDSolver):

Parameter	No.1	No.2	No.3	No.4	Mean	SD	RSD(%)
α	-6.757	-7.343	-8.782	-6.971	-7.463	0.912	-12.219
β	4.590	5.005	6.017	4.815	5.107	0.630	12.341

Number of dissolution data points (N), degrees of freedom (df), and selected goodness of fit criteria – Pearson correlation coefficient (R), coefficient of determination (R^2), adjusted coefficient of determination ($R^2_{adjusted}$), and residual sum of squares (RSS) (manual calculation in MS Excel):

Parameter	No.1	No.2	No.3	No.4
N	7	7	7	7
df	5	5	5	5
R	0.99586915	0.98895521	0.97795797	0.99258563
R^2	0.99175536	0.97803242	0.95640179	0.98522623
$R^2_{adjusted}$	0.99010643	0.9736389	0.94768215	0.98227147
RSS	56.5754647	172.209115	453.960433	109.233162

Graphical abstract of model fit presented as mean ± 1 SD of the fraction % of released carvedilol:

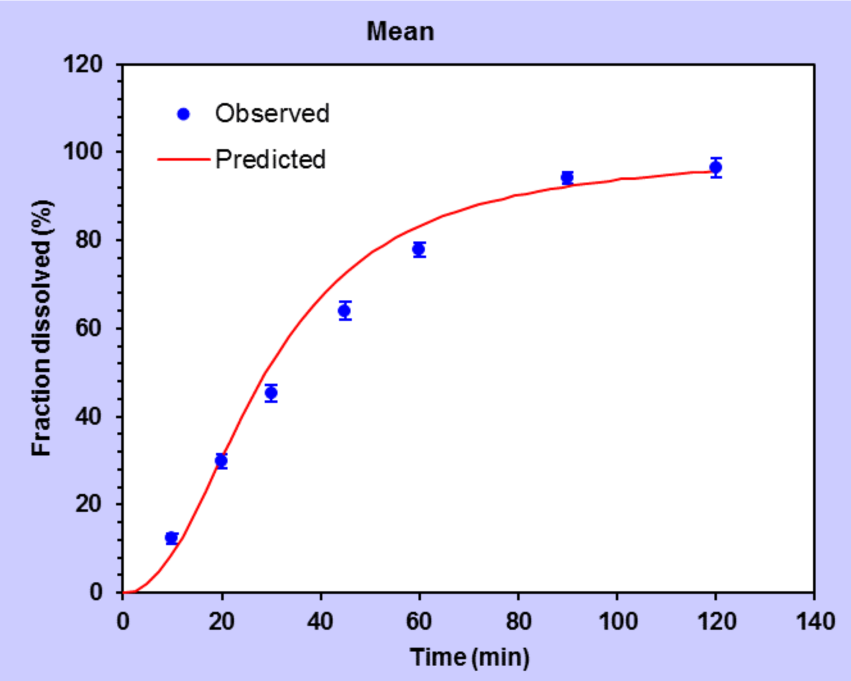

Supplementary materials – Model fitting summary – Polyglykol® 8000 P (modelling the entire relevant dissolution data)

Graphical abstract of model fit presented as the fraction % of released carvedilol per tested tablet:

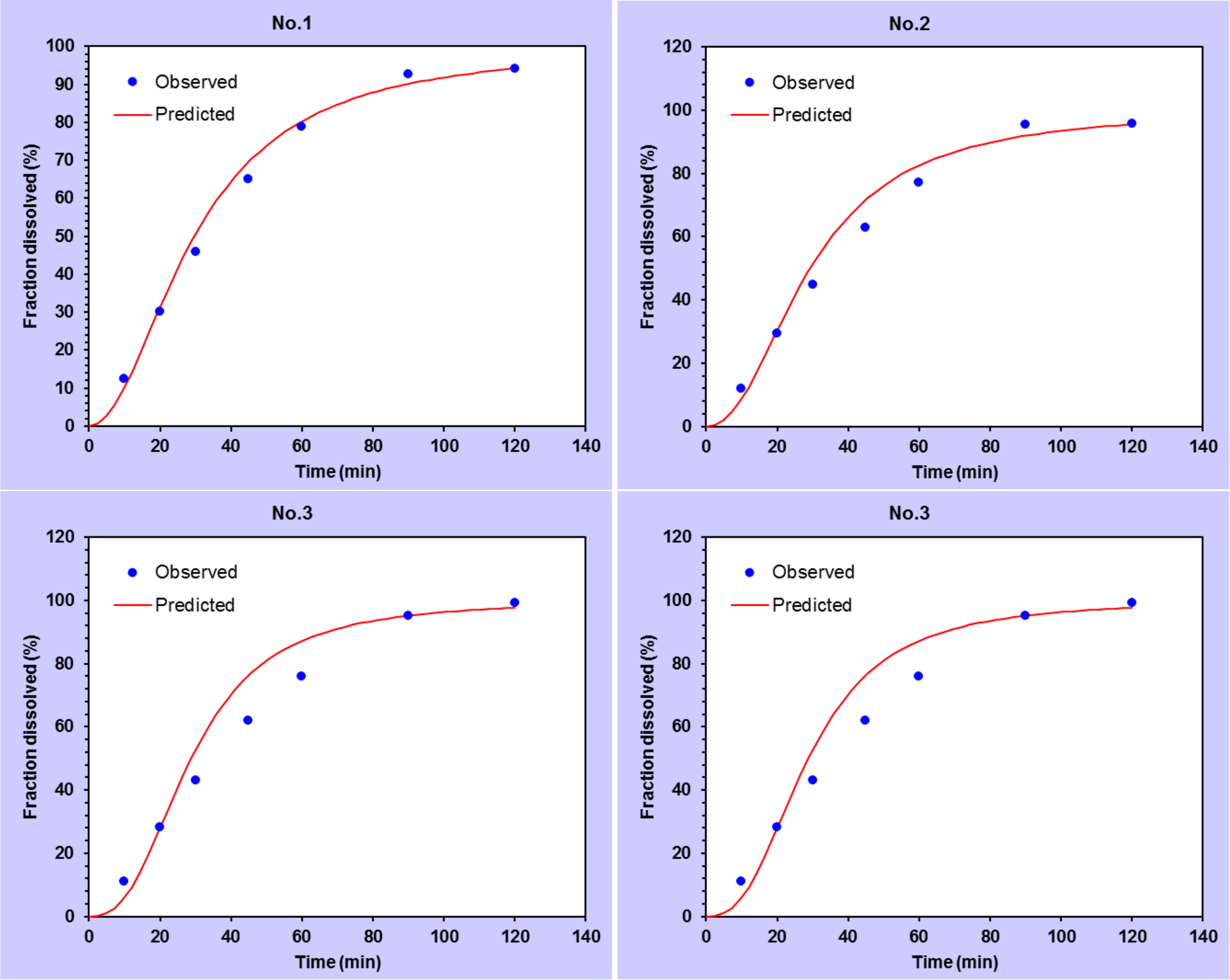

Supplementary materials – Model fitting summary – Polyglykol® 8000 P (modelling the entire relevant dissolution data)

Model: **Logistic_2**

Model equation: $F = F_{max} \cdot \frac{e^{\alpha+\beta \cdot \log(t)}}{1+e^{\alpha+\beta \cdot \log(t)}}$

Fitted model parameters per tested tablet (N = 4) with statistics – mean, standard deviation (SD), and relative standard deviation expressed in % (RSD%) (output from DDSolver):

Parameter	No.1	No.2	No.3	No.4	Mean	SD	RSD(%)
α	-6.983	-7.804	-7.179	-6.665	-7.158	0.480	-6.708
β	4.779	5.211	4.760	4.564	4.829	0.273	5.654
F_{max}	98.832	95.603	104.185	101.347	99.992	3.653	3.653

Number of dissolution data points (N), degrees of freedom (df), and selected goodness of fit criteria – Pearson correlation coefficient (R), coefficient of determination (R^2), adjusted coefficient of determination ($R^2_{adjusted}$), and residual sum of squares (RSS) (manual calculation in MS Excel):

Parameter	No.1	No.2	No.3	No.4
N	7	7	7	7
df	4	4	4	4
R	0.99451626	0.9922822	0.99215269	0.99472169
R^2	0.9890626	0.98462396	0.98436695	0.98947124
$R^2_{adjusted}$	0.9835939	0.97693594	0.97655043	0.98420687
RSS	77.4525515	143.50148	128.347504	74.3118474

Graphical abstract of model fit presented as mean ± 1 SD of the fraction % of released carvedilol:

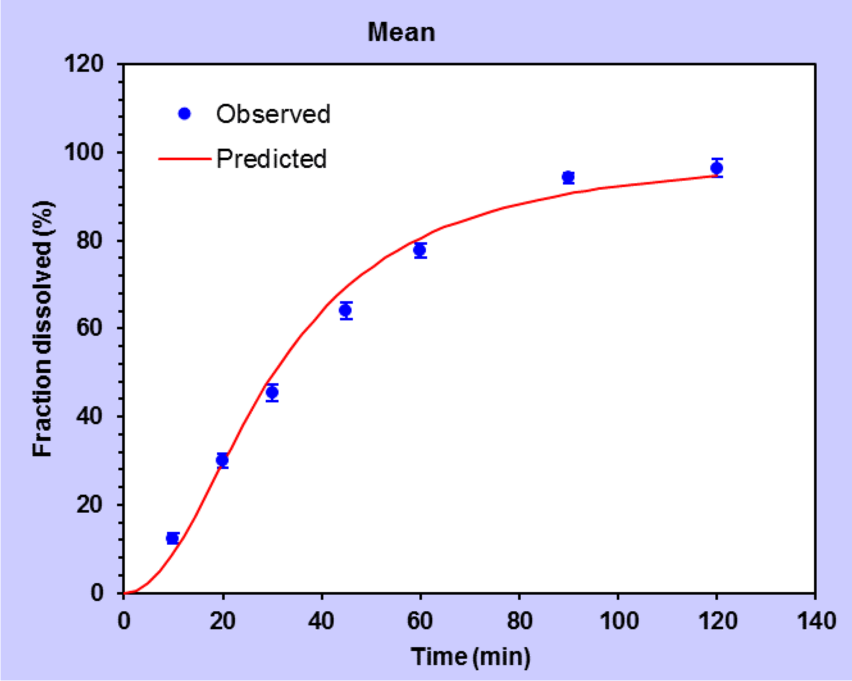

Supplementary materials – Model fitting summary – Polyglykol® 8000 P (modelling the entire relevant dissolution data)

Graphical abstract of model fit presented as the fraction % of released carvedilol per tested tablet:

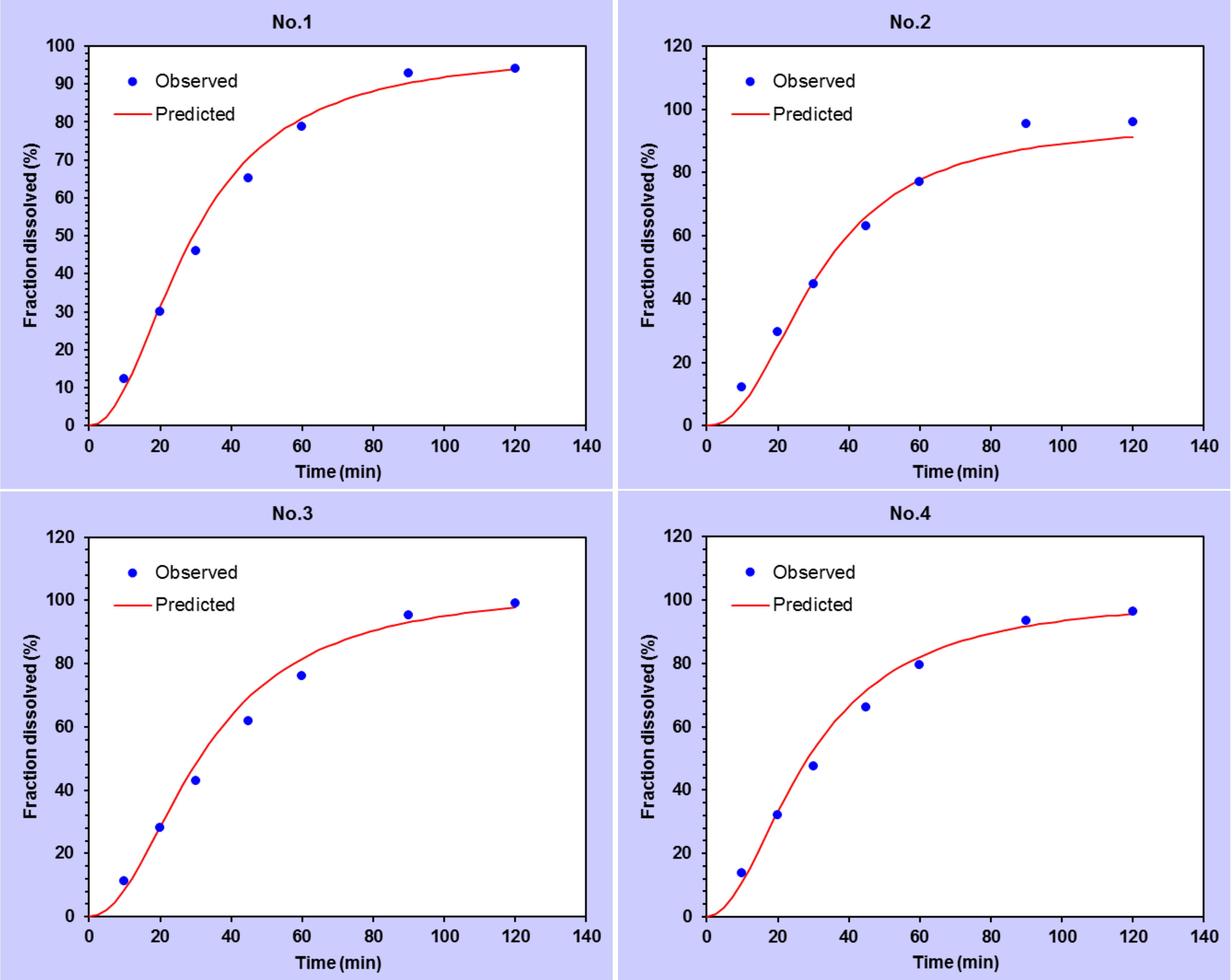

Supplementary materials – Model fitting summary – Polyglykol® 8000 P (modelling the entire relevant dissolution data)

Model: **Logistic_3**

Model equation: $F = F_{max} \cdot \frac{1}{1+e^{-k\cdot(t-\gamma)}}$

Fitted model parameters per tested tablet (N = 4) with statistics – mean, standard deviation (SD), and relative standard deviation expressed in % (RSD%) (output from DDSolver):

Parameter	No.1	No.2	No.3	No.4	Mean	SD	RSD(%)
k	0.066	0.045	0.045	0.064	0.055	0.012	21.002
γ	37.907	39.495	43.173	37.897	39.618	2.486	6.275
F_{max}	98.832	100.718	104.185	101.347	101.270	2.218	2.190

Number of dissolution data points (N), degrees of freedom (df), and selected goodness of fit criteria – Pearson correlation coefficient (R), coefficient of determination (R^2), adjusted coefficient of determination ($R^2_{adjusted}$), and residual sum of squares (RSS) (manual calculation in MS Excel):

Parameter	No.1	No.2	No.3	No.4
N	7	7	7	7
df	4	4	4	4
R	0.99177861	0.98755005	0.98753114	0.99140436
R^2	0.98362482	0.97525509	0.97521775	0.9828826
$R^2_{adjusted}$	0.97543722	0.96288264	0.96282663	0.97432391
RSS	183.522604	186.831534	195.542366	203.768253

Graphical abstract of model fit presented as mean ± 1 SD of the fraction % of released carvedilol:

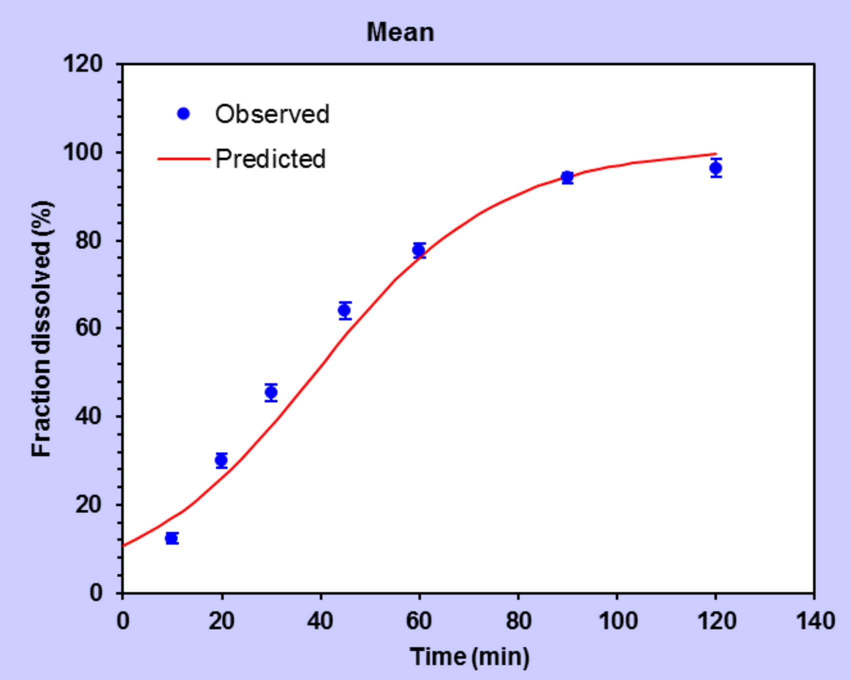

Supplementary materials – Model fitting summary – Polyglykol® 8000 P (modelling the entire relevant dissolution data)

Graphical abstract of model fit presented as the fraction % of released carvedilol per tested tablet:

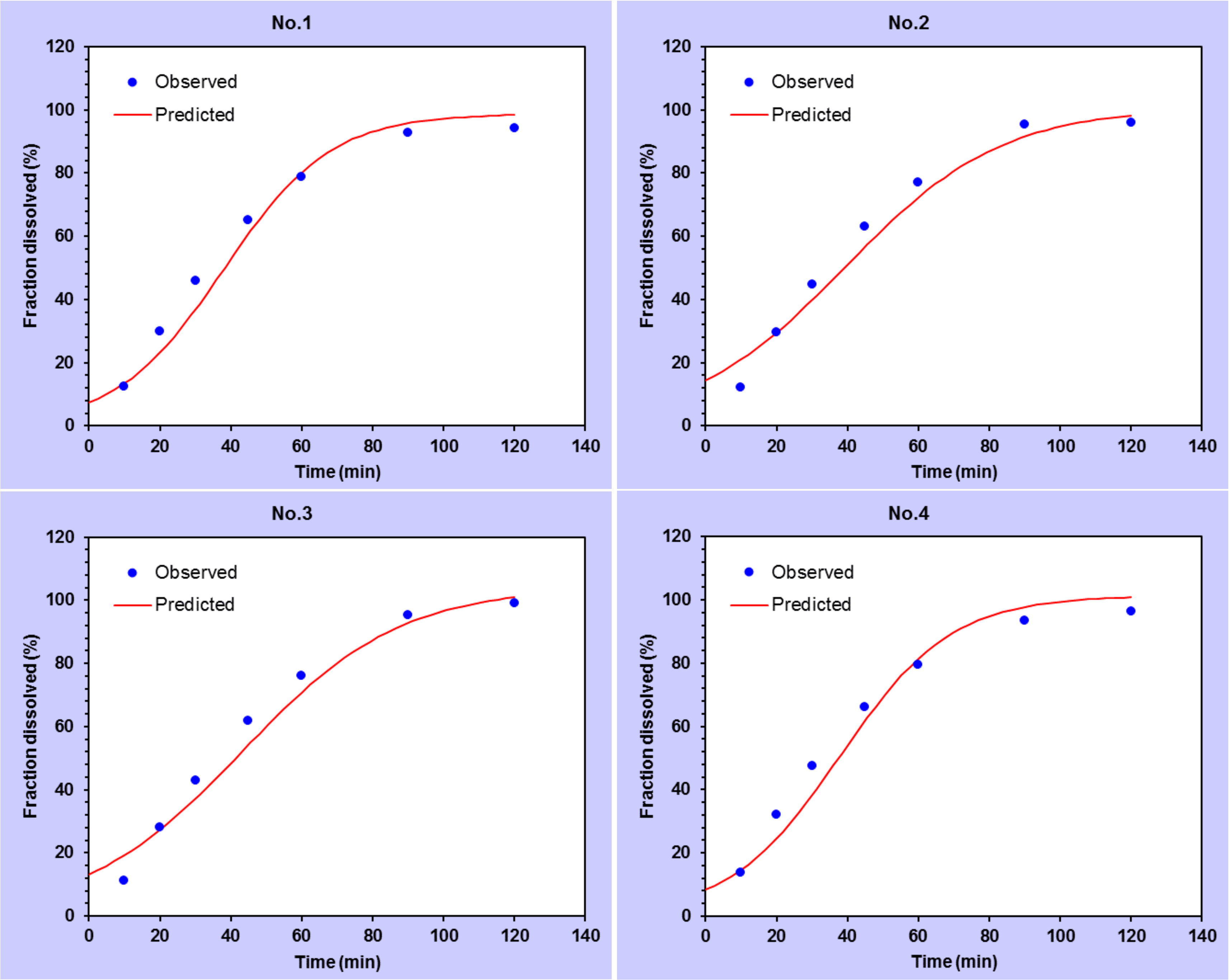

Supplementary materials – Model fitting summary – Polyglykol® 8000 P (modelling the entire relevant dissolution data)

Model: **Gompertz_1**

Model equation: $F = 100 \cdot e^{-\alpha \cdot e^{-\beta \cdot \log(t)}}$

Fitted model parameters per tested tablet (N = 4) with statistics – mean, standard deviation (SD), and relative standard deviation expressed in % (RSD%) (output from DDSolver):

Parameter	No.1	No.2	No.3	No.4	Mean	SD	RSD(%)
α	100.700	259.123	973.572	210.573	385.992	397.286	102.926
β	3.495	3.885	4.836	3.780	3.999	0.582	14.547

Number of dissolution data points (N), degrees of freedom (df), and selected goodness of fit criteria – Pearson correlation coefficient (R), coefficient of determination (R^2), adjusted coefficient of determination ($R^2_{adjusted}$), and residual sum of squares (RSS) (manual calculation in MS Excel):

Parameter	No.1	No.2	No.3	No.4
N	7	7	7	7
df	5	5	5	5
R	0.98103773	0.99051933	0.97959205	0.99432759
R^2	0.96243502	0.98112855	0.95960058	0.98868735
$R^2_{adjusted}$	0.95492202	0.97735425	0.95152069	0.98642482
RSS	265.963654	324.558201	448.429169	343.296629

Graphical abstract of model fit presented as mean ± 1 SD of the fraction % of released carvedilol:

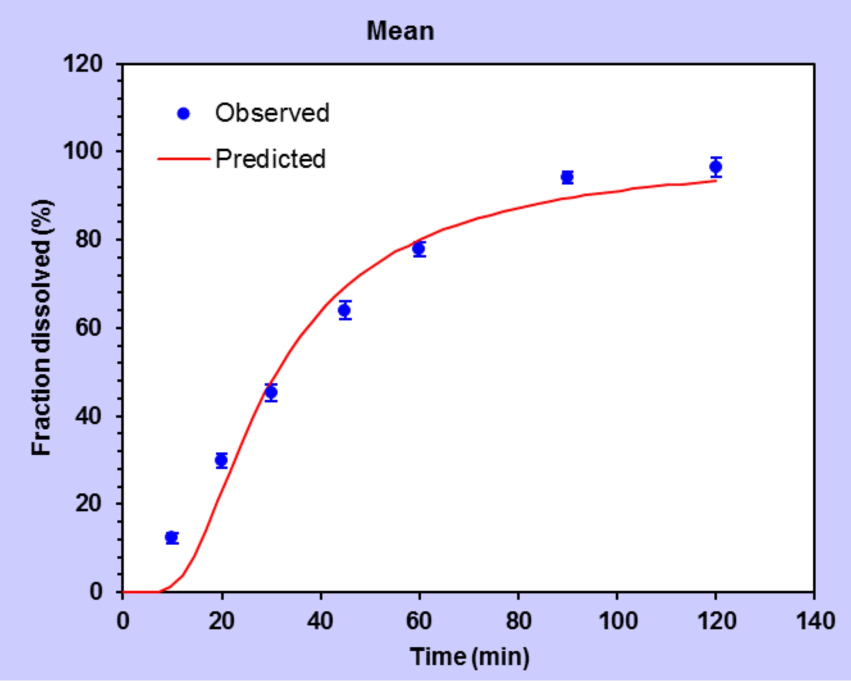

Supplementary materials – Model fitting summary – Polyglykol® 8000 P (modelling the entire relevant dissolution data)

Graphical abstract of model fit presented as the fraction % of released carvedilol per tested tablet:

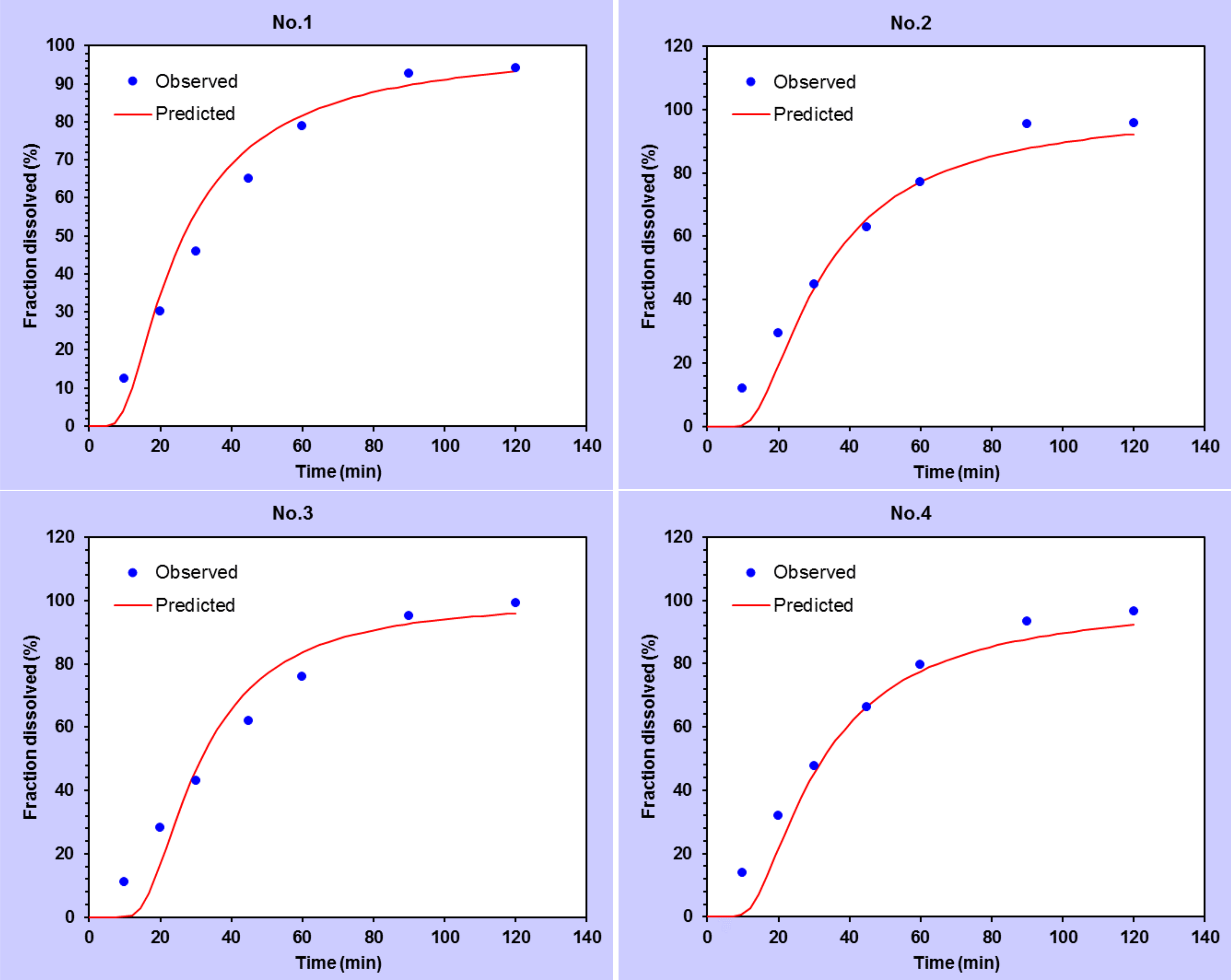

Supplementary materials – Model fitting summary – Polyglykol® 8000 P (modelling the entire relevant dissolution data)

Model: **Gompertz_2**

Model equation: $F = F_{max} \cdot e^{-\alpha \cdot e^{-\beta \cdot \log(t)}}$

Fitted model parameters per tested tablet (N = 4) with statistics – mean, standard deviation (SD), and relative standard deviation expressed in % (RSD%) (output from DDSolver):

Parameter	No.1	No.2	No.3	No.4	Mean	SD	RSD(%)
α	127.488	215.900	189.573	102.291	158.813	52.855	33.282
β	3.686	3.739	3.572	3.527	3.631	0.099	2.714
F_{max}	98.832	100.718	104.185	101.347	101.270	2.218	2.190

Number of dissolution data points (N), degrees of freedom (df), and selected goodness of fit criteria – Pearson correlation coefficient (R), coefficient of determination (R^2), adjusted coefficient of determination ($R^2_{adjusted}$), and residual sum of squares (RSS) (manual calculation in MS Excel):

Parameter	No.1	No.2	No.3	No.4
N	7	7	7	7
df	4	4	4	4
R	0.9783665	0.99186429	0.99333025	0.97909702
R^2	0.95720101	0.98379476	0.98670499	0.95863098
$R^2_{adjusted}$	0.93580151	0.97569215	0.98005749	0.93794647
RSS	312.799381	334.521135	353.325754	302.305334

Graphical abstract of model fit presented as mean ± 1 SD of the fraction % of released carvedilol:

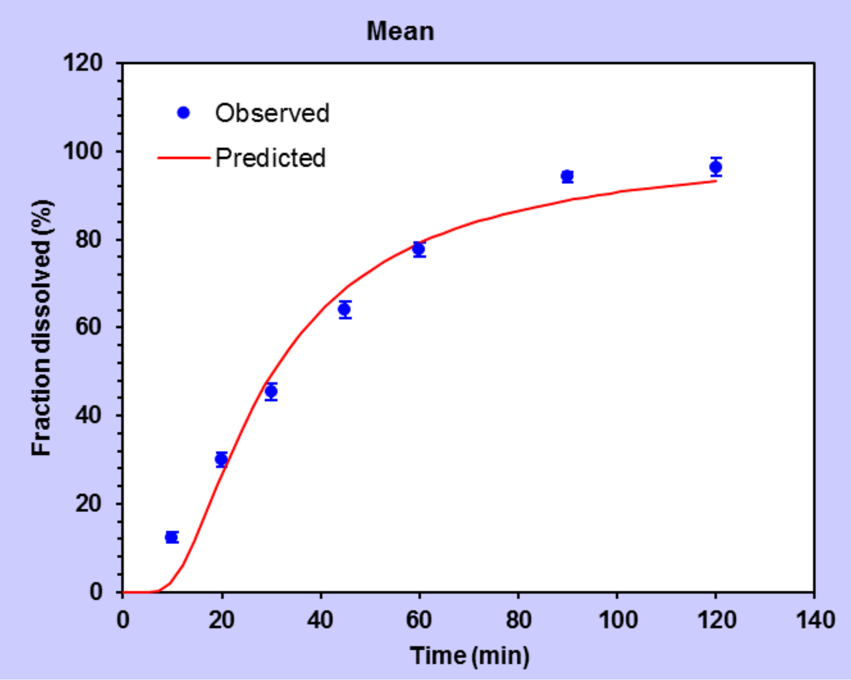

Supplementary materials – Model fitting summary – Polyglykol® 8000 P (modelling the entire relevant dissolution data)

Graphical abstract of model fit presented as the fraction % of released carvedilol per tested tablet:

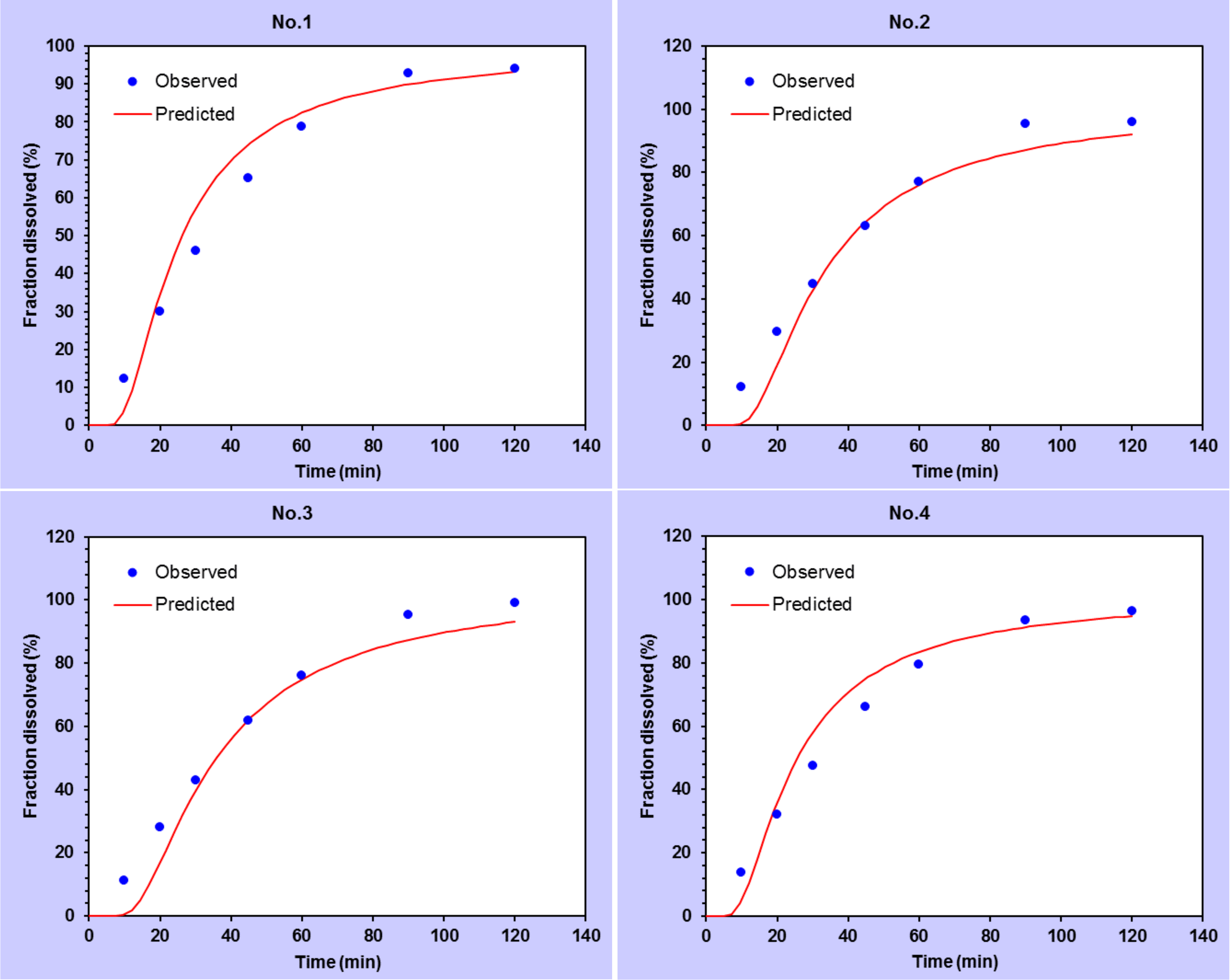

Supplementary materials – Model fitting summary – Polyglykol® 8000 P (modelling the entire relevant dissolution data)

Model: **Gompertz_3**

Model equation: $F = F_{max} \cdot e^{-e^{-k\cdot(t-\gamma)}}$

Fitted model parameters per tested tablet (N = 4) with statistics – mean, standard deviation (SD), and relative standard deviation expressed in % (RSD%) (output from DDSolver):

Parameter	No.1	No.2	No.3	No.4	Mean	SD	RSD(%)
k	0.044	0.036	0.035	0.042	0.039	0.004	11.034
γ	25.030	25.068	28.020	24.892	25.752	1.514	5.877
F_{max}	93.684	100.718	104.185	96.068	98.664	4.699	4.762

Number of dissolution data points (N), degrees of freedom (df), and selected goodness of fit criteria – Pearson correlation coefficient (R), coefficient of determination (R^2), adjusted coefficient of determination ($R^2_{adjusted}$), and residual sum of squares (RSS) (manual calculation in MS Excel):

Parameter	No.1	No.2	No.3	No.4
N	7	7	7	7
df	4	4	4	4
R	0.99868445	0.99721674	0.9977925	0.99853601
R^2	0.99737063	0.99444123	0.99558986	0.99707416
$R^2_{adjusted}$	0.99605594	0.99166185	0.9933848	0.99561125
RSS	71.7099782	53.1875233	36.5660903	74.5274804

Graphical abstract of model fit presented as mean ± 1 SD of the fraction % of released carvedilol:

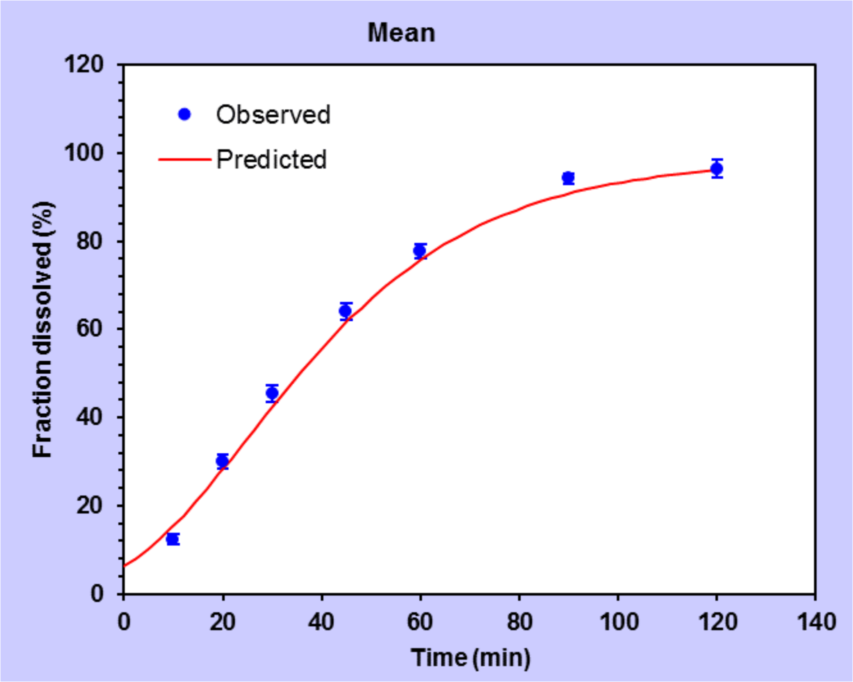

Supplementary materials – Model fitting summary – Polyglykol® 8000 P (modelling the entire relevant dissolution data)

Graphical abstract of model fit presented as the fraction % of released carvedilol per tested tablet:

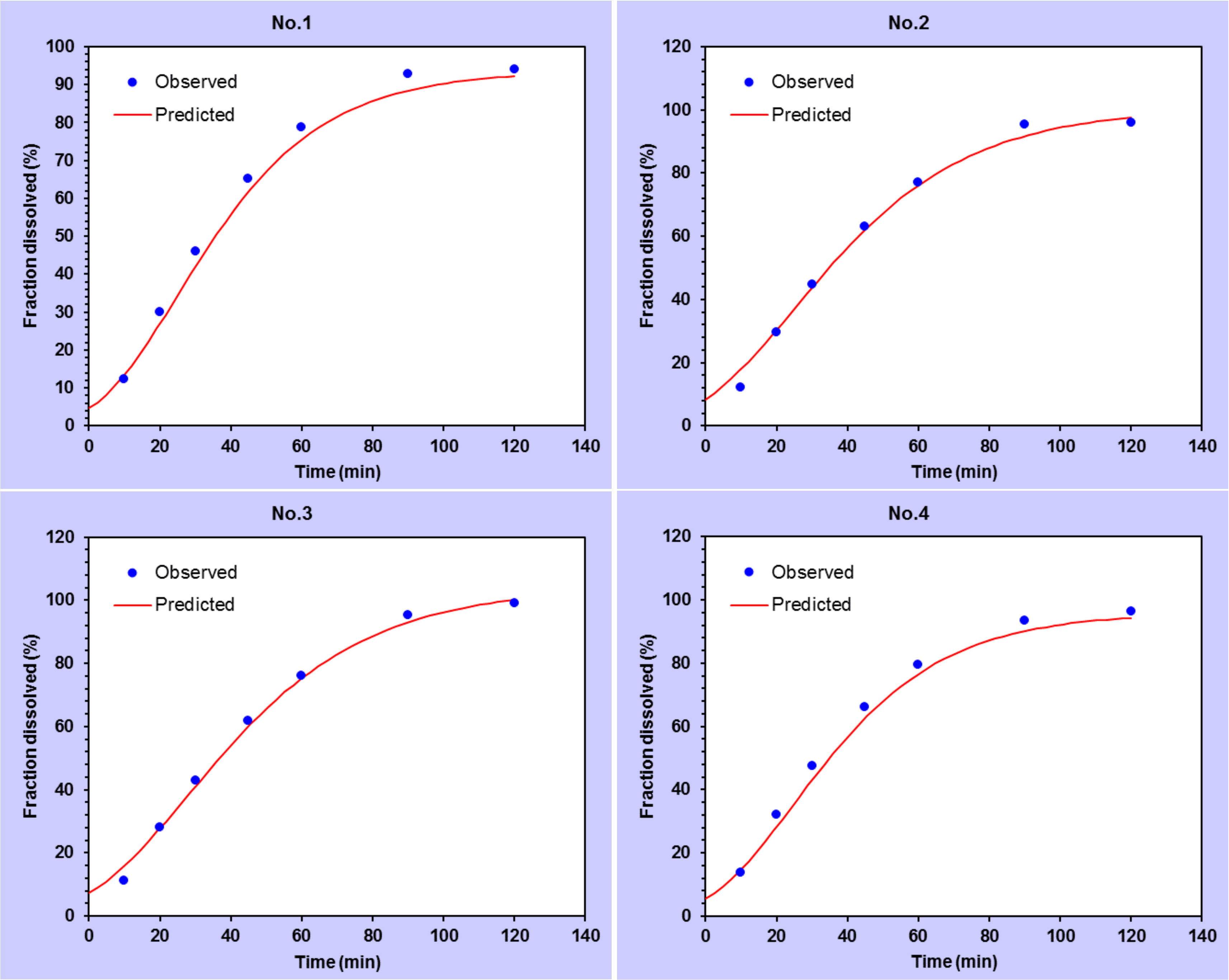

Supplementary materials – Model fitting summary – Polyglykol® 8000 P (modelling the entire relevant dissolution data)

Model: **Gompertz_4**

Model equation: $F = F_{max} \cdot e^{-\beta \cdot e^{-k \cdot t}}$

Fitted model parameters per tested tablet (N = 4) with statistics – mean, standard deviation (SD), and relative standard deviation expressed in % (RSD%) (output from DDSolver):

Parameter	No.1	No.2	No.3	No.4	Mean	SD	RSD(%)
k	0.035	0.040	0.035	0.038	0.037	0.002	6.284
β	2.287	2.960	2.675	2.555	2.619	0.279	10.666
F_{max}	98.832	99.815	104.185	100.027	100.715	2.371	2.355

Number of dissolution data points (N), degrees of freedom (df), and selected goodness of fit criteria – Pearson correlation coefficient (R), coefficient of determination (R^2), adjusted coefficient of determination ($R^2_{adjusted}$), and residual sum of squares (RSS) (manual calculation in MS Excel):

Parameter	No.1	No.2	No.3	No.4
N	7	7	7	7
df	4	4	4	4
R	0.99526975	0.99757531	0.9977925	0.99707704
R^2	0.99056188	0.9951565	0.99558986	0.99416263
$R^2_{adjusted}$	0.98584283	0.99273475	0.9933848	0.99124395
RSS	92.9964826	43.1193125	36.5660903	44.7127456

Graphical abstract of model fit presented as mean ± 1 SD of the fraction % of released carvedilol:

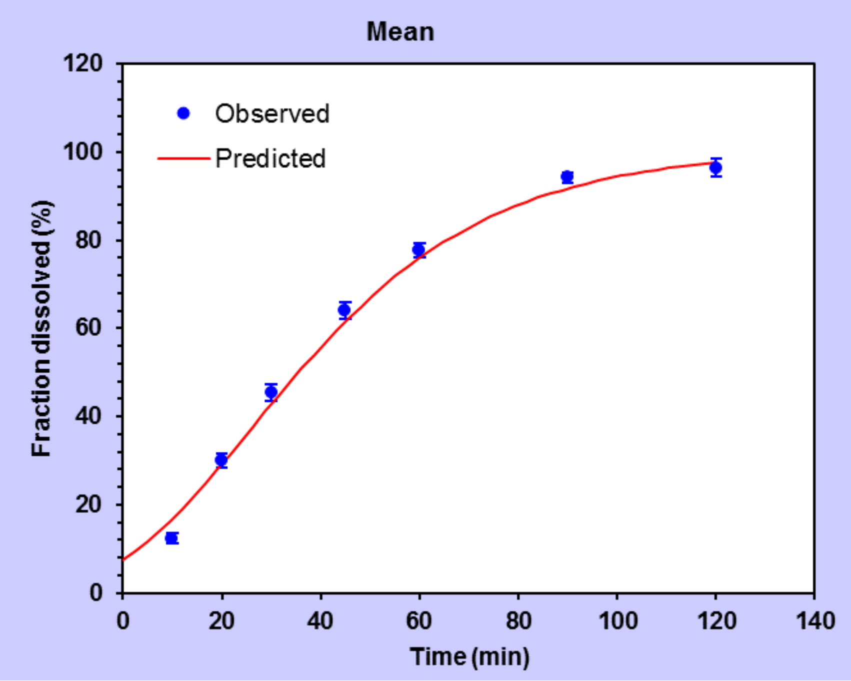

Supplementary materials – Model fitting summary – Polyglykol® 8000 P (modelling the entire relevant dissolution data)

Graphical abstract of model fit presented as the fraction % of released carvedilol per tested tablet:

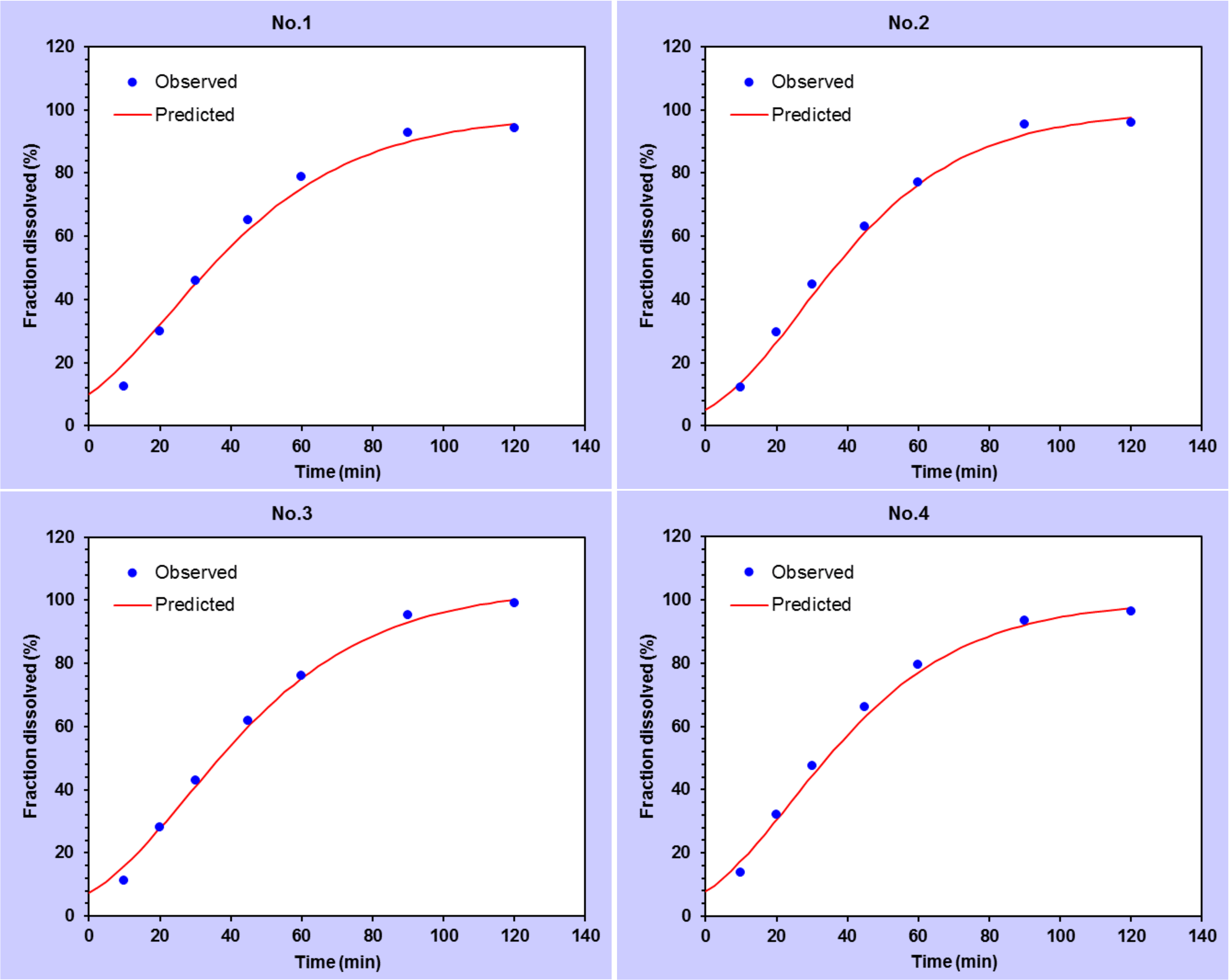

Supplementary materials – Model fitting summary – Polyglykol® 8000 P (modelling the entire relevant dissolution data)

Model: **Probit_1**

Model equation: $F = 100 \cdot \phi[\alpha + \beta \cdot \log(t)]$

Fitted model parameters per tested tablet (N = 4) with statistics – mean, standard deviation (SD), and relative standard deviation expressed in % (RSD%) (output from DDSolver):

Parameter	No.1	No.2	No.3	No.4	Mean	SD	RSD(%)
α	-3.935	-4.214	-4.819	-4.006	-4.243	0.401	-9.462
β	2.667	2.860	3.278	2.757	2.890	0.270	9.343

Number of dissolution data points (N), degrees of freedom (df), and selected goodness of fit criteria – Pearson correlation coefficient (R), coefficient of determination (R^2), adjusted coefficient of determination ($R^2_{adjusted}$), and residual sum of squares (RSS) (manual calculation in MS Excel):

Parameter	No.1	No.2	No.3	No.4
N	7	7	7	7
df	5	5	5	5
R	0.9966183	0.99183661	0.98538095	0.99487887
R^2	0.99324804	0.98373985	0.97097562	0.98978396
$R^2_{adjusted}$	0.99189764	0.98048782	0.96517075	0.98774075
RSS	43.9223781	118.75441	274.476102	69.8410119

Graphical abstract of model fit presented as mean ± 1 SD of the fraction % of released carvedilol:

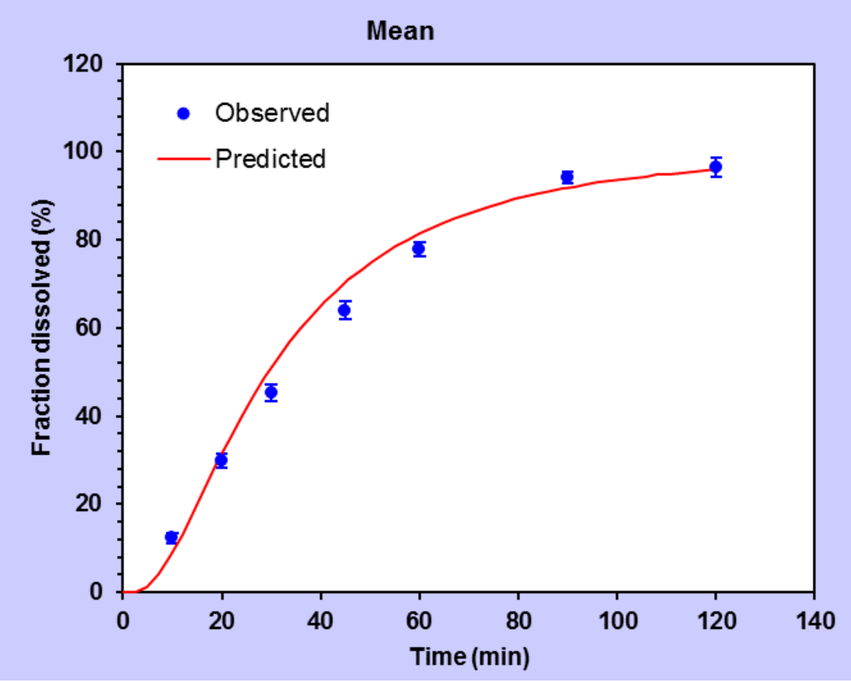

Supplementary materials – Model fitting summary – Polyglykol® 8000 P (modelling the entire relevant dissolution data)

Graphical abstract of model fit presented as the fraction % of released carvedilol per tested tablet:

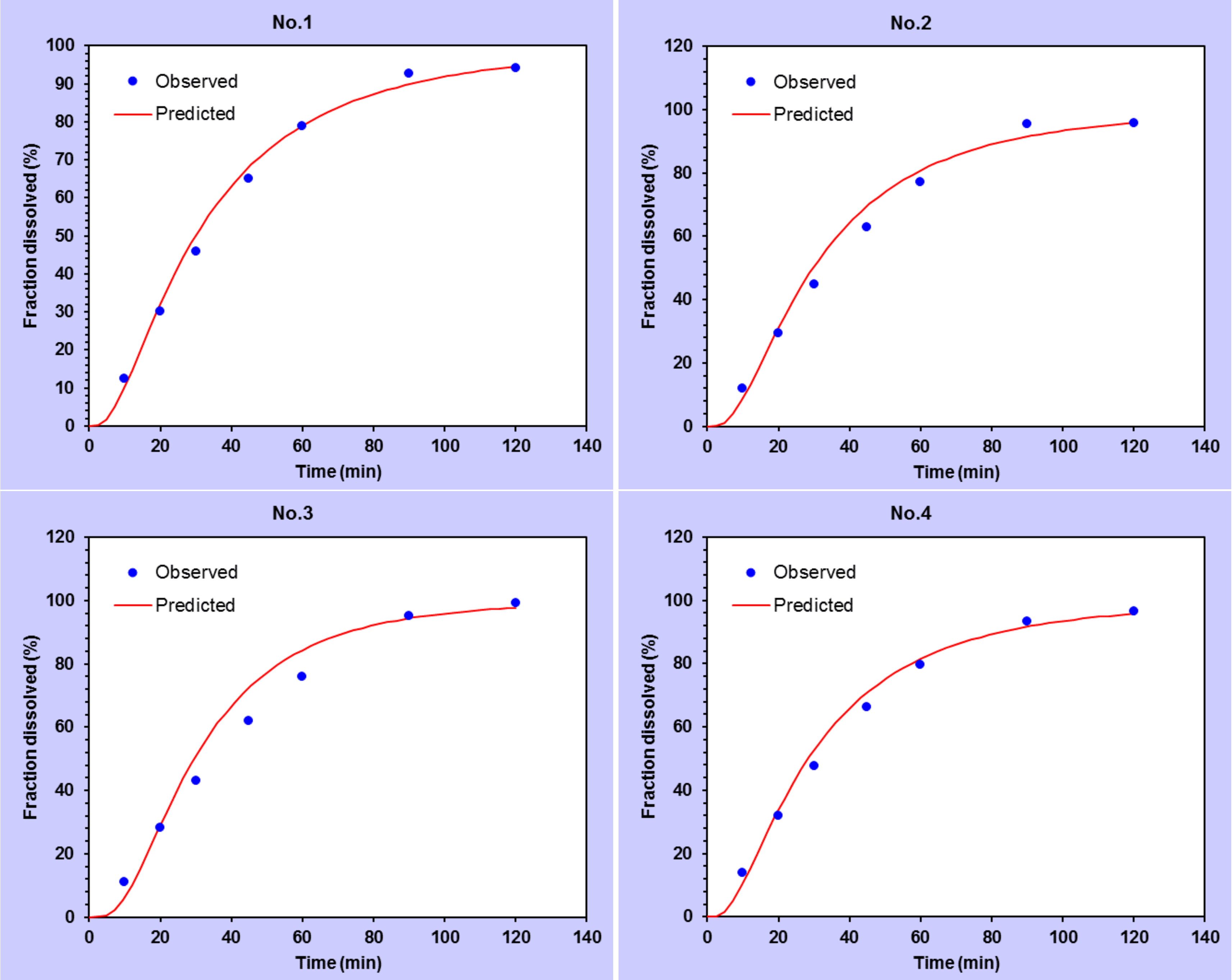

Supplementary materials – Model fitting summary – Polyglykol® 8000 P (modelling the entire relevant dissolution data)

Model: **Probit_2**

Model equation: $F = F_{max} \cdot \phi[\alpha + \beta \cdot \log(t)]$

Fitted model parameters per tested tablet (N = 4) with statistics – mean, standard deviation (SD), and relative standard deviation expressed in % (RSD%) (output from DDSolver):

Parameter	No.1	No.2	No.3	No.4	Mean	SD	RSD(%)
α	-4.038	-4.135	-4.162	-3.871	-4.052	0.132	-3.256
β	2.755	2.794	2.752	2.643	2.736	0.065	2.373
F_{max}	98.832	100.718	104.185	101.347	101.270	2.218	2.190

Number of dissolution data points (N), degrees of freedom (df), and selected goodness of fit criteria – Pearson correlation coefficient (R), coefficient of determination (R^2), adjusted coefficient of determination ($R^2_{adjusted}$), and residual sum of squares (RSS) (manual calculation in MS Excel):

Parameter	No.1	No.2	No.3	No.4
N	7	7	7	7
df	4	4	4	4
R	0.99585943	0.99275988	0.99389306	0.99614985
R^2	0.991736	0.98557218	0.98782341	0.99231452
$R^2_{adjusted}$	0.987604	0.97835827	0.98173512	0.98847178
RSS	54.8108362	103.342408	94.2543524	50.7164

Graphical abstract of model fit presented as mean ± 1 SD of the fraction % of released carvedilol:

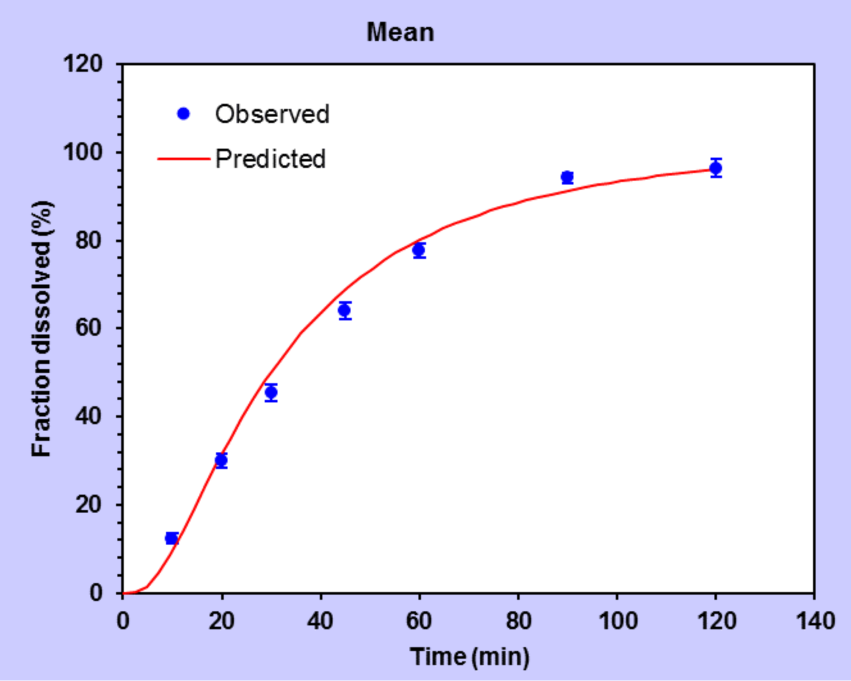

Supplementary materials – Model fitting summary – Polyglykol® 8000 P (modelling the entire relevant dissolution data)

Graphical abstract of model fit presented as the fraction % of released carvedilol per tested tablet:

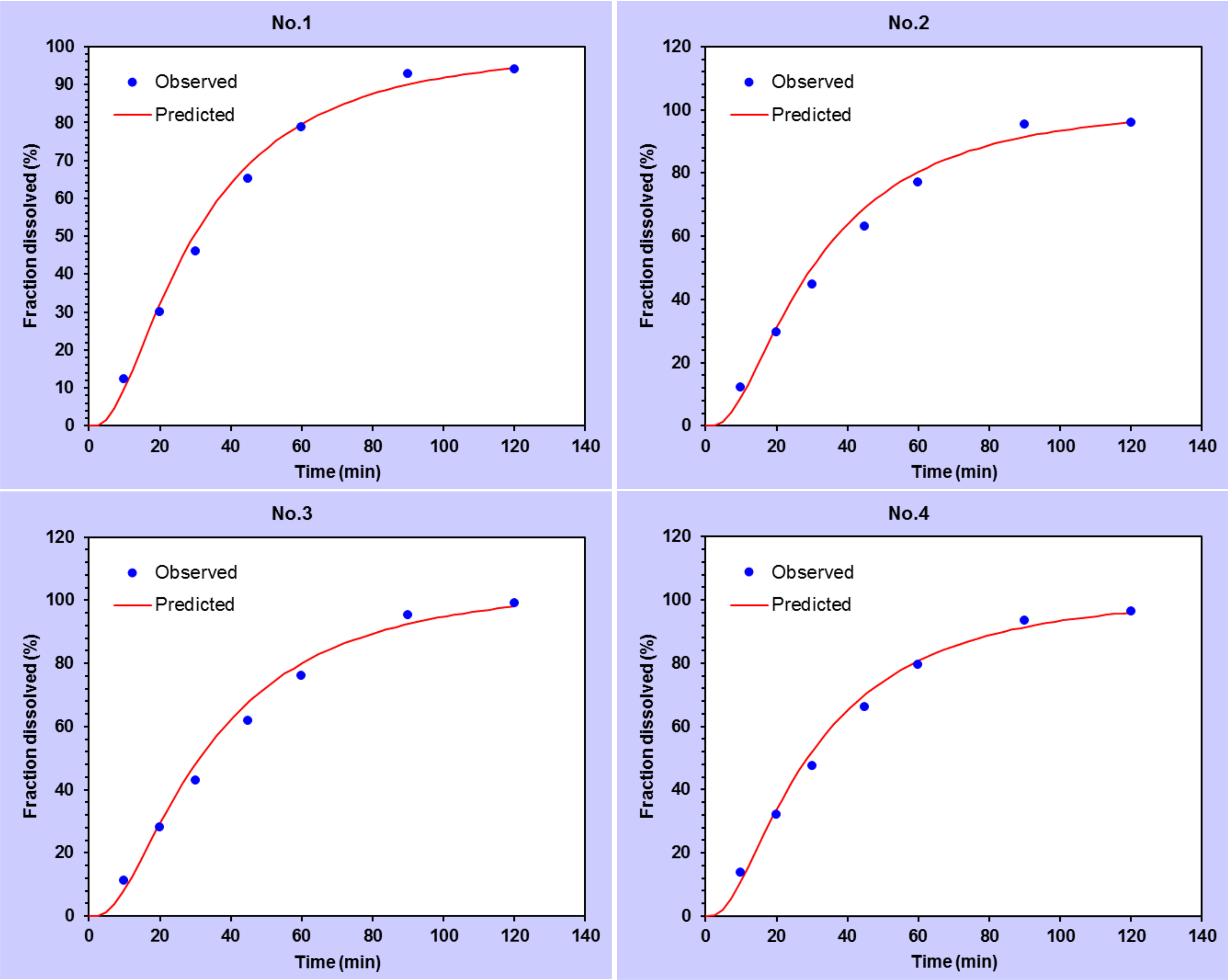

Supplementary materials – Model fitting summary – Polyglykol® 8000 P (modelling up to app. 60 % of carvedilol released)

Model: **Zero–order**

Model equation: $F = k_0 \cdot t$

Fitted model parameters per tested tablet (N = 4) with statistics – mean, standard deviation (SD), and relative standard deviation expressed in % (RSD%) (output from DDSolver):

Parameter	No.1	No.2	No.3	No.4	Mean	SD	RSD(%)
k_0	1.471	1.428	1.387	1.515	1.450	0.055	3.802

Number of dissolution data points (N), degrees of freedom (df), and selected goodness of fit criteria – Pearson correlation coefficient (R), coefficient of determination (R^2), adjusted coefficient of determination ($R^2_{adjusted}$), and residual sum of squares (RSS) (manual calculation in MS Excel):

Parameter	No.1	No.2	No.3	No.4
N	4	4	4	4
df	3	3	3	3
R	0.996875343	0.996085399	0.997207797	0.995957165
R^2	0.993760449	0.992186121	0.994423391	0.991930674
$R^2_{adjusted}$	0.993760449	0.992186121	0.994423391	0.991930674
RSS	10.29435359	11.28288015	10.35417069	12.6424979

Graphical abstract of model fit presented as mean ± 1 SD of the fraction % of released carvedilol:

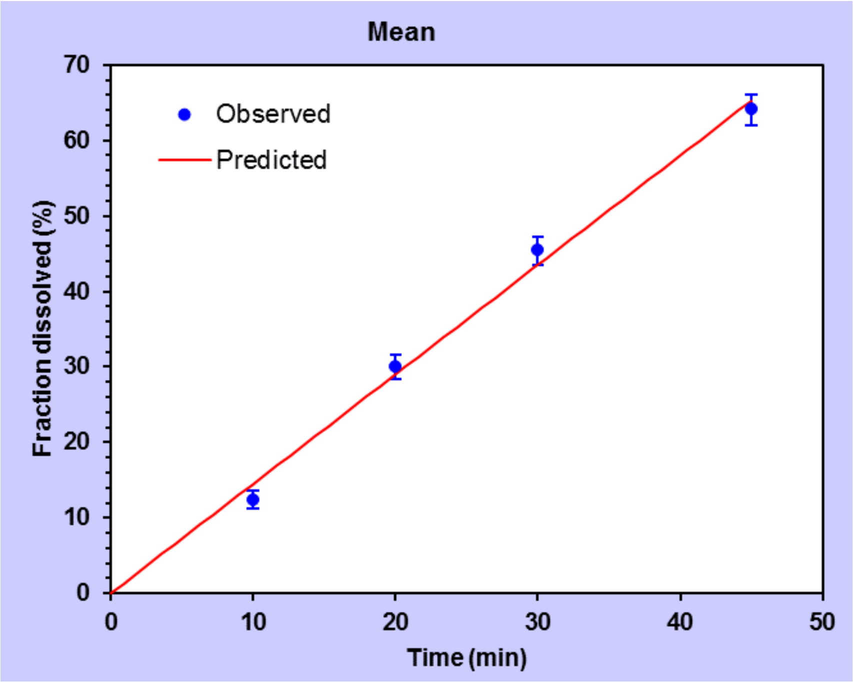

Supplementary materials – Model fitting summary – Polyglykol® 8000 P (modelling up to app. 60 % of carvedilol released)

Graphical abstract of model fit presented as the fraction % of released carvedilol per tested tablet:

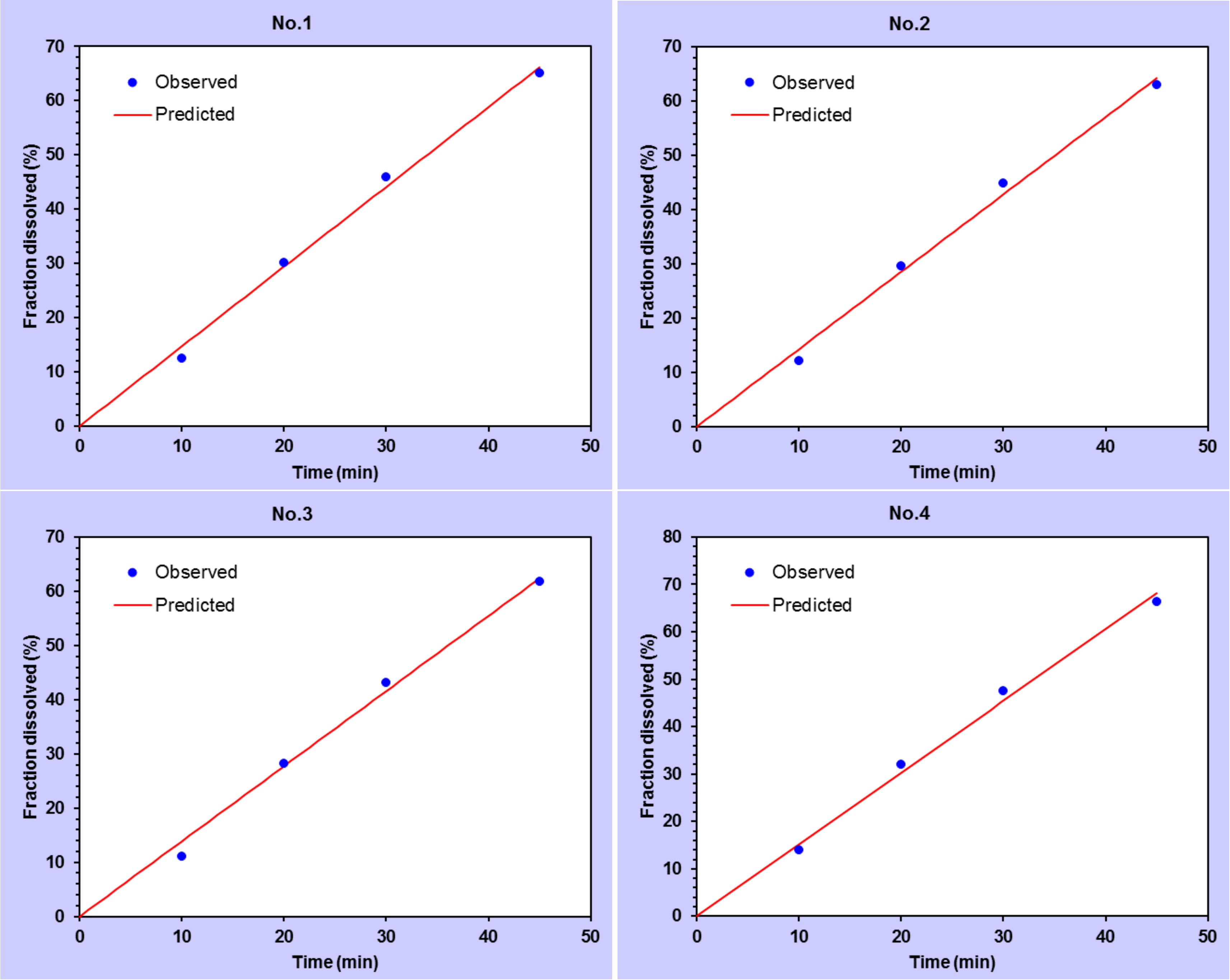

Supplementary materials – Model fitting summary – Polyglykol® 8000 P (modelling up to app. 60 % of carvedilol released)

Model: **Zero–order with T_{lag}**

Model equation: $F = k_0 \cdot (t - T_{lag})$

Fitted model parameters per tested tablet (N = 4) with statistics – mean, standard deviation (SD), and relative standard deviation expressed in % (RSD%) (output from DDSolver):

Parameter	No.1	No.2	No.3	No.4	Mean	SD	RSD(%)
k_0	1.502	1.446	1.442	1.488	1.469	0.030	2.045
T_{lag}	0.682	0.411	1.254	-0.590	0.439	0.771	175.535

Number of dissolution data points (N), degrees of freedom (df), and selected goodness of fit criteria – Pearson correlation coefficient (R), coefficient of determination (R^2), adjusted coefficient of determination ($R^2_{adjusted}$), and residual sum of squares (RSS) (manual calculation in MS Excel):

Parameter	No.1	No.2	No.3	No.4
N	4	4	4	4
df	2	2	2	2
R	0.996875343	0.996085399	0.997207797	0.995957165
R^2	0.993760449	0.992186121	0.994423391	0.991930674
$R^2_{adjusted}$	0.990640673	0.988279182	0.991635086	0.987896011
RSS	9.47410694	11.00768253	7.799628508	12.04069588

Graphical abstract of model fit presented as mean ± 1 SD of the fraction % of released carvedilol:

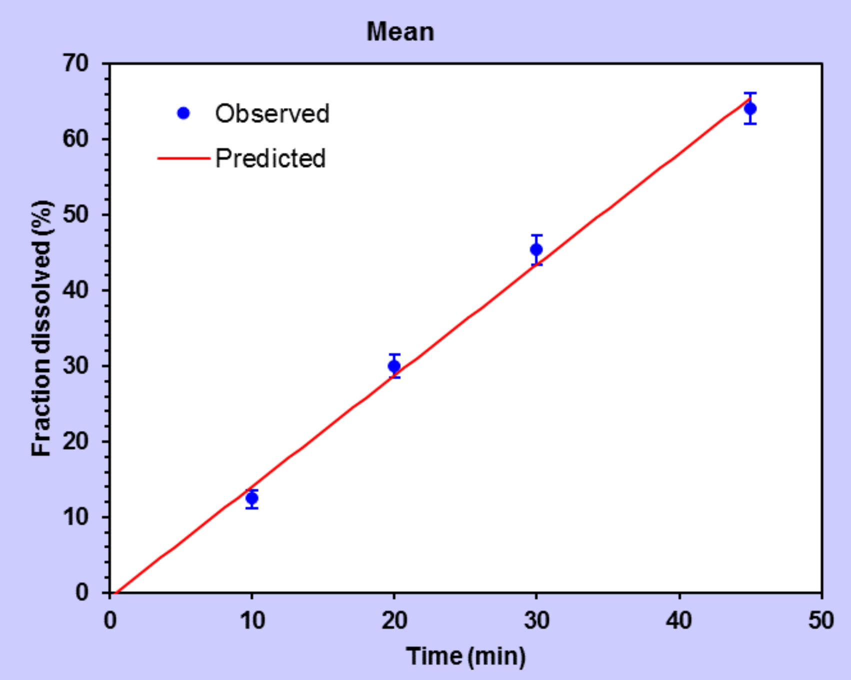

Supplementary materials – Model fitting summary – Polyglykol® 8000 P (modelling up to app. 60 % of carvedilol released)

Graphical abstract of model fit presented as the fraction % of released carvedilol per tested tablet:

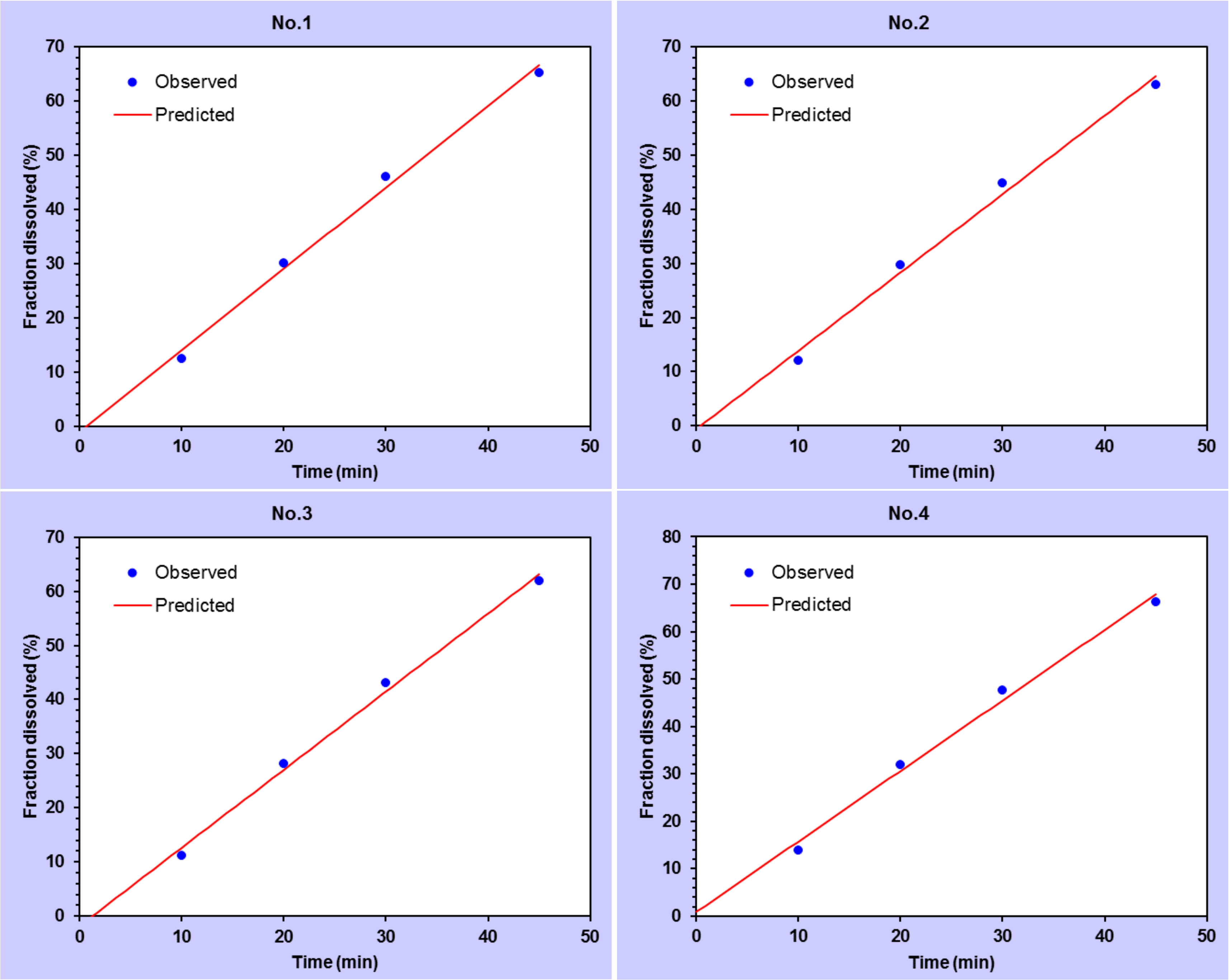

Supplementary materials – Model fitting summary – Polyglykol® 8000 P (modelling up to app. 60 % of carvedilol released)

Model: **Zero–order with F_0**

Model equation: $F = F_0 + k_0 \cdot t$

Fitted model parameters per tested tablet (N = 4) with statistics – mean, standard deviation (SD), and relative standard deviation expressed in % (RSD%) (output from DDSolver):

Parameter	No.1	No.2	No.3	No.4	Mean	SD	RSD(%)
k_0	1.502	1.446	1.442	1.488	1.469	0.030	2.045
F_0	-1.025	-0.594	-1.809	0.878	-0.637	1.128	-177.053

Number of dissolution data points (N), degrees of freedom (df), and selected goodness of fit criteria – Pearson correlation coefficient (R), coefficient of determination (R^2), adjusted coefficient of determination ($R^2_{adjusted}$), and residual sum of squares (RSS) (manual calculation in MS Excel):

Parameter	No.1	No.2	No.3	No.4
N	4	4	4	4
df	2	2	2	2
R	0.996875343	0.996085399	0.997207797	0.995957165
R^2	0.993760449	0.992186121	0.994423391	0.991930674
$R^2_{adjusted}$	0.990640673	0.988279182	0.991635086	0.987896011
RSS	9.47410694	11.00768253	7.799628508	12.04069588

Graphical abstract of model fit presented as mean ± 1 SD of the fraction % of released carvedilol:

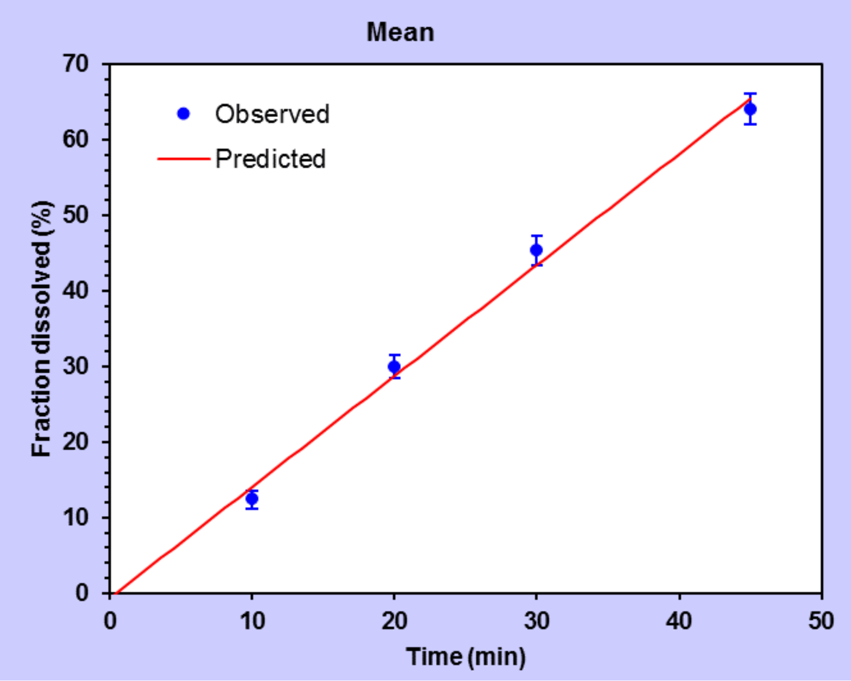

Supplementary materials – Model fitting summary – Polyglykol® 8000 P (modelling up to app. 60 % of carvedilol released)

Graphical abstract of model fit presented as the fraction % of released carvedilol per tested tablet:

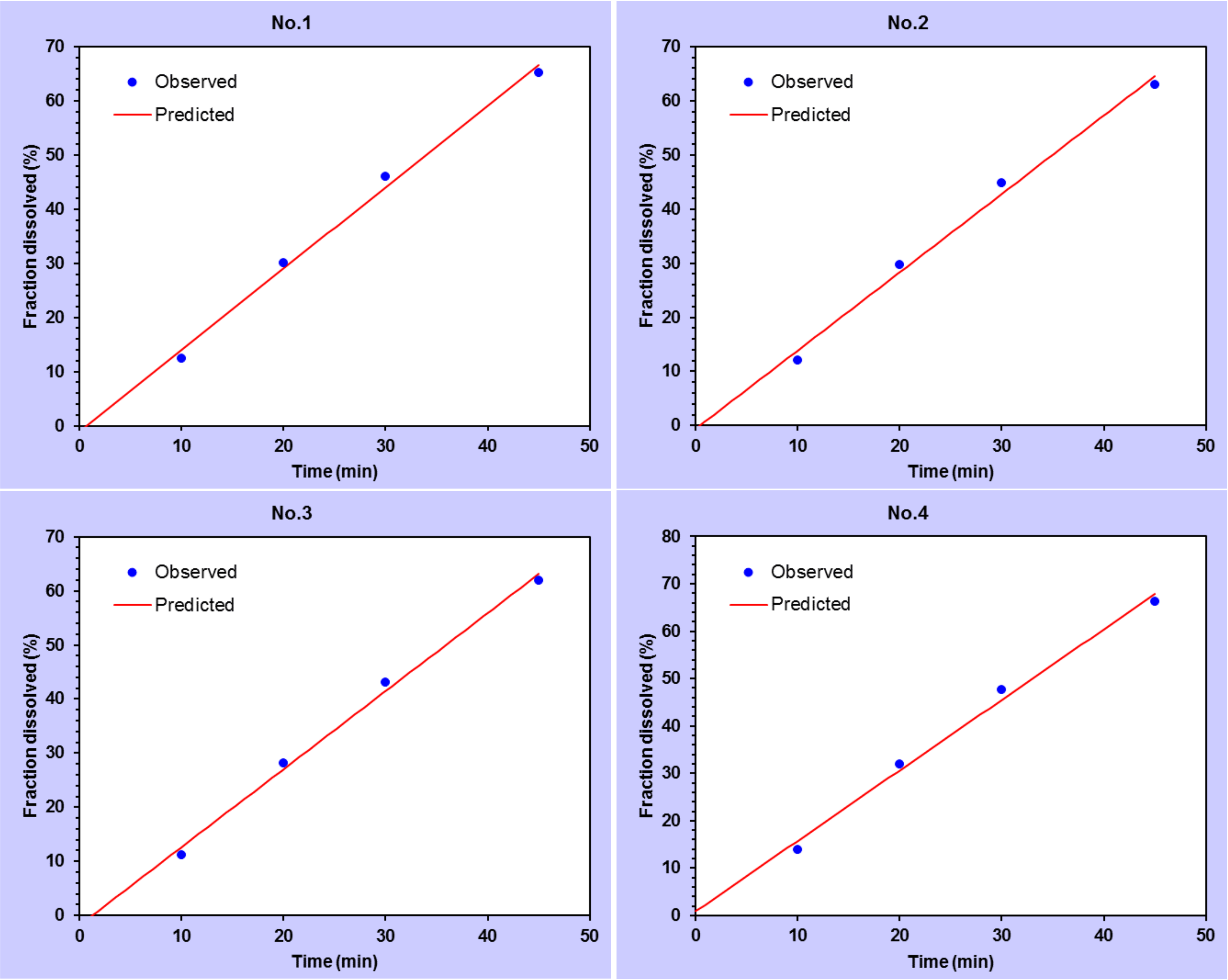

Supplementary materials – Model fitting summary – Polyglykol® 8000 P (modelling up to app. 60 % of carvedilol released)

Model: **First–order**

Model equation: $F = 100 \cdot (1 - e^{-k_1 \cdot t})$

Fitted model parameters per tested tablet (N = 4) with statistics – mean, standard deviation (SD), and relative standard deviation expressed in % (RSD%) (output from DDSolver):

Parameter	No.1	No.2	No.3	No.4	Mean	SD	RSD(%)
k_1	0.022	0.021	0.020	0.023	0.021	0.001	5.664

Number of dissolution data points (N), degrees of freedom (df), and selected goodness of fit criteria – Pearson correlation coefficient (R), coefficient of determination (R^2), adjusted coefficient of determination ($R^2_{adjusted}$), and residual sum of squares (RSS) (manual calculation in MS Excel):

Parameter	No.1	No.2	No.3	No.4
N	4	4	4	4
df	3	3	3	3
R	0.99924951	0.999733012	0.999491845	0.999497644
R^2	0.998499582	0.999466096	0.998983947	0.99899554
$R^2_{adjusted}$	0.998499582	0.999466096	0.998983947	0.99899554
RSS	88.63007921	69.38018319	79.91945882	68.15515416

Graphical abstract of model fit presented as mean ± 1 SD of the fraction % of released carvedilol:

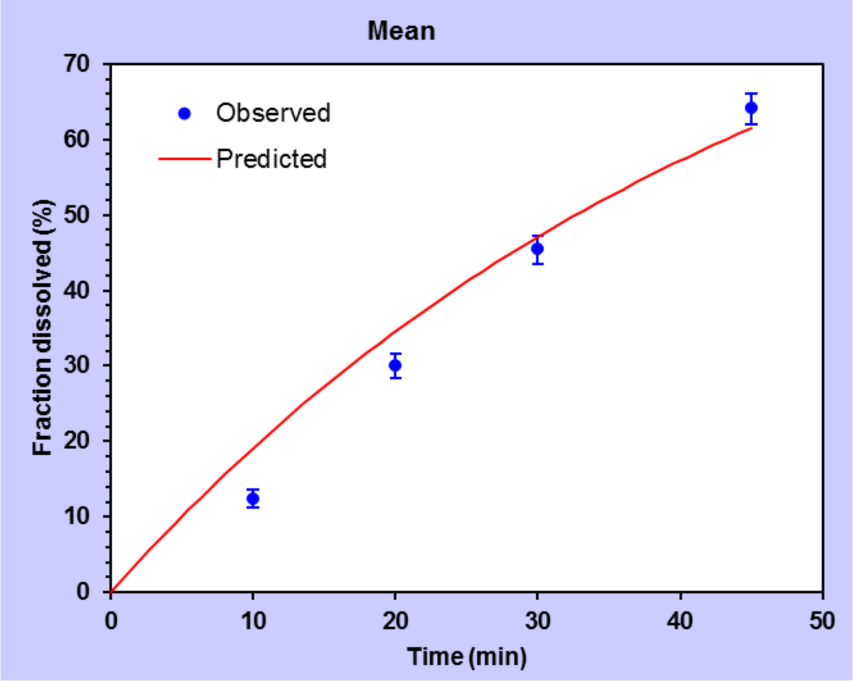

Supplementary materials – Model fitting summary – Polyglykol® 8000 P (modelling up to app. 60 % of carvedilol released)

Graphical abstract of model fit presented as the fraction % of released carvedilol per tested tablet:

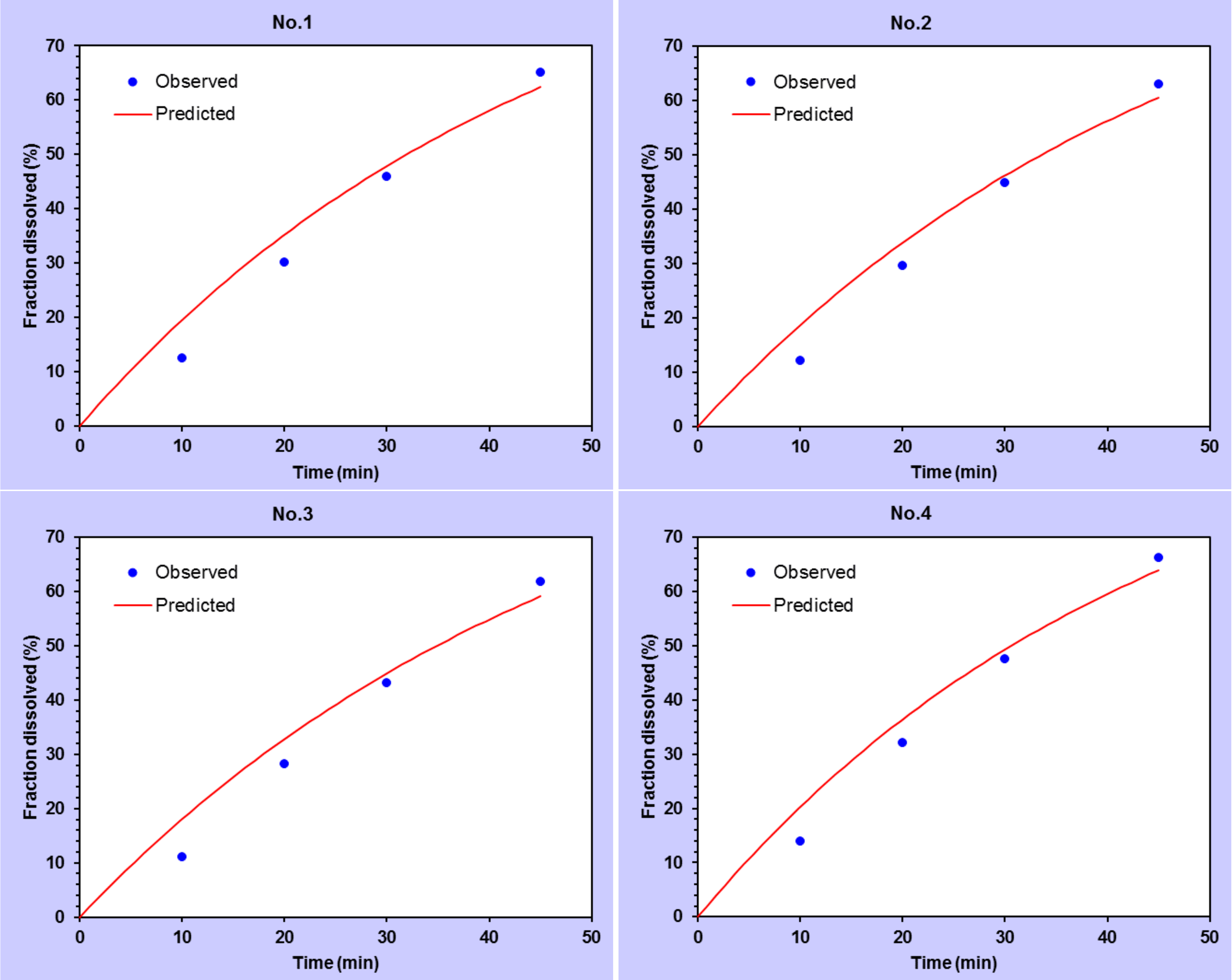

Supplementary materials – Model fitting summary – Polyglykol® 8000 P (modelling up to app. 60 % of carvedilol released)

Model: **First–order with T_{lag}**

Model equation: $F = 100 \cdot \left[1 - e^{-k_1 \cdot (t - T_{lag})}\right]$

Fitted model parameters per tested tablet (N = 4) with statistics – mean, standard deviation (SD), and relative standard deviation expressed in % (RSD%) (output from DDSolver):

Parameter	No.1	No.2	No.3	No.4	Mean	SD	RSD(%)
k_1	0.026	0.024	0.024	0.027	0.025	0.002	6.243
T_{lag}	5.830	4.675	5.855	5.131	5.373	0.574	10.677

Number of dissolution data points (N), degrees of freedom (df), and selected goodness of fit criteria – Pearson correlation coefficient (R), coefficient of determination (R^2), adjusted coefficient of determination ($R^2_{adjusted}$), and residual sum of squares (RSS) (manual calculation in MS Excel):

Parameter	No.1	No.2	No.3	No.4
N	4	4	4	4
df	2	2	2	2
R	0.997982761	0.999241878	0.998491059	0.998546573
R^2	0.995969591	0.998484331	0.996984394	0.997095259
$R^2_{adjusted}$	0.993954387	0.997726497	0.995476592	0.995642888
RSS	7.166555677	2.946947401	4.849099027	5.117697337

Graphical abstract of model fit presented as mean ± 1 SD of the fraction % of released carvedilol:

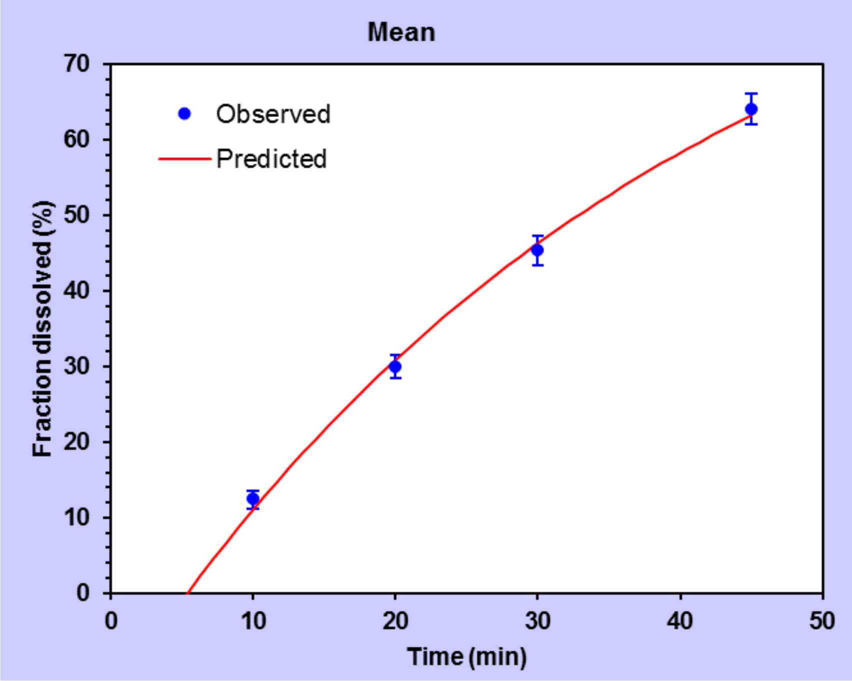

Supplementary materials – Model fitting summary – Polyglykol® 8000 P (modelling up to app. 60 % of carvedilol released)

Graphical abstract of model fit presented as the fraction % of released carvedilol per tested tablet:

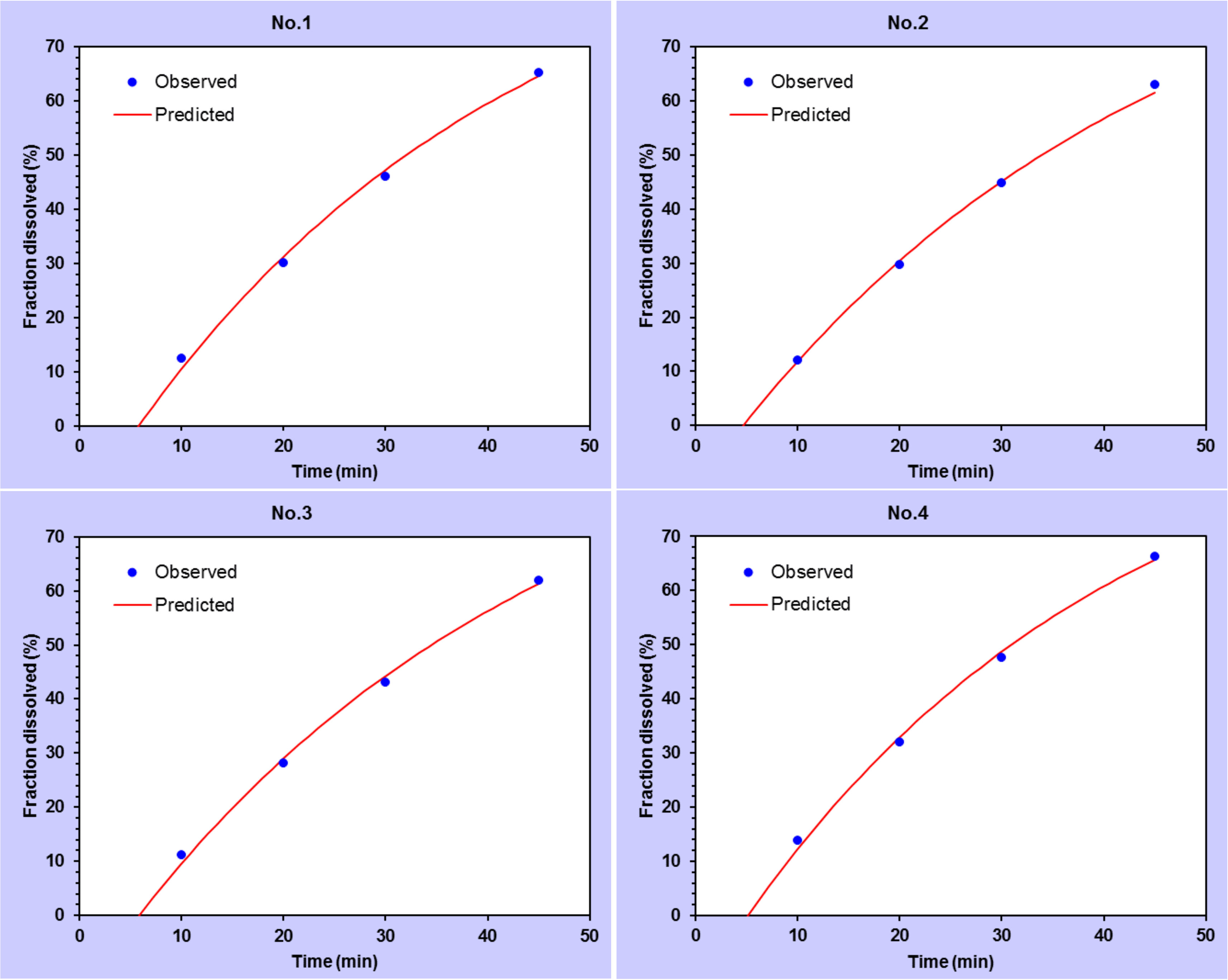

Supplementary materials – Model fitting summary – Polyglykol® 8000 P (modelling up to app. 60 % of carvedilol released)

Model: **First–order with F_{max}**

Model equation: $F = F_{max} \cdot (1 - e^{-k_1 \cdot t})$

Fitted model parameters per tested tablet (N = 4) with statistics – mean, standard deviation (SD), and relative standard deviation expressed in % (RSD%) (output from DDSolver):

Parameter	No.1	No.2	No.3	No.4	Mean	SD	RSD(%)
k_1	0.054	0.054	0.053	0.054	0.054	0.000	0.725
F_{max}	68.412	66.104	64.927	69.567	67.253	2.116	3.146

Number of dissolution data points (N), degrees of freedom (df), and selected goodness of fit criteria – Pearson correlation coefficient (R), coefficient of determination (R^2), adjusted coefficient of determination ($R^2_{adjusted}$), and residual sum of squares (RSS) (manual calculation in MS Excel):

Parameter	No.1	No.2	No.3	No.4
N	4	4	4	4
df	2	2	2	2
R	0.980184438	0.981921789	0.979883972	0.981901665
R^2	0.960761533	0.9641704	0.960172598	0.96413088
$R^2_{adjusted}$	0.9411423	0.9462556	0.940258897	0.94619632
RSS	566.5680689	511.6675842	541.6686598	509.4174526

Graphical abstract of model fit presented as mean ± 1 SD of the fraction % of released carvedilol:

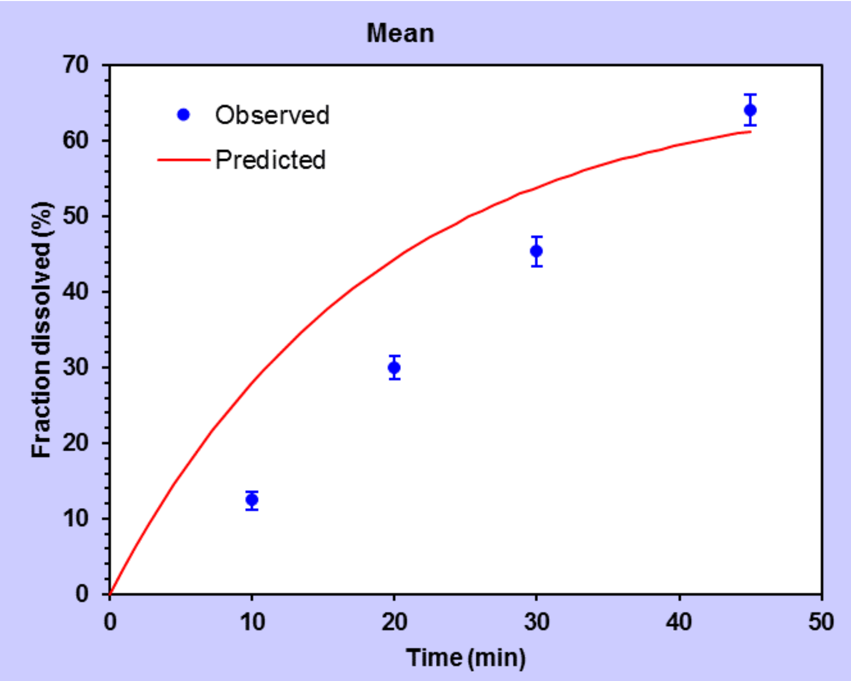

Supplementary materials – Model fitting summary – Polyglykol® 8000 P (modelling up to app. 60 % of carvedilol released)

Graphical abstract of model fit presented as the fraction % of released carvedilol per tested tablet:

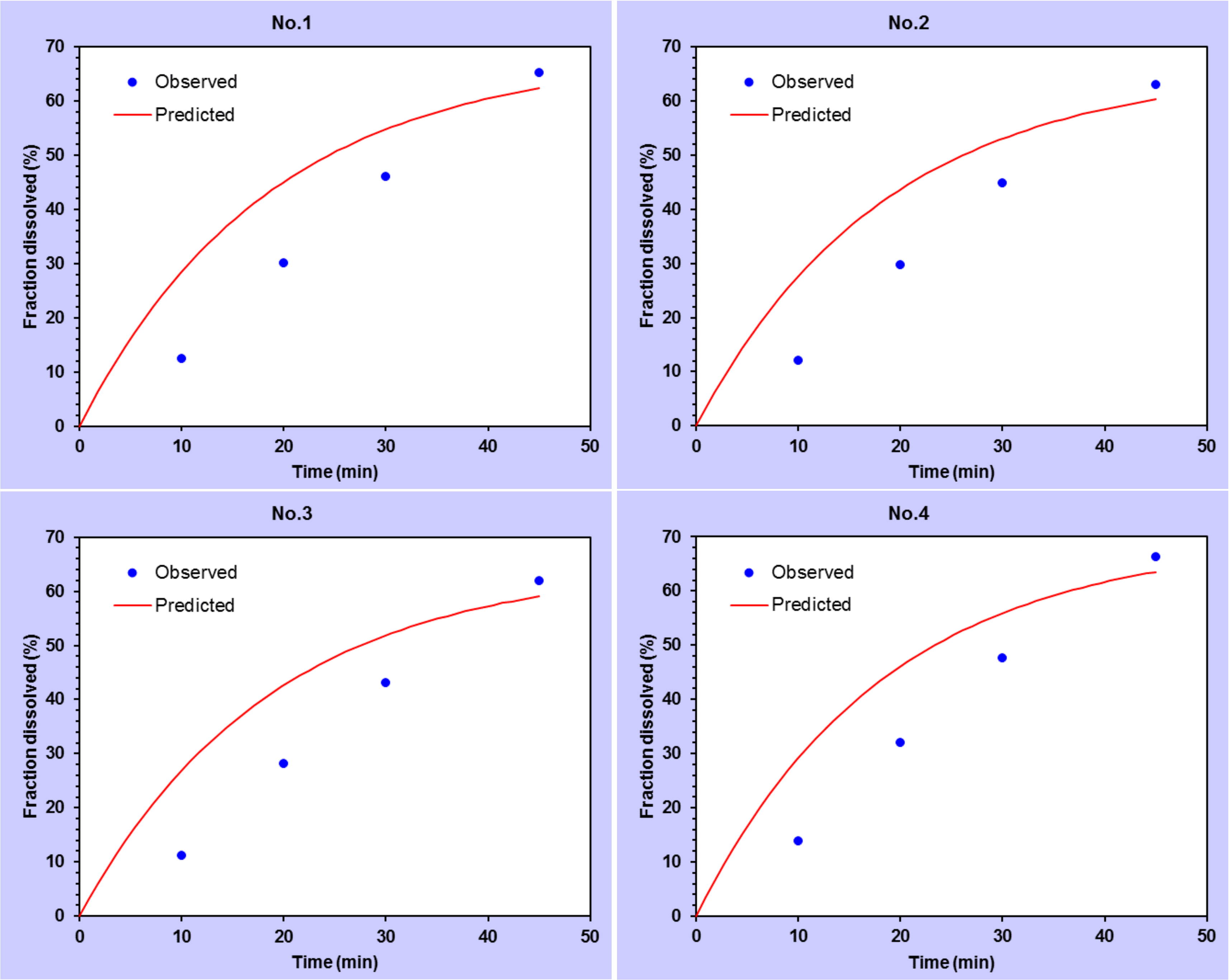

Supplementary materials – Model fitting summary – Polyglykol® 8000 P (modelling up to app. 60 % of carvedilol released)

Model: **First–order with T_{lag} and F_{max}**

Model equation: $F = F_{max} \cdot \left[1 - e^{-k_1 \cdot (t - T_{lag})}\right]$

Fitted model parameters per tested tablet (N = 4) with statistics – mean, standard deviation (SD), and relative standard deviation expressed in % (RSD%) (output from DDSolver):

Parameter	No.1	No.2	No.3	No.4	Mean	SD	RSD(%)
k_1	0.081	0.081	0.082	0.082	0.081	0.000	0.292
T_{lag}	11.070	10.952	11.266	8.425	10.428	1.342	12.868
F_{max}	68.412	66.104	64.927	58.938	64.596	4.040	6.254

Number of dissolution data points (N), degrees of freedom (df), and selected goodness of fit criteria – Pearson correlation coefficient (R), coefficient of determination (R^2), adjusted coefficient of determination ($R^2_{adjusted}$), and residual sum of squares (RSS) (manual calculation in MS Excel):

Parameter	No.1	No.2	No.3	No.4
N	4	4	4	4
df	1	1	1	1
R	0.950537486	0.95373504	0.949419286	0.953637088
R^2	0.903521513	0.909610527	0.901396981	0.909423695
$R^2_{adjusted}$	0.710564539	0.72883158	0.704190942	0.728271085
RSS	435.3804832	380.2023814	415.5204005	169.7153459

Graphical abstract of model fit presented as mean ± 1 SD of the fraction % of released carvedilol:

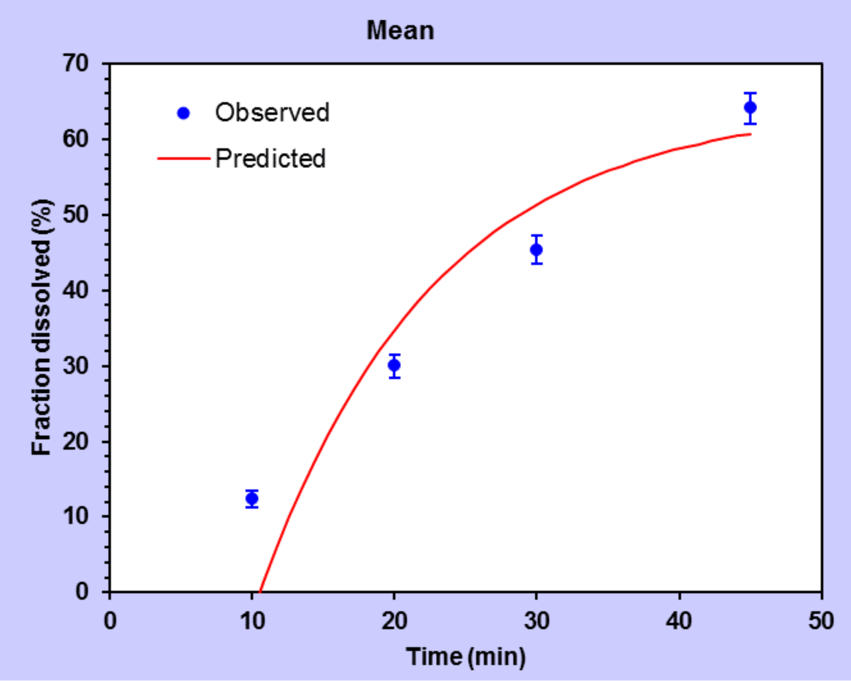

Supplementary materials – Model fitting summary – Polyglykol® 8000 P (modelling up to app. 60 % of carvedilol released)

Graphical abstract of model fit presented as the fraction % of released carvedilol per tested tablet:

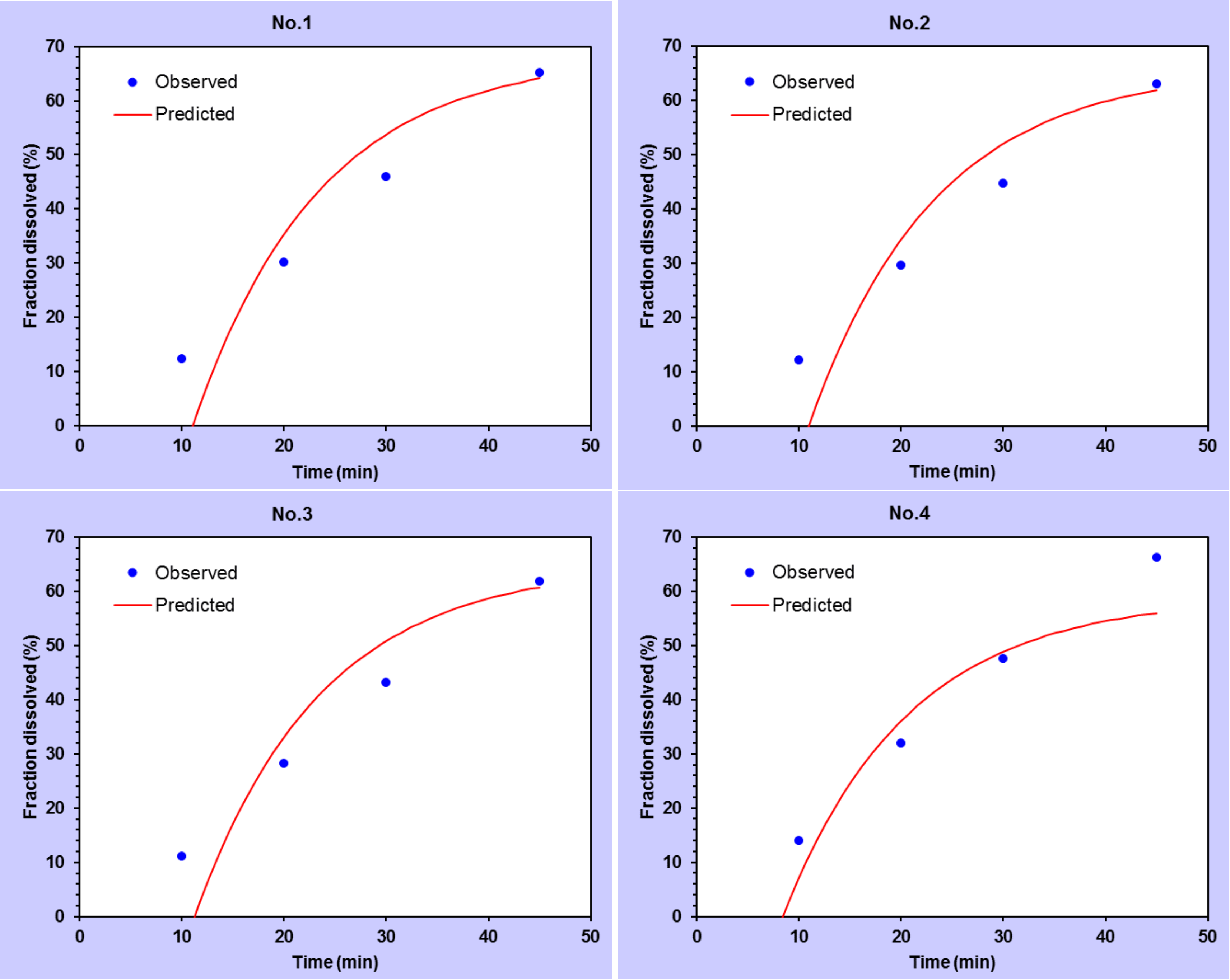

Supplementary materials – Model fitting summary – Polyglykol® 8000 P (modelling up to app. 60 % of carvedilol released)

Model: **Higuchi**

Model equation: $F = k_H \cdot t^{0.5}$

Fitted model parameters per tested tablet (N = 4) with statistics – mean, standard deviation (SD), and relative standard deviation expressed in % (RSD%) (output from DDSolver):

Parameter	No.1	No.2	No.3	No.4	Mean	SD	RSD(%)
k_H	8.216	7.983	7.732	8.496	8.107	0.326	4.023

Number of dissolution data points (N), degrees of freedom (df), and selected goodness of fit criteria – Pearson correlation coefficient (R), coefficient of determination (R^2), adjusted coefficient of determination ($R^2_{adjusted}$), and residual sum of squares (RSS) (manual calculation in MS Excel):

Parameter	No.1	No.2	No.3	No.4
N	4	4	4	4
df	3	3	3	3
R	0.999283323	0.999607956	0.999253813	0.999655974
R^2	0.99856716	0.999216066	0.998508182	0.999312067
$R^2_{adjusted}$	0.99856716	0.999216066	0.998508182	0.999312067
RSS	330.5169591	299.7201915	319.0367542	291.1089581

Graphical abstract of model fit presented as mean ± 1 SD of the fraction % of released carvedilol:

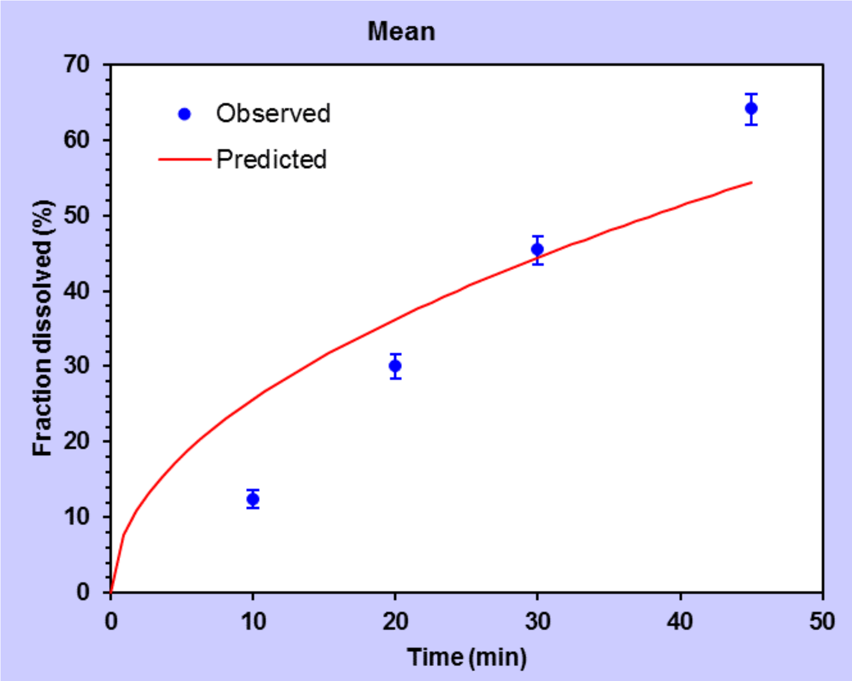

Supplementary materials – Model fitting summary – Polyglykol® 8000 P (modelling up to app. 60 % of carvedilol released)

Graphical abstract of model fit presented as the fraction % of released carvedilol per tested tablet:

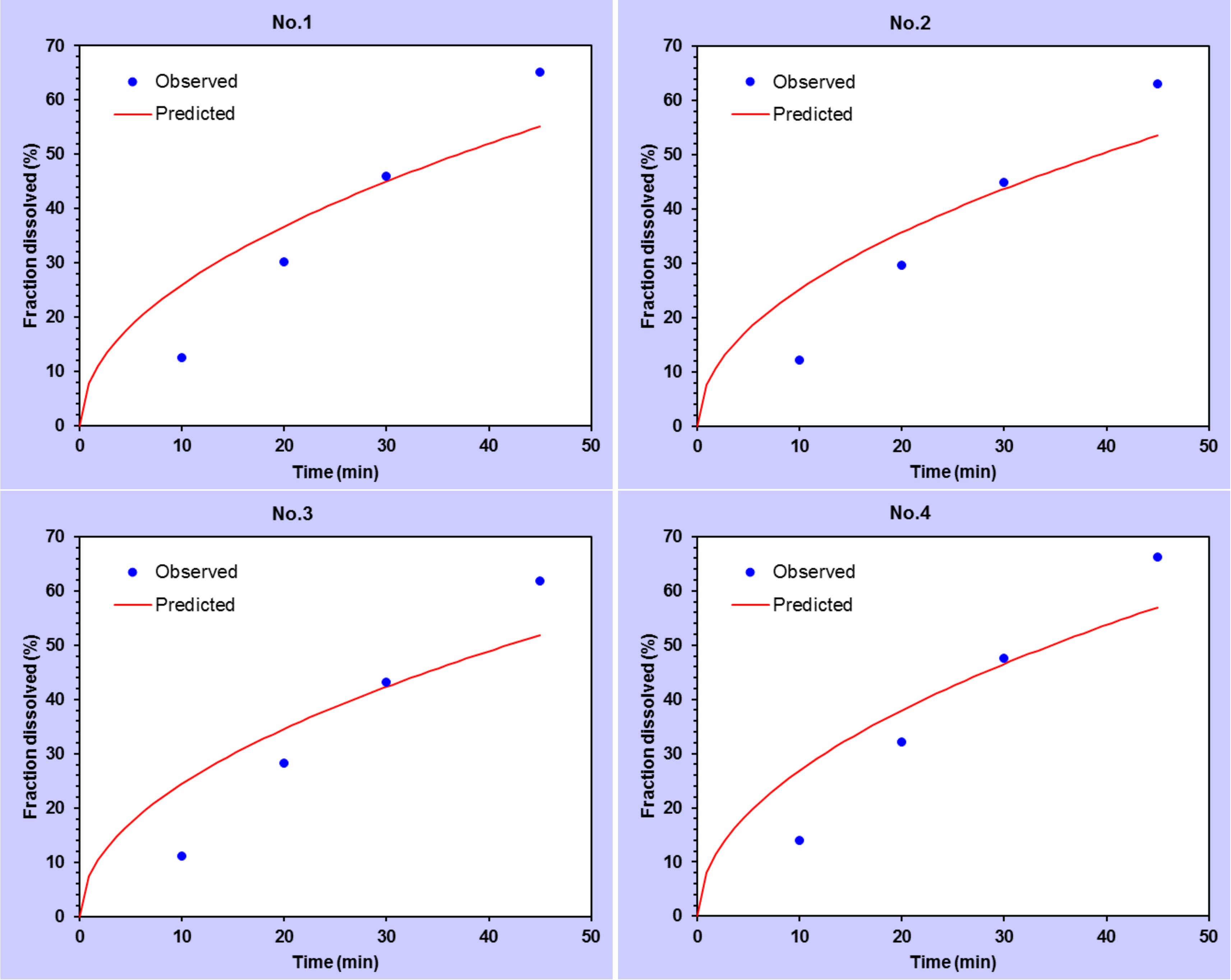

Supplementary materials – Model fitting summary – Polyglykol® 8000 P (modelling up to app. 60 % of carvedilol released)

Model: **Higuchi with T_{lag}**

Model equation: $F = k_H \cdot (t - T_{lag})^{0.5}$

Fitted model parameters per tested tablet (N = 4) with statistics – mean, standard deviation (SD), and relative standard deviation expressed in % (RSD%) (output from DDSolver):

Parameter	No.1	No.2	No.3	No.4	Mean	SD	RSD(%)
k_H	10.894	10.518	10.353	11.023	10.697	0.314	2.933
T_{lag}	10.623	10.453	10.865	10.058	10.500	0.340	3.234

Number of dissolution data points (N), degrees of freedom (df), and selected goodness of fit criteria – Pearson correlation coefficient (R), coefficient of determination (R^2), adjusted coefficient of determination ($R^2_{adjusted}$), and residual sum of squares (RSS) (manual calculation in MS Excel):

Parameter	No.1	No.2	No.3	No.4
N	4	4	4	4
df	2	2	2	2
R	0.976783002	0.978135484	0.977683445	0.976726228
R^2	0.954105032	0.956749025	0.955864919	0.953994123
$R^2_{adjusted}$	0.931157549	0.935123537	0.933797379	0.930991185
RSS	169.9408416	159.083176	139.5254806	204.7143237

Graphical abstract of model fit presented as mean ± 1 SD of the fraction % of released carvedilol:

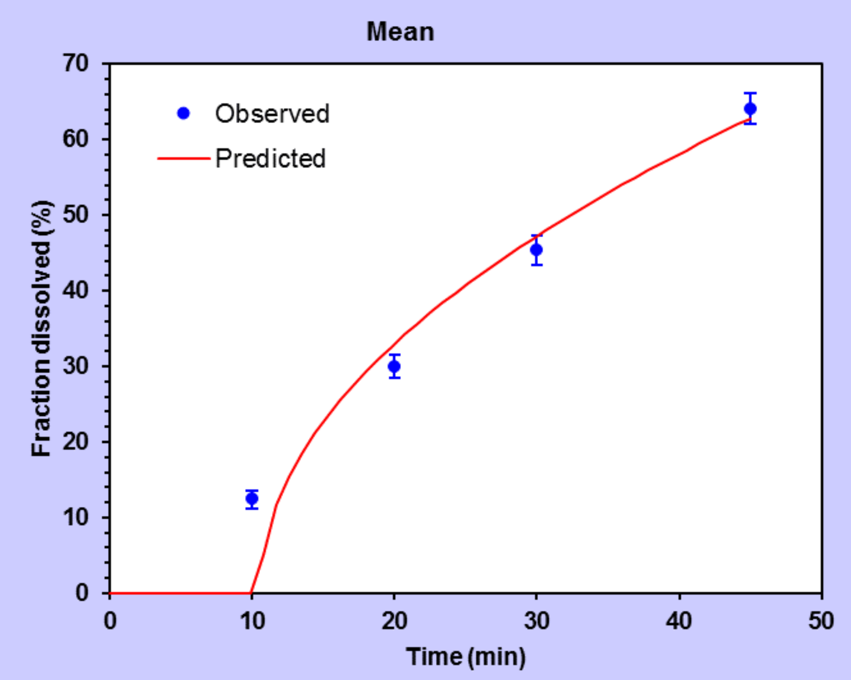

Supplementary materials – Model fitting summary – Polyglykol® 8000 P (modelling up to app. 60 % of carvedilol released)

Graphical abstract of model fit presented as the fraction % of released carvedilol per tested tablet:

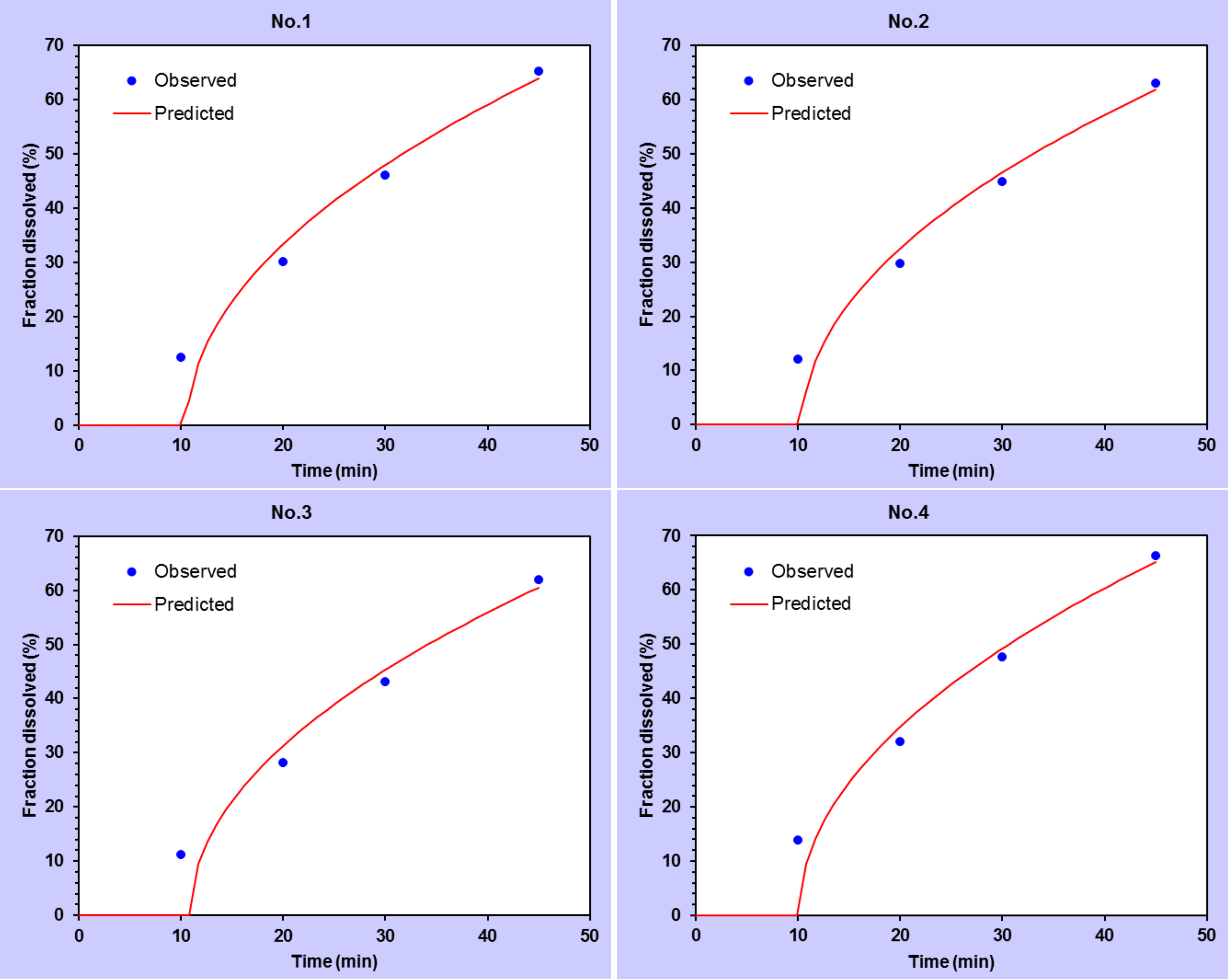

Supplementary materials – Model fitting summary – Polyglykol® 8000 P (modelling up to app. 60 % of carvedilol released)

Model: **Higuchi with F_0**

Model equation: $F = F_0 + k_H \cdot t^{0.5}$

Fitted model parameters per tested tablet (N = 4) with statistics – mean, standard deviation (SD), and relative standard deviation expressed in % (RSD%) (output from DDSolver):

Parameter	No.1	No.2	No.3	No.4	Mean	SD	RSD(%)
k_H	14.939	14.395	14.338	14.815	14.622	0.300	2.054
F_0	-35.619	-33.968	-34.996	-33.480	-34.516	0.970	-2.809

Number of dissolution data points (N), degrees of freedom (df), and selected goodness of fit criteria – Pearson correlation coefficient (R), coefficient of determination (R^2), adjusted coefficient of determination ($R^2_{adjusted}$), and residual sum of squares (RSS) (manual calculation in MS Excel):

Parameter	No.1	No.2	No.3	No.4
N	4	4	4	4
df	2	2	2	2
R	0.999283323	0.999607956	0.999253813	0.999655974
R^2	0.99856716	0.999216066	0.998508182	0.999312067
$R^2_{adjusted}$	0.99785074	0.998824098	0.997762273	0.9989681
RSS	2.175618058	1.104355663	2.086505603	1.026504086

Graphical abstract of model fit presented as mean ± 1 SD of the fraction % of released carvedilol:

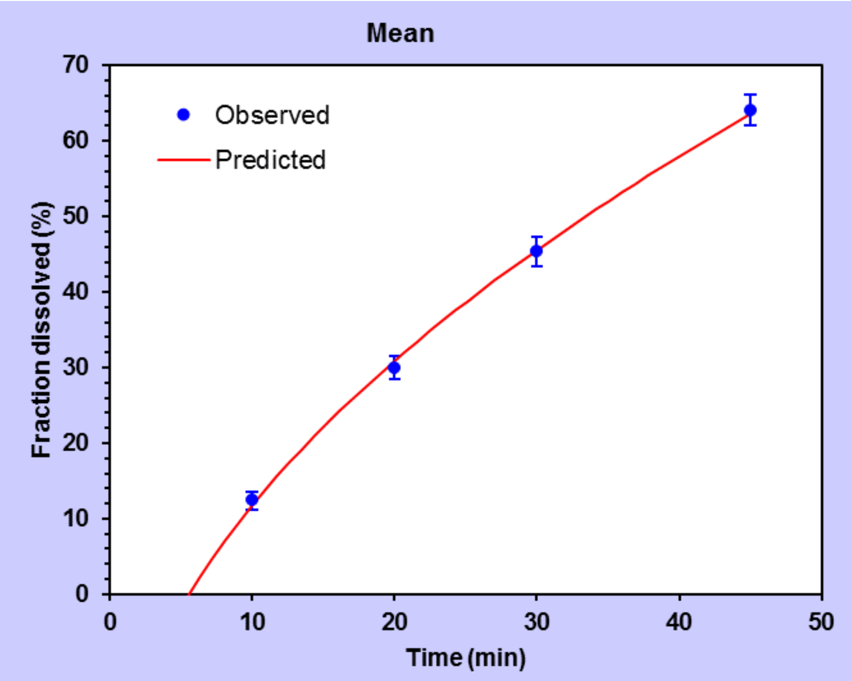

Supplementary materials – Model fitting summary – Polyglykol® 8000 P (modelling up to app. 60 % of carvedilol released)

Graphical abstract of model fit presented as the fraction % of released carvedilol per tested tablet:

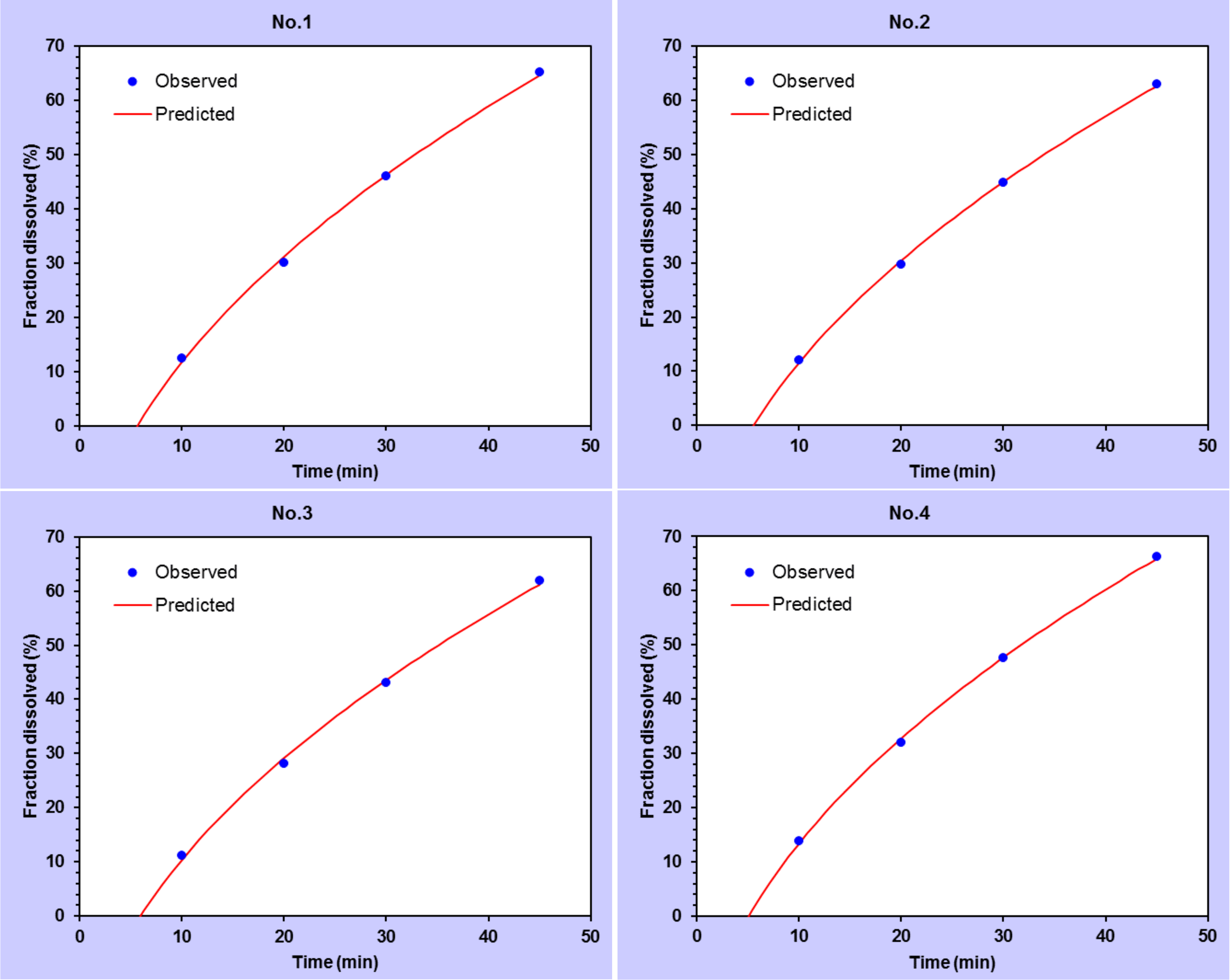

Supplementary materials – Model fitting summary – Polyglykol® 8000 P (modelling up to app. 60 % of carvedilol released)

Model: **Korsmeyer–Peppas**

Model equation: $F = k_{KP} \cdot t^n$

Fitted model parameters per tested tablet (N = 4) with statistics – mean, standard deviation (SD), and relative standard deviation expressed in % (RSD%) (output from DDSolver):

Parameter	No.1	No.2	No.3	No.4	Mean	SD	RSD(%)
k_{KP}	1.203	1.197	0.994	1.564	1.239	0.237	19.141
n	1.047	1.041	1.082	0.985	1.039	0.040	3.878

Number of dissolution data points (N), degrees of freedom (df), and selected goodness of fit criteria – Pearson correlation coefficient (R), coefficient of determination (R^2), adjusted coefficient of determination ($R^2_{adjusted}$), and residual sum of squares (RSS) (manual calculation in MS Excel):

Parameter	No.1	No.2	No.3	No.4
N	4	4	4	4
df	2	2	2	2
R	0.996035879	0.995277782	0.995739152	0.996242159
R^2	0.992087472	0.990577864	0.99149646	0.99249844
$R^2_{adjusted}$	0.988131207	0.985866796	0.987244689	0.98874766
RSS	20.26692867	20.06811776	21.99245231	14.90137075

Graphical abstract of model fit presented as mean ± 1 SD of the fraction % of released carvedilol:

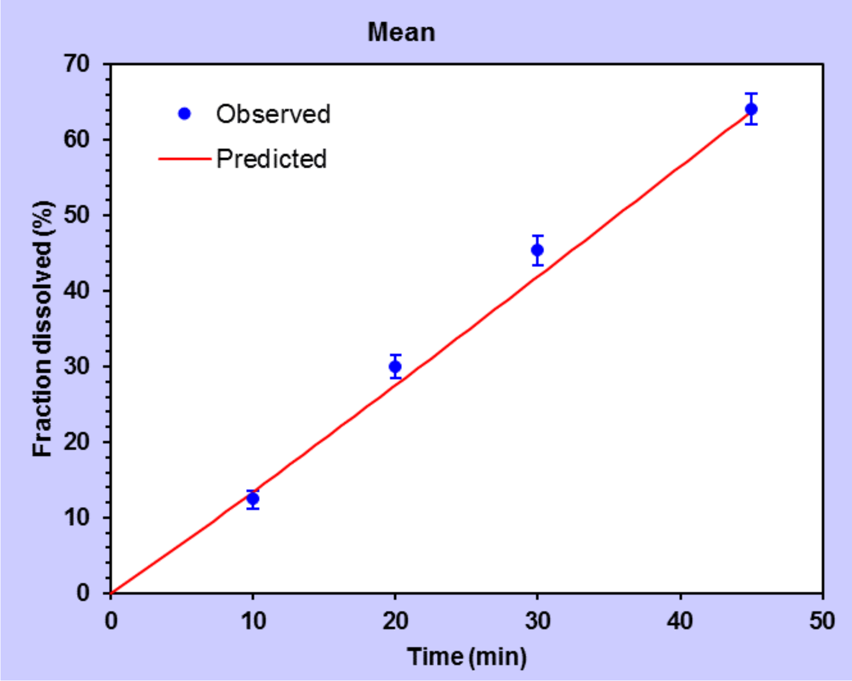

Supplementary materials – Model fitting summary – Polyglykol® 8000 P (modelling up to app. 60 % of carvedilol released)

Graphical abstract of model fit presented as the fraction % of released carvedilol per tested tablet:

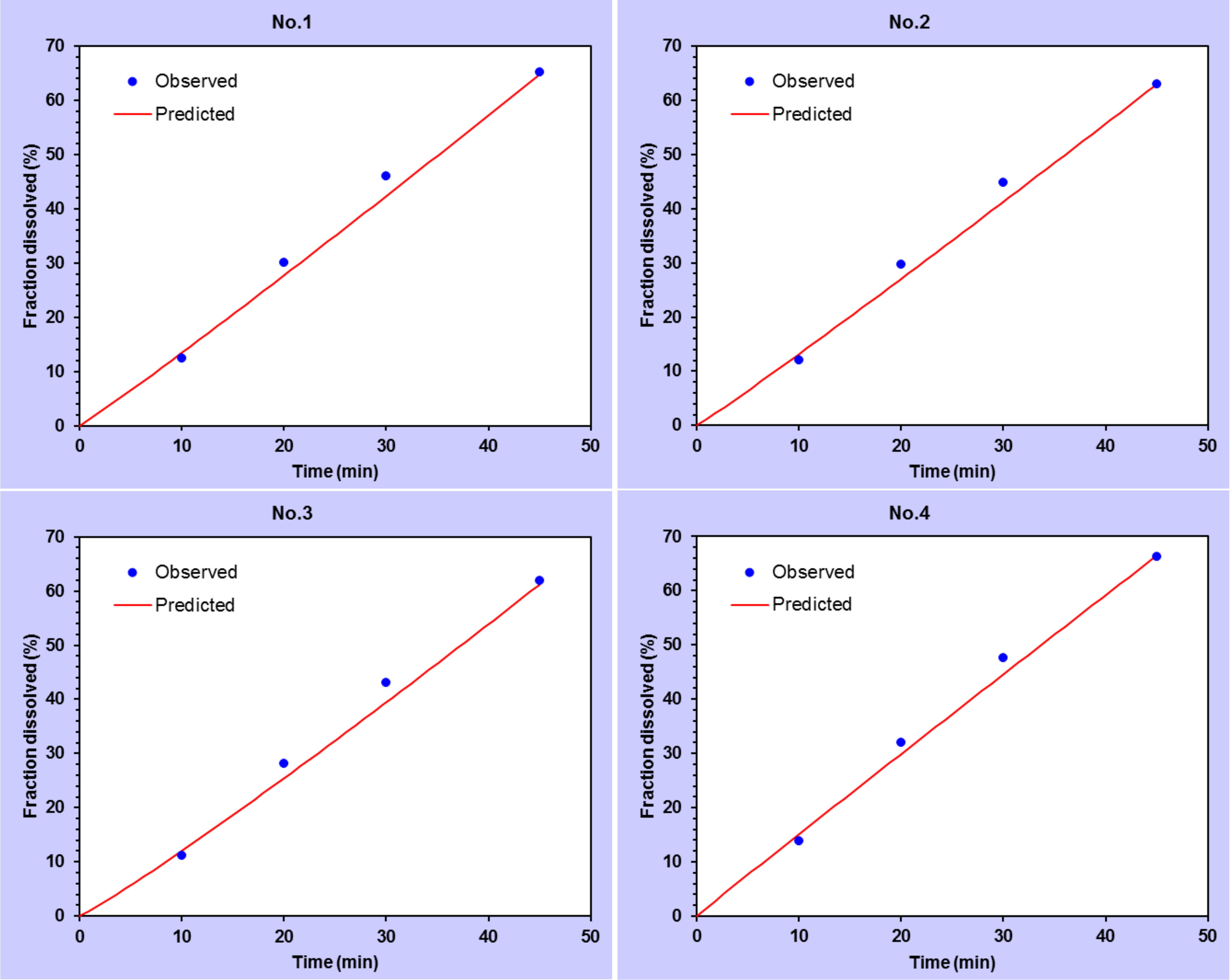

Supplementary materials – Model fitting summary – Polyglykol® 8000 P (modelling up to app. 60 % of carvedilol released)

Model: **Korsmeyer–Peppas with T_{lag}**

Model equation: $F = k_{KP} \cdot \left(t - T_{lag}\right)^{n}$

Fitted model parameters per tested tablet (N = 4) with statistics – mean, standard deviation (SD), and relative standard deviation expressed in % (RSD%) (output from DDSolver):

Parameter	No.1	No.2	No.3	No.4	Mean	SD	RSD(%)
k_{KP}	2.731	2.699	2.497	3.547	2.869	0.464	16.171
n	0.853	0.849	0.868	0.792	0.840	0.034	3.992
T_{lag}	3.809	3.809	4.415	4.495	4.132	0.374	9.060

Number of dissolution data points (N), degrees of freedom (df), and selected goodness of fit criteria – Pearson correlation coefficient (R), coefficient of determination (R^2), adjusted coefficient of determination ($R^2_{adjusted}$), and residual sum of squares (RSS) (manual calculation in MS Excel):

Parameter	No.1	No.2	No.3	No.4
N	4	4	4	4
df	1	1	1	1
R	0.999145146	0.998803551	0.999250814	0.99944526
R^2	0.998291023	0.997608534	0.99850219	0.998890829
$R^2_{adjusted}$	0.994873068	0.992825603	0.99550657	0.996672486
RSS	3.633497051	4.099018362	3.398200154	3.28906659

Graphical abstract of model fit presented as mean ± 1 SD of the fraction % of released carvedilol:

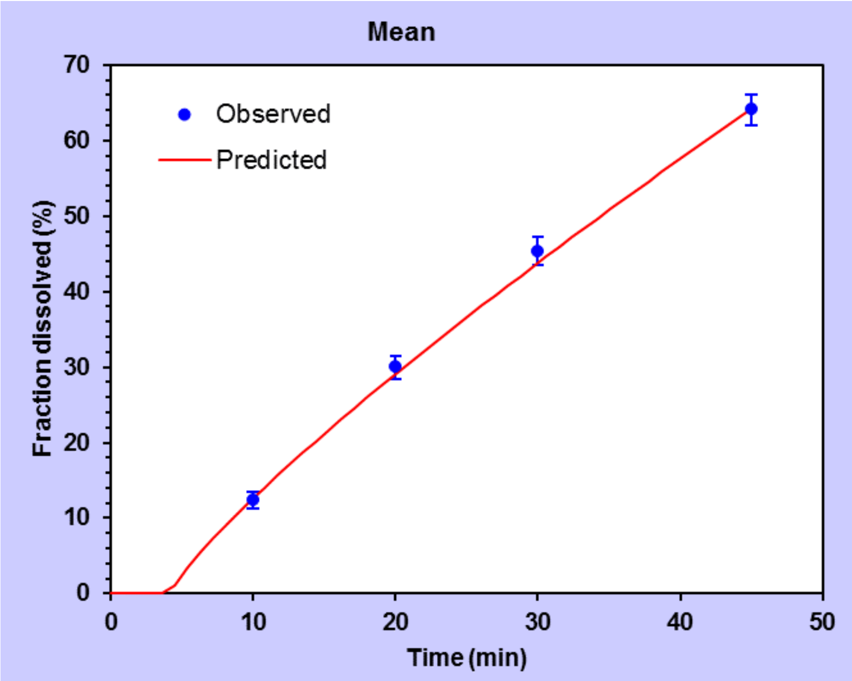

Supplementary materials – Model fitting summary – Polyglykol® 8000 P (modelling up to app. 60 % of carvedilol released)

Graphical abstract of model fit presented as the fraction % of released carvedilol per tested tablet:

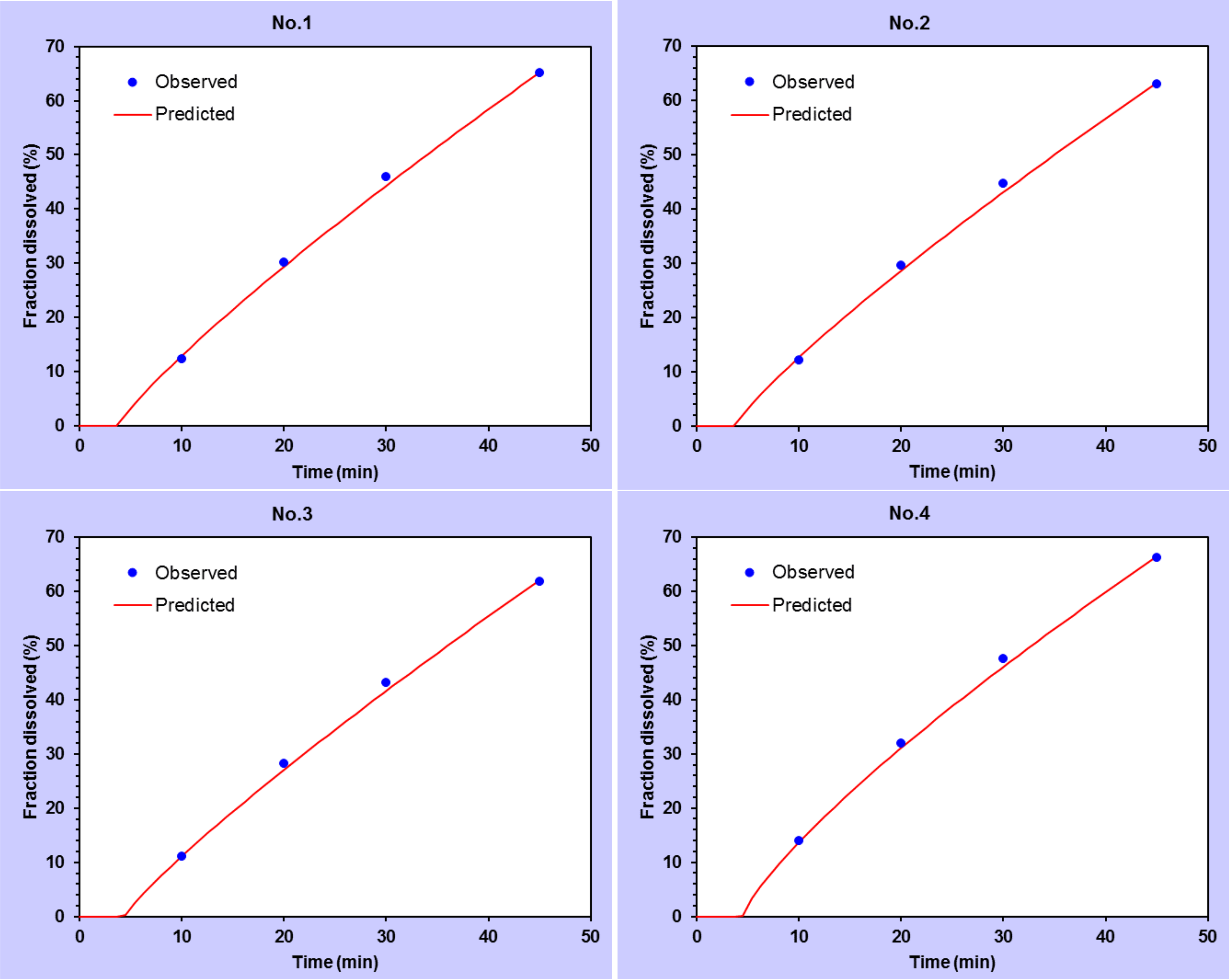

Supplementary materials – Model fitting summary – Polyglykol® 8000 P (modelling up to app. 60 % of carvedilol released)

Model: **Korsmeyer–Peppas with F_0**

Model equation: $F = F_0 + k_{KP} \cdot t^n$

Fitted model parameters per tested tablet (N = 4) with statistics – mean, standard deviation (SD), and relative standard deviation expressed in % (RSD%) (output from DDSolver):

Parameter	No.1	No.2	No.3	No.4	Mean	SD	RSD(%)
k_{KP}	0.324	0.323	0.267	0.426	0.335	0.066	19.804
n	1.403	1.396	1.444	1.332	1.394	0.046	3.328
F_0	4.959	4.839	4.439	5.559	4.949	0.464	9.366

Number of dissolution data points (N), degrees of freedom (df), and selected goodness of fit criteria – Pearson correlation coefficient (R), coefficient of determination (R^2), adjusted coefficient of determination ($R^2_{adjusted}$), and residual sum of squares (RSS) (manual calculation in MS Excel):

Parameter	No.1	No.2	No.3	No.4
N	4	4	4	4
df	1	1	1	1
R	0.986904982	0.985555933	0.986285444	0.987404821
R^2	0.973981443	0.971320498	0.972758976	0.974968281
$R^2_{adjusted}$	0.92194433	0.913961494	0.918276929	0.924904842
RSS	75.94240508	77.55471084	76.76161338	68.36473423

Graphical abstract of model fit presented as mean ± 1 SD of the fraction % of released carvedilol:

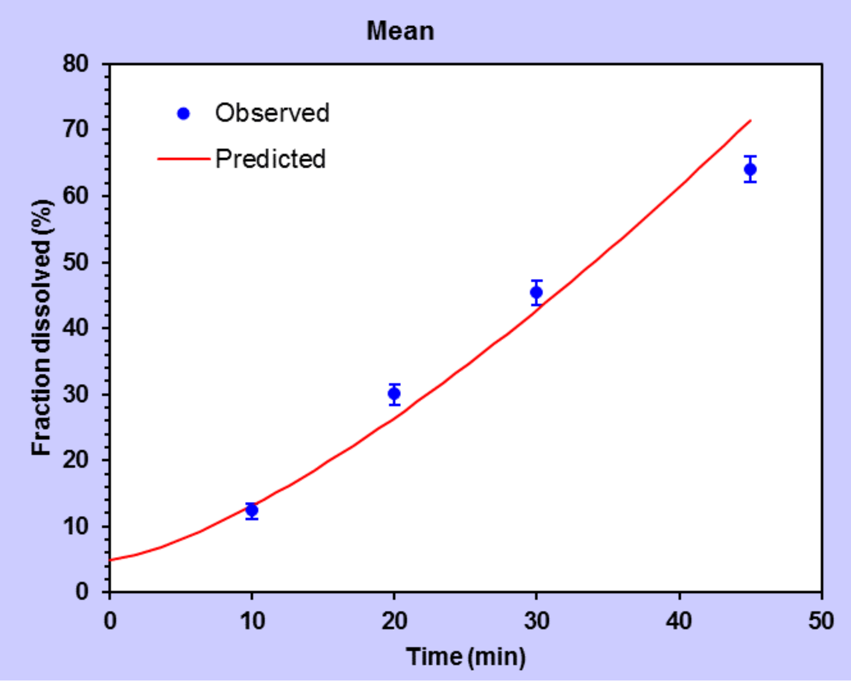

Supplementary materials – Model fitting summary – Polyglykol® 8000 P (modelling up to app. 60 % of carvedilol released)

Graphical abstract of model fit presented as the fraction % of released carvedilol per tested tablet:

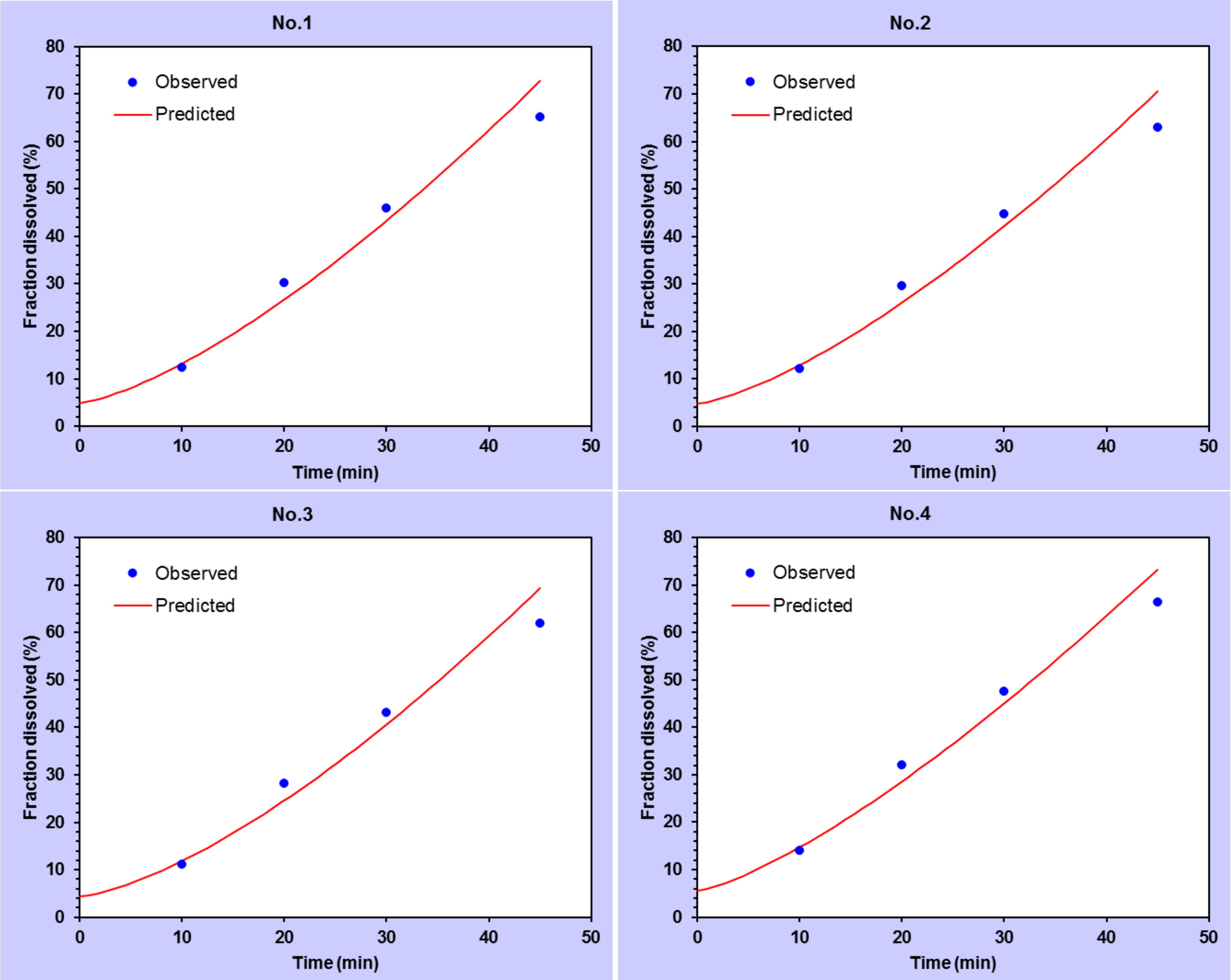

Supplementary materials – Model fitting summary – Polyglykol® 8000 P (modelling up to app. 60 % of carvedilol released)

Model: **Hixson–Crowell**

Model equation: $F = 100 \cdot [1 - (1 - k_{HC} \cdot t)^3]$

Fitted model parameters per tested tablet (N = 4) with statistics – mean, standard deviation (SD), and relative standard deviation expressed in % (RSD%) (output from DDSolver):

Parameter	No.1	No.2	No.3	No.4	Mean	SD	RSD(%)
k_{HC}	0.006	0.006	0.006	0.007	0.006	0.000	4.955

Number of dissolution data points (N), degrees of freedom (df), and selected goodness of fit criteria – Pearson correlation coefficient (R), coefficient of determination (R^2), adjusted coefficient of determination ($R^2_{adjusted}$), and residual sum of squares (RSS) (manual calculation in MS Excel):

Parameter	No.1	No.2	No.3	No.4
N	4	4	4	4
df	3	3	3	3
R	0.999978984	0.999937661	0.999999411	0.99998756
R^2	0.999957969	0.999875326	0.999998822	0.99997512
$R^2_{adjusted}$	0.999957969	0.999875326	0.999998822	0.99997512
RSS	42.64635127	33.15282499	41.82010687	27.44891504

Graphical abstract of model fit presented as mean ± 1 SD of the fraction % of released carvedilol:

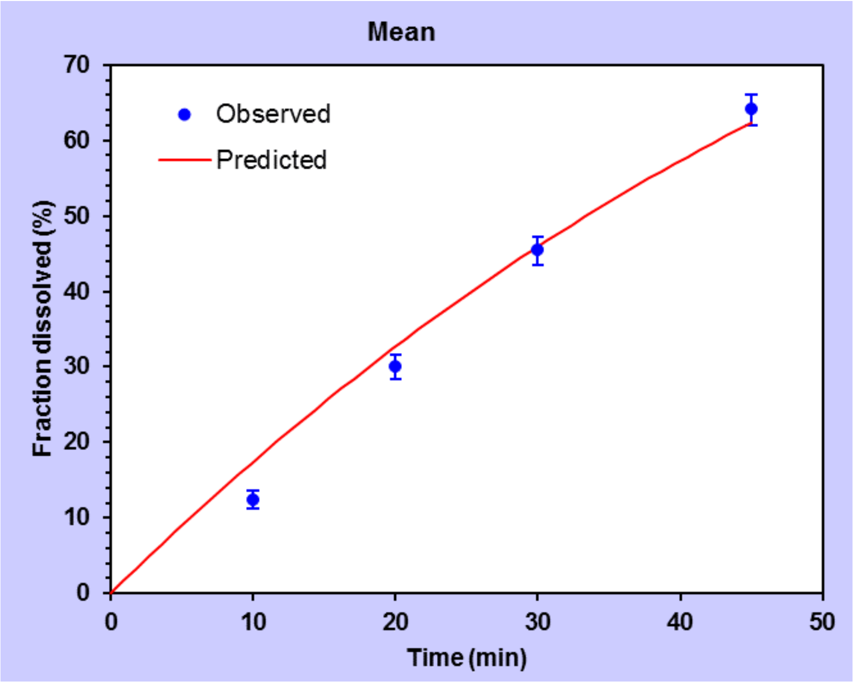

Supplementary materials – Model fitting summary – Polyglykol® 8000 P (modelling up to app. 60 % of carvedilol released)

Graphical abstract of model fit presented as the fraction % of released carvedilol per tested tablet:

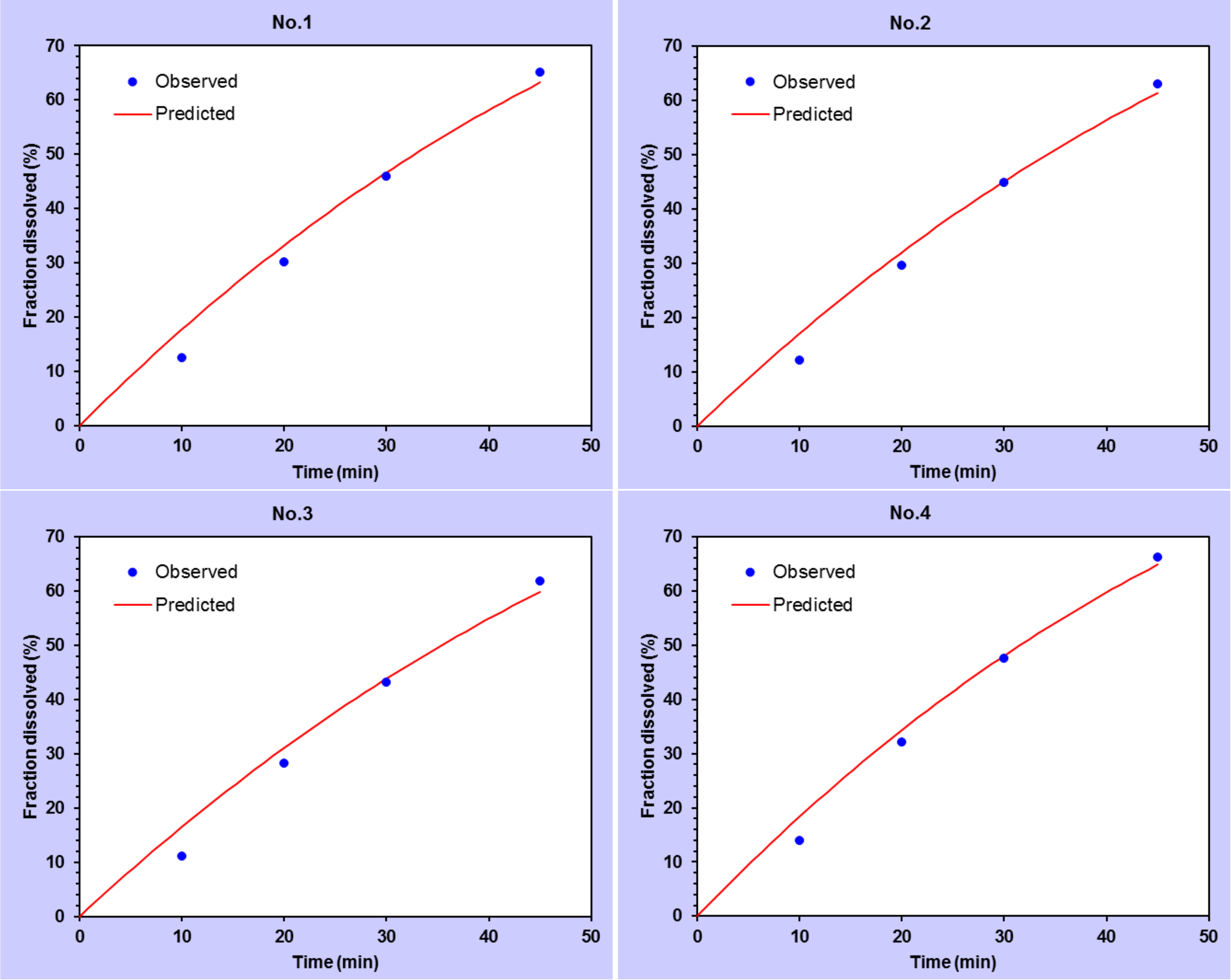

Supplementary materials – Model fitting summary – Polyglykol® 8000 P (modelling up to app. 60 % of carvedilol released)

Model: **Hixson–Crowell with T_{lag}**

Model equation: $F = 100 \cdot \left\{1 - \left[1 - k_{HC} \cdot \left(t - T_{lag}\right)\right]^3\right\}$

Fitted model parameters per tested tablet (N = 4) with statistics – mean, standard deviation (SD), and relative standard deviation expressed in % (RSD%) (output from DDSolver):

Parameter	No.1	No.2	No.3	No.4	Mean	SD	RSD(%)
k_{HC}	0.007	0.007	0.007	0.007	0.007	0.000	3.908
T_{lag}	4.261	3.862	4.441	3.399	3.991	0.463	11.596

Number of dissolution data points (N), degrees of freedom (df), and selected goodness of fit criteria – Pearson correlation coefficient (R), coefficient of determination (R^2), adjusted coefficient of determination ($R^2_{adjusted}$), and residual sum of squares (RSS) (manual calculation in MS Excel):

Parameter	No.1	No.2	No.3	No.4
N	4	4	4	4
df	2	2	2	2
R	0.999878895	0.999995997	0.999941642	0.999987607
R^2	0.999757805	0.999991994	0.999883287	0.999975214
$R^2_{adjusted}$	0.999636707	0.999987992	0.999824931	0.999962821
RSS	0.390449629	0.011405368	0.173917195	0.039425116

Graphical abstract of model fit presented as mean ± 1 SD of the fraction % of released carvedilol:

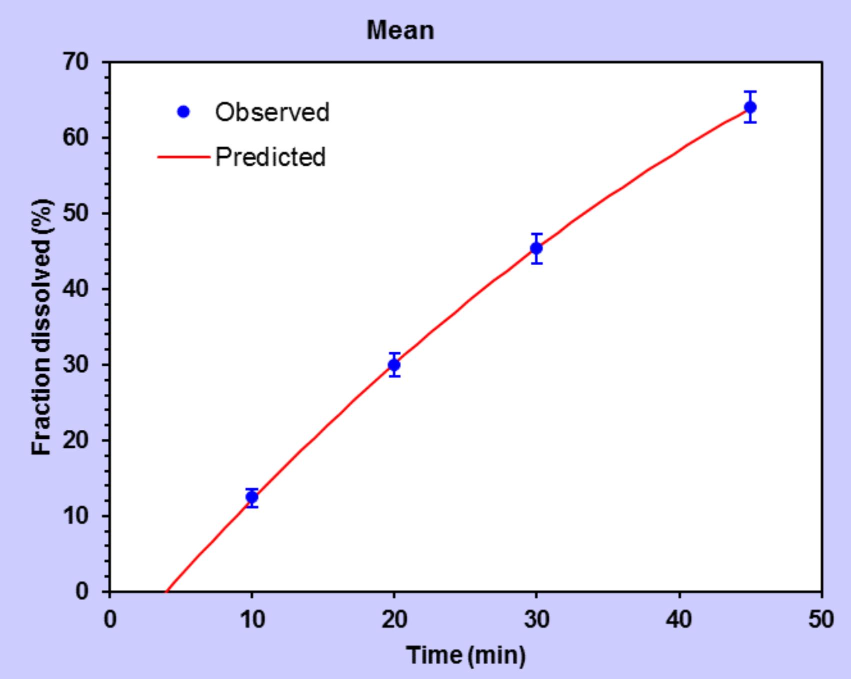

Supplementary materials – Model fitting summary – Polyglykol® 8000 P (modelling up to app. 60 % of carvedilol released)

Graphical abstract of model fit presented as the fraction % of released carvedilol per tested tablet:

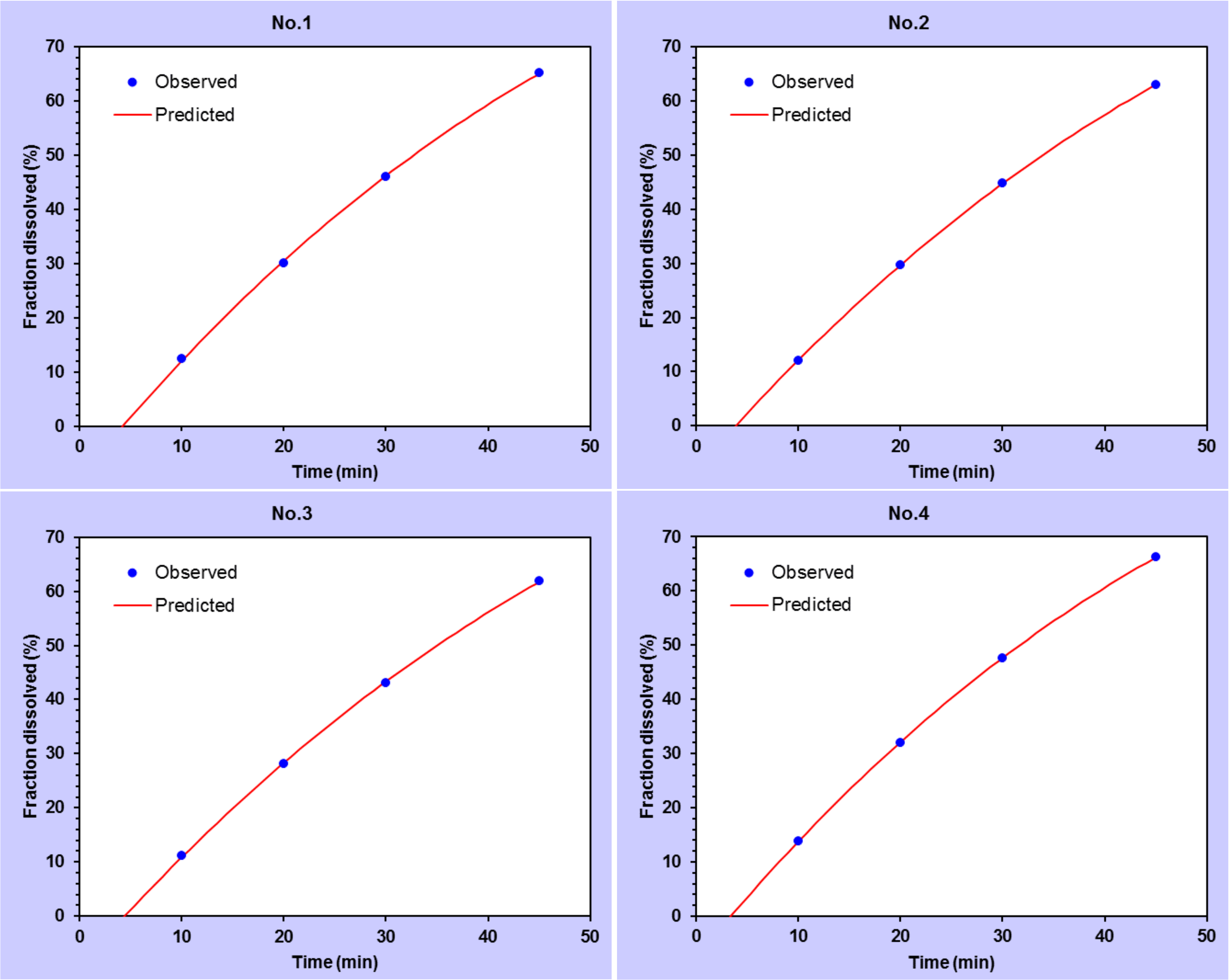

Supplementary materials – Model fitting summary – Polyglykol® 8000 P (modelling up to app. 60 % of carvedilol released)

Model: **Hopfenberg**

Model equation: $F = 100 \cdot [1 - (1 - k_{HB} \cdot t)^n]$

Fitted model parameters per tested tablet (N = 4) with statistics – mean, standard deviation (SD), and relative standard deviation expressed in % (RSD%) (output from DDSolver):

Parameter	No.1	No.2	No.3	No.4	Mean	SD	RSD(%)
k_{HB}	0.015	0.014	0.014	0.015	0.014	0.001	3.802
n	1.000	1.000	1.000	1.000	1.000	0.000	0.000

Number of dissolution data points (N), degrees of freedom (df), and selected goodness of fit criteria – Pearson correlation coefficient (R), coefficient of determination (R^2), adjusted coefficient of determination ($R^2_{adjusted}$), and residual sum of squares (RSS) (manual calculation in MS Excel):

Parameter	No.1	No.2	No.3	No.4
N	4	4	4	4
df	2	2	2	2
R	0.996875343	0.996085399	0.997207797	0.995957165
R^2	0.993760449	0.992186121	0.994423391	0.991930674
$R^2_{adjusted}$	0.990640673	0.988279182	0.991635086	0.987896011
RSS	10.29435359	11.28288015	10.35417069	12.6424979

Graphical abstract of model fit presented as mean ± 1 SD of the fraction % of released carvedilol:

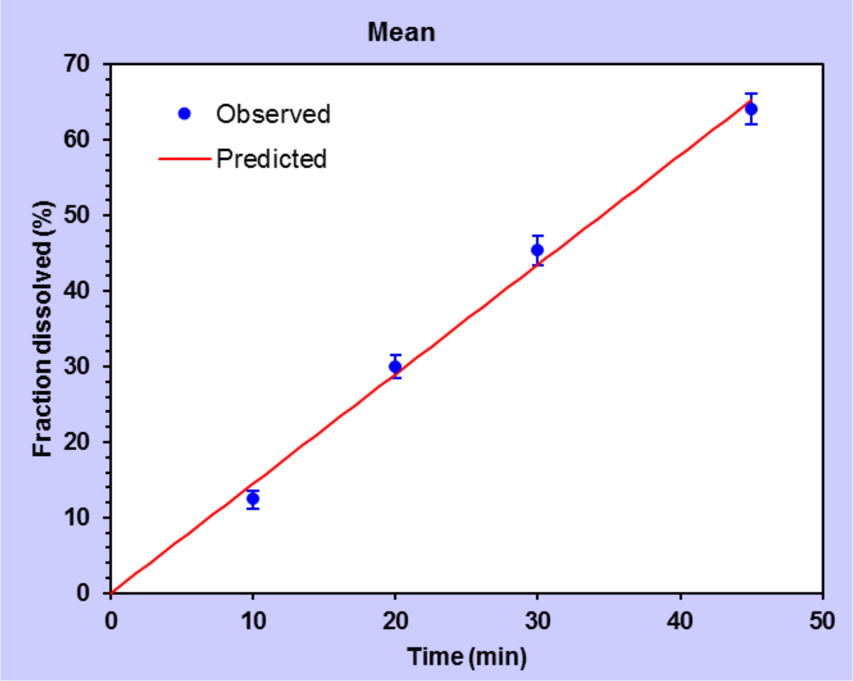

Supplementary materials – Model fitting summary – Polyglykol® 8000 P (modelling up to app. 60 % of carvedilol released)

Graphical abstract of model fit presented as the fraction % of released carvedilol per tested tablet:

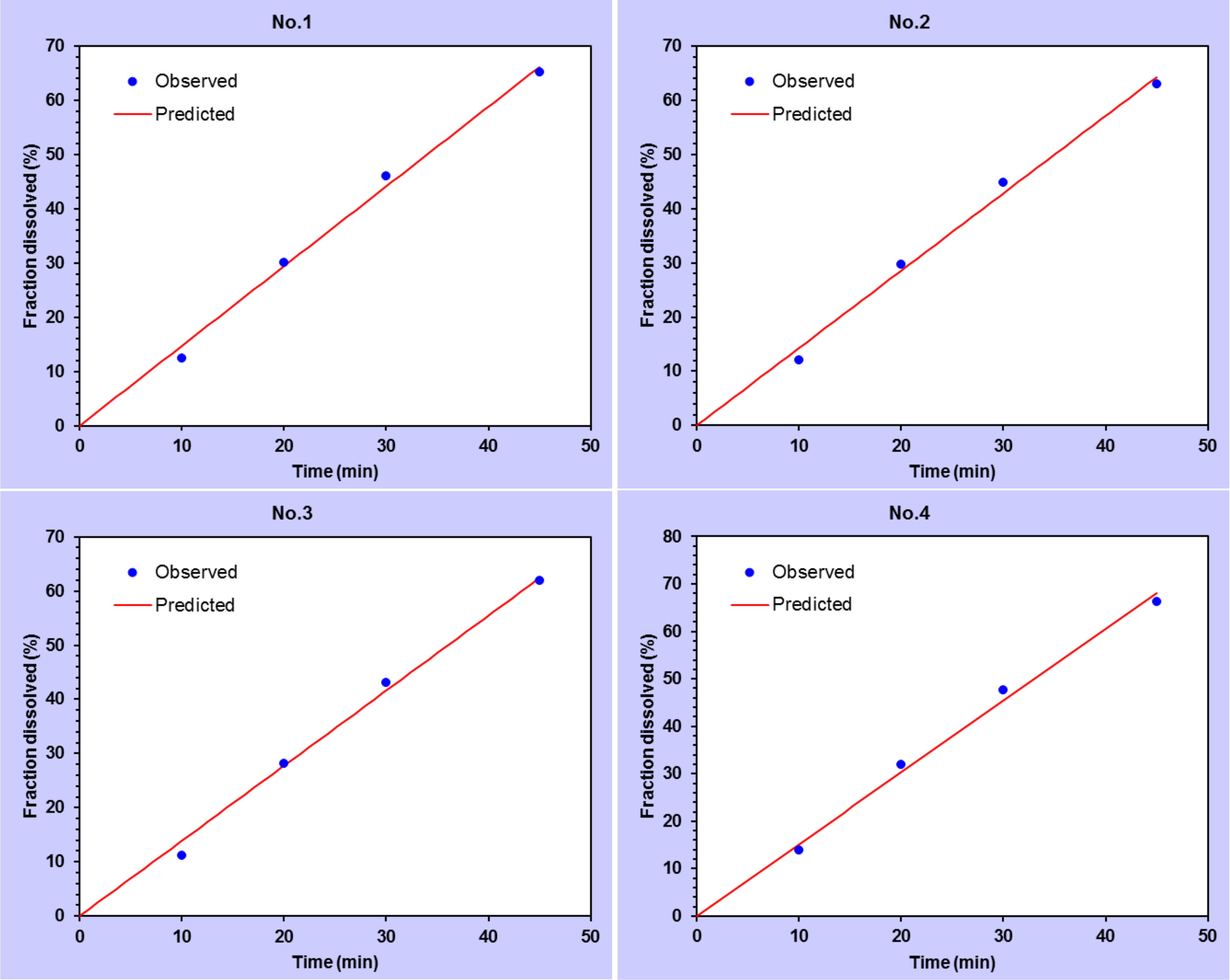

Supplementary materials – Model fitting summary – Polyglykol® 8000 P (modelling up to app. 60 % of carvedilol released)

Model: **Hopfenberg with T_{lag}**

Model equation: $F = 100 \cdot \{1 - [1 - k_{HB} \cdot (t - T_{lag})]^n\}$

Fitted model parameters per tested tablet (N = 4) with statistics – mean, standard deviation (SD), and relative standard deviation expressed in % (RSD%) (output from DDSolver):

Parameter	No.1	No.2	No.3	No.4	Mean	SD	RSD(%)
k_{HB}	0.010	0.007	0.007	0.007	0.008	0.001	15.828
n	2.104	3.000	2.782	3.000	2.722	0.424	15.585
T_{lag}	3.586	3.862	4.209	3.399	3.764	0.352	9.357

Number of dissolution data points (N), degrees of freedom (df), and selected goodness of fit criteria – Pearson correlation coefficient (R), coefficient of determination (R^2), adjusted coefficient of determination ($R^2_{adjusted}$), and residual sum of squares (RSS) (manual calculation in MS Excel):

Parameter	No.1	No.2	No.3	No.4
N	4	4	4	4
df	1	1	1	1
R	0.999970107	0.999995997	0.999974943	0.999987607
R^2	0.999940215	0.999991994	0.999949887	0.999975214
$R^2_{adjusted}$	0.999820646	0.999975983	0.999849662	0.999925643
RSS	0.158316549	0.011405368	0.088194989	0.039425116

Graphical abstract of model fit presented as mean ± 1 SD of the fraction % of released carvedilol:

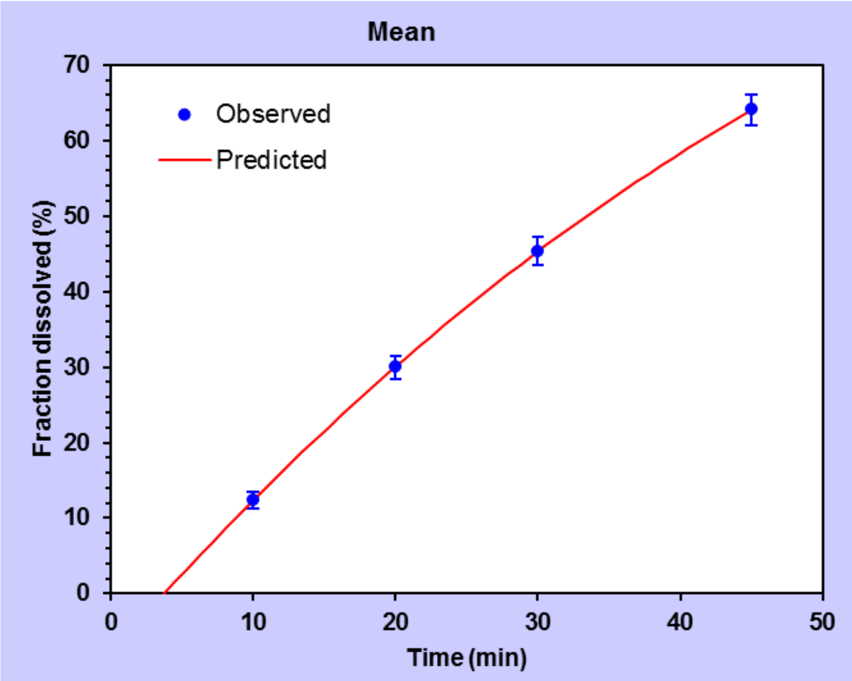

Supplementary materials – Model fitting summary – Polyglykol® 8000 P (modelling up to app. 60 % of carvedilol released)

Graphical abstract of model fit presented as the fraction % of released carvedilol per tested tablet:

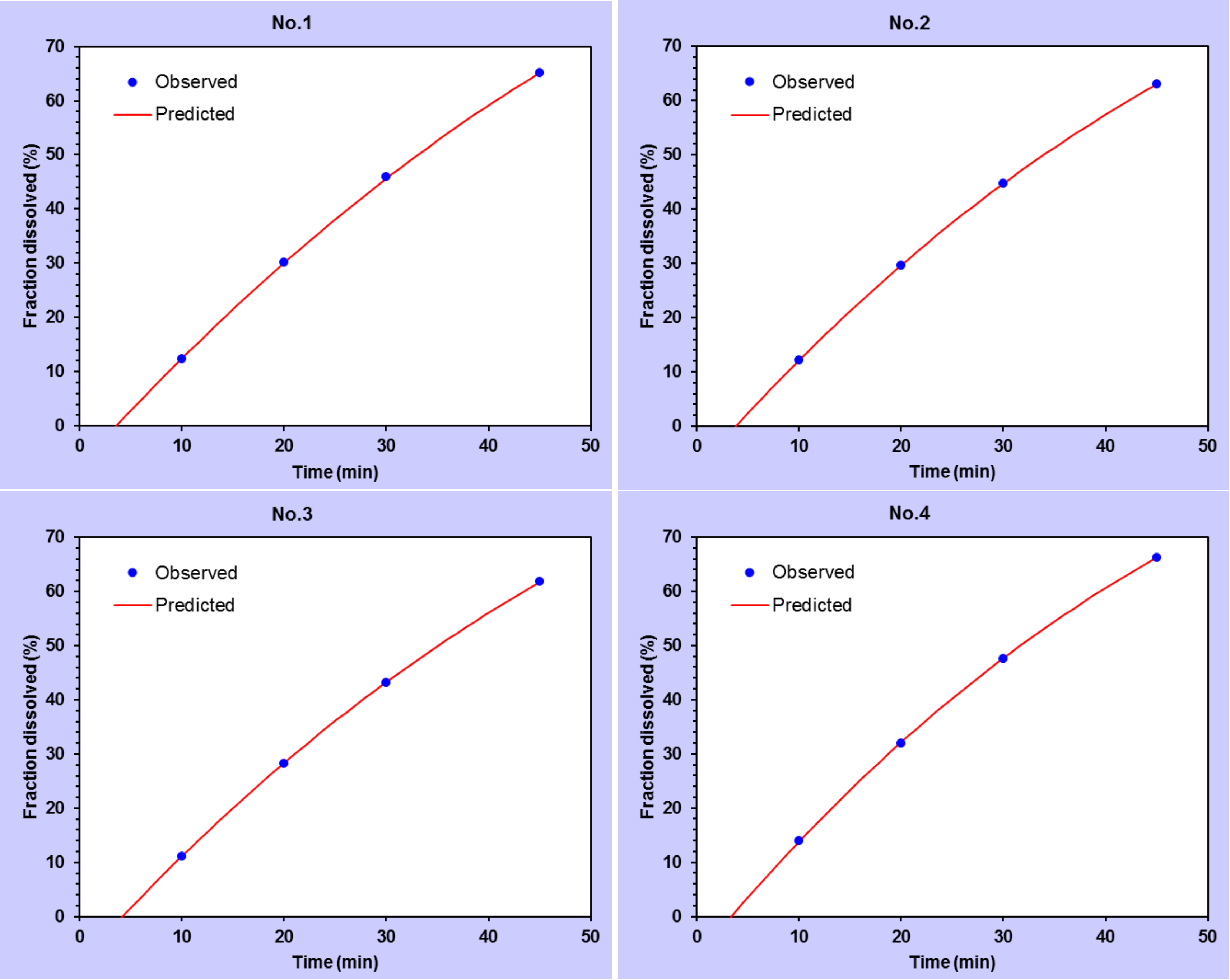

Supplementary materials – Model fitting summary – Polyglykol® 8000 P (modelling up to app. 60 % of carvedilol released)

Model: **Baker–Lonsdale**

Model equation: $\frac{3}{2} \cdot \left[1 - \left(1 - \frac{F}{100}\right)^{\frac{2}{3}}\right] - \frac{F}{100} = k_{BL} \cdot t$

Fitted model parameters per tested tablet (N = 4) with statistics – mean, standard deviation (SD), and relative standard deviation expressed in % (RSD%) (output from DDSolver):

Parameter	No.1	No.2	No.3	No.4	Mean	SD	RSD(%)
k_{BL}	0.003	0.003	0.003	0.003	0.003	0.000	7.827

Number of dissolution data points (N), degrees of freedom (df), and selected goodness of fit criteria – Pearson correlation coefficient (R), coefficient of determination (R^2), adjusted coefficient of determination ($R^2_{adjusted}$), and residual sum of squares (RSS) (manual calculation in MS Excel):

Parameter	No.1	No.2	No.3	No.4
N	4	4	4	4
df	3	3	3	3
R	0.9962169	0.997302837	0.996448508	0.997065635
R^2	0.992448112	0.994612948	0.99290963	0.99413988
$R^2_{adjusted}$	0.992448112	0.994612948	0.99290963	0.99413988
RSS	1405.99504	1255.672019	1310.664754	1293.346076

Graphical abstract of model fit presented as mean ± 1 SD of the fraction % of released carvedilol:

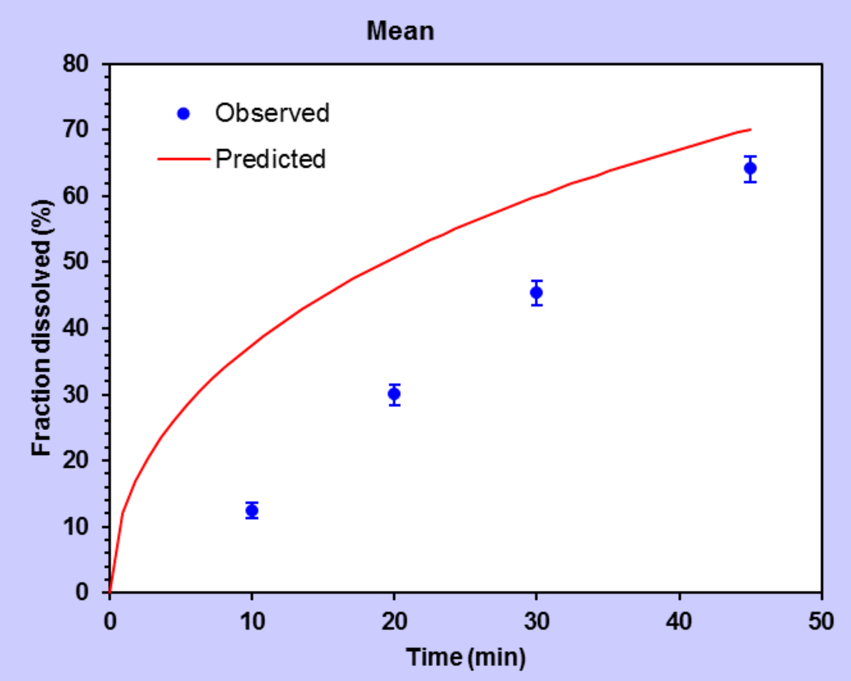

Supplementary materials – Model fitting summary – Polyglykol® 8000 P (modelling up to app. 60 % of carvedilol released)

Graphical abstract of model fit presented as the fraction % of released carvedilol per tested tablet:

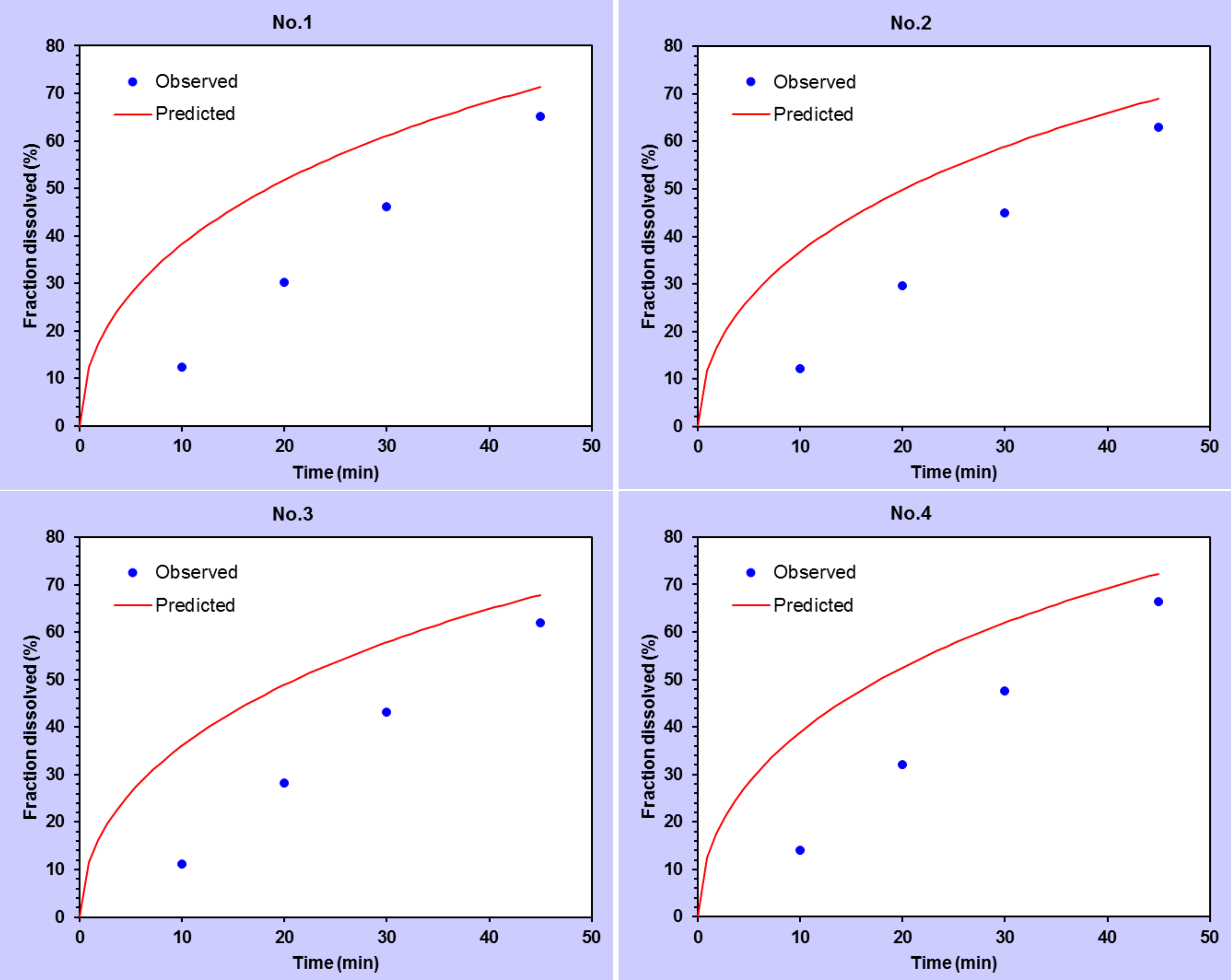

Supplementary materials – Model fitting summary – Polyglykol® 8000 P (modelling up to app. 60 % of carvedilol released)

Model: **Baker–Lonsdale with T_{lag}**

Model equation: $\frac{3}{2}\cdot\left[1-\left(1-\frac{F}{100}\right)^{\frac{2}{3}}\right]-\frac{F}{100}=k_{BL}\cdot\left(t-T_{lag}\right)$

Fitted model parameters per tested tablet (N = 4) with statistics – mean, standard deviation (SD), and relative standard deviation expressed in % (RSD%) (output from DDSolver):

Parameter	No.1	No.2	No.3	No.4	Mean	SD	RSD(%)
k_{BL}	0.003	0.003	0.003	0.003	0.003	0.000	7.827
T_{lag}	11.919	11.706	12.026	11.496	11.787	0.235	1.996

Number of dissolution data points (N), degrees of freedom (df), and selected goodness of fit criteria – Pearson correlation coefficient (R), coefficient of determination (R^2), adjusted coefficient of determination ($R^2_{adjusted}$), and residual sum of squares (RSS) (manual calculation in MS Excel):

Parameter	No.1	No.2	No.3	No.4
N	4	4	4	4
df	2	2	2	2
R	0.968856948	0.970896242	0.970393095	0.968815607
R^2	0.938683786	0.942639513	0.941662759	0.938603681
$R^2_{adjusted}$	0.908025679	0.913959269	0.912494139	0.907905521
RSS	191.748158	176.6384738	158.512709	224.1473065

Graphical abstract of model fit presented as mean ± 1 SD of the fraction % of released carvedilol:

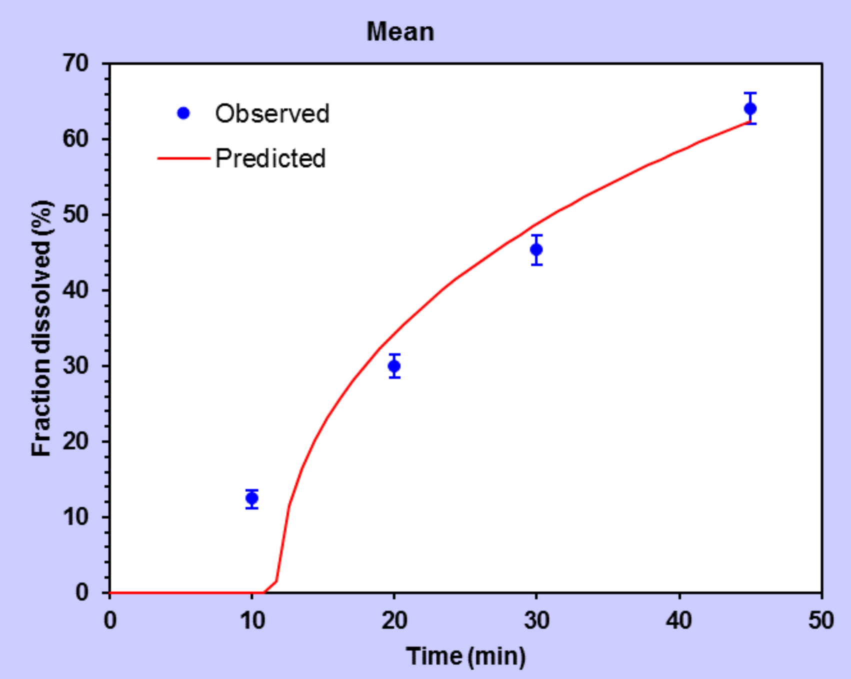

Supplementary materials – Model fitting summary – Polyglykol® 8000 P (modelling up to app. 60 % of carvedilol released)

Graphical abstract of model fit presented as the fraction % of released carvedilol per tested tablet:

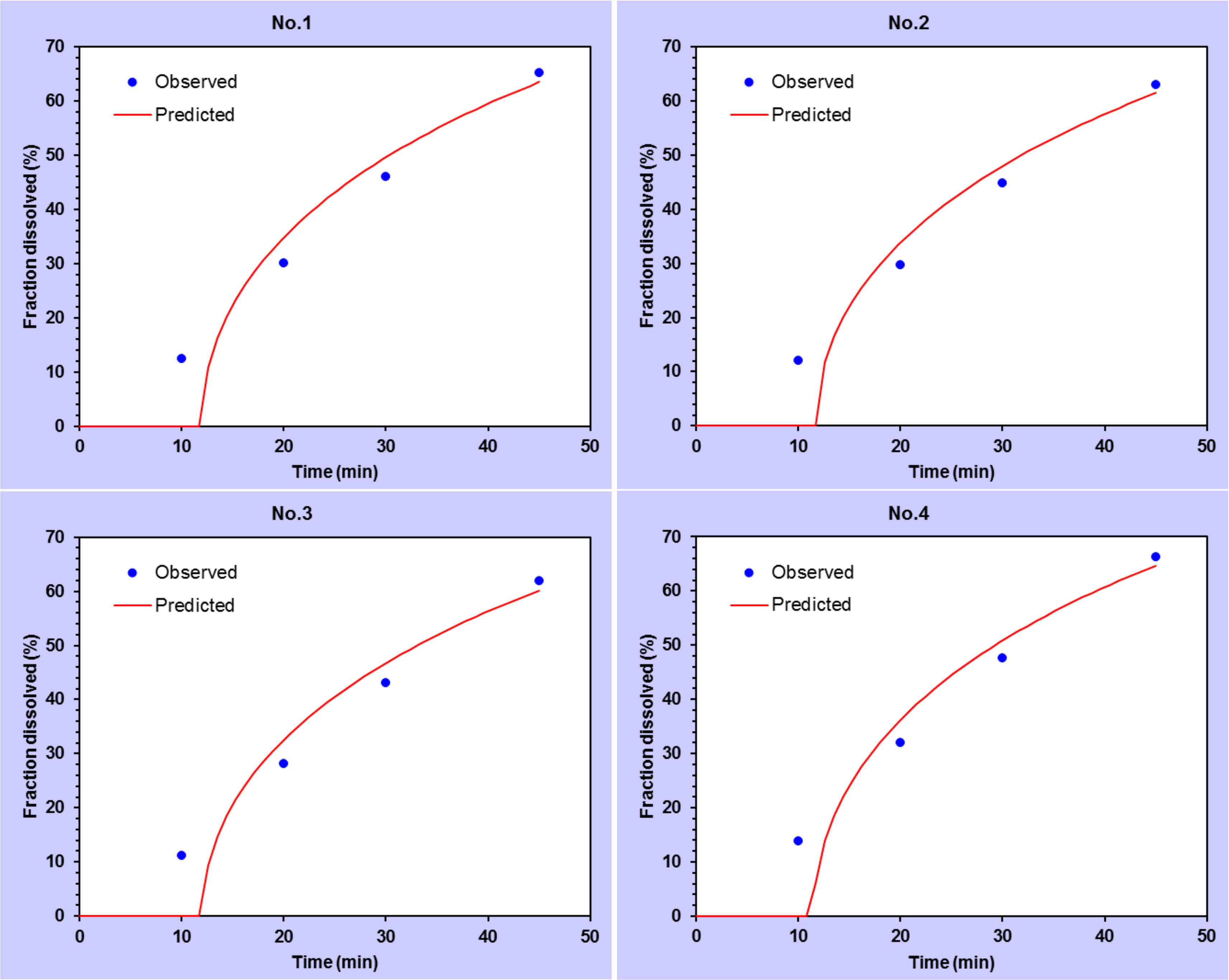

Supplementary materials – Model fitting summary – Polyglykol® 8000 P (modelling up to app. 60 % of carvedilol released)

Model: **Makoid–Banakar**

Model equation: $F = k_{MB} \cdot t^n \cdot e^{-k \cdot t}$

Fitted model parameters per tested tablet (N = 4) with statistics – mean, standard deviation (SD), and relative standard deviation expressed in % (RSD%) (output from DDSolver):

Parameter	No.1	No.2	No.3	No.4	Mean	SD	RSD(%)
k_{MB}	0.429	0.398	0.325	0.614	0.442	0.123	27.864
n	1.544	1.573	1.626	1.428	1.543	0.084	5.432
k	0.019	0.021	0.021	0.017	0.019	0.002	9.867

Number of dissolution data points (N), degrees of freedom (df), and selected goodness of fit criteria – Pearson correlation coefficient (R), coefficient of determination (R^2), adjusted coefficient of determination ($R^2_{adjusted}$), and residual sum of squares (RSS) (manual calculation in MS Excel):

Parameter	No.1	No.2	No.3	No.4
N	4	4	4	4
df	1	1	1	1
R	0.999959259	0.999907387	0.99981706	0.999956645
R^2	0.999918519	0.999814783	0.999634153	0.999913292
$R^2_{adjusted}$	0.999755557	0.99944435	0.998902459	0.999739875
RSS	0.123744343	0.260955406	0.511766744	0.154682737

Graphical abstract of model fit presented as mean ± 1 SD of the fraction % of released carvedilol:

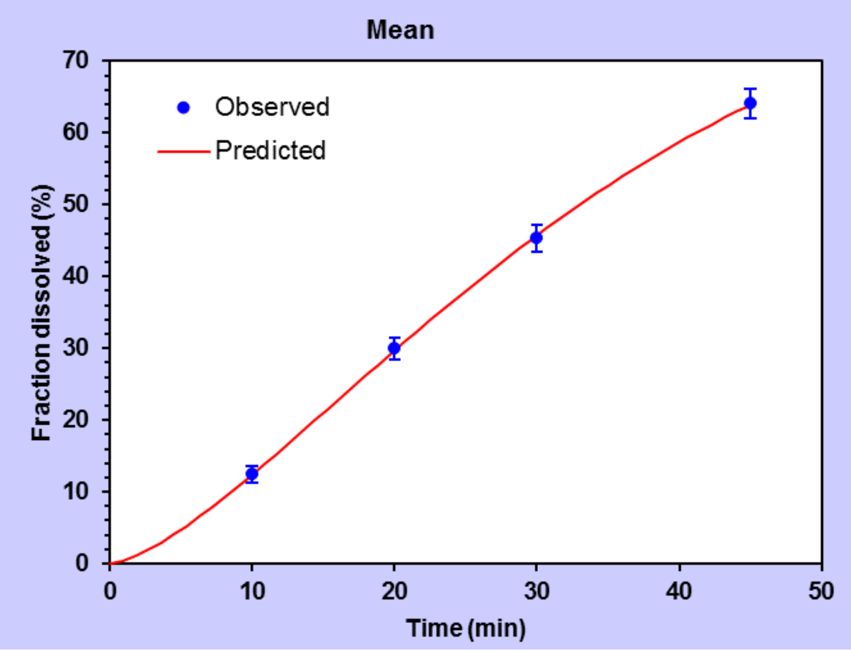

Supplementary materials – Model fitting summary – Polyglykol® 8000 P (modelling up to app. 60 % of carvedilol released)

Graphical abstract of model fit presented as the fraction % of released carvedilol per tested tablet:

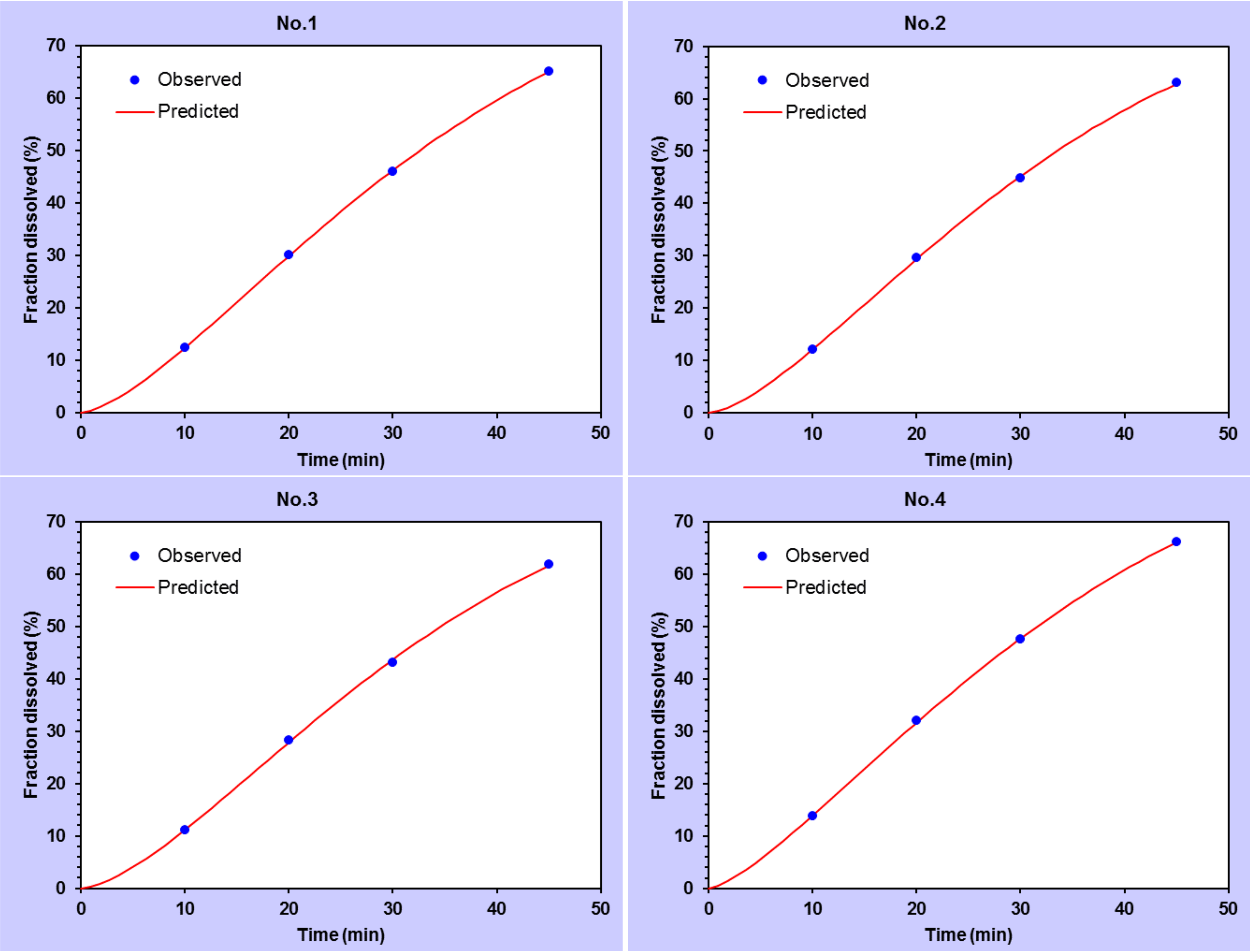

Supplementary materials – Model fitting summary – Polyglykol® 8000 P (modelling up to app. 60 % of carvedilol released)

Model: **Makoid–Banakar with T_{lag}**

Model equation: $F = k_{MB} \cdot (t - T_{lag})^n \cdot e^{-k \cdot (t - T_{lag})}$

Fitted model parameters per tested tablet (N = 4) with statistics – mean, standard deviation (SD), and relative standard deviation expressed in % (RSD%) (output from DDSolver):

Parameter	No.1	No.2	No.3	No.4	Mean	SD	RSD(%)
k_{MB}	/	/	/	/	/	/	/
n	/	/	/	/	/	/	/
k	/	/	/	/	/	/	/
T_{lag}	/	/	/	/	/	/	/

Number of dissolution data points (N), degrees of freedom (df), and selected goodness of fit criteria – Pearson correlation coefficient (R), coefficient of determination (R^2), adjusted coefficient of determination ($R^2_{adjusted}$), and residual sum of squares (RSS) (manual calculation in MS Excel):

Parameter	No.1	No.2	No.3	No.4
N	/	/	/	/
df	/	/	/	/
R	/	/	/	/
R^2	/	/	/	/
$R^2_{adjusted}$	/	/	/	/
RSS	/	/	/	/

Graphical abstract of model fit presented as mean ± 1 SD of the fraction % of released carvedilol: /

Graphical abstract of model fit presented as the fraction % of released carvedilol per tested tablet: /

Note: model could not be fitted to experimental dissolution data due too few data points being available for fitting

Supplementary materials – Model fitting summary – Polyglykol® 8000 P (modelling up to app. 60 % of carvedilol released)

Model: **Peppas–Sahlin_1**

Model equation: $F = k_1 \cdot t^m + k_2 \cdot t^{2m}$

Fitted model parameters per tested tablet (N = 4) with statistics – mean, standard deviation (SD), and relative standard deviation expressed in % (RSD%) (output from DDSolver):

Parameter	No.1	No.2	No.3	No.4	Mean	SD	RSD(%)
k_1	-2.249	-1.946	-2.575	-1.262	-2.008	0.559	-27.857
k_2	2.561	2.437	2.508	2.421	2.482	0.065	2.617
m	0.450	0.450	0.450	0.450	0.450	0.000	0.000

Number of dissolution data points (N), degrees of freedom (df), and selected goodness of fit criteria – Pearson correlation coefficient (R), coefficient of determination (R^2), adjusted coefficient of determination ($R^2_{adjusted}$), and residual sum of squares (RSS) (manual calculation in MS Excel):

Parameter	No.1	No.2	No.3	No.4
N	4	4	4	4
df	1	1	1	1
R	0.997607591	0.997004835	0.997751782	0.997192263
R^2	0.995220905	0.99401864	0.995508618	0.99439241
$R^2_{adjusted}$	0.985662716	0.98205592	0.986525855	0.98317723
RSS	7.34594823	8.534139907	6.359203187	8.481648931

Graphical abstract of model fit presented as mean ± 1 SD of the fraction % of released carvedilol:

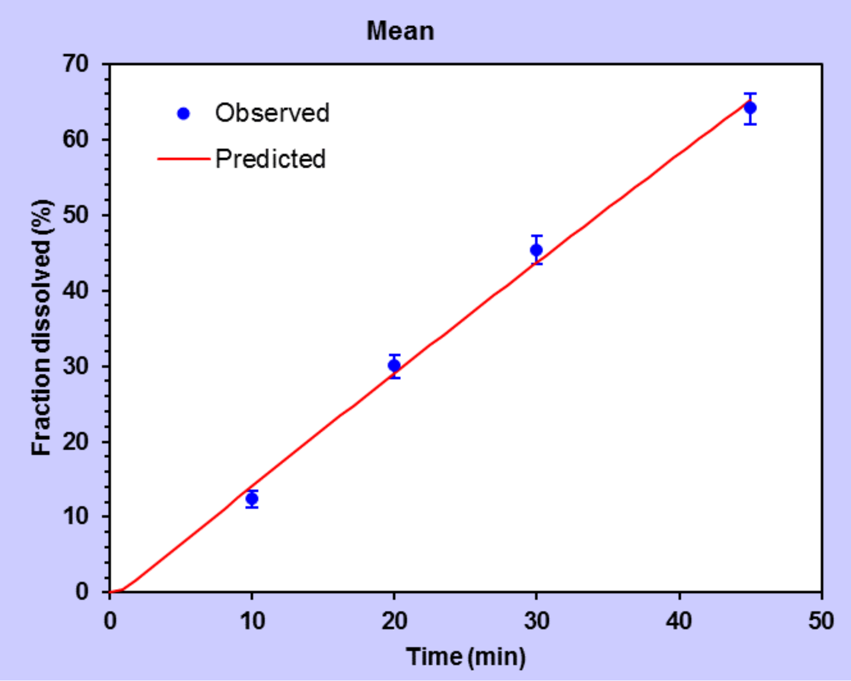

Supplementary materials – Model fitting summary – Polyglykol® 8000 P (modelling up to app. 60 % of carvedilol released)

Graphical abstract of model fit presented as the fraction % of released carvedilol per tested tablet:

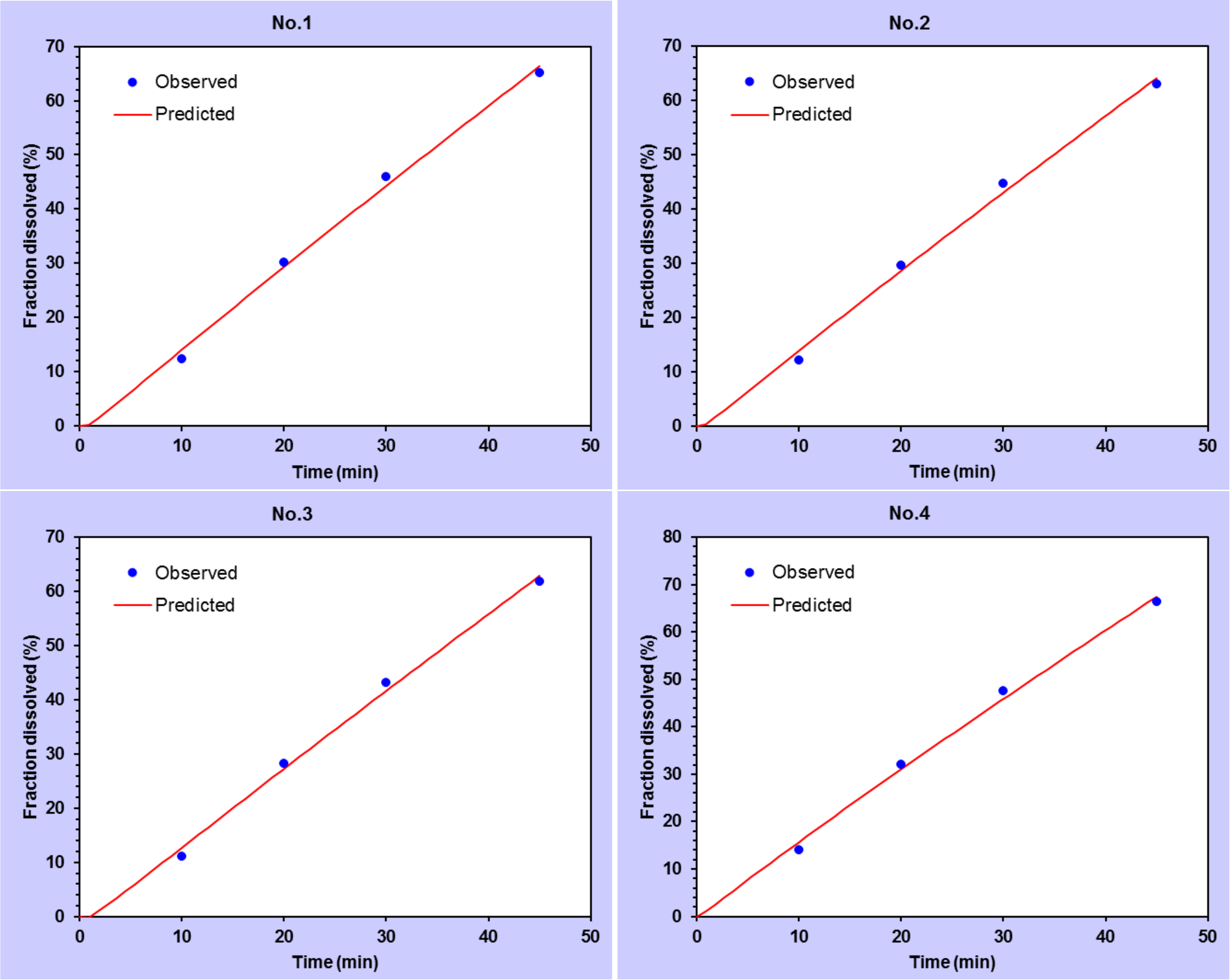

Supplementary materials – Model fitting summary – Polyglykol® 8000 P (modelling up to app. 60 % of carvedilol released)

Model: **Peppas–Sahlin_1 with T_{lag}**

Model equation: $F = k_1 \cdot (t - T_{lag})^m + k_2 \cdot (t - T_{lag})^{2m}$

Fitted model parameters per tested tablet (N = 4) with statistics – mean, standard deviation (SD), and relative standard deviation expressed in % (RSD%) (output from DDSolver):

Parameter	No.1	No.2	No.3	No.4	Mean	SD	RSD(%)
k_1	/	/	/	/	/	/	/
k_2	/	/	/	/	/	/	/
m	/	/	/	/	/	/	/
T_{lag}	/	/	/	/	/	/	/

Number of dissolution data points (N), degrees of freedom (df), and selected goodness of fit criteria – Pearson correlation coefficient (R), coefficient of determination (R^2), adjusted coefficient of determination ($R^2_{adjusted}$), and residual sum of squares (RSS) (manual calculation in MS Excel):

Parameter	No.1	No.2	No.3	No.4
N	/	/	/	/
df	/	/	/	/
R	/	/	/	/
R^2	/	/	/	/
$R^2_{adjusted}$	/	/	/	/
RSS	/	/	/	/

Graphical abstract of model fit presented as mean ± 1 SD of the fraction % of released carvedilol: /

Graphical abstract of model fit presented as the fraction % of released carvedilol per tested tablet: /

Note: model could not be fitted to experimental dissolution data due too few data points being available for fitting

Supplementary materials – Model fitting summary – Polyglykol® 8000 P (modelling up to app. 60 % of carvedilol released)

Model: **Peppas–Sahlin_2**

Model equation: $F = k_1 \cdot t^{0.5} + k_2 \cdot t$

Fitted model parameters per tested tablet (N = 4) with statistics – mean, standard deviation (SD), and relative standard deviation expressed in % (RSD%) (output from DDSolver):

Parameter	No.1	No.2	No.3	No.4	Mean	SD	RSD(%)
k_1	-0.228	-0.029	-0.575	0.602	-0.057	0.494	-861.893
k_2	1.510	1.432	1.485	1.411	1.460	0.046	3.125

Number of dissolution data points (N), degrees of freedom (df), and selected goodness of fit criteria – Pearson correlation coefficient (R), coefficient of determination (R^2), adjusted coefficient of determination ($R^2_{adjusted}$), and residual sum of squares (RSS) (manual calculation in MS Excel):

Parameter	No.1	No.2	No.3	No.4
N	4	4	4	4
df	2	2	2	2
R	0.996740114	0.99606567	0.996861123	0.996356326
R^2	0.993490856	0.992146818	0.993732099	0.992725929
$R^2_{adjusted}$	0.990236284	0.988220227	0.990598149	0.989088894
RSS	10.07017921	11.27934722	8.930511941	11.0800359

Graphical abstract of model fit presented as mean ± 1 SD of the fraction % of released carvedilol:

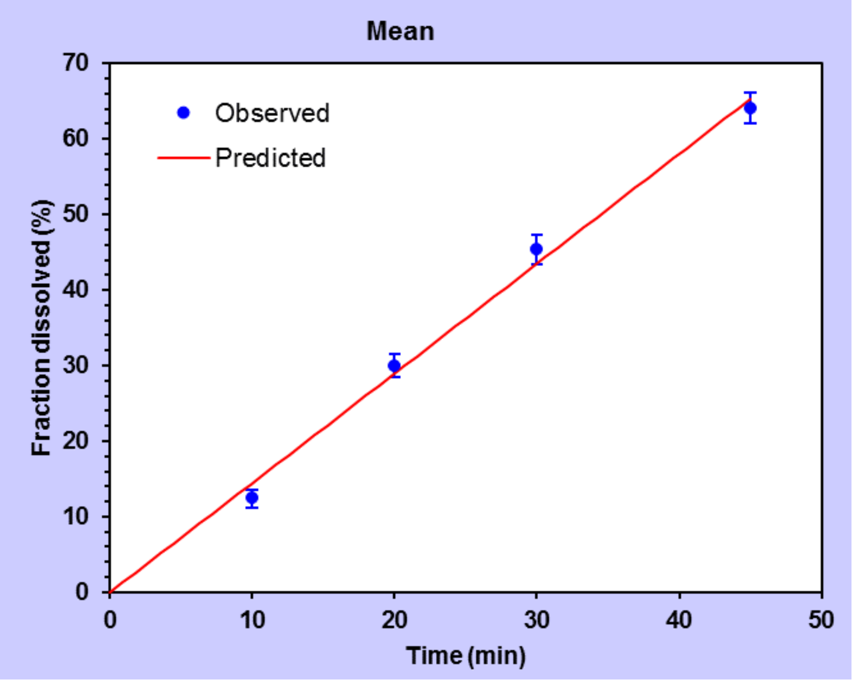

Supplementary materials – Model fitting summary – Polyglykol® 8000 P (modelling up to app. 60 % of carvedilol released)

Graphical abstract of model fit presented as the fraction % of released carvedilol per tested tablet:

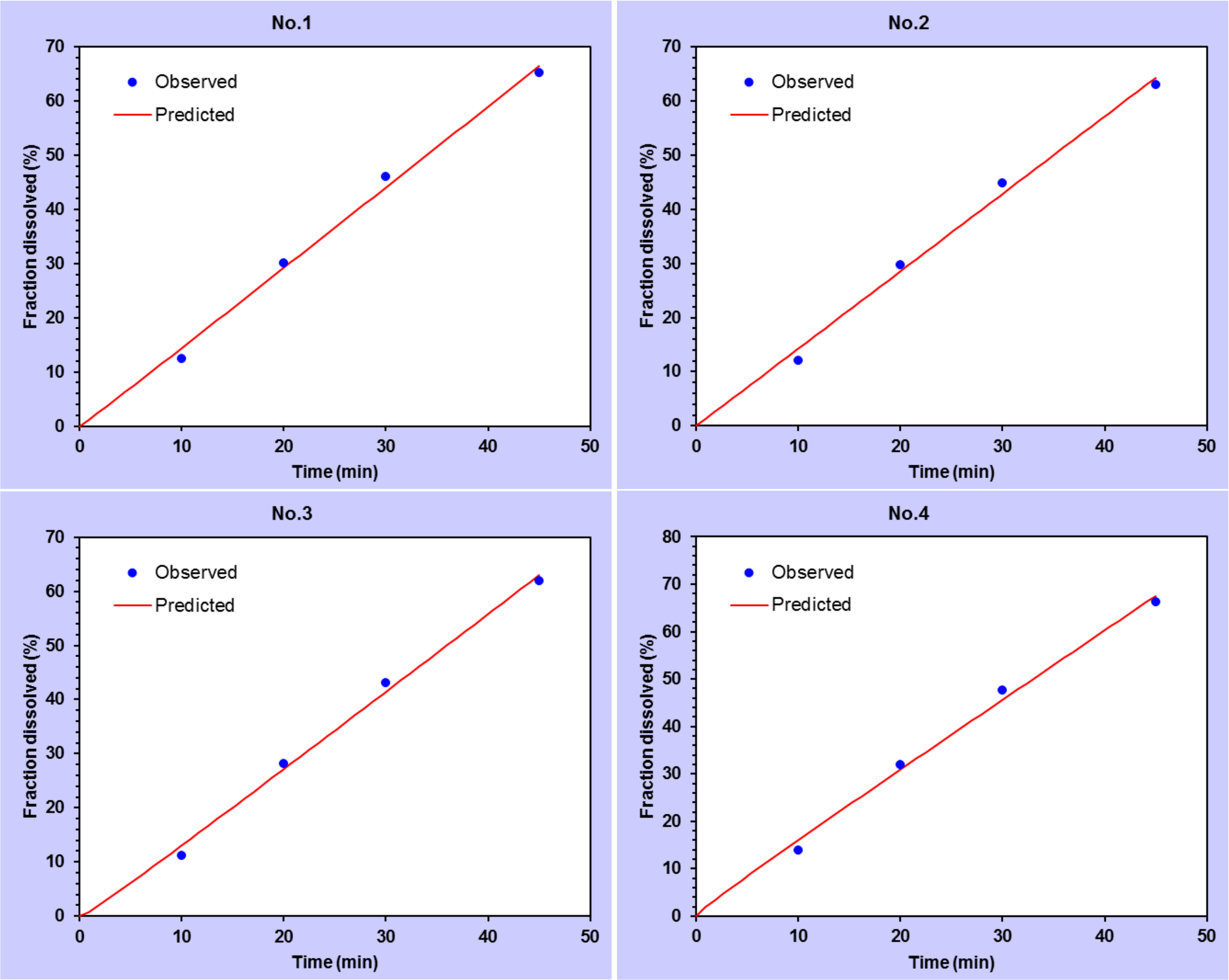

Supplementary materials – Model fitting summary – Polyglykol® 8000 P (modelling up to app. 60 % of carvedilol released)

Model: **Peppas–Sahlin_2 with T_{lag}**

Model equation: $F = k_1 \cdot (t - T_{lag})^{0.5} + k_2 \cdot (t - T_{lag})$

Fitted model parameters per tested tablet (N = 4) with statistics – mean, standard deviation (SD), and relative standard deviation expressed in % (RSD%) (output from DDSolver):

Parameter	No.1	No.2	No.3	No.4	Mean	SD	RSD(%)
k_1	2.665	2.788	2.154	3.586	2.798	0.593	21.183
k_2	1.193	1.121	1.190	1.076	1.145	0.057	4.960
T_{lag}	4.000	4.000	4.000	4.000	4.000	0.000	0.000

Number of dissolution data points (N), degrees of freedom (df), and selected goodness of fit criteria – Pearson correlation coefficient (R), coefficient of determination (R^2), adjusted coefficient of determination ($R^2_{adjusted}$), and residual sum of squares (RSS) (manual calculation in MS Excel):

Parameter	No.1	No.2	No.3	No.4
N	4	4	4	4
df	1	1	1	1
R	0.998632668	0.998264843	0.998669211	0.998595725
R^2	0.997267205	0.996532696	0.997340193	0.997193422
$R^2_{adjusted}$	0.991801614	0.989598088	0.99202058	0.991580267
RSS	4.33441136	5.117755631	3.893869281	4.398387429

Graphical abstract of model fit presented as mean ± 1 SD of the fraction % of released carvedilol:

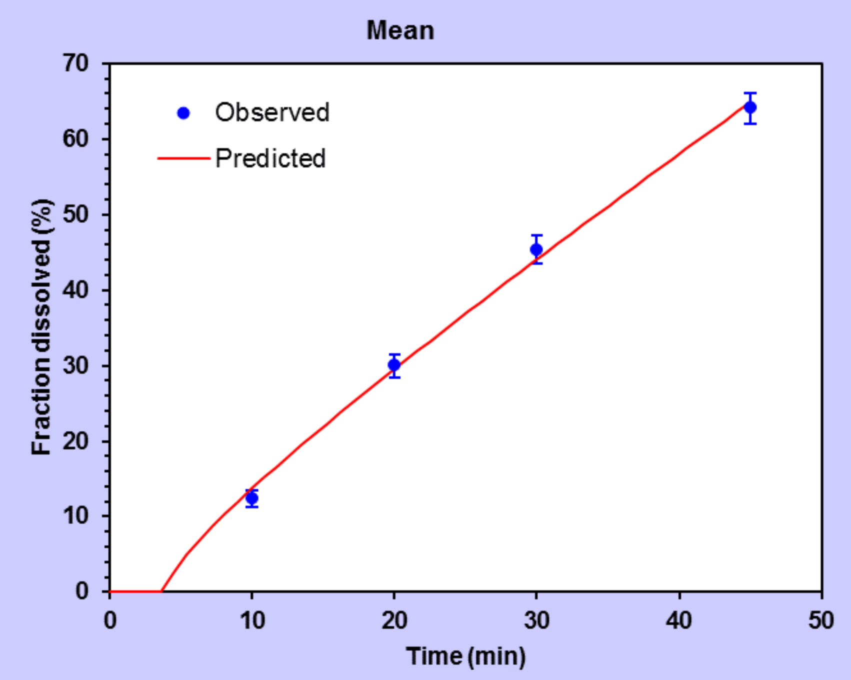

Supplementary materials – Model fitting summary – Polyglykol® 8000 P (modelling up to app. 60 % of carvedilol released)

Graphical abstract of model fit presented as the fraction % of released carvedilol per tested tablet:

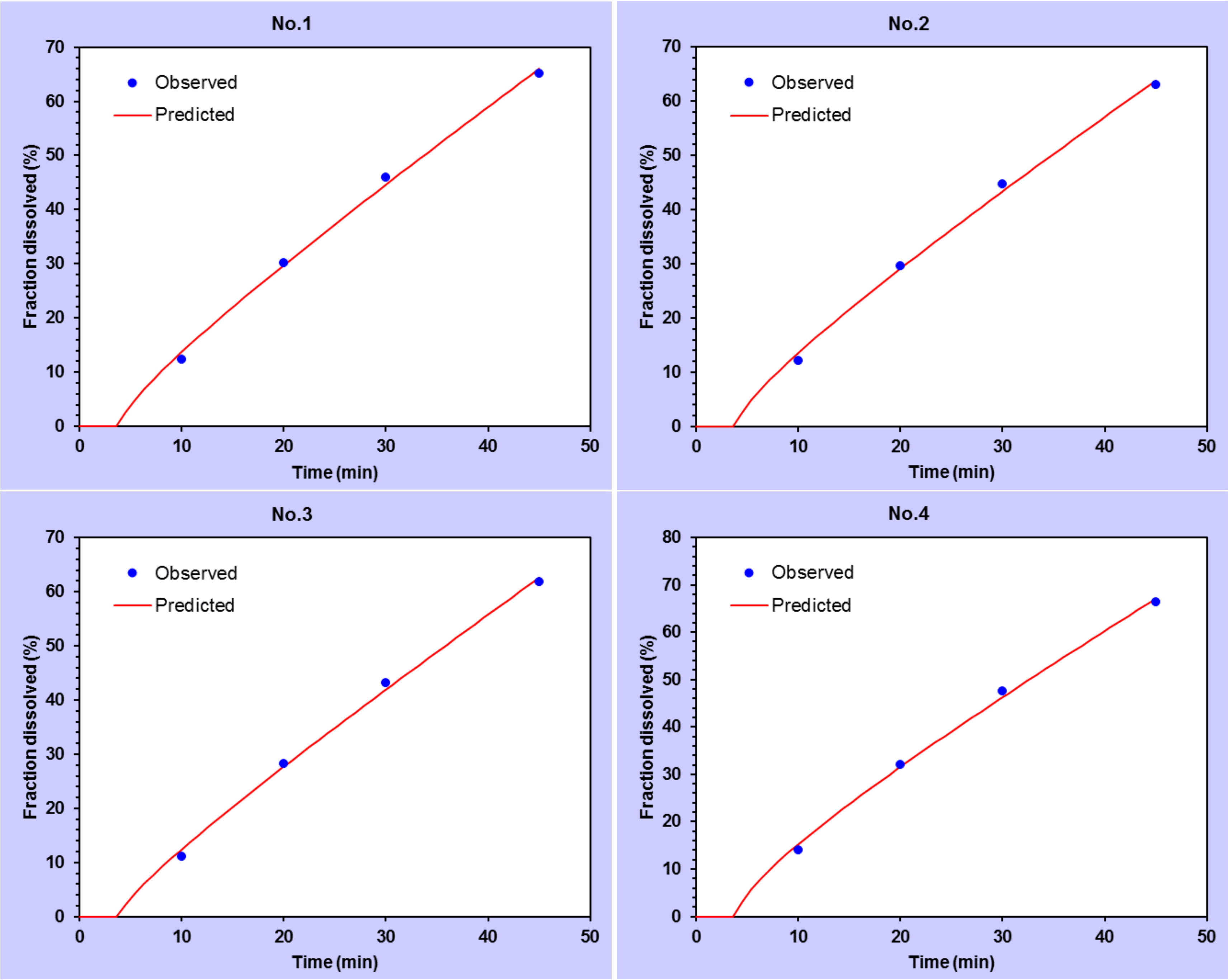

Supplementary materials – Model fitting summary – Polyglykol® 8000 P (modelling up to app. 60 % of carvedilol released)

Model: **Quadratic**

Model equation: $F = 100 \cdot (k_1 \cdot t^2 + k_2 \cdot t)$

Fitted model parameters per tested tablet (N = 4) with statistics – mean, standard deviation (SD), and relative standard deviation expressed in % (RSD%) (output from DDSolver):

Parameter	No.1	No.2	No.3	No.4	Mean	SD	RSD(%)
k_1	0.000	0.000	0.000	0.000	0.000	0.000	-106.293
k_2	0.015	0.015	0.014	0.017	0.015	0.001	7.538

Number of dissolution data points (N), degrees of freedom (df), and selected goodness of fit criteria – Pearson correlation coefficient (R), coefficient of determination (R^2), adjusted coefficient of determination ($R^2_{adjusted}$), and residual sum of squares (RSS) (manual calculation in MS Excel):

Parameter	No.1	No.2	No.3	No.4
N	4	4	4	4
df	2	2	2	2
R	0.997435707	0.997188656	0.997077003	0.998089278
R^2	0.99487799	0.994385216	0.99416255	0.996182207
$R^2_{adjusted}$	0.992316986	0.991577824	0.991243826	0.994273311
RSS	9.923598188	10.1596774	10.33581642	7.423088032

Graphical abstract of model fit presented as mean ± 1 SD of the fraction % of released carvedilol:

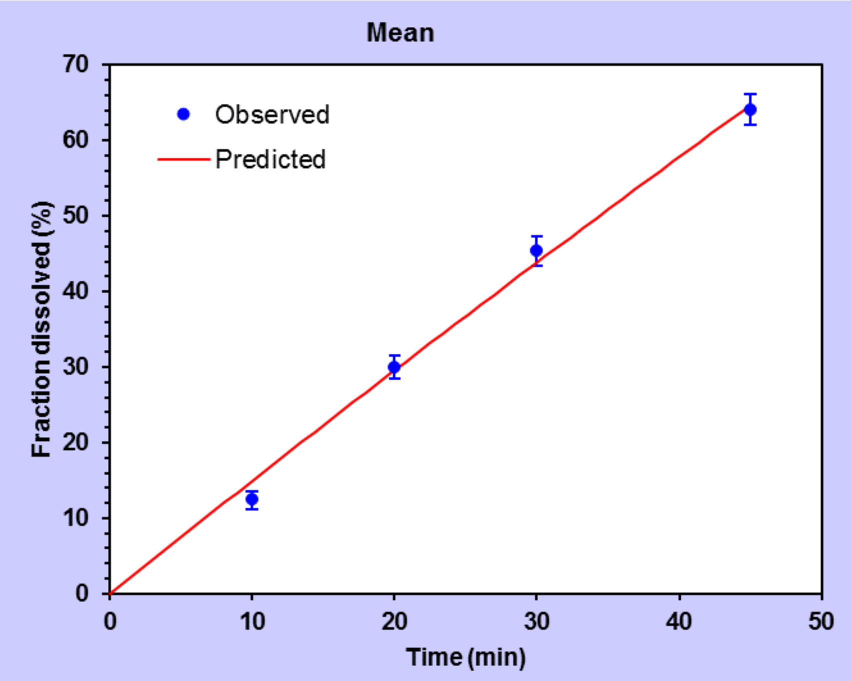

Supplementary materials – Model fitting summary – Polyglykol® 8000 P (modelling up to app. 60 % of carvedilol released)

Graphical abstract of model fit presented as the fraction % of released carvedilol per tested tablet:

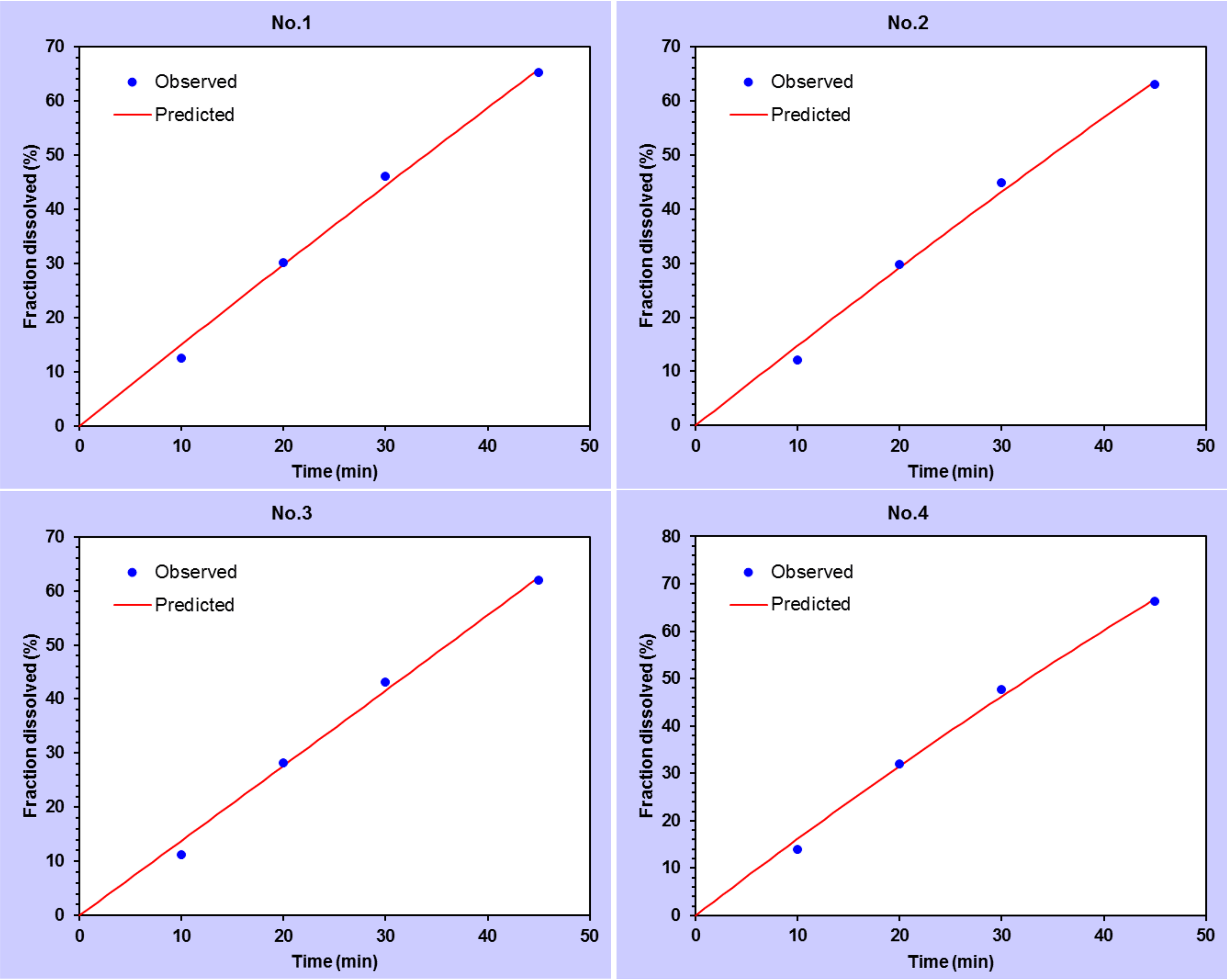

Supplementary materials – Model fitting summary – Polyglykol® 8000 P (modelling up to app. 60 % of carvedilol released)

Model: **Quadratic with T_{lag}**

Model equation: $F = 100 \cdot \left[k_1 \cdot (t - T_{lag})^2 + k_2 \cdot (t - T_{lag})\right]$

Fitted model parameters per tested tablet (N = 4) with statistics – mean, standard deviation (SD), and relative standard deviation expressed in % (RSD%) (output from DDSolver):

Parameter	No.1	No.2	No.3	No.4	Mean	SD	RSD(%)
k_1	0.000	0.000	0.000	0.000	0.000	0.000	-18.981
k_2	0.021	0.020	0.019	0.023	0.021	0.001	6.779
T_{lag}	4.000	3.841	4.000	4.000	3.960	0.079	2.001

Number of dissolution data points (N), degrees of freedom (df), and selected goodness of fit criteria – Pearson correlation coefficient (R), coefficient of determination (R^2), adjusted coefficient of determination ($R^2_{adjusted}$), and residual sum of squares (RSS) (manual calculation in MS Excel):

Parameter	No.1	No.2	No.3	No.4
N	4	4	4	4
df	1	1	1	1
R	0.999968912	0.99999941	0.999997451	0.999784649
R^2	0.999937824	0.99999882	0.999994902	0.999569345
$R^2_{adjusted}$	0.999813473	0.999996459	0.999984705	0.998708036
RSS	0.135515896	0.020911361	0.007515927	1.10385662

Graphical abstract of model fit presented as mean ± 1 SD of the fraction % of released carvedilol:

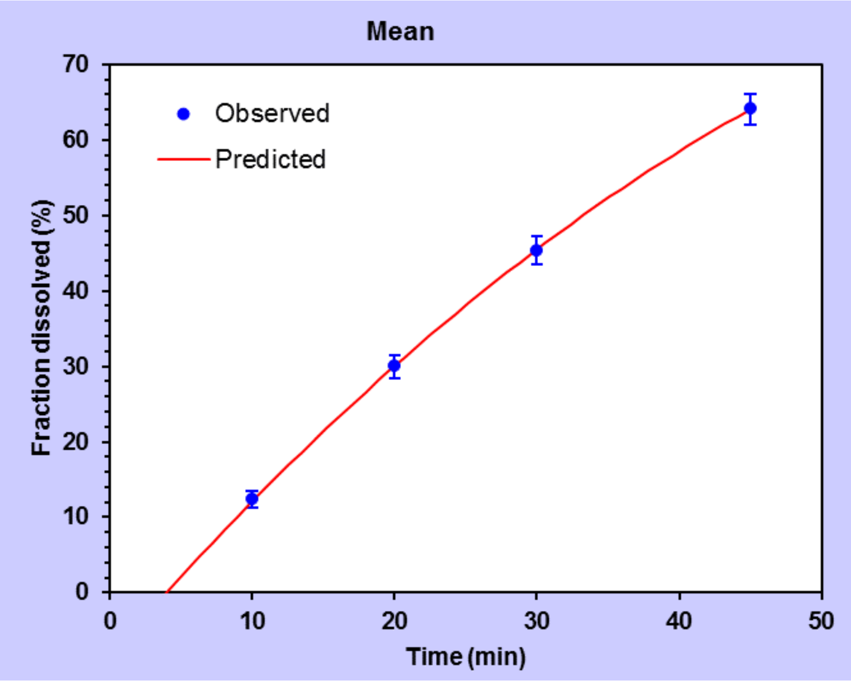

Supplementary materials – Model fitting summary – Polyglykol® 8000 P (modelling up to app. 60 % of carvedilol released)

Graphical abstract of model fit presented as the fraction % of released carvedilol per tested tablet:

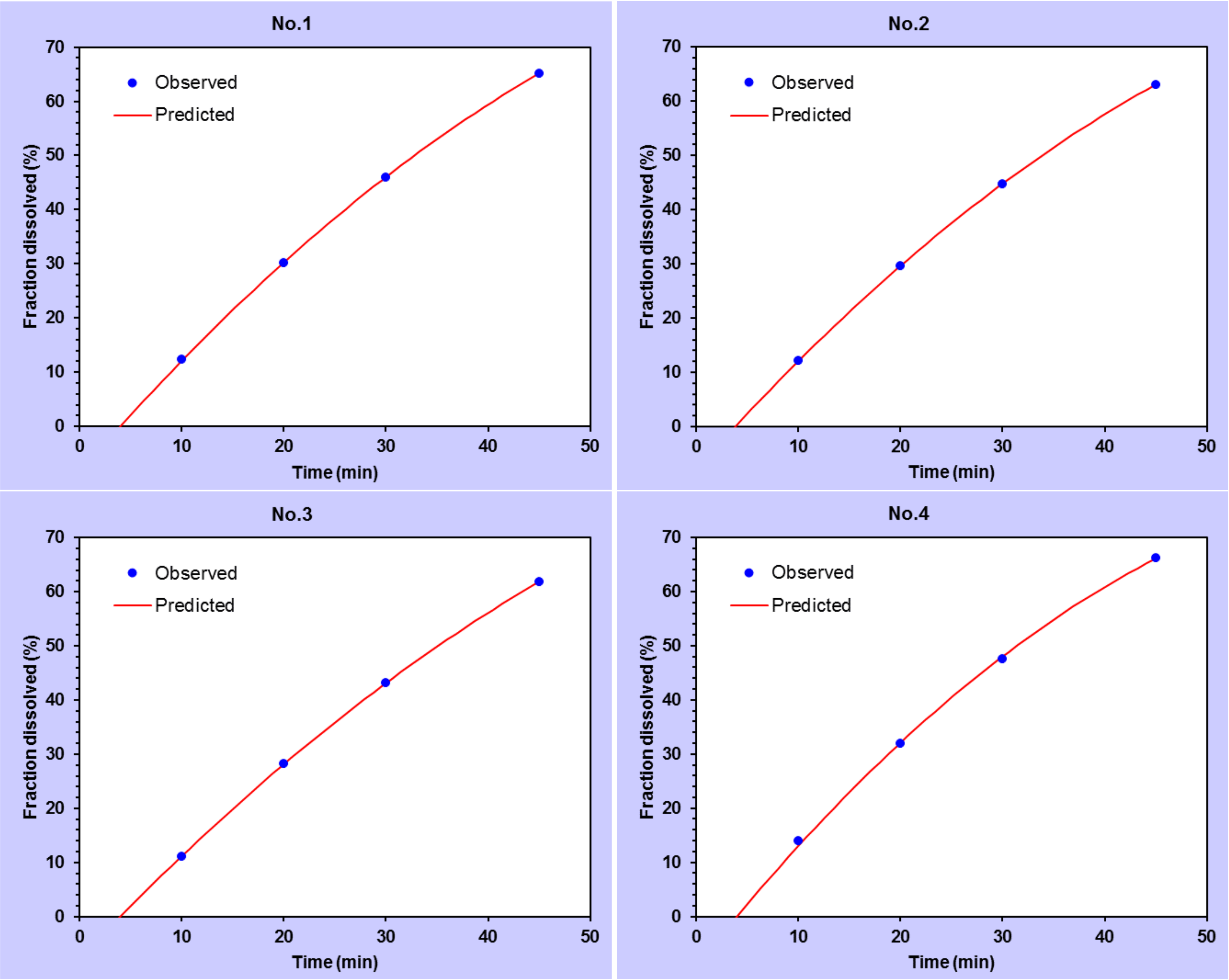

Supplementary materials – Model fitting summary – Polyglykol® 8000 P (modelling up to app. 60 % of carvedilol released)

Model: **Weibull_1**

Model equation: $F = 100 \cdot \left[1 - e^{-\frac{(t-T_i)^{\beta}}{\alpha}}\right]$

Fitted model parameters per tested tablet (N = 4) with statistics – mean, standard deviation (SD), and relative standard deviation expressed in % (RSD%) (output from DDSolver):

Parameter	No.1	No.2	No.3	No.4	Mean	SD	RSD(%)
α	52.987	57.316	60.683	42.962	53.487	7.691	14.379
β	1.075	1.084	1.089	1.026	1.069	0.029	2.722
T_i	4.000	4.057	4.000	4.000	4.014	0.029	0.716

Number of dissolution data points (N), degrees of freedom (df), and selected goodness of fit criteria – Pearson correlation coefficient (R), coefficient of determination (R^2), adjusted coefficient of determination ($R^2_{adjusted}$), and residual sum of squares (RSS) (manual calculation in MS Excel):

Parameter	No.1	No.2	No.3	No.4
N	4	4	4	4
df	1	1	1	1
R	0.999288643	0.999806095	0.999620518	0.999140036
R^2	0.998577793	0.999612228	0.999241179	0.998280811
$R^2_{adjusted}$	0.995733379	0.998836683	0.997723537	0.994842433
RSS	2.605162057	0.95080326	1.272318223	2.993018929

Graphical abstract of model fit presented as mean ± 1 SD of the fraction % of released carvedilol:

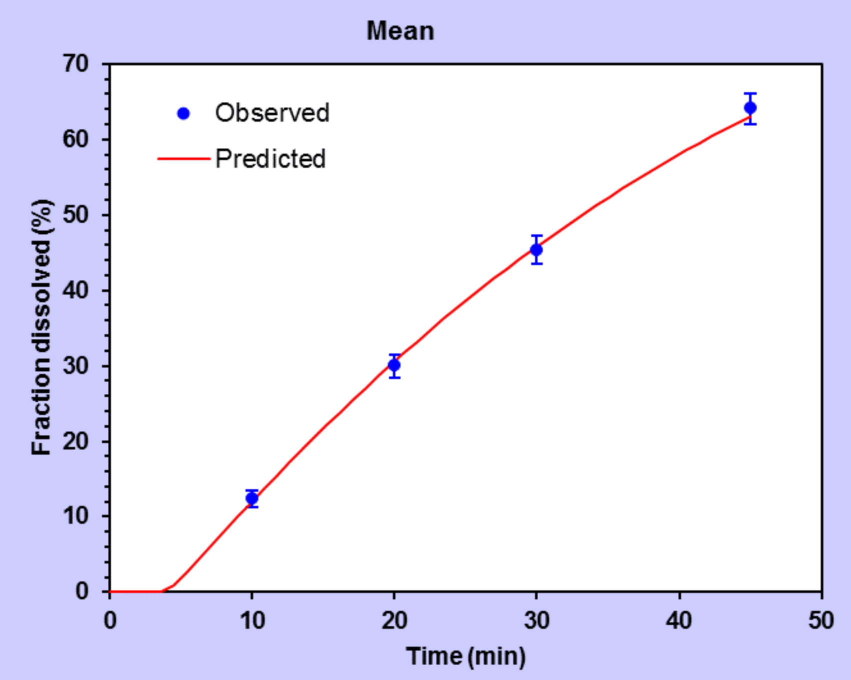

Supplementary materials – Model fitting summary – Polyglykol® 8000 P (modelling up to app. 60 % of carvedilol released)

Graphical abstract of model fit presented as the fraction % of released carvedilol per tested tablet:

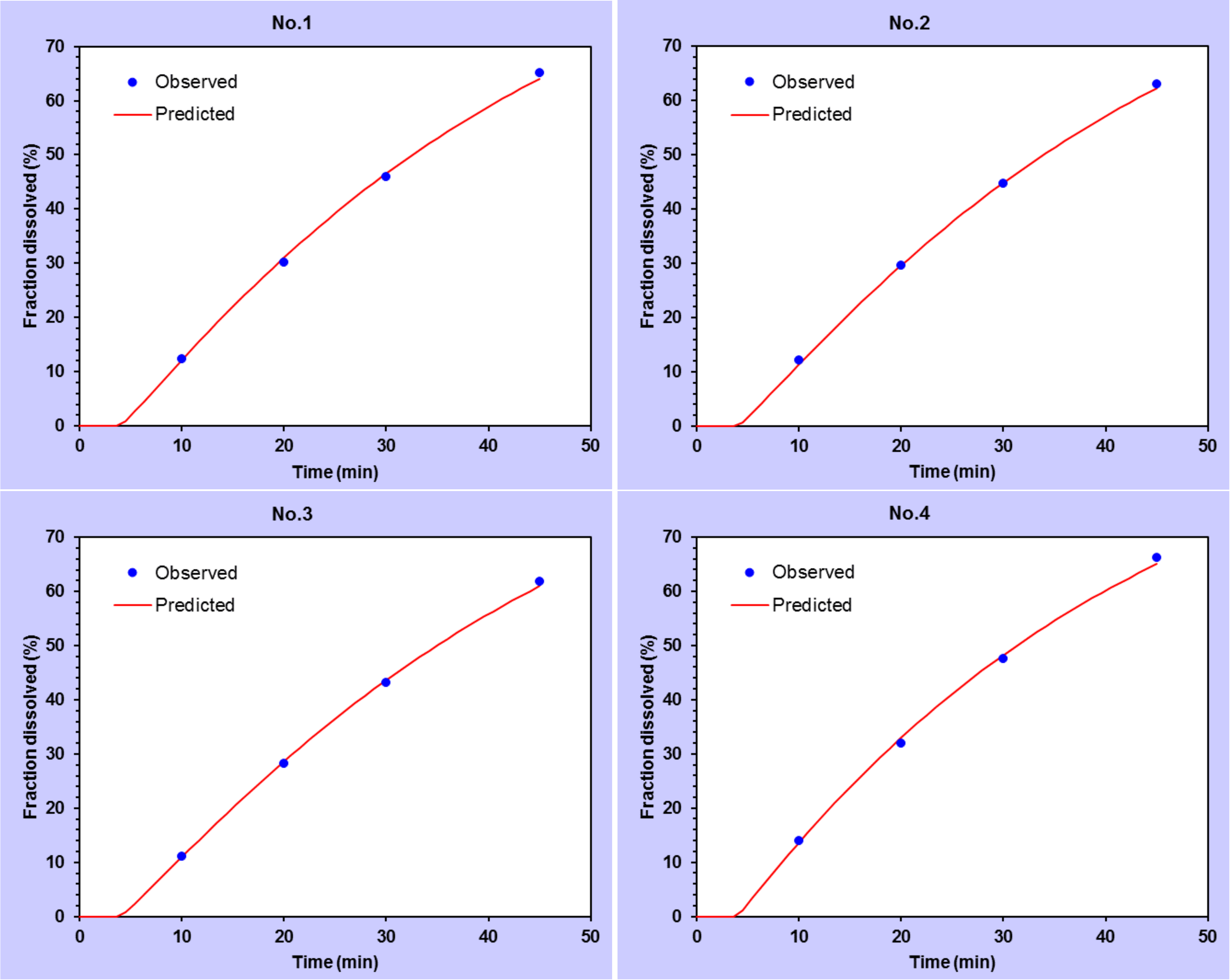

Supplementary materials – Model fitting summary – Polyglykol® 8000 P (modelling up to app. 60 % of carvedilol released)

Model: **Weibull_2**

Model equation: $F = 100 \cdot \left(1 - e^{-\frac{t^{\beta}}{\alpha}}\right)$

Fitted model parameters per tested tablet (N = 4) with statistics – mean, standard deviation (SD), and relative standard deviation expressed in % (RSD%) (output from DDSolver):

Parameter	No.1	No.2	No.3	No.4	Mean	SD	RSD(%)
α	178.754	173.226	207.403	137.227	174.152	28.817	16.547
β	1.380	1.359	1.398	1.318	1.364	0.035	2.534

Number of dissolution data points (N), degrees of freedom (df), and selected goodness of fit criteria – Pearson correlation coefficient (R), coefficient of determination (R^2), adjusted coefficient of determination ($R^2_{adjusted}$), and residual sum of squares (RSS) (manual calculation in MS Excel):

Parameter	No.1	No.2	No.3	No.4
N	4	4	4	4
df	2	2	2	2
R	0.999802087	0.999471815	0.99948937	0.999856767
R^2	0.999604213	0.998943908	0.998979	0.999713554
$R^2_{adjusted}$	0.999406319	0.998415862	0.9984685	0.999570331
RSS	0.745685745	1.856235723	1.840035787	0.512732534

Graphical abstract of model fit presented as mean ± 1 SD of the fraction % of released carvedilol:

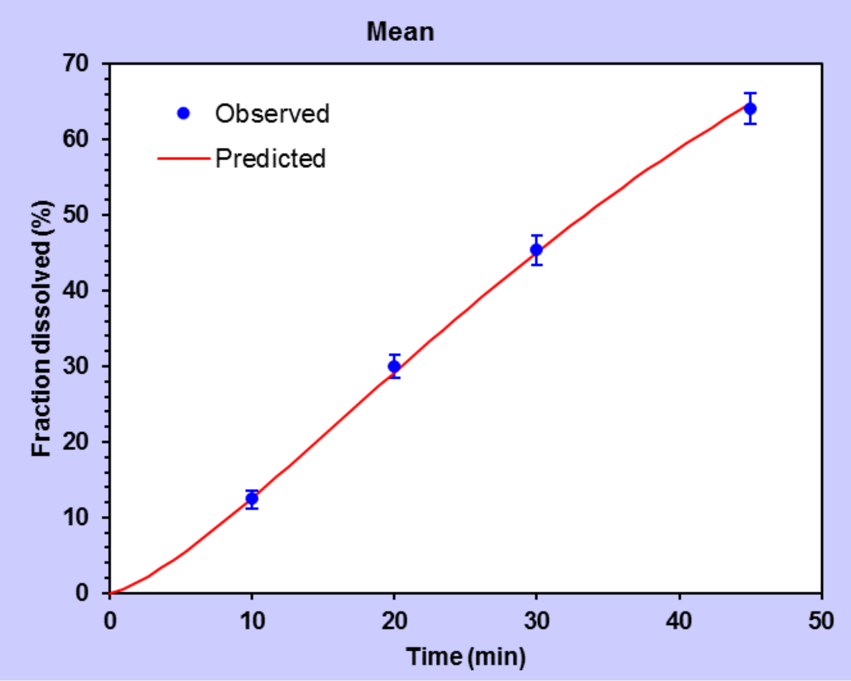

Supplementary materials – Model fitting summary – Polyglykol® 8000 P (modelling up to app. 60 % of carvedilol released)

Graphical abstract of model fit presented as the fraction % of released carvedilol per tested tablet:

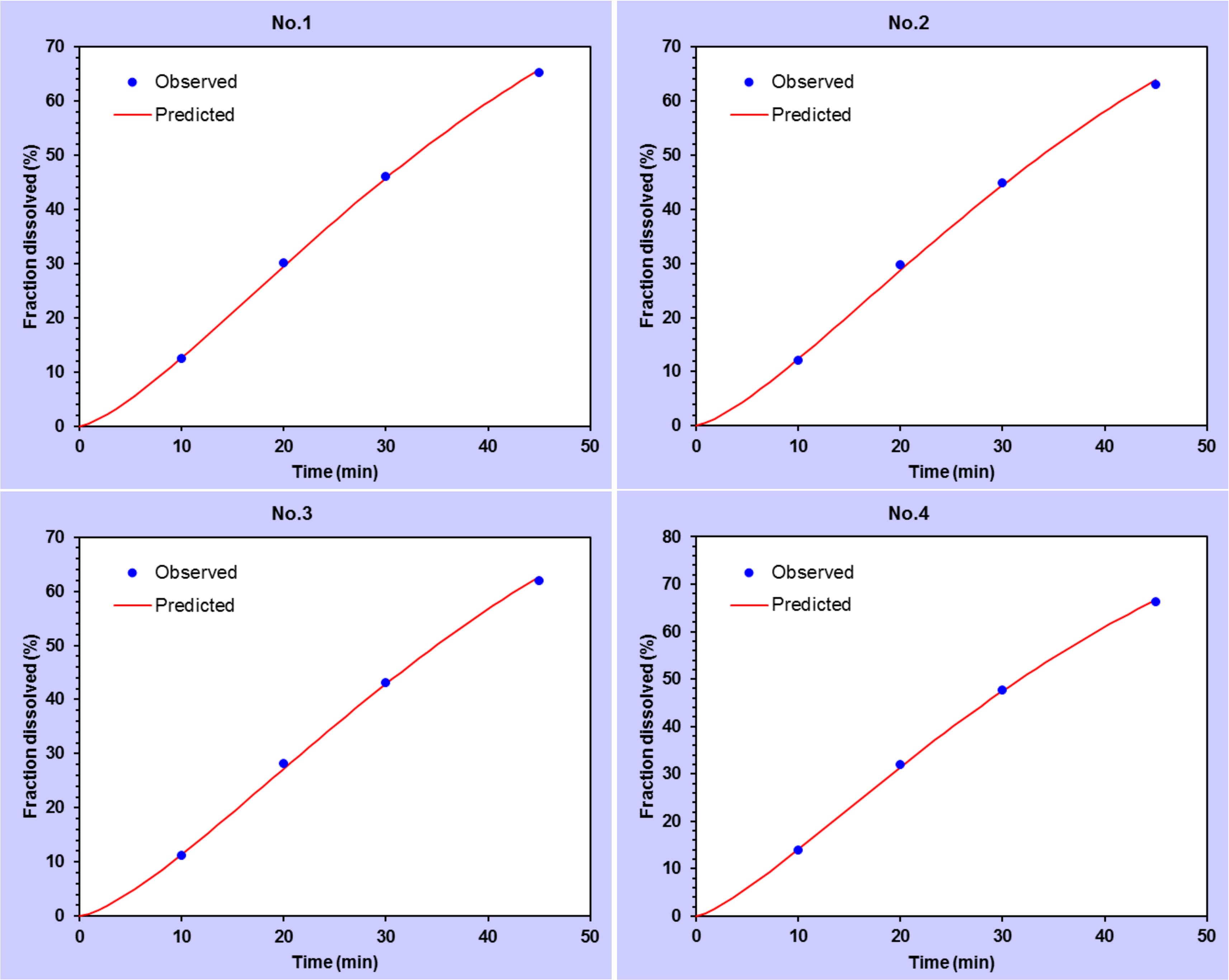

Supplementary materials – Model fitting summary – Polyglykol® 8000 P (modelling up to app. 60 % of carvedilol released)

Model: **Weibull_3**

Model equation: $F = F_{max} \cdot \left(1 - e^{-\frac{t^{\beta}}{\alpha}}\right)$

Fitted model parameters per tested tablet (N = 4) with statistics – mean, standard deviation (SD), and relative standard deviation expressed in % (RSD%) (output from DDSolver):

Parameter	No.1	No.2	No.3	No.4	Mean	SD	RSD(%)
α	419.614	406.050	487.833	318.856	408.088	69.423	17.012
β	1.792	1.785	1.832	1.719	1.782	0.047	2.620
F_{max}	70.669	68.285	67.069	71.861	69.471	2.185	3.146

Number of dissolution data points (N), degrees of freedom (df), and selected goodness of fit criteria – Pearson correlation coefficient (R), coefficient of determination (R^2), adjusted coefficient of determination ($R^2_{adjusted}$), and residual sum of squares (RSS) (manual calculation in MS Excel):

Parameter	No.1	No.2	No.3	No.4
N	4	4	4	4
df	1	1	1	1
R	0.99852787	0.998724805	0.998359513	0.998447329
R^2	0.997057907	0.997451236	0.996721718	0.996897069
$R^2_{adjusted}$	0.991173722	0.992353708	0.990165154	0.990691208
RSS	16.45281708	15.49332801	14.74787288	19.0646752

Graphical abstract of model fit presented as mean ± 1 SD of the fraction % of released carvedilol:

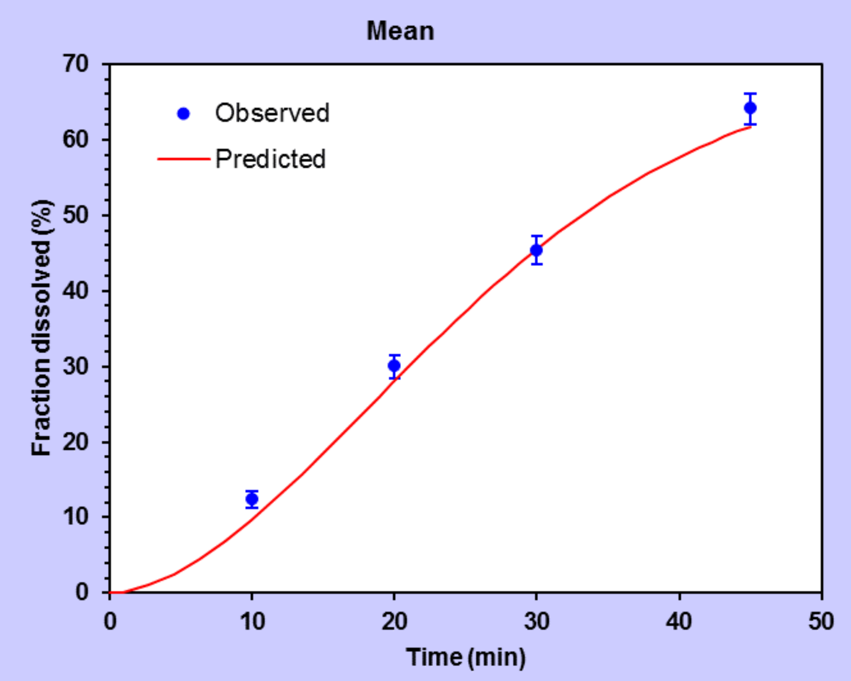

Supplementary materials – Model fitting summary – Polyglykol® 8000 P (modelling up to app. 60 % of carvedilol released)

Graphical abstract of model fit presented as the fraction % of released carvedilol per tested tablet:

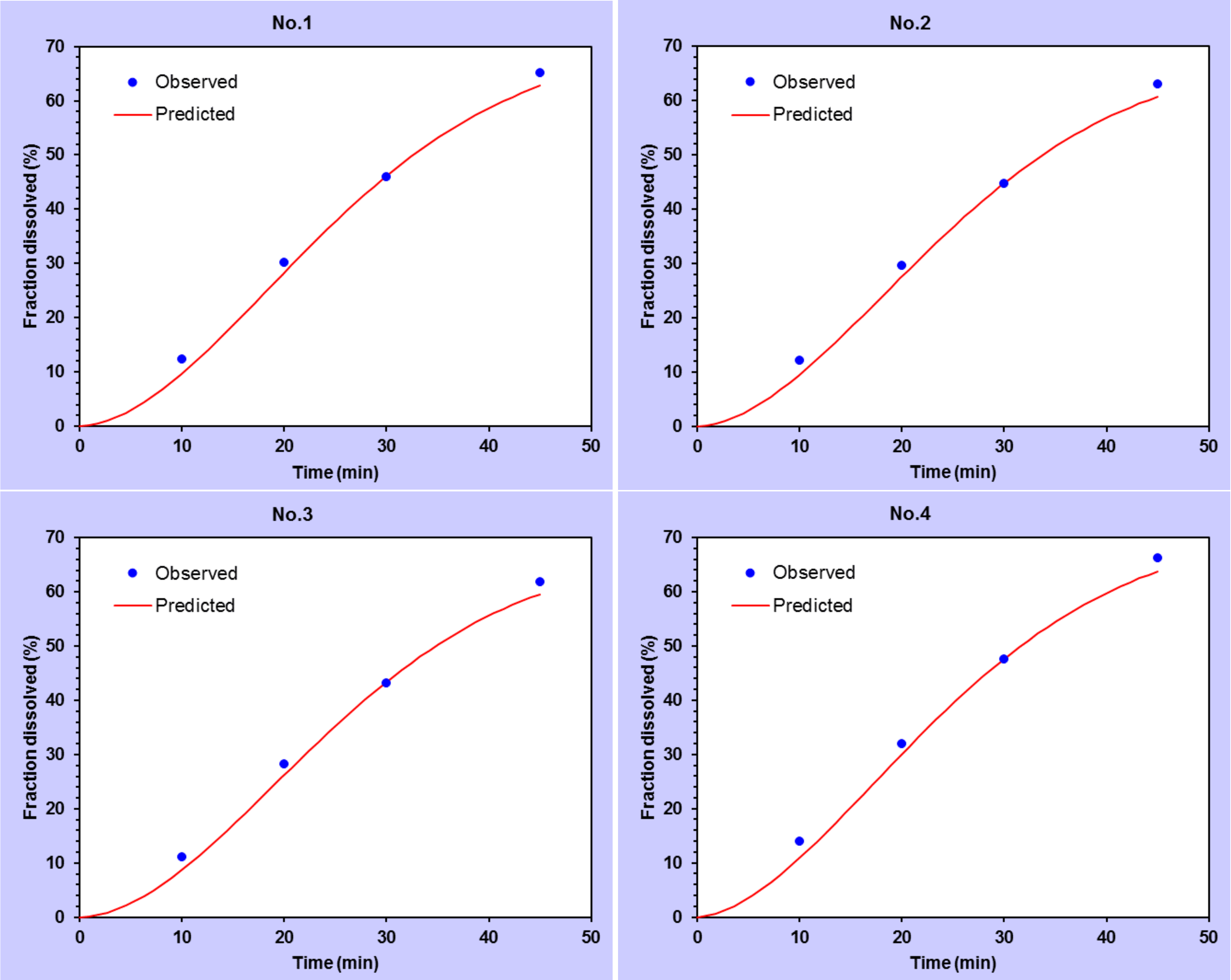

Supplementary materials – Model fitting summary – Polyglykol® 8000 P (modelling up to app. 60 % of carvedilol released)

Model: **Weibull_4**

Model equation: $F = F_{max} \cdot \left[1 - e^{-\frac{(t-T_i)^\beta}{\alpha}}\right]$

Fitted model parameters per tested tablet (N = 4) with statistics – mean, standard deviation (SD), and relative standard deviation expressed in % (RSD%) (output from DDSolver):

Parameter	No.1	No.2	No.3	No.4	Mean	SD	RSD(%)
α	/	/	/	/	/	/	/
β	/	/	/	/	/	/	/
T_i	/	/	/	/	/	/	/
F_{max}	/	/	/	/	/	/	/

Number of dissolution data points (N), degrees of freedom (df), and selected goodness of fit criteria – Pearson correlation coefficient (R), coefficient of determination (R^2), adjusted coefficient of determination ($R^2_{adjusted}$), and residual sum of squares (RSS) (manual calculation in MS Excel):

Parameter	No.1	No.2	No.3	No.4
N	/	/	/	/
df	/	/	/	/
R	/	/	/	/
R^2	/	/	/	/
$R^2_{adjusted}$	/	/	/	/
RSS	/	/	/	/

Graphical abstract of model fit presented as mean ± 1 SD of the fraction % of released carvedilol: /

Graphical abstract of model fit presented as the fraction % of released carvedilol per tested tablet: /

Note: model could not be fitted to experimental dissolution data due too few data points being available for fitting

Supplementary materials – Model fitting summary – Polyglykol® 8000 P (modelling up to app. 60 % of carvedilol released)

Model: **Logistic_1**

Model equation: $F = 100 \cdot \frac{e^{\alpha+\beta\cdot\log(t)}}{1+e^{\alpha+\beta\cdot\log(t)}}$

Fitted model parameters per tested tablet (N = 4) with statistics – mean, standard deviation (SD), and relative standard deviation expressed in % (RSD%) (output from DDSolver):

Parameter	No.1	No.2	No.3	No.4	Mean	SD	RSD(%)
α	-5.911	-5.828	-5.994	-5.652	-5.846	0.146	-2.499
β	3.923	3.826	3.897	3.795	3.860	0.060	1.555

Number of dissolution data points (N), degrees of freedom (df), and selected goodness of fit criteria – Pearson correlation coefficient (R), coefficient of determination (R^2), adjusted coefficient of determination ($R^2_{adjusted}$), and residual sum of squares (RSS) (manual calculation in MS Excel):

Parameter	No.1	No.2	No.3	No.4
N	4	4	4	4
df	2	2	2	2
R	0.998971091	0.999517866	0.999405197	0.998767782
R^2	0.99794324	0.999035964	0.998810748	0.997537082
$R^2_{adjusted}$	0.99691486	0.998553946	0.998216121	0.996305622
RSS	3.348021731	1.440954707	1.749889319	3.881122263

Graphical abstract of model fit presented as mean ± 1 SD of the fraction % of released carvedilol:

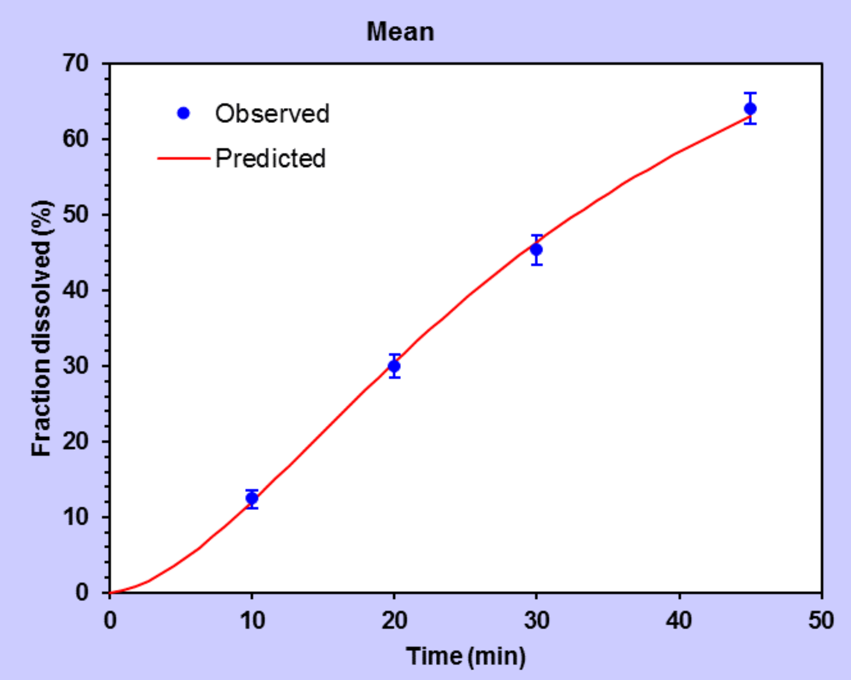

Supplementary materials – Model fitting summary – Polyglykol® 8000 P (modelling up to app. 60 % of carvedilol released)

Graphical abstract of model fit presented as the fraction % of released carvedilol per tested tablet:

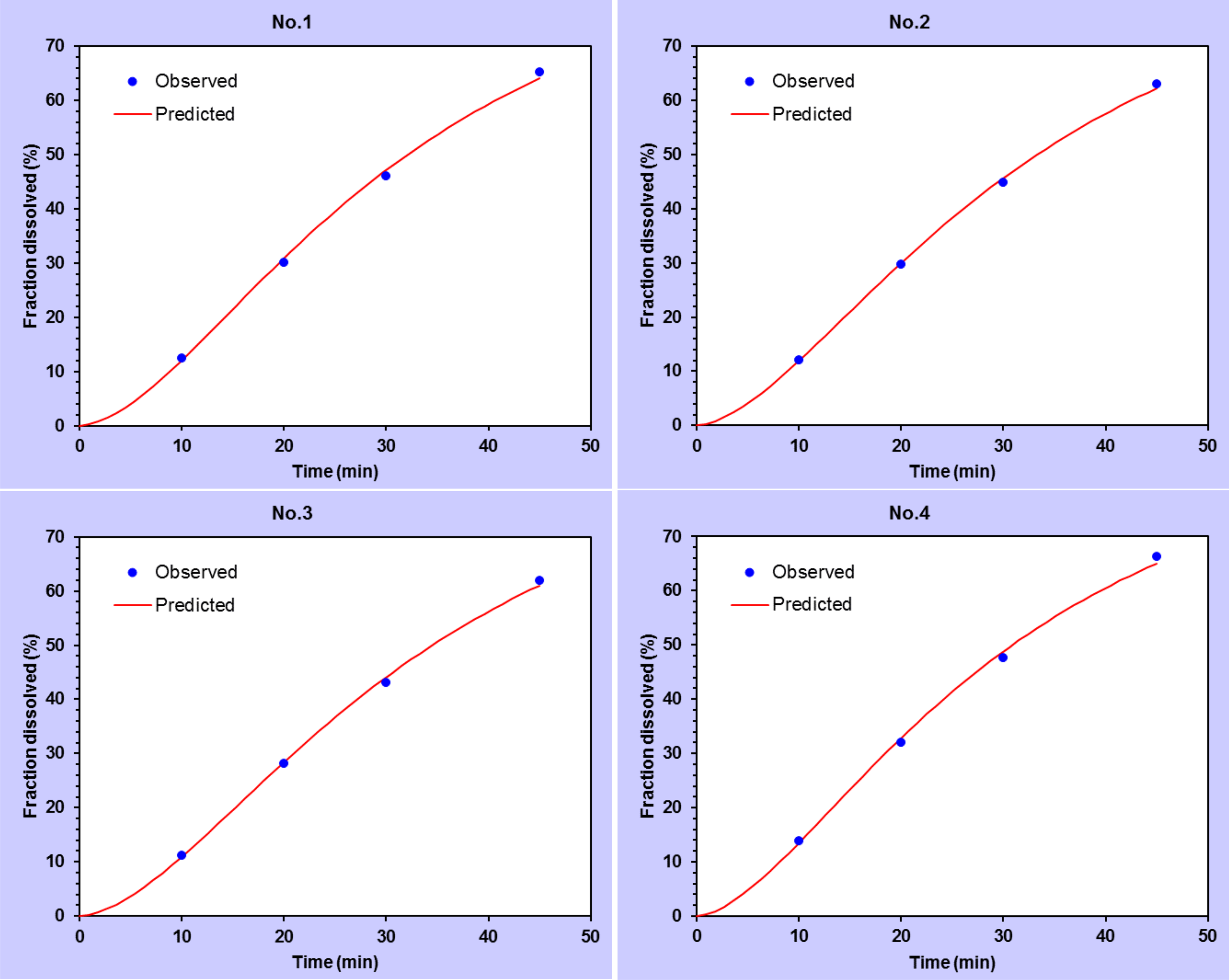

Supplementary materials – Model fitting summary – Polyglykol® 8000 P (modelling up to app. 60 % of carvedilol released)

Model: **Logistic_2**

Model equation: $F = F_{max} \cdot \frac{e^{\alpha+\beta\cdot\log(t)}}{1+e^{\alpha+\beta\cdot\log(t)}}$

Fitted model parameters per tested tablet (N = 4) with statistics – mean, standard deviation (SD), and relative standard deviation expressed in % (RSD%) (output from DDSolver):

Parameter	No.1	No.2	No.3	No.4	Mean	SD	RSD(%)
α	-9.687	-9.646	-9.874	-9.336	-9.636	0.223	-2.315
β	6.930	6.915	7.030	6.743	6.905	0.119	1.730
F_{max}	75.043	72.511	71.220	76.309	73.770	2.321	3.146

Number of dissolution data points (N), degrees of freedom (df), and selected goodness of fit criteria – Pearson correlation coefficient (R), coefficient of determination (R^2), adjusted coefficient of determination ($R^2_{adjusted}$), and residual sum of squares (RSS) (manual calculation in MS Excel):

Parameter	No.1	No.2	No.3	No.4
N	4	4	4	4
df	1	1	1	1
R	0.99570148	0.99592728	0.995544727	0.99508134
R^2	0.991421436	0.991871146	0.991109303	0.990186872
$R^2_{adjusted}$	0.974264309	0.975613438	0.973327909	0.970560617
RSS	88.52285696	83.86306562	79.89732104	97.32394793

Graphical abstract of model fit presented as mean ± 1 SD of the fraction % of released carvedilol:

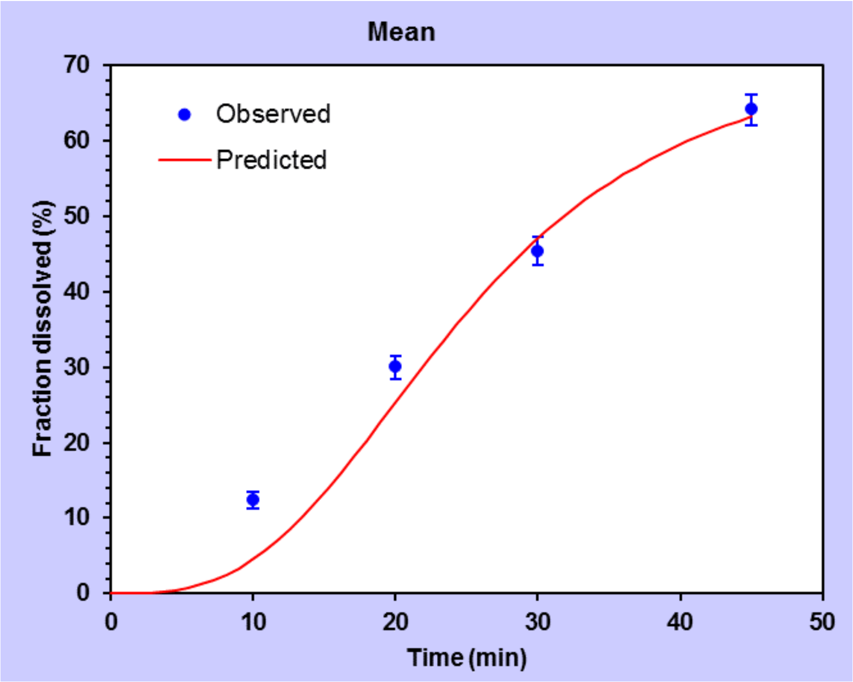

Supplementary materials – Model fitting summary – Polyglykol® 8000 P (modelling up to app. 60 % of carvedilol released)

Graphical abstract of model fit presented as the fraction % of released carvedilol per tested tablet:

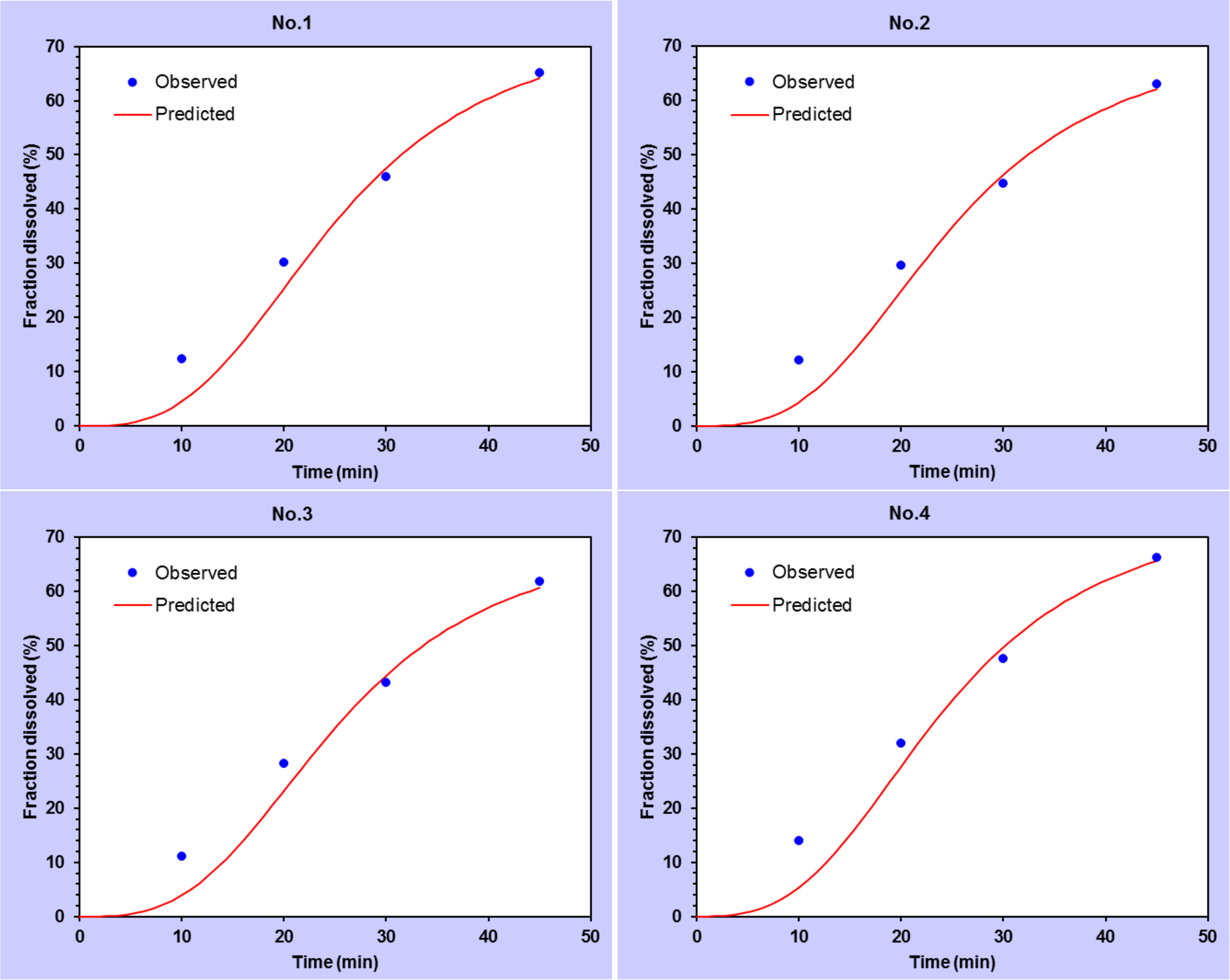

Supplementary materials – Model fitting summary – Polyglykol® 8000 P (modelling up to app. 60 % of carvedilol released)

Model: **Logistic_3**

Model equation: $F = F_{max} \cdot \frac{1}{1+e^{-k\cdot(t-\gamma)}}$

Fitted model parameters per tested tablet (N = 4) with statistics – mean, standard deviation (SD), and relative standard deviation expressed in % (RSD%) (output from DDSolver):

Parameter	No.1	No.2	No.3	No.4	Mean	SD	RSD(%)
k	0.127	0.126	0.129	0.124	0.126	0.002	1.676
γ	22.382	22.234	22.694	21.760	22.268	0.389	1.747
F_{max}	68.412	66.104	64.927	69.567	67.253	2.116	3.146

Number of dissolution data points (N), degrees of freedom (df), and selected goodness of fit criteria – Pearson correlation coefficient (R), coefficient of determination (R^2), adjusted coefficient of determination ($R^2_{adjusted}$), and residual sum of squares (RSS) (manual calculation in MS Excel):

Parameter	No.1	No.2	No.3	No.4
N	4	4	4	4
df	1	1	1	1
R	0.996038779	0.996186203	0.995414079	0.996110409
R^2	0.99209325	0.992386952	0.990849189	0.992235947
$R^2_{adjusted}$	0.97627975	0.977160855	0.972547568	0.976707841
RSS	14.45599736	12.74873605	15.15078488	14.04968283

Graphical abstract of model fit presented as mean ± 1 SD of the fraction % of released carvedilol:

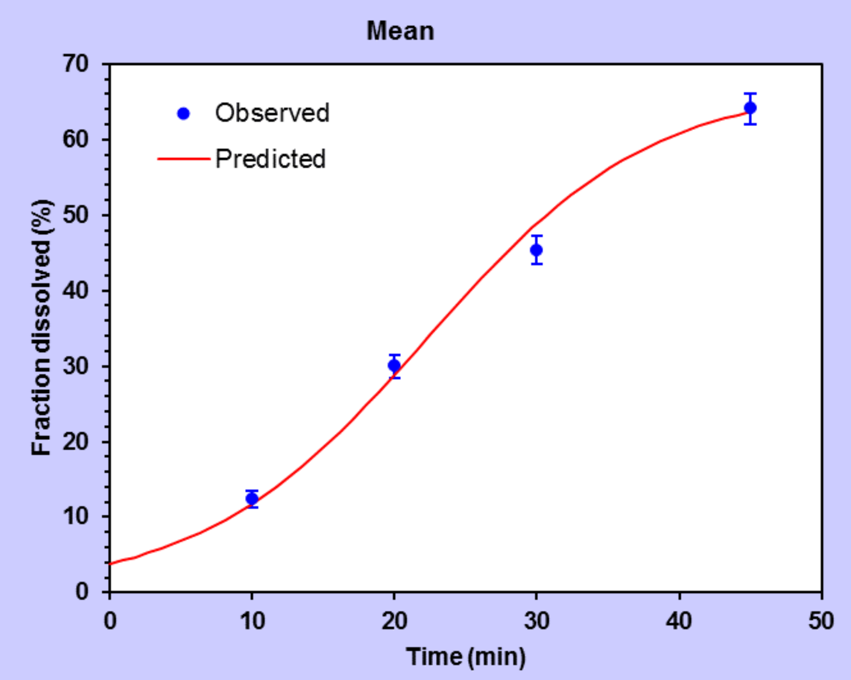

Supplementary materials – Model fitting summary – Polyglykol® 8000 P (modelling up to app. 60 % of carvedilol released)

Graphical abstract of model fit presented as the fraction % of released carvedilol per tested tablet:

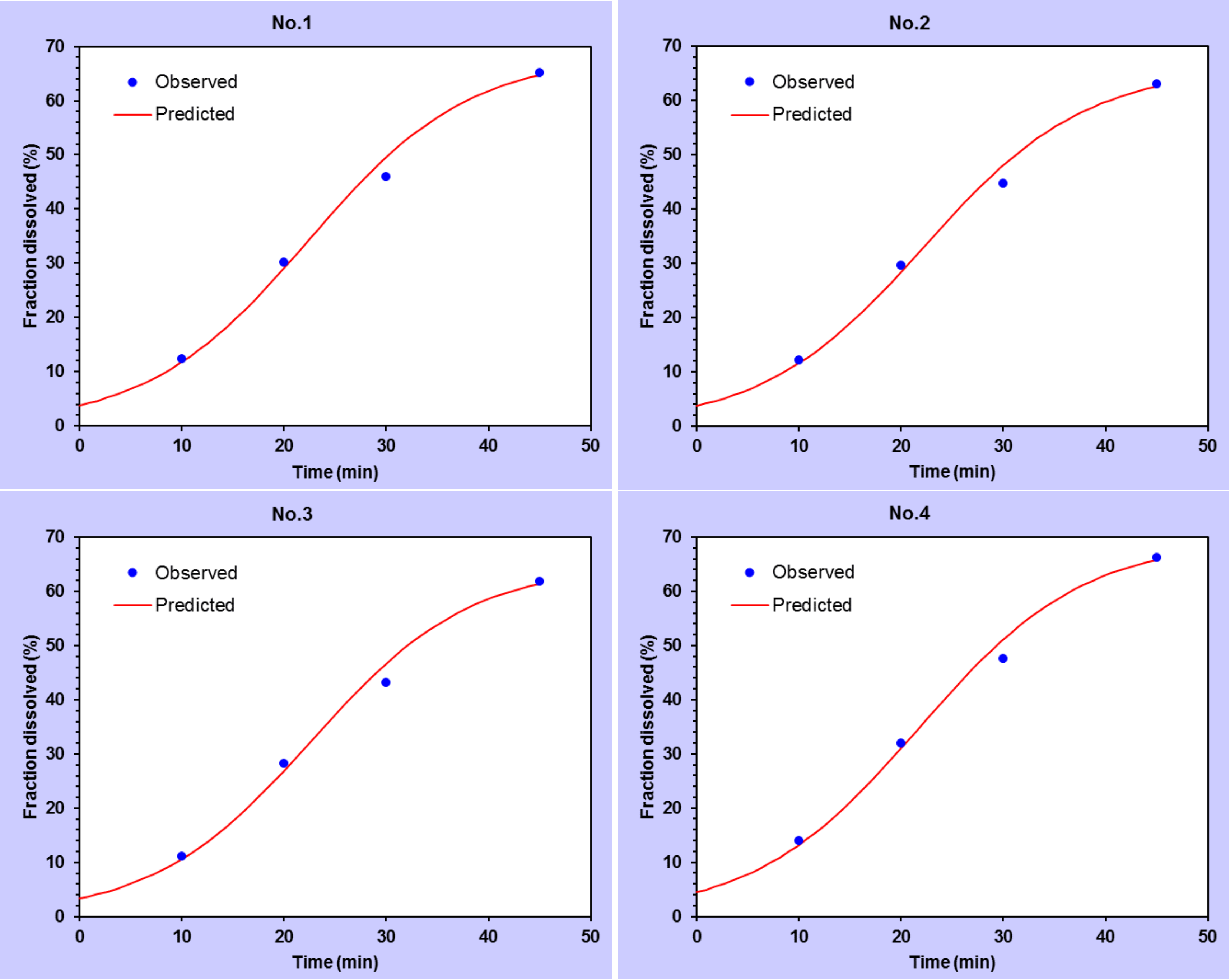

Supplementary materials – Model fitting summary – Polyglykol® 8000 P (modelling up to app. 60 % of carvedilol released)

Model: **Gompertz_1**

Model equation: $F = 100 \cdot e^{-\alpha \cdot e^{-\beta \cdot \log(t)}}$

Fitted model parameters per tested tablet (N = 4) with statistics – mean, standard deviation (SD), and relative standard deviation expressed in % (RSD%) (output from DDSolver):

Parameter	No.1	No.2	No.3	No.4	Mean	SD	RSD(%)
α	24.247	22.091	22.974	22.312	22.906	0.970	4.233
β	2.381	2.288	2.286	2.356	2.328	0.048	2.072

Number of dissolution data points (N), degrees of freedom (df), and selected goodness of fit criteria – Pearson correlation coefficient (R), coefficient of determination (R^2), adjusted coefficient of determination ($R^2_{adjusted}$), and residual sum of squares (RSS) (manual calculation in MS Excel):

Parameter	No.1	No.2	No.3	No.4
N	4	4	4	4
df	2	2	2	2
R	0.990245614	0.992360684	0.991876107	0.990312342
R^2	0.980586376	0.984779727	0.983818211	0.980718534
$R^2_{adjusted}$	0.970879564	0.977169591	0.975727316	0.971077801
RSS	30.08979403	21.87200881	23.16121311	29.27669858

Graphical abstract of model fit presented as mean ± 1 SD of the fraction % of released carvedilol:

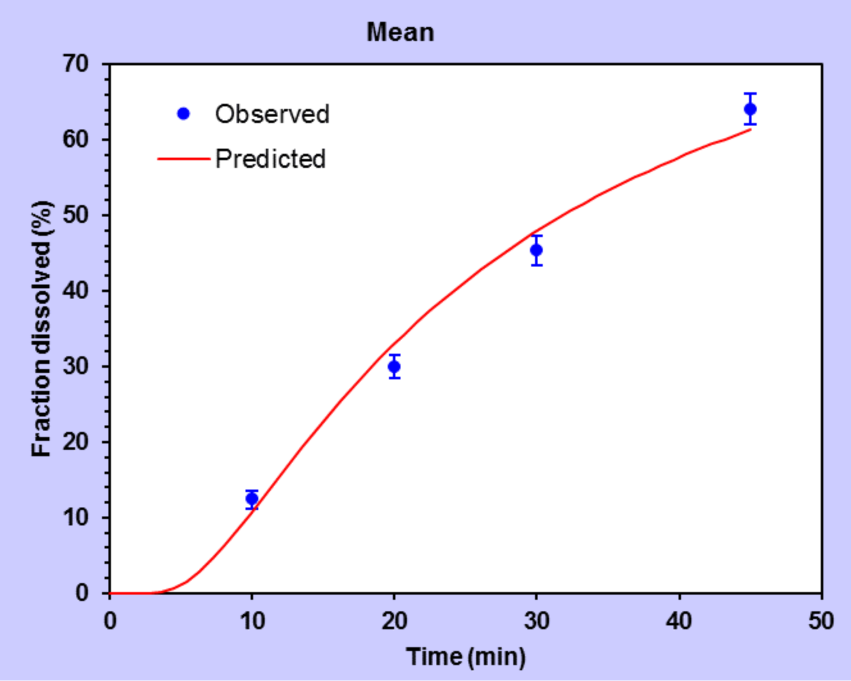

Supplementary materials – Model fitting summary – Polyglykol® 8000 P (modelling up to app. 60 % of carvedilol released)

Graphical abstract of model fit presented as the fraction % of released carvedilol per tested tablet:

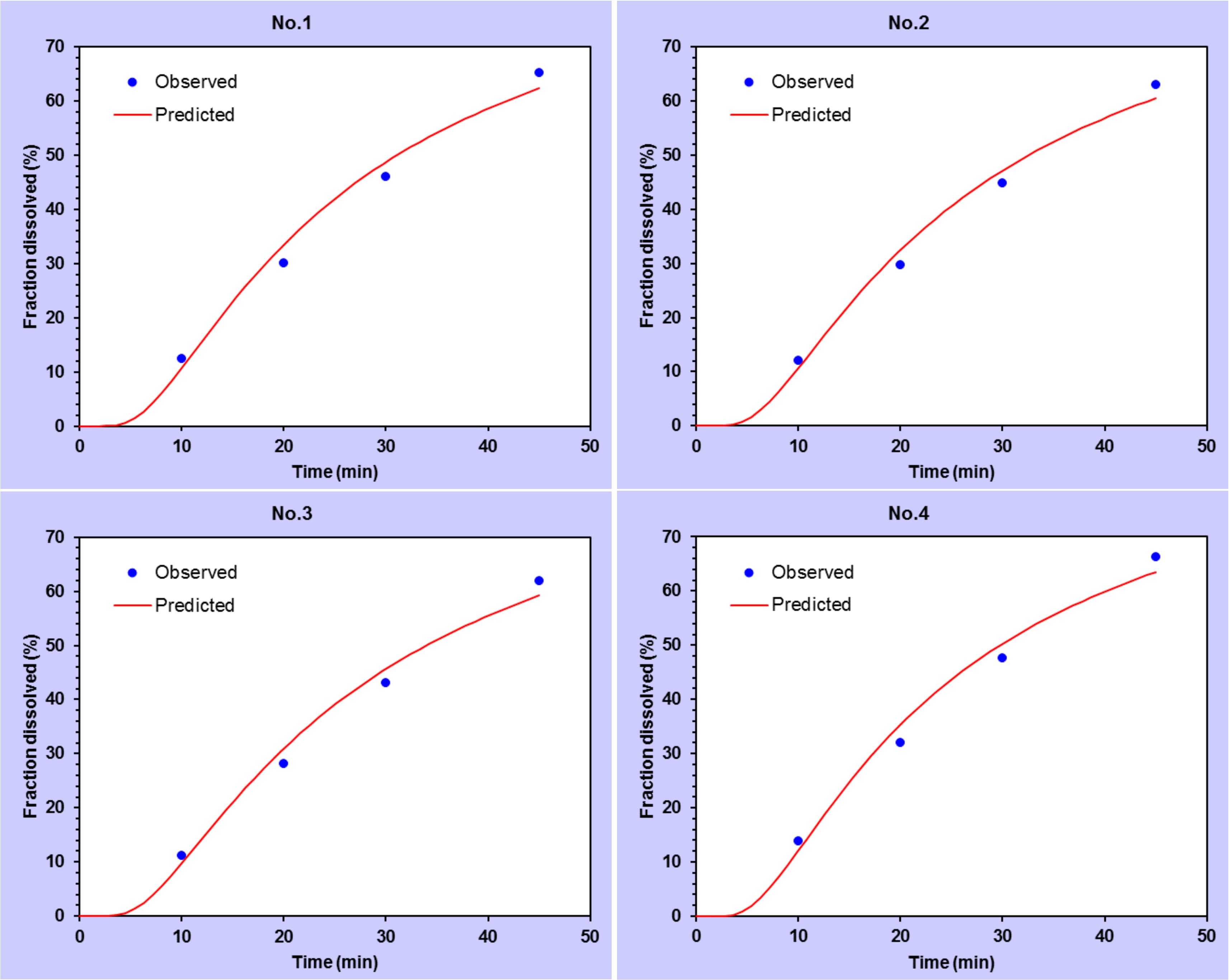

Supplementary materials – Model fitting summary – Polyglykol® 8000 P (modelling up to app. 60 % of carvedilol released)

Model: **Gompertz_2**

Model equation: $F = F_{max} \cdot e^{-\alpha \cdot e^{-\beta \cdot \log(t)}}$

Fitted model parameters per tested tablet (N = 4) with statistics – mean, standard deviation (SD), and relative standard deviation expressed in % (RSD%) (output from DDSolver):

Parameter	No.1	No.2	No.3	No.4	Mean	SD	RSD(%)
α	603.782	593.528	649.856	519.845	591.753	53.835	9.097
β	5.081	5.077	5.120	5.000	5.069	0.050	0.991
F_{max}	68.412	66.104	64.927	69.567	67.253	2.116	3.146

Number of dissolution data points (N), degrees of freedom (df), and selected goodness of fit criteria – Pearson correlation coefficient (R), coefficient of determination (R^2), adjusted coefficient of determination ($R^2_{adjusted}$), and residual sum of squares (RSS) (manual calculation in MS Excel):

Parameter	No.1	No.2	No.3	No.4
N	4	4	4	4
df	1	1	1	1
R	0.974507419	0.975892695	0.974988643	0.97430349
R^2	0.94966471	0.952366551	0.950602854	0.94926729
$R^2_{adjusted}$	0.848994129	0.857099654	0.851808562	0.847801871
RSS	155.6105136	144.1855162	133.1775169	176.3262096

Graphical abstract of model fit presented as mean ± 1 SD of the fraction % of released carvedilol:

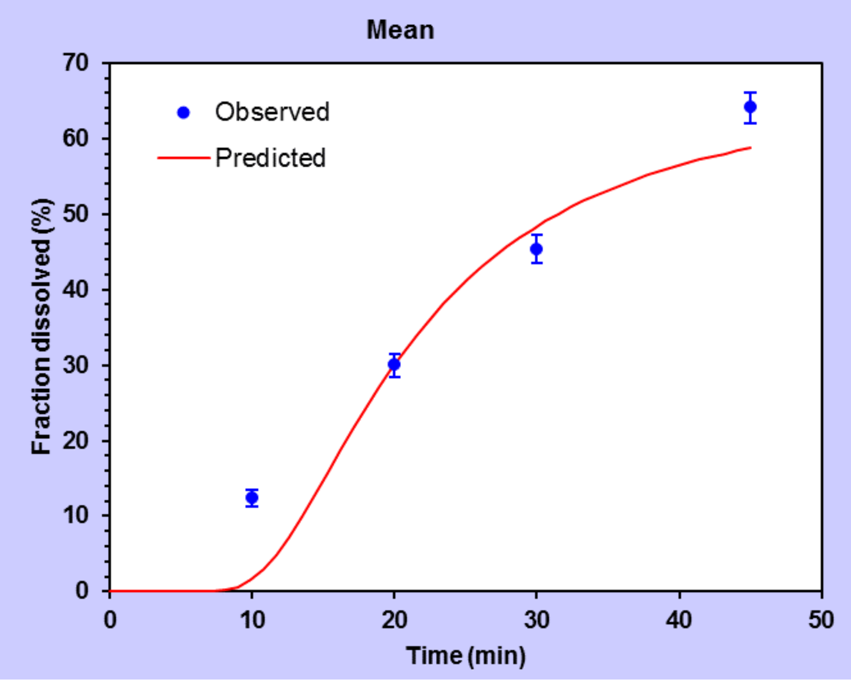

Supplementary materials – Model fitting summary – Polyglykol® 8000 P (modelling up to app. 60 % of carvedilol released)

Graphical abstract of model fit presented as the fraction % of released carvedilol per tested tablet:

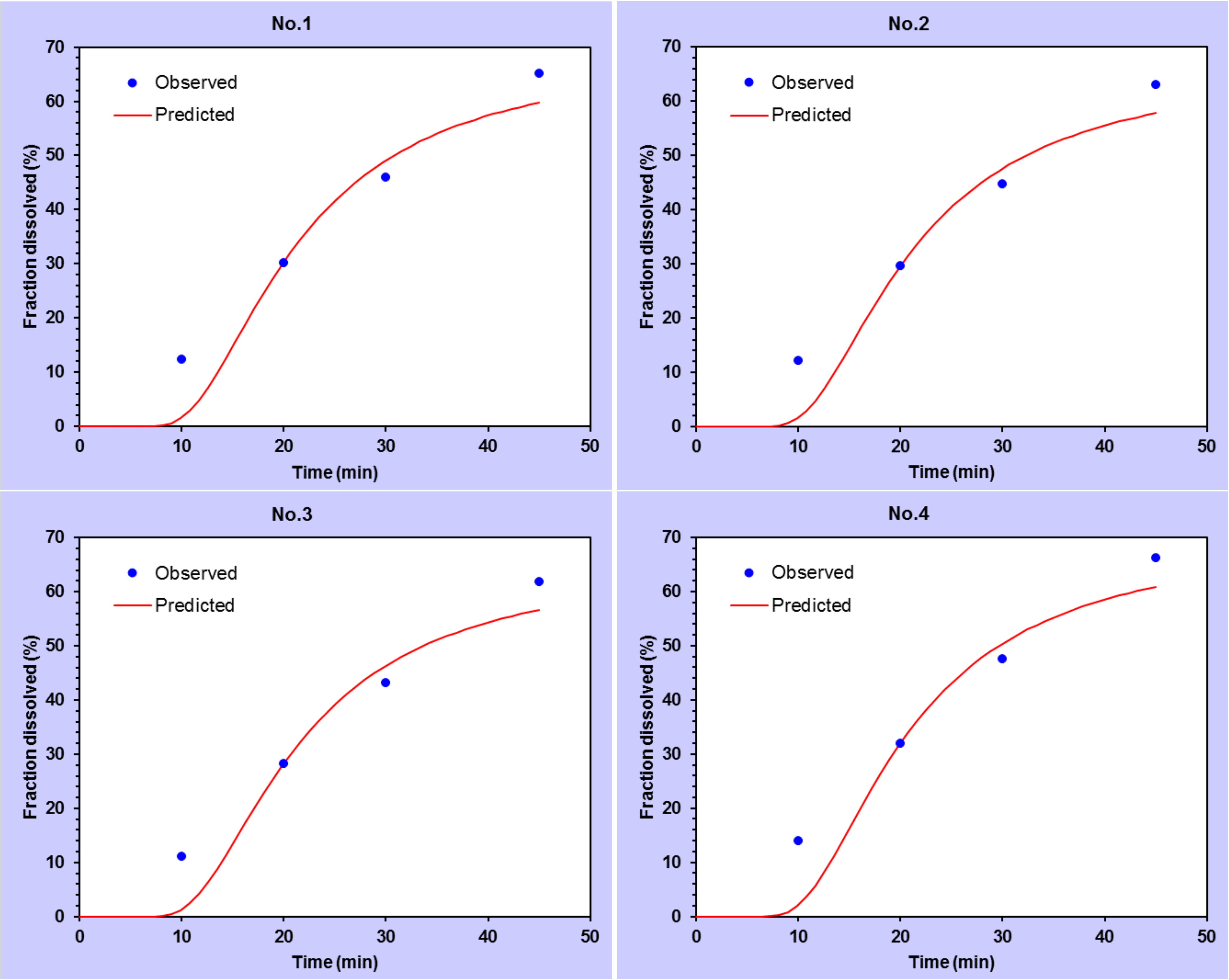

Supplementary materials – Model fitting summary – Polyglykol® 8000 P (modelling up to app. 60 % of carvedilol released)

Model: **Gompertz_3**

Model equation: $F = F_{max} \cdot e^{-e^{-k\cdot(t-\gamma)}}$

Fitted model parameters per tested tablet (N = 4) with statistics – mean, standard deviation (SD), and relative standard deviation expressed in % (RSD%) (output from DDSolver):

Parameter	No.1	No.2	No.3	No.4	Mean	SD	RSD(%)
k	0.101	0.072	0.102	0.071	0.086	0.017	20.234
γ	17.330	16.688	17.600	16.284	16.975	0.599	3.527
F_{max}	68.412	65.798	64.927	69.245	67.096	2.061	3.071

Number of dissolution data points (N), degrees of freedom (df), and selected goodness of fit criteria – Pearson correlation coefficient (R), coefficient of determination (R^2), adjusted coefficient of determination ($R^2_{adjusted}$), and residual sum of squares (RSS) (manual calculation in MS Excel):

Parameter	No.1	No.2	No.3	No.4
N	4	4	4	4
df	1	1	1	1
R	0.9877938	0.997221854	0.987437416	0.997190135
R^2	0.97573659	0.994451426	0.975032651	0.994388164
$R^2_{adjusted}$	0.927209771	0.983354278	0.925097952	0.983164493
RSS	53.75717584	28.68329499	50.5446242	31.17008792

Graphical abstract of model fit presented as mean ± 1 SD of the fraction % of released carvedilol:

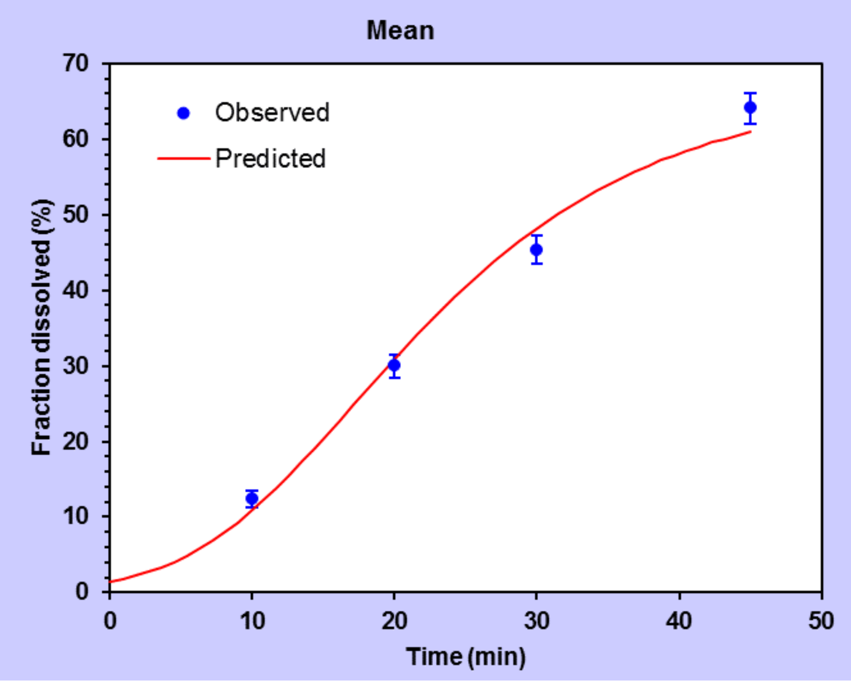

Supplementary materials – Model fitting summary – Polyglykol® 8000 P (modelling up to app. 60 % of carvedilol released)

Graphical abstract of model fit presented as the fraction % of released carvedilol per tested tablet:

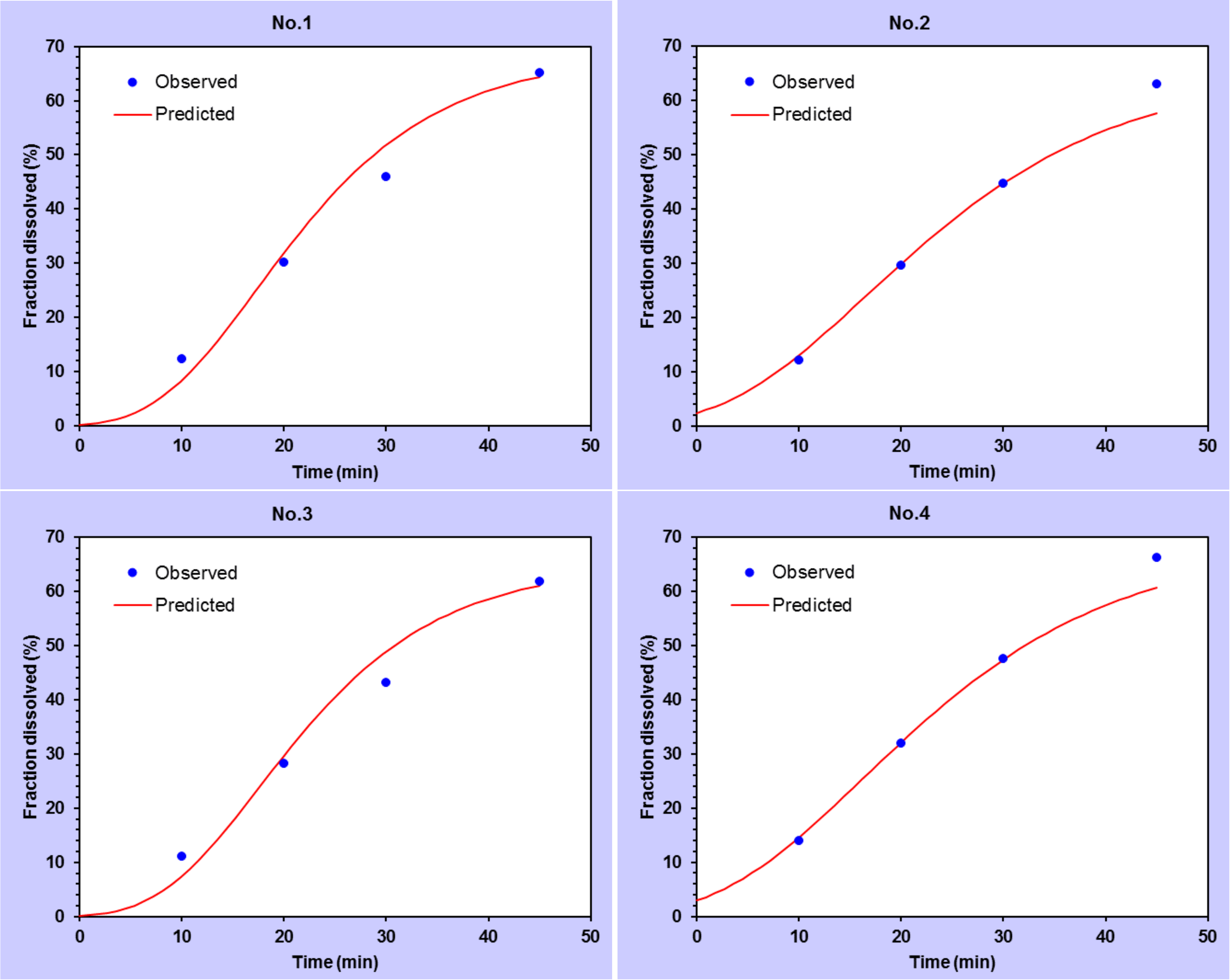

Supplementary materials – Model fitting summary – Polyglykol® 8000 P (modelling up to app. 60 % of carvedilol released)

Model: **Gompertz_4**

Model equation: $F = F_{max} \cdot e^{-\beta \cdot e^{-k \cdot t}}$

Fitted model parameters per tested tablet (N = 4) with statistics – mean, standard deviation (SD), and relative standard deviation expressed in % (RSD%) (output from DDSolver):

Parameter	No.1	No.2	No.3	No.4	Mean	SD	RSD(%)
k	0.101	0.101	0.102	0.099	0.101	0.001	1.028
β	5.759	5.656	6.000	5.293	5.677	0.294	5.177
F_{max}	68.412	66.104	64.927	69.567	67.253	2.116	3.146

Number of dissolution data points (N), degrees of freedom (df), and selected goodness of fit criteria – Pearson correlation coefficient (R), coefficient of determination (R^2), adjusted coefficient of determination ($R^2_{adjusted}$), and residual sum of squares (RSS) (manual calculation in MS Excel):

Parameter	No.1	No.2	No.3	No.4
N	4	4	4	4
df	1	1	1	1
R	0.9877938	0.988785765	0.987437416	0.988365173
R^2	0.97573659	0.977697289	0.975032651	0.976865716
$R^2_{adjusted}$	0.927209771	0.933091868	0.925097952	0.930597148
RSS	53.75717584	46.14785752	50.5446242	51.42460881

Graphical abstract of model fit presented as mean ± 1 SD of the fraction % of released carvedilol:

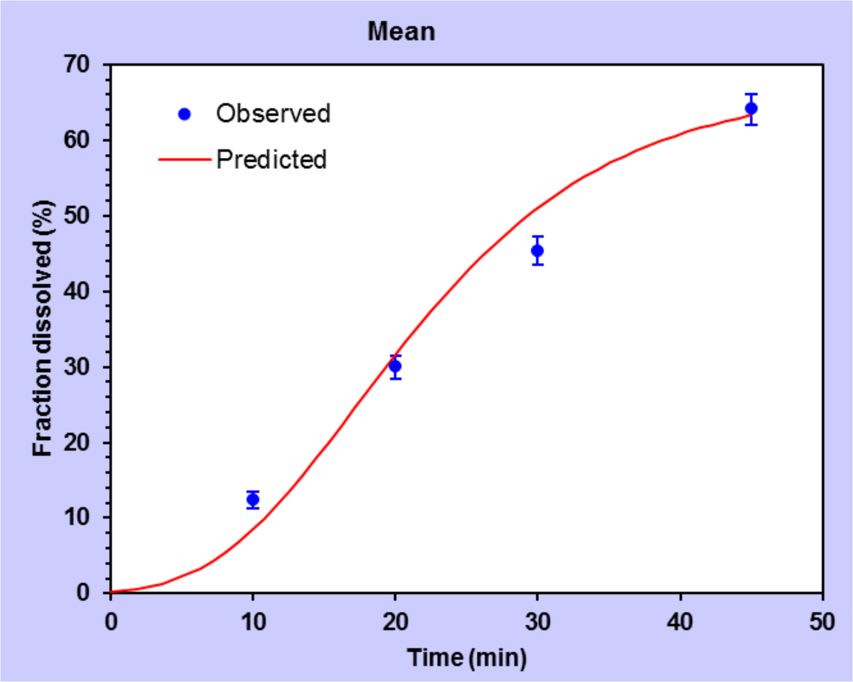

Supplementary materials – Model fitting summary – Polyglykol® 8000 P (modelling up to app. 60 % of carvedilol released)

Graphical abstract of model fit presented as the fraction % of released carvedilol per tested tablet:

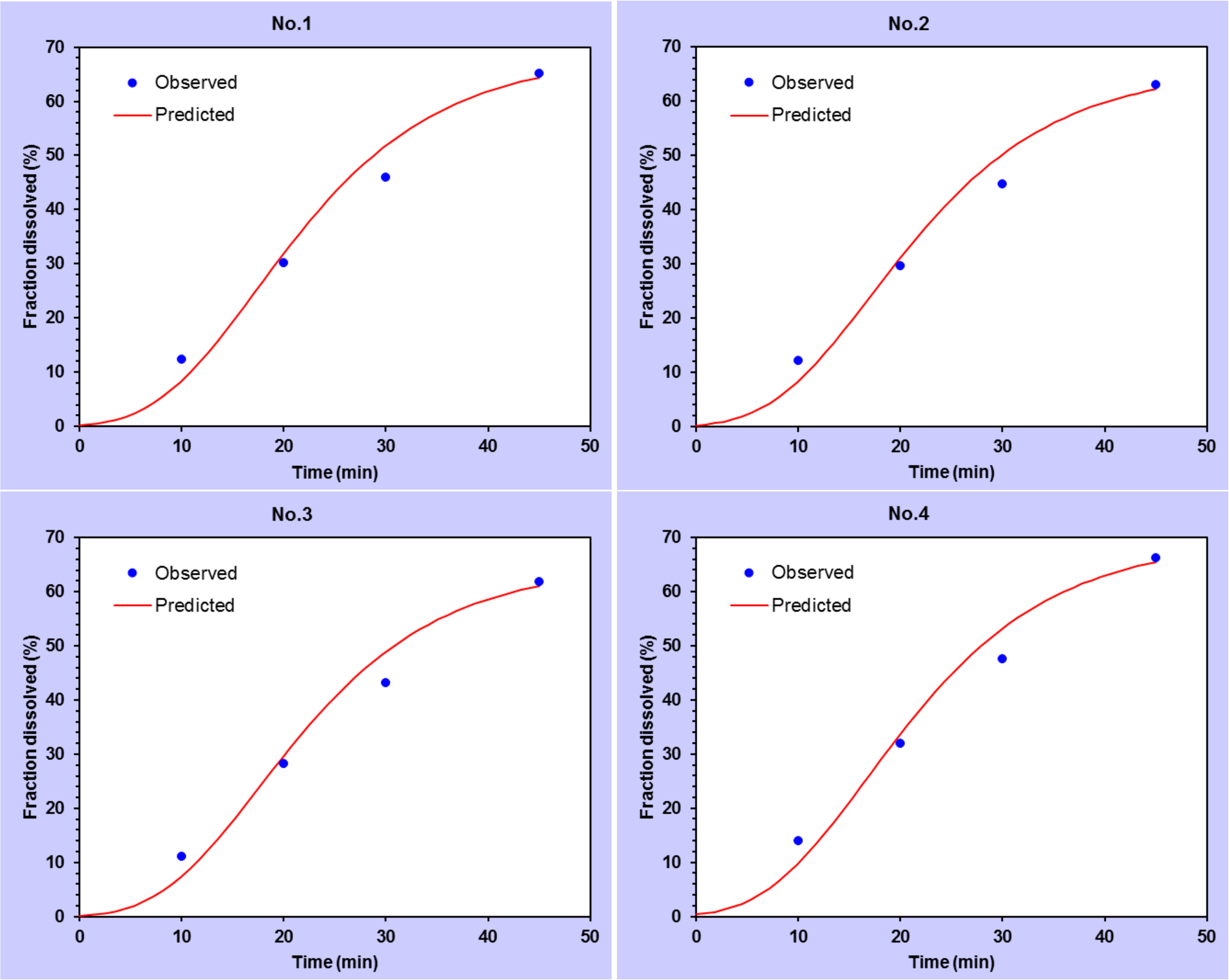

Supplementary materials – Model fitting summary – Polyglykol® 8000 P (modelling up to app. 60 % of carvedilol released)

Model: **Probit_1**

Model equation: $F = 100 \cdot \phi[\alpha + \beta \cdot \log(t)]$

Fitted model parameters per tested tablet (N = 4) with statistics – mean, standard deviation (SD), and relative standard deviation expressed in % (RSD%) (output from DDSolver):

Parameter	No.1	No.2	No.3	No.4	Mean	SD	RSD(%)
α	-3.533	-3.476	-3.557	-3.399	-3.491	0.070	-2.009
β	2.346	2.282	2.311	2.283	2.306	0.030	1.305

Number of dissolution data points (N), degrees of freedom (df), and selected goodness of fit criteria – Pearson correlation coefficient (R), coefficient of determination (R^2), adjusted coefficient of determination ($R^2_{adjusted}$), and residual sum of squares (RSS) (manual calculation in MS Excel):

Parameter	No.1	No.2	No.3	No.4
N	4	4	4	4
df	2	2	2	2
R	0.99784104	0.998660208	0.998435734	0.997767923
R^2	0.99568674	0.997322211	0.996873915	0.995540828
$R^2_{adjusted}$	0.99353011	0.995983316	0.995310873	0.993311243
RSS	6.957406509	4.015429614	4.67195853	6.968777404

Graphical abstract of model fit presented as mean ± 1 SD of the fraction % of released carvedilol:

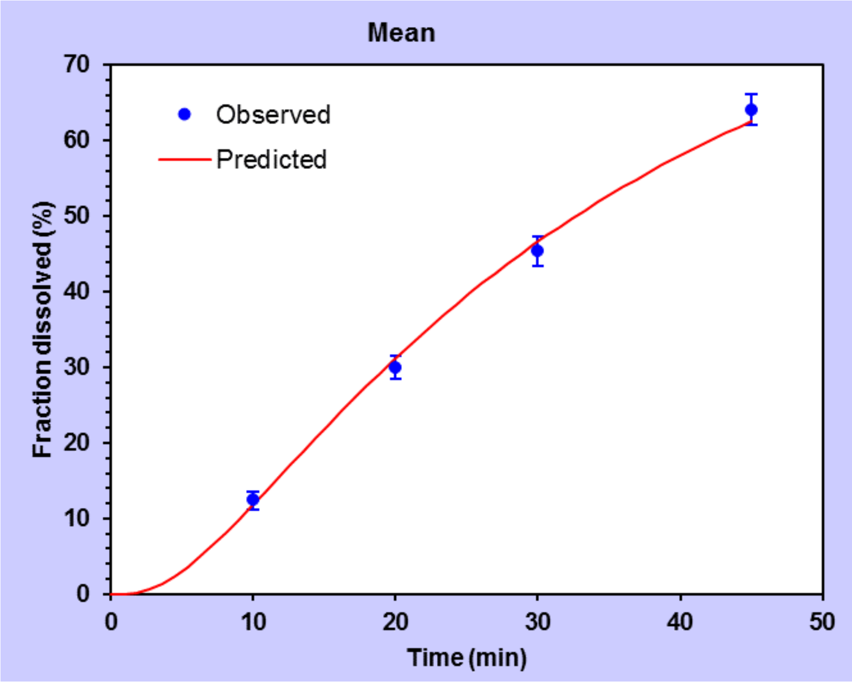

Supplementary materials – Model fitting summary – Polyglykol® 8000 P (modelling up to app. 60 % of carvedilol released)

Graphical abstract of model fit presented as the fraction % of released carvedilol per tested tablet:

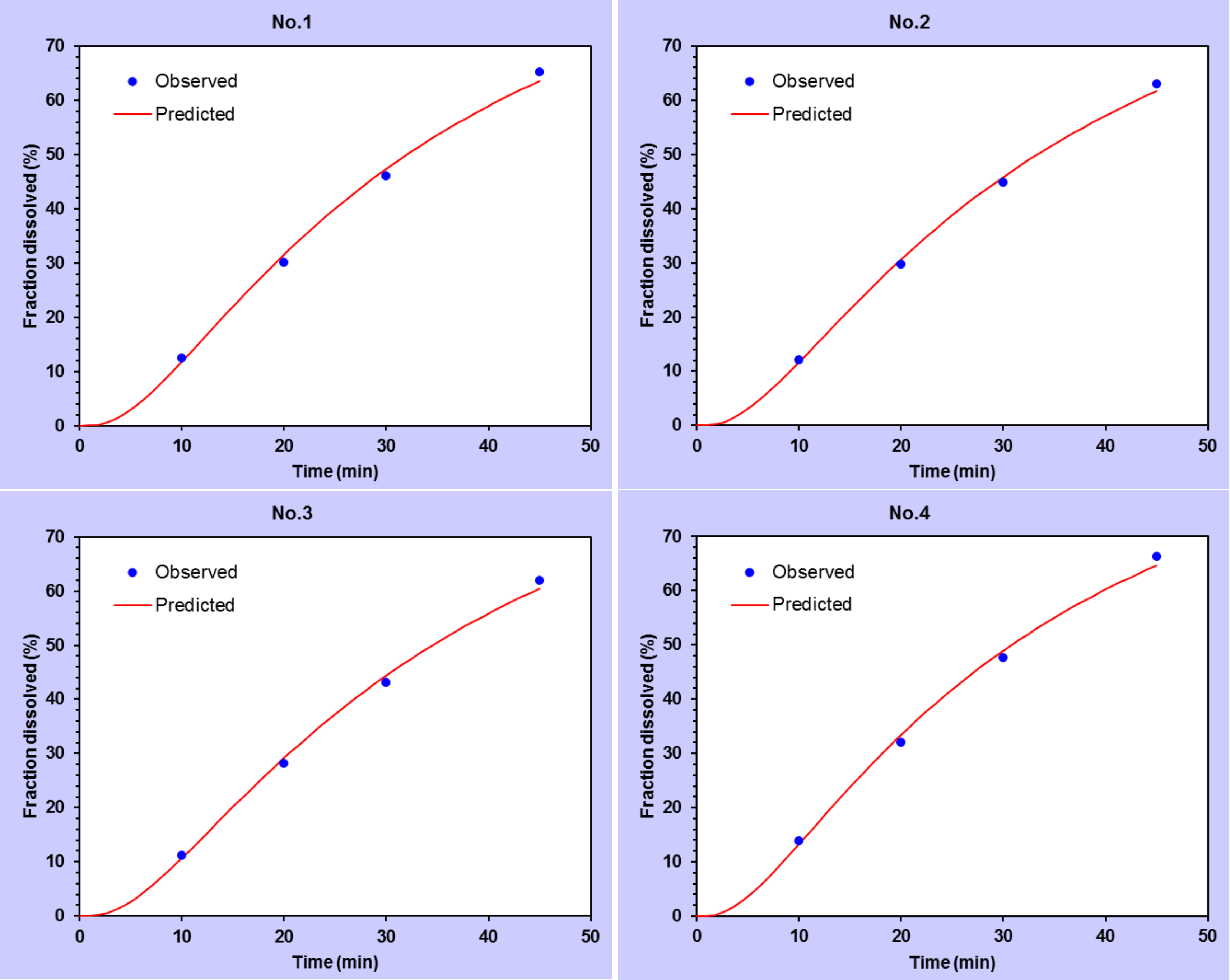

Supplementary materials – Model fitting summary – Polyglykol® 8000 P (modelling up to app. 60 % of carvedilol released)

Model: **Probit_2**

Model equation: $F = F_{max} \cdot \phi[\alpha + \beta \cdot \log(t)]$

Fitted model parameters per tested tablet (N = 4) with statistics – mean, standard deviation (SD), and relative standard deviation expressed in % (RSD%) (output from DDSolver):

Parameter	No.1	No.2	No.3	No.4	Mean	SD	RSD(%)
α	-4.873	-4.853	-4.964	-4.699	-4.847	0.110	-2.271
β	3.783	3.775	3.835	3.682	3.769	0.064	1.686
F_{max}	68.412	66.104	64.927	69.567	67.253	2.116	3.146

Number of dissolution data points (N), degrees of freedom (df), and selected goodness of fit criteria – Pearson correlation coefficient (R), coefficient of determination (R^2), adjusted coefficient of determination ($R^2_{adjusted}$), and residual sum of squares (RSS) (manual calculation in MS Excel):

Parameter	No.1	No.2	No.3	No.4
N	4	4	4	4
df	1	1	1	1
R	0.977160263	0.978587279	0.977461835	0.977431729
R^2	0.95484218	0.957633062	0.955431638	0.955372785
$R^2_{adjusted}$	0.864526541	0.872899185	0.866294915	0.866118354
RSS	82.64873718	72.09786098	75.33446612	80.41866134

Graphical abstract of model fit presented as mean ± 1 SD of the fraction % of released carvedilol:

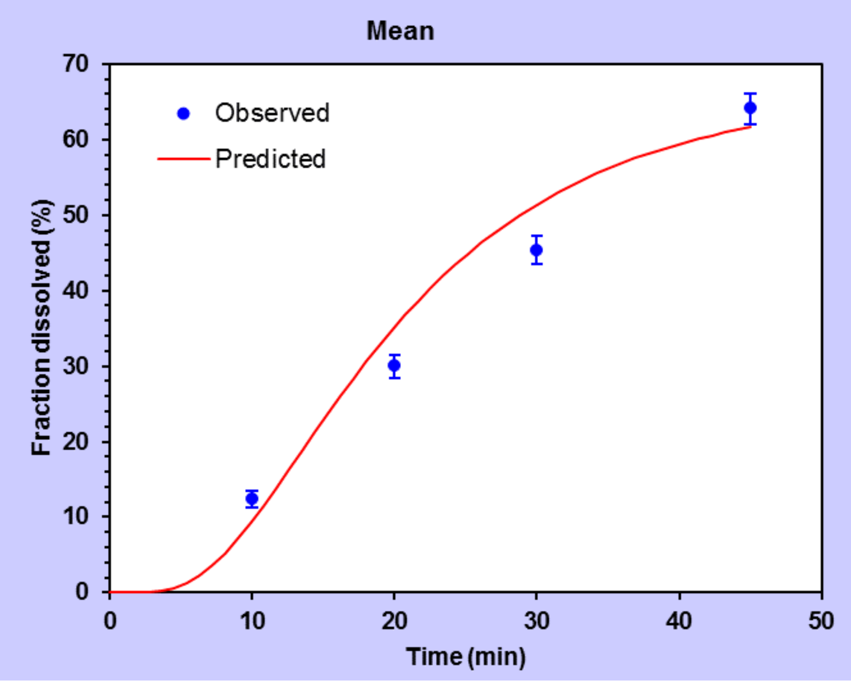

Supplementary materials – Model fitting summary – Polyglykol® 8000 P (modelling up to app. 60 % of carvedilol released)

Graphical abstract of model fit presented as the fraction % of released carvedilol per tested tablet:

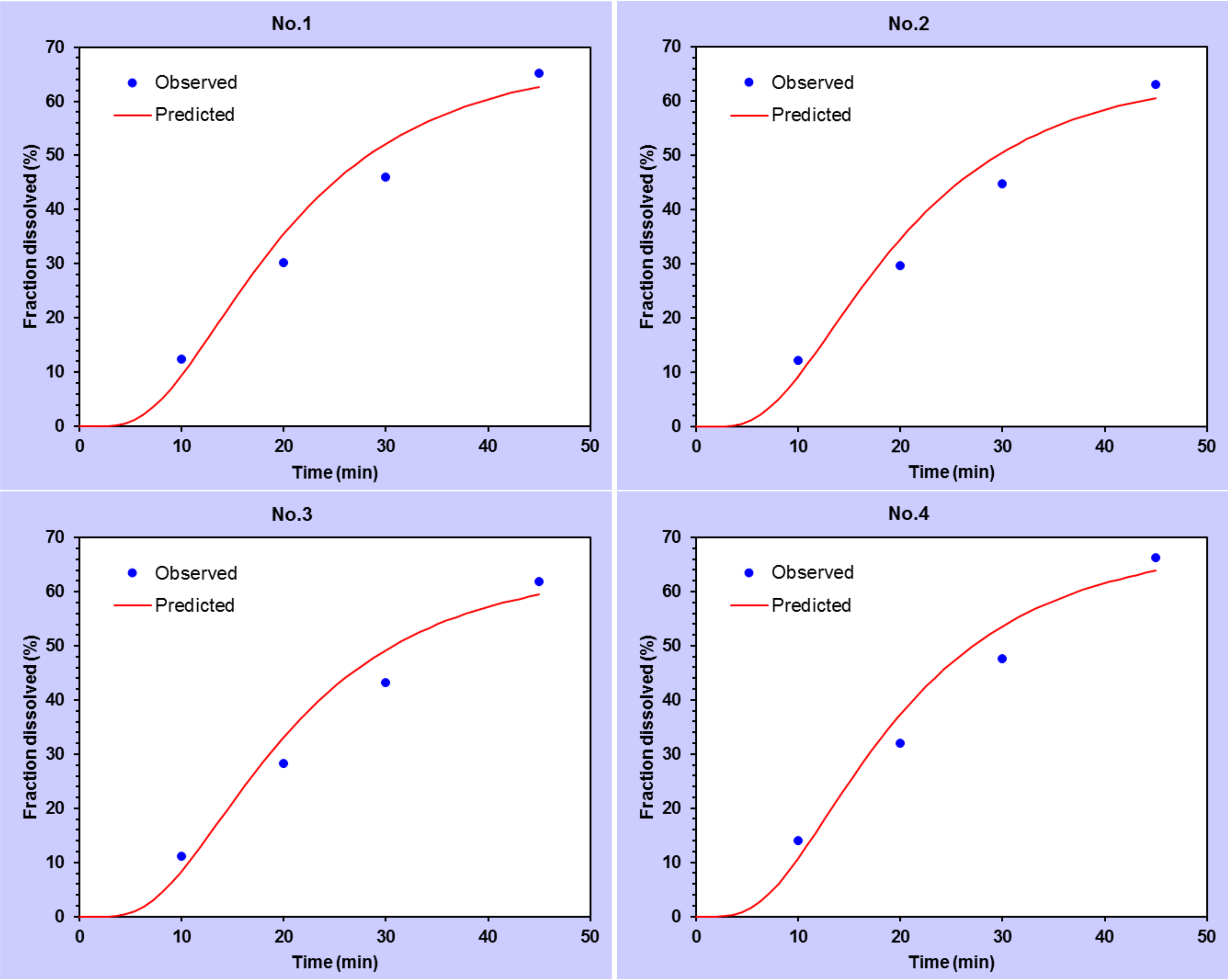

Supplementary materials – Model fitting summary – Polyglykol® 8000 P (modelling up to app. 75 % of carvedilol released)

Model: **Zero–order**

Model equation: $F = k_0 \cdot t$

Fitted model parameters per tested tablet (N = 4) with statistics – mean, standard deviation (SD), and relative standard deviation expressed in % (RSD%) (output from DDSolver):

Parameter	No.1	No.2	No.3	No.4	Mean	SD	RSD(%)
k_0	1.390	1.354	1.325	1.418	1.372	0.041	2.960

Number of dissolution data points (N), degrees of freedom (df), and selected goodness of fit criteria – Pearson correlation coefficient (R), coefficient of determination (R^2), adjusted coefficient of determination ($R^2_{adjusted}$), and residual sum of squares (RSS) (manual calculation in MS Excel):

Parameter	No.1	No.2	No.3	No.4
N	5	5	5	5
df	4	4	4	4
R	0.991965381	0.992631525	0.993704543	0.99082164
R^2	0.983995318	0.985317345	0.987448719	0.981727522
$R^2_{adjusted}$	0.983995318	0.985317345	0.987448719	0.981727522
RSS	53.50725542	47.52422132	35.67836611	75.45426118

Graphical abstract of model fit presented as mean ± 1 SD of the fraction % of released carvedilol:

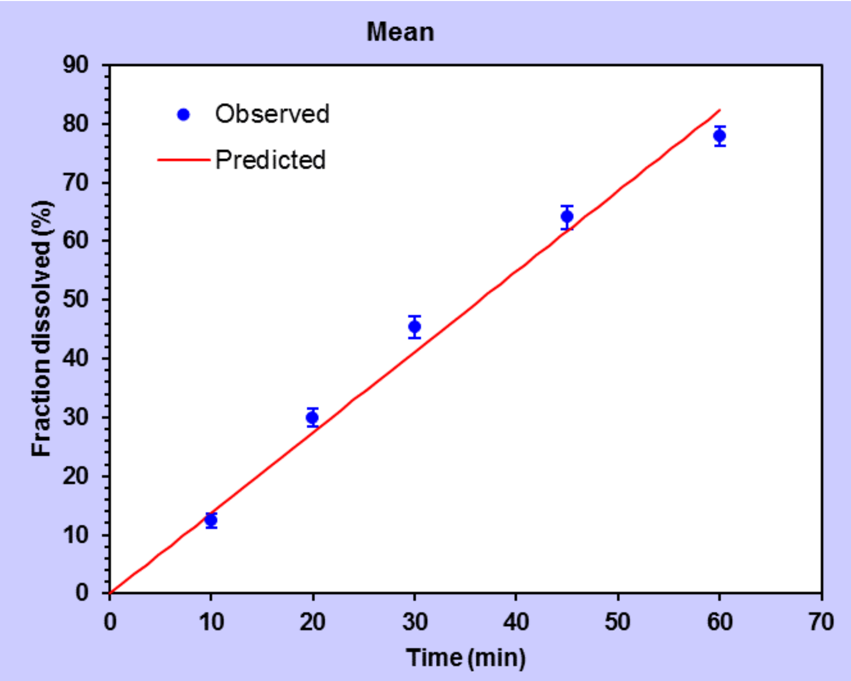

Supplementary materials – Model fitting summary – Polyglykol® 8000 P (modelling up to app. 75 % of carvedilol released)

Graphical abstract of model fit presented as the fraction % of released carvedilol per tested tablet:

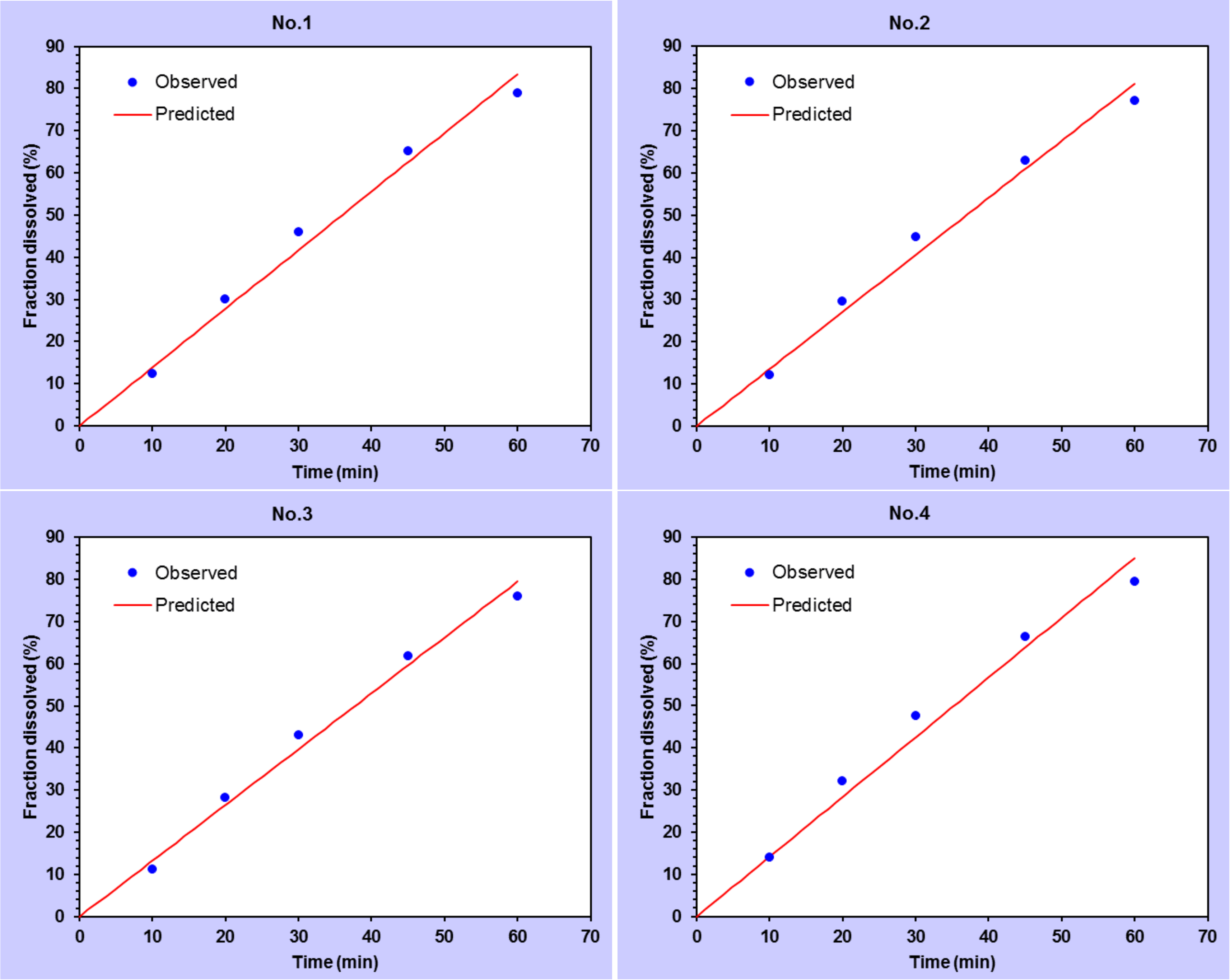

Supplementary materials – Model fitting summary – Polyglykol® 8000 P (modelling up to app. 75 % of carvedilol released)

Model: **Zero–order with T_{lag}**

Model equation: $F = k_0 \cdot (t - T_{lag})$

Fitted model parameters per tested tablet (N = 4) with statistics – mean, standard deviation (SD), and relative standard deviation expressed in % (RSD%) (output from DDSolver):

Parameter	No.1	No.2	No.3	No.4	Mean	SD	RSD(%)
k_0	1.327	1.290	1.293	1.306	1.304	0.017	1.278
T_{lag}	-2.046	-2.113	-1.057	-3.629	-2.211	1.061	-47.991

Number of dissolution data points (N), degrees of freedom (df), and selected goodness of fit criteria – Pearson correlation coefficient (R), coefficient of determination (R^2), adjusted coefficient of determination ($R^2_{adjusted}$), and residual sum of squares (RSS) (manual calculation in MS Excel):

Parameter	No.1	No.2	No.3	No.4
N	5	5	5	5
df	3	3	3	3
R	0.991965381	0.992631525	0.993704543	0.99082164
R^2	0.983995318	0.985317345	0.987448719	0.981727522
$R^2_{adjusted}$	0.978660424	0.980423127	0.983264959	0.975636696
RSS	45.22133414	39.17126138	33.57757813	50.18447002

Graphical abstract of model fit presented as mean ± 1 SD of the fraction % of released carvedilol:

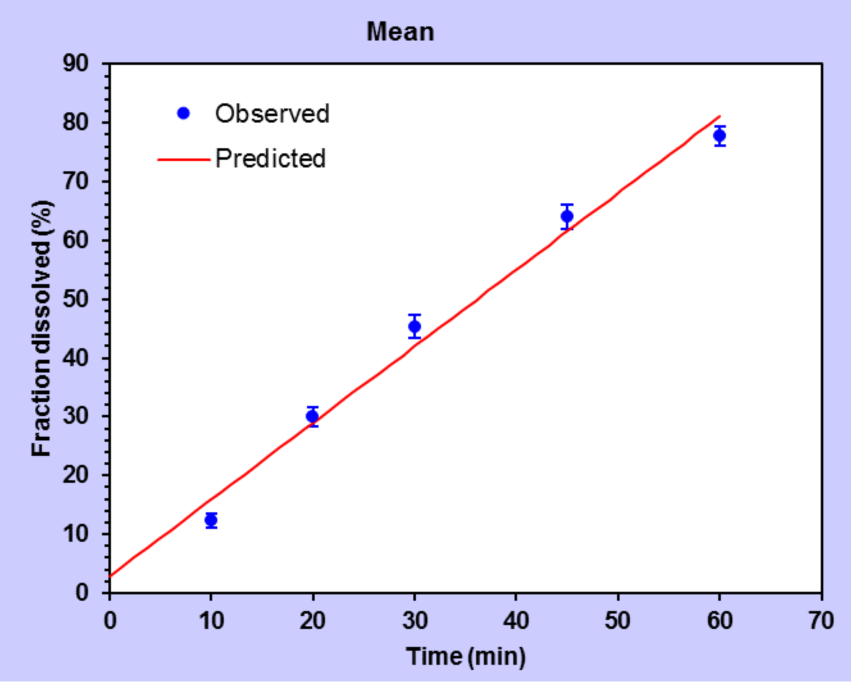

Supplementary materials – Model fitting summary – Polyglykol® 8000 P (modelling up to app. 75 % of carvedilol released)

Graphical abstract of model fit presented as the fraction % of released carvedilol per tested tablet:

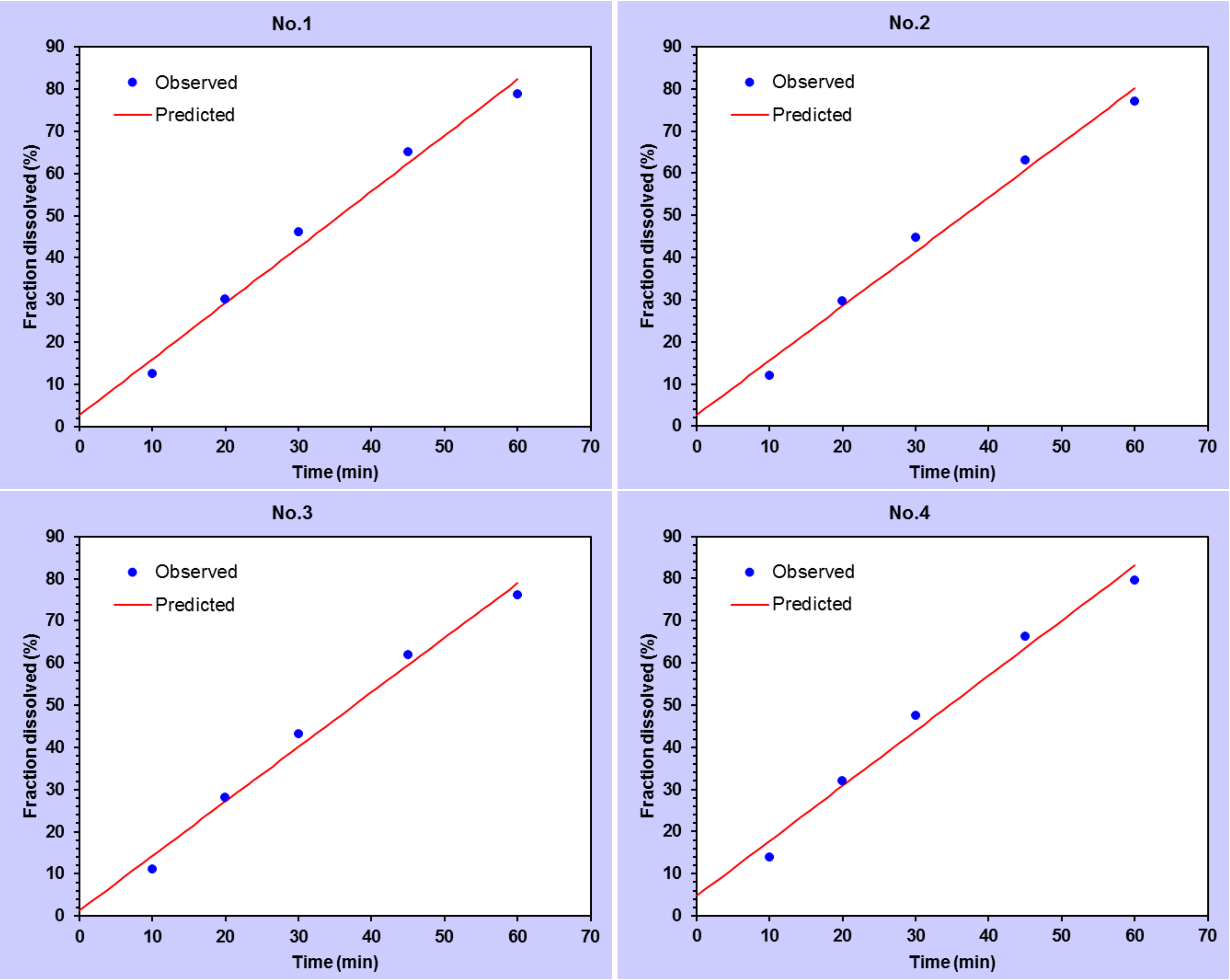

Supplementary materials – Model fitting summary – Polyglykol® 8000 P (modelling up to app. 75 % of carvedilol released)

Model: **Zero–order with F_0**

Model equation: $F = F_0 + k_0 \cdot t$

Fitted model parameters per tested tablet (N = 4) with statistics – mean, standard deviation (SD), and relative standard deviation expressed in % (RSD%) (output from DDSolver):

Parameter	No.1	No.2	No.3	No.4	Mean	SD	RSD(%)
k_0	1.327	1.290	1.293	1.306	1.304	0.017	1.278
F_0	2.714	2.725	1.367	4.740	2.887	1.391	48.174

Number of dissolution data points (N), degrees of freedom (df), and selected goodness of fit criteria – Pearson correlation coefficient (R), coefficient of determination (R^2), adjusted coefficient of determination ($R^2_{adjusted}$), and residual sum of squares (RSS) (manual calculation in MS Excel):

Parameter	No.1	No.2	No.3	No.4
N	5	5	5	5
df	3	3	3	3
R	0.991965381	0.992631525	0.993704543	0.99082164
R^2	0.983995318	0.985317345	0.987448719	0.981727522
$R^2_{adjusted}$	0.978660424	0.980423127	0.983264959	0.975636696
RSS	45.22133414	39.17126138	33.57757813	50.18447002

Graphical abstract of model fit presented as mean ± 1 SD of the fraction % of released carvedilol:

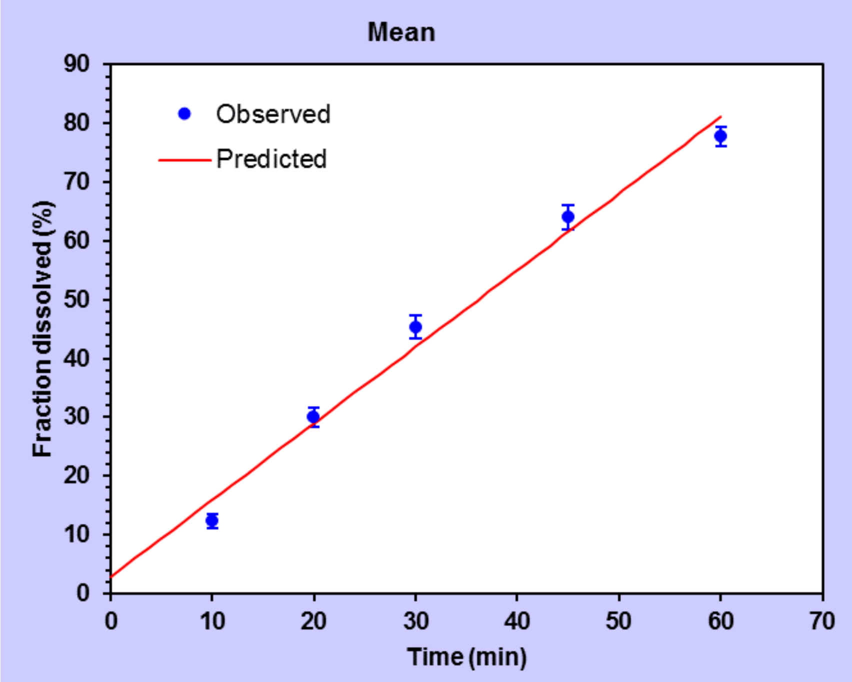

Supplementary materials – Model fitting summary – Polyglykol® 8000 P (modelling up to app. 75 % of carvedilol released)

Graphical abstract of model fit presented as the fraction % of released carvedilol per tested tablet:

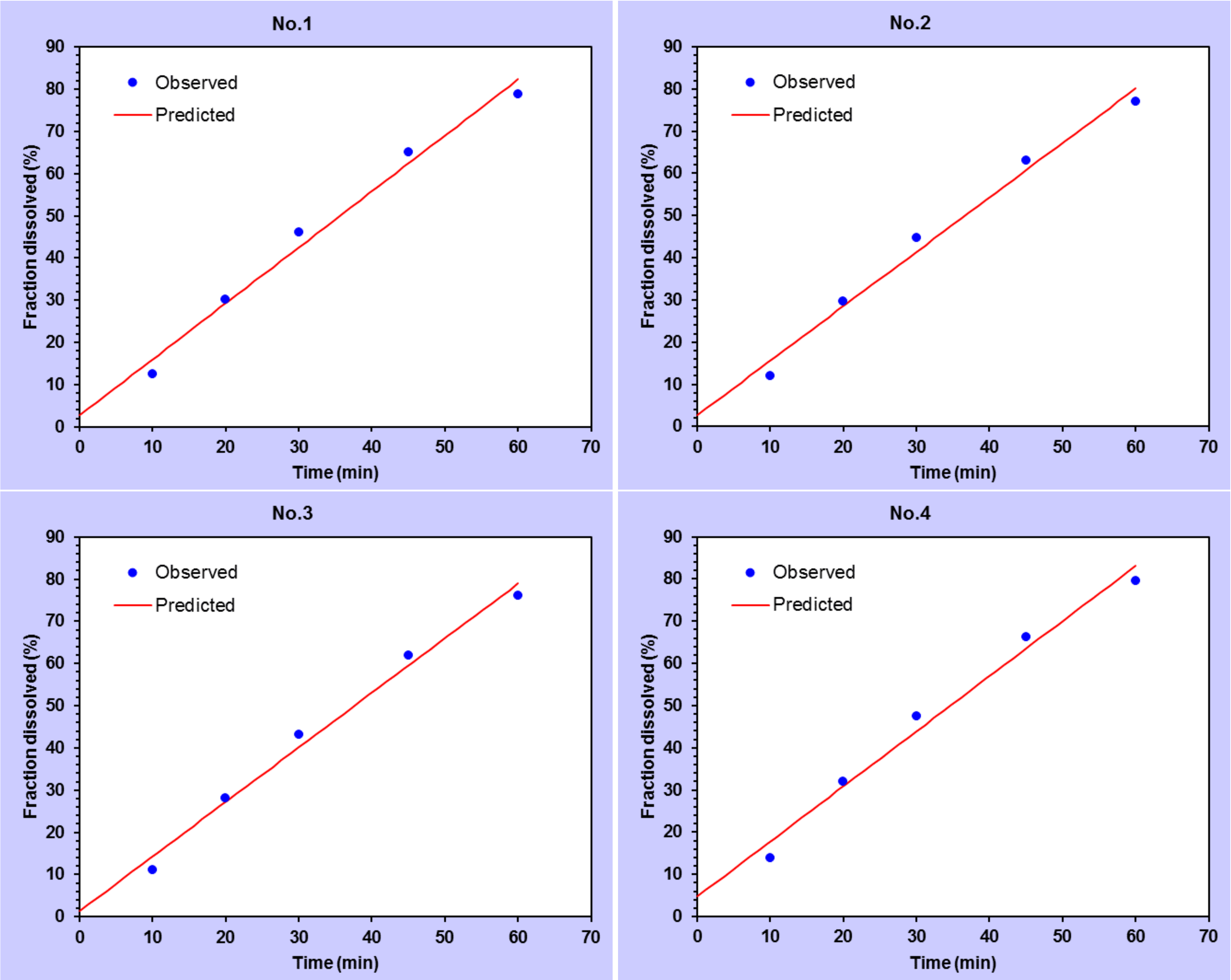

Supplementary materials – Model fitting summary – Polyglykol® 8000 P (modelling up to app. 75 % of carvedilol released)

Model: **First–order**

Model equation: $F = 100 \cdot (1 - e^{-k_1 \cdot t})$

Fitted model parameters per tested tablet (N = 4) with statistics – mean, standard deviation (SD), and relative standard deviation expressed in % (RSD%) (output from DDSolver):

Parameter	No.1	No.2	No.3	No.4	Mean	SD	RSD(%)
k_1	0.021	0.020	0.019	0.022	0.020	0.001	5.197

Number of dissolution data points (N), degrees of freedom (df), and selected goodness of fit criteria – Pearson correlation coefficient (R), coefficient of determination (R^2), adjusted coefficient of determination ($R^2_{adjusted}$), and residual sum of squares (RSS) (manual calculation in MS Excel):

Parameter	No.1	No.2	No.3	No.4
N	5	5	5	5
df	4	4	4	4
R	0.999589021	0.999767684	0.999630053	0.999753785
R^2	0.99917821	0.999535421	0.999260242	0.99950763
$R^2_{adjusted}$	0.99917821	0.999535421	0.999260242	0.99950763
RSS	131.0931767	116.0347666	129.6711087	106.276087

Graphical abstract of model fit presented as mean ± 1 SD of the fraction % of released carvedilol:

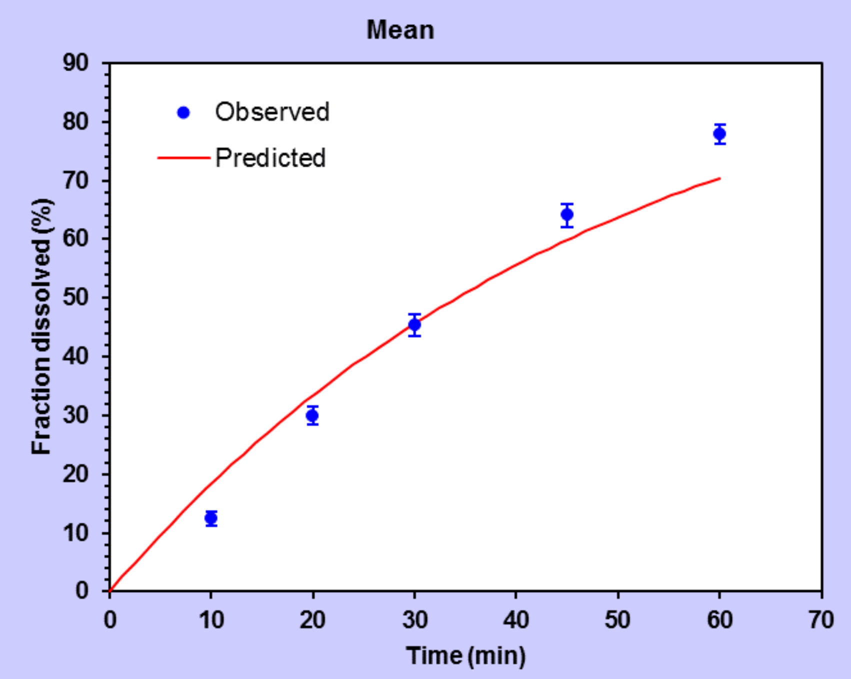

Supplementary materials – Model fitting summary – Polyglykol® 8000 P (modelling up to app. 75 % of carvedilol released)

Graphical abstract of model fit presented as the fraction % of released carvedilol per tested tablet:

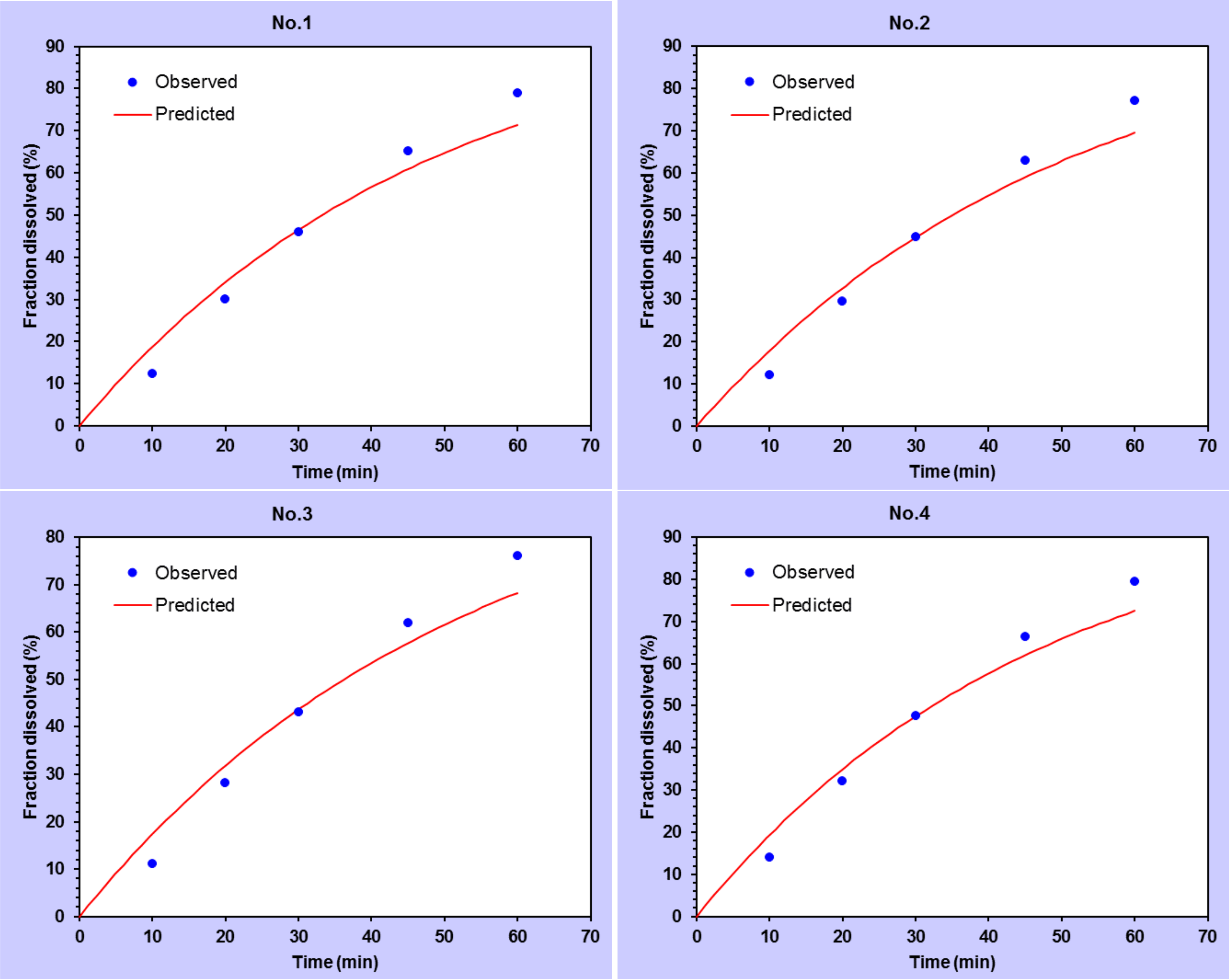

Supplementary materials – Model fitting summary – Polyglykol® 8000 P (modelling up to app. 75 % of carvedilol released)

Model: **First–order with T_{lag}**

Model equation: $F = 100 \cdot \left[1 - e^{-k_1 \cdot (t - T_{lag})}\right]$

Fitted model parameters per tested tablet (N = 4) with statistics – mean, standard deviation (SD), and relative standard deviation expressed in % (RSD%) (output from DDSolver):

Parameter	No.1	No.2	No.3	No.4	Mean	SD	RSD(%)
k_1	0.026	0.027	0.024	0.029	0.026	0.002	7.568
T_{lag}	5.991	6.587	6.062	6.219	6.215	0.266	4.276

Number of dissolution data points (N), degrees of freedom (df), and selected goodness of fit criteria – Pearson correlation coefficient (R), coefficient of determination (R^2), adjusted coefficient of determination ($R^2_{adjusted}$), and residual sum of squares (RSS) (manual calculation in MS Excel):

Parameter	No.1	No.2	No.3	No.4
N	5	5	5	5
df	3	3	3	3
R	0.998014999	0.997545082	0.998168246	0.997458561
R^2	0.996033939	0.99509619	0.996339847	0.994923581
$R^2_{adjusted}$	0.994711918	0.993461587	0.995119795	0.993231441
RSS	19.54231634	17.51690594	18.32680381	18.97480416

Graphical abstract of model fit presented as mean ± 1 SD of the fraction % of released carvedilol:

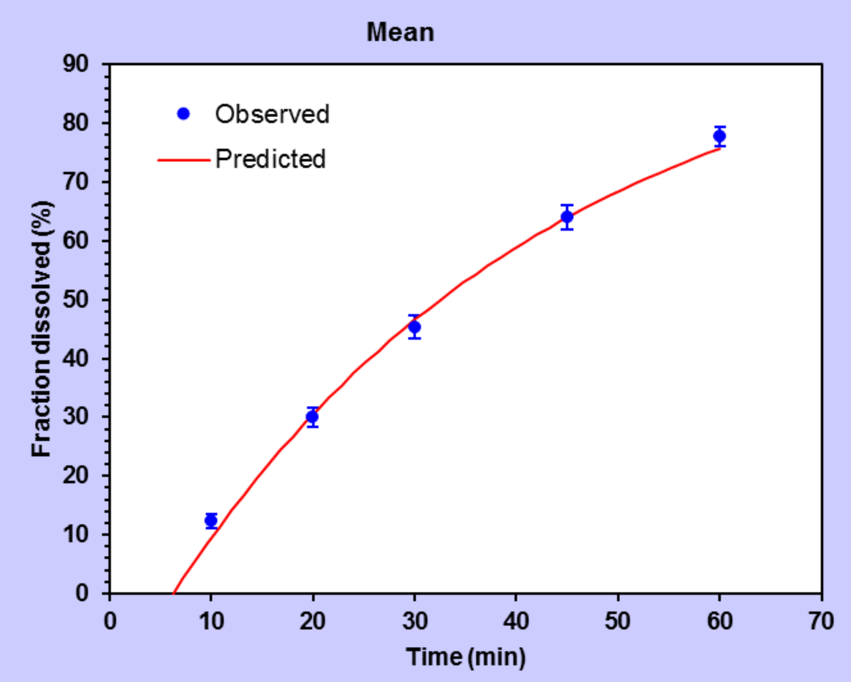

Supplementary materials – Model fitting summary – Polyglykol® 8000 P (modelling up to app. 75 % of carvedilol released)

Graphical abstract of model fit presented as the fraction % of released carvedilol per tested tablet:

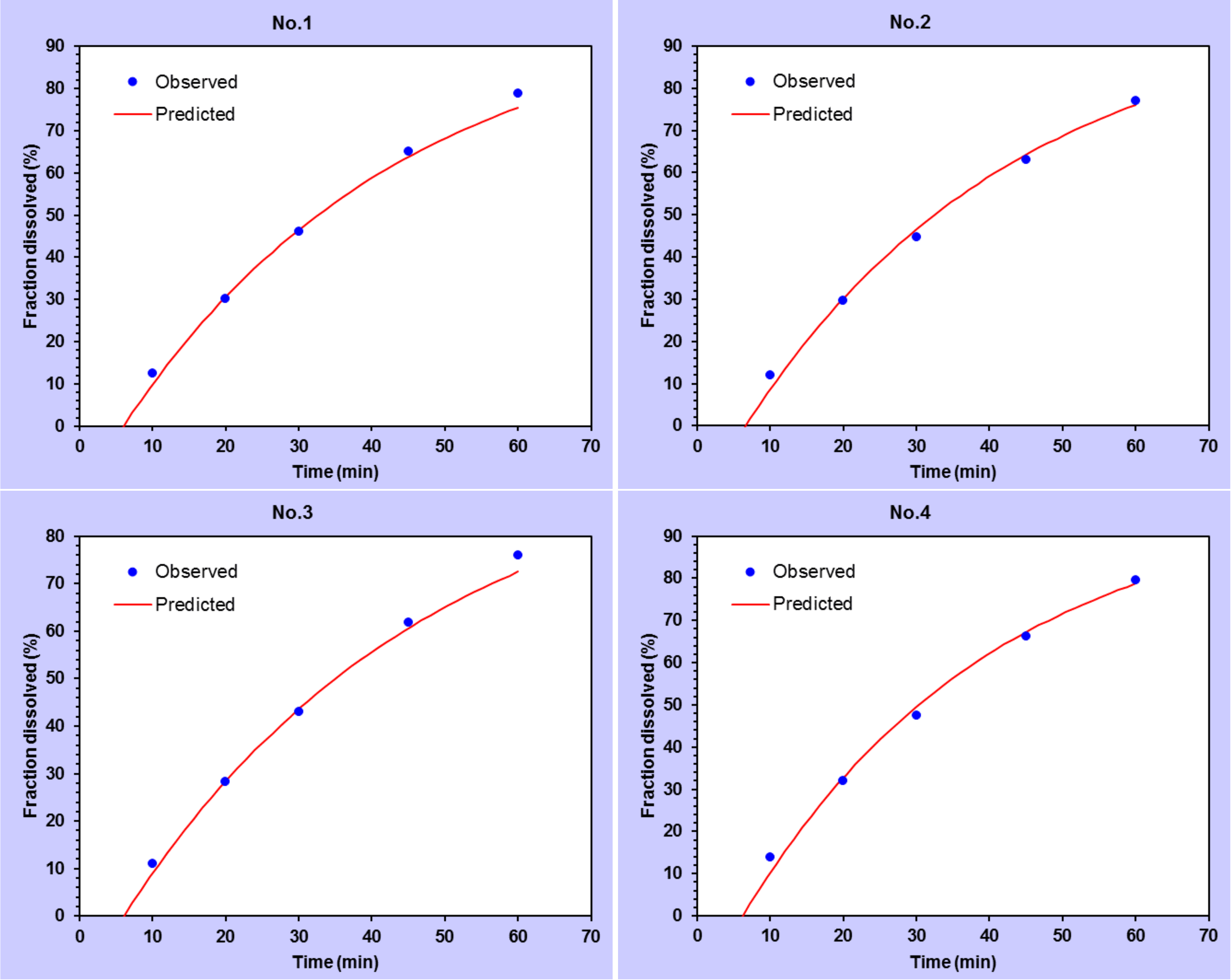

Supplementary materials – Model fitting summary – Polyglykol® 8000 P (modelling up to app. 75 % of carvedilol released)

Model: **First–order with F_{max}**

Model equation: $F = F_{max} \cdot (1 - e^{-k_1 \cdot t})$

Fitted model parameters per tested tablet (N = 4) with statistics – mean, standard deviation (SD), and relative standard deviation expressed in % (RSD%) (output from DDSolver):

Parameter	No.1	No.2	No.3	No.4	Mean	SD	RSD(%)
k_1	0.042	0.042	0.040	0.043	0.042	0.001	2.401
F_{max}	67.249	65.715	79.794	67.846	70.151	6.491	9.253

Number of dissolution data points (N), degrees of freedom (df), and selected goodness of fit criteria – Pearson correlation coefficient (R), coefficient of determination (R^2), adjusted coefficient of determination ($R^2_{adjusted}$), and residual sum of squares (RSS) (manual calculation in MS Excel):

Parameter	No.1	No.2	No.3	No.4
N	5	5	5	5
df	3	3	3	3
R	0.985665753	0.98559615	0.985561834	0.986865331
R^2	0.971536977	0.971399772	0.971332128	0.973903182
$R^2_{adjusted}$	0.962049303	0.961866362	0.961776171	0.965204242
RSS	538.8156142	501.0186568	694.6245964	500.0313385

Graphical abstract of model fit presented as mean ± 1 SD of the fraction % of released carvedilol:

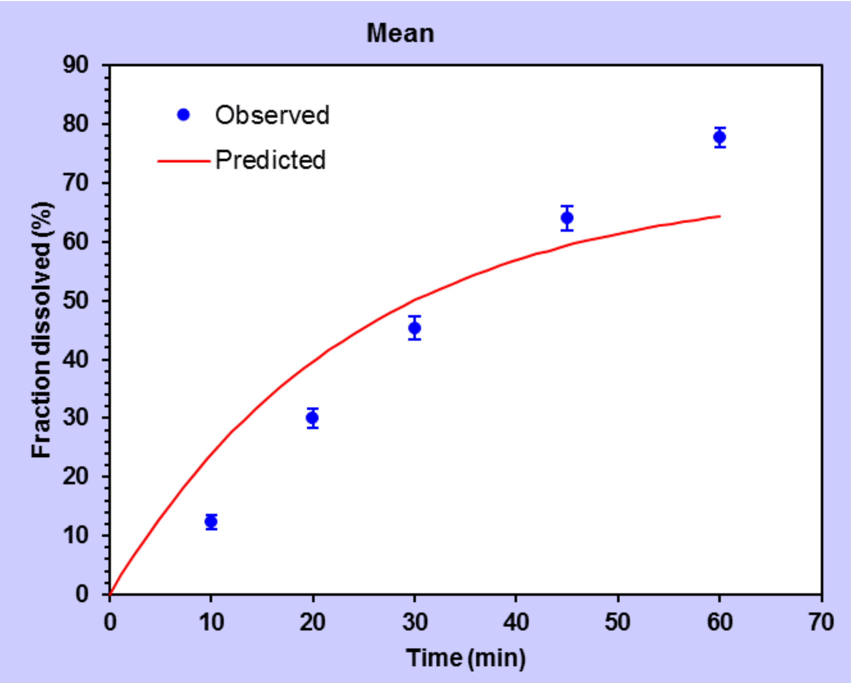

Supplementary materials – Model fitting summary – Polyglykol® 8000 P (modelling up to app. 75 % of carvedilol released)

Graphical abstract of model fit presented as the fraction % of released carvedilol per tested tablet:

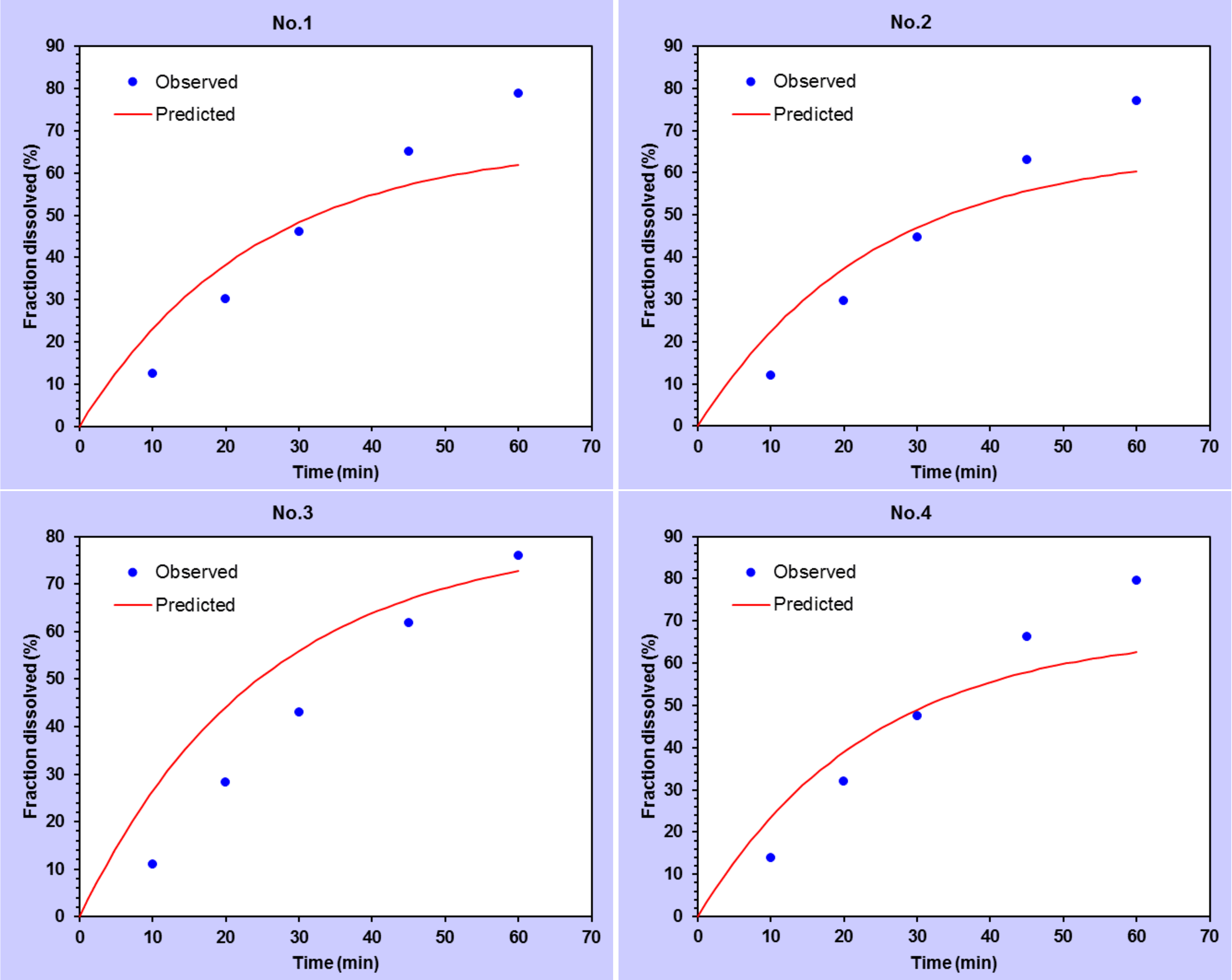

Supplementary materials – Model fitting summary – Polyglykol® 8000 P (modelling up to app. 75 % of carvedilol released)

Model: **First–order with T_{lag} and F_{max}**

Model equation: $F = F_{max} \cdot \left[1 - e^{-k_1 \cdot (t - T_{lag})}\right]$

Fitted model parameters per tested tablet (N = 4) with statistics – mean, standard deviation (SD), and relative standard deviation expressed in % (RSD%) (output from DDSolver):

Parameter	No.1	No.2	No.3	No.4	Mean	SD	RSD(%)
k_1	0.084	0.084	0.084	0.056	0.077	0.014	18.366
T_{lag}	7.715	7.730	7.987	11.020	8.613	1.609	18.685
F_{max}	68.974	67.400	66.495	83.503	71.593	8.006	11.182

Number of dissolution data points (N), degrees of freedom (df), and selected goodness of fit criteria – Pearson correlation coefficient (R), coefficient of determination (R^2), adjusted coefficient of determination ($R^2_{adjusted}$), and residual sum of squares (RSS) (manual calculation in MS Excel):

Parameter	No.1	No.2	No.3	No.4
N	5	5	5	5
df	2	2	2	2
R	0.92419528	0.924671308	0.919993309	0.971140115
R^2	0.854136916	0.855017028	0.846387688	0.943113123
$R^2_{adjusted}$	0.708273833	0.710034056	0.692775377	0.886226245
RSS	476.7636732	447.8633209	478.5283172	426.3225141

Graphical abstract of model fit presented as mean ± 1 SD of the fraction % of released carvedilol:

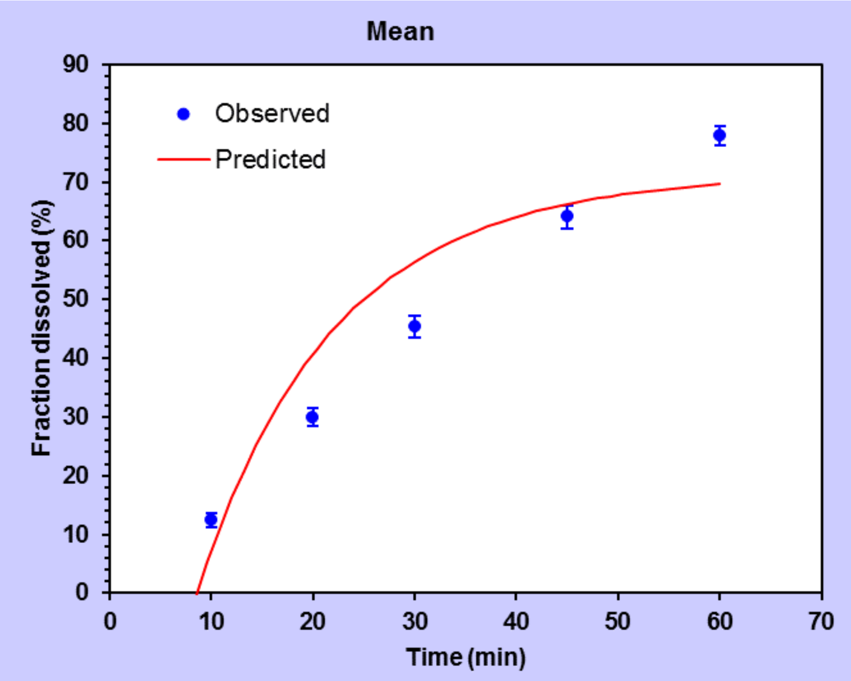

Supplementary materials – Model fitting summary – Polyglykol® 8000 P (modelling up to app. 75 % of carvedilol released)

Graphical abstract of model fit presented as the fraction % of released carvedilol per tested tablet:

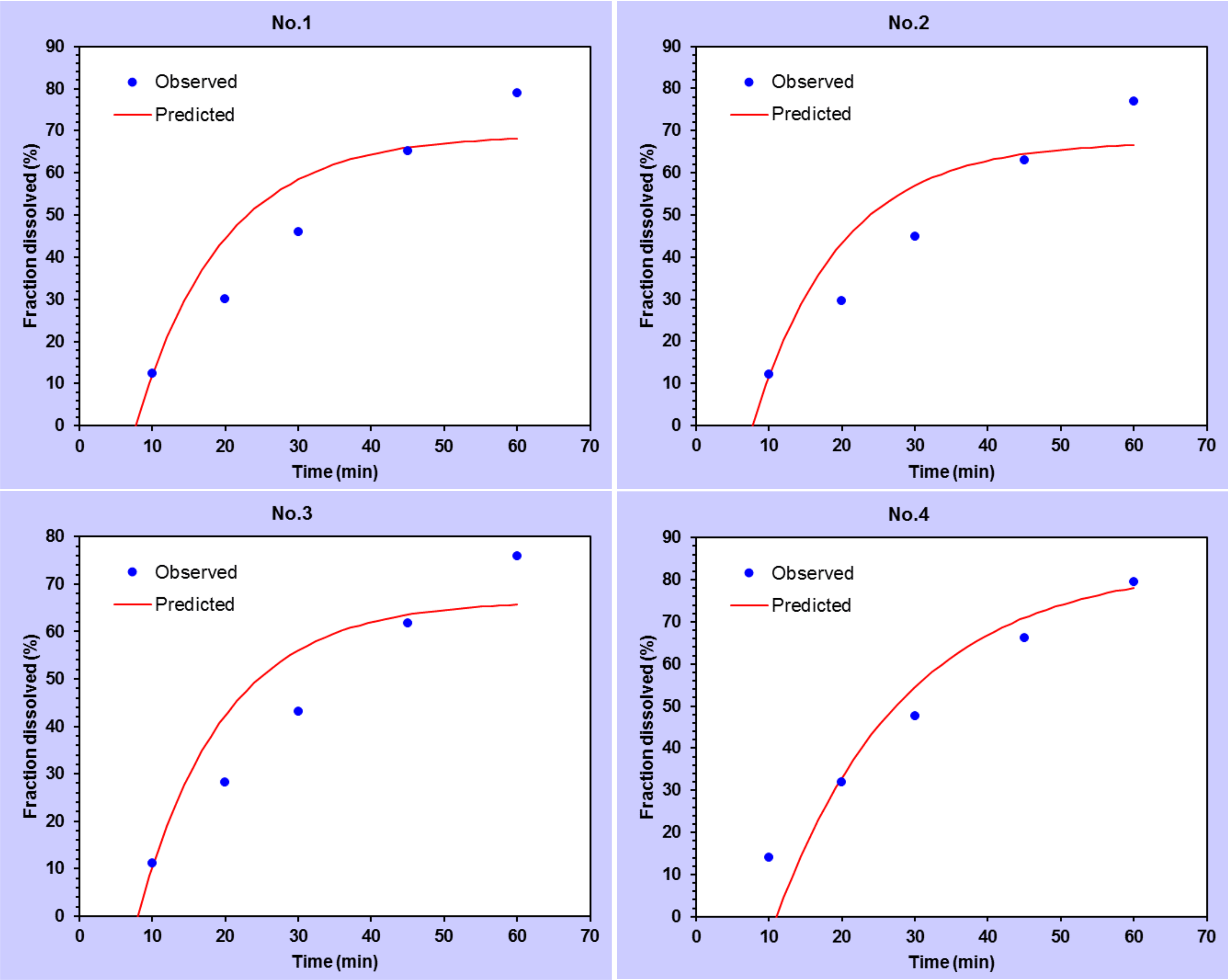

Supplementary materials – Model fitting summary – Polyglykol® 8000 P (modelling up to app. 75 % of carvedilol released)

Model: **Higuchi**

Model equation: $F = k_H \cdot t^{0.5}$

Fitted model parameters per tested tablet (N = 4) with statistics – mean, standard deviation (SD), and relative standard deviation expressed in % (RSD%) (output from DDSolver):

Parameter	No.1	No.2	No.3	No.4	Mean	SD	RSD(%)
k_H	8.929	8.696	8.488	9.140	8.813	0.283	3.206

Number of dissolution data points (N), degrees of freedom (df), and selected goodness of fit criteria – Pearson correlation coefficient (R), coefficient of determination (R^2), adjusted coefficient of determination ($R^2_{adjusted}$), and residual sum of squares (RSS) (manual calculation in MS Excel):

Parameter	No.1	No.2	No.3	No.4
N	5	5	5	5
df	4	4	4	4
R	0.999494881	0.999773225	0.999609574	0.999579482
R^2	0.998990017	0.999546502	0.9992193	0.999159141
$R^2_{adjusted}$	0.998990017	0.999546502	0.9992193	0.999159141
RSS	477.2692495	446.6495351	484.0414741	410.8713418

Graphical abstract of model fit presented as mean ± 1 SD of the fraction % of released carvedilol:

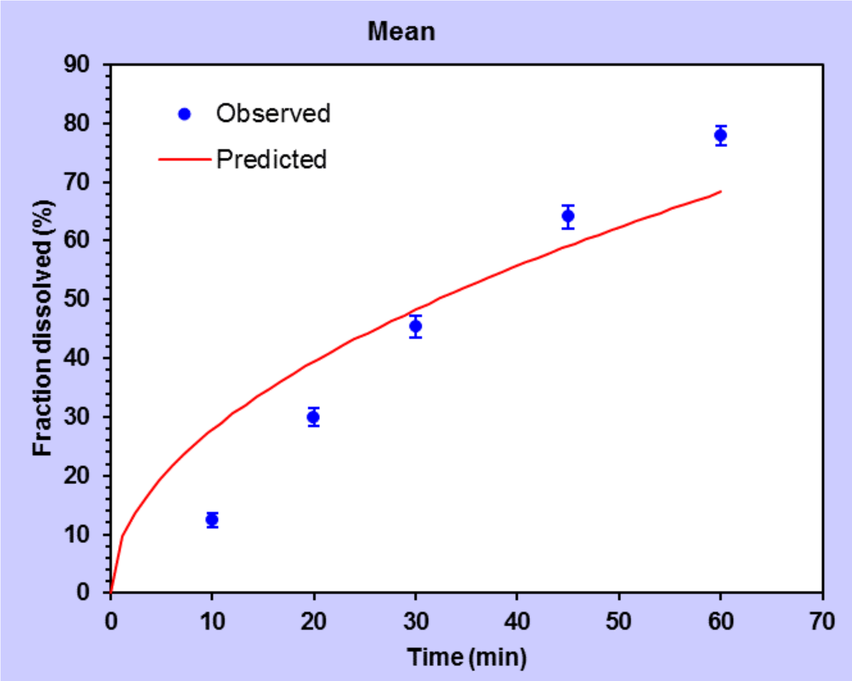

Supplementary materials – Model fitting summary – Polyglykol® 8000 P (modelling up to app. 75 % of carvedilol released)

Graphical abstract of model fit presented as the fraction % of released carvedilol per tested tablet:

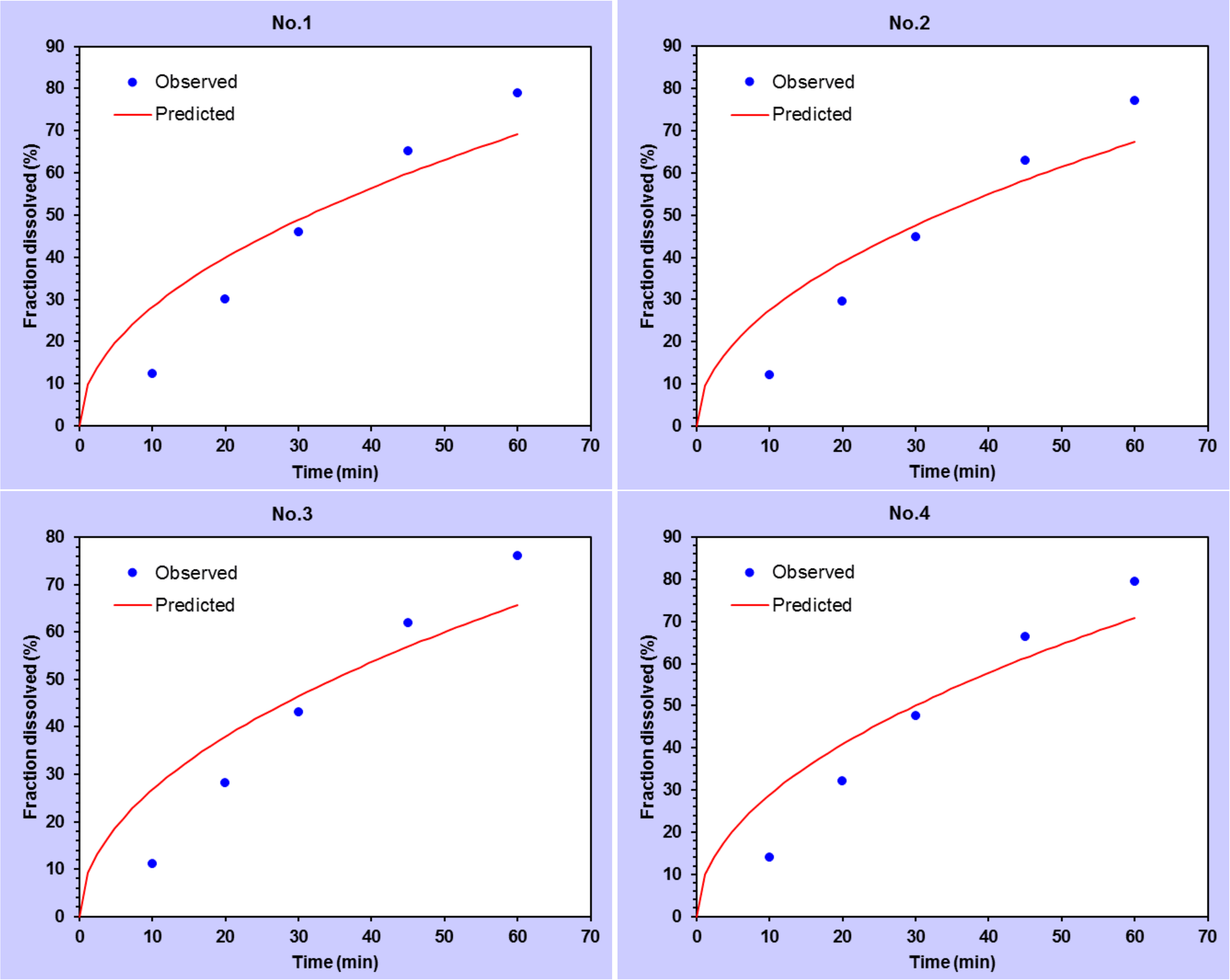

Supplementary materials – Model fitting summary – Polyglykol® 8000 P (modelling up to app. 75 % of carvedilol released)

Model: **Higuchi with T_{lag}**

Model equation: $F = k_H \cdot (t - T_{lag})^{0.5}$

Fitted model parameters per tested tablet (N = 4) with statistics – mean, standard deviation (SD), and relative standard deviation expressed in % (RSD%) (output from DDSolver):

Parameter	No.1	No.2	No.3	No.4	Mean	SD	RSD(%)
k_H	11.168	10.879	10.765	11.220	11.008	0.221	2.006
T_{lag}	11.140	11.160	11.647	10.449	11.099	0.493	4.441

Number of dissolution data points (N), degrees of freedom (df), and selected goodness of fit criteria – Pearson correlation coefficient (R), coefficient of determination (R^2), adjusted coefficient of determination ($R^2_{adjusted}$), and residual sum of squares (RSS) (manual calculation in MS Excel):

Parameter	No.1	No.2	No.3	No.4
N	5	5	5	5
df	3	3	3	3
R	0.983851445	0.984294884	0.984379347	0.983187668
R^2	0.967963665	0.968836419	0.969002698	0.96665799
$R^2_{adjusted}$	0.957284887	0.958448559	0.958670264	0.955543986
RSS	170.6080671	161.0180163	142.3911028	204.8798669

Graphical abstract of model fit presented as mean ± 1 SD of the fraction % of released carvedilol:

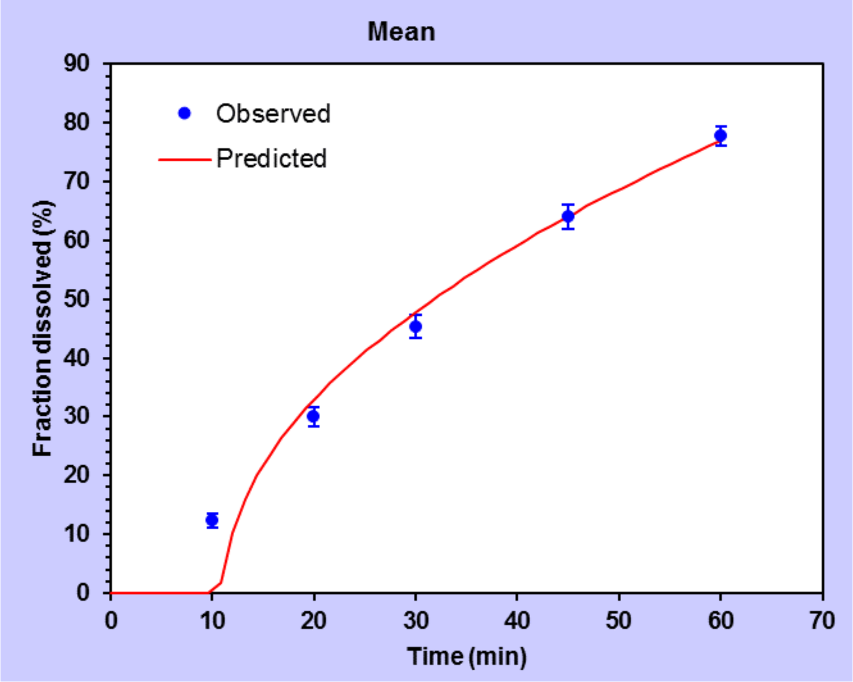

Supplementary materials – Model fitting summary – Polyglykol® 8000 P (modelling up to app. 75 % of carvedilol released)

Graphical abstract of model fit presented as the fraction % of released carvedilol per tested tablet:

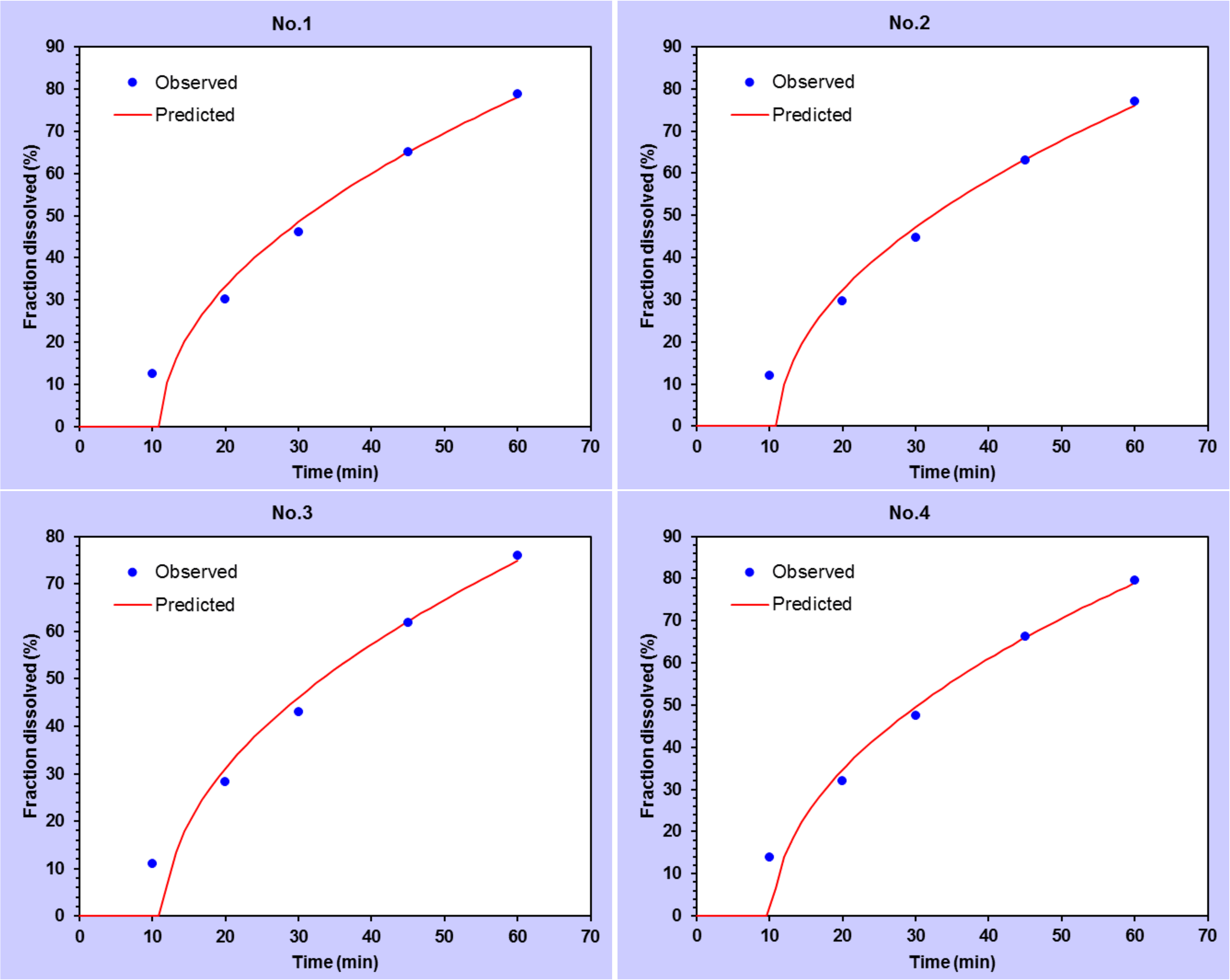

Supplementary materials – Model fitting summary – Polyglykol® 8000 P (modelling up to app. 75 % of carvedilol released)

Model: **Higuchi with F_0**

Model equation: $F = F_0 + k_H \cdot t^{0.5}$

Fitted model parameters per tested tablet (N = 4) with statistics – mean, standard deviation (SD), and relative standard deviation expressed in % (RSD%) (output from DDSolver):

Parameter	No.1	No.2	No.3	No.4	Mean	SD	RSD(%)
k_H	14.721	14.308	14.326	14.515	14.467	0.193	1.335
F_0	-34.669	-33.594	-34.944	-32.173	-33.845	1.258	-3.716

Number of dissolution data points (N), degrees of freedom (df), and selected goodness of fit criteria – Pearson correlation coefficient (R), coefficient of determination (R^2), adjusted coefficient of determination ($R^2_{adjusted}$), and residual sum of squares (RSS) (manual calculation in MS Excel):

Parameter	No.1	No.2	No.3	No.4
N	5	5	5	5
df	3	3	3	3
R	0.999494881	0.999773225	0.999609574	0.999579482
R^2	0.998990017	0.999546502	0.9992193	0.999159141
$R^2_{adjusted}$	0.998653357	0.999395336	0.998959067	0.998878855
RSS	2.853712447	1.209869621	2.08855321	2.309377283

Graphical abstract of model fit presented as mean ± 1 SD of the fraction % of released carvedilol:

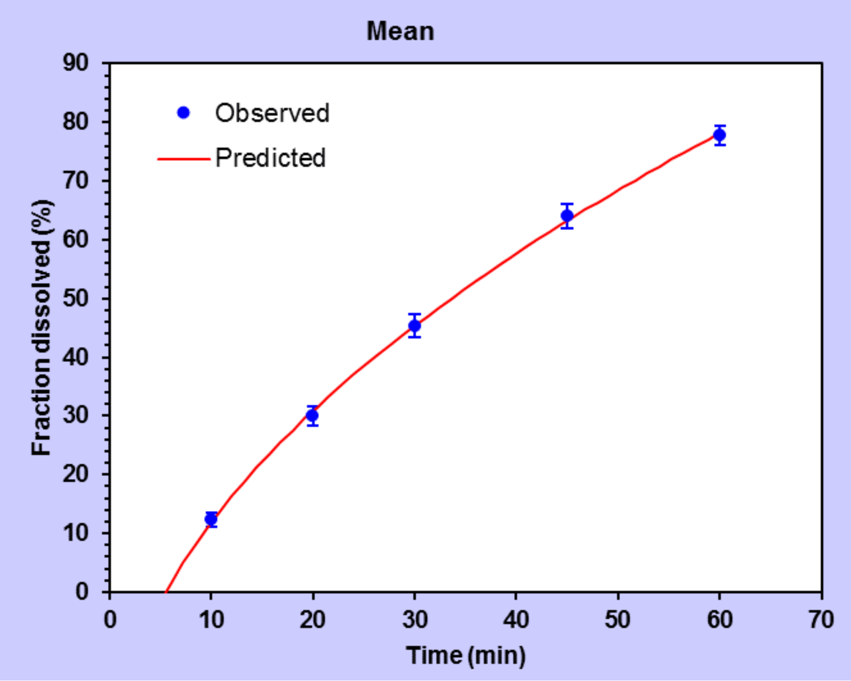

Supplementary materials – Model fitting summary – Polyglykol® 8000 P (modelling up to app. 75 % of carvedilol released)

Graphical abstract of model fit presented as the fraction % of released carvedilol per tested tablet:

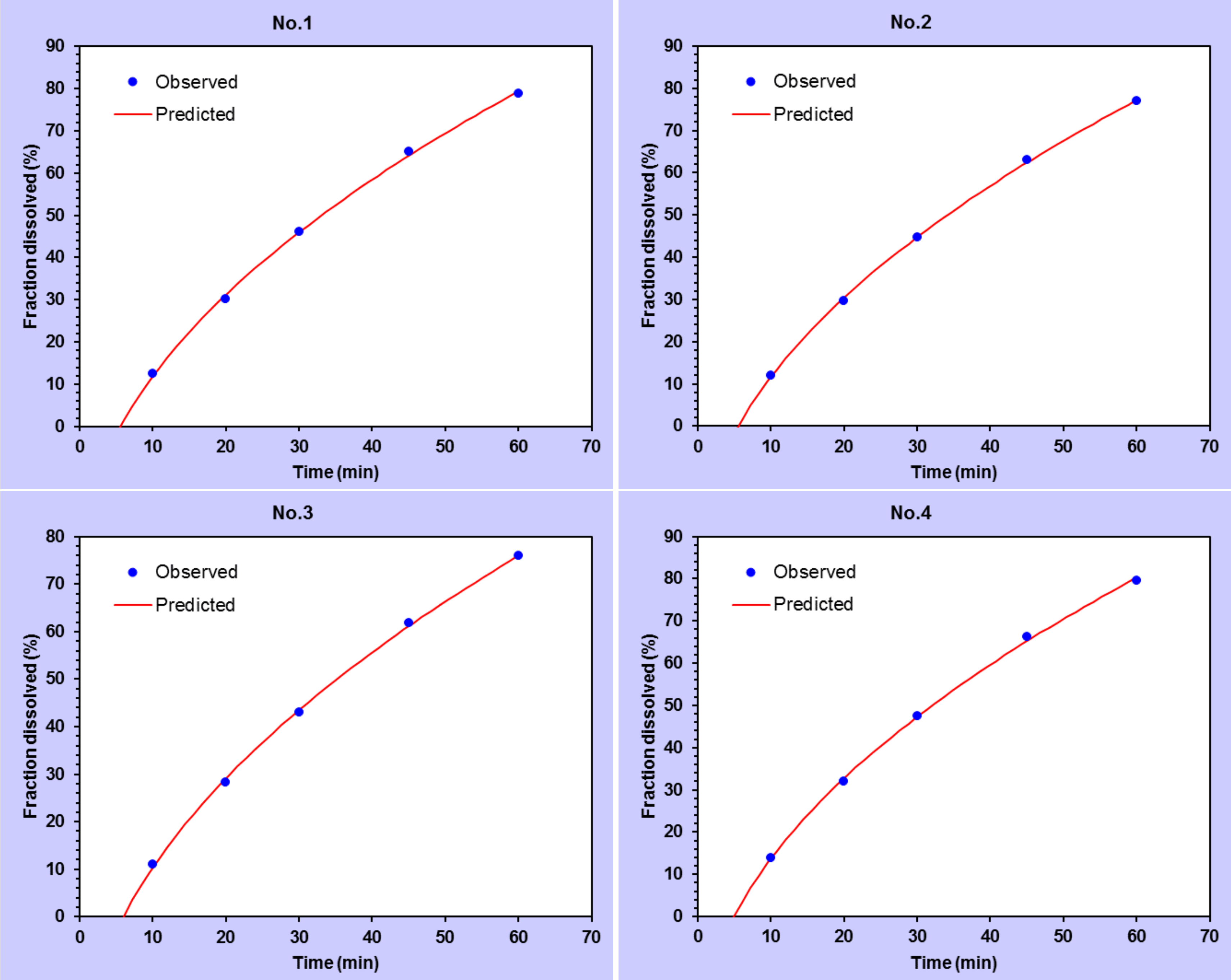

Supplementary materials – Model fitting summary – Polyglykol® 8000 P (modelling up to app. 75 % of carvedilol released)

Model: **Korsmeyer–Peppas**

Model equation: $F = k_{KP} \cdot t^n$

Fitted model parameters per tested tablet (N = 4) with statistics – mean, standard deviation (SD), and relative standard deviation expressed in % (RSD%) (output from DDSolver):

Parameter	No.1	No.2	No.3	No.4	Mean	SD	RSD(%)
k_{KP}	1.238	1.220	1.022	1.582	1.266	0.232	18.361
n	1.038	1.034	1.075	0.978	1.031	0.040	3.893

Number of dissolution data points (N), degrees of freedom (df), and selected goodness of fit criteria – Pearson correlation coefficient (R), coefficient of determination (R^2), adjusted coefficient of determination ($R^2_{adjusted}$), and residual sum of squares (RSS) (manual calculation in MS Excel):

Parameter	No.1	No.2	No.3	No.4
N	5	5	5	5
df	3	3	3	3
R	0.990823081	0.991620937	0.99159047	0.991503877
R^2	0.981730379	0.983312083	0.983251661	0.983079938
$R^2_{adjusted}$	0.975640505	0.977749444	0.977668881	0.977439917
RSS	83.33397139	74.07041456	76.6787857	72.24718174

Graphical abstract of model fit presented as mean ± 1 SD of the fraction % of released carvedilol:

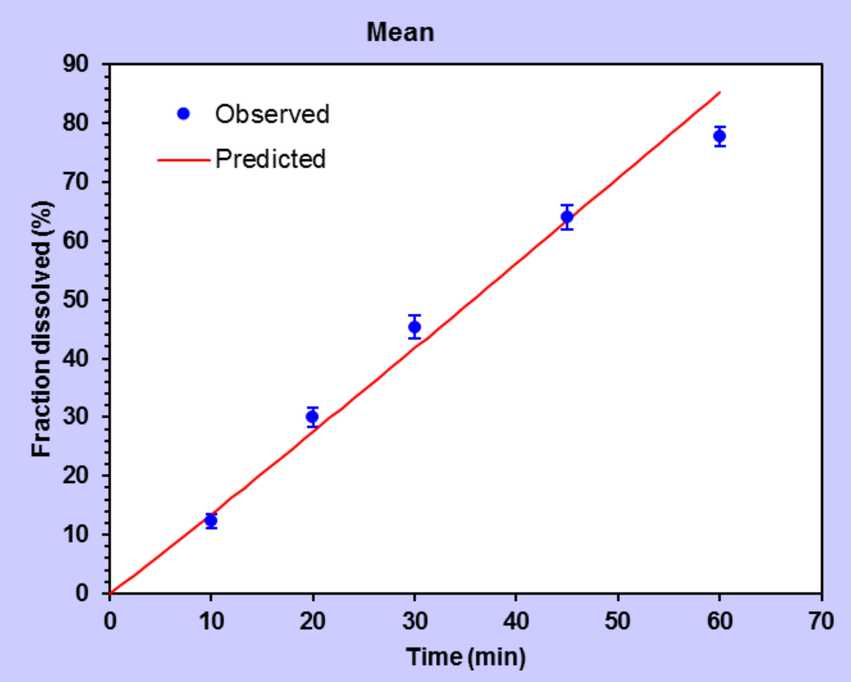

Supplementary materials – Model fitting summary – Polyglykol® 8000 P (modelling up to app. 75 % of carvedilol released)

Graphical abstract of model fit presented as the fraction % of released carvedilol per tested tablet:

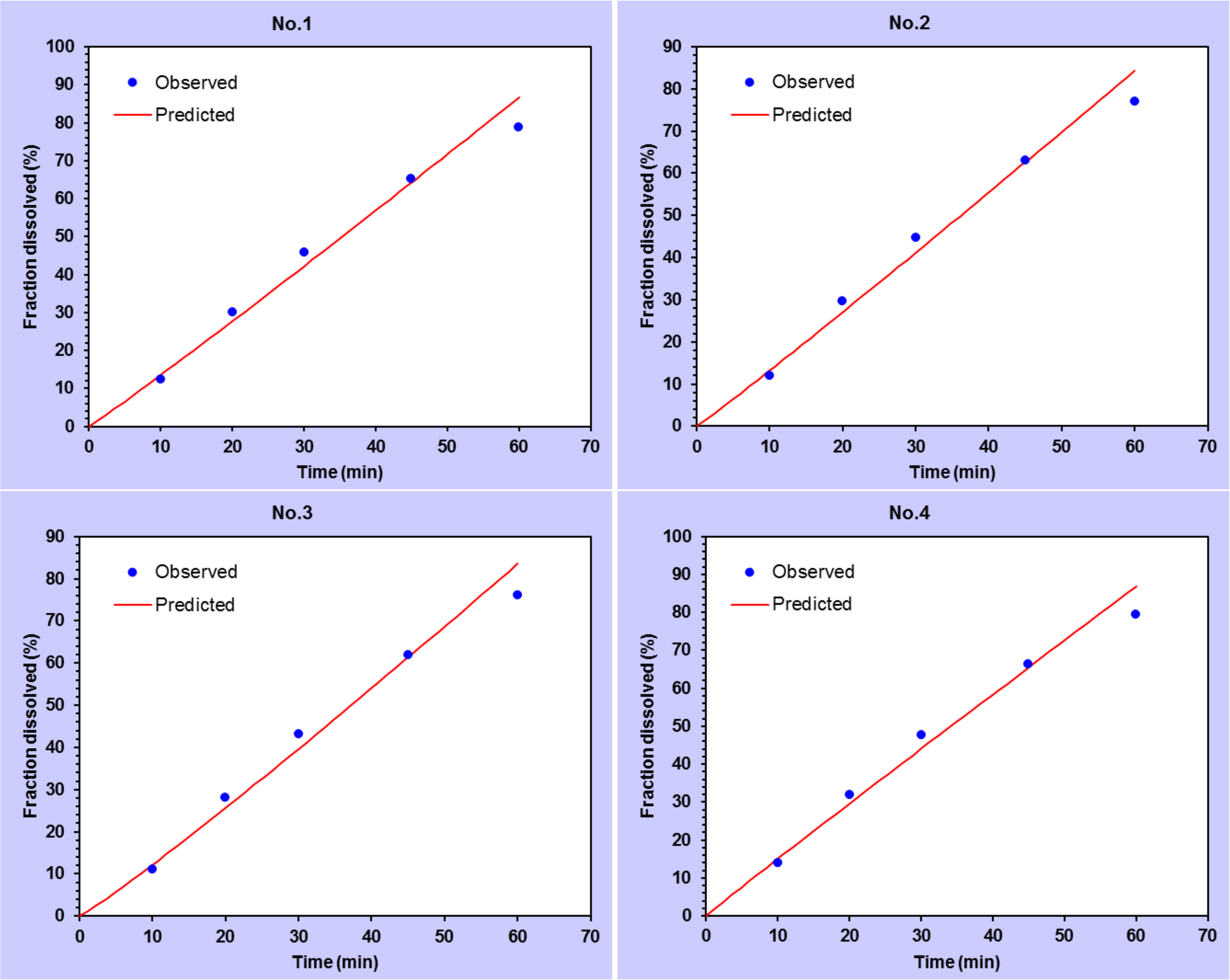

Supplementary materials – Model fitting summary – Polyglykol® 8000 P (modelling up to app. 75 % of carvedilol released)

Model: **Korsmeyer–Peppas with T_{lag}**

Model equation: $F = k_{KP} \cdot \left(t - T_{lag}\right)^n$

Fitted model parameters per tested tablet (N = 4) with statistics – mean, standard deviation (SD), and relative standard deviation expressed in % (RSD%) (output from DDSolver):

Parameter	No.1	No.2	No.3	No.4	Mean	SD	RSD(%)
k_{KP}	2.862	2.813	2.436	3.486	2.899	0.435	15.014
n	0.838	0.835	0.868	0.789	0.833	0.033	3.905
T_{lag}	4.000	4.000	4.000	4.000	4.000	0.000	0.000

Number of dissolution data points (N), degrees of freedom (df), and selected goodness of fit criteria – Pearson correlation coefficient (R), coefficient of determination (R^2), adjusted coefficient of determination ($R^2_{adjusted}$), and residual sum of squares (RSS) (manual calculation in MS Excel):

Parameter	No.1	No.2	No.3	No.4
N	5	5	5	5
df	2	2	2	2
R	0.996634054	0.997198956	0.997151664	0.997072588
R^2	0.993279438	0.994405758	0.994311442	0.994153747
$R^2_{adjusted}$	0.986558877	0.988811516	0.988622883	0.988307493
RSS	27.28154685	22.72210696	23.59764354	22.4334579

Graphical abstract of model fit presented as mean ± 1 SD of the fraction % of released carvedilol:

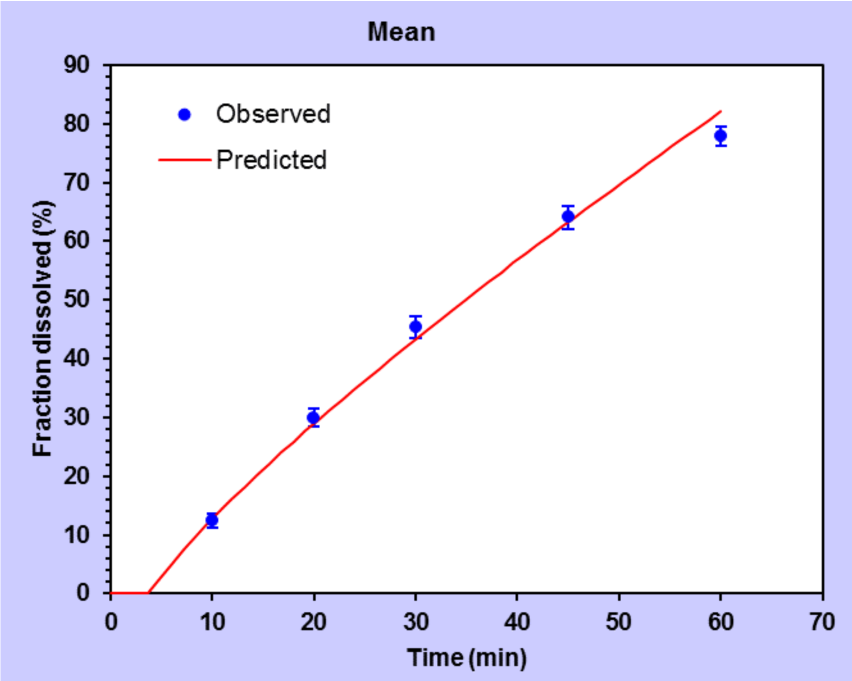

Supplementary materials – Model fitting summary – Polyglykol® 8000 P (modelling up to app. 75 % of carvedilol released)

Graphical abstract of model fit presented as the fraction % of released carvedilol per tested tablet:

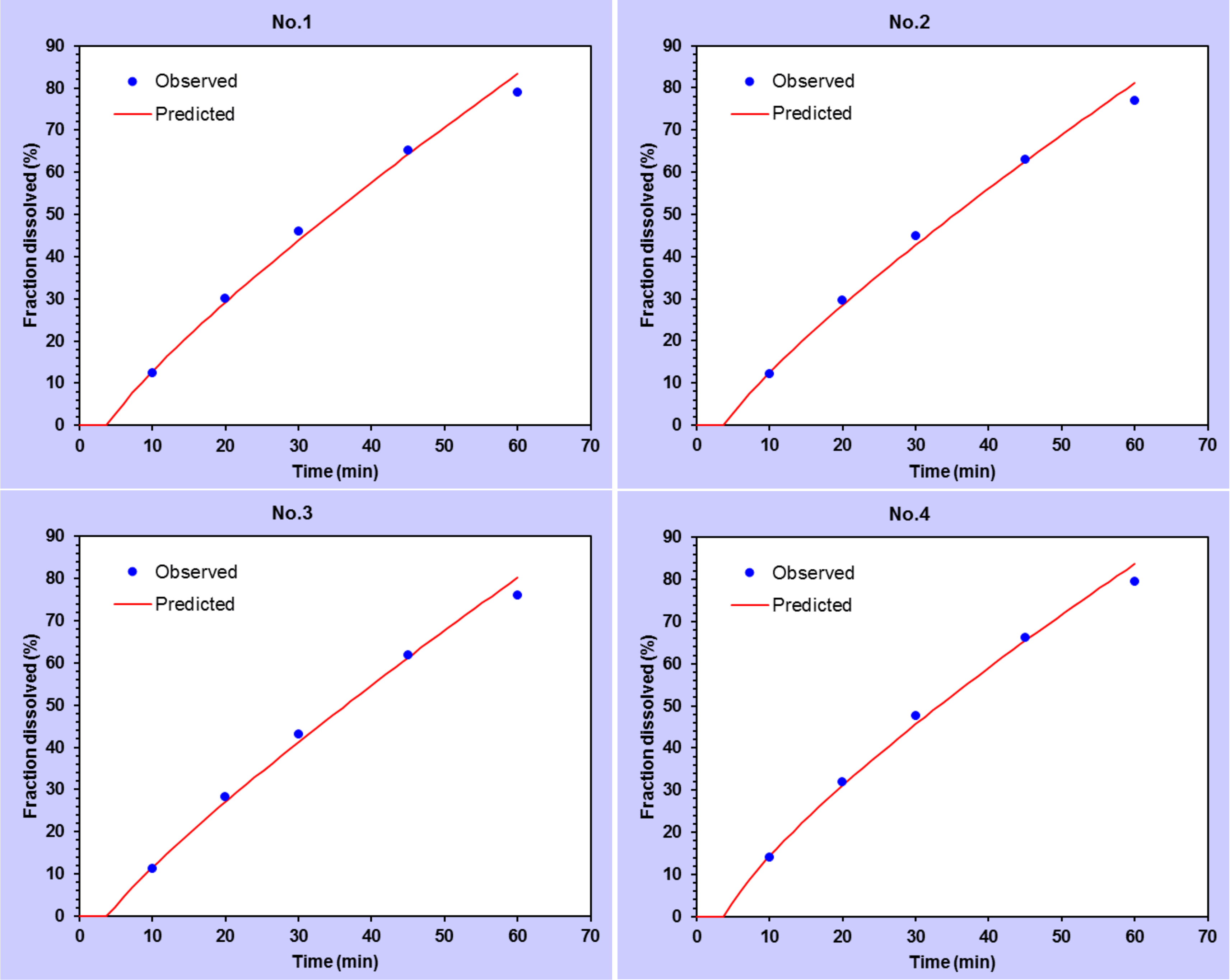

Supplementary materials – Model fitting summary – Polyglykol® 8000 P (modelling up to app. 75 % of carvedilol released)

Model: **Korsmeyer–Peppas with F_0**

Model equation: $F = F_0 + k_{KP} \cdot t^n$

Fitted model parameters per tested tablet (N = 4) with statistics – mean, standard deviation (SD), and relative standard deviation expressed in % (RSD%) (output from DDSolver):

Parameter	No.1	No.2	No.3	No.4	Mean	SD	RSD(%)
k_{KP}	0.458	0.453	0.378	0.588	0.469	0.087	18.627
n	1.280	1.276	1.319	1.216	1.273	0.042	3.332
F_0	4.959	4.839	4.439	5.559	4.949	0.464	9.366

Number of dissolution data points (N), degrees of freedom (df), and selected goodness of fit criteria – Pearson correlation coefficient (R), coefficient of determination (R^2), adjusted coefficient of determination ($R^2_{adjusted}$), and residual sum of squares (RSS) (manual calculation in MS Excel):

Parameter	No.1	No.2	No.3	No.4
N	5	5	5	5
df	2	2	2	2
R	0.982015933	0.983100968	0.983095659	0.982938812
R^2	0.964355293	0.966487514	0.966477076	0.966168708
$R^2_{adjusted}$	0.928710585	0.932975027	0.932954151	0.932337416
RSS	205.3348386	187.3076475	194.5931424	180.7163271

Graphical abstract of model fit presented as mean ± 1 SD of the fraction % of released carvedilol:

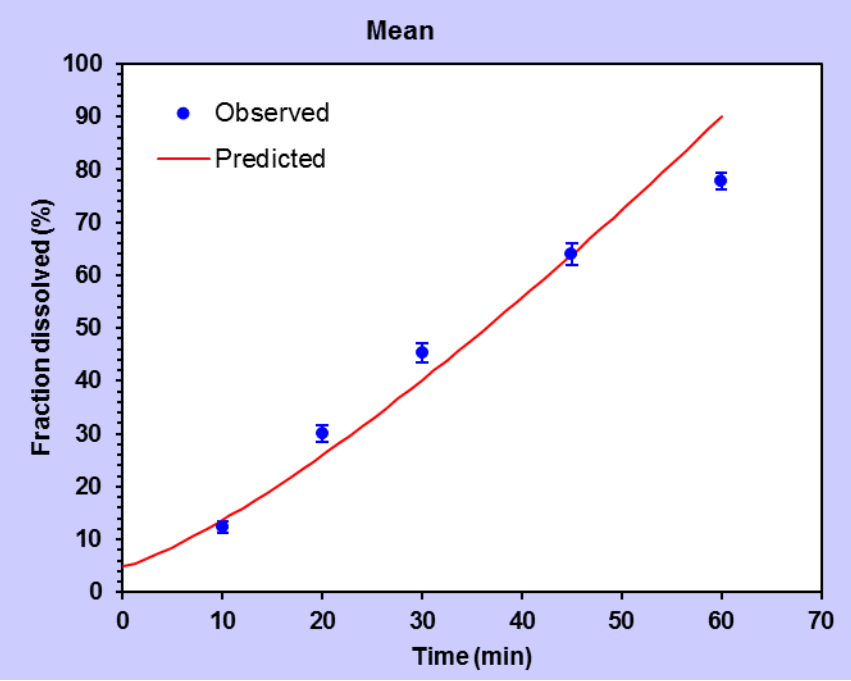

Supplementary materials – Model fitting summary – Polyglykol® 8000 P (modelling up to app. 75 % of carvedilol released)

Graphical abstract of model fit presented as the fraction % of released carvedilol per tested tablet:

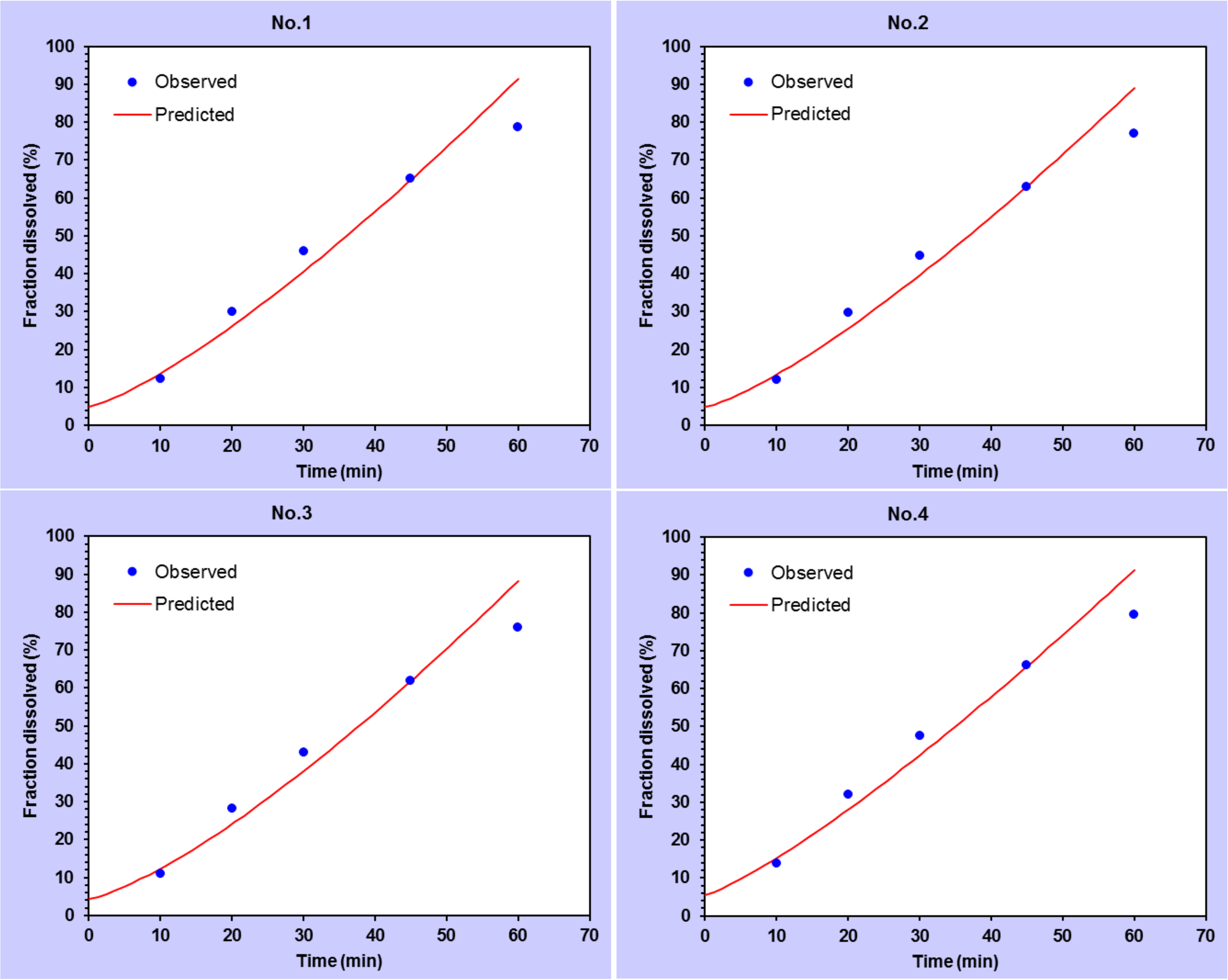

Supplementary materials – Model fitting summary – Polyglykol® 8000 P (modelling up to app. 75 % of carvedilol released)

Model: **Hixson–Crowell**

Model equation: $F = 100 \cdot [1 - (1 - k_{HC} \cdot t)^3]$

Fitted model parameters per tested tablet (N = 4) with statistics – mean, standard deviation (SD), and relative standard deviation expressed in % (RSD%) (output from DDSolver):

Parameter	No.1	No.2	No.3	No.4	Mean	SD	RSD(%)
k_{HC}	0.007	0.006	0.006	0.007	0.006	0.000	4.298

Number of dissolution data points (N), degrees of freedom (df), and selected goodness of fit criteria – Pearson correlation coefficient (R), coefficient of determination (R^2), adjusted coefficient of determination ($R^2_{adjusted}$), and residual sum of squares (RSS) (manual calculation in MS Excel):

Parameter	No.1	No.2	No.3	No.4
N	5	5	5	5
df	4	4	4	4
R	0.999942125	0.999971852	0.999992032	0.999935258
R^2	0.999884254	0.999943705	0.999984064	0.999870519
$R^2_{adjusted}$	0.999884254	0.999943705	0.999984064	0.999870519
RSS	5	5	5	5

Graphical abstract of model fit presented as mean ± 1 SD of the fraction % of released carvedilol:

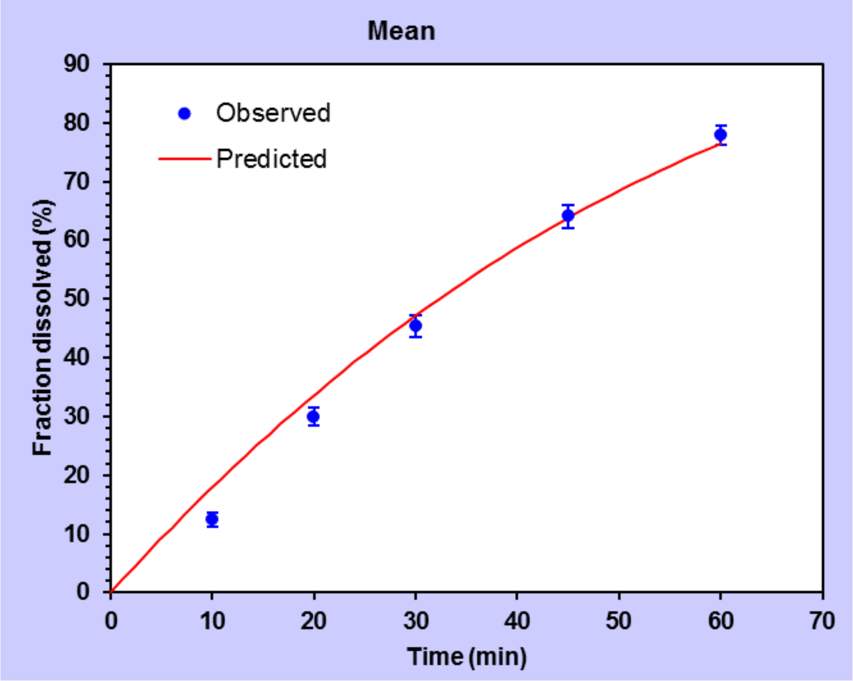

Supplementary materials – Model fitting summary – Polyglykol® 8000 P (modelling up to app. 75 % of carvedilol released)

Graphical abstract of model fit presented as the fraction % of released carvedilol per tested tablet:

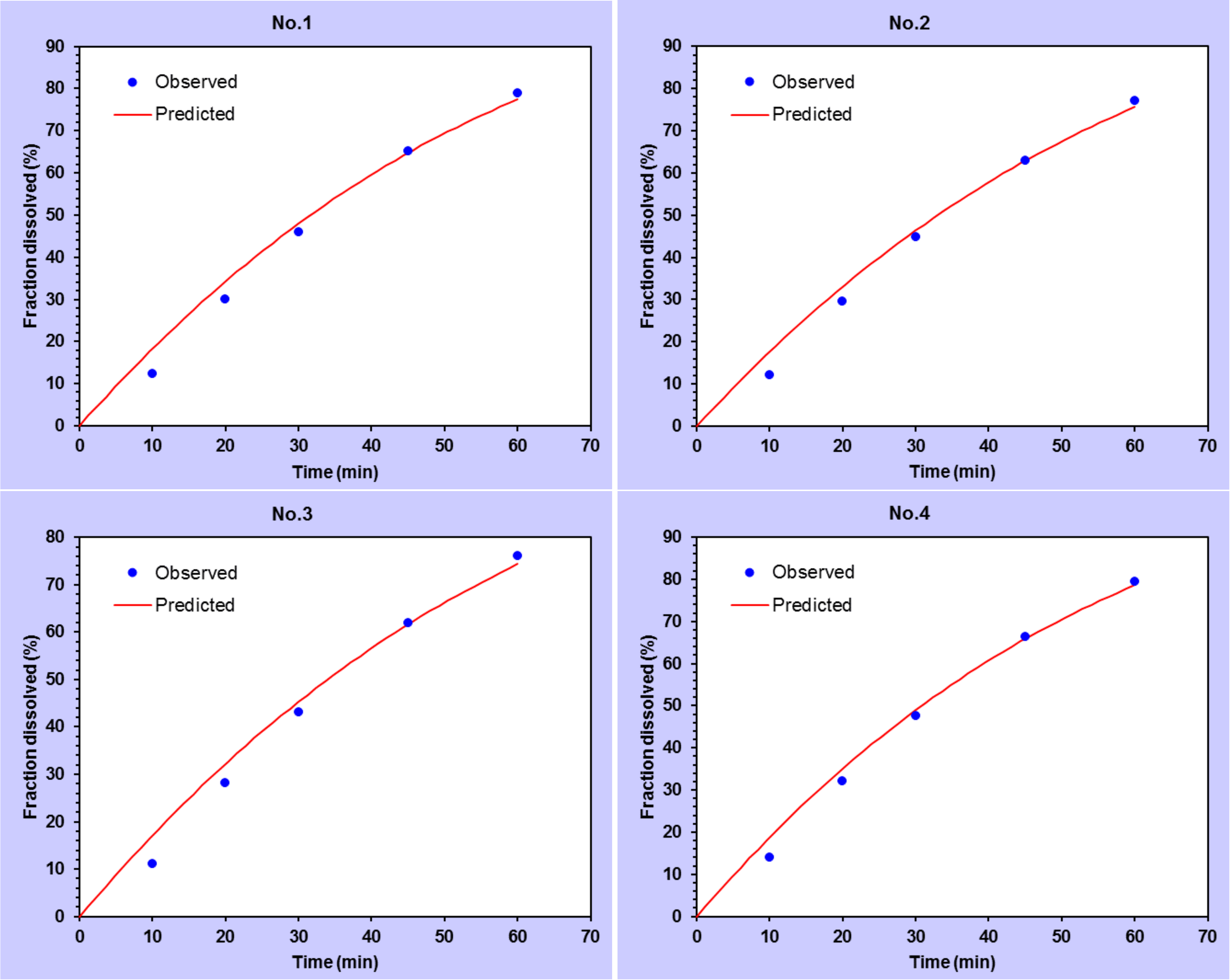

Supplementary materials – Model fitting summary – Polyglykol® 8000 P (modelling up to app. 75 % of carvedilol released)

Model: **Hixson–Crowell with T_{lag}**

Model equation: $F = 100 \cdot \left\{1 - \left[1 - k_{HC} \cdot (t - T_{lag})\right]^3\right\}$

Fitted model parameters per tested tablet (N = 4) with statistics – mean, standard deviation (SD), and relative standard deviation expressed in % (RSD%) (output from DDSolver):

Parameter	No.1	No.2	No.3	No.4	Mean	SD	RSD(%)
k_{HC}	0.007	0.007	0.007	0.007	0.007	0.000	3.287
T_{lag}	4.260	3.981	4.589	3.304	4.034	0.546	13.539

Number of dissolution data points (N), degrees of freedom (df), and selected goodness of fit criteria – Pearson correlation coefficient (R), coefficient of determination (R^2), adjusted coefficient of determination ($R^2_{adjusted}$), and residual sum of squares (RSS) (manual calculation in MS Excel):

Parameter	No.1	No.2	No.3	No.4
N	5	5	5	5
df	3	3	3	3
R	0.999933272	0.999987459	0.999936479	0.99999009
R^2	0.999866548	0.999974918	0.999872961	0.999980181
$R^2_{adjusted}$	0.999822063	0.999966557	0.999830615	0.999973575
RSS	0.389387313	0.074174981	0.377964955	0.056115804

Graphical abstract of model fit presented as mean ± 1 SD of the fraction % of released carvedilol:

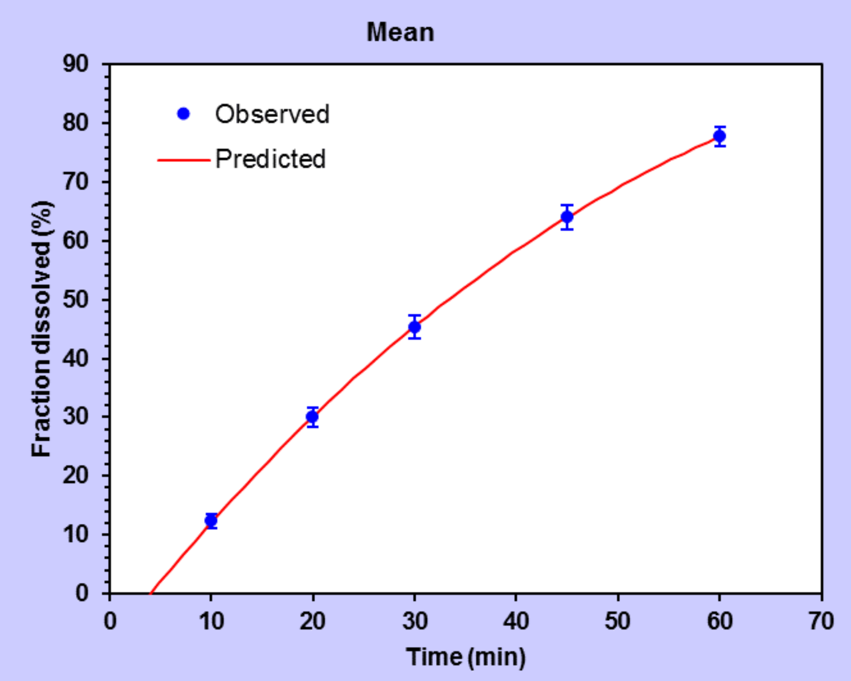

Supplementary materials – Model fitting summary – Polyglykol® 8000 P (modelling up to app. 75 % of carvedilol released)

Graphical abstract of model fit presented as the fraction % of released carvedilol per tested tablet:

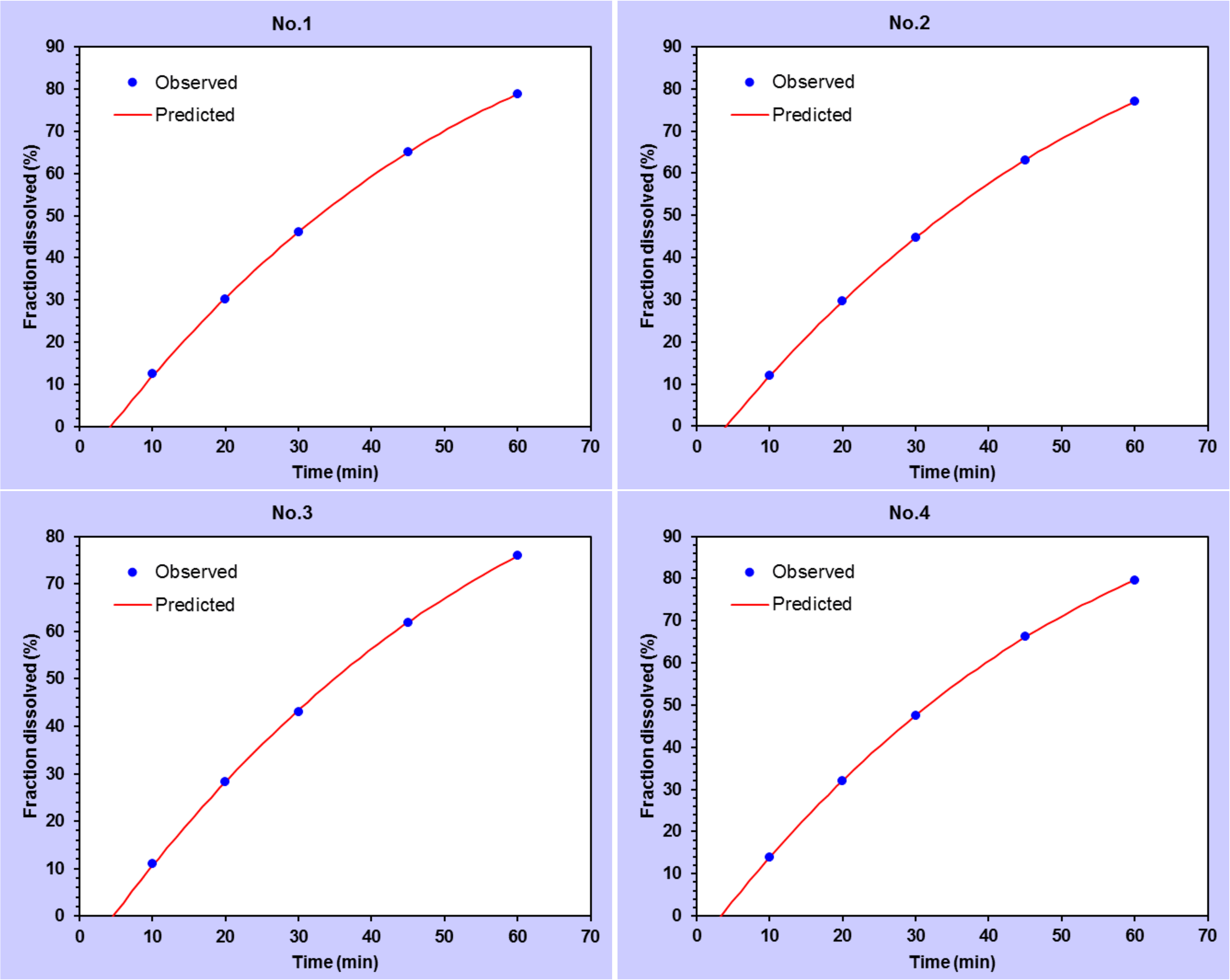

Supplementary materials – Model fitting summary – Polyglykol® 8000 P (modelling up to app. 75 % of carvedilol released)

Model: **Hopfenberg**

Model equation: $F = 100 \cdot [1 - (1 - k_{HB} \cdot t)^n]$

Fitted model parameters per tested tablet (N = 4) with statistics – mean, standard deviation (SD), and relative standard deviation expressed in % (RSD%) (output from DDSolver):

Parameter	No.1	No.2	No.3	No.4	Mean	SD	RSD(%)
k_{HB}	0.009	0.009	0.008	0.009	0.009	0.000	3.909
n	2.000	2.000	2.000	2.000	2.000	0.000	0.000

Number of dissolution data points (N), degrees of freedom (df), and selected goodness of fit criteria – Pearson correlation coefficient (R), coefficient of determination (R^2), adjusted coefficient of determination ($R^2_{adjusted}$), and residual sum of squares (RSS) (manual calculation in MS Excel):

Parameter	No.1	No.2	No.3	No.4
N	5	5	5	5
df	3	3	3	3
R	0.999445447	0.999452843	0.999642473	0.999260317
R^2	0.998891201	0.998905985	0.999285075	0.998521181
$R^2_{adjusted}$	0.998521602	0.998541313	0.999046766	0.998028241
RSS	28.78390666	22.94206244	32.3867935	15.00106968

Graphical abstract of model fit presented as mean ± 1 SD of the fraction % of released carvedilol:

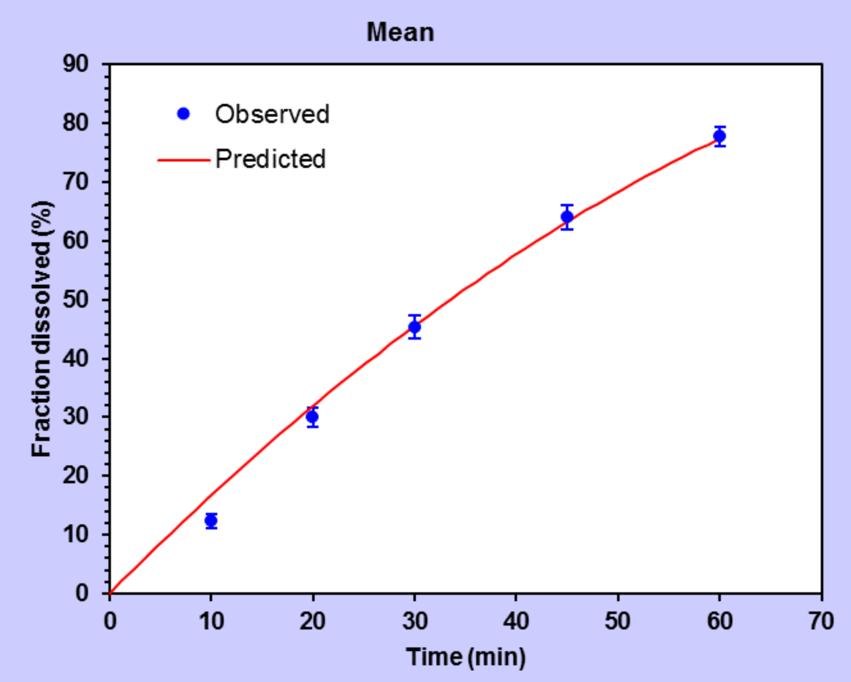

Supplementary materials – Model fitting summary – Polyglykol® 8000 P (modelling up to app. 75 % of carvedilol released)

Graphical abstract of model fit presented as the fraction % of released carvedilol per tested tablet:

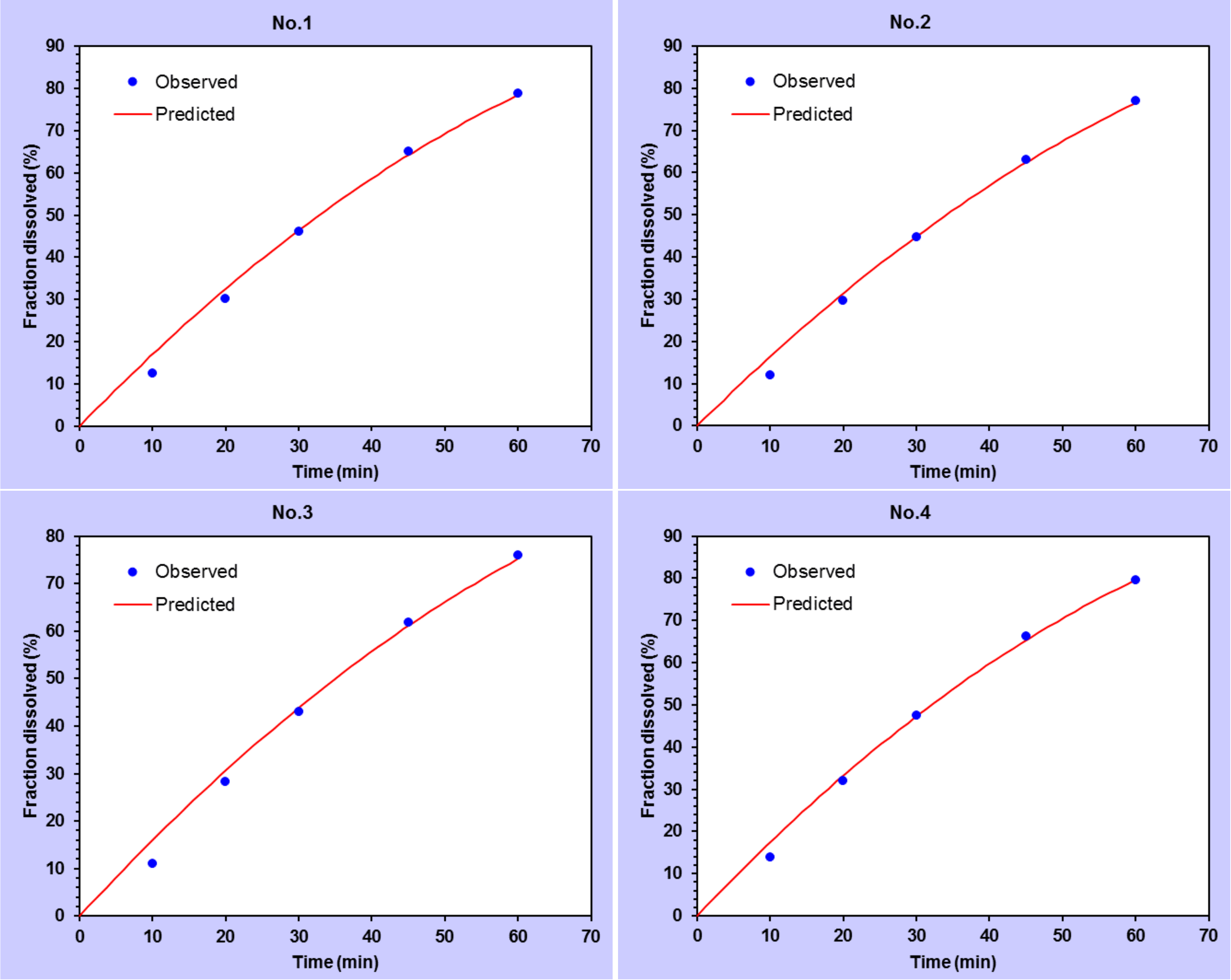

Supplementary materials – Model fitting summary – Polyglykol® 8000 P (modelling up to app. 75 % of carvedilol released)

Model: **Hopfenberg with T_{lag}**

Model equation: $F = 100 \cdot \{1 - [1 - k_{HB} \cdot (t - T_{lag})]^n\}$

Fitted model parameters per tested tablet (N = 4) with statistics – mean, standard deviation (SD), and relative standard deviation expressed in % (RSD%) (output from DDSolver):

Parameter	No.1	No.2	No.3	No.4	Mean	SD	RSD(%)
k_{HB}	0.007	0.007	0.007	0.007	0.007	0.000	3.287
n	3.000	3.000	3.000	3.000	3.000	0.000	0.000
T_{lag}	4.260	3.981	4.589	3.304	4.034	0.546	13.539

Number of dissolution data points (N), degrees of freedom (df), and selected goodness of fit criteria – Pearson correlation coefficient (R), coefficient of determination (R^2), adjusted coefficient of determination ($R^2_{adjusted}$), and residual sum of squares (RSS) (manual calculation in MS Excel):

Parameter	No.1	No.2	No.3	No.4
N	5	5	5	5
df	2	2	2	2
R	0.999933272	0.999987459	0.999936479	0.99999009
R^2	0.999866548	0.999974918	0.999872961	0.999980181
$R^2_{adjusted}$	0.999733095	0.999949836	0.999745923	0.999960362
RSS	0.389387313	0.074174981	0.377964955	0.056115804

Graphical abstract of model fit presented as mean ± 1 SD of the fraction % of released carvedilol:

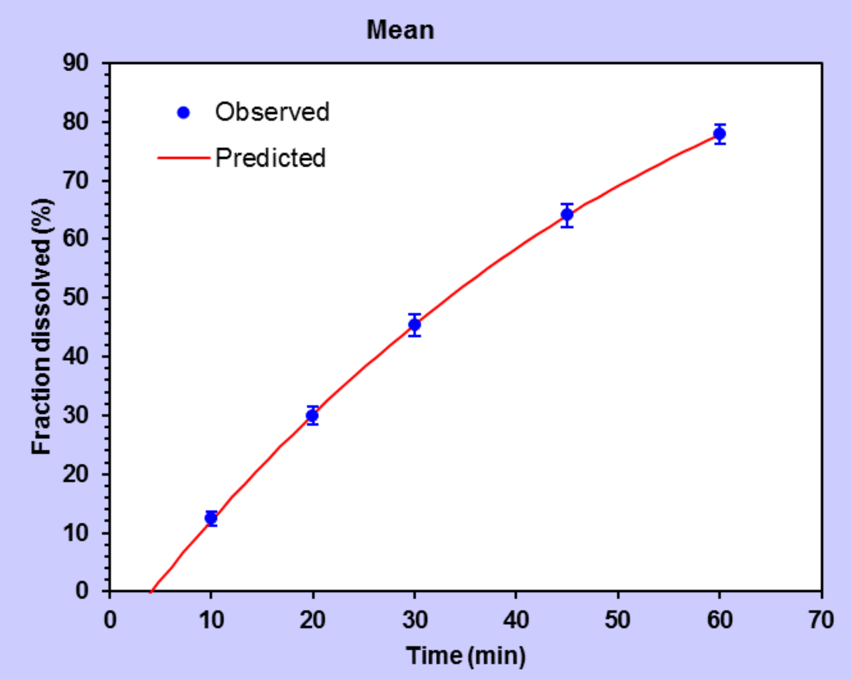

Supplementary materials – Model fitting summary – Polyglykol® 8000 P (modelling up to app. 75 % of carvedilol released)

Graphical abstract of model fit presented as the fraction % of released carvedilol per tested tablet:

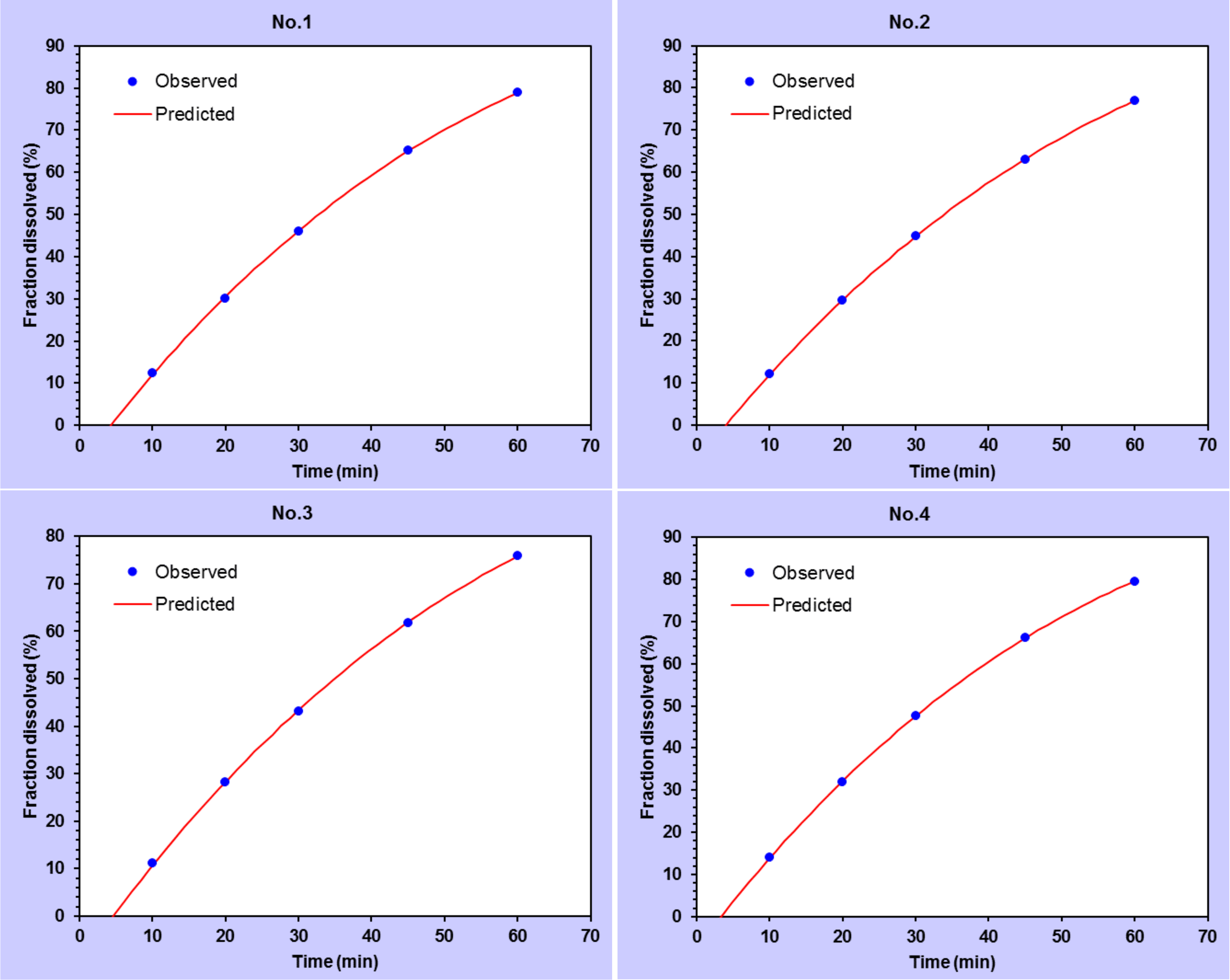

Supplementary materials – Model fitting summary – Polyglykol® 8000 P (modelling up to app. 75 % of carvedilol released)

Model: **Baker–Lonsdale**

Model equation: $\frac{3}{2}\cdot\left[1-\left(1-\frac{F}{100}\right)^{\frac{2}{3}}\right]-\frac{F}{100}=k_{BL}\cdot t$

Fitted model parameters per tested tablet (N = 4) with statistics – mean, standard deviation (SD), and relative standard deviation expressed in % (RSD%) (output from DDSolver):

Parameter	No.1	No.2	No.3	No.4	Mean	SD	RSD(%)
k_{BL}	0.004	0.003	0.003	0.004	0.003	0.000	6.159

Number of dissolution data points (N), degrees of freedom (df), and selected goodness of fit criteria – Pearson correlation coefficient (R), coefficient of determination (R^2), adjusted coefficient of determination ($R^2_{adjusted}$), and residual sum of squares (RSS) (manual calculation in MS Excel):

Parameter	No.1	No.2	No.3	No.4
N	5	5	5	5
df	4	4	4	4
R	0.996222354	0.996652455	0.99595334	0.996968771
R^2	0.992458978	0.993316117	0.991923055	0.993946731
$R^2_{adjusted}$	0.992458978	0.993316117	0.991923055	0.993946731
RSS	2043.861843	1903.17998	1992.924625	1858.859436

Graphical abstract of model fit presented as mean ± 1 SD of the fraction % of released carvedilol:

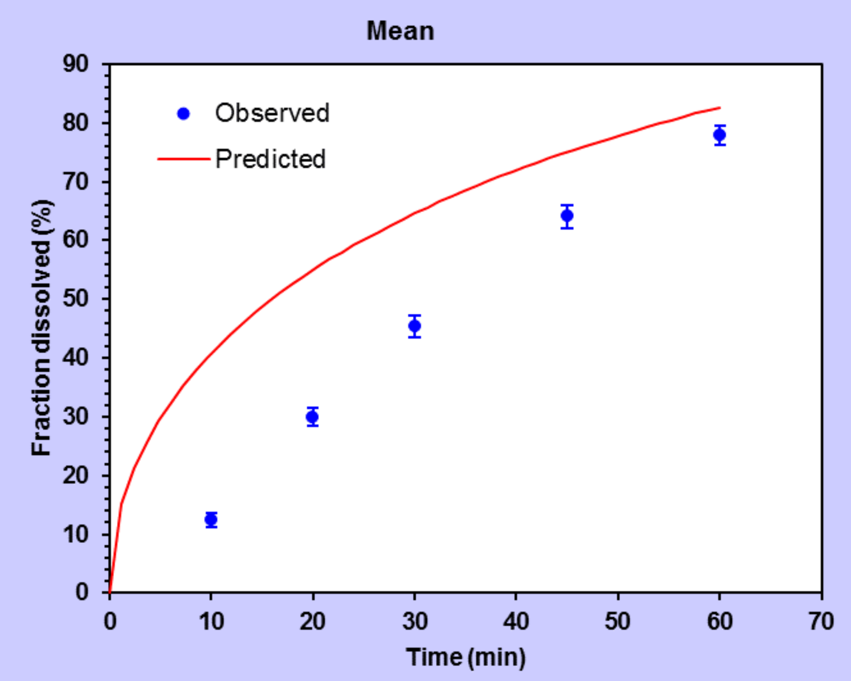

Supplementary materials – Model fitting summary – Polyglykol® 8000 P (modelling up to app. 75 % of carvedilol released)

Graphical abstract of model fit presented as the fraction % of released carvedilol per tested tablet:

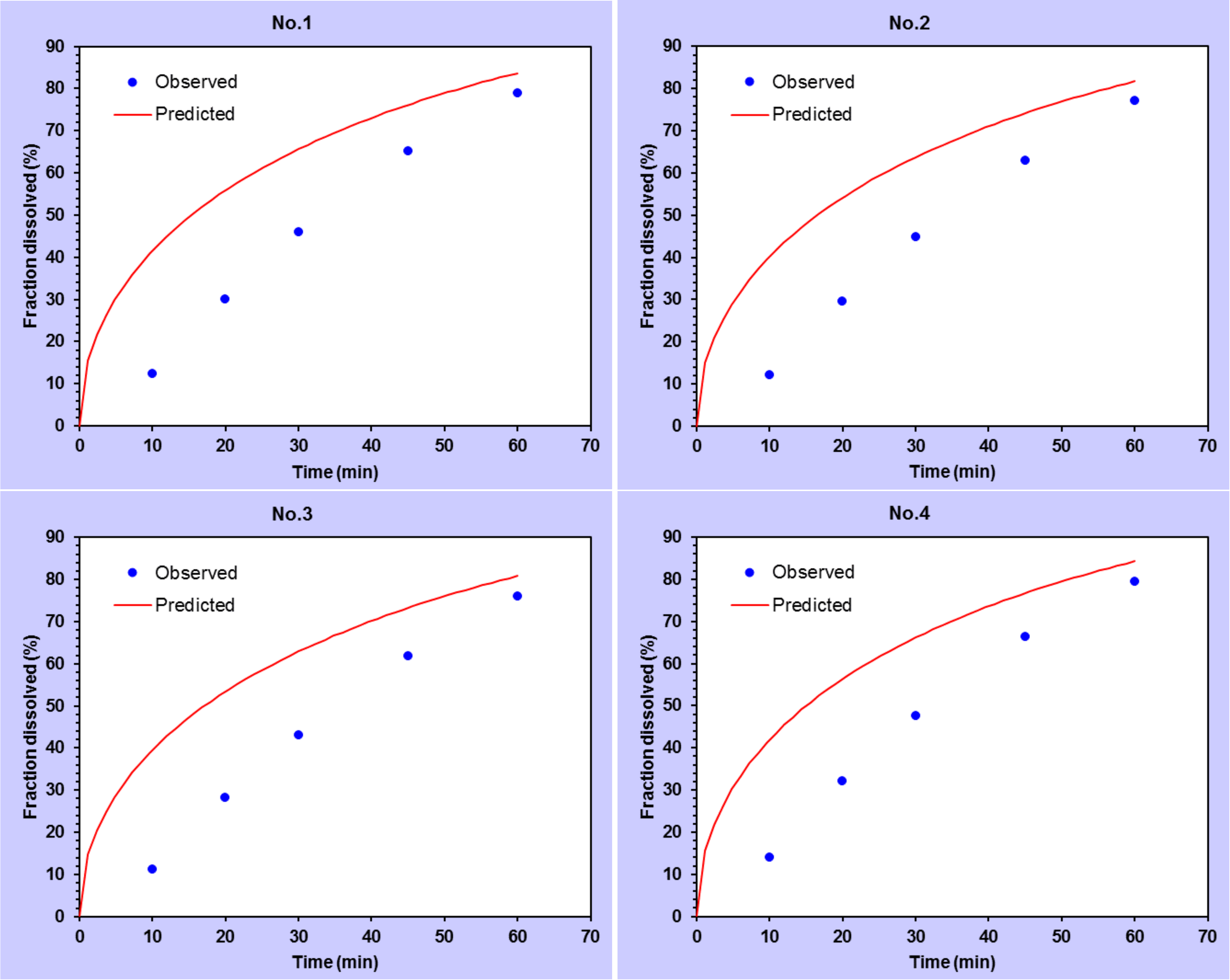

Supplementary materials – Model fitting summary – Polyglykol® 8000 P (modelling up to app. 75 % of carvedilol released)

Model: **Baker–Lonsdale with T_{lag}**

Model equation: $\frac{3}{2}\cdot\left[1-\left(1-\frac{F}{100}\right)^{\frac{2}{3}}\right]-\frac{F}{100}=k_{BL}\cdot(t-T_{lag})$

Fitted model parameters per tested tablet (N = 4) with statistics – mean, standard deviation (SD), and relative standard deviation expressed in % (RSD%) (output from DDSolver):

Parameter	No.1	No.2	No.3	No.4	Mean	SD	RSD(%)
k_{BL}	0.004	0.004	0.004	0.004	0.004	0.000	6.159
T_{lag}	16.867	16.799	17.201	16.273	16.785	0.384	2.287

Number of dissolution data points (N), degrees of freedom (df), and selected goodness of fit criteria – Pearson correlation coefficient (R), coefficient of determination (R^2), adjusted coefficient of determination ($R^2_{adjusted}$), and residual sum of squares (RSS) (manual calculation in MS Excel):

Parameter	No.1	No.2	No.3	No.4
N	5	5	5	5
df	3	3	3	3
R	0.991605342	0.991513544	0.991476062	0.990622029
R^2	0.983281154	0.983099108	0.983024781	0.981332005
$R^2_{adjusted}$	0.977708205	0.977465478	0.977366375	0.97510934
RSS	185.9543118	180.0157755	163.7562148	219.4000566

Graphical abstract of model fit presented as mean ± 1 SD of the fraction % of released carvedilol:

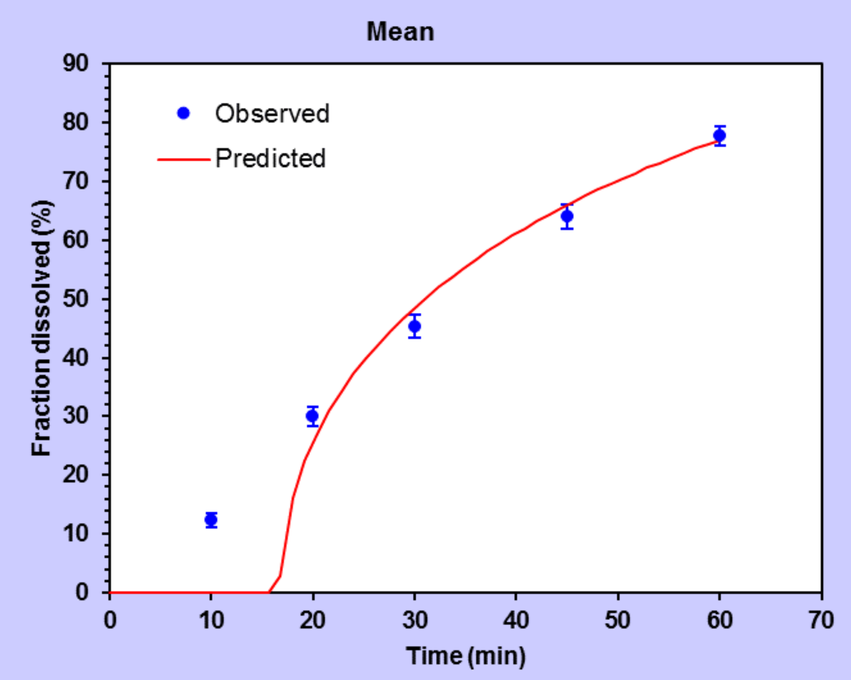

Supplementary materials – Model fitting summary – Polyglykol® 8000 P (modelling up to app. 75 % of carvedilol released)

Graphical abstract of model fit presented as the fraction % of released carvedilol per tested tablet:

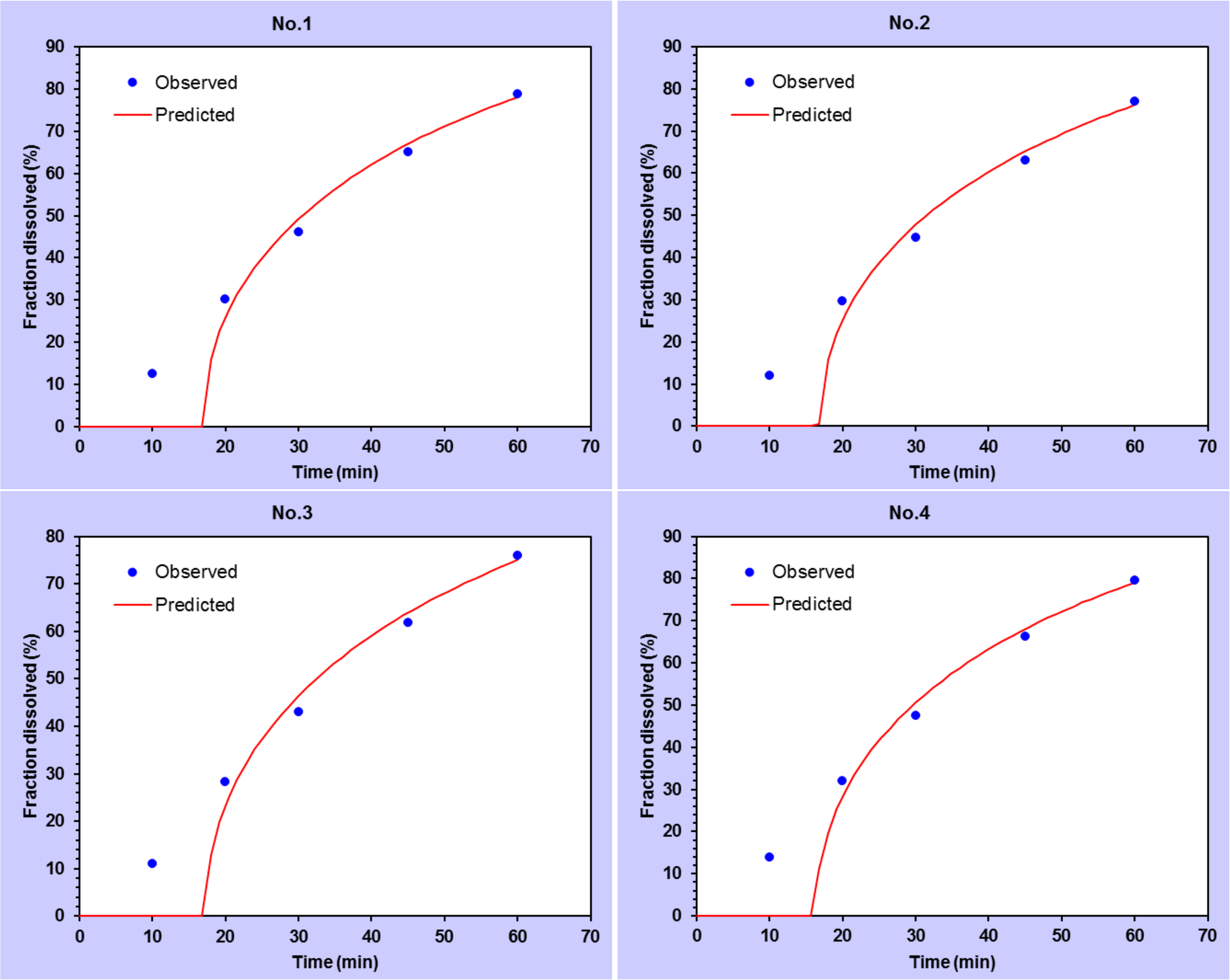

Supplementary materials – Model fitting summary – Polyglykol® 8000 P (modelling up to app. 75 % of carvedilol released)

Model: **Makoid–Banakar**

Model equation: $F = k_{MB} \cdot t^n \cdot e^{-k \cdot t}$

Fitted model parameters per tested tablet (N = 4) with statistics – mean, standard deviation (SD), and relative standard deviation expressed in % (RSD%) (output from DDSolver):

Parameter	No.1	No.2	No.3	No.4	Mean	SD	RSD(%)
k_{MB}	0.462	0.455	0.368	0.639	0.481	0.114	23.643
n	1.505	1.502	1.559	1.407	1.493	0.063	4.230
k	0.017	0.017	0.018	0.016	0.017	0.001	5.012

Number of dissolution data points (N), degrees of freedom (df), and selected goodness of fit criteria – Pearson correlation coefficient (R), coefficient of determination (R^2), adjusted coefficient of determination ($R^2_{adjusted}$), and residual sum of squares (RSS) (manual calculation in MS Excel):

Parameter	No.1	No.2	No.3	No.4
N	5	5	5	5
df	2	2	2	2
R	0.999882978	0.999619015	0.999660225	0.999876841
R^2	0.999765969	0.999238174	0.999320566	0.999753696
$R^2_{adjusted}$	0.999531938	0.998476349	0.998641131	0.999507392
RSS	0.661982632	2.034533768	1.818919064	0.677288809

Graphical abstract of model fit presented as mean ± 1 SD of the fraction % of released carvedilol:

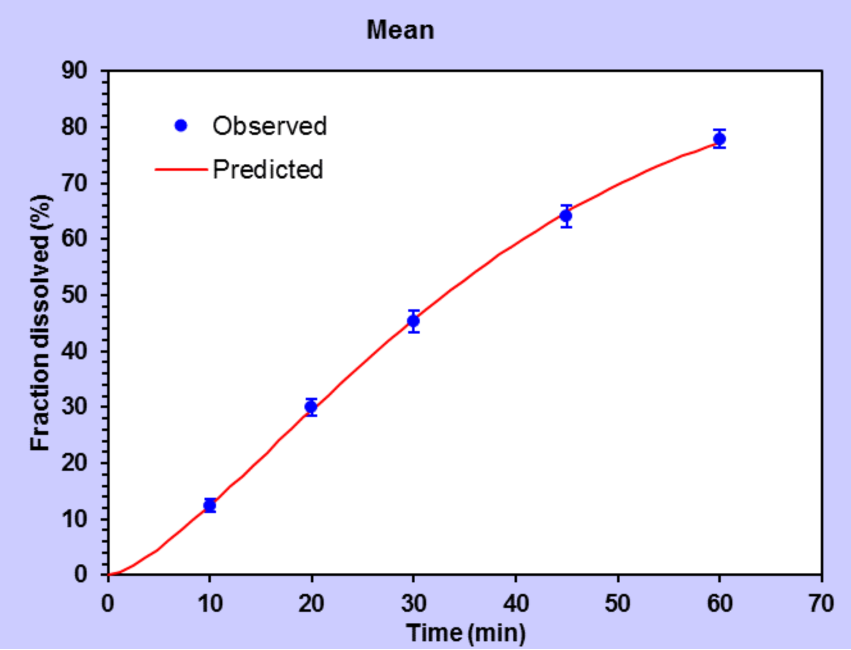

Supplementary materials – Model fitting summary – Polyglykol® 8000 P (modelling up to app. 75 % of carvedilol released)

Graphical abstract of model fit presented as the fraction % of released carvedilol per tested tablet:

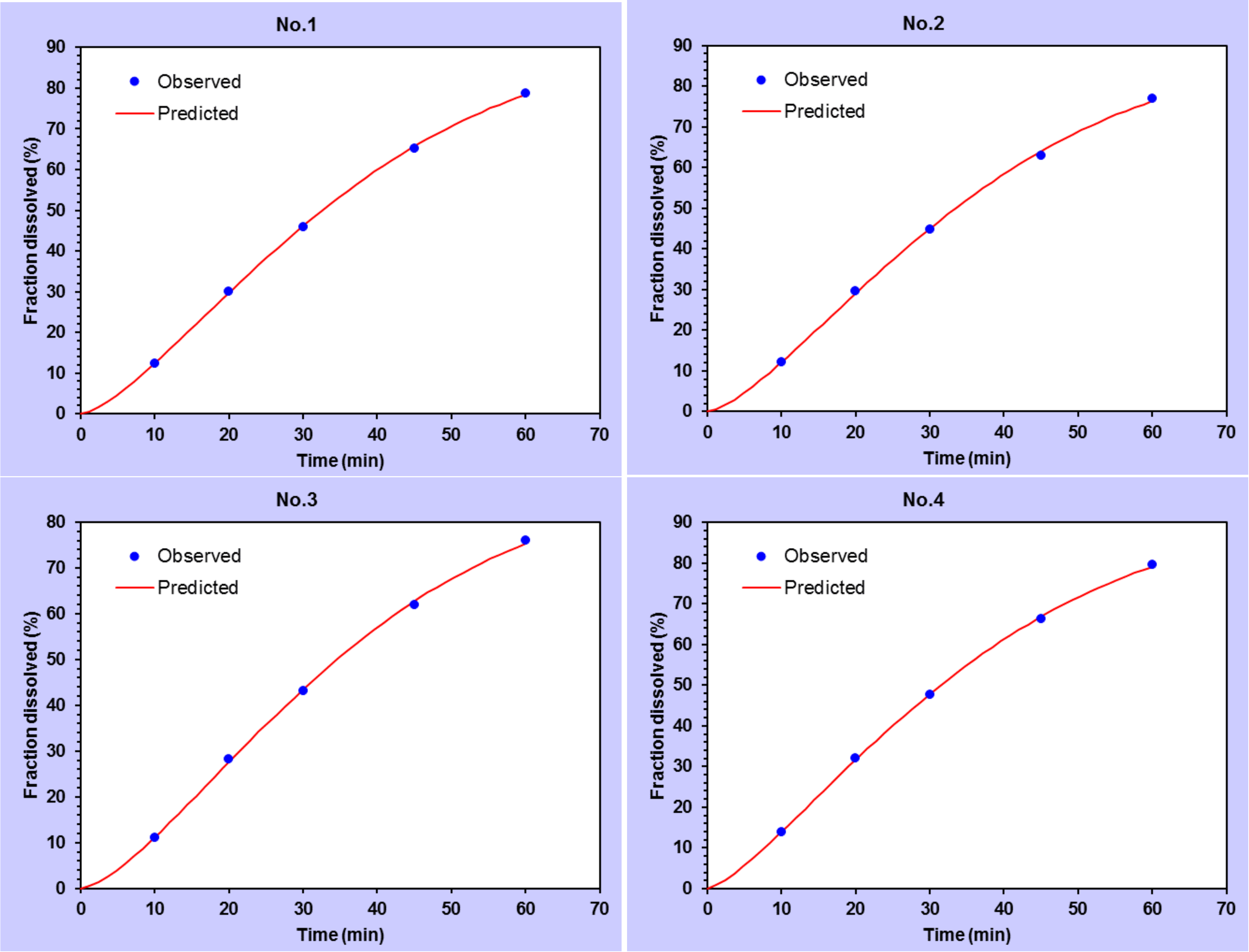

Supplementary materials – Model fitting summary – Polyglykol® 8000 P (modelling up to app. 75 % of carvedilol released)

Model: **Makoid–Banakar with T_{lag}**

Model equation: $F = k_{MB} \cdot (t - T_{lag})^n \cdot e^{-k \cdot (t - T_{lag})}$

Fitted model parameters per tested tablet (N = 4) with statistics – mean, standard deviation (SD), and relative standard deviation expressed in % (RSD%) (output from DDSolver):

Parameter	No.1	No.2	No.3	No.4	Mean	SD	RSD(%)
k_{MB}	2.194	2.150	1.845	2.747	2.234	0.376	16.806
n	0.988	0.987	1.026	0.924	0.981	0.042	4.274
k	0.007	0.007	0.007	0.006	0.007	0.000	6.452
T_{lag}	4.000	4.000	4.000	4.000	4.000	0.000	0.000

Number of dissolution data points (N), degrees of freedom (df), and selected goodness of fit criteria – Pearson correlation coefficient (R), coefficient of determination (R^2), adjusted coefficient of determination ($R^2_{adjusted}$), and residual sum of squares (RSS) (manual calculation in MS Excel):

Parameter	No.1	No.2	No.3	No.4
N	5	5	5	5
df	1	1	1	1
R	0.999904878	0.999995656	0.999992193	0.999921367
R^2	0.999809765	0.999991312	0.999984385	0.999842741
$R^2_{adjusted}$	0.999239059	0.999965248	0.999937541	0.999370963
RSS	0.537963582	0.023202474	0.041778909	0.432106565

Graphical abstract of model fit presented as mean ± 1 SD of the fraction % of released carvedilol:

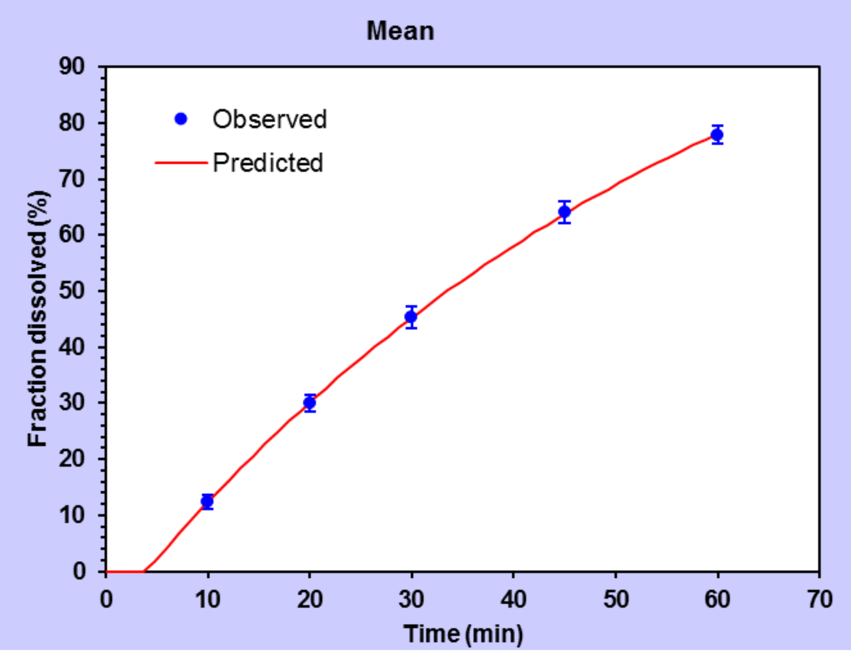

Supplementary materials – Model fitting summary – Polyglykol® 8000 P (modelling up to app. 75 % of carvedilol released)

Graphical abstract of model fit presented as the fraction % of released carvedilol per tested tablet:

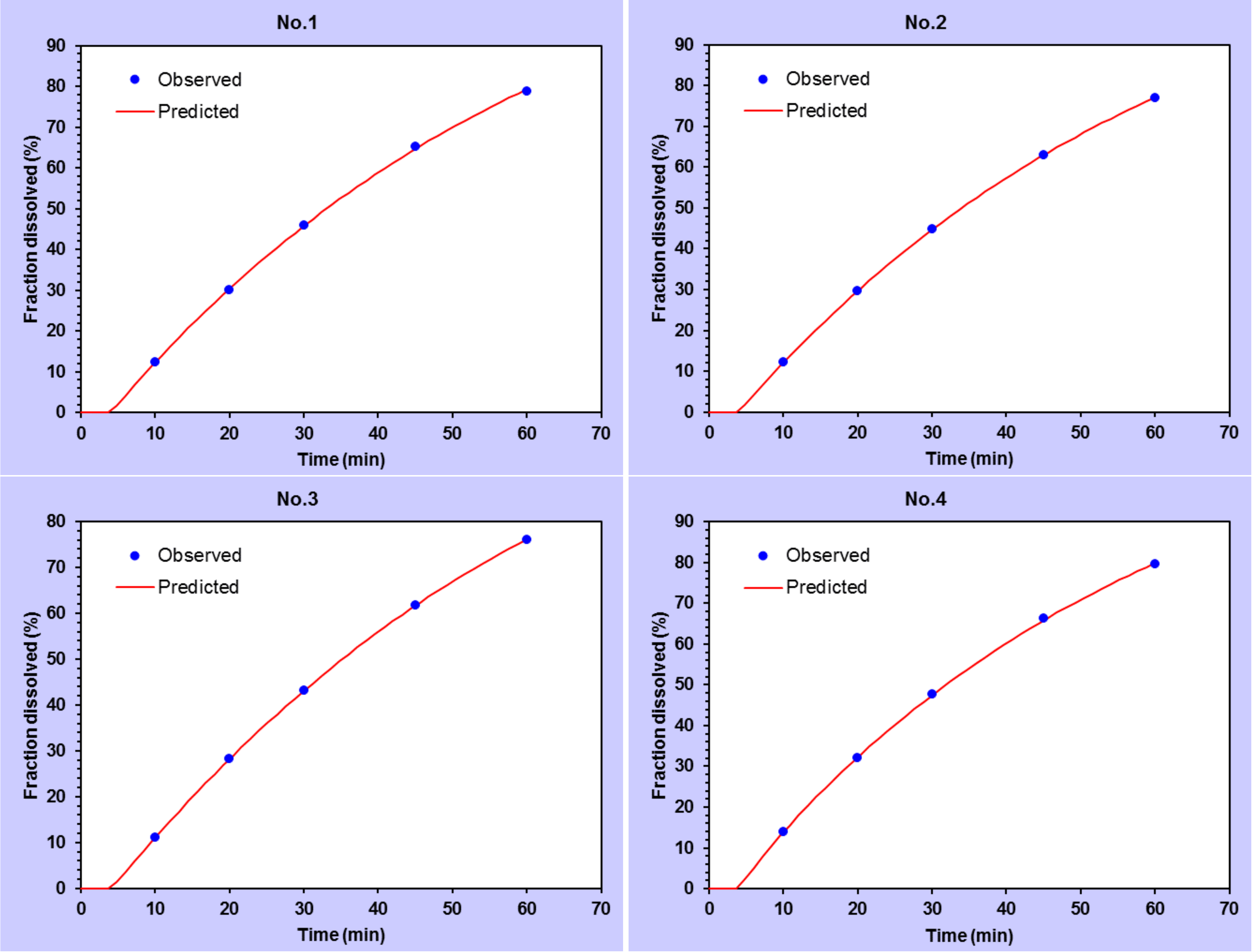

Supplementary materials – Model fitting summary – Polyglykol® 8000 P (modelling up to app. 75 % of carvedilol released)

Model: **Peppas–Sahlin_1**

Model equation: $F = k_1 \cdot t^m + k_2 \cdot t^{2m}$

Fitted model parameters per tested tablet (N = 4) with statistics – mean, standard deviation (SD), and relative standard deviation expressed in % (RSD%) (output from DDSolver):

Parameter	No.1	No.2	No.3	No.4	Mean	SD	RSD(%)
k_1	-0.309	-0.270	-0.922	0.666	-0.209	0.655	-313.791
k_2	2.096	2.035	2.111	1.958	2.050	0.070	3.391
m	0.450	0.450	0.450	0.450	0.450	0.000	0.000

Number of dissolution data points (N), degrees of freedom (df), and selected goodness of fit criteria – Pearson correlation coefficient (R), coefficient of determination (R^2), adjusted coefficient of determination ($R^2_{adjusted}$), and residual sum of squares (RSS) (manual calculation in MS Excel):

Parameter	No.1	No.2	No.3	No.4
N	5	5	5	5
df	2	2	2	2
R	0.994466066	0.995066847	0.9955681	0.994165398
R^2	0.988962757	0.99015803	0.991155842	0.988364839
$R^2_{adjusted}$	0.977925515	0.98031606	0.982311685	0.976729677
RSS	31.78356873	26.78889463	24.10695018	32.63168012

Graphical abstract of model fit presented as mean ± 1 SD of the fraction % of released carvedilol:

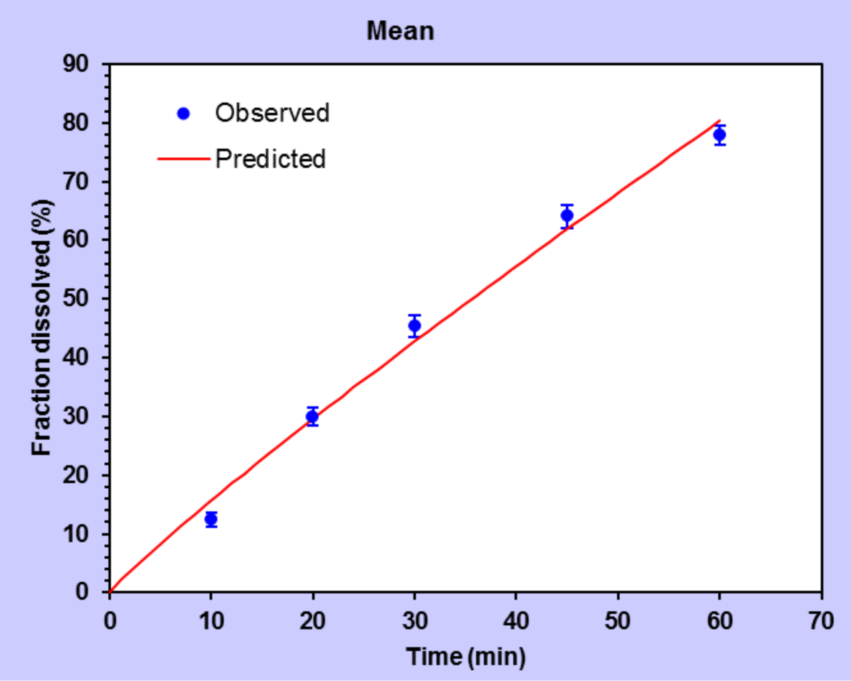

Supplementary materials – Model fitting summary – Polyglykol® 8000 P (modelling up to app. 75 % of carvedilol released)

Graphical abstract of model fit presented as the fraction % of released carvedilol per tested tablet:

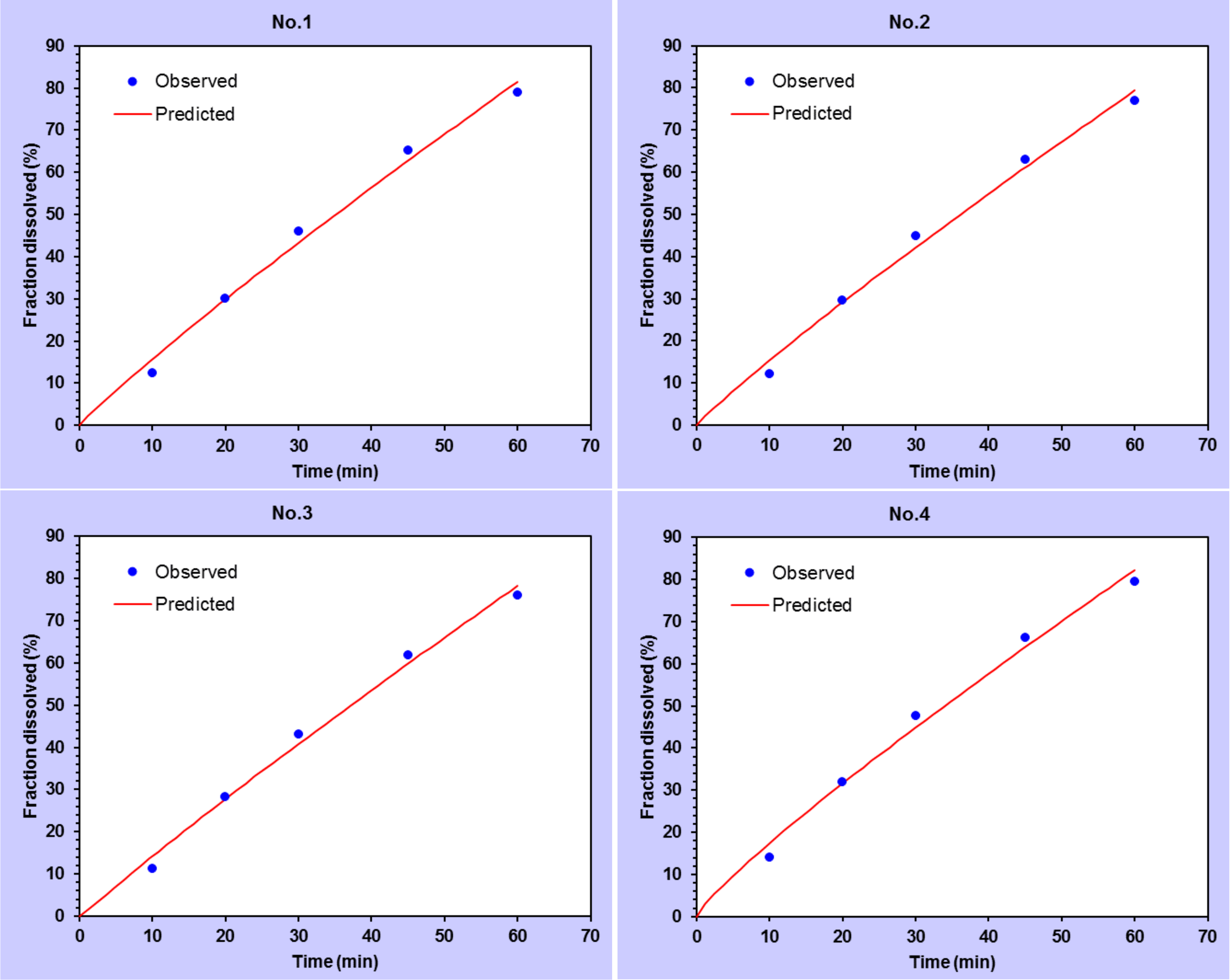

Supplementary materials – Model fitting summary – Polyglykol® 8000 P (modelling up to app. 75 % of carvedilol released)

Model: **Peppas–Sahlin_1 with T_{lag}**

Model equation: $F = k_1 \cdot (t - T_{lag})^m + k_2 \cdot (t - T_{lag})^{2m}$

Fitted model parameters per tested tablet (N = 4) with statistics – mean, standard deviation (SD), and relative standard deviation expressed in % (RSD%) (output from DDSolver):

Parameter	No.1	No.2	No.3	No.4	Mean	SD	RSD(%)
k_1	2.782	2.739	2.018	3.820	2.840	0.742	26.117
k_2	1.707	1.656	1.747	1.554	1.666	0.083	4.995
m	0.450	0.450	0.450	0.450	0.450	0.000	0.000
T_{lag}	4.000	4.000	4.000	4.000	4.000	0.000	0.000

Number of dissolution data points (N), degrees of freedom (df), and selected goodness of fit criteria – Pearson correlation coefficient (R), coefficient of determination (R^2), adjusted coefficient of determination ($R^2_{adjusted}$), and residual sum of squares (RSS) (manual calculation in MS Excel):

Parameter	No.1	No.2	No.3	No.4
N	5	5	5	5
df	1	1	1	1
R	0.996898898	0.997432987	0.997636596	0.996925833
R^2	0.993807412	0.994872564	0.995278778	0.993861116
$R^2_{adjusted}$	0.97522965	0.979490256	0.981115114	0.975444462
RSS	18.16771686	14.26652426	13.12511473	17.58657435

Graphical abstract of model fit presented as mean ± 1 SD of the fraction % of released carvedilol:

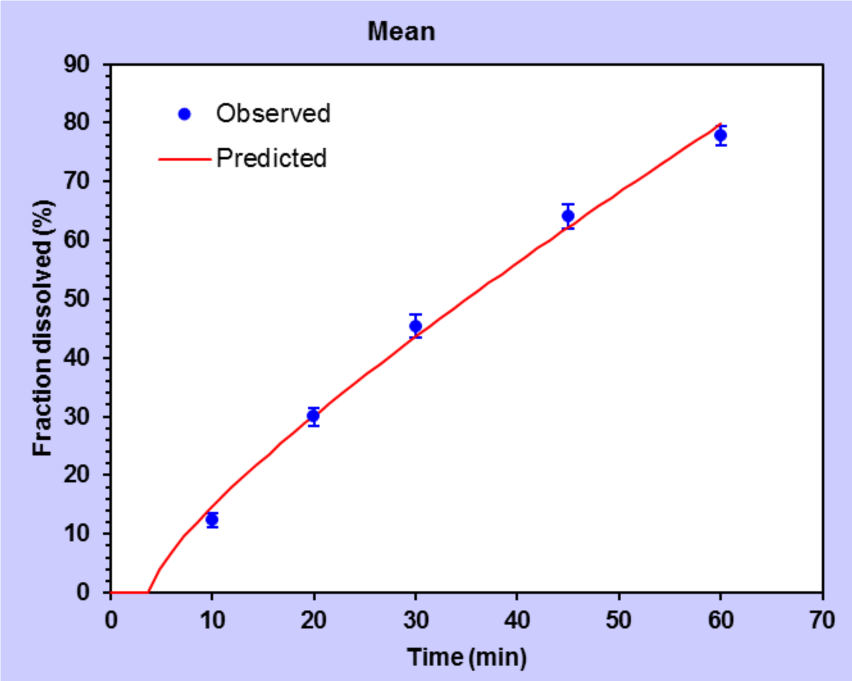

Supplementary materials – Model fitting summary – Polyglykol® 8000 P (modelling up to app. 75 % of carvedilol released)

Graphical abstract of model fit presented as the fraction % of released carvedilol per tested tablet:

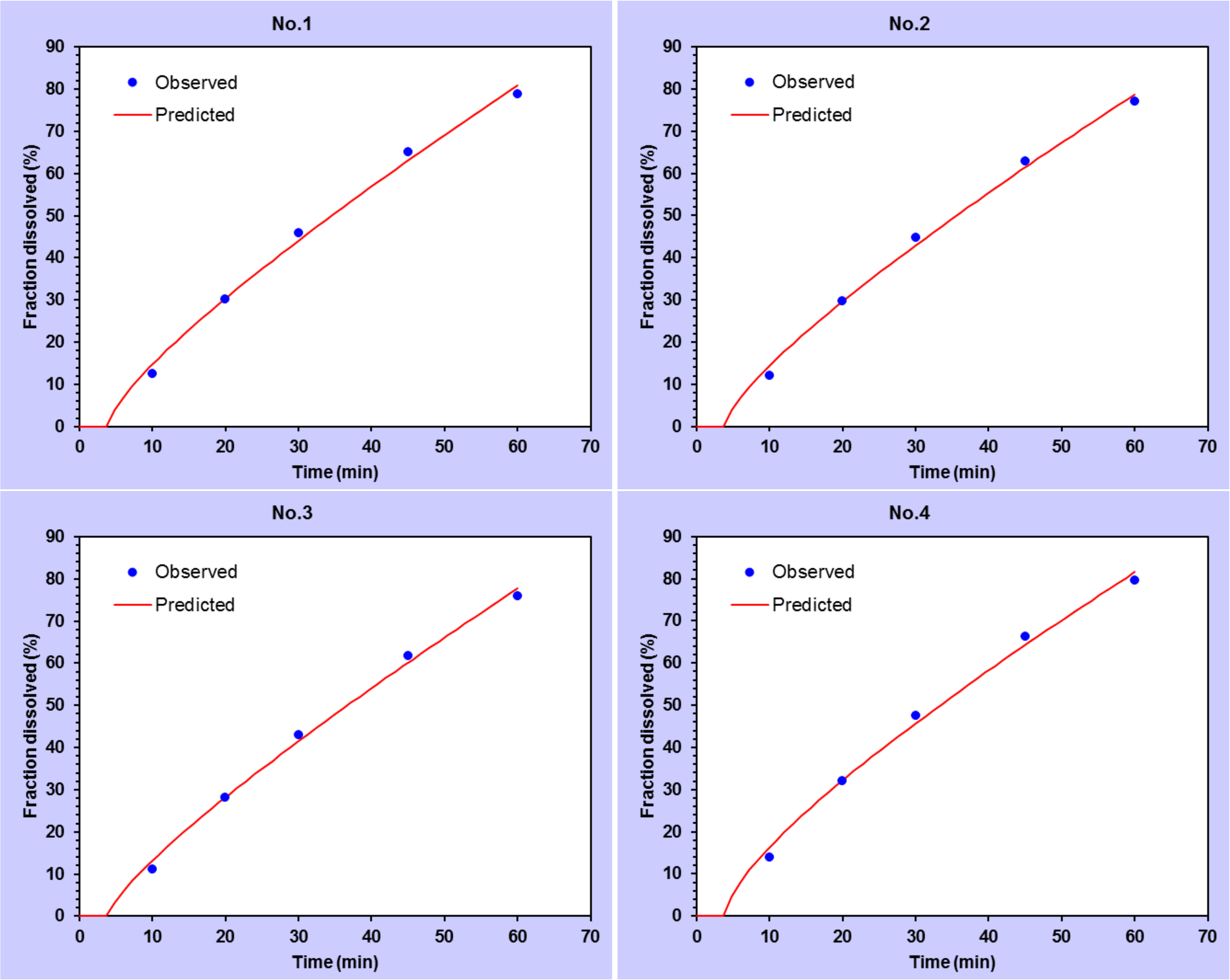

Supplementary materials – Model fitting summary – Polyglykol® 8000 P (modelling up to app. 75 % of carvedilol released)

Model: **Peppas–Sahlin_2**

Model equation: $F = k_1 \cdot t^{0.5} + k_2 \cdot t$

Fitted model parameters per tested tablet (N = 4) with statistics – mean, standard deviation (SD), and relative standard deviation expressed in % (RSD%) (output from DDSolver):

Parameter	No.1	No.2	No.3	No.4	Mean	SD	RSD(%)
k_1	1.432	1.416	0.865	2.233	1.487	0.563	37.890
k_2	1.176	1.142	1.196	1.083	1.149	0.049	4.283

Number of dissolution data points (N), degrees of freedom (df), and selected goodness of fit criteria – Pearson correlation coefficient (R), coefficient of determination (R^2), adjusted coefficient of determination ($R^2_{adjusted}$), and residual sum of squares (RSS) (manual calculation in MS Excel):

Parameter	No.1	No.2	No.3	No.4
N	5	5	5	5
df	3	3	3	3
R	0.993410299	0.994059638	0.994517565	0.993232356
R^2	0.986864021	0.988154565	0.989065188	0.986510513
$R^2_{adjusted}$	0.982485362	0.984206086	0.98542025	0.982014018
RSS	38.20641178	32.57338994	30.09447643	38.24432247

Graphical abstract of model fit presented as mean ± 1 SD of the fraction % of released carvedilol:

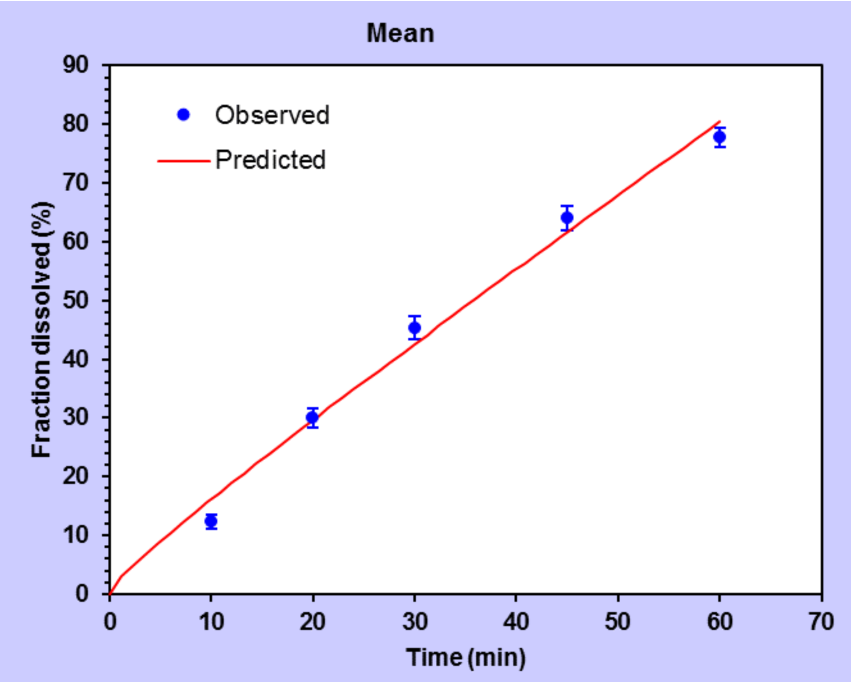

Supplementary materials – Model fitting summary – Polyglykol® 8000 P (modelling up to app. 75 % of carvedilol released)

Graphical abstract of model fit presented as the fraction % of released carvedilol per tested tablet:

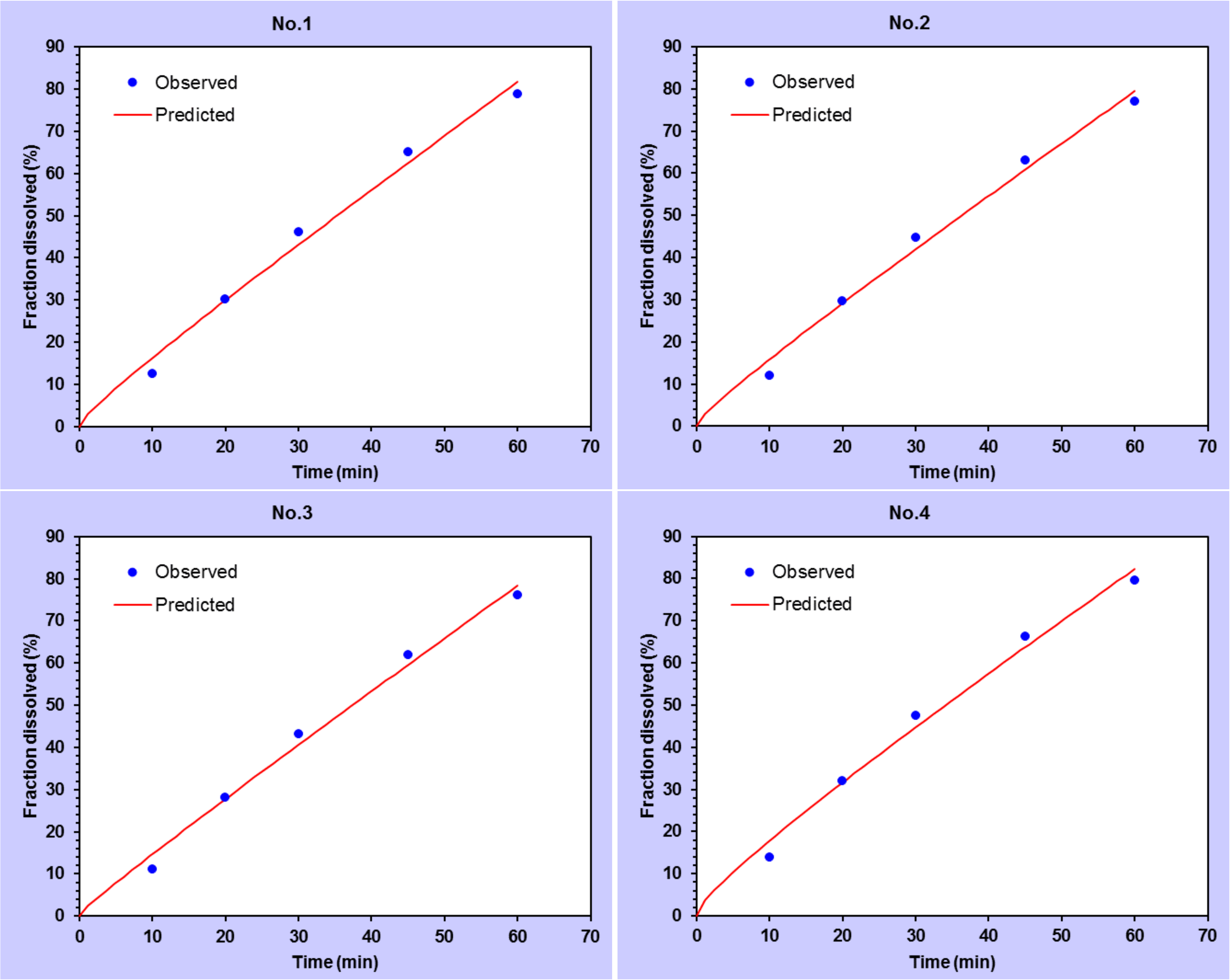

Supplementary materials – Model fitting summary – Polyglykol® 8000 P (modelling up to app. 75 % of carvedilol released)

Model: **Peppas–Sahlin_2 with T_{lag}**

Model equation: $F = k_1 \cdot (t - T_{lag})^{0.5} + k_2 \cdot (t - T_{lag})$

Fitted model parameters per tested tablet (N = 4) with statistics – mean, standard deviation (SD), and relative standard deviation expressed in % (RSD%) (output from DDSolver):

Parameter	No.1	No.2	No.3	No.4	Mean	SD	RSD(%)
k_1	4.648	4.549	3.244	4.803	4.311	0.719	16.684
k_2	0.859	0.833	0.956	0.815	0.866	0.063	7.275
T_{lag}	4.773	4.773	4.000	4.000	4.387	0.446	10.176

Number of dissolution data points (N), degrees of freedom (df), and selected goodness of fit criteria – Pearson correlation coefficient (R), coefficient of determination (R^2), adjusted coefficient of determination ($R^2_{adjusted}$), and residual sum of squares (RSS) (manual calculation in MS Excel):

Parameter	No.1	No.2	No.3	No.4
N	5	5	5	5
df	2	2	2	2
R	0.997184912	0.997728391	0.997126333	0.996605437
R^2	0.994377749	0.995461943	0.994260923	0.993222396
$R^2_{adjusted}$	0.988755497	0.990923885	0.988521847	0.986444793
RSS	20.70047671	16.78673056	16.27823544	19.8432926

Graphical abstract of model fit presented as mean ± 1 SD of the fraction % of released carvedilol:

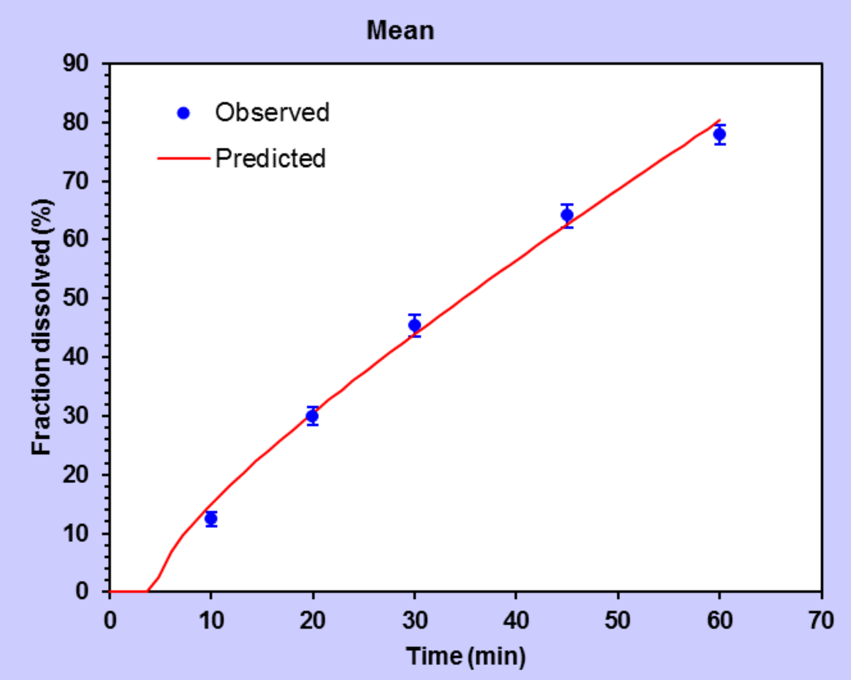

Supplementary materials – Model fitting summary – Polyglykol® 8000 P (modelling up to app. 75 % of carvedilol released)

Graphical abstract of model fit presented as the fraction % of released carvedilol per tested tablet:

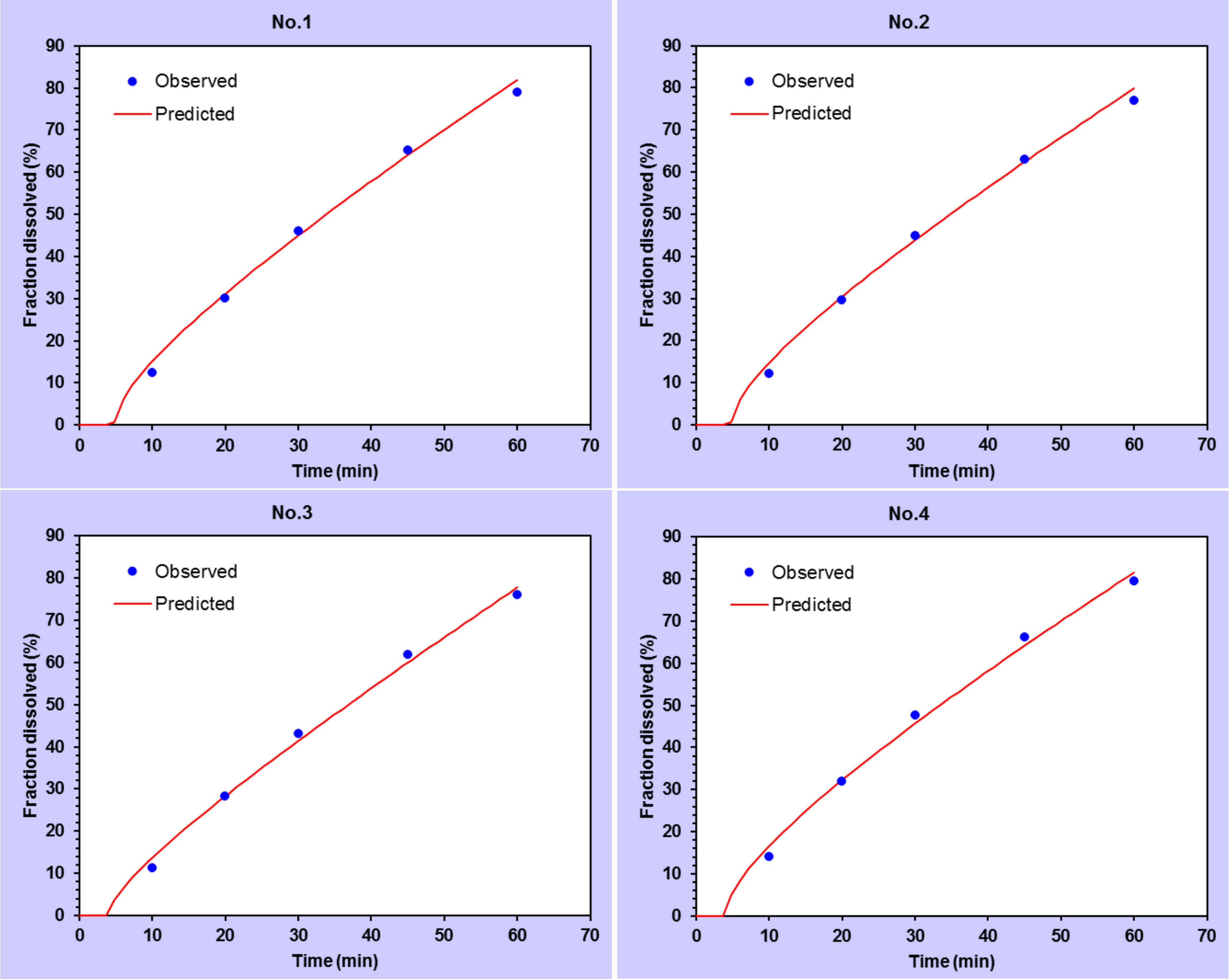

Supplementary materials – Model fitting summary – Polyglykol® 8000 P (modelling up to app. 75 % of carvedilol released)

Model: **Quadratic**

Model equation: $F = 100 \cdot (k_1 \cdot t^2 + k_2 \cdot t)$

Fitted model parameters per tested tablet (N = 4) with statistics – mean, standard deviation (SD), and relative standard deviation expressed in % (RSD%) (output from DDSolver):

Parameter	No.1	No.2	No.3	No.4	Mean	SD	RSD(%)
k_1	0.000	0.000	0.000	0.000	0.000	0.000	-25.975
k_2	0.016	0.016	0.015	0.018	0.016	0.001	6.486

Number of dissolution data points (N), degrees of freedom (df), and selected goodness of fit criteria – Pearson correlation coefficient (R), coefficient of determination (R^2), adjusted coefficient of determination ($R^2_{adjusted}$), and residual sum of squares (RSS) (manual calculation in MS Excel):

Parameter	No.1	No.2	No.3	No.4
N	5	5	5	5
df	3	3	3	3
R	0.997680907	0.99797398	0.997598035	0.998442165
R^2	0.995367193	0.995952065	0.995201839	0.996886757
$R^2_{adjusted}$	0.993822924	0.994602754	0.993602452	0.99584901
RSS	18.79940865	15.46353439	18.12113878	12.61528043

Graphical abstract of model fit presented as mean ± 1 SD of the fraction % of released carvedilol:

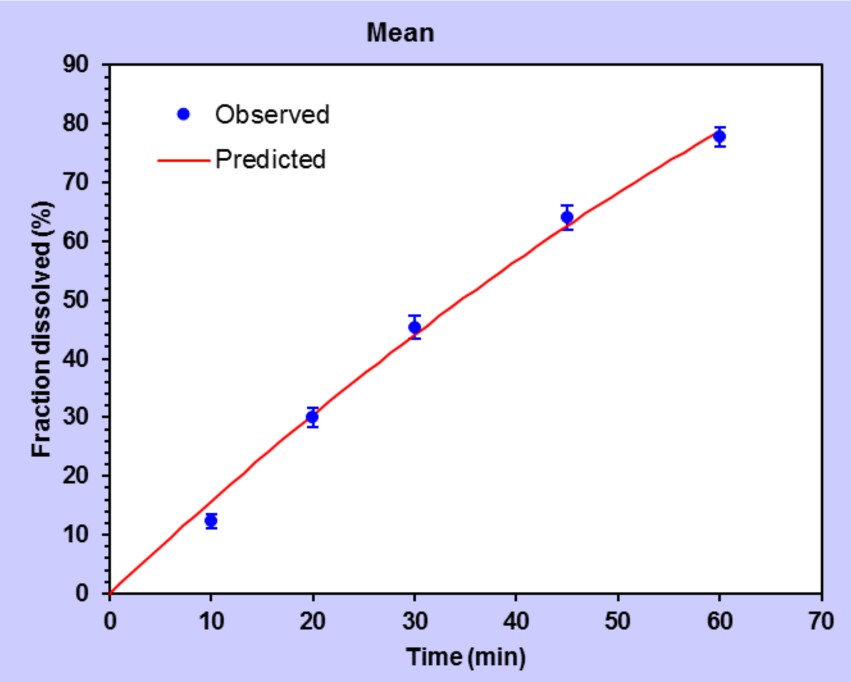

Supplementary materials – Model fitting summary – Polyglykol® 8000 P (modelling up to app. 75 % of carvedilol released)

Graphical abstract of model fit presented as the fraction % of released carvedilol per tested tablet:

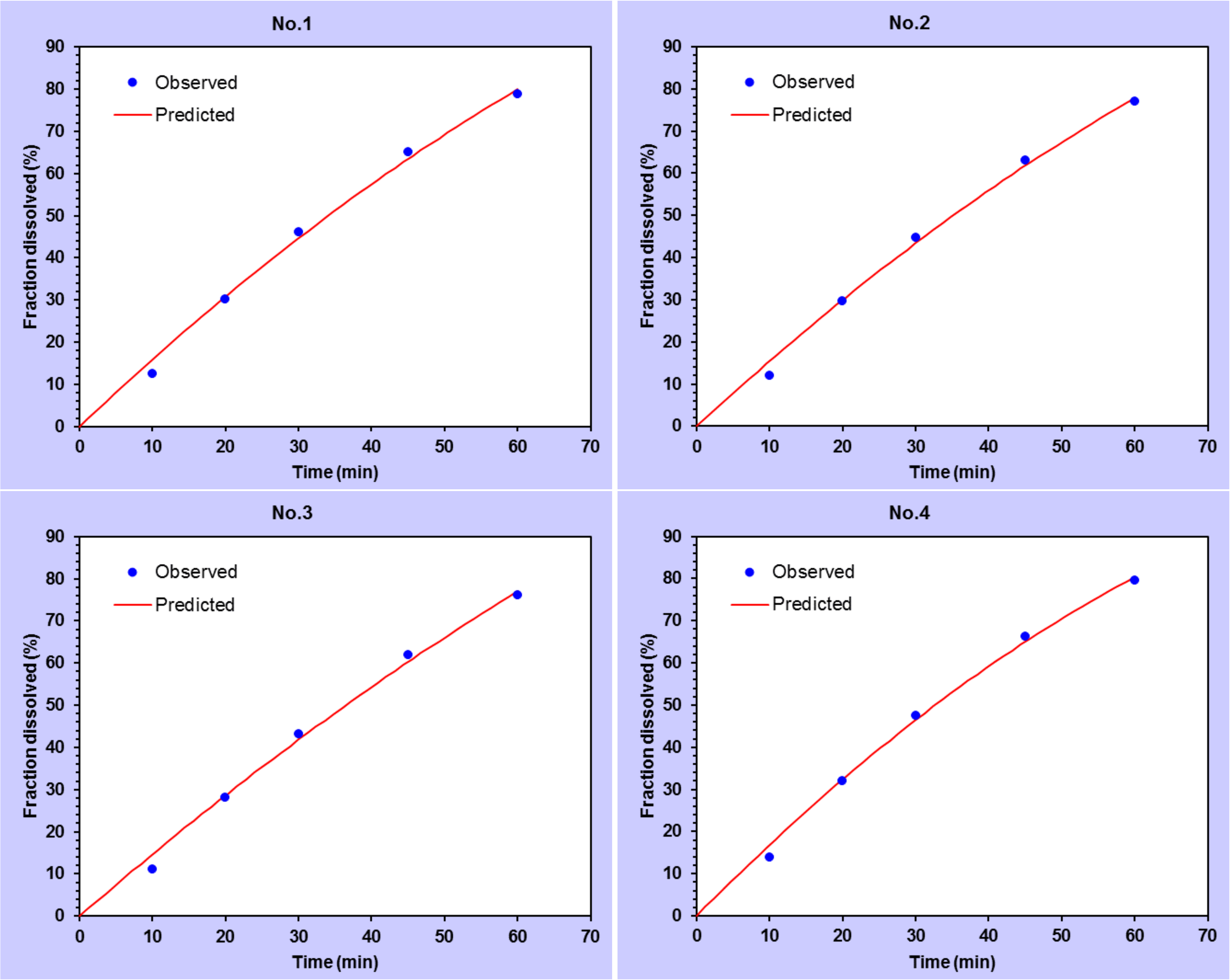

Supplementary materials – Model fitting summary – Polyglykol® 8000 P (modelling up to app. 75 % of carvedilol released)

Model: **Quadratic with T_{lag}**

Model equation: $F = 100 \cdot \left[k_1 \cdot \left(t - T_{lag}\right)^2 + k_2 \cdot \left(t - T_{lag}\right)\right]$

Fitted model parameters per tested tablet (N = 4) with statistics – mean, standard deviation (SD), and relative standard deviation expressed in % (RSD%) (output from DDSolver):

Parameter	No.1	No.2	No.3	No.4	Mean	SD	RSD(%)
k_1	0.000	0.000	0.000	0.000	0.000	0.000	-14.306
k_2	0.021	0.020	0.019	0.022	0.021	0.001	5.894
T_{lag}	4.000	4.000	4.000	4.000	4.000	0.000	0.000

Number of dissolution data points (N), degrees of freedom (df), and selected goodness of fit criteria – Pearson correlation coefficient (R), coefficient of determination (R^2), adjusted coefficient of determination ($R^2_{adjusted}$), and residual sum of squares (RSS) (manual calculation in MS Excel):

Parameter	No.1	No.2	No.3	No.4
N	5	5	5	5
df	2	2	2	2
R	0.999981806	0.999947247	0.999998636	0.999834236
R^2	0.999963613	0.999894496	0.999997271	0.9996685
$R^2_{adjusted}$	0.999927227	0.999788993	0.999994542	0.999337001
RSS	0.135516655	0.421150398	0.007957013	1.795107009

Graphical abstract of model fit presented as mean ± 1 SD of the fraction % of released carvedilol:

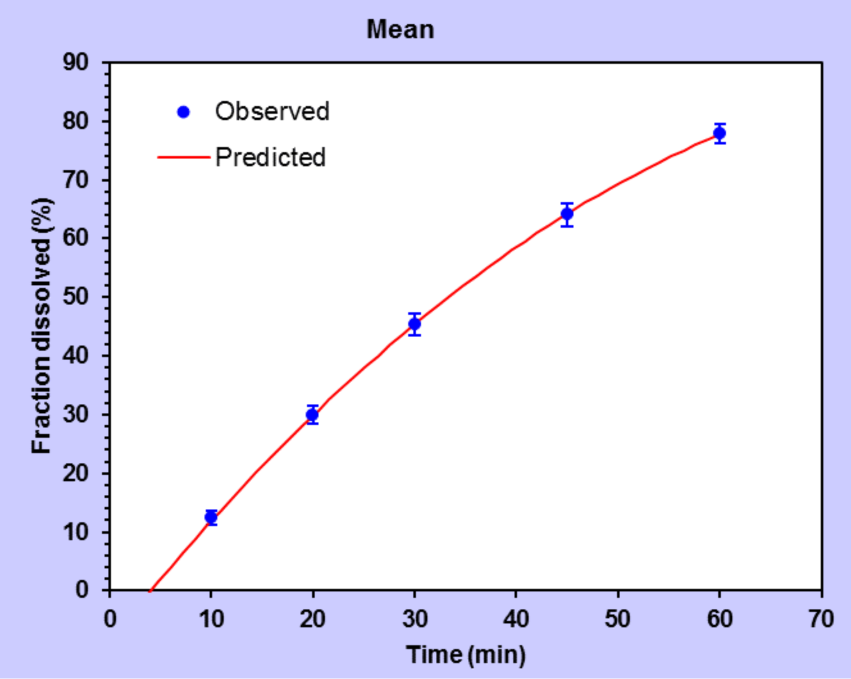

Supplementary materials – Model fitting summary – Polyglykol® 8000 P (modelling up to app. 75 % of carvedilol released)

Graphical abstract of model fit presented as the fraction % of released carvedilol per tested tablet:

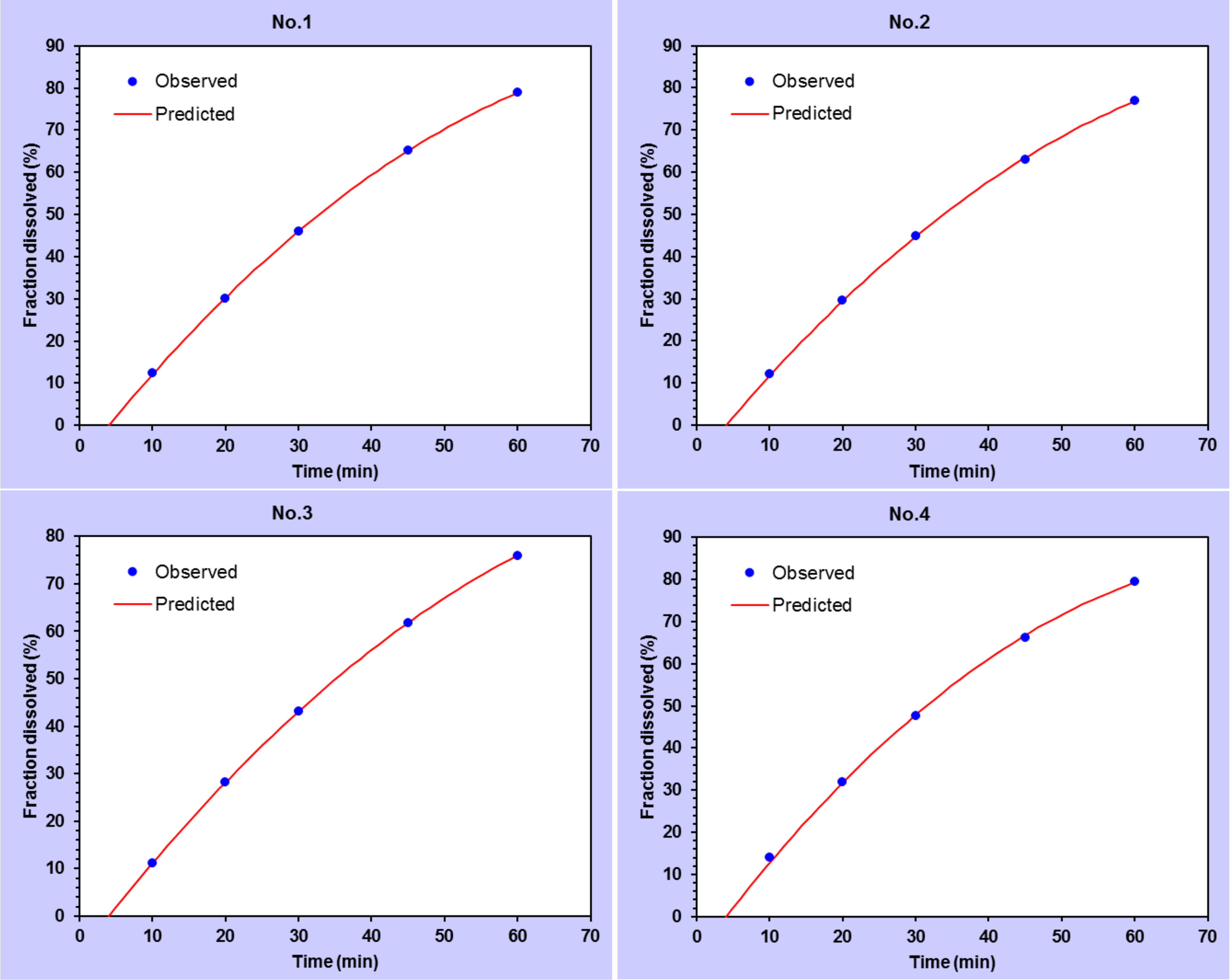

Supplementary materials – Model fitting summary – Polyglykol® 8000 P (modelling up to app. 75 % of carvedilol released)

Model: **Weibull_1**

Model equation: $F = 100 \cdot \left[1 - e^{-\frac{(t-T_i)^{\beta}}{\alpha}}\right]$

Fitted model parameters per tested tablet (N = 4) with statistics – mean, standard deviation (SD), and relative standard deviation expressed in % (RSD%) (output from DDSolver):

Parameter	No.1	No.2	No.3	No.4	Mean	SD	RSD(%)
α	56.289	55.705	64.136	45.927	55.514	7.459	13.436
β	1.100	1.084	1.112	1.053	1.087	0.025	2.336
T_i	4.000	4.000	4.000	4.000	4.000	0.000	0.000

Number of dissolution data points (N), degrees of freedom (df), and selected goodness of fit criteria – Pearson correlation coefficient (R), coefficient of determination (R^2), adjusted coefficient of determination ($R^2_{adjusted}$), and residual sum of squares (RSS) (manual calculation in MS Excel):

Parameter	No.1	No.2	No.3	No.4
N	5	5	5	5
df	2	2	2	2
R	0.999230963	0.99931458	0.999393894	0.998999687
R^2	0.998462518	0.99862963	0.998788155	0.998000375
$R^2_{adjusted}$	0.996925036	0.99725926	0.997576309	0.99600075
RSS	5.203560679	4.250235924	3.846909332	6.363682709

Graphical abstract of model fit presented as mean ± 1 SD of the fraction % of released carvedilol:

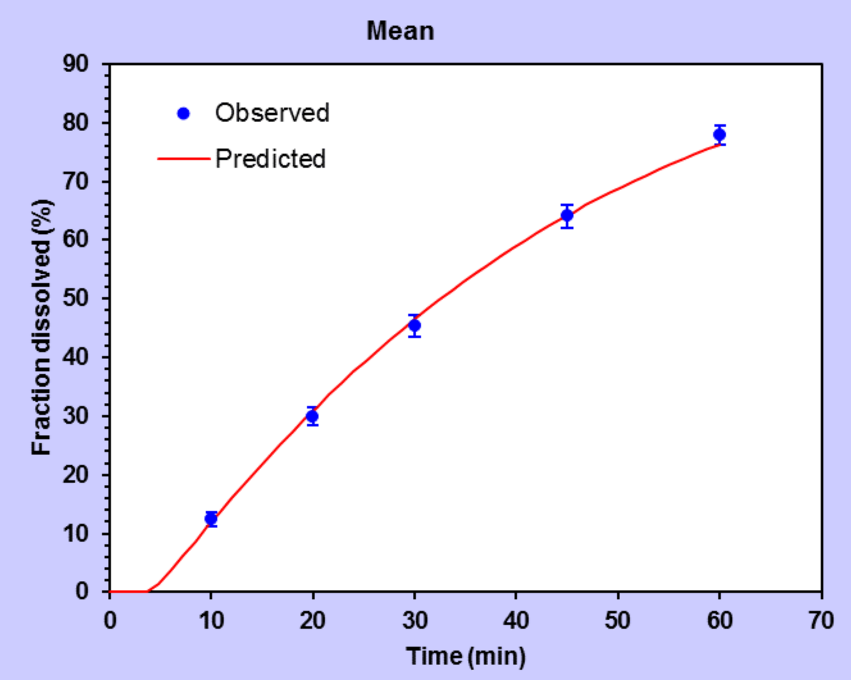

Supplementary materials – Model fitting summary – Polyglykol® 8000 P (modelling up to app. 75 % of carvedilol released)

Graphical abstract of model fit presented as the fraction % of released carvedilol per tested tablet:

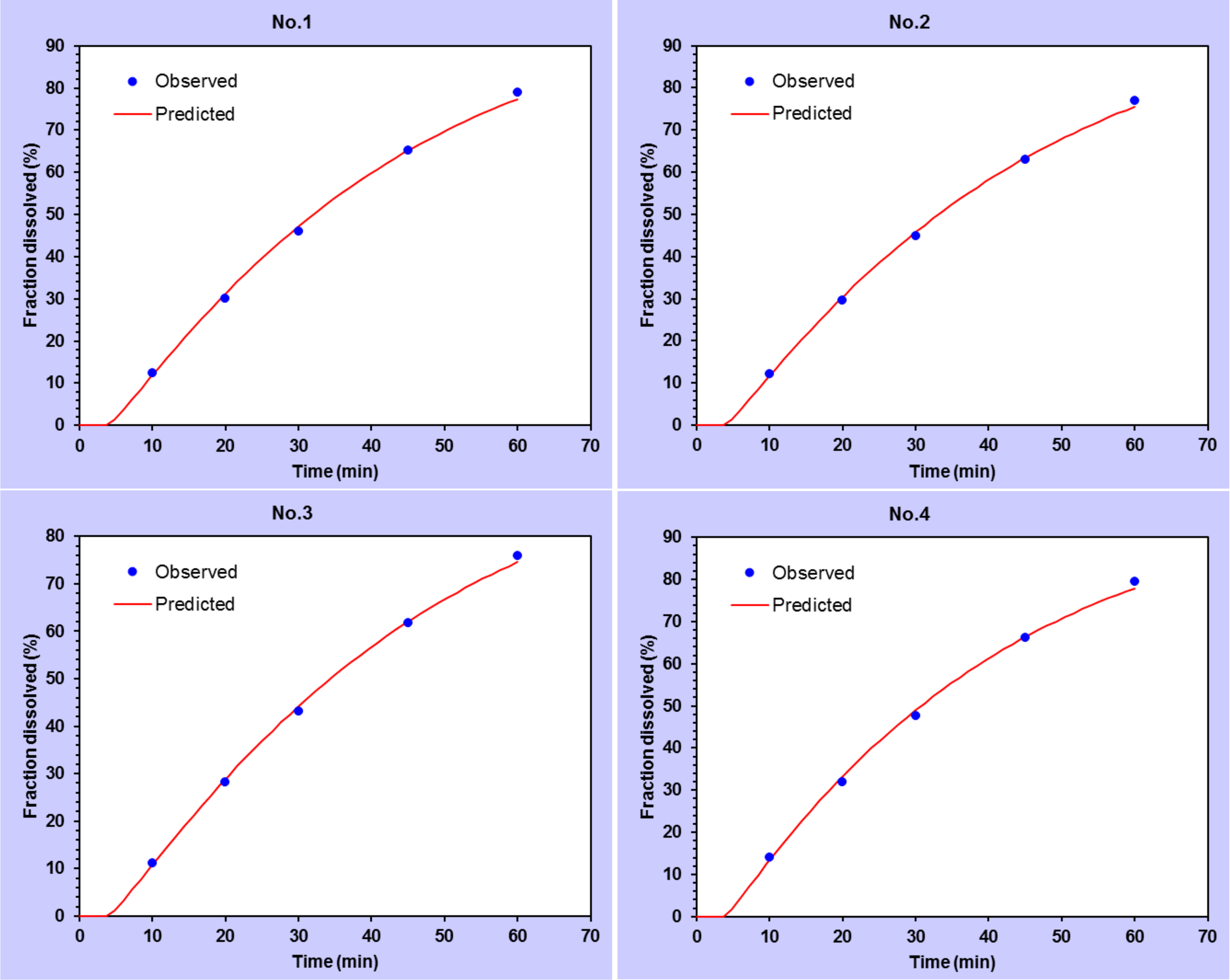

Supplementary materials – Model fitting summary – Polyglykol® 8000 P (modelling up to app. 75 % of carvedilol released)

Model: **Weibull_2**

Model equation: $F = 100 \cdot \left(1 - e^{-\frac{t^{\beta}}{\alpha}}\right)$

Fitted model parameters per tested tablet (N = 4) with statistics – mean, standard deviation (SD), and relative standard deviation expressed in % (RSD%) (output from DDSolver):

Parameter	No.1	No.2	No.3	No.4	Mean	SD	RSD(%)
α	162.996	168.861	200.003	135.396	166.814	26.504	15.888
β	1.351	1.350	1.385	1.313	1.350	0.029	2.179

Number of dissolution data points (N), degrees of freedom (df), and selected goodness of fit criteria – Pearson correlation coefficient (R), coefficient of determination (R^2), adjusted coefficient of determination ($R^2_{adjusted}$), and residual sum of squares (RSS) (manual calculation in MS Excel):

Parameter	No.1	No.2	No.3	No.4
N	5	5	5	5
df	3	3	3	3
R	0.999908242	0.999727646	0.999728381	0.999923146
R^2	0.999816493	0.999455366	0.999456835	0.999846298
$R^2_{adjusted}$	0.999755325	0.999273822	0.99927578	0.999795064
RSS	0.592951173	1.770531414	1.861422638	0.496730409

Graphical abstract of model fit presented as mean ± 1 SD of the fraction % of released carvedilol:

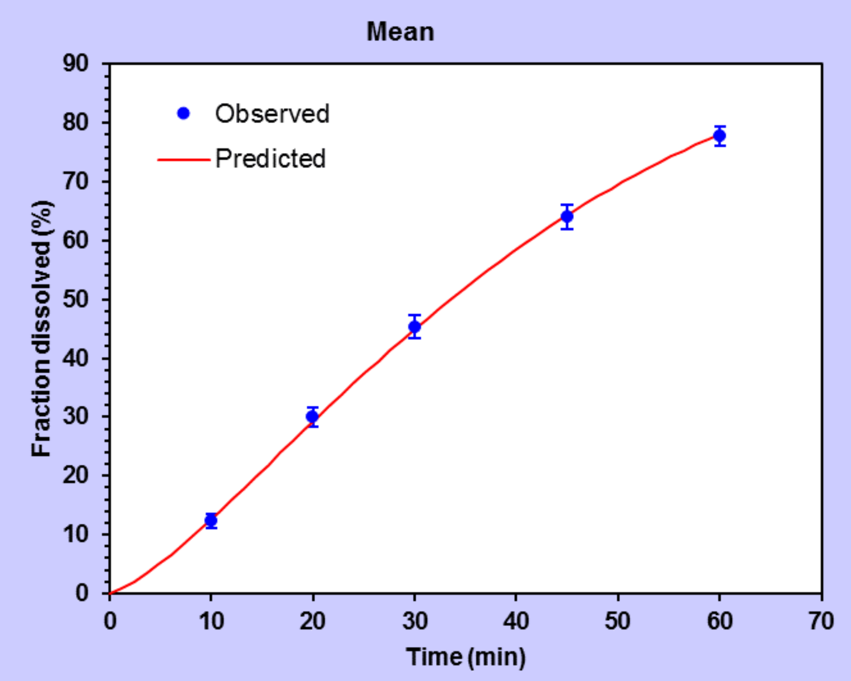

Supplementary materials – Model fitting summary – Polyglykol® 8000 P (modelling up to app. 75 % of carvedilol released)

Graphical abstract of model fit presented as the fraction % of released carvedilol per tested tablet:

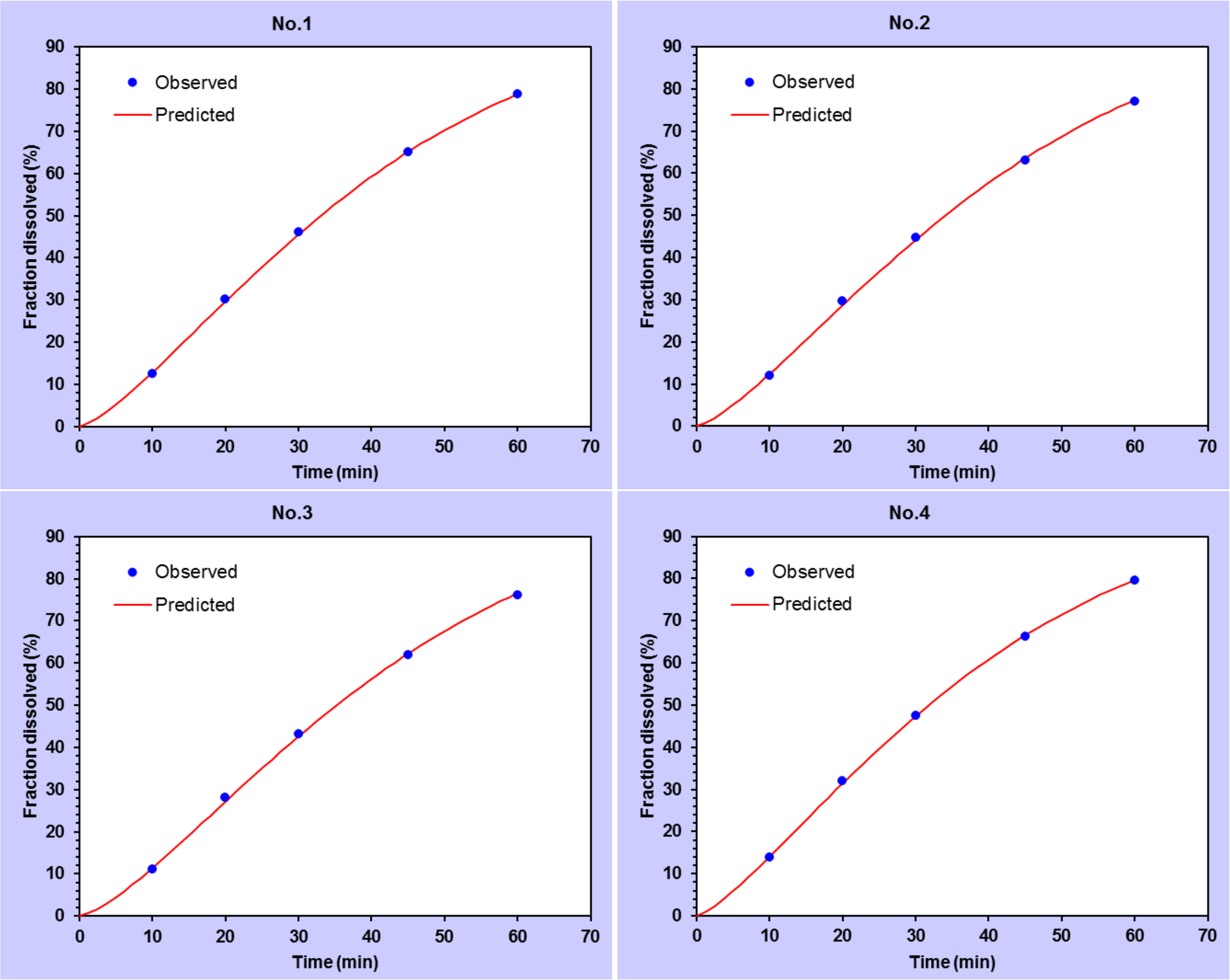

Supplementary materials – Model fitting summary – Polyglykol® 8000 P (modelling up to app. 75 % of carvedilol released)

Model: **Weibull_3**

Model equation: $F = F_{max} \cdot \left(1 - e^{-\frac{t^{\beta}}{\alpha}}\right)$

Fitted model parameters per tested tablet (N = 4) with statistics – mean, standard deviation (SD), and relative standard deviation expressed in % (RSD%) (output from DDSolver):

Parameter	No.1	No.2	No.3	No.4	Mean	SD	RSD(%)
α	258.124	253.264	302.902	199.178	253.367	42.477	16.765
β	1.596	1.589	1.632	1.532	1.587	0.041	2.602
F_{max}	82.768	80.880	79.794	83.503	81.736	1.702	2.082

Number of dissolution data points (N), degrees of freedom (df), and selected goodness of fit criteria – Pearson correlation coefficient (R), coefficient of determination (R^2), adjusted coefficient of determination ($R^2_{adjusted}$), and residual sum of squares (RSS) (manual calculation in MS Excel):

Parameter	No.1	No.2	No.3	No.4
N	5	5	5	5
df	2	2	2	2
R	0.997534979	0.997036215	0.997001599	0.997320054
R^2	0.995076034	0.994081214	0.994012189	0.994647289
$R^2_{adjusted}$	0.990152069	0.988162429	0.988024378	0.989294578
RSS	15.60118163	17.60414498	17.84929107	16.43481662

Graphical abstract of model fit presented as mean ± 1 SD of the fraction % of released carvedilol:

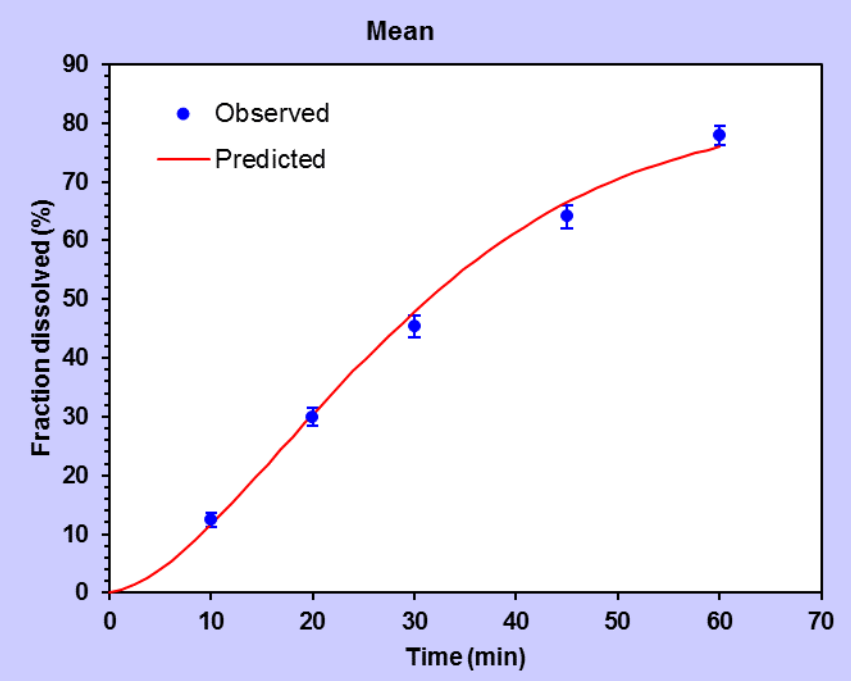

Supplementary materials – Model fitting summary – Polyglykol® 8000 P (modelling up to app. 75 % of carvedilol released)

Graphical abstract of model fit presented as the fraction % of released carvedilol per tested tablet:

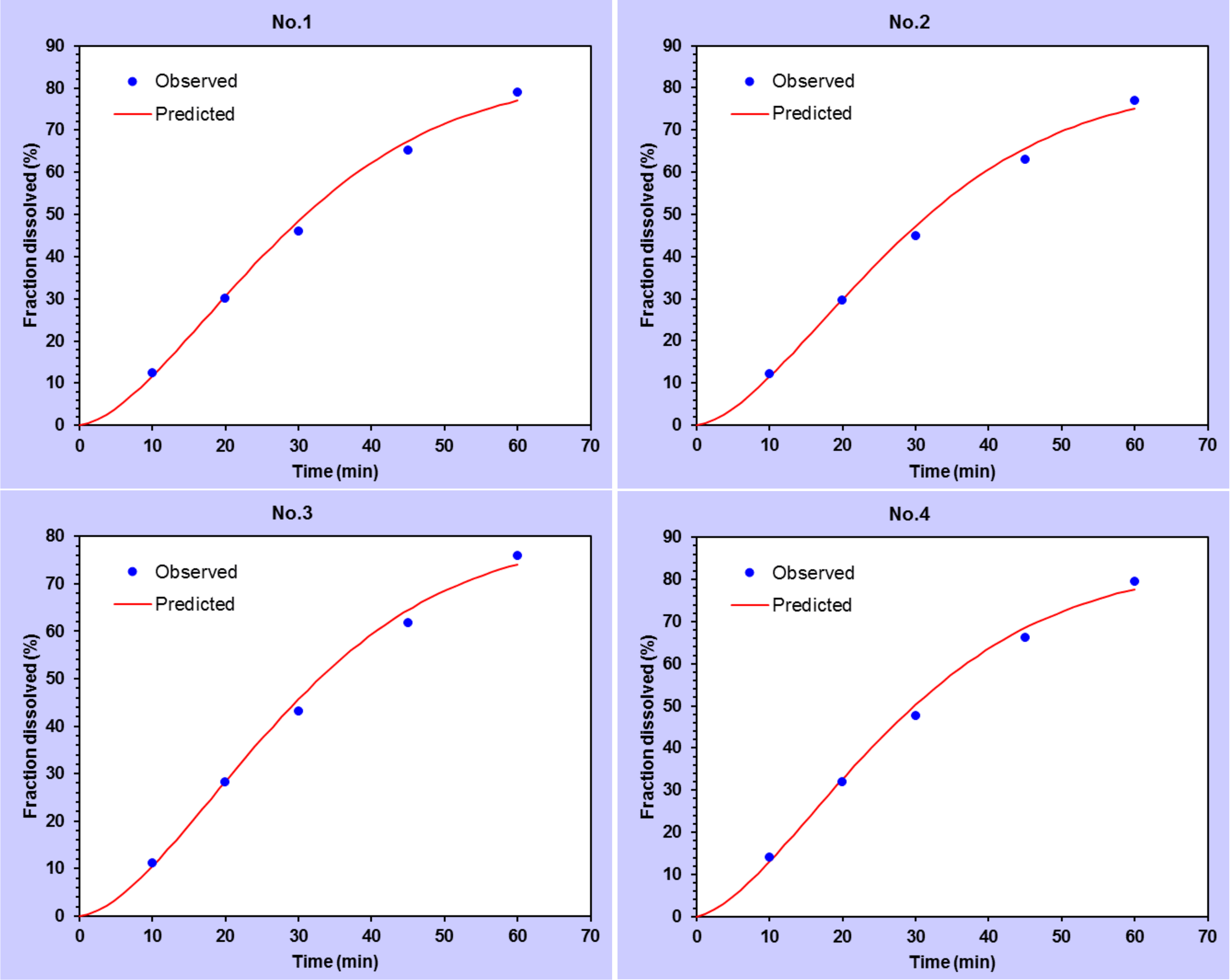

Supplementary materials – Model fitting summary – Polyglykol® 8000 P (modelling up to app. 75 % of carvedilol released)

Model: **Weibull_4**

Model equation: $F = F_{max} \cdot \left[1 - e^{-\frac{(t-T_i)^\beta}{\alpha}}\right]$

Fitted model parameters per tested tablet (N = 4) with statistics – mean, standard deviation (SD), and relative standard deviation expressed in % (RSD%) (output from DDSolver):

Parameter	No.1	No.2	No.3	No.4	Mean	SD	RSD(%)
α	68.089	67.238	77.568	55.347	67.060	9.105	13.577
β	1.274	1.269	1.304	1.223	1.268	0.033	2.620
T_i	4.000	4.000	4.000	4.000	4.000	0.000	0.000
F_{max}	82.768	80.880	79.794	83.503	81.736	1.702	2.082

Number of dissolution data points (N), degrees of freedom (df), and selected goodness of fit criteria – Pearson correlation coefficient (R), coefficient of determination (R^2), adjusted coefficient of determination ($R^2_{adjusted}$), and residual sum of squares (RSS) (manual calculation in MS Excel):

Parameter	No.1	No.2	No.3	No.4
N	5	5	5	5
df	1	1	1	1
R	0.993785525	0.993412055	0.993381699	0.99332401
R^2	0.987609671	0.986867511	0.9868072	0.986692589
$R^2_{adjusted}$	0.950438682	0.947470044	0.947228799	0.946770355
RSS	39.47767328	39.34249602	39.75442263	40.86859843

Graphical abstract of model fit presented as mean ± 1 SD of the fraction % of released carvedilol:

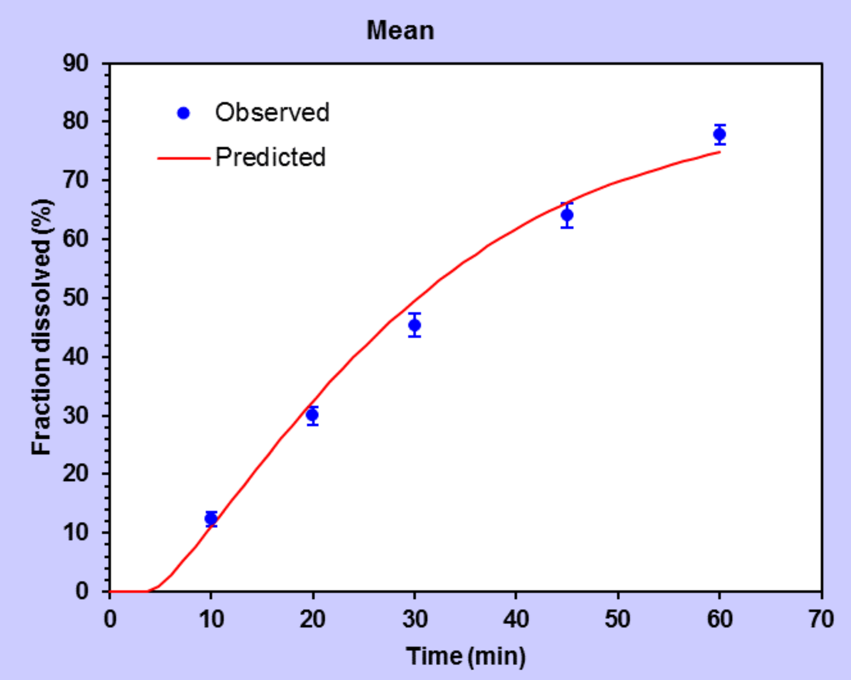

Supplementary materials – Model fitting summary – Polyglykol® 8000 P (modelling up to app. 75 % of carvedilol released)

Graphical abstract of model fit presented as the fraction % of released carvedilol per tested tablet:

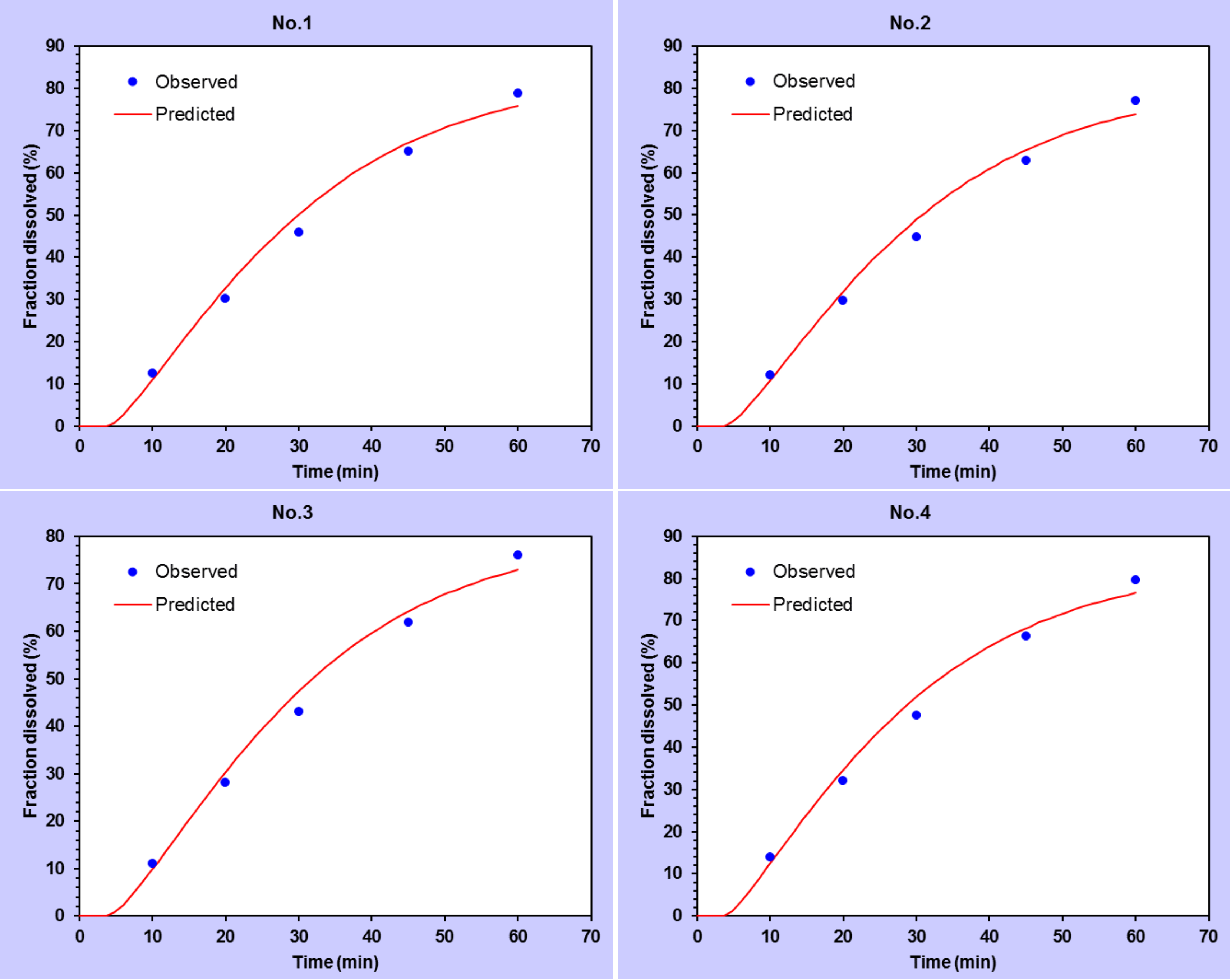

Supplementary materials – Model fitting summary – Polyglykol® 8000 P (modelling up to app. 75 % of carvedilol released)

Model: **Logistic_1**

Model equation: $F = 100 \cdot \frac{e^{\alpha+\beta\cdot\log(t)}}{1+e^{\alpha+\beta\cdot\log(t)}}$

Fitted model parameters per tested tablet (N = 4) with statistics – mean, standard deviation (SD), and relative standard deviation expressed in % (RSD%) (output from DDSolver):

Parameter	No.1	No.2	No.3	No.4	Mean	SD	RSD(%)
α	-6.184	-6.084	-6.233	-5.940	-6.110	0.129	-2.116
β	4.147	4.037	4.092	4.030	4.077	0.055	1.342

Number of dissolution data points (N), degrees of freedom (df), and selected goodness of fit criteria – Pearson correlation coefficient (R), coefficient of determination (R^2), adjusted coefficient of determination ($R^2_{adjusted}$), and residual sum of squares (RSS) (manual calculation in MS Excel):

Parameter	No.1	No.2	No.3	No.4
N	5	5	5	5
df	3	3	3	3
R	0.99760543	0.997846228	0.998021797	0.997208999
R^2	0.995216594	0.995697096	0.996047508	0.994425787
$R^2_{adjusted}$	0.993622125	0.994262794	0.994730011	0.992567716
RSS	14.43179172	12.09511086	11.18273467	16.20260732

Graphical abstract of model fit presented as mean ± 1 SD of the fraction % of released carvedilol:

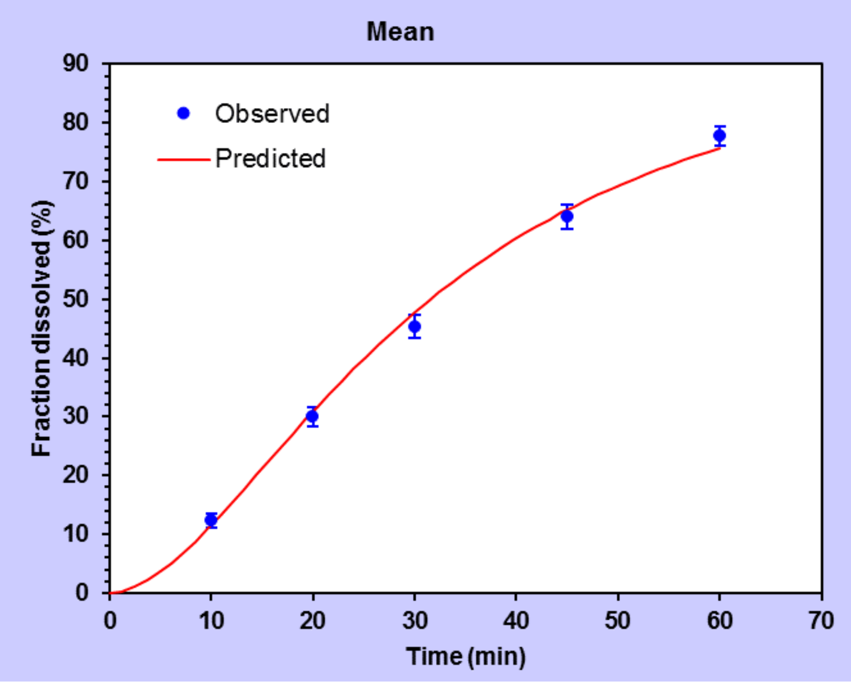

Supplementary materials – Model fitting summary – Polyglykol® 8000 P (modelling up to app. 75 % of carvedilol released)

Graphical abstract of model fit presented as the fraction % of released carvedilol per tested tablet:

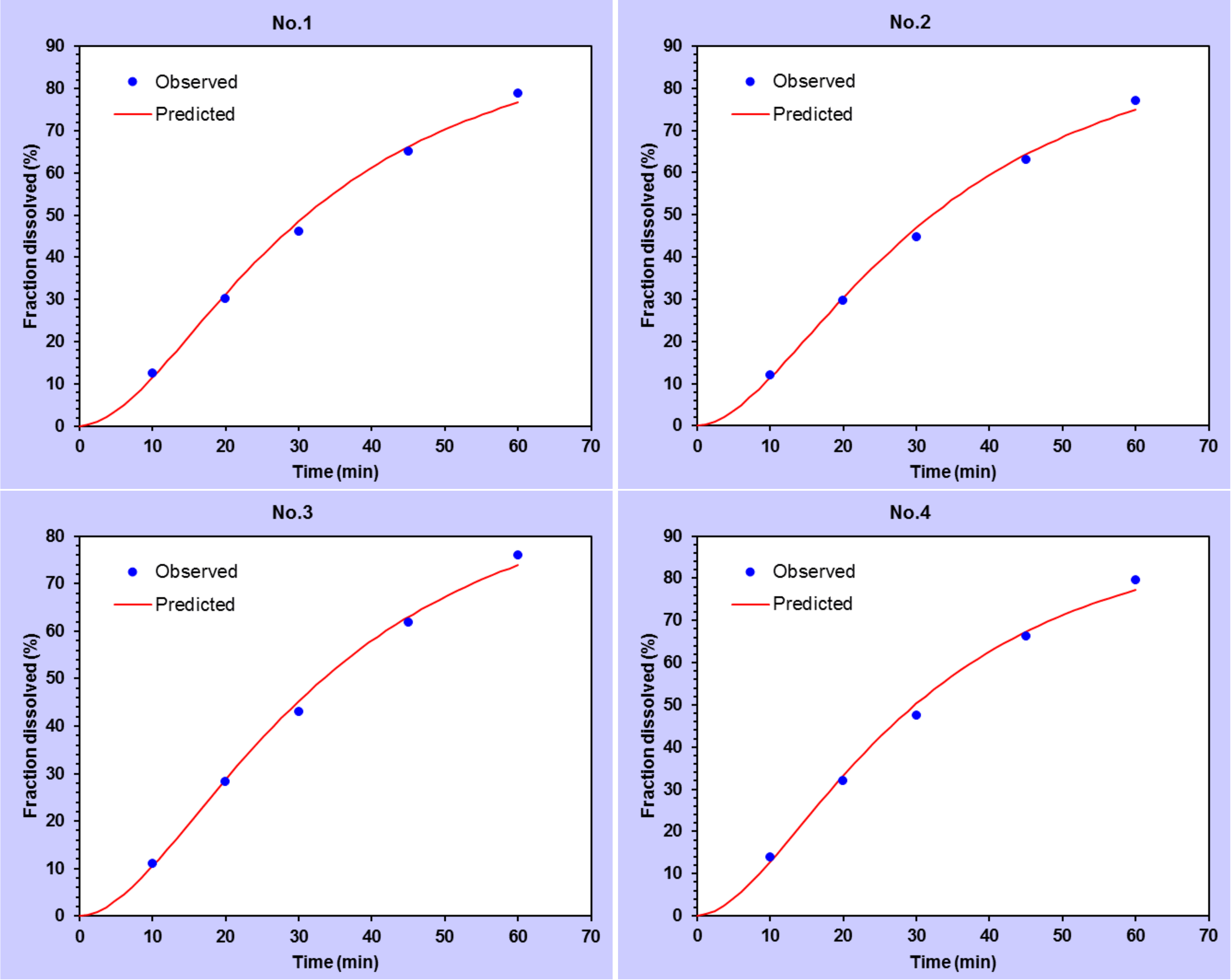

Supplementary materials – Model fitting summary – Polyglykol® 8000 P (modelling up to app. 75 % of carvedilol released)

Model: **Logistic_2**

Model equation: $F = F_{max} \cdot \frac{e^{\alpha+\beta \cdot \log(t)}}{1+e^{\alpha+\beta \cdot \log(t)}}$

Fitted model parameters per tested tablet (N = 4) with statistics – mean, standard deviation (SD), and relative standard deviation expressed in % (RSD%) (output from DDSolver):

Parameter	No.1	No.2	No.3	No.4	Mean	SD	RSD(%)
α	-7.794	-7.758	-7.952	-7.513	-7.754	0.182	-2.343
β	5.715	5.683	5.787	5.562	5.687	0.094	1.652
F_{max}	82.768	80.880	79.794	83.503	81.736	1.702	2.082

Number of dissolution data points (N), degrees of freedom (df), and selected goodness of fit criteria – Pearson correlation coefficient (R), coefficient of determination (R^2), adjusted coefficient of determination ($R^2_{adjusted}$), and residual sum of squares (RSS) (manual calculation in MS Excel):

Parameter	No.1	No.2	No.3	No.4
N	5	5	5	5
df	2	2	2	2
R	0.982497483	0.982090769	0.981798887	0.981949608
R^2	0.965301305	0.964502278	0.963929055	0.964225033
$R^2_{adjusted}$	0.93060261	0.929004556	0.92785811	0.928450065
RSS	124.1401191	120.6744195	122.956847	123.8671163

Graphical abstract of model fit presented as mean ± 1 SD of the fraction % of released carvedilol:

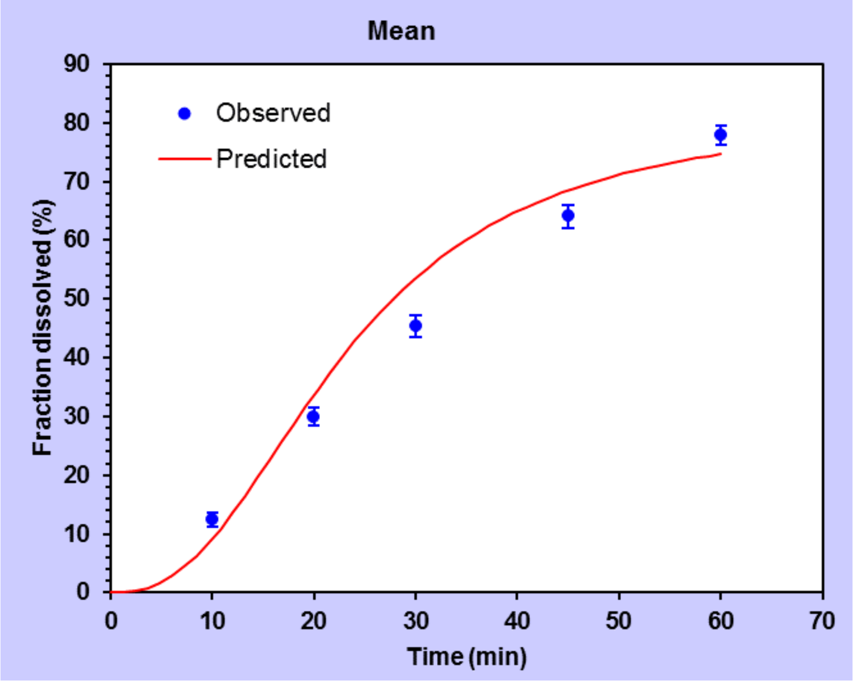

Supplementary materials – Model fitting summary – Polyglykol® 8000 P (modelling up to app. 75 % of carvedilol released)

Graphical abstract of model fit presented as the fraction % of released carvedilol per tested tablet:

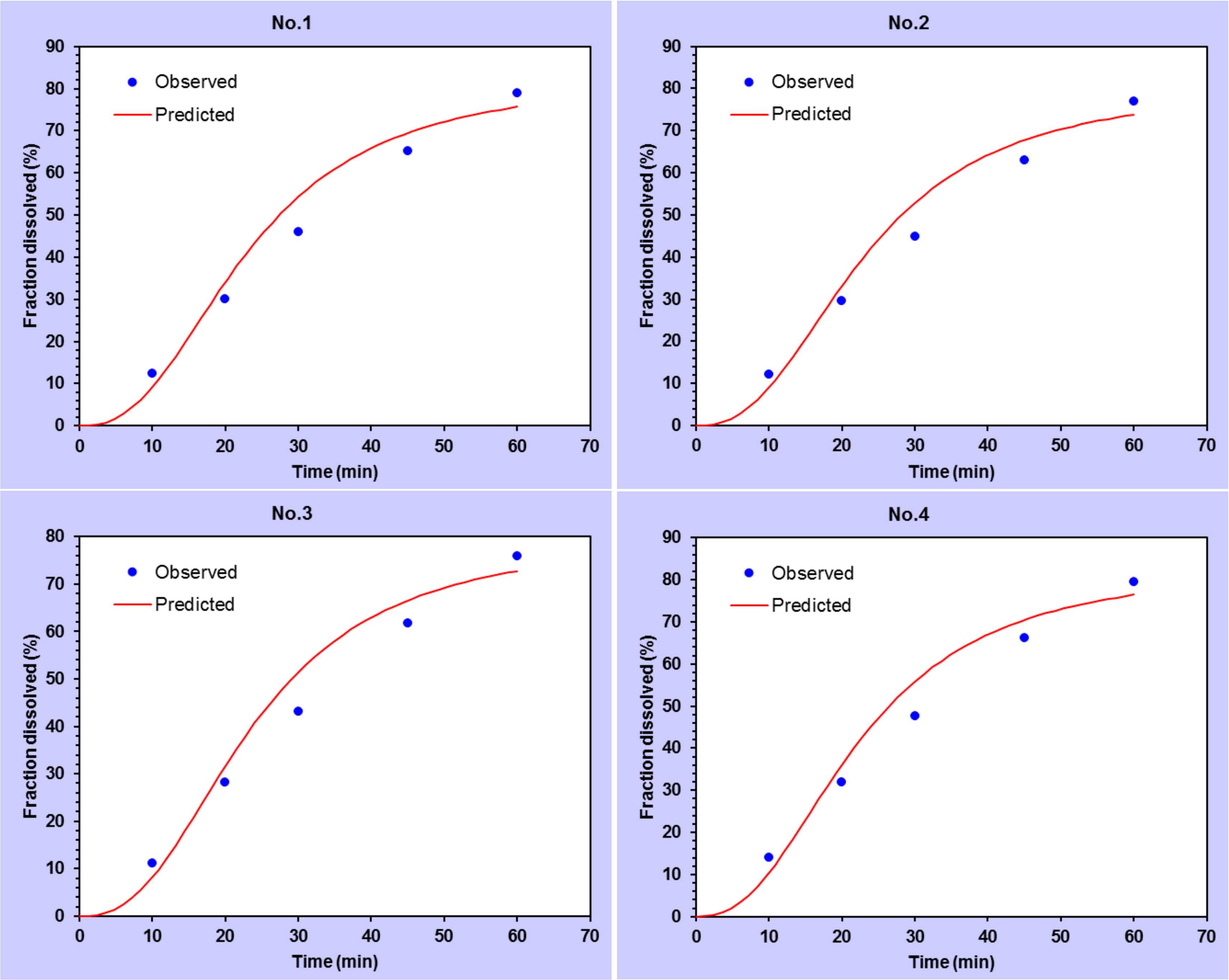

Supplementary materials – Model fitting summary – Polyglykol® 8000 P (modelling up to app. 75 % of carvedilol released)

Model: **Logistic_3**

Model equation: $F = F_{max} \cdot \frac{1}{1+e^{-k\cdot(t-\gamma)}}$

Fitted model parameters per tested tablet (N = 4) with statistics – mean, standard deviation (SD), and relative standard deviation expressed in % (RSD%) (output from DDSolver):

Parameter	No.1	No.2	No.3	No.4	Mean	SD	RSD(%)
k	0.091	0.090	0.092	0.088	0.090	0.001	1.630
γ	28.075	28.165	28.721	27.255	28.054	0.604	2.155
F_{max}	82.768	80.880	79.794	83.503	81.736	1.702	2.082

Number of dissolution data points (N), degrees of freedom (df), and selected goodness of fit criteria – Pearson correlation coefficient (R), coefficient of determination (R^2), adjusted coefficient of determination ($R^2_{adjusted}$), and residual sum of squares (RSS) (manual calculation in MS Excel):

Parameter	No.1	No.2	No.3	No.4
N	5	5	5	5
df	2	2	2	2
R	0.996642591	0.995760259	0.995853313	0.996719652
R^2	0.993296453	0.991538494	0.991723821	0.993450065
$R^2_{adjusted}$	0.986592907	0.983076988	0.983447643	0.98690013
RSS	21.09872888	25.3521	25.07335005	19.90196389

Graphical abstract of model fit presented as mean ± 1 SD of the fraction % of released carvedilol:

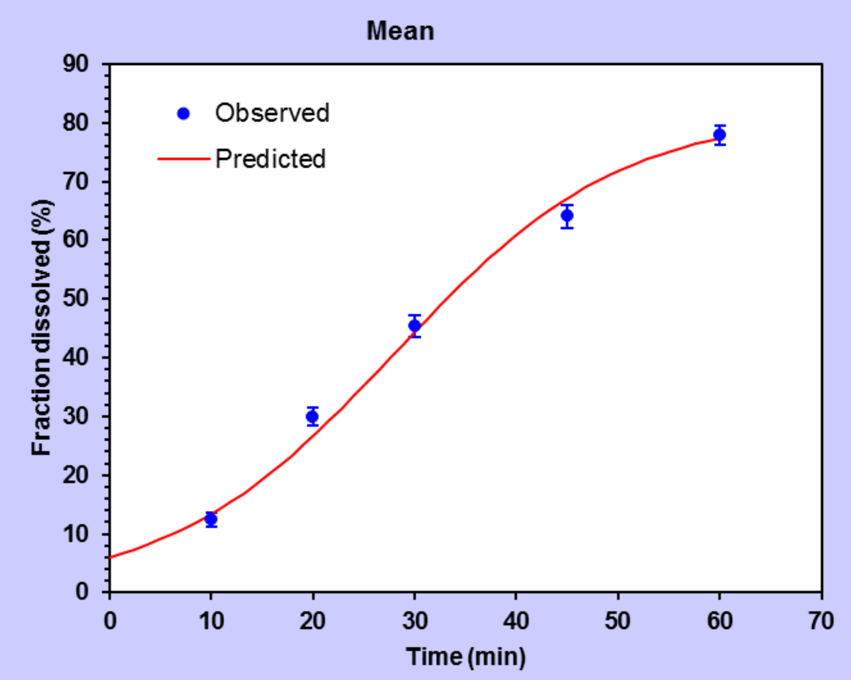

Supplementary materials – Model fitting summary – Polyglykol® 8000 P (modelling up to app. 75 % of carvedilol released)

Graphical abstract of model fit presented as the fraction % of released carvedilol per tested tablet:

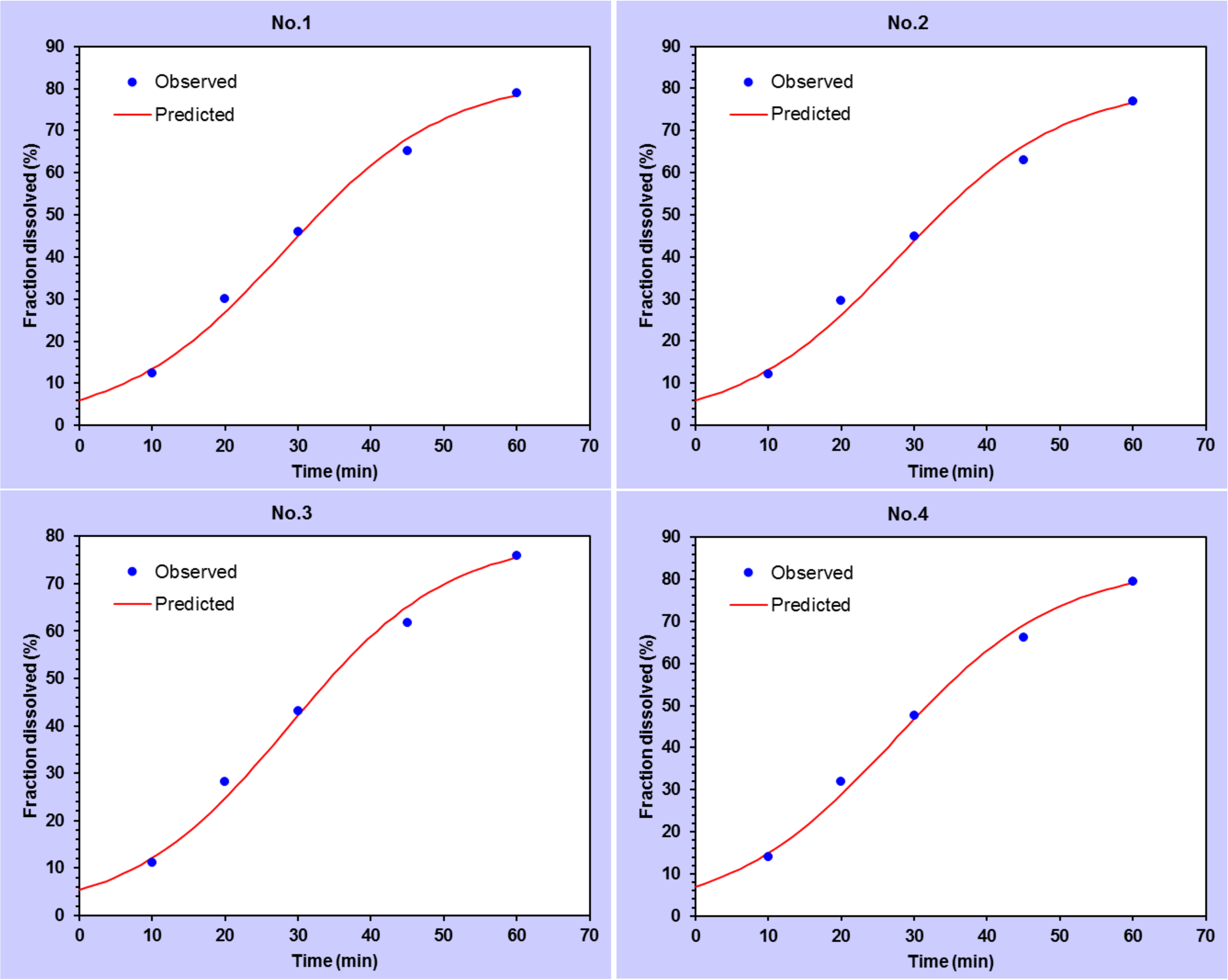

Supplementary materials – Model fitting summary – Polyglykol® 8000 P (modelling up to app. 75 % of carvedilol released)

Model: **Gompertz_1**

Model equation: $F = 100 \cdot e^{-\alpha \cdot e^{-\beta \cdot \log(t)}}$

Fitted model parameters per tested tablet (N = 4) with statistics – mean, standard deviation (SD), and relative standard deviation expressed in % (RSD%) (output from DDSolver):

Parameter	No.1	No.2	No.3	No.4	Mean	SD	RSD(%)
α	37.168	33.157	34.154	34.212	34.673	1.733	4.997
β	2.732	2.621	2.611	2.706	2.668	0.060	2.264

Number of dissolution data points (N), degrees of freedom (df), and selected goodness of fit criteria – Pearson correlation coefficient (R), coefficient of determination (R^2), adjusted coefficient of determination ($R^2_{adjusted}$), and residual sum of squares (RSS) (manual calculation in MS Excel):

Parameter	No.1	No.2	No.3	No.4
N	5	5	5	5
df	3	3	3	3
R	0.985806972	0.987022766	0.987073888	0.985516147
R^2	0.971815386	0.97421394	0.97431486	0.971242077
$R^2_{adjusted}$	0.962420514	0.965618587	0.965753146	0.961656102
RSS	83.56742264	71.7854277	71.71090831	82.90045892

Graphical abstract of model fit presented as mean ± 1 SD of the fraction % of released carvedilol:

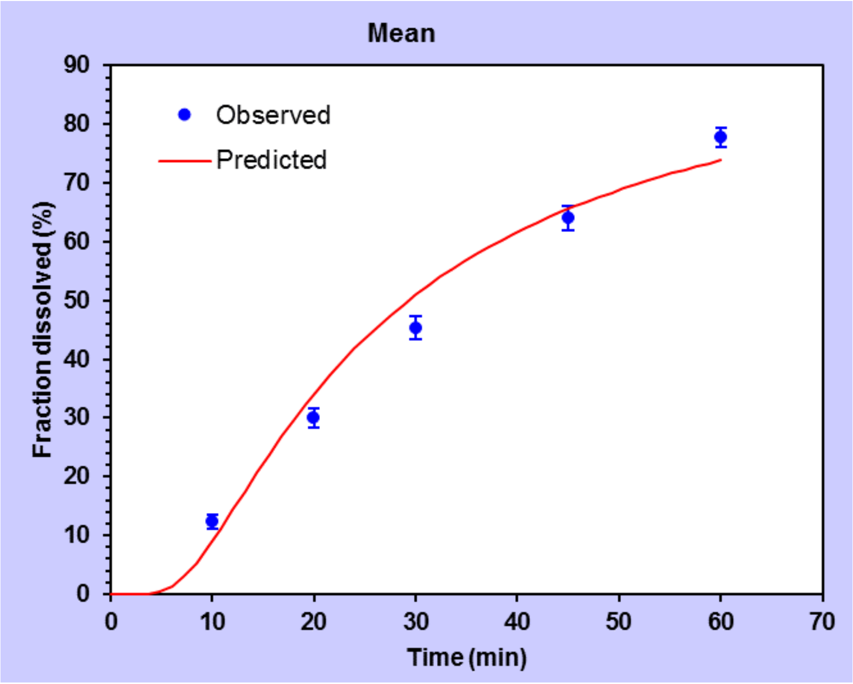

Supplementary materials – Model fitting summary – Polyglykol® 8000 P (modelling up to app. 75 % of carvedilol released)

Graphical abstract of model fit presented as the fraction % of released carvedilol per tested tablet:

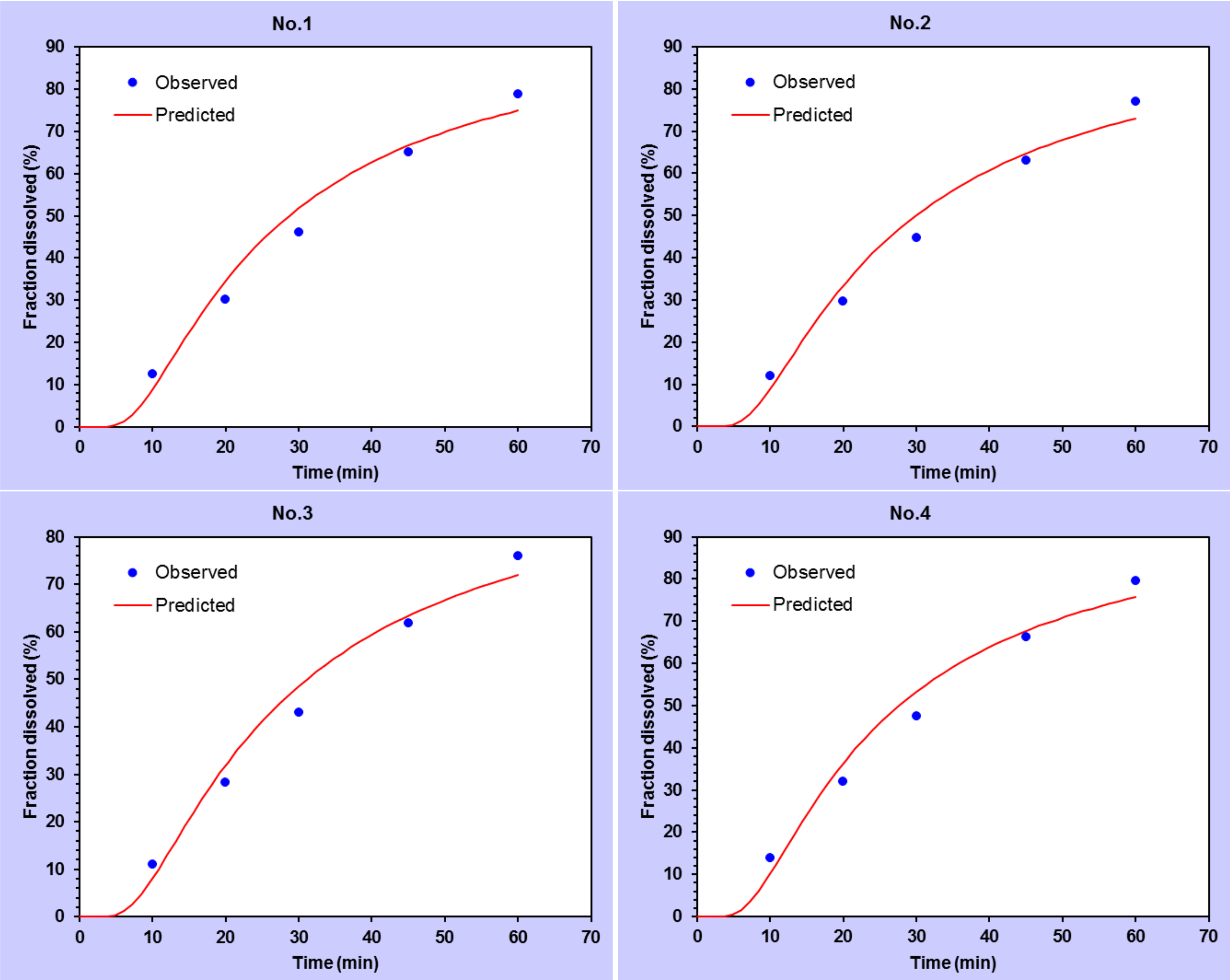

Supplementary materials – Model fitting summary – Polyglykol® 8000 P (modelling up to app. 75 % of carvedilol released)

Model: **Gompertz_2**

Model equation: $F = F_{max} \cdot e^{-\alpha \cdot e^{-\beta \cdot \log(t)}}$

Fitted model parameters per tested tablet (N = 4) with statistics – mean, standard deviation (SD), and relative standard deviation expressed in % (RSD%) (output from DDSolver):

Parameter	No.1	No.2	No.3	No.4	Mean	SD	RSD(%)
α	326.840	316.830	346.311	286.889	319.218	24.786	7.764
β	4.334	4.306	4.350	4.270	4.315	0.035	0.813
F_{max}	82.768	80.880	79.794	83.503	81.736	1.702	2.082

Number of dissolution data points (N), degrees of freedom (df), and selected goodness of fit criteria – Pearson correlation coefficient (R), coefficient of determination (R^2), adjusted coefficient of determination ($R^2_{adjusted}$), and residual sum of squares (RSS) (manual calculation in MS Excel):

Parameter	No.1	No.2	No.3	No.4
N	5	5	5	5
df	2	2	2	2
R	0.985216579	0.984768401	0.98443301	0.984690453
R^2	0.970651707	0.969768803	0.969108352	0.969615288
$R^2_{adjusted}$	0.941303414	0.939537607	0.938216703	0.939230576
RSS	204.1396504	198.9355886	183.541094	230.6864478

Graphical abstract of model fit presented as mean ± 1 SD of the fraction % of released carvedilol:

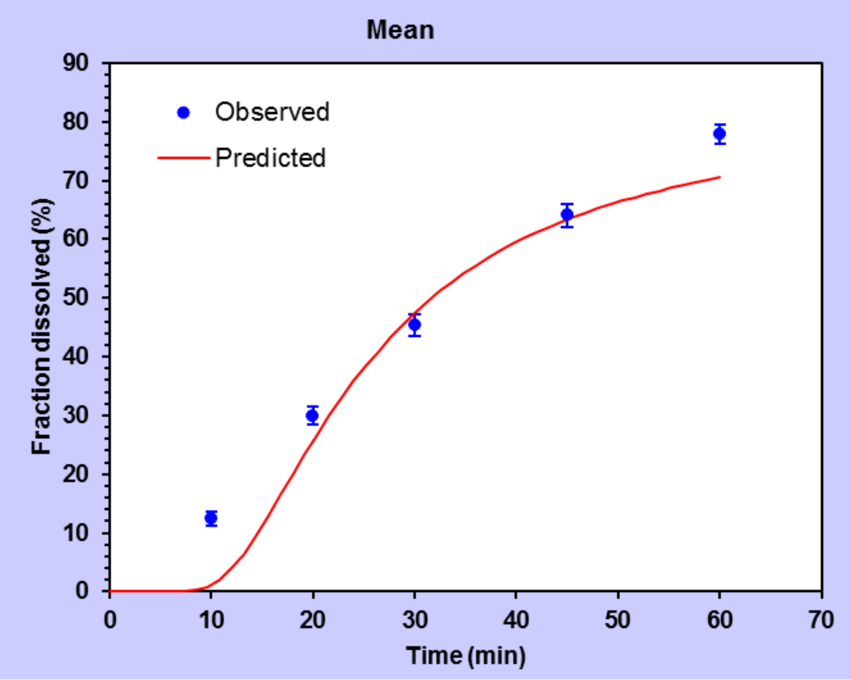

Supplementary materials – Model fitting summary – Polyglykol® 8000 P (modelling up to app. 75 % of carvedilol released)

Graphical abstract of model fit presented as the fraction % of released carvedilol per tested tablet:

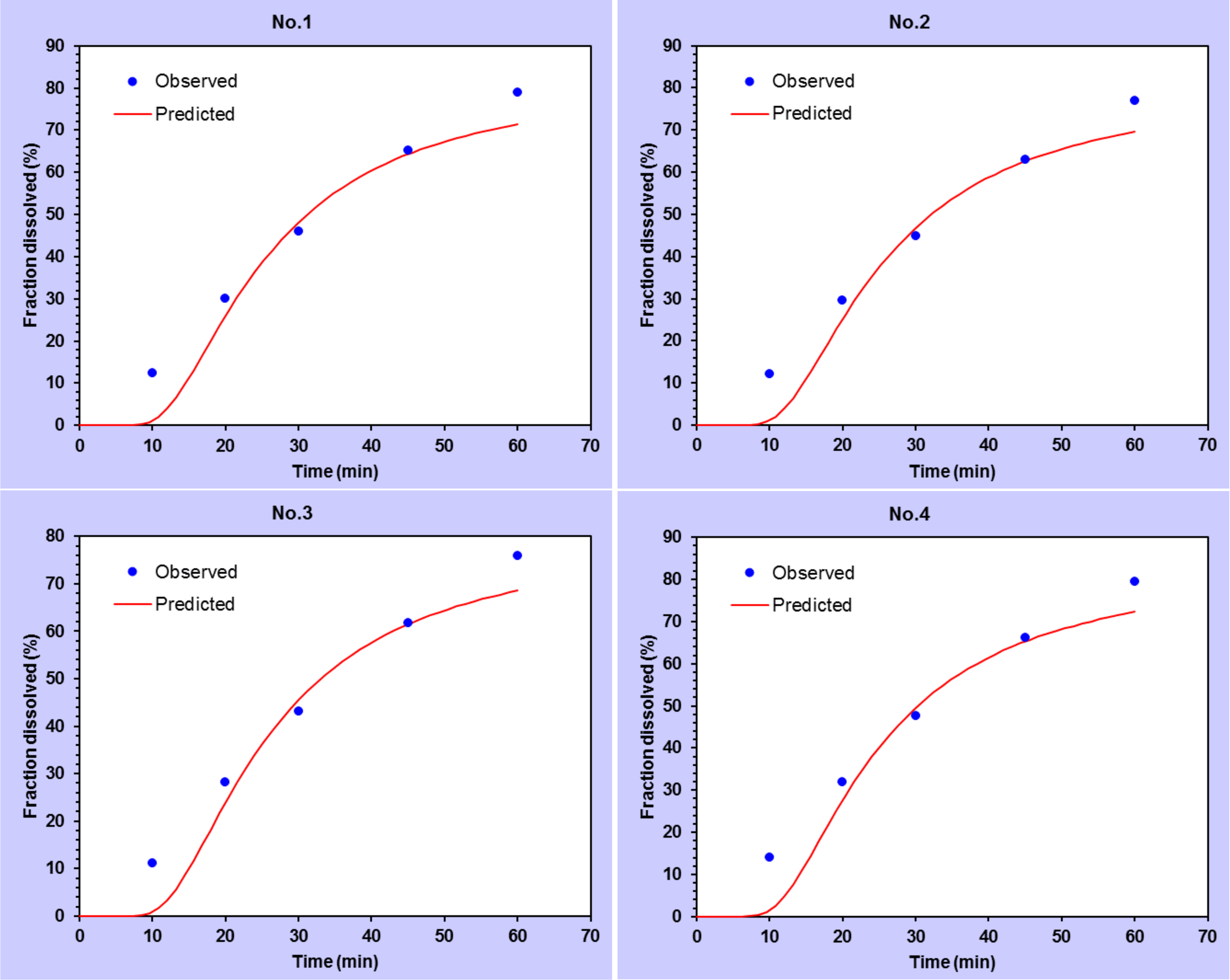

Supplementary materials – Model fitting summary – Polyglykol® 8000 P (modelling up to app. 75 % of carvedilol released)

Model: **Gompertz_3**

Model equation: $F = F_{max} \cdot e^{-e^{-k\cdot(t-\gamma)}}$

Fitted model parameters per tested tablet (N = 4) with statistics – mean, standard deviation (SD), and relative standard deviation expressed in % (RSD%) (output from DDSolver):

Parameter	No.1	No.2	No.3	No.4	Mean	SD	RSD(%)
k	0.071	0.071	0.071	0.070	0.071	0.001	0.901
γ	20.788	20.853	21.354	20.057	20.763	0.534	2.573
F_{max}	82.768	80.880	79.794	83.503	81.736	1.702	2.082

Number of dissolution data points (N), degrees of freedom (df), and selected goodness of fit criteria – Pearson correlation coefficient (R), coefficient of determination (R^2), adjusted coefficient of determination ($R^2_{adjusted}$), and residual sum of squares (RSS) (manual calculation in MS Excel):

Parameter	No.1	No.2	No.3	No.4
N	5	5	5	5
df	2	2	2	2
R	0.995683584	0.994972728	0.994586343	0.995845697
R^2	0.991385799	0.98997073	0.989201993	0.991708653
$R^2_{adjusted}$	0.982771598	0.97994146	0.978403987	0.983417306
RSS	36.68542426	40.33755826	42.77450524	34.89187942

Graphical abstract of model fit presented as mean ± 1 SD of the fraction % of released carvedilol:

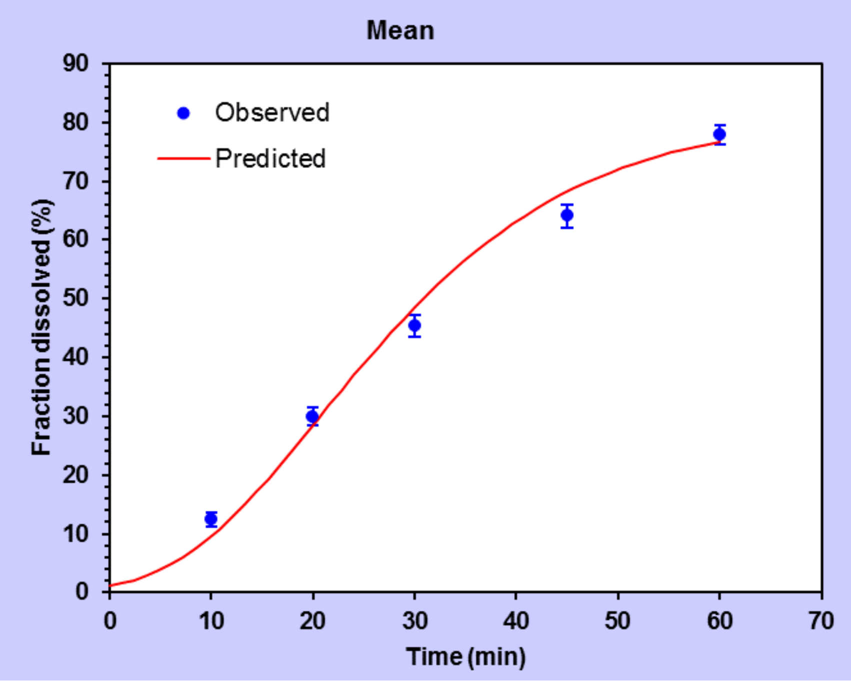

Supplementary materials – Model fitting summary – Polyglykol® 8000 P (modelling up to app. 75 % of carvedilol released)

Graphical abstract of model fit presented as the fraction % of released carvedilol per tested tablet:

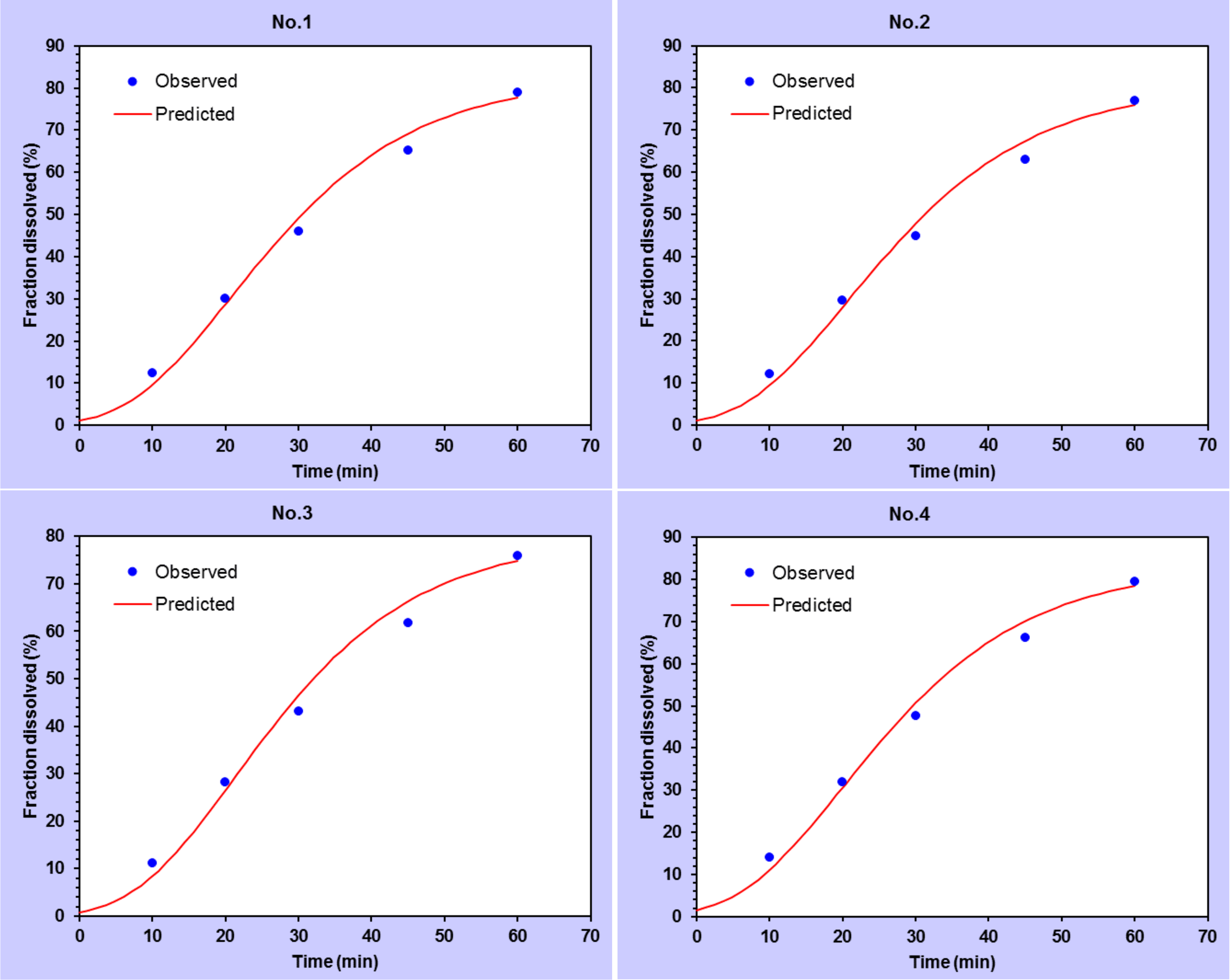

Supplementary materials – Model fitting summary – Polyglykol® 8000 P (modelling up to app. 75 % of carvedilol released)

Model: **Gompertz_4**

Model equation: $F = F_{max} \cdot e^{-\beta \cdot e^{-k \cdot t}}$

Fitted model parameters per tested tablet (N = 4) with statistics – mean, standard deviation (SD), and relative standard deviation expressed in % (RSD%) (output from DDSolver):

Parameter	No.1	No.2	No.3	No.4	Mean	SD	RSD(%)
k	0.071	0.071	0.071	0.070	0.071	0.001	0.901
β	4.365	4.351	4.583	4.055	4.339	0.217	4.999
F_{max}	82.768	80.880	79.794	83.503	81.736	1.702	2.082

Number of dissolution data points (N), degrees of freedom (df), and selected goodness of fit criteria – Pearson correlation coefficient (R), coefficient of determination (R^2), adjusted coefficient of determination ($R^2_{adjusted}$), and residual sum of squares (RSS) (manual calculation in MS Excel):

Parameter	No.1	No.2	No.3	No.4
N	5	5	5	5
df	2	2	2	2
R	0.995683584	0.994972728	0.994586343	0.995845697
R^2	0.991385799	0.98997073	0.989201993	0.991708653
$R^2_{adjusted}$	0.982771598	0.97994146	0.978403987	0.983417306
RSS	36.68542426	40.33755826	42.77450524	34.89187942

Graphical abstract of model fit presented as mean ± 1 SD of the fraction % of released carvedilol:

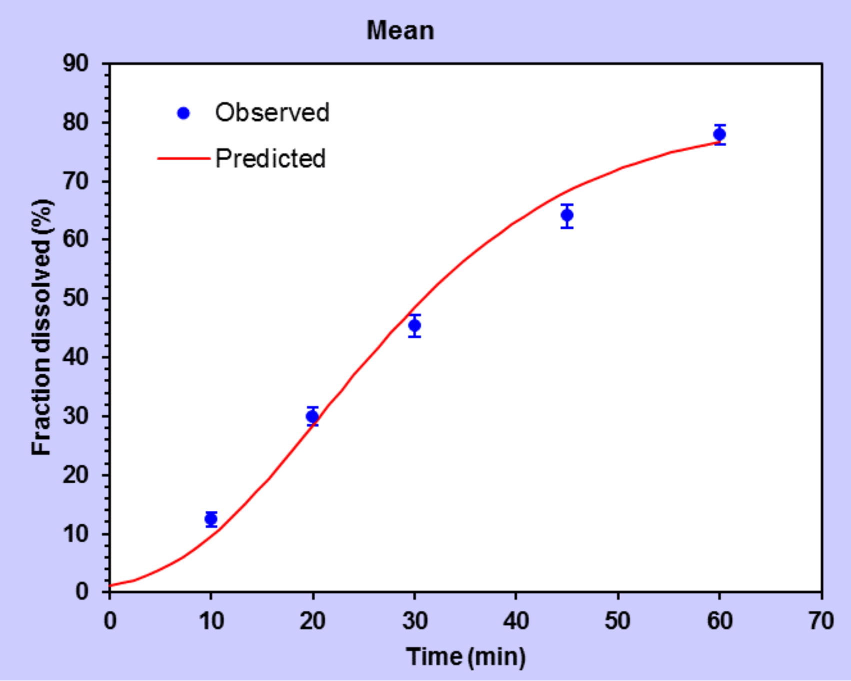

Supplementary materials – Model fitting summary – Polyglykol® 8000 P (modelling up to app. 75 % of carvedilol released)

Graphical abstract of model fit presented as the fraction % of released carvedilol per tested tablet:

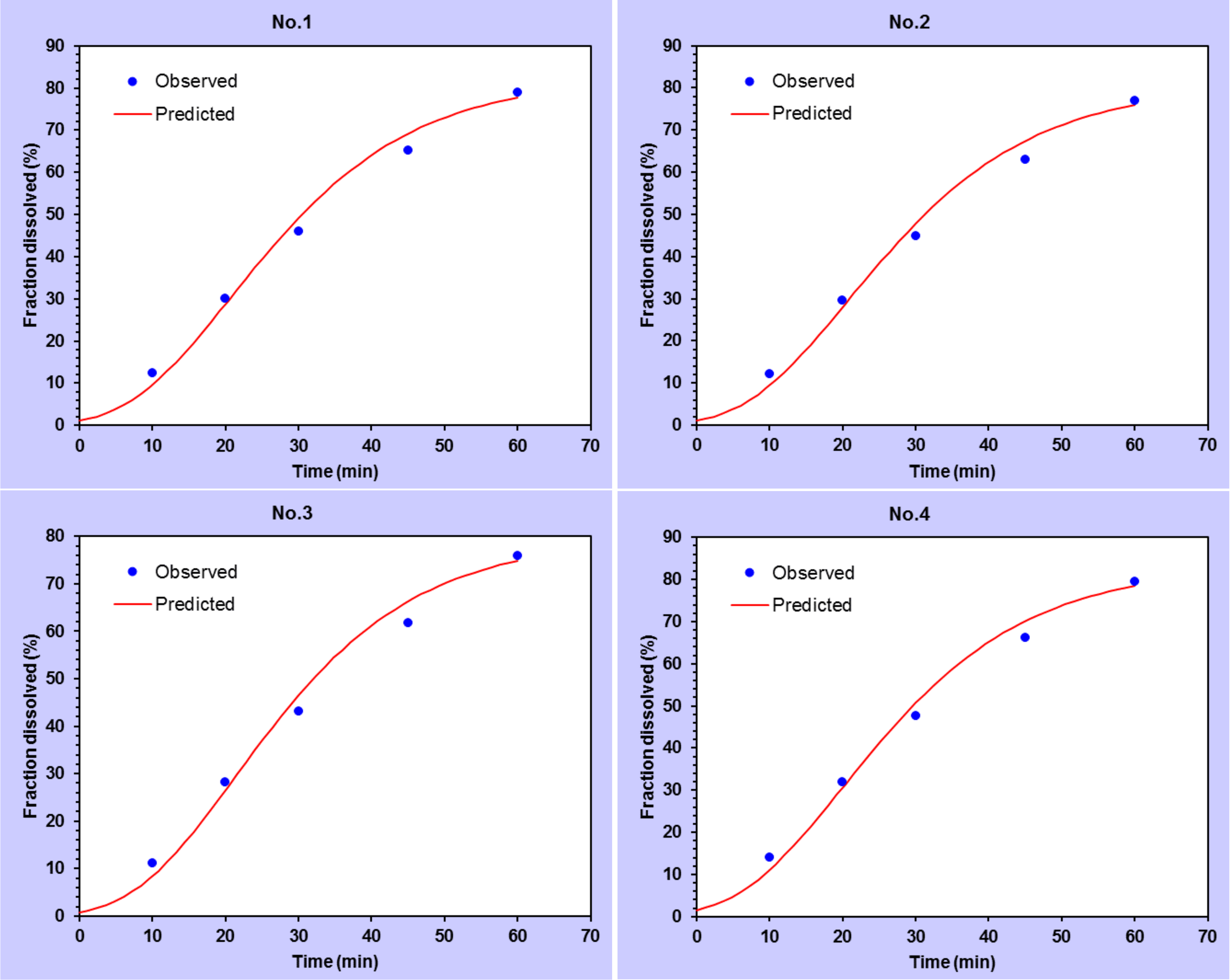

Supplementary materials – Model fitting summary – Polyglykol® 8000 P (modelling up to app. 75 % of carvedilol released)

Model: **Probit_1**

Model equation: $F = 100 \cdot \phi[\alpha + \beta \cdot \log(t)]$

Fitted model parameters per tested tablet (N = 4) with statistics – mean, standard deviation (SD), and relative standard deviation expressed in % (RSD%) (output from DDSolver):

Parameter	No.1	No.2	No.3	No.4	Mean	SD	RSD(%)
α	-3.710	-3.648	-3.724	-3.578	-3.665	0.066	-1.814
β	2.491	2.424	2.449	2.430	2.448	0.030	1.242

Number of dissolution data points (N), degrees of freedom (df), and selected goodness of fit criteria – Pearson correlation coefficient (R), coefficient of determination (R^2), adjusted coefficient of determination ($R^2_{adjusted}$), and residual sum of squares (RSS) (manual calculation in MS Excel):

Parameter	No.1	No.2	No.3	No.4
N	5	5	5	5
df	3	3	3	3
R	0.996761264	0.997047033	0.997085056	0.99653226
R^2	0.993533017	0.994102785	0.994178609	0.993076545
$R^2_{adjusted}$	0.991377356	0.992137047	0.992238146	0.990768727
RSS	19.35592646	16.57306282	16.53950684	19.91809546

Graphical abstract of model fit presented as mean ± 1 SD of the fraction % of released carvedilol:

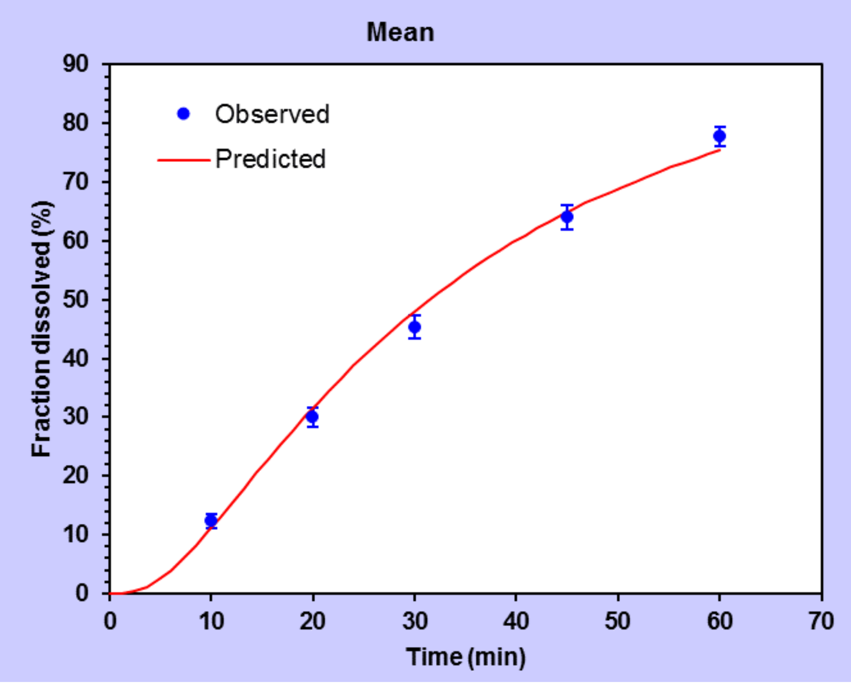

Supplementary materials – Model fitting summary – Polyglykol® 8000 P (modelling up to app. 75 % of carvedilol released)

Graphical abstract of model fit presented as the fraction % of released carvedilol per tested tablet:

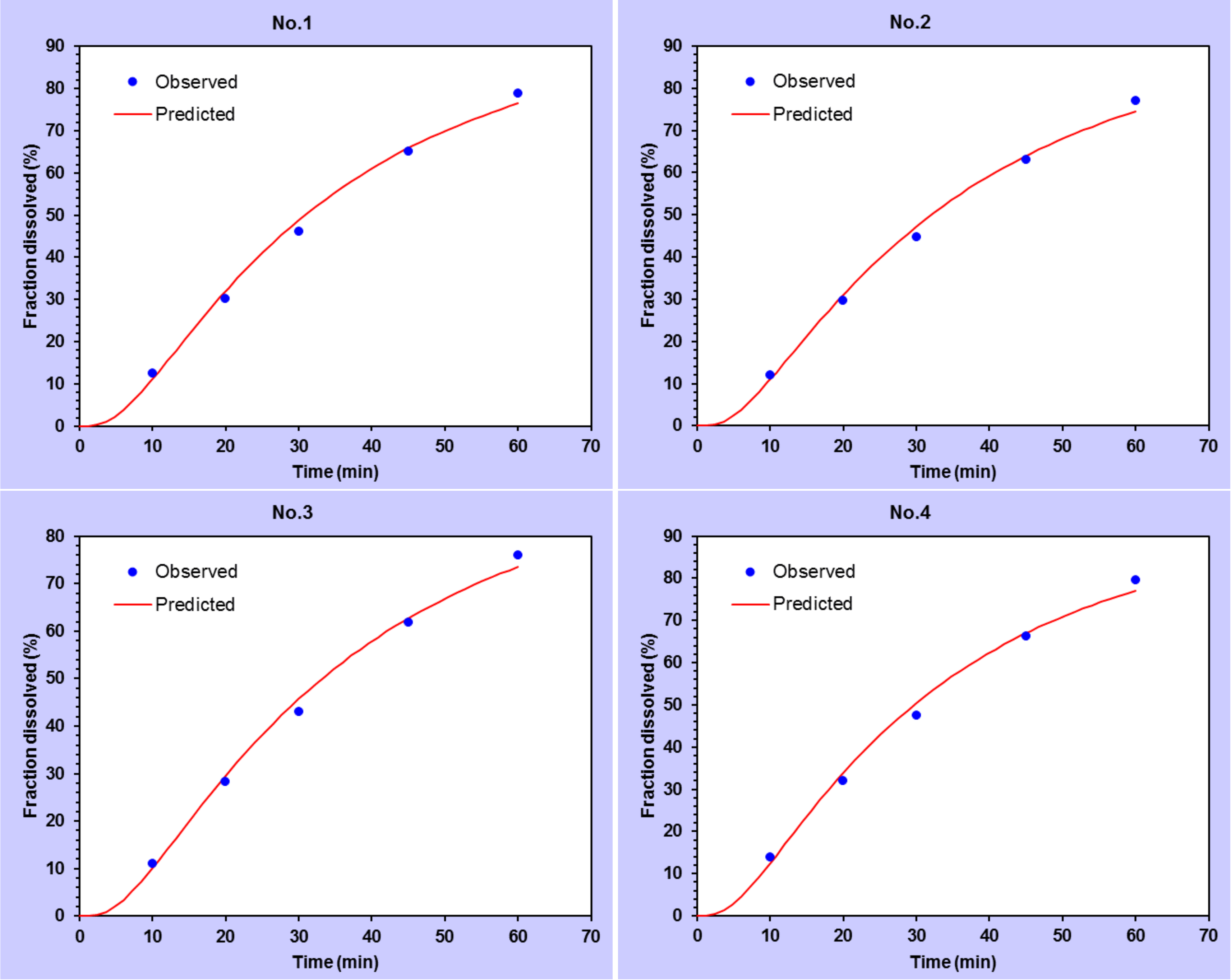

Supplementary materials – Model fitting summary – Polyglykol® 8000 P (modelling up to app. 75 % of carvedilol released)

Model: **Probit_2**

Model equation: $F = F_{max} \cdot \phi[\alpha + \beta \cdot \log(t)]$

Fitted model parameters per tested tablet (N = 4) with statistics – mean, standard deviation (SD), and relative standard deviation expressed in % (RSD%) (output from DDSolver):

Parameter	No.1	No.2	No.3	No.4	Mean	SD	RSD(%)
α	-4.543	-4.522	-4.630	-4.384	-4.520	0.102	-2.253
β	3.320	3.301	3.359	3.234	3.303	0.052	1.571
F_{max}	82.768	80.880	79.794	83.503	81.736	1.702	2.082

Number of dissolution data points (N), degrees of freedom (df), and selected goodness of fit criteria – Pearson correlation coefficient (R), coefficient of determination (R^2), adjusted coefficient of determination ($R^2_{adjusted}$), and residual sum of squares (RSS) (manual calculation in MS Excel):

Parameter	No.1	No.2	No.3	No.4
N	5	5	5	5
df	2	2	2	2
R	0.985206782	0.984877144	0.984449531	0.985002434
R^2	0.970632403	0.969982989	0.969140878	0.970229795
$R^2_{adjusted}$	0.941264807	0.939965978	0.938281757	0.940459589
RSS	97.85814493	94.91547078	97.85076191	96.16367555

Graphical abstract of model fit presented as mean ± 1 SD of the fraction % of released carvedilol:

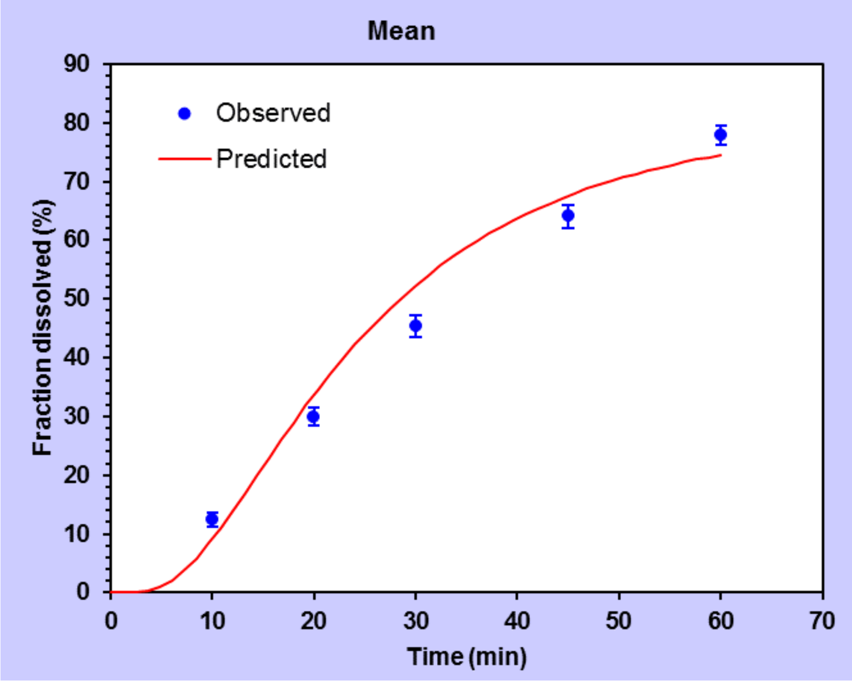

Supplementary materials – Model fitting summary – Polyglykol® 8000 P (modelling up to app. 75 % of carvedilol released)

Graphical abstract of model fit presented as the fraction % of released carvedilol per tested tablet:

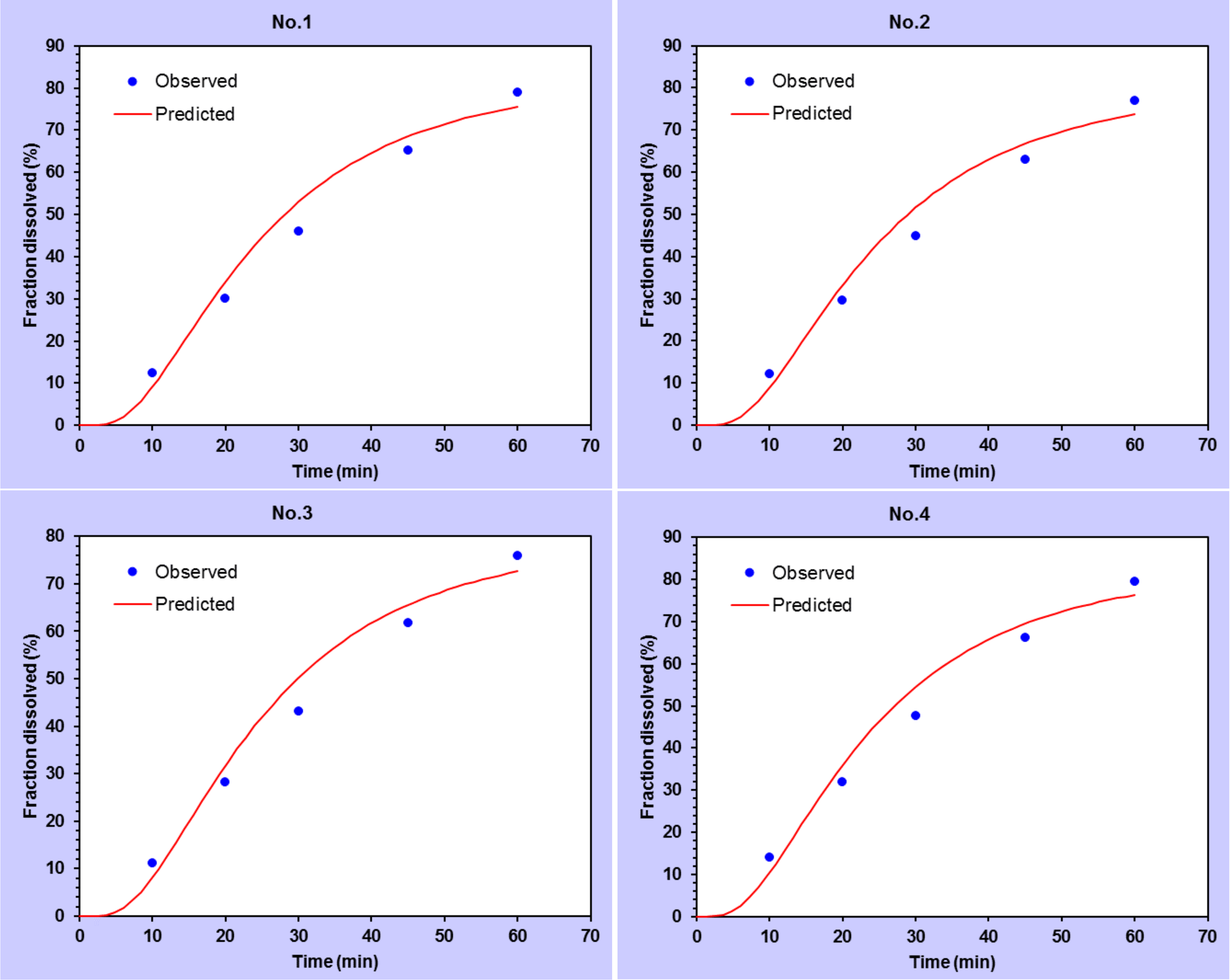

Supplement: Supplementary file 1 [file pharmaceutics-16-00498-s001.zip › Supplementary materials_Model fitting summary_Polyglykol® 8000 P.pdf]
