# Supplementary material for: Comparative Fitting of Mathematical Models to Carvedilol Release Profiles Obtained from Hypromellose Matrix Tablets
Source: Pharmaceutics. 2024 Apr 4;16(4):498. doi: 10.3390/pharmaceutics16040498 (PMC11053526; doi:10.3390/pharmaceutics16040498)

Supplementary materials – Model fitting summary – Polyox™ WSR N-80 (LEO NF Grade) (modelling the entire relevant dissolution data)

Model: **Zero–order**

Model equation: $F = k_0 \cdot t$

Fitted model parameters per tested tablet (N = 4) with statistics – mean, standard deviation (SD), and relative standard deviation expressed in % (RSD%) (output from DDSolver):

Parameter	No.1	No.2	No.3	No.4	Mean	SD	RSD(%)
k_0	0.370	0.367	0.393	0.385	0.379	0.012	3.298

Number of dissolution data points (N), degrees of freedom (df), and selected goodness of fit criteria – Pearson correlation coefficient (R), coefficient of determination (R^2), adjusted coefficient of determination ($R^2_{adjusted}$), and residual sum of squares (RSS) (manual calculation in MS Excel):

Parameter	No.1	No.2	No.3	No.4
N	13	13	13	13
df	12	12	12	12
R	0.98200221	0.98378276	0.97382153	0.97458471
R^2	0.96432834	0.96782853	0.94832836	0.94981536
$R^2_{adjusted}$	0.96432834	0.96782853	0.94832836	0.94981536
RSS	599.067355	592.198721	1023.85698	985.913702

Graphical abstract of model fit presented as mean ± 1 SD of the fraction % of released carvedilol:

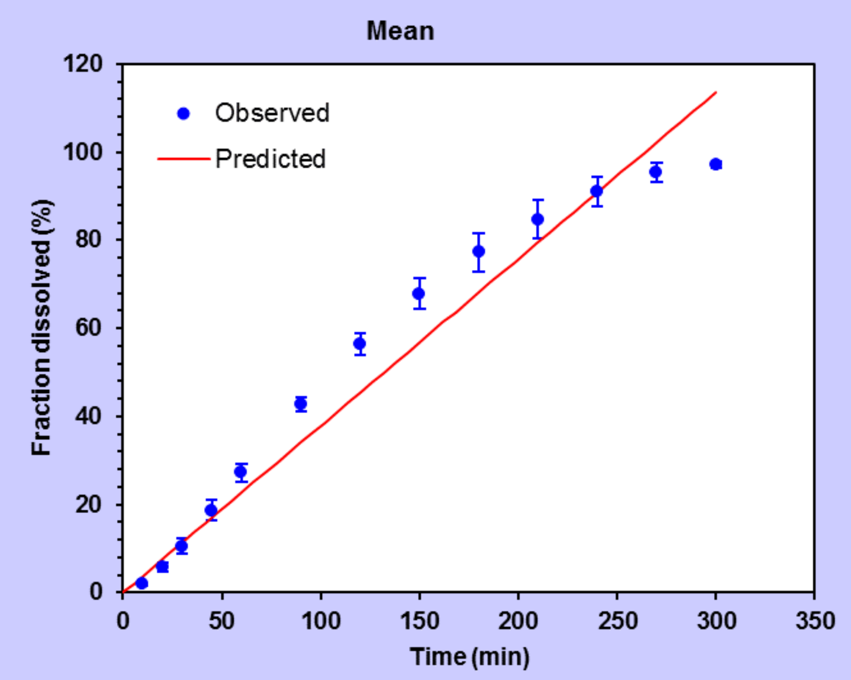

Supplementary materials – Model fitting summary – Polyox^{TM} WSR N-80 (LEO NF Grade) (modelling the entire relevant dissolution data)

Graphical abstract of model fit presented as the fraction % of released carvedilol per tested tablet:

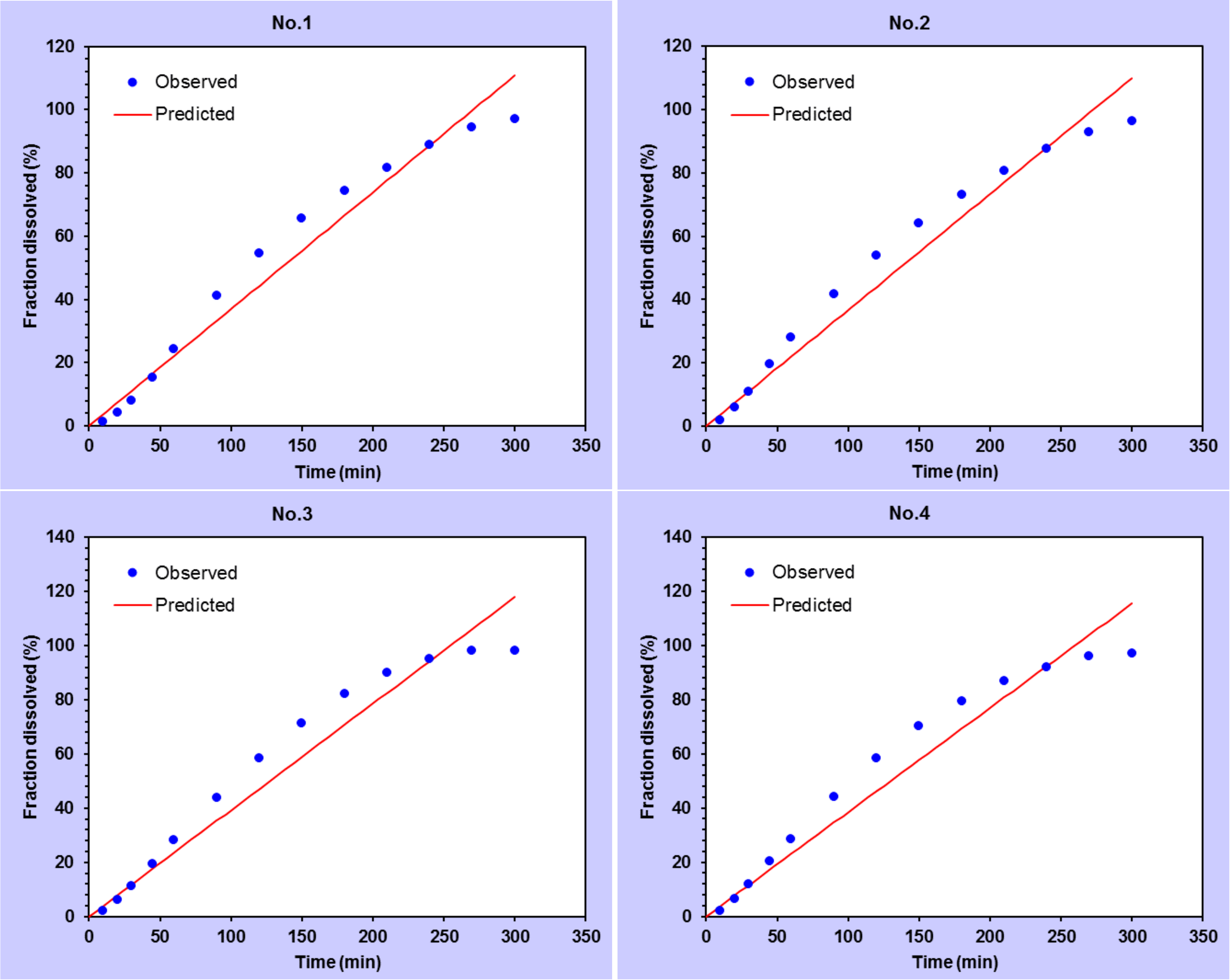

Supplementary materials – Model fitting summary – Polyox™ WSR N-80 (LEO NF Grade) (modelling the entire relevant dissolution data)

Model: **Zero–order with T_{lag}**

Model equation: $F = k_0 \cdot (t - T_{lag})$

Fitted model parameters per tested tablet (N = 4) with statistics – mean, standard deviation (SD), and relative standard deviation expressed in % (RSD%) (output from DDSolver):

Parameter	No.1	No.2	No.3	No.4	Mean	SD	RSD(%)
k_0	0.355	0.340	0.364	0.352	0.353	0.010	2.752
T_{lag}	-8.272	-15.827	-16.321	-18.976	-14.849	4.598	-30.964

Number of dissolution data points (N), degrees of freedom (df), and selected goodness of fit criteria – Pearson correlation coefficient (R), coefficient of determination (R^2), adjusted coefficient of determination ($R^2_{adjusted}$), and residual sum of squares (RSS) (manual calculation in MS Excel):

Parameter	No.1	No.2	No.3	No.4
N	13	13	13	13
df	11	11	11	11
R	0.98200221	0.98378276	0.97382153	0.97458471
R^2	0.96432834	0.96782853	0.94832836	0.94981536
$R^2_{adjusted}$	0.96108547	0.96490385	0.94363094	0.94525312
RSS	560.45629	462.410312	866.143049	786.309404

Graphical abstract of model fit presented as mean ± 1 SD of the fraction % of released carvedilol:

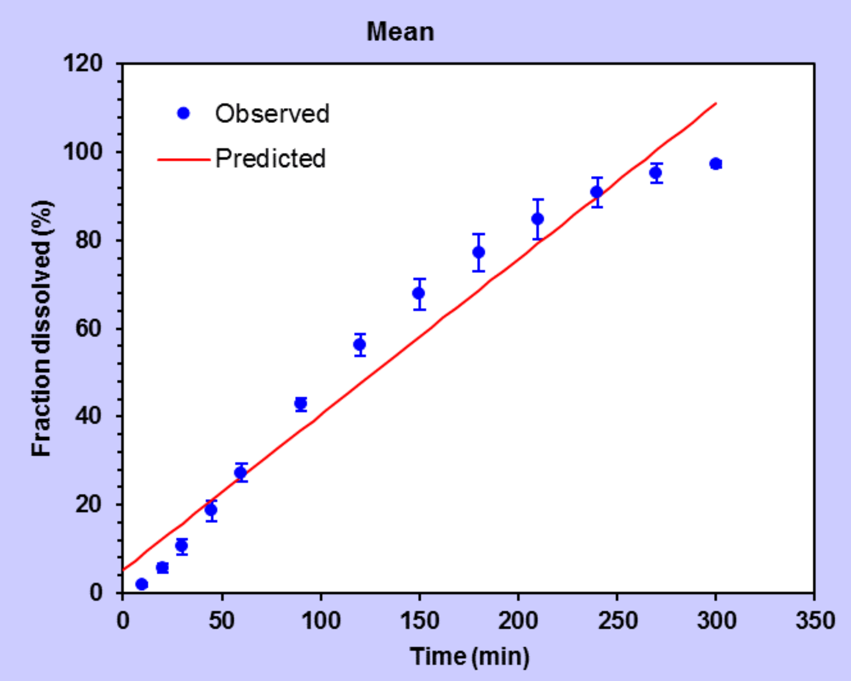

Supplementary materials – Model fitting summary – Polyox^{TM} WSR N-80 (LEO NF Grade) (modelling the entire relevant dissolution data)

Graphical abstract of model fit presented as the fraction % of released carvedilol per tested tablet:

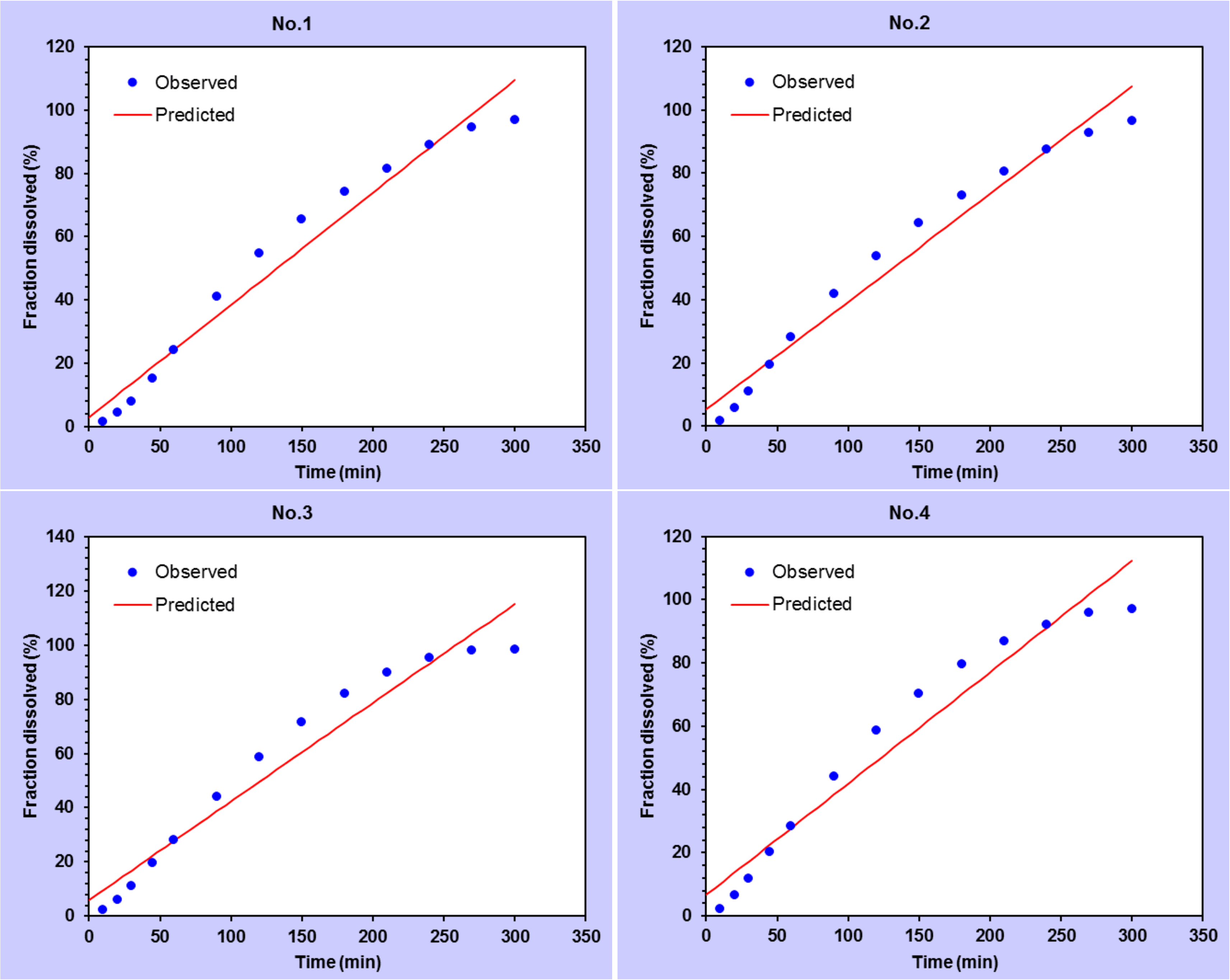

Supplementary materials – Model fitting summary – Polyox™ WSR N-80 (LEO NF Grade) (modelling the entire relevant dissolution data)

Model: **Zero–order with F_0**

Model equation: $F = F_0 + k_0 \cdot t$

Fitted model parameters per tested tablet (N = 4) with statistics – mean, standard deviation (SD), and relative standard deviation expressed in % (RSD%) (output from DDSolver):

Parameter	No.1	No.2	No.3	No.4	Mean	SD	RSD(%)
k_0	0.355	0.340	0.364	0.352	0.353	0.010	2.752
F_0	2.938	5.386	5.937	6.679	5.235	1.621	30.958

Number of dissolution data points (N), degrees of freedom (df), and selected goodness of fit criteria – Pearson correlation coefficient (R), coefficient of determination (R^2), adjusted coefficient of determination ($R^2_{adjusted}$), and residual sum of squares (RSS) (manual calculation in MS Excel):

Parameter	No.1	No.2	No.3	No.4
N	13	13	13	13
df	11	11	11	11
R	0.98200221	0.98378276	0.97382153	0.97458471
R^2	0.96432834	0.96782853	0.94832836	0.94981536
$R^2_{adjusted}$	0.96108547	0.96490385	0.94363094	0.94525312
RSS	560.45629	462.410312	866.143049	786.309404

Graphical abstract of model fit presented as mean ± 1 SD of the fraction % of released carvedilol:

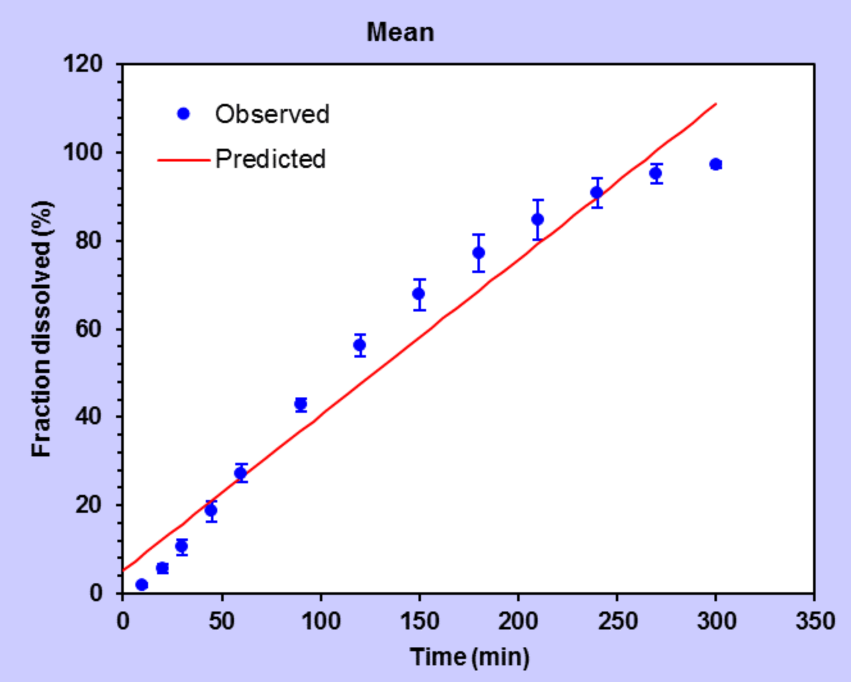

Supplementary materials – Model fitting summary – Polyox™ WSR N-80 (LEO NF Grade) (modelling the entire relevant dissolution data)

Graphical abstract of model fit presented as the fraction % of released carvedilol per tested tablet:

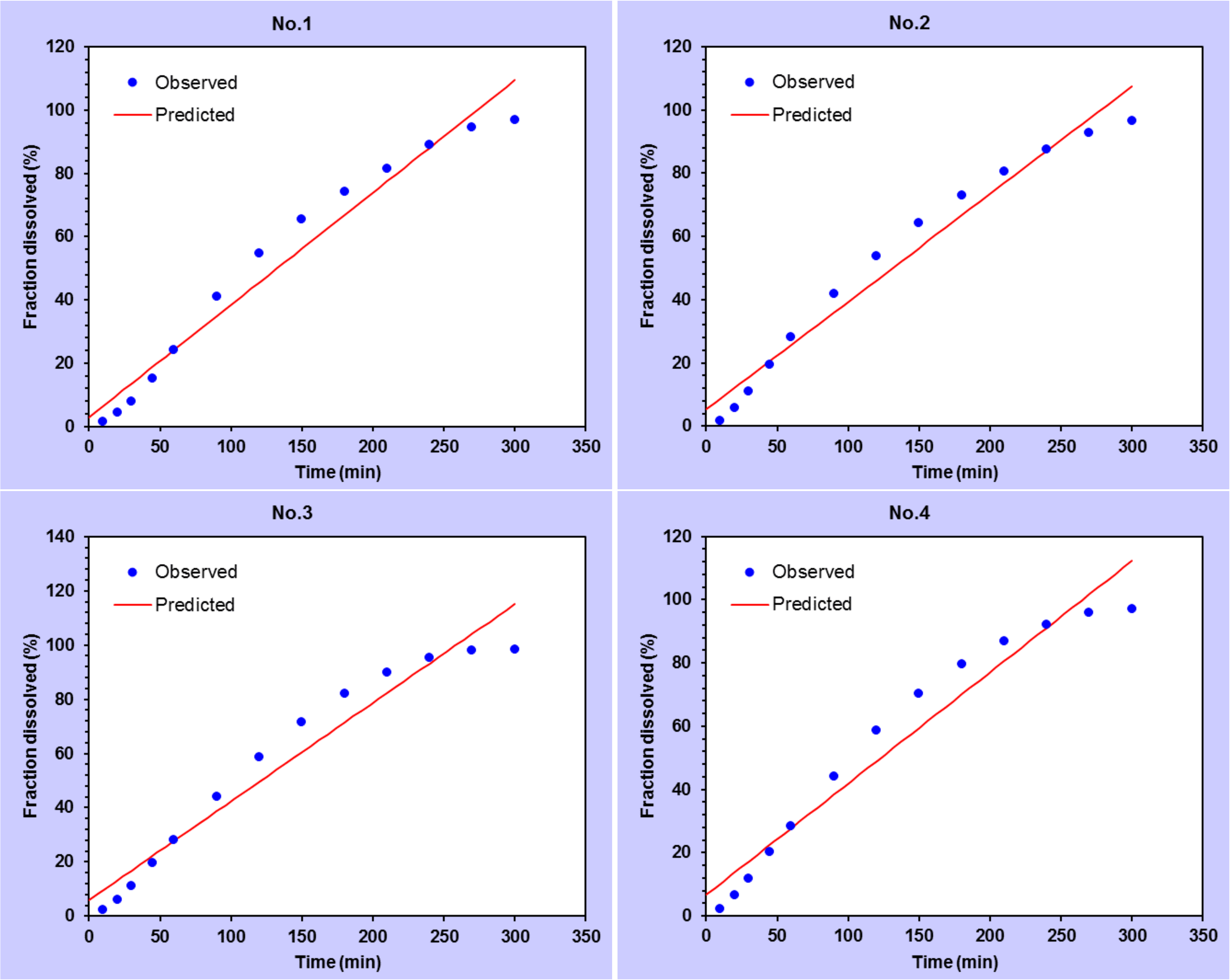

Supplementary materials – Model fitting summary – Polyox™ WSR N-80 (LEO NF Grade) (modelling the entire relevant dissolution data)

Model: **First–order**

Model equation: $F = 100 \cdot (1 - e^{-k_1 \cdot t})$

Fitted model parameters per tested tablet (N = 4) with statistics – mean, standard deviation (SD), and relative standard deviation expressed in % (RSD%) (output from DDSolver):

Parameter	No.1	No.2	No.3	No.4	Mean	SD	RSD(%)
k_1	0.007	0.007	0.012	0.008	0.008	0.002	28.954

Number of dissolution data points (N), degrees of freedom (df), and selected goodness of fit criteria – Pearson correlation coefficient (R), coefficient of determination (R^2), adjusted coefficient of determination ($R^2_{adjusted}$), and residual sum of squares (RSS) (manual calculation in MS Excel):

Parameter	No.1	No.2	No.3	No.4
N	13	13	13	13
df	12	12	12	12
R	0.99591473	0.99797244	0.97994133	0.99679577
R^2	0.99184615	0.99594899	0.96028501	0.99360181
$R^2_{adjusted}$	0.99184615	0.99594899	0.96028501	0.99360181
RSS	765.725403	474.453351	2690.08221	605.058625

Graphical abstract of model fit presented as mean ± 1 SD of the fraction % of released carvedilol:

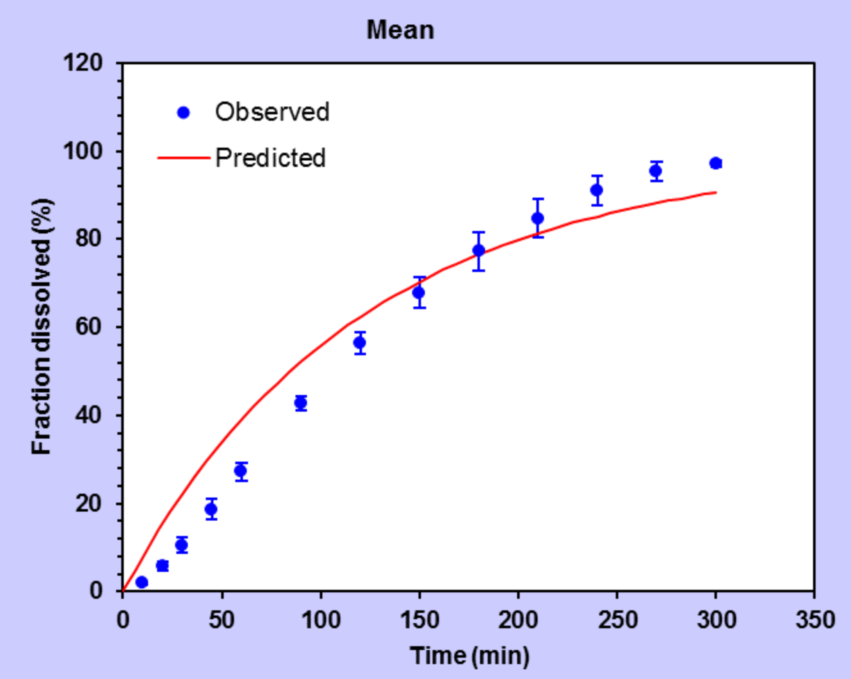

Supplementary materials – Model fitting summary – Polyox^{TM} WSR N-80 (LEO NF Grade) (modelling the entire relevant dissolution data)

Graphical abstract of model fit presented as the fraction % of released carvedilol per tested tablet:

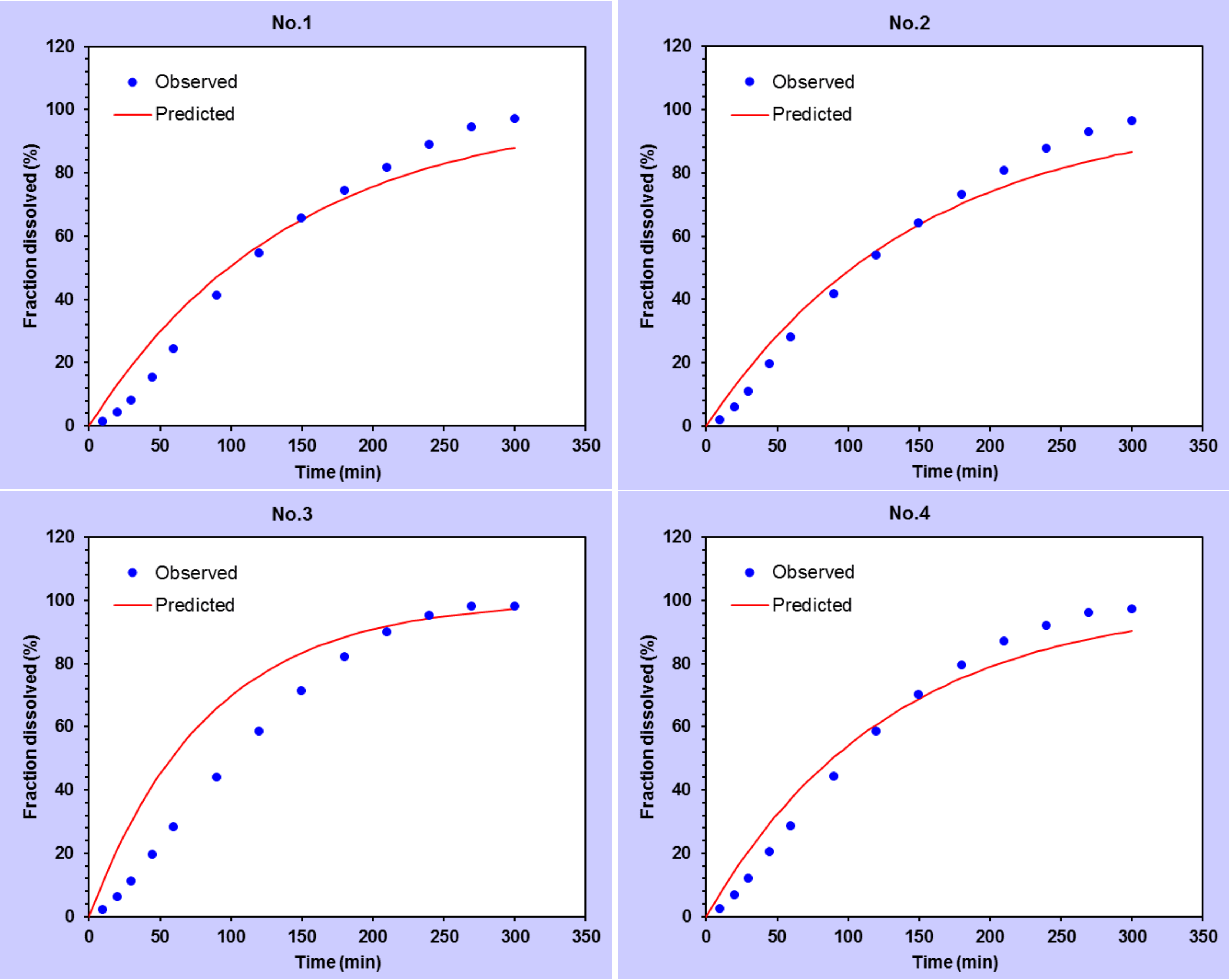

Supplementary materials – Model fitting summary – Polyox™ WSR N-80 (LEO NF Grade) (modelling the entire relevant dissolution data)

Model: **First–order with T_{lag}**

Model equation: $F = 100 \cdot \left[1 - e^{-k_1 \cdot (t - T_{lag})}\right]$

Fitted model parameters per tested tablet (N = 4) with statistics – mean, standard deviation (SD), and relative standard deviation expressed in % (RSD%) (output from DDSolver):

Parameter	No.1	No.2	No.3	No.4	Mean	SD	RSD(%)
k_1	0.008	0.008	0.014	0.010	0.010	0.003	28.887
T_{lag}	8.143	7.261	33.490	21.108	17.500	12.398	70.842

Number of dissolution data points (N), degrees of freedom (df), and selected goodness of fit criteria – Pearson correlation coefficient (R), coefficient of determination (R^2), adjusted coefficient of determination ($R^2_{adjusted}$), and residual sum of squares (RSS) (manual calculation in MS Excel):

Parameter	No.1	No.2	No.3	No.4
N	13	13	13	13
df	11	11	11	11
R	0.99194671	0.99522648	0.96691752	0.99050033
R^2	0.98395827	0.99047575	0.93492949	0.98109091
$R^2_{adjusted}$	0.98249993	0.9896099	0.92901399	0.9793719
RSS	566.27757	276.80482	3275.04179	379.528614

Graphical abstract of model fit presented as mean ± 1 SD of the fraction % of released carvedilol:

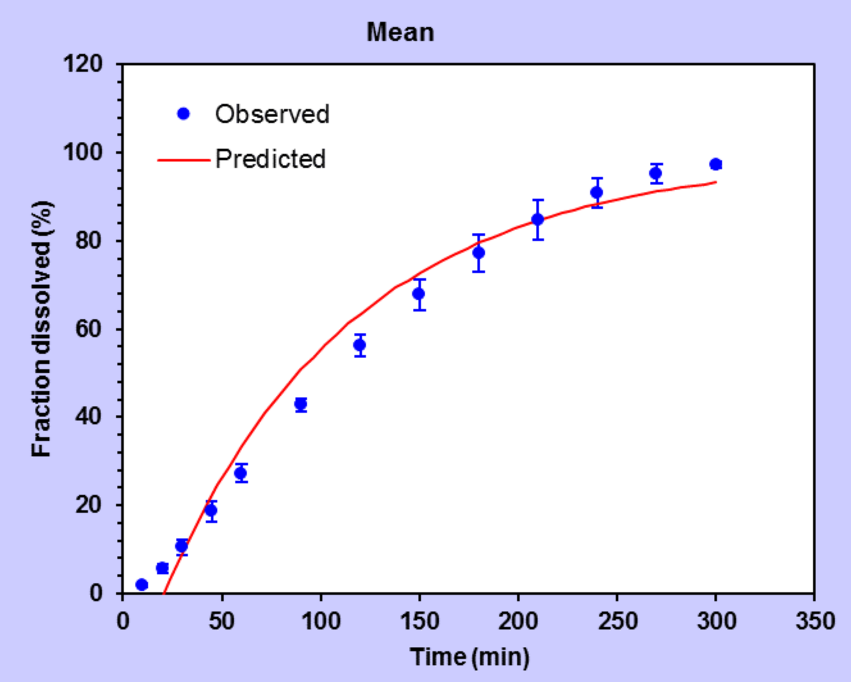

Supplementary materials – Model fitting summary – Polyox™ WSR N-80 (LEO NF Grade) (modelling the entire relevant dissolution data)

Graphical abstract of model fit presented as the fraction % of released carvedilol per tested tablet:

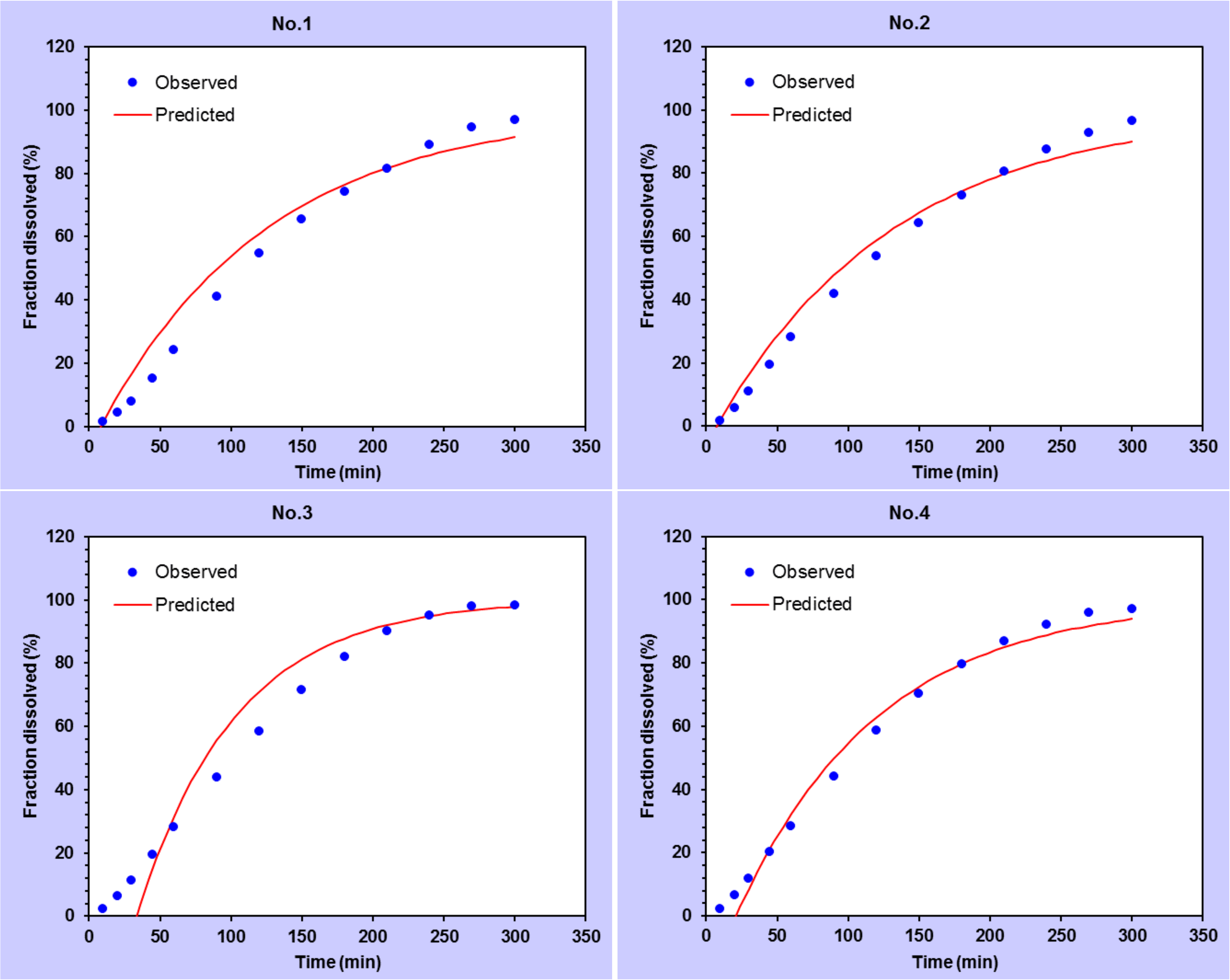

Supplementary materials – Model fitting summary – Polyox™ WSR N-80 (LEO NF Grade) (modelling the entire relevant dissolution data)

Model: **First–order with F_{max}**

Model equation: $F = F_{max} \cdot (1 - e^{-k_1 \cdot t})$

Fitted model parameters per tested tablet (N = 4) with statistics – mean, standard deviation (SD), and relative standard deviation expressed in % (RSD%) (output from DDSolver):

Parameter	No.1	No.2	No.3	No.4	Mean	SD	RSD(%)
k_1	0.009	0.008	0.010	0.009	0.009	0.001	6.912
F_{max}	101.768	101.378	103.155	101.977	102.070	0.765	0.749

Number of dissolution data points (N), degrees of freedom (df), and selected goodness of fit criteria – Pearson correlation coefficient (R), coefficient of determination (R^2), adjusted coefficient of determination ($R^2_{adjusted}$), and residual sum of squares (RSS) (manual calculation in MS Excel):

Parameter	No.1	No.2	No.3	No.4
N	13	13	13	13
df	11	11	11	11
R	0.99109946	0.99340704	0.98952049	0.99271833
R^2	0.98227814	0.98685755	0.97915081	0.98548968
$R^2_{adjusted}$	0.98066706	0.98566278	0.97725543	0.98417056
RSS	1471.29579	985.674474	1562.90049	1159.71147

Graphical abstract of model fit presented as mean ± 1 SD of the fraction % of released carvedilol:

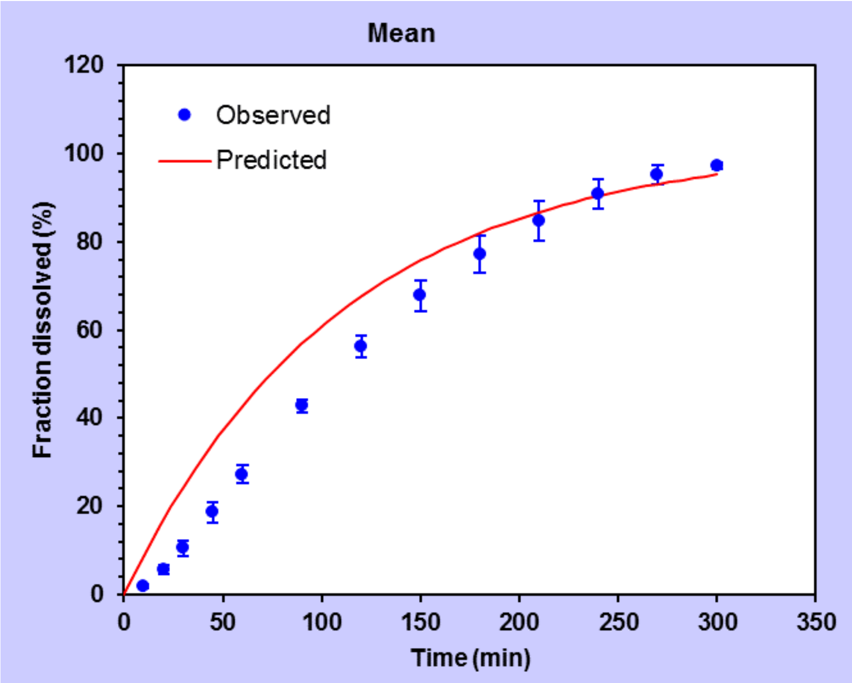

Supplementary materials – Model fitting summary – Polyox™ WSR N-80 (LEO NF Grade) (modelling the entire relevant dissolution data)

Graphical abstract of model fit presented as the fraction % of released carvedilol per tested tablet:

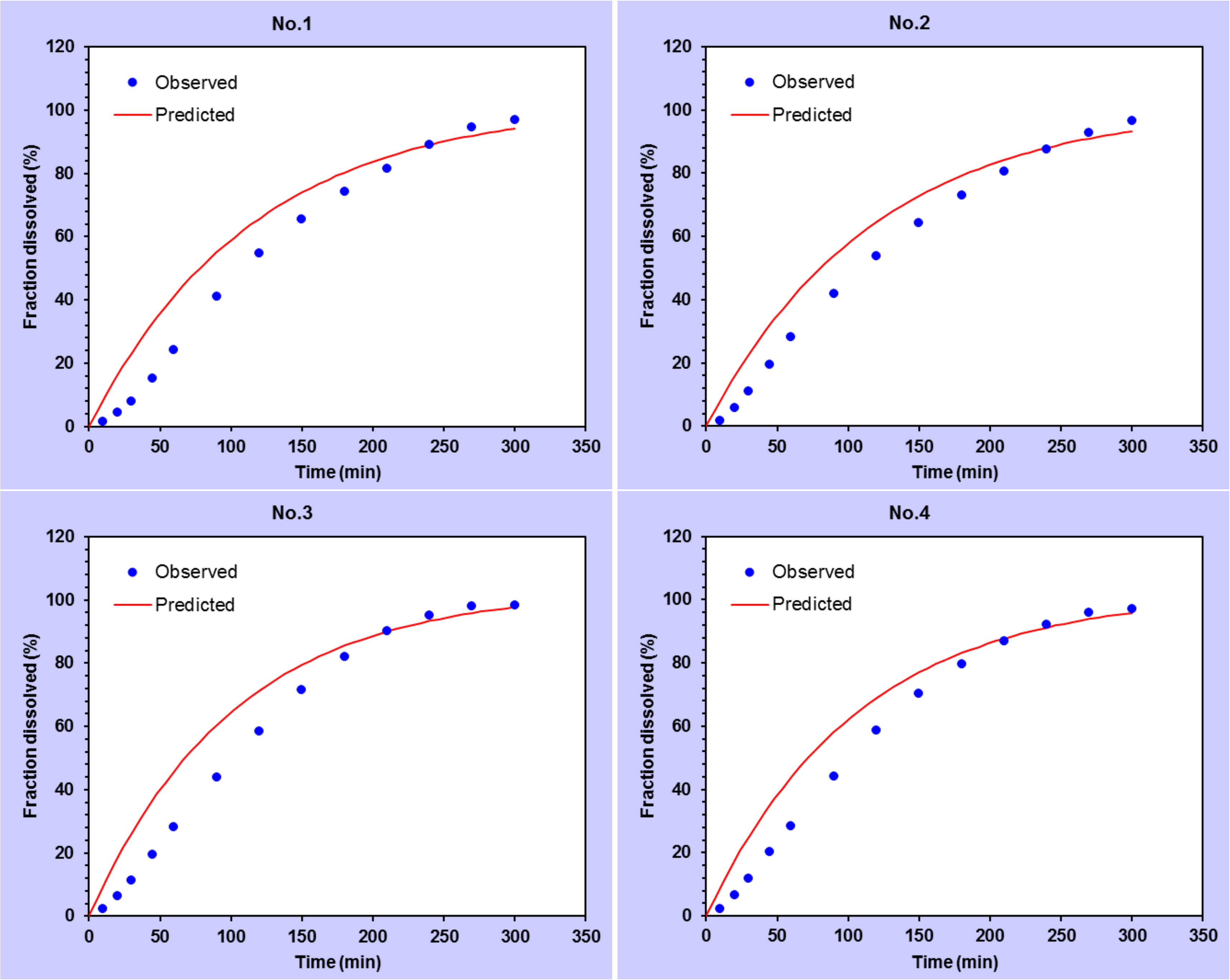

Supplementary materials – Model fitting summary – Polyox™ WSR N-80 (LEO NF Grade) (modelling the entire relevant dissolution data)

Model: **First–order with T_{lag} and F_{max}**

Model equation: $F = F_{max} \cdot \left[1 - e^{-k_1 \cdot (t - T_{lag})}\right]$

Fitted model parameters per tested tablet (N = 4) with statistics – mean, standard deviation (SD), and relative standard deviation expressed in % (RSD%) (output from DDSolver):

Parameter	No.1	No.2	No.3	No.4	Mean	SD	RSD(%)
k_1	0.011	0.010	0.011	0.011	0.011	0.001	6.259
T_{lag}	14.574	26.739	26.570	25.028	23.228	5.820	25.057
F_{max}	97.057	101.378	103.155	101.977	100.892	2.661	2.638

Number of dissolution data points (N), degrees of freedom (df), and selected goodness of fit criteria – Pearson correlation coefficient (R), coefficient of determination (R^2), adjusted coefficient of determination ($R^2_{adjusted}$), and residual sum of squares (RSS) (manual calculation in MS Excel):

Parameter	No.1	No.2	No.3	No.4
N	13	13	13	13
df	10	10	10	10
R	0.98066325	0.98849186	0.98330974	0.98787092
R^2	0.96170041	0.97711615	0.96689804	0.97588895
$R^2_{adjusted}$	0.9540405	0.97253938	0.96027764	0.97106674
RSS	949.405829	787.075687	1019.76217	730.852041

Graphical abstract of model fit presented as mean ± 1 SD of the fraction % of released carvedilol:

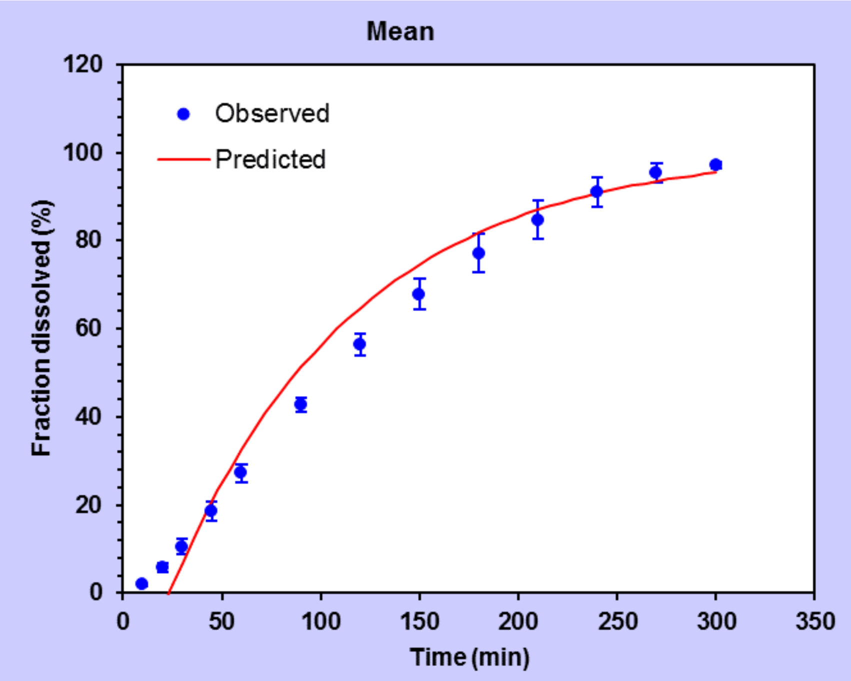

Supplementary materials – Model fitting summary – Polyox™ WSR N-80 (LEO NF Grade) (modelling the entire relevant dissolution data)

Graphical abstract of model fit presented as the fraction % of released carvedilol per tested tablet:

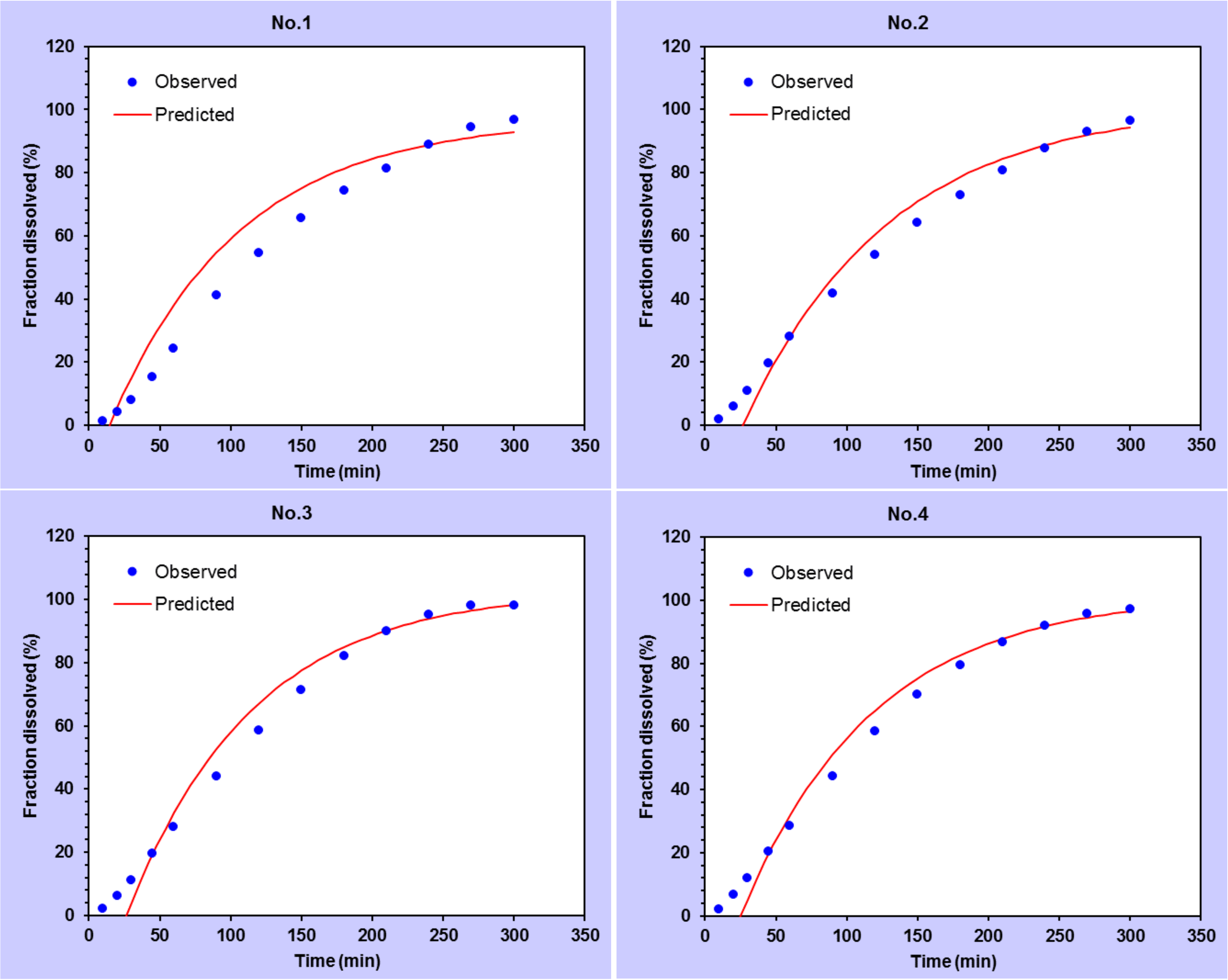

Supplementary materials – Model fitting summary – Polyox™ WSR N-80 (LEO NF Grade) (modelling the entire relevant dissolution data)

Model: **Higuchi**

Model equation: $F = k_H \cdot t^{0.5}$

Fitted model parameters per tested tablet (N = 4) with statistics – mean, standard deviation (SD), and relative standard deviation expressed in % (RSD%) (output from DDSolver):

Parameter	No.1	No.2	No.3	No.4	Mean	SD	RSD(%)
k_H	5.181	5.171	5.548	5.444	5.336	0.189	3.551

Number of dissolution data points (N), degrees of freedom (df), and selected goodness of fit criteria – Pearson correlation coefficient (R), coefficient of determination (R^2), adjusted coefficient of determination ($R^2_{adjusted}$), and residual sum of squares (RSS) (manual calculation in MS Excel):

Parameter	No.1	No.2	No.3	No.4
N	13	13	13	13
df	12	12	12	12
R	0.99489497	0.99774195	0.99260598	0.99425902
R^2	0.98981601	0.99548901	0.98526664	0.98855099
$R^2_{adjusted}$	0.98981601	0.99548901	0.98526664	0.98855099
RSS	1980.43634	1457.04944	1864.51237	1598.81263

Graphical abstract of model fit presented as mean ± 1 SD of the fraction % of released carvedilol:

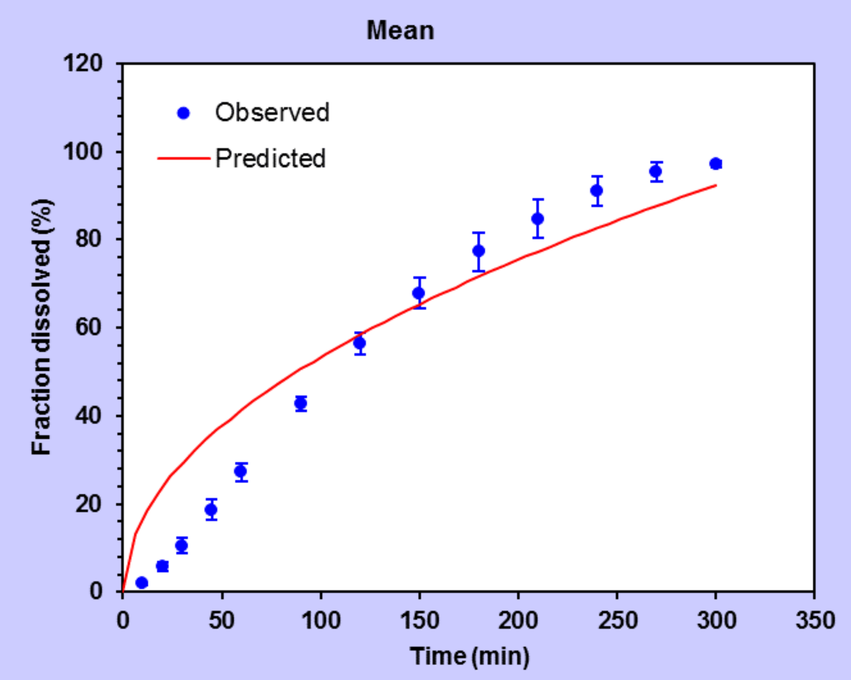

Supplementary materials – Model fitting summary – Polyox™ WSR N-80 (LEO NF Grade) (modelling the entire relevant dissolution data)

Graphical abstract of model fit presented as the fraction % of released carvedilol per tested tablet:

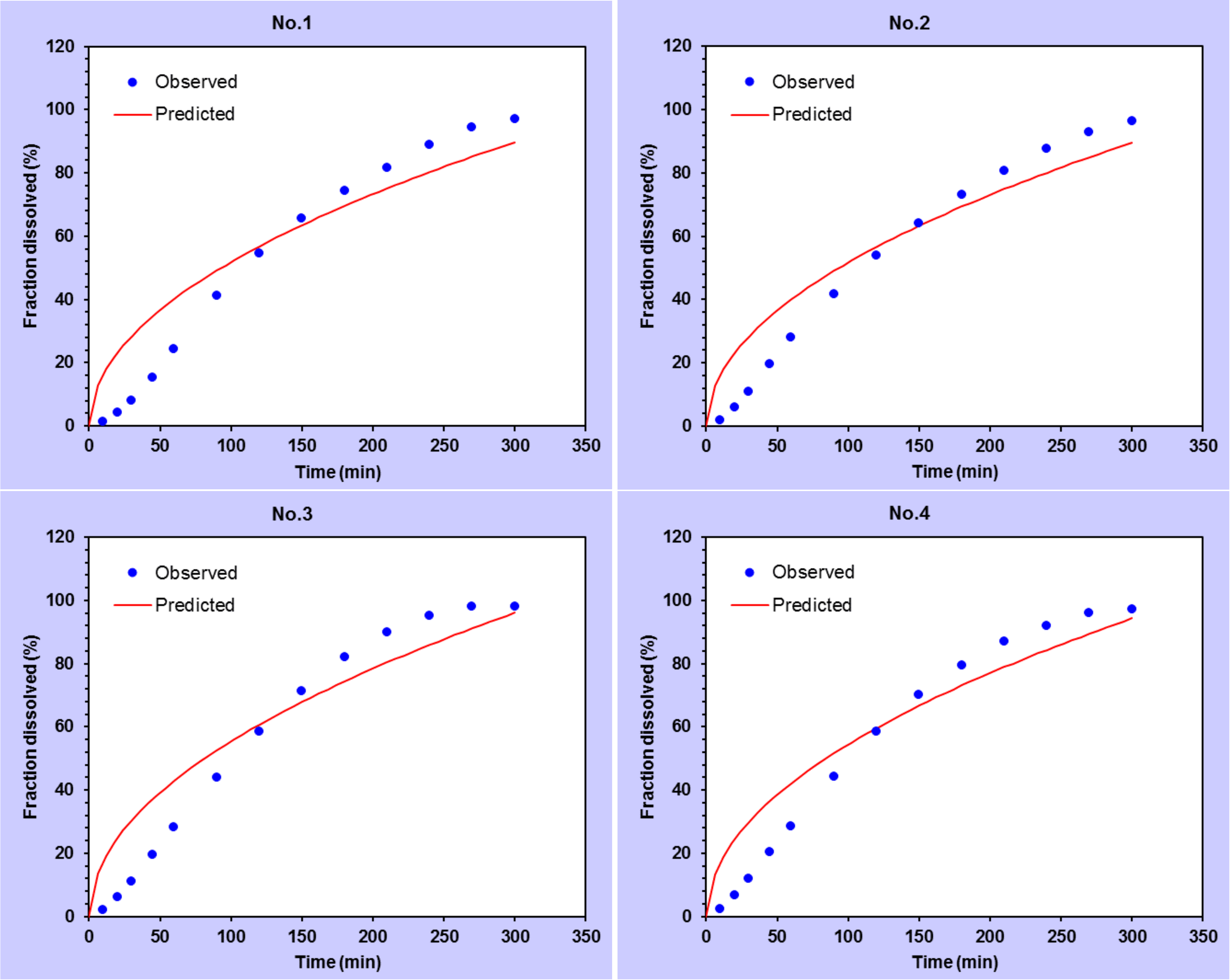

Supplementary materials – Model fitting summary – Polyox™ WSR N-80 (LEO NF Grade) (modelling the entire relevant dissolution data)

Model: **Higuchi with T_{lag}**

Model equation: $F = k_H \cdot (t - T_{lag})^{0.5}$

Fitted model parameters per tested tablet (N = 4) with statistics – mean, standard deviation (SD), and relative standard deviation expressed in % (RSD%) (output from DDSolver):

Parameter	No.1	No.2	No.3	No.4	Mean	SD	RSD(%)
k_H	5.993	5.897	6.259	6.105	6.063	0.156	2.567
T_{lag}	29.269	27.436	24.771	23.893	26.342	2.465	9.358

Number of dissolution data points (N), degrees of freedom (df), and selected goodness of fit criteria – Pearson correlation coefficient (R), coefficient of determination (R^2), adjusted coefficient of determination ($R^2_{adjusted}$), and residual sum of squares (RSS) (manual calculation in MS Excel):

Parameter	No.1	No.2	No.3	No.4
N	13	13	13	13
df	11	11	11	11
R	0.99316862	0.99542013	0.9901928	0.99168302
R^2	0.98638391	0.99086123	0.98048178	0.98343521
$R^2_{adjusted}$	0.98514609	0.99003043	0.97870739	0.98192932
RSS	228.732352	136.545184	343.262661	268.510298

Graphical abstract of model fit presented as mean ± 1 SD of the fraction % of released carvedilol:

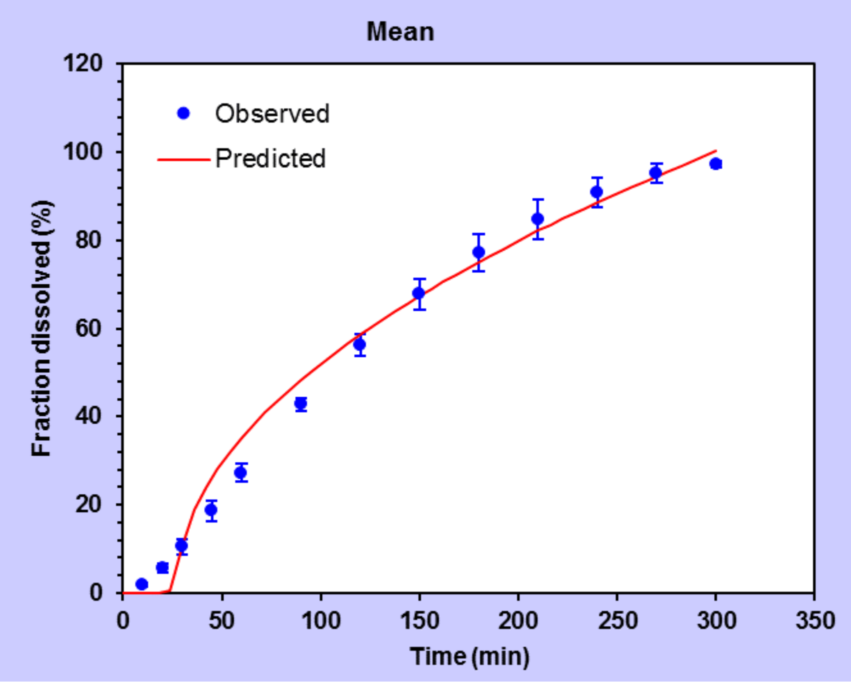

Supplementary materials – Model fitting summary – Polyox™ WSR N-80 (LEO NF Grade) (modelling the entire relevant dissolution data)

Graphical abstract of model fit presented as the fraction % of released carvedilol per tested tablet:

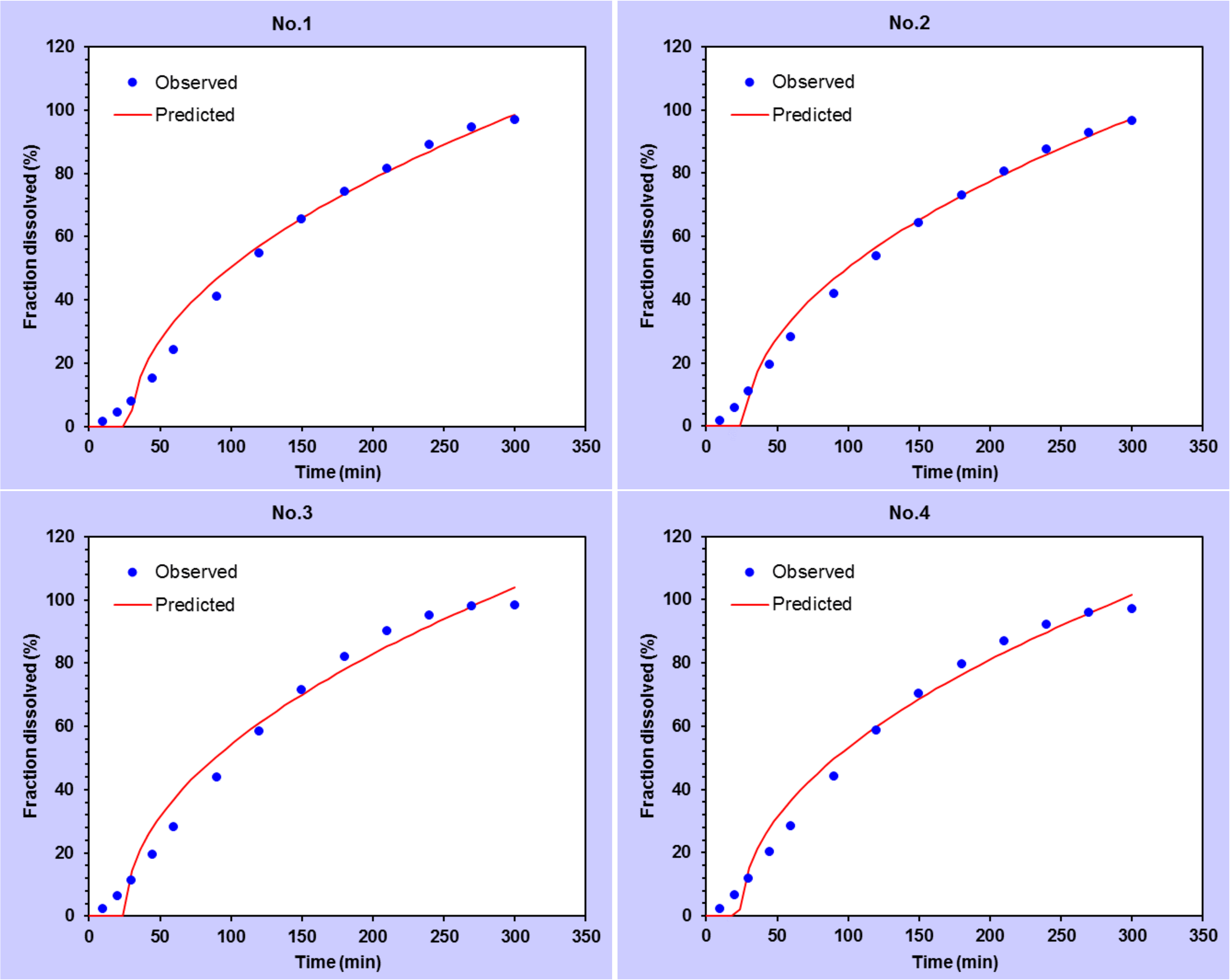

Supplementary materials – Model fitting summary – Polyox™ WSR N-80 (LEO NF Grade) (modelling the entire relevant dissolution data)

Model: **Higuchi with F_0**

Model equation: $F = F_0 + k_H \cdot t^{0.5}$

Fitted model parameters per tested tablet (N = 4) with statistics – mean, standard deviation (SD), and relative standard deviation expressed in % (RSD%) (output from DDSolver):

Parameter	No.1	No.2	No.3	No.4	Mean	SD	RSD(%)
k_H	7.552	7.244	7.783	7.537	7.529	0.221	2.932
F_0	-29.765	-26.030	-28.057	-26.283	-27.534	1.740	-6.318

Number of dissolution data points (N), degrees of freedom (df), and selected goodness of fit criteria – Pearson correlation coefficient (R), coefficient of determination (R^2), adjusted coefficient of determination ($R^2_{adjusted}$), and residual sum of squares (RSS) (manual calculation in MS Excel):

Parameter	No.1	No.2	No.3	No.4
N	13	13	13	13
df	11	11	11	11
R	0.99489497	0.99774195	0.99260598	0.99425902
R^2	0.98981601	0.99548901	0.98526664	0.98855099
$R^2_{adjusted}$	0.98889019	0.99507892	0.98392725	0.98751017
RSS	160.006059	64.8378607	246.967139	179.386873

Graphical abstract of model fit presented as mean ± 1 SD of the fraction % of released carvedilol:

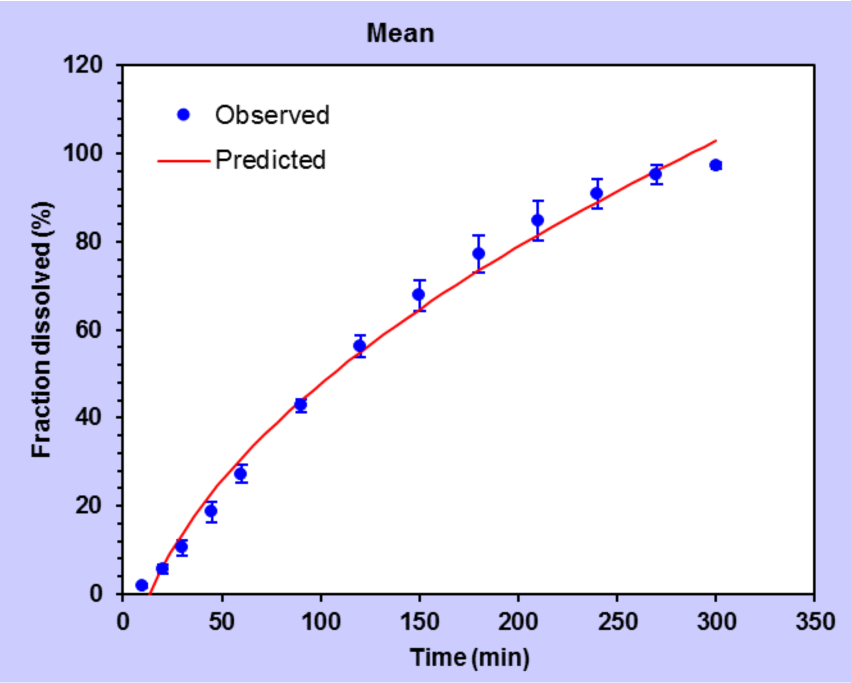

Supplementary materials – Model fitting summary – Polyox™ WSR N-80 (LEO NF Grade) (modelling the entire relevant dissolution data)

Graphical abstract of model fit presented as the fraction % of released carvedilol per tested tablet:

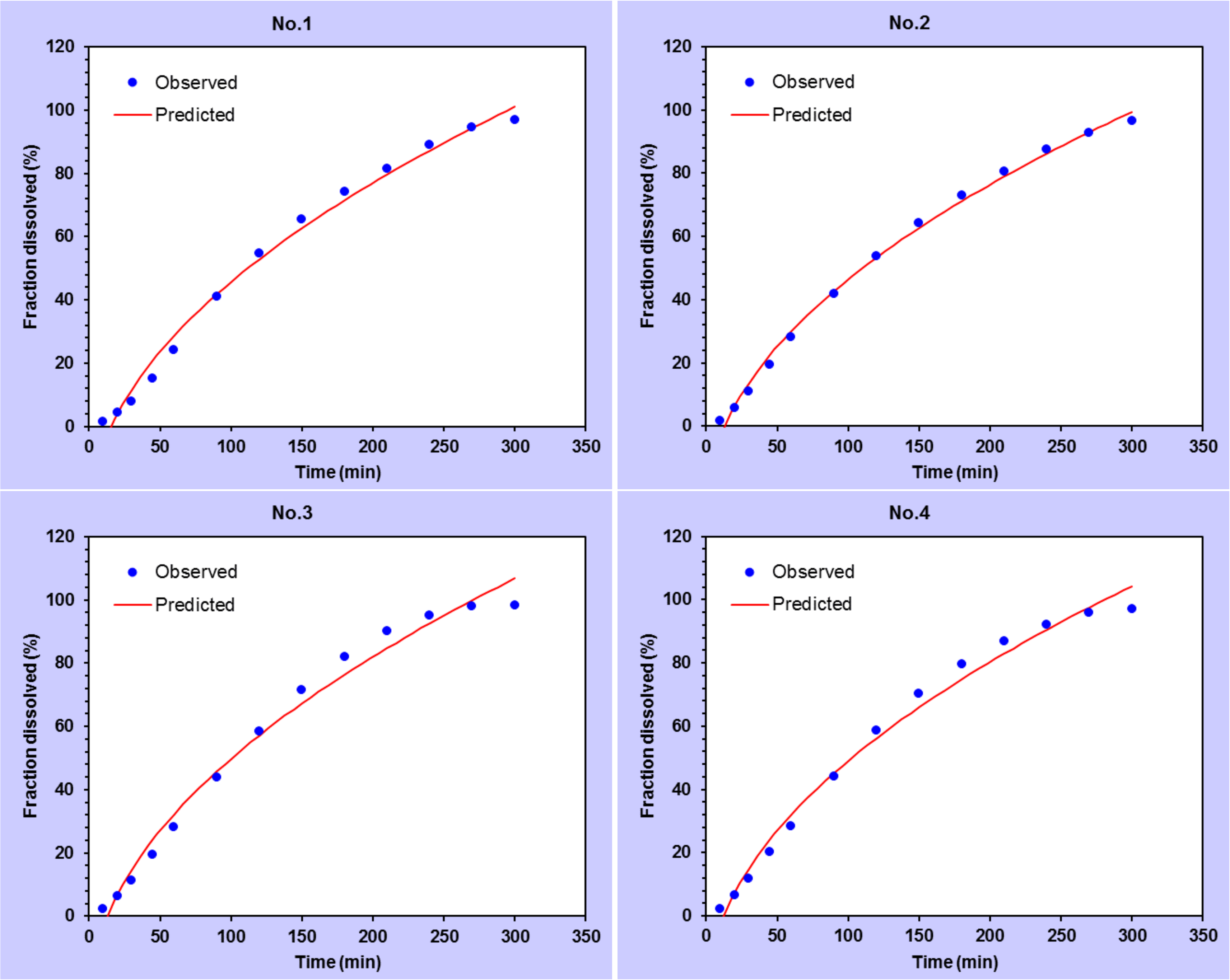

Supplementary materials – Model fitting summary – Polyox™ WSR N-80 (LEO NF Grade) (modelling the entire relevant dissolution data)

Model: **Korsmeyer–Peppas**

Model equation: $F = k_{KP} \cdot t^n$

Fitted model parameters per tested tablet (N = 4) with statistics – mean, standard deviation (SD), and relative standard deviation expressed in % (RSD%) (output from DDSolver):

Parameter	No.1	No.2	No.3	No.4	Mean	SD	RSD(%)
k_{KP}	0.155	0.289	0.319	0.354	0.279	0.087	31.089
n	1.160	1.050	1.043	1.022	1.069	0.062	5.811

Number of dissolution data points (N), degrees of freedom (df), and selected goodness of fit criteria – Pearson correlation coefficient (R), coefficient of determination (R^2), adjusted coefficient of determination ($R^2_{adjusted}$), and residual sum of squares (RSS) (manual calculation in MS Excel):

Parameter	No.1	No.2	No.3	No.4
N	13	13	13	13
df	11	11	11	11
R	0.97229352	0.98094212	0.97091031	0.97311321
R^2	0.94535469	0.96224744	0.94266683	0.94694931
$R^2_{adjusted}$	0.94038693	0.95881538	0.93745472	0.94212652
RSS	1159.61128	816.345392	1254.70852	1148.73773

Graphical abstract of model fit presented as mean ± 1 SD of the fraction % of released carvedilol:

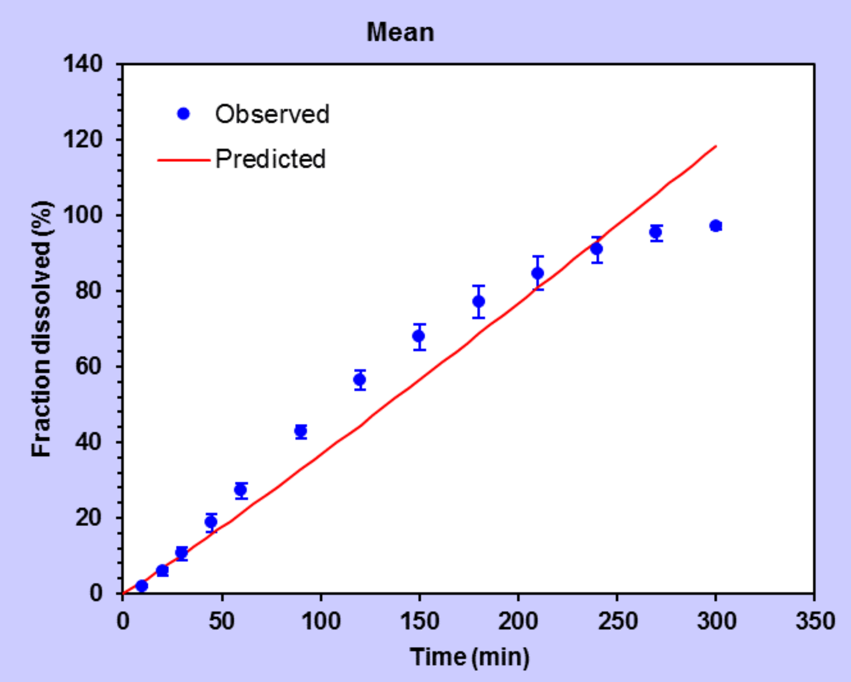

Supplementary materials – Model fitting summary – Polyox™ WSR N-80 (LEO NF Grade) (modelling the entire relevant dissolution data)

Graphical abstract of model fit presented as the fraction % of released carvedilol per tested tablet:

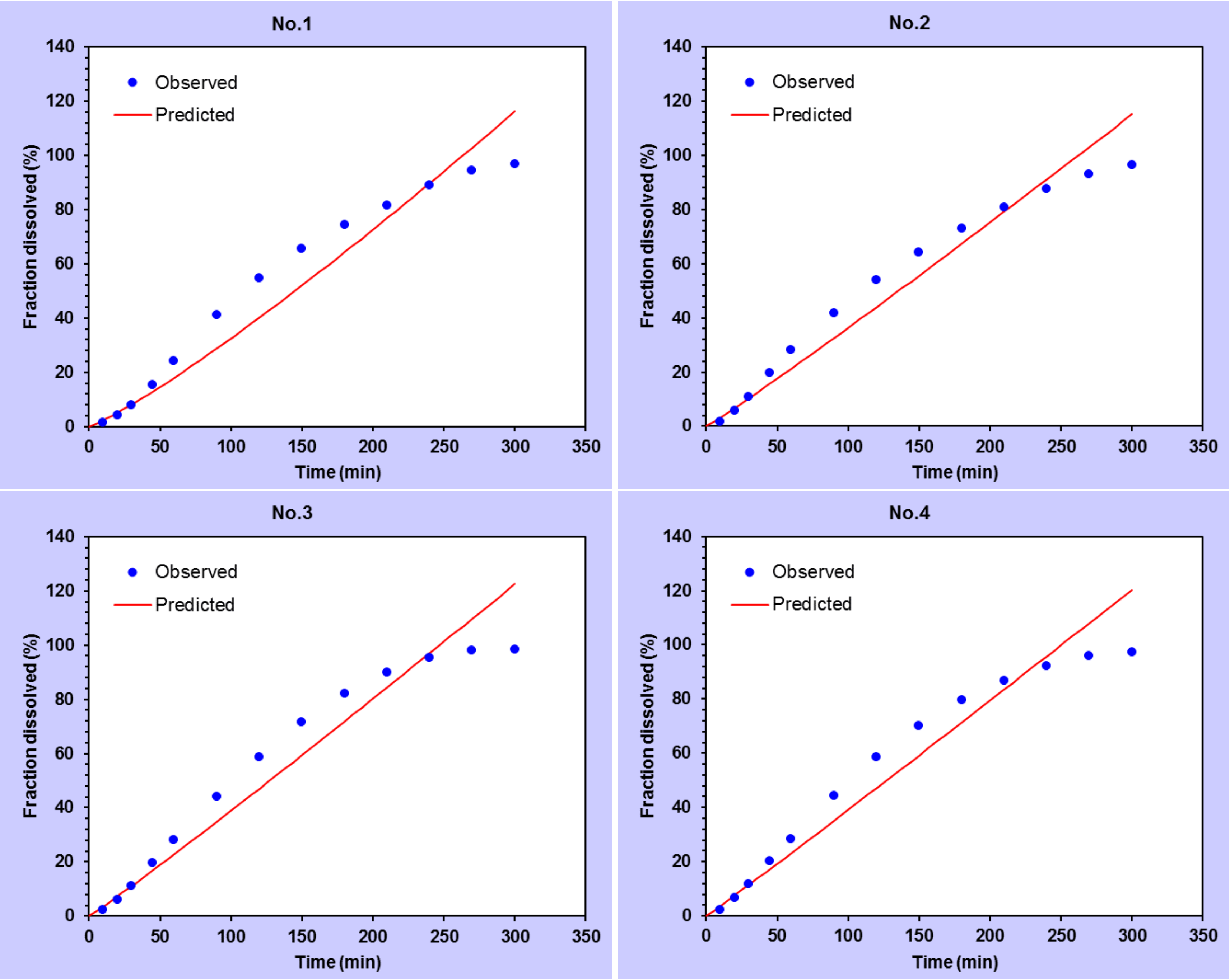

Supplementary materials – Model fitting summary – Polyox™ WSR N-80 (LEO NF Grade) (modelling the entire relevant dissolution data)

Model: **Korsmeyer–Peppas with T_{lag}**

Model equation: $F = k_{KP} \cdot (t - T_{lag})^n$

Fitted model parameters per tested tablet (N = 4) with statistics – mean, standard deviation (SD), and relative standard deviation expressed in % (RSD%) (output from DDSolver):

Parameter	No.1	No.2	No.3	No.4	Mean	SD	RSD(%)
k_{KP}	0.240	0.380	0.423	0.462	0.376	0.097	25.735
n	1.062	1.014	1.005	0.986	1.017	0.032	3.173
T_{lag}	4.919	6.000	6.000	6.000	5.730	0.541	9.433

Number of dissolution data points (N), degrees of freedom (df), and selected goodness of fit criteria – Pearson correlation coefficient (R), coefficient of determination (R^2), adjusted coefficient of determination ($R^2_{adjusted}$), and residual sum of squares (RSS) (manual calculation in MS Excel):

Parameter	No.1	No.2	No.3	No.4
N	13	13	13	13
df	10	10	10	10
R	0.97827774	0.9829589	0.97345003	0.97560528
R^2	0.95702734	0.96620821	0.94760495	0.95180565
$R^2_{adjusted}$	0.94843281	0.95944985	0.93712594	0.94216678
RSS	1507.34415	1123.44393	1510.45611	1398.61594

Graphical abstract of model fit presented as mean ± 1 SD of the fraction % of released carvedilol:

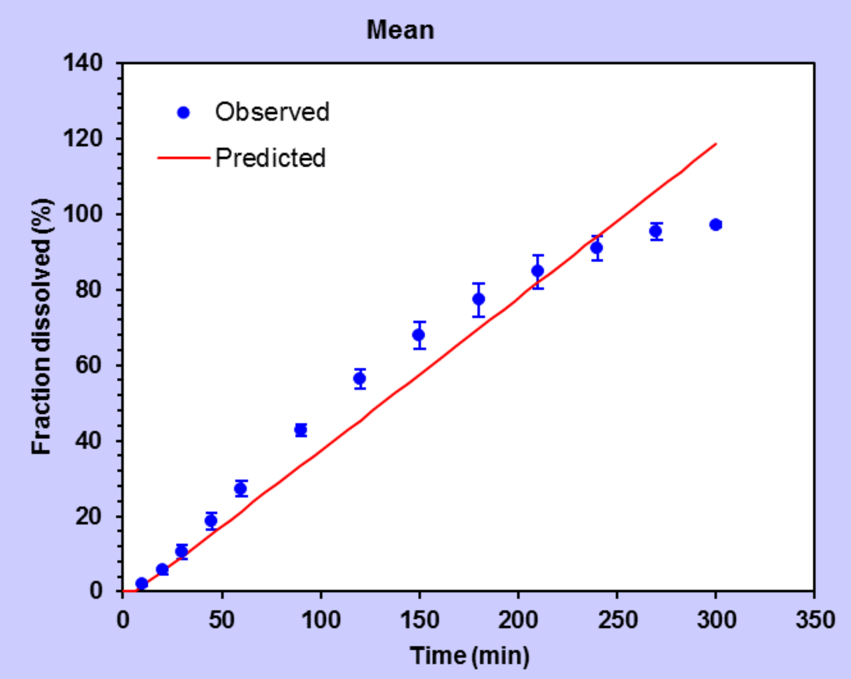

Supplementary materials – Model fitting summary – Polyox™ WSR N-80 (LEO NF Grade) (modelling the entire relevant dissolution data)

Graphical abstract of model fit presented as the fraction % of released carvedilol per tested tablet:

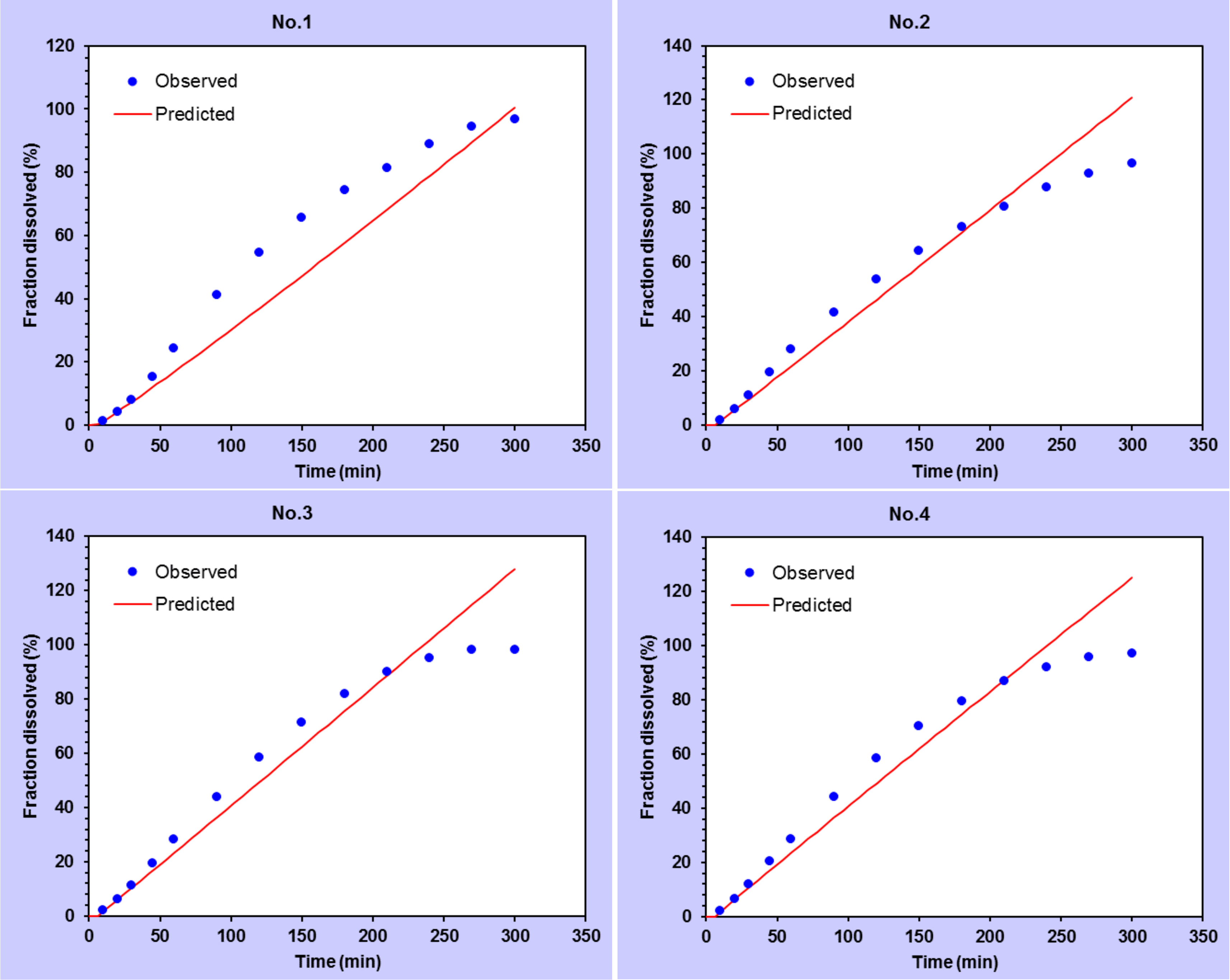

Supplementary materials – Model fitting summary – Polyox™ WSR N-80 (LEO NF Grade) (modelling the entire relevant dissolution data)

Model: **Korsmeyer–Peppas with F_0**

Model equation: $F = F_0 + k_{KP} \cdot t^n$

Fitted model parameters per tested tablet (N = 4) with statistics – mean, standard deviation (SD), and relative standard deviation expressed in % (RSD%) (output from DDSolver):

Parameter	No.1	No.2	No.3	No.4	Mean	SD	RSD(%)
k_{KP}	0.070	0.130	0.141	0.158	0.125	0.039	30.870
n	1.336	1.217	1.213	1.188	1.239	0.066	5.356
F_0	0.560	0.720	0.880	0.880	0.760	0.153	20.157

Number of dissolution data points (N), degrees of freedom (df), and selected goodness of fit criteria – Pearson correlation coefficient (R), coefficient of determination (R^2), adjusted coefficient of determination ($R^2_{adjusted}$), and residual sum of squares (RSS) (manual calculation in MS Excel):

Parameter	No.1	No.2	No.3	No.4
N	13	13	13	13
df	10	10	10	10
R	0.95986918	0.97030175	0.95825822	0.96082482
R^2	0.92134884	0.94148548	0.91825882	0.92318433
$R^2_{adjusted}$	0.90561861	0.92978258	0.90191059	0.9078212
RSS	3762.98142	2799.76168	3472.68918	3231.35639

Graphical abstract of model fit presented as mean ± 1 SD of the fraction % of released carvedilol:

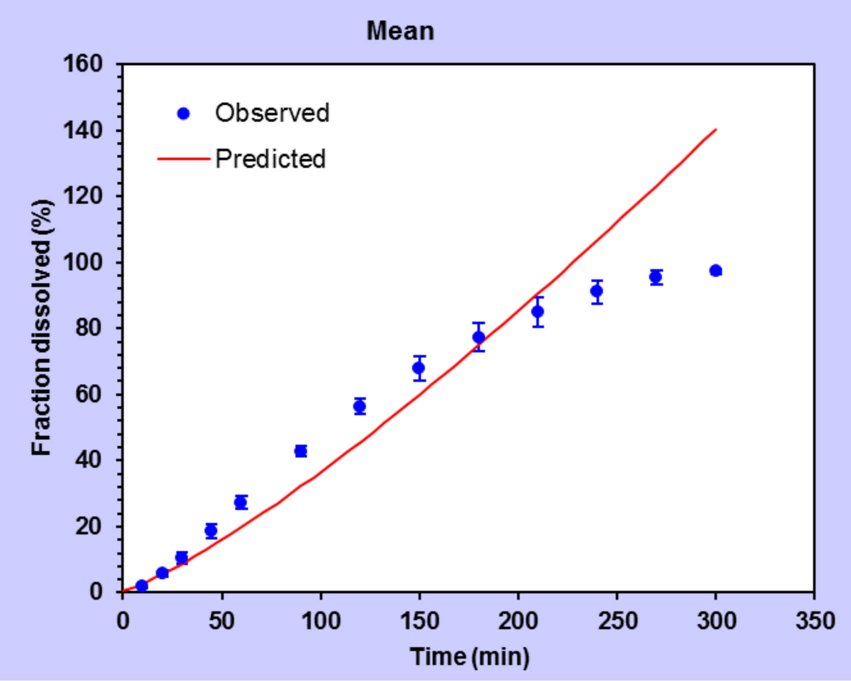

Supplementary materials – Model fitting summary – Polyox™ WSR N-80 (LEO NF Grade) (modelling the entire relevant dissolution data)

Graphical abstract of model fit presented as the fraction % of released carvedilol per tested tablet:

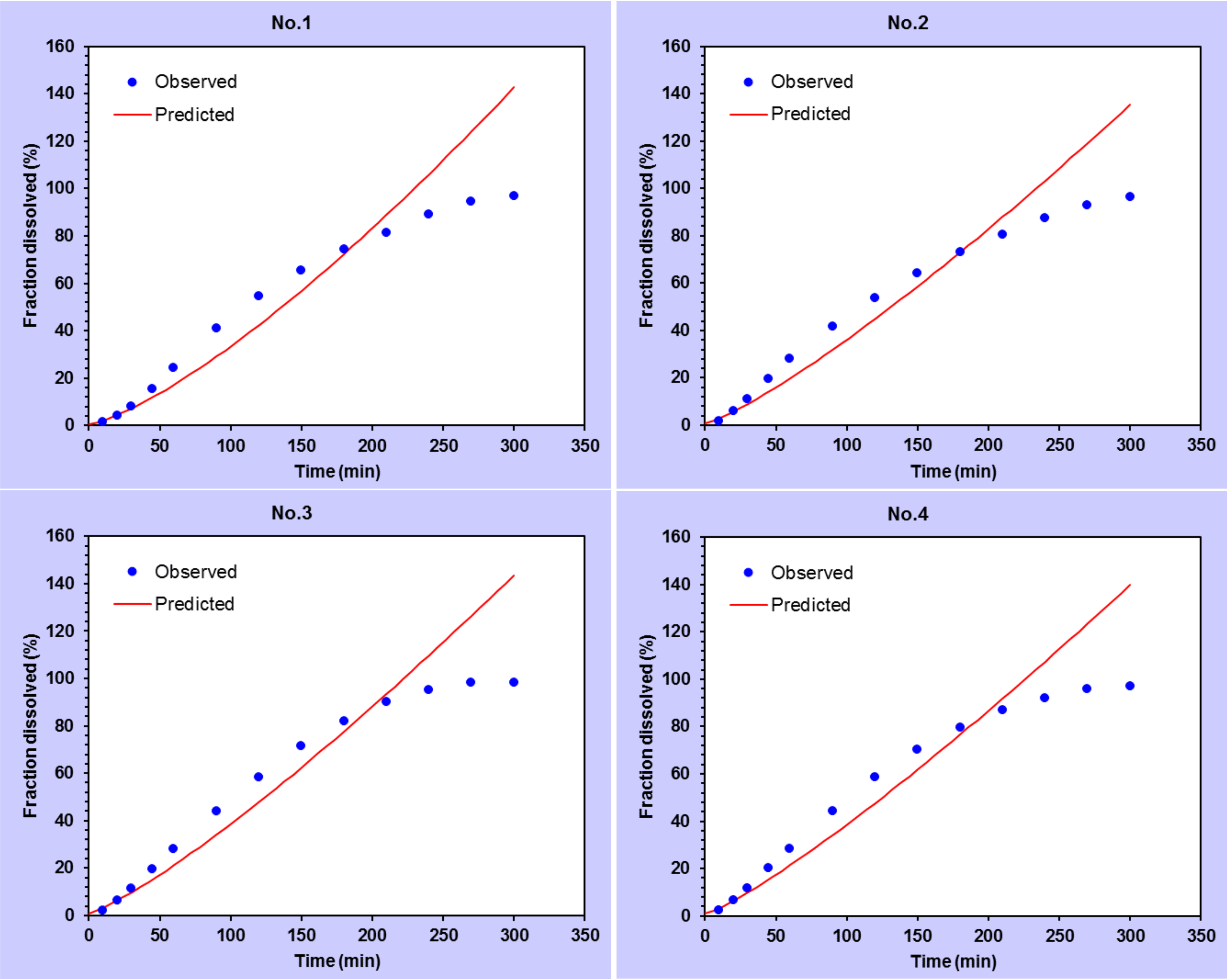

Supplementary materials – Model fitting summary – Polyox™ WSR N-80 (LEO NF Grade) (modelling the entire relevant dissolution data)

Model: **Hixson–Crowell**

Model equation: $F = 100 \cdot [1 - (1 - k_{HC} \cdot t)^3]$

Fitted model parameters per tested tablet (N = 4) with statistics – mean, standard deviation (SD), and relative standard deviation expressed in % (RSD%) (output from DDSolver):

Parameter	No.1	No.2	No.3	No.4	Mean	SD	RSD(%)
k_{HC}	0.002	0.002	0.002	0.002	0.002	0.000	8.061

Number of dissolution data points (N), degrees of freedom (df), and selected goodness of fit criteria – Pearson correlation coefficient (R), coefficient of determination (R^2), adjusted coefficient of determination ($R^2_{adjusted}$), and residual sum of squares (RSS) (manual calculation in MS Excel):

Parameter	No.1	No.2	No.3	No.4
N	13	13	13	13
df	12	12	12	12
R	0.99886308	0.99986489	0.99838898	0.99910785
R^2	0.99772745	0.9997298	0.99678055	0.99821649
$R^2_{adjusted}$	0.99772745	0.9997298	0.99678055	0.99821649
RSS	355.88421	196.608261	342.27942	256.677783

Graphical abstract of model fit presented as mean ± 1 SD of the fraction % of released carvedilol:

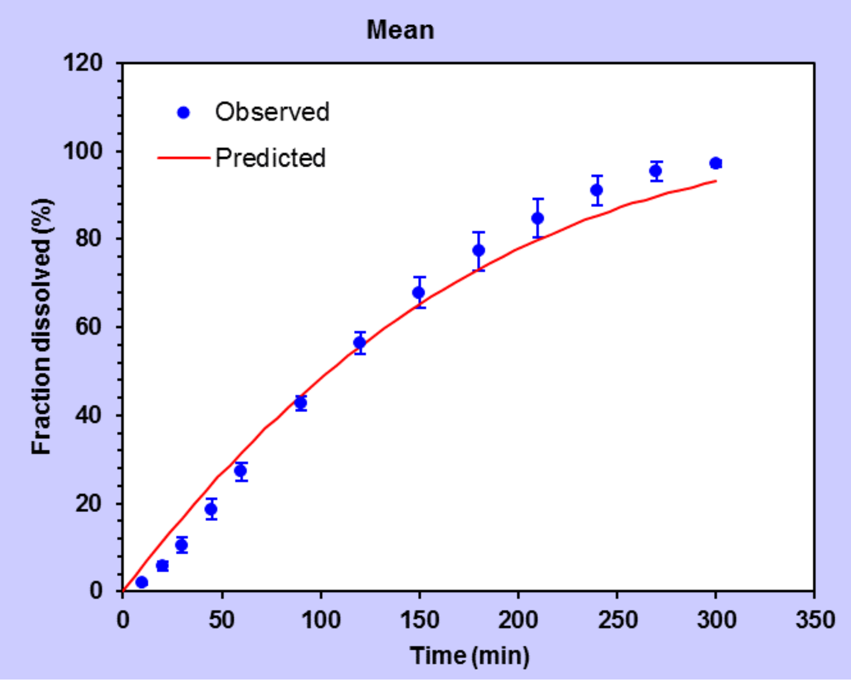

Supplementary materials – Model fitting summary – Polyox™ WSR N-80 (LEO NF Grade) (modelling the entire relevant dissolution data)

Graphical abstract of model fit presented as the fraction % of released carvedilol per tested tablet:

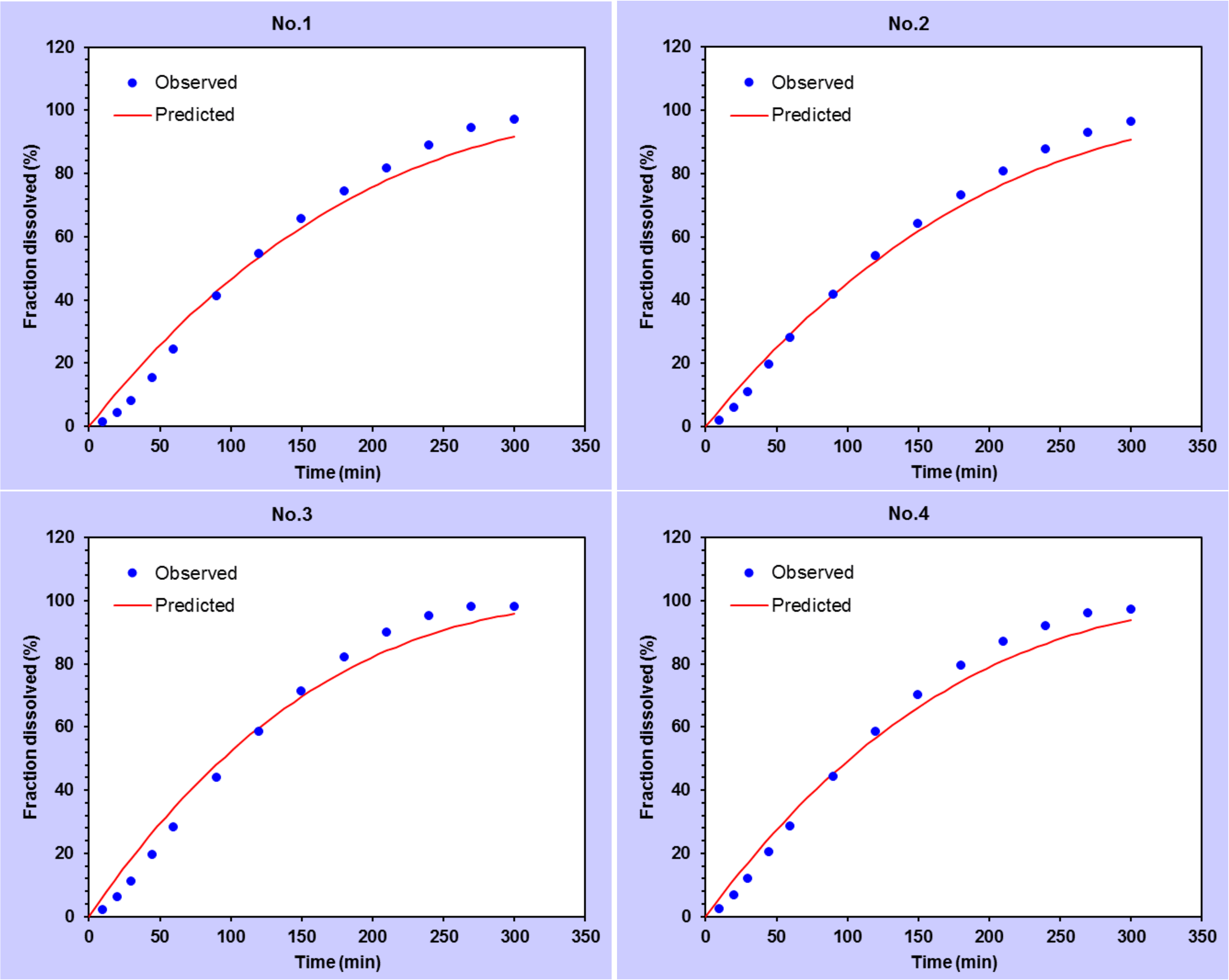

Supplementary materials – Model fitting summary – Polyox™ WSR N-80 (LEO NF Grade) (modelling the entire relevant dissolution data)

Model: **Hixson–Crowell with T_{lag}**

Model equation: $F = 100 \cdot \left\{1 - \left[1 - k_{HC} \cdot \left(t - T_{lag}\right)\right]^3\right\}$

Fitted model parameters per tested tablet (N = 4) with statistics – mean, standard deviation (SD), and relative standard deviation expressed in % (RSD%) (output from DDSolver):

Parameter	No.1	No.2	No.3	No.4	Mean	SD	RSD(%)
k_{HC}	0.002	0.002	0.003	0.002	0.002	0.000	8.320
T_{lag}	19.310	14.757	17.701	13.730	16.375	2.581	15.763

Number of dissolution data points (N), degrees of freedom (df), and selected goodness of fit criteria – Pearson correlation coefficient (R), coefficient of determination (R^2), adjusted coefficient of determination ($R^2_{adjusted}$), and residual sum of squares (RSS) (manual calculation in MS Excel):

Parameter	No.1	No.2	No.3	No.4
N	13	13	13	13
df	11	11	11	11
R	0.99741282	0.99886925	0.99626267	0.99868834
R^2	0.99483232	0.99773977	0.99253932	0.99737841
$R^2_{adjusted}$	0.99436254	0.9975343	0.99186107	0.99714008
RSS	106.623568	52.2331031	152.727023	45.4703104

Graphical abstract of model fit presented as mean ± 1 SD of the fraction % of released carvedilol:

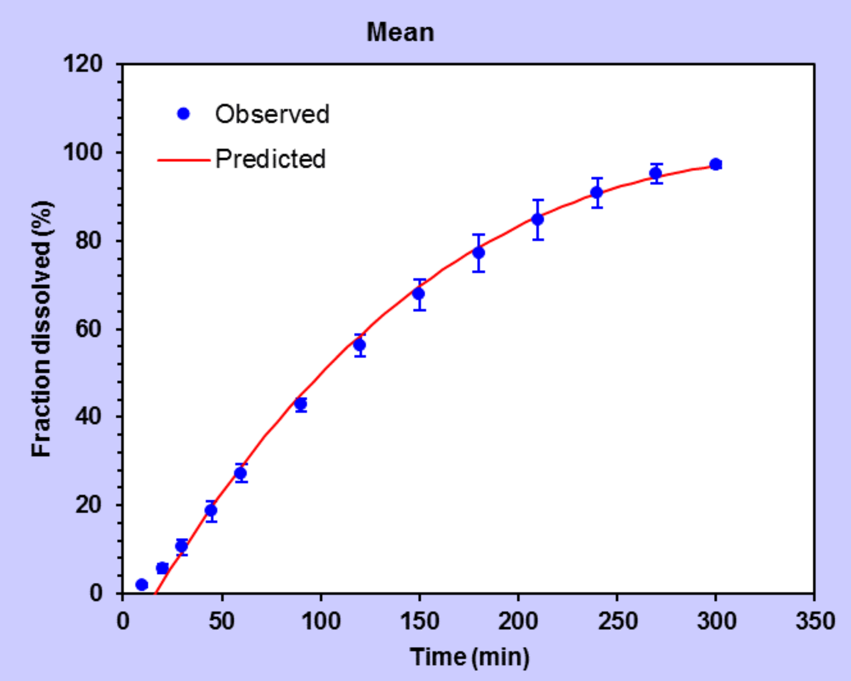

Supplementary materials – Model fitting summary – Polyox^{TM} WSR N-80 (LEO NF Grade) (modelling the entire relevant dissolution data)

Graphical abstract of model fit presented as the fraction % of released carvedilol per tested tablet:

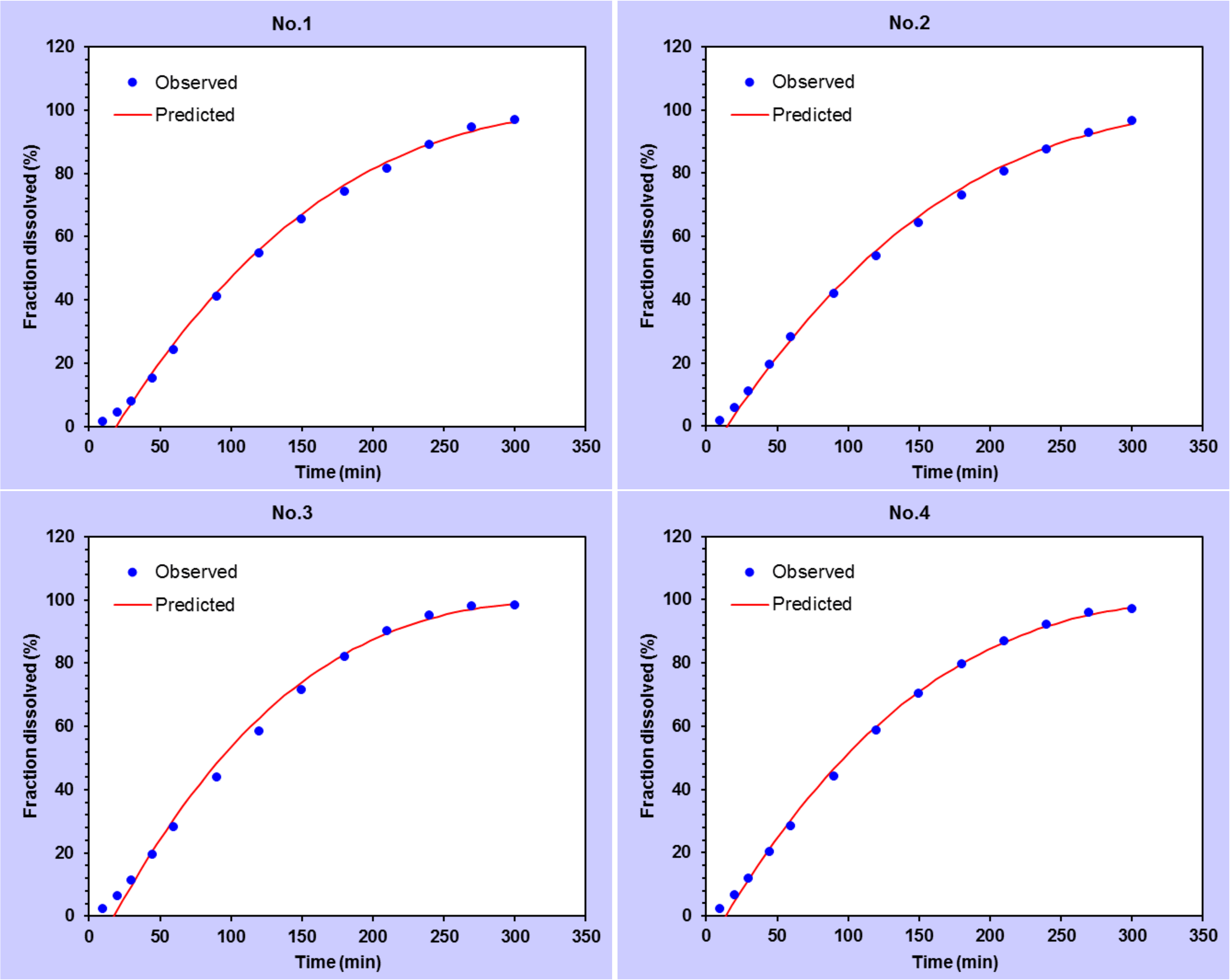

Supplementary materials – Model fitting summary – Polyox[TM] WSR N-80 (LEO NF Grade) (modelling the entire relevant dissolution data)

Model: **Hopfenberg**

Model equation: $F = 100 \cdot [1 - (1 - k_{HB} \cdot t)^n]$

Fitted model parameters per tested tablet (N = 4) with statistics – mean, standard deviation (SD), and relative standard deviation expressed in % (RSD%) (output from DDSolver):

Parameter	No.1	No.2	No.3	No.4	Mean	SD	RSD(%)
k_{HB}	0.003	0.003	0.003	0.003	0.003	0.000	6.368
n	2.000	2.000	2.000	2.000	2.000	0.000	0.000

Number of dissolution data points (N), degrees of freedom (df), and selected goodness of fit criteria – Pearson correlation coefficient (R), coefficient of determination (R^2), adjusted coefficient of determination ($R^2_{adjusted}$), and residual sum of squares (RSS) (manual calculation in MS Excel):

Parameter	No.1	No.2	No.3	No.4
N	13	13	13	13
df	11	11	11	11
R	0.99901199	0.99975662	0.99910974	0.999154
R^2	0.99802495	0.9995133	0.99822028	0.99830871
$R^2_{adjusted}$	0.9978454	0.99946905	0.99805849	0.99815495
RSS	222.720489	67.0920656	195.006388	106.141254

Graphical abstract of model fit presented as mean ± 1 SD of the fraction % of released carvedilol:

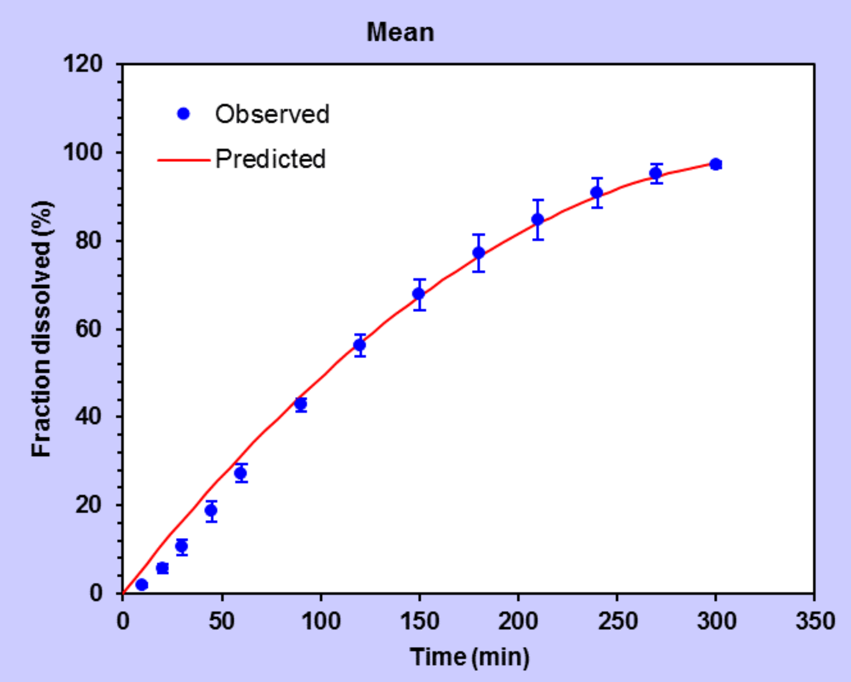

Supplementary materials – Model fitting summary – Polyox^{TM} WSR N-80 (LEO NF Grade) (modelling the entire relevant dissolution data)

Graphical abstract of model fit presented as the fraction % of released carvedilol per tested tablet:

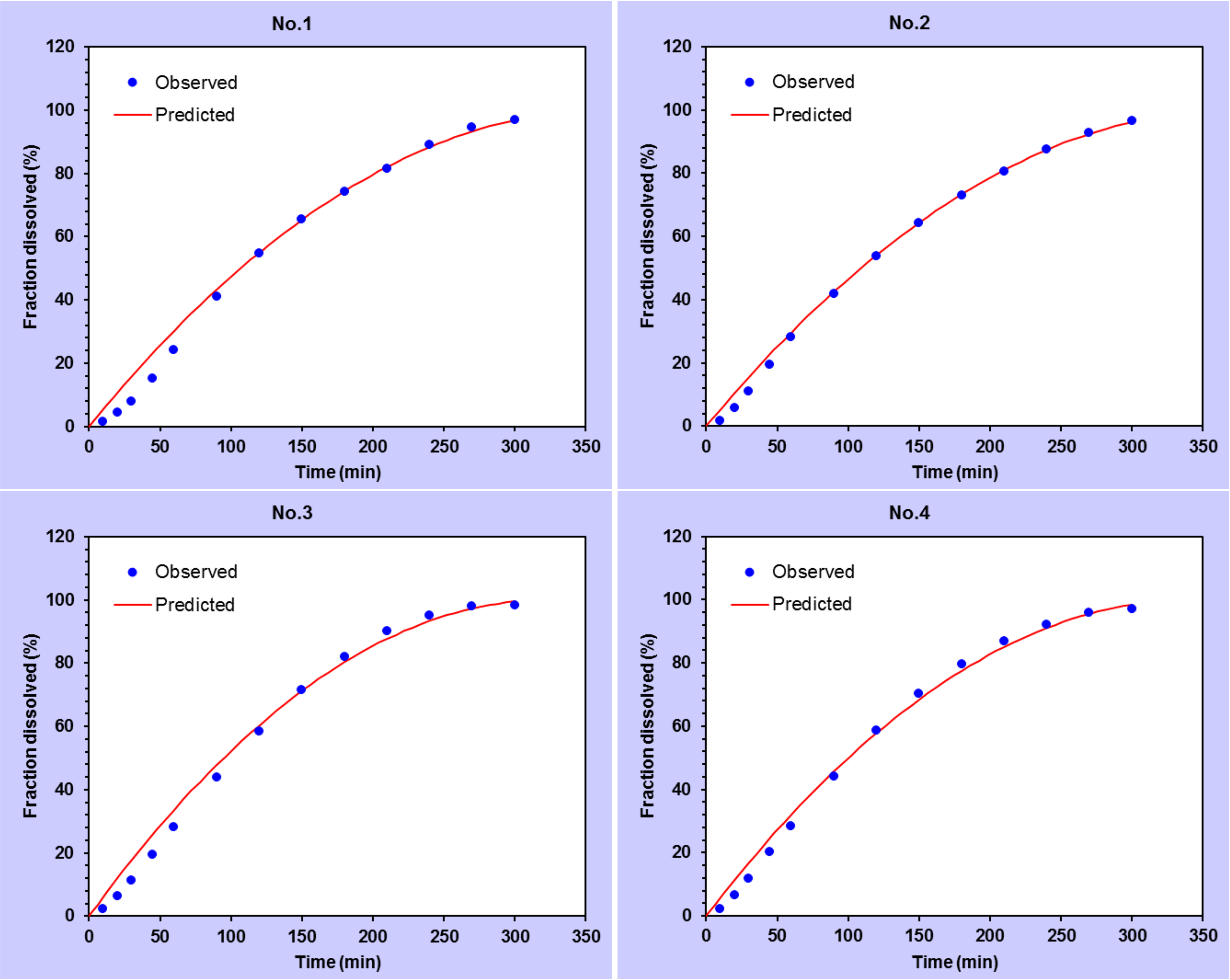

Supplementary materials – Model fitting summary – Polyox™ WSR N-80 (LEO NF Grade) (modelling the entire relevant dissolution data)

Model: **Hopfenberg with T_{lag}**

Model equation: $F = 100 \cdot \{1 - [1 - k_{HB} \cdot (t - T_{lag})]^n\}$

Fitted model parameters per tested tablet (N = 4) with statistics – mean, standard deviation (SD), and relative standard deviation expressed in % (RSD%) (output from DDSolver):

Parameter	No.1	No.2	No.3	No.4	Mean	SD	RSD(%)
k_{HB}	0.003	0.003	0.003	0.003	0.003	0.000	6.326
n	2.000	2.000	2.000	2.000	2.000	0.000	0.000
T_{lag}	12.424	7.250	9.284	5.739	8.674	2.891	33.332

Number of dissolution data points (N), degrees of freedom (df), and selected goodness of fit criteria – Pearson correlation coefficient (R), coefficient of determination (R^2), adjusted coefficient of determination ($R^2_{adjusted}$), and residual sum of squares (RSS) (manual calculation in MS Excel):

Parameter	No.1	No.2	No.3	No.4
N	13	13	13	13
df	10	10	10	10
R	0.99908222	0.99980397	0.99918441	0.99927474
R^2	0.99816527	0.99960797	0.99836949	0.99855
$R^2_{adjusted}$	0.99779833	0.99952957	0.99804339	0.99826
RSS	29.2074083	5.85004813	33.2254851	33.180409

Graphical abstract of model fit presented as mean ± 1 SD of the fraction % of released carvedilol:

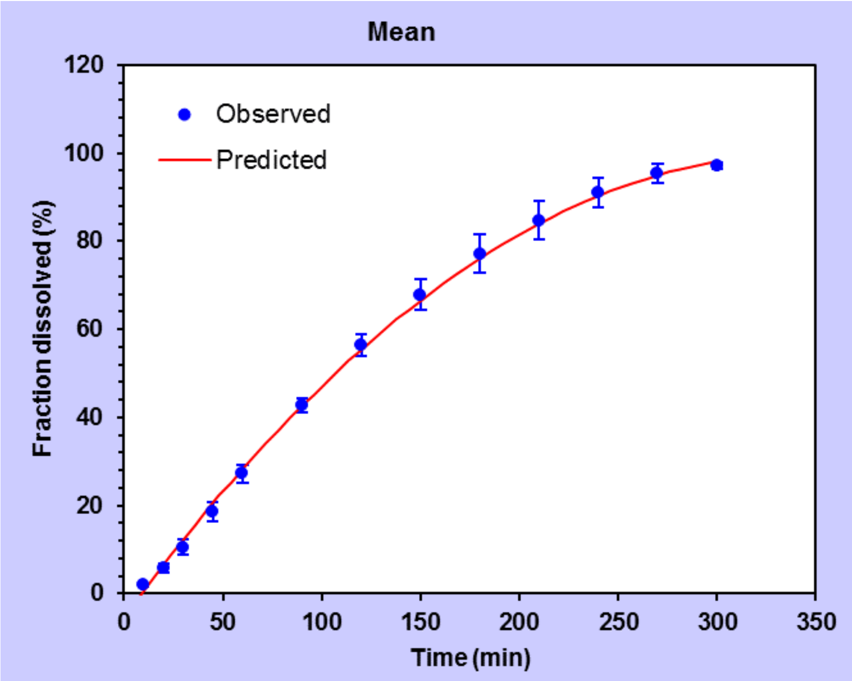

Supplementary materials – Model fitting summary – Polyox™ WSR N-80 (LEO NF Grade) (modelling the entire relevant dissolution data)

Graphical abstract of model fit presented as the fraction % of released carvedilol per tested tablet:

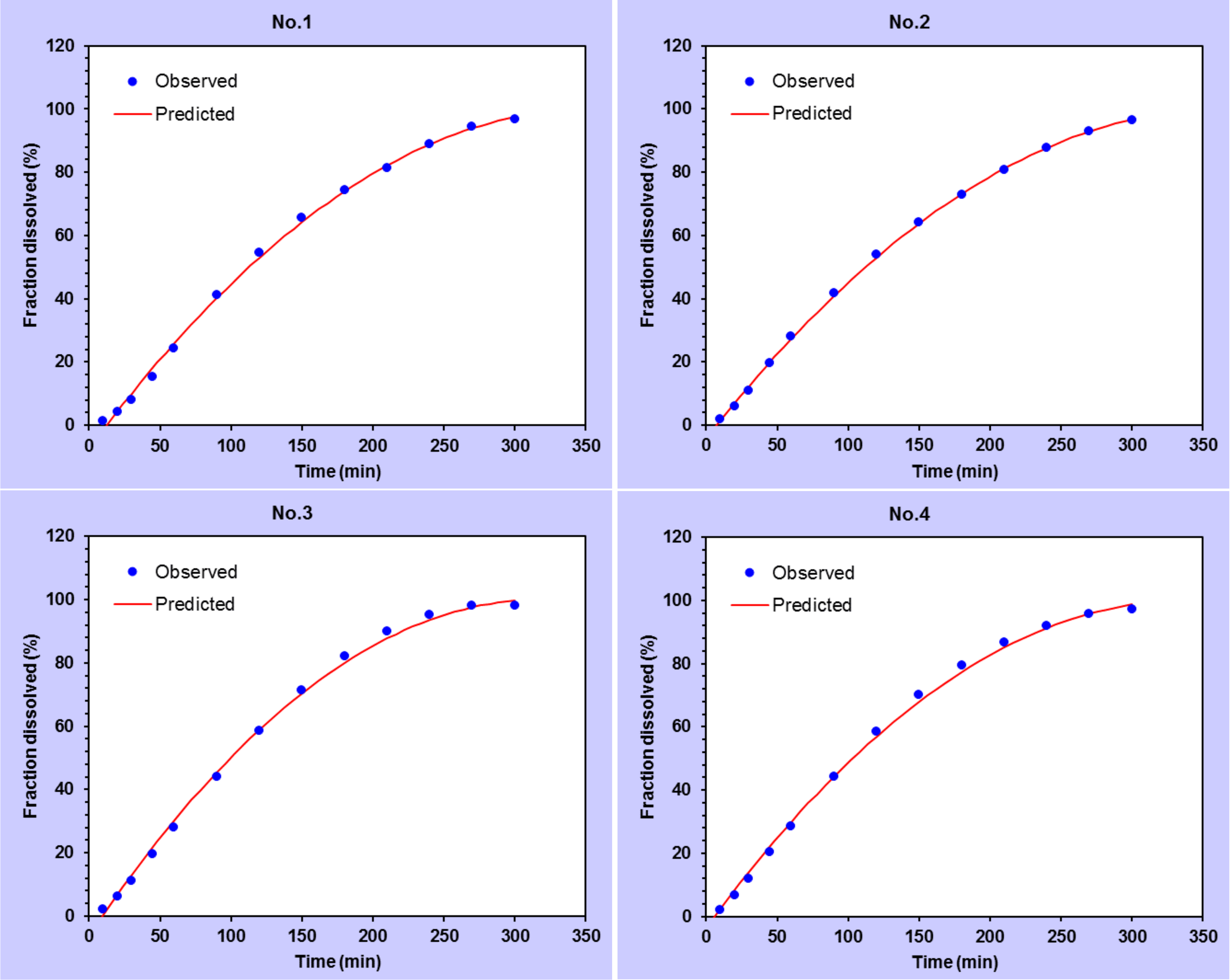

Supplementary materials – Model fitting summary – Polyox^{TM} WSR N-80 (LEO NF Grade) (modelling the entire relevant dissolution data)

Model: **Baker–Lonsdale**

Model equation: $\frac{3}{2} \cdot \left[1 - \left(1 - \frac{F}{100}\right)^{\frac{2}{3}}\right] - \frac{F}{100} = k_{BL} \cdot t$

Fitted model parameters per tested tablet (N = 4) with statistics – mean, standard deviation (SD), and relative standard deviation expressed in % (RSD%) (output from DDSolver):

Parameter	No.1	No.2	No.3	No.4	Mean	SD	RSD(%)
k_{BL}	0.001	0.001	0.001	0.001	0.001	0.000	10.386

Number of dissolution data points (N), degrees of freedom (df), and selected goodness of fit criteria – Pearson correlation coefficient (R), coefficient of determination (R^2), adjusted coefficient of determination ($R^2_{adjusted}$), and residual sum of squares (RSS) (manual calculation in MS Excel):

Parameter	No.1	No.2	No.3	No.4
N	13	13	13	13
df	12	12	12	12
R	0.99058808	0.99411979	0.99021015	0.99278345
R^2	0.98126473	0.98827417	0.98051614	0.98561898
$R^2_{adjusted}$	0.98126473	0.98827417	0.98051614	0.98561898
RSS	3177.08783	2511.62478	3230.60797	2801.12034

Graphical abstract of model fit presented as mean ± 1 SD of the fraction % of released carvedilol:

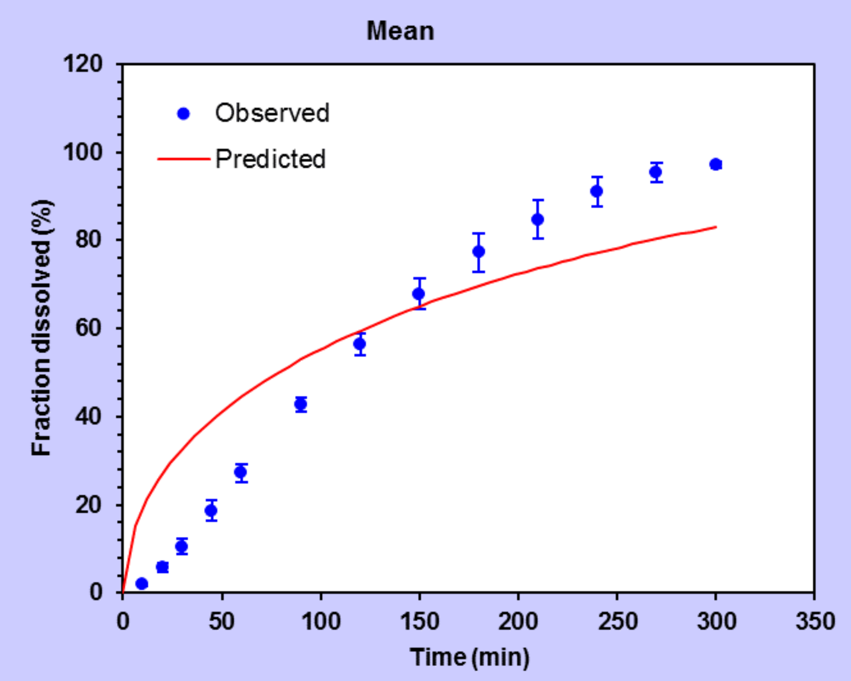

Supplementary materials – Model fitting summary – Polyox^{TM} WSR N-80 (LEO NF Grade) (modelling the entire relevant dissolution data)

Graphical abstract of model fit presented as the fraction % of released carvedilol per tested tablet:

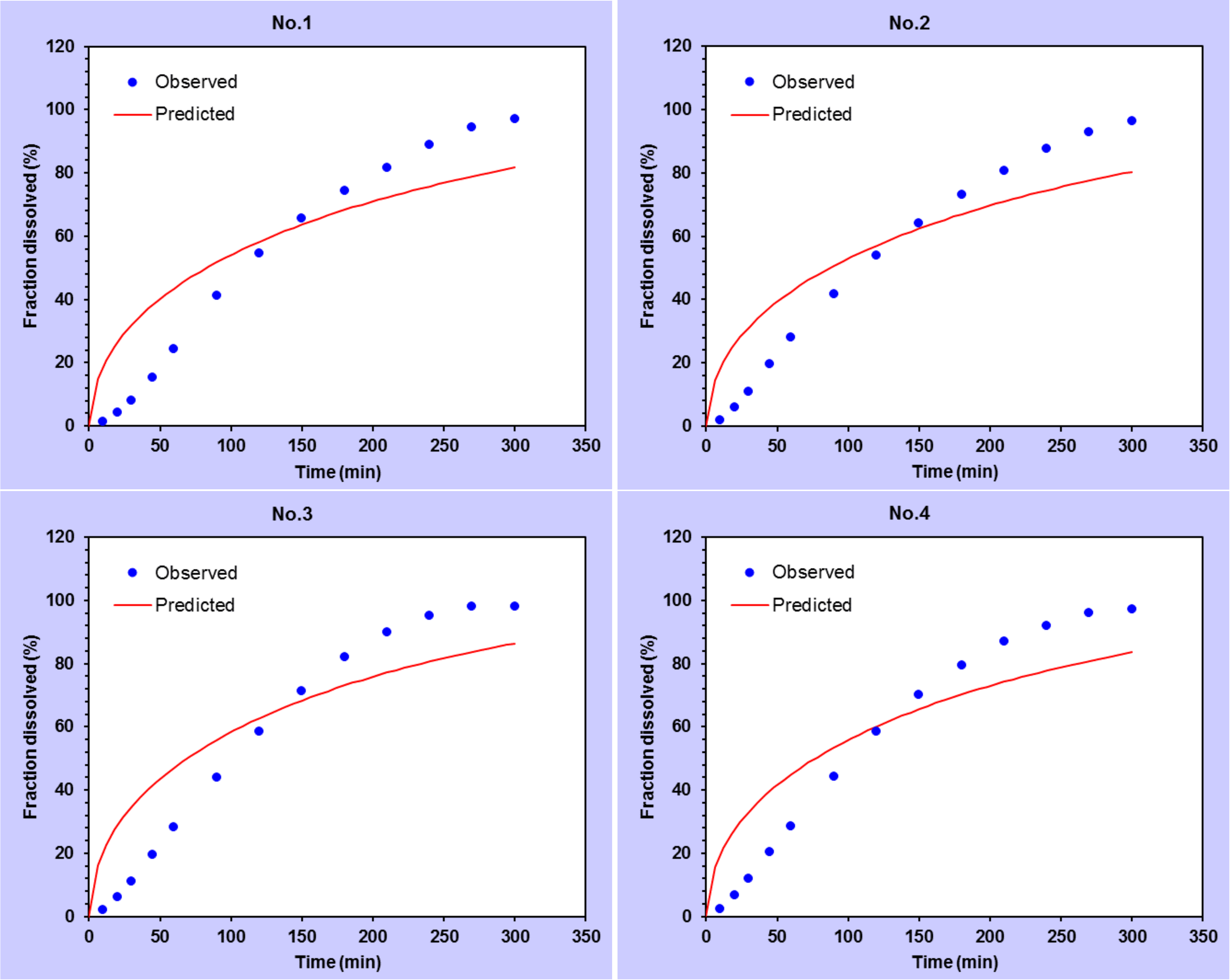

Supplementary materials – Model fitting summary – Polyox™ WSR N-80 (LEO NF Grade) (modelling the entire relevant dissolution data)

Model: **Baker–Lonsdale with T_{lag}**

Model equation: $\frac{3}{2} \cdot \left[1 - \left(1 - \frac{F}{100}\right)^{\frac{2}{3}}\right] - \frac{F}{100} = k_{BL} \cdot (t - T_{lag})$

Fitted model parameters per tested tablet (N = 4) with statistics – mean, standard deviation (SD), and relative standard deviation expressed in % (RSD%) (output from DDSolver):

Parameter	No.1	No.2	No.3	No.4	Mean	SD	RSD(%)
k_{BL}	0.001	0.001	0.002	0.001	0.001	0.000	10.386
T_{lag}	42.244	41.140	39.515	38.233	40.283	1.768	4.389

Number of dissolution data points (N), degrees of freedom (df), and selected goodness of fit criteria – Pearson correlation coefficient (R), coefficient of determination (R^2), adjusted coefficient of determination ($R^2_{adjusted}$), and residual sum of squares (RSS) (manual calculation in MS Excel):

Parameter	No.1	No.2	No.3	No.4
N	13	13	13	13
df	11	11	11	11
R	0.98643062	0.98571513	0.98176914	0.98414172
R^2	0.97304537	0.97163431	0.96387063	0.96853492
$R^2_{adjusted}$	0.97059495	0.96905561	0.96058615	0.96567446
RSS	532.773384	529.704944	727.518099	589.337149

Graphical abstract of model fit presented as mean ± 1 SD of the fraction % of released carvedilol:

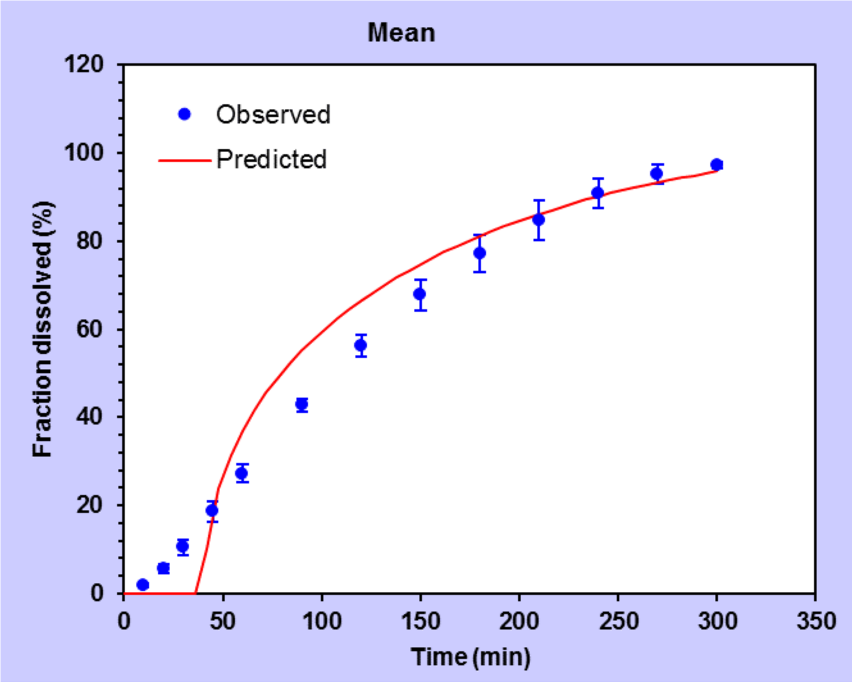

Supplementary materials – Model fitting summary – Polyox™ WSR N-80 (LEO NF Grade) (modelling the entire relevant dissolution data)

Graphical abstract of model fit presented as the fraction % of released carvedilol per tested tablet:

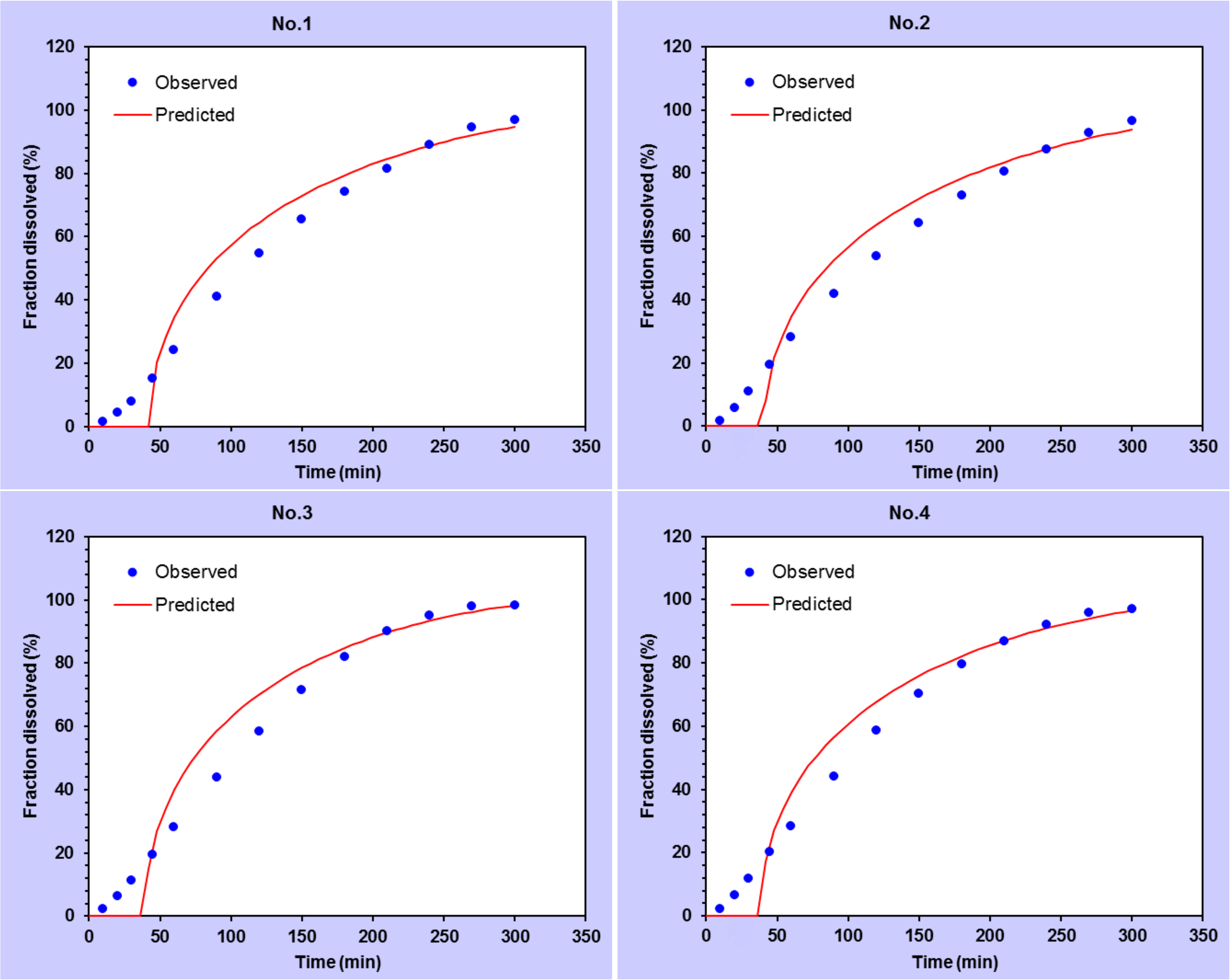

Supplementary materials – Model fitting summary – Polyox™ WSR N-80 (LEO NF Grade) (modelling the entire relevant dissolution data)

Model: **Makoid–Banakar**

Model equation: $F = k_{MB} \cdot t^n \cdot e^{-k \cdot t}$

Fitted model parameters per tested tablet (N = 4) with statistics – mean, standard deviation (SD), and relative standard deviation expressed in % (RSD%) (output from DDSolver):

Parameter	No.1	No.2	No.3	No.4	Mean	SD	RSD(%)
k_{MB}	0.027	0.053	0.066	0.069	0.054	0.019	35.310
n	1.730	1.557	1.549	1.523	1.590	0.094	5.936
k	0.006	0.005	0.005	0.005	0.005	0.001	10.238

Number of dissolution data points (N), degrees of freedom (df), and selected goodness of fit criteria – Pearson correlation coefficient (R), coefficient of determination (R^2), adjusted coefficient of determination ($R^2_{adjusted}$), and residual sum of squares (RSS) (manual calculation in MS Excel):

Parameter	No.1	No.2	No.3	No.4
N	13	13	13	13
df	10	10	10	10
R	0.99864644	0.99875209	0.99988186	0.99983281
R^2	0.99729472	0.99750574	0.99976373	0.99966565
$R^2_{adjusted}$	0.99675367	0.99700689	0.99971648	0.99959878
RSS	43.0852458	66.8274137	3.98456095	16.8806906

Graphical abstract of model fit presented as mean ± 1 SD of the fraction % of released carvedilol:

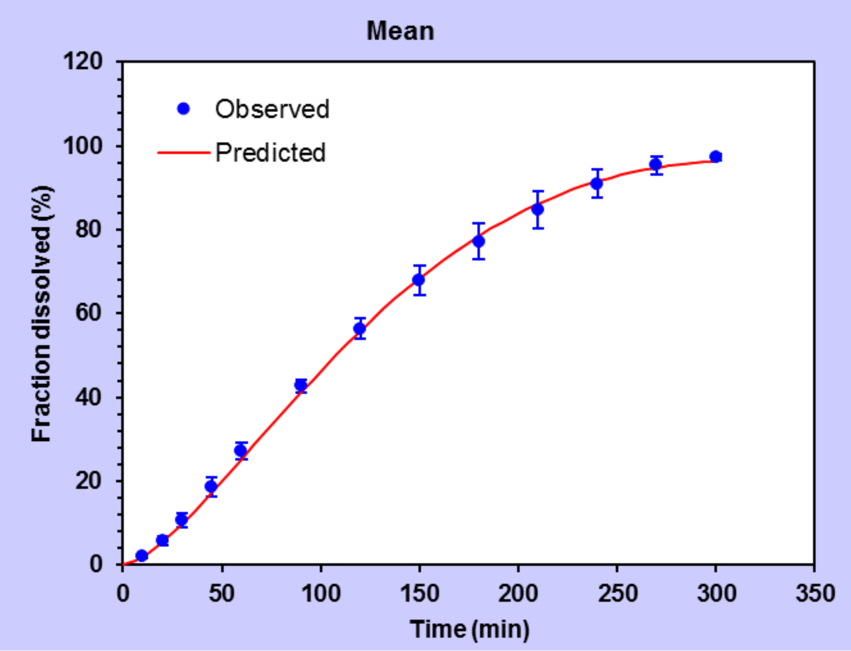

Supplementary materials – Model fitting summary – Polyox™ WSR N-80 (LEO NF Grade) (modelling the entire relevant dissolution data)

Graphical abstract of model fit presented as the fraction % of released carvedilol per tested tablet:

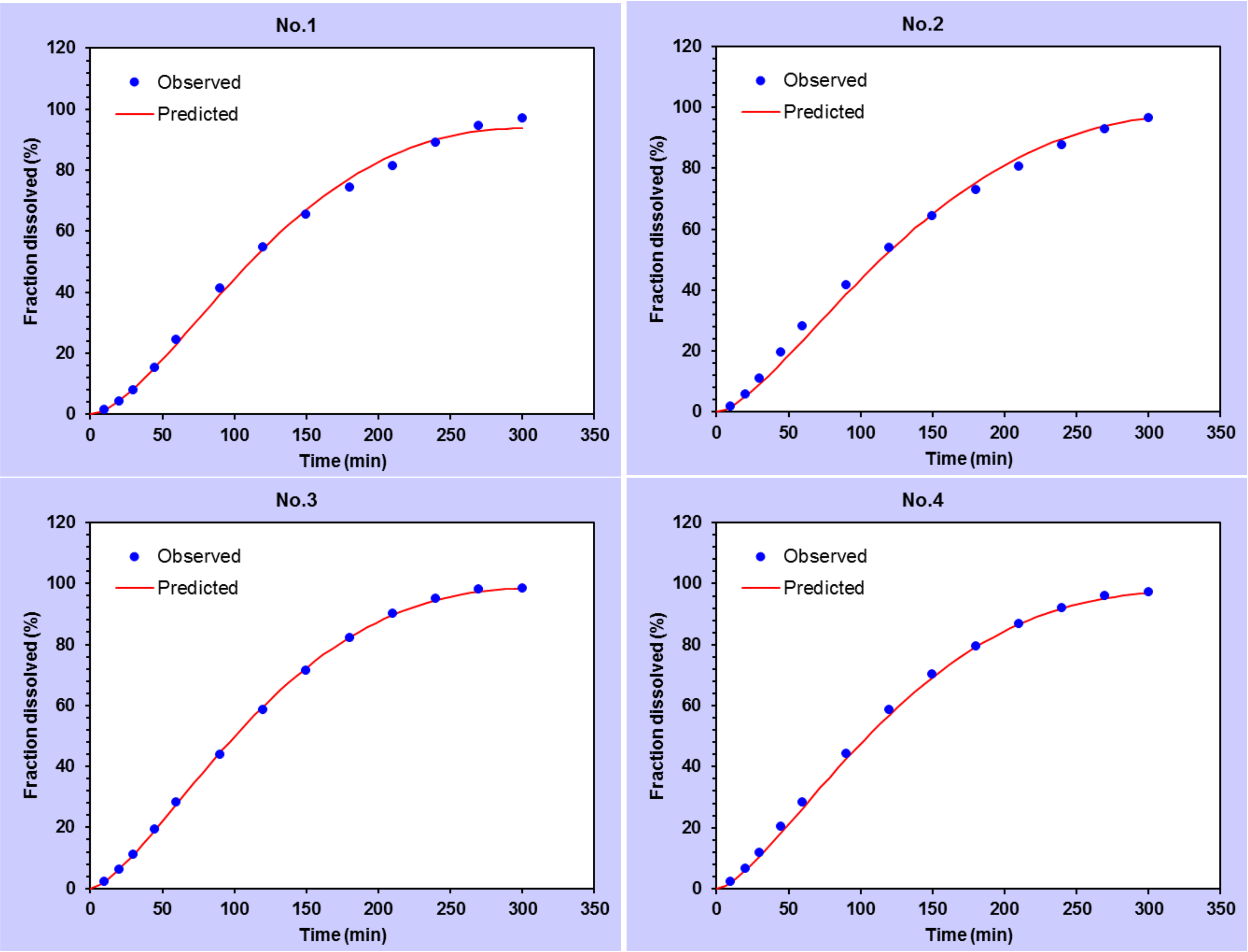

Supplementary materials – Model fitting summary – Polyox™ WSR N-80 (LEO NF Grade) (modelling the entire relevant dissolution data)

Model: **Makoid–Banakar with T_{lag}**

Model equation: $F = k_{MB} \cdot (t - T_{lag})^n \cdot e^{-k\cdot(t-T_{lag})}$

Fitted model parameters per tested tablet (N = 4) with statistics – mean, standard deviation (SD), and relative standard deviation expressed in % (RSD%) (output from DDSolver):

Parameter	No.1	No.2	No.3	No.4	Mean	SD	RSD(%)
k_{MB}	0.116	0.181	0.230	0.232	0.190	0.054	28.629
n	1.383	1.292	1.250	1.243	1.292	0.064	4.988
k	0.004	0.004	0.004	0.003	0.004	0.000	4.675
T_{lag}	4.718	4.000	4.718	4.000	4.359	0.414	9.509

Number of dissolution data points (N), degrees of freedom (df), and selected goodness of fit criteria – Pearson correlation coefficient (R), coefficient of determination (R^2), adjusted coefficient of determination ($R^2_{adjusted}$), and residual sum of squares (RSS) (manual calculation in MS Excel):

Parameter	No.1	No.2	No.3	No.4
N	13	13	13	13
df	9	9	9	9
R	0.99950426	0.99929357	0.99944604	0.99969792
R^2	0.99900876	0.99858764	0.99889238	0.99939594
$R^2_{adjusted}$	0.99867835	0.99811685	0.99852317	0.99919458
RSS	25.3401926	20.3955166	39.0347818	9.57130115

Graphical abstract of model fit presented as mean ± 1 SD of the fraction % of released carvedilol:

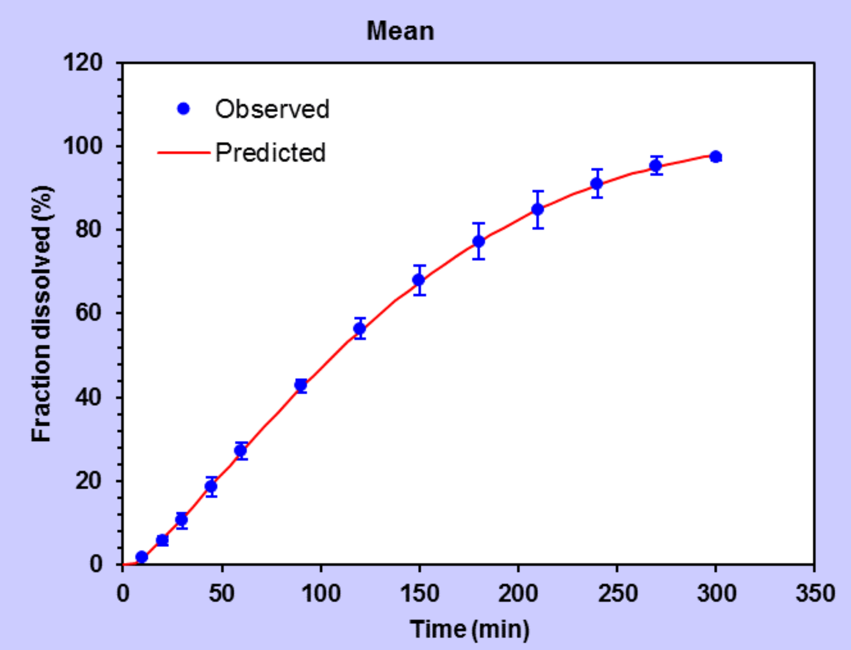

Supplementary materials – Model fitting summary – Polyox™ WSR N-80 (LEO NF Grade) (modelling the entire relevant dissolution data)

Graphical abstract of model fit presented as the fraction % of released carvedilol per tested tablet:

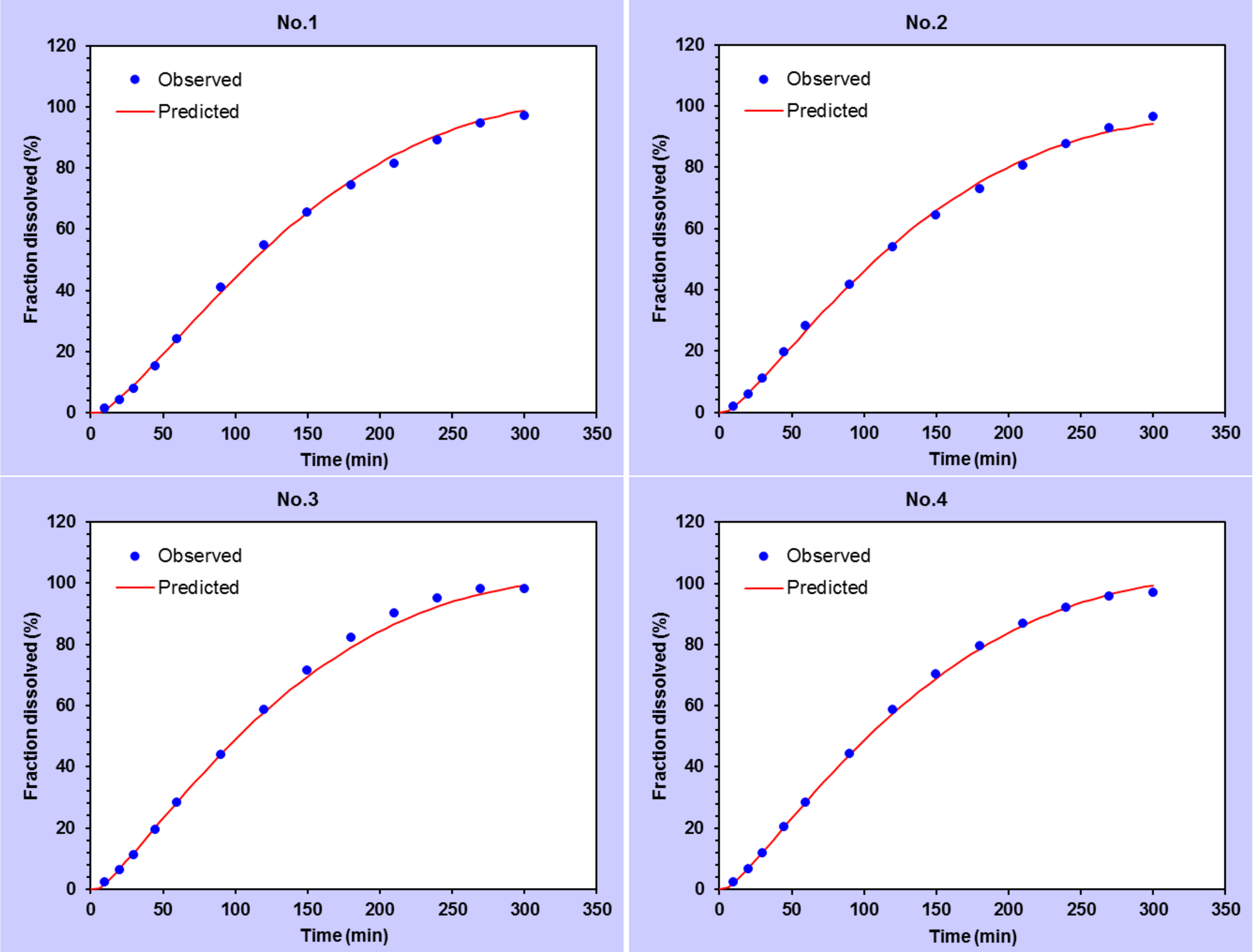

Supplementary materials – Model fitting summary – Polyox™ WSR N-80 (LEO NF Grade) (modelling the entire relevant dissolution data)

Model: **Peppas–Sahlin_1**

Model equation: $F = k_1 \cdot t^m + k_2 \cdot t^{2m}$

Fitted model parameters per tested tablet (N = 4) with statistics – mean, standard deviation (SD), and relative standard deviation expressed in % (RSD%) (output from DDSolver):

Parameter	No.1	No.2	No.3	No.4	Mean	SD	RSD(%)
k_1	0.364	1.069	1.299	1.531	1.066	0.504	47.332
k_2	0.602	0.534	0.558	0.523	0.554	0.035	6.305
m	0.450	0.450	0.450	0.450	0.450	0.000	0.000

Number of dissolution data points (N), degrees of freedom (df), and selected goodness of fit criteria – Pearson correlation coefficient (R), coefficient of determination (R^2), adjusted coefficient of determination ($R^2_{adjusted}$), and residual sum of squares (RSS) (manual calculation in MS Excel):

Parameter	No.1	No.2	No.3	No.4
N	13	13	13	13
df	10	10	10	10
R	0.98773059	0.99108671	0.98298176	0.98460059
R^2	0.97561172	0.98225286	0.96625314	0.96943831
$R^2_{adjusted}$	0.97073406	0.97870343	0.95950376	0.96332598
RSS	401.055572	271.431753	593.325318	505.895464

Graphical abstract of model fit presented as mean ± 1 SD of the fraction % of released carvedilol:

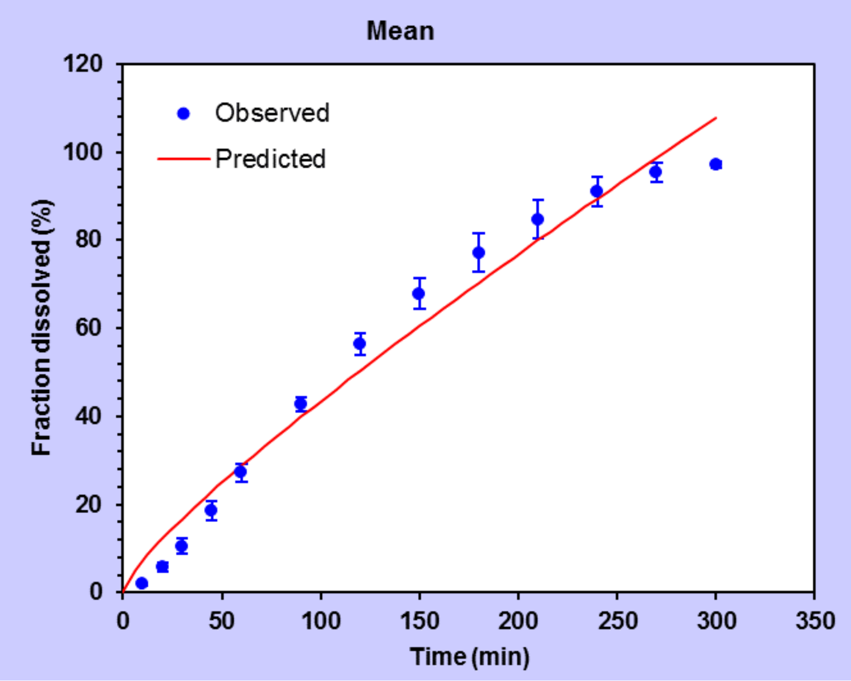

Supplementary materials – Model fitting summary – Polyox™ WSR N-80 (LEO NF Grade) (modelling the entire relevant dissolution data)

Graphical abstract of model fit presented as the fraction % of released carvedilol per tested tablet:

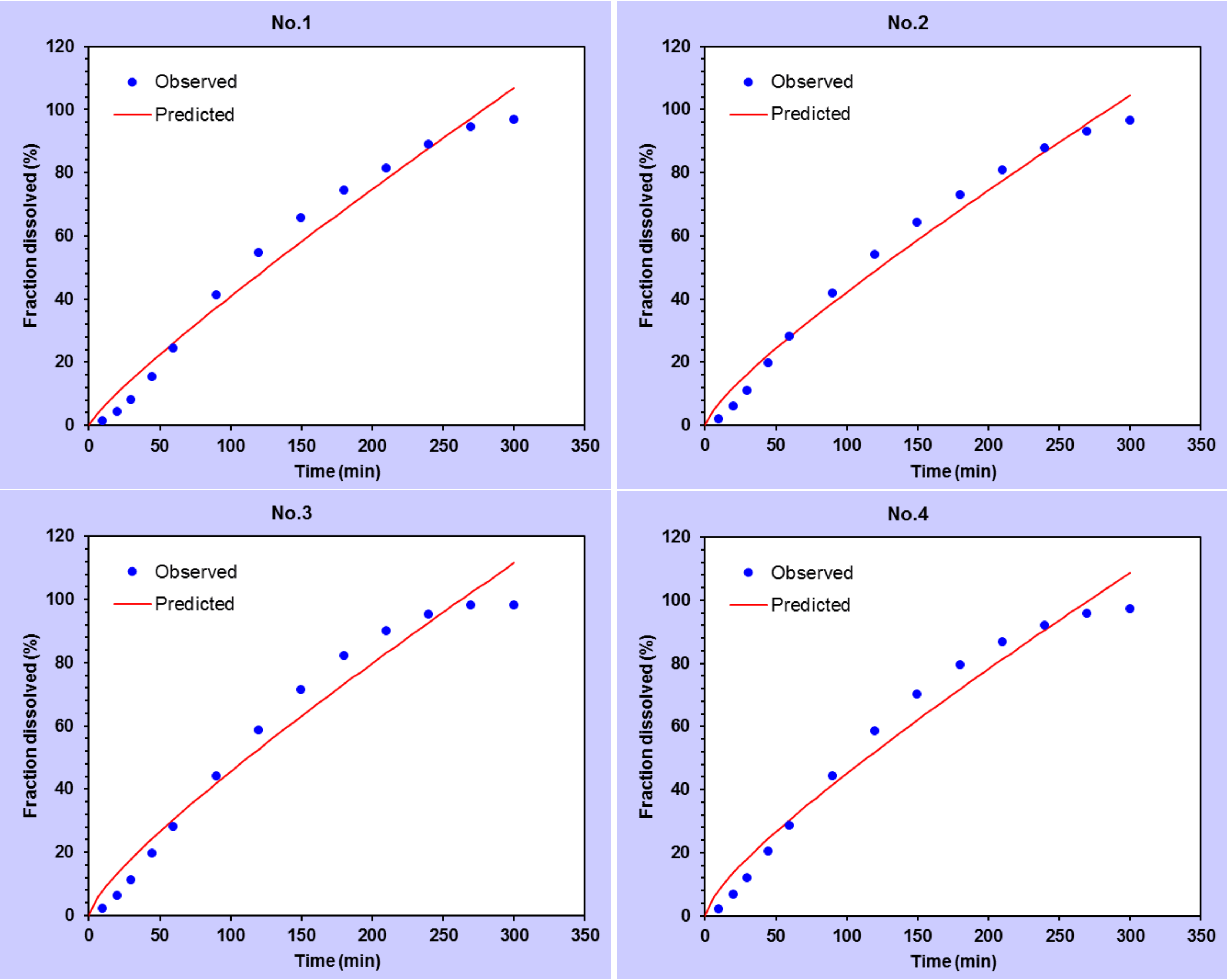

Supplementary materials – Model fitting summary – Polyox[TM] WSR N-80 (LEO NF Grade) (modelling the entire relevant dissolution data)

Model: **Peppas–Sahlin_1 with T_{lag}**

Model equation: $F = k_1 \cdot (t - T_{lag})^m + k_2 \cdot (t - T_{lag})^{2m}$

Fitted model parameters per tested tablet (N = 4) with statistics – mean, standard deviation (SD), and relative standard deviation expressed in % (RSD%) (output from DDSolver):

Parameter	No.1	No.2	No.3	No.4	Mean	SD	RSD(%)
k_1	0.895	1.611	1.892	2.119	1.629	0.532	32.638
k_2	0.566	0.496	0.516	0.481	0.515	0.037	7.139
m	0.450	0.450	0.450	0.450	0.450	0.000	0.000
T_{lag}	6.000	6.000	6.000	6.000	6.000	0.000	0.000

Number of dissolution data points (N), degrees of freedom (df), and selected goodness of fit criteria – Pearson correlation coefficient (R), coefficient of determination (R^2), adjusted coefficient of determination ($R^2_{adjusted}$), and residual sum of squares (RSS) (manual calculation in MS Excel):

Parameter	No.1	No.2	No.3	No.4
N	13	13	13	13
df	9	9	9	9
R	0.98918092	0.99276832	0.9850036	0.98677852
R^2	0.9784789	0.98558895	0.9702321	0.97373184
$R^2_{adjusted}$	0.9713052	0.98078526	0.96030947	0.96497579
RSS	348.275529	215.624515	514.145155	426.15549

Graphical abstract of model fit presented as mean ± 1 SD of the fraction % of released carvedilol:

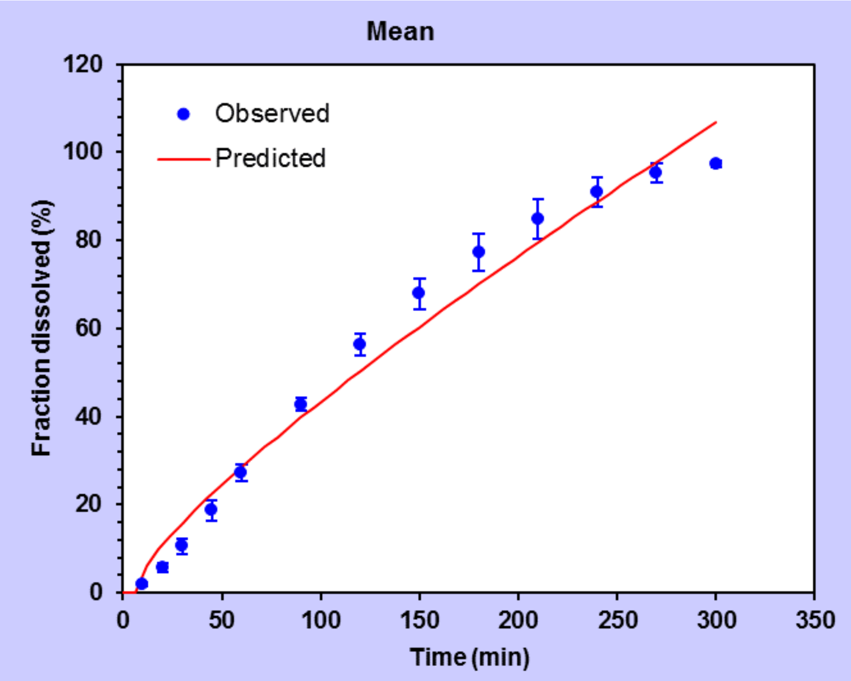

Supplementary materials – Model fitting summary – Polyox™ WSR N-80 (LEO NF Grade) (modelling the entire relevant dissolution data)

Graphical abstract of model fit presented as the fraction % of released carvedilol per tested tablet:

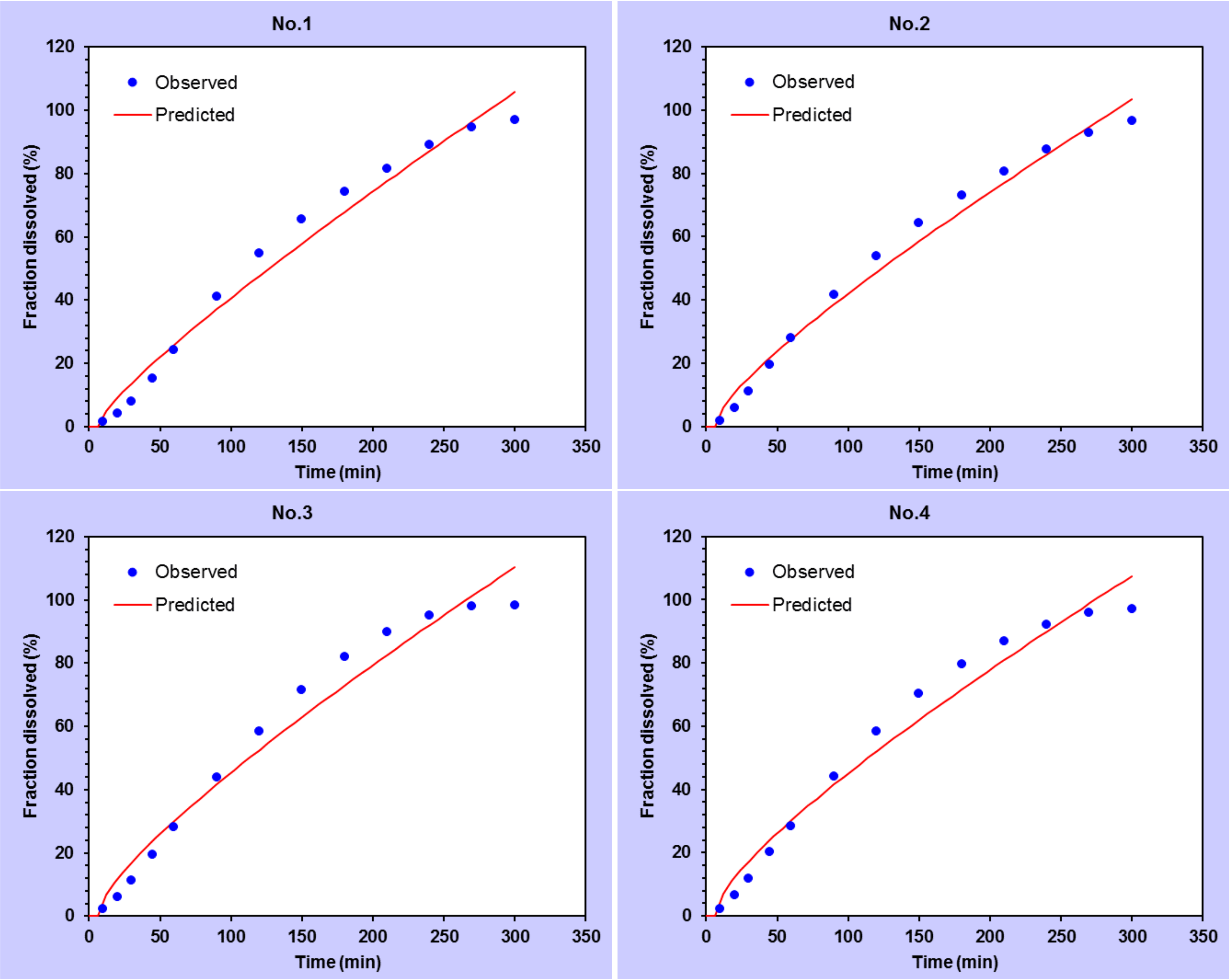

Supplementary materials – Model fitting summary – Polyox™ WSR N-80 (LEO NF Grade) (modelling the entire relevant dissolution data)

Model: **Peppas–Sahlin_2**

Model equation: $F = k_1 \cdot t^{0.5} + k_2 \cdot t$

Fitted model parameters per tested tablet (N = 4) with statistics – mean, standard deviation (SD), and relative standard deviation expressed in % (RSD%) (output from DDSolver):

Parameter	No.1	No.2	No.3	No.4	Mean	SD	RSD(%)
k_1	1.243	1.751	2.023	2.173	1.797	0.409	22.746
k_2	0.285	0.247	0.255	0.236	0.256	0.021	8.089

Number of dissolution data points (N), degrees of freedom (df), and selected goodness of fit criteria – Pearson correlation coefficient (R), coefficient of determination (R^2), adjusted coefficient of determination ($R^2_{adjusted}$), and residual sum of squares (RSS) (manual calculation in MS Excel):

Parameter	No.1	No.2	No.3	No.4
N	13	13	13	13
df	11	11	11	11
R	0.98642308	0.99022962	0.98204059	0.98383844
R^2	0.97303049	0.98055471	0.96440372	0.96793808
$R^2_{adjusted}$	0.97057872	0.97878695	0.9611677	0.96502336
RSS	455.390169	307.101844	643.104615	546.903576

Graphical abstract of model fit presented as mean ± 1 SD of the fraction % of released carvedilol:

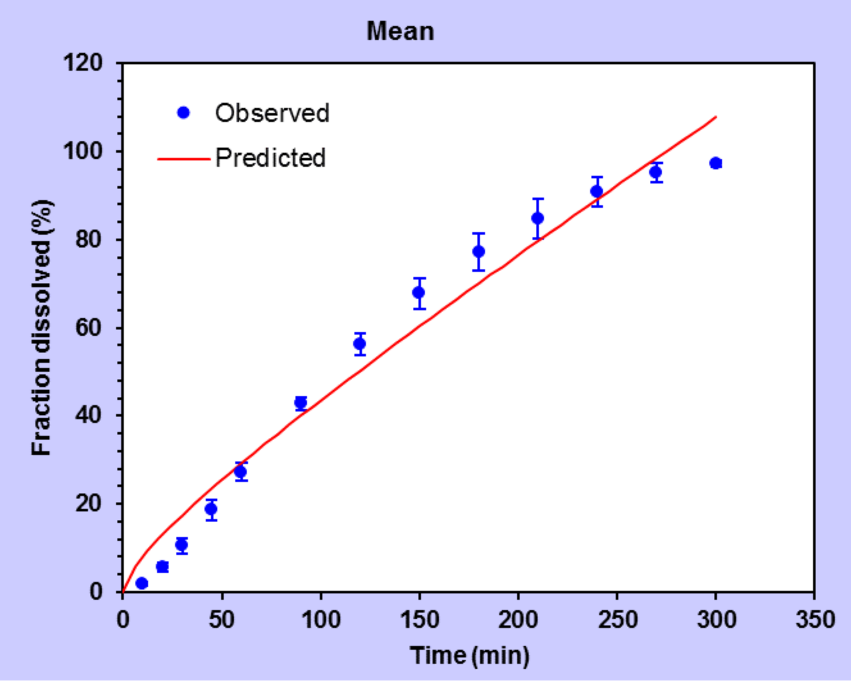

Supplementary materials – Model fitting summary – Polyox^{TM} WSR N-80 (LEO NF Grade) (modelling the entire relevant dissolution data)

Graphical abstract of model fit presented as the fraction % of released carvedilol per tested tablet:

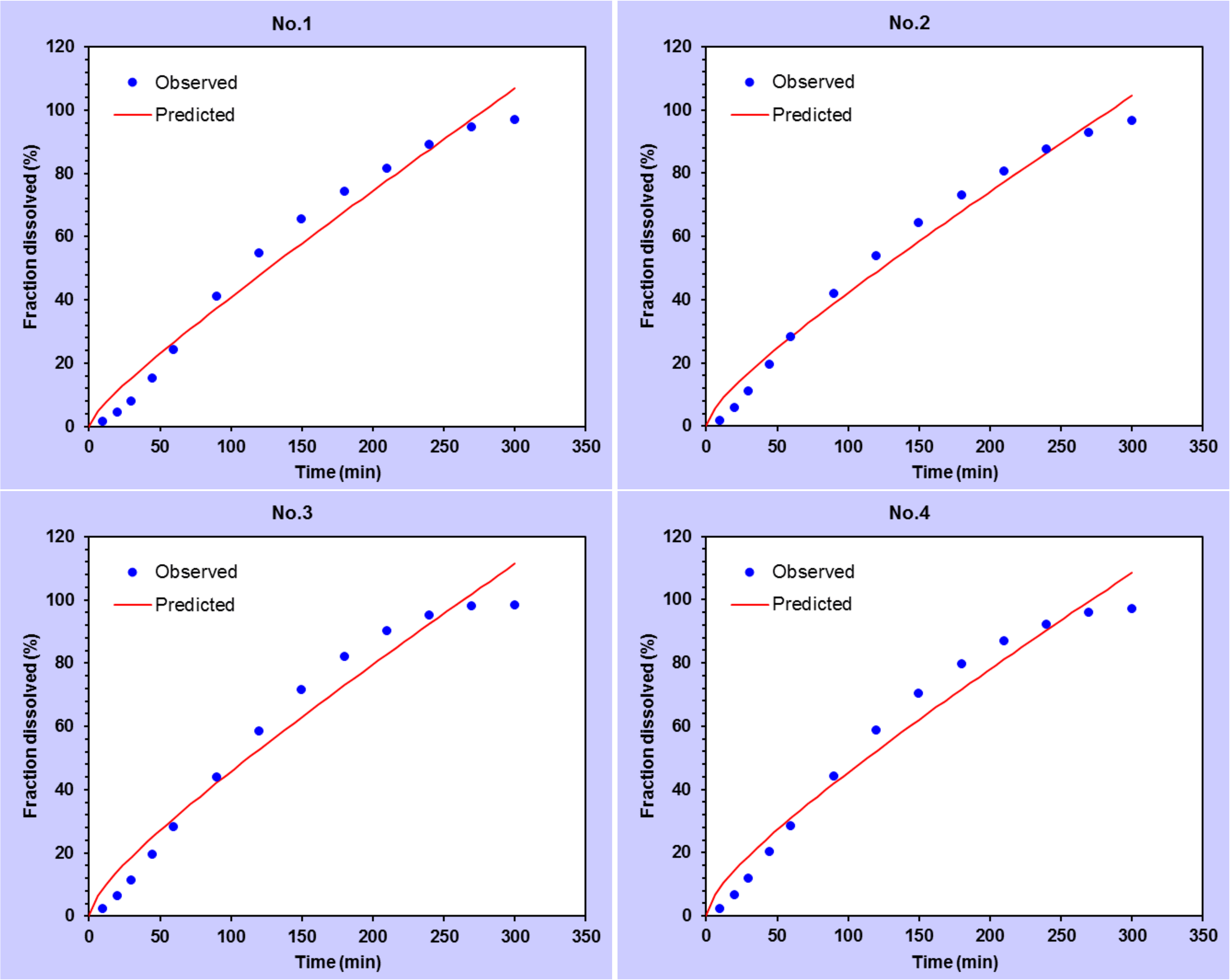

Supplementary materials – Model fitting summary – Polyox™ WSR N-80 (LEO NF Grade) (modelling the entire relevant dissolution data)

Model: **Peppas–Sahlin_2 with T_{lag}**

Model equation: $F = k_1 \cdot (t - T_{lag})^{0.5} + k_2 \cdot (t - T_{lag})$

Fitted model parameters per tested tablet (N = 4) with statistics – mean, standard deviation (SD), and relative standard deviation expressed in % (RSD%) (output from DDSolver):

Parameter	No.1	No.2	No.3	No.4	Mean	SD	RSD(%)
k_1	1.654	2.169	2.482	2.627	2.233	0.431	19.305
k_2	0.263	0.225	0.230	0.212	0.232	0.022	9.419
T_{lag}	6.000	6.000	6.000	6.000	6.000	0.000	0.000

Number of dissolution data points (N), degrees of freedom (df), and selected goodness of fit criteria – Pearson correlation coefficient (R), coefficient of determination (R^2), adjusted coefficient of determination ($R^2_{adjusted}$), and residual sum of squares (RSS) (manual calculation in MS Excel):

Parameter	No.1	No.2	No.3	No.4
N	13	13	13	13
df	10	10	10	10
R	0.98824822	0.99226603	0.98448612	0.98643413
R^2	0.97663455	0.98459187	0.96921293	0.9730523
$R^2_{adjusted}$	0.97196146	0.98151025	0.96305552	0.96766276
RSS	386.240764	236.319252	543.346155	447.774676

Graphical abstract of model fit presented as mean ± 1 SD of the fraction % of released carvedilol:

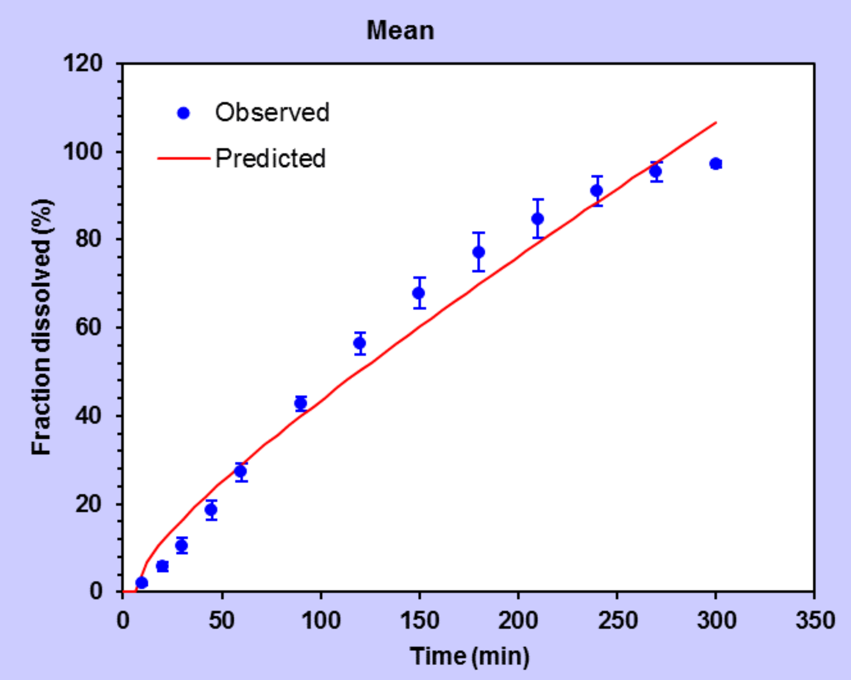

Supplementary materials – Model fitting summary – Polyox™ WSR N-80 (LEO NF Grade) (modelling the entire relevant dissolution data)

Graphical abstract of model fit presented as the fraction % of released carvedilol per tested tablet:

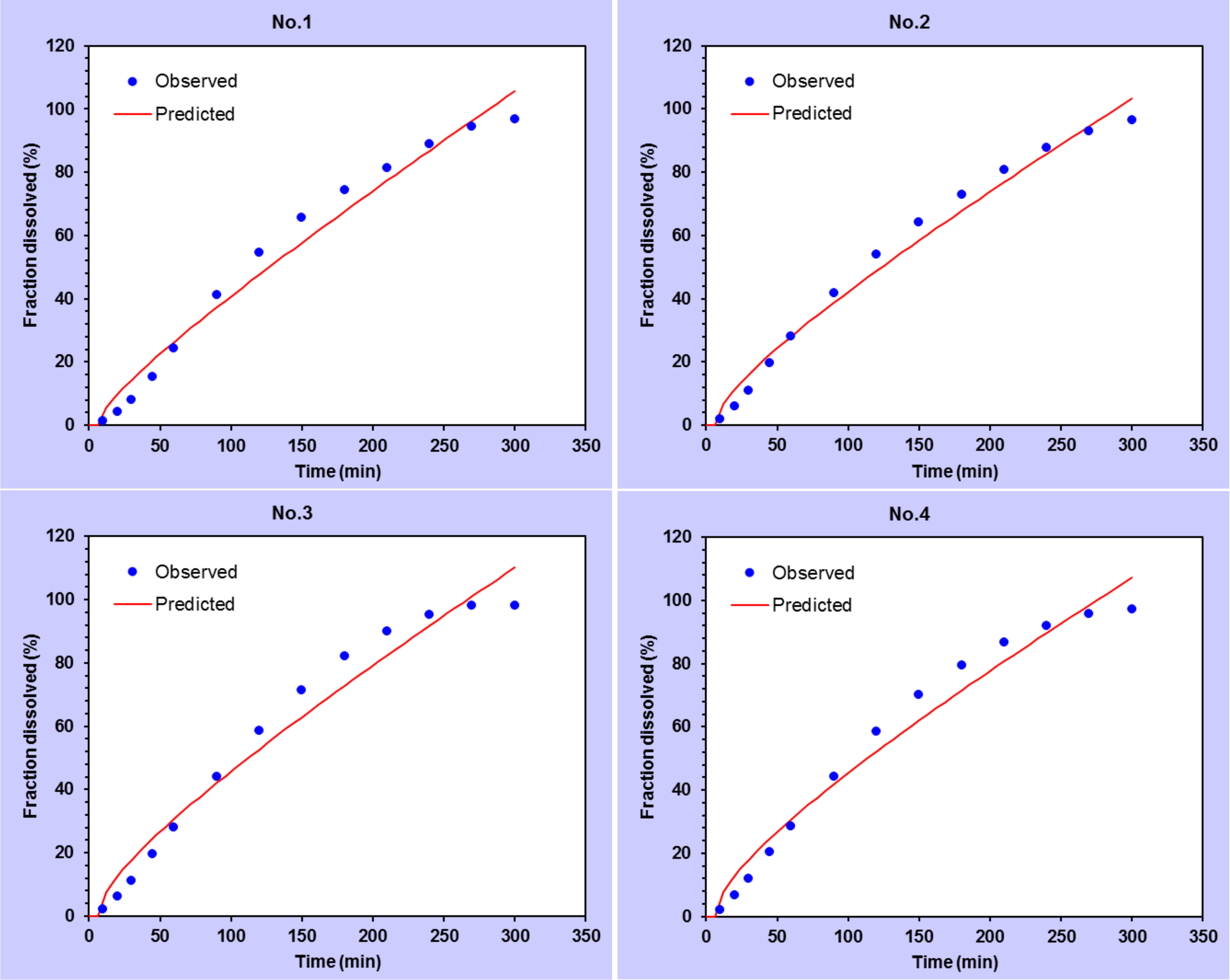

Supplementary materials – Model fitting summary – Polyox™ WSR N-80 (LEO NF Grade) (modelling the entire relevant dissolution data)

Model: **Quadratic**

Model equation: $F = 100 \cdot (k_1 \cdot t^2 + k_2 \cdot t)$

Fitted model parameters per tested tablet (N = 4) with statistics – mean, standard deviation (SD), and relative standard deviation expressed in % (RSD%) (output from DDSolver):

Parameter	No.1	No.2	No.3	No.4	Mean	SD	RSD(%)
k_1	0.000	0.000	0.000	0.000	0.000	0.000	-17.474
k_2	0.005	0.005	0.006	0.006	0.005	0.000	7.421

Number of dissolution data points (N), degrees of freedom (df), and selected goodness of fit criteria – Pearson correlation coefficient (R), coefficient of determination (R^2), adjusted coefficient of determination ($R^2_{adjusted}$), and residual sum of squares (RSS) (manual calculation in MS Excel):

Parameter	No.1	No.2	No.3	No.4
N	13	13	13	13
df	11	11	11	11
R	0.99731954	0.99918431	0.99779643	0.99865396
R^2	0.99464626	0.99836929	0.99559771	0.99730974
$R^2_{adjusted}$	0.99415956	0.99822105	0.99519751	0.99706517
RSS	169.446504	54.0310588	159.731275	102.699266

Graphical abstract of model fit presented as mean ± 1 SD of the fraction % of released carvedilol:

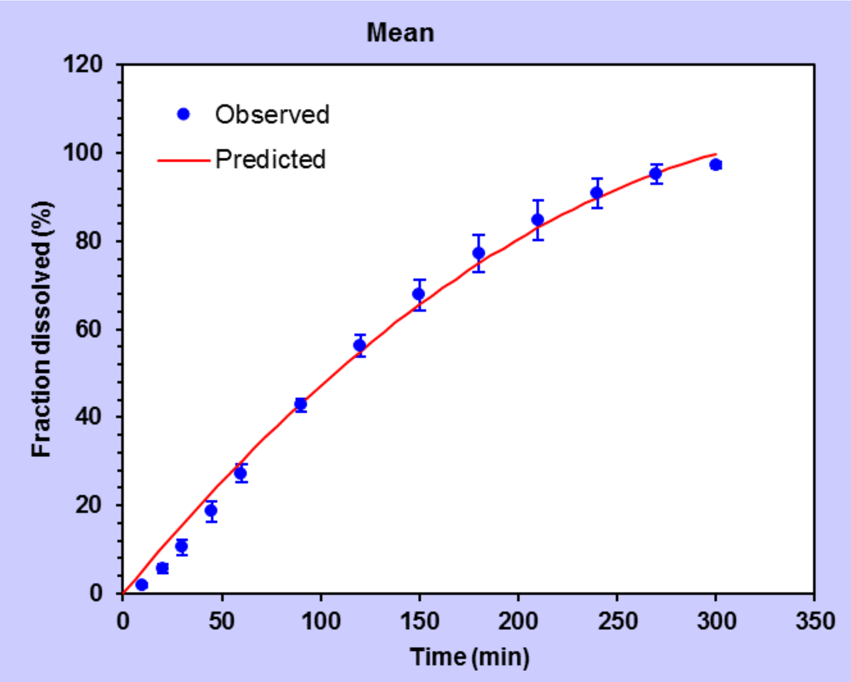

Supplementary materials – Model fitting summary – Polyox™ WSR N-80 (LEO NF Grade) (modelling the entire relevant dissolution data)

Graphical abstract of model fit presented as the fraction % of released carvedilol per tested tablet:

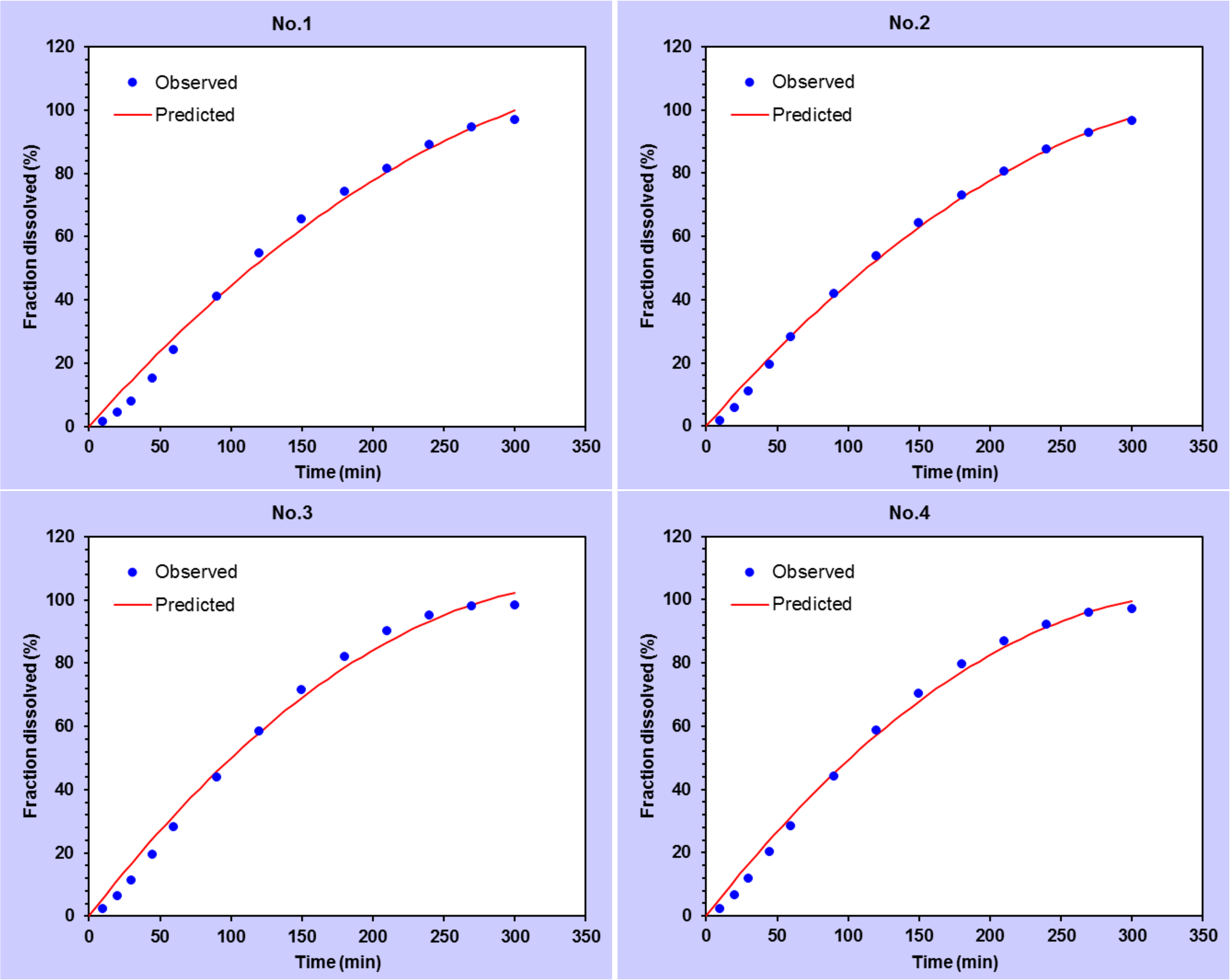

Supplementary materials – Model fitting summary – Polyox™ WSR N-80 (LEO NF Grade) (modelling the entire relevant dissolution data)

Model: **Quadratic with T_{lag}**

Model equation: $F = 100 \cdot \left[k_1 \cdot (t - T_{lag})^2 + k_2 \cdot (t - T_{lag})\right]$

Fitted model parameters per tested tablet (N = 4) with statistics – mean, standard deviation (SD), and relative standard deviation expressed in % (RSD%) (output from DDSolver):

Parameter	No.1	No.2	No.3	No.4	Mean	SD	RSD(%)
k_1	0.000	0.000	0.000	0.000	0.000	0.000	-16.129
k_2	0.005	0.005	0.006	0.006	0.006	0.000	7.308
T_{lag}	6.000	6.000	6.000	6.000	6.000	0.000	0.000

Number of dissolution data points (N), degrees of freedom (df), and selected goodness of fit criteria – Pearson correlation coefficient (R), coefficient of determination (R^2), adjusted coefficient of determination ($R^2_{adjusted}$), and residual sum of squares (RSS) (manual calculation in MS Excel):

Parameter	No.1	No.2	No.3	No.4
N	13	13	13	13
df	10	10	10	10
R	0.99822388	0.99964903	0.99858431	0.99929543
R^2	0.99645092	0.99929818	0.99717062	0.99859135
$R^2_{adjusted}$	0.9957411	0.99915782	0.99660474	0.99830962
RSS	82.4043374	13.5298931	66.8250755	31.3420254

Graphical abstract of model fit presented as mean ± 1 SD of the fraction % of released carvedilol:

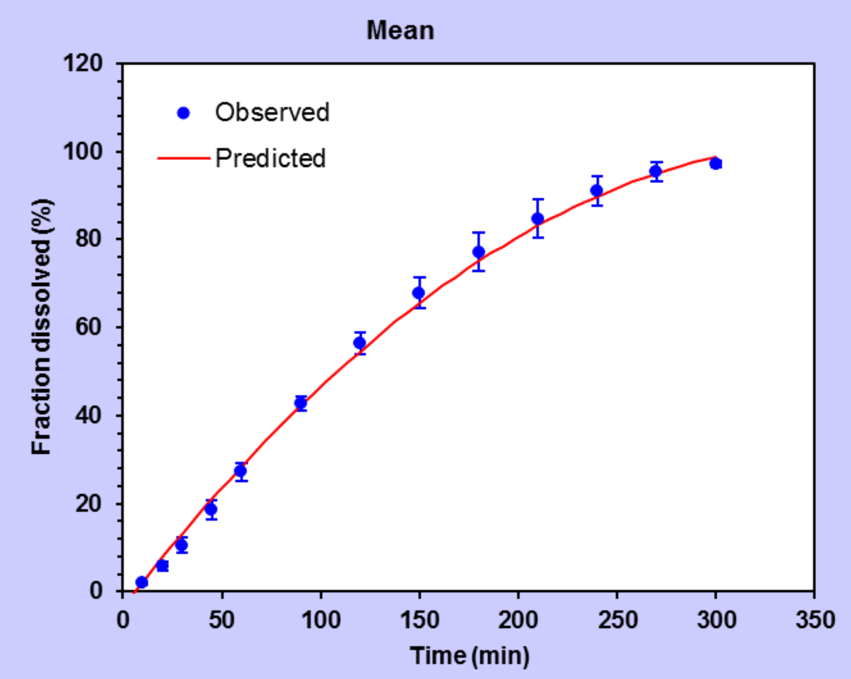

Supplementary materials – Model fitting summary – Polyox™ WSR N-80 (LEO NF Grade) (modelling the entire relevant dissolution data)

Graphical abstract of model fit presented as the fraction % of released carvedilol per tested tablet:

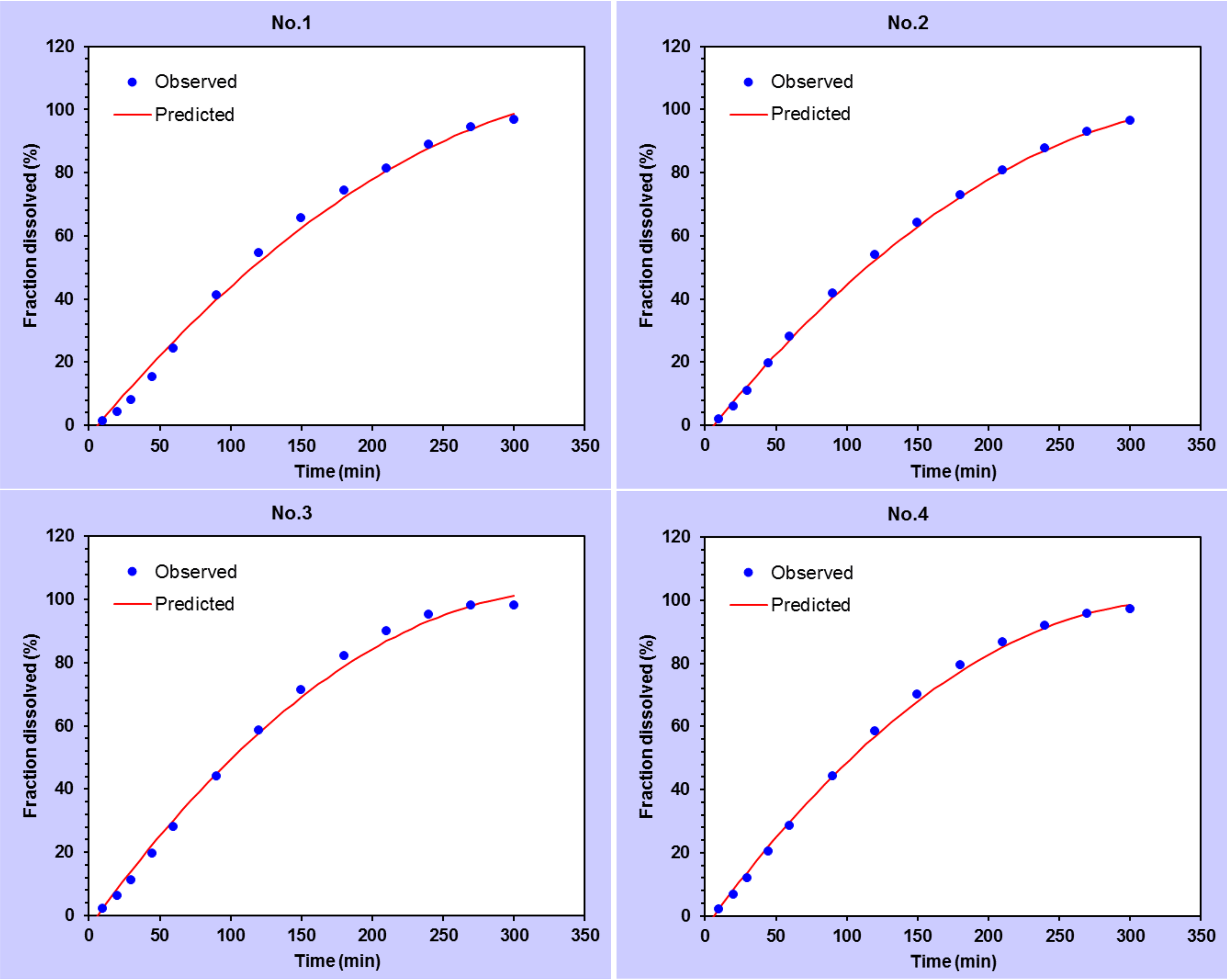

Supplementary materials – Model fitting summary – Polyox™ WSR N-80 (LEO NF Grade) (modelling the entire relevant dissolution data)

Model: **Weibull_1**

Model equation: $F = 100 \cdot \left[1 - e^{-\frac{(t-T_i)^\beta}{\alpha}}\right]$

Fitted model parameters per tested tablet (N = 4) with statistics – mean, standard deviation (SD), and relative standard deviation expressed in % (RSD%) (output from DDSolver):

Parameter	No.1	No.2	No.3	No.4	Mean	SD	RSD(%)
α	1108.126	603.174	685.833	553.491	737.656	252.940	34.290
β	1.427	1.307	1.375	1.317	1.356	0.056	4.115
T_i	6.000	6.000	6.000	6.000	6.000	0.000	0.000

Number of dissolution data points (N), degrees of freedom (df), and selected goodness of fit criteria – Pearson correlation coefficient (R), coefficient of determination (R^2), adjusted coefficient of determination ($R^2_{adjusted}$), and residual sum of squares (RSS) (manual calculation in MS Excel):

Parameter	No.1	No.2	No.3	No.4
N	13	13	13	13
df	10	10	10	10
R	0.99947003	0.99885166	0.99835889	0.99941305
R^2	0.99894034	0.99770463	0.99672048	0.99882644
$R^2_{adjusted}$	0.99872841	0.99724556	0.99606458	0.99859173
RSS	18.3355242	35.4085886	57.3045914	18.9085447

Graphical abstract of model fit presented as mean ± 1 SD of the fraction % of released carvedilol:

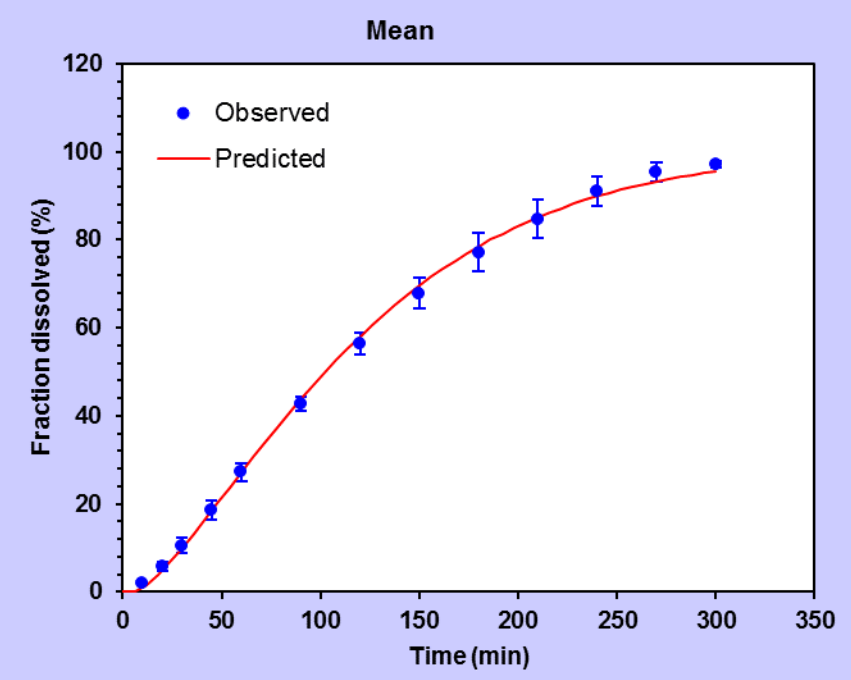

Supplementary materials – Model fitting summary – Polyox™ WSR N-80 (LEO NF Grade) (modelling the entire relevant dissolution data)

Graphical abstract of model fit presented as the fraction % of released carvedilol per tested tablet:

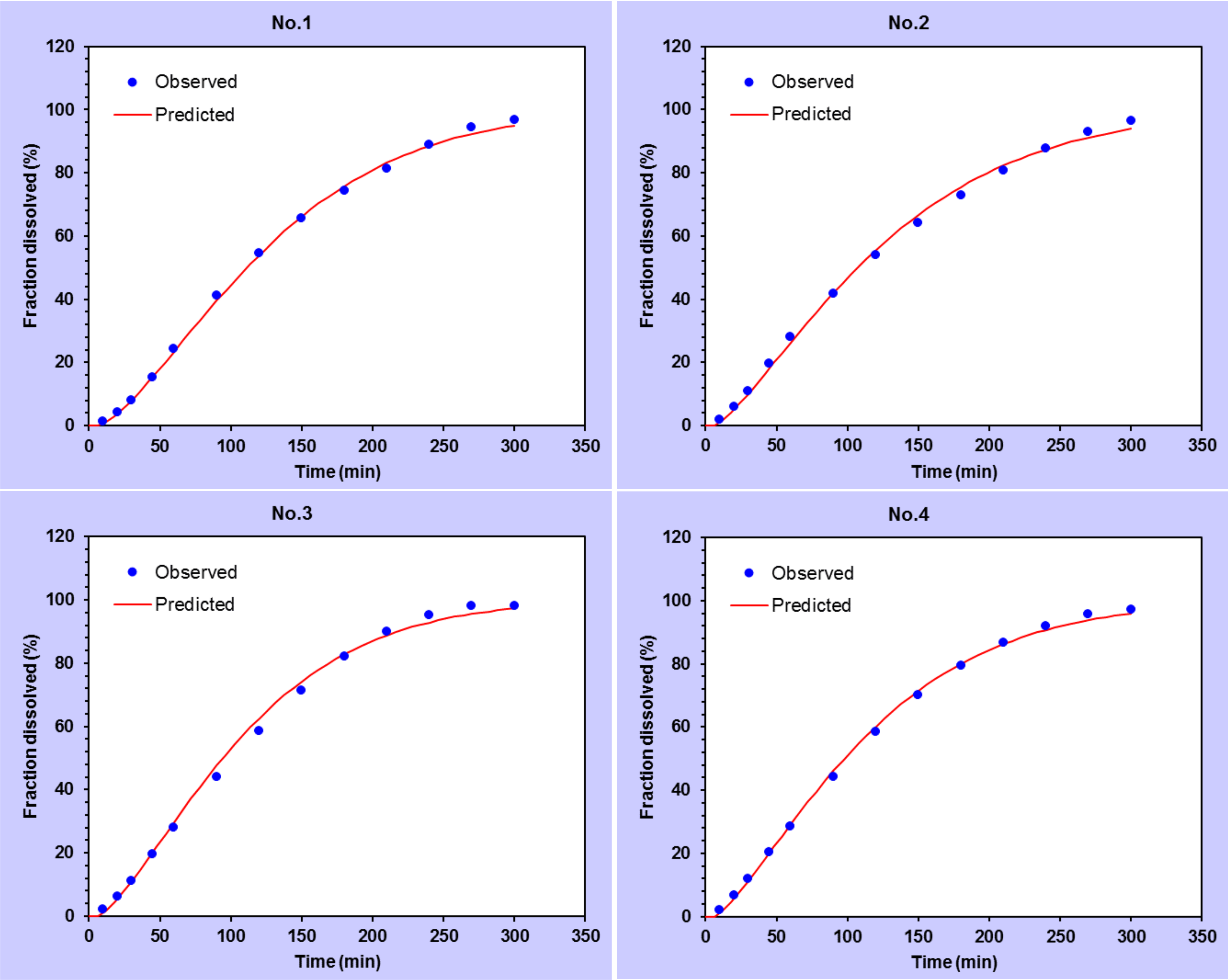

Supplementary materials – Model fitting summary – Polyox™ WSR N-80 (LEO NF Grade) (modelling the entire relevant dissolution data)

Model: **Weibull_2**

Model equation: $F = 100 \cdot \left(1 - e^{-\frac{t^{\beta}}{\alpha}}\right)$

Fitted model parameters per tested tablet (N = 4) with statistics – mean, standard deviation (SD), and relative standard deviation expressed in % (RSD%) (output from DDSolver):

Parameter	No.1	No.2	No.3	No.4	Mean	SD	RSD(%)
α	2639.943	1322.361	1601.376	1233.272	1699.238	646.442	38.043
β	1.590	1.455	1.536	1.468	1.512	0.063	4.179

Number of dissolution data points (N), degrees of freedom (df), and selected goodness of fit criteria – Pearson correlation coefficient (R), coefficient of determination (R^2), adjusted coefficient of determination ($R^2_{adjusted}$), and residual sum of squares (RSS) (manual calculation in MS Excel):

Parameter	No.1	No.2	No.3	No.4
N	13	13	13	13
df	11	11	11	11
R	0.99916523	0.99877167	0.99904604	0.9997516
R^2	0.99833116	0.99754485	0.99809298	0.99950327
$R^2_{adjusted}$	0.99817945	0.99732166	0.99791962	0.99945811
RSS	30.3177223	45.4907077	37.1442537	9.03255543

Graphical abstract of model fit presented as mean ± 1 SD of the fraction % of released carvedilol:

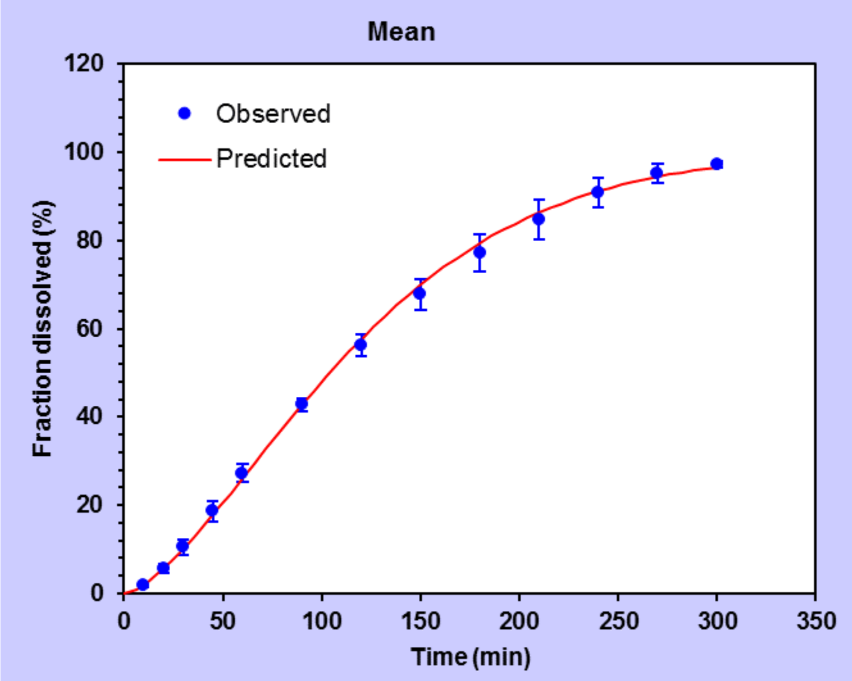

Supplementary materials – Model fitting summary – Polyox™ WSR N-80 (LEO NF Grade) (modelling the entire relevant dissolution data)

Graphical abstract of model fit presented as the fraction % of released carvedilol per tested tablet:

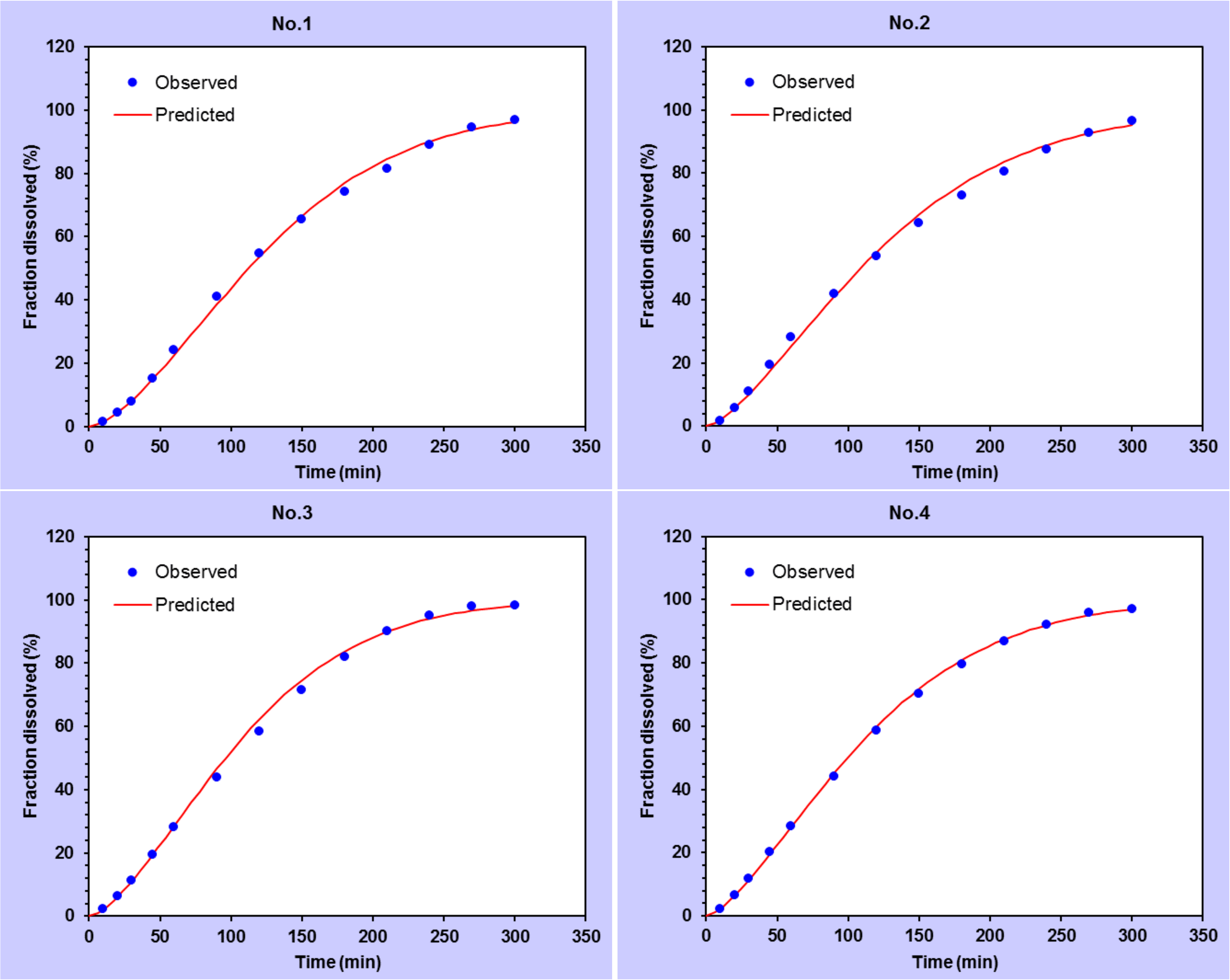

Supplementary materials – Model fitting summary – Polyox™ WSR N-80 (LEO NF Grade) (modelling the entire relevant dissolution data)

Model: **Weibull_3**

Model equation: $F = F_{max} \cdot \left(1 - e^{-\frac{t^{\beta}}{\alpha}}\right)$

Fitted model parameters per tested tablet (N = 4) with statistics – mean, standard deviation (SD), and relative standard deviation expressed in % (RSD%) (output from DDSolver):

Parameter	No.1	No.2	No.3	No.4	Mean	SD	RSD(%)
α	2489.582	1269.967	1350.807	1140.592	1562.737	623.933	39.926
β	1.568	1.439	1.476	1.439	1.480	0.061	4.124
F_{max}	101.768	101.378	103.155	101.977	102.070	0.765	0.749

Number of dissolution data points (N), degrees of freedom (df), and selected goodness of fit criteria – Pearson correlation coefficient (R), coefficient of determination (R^2), adjusted coefficient of determination ($R^2_{adjusted}$), and residual sum of squares (RSS) (manual calculation in MS Excel):

Parameter	No.1	No.2	No.3	No.4
N	13	13	13	13
df	10	10	10	10
R	0.99918332	0.99899824	0.99974072	0.99992172
R^2	0.99836732	0.99799748	0.99948151	0.99984344
$R^2_{adjusted}$	0.99804078	0.99759698	0.99937781	0.99981213
RSS	30.6500719	38.9124354	8.69818369	3.42355371

Graphical abstract of model fit presented as mean ± 1 SD of the fraction % of released carvedilol:

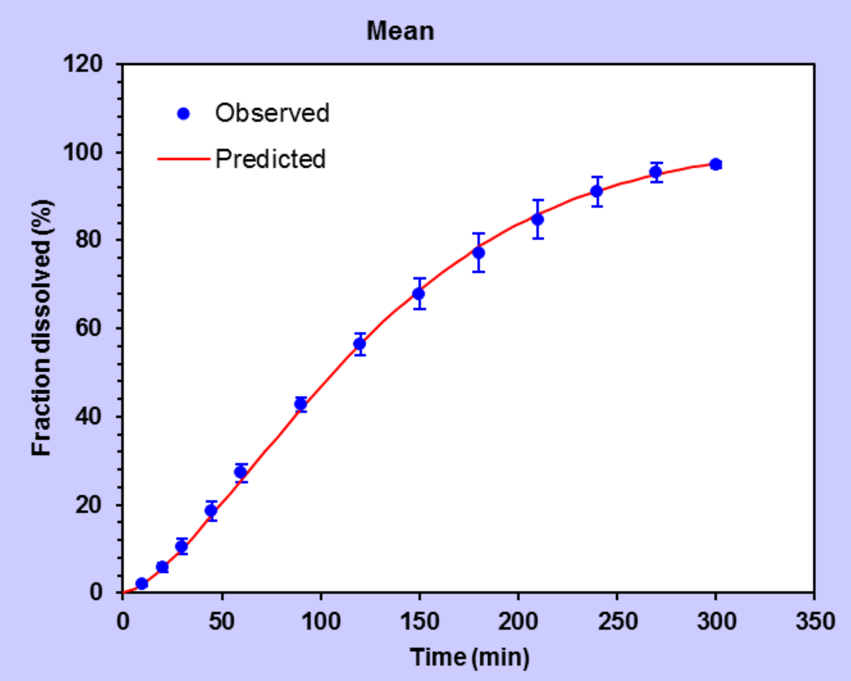

Supplementary materials – Model fitting summary – Polyox^{TM} WSR N-80 (LEO NF Grade) (modelling the entire relevant dissolution data)

Graphical abstract of model fit presented as the fraction % of released carvedilol per tested tablet:

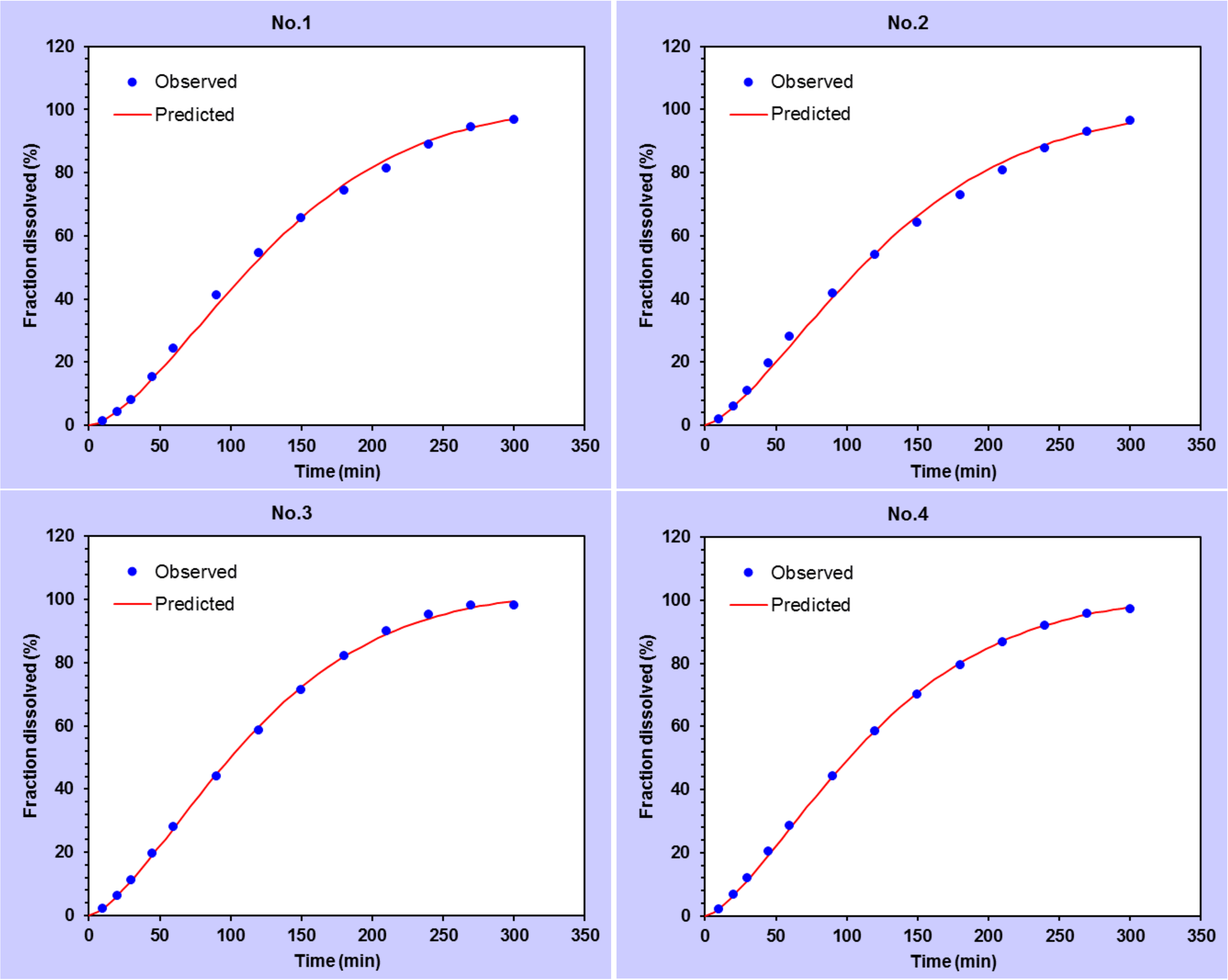

Supplementary materials – Model fitting summary – Polyox™ WSR N-80 (LEO NF Grade) (modelling the entire relevant dissolution data)

Model: **Weibull_4**

Model equation: $F = F_{max} \cdot \left[1 - e^{-\frac{(t-T_i)^\beta}{\alpha}}\right]$

Fitted model parameters per tested tablet (N = 4) with statistics – mean, standard deviation (SD), and relative standard deviation expressed in % (RSD%) (output from DDSolver):

Parameter	No.1	No.2	No.3	No.4	Mean	SD	RSD(%)
α	1060.957	585.559	602.107	575.353	705.994	236.899	33.555
β	1.407	1.293	1.324	1.301	1.331	0.052	3.937
T_i	4.000	6.000	6.000	4.760	5.190	0.985	18.988
F_{max}	101.768	101.378	103.155	103.074	102.344	0.904	0.884

Number of dissolution data points (N), degrees of freedom (df), and selected goodness of fit criteria – Pearson correlation coefficient (R), coefficient of determination (R^2), adjusted coefficient of determination ($R^2_{adjusted}$), and residual sum of squares (RSS) (manual calculation in MS Excel):

Parameter	No.1	No.2	No.3	No.4
N	13	13	13	13
df	9	9	9	9
R	0.99959862	0.99916374	0.99933228	0.99990267
R^2	0.99919741	0.99832818	0.998665	0.99980535
$R^2_{adjusted}$	0.99892988	0.99777091	0.99822	0.99974046
RSS	13.3432743	27.426582	26.6575113	9.47048464

Graphical abstract of model fit presented as mean ± 1 SD of the fraction % of released carvedilol:

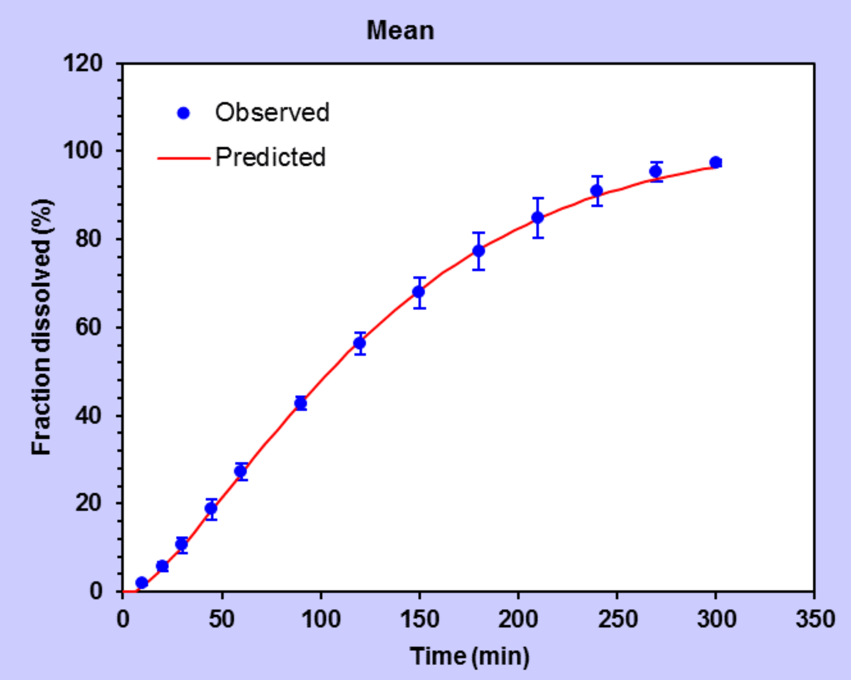

Supplementary materials – Model fitting summary – Polyox™ WSR N-80 (LEO NF Grade) (modelling the entire relevant dissolution data)

Graphical abstract of model fit presented as the fraction % of released carvedilol per tested tablet:

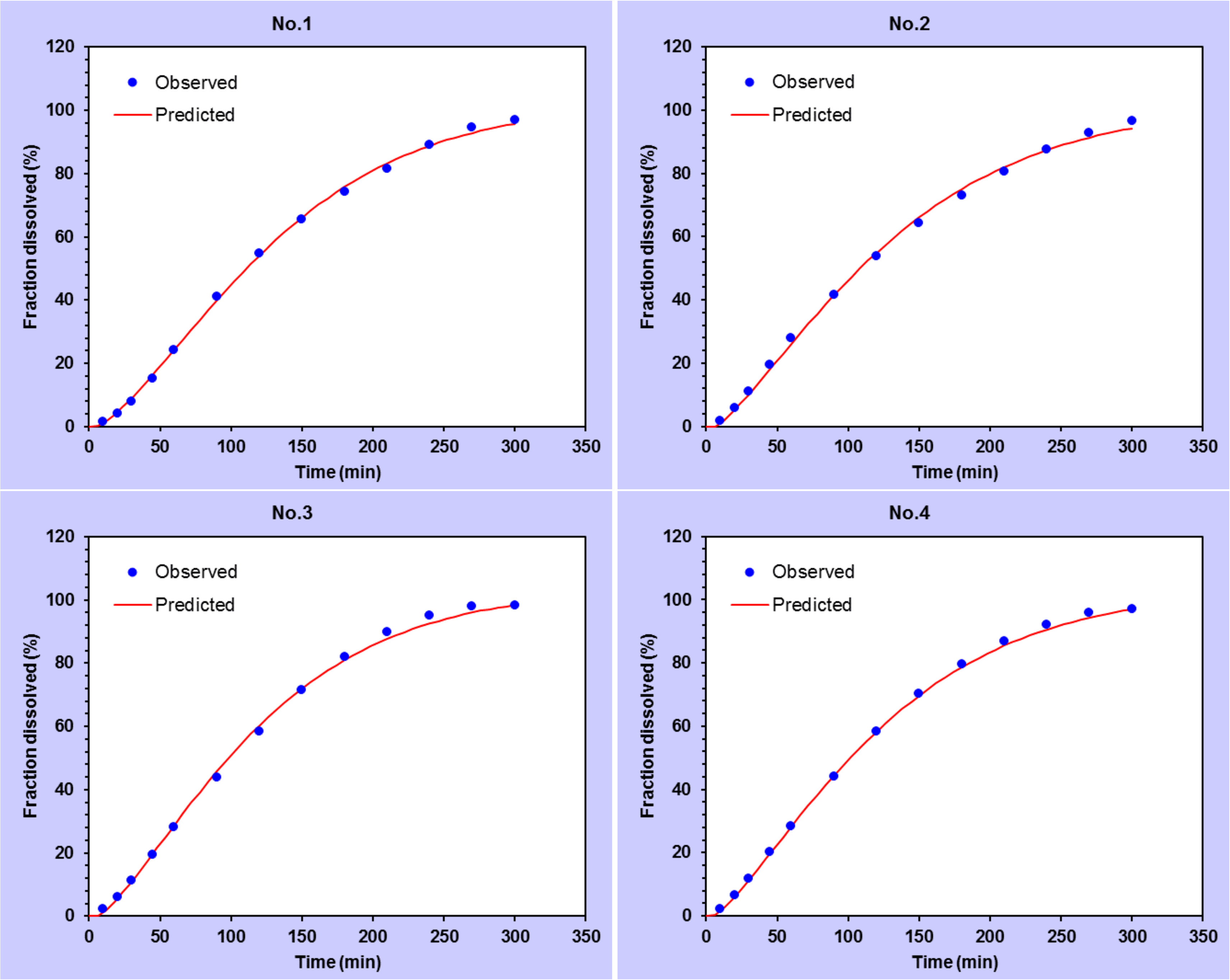

Supplementary materials – Model fitting summary – Polyox™ WSR N-80 (LEO NF Grade) (modelling the entire relevant dissolution data)

Model: **Logistic_1**

Model equation: $F = 100 \cdot \frac{e^{\alpha+\beta\cdot\log(t)}}{1+e^{\alpha+\beta\cdot\log(t)}}$

Fitted model parameters per tested tablet (N = 4) with statistics – mean, standard deviation (SD), and relative standard deviation expressed in % (RSD%) (output from DDSolver):

Parameter	No.1	No.2	No.3	No.4	Mean	SD	RSD(%)
α	-11.104	-10.152	-9.719	-9.580	-10.139	0.688	-6.786
β	5.355	4.923	5.184	4.800	5.066	0.251	4.946

Number of dissolution data points (N), degrees of freedom (df), and selected goodness of fit criteria – Pearson correlation coefficient (R), coefficient of determination (R^2), adjusted coefficient of determination ($R^2_{adjusted}$), and residual sum of squares (RSS) (manual calculation in MS Excel):

Parameter	No.1	No.2	No.3	No.4
N	13	13	13	13
df	11	11	11	11
R	0.99808746	0.99665901	0.98252678	0.99750597
R^2	0.99617858	0.99332918	0.96535888	0.99501816
$R^2_{adjusted}$	0.99583118	0.99272275	0.96220969	0.99456527
RSS	315.768883	344.831839	808.738761	193.583585

Graphical abstract of model fit presented as mean ± 1 SD of the fraction % of released carvedilol:

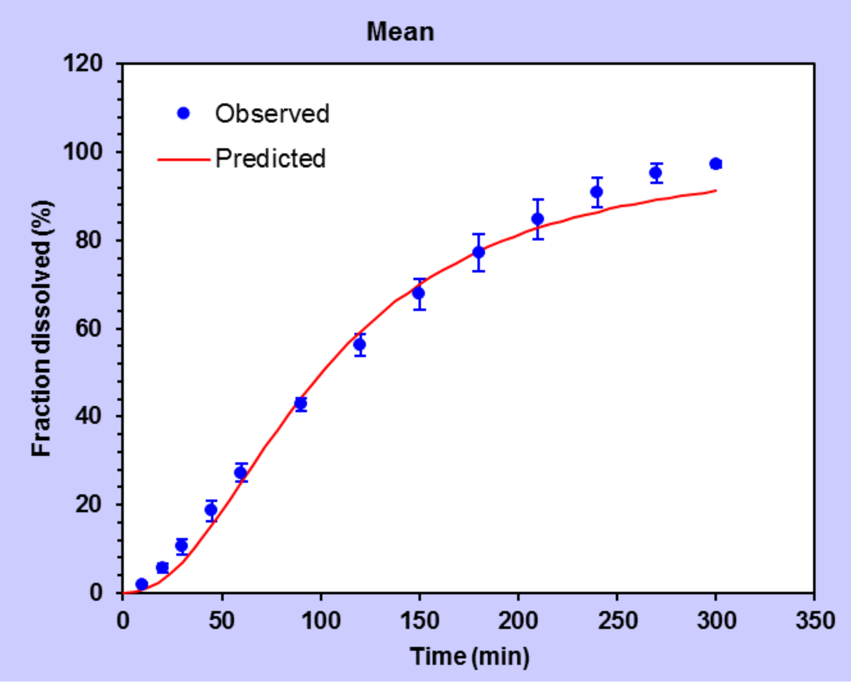

Supplementary materials – Model fitting summary – Polyox^{TM} WSR N-80 (LEO NF Grade) (modelling the entire relevant dissolution data)

Graphical abstract of model fit presented as the fraction % of released carvedilol per tested tablet:

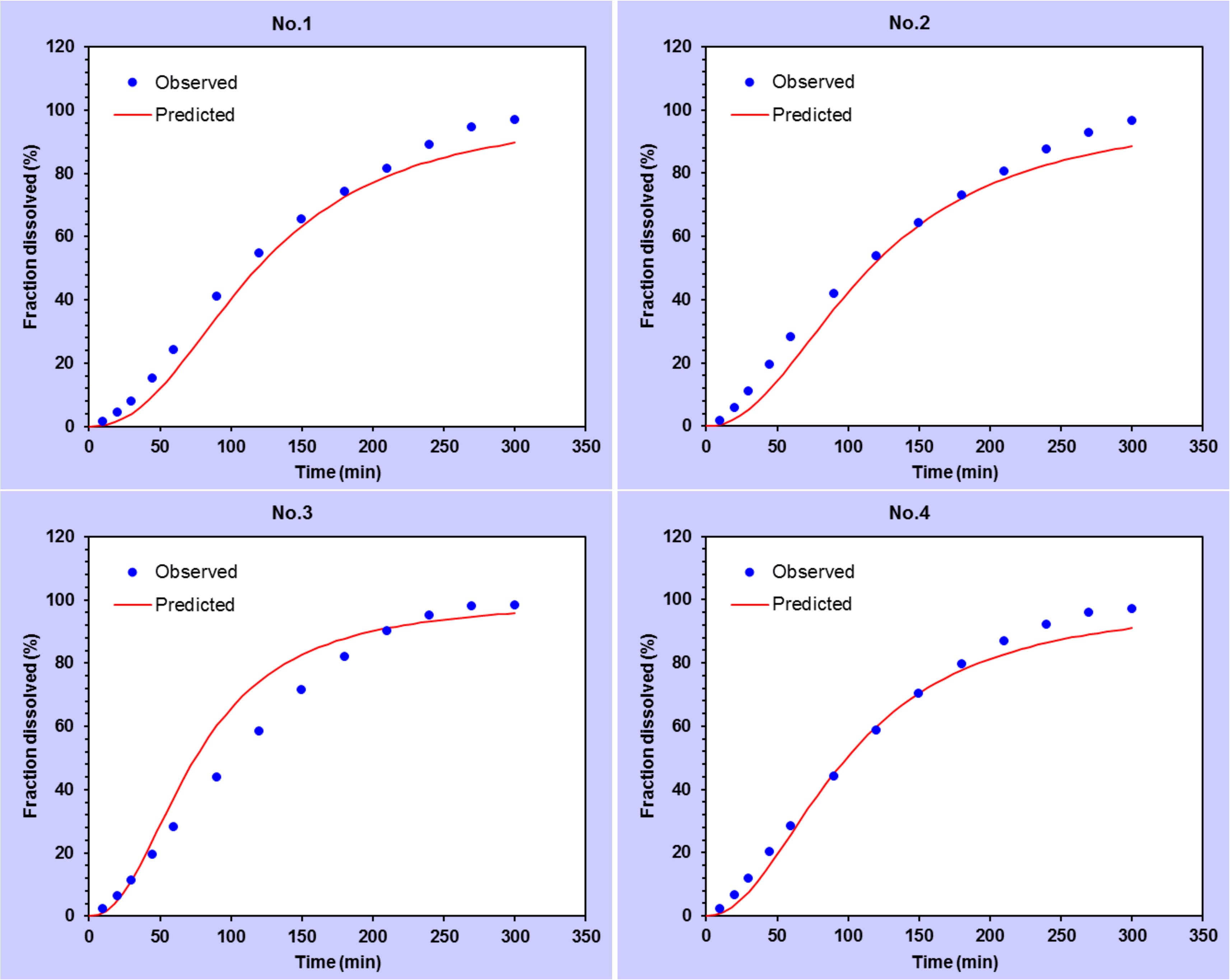

Supplementary materials – Model fitting summary – Polyox™ WSR N-80 (LEO NF Grade) (modelling the entire relevant dissolution data)

Model: **Logistic_2**

Model equation: $F = F_{max} \cdot \frac{e^{\alpha+\beta\cdot\log(t)}}{1+e^{\alpha+\beta\cdot\log(t)}}$

Fitted model parameters per tested tablet (N = 4) with statistics – mean, standard deviation (SD), and relative standard deviation expressed in % (RSD%) (output from DDSolver):

Parameter	No.1	No.2	No.3	No.4	Mean	SD	RSD(%)
α	-10.320	-9.454	-9.563	-9.267	-9.651	0.463	-4.793
β	5.088	4.725	4.609	4.453	4.719	0.270	5.731
F_{max}	97.211	95.497	115.912	114.588	105.802	10.945	10.345

Number of dissolution data points (N), degrees of freedom (df), and selected goodness of fit criteria – Pearson correlation coefficient (R), coefficient of determination (R^2), adjusted coefficient of determination ($R^2_{adjusted}$), and residual sum of squares (RSS) (manual calculation in MS Excel):

Parameter	No.1	No.2	No.3	No.4
N	13	13	13	13
df	10	10	10	10
R	0.9972361	0.99414214	0.9991993	0.99941659
R^2	0.99447984	0.9883186	0.99839925	0.99883353
$R^2_{adjusted}$	0.99337581	0.98598232	0.99807909	0.99860023
RSS	212.244756	273.923161	93.0935981	104.329884

Graphical abstract of model fit presented as mean ± 1 SD of the fraction % of released carvedilol:

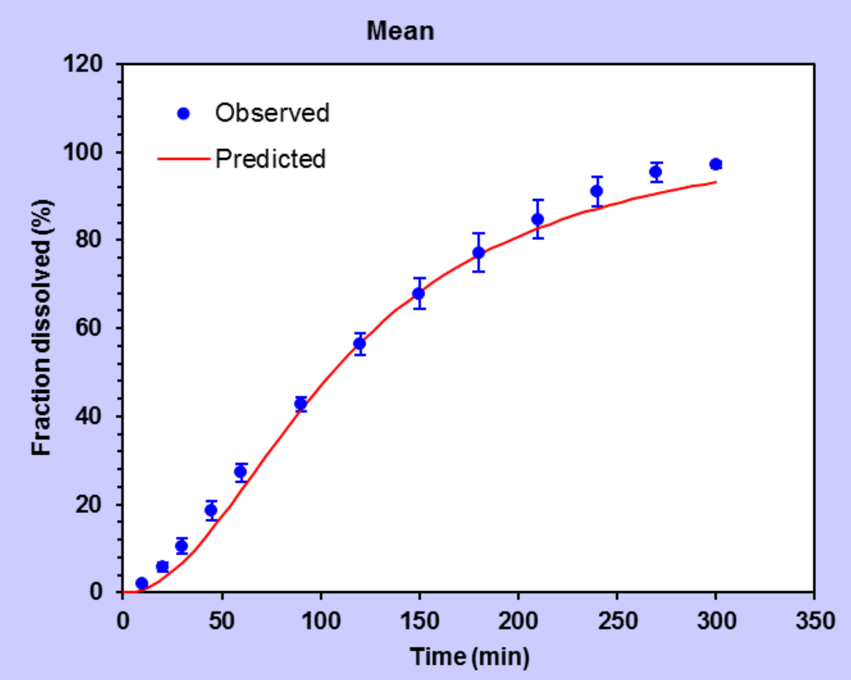

Graphical abstract of model fit presented as the fraction % of released carvedilol per tested tablet:

Supplementary materials – Model fitting summary – Polyox™ WSR N-80 (LEO NF Grade) (modelling the entire relevant dissolution data)

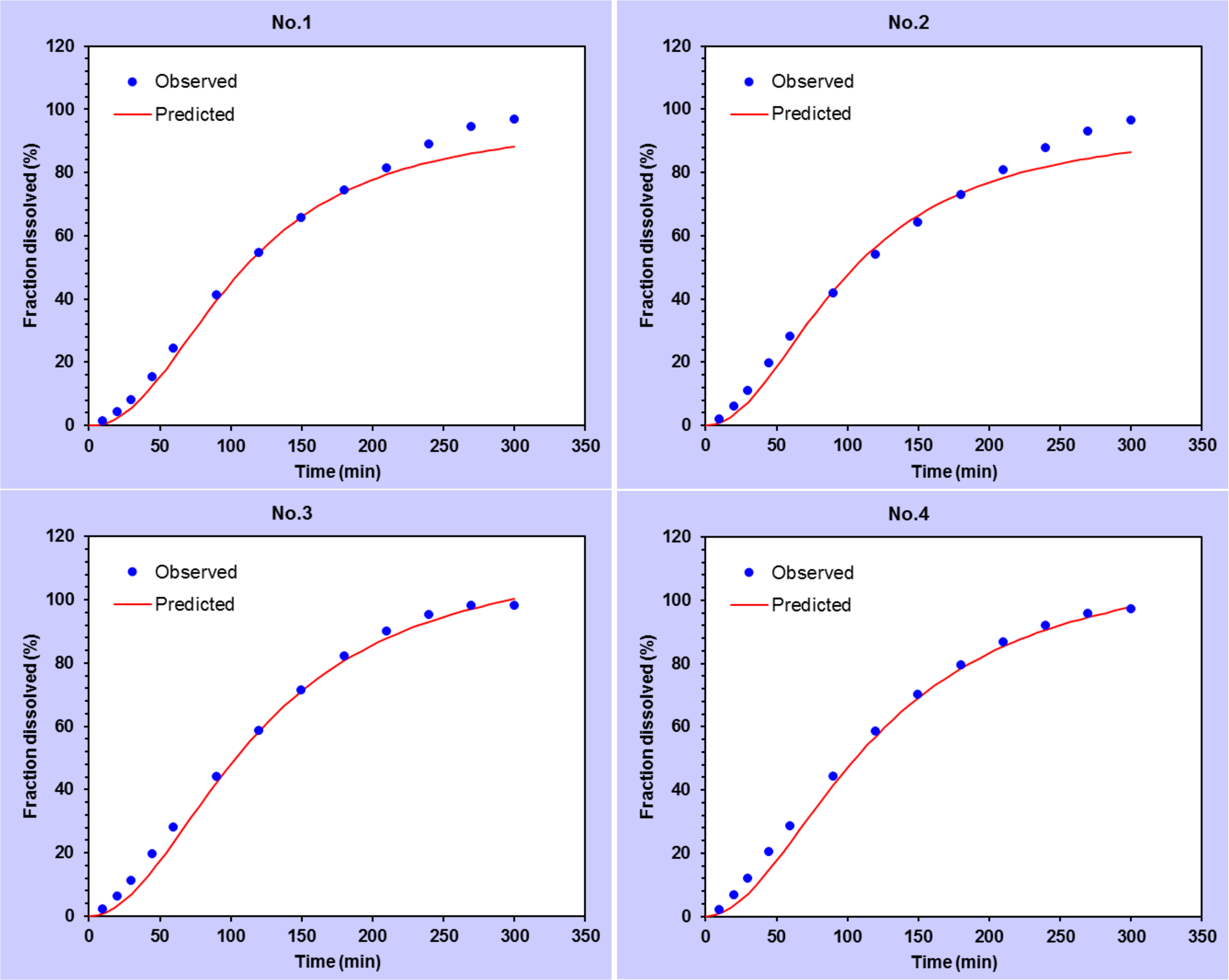

Supplementary materials – Model fitting summary – Polyox™ WSR N-80 (LEO NF Grade) (modelling the entire relevant dissolution data)

Model: **Logistic_3**

Model equation: $F = F_{max} \cdot \frac{1}{1+e^{-k\cdot(t-\gamma)}}$

Fitted model parameters per tested tablet (N = 4) with statistics – mean, standard deviation (SD), and relative standard deviation expressed in % (RSD%) (output from DDSolver):

Parameter	No.1	No.2	No.3	No.4	Mean	SD	RSD(%)
k	0.022	0.020	0.022	0.021	0.021	0.001	3.507
γ	107.278	138.135	127.791	129.097	125.575	13.037	10.382
F_{max}	92.816	101.378	103.155	101.977	99.832	4.735	4.743

Number of dissolution data points (N), degrees of freedom (df), and selected goodness of fit criteria – Pearson correlation coefficient (R), coefficient of determination (R^2), adjusted coefficient of determination ($R^2_{adjusted}$), and residual sum of squares (RSS) (manual calculation in MS Excel):

Parameter	No.1	No.2	No.3	No.4
N	13	13	13	13
df	10	10	10	10
R	0.9959929	0.98411346	0.9887376	0.98640856
R^2	0.99200187	0.96847929	0.97760204	0.97300184
$R^2_{adjusted}$	0.99040224	0.96217515	0.97312245	0.96760221
RSS	273.754326	605.087714	502.933925	557.7767

Graphical abstract of model fit presented as mean ± 1 SD of the fraction % of released carvedilol:

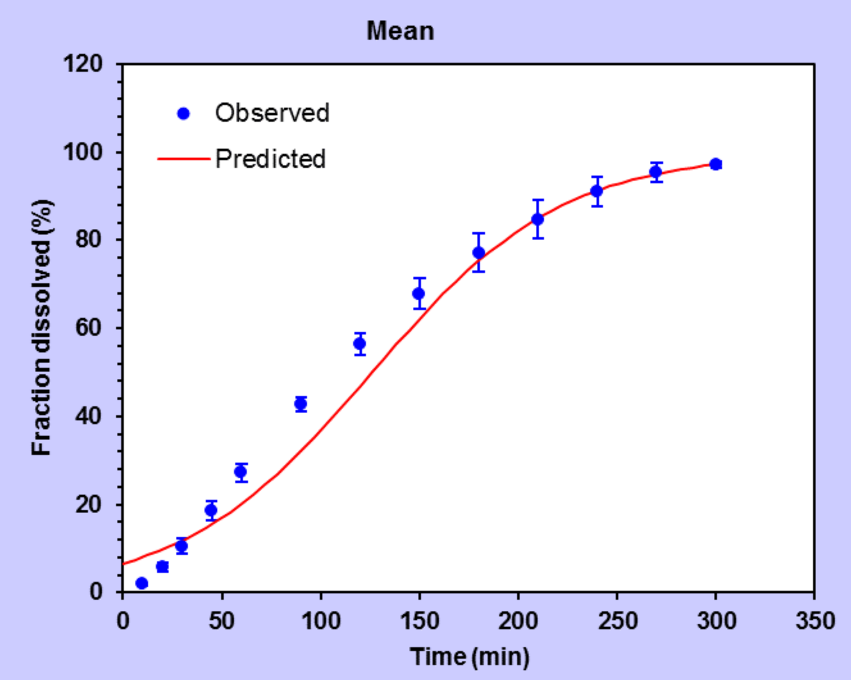

Supplementary materials – Model fitting summary – Polyox^{TM} WSR N-80 (LEO NF Grade) (modelling the entire relevant dissolution data)

Graphical abstract of model fit presented as the fraction % of released carvedilol per tested tablet:

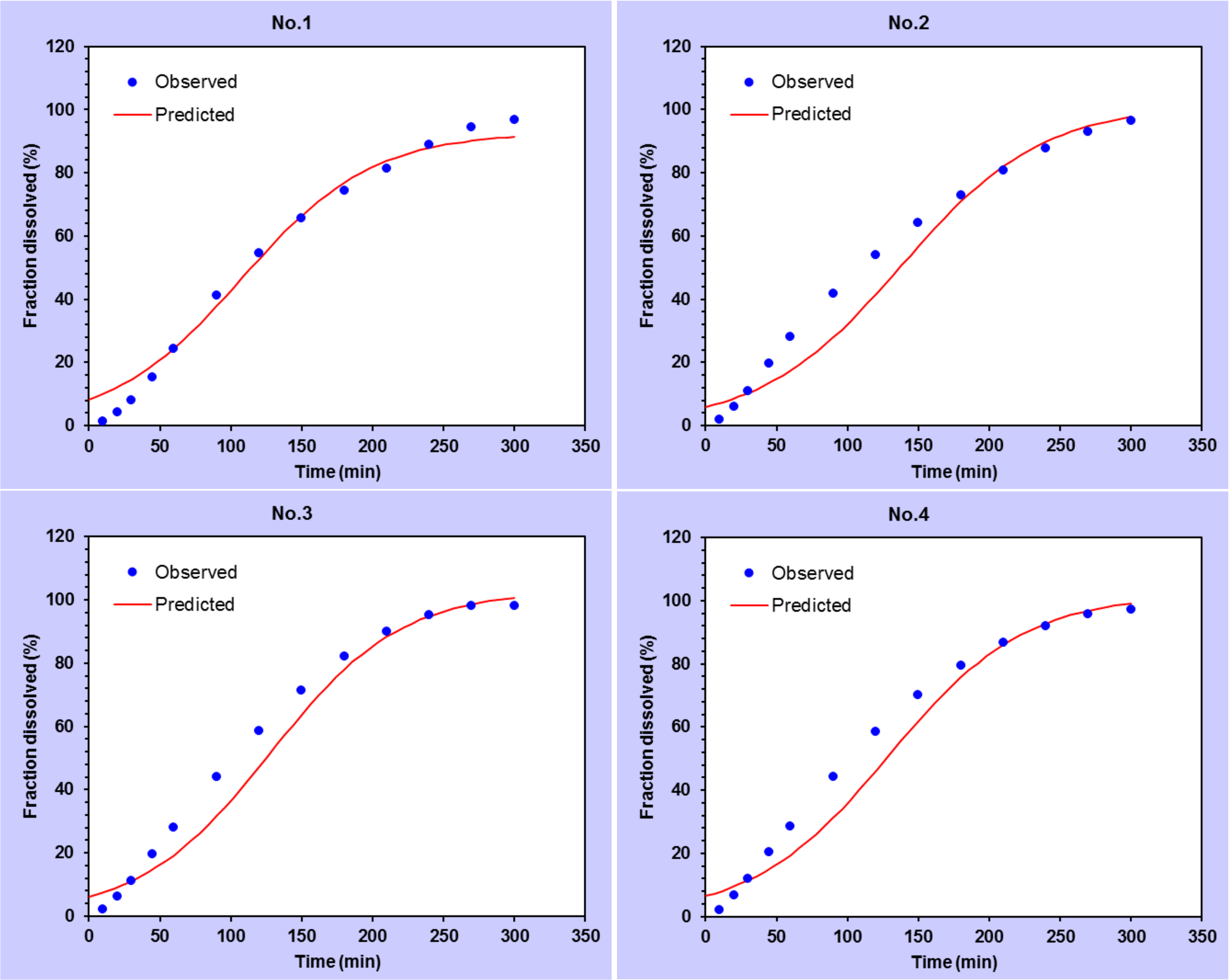

Supplementary materials – Model fitting summary – Polyox™ WSR N-80 (LEO NF Grade) (modelling the entire relevant dissolution data)

Model: **Gompertz_1**

Model equation: $F = 100 \cdot e^{-\alpha \cdot e^{-\beta \cdot \log(t)}}$

Fitted model parameters per tested tablet (N = 4) with statistics – mean, standard deviation (SD), and relative standard deviation expressed in % (RSD%) (output from DDSolver):

Parameter	No.1	No.2	No.3	No.4	Mean	SD	RSD(%)
α	321.189	217.985	508.812	323.106	342.773	121.097	35.329
β	3.086	2.883	3.362	3.186	3.129	0.200	6.397

Number of dissolution data points (N), degrees of freedom (df), and selected goodness of fit criteria – Pearson correlation coefficient (R), coefficient of determination (R^2), adjusted coefficient of determination ($R^2_{adjusted}$), and residual sum of squares (RSS) (manual calculation in MS Excel):

Parameter	No.1	No.2	No.3	No.4
N	13	13	13	13
df	11	11	11	11
R	0.99024926	0.98890909	0.98985625	0.98695784
R^2	0.98059359	0.97794118	0.9798154	0.97408578
$R^2_{adjusted}$	0.97882937	0.97593583	0.97798043	0.97172994
RSS	407.626808	482.464526	625.908386	456.356351

Graphical abstract of model fit presented as mean ± 1 SD of the fraction % of released carvedilol:

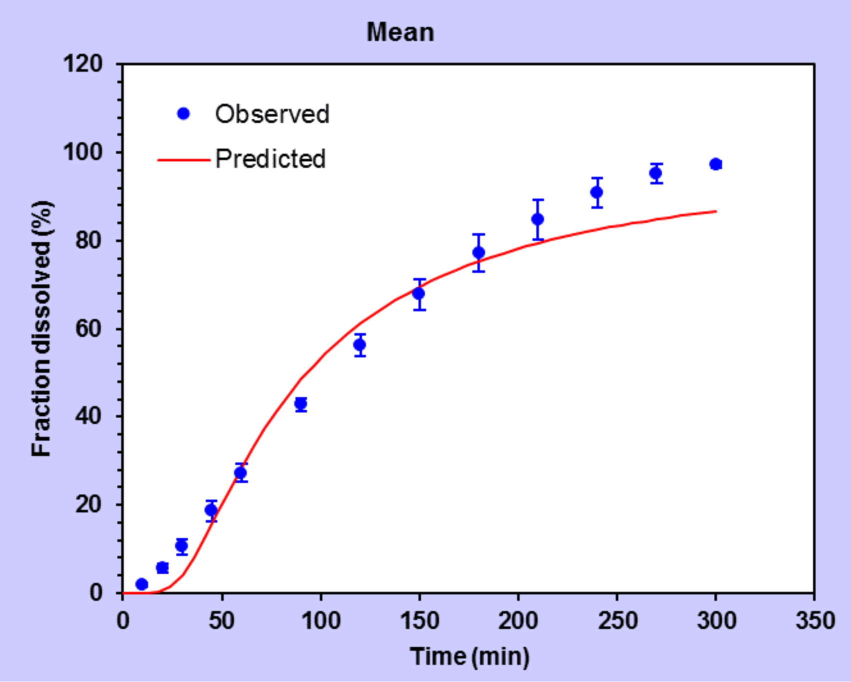

Supplementary materials – Model fitting summary – Polyox^{TM} WSR N-80 (LEO NF Grade) (modelling the entire relevant dissolution data)

Graphical abstract of model fit presented as the fraction % of released carvedilol per tested tablet:

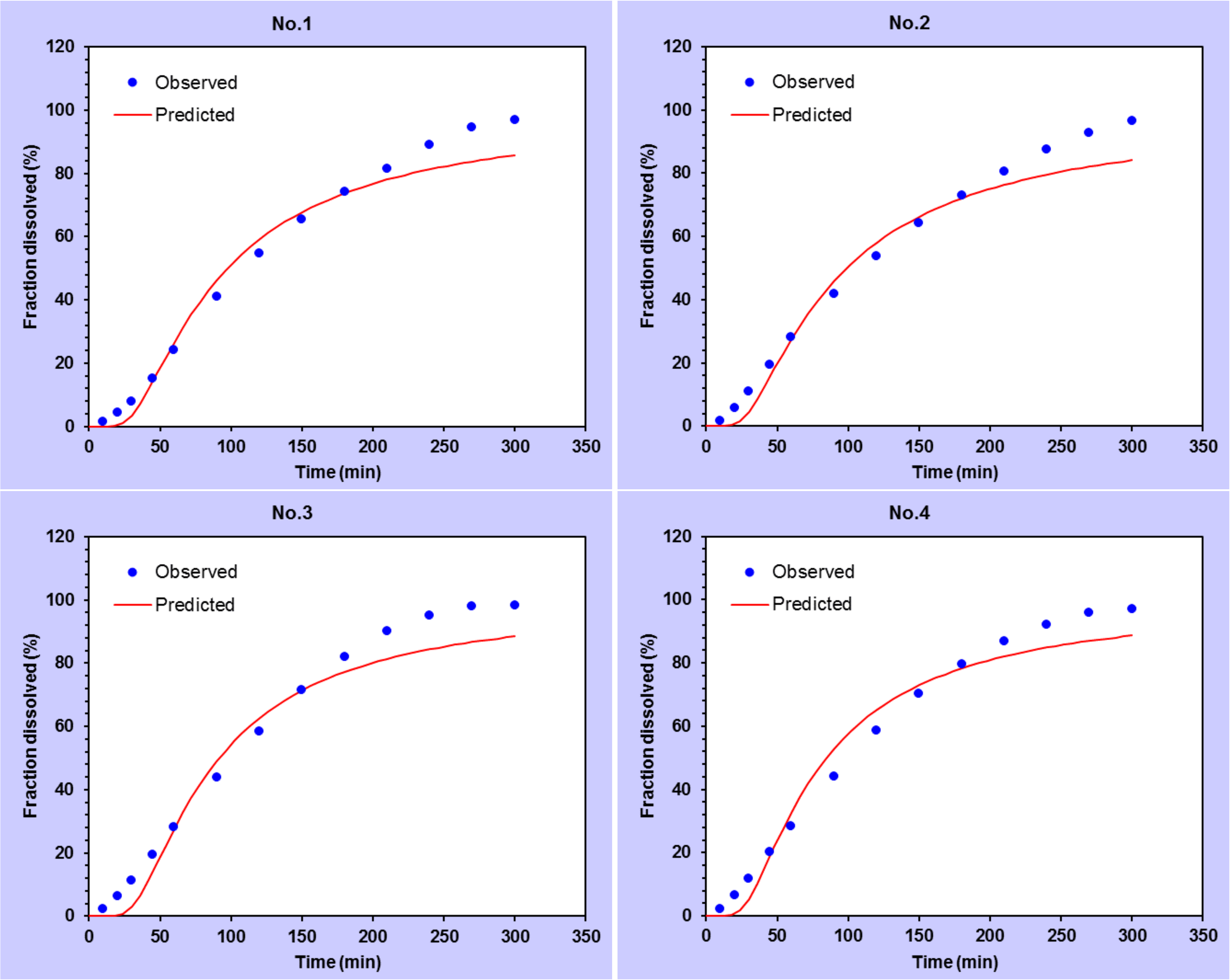

Supplementary materials – Model fitting summary – Polyox™ WSR N-80 (LEO NF Grade) (modelling the entire relevant dissolution data)

Model: **Gompertz_2**

Model equation: $F = F_{max} \cdot e^{-\alpha \cdot e^{-\beta \cdot \log(t)}}$

Fitted model parameters per tested tablet (N = 4) with statistics – mean, standard deviation (SD), and relative standard deviation expressed in % (RSD%) (output from DDSolver):

Parameter	No.1	No.2	No.3	No.4	Mean	SD	RSD(%)
α	247.177	181.564	274.833	188.152	222.931	45.470	20.396
β	2.907	2.757	2.902	2.715	2.820	0.099	3.498
F_{max}	101.768	101.378	115.434	114.134	108.179	7.647	7.069

Number of dissolution data points (N), degrees of freedom (df), and selected goodness of fit criteria – Pearson correlation coefficient (R), coefficient of determination (R^2), adjusted coefficient of determination ($R^2_{adjusted}$), and residual sum of squares (RSS) (manual calculation in MS Excel):

Parameter	No.1	No.2	No.3	No.4
N	13	13	13	13
df	10	10	10	10
R	0.99275524	0.9907681	0.9962706	0.99672039
R^2	0.98556296	0.98162142	0.99255512	0.99345153
$R^2_{adjusted}$	0.98267555	0.97794571	0.99106614	0.99214183
RSS	470.156897	544.123246	381.554322	355.333675

Graphical abstract of model fit presented as mean ± 1 SD of the fraction % of released carvedilol:

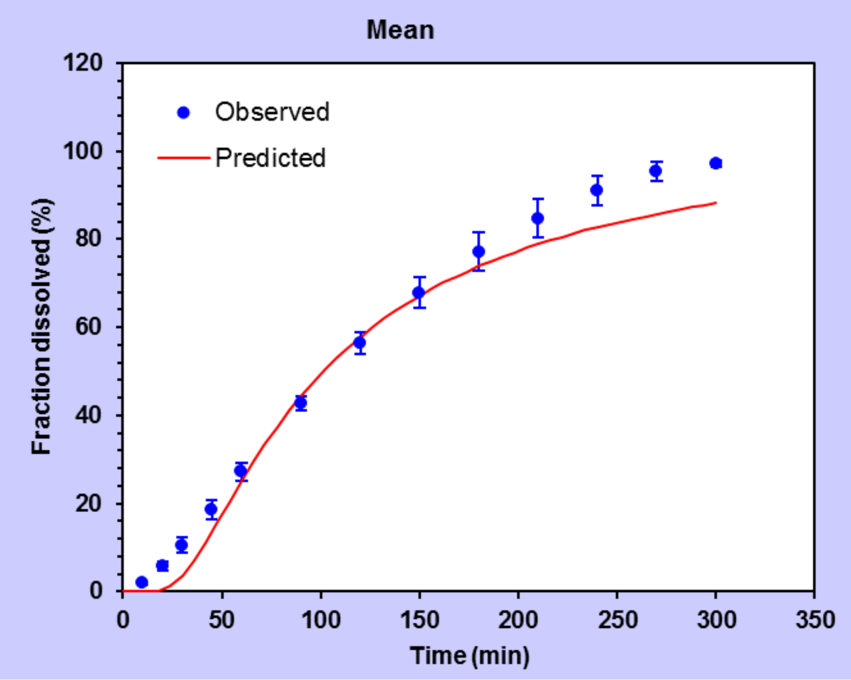

Supplementary materials – Model fitting summary – Polyox^{TM} WSR N-80 (LEO NF Grade) (modelling the entire relevant dissolution data)

Graphical abstract of model fit presented as the fraction % of released carvedilol per tested tablet:

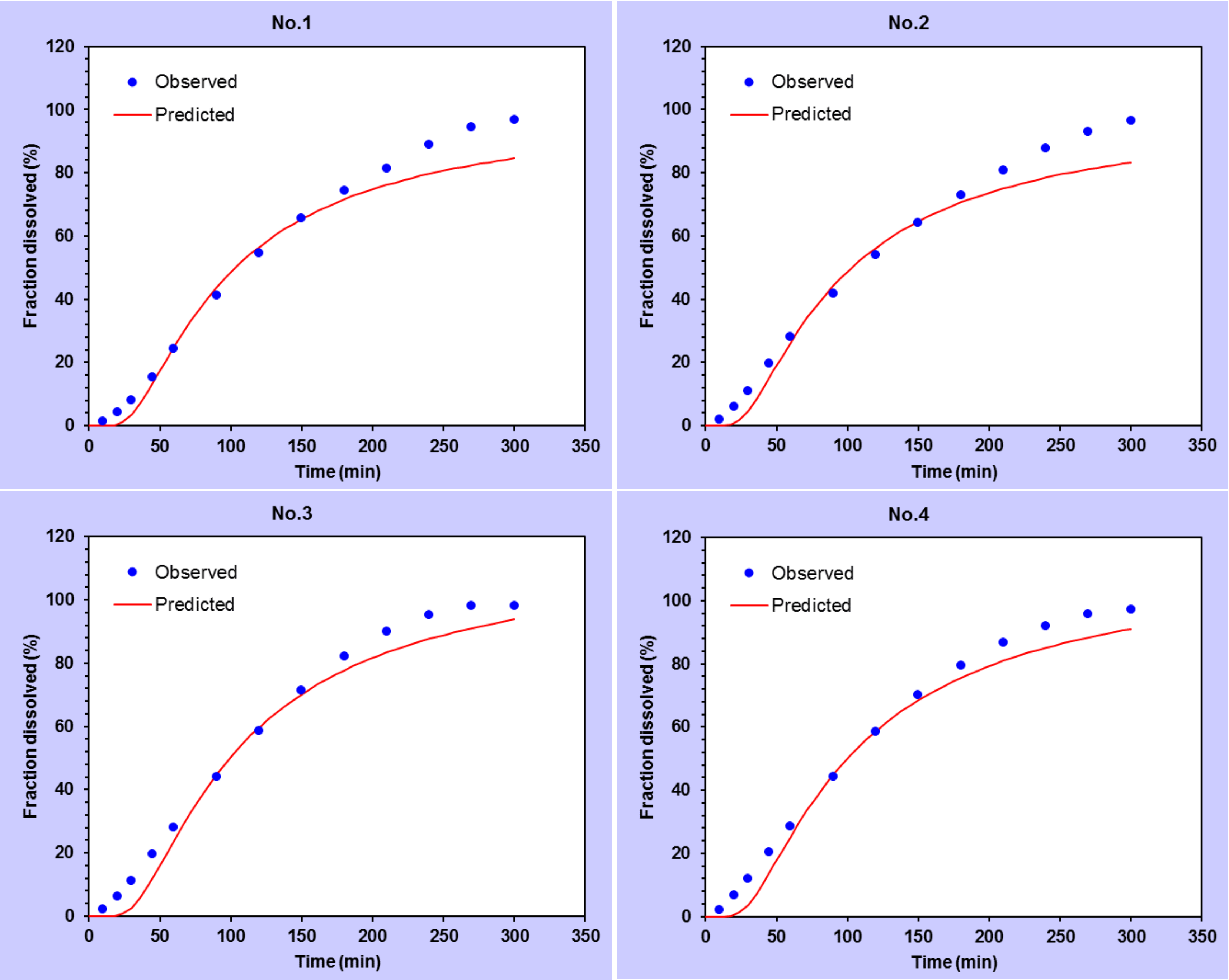

Supplementary materials – Model fitting summary – Polyox™ WSR N-80 (LEO NF Grade) (modelling the entire relevant dissolution data)

Model: **Gompertz_3**

Model equation: $F = F_{max} \cdot e^{-e^{-k\cdot(t-\gamma)}}$

Fitted model parameters per tested tablet (N = 4) with statistics – mean, standard deviation (SD), and relative standard deviation expressed in % (RSD%) (output from DDSolver):

Parameter	No.1	No.2	No.3	No.4	Mean	SD	RSD(%)
k	0.014	0.014	0.015	0.016	0.015	0.001	6.591
γ	94.729	91.474	83.643	77.468	86.828	7.784	8.965
F_{max}	101.768	101.378	103.155	100.706	101.752	1.033	1.015

Number of dissolution data points (N), degrees of freedom (df), and selected goodness of fit criteria – Pearson correlation coefficient (R), coefficient of determination (R^2), adjusted coefficient of determination ($R^2_{adjusted}$), and residual sum of squares (RSS) (manual calculation in MS Excel):

Parameter	No.1	No.2	No.3	No.4
N	13	13	13	13
df	10	10	10	10
R	0.99728861	0.9965355	0.99901047	0.99900526
R^2	0.99458457	0.993083	0.99802191	0.9980115
$R^2_{adjusted}$	0.99350149	0.99169961	0.99762629	0.9976138
RSS	98.9970195	117.943962	36.6481002	52.6495972

Graphical abstract of model fit presented as mean ± 1 SD of the fraction % of released carvedilol:

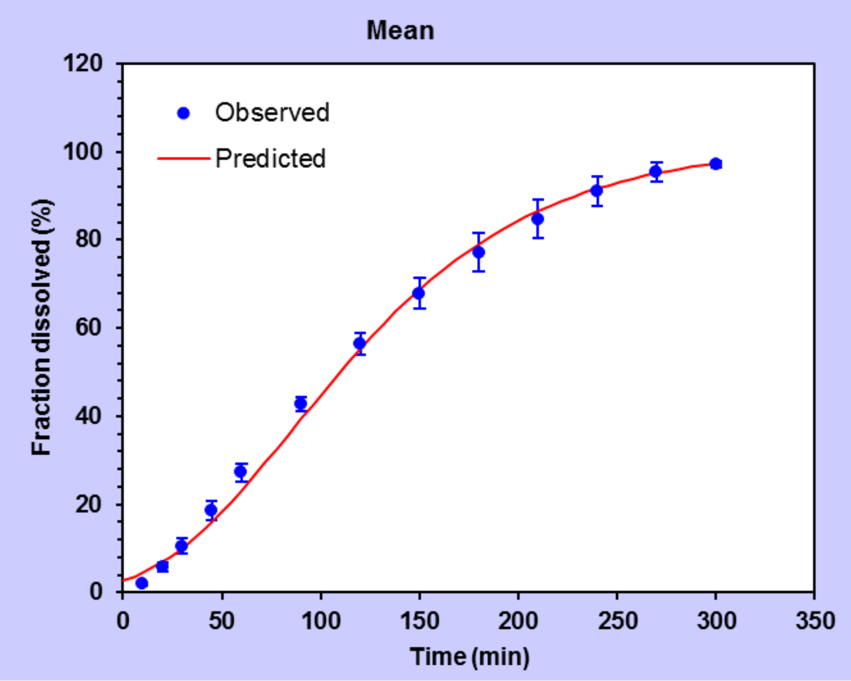

Supplementary materials – Model fitting summary – Polyox™ WSR N-80 (LEO NF Grade) (modelling the entire relevant dissolution data)

Graphical abstract of model fit presented as the fraction % of released carvedilol per tested tablet:

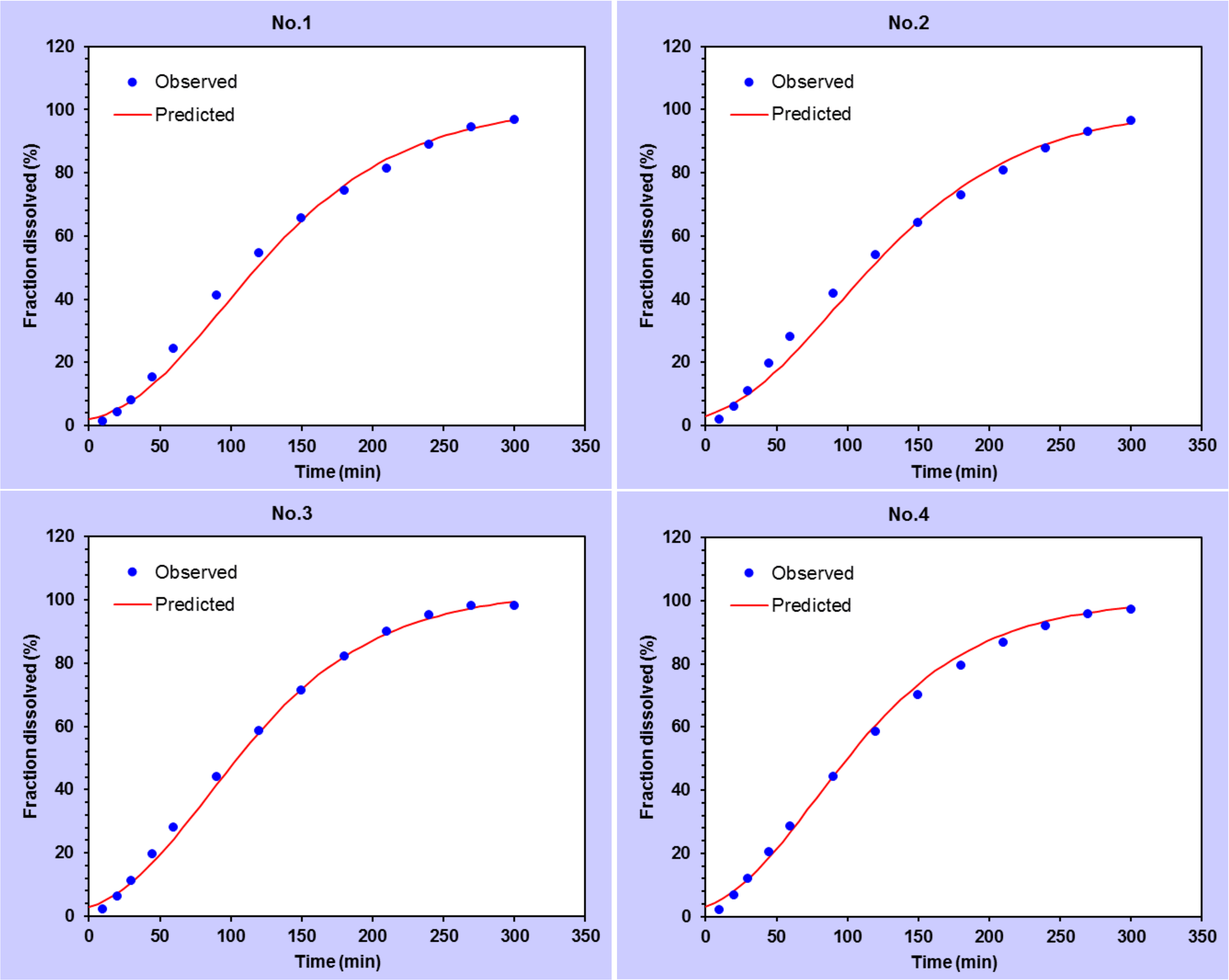

Supplementary materials – Model fitting summary – Polyox™ WSR N-80 (LEO NF Grade) (modelling the entire relevant dissolution data)

Model: **Gompertz_4**

Model equation: $F = F_{max} \cdot e^{-\beta \cdot e^{-k \cdot t}}$

Fitted model parameters per tested tablet (N = 4) with statistics – mean, standard deviation (SD), and relative standard deviation expressed in % (RSD%) (output from DDSolver):

Parameter	No.1	No.2	No.3	No.4	Mean	SD	RSD(%)
k	0.014	0.014	0.015	0.015	0.015	0.001	4.493
β	3.943	3.511	3.602	3.433	3.622	0.225	6.215
F_{max}	101.768	101.378	103.155	101.977	102.070	0.765	0.749

Number of dissolution data points (N), degrees of freedom (df), and selected goodness of fit criteria – Pearson correlation coefficient (R), coefficient of determination (R^2), adjusted coefficient of determination ($R^2_{adjusted}$), and residual sum of squares (RSS) (manual calculation in MS Excel):

Parameter	No.1	No.2	No.3	No.4
N	13	13	13	13
df	10	10	10	10
R	0.99728861	0.9965355	0.99901047	0.99849326
R^2	0.99458457	0.993083	0.99802191	0.99698878
$R^2_{adjusted}$	0.99350149	0.99169961	0.99762629	0.99638654
RSS	98.9970195	117.943962	36.6481002	54.0079202

Graphical abstract of model fit presented as mean ± 1 SD of the fraction % of released carvedilol:

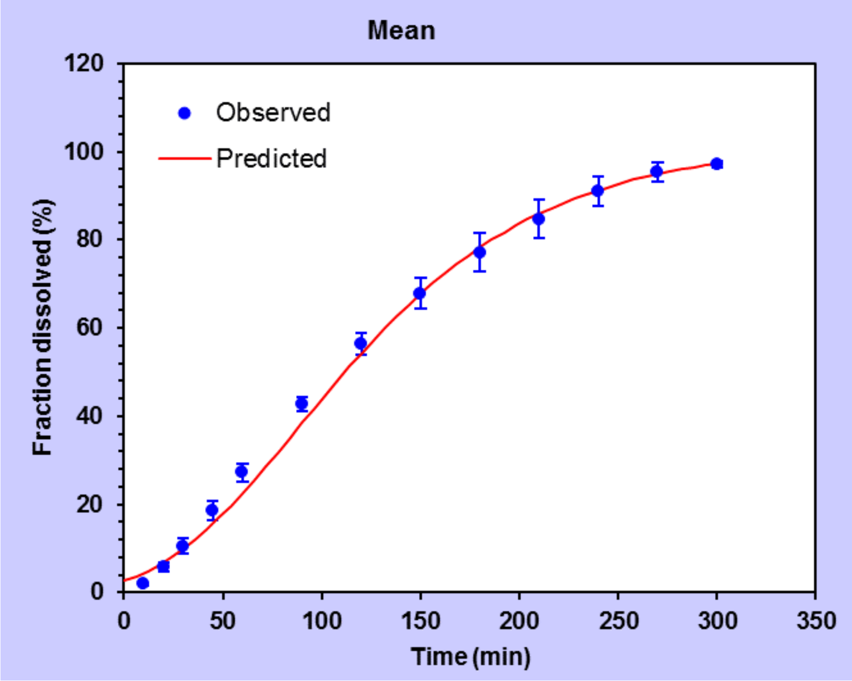

Supplementary materials – Model fitting summary – Polyox™ WSR N-80 (LEO NF Grade) (modelling the entire relevant dissolution data)

Graphical abstract of model fit presented as the fraction % of released carvedilol per tested tablet:

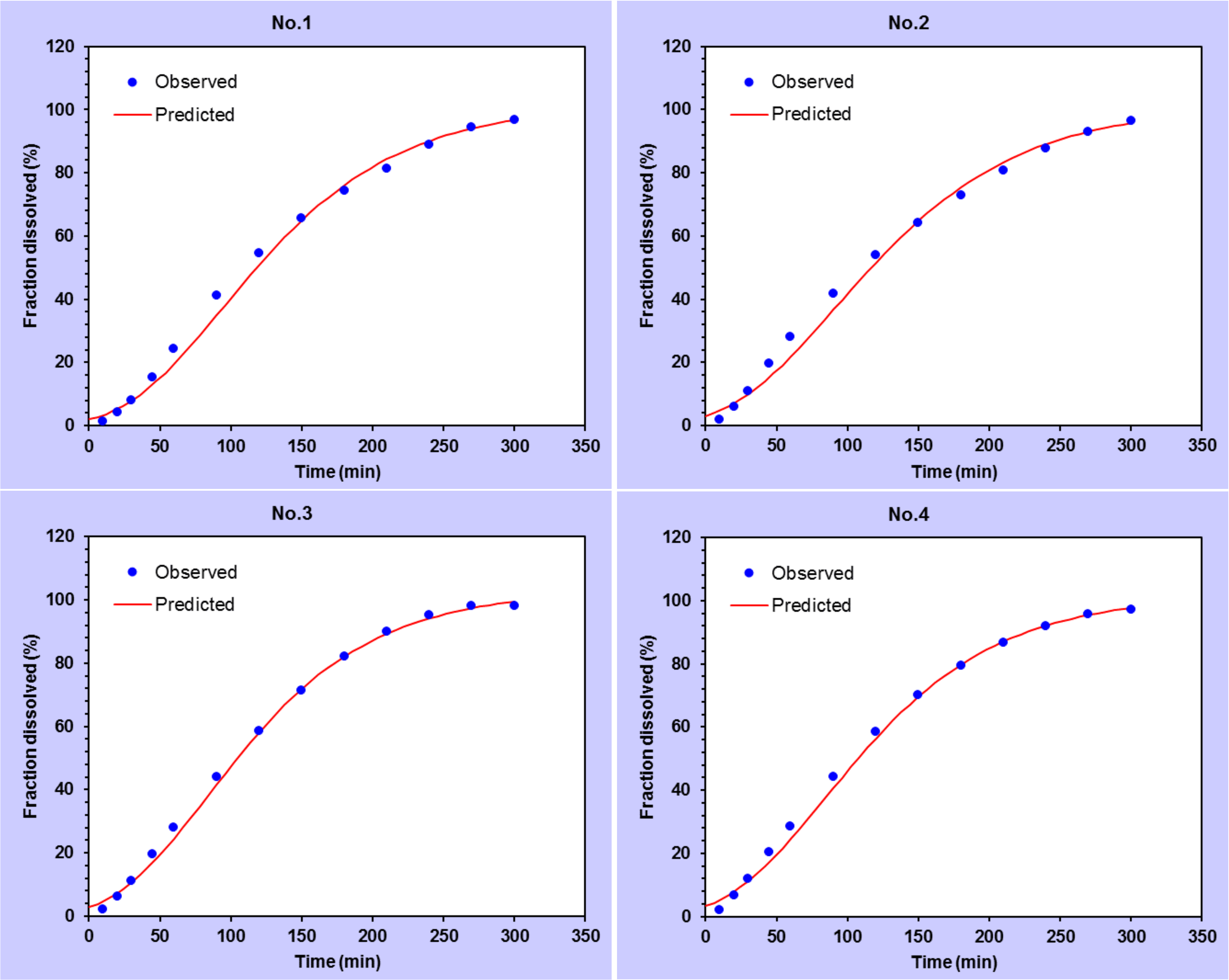

Supplementary materials – Model fitting summary – Polyox™ WSR N-80 (LEO NF Grade) (modelling the entire relevant dissolution data)

Model: **Probit_1**

Model equation: $F = 100 \cdot \phi[\alpha + \beta \cdot \log(t)]$

Fitted model parameters per tested tablet (N = 4) with statistics – mean, standard deviation (SD), and relative standard deviation expressed in % (RSD%) (output from DDSolver):

Parameter	No.1	No.2	No.3	No.4	Mean	SD	RSD(%)
α	-6.121	-4.918	-5.362	-5.822	-5.556	0.527	-9.493
β	2.960	2.523	2.854	2.900	2.809	0.196	6.971

Number of dissolution data points (N), degrees of freedom (df), and selected goodness of fit criteria – Pearson correlation coefficient (R), coefficient of determination (R^2), adjusted coefficient of determination ($R^2_{adjusted}$), and residual sum of squares (RSS) (manual calculation in MS Excel):

Parameter	No.1	No.2	No.3	No.4
N	13	13	13	13
df	11	11	11	11
R	0.99898979	0.99061681	0.98502224	0.99818624
R^2	0.9979806	0.98132167	0.97026881	0.99637577
$R^2_{adjusted}$	0.99779702	0.97962363	0.96756597	0.99604629
RSS	321.975231	330.275279	665.671014	245.531978

Graphical abstract of model fit presented as mean ± 1 SD of the fraction % of released carvedilol:

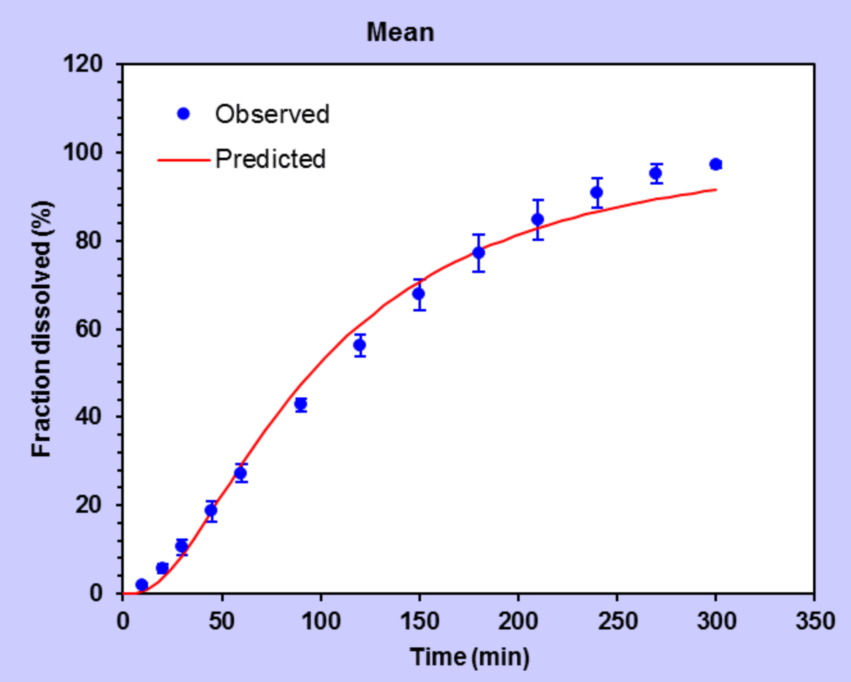

Supplementary materials – Model fitting summary – Polyox^{TM} WSR N-80 (LEO NF Grade) (modelling the entire relevant dissolution data)

Graphical abstract of model fit presented as the fraction % of released carvedilol per tested tablet:

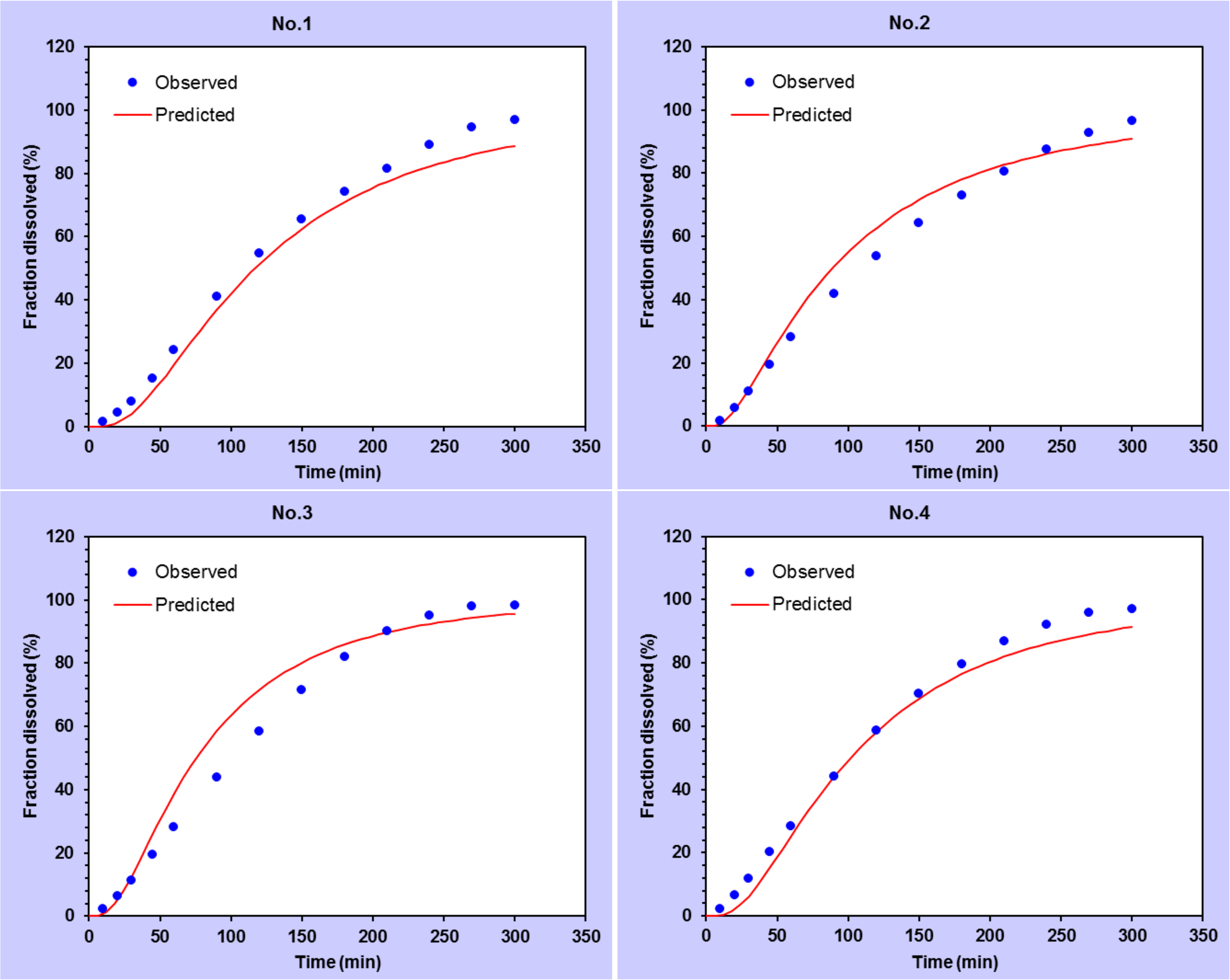

Supplementary materials – Model fitting summary – Polyox™ WSR N-80 (LEO NF Grade) (modelling the entire relevant dissolution data)

Model: **Probit_2**

Model equation: $F = F_{max} \cdot \phi[\alpha + \beta \cdot \log(t)]$

Fitted model parameters per tested tablet (N = 4) with statistics – mean, standard deviation (SD), and relative standard deviation expressed in % (RSD%) (output from DDSolver):

Parameter	No.1	No.2	No.3	No.4	Mean	SD	RSD(%)
α	-5.709	-4.836	-5.360	-5.210	-5.279	0.362	-6.850
β	2.825	2.462	2.588	2.509	2.596	0.161	6.218
F_{max}	97.211	101.378	115.912	114.588	107.272	9.383	8.747

Number of dissolution data points (N), degrees of freedom (df), and selected goodness of fit criteria – Pearson correlation coefficient (R), coefficient of determination (R^2), adjusted coefficient of determination ($R^2_{adjusted}$), and residual sum of squares (RSS) (manual calculation in MS Excel):

Parameter	No.1	No.2	No.3	No.4
N	13	13	13	13
df	10	10	10	10
R	0.99719354	0.99185084	0.99900547	0.99952536
R^2	0.99439496	0.98376808	0.99801194	0.99905094
$R^2_{adjusted}$	0.99327395	0.9805217	0.99761432	0.99886112
RSS	244.148467	280.674614	93.8327657	91.9232681

Graphical abstract of model fit presented as mean ± 1 SD of the fraction % of released carvedilol:

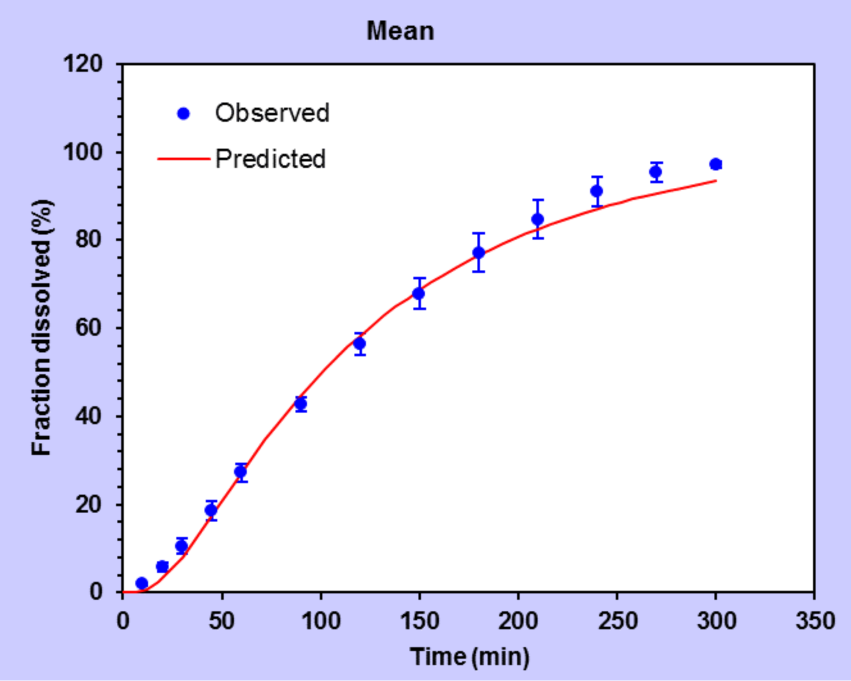

Supplementary materials – Model fitting summary – Polyox^{TM} WSR N-80 (LEO NF Grade) (modelling the entire relevant dissolution data)

Graphical abstract of model fit presented as the fraction % of released carvedilol per tested tablet:

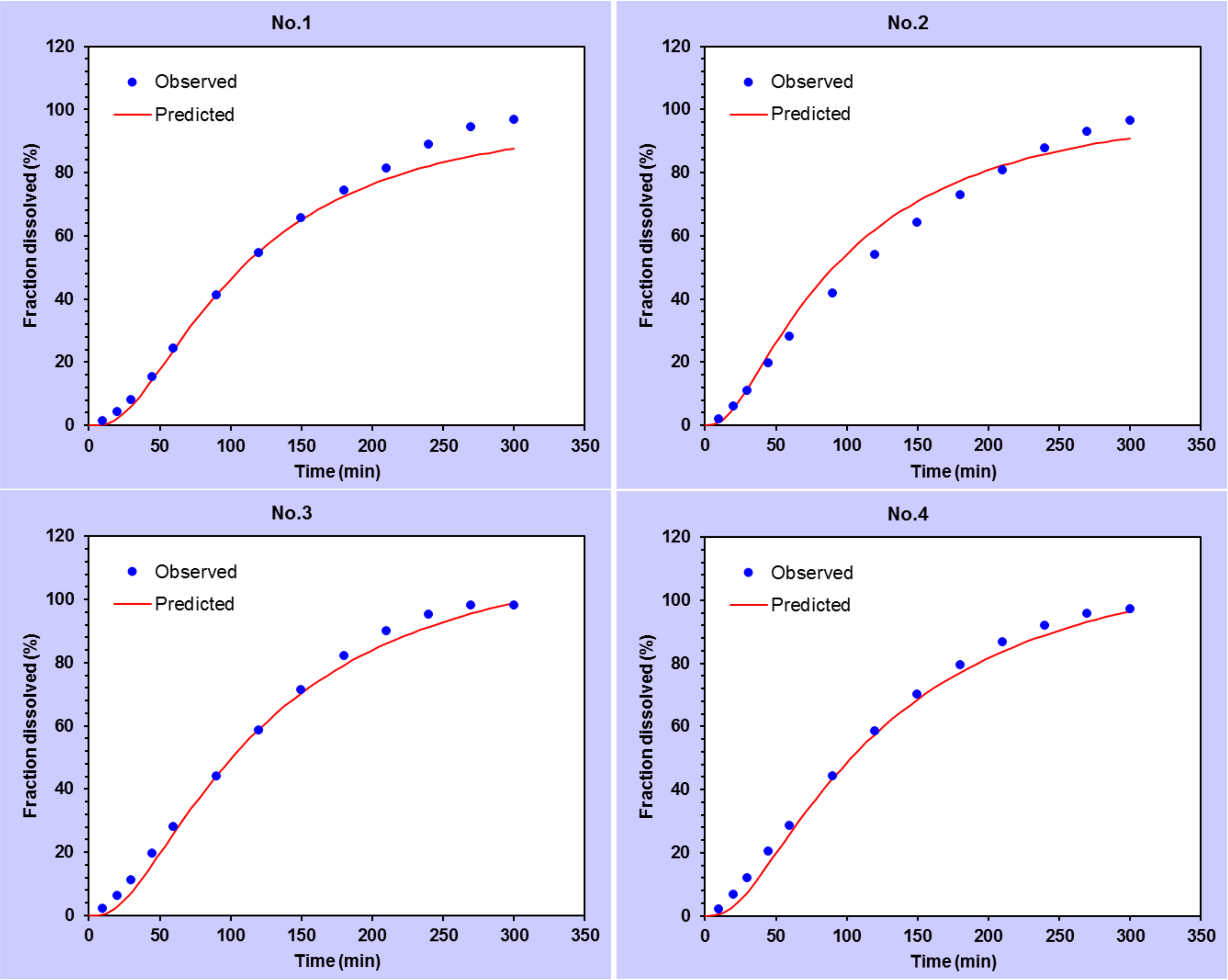

Supplementary materials – Model fitting summary – Polyox™ WSR N-80 (LEO NF Grade) (modelling up to app. 60 % of carvedilol released)

Model: **Zero–order**

Model equation: $F = k_0 \cdot t$

Fitted model parameters per tested tablet (N = 4) with statistics – mean, standard deviation (SD), and relative standard deviation expressed in % (RSD%) (output from DDSolver):

Parameter	No.1	No.2	No.3	No.4	Mean	SD	RSD(%)
k_0	0.432	0.449	0.475	0.479	0.459	0.022	4.869

Number of dissolution data points (N), degrees of freedom (df), and selected goodness of fit criteria – Pearson correlation coefficient (R), coefficient of determination (R^2), adjusted coefficient of determination ($R^2_{adjusted}$), and residual sum of squares (RSS) (manual calculation in MS Excel):

Parameter	No.1	No.2	No.3	No.4
N	7	7	7	7
df	6	6	6	6
R	0.99764176	0.998174355	0.99954146	0.999675136
R^2	0.995289081	0.996352044	0.99908313	0.999350377
$R^2_{adjusted}$	0.995289081	0.996352044	0.99908313	0.999350377
RSS	84.91380011	26.3043648	34.05182712	25.77752023

Graphical abstract of model fit presented as mean ± 1 SD of the fraction % of released carvedilol:

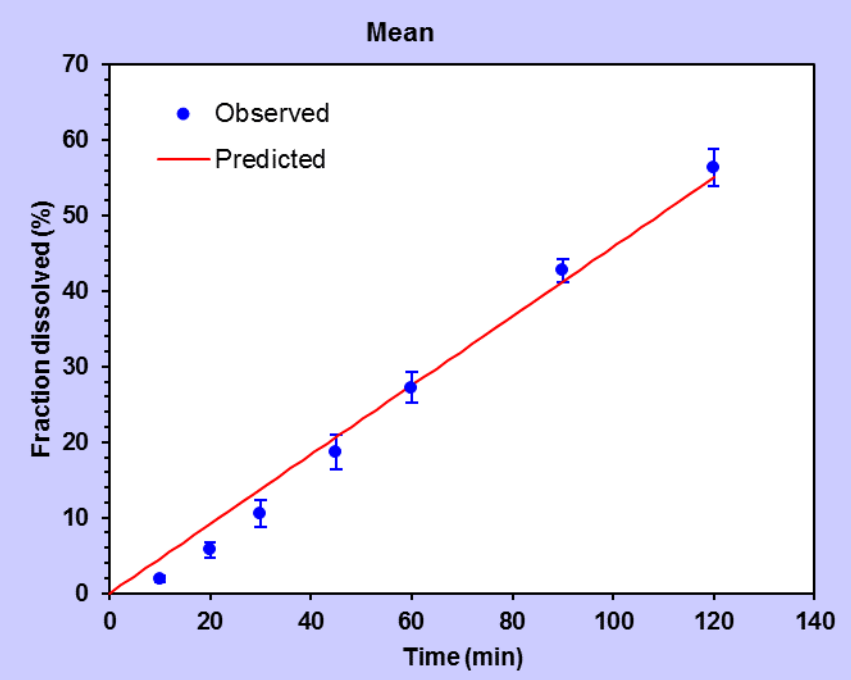

Supplementary materials – Model fitting summary – Polyox™ WSR N-80 (LEO NF Grade) (modelling up to app. 60 % of carvedilol released)

Graphical abstract of model fit presented as the fraction % of released carvedilol per tested tablet:

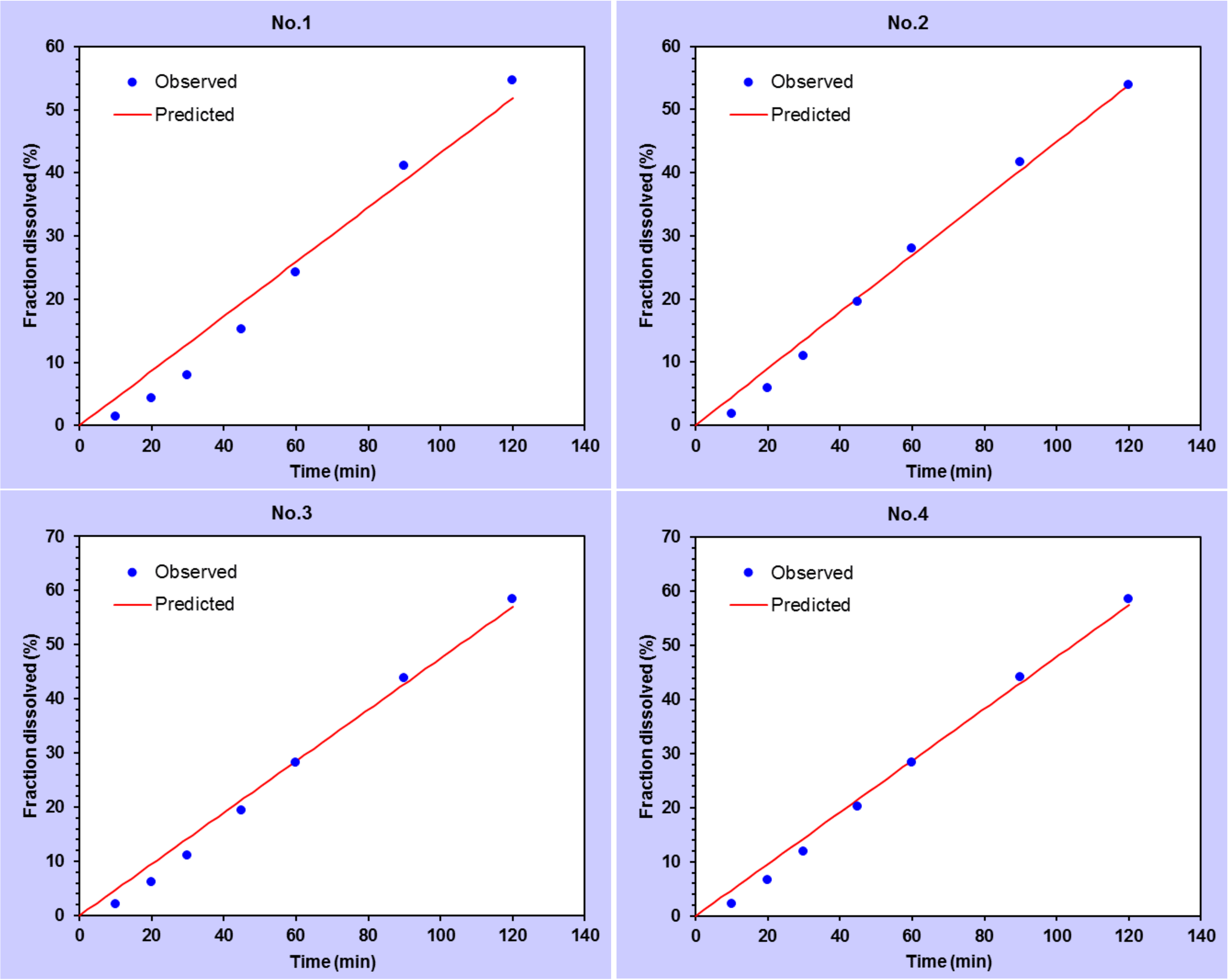

Supplementary materials – Model fitting summary – Polyox™ WSR N-80 (LEO NF Grade) (modelling up to app. 60 % of carvedilol released)

Model: **Zero–order with T_{lag}**

Model equation: $F = k_0 \cdot (t - T_{lag})$

Fitted model parameters per tested tablet (N = 4) with statistics – mean, standard deviation (SD), and relative standard deviation expressed in % (RSD%) (output from DDSolver):

Parameter	No.1	No.2	No.3	No.4	Mean	SD	RSD(%)
k_0	0.504	0.485	0.523	0.520	0.508	0.017	3.417
T_{lag}	11.368	5.874	7.200	6.309	7.688	2.515	32.714

Number of dissolution data points (N), degrees of freedom (df), and selected goodness of fit criteria – Pearson correlation coefficient (R), coefficient of determination (R^2), adjusted coefficient of determination ($R^2_{adjusted}$), and residual sum of squares (RSS) (manual calculation in MS Excel):

Parameter	No.1	No.2	No.3	No.4
N	7	7	7	7
df	5	5	5	5
R	0.99764176	0.998174355	0.99954146	0.999675136
R^2	0.995289081	0.996352044	0.99908313	0.999350377
$R^2_{adjusted}$	0.994346898	0.995622452	0.998899756	0.999220453
RSS	11.36175933	8.13265667	2.366207878	1.661180631

Graphical abstract of model fit presented as mean ± 1 SD of the fraction % of released carvedilol:

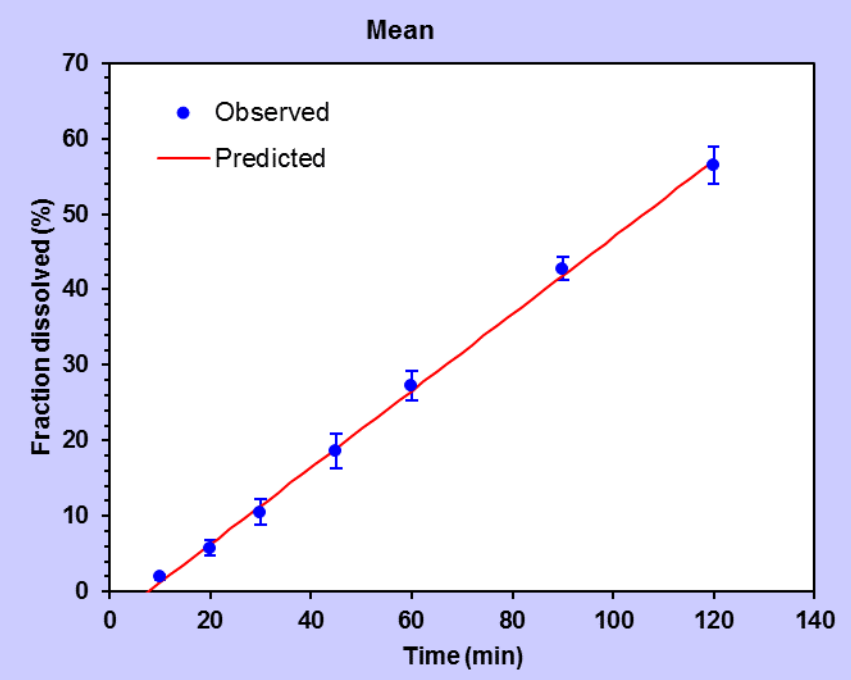

Supplementary materials – Model fitting summary – Polyox™ WSR N-80 (LEO NF Grade) (modelling up to app. 60 % of carvedilol released)

Graphical abstract of model fit presented as the fraction % of released carvedilol per tested tablet:

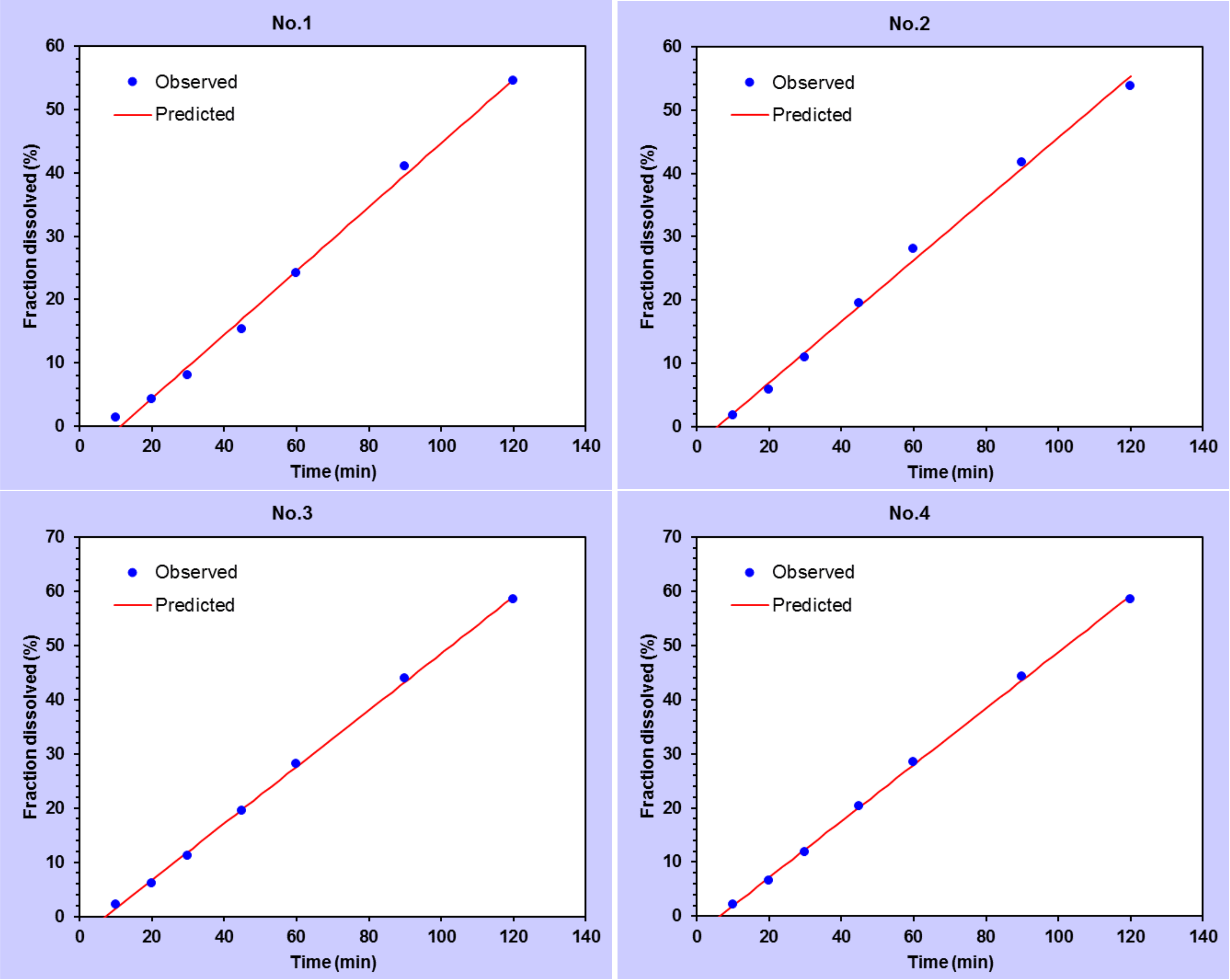

Supplementary materials – Model fitting summary – Polyox™ WSR N-80 (LEO NF Grade) (modelling up to app. 60 % of carvedilol released)

Model: **Zero–order with F_0**

Model equation: $F = F_0 + k_0 \cdot t$

Fitted model parameters per tested tablet (N = 4) with statistics – mean, standard deviation (SD), and relative standard deviation expressed in % (RSD%) (output from DDSolver):

Parameter	No.1	No.2	No.3	No.4	Mean	SD	RSD(%)
k_0	0.504	0.485	0.523	0.520	0.508	0.017	3.417
F_0	-5.734	-2.850	-3.763	-3.283	-3.908	1.273	-32.586

Number of dissolution data points (N), degrees of freedom (df), and selected goodness of fit criteria – Pearson correlation coefficient (R), coefficient of determination (R^2), adjusted coefficient of determination ($R^2_{adjusted}$), and residual sum of squares (RSS) (manual calculation in MS Excel):

Parameter	No.1	No.2	No.3	No.4
N	7	7	7	7
df	5	5	5	5
R	0.99764176	0.998174355	0.99954146	0.999675136
R^2	0.995289081	0.996352044	0.99908313	0.999350377
$R^2_{adjusted}$	0.994346898	0.995622452	0.998899756	0.999220453
RSS	11.36175933	8.13265667	2.366207878	1.661180631

Graphical abstract of model fit presented as mean ± 1 SD of the fraction % of released carvedilol:

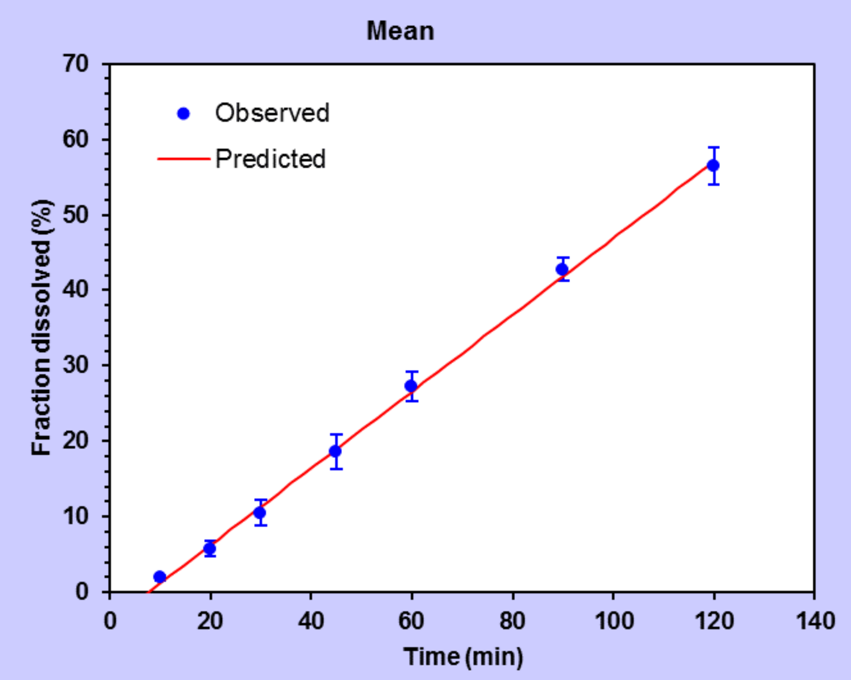

Supplementary materials – Model fitting summary – Polyox™ WSR N-80 (LEO NF Grade) (modelling up to app. 60 % of carvedilol released)

Graphical abstract of model fit presented as the fraction % of released carvedilol per tested tablet:

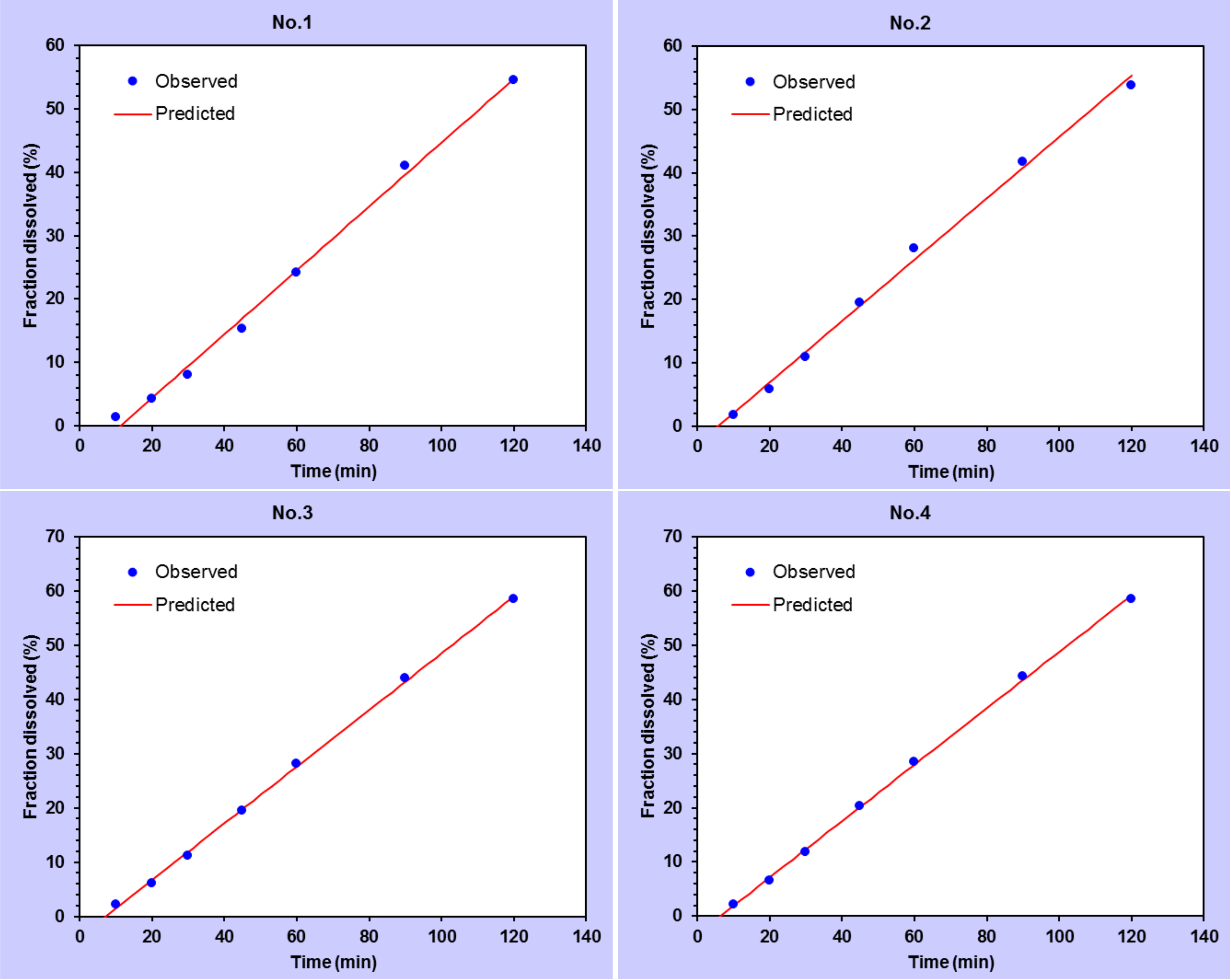

Supplementary materials – Model fitting summary – Polyox™ WSR N-80 (LEO NF Grade) (modelling up to app. 60 % of carvedilol released)

Model: **First–order**

Model equation: $F = 100 \cdot (1 - e^{-k_1 \cdot t})$

Fitted model parameters per tested tablet (N = 4) with statistics – mean, standard deviation (SD), and relative standard deviation expressed in % (RSD%) (output from DDSolver):

Parameter	No.1	No.2	No.3	No.4	Mean	SD	RSD(%)
k_1	0.006	0.006	0.007	0.007	0.006	0.000	6.430

Number of dissolution data points (N), degrees of freedom (df), and selected goodness of fit criteria – Pearson correlation coefficient (R), coefficient of determination (R^2), adjusted coefficient of determination ($R^2_{adjusted}$), and residual sum of squares (RSS) (manual calculation in MS Excel):

Parameter	No.1	No.2	No.3	No.4
N	7	7	7	7
df	6	6	6	6
R	0.990780019	0.998298401	0.994800079	0.995837796
R^2	0.981645046	0.996599698	0.989627196	0.991692916
$R^2_{adjusted}$	0.981645046	0.996599698	0.989627196	0.991692916
RSS	227.9531773	100.8400671	166.6729946	146.8993694

Graphical abstract of model fit presented as mean ± 1 SD of the fraction % of released carvedilol:

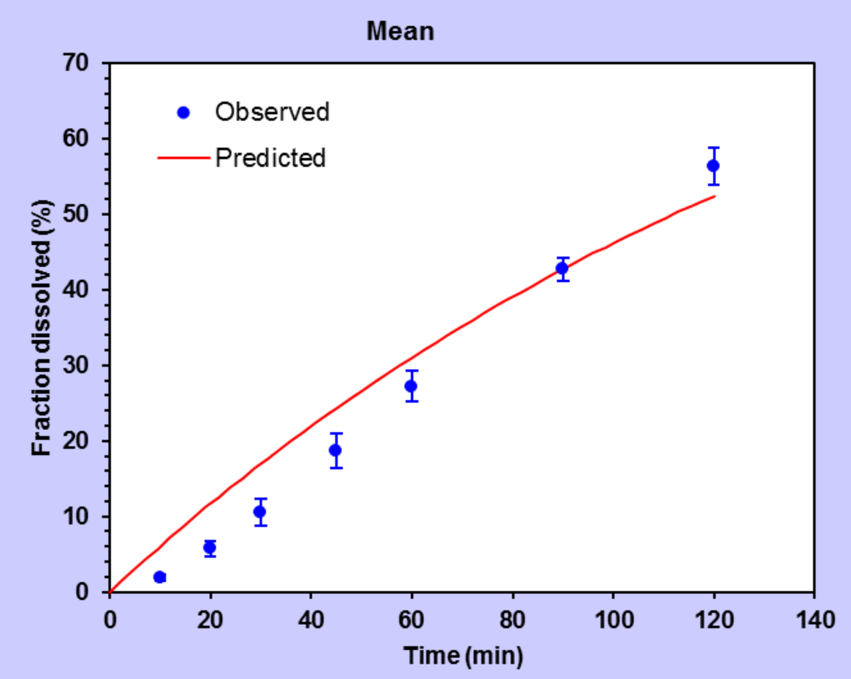

Supplementary materials – Model fitting summary – Polyox™ WSR N-80 (LEO NF Grade) (modelling up to app. 60 % of carvedilol released)

Graphical abstract of model fit presented as the fraction % of released carvedilol per tested tablet:

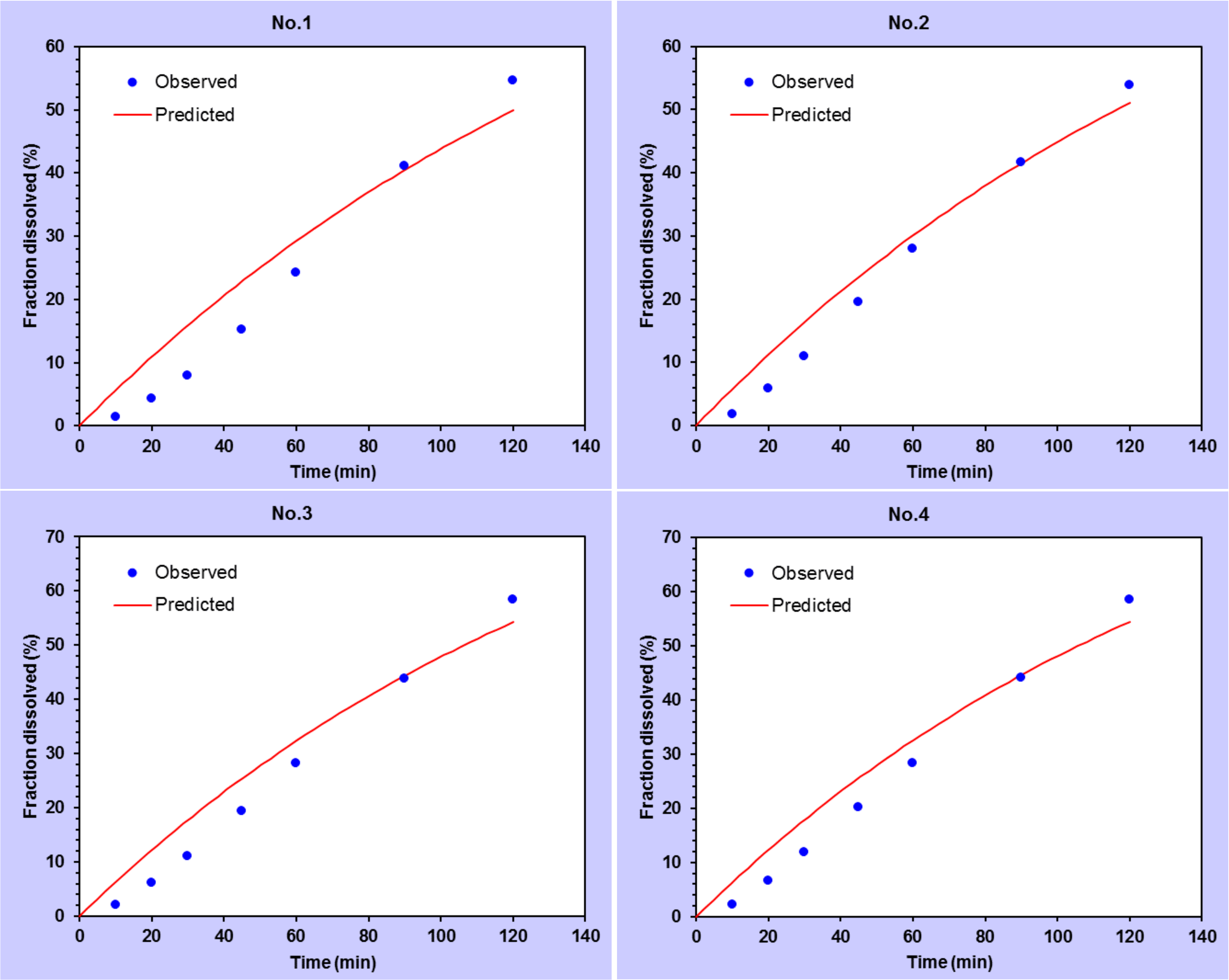

Supplementary materials – Model fitting summary – Polyox™ WSR N-80 (LEO NF Grade) (modelling up to app. 60 % of carvedilol released)

Model: **First–order with T_{lag}**

Model equation: $F = 100 \cdot \left[1 - e^{-k_1 \cdot (t - T_{lag})}\right]$

Fitted model parameters per tested tablet (N = 4) with statistics – mean, standard deviation (SD), and relative standard deviation expressed in % (RSD%) (output from DDSolver):

Parameter	No.1	No.2	No.3	No.4	Mean	SD	RSD(%)
k_1	0.007	0.007	0.008	0.008	0.007	0.000	5.753
T_{lag}	15.872	11.416	13.219	12.558	13.266	1.890	14.246

Number of dissolution data points (N), degrees of freedom (df), and selected goodness of fit criteria – Pearson correlation coefficient (R), coefficient of determination (R^2), adjusted coefficient of determination ($R^2_{adjusted}$), and residual sum of squares (RSS) (manual calculation in MS Excel):

Parameter	No.1	No.2	No.3	No.4
N	7	7	7	7
df	5	5	5	5
R	0.9877412	0.997453205	0.99260841	0.993947664
R^2	0.975632677	0.994912897	0.985271456	0.987931959
$R^2_{adjusted}$	0.970759213	0.993895476	0.982325747	0.985518351
RSS	63.61313729	12.0897212	42.38792593	34.54625054

Graphical abstract of model fit presented as mean ± 1 SD of the fraction % of released carvedilol:

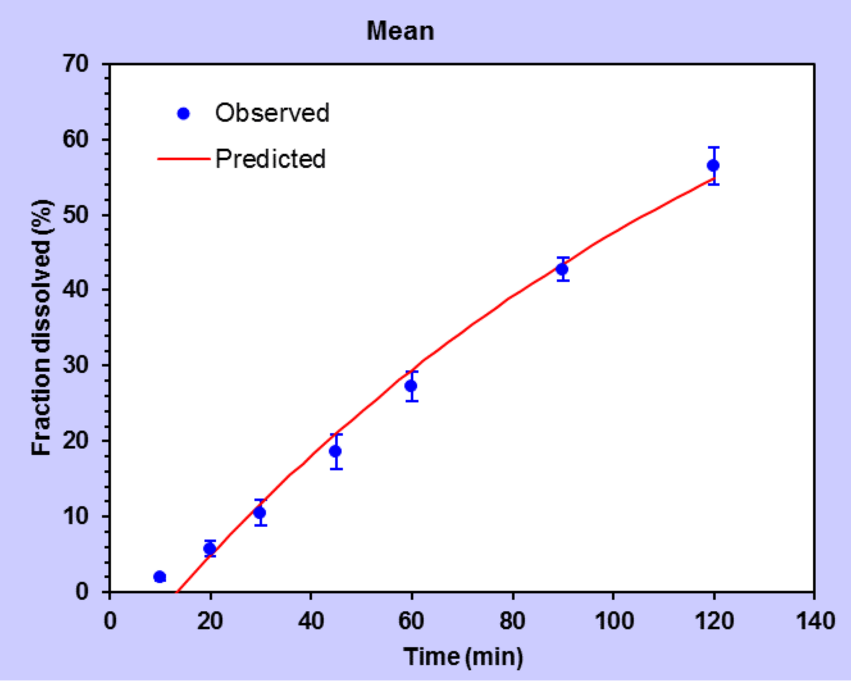

Supplementary materials – Model fitting summary – Polyox™ WSR N-80 (LEO NF Grade) (modelling up to app. 60 % of carvedilol released)

Graphical abstract of model fit presented as the fraction % of released carvedilol per tested tablet:

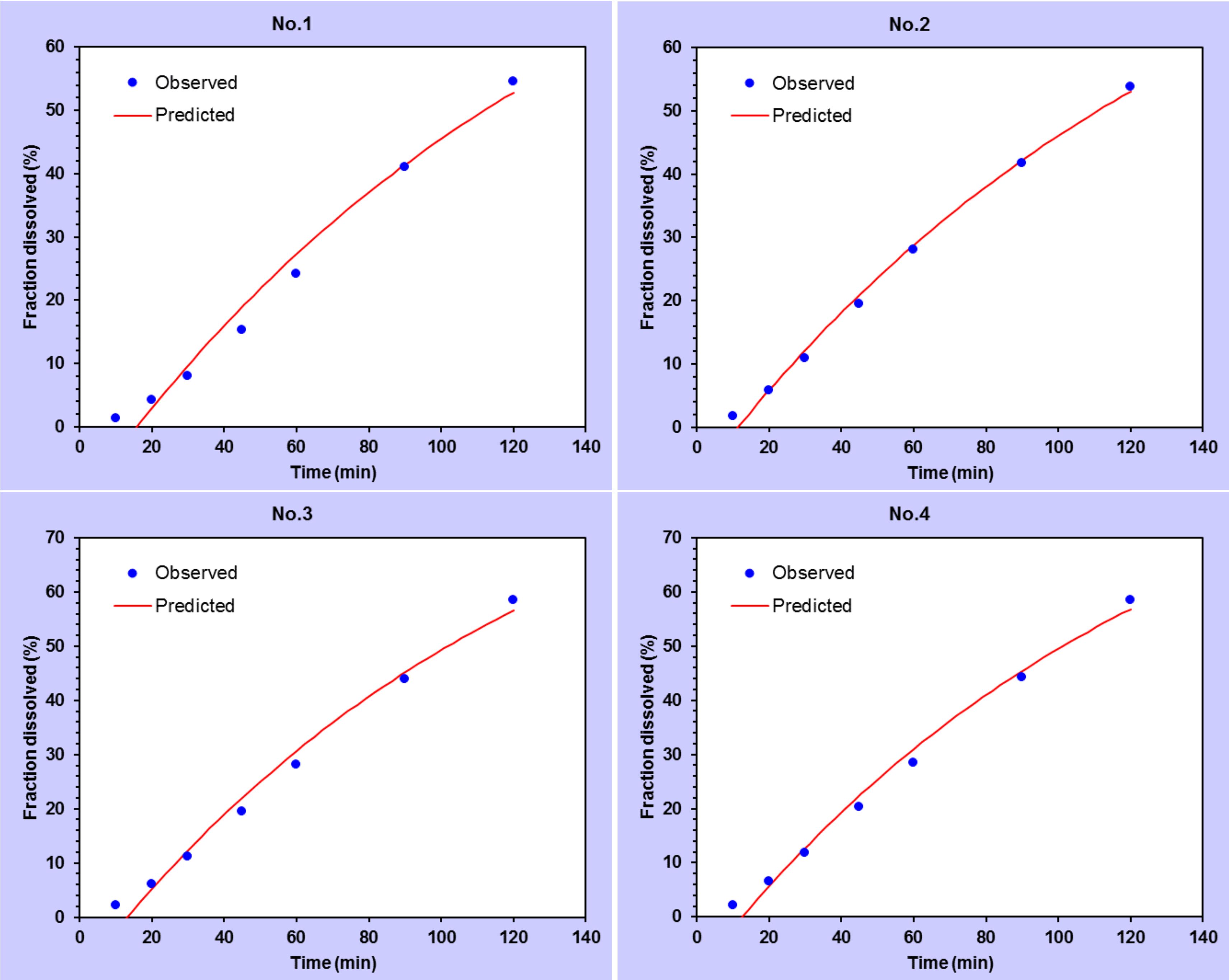

Supplementary materials – Model fitting summary – Polyox™ WSR N-80 (LEO NF Grade) (modelling up to app. 60 % of carvedilol released)

Model: **First–order with F_{max}**

Model equation: $F = F_{max} \cdot (1 - e^{-k_1 \cdot t})$

Fitted model parameters per tested tablet (N = 4) with statistics – mean, standard deviation (SD), and relative standard deviation expressed in % (RSD%) (output from DDSolver):

Parameter	No.1	No.2	No.3	No.4	Mean	SD	RSD(%)
k_1	0.018	0.019	0.018	0.018	0.018	0.000	1.744
F_{max}	57.380	56.595	61.416	61.464	59.214	2.591	4.375

Number of dissolution data points (N), degrees of freedom (df), and selected goodness of fit criteria – Pearson correlation coefficient (R), coefficient of determination (R^2), adjusted coefficient of determination ($R^2_{adjusted}$), and residual sum of squares (RSS) (manual calculation in MS Excel):

Parameter	No.1	No.2	No.3	No.4
N	7	7	7	7
df	5	5	5	5
R	0.951660715	0.971587427	0.962745804	0.965324668
R^2	0.905658116	0.943982127	0.926879484	0.931851715
$R^2_{adjusted}$	0.886789739	0.932778553	0.912255381	0.918222058
RSS	991.5743821	677.8495802	879.4221086	832.7078676

Graphical abstract of model fit presented as mean ± 1 SD of the fraction % of released carvedilol:

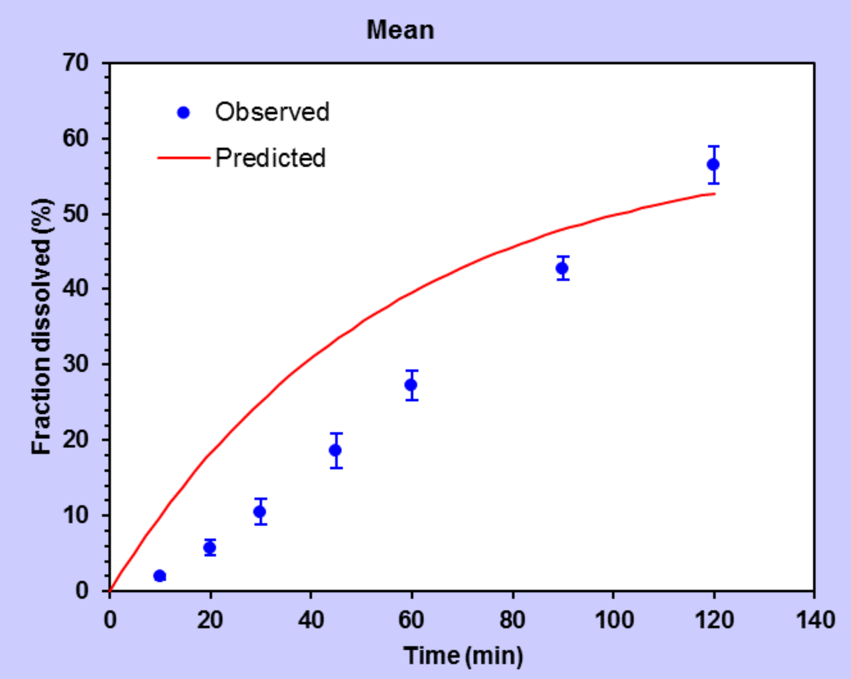

Supplementary materials – Model fitting summary – Polyox^{TM} WSR N-80 (LEO NF Grade) (modelling up to app. 60 % of carvedilol released)

Graphical abstract of model fit presented as the fraction % of released carvedilol per tested tablet:

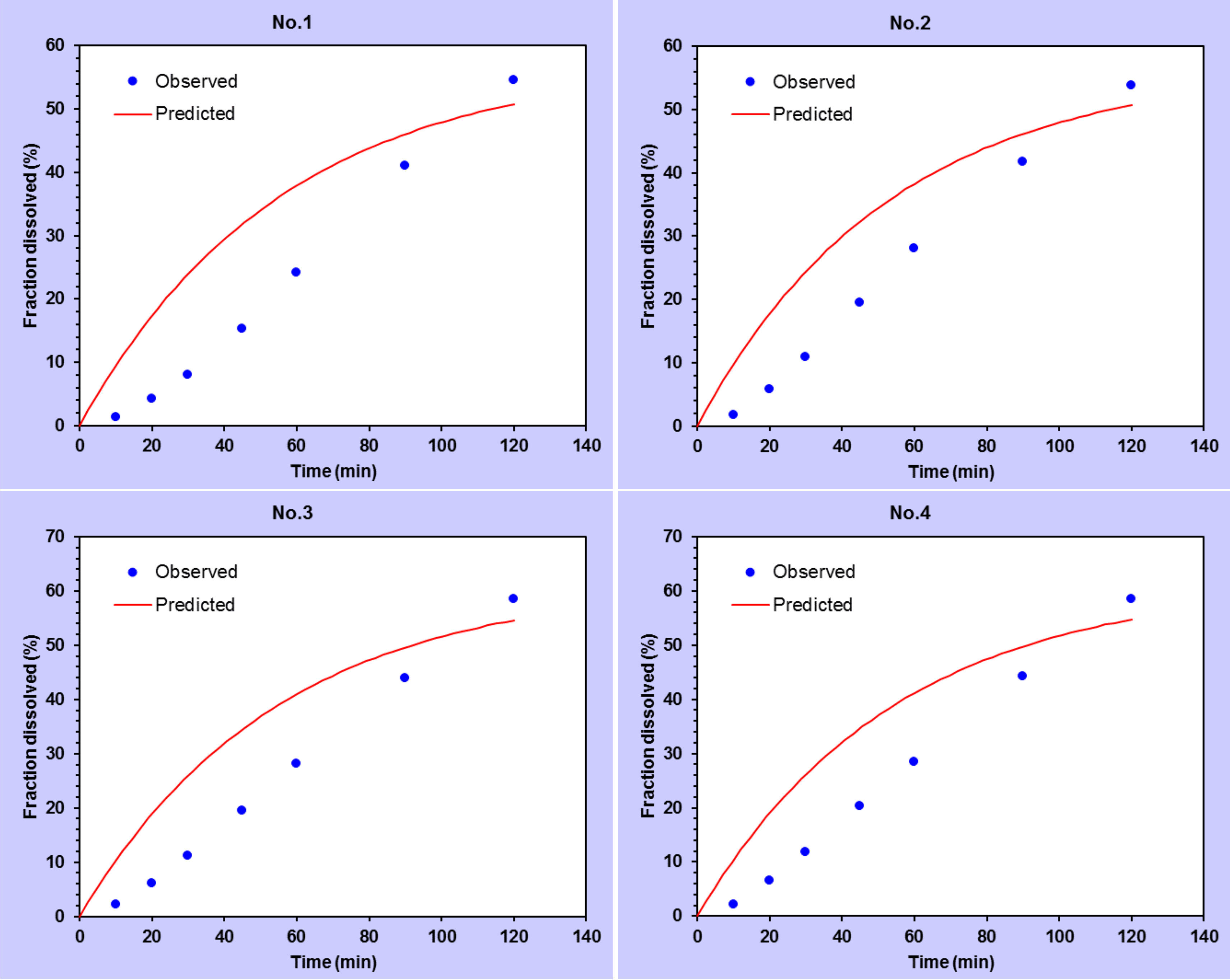

Supplementary materials – Model fitting summary – Polyox™ WSR N-80 (LEO NF Grade) (modelling up to app. 60 % of carvedilol released)

Model: **First–order with T_{lag} and F_{max}**

Model equation: $F = F_{max} \cdot \left[1 - e^{-k_1 \cdot (t - T_{lag})}\right]$

Fitted model parameters per tested tablet (N = 4) with statistics – mean, standard deviation (SD), and relative standard deviation expressed in % (RSD%) (output from DDSolver):

Parameter	No.1	No.2	No.3	No.4	Mean	SD	RSD(%)
k_1	0.040	0.040	0.039	0.039	0.040	0.000	0.717
T_{lag}	16.882	15.110	15.694	15.427	15.778	0.773	4.902
F_{max}	38.253	37.730	40.944	40.976	39.476	1.727	4.375

Number of dissolution data points (N), degrees of freedom (df), and selected goodness of fit criteria – Pearson correlation coefficient (R), coefficient of determination (R^2), adjusted coefficient of determination ($R^2_{adjusted}$), and residual sum of squares (RSS) (manual calculation in MS Excel):

Parameter	No.1	No.2	No.3	No.4
N	7	7	7	7
df	4	4	4	4
R	0.845476903	0.887223149	0.870360824	0.876236939
R^2	0.714831194	0.787164917	0.757527964	0.767791174
$R^2_{adjusted}$	0.57224679	0.680747375	0.636291946	0.65168676
RSS	714.285733	512.8256028	660.8403522	632.0390101

Graphical abstract of model fit presented as mean ± 1 SD of the fraction % of released carvedilol:

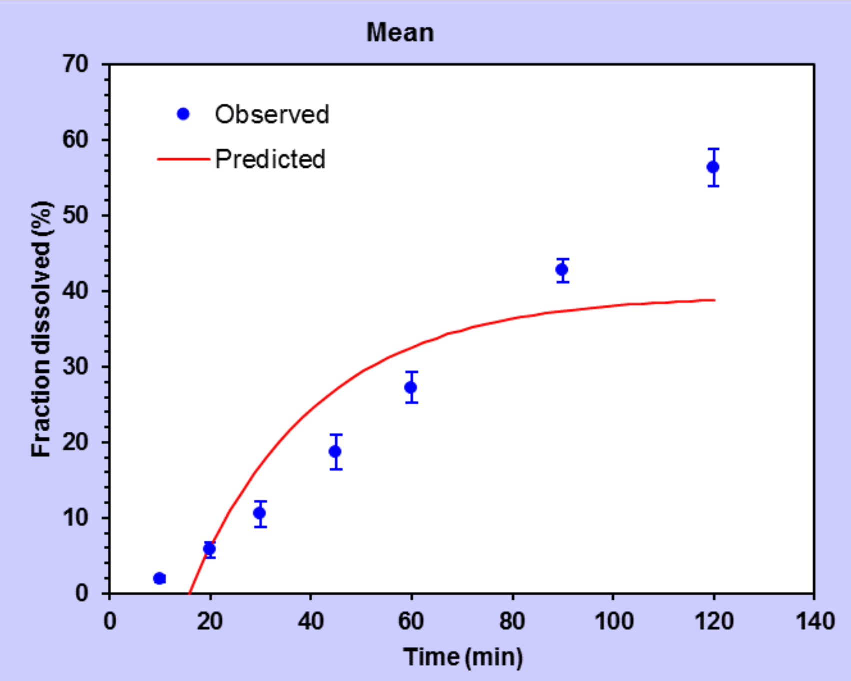

Supplementary materials – Model fitting summary – Polyox™ WSR N-80 (LEO NF Grade) (modelling up to app. 60 % of carvedilol released)

Graphical abstract of model fit presented as the fraction % of released carvedilol per tested tablet:

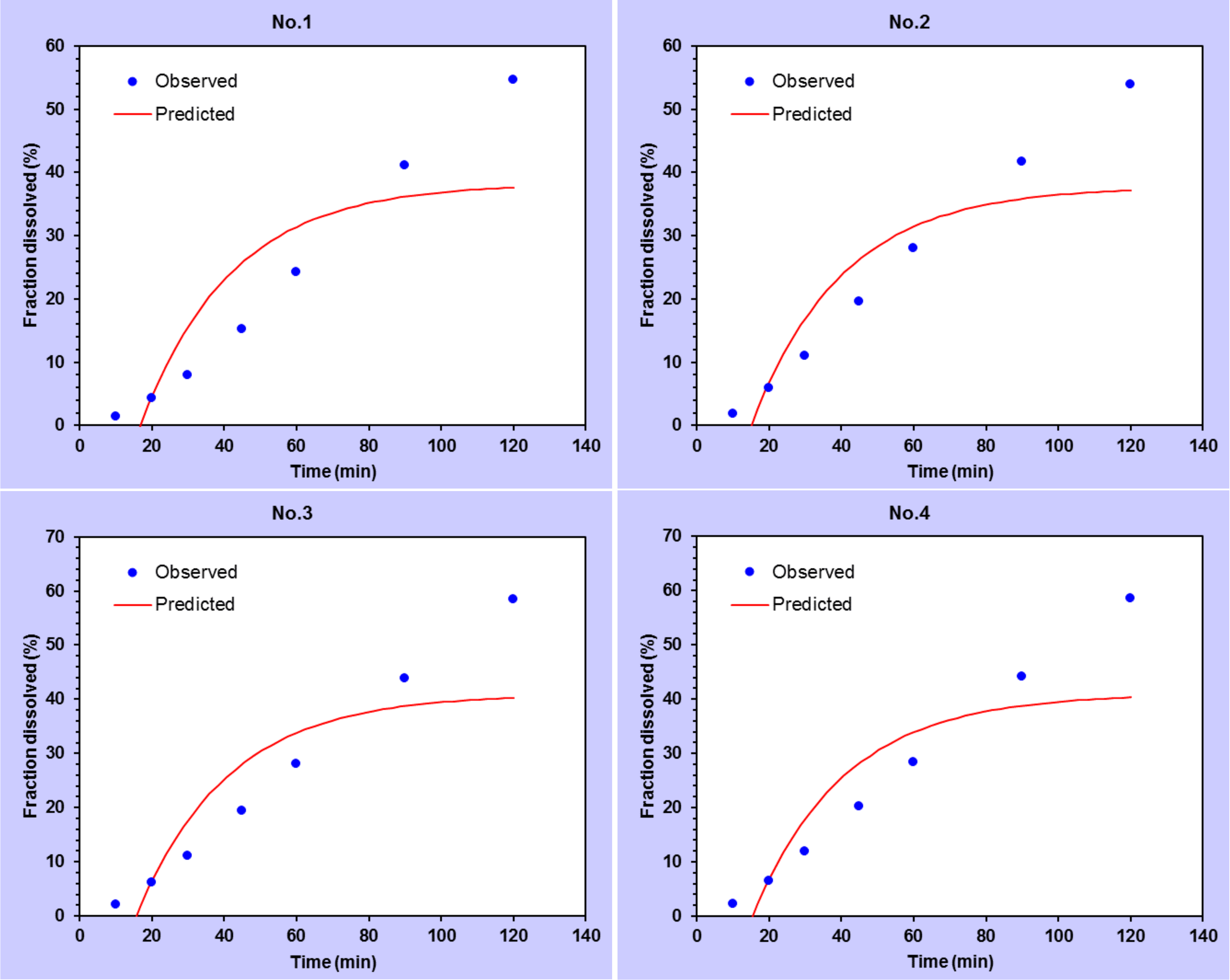

Supplementary materials – Model fitting summary – Polyox™ WSR N-80 (LEO NF Grade) (modelling up to app. 60 % of carvedilol released)

Model: **Higuchi**

Model equation: $F = k_H \cdot t^{0.5}$

Fitted model parameters per tested tablet (N = 4) with statistics – mean, standard deviation (SD), and relative standard deviation expressed in % (RSD%) (output from DDSolver):

Parameter	No.1	No.2	No.3	No.4	Mean	SD	RSD(%)
k_H	3.591	3.807	4.007	4.050	3.864	0.210	5.444

Number of dissolution data points (N), degrees of freedom (df), and selected goodness of fit criteria – Pearson correlation coefficient (R), coefficient of determination (R^2), adjusted coefficient of determination ($R^2_{adjusted}$), and residual sum of squares (RSS) (manual calculation in MS Excel):

Parameter	No.1	No.2	No.3	No.4
N	7	7	7	7
df	6	6	6	6
R	0.977434723	0.99122316	0.985927378	0.987879214
R^2	0.955378637	0.982523353	0.972052794	0.975905341
$R^2_{adjusted}$	0.955378637	0.982523353	0.972052794	0.975905341
RSS	747.9380262	543.6369063	673.7129318	641.2723974

Graphical abstract of model fit presented as mean ± 1 SD of the fraction % of released carvedilol:

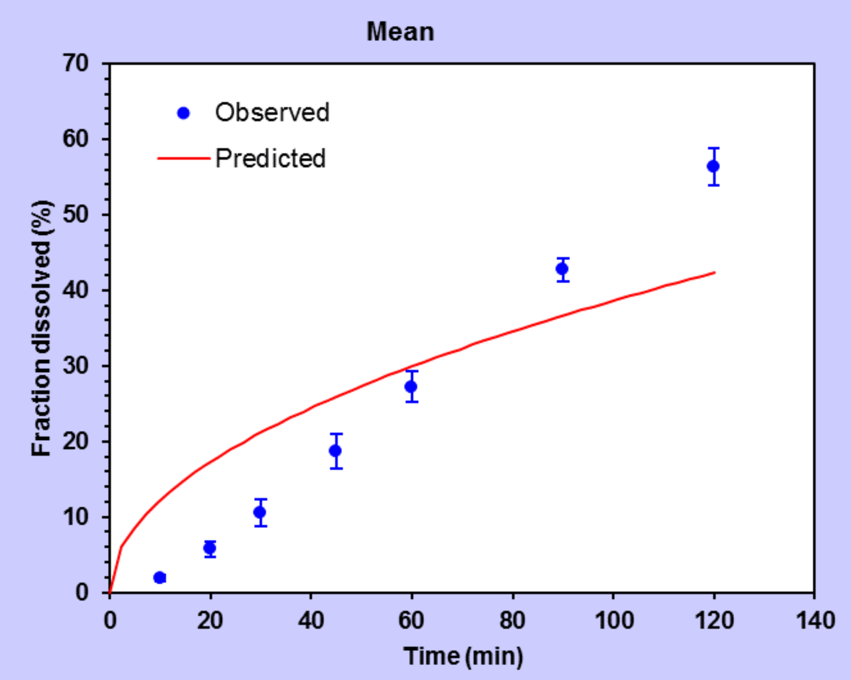

Supplementary materials – Model fitting summary – Polyox™ WSR N-80 (LEO NF Grade) (modelling up to app. 60 % of carvedilol released)

Graphical abstract of model fit presented as the fraction % of released carvedilol per tested tablet:

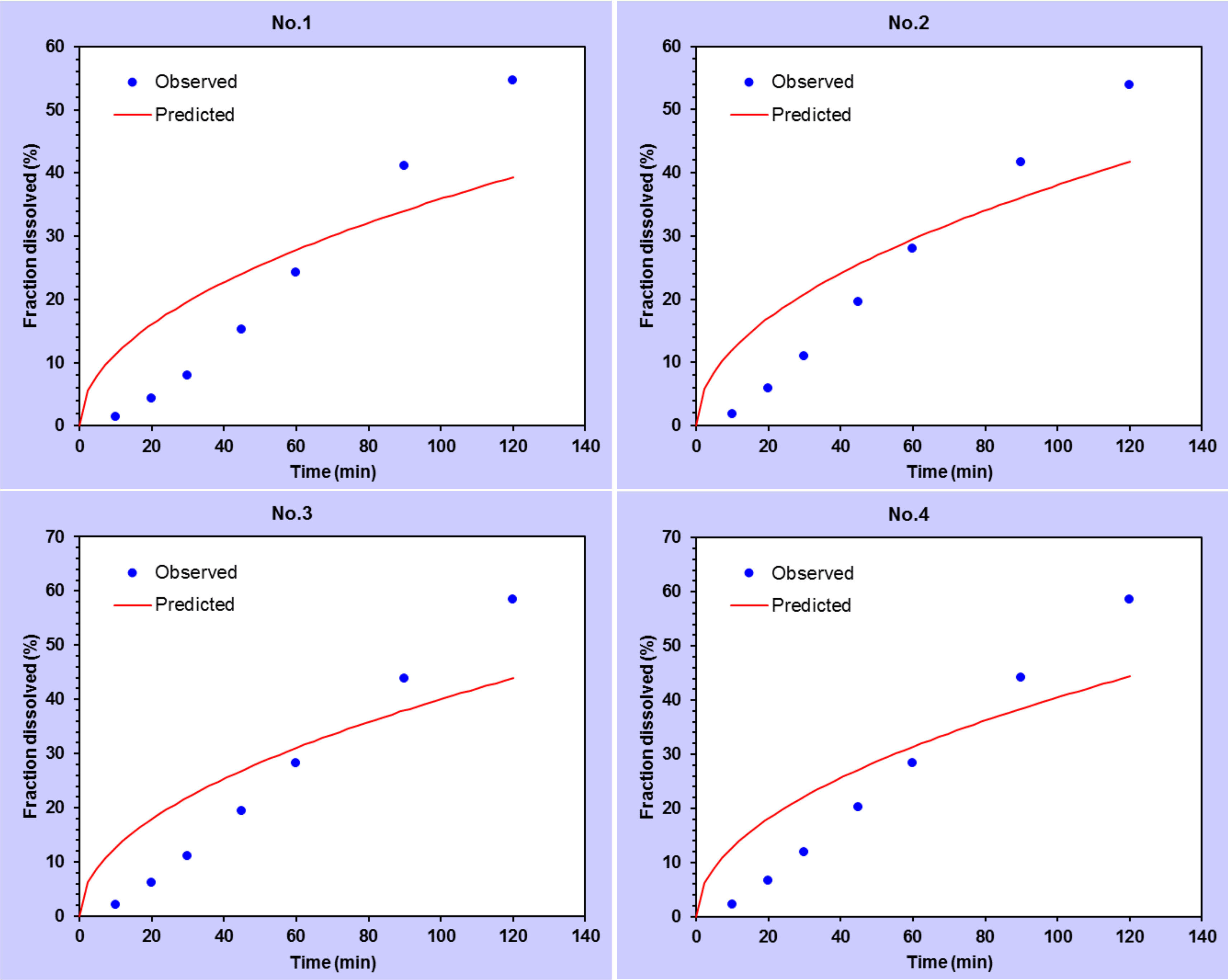

Supplementary materials – Model fitting summary – Polyox™ WSR N-80 (LEO NF Grade) (modelling up to app. 60 % of carvedilol released)

Model: **Higuchi with T_{lag}**

Model equation: $F = k_H \cdot (t - T_{lag})^{0.5}$

Fitted model parameters per tested tablet (N = 4) with statistics – mean, standard deviation (SD), and relative standard deviation expressed in % (RSD%) (output from DDSolver):

Parameter	No.1	No.2	No.3	No.4	Mean	SD	RSD(%)
k_H	5.245	5.189	5.591	5.596	5.405	0.219	4.047
T_{lag}	36.857	21.855	22.984	22.589	26.071	7.206	27.639

Number of dissolution data points (N), degrees of freedom (df), and selected goodness of fit criteria – Pearson correlation coefficient (R), coefficient of determination (R^2), adjusted coefficient of determination ($R^2_{adjusted}$), and residual sum of squares (RSS) (manual calculation in MS Excel):

Parameter	No.1	No.2	No.3	No.4
N	7	7	7	7
df	5	5	5	5
R	0.986161129	0.978653192	0.973435901	0.974333428
R^2	0.972513773	0.95776207	0.947577454	0.949325629
$R^2_{adjusted}$	0.967016528	0.949314484	0.937092945	0.939190755
RSS	140.2826676	104.7125622	151.0873063	145.3575257

Graphical abstract of model fit presented as mean ± 1 SD of the fraction % of released carvedilol:

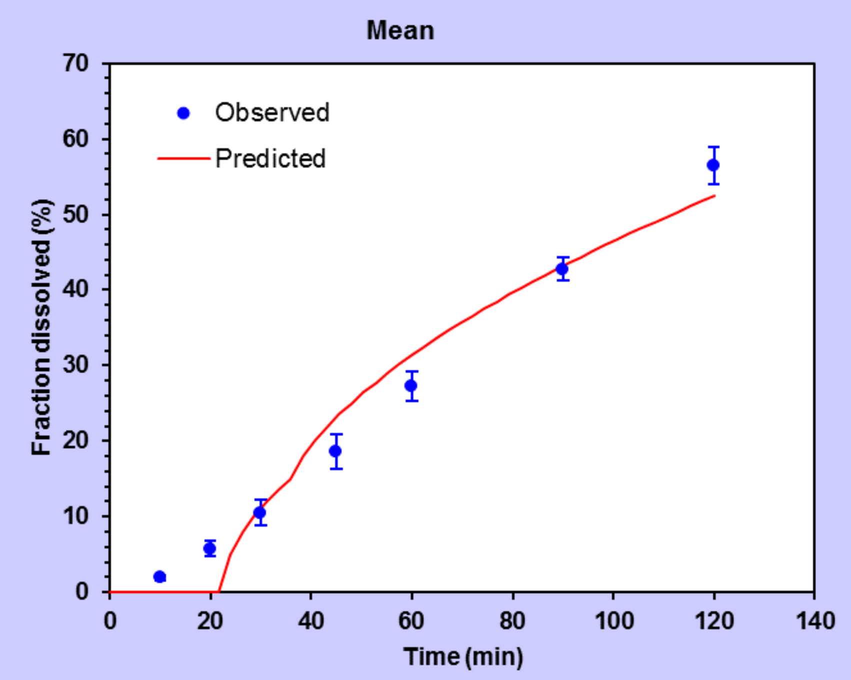

Supplementary materials – Model fitting summary – Polyox™ WSR N-80 (LEO NF Grade) (modelling up to app. 60 % of carvedilol released)

Graphical abstract of model fit presented as the fraction % of released carvedilol per tested tablet:

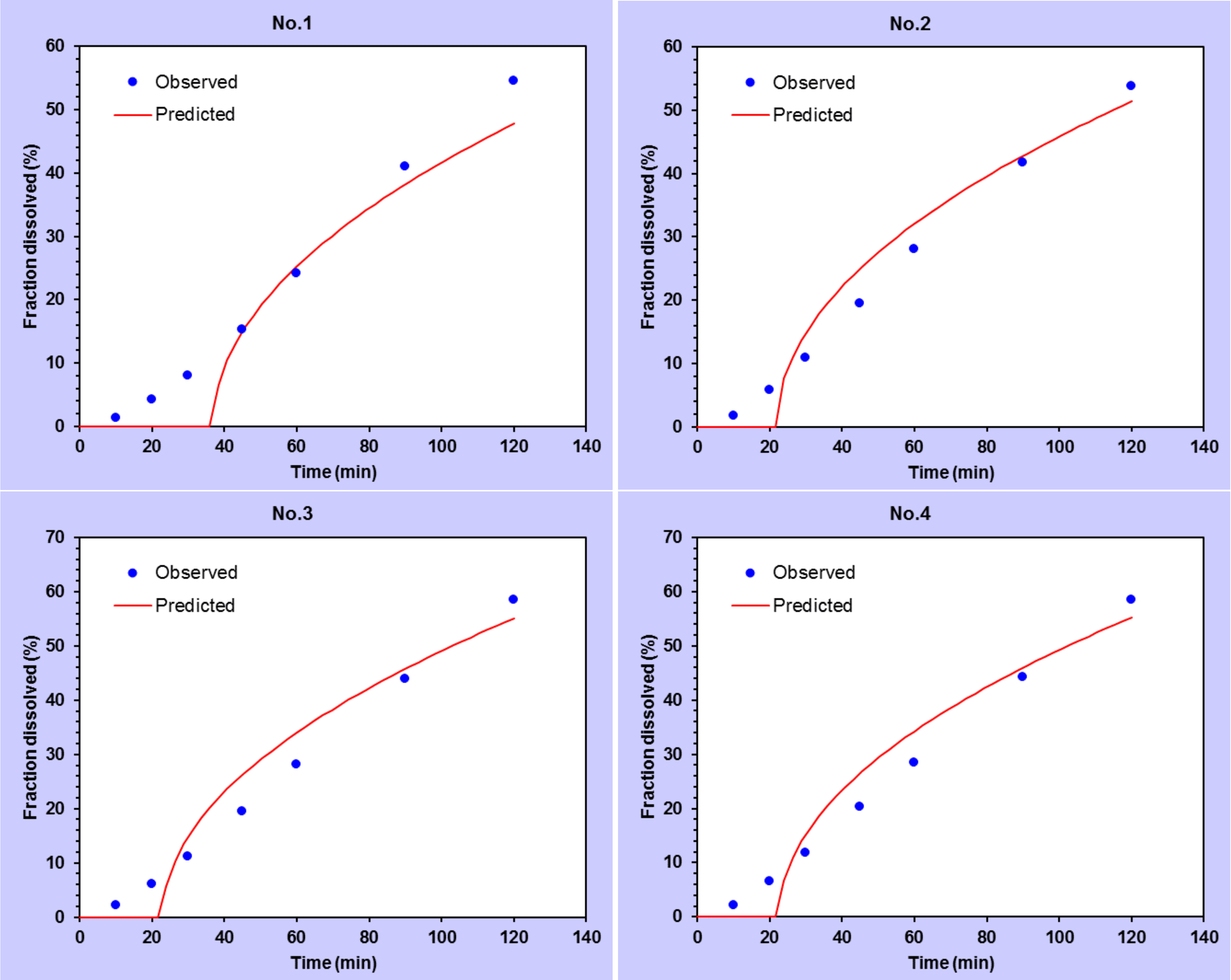

Supplementary materials – Model fitting summary – Polyox™ WSR N-80 (LEO NF Grade) (modelling up to app. 60 % of carvedilol released)

Model: **Higuchi with F_0**

Model equation: $F = F_0 + k_H \cdot t^{0.5}$

Fitted model parameters per tested tablet (N = 4) with statistics – mean, standard deviation (SD), and relative standard deviation expressed in % (RSD%) (output from DDSolver):

Parameter	No.1	No.2	No.3	No.4	Mean	SD	RSD(%)
k_H	7.096	6.919	7.404	7.385	7.201	0.235	3.265
F_0	-27.379	-24.307	-26.538	-26.050	-26.069	1.296	-4.973

Number of dissolution data points (N), degrees of freedom (df), and selected goodness of fit criteria – Pearson correlation coefficient (R), coefficient of determination (R^2), adjusted coefficient of determination ($R^2_{adjusted}$), and residual sum of squares (RSS) (manual calculation in MS Excel):

Parameter	No.1	No.2	No.3	No.4
N	7	7	7	7
df	5	5	5	5
R	0.977434723	0.99122316	0.985927378	0.987879214
R^2	0.955378637	0.982523353	0.972052794	0.975905341
$R^2_{adjusted}$	0.946454364	0.979028024	0.966463353	0.971086409
RSS	107.6174788	38.9619708	72.12463705	61.61356762

Graphical abstract of model fit presented as mean ± 1 SD of the fraction % of released carvedilol:

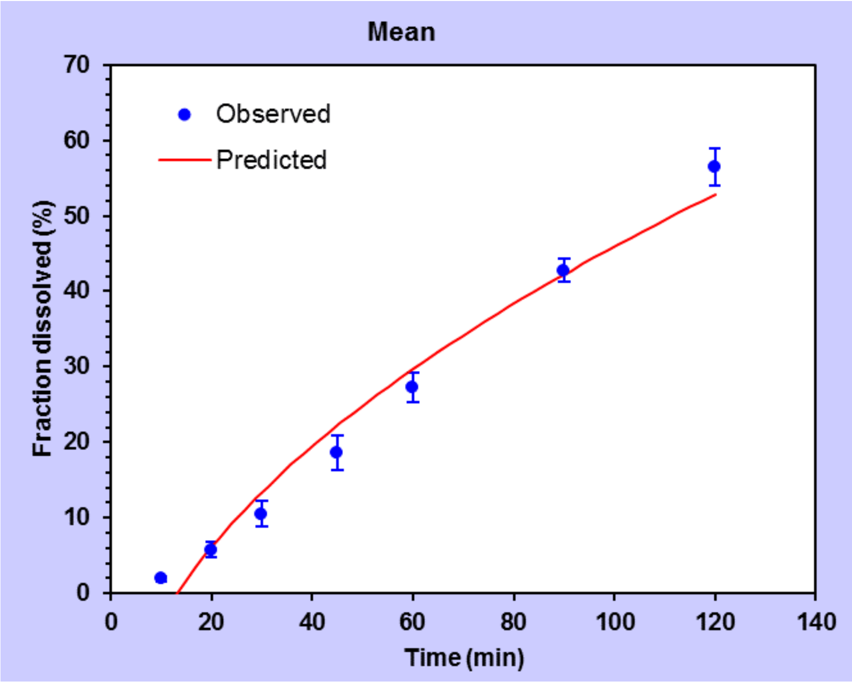

Supplementary materials – Model fitting summary – Polyox™ WSR N-80 (LEO NF Grade) (modelling up to app. 60 % of carvedilol released)

Graphical abstract of model fit presented as the fraction % of released carvedilol per tested tablet:

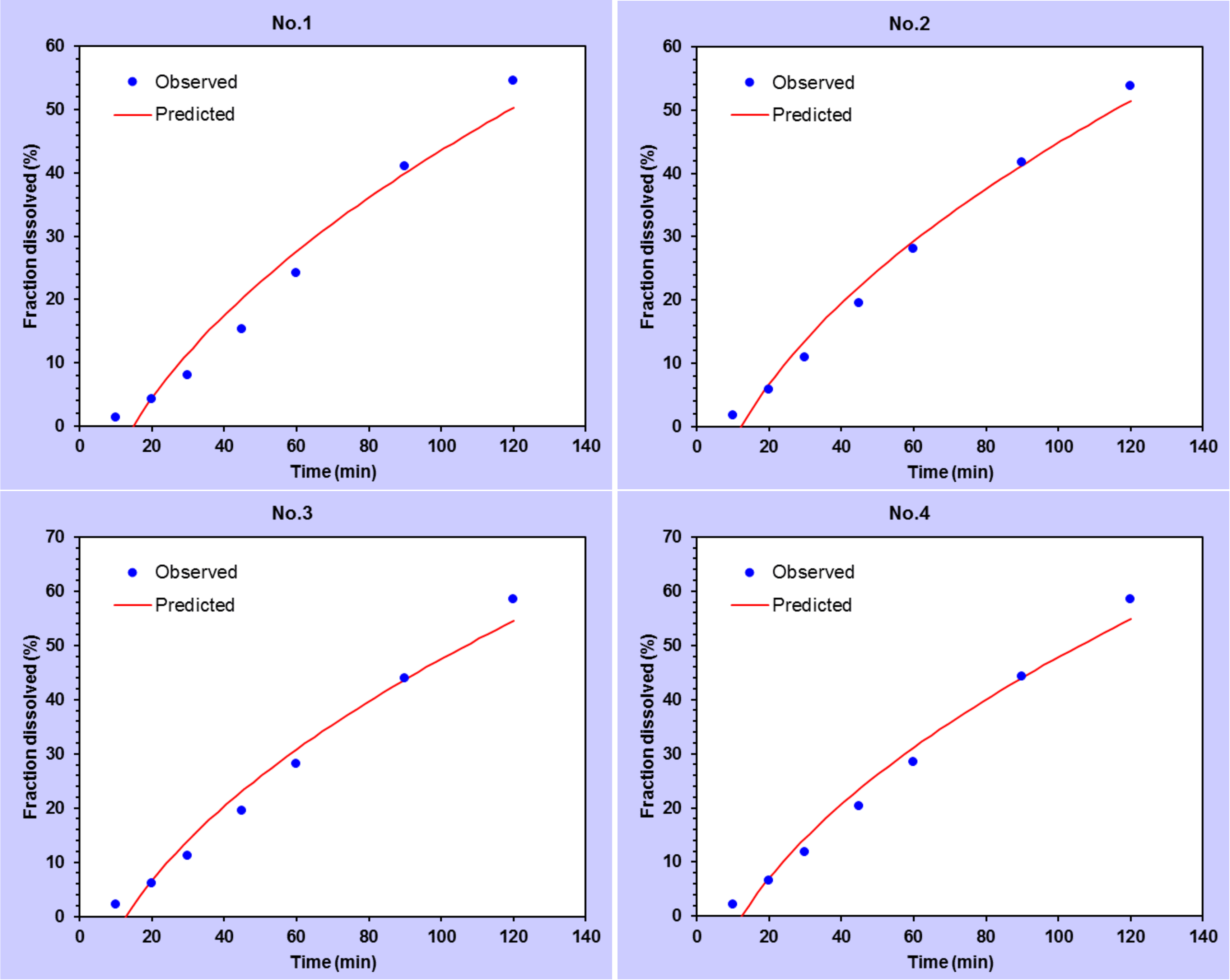

Supplementary materials – Model fitting summary – Polyox™ WSR N-80 (LEO NF Grade) (modelling up to app. 60 % of carvedilol released)

Model: **Korsmeyer–Peppas**

Model equation: $F = k_{KP} \cdot t^n$

Fitted model parameters per tested tablet (N = 4) with statistics – mean, standard deviation (SD), and relative standard deviation expressed in % (RSD%) (output from DDSolver):

Parameter	No.1	No.2	No.3	No.4	Mean	SD	RSD(%)
k_{KP}	0.057	0.115	0.134	0.149	0.114	0.040	35.372
n	1.434	1.273	1.270	1.273	1.312	0.081	6.186

Number of dissolution data points (N), degrees of freedom (df), and selected goodness of fit criteria – Pearson correlation coefficient (R), coefficient of determination (R^2), adjusted coefficient of determination ($R^2_{adjusted}$), and residual sum of squares (RSS) (manual calculation in MS Excel):

Parameter	No.1	No.2	No.3	No.4
N	7	7	7	7
df	5	5	5	5
R	0.994958753	0.991940544	0.996655076	0.995939901
R^2	0.989942921	0.983946042	0.993321341	0.991896286
$R^2_{adjusted}$	0.987931505	0.980735251	0.99198561	0.990275543
RSS	40.67871402	132.4973336	33.1811816	65.99038147

Graphical abstract of model fit presented as mean ± 1 SD of the fraction % of released carvedilol:

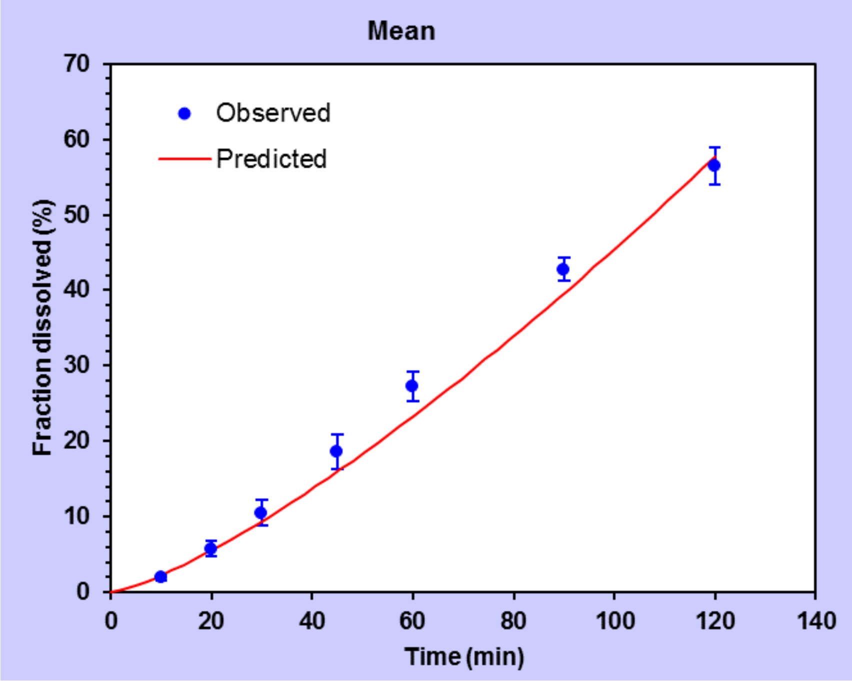

Supplementary materials – Model fitting summary – Polyox™ WSR N-80 (LEO NF Grade) (modelling up to app. 60 % of carvedilol released)

Graphical abstract of model fit presented as the fraction % of released carvedilol per tested tablet:

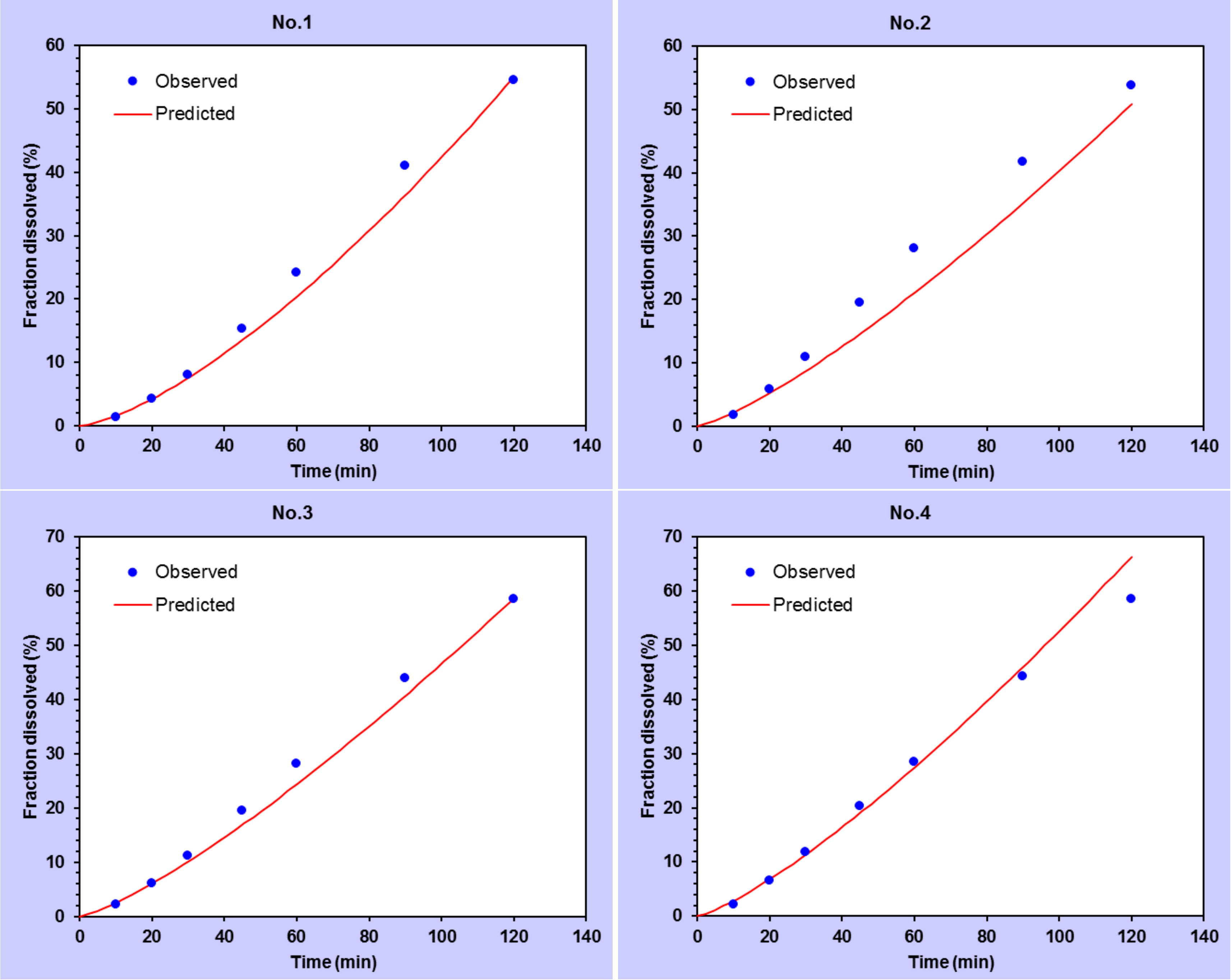

Supplementary materials – Model fitting summary – Polyox™ WSR N-80 (LEO NF Grade) (modelling up to app. 60 % of carvedilol released)

Model: **Korsmeyer–Peppas with T_{lag}**

Model equation: $F = k_{KP} \cdot (t - T_{lag})^n$

Fitted model parameters per tested tablet (N = 4) with statistics – mean, standard deviation (SD), and relative standard deviation expressed in % (RSD%) (output from DDSolver):

Parameter	No.1	No.2	No.3	No.4	Mean	SD	RSD(%)
k_{KP}	0.136	0.260	0.287	0.302	0.246	0.076	30.747
n	1.271	1.124	1.128	1.120	1.161	0.074	6.354
T_{lag}	4.000	4.866	4.000	4.000	4.216	0.433	10.265

Number of dissolution data points (N), degrees of freedom (df), and selected goodness of fit criteria – Pearson correlation coefficient (R), coefficient of determination (R^2), adjusted coefficient of determination ($R^2_{adjusted}$), and residual sum of squares (RSS) (manual calculation in MS Excel):

Parameter	No.1	No.2	No.3	No.4
N	7	7	7	7
df	4	4	4	4
R	0.997345156	0.995663926	0.998741873	0.998558821
R^2	0.99469736	0.991346653	0.997485329	0.997119718
$R^2_{adjusted}$	0.99204604	0.987019979	0.996227993	0.995679578
RSS	13.49517451	41.74196794	8.808594182	14.37562906

Graphical abstract of model fit presented as mean ± 1 SD of the fraction % of released carvedilol:

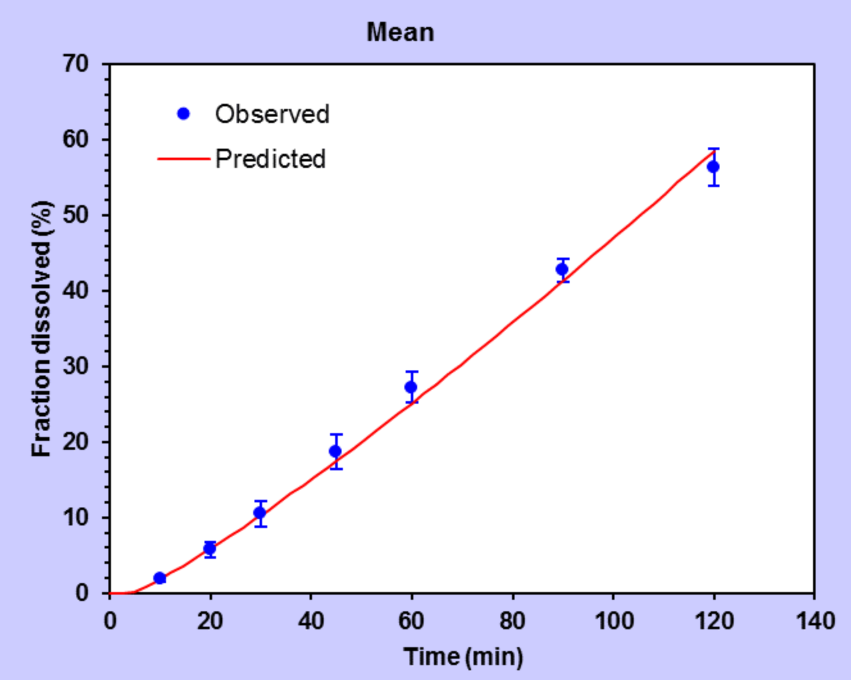

Supplementary materials – Model fitting summary – Polyox™ WSR N-80 (LEO NF Grade) (modelling up to app. 60 % of carvedilol released)

Graphical abstract of model fit presented as the fraction % of released carvedilol per tested tablet:

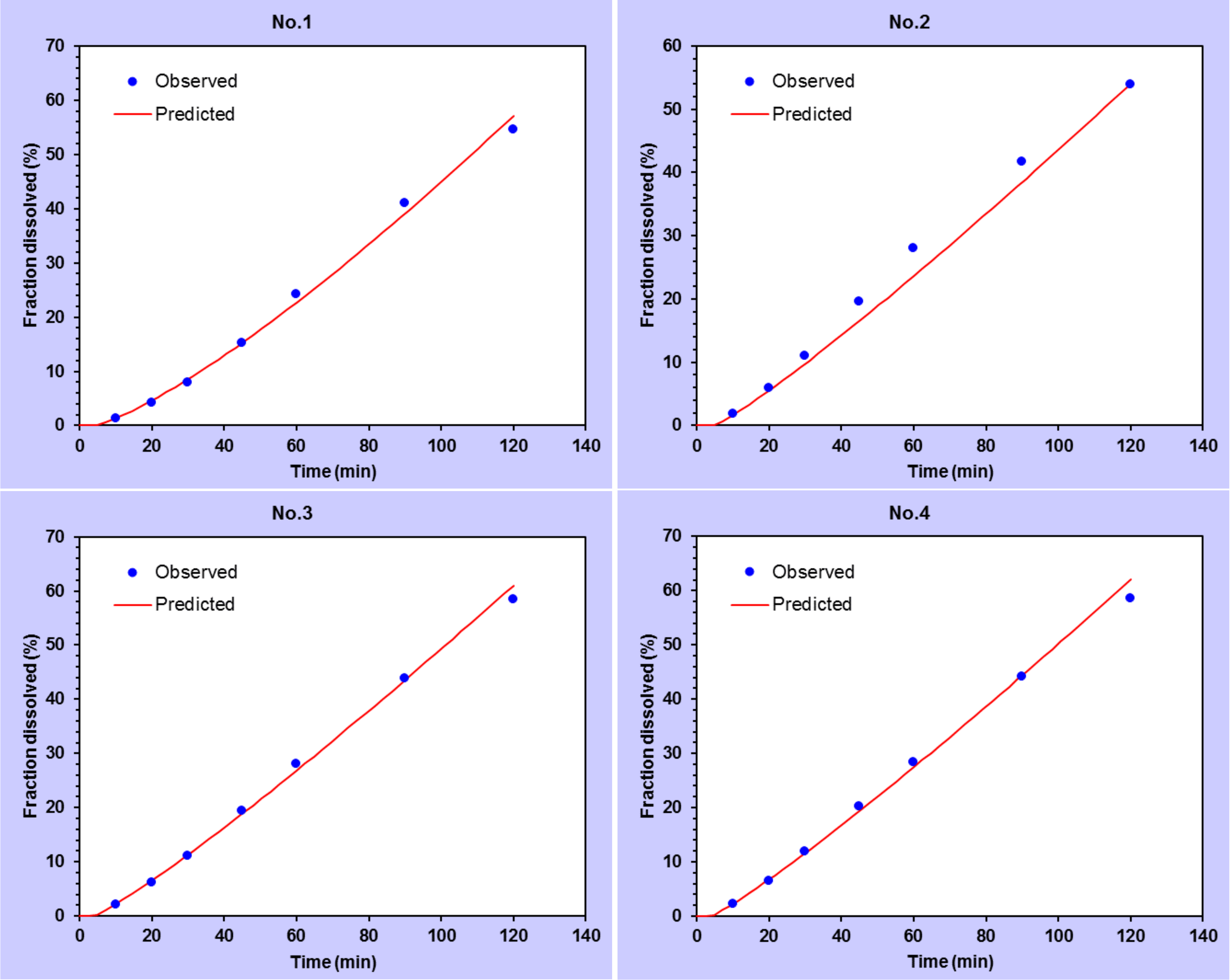

Supplementary materials – Model fitting summary – Polyox[TM] WSR N-80 (LEO NF Grade) (modelling up to app. 60 % of carvedilol released)

Model: **Korsmeyer–Peppas with F_0**

Model equation: $F = F_0 + k_{KP} \cdot t^n$

Fitted model parameters per tested tablet (N = 4) with statistics – mean, standard deviation (SD), and relative standard deviation expressed in % (RSD%) (output from DDSolver):

Parameter	No.1	No.2	No.3	No.4	Mean	SD	RSD(%)
k_{KP}	0.022	0.044	0.053	0.057	0.044	0.016	35.328
n	1.675	1.542	1.503	1.490	1.552	0.084	5.433
F_0	0.560	0.720	0.880	0.880	0.760	0.153	20.157

Number of dissolution data points (N), degrees of freedom (df), and selected goodness of fit criteria – Pearson correlation coefficient (R), coefficient of determination (R^2), adjusted coefficient of determination ($R^2_{adjusted}$), and residual sum of squares (RSS) (manual calculation in MS Excel):

Parameter	No.1	No.2	No.3	No.4
N	7	7	7	7
df	4	4	4	4
R	0.988537302	0.98159534	0.990424894	0.989662469
R^2	0.977205996	0.963529411	0.980941472	0.979431803
$R^2_{adjusted}$	0.965808995	0.945294117	0.971412207	0.969147705
RSS	186.936365	325.9526009	187.4159937	219.8768353

Graphical abstract of model fit presented as mean ± 1 SD of the fraction % of released carvedilol:

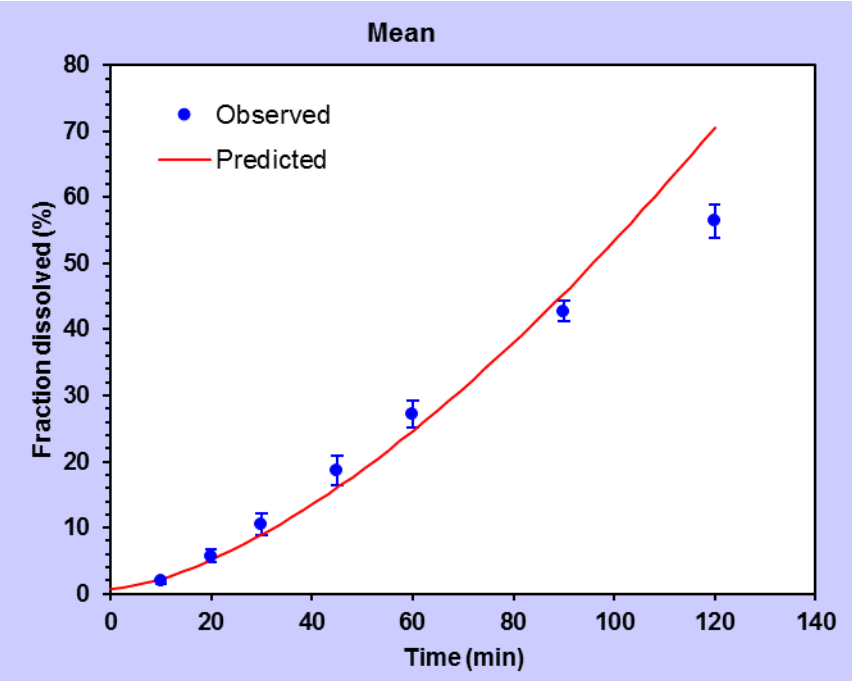

Supplementary materials – Model fitting summary – Polyox™ WSR N-80 (LEO NF Grade) (modelling up to app. 60 % of carvedilol released)

Graphical abstract of model fit presented as the fraction % of released carvedilol per tested tablet:

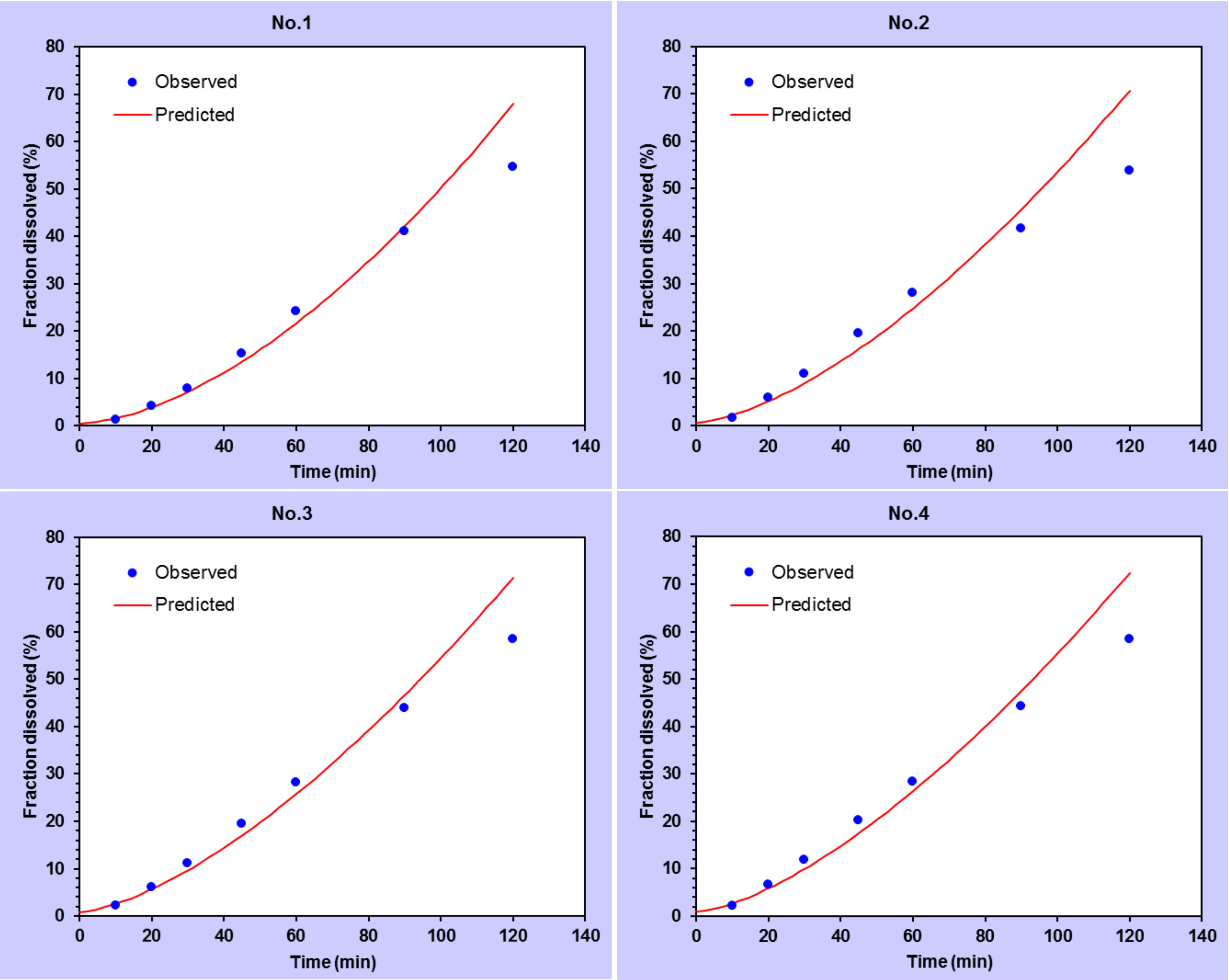

Supplementary materials – Model fitting summary – Polyox[TM] WSR N-80 (LEO NF Grade) (modelling up to app. 60 % of carvedilol released)

Model: **Hixson–Crowell**

Model equation: $F = 100 \cdot [1 - (1 - k_{HC} \cdot t)^3]$

Fitted model parameters per tested tablet (N = 4) with statistics – mean, standard deviation (SD), and relative standard deviation expressed in % (RSD%) (output from DDSolver):

Parameter	No.1	No.2	No.3	No.4	Mean	SD	RSD(%)
k_{HC}	0.002	0.002	0.002	0.002	0.002	0.000	5.835

Number of dissolution data points (N), degrees of freedom (df), and selected goodness of fit criteria – Pearson correlation coefficient (R), coefficient of determination (R^2), adjusted coefficient of determination ($R^2_{adjusted}$), and residual sum of squares (RSS) (manual calculation in MS Excel):

Parameter	No.1	No.2	No.3	No.4
N	7	7	7	7
df	6	6	6	6
R	0.993988386	0.999250433	0.997486214	0.998230445
R^2	0.988012912	0.998501427	0.994978748	0.996464021
$R^2_{adjusted}$	0.988012912	0.998501427	0.994978748	0.996464021
RSS	168.5820088	64.97288923	106.9020539	90.95156237

Graphical abstract of model fit presented as mean ± 1 SD of the fraction % of released carvedilol:

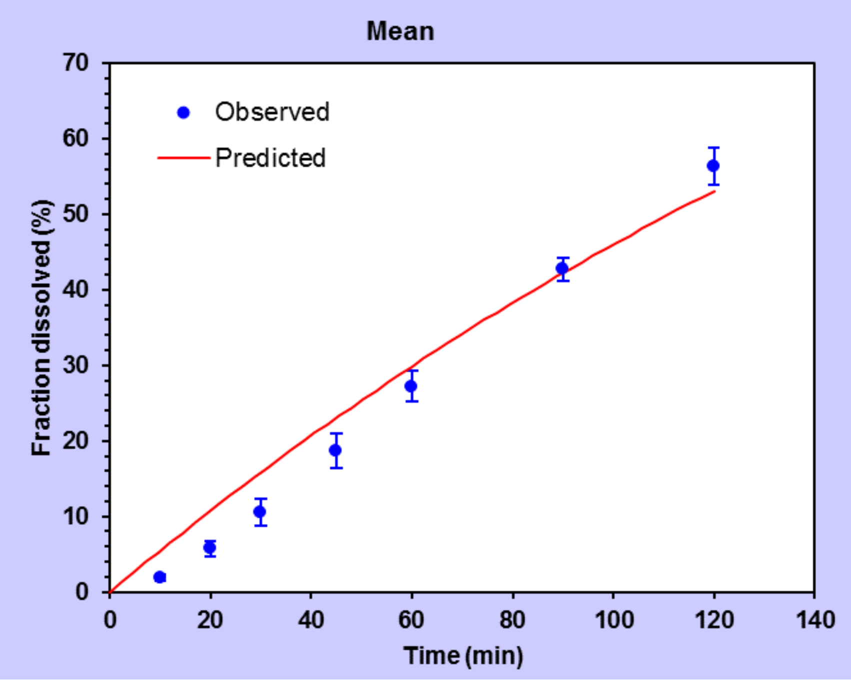

Supplementary materials – Model fitting summary – Polyox™ WSR N-80 (LEO NF Grade) (modelling up to app. 60 % of carvedilol released)

Graphical abstract of model fit presented as the fraction % of released carvedilol per tested tablet:

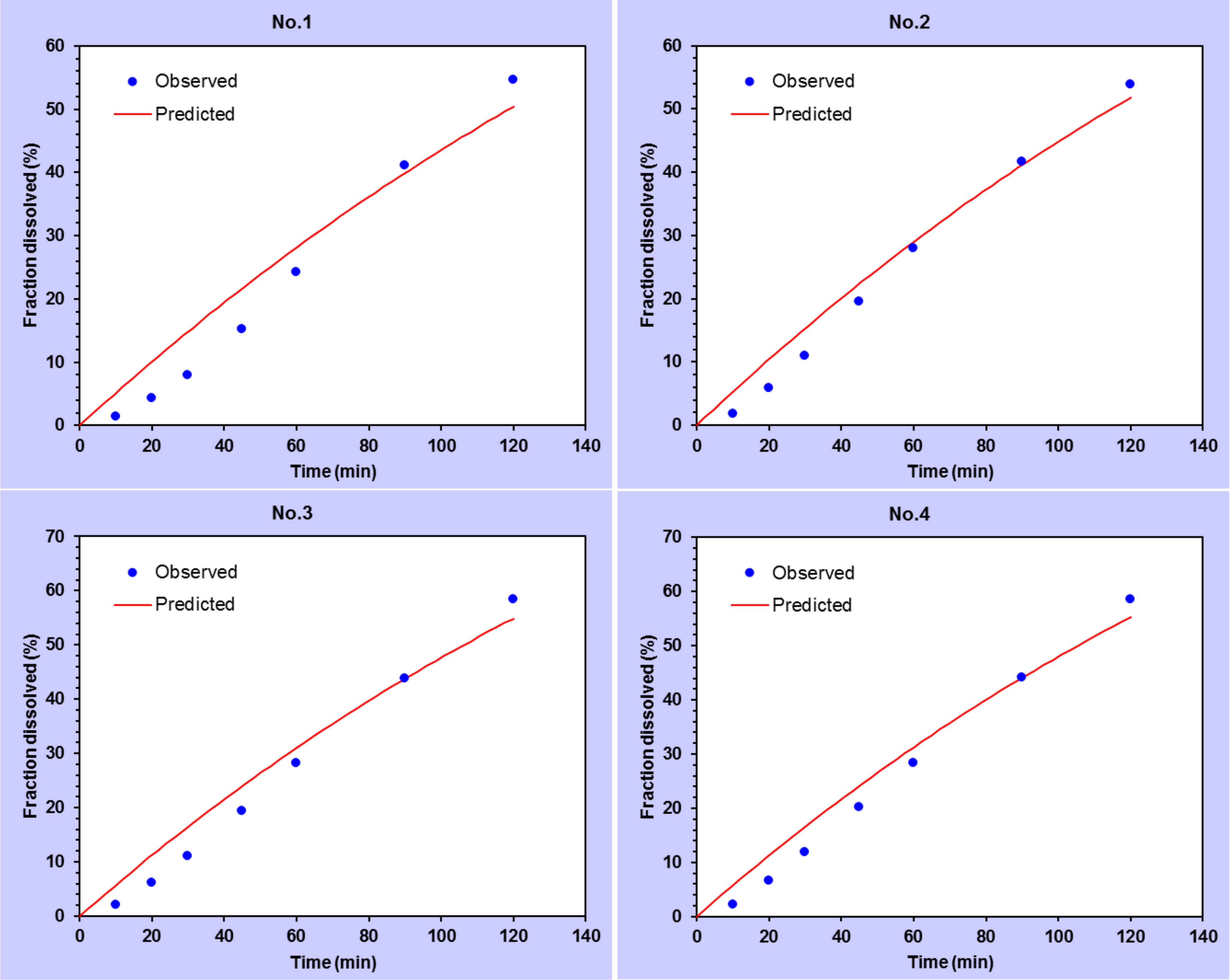

Supplementary materials – Model fitting summary – Polyox™ WSR N-80 (LEO NF Grade) (modelling up to app. 60 % of carvedilol released)

Model: **Hixson–Crowell with T_{lag}**

Model equation: $F = 100 \cdot \left\{1 - \left[1 - k_{HC} \cdot \left(t - T_{lag}\right)\right]^3\right\}$

Fitted model parameters per tested tablet (N = 4) with statistics – mean, standard deviation (SD), and relative standard deviation expressed in % (RSD%) (output from DDSolver):

Parameter	No.1	No.2	No.3	No.4	Mean	SD	RSD(%)
k_{HC}	0.002	0.002	0.002	0.002	0.002	0.000	4.885
T_{lag}	14.428	9.643	11.303	10.571	11.486	2.075	18.069

Number of dissolution data points (N), degrees of freedom (df), and selected goodness of fit criteria – Pearson correlation coefficient (R), coefficient of determination (R^2), adjusted coefficient of determination ($R^2_{adjusted}$), and residual sum of squares (RSS) (manual calculation in MS Excel):

Parameter	No.1	No.2	No.3	No.4
N	7	7	7	7
df	5	5	5	5
R	0.992709189	0.999087468	0.996713991	0.997604475
R^2	0.985471533	0.998175769	0.99343878	0.995214689
$R^2_{adjusted}$	0.98256584	0.997810923	0.992126536	0.994257626
RSS	36.14946739	4.110824474	17.72155018	12.84278766

Graphical abstract of model fit presented as mean ± 1 SD of the fraction % of released carvedilol:

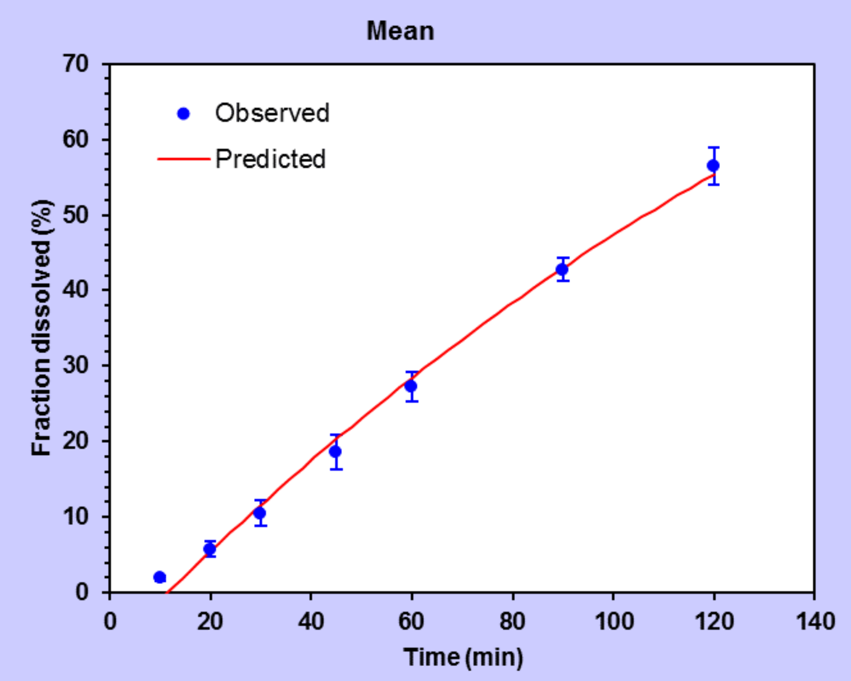

Supplementary materials – Model fitting summary – Polyox^{TM} WSR N-80 (LEO NF Grade) (modelling up to app. 60 % of carvedilol released)

Graphical abstract of model fit presented as the fraction % of released carvedilol per tested tablet:

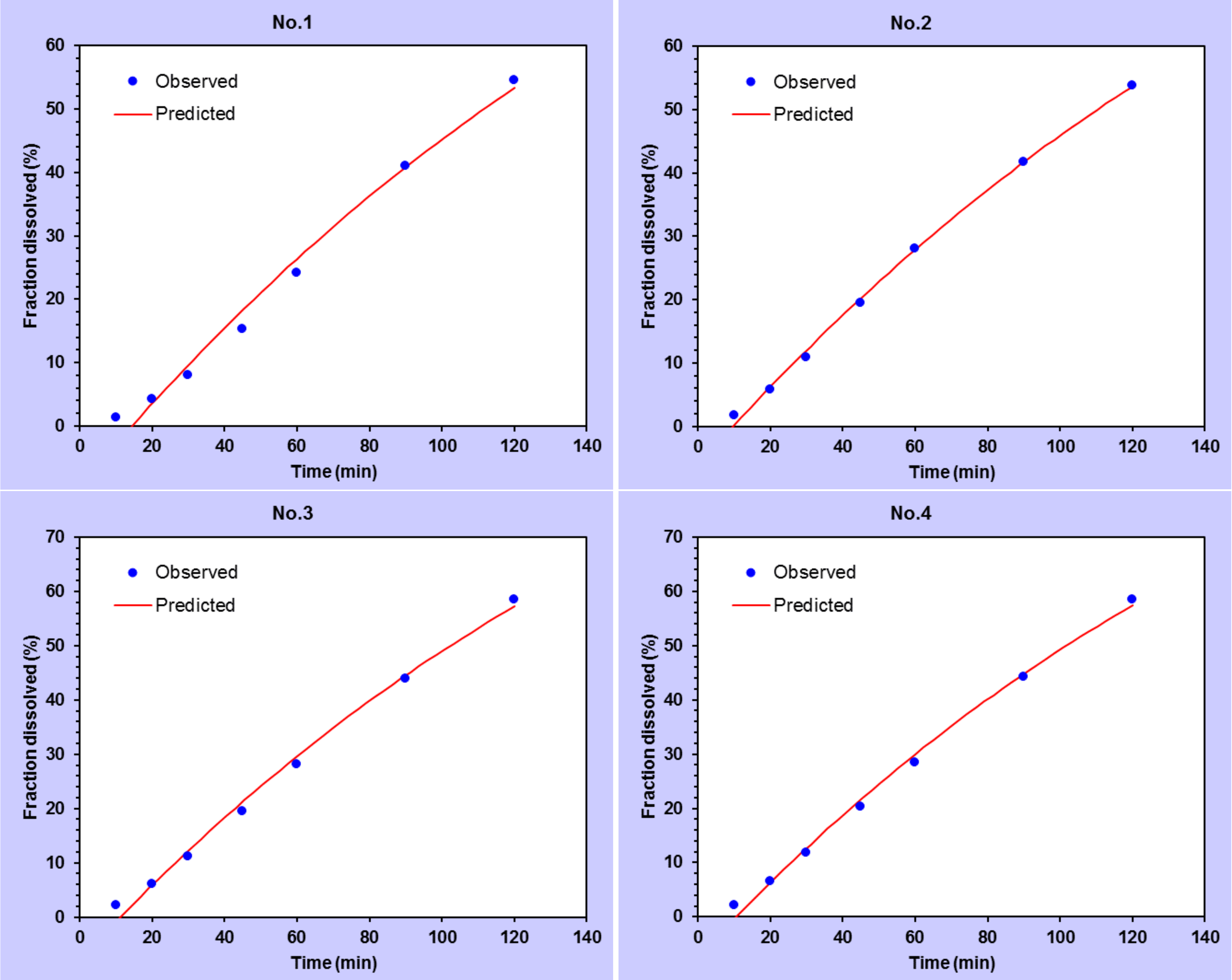

Supplementary materials – Model fitting summary – Polyox™ WSR N-80 (LEO NF Grade) (modelling up to app. 60 % of carvedilol released)

Model: **Hopfenberg**

Model equation: $F = 100 \cdot [1 - (1 - k_{HB} \cdot t)^n]$

Fitted model parameters per tested tablet (N = 4) with statistics – mean, standard deviation (SD), and relative standard deviation expressed in % (RSD%) (output from DDSolver):

Parameter	No.1	No.2	No.3	No.4	Mean	SD	RSD(%)
k_{HB}	0.004	0.004	0.005	0.005	0.005	0.000	4.869
n	1.000	1.000	1.000	1.000	1.000	0.000	0.000

Number of dissolution data points (N), degrees of freedom (df), and selected goodness of fit criteria – Pearson correlation coefficient (R), coefficient of determination (R^2), adjusted coefficient of determination ($R^2_{adjusted}$), and residual sum of squares (RSS) (manual calculation in MS Excel):

Parameter	No.1	No.2	No.3	No.4
N	7	7	7	7
df	5	5	5	5
R	0.99764176	0.998174355	0.99954146	0.999675136
R^2	0.995289081	0.996352044	0.99908313	0.999350377
$R^2_{adjusted}$	0.994346898	0.995622452	0.998899756	0.999220453
RSS	84.91380011	26.3043648	34.05182712	25.77752023

Graphical abstract of model fit presented as mean ± 1 SD of the fraction % of released carvedilol:

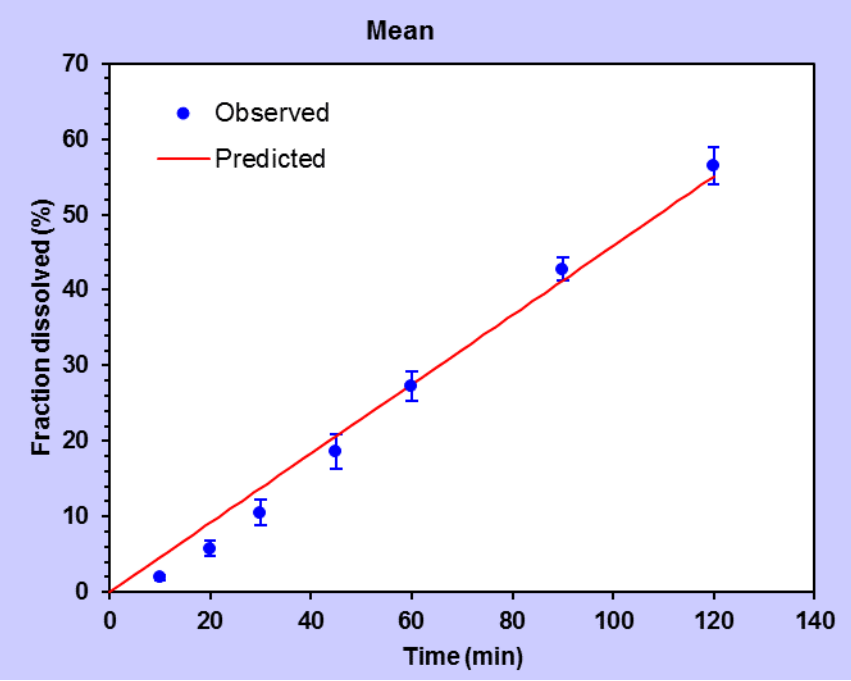

Supplementary materials – Model fitting summary – Polyox^{TM} WSR N-80 (LEO NF Grade) (modelling up to app. 60 % of carvedilol released)

Graphical abstract of model fit presented as the fraction % of released carvedilol per tested tablet:

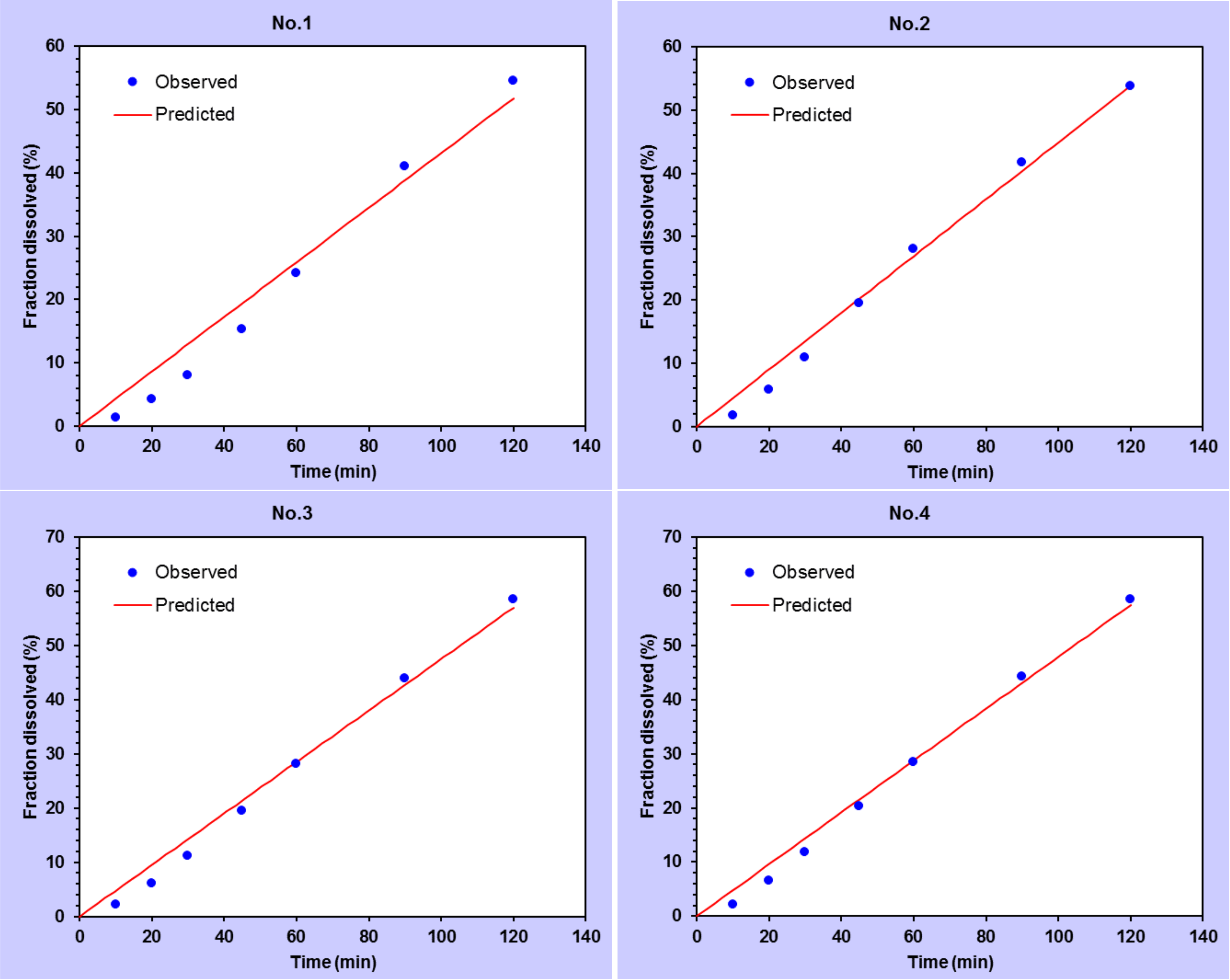

Supplementary materials – Model fitting summary – Polyox™ WSR N-80 (LEO NF Grade) (modelling up to app. 60 % of carvedilol released)

Model: **Hopfenberg with T_{lag}**

Model equation: $F = 100 \cdot \{1 - [1 - k_{HB} \cdot (t - T_{lag})]^n\}$

Fitted model parameters per tested tablet (N = 4) with statistics – mean, standard deviation (SD), and relative standard deviation expressed in % (RSD%) (output from DDSolver):

Parameter	No.1	No.2	No.3	No.4	Mean	SD	RSD(%)
k_{HB}	0.005	0.003	0.005	0.005	0.005	0.001	24.717
n	1.000	2.000	1.000	1.000	1.250	0.500	40.000
T_{lag}	11.368	8.728	7.200	6.309	8.401	2.216	26.377

Number of dissolution data points (N), degrees of freedom (df), and selected goodness of fit criteria – Pearson correlation coefficient (R), coefficient of determination (R^2), adjusted coefficient of determination ($R^2_{adjusted}$), and residual sum of squares (RSS) (manual calculation in MS Excel):

Parameter	No.1	No.2	No.3	No.4
N	7	7	7	7
df	4	4	4	4
R	0.99764176	0.999369642	0.99954146	0.999675136
R^2	0.995289081	0.998739682	0.99908313	0.999350377
$R^2_{adjusted}$	0.992933622	0.998109523	0.998624695	0.999025566
RSS	11.36175933	2.810183453	2.366207878	1.661180631

Graphical abstract of model fit presented as mean ± 1 SD of the fraction % of released carvedilol:

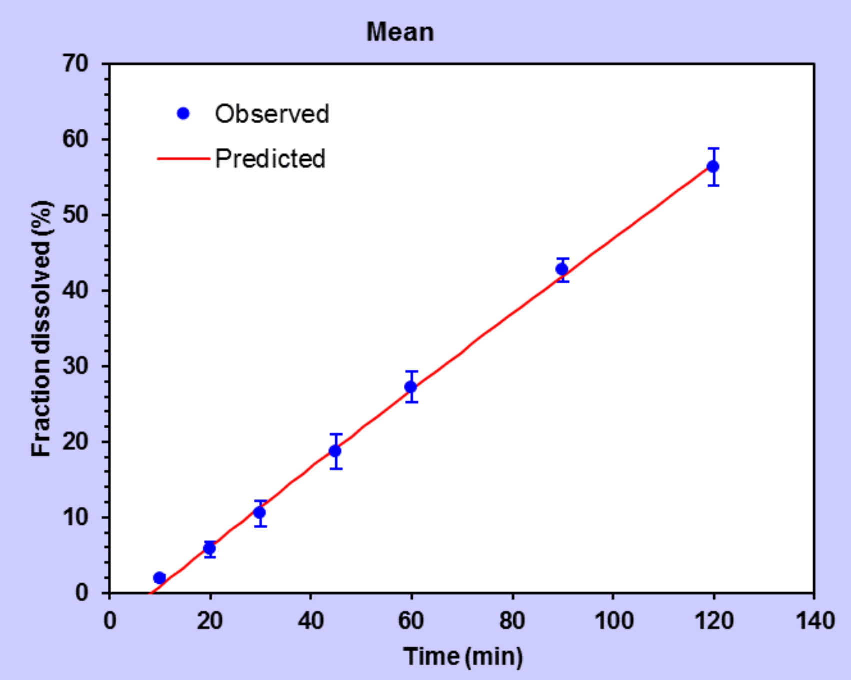

Supplementary materials – Model fitting summary – Polyox™ WSR N-80 (LEO NF Grade) (modelling up to app. 60 % of carvedilol released)

Graphical abstract of model fit presented as the fraction % of released carvedilol per tested tablet:

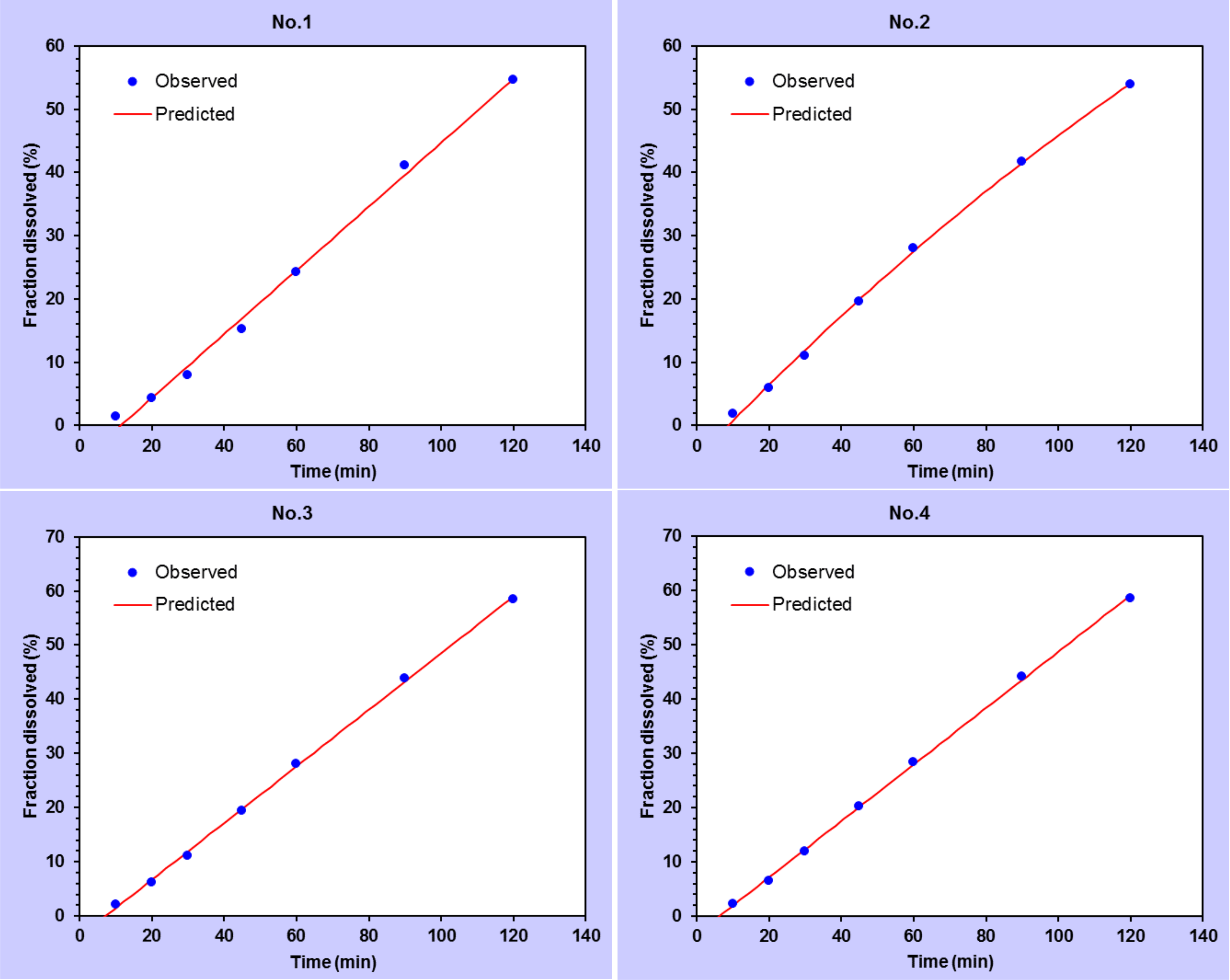

Supplementary materials – Model fitting summary – Polyox™ WSR N-80 (LEO NF Grade) (modelling up to app. 60 % of carvedilol released)

Model: **Baker–Lonsdale**

Model equation: $\frac{3}{2}\cdot\left[1-\left(1-\frac{F}{100}\right)^{\frac{2}{3}}\right]-\frac{F}{100}=k_{BL}\cdot t$

Fitted model parameters per tested tablet (N = 4) with statistics – mean, standard deviation (SD), and relative standard deviation expressed in % (RSD%) (output from DDSolver):

Parameter	No.1	No.2	No.3	No.4	Mean	SD	RSD(%)
k_{BL}	0.001	0.001	0.001	0.001	0.001	0.000	9.889

Number of dissolution data points (N), degrees of freedom (df), and selected goodness of fit criteria – Pearson correlation coefficient (R), coefficient of determination (R^2), adjusted coefficient of determination ($R^2_{adjusted}$), and residual sum of squares (RSS) (manual calculation in MS Excel):

Parameter	No.1	No.2	No.3	No.4
N	7	7	7	7
df	6	6	6	6
R	0.968368779	0.985533887	0.977566481	0.979999167
R^2	0.937738092	0.971277043	0.955636225	0.960398367
$R^2_{adjusted}$	0.937738092	0.971277043	0.955636225	0.960398367
RSS	2127.595495	1557.410286	2027.961301	1943.948883

Graphical abstract of model fit presented as mean ± 1 SD of the fraction % of released carvedilol:

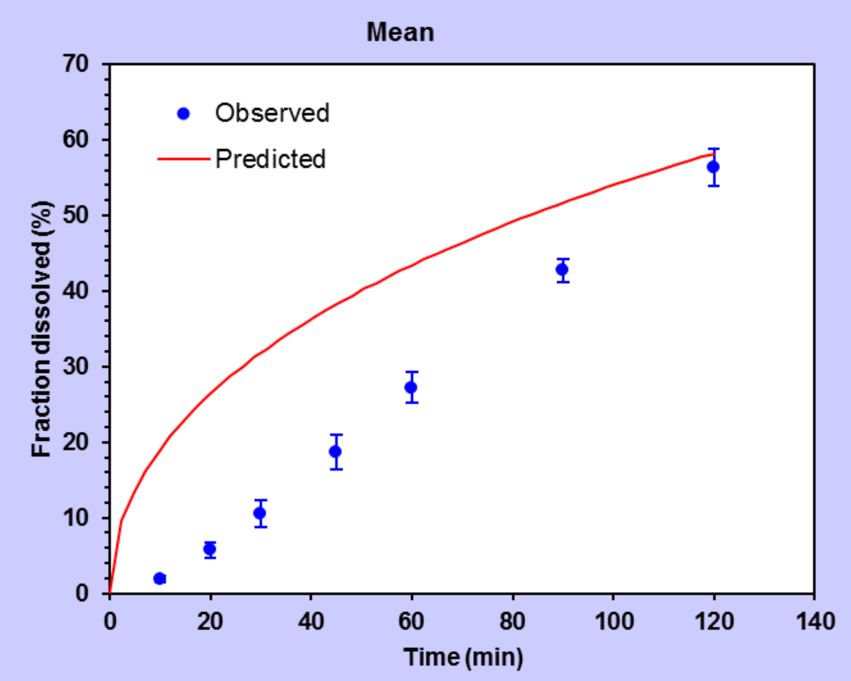

Supplementary materials – Model fitting summary – Polyox™ WSR N-80 (LEO NF Grade) (modelling up to app. 60 % of carvedilol released)

Graphical abstract of model fit presented as the fraction % of released carvedilol per tested tablet:

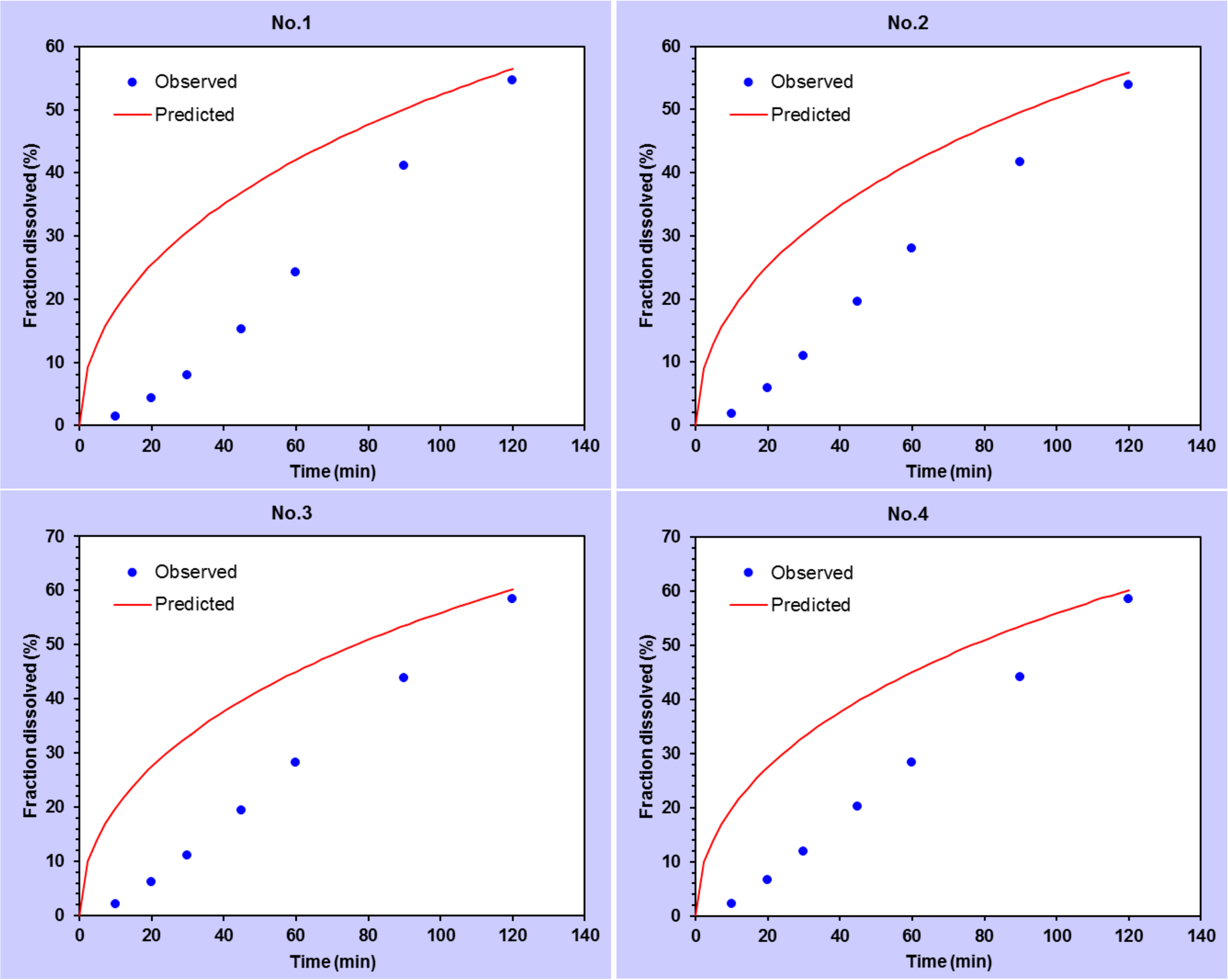

Supplementary materials – Model fitting summary – Polyox™ WSR N-80 (LEO NF Grade) (modelling up to app. 60 % of carvedilol released)

Model: **Baker–Lonsdale with T_{lag}**

Model equation: $\frac{3}{2}\cdot\left[1-\left(1-\frac{F}{100}\right)^{\frac{2}{3}}\right]-\frac{F}{100}=k_{BL}\cdot\left(t-T_{lag}\right)$

Fitted model parameters per tested tablet (N = 4) with statistics – mean, standard deviation (SD), and relative standard deviation expressed in % (RSD%) (output from DDSolver):

Parameter	No.1	No.2	No.3	No.4	Mean	SD	RSD(%)
k_{BL}	0.001	0.001	0.001	0.001	0.001	0.000	9.889
T_{lag}	38.563	23.323	24.519	24.191	27.649	7.294	26.380

Number of dissolution data points (N), degrees of freedom (df), and selected goodness of fit criteria – Pearson correlation coefficient (R), coefficient of determination (R^2), adjusted coefficient of determination ($R^2_{adjusted}$), and residual sum of squares (RSS) (manual calculation in MS Excel):

Parameter	No.1	No.2	No.3	No.4
N	7	7	7	7
df	5	5	5	5
R	0.984168604	0.973406668	0.966408212	0.967485813
R^2	0.968587841	0.947520541	0.933944832	0.936028798
$R^2_{adjusted}$	0.96230541	0.93702465	0.920733799	0.923234558
RSS	136.3789943	136.5117155	202.7525906	194.5261491

Graphical abstract of model fit presented as mean ± 1 SD of the fraction % of released carvedilol:

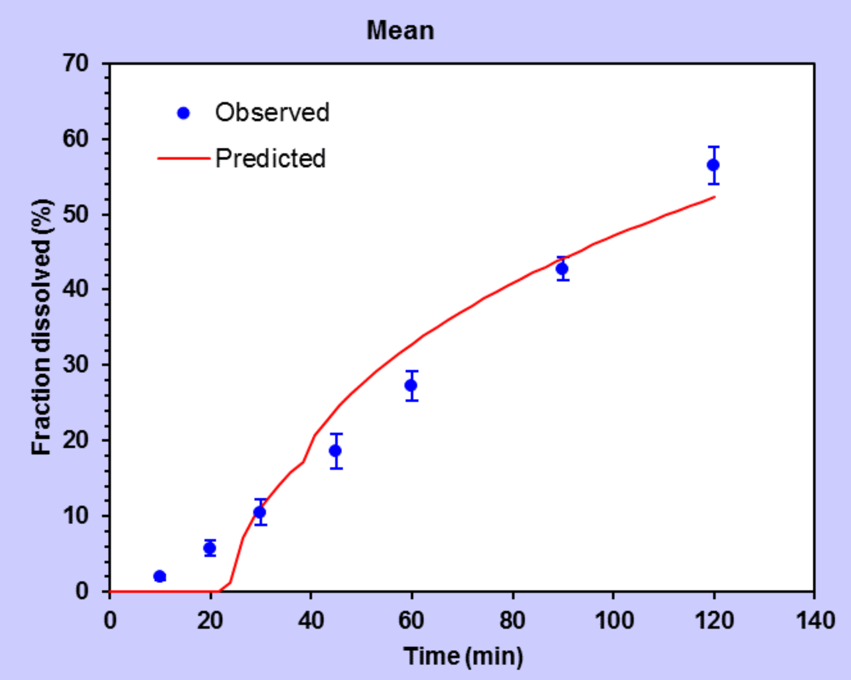

Supplementary materials – Model fitting summary – Polyox^{TM} WSR N-80 (LEO NF Grade) (modelling up to app. 60 % of carvedilol released)

Graphical abstract of model fit presented as the fraction % of released carvedilol per tested tablet:

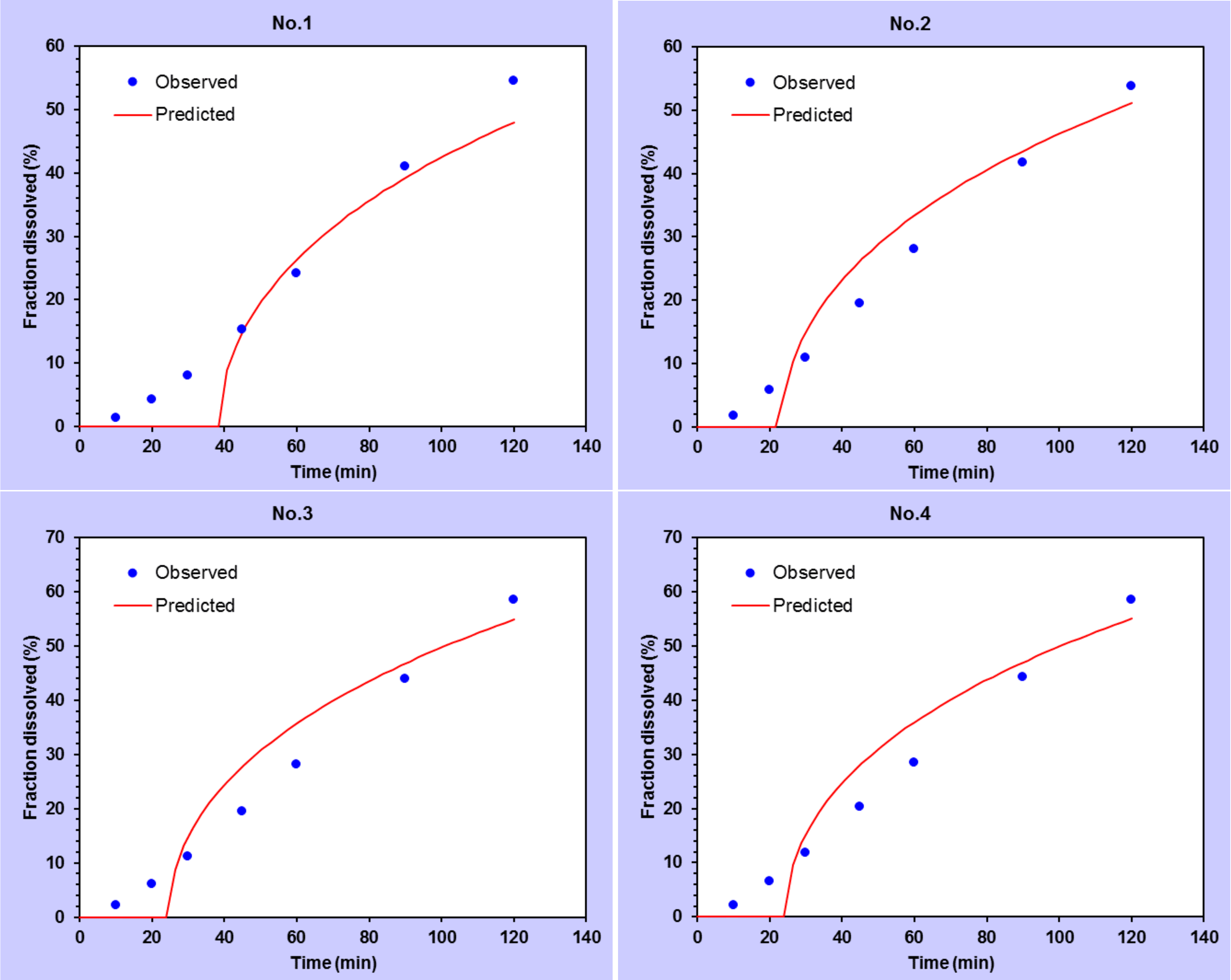

Supplementary materials – Model fitting summary – Polyox™ WSR N-80 (LEO NF Grade) (modelling up to app. 60 % of carvedilol released)

Model: **Makoid–Banakar**

Model equation: $F = k_{MB} \cdot t^n \cdot e^{-k \cdot t}$

Fitted model parameters per tested tablet (N = 4) with statistics – mean, standard deviation (SD), and relative standard deviation expressed in % (RSD%) (output from DDSolver):

Parameter	No.1	No.2	No.3	No.4	Mean	SD	RSD(%)
k_{MB}	0.027	0.031	0.059	0.055	0.043	0.017	38.459
n	1.734	1.808	1.595	1.643	1.695	0.095	5.601
k	0.005	0.010	0.006	0.008	0.007	0.002	27.596

Number of dissolution data points (N), degrees of freedom (df), and selected goodness of fit criteria – Pearson correlation coefficient (R), coefficient of determination (R^2), adjusted coefficient of determination ($R^2_{adjusted}$), and residual sum of squares (RSS) (manual calculation in MS Excel):

Parameter	No.1	No.2	No.3	No.4
N	7	7	7	7
df	4	4	4	4
R	0.999259041	0.999592462	0.999946548	0.999596486
R^2	0.998518631	0.999185089	0.999893099	0.999193134
$R^2_{adjusted}$	0.997777947	0.998777634	0.999839649	0.998789701
RSS	3.760405908	2.463826592	0.275889293	2.104042558

Graphical abstract of model fit presented as mean ± 1 SD of the fraction % of released carvedilol:

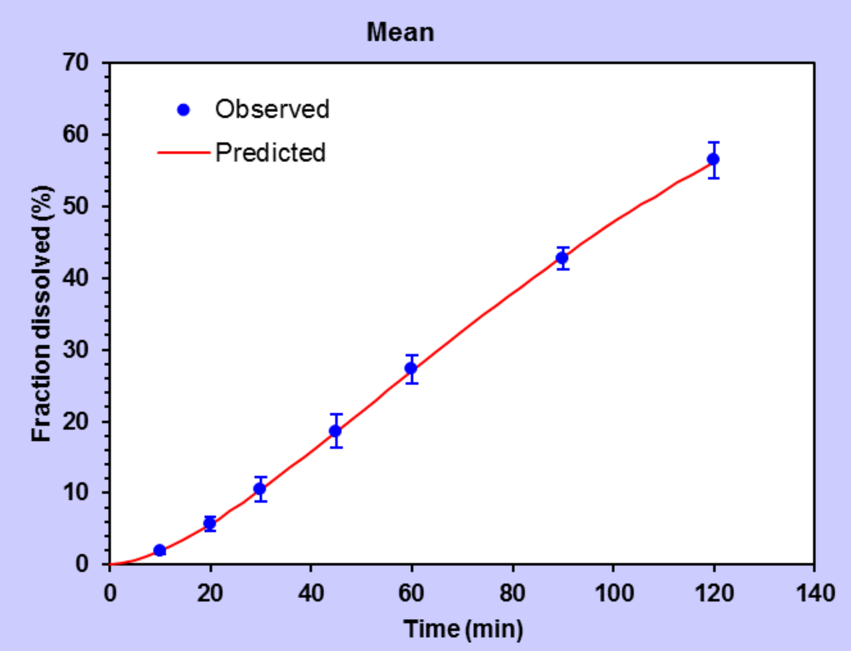

Supplementary materials – Model fitting summary – Polyox™ WSR N-80 (LEO NF Grade) (modelling up to app. 60 % of carvedilol released)

Graphical abstract of model fit presented as the fraction % of released carvedilol per tested tablet:

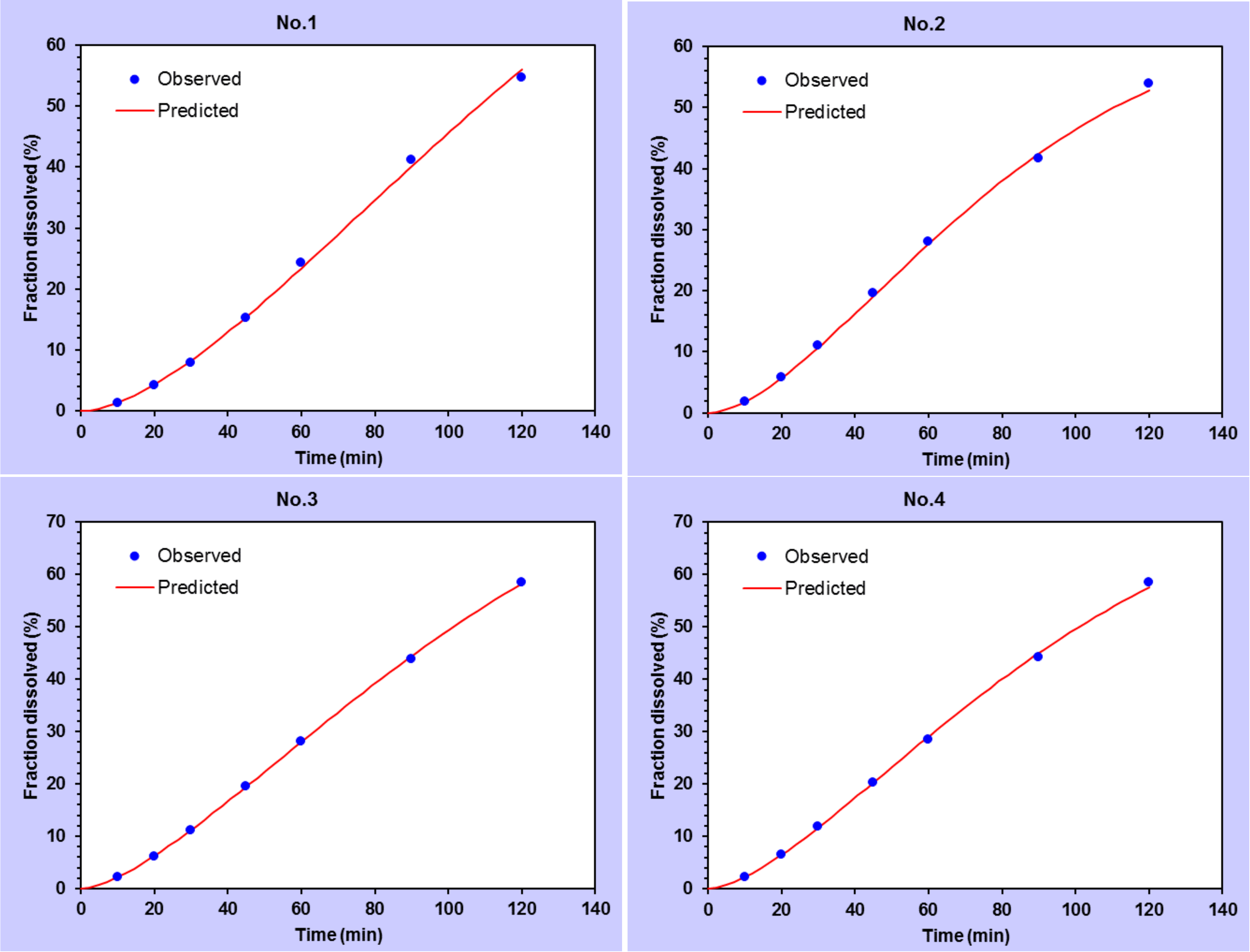

Supplementary materials – Model fitting summary – Polyox™ WSR N-80 (LEO NF Grade) (modelling up to app. 60 % of carvedilol released)

Model: **Makoid–Banakar with T_{lag}**

Model equation: $F = k_{MB} \cdot (t - T_{lag})^n \cdot e^{-k \cdot (t - T_{lag})}$

Fitted model parameters per tested tablet (N = 4) with statistics – mean, standard deviation (SD), and relative standard deviation expressed in % (RSD%) (output from DDSolver):

Parameter	No.1	No.2	No.3	No.4	Mean	SD	RSD(%)
k_{MB}	0.141	0.168	0.270	0.263	0.211	0.066	31.170
n	1.254	1.324	1.156	1.195	1.232	0.073	5.950
k	0.000	0.004	0.001	0.002	0.002	0.002	120.057
T_{lag}	4.000	4.000	4.000	4.626	4.156	0.313	7.527

Number of dissolution data points (N), degrees of freedom (df), and selected goodness of fit criteria – Pearson correlation coefficient (R), coefficient of determination (R^2), adjusted coefficient of determination ($R^2_{adjusted}$), and residual sum of squares (RSS) (manual calculation in MS Excel):

Parameter	No.1	No.2	No.3	No.4
N	7	7	7	7
df	3	3	3	3
R	0.996801178	0.999653659	0.999331097	0.999948332
R^2	0.993612588	0.999307437	0.998662642	0.999896666
$R^2_{adjusted}$	0.987225176	0.998614875	0.997325284	0.999793333
RSS	17.30987727	1.647857849	3.823311128	0.650078248

Graphical abstract of model fit presented as mean ± 1 SD of the fraction % of released carvedilol:

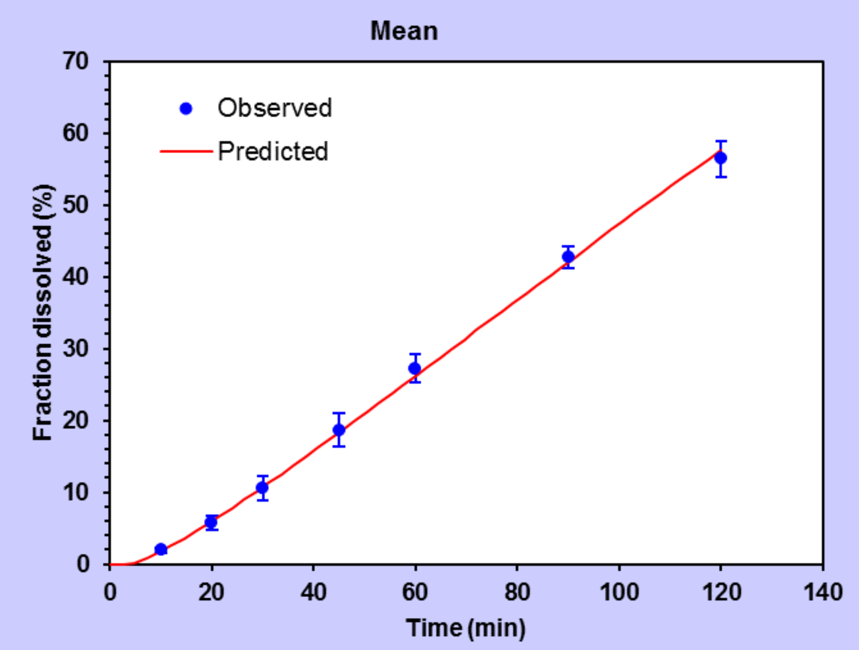

Supplementary materials – Model fitting summary – Polyox^{TM} WSR N-80 (LEO NF Grade) (modelling up to app. 60 % of carvedilol released)

Graphical abstract of model fit presented as the fraction % of released carvedilol per tested tablet:

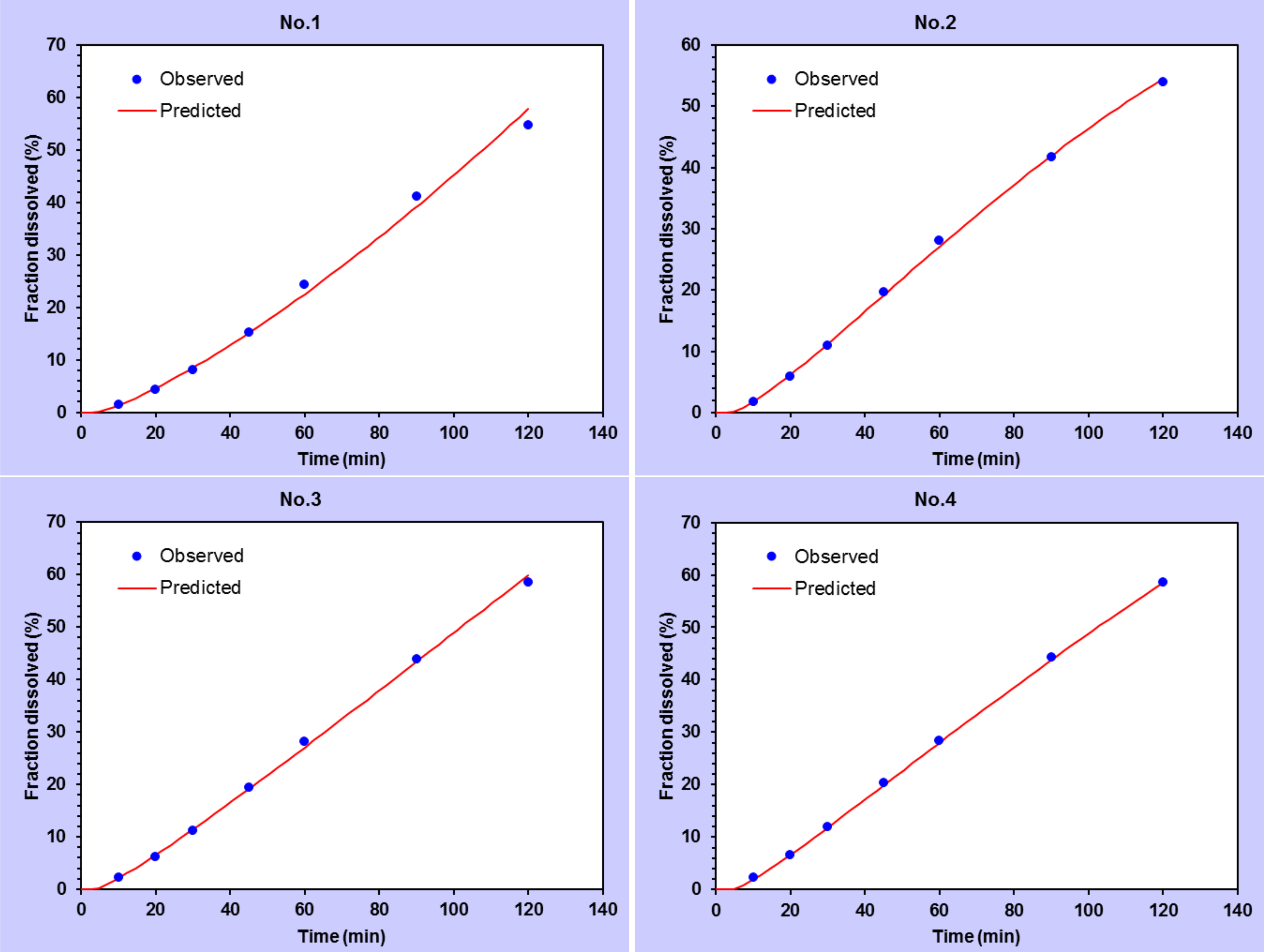

Supplementary materials – Model fitting summary – Polyox™ WSR N-80 (LEO NF Grade) (modelling up to app. 60 % of carvedilol released)

Model: **Peppas–Sahlin_1**

Model equation: $F = k_1 \cdot t^m + k_2 \cdot t^{2m}$

Fitted model parameters per tested tablet (N = 4) with statistics – mean, standard deviation (SD), and relative standard deviation expressed in % (RSD%) (output from DDSolver):

Parameter	No.1	No.2	No.3	No.4	Mean	SD	RSD(%)
k_1	-3.190	-1.913	-2.410	-2.203	-2.429	0.547	-22.504
k_2	1.112	0.965	1.073	1.051	1.050	0.062	5.943
m	0.450	0.450	0.450	0.450	0.450	0.000	0.000

Number of dissolution data points (N), degrees of freedom (df), and selected goodness of fit criteria – Pearson correlation coefficient (R), coefficient of determination (R^2), adjusted coefficient of determination ($R^2_{adjusted}$), and residual sum of squares (RSS) (manual calculation in MS Excel):

Parameter	No.1	No.2	No.3	No.4
N	7	7	7	7
df	4	4	4	4
R	0.99848082	0.998311621	0.999651608	0.999733808
R^2	0.996963948	0.996626093	0.999303337	0.999467686
$R^2_{adjusted}$	0.995445923	0.994939139	0.998955006	0.999201529
RSS	7.37087159	7.615804676	1.797982014	1.368387737

Graphical abstract of model fit presented as mean ± 1 SD of the fraction % of released carvedilol:

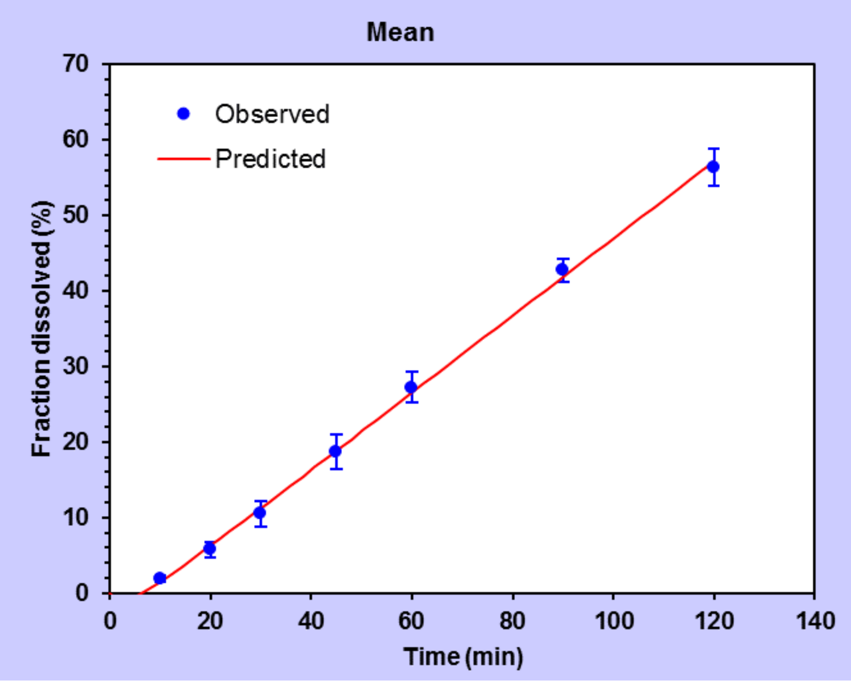

Supplementary materials – Model fitting summary – Polyox™ WSR N-80 (LEO NF Grade) (modelling up to app. 60 % of carvedilol released)

Graphical abstract of model fit presented as the fraction % of released carvedilol per tested tablet:

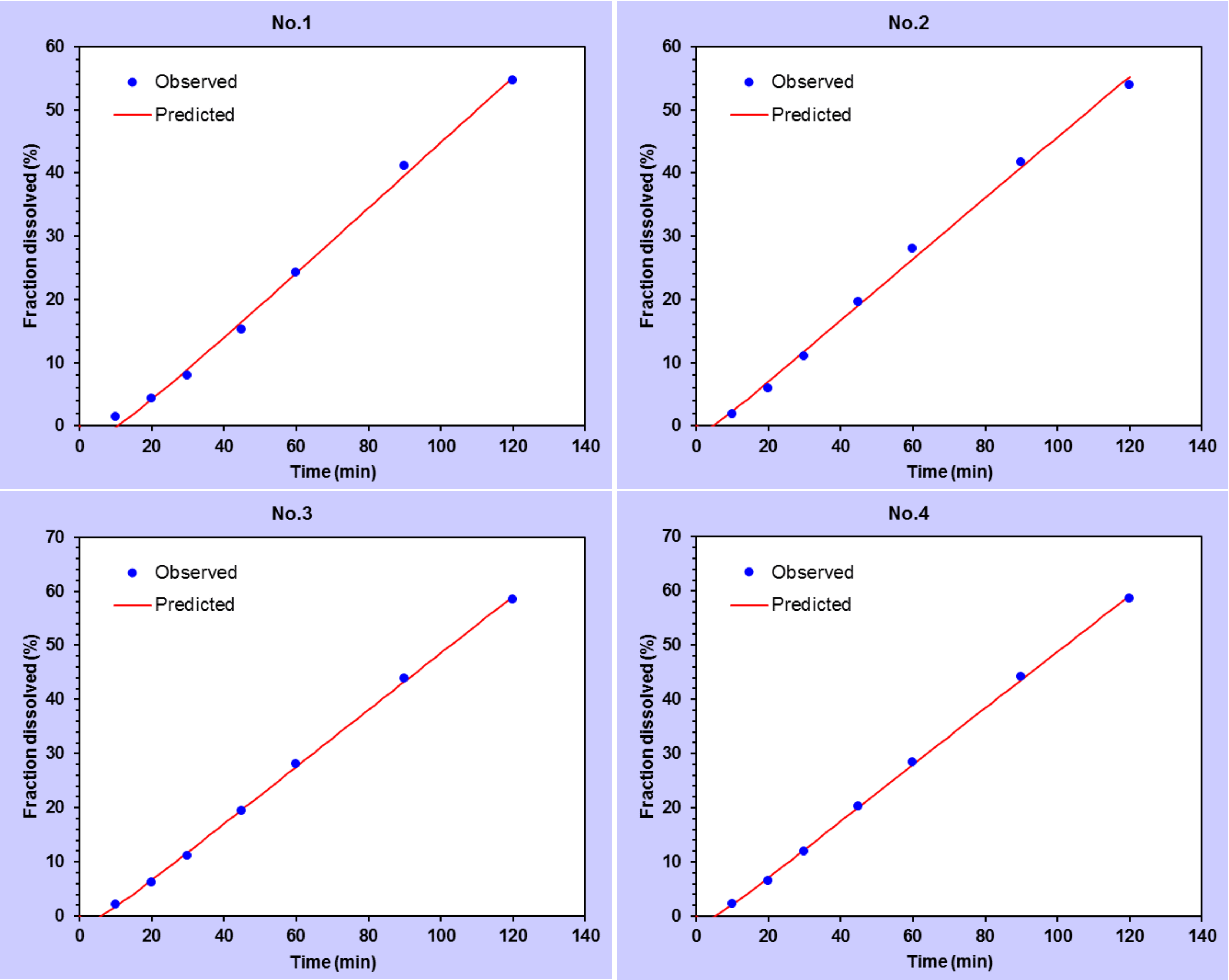

Supplementary materials – Model fitting summary – Polyox^{TM} WSR N-80 (LEO NF Grade) (modelling up to app. 60 % of carvedilol released)

Model: **Peppas–Sahlin_1 with T_{lag}**

Model equation: $F = k_1 \cdot (t - T_{lag})^m + k_2 \cdot (t - T_{lag})^{2m}$

Fitted model parameters per tested tablet (N = 4) with statistics – mean, standard deviation (SD), and relative standard deviation expressed in % (RSD%) (output from DDSolver):

Parameter	No.1	No.2	No.3	No.4	Mean	SD	RSD(%)
k_1	-2.533	-1.166	-1.647	-1.423	-1.692	0.594	-35.089
k_2	1.062	0.900	1.010	0.985	0.989	0.068	6.829
m	0.450	0.450	0.450	0.450	0.450	0.000	0.000
T_{lag}	4.000	4.000	4.000	4.000	4.000	0.000	0.000

Number of dissolution data points (N), degrees of freedom (df), and selected goodness of fit criteria – Pearson correlation coefficient (R), coefficient of determination (R^2), adjusted coefficient of determination ($R^2_{adjusted}$), and residual sum of squares (RSS) (manual calculation in MS Excel):

Parameter	No.1	No.2	No.3	No.4
N	7	7	7	7
df	3	3	3	3
R	0.99839838	0.998689251	0.999641589	0.99979517
R^2	0.996799325	0.997380221	0.999283306	0.999590382
$R^2_{adjusted}$	0.99359865	0.994760441	0.998566611	0.999180764
RSS	7.851079453	5.908794736	1.862282925	1.047897473

Graphical abstract of model fit presented as mean ± 1 SD of the fraction % of released carvedilol:

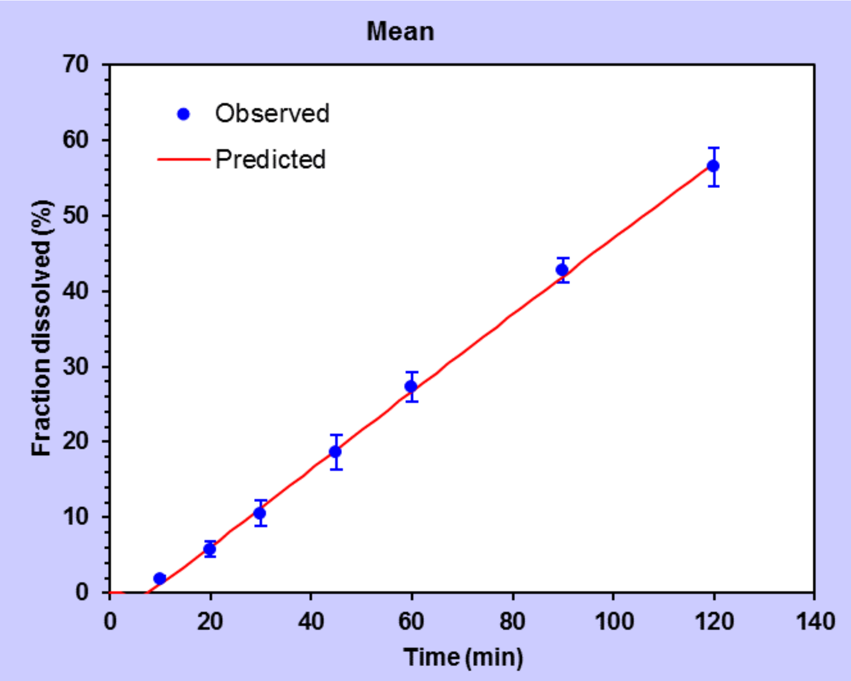

Supplementary materials – Model fitting summary – Polyox™ WSR N-80 (LEO NF Grade) (modelling up to app. 60 % of carvedilol released)

Graphical abstract of model fit presented as the fraction % of released carvedilol per tested tablet:

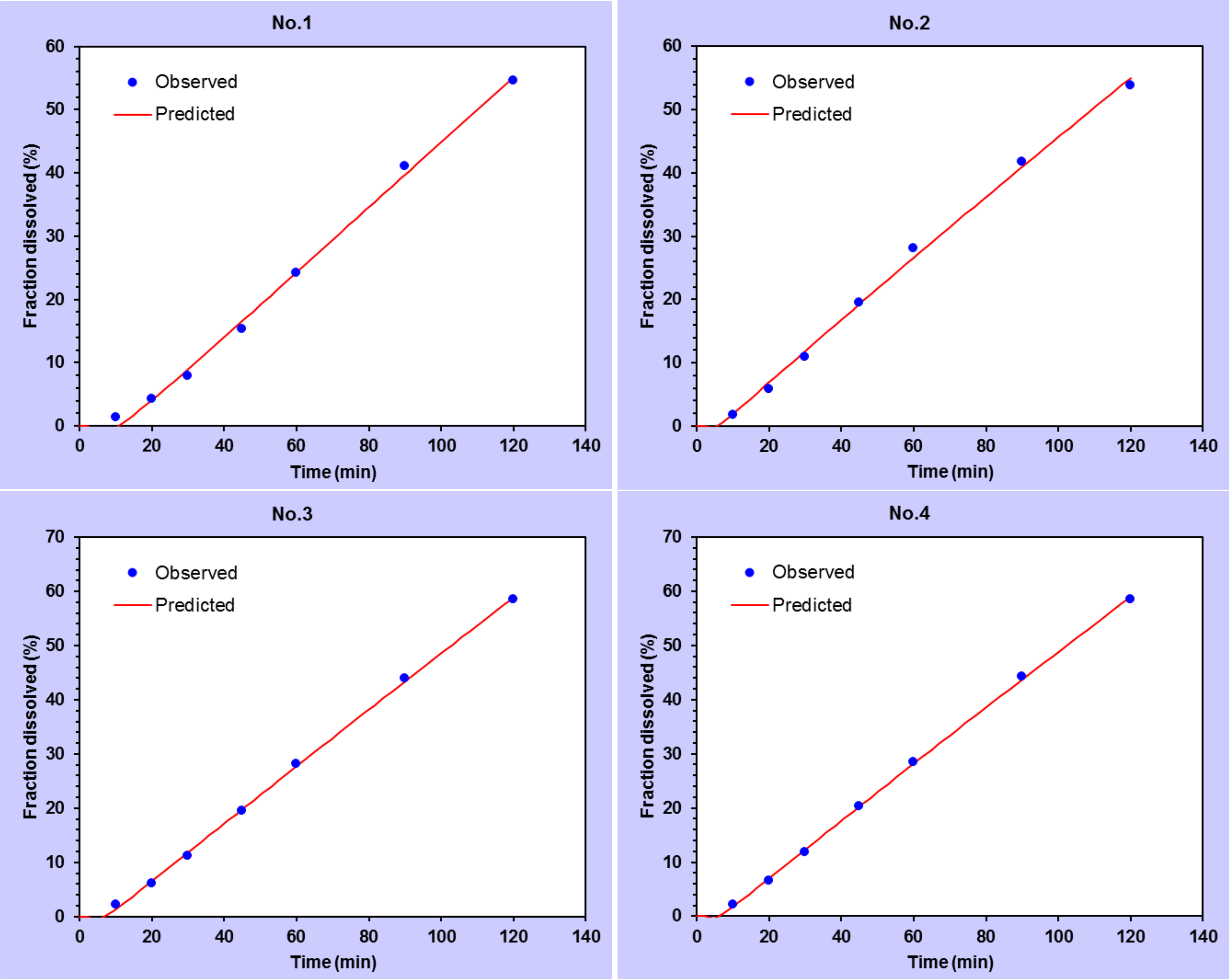

Supplementary materials – Model fitting summary – Polyox™ WSR N-80 (LEO NF Grade) (modelling up to app. 60 % of carvedilol released)

Model: **Peppas–Sahlin_2**

Model equation: $F = k_1 \cdot t^{0.5} + k_2 \cdot t$

Fitted model parameters per tested tablet (N = 4) with statistics – mean, standard deviation (SD), and relative standard deviation expressed in % (RSD%) (output from DDSolver):

Parameter	No.1	No.2	No.3	No.4	Mean	SD	RSD(%)
k_1	-1.817	-0.772	-1.135	-0.965	-1.172	0.455	-38.795
k_2	0.630	0.533	0.599	0.584	0.586	0.040	6.870

Number of dissolution data points (N), degrees of freedom (df), and selected goodness of fit criteria – Pearson correlation coefficient (R), coefficient of determination (R^2), adjusted coefficient of determination ($R^2_{adjusted}$), and residual sum of squares (RSS) (manual calculation in MS Excel):

Parameter	No.1	No.2	No.3	No.4
N	7	7	7	7
df	5	5	5	5
R	0.998643444	0.997337808	0.999336967	0.999300779
R^2	0.997288727	0.994682703	0.998674374	0.998602046
$R^2_{adjusted}$	0.996746473	0.993619244	0.998409249	0.998322455
RSS	6.541131298	12.16733908	3.46851366	3.666596901

Graphical abstract of model fit presented as mean ± 1 SD of the fraction % of released carvedilol:

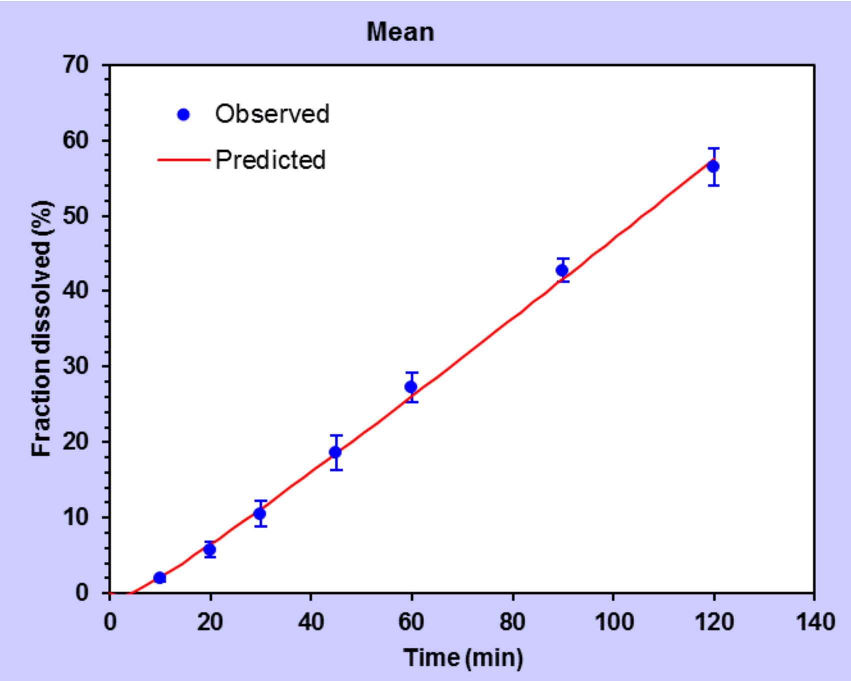

Supplementary materials – Model fitting summary – Polyox™ WSR N-80 (LEO NF Grade) (modelling up to app. 60 % of carvedilol released)

Graphical abstract of model fit presented as the fraction % of released carvedilol per tested tablet:

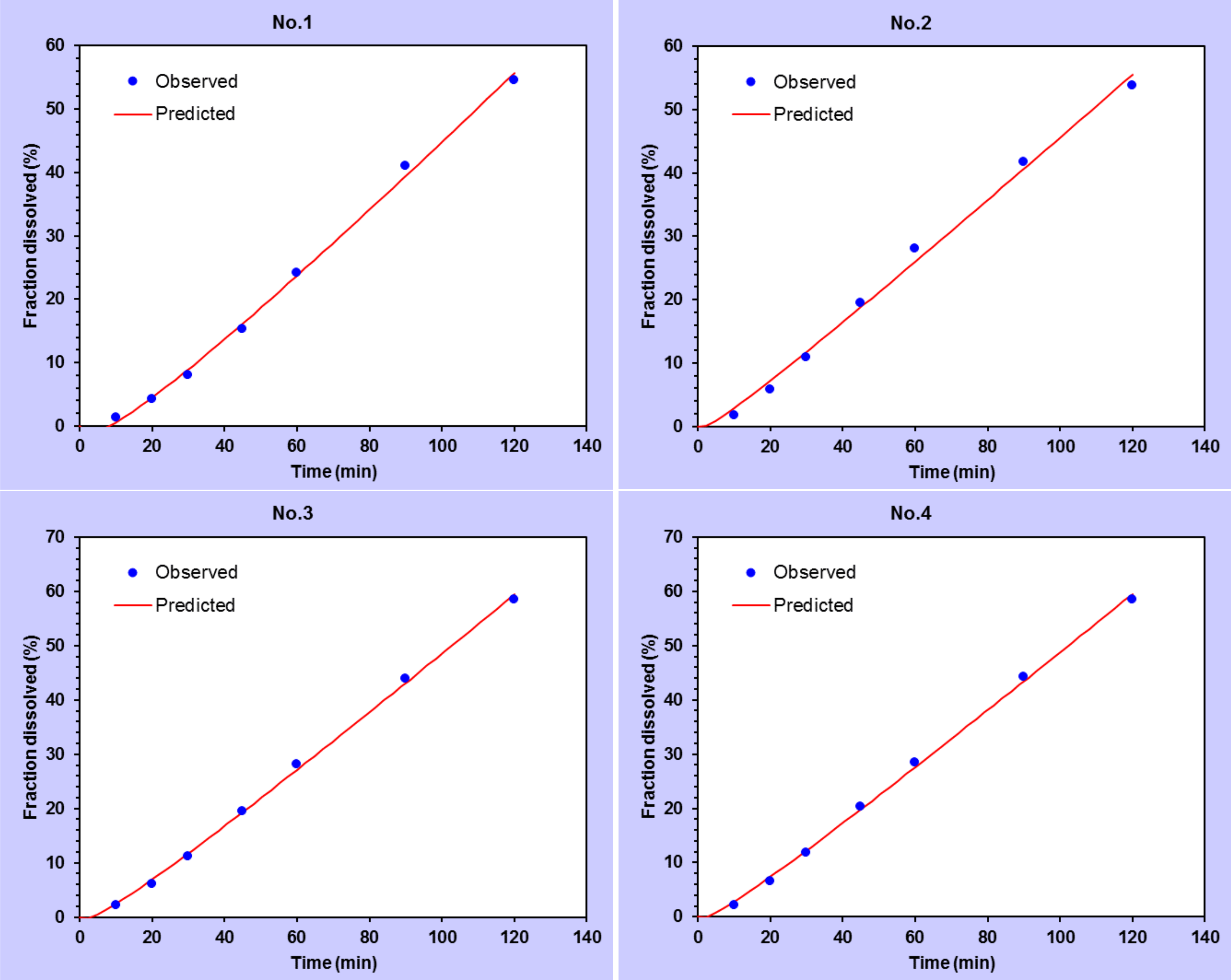

Supplementary materials – Model fitting summary – Polyox™ WSR N-80 (LEO NF Grade) (modelling up to app. 60 % of carvedilol released)

Model: **Peppas–Sahlin_2 with T_{lag}**

Model equation: $F = k_1 \cdot (t - T_{lag})^{0.5} + k_2 \cdot (t - T_{lag})$

Fitted model parameters per tested tablet (N = 4) with statistics – mean, standard deviation (SD), and relative standard deviation expressed in % (RSD%) (output from DDSolver):

Parameter	No.1	No.2	No.3	No.4	Mean	SD	RSD(%)
k_1	-1.318	-0.192	-0.548	-0.364	-0.605	0.497	-82.070
k_2	0.601	0.495	0.561	0.545	0.551	0.044	8.040
T_{lag}	4.000	4.000	4.000	4.000	4.000	0.000	0.000

Number of dissolution data points (N), degrees of freedom (df), and selected goodness of fit criteria – Pearson correlation coefficient (R), coefficient of determination (R^2), adjusted coefficient of determination ($R^2_{adjusted}$), and residual sum of squares (RSS) (manual calculation in MS Excel):

Parameter	No.1	No.2	No.3	No.4
N	7	7	7	7
df	4	4	4	4
R	0.998671405	0.997967751	0.99951311	0.999568675
R^2	0.997344575	0.995939632	0.999026457	0.999137537
$R^2_{adjusted}$	0.996016863	0.993909448	0.998539685	0.998706305
RSS	6.425156122	9.353079042	2.52909487	2.256923219

Graphical abstract of model fit presented as mean ± 1 SD of the fraction % of released carvedilol:

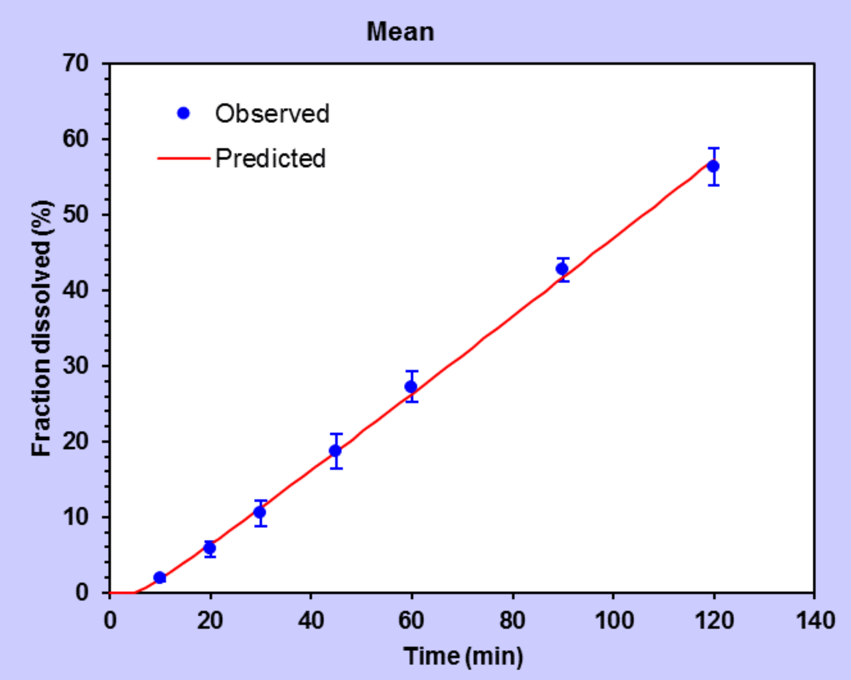

Supplementary materials – Model fitting summary – Polyox™ WSR N-80 (LEO NF Grade) (modelling up to app. 60 % of carvedilol released)

Graphical abstract of model fit presented as the fraction % of released carvedilol per tested tablet:

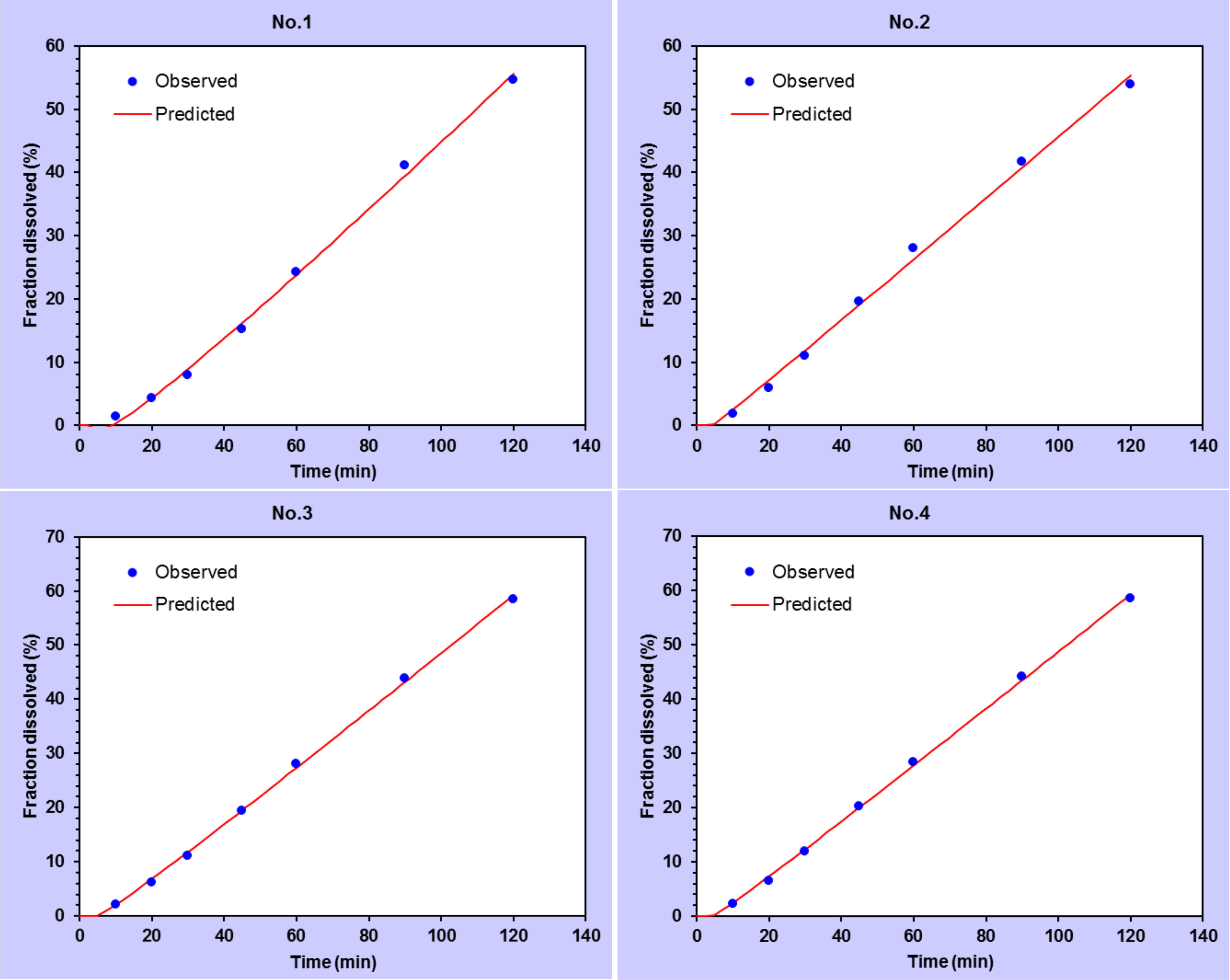

Supplementary materials – Model fitting summary – Polyox™ WSR N-80 (LEO NF Grade) (modelling up to app. 60 % of carvedilol released)

Model: **Quadratic**

Model equation: $F = 100 \cdot (k_1 \cdot t^2 + k_2 \cdot t)$

Fitted model parameters per tested tablet (N = 4) with statistics – mean, standard deviation (SD), and relative standard deviation expressed in % (RSD%) (output from DDSolver):

Parameter	No.1	No.2	No.3	No.4	Mean	SD	RSD(%)
k_1	0.000	0.000	0.000	0.000	0.000	0.000	54.858
k_2	0.003	0.004	0.004	0.004	0.004	0.001	16.027

Number of dissolution data points (N), degrees of freedom (df), and selected goodness of fit criteria – Pearson correlation coefficient (R), coefficient of determination (R^2), adjusted coefficient of determination ($R^2_{adjusted}$), and residual sum of squares (RSS) (manual calculation in MS Excel):

Parameter	No.1	No.2	No.3	No.4
N	7	7	7	7
df	5	5	5	5
R	0.995676275	0.996487666	0.997859493	0.998140701
R^2	0.991371245	0.992987668	0.995723567	0.996284858
$R^2_{adjusted}$	0.989645494	0.991585201	0.994868281	0.99554183
RSS	25.39987066	22.21062425	15.11736659	13.54617566

Graphical abstract of model fit presented as mean ± 1 SD of the fraction % of released carvedilol:

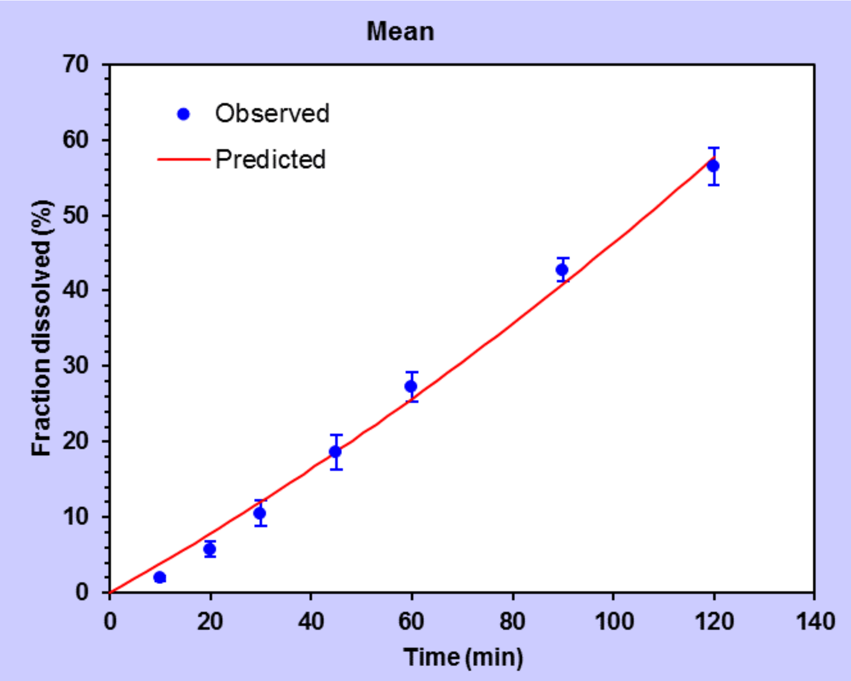

Supplementary materials – Model fitting summary – Polyox^{TM} WSR N-80 (LEO NF Grade) (modelling up to app. 60 % of carvedilol released)

Graphical abstract of model fit presented as the fraction % of released carvedilol per tested tablet:

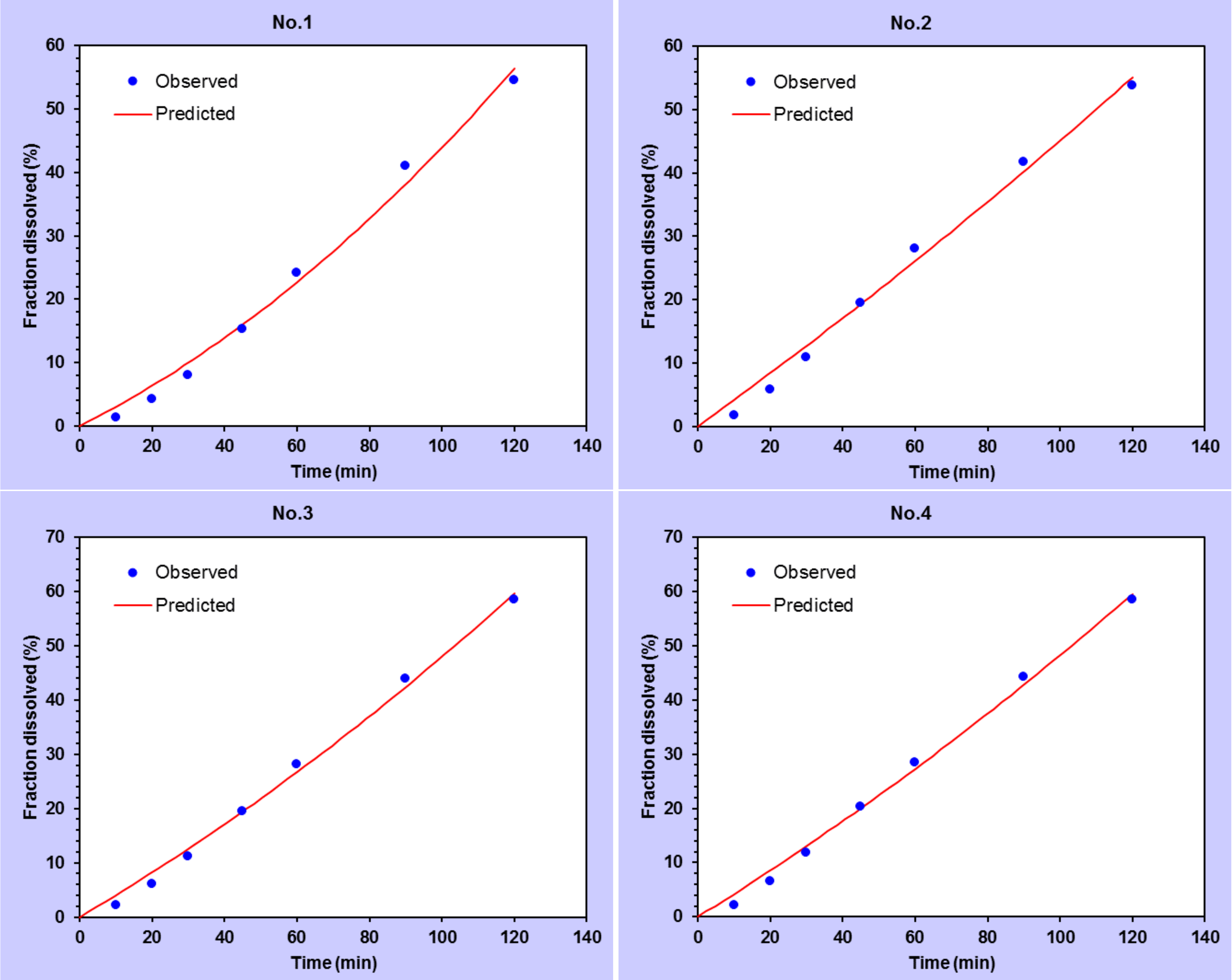

Supplementary materials – Model fitting summary – Polyox™ WSR N-80 (LEO NF Grade) (modelling up to app. 60 % of carvedilol released)

Model: **Quadratic with T_{lag}**

Model equation: $F = 100 \cdot \left[k_1 \cdot (t - T_{lag})^2 + k_2 \cdot (t - T_{lag})\right]$

Fitted model parameters per tested tablet (N = 4) with statistics – mean, standard deviation (SD), and relative standard deviation expressed in % (RSD%) (output from DDSolver):

Parameter	No.1	No.2	No.3	No.4	Mean	SD	RSD(%)
k_1	0.000	0.000	0.000	0.000	0.000	0.000	128.800
k_2	0.003	0.005	0.005	0.005	0.004	0.001	14.629
T_{lag}	4.000	4.000	4.000	4.000	4.000	0.000	0.000

Number of dissolution data points (N), degrees of freedom (df), and selected goodness of fit criteria – Pearson correlation coefficient (R), coefficient of determination (R^2), adjusted coefficient of determination ($R^2_{adjusted}$), and residual sum of squares (RSS) (manual calculation in MS Excel):

Parameter	No.1	No.2	No.3	No.4
N	7	7	7	7
df	4	4	4	4
R	0.997055455	0.998468348	0.999132564	0.999415818
R^2	0.994119581	0.996939043	0.99826588	0.998831977
$R^2_{adjusted}$	0.991179372	0.995408564	0.99739882	0.998247966
RSS	17.08791169	9.984618332	6.010071649	4.339946362

Graphical abstract of model fit presented as mean ± 1 SD of the fraction % of released carvedilol:

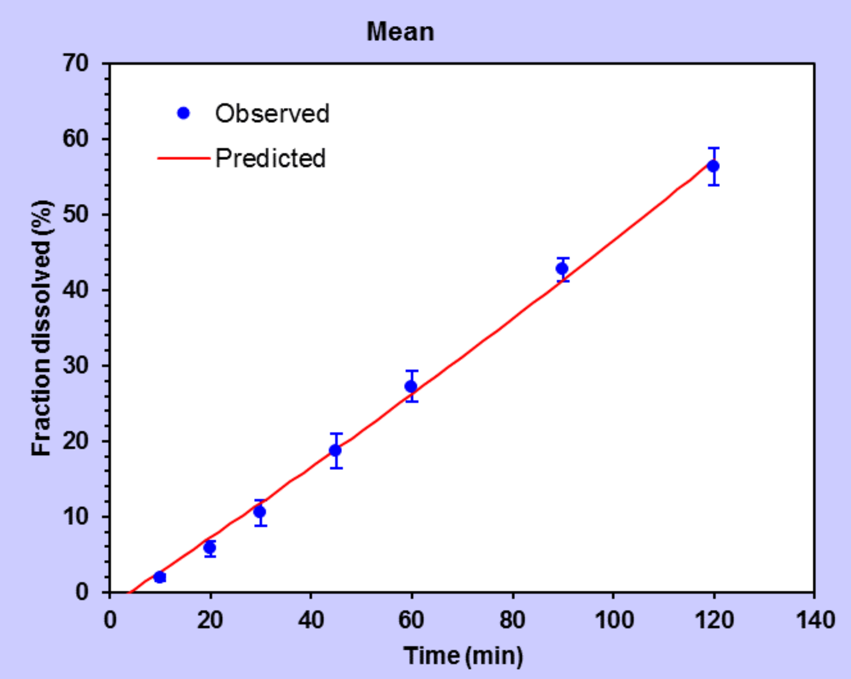

Supplementary materials – Model fitting summary – Polyox™ WSR N-80 (LEO NF Grade) (modelling up to app. 60 % of carvedilol released)

Graphical abstract of model fit presented as the fraction % of released carvedilol per tested tablet:

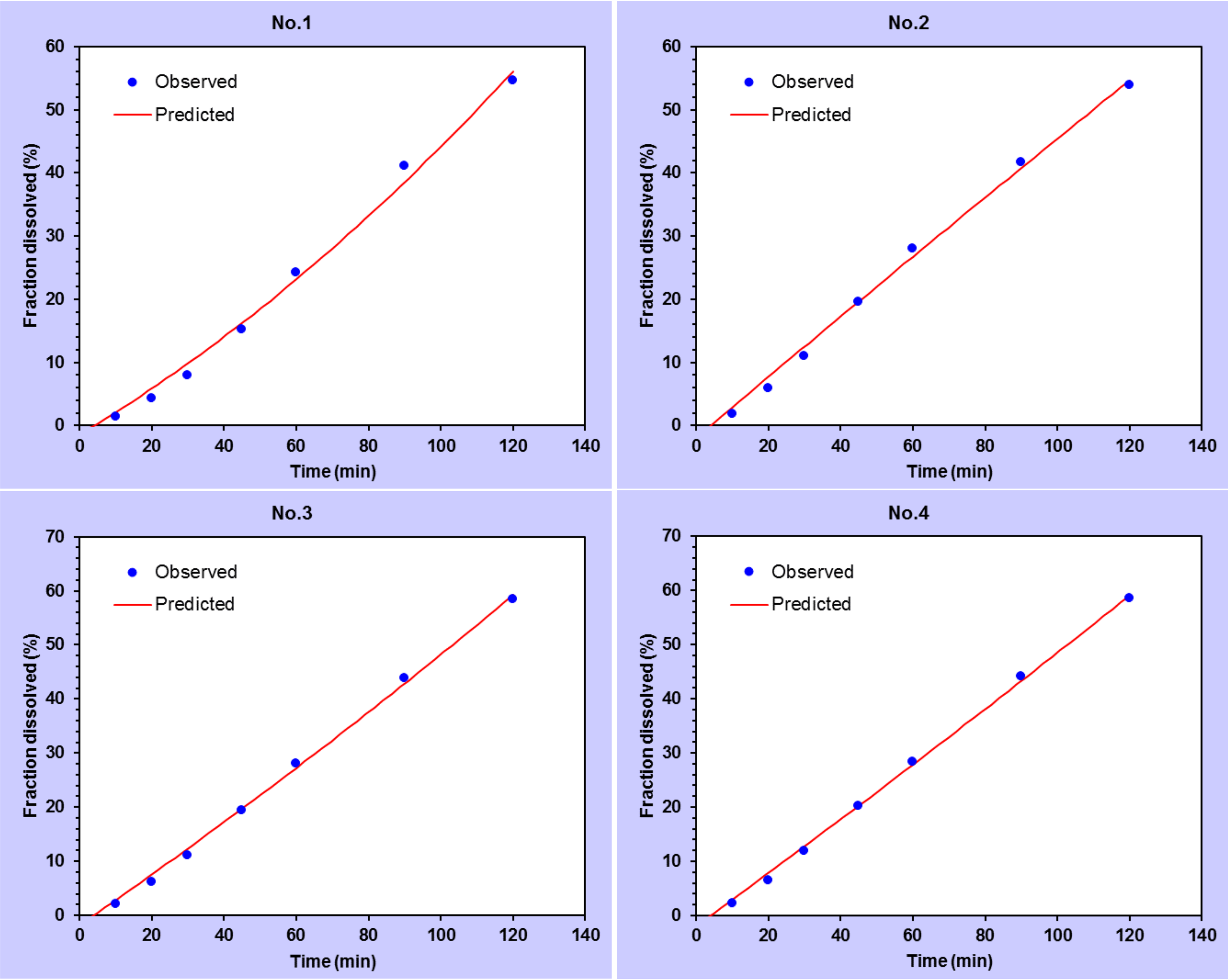

Supplementary materials – Model fitting summary – Polyox™ WSR N-80 (LEO NF Grade) (modelling up to app. 60 % of carvedilol released)

Model: **Weibull_1**

Model equation: $F = 100 \cdot \left[1 - e^{-\frac{(t-T_i)^\beta}{\alpha}}\right]$

Fitted model parameters per tested tablet (N = 4) with statistics – mean, standard deviation (SD), and relative standard deviation expressed in % (RSD%) (output from DDSolver):

Parameter	No.1	No.2	No.3	No.4	Mean	SD	RSD(%)
α	940.369	551.251	466.004	471.260	607.221	225.498	37.136
β	1.398	1.281	1.252	1.269	1.300	0.067	5.124
T_i	5.043	4.000	4.000	4.829	4.468	0.548	12.256

Number of dissolution data points (N), degrees of freedom (df), and selected goodness of fit criteria – Pearson correlation coefficient (R), coefficient of determination (R^2), adjusted coefficient of determination ($R^2_{adjusted}$), and residual sum of squares (RSS) (manual calculation in MS Excel):

Parameter	No.1	No.2	No.3	No.4
N	7	7	7	7
df	4	4	4	4
R	0.999442498	0.999459008	0.999550262	0.999793812
R^2	0.998885307	0.99891831	0.999100727	0.999587666
$R^2_{adjusted}$	0.99832796	0.998377464	0.99865109	0.999381499
RSS	5.281218641	2.915106255	6.929577455	1.386684251

Graphical abstract of model fit presented as mean ± 1 SD of the fraction % of released carvedilol:

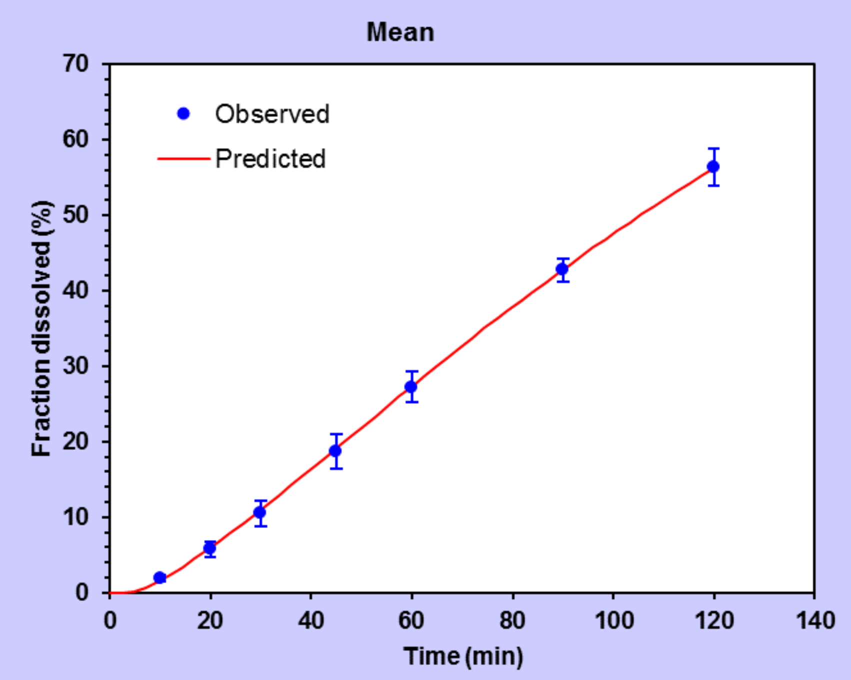

Supplementary materials – Model fitting summary – Polyox™ WSR N-80 (LEO NF Grade) (modelling up to app. 60 % of carvedilol released)

Graphical abstract of model fit presented as the fraction % of released carvedilol per tested tablet:

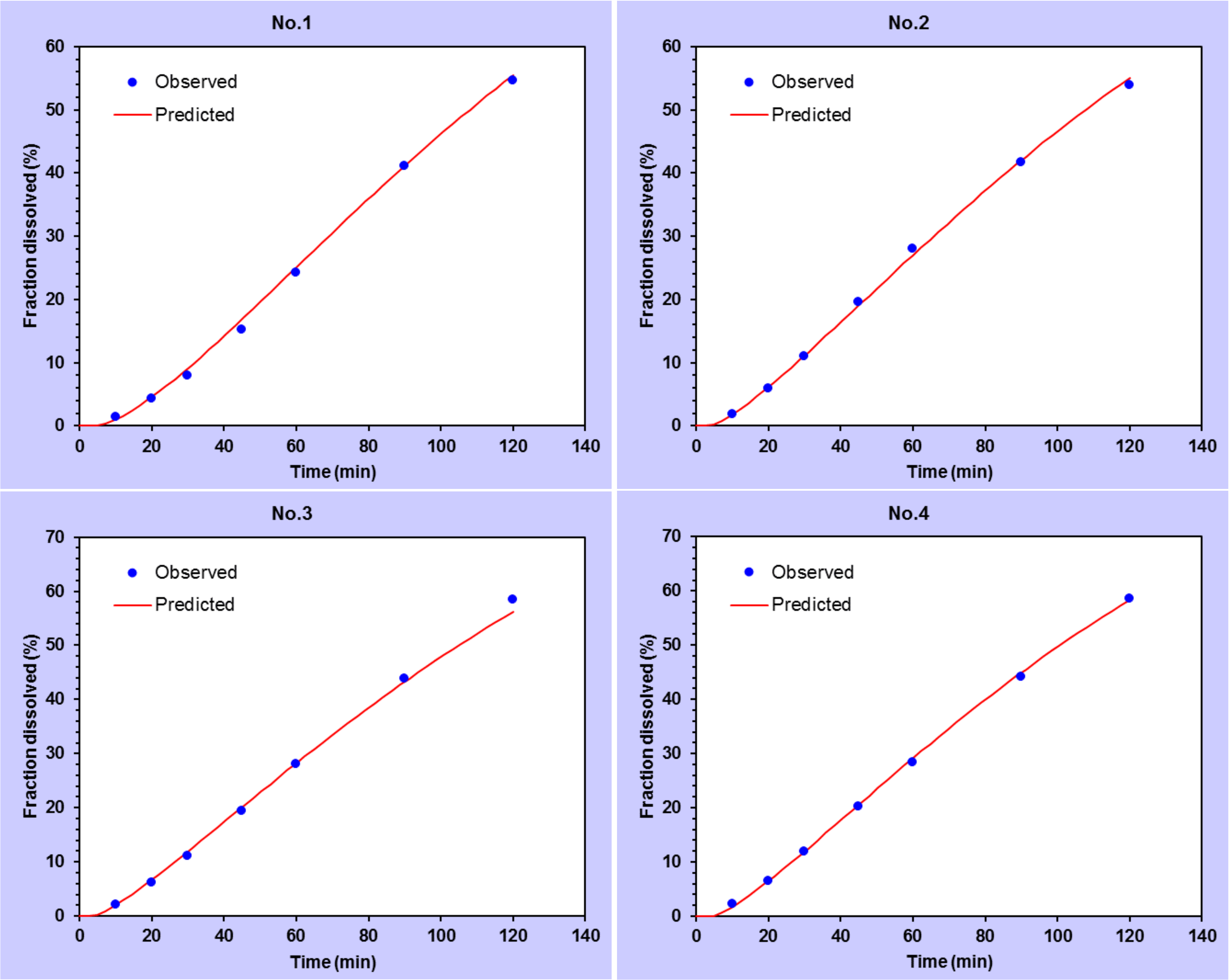

Supplementary materials – Model fitting summary – Polyox™ WSR N-80 (LEO NF Grade) (modelling up to app. 60 % of carvedilol released)

Model: **Weibull_2**

Model equation: $F = 100 \cdot \left(1 - e^{-\frac{t^{\beta}}{\alpha}}\right)$

Fitted model parameters per tested tablet (N = 4) with statistics – mean, standard deviation (SD), and relative standard deviation expressed in % (RSD%) (output from DDSolver):

Parameter	No.1	No.2	No.3	No.4	Mean	SD	RSD(%)
α	3075.570	1563.541	1315.211	1231.246	1796.392	864.376	48.117
β	1.638	1.507	1.479	1.468	1.523	0.078	5.143

Number of dissolution data points (N), degrees of freedom (df), and selected goodness of fit criteria – Pearson correlation coefficient (R), coefficient of determination (R^2), adjusted coefficient of determination ($R^2_{adjusted}$), and residual sum of squares (RSS) (manual calculation in MS Excel):

Parameter	No.1	No.2	No.3	No.4
N	7	7	7	7
df	5	5	5	5
R	0.999256335	0.997305776	0.999830642	0.999697366
R^2	0.998513222	0.994618811	0.999661314	0.999394823
$R^2_{adjusted}$	0.998215867	0.993542573	0.999593576	0.999273787
RSS	4.12315434	25.8352361	1.725679099	4.21792707

Graphical abstract of model fit presented as mean ± 1 SD of the fraction % of released carvedilol:

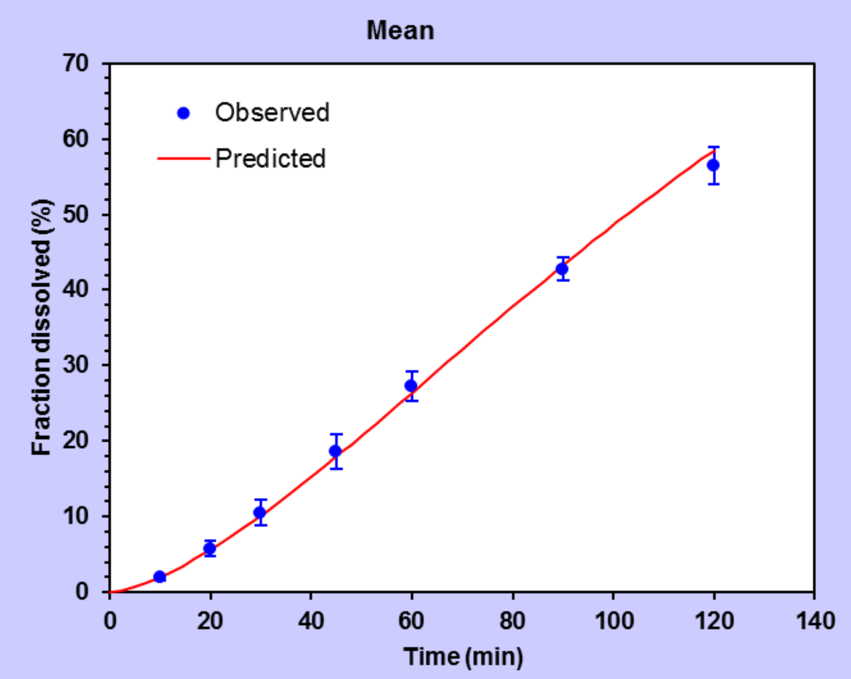

Supplementary materials – Model fitting summary – Polyox™ WSR N-80 (LEO NF Grade) (modelling up to app. 60 % of carvedilol released)

Graphical abstract of model fit presented as the fraction % of released carvedilol per tested tablet:

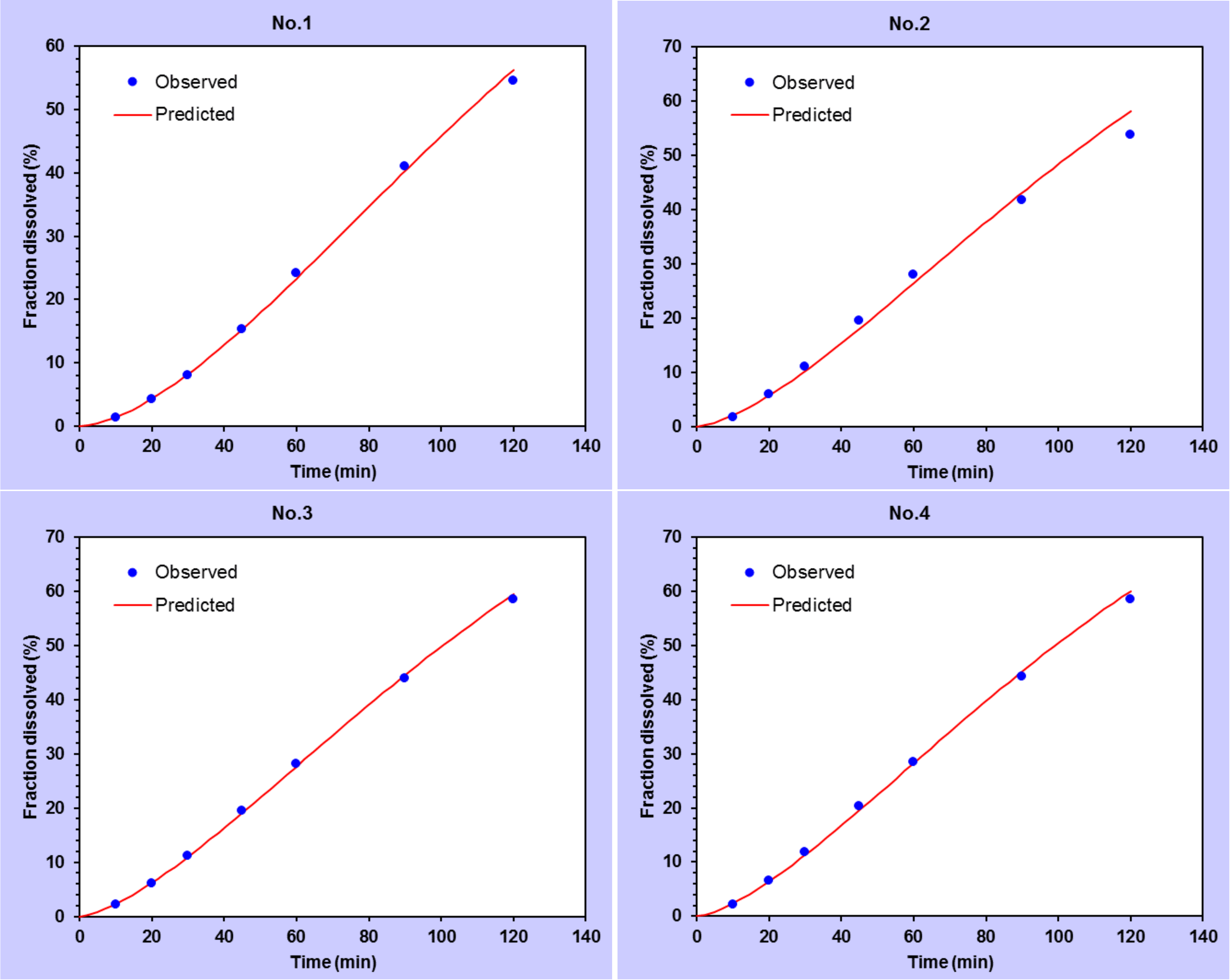

Supplementary materials – Model fitting summary – Polyox™ WSR N-80 (LEO NF Grade) (modelling up to app. 60 % of carvedilol released)

Model: **Weibull_3**

Model equation: $F = F_{max} \cdot \left(1 - e^{-\frac{t^{\beta}}{\alpha}}\right)$

Fitted model parameters per tested tablet (N = 4) with statistics – mean, standard deviation (SD), and relative standard deviation expressed in % (RSD%) (output from DDSolver):

Parameter	No.1	No.2	No.3	No.4	Mean	SD	RSD(%)
α	4097.758	1871.673	1787.654	1673.675	2357.690	1162.879	49.323
β	1.826	1.767	1.658	1.649	1.725	0.086	4.989
F_{max}	65.997	56.595	69.594	69.648	65.458	6.151	9.397

Number of dissolution data points (N), degrees of freedom (df), and selected goodness of fit criteria – Pearson correlation coefficient (R), coefficient of determination (R^2), adjusted coefficient of determination ($R^2_{adjusted}$), and residual sum of squares (RSS) (manual calculation in MS Excel):

Parameter	No.1	No.2	No.3	No.4
N	7	7	7	7
df	4	4	4	4
R	0.999912832	0.997473696	0.999393454	0.999243395
R^2	0.999825671	0.994953775	0.998787277	0.998487362
$R^2_{adjusted}$	0.999738507	0.992430662	0.998180915	0.997731043
RSS	14.36530498	12.30602722	15.60347626	14.05005487

Graphical abstract of model fit presented as mean ± 1 SD of the fraction % of released carvedilol:

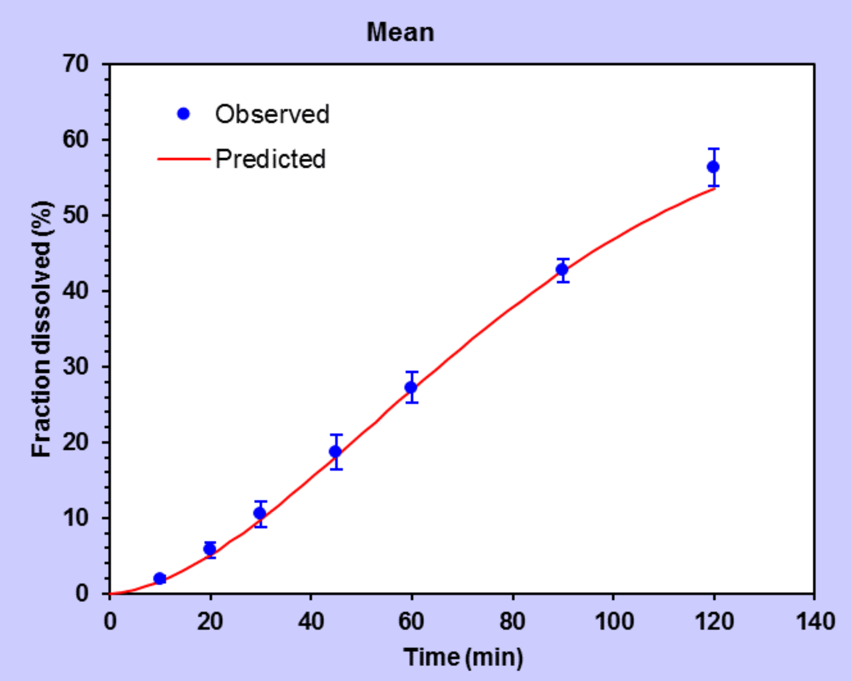

Supplementary materials – Model fitting summary – Polyox™ WSR N-80 (LEO NF Grade) (modelling up to app. 60 % of carvedilol released)

Graphical abstract of model fit presented as the fraction % of released carvedilol per tested tablet:

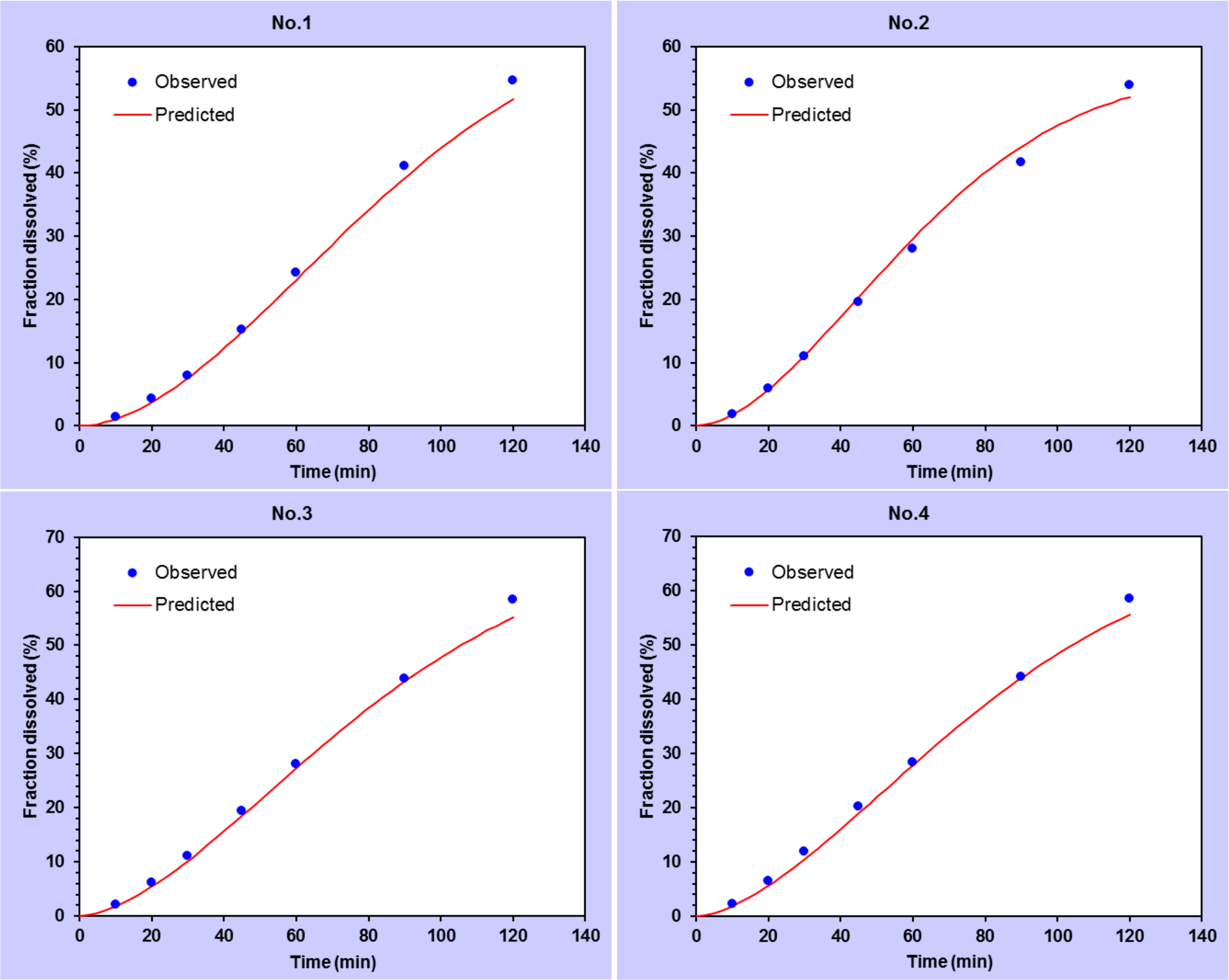

Supplementary materials – Model fitting summary – Polyox™ WSR N-80 (LEO NF Grade) (modelling up to app. 60 % of carvedilol released)

Model: **Weibull_4**

Model equation: $F = F_{max} \cdot \left[1 - e^{-\frac{(t-T_i)^\beta}{\alpha}}\right]$

Fitted model parameters per tested tablet (N = 4) with statistics – mean, standard deviation (SD), and relative standard deviation expressed in % (RSD%) (output from DDSolver):

Parameter	No.1	No.2	No.3	No.4	Mean	SD	RSD(%)
α	1026.997	582.133	469.879	445.757	631.191	270.479	42.852
β	1.573	1.488	1.445	1.439	1.486	0.062	4.154
T_i	4.550	4.660	6.000	6.000	5.303	0.807	15.210
F_{max}	66.116	61.271	61.416	61.464	62.567	2.368	3.784

Number of dissolution data points (N), degrees of freedom (df), and selected goodness of fit criteria – Pearson correlation coefficient (R), coefficient of determination (R^2), adjusted coefficient of determination ($R^2_{adjusted}$), and residual sum of squares (RSS) (manual calculation in MS Excel):

Parameter	No.1	No.2	No.3	No.4
N	7	7	7	7
df	3	3	3	3
R	0.996721291	0.99787687	0.993529876	0.993614039
R^2	0.993453332	0.995758248	0.987101614	0.987268859
$R^2_{adjusted}$	0.986906664	0.991516497	0.974203228	0.974537718
RSS	29.17948071	14.21213863	38.79449364	35.91196909

Graphical abstract of model fit presented as mean ± 1 SD of the fraction % of released carvedilol:

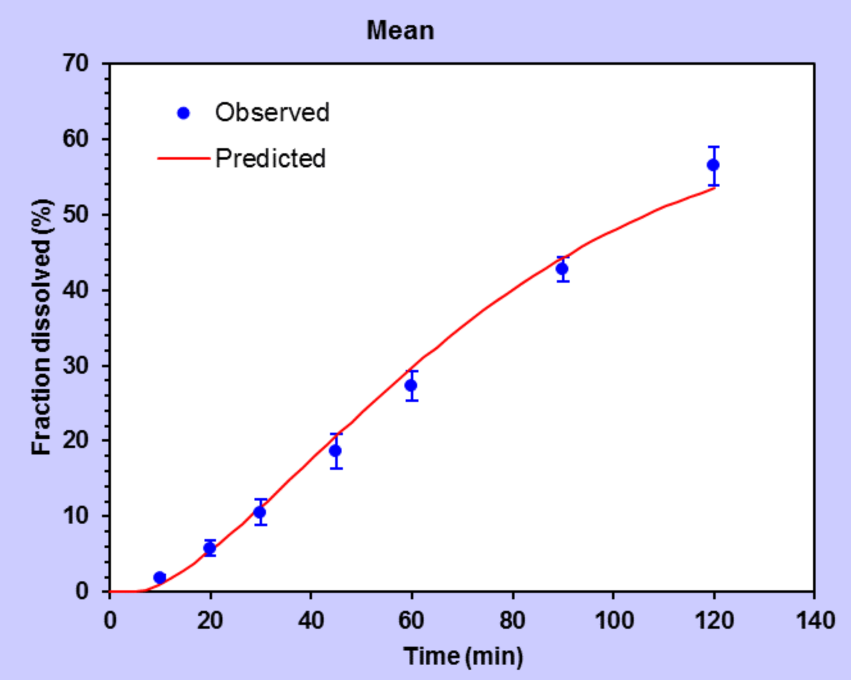

Supplementary materials – Model fitting summary – Polyox^{TM} WSR N-80 (LEO NF Grade) (modelling up to app. 60 % of carvedilol released)

Graphical abstract of model fit presented as the fraction % of released carvedilol per tested tablet:

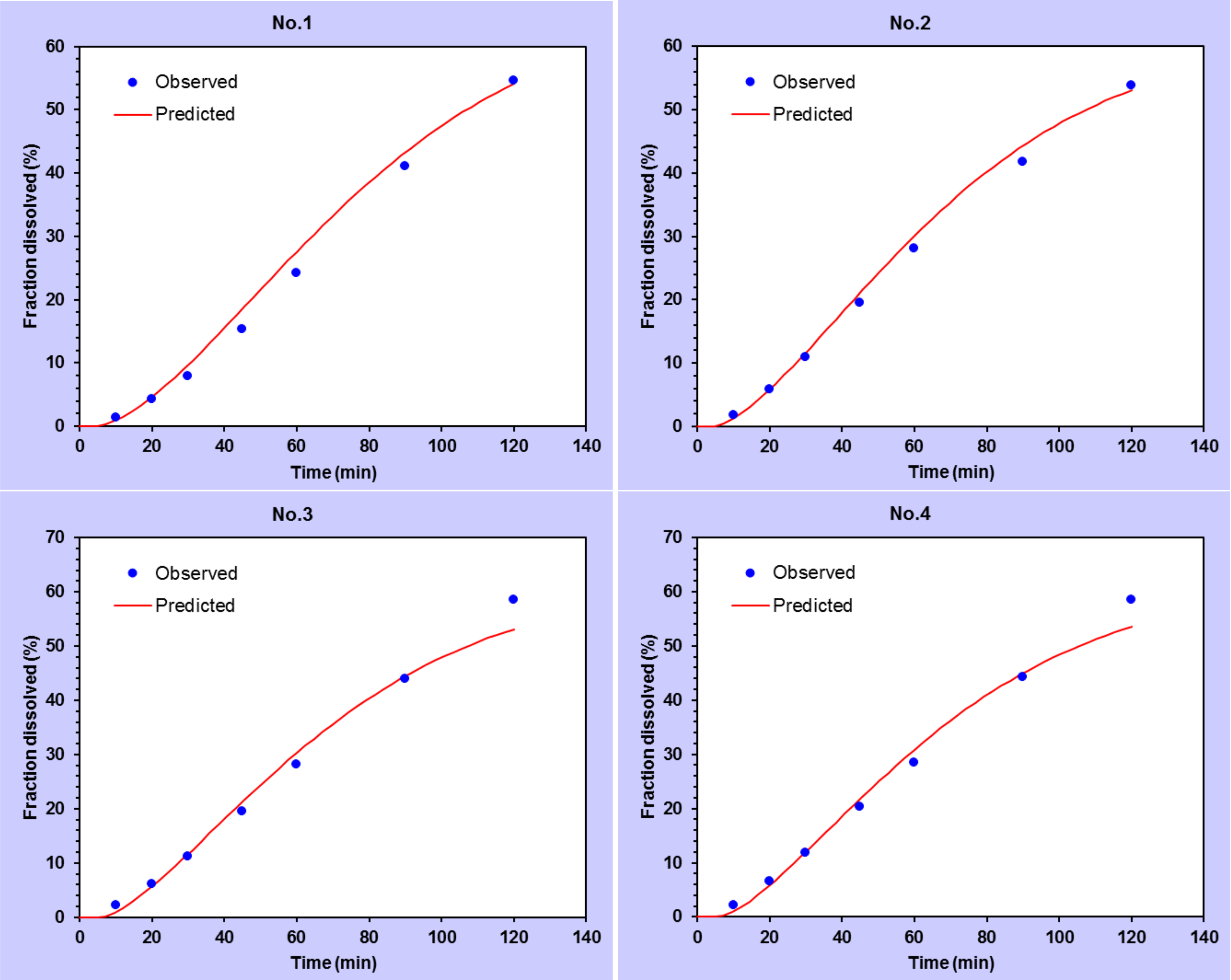

Supplementary materials – Model fitting summary – Polyox™ WSR N-80 (LEO NF Grade) (modelling up to app. 60 % of carvedilol released)

Model: **Logistic_1**

Model equation: $F = 100 \cdot \frac{e^{\alpha+\beta \cdot \log(t)}}{1+e^{\alpha+\beta \cdot \log(t)}}$

Fitted model parameters per tested tablet (N = 4) with statistics – mean, standard deviation (SD), and relative standard deviation expressed in % (RSD%) (output from DDSolver):

Parameter	No.1	No.2	No.3	No.4	Mean	SD	RSD(%)
α	-9.063	-7.348	-7.654	-7.586	-7.913	0.778	-9.834
β	4.444	3.591	3.801	3.778	3.904	0.372	9.530

Number of dissolution data points (N), degrees of freedom (df), and selected goodness of fit criteria – Pearson correlation coefficient (R), coefficient of determination (R^2), adjusted coefficient of determination ($R^2_{adjusted}$), and residual sum of squares (RSS) (manual calculation in MS Excel):

Parameter	No.1	No.2	No.3	No.4
N	7	7	7	7
df	5	5	5	5
R	0.999957357	0.99989021	0.99900717	0.999053151
R^2	0.999914716	0.999780431	0.998015326	0.998107199
$R^2_{adjusted}$	0.999897659	0.999736518	0.997618392	0.997728639
RSS	1.306738659	1.795262947	6.850754034	5.411933175

Graphical abstract of model fit presented as mean ± 1 SD of the fraction % of released carvedilol:

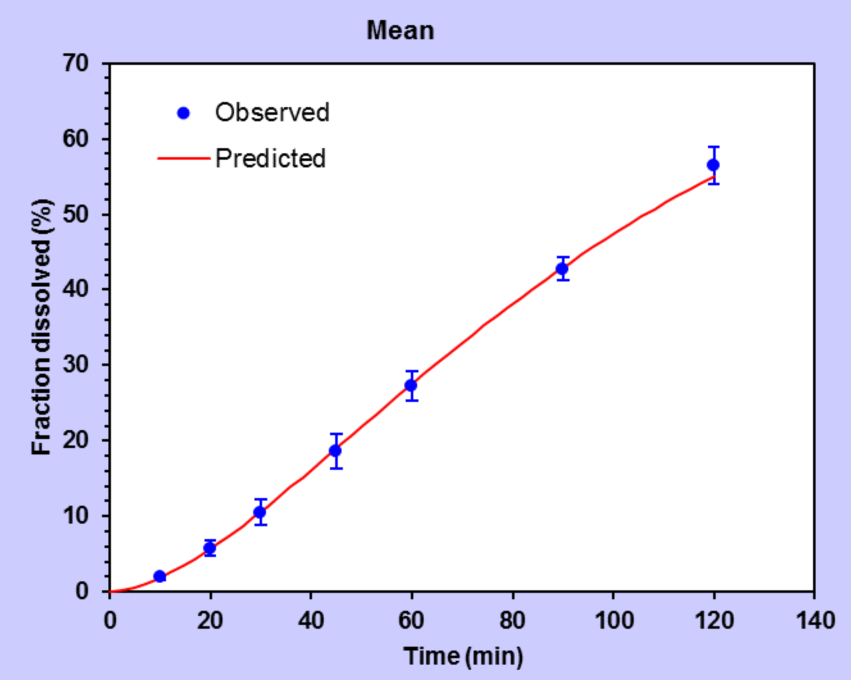

Supplementary materials – Model fitting summary – Polyox™ WSR N-80 (LEO NF Grade) (modelling up to app. 60 % of carvedilol released)

Graphical abstract of model fit presented as the fraction % of released carvedilol per tested tablet:

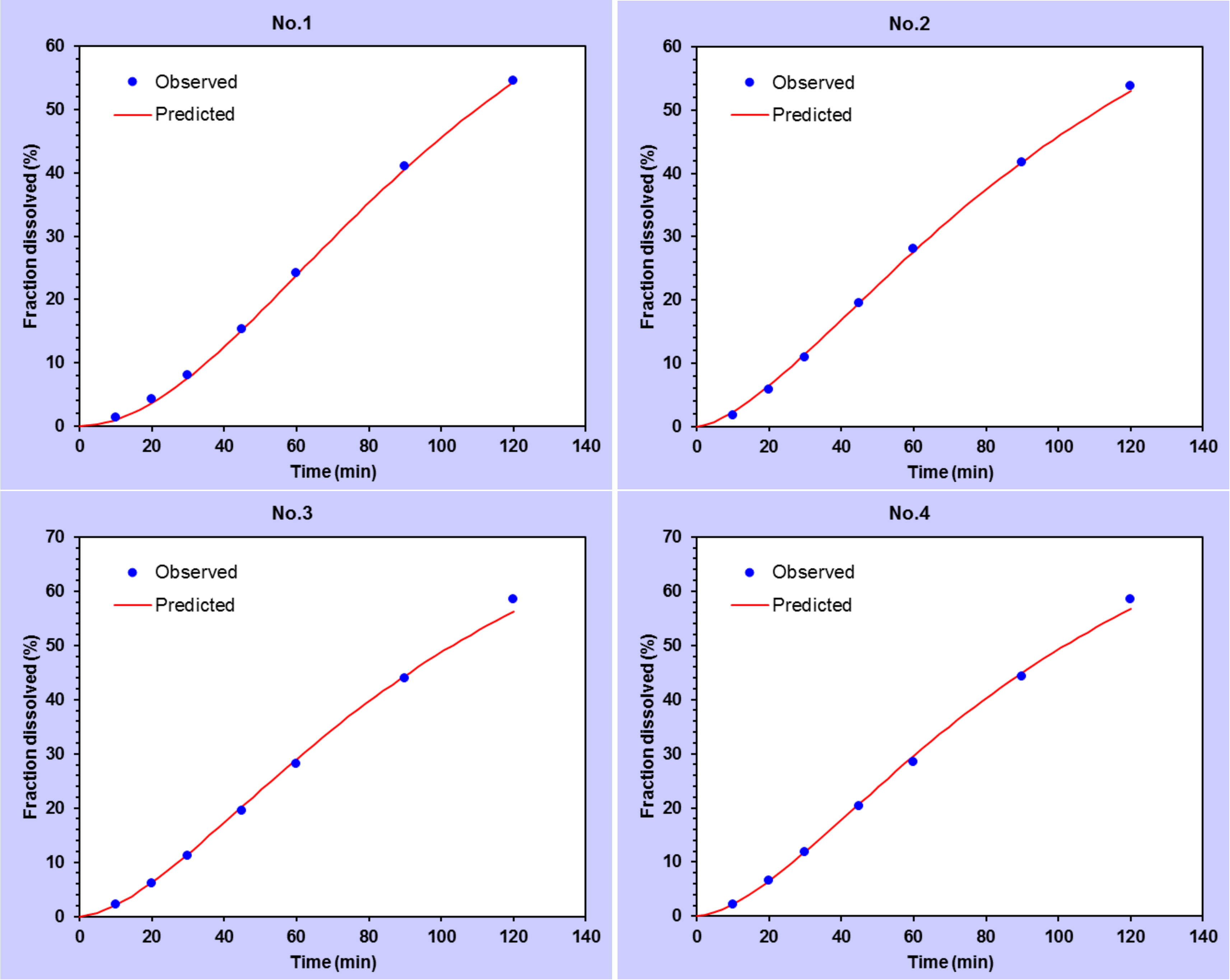

Supplementary materials – Model fitting summary – Polyox™ WSR N-80 (LEO NF Grade) (modelling up to app. 60 % of carvedilol released)

Model: **Logistic_2**

Model equation: $F = F_{max} \cdot \frac{e^{\alpha+\beta\cdot\log(t)}}{1+e^{\alpha+\beta\cdot\log(t)}}$

Fitted model parameters per tested tablet (N = 4) with statistics – mean, standard deviation (SD), and relative standard deviation expressed in % (RSD%) (output from DDSolver):

Parameter	No.1	No.2	No.3	No.4	Mean	SD	RSD(%)
α	-11.618	-10.689	-9.092	-9.025	-10.106	1.268	-12.545
β	6.024	5.865	5.306	5.285	5.620	0.381	6.772
F_{max}	68.448	67.804	61.416	61.464	64.783	3.869	5.973

Number of dissolution data points (N), degrees of freedom (df), and selected goodness of fit criteria – Pearson correlation coefficient (R), coefficient of determination (R^2), adjusted coefficient of determination ($R^2_{adjusted}$), and residual sum of squares (RSS) (manual calculation in MS Excel):

Parameter	No.1	No.2	No.3	No.4
N	7	7	7	7
df	4	4	4	4
R	0.998962322	0.997042679	0.977614494	0.977838816
R^2	0.997925721	0.994094103	0.9557301	0.95616875
$R^2_{adjusted}$	0.996888582	0.991141155	0.933595149	0.934253125
RSS	115.6078405	47.07448996	146.1883047	143.0989094

Graphical abstract of model fit presented as mean ± 1 SD of the fraction % of released carvedilol:

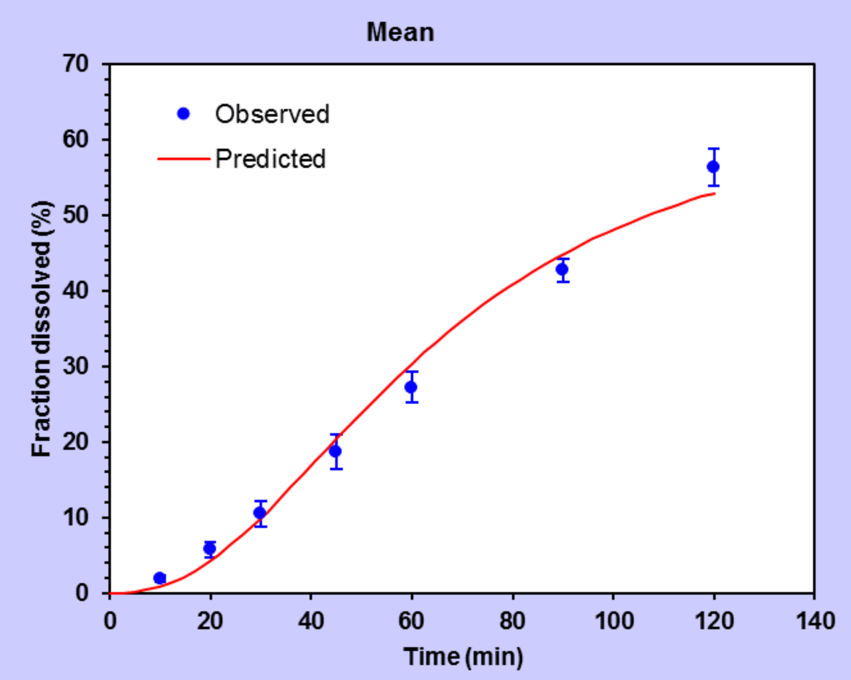

Graphical abstract of model fit presented as the fraction % of released carvedilol per tested tablet:

Supplementary materials – Model fitting summary – Polyox^{TM} WSR N-80 (LEO NF Grade) (modelling up to app. 60 % of carvedilol released)

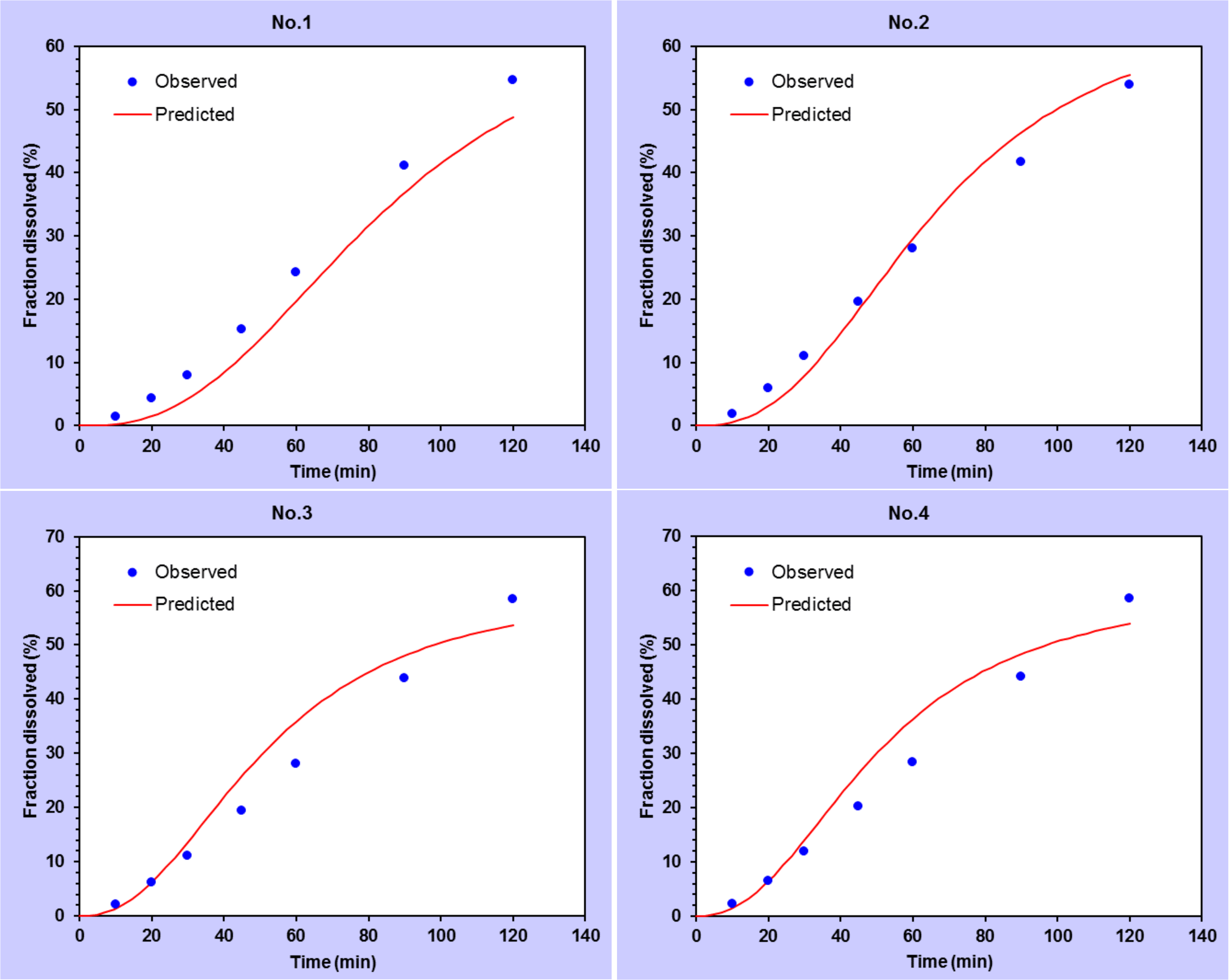

Supplementary materials – Model fitting summary – Polyox™ WSR N-80 (LEO NF Grade) (modelling up to app. 60 % of carvedilol released)

Model: **Logistic_3**

Model equation: $F = F_{max} \cdot \frac{1}{1+e^{-k\cdot(t-\gamma)}}$

Fitted model parameters per tested tablet (N = 4) with statistics – mean, standard deviation (SD), and relative standard deviation expressed in % (RSD%) (output from DDSolver):

Parameter	No.1	No.2	No.3	No.4	Mean	SD	RSD(%)
k	0.056	0.053	0.052	0.052	0.053	0.002	3.718
γ	67.433	63.378	64.553	63.985	64.837	1.796	2.769
F_{max}	57.380	56.595	61.416	61.464	59.214	2.591	4.375

Number of dissolution data points (N), degrees of freedom (df), and selected goodness of fit criteria – Pearson correlation coefficient (R), coefficient of determination (R^2), adjusted coefficient of determination ($R^2_{adjusted}$), and residual sum of squares (RSS) (manual calculation in MS Excel):

Parameter	No.1	No.2	No.3	No.4
N	7	7	7	7
df	4	4	4	4
R	0.995919211	0.992154253	0.993803465	0.993154988
R^2	0.991855075	0.984370061	0.987645327	0.98635683
$R^2_{adjusted}$	0.987782613	0.976555092	0.981467991	0.979535246
RSS	25.40908622	44.19275322	39.95971476	44.02706614

Graphical abstract of model fit presented as mean ± 1 SD of the fraction % of released carvedilol:

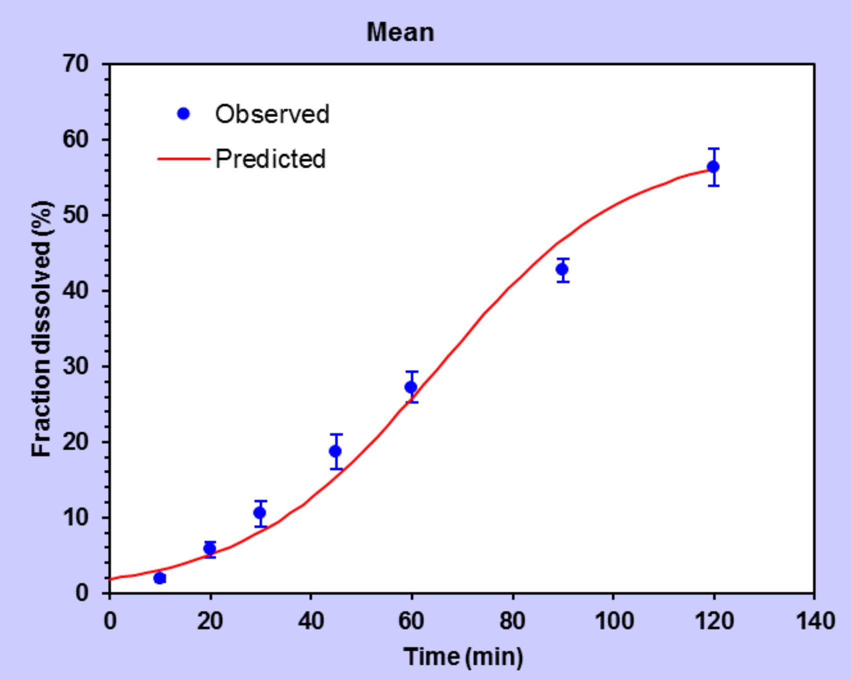

Supplementary materials – Model fitting summary – Polyox™ WSR N-80 (LEO NF Grade) (modelling up to app. 60 % of carvedilol released)

Graphical abstract of model fit presented as the fraction % of released carvedilol per tested tablet:

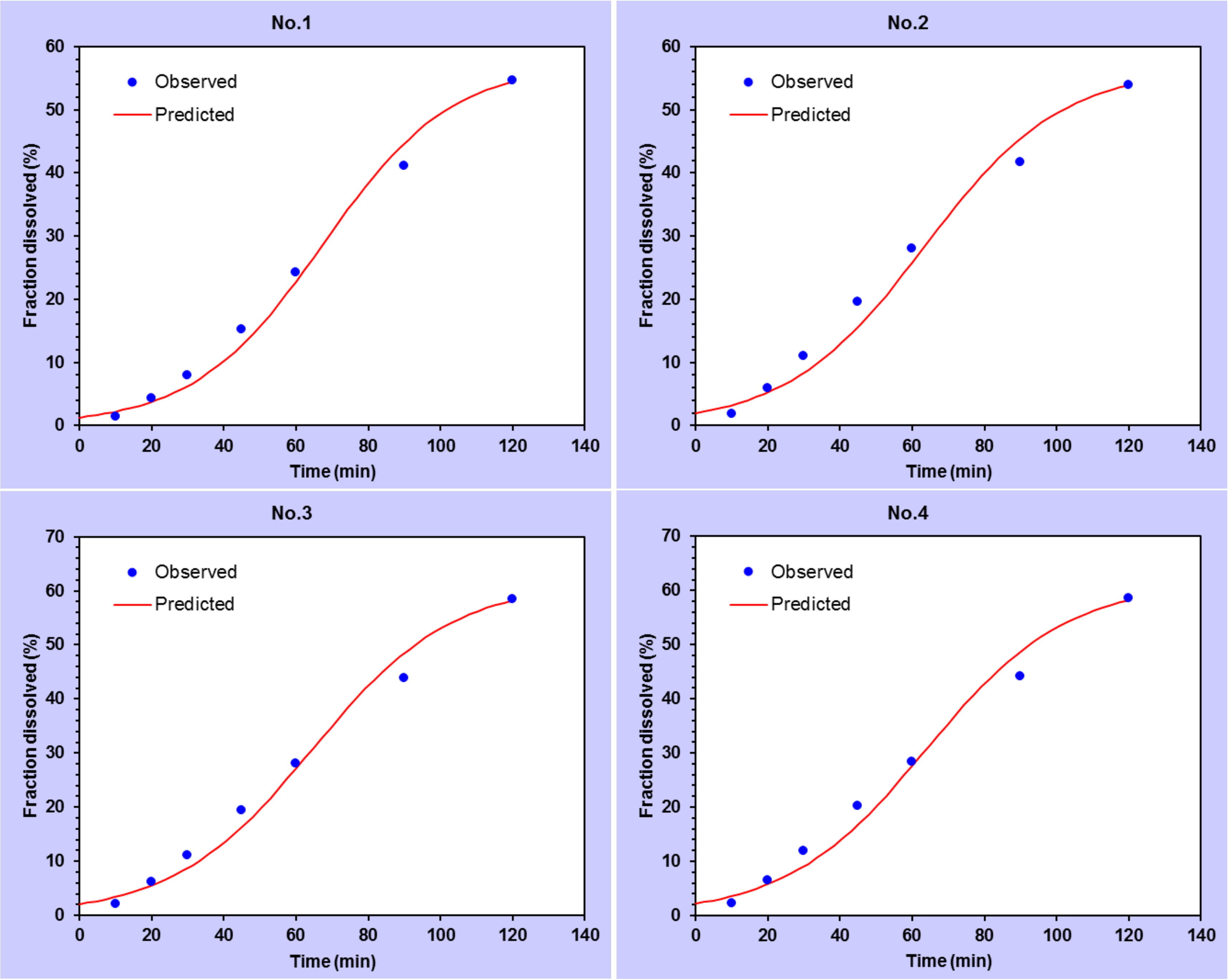

Supplementary materials – Model fitting summary – Polyox™ WSR N-80 (LEO NF Grade) (modelling up to app. 60 % of carvedilol released)

Model: **Gompertz_1**

Model equation: $F = 100 \cdot e^{-\alpha \cdot e^{-\beta \cdot \log(t)}}$

Fitted model parameters per tested tablet (N = 4) with statistics – mean, standard deviation (SD), and relative standard deviation expressed in % (RSD%) (output from DDSolver):

Parameter	No.1	No.2	No.3	No.4	Mean	SD	RSD(%)
α	31.470	25.716	27.199	26.607	27.748	2.555	9.209
β	1.802	1.725	1.785	1.779	1.773	0.033	1.879

Number of dissolution data points (N), degrees of freedom (df), and selected goodness of fit criteria – Pearson correlation coefficient (R), coefficient of determination (R^2), adjusted coefficient of determination ($R^2_{adjusted}$), and residual sum of squares (RSS) (manual calculation in MS Excel):

Parameter	No.1	No.2	No.3	No.4
N	7	7	7	7
df	5	5	5	5
R	0.984737793	0.992393031	0.984599805	0.985513252
R^2	0.969708521	0.984843928	0.969436776	0.97123637
$R^2_{adjusted}$	0.963650226	0.981812714	0.963324131	0.965483644
RSS	100.4540383	44.25542365	97.70230971	89.27237625

Graphical abstract of model fit presented as mean ± 1 SD of the fraction % of released carvedilol:

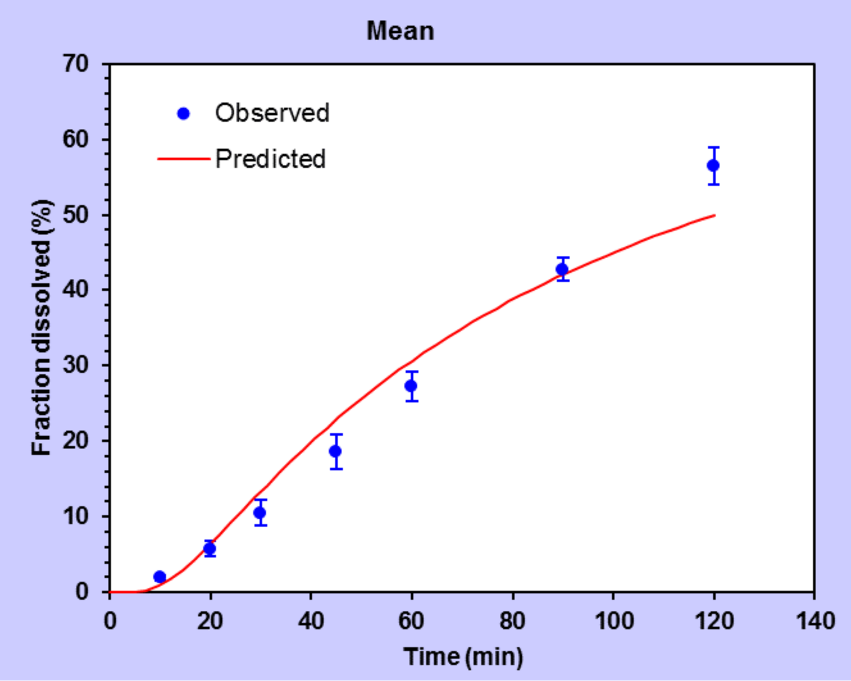

Supplementary materials – Model fitting summary – Polyox™ WSR N-80 (LEO NF Grade) (modelling up to app. 60 % of carvedilol released)

Graphical abstract of model fit presented as the fraction % of released carvedilol per tested tablet:

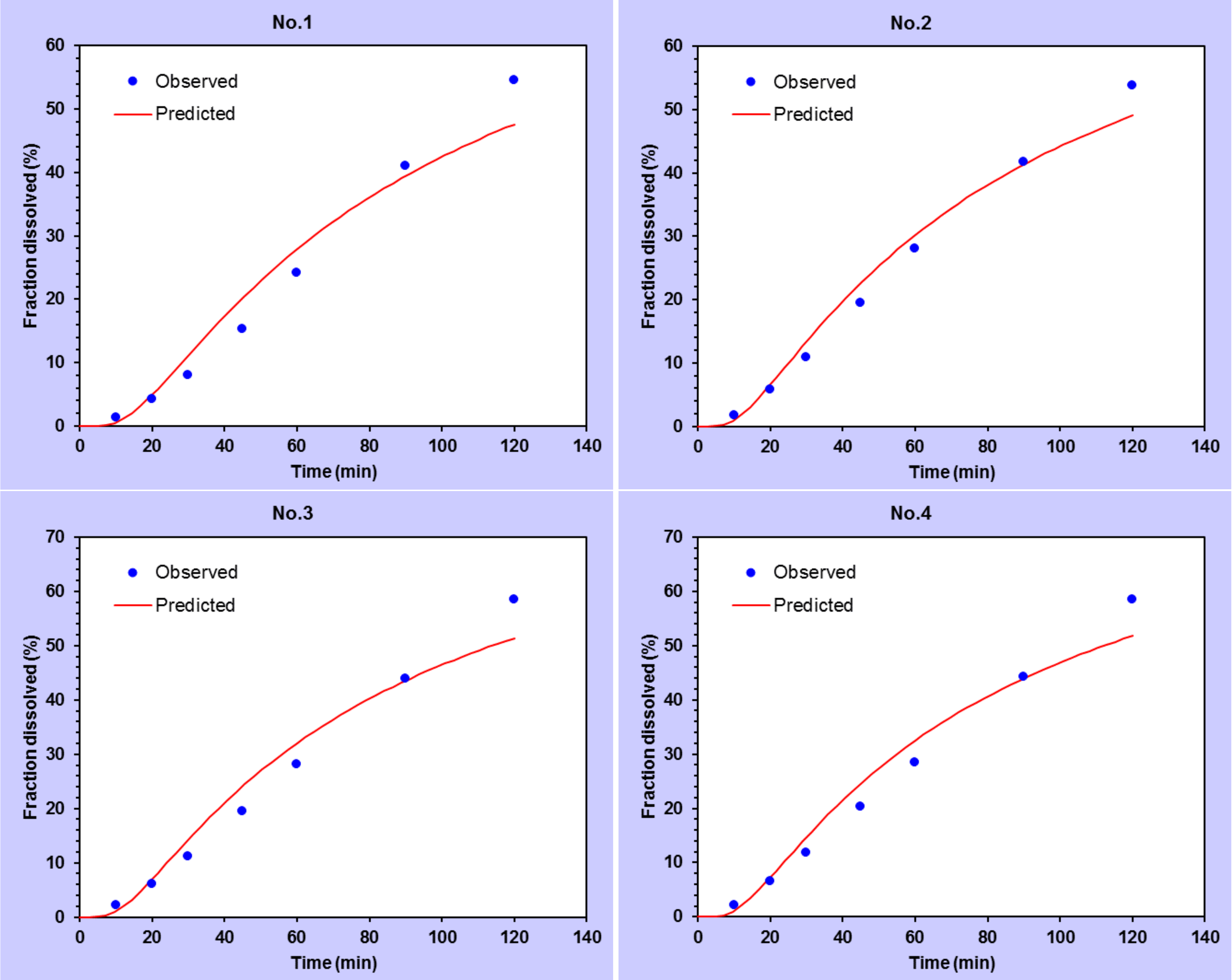

Supplementary materials – Model fitting summary – Polyox™ WSR N-80 (LEO NF Grade) (modelling up to app. 60 % of carvedilol released)

Model: **Gompertz_2**

Model equation: $F = F_{max} \cdot e^{-\alpha \cdot e^{-\beta \cdot \log(t)}}$

Fitted model parameters per tested tablet (N = 4) with statistics – mean, standard deviation (SD), and relative standard deviation expressed in % (RSD%) (output from DDSolver):

Parameter	No.1	No.2	No.3	No.4	Mean	SD	RSD(%)
α	294.634	243.059	225.314	219.877	245.721	34.078	13.869
β	3.254	3.218	3.145	3.141	3.189	0.056	1.752
F_{max}	65.150	64.259	68.536	68.590	66.634	2.257	3.387

Number of dissolution data points (N), degrees of freedom (df), and selected goodness of fit criteria – Pearson correlation coefficient (R), coefficient of determination (R^2), adjusted coefficient of determination ($R^2_{adjusted}$), and residual sum of squares (RSS) (manual calculation in MS Excel):

Parameter	No.1	No.2	No.3	No.4
N	7	7	7	7
df	4	4	4	4
R	0.987121585	0.991167417	0.986856405	0.986736243
R^2	0.974409023	0.982412848	0.973885563	0.973648414
$R^2_{adjusted}$	0.961613534	0.973619271	0.960828345	0.960472621
RSS	96.75624091	73.5918383	123.4914282	124.1964294

Graphical abstract of model fit presented as mean ± 1 SD of the fraction % of released carvedilol:

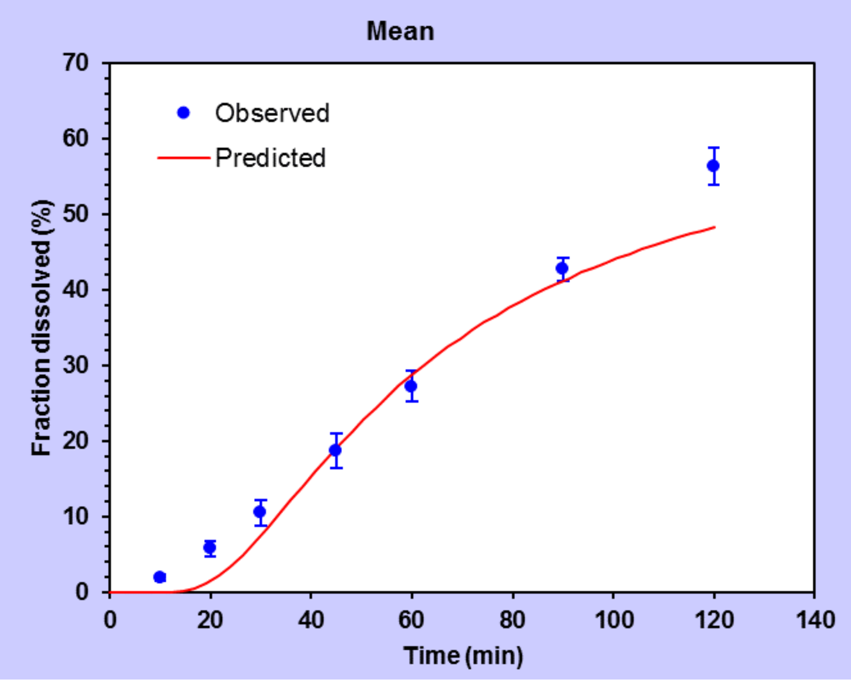

Supplementary materials – Model fitting summary – Polyox^{TM} WSR N-80 (LEO NF Grade) (modelling up to app. 60 % of carvedilol released)

Graphical abstract of model fit presented as the fraction % of released carvedilol per tested tablet:

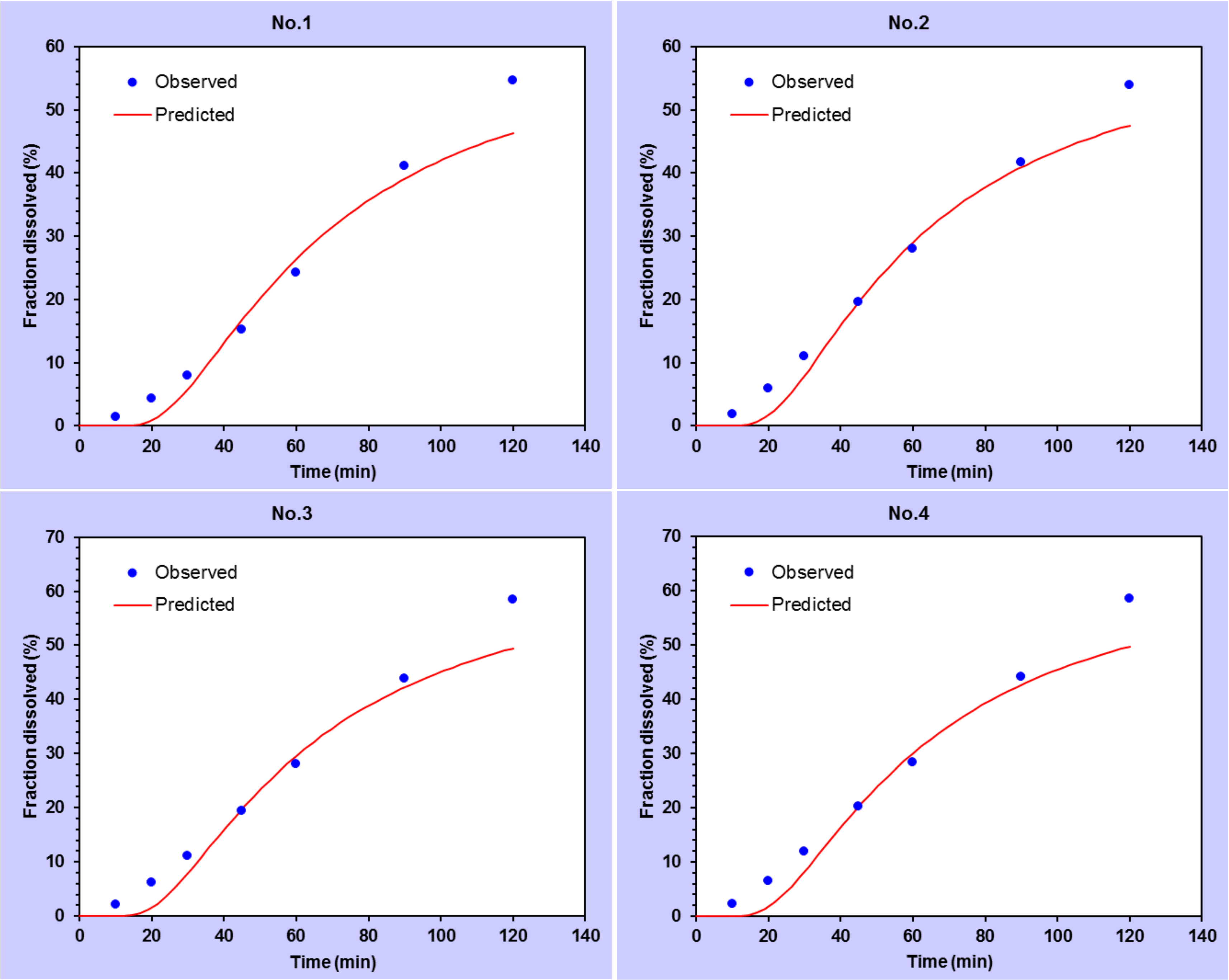

Supplementary materials – Model fitting summary – Polyox[TM] WSR N-80 (LEO NF Grade) (modelling up to app. 60 % of carvedilol released)

Model: **Gompertz_3**

Model equation: $F = F_{max} \cdot e^{-e^{-k\cdot(t-\gamma)}}$

Fitted model parameters per tested tablet (N = 4) with statistics – mean, standard deviation (SD), and relative standard deviation expressed in % (RSD%) (output from DDSolver):

Parameter	No.1	No.2	No.3	No.4	Mean	SD	RSD(%)
k	0.037	0.036	0.036	0.035	0.036	0.001	1.914
γ	49.532	45.803	46.910	46.378	47.156	1.647	3.493
F_{max}	57.380	56.595	61.416	61.464	59.214	2.591	4.375

Number of dissolution data points (N), degrees of freedom (df), and selected goodness of fit criteria – Pearson correlation coefficient (R), coefficient of determination (R^2), adjusted coefficient of determination ($R^2_{adjusted}$), and residual sum of squares (RSS) (manual calculation in MS Excel):

Parameter	No.1	No.2	No.3	No.4
N	7	7	7	7
df	4	4	4	4
R	0.991773673	0.994646057	0.991551298	0.991438628
R^2	0.983615019	0.989320779	0.983173977	0.982950554
$R^2_{adjusted}$	0.975422528	0.983981169	0.974760966	0.974425831
RSS	56.00338289	33.89333924	62.00804561	61.80797757

Graphical abstract of model fit presented as mean ± 1 SD of the fraction % of released carvedilol:

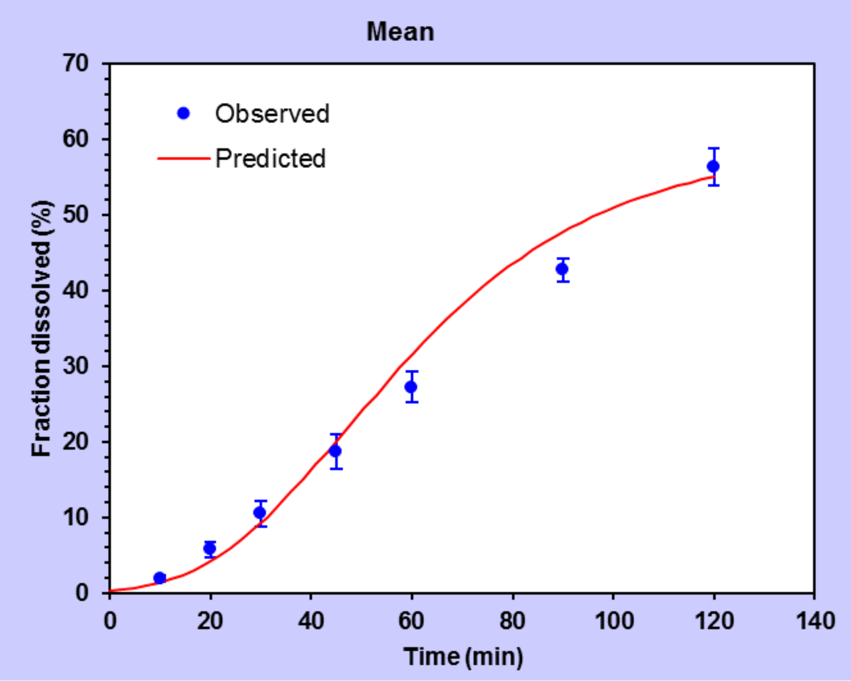

Supplementary materials – Model fitting summary – Polyox™ WSR N-80 (LEO NF Grade) (modelling up to app. 60 % of carvedilol released)

Graphical abstract of model fit presented as the fraction % of released carvedilol per tested tablet:

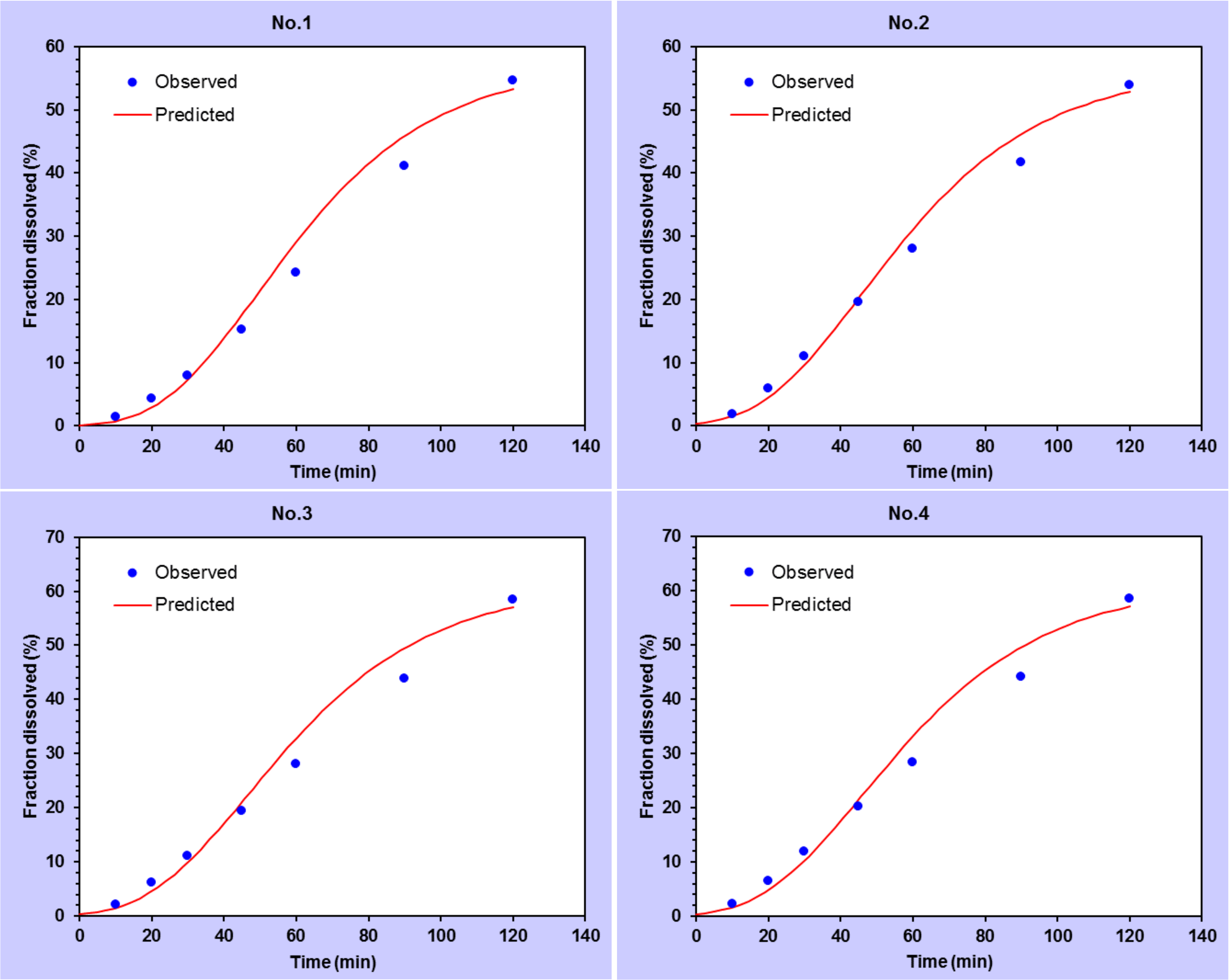

Supplementary materials – Model fitting summary – Polyox™ WSR N-80 (LEO NF Grade) (modelling up to app. 60 % of carvedilol released)

Model: **Gompertz_4**

Model equation: $F = F_{max} \cdot e^{-\beta \cdot e^{-k \cdot t}}$

Fitted model parameters per tested tablet (N = 4) with statistics – mean, standard deviation (SD), and relative standard deviation expressed in % (RSD%) (output from DDSolver):

Parameter	No.1	No.2	No.3	No.4	Mean	SD	RSD(%)
k	0.037	0.036	0.036	0.035	0.036	0.001	1.914
β	6.251	5.202	5.317	5.182	5.488	0.512	9.336
F_{max}	57.380	56.595	61.416	61.464	59.214	2.591	4.375

Number of dissolution data points (N), degrees of freedom (df), and selected goodness of fit criteria – Pearson correlation coefficient (R), coefficient of determination (R^2), adjusted coefficient of determination ($R^2_{adjusted}$), and residual sum of squares (RSS) (manual calculation in MS Excel):

Parameter	No.1	No.2	No.3	No.4
N	7	7	7	7
df	4	4	4	4
R	0.991773673	0.994646057	0.991551298	0.991438628
R^2	0.983615019	0.989320779	0.983173977	0.982950554
$R^2_{adjusted}$	0.975422528	0.983981169	0.974760966	0.974425831
RSS	56.00338289	33.89333924	62.00804561	61.80797757

Graphical abstract of model fit presented as mean ± 1 SD of the fraction % of released carvedilol:

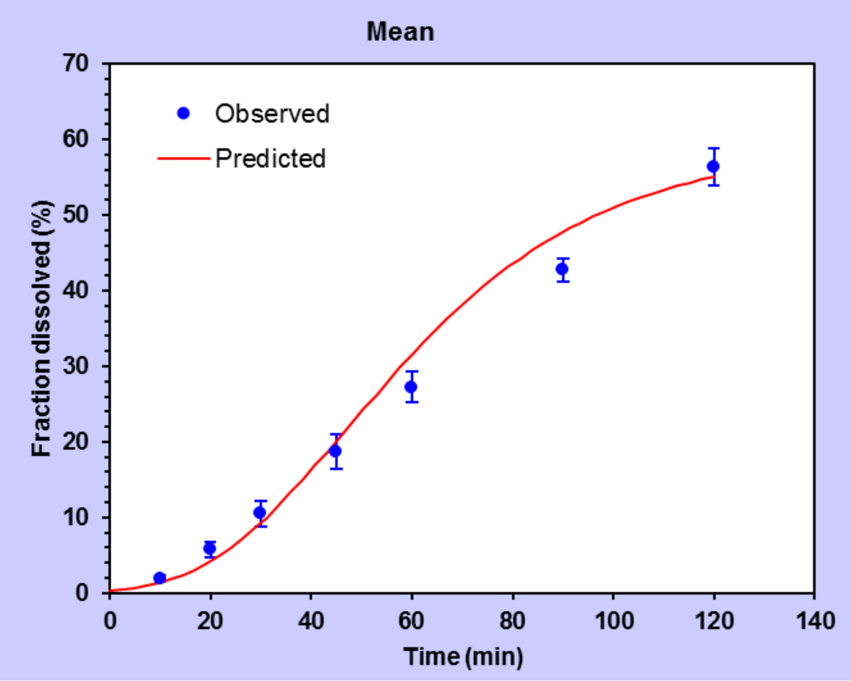

Supplementary materials – Model fitting summary – Polyox™ WSR N-80 (LEO NF Grade) (modelling up to app. 60 % of carvedilol released)

Graphical abstract of model fit presented as the fraction % of released carvedilol per tested tablet:

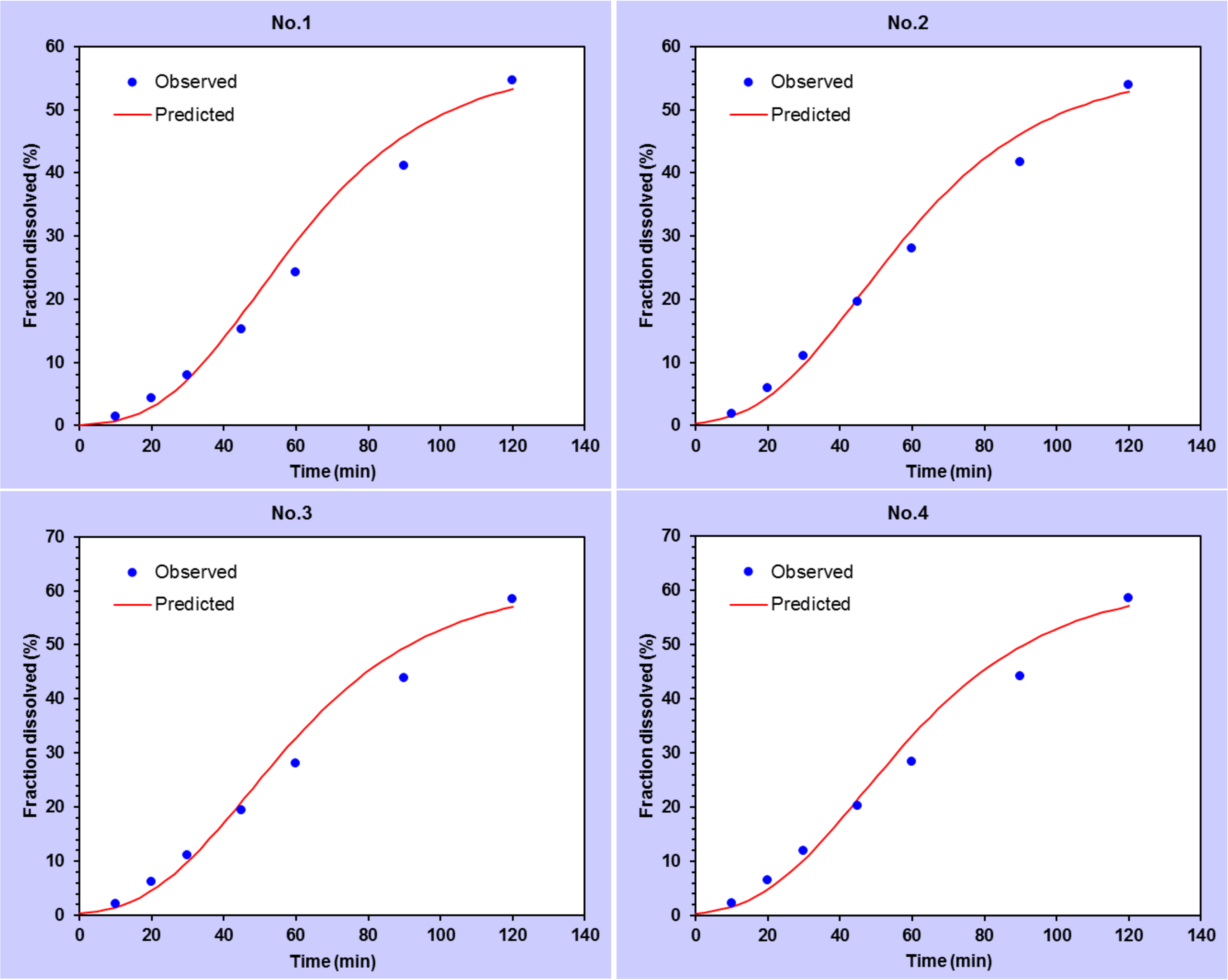

Supplementary materials – Model fitting summary – Polyox™ WSR N-80 (LEO NF Grade) (modelling up to app. 60 % of carvedilol released)

Model: **Probit_1**

Model equation: $F = 100 \cdot \phi[\alpha + \beta \cdot \log(t)]$

Fitted model parameters per tested tablet (N = 4) with statistics – mean, standard deviation (SD), and relative standard deviation expressed in % (RSD%) (output from DDSolver):

Parameter	No.1	No.2	No.3	No.4	Mean	SD	RSD(%)
α	-4.494	-4.192	-4.487	-4.452	-4.406	0.144	-3.267
β	2.162	2.038	2.249	2.238	2.172	0.098	4.491

Number of dissolution data points (N), degrees of freedom (df), and selected goodness of fit criteria – Pearson correlation coefficient (R), coefficient of determination (R^2), adjusted coefficient of determination ($R^2_{adjusted}$), and residual sum of squares (RSS) (manual calculation in MS Excel):

Parameter	No.1	No.2	No.3	No.4
N	7	7	7	7
df	5	5	5	5
R	0.995885914	0.999111175	0.996568928	0.99682387
R^2	0.991788753	0.99822314	0.993149628	0.993657827
$R^2_{adjusted}$	0.990146504	0.997867768	0.991779554	0.992389393
RSS	36.33108312	6.401935261	25.57765633	25.76416381

Graphical abstract of model fit presented as mean ± 1 SD of the fraction % of released carvedilol:

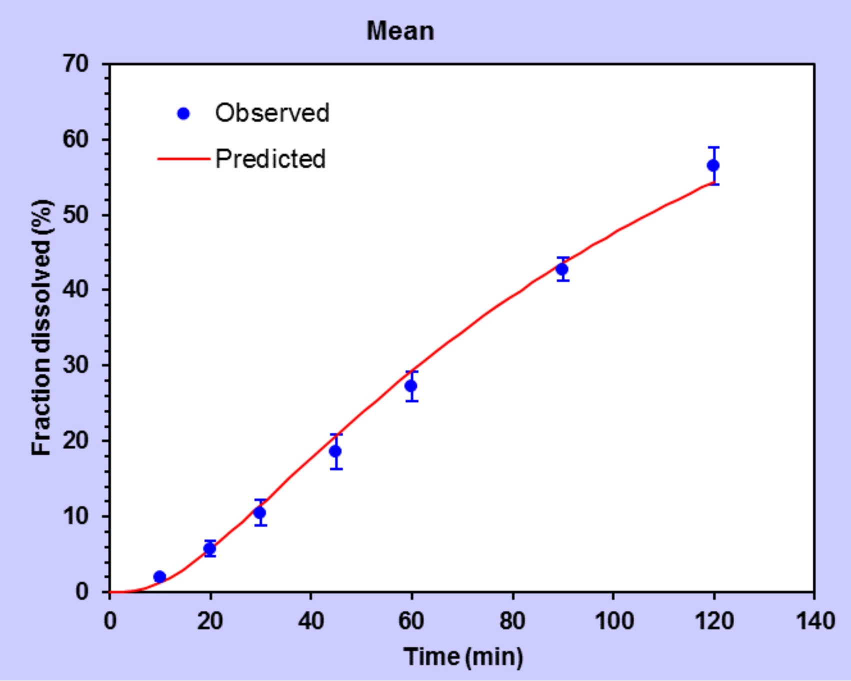

Supplementary materials – Model fitting summary – Polyox^{TM} WSR N-80 (LEO NF Grade) (modelling up to app. 60 % of carvedilol released)

Graphical abstract of model fit presented as the fraction % of released carvedilol per tested tablet:

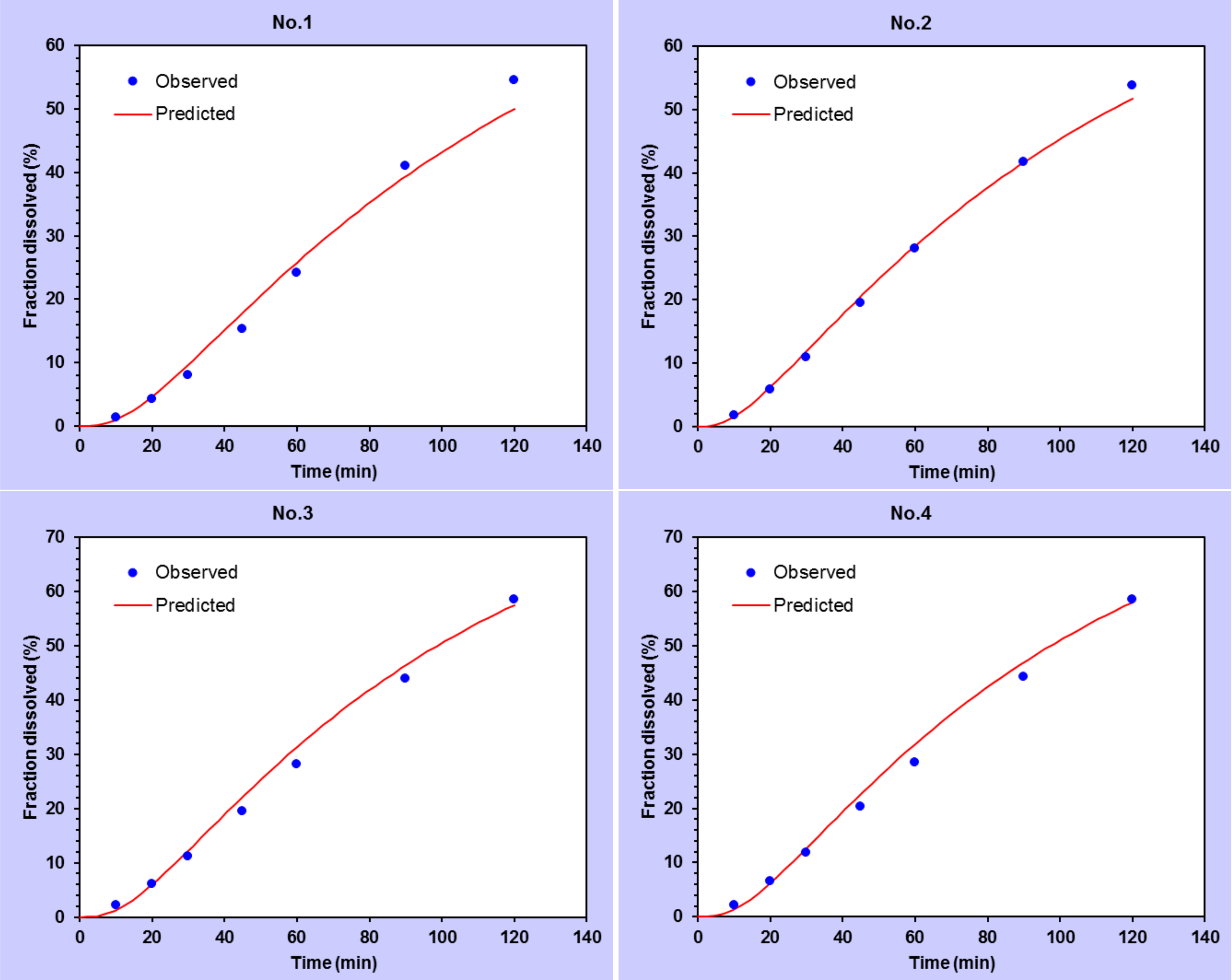

Supplementary materials – Model fitting summary – Polyox™ WSR N-80 (LEO NF Grade) (modelling up to app. 60 % of carvedilol released)

Model: **Probit_2**

Model equation: $F = F_{max} \cdot \phi[\alpha + \beta \cdot \log(t)]$

Fitted model parameters per tested tablet (N = 4) with statistics – mean, standard deviation (SD), and relative standard deviation expressed in % (RSD%) (output from DDSolver):

Parameter	No.1	No.2	No.3	No.4	Mean	SD	RSD(%)
α	-6.240	-5.205	-5.638	-5.085	-5.542	0.522	-9.422
β	3.310	3.056	3.249	2.971	3.147	0.159	5.067
F_{max}	62.074	56.595	64.929	61.464	61.265	3.461	5.648

Number of dissolution data points (N), degrees of freedom (df), and selected goodness of fit criteria – Pearson correlation coefficient (R), coefficient of determination (R^2), adjusted coefficient of determination ($R^2_{adjusted}$), and residual sum of squares (RSS) (manual calculation in MS Excel):

Parameter	No.1	No.2	No.3	No.4
N	7	7	7	7
df	4	4	4	4
R	0.997222042	0.983474872	0.981891509	0.977415461
R^2	0.9944518	0.967222824	0.964110936	0.955340983
$R^2_{adjusted}$	0.9916777	0.950834236	0.946166403	0.933011474
RSS	116.5227045	90.2157208	142.3869876	137.9324546

Graphical abstract of model fit presented as mean ± 1 SD of the fraction % of released carvedilol:

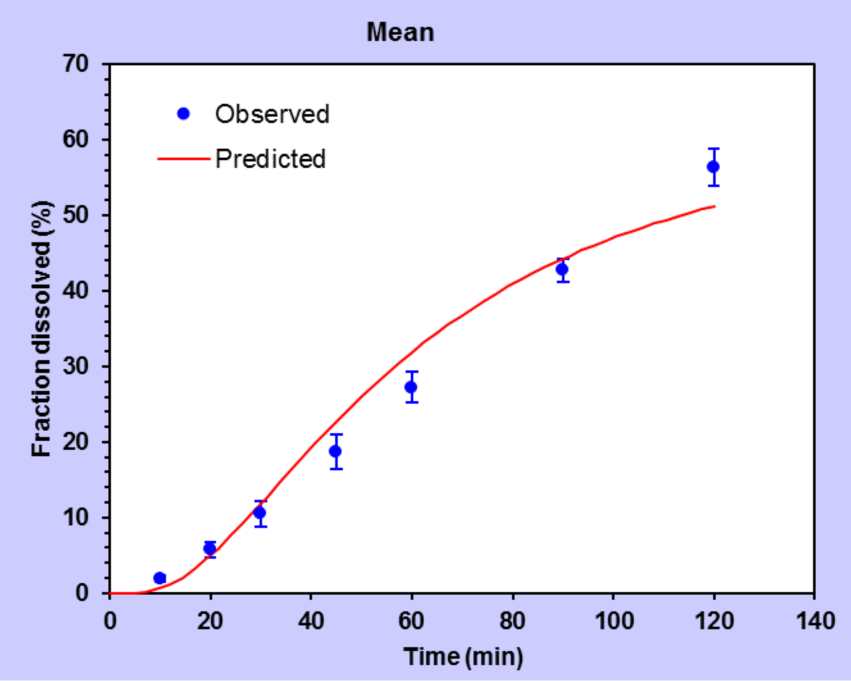

Supplementary materials – Model fitting summary – Polyox™ WSR N-80 (LEO NF Grade) (modelling up to app. 60 % of carvedilol released)

Graphical abstract of model fit presented as the fraction % of released carvedilol per tested tablet:

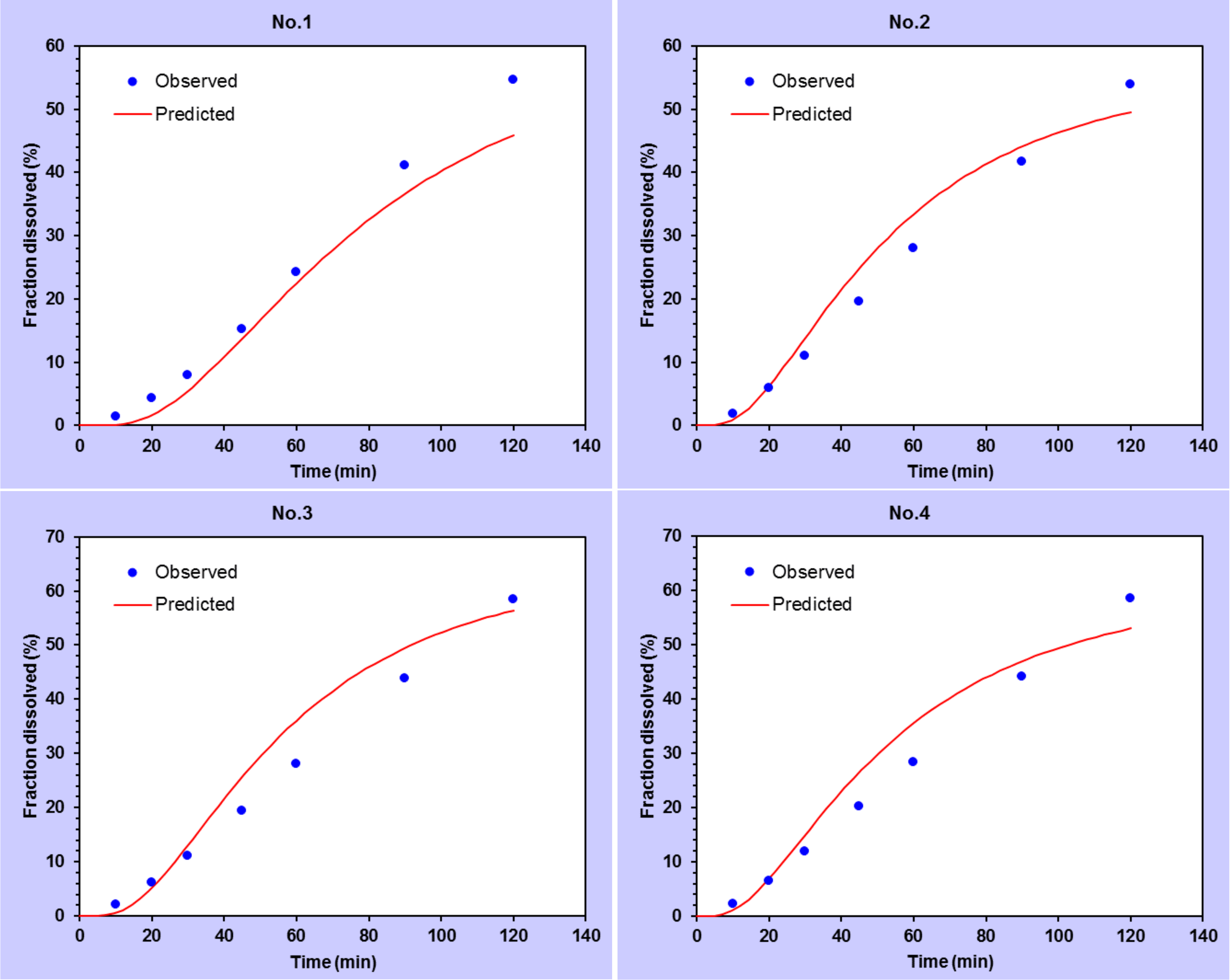

Supplement: Supplementary file 1 [file pharmaceutics-16-00498-s001.zip › Supplementary materials_Model fitting summary_PolyoxTM WSR N-80.pdf]
