# Supplementary material for: Comparative Fitting of Mathematical Models to Carvedilol Release Profiles Obtained from Hypromellose Matrix Tablets
Source: Pharmaceutics. 2024 Apr 4;16(4):498. doi: 10.3390/pharmaceutics16040498 (PMC11053526; doi:10.3390/pharmaceutics16040498)

Supplementary materials – Model fitting summary – Starch 1500® sample with larger particle size (modelling the entire relevant dissolution data)

Model: **Zero–order**

Model equation: $F = k_0 \cdot t$

Fitted model parameters per tested tablet (N = 4) with statistics – mean, standard deviation (SD), and relative standard deviation expressed in % (RSD%) (output from DDSolver):

Parameter	No.1	No.2	No.3	No.4	Mean	SD	RSD(%)
k_0	0.060	0.056	0.062	0.063	0.060	0.003	5.219

Number of dissolution data points (N), degrees of freedom (df), and selected goodness of fit criteria – Pearson correlation coefficient (R), coefficient of determination (R^2), adjusted coefficient of determination ($R^2_{adjusted}$), and residual sum of squares (RSS) (manual calculation in MS Excel):

Parameter	No.1	No.2	No.3	No.4
N	33	33	33	33
df	32	32	32	32
R	0.995072104	0.9954781	0.997057195	0.993382008
R^2	0.990168492	0.990976648	0.99412305	0.986807813
$R^2_{adjusted}$	0.990168492	0.990976648	0.99412305	0.986807813
RSS	1897.358166	1497.402125	1366.838828	1897.643031

Graphical abstract of model fit presented as mean ± 1 SD of the fraction % of released carvedilol:

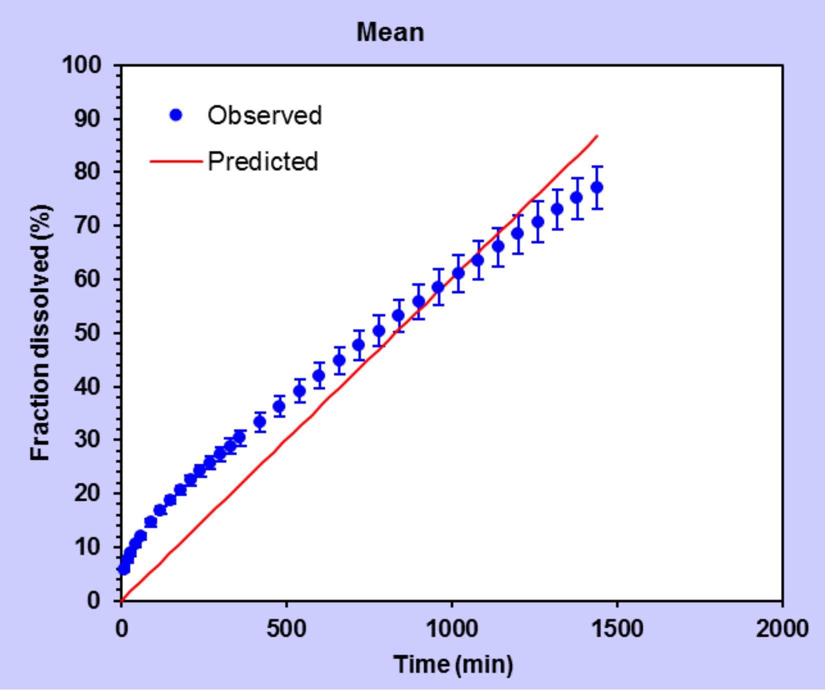

Supplementary materials – Model fitting summary – Starch 1500® sample with larger particle size (modelling the entire relevant dissolution data)

Graphical abstract of model fit presented as the fraction % of released carvedilol per tested tablet:

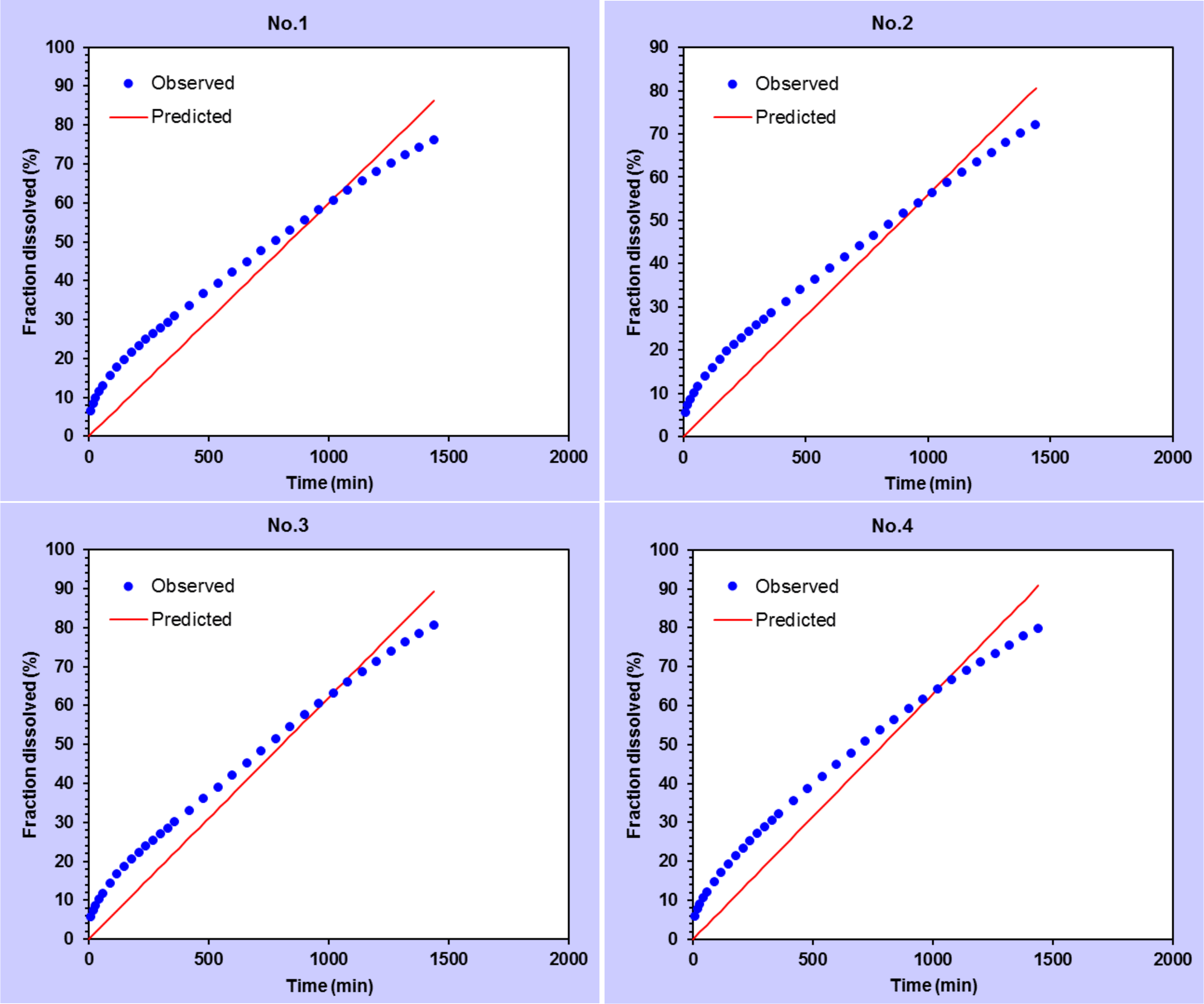

Supplementary materials – Model fitting summary – Starch 1500® sample with larger particle size (modelling the entire relevant dissolution data)

Model: **Zero–order with T_{lag}**

Model equation: $F = k_0 \cdot (t - T_{lag})$

Fitted model parameters per tested tablet (N = 4) with statistics – mean, standard deviation (SD), and relative standard deviation expressed in % (RSD%) (output from DDSolver):

Parameter	No.1	No.2	No.3	No.4	Mean	SD	RSD(%)
k_0	0.047	0.045	0.051	0.051	0.048	0.003	6.360
T_{lag}	-250.249	-234.375	-195.663	-227.778	-227.016	22.931	-10.101

Number of dissolution data points (N), degrees of freedom (df), and selected goodness of fit criteria – Pearson correlation coefficient (R), coefficient of determination (R^2), adjusted coefficient of determination ($R^2_{adjusted}$), and residual sum of squares (RSS) (manual calculation in MS Excel):

Parameter	No.1	No.2	No.3	No.4
N	33	33	33	33
df	31	31	31	31
R	0.995072104	0.9954781	0.997057195	0.993382008
R^2	0.990168492	0.990976648	0.99412305	0.986807813
$R^2_{adjusted}$	0.989851347	0.990685572	0.993933471	0.986382259
RSS	150.252675	123.5279092	105.6372599	232.6998992

Graphical abstract of model fit presented as mean ± 1 SD of the fraction % of released carvedilol:

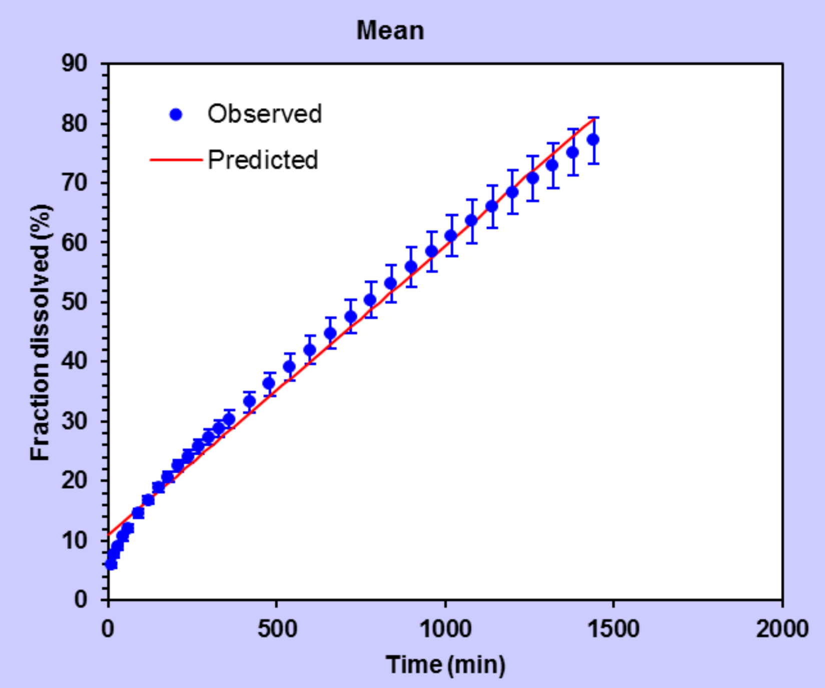

Supplementary materials – Model fitting summary – Starch 1500® sample with larger particle size (modelling the entire relevant dissolution data)

Graphical abstract of model fit presented as the fraction % of released carvedilol per tested tablet:

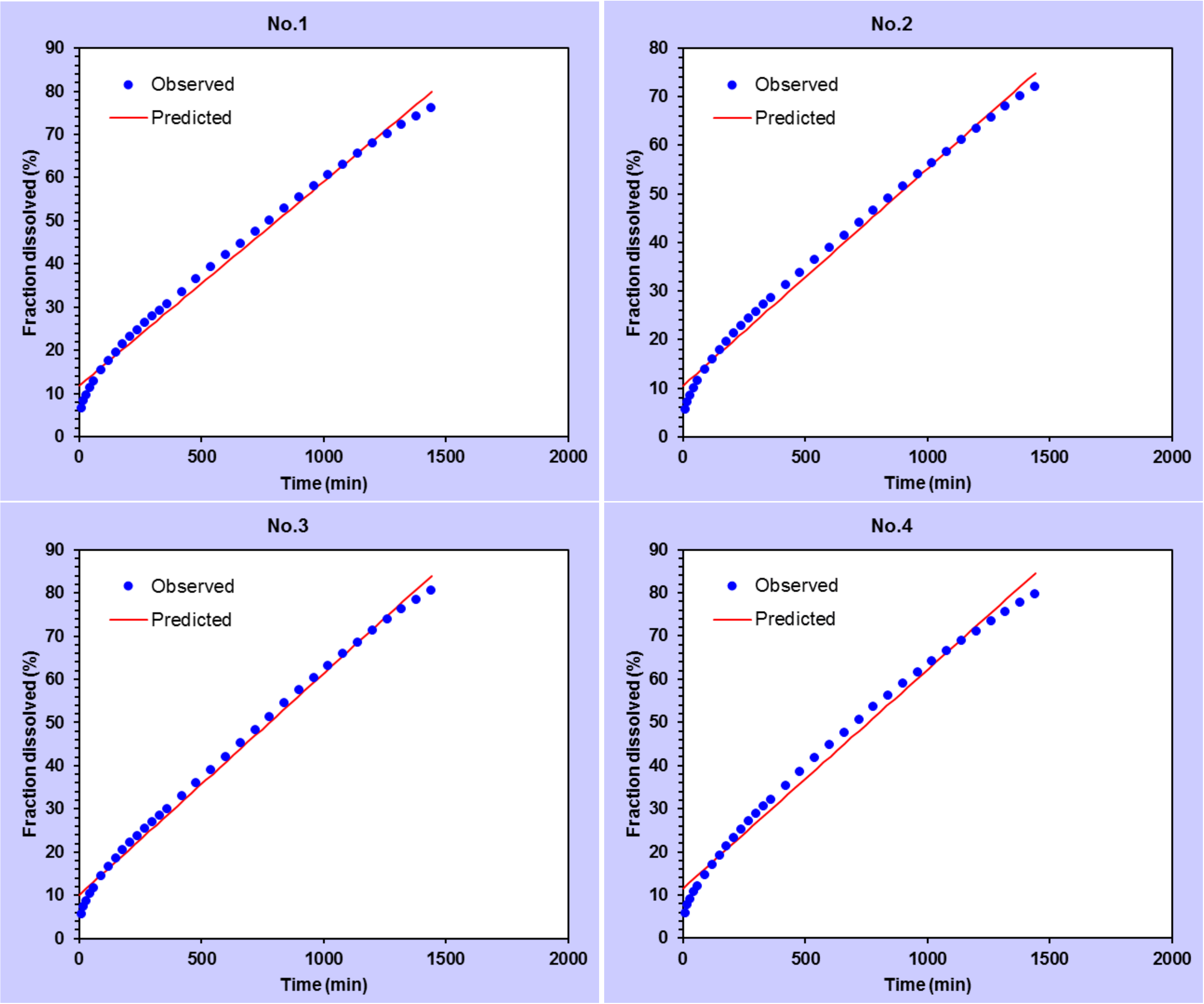

Supplementary materials – Model fitting summary – Starch 1500® sample with larger particle size (modelling the entire relevant dissolution data)

Model: **Zero–order with F_0**

Model equation: $F = F_0 + k_0 \cdot t$

Fitted model parameters per tested tablet (N = 4) with statistics – mean, standard deviation (SD), and relative standard deviation expressed in % (RSD%) (output from DDSolver):

Parameter	No.1	No.2	No.3	No.4	Mean	SD	RSD(%)
k_0	0.047	0.045	0.051	0.051	0.048	0.003	6.360
F_0	11.821	10.483	10.044	11.540	10.972	0.846	7.706

Number of dissolution data points (N), degrees of freedom (df), and selected goodness of fit criteria – Pearson correlation coefficient (R), coefficient of determination (R^2), adjusted coefficient of determination ($R^2_{adjusted}$), and residual sum of squares (RSS) (manual calculation in MS Excel):

Parameter	No.1	No.2	No.3	No.4
N	33	33	33	33
df	31	31	31	31
R	0.995072104	0.9954781	0.997057195	0.993382008
R^2	0.990168492	0.990976648	0.99412305	0.986807813
$R^2_{adjusted}$	0.989851347	0.990685572	0.993933471	0.986382259
RSS	150.252675	123.5279092	105.6372599	232.6998992

Graphical abstract of model fit presented as mean ± 1 SD of the fraction % of released carvedilol:

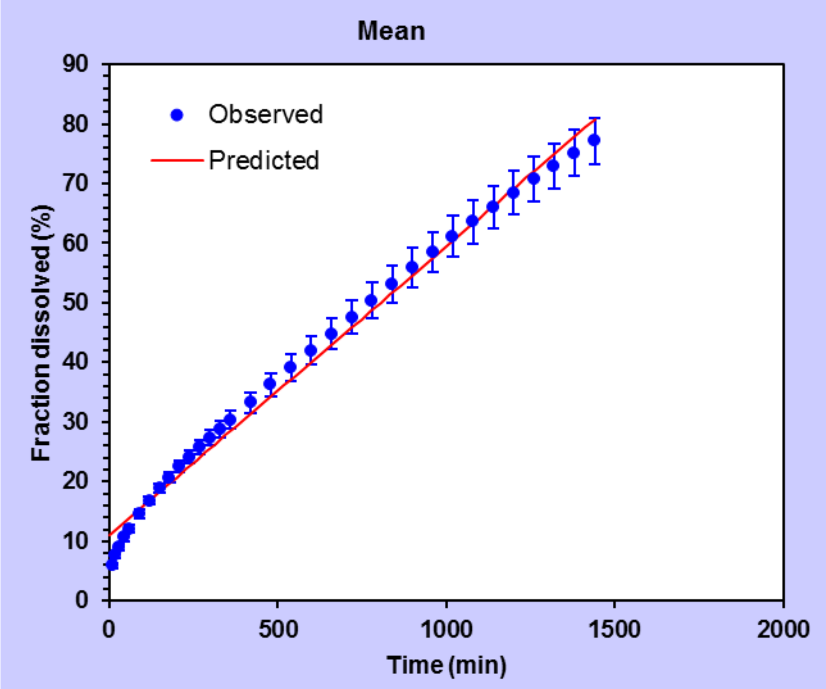

Supplementary materials – Model fitting summary – Starch 1500® sample with larger particle size (modelling the entire relevant dissolution data)

Graphical abstract of model fit presented as the fraction % of released carvedilol per tested tablet:

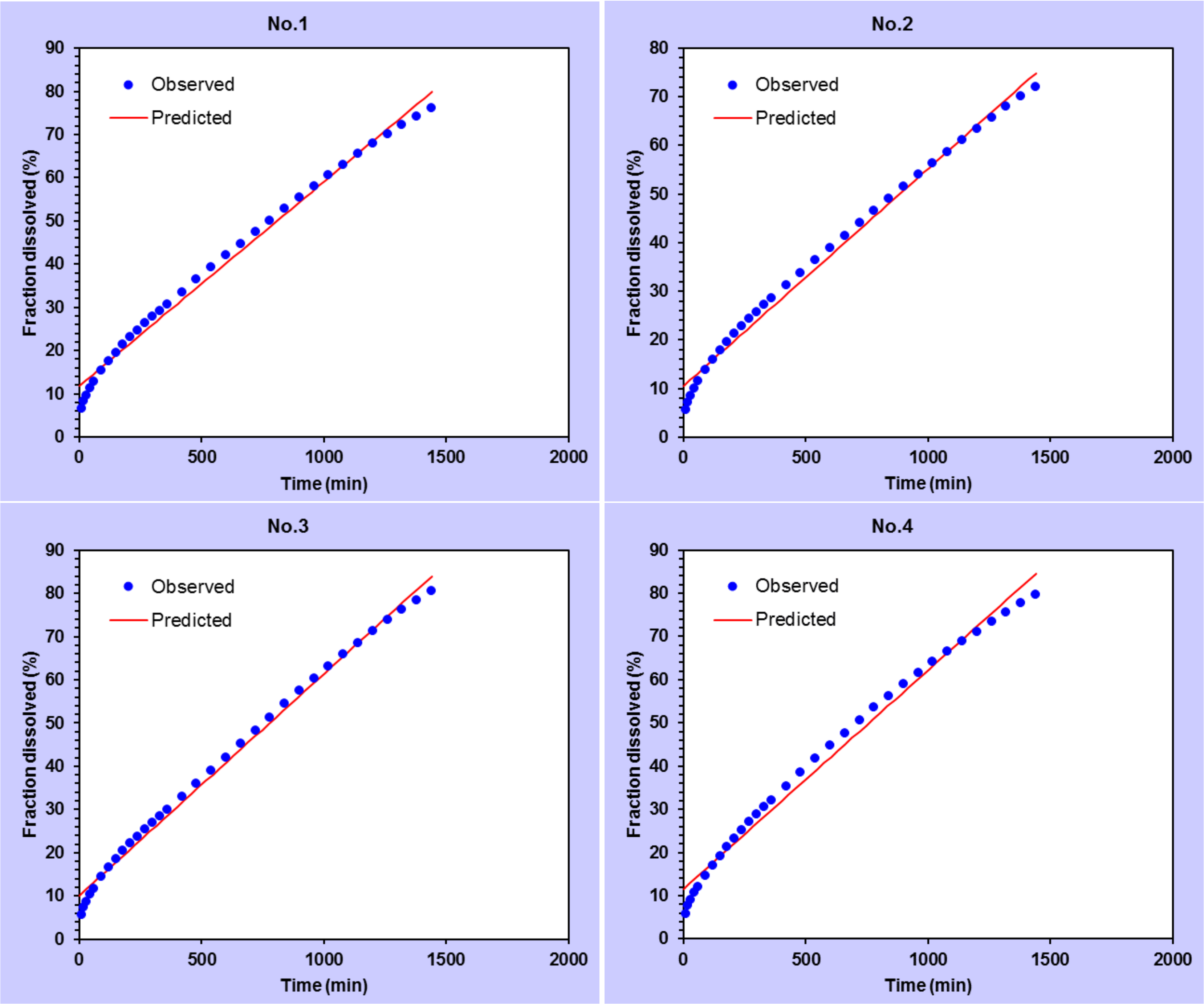

Supplementary materials – Model fitting summary – Starch 1500® sample with larger particle size (modelling the entire relevant dissolution data)

Model: **First–order**

Model equation: $F = 100 \cdot (1 - e^{-k_1 \cdot t})$

Fitted model parameters per tested tablet (N = 4) with statistics – mean, standard deviation (SD), and relative standard deviation expressed in % (RSD%) (output from DDSolver):

Parameter	No.1	No.2	No.3	No.4	Mean	SD	RSD(%)
k_1	0.001	0.001	0.001	0.001	0.001	0.000	9.259

Number of dissolution data points (N), degrees of freedom (df), and selected goodness of fit criteria – Pearson correlation coefficient (R), coefficient of determination (R^2), adjusted coefficient of determination ($R^2_{adjusted}$), and residual sum of squares (RSS) (manual calculation in MS Excel):

Parameter	No.1	No.2	No.3	No.4
N	33	33	33	33
df	32	32	32	32
R	0.996333145	0.996885867	0.993254656	0.997225015
R^2	0.992679736	0.993781432	0.986554811	0.99445773
$R^2_{adjusted}$	0.992679736	0.993781432	0.986554811	0.99445773
RSS	508.388988	431.295017	389.7794656	338.7963686

Graphical abstract of model fit presented as mean ± 1 SD of the fraction % of released carvedilol:

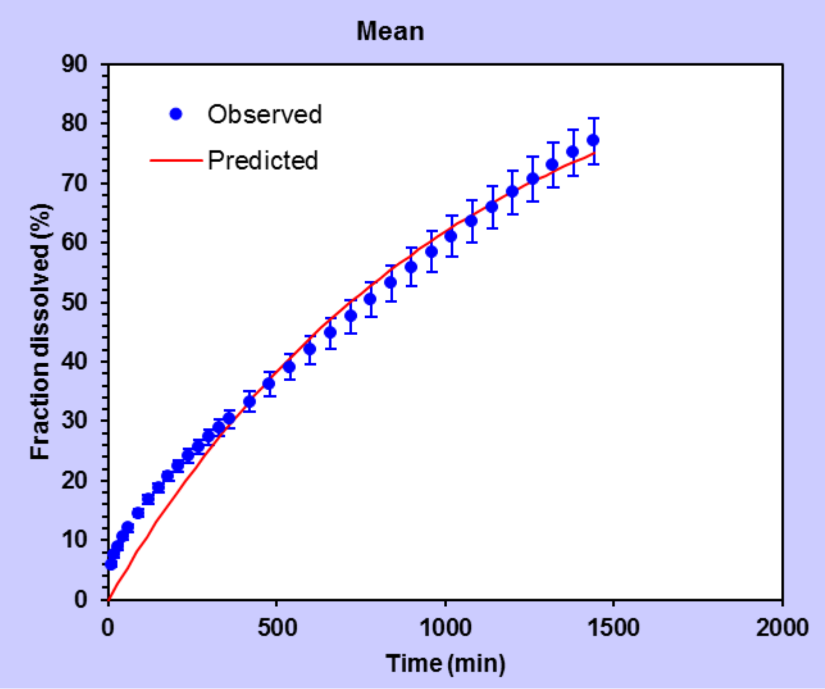

Supplementary materials – Model fitting summary – Starch 1500® sample with larger particle size (modelling the entire relevant dissolution data)

Graphical abstract of model fit presented as the fraction % of released carvedilol per tested tablet:

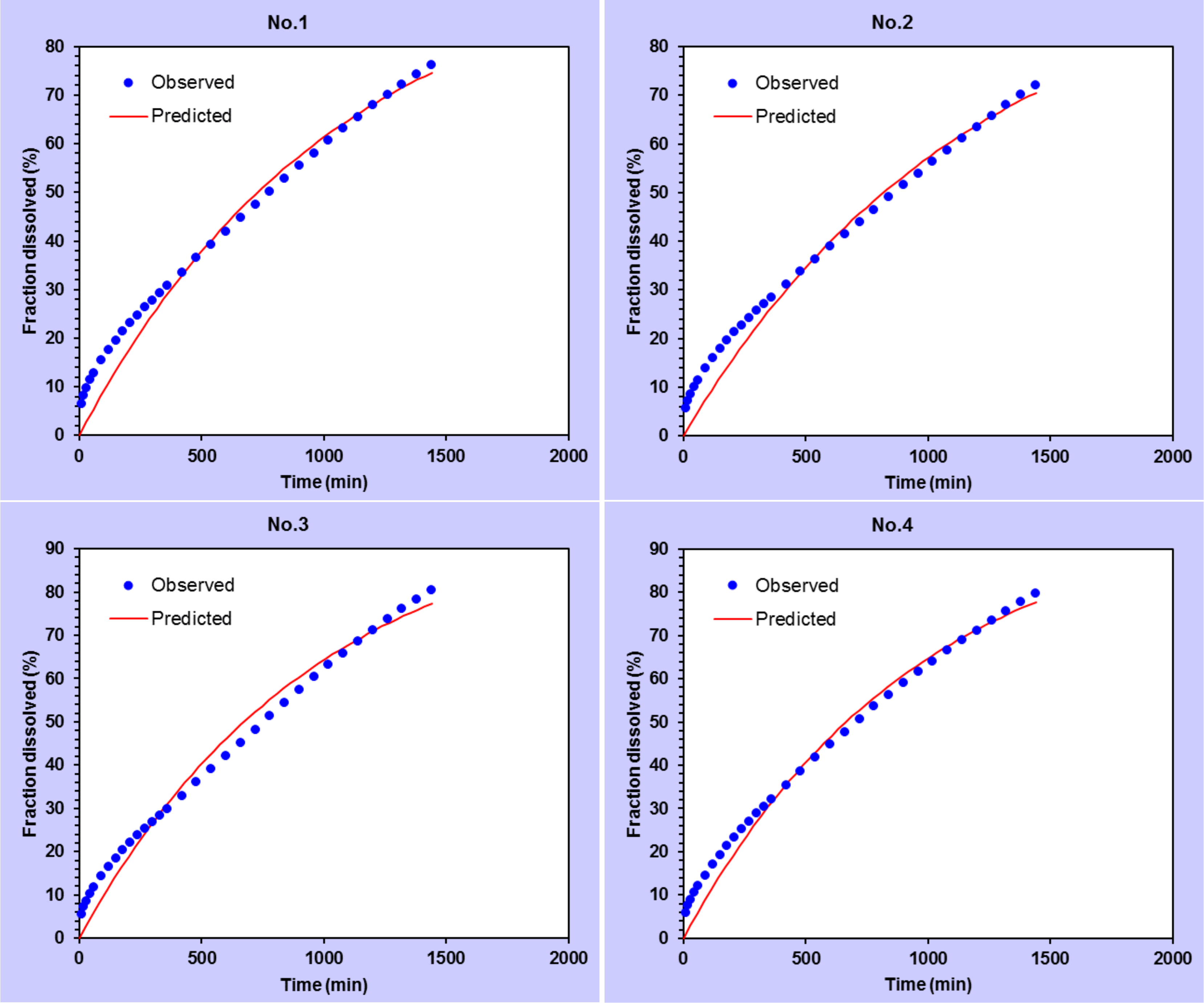

Supplementary materials – Model fitting summary – Starch 1500® sample with larger particle size (modelling the entire relevant dissolution data)

Model: **First–order with T_{lag}**

Model equation: $F = 100 \cdot \left[1 - e^{-k_1 \cdot (t - T_{lag})}\right]$

Fitted model parameters per tested tablet (N = 4) with statistics – mean, standard deviation (SD), and relative standard deviation expressed in % (RSD%) (output from DDSolver):

Parameter	No.1	No.2	No.3	No.4	Mean	SD	RSD(%)
k_1	0.001	0.001	0.001	0.001	0.001	0.000	10.354
T_{lag}	-62.714	-68.525	-20.518	-49.220	-50.244	21.404	-42.600

Number of dissolution data points (N), degrees of freedom (df), and selected goodness of fit criteria – Pearson correlation coefficient (R), coefficient of determination (R^2), adjusted coefficient of determination ($R^2_{adjusted}$), and residual sum of squares (RSS) (manual calculation in MS Excel):

Parameter	No.1	No.2	No.3	No.4
N	33	33	33	33
df	31	31	31	31
R	0.997004817	0.997440389	0.993515976	0.998228858
R^2	0.994018606	0.994887329	0.987073994	0.996460852
$R^2_{adjusted}$	0.993825658	0.994722405	0.986657026	0.996346686
RSS	110.6026707	80.19056801	292.8328519	106.949454

Graphical abstract of model fit presented as mean ± 1 SD of the fraction % of released carvedilol:

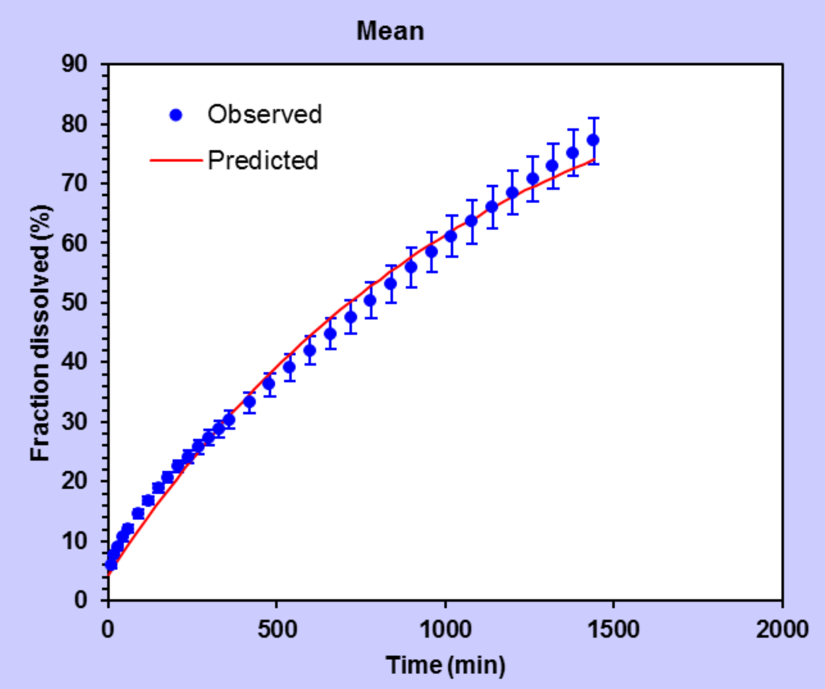

Supplementary materials – Model fitting summary – Starch 1500® sample with larger particle size (modelling the entire relevant dissolution data)

Graphical abstract of model fit presented as the fraction % of released carvedilol per tested tablet:

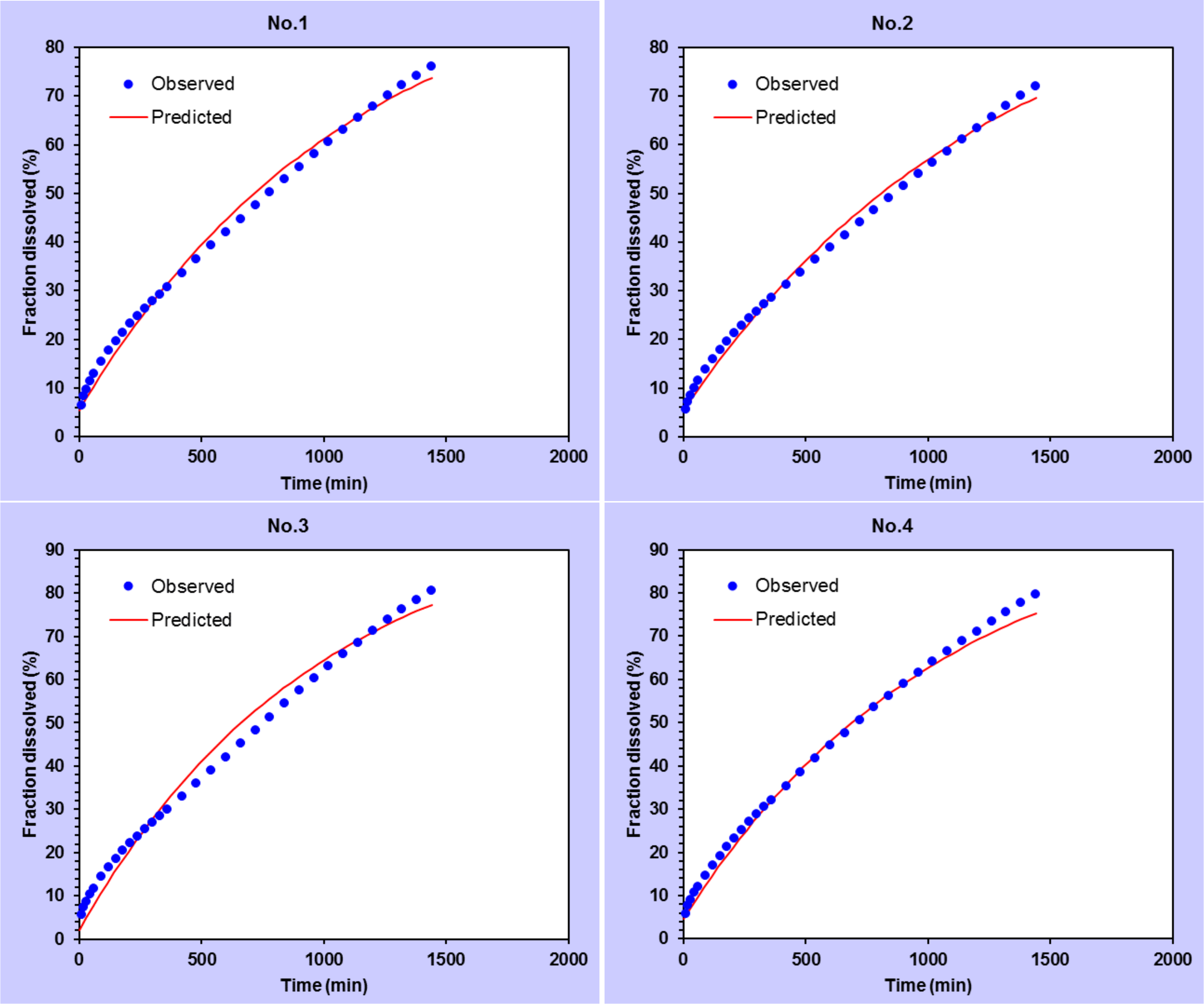

Supplementary materials – Model fitting summary – Starch 1500® sample with larger particle size (modelling the entire relevant dissolution data)

Model: **First–order with F_{max}**

Model equation: $F = F_{max} \cdot (1 - e^{-k_1 \cdot t})$

Fitted model parameters per tested tablet (N = 4) with statistics – mean, standard deviation (SD), and relative standard deviation expressed in % (RSD%) (output from DDSolver):

Parameter	No.1	No.2	No.3	No.4	Mean	SD	RSD(%)
k_1	0.002	0.002	0.002	0.002	0.002	0.000	1.740
F_{max}	79.982	75.665	84.550	83.708	80.976	4.059	5.013

Number of dissolution data points (N), degrees of freedom (df), and selected goodness of fit criteria – Pearson correlation coefficient (R), coefficient of determination (R^2), adjusted coefficient of determination ($R^2_{adjusted}$), and residual sum of squares (RSS) (manual calculation in MS Excel):

Parameter	No.1	No.2	No.3	No.4
N	33	33	33	33
df	31	31	31	31
R	0.984175974	0.98403831	0.981136004	0.987560214
R^2	0.968602347	0.968331396	0.962627858	0.975275177
$R^2_{adjusted}$	0.96758952	0.967309828	0.961422305	0.974477602
RSS	701.040417	623.5424053	931.6976826	614.0524426

Graphical abstract of model fit presented as mean ± 1 SD of the fraction % of released carvedilol:

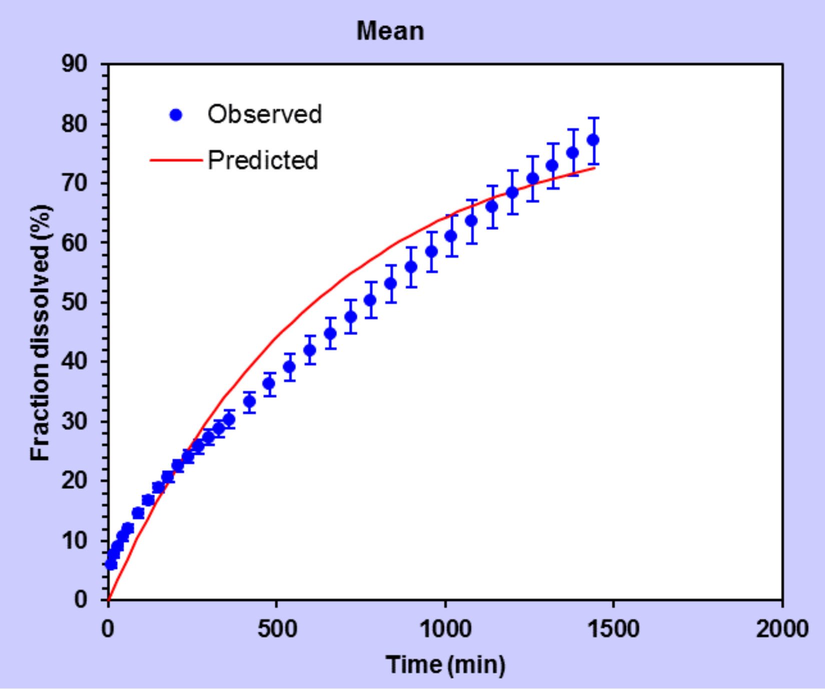

Supplementary materials – Model fitting summary – Starch 1500® sample with larger particle size (modelling the entire relevant dissolution data)

Graphical abstract of model fit presented as the fraction % of released carvedilol per tested tablet:

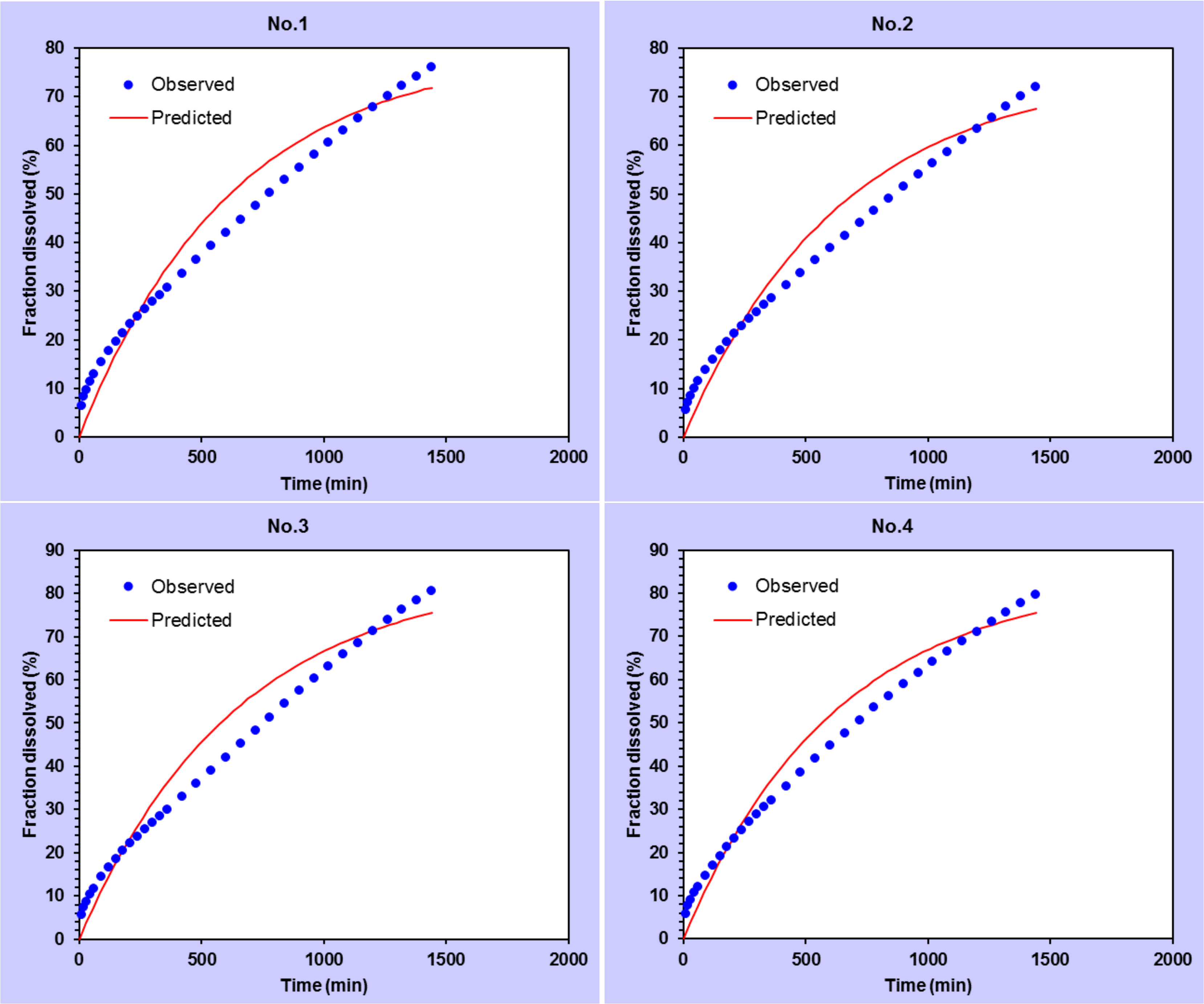

Supplementary materials – Model fitting summary – Starch 1500® sample with larger particle size (modelling the entire relevant dissolution data)

Model: **First–order with T_{lag} and F_{max}**

Model equation: $F = F_{max} \cdot \left[1 - e^{-k_1 \cdot (t - T_{lag})}\right]$

Fitted model parameters per tested tablet (N = 4) with statistics – mean, standard deviation (SD), and relative standard deviation expressed in % (RSD%) (output from DDSolver):

Parameter	No.1	No.2	No.3	No.4	Mean	SD	RSD(%)
k_1	0.002	0.002	0.002	0.002	0.002	0.000	1.431
T_{lag}	37.178	42.242	60.432	43.548	45.850	10.102	22.033
F_{max}	79.982	75.665	84.550	83.708	80.976	4.059	5.013

Number of dissolution data points (N), degrees of freedom (df), and selected goodness of fit criteria – Pearson correlation coefficient (R), coefficient of determination (R^2), adjusted coefficient of determination ($R^2_{adjusted}$), and residual sum of squares (RSS) (manual calculation in MS Excel):

Parameter	No.1	No.2	No.3	No.4
N	33	33	33	33
df	30	30	30	30
R	0.982487051	0.982152054	0.978034158	0.985728245
R^2	0.965280806	0.964622656	0.956550813	0.971660174
$R^2_{adjusted}$	0.962966193	0.962264167	0.953654201	0.969770852
RSS	1132.969094	1037.831806	1641.457748	1092.734945

Graphical abstract of model fit presented as mean ± 1 SD of the fraction % of released carvedilol:

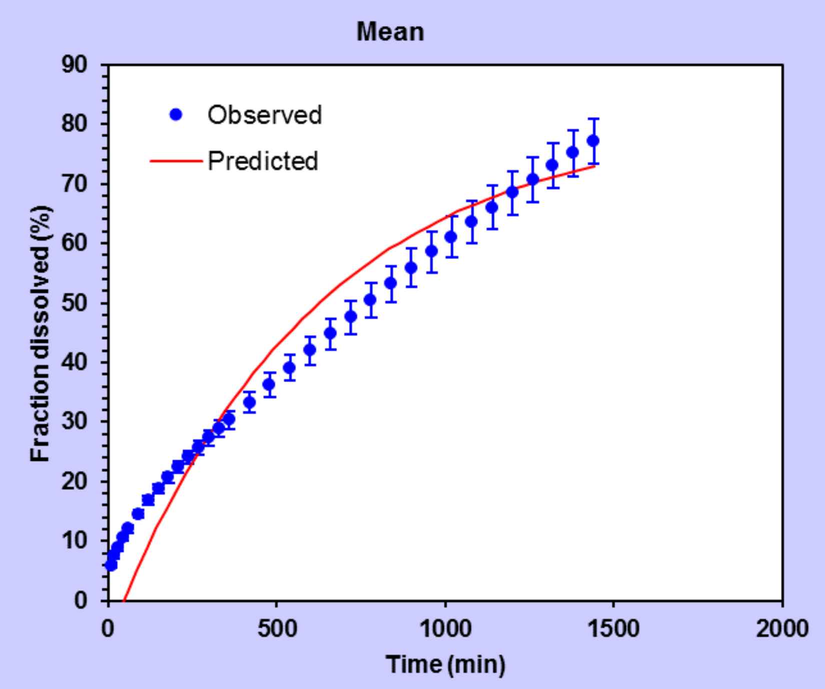

Supplementary materials – Model fitting summary – Starch 1500® sample with larger particle size (modelling the entire relevant dissolution data)

Graphical abstract of model fit presented as the fraction % of released carvedilol per tested tablet:

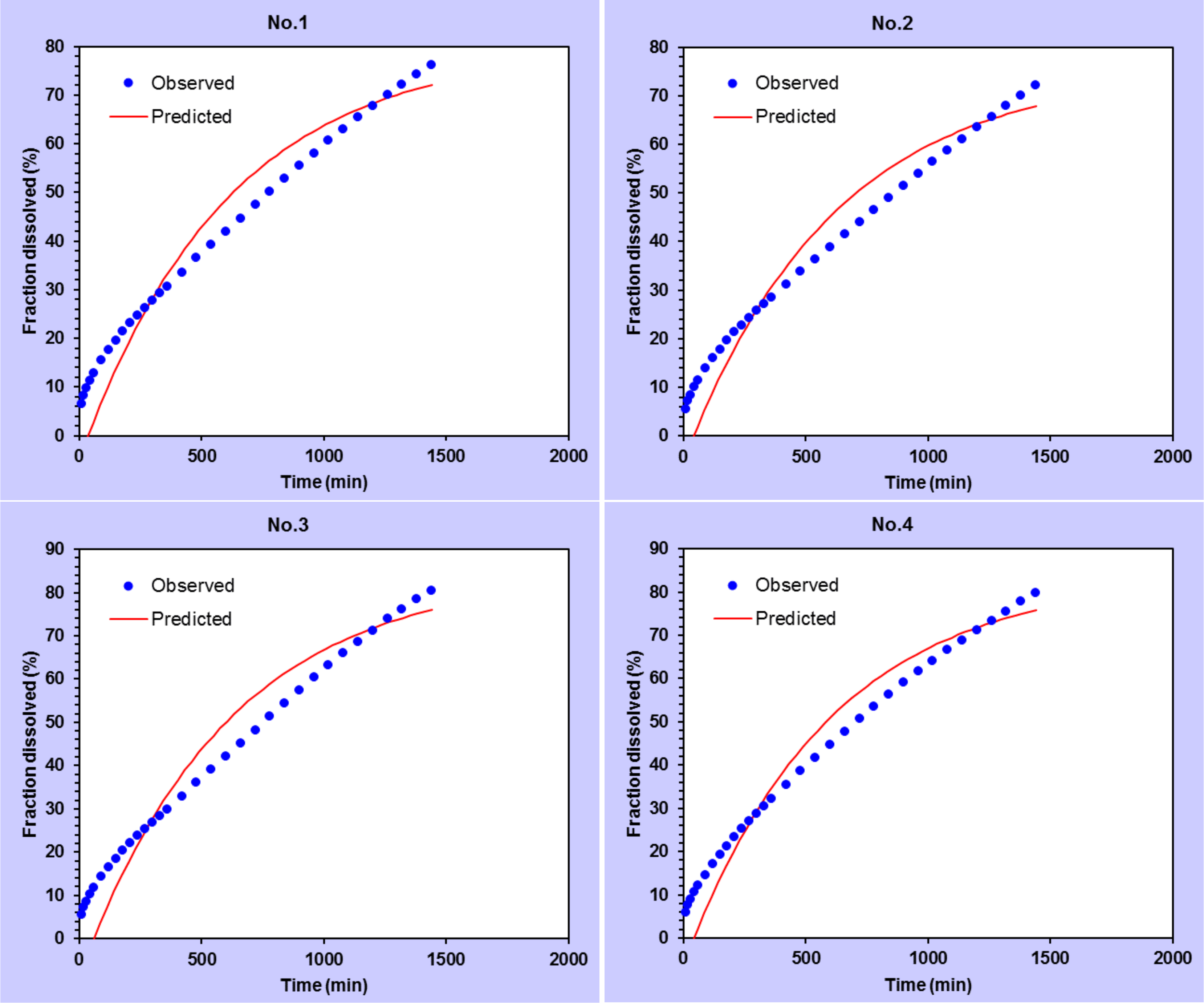

Supplementary materials – Model fitting summary – Starch 1500® sample with larger particle size (modelling the entire relevant dissolution data)

Model: **Higuchi**

Model equation: $F = k_H \cdot t^{0.5}$

Fitted model parameters per tested tablet (N = 4) with statistics – mean, standard deviation (SD), and relative standard deviation expressed in % (RSD%) (output from DDSolver):

Parameter	No.1	No.2	No.3	No.4	Mean	SD	RSD(%)
k_H	1.853	1.727	1.906	1.946	1.858	0.095	5.122

Number of dissolution data points (N), degrees of freedom (df), and selected goodness of fit criteria – Pearson correlation coefficient (R), coefficient of determination (R^2), adjusted coefficient of determination ($R^2_{adjusted}$), and residual sum of squares (RSS) (manual calculation in MS Excel):

Parameter	No.1	No.2	No.3	No.4
N	33	33	33	33
df	32	32	32	32
R	0.993082424	0.99261338	0.98992119	0.994747611
R^2	0.986212701	0.985281321	0.979943963	0.989522809
$R^2_{adjusted}$	0.986212701	0.985281321	0.979943963	0.989522809
RSS	354.5554464	371.6733963	738.52236	444.6606677

Graphical abstract of model fit presented as mean ± 1 SD of the fraction % of released carvedilol:

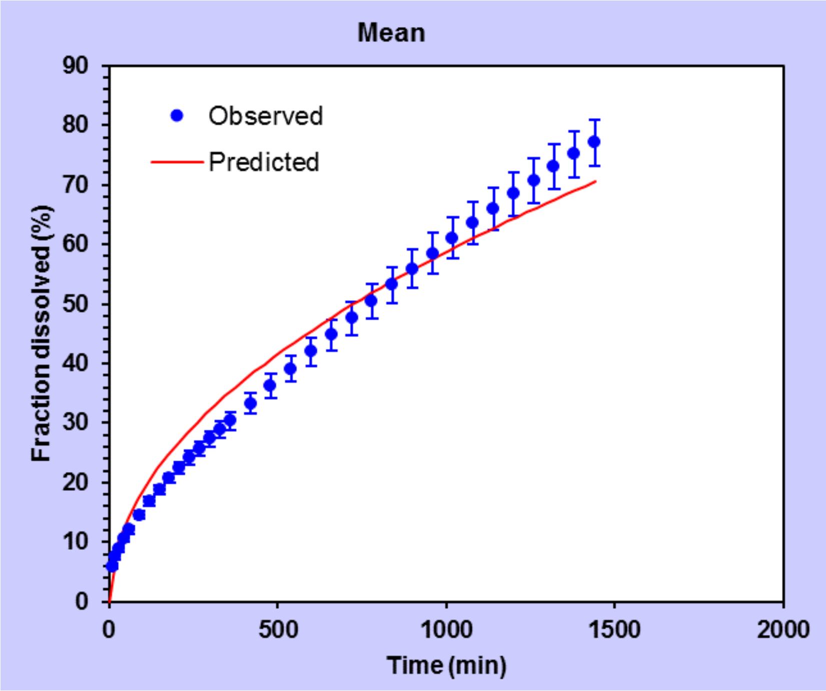

Supplementary materials – Model fitting summary – Starch 1500® sample with larger particle size (modelling the entire relevant dissolution data)

Graphical abstract of model fit presented as the fraction % of released carvedilol per tested tablet:

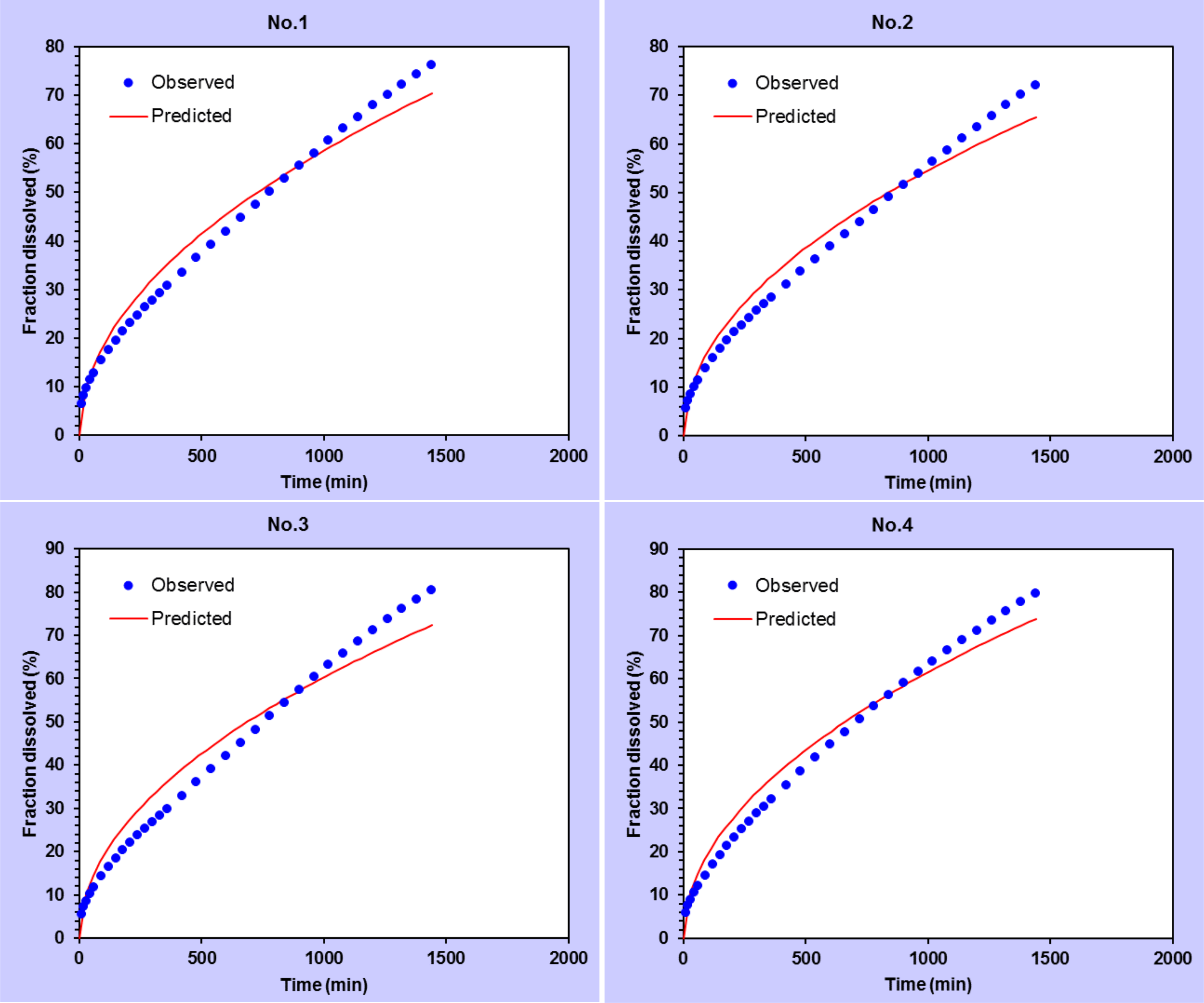

Supplementary materials – Model fitting summary – Starch 1500® sample with larger particle size (modelling the entire relevant dissolution data)

Model: **Higuchi with T_{lag}**

Model equation: $F = k_H \cdot (t - T_{lag})^{0.5}$

Fitted model parameters per tested tablet (N = 4) with statistics – mean, standard deviation (SD), and relative standard deviation expressed in % (RSD%) (output from DDSolver):

Parameter	No.1	No.2	No.3	No.4	Mean	SD	RSD(%)
k_H	1.993	1.873	2.108	2.101	2.019	0.111	5.489
T_{lag}	76.130	83.369	100.855	79.542	84.974	10.993	12.936

Number of dissolution data points (N), degrees of freedom (df), and selected goodness of fit criteria – Pearson correlation coefficient (R), coefficient of determination (R^2), adjusted coefficient of determination ($R^2_{adjusted}$), and residual sum of squares (RSS) (manual calculation in MS Excel):

Parameter	No.1	No.2	No.3	No.4
N	33	33	33	33
df	31	31	31	31
R	0.985737844	0.985606514	0.982393686	0.98870775
R^2	0.971679097	0.971420201	0.965097354	0.977543015
$R^2_{adjusted}$	0.970765519	0.970498272	0.963971462	0.976818596
RSS	720.682112	634.3218651	922.401795	621.3413407

Graphical abstract of model fit presented as mean ± 1 SD of the fraction % of released carvedilol:

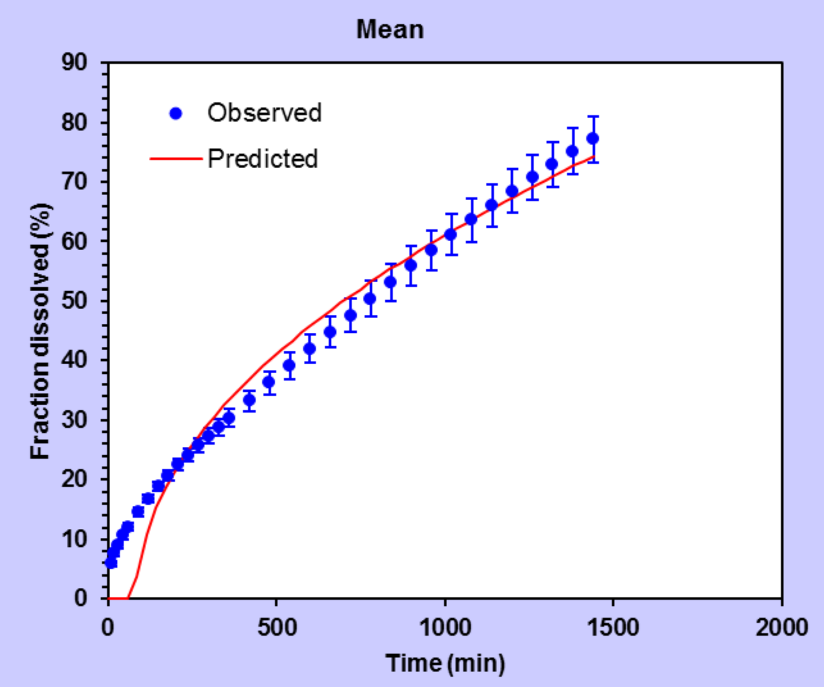

Supplementary materials – Model fitting summary – Starch 1500® sample with larger particle size (modelling the entire relevant dissolution data)

Graphical abstract of model fit presented as the fraction % of released carvedilol per tested tablet:

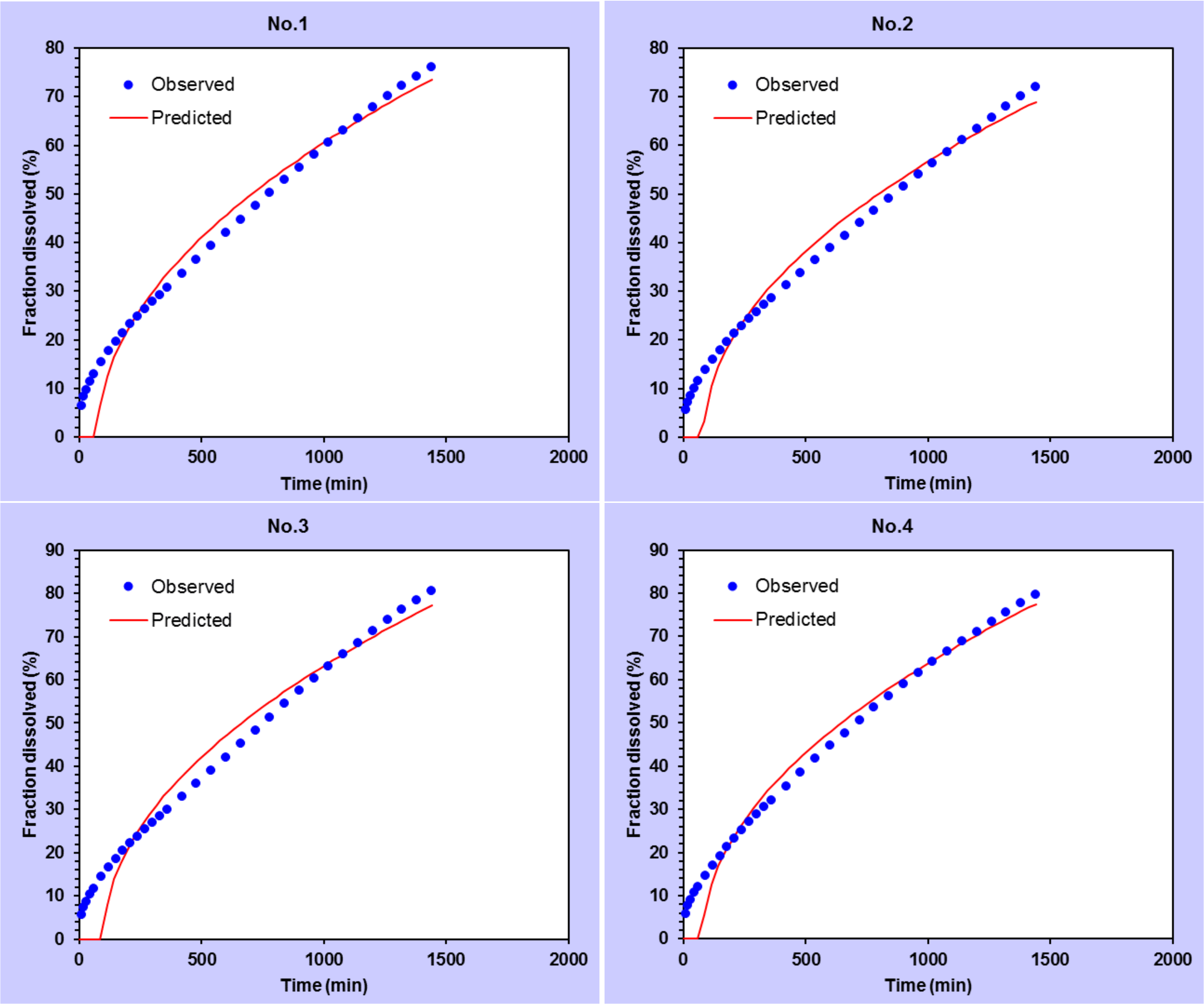

Supplementary materials – Model fitting summary – Starch 1500® sample with larger particle size (modelling the entire relevant dissolution data)

Model: **Higuchi with F_0**

Model equation: $F = F_0 + k_H \cdot t^{0.5}$

Fitted model parameters per tested tablet (N = 4) with statistics – mean, standard deviation (SD), and relative standard deviation expressed in % (RSD%) (output from DDSolver):

Parameter	No.1	No.2	No.3	No.4	Mean	SD	RSD(%)
k_H	2.031	1.921	2.196	2.186	2.084	0.132	6.323
F_0	-4.781	-5.200	-7.750	-6.425	-6.039	1.337	-22.142

Number of dissolution data points (N), degrees of freedom (df), and selected goodness of fit criteria – Pearson correlation coefficient (R), coefficient of determination (R^2), adjusted coefficient of determination ($R^2_{adjusted}$), and residual sum of squares (RSS) (manual calculation in MS Excel):

Parameter	No.1	No.2	No.3	No.4
N	33	33	33	33
df	31	31	31	31
R	0.993082424	0.99261338	0.98992119	0.994747611
R^2	0.986212701	0.985281321	0.979943963	0.989522809
$R^2_{adjusted}$	0.985767949	0.984806525	0.979296994	0.989184835
RSS	210.7081275	201.4957997	360.5041297	184.8094913

Graphical abstract of model fit presented as mean ± 1 SD of the fraction % of released carvedilol:

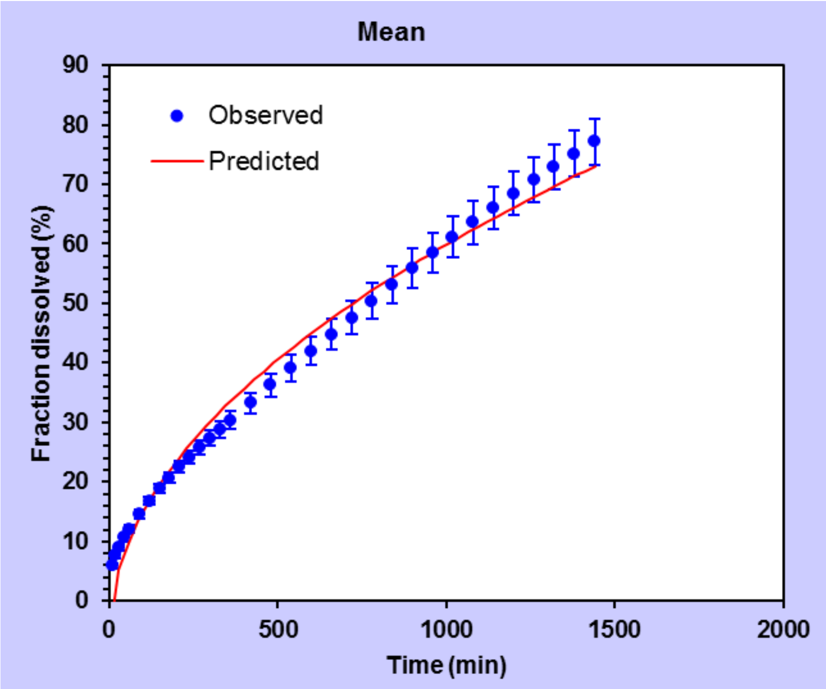

Supplementary materials – Model fitting summary – Starch 1500® sample with larger particle size (modelling the entire relevant dissolution data)

Graphical abstract of model fit presented as the fraction % of released carvedilol per tested tablet:

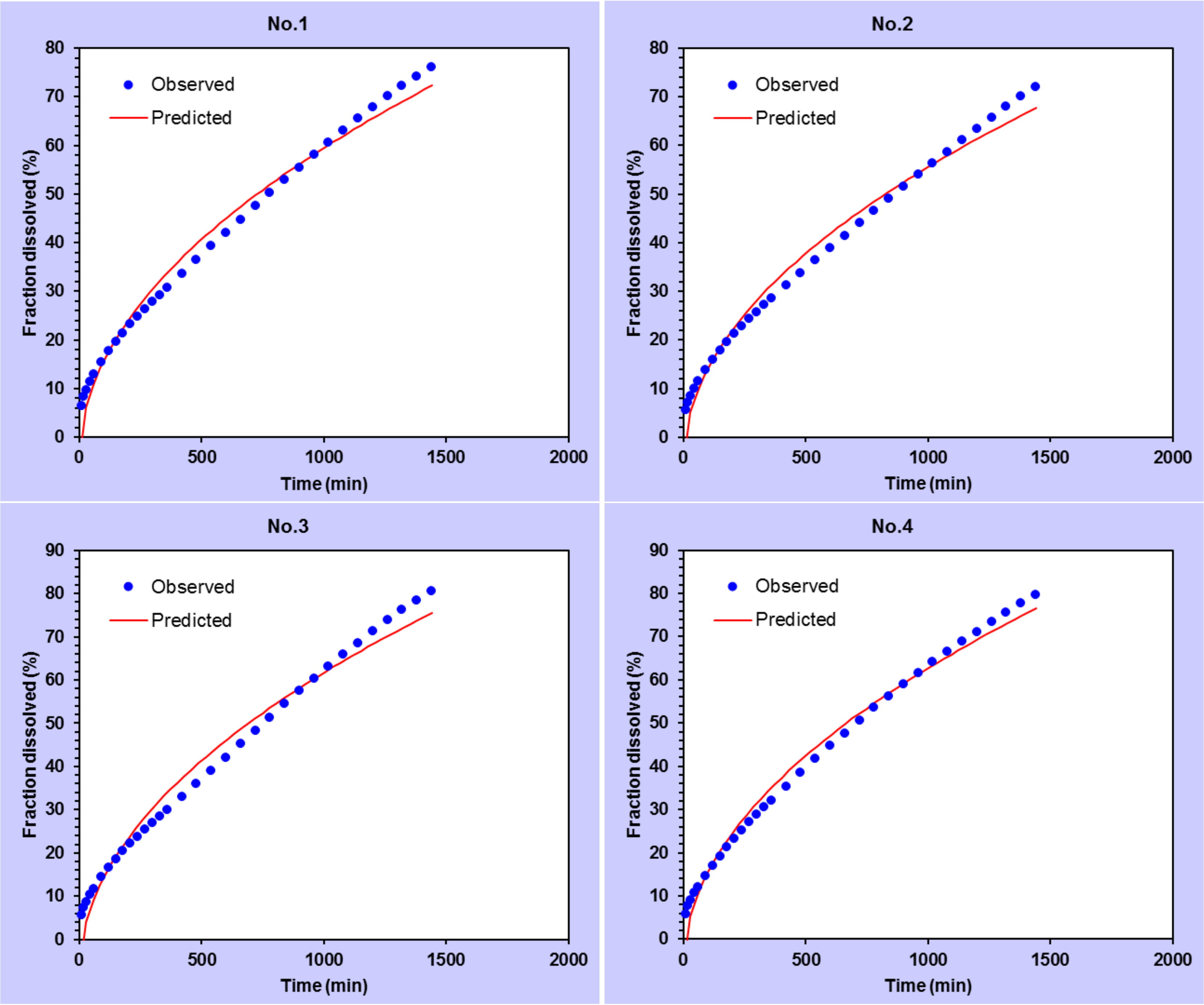

Supplementary materials – Model fitting summary – Starch 1500® sample with larger particle size (modelling the entire relevant dissolution data)

Model: **Korsmeyer–Peppas**

Model equation: $F = k_{KP} \cdot t^n$

Fitted model parameters per tested tablet (N = 4) with statistics – mean, standard deviation (SD), and relative standard deviation expressed in % (RSD%) (output from DDSolver):

Parameter	No.1	No.2	No.3	No.4	Mean	SD	RSD(%)
k_{KP}	1.569	1.307	1.196	1.302	1.344	0.159	11.825
n	0.520	0.537	0.564	0.556	0.544	0.020	3.613

Number of dissolution data points (N), degrees of freedom (df), and selected goodness of fit criteria – Pearson correlation coefficient (R), coefficient of determination (R^2), adjusted coefficient of determination ($R^2_{adjusted}$), and residual sum of squares (RSS) (manual calculation in MS Excel):

Parameter	No.1	No.2	No.3	No.4
N	33	33	33	33
df	31	31	31	31
R	0.994206632	0.994656714	0.993873063	0.997238187
R^2	0.988446827	0.989341979	0.987783665	0.994484001
$R^2_{adjusted}$	0.988074144	0.988998172	0.98738959	0.994306066
RSS	328.780796	271.2901337	435.2020794	221.7236074

Graphical abstract of model fit presented as mean ± 1 SD of the fraction % of released carvedilol:

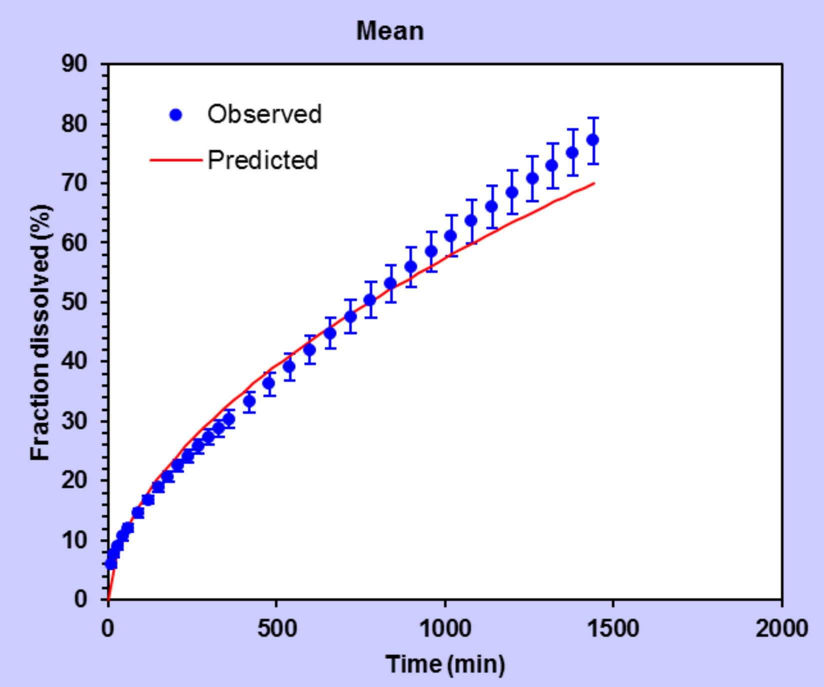

Supplementary materials – Model fitting summary – Starch 1500® sample with larger particle size (modelling the entire relevant dissolution data)

Graphical abstract of model fit presented as the fraction % of released carvedilol per tested tablet:

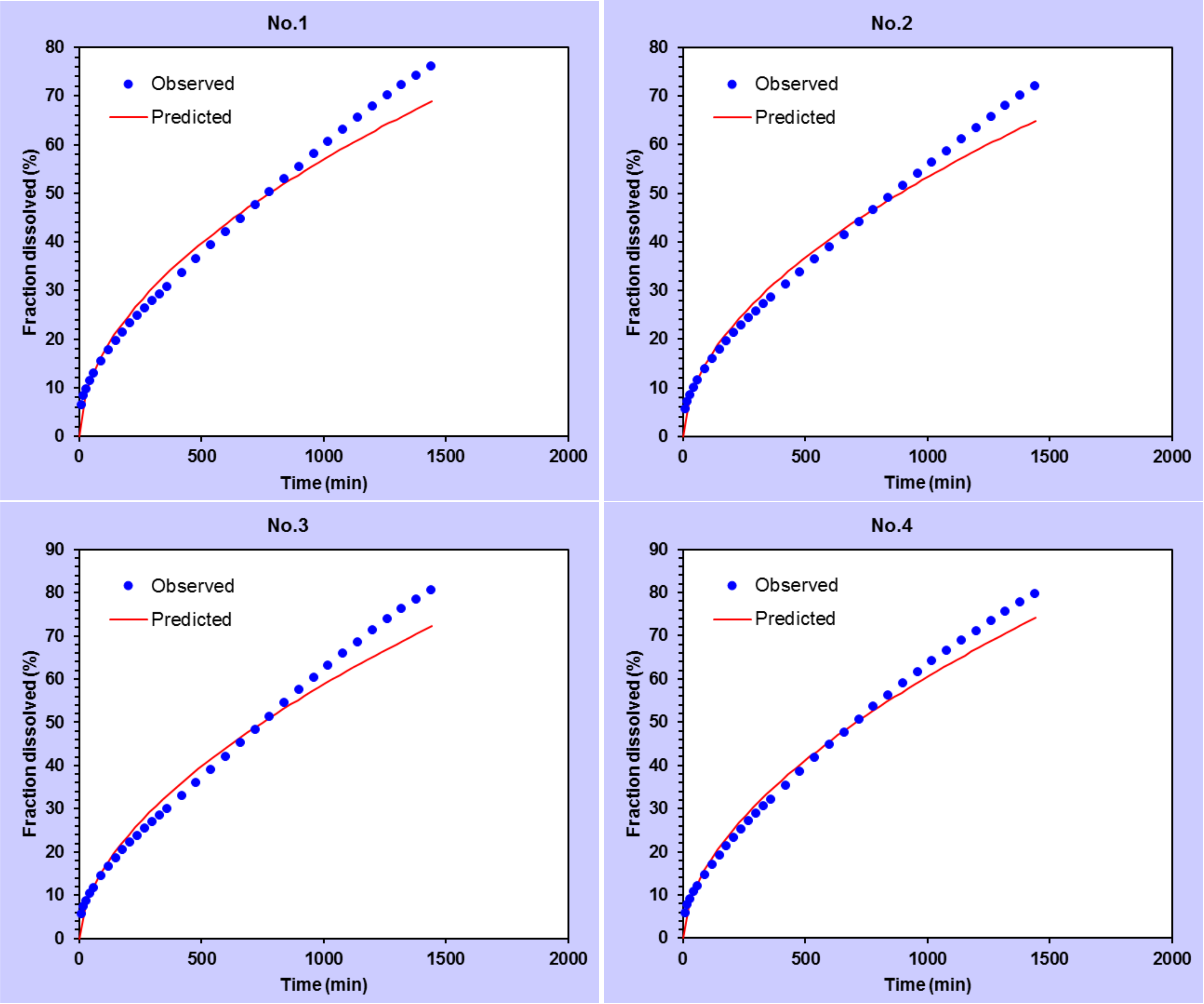

Supplementary materials – Model fitting summary – Starch 1500® sample with larger particle size (modelling the entire relevant dissolution data)

Model: **Korsmeyer–Peppas with T_{lag}**

Model equation: $F = k_{KP} \cdot (t - T_{lag})^n$

Fitted model parameters per tested tablet (N = 4) with statistics – mean, standard deviation (SD), and relative standard deviation expressed in % (RSD%) (output from DDSolver):

Parameter	No.1	No.2	No.3	No.4	Mean	SD	RSD(%)
k_{KP}	1.912	1.601	1.482	1.619	1.654	0.183	11.042
n	0.489	0.506	0.531	0.535	0.515	0.021	4.171
T_{lag}	4.000	4.000	4.000	4.939	4.235	0.470	11.092

Number of dissolution data points (N), degrees of freedom (df), and selected goodness of fit criteria – Pearson correlation coefficient (R), coefficient of determination (R^2), adjusted coefficient of determination ($R^2_{adjusted}$), and residual sum of squares (RSS) (manual calculation in MS Excel):

Parameter	No.1	No.2	No.3	No.4
N	33	33	33	33
df	30	30	30	30
R	0.991600894	0.992200876	0.991254293	0.995724737
R^2	0.983272333	0.984462579	0.982585073	0.991467752
$R^2_{adjusted}$	0.982157155	0.983426751	0.981424078	0.990898936
RSS	505.8954366	424.9892204	661.0847825	378.0711511

Graphical abstract of model fit presented as mean ± 1 SD of the fraction % of released carvedilol:

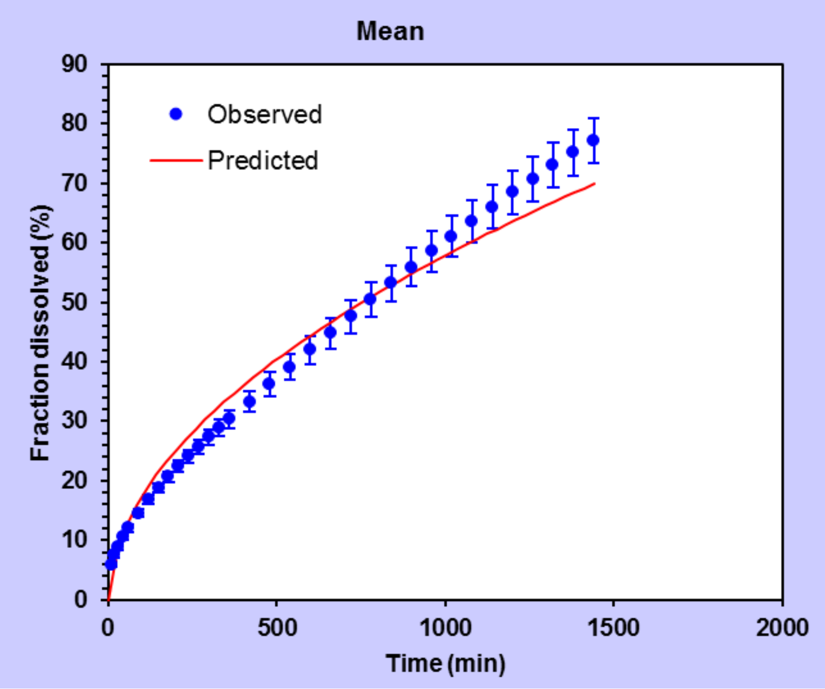

Supplementary materials – Model fitting summary – Starch 1500® sample with larger particle size (modelling the entire relevant dissolution data)

Graphical abstract of model fit presented as the fraction % of released carvedilol per tested tablet:

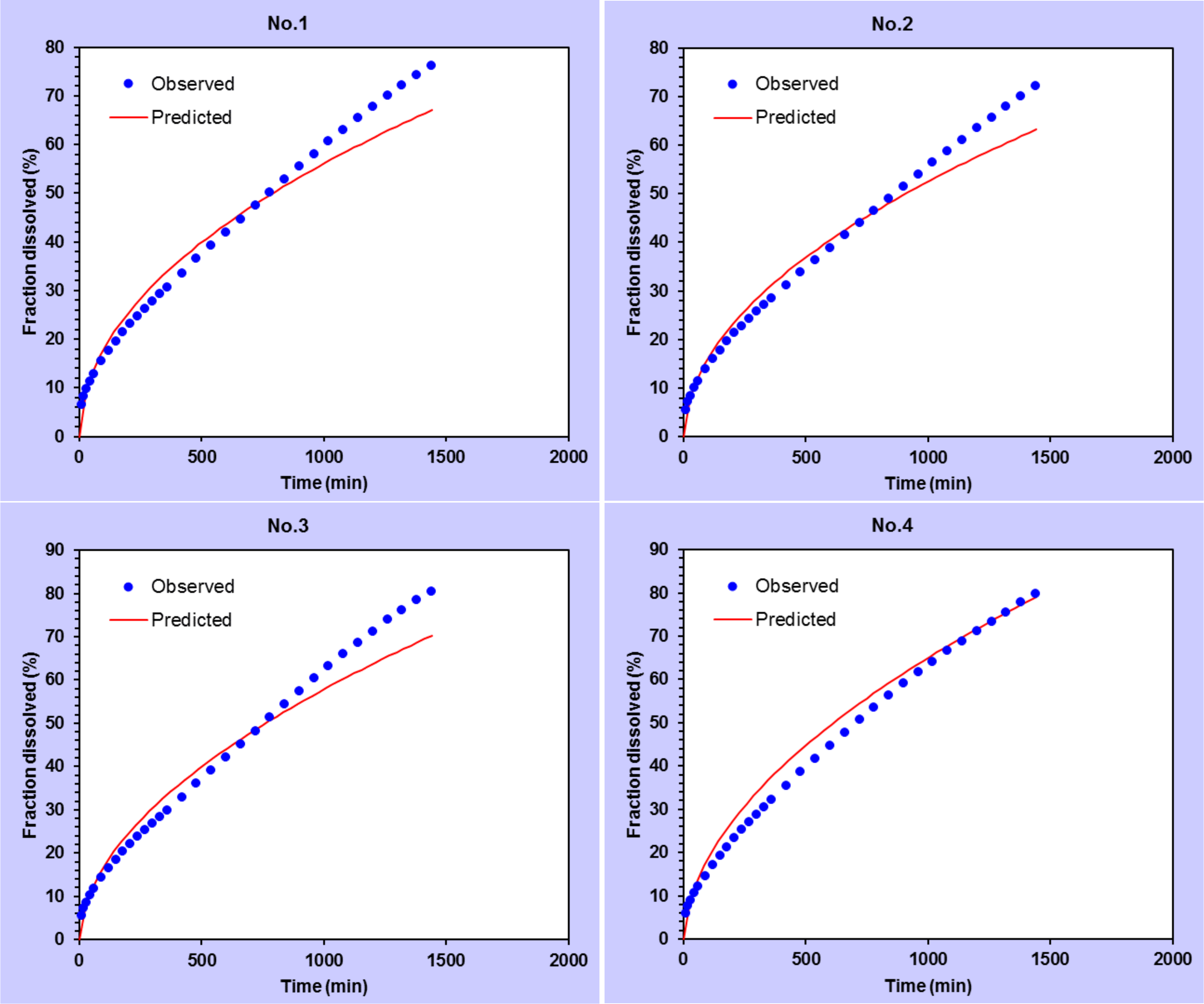

Supplementary materials – Model fitting summary – Starch 1500® sample with larger particle size (modelling the entire relevant dissolution data)

Model: **Korsmeyer–Peppas with F_0**

Model equation: $F = F_0 + k_{KP} \cdot t^n$

Fitted model parameters per tested tablet (N = 4) with statistics – mean, standard deviation (SD), and relative standard deviation expressed in % (RSD%) (output from DDSolver):

Parameter	No.1	No.2	No.3	No.4	Mean	SD	RSD(%)
k_{KP}	0.883	0.742	0.682	0.740	0.762	0.085	11.227
n	0.599	0.615	0.642	0.634	0.623	0.019	3.127
F_0	2.599	2.240	2.239	2.360	2.360	0.170	7.189

Number of dissolution data points (N), degrees of freedom (df), and selected goodness of fit criteria – Pearson correlation coefficient (R), coefficient of determination (R^2), adjusted coefficient of determination ($R^2_{adjusted}$), and residual sum of squares (RSS) (manual calculation in MS Excel):

Parameter	No.1	No.2	No.3	No.4
N	33	33	33	33
df	30	30	30	30
R	0.997549984	0.997755899	0.997200694	0.999316914
R^2	0.99510597	0.995516833	0.994409224	0.998634295
$R^2_{adjusted}$	0.994779702	0.995217956	0.994036505	0.998543248
RSS	143.2637551	112.2649938	201.9302665	62.26984242

Graphical abstract of model fit presented as mean ± 1 SD of the fraction % of released carvedilol:

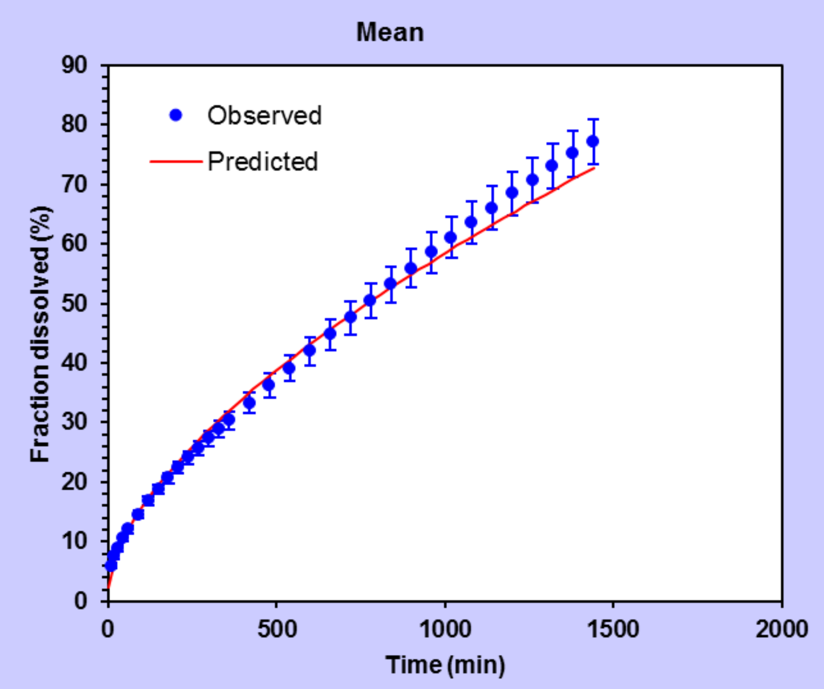

Supplementary materials – Model fitting summary – Starch 1500® sample with larger particle size (modelling the entire relevant dissolution data)

Graphical abstract of model fit presented as the fraction % of released carvedilol per tested tablet:

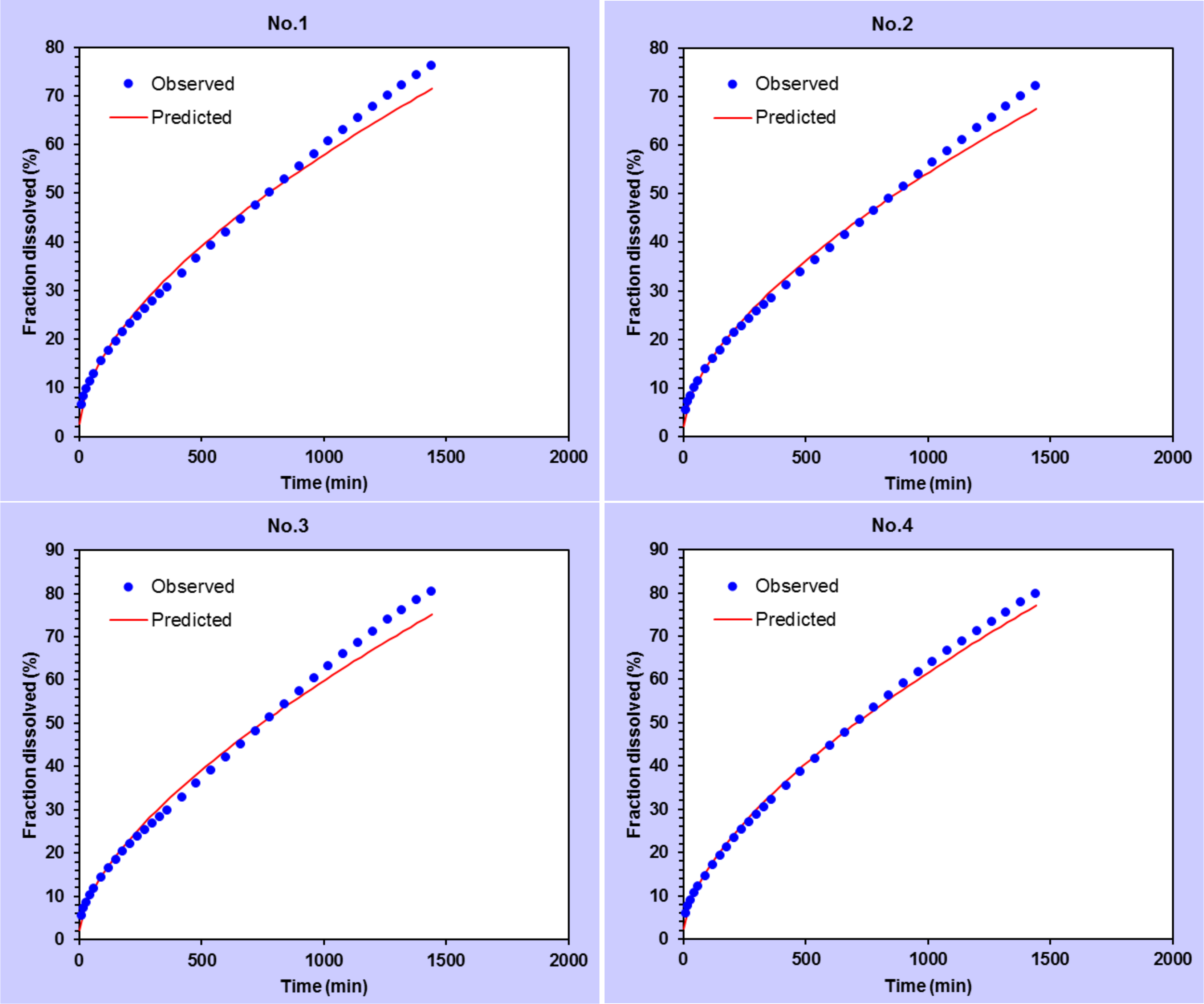

Supplementary materials – Model fitting summary – Starch 1500® sample with larger particle size (modelling the entire relevant dissolution data)

Model: **Hixson–Crowell**

Model equation: $F = 100 \cdot [1 - (1 - k_{HC} \cdot t)^3]$

Fitted model parameters per tested tablet (N = 4) with statistics – mean, standard deviation (SD), and relative standard deviation expressed in % (RSD%) (output from DDSolver):

Parameter	No.1	No.2	No.3	No.4	Mean	SD	RSD(%)
k_{HC}	0.0003	0.0002	0.0003	0.0003	0.0003	0.0000	7.6669

Number of dissolution data points (N), degrees of freedom (df), and selected goodness of fit criteria – Pearson correlation coefficient (R), coefficient of determination (R^2), adjusted coefficient of determination ($R^2_{adjusted}$), and residual sum of squares (RSS) (manual calculation in MS Excel):

Parameter	No.1	No.2	No.3	No.4
N	33	33	33	33
df	32	32	32	32
R	0.998613685	0.99855407	0.997616476	0.999298992
R^2	0.997229291	0.997110231	0.995238634	0.998598476
$R^2_{adjusted}$	0.997229291	0.997110231	0.995238634	0.998598476
RSS	760.6102869	636.6795095	448.7450184	574.1824315

Graphical abstract of model fit presented as mean ± 1 SD of the fraction % of released carvedilol:

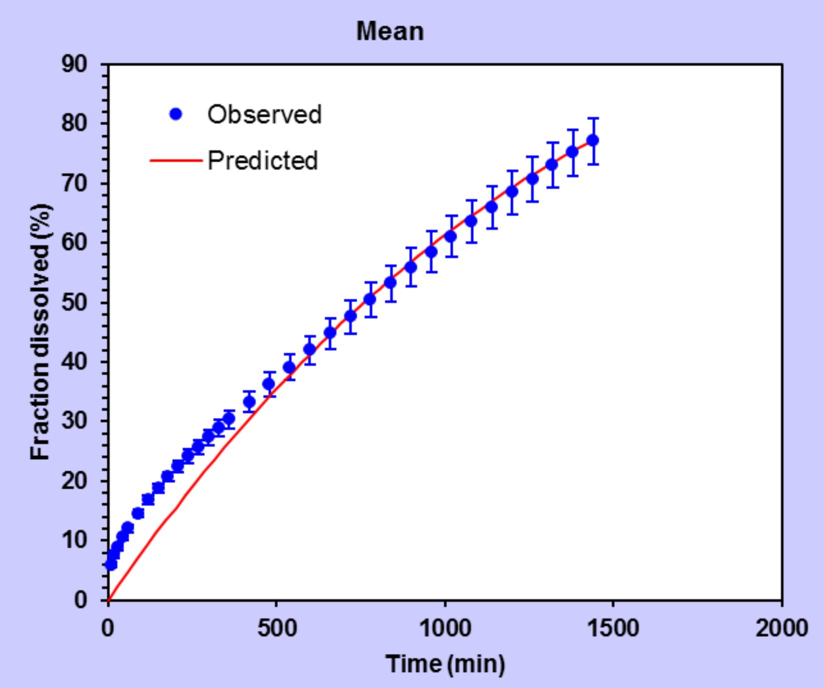

Supplementary materials – Model fitting summary – Starch 1500® sample with larger particle size (modelling the entire relevant dissolution data)

Graphical abstract of model fit presented as the fraction % of released carvedilol per tested tablet:

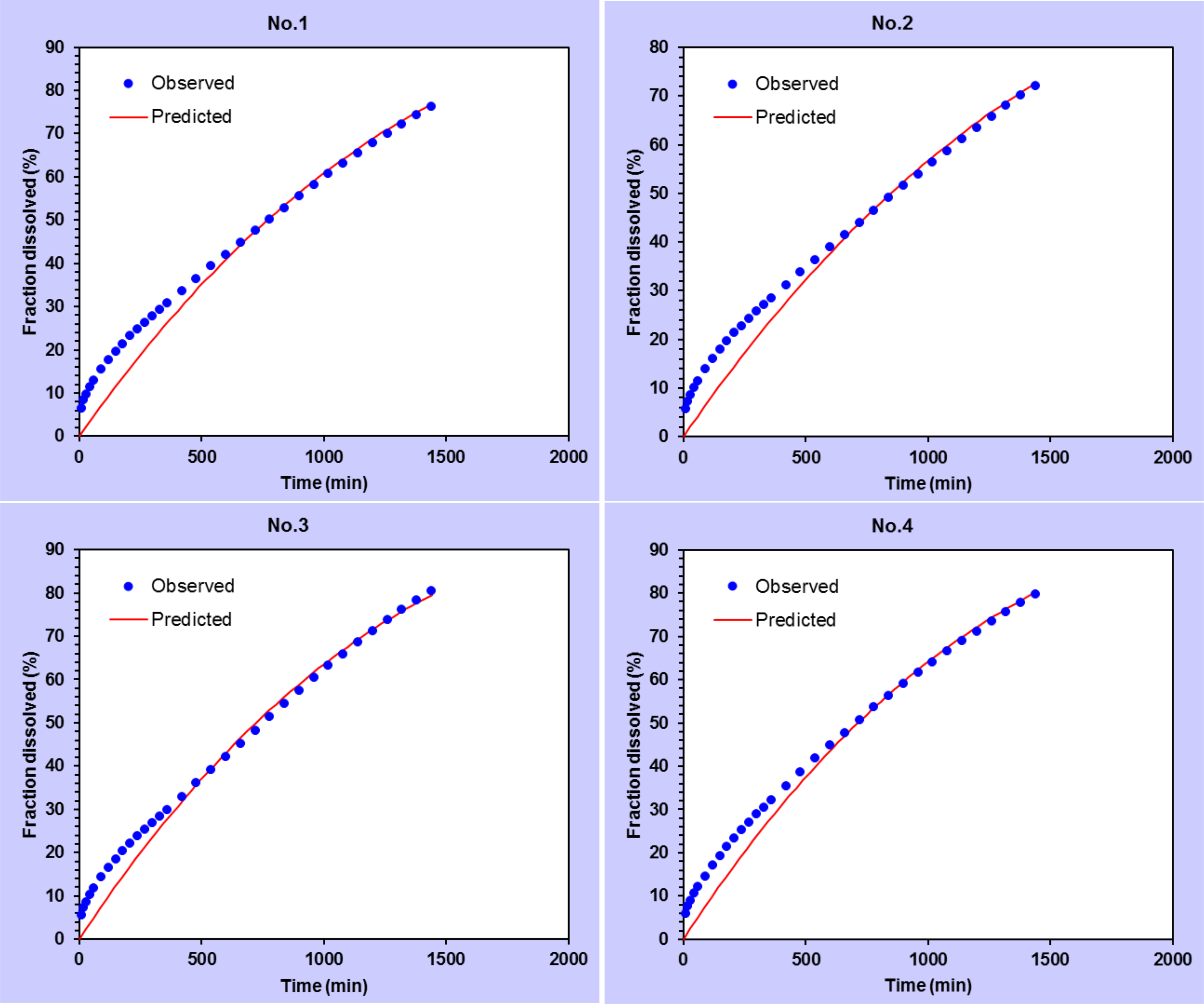

Supplementary materials – Model fitting summary – Starch 1500® sample with larger particle size (modelling the entire relevant dissolution data)

Model: **Hixson–Crowell with T_{lag}**

Model equation: $F = 100 \cdot \left\{1 - \left[1 - k_{HC} \cdot \left(t - T_{lag}\right)\right]^3\right\}$

Fitted model parameters per tested tablet (N = 4) with statistics – mean, standard deviation (SD), and relative standard deviation expressed in % (RSD%) (output from DDSolver):

Parameter	No.1	No.2	No.3	No.4	Mean	SD	RSD(%)
k_{HC}	0.000	0.000	0.000	0.000	0.000	0.000	9.485
T_{lag}	-119.697	-119.429	-69.059	-95.939	-101.031	24.049	-23.803

Number of dissolution data points (N), degrees of freedom (df), and selected goodness of fit criteria – Pearson correlation coefficient (R), coefficient of determination (R^2), adjusted coefficient of determination ($R^2_{adjusted}$), and residual sum of squares (RSS) (manual calculation in MS Excel):

Parameter	No.1	No.2	No.3	No.4
N	33	33	33	33
df	31	31	31	31
R	0.99885661	0.998722039	0.998019792	0.999410444
R^2	0.997714527	0.997445711	0.996043505	0.998821236
$R^2_{adjusted}$	0.997640802	0.997363314	0.995915876	0.998783211
RSS	35.55819945	35.26439825	78.38786862	21.04950139

Graphical abstract of model fit presented as mean ± 1 SD of the fraction % of released carvedilol:

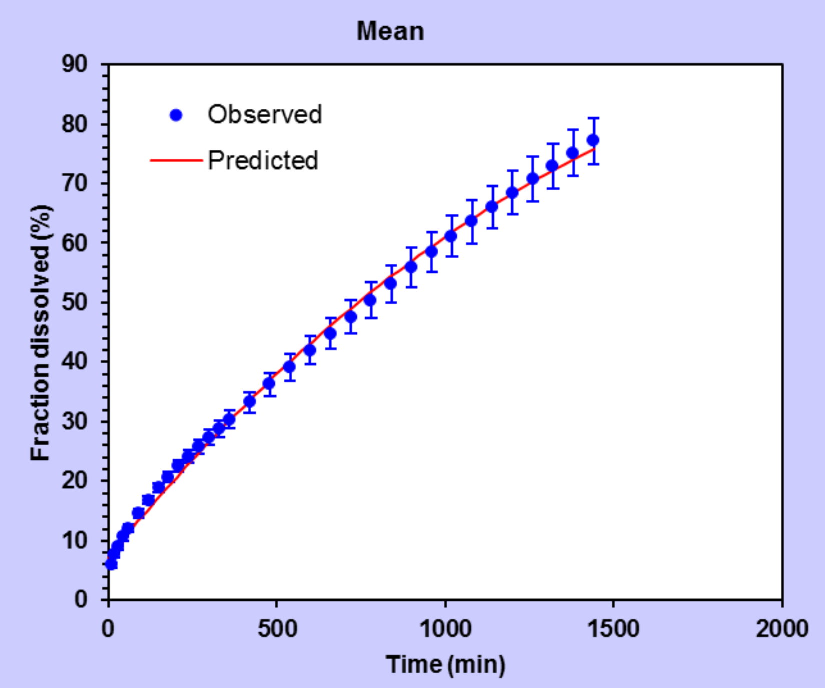

Supplementary materials – Model fitting summary – Starch 1500® sample with larger particle size (modelling the entire relevant dissolution data)

Graphical abstract of model fit presented as the fraction % of released carvedilol per tested tablet:

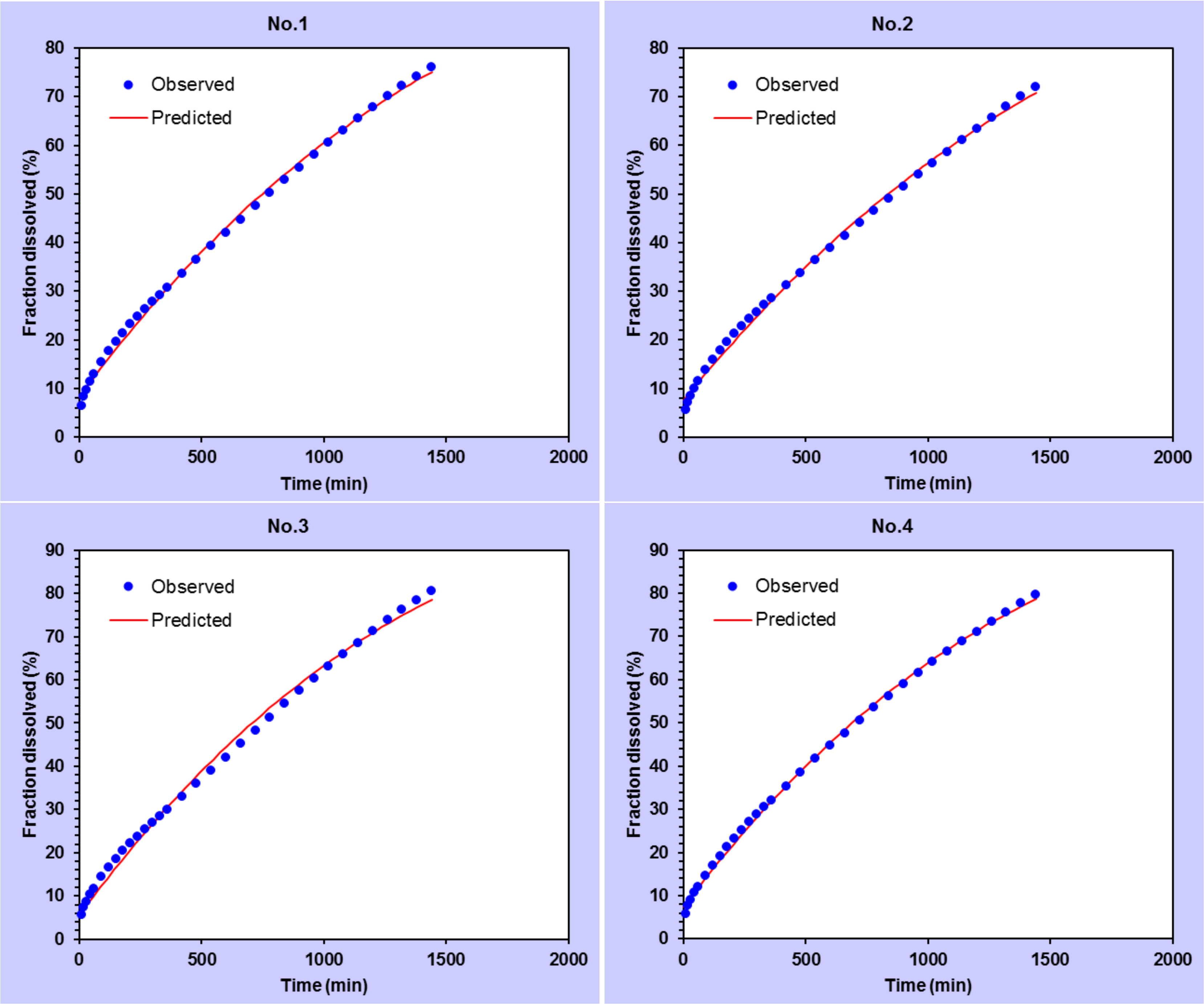

Supplementary materials – Model fitting summary – Starch 1500® sample with larger particle size (modelling the entire relevant dissolution data)

Model: **Hopfenberg**

Model equation: $F = 100 \cdot [1 - (1 - k_{HB} \cdot t)^n]$

Fitted model parameters per tested tablet (N = 4) with statistics – mean, standard deviation (SD), and relative standard deviation expressed in % (RSD%) (output from DDSolver):

Parameter	No.1	No.2	No.3	No.4	Mean	SD	RSD(%)
k_{HB}	0.0002	0.0002	0.0003	0.0002	0.0002	0.0000	15.5444
n	4.1250	3.0000	3.0000	4.1250	3.5625	0.6495	18.2321

Number of dissolution data points (N), degrees of freedom (df), and selected goodness of fit criteria – Pearson correlation coefficient (R), coefficient of determination (R^2), adjusted coefficient of determination ($R^2_{adjusted}$), and residual sum of squares (RSS) (manual calculation in MS Excel):

Parameter	No.1	No.2	No.3	No.4
N	33	33	33	33
df	31	31	31	31
R	0.998284823	0.99855407	0.997616476	0.99908594
R^2	0.996572588	0.997110231	0.995238634	0.998172715
$R^2_{adjusted}$	0.996462026	0.997017012	0.995085041	0.99811377
RSS	700.4231337	636.6795095	448.7450184	521.0763011

Graphical abstract of model fit presented as mean ± 1 SD of the fraction % of released carvedilol:

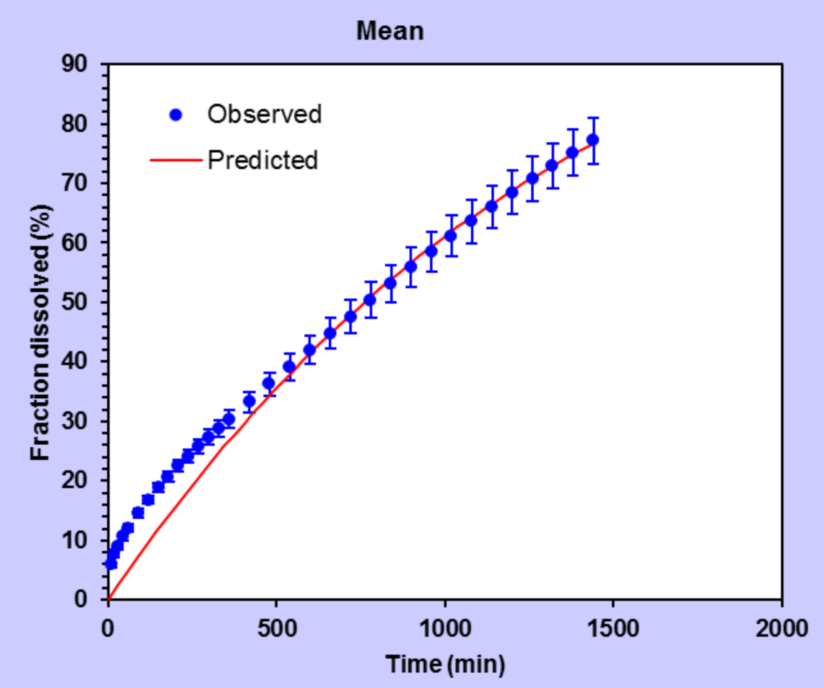

Supplementary materials – Model fitting summary – Starch 1500® sample with larger particle size (modelling the entire relevant dissolution data)

Graphical abstract of model fit presented as the fraction % of released carvedilol per tested tablet:

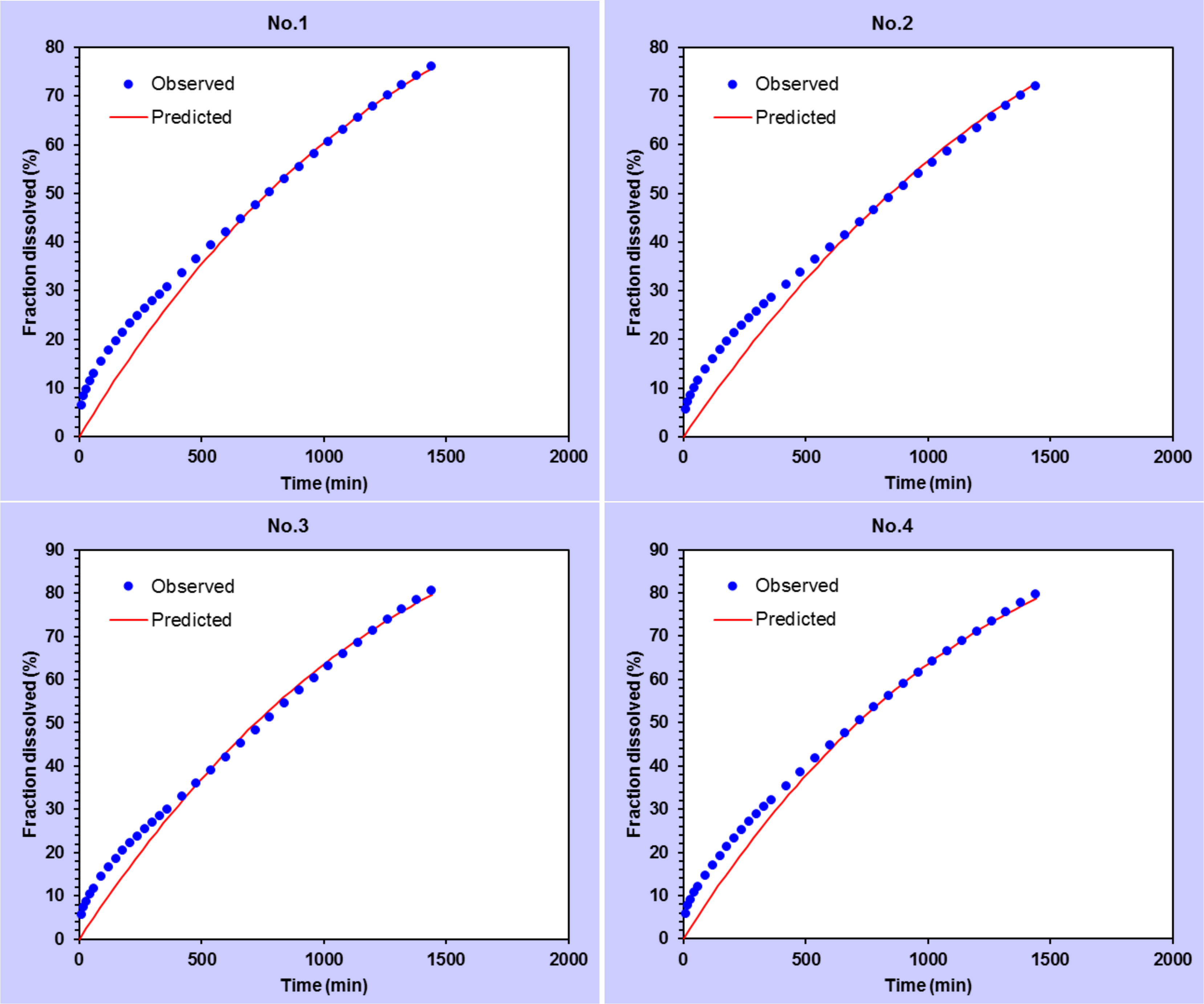

Supplementary materials – Model fitting summary – Starch 1500® sample with larger particle size (modelling the entire relevant dissolution data)

Model: **Hopfenberg with T_{lag}**

Model equation: $F = 100 \cdot \{1 - [1 - k_{HB} \cdot (t - T_{lag})]^n\}$

Fitted model parameters per tested tablet (N = 4) with statistics – mean, standard deviation (SD), and relative standard deviation expressed in % (RSD%) (output from DDSolver):

Parameter	No.1	No.2	No.3	No.4	Mean	SD	RSD(%)
k_{HB}	0.0003	0.0002	0.0004	0.0003	0.0003	0.0001	29.0683
n	2.0000	3.0000	1.5417	3.0000	2.3854	0.7339	30.7667
T_{lag}	-150.2602	-119.4292	-125.5083	-95.9386	-122.7841	22.3181	-18.1767

Number of dissolution data points (N), degrees of freedom (df), and selected goodness of fit criteria – Pearson correlation coefficient (R), coefficient of determination (R^2), adjusted coefficient of determination ($R^2_{adjusted}$), and residual sum of squares (RSS) (manual calculation in MS Excel):

Parameter	No.1	No.2	No.3	No.4
N	33	33	33	33
df	30	30	30	30
R	0.99885214	0.998722039	0.999200516	0.999410444
R^2	0.997705598	0.997445711	0.99840167	0.998821236
$R^2_{adjusted}$	0.997552638	0.997275425	0.998295115	0.998742652
RSS	35.0977501	35.26439825	33.78514482	21.04950139

Graphical abstract of model fit presented as mean ± 1 SD of the fraction % of released carvedilol:

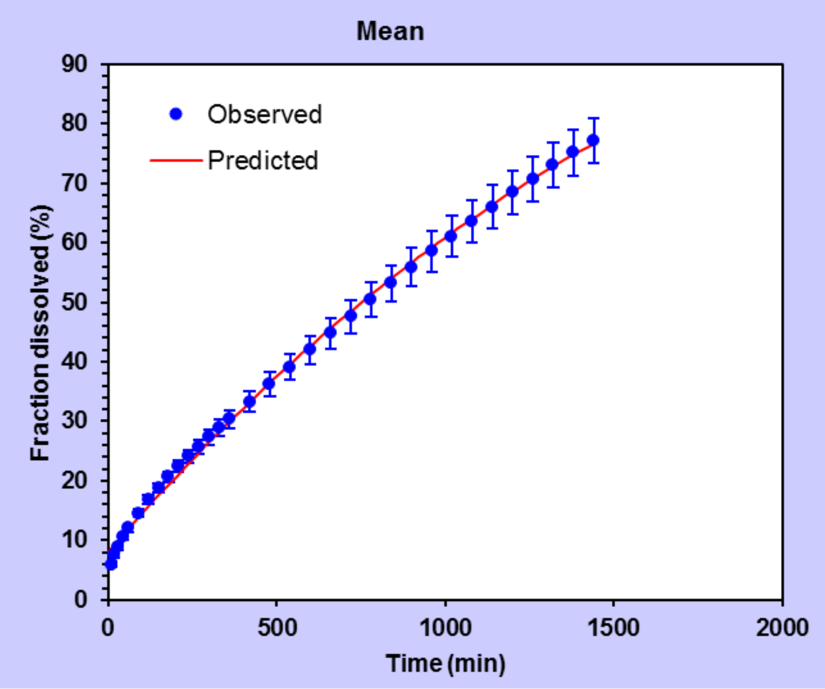

Supplementary materials – Model fitting summary – Starch 1500® sample with larger particle size (modelling the entire relevant dissolution data)

Graphical abstract of model fit presented as the fraction % of released carvedilol per tested tablet:

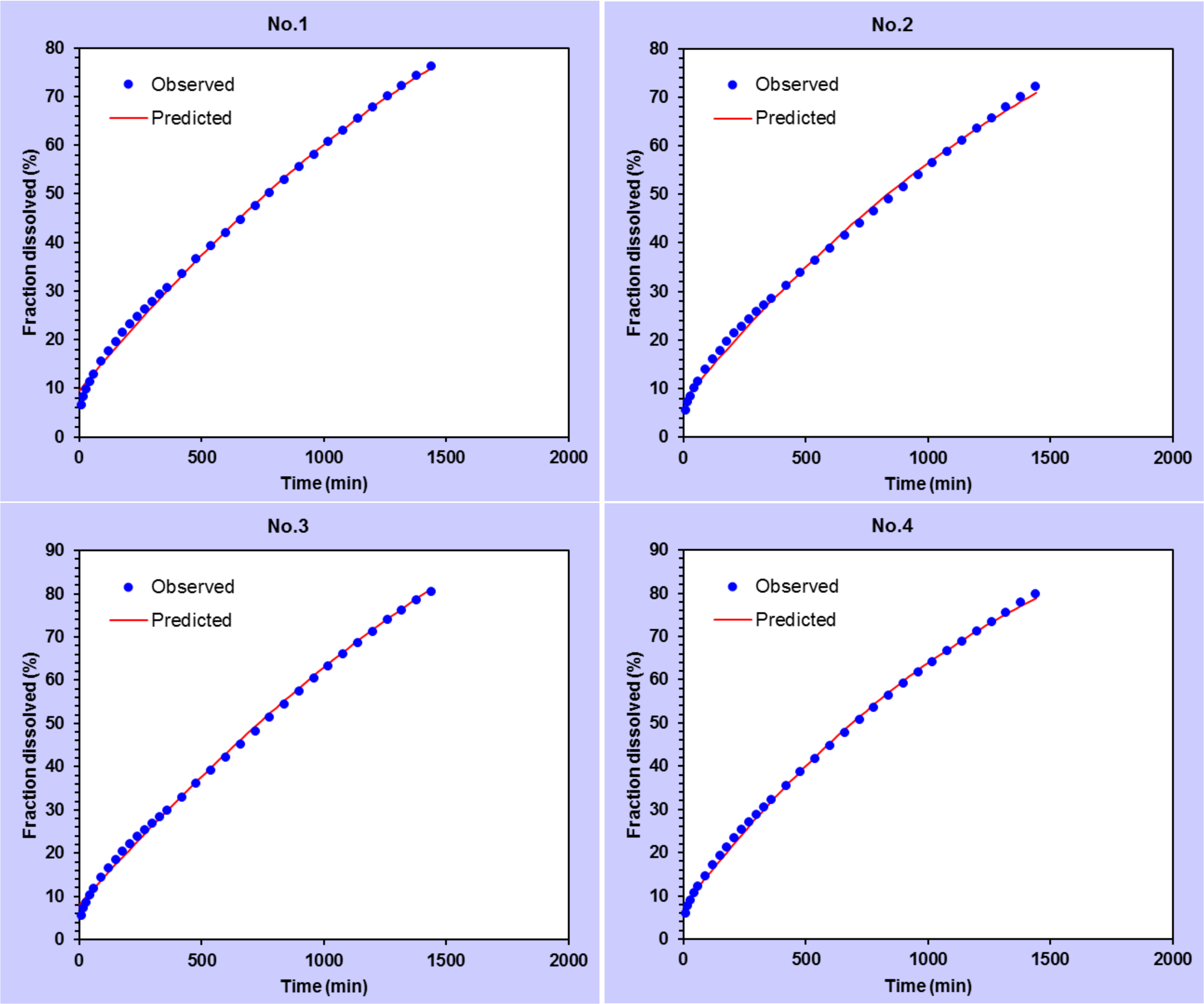

Supplementary materials – Model fitting summary – Starch 1500® sample with larger particle size (modelling the entire relevant dissolution data)

Model: **Baker–Lonsdale**

Model equation: $\frac{3}{2}\cdot\left[1-\left(1-\frac{F}{100}\right)^{\frac{2}{3}}\right]-\frac{F}{100}=k_{BL}\cdot t$

Fitted model parameters per tested tablet (N = 4) with statistics – mean, standard deviation (SD), and relative standard deviation expressed in % (RSD%) (output from DDSolver):

Parameter	No.1	No.2	No.3	No.4	Mean	SD	RSD(%)
k_{BL}	0.0001	0.0001	0.0001	0.0001	0.0001	0.0000	33.8665

Number of dissolution data points (N), degrees of freedom (df), and selected goodness of fit criteria – Pearson correlation coefficient (R), coefficient of determination (R^2), adjusted coefficient of determination ($R^2_{adjusted}$), and residual sum of squares (RSS) (manual calculation in MS Excel):

Parameter	No.1	No.2	No.3	No.4
N	33	33	33	33
df	32	32	32	32
R	0.984507551	0.984874057	0.975597385	0.988534548
R^2	0.969255118	0.969976907	0.951790258	0.977200552
$R^2_{adjusted}$	0.969255118	0.969976907	0.951790258	0.977200552
RSS	868.5454343	802.0159369	3698.029151	2418.736871

Graphical abstract of model fit presented as mean ± 1 SD of the fraction % of released carvedilol:

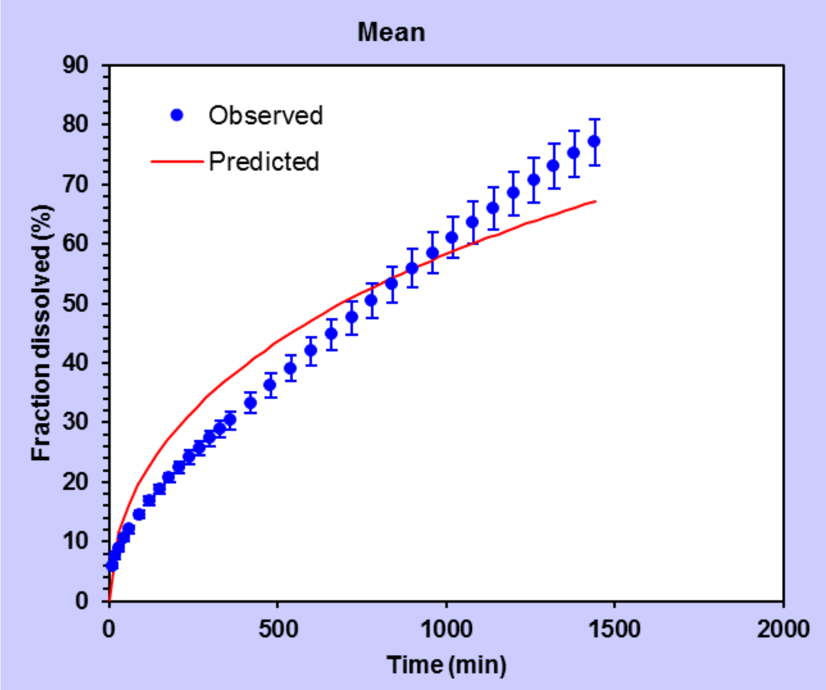

Supplementary materials – Model fitting summary – Starch 1500® sample with larger particle size (modelling the entire relevant dissolution data)

Graphical abstract of model fit presented as the fraction % of released carvedilol per tested tablet:

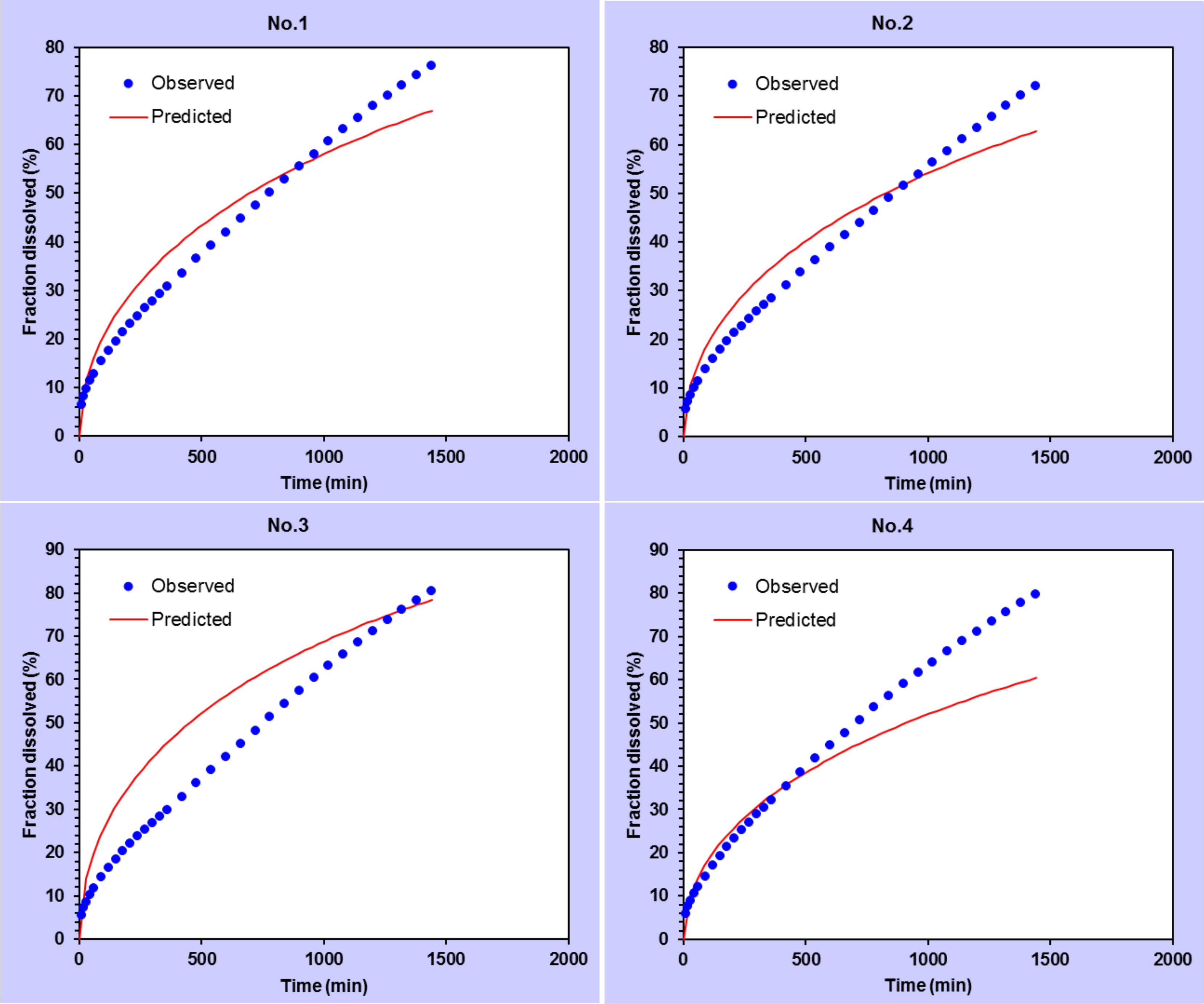

Supplementary materials – Model fitting summary – Starch 1500® sample with larger particle size (modelling the entire relevant dissolution data)

Model: **Baker–Lonsdale with T_{lag}**

Model equation: $\frac{3}{2}\cdot\left[1-\left(1-\frac{F}{100}\right)^{\frac{2}{3}}\right]-\frac{F}{100}=k_{BL}\cdot\left(t-T_{lag}\right)$

Fitted model parameters per tested tablet (N = 4) with statistics – mean, standard deviation (SD), and relative standard deviation expressed in % (RSD%) (output from DDSolver):

Parameter	No.1	No.2	No.3	No.4	Mean	SD	RSD(%)
k_{BL}	0.0001	0.0001	0.0001	0.0001	0.0001	0.0000	14.2981
T_{lag}	123.5312	125.3574	147.1995	128.5374	131.1563	10.8936	8.3058

Number of dissolution data points (N), degrees of freedom (df), and selected goodness of fit criteria – Pearson correlation coefficient (R), coefficient of determination (R^2), adjusted coefficient of determination ($R^2_{adjusted}$), and residual sum of squares (RSS) (manual calculation in MS Excel):

Parameter	No.1	No.2	No.3	No.4
N	33	33	33	33
df	31	31	31	31
R	0.972716557	0.973859885	0.969229894	0.976807546
R^2	0.946177501	0.948403075	0.939406587	0.954152982
$R^2_{adjusted}$	0.944441291	0.946738658	0.937451961	0.952674046
RSS	1460.676561	1208.902518	1711.874351	1355.651633

Graphical abstract of model fit presented as mean ± 1 SD of the fraction % of released carvedilol:

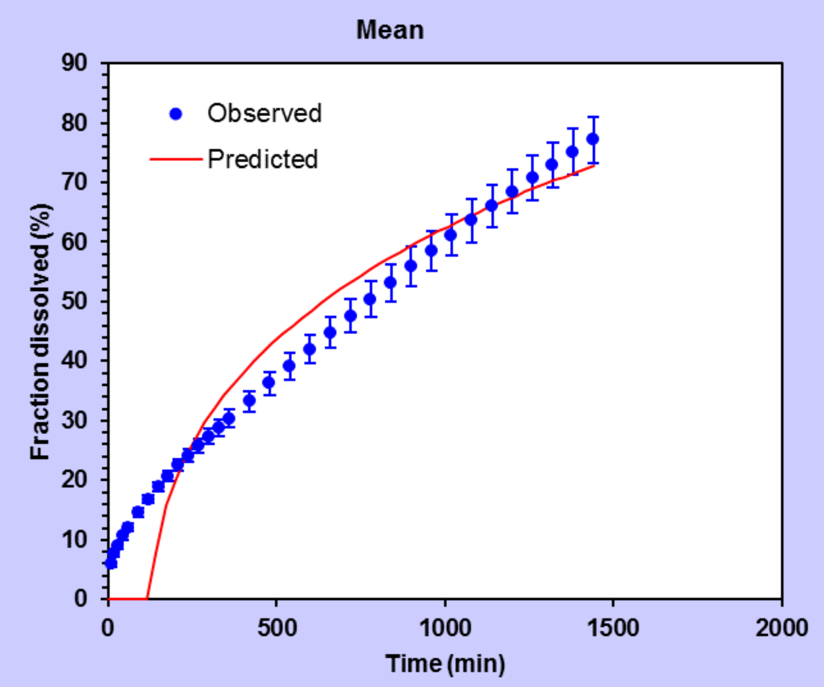

Supplementary materials – Model fitting summary – Starch 1500® sample with larger particle size (modelling the entire relevant dissolution data)

Graphical abstract of model fit presented as the fraction % of released carvedilol per tested tablet:

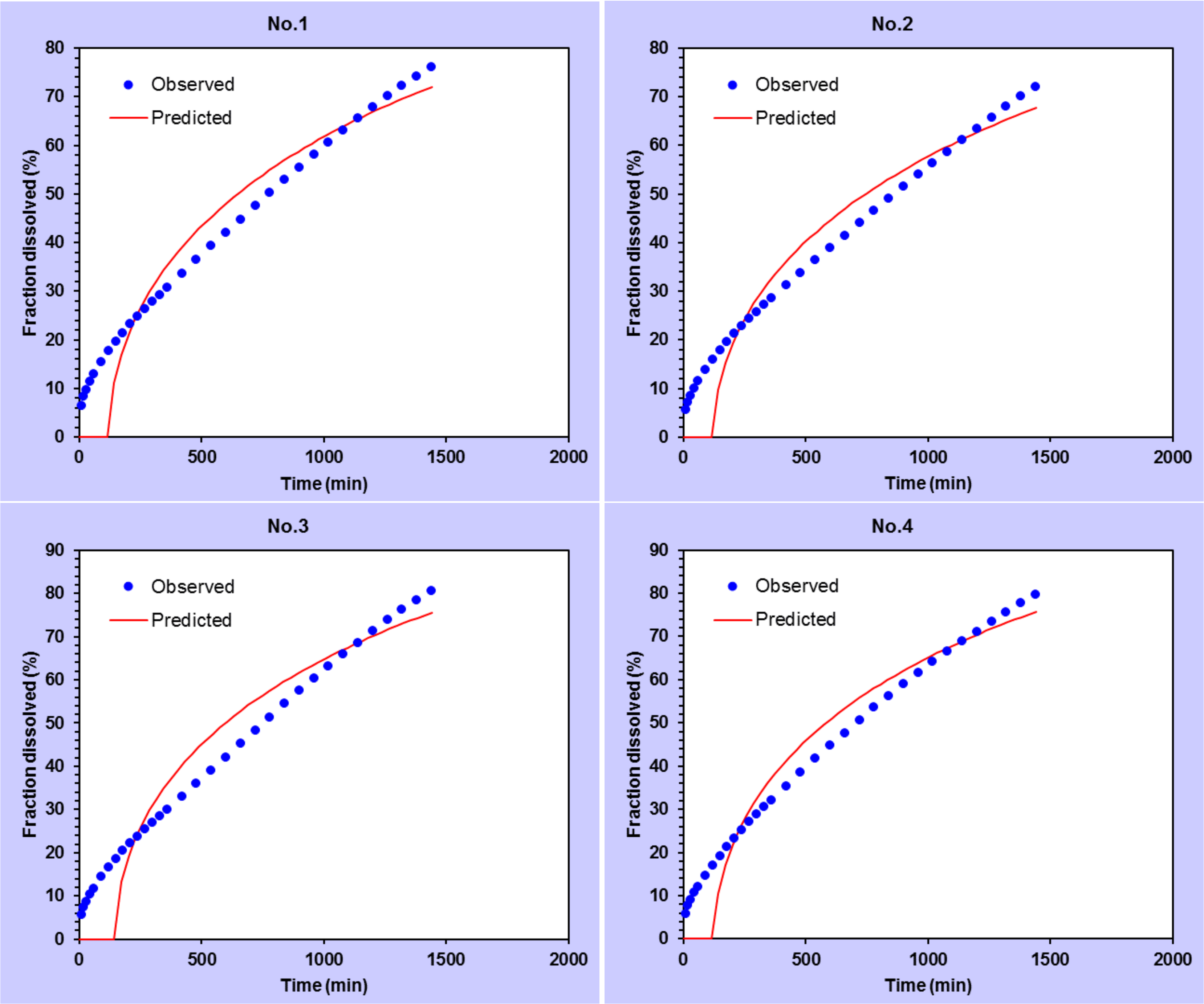

Supplementary materials – Model fitting summary – Starch 1500® sample with larger particle size (modelling the entire relevant dissolution data)

Model: **Makoid–Banakar**

Model equation: $F = k_{MB} \cdot t^n \cdot e^{-k \cdot t}$

Fitted model parameters per tested tablet (N = 4) with statistics – mean, standard deviation (SD), and relative standard deviation expressed in % (RSD%) (output from DDSolver):

Parameter	No.1	No.2	No.3	No.4	Mean	SD	RSD(%)
k_{MB}	2.3567	1.9263	1.8775	1.8472	2.0019	0.2388	11.9260
n	0.4155	0.4374	0.4481	0.4659	0.4417	0.0210	4.7644
k	-0.0003	-0.0003	-0.0004	-0.0003	-0.0003	0.0000	-10.5469

Number of dissolution data points (N), degrees of freedom (df), and selected goodness of fit criteria – Pearson correlation coefficient (R), coefficient of determination (R^2), adjusted coefficient of determination ($R^2_{adjusted}$), and residual sum of squares (RSS) (manual calculation in MS Excel):

Parameter	No.1	No.2	No.3	No.4
N	33	33	33	33
df	30	30	30	30
R	0.999078366	0.999518024	0.998817008	0.998443721
R^2	0.998157582	0.999036281	0.997635416	0.996889865
$R^2_{adjusted}$	0.998034754	0.998972033	0.997477777	0.996682523
RSS	29.11474568	13.73225188	44.18243383	57.23086251

Graphical abstract of model fit presented as mean ± 1 SD of the fraction % of released carvedilol:

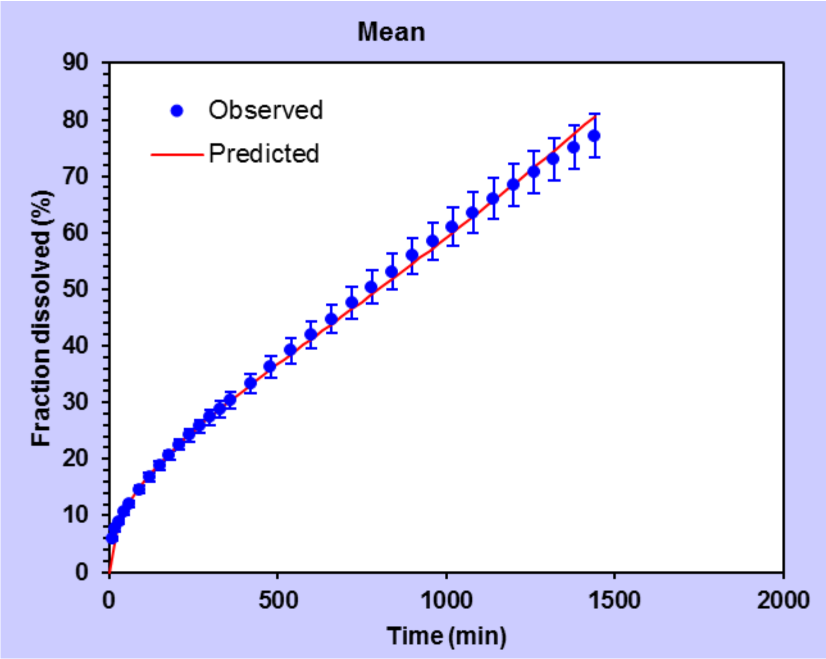

Supplementary materials – Model fitting summary – Starch 1500® sample with larger particle size (modelling the entire relevant dissolution data)

Graphical abstract of model fit presented as the fraction % of released carvedilol per tested tablet:

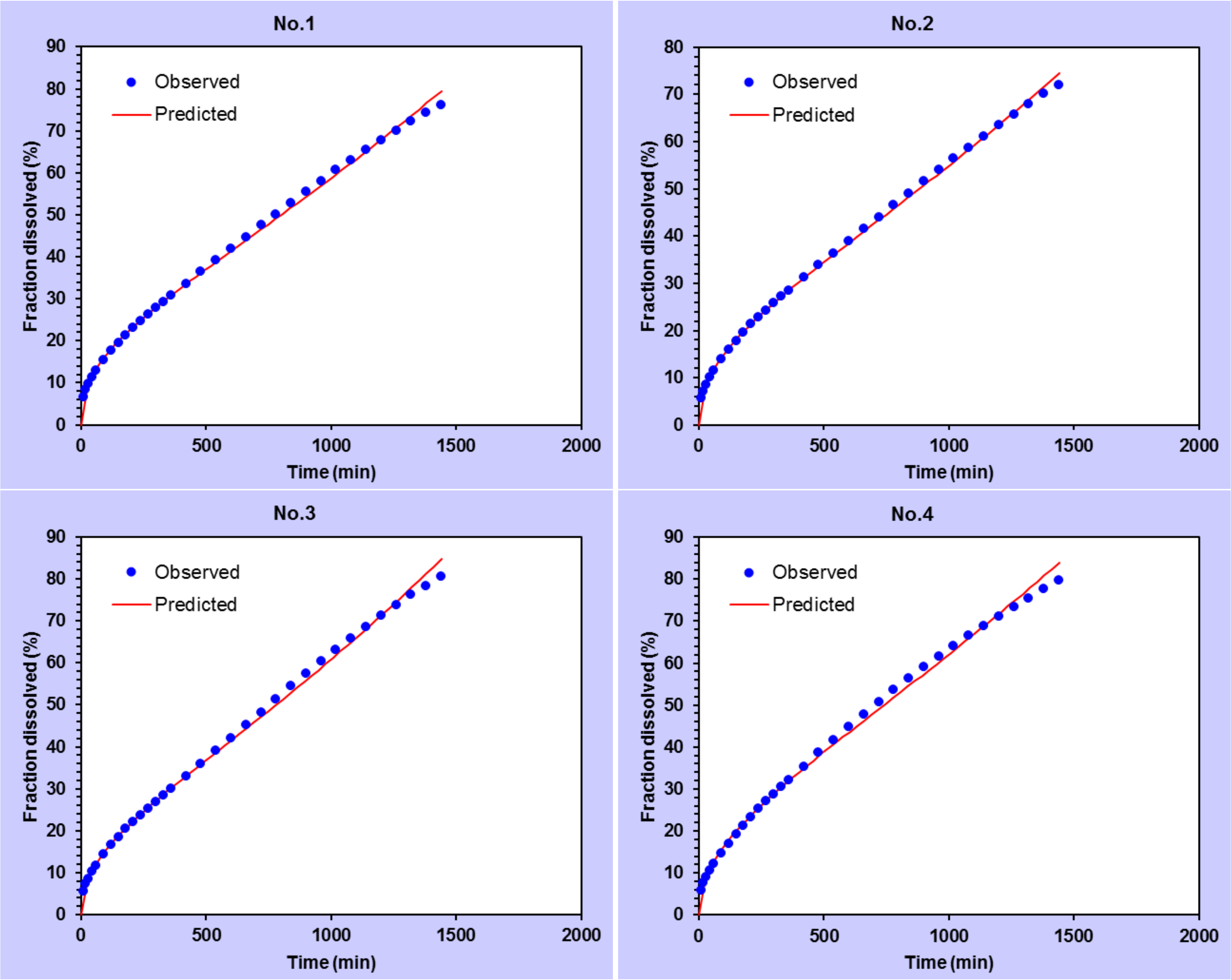

Supplementary materials – Model fitting summary – Starch 1500® sample with larger particle size (modelling the entire relevant dissolution data)

Model: **Makoid–Banakar with T_{lag}**

Model equation: $F = k_{MB} \cdot (t - T_{lag})^n \cdot e^{-k \cdot (t - T_{lag})}$

Fitted model parameters per tested tablet (N = 4) with statistics – mean, standard deviation (SD), and relative standard deviation expressed in % (RSD%) (output from DDSolver):

Parameter	No.1	No.2	No.3	No.4	Mean	SD	RSD(%)
k_{MB}	2.9741	2.4596	2.4018	2.4044	2.5600	0.2774	10.8349
n	0.3707	0.3902	0.4058	0.4149	0.3954	0.0194	4.8988
k	-0.0004	-0.0004	-0.0004	-0.0004	-0.0004	0.0000	-3.8063
T_{lag}	4.0000	4.0000	5.2209	4.0000	4.3052	0.6105	14.1796

Number of dissolution data points (N), degrees of freedom (df), and selected goodness of fit criteria – Pearson correlation coefficient (R), coefficient of determination (R^2), adjusted coefficient of determination ($R^2_{adjusted}$), and residual sum of squares (RSS) (manual calculation in MS Excel):

Parameter	No.1	No.2	No.3	No.4
N	33	33	33	33
df	29	29	29	29
R	0.998216879	0.998799803	0.998686493	0.99714166
R^2	0.996436937	0.997601046	0.997374712	0.99429149
$R^2_{adjusted}$	0.996068345	0.997352879	0.99710313	0.993700955
RSS	57.61980643	35.09995388	68.79842142	107.3375062

Graphical abstract of model fit presented as mean ± 1 SD of the fraction % of released carvedilol:

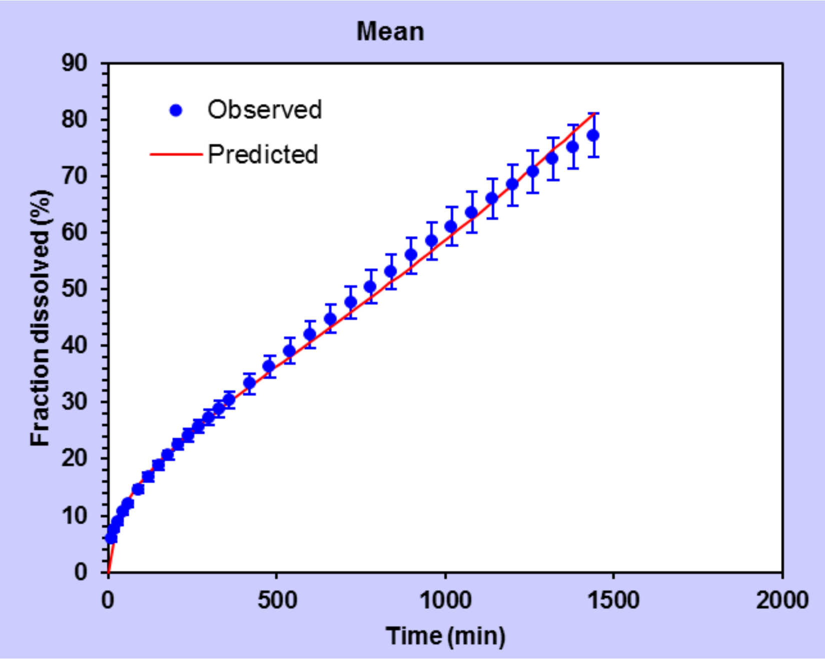

Supplementary materials – Model fitting summary – Starch 1500® sample with larger particle size (modelling the entire relevant dissolution data)

Graphical abstract of model fit presented as the fraction % of released carvedilol per tested tablet:

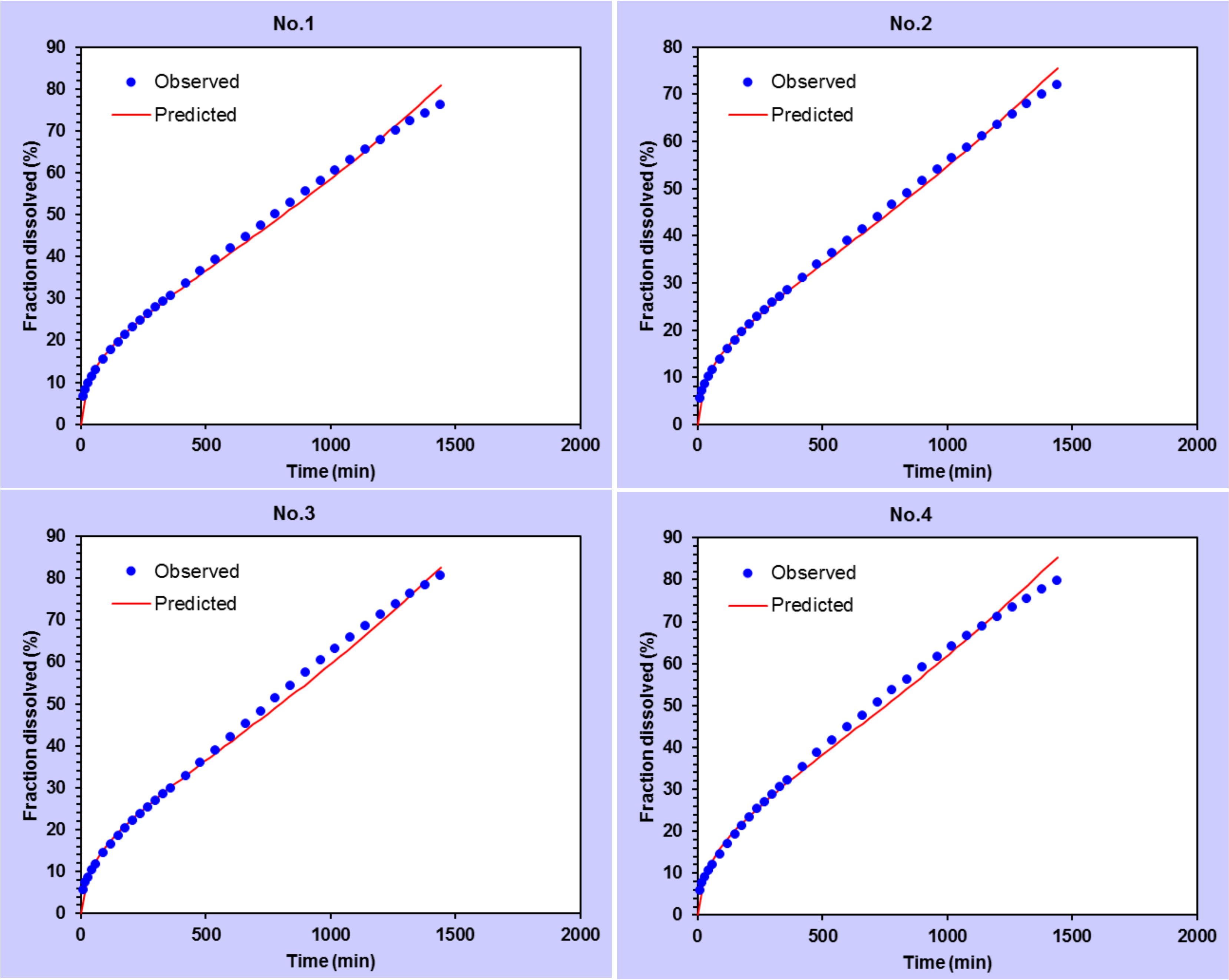

Supplementary materials – Model fitting summary – Starch 1500® sample with larger particle size (modelling the entire relevant dissolution data)

Model: **Peppas–Sahlin_1**

Model equation: $F = k_1 \cdot t^m + k_2 \cdot t^{2m}$

Fitted model parameters per tested tablet (N = 4) with statistics – mean, standard deviation (SD), and relative standard deviation expressed in % (RSD%) (output from DDSolver):

Parameter	No.1	No.2	No.3	No.4	Mean	SD	RSD(%)
k_1	1.483	1.300	1.177	1.461	1.356	0.144	10.648
k_2	0.053	0.053	0.071	0.060	0.059	0.009	14.367
m	0.450	0.450	0.450	0.450	0.450	0.000	0.000

Number of dissolution data points (N), degrees of freedom (df), and selected goodness of fit criteria – Pearson correlation coefficient (R), coefficient of determination (R^2), adjusted coefficient of determination ($R^2_{adjusted}$), and residual sum of squares (RSS) (manual calculation in MS Excel):

Parameter	No.1	No.2	No.3	No.4
N	33	33	33	33
df	30	30	30	30
R	0.999254214	0.999362045	0.999237515	0.999793809
R^2	0.998508985	0.998724498	0.998475612	0.99958766
$R^2_{adjusted}$	0.998409584	0.998639464	0.998373986	0.999560171
RSS	27.32832705	20.69060343	31.5718643	8.009730074

Graphical abstract of model fit presented as mean ± 1 SD of the fraction % of released carvedilol:

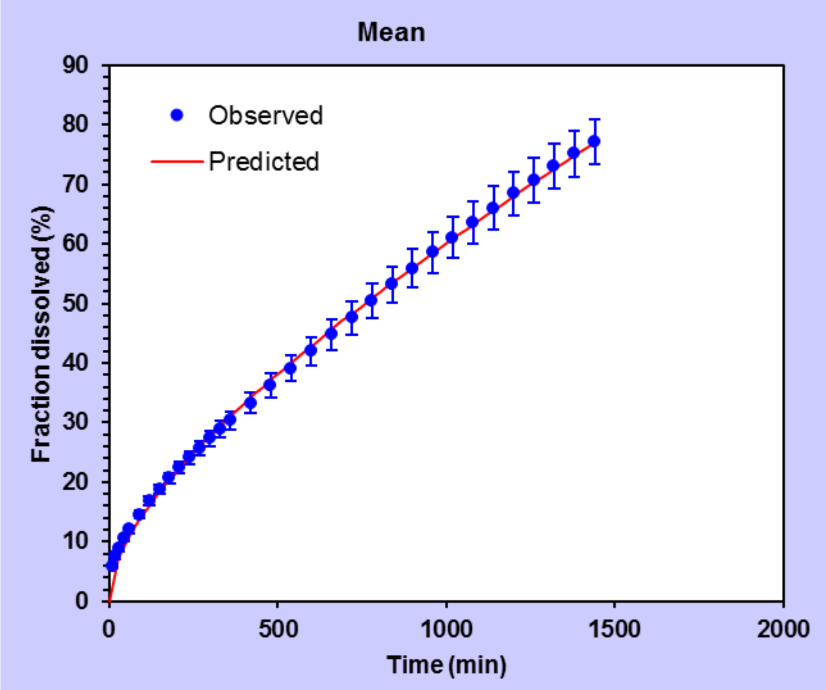

Supplementary materials – Model fitting summary – Starch 1500® sample with larger particle size (modelling the entire relevant dissolution data)

Graphical abstract of model fit presented as the fraction % of released carvedilol per tested tablet:

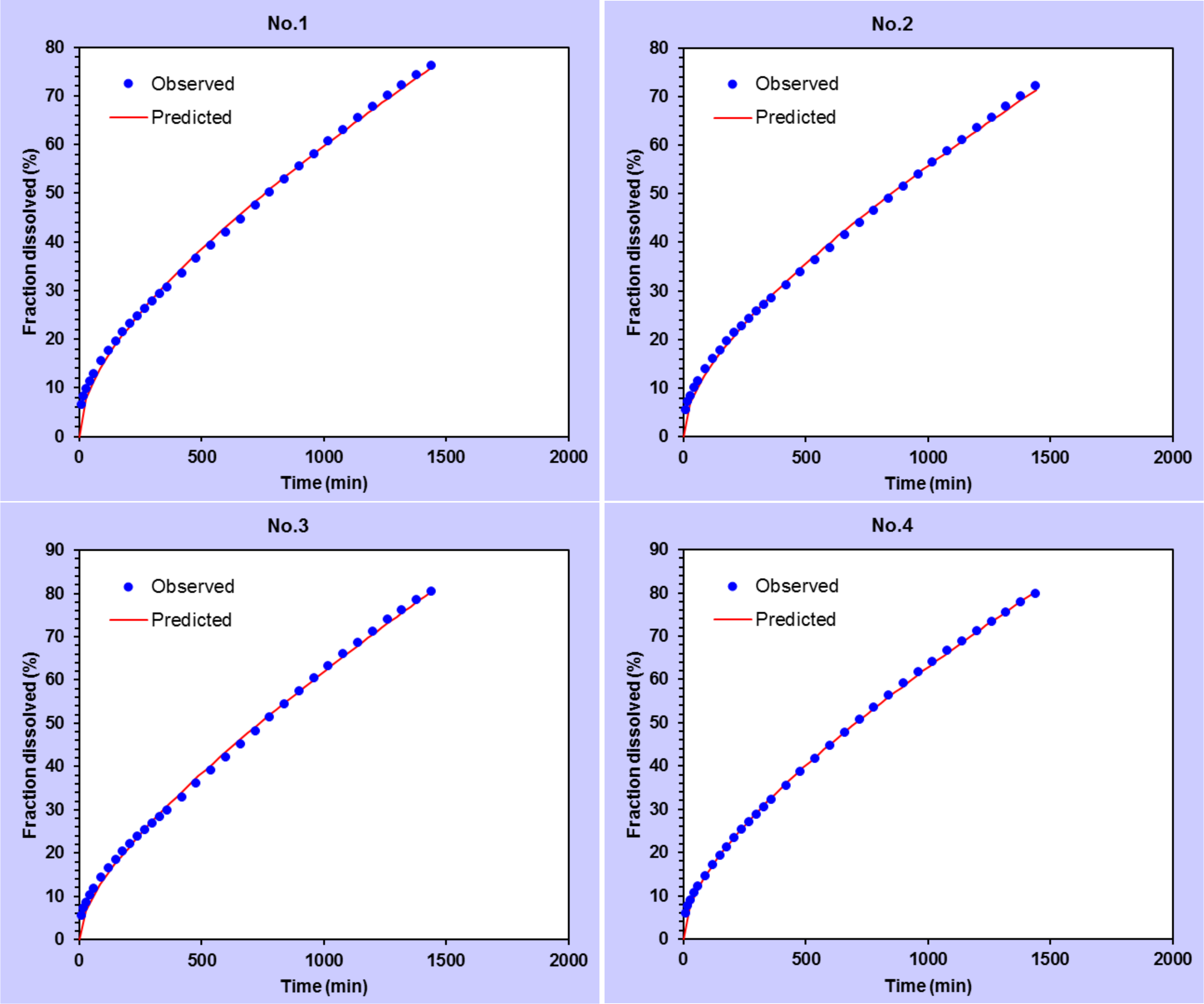

Supplementary materials – Model fitting summary – Starch 1500® sample with larger particle size (modelling the entire relevant dissolution data)

Model: **Peppas–Sahlin_1 with T_{lag}**

Model equation: $F = k_1 \cdot (t - T_{lag})^m + k_2 \cdot (t - T_{lag})^{2m}$

Fitted model parameters per tested tablet (N = 4) with statistics – mean, standard deviation (SD), and relative standard deviation expressed in % (RSD%) (output from DDSolver):

Parameter	No.1	No.2	No.3	No.4	Mean	SD	RSD(%)
k_1	1.527	1.341	1.220	1.509	1.399	0.146	10.423
k_2	0.051	0.052	0.069	0.058	0.058	0.009	14.767
m	0.450	0.450	0.450	0.450	0.450	0.000	0.000
T_{lag}	4.000	4.000	4.000	4.000	4.000	0.000	0.000

Number of dissolution data points (N), degrees of freedom (df), and selected goodness of fit criteria – Pearson correlation coefficient (R), coefficient of determination (R^2), adjusted coefficient of determination ($R^2_{adjusted}$), and residual sum of squares (RSS) (manual calculation in MS Excel):

Parameter	No.1	No.2	No.3	No.4
N	33	33	33	33
df	29	29	29	29
R	0.998960252	0.999114448	0.999016966	0.999634693
R^2	0.997921584	0.99822968	0.998034898	0.99926952
$R^2_{adjusted}$	0.997706576	0.998046543	0.997831611	0.999193953
RSS	39.55476357	29.87220891	42.14192914	15.04347734

Graphical abstract of model fit presented as mean ± 1 SD of the fraction % of released carvedilol:

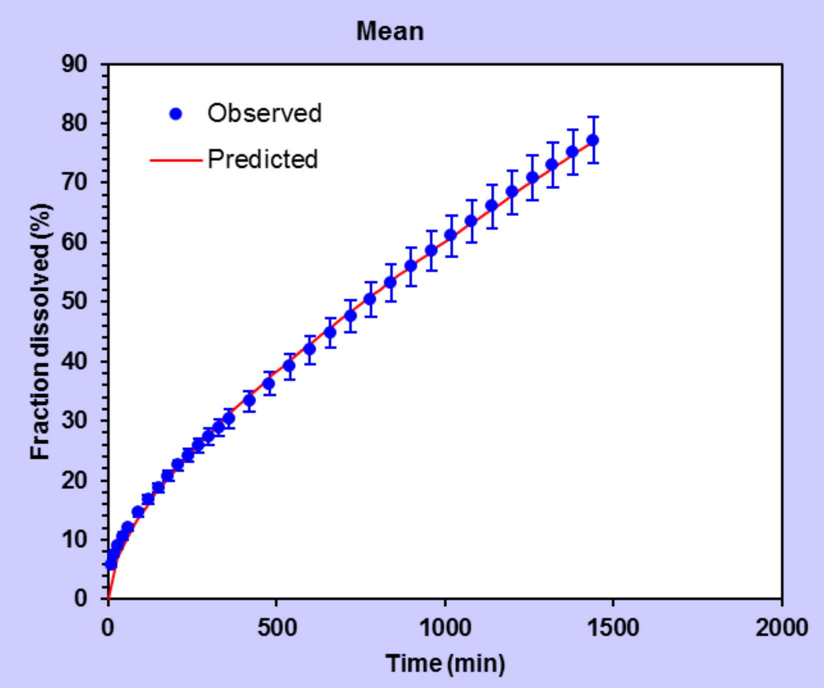

Supplementary materials – Model fitting summary – Starch 1500® sample with larger particle size (modelling the entire relevant dissolution data)

Graphical abstract of model fit presented as the fraction % of released carvedilol per tested tablet:

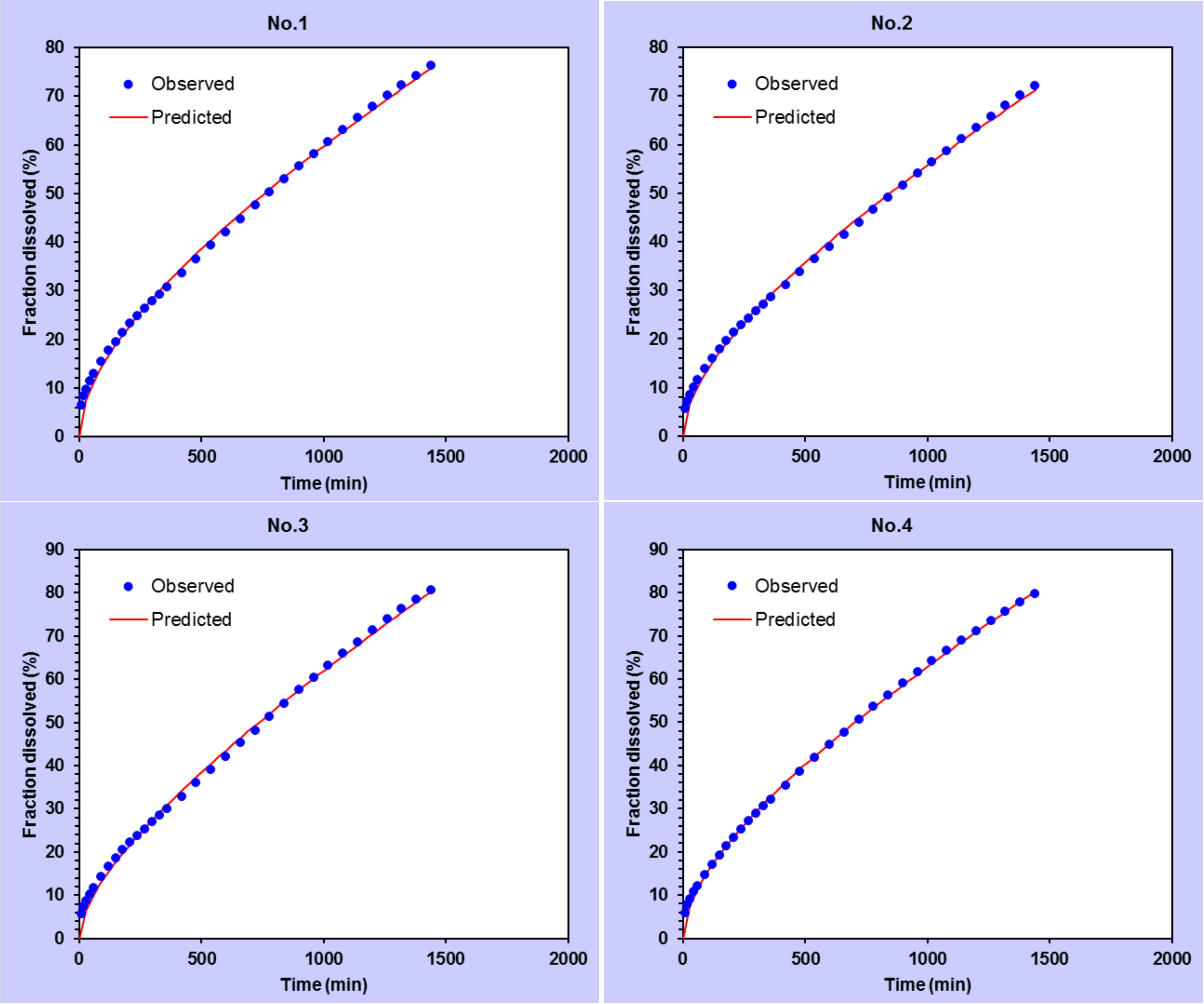

Supplementary materials – Model fitting summary – Starch 1500® sample with larger particle size (modelling the entire relevant dissolution data)

Model: **Peppas–Sahlin_2**

Model equation: $F = k_1 \cdot t^{0.5} + k_2 \cdot t$

Fitted model parameters per tested tablet (N = 4) with statistics – mean, standard deviation (SD), and relative standard deviation expressed in % (RSD%) (output from DDSolver):

Parameter	No.1	No.2	No.3	No.4	Mean	SD	RSD(%)
k_1	1.319	1.173	1.117	1.327	1.234	0.106	8.556
k_2	0.018	0.019	0.027	0.021	0.021	0.004	18.632

Number of dissolution data points (N), degrees of freedom (df), and selected goodness of fit criteria – Pearson correlation coefficient (R), coefficient of determination (R^2), adjusted coefficient of determination ($R^2_{adjusted}$), and residual sum of squares (RSS) (manual calculation in MS Excel):

Parameter	No.1	No.2	No.3	No.4
N	33	33	33	33
df	31	31	31	31
R	0.999197148	0.999350739	0.999307143	0.999756595
R^2	0.998394941	0.9987019	0.998614767	0.99951325
$R^2_{adjusted}$	0.998343165	0.998660026	0.998570082	0.999497548
RSS	31.47932487	22.59773957	30.15869799	9.751708918

Graphical abstract of model fit presented as mean ± 1 SD of the fraction % of released carvedilol:

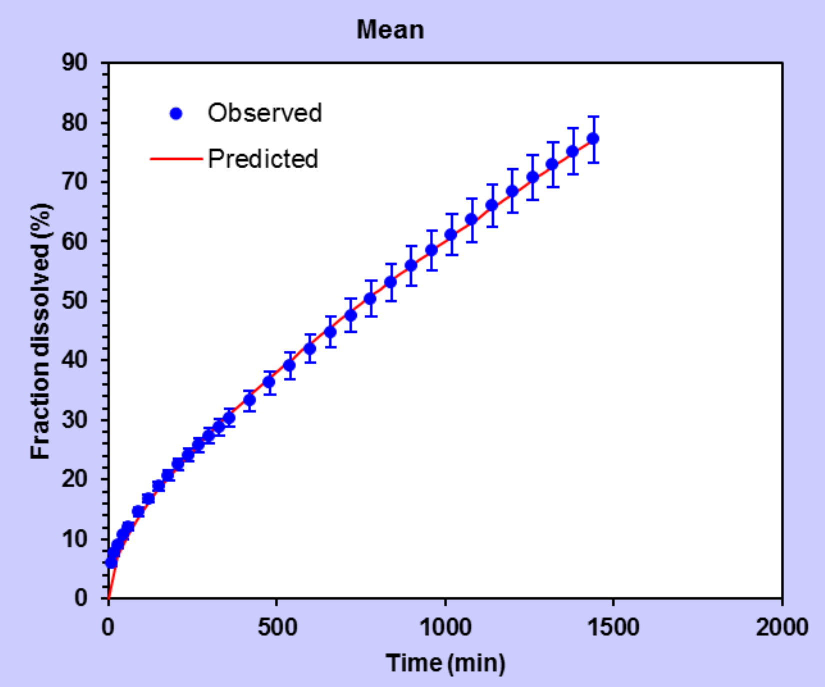

Supplementary materials – Model fitting summary – Starch 1500® sample with larger particle size (modelling the entire relevant dissolution data)

Graphical abstract of model fit presented as the fraction % of released carvedilol per tested tablet:

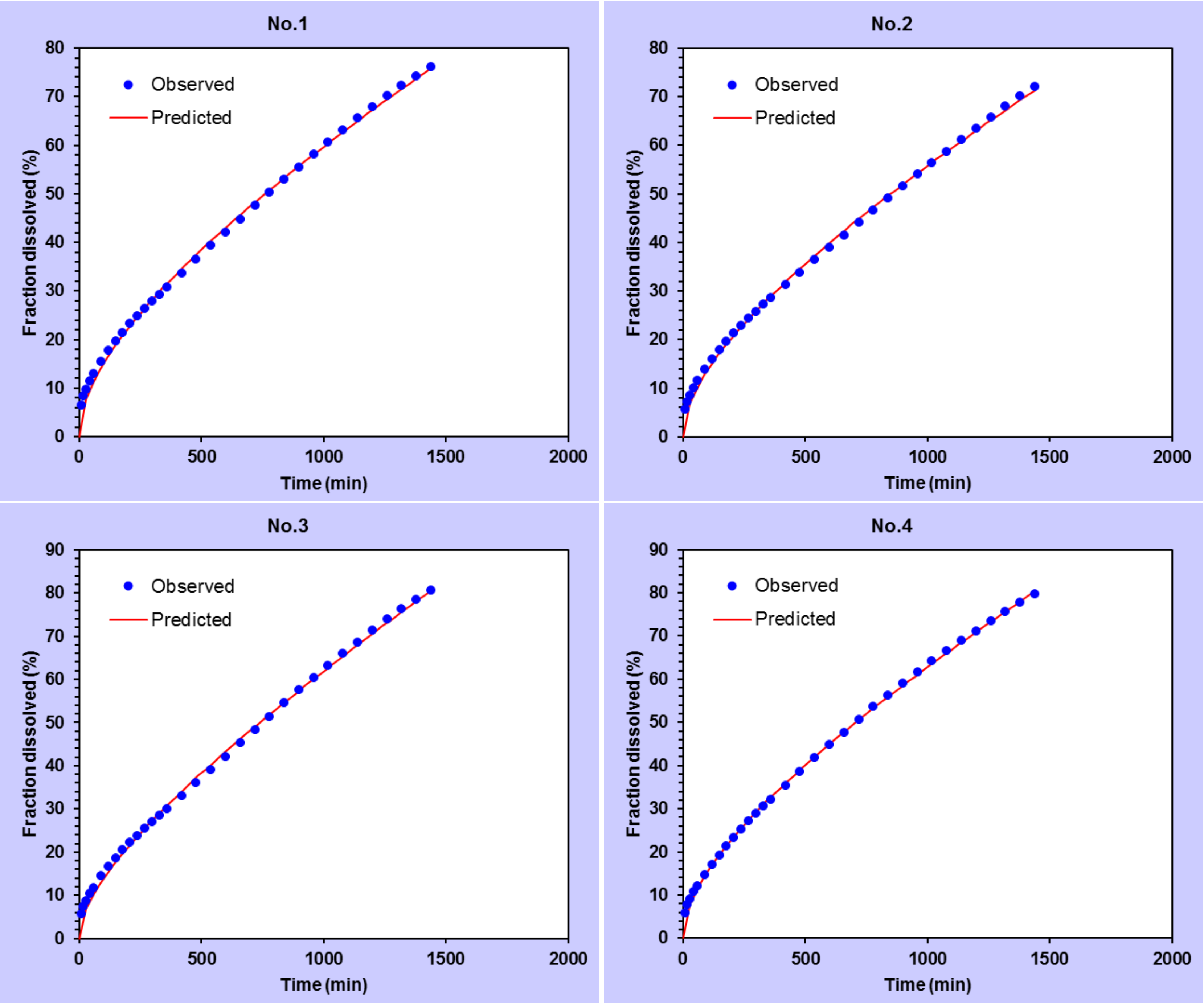

Supplementary materials – Model fitting summary – Starch 1500® sample with larger particle size (modelling the entire relevant dissolution data)

Model: **Peppas–Sahlin_2 with T_{lag}**

Model equation: $F = k_1 \cdot (t - T_{lag})^{0.5} + k_2 \cdot (t - T_{lag})$

Fitted model parameters per tested tablet (N = 4) with statistics – mean, standard deviation (SD), and relative standard deviation expressed in % (RSD%) (output from DDSolver):

Parameter	No.1	No.2	No.3	No.4	Mean	SD	RSD(%)
k_1	1.349	1.200	1.146	1.359	1.263	0.107	8.451
k_2	0.017	0.018	0.026	0.020	0.020	0.004	19.334
T_{lag}	4.000	4.000	4.000	4.000	4.000	0.000	0.000

Number of dissolution data points (N), degrees of freedom (df), and selected goodness of fit criteria – Pearson correlation coefficient (R), coefficient of determination (R^2), adjusted coefficient of determination ($R^2_{adjusted}$), and residual sum of squares (RSS) (manual calculation in MS Excel):

Parameter	No.1	No.2	No.3	No.4
N	33	33	33	33
df	30	30	30	30
R	0.99888353	0.999087811	0.999084682	0.999580607
R^2	0.997768307	0.998176454	0.998170202	0.999161391
$R^2_{adjusted}$	0.997619527	0.998054884	0.998048215	0.999105483
RSS	45.65682217	33.1792109	41.59210155	18.01916362

Graphical abstract of model fit presented as mean ± 1 SD of the fraction % of released carvedilol:

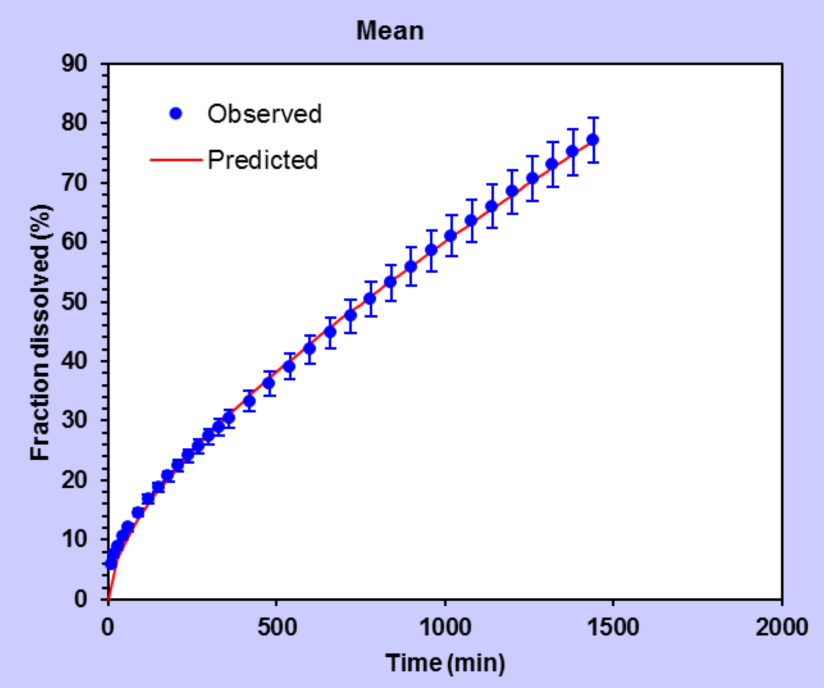

Supplementary materials – Model fitting summary – Starch 1500® sample with larger particle size (modelling the entire relevant dissolution data)

Graphical abstract of model fit presented as the fraction % of released carvedilol per tested tablet:

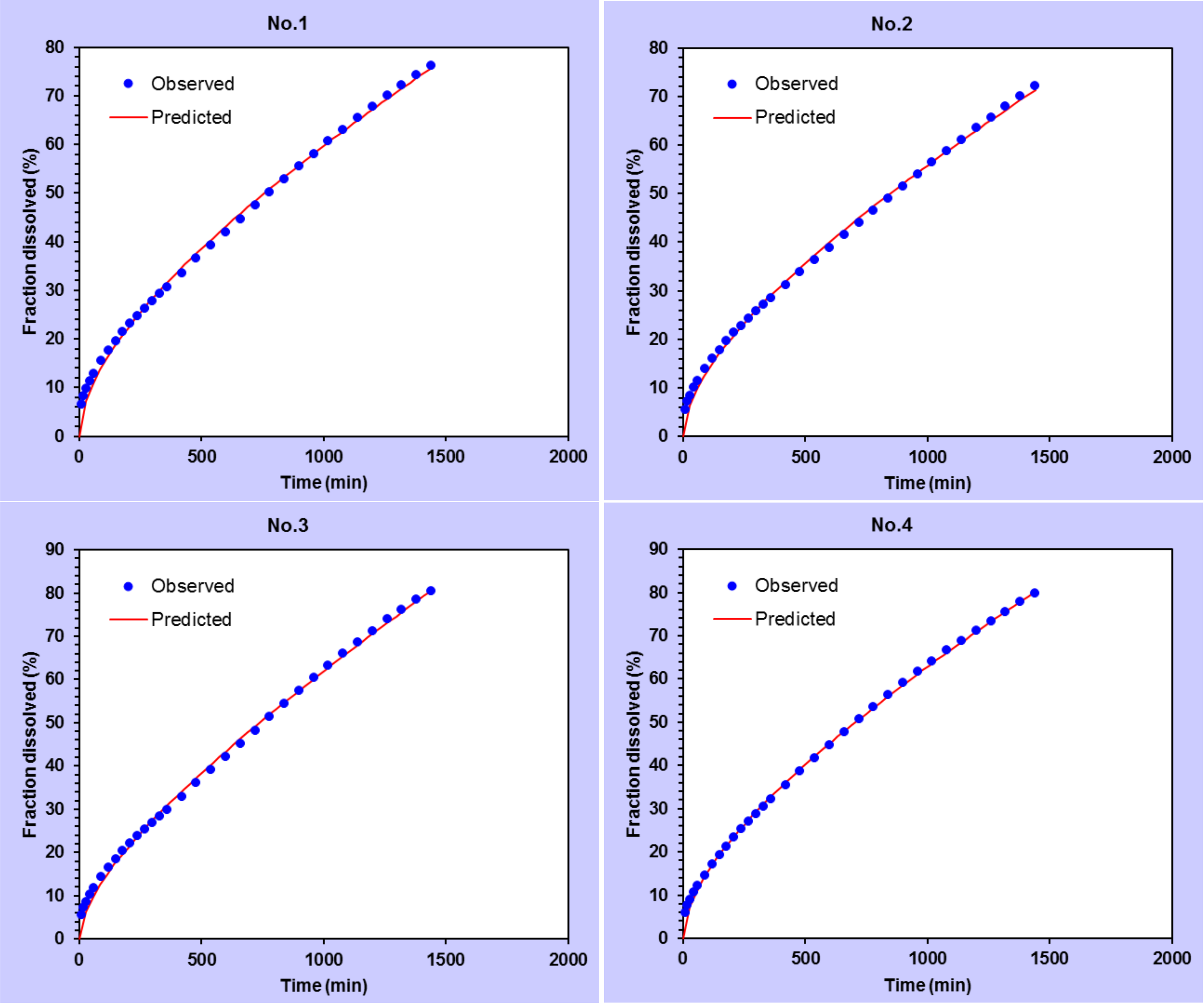

Supplementary materials – Model fitting summary – Starch 1500® sample with larger particle size (modelling the entire relevant dissolution data)

Model: **Quadratic**

Model equation: $F = 100 \cdot (k_1 \cdot t^2 + k_2 \cdot t)$

Fitted model parameters per tested tablet (N = 4) with statistics – mean, standard deviation (SD), and relative standard deviation expressed in % (RSD%) (output from DDSolver):

Parameter	No.1	No.2	No.3	No.4	Mean	SD	RSD(%)
k_1	-0.0000003	-0.0000002	-0.0000002	-0.0000003	-0.0000003	0.0000000	-10.5598913
k_2	0.0009071	0.0008315	0.0008808	0.0009562	0.0008939	0.0000520	5.8182273

Number of dissolution data points (N), degrees of freedom (df), and selected goodness of fit criteria – Pearson correlation coefficient (R), coefficient of determination (R^2), adjusted coefficient of determination ($R^2_{adjusted}$), and residual sum of squares (RSS) (manual calculation in MS Excel):

Parameter	No.1	No.2	No.3	No.4
N	33	33	33	33
df	31	31	31	31
R	0.993352273	0.993622927	0.995430838	0.995714521
R^2	0.986748738	0.987286521	0.990882554	0.991447407
$R^2_{adjusted}$	0.986321278	0.986876409	0.990588443	0.991171517
RSS	613.3054145	496.8869198	453.3639159	460.8691421

Graphical abstract of model fit presented as mean ± 1 SD of the fraction % of released carvedilol:

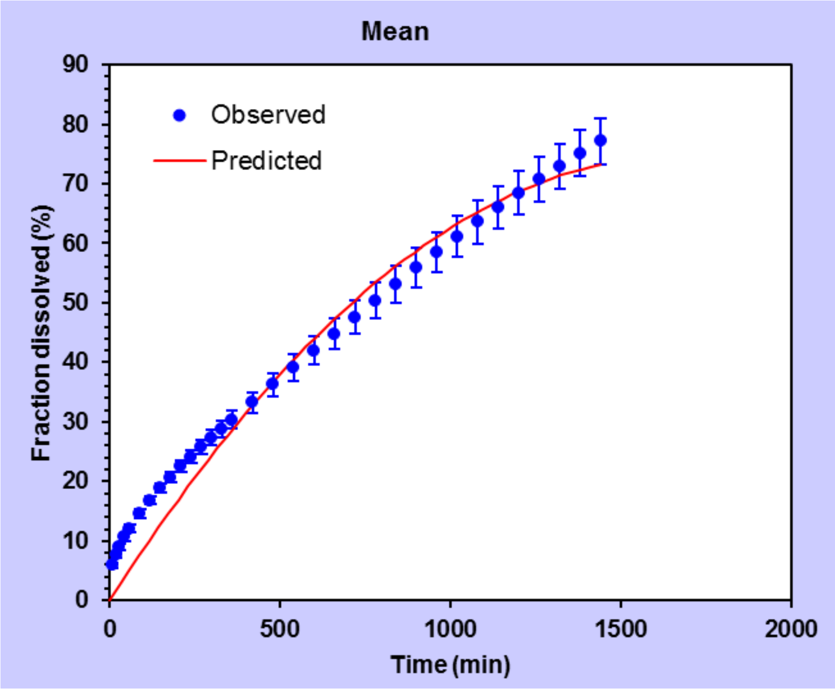

Supplementary materials – Model fitting summary – Starch 1500® sample with larger particle size (modelling the entire relevant dissolution data)

Graphical abstract of model fit presented as the fraction % of released carvedilol per tested tablet:

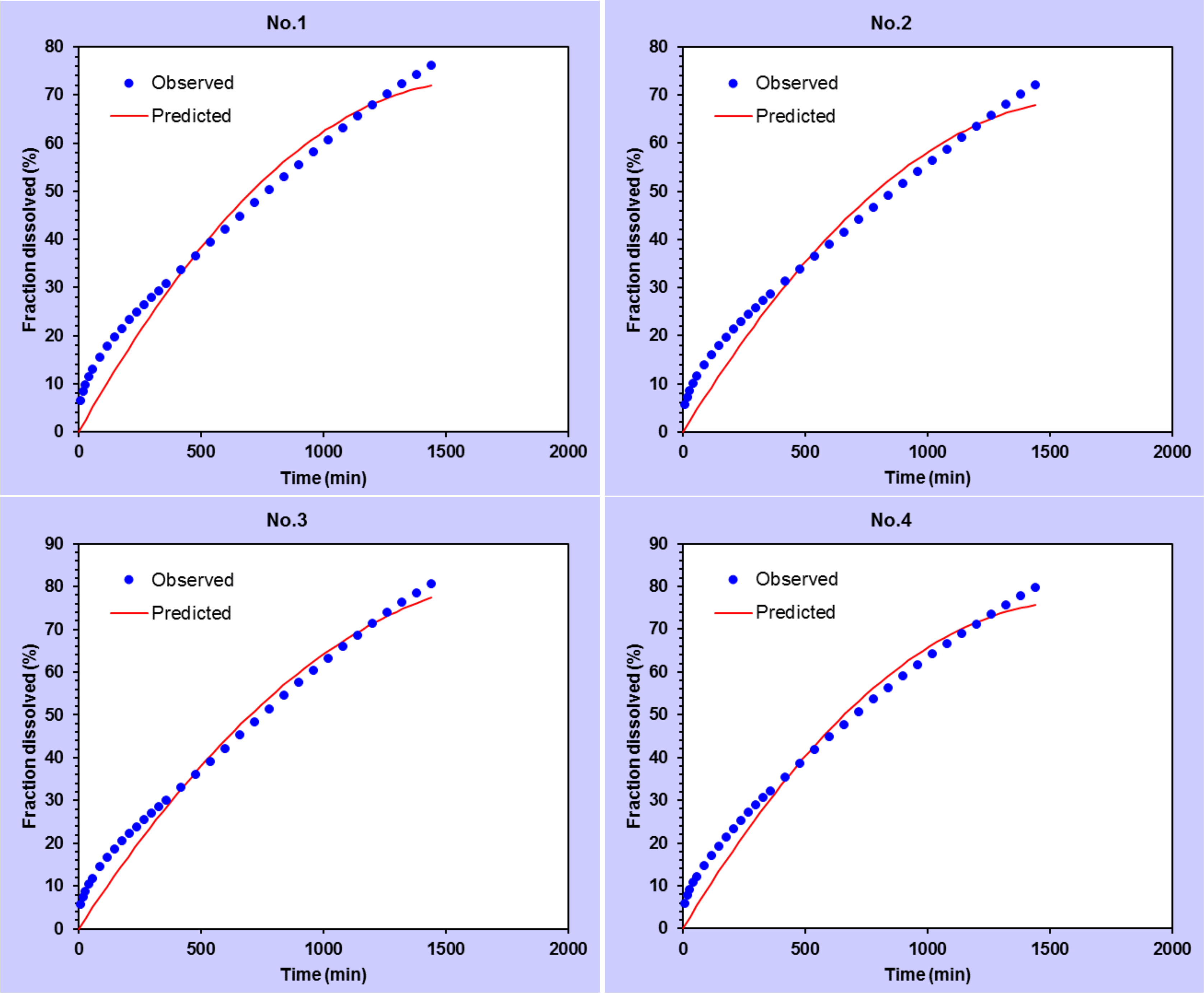

Supplementary materials – Model fitting summary – Starch 1500® sample with larger particle size (modelling the entire relevant dissolution data)

Model: **Quadratic with T_{lag}**

Model equation: $F = 100 \cdot \left[k_1 \cdot \left(t - T_{lag}\right)^2 + k_2 \cdot \left(t - T_{lag}\right)\right]$

Fitted model parameters per tested tablet (N = 4) with statistics – mean, standard deviation (SD), and relative standard deviation expressed in % (RSD%) (output from DDSolver):

Parameter	No.1	No.2	No.3	No.4	Mean	SD	RSD(%)
k_1	-0.0000003	-0.0000003	-0.0000002	-0.0000003	-0.0000003	0.0000000	-10.4682580
k_2	0.0009140	0.0008380	0.0008880	0.0009638	0.0009009	0.0000525	5.8226271
T_{lag}	4.0000000	4.0000000	4.0000000	4.0000000	4.0000000	0.0000000	0.0000000

Number of dissolution data points (N), degrees of freedom (df), and selected goodness of fit criteria – Pearson correlation coefficient (R), coefficient of determination (R^2), adjusted coefficient of determination ($R^2_{adjusted}$), and residual sum of squares (RSS) (manual calculation in MS Excel):

Parameter	No.1	No.2	No.3	No.4
N	33	33	33	33
df	30	30	30	30
R	0.993096985	0.993374864	0.995206746	0.995488447
R^2	0.986241622	0.986793621	0.990436467	0.990997247
$R^2_{adjusted}$	0.985324397	0.985913196	0.989798898	0.990397064
RSS	660.8587243	535.8006785	493.0873132	504.5791089

Graphical abstract of model fit presented as mean ± 1 SD of the fraction % of released carvedilol:

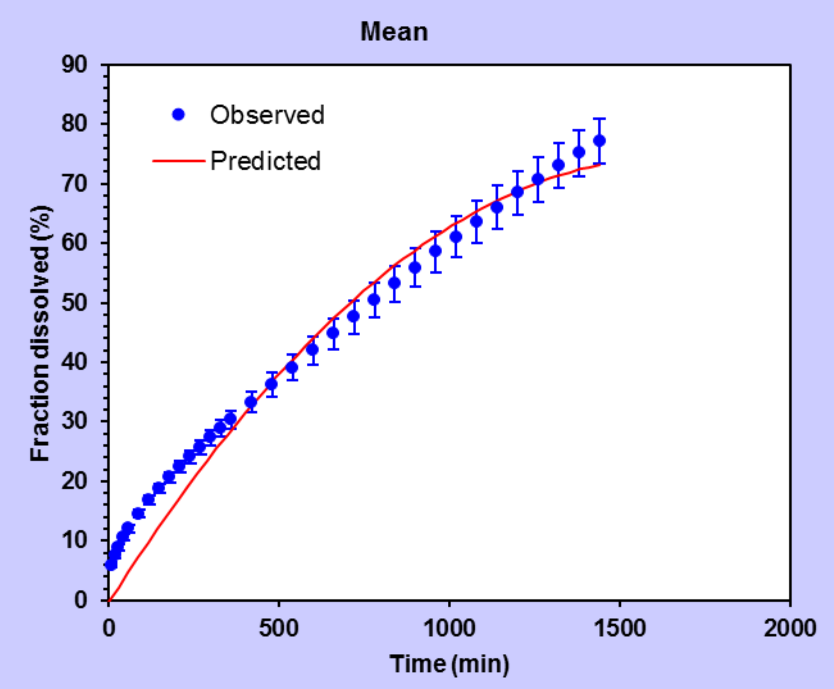

Supplementary materials – Model fitting summary – Starch 1500® sample with larger particle size (modelling the entire relevant dissolution data)

Graphical abstract of model fit presented as the fraction % of released carvedilol per tested tablet:

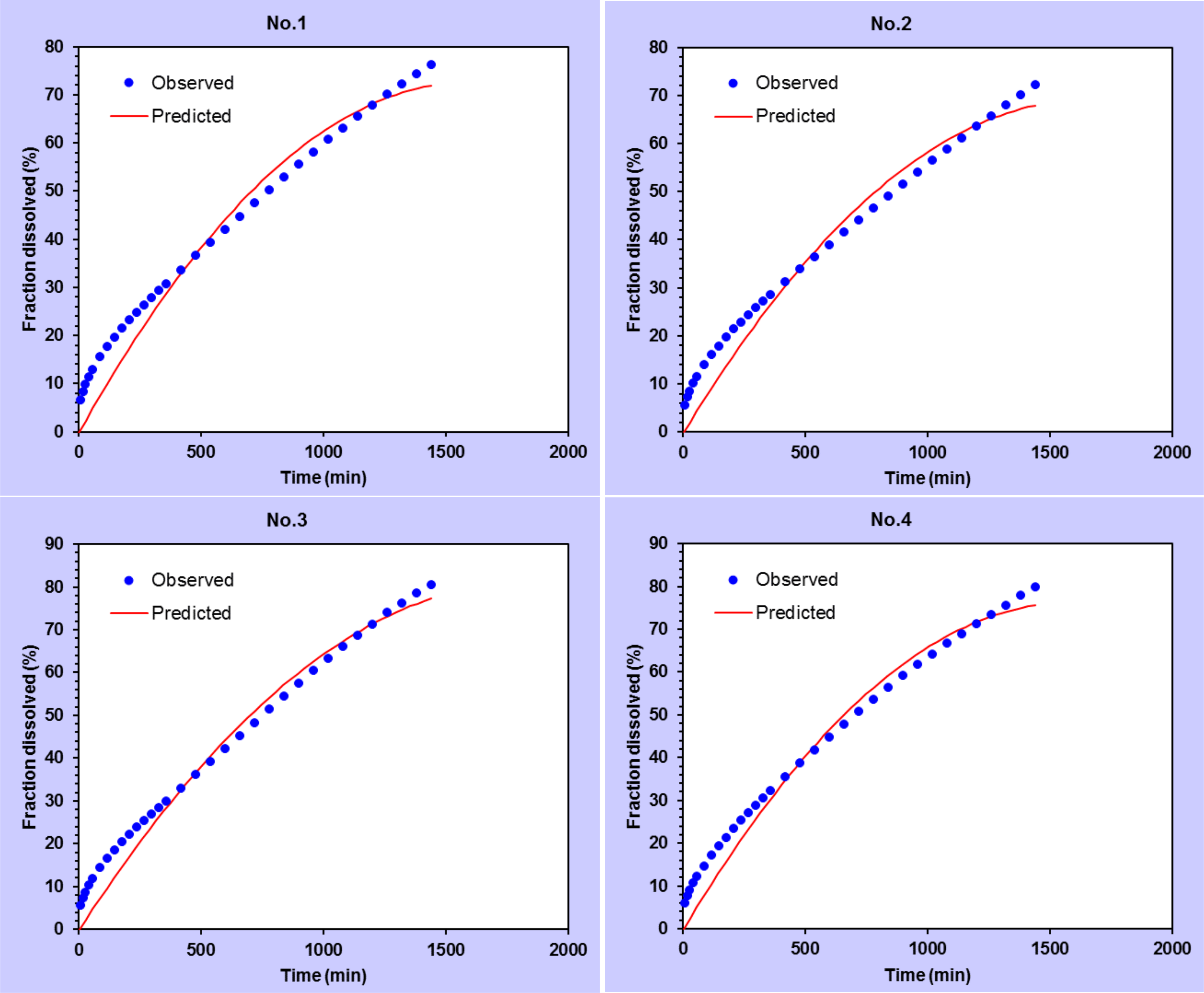

Supplementary materials – Model fitting summary – Starch 1500® sample with larger particle size (modelling the entire relevant dissolution data)

Model: **Weibull_1**

Model equation: $F = 100 \cdot \left[1 - e^{-\frac{(t-T_i)^{\beta}}{\alpha}}\right]$

Fitted model parameters per tested tablet (N = 4) with statistics – mean, standard deviation (SD), and relative standard deviation expressed in % (RSD%) (output from DDSolver):

Parameter	No.1	No.2	No.3	No.4	Mean	SD	RSD(%)
α	75.716	87.220	103.221	94.904	90.265	11.695	12.956
β	0.600	0.605	0.652	0.646	0.626	0.027	4.353
T_i	6.000	6.000	6.000	6.000	6.000	0.000	0.000

Number of dissolution data points (N), degrees of freedom (df), and selected goodness of fit criteria – Pearson correlation coefficient (R), coefficient of determination (R^2), adjusted coefficient of determination ($R^2_{adjusted}$), and residual sum of squares (RSS) (manual calculation in MS Excel):

Parameter	No.1	No.2	No.3	No.4
N	33	33	33	33
df	30	30	30	30
R	0.978930233	0.98112408	0.977217141	0.983619052
R^2	0.958304402	0.962604461	0.95495334	0.967506439
$R^2_{adjusted}$	0.955524695	0.960111425	0.95195023	0.965340202
RSS	863.6128851	719.6757653	1117.198902	813.0972299

Graphical abstract of model fit presented as mean ± 1 SD of the fraction % of released carvedilol:

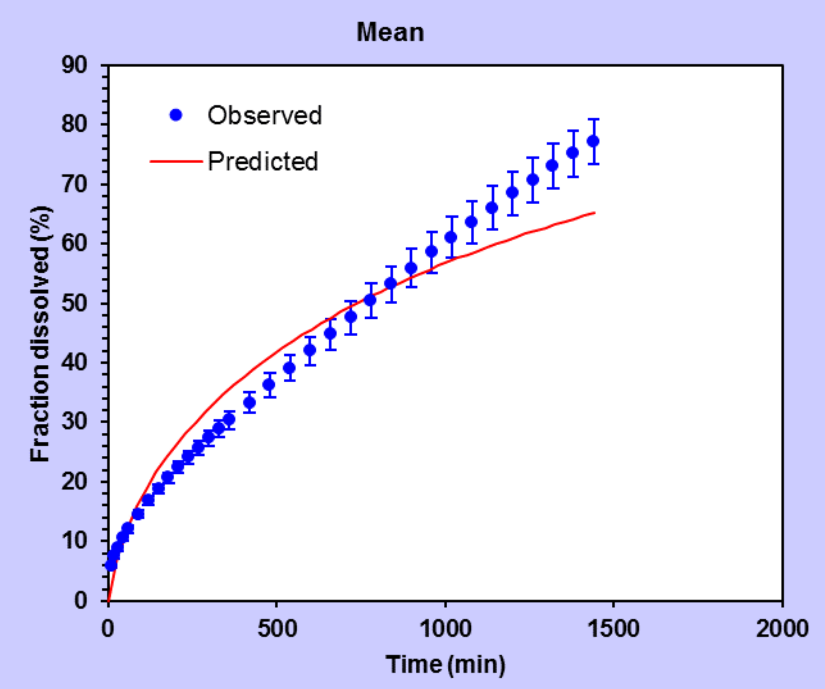

Supplementary materials – Model fitting summary – Starch 1500® sample with larger particle size (modelling the entire relevant dissolution data)

Graphical abstract of model fit presented as the fraction % of released carvedilol per tested tablet:

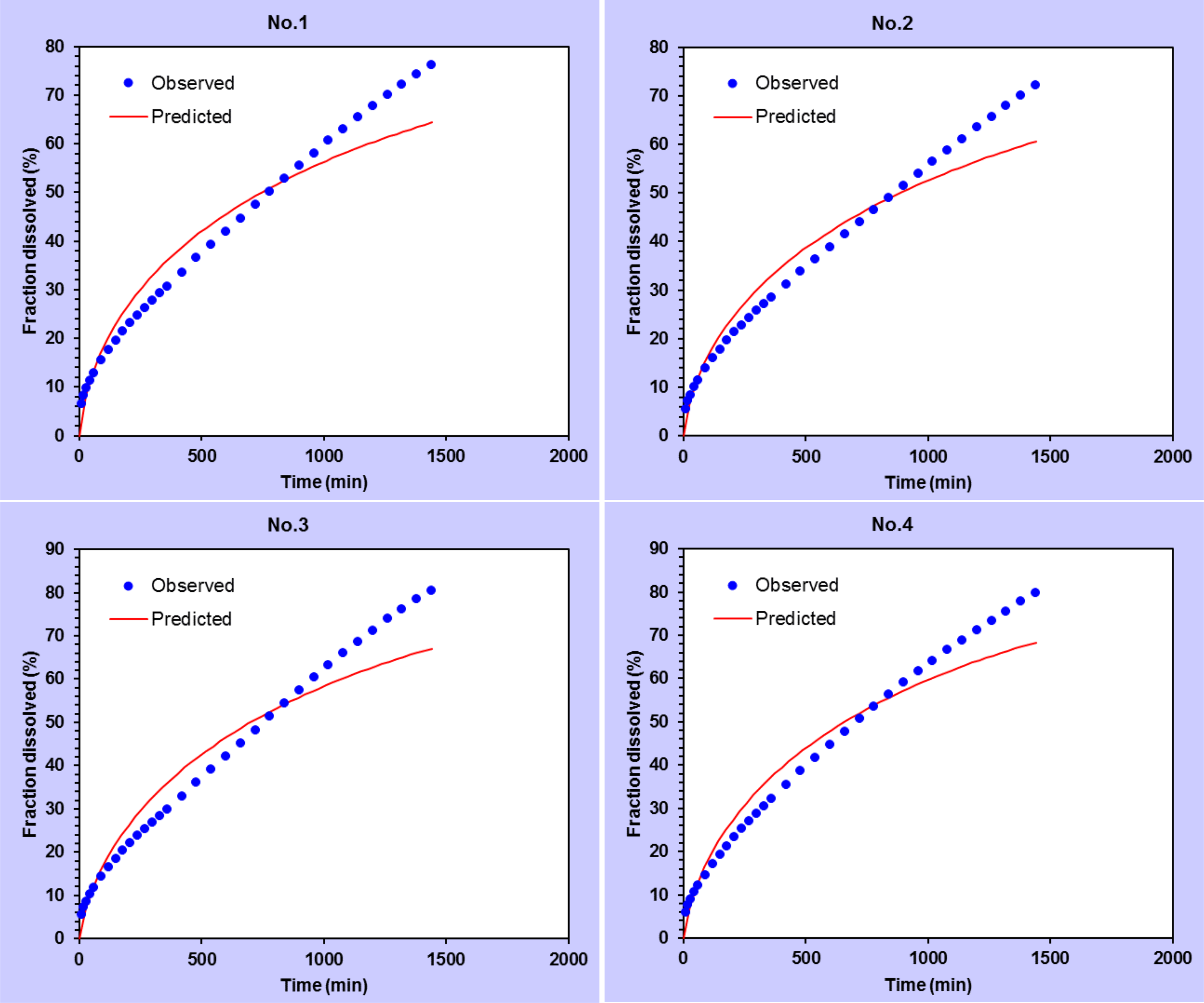

Supplementary materials – Model fitting summary – Starch 1500® sample with larger particle size (modelling the entire relevant dissolution data)

Model: **Weibull_2**

Model equation: $F = 100 \cdot \left(1 - e^{-\frac{t^{\beta}}{\alpha}}\right)$

Fitted model parameters per tested tablet (N = 4) with statistics – mean, standard deviation (SD), and relative standard deviation expressed in % (RSD%) (output from DDSolver):

Parameter	No.1	No.2	No.3	No.4	Mean	SD	RSD(%)
α	97.269	112.082	135.691	124.248	117.323	16.482	14.049
β	0.639	0.644	0.695	0.688	0.666	0.029	4.372

Number of dissolution data points (N), degrees of freedom (df), and selected goodness of fit criteria – Pearson correlation coefficient (R), coefficient of determination (R^2), adjusted coefficient of determination ($R^2_{adjusted}$), and residual sum of squares (RSS) (manual calculation in MS Excel):

Parameter	No.1	No.2	No.3	No.4
N	33	33	33	33
df	31	31	31	31
R	0.983318322	0.985177288	0.981470604	0.987411122
R^2	0.966914922	0.970574289	0.963284546	0.974980725
$R^2_{adjusted}$	0.965847662	0.969625072	0.962100176	0.974173651
RSS	680.2675854	558.2808338	896.3040151	619.8654642

Graphical abstract of model fit presented as mean ± 1 SD of the fraction % of released carvedilol:

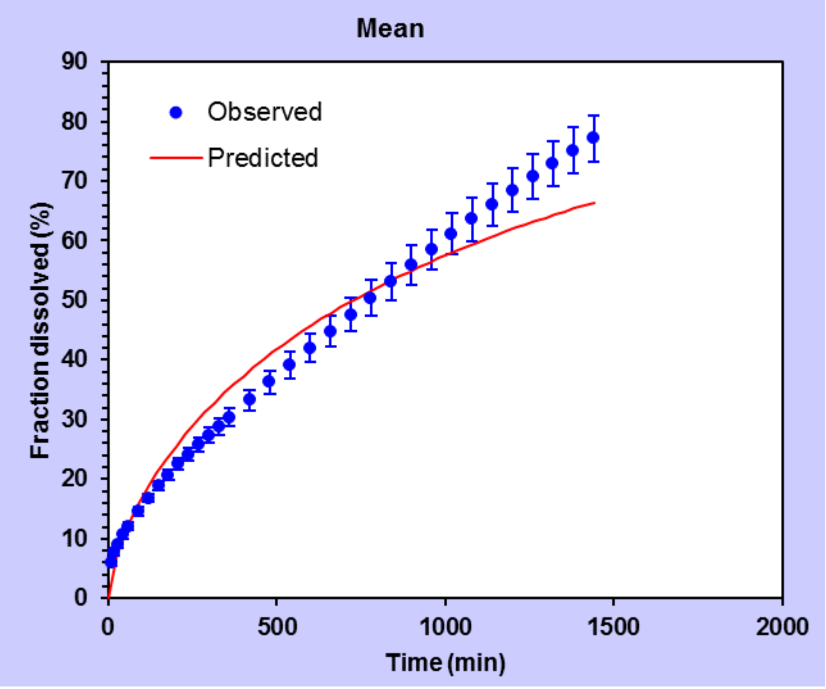

Supplementary materials – Model fitting summary – Starch 1500® sample with larger particle size (modelling the entire relevant dissolution data)

Graphical abstract of model fit presented as the fraction % of released carvedilol per tested tablet:

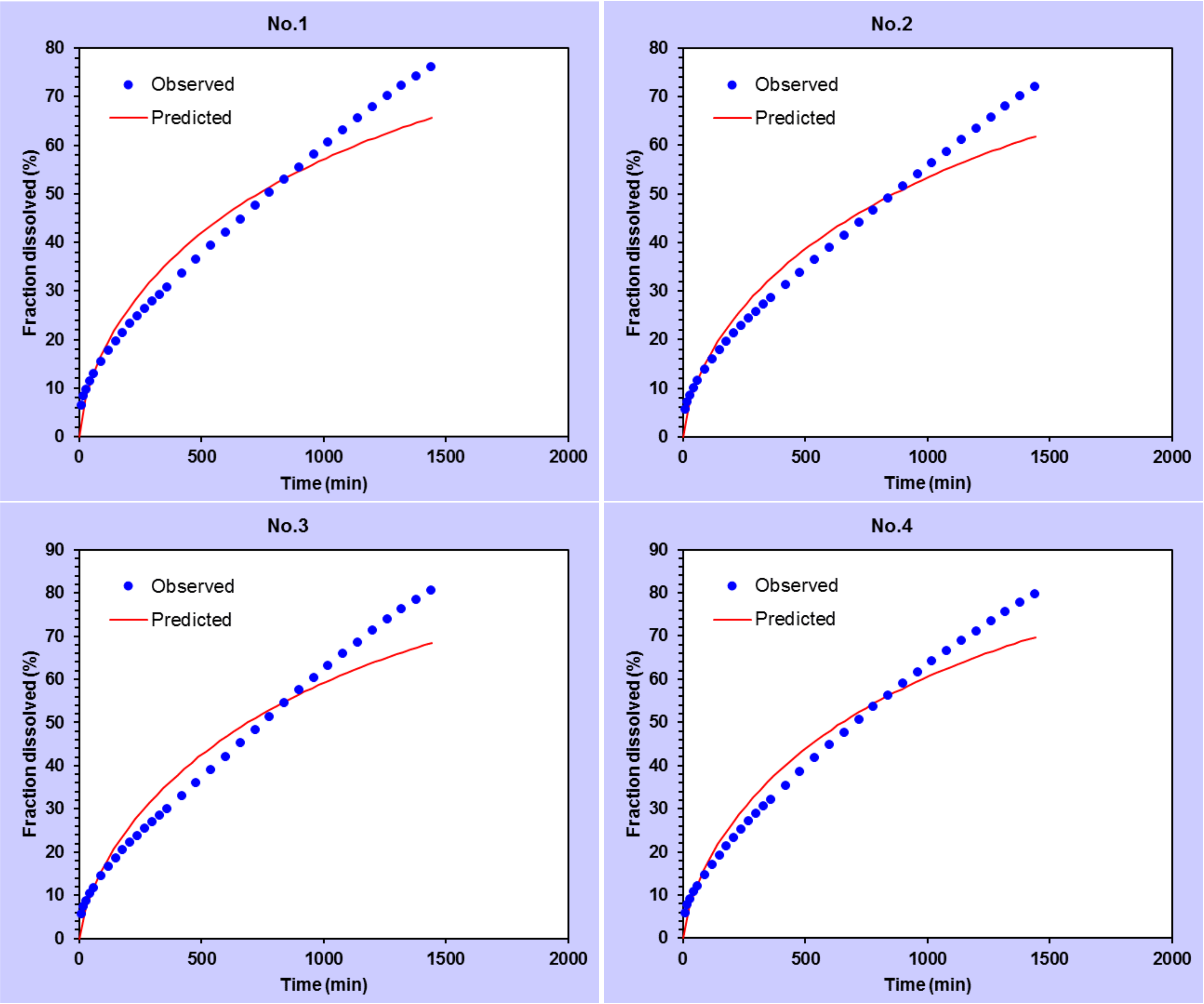

Supplementary materials – Model fitting summary – Starch 1500® sample with larger particle size (modelling the entire relevant dissolution data)

Model: **Weibull_3**

Model equation: $F = F_{max} \cdot \left(1 - e^{-\frac{t^{\beta}}{\alpha}}\right)$

Fitted model parameters per tested tablet (N = 4) with statistics – mean, standard deviation (SD), and relative standard deviation expressed in % (RSD%) (output from DDSolver):

Parameter	No.1	No.2	No.3	No.4	Mean	SD	RSD(%)
α	135.793	117.373	146.180	175.704	143.763	24.399	16.972
β	0.721	0.724	0.754	0.762	0.740	0.021	2.781
F_{max}	83.531	75.665	84.550	87.422	82.792	5.029	6.074

Number of dissolution data points (N), degrees of freedom (df), and selected goodness of fit criteria – Pearson correlation coefficient (R), coefficient of determination (R^2), adjusted coefficient of determination ($R^2_{adjusted}$), and residual sum of squares (RSS) (manual calculation in MS Excel):

Parameter	No.1	No.2	No.3	No.4
N	33	33	33	33
df	30	30	30	30
R	0.98268145	0.9759995	0.974723277	0.987705389
R^2	0.965662832	0.952575023	0.950085467	0.975561935
$R^2_{adjusted}$	0.963373687	0.949413358	0.946757831	0.973932731
RSS	883.8109271	765.718577	1080.749423	804.526398

Graphical abstract of model fit presented as mean ± 1 SD of the fraction % of released carvedilol:

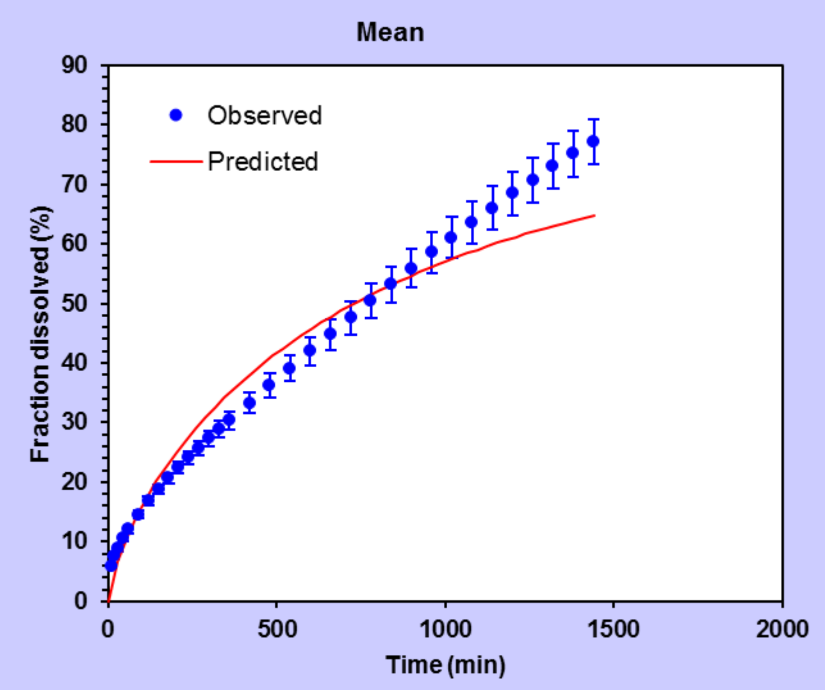

Supplementary materials – Model fitting summary – Starch 1500® sample with larger particle size (modelling the entire relevant dissolution data)

Graphical abstract of model fit presented as the fraction % of released carvedilol per tested tablet:

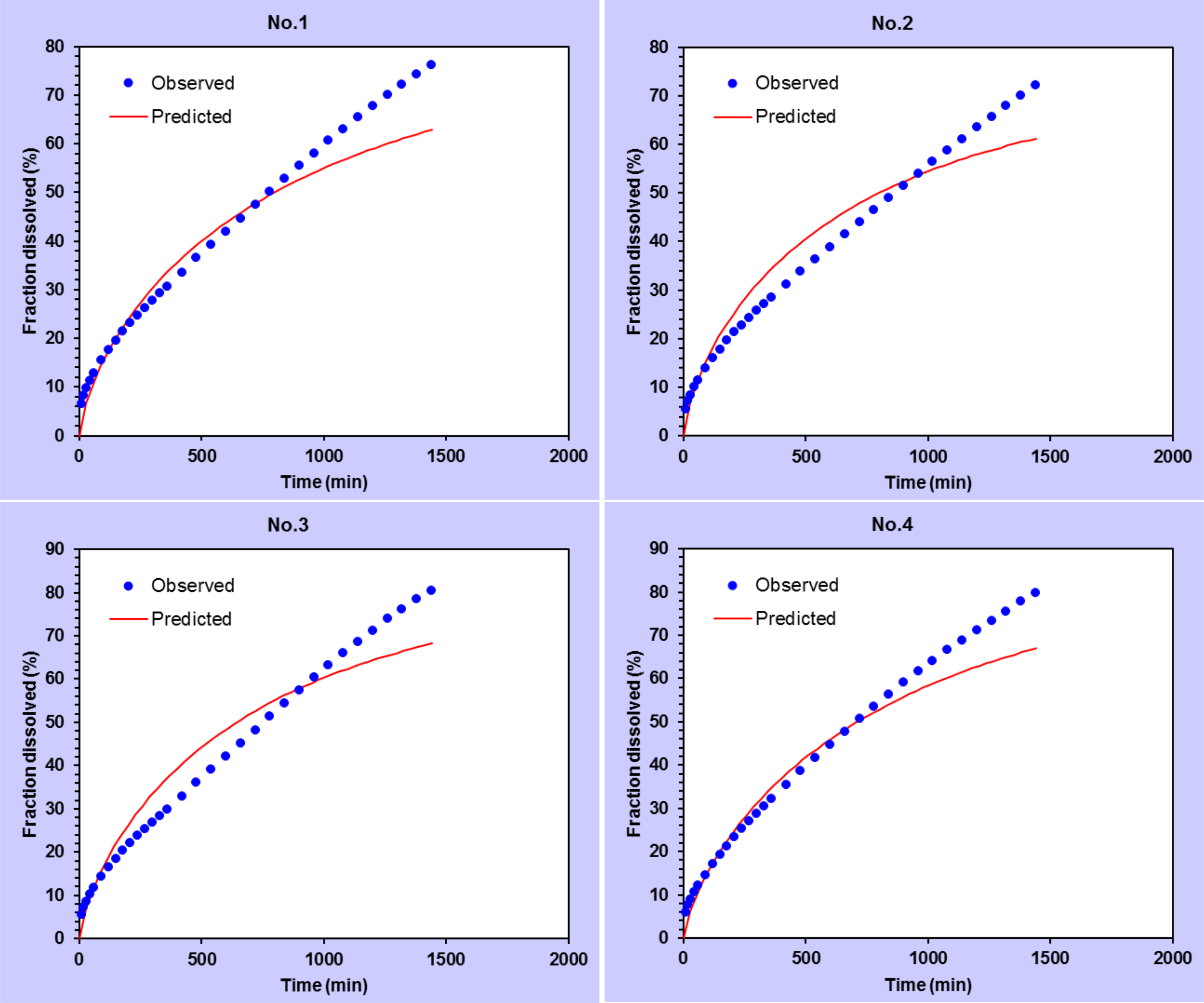

Supplementary materials – Model fitting summary – Starch 1500® sample with larger particle size (modelling the entire relevant dissolution data)

Model: **Weibull_4**

Model equation: $F = F_{max} \cdot \left[1 - e^{-\frac{(t-T_i)^{\beta}}{\alpha}}\right]$

Fitted model parameters per tested tablet (N = 4) with statistics – mean, standard deviation (SD), and relative standard deviation expressed in % (RSD%) (output from DDSolver):

Parameter	No.1	No.2	No.3	No.4	Mean	SD	RSD(%)
α	78.375	87.943	108.139	99.913	93.592	13.103	14.000
β	0.666	0.680	0.707	0.704	0.689	0.020	2.847
T_i	6.000	6.000	6.000	6.000	6.000	0.000	0.000
F_{max}	79.982	75.665	84.550	83.708	80.976	4.059	5.013

Number of dissolution data points (N), degrees of freedom (df), and selected goodness of fit criteria – Pearson correlation coefficient (R), coefficient of determination (R^2), adjusted coefficient of determination ($R^2_{adjusted}$), and residual sum of squares (RSS) (manual calculation in MS Excel):

Parameter	No.1	No.2	No.3	No.4
N	33	33	33	33
df	29	29	29	29
R	0.970272209	0.971635609	0.97033833	0.976807245
R^2	0.94142816	0.944075756	0.941556475	0.954152394
$R^2_{adjusted}$	0.935369004	0.93829049	0.935510594	0.949409538
RSS	1052.070282	907.4165154	1276.348351	989.7775234

Graphical abstract of model fit presented as mean ± 1 SD of the fraction % of released carvedilol:

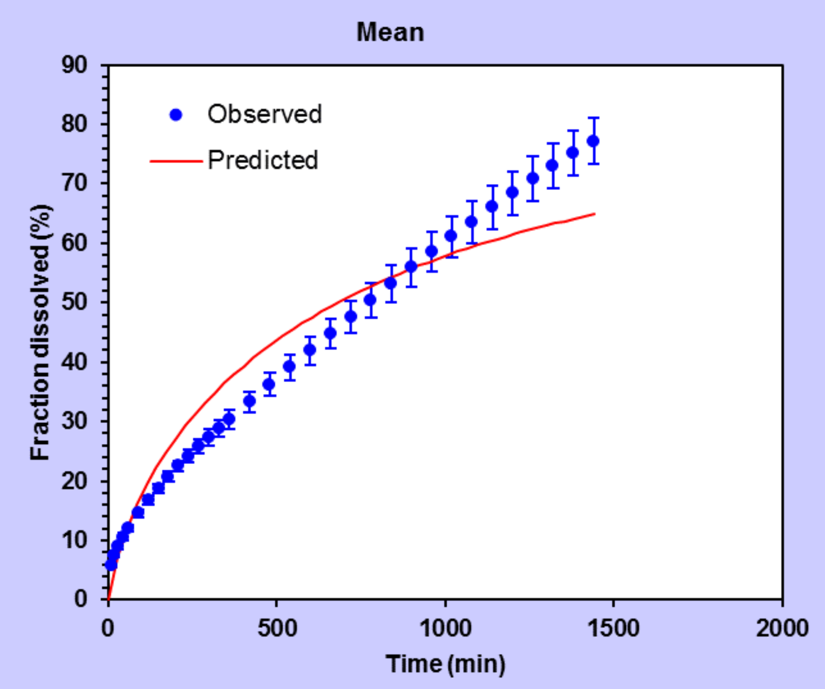

Supplementary materials – Model fitting summary – Starch 1500® sample with larger particle size (modelling the entire relevant dissolution data)

Graphical abstract of model fit presented as the fraction % of released carvedilol per tested tablet:

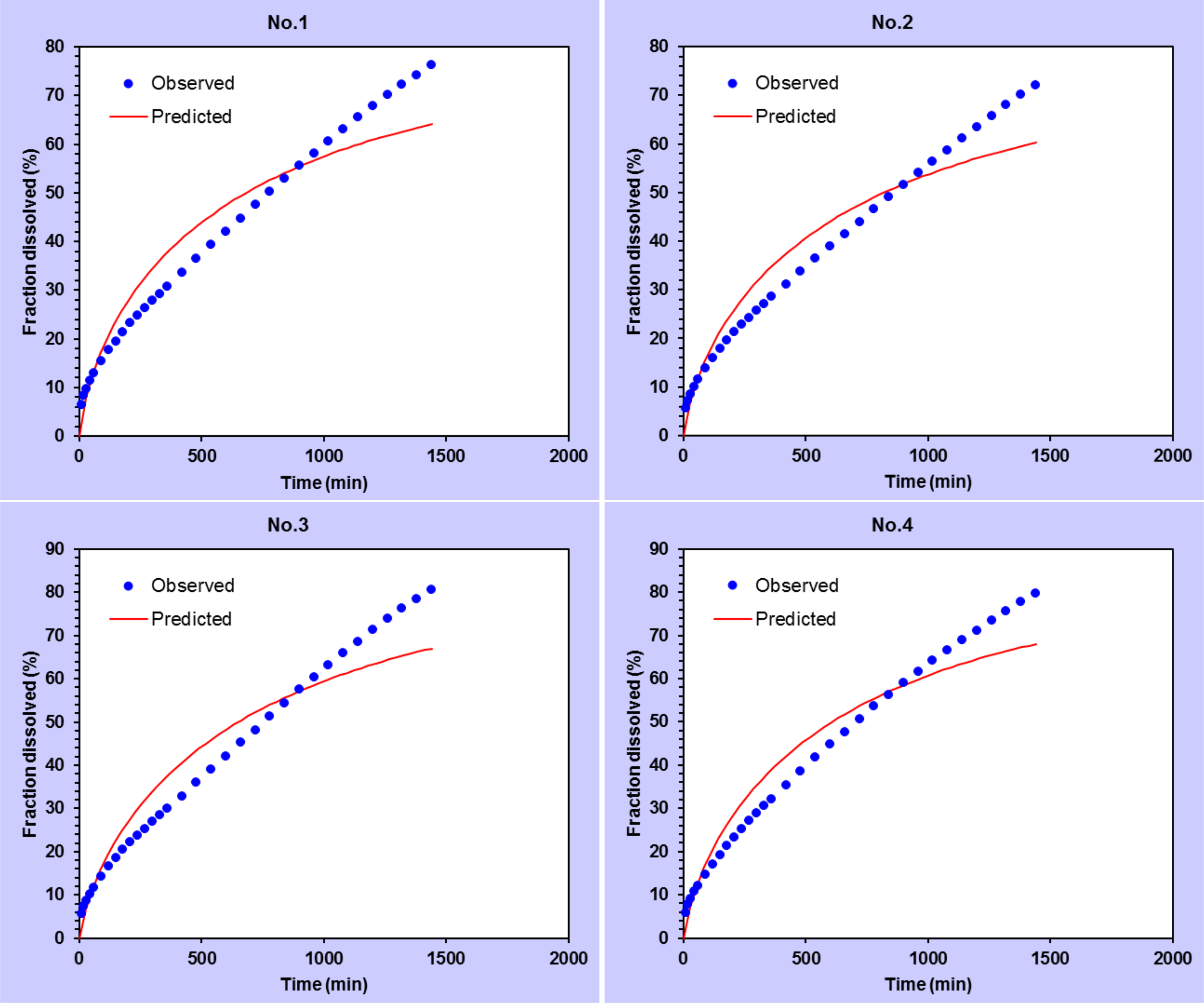

Supplementary materials – Model fitting summary – Starch 1500® sample with larger particle size (modelling the entire relevant dissolution data)

Model: **Logistic_1**

Model equation: $F = 100 \cdot \frac{e^{\alpha+\beta \cdot \log(t)}}{1+e^{\alpha+\beta \cdot \log(t)}}$

Fitted model parameters per tested tablet (N = 4) with statistics – mean, standard deviation (SD), and relative standard deviation expressed in % (RSD%) (output from DDSolver):

Parameter	No.1	No.2	No.3	No.4	Mean	SD	RSD(%)
α	-5.113	-5.190	-5.533	-5.440	-5.319	0.200	-3.753
β	1.804	1.777	1.976	1.961	1.879	0.103	5.493

Number of dissolution data points (N), degrees of freedom (df), and selected goodness of fit criteria – Pearson correlation coefficient (R), coefficient of determination (R^2), adjusted coefficient of determination ($R^2_{adjusted}$), and residual sum of squares (RSS) (manual calculation in MS Excel):

Parameter	No.1	No.2	No.3	No.4
N	33	33	33	33
df	31	31	31	31
R	0.970874553	0.974425325	0.967183462	0.974829156
R^2	0.942597397	0.949504715	0.935443849	0.950291884
$R^2_{adjusted}$	0.9407457	0.947875835	0.933361392	0.948688397
RSS	1035.611106	839.9672137	1371.589663	1053.102069

Graphical abstract of model fit presented as mean ± 1 SD of the fraction % of released carvedilol:

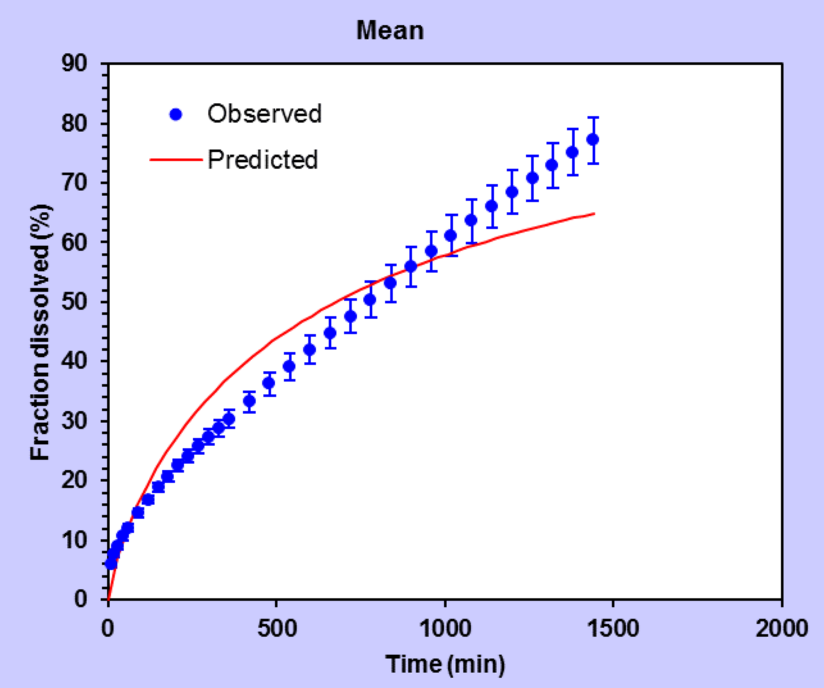

Supplementary materials – Model fitting summary – Starch 1500® sample with larger particle size (modelling the entire relevant dissolution data)

Graphical abstract of model fit presented as the fraction % of released carvedilol per tested tablet:

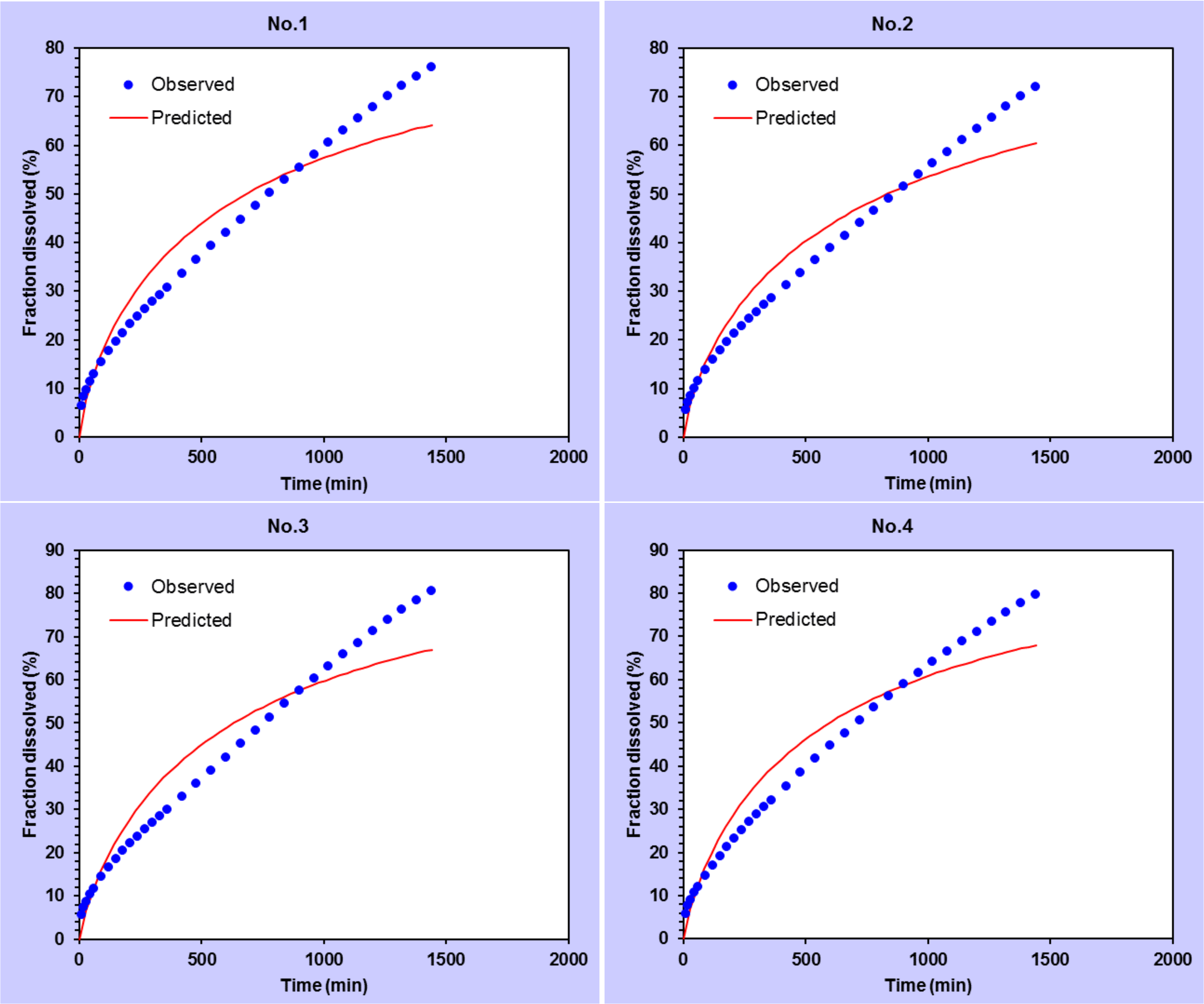

Supplementary materials – Model fitting summary – Starch 1500® sample with larger particle size (modelling the entire relevant dissolution data)

Model: **Logistic_2**

Model equation: $F = F_{max} \cdot \frac{e^{\alpha+\beta \cdot \log(t)}}{1+e^{\alpha+\beta \cdot \log(t)}}$

Fitted model parameters per tested tablet (N = 4) with statistics – mean, standard deviation (SD), and relative standard deviation expressed in % (RSD%) (output from DDSolver):

Parameter	No.1	No.2	No.3	No.4	Mean	SD	RSD(%)
α	-5.711	-5.810	-6.054	-6.000	-5.893	0.161	-2.725
β	2.259	2.277	2.351	2.365	2.313	0.053	2.294
F_{max}	79.982	75.665	84.550	83.708	80.976	4.059	5.013

Number of dissolution data points (N), degrees of freedom (df), and selected goodness of fit criteria – Pearson correlation coefficient (R), coefficient of determination (R^2), adjusted coefficient of determination ($R^2_{adjusted}$), and residual sum of squares (RSS) (manual calculation in MS Excel):

Parameter	No.1	No.2	No.3	No.4
N	33	33	33	33
df	30	30	30	30
R	0.951529003	0.953525454	0.95131832	0.958892319
R^2	0.905407444	0.909210792	0.905006546	0.91947448
$R^2_{adjusted}$	0.899101273	0.903158178	0.898673649	0.914106112
RSS	1652.746782	1423.191474	1968.95744	1655.527669

Graphical abstract of model fit presented as mean ± 1 SD of the fraction % of released carvedilol:

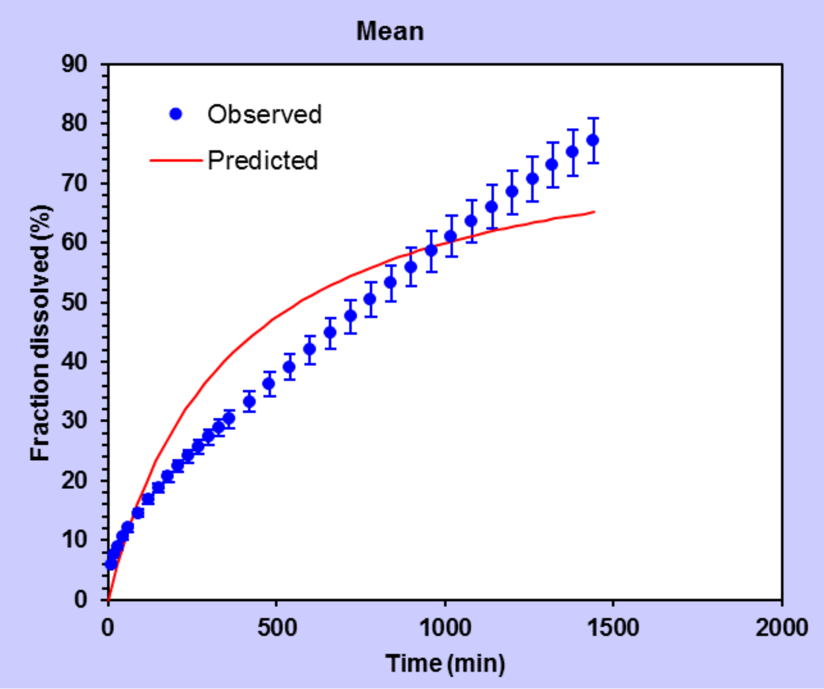

Supplementary materials – Model fitting summary – Starch 1500® sample with larger particle size (modelling the entire relevant dissolution data)

Graphical abstract of model fit presented as the fraction % of released carvedilol per tested tablet:

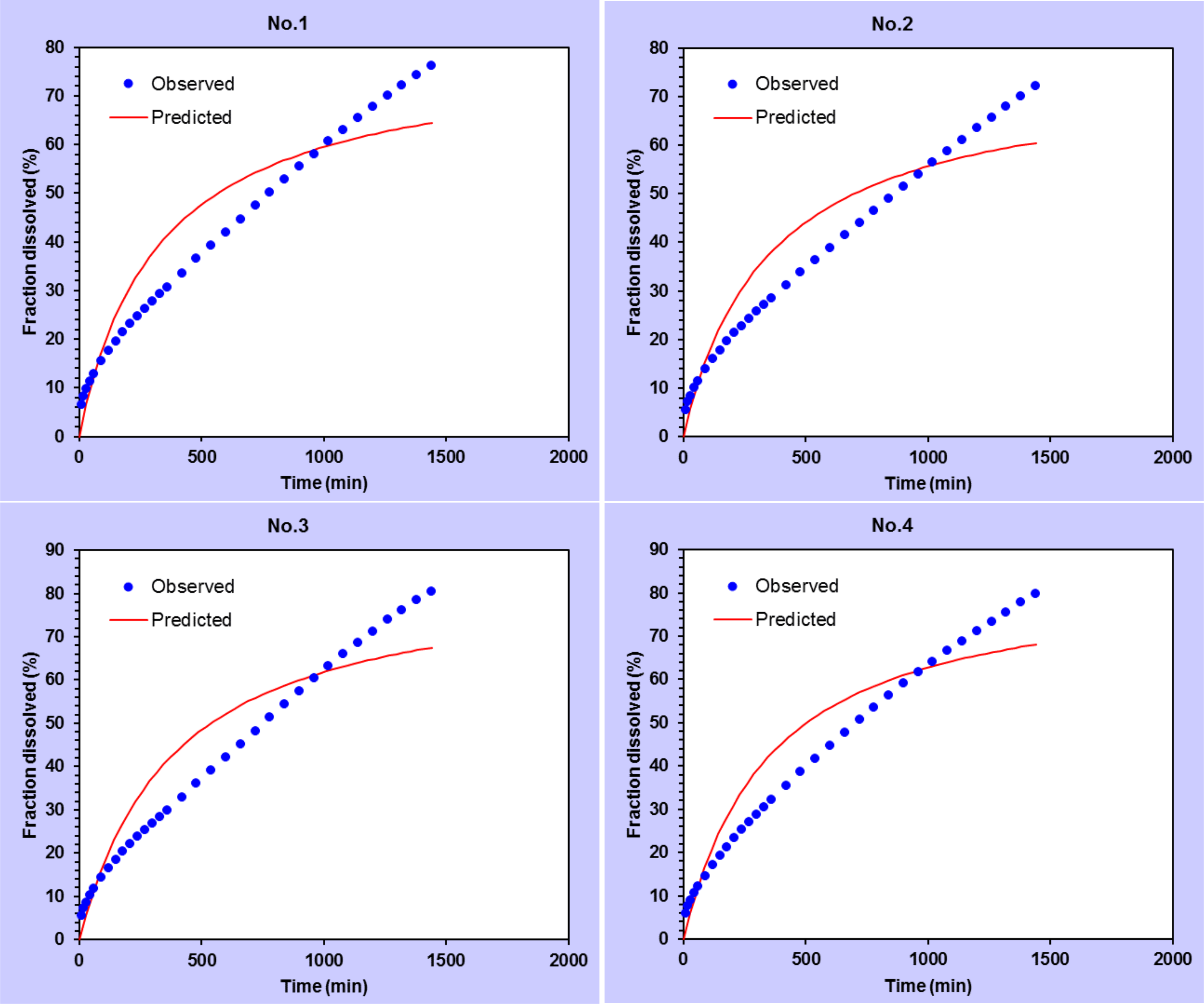

Supplementary materials – Model fitting summary – Starch 1500® sample with larger particle size (modelling the entire relevant dissolution data)

Model: **Logistic_3**

Model equation: $F = F_{max} \cdot \frac{1}{1+e^{-k\cdot(t-\gamma)}}$

Fitted model parameters per tested tablet (N = 4) with statistics – mean, standard deviation (SD), and relative standard deviation expressed in % (RSD%) (output from DDSolver):

Parameter	No.1	No.2	No.3	No.4	Mean	SD	RSD(%)
k	0.003	0.003	0.003	0.003	0.003	0.000	2.045
γ	583.324	600.822	617.807	589.639	597.898	15.116	2.528
F_{max}	79.982	75.665	84.550	83.708	80.976	4.059	5.013

Number of dissolution data points (N), degrees of freedom (df), and selected goodness of fit criteria – Pearson correlation coefficient (R), coefficient of determination (R^2), adjusted coefficient of determination ($R^2_{adjusted}$), and residual sum of squares (RSS) (manual calculation in MS Excel):

Parameter	No.1	No.2	No.3	No.4
N	33	33	33	33
df	30	30	30	30
R	0.992975798	0.992013538	0.993745682	0.992347891
R^2	0.986000935	0.98409086	0.98753048	0.984754336
$R^2_{adjusted}$	0.985067664	0.983030251	0.986699178	0.983737958
RSS	237.5674151	244.5734646	255.5980009	299.8279234

Graphical abstract of model fit presented as mean ± 1 SD of the fraction % of released carvedilol:

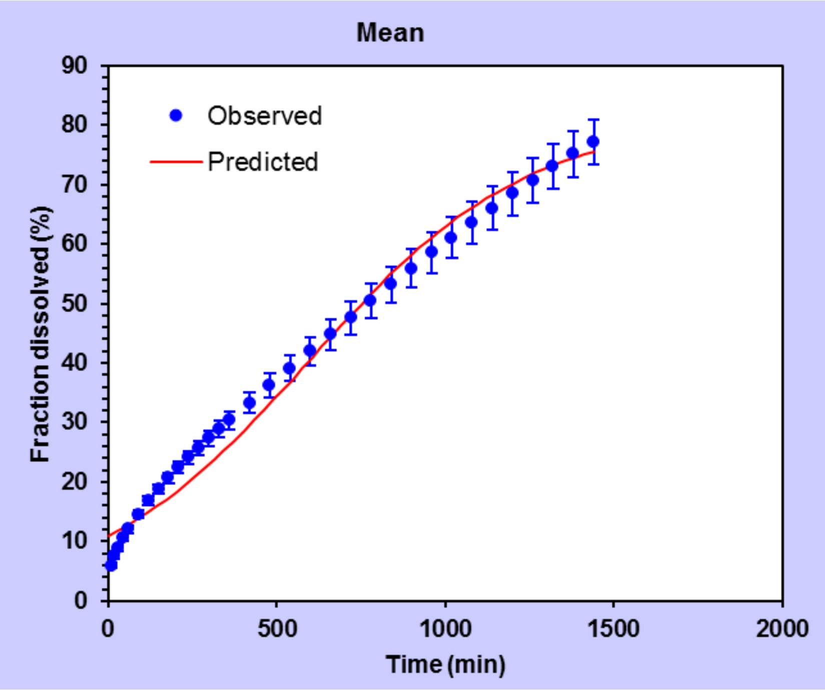

Supplementary materials – Model fitting summary – Starch 1500® sample with larger particle size (modelling the entire relevant dissolution data)

Graphical abstract of model fit presented as the fraction % of released carvedilol per tested tablet:

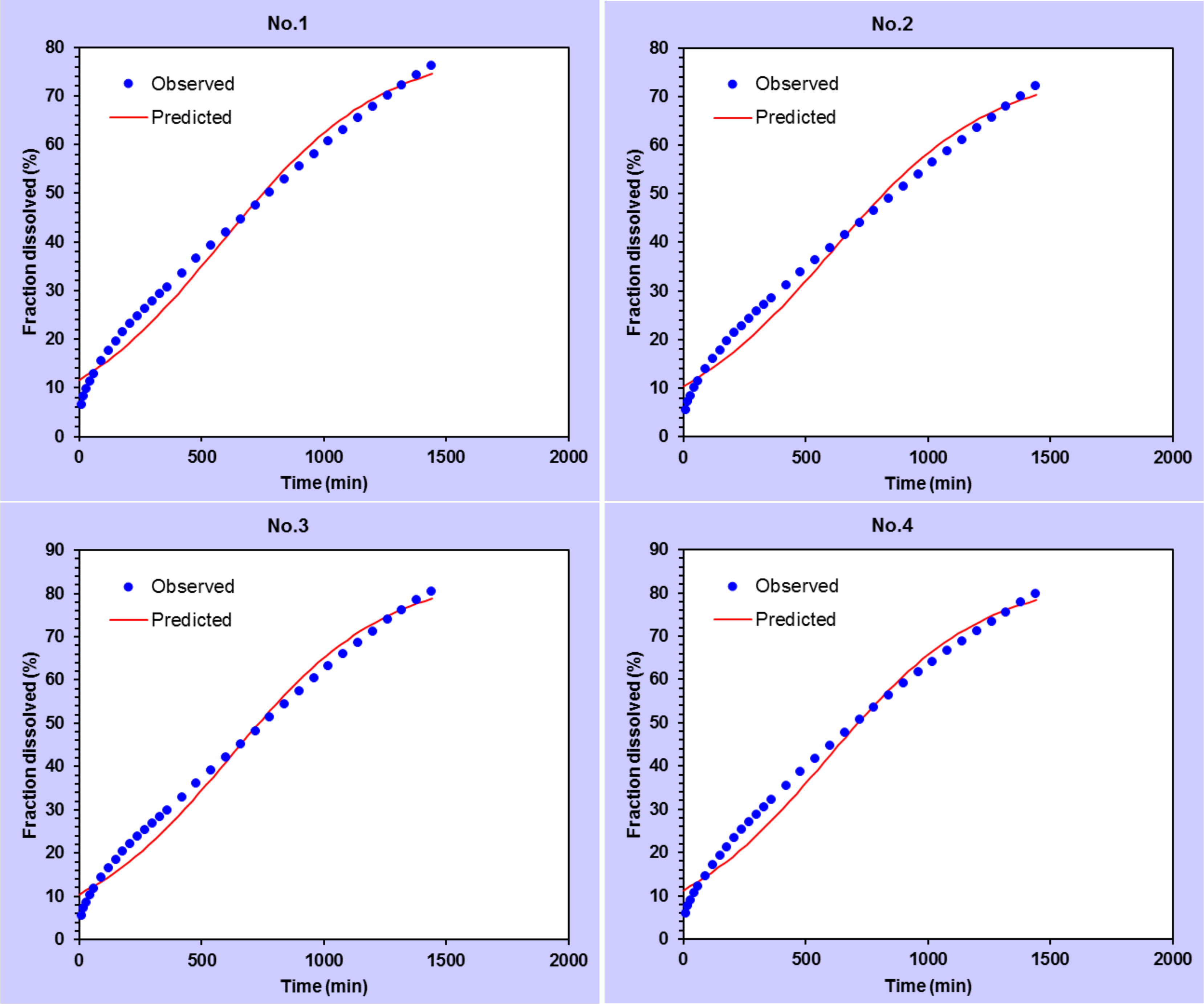

Supplementary materials – Model fitting summary – Starch 1500® sample with larger particle size (modelling the entire relevant dissolution data)

Model: **Gompertz_1**

Model equation: $F = 100 \cdot e^{-\alpha \cdot e^{-\beta \cdot \log(t)}}$

Fitted model parameters per tested tablet (N = 4) with statistics – mean, standard deviation (SD), and relative standard deviation expressed in % (RSD%) (output from DDSolver):

Parameter	No.1	No.2	No.3	No.4	Mean	SD	RSD(%)
α	13.860	13.101	17.675	17.087	15.431	2.286	14.814
β	1.072	1.013	1.179	1.178	1.111	0.082	7.397

Number of dissolution data points (N), degrees of freedom (df), and selected goodness of fit criteria – Pearson correlation coefficient (R), coefficient of determination (R^2), adjusted coefficient of determination ($R^2_{adjusted}$), and residual sum of squares (RSS) (manual calculation in MS Excel):

Parameter	No.1	No.2	No.3	No.4
N	33	33	33	33
df	31	31	31	31
R	0.943229064	0.947942641	0.936776097	0.946955965
R^2	0.889681067	0.89859525	0.877549456	0.8967256
$R^2_{adjusted}$	0.886122391	0.895324129	0.873599438	0.893394168
RSS	1824.4597	1520.419305	2416.057432	2007.707895

Graphical abstract of model fit presented as mean ± 1 SD of the fraction % of released carvedilol:

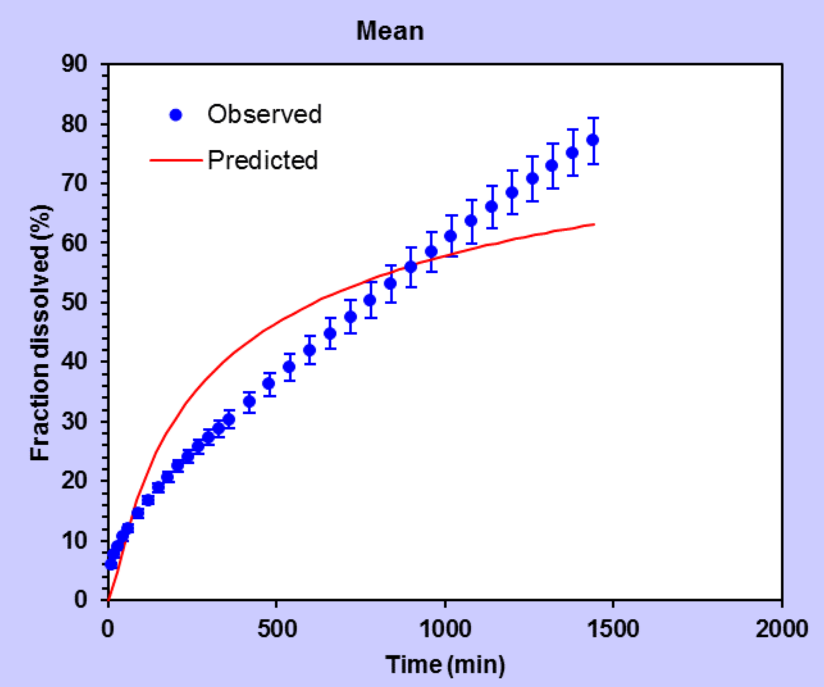

Supplementary materials – Model fitting summary – Starch 1500® sample with larger particle size (modelling the entire relevant dissolution data)

Graphical abstract of model fit presented as the fraction % of released carvedilol per tested tablet:

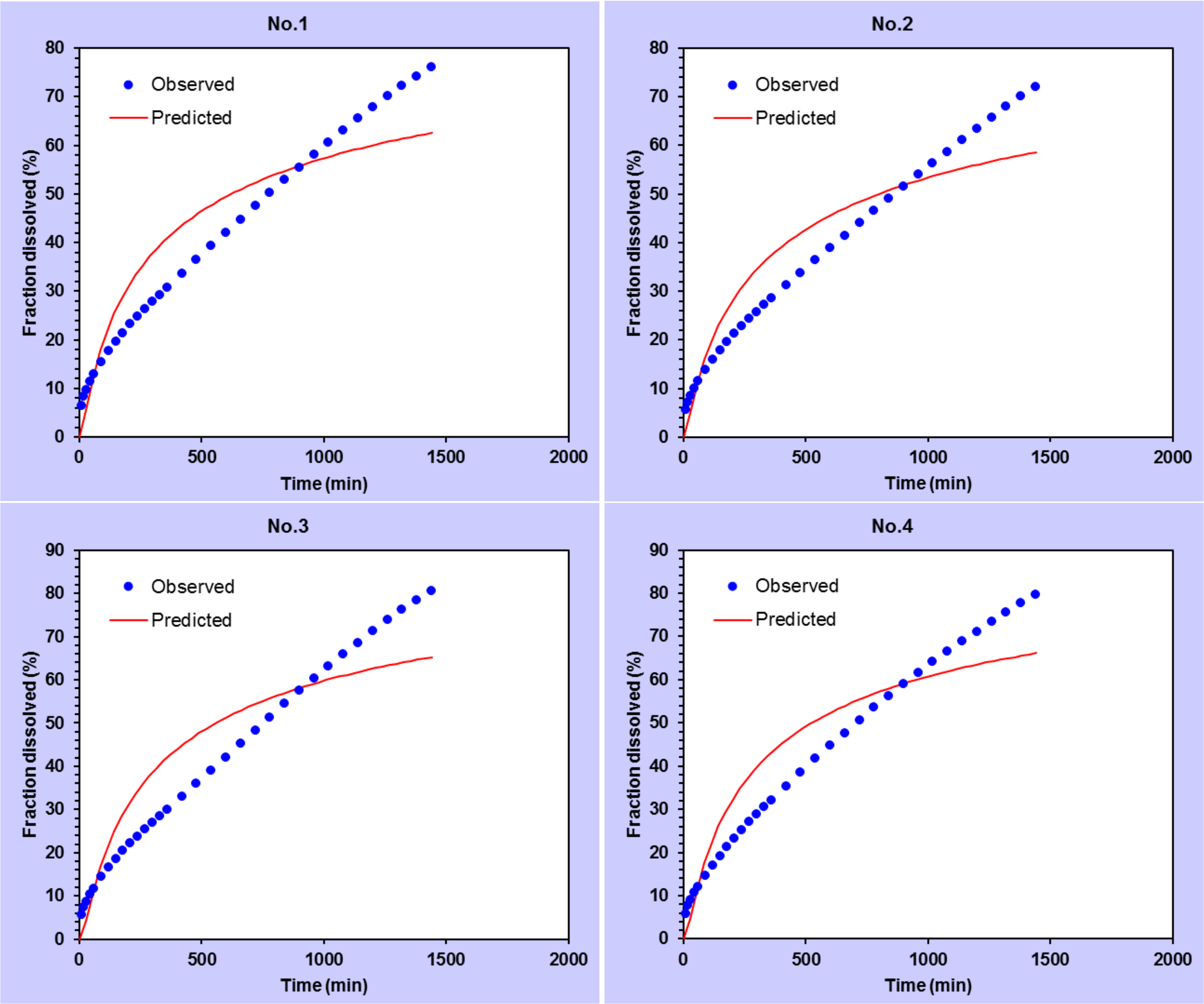

Supplementary materials – Model fitting summary – Starch 1500® sample with larger particle size (modelling the entire relevant dissolution data)

Model: **Gompertz_2**

Model equation: $F = F_{max} \cdot e^{-\alpha \cdot e^{-\beta \cdot \log(t)}}$

Fitted model parameters per tested tablet (N = 4) with statistics – mean, standard deviation (SD), and relative standard deviation expressed in % (RSD%) (output from DDSolver):

Parameter	No.1	No.2	No.3	No.4	Mean	SD	RSD(%)
α	45.329	45.904	51.345	39.834	45.603	4.705	10.318
β	1.548	1.538	1.571	1.496	1.538	0.031	2.046
F_{max}	79.982	75.665	84.550	105.791	86.497	13.364	15.451

Number of dissolution data points (N), degrees of freedom (df), and selected goodness of fit criteria – Pearson correlation coefficient (R), coefficient of determination (R^2), adjusted coefficient of determination ($R^2_{adjusted}$), and residual sum of squares (RSS) (manual calculation in MS Excel):

Parameter	No.1	No.2	No.3	No.4
N	33	33	33	33
df	30	30	30	30
R	0.956613774	0.958188606	0.955595674	0.960807249
R^2	0.915109912	0.918125404	0.913163092	0.92315057
$R^2_{adjusted}$	0.909450572	0.912667098	0.907373964	0.918027275
RSS	2260.661571	2038.387713	2648.412851	2047.455215

Graphical abstract of model fit presented as mean ± 1 SD of the fraction % of released carvedilol:

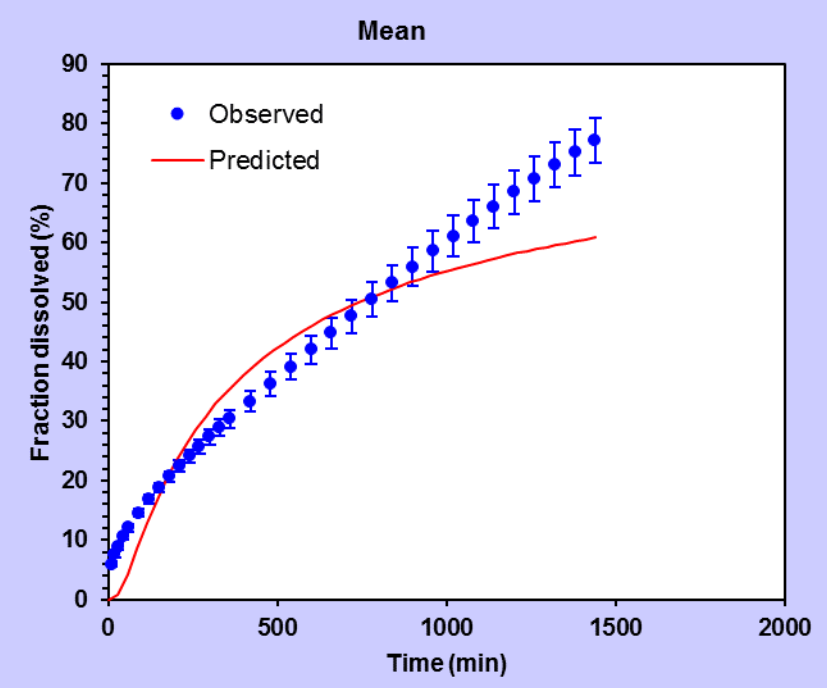

Supplementary materials – Model fitting summary – Starch 1500® sample with larger particle size (modelling the entire relevant dissolution data)

Graphical abstract of model fit presented as the fraction % of released carvedilol per tested tablet:

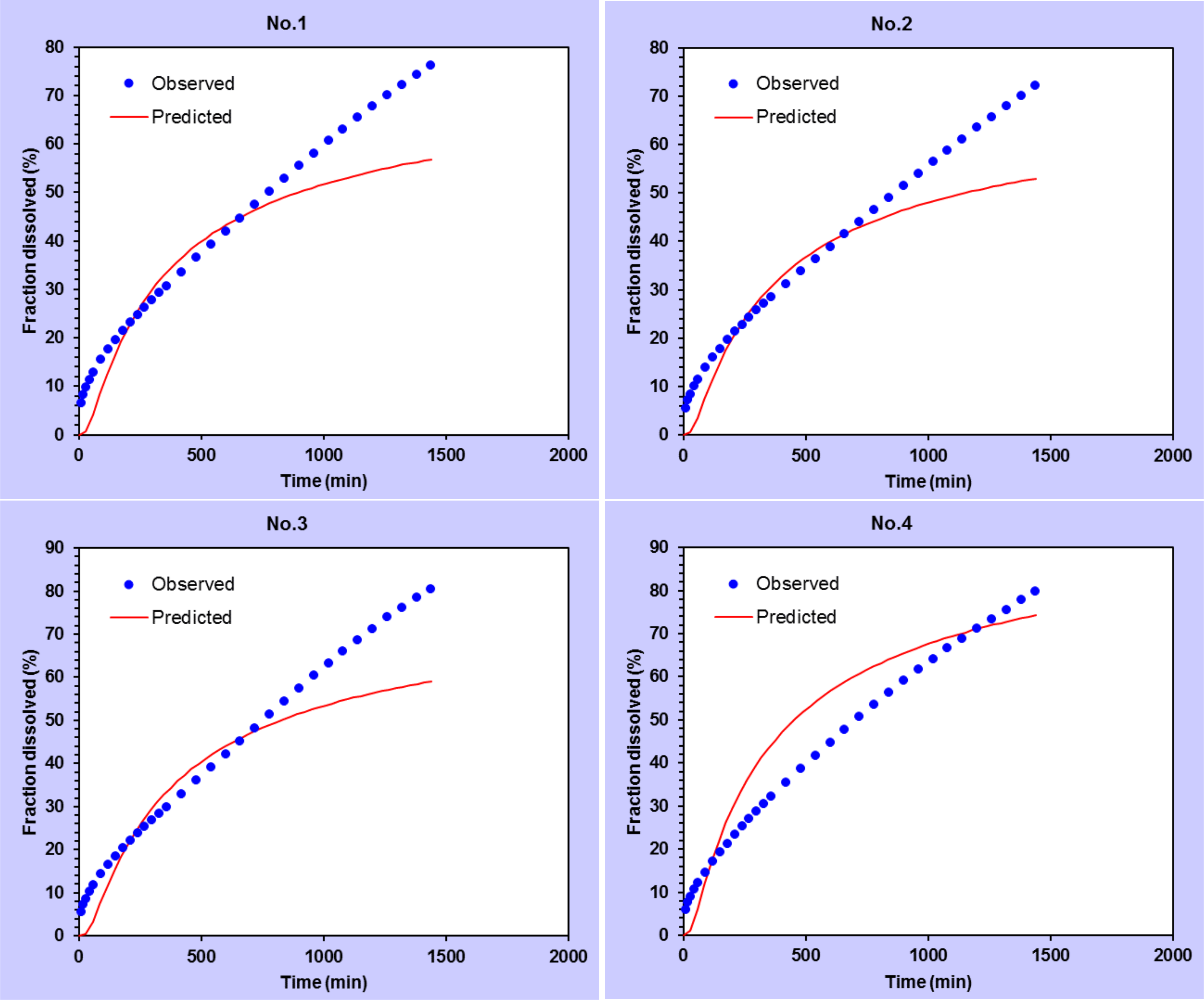

Supplementary materials – Model fitting summary – Starch 1500® sample with larger particle size (modelling the entire relevant dissolution data)

Model: **Gompertz_3**

Model equation: $F = F_{max} \cdot e^{-e^{-k\cdot(t-\gamma)}}$

Fitted model parameters per tested tablet (N = 4) with statistics – mean, standard deviation (SD), and relative standard deviation expressed in % (RSD%) (output from DDSolver):

Parameter	No.1	No.2	No.3	No.4	Mean	SD	RSD(%)
k	0.002	0.002	0.002	0.002	0.002	0.000	12.152
γ	358.029	372.341	390.675	420.874	385.480	27.117	7.034
F_{max}	79.982	75.665	84.550	90.184	82.595	6.225	7.537

Number of dissolution data points (N), degrees of freedom (df), and selected goodness of fit criteria – Pearson correlation coefficient (R), coefficient of determination (R^2), adjusted coefficient of determination ($R^2_{adjusted}$), and residual sum of squares (RSS) (manual calculation in MS Excel):

Parameter	No.1	No.2	No.3	No.4
N	33	33	33	33
df	30	30	30	30
R	0.99432542	0.993672972	0.994064435	0.996853206
R^2	0.98868304	0.987385976	0.988164101	0.993716313
$R^2_{adjusted}$	0.987928576	0.986545041	0.987375042	0.993297401
RSS	217.514143	216.3510071	270.9352355	172.0507274

Graphical abstract of model fit presented as mean ± 1 SD of the fraction % of released carvedilol:

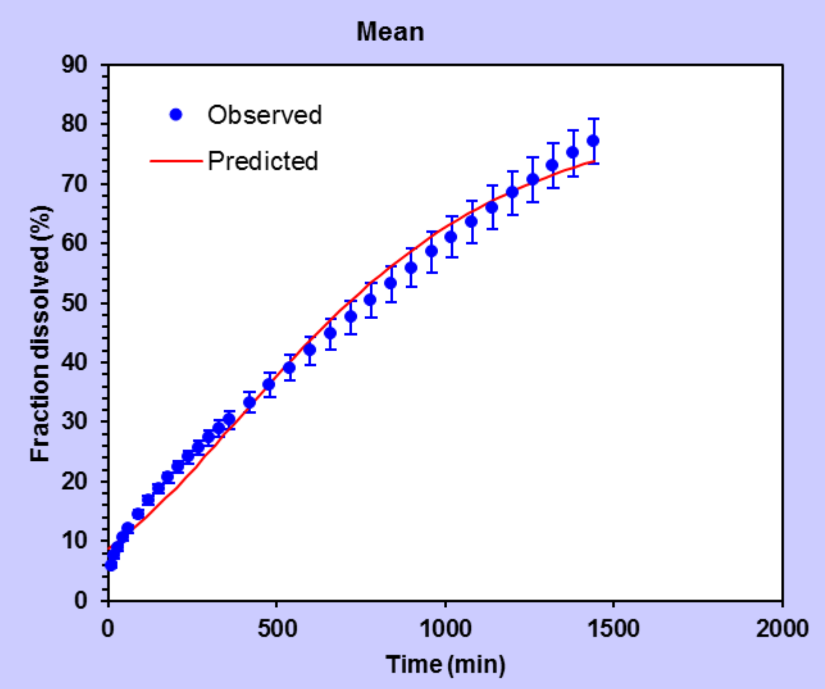

Supplementary materials – Model fitting summary – Starch 1500® sample with larger particle size (modelling the entire relevant dissolution data)

Graphical abstract of model fit presented as the fraction % of released carvedilol per tested tablet:

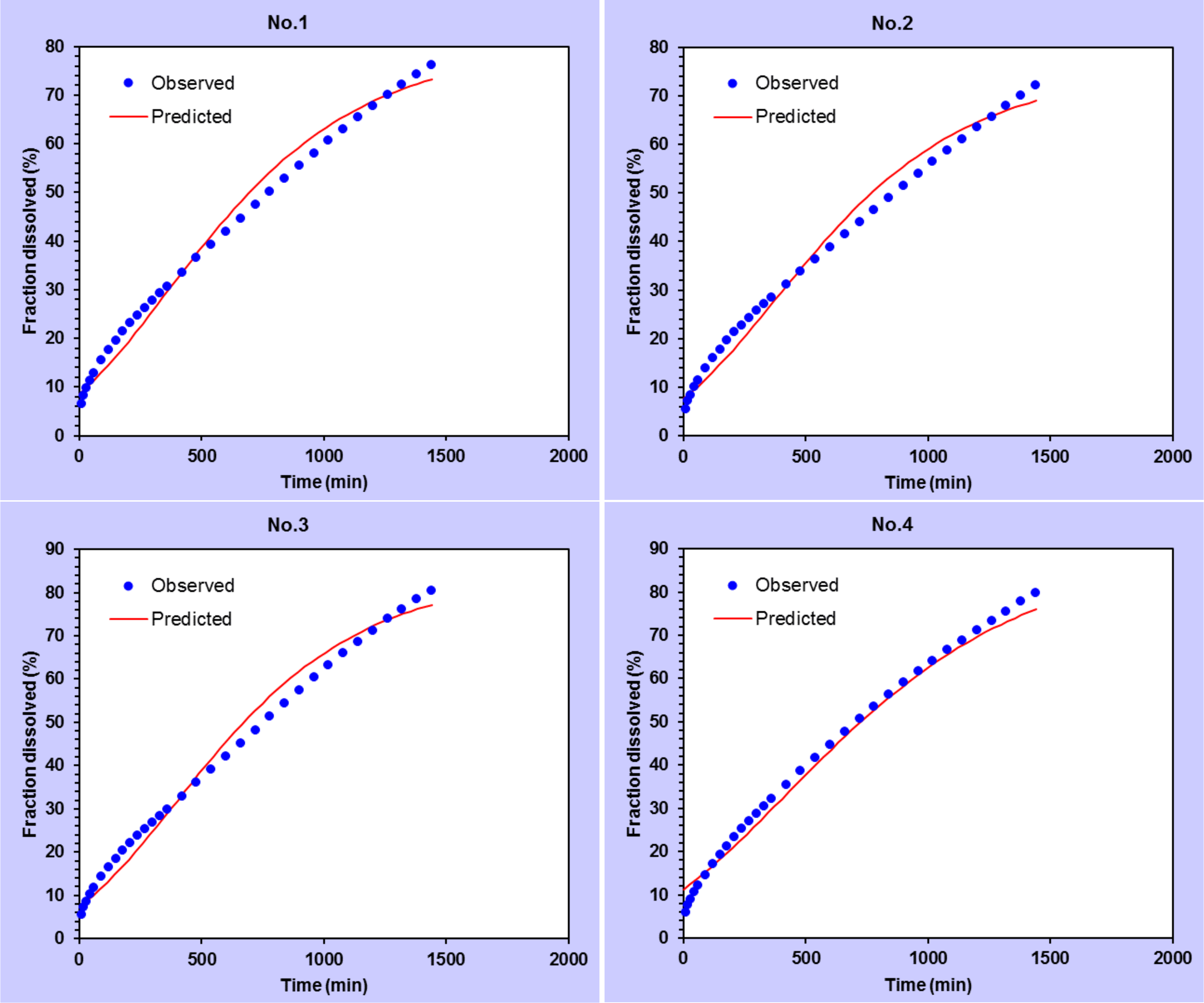

Supplementary materials – Model fitting summary – Starch 1500® sample with larger particle size (modelling the entire relevant dissolution data)

Model: **Gompertz_4**

Model equation: $F = F_{max} \cdot e^{-\beta \cdot e^{-k \cdot t}}$

Fitted model parameters per tested tablet (N = 4) with statistics – mean, standard deviation (SD), and relative standard deviation expressed in % (RSD%) (output from DDSolver):

Parameter	No.1	No.2	No.3	No.4	Mean	SD	RSD(%)
k	0.002	0.002	0.002	0.002	0.002	0.000	1.539
β	2.230	2.287	2.440	2.311	2.317	0.089	3.829
F_{max}	79.982	75.665	84.550	83.708	80.976	4.059	5.013

Number of dissolution data points (N), degrees of freedom (df), and selected goodness of fit criteria – Pearson correlation coefficient (R), coefficient of determination (R^2), adjusted coefficient of determination ($R^2_{adjusted}$), and residual sum of squares (RSS) (manual calculation in MS Excel):

Parameter	No.1	No.2	No.3	No.4
N	33	33	33	33
df	30	30	30	30
R	0.99432542	0.993672972	0.994064435	0.995782448
R^2	0.98868304	0.987385976	0.988164101	0.991582684
$R^2_{adjusted}$	0.987928576	0.986545041	0.987375042	0.99102153
RSS	217.514143	216.3510071	270.9352355	182.7694847

Graphical abstract of model fit presented as mean ± 1 SD of the fraction % of released carvedilol:

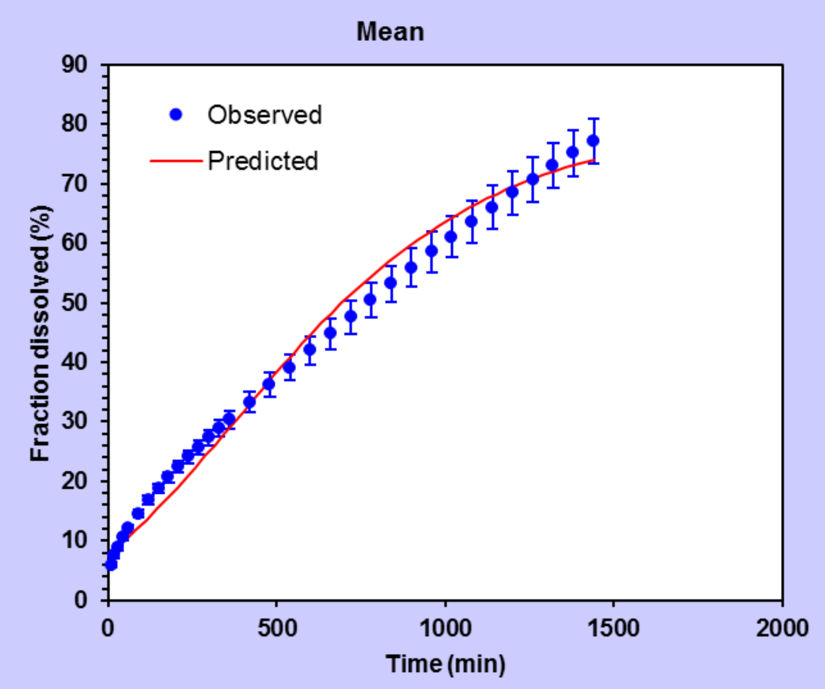

Supplementary materials – Model fitting summary – Starch 1500® sample with larger particle size (modelling the entire relevant dissolution data)

Graphical abstract of model fit presented as the fraction % of released carvedilol per tested tablet:

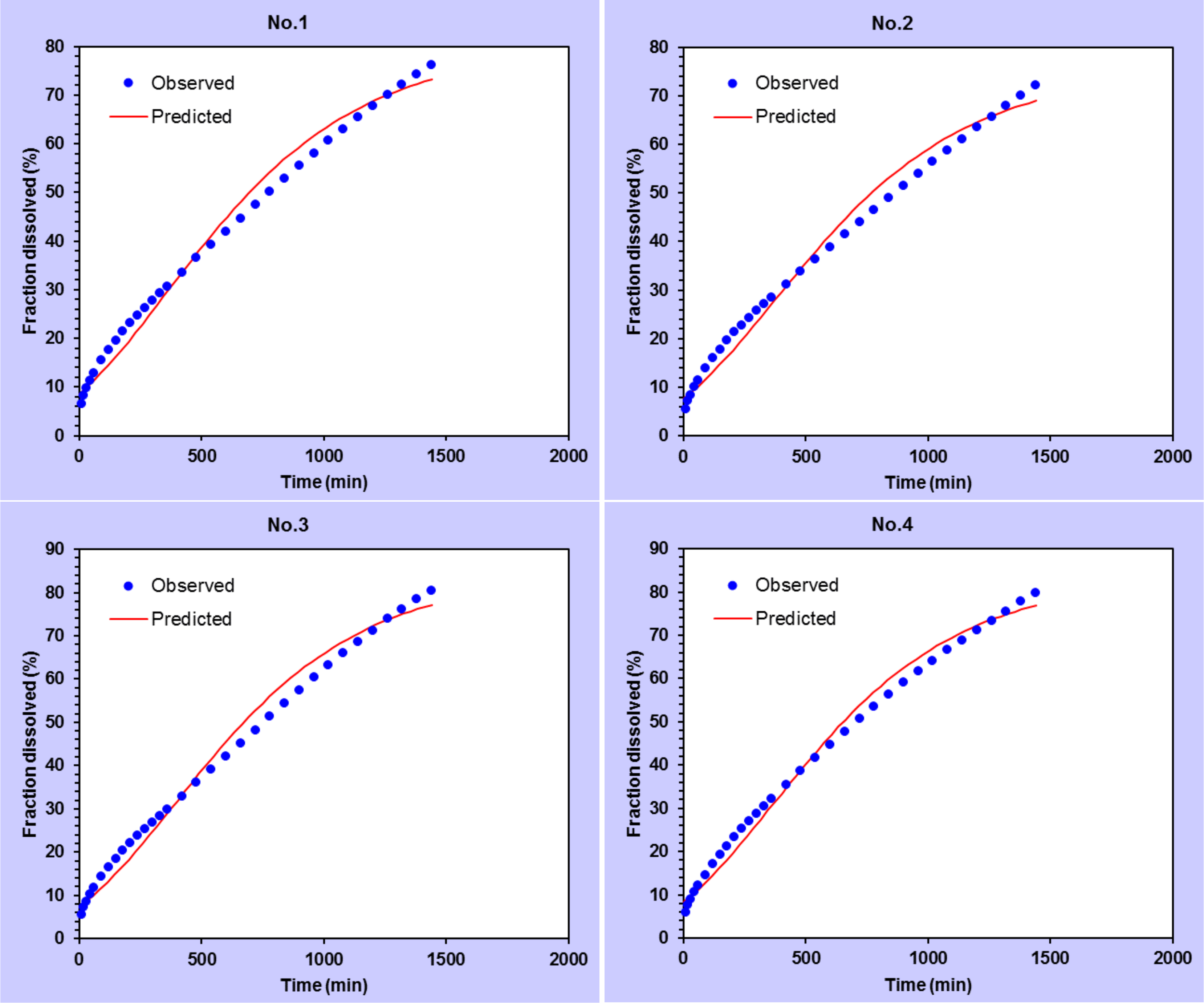

Supplementary materials – Model fitting summary – Starch 1500® sample with larger particle size (modelling the entire relevant dissolution data)

Model: **Probit_1**

Model equation: $F = 100 \cdot \phi[\alpha + \beta \cdot \log(t)]$

Fitted model parameters per tested tablet (N = 4) with statistics – mean, standard deviation (SD), and relative standard deviation expressed in % (RSD%) (output from DDSolver):

Parameter	No.1	No.2	No.3	No.4	Mean	SD	RSD(%)
α	-3.015	-3.034	-3.245	-3.200	-3.124	0.116	-3.711
β	1.064	1.038	1.159	1.154	1.104	0.062	5.622

Number of dissolution data points (N), degrees of freedom (df), and selected goodness of fit criteria – Pearson correlation coefficient (R), coefficient of determination (R^2), adjusted coefficient of determination ($R^2_{adjusted}$), and residual sum of squares (RSS) (manual calculation in MS Excel):

Parameter	No.1	No.2	No.3	No.4
N	33	33	33	33
df	31	31	31	31
R	0.964527079	0.967583842	0.960480499	0.968928722
R^2	0.930312486	0.936218491	0.922522789	0.938822867
$R^2_{adjusted}$	0.928064502	0.934161023	0.920023524	0.936849411
RSS	1230.728772	1033.554745	1620.169659	1271.15536

Graphical abstract of model fit presented as mean ± 1 SD of the fraction % of released carvedilol:

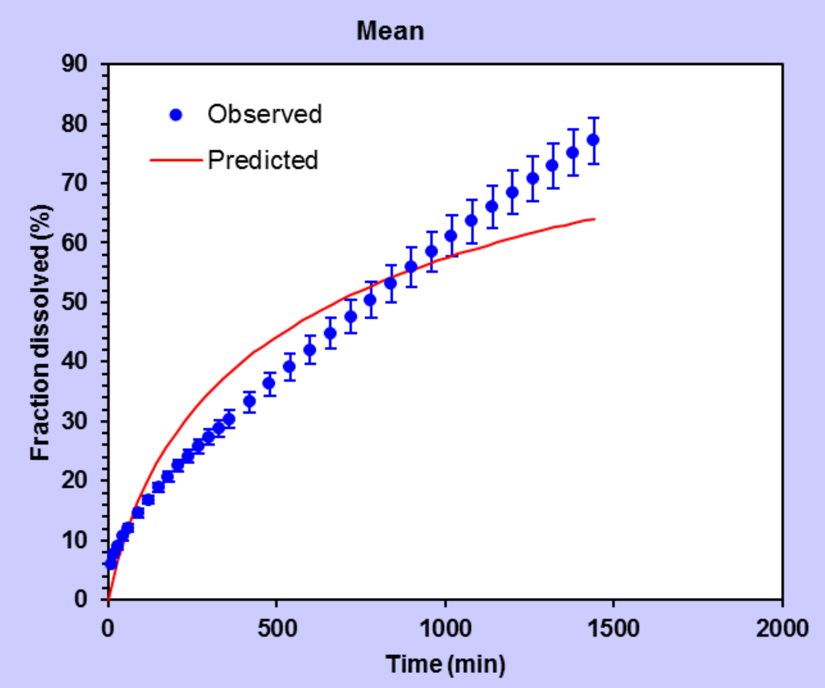

Supplementary materials – Model fitting summary – Starch 1500® sample with larger particle size (modelling the entire relevant dissolution data)

Graphical abstract of model fit presented as the fraction % of released carvedilol per tested tablet:

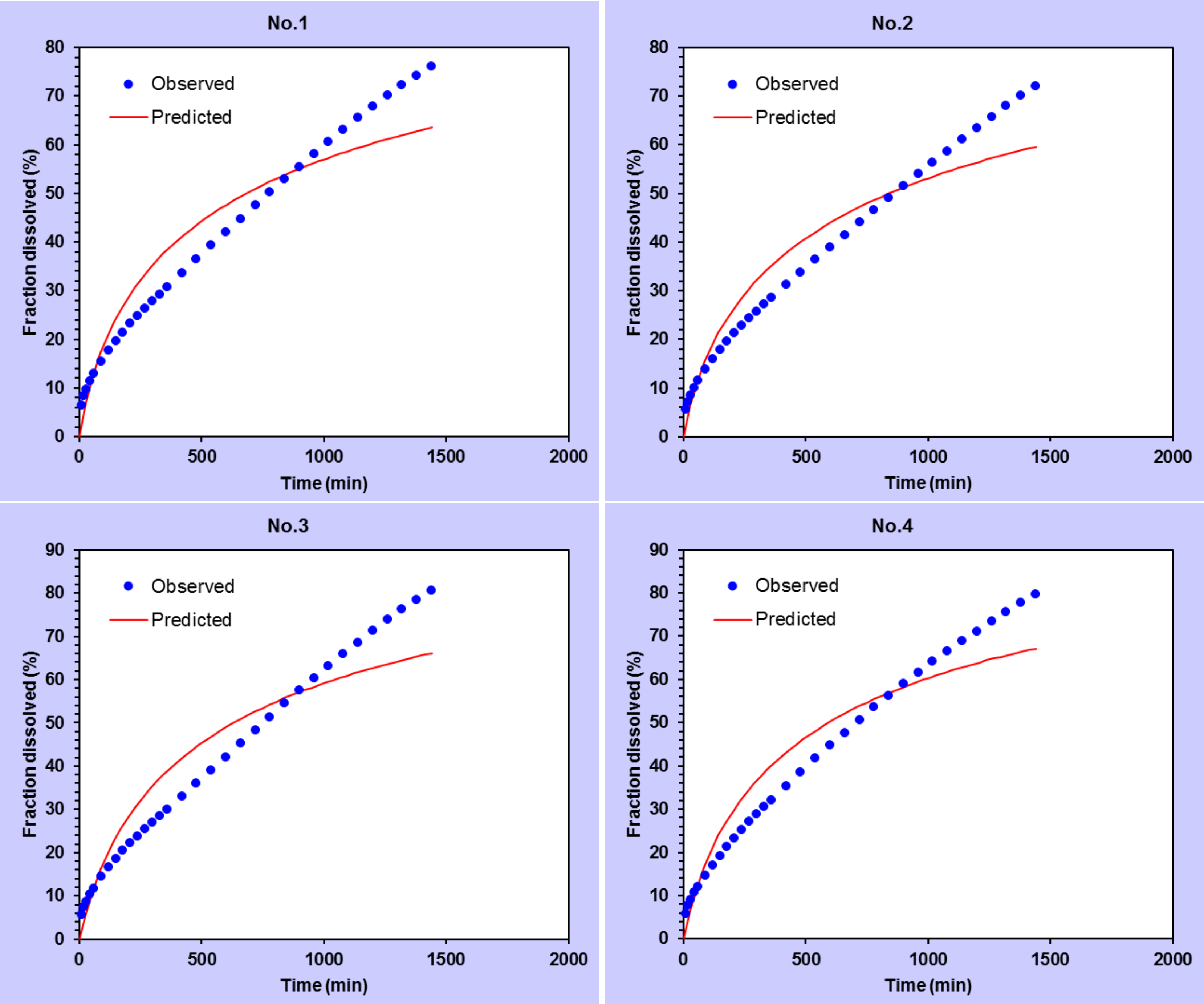

Supplementary materials – Model fitting summary – Starch 1500® sample with larger particle size (modelling the entire relevant dissolution data)

Model: **Probit_2**

Model equation: $F = F_{max} \cdot \phi[\alpha + \beta \cdot \log(t)]$

Fitted model parameters per tested tablet (N = 4) with statistics – mean, standard deviation (SD), and relative standard deviation expressed in % (RSD%) (output from DDSolver):

Parameter	No.1	No.2	No.3	No.4	Mean	SD	RSD(%)
α	-3.373	-3.423	-3.553	-3.528	-3.469	0.085	-2.463
β	1.332	1.340	1.378	1.389	1.360	0.028	2.079
F_{max}	79.982	75.665	84.550	83.708	80.976	4.059	5.013

Number of dissolution data points (N), degrees of freedom (df), and selected goodness of fit criteria – Pearson correlation coefficient (R), coefficient of determination (R^2), adjusted coefficient of determination ($R^2_{adjusted}$), and residual sum of squares (RSS) (manual calculation in MS Excel):

Parameter	No.1	No.2	No.3	No.4
N	33	33	33	33
df	30	30	30	30
R	0.949342644	0.950934931	0.948188523	0.956430311
R^2	0.901251457	0.904277243	0.899061475	0.91475894
$R^2_{adjusted}$	0.89466822	0.897895726	0.89233224	0.909076203
RSS	1677.515145	1462.22197	2043.196233	1706.135887

Graphical abstract of model fit presented as mean ± 1 SD of the fraction % of released carvedilol:

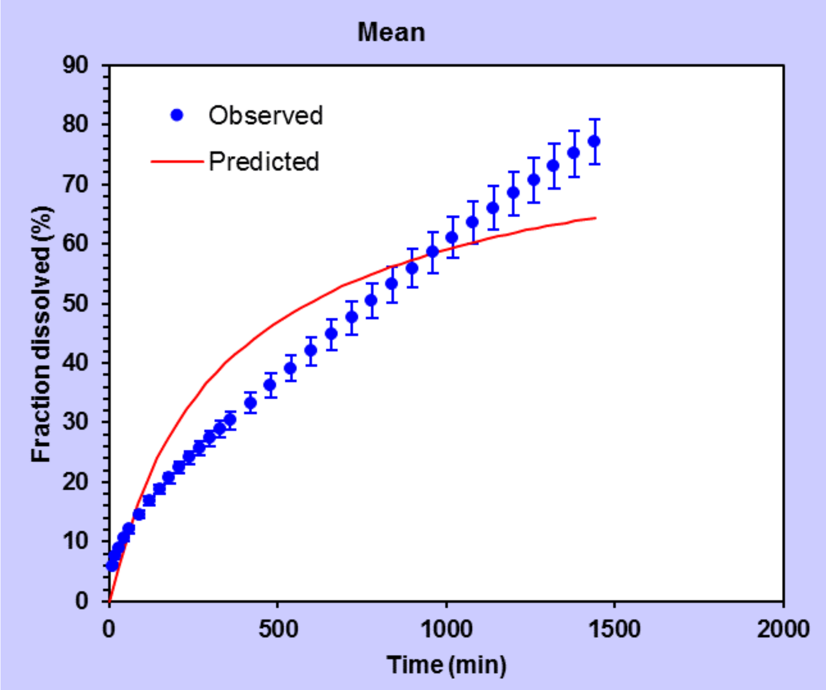

Supplementary materials – Model fitting summary – Starch 1500® sample with larger particle size (modelling the entire relevant dissolution data)

Graphical abstract of model fit presented as the fraction % of released carvedilol per tested tablet:

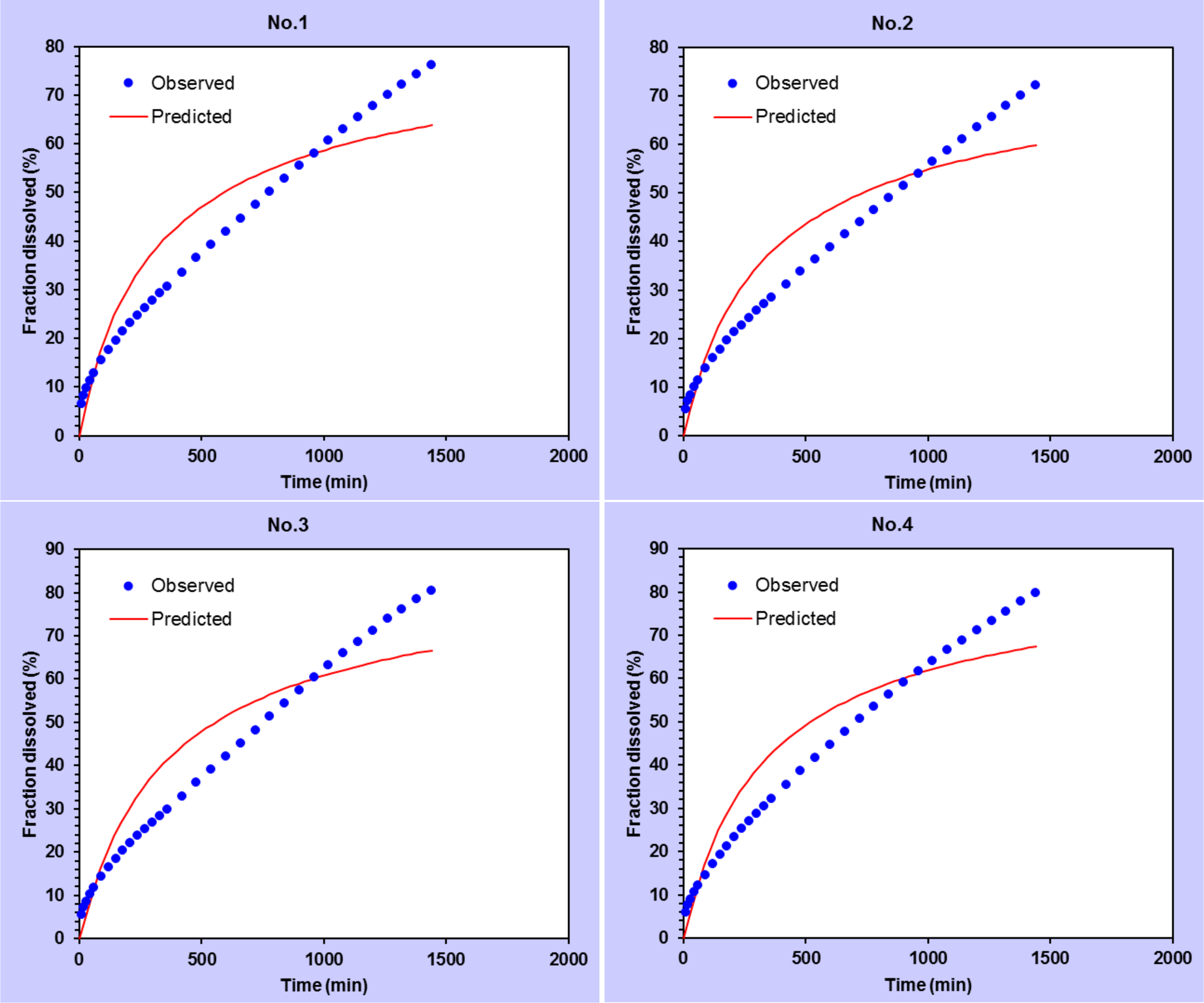

Supplementary materials – Model fitting summary – Starch 1500® sample with larger particle size (modelling up to app. 60 % of carvedilol released)

Model: **Zero–order**

Model equation: $F = k_0 \cdot t$

Fitted model parameters per tested tablet (N = 4) with statistics – mean, standard deviation (SD), and relative standard deviation expressed in % (RSD%) (output from DDSolver):

Parameter	No.1	No.2	No.3	No.4	Mean	SD	RSD(%)
k_0	0.068	0.063	0.069	0.071	0.068	0.004	5.411

Number of dissolution data points (N), degrees of freedom (df), and selected goodness of fit criteria – Pearson correlation coefficient (R), coefficient of determination (R^2), adjusted coefficient of determination ($R^2_{adjusted}$), and residual sum of squares (RSS) (manual calculation in MS Excel):

Parameter	No.1	No.2	No.3	No.4
N	26	26	26	26
df	25	25	25	25
R	0.994002148	0.993558444	0.99643135	0.994081863
R^2	0.98804027	0.987158381	0.992875435	0.98819875
$R^2_{adjusted}$	0.98804027	0.987158381	0.992875435	0.98819875
RSS	1226.771572	989.3140173	883.695152	1086.252966

Graphical abstract of model fit presented as mean ± 1 SD of the fraction % of released carvedilol:

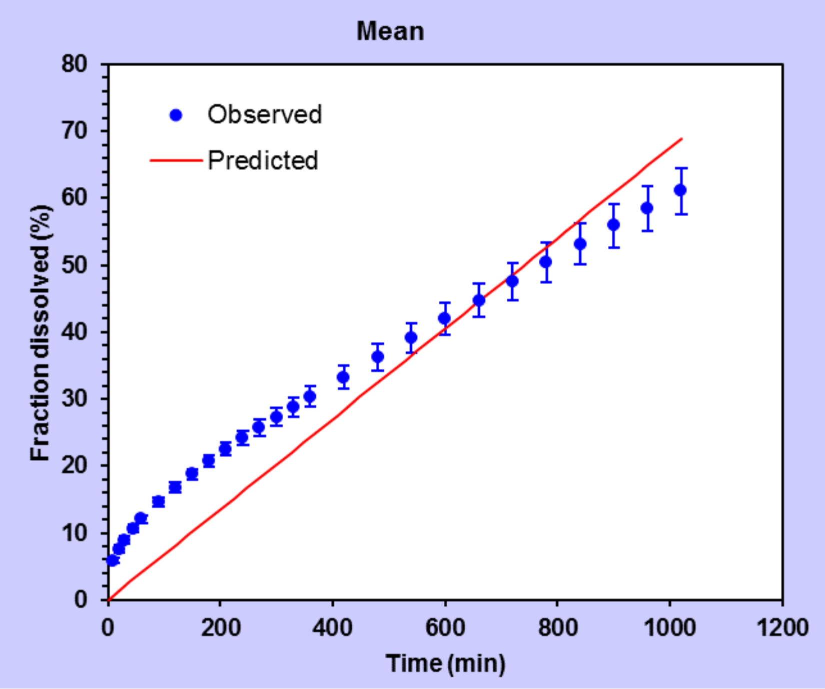

Supplementary materials – Model fitting summary – Starch 1500® sample with larger particle size (modelling up to app. 60 % of carvedilol released)

Graphical abstract of model fit presented as the fraction % of released carvedilol per tested tablet:

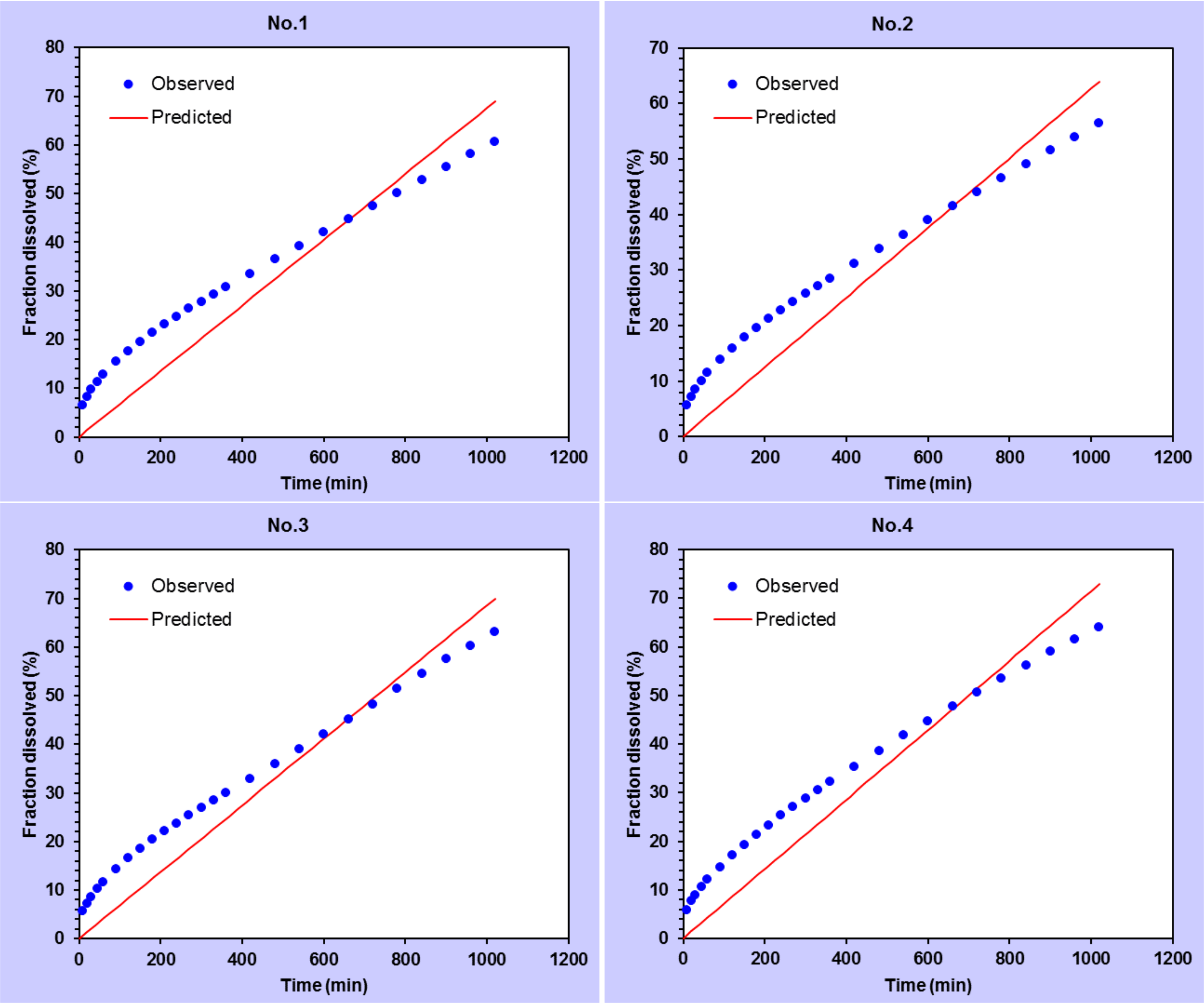

Supplementary materials – Model fitting summary – Starch 1500® sample with larger particle size (modelling up to app. 60 % of carvedilol released)

Model: **Zero–order with T_{lag}**

Model equation: $F = k_0 \cdot (t - T_{lag})$

Fitted model parameters per tested tablet (N = 4) with statistics – mean, standard deviation (SD), and relative standard deviation expressed in % (RSD%) (output from DDSolver):

Parameter	No.1	No.2	No.3	No.4	Mean	SD	RSD(%)
k_0	0.051	0.048	0.055	0.056	0.052	0.004	6.968
T_{lag}	-209.295	-198.958	-166.726	-176.999	-187.995	19.555	-10.402

Number of dissolution data points (N), degrees of freedom (df), and selected goodness of fit criteria – Pearson correlation coefficient (R), coefficient of determination (R^2), adjusted coefficient of determination ($R^2_{adjusted}$), and residual sum of squares (RSS) (manual calculation in MS Excel):

Parameter	No.1	No.2	No.3	No.4
N	26	26	26	26
df	24	24	24	24
R	0.994002148	0.993558444	0.99643135	0.994081863
R^2	0.98804027	0.987158381	0.992875435	0.98819875
$R^2_{adjusted}$	0.987541948	0.986623314	0.992578578	0.987707032
RSS	81.38956875	77.08999968	55.00894882	96.97301133

Graphical abstract of model fit presented as mean ± 1 SD of the fraction % of released carvedilol:

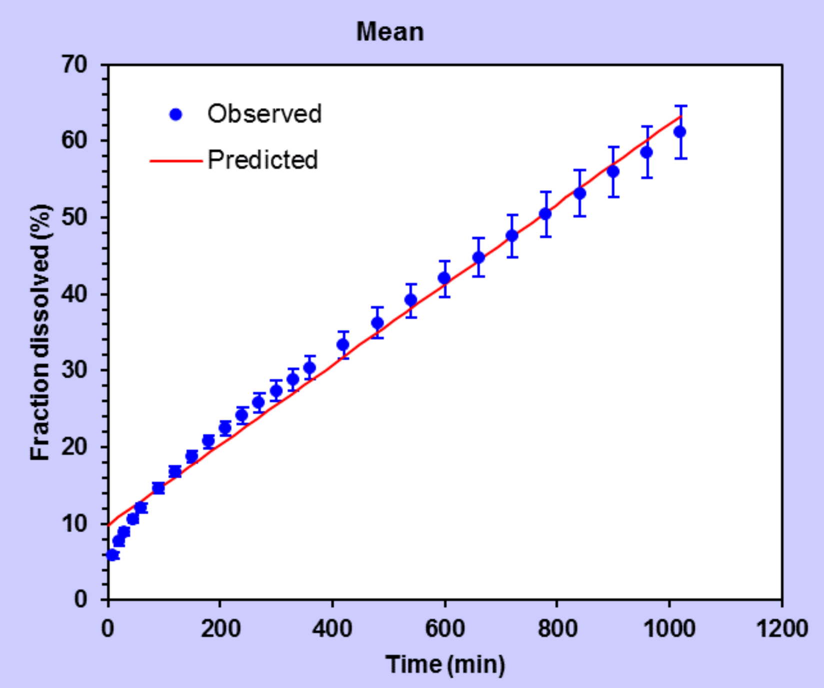

Supplementary materials – Model fitting summary – Starch 1500® sample with larger particle size (modelling up to app. 60 % of carvedilol released)

Graphical abstract of model fit presented as the fraction % of released carvedilol per tested tablet:

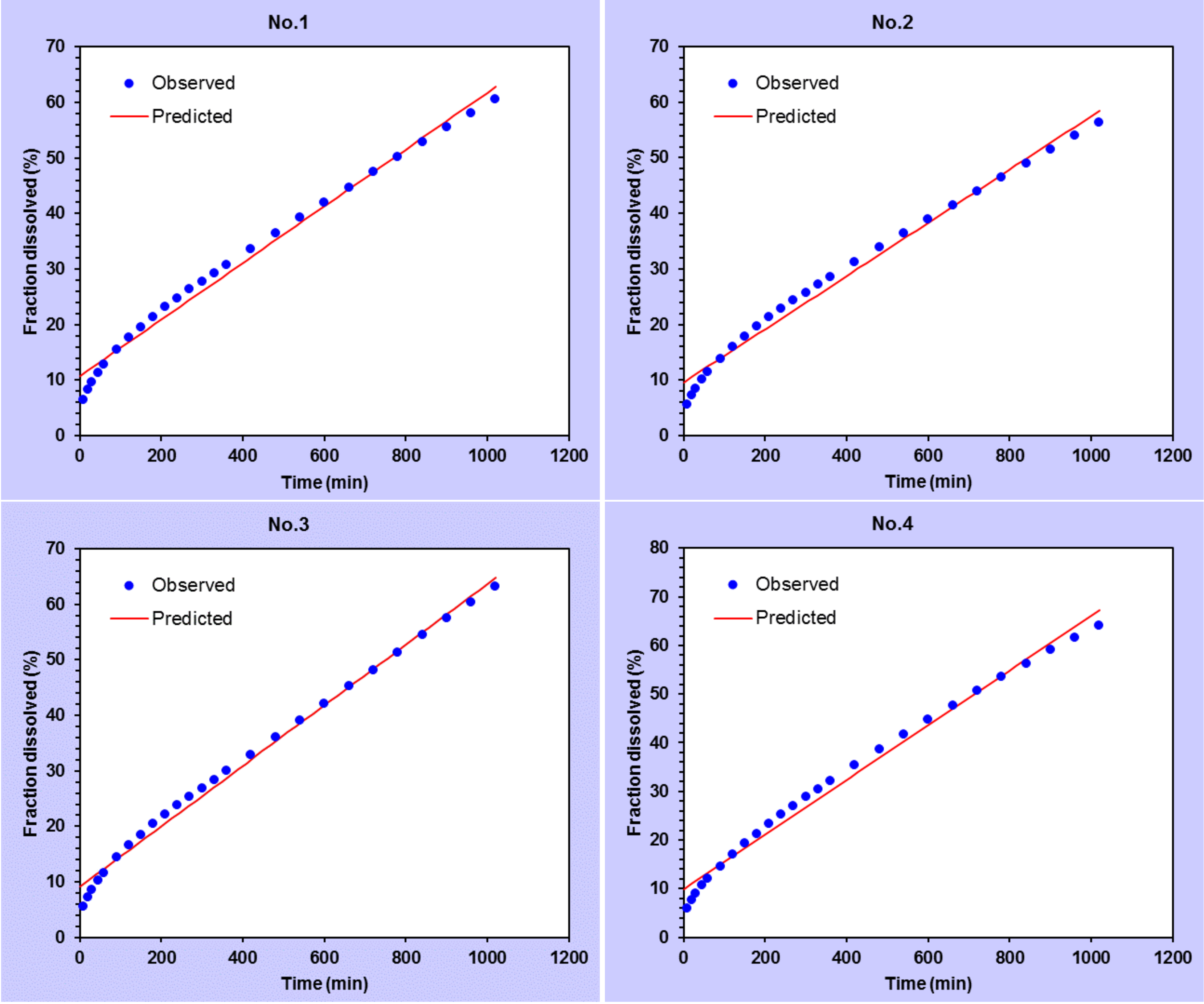

Supplementary materials – Model fitting summary – Starch 1500® sample with larger particle size (modelling up to app. 60 % of carvedilol released)

Model: **Zero–order with F_0**

Model equation: $F = F_0 + k_0 \cdot t$

Fitted model parameters per tested tablet (N = 4) with statistics – mean, standard deviation (SD), and relative standard deviation expressed in % (RSD%) (output from DDSolver):

Parameter	No.1	No.2	No.3	No.4	Mean	SD	RSD(%)
k_0	0.051	0.048	0.055	0.056	0.052	0.004	6.968
F_0	10.689	9.539	9.092	9.934	9.814	0.677	6.903

Number of dissolution data points (N), degrees of freedom (df), and selected goodness of fit criteria – Pearson correlation coefficient (R), coefficient of determination (R^2), adjusted coefficient of determination ($R^2_{adjusted}$), and residual sum of squares (RSS) (manual calculation in MS Excel):

Parameter	No.1	No.2	No.3	No.4
N	26	26	26	26
df	24	24	24	24
R	0.994002148	0.993558444	0.99643135	0.994081863
R^2	0.98804027	0.987158381	0.992875435	0.98819875
$R^2_{adjusted}$	0.987541948	0.986623314	0.992578578	0.987707032
RSS	81.38956875	77.08999968	55.00894882	96.97301133

Graphical abstract of model fit presented as mean ± 1 SD of the fraction % of released carvedilol:

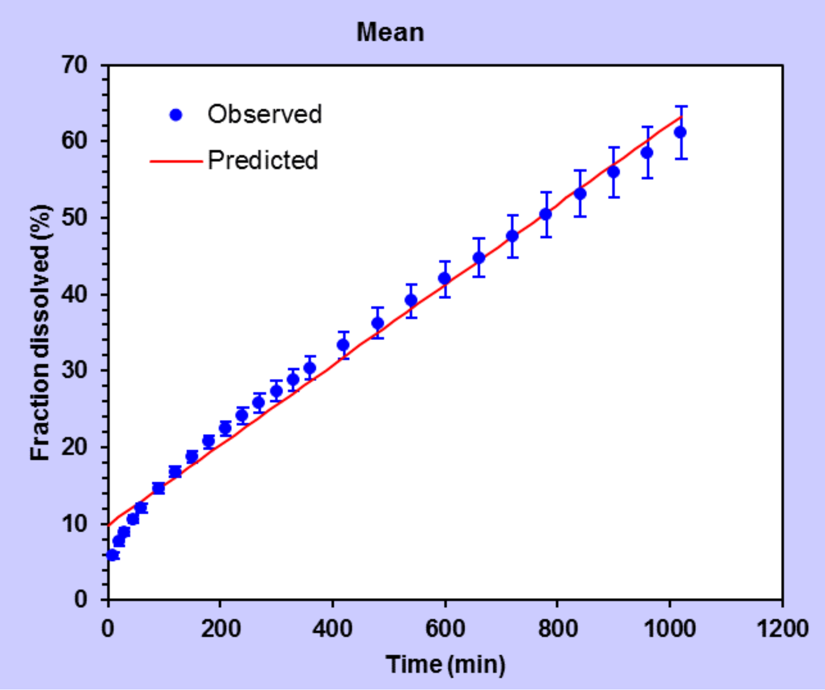

Supplementary materials – Model fitting summary – Starch 1500® sample with larger particle size (modelling up to app. 60 % of carvedilol released)

Graphical abstract of model fit presented as the fraction % of released carvedilol per tested tablet:

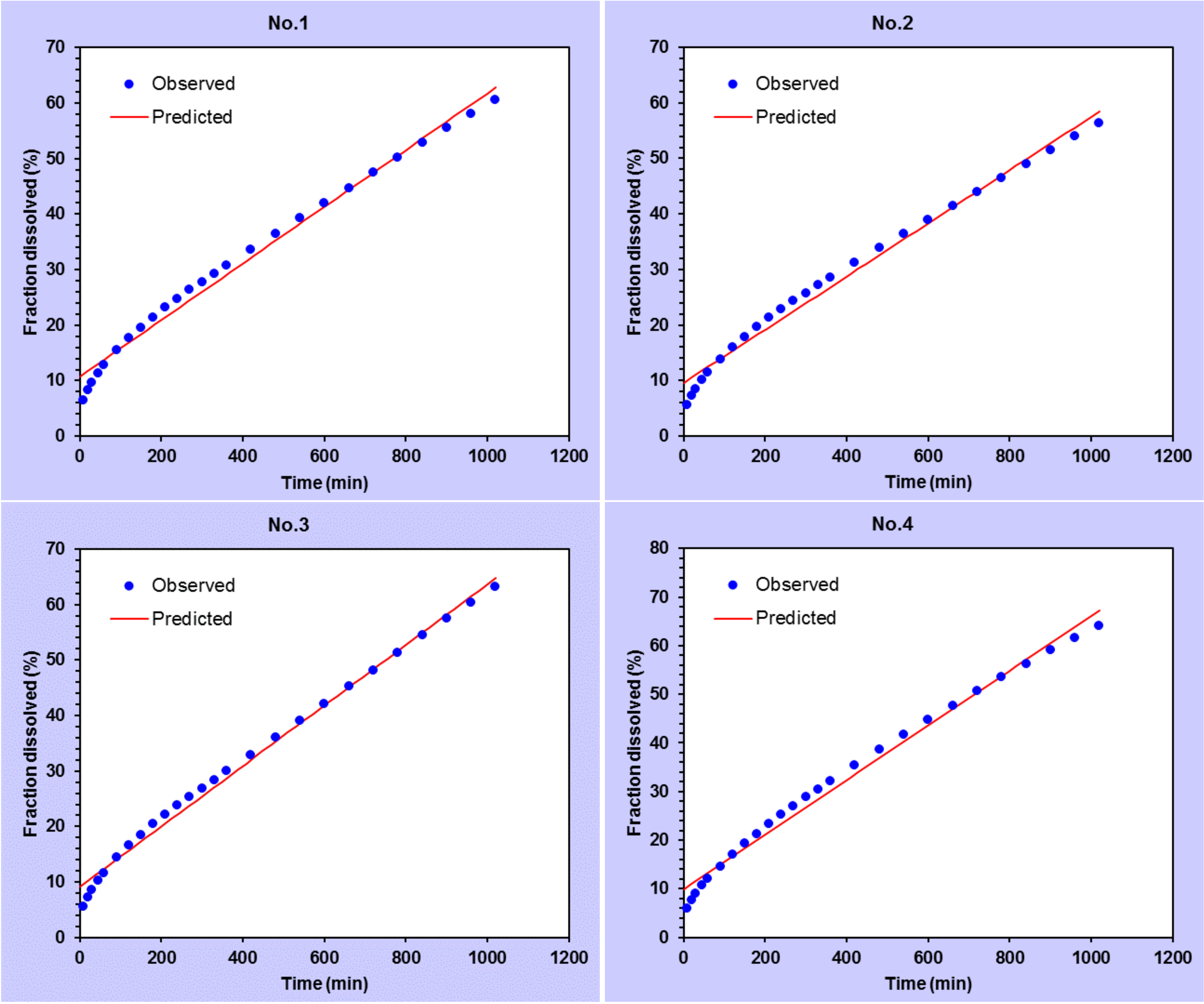

Supplementary materials – Model fitting summary – Starch 1500® sample with larger particle size (modelling up to app. 60 % of carvedilol released)

Model: **First–order**

Model equation: $F = 100 \cdot (1 - e^{-k_1 \cdot t})$

Fitted model parameters per tested tablet (N = 4) with statistics – mean, standard deviation (SD), and relative standard deviation expressed in % (RSD%) (output from DDSolver):

Parameter	No.1	No.2	No.3	No.4	Mean	SD	RSD(%)
k_1	0.001	0.001	0.001	0.001	0.001	0.000	7.891

Number of dissolution data points (N), degrees of freedom (df), and selected goodness of fit criteria – Pearson correlation coefficient (R), coefficient of determination (R^2), adjusted coefficient of determination ($R^2_{adjusted}$), and residual sum of squares (RSS) (manual calculation in MS Excel):

Parameter	No.1	No.2	No.3	No.4
N	26	26	26	26
df	25	25	25	25
R	0.998196474	0.998415975	0.997107948	0.998956514
R^2	0.9963962	0.996834459	0.99422426	0.997914117
$R^2_{adjusted}$	0.9963962	0.996834459	0.99422426	0.997914117
RSS	520.8199837	436.6117487	328.3383827	345.6060408

Graphical abstract of model fit presented as mean ± 1 SD of the fraction % of released carvedilol:

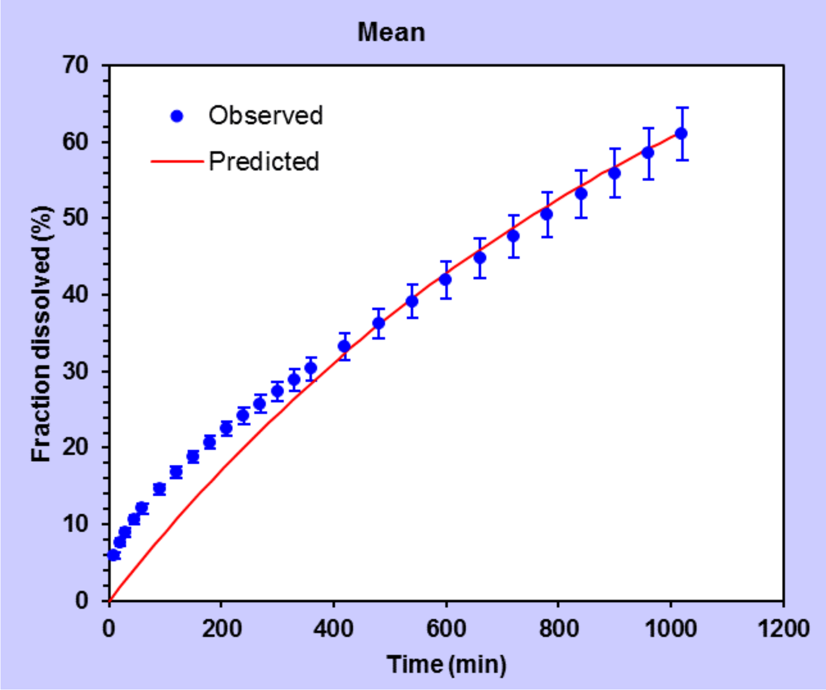

Supplementary materials – Model fitting summary – Starch 1500® sample with larger particle size (modelling up to app. 60 % of carvedilol released)

Graphical abstract of model fit presented as the fraction % of released carvedilol per tested tablet:

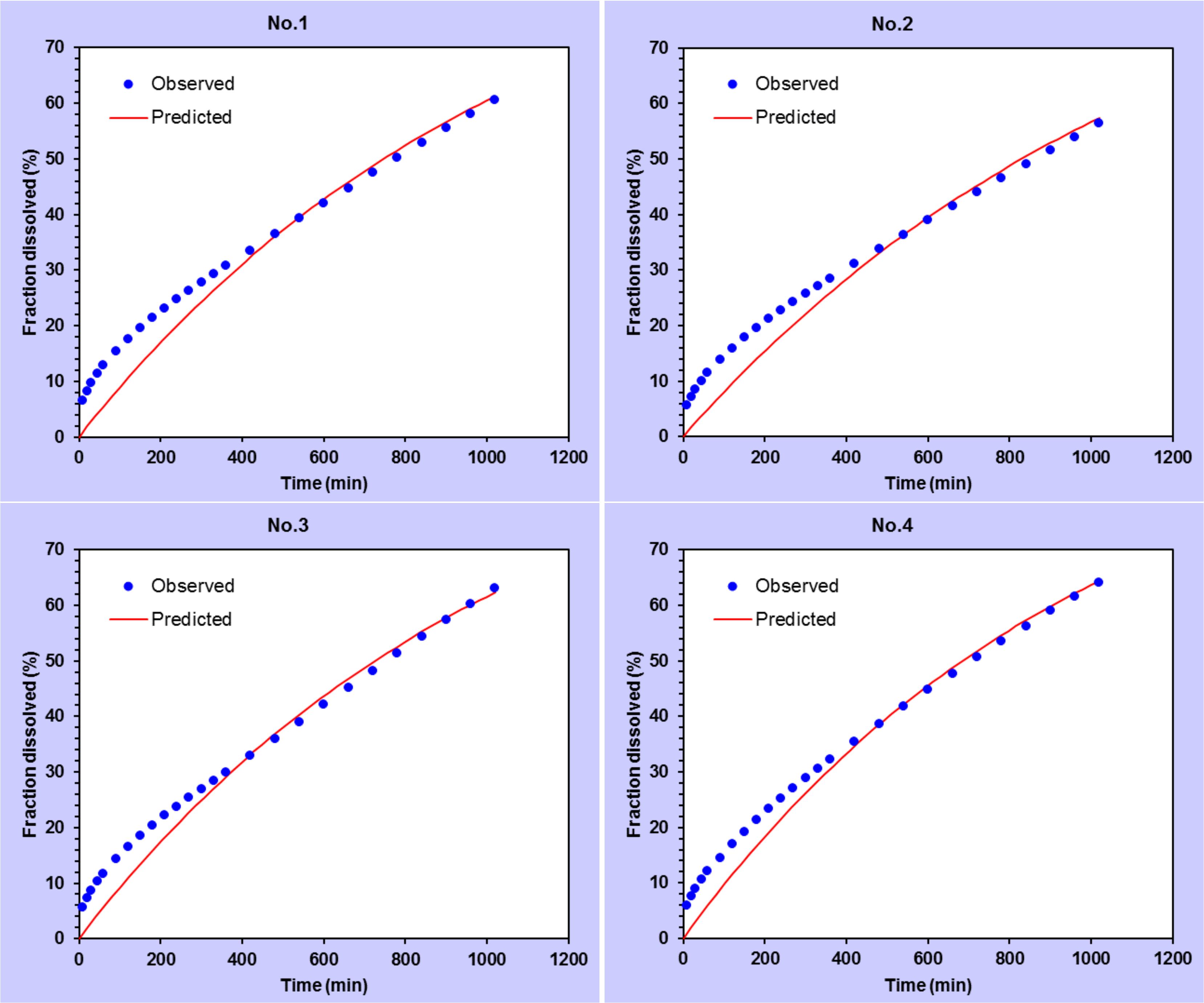

Supplementary materials – Model fitting summary – Starch 1500® sample with larger particle size (modelling up to app. 60 % of carvedilol released)

Model: **First–order with T_{lag}**

Model equation: $F = 100 \cdot \left[1 - e^{-k_1 \cdot (t - T_{lag})}\right]$

Fitted model parameters per tested tablet (N = 4) with statistics – mean, standard deviation (SD), and relative standard deviation expressed in % (RSD%) (output from DDSolver):

Parameter	No.1	No.2	No.3	No.4	Mean	SD	RSD(%)
k_1	0.001	0.001	0.001	0.001	0.001	0.000	10.082
T_{lag}	-102.714	-105.164	-66.925	-72.799	-86.900	19.845	-22.837

Number of dissolution data points (N), degrees of freedom (df), and selected goodness of fit criteria – Pearson correlation coefficient (R), coefficient of determination (R^2), adjusted coefficient of determination ($R^2_{adjusted}$), and residual sum of squares (RSS) (manual calculation in MS Excel):

Parameter	No.1	No.2	No.3	No.4
N	26	26	26	26
df	24	24	24	24
R	0.99842688	0.998403691	0.997649964	0.999208409
R^2	0.996856234	0.996809931	0.995305451	0.998417445
$R^2_{adjusted}$	0.996725243	0.996677012	0.995109845	0.998351505
RSS	21.55055097	19.15072612	38.71135859	13.42471288

Graphical abstract of model fit presented as mean ± 1 SD of the fraction % of released carvedilol:

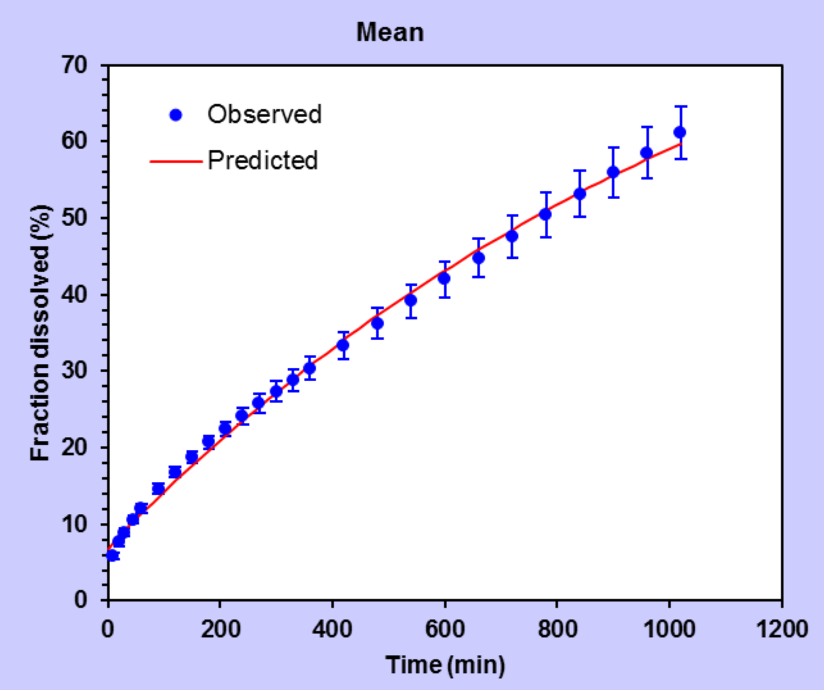

Supplementary materials – Model fitting summary – Starch 1500® sample with larger particle size (modelling up to app. 60 % of carvedilol released)

Graphical abstract of model fit presented as the fraction % of released carvedilol per tested tablet:

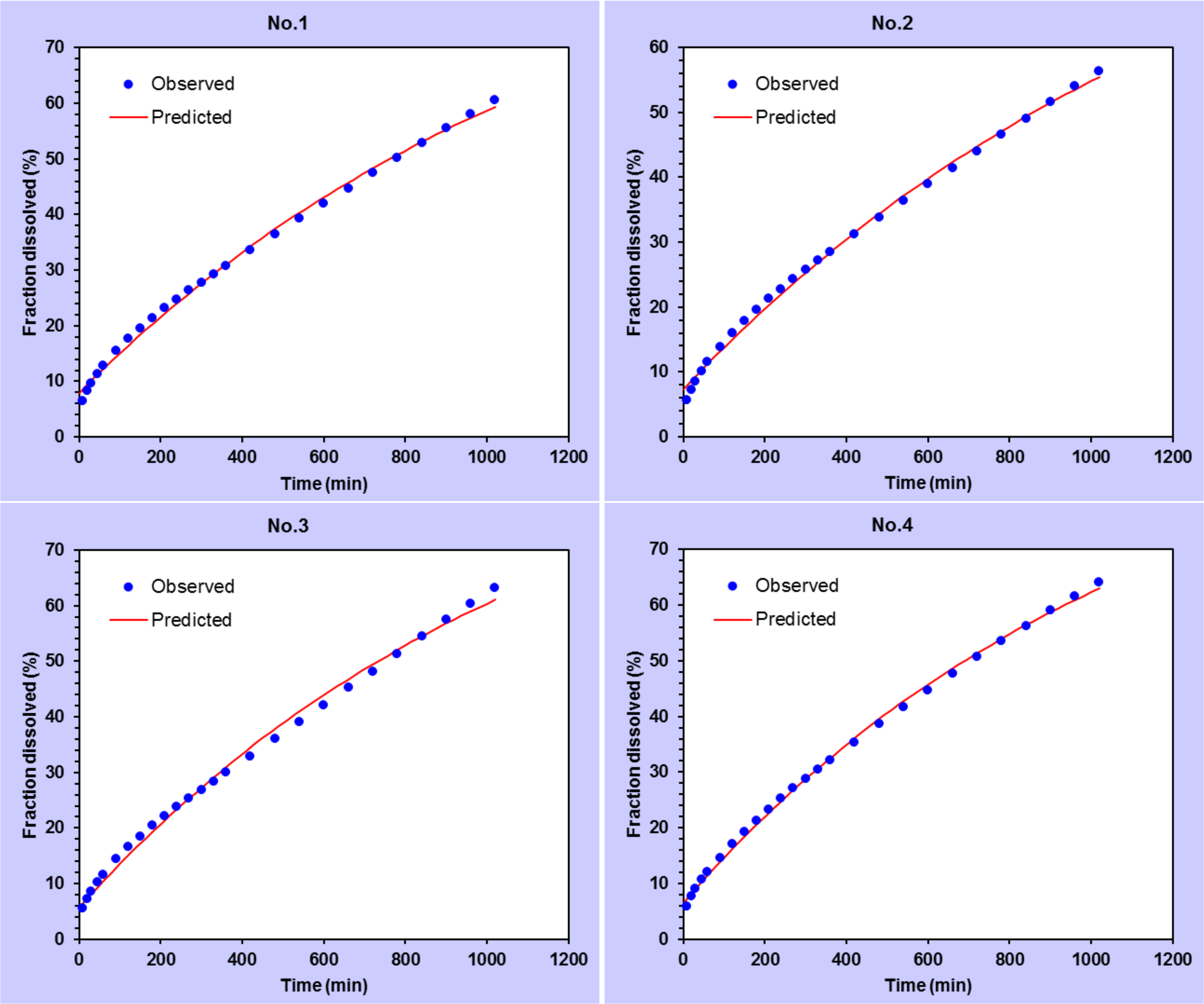

Supplementary materials – Model fitting summary – Starch 1500® sample with larger particle size (modelling up to app. 60 % of carvedilol released)

Model: **First–order with F_{max}**

Model equation: $F = F_{max} \cdot (1 - e^{-k_1 \cdot t})$

Fitted model parameters per tested tablet (N = 4) with statistics – mean, standard deviation (SD), and relative standard deviation expressed in % (RSD%) (output from DDSolver):

Parameter	No.1	No.2	No.3	No.4	Mean	SD	RSD(%)
k_1	0.002	0.002	0.002	0.002	0.002	0.000	1.926
F_{max}	63.665	59.218	66.331	67.319	64.133	3.622	5.648

Number of dissolution data points (N), degrees of freedom (df), and selected goodness of fit criteria – Pearson correlation coefficient (R), coefficient of determination (R^2), adjusted coefficient of determination ($R^2_{adjusted}$), and residual sum of squares (RSS) (manual calculation in MS Excel):

Parameter	No.1	No.2	No.3	No.4
N	26	26	26	26
df	24	24	24	24
R	0.983761534	0.984742169	0.980208944	0.985035219
R^2	0.967786755	0.96971714	0.960809574	0.970294383
$R^2_{adjusted}$	0.966444537	0.968455354	0.95917664	0.969056649
RSS	373.8825706	297.7238487	444.1955109	363.1129367

Graphical abstract of model fit presented as mean ± 1 SD of the fraction % of released carvedilol:

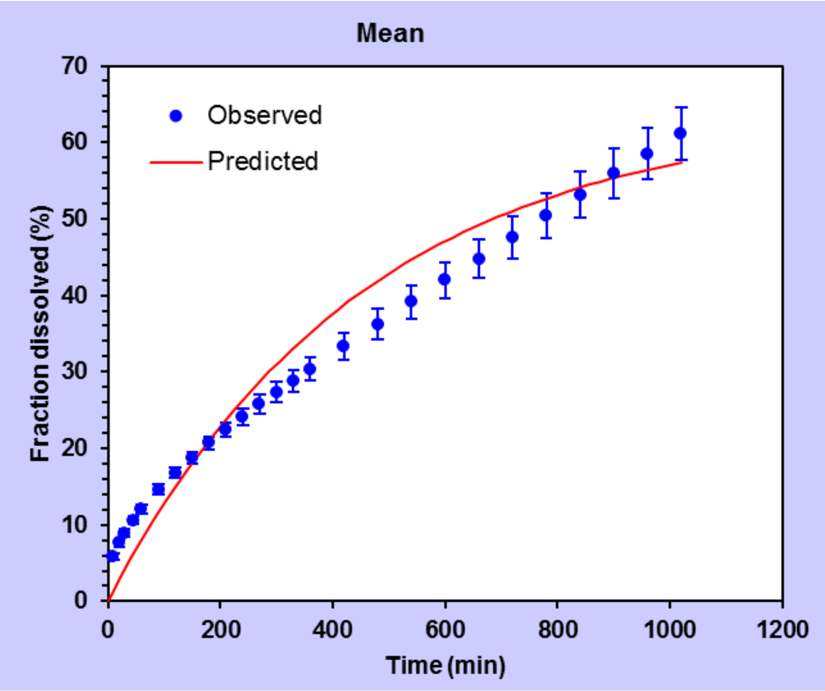

Supplementary materials – Model fitting summary – Starch 1500® sample with larger particle size (modelling up to app. 60 % of carvedilol released)

Graphical abstract of model fit presented as the fraction % of released carvedilol per tested tablet:

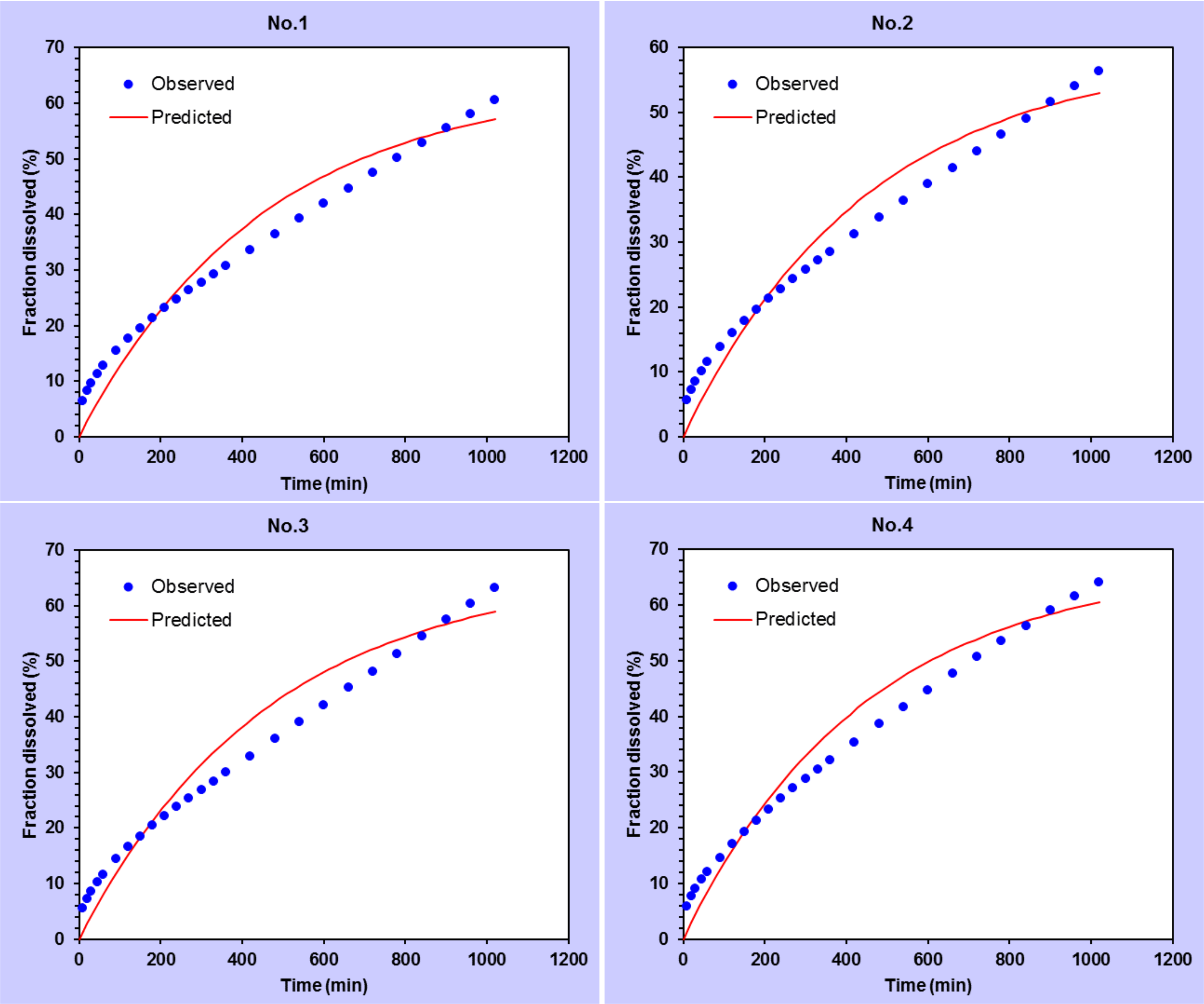

Supplementary materials – Model fitting summary – Starch 1500® sample with larger particle size (modelling up to app. 60 % of carvedilol released)

Model: **First–order with T_{lag} and F_{max}**

Model equation: $F = F_{max} \cdot \left[1 - e^{-k_1 \cdot (t - T_{lag})}\right]$

Fitted model parameters per tested tablet (N = 4) with statistics – mean, standard deviation (SD), and relative standard deviation expressed in % (RSD%) (output from DDSolver):

Parameter	No.1	No.2	No.3	No.4	Mean	SD	RSD(%)
k_1	0.002	0.002	0.002	0.002	0.002	0.000	1.636
T_{lag}	15.276	18.743	33.243	28.383	23.911	8.334	34.852
F_{max}	63.665	59.218	66.331	67.319	64.133	3.622	5.648

Number of dissolution data points (N), degrees of freedom (df), and selected goodness of fit criteria – Pearson correlation coefficient (R), coefficient of determination (R^2), adjusted coefficient of determination ($R^2_{adjusted}$), and residual sum of squares (RSS) (manual calculation in MS Excel):

Parameter	No.1	No.2	No.3	No.4
N	26	26	26	26
df	23	23	23	23
R	0.98276775	0.983558678	0.977757275	0.983116302
R^2	0.965832451	0.967387673	0.95600929	0.966517663
$R^2_{adjusted}$	0.96286136	0.964551818	0.95218401	0.963606156
RSS	508.0835265	432.7124677	733.068057	611.5182848

Graphical abstract of model fit presented as mean ± 1 SD of the fraction % of released carvedilol:

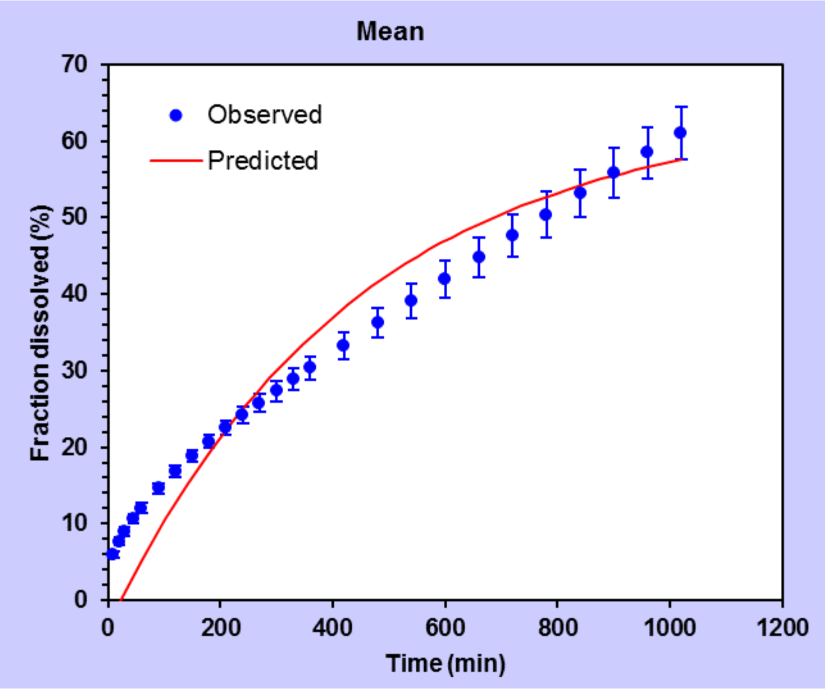

Supplementary materials – Model fitting summary – Starch 1500® sample with larger particle size (modelling up to app. 60 % of carvedilol released)

Graphical abstract of model fit presented as the fraction % of released carvedilol per tested tablet:

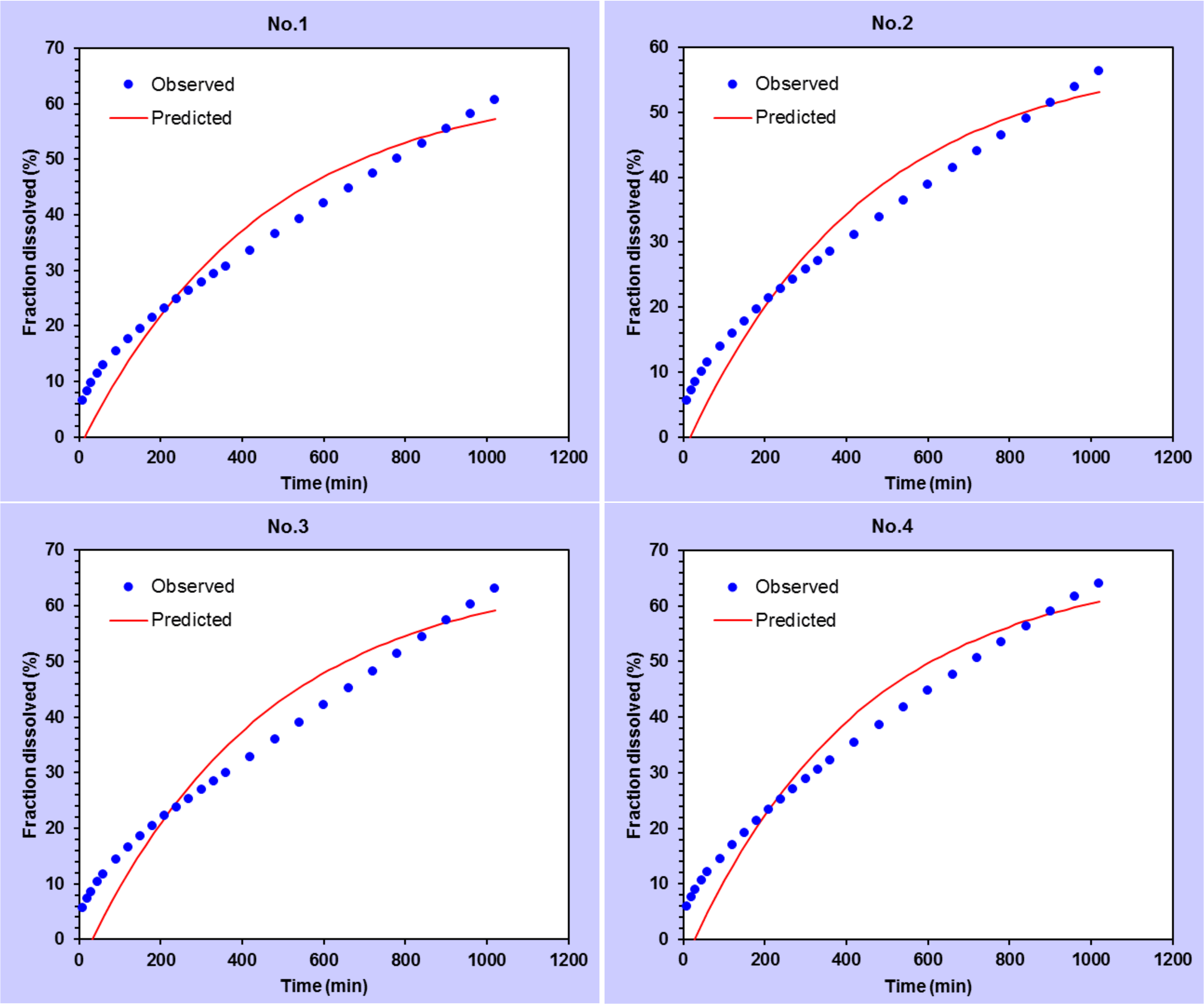

Supplementary materials – Model fitting summary – Starch 1500® sample with larger particle size (modelling up to app. 60 % of carvedilol released)

Model: **Higuchi**

Model equation: $F = k_H \cdot t^{0.5}$

Fitted model parameters per tested tablet (N = 4) with statistics – mean, standard deviation (SD), and relative standard deviation expressed in % (RSD%) (output from DDSolver):

Parameter	No.1	No.2	No.3	No.4	Mean	SD	RSD(%)
k_H	1.750	1.621	1.762	1.842	1.744	0.091	5.234

Number of dissolution data points (N), degrees of freedom (df), and selected goodness of fit criteria – Pearson correlation coefficient (R), coefficient of determination (R^2), adjusted coefficient of determination ($R^2_{adjusted}$), and residual sum of squares (RSS) (manual calculation in MS Excel):

Parameter	No.1	No.2	No.3	No.4
N	26	26	26	26
df	25	25	25	25
R	0.993788259	0.994267109	0.990281302	0.993996087
R^2	0.987615104	0.988567084	0.980657057	0.988028221
$R^2_{adjusted}$	0.987615104	0.988567084	0.980657057	0.988028221
RSS	112.3709602	106.2873463	255.9498109	195.2956792

Graphical abstract of model fit presented as mean ± 1 SD of the fraction % of released carvedilol:

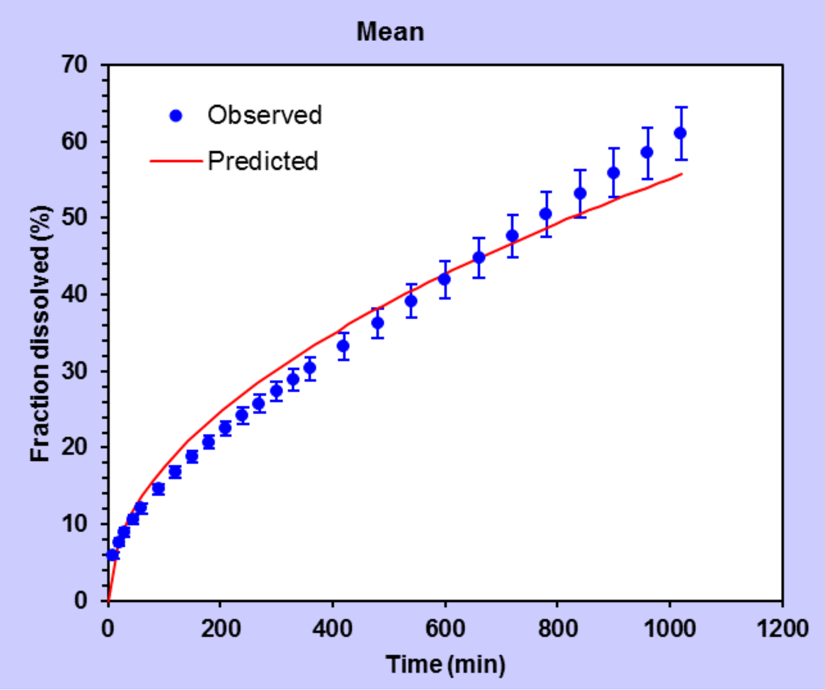

Supplementary materials – Model fitting summary – Starch 1500® sample with larger particle size (modelling up to app. 60 % of carvedilol released)

Graphical abstract of model fit presented as the fraction % of released carvedilol per tested tablet:

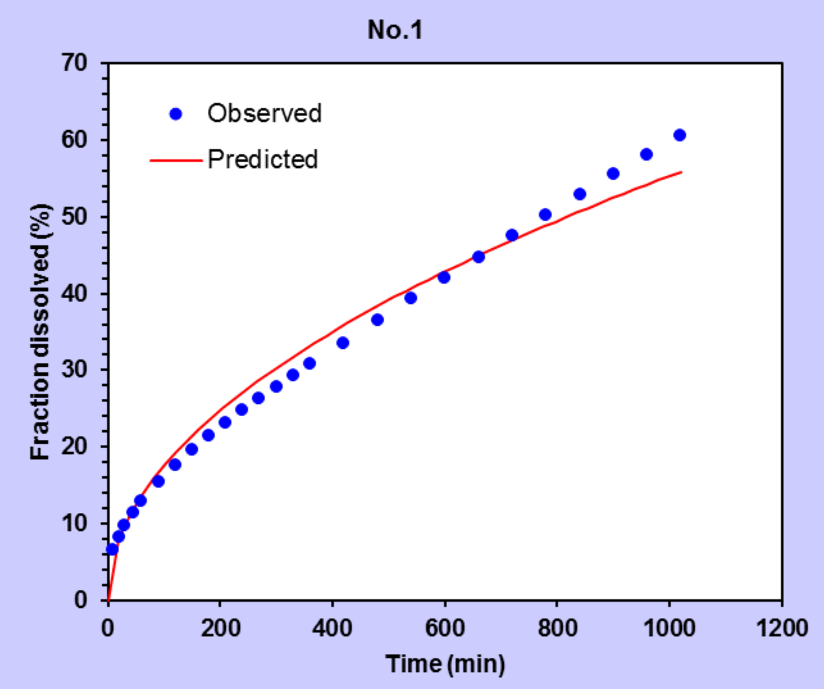

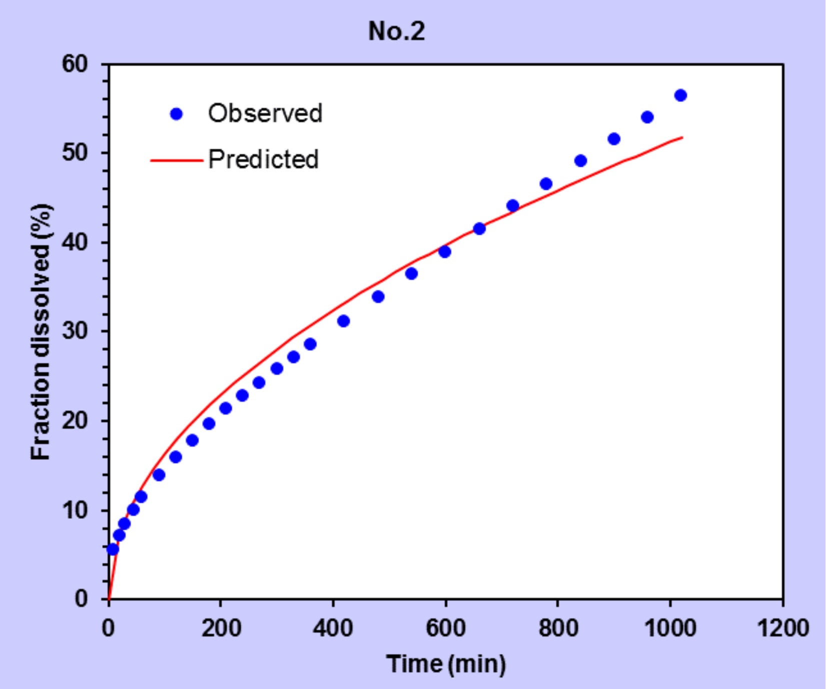

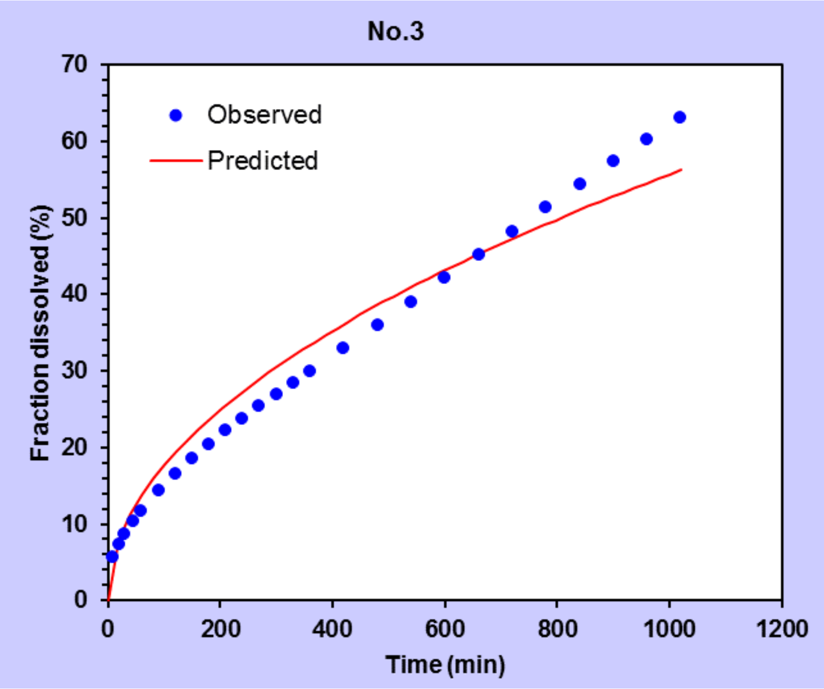

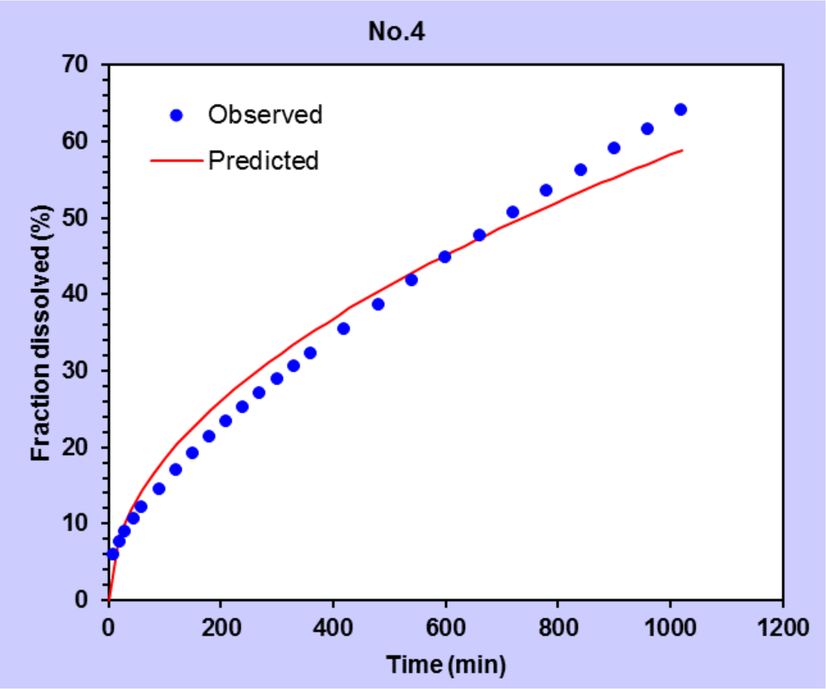

Supplementary materials – Model fitting summary – Starch 1500® sample with larger particle size (modelling up to app. 60 % of carvedilol released)

Model: **Higuchi with T_{lag}**

Model equation: $F = k_H \cdot (t - T_{lag})^{0.5}$

Fitted model parameters per tested tablet (N = 4) with statistics – mean, standard deviation (SD), and relative standard deviation expressed in % (RSD%) (output from DDSolver):

Parameter	No.1	No.2	No.3	No.4	Mean	SD	RSD(%)
k_H	1.859	1.730	1.932	1.990	1.878	0.112	5.964
T_{lag}	43.979	47.226	64.347	55.168	52.680	9.088	17.251

Number of dissolution data points (N), degrees of freedom (df), and selected goodness of fit criteria – Pearson correlation coefficient (R), coefficient of determination (R^2), adjusted coefficient of determination ($R^2_{adjusted}$), and residual sum of squares (RSS) (manual calculation in MS Excel):

Parameter	No.1	No.2	No.3	No.4
N	26	26	26	26
df	24	24	24	24
R	0.985022932	0.985024819	0.979713873	0.985420715
R^2	0.970270176	0.970273893	0.959839274	0.971053985
$R^2_{adjusted}$	0.969031433	0.969035306	0.95816591	0.969847901
RSS	387.5870814	338.2943246	532.7599676	418.0775539

Graphical abstract of model fit presented as mean ± 1 SD of the fraction % of released carvedilol:

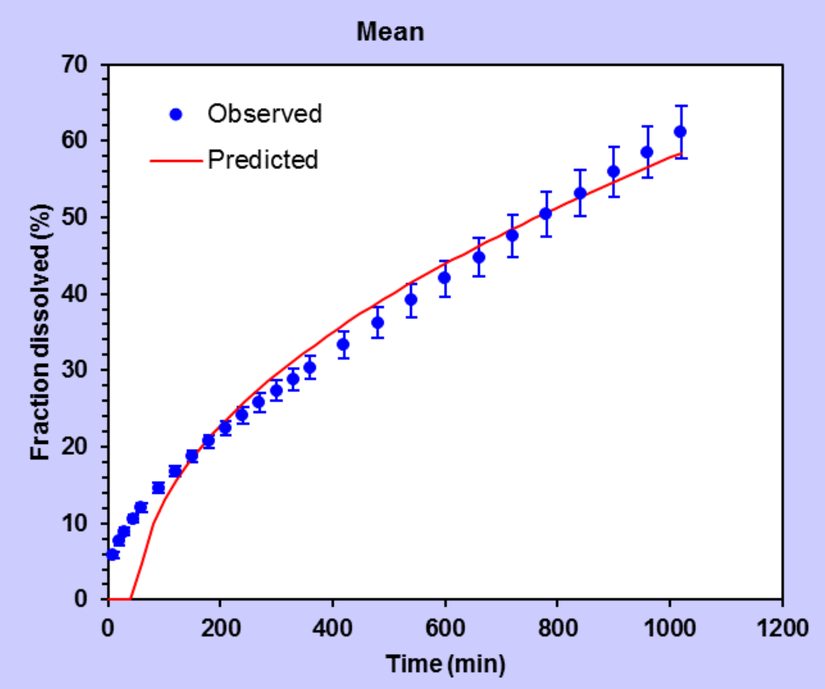

Supplementary materials – Model fitting summary – Starch 1500® sample with larger particle size (modelling up to app. 60 % of carvedilol released)

Graphical abstract of model fit presented as the fraction % of released carvedilol per tested tablet:

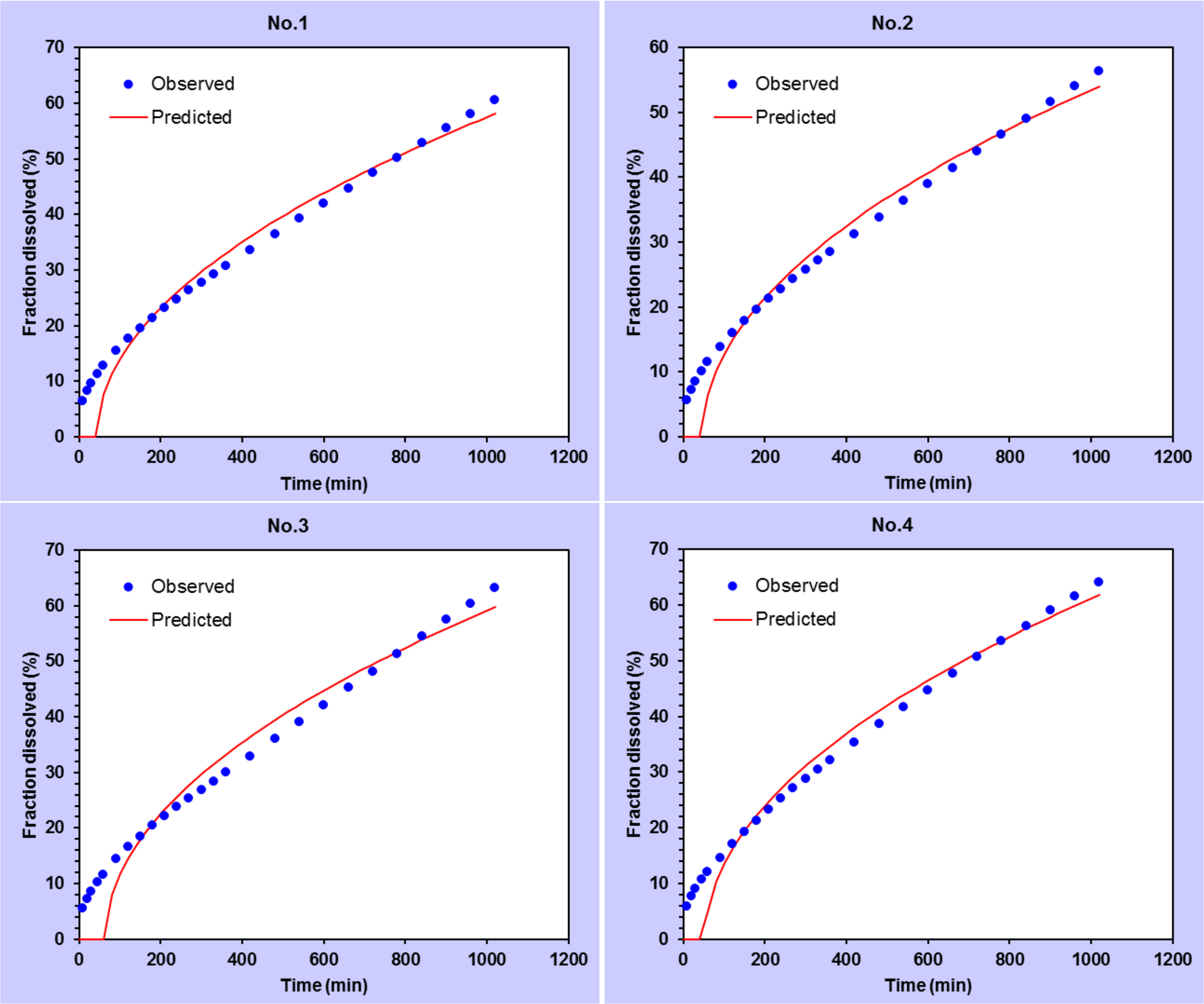

Supplementary materials – Model fitting summary – Starch 1500® sample with larger particle size (modelling up to app. 60 % of carvedilol released)

Model: **Higuchi with F_0**

Model equation: $F = F_0 + k_H \cdot t^{0.5}$

Fitted model parameters per tested tablet (N = 4) with statistics – mean, standard deviation (SD), and relative standard deviation expressed in % (RSD%) (output from DDSolver):

Parameter	No.1	No.2	No.3	No.4	Mean	SD	RSD(%)
k_H	1.859	1.746	1.973	2.043	1.905	0.130	6.833
F_0	-2.396	-2.774	-4.667	-4.450	-3.571	1.153	-32.295

Number of dissolution data points (N), degrees of freedom (df), and selected goodness of fit criteria – Pearson correlation coefficient (R), coefficient of determination (R^2), adjusted coefficient of determination ($R^2_{adjusted}$), and residual sum of squares (RSS) (manual calculation in MS Excel):

Parameter	No.1	No.2	No.3	No.4
N	26	26	26	26
df	24	24	24	24
R	0.993788259	0.994267109	0.990281302	0.993996087
R^2	0.987615104	0.988567084	0.980657057	0.988028221
$R^2_{adjusted}$	0.987099066	0.988090712	0.979851101	0.987529397
RSS	84.28295496	68.63336466	149.3473563	98.37428497

Graphical abstract of model fit presented as mean ± 1 SD of the fraction % of released carvedilol:

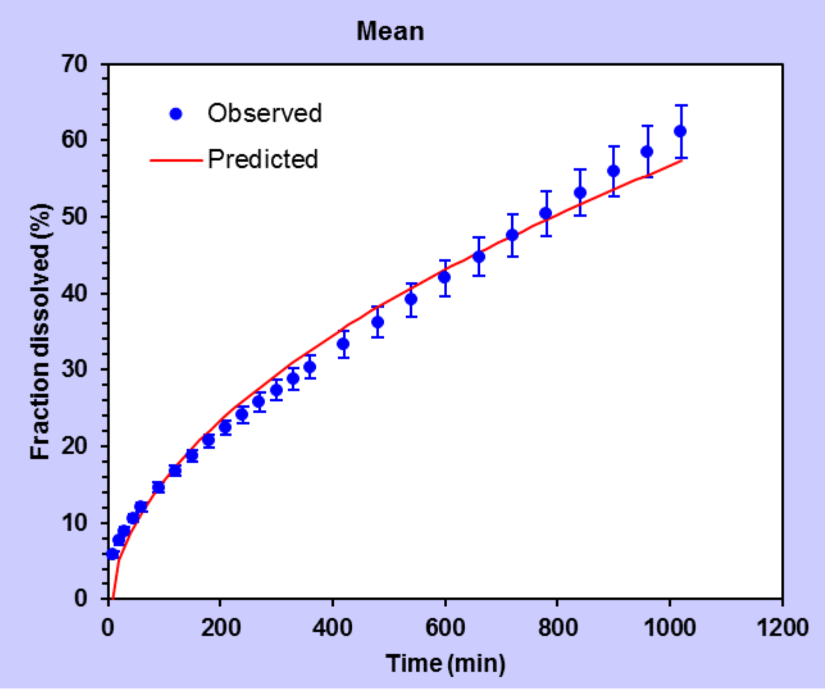

Supplementary materials – Model fitting summary – Starch 1500® sample with larger particle size (modelling up to app. 60 % of carvedilol released)

Graphical abstract of model fit presented as the fraction % of released carvedilol per tested tablet:

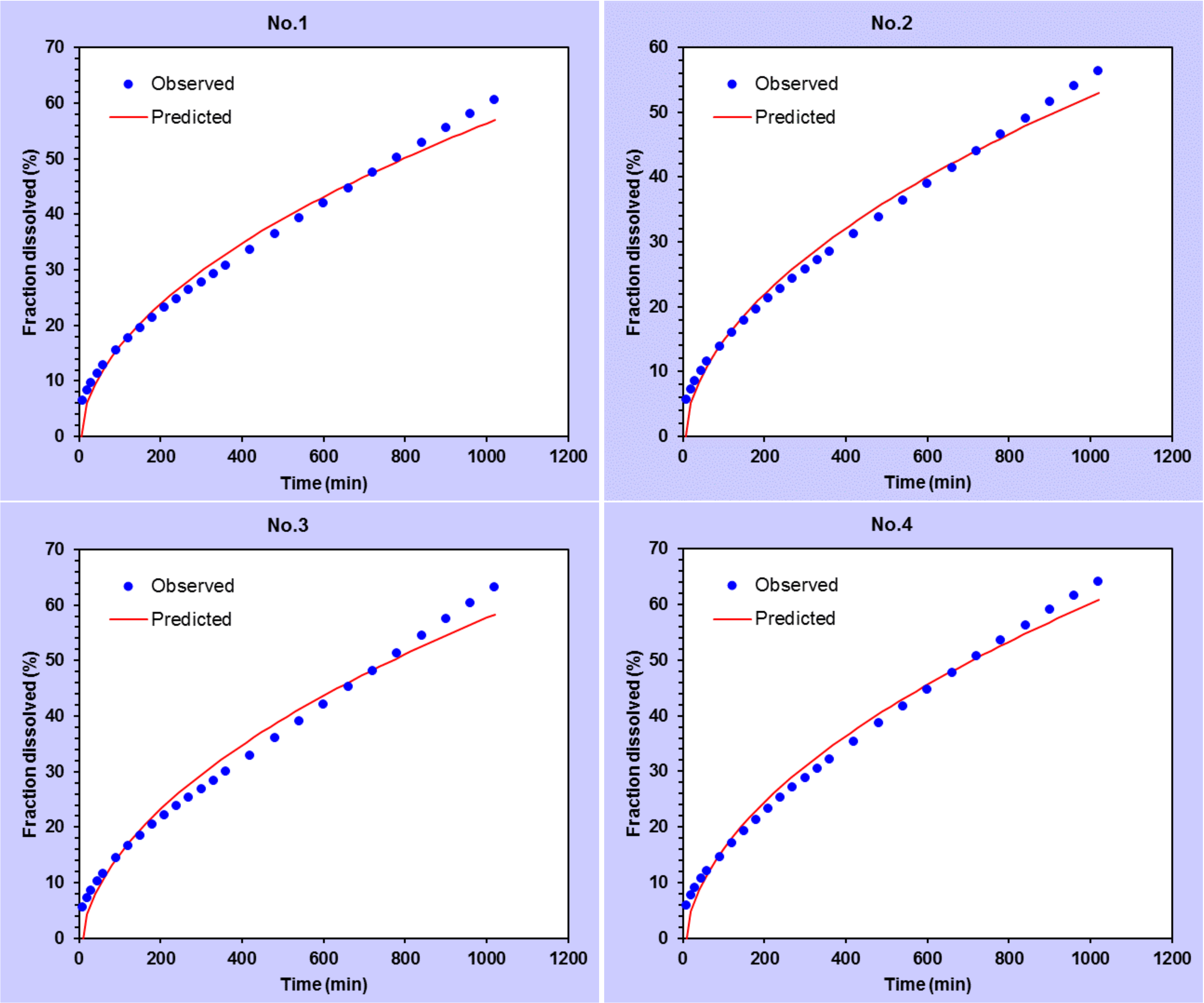

Supplementary materials – Model fitting summary – Starch 1500® sample with larger particle size (modelling up to app. 60 % of carvedilol released)

Model: **Korsmeyer–Peppas**

Model equation: $F = k_{KP} \cdot t^n$

Fitted model parameters per tested tablet (N = 4) with statistics – mean, standard deviation (SD), and relative standard deviation expressed in % (RSD%) (output from DDSolver):

Parameter	No.1	No.2	No.3	No.4	Mean	SD	RSD(%)
k_{KP}	1.755	1.459	1.353	1.416	1.496	0.178	11.902
n	0.495	0.513	0.537	0.537	0.520	0.020	3.892

Number of dissolution data points (N), degrees of freedom (df), and selected goodness of fit criteria – Pearson correlation coefficient (R), coefficient of determination (R^2), adjusted coefficient of determination ($R^2_{adjusted}$), and residual sum of squares (RSS) (manual calculation in MS Excel):

Parameter	No.1	No.2	No.3	No.4
N	26	26	26	26
df	24	24	24	24
R	0.993528187	0.994913642	0.99257779	0.995873215
R^2	0.987098259	0.989853155	0.98521067	0.991763461
$R^2_{adjusted}$	0.986560686	0.98943037	0.984594447	0.991420272
RSS	143.7098927	102.3724236	192.9116758	130.6802252

Graphical abstract of model fit presented as mean ± 1 SD of the fraction % of released carvedilol:

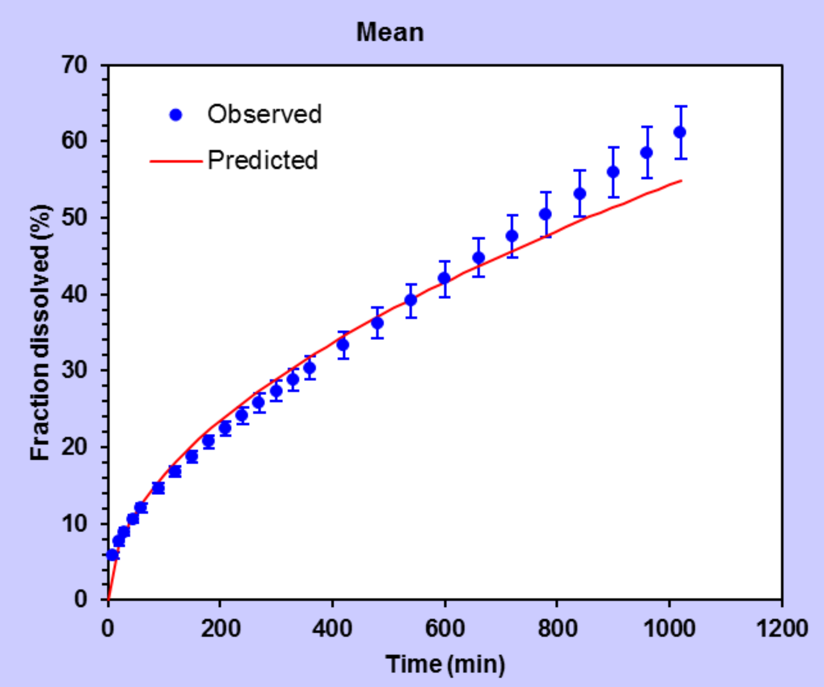

Supplementary materials – Model fitting summary – Starch 1500® sample with larger particle size (modelling up to app. 60 % of carvedilol released)

Graphical abstract of model fit presented as the fraction % of released carvedilol per tested tablet:

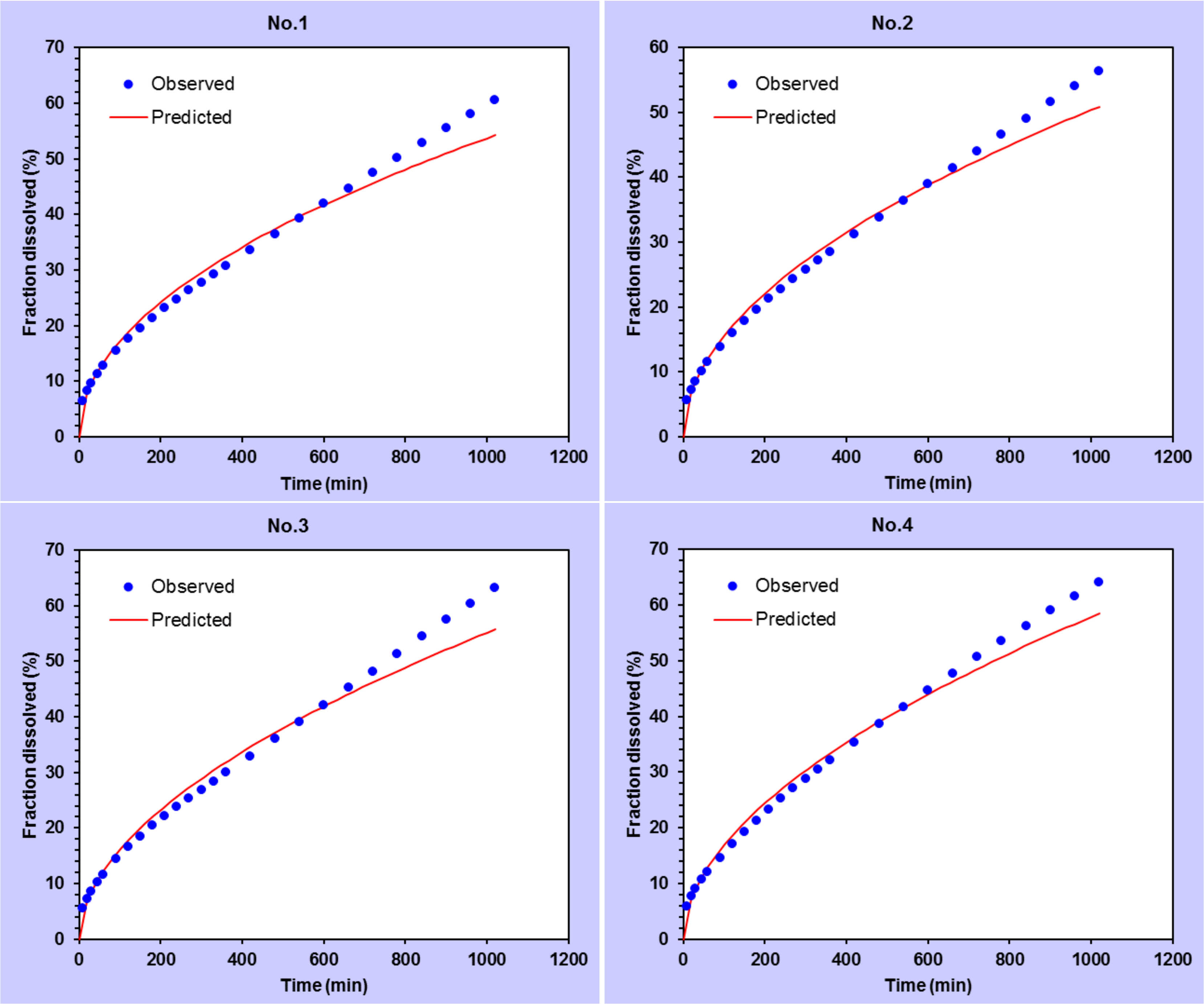

Supplementary materials – Model fitting summary – Starch 1500® sample with larger particle size (modelling up to app. 60 % of carvedilol released)

Model: **Korsmeyer–Peppas with T_{lag}**

Model equation: $F = k_{KP} \cdot \left(t - T_{lag}\right)^n$

Fitted model parameters per tested tablet (N = 4) with statistics – mean, standard deviation (SD), and relative standard deviation expressed in % (RSD%) (output from DDSolver):

Parameter	No.1	No.2	No.3	No.4	Mean	SD	RSD(%)
k_{KP}	2.161	1.808	1.696	1.776	1.861	0.206	11.076
n	0.461	0.478	0.500	0.500	0.485	0.019	3.884
T_{lag}	4.000	4.000	4.000	4.000	4.000	0.000	0.000

Number of dissolution data points (N), degrees of freedom (df), and selected goodness of fit criteria – Pearson correlation coefficient (R), coefficient of determination (R^2), adjusted coefficient of determination ($R^2_{adjusted}$), and residual sum of squares (RSS) (manual calculation in MS Excel):

Parameter	No.1	No.2	No.3	No.4
N	26	26	26	26
df	23	23	23	23
R	0.990241874	0.991978446	0.989187111	0.993051374
R^2	0.980578969	0.984021238	0.978491141	0.986151031
$R^2_{adjusted}$	0.978890184	0.98263178	0.976620805	0.984946773
RSS	230.0662771	173.2772782	300.0531241	231.2914544

Graphical abstract of model fit presented as mean ± 1 SD of the fraction % of released carvedilol:

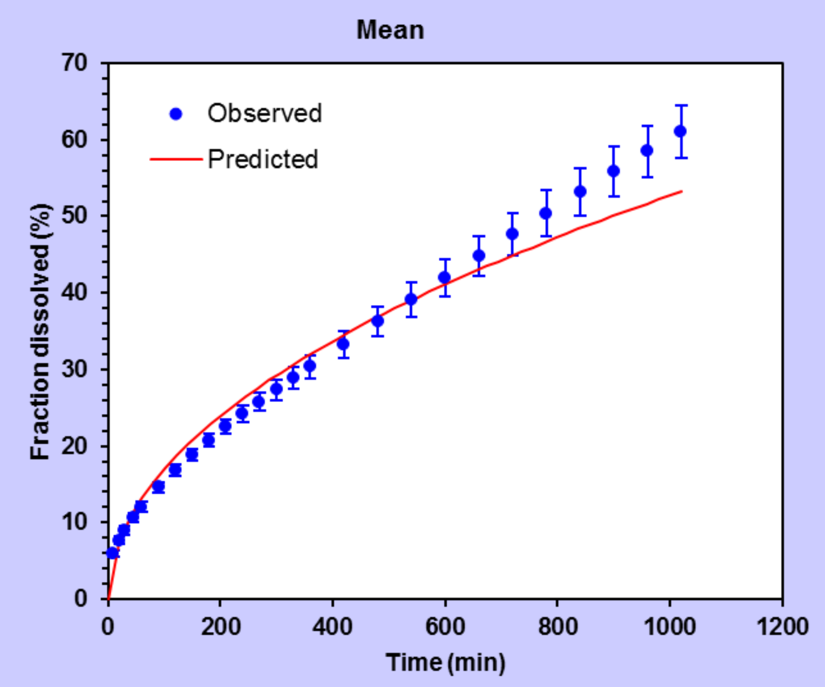

Supplementary materials – Model fitting summary – Starch 1500® sample with larger particle size (modelling up to app. 60 % of carvedilol released)

Graphical abstract of model fit presented as the fraction % of released carvedilol per tested tablet:

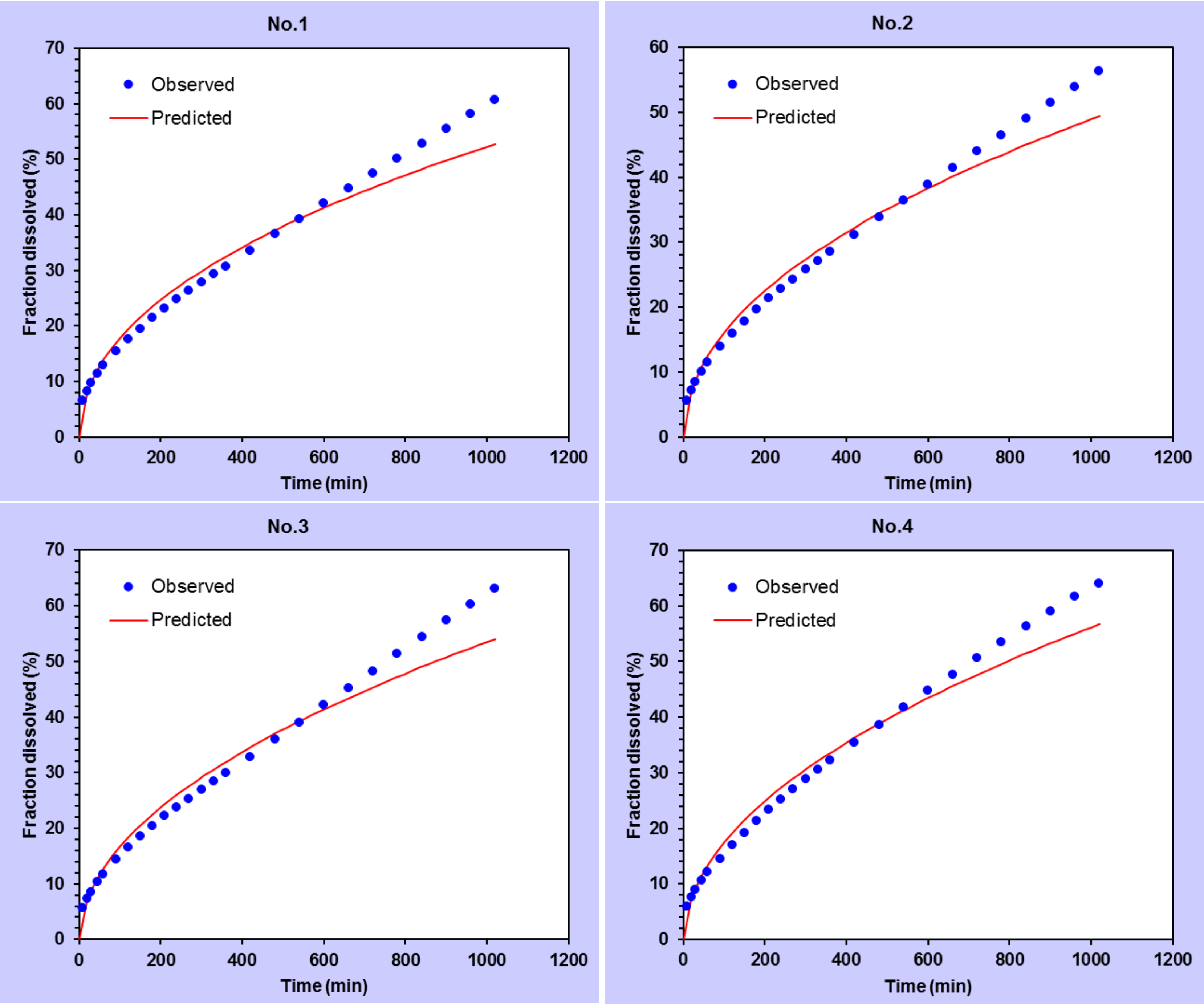

Supplementary materials – Model fitting summary – Starch 1500® sample with larger particle size (modelling up to app. 60 % of carvedilol released)

Model: **Korsmeyer–Peppas with F_0**

Model equation: $F = F_0 + k_{KP} \cdot t^n$

Fitted model parameters per tested tablet (N = 4) with statistics – mean, standard deviation (SD), and relative standard deviation expressed in % (RSD%) (output from DDSolver):

Parameter	No.1	No.2	No.3	No.4	Mean	SD	RSD(%)
k_{KP}	0.953	0.798	0.743	0.774	0.817	0.093	11.442
n	0.582	0.599	0.623	0.625	0.607	0.020	3.363
F_0	2.599	2.240	2.239	2.360	2.360	0.170	7.189

Number of dissolution data points (N), degrees of freedom (df), and selected goodness of fit criteria – Pearson correlation coefficient (R), coefficient of determination (R^2), adjusted coefficient of determination ($R^2_{adjusted}$), and residual sum of squares (RSS) (manual calculation in MS Excel):

Parameter	No.1	No.2	No.3	No.4
N	26	26	26	26
df	23	23	23	23
R	0.997346541	0.998151125	0.99657974	0.998816525
R^2	0.994700123	0.996305668	0.993171178	0.99763445
$R^2_{adjusted}$	0.994239264	0.995984422	0.992577367	0.99742875
RSS	58.77565058	35.65758018	86.73090041	40.72071888

Graphical abstract of model fit presented as mean ± 1 SD of the fraction % of released carvedilol:

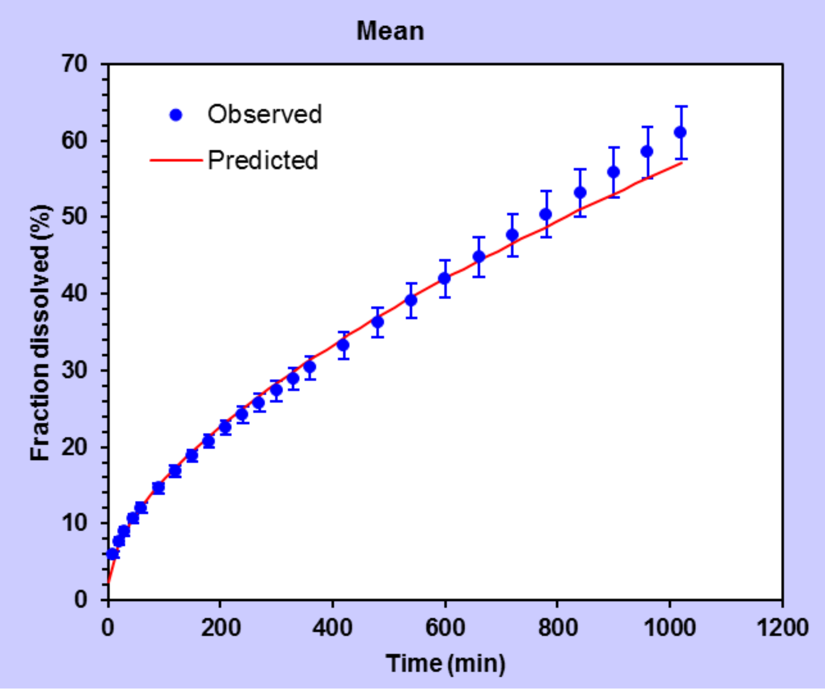

Supplementary materials – Model fitting summary – Starch 1500® sample with larger particle size (modelling up to app. 60 % of carvedilol released)

Graphical abstract of model fit presented as the fraction % of released carvedilol per tested tablet:

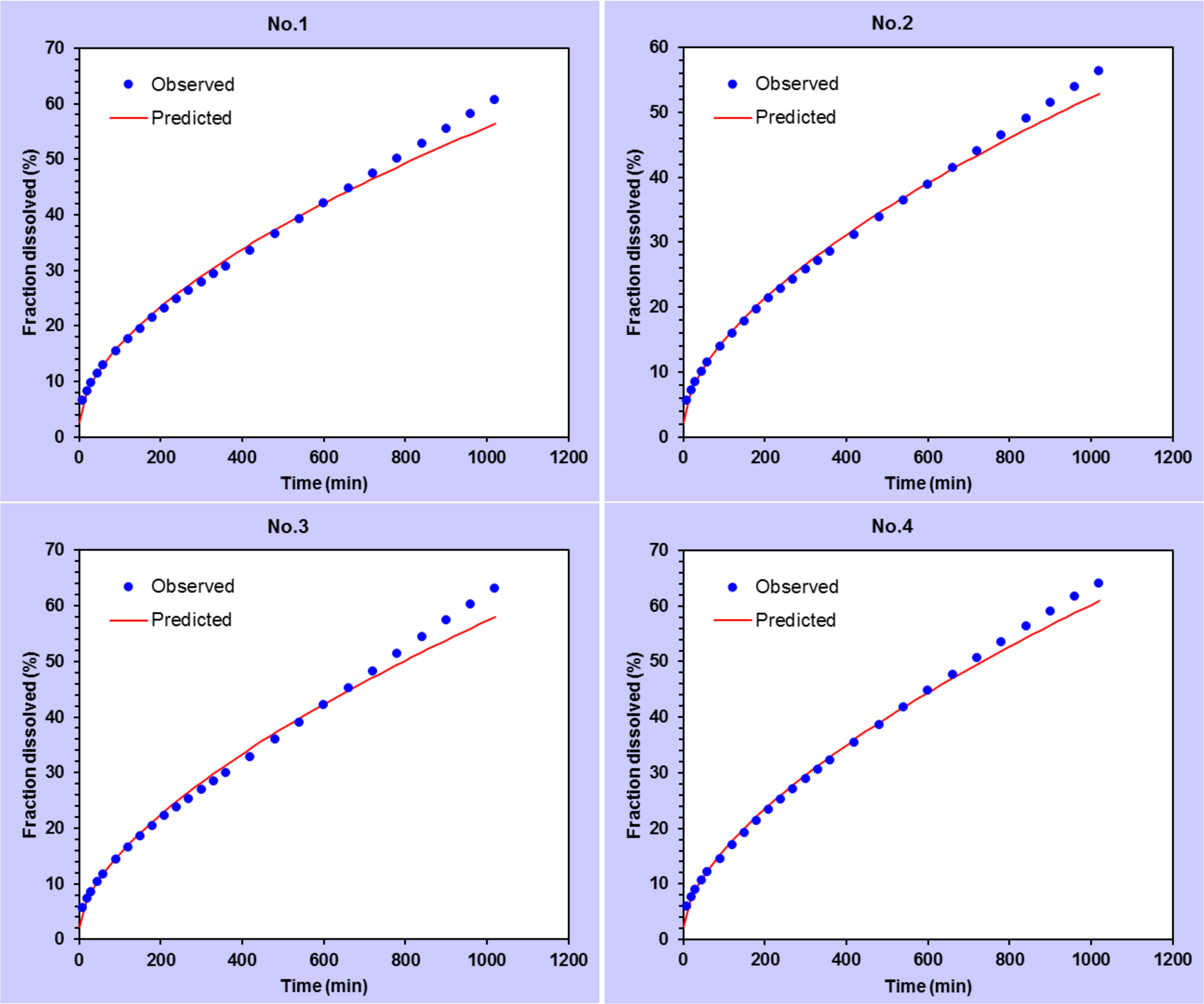

Supplementary materials – Model fitting summary – Starch 1500® sample with larger particle size (modelling up to app. 60 % of carvedilol released)

Model: **Hixson–Crowell**

Model equation: $F = 100 \cdot [1 - (1 - k_{HC} \cdot t)^3]$

Fitted model parameters per tested tablet (N = 4) with statistics – mean, standard deviation (SD), and relative standard deviation expressed in % (RSD%) (output from DDSolver):

Parameter	No.1	No.2	No.3	No.4	Mean	SD	RSD(%)
k_{HC}	0.0003	0.0003	0.0003	0.0003	0.0003	0.0000	6.9707

Number of dissolution data points (N), degrees of freedom (df), and selected goodness of fit criteria – Pearson correlation coefficient (R), coefficient of determination (R^2), adjusted coefficient of determination ($R^2_{adjusted}$), and residual sum of squares (RSS) (manual calculation in MS Excel):

Parameter	No.1	No.2	No.3	No.4
N	26	26	26	26
df	25	25	25	25
R	0.99824937	0.997996937	0.998345779	0.999122922
R^2	0.996501805	0.995997886	0.996694294	0.998246612
$R^2_{adjusted}$	0.996501805	0.995997886	0.996694294	0.998246612
RSS	695.0185951	576.5292894	450.4412103	512.2840694

Graphical abstract of model fit presented as mean ± 1 SD of the fraction % of released carvedilol:

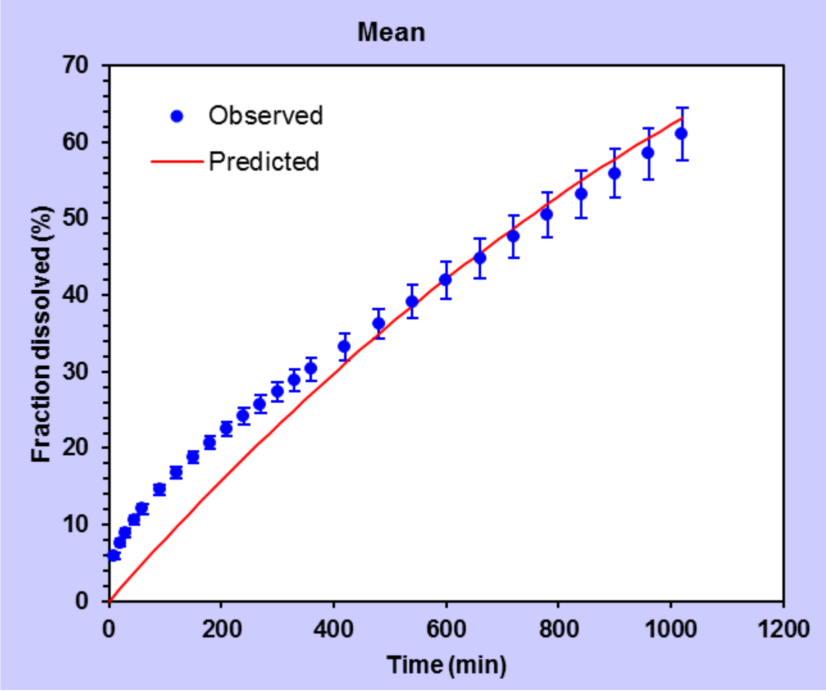

Supplementary materials – Model fitting summary – Starch 1500® sample with larger particle size (modelling up to app. 60 % of carvedilol released)

Graphical abstract of model fit presented as the fraction % of released carvedilol per tested tablet:

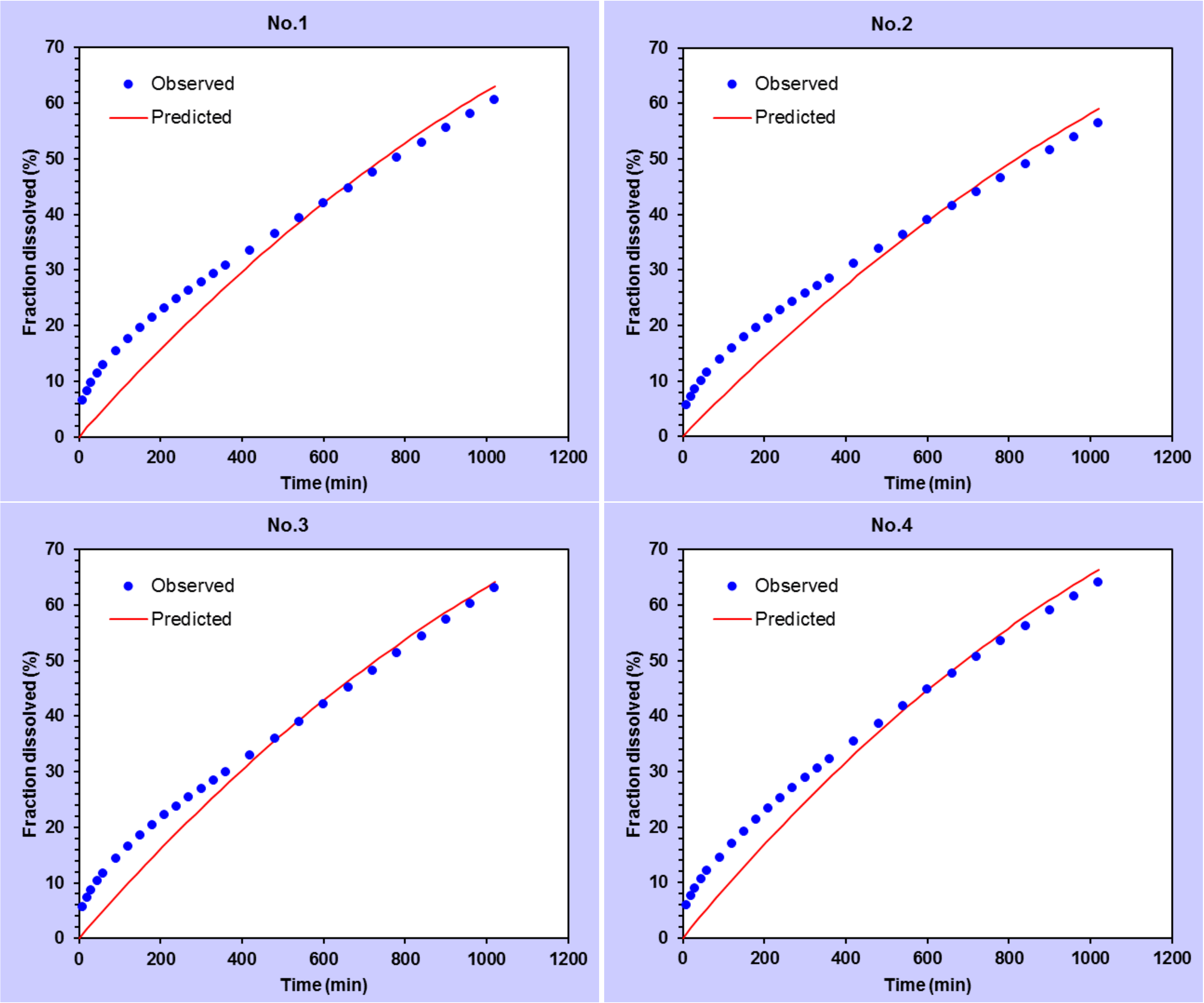

Supplementary materials – Model fitting summary – Starch 1500® sample with larger particle size (modelling up to app. 60 % of carvedilol released)

Model: **Hixson–Crowell with T_{lag}**

Model equation: $F = 100 \cdot \left\{1 - \left[1 - k_{HC} \cdot \left(t - T_{lag}\right)\right]^3\right\}$

Fitted model parameters per tested tablet (N = 4) with statistics – mean, standard deviation (SD), and relative standard deviation expressed in % (RSD%) (output from DDSolver):

Parameter	No.1	No.2	No.3	No.4	Mean	SD	RSD(%)
k_{HC}	0.000	0.000	0.000	0.000	0.000	0.000	8.958
T_{lag}	-135.771	-134.490	-97.940	-105.076	-118.319	19.636	-16.596

Number of dissolution data points (N), degrees of freedom (df), and selected goodness of fit criteria – Pearson correlation coefficient (R), coefficient of determination (R^2), adjusted coefficient of determination ($R^2_{adjusted}$), and residual sum of squares (RSS) (manual calculation in MS Excel):

Parameter	No.1	No.2	No.3	No.4
N	26	26	26	26
df	24	24	24	24
R	0.998016632	0.997643728	0.998495856	0.998923349
R^2	0.996037198	0.995293008	0.996993975	0.997847856
$R^2_{adjusted}$	0.995872081	0.995096883	0.996868724	0.997758184
RSS	27.08565631	28.46788748	23.29077612	17.86062329

Graphical abstract of model fit presented as mean ± 1 SD of the fraction % of released carvedilol:

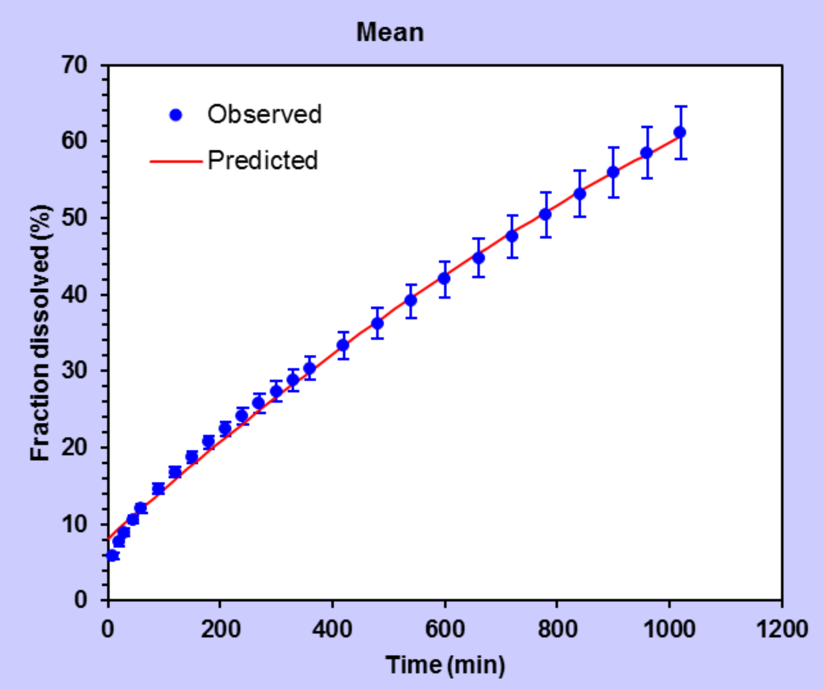

Supplementary materials – Model fitting summary – Starch 1500® sample with larger particle size (modelling up to app. 60 % of carvedilol released)

Graphical abstract of model fit presented as the fraction % of released carvedilol per tested tablet:

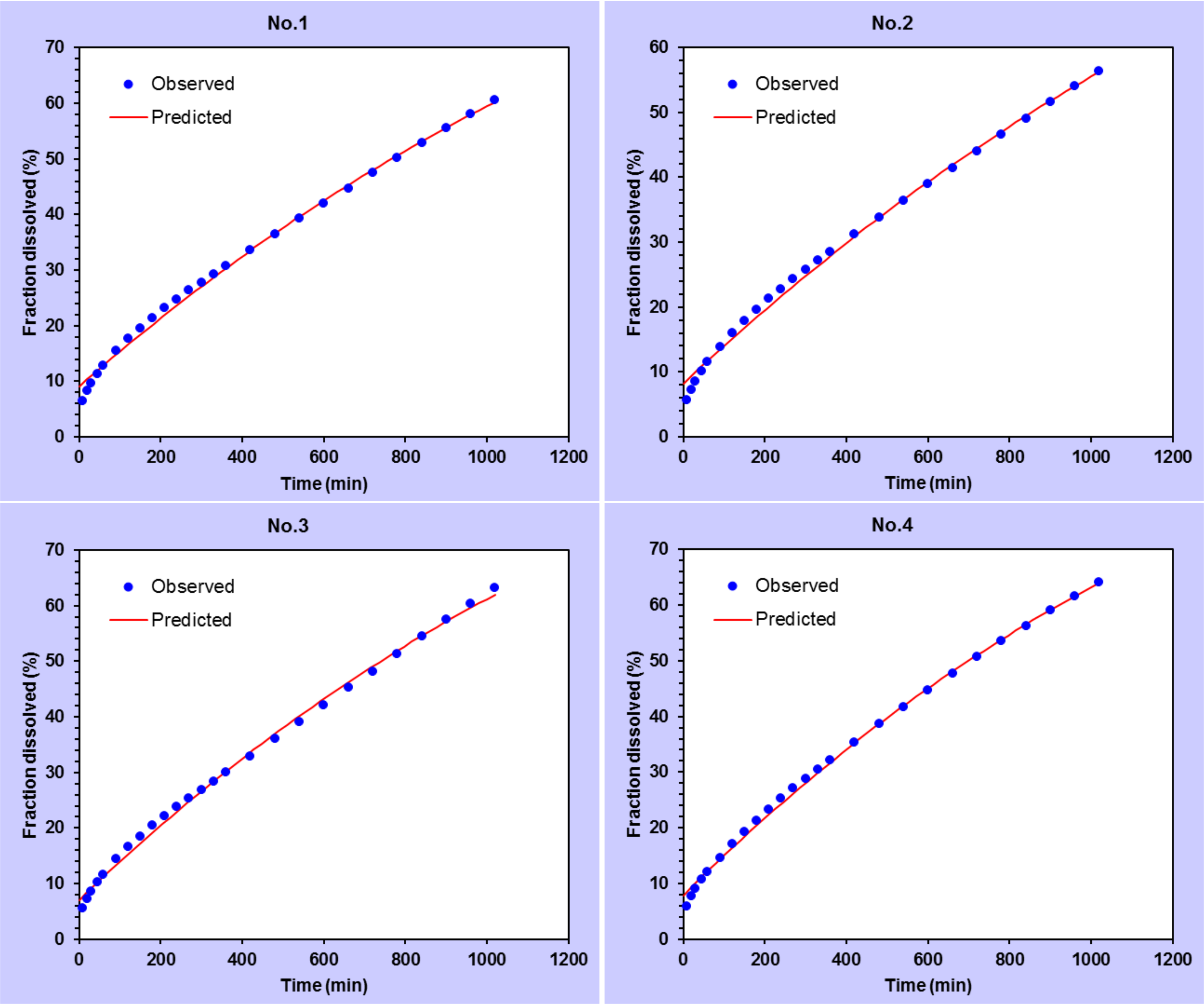

Supplementary materials – Model fitting summary – Starch 1500® sample with larger particle size (modelling up to app. 60 % of carvedilol released)

Model: **Hopfenberg**

Model equation: $F = 100 \cdot [1 - (1 - k_{HB} \cdot t)^n]$

Fitted model parameters per tested tablet (N = 4) with statistics – mean, standard deviation (SD), and relative standard deviation expressed in % (RSD%) (output from DDSolver):

Parameter	No.1	No.2	No.3	No.4	Mean	SD	RSD(%)
k_{HB}	0.0003	0.0003	0.0003	0.0003	0.0003	0.0000	6.9707
n	3.0000	3.0000	3.0000	3.0000	3.0000	0.0000	0.0000

Number of dissolution data points (N), degrees of freedom (df), and selected goodness of fit criteria – Pearson correlation coefficient (R), coefficient of determination (R^2), adjusted coefficient of determination ($R^2_{adjusted}$), and residual sum of squares (RSS) (manual calculation in MS Excel):

Parameter	No.1	No.2	No.3	No.4
N	26	26	26	26
df	24	24	24	24
R	0.99824937	0.997996937	0.998345779	0.999122922
R^2	0.996501805	0.995997886	0.996694294	0.998246612
$R^2_{adjusted}$	0.996356047	0.995831131	0.996556556	0.998173555
RSS	695.0185951	576.5292894	450.4412103	512.2840694

Graphical abstract of model fit presented as mean ± 1 SD of the fraction % of released carvedilol:

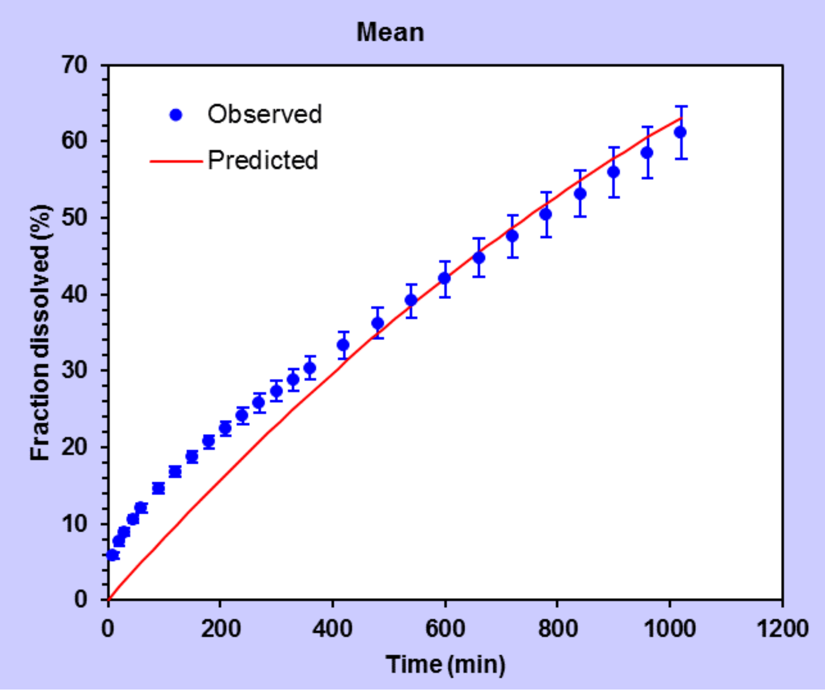

Supplementary materials – Model fitting summary – Starch 1500® sample with larger particle size (modelling up to app. 60 % of carvedilol released)

Graphical abstract of model fit presented as the fraction % of released carvedilol per tested tablet:

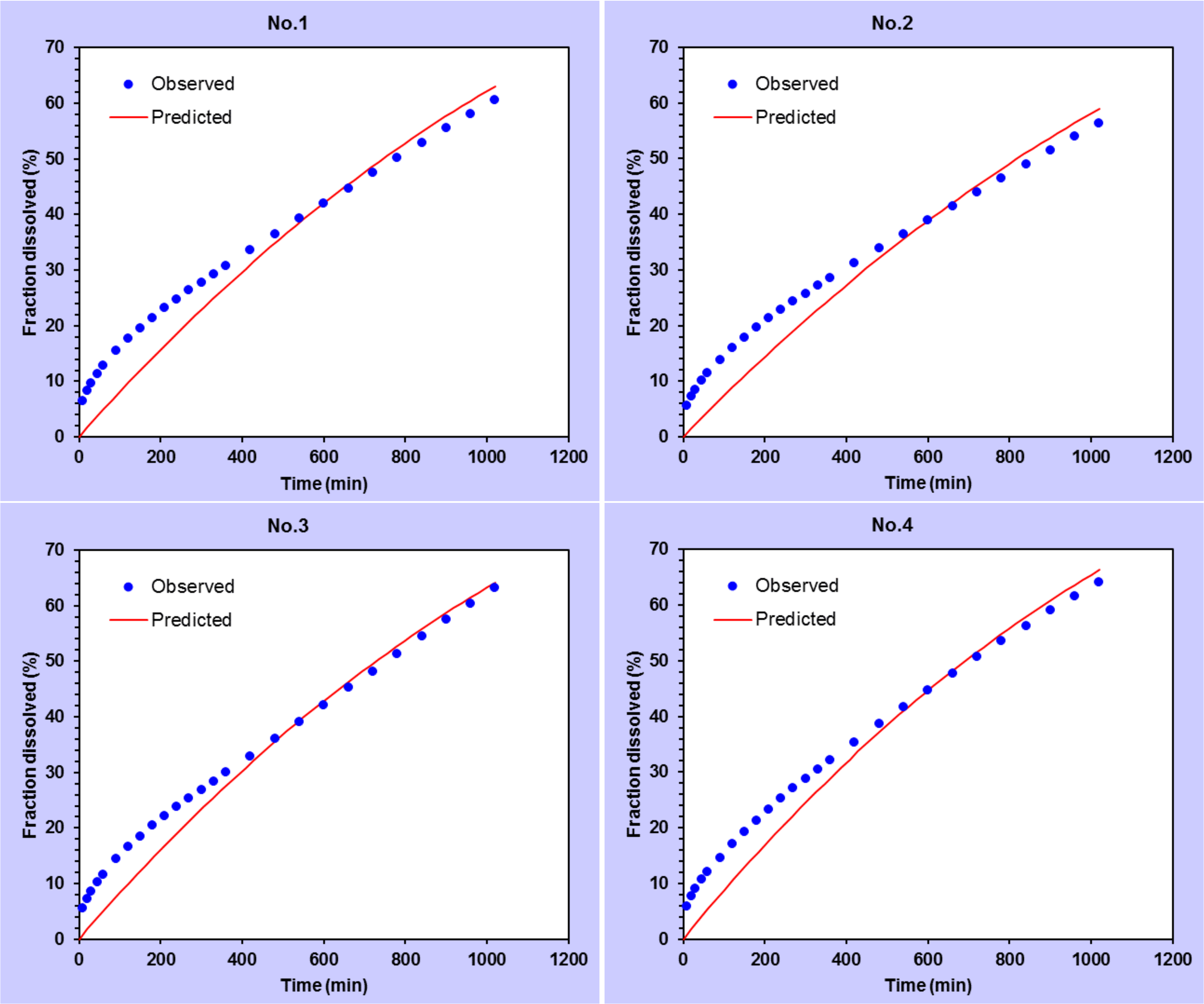

Supplementary materials – Model fitting summary – Starch 1500® sample with larger particle size (modelling up to app. 60 % of carvedilol released)

Model: **Hopfenberg with T_{lag}**

Model equation: $F = 100 \cdot \{1 - [1 - k_{HB} \cdot (t - T_{lag})]^n\}$

Fitted model parameters per tested tablet (N = 4) with statistics – mean, standard deviation (SD), and relative standard deviation expressed in % (RSD%) (output from DDSolver):

Parameter	No.1	No.2	No.3	No.4	Mean	SD	RSD(%)
k_{HB}	0.0002	0.0002	0.0002	0.0003	0.0002	0.0000	8.9582
n	3.0000	3.0000	3.0000	3.0000	3.0000	0.0000	0.0000
T_{lag}	-135.7712	-134.4897	-97.9400	-105.0763	-118.3193	19.6362	-16.5960

Number of dissolution data points (N), degrees of freedom (df), and selected goodness of fit criteria – Pearson correlation coefficient (R), coefficient of determination (R^2), adjusted coefficient of determination ($R^2_{adjusted}$), and residual sum of squares (RSS) (manual calculation in MS Excel):

Parameter	No.1	No.2	No.3	No.4
N	26	26	26	26
df	23	23	23	23
R	0.998016632	0.997643728	0.998495856	0.998923349
R^2	0.996037198	0.995293008	0.996993975	0.997847856
$R^2_{adjusted}$	0.995692606	0.994883704	0.996732581	0.997660713
RSS	27.08565631	28.46788748	23.29077612	17.86062329

Graphical abstract of model fit presented as mean ± 1 SD of the fraction % of released carvedilol:

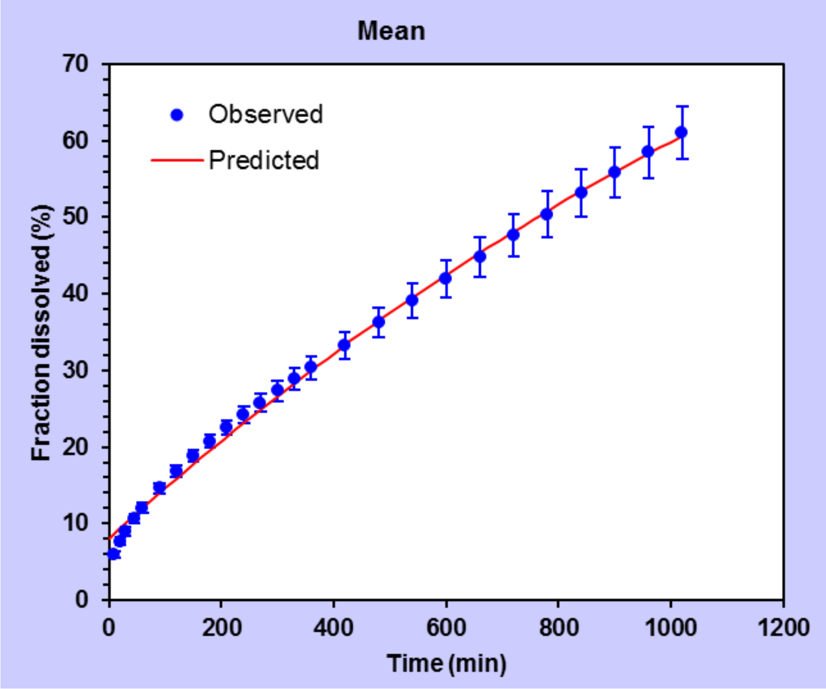

Supplementary materials – Model fitting summary – Starch 1500® sample with larger particle size (modelling up to app. 60 % of carvedilol released)

Graphical abstract of model fit presented as the fraction % of released carvedilol per tested tablet:

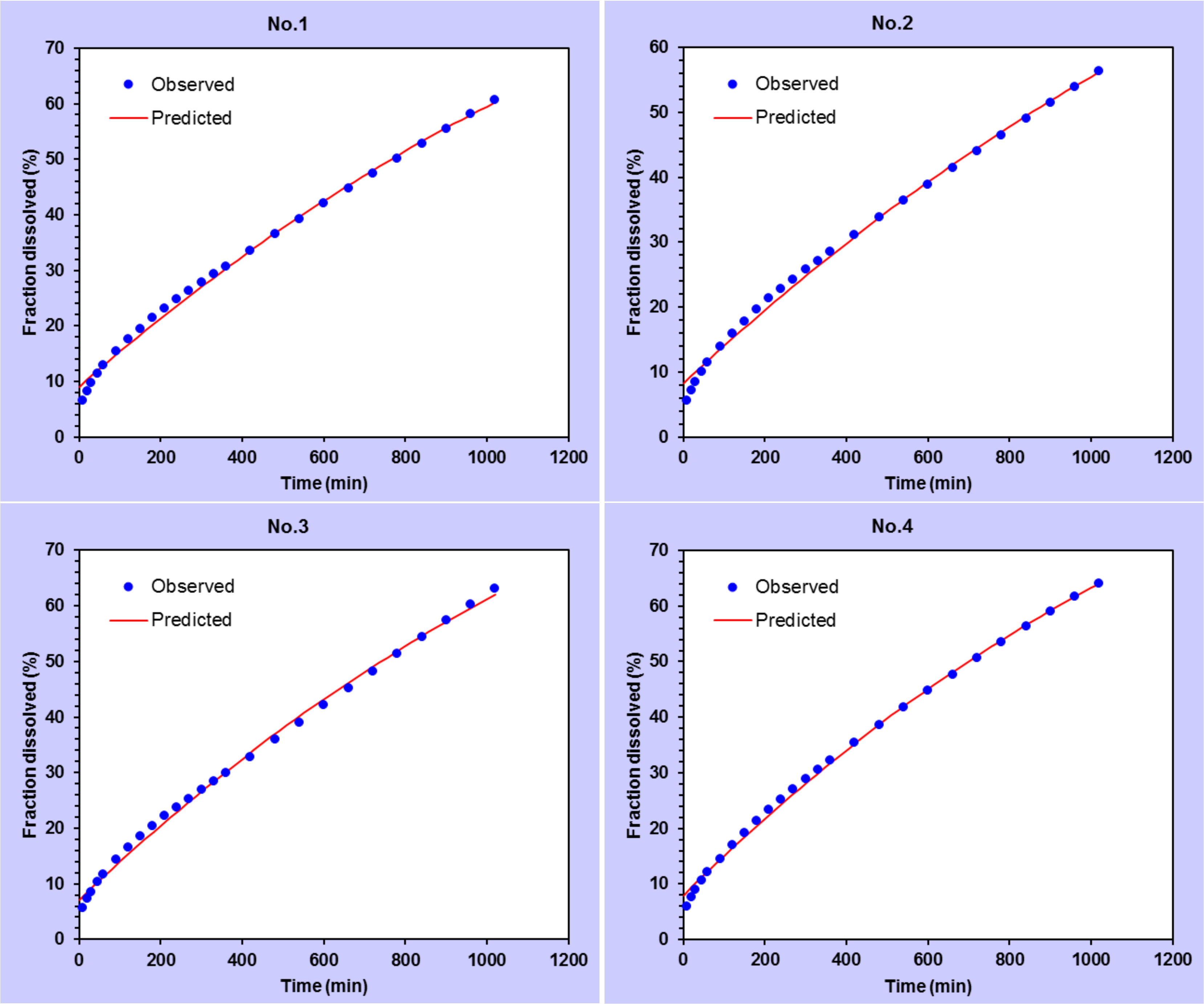

Supplementary materials – Model fitting summary – Starch 1500® sample with larger particle size (modelling up to app. 60 % of carvedilol released)

Model: **Baker–Lonsdale**

Model equation: $\frac{3}{2}\cdot\left[1-\left(1-\frac{F}{100}\right)^{\frac{2}{3}}\right]-\frac{F}{100}=k_{BL}\cdot t$

Fitted model parameters per tested tablet (N = 4) with statistics – mean, standard deviation (SD), and relative standard deviation expressed in % (RSD%) (output from DDSolver):

Parameter	No.1	No.2	No.3	No.4	Mean	SD	RSD(%)
k_{BL}	0.00006	0.00005	0.00007	0.00007	0.00006	0.00001	14.05919

Number of dissolution data points (N), degrees of freedom (df), and selected goodness of fit criteria – Pearson correlation coefficient (R), coefficient of determination (R^2), adjusted coefficient of determination ($R^2_{adjusted}$), and residual sum of squares (RSS) (manual calculation in MS Excel):

Parameter	No.1	No.2	No.3	No.4
N	26	26	26	26
df	25	25	25	25
R	0.988504803	0.989744311	0.983489746	0.988094444
R^2	0.977141746	0.979593802	0.96725208	0.976330631
$R^2_{adjusted}$	0.977141746	0.979593802	0.96725208	0.976330631
RSS	264.6893514	233.9832452	465.0590498	405.8385642

Graphical abstract of model fit presented as mean ± 1 SD of the fraction % of released carvedilol:

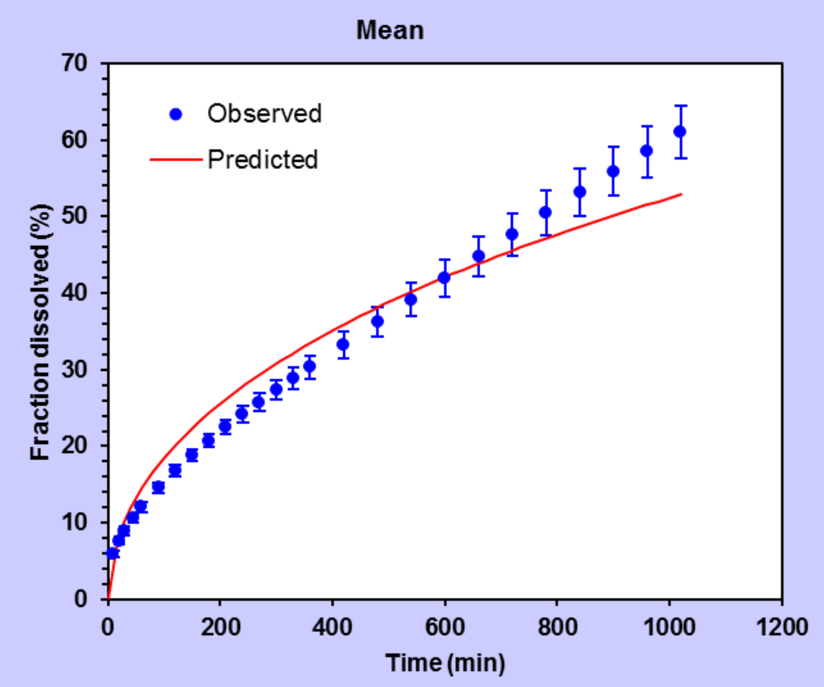

Supplementary materials – Model fitting summary – Starch 1500® sample with larger particle size (modelling up to app. 60 % of carvedilol released)

Graphical abstract of model fit presented as the fraction % of released carvedilol per tested tablet:

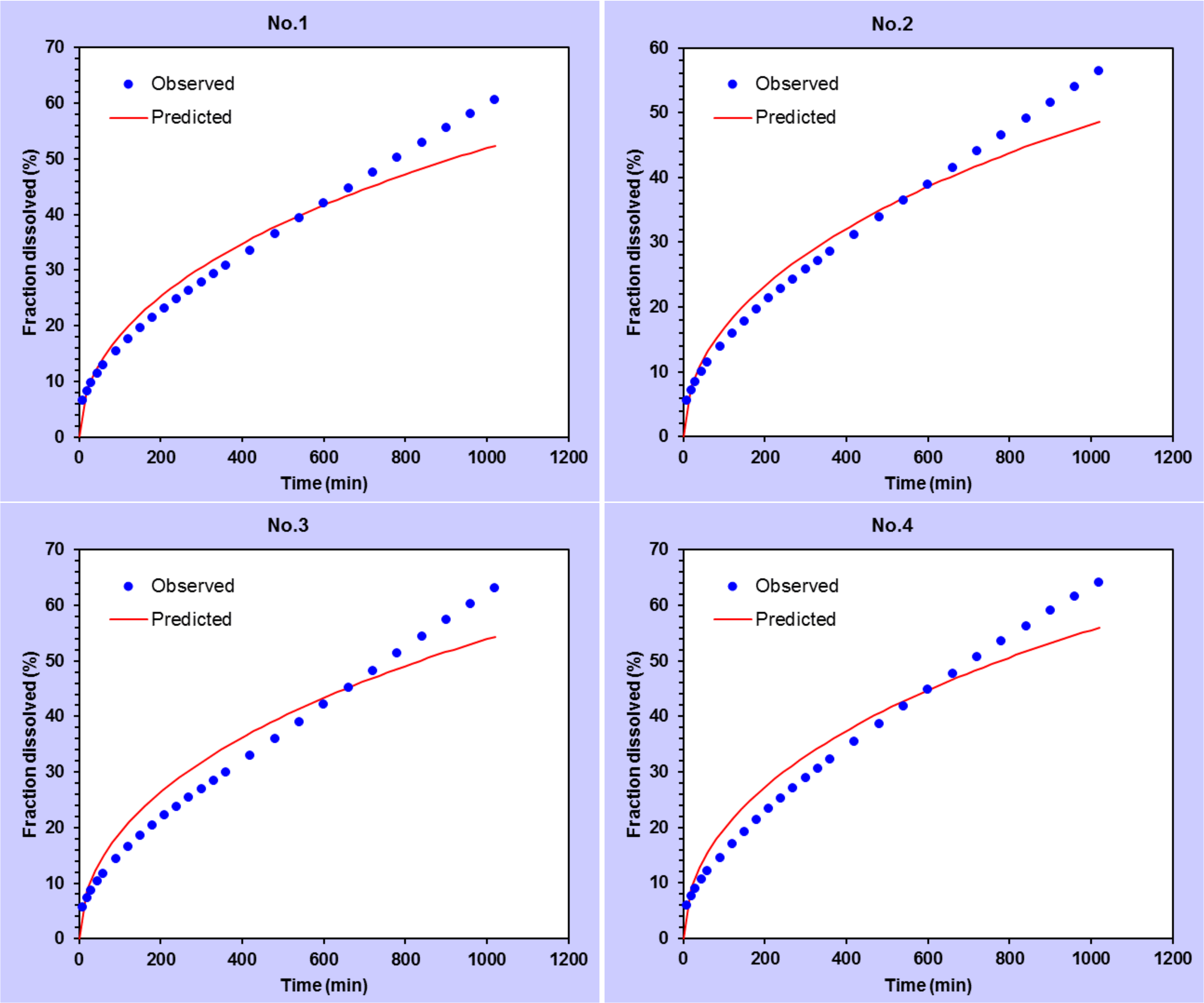

Supplementary materials – Model fitting summary – Starch 1500® sample with larger particle size (modelling up to app. 60 % of carvedilol released)

Model: **Baker–Lonsdale with T_{lag}**

Model equation: $$\frac{3}{2}\cdot\left[1-\left(1-\frac{F}{100}\right)^{\frac{2}{3}}\right]-\frac{F}{100}=k_{BL}\cdot\left(t-T_{lag}\right)$$

Fitted model parameters per tested tablet (N = 4) with statistics – mean, standard deviation (SD), and relative standard deviation expressed in % (RSD%) (output from DDSolver):

Parameter	No.1	No.2	No.3	No.4	Mean	SD	RSD(%)
k_{BL}	0.00008	0.00007	0.00009	0.00009	0.00008	0.00001	14.05919
T_{lag}	70.34993	70.49680	89.14168	81.28866	77.81927	9.12219	11.72228

Number of dissolution data points (N), degrees of freedom (df), and selected goodness of fit criteria – Pearson correlation coefficient (R), coefficient of determination (R^2), adjusted coefficient of determination ($R^2_{adjusted}$), and residual sum of squares (RSS) (manual calculation in MS Excel):

Parameter	No.1	No.2	No.3	No.4
N	26	26	26	26
df	24	24	24	24
R	0.975592403	0.977752292	0.971020808	0.977835562
R^2	0.951780537	0.955999544	0.94288141	0.956162386
$R^2_{adjusted}$	0.949771393	0.954166191	0.940501469	0.954335818
RSS	657.4298271	513.1370364	771.893159	644.1919998

Graphical abstract of model fit presented as mean ± 1 SD of the fraction % of released carvedilol:

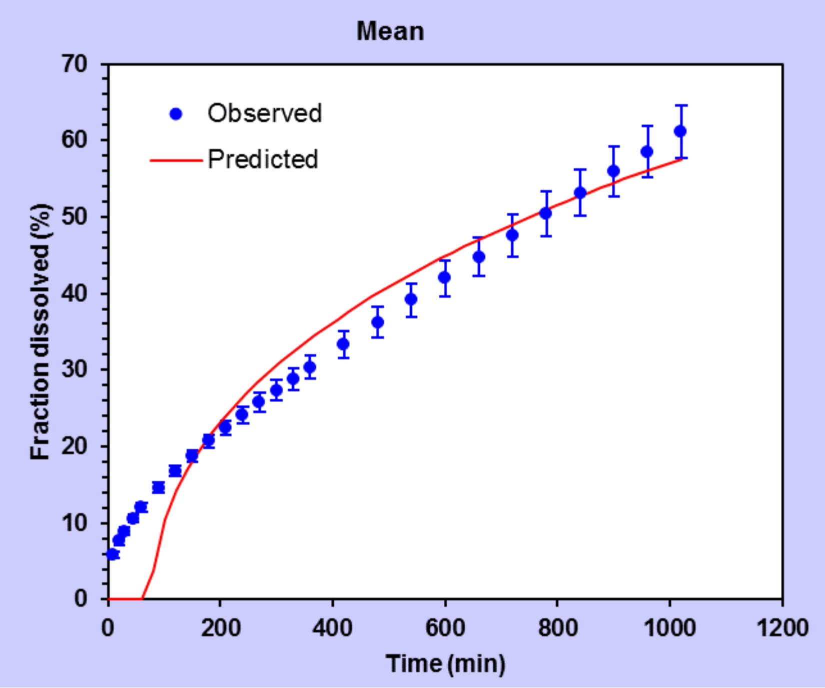

Supplementary materials – Model fitting summary – Starch 1500® sample with larger particle size (modelling up to app. 60 % of carvedilol released)

Graphical abstract of model fit presented as the fraction % of released carvedilol per tested tablet:

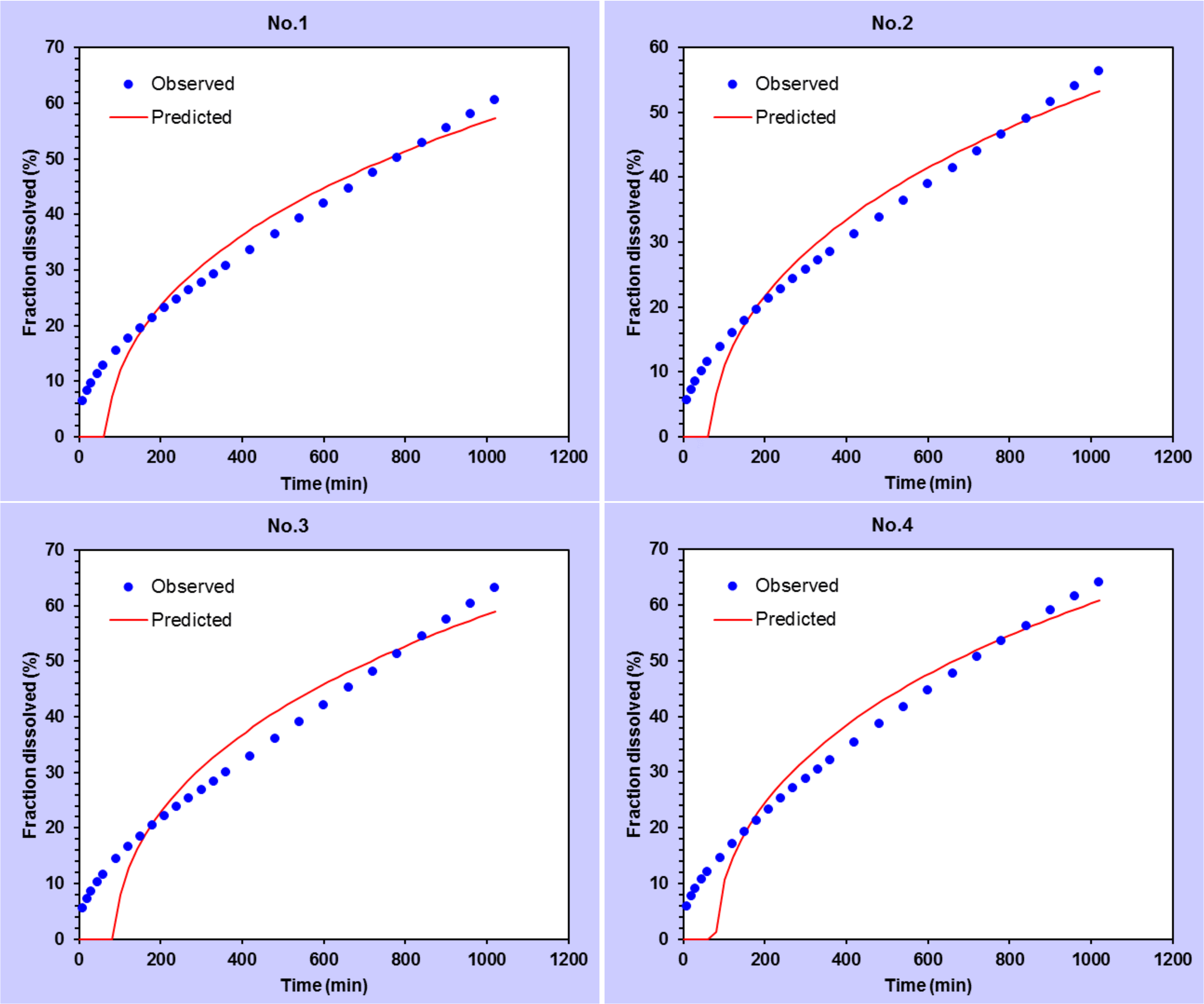

Supplementary materials – Model fitting summary – Starch 1500® sample with larger particle size (modelling up to app. 60 % of carvedilol released)

Model: **Makoid–Banakar**

Model equation: $F = k_{MB} \cdot t^{n} \cdot e^{-k \cdot t}$

Fitted model parameters per tested tablet (N = 4) with statistics – mean, standard deviation (SD), and relative standard deviation expressed in % (RSD%) (output from DDSolver):

Parameter	No.1	No.2	No.3	No.4	Mean	SD	RSD(%)
k_{MB}	2.4880	2.0070	1.9977	1.9992	2.1230	0.2434	11.4634
n	0.3997	0.4254	0.4300	0.4428	0.4245	0.0181	4.2527
k	-0.0004	-0.0004	-0.0005	-0.0004	-0.0004	0.0000	-8.3070

Number of dissolution data points (N), degrees of freedom (df), and selected goodness of fit criteria – Pearson correlation coefficient (R), coefficient of determination (R^2), adjusted coefficient of determination ($R^2_{adjusted}$), and residual sum of squares (RSS) (manual calculation in MS Excel):

Parameter	No.1	No.2	No.3	No.4
N	26	26	26	26
df	23	23	23	23
R	0.999805997	0.999851669	0.999848296	0.999180937
R^2	0.999612032	0.999703359	0.999696614	0.998362545
$R^2_{adjusted}$	0.999578296	0.999677564	0.999670233	0.998220157
RSS	2.729635986	1.85455783	2.429555636	14.02920148

Graphical abstract of model fit presented as mean ± 1 SD of the fraction % of released carvedilol:

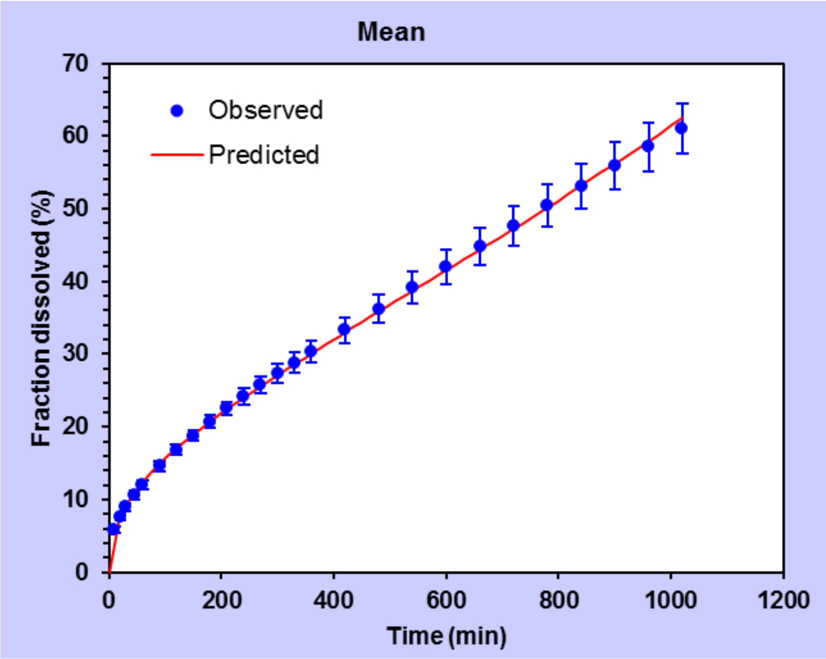

Supplementary materials – Model fitting summary – Starch 1500® sample with larger particle size (modelling up to app. 60 % of carvedilol released)

Graphical abstract of model fit presented as the fraction % of released carvedilol per tested tablet:

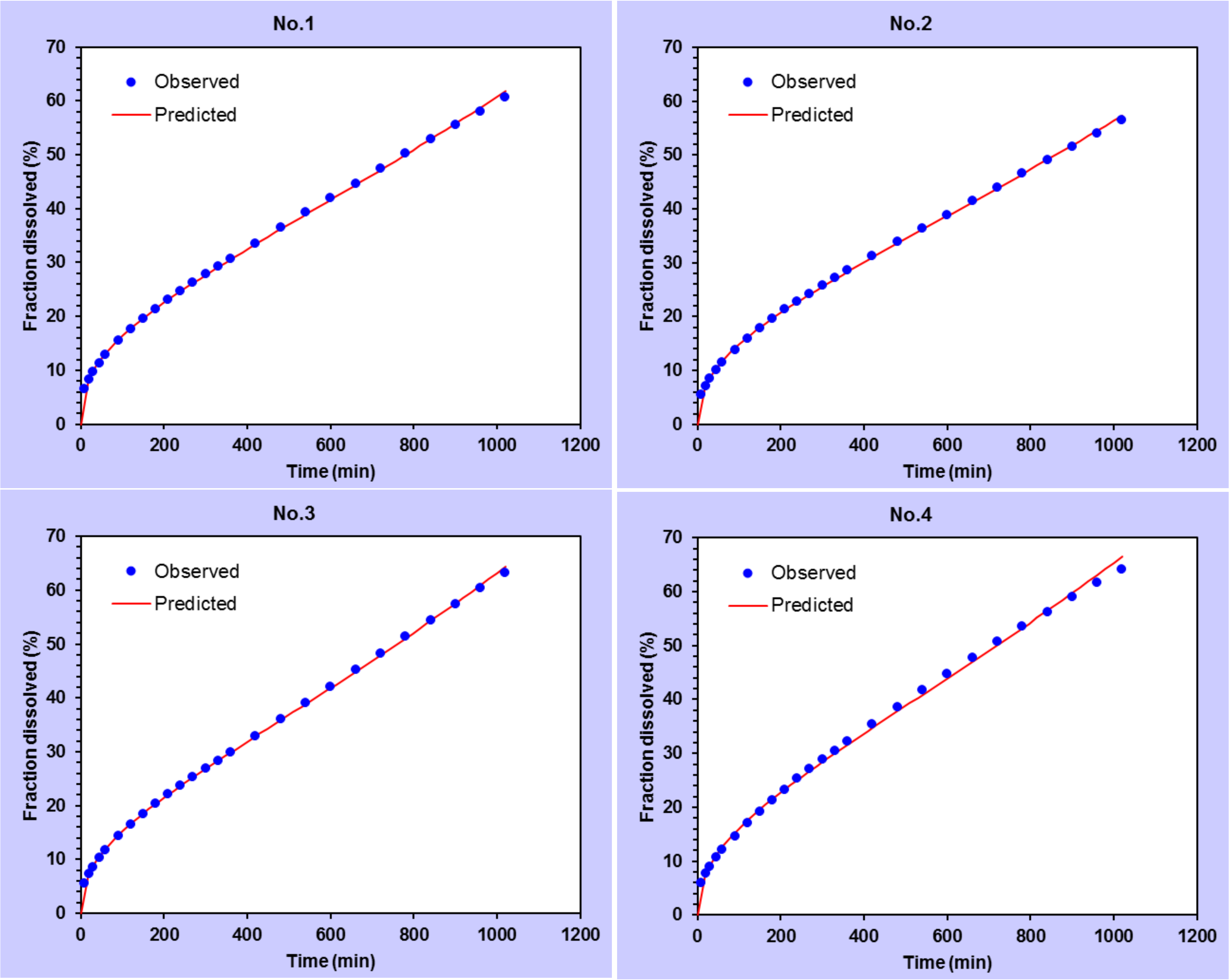

Supplementary materials – Model fitting summary – Starch 1500® sample with larger particle size (modelling up to app. 60 % of carvedilol released)

Model: **Makoid–Banakar with T_{lag}**

Model equation: $F = k_{MB} \cdot (t - T_{lag})^n \cdot e^{-k \cdot (t - T_{lag})}$

Fitted model parameters per tested tablet (N = 4) with statistics – mean, standard deviation (SD), and relative standard deviation expressed in % (RSD%) (output from DDSolver):

Parameter	No.1	No.2	No.3	No.4	Mean	SD	RSD(%)
k_{MB}	3.1522	2.5971	2.5909	2.5313	2.7179	0.2910	10.7083
n	0.3618	0.3732	0.3775	0.3965	0.3772	0.0144	3.8281
k	-0.0005	-0.0005	-0.0006	-0.0005	-0.0005	0.0001	-12.0663
T_{lag}	5.2765	4.0000	4.0000	5.2392	4.6289	0.7264	15.6924

Number of dissolution data points (N), degrees of freedom (df), and selected goodness of fit criteria – Pearson correlation coefficient (R), coefficient of determination (R^2), adjusted coefficient of determination ($R^2_{adjusted}$), and residual sum of squares (RSS) (manual calculation in MS Excel):

Parameter	No.1	No.2	No.3	No.4
N	26	26	26	26
df	22	22	22	22
R	0.999614802	0.999285434	0.999371967	0.998802877
R^2	0.999229753	0.998571378	0.998744329	0.997607186
$R^2_{adjusted}$	0.99912472	0.998376566	0.998573102	0.997280894
RSS	7.371155682	9.114699708	10.44431063	32.3673064

Graphical abstract of model fit presented as mean ± 1 SD of the fraction % of released carvedilol:

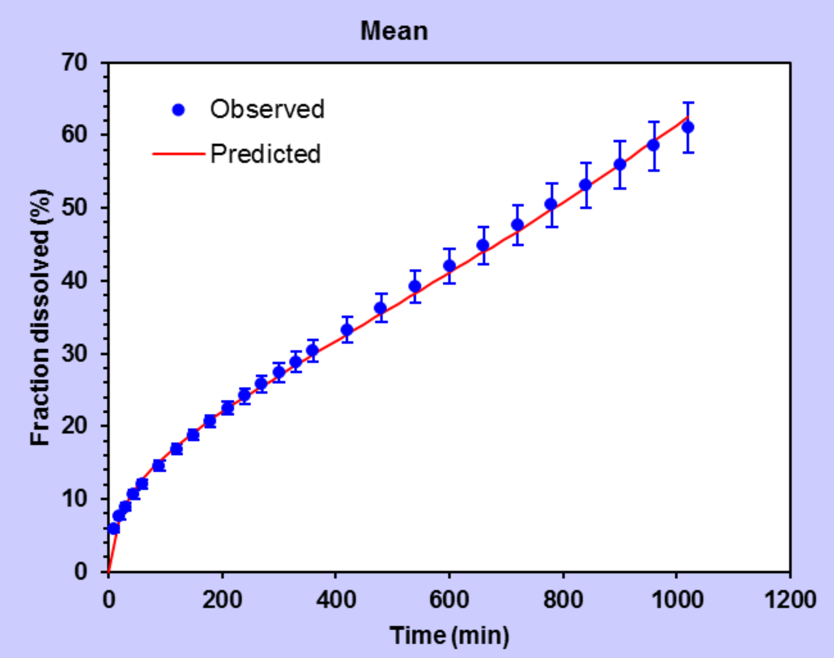

Supplementary materials – Model fitting summary – Starch 1500® sample with larger particle size (modelling up to app. 60 % of carvedilol released)

Graphical abstract of model fit presented as the fraction % of released carvedilol per tested tablet:

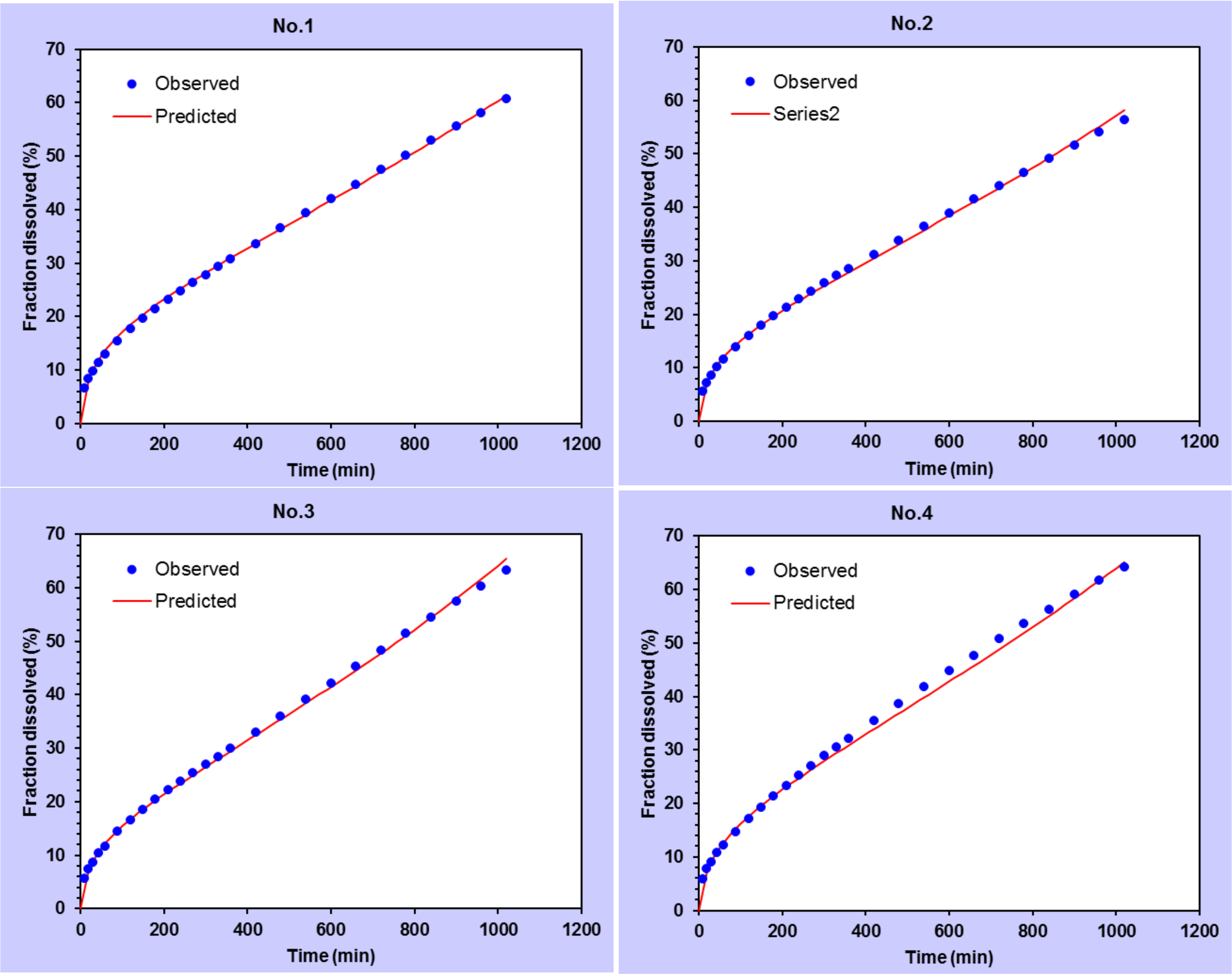

Supplementary materials – Model fitting summary – Starch 1500® sample with larger particle size (modelling up to app. 60 % of carvedilol released)

Model: **Peppas–Sahlin_1**

Model equation: $F = k_1 \cdot t^m + k_2 \cdot t^{2m}$

Fitted model parameters per tested tablet (N = 4) with statistics – mean, standard deviation (SD), and relative standard deviation expressed in % (RSD%) (output from DDSolver):

Parameter	No.1	No.2	No.3	No.4	Mean	SD	RSD(%)
k_1	1.580	1.410	1.272	1.445	1.427	0.127	8.878
k_2	0.046	0.046	0.065	0.061	0.055	0.010	17.903
m	0.450	0.450	0.450	0.450	0.450	0.000	0.000

Number of dissolution data points (N), degrees of freedom (df), and selected goodness of fit criteria – Pearson correlation coefficient (R), coefficient of determination (R^2), adjusted coefficient of determination ($R^2_{adjusted}$), and residual sum of squares (RSS) (manual calculation in MS Excel):

Parameter	No.1	No.2	No.3	No.4
N	26	26	26	26
df	23	23	23	23
R	0.998802009	0.999213452	0.998673801	0.999643667
R^2	0.997605453	0.998427523	0.997349362	0.999287462
$R^2_{adjusted}$	0.997397232	0.998290786	0.997118871	0.999225502
RSS	19.57424395	11.18702281	23.53478918	7.107306578

Graphical abstract of model fit presented as mean ± 1 SD of the fraction % of released carvedilol:

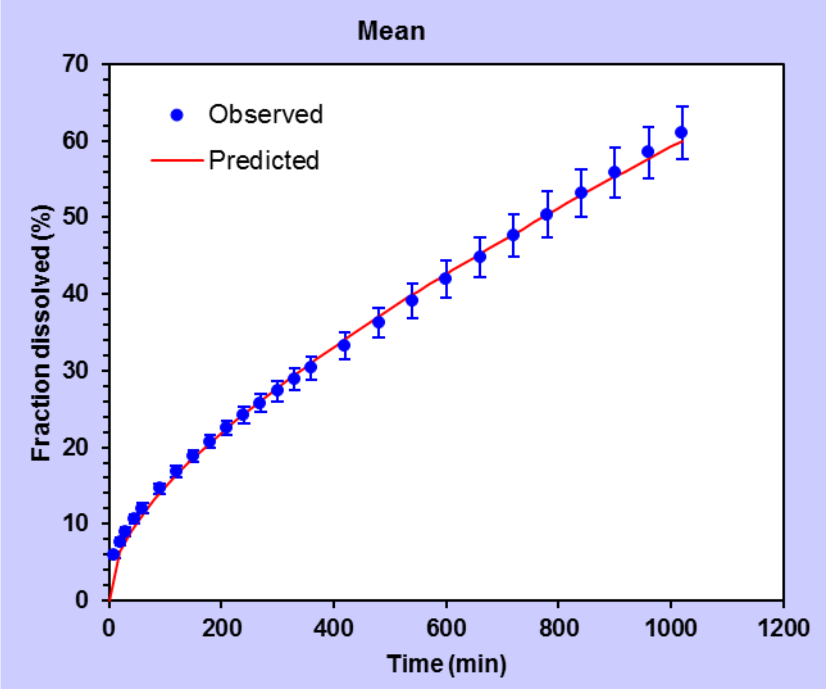

Supplementary materials – Model fitting summary – Starch 1500® sample with larger particle size (modelling up to app. 60 % of carvedilol released)

Graphical abstract of model fit presented as the fraction % of released carvedilol per tested tablet:

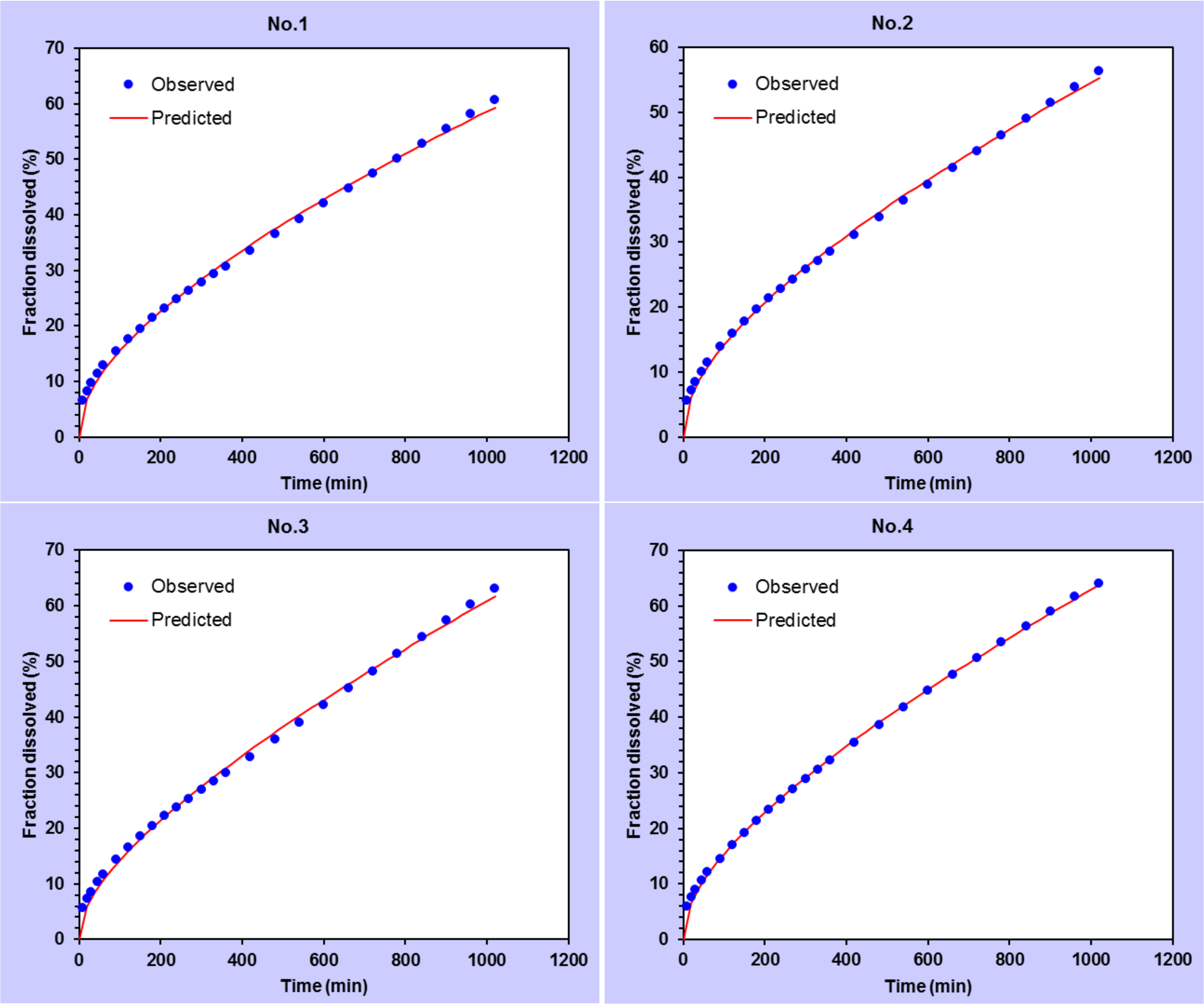

Supplementary materials – Model fitting summary – Starch 1500® sample with larger particle size (modelling up to app. 60 % of carvedilol released)

Model: **Peppas–Sahlin_1 with T_{lag}**

Model equation: $F = k_1 \cdot (t - T_{lag})^m + k_2 \cdot (t - T_{lag})^{2m}$

Fitted model parameters per tested tablet (N = 4) with statistics – mean, standard deviation (SD), and relative standard deviation expressed in % (RSD%) (output from DDSolver):

Parameter	No.1	No.2	No.3	No.4	Mean	SD	RSD(%)
k_1	1.638	1.464	1.328	1.505	1.484	0.128	8.606
k_2	0.044	0.044	0.062	0.058	0.052	0.010	18.721
m	0.450	0.450	0.450	0.450	0.450	0.000	0.000
T_{lag}	4.000	4.000	4.000	4.000	4.000	0.000	0.000

Number of dissolution data points (N), degrees of freedom (df), and selected goodness of fit criteria – Pearson correlation coefficient (R), coefficient of determination (R^2), adjusted coefficient of determination ($R^2_{adjusted}$), and residual sum of squares (RSS) (manual calculation in MS Excel):

Parameter	No.1	No.2	No.3	No.4
N	26	26	26	26
df	22	22	22	22
R	0.998261577	0.998783268	0.998254848	0.999303252
R^2	0.996526176	0.997568017	0.996512741	0.99860699
$R^2_{adjusted}$	0.996052473	0.997236383	0.996037205	0.998417034
RSS	29.78044217	18.21773084	32.32377679	14.40400139

Graphical abstract of model fit presented as mean ± 1 SD of the fraction % of released carvedilol:

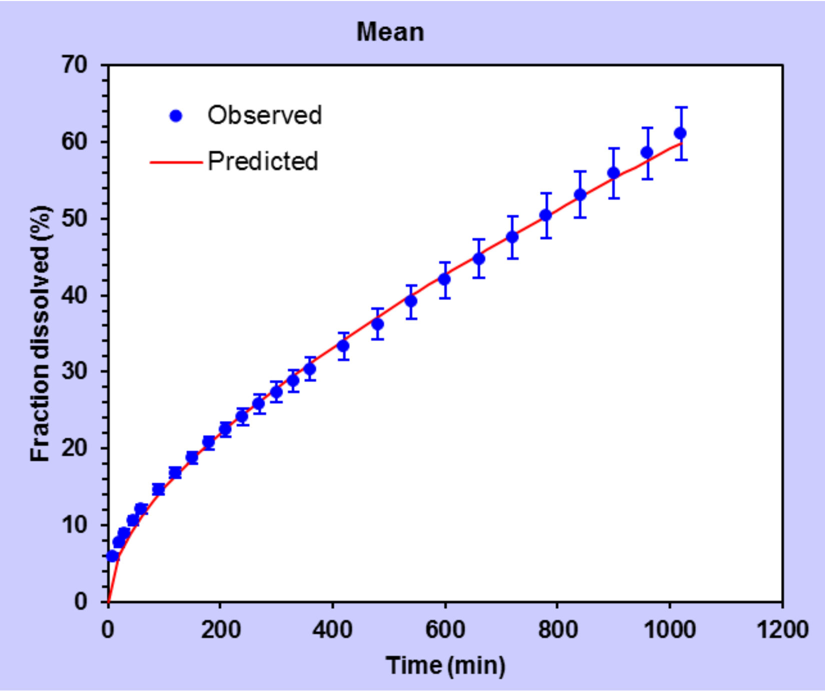

Supplementary materials – Model fitting summary – Starch 1500® sample with larger particle size (modelling up to app. 60 % of carvedilol released)

Graphical abstract of model fit presented as the fraction % of released carvedilol per tested tablet:

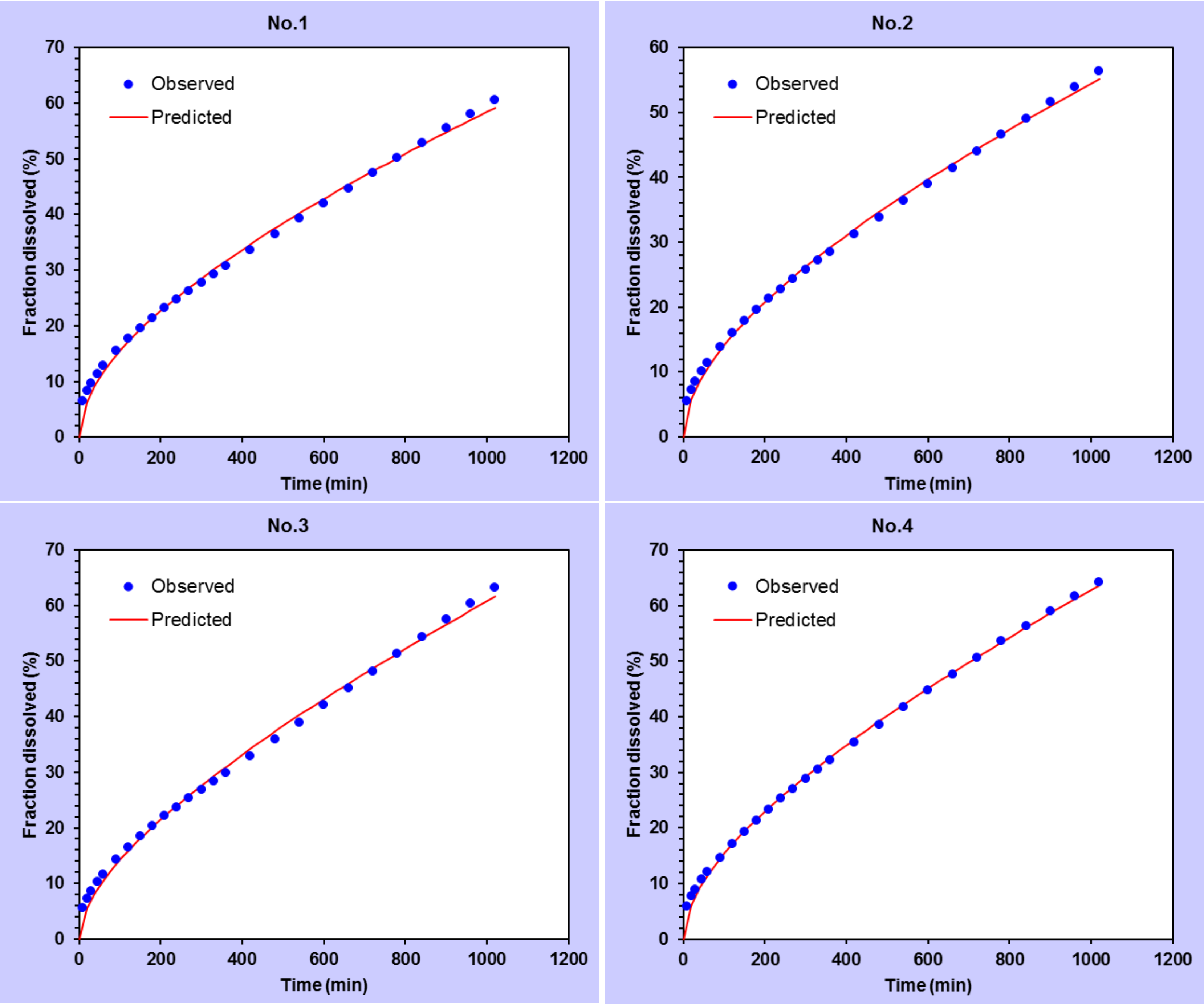

Supplementary materials – Model fitting summary – Starch 1500® sample with larger particle size (modelling up to app. 60 % of carvedilol released)

Model: **Peppas–Sahlin_2**

Model equation: $F = k_1 \cdot t^{0.5} + k_2 \cdot t$

Fitted model parameters per tested tablet (N = 4) with statistics – mean, standard deviation (SD), and relative standard deviation expressed in % (RSD%) (output from DDSolver):

Parameter	No.1	No.2	No.3	No.4	Mean	SD	RSD(%)
k_1	1.386	1.249	1.172	1.312	1.280	0.091	7.135
k_2	0.015	0.015	0.024	0.021	0.019	0.005	24.415

Number of dissolution data points (N), degrees of freedom (df), and selected goodness of fit criteria – Pearson correlation coefficient (R), coefficient of determination (R^2), adjusted coefficient of determination ($R^2_{adjusted}$), and residual sum of squares (RSS) (manual calculation in MS Excel):

Parameter	No.1	No.2	No.3	No.4
N	26	26	26	26
df	24	24	24	24
R	0.998627736	0.99909399	0.998713682	0.99959034
R^2	0.997257354	0.998188801	0.997429019	0.999180848
$R^2_{adjusted}$	0.997143077	0.998113335	0.997321894	0.999146717
RSS	24.18000116	13.88990904	24.29737107	8.730772586

Graphical abstract of model fit presented as mean ± 1 SD of the fraction % of released carvedilol:

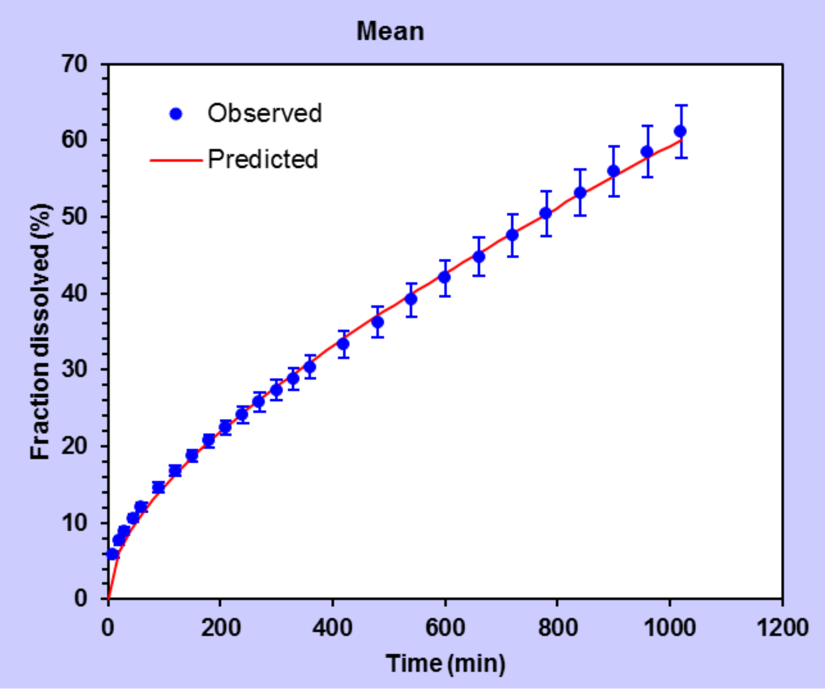

Supplementary materials – Model fitting summary – Starch 1500® sample with larger particle size (modelling up to app. 60 % of carvedilol released)

Graphical abstract of model fit presented as the fraction % of released carvedilol per tested tablet:

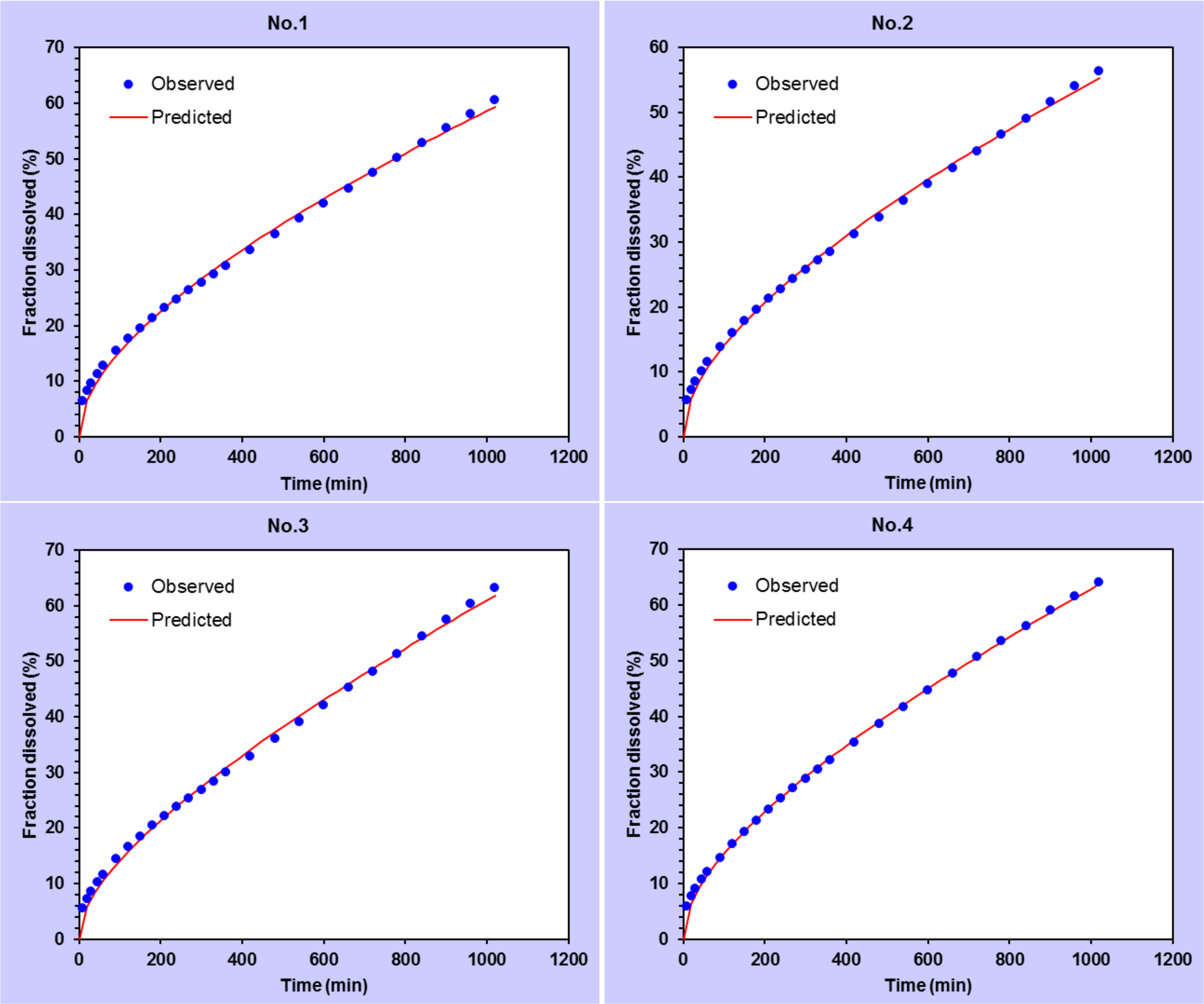

Supplementary materials – Model fitting summary – Starch 1500® sample with larger particle size (modelling up to app. 60 % of carvedilol released)

Model: **Peppas–Sahlin_2 with T_{lag}**

Model equation: $F = k_1 \cdot (t - T_{lag})^{0.5} + k_2 \cdot (t - T_{lag})$

Fitted model parameters per tested tablet (N = 4) with statistics – mean, standard deviation (SD), and relative standard deviation expressed in % (RSD%) (output from DDSolver):

Parameter	No.1	No.2	No.3	No.4	Mean	SD	RSD(%)
k_1	1.426	1.285	1.210	1.354	1.319	0.092	6.995
k_2	0.014	0.014	0.023	0.020	0.018	0.005	26.020
T_{lag}	4.000	4.000	4.000	4.000	4.000	0.000	0.000

Number of dissolution data points (N), degrees of freedom (df), and selected goodness of fit criteria – Pearson correlation coefficient (R), coefficient of determination (R^2), adjusted coefficient of determination ($R^2_{adjusted}$), and residual sum of squares (RSS) (manual calculation in MS Excel):

Parameter	No.1	No.2	No.3	No.4
N	26	26	26	26
df	23	23	23	23
R	0.998038109	0.998617072	0.998276328	0.999218708
R^2	0.996080067	0.997236056	0.996555627	0.998438026
$R^2_{adjusted}$	0.995739203	0.996995713	0.996256116	0.998302202
RSS	36.4613272	22.44701863	34.29610328	17.33754781

Graphical abstract of model fit presented as mean ± 1 SD of the fraction % of released carvedilol:

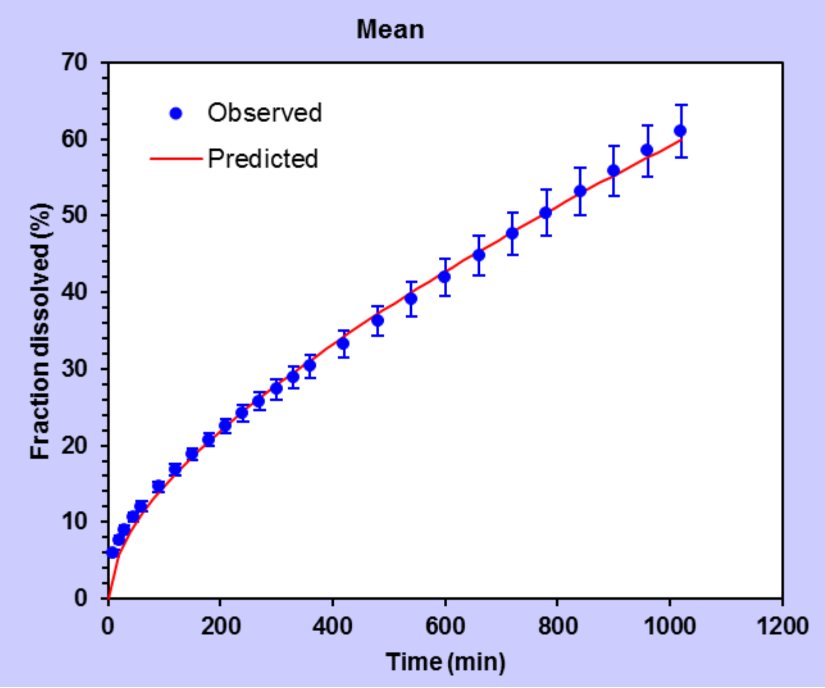

Supplementary materials – Model fitting summary – Starch 1500® sample with larger particle size (modelling up to app. 60 % of carvedilol released)

Graphical abstract of model fit presented as the fraction % of released carvedilol per tested tablet:

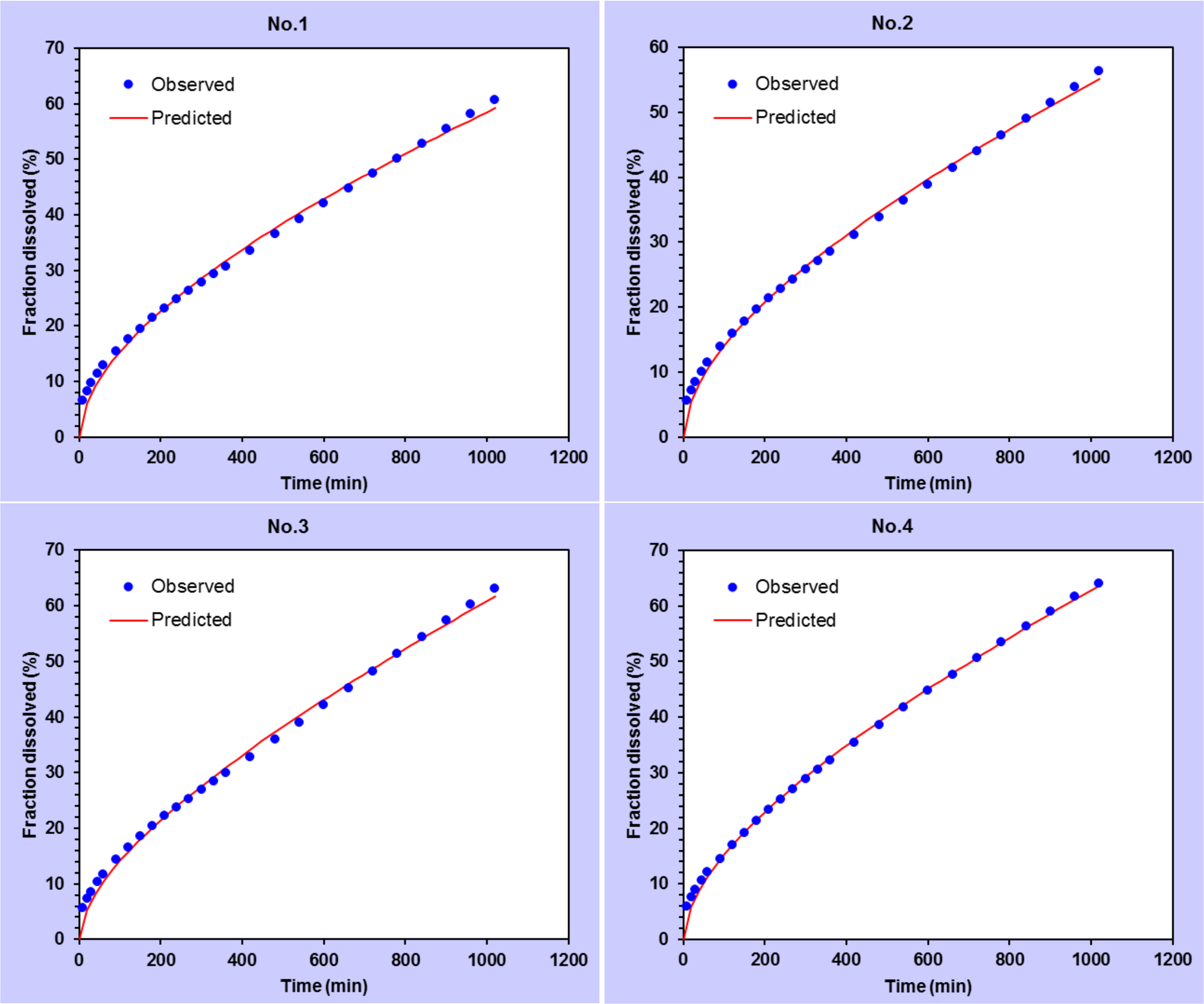

Supplementary materials – Model fitting summary – Starch 1500® sample with larger particle size (modelling up to app. 60 % of carvedilol released)

Model: **Quadratic**

Model equation: $F = 100 \cdot (k_1 \cdot t^2 + k_2 \cdot t)$

Fitted model parameters per tested tablet (N = 4) with statistics – mean, standard deviation (SD), and relative standard deviation expressed in % (RSD%) (output from DDSolver):

Parameter	No.1	No.2	No.3	No.4	Mean	SD	RSD(%)
k_1	-0.0000005	-0.0000004	-0.0000004	-0.0000005	-0.0000005	0.0000000	-8.3155756
k_2	0.0010536	0.0009705	0.0009995	0.0010859	0.0010274	0.0000520	5.0643908

Number of dissolution data points (N), degrees of freedom (df), and selected goodness of fit criteria – Pearson correlation coefficient (R), coefficient of determination (R^2), adjusted coefficient of determination ($R^2_{adjusted}$), and residual sum of squares (RSS) (manual calculation in MS Excel):

Parameter	No.1	No.2	No.3	No.4
N	26	26	26	26
df	24	24	24	24
R	0.98986842	0.990906225	0.992192371	0.993768992
R^2	0.979839489	0.981895147	0.984445702	0.987576809
$R^2_{adjusted}$	0.978999467	0.981140778	0.983797606	0.987059176
RSS	418.1708356	320.0990662	325.9898757	305.6805309

Graphical abstract of model fit presented as mean ± 1 SD of the fraction % of released carvedilol:

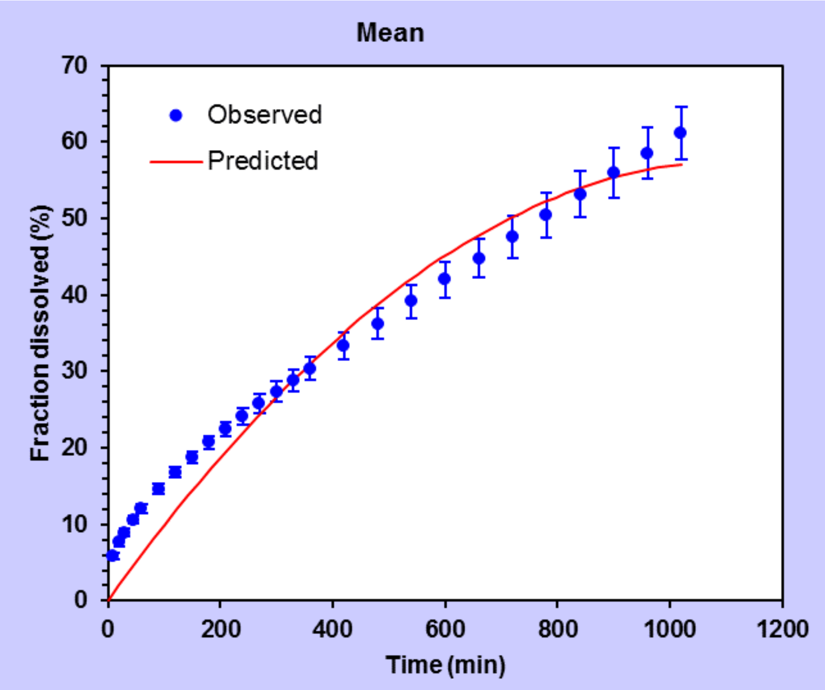

Supplementary materials – Model fitting summary – Starch 1500® sample with larger particle size (modelling up to app. 60 % of carvedilol released)

Graphical abstract of model fit presented as the fraction % of released carvedilol per tested tablet:

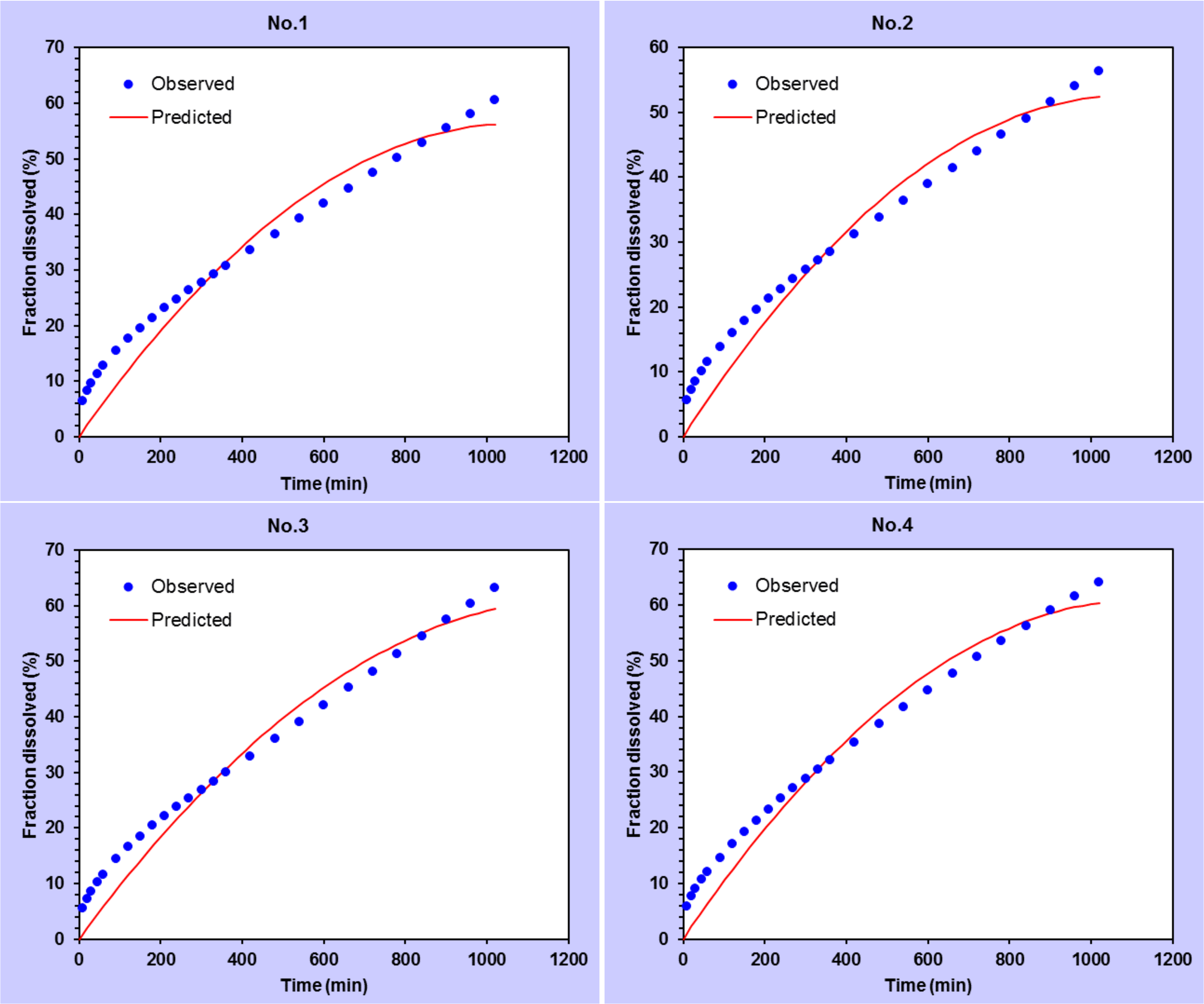

Supplementary materials – Model fitting summary – Starch 1500® sample with larger particle size (modelling up to app. 60 % of carvedilol released)

Model: **Quadratic with T_{lag}**

Model equation: $F = 100 \cdot \left[k_1 \cdot (t - T_{lag})^2 + k_2 \cdot (t - T_{lag})\right]$

Fitted model parameters per tested tablet (N = 4) with statistics – mean, standard deviation (SD), and relative standard deviation expressed in % (RSD%) (output from DDSolver):

Parameter	No.1	No.2	No.3	No.4	Mean	SD	RSD(%)
k_1	-0.0000005	-0.0000005	-0.0000004	-0.0000005	-0.0000005	0.0000000	-8.2049075
k_2	0.0010645	0.0009808	0.0010106	0.0010979	0.0010385	0.0000527	5.0709676
T_{lag}	4.0000000	4.0000000	4.0000000	4.0000000	4.0000000	0.0000000	0.0000000

Number of dissolution data points (N), degrees of freedom (df), and selected goodness of fit criteria – Pearson correlation coefficient (R), coefficient of determination (R^2), adjusted coefficient of determination ($R^2_{adjusted}$), and residual sum of squares (RSS) (manual calculation in MS Excel):

Parameter	No.1	No.2	No.3	No.4
N	26	26	26	26
df	23	23	23	23
R	0.989431917	0.990484182	0.991791006	0.993381064
R^2	0.978975519	0.981058914	0.9836494	0.986805939
$R^2_{adjusted}$	0.977147303	0.979411863	0.982227609	0.985658629
RSS	458.6700053	352.5905259	359.7672779	341.7351069

Graphical abstract of model fit presented as mean ± 1 SD of the fraction % of released carvedilol:

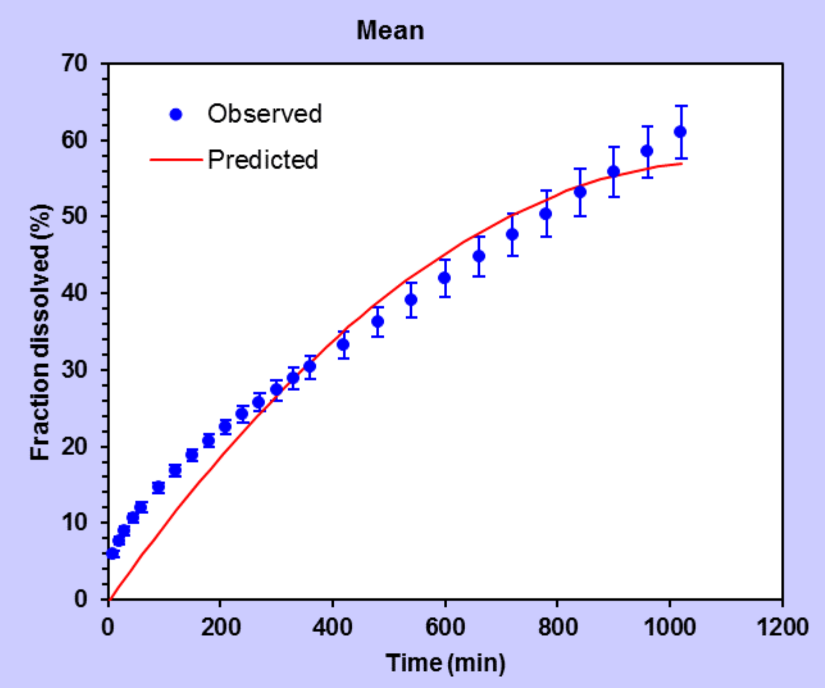

Supplementary materials – Model fitting summary – Starch 1500® sample with larger particle size (modelling up to app. 60 % of carvedilol released)

Graphical abstract of model fit presented as the fraction % of released carvedilol per tested tablet:

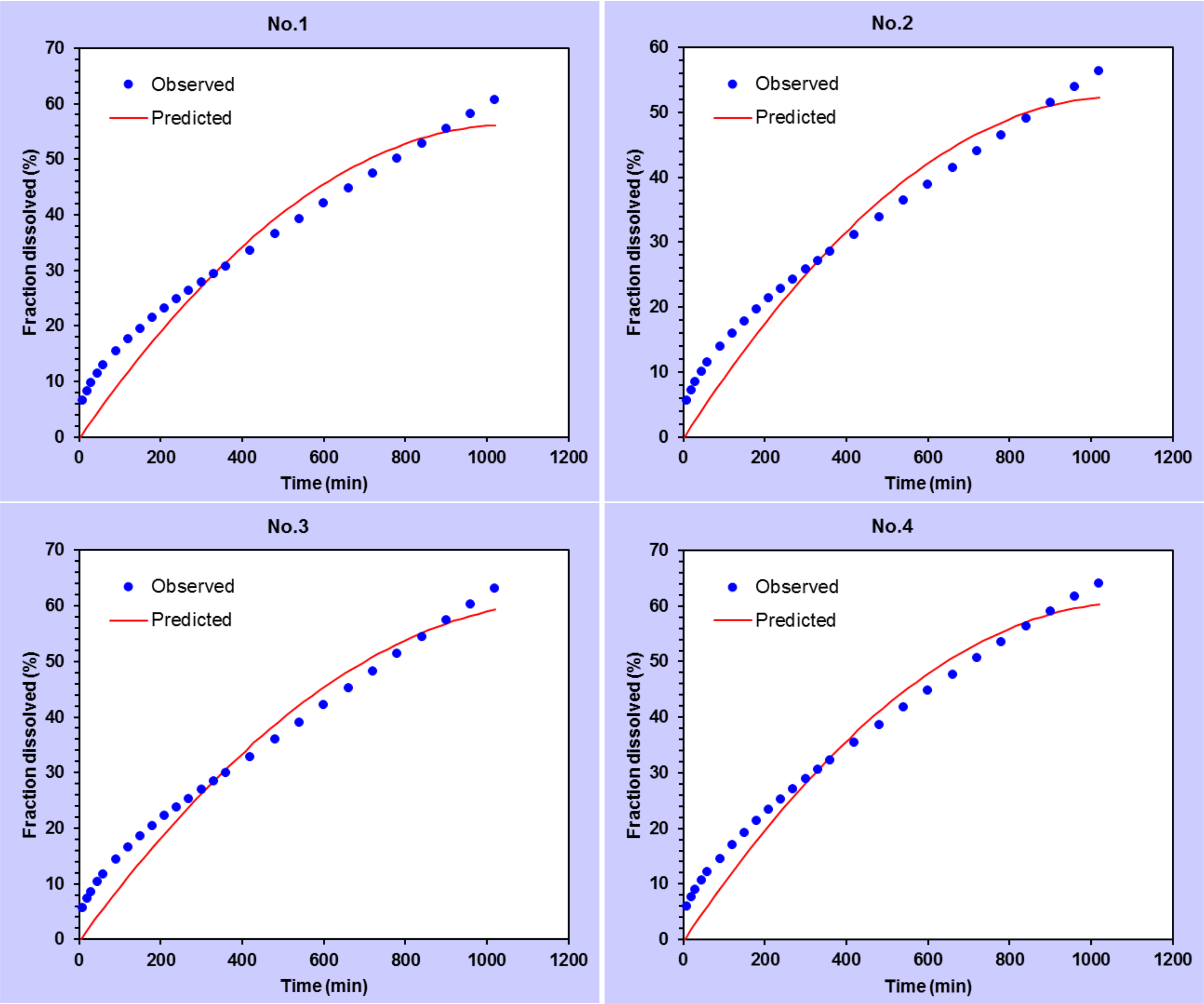

Supplementary materials – Model fitting summary – Starch 1500® sample with larger particle size (modelling up to app. 60 % of carvedilol released)

Model: **Weibull_1**

Model equation: $F = 100 \cdot \left[1 - e^{-\frac{(t-T_i)^{\beta}}{\alpha}}\right]$

Fitted model parameters per tested tablet (N = 4) with statistics – mean, standard deviation (SD), and relative standard deviation expressed in % (RSD%) (output from DDSolver):

Parameter	No.1	No.2	No.3	No.4	Mean	SD	RSD(%)
α	57.640	67.611	75.256	72.844	68.338	7.813	11.433
β	0.537	0.547	0.580	0.585	0.563	0.024	4.253
T_i	4.000	4.000	6.000	6.000	5.000	1.155	23.094

Number of dissolution data points (N), degrees of freedom (df), and selected goodness of fit criteria – Pearson correlation coefficient (R), coefficient of determination (R^2), adjusted coefficient of determination ($R^2_{adjusted}$), and residual sum of squares (RSS) (manual calculation in MS Excel):

Parameter	No.1	No.2	No.3	No.4
N	26	26	26	26
df	23	23	23	23
R	0.981900311	0.984981409	0.979074287	0.983884548
R^2	0.964128221	0.970188377	0.95858646	0.968028803
$R^2_{adjusted}$	0.961008935	0.967596062	0.954985283	0.965248699
RSS	346.8287276	265.2709527	444.7307219	379.2354778

Graphical abstract of model fit presented as mean ± 1 SD of the fraction % of released carvedilol:

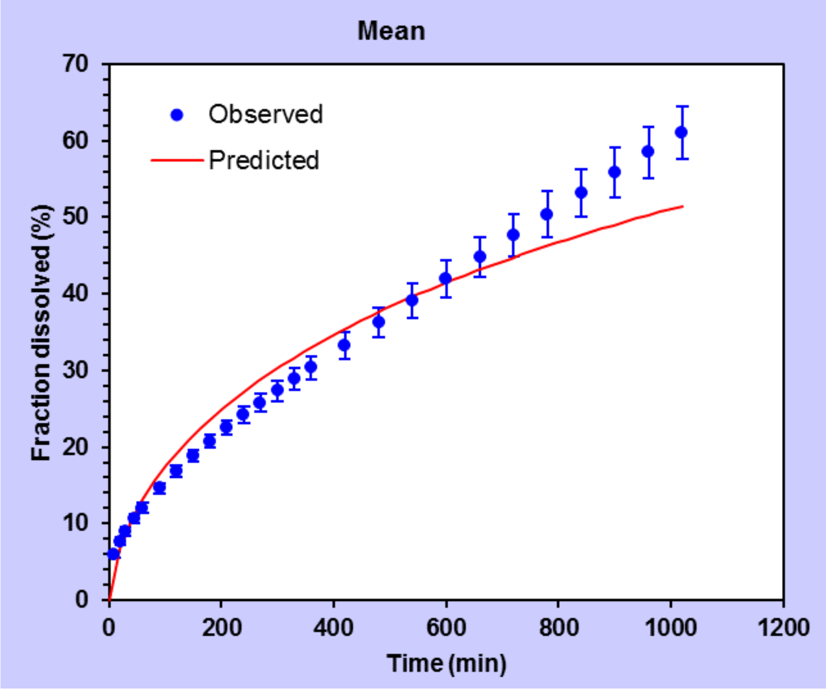

Supplementary materials – Model fitting summary – Starch 1500® sample with larger particle size (modelling up to app. 60 % of carvedilol released)

Graphical abstract of model fit presented as the fraction % of released carvedilol per tested tablet:

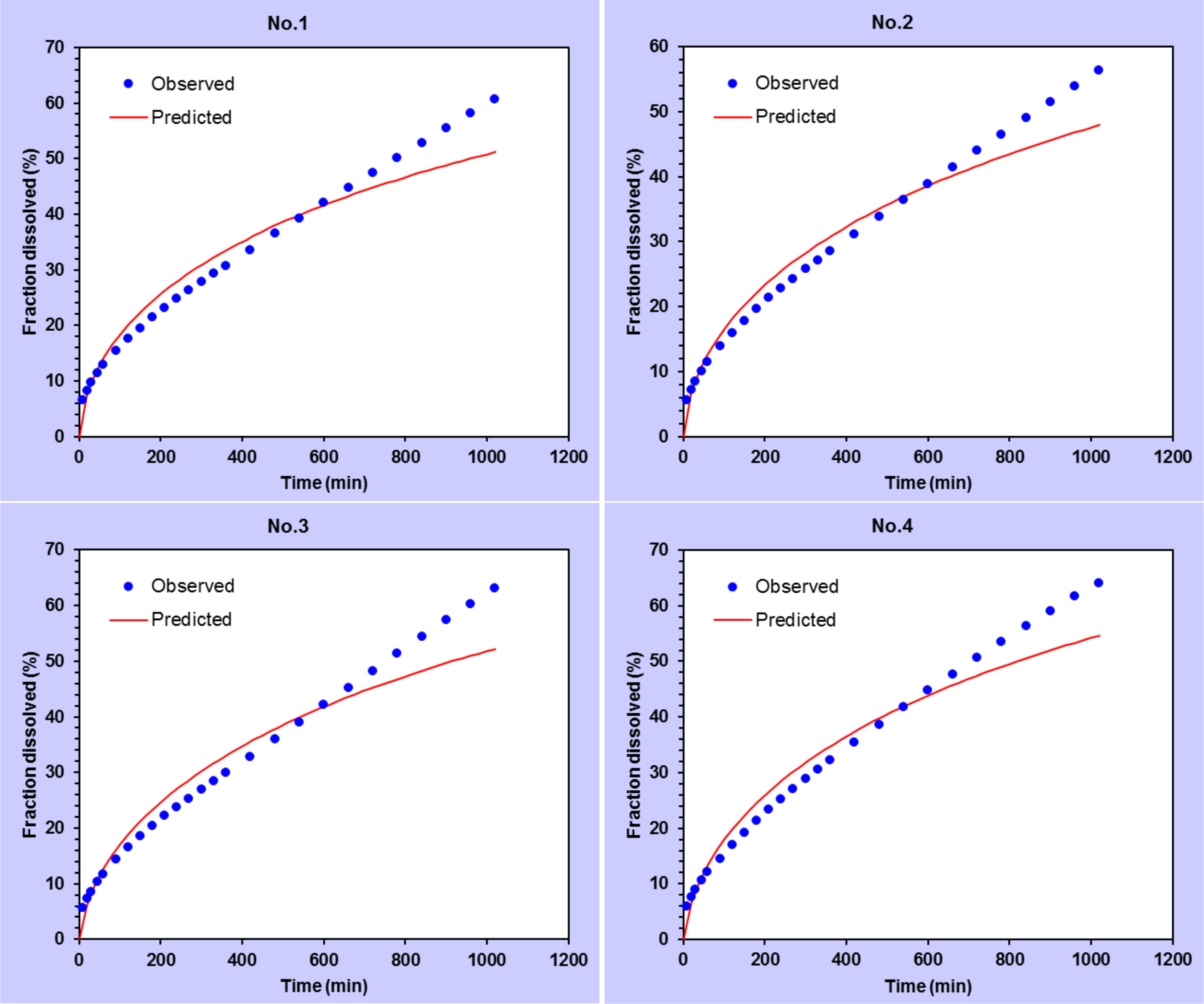

Supplementary materials – Model fitting summary – Starch 1500® sample with larger particle size (modelling up to app. 60 % of carvedilol released)

Model: **Weibull_2**

Model equation: $F = 100 \cdot \left(1 - e^{-\frac{t^{\beta}}{\alpha}}\right)$

Fitted model parameters per tested tablet (N = 4) with statistics – mean, standard deviation (SD), and relative standard deviation expressed in % (RSD%) (output from DDSolver):

Parameter	No.1	No.2	No.3	No.4	Mean	SD	RSD(%)
α	73.896	86.915	98.458	95.557	88.707	11.024	12.428
β	0.578	0.588	0.624	0.630	0.605	0.026	4.275

Number of dissolution data points (N), degrees of freedom (df), and selected goodness of fit criteria – Pearson correlation coefficient (R), coefficient of determination (R^2), adjusted coefficient of determination ($R^2_{adjusted}$), and residual sum of squares (RSS) (manual calculation in MS Excel):

Parameter	No.1	No.2	No.3	No.4
N	26	26	26	26
df	24	24	24	24
R	0.986146919	0.988827417	0.984270037	0.988674423
R^2	0.972485747	0.977779661	0.968787506	0.977477115
$R^2_{adjusted}$	0.971339319	0.976853813	0.967486986	0.976538661
RSS	254.231732	187.5390791	333.2803227	268.7963088

Graphical abstract of model fit presented as mean ± 1 SD of the fraction % of released carvedilol:

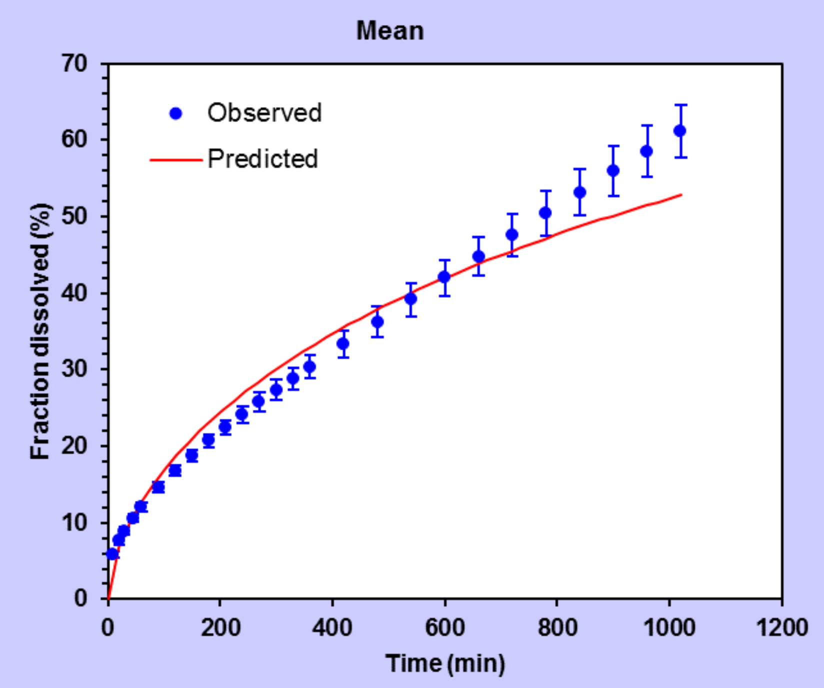

Supplementary materials – Model fitting summary – Starch 1500® sample with larger particle size (modelling up to app. 60 % of carvedilol released)

Graphical abstract of model fit presented as the fraction % of released carvedilol per tested tablet:

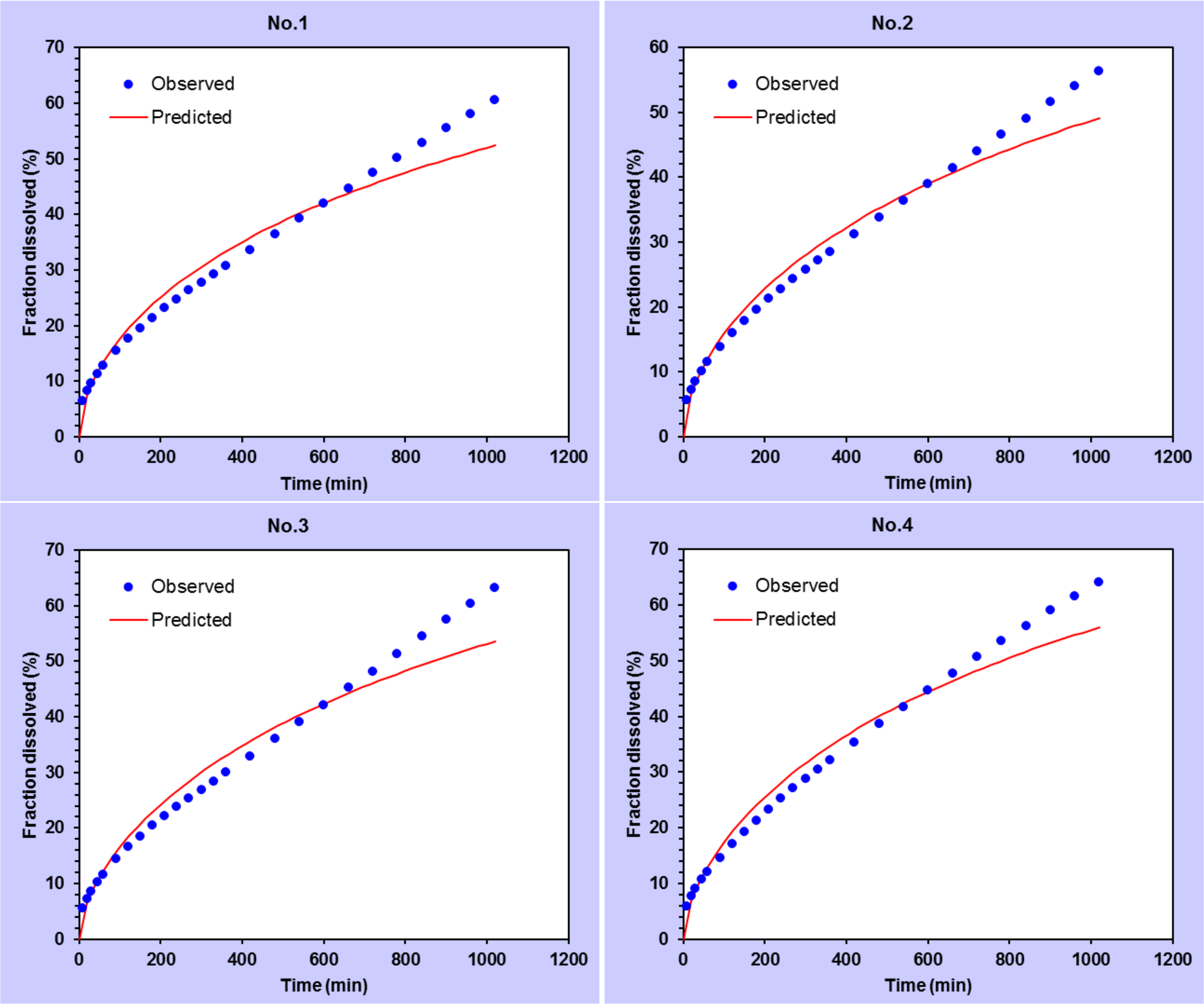

Supplementary materials – Model fitting summary – Starch 1500® sample with larger particle size (modelling up to app. 60 % of carvedilol released)

Model: **Weibull_3**

Model equation: $F = F_{max} \cdot \left(1 - e^{-\frac{t^{\beta}}{\alpha}}\right)$

Fitted model parameters per tested tablet (N = 4) with statistics – mean, standard deviation (SD), and relative standard deviation expressed in % (RSD%) (output from DDSolver):

Parameter	No.1	No.2	No.3	No.4	Mean	SD	RSD(%)
α	69.925	78.788	95.117	105.162	87.248	15.859	18.177
β	0.686	0.704	0.724	0.690	0.701	0.017	2.483
F_{max}	63.665	59.218	66.331	79.019	67.058	8.497	12.670

Number of dissolution data points (N), degrees of freedom (df), and selected goodness of fit criteria – Pearson correlation coefficient (R), coefficient of determination (R^2), adjusted coefficient of determination ($R^2_{adjusted}$), and residual sum of squares (RSS) (manual calculation in MS Excel):

Parameter	No.1	No.2	No.3	No.4
N	26	26	26	26
df	23	23	23	23
R	0.973491768	0.97591504	0.972413579	0.98673411
R^2	0.947686222	0.952410165	0.945588169	0.973644204
$R^2_{adjusted}$	0.943137198	0.948271918	0.940856706	0.971352395
RSS	401.316219	324.4814481	482.5145785	391.6405255

Graphical abstract of model fit presented as mean ± 1 SD of the fraction % of released carvedilol:

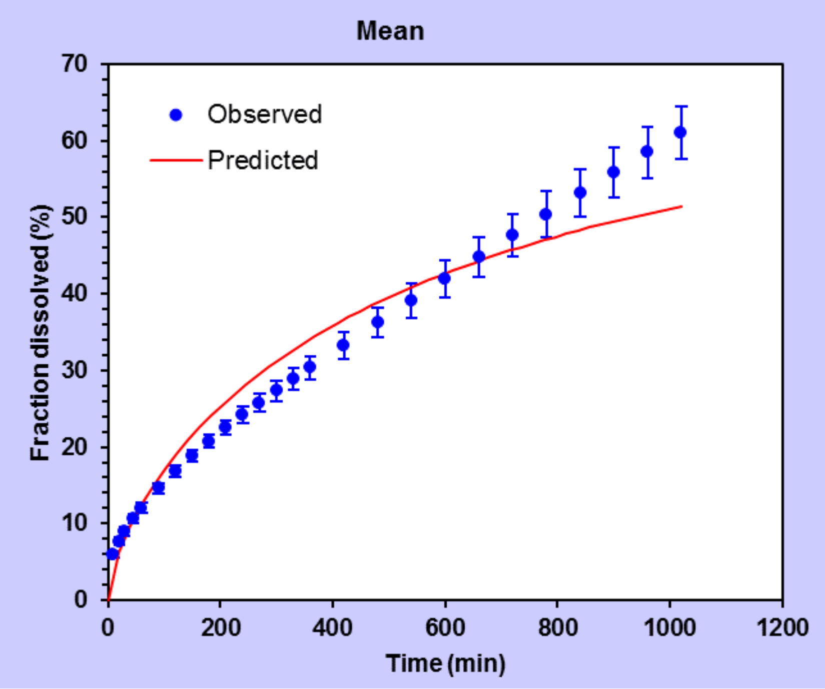

Supplementary materials – Model fitting summary – Starch 1500® sample with larger particle size (modelling up to app. 60 % of carvedilol released)

Graphical abstract of model fit presented as the fraction % of released carvedilol per tested tablet:

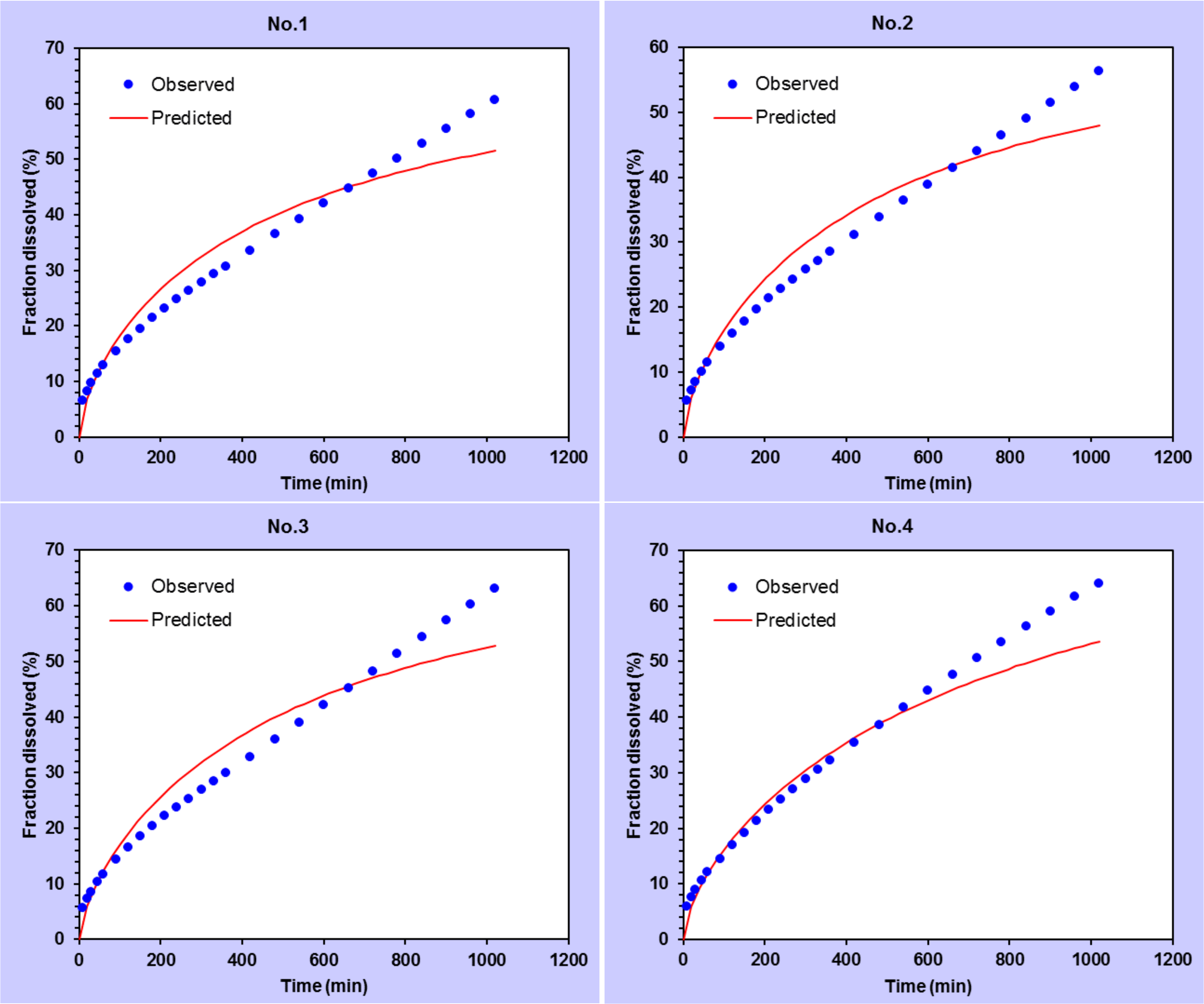

Supplementary materials – Model fitting summary – Starch 1500® sample with larger particle size (modelling up to app. 60 % of carvedilol released)

Model: **Weibull_4**

Model equation: $F = F_{max} \cdot \left[1 - e^{-\frac{(t-T_i)^\beta}{\alpha}} \right]$

Fitted model parameters per tested tablet (N = 4) with statistics – mean, standard deviation (SD), and relative standard deviation expressed in % (RSD%) (output from DDSolver):

Parameter	No.1	No.2	No.3	No.4	Mean	SD	RSD(%)
α	51.617	57.768	69.044	68.541	61.742	8.521	13.802
β	0.636	0.653	0.672	0.680	0.660	0.020	2.999
T_i	6.000	6.000	6.000	6.000	6.000	0.000	0.000
F_{max}	63.665	59.218	66.331	67.319	64.133	3.622	5.648

Number of dissolution data points (N), degrees of freedom (df), and selected goodness of fit criteria – Pearson correlation coefficient (R), coefficient of determination (R^2), adjusted coefficient of determination ($R^2_{adjusted}$), and residual sum of squares (RSS) (manual calculation in MS Excel):

Parameter	No.1	No.2	No.3	No.4
N	26	26	26	26
df	22	22	22	22
R	0.967545795	0.970385851	0.96677232	0.972111397
R^2	0.936144865	0.9416487	0.934648719	0.945000567
$R^2_{adjusted}$	0.927437347	0.933691705	0.925737181	0.937500645
RSS	484.1854842	394.0672307	577.6876449	522.9650888

Graphical abstract of model fit presented as mean ± 1 SD of the fraction % of released carvedilol:

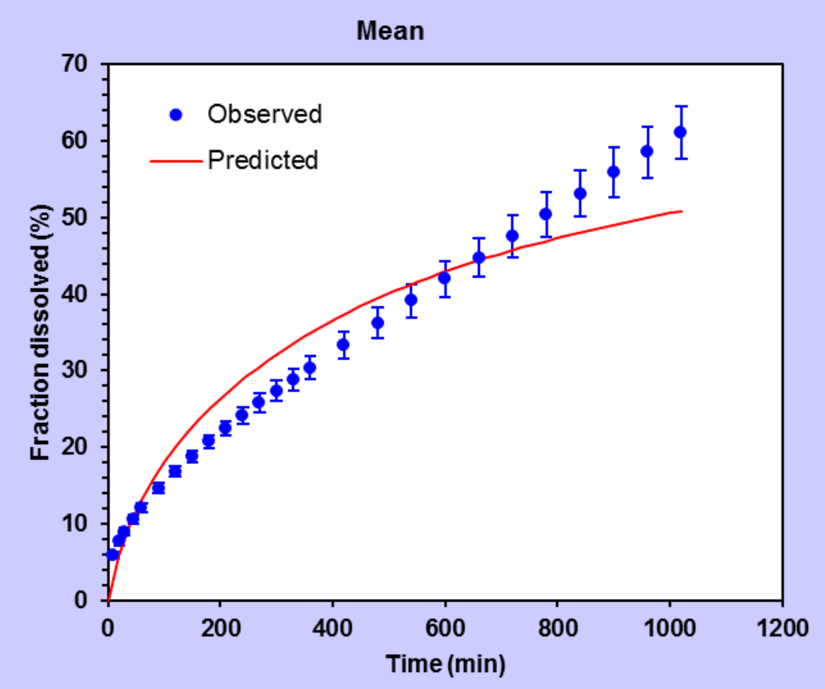

Supplementary materials – Model fitting summary – Starch 1500® sample with larger particle size (modelling up to app. 60 % of carvedilol released)

Graphical abstract of model fit presented as the fraction % of released carvedilol per tested tablet:

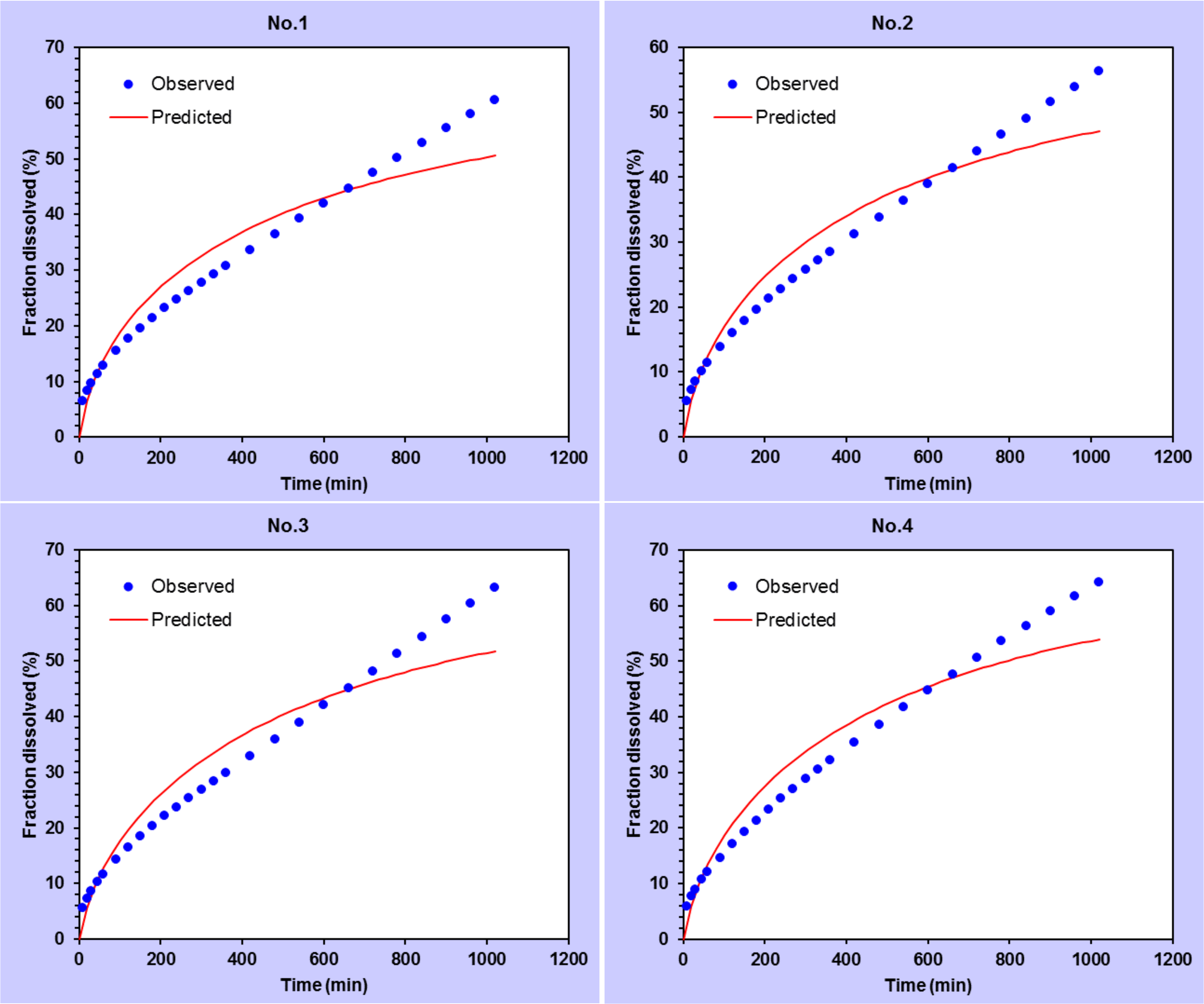

Supplementary materials – Model fitting summary – Starch 1500® sample with larger particle size (modelling up to app. 60 % of carvedilol released)

Model: **Logistic_1**

Model equation: $F = 100 \cdot \frac{e^{\alpha+\beta \cdot \log(t)}}{1+e^{\alpha+\beta \cdot \log(t)}}$

Fitted model parameters per tested tablet (N = 4) with statistics – mean, standard deviation (SD), and relative standard deviation expressed in % (RSD%) (output from DDSolver):

Parameter	No.1	No.2	No.3	No.4	Mean	SD	RSD(%)
α	-4.611	-5.241	-4.931	-4.922	-4.926	0.257	-5.225
β	1.550	1.780	1.671	1.698	1.674	0.095	5.695

Number of dissolution data points (N), degrees of freedom (df), and selected goodness of fit criteria – Pearson correlation coefficient (R), coefficient of determination (R^2), adjusted coefficient of determination ($R^2_{adjusted}$), and residual sum of squares (RSS) (manual calculation in MS Excel):

Parameter	No.1	No.2	No.3	No.4
N	26	26	26	26
df	24	24	24	24
R	0.978072994	0.984812057	0.97529129	0.980232672
R^2	0.956626782	0.969854787	0.951193101	0.960856092
$R^2_{adjusted}$	0.954819565	0.968598736	0.94915948	0.959225096
RSS	360.2529259	257.7315999	465.2455519	405.6729141

Graphical abstract of model fit presented as mean ± 1 SD of the fraction % of released carvedilol:

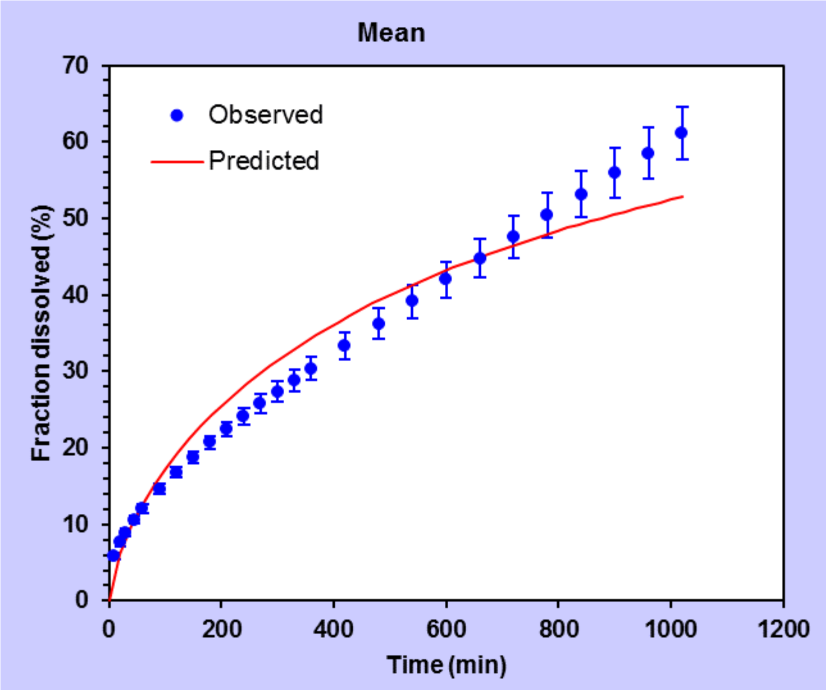

Supplementary materials – Model fitting summary – Starch 1500® sample with larger particle size (modelling up to app. 60 % of carvedilol released)

Graphical abstract of model fit presented as the fraction % of released carvedilol per tested tablet:

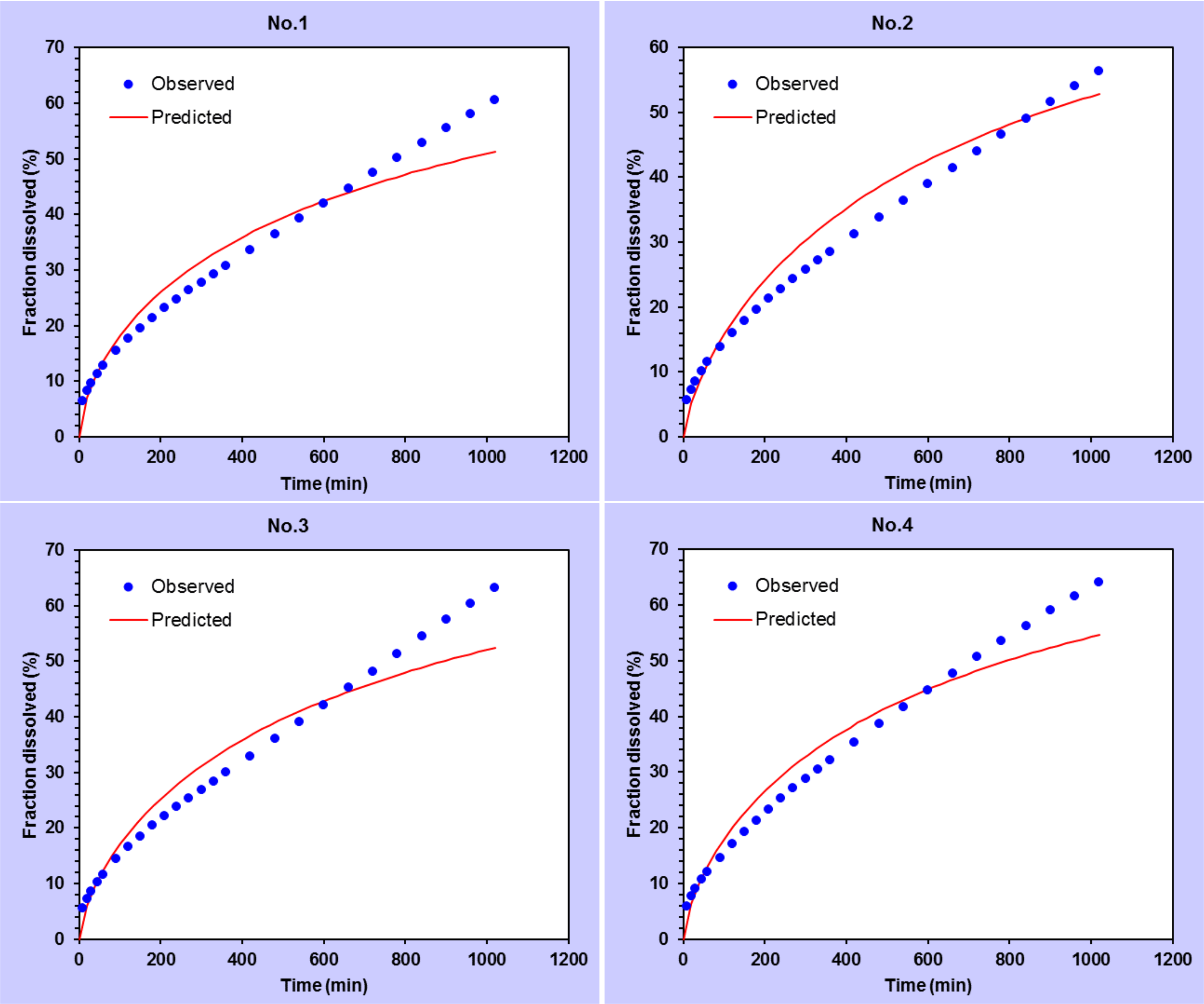

Supplementary materials – Model fitting summary – Starch 1500® sample with larger particle size (modelling up to app. 60 % of carvedilol released)

Model: **Logistic_2**

Model equation: $F = F_{max} \cdot \frac{e^{\alpha+\beta\cdot\log(t)}}{1+e^{\alpha+\beta\cdot\log(t)}}$

Fitted model parameters per tested tablet (N = 4) with statistics – mean, standard deviation (SD), and relative standard deviation expressed in % (RSD%) (output from DDSolver):

Parameter	No.1	No.2	No.3	No.4	Mean	SD	RSD(%)
α	-5.236	-5.360	-5.539	-5.585	-5.430	0.162	-2.977
β	2.202	2.244	2.278	2.329	2.263	0.054	2.387
F_{max}	63.665	59.218	66.331	67.319	64.133	3.622	5.648

Number of dissolution data points (N), degrees of freedom (df), and selected goodness of fit criteria – Pearson correlation coefficient (R), coefficient of determination (R^2), adjusted coefficient of determination ($R^2_{adjusted}$), and residual sum of squares (RSS) (manual calculation in MS Excel):

Parameter	No.1	No.2	No.3	No.4
N	26	26	26	26
df	23	23	23	23
R	0.95018388	0.953335926	0.949132943	0.954570809
R^2	0.902849405	0.908849388	0.900853344	0.91120543
$R^2_{adjusted}$	0.894401527	0.900923248	0.892231896	0.903484163
RSS	741.0895101	616.5062137	862.1064129	831.4730391

Graphical abstract of model fit presented as mean ± 1 SD of the fraction % of released carvedilol:

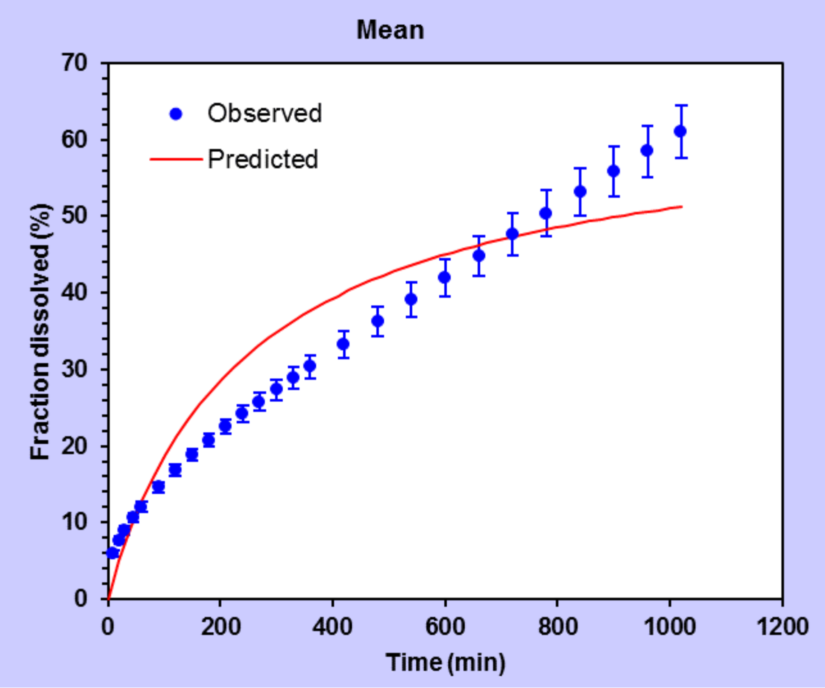

Supplementary materials – Model fitting summary – Starch 1500® sample with larger particle size (modelling up to app. 60 % of carvedilol released)

Graphical abstract of model fit presented as the fraction % of released carvedilol per tested tablet:

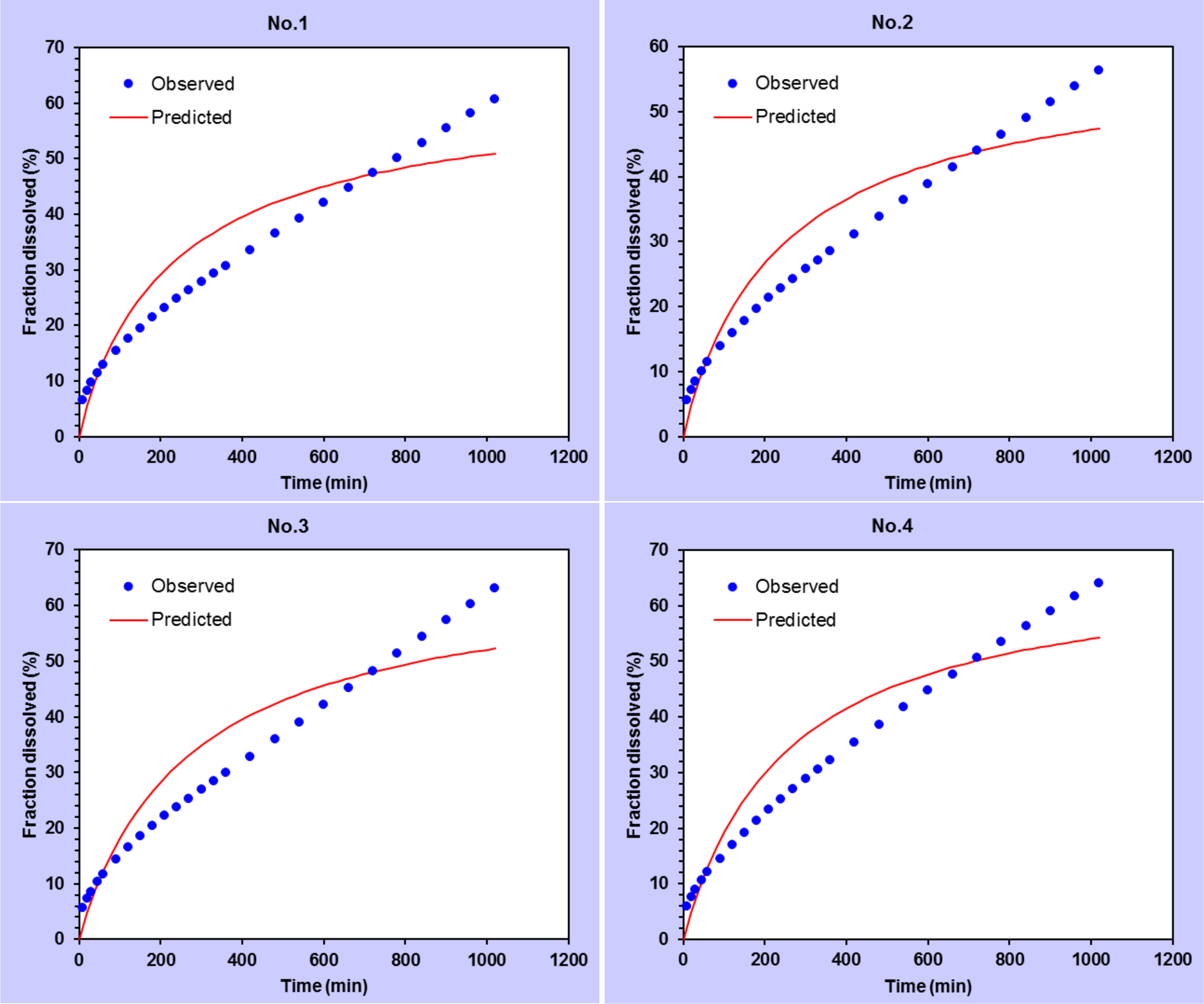

Supplementary materials – Model fitting summary – Starch 1500® sample with larger particle size (modelling up to app. 60 % of carvedilol released)

Model: **Logistic_3**

Model equation: $F = F_{max} \cdot \frac{1}{1+e^{-k\cdot(t-\gamma)}}$

Fitted model parameters per tested tablet (N = 4) with statistics – mean, standard deviation (SD), and relative standard deviation expressed in % (RSD%) (output from DDSolver):

Parameter	No.1	No.2	No.3	No.4	Mean	SD	RSD(%)
k	0.004	0.004	0.004	0.004	0.004	0.000	2.232
γ	402.005	407.817	430.660	412.546	413.257	12.377	2.995
F_{max}	63.665	59.218	66.331	67.319	64.133	3.622	5.648

Number of dissolution data points (N), degrees of freedom (df), and selected goodness of fit criteria – Pearson correlation coefficient (R), coefficient of determination (R^2), adjusted coefficient of determination ($R^2_{adjusted}$), and residual sum of squares (RSS) (manual calculation in MS Excel):

Parameter	No.1	No.2	No.3	No.4
N	26	26	26	26
df	23	23	23	23
R	0.991393426	0.990617738	0.991549922	0.992079767
R^2	0.982860925	0.981323504	0.983171247	0.984222265
$R^2_{adjusted}$	0.981370571	0.979699461	0.981707877	0.982850288
RSS	127.9581493	123.3145695	146.4939936	142.9215332

Graphical abstract of model fit presented as mean ± 1 SD of the fraction % of released carvedilol:

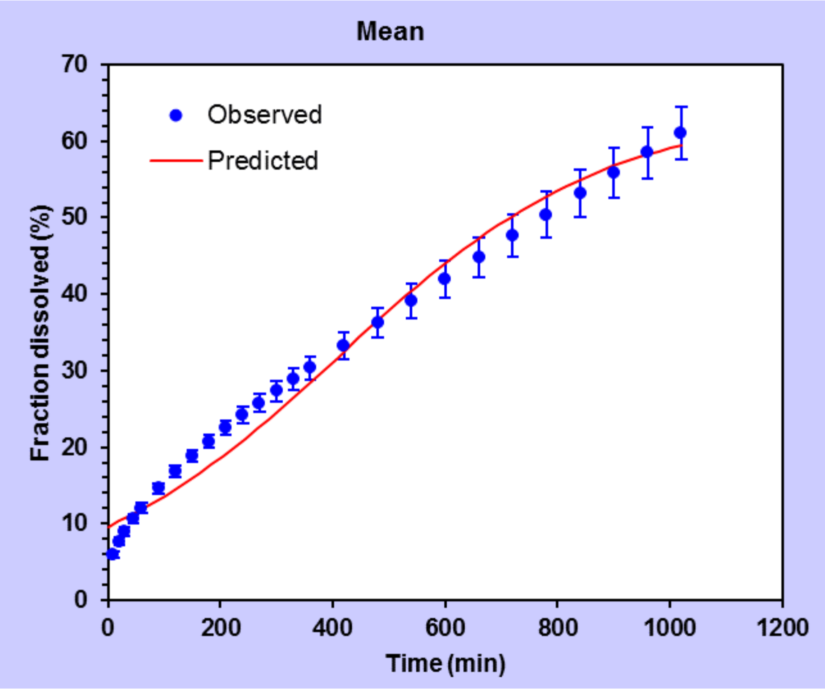

Supplementary materials – Model fitting summary – Starch 1500® sample with larger particle size (modelling up to app. 60 % of carvedilol released)

Graphical abstract of model fit presented as the fraction % of released carvedilol per tested tablet:

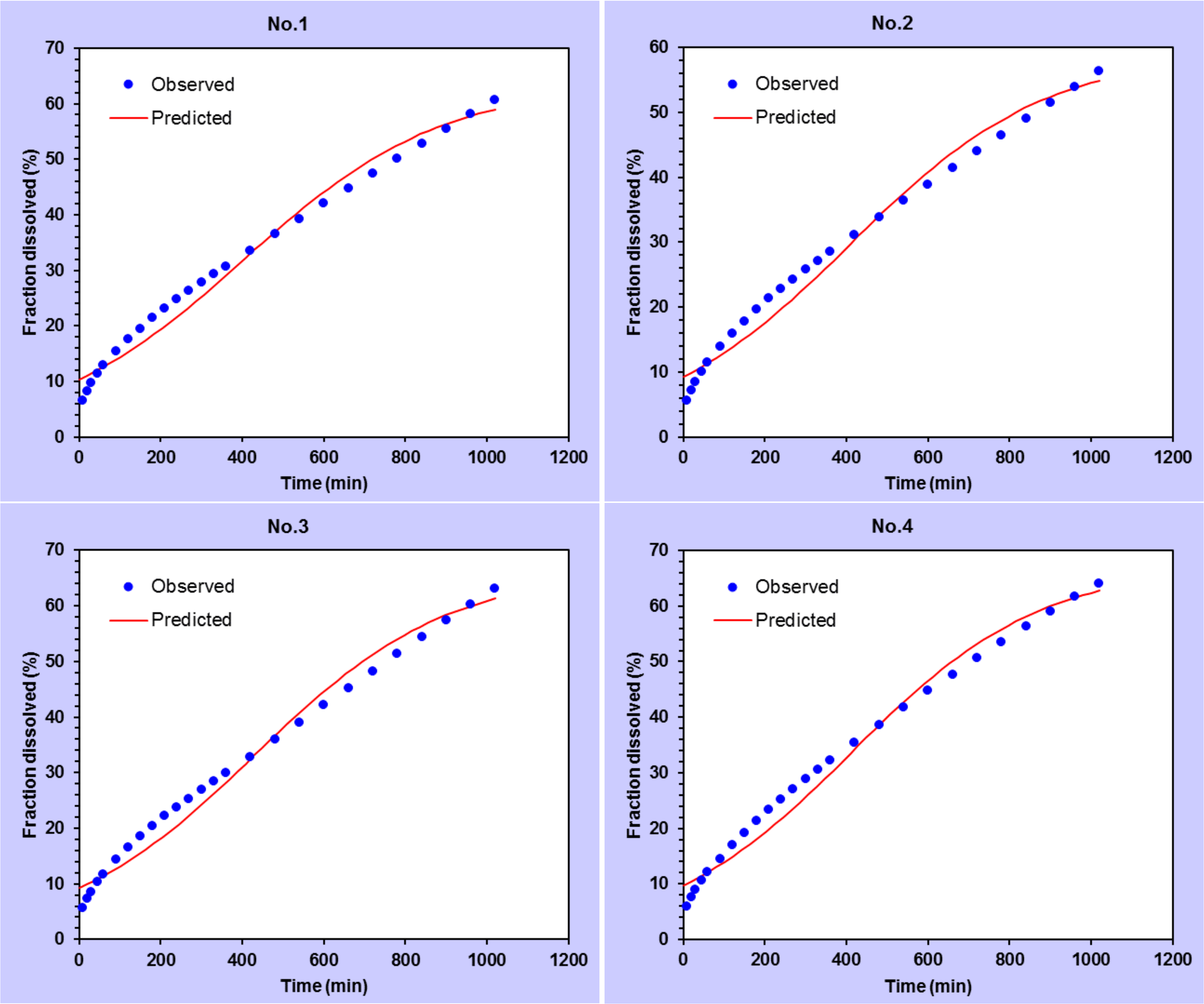

Supplementary materials – Model fitting summary – Starch 1500® sample with larger particle size (modelling up to app. 60 % of carvedilol released)

Model: **Gompertz_1**

Model equation: $F = 100 \cdot e^{-\alpha \cdot e^{-\beta \cdot \log(t)}}$

Fitted model parameters per tested tablet (N = 4) with statistics – mean, standard deviation (SD), and relative standard deviation expressed in % (RSD%) (output from DDSolver):

Parameter	No.1	No.2	No.3	No.4	Mean	SD	RSD(%)
α	8.746	8.696	10.115	10.403	9.490	0.896	9.443
β	0.839	0.805	0.896	0.927	0.867	0.055	6.326

Number of dissolution data points (N), degrees of freedom (df), and selected goodness of fit criteria – Pearson correlation coefficient (R), coefficient of determination (R^2), adjusted coefficient of determination ($R^2_{adjusted}$), and residual sum of squares (RSS) (manual calculation in MS Excel):

Parameter	No.1	No.2	No.3	No.4
N	26	26	26	26
df	24	24	24	24
R	0.955027522	0.96026791	0.949938026	0.956058924
R^2	0.912077568	0.922114458	0.902382253	0.914048666
$R^2_{adjusted}$	0.908414133	0.918869228	0.898314847	0.910467361
RSS	645.7130973	514.6429877	821.8458557	773.912032

Graphical abstract of model fit presented as mean ± 1 SD of the fraction % of released carvedilol:

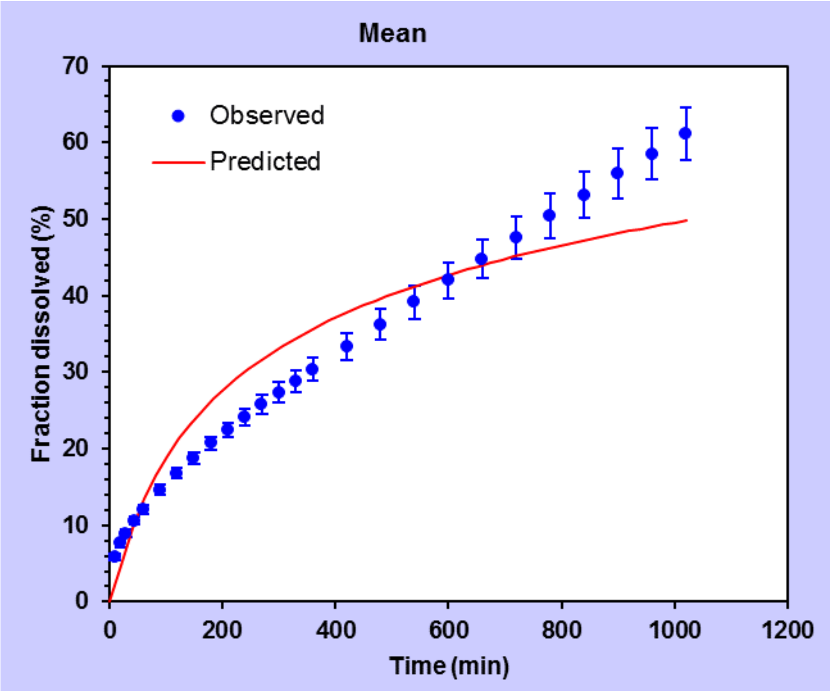

Supplementary materials – Model fitting summary – Starch 1500® sample with larger particle size (modelling up to app. 60 % of carvedilol released)

Graphical abstract of model fit presented as the fraction % of released carvedilol per tested tablet:

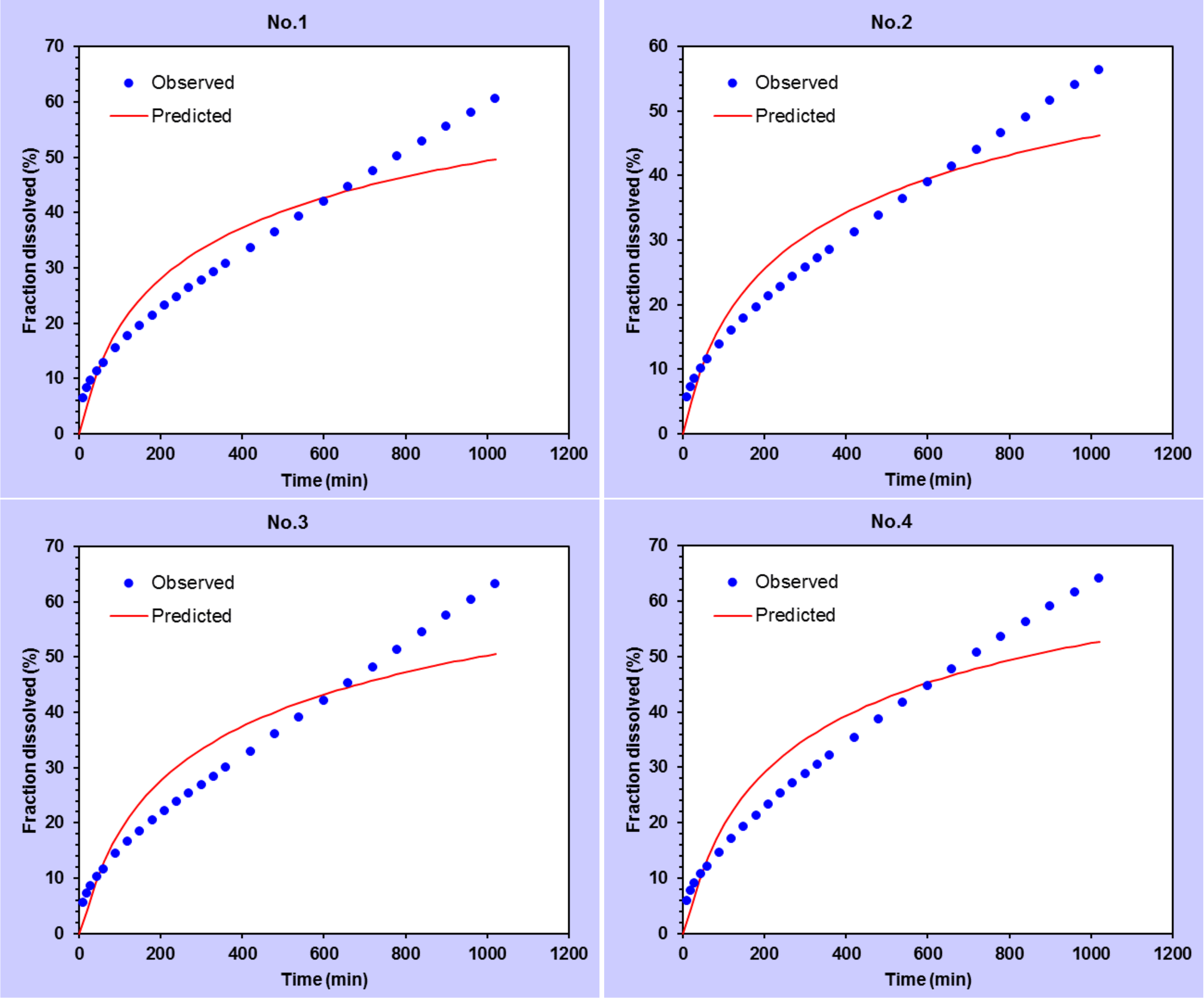

Supplementary materials – Model fitting summary – Starch 1500® sample with larger particle size (modelling up to app. 60 % of carvedilol released)

Model: **Gompertz_2**

Model equation: $F = F_{max} \cdot e^{-\alpha \cdot e^{-\beta \cdot \log(t)}}$

Fitted model parameters per tested tablet (N = 4) with statistics – mean, standard deviation (SD), and relative standard deviation expressed in % (RSD%) (output from DDSolver):

Parameter	No.1	No.2	No.3	No.4	Mean	SD	RSD(%)
α	35.054	36.896	38.912	41.723	38.146	2.858	7.492
β	1.530	1.546	1.542	1.595	1.553	0.029	1.851
F_{max}	63.665	59.218	66.331	67.319	64.133	3.622	5.648

Number of dissolution data points (N), degrees of freedom (df), and selected goodness of fit criteria – Pearson correlation coefficient (R), coefficient of determination (R^2), adjusted coefficient of determination ($R^2_{adjusted}$), and residual sum of squares (RSS) (manual calculation in MS Excel):

Parameter	No.1	No.2	No.3	No.4
N	26	26	26	26
df	23	23	23	23
R	0.955658019	0.958460595	0.954075558	0.95962324
R^2	0.91328225	0.918646713	0.91026017	0.920876763
$R^2_{adjusted}$	0.905741576	0.911572514	0.902456707	0.913996481
RSS	1148.274236	967.7546557	1291.979577	1217.274416

Graphical abstract of model fit presented as mean ± 1 SD of the fraction % of released carvedilol:

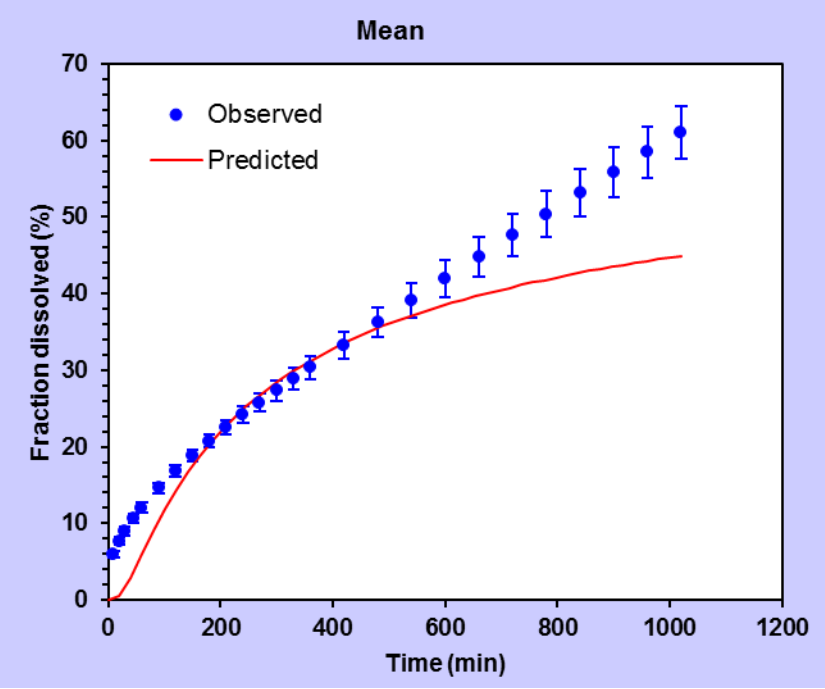

Supplementary materials – Model fitting summary – Starch 1500® sample with larger particle size (modelling up to app. 60 % of carvedilol released)

Graphical abstract of model fit presented as the fraction % of released carvedilol per tested tablet:

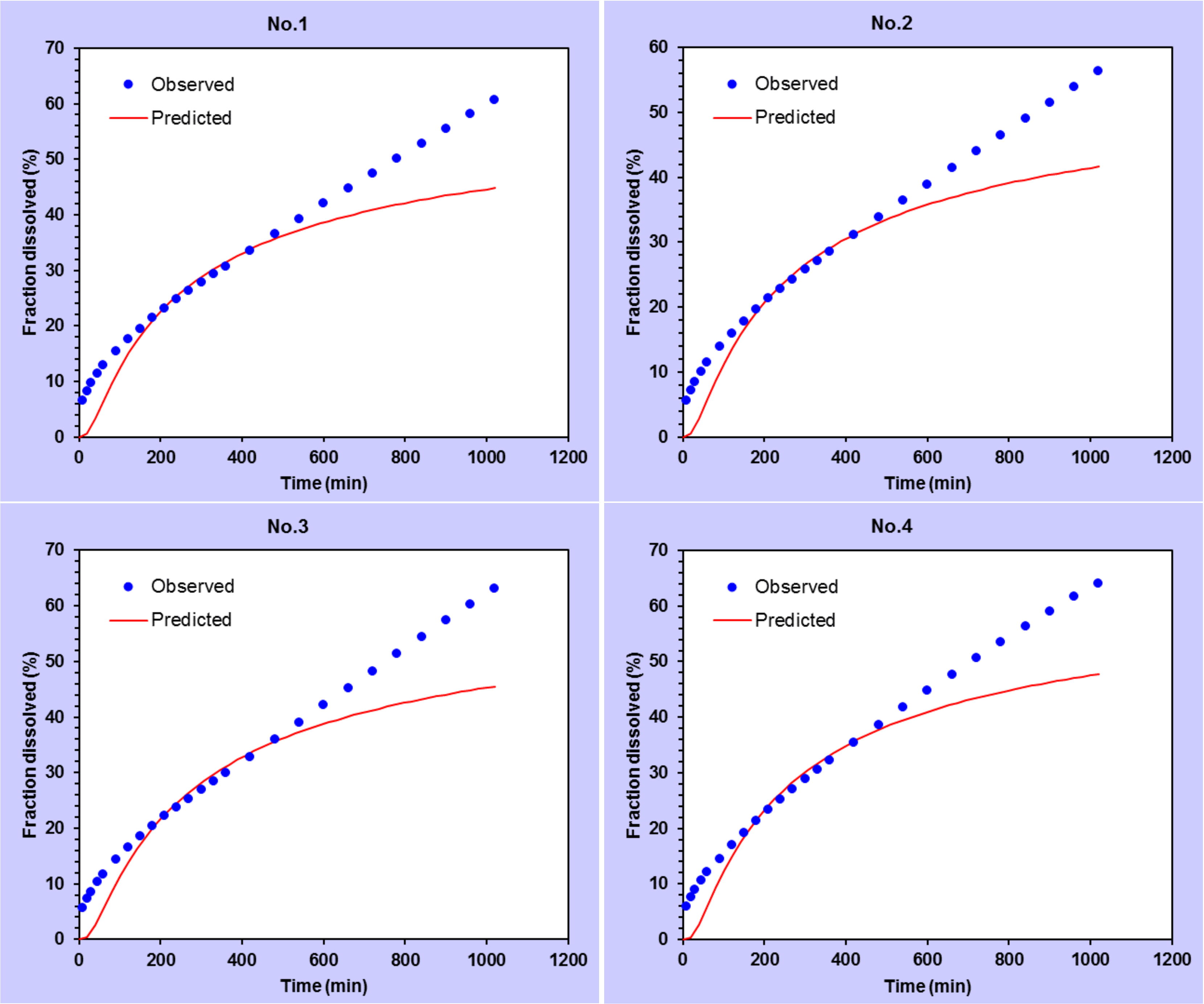

Supplementary materials – Model fitting summary – Starch 1500® sample with larger particle size (modelling up to app. 60 % of carvedilol released)

Model: **Gompertz_3**

Model equation: $F = F_{max} \cdot e^{-e^{-k \cdot (t-\gamma)}}$

Fitted model parameters per tested tablet (N = 4) with statistics – mean, standard deviation (SD), and relative standard deviation expressed in % (RSD%) (output from DDSolver):

Parameter	No.1	No.2	No.3	No.4	Mean	SD	RSD(%)
k	0.003	0.003	0.003	0.003	0.003	0.000	1.724
γ	242.110	247.515	268.696	254.296	253.154	11.498	4.542
F_{max}	63.665	59.218	66.331	67.319	64.133	3.622	5.648

Number of dissolution data points (N), degrees of freedom (df), and selected goodness of fit criteria – Pearson correlation coefficient (R), coefficient of determination (R^2), adjusted coefficient of determination ($R^2_{adjusted}$), and residual sum of squares (RSS) (manual calculation in MS Excel):

Parameter	No.1	No.2	No.3	No.4
N	26	26	26	26
df	23	23	23	23
R	0.993428347	0.993438074	0.99243911	0.995007911
R^2	0.986899881	0.986919207	0.984935388	0.990040744
$R^2_{adjusted}$	0.98576074	0.985781747	0.983625422	0.989174721
RSS	112.6363788	98.06211245	149.2334399	103.7105821

Graphical abstract of model fit presented as mean ± 1 SD of the fraction % of released carvedilol:

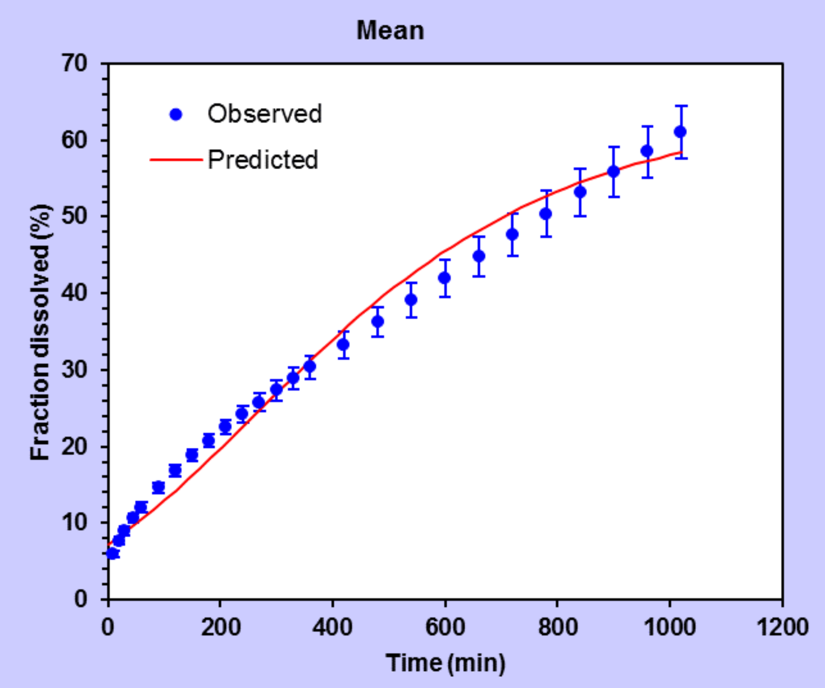

Supplementary materials – Model fitting summary – Starch 1500® sample with larger particle size (modelling up to app. 60 % of carvedilol released)

Graphical abstract of model fit presented as the fraction % of released carvedilol per tested tablet:

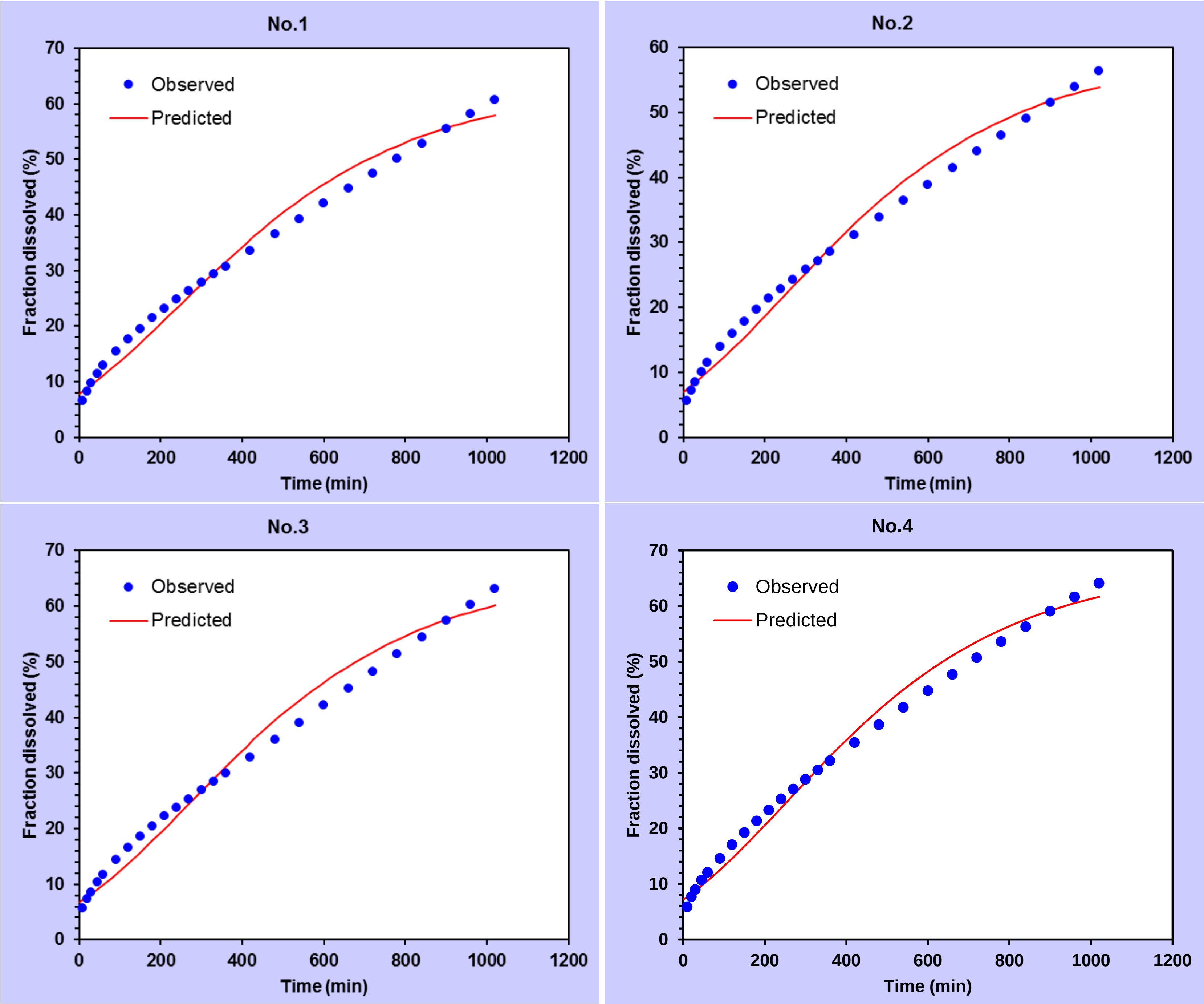

Supplementary materials – Model fitting summary – Starch 1500® sample with larger particle size (modelling up to app. 60 % of carvedilol released)

Model: **Gompertz_4**

Model equation: $F = F_{max} \cdot e^{-\beta \cdot e^{-k \cdot t}}$

Fitted model parameters per tested tablet (N = 4) with statistics – mean, standard deviation (SD), and relative standard deviation expressed in % (RSD%) (output from DDSolver):

Parameter	No.1	No.2	No.3	No.4	Mean	SD	RSD(%)
k	0.003	0.003	0.003	0.003	0.003	0.000	1.724
β	2.092	2.138	2.293	2.240	2.191	0.092	4.189
F_{max}	63.665	59.218	66.331	67.319	64.133	3.622	5.648

Number of dissolution data points (N), degrees of freedom (df), and selected goodness of fit criteria – Pearson correlation coefficient (R), coefficient of determination (R^2), adjusted coefficient of determination ($R^2_{adjusted}$), and residual sum of squares (RSS) (manual calculation in MS Excel):

Parameter	No.1	No.2	No.3	No.4
N	26	26	26	26
df	23	23	23	23
R	0.993428347	0.993438074	0.99243911	0.995007911
R^2	0.986899881	0.986919207	0.984935388	0.990040744
$R^2_{adjusted}$	0.98576074	0.985781747	0.983625422	0.989174721
RSS	112.6363788	98.06211245	149.2334399	103.7105821

Graphical abstract of model fit presented as mean ± 1 SD of the fraction % of released carvedilol:

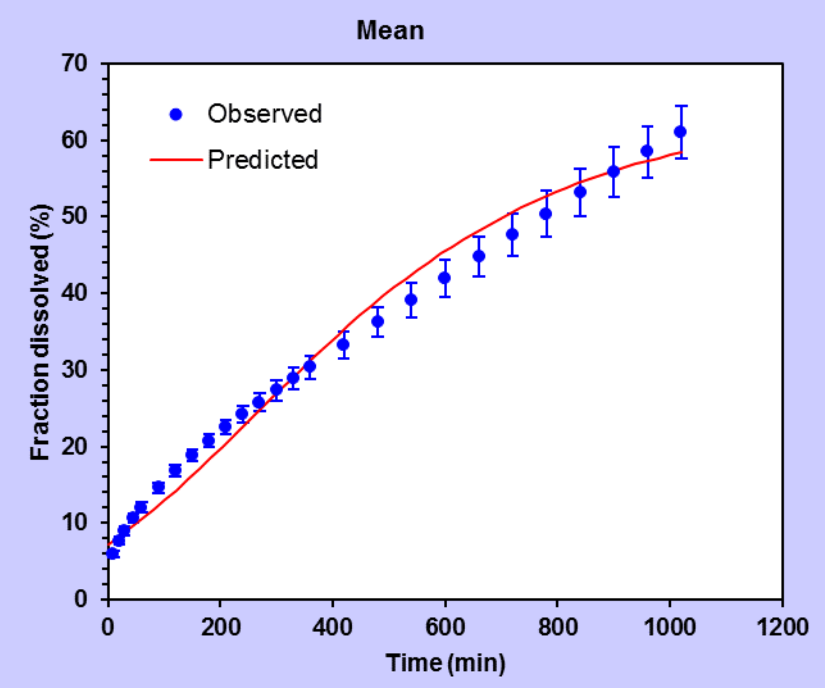

Supplementary materials – Model fitting summary – Starch 1500® sample with larger particle size (modelling up to app. 60 % of carvedilol released)

Graphical abstract of model fit presented as the fraction % of released carvedilol per tested tablet:

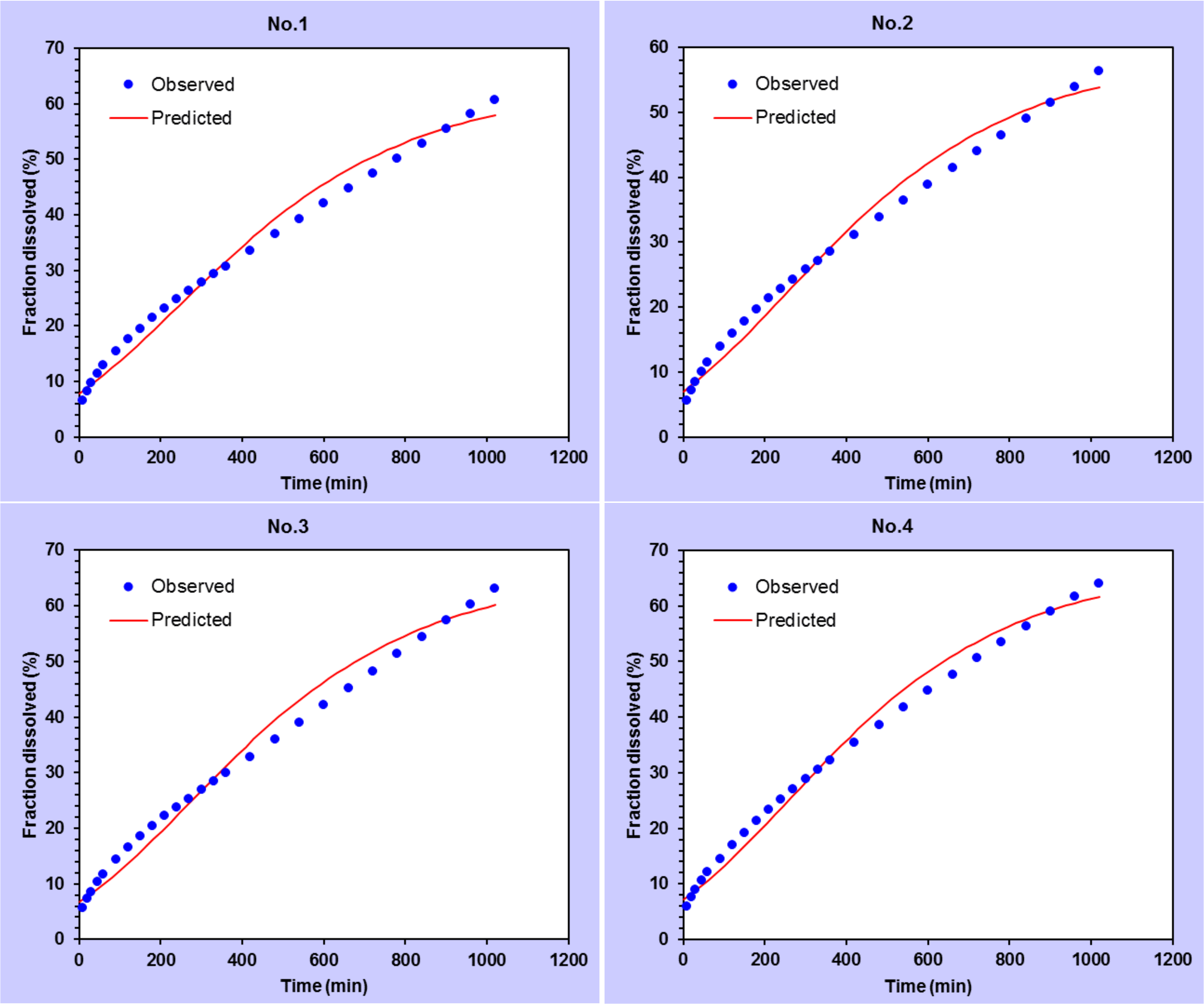

Supplementary materials – Model fitting summary – Starch 1500® sample with larger particle size (modelling up to app. 60 % of carvedilol released)

Model: **Probit_1**

Model equation: $F = 100 \cdot \phi[\alpha + \beta \cdot \log(t)]$

Fitted model parameters per tested tablet (N = 4) with statistics – mean, standard deviation (SD), and relative standard deviation expressed in % (RSD%) (output from DDSolver):

Parameter	No.1	No.2	No.3	No.4	Mean	SD	RSD(%)
α	-2.971	-2.735	-3.158	-2.863	-2.932	0.179	-6.109
β	1.031	0.886	1.105	0.983	1.001	0.092	9.172

Number of dissolution data points (N), degrees of freedom (df), and selected goodness of fit criteria – Pearson correlation coefficient (R), coefficient of determination (R^2), adjusted coefficient of determination ($R^2_{adjusted}$), and residual sum of squares (RSS) (manual calculation in MS Excel):

Parameter	No.1	No.2	No.3	No.4
N	26	26	26	26
df	24	24	24	24
R	0.974187082	0.975077119	0.970560915	0.973243379
R^2	0.949040471	0.950775388	0.941988489	0.947202674
$R^2_{adjusted}$	0.946917157	0.948724362	0.939571343	0.945002786
RSS	436.6217613	356.0899957	560.9493417	522.7658642

Graphical abstract of model fit presented as mean ± 1 SD of the fraction % of released carvedilol:

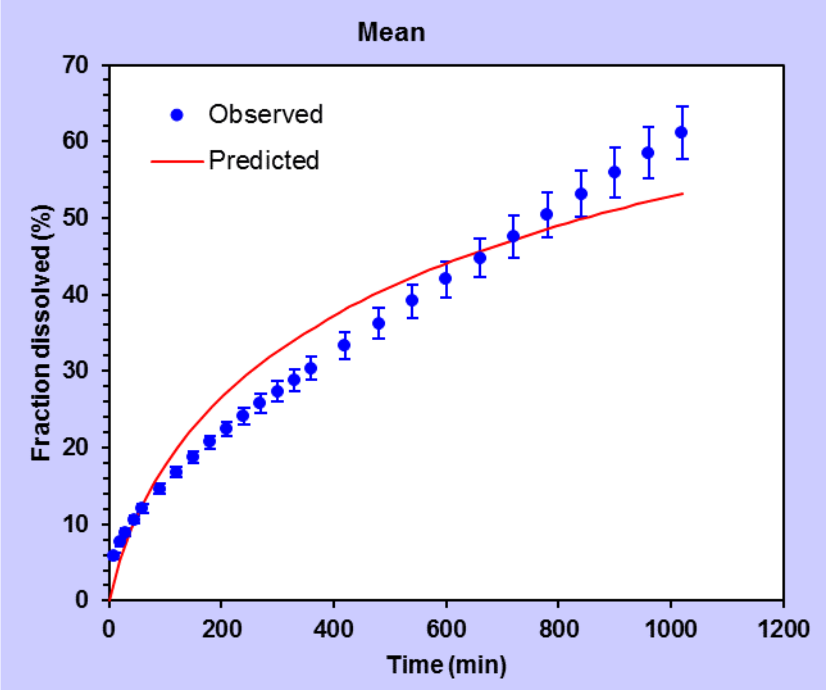

Supplementary materials – Model fitting summary – Starch 1500® sample with larger particle size (modelling up to app. 60 % of carvedilol released)

Graphical abstract of model fit presented as the fraction % of released carvedilol per tested tablet:

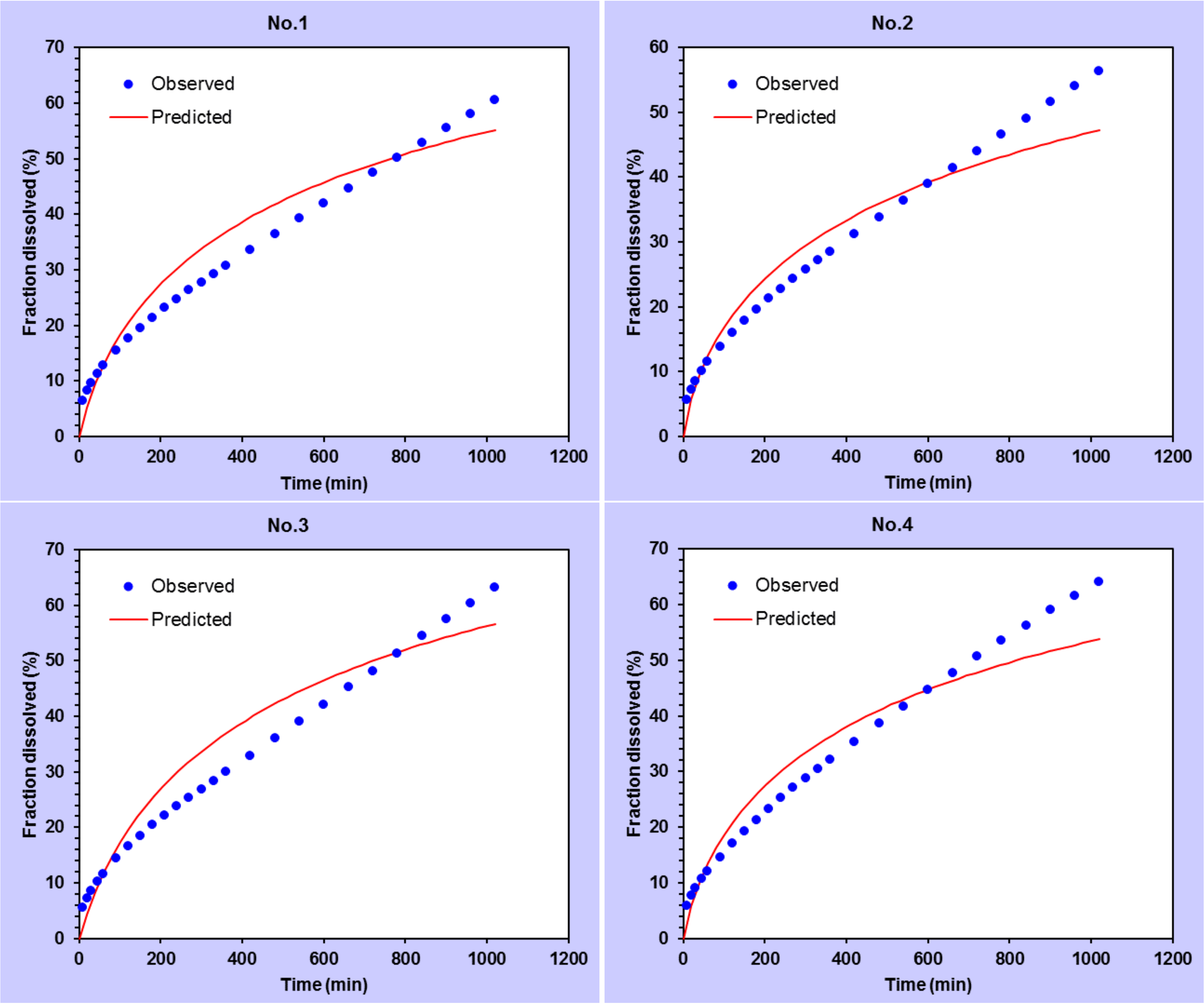

Supplementary materials – Model fitting summary – Starch 1500® sample with larger particle size (modelling up to app. 60 % of carvedilol released)

Model: **Probit_2**

Model equation: $F = F_{max} \cdot \phi[\alpha + \beta \cdot \log(t)]$

Fitted model parameters per tested tablet (N = 4) with statistics – mean, standard deviation (SD), and relative standard deviation expressed in % (RSD%) (output from DDSolver):

Parameter	No.1	No.2	No.3	No.4	Mean	SD	RSD(%)
α	-3.109	-3.176	-3.270	-3.300	-3.214	0.088	-2.729
β	1.304	1.327	1.342	1.374	1.337	0.029	2.181
F_{max}	63.665	59.218	66.331	67.319	64.133	3.622	5.648

Number of dissolution data points (N), degrees of freedom (df), and selected goodness of fit criteria – Pearson correlation coefficient (R), coefficient of determination (R^2), adjusted coefficient of determination ($R^2_{adjusted}$), and residual sum of squares (RSS) (manual calculation in MS Excel):

Parameter	No.1	No.2	No.3	No.4
N	26	26	26	26
df	23	23	23	23
R	0.948605014	0.951569833	0.946520527	0.952490571
R^2	0.899851472	0.905485146	0.895901108	0.907238289
$R^2_{adjusted}$	0.891142905	0.897266463	0.886849031	0.899172053
RSS	739.7040171	619.2107777	879.7515212	841.550158

Graphical abstract of model fit presented as mean ± 1 SD of the fraction % of released carvedilol:

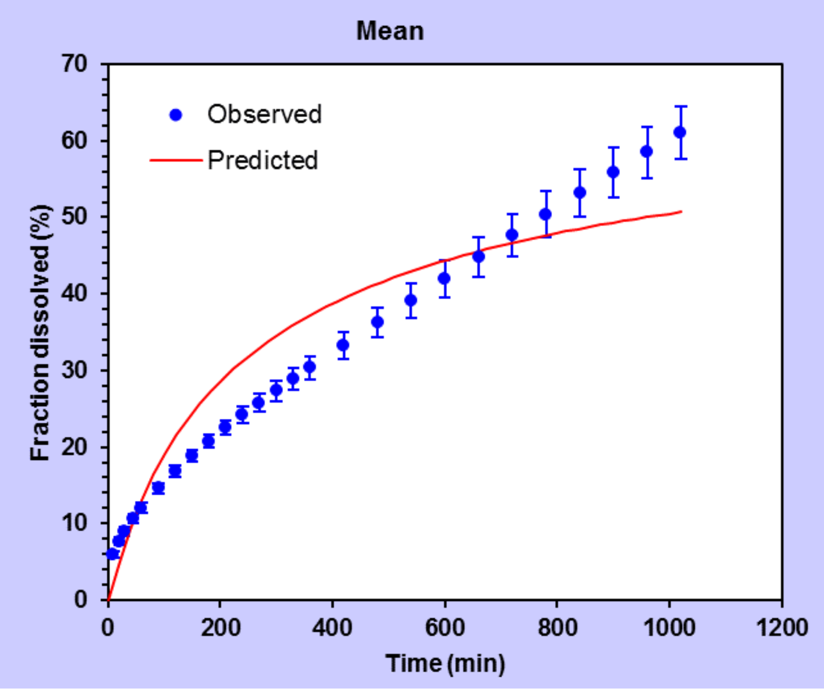

Supplementary materials – Model fitting summary – Starch 1500® sample with larger particle size (modelling up to app. 60 % of carvedilol released)

Graphical abstract of model fit presented as the fraction % of released carvedilol per tested tablet:

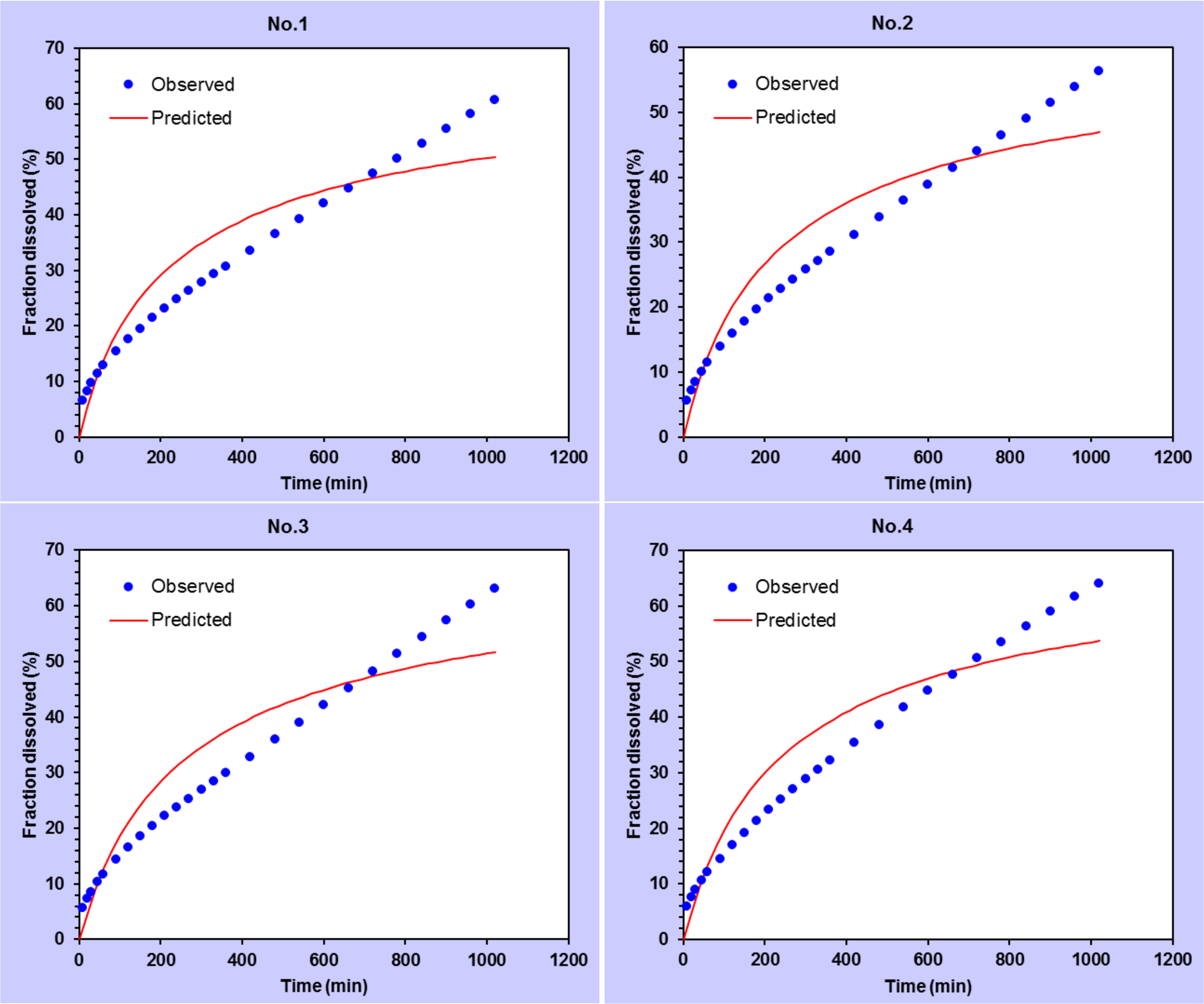

Supplement: Supplementary file 1 [file pharmaceutics-16-00498-s001.zip › Supplementary materials_Model fitting summary_Starch 1500® sample with larger particle size.pdf]
