# Supplementary material for: Comparative Fitting of Mathematical Models to Carvedilol Release Profiles Obtained from Hypromellose Matrix Tablets
Source: Pharmaceutics. 2024 Apr 4;16(4):498. doi: 10.3390/pharmaceutics16040498 (PMC11053526; doi:10.3390/pharmaceutics16040498)

Supplementary materials – Model fitting summary – Starch 1500® sample with smaller particle size (modelling the entire relevant dissolution data)

Model: **Zero–order**

Model equation: $F = k_0 \cdot t$

Fitted model parameters per tested tablet (N = 4) with statistics – mean, standard deviation (SD), and relative standard deviation expressed in % (RSD%) (output from DDSolver):

Parameter	No.1	No.2	No.3	No.4	Mean	SD	RSD(%)
k_0	0.061	0.060	0.068	0.066	0.064	0.004	6.468

Number of dissolution data points (N), degrees of freedom (df), and selected goodness of fit criteria – Pearson correlation coefficient (R), coefficient of determination (R^2), adjusted coefficient of determination ($R^2_{adjusted}$), and residual sum of squares (RSS) (manual calculation in MS Excel):

Parameter	No.1	No.2	No.3	No.4
N	33	33	33	33
df	32	32	32	32
R	0.993998144	0.993484242	0.993797377	0.994139149
R^2	0.988032311	0.987010939	0.987633226	0.988312647
$R^2_{adjusted}$	0.988032311	0.987010939	0.987633226	0.988312647
RSS	2786.008881	2415.940076	5050.179276	3151.740244

Graphical abstract of model fit presented as mean ± 1 SD of the fraction % of released carvedilol:

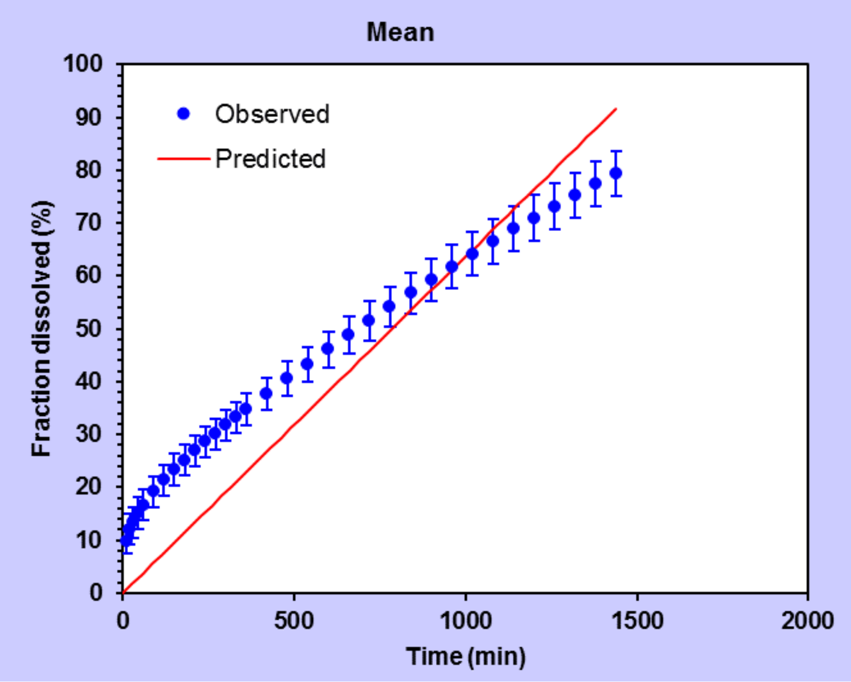

Supplementary materials – Model fitting summary – Starch 1500® sample with smaller particle size (modelling the entire relevant dissolution data)

Graphical abstract of model fit presented as the fraction % of released carvedilol per tested tablet:

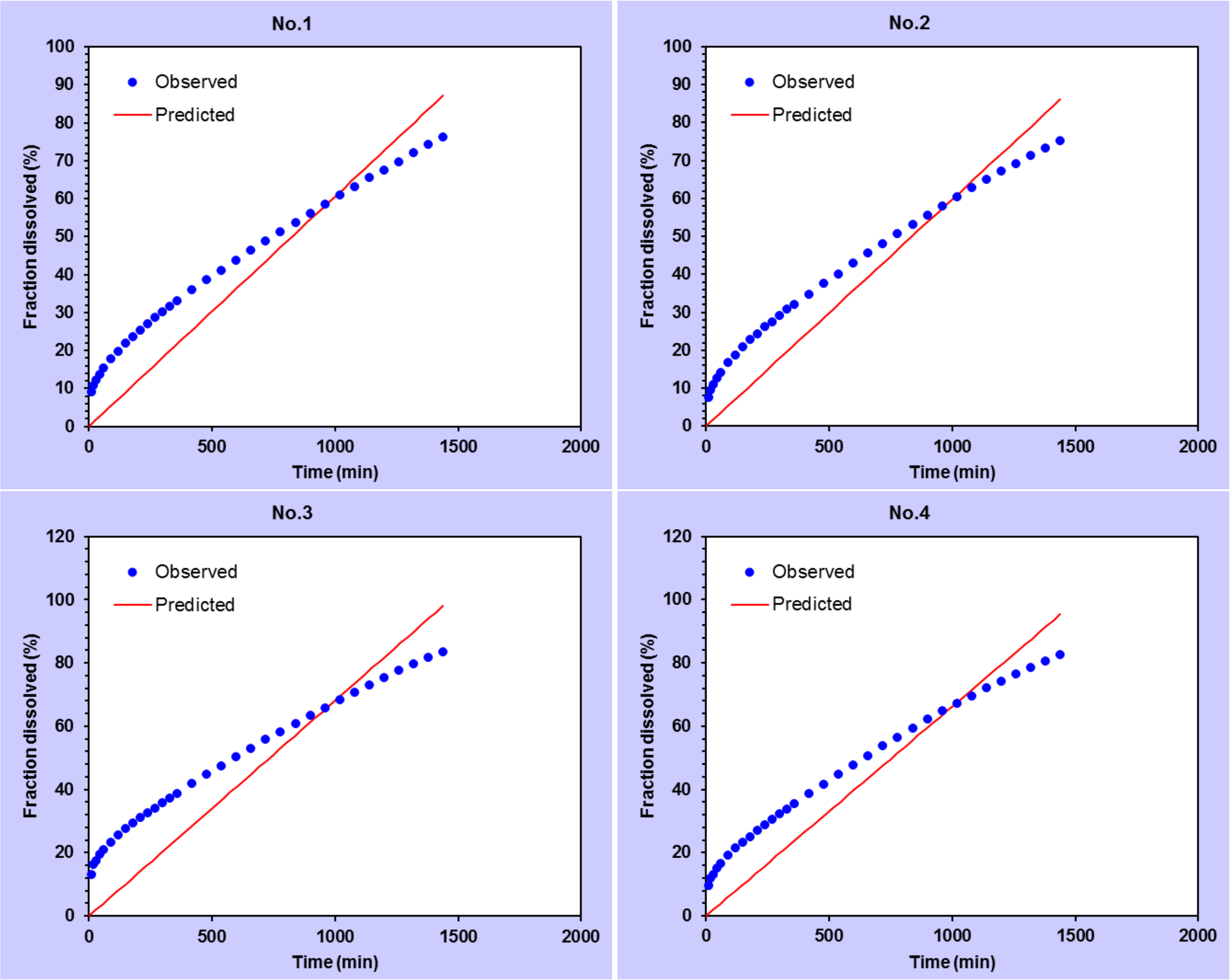

Supplementary materials – Model fitting summary – Starch 1500® sample with smaller particle size (modelling the entire relevant dissolution data)

Model: **Zero–order with T_{lag}**

Model equation: $F = k_0 \cdot (t - T_{lag})$

Fitted model parameters per tested tablet (N = 4) with statistics – mean, standard deviation (SD), and relative standard deviation expressed in % (RSD%) (output from DDSolver):

Parameter	No.1	No.2	No.3	No.4	Mean	SD	RSD(%)
k_0	0.045	0.046	0.047	0.050	0.047	0.002	4.514
T_{lag}	-320.972	-293.569	-419.328	-308.845	-335.679	56.882	-16.945

Number of dissolution data points (N), degrees of freedom (df), and selected goodness of fit criteria – Pearson correlation coefficient (R), coefficient of determination (R^2), adjusted coefficient of determination ($R^2_{adjusted}$), and residual sum of squares (RSS) (manual calculation in MS Excel):

Parameter	No.1	No.2	No.3	No.4
N	33	33	33	33
df	31	31	31	31
R	0.993998144	0.993484242	0.993797377	0.994139149
R^2	0.988032311	0.987010939	0.987633226	0.988312647
$R^2_{adjusted}$	0.987646257	0.986591937	0.987234298	0.987935636
RSS	167.0230413	184.7987089	187.8181962	198.5920342

Graphical abstract of model fit presented as mean ± 1 SD of the fraction % of released carvedilol:

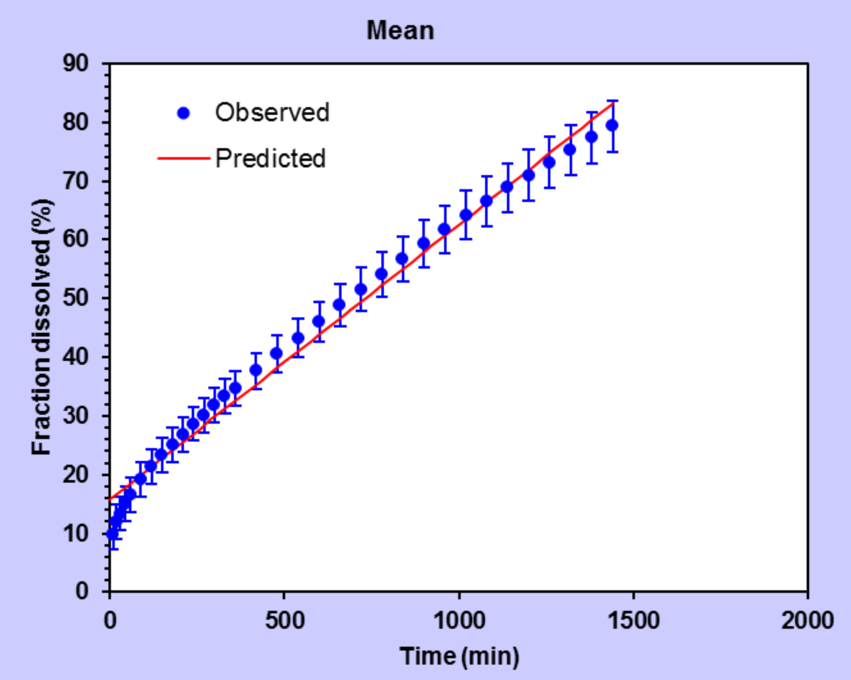

Supplementary materials – Model fitting summary – Starch 1500® sample with smaller particle size (modelling the entire relevant dissolution data)

Graphical abstract of model fit presented as the fraction % of released carvedilol per tested tablet:

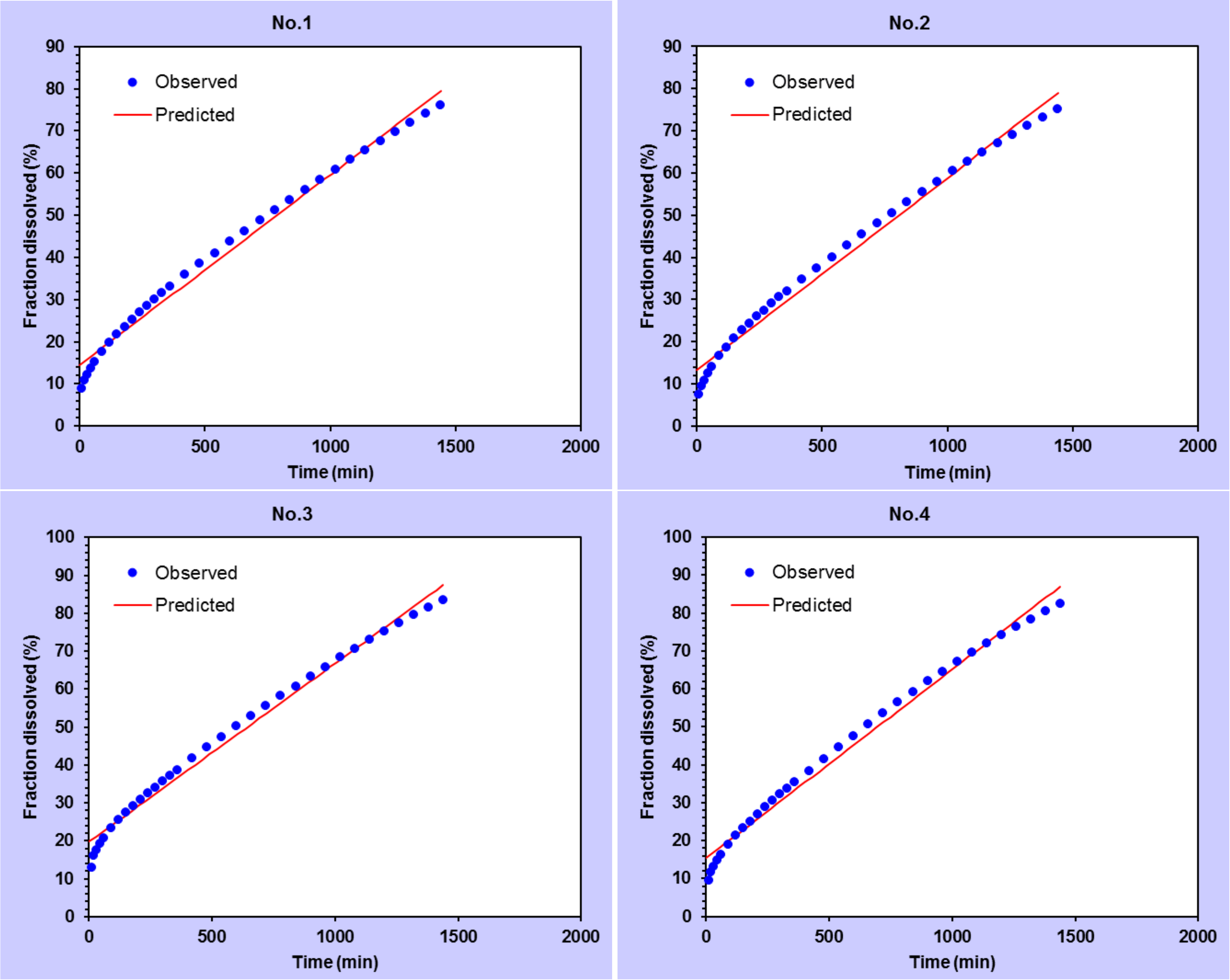

Supplementary materials – Model fitting summary – Starch 1500® sample with smaller particle size (modelling the entire relevant dissolution data)

Model: **Zero–order with F_0**

Model equation: $F = F_0 + k_0 \cdot t$

Fitted model parameters per tested tablet (N = 4) with statistics – mean, standard deviation (SD), and relative standard deviation expressed in % (RSD%) (output from DDSolver):

Parameter	No.1	No.2	No.3	No.4	Mean	SD	RSD(%)
k_0	0.045	0.046	0.047	0.050	0.047	0.002	4.514
F_0	14.474	13.359	19.721	15.369	15.731	2.785	17.701

Number of dissolution data points (N), degrees of freedom (df), and selected goodness of fit criteria – Pearson correlation coefficient (R), coefficient of determination (R^2), adjusted coefficient of determination ($R^2_{adjusted}$), and residual sum of squares (RSS) (manual calculation in MS Excel):

Parameter	No.1	No.2	No.3	No.4
N	33	33	33	33
df	31	31	31	31
R	0.993998144	0.993484242	0.993797377	0.994139149
R^2	0.988032311	0.987010939	0.987633226	0.988312647
$R^2_{adjusted}$	0.987646257	0.986591937	0.987234298	0.987935636
RSS	167.0230413	184.7987089	187.8181962	198.5920342

Graphical abstract of model fit presented as mean ± 1 SD of the fraction % of released carvedilol:

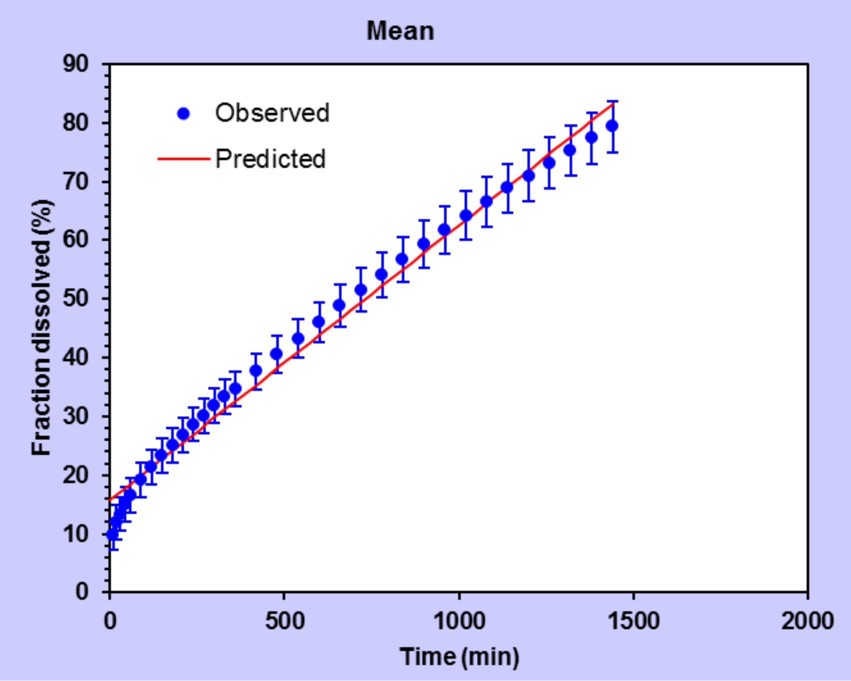

Supplementary materials – Model fitting summary – Starch 1500® sample with smaller particle size (modelling the entire relevant dissolution data)

Graphical abstract of model fit presented as the fraction % of released carvedilol per tested tablet:

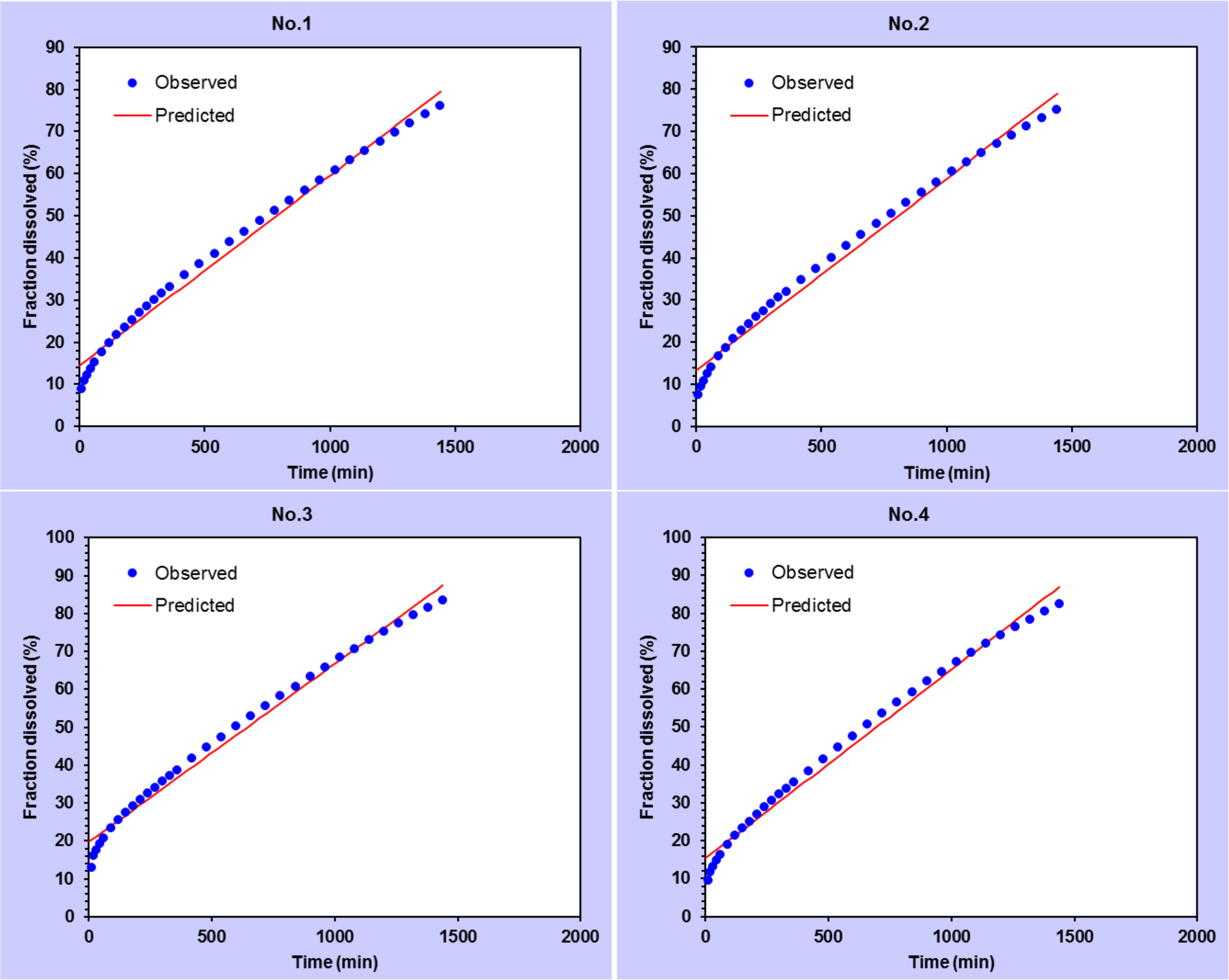

Supplementary materials – Model fitting summary – Starch 1500® sample with smaller particle size (modelling the entire relevant dissolution data)

Model: **First–order**

Model equation: $F = 100 \cdot (1 - e^{-k_1 \cdot t})$

Fitted model parameters per tested tablet (N = 4) with statistics – mean, standard deviation (SD), and relative standard deviation expressed in % (RSD%) (output from DDSolver):

Parameter	No.1	No.2	No.3	No.4	Mean	SD	RSD(%)
k_1	0.001	0.001	0.001	0.001	0.001	0.000	11.807

Number of dissolution data points (N), degrees of freedom (df), and selected goodness of fit criteria – Pearson correlation coefficient (R), coefficient of determination (R^2), adjusted coefficient of determination ($R^2_{adjusted}$), and residual sum of squares (RSS) (manual calculation in MS Excel):

Parameter	No.1	No.2	No.3	No.4
N	33	33	33	33
df	32	32	32	32
R	0.996776116	0.997243651	0.993836505	0.995112351
R^2	0.993562625	0.9944949	0.987710998	0.990248592
$R^2_{adjusted}$	0.993562625	0.9944949	0.987710998	0.990248592
RSS	918.6060554	725.3555229	1760.086958	843.3676552

Graphical abstract of model fit presented as mean ± 1 SD of the fraction % of released carvedilol:

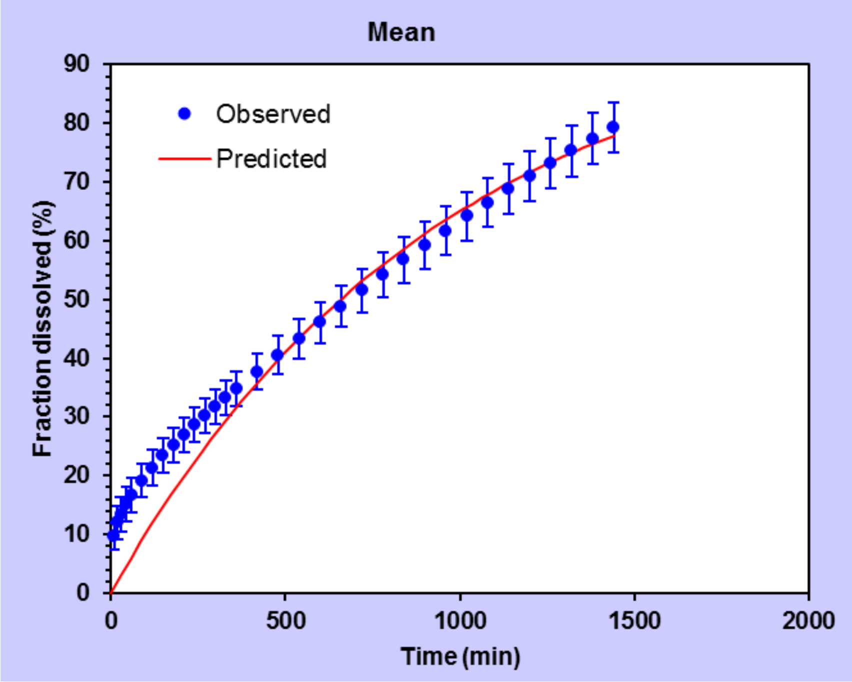

Supplementary materials – Model fitting summary – Starch 1500® sample with smaller particle size (modelling the entire relevant dissolution data)

Graphical abstract of model fit presented as the fraction % of released carvedilol per tested tablet:

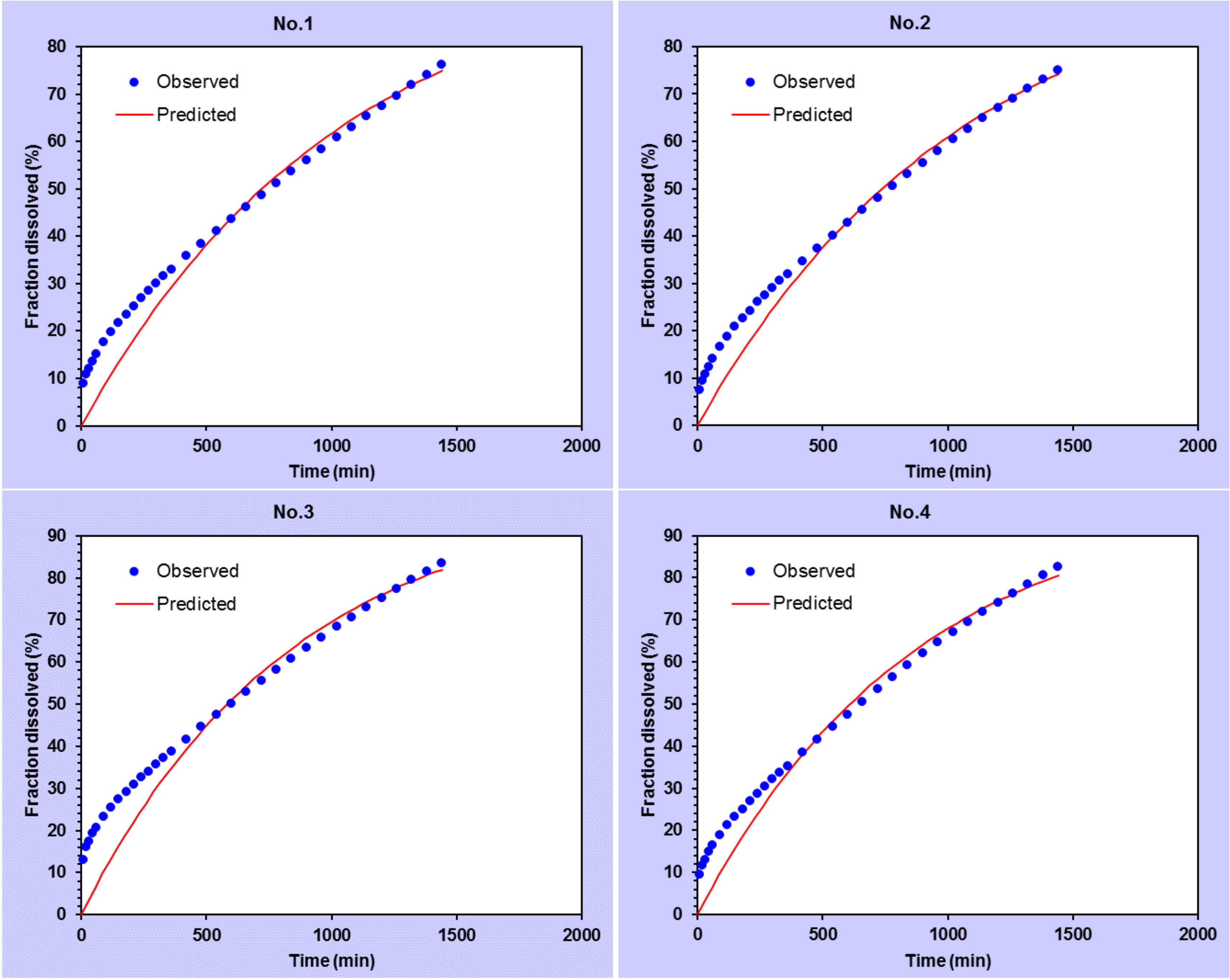

Supplementary materials – Model fitting summary – Starch 1500® sample with smaller particle size (modelling the entire relevant dissolution data)

Model: **First–order with T_{lag}**

Model equation: $F = 100 \cdot \left[1 - e^{-k_1 \cdot (t - T_{lag})}\right]$

Fitted model parameters per tested tablet (N = 4) with statistics – mean, standard deviation (SD), and relative standard deviation expressed in % (RSD%) (output from DDSolver):

Parameter	No.1	No.2	No.3	No.4	Mean	SD	RSD(%)
k_1	0.001	0.001	0.001	0.001	0.001	0.000	14.026
T_{lag}	-105.442	-119.917	-118.454	-67.982	-102.949	24.202	-23.509

Number of dissolution data points (N), degrees of freedom (df), and selected goodness of fit criteria – Pearson correlation coefficient (R), coefficient of determination (R^2), adjusted coefficient of determination ($R^2_{adjusted}$), and residual sum of squares (RSS) (manual calculation in MS Excel):

Parameter	No.1	No.2	No.3	No.4
N	33	33	33	33
df	31	31	31	31
R	0.997673255	0.998280858	0.995730919	0.996174353
R^2	0.995351923	0.996564672	0.991480063	0.992363341
$R^2_{adjusted}$	0.995201985	0.996453855	0.991205227	0.992116997
RSS	76.2690717	66.0359051	171.7762106	174.4747966

Graphical abstract of model fit presented as mean ± 1 SD of the fraction % of released carvedilol:

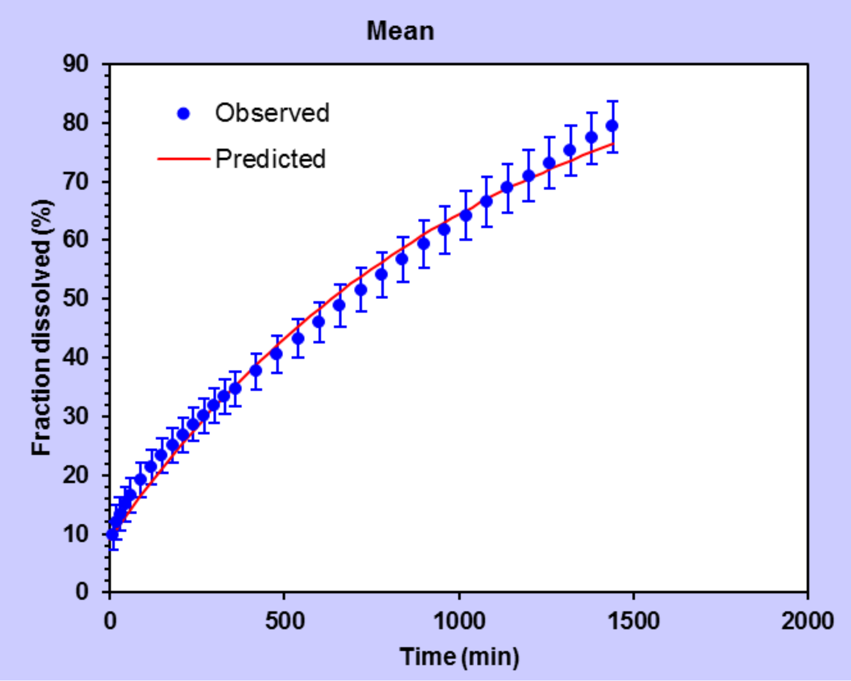

Supplementary materials – Model fitting summary – Starch 1500® sample with smaller particle size (modelling the entire relevant dissolution data)

Graphical abstract of model fit presented as the fraction % of released carvedilol per tested tablet:

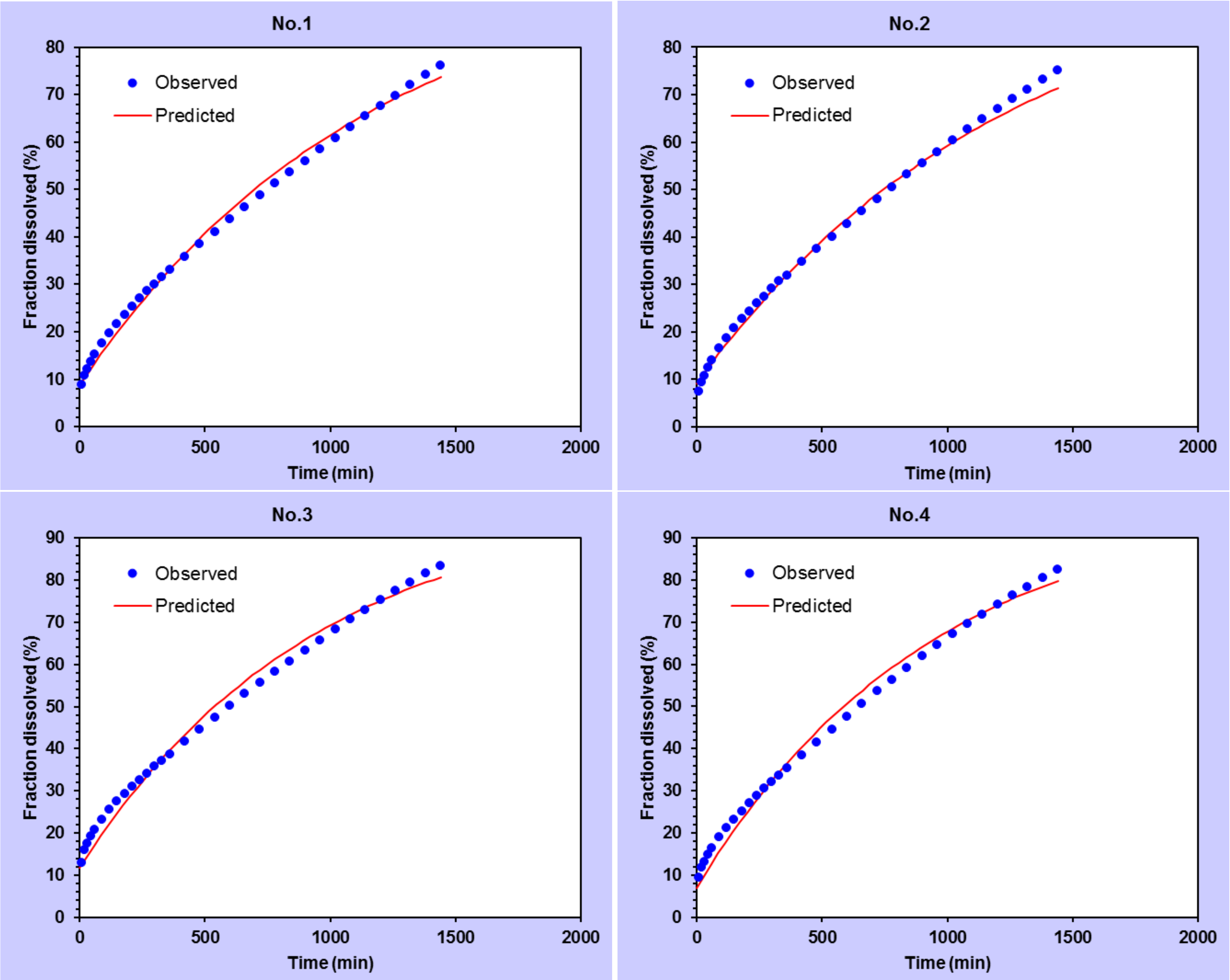

Supplementary materials – Model fitting summary – Starch 1500® sample with smaller particle size (modelling the entire relevant dissolution data)

Model: **First–order with F_{max}**

Model equation: $F = F_{max} \cdot (1 - e^{-k_1 \cdot t})$

Fitted model parameters per tested tablet (N = 4) with statistics – mean, standard deviation (SD), and relative standard deviation expressed in % (RSD%) (output from DDSolver):

Parameter	No.1	No.2	No.3	No.4	Mean	SD	RSD(%)
k_1	0.002	0.002	0.002	0.002	0.002	0.000	1.882
F_{max}	79.977	78.866	87.696	86.651	83.297	4.519	5.425

Number of dissolution data points (N), degrees of freedom (df), and selected goodness of fit criteria – Pearson correlation coefficient (R), coefficient of determination (R^2), adjusted coefficient of determination ($R^2_{adjusted}$), and residual sum of squares (RSS) (manual calculation in MS Excel):

Parameter	No.1	No.2	No.3	No.4
N	33	33	33	33
df	31	31	31	31
R	0.985597473	0.986000527	0.983956045	0.985149888
R^2	0.971402378	0.972197039	0.968169498	0.970520302
$R^2_{adjusted}$	0.970479874	0.97130017	0.967142707	0.969569344
RSS	777.5256943	670.0970126	1452.431138	883.3996718

Graphical abstract of model fit presented as mean ± 1 SD of the fraction % of released carvedilol:

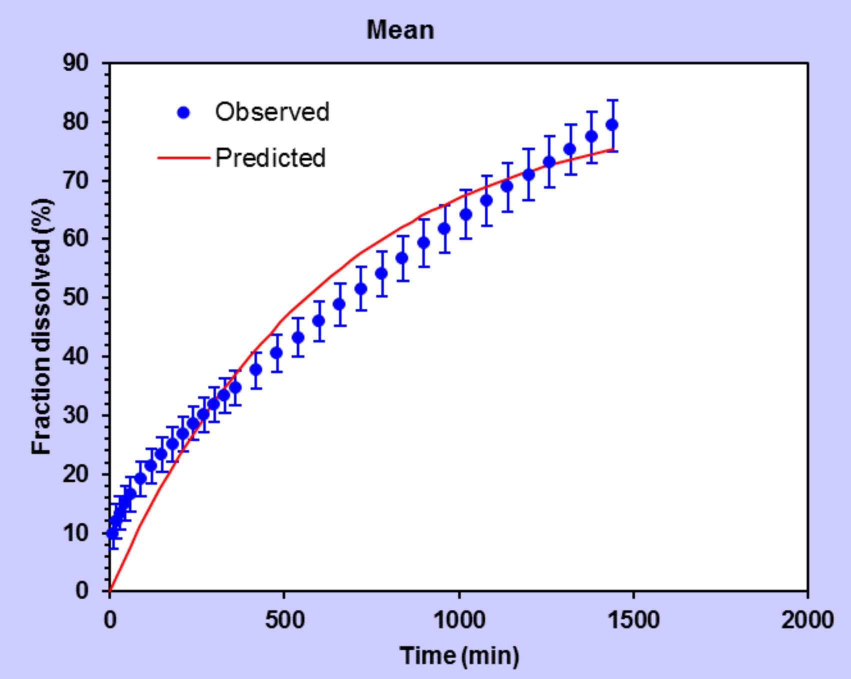

Supplementary materials – Model fitting summary – Starch 1500® sample with smaller particle size (modelling the entire relevant dissolution data)

Graphical abstract of model fit presented as the fraction % of released carvedilol per tested tablet:

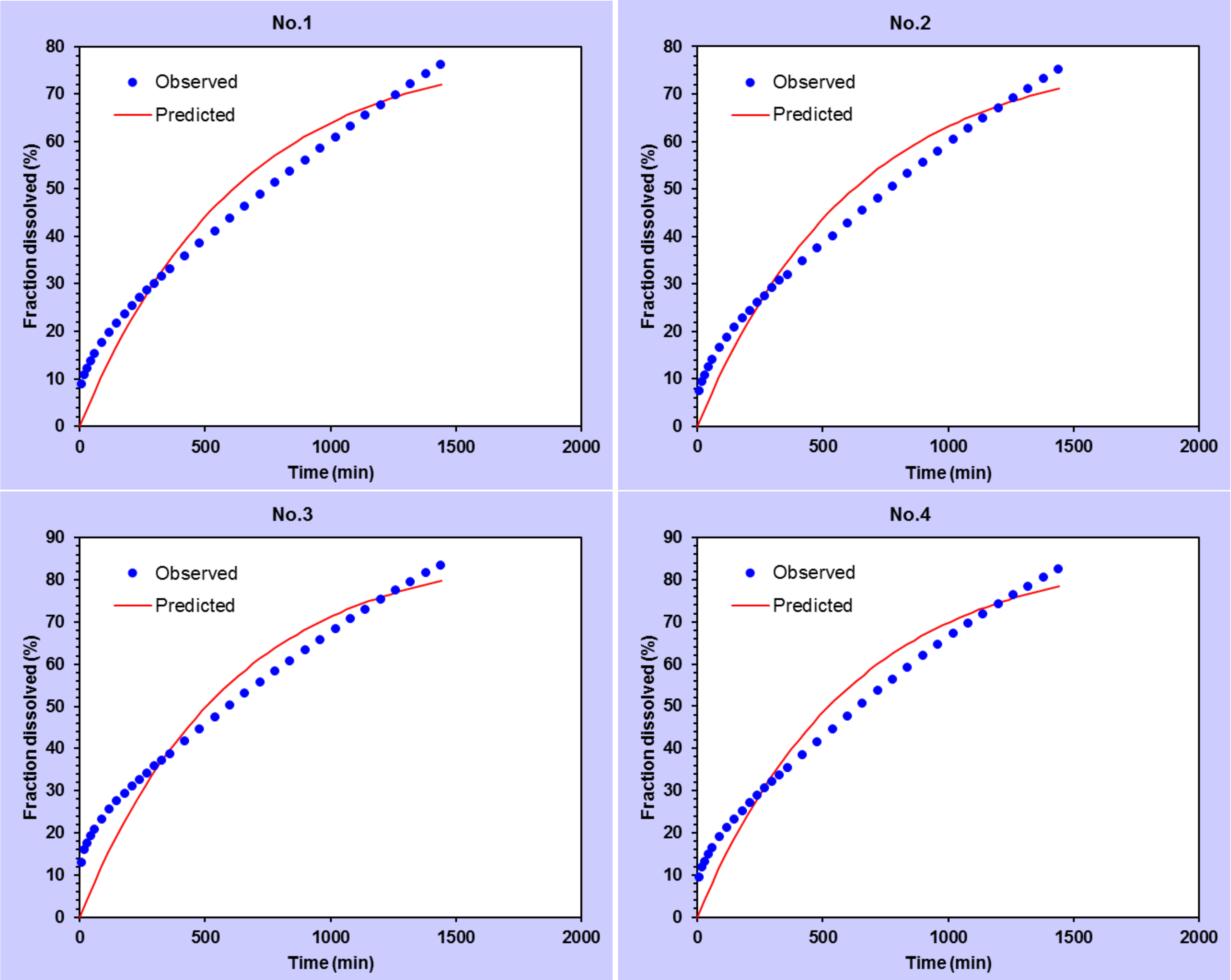

Supplementary materials – Model fitting summary – Starch 1500® sample with smaller particle size (modelling the entire relevant dissolution data)

Model: **First–order with T_{lag} and F_{max}**

Model equation: $F = F_{max} \cdot \left[1 - e^{-k_1 \cdot (t - T_{lag})}\right]$

Fitted model parameters per tested tablet (N = 4) with statistics – mean, standard deviation (SD), and relative standard deviation expressed in % (RSD%) (output from DDSolver):

Parameter	No.1	No.2	No.3	No.4	Mean	SD	RSD(%)
k_1	0.002	0.002	0.002	0.002	0.002	0.000	1.310
T_{lag}	7.262	18.496	-39.920	13.948	-0.053	26.975	-50528.875
F_{max}	79.977	78.866	87.696	86.651	83.297	4.519	5.425

Number of dissolution data points (N), degrees of freedom (df), and selected goodness of fit criteria – Pearson correlation coefficient (R), coefficient of determination (R^2), adjusted coefficient of determination ($R^2_{adjusted}$), and residual sum of squares (RSS) (manual calculation in MS Excel):

Parameter	No.1	No.2	No.3	No.4
N	33	33	33	33
df	30	30	30	30
R	0.985300976	0.985236314	0.985083645	0.984535594
R^2	0.970818013	0.970690594	0.970389787	0.969310335
$R^2_{adjusted}$	0.968872547	0.968736634	0.968415773	0.967264357
RSS	873.1823362	893.2259131	799.9017203	1099.619312

Graphical abstract of model fit presented as mean ± 1 SD of the fraction % of released carvedilol:

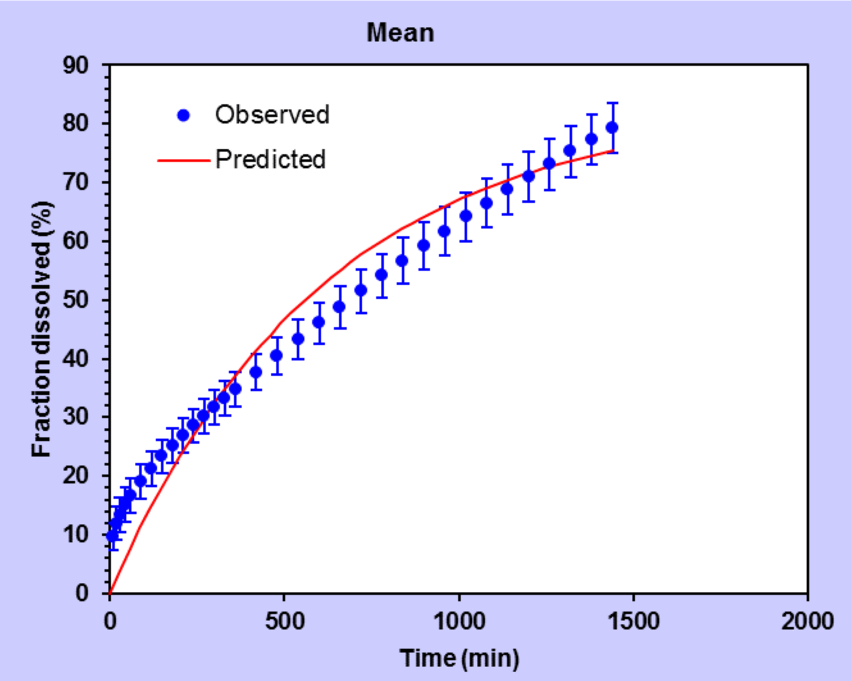

Supplementary materials – Model fitting summary – Starch 1500® sample with smaller particle size (modelling the entire relevant dissolution data)

Graphical abstract of model fit presented as the fraction % of released carvedilol per tested tablet:

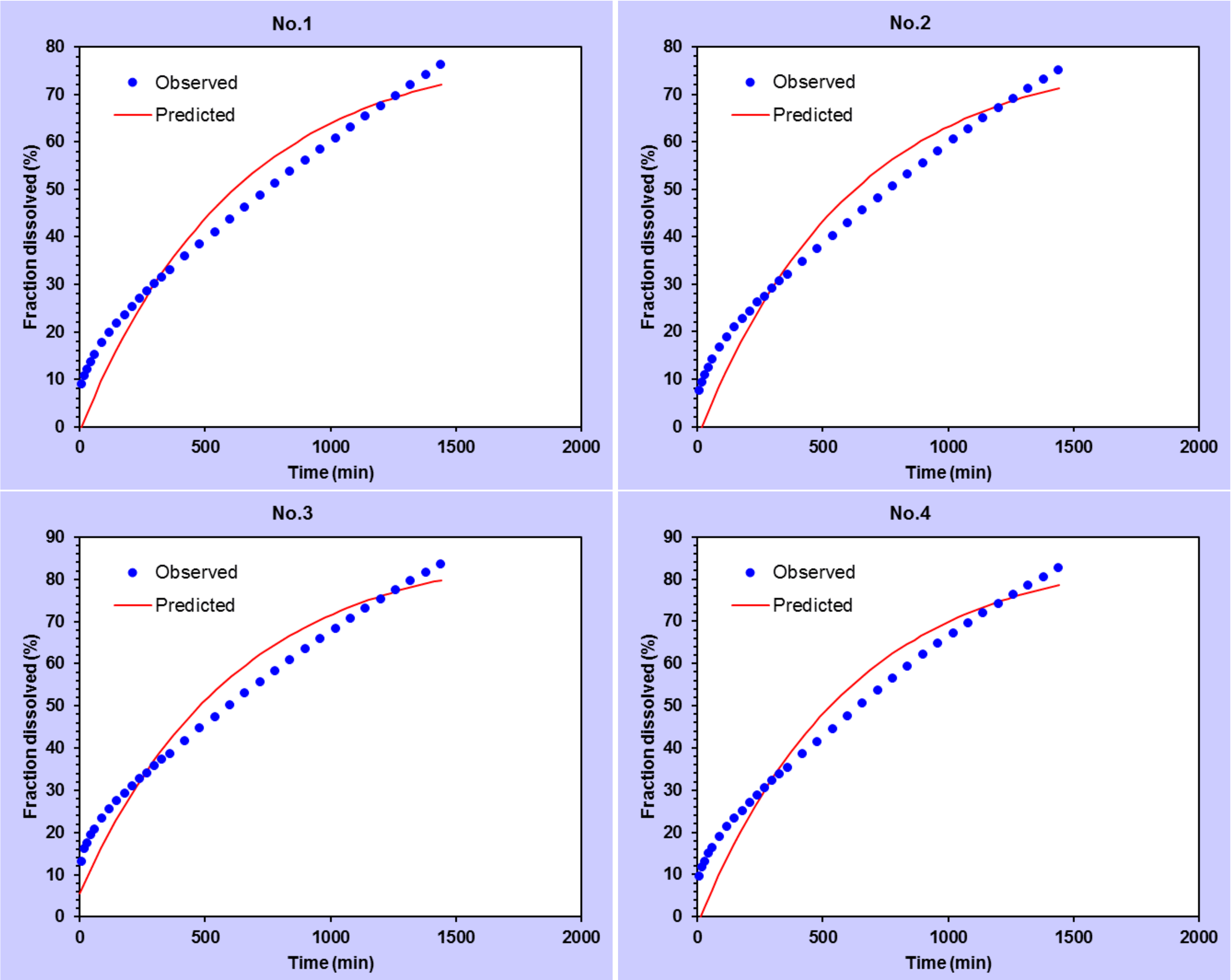

Supplementary materials – Model fitting summary – Starch 1500® sample with smaller particle size (modelling the entire relevant dissolution data)

Model: **Higuchi**

Model equation: $F = k_H \cdot t^{0.5}$

Fitted model parameters per tested tablet (N = 4) with statistics – mean, standard deviation (SD), and relative standard deviation expressed in % (RSD%) (output from DDSolver):

Parameter	No.1	No.2	No.3	No.4	Mean	SD	RSD(%)
k_H	1.888	1.859	2.142	2.061	1.988	0.136	6.850

Number of dissolution data points (N), degrees of freedom (df), and selected goodness of fit criteria – Pearson correlation coefficient (R), coefficient of determination (R^2), adjusted coefficient of determination ($R^2_{adjusted}$), and residual sum of squares (RSS) (manual calculation in MS Excel):

Parameter	No.1	No.2	No.3	No.4
N	33	33	33	33
df	32	32	32	32
R	0.994392538	0.994778261	0.994352932	0.993956127
R^2	0.98881652	0.989583788	0.988737753	0.987948782
$R^2_{adjusted}$	0.98881652	0.989583788	0.988737753	0.987948782
RSS	169.7778794	196.6358638	230.7480849	235.3646635

Graphical abstract of model fit presented as mean ± 1 SD of the fraction % of released carvedilol:

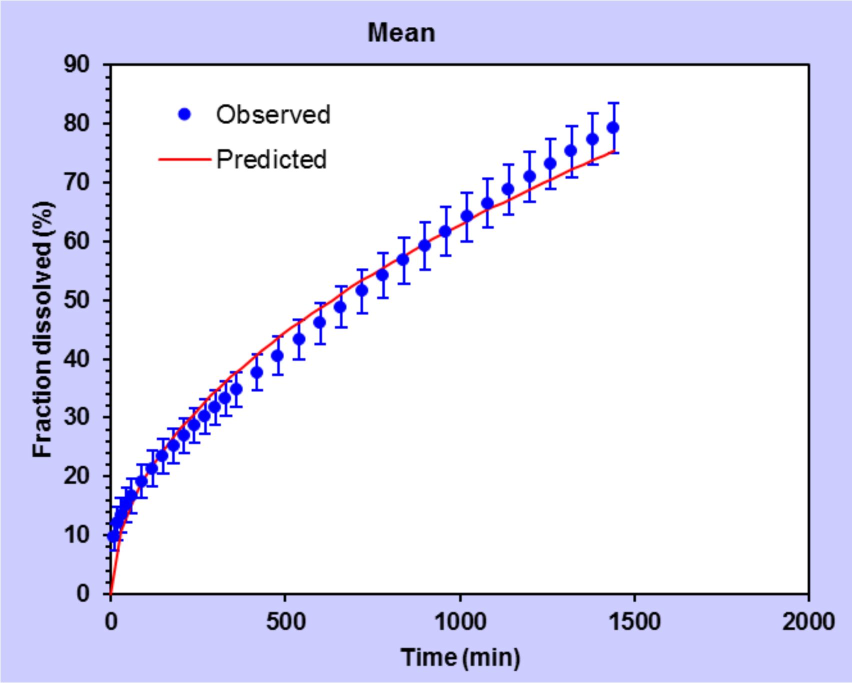

Supplementary materials – Model fitting summary – Starch 1500® sample with smaller particle size (modelling the entire relevant dissolution data)

Graphical abstract of model fit presented as the fraction % of released carvedilol per tested tablet:

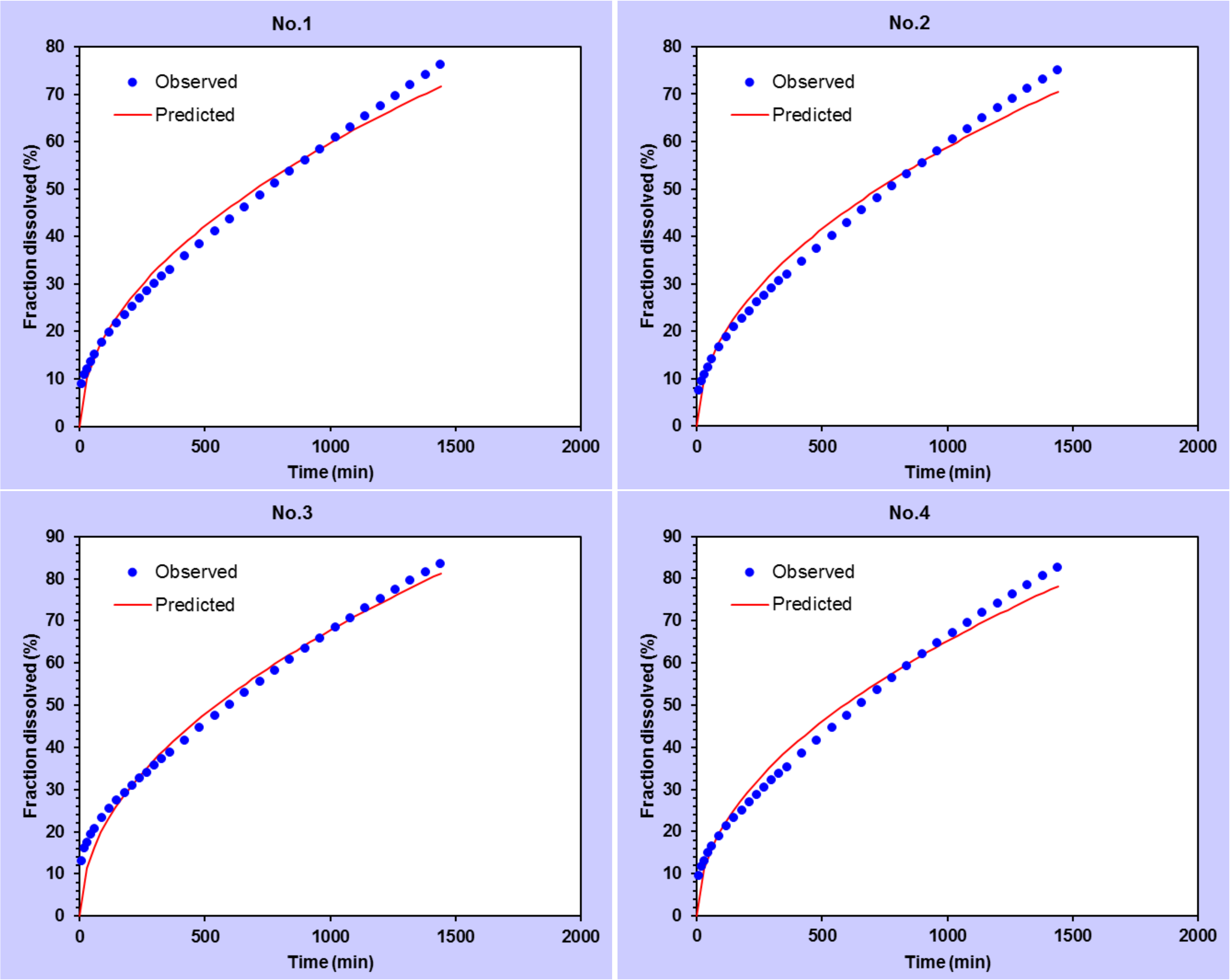

Supplementary materials – Model fitting summary – Starch 1500® sample with smaller particle size (modelling the entire relevant dissolution data)

Model: **Higuchi with T_{lag}**

Model equation: $F = k_H \cdot (t - T_{lag})^{0.5}$

Fitted model parameters per tested tablet (N = 4) with statistics – mean, standard deviation (SD), and relative standard deviation expressed in % (RSD%) (output from DDSolver):

Parameter	No.1	No.2	No.3	No.4	Mean	SD	RSD(%)
k_H	1.973	1.960	2.160	2.162	2.064	0.112	5.450
T_{lag}	47.321	56.614	7.989	51.463	40.847	22.233	54.430

Number of dissolution data points (N), degrees of freedom (df), and selected goodness of fit criteria – Pearson correlation coefficient (R), coefficient of determination (R^2), adjusted coefficient of determination ($R^2_{adjusted}$), and residual sum of squares (RSS) (manual calculation in MS Excel):

Parameter	No.1	No.2	No.3	No.4
N	33	33	33	33
df	31	31	31	31
R	0.986546597	0.987407249	0.992695699	0.985956845
R^2	0.973274189	0.974973075	0.98544475	0.9721109
$R^2_{adjusted}$	0.972412066	0.974165755	0.984975226	0.971211252
RSS	706.4583929	650.0364341	371.3870949	858.1368238

Graphical abstract of model fit presented as mean ± 1 SD of the fraction % of released carvedilol:

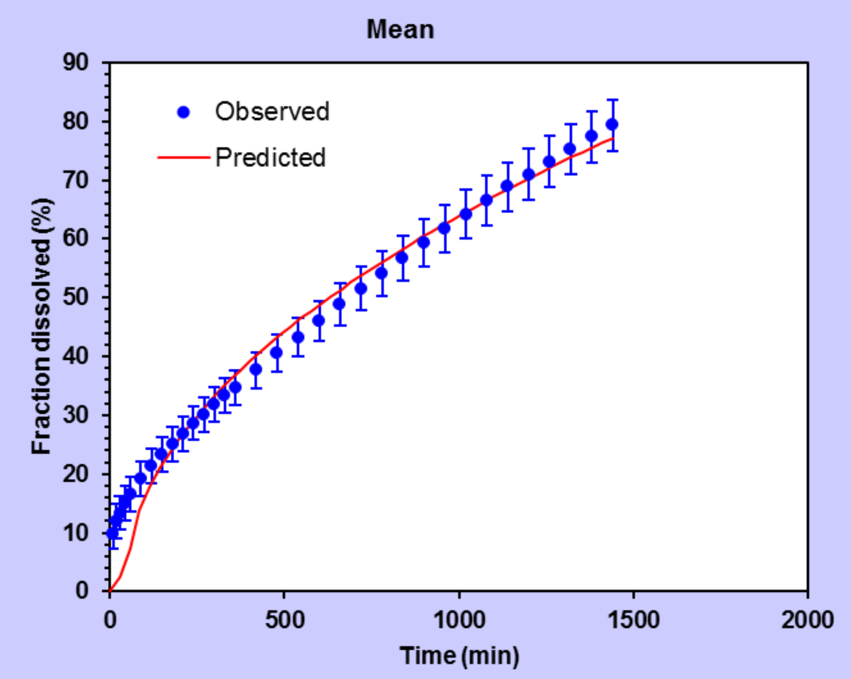

Supplementary materials – Model fitting summary – Starch 1500® sample with smaller particle size (modelling the entire relevant dissolution data)

Graphical abstract of model fit presented as the fraction % of released carvedilol per tested tablet:

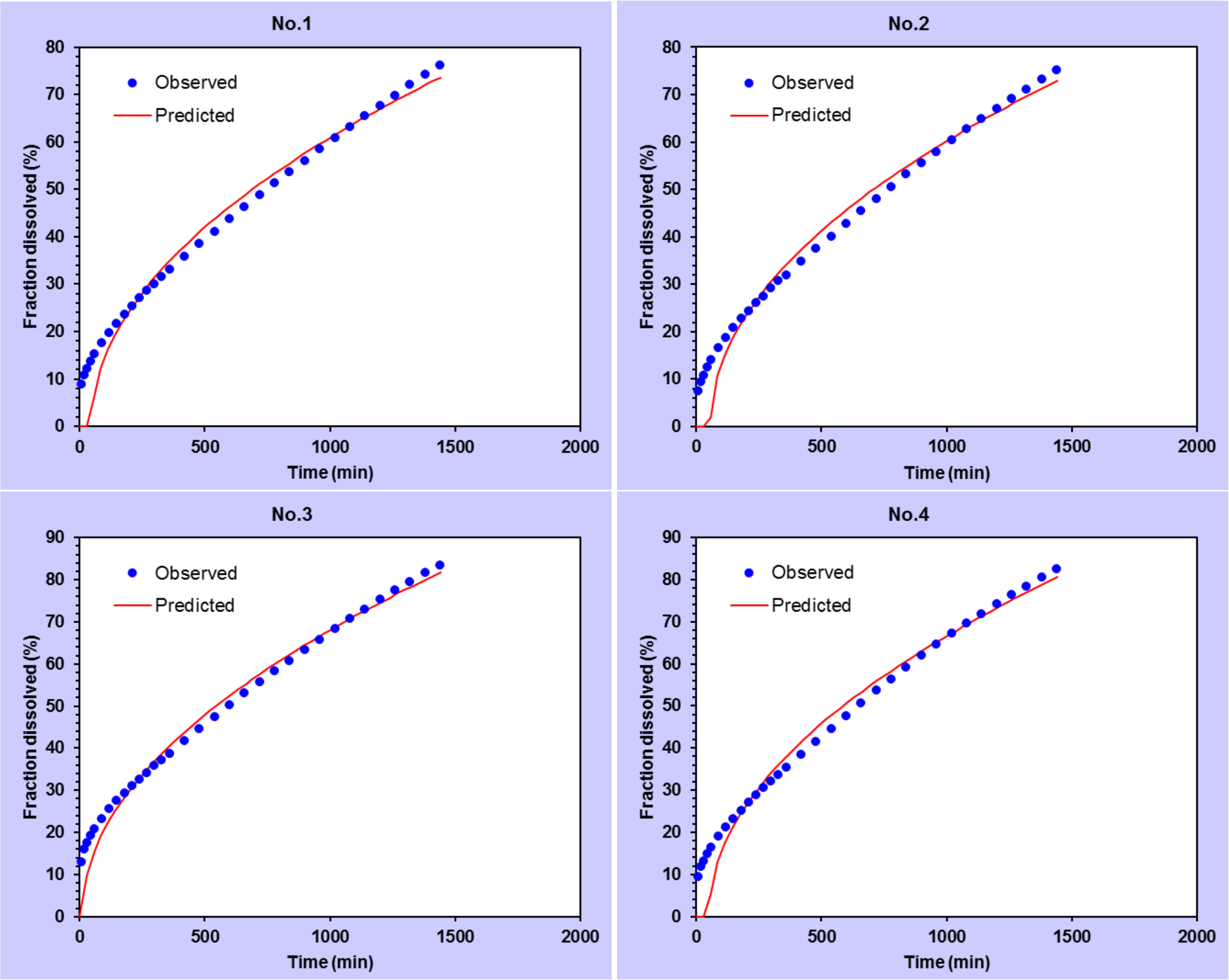

Supplementary materials – Model fitting summary – Starch 1500® sample with smaller particle size (modelling the entire relevant dissolution data)

Model: **Higuchi with F_0**

Model equation: $F = F_0 + k_H \cdot t^{0.5}$

Fitted model parameters per tested tablet (N = 4) with statistics – mean, standard deviation (SD), and relative standard deviation expressed in % (RSD%) (output from DDSolver):

Parameter	No.1	No.2	No.3	No.4	Mean	SD	RSD(%)
k_H	1.944	1.963	2.027	2.144	2.019	0.090	4.467
F_0	-1.475	-2.774	3.080	-2.205	-0.844	2.669	-316.415

Number of dissolution data points (N), degrees of freedom (df), and selected goodness of fit criteria – Pearson correlation coefficient (R), coefficient of determination (R^2), adjusted coefficient of determination ($R^2_{adjusted}$), and residual sum of squares (RSS) (manual calculation in MS Excel):

Parameter	No.1	No.2	No.3	No.4
N	33	33	33	33
df	31	31	31	31
R	0.994392538	0.994778261	0.994352932	0.993956127
R^2	0.98881652	0.989583788	0.988737753	0.987948782
$R^2_{adjusted}$	0.988455762	0.989247781	0.988374455	0.987560033
RSS	156.0784994	148.19412	171.0433887	204.7748474

Graphical abstract of model fit presented as mean ± 1 SD of the fraction % of released carvedilol:

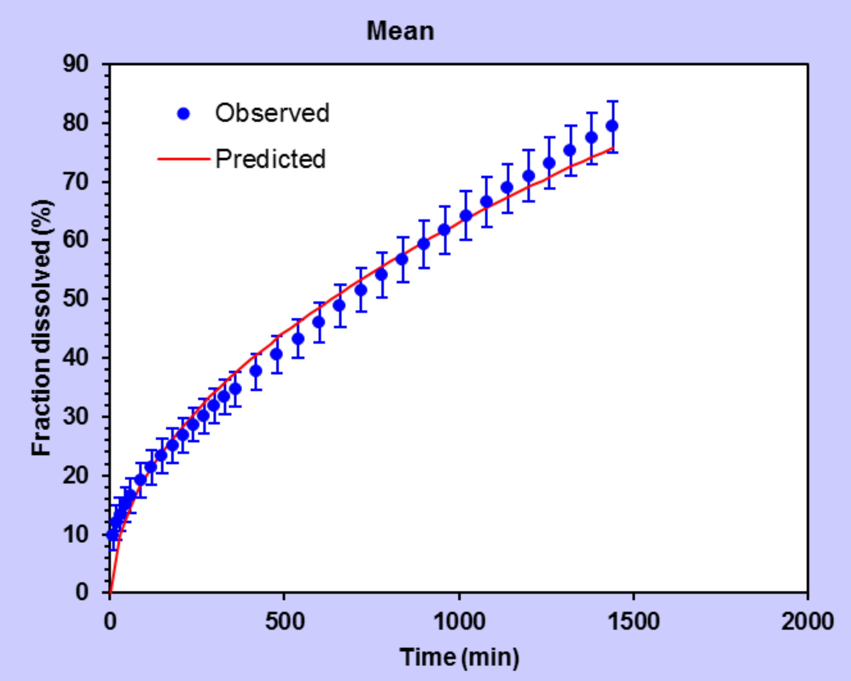

Supplementary materials – Model fitting summary – Starch 1500® sample with smaller particle size (modelling the entire relevant dissolution data)

Graphical abstract of model fit presented as the fraction % of released carvedilol per tested tablet:

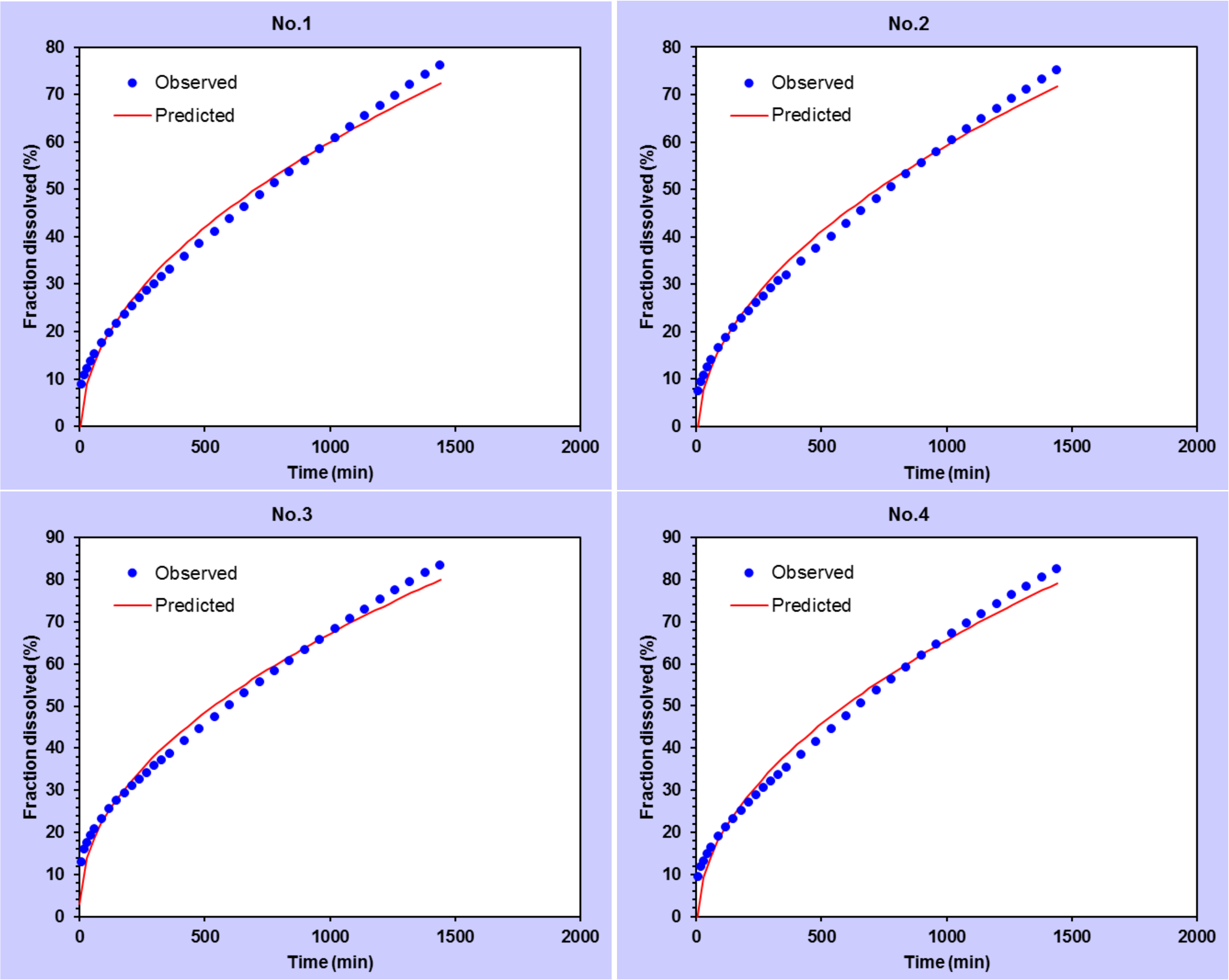

Supplementary materials – Model fitting summary – Starch 1500® sample with smaller particle size (modelling the entire relevant dissolution data)

Model: **Korsmeyer–Peppas**

Model equation: $F = k_{KP} \cdot t^n$

Fitted model parameters per tested tablet (N = 4) with statistics – mean, standard deviation (SD), and relative standard deviation expressed in % (RSD%) (output from DDSolver):

Parameter	No.1	No.2	No.3	No.4	Mean	SD	RSD(%)
k_{KP}	2.398	1.956	4.235	2.521	2.777	1.001	36.047
n	0.459	0.488	0.393	0.465	0.451	0.041	9.034

Number of dissolution data points (N), degrees of freedom (df), and selected goodness of fit criteria – Pearson correlation coefficient (R), coefficient of determination (R^2), adjusted coefficient of determination ($R^2_{adjusted}$), and residual sum of squares (RSS) (manual calculation in MS Excel):

Parameter	No.1	No.2	No.3	No.4
N	33	33	33	33
df	31	31	31	31
R	0.991964585	0.994137572	0.986845673	0.991786197
R^2	0.983993738	0.988309513	0.973864383	0.98363986
$R^2_{adjusted}$	0.983477407	0.987932401	0.973021298	0.983112114
RSS	384.9708365	297.2580873	602.7599637	495.4542745

Graphical abstract of model fit presented as mean ± 1 SD of the fraction % of released carvedilol:

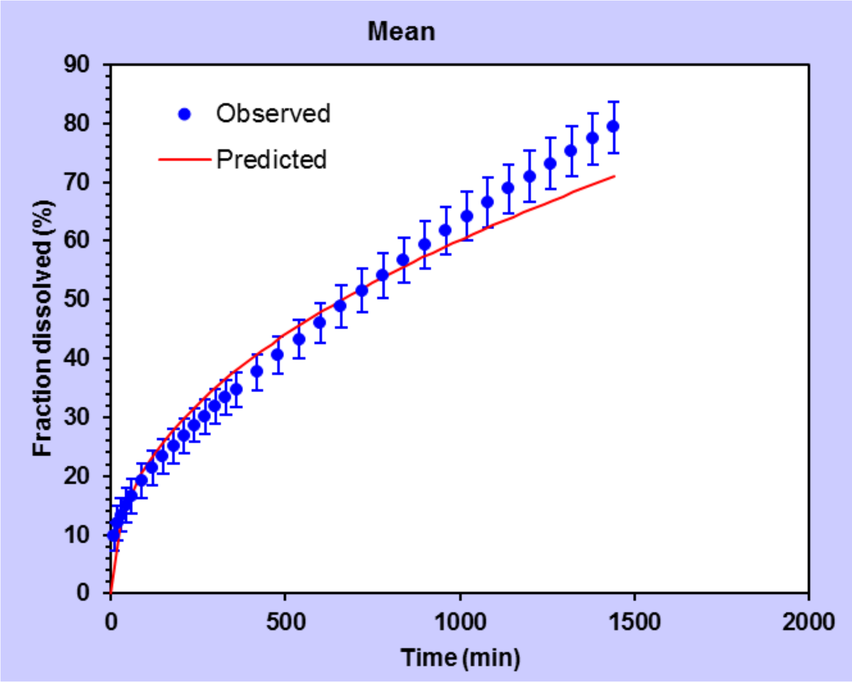

Supplementary materials – Model fitting summary – Starch 1500® sample with smaller particle size (modelling the entire relevant dissolution data)

Graphical abstract of model fit presented as the fraction % of released carvedilol per tested tablet:

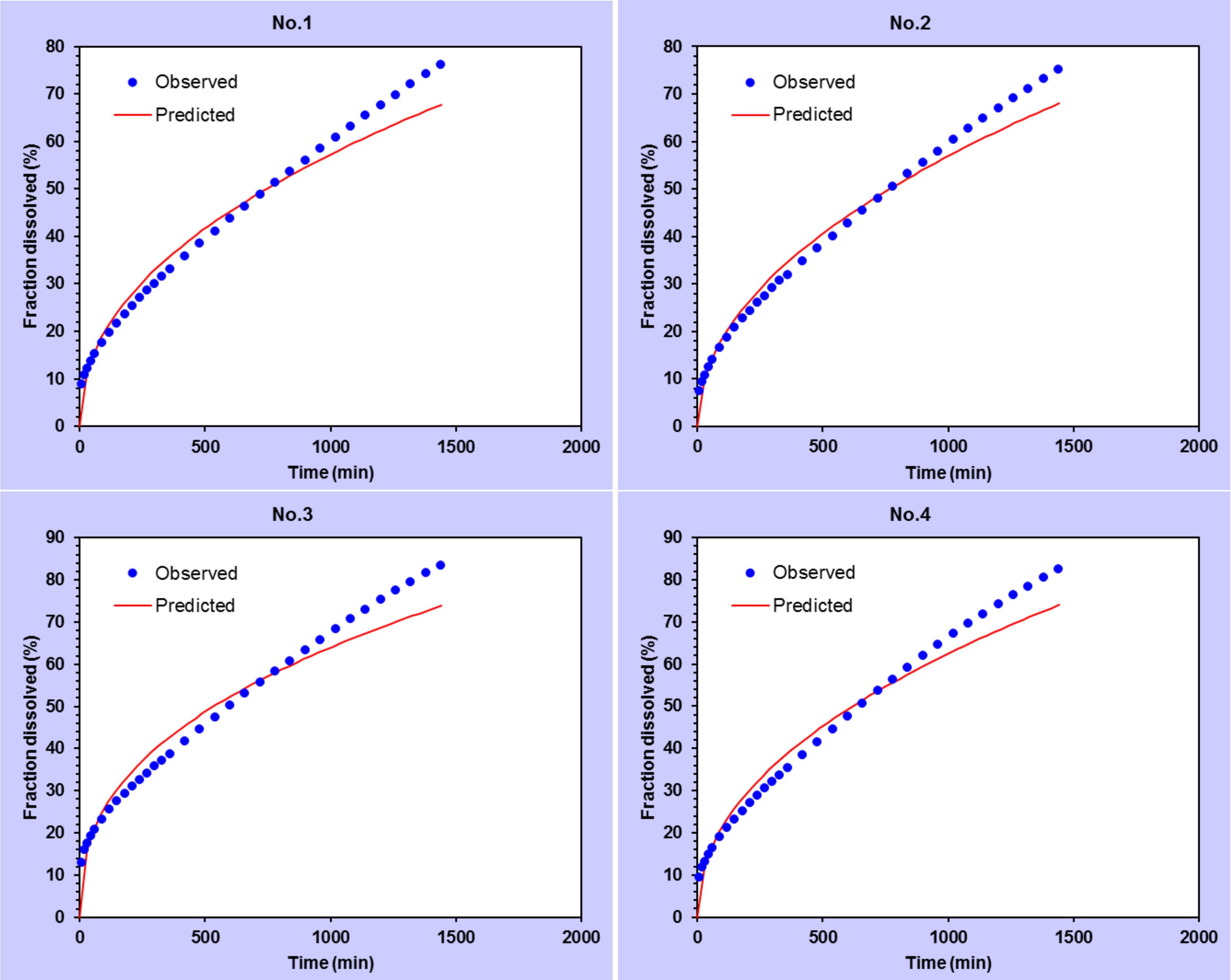

Supplementary materials – Model fitting summary – Starch 1500® sample with smaller particle size (modelling the entire relevant dissolution data)

Model: **Korsmeyer–Peppas with T_{lag}**

Model equation: $F = k_{KP} \cdot (t - T_{lag})^n$

Fitted model parameters per tested tablet (N = 4) with statistics – mean, standard deviation (SD), and relative standard deviation expressed in % (RSD%) (output from DDSolver):

Parameter	No.1	No.2	No.3	No.4	Mean	SD	RSD(%)
k_{KP}	2.863	2.356	4.935	3.017	3.293	1.131	34.334
n	0.432	0.459	0.369	0.437	0.424	0.039	9.089
T_{lag}	4.000	4.000	4.000	4.000	4.000	0.000	0.000

Number of dissolution data points (N), degrees of freedom (df), and selected goodness of fit criteria – Pearson correlation coefficient (R), coefficient of determination (R^2), adjusted coefficient of determination ($R^2_{adjusted}$), and residual sum of squares (RSS) (manual calculation in MS Excel):

Parameter	No.1	No.2	No.3	No.4
N	No.1	No.2	No.3	No.4
df	33	33	33	33
R	30	30	30	30
R^2	0.988783585	0.991460413	0.982769989	0.988537523
$R^2_{adjusted}$	0.977692978	0.98299375	0.965836852	0.977206435
RSS	560.2859056	457.2830544	811.8335583	716.7212725

Graphical abstract of model fit presented as mean ± 1 SD of the fraction % of released carvedilol:

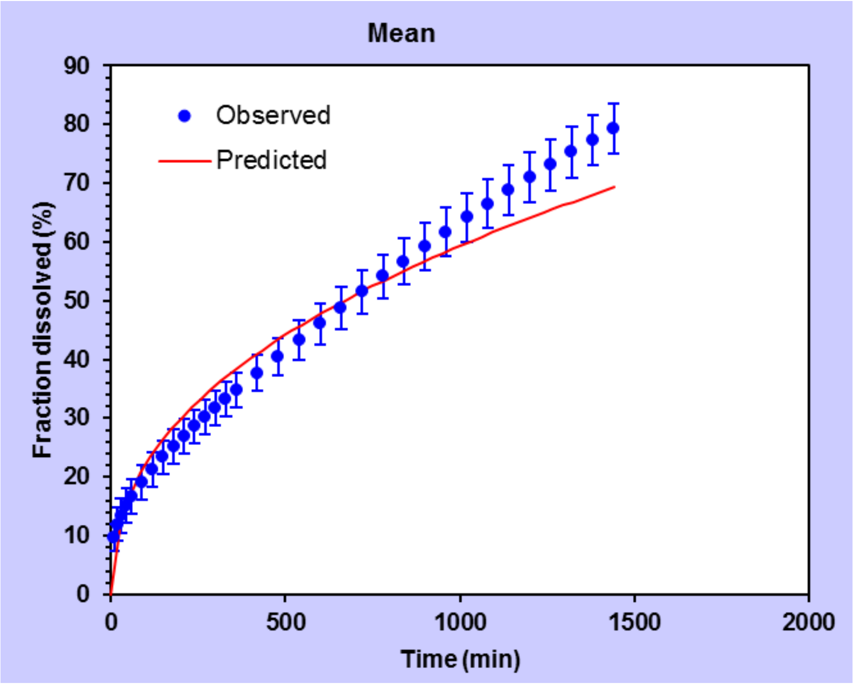

Supplementary materials – Model fitting summary – Starch 1500® sample with smaller particle size (modelling the entire relevant dissolution data)

Graphical abstract of model fit presented as the fraction % of released carvedilol per tested tablet:

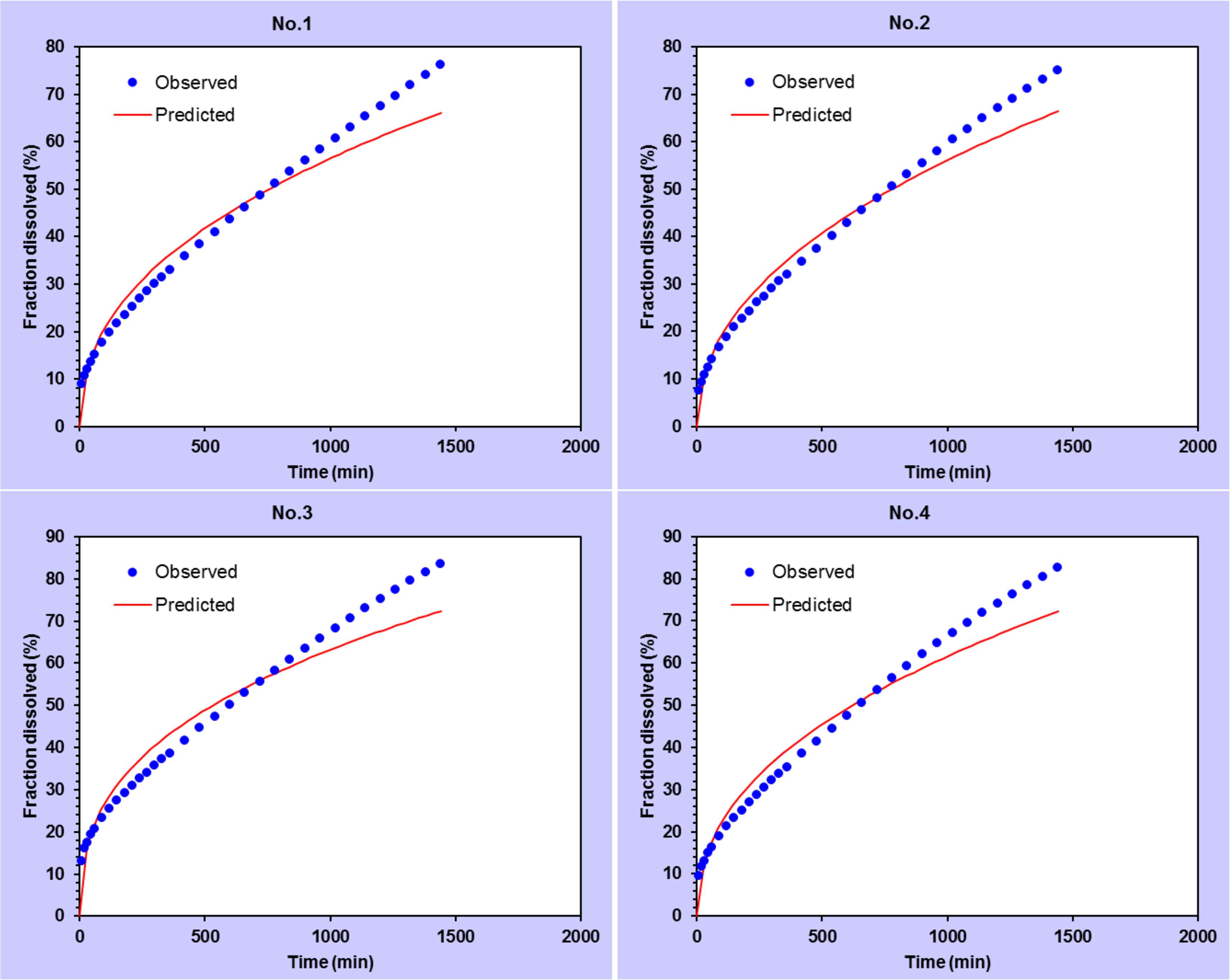

Supplementary materials – Model fitting summary – Starch 1500® sample with smaller particle size (modelling the entire relevant dissolution data)

Model: **Korsmeyer–Peppas with F_0**

Model equation: $F = F_0 + k_{KP} \cdot t^n$

Fitted model parameters per tested tablet (N = 4) with statistics – mean, standard deviation (SD), and relative standard deviation expressed in % (RSD%) (output from DDSolver):

Parameter	No.1	No.2	No.3	No.4	Mean	SD	RSD(%)
k_{KP}	1.290	1.084	2.262	1.362	1.500	0.522	34.801
n	0.542	0.568	0.473	0.547	0.533	0.041	7.727
F_0	3.559	2.999	5.199	3.799	3.889	0.935	24.046

Number of dissolution data points (N), degrees of freedom (df), and selected goodness of fit criteria – Pearson correlation coefficient (R), coefficient of determination (R^2), adjusted coefficient of determination ($R^2_{adjusted}$), and residual sum of squares (RSS) (manual calculation in MS Excel):

Parameter	No.1	No.2	No.3	No.4
N	33	33	33	33
df	30	30	30	30
R	0.996386967	0.997617068	0.992817274	0.996253064
R^2	0.992786989	0.995239814	0.98568614	0.992520168
$R^2_{adjusted}$	0.992306122	0.994922468	0.984731883	0.992021512
RSS	192.3609036	127.5462049	368.2073566	256.8350218

Graphical abstract of model fit presented as mean ± 1 SD of the fraction % of released carvedilol:

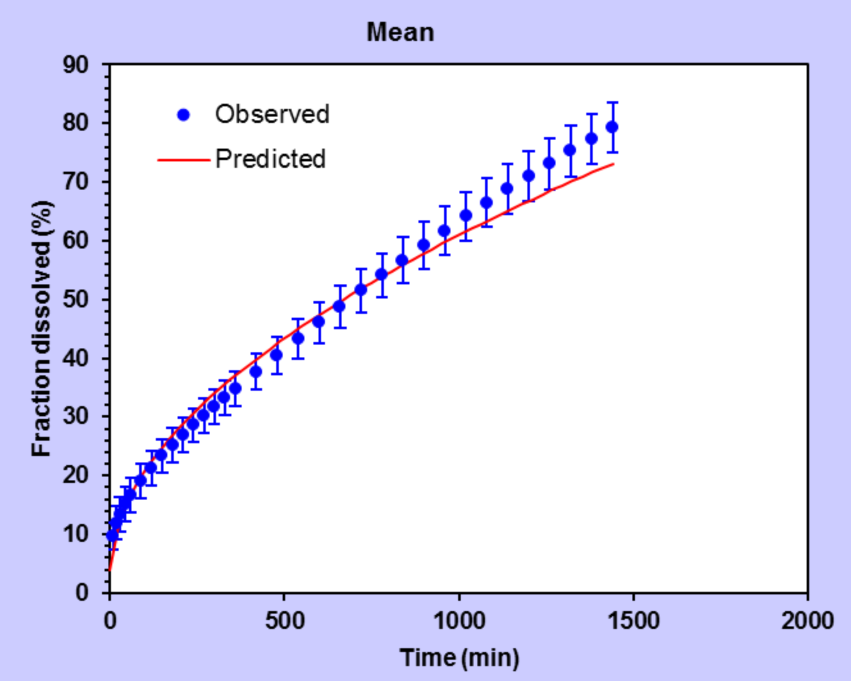

Supplementary materials – Model fitting summary – Starch 1500® sample with smaller particle size (modelling the entire relevant dissolution data)

Graphical abstract of model fit presented as the fraction % of released carvedilol per tested tablet:

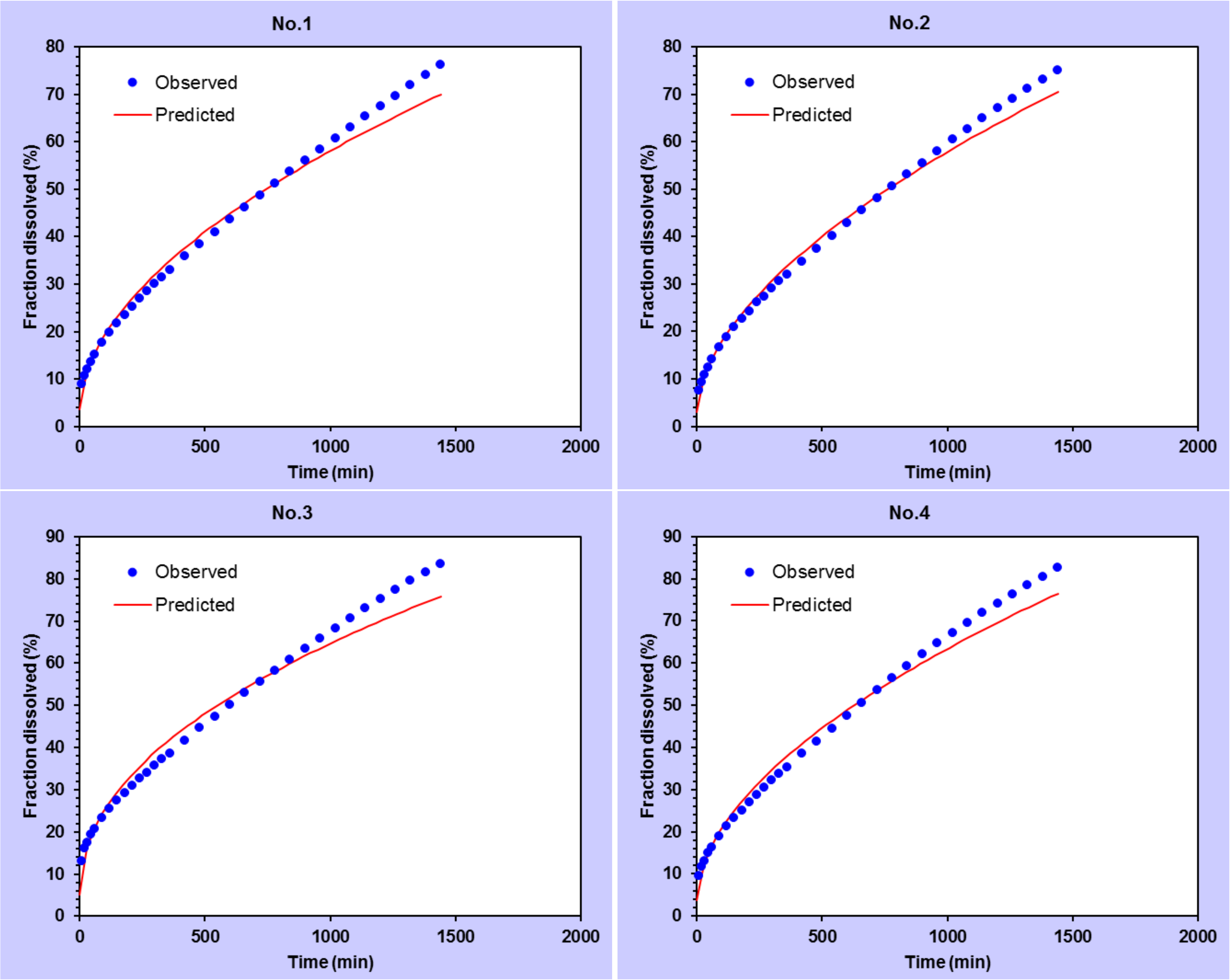

Supplementary materials – Model fitting summary – Starch 1500® sample with smaller particle size (modelling the entire relevant dissolution data)

Model: **Hixson–Crowell**

Model equation: $F = 100 \cdot [1 - (1 - k_{HC} \cdot t)^3]$

Fitted model parameters per tested tablet (N = 4) with statistics – mean, standard deviation (SD), and relative standard deviation expressed in % (RSD%) (output from DDSolver):

Parameter	No.1	No.2	No.3	No.4	Mean	SD	RSD(%)
k_{HC}	0.0003	0.0003	0.0003	0.0003	0.0003	0.0000	9.6681

Number of dissolution data points (N), degrees of freedom (df), and selected goodness of fit criteria – Pearson correlation coefficient (R), coefficient of determination (R^2), adjusted coefficient of determination ($R^2_{adjusted}$), and residual sum of squares (RSS) (manual calculation in MS Excel):

Parameter	No.1	No.2	No.3	No.4
N	33	33	33	33
df	32	32	32	32
R	0.998553683	0.998665008	0.997777317	0.998630909
R^2	0.997109458	0.997331798	0.995559575	0.997263693
$R^2_{adjusted}$	0.997109458	0.997331798	0.995559575	0.997263693
RSS	1326.00153	1082.42654	2484.112493	1266.144462

Graphical abstract of model fit presented as mean ± 1 SD of the fraction % of released carvedilol:

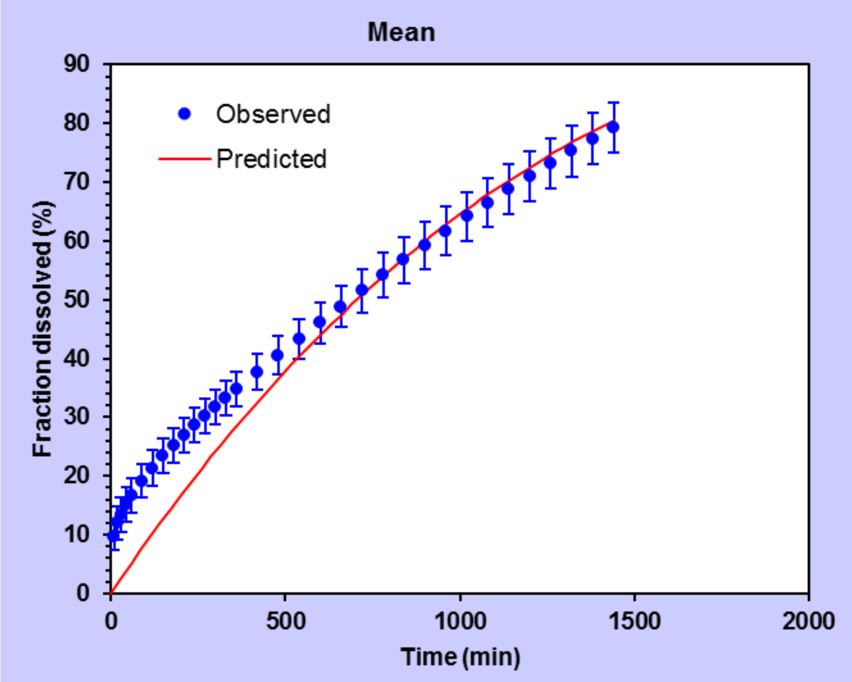

Supplementary materials – Model fitting summary – Starch 1500® sample with smaller particle size (modelling the entire relevant dissolution data)

Graphical abstract of model fit presented as the fraction % of released carvedilol per tested tablet:

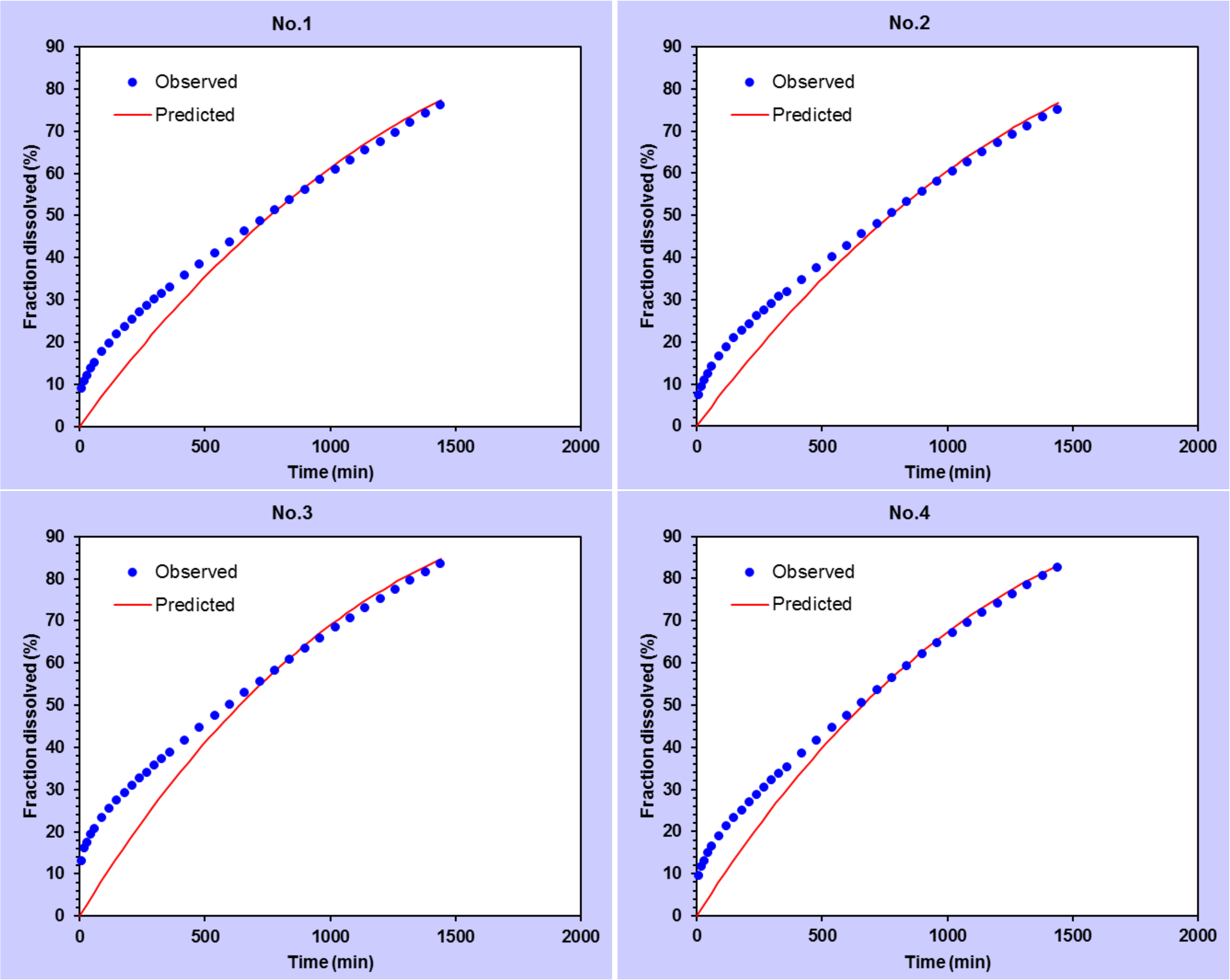

Supplementary materials – Model fitting summary – Starch 1500® sample with smaller particle size (modelling the entire relevant dissolution data)

Model: **Hixson–Crowell with T_{lag}**

Model equation: $F = 100 \cdot \left\{1 - \left[1 - k_{HC} \cdot \left(t - T_{lag}\right)\right]^3\right\}$

Fitted model parameters per tested tablet (N = 4) with statistics – mean, standard deviation (SD), and relative standard deviation expressed in % (RSD%) (output from DDSolver):

Parameter	No.1	No.2	No.3	No.4	Mean	SD	RSD(%)
k_{HC}	0.0002	0.0002	0.0003	0.0003	0.0002	0.0000	9.0136
T_{lag}	-170.3735	-154.1298	-206.3073	-139.6243	-167.6087	28.6940	-17.1196

Number of dissolution data points (N), degrees of freedom (df), and selected goodness of fit criteria – Pearson correlation coefficient (R), coefficient of determination (R^2), adjusted coefficient of determination ($R^2_{adjusted}$), and residual sum of squares (RSS) (manual calculation in MS Excel):

Parameter	No.1	No.2	No.3	No.4
N	33	33	33	33
df	31	31	31	31
R	0.998724808	0.998724423	0.998509014	0.999068818
R^2	0.997451242	0.997450472	0.99702025	0.998138503
$R^2_{adjusted}$	0.997369024	0.99736823	0.996924129	0.998078454
RSS	35.69035697	36.27932589	47.01425148	33.21242127

Graphical abstract of model fit presented as mean ± 1 SD of the fraction % of released carvedilol:

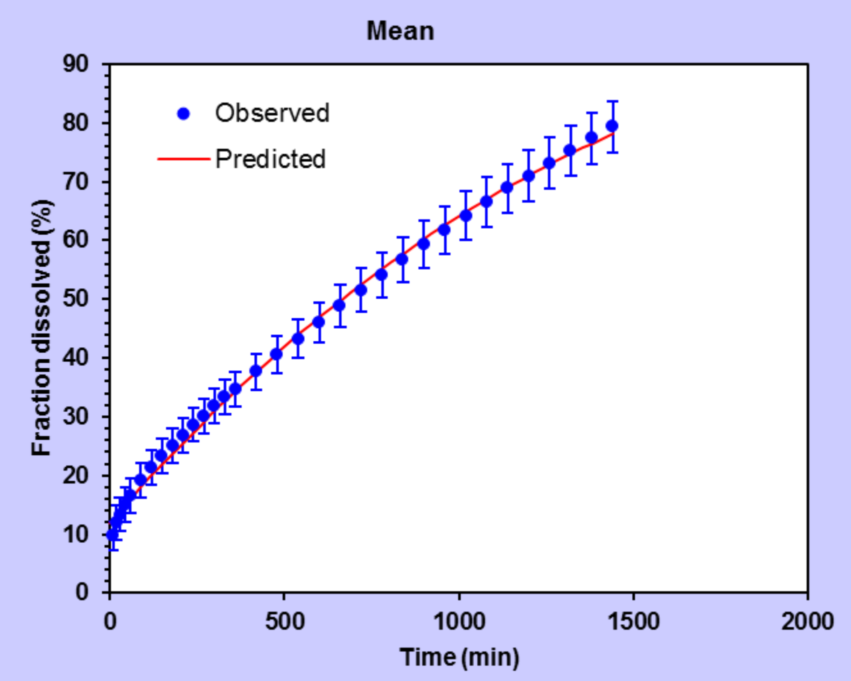

Supplementary materials – Model fitting summary – Starch 1500® sample with smaller particle size (modelling the entire relevant dissolution data)

Graphical abstract of model fit presented as the fraction % of released carvedilol per tested tablet:

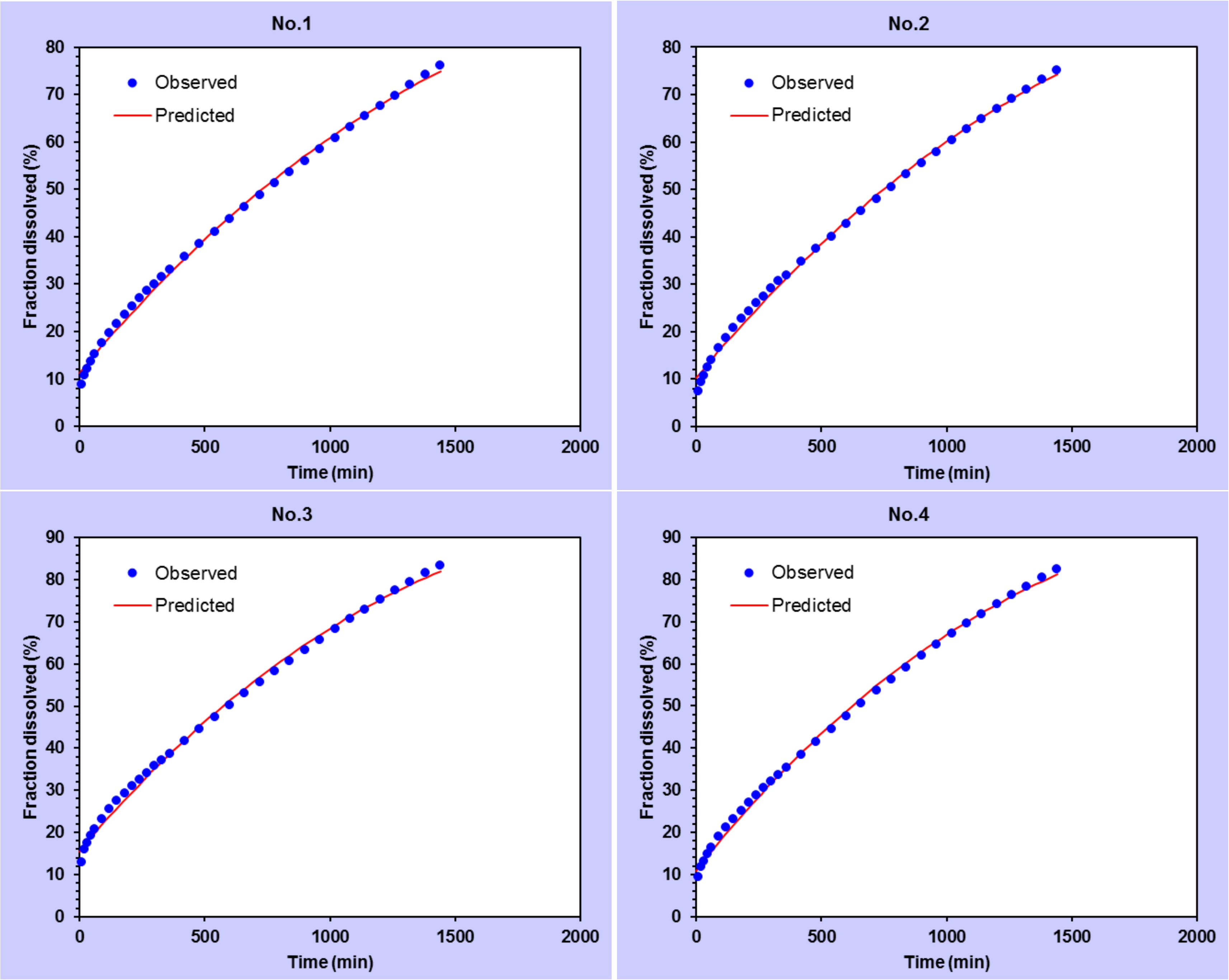

Supplementary materials – Model fitting summary – Starch 1500® sample with smaller particle size (modelling the entire relevant dissolution data)

Model: **Hopfenberg**

Model equation: $F = 100 \cdot [1 - (1 - k_{HB} \cdot t)^n]$

Fitted model parameters per tested tablet (N = 4) with statistics – mean, standard deviation (SD), and relative standard deviation expressed in % (RSD%) (output from DDSolver):

Parameter	No.1	No.2	No.3	No.4	Mean	SD	RSD(%)
k_{HB}	0.0002	0.0002	0.0003	0.0002	0.0002	0.0001	23.9398
n	4.1250	4.1250	3.0000	4.1250	3.8438	0.5625	14.6341

Number of dissolution data points (N), degrees of freedom (df), and selected goodness of fit criteria – Pearson correlation coefficient (R), coefficient of determination (R^2), adjusted coefficient of determination ($R^2_{adjusted}$), and residual sum of squares (RSS) (manual calculation in MS Excel):

Parameter	No.1	No.2	No.3	No.4
N	33	33	33	33
df	31	31	31	31
R	0.998344253	0.998532189	0.997777317	0.998162096
R^2	0.996691247	0.997066532	0.995559575	0.99632757
$R^2_{adjusted}$	0.996584513	0.996971905	0.995416336	0.996209104
RSS	1236.691653	1001.575925	2484.112493	1187.874674

Graphical abstract of model fit presented as mean ± 1 SD of the fraction % of released carvedilol:

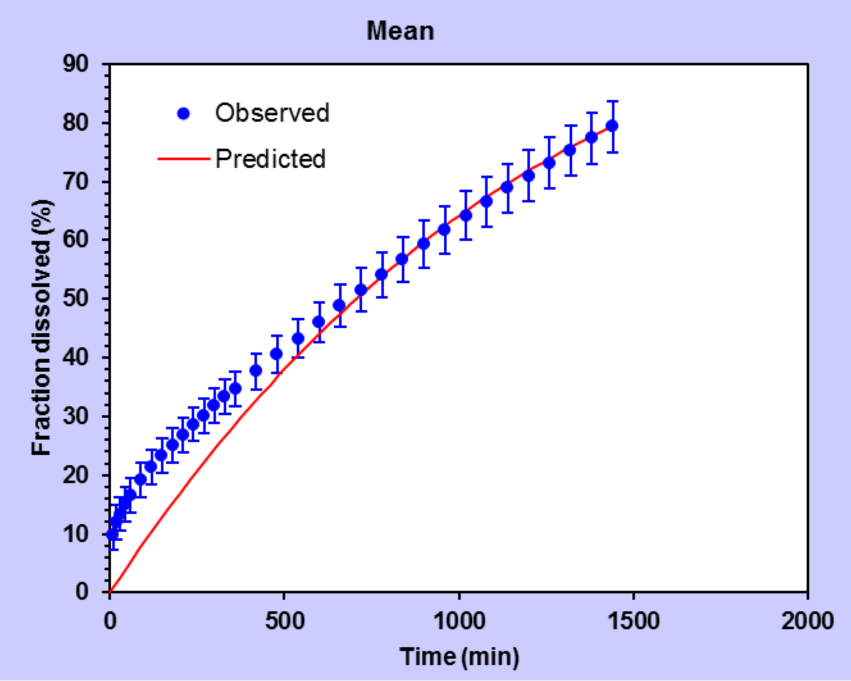

Supplementary materials – Model fitting summary – Starch 1500® sample with smaller particle size (modelling the entire relevant dissolution data)

Graphical abstract of model fit presented as the fraction % of released carvedilol per tested tablet:

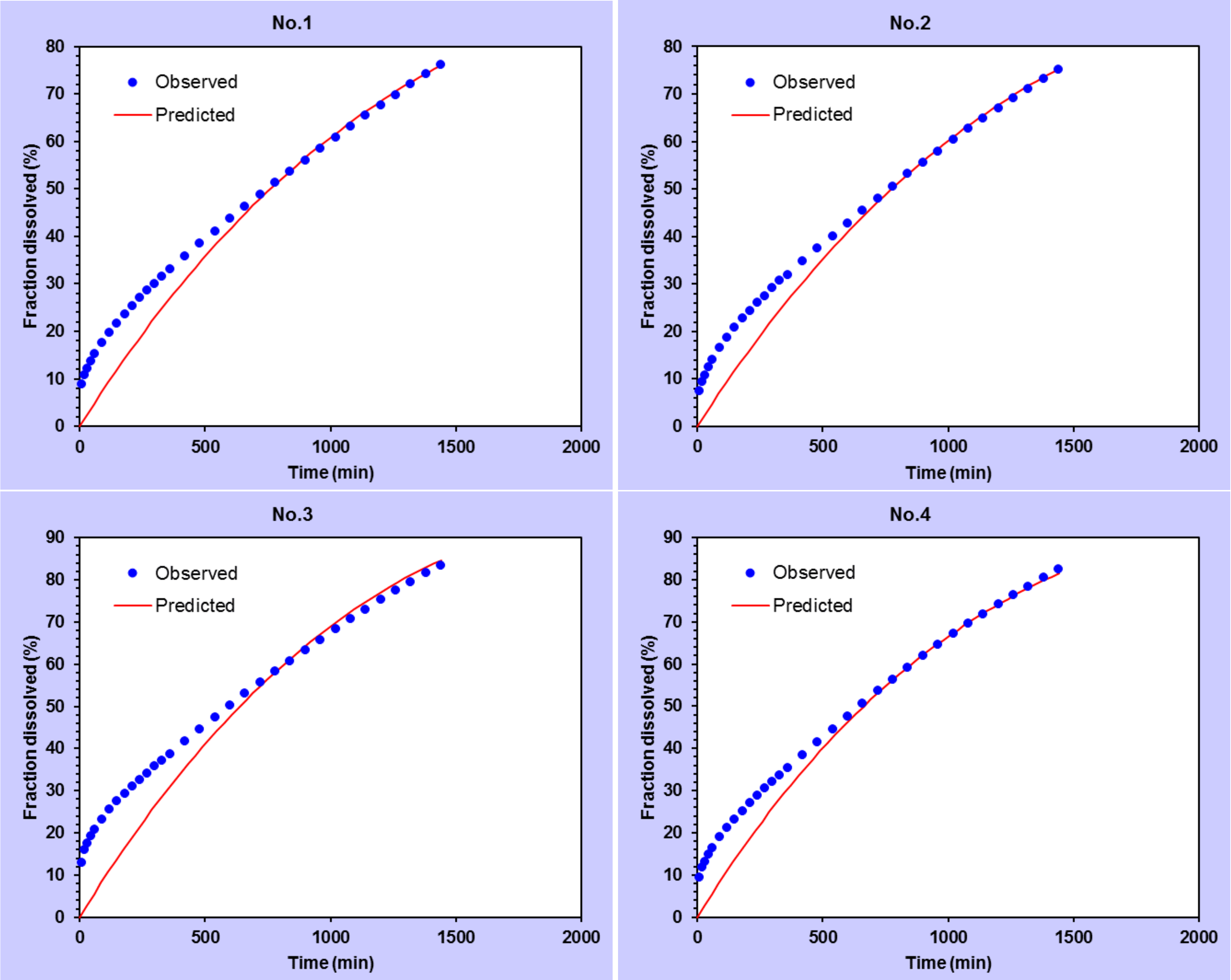

Supplementary materials – Model fitting summary – Starch 1500® sample with smaller particle size (modelling the entire relevant dissolution data)

Model: **Hopfenberg with T_{lag}**

Model equation: $F = 100 \cdot \{1 - [1 - k_{HB} \cdot (t - T_{lag})]^n\}$

Fitted model parameters per tested tablet (N = 4) with statistics – mean, standard deviation (SD), and relative standard deviation expressed in % (RSD%) (output from DDSolver):

Parameter	No.1	No.2	No.3	No.4	Mean	SD	RSD(%)
k_{HB}	0.0002	0.0002	0.0003	0.0004	0.0003	0.0001	24.4350
n	3.0000	3.0000	2.0000	2.0000	2.5000	0.5774	23.0940
T_{lag}	-170.3735	-154.1298	-254.7900	-178.6674	-189.4902	44.7102	-23.5950

Number of dissolution data points (N), degrees of freedom (df), and selected goodness of fit criteria – Pearson correlation coefficient (R), coefficient of determination (R^2), adjusted coefficient of determination ($R^2_{adjusted}$), and residual sum of squares (RSS) (manual calculation in MS Excel):

Parameter	No.1	No.2	No.3	No.4
N	33	33	33	33
df	30	30	30	30
R	0.998724808	0.998724423	0.998603941	0.999167566
R^2	0.997451242	0.997450472	0.997209832	0.998335826
$R^2_{adjusted}$	0.997281325	0.997280504	0.99702382	0.998224881
RSS	35.69035697	36.27932589	42.38168925	28.31706777

Graphical abstract of model fit presented as mean ± 1 SD of the fraction % of released carvedilol:

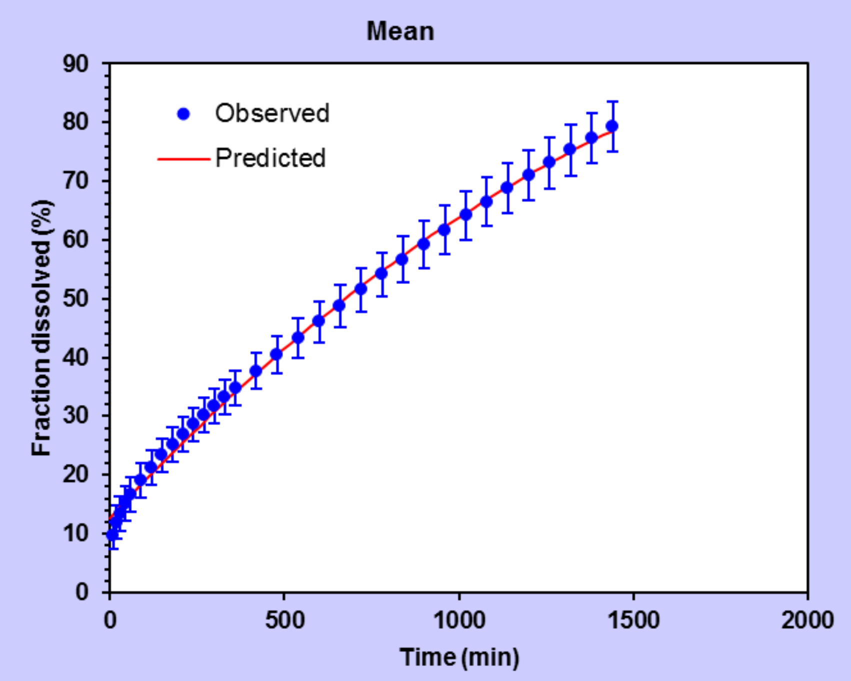

Supplementary materials – Model fitting summary – Starch 1500® sample with smaller particle size (modelling the entire relevant dissolution data)

Graphical abstract of model fit presented as the fraction % of released carvedilol per tested tablet:

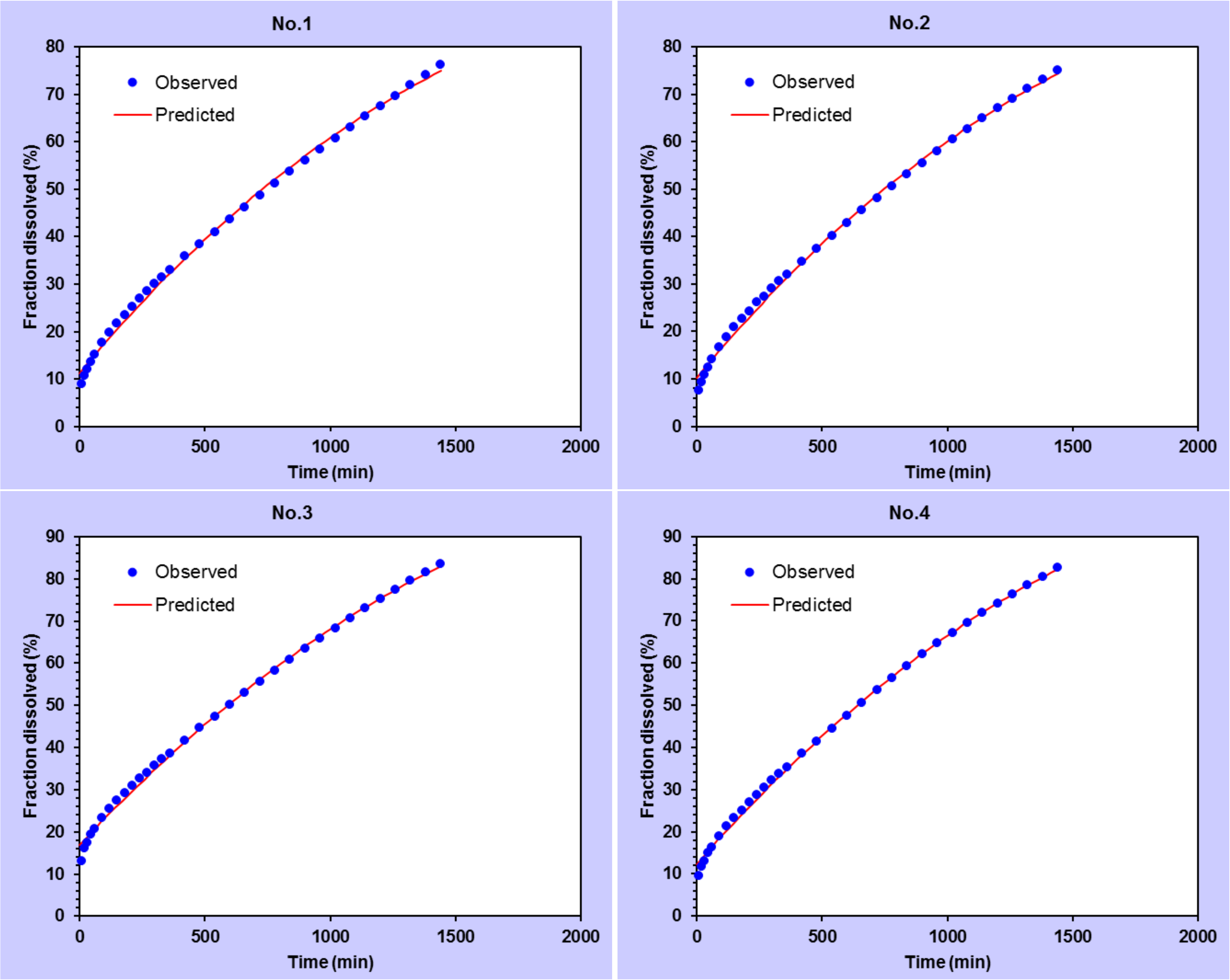

Supplementary materials – Model fitting summary – Starch 1500® sample with smaller particle size (modelling the entire relevant dissolution data)

Model: **Baker–Lonsdale**

Model equation: $\frac{3}{2}\cdot\left[1-\left(1-\frac{F}{100}\right)^{\frac{2}{3}}\right]-\frac{F}{100}=k_{BL}\cdot t$

Fitted model parameters per tested tablet (N = 4) with statistics – mean, standard deviation (SD), and relative standard deviation expressed in % (RSD%) (output from DDSolver):

Parameter	No.1	No.2	No.3	No.4	Mean	SD	RSD(%)
k_{BL}	0.0001	0.0001	0.0001	0.0001	0.0001	0.0000	15.9144

Number of dissolution data points (N), degrees of freedom (df), and selected goodness of fit criteria – Pearson correlation coefficient (R), coefficient of determination (R^2), adjusted coefficient of determination ($R^2_{adjusted}$), and residual sum of squares (RSS) (manual calculation in MS Excel):

Parameter	No.1	No.2	No.3	No.4
N	33	33	33	33
df	32	32	32	32
R	0.986564309	0.987333751	0.984544587	0.984106147
R^2	0.973309136	0.974827936	0.969328044	0.968464908
$R^2_{adjusted}$	0.973309136	0.974827936	0.969328044	0.968464908
RSS	540.7987187	592.275874	585.8940696	785.6512651

Graphical abstract of model fit presented as mean ± 1 SD of the fraction % of released carvedilol:

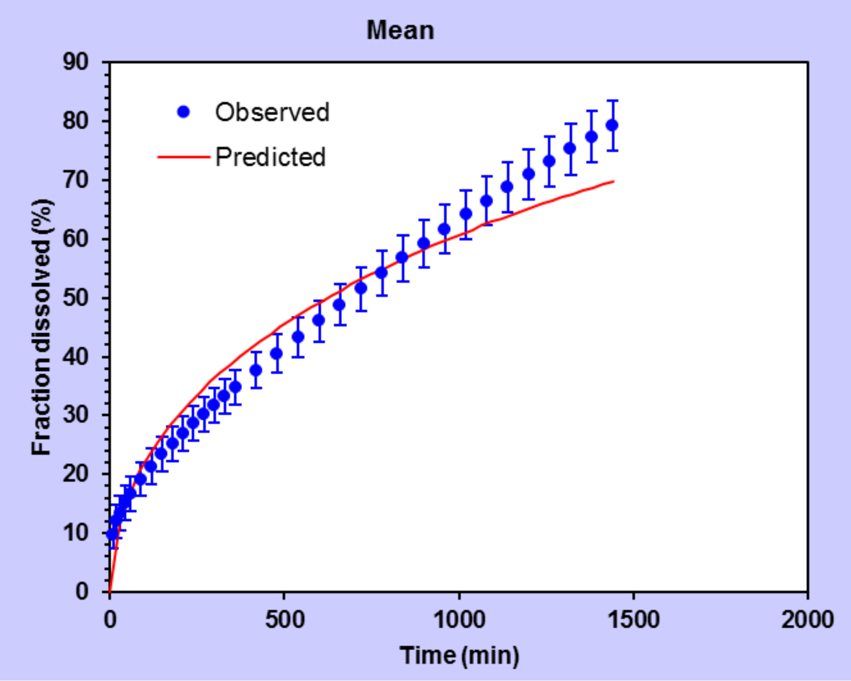

Supplementary materials – Model fitting summary – Starch 1500® sample with smaller particle size (modelling the entire relevant dissolution data)

Graphical abstract of model fit presented as the fraction % of released carvedilol per tested tablet:

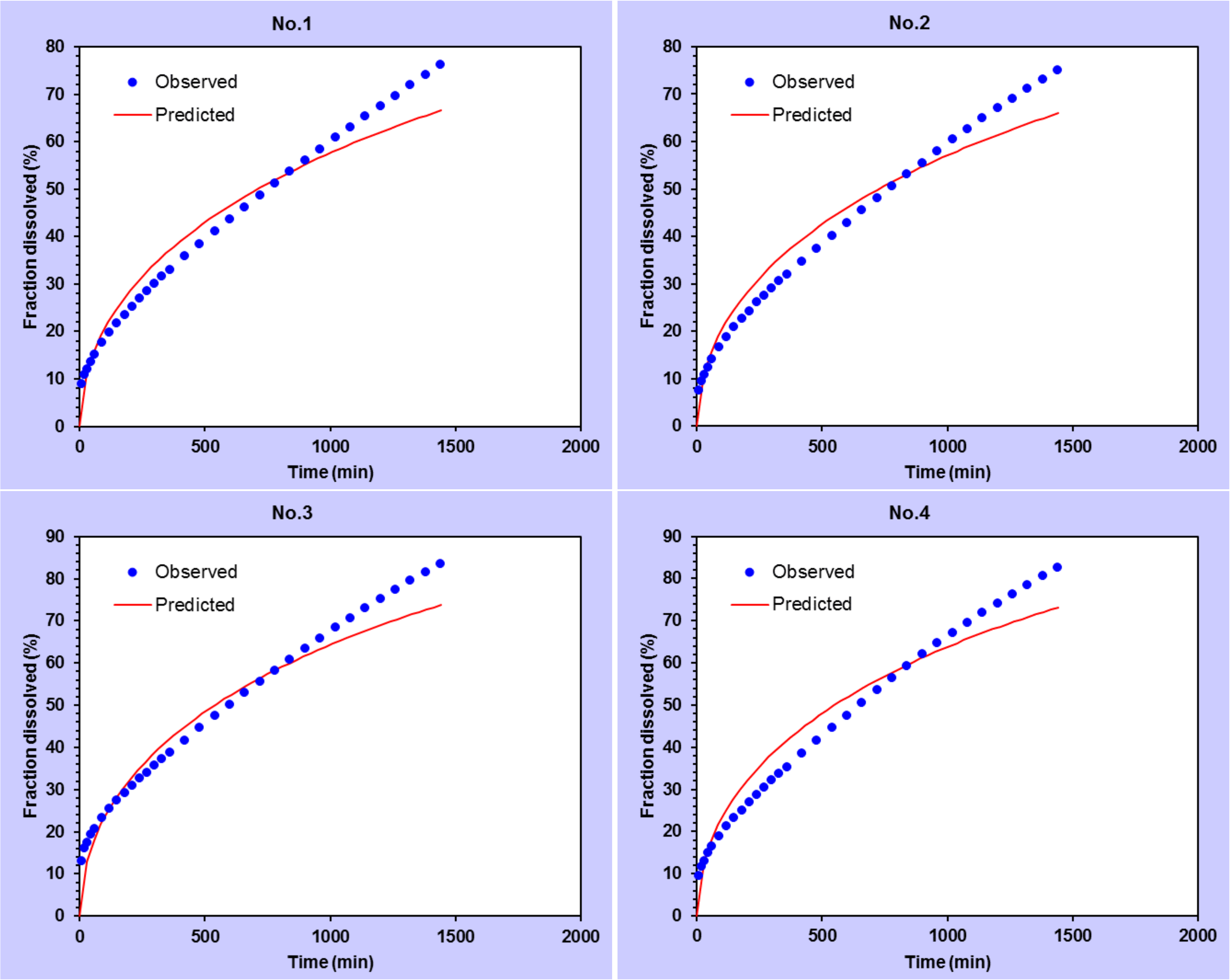

Supplementary materials – Model fitting summary – Starch 1500® sample with smaller particle size (modelling the entire relevant dissolution data)

Model: **Baker–Lonsdale with T_{lag}**

Model equation: $\frac{3}{2} \cdot \left[1 - \left(1 - \frac{F}{100}\right)^{\frac{2}{3}}\right] - \frac{F}{100} = k_{BL} \cdot (t - T_{lag})$

Fitted model parameters per tested tablet (N = 4) with statistics – mean, standard deviation (SD), and relative standard deviation expressed in % (RSD%) (output from DDSolver):

Parameter	No.1	No.2	No.3	No.4	Mean	SD	RSD(%)
k_{BL}	0.0001	0.0001	0.0001	0.0001	0.0001	0.0000	15.9144
T_{lag}	101.3340	106.9699	83.1351	112.0498	100.8722	12.6087	12.4997

Number of dissolution data points (N), degrees of freedom (df), and selected goodness of fit criteria – Pearson correlation coefficient (R), coefficient of determination (R^2), adjusted coefficient of determination ($R^2_{adjusted}$), and residual sum of squares (RSS) (manual calculation in MS Excel):

Parameter	No.1	No.2	No.3	No.4
N	33	33	33	33
df	31	31	31	31
R	0.974942084	0.97643033	0.970131039	0.972831253
R^2	0.950512068	0.95341619	0.941154233	0.946400646
$R^2_{adjusted}$	0.948915683	0.951913487	0.939255982	0.944671635
RSS	1434.726002	1280.207255	2170.135657	1825.774846

Graphical abstract of model fit presented as mean ± 1 SD of the fraction % of released carvedilol:

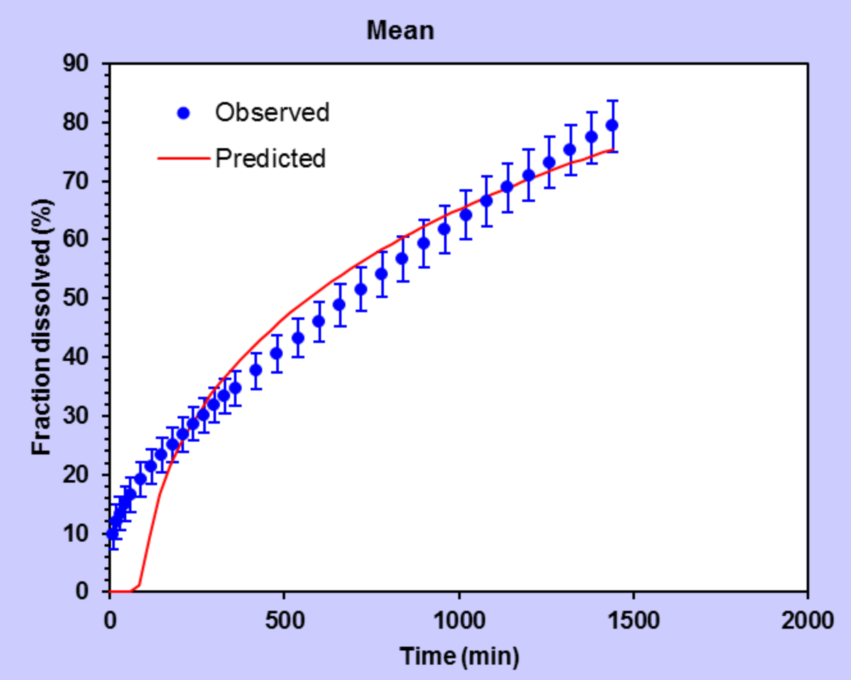

Supplementary materials – Model fitting summary – Starch 1500® sample with smaller particle size (modelling the entire relevant dissolution data)

Graphical abstract of model fit presented as the fraction % of released carvedilol per tested tablet:

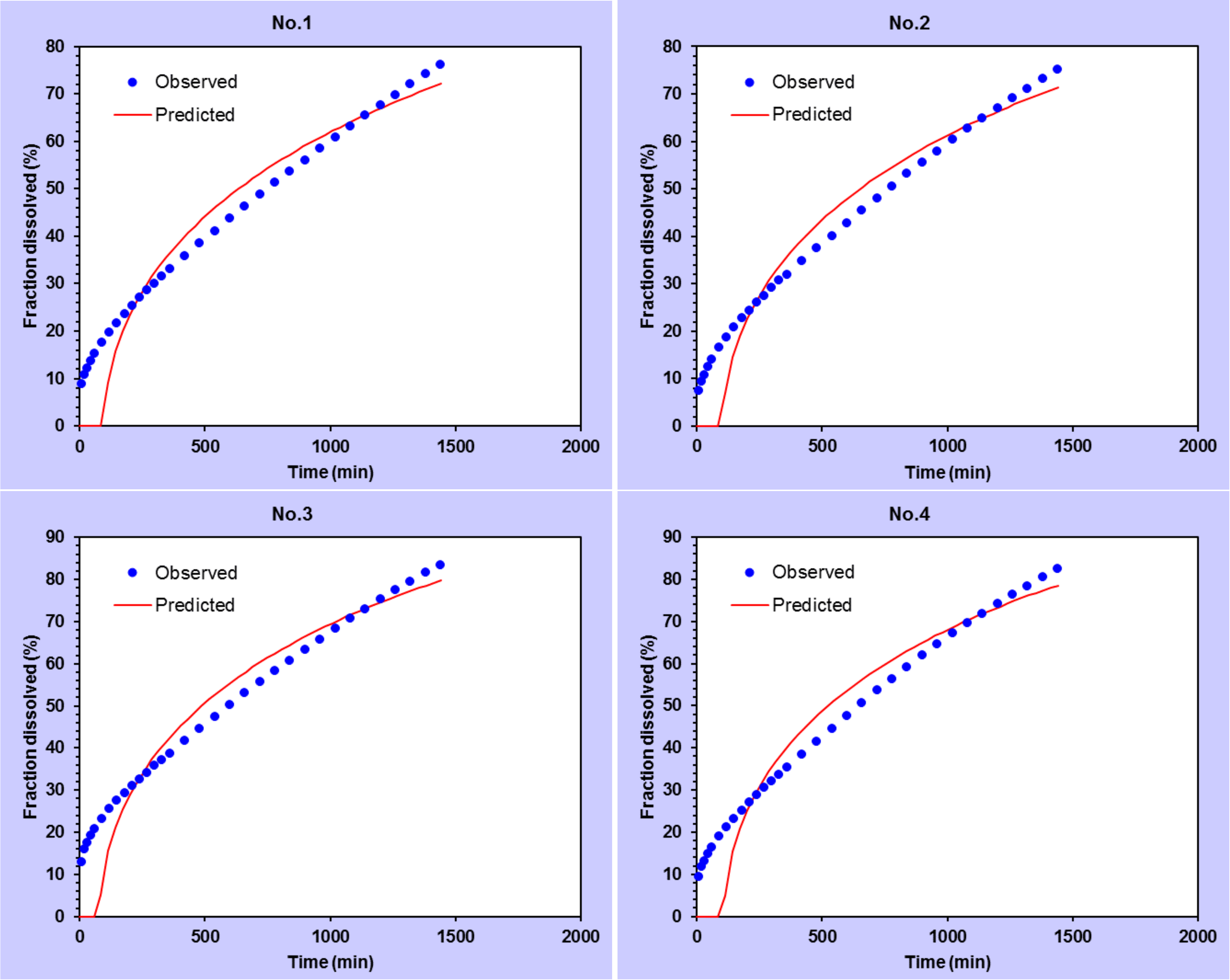

Supplementary materials – Model fitting summary – Starch 1500® sample with smaller particle size (modelling the entire relevant dissolution data)

Model: **Makoid–Banakar**

Model equation: $F = k_{MB} \cdot t^n \cdot e^{-k \cdot t}$

Fitted model parameters per tested tablet (N = 4) with statistics – mean, standard deviation (SD), and relative standard deviation expressed in % (RSD%) (output from DDSolver):

Parameter	No.1	No.2	No.3	No.4	Mean	SD	RSD(%)
k_{MB}	3.7239	2.8874	6.8099	4.0223	4.3609	1.7019	39.0267
n	0.3464	0.3881	0.2710	0.3447	0.3376	0.0487	14.4163
k	-0.0004	-0.0003	-0.0004	-0.0004	-0.0004	0.0000	-8.7691

Number of dissolution data points (N), degrees of freedom (df), and selected goodness of fit criteria – Pearson correlation coefficient (R), coefficient of determination (R^2), adjusted coefficient of determination ($R^2_{adjusted}$), and residual sum of squares (RSS) (manual calculation in MS Excel):

Parameter	No.1	No.2	No.3	No.4
N	33	33	33	33
df	30	30	30	30
R	0.998970404	0.999107527	0.998521648	0.997852605
R^2	0.997941867	0.99821585	0.997045482	0.995709822
$R^2_{adjusted}$	0.997804659	0.998096907	0.996848515	0.99542381
RSS	29.90155112	26.17472028	46.08098632	75.53530399

Graphical abstract of model fit presented as mean ± 1 SD of the fraction % of released carvedilol:

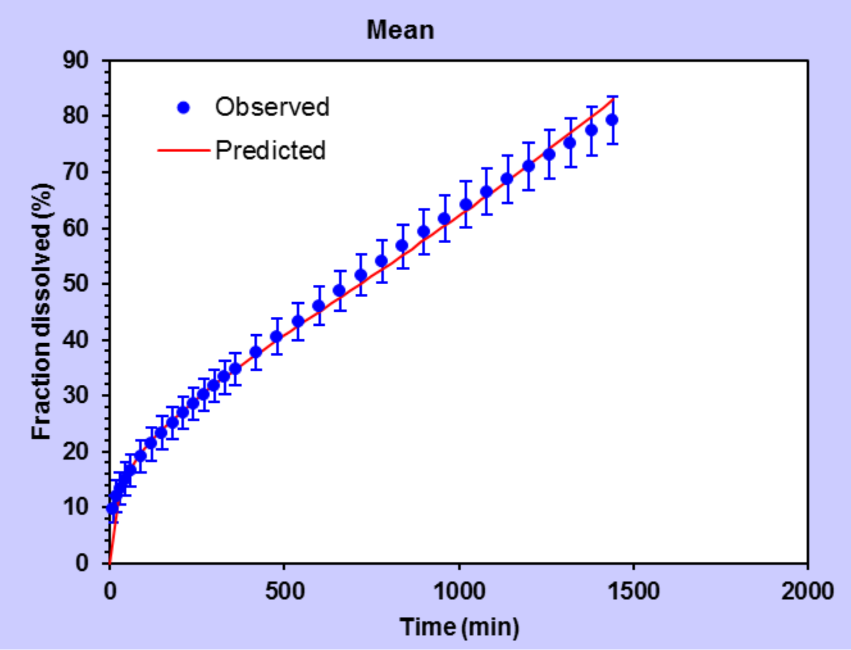

Supplementary materials – Model fitting summary – Starch 1500® sample with smaller particle size (modelling the entire relevant dissolution data)

Graphical abstract of model fit presented as the fraction % of released carvedilol per tested tablet:

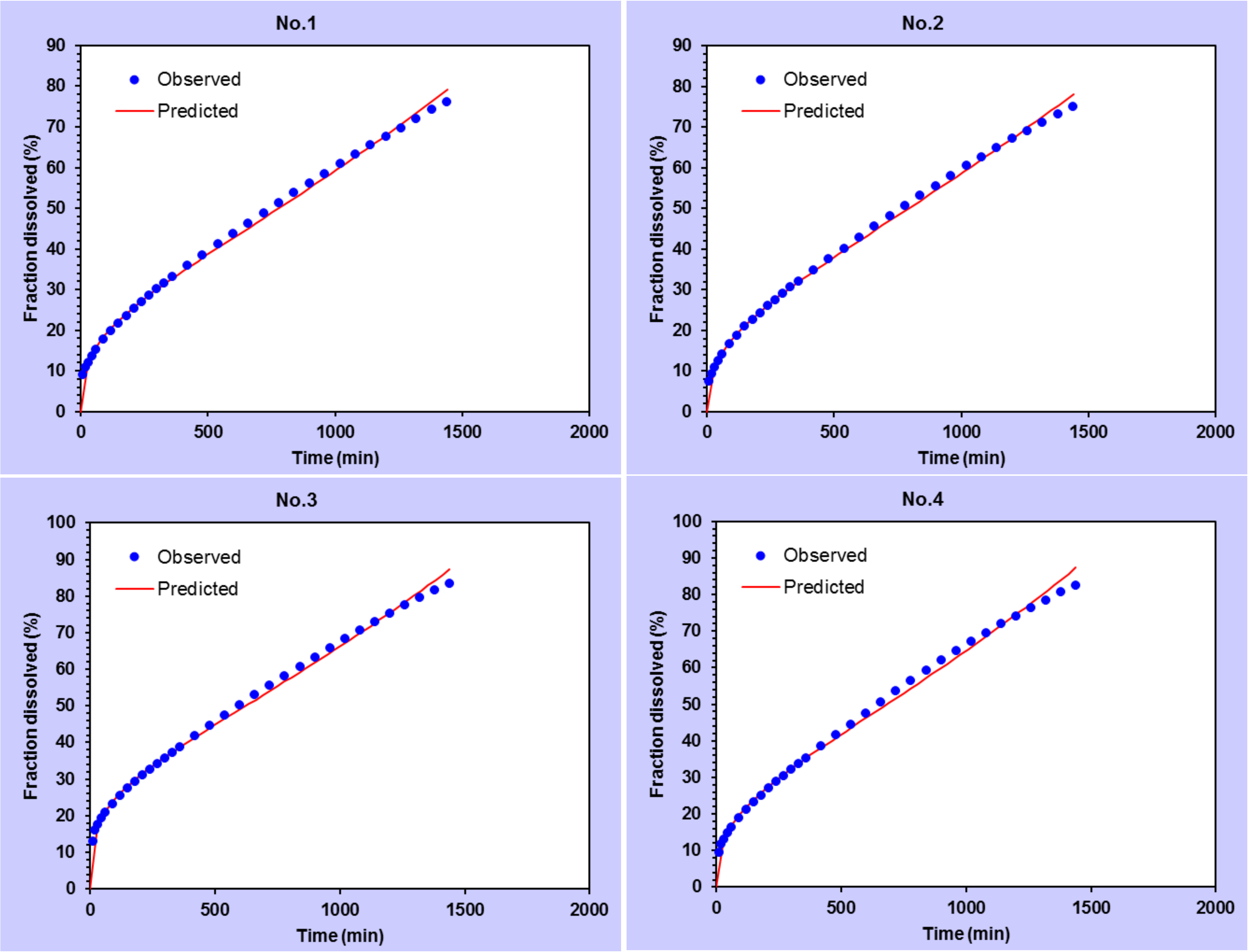

Supplementary materials – Model fitting summary – Starch 1500® sample with smaller particle size (modelling the entire relevant dissolution data)

Model: **Makoid–Banakar with T_{lag}**

Model equation: $F = k_{MB} \cdot (t - T_{lag})^n \cdot e^{-k\cdot(t-T_{lag})}$

Fitted model parameters per tested tablet (N = 4) with statistics – mean, standard deviation (SD), and relative standard deviation expressed in % (RSD%) (output from DDSolver):

Parameter	No.1	No.2	No.3	No.4	Mean	SD	RSD(%)
k_{MB}	4.5355	3.5590	7.9310	4.8938	5.2298	1.8871	36.0833
n	0.3083	0.3538	0.2418	0.3069	0.3027	0.0461	15.2279
k	-0.0004	-0.0004	-0.0005	-0.0005	-0.0004	0.0000	-9.6786
T_{lag}	4.0000	5.1337	4.0000	4.0000	4.2834	0.5668	13.2336

Number of dissolution data points (N), degrees of freedom (df), and selected goodness of fit criteria – Pearson correlation coefficient (R), coefficient of determination (R^2), adjusted coefficient of determination ($R^2_{adjusted}$), and residual sum of squares (RSS) (manual calculation in MS Excel):

Parameter	No.1	No.2	No.3	No.4
N	33	33	33	33
df	29	29	29	29
R	0.998074851	0.998735616	0.997754412	0.996729962
R^2	0.996153409	0.997472831	0.995513867	0.993470617
$R^2_{adjusted}$	0.995755486	0.9972114	0.995049784	0.992795163
RSS	56.81425315	49.28767599	70.80986135	116.8370278

Graphical abstract of model fit presented as mean ± 1 SD of the fraction % of released carvedilol:

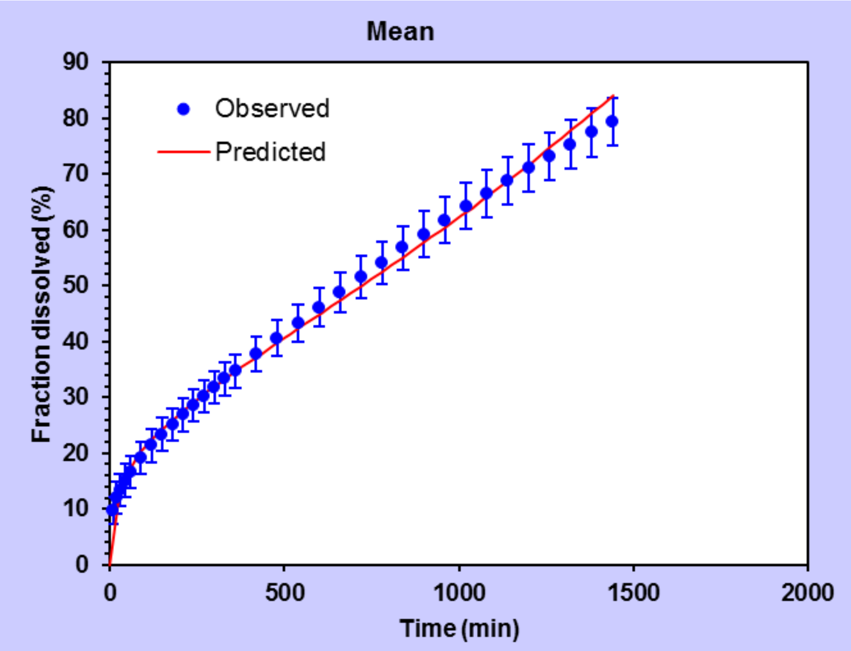

Supplementary materials – Model fitting summary – Starch 1500® sample with smaller particle size (modelling the entire relevant dissolution data)

Graphical abstract of model fit presented as the fraction % of released carvedilol per tested tablet:

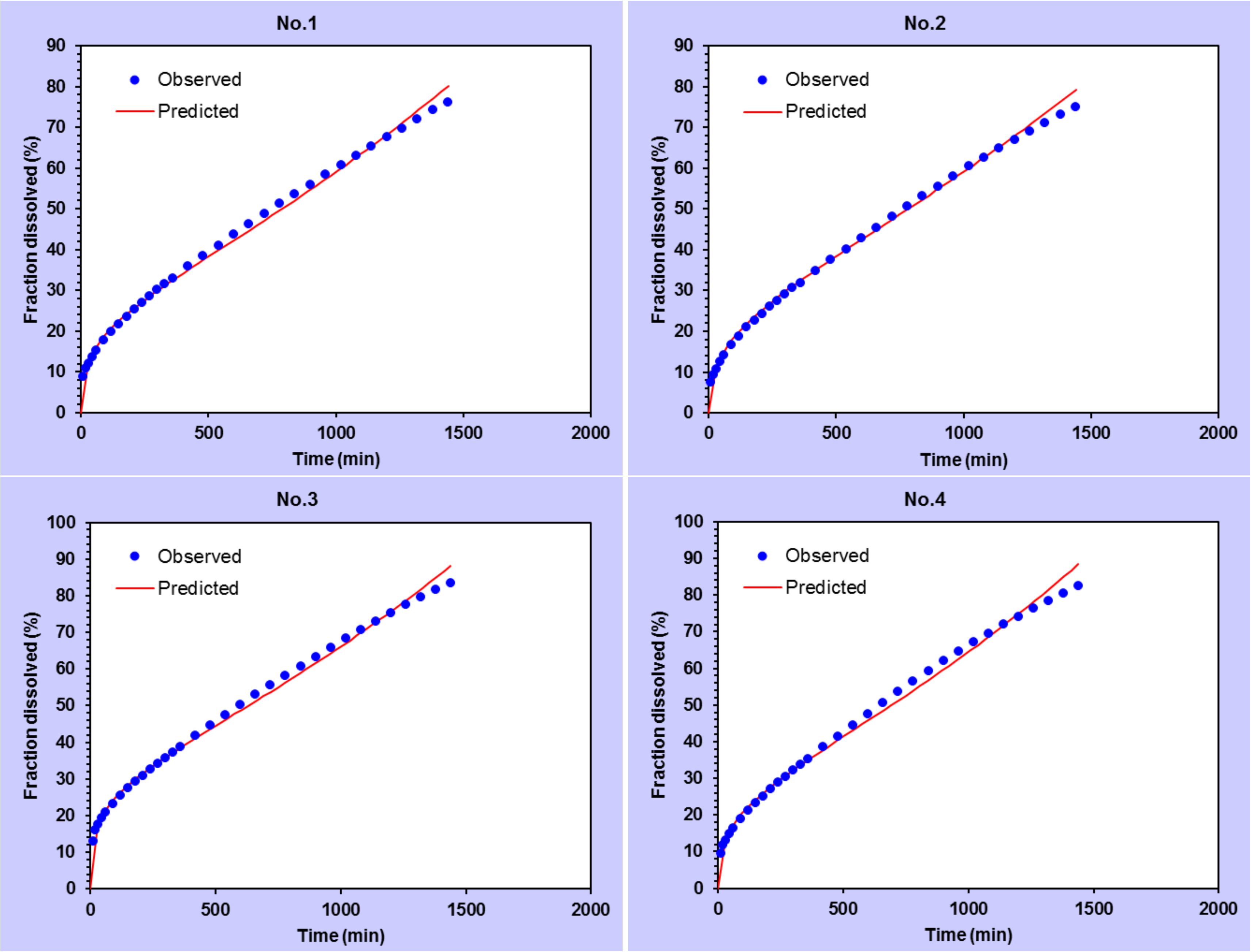

Supplementary materials – Model fitting summary – Starch 1500® sample with smaller particle size (modelling the entire relevant dissolution data)

Model: **Peppas–Sahlin_1**

Model equation: $F = k_1 \cdot t^m + k_2 \cdot t^{2m}$

Fitted model parameters per tested tablet (N = 4) with statistics – mean, standard deviation (SD), and relative standard deviation expressed in % (RSD%) (output from DDSolver):

Parameter	No.1	No.2	No.3	No.4	Mean	SD	RSD(%)
k_1	1.877	1.729	2.599	1.981	2.046	0.383	18.706
k_2	0.036	0.042	0.019	0.043	0.035	0.011	31.713
m	0.450	0.450	0.450	0.450	0.450	0.000	0.000

Number of dissolution data points (N), degrees of freedom (df), and selected goodness of fit criteria – Pearson correlation coefficient (R), coefficient of determination (R^2), adjusted coefficient of determination ($R^2_{adjusted}$), and residual sum of squares (RSS) (manual calculation in MS Excel):

Parameter	No.1	No.2	No.3	No.4
N	33	33	33	33
df	30	30	30	30
R	0.998450786	0.999107759	0.995484938	0.998443797
R^2	0.996903972	0.998216315	0.990990262	0.996890015
$R^2_{adjusted}$	0.99669757	0.998097402	0.990389612	0.996682683
RSS	54.59458189	31.33617197	179.0720279	65.36167848

Graphical abstract of model fit presented as mean ± 1 SD of the fraction % of released carvedilol:

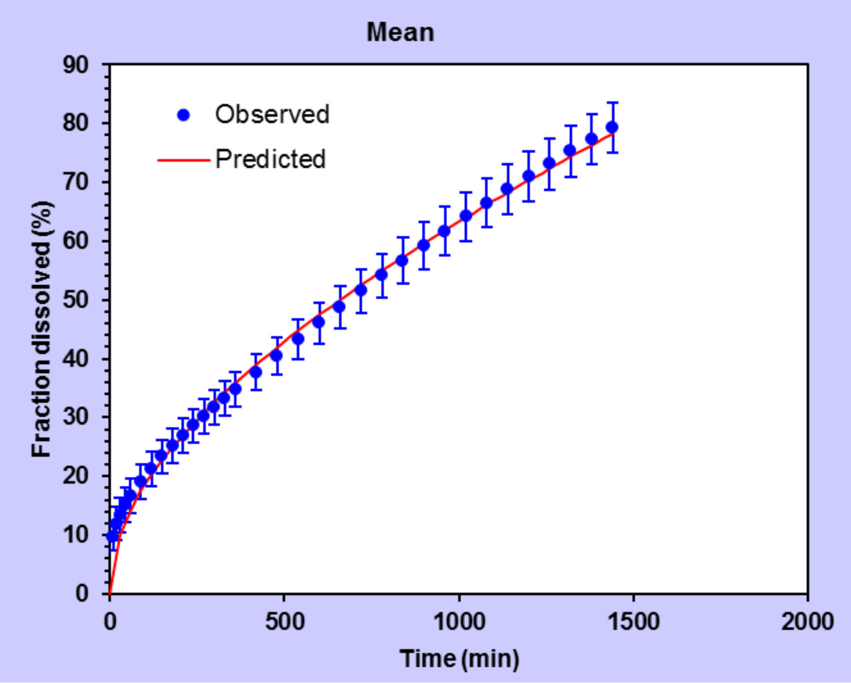

Supplementary materials – Model fitting summary – Starch 1500® sample with smaller particle size (modelling the entire relevant dissolution data)

Graphical abstract of model fit presented as the fraction % of released carvedilol per tested tablet:

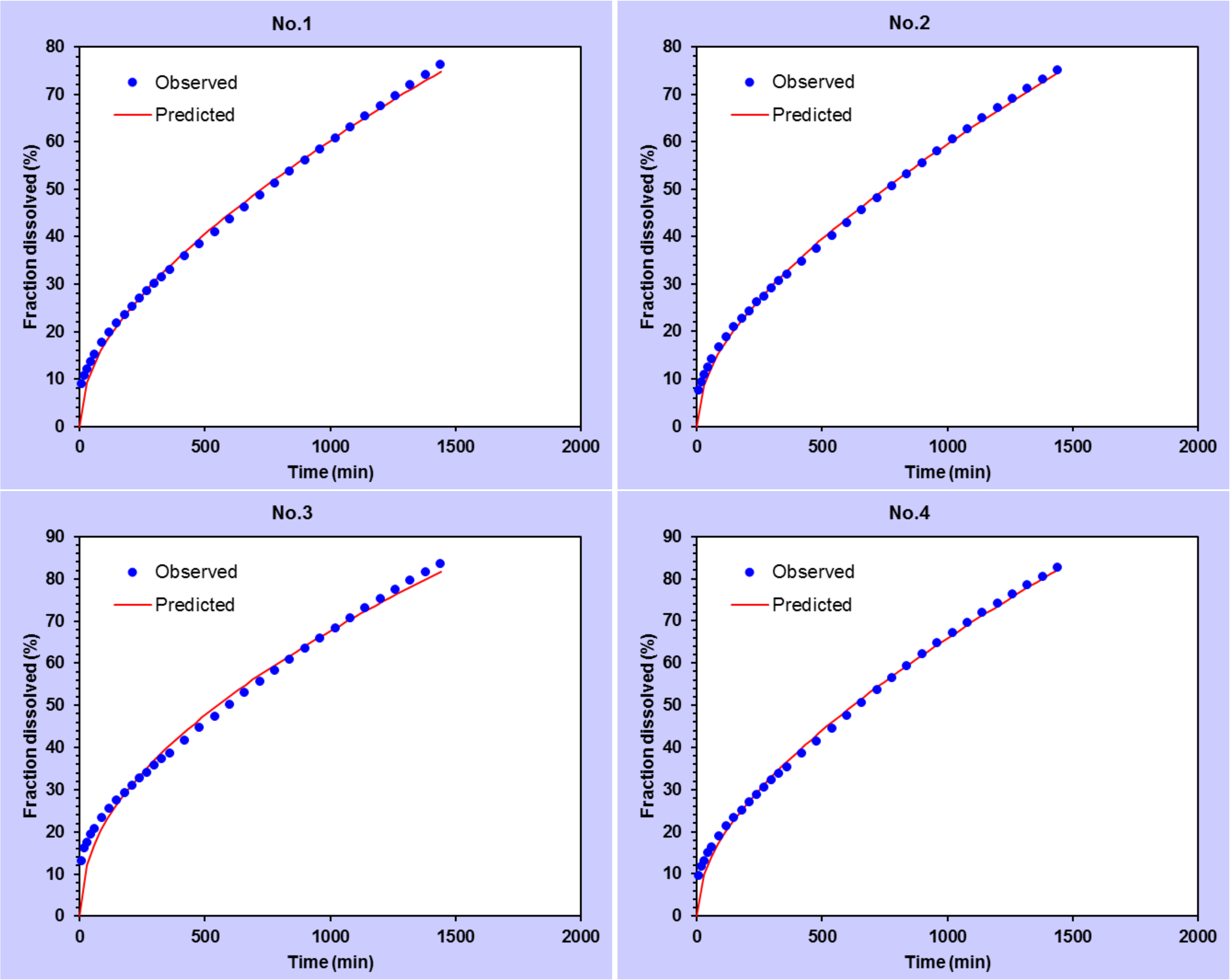

Supplementary materials – Model fitting summary – Starch 1500® sample with smaller particle size (modelling the entire relevant dissolution data)

Model: **Peppas–Sahlin_1 with T_{lag}**

Model equation: $F = k_1 \cdot (t - T_{lag})^m + k_2 \cdot (t - T_{lag})^{2m}$

Fitted model parameters per tested tablet (N = 4) with statistics – mean, standard deviation (SD), and relative standard deviation expressed in % (RSD%) (output from DDSolver):

Parameter	No.1	No.2	No.3	No.4	Mean	SD	RSD(%)
k_1	1.921	1.773	2.648	2.029	2.093	0.385	18.379
k_2	0.035	0.040	0.017	0.041	0.033	0.011	33.601
m	0.450	0.450	0.450	0.450	0.450	0.000	0.000
T_{lag}	4.000	4.000	4.000	4.000	4.000	0.000	0.000

Number of dissolution data points (N), degrees of freedom (df), and selected goodness of fit criteria – Pearson correlation coefficient (R), coefficient of determination (R^2), adjusted coefficient of determination ($R^2_{adjusted}$), and residual sum of squares (RSS) (manual calculation in MS Excel):

Parameter	No.1	No.2	No.3	No.4
N	33	33	33	33
df	29	29	29	29
R	0.997911687	0.998724267	0.994467243	0.997922195
R^2	0.995827736	0.997450161	0.988965098	0.995848707
$R^2_{adjusted}$	0.995396122	0.997186384	0.987823557	0.995419263
RSS	76.11119671	46.49258547	227.7738801	90.32824663

Graphical abstract of model fit presented as mean ± 1 SD of the fraction % of released carvedilol:

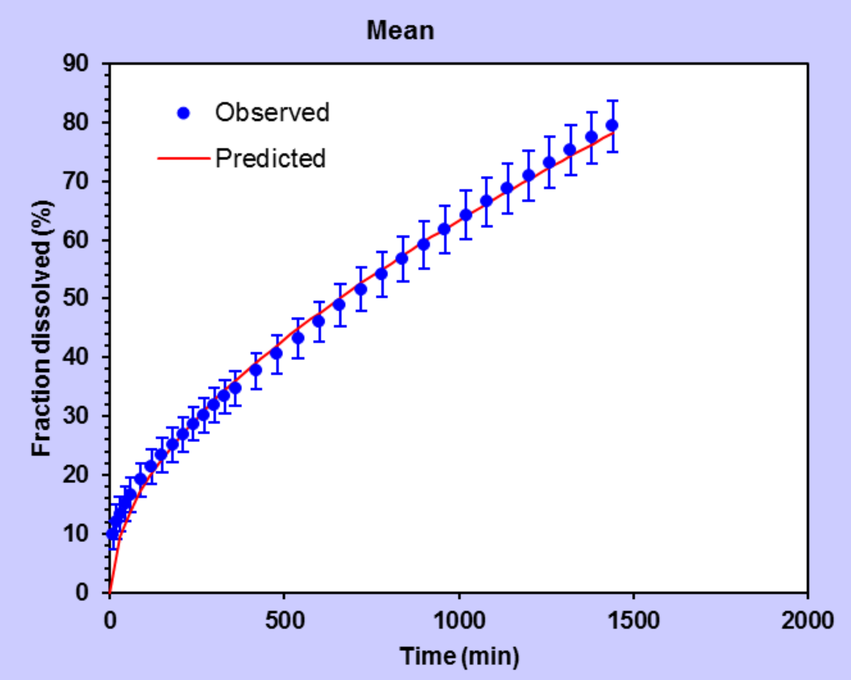

Supplementary materials – Model fitting summary – Starch 1500® sample with smaller particle size (modelling the entire relevant dissolution data)

Graphical abstract of model fit presented as the fraction % of released carvedilol per tested tablet:

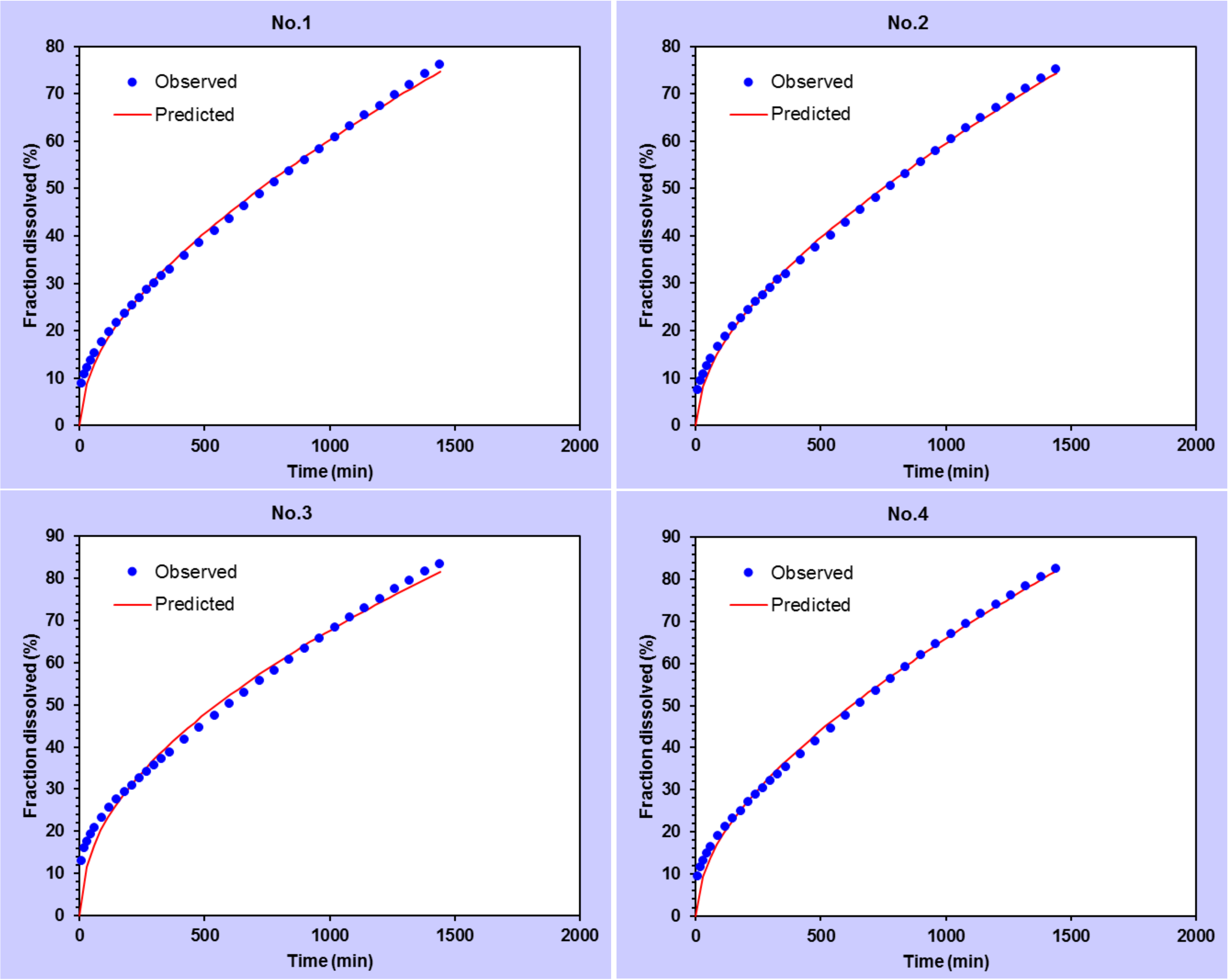

Supplementary materials – Model fitting summary – Starch 1500® sample with smaller particle size (modelling the entire relevant dissolution data)

Model: **Peppas–Sahlin_2**

Model equation: $F = k_1 \cdot t^{0.5} + k_2 \cdot t$

Fitted model parameters per tested tablet (N = 4) with statistics – mean, standard deviation (SD), and relative standard deviation expressed in % (RSD%) (output from DDSolver):

Parameter	No.1	No.2	No.3	No.4	Mean	SD	RSD(%)
k_1	1.592	1.489	2.120	1.692	1.723	0.277	16.103
k_2	0.010	0.012	0.001	0.012	0.009	0.006	62.495

Number of dissolution data points (N), degrees of freedom (df), and selected goodness of fit criteria – Pearson correlation coefficient (R), coefficient of determination (R^2), adjusted coefficient of determination ($R^2_{adjusted}$), and residual sum of squares (RSS) (manual calculation in MS Excel):

Parameter	No.1	No.2	No.3	No.4
N	No.1	No.2	No.3	No.4
df	33	33	33	33
R	31	31	31	31
R^2	0.998169522	0.99893333	0.994679744	0.998206433
$R^2_{adjusted}$	0.996342396	0.997867798	0.989387794	0.996416082
RSS	69.7913388	40.38669382	230.1977242	80.88311535

Graphical abstract of model fit presented as mean ± 1 SD of the fraction % of released carvedilol:

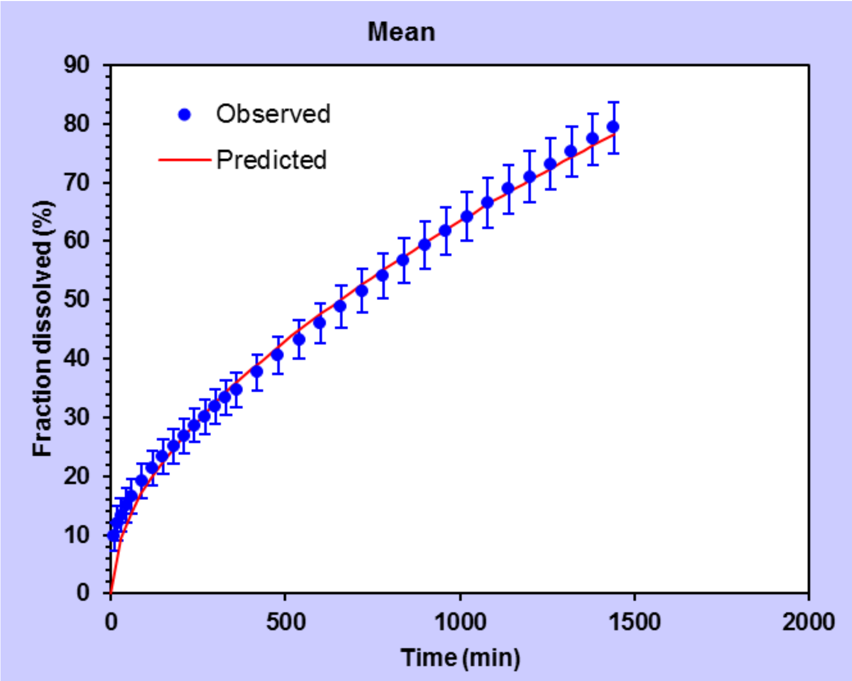

Supplementary materials – Model fitting summary – Starch 1500® sample with smaller particle size (modelling the entire relevant dissolution data)

Graphical abstract of model fit presented as the fraction % of released carvedilol per tested tablet:

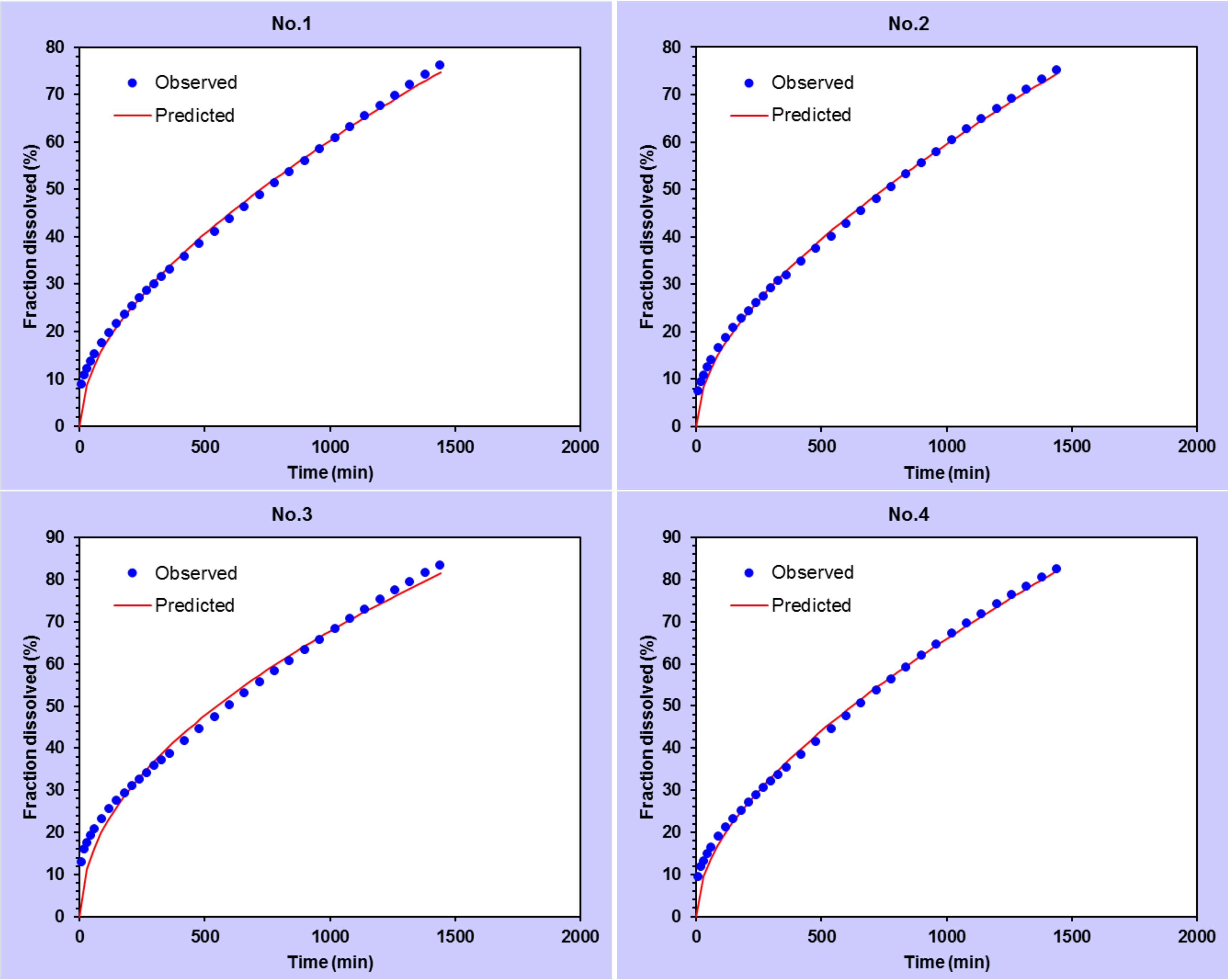

Supplementary materials – Model fitting summary – Starch 1500® sample with smaller particle size (modelling the entire relevant dissolution data)

Model: **Peppas–Sahlin_2 with T_{lag}**

Model equation: $F = k_1 \cdot (t - T_{lag})^{0.5} + k_2 \cdot (t - T_{lag})$

Fitted model parameters per tested tablet (N = 4) with statistics – mean, standard deviation (SD), and relative standard deviation expressed in % (RSD%) (output from DDSolver):

Parameter	No.1	No.2	No.3	No.4	Mean	SD	RSD(%)
k_1	1.621	1.518	2.152	1.724	1.754	0.278	15.875
k_2	0.009	0.012	0.000	0.012	0.008	0.006	68.986
T_{lag}	4.000	4.000	4.000	4.000	4.000	0.000	0.000

Number of dissolution data points (N), degrees of freedom (df), and selected goodness of fit criteria – Pearson correlation coefficient (R), coefficient of determination (R^2), adjusted coefficient of determination ($R^2_{adjusted}$), and residual sum of squares (RSS) (manual calculation in MS Excel):

Parameter	No.1	No.2	No.3	No.4
N	33	33	33	33
df	30	30	30	30
R	0.997596945	0.998516243	0.993636732	0.997654619
R^2	0.995199665	0.997034687	0.987313954	0.99531474
$R^2_{adjusted}$	0.994879643	0.996837	0.986468218	0.995002389
RSS	95.09904578	58.50436919	286.9612441	109.9260843

Graphical abstract of model fit presented as mean ± 1 SD of the fraction % of released carvedilol:

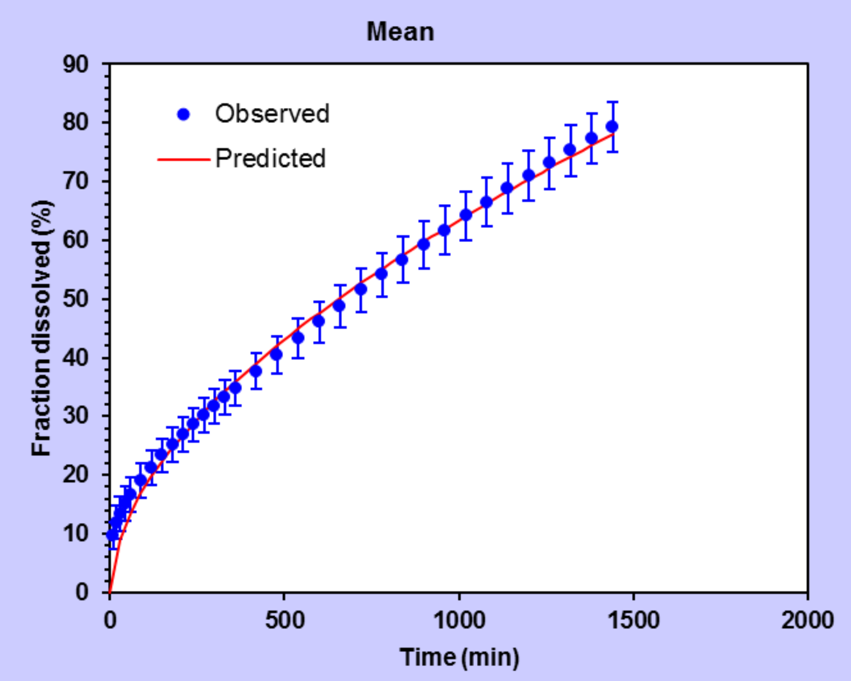

Supplementary materials – Model fitting summary – Starch 1500® sample with smaller particle size (modelling the entire relevant dissolution data)

Graphical abstract of model fit presented as the fraction % of released carvedilol per tested tablet:

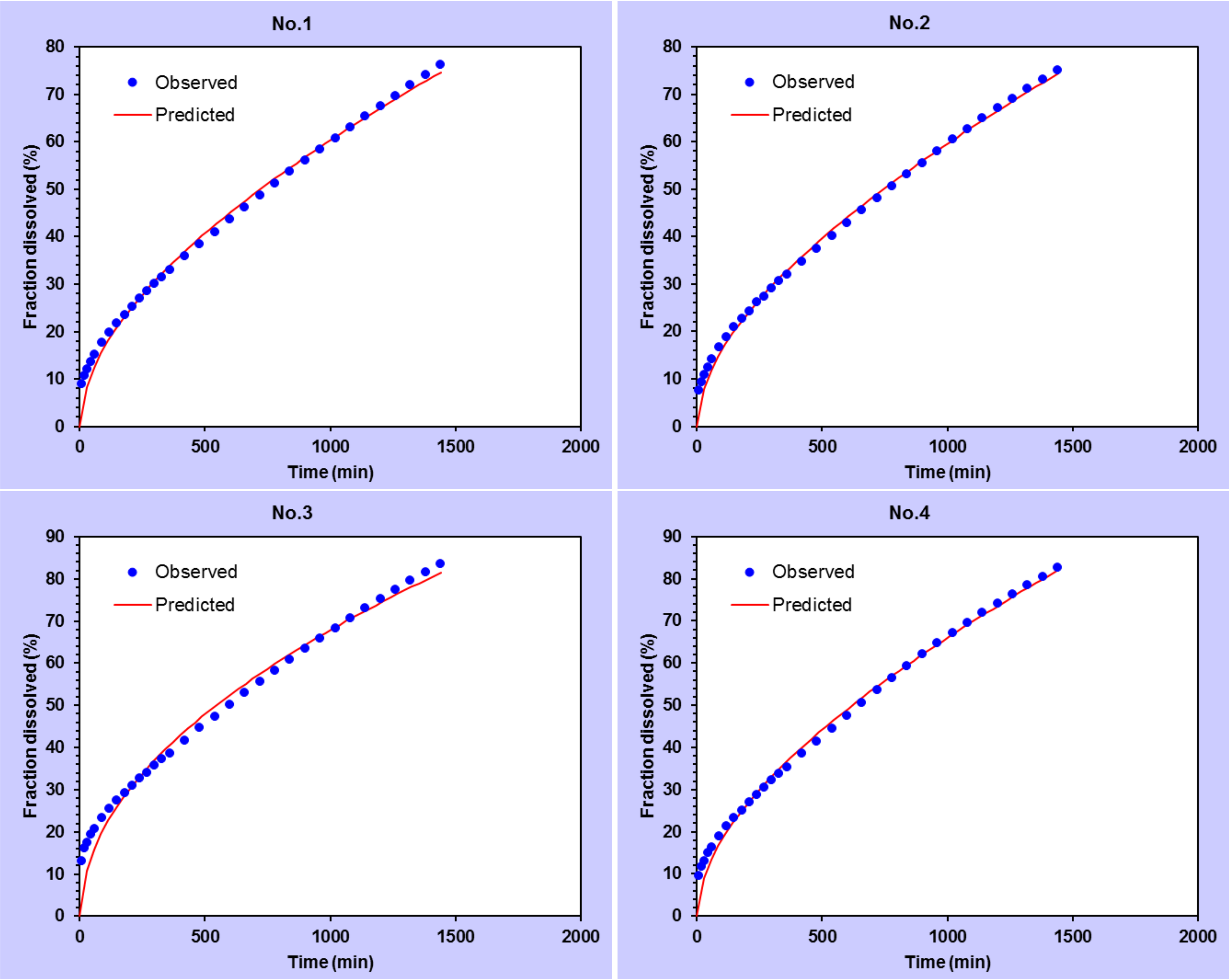

Supplementary materials – Model fitting summary – Starch 1500® sample with smaller particle size (modelling the entire relevant dissolution data)

Model: **Quadratic**

Model equation: $F = 100 \cdot (k_1 \cdot t^2 + k_2 \cdot t)$

Fitted model parameters per tested tablet (N = 4) with statistics – mean, standard deviation (SD), and relative standard deviation expressed in % (RSD%) (output from DDSolver):

Parameter	No.1	No.2	No.3	No.4	Mean	SD	RSD(%)
k_1	-0.0000003	-0.0000003	-0.0000004	-0.0000004	-0.0000004	0.0000001	-14.4058606
k_2	0.0009710	0.0009454	0.0011576	0.0010569	0.0010327	0.0000959	9.2907518

Number of dissolution data points (N), degrees of freedom (df), and selected goodness of fit criteria – Pearson correlation coefficient (R), coefficient of determination (R^2), adjusted coefficient of determination ($R^2_{adjusted}$), and residual sum of squares (RSS) (manual calculation in MS Excel):

Parameter	No.1	No.2	No.3	No.4
N	33	33	33	33
df	31	31	31	31
R	0.98963986	0.99148868	0.98362754	0.991215958
R^2	0.979387053	0.983049803	0.967523138	0.982509076
$R^2_{adjusted}$	0.978722119	0.982503022	0.966475498	0.981944852
RSS	983.8317786	785.285069	1984.902557	1045.668327

Graphical abstract of model fit presented as mean ± 1 SD of the fraction % of released carvedilol:

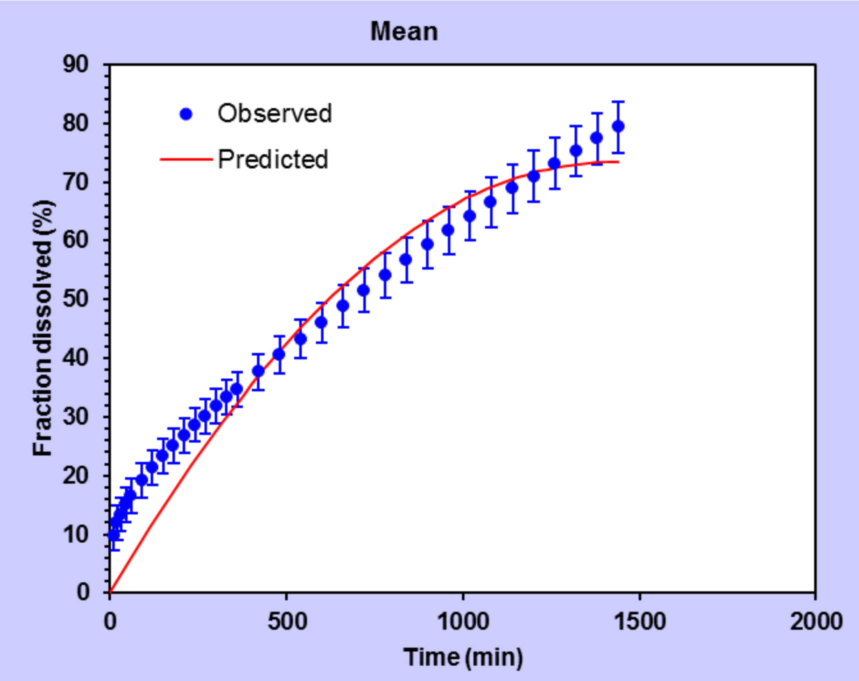

Supplementary materials – Model fitting summary – Starch 1500® sample with smaller particle size (modelling the entire relevant dissolution data)

Graphical abstract of model fit presented as the fraction % of released carvedilol per tested tablet:

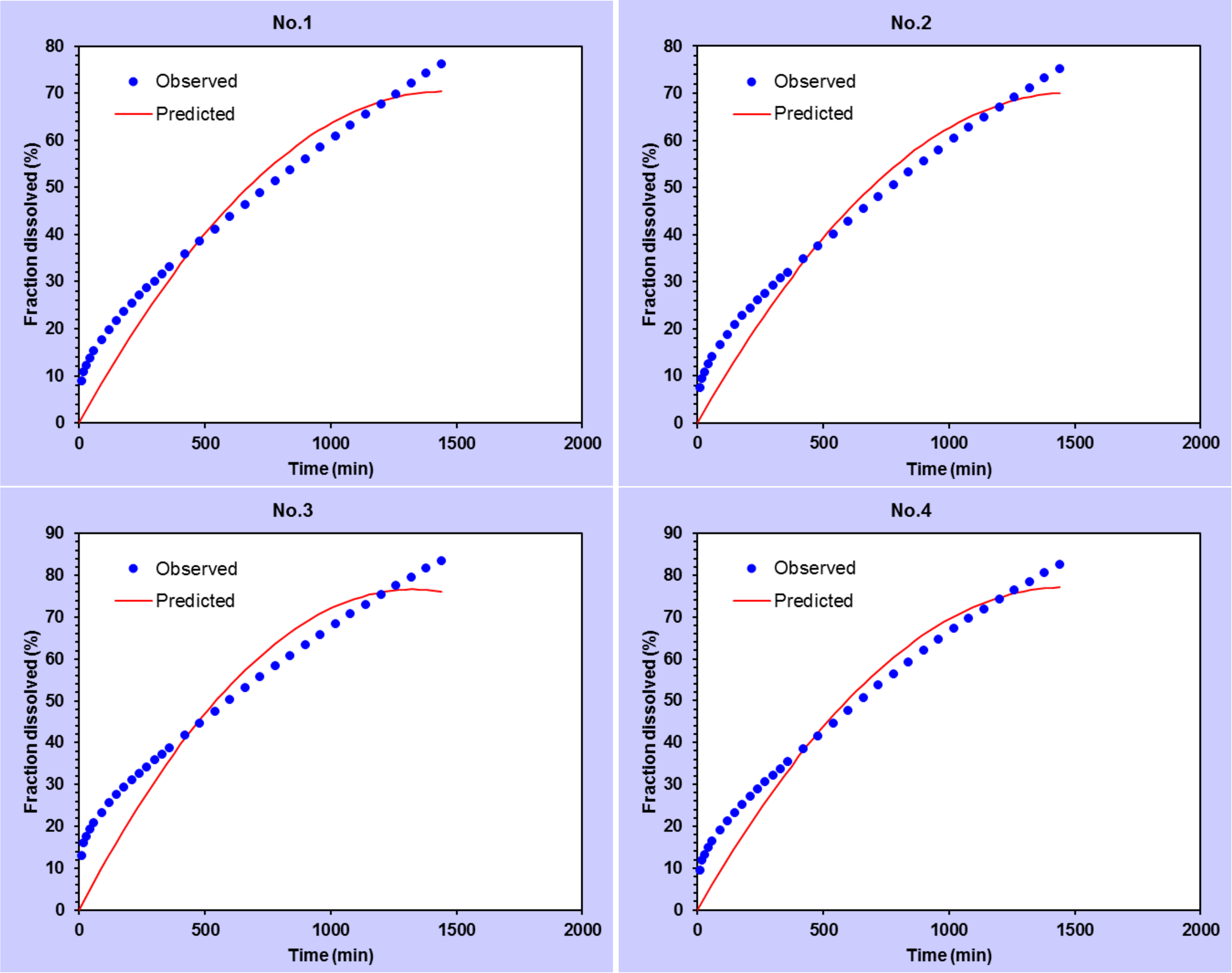

Supplementary materials – Model fitting summary – Starch 1500® sample with smaller particle size (modelling the entire relevant dissolution data)

Model: **Quadratic with T_{lag}**

Model equation: $F = 100 \cdot \left[k_1 \cdot (t - T_{lag})^2 + k_2 \cdot (t - T_{lag})\right]$

Fitted model parameters per tested tablet (N = 4) with statistics – mean, standard deviation (SD), and relative standard deviation expressed in % (RSD%) (output from DDSolver):

Parameter	No.1	No.2	No.3	No.4	Mean	SD	RSD(%)
k_1	-0.0000003	-0.0000003	-0.0000004	-0.0000004	-0.0000004	0.0000001	-14.2484838
k_2	0.0009777	0.0009522	0.0011646	0.0010643	0.0010397	0.0000961	9.2457399
T_{lag}	4.0000000	4.0000000	4.0000000	4.0000000	4.0000000	0.0000000	0.0000000

Number of dissolution data points (N), degrees of freedom (df), and selected goodness of fit criteria – Pearson correlation coefficient (R), coefficient of determination (R^2), adjusted coefficient of determination ($R^2_{adjusted}$), and residual sum of squares (RSS) (manual calculation in MS Excel):

Parameter	No.1	No.2	No.3	No.4
N	33	33	33	33
df	30	30	30	30
R	0.989356437	0.99121679	0.983342121	0.990937807
R^2	0.978826159	0.982510724	0.966961727	0.981957738
$R^2_{adjusted}$	0.97741457	0.981344772	0.964759175	0.980754921
RSS	1048.59437	841.394357	2095.286865	1118.926747

Graphical abstract of model fit presented as mean ± 1 SD of the fraction % of released carvedilol:

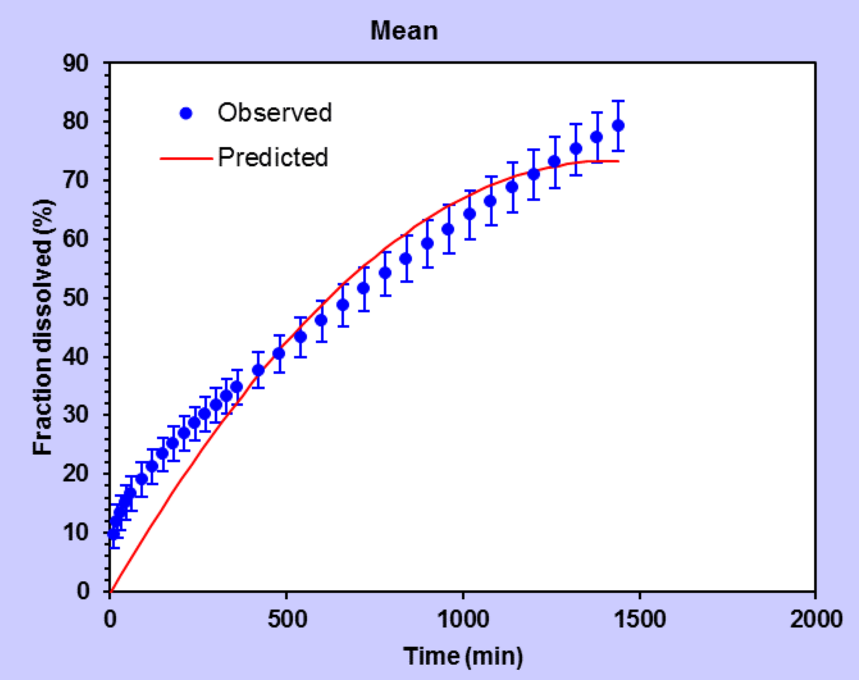

Supplementary materials – Model fitting summary – Starch 1500® sample with smaller particle size (modelling the entire relevant dissolution data)

Graphical abstract of model fit presented as the fraction % of released carvedilol per tested tablet:

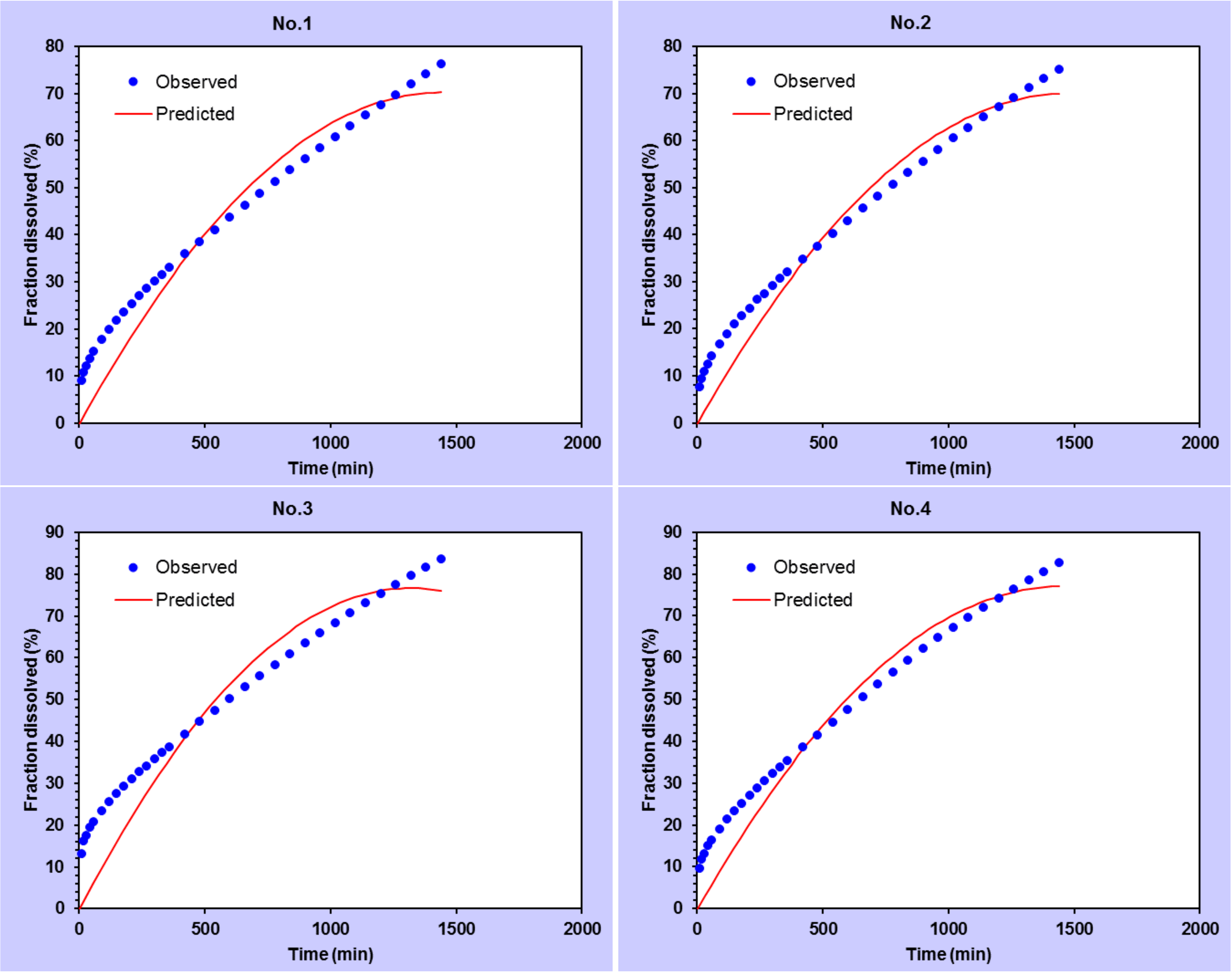

Supplementary materials – Model fitting summary – Starch 1500® sample with smaller particle size (modelling the entire relevant dissolution data)

Model: **Weibull_1**

Model equation: $F = 100 \cdot \left[1 - e^{-\frac{(t-T_i)^{\beta}}{\alpha}}\right]$

Fitted model parameters per tested tablet (N = 4) with statistics – mean, standard deviation (SD), and relative standard deviation expressed in % (RSD%) (output from DDSolver):

Parameter	No.1	No.2	No.3	No.4	Mean	SD	RSD(%)
α	49.254	60.174	29.831	50.345	47.401	12.701	26.795
β	0.539	0.566	0.495	0.564	0.541	0.033	6.136
T_i	4.000	6.000	4.000	4.000	4.500	1.000	22.222

Number of dissolution data points (N), degrees of freedom (df), and selected goodness of fit criteria – Pearson correlation coefficient (R), coefficient of determination (R^2), adjusted coefficient of determination ($R^2_{adjusted}$), and residual sum of squares (RSS) (manual calculation in MS Excel):

Parameter	No.1	No.2	No.3	No.4
N	33	33	33	33
df	30	30	30	30
R	0.976087016	0.978962441	0.964964271	0.973230858
R^2	0.952745863	0.958367462	0.931156045	0.947178304
$R^2_{adjusted}$	0.949595587	0.955591959	0.926566448	0.943656857
RSS	880.7265932	781.6433481	1242.569636	1164.395982

Graphical abstract of model fit presented as mean ± 1 SD of the fraction % of released carvedilol:

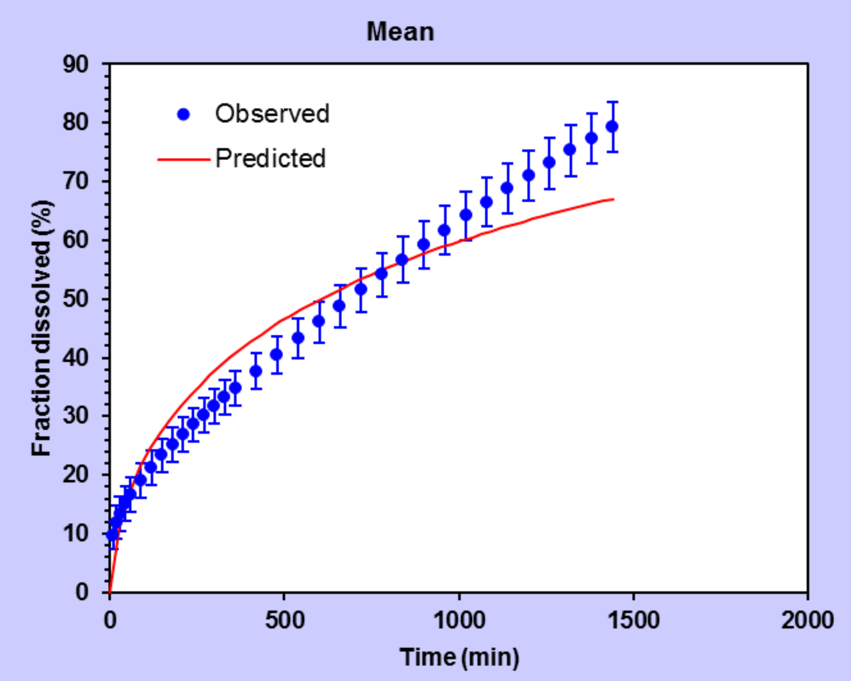

Supplementary materials – Model fitting summary – Starch 1500® sample with smaller particle size (modelling the entire relevant dissolution data)

Graphical abstract of model fit presented as the fraction % of released carvedilol per tested tablet:

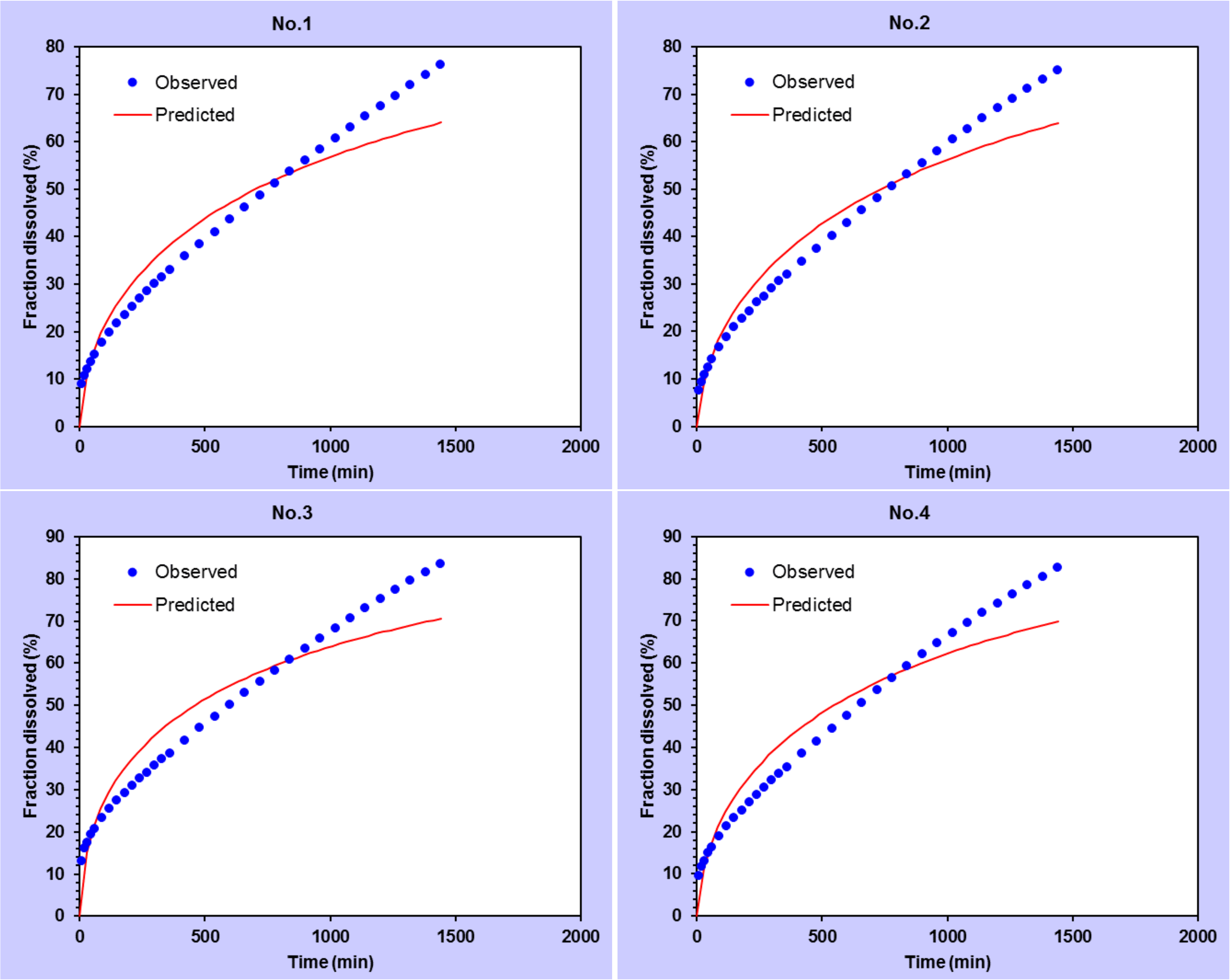

Supplementary materials – Model fitting summary – Starch 1500® sample with smaller particle size (modelling the entire relevant dissolution data)

Model: **Weibull_2**

Model equation: $F = 100 \cdot \left(1 - e^{-\frac{t^{\beta}}{\alpha}}\right)$

Fitted model parameters per tested tablet (N = 4) with statistics – mean, standard deviation (SD), and relative standard deviation expressed in % (RSD%) (output from DDSolver):

Parameter	No.1	No.2	No.3	No.4	Mean	SD	RSD(%)
α	61.914	76.264	36.925	64.075	59.795	16.503	27.599
β	0.575	0.603	0.528	0.601	0.577	0.035	6.075

Number of dissolution data points (N), degrees of freedom (df), and selected goodness of fit criteria – Pearson correlation coefficient (R), coefficient of determination (R^2), adjusted coefficient of determination ($R^2_{adjusted}$), and residual sum of squares (RSS) (manual calculation in MS Excel):

Parameter	No.1	No.2	No.3	No.4
N	33	33	33	33
df	31	31	31	31
R	0.980334987	0.983578977	0.970249935	0.977702377
R^2	0.961056686	0.967427605	0.941384936	0.955901938
$R^2_{adjusted}$	0.95980045	0.966376882	0.939494128	0.95447942
RSS	701.1849685	611.6403987	1034.73177	942.3638805

Graphical abstract of model fit presented as mean ± 1 SD of the fraction % of released carvedilol:

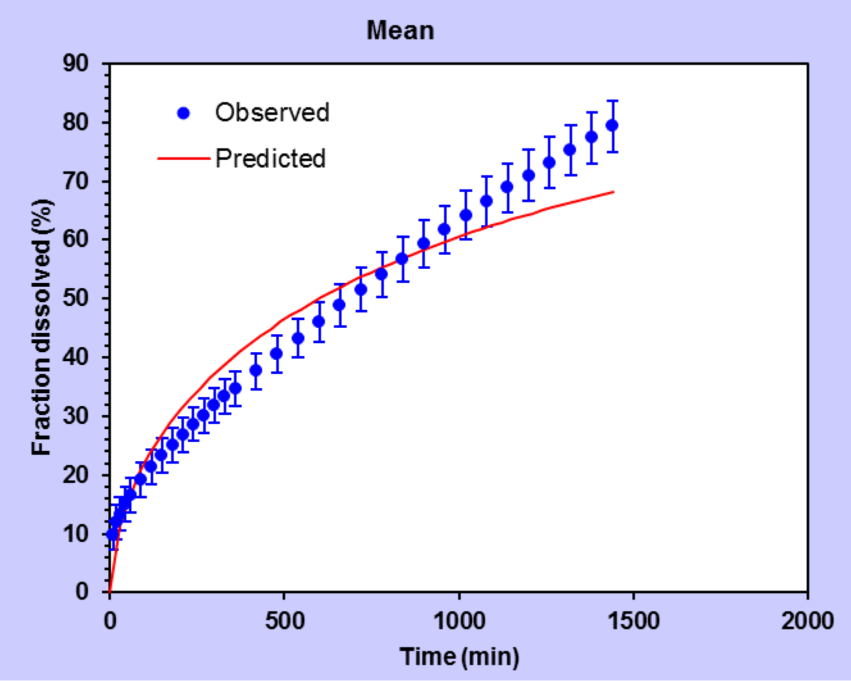

Supplementary materials – Model fitting summary – Starch 1500® sample with smaller particle size (modelling the entire relevant dissolution data)

Graphical abstract of model fit presented as the fraction % of released carvedilol per tested tablet:

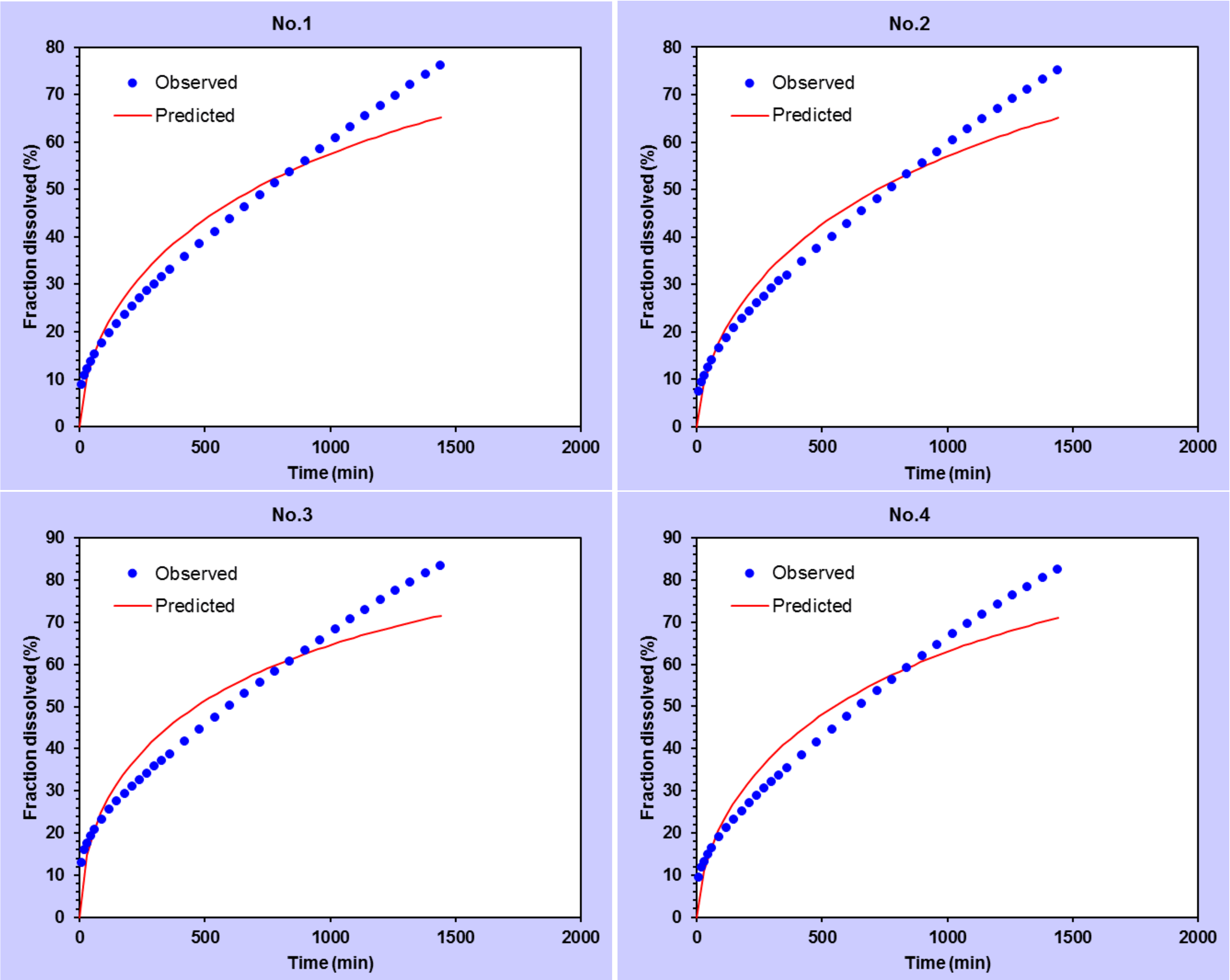

Supplementary materials – Model fitting summary – Starch 1500® sample with smaller particle size (modelling the entire relevant dissolution data)

Model: **Weibull_3**

Model equation: $F = F_{max} \cdot \left(1 - e^{-\frac{t^{\beta}}{\alpha}}\right)$

Fitted model parameters per tested tablet (N = 4) with statistics – mean, standard deviation (SD), and relative standard deviation expressed in % (RSD%) (output from DDSolver):

Parameter	No.1	No.2	No.3	No.4	Mean	SD	RSD(%)
α	65.704	105.976	39.662	90.277	75.405	29.025	38.493
β	0.646	0.688	0.579	0.666	0.645	0.047	7.317
F_{max}	79.977	82.365	87.696	90.495	85.133	4.815	5.656

Number of dissolution data points (N), degrees of freedom (df), and selected goodness of fit criteria – Pearson correlation coefficient (R), coefficient of determination (R^2), adjusted coefficient of determination ($R^2_{adjusted}$), and residual sum of squares (RSS) (manual calculation in MS Excel):

Parameter	No.1	No.2	No.3	No.4
N	33	33	33	33
df	30	30	30	30
R	0.971501126	0.982648195	0.962951	0.979649533
R^2	0.943814437	0.965597476	0.927274629	0.959713208
$R^2_{adjusted}$	0.940068733	0.963303974	0.922426271	0.957027422
RSS	897.7146245	821.2506042	1212.895192	1112.307855

Graphical abstract of model fit presented as mean ± 1 SD of the fraction % of released carvedilol:

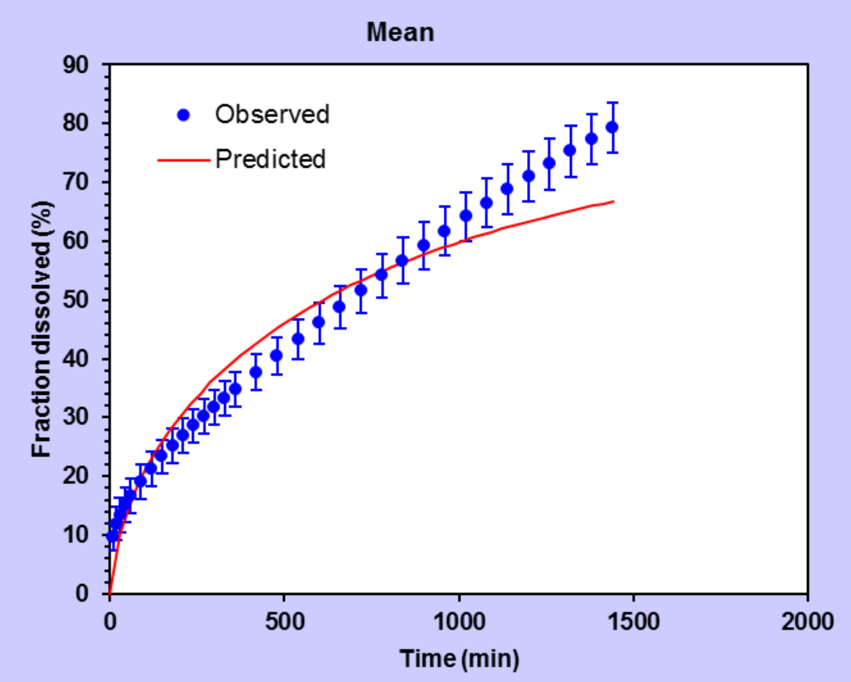

Supplementary materials – Model fitting summary – Starch 1500® sample with smaller particle size (modelling the entire relevant dissolution data)

Graphical abstract of model fit presented as the fraction % of released carvedilol per tested tablet:

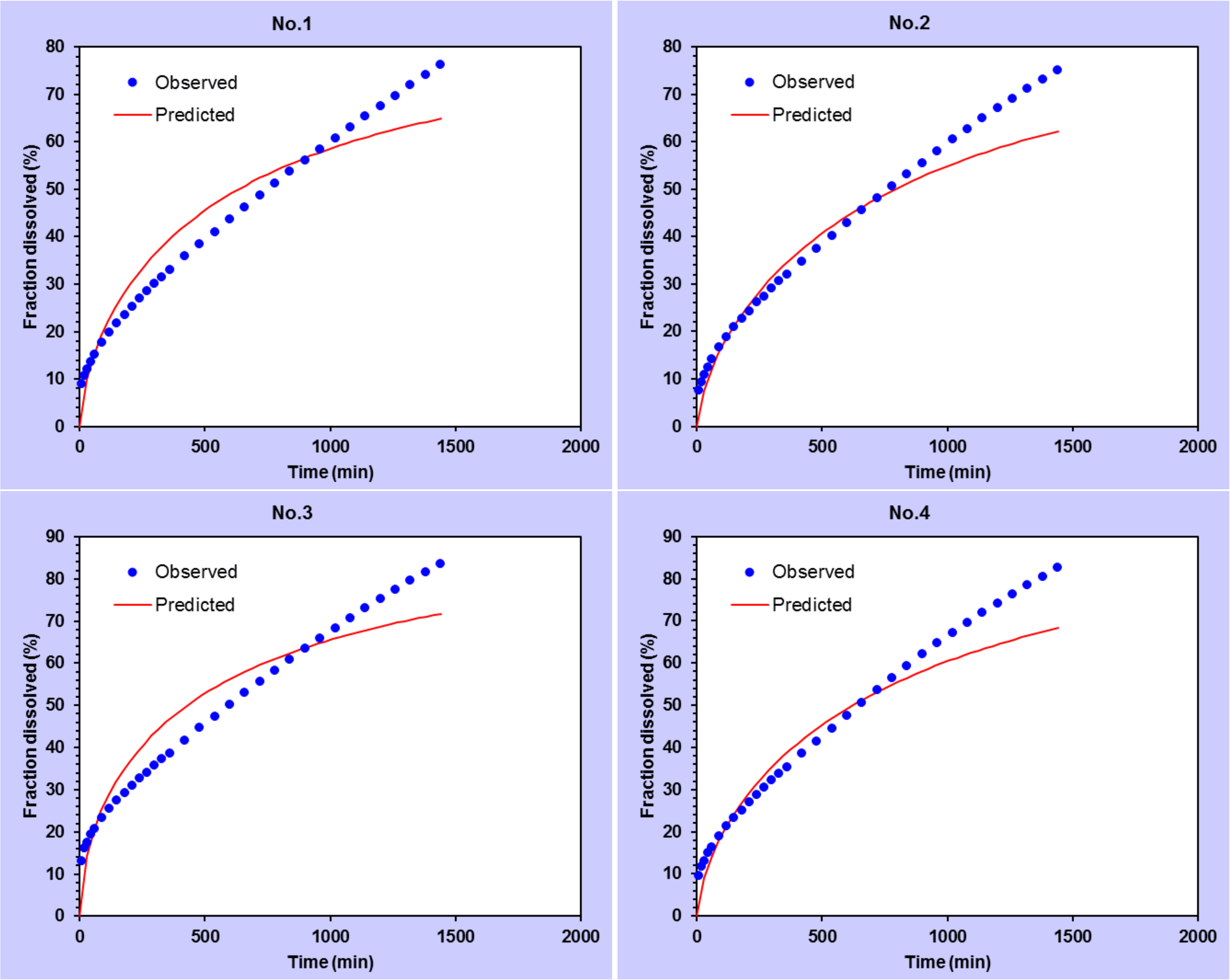

Supplementary materials – Model fitting summary – Starch 1500® sample with smaller particle size (modelling the entire relevant dissolution data)

Model: **Weibull_4**

Model equation: $F = F_{max} \cdot \left[1 - e^{-\frac{(t-T_i)^{\beta}}{\alpha}}\right]$

Fitted model parameters per tested tablet (N = 4) with statistics – mean, standard deviation (SD), and relative standard deviation expressed in % (RSD%) (output from DDSolver):

Parameter	No.1	No.2	No.3	No.4	Mean	SD	RSD(%)
α	50.552	61.914	31.278	53.022	49.191	12.901	26.226
β	0.605	0.636	0.542	0.614	0.599	0.040	6.733
T_i	6.000	6.000	4.000	6.000	5.500	1.000	18.182
F_{max}	79.977	78.866	87.696	86.651	83.297	4.519	5.425

Number of dissolution data points (N), degrees of freedom (df), and selected goodness of fit criteria – Pearson correlation coefficient (R), coefficient of determination (R^2), adjusted coefficient of determination ($R^2_{adjusted}$), and residual sum of squares (RSS) (manual calculation in MS Excel):

Parameter	No.1	No.2	No.3	No.4
N	33	33	33	33
df	29	29	29	29
R	0.965910065	0.969716884	0.957482804	0.965185992
R^2	0.932982255	0.940350835	0.916773321	0.931583999
$R^2_{adjusted}$	0.926049384	0.934180232	0.908163664	0.924506482
RSS	1062.436486	976.4983661	1411.188296	1329.440292

Graphical abstract of model fit presented as mean ± 1 SD of the fraction % of released carvedilol:

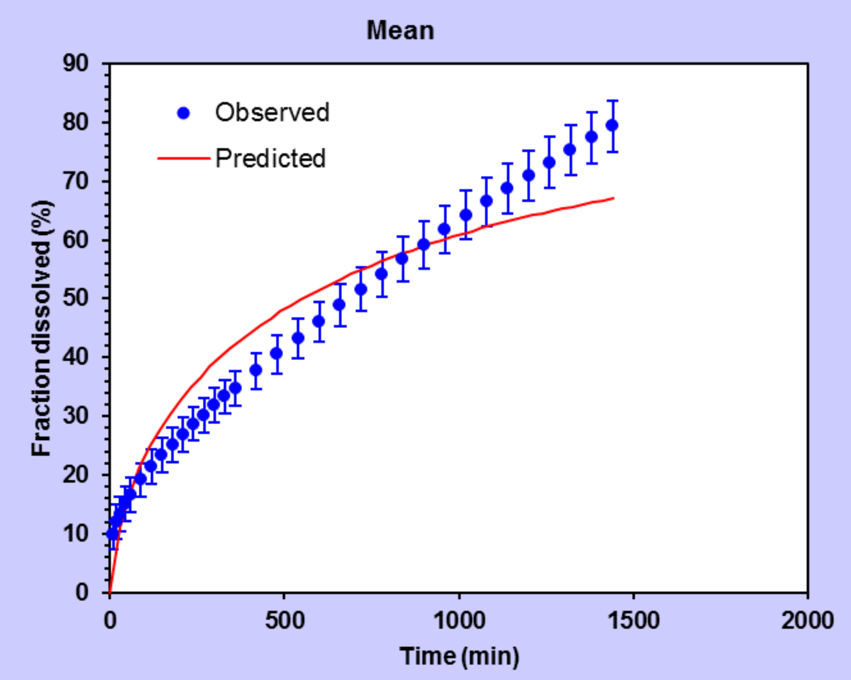

Supplementary materials – Model fitting summary – Starch 1500® sample with smaller particle size (modelling the entire relevant dissolution data)

Graphical abstract of model fit presented as the fraction % of released carvedilol per tested tablet:

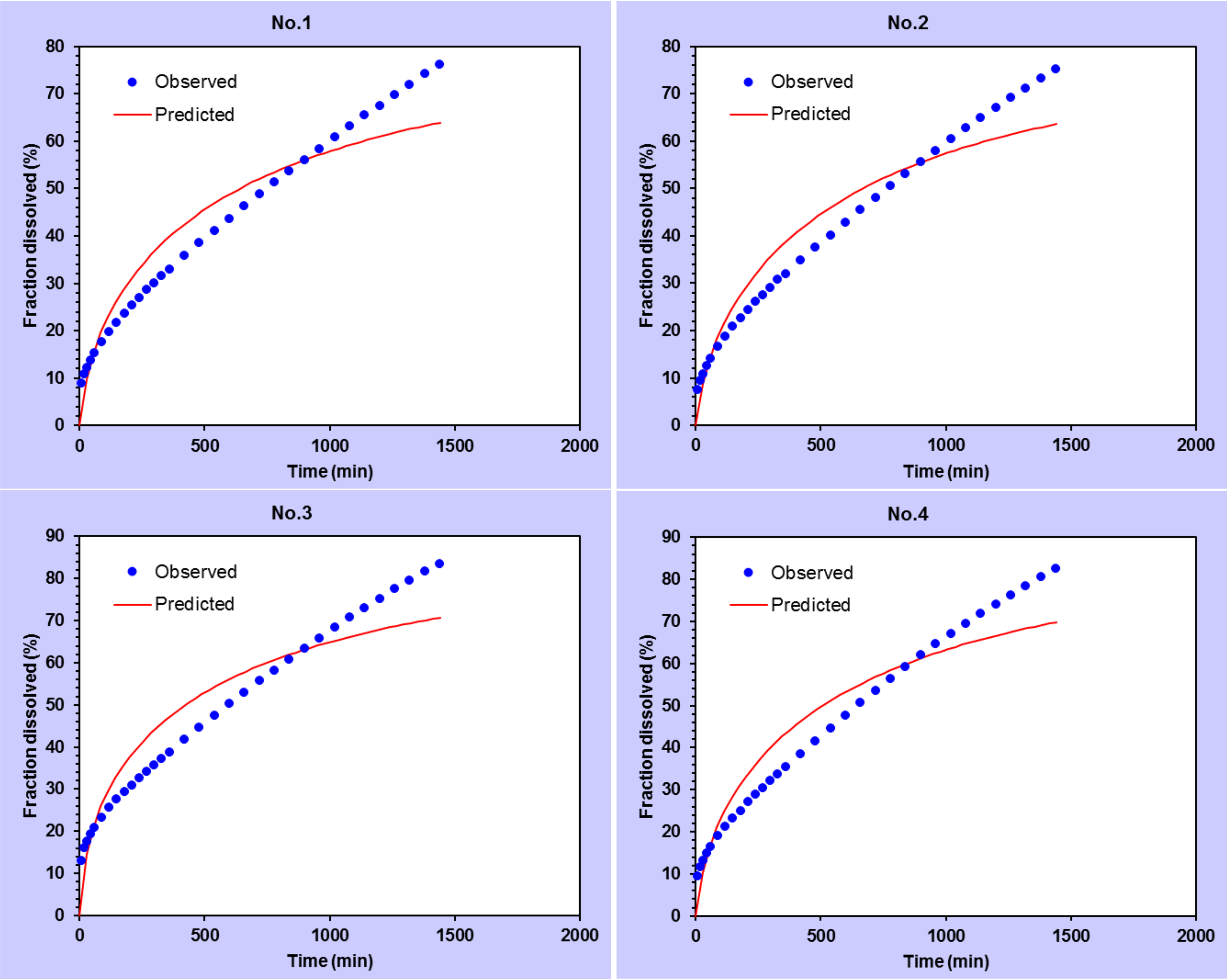

Supplementary materials – Model fitting summary – Starch 1500® sample with smaller particle size (modelling the entire relevant dissolution data)

Model: **Logistic_1**

Model equation: $F = 100 \cdot \frac{e^{\alpha+\beta \cdot \log(t)}}{1+e^{\alpha+\beta \cdot \log(t)}}$

Fitted model parameters per tested tablet (N = 4) with statistics – mean, standard deviation (SD), and relative standard deviation expressed in % (RSD%) (output from DDSolver):

Parameter	No.1	No.2	No.3	No.4	Mean	SD	RSD(%)
α	-4.632	-4.841	-4.224	-5.512	-4.802	0.538	-11.206
β	1.649	1.711	1.618	1.974	1.738	0.162	9.317

Number of dissolution data points (N), degrees of freedom (df), and selected goodness of fit criteria – Pearson correlation coefficient (R), coefficient of determination (R^2), adjusted coefficient of determination ($R^2_{adjusted}$), and residual sum of squares (RSS) (manual calculation in MS Excel):

Parameter	No.1	No.2	No.3	No.4
N	33	33	33	33
df	31	31	31	31
R	0.967533857	0.971568256	0.952542679	0.974473643
R^2	0.936121764	0.943944875	0.907337556	0.949598881
$R^2_{adjusted}$	0.934061176	0.942136646	0.904348445	0.947973039
RSS	1022.047463	930.2383008	1509.916887	1329.379794

Graphical abstract of model fit presented as mean ± 1 SD of the fraction % of released carvedilol:

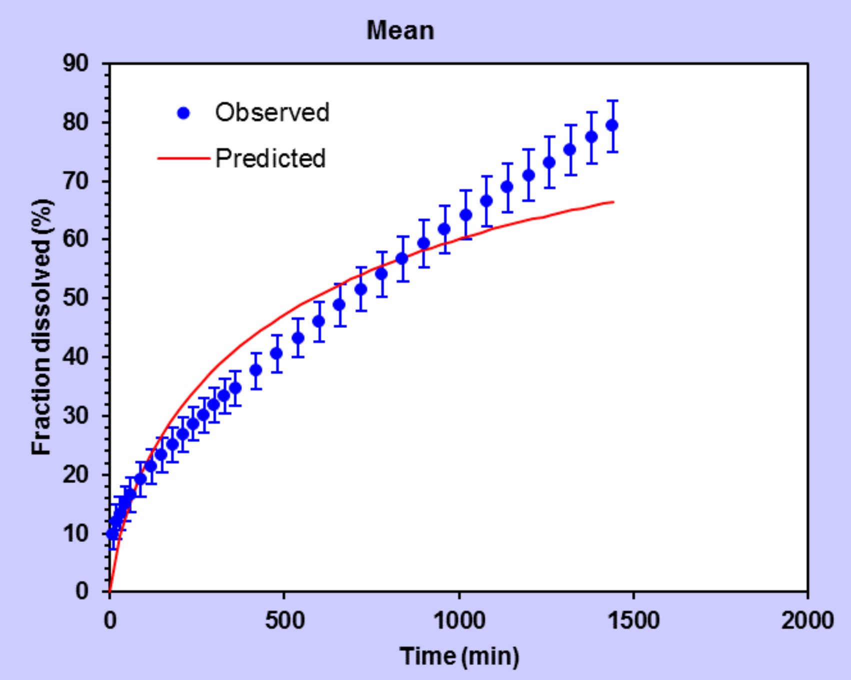

Supplementary materials – Model fitting summary – Starch 1500® sample with smaller particle size (modelling the entire relevant dissolution data)

Graphical abstract of model fit presented as the fraction % of released carvedilol per tested tablet:

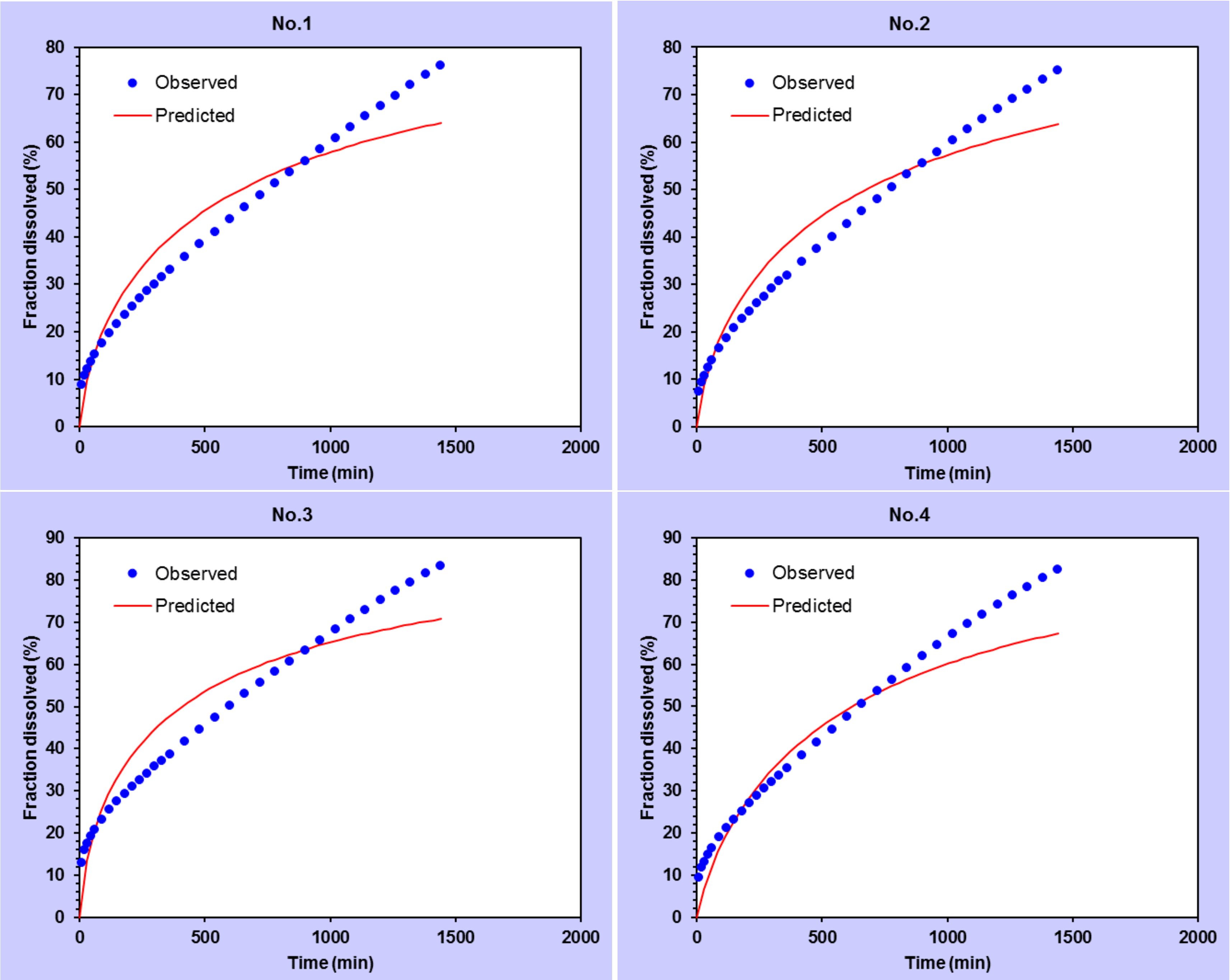

Supplementary materials – Model fitting summary – Starch 1500® sample with smaller particle size (modelling the entire relevant dissolution data)

Model: **Logistic_2**

Model equation: $F = F_{max} \cdot \frac{e^{\alpha+\beta\cdot\log(t)}}{1+e^{\alpha+\beta\cdot\log(t)}}$

Fitted model parameters per tested tablet (N = 4) with statistics – mean, standard deviation (SD), and relative standard deviation expressed in % (RSD%) (output from DDSolver):

Parameter	No.1	No.2	No.3	No.4	Mean	SD	RSD(%)
α	-5.209	-5.452	-4.693	-5.299	-5.163	0.329	-6.378
β	2.099	2.186	1.955	2.141	2.095	0.100	4.783
F_{max}	79.977	78.866	87.696	86.651	83.297	4.519	5.425

Number of dissolution data points (N), degrees of freedom (df), and selected goodness of fit criteria – Pearson correlation coefficient (R), coefficient of determination (R^2), adjusted coefficient of determination ($R^2_{adjusted}$), and residual sum of squares (RSS) (manual calculation in MS Excel):

Parameter	No.1	No.2	No.3	No.4
N	33	33	33	33
df	30	30	30	30
R	0.947649305	0.951221408	0.93622214	0.945906718
R^2	0.898039205	0.904822167	0.876511896	0.894739519
$R^2_{adjusted}$	0.891241818	0.898476978	0.868279356	0.887722154
RSS	1599.04005	1536.20046	2068.407741	2017.318193

Graphical abstract of model fit presented as mean ± 1 SD of the fraction % of released carvedilol:

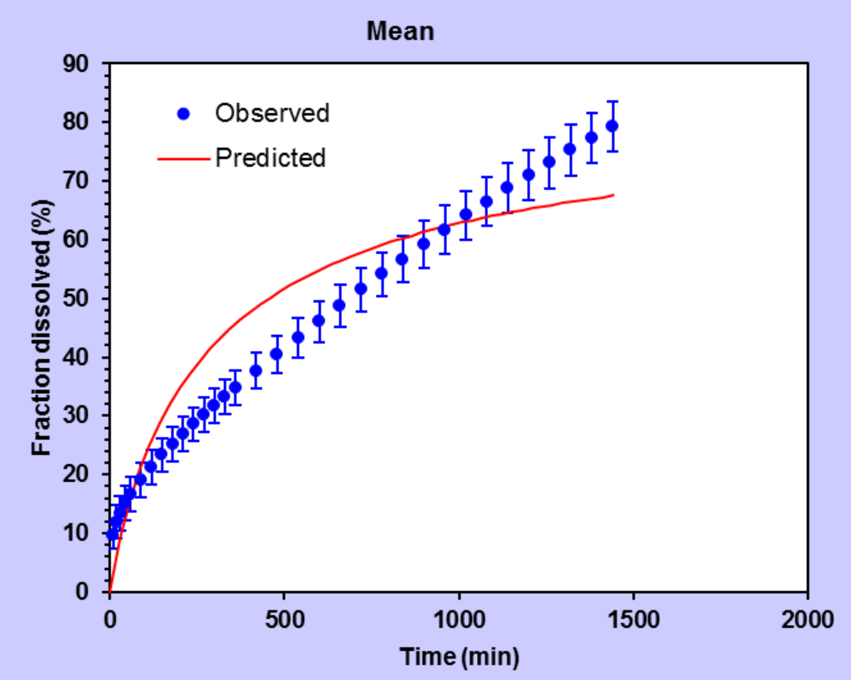

Supplementary materials – Model fitting summary – Starch 1500® sample with smaller particle size (modelling the entire relevant dissolution data)

Graphical abstract of model fit presented as the fraction % of released carvedilol per tested tablet:

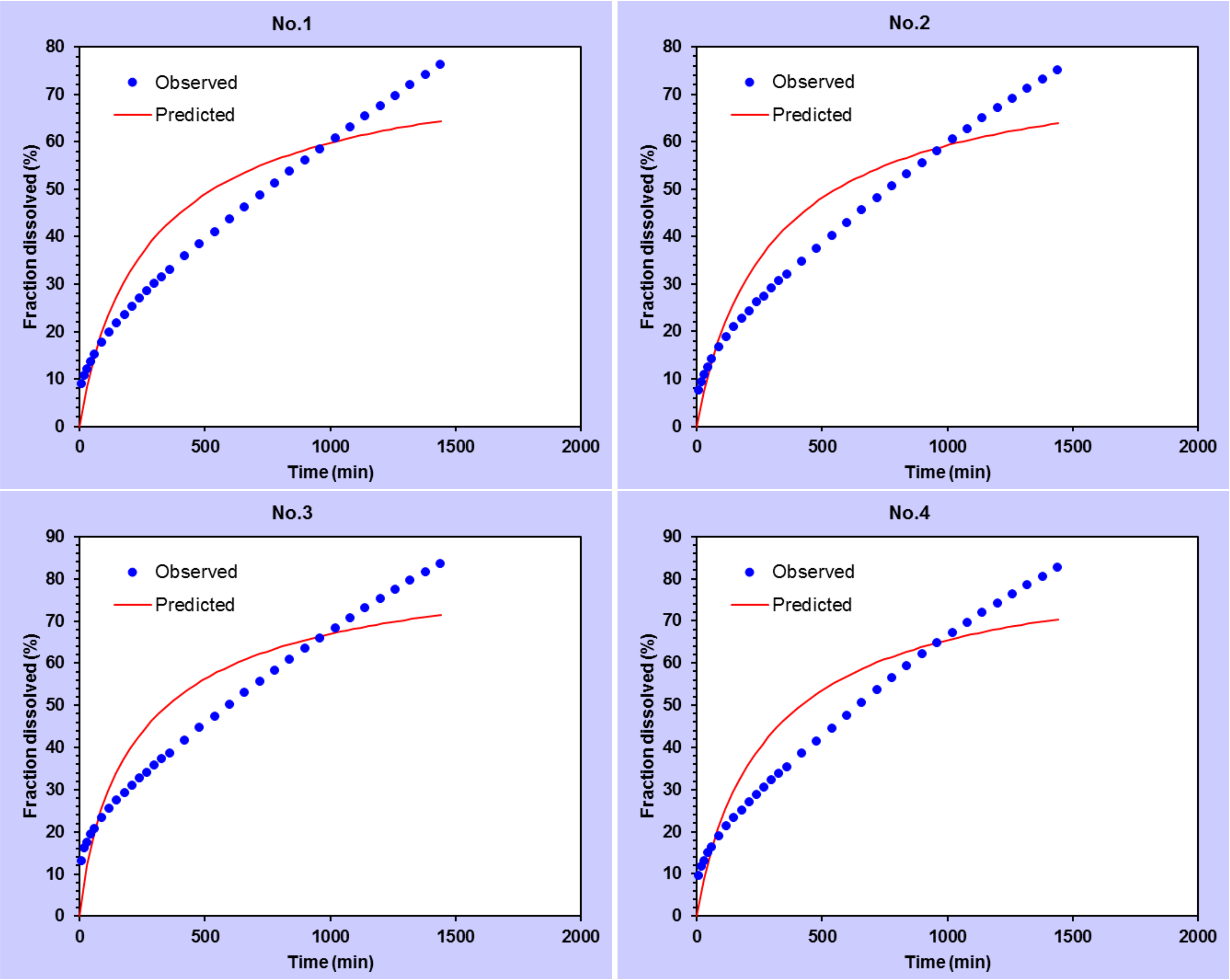

Supplementary materials – Model fitting summary – Starch 1500® sample with smaller particle size (modelling the entire relevant dissolution data)

Model: **Logistic_3**

Model equation: $F = F_{max} \cdot \frac{1}{1+e^{-k\cdot(t-\gamma)}}$

Fitted model parameters per tested tablet (N = 4) with statistics – mean, standard deviation (SD), and relative standard deviation expressed in % (RSD%) (output from DDSolver):

Parameter	No.1	No.2	No.3	No.4	Mean	SD	RSD(%)
k	0.003	0.003	0.003	0.003	0.003	0.000	3.787
γ	549.170	558.277	491.444	544.087	535.744	30.111	5.620
F_{max}	79.977	78.866	87.696	86.651	83.297	4.519	5.425

Number of dissolution data points (N), degrees of freedom (df), and selected goodness of fit criteria – Pearson correlation coefficient (R), coefficient of determination (R^2), adjusted coefficient of determination ($R^2_{adjusted}$), and residual sum of squares (RSS) (manual calculation in MS Excel):

Parameter	No.1	No.2	No.3	No.4
N	33	33	33	33
df	30	30	30	30
R	0.992974239	0.99263705	0.994706531	0.994654325
R^2	0.98599784	0.985328313	0.989441082	0.989337227
$R^2_{adjusted}$	0.985064363	0.9843502	0.988737154	0.988626376
RSS	212.1607142	227.5547445	172.8825366	195.8724983

Graphical abstract of model fit presented as mean ± 1 SD of the fraction % of released carvedilol:

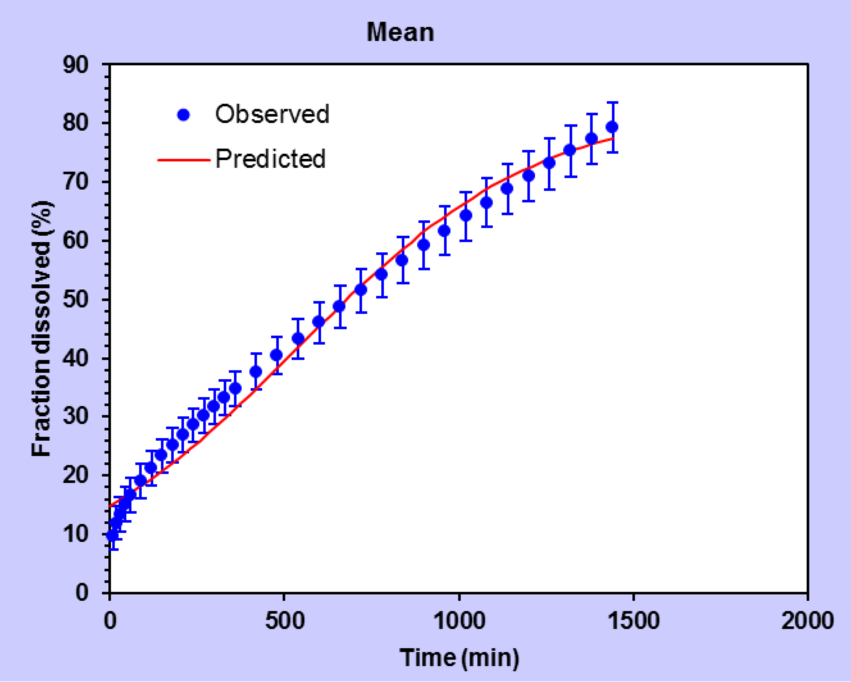

Supplementary materials – Model fitting summary – Starch 1500® sample with smaller particle size (modelling the entire relevant dissolution data)

Graphical abstract of model fit presented as the fraction % of released carvedilol per tested tablet:

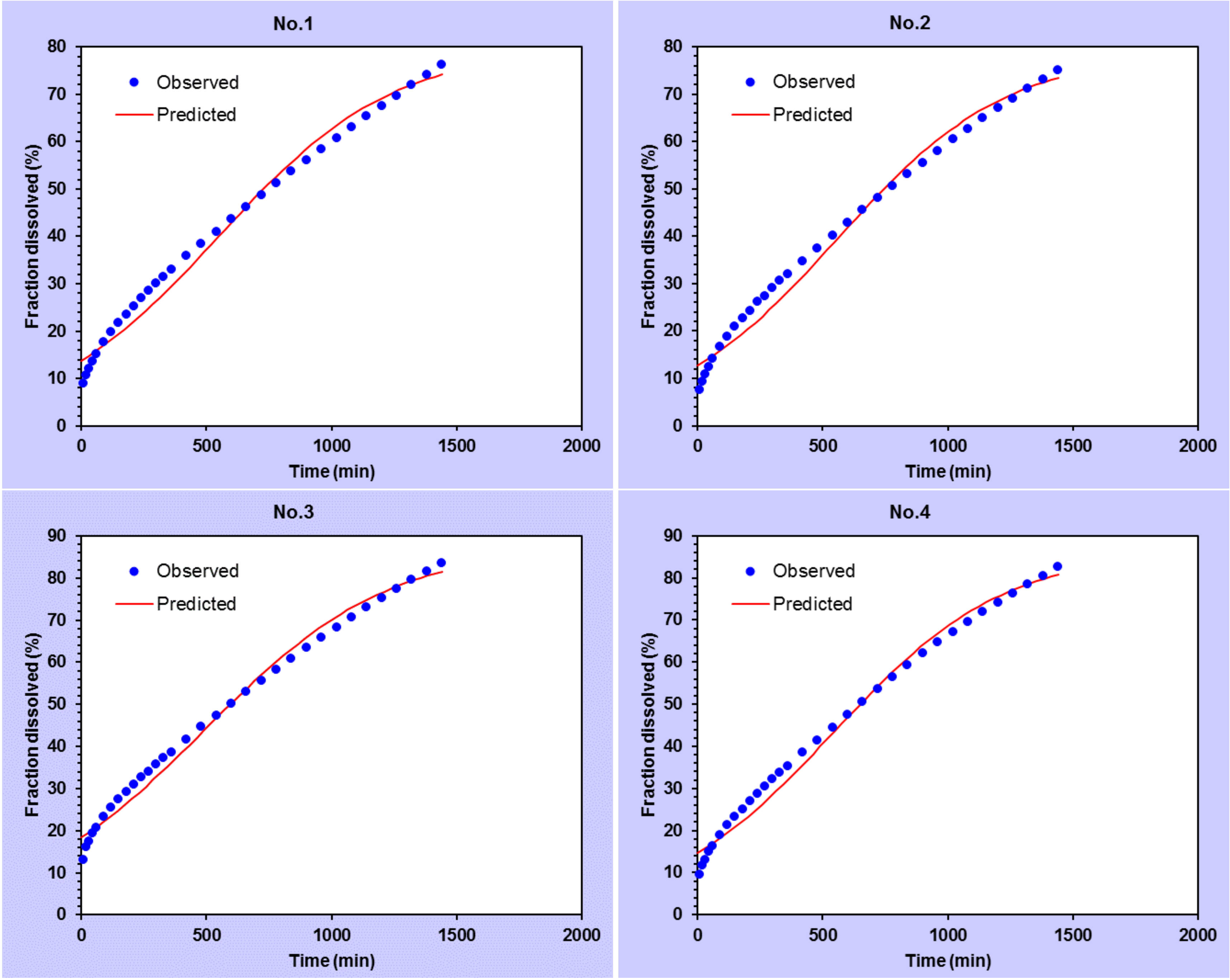

Supplementary materials – Model fitting summary – Starch 1500® sample with smaller particle size (modelling the entire relevant dissolution data)

Model: **Gompertz_1**

Model equation: $F = 100 \cdot e^{-\alpha \cdot e^{-\beta \cdot \log(t)}}$

Fitted model parameters per tested tablet (N = 4) with statistics – mean, standard deviation (SD), and relative standard deviation expressed in % (RSD%) (output from DDSolver):

Parameter	No.1	No.2	No.3	No.4	Mean	SD	RSD(%)
α	11.386	12.239	11.093	14.087	12.201	1.348	11.047
β	1.012	1.029	1.089	1.148	1.069	0.062	5.770

Number of dissolution data points (N), degrees of freedom (df), and selected goodness of fit criteria – Pearson correlation coefficient (R), coefficient of determination (R^2), adjusted coefficient of determination ($R^2_{adjusted}$), and residual sum of squares (RSS) (manual calculation in MS Excel):

Parameter	No.1	No.2	No.3	No.4
N	33	33	33	33
df	31	31	31	31
R	0.940642836	0.945156543	0.923352547	0.932245145
R^2	0.884808945	0.893320891	0.852579927	0.86908101
$R^2_{adjusted}$	0.881093105	0.889879629	0.847824441	0.864857817
RSS	1700.707261	1623.625857	2333.381894	2363.463806

Graphical abstract of model fit presented as mean ± 1 SD of the fraction % of released carvedilol:

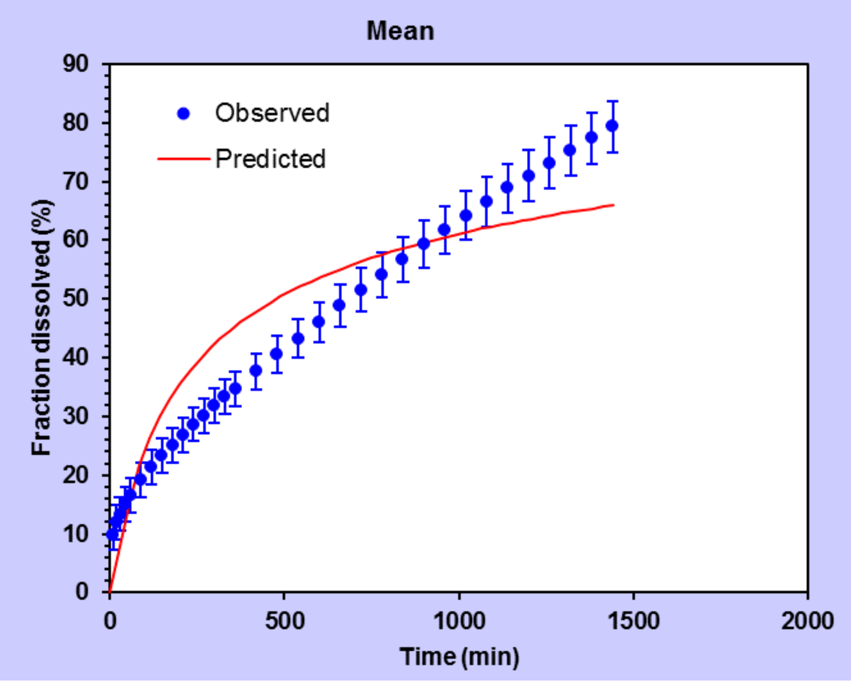

Supplementary materials – Model fitting summary – Starch 1500® sample with smaller particle size (modelling the entire relevant dissolution data)

Graphical abstract of model fit presented as the fraction % of released carvedilol per tested tablet:

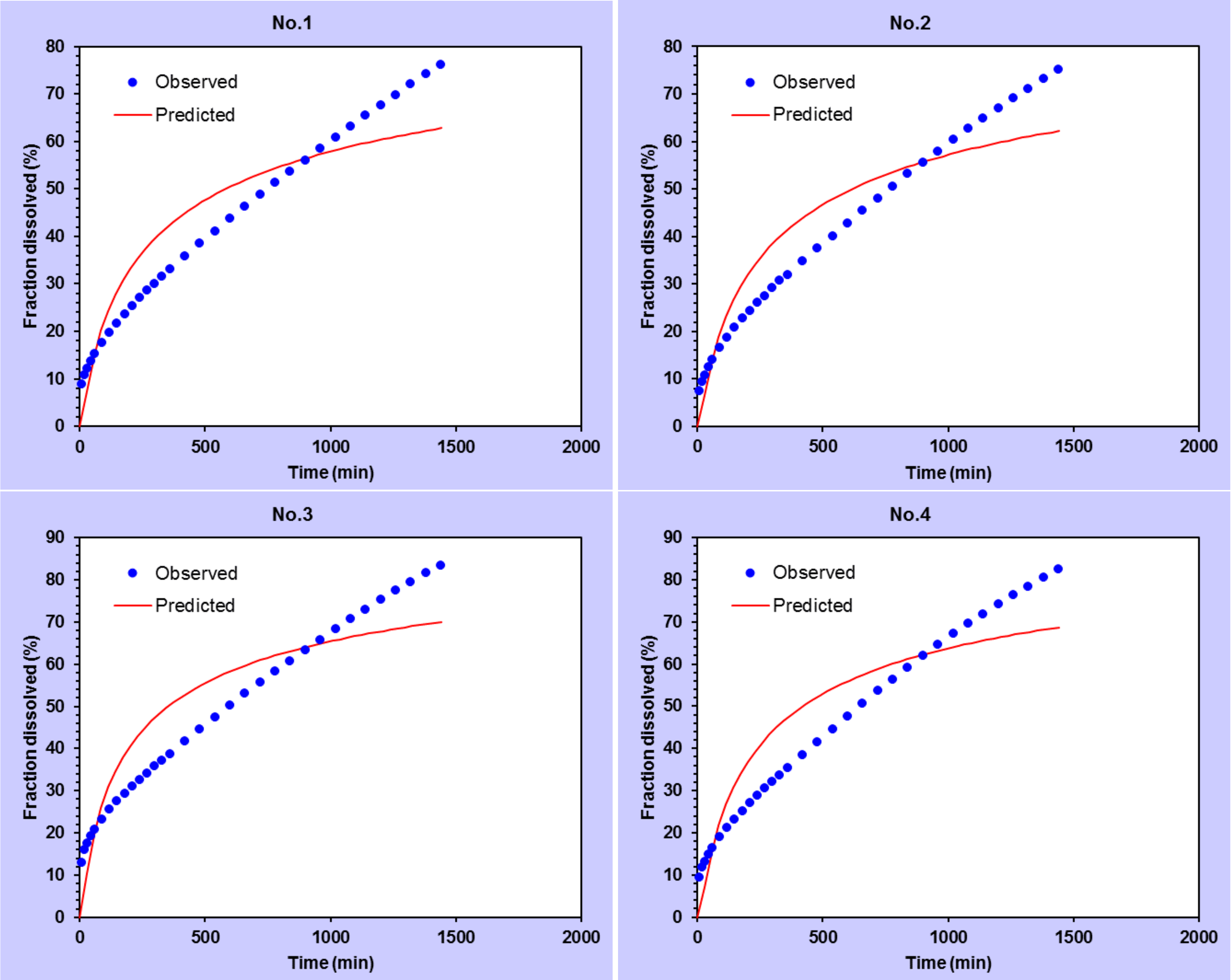

Supplementary materials – Model fitting summary – Starch 1500® sample with smaller particle size (modelling the entire relevant dissolution data)

Model: **Gompertz_2**

Model equation: $F = F_{max} \cdot e^{-\alpha \cdot e^{-\beta \cdot \log(t)}}$

Fitted model parameters per tested tablet (N = 4) with statistics – mean, standard deviation (SD), and relative standard deviation expressed in % (RSD%) (output from DDSolver):

Parameter	No.1	No.2	No.3	No.4	Mean	SD	RSD(%)
α	36.234	40.858	29.339	38.960	36.348	5.043	13.875
β	1.480	1.524	1.435	1.515	1.489	0.041	2.729
F_{max}	79.977	78.866	87.696	86.651	83.297	4.519	5.425

Number of dissolution data points (N), degrees of freedom (df), and selected goodness of fit criteria – Pearson correlation coefficient (R), coefficient of determination (R^2), adjusted coefficient of determination ($R^2_{adjusted}$), and residual sum of squares (RSS) (manual calculation in MS Excel):

Parameter	No.1	No.2	No.3	No.4
N	33	33	33	33
df	30	30	30	30
R	0.954155216	0.95688742	0.943916809	0.95249454
R^2	0.910412177	0.915633534	0.890978942	0.907245849
$R^2_{adjusted}$	0.904439655	0.910009103	0.883710871	0.901062239
RSS	2307.040701	2162.910101	2979.982185	2705.422183

Graphical abstract of model fit presented as mean ± 1 SD of the fraction % of released carvedilol:

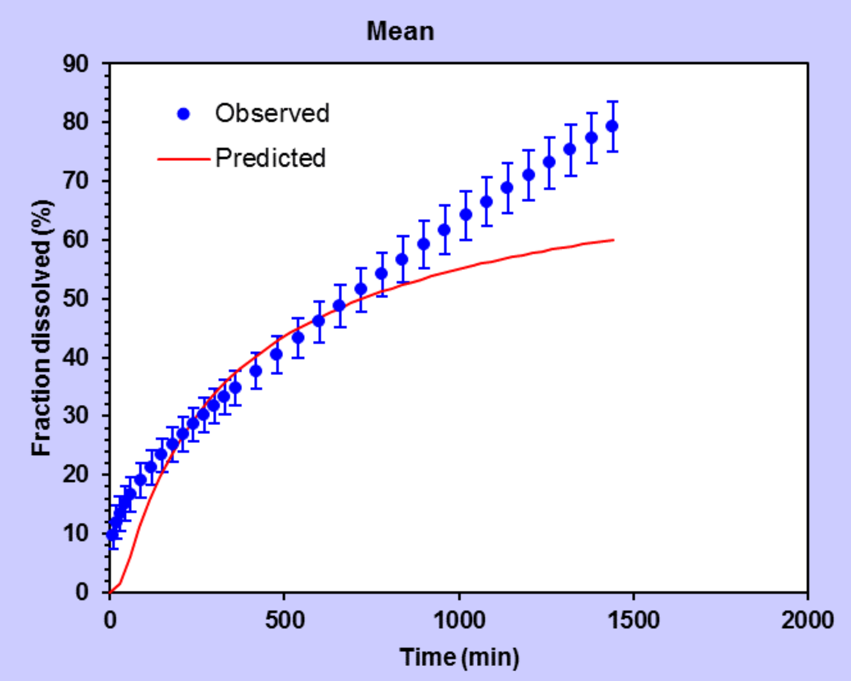

Supplementary materials – Model fitting summary – Starch 1500® sample with smaller particle size (modelling the entire relevant dissolution data)

Graphical abstract of model fit presented as the fraction % of released carvedilol per tested tablet:

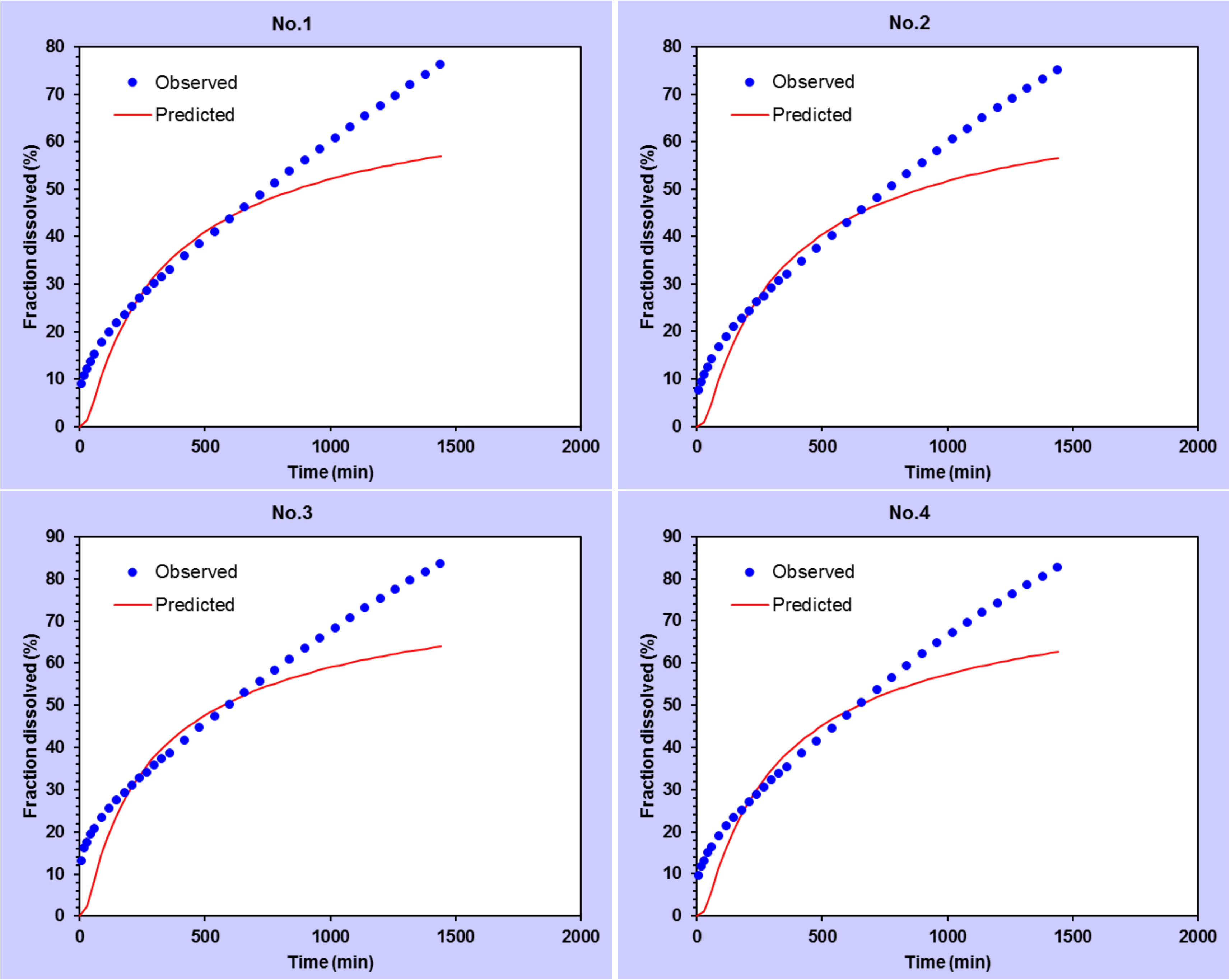

Supplementary materials – Model fitting summary – Starch 1500® sample with smaller particle size (modelling the entire relevant dissolution data)

Model: **Gompertz_3**

Model equation: $F = F_{max} \cdot e^{-e^{-k \cdot (t-\gamma)}}$

Fitted model parameters per tested tablet (N = 4) with statistics – mean, standard deviation (SD), and relative standard deviation expressed in % (RSD%) (output from DDSolver):

Parameter	No.1	No.2	No.3	No.4	Mean	SD	RSD(%)
k	0.002	0.002	0.002	0.002	0.002	0.000	16.026
γ	323.603	333.933	331.383	322.440	327.840	5.680	1.733
F_{max}	79.977	78.866	96.899	86.651	85.598	8.281	9.674

Number of dissolution data points (N), degrees of freedom (df), and selected goodness of fit criteria – Pearson correlation coefficient (R), coefficient of determination (R^2), adjusted coefficient of determination ($R^2_{adjusted}$), and residual sum of squares (RSS) (manual calculation in MS Excel):

Parameter	No.1	No.2	No.3	No.4
N	33	33	33	33
df	30	30	30	30
R	0.994516245	0.99456846	0.997160097	0.995656048
R^2	0.989062562	0.989166421	0.99432826	0.991330966
$R^2_{adjusted}$	0.9883334	0.988444182	0.993950144	0.99075303
RSS	192.969971	191.0973039	99.12901738	188.79474

Graphical abstract of model fit presented as mean ± 1 SD of the fraction % of released carvedilol:

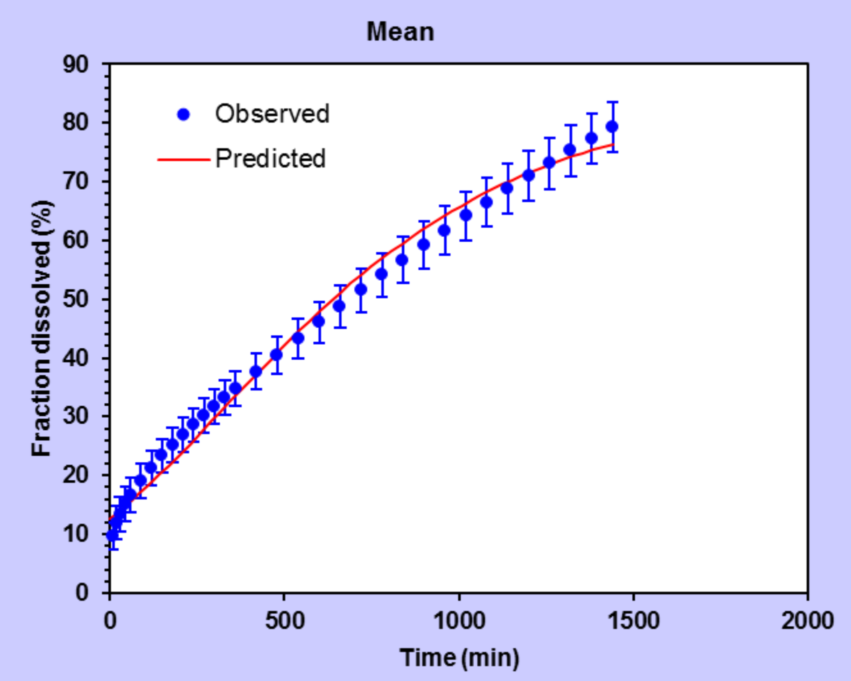

Supplementary materials – Model fitting summary – Starch 1500® sample with smaller particle size (modelling the entire relevant dissolution data)

Graphical abstract of model fit presented as the fraction % of released carvedilol per tested tablet:

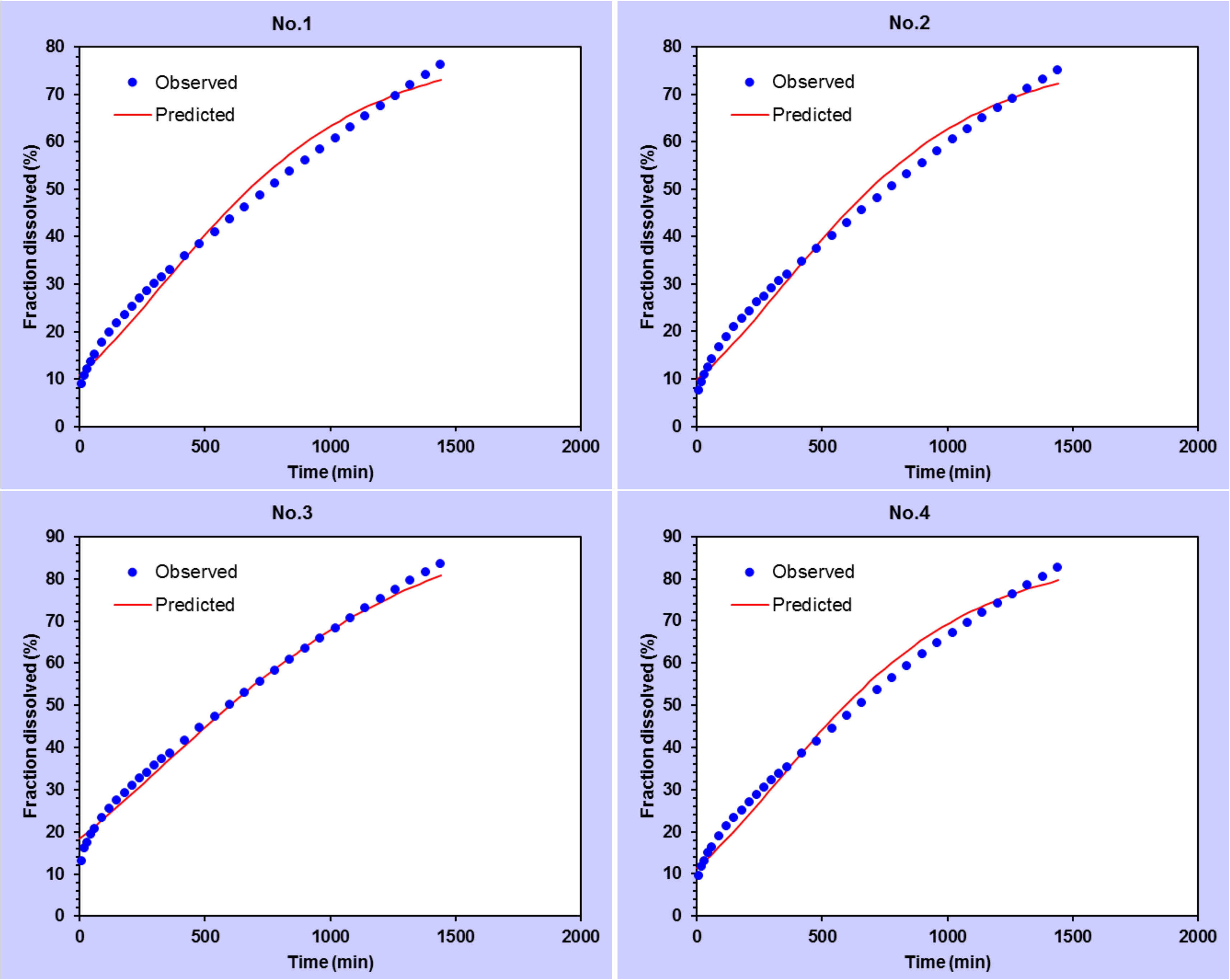

Supplementary materials – Model fitting summary – Starch 1500® sample with smaller particle size (modelling the entire relevant dissolution data)

Model: **Gompertz_4**

Model equation: $F = F_{max} \cdot e^{-\beta \cdot e^{-k \cdot t}}$

Fitted model parameters per tested tablet (N = 4) with statistics – mean, standard deviation (SD), and relative standard deviation expressed in % (RSD%) (output from DDSolver):

Parameter	No.1	No.2	No.3	No.4	Mean	SD	RSD(%)
k	0.002	0.002	0.002	0.002	0.002	0.000	2.295
β	2.003	2.085	1.768	2.035	1.973	0.140	7.118
F_{max}	79.977	78.866	87.696	86.651	83.297	4.519	5.425

Number of dissolution data points (N), degrees of freedom (df), and selected goodness of fit criteria – Pearson correlation coefficient (R), coefficient of determination (R^2), adjusted coefficient of determination ($R^2_{adjusted}$), and residual sum of squares (RSS) (manual calculation in MS Excel):

Parameter	No.1	No.2	No.3	No.4
N	33	33	33	33
df	30	30	30	30
R	0.994516245	0.99456846	0.994731016	0.995656048
R^2	0.989062562	0.989166421	0.989489795	0.991330966
$R^2_{adjusted}$	0.9883334	0.988444182	0.988789115	0.99075303
RSS	192.969971	191.0973039	208.3013706	188.79474

Graphical abstract of model fit presented as mean ± 1 SD of the fraction % of released carvedilol:

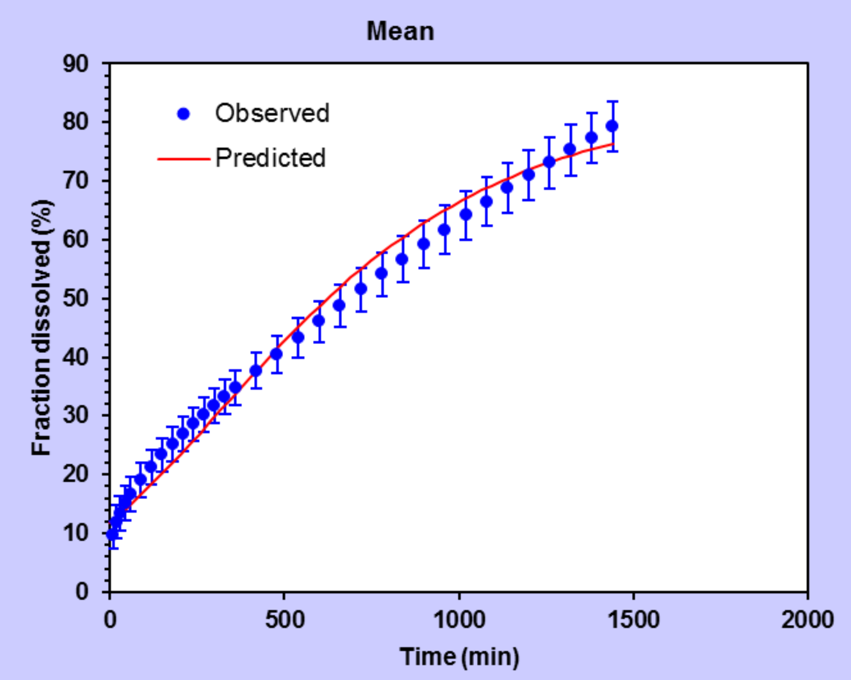

Supplementary materials – Model fitting summary – Starch 1500® sample with smaller particle size (modelling the entire relevant dissolution data)

Graphical abstract of model fit presented as the fraction % of released carvedilol per tested tablet:

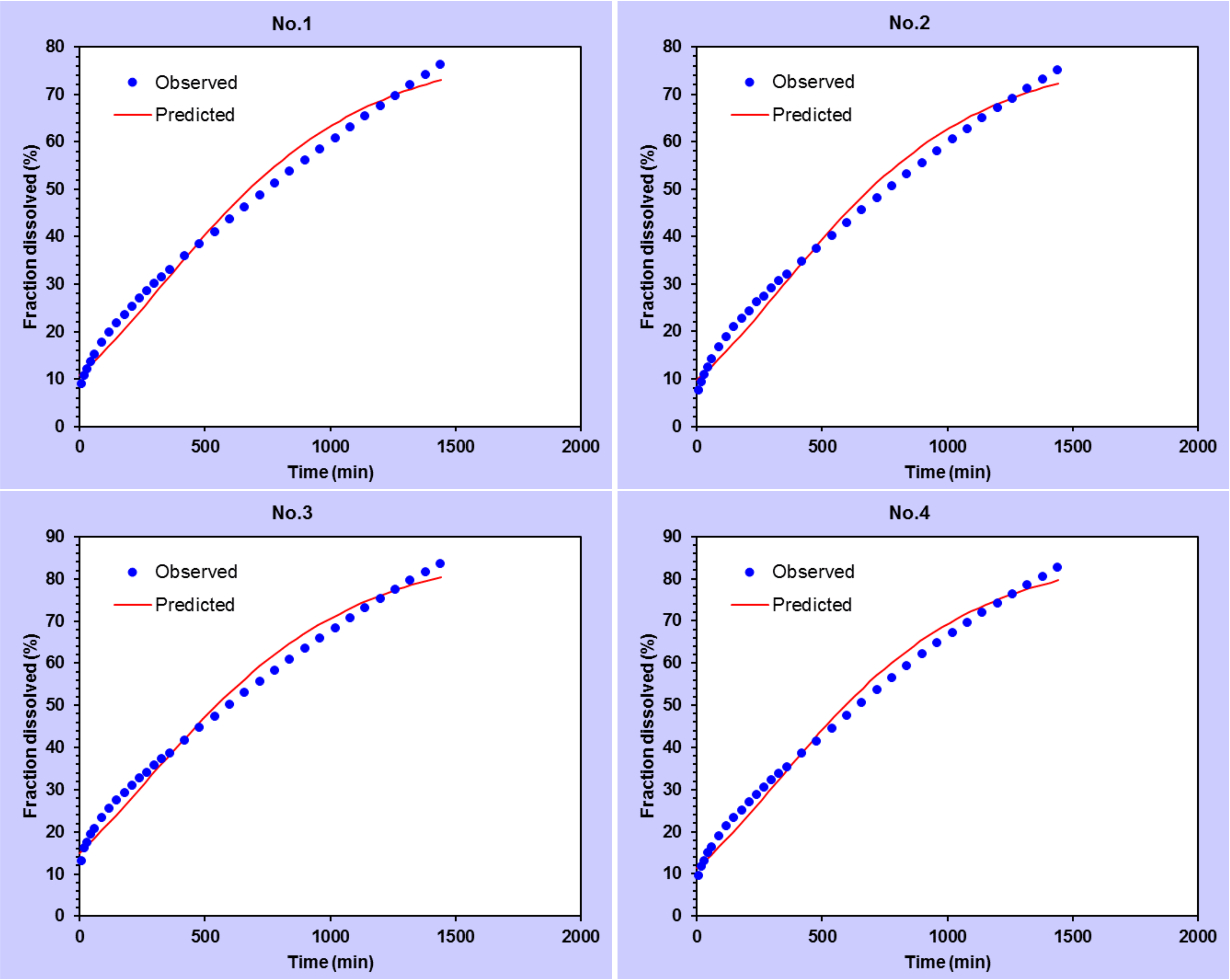

Supplementary materials – Model fitting summary – Starch 1500® sample with smaller particle size (modelling the entire relevant dissolution data)

Model: **Probit_1**

Model equation: $F = 100 \cdot \phi[\alpha + \beta \cdot \log(t)]$

Fitted model parameters per tested tablet (N = 4) with statistics – mean, standard deviation (SD), and relative standard deviation expressed in % (RSD%) (output from DDSolver):

Parameter	No.1	No.2	No.3	No.4	Mean	SD	RSD(%)
α	-2.764	-2.871	-2.559	-3.302	-2.874	0.313	-10.895
β	0.985	1.015	0.980	1.183	1.041	0.096	9.242

Number of dissolution data points (N), degrees of freedom (df), and selected goodness of fit criteria – Pearson correlation coefficient (R), coefficient of determination (R^2), adjusted coefficient of determination ($R^2_{adjusted}$), and residual sum of squares (RSS) (manual calculation in MS Excel):

Parameter	No.1	No.2	No.3	No.4
N	33	33	33	33
df	31	31	31	31
R	0.961745355	0.965642523	0.948616067	0.969481605
R^2	0.924954127	0.932465483	0.899872443	0.939894582
$R^2_{adjusted}$	0.922533293	0.93028695	0.896642521	0.937955698
RSS	1174.28931	1095.784681	1609.700325	1496.505073

Graphical abstract of model fit presented as mean ± 1 SD of the fraction % of released carvedilol:

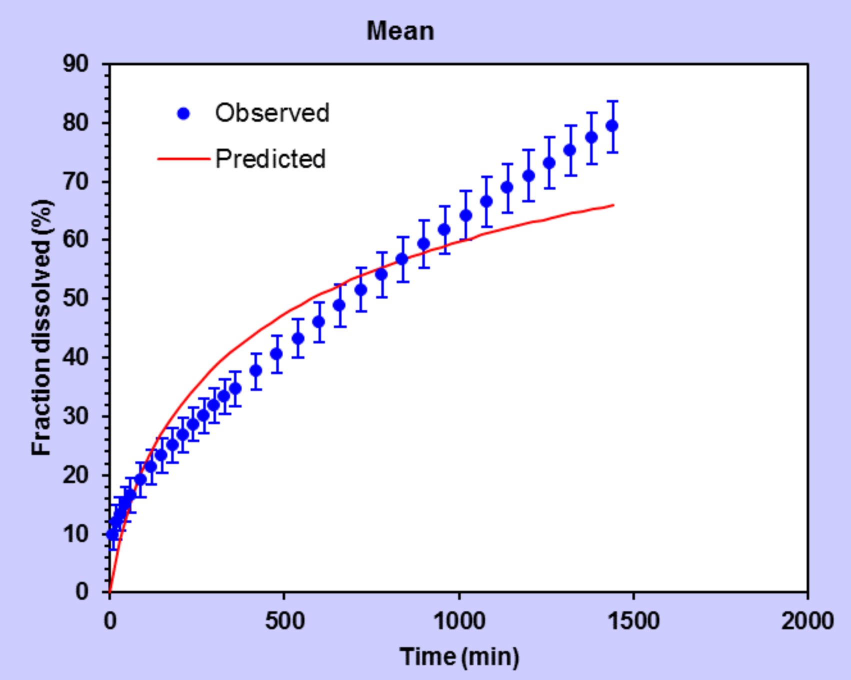

Supplementary materials – Model fitting summary – Starch 1500® sample with smaller particle size (modelling the entire relevant dissolution data)

Graphical abstract of model fit presented as the fraction % of released carvedilol per tested tablet:

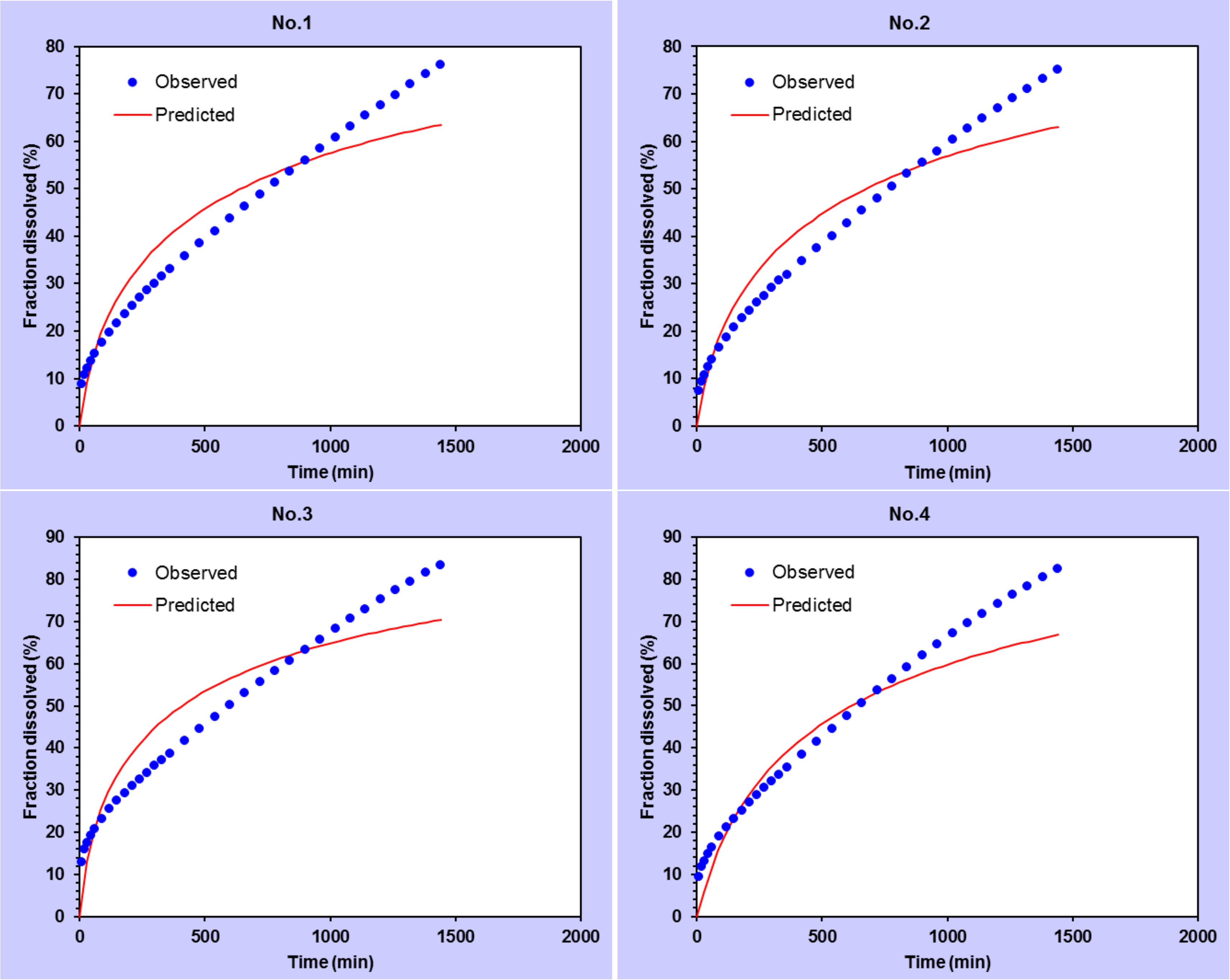

Supplementary materials – Model fitting summary – Starch 1500® sample with smaller particle size (modelling the entire relevant dissolution data)

Model: **Probit_2**

Model equation: $F = F_{max} \cdot \phi[\alpha + \beta \cdot \log(t)]$

Fitted model parameters per tested tablet (N = 4) with statistics – mean, standard deviation (SD), and relative standard deviation expressed in % (RSD%) (output from DDSolver):

Parameter	No.1	No.2	No.3	No.4	Mean	SD	RSD(%)
α	-3.102	-3.234	-2.811	-3.153	-3.075	0.184	-6.000
β	1.247	1.294	1.167	1.271	1.245	0.055	4.450
F_{max}	79.977	78.866	87.696	86.651	83.297	4.519	5.425

Number of dissolution data points (N), degrees of freedom (df), and selected goodness of fit criteria – Pearson correlation coefficient (R), coefficient of determination (R^2), adjusted coefficient of determination ($R^2_{adjusted}$), and residual sum of squares (RSS) (manual calculation in MS Excel):

Parameter	No.1	No.2	No.3	No.4
N	33	33	33	33
df	30	30	30	30
R	0.946138148	0.949624377	0.936130137	0.944514445
R^2	0.895177395	0.901786457	0.876339633	0.892107537
$R^2_{adjusted}$	0.888189221	0.895238888	0.868095609	0.884914706
RSS	1594.311954	1537.963783	2005.099379	2004.539198

Graphical abstract of model fit presented as mean ± 1 SD of the fraction % of released carvedilol:

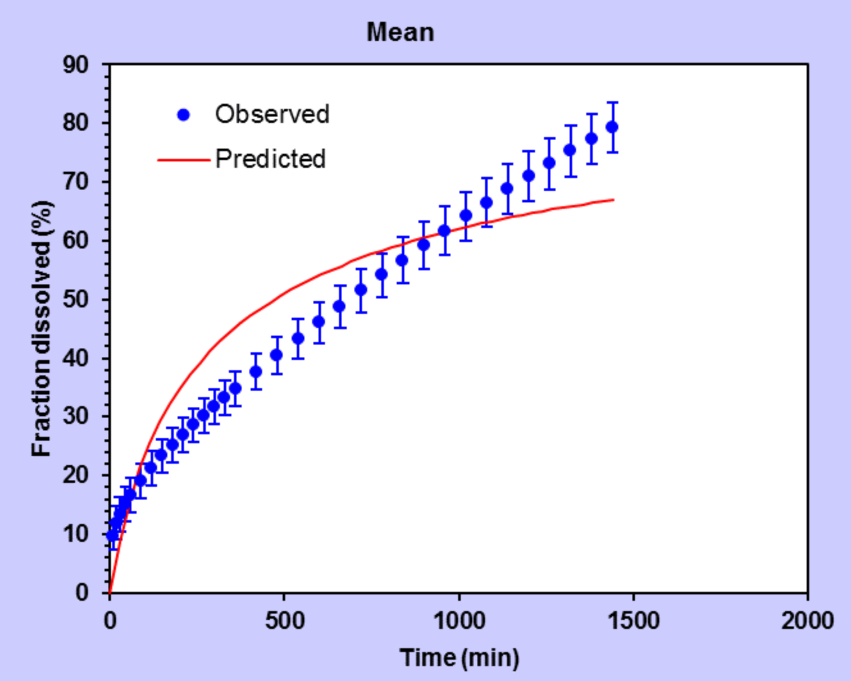

Supplementary materials – Model fitting summary – Starch 1500® sample with smaller particle size (modelling the entire relevant dissolution data)

Graphical abstract of model fit presented as the fraction % of released carvedilol per tested tablet:

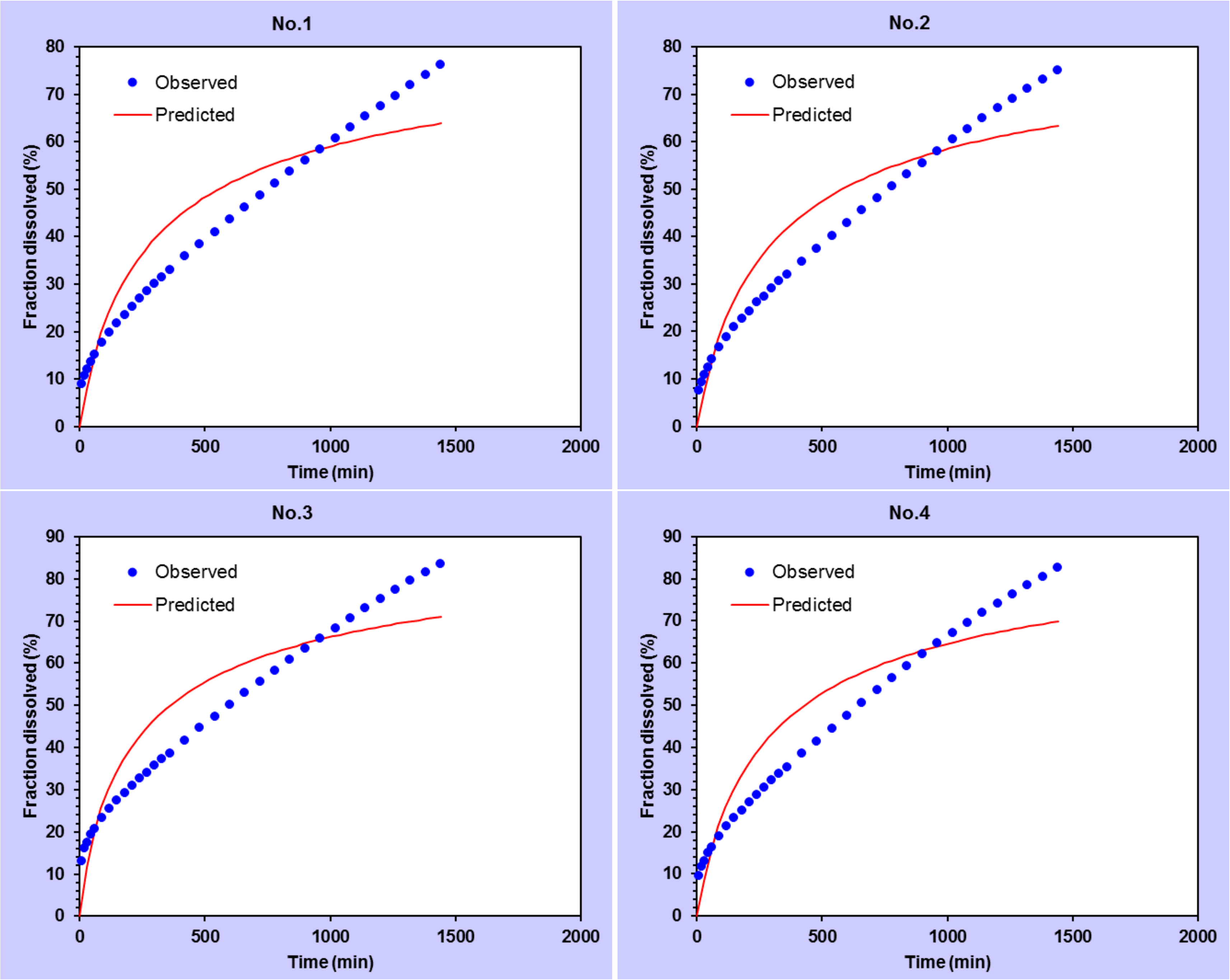

Supplementary materials – Model fitting summary – Starch 1500® sample with smaller particle size (modelling up to app. 60 % of carvedilol released)

Model: **Zero–order**

Model equation: $F = k_0 \cdot t$

Fitted model parameters per tested tablet (N = 4) with statistics – mean, standard deviation (SD), and relative standard deviation expressed in % (RSD%) (output from DDSolver):

Parameter	No.1	No.2	No.3	No.4	Mean	SD	RSD(%)
k_0	0.074	0.072	0.085	0.080	0.078	0.006	7.689

Number of dissolution data points (N), degrees of freedom (df), and selected goodness of fit criteria – Pearson correlation coefficient (R), coefficient of determination (R^2), adjusted coefficient of determination ($R^2_{adjusted}$), and residual sum of squares (RSS) (manual calculation in MS Excel):

Parameter	No.1	No.2	No.3	No.4
N	24	24	24	24
df	23	23	23	23
R	0.991428944	0.99123403	0.99173583	0.994073461
R^2	0.982931351	0.982544903	0.983539957	0.988182046
$R^2_{adjusted}$	0.982931351	0.982544903	0.983539957	0.988182046
RSS	1624.605433	1354.313774	3096.965132	1759.763179

Graphical abstract of model fit presented as mean ± 1 SD of the fraction % of released carvedilol:

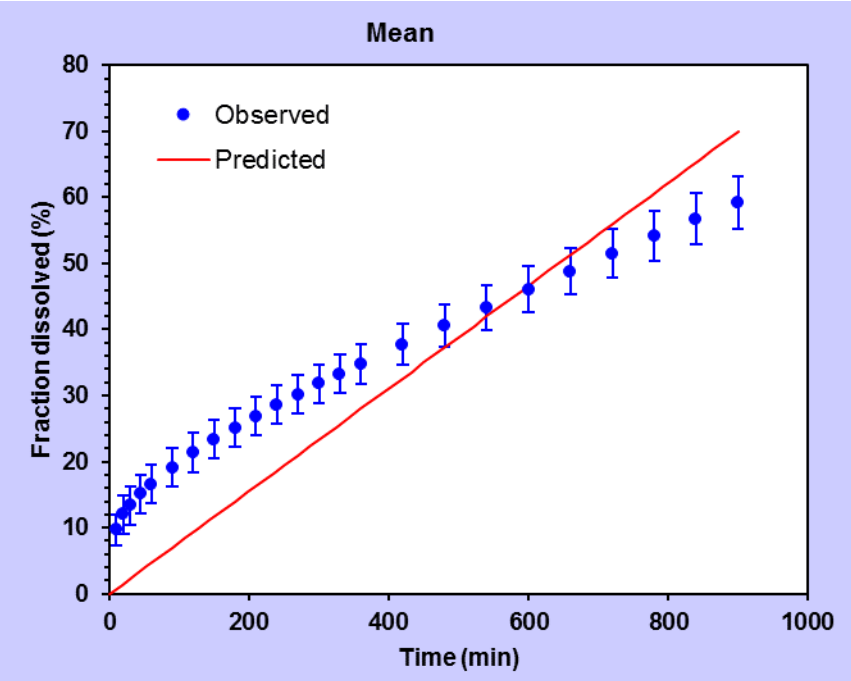

Supplementary materials – Model fitting summary – Starch 1500® sample with smaller particle size (modelling up to app. 60 % of carvedilol released)

Graphical abstract of model fit presented as the fraction % of released carvedilol per tested tablet:

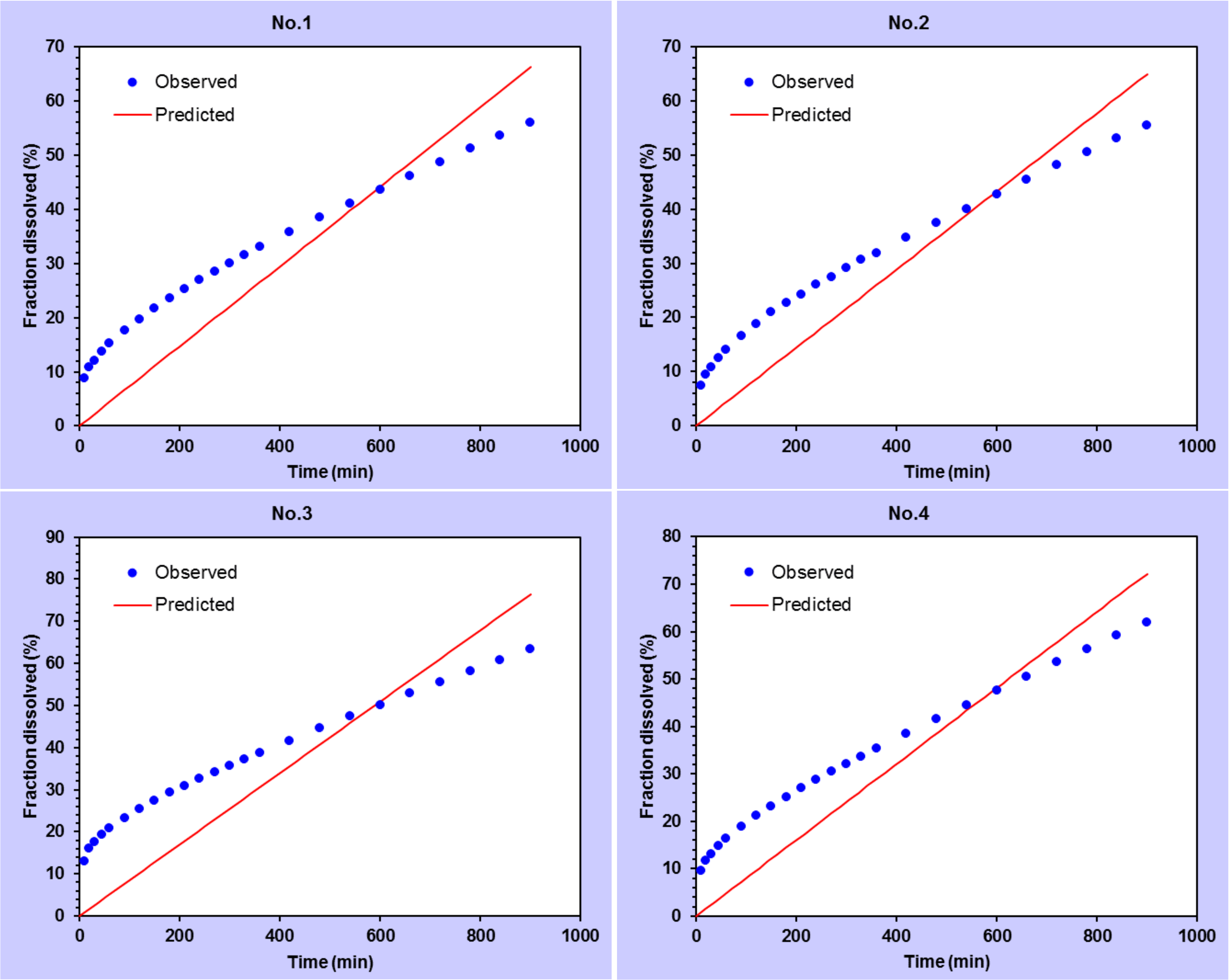

Supplementary materials – Model fitting summary – Starch 1500® sample with smaller particle size (modelling up to app. 60 % of carvedilol released)

Model: **Zero–order with T_{lag}**

Model equation: $F = k_0 \cdot (t - T_{lag})$

Fitted model parameters per tested tablet (N = 4) with statistics – mean, standard deviation (SD), and relative standard deviation expressed in % (RSD%) (output from DDSolver):

Parameter	No.1	No.2	No.3	No.4	Mean	SD	RSD(%)
k_0	0.051	0.051	0.053	0.056	0.053	0.003	4.737
T_{lag}	-255.232	-228.329	-341.056	-240.486	-266.276	51.053	-19.173

Number of dissolution data points (N), degrees of freedom (df), and selected goodness of fit criteria – Pearson correlation coefficient (R), coefficient of determination (R^2), adjusted coefficient of determination ($R^2_{adjusted}$), and residual sum of squares (RSS) (manual calculation in MS Excel):

Parameter	No.1	No.2	No.3	No.4
N	24	24	24	24
df	22	22	22	22
R	0.991428944	0.99123403	0.99173583	0.994073461
R^2	0.982931351	0.982544903	0.983539957	0.988182046
$R^2_{adjusted}$	0.982155504	0.981751489	0.982791773	0.987644866
RSS	80.99080569	85.12663445	85.28780493	68.82511921

Graphical abstract of model fit presented as mean ± 1 SD of the fraction % of released carvedilol:

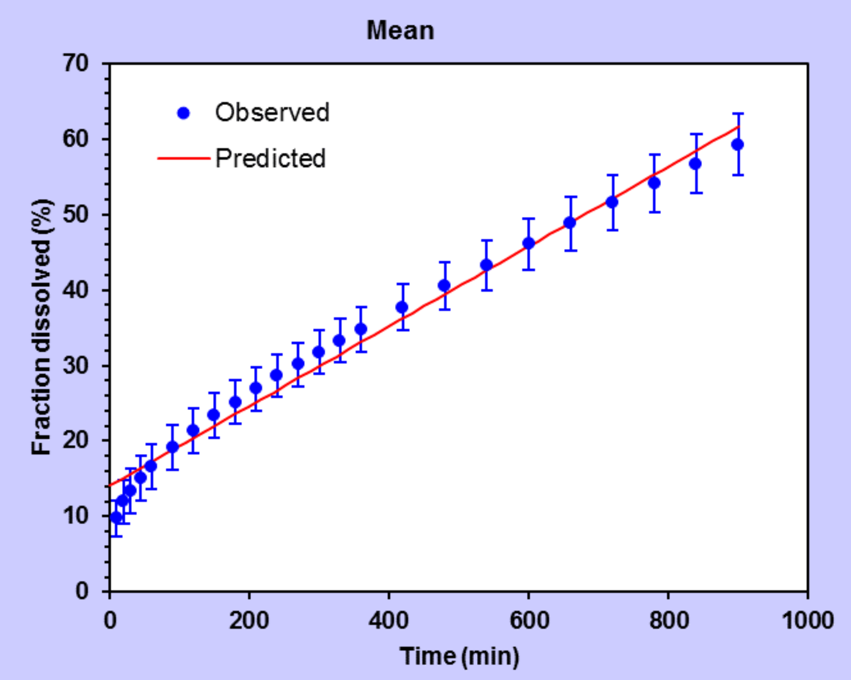

Supplementary materials – Model fitting summary – Starch 1500® sample with smaller particle size (modelling up to app. 60 % of carvedilol released)

Graphical abstract of model fit presented as the fraction % of released carvedilol per tested tablet:

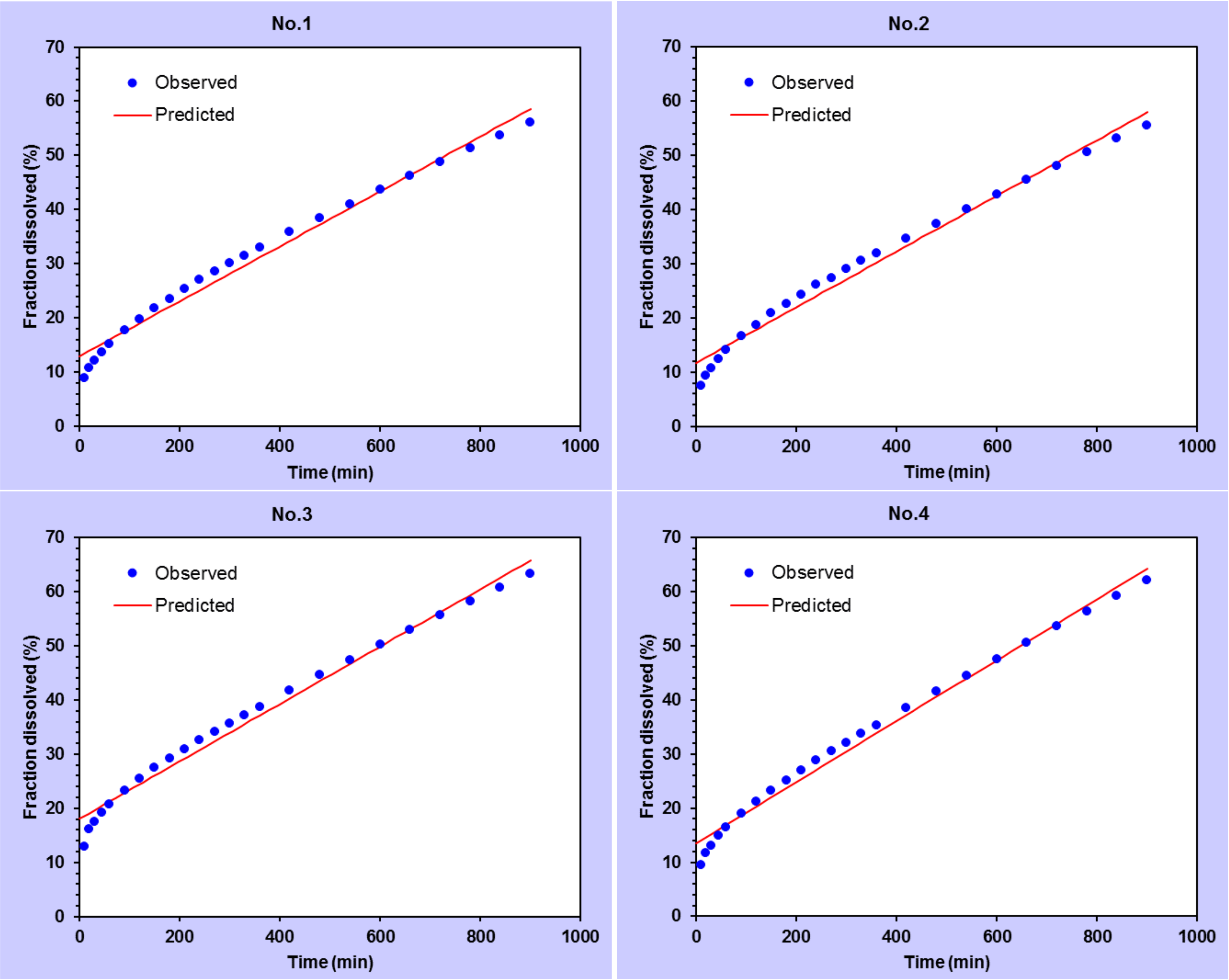

Supplementary materials – Model fitting summary – Starch 1500® sample with smaller particle size (modelling up to app. 60 % of carvedilol released)

Model: **Zero–order with F_0**

Model equation: $F = F_0 + k_0 \cdot t$

Fitted model parameters per tested tablet (N = 4) with statistics – mean, standard deviation (SD), and relative standard deviation expressed in % (RSD%) (output from DDSolver):

Parameter	No.1	No.2	No.3	No.4	Mean	SD	RSD(%)
k_0	0.051	0.051	0.053	0.056	0.053	0.003	4.737
F_0	12.937	11.731	18.071	13.540	14.070	2.771	19.697

Number of dissolution data points (N), degrees of freedom (df), and selected goodness of fit criteria – Pearson correlation coefficient (R), coefficient of determination (R^2), adjusted coefficient of determination ($R^2_{adjusted}$), and residual sum of squares (RSS) (manual calculation in MS Excel):

Parameter	No.1	No.2	No.3	No.4
N	24	24	24	24
df	22	22	22	22
R	0.991428944	0.99123403	0.99173583	0.994073461
R^2	0.982931351	0.982544903	0.983539957	0.988182046
$R^2_{adjusted}$	0.982155504	0.981751489	0.982791773	0.987644866
RSS	80.99080569	85.12663445	85.28780493	68.82511921

Graphical abstract of model fit presented as mean ± 1 SD of the fraction % of released carvedilol:

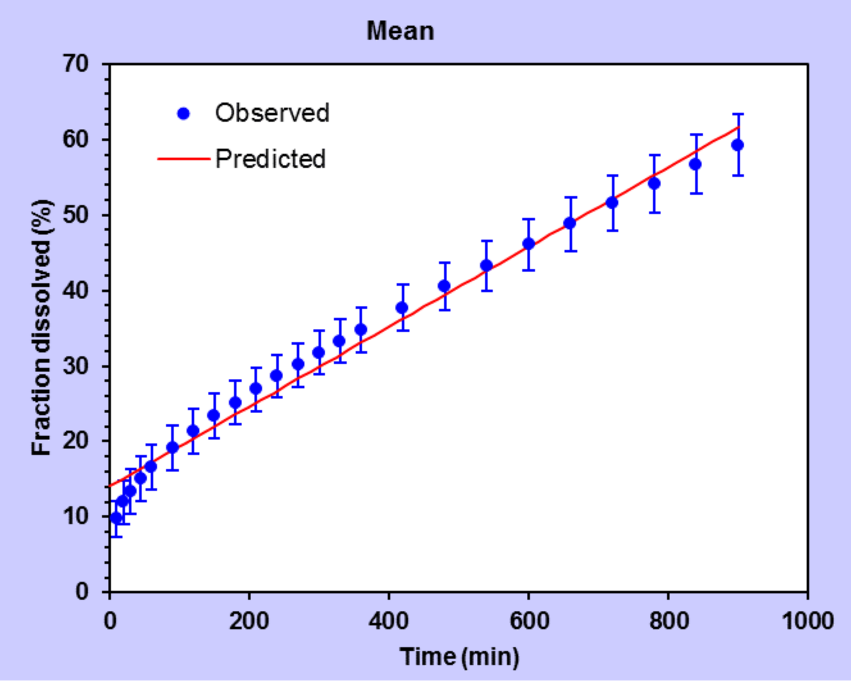

Supplementary materials – Model fitting summary – Starch 1500® sample with smaller particle size (modelling up to app. 60 % of carvedilol released)

Graphical abstract of model fit presented as the fraction % of released carvedilol per tested tablet:

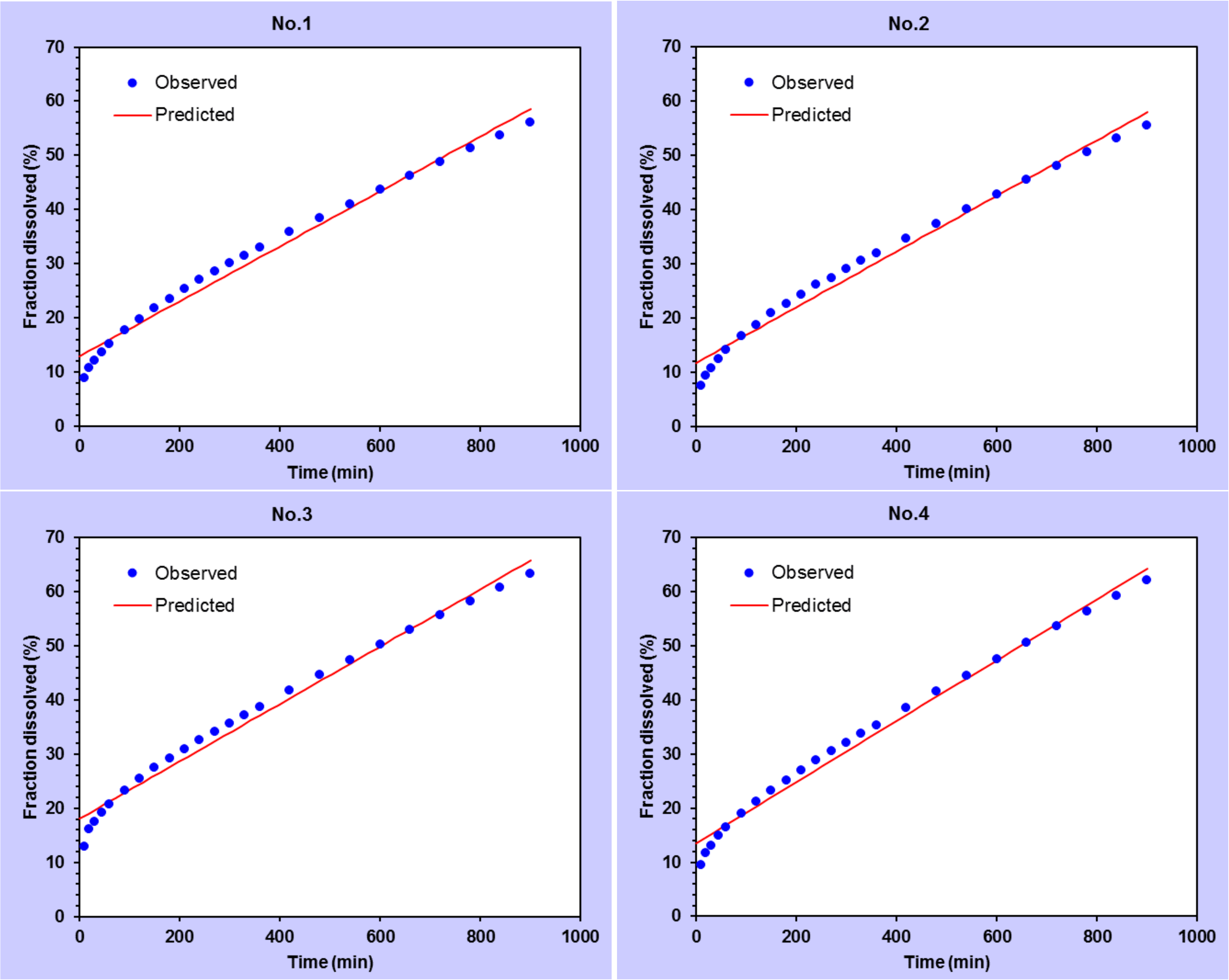

Supplementary materials – Model fitting summary – Starch 1500® sample with smaller particle size (modelling up to app. 60 % of carvedilol released)

Model: **First–order**

Model equation: $F = 100 \cdot (1 - e^{-k_1 \cdot t})$

Fitted model parameters per tested tablet (N = 4) with statistics – mean, standard deviation (SD), and relative standard deviation expressed in % (RSD%) (output from DDSolver):

Parameter	No.1	No.2	No.3	No.4	Mean	SD	RSD(%)
k_1	0.001	0.001	0.001	0.001	0.001	0.000	10.788

Number of dissolution data points (N), degrees of freedom (df), and selected goodness of fit criteria – Pearson correlation coefficient (R), coefficient of determination (R^2), adjusted coefficient of determination ($R^2_{adjusted}$), and residual sum of squares (RSS) (manual calculation in MS Excel):

Parameter	No.1	No.2	No.3	No.4
N	24	24	24	24
df	23	23	23	23
R	0.998598939	0.998178661	0.997567873	0.998104896
R^2	0.997199841	0.996360638	0.995141661	0.996213384
$R^2_{adjusted}$	0.997199841	0.996360638	0.995141661	0.996213384
RSS	864.1379076	688.4836729	1712.354447	853.9236349

Graphical abstract of model fit presented as mean ± 1 SD of the fraction % of released carvedilol:

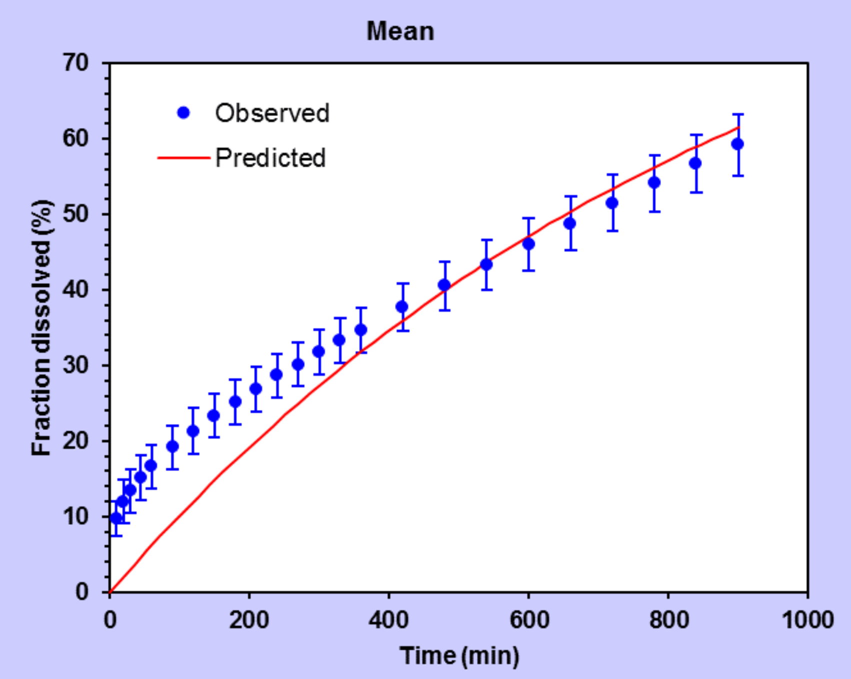

Supplementary materials – Model fitting summary – Starch 1500® sample with smaller particle size (modelling up to app. 60 % of carvedilol released)

Graphical abstract of model fit presented as the fraction % of released carvedilol per tested tablet:

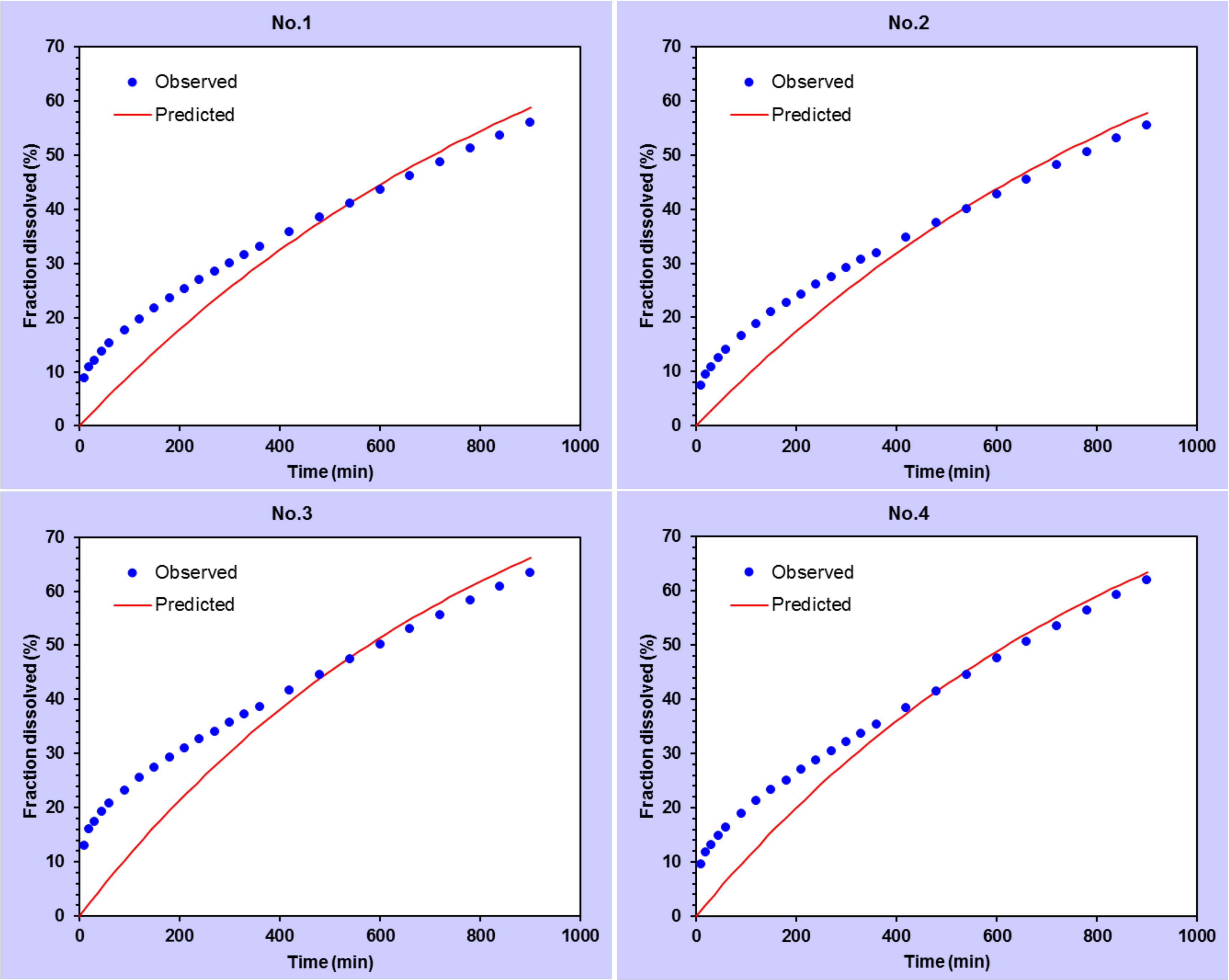

Supplementary materials – Model fitting summary – Starch 1500® sample with smaller particle size (modelling up to app. 60 % of carvedilol released)

Model: **First–order with T_{lag}**

Model equation: $F = 100 \cdot \left[1 - e^{-k_1 \cdot (t - T_{lag})}\right]$

Fitted model parameters per tested tablet (N = 4) with statistics – mean, standard deviation (SD), and relative standard deviation expressed in % (RSD%) (output from DDSolver):

Parameter	No.1	No.2	No.3	No.4	Mean	SD	RSD(%)
k_1	0.001	0.001	0.001	0.001	0.001	0.000	9.072
T_{lag}	-150.044	-132.192	-187.646	-124.480	-148.590	28.152	-18.946

Number of dissolution data points (N), degrees of freedom (df), and selected goodness of fit criteria – Pearson correlation coefficient (R), coefficient of determination (R^2), adjusted coefficient of determination ($R^2_{adjusted}$), and residual sum of squares (RSS) (manual calculation in MS Excel):

Parameter	No.1	No.2	No.3	No.4
N	24	24	24	24
df	22	22	22	22
R	0.99813817	0.997774655	0.997835858	0.99855868
R^2	0.996279806	0.995554262	0.9956764	0.997119437
$R^2_{adjusted}$	0.996110706	0.995352183	0.995479873	0.996988502
RSS	17.87017043	21.86674333	22.40329768	16.94433339

Graphical abstract of model fit presented as mean ± 1 SD of the fraction % of released carvedilol:

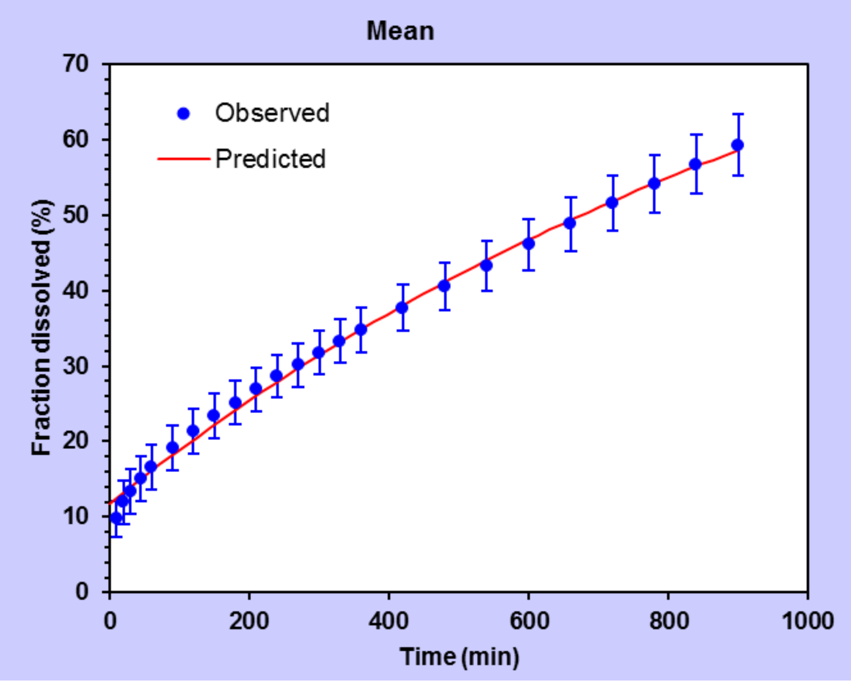

Supplementary materials – Model fitting summary – Starch 1500® sample with smaller particle size (modelling up to app. 60 % of carvedilol released)

Graphical abstract of model fit presented as the fraction % of released carvedilol per tested tablet:

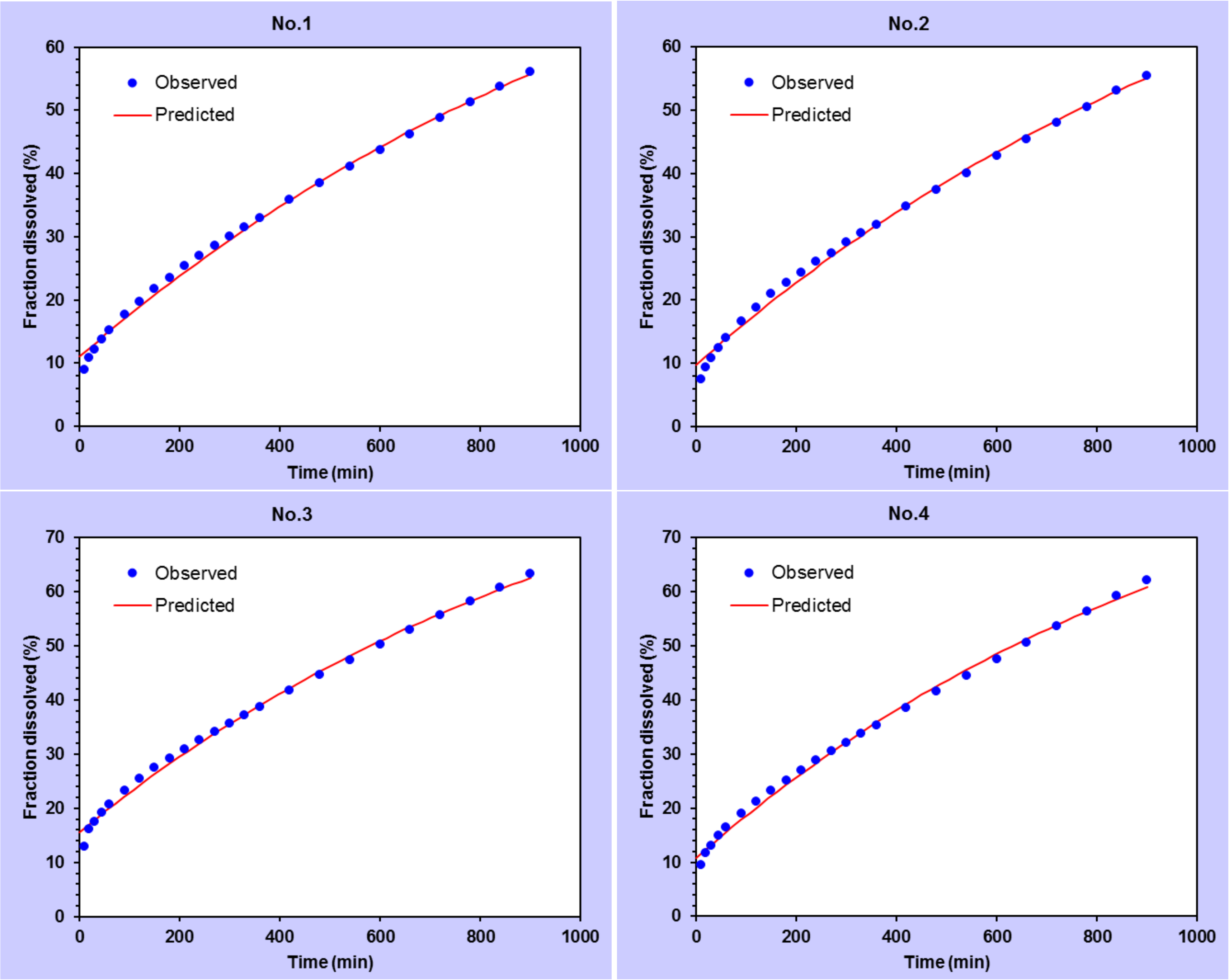

Supplementary materials – Model fitting summary – Starch 1500® sample with smaller particle size (modelling up to app. 60 % of carvedilol released)

Model: **First–order with F_{max}**

Model equation: $F = F_{max} \cdot (1 - e^{-k_1 \cdot t})$

Fitted model parameters per tested tablet (N = 4) with statistics – mean, standard deviation (SD), and relative standard deviation expressed in % (RSD%) (output from DDSolver):

Parameter	No.1	No.2	No.3	No.4	Mean	SD	RSD(%)
k_1	0.003	0.003	0.003	0.003	0.003	0.000	2.224
F_{max}	58.812	58.295	66.520	65.128	62.188	4.241	6.820

Number of dissolution data points (N), degrees of freedom (df), and selected goodness of fit criteria – Pearson correlation coefficient (R), coefficient of determination (R^2), adjusted coefficient of determination ($R^2_{adjusted}$), and residual sum of squares (RSS) (manual calculation in MS Excel):

Parameter	No.1	No.2	No.3	No.4
N	24	24	24	24
df	22	22	22	22
R	0.985146102	0.98530555	0.981831001	0.981767174
R^2	0.970512843	0.970827026	0.963992114	0.963866784
$R^2_{adjusted}$	0.969172518	0.969500982	0.962355392	0.962224365
RSS	406.1779516	332.6334141	939.8510732	510.6578432

Graphical abstract of model fit presented as mean ± 1 SD of the fraction % of released carvedilol:

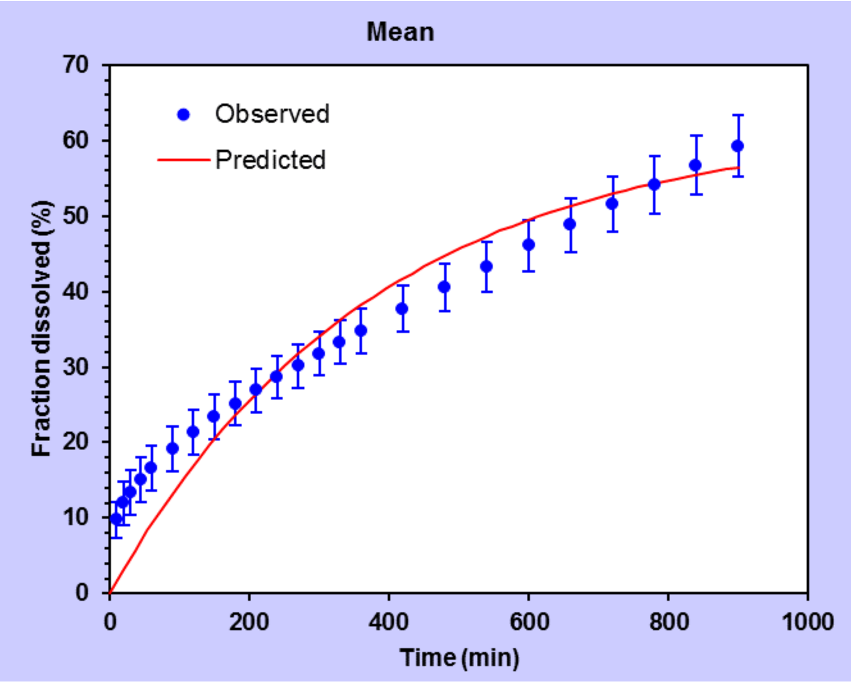

Supplementary materials – Model fitting summary – Starch 1500® sample with smaller particle size (modelling up to app. 60 % of carvedilol released)

Graphical abstract of model fit presented as the fraction % of released carvedilol per tested tablet:

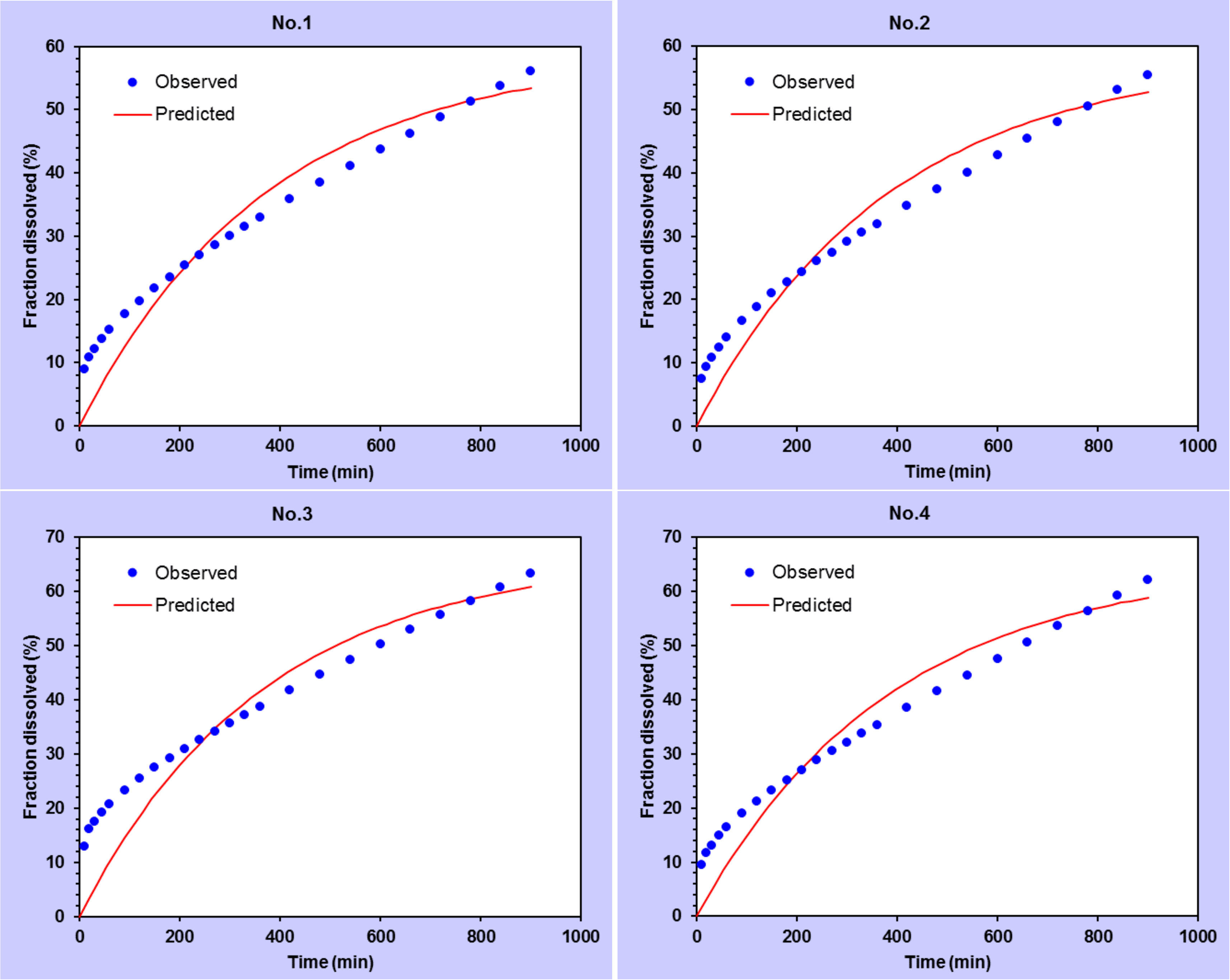

Supplementary materials – Model fitting summary – Starch 1500® sample with smaller particle size (modelling up to app. 60 % of carvedilol released)

Model: **First–order with T_{lag} and F_{max}**

Model equation: $F = F_{max} \cdot \left[1 - e^{-k_1 \cdot (t - T_{lag})}\right]$

Fitted model parameters per tested tablet (N = 4) with statistics – mean, standard deviation (SD), and relative standard deviation expressed in % (RSD%) (output from DDSolver):

Parameter	No.1	No.2	No.3	No.4	Mean	SD	RSD(%)
k_1	0.003	0.003	0.003	0.003	0.003	0.000	1.204
T_{lag}	-17.001	-2.448	-62.221	-7.611	-22.320	27.274	-122.197
F_{max}	58.812	58.295	66.520	65.128	62.188	4.241	6.820

Number of dissolution data points (N), degrees of freedom (df), and selected goodness of fit criteria – Pearson correlation coefficient (R), coefficient of determination (R^2), adjusted coefficient of determination ($R^2_{adjusted}$), and residual sum of squares (RSS) (manual calculation in MS Excel):

Parameter	No.1	No.2	No.3	No.4
N	24	24	24	24
df	21	21	21	21
R	0.985943192	0.985419114	0.984809197	0.982165905
R^2	0.972083979	0.971050829	0.969849154	0.964649864
$R^2_{adjusted}$	0.96942531	0.968293765	0.966977645	0.961283184
RSS	264.6007988	312.6726381	263.3687407	429.3602146

Graphical abstract of model fit presented as mean ± 1 SD of the fraction % of released carvedilol:

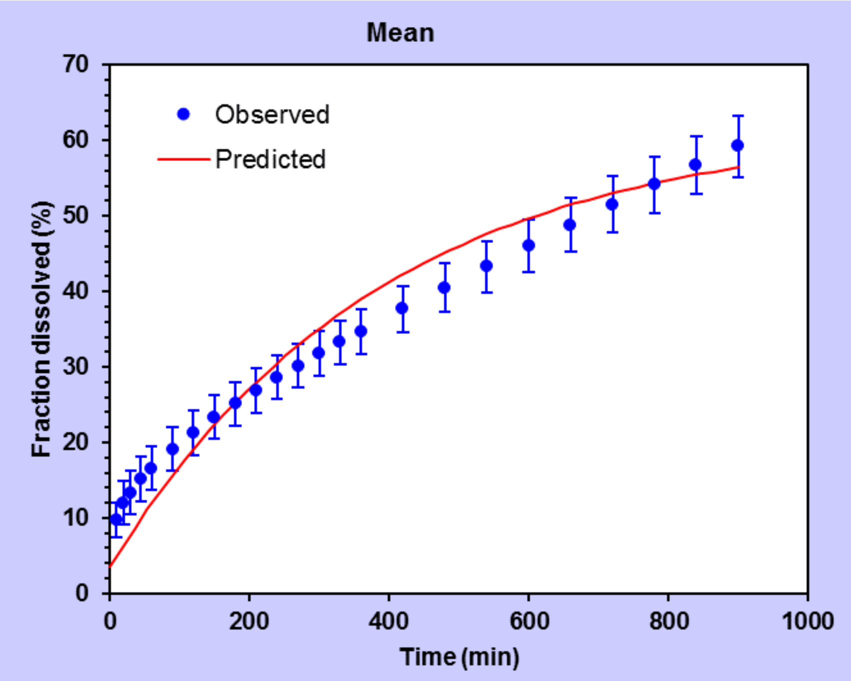

Supplementary materials – Model fitting summary – Starch 1500® sample with smaller particle size (modelling up to app. 60 % of carvedilol released)

Graphical abstract of model fit presented as the fraction % of released carvedilol per tested tablet:

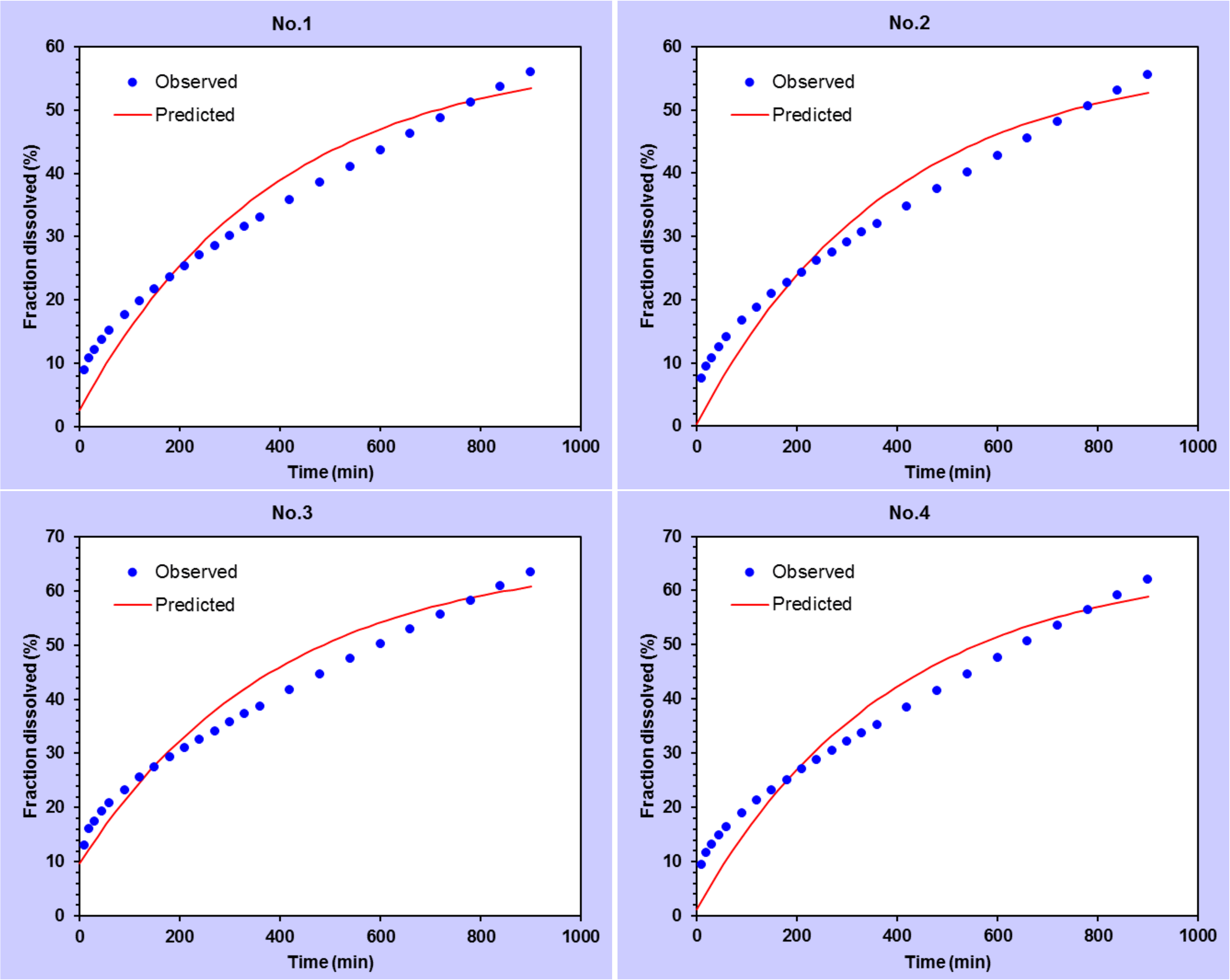

Supplementary materials – Model fitting summary – Starch 1500® sample with smaller particle size (modelling up to app. 60 % of carvedilol released)

Model: **Higuchi**

Model equation: $F = k_H \cdot t^{0.5}$

Fitted model parameters per tested tablet (N = 4) with statistics – mean, standard deviation (SD), and relative standard deviation expressed in % (RSD%) (output from DDSolver):

Parameter	No.1	No.2	No.3	No.4	Mean	SD	RSD(%)
k_H	1.803	1.760	2.104	1.960	1.907	0.157	8.238

Number of dissolution data points (N), degrees of freedom (df), and selected goodness of fit criteria – Pearson correlation coefficient (R), coefficient of determination (R^2), adjusted coefficient of determination ($R^2_{adjusted}$), and residual sum of squares (RSS) (manual calculation in MS Excel):

Parameter	No.1	No.2	No.3	No.4
N	24	24	24	24
df	23	23	23	23
R	0.996053723	0.996032084	0.995319519	0.993647506
R^2	0.992123019	0.992079912	0.990660945	0.987335366
$R^2_{adjusted}$	0.992123019	0.992079912	0.990660945	0.987335366
RSS	44.24561963	38.69905214	202.2468066	76.02953003

Graphical abstract of model fit presented as mean ± 1 SD of the fraction % of released carvedilol:

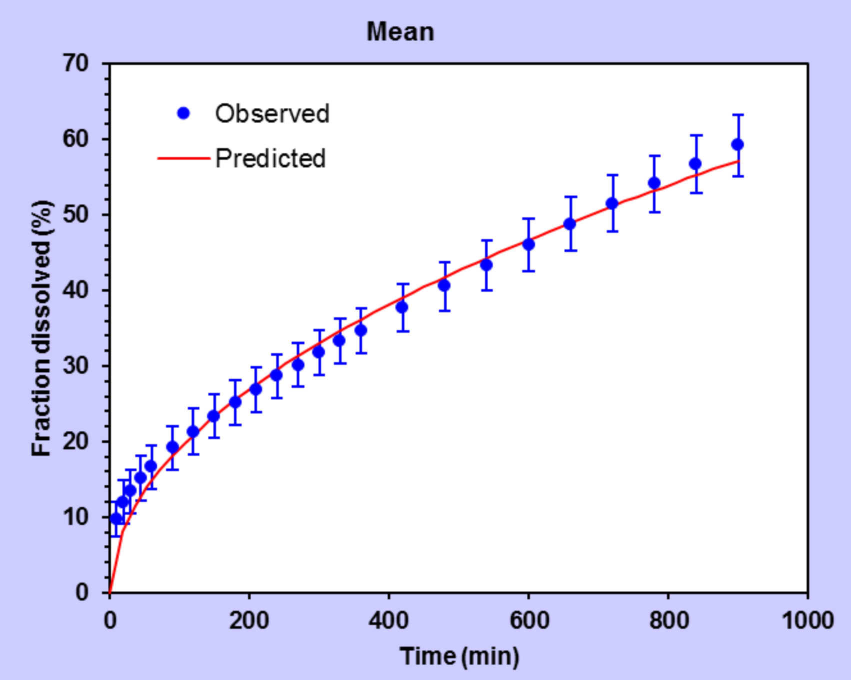

Supplementary materials – Model fitting summary – Starch 1500® sample with smaller particle size (modelling up to app. 60 % of carvedilol released)

Graphical abstract of model fit presented as the fraction % of released carvedilol per tested tablet:

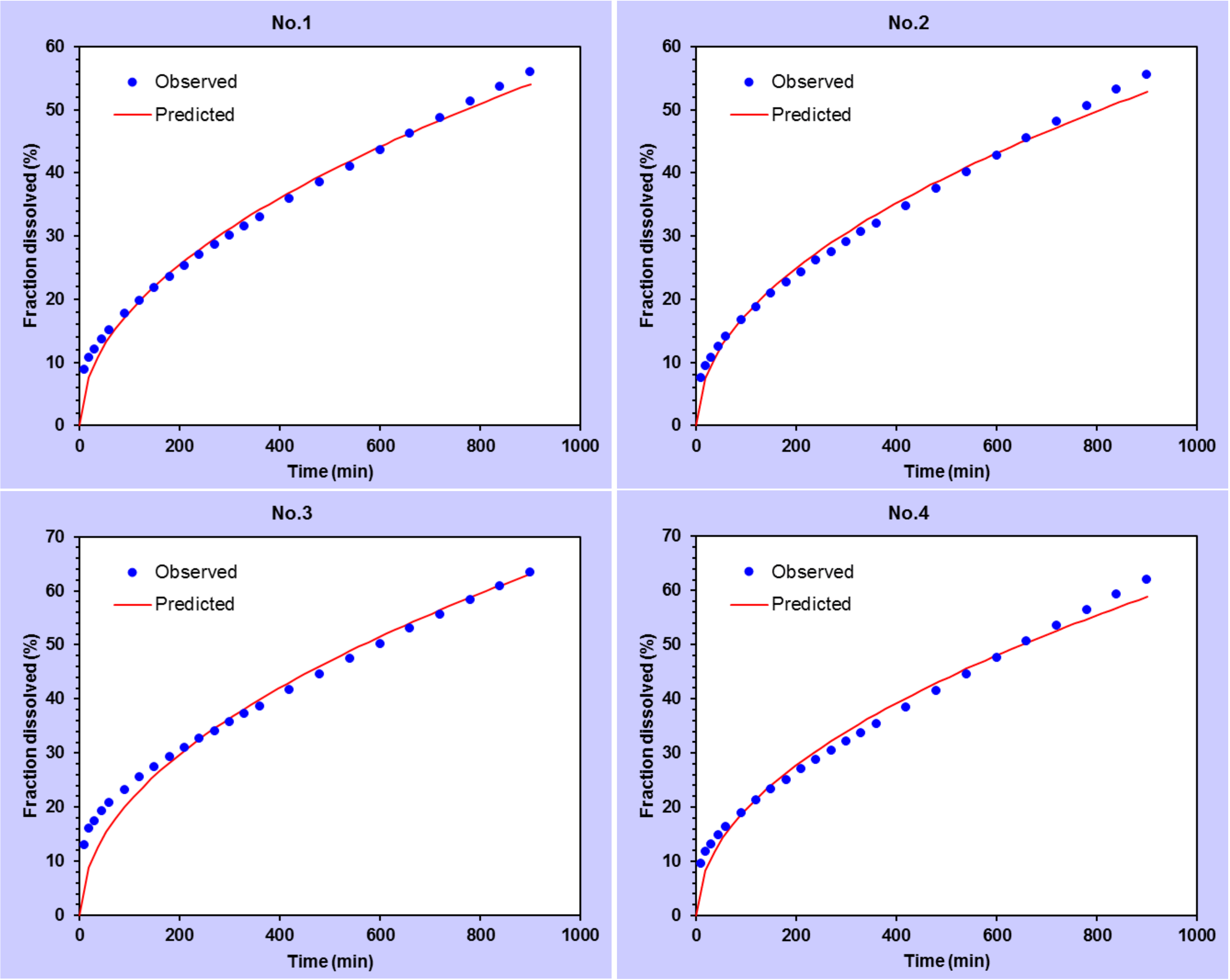

Supplementary materials – Model fitting summary – Starch 1500® sample with smaller particle size (modelling up to app. 60 % of carvedilol released)

Model: **Higuchi with T_{lag}**

Model equation: $F = k_H \cdot (t - T_{lag})^{0.5}$

Fitted model parameters per tested tablet (N = 4) with statistics – mean, standard deviation (SD), and relative standard deviation expressed in % (RSD%) (output from DDSolver):

Parameter	No.1	No.2	No.3	No.4	Mean	SD	RSD(%)
k_H	1.827	1.813	2.034	2.015	1.922	0.119	6.172
T_{lag}	8.516	19.476	-39.420	17.849	1.605	27.773	1730.125

Number of dissolution data points (N), degrees of freedom (df), and selected goodness of fit criteria – Pearson correlation coefficient (R), coefficient of determination (R^2), adjusted coefficient of determination ($R^2_{adjusted}$), and residual sum of squares (RSS) (manual calculation in MS Excel):

Parameter	No.1	No.2	No.3	No.4
N	24	24	24	24
df	22	22	22	22
R	0.993615554	0.990175006	0.997959599	0.98759448
R^2	0.987271869	0.980446543	0.995923362	0.975342858
$R^2_{adjusted}$	0.986693318	0.97955775	0.99573806	0.974222079
RSS	106.2913525	185.7417357	33.21184372	271.6214565

Graphical abstract of model fit presented as mean ± 1 SD of the fraction % of released carvedilol:

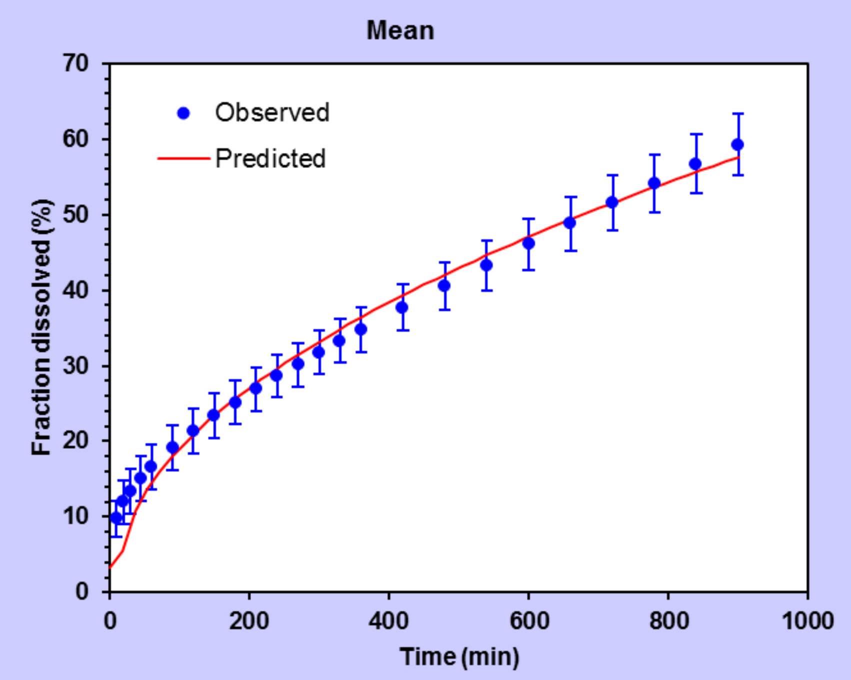

Supplementary materials – Model fitting summary – Starch 1500® sample with smaller particle size (modelling up to app. 60 % of carvedilol released)

Graphical abstract of model fit presented as the fraction % of released carvedilol per tested tablet:

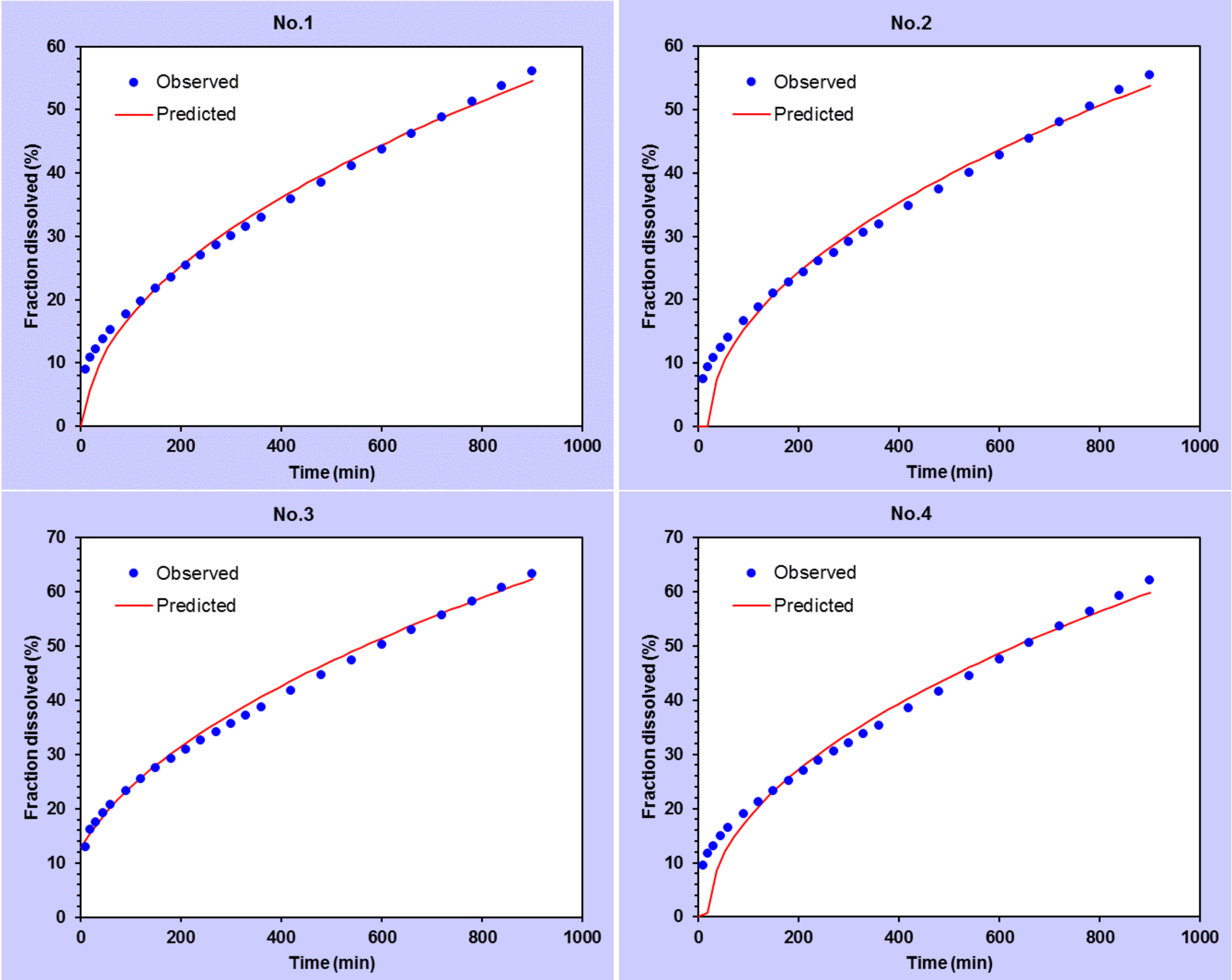

Supplementary materials – Model fitting summary – Starch 1500® sample with smaller particle size (modelling up to app. 60 % of carvedilol released)

Model: **Higuchi with F_0**

Model equation: $F = F_0 + k_H \cdot t^{0.5}$

Fitted model parameters per tested tablet (N = 4) with statistics – mean, standard deviation (SD), and relative standard deviation expressed in % (RSD%) (output from DDSolver):

Parameter	No.1	No.2	No.3	No.4	Mean	SD	RSD(%)
k_H	1.743	1.767	1.820	1.926	1.814	0.081	4.490
F_0	1.242	-0.129	5.878	0.714	1.926	2.694	139.847

Number of dissolution data points (N), degrees of freedom (df), and selected goodness of fit criteria – Pearson correlation coefficient (R), coefficient of determination (R^2), adjusted coefficient of determination ($R^2_{adjusted}$), and residual sum of squares (RSS) (manual calculation in MS Excel):

Parameter	No.1	No.2	No.3	No.4
N	24	24	24	24
df	22	22	22	22
R	0.996053723	0.996032084	0.995319519	0.993647506
R^2	0.992123019	0.992079912	0.990660945	0.987335366
$R^2_{adjusted}$	0.991764974	0.991719908	0.990236443	0.986759701
RSS	37.37630734	38.62541958	48.39036371	73.75599357

Graphical abstract of model fit presented as mean ± 1 SD of the fraction % of released carvedilol:

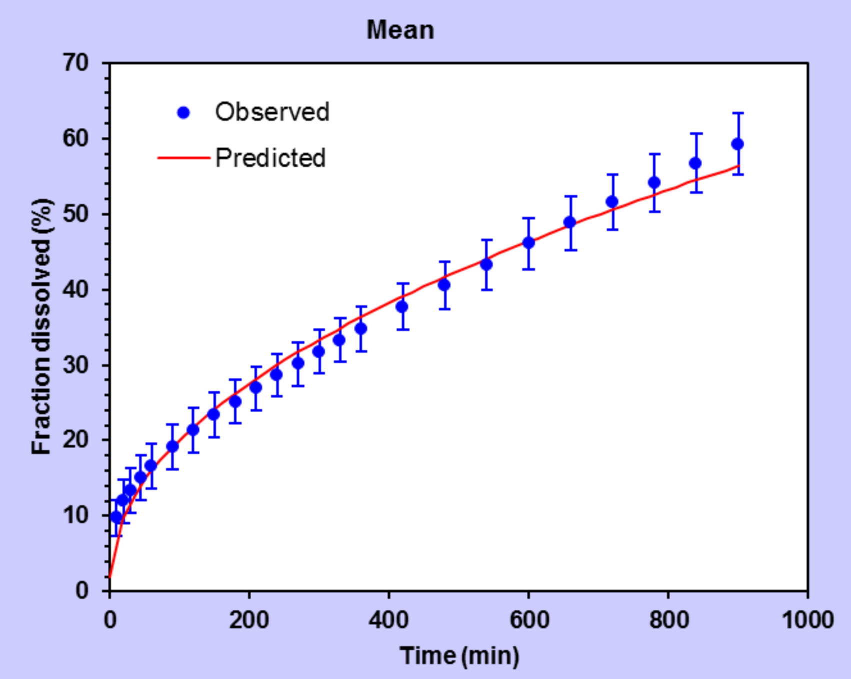

Supplementary materials – Model fitting summary – Starch 1500® sample with smaller particle size (modelling up to app. 60 % of carvedilol released)

Graphical abstract of model fit presented as the fraction % of released carvedilol per tested tablet:

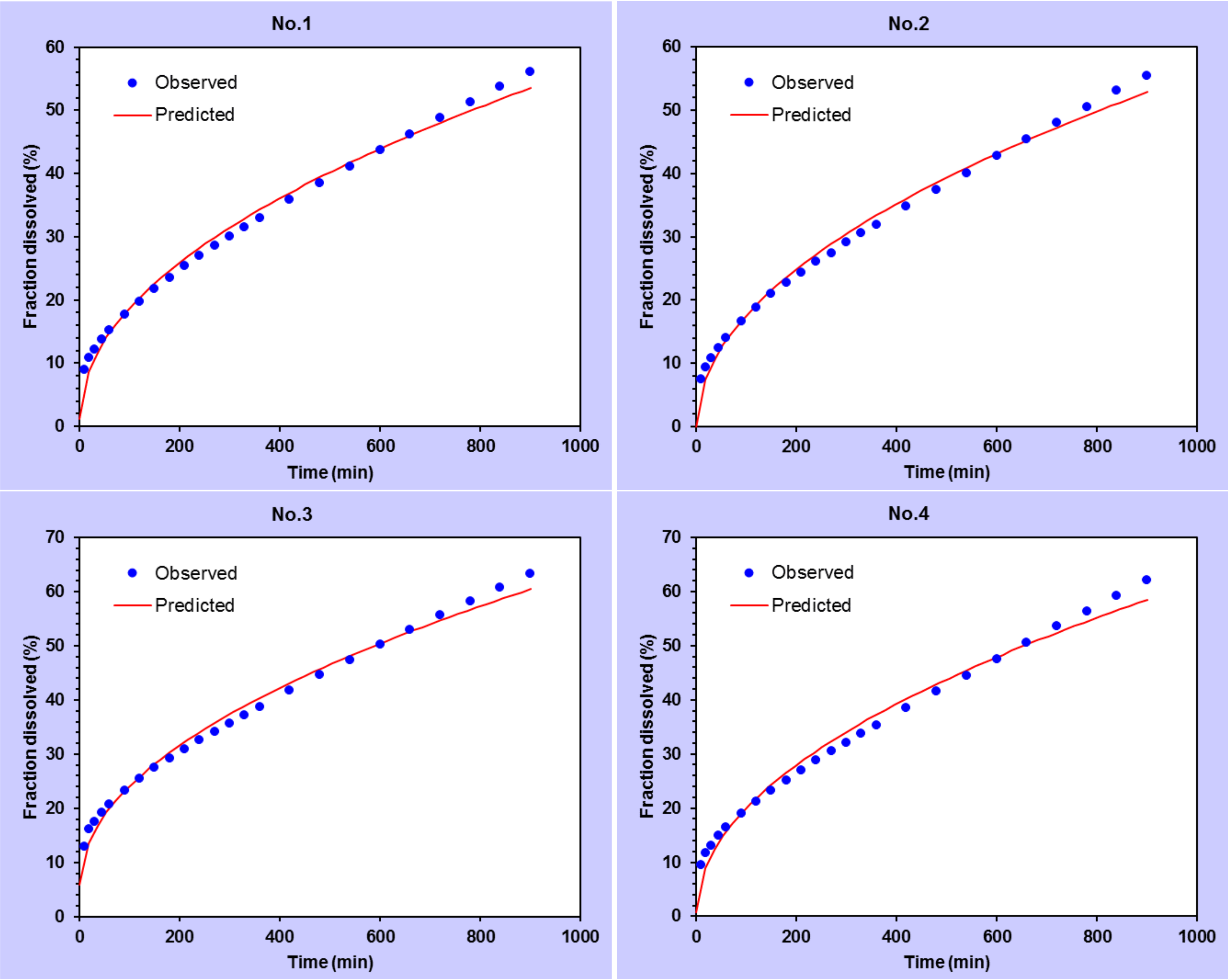

Supplementary materials – Model fitting summary – Starch 1500® sample with smaller particle size (modelling up to app. 60 % of carvedilol released)

Model: **Korsmeyer–Peppas**

Model equation: $F = k_{KP} \cdot t^n$

Fitted model parameters per tested tablet (N = 4) with statistics – mean, standard deviation (SD), and relative standard deviation expressed in % (RSD%) (output from DDSolver):

Parameter	No.1	No.2	No.3	No.4	Mean	SD	RSD(%)
k_{KP}	2.815	2.259	5.051	2.977	3.276	1.223	37.332
n	0.424	0.456	0.354	0.428	0.415	0.043	10.460

Number of dissolution data points (N), degrees of freedom (df), and selected goodness of fit criteria – Pearson correlation coefficient (R), coefficient of determination (R^2), adjusted coefficient of determination ($R^2_{adjusted}$), and residual sum of squares (RSS) (manual calculation in MS Excel):

Parameter	No.1	No.2	No.3	No.4
N	24	24	24	24
df	22	22	22	22
R	0.991831665	0.993852652	0.985555895	0.988888267
R^2	0.983730052	0.987743094	0.971320422	0.977900005
$R^2_{adjusted}$	0.982990509	0.987185962	0.970016805	0.97689546
RSS	117.0836002	92.03044094	196.8374256	191.4707939

Graphical abstract of model fit presented as mean ± 1 SD of the fraction % of released carvedilol:

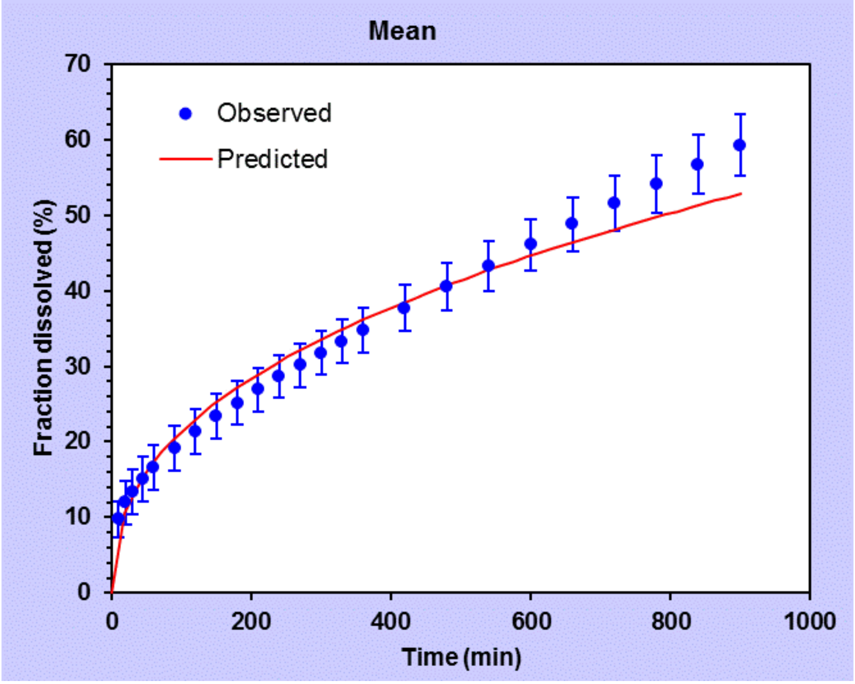

Supplementary materials – Model fitting summary – Starch 1500® sample with smaller particle size (modelling up to app. 60 % of carvedilol released)

Graphical abstract of model fit presented as the fraction % of released carvedilol per tested tablet:

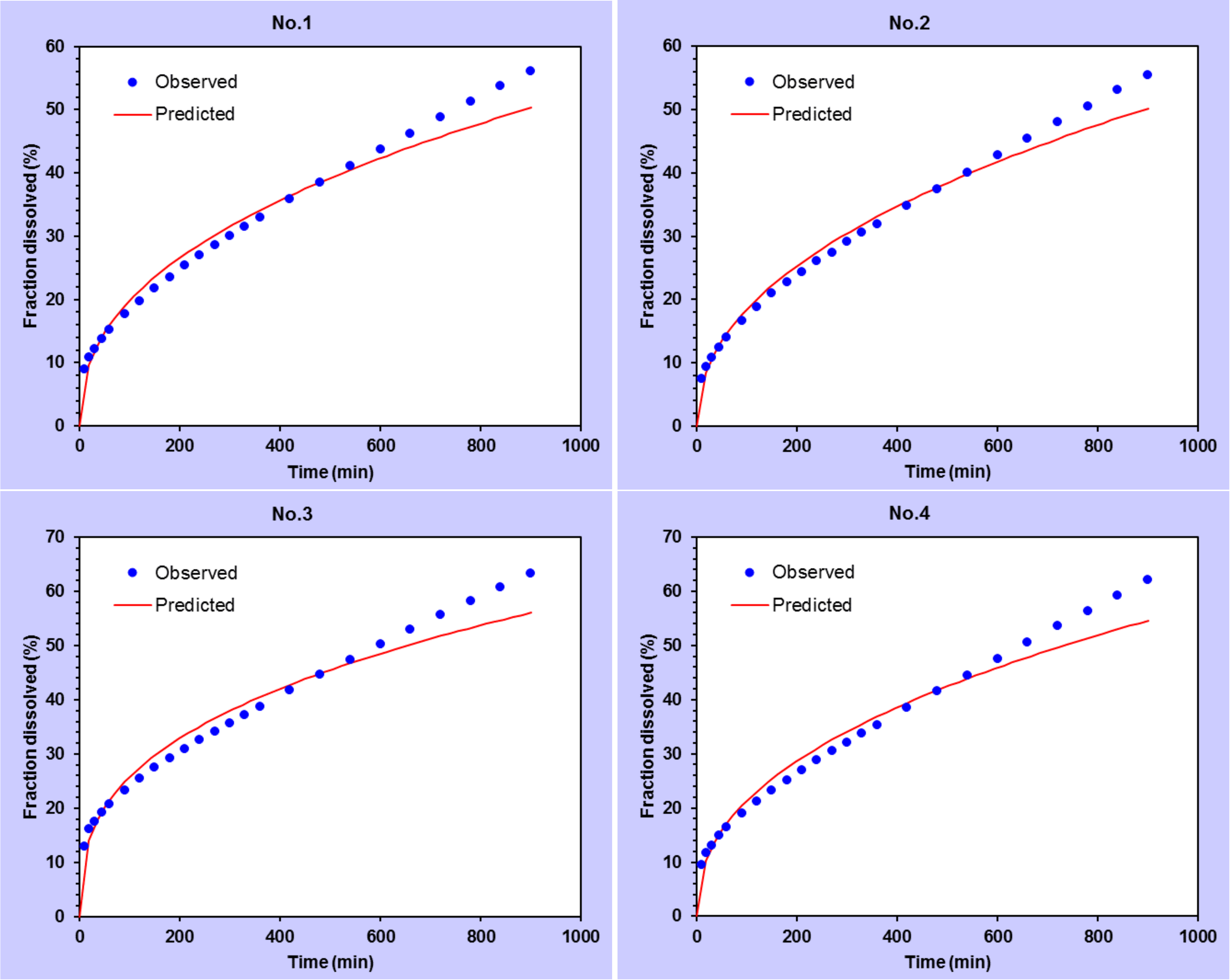

Supplementary materials – Model fitting summary – Starch 1500® sample with smaller particle size (modelling up to app. 60 % of carvedilol released)

Model: **Korsmeyer–Peppas with T_{lag}**

Model equation: $F = k_{KP} \cdot (t - T_{lag})^n$

Fitted model parameters per tested tablet (N = 4) with statistics – mean, standard deviation (SD), and relative standard deviation expressed in % (RSD%) (output from DDSolver):

Parameter	No.1	No.2	No.3	No.4	Mean	SD	RSD(%)
k_{KP}	3.396	2.757	5.910	3.599	3.916	1.377	35.166
n	0.393	0.423	0.328	0.396	0.385	0.040	10.505
T_{lag}	4.000	4.000	4.000	4.000	4.000	0.000	0.000

Number of dissolution data points (N), degrees of freedom (df), and selected goodness of fit criteria – Pearson correlation coefficient (R), coefficient of determination (R^2), adjusted coefficient of determination ($R^2_{adjusted}$), and residual sum of squares (RSS) (manual calculation in MS Excel):

Parameter	No.1	No.2	No.3	No.4
N	24	24	24	24
df	21	21	21	21
R	0.987644737	0.990330515	0.980291229	0.984215868
R^2	0.975442126	0.980754528	0.960970893	0.968680875
$R^2_{adjusted}$	0.973103281	0.978921626	0.957253835	0.965698101
RSS	183.0728355	152.4534307	274.9967567	280.7879618

Graphical abstract of model fit presented as mean ± 1 SD of the fraction % of released carvedilol:

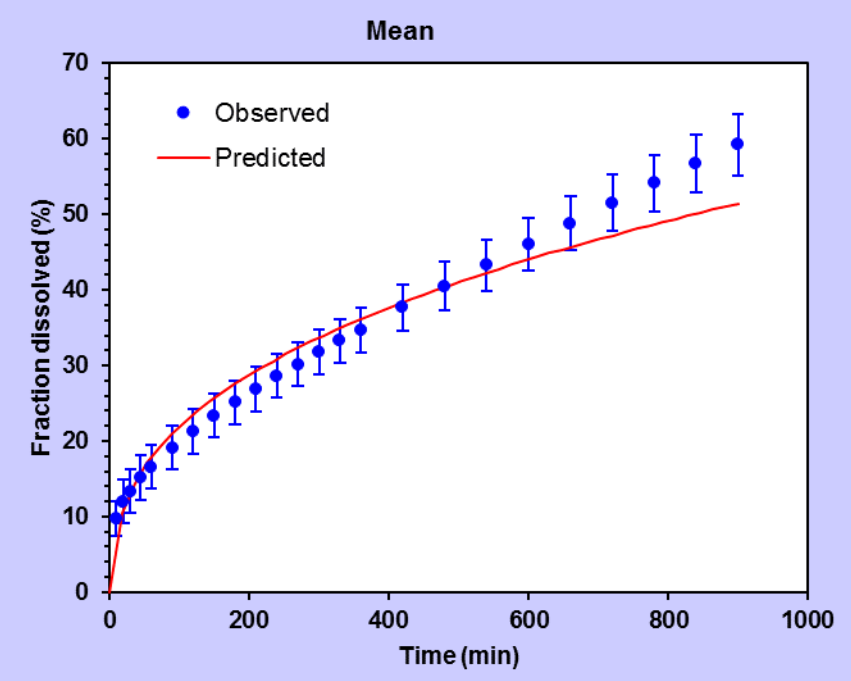

Supplementary materials – Model fitting summary – Starch 1500® sample with smaller particle size (modelling up to app. 60 % of carvedilol released)

Graphical abstract of model fit presented as the fraction % of released carvedilol per tested tablet:

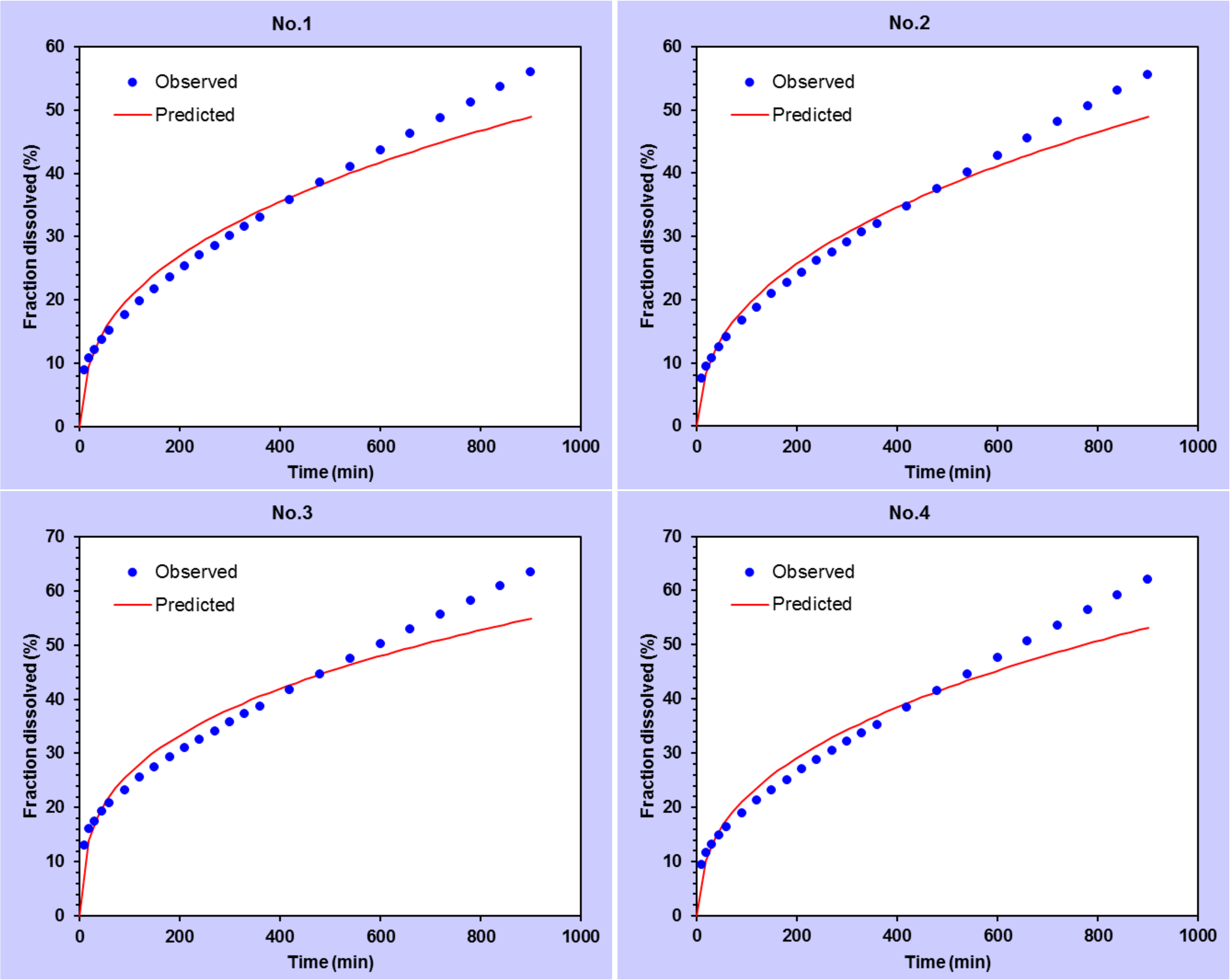

Supplementary materials – Model fitting summary – Starch 1500® sample with smaller particle size (modelling up to app. 60 % of carvedilol released)

Model: **Korsmeyer–Peppas with F_0**

Model equation: $F = F_0 + k_{KP} \cdot t^n$

Fitted model parameters per tested tablet (N = 4) with statistics – mean, standard deviation (SD), and relative standard deviation expressed in % (RSD%) (output from DDSolver):

Parameter	No.1	No.2	No.3	No.4	Mean	SD	RSD(%)
k_{KP}	1.456	1.197	2.631	1.548	1.708	0.633	37.057
n	0.515	0.546	0.440	0.518	0.505	0.046	9.035
F_0	3.559	2.999	5.199	3.799	3.889	0.935	24.046

Number of dissolution data points (N), degrees of freedom (df), and selected goodness of fit criteria – Pearson correlation coefficient (R), coefficient of determination (R^2), adjusted coefficient of determination ($R^2_{adjusted}$), and residual sum of squares (RSS) (manual calculation in MS Excel):

Parameter	No.1	No.2	No.3	No.4
N	24	24	24	24
df	21	21	21	21
R	0.996691754	0.997731071	0.992069388	0.994613548
R^2	0.993394453	0.99546729	0.98420167	0.989256109
$R^2_{adjusted}$	0.992765353	0.995035603	0.982697067	0.988232882
RSS	52.72229779	34.75849199	116.9737286	101.204368

Graphical abstract of model fit presented as mean ± 1 SD of the fraction % of released carvedilol:

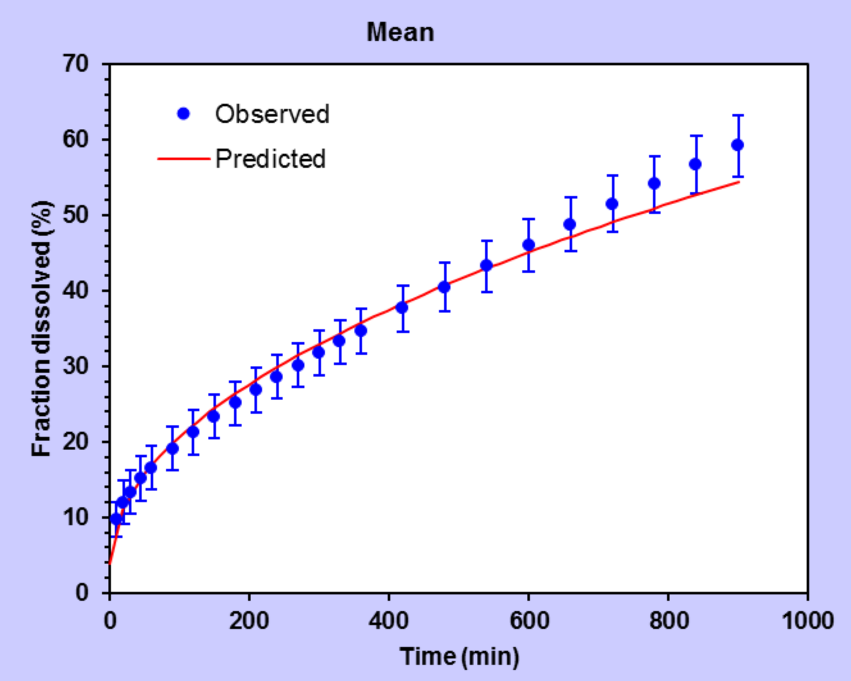

Supplementary materials – Model fitting summary – Starch 1500® sample with smaller particle size (modelling up to app. 60 % of carvedilol released)

Graphical abstract of model fit presented as the fraction % of released carvedilol per tested tablet:

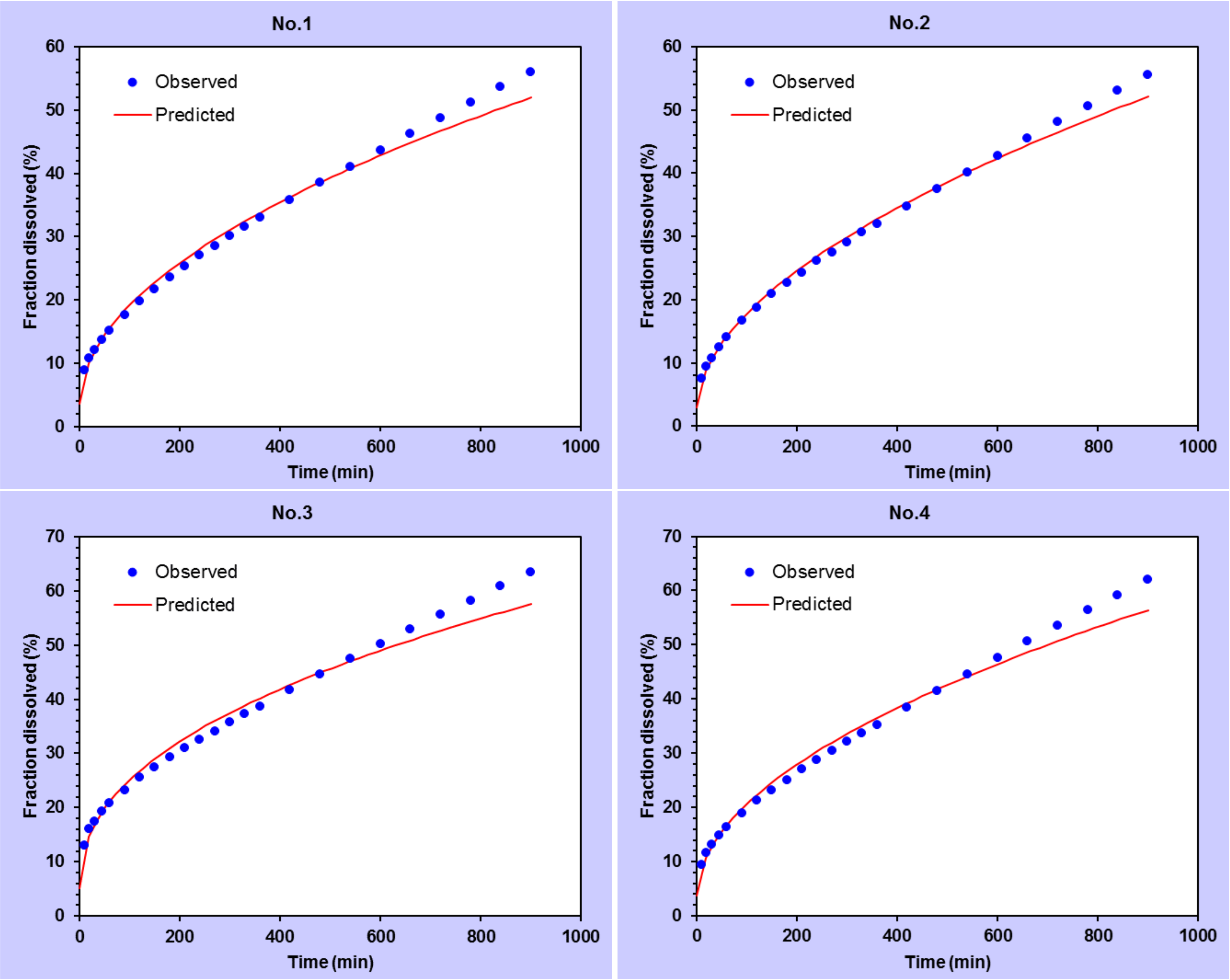

Supplementary materials – Model fitting summary – Starch 1500® sample with smaller particle size (modelling up to app. 60 % of carvedilol released)

Model: **Hixson–Crowell**

Model equation: $F = 100 \cdot [1 - (1 - k_{HC} \cdot t)^3]$

Fitted model parameters per tested tablet (N = 4) with statistics – mean, standard deviation (SD), and relative standard deviation expressed in % (RSD%) (output from DDSolver):

Parameter	No.1	No.2	No.3	No.4	Mean	SD	RSD(%)
k_{HC}	0.0003	0.0003	0.0004	0.0003	0.0003	0.0000	9.6452

Number of dissolution data points (N), degrees of freedom (df), and selected goodness of fit criteria – Pearson correlation coefficient (R), coefficient of determination (R^2), adjusted coefficient of determination ($R^2_{adjusted}$), and residual sum of squares (RSS) (manual calculation in MS Excel):

Parameter	No.1	No.2	No.3	No.4
N	24	24	24	24
df	23	23	23	23
R	0.997622219	0.997186531	0.997588698	0.998460891
R^2	0.995250092	0.994380977	0.995183211	0.996924151
$R^2_{adjusted}$	0.995250092	0.994380977	0.995183211	0.996924151
RSS	1065.574744	863.5226934	2077.079084	1083.729964

Graphical abstract of model fit presented as mean ± 1 SD of the fraction % of released carvedilol:

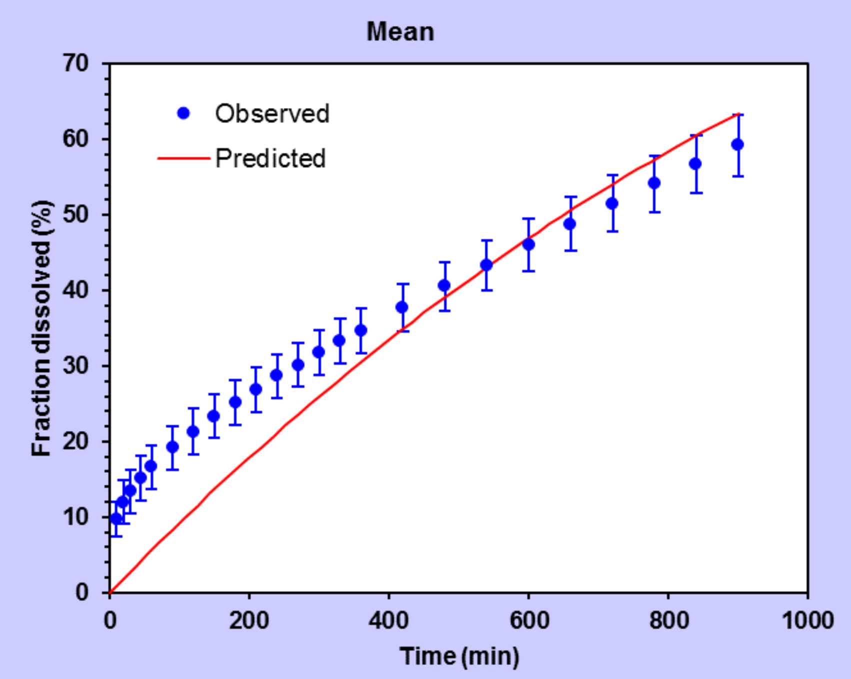

Supplementary materials – Model fitting summary – Starch 1500® sample with smaller particle size (modelling up to app. 60 % of carvedilol released)

Graphical abstract of model fit presented as the fraction % of released carvedilol per tested tablet:

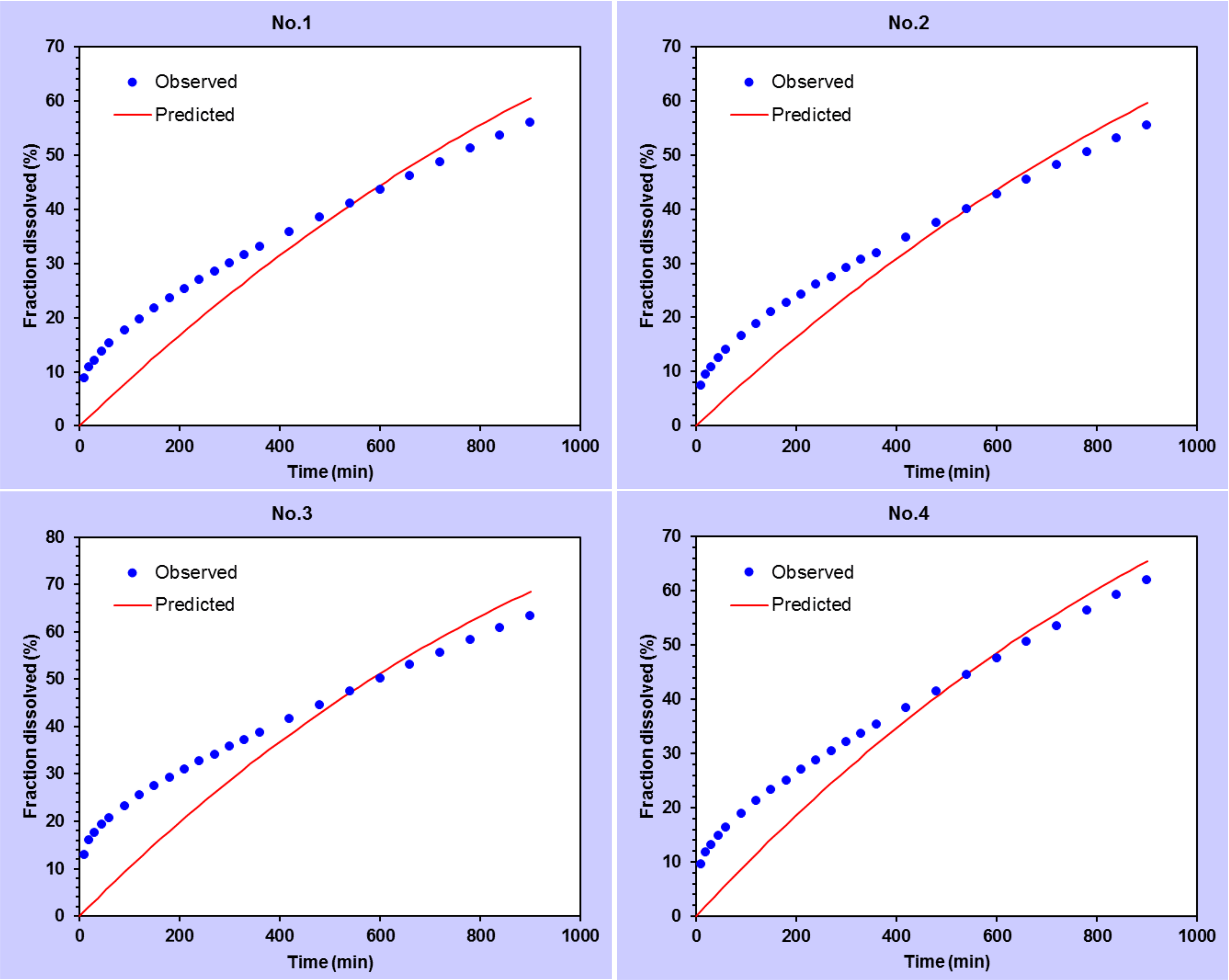

Supplementary materials – Model fitting summary – Starch 1500® sample with smaller particle size (modelling up to app. 60 % of carvedilol released)

Model: **Hixson–Crowell with T_{lag}**

Model equation: $F = 100 \cdot \left\{1 - \left[1 - k_{HC} \cdot \left(t - T_{lag}\right)\right]^3\right\}$

Fitted model parameters per tested tablet (N = 4) with statistics – mean, standard deviation (SD), and relative standard deviation expressed in % (RSD%) (output from DDSolver):

Parameter	No.1	No.2	No.3	No.4	Mean	SD	RSD(%)
k_{HC}	0.0002	0.0002	0.0003	0.0003	0.0002	0.0000	7.3922
T_{lag}	-182.5988	-162.0877	-233.9835	-160.0667	-184.6842	34.4064	-18.6298

Number of dissolution data points (N), degrees of freedom (df), and selected goodness of fit criteria – Pearson correlation coefficient (R), coefficient of determination (R^2), adjusted coefficient of determination ($R^2_{adjusted}$), and residual sum of squares (RSS) (manual calculation in MS Excel):

Parameter	No.1	No.2	No.3	No.4
N	24	24	24	24
df	22	22	22	22
R	0.996730541	0.996408904	0.996897064	0.998204211
R^2	0.993471771	0.992830703	0.993803756	0.996411647
$R^2_{adjusted}$	0.993175034	0.992504826	0.993522109	0.99624854
RSS	31.45698198	35.41915319	32.40311253	20.99802197

Graphical abstract of model fit presented as mean ± 1 SD of the fraction % of released carvedilol:

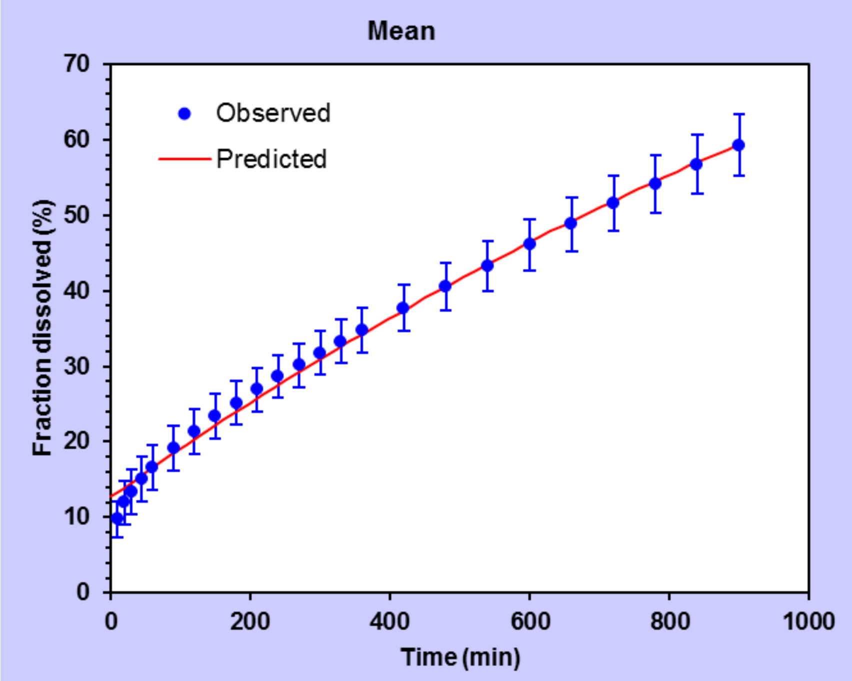

Supplementary materials – Model fitting summary – Starch 1500® sample with smaller particle size (modelling up to app. 60 % of carvedilol released)

Graphical abstract of model fit presented as the fraction % of released carvedilol per tested tablet:

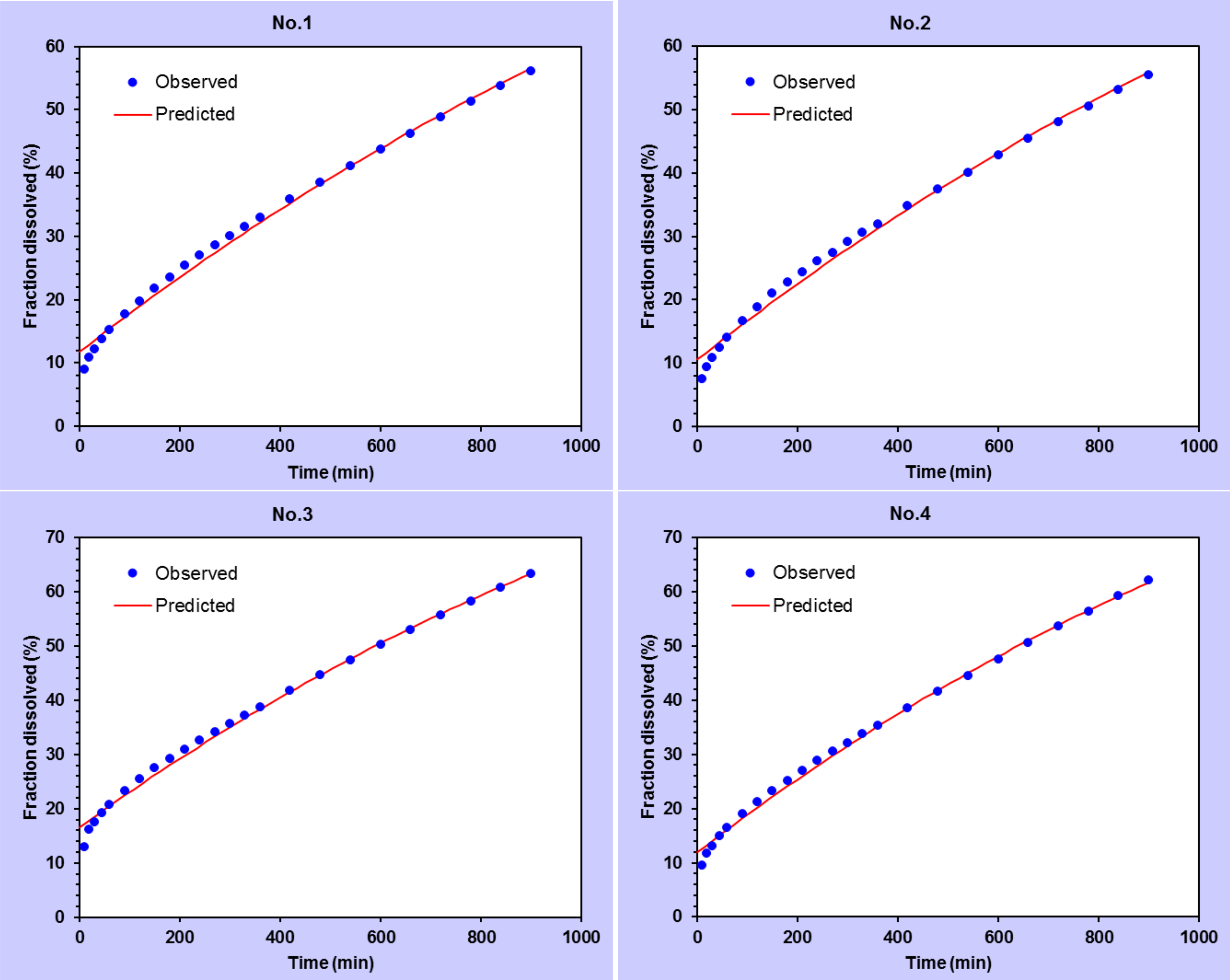

Supplementary materials – Model fitting summary – Starch 1500® sample with smaller particle size (modelling up to app. 60 % of carvedilol released)

Model: **Hopfenberg**

Model equation: $F = 100 \cdot [1 - (1 - k_{HB} \cdot t)^n]$

Fitted model parameters per tested tablet (N = 4) with statistics – mean, standard deviation (SD), and relative standard deviation expressed in % (RSD%) (output from DDSolver):

Parameter	No.1	No.2	No.3	No.4	Mean	SD	RSD(%)
k_{HB}	0.0003	0.0003	0.0004	0.0002	0.0003	0.0000	14.7023
n	3.0000	3.0000	3.0000	4.1250	3.2813	0.5625	17.1429

Number of dissolution data points (N), degrees of freedom (df), and selected goodness of fit criteria – Pearson correlation coefficient (R), coefficient of determination (R^2), adjusted coefficient of determination ($R^2_{adjusted}$), and residual sum of squares (RSS) (manual calculation in MS Excel):

Parameter	No.1	No.2	No.3	No.4
N	24	24	24	24
df	22	22	22	22
R	0.997622219	0.997186531	0.997588698	0.998503414
R^2	0.995250092	0.994380977	0.995183211	0.997009068
$R^2_{adjusted}$	0.995034187	0.994125567	0.994964266	0.996873117
RSS	1065.574744	863.5226934	2077.079084	1016.316061

Graphical abstract of model fit presented as mean ± 1 SD of the fraction % of released carvedilol:

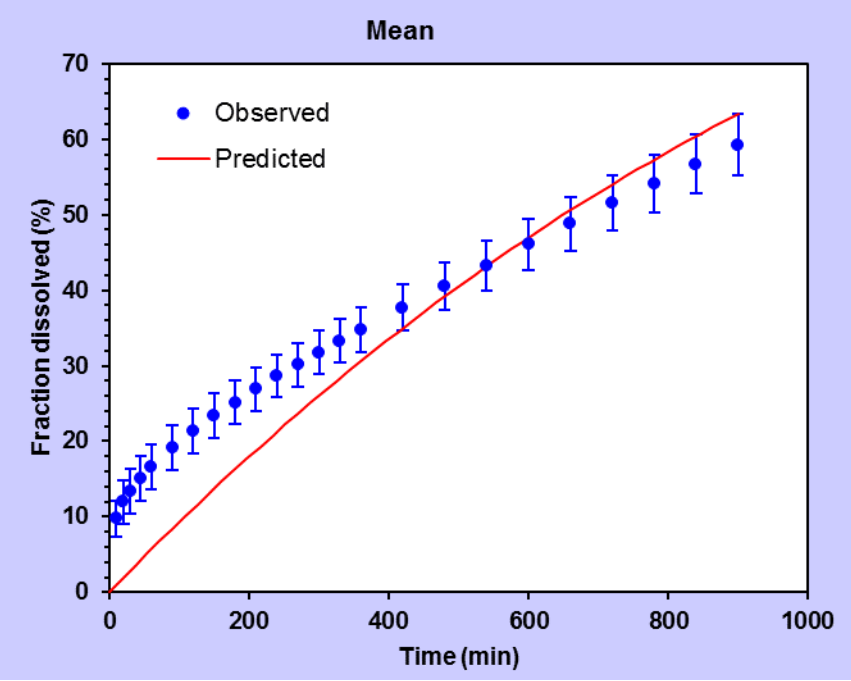

Supplementary materials – Model fitting summary – Starch 1500® sample with smaller particle size (modelling up to app. 60 % of carvedilol released)

Graphical abstract of model fit presented as the fraction % of released carvedilol per tested tablet:

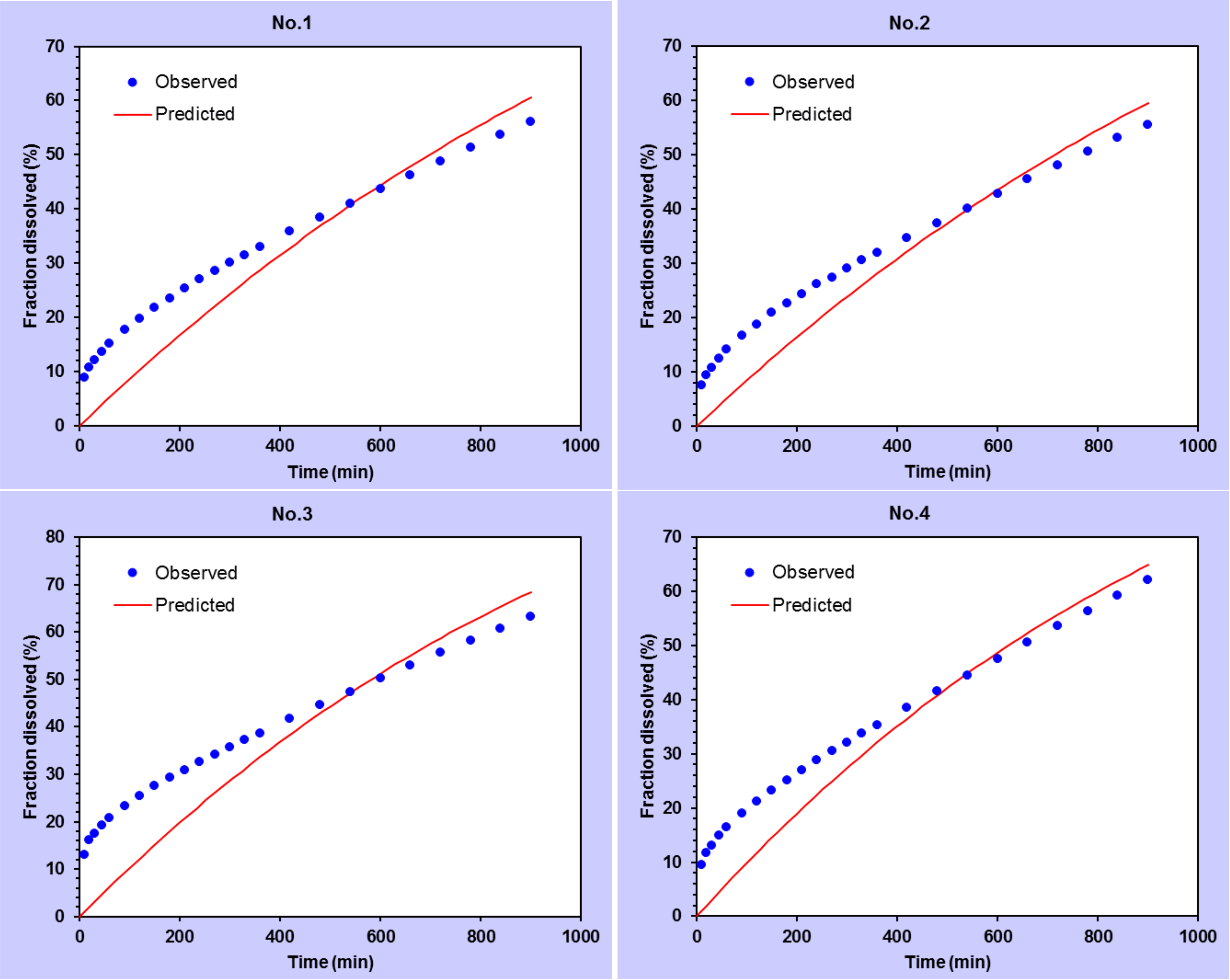

Supplementary materials – Model fitting summary – Starch 1500® sample with smaller particle size (modelling up to app. 60 % of carvedilol released)

Model: **Hopfenberg with T_{lag}**

Model equation: $F = 100 \cdot \{1 - [1 - k_{HB} \cdot (t - T_{lag})]^n\}$

Fitted model parameters per tested tablet (N = 4) with statistics – mean, standard deviation (SD), and relative standard deviation expressed in % (RSD%) (output from DDSolver):

Parameter	No.1	No.2	No.3	No.4	Mean	SD	RSD(%)
k_{HB}	0.0002	0.0002	0.0003	0.0003	0.0002	0.0000	7.3922
n	3.0000	3.0000	3.0000	3.0000	3.0000	0.0000	0.0000
T_{lag}	-182.5988	-162.0877	-233.9835	-160.0667	-184.6842	34.4064	-18.6298

Number of dissolution data points (N), degrees of freedom (df), and selected goodness of fit criteria – Pearson correlation coefficient (R), coefficient of determination (R^2), adjusted coefficient of determination ($R^2_{adjusted}$), and residual sum of squares (RSS) (manual calculation in MS Excel):

Parameter	No.1	No.2	No.3	No.4
N	24	24	24	24
df	21	21	21	21
R	0.996730541	0.996408904	0.996897064	0.998204211
R^2	0.993471771	0.992830703	0.993803756	0.996411647
$R^2_{adjusted}$	0.992850035	0.992147913	0.993213638	0.996069899
RSS	31.45698198	35.41915319	32.40311253	20.99802197

Graphical abstract of model fit presented as mean ± 1 SD of the fraction % of released carvedilol:

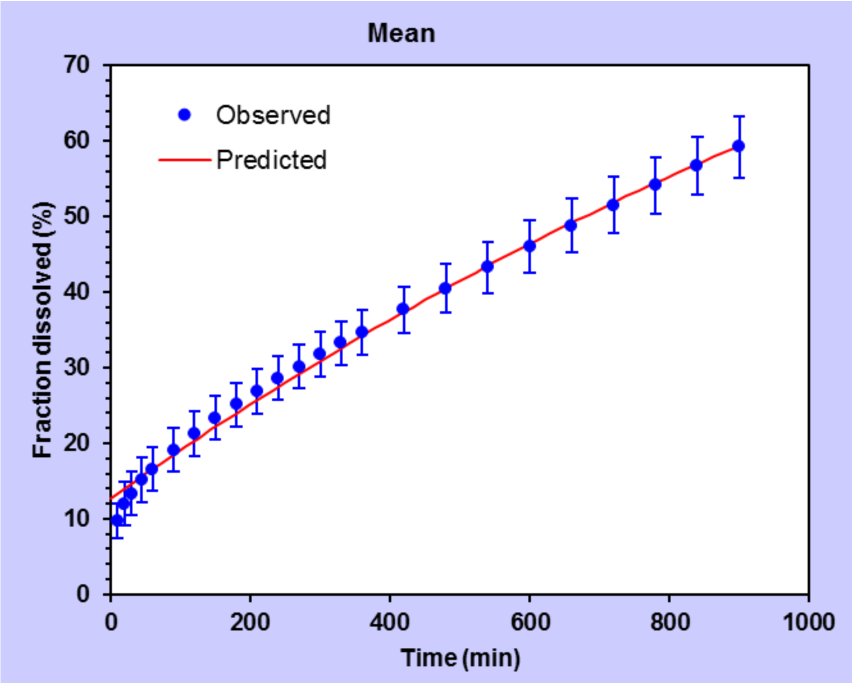

Supplementary materials – Model fitting summary – Starch 1500® sample with smaller particle size (modelling up to app. 60 % of carvedilol released)

Graphical abstract of model fit presented as the fraction % of released carvedilol per tested tablet:

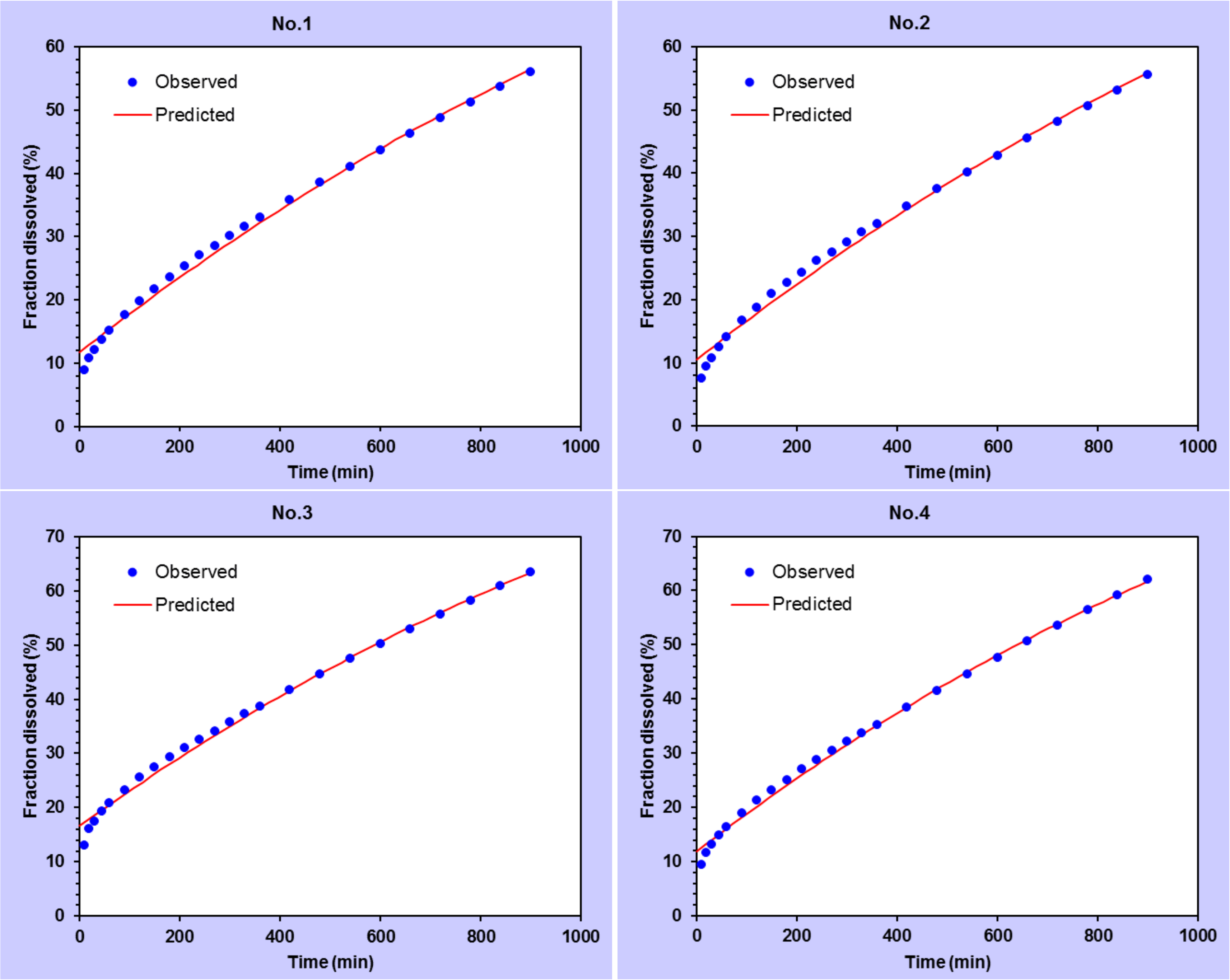

Supplementary materials – Model fitting summary – Starch 1500® sample with smaller particle size (modelling up to app. 60 % of carvedilol released)

Model: **Baker–Lonsdale**

Model equation: $\frac{3}{2}\cdot\left[1-\left(1-\frac{F}{100}\right)^{\frac{2}{3}}\right]-\frac{F}{100}=k_{BL}\cdot t$

Fitted model parameters per tested tablet (N = 4) with statistics – mean, standard deviation (SD), and relative standard deviation expressed in % (RSD%) (output from DDSolver):

Parameter	No.1	No.2	No.3	No.4	Mean	SD	RSD(%)
k_{BL}	0.00008	0.00006	0.00010	0.00007	0.00008	0.00002	24.32829

Number of dissolution data points (N), degrees of freedom (df), and selected goodness of fit criteria – Pearson correlation coefficient (R), coefficient of determination (R^2), adjusted coefficient of determination ($R^2_{adjusted}$), and residual sum of squares (RSS) (manual calculation in MS Excel):

Parameter	No.1	No.2	No.3	No.4
N	24	24	24	24
df	23	23	23	23
R	0.991268096	0.992228335	0.989139686	0.988064193
R^2	0.982612437	0.984517069	0.978397319	0.976270849
$R^2_{adjusted}$	0.982612437	0.984517069	0.978397319	0.976270849
RSS	159.5074333	180.9361689	168.2850533	234.0190265

Graphical abstract of model fit presented as mean ± 1 SD of the fraction % of released carvedilol:

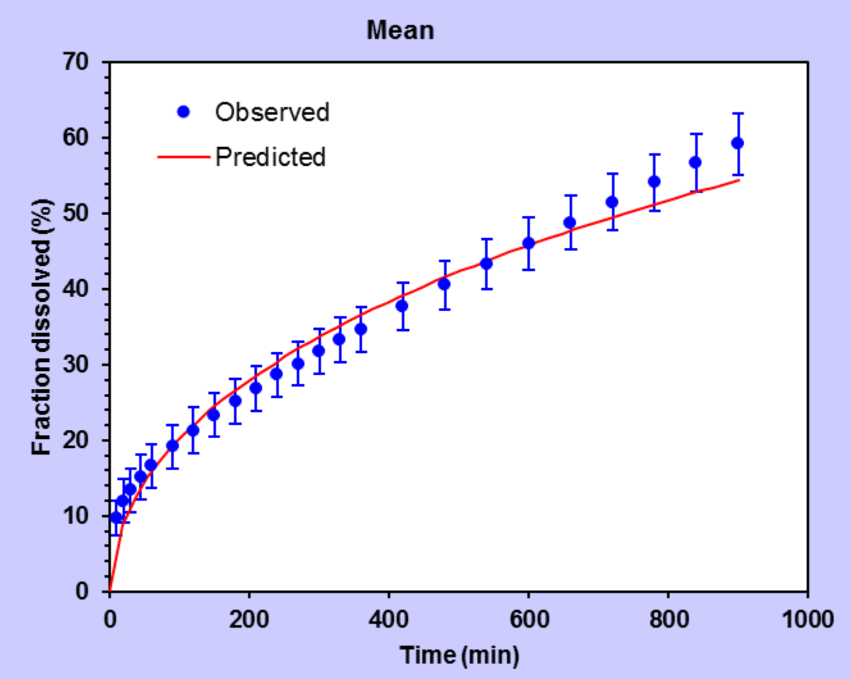

Supplementary materials – Model fitting summary – Starch 1500® sample with smaller particle size (modelling up to app. 60 % of carvedilol released)

Graphical abstract of model fit presented as the fraction % of released carvedilol per tested tablet:

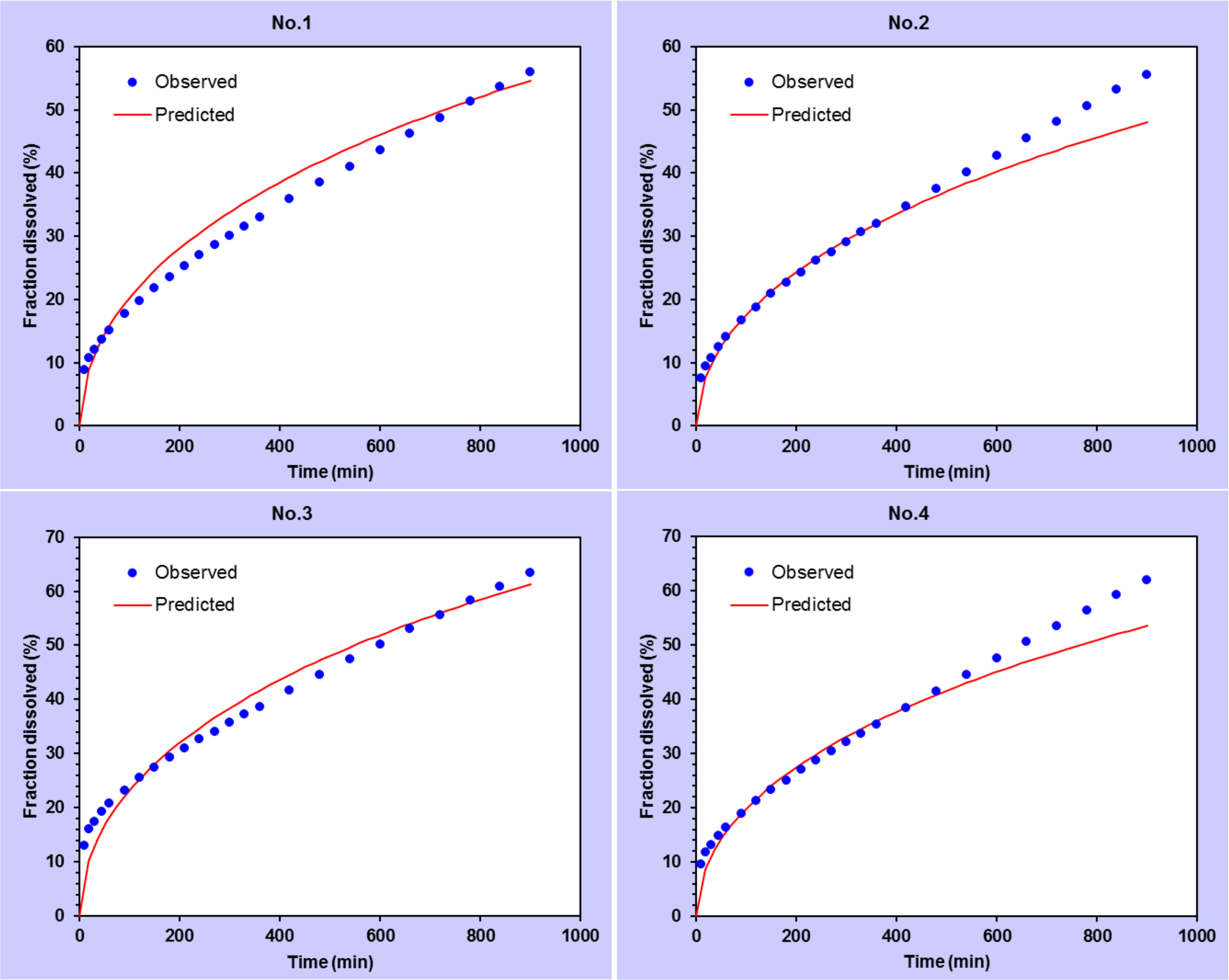

Supplementary materials – Model fitting summary – Starch 1500® sample with smaller particle size (modelling up to app. 60 % of carvedilol released)

Model: **Baker–Lonsdale with T_{lag}**

Model equation: $\frac{3}{2}\cdot\left[1-\left(1-\frac{F}{100}\right)^{\frac{2}{3}}\right]-\frac{F}{100}=k_{BL}\cdot\left(t-T_{lag}\right)$

Fitted model parameters per tested tablet (N = 4) with statistics – mean, standard deviation (SD), and relative standard deviation expressed in % (RSD%) (output from DDSolver):

Parameter	No.1	No.2	No.3	No.4	Mean	SD	RSD(%)
k_{BL}	0.00008	0.00007	0.00010	0.00010	0.00009	0.00001	15.68411
T_{lag}	34.16443	43.16978	11.05502	46.19456	33.64595	15.90377	47.26801

Number of dissolution data points (N), degrees of freedom (df), and selected goodness of fit criteria – Pearson correlation coefficient (R), coefficient of determination (R^2), adjusted coefficient of determination ($R^2_{adjusted}$), and residual sum of squares (RSS) (manual calculation in MS Excel):

Parameter	No.1	No.2	No.3	No.4
N	24	24	24	24
df	22	22	22	22
R	0.979372304	0.980193911	0.982390049	0.971672793
R^2	0.959170109	0.960780104	0.965090208	0.944148016
$R^2_{adjusted}$	0.957314205	0.958997381	0.963503399	0.941609289
RSS	455.816251	443.4645674	399.8239174	777.8518596

Graphical abstract of model fit presented as mean ± 1 SD of the fraction % of released carvedilol:

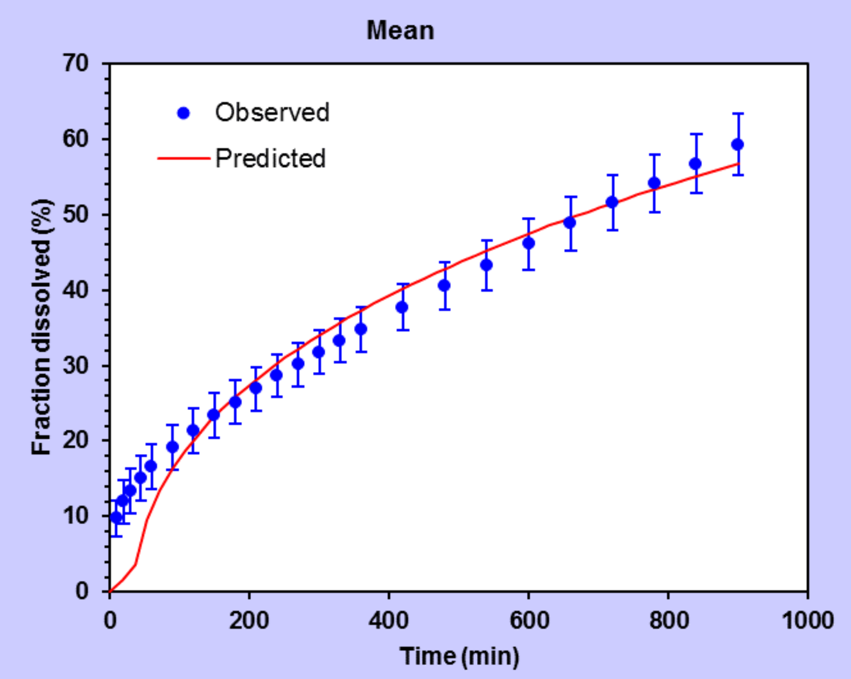

Supplementary materials – Model fitting summary – Starch 1500® sample with smaller particle size (modelling up to app. 60 % of carvedilol released)

Graphical abstract of model fit presented as the fraction % of released carvedilol per tested tablet:

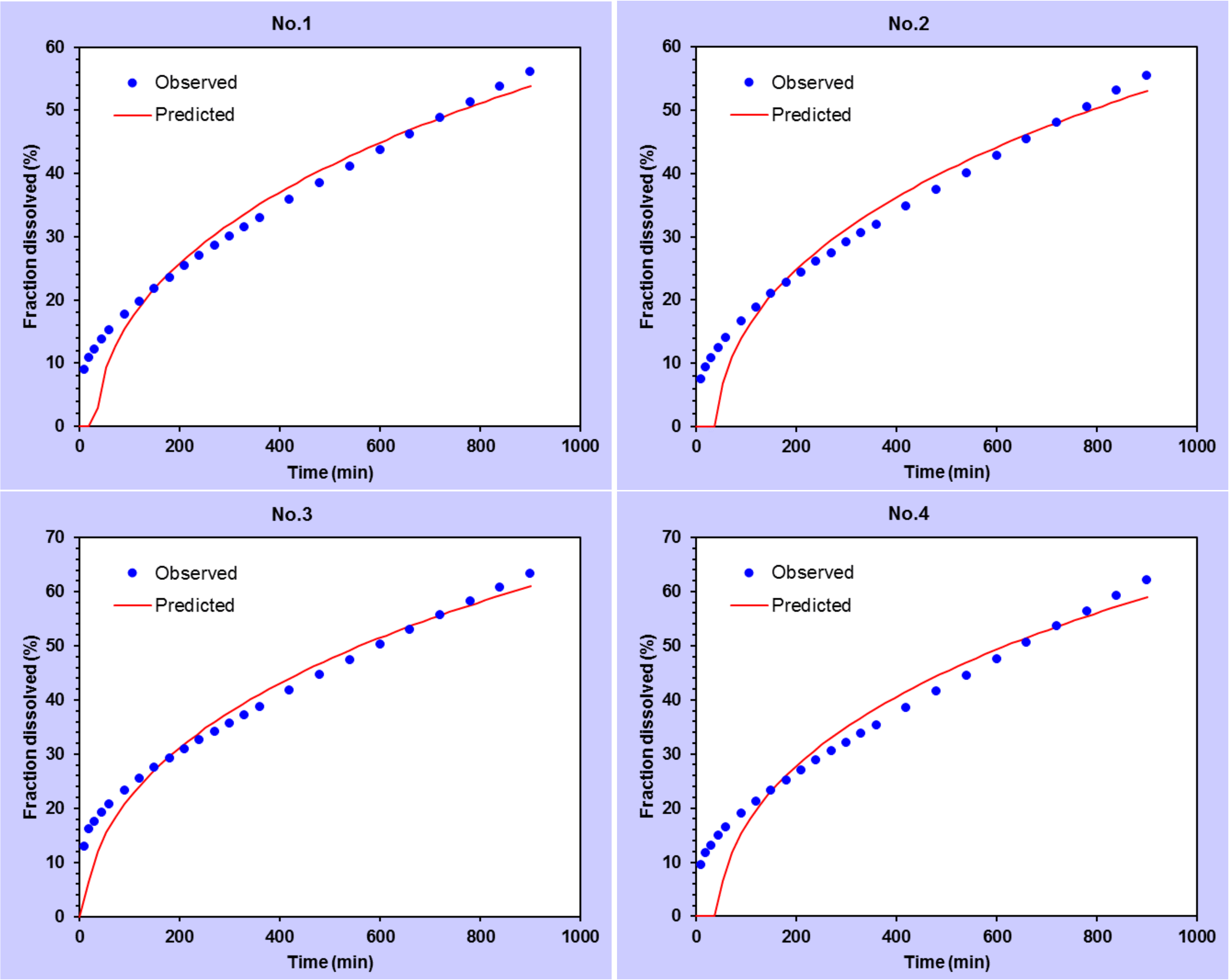

Supplementary materials – Model fitting summary – Starch 1500® sample with smaller particle size (modelling up to app. 60 % of carvedilol released)

Model: **Makoid–Banakar**

Model equation: $F = k_{MB} \cdot t^n \cdot e^{-k \cdot t}$

Fitted model parameters per tested tablet (N = 4) with statistics – mean, standard deviation (SD), and relative standard deviation expressed in % (RSD%) (output from DDSolver):

Parameter	No.1	No.2	No.3	No.4	Mean	SD	RSD(%)
k_{MB}	4.0154	3.0824	7.3394	4.4586	4.7239	1.8355	38.8562
n	0.3241	0.3688	0.2490	0.3144	0.3141	0.0494	15.7429
k	-0.0005	-0.0004	-0.0005	-0.0006	-0.0005	0.0001	-10.7904

Number of dissolution data points (N), degrees of freedom (df), and selected goodness of fit criteria – Pearson correlation coefficient (R), coefficient of determination (R^2), adjusted coefficient of determination ($R^2_{adjusted}$), and residual sum of squares (RSS) (manual calculation in MS Excel):

Parameter	No.1	No.2	No.3	No.4
N	24	24	24	24
df	21	21	21	21
R	0.999400652	0.999782046	0.999664838	0.99954803
R^2	0.998801663	0.99956414	0.999329788	0.999096265
$R^2_{adjusted}$	0.998687536	0.99952263	0.999265959	0.999010195
RSS	5.839635171	2.180560956	3.514480459	5.417981009

Graphical abstract of model fit presented as mean ± 1 SD of the fraction % of released carvedilol:

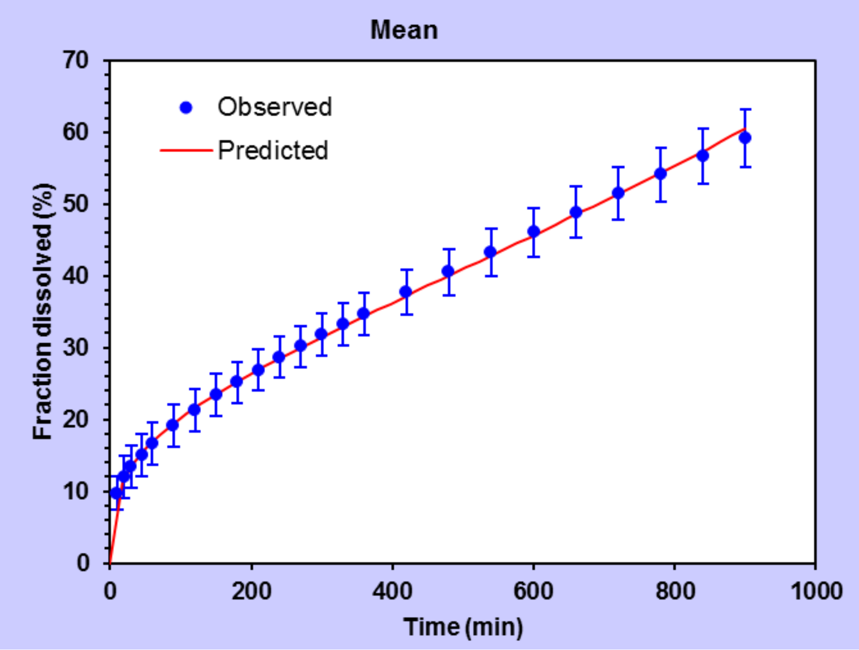

Supplementary materials – Model fitting summary – Starch 1500® sample with smaller particle size (modelling up to app. 60 % of carvedilol released)

Graphical abstract of model fit presented as the fraction % of released carvedilol per tested tablet:

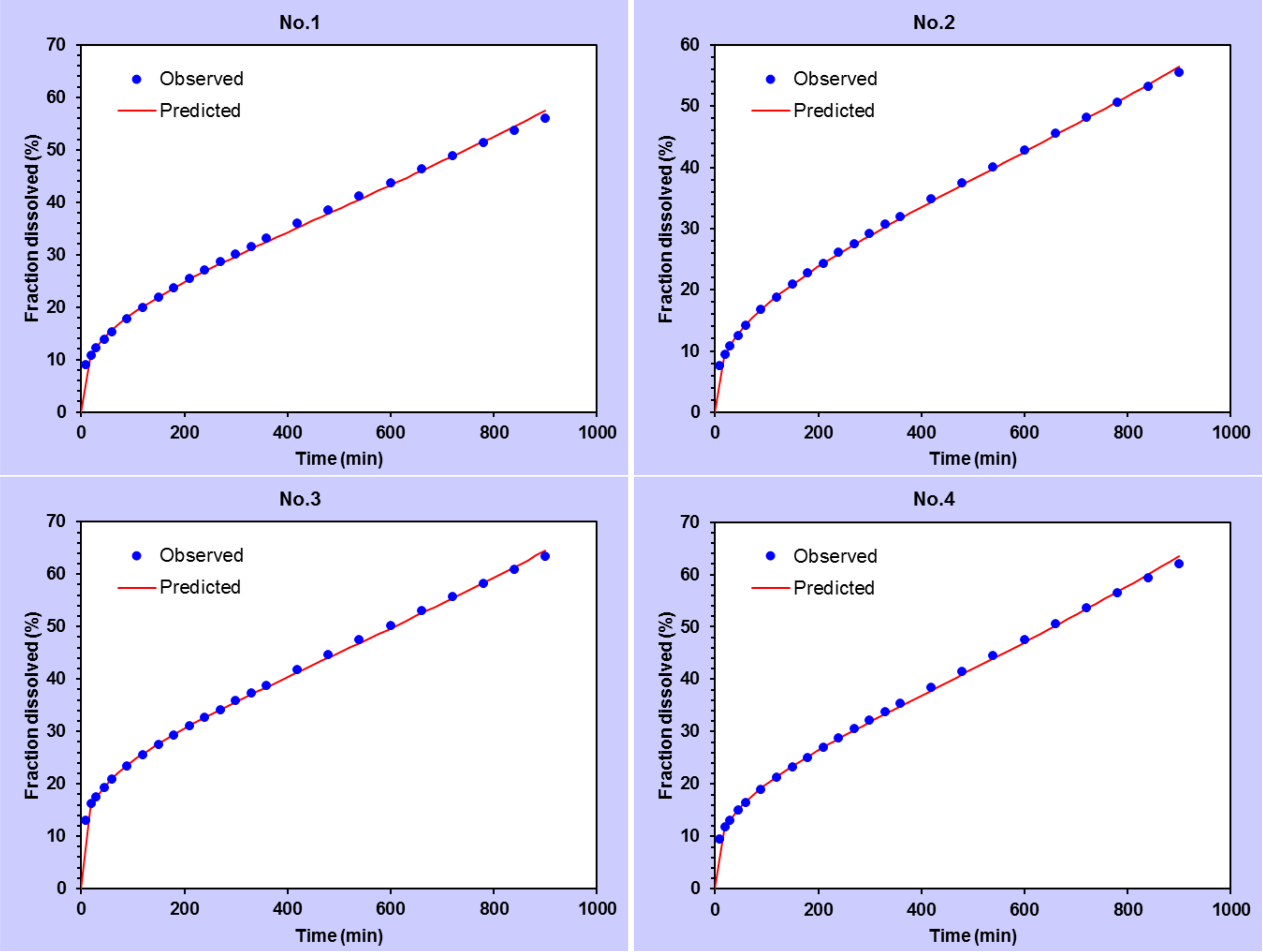

Supplementary materials – Model fitting summary – Starch 1500® sample with smaller particle size (modelling up to app. 60 % of carvedilol released)

Model: **Makoid–Banakar with T_{lag}**

Model equation: $F = k_{MB} \cdot (t - T_{lag})^n \cdot e^{-k \cdot (t - T_{lag})}$

Fitted model parameters per tested tablet (N = 4) with statistics – mean, standard deviation (SD), and relative standard deviation expressed in % (RSD%) (output from DDSolver):

Parameter	No.1	No.2	No.3	No.4	Mean	SD	RSD(%)
k_{MB}	4.9307	3.8824	8.5626	5.4328	5.7022	2.0134	35.3095
n	0.2818	0.3213	0.2176	0.2738	0.2736	0.0427	15.6226
k	-0.0006	-0.0006	-0.0006	-0.0007	-0.0006	0.0000	-7.6136
T_{lag}	4.0000	4.0000	4.0000	4.0000	4.0000	0.0000	0.0000

Number of dissolution data points (N), degrees of freedom (df), and selected goodness of fit criteria – Pearson correlation coefficient (R), coefficient of determination (R^2), adjusted coefficient of determination ($R^2_{adjusted}$), and residual sum of squares (RSS) (manual calculation in MS Excel):

Parameter	No.1	No.2	No.3	No.4
N	24	24	24	24
df	20	20	20	20
R	0.998572717	0.999165426	0.999253936	0.998902548
R^2	0.997147471	0.998331548	0.998508428	0.997806301
$R^2_{adjusted}$	0.996719591	0.99808128	0.998284692	0.997477246
RSS	14.09388691	8.485448461	7.919266379	13.37635991

Graphical abstract of model fit presented as mean ± 1 SD of the fraction % of released carvedilol:

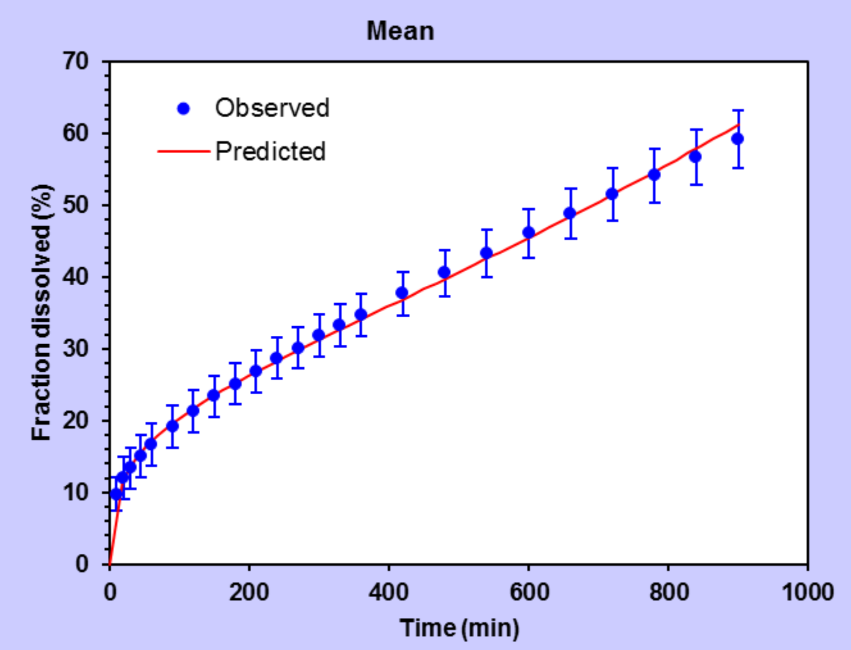

Supplementary materials – Model fitting summary – Starch 1500® sample with smaller particle size (modelling up to app. 60 % of carvedilol released)

Graphical abstract of model fit presented as the fraction % of released carvedilol per tested tablet:

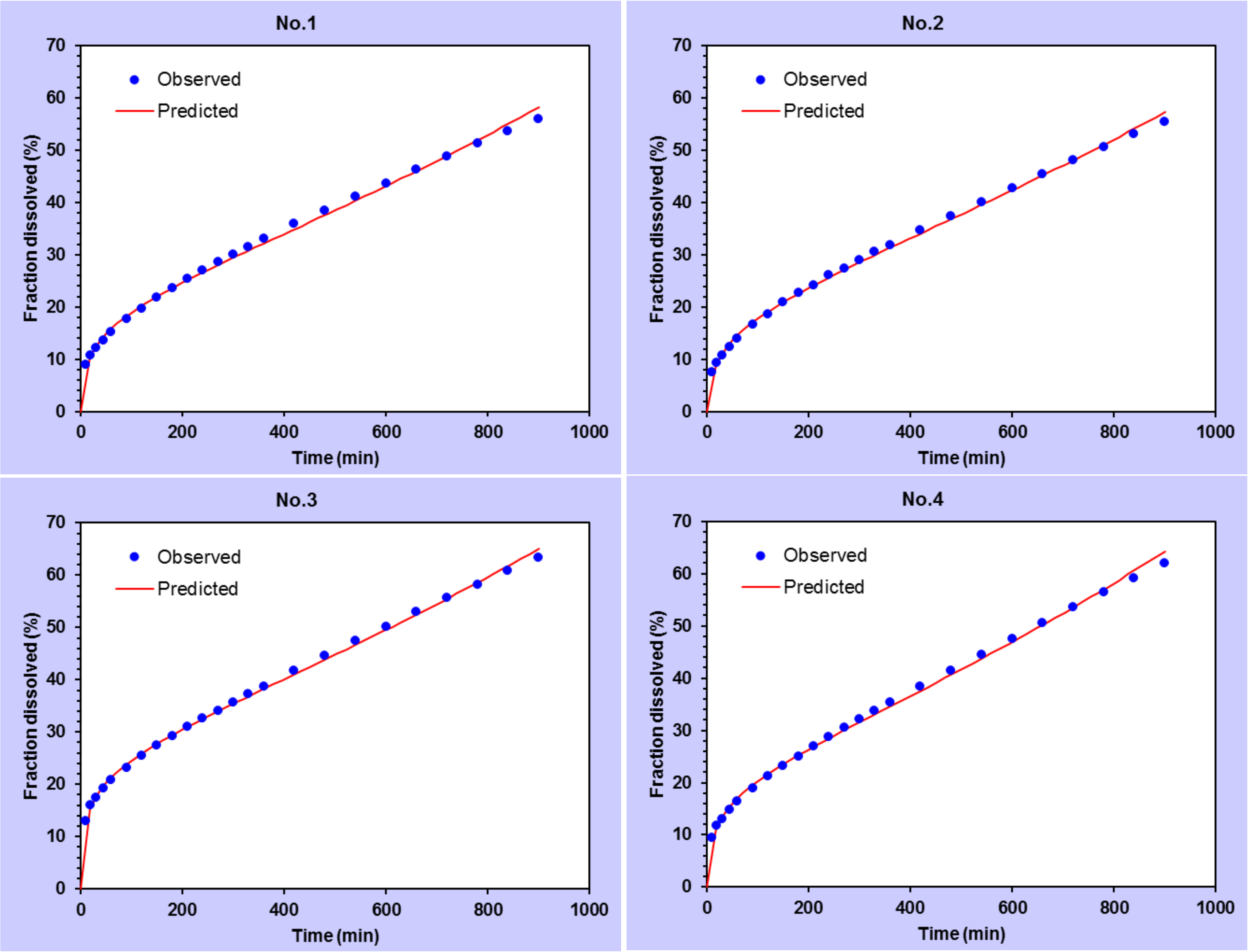

Supplementary materials – Model fitting summary – Starch 1500® sample with smaller particle size (modelling up to app. 60 % of carvedilol released)

Model: **Peppas–Sahlin_1**

Model equation: $F = k_1 \cdot t^m + k_2 \cdot t^{2m}$

Fitted model parameters per tested tablet (N = 4) with statistics – mean, standard deviation (SD), and relative standard deviation expressed in % (RSD%) (output from DDSolver):

Parameter	No.1	No.2	No.3	No.4	Mean	SD	RSD(%)
k_1	2.089	1.885	2.947	2.146	2.267	0.467	20.614
k_2	0.022	0.031	-0.005	0.032	0.020	0.017	85.802
m	0.450	0.450	0.450	0.450	0.450	0.000	0.000

Number of dissolution data points (N), degrees of freedom (df), and selected goodness of fit criteria – Pearson correlation coefficient (R), coefficient of determination (R^2), adjusted coefficient of determination ($R^2_{adjusted}$), and residual sum of squares (RSS) (manual calculation in MS Excel):

Parameter	No.1	No.2	No.3	No.4
N	24	24	24	24
df	21	21	21	21
R	0.997455967	0.998468408	0.991624657	0.996554172
R^2	0.994918406	0.996939162	0.983319461	0.993120218
$R^2_{adjusted}$	0.994434445	0.996647653	0.981730838	0.992465001
RSS	30.96858436	18.56798134	116.2510214	50.5024748

Graphical abstract of model fit presented as mean ± 1 SD of the fraction % of released carvedilol:

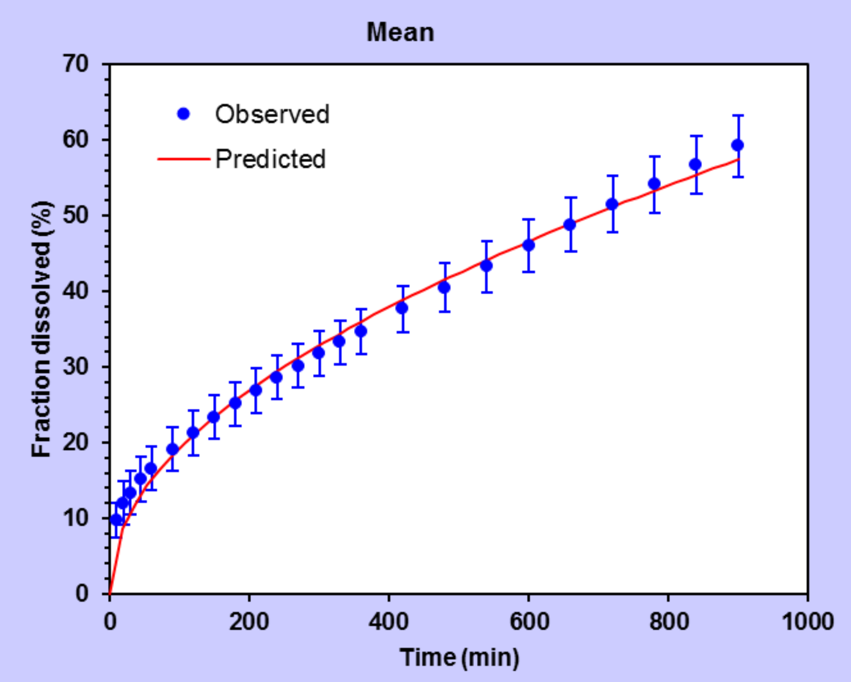

Supplementary materials – Model fitting summary – Starch 1500® sample with smaller particle size (modelling up to app. 60 % of carvedilol released)

Graphical abstract of model fit presented as the fraction % of released carvedilol per tested tablet:

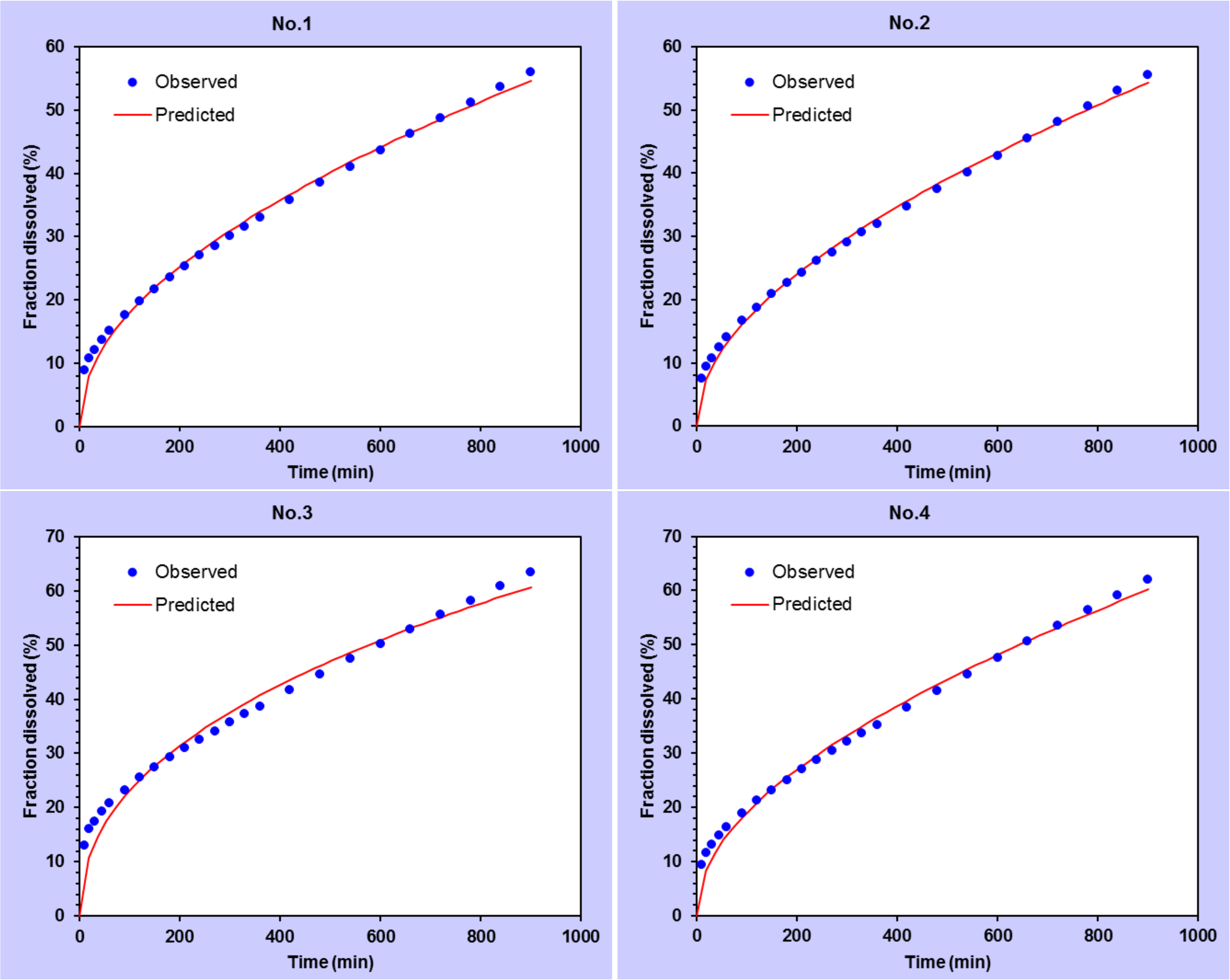

Supplementary materials – Model fitting summary – Starch 1500® sample with smaller particle size (modelling up to app. 60 % of carvedilol released)

Model: **Peppas–Sahlin_1 with T_{lag}**

Model equation: $F = k_1 \cdot (t - T_{lag})^m + k_2 \cdot (t - T_{lag})^{2m}$

Fitted model parameters per tested tablet (N = 4) with statistics – mean, standard deviation (SD), and relative standard deviation expressed in % (RSD%) (output from DDSolver):

Parameter	No.1	No.2	No.3	No.4	Mean	SD	RSD(%)
k_1	2.156	1.952	3.024	2.217	2.337	0.472	20.176
k_2	0.019	0.028	-0.009	0.028	0.016	0.017	104.757
m	0.450	0.450	0.450	0.450	0.450	0.000	0.000
T_{lag}	4.000	4.000	4.000	4.000	4.000	0.000	0.000

Number of dissolution data points (N), degrees of freedom (df), and selected goodness of fit criteria – Pearson correlation coefficient (R), coefficient of determination (R^2), adjusted coefficient of determination ($R^2_{adjusted}$), and residual sum of squares (RSS) (manual calculation in MS Excel):

Parameter	No.1	No.2	No.3	No.4
N	24	24	24	24
df	20	20	20	20
R	0.99624689	0.997622255	0.989340445	0.995322791
R^2	0.992507867	0.995250163	0.978794516	0.990667458
$R^2_{adjusted}$	0.991384047	0.994537688	0.975613694	0.989267577
RSS	47.90287485	30.35476909	156.4848243	71.83532871

Graphical abstract of model fit presented as mean ± 1 SD of the fraction % of released carvedilol:

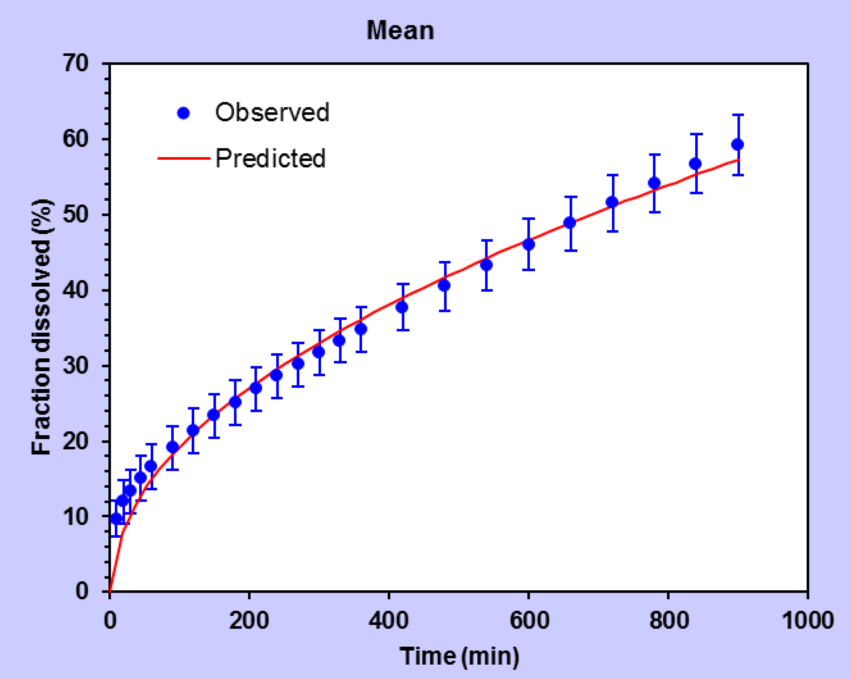

Supplementary materials – Model fitting summary – Starch 1500® sample with smaller particle size (modelling up to app. 60 % of carvedilol released)

Graphical abstract of model fit presented as the fraction % of released carvedilol per tested tablet:

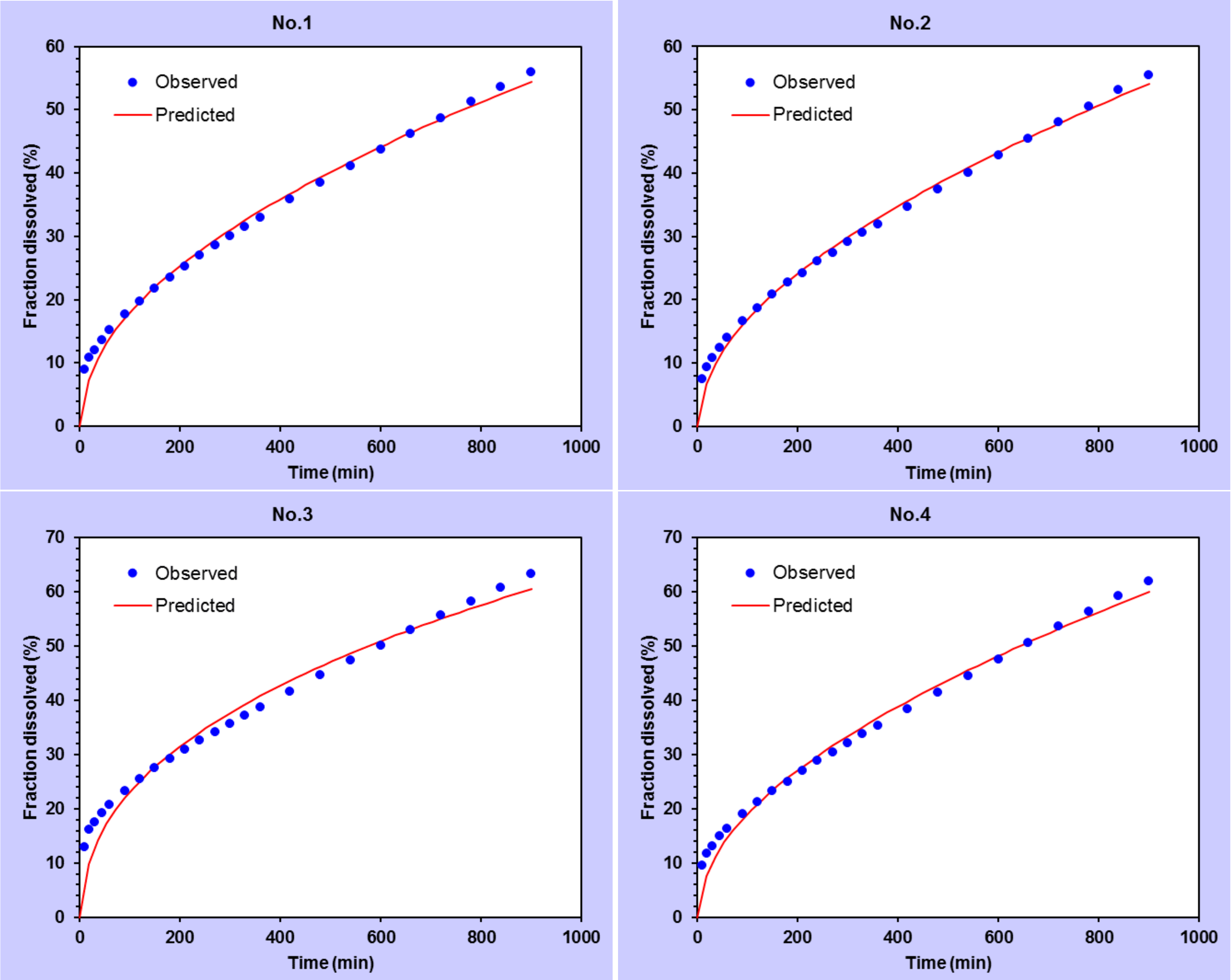

Supplementary materials – Model fitting summary – Starch 1500® sample with smaller particle size (modelling up to app. 60 % of carvedilol released)

Model: **Peppas–Sahlin_2**

Model equation: $F = k_1 \cdot t^{0.5} + k_2 \cdot t$

Fitted model parameters per tested tablet (N = 4) with statistics – mean, standard deviation (SD), and relative standard deviation expressed in % (RSD%) (output from DDSolver):

Parameter	No.1	No.2	No.3	No.4	Mean	SD	RSD(%)
k_1	1.755	1.608	2.393	1.817	1.893	0.344	18.192
k_2	0.002	0.007	-0.013	0.006	0.001	0.009	1555.901

Number of dissolution data points (N), degrees of freedom (df), and selected goodness of fit criteria – Pearson correlation coefficient (R), coefficient of determination (R^2), adjusted coefficient of determination ($R^2_{adjusted}$), and residual sum of squares (RSS) (manual calculation in MS Excel):

Parameter	No.1	No.2	No.3	No.4
N	24	24	24	24
df	22	22	22	22
R	0.996760451	0.998021147	0.98980424	0.99593241
R^2	0.993531397	0.996046211	0.979712433	0.991881366
$R^2_{adjusted}$	0.99323737	0.995866493	0.978790271	0.991512337
RSS	43.01527233	26.08352567	156.4426308	64.78591264

Graphical abstract of model fit presented as mean ± 1 SD of the fraction % of released carvedilol:

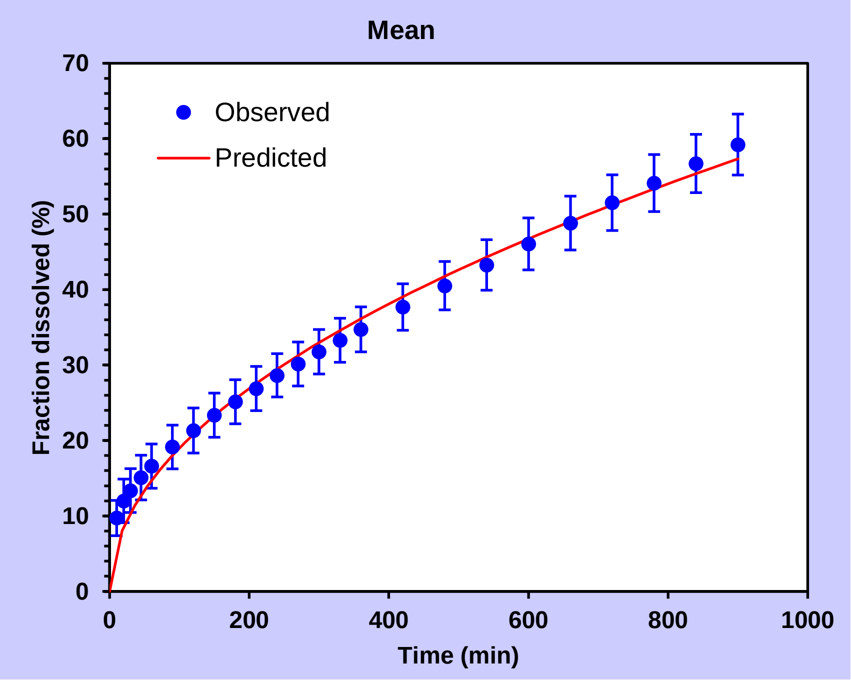

Supplementary materials – Model fitting summary – Starch 1500® sample with smaller particle size (modelling up to app. 60 % of carvedilol released)

Graphical abstract of model fit presented as the fraction % of released carvedilol per tested tablet:

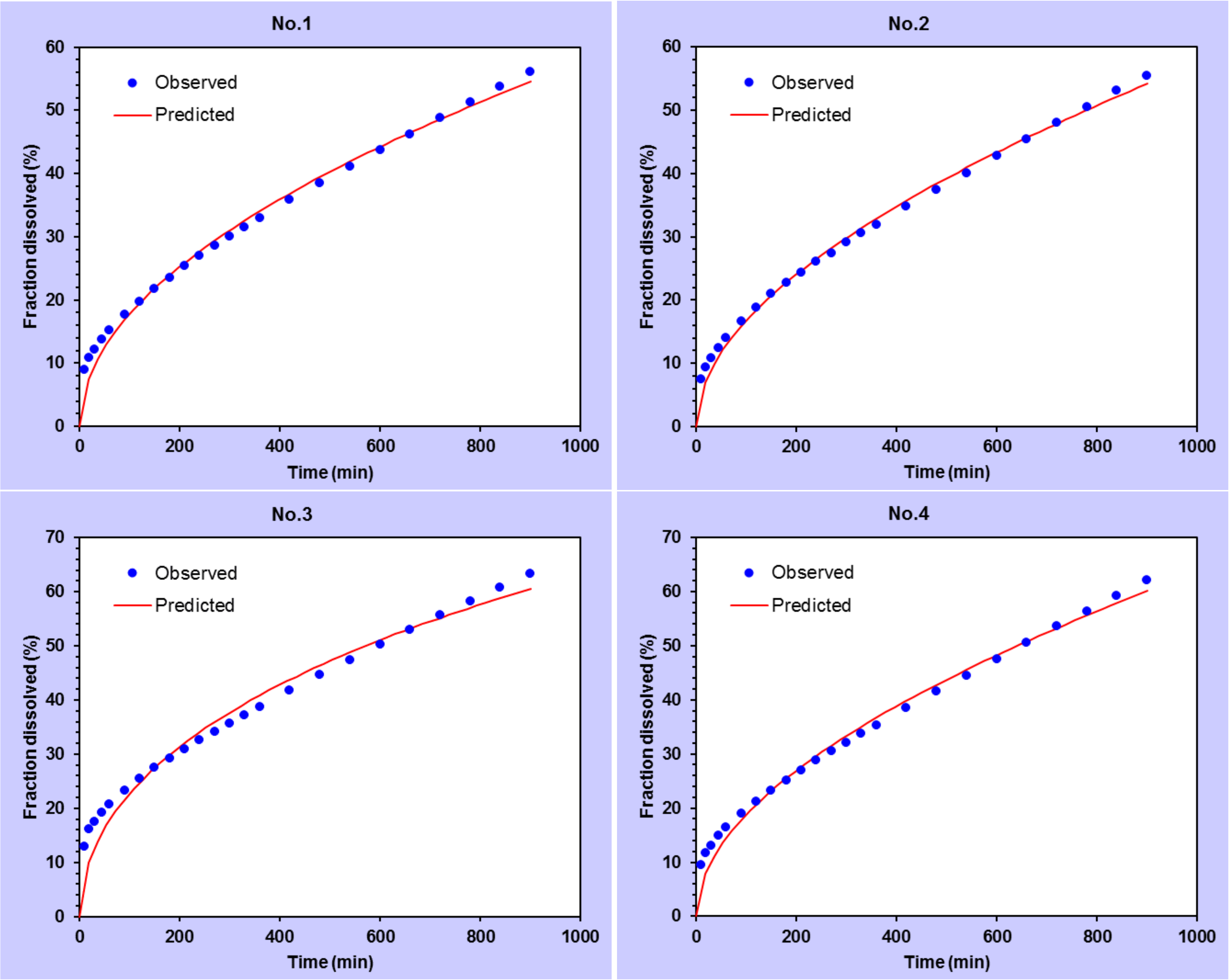

Supplementary materials – Model fitting summary – Starch 1500® sample with smaller particle size (modelling up to app. 60 % of carvedilol released)

Model: **Peppas–Sahlin_2 with T_{lag}**

Model equation: $F = k_1 \cdot (t - T_{lag})^{0.5} + k_2 \cdot (t - T_{lag})$

Fitted model parameters per tested tablet (N = 4) with statistics – mean, standard deviation (SD), and relative standard deviation expressed in % (RSD%) (output from DDSolver):

Parameter	No.1	No.2	No.3	No.4	Mean	SD	RSD(%)
k_1	1.800	1.654	2.444	1.865	1.941	0.347	17.870
k_2	0.001	0.005	-0.014	0.005	-0.001	0.009	-886.716
T_{lag}	4.000	4.000	4.000	4.000	4.000	0.000	0.000

Number of dissolution data points (N), degrees of freedom (df), and selected goodness of fit criteria – Pearson correlation coefficient (R), coefficient of determination (R^2), adjusted coefficient of determination ($R^2_{adjusted}$), and residual sum of squares (RSS) (manual calculation in MS Excel):

Parameter	No.1	No.2	No.3	No.4
N	24	24	24	24
df	21	21	21	21
R	0.99545603	0.997080202	0.9874681	0.994629879
R^2	0.990932708	0.99416893	0.975093248	0.989288596
$R^2_{adjusted}$	0.990069156	0.99361359	0.972721176	0.988268462
RSS	63.70144467	40.78669105	204.8815212	90.24607795

Graphical abstract of model fit presented as mean ± 1 SD of the fraction % of released carvedilol:

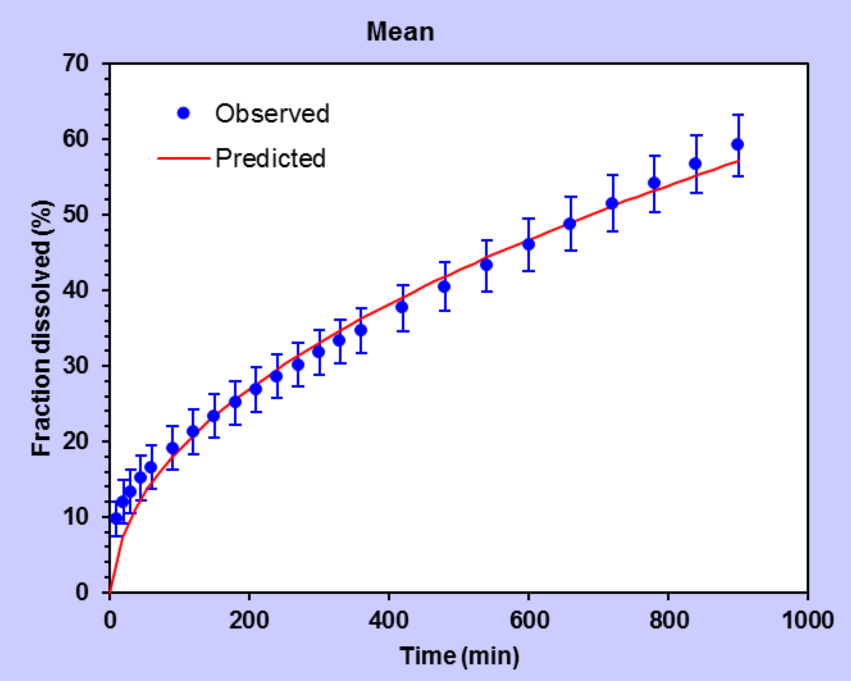

Supplementary materials – Model fitting summary – Starch 1500® sample with smaller particle size (modelling up to app. 60 % of carvedilol released)

Graphical abstract of model fit presented as the fraction % of released carvedilol per tested tablet:

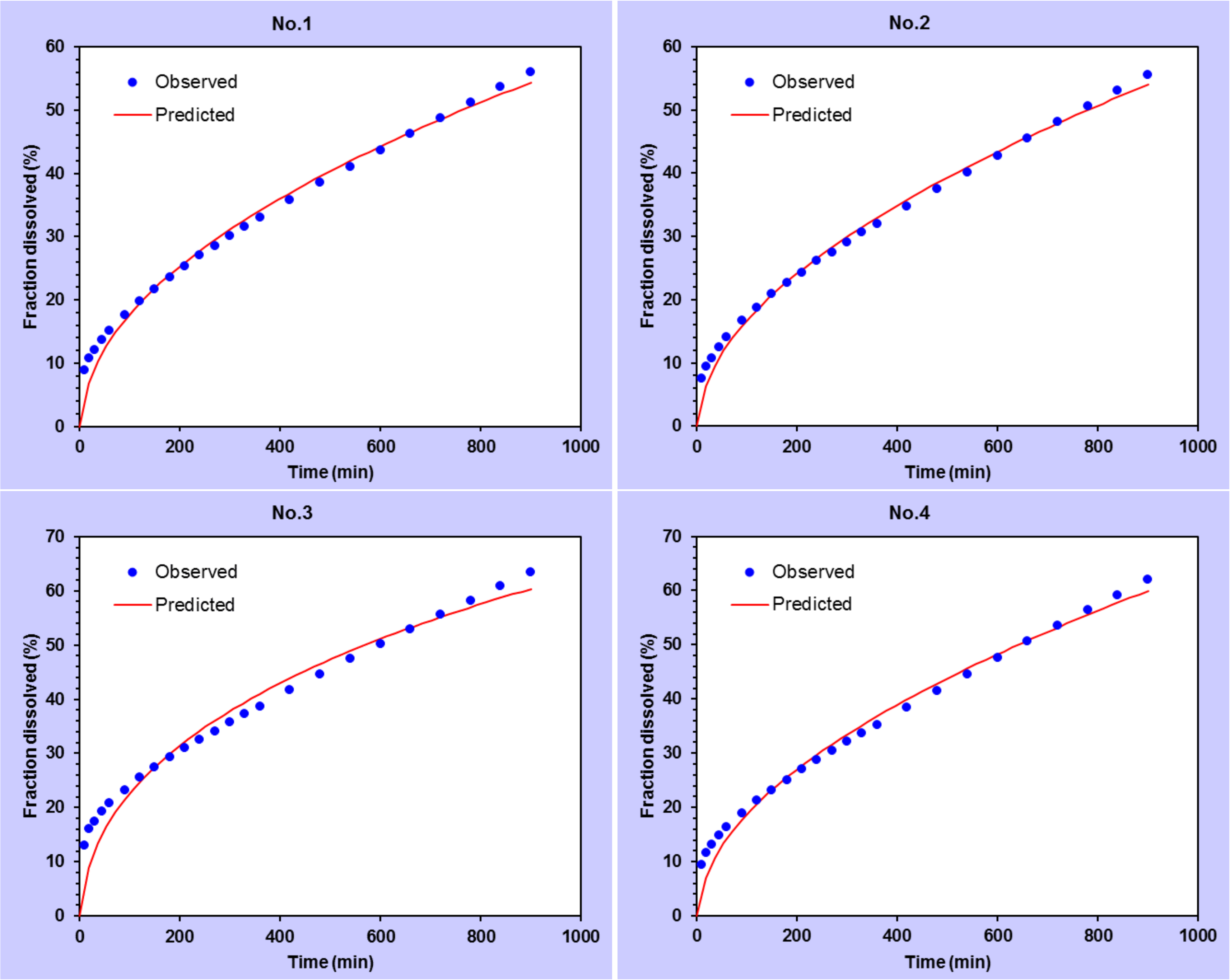

Supplementary materials – Model fitting summary – Starch 1500® sample with smaller particle size (modelling up to app. 60 % of carvedilol released)

Model: **Quadratic**

Model equation: $F = 100 \cdot (k_1 \cdot t^2 + k_2 \cdot t)$

Fitted model parameters per tested tablet (N = 4) with statistics – mean, standard deviation (SD), and relative standard deviation expressed in % (RSD%) (output from DDSolver):

Parameter	No.1	No.2	No.3	No.4	Mean	SD	RSD(%)
k_1	-0.0000007	-0.0000007	-0.0000010	-0.0000008	-0.0000008	0.0000001	-16.3671319
k_2	0.0012371	0.0011839	0.0015106	0.0013124	0.0013110	0.0001431	10.9164693

Number of dissolution data points (N), degrees of freedom (df), and selected goodness of fit criteria – Pearson correlation coefficient (R), coefficient of determination (R^2), adjusted coefficient of determination ($R^2_{adjusted}$), and residual sum of squares (RSS) (manual calculation in MS Excel):

Parameter	No.1	No.2	No.3	No.4
N	24	24	24	24
df	22	22	22	22
R	0.984180759	0.986421117	0.972582188	0.984006507
R^2	0.968611767	0.97302662	0.945916112	0.968268805
$R^2_{adjusted}$	0.967185029	0.971800557	0.943457754	0.966826478
RSS	566.7517771	453.7903313	1258.028181	666.1148725

Graphical abstract of model fit presented as mean ± 1 SD of the fraction % of released carvedilol:

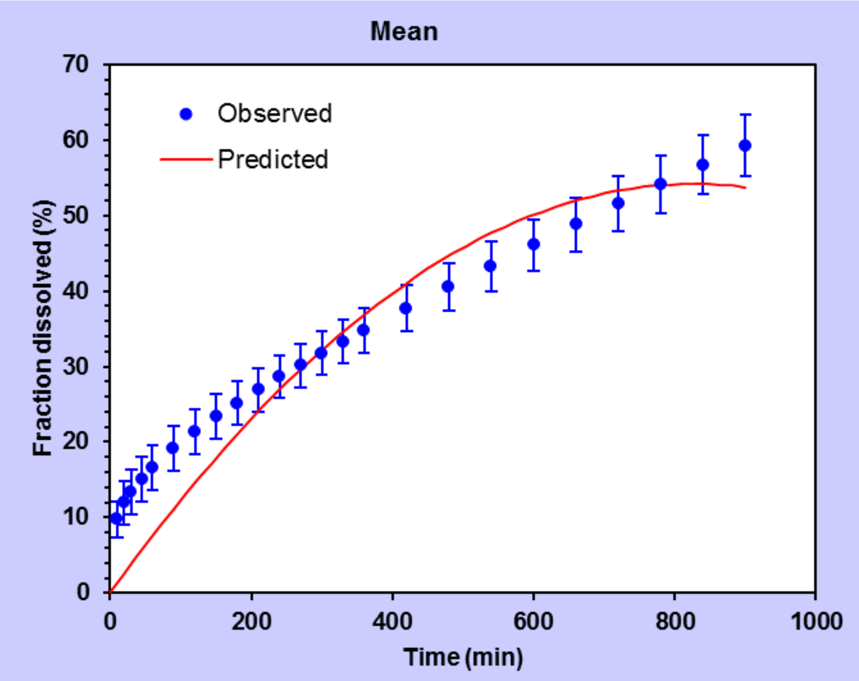

Supplementary materials – Model fitting summary – Starch 1500® sample with smaller particle size (modelling up to app. 60 % of carvedilol released)

Graphical abstract of model fit presented as the fraction % of released carvedilol per tested tablet:

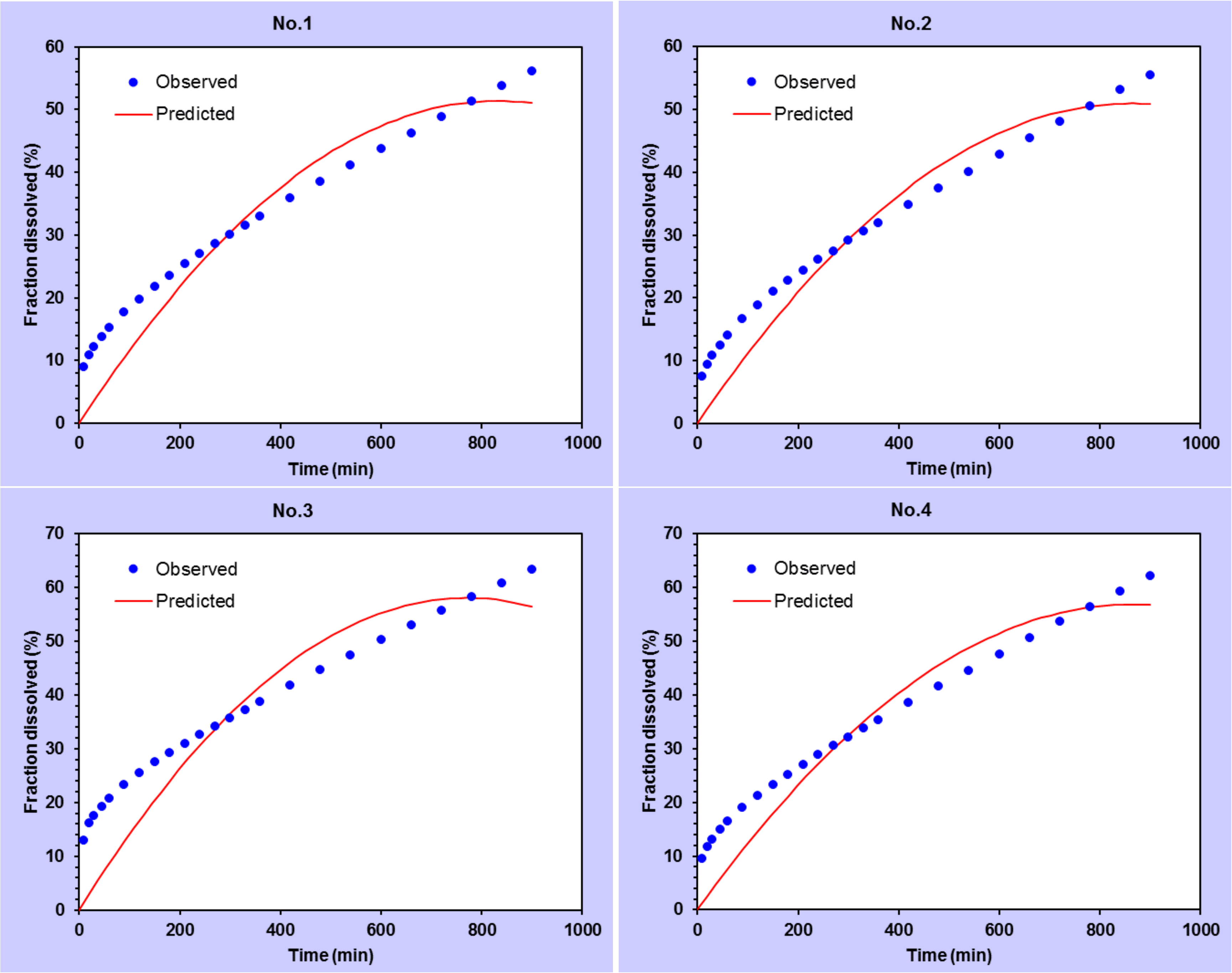

Supplementary materials – Model fitting summary – Starch 1500® sample with smaller particle size (modelling up to app. 60 % of carvedilol released)

Model: **Quadratic with T_{lag}**

Model equation: $F = 100 \cdot \left[k_1 \cdot (t - T_{lag})^2 + k_2 \cdot (t - T_{lag})\right]$

Fitted model parameters per tested tablet (N = 4) with statistics – mean, standard deviation (SD), and relative standard deviation expressed in % (RSD%) (output from DDSolver):

Parameter	No.1	No.2	No.3	No.4	Mean	SD	RSD(%)
k_1	-0.0000008	-0.0000007	-0.0000010	-0.0000008	-0.0000008	0.0000001	-16.1167404
k_2	0.0012501	0.0011969	0.0015243	0.0013262	0.0013244	0.0001435	10.8317261
T_{lag}	4.0000000	4.0000000	4.0000000	4.0000000	4.0000000	0.0000000	0.0000000

Number of dissolution data points (N), degrees of freedom (df), and selected goodness of fit criteria – Pearson correlation coefficient (R), coefficient of determination (R^2), adjusted coefficient of determination ($R^2_{adjusted}$), and residual sum of squares (RSS) (manual calculation in MS Excel):

Parameter	No.1	No.2	No.3	No.4
N	24	24	24	24
df	21	21	21	21
R	0.983648124	0.985893849	0.972108219	0.983461284
R^2	0.967563631	0.971986681	0.944994389	0.967196097
$R^2_{adjusted}$	0.964474453	0.969318746	0.939755759	0.964071916
RSS	620.5621494	499.5328056	1355.905885	728.0151781

Graphical abstract of model fit presented as mean ± 1 SD of the fraction % of released carvedilol:

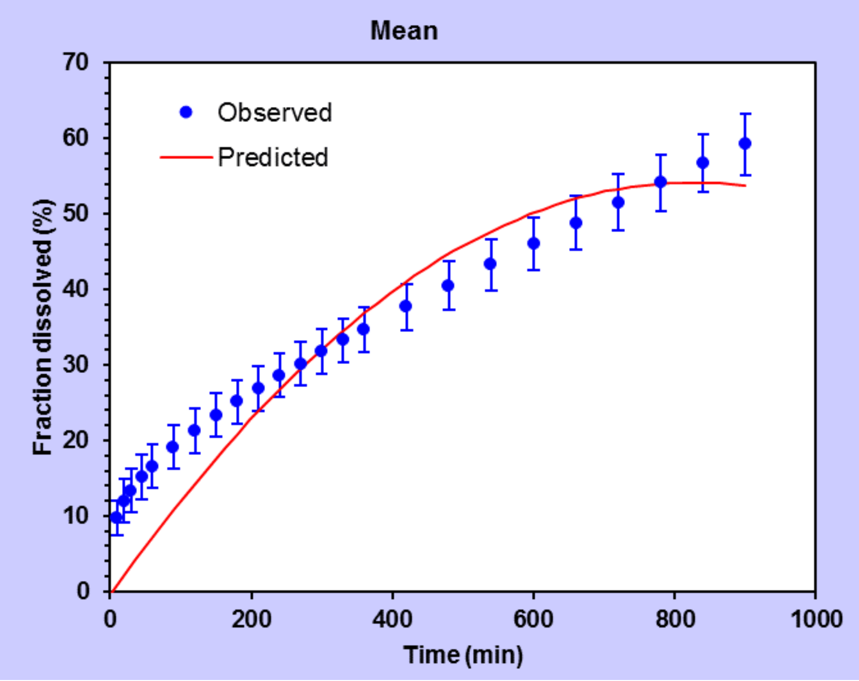

Supplementary materials – Model fitting summary – Starch 1500® sample with smaller particle size (modelling up to app. 60 % of carvedilol released)

Graphical abstract of model fit presented as the fraction % of released carvedilol per tested tablet:

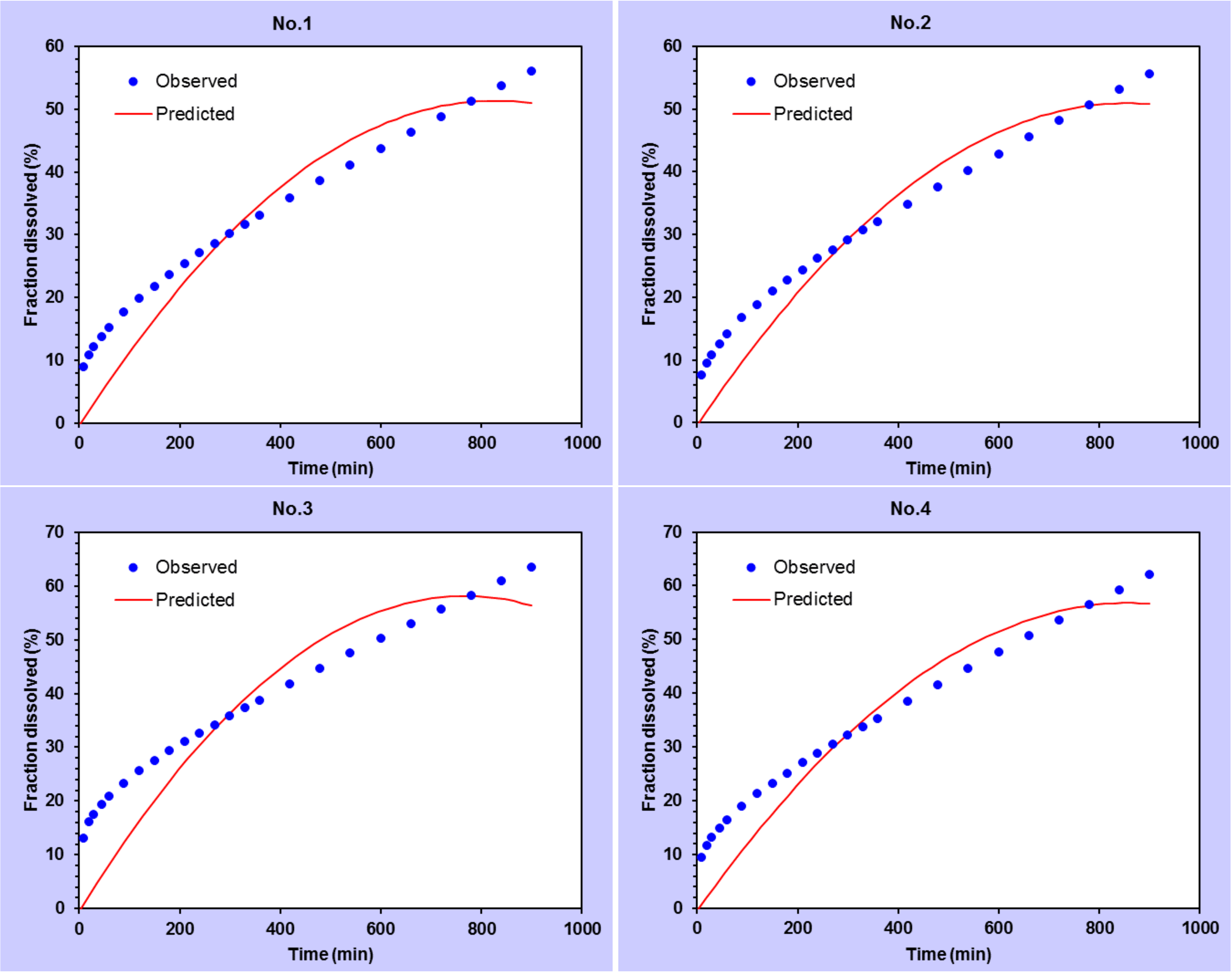

Supplementary materials – Model fitting summary – Starch 1500® sample with smaller particle size (modelling up to app. 60 % of carvedilol released)

Model: **Weibull_1**

Model equation: $F = 100 \cdot \left[1 - e^{-\frac{(t-T_i)^{\beta}}{\alpha}}\right]$

Fitted model parameters per tested tablet (N = 4) with statistics – mean, standard deviation (SD), and relative standard deviation expressed in % (RSD%) (output from DDSolver):

Parameter	No.1	No.2	No.3	No.4	Mean	SD	RSD(%)
α	38.570	43.220	20.054	33.869	33.928	10.006	29.493
β	0.490	0.490	0.403	0.473	0.464	0.041	8.882
T_i	4.653	4.000	4.000	4.000	4.163	0.326	7.840

Number of dissolution data points (N), degrees of freedom (df), and selected goodness of fit criteria – Pearson correlation coefficient (R), coefficient of determination (R^2), adjusted coefficient of determination ($R^2_{adjusted}$), and residual sum of squares (RSS) (manual calculation in MS Excel):

Parameter	No.1	No.2	No.3	No.4
N	23	23	23	23
df	20	20	20	20
R	0.982796137	0.984647746	0.971478809	0.976469637
R^2	0.965888247	0.969531185	0.943771076	0.953492953
$R^2_{adjusted}$	0.962477071	0.966484303	0.938148183	0.948842248
RSS	236.8637107	224.3881241	370.0886605	383.3587544

Graphical abstract of model fit presented as mean ± 1 SD of the fraction % of released carvedilol:

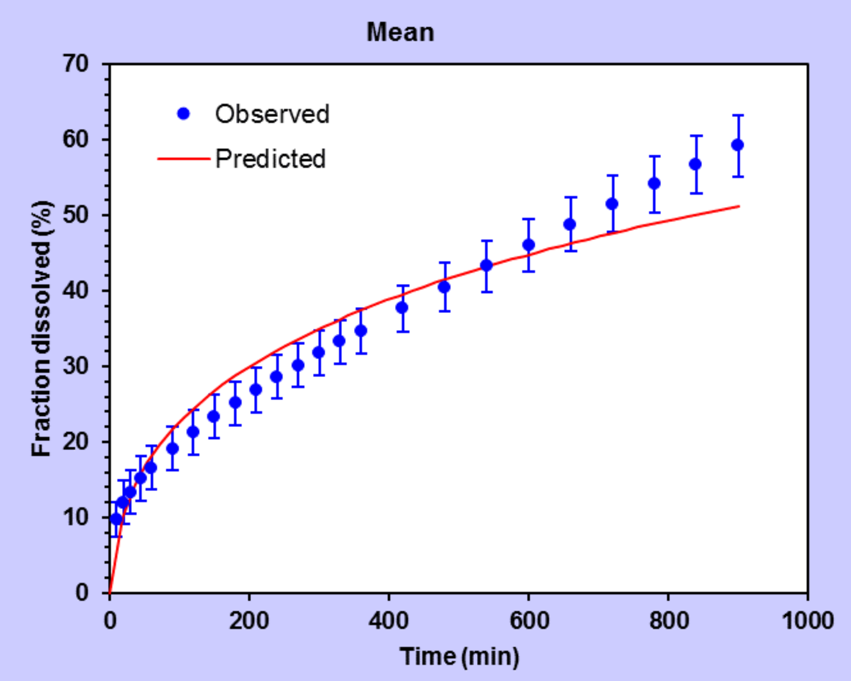

Supplementary materials – Model fitting summary – Starch 1500® sample with smaller particle size (modelling up to app. 60 % of carvedilol released)

Graphical abstract of model fit presented as the fraction % of released carvedilol per tested tablet:

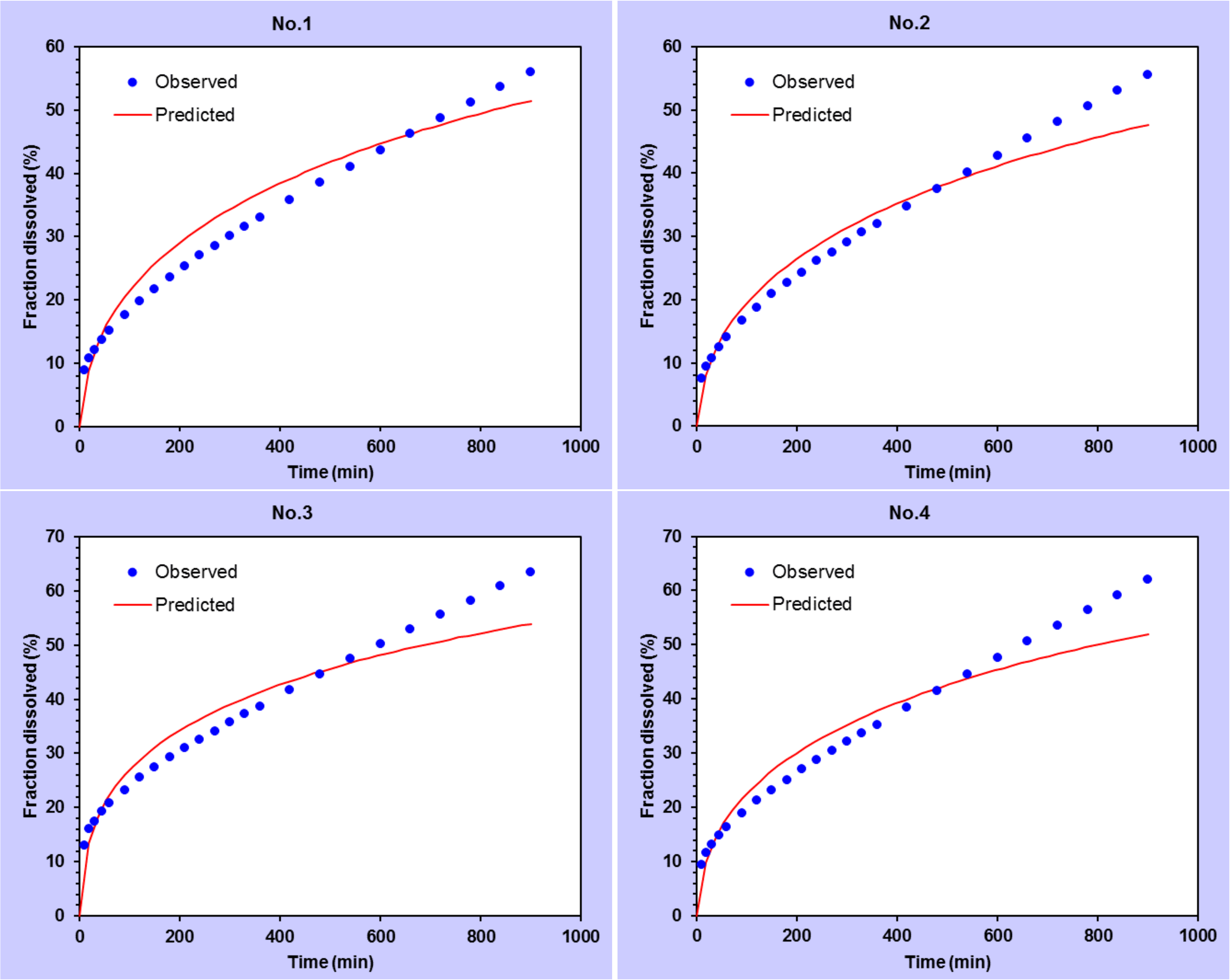

Supplementary materials – Model fitting summary – Starch 1500® sample with smaller particle size (modelling up to app. 60 % of carvedilol released)

Model: **Weibull_2**

Model equation: $F = 100 \cdot \left(1 - e^{-\frac{t^{\beta}}{\alpha}}\right)$

Fitted model parameters per tested tablet (N = 4) with statistics – mean, standard deviation (SD), and relative standard deviation expressed in % (RSD%) (output from DDSolver):

Parameter	No.1	No.2	No.3	No.4	Mean	SD	RSD(%)
α	50.872	63.888	24.443	49.864	47.267	16.502	34.912
β	0.532	0.566	0.436	0.547	0.520	0.058	11.108

Number of dissolution data points (N), degrees of freedom (df), and selected goodness of fit criteria – Pearson correlation coefficient (R), coefficient of determination (R^2), adjusted coefficient of determination ($R^2_{adjusted}$), and residual sum of squares (RSS) (manual calculation in MS Excel):

Parameter	No.1	No.2	No.3	No.4
N	24	24	24	24
df	22	22	22	22
R	0.986530176	0.988852652	0.975775399	0.981425823
R^2	0.973241788	0.977829568	0.952137629	0.963196645
$R^2_{adjusted}$	0.972025505	0.976821821	0.949962067	0.961523765
RSS	151.2087925	147.6725199	288.7412459	251.4449868

Graphical abstract of model fit presented as mean ± 1 SD of the fraction % of released carvedilol:

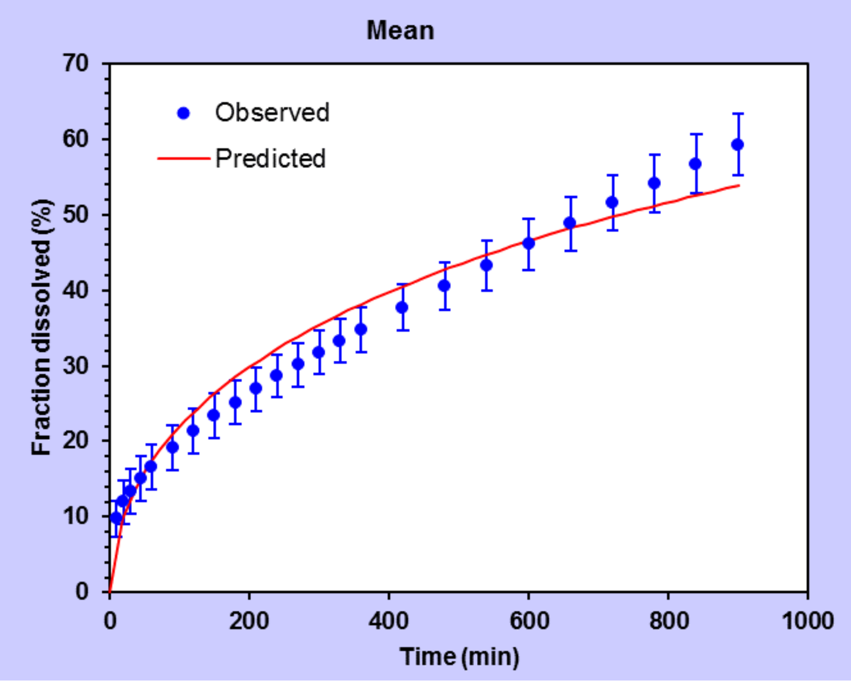

Supplementary materials – Model fitting summary – Starch 1500® sample with smaller particle size (modelling up to app. 60 % of carvedilol released)

Graphical abstract of model fit presented as the fraction % of released carvedilol per tested tablet:

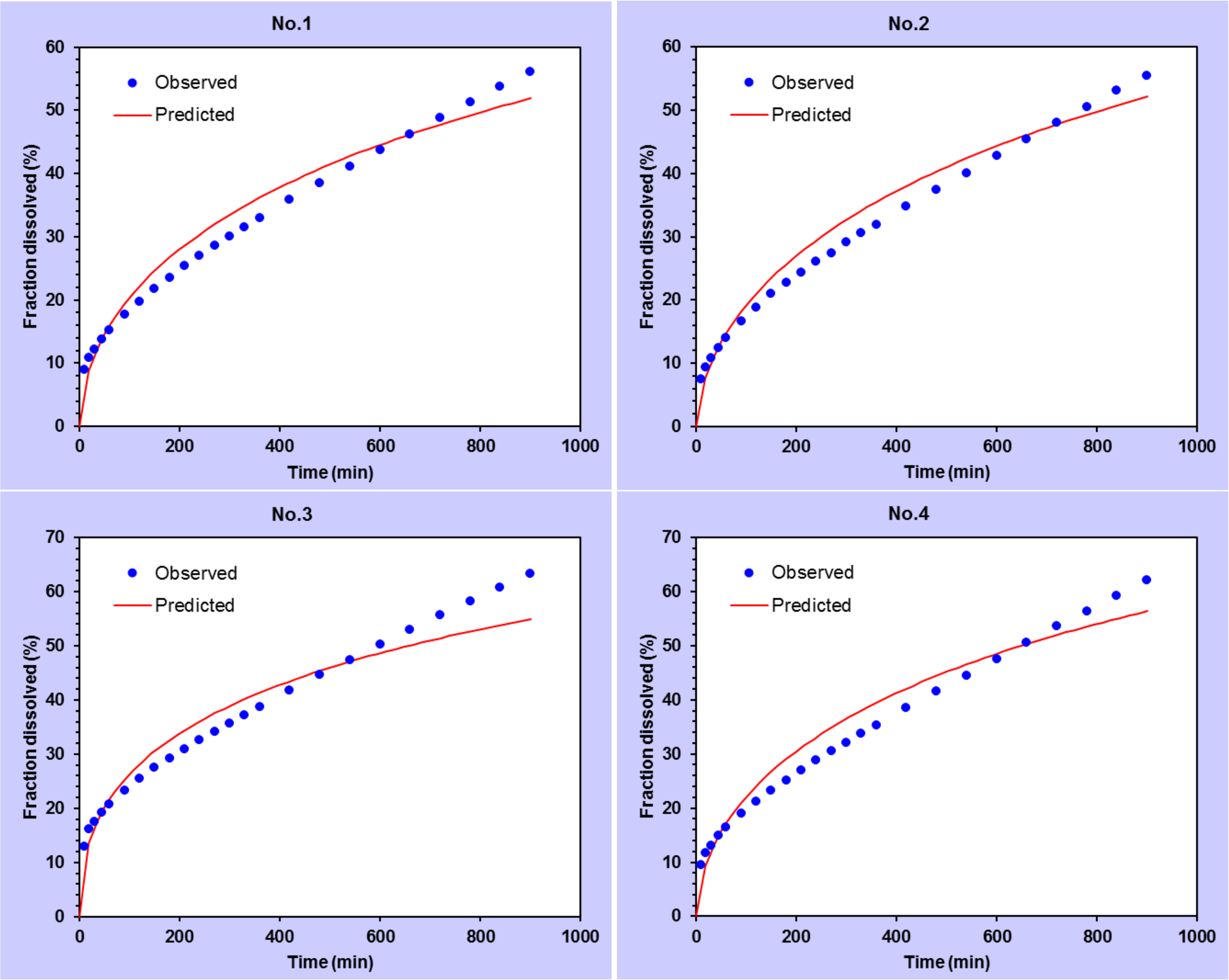

Supplementary materials – Model fitting summary – Starch 1500® sample with smaller particle size (modelling up to app. 60 % of carvedilol released)

Model: **Weibull_3**

Model equation: $F = F_{max} \cdot \left(1 - e^{-\frac{t^{\beta}}{\alpha}}\right)$

Fitted model parameters per tested tablet (N = 4) with statistics – mean, standard deviation (SD), and relative standard deviation expressed in % (RSD%) (output from DDSolver):

Parameter	No.1	No.2	No.3	No.4	Mean	SD	RSD(%)
α	38.873	48.735	23.143	40.404	37.789	10.682	28.268
β	0.617	0.650	0.541	0.617	0.606	0.046	7.642
F_{max}	58.812	58.295	66.520	65.128	62.188	4.241	6.820

Number of dissolution data points (N), degrees of freedom (df), and selected goodness of fit criteria – Pearson correlation coefficient (R), coefficient of determination (R^2), adjusted coefficient of determination ($R^2_{adjusted}$), and residual sum of squares (RSS) (manual calculation in MS Excel):

Parameter	No.1	No.2	No.3	No.4
N	24	24	24	24
df	21	21	21	21
R	0.969992209	0.973375352	0.960235219	0.965893781
R^2	0.940884886	0.947459576	0.922051675	0.932950797
$R^2_{adjusted}$	0.935254875	0.942455726	0.914628025	0.926565158
RSS	305.0832939	281.1605545	424.4328271	424.7303759

Graphical abstract of model fit presented as mean ± 1 SD of the fraction % of released carvedilol:

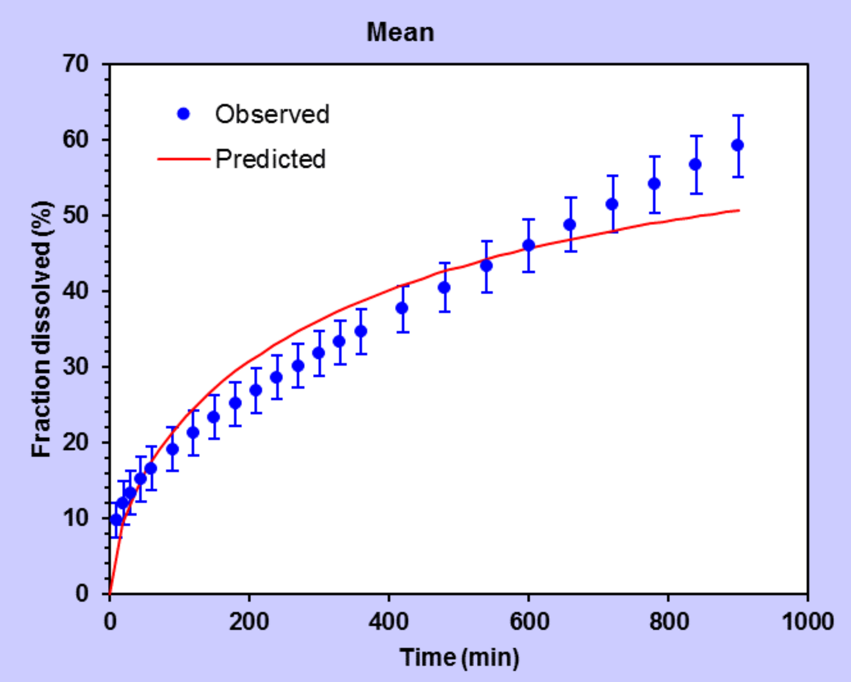

Supplementary materials – Model fitting summary – Starch 1500® sample with smaller particle size (modelling up to app. 60 % of carvedilol released)

Graphical abstract of model fit presented as the fraction % of released carvedilol per tested tablet:

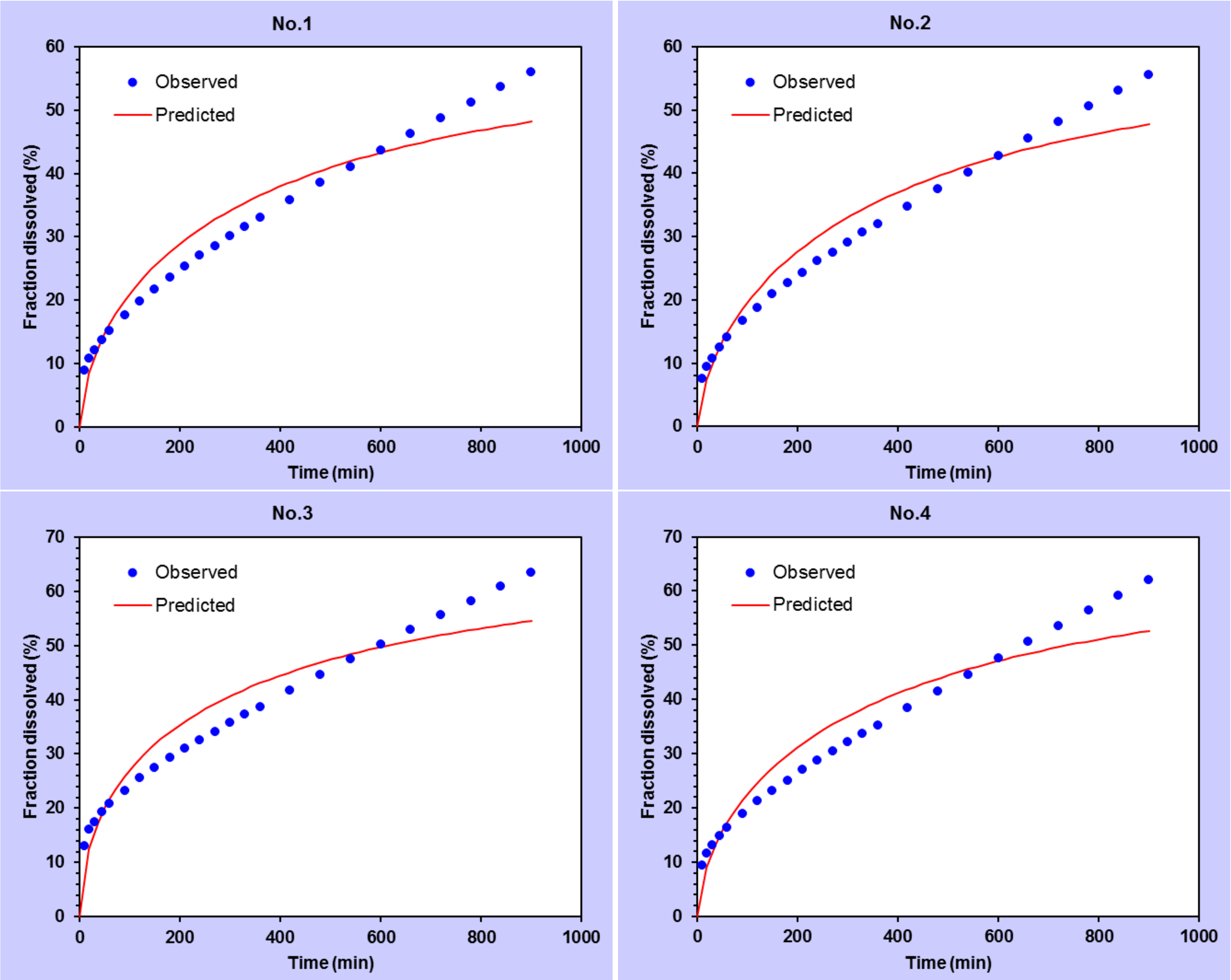

Supplementary materials – Model fitting summary – Starch 1500® sample with smaller particle size (modelling up to app. 60 % of carvedilol released)

Model: **Weibull_4**

Model equation: $F = F_{max} \cdot \left[1 - e^{-\frac{(t-T_i)^\beta}{\alpha}}\right]$

Fitted model parameters per tested tablet (N = 4) with statistics – mean, standard deviation (SD), and relative standard deviation expressed in % (RSD%) (output from DDSolver):

Parameter	No.1	No.2	No.3	No.4	Mean	SD	RSD(%)
α	29.173	36.136	17.972	30.302	28.395	7.590	26.729
β	0.569	0.600	0.499	0.569	0.559	0.043	7.696
T_i	4.000	6.000	4.000	4.000	4.500	1.000	22.222
F_{max}	58.812	58.295	66.520	65.128	62.188	4.241	6.820

Number of dissolution data points (N), degrees of freedom (df), and selected goodness of fit criteria – Pearson correlation coefficient (R), coefficient of determination (R^2), adjusted coefficient of determination ($R^2_{adjusted}$), and residual sum of squares (RSS) (manual calculation in MS Excel):

Parameter	No.1	No.2	No.3	No.4
N	24	24	24	24
df	20	20	20	20
R	0.963994503	0.966572505	0.953259565	0.959655886
R^2	0.929285403	0.934262407	0.908703799	0.920939419
$R^2_{adjusted}$	0.918678213	0.924401768	0.895009369	0.909080332
RSS	371.5613633	344.0325502	502.1011484	509.7922246

Graphical abstract of model fit presented as mean ± 1 SD of the fraction % of released carvedilol:

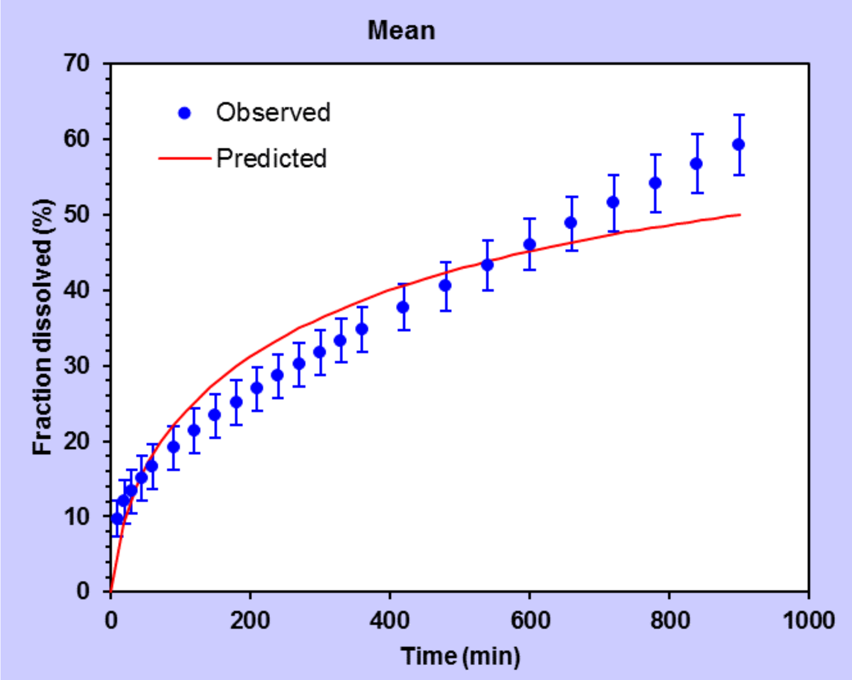

Supplementary materials – Model fitting summary – Starch 1500® sample with smaller particle size (modelling up to app. 60 % of carvedilol released)

Graphical abstract of model fit presented as the fraction % of released carvedilol per tested tablet:

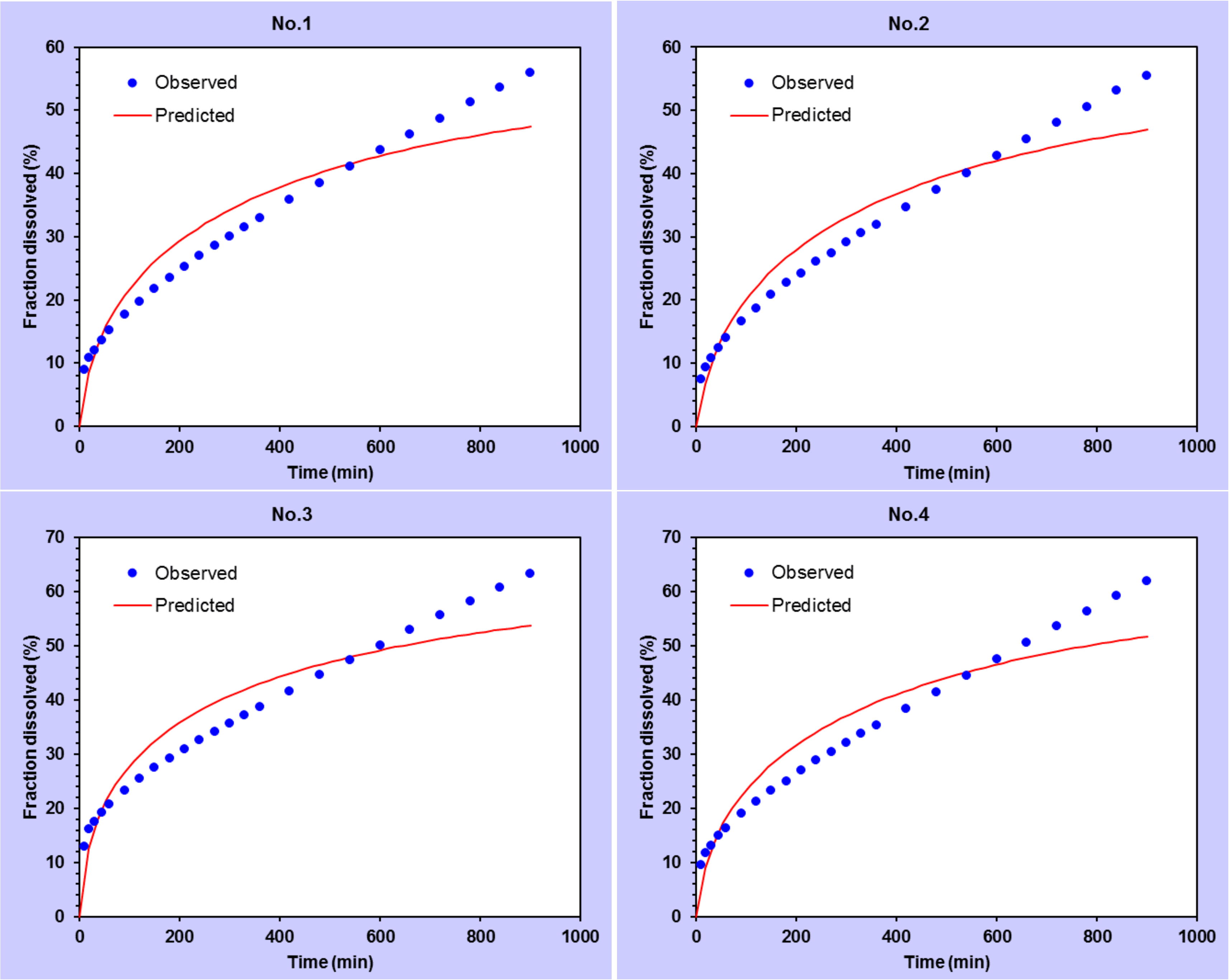

Supplementary materials – Model fitting summary – Starch 1500® sample with smaller particle size (modelling up to app. 60 % of carvedilol released)

Model: **Logistic_1**

Model equation: $F = 100 \cdot \frac{e^{\alpha+\beta \cdot \log(t)}}{1+e^{\alpha+\beta \cdot \log(t)}}$

Fitted model parameters per tested tablet (N = 4) with statistics – mean, standard deviation (SD), and relative standard deviation expressed in % (RSD%) (output from DDSolver):

Parameter	No.1	No.2	No.3	No.4	Mean	SD	RSD(%)
α	-4.016	-4.699	-3.462	-4.045	-4.056	0.506	-12.480
β	1.335	1.619	1.229	1.400	1.396	0.165	11.800

Number of dissolution data points (N), degrees of freedom (df), and selected goodness of fit criteria – Pearson correlation coefficient (R), coefficient of determination (R^2), adjusted coefficient of determination ($R^2_{adjusted}$), and residual sum of squares (RSS) (manual calculation in MS Excel):

Parameter	No.1	No.2	No.3	No.4
N	24	24	24	24
df	22	22	22	22
R	0.977771386	0.98367678	0.965892866	0.970731485
R^2	0.956036882	0.967620007	0.932949028	0.942319616
$R^2_{adjusted}$	0.954038559	0.966148189	0.929901256	0.93969778
RSS	246.9973926	211.4450688	376.8792332	386.4102218

Graphical abstract of model fit presented as mean ± 1 SD of the fraction % of released carvedilol:

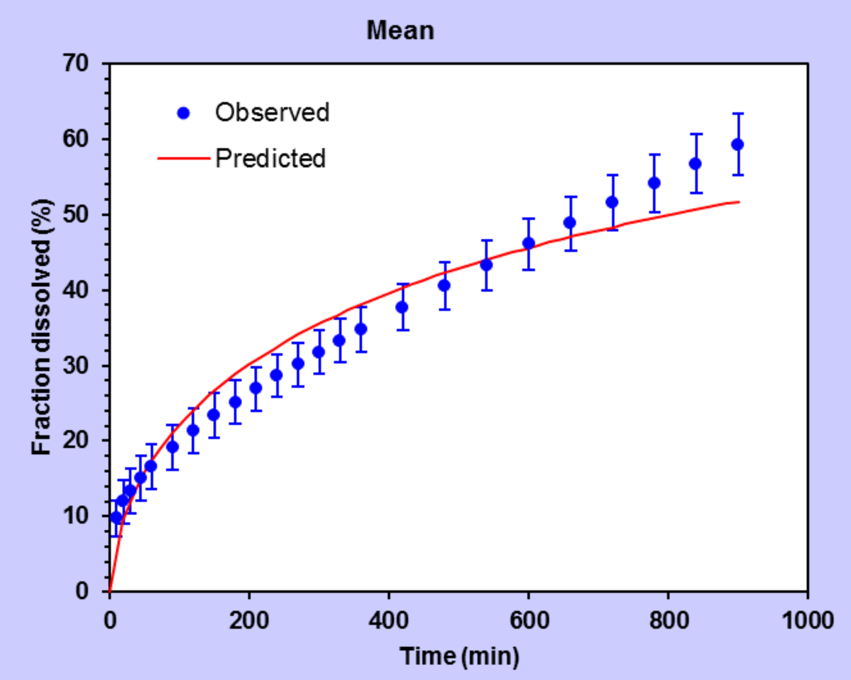

Supplementary materials – Model fitting summary – Starch 1500® sample with smaller particle size (modelling up to app. 60 % of carvedilol released)

Graphical abstract of model fit presented as the fraction % of released carvedilol per tested tablet:

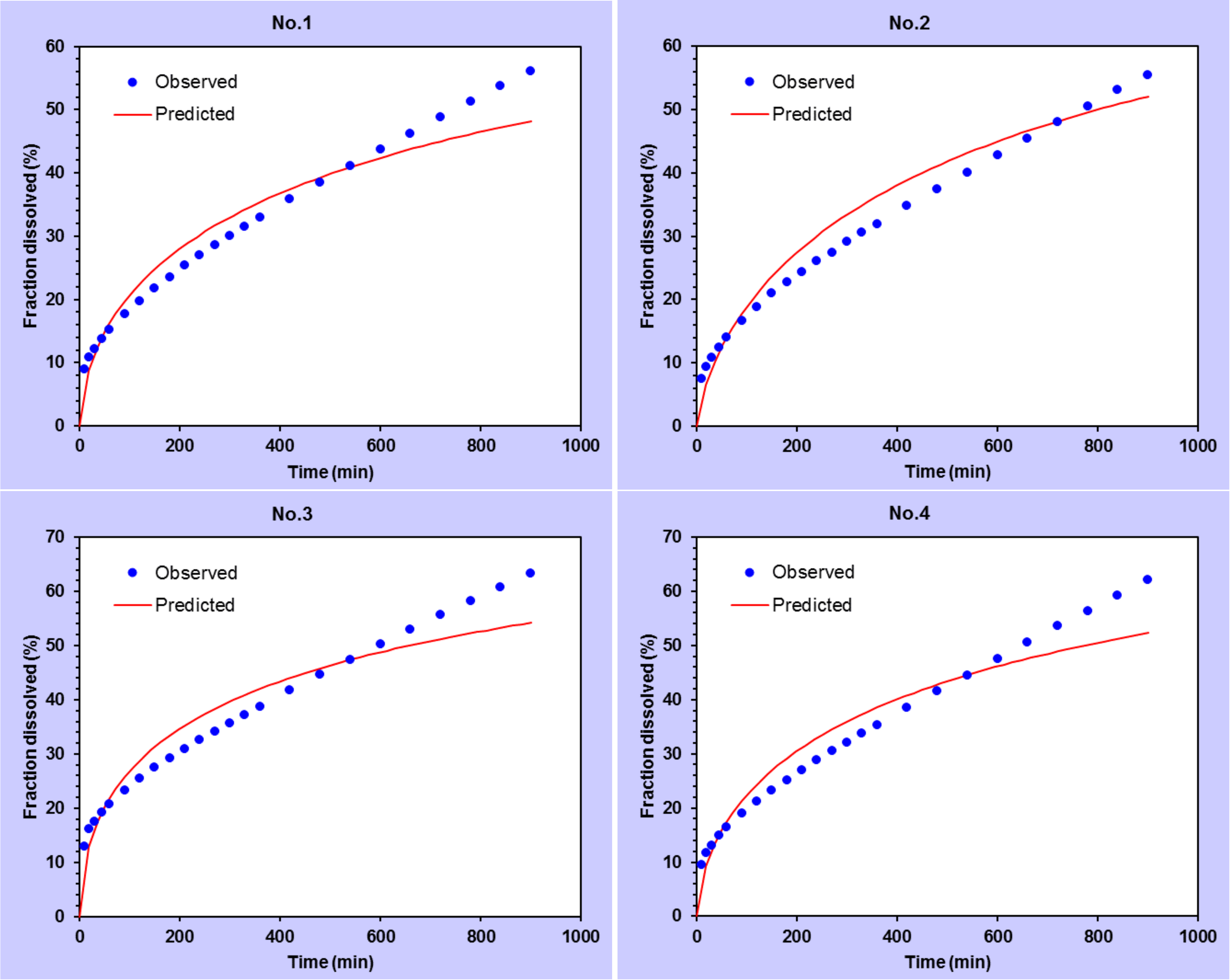

Supplementary materials – Model fitting summary – Starch 1500® sample with smaller particle size (modelling up to app. 60 % of carvedilol released)

Model: **Logistic_2**

Model equation: $F = F_{max} \cdot \frac{e^{\alpha+\beta \cdot \log(t)}}{1+e^{\alpha+\beta \cdot \log(t)}}$

Fitted model parameters per tested tablet (N = 4) with statistics – mean, standard deviation (SD), and relative standard deviation expressed in % (RSD%) (output from DDSolver):

Parameter	No.1	No.2	No.3	No.4	Mean	SD	RSD(%)
α	-4.629	-4.865	-4.336	-4.650	-4.620	0.217	-4.704
β	2.065	2.140	1.557	2.051	1.953	0.267	13.676
F_{max}	58.812	58.295	95.776	65.128	69.503	17.789	25.595

Number of dissolution data points (N), degrees of freedom (df), and selected goodness of fit criteria – Pearson correlation coefficient (R), coefficient of determination (R^2), adjusted coefficient of determination ($R^2_{adjusted}$), and residual sum of squares (RSS) (manual calculation in MS Excel):

Parameter	No.1	No.2	No.3	No.4
N	24	24	24	24
df	21	21	21	21
R	0.945389856	0.949884595	0.975593289	0.940670086
R^2	0.893761979	0.902280745	0.951782266	0.88486021
$R^2_{adjusted}$	0.883644072	0.892974149	0.947190101	0.873894516
RSS	558.2137801	530.9931479	444.4944238	739.4673679

Graphical abstract of model fit presented as mean ± 1 SD of the fraction % of released carvedilol:

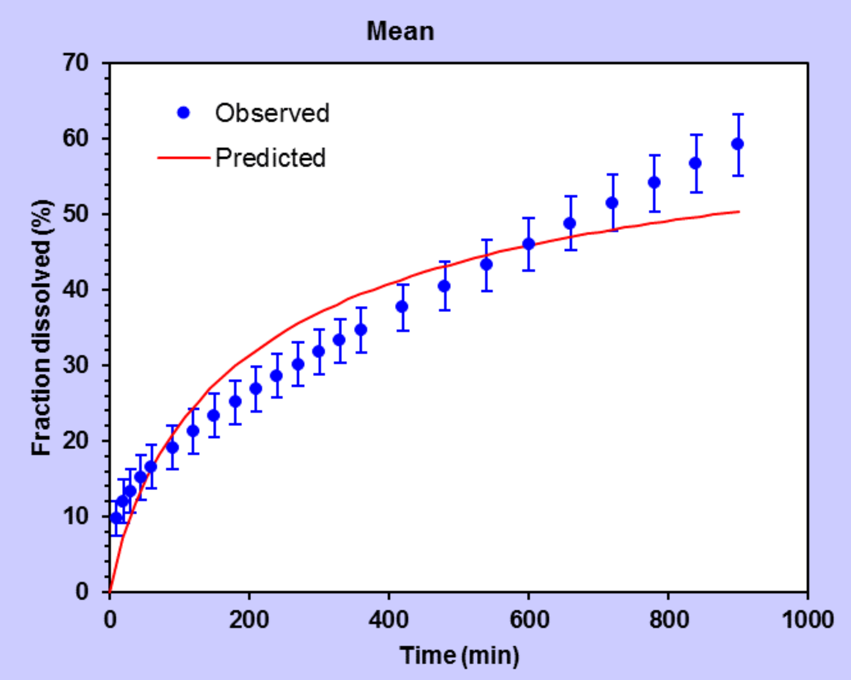

Supplementary materials – Model fitting summary – Starch 1500® sample with smaller particle size (modelling up to app. 60 % of carvedilol released)

Graphical abstract of model fit presented as the fraction % of released carvedilol per tested tablet:

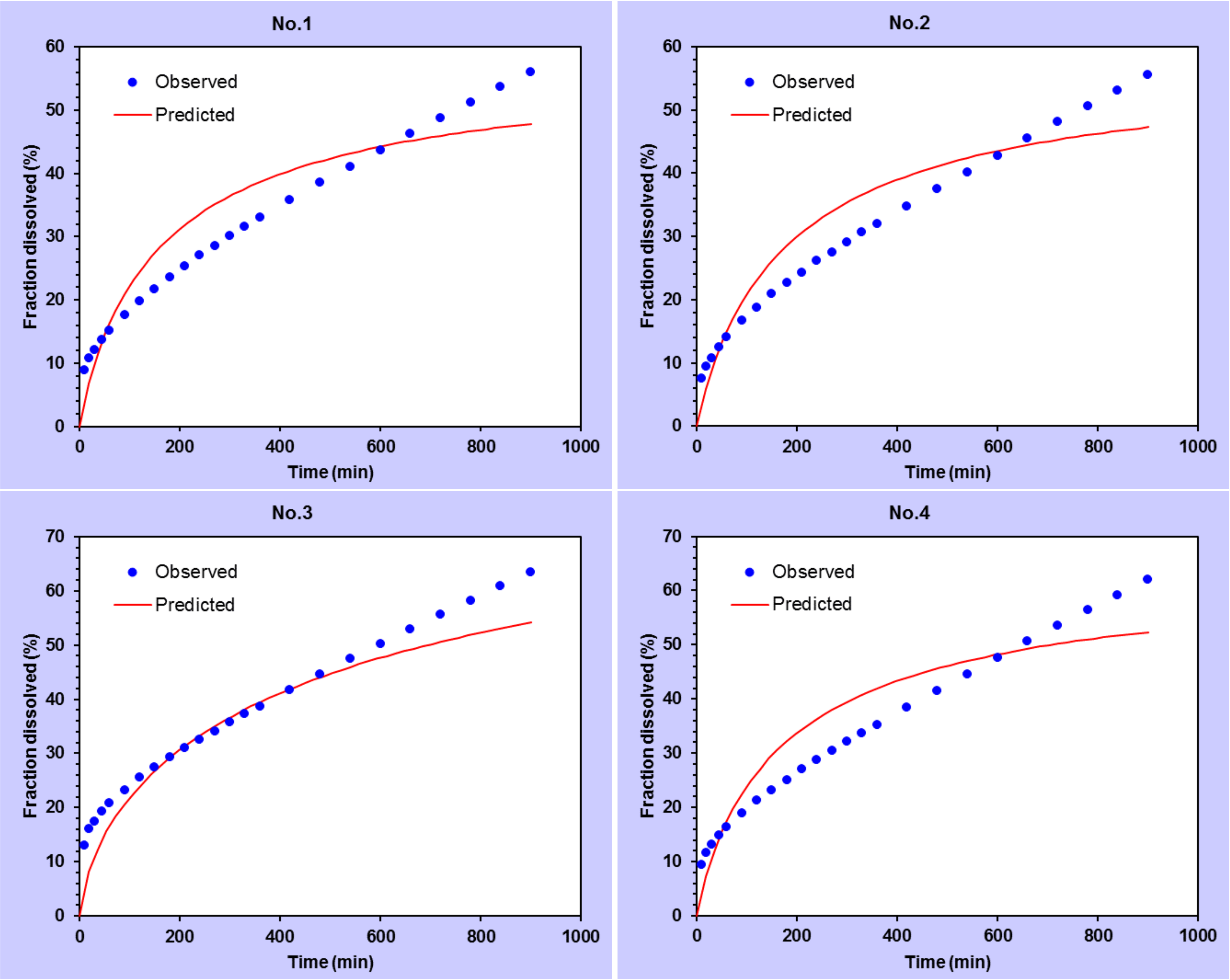

Supplementary materials – Model fitting summary – Starch 1500® sample with smaller particle size (modelling up to app. 60 % of carvedilol released)

Model: **Logistic_3**

Model equation: $F = F_{max} \cdot \frac{1}{1+e^{-k\cdot(t-\gamma)}}$

Fitted model parameters per tested tablet (N = 4) with statistics – mean, standard deviation (SD), and relative standard deviation expressed in % (RSD%) (output from DDSolver):

Parameter	No.1	No.2	No.3	No.4	Mean	SD	RSD(%)
k	0.004	0.004	0.004	0.004	0.004	0.000	4.439
γ	312.095	327.154	274.064	324.241	309.389	24.436	7.898
F_{max}	58.812	58.295	66.520	65.128	62.188	4.241	6.820

Number of dissolution data points (N), degrees of freedom (df), and selected goodness of fit criteria – Pearson correlation coefficient (R), coefficient of determination (R^2), adjusted coefficient of determination ($R^2_{adjusted}$), and residual sum of squares (RSS) (manual calculation in MS Excel):

Parameter	No.1	No.2	No.3	No.4
N	24	24	24	24
df	21	21	21	21
R	0.992816804	0.991286459	0.993490256	0.993341071
R^2	0.985685206	0.982648843	0.987022889	0.986726484
$R^2_{adjusted}$	0.984321892	0.980996352	0.985786973	0.98546234
RSS	72.30497786	90.61154774	72.4526761	84.12920038

Graphical abstract of model fit presented as mean ± 1 SD of the fraction % of released carvedilol:

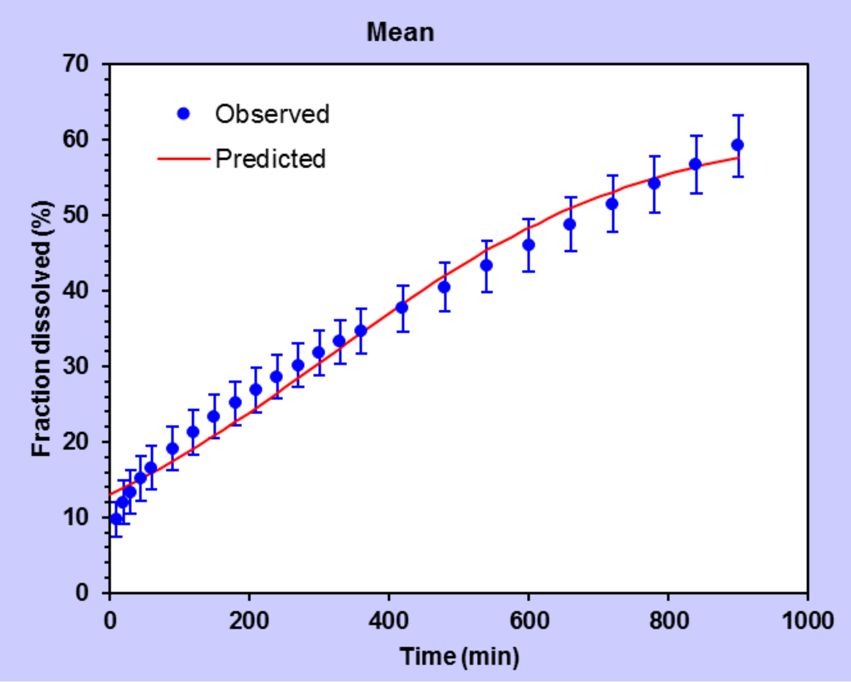

Supplementary materials – Model fitting summary – Starch 1500® sample with smaller particle size (modelling up to app. 60 % of carvedilol released)

Graphical abstract of model fit presented as the fraction % of released carvedilol per tested tablet:

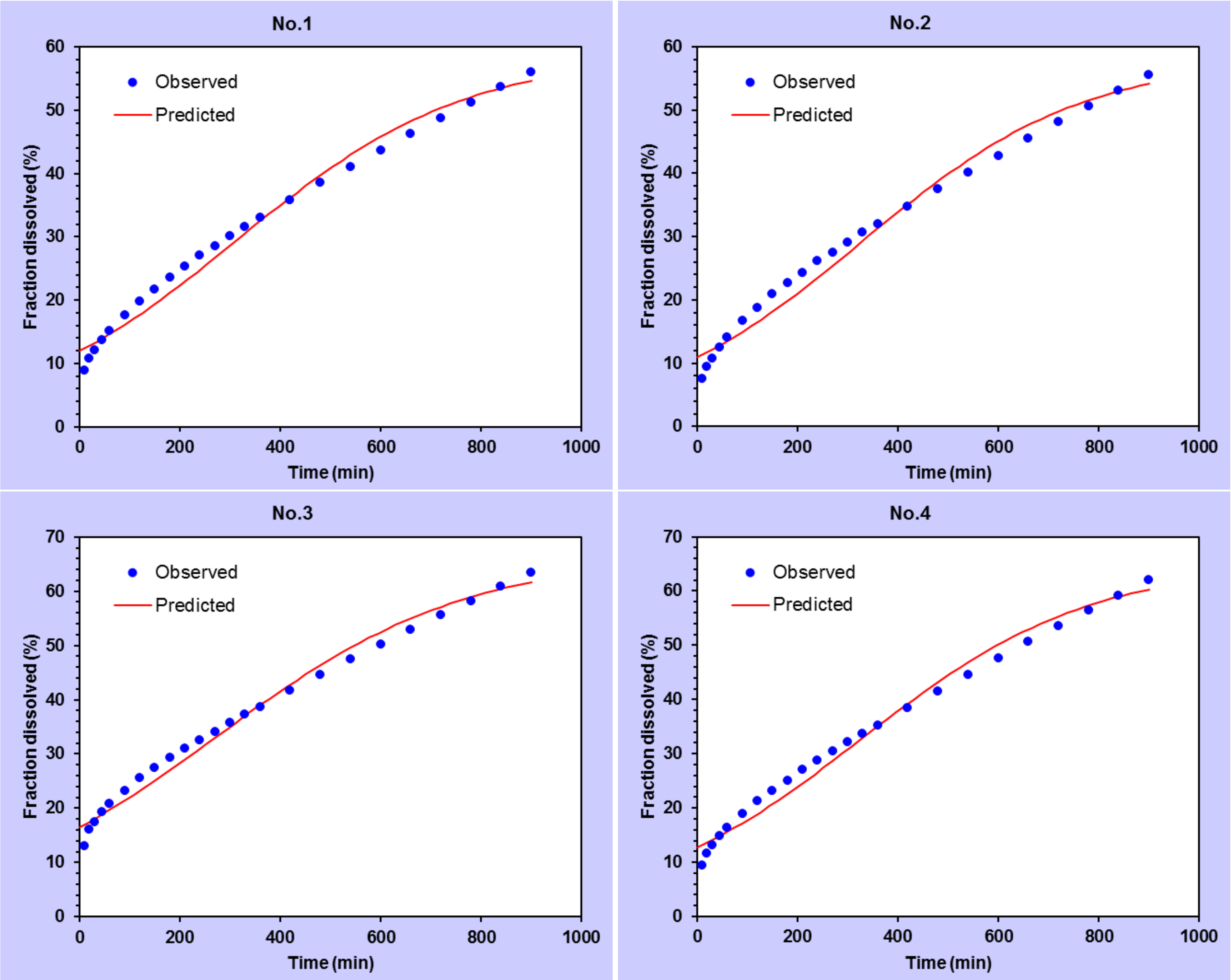

Supplementary materials – Model fitting summary – Starch 1500® sample with smaller particle size (modelling up to app. 60 % of carvedilol released)

Model: **Gompertz_1**

Model equation: $F = 100 \cdot e^{-\alpha \cdot e^{-\beta \cdot \log(t)}}$

Fitted model parameters per tested tablet (N = 4) with statistics – mean, standard deviation (SD), and relative standard deviation expressed in % (RSD%) (output from DDSolver):

Parameter	No.1	No.2	No.3	No.4	Mean	SD	RSD(%)
α	6.587	7.168	5.614	7.109	6.620	0.719	10.866
β	0.733	0.756	0.741	0.799	0.757	0.029	3.871

Number of dissolution data points (N), degrees of freedom (df), and selected goodness of fit criteria – Pearson correlation coefficient (R), coefficient of determination (R^2), adjusted coefficient of determination ($R^2_{adjusted}$), and residual sum of squares (RSS) (manual calculation in MS Excel):

Parameter	No.1	No.2	No.3	No.4
N	24	24	24	24
df	22	22	22	22
R	0.957191441	0.960572186	0.94439187	0.947592169
R^2	0.916215456	0.922698924	0.891876004	0.897930919
$R^2_{adjusted}$	0.912407067	0.919185239	0.886961277	0.893291416
RSS	419.133105	402.3161577	569.8565259	619.486061

Graphical abstract of model fit presented as mean ± 1 SD of the fraction % of released carvedilol:

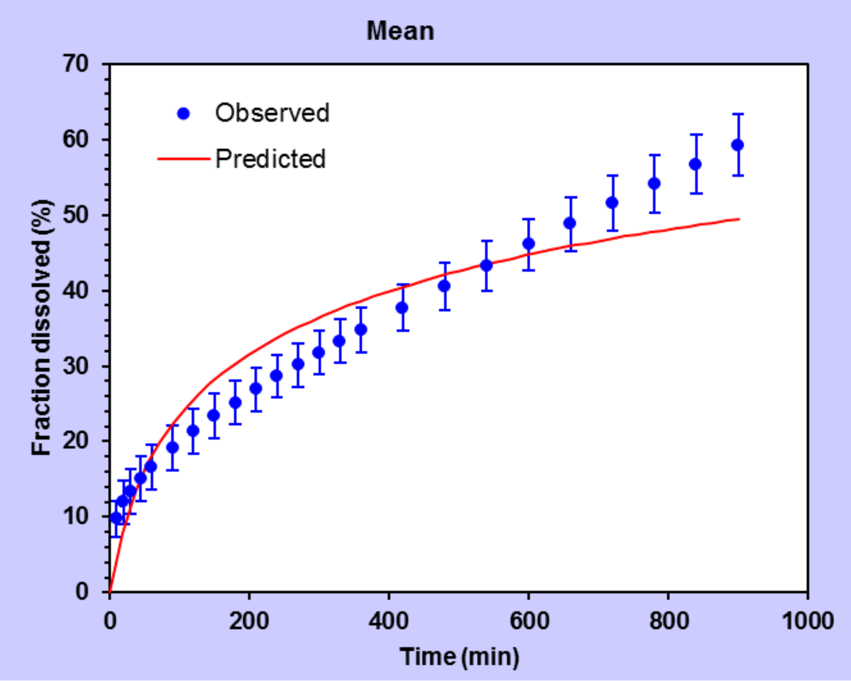

Supplementary materials – Model fitting summary – Starch 1500® sample with smaller particle size (modelling up to app. 60 % of carvedilol released)

Graphical abstract of model fit presented as the fraction % of released carvedilol per tested tablet:

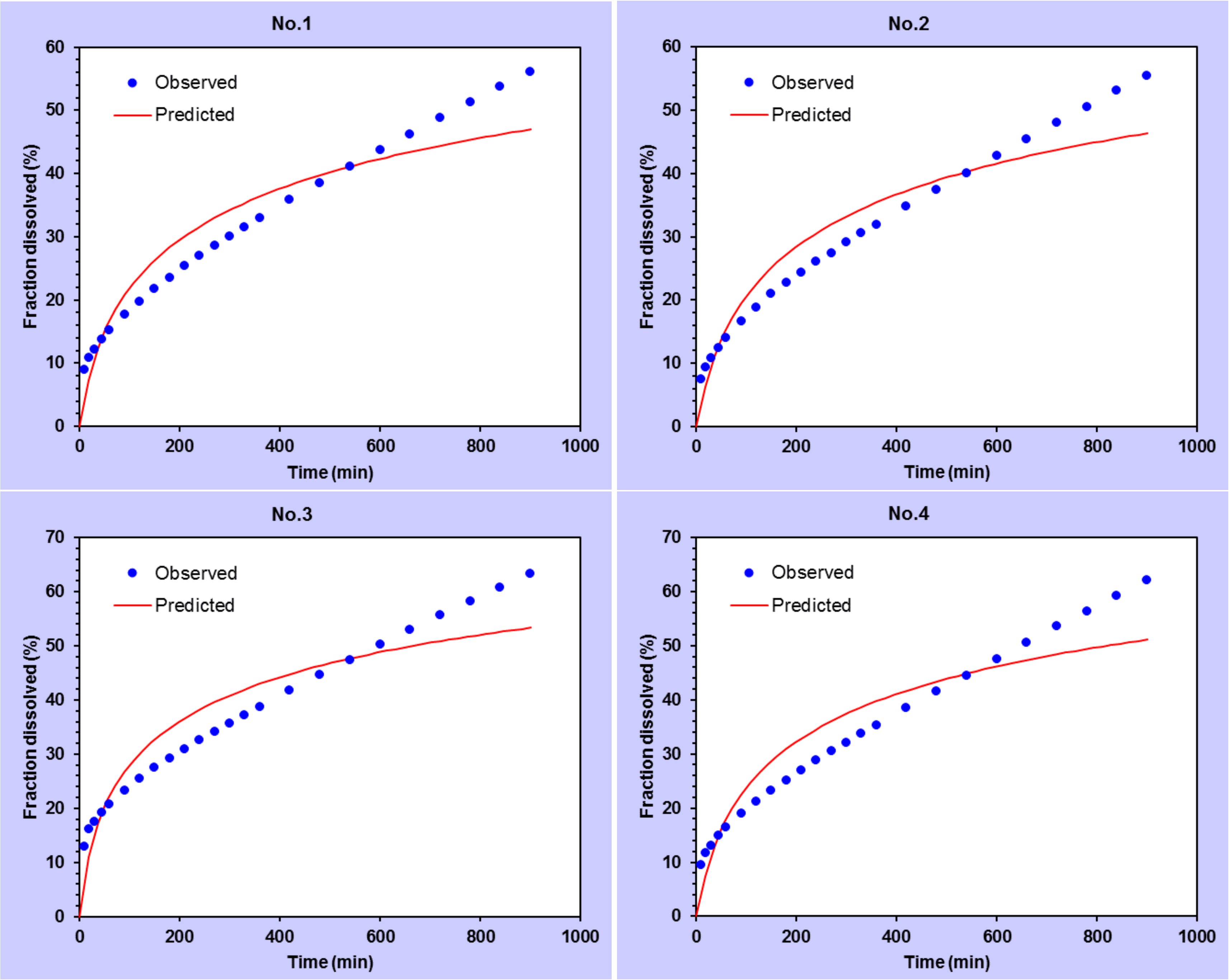

Supplementary materials – Model fitting summary – Starch 1500® sample with smaller particle size (modelling up to app. 60 % of carvedilol released)

Model: **Gompertz_2**

Model equation: $F = F_{max} \cdot e^{-\alpha \cdot e^{-\beta \cdot \log(t)}}$

Fitted model parameters per tested tablet (N = 4) with statistics – mean, standard deviation (SD), and relative standard deviation expressed in % (RSD%) (output from DDSolver):

Parameter	No.1	No.2	No.3	No.4	Mean	SD	RSD(%)
α	18.174	20.102	13.625	18.016	17.479	2.739	15.669
β	1.503	1.529	1.417	1.482	1.483	0.048	3.226
F_{max}	58.812	58.295	66.520	65.128	62.188	4.241	6.820

Number of dissolution data points (N), degrees of freedom (df), and selected goodness of fit criteria – Pearson correlation coefficient (R), coefficient of determination (R^2), adjusted coefficient of determination ($R^2_{adjusted}$), and residual sum of squares (RSS) (manual calculation in MS Excel):

Parameter	No.1	No.2	No.3	No.4
N	24	24	24	24
df	21	21	21	21
R	0.91519225	0.920147266	0.903726571	0.909388716
R^2	0.837576854	0.846670991	0.816721715	0.826987837
$R^2_{adjusted}$	0.822107983	0.832068228	0.799266641	0.810510489
RSS	891.113316	865.301475	1111.956996	1160.604773

Graphical abstract of model fit presented as mean ± 1 SD of the fraction % of released carvedilol:

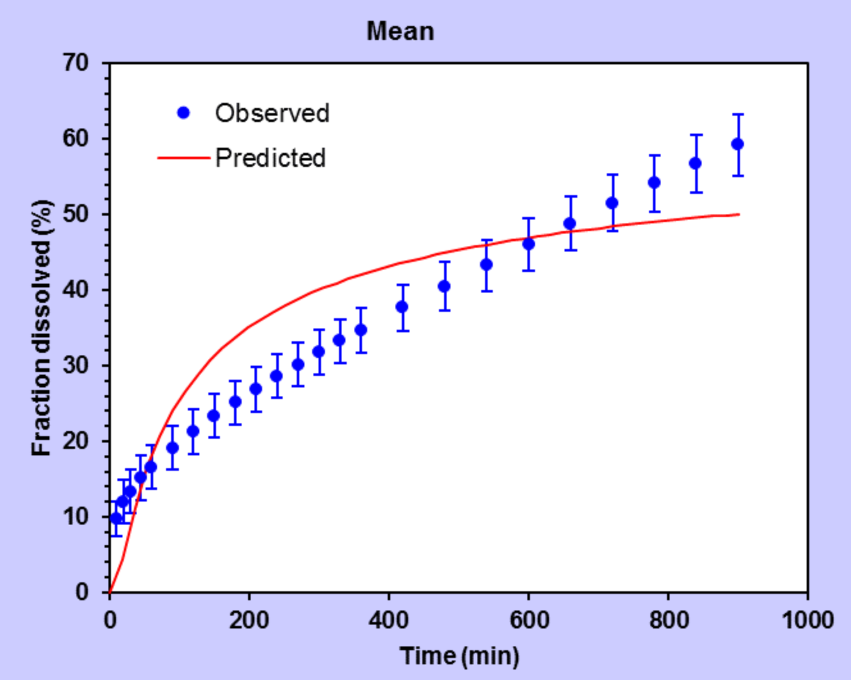

Supplementary materials – Model fitting summary – Starch 1500® sample with smaller particle size (modelling up to app. 60 % of carvedilol released)

Graphical abstract of model fit presented as the fraction % of released carvedilol per tested tablet:

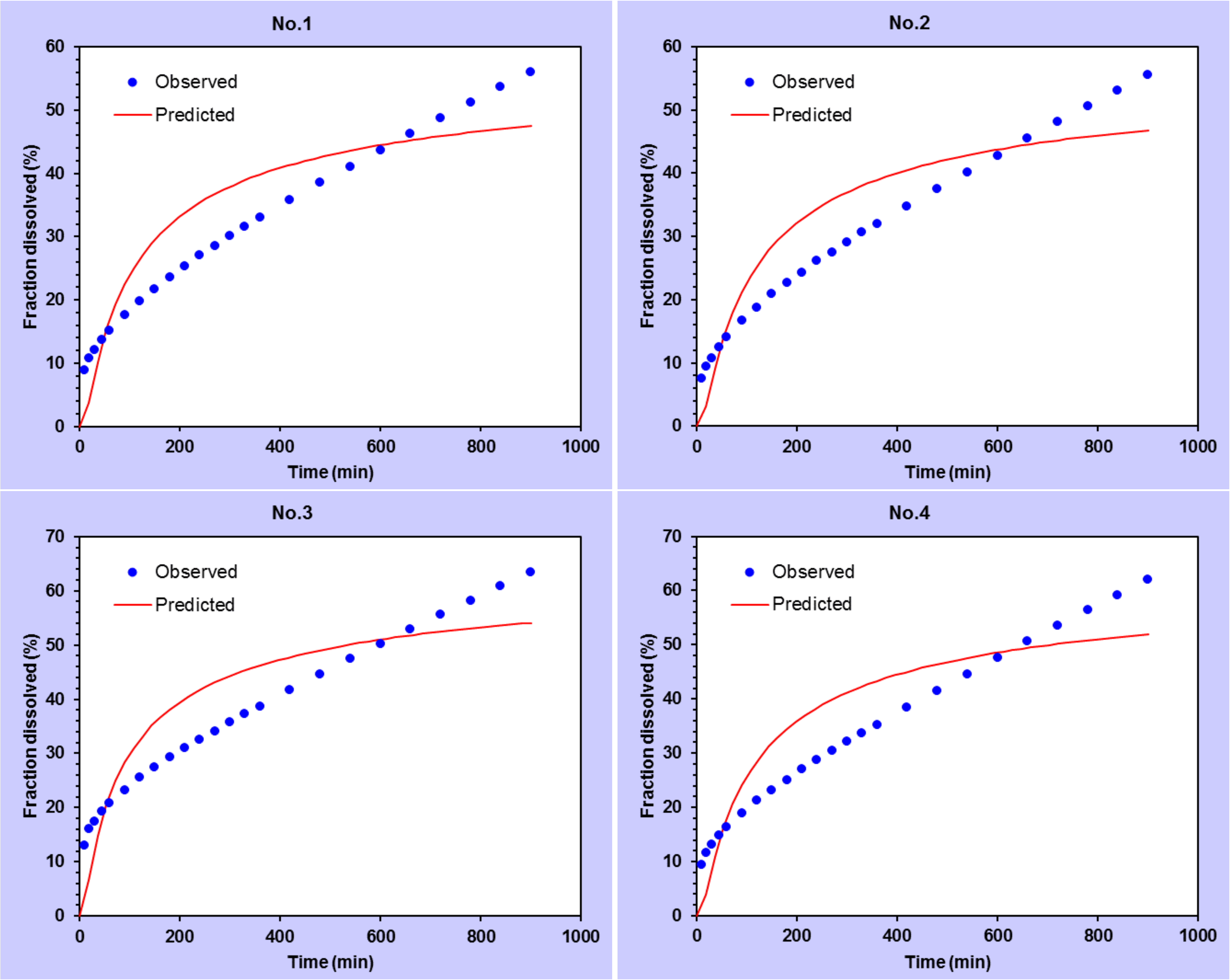

Supplementary materials – Model fitting summary – Starch 1500® sample with smaller particle size (modelling up to app. 60 % of carvedilol released)

Model: **Gompertz_3**

Model equation: $F = F_{max} \cdot e^{-e^{-k\cdot(t-\gamma)}}$

Fitted model parameters per tested tablet (N = 4) with statistics – mean, standard deviation (SD), and relative standard deviation expressed in % (RSD%) (output from DDSolver):

Parameter	No.1	No.2	No.3	No.4	Mean	SD	RSD(%)
k	0.002	0.003	0.003	0.003	0.003	0.001	17.588
γ	213.979	189.203	136.508	185.794	181.371	32.439	17.885
F_{max}	64.983	58.295	69.142	65.128	64.387	4.496	6.982

Number of dissolution data points (N), degrees of freedom (df), and selected goodness of fit criteria – Pearson correlation coefficient (R), coefficient of determination (R^2), adjusted coefficient of determination ($R^2_{adjusted}$), and residual sum of squares (RSS) (manual calculation in MS Excel):

Parameter	No.1	No.2	No.3	No.4
N	24	24	24	24
df	21	21	21	21
R	0.99603432	0.993811564	0.995976066	0.993715935
R^2	0.992084367	0.987661424	0.991968325	0.98747136
$R^2_{adjusted}$	0.991330497	0.986486322	0.991203403	0.986278157
RSS	42.5460999	75.66298242	56.24588818	97.01358277

Graphical abstract of model fit presented as mean ± 1 SD of the fraction % of released carvedilol:

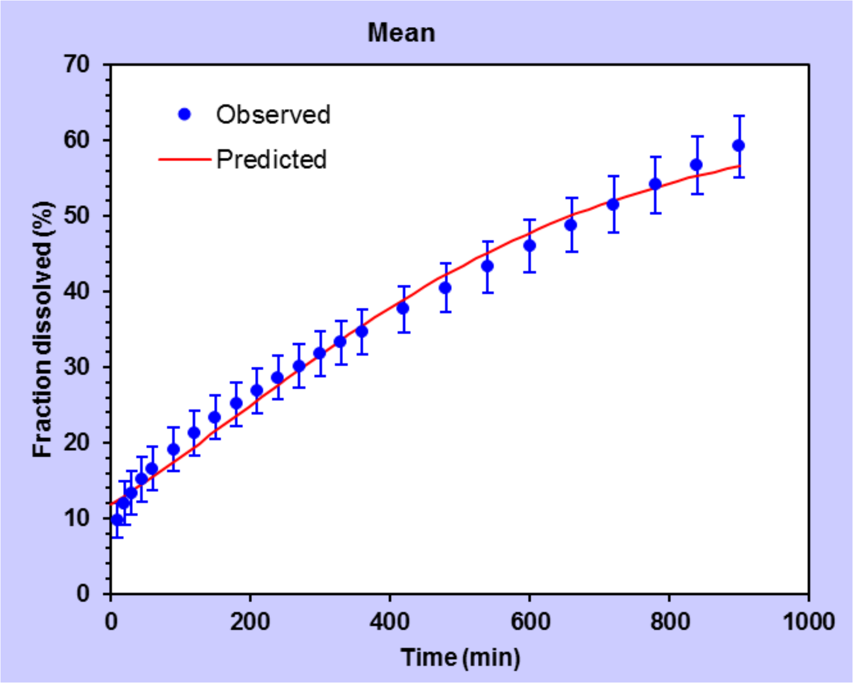

Supplementary materials – Model fitting summary – Starch 1500® sample with smaller particle size (modelling up to app. 60 % of carvedilol released)

Graphical abstract of model fit presented as the fraction % of released carvedilol per tested tablet:

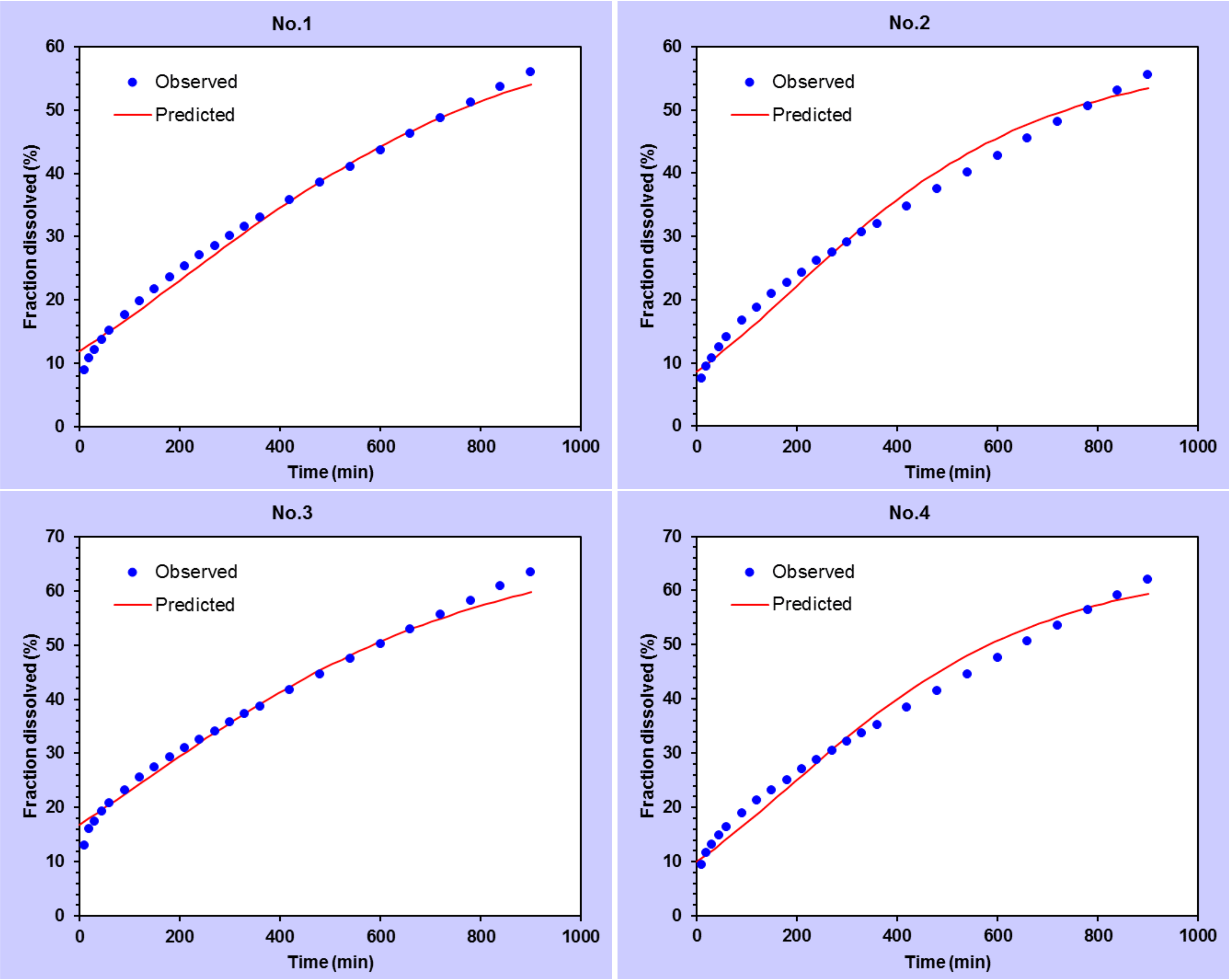

Supplementary materials – Model fitting summary – Starch 1500® sample with smaller particle size (modelling up to app. 60 % of carvedilol released)

Model: **Gompertz_4**

Model equation: $F = F_{max} \cdot e^{-\beta \cdot e^{-k \cdot t}}$

Fitted model parameters per tested tablet (N = 4) with statistics – mean, standard deviation (SD), and relative standard deviation expressed in % (RSD%) (output from DDSolver):

Parameter	No.1	No.2	No.3	No.4	Mean	SD	RSD(%)
k	0.003	0.003	0.003	0.003	0.003	0.000	2.725
β	1.807	1.909	1.560	1.864	1.785	0.156	8.741
F_{max}	58.812	58.295	66.520	65.128	62.188	4.241	6.820

Number of dissolution data points (N), degrees of freedom (df), and selected goodness of fit criteria – Pearson correlation coefficient (R), coefficient of determination (R^2), adjusted coefficient of determination ($R^2_{adjusted}$), and residual sum of squares (RSS) (manual calculation in MS Excel):

Parameter	No.1	No.2	No.3	No.4
N	24	24	24	24
df	21	21	21	21
R	0.99467125	0.993811564	0.993446998	0.993715935
R^2	0.989370895	0.987661424	0.986936938	0.98747136
$R^2_{adjusted}$	0.9883586	0.986486322	0.985692837	0.986278157
RSS	65.34792754	75.66298242	89.65459684	97.01358277

Graphical abstract of model fit presented as mean ± 1 SD of the fraction % of released carvedilol:

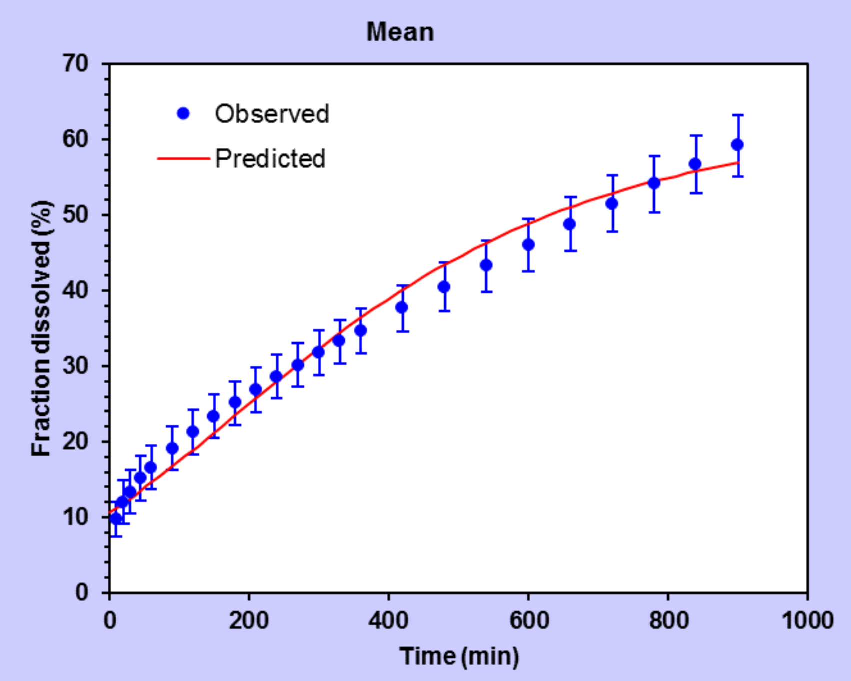

Supplementary materials – Model fitting summary – Starch 1500® sample with smaller particle size (modelling up to app. 60 % of carvedilol released)

Graphical abstract of model fit presented as the fraction % of released carvedilol per tested tablet:

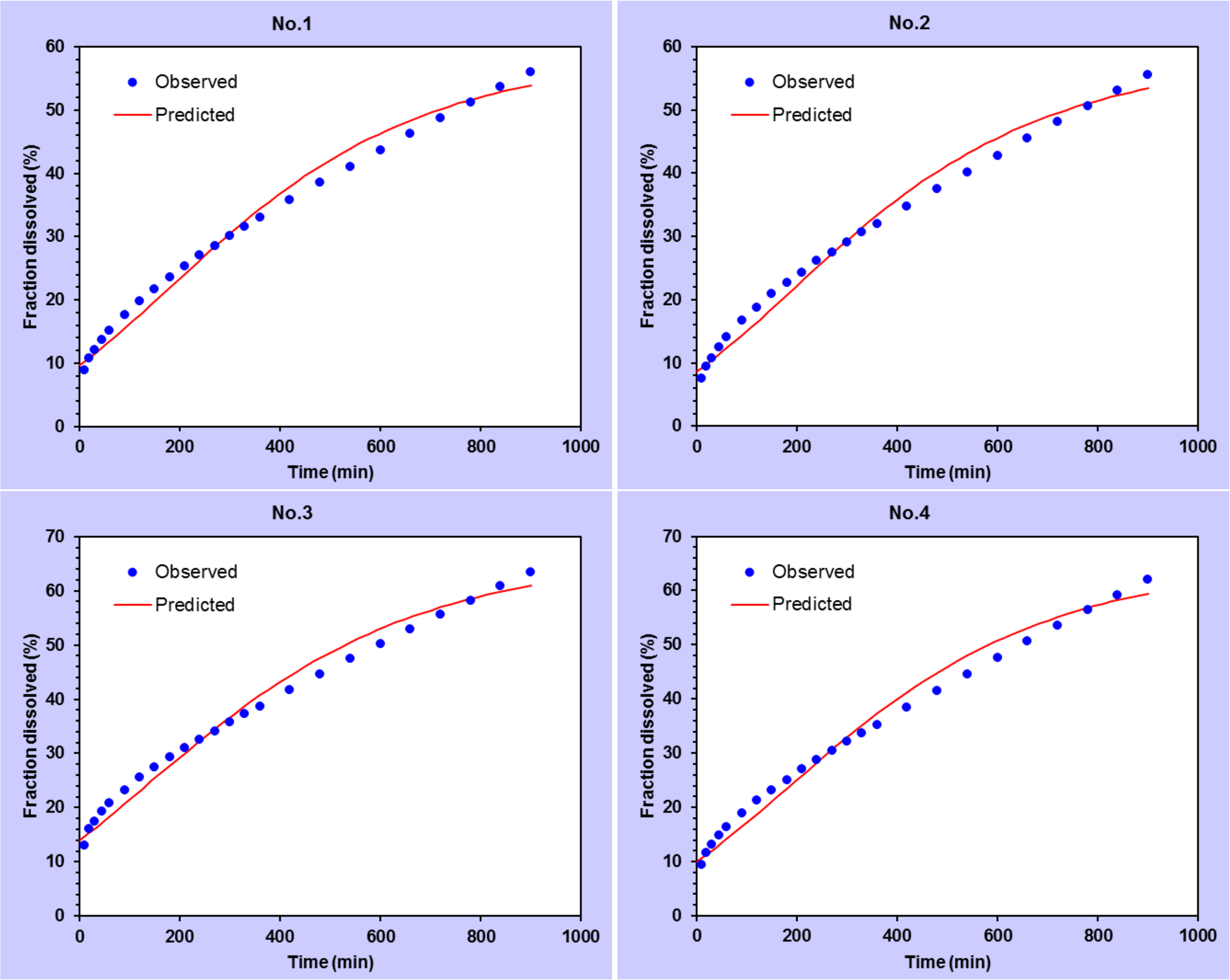

Supplementary materials – Model fitting summary – Starch 1500® sample with smaller particle size (modelling up to app. 60 % of carvedilol released)

Model: **Probit_1**

Model equation: $F = 100 \cdot \phi[\alpha + \beta \cdot \log(t)]$

Fitted model parameters per tested tablet (N = 4) with statistics – mean, standard deviation (SD), and relative standard deviation expressed in % (RSD%) (output from DDSolver):

Parameter	No.1	No.2	No.3	No.4	Mean	SD	RSD(%)
α	-2.366	-2.484	-2.088	-2.653	-2.398	0.238	-9.917
β	0.781	0.817	0.740	0.950	0.822	0.091	11.095

Number of dissolution data points (N), degrees of freedom (df), and selected goodness of fit criteria – Pearson correlation coefficient (R), coefficient of determination (R^2), adjusted coefficient of determination ($R^2_{adjusted}$), and residual sum of squares (RSS) (manual calculation in MS Excel):

Parameter	No.1	No.2	No.3	No.4
N	24	24	24	24
df	22	22	22	22
R	0.971565196	0.974578123	0.960686362	0.967131731
R^2	0.94393893	0.949802518	0.922918285	0.935343784
$R^2_{adjusted}$	0.9413907	0.947520814	0.919414571	0.932404866
RSS	301.8539309	282.2956421	424.3094411	454.0552181

Graphical abstract of model fit presented as mean ± 1 SD of the fraction % of released carvedilol:

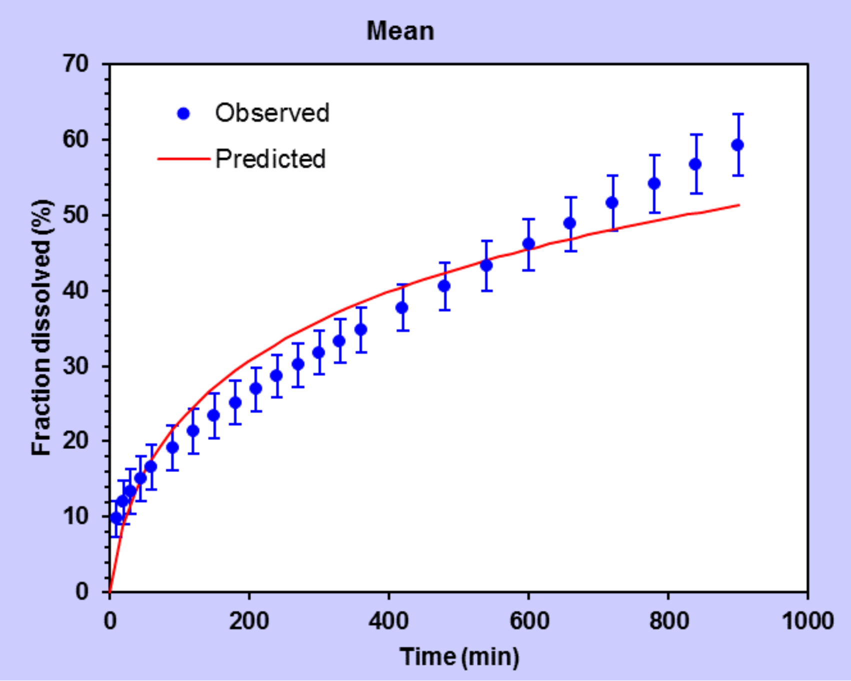

Supplementary materials – Model fitting summary – Starch 1500® sample with smaller particle size (modelling up to app. 60 % of carvedilol released)

Graphical abstract of model fit presented as the fraction % of released carvedilol per tested tablet:

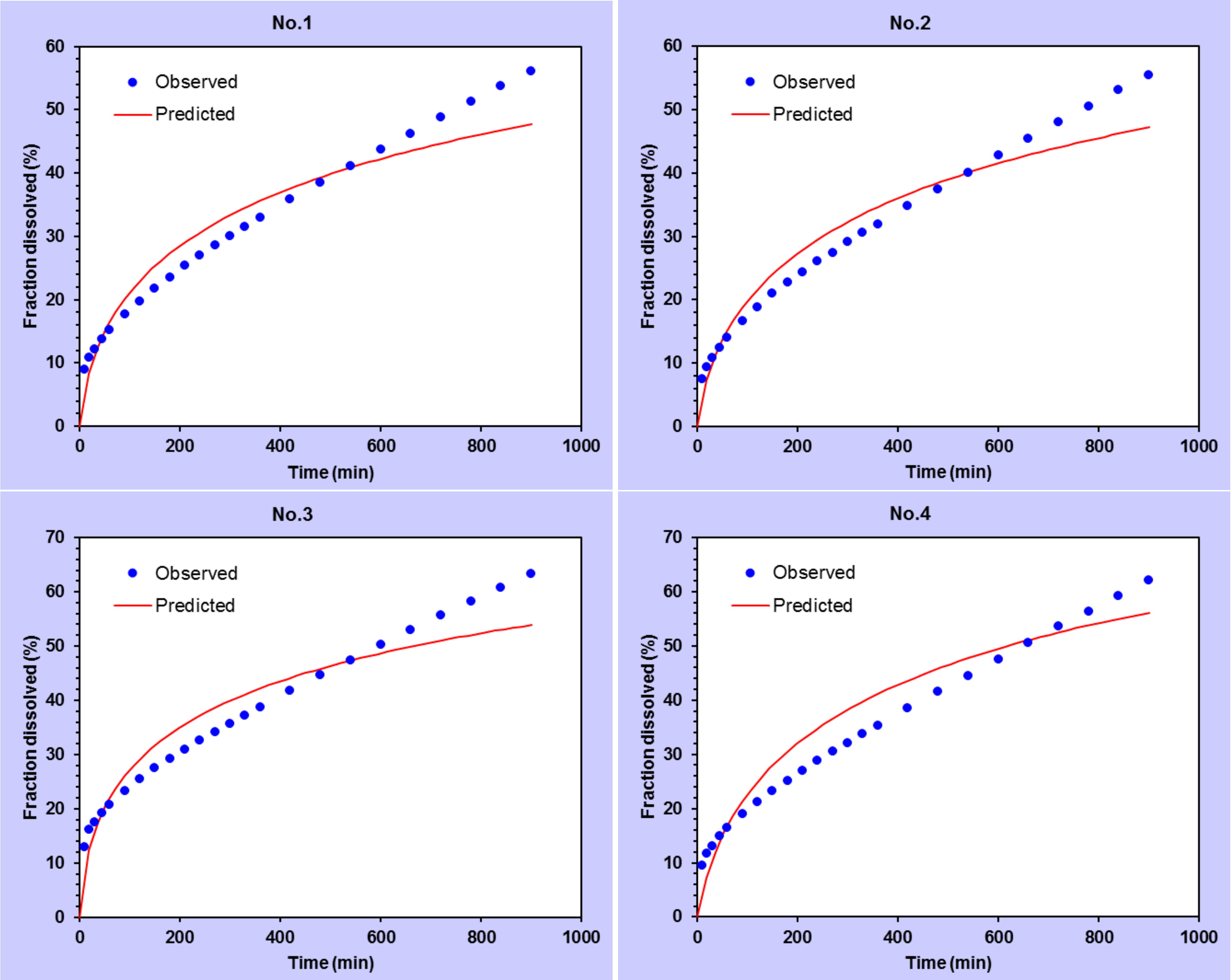

Supplementary materials – Model fitting summary – Starch 1500® sample with smaller particle size (modelling up to app. 60 % of carvedilol released)

Model: **Probit_2**

Model equation: $F = F_{max} \cdot \phi[\alpha + \beta \cdot \log(t)]$

Fitted model parameters per tested tablet (N = 4) with statistics – mean, standard deviation (SD), and relative standard deviation expressed in % (RSD%) (output from DDSolver):

Parameter	No.1	No.2	No.3	No.4	Mean	SD	RSD(%)
α	-2.770	-2.902	-2.434	-2.782	-2.722	0.201	-7.381
β	1.232	1.273	1.124	1.223	1.213	0.063	5.214
F_{max}	58.812	58.295	66.520	65.128	62.188	4.241	6.820

Number of dissolution data points (N), degrees of freedom (df), and selected goodness of fit criteria – Pearson correlation coefficient (R), coefficient of determination (R^2), adjusted coefficient of determination ($R^2_{adjusted}$), and residual sum of squares (RSS) (manual calculation in MS Excel):

Parameter	No.1	No.2	No.3	No.4
N	24	24	24	24
df	21	21	21	21
R	0.945403084	0.949468132	0.935076596	0.940289018
R^2	0.893786991	0.901489734	0.874368241	0.884143437
$R^2_{adjusted}$	0.883671466	0.892107804	0.862403312	0.873109478
RSS	537.982841	516.0148981	688.3731748	718.5501187

Graphical abstract of model fit presented as mean ± 1 SD of the fraction % of released carvedilol:

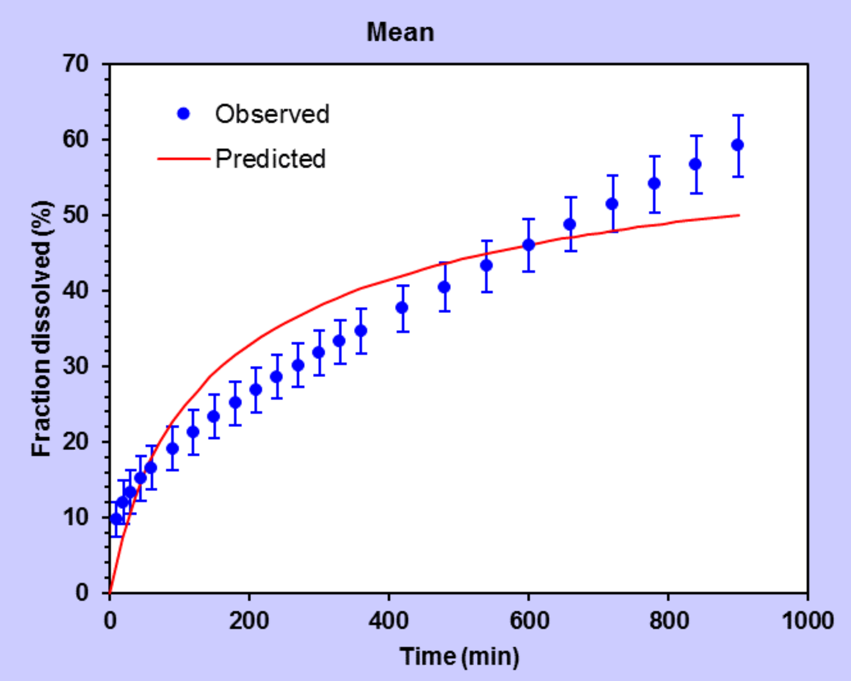

Supplementary materials – Model fitting summary – Starch 1500® sample with smaller particle size (modelling up to app. 60 % of carvedilol released)

Graphical abstract of model fit presented as the fraction % of released carvedilol per tested tablet:

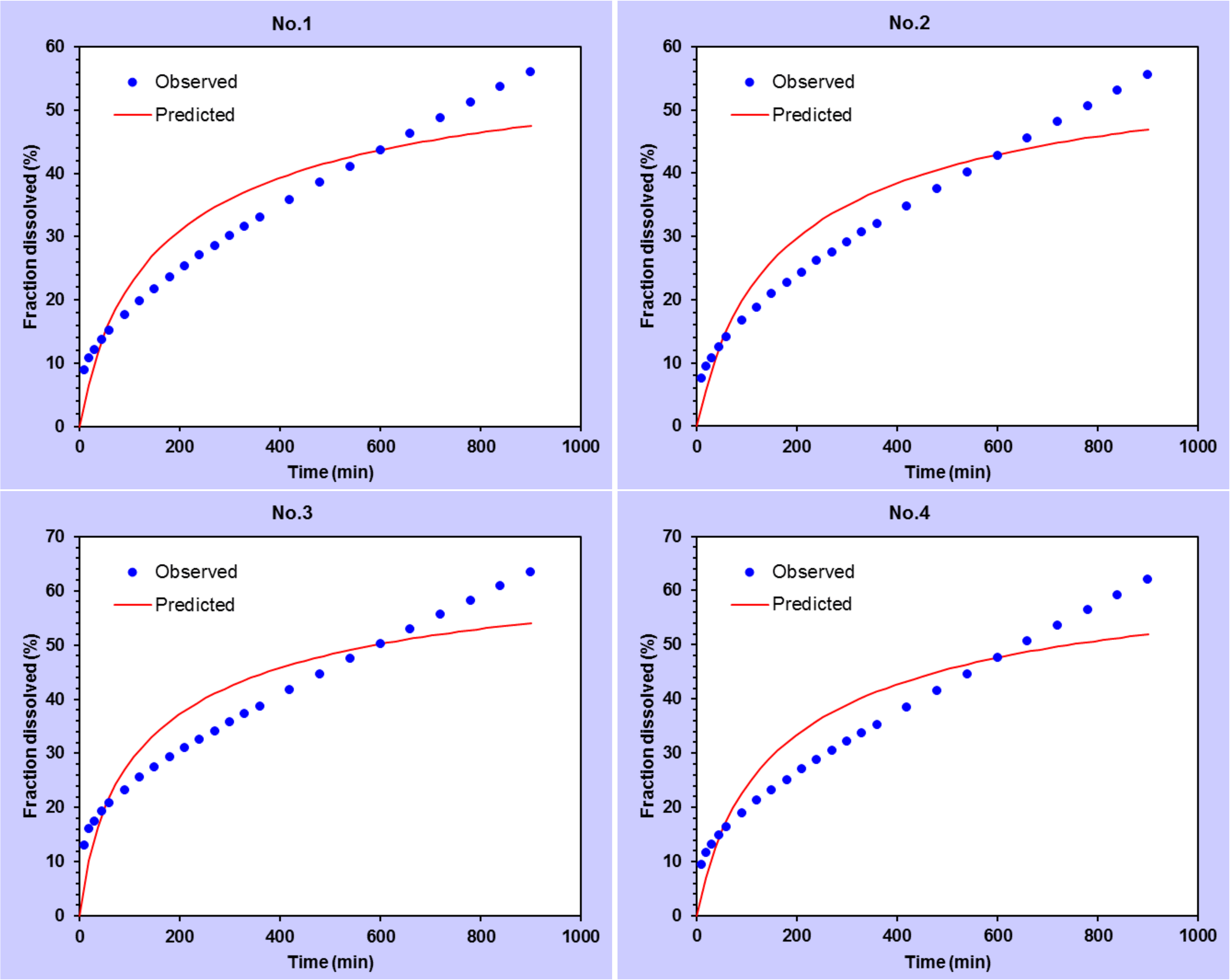

Supplement: Supplementary file 1 [file pharmaceutics-16-00498-s001.zip › Supplementary materials_Model fitting summary_Starch 1500® sample with smaller particle size.pdf]
