# Supplementary material for: Comparative Fitting of Mathematical Models to Carvedilol Release Profiles Obtained from Hypromellose Matrix Tablets
Source: Pharmaceutics. 2024 Apr 4;16(4):498. doi: 10.3390/pharmaceutics16040498 (PMC11053526; doi:10.3390/pharmaceutics16040498)

Supplementary materials – Model fitting summary – SuperTab® 11SD (modelling the entire relevant dissolution data)

Model: **Zero–order**

Model equation: $F = k_0 \cdot t$

Fitted model parameters per tested tablet (N = 4) with statistics – mean, standard deviation (SD), and relative standard deviation expressed in % (RSD%) (output from DDSolver):

Parameter	No.1	No.2	No.3	No.4	Mean	SD	RSD(%)
k_0	0.188	0.189	0.178	0.178	0.183	0.006	3.423

Number of dissolution data points (N), degrees of freedom (df), and selected goodness of fit criteria – Pearson correlation coefficient (R), coefficient of determination (R^2), adjusted coefficient of determination ($R^2_{adjusted}$), and residual sum of squares (RSS) (manual calculation in MS Excel):

Parameter	No.1	No.2	No.3	No.4
N	21	21	21	21
df	20	20	20	20
R	0.973961665	0.974833982	0.978887496	0.978588341
R^2	0.948601324	0.950301293	0.958220731	0.95763514
$R^2_{adjusted}$	0.948601324	0.950301293	0.958220731	0.95763514
RSS	13295.57082	16847.5385	10808.73417	10038.18552

Graphical abstract of model fit presented as mean ± 1 SD of the fraction % of released carvedilol:

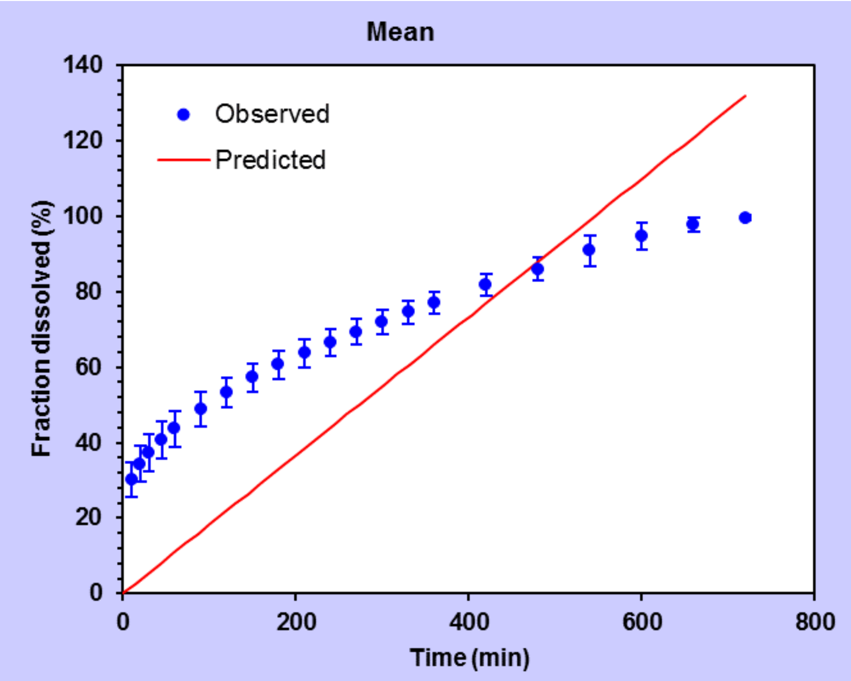

Supplementary materials – Model fitting summary – SuperTab® 11SD (modelling the entire relevant dissolution data)

Graphical abstract of model fit presented as the fraction % of released carvedilol per tested tablet:

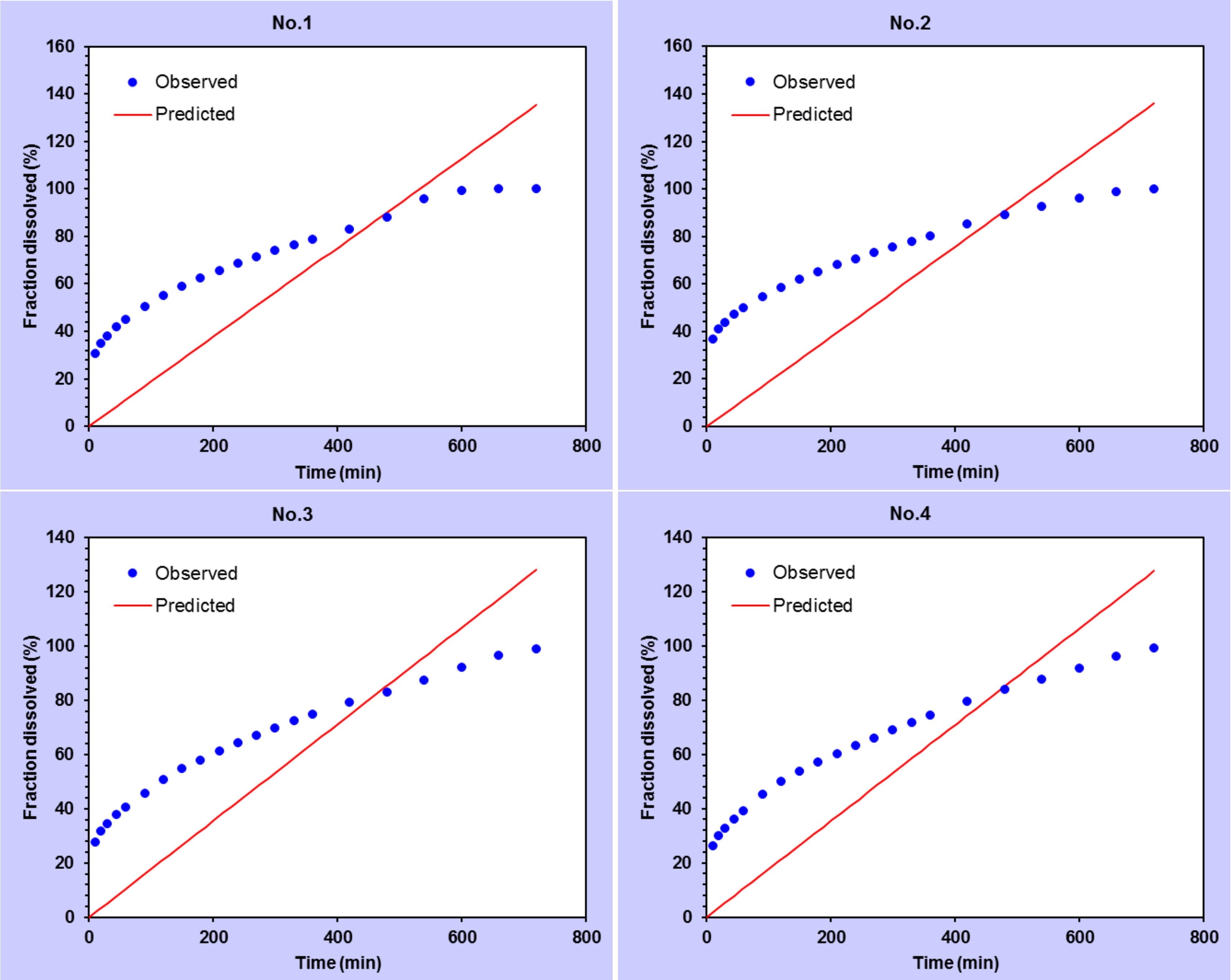

Supplementary materials – Model fitting summary – SuperTab® 11SD (modelling the entire relevant dissolution data)

Model: **Zero–order with T_{lag}**

Model equation: $F = k_0 \cdot (t - T_{lag})$

Fitted model parameters per tested tablet (N = 4) with statistics – mean, standard deviation (SD), and relative standard deviation expressed in % (RSD%) (output from DDSolver):

Parameter	No.1	No.2	No.3	No.4	Mean	SD	RSD(%)
k_0	0.098	0.087	0.097	0.099	0.095	0.006	6.017
T_{lag}	-410.867	-526.495	-375.516	-351.354	-416.058	77.575	-18.645

Number of dissolution data points (N), degrees of freedom (df), and selected goodness of fit criteria – Pearson correlation coefficient (R), coefficient of determination (R^2), adjusted coefficient of determination ($R^2_{adjusted}$), and residual sum of squares (RSS) (manual calculation in MS Excel):

Parameter	No.1	No.2	No.3	No.4
N	21	21	21	21
df	19	19	19	19
R	0.973961665	0.974833982	0.978887496	0.978588341
R^2	0.948601324	0.950301293	0.958220731	0.95763514
$R^2_{adjusted}$	0.945896131	0.947685571	0.956021822	0.955405411
RSS	508.147309	384.5433049	398.5098307	426.4411826

Graphical abstract of model fit presented as mean ± 1 SD of the fraction % of released carvedilol:

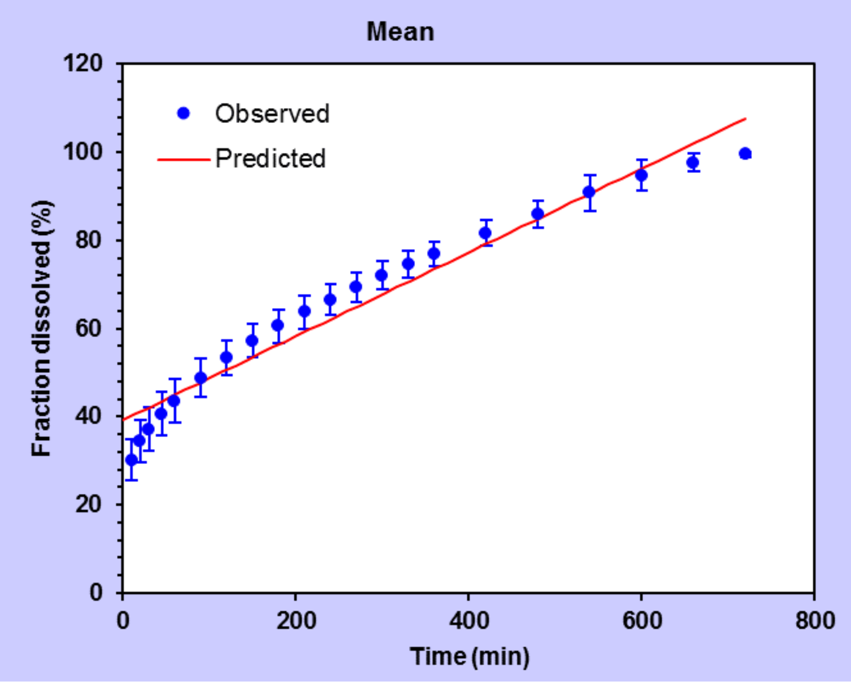

Supplementary materials – Model fitting summary – SuperTab® 11SD (modelling the entire relevant dissolution data)

Graphical abstract of model fit presented as the fraction % of released carvedilol per tested tablet:

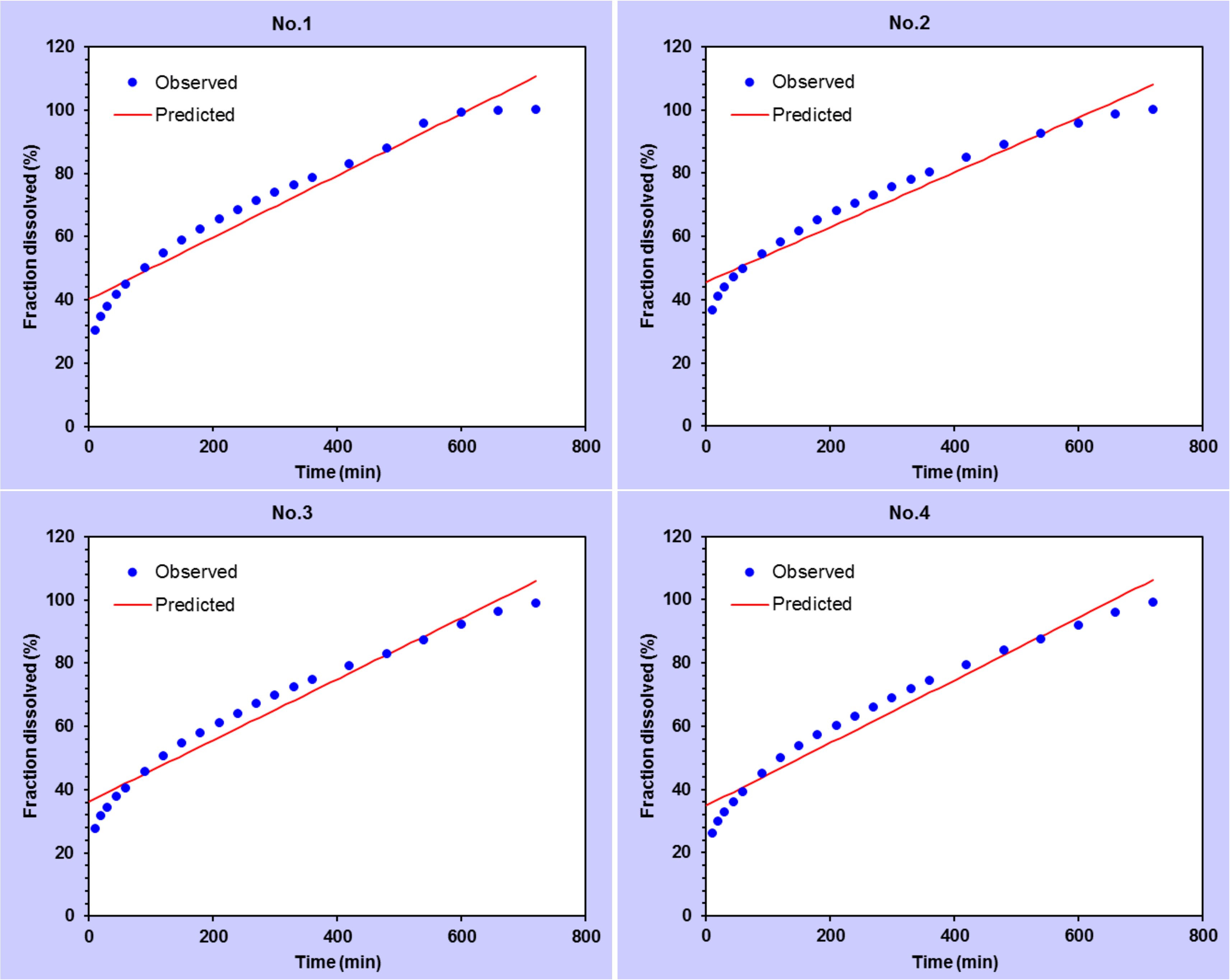

Supplementary materials – Model fitting summary – SuperTab® 11SD (modelling the entire relevant dissolution data)

Model: **Zero–order with F_0**

Model equation: $F = F_0 + k_0 \cdot t$

Fitted model parameters per tested tablet (N = 4) with statistics – mean, standard deviation (SD), and relative standard deviation expressed in % (RSD%) (output from DDSolver):

Parameter	No.1	No.2	No.3	No.4	Mean	SD	RSD(%)
k_0	0.098	0.087	0.097	0.099	0.095	0.006	6.017
F_0	40.221	45.637	36.290	34.871	39.255	4.819	12.276

Number of dissolution data points (N), degrees of freedom (df), and selected goodness of fit criteria – Pearson correlation coefficient (R), coefficient of determination (R^2), adjusted coefficient of determination ($R^2_{adjusted}$), and residual sum of squares (RSS) (manual calculation in MS Excel):

Parameter	No.1	No.2	No.3	No.4
N	21	21	21	21
df	19	19	19	19
R	0.973961665	0.974833982	0.978887496	0.978588341
R^2	0.948601324	0.950301293	0.958220731	0.95763514
$R^2_{adjusted}$	0.945896131	0.947685571	0.956021822	0.955405411
RSS	508.147309	384.5433049	398.5098307	426.4411826

Graphical abstract of model fit presented as mean ± 1 SD of the fraction % of released carvedilol:

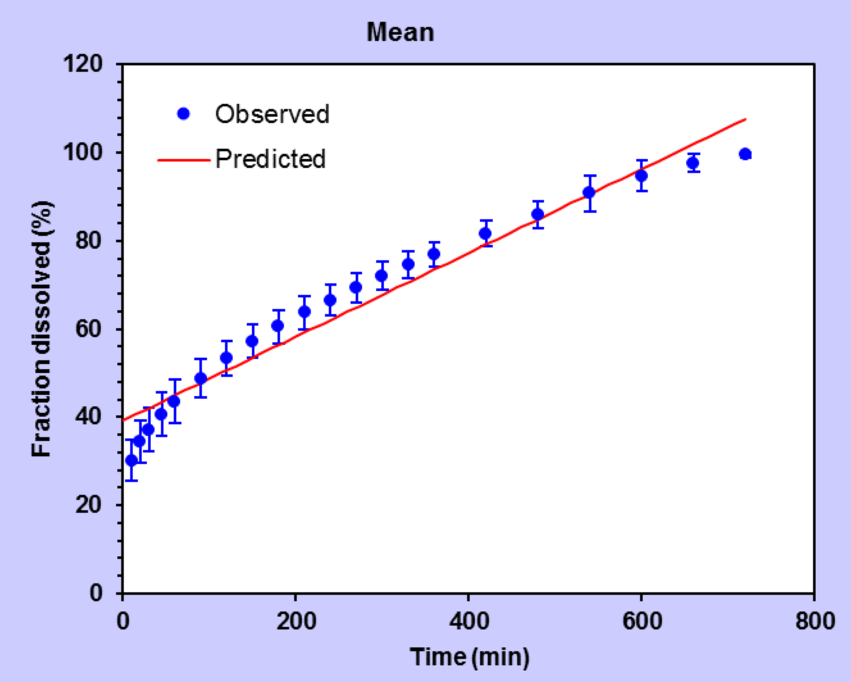

Supplementary materials – Model fitting summary – SuperTab® 11SD (modelling the entire relevant dissolution data)

Graphical abstract of model fit presented as the fraction % of released carvedilol per tested tablet:

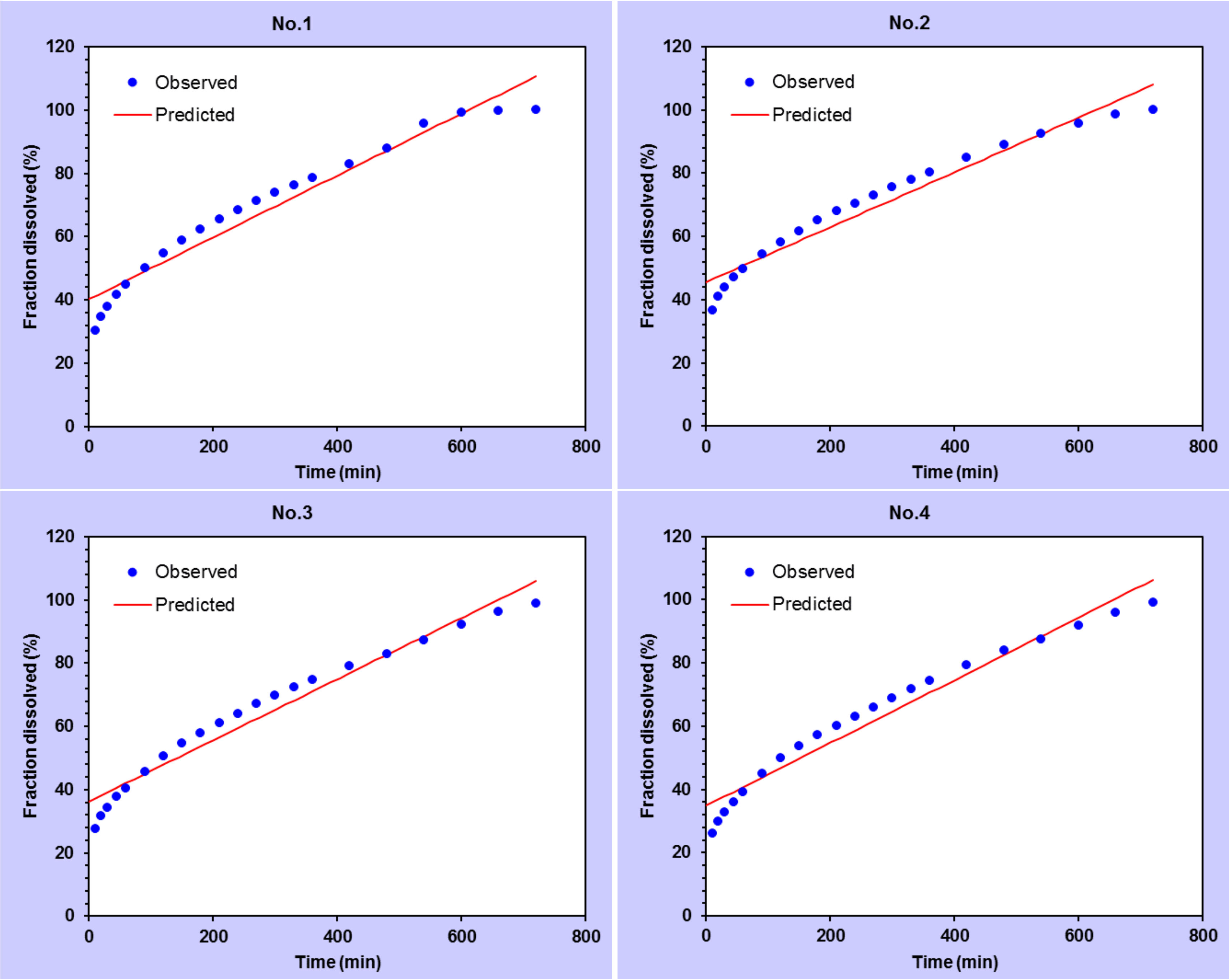

Supplementary materials – Model fitting summary – SuperTab® 11SD (modelling the entire relevant dissolution data)

Model: **First–order**

Model equation: $F = 100 \cdot (1 - e^{-k_1 \cdot t})$

Fitted model parameters per tested tablet (N = 4) with statistics – mean, standard deviation (SD), and relative standard deviation expressed in % (RSD%) (output from DDSolver):

Parameter	No.1	No.2	No.3	No.4	Mean	SD	RSD(%)
k_1	0.007	0.007	0.005	0.005	0.006	0.001	20.748

Number of dissolution data points (N), degrees of freedom (df), and selected goodness of fit criteria – Pearson correlation coefficient (R), coefficient of determination (R^2), adjusted coefficient of determination ($R^2_{adjusted}$), and residual sum of squares (RSS) (manual calculation in MS Excel):

Parameter	No.1	No.2	No.3	No.4
N	21	21	21	21
df	20	20	20	20
R	0.953511651	0.95908982	0.980860893	0.980851024
R^2	0.909184468	0.919853283	0.962088092	0.962068731
$R^2_{adjusted}$	0.909184468	0.919853283	0.962088092	0.962068731
RSS	3047.835405	4001.223231	2590.05229	2269.575519

Graphical abstract of model fit presented as mean ± 1 SD of the fraction % of released carvedilol:

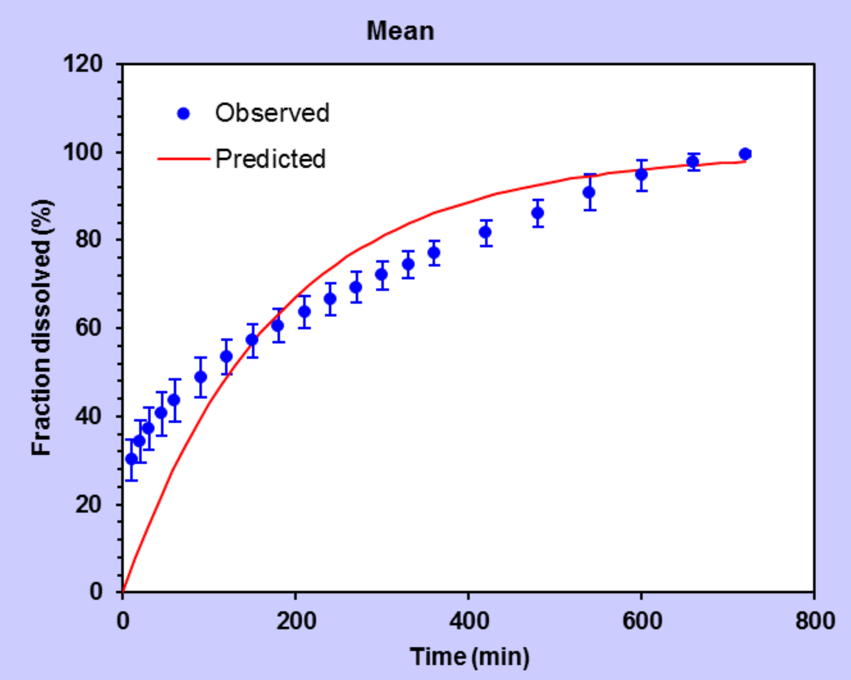

Supplementary materials – Model fitting summary – SuperTab® 11SD (modelling the entire relevant dissolution data)

Graphical abstract of model fit presented as the fraction % of released carvedilol per tested tablet:

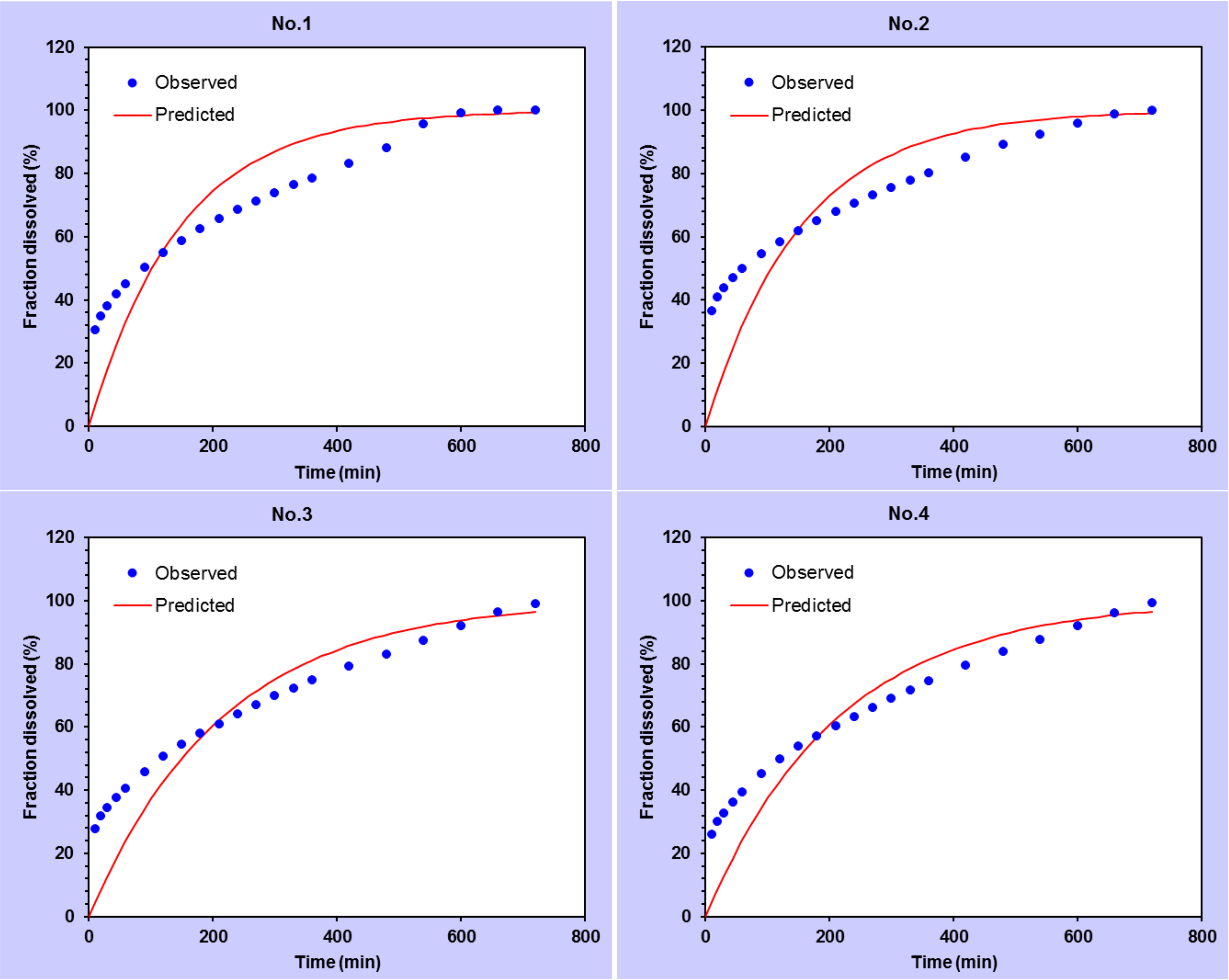

Model: **First–order with T_{lag}**

Model equation: $F = 100 \cdot \left[1 - e^{-k_1 \cdot (t - T_{lag})}\right]$

Fitted model parameters per tested tablet (N = 4) with statistics – mean, standard deviation (SD), and relative standard deviation expressed in % (RSD%) (output from DDSolver):

Parameter	No.1	No.2	No.3	No.4	Mean	SD	RSD(%)
k_1	/	/	/	/	/	/	/
T_{lag}	/	/	/	/	/	/	/

Number of dissolution data points (N), degrees of freedom (df), and selected goodness of fit criteria – Pearson correlation coefficient (R), coefficient of determination (R^2), adjusted coefficient of determination ($R^2_{adjusted}$), and residual sum of squares (RSS) (manual calculation in MS Excel):

Parameter	No.1	No.2	No.3	No.4
N	/	/	/	/
df	/	/	/	/
R	/	/	/	/
R^2	/	/	/	/
$R^2_{adjusted}$	/	/	/	/
RSS	/	/	/	/

Graphical abstract of model fit presented as mean ± 1 SD of the fraction % of released carvedilol: /

Graphical abstract of model fit presented as the fraction % of released carvedilol per tested tablet: /

Note: the model could not be fitted

Supplementary materials – Model fitting summary – SuperTab® 11SD (modelling the entire relevant dissolution data)

Model: **First–order with F_{max}**

Model equation: $F = F_{max} \cdot (1 - e^{-k_1 \cdot t})$

Fitted model parameters per tested tablet (N = 4) with statistics – mean, standard deviation (SD), and relative standard deviation expressed in % (RSD%) (output from DDSolver):

Parameter	No.1	No.2	No.3	No.4	Mean	SD	RSD(%)
k_1	0.004	0.006	0.004	0.004	0.005	0.001	26.290
F_{max}	105.109	104.976	103.833	104.076	104.498	0.638	0.610

Number of dissolution data points (N), degrees of freedom (df), and selected goodness of fit criteria – Pearson correlation coefficient (R), coefficient of determination (R^2), adjusted coefficient of determination ($R^2_{adjusted}$), and residual sum of squares (RSS) (manual calculation in MS Excel):

Parameter	No.1	No.2	No.3	No.4
N	21	21	21	21
df	19	19	19	19
R	0.984730719	0.962073039	0.989596207	0.990267888
R^2	0.96969459	0.925584533	0.979300652	0.980630491
$R^2_{adjusted}$	0.968099568	0.921667929	0.978211213	0.979611043
RSS	3523.552649	4643.253804	3088.111531	2712.671388

Graphical abstract of model fit presented as mean ± 1 SD of the fraction % of released carvedilol:

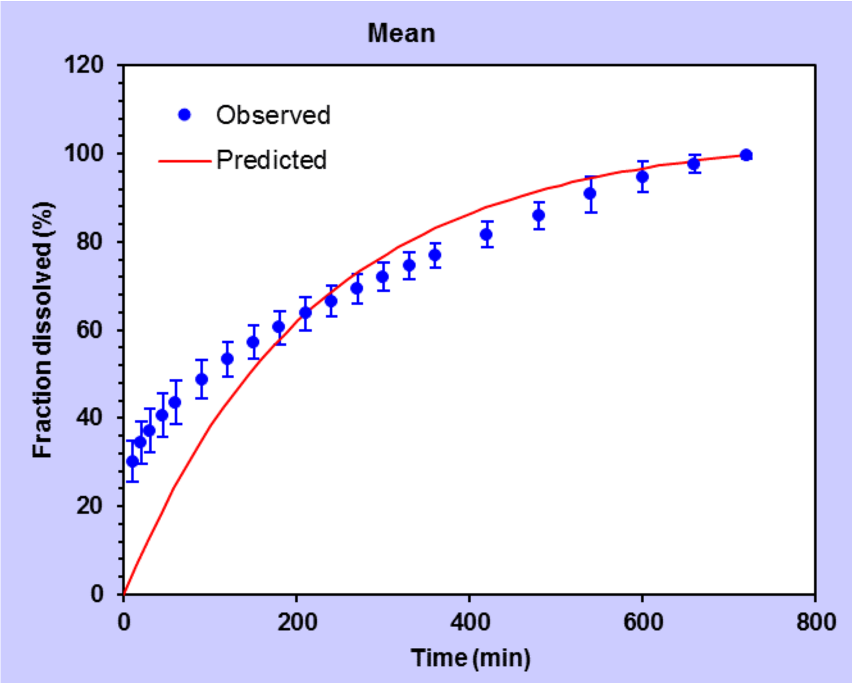

Supplementary materials – Model fitting summary – SuperTab® 11SD (modelling the entire relevant dissolution data)

Graphical abstract of model fit presented as the fraction % of released carvedilol per tested tablet:

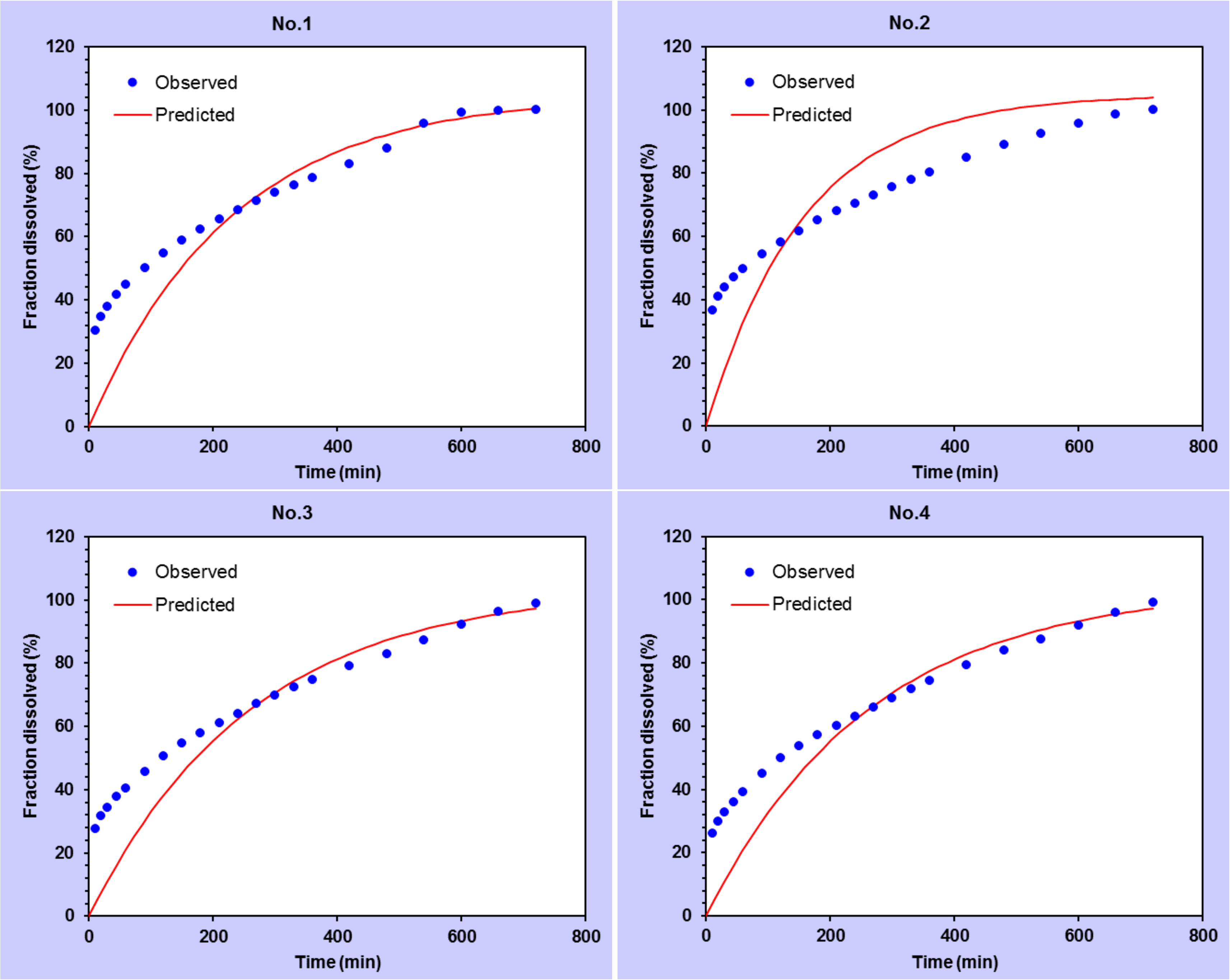

Supplementary materials – Model fitting summary – SuperTab® 11SD (modelling the entire relevant dissolution data)

Model: **First–order with T_{lag} and F_{max}**

Model equation: $F = F_{max} \cdot \left[1 - e^{-k_1 \cdot (t - T_{lag})}\right]$

Fitted model parameters per tested tablet (N = 4) with statistics – mean, standard deviation (SD), and relative standard deviation expressed in % (RSD%) (output from DDSolver):

Parameter	No.1	No.2	No.3	No.4	Mean	SD	RSD(%)
k_1	0.004	0.003	0.003	0.003	0.003	0.000	6.896
T_{lag}	-58.281	-141.211	-64.493	-58.136	-80.531	40.562	-50.368
F_{max}	105.109	101.857	103.833	104.076	103.719	1.359	1.310

Number of dissolution data points (N), degrees of freedom (df), and selected goodness of fit criteria – Pearson correlation coefficient (R), coefficient of determination (R^2), adjusted coefficient of determination ($R^2_{adjusted}$), and residual sum of squares (RSS) (manual calculation in MS Excel):

Parameter	No.1	No.2	No.3	No.4
N	21	21	21	21
df	18	18	18	18
R	0.989772949	0.994514822	0.993581999	0.993797501
R^2	0.979650492	0.989059732	0.987205188	0.987633473
$R^2_{adjusted}$	0.977389435	0.987844146	0.985783542	0.986259415
RSS	442.6640138	157.0440662	289.859522	291.3584892

Graphical abstract of model fit presented as mean ± 1 SD of the fraction % of released carvedilol:

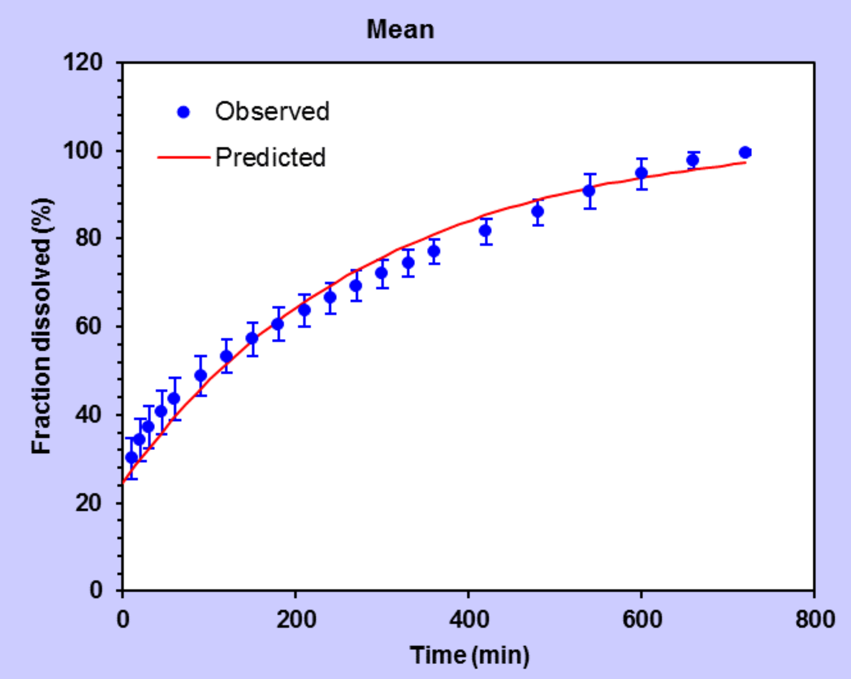

Supplementary materials – Model fitting summary – SuperTab® 11SD (modelling the entire relevant dissolution data)

Graphical abstract of model fit presented as the fraction % of released carvedilol per tested tablet:

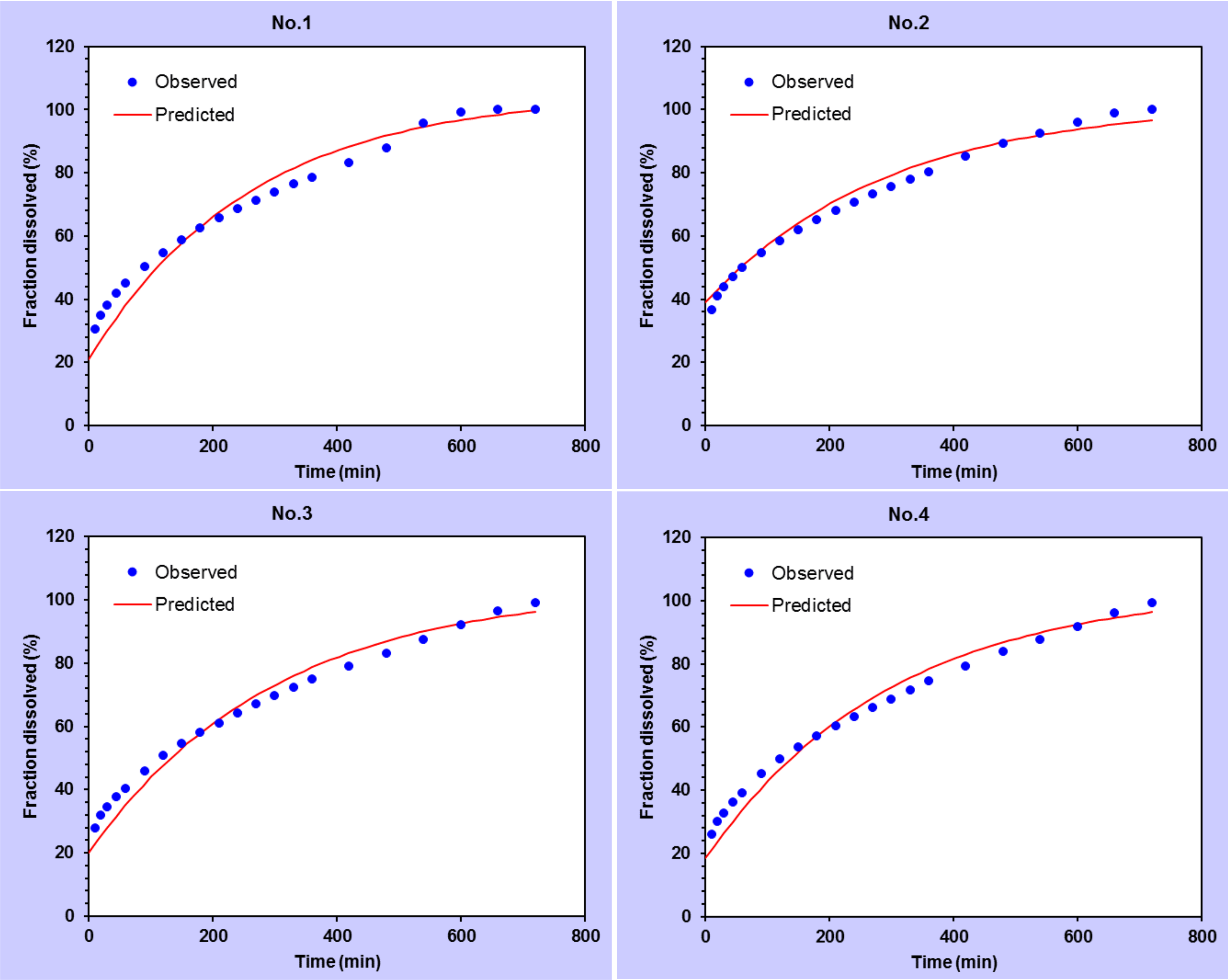

Supplementary materials – Model fitting summary – SuperTab® 11SD (modelling the entire relevant dissolution data)

Model: **Higuchi**

Model equation: $F = k_H \cdot t^{0.5}$

Fitted model parameters per tested tablet (N = 4) with statistics – mean, standard deviation (SD), and relative standard deviation expressed in % (RSD%) (output from DDSolver):

Parameter	No.1	No.2	No.3	No.4	Mean	SD	RSD(%)
k_H	4.215	4.277	3.973	3.950	4.104	0.166	4.053

Number of dissolution data points (N), degrees of freedom (df), and selected goodness of fit criteria – Pearson correlation coefficient (R), coefficient of determination (R^2), adjusted coefficient of determination ($R^2_{adjusted}$), and residual sum of squares (RSS) (manual calculation in MS Excel):

Parameter	No.1	No.2	No.3	No.4
N	21	21	21	21
df	20	20	20	20
R	0.99833037	0.999647532	0.999777929	0.999861929
R^2	0.996663528	0.999295188	0.999555908	0.999723877
$R^2_{adjusted}$	0.996663528	0.999295188	0.999555908	0.999723877
RSS	1690.636193	3079.214891	1152.863132	910.1353314

Graphical abstract of model fit presented as mean ± 1 SD of the fraction % of released carvedilol:

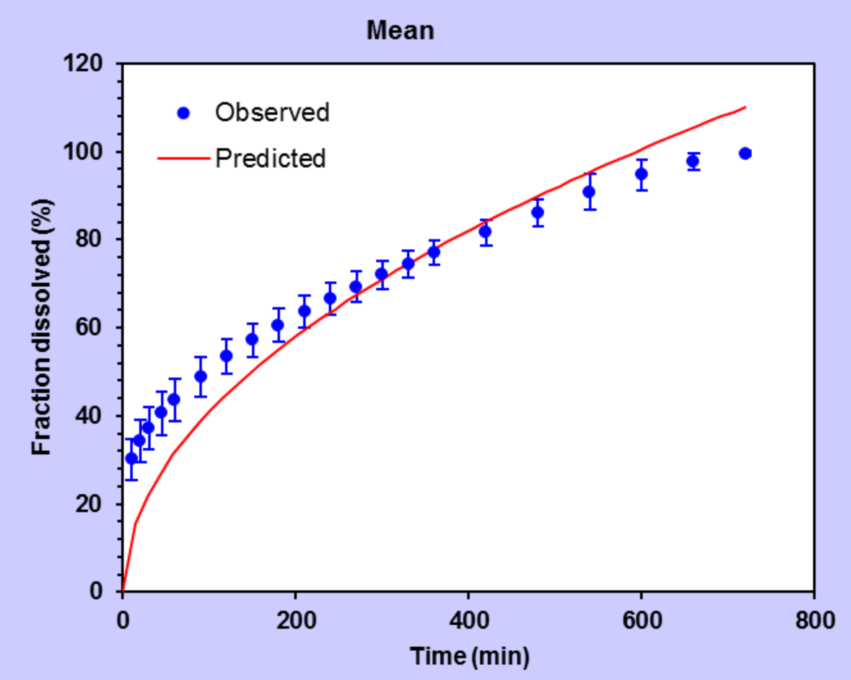

Supplementary materials – Model fitting summary – SuperTab® 11SD (modelling the entire relevant dissolution data)

Graphical abstract of model fit presented as the fraction % of released carvedilol per tested tablet:

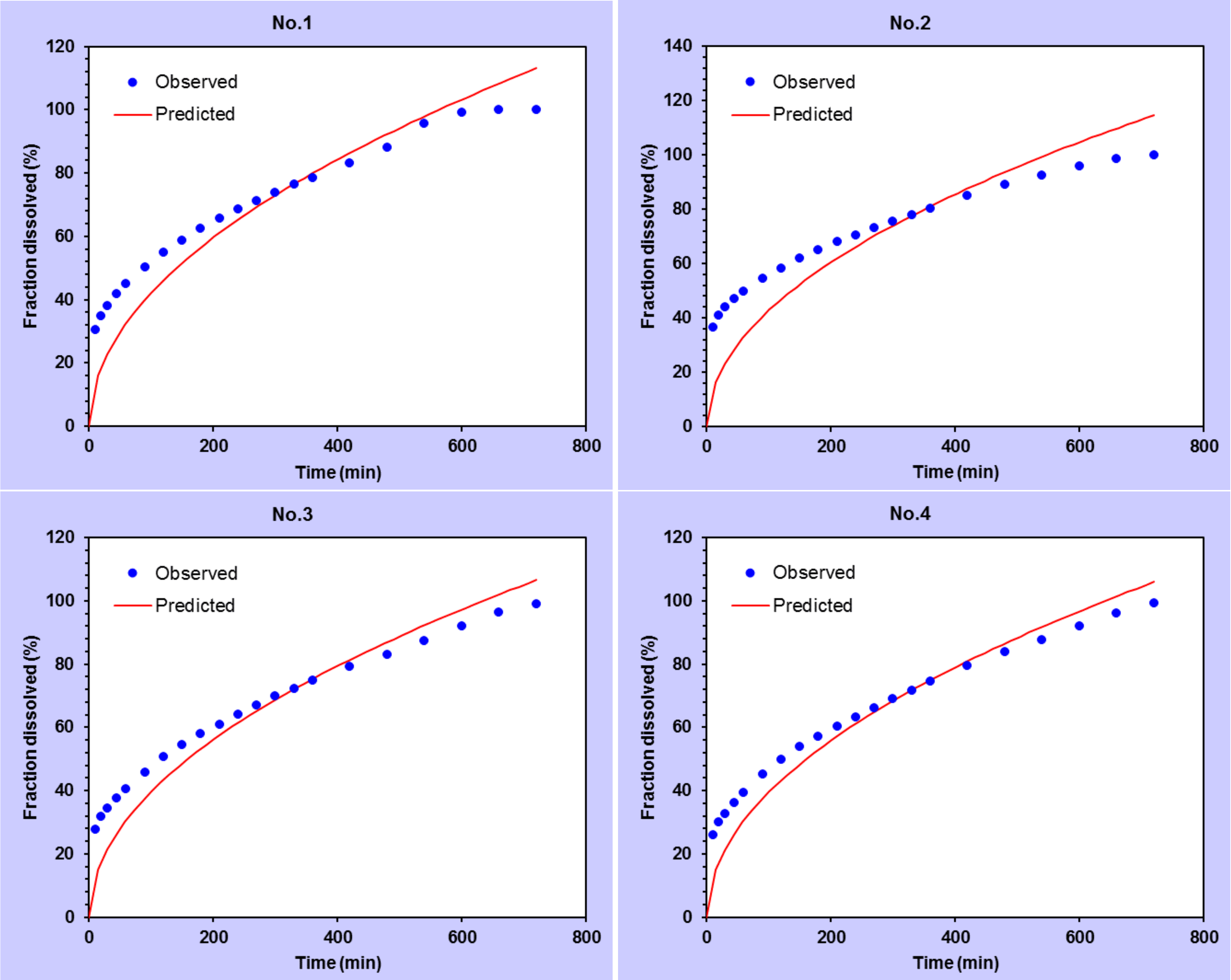

Supplementary materials – Model fitting summary – SuperTab® 11SD (modelling the entire relevant dissolution data)

Model: **Higuchi with T_{lag}**

Model equation: $F = k_H \cdot (t - T_{lag})^{0.5}$

Fitted model parameters per tested tablet (N = 4) with statistics – mean, standard deviation (SD), and relative standard deviation expressed in % (RSD%) (output from DDSolver):

Parameter	No.1	No.2	No.3	No.4	Mean	SD	RSD(%)
k_H	3.680	3.513	3.549	3.577	3.580	0.072	2.001
T_{lag}	-92.646	-145.900	-74.783	-64.321	-94.413	36.262	-38.408

Number of dissolution data points (N), degrees of freedom (df), and selected goodness of fit criteria – Pearson correlation coefficient (R), coefficient of determination (R^2), adjusted coefficient of determination ($R^2_{adjusted}$), and residual sum of squares (RSS) (manual calculation in MS Excel):

Parameter	No.1	No.2	No.3	No.4
N	21	21	21	21
df	19	19	19	19
R	0.99421244	0.993294861	0.997615757	0.997982234
R^2	0.988458377	0.98663468	0.995237198	0.99596854
$R^2_{adjusted}$	0.987850923	0.985931242	0.994986524	0.995756358
RSS	128.5075059	115.6942294	53.17172651	48.53430073

Graphical abstract of model fit presented as mean ± 1 SD of the fraction % of released carvedilol:

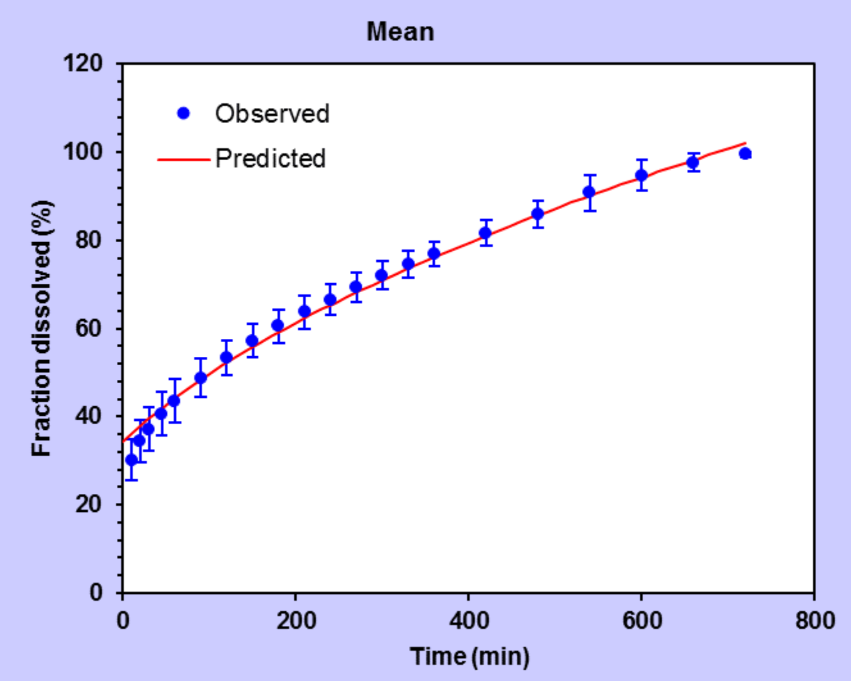

Supplementary materials – Model fitting summary – SuperTab® 11SD (modelling the entire relevant dissolution data)

Graphical abstract of model fit presented as the fraction % of released carvedilol per tested tablet:

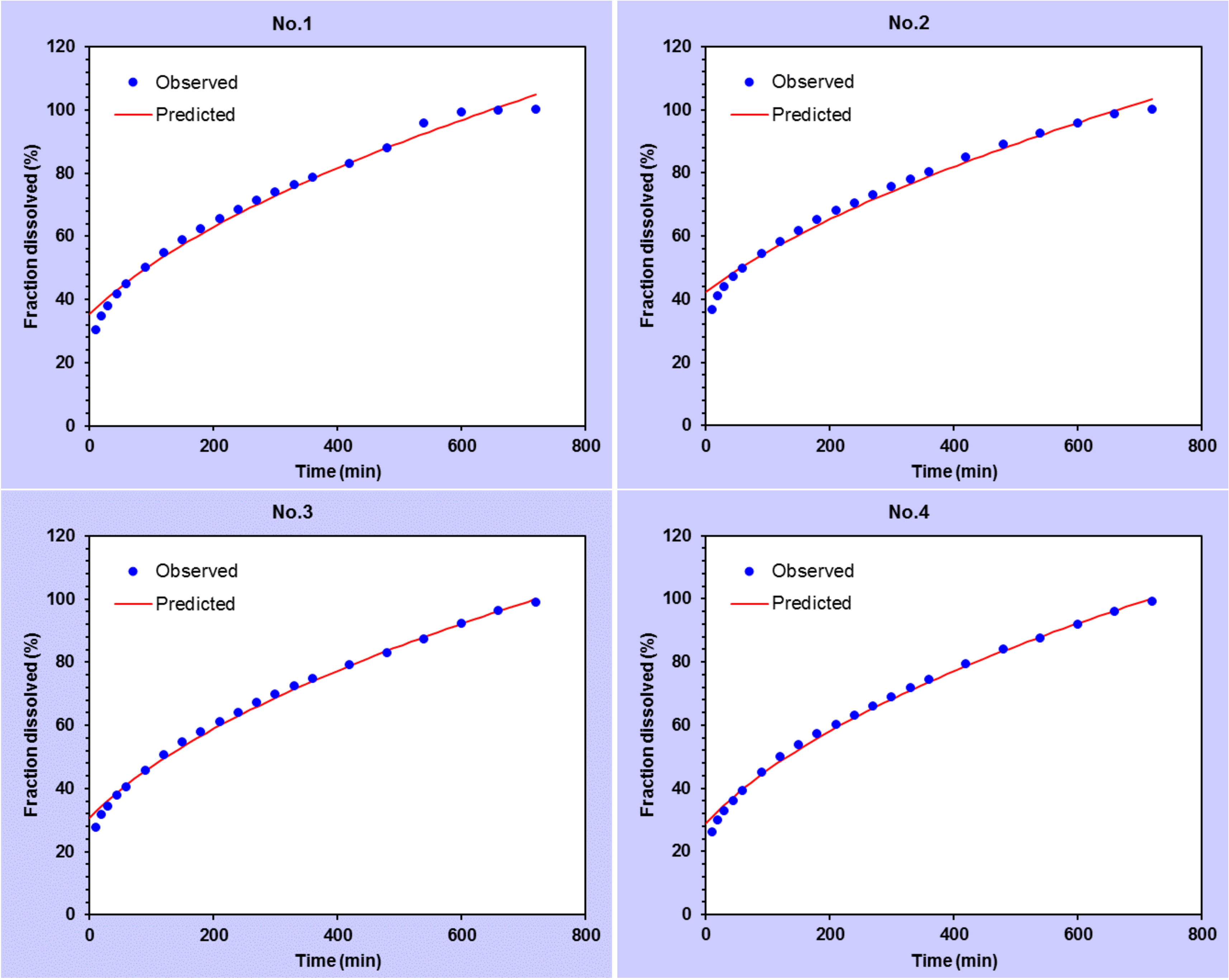

Supplementary materials – Model fitting summary – SuperTab® 11SD (modelling the entire relevant dissolution data)

Model: **Higuchi with F_0**

Model equation: $F = F_0 + k_H \cdot t^{0.5}$

Fitted model parameters per tested tablet (N = 4) with statistics – mean, standard deviation (SD), and relative standard deviation expressed in % (RSD%) (output from DDSolver):

Parameter	No.1	No.2	No.3	No.4	Mean	SD	RSD(%)
k_H	3.073	2.722	3.023	3.105	2.981	0.176	5.897
F_0	21.008	28.607	17.488	15.543	20.661	5.760	27.878

Number of dissolution data points (N), degrees of freedom (df), and selected goodness of fit criteria – Pearson correlation coefficient (R), coefficient of determination (R^2), adjusted coefficient of determination ($R^2_{adjusted}$), and residual sum of squares (RSS) (manual calculation in MS Excel):

Parameter	No.1	No.2	No.3	No.4
N	21	21	21	21
df	19	19	19	19
R	0.99833037	0.999647532	0.999777929	0.999861929
R^2	0.996663528	0.999295188	0.999555908	0.999723877
$R^2_{adjusted}$	0.996487924	0.999258093	0.999532535	0.999709344
RSS	32.98566212	5.453474092	4.23595396	2.779435826

Graphical abstract of model fit presented as mean ± 1 SD of the fraction % of released carvedilol:

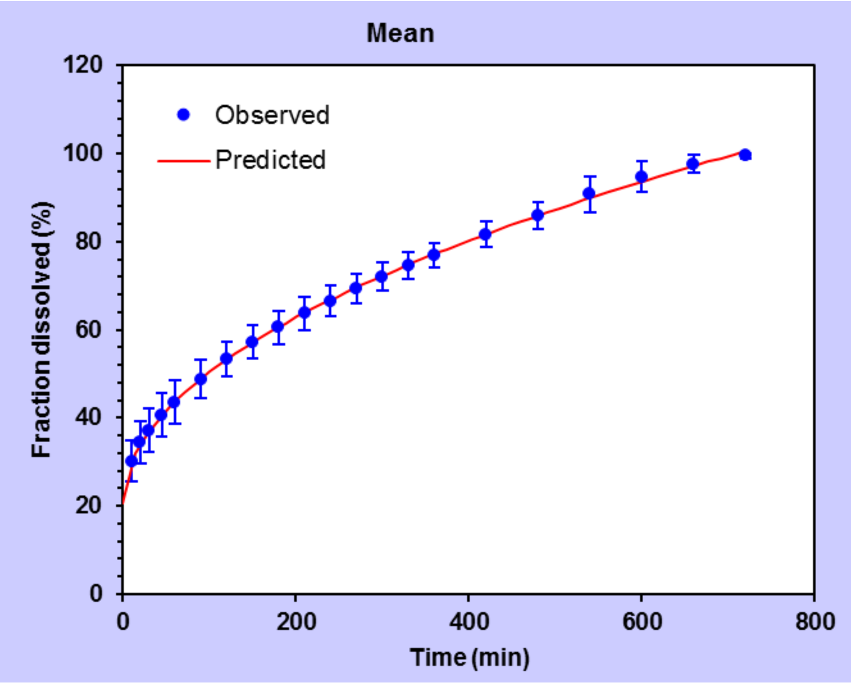

Supplementary materials – Model fitting summary – SuperTab® 11SD (modelling the entire relevant dissolution data)

Graphical abstract of model fit presented as the fraction % of released carvedilol per tested tablet:

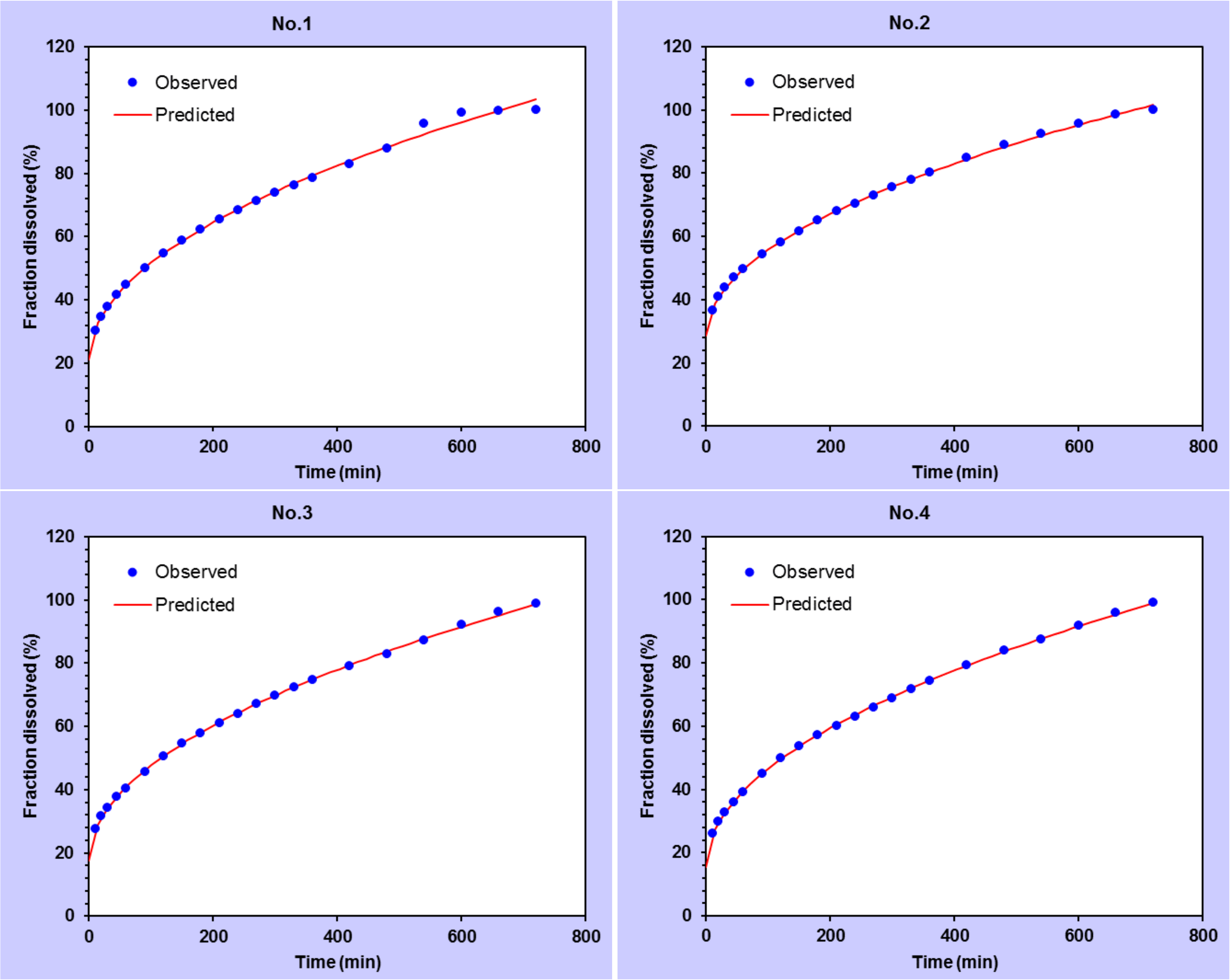

Supplementary materials – Model fitting summary – SuperTab® 11SD (modelling the entire relevant dissolution data)

Model: **Korsmeyer–Peppas**

Model equation: $F = k_{KP} \cdot t^n$

Fitted model parameters per tested tablet (N = 4) with statistics – mean, standard deviation (SD), and relative standard deviation expressed in % (RSD%) (output from DDSolver):

Parameter	No.1	No.2	No.3	No.4	Mean	SD	RSD(%)
k_{KP}	13.944	18.828	11.940	10.830	13.886	3.538	25.480
n	0.295	0.247	0.312	0.327	0.295	0.035	11.813

Number of dissolution data points (N), degrees of freedom (df), and selected goodness of fit criteria – Pearson correlation coefficient (R), coefficient of determination (R^2), adjusted coefficient of determination ($R^2_{adjusted}$), and residual sum of squares (RSS) (manual calculation in MS Excel):

Parameter	No.1	No.2	No.3	No.4
N	21	21	21	21
df	19	19	19	19
R	0.992762278	0.99116479	0.993607302	0.994743872
R^2	0.985576941	0.98240764	0.98725547	0.98951537
$R^2_{adjusted}$	0.984817833	0.981481727	0.986584705	0.988963548
RSS	178.3720751	159.3006842	155.5227702	138.6297761

Graphical abstract of model fit presented as mean ± 1 SD of the fraction % of released carvedilol:

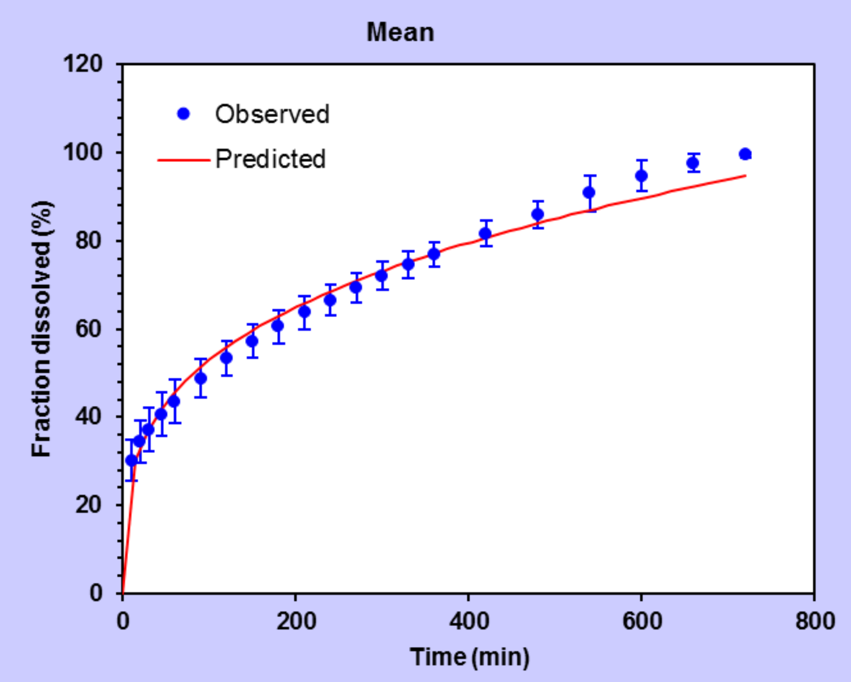

Supplementary materials – Model fitting summary – SuperTab® 11SD (modelling the entire relevant dissolution data)

Graphical abstract of model fit presented as the fraction % of released carvedilol per tested tablet:

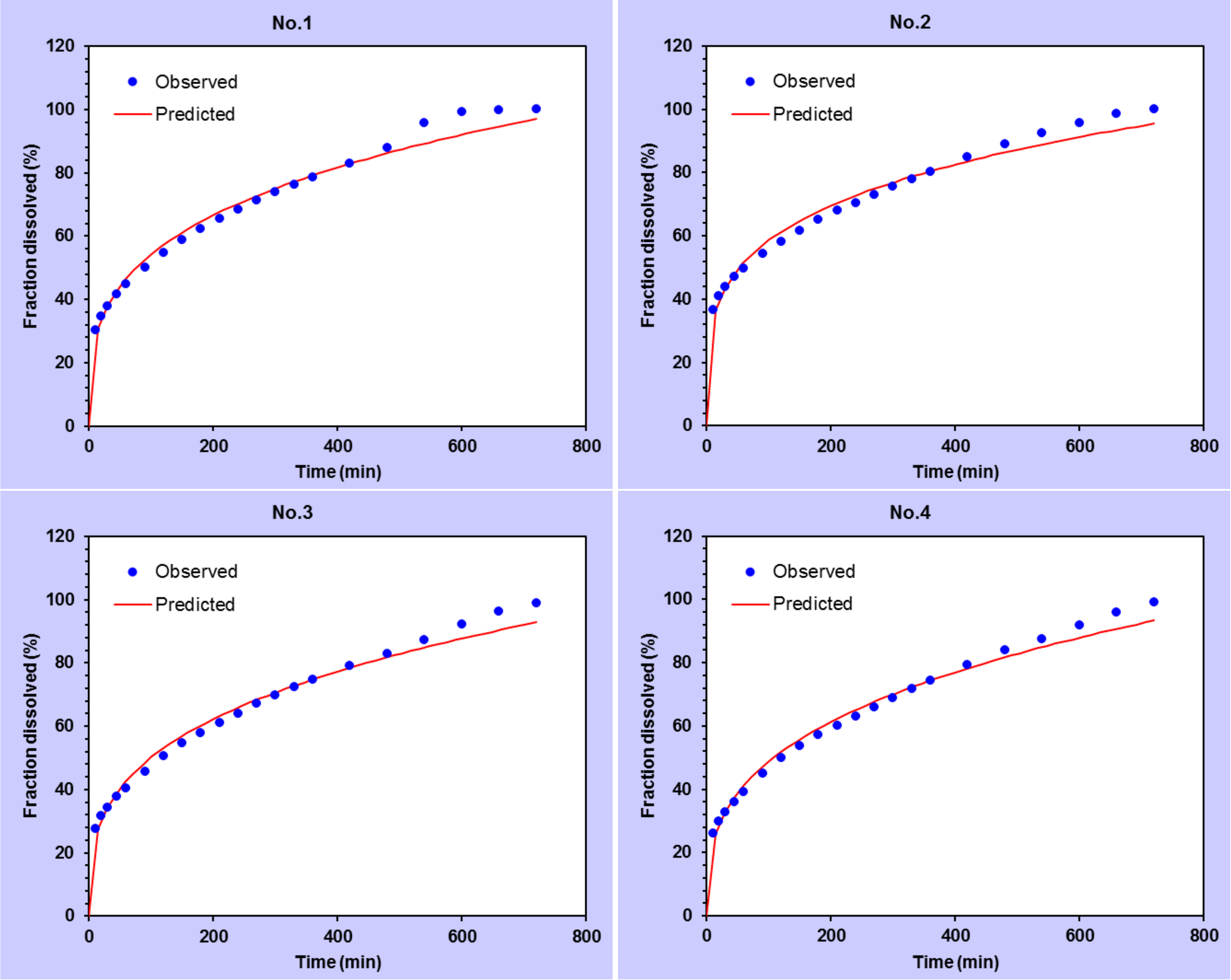

Supplementary materials – Model fitting summary – SuperTab® 11SD (modelling the entire relevant dissolution data)

Model: **Korsmeyer–Peppas with T_{lag}**

Model equation: $F = k_{KP} \cdot \left(t - T_{lag}\right)^{n}$

Fitted model parameters per tested tablet (N = 4) with statistics – mean, standard deviation (SD), and relative standard deviation expressed in % (RSD%) (output from DDSolver):

Parameter	No.1	No.2	No.3	No.4	Mean	SD	RSD(%)
k_{KP}	15.927	21.158	13.829	12.630	15.886	3.770	23.729
n	0.273	0.227	0.287	0.301	0.272	0.032	11.823
T_{lag}	4.000	4.000	4.000	4.000	4.000	0.000	0.000

Number of dissolution data points (N), degrees of freedom (df), and selected goodness of fit criteria – Pearson correlation coefficient (R), coefficient of determination (R^2), adjusted coefficient of determination ($R^2_{adjusted}$), and residual sum of squares (RSS) (manual calculation in MS Excel):

Parameter	No.1	No.2	No.3	No.4
N	21	21	21	21
df	18	18	18	18
R	0.988523909	0.985819652	0.989043122	0.990551782
R^2	0.977179519	0.971840387	0.978206297	0.981192832
$R^2_{adjusted}$	0.97464391	0.968711541	0.975784775	0.979103147
RSS	280.360488	258.6715336	271.4854154	254.3107216

Graphical abstract of model fit presented as mean ± 1 SD of the fraction % of released carvedilol:

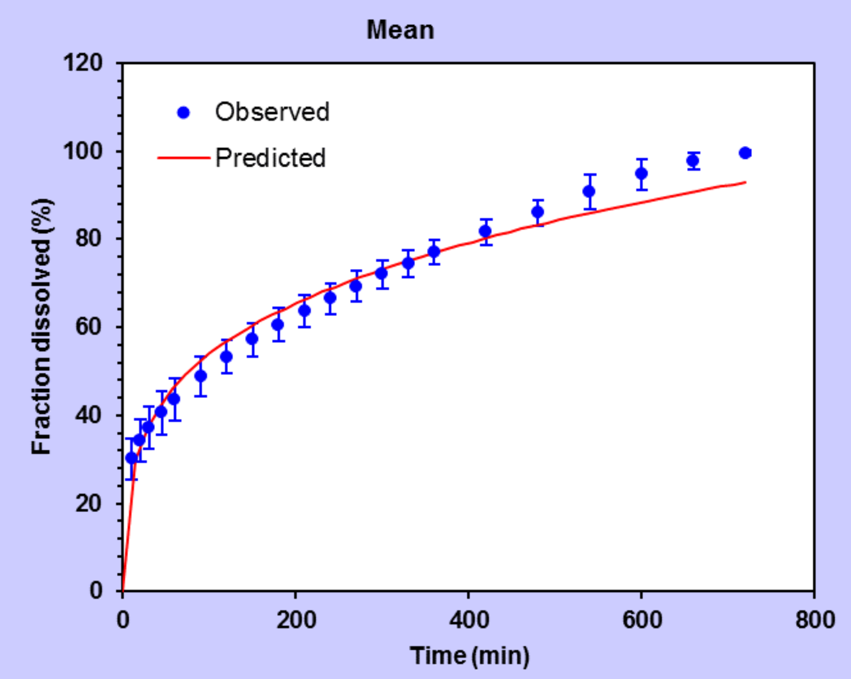

Supplementary materials – Model fitting summary – SuperTab® 11SD (modelling the entire relevant dissolution data)

Graphical abstract of model fit presented as the fraction % of released carvedilol per tested tablet:

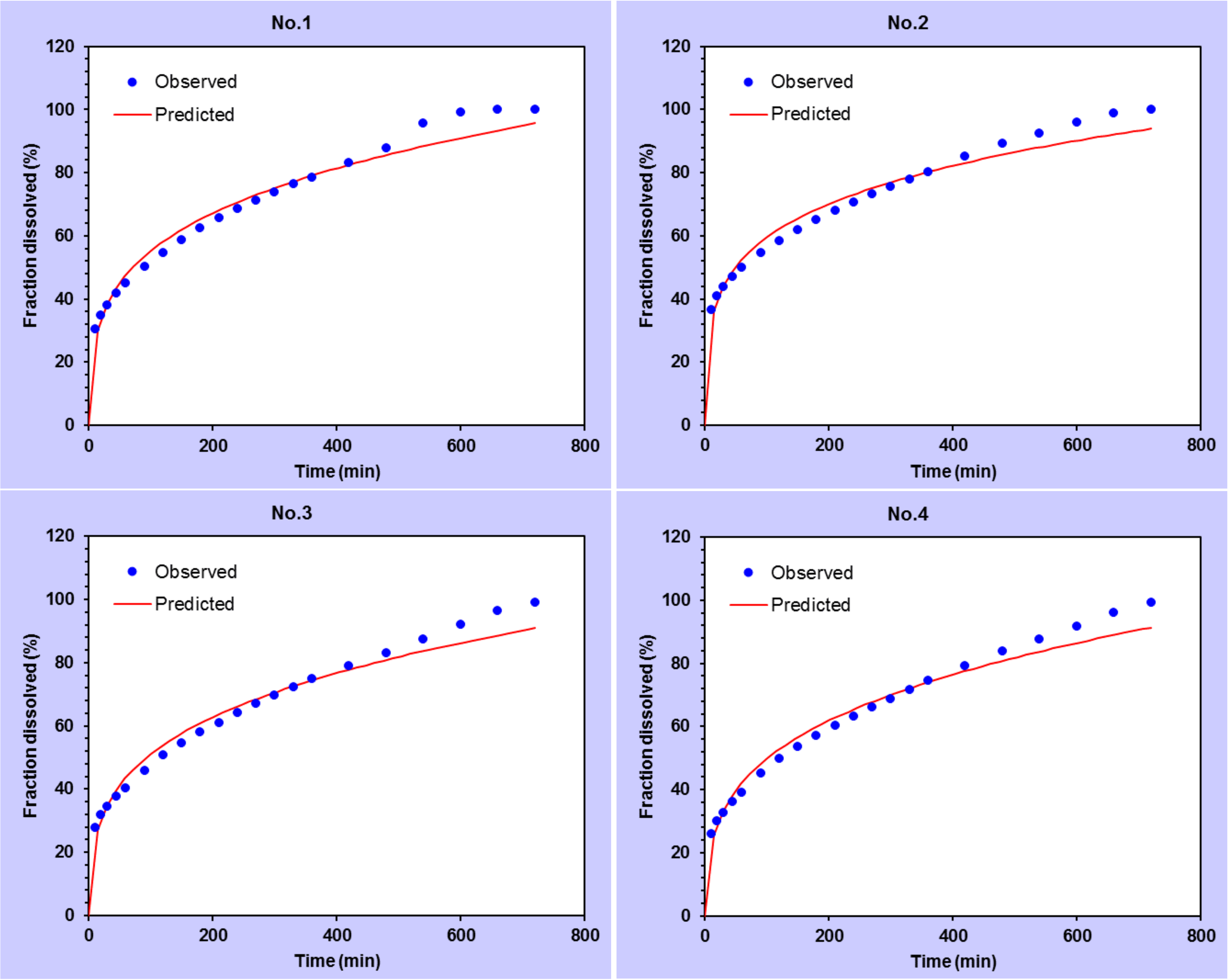

Supplementary materials – Model fitting summary – SuperTab® 11SD (modelling the entire relevant dissolution data)

Model: **Korsmeyer–Peppas with F_0**

Model equation: $F = F_0 + k_{KP} \cdot t^n$

Fitted model parameters per tested tablet (N = 4) with statistics – mean, standard deviation (SD), and relative standard deviation expressed in % (RSD%) (output from DDSolver):

Parameter	No.1	No.2	No.3	No.4	Mean	SD	RSD(%)
k_{KP}	6.990	5.891	5.972	5.811	6.166	0.553	8.969
n	0.384	0.405	0.402	0.415	0.401	0.013	3.144
F_0	12.157	16.933	11.038	8.263	12.098	3.615	29.885

Number of dissolution data points (N), degrees of freedom (df), and selected goodness of fit criteria – Pearson correlation coefficient (R), coefficient of determination (R^2), adjusted coefficient of determination ($R^2_{adjusted}$), and residual sum of squares (RSS) (manual calculation in MS Excel):

Parameter	No.1	No.2	No.3	No.4
N	21	21	21	21
df	18	18	18	18
R	0.996682413	0.998662112	0.997841433	0.998395234
R^2	0.993375833	0.997326015	0.995687525	0.996793043
$R^2_{adjusted}$	0.992639815	0.997028905	0.995208361	0.996436714
RSS	80.06330643	69.67087186	60.0131367	32.45392016

Graphical abstract of model fit presented as mean ± 1 SD of the fraction % of released carvedilol:

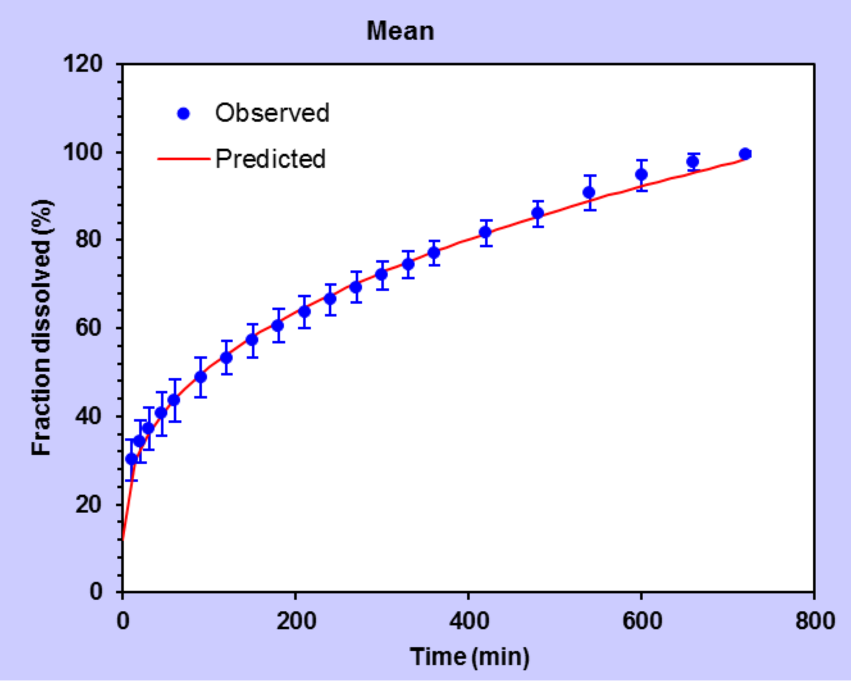

Supplementary materials – Model fitting summary – SuperTab® 11SD (modelling the entire relevant dissolution data)

Graphical abstract of model fit presented as the fraction % of released carvedilol per tested tablet:

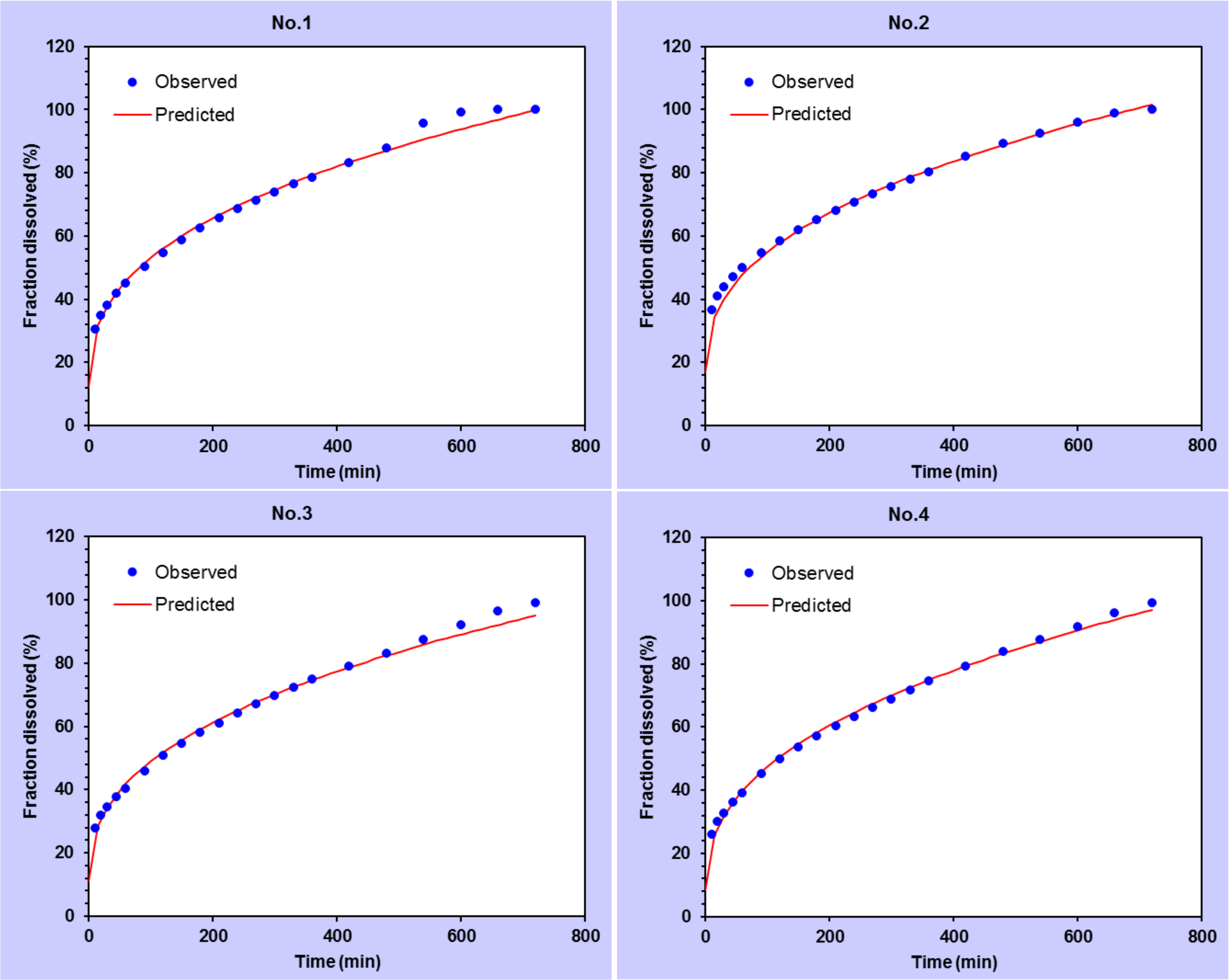

Supplementary materials – Model fitting summary – SuperTab® 11SD (modelling the entire relevant dissolution data)

Model: **Hixson–Crowell**

Model equation: $F = 100 \cdot [1 - (1 - k_{HC} \cdot t)^3]$

Fitted model parameters per tested tablet (N = 4) with statistics – mean, standard deviation (SD), and relative standard deviation expressed in % (RSD%) (output from DDSolver):

Parameter	No.1	No.2	No.3	No.4	Mean	SD	RSD(%)
k_{HC}	0.001	0.002	0.002	0.001	0.001	0.000	23.868

Number of dissolution data points (N), degrees of freedom (df), and selected goodness of fit criteria – Pearson correlation coefficient (R), coefficient of determination (R^2), adjusted coefficient of determination ($R^2_{adjusted}$), and residual sum of squares (RSS) (manual calculation in MS Excel):

Parameter	No.1	No.2	No.3	No.4
N	21	21	21	21
df	20	20	20	20
R	0.985668253	0.956126375	0.966949318	0.994269568
R^2	0.971541904	0.914177645	0.934990983	0.988571974
$R^2_{adjusted}$	0.971541904	0.914177645	0.934990983	0.988571974
RSS	4421.276155	5451.528779	4054.190951	3782.335396

Graphical abstract of model fit presented as mean ± 1 SD of the fraction % of released carvedilol:

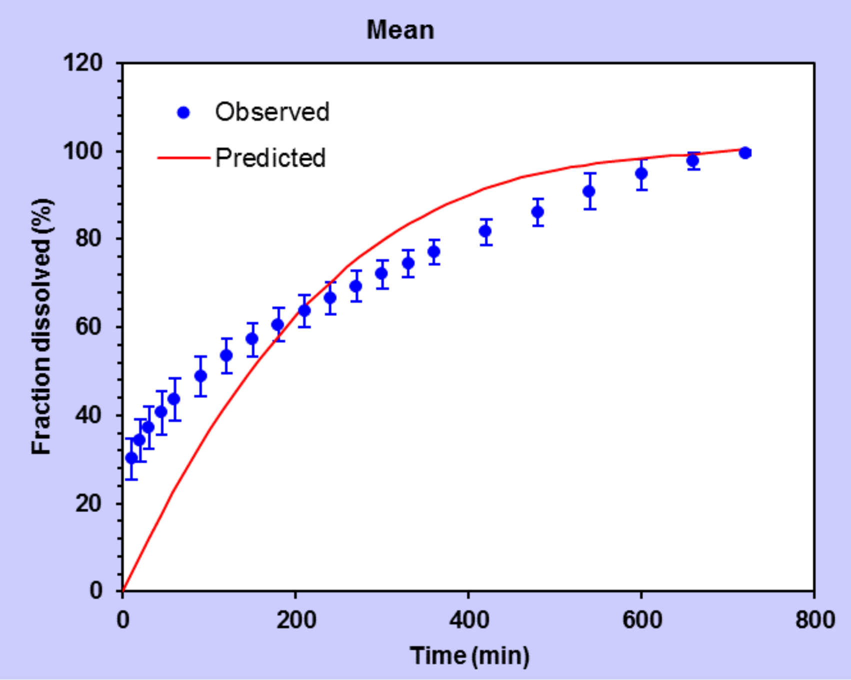

Supplementary materials – Model fitting summary – SuperTab® 11SD (modelling the entire relevant dissolution data)

Graphical abstract of model fit presented as the fraction % of released carvedilol per tested tablet:

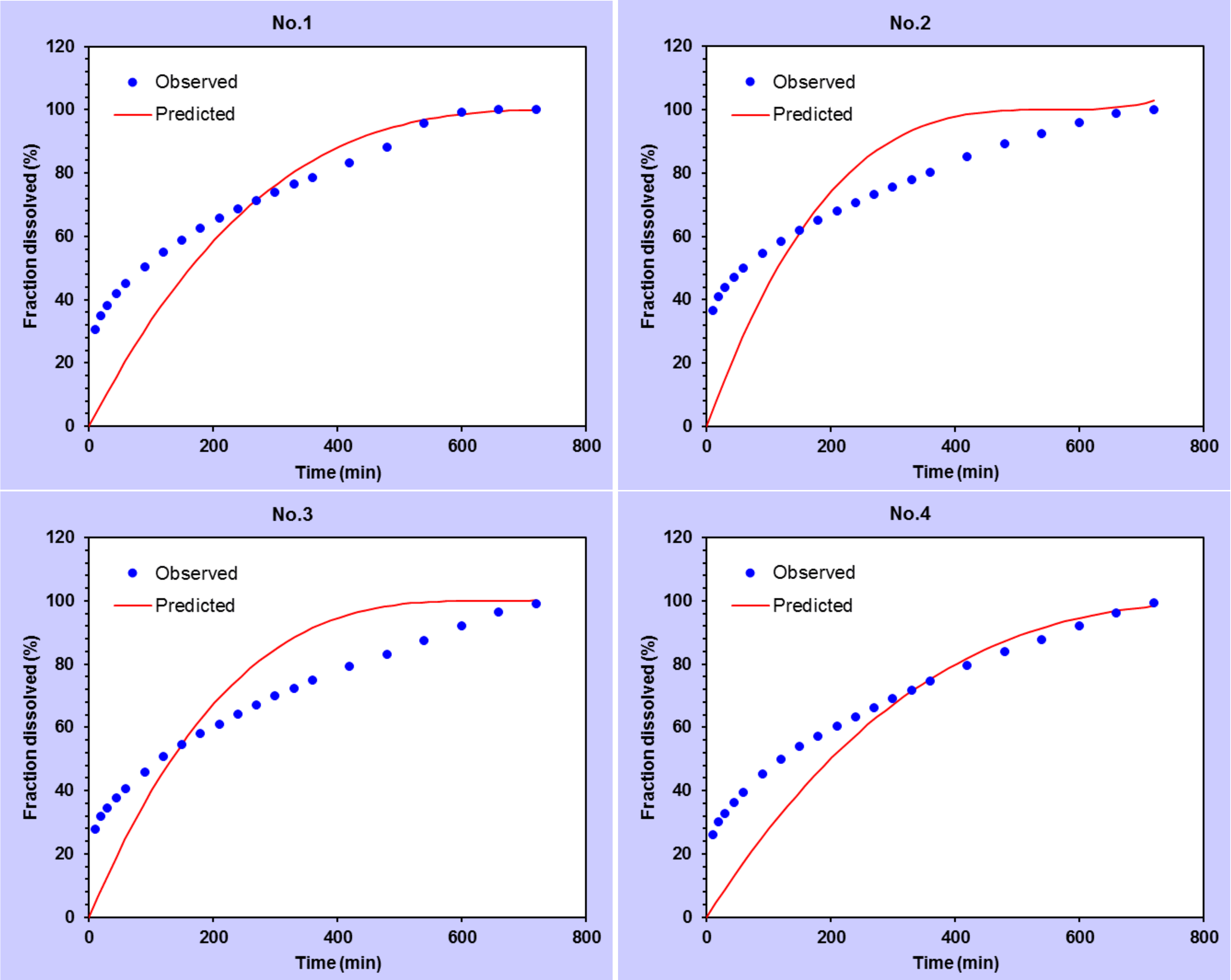

Supplementary materials – Model fitting summary – SuperTab® 11SD (modelling the entire relevant dissolution data)

Model: **Hixson–Crowell with T_{lag}**

Model equation: $F = 100 \cdot \left\{1 - \left[1 - k_{HC} \cdot \left(t - T_{lag}\right)\right]^3\right\}$

Fitted model parameters per tested tablet (N = 4) with statistics – mean, standard deviation (SD), and relative standard deviation expressed in % (RSD%) (output from DDSolver):

Parameter	No.1	No.2	No.3	No.4	Mean	SD	RSD(%)
k_{HC}	0.001	0.001	0.001	0.001	0.001	0.000	8.168
T_{lag}	-109.039	-171.909	-117.015	-129.105	-131.767	28.004	-21.252

Number of dissolution data points (N), degrees of freedom (df), and selected goodness of fit criteria – Pearson correlation coefficient (R), coefficient of determination (R^2), adjusted coefficient of determination ($R^2_{adjusted}$), and residual sum of squares (RSS) (manual calculation in MS Excel):

Parameter	No.1	No.2	No.3	No.4
N	21	21	21	21
df	19	19	19	19
R	0.994262881	0.997137123	0.996534977	0.997342694
R^2	0.988558678	0.994282442	0.993081961	0.99469245
$R^2_{adjusted}$	0.987956503	0.993981518	0.992717853	0.994413105
RSS	193.4201022	101.9432126	104.9005024	58.43652751

Graphical abstract of model fit presented as mean ± 1 SD of the fraction % of released carvedilol:

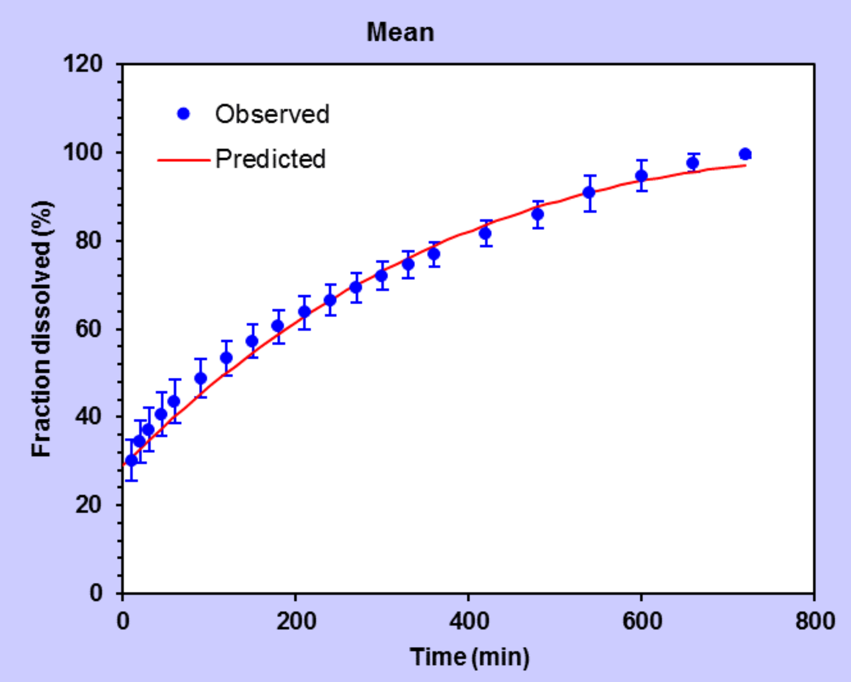

Supplementary materials – Model fitting summary – SuperTab® 11SD (modelling the entire relevant dissolution data)

Graphical abstract of model fit presented as the fraction % of released carvedilol per tested tablet:

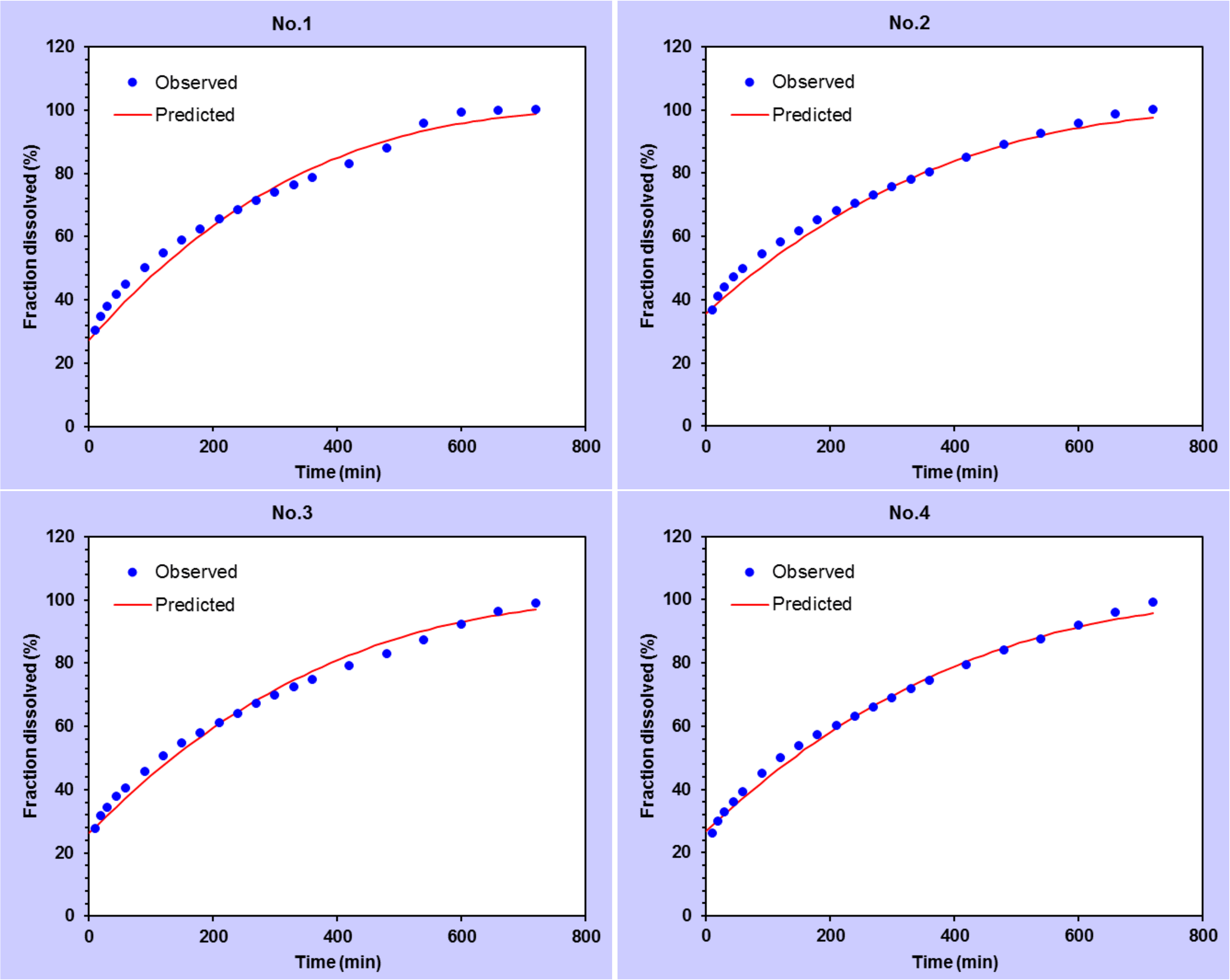

Supplementary materials – Model fitting summary – SuperTab® 11SD (modelling the entire relevant dissolution data)

Model: **Hopfenberg**

Model equation: $F = 100 \cdot [1 - (1 - k_{HB} \cdot t)^n]$

Fitted model parameters per tested tablet (N = 4) with statistics – mean, standard deviation (SD), and relative standard deviation expressed in % (RSD%) (output from DDSolver):

Parameter	No.1	No.2	No.3	No.4	Mean	SD	RSD(%)
k_{HB}	0.001	0.001	0.001	0.001	0.001	0.000	10.490
n	4.500	4.500	4.500	4.500	4.500	0.000	0.000

Number of dissolution data points (N), degrees of freedom (df), and selected goodness of fit criteria – Pearson correlation coefficient (R), coefficient of determination (R^2), adjusted coefficient of determination ($R^2_{adjusted}$), and residual sum of squares (RSS) (manual calculation in MS Excel):

Parameter	No.1	No.2	No.3	No.4
N	21	21	21	21
df	19	19	19	19
R	0.956011039	0.963381845	0.974200933	0.974674407
R^2	0.913957107	0.928104579	0.949067458	0.949990199
$R^2_{adjusted}$	0.909428534	0.92432061	0.946386797	0.947358104
RSS	3751.556612	4919.842571	3357.279471	3098.337949

Graphical abstract of model fit presented as mean ± 1 SD of the fraction % of released carvedilol:

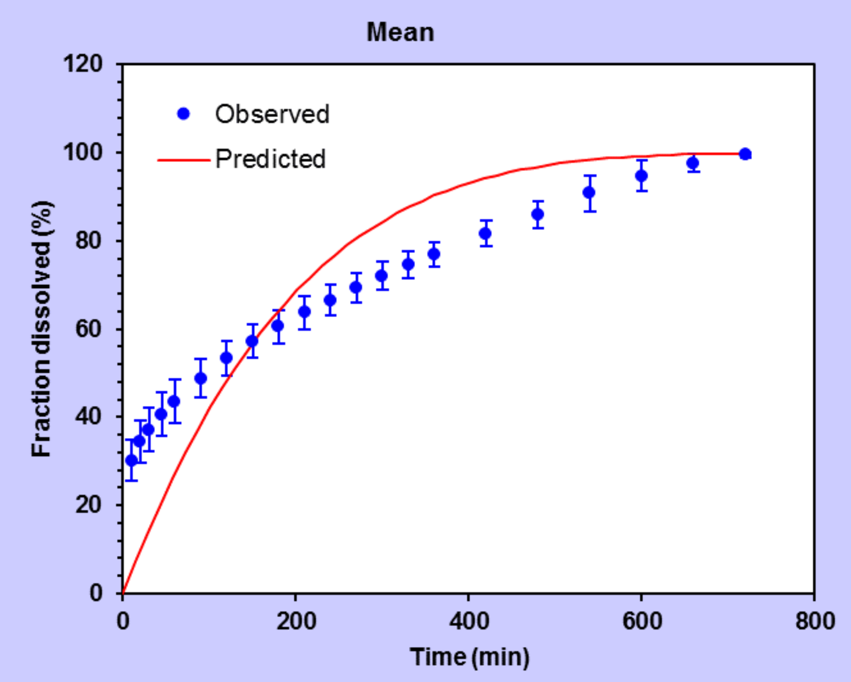

Supplementary materials – Model fitting summary – SuperTab® 11SD (modelling the entire relevant dissolution data)

Graphical abstract of model fit presented as the fraction % of released carvedilol per tested tablet:

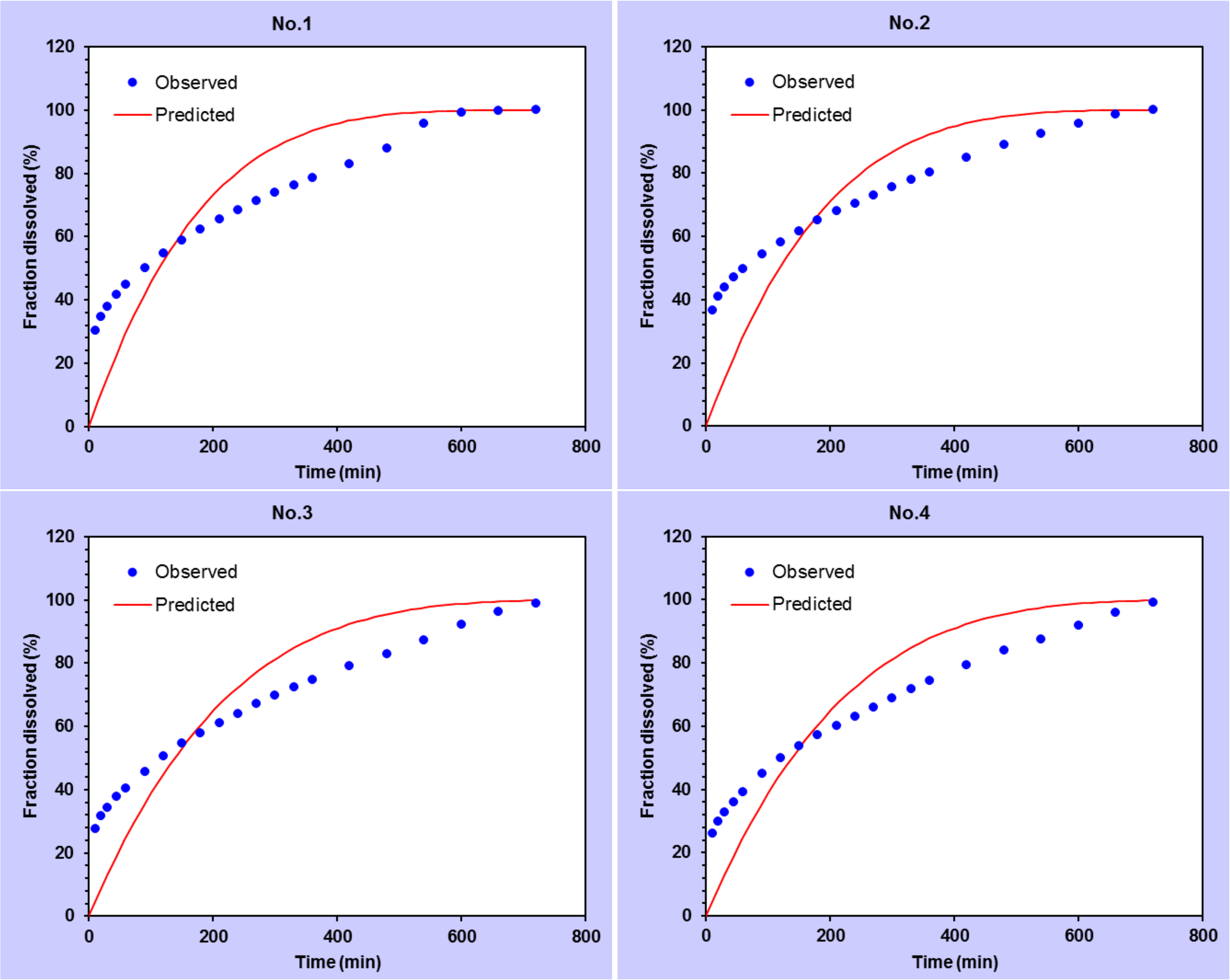

Supplementary materials – Model fitting summary – SuperTab® 11SD (modelling the entire relevant dissolution data)

Model: **Hopfenberg with T_{lag}**

Model equation: $F = 100 \cdot \{1 - [1 - k_{HB} \cdot (t - T_{lag})]^n\}$

Fitted model parameters per tested tablet (N = 4) with statistics – mean, standard deviation (SD), and relative standard deviation expressed in % (RSD%) (output from DDSolver):

Parameter	No.1	No.2	No.3	No.4	Mean	SD	RSD(%)
k_{HB}	0.001	0.001	0.001	0.001	0.001	0.000	5.674
n	2.527	2.646	2.000	2.000	2.293	0.342	14.915
T_{lag}	-138.647	-200.426	-173.958	-160.336	-168.342	25.864	-15.364

Number of dissolution data points (N), degrees of freedom (df), and selected goodness of fit criteria – Pearson correlation coefficient (R), coefficient of determination (R^2), adjusted coefficient of determination ($R^2_{adjusted}$), and residual sum of squares (RSS) (manual calculation in MS Excel):

Parameter	No.1	No.2	No.3	No.4
N	21	21	21	21
df	18	18	18	18
R	0.995504029	0.99710422	0.996585192	0.996785437
R^2	0.991028271	0.994216825	0.993182045	0.993581207
$R^2_{adjusted}$	0.990031412	0.99357425	0.992424495	0.992868008
RSS	114.9867022	47.45445222	65.81512119	65.40979198

Graphical abstract of model fit presented as mean ± 1 SD of the fraction % of released carvedilol:

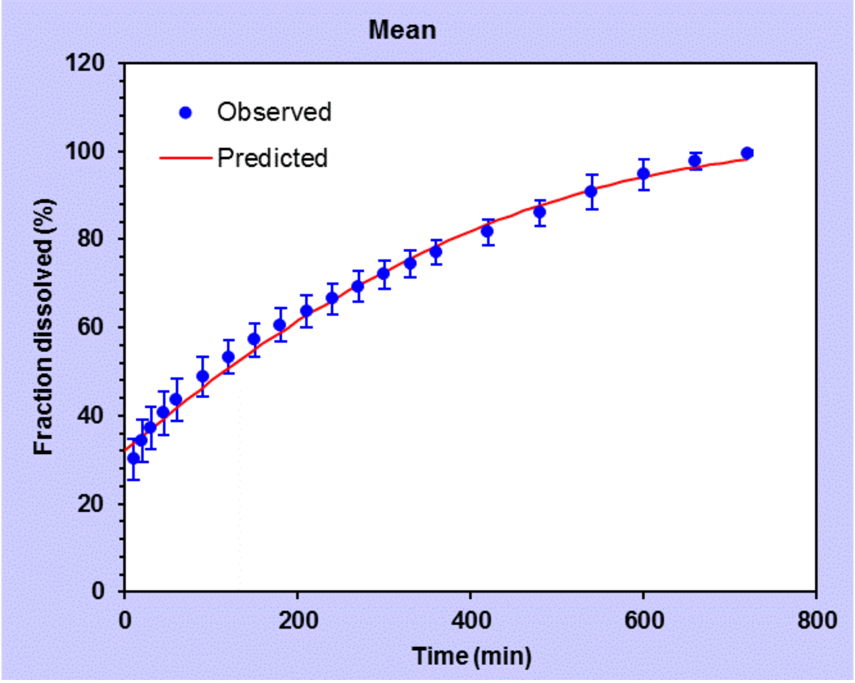

Supplementary materials – Model fitting summary – SuperTab® 11SD (modelling the entire relevant dissolution data)

Graphical abstract of model fit presented as the fraction % of released carvedilol per tested tablet:

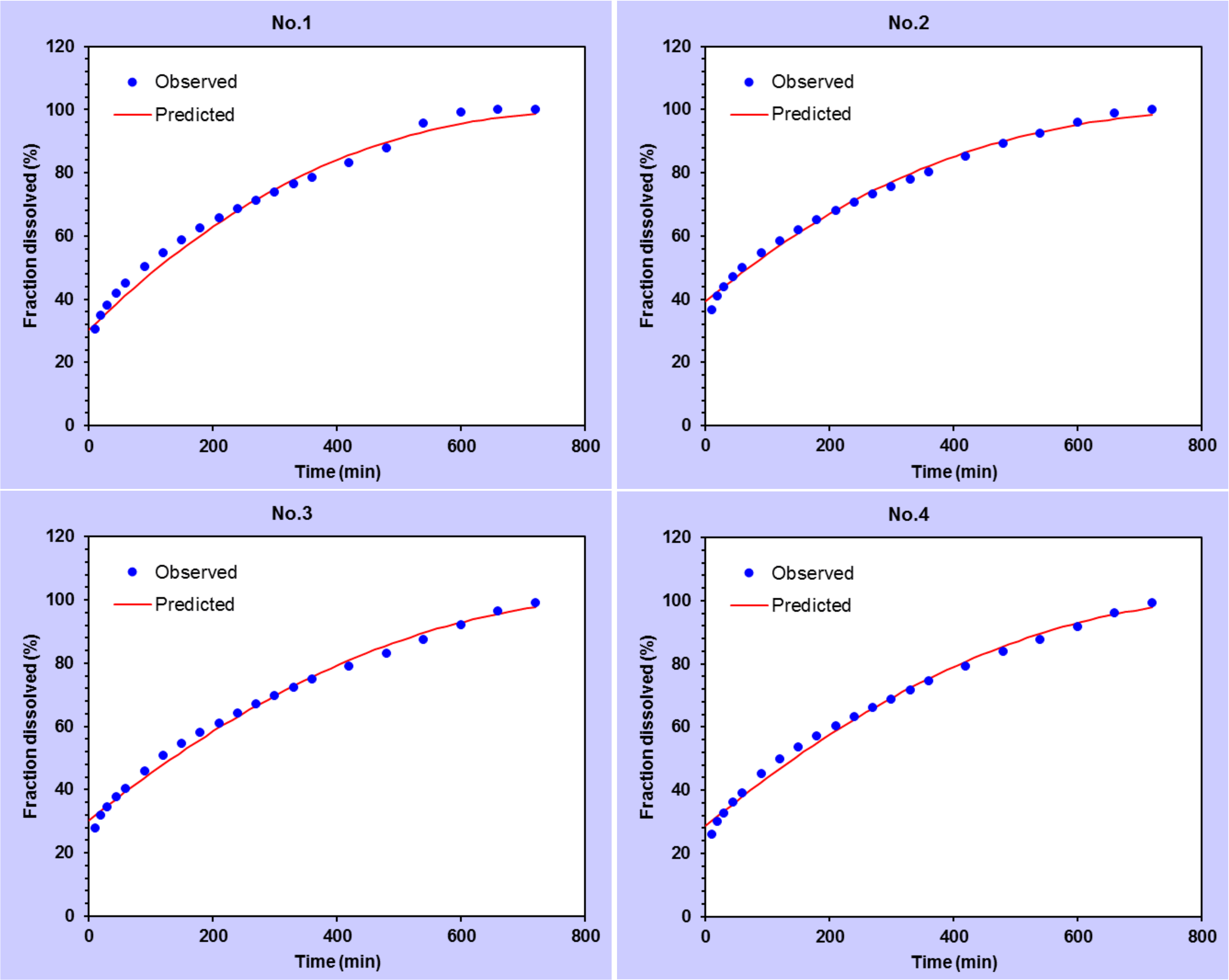

Supplementary materials – Model fitting summary – SuperTab® 11SD (modelling the entire relevant dissolution data)

Model: **Baker–Lonsdale**

Model equation: $\frac{3}{2}\cdot\left[1-\left(1-\frac{F}{100}\right)^{\frac{2}{3}}\right]-\frac{F}{100}=k_{BL}\cdot t$

Fitted model parameters per tested tablet (N = 4) with statistics – mean, standard deviation (SD), and relative standard deviation expressed in % (RSD%) (output from DDSolver):

Parameter	No.1	No.2	No.3	No.4	Mean	SD	RSD(%)
k_{BL}	0.0007	0.0006	0.0005	0.0004	0.0005	0.0001	20.5807

Number of dissolution data points (N), degrees of freedom (df), and selected goodness of fit criteria – Pearson correlation coefficient (R), coefficient of determination (R^2), adjusted coefficient of determination ($R^2_{adjusted}$), and residual sum of squares (RSS) (manual calculation in MS Excel):

Parameter	No.1	No.2	No.3	No.4
N	21	21	21	21
df	20	20	20	20
R	0.979824944	0.985265821	0.985859828	0.991280318
R^2	0.96005692	0.970748737	0.9719196	0.982636669
$R^2_{adjusted}$	0.96005692	0.970748737	0.9719196	0.982636669
RSS	733.2385357	1052.613613	513.6127818	522.0462342

Graphical abstract of model fit presented as mean ± 1 SD of the fraction % of released carvedilol:

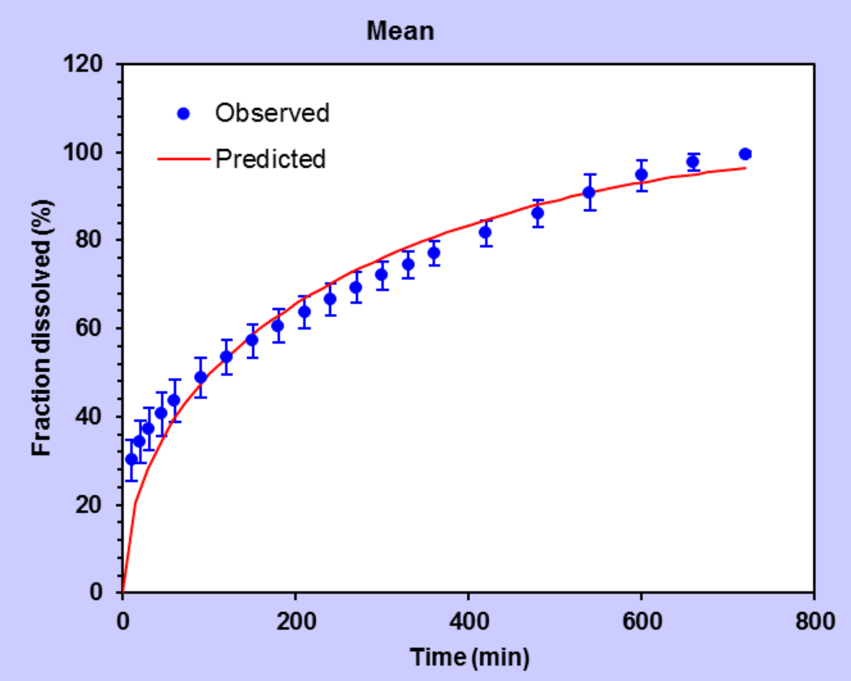

Supplementary materials – Model fitting summary – SuperTab® 11SD (modelling the entire relevant dissolution data)

Graphical abstract of model fit presented as the fraction % of released carvedilol per tested tablet:

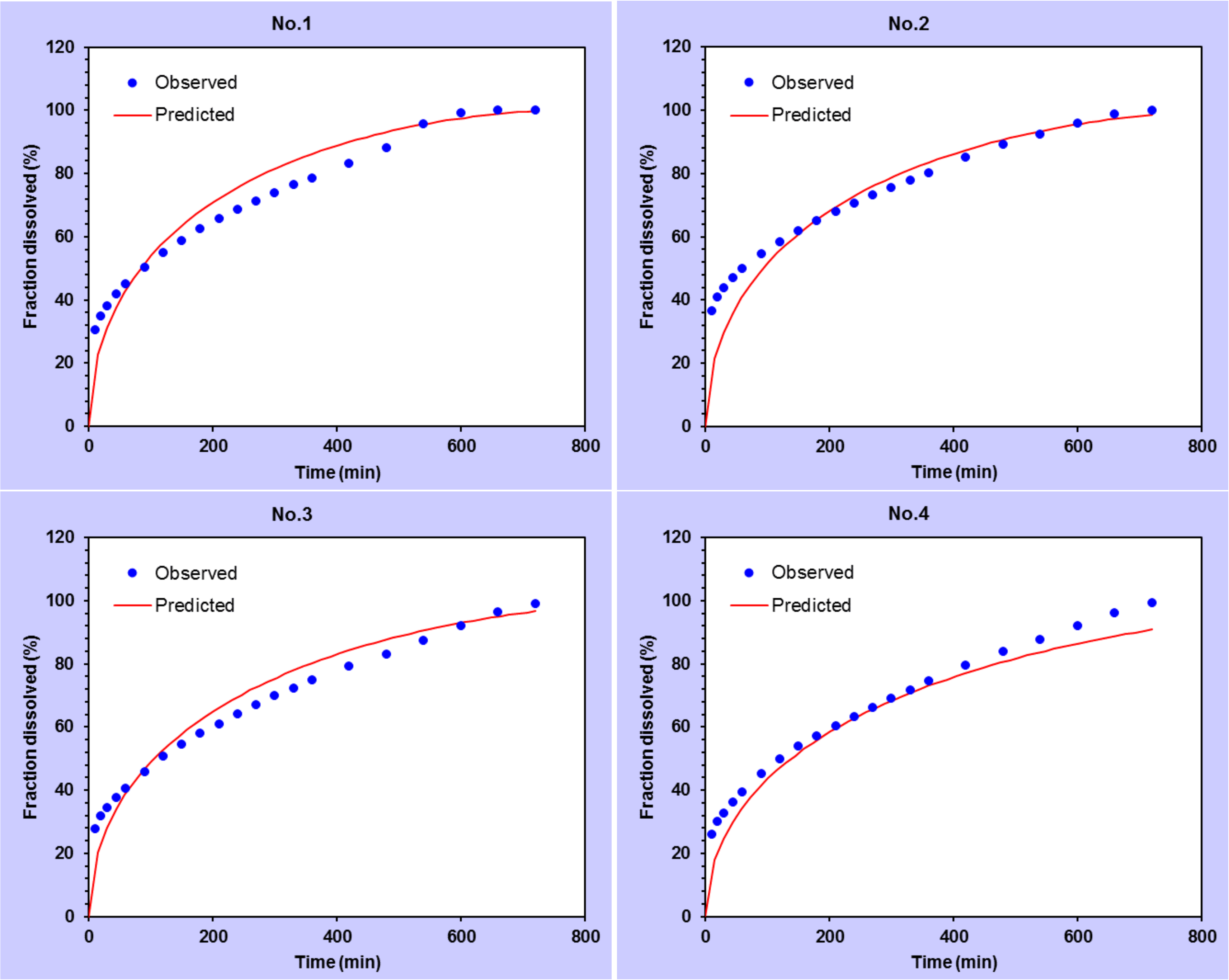

Supplementary materials – Model fitting summary – SuperTab® 11SD (modelling the entire relevant dissolution data)

Model: **Baker–Lonsdale with T_{lag}**

Model equation: $\frac{3}{2}\cdot\left[1-\left(1-\frac{F}{100}\right)^{\frac{2}{3}}\right]-\frac{F}{100}=k_{BL}\cdot\left(t-T_{lag}\right)$

Fitted model parameters per tested tablet (N = 4) with statistics – mean, standard deviation (SD), and relative standard deviation expressed in % (RSD%) (output from DDSolver):

Parameter	No.1	No.2	No.3	No.4	Mean	SD	RSD(%)
k_{BL}	0.0007	0.0006	0.0005	0.0005	0.0006	0.0001	10.9797
T_{lag}	24.1720	-8.4316	20.6270	26.5252	15.7232	16.2846	103.5710

Number of dissolution data points (N), degrees of freedom (df), and selected goodness of fit criteria – Pearson correlation coefficient (R), coefficient of determination (R^2), adjusted coefficient of determination ($R^2_{adjusted}$), and residual sum of squares (RSS) (manual calculation in MS Excel):

Parameter	No.1	No.2	No.3	No.4
N	21	21	21	21
df	19	19	19	19
R	0.964117508	0.987872027	0.969434889	0.971555031
R^2	0.929522568	0.975891141	0.939804004	0.943919177
$R^2_{adjusted}$	0.92581323	0.974622254	0.936635793	0.940967555
RSS	3250.584037	584.7799942	2426.320355	2490.36837

Graphical abstract of model fit presented as mean ± 1 SD of the fraction % of released carvedilol:

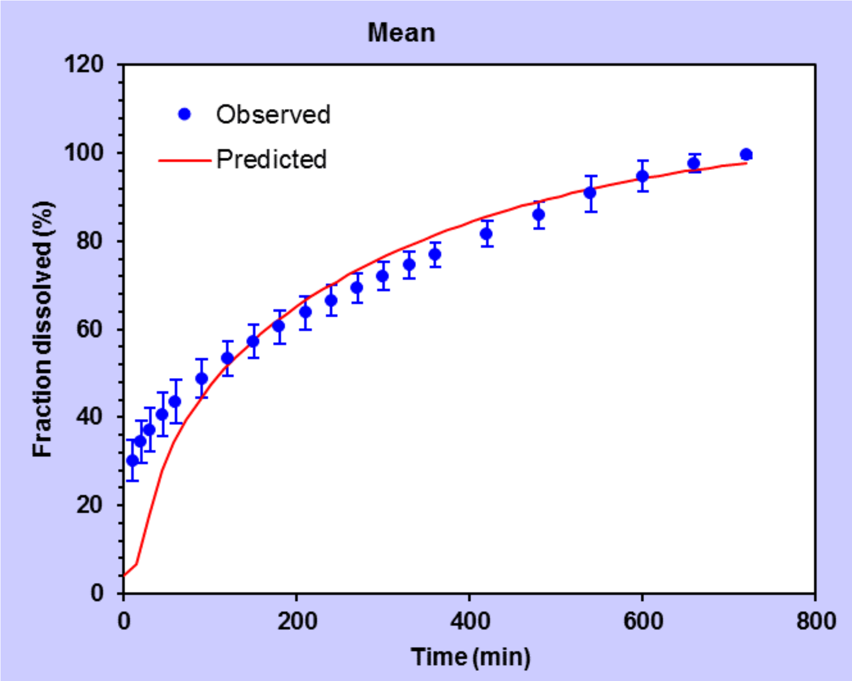

Supplementary materials – Model fitting summary – SuperTab® 11SD (modelling the entire relevant dissolution data)

Graphical abstract of model fit presented as the fraction % of released carvedilol per tested tablet:

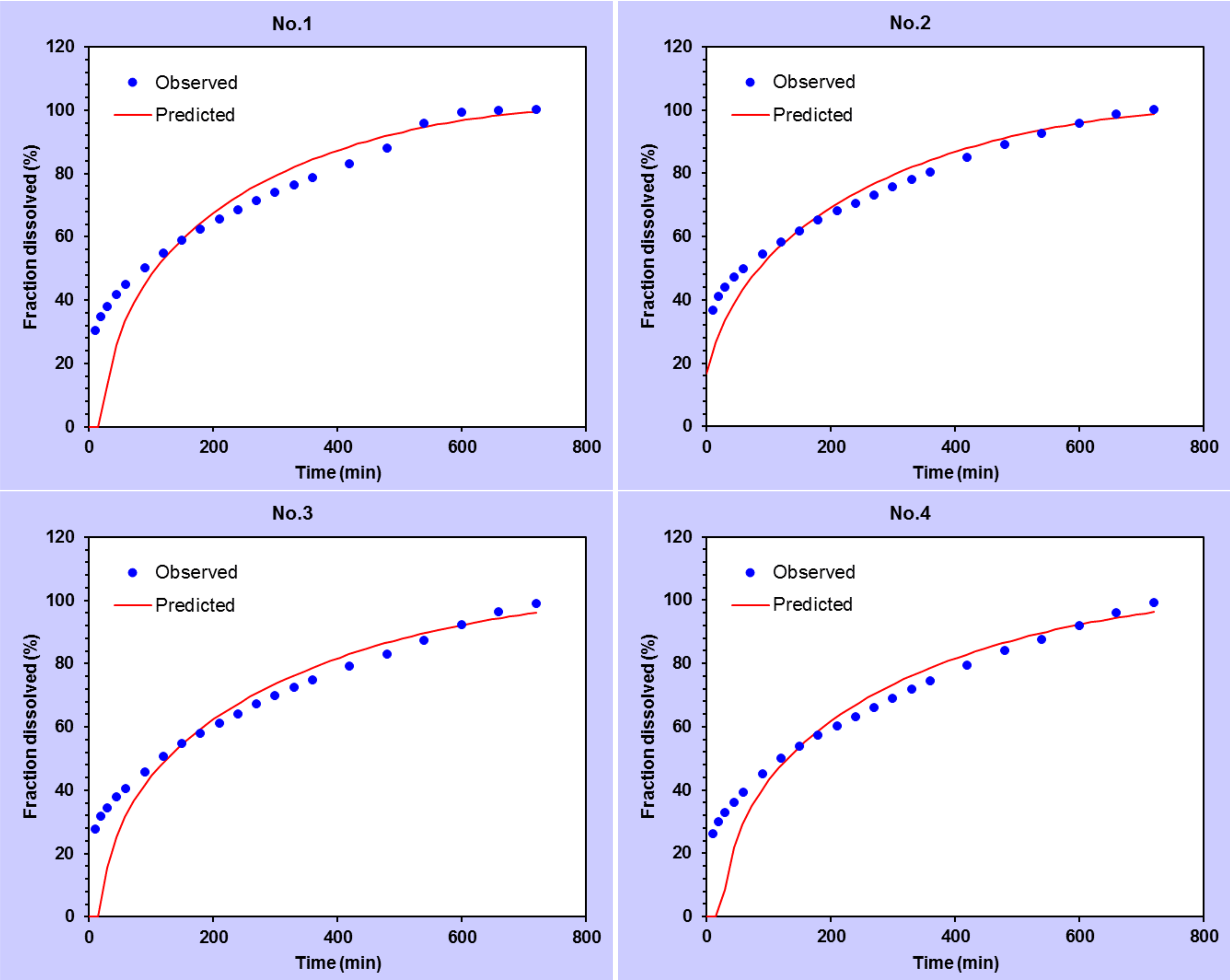

Supplementary materials – Model fitting summary – SuperTab® 11SD (modelling the entire relevant dissolution data)

Model: **Makoid–Banakar**

Model equation: $F = k_{MB} \cdot t^{n} \cdot e^{-k \cdot t}$

Fitted model parameters per tested tablet (N = 4) with statistics – mean, standard deviation (SD), and relative standard deviation expressed in % (RSD%) (output from DDSolver):

Parameter	No.1	No.2	No.3	No.4	Mean	SD	RSD(%)
k_{MB}	16.9801	23.2149	15.0319	13.5096	17.1841	4.2640	24.8138
n	0.2371	0.1854	0.2442	0.2625	0.2323	0.0331	14.2319
k	-0.0004	-0.0004	-0.0004	-0.0004	-0.0004	0.0000	-5.6238

Number of dissolution data points (N), degrees of freedom (df), and selected goodness of fit criteria – Pearson correlation coefficient (R), coefficient of determination (R^2), adjusted coefficient of determination ($R^2_{adjusted}$), and residual sum of squares (RSS) (manual calculation in MS Excel):

Parameter	No.1	No.2	No.3	No.4
N	21	21	21	21
df	18	18	18	18
R	0.99728162	0.998445054	0.998981394	0.999065108
R^2	0.994570629	0.996892526	0.997963825	0.99813109
$R^2_{adjusted}$	0.993967365	0.996547251	0.997737583	0.997923433
RSS	53.72097331	24.08798799	19.51800577	18.91281027

Graphical abstract of model fit presented as mean ± 1 SD of the fraction % of released carvedilol:

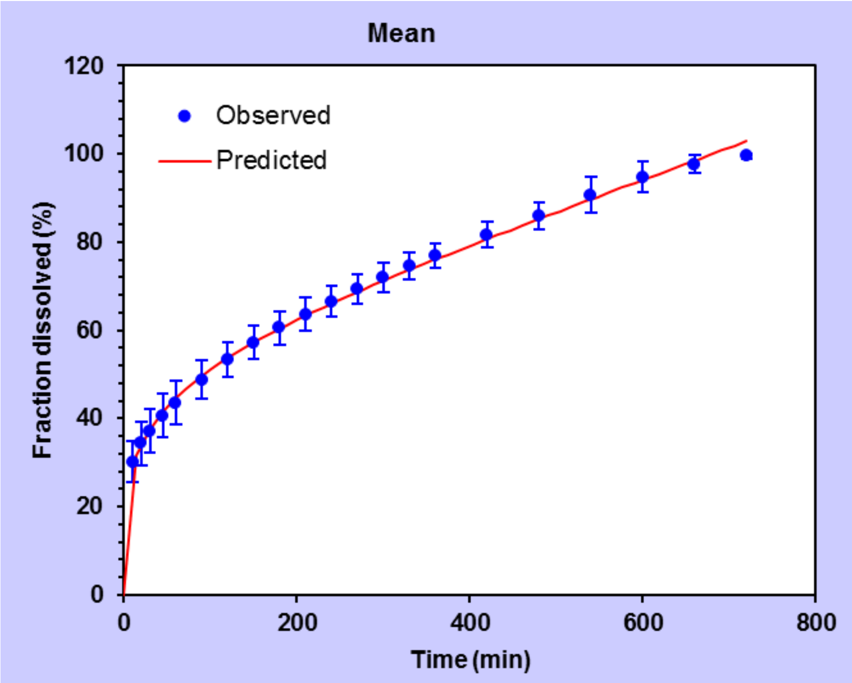

Supplementary materials – Model fitting summary – SuperTab® 11SD (modelling the entire relevant dissolution data)

Graphical abstract of model fit presented as the fraction % of released carvedilol per tested tablet:

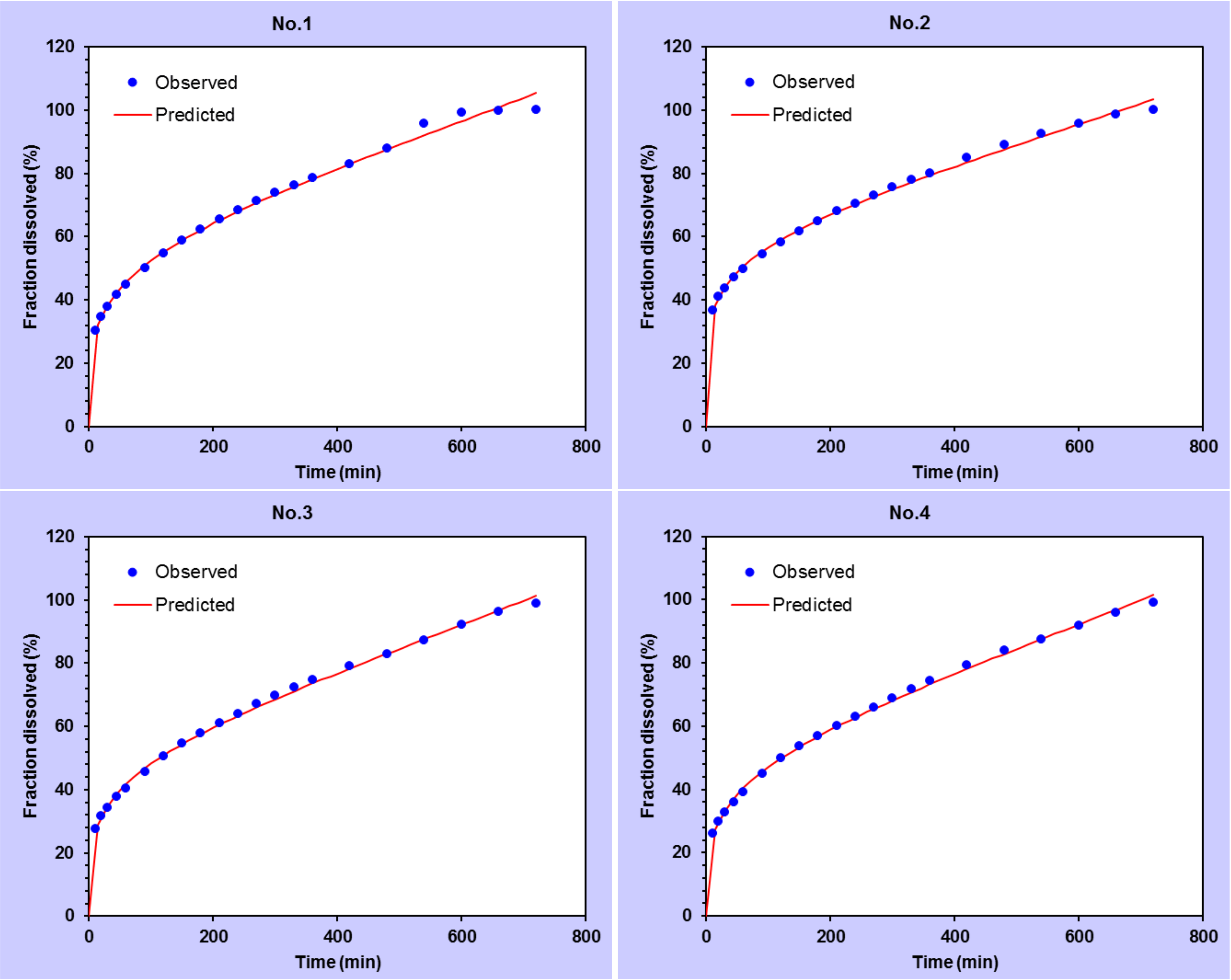

Supplementary materials – Model fitting summary – SuperTab® 11SD (modelling the entire relevant dissolution data)

Model: **Makoid–Banakar with T_{lag}**

Model equation: $F = k_{MB} \cdot (t - T_{lag})^n \cdot e^{-k \cdot (t - T_{lag})}$

Fitted model parameters per tested tablet (N = 4) with statistics – mean, standard deviation (SD), and relative standard deviation expressed in % (RSD%) (output from DDSolver):

Parameter	No.1	No.2	No.3	No.4	Mean	SD	RSD(%)
k_{MB}	19.8966	26.2933	17.7232	16.1231	20.0091	4.4658	22.3189
n	0.2033	0.1589	0.2090	0.2247	0.1990	0.0282	14.1815
k	-0.0005	-0.0005	-0.0005	-0.0005	-0.0005	0.0000	-6.5994
T_{lag}	4.0000	4.0000	4.0000	4.0000	4.0000	0.0000	0.0000

Number of dissolution data points (N), degrees of freedom (df), and selected goodness of fit criteria – Pearson correlation coefficient (R), coefficient of determination (R^2), adjusted coefficient of determination ($R^2_{adjusted}$), and residual sum of squares (RSS) (manual calculation in MS Excel):

Parameter	No.1	No.2	No.3	No.4
N	21	21	21	21
df	17	17	17	17
R	0.996139387	0.99736819	0.997850328	0.997899854
R^2	0.992293678	0.994743307	0.995705278	0.995804118
$R^2_{adjusted}$	0.990933738	0.993815656	0.994947386	0.995063669
RSS	76.42989311	40.84624664	41.37529501	42.69173525

Graphical abstract of model fit presented as mean ± 1 SD of the fraction % of released carvedilol:

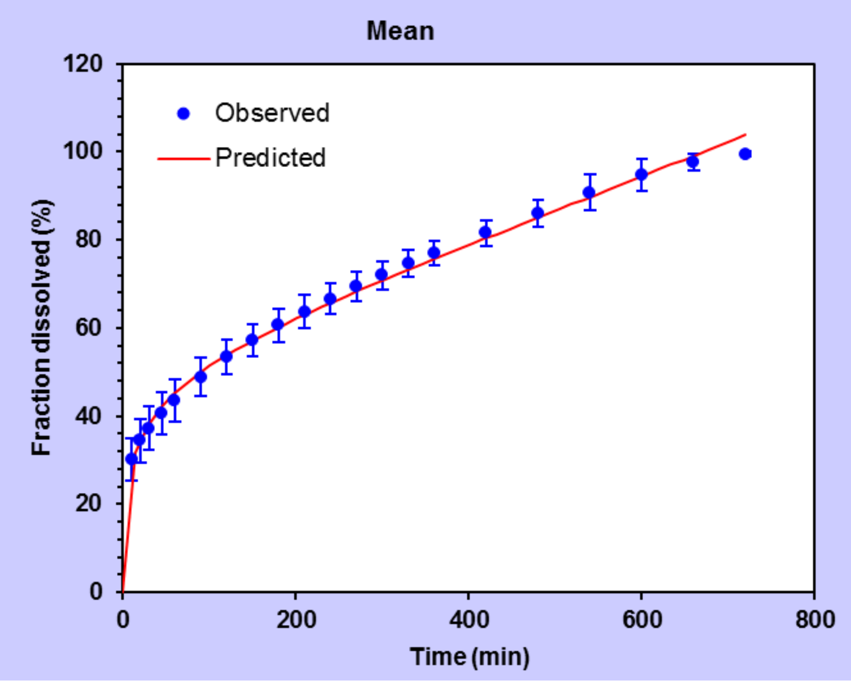

Supplementary materials – Model fitting summary – SuperTab® 11SD (modelling the entire relevant dissolution data)

Graphical abstract of model fit presented as the fraction % of released carvedilol per tested tablet:

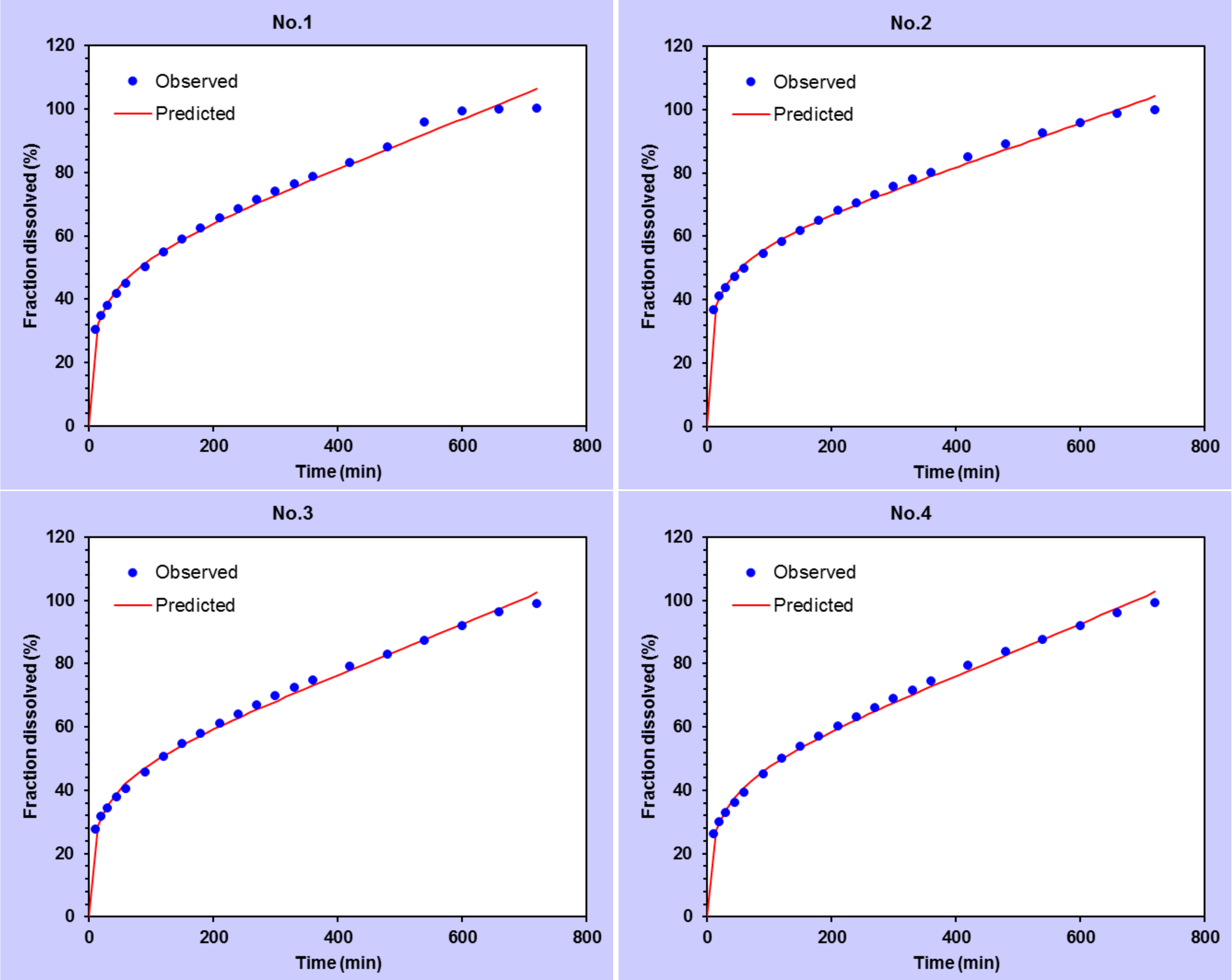

Supplementary materials – Model fitting summary – SuperTab® 11SD (modelling the entire relevant dissolution data)

Model: **Peppas–Sahlin_1**

Model equation: $F = k_1 \cdot t^m + k_2 \cdot t^{2m}$

Fitted model parameters per tested tablet (N = 4) with statistics – mean, standard deviation (SD), and relative standard deviation expressed in % (RSD%) (output from DDSolver):

Parameter	No.1	No.2	No.3	No.4	Mean	SD	RSD(%)
k_1	7.512	8.523	6.744	7.315	7.524	0.742	9.860
k_2	-0.123	-0.185	-0.093	-0.086	-0.122	0.045	-36.834
m	0.450	0.450	0.450	0.419	0.442	0.015	3.492

Number of dissolution data points (N), degrees of freedom (df), and selected goodness of fit criteria – Pearson correlation coefficient (R), coefficient of determination (R^2), adjusted coefficient of determination ($R^2_{adjusted}$), and residual sum of squares (RSS) (manual calculation in MS Excel):

Parameter	No.1	No.2	No.3	No.4
N	21	21	21	21
df	18	18	18	18
R	0.988467699	0.977400735	0.991374809	0.994061896
R^2	0.977068392	0.955312197	0.982824012	0.988159053
$R^2_{adjusted}$	0.974520436	0.950346886	0.980915569	0.986843393
RSS	330.773851	588.853022	241.9387824	179.0158452

Graphical abstract of model fit presented as mean ± 1 SD of the fraction % of released carvedilol:

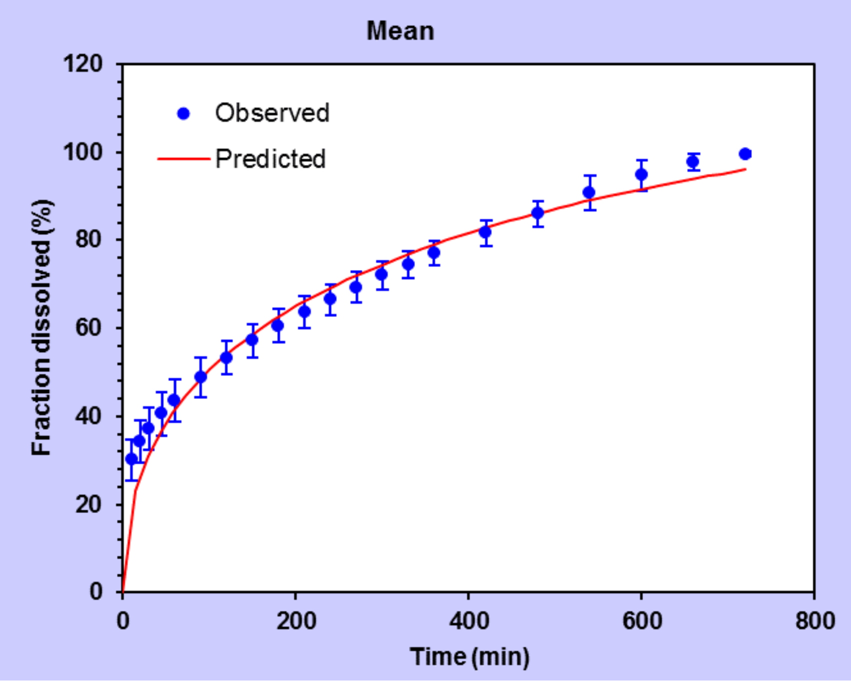

Supplementary materials – Model fitting summary – SuperTab® 11SD (modelling the entire relevant dissolution data)

Graphical abstract of model fit presented as the fraction % of released carvedilol per tested tablet:

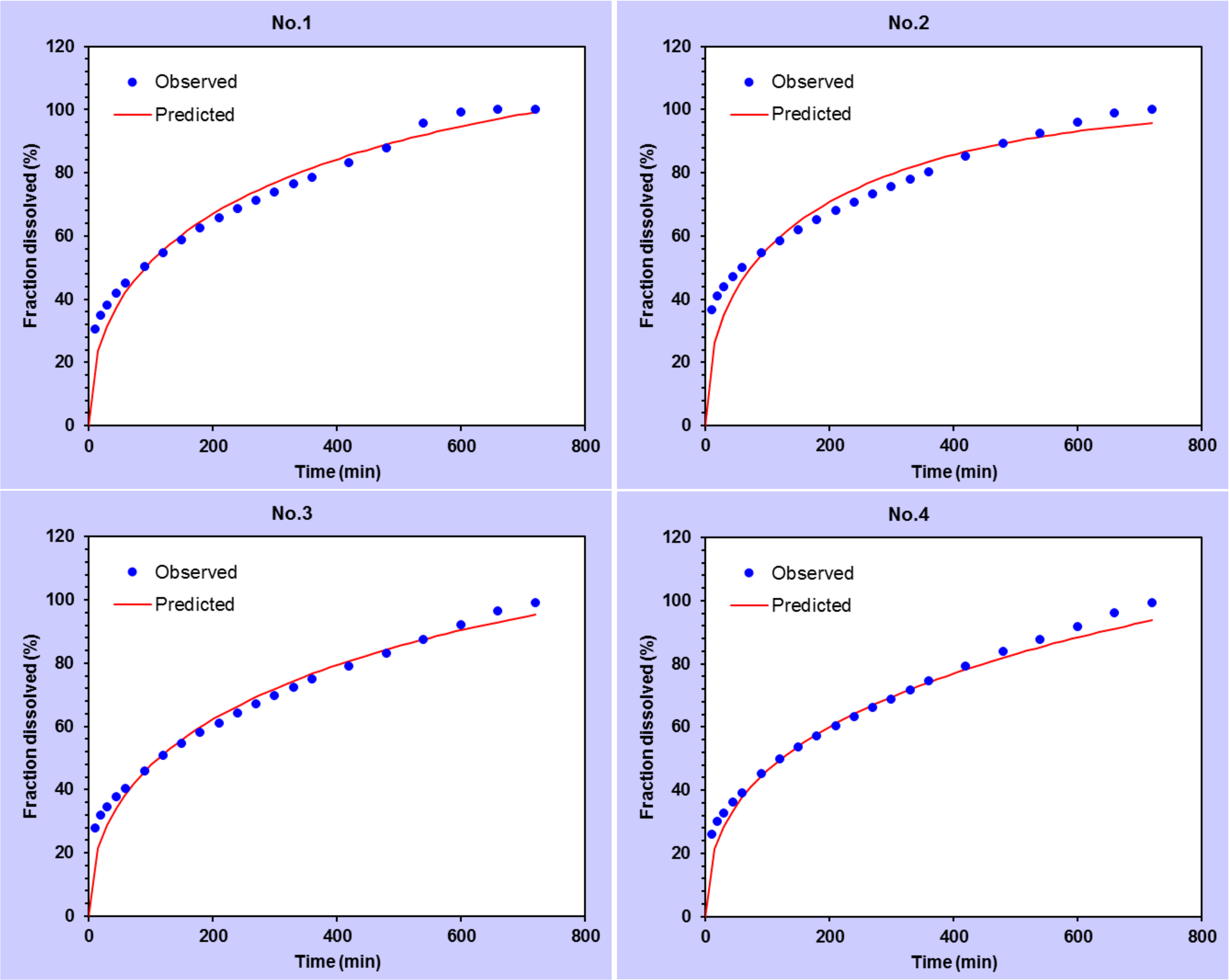

Supplementary materials – Model fitting summary – SuperTab® 11SD (modelling the entire relevant dissolution data)

Model: **Peppas–Sahlin_1 with T_{lag}**

Model equation: $F = k_1 \cdot (t - T_{lag})^m + k_2 \cdot (t - T_{lag})^{2m}$

Fitted model parameters per tested tablet (N = 4) with statistics – mean, standard deviation (SD), and relative standard deviation expressed in % (RSD%) (output from DDSolver):

Parameter	No.1	No.2	No.3	No.4	Mean	SD	RSD(%)
k_1	7.715	8.722	6.933	6.673	7.511	0.921	12.262
k_2	-0.134	-0.196	-0.104	-0.089	-0.131	0.048	-36.422
m	0.450	0.450	0.450	0.450	0.450	0.000	0.000
T_{lag}	4.000	4.000	4.000	4.000	4.000	0.000	0.000

Number of dissolution data points (N), degrees of freedom (df), and selected goodness of fit criteria – Pearson correlation coefficient (R), coefficient of determination (R^2), adjusted coefficient of determination ($R^2_{adjusted}$), and residual sum of squares (RSS) (manual calculation in MS Excel):

Parameter	No.1	No.2	No.3	No.4
N	21	21	21	21
df	17	17	17	17
R	0.984213685	0.970884793	0.987549065	0.990334027
R^2	0.968676578	0.942617281	0.975253156	0.980761486
$R^2_{adjusted}$	0.963148916	0.932490918	0.970886066	0.977366454
RSS	494.6910164	834.3002637	376.5126278	301.7007899

Graphical abstract of model fit presented as mean ± 1 SD of the fraction % of released carvedilol:

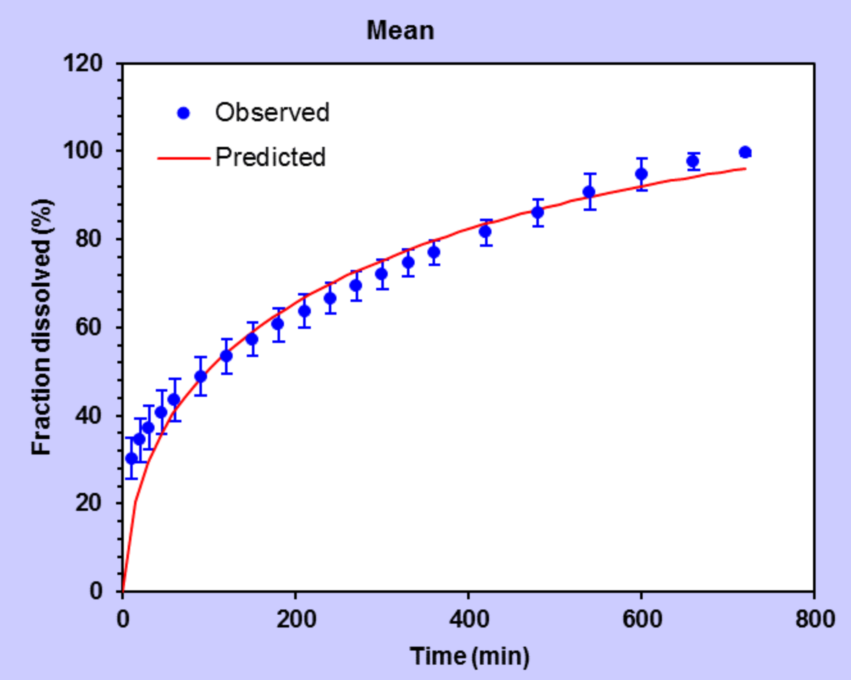

Supplementary materials – Model fitting summary – SuperTab® 11SD (modelling the entire relevant dissolution data)

Graphical abstract of model fit presented as the fraction % of released carvedilol per tested tablet:

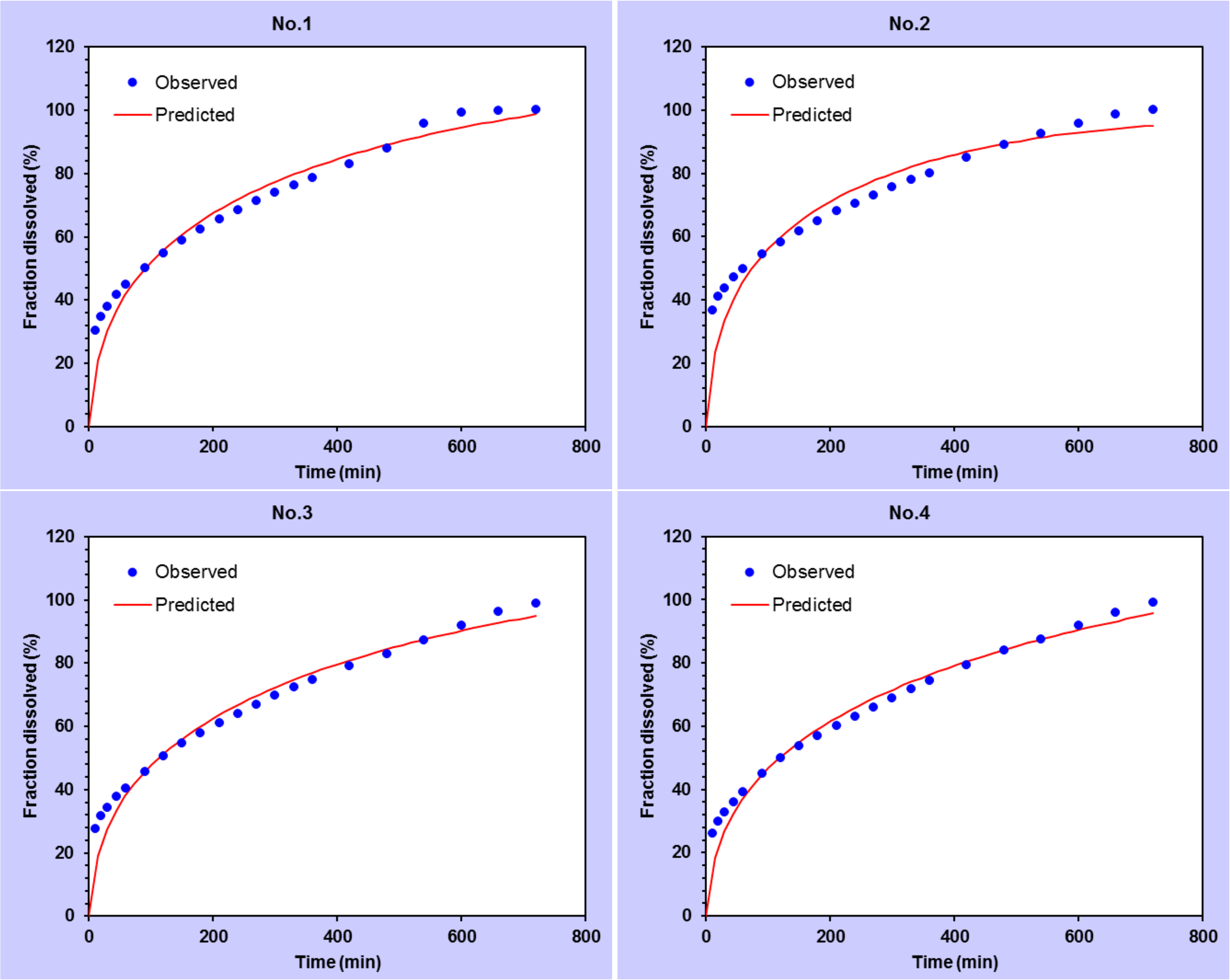

Supplementary materials – Model fitting summary – SuperTab® 11SD (modelling the entire relevant dissolution data)

Model: **Peppas–Sahlin_2**

Model equation: $F = k_1 \cdot t^{0.5} + k_2 \cdot t$

Fitted model parameters per tested tablet (N = 4) with statistics – mean, standard deviation (SD), and relative standard deviation expressed in % (RSD%) (output from DDSolver):

Parameter	No.1	No.2	No.3	No.4	Mean	SD	RSD(%)
k_1	5.983	6.685	5.402	5.220	5.823	0.660	11.344
k_2	-0.086	-0.118	-0.070	-0.062	-0.084	0.025	-29.333

Number of dissolution data points (N), degrees of freedom (df), and selected goodness of fit criteria – Pearson correlation coefficient (R), coefficient of determination (R^2), adjusted coefficient of determination ($R^2_{adjusted}$), and residual sum of squares (RSS) (manual calculation in MS Excel):

Parameter	No.1	No.2	No.3	No.4
N	21	21	21	21
df	19	19	19	19
R	0.984895806	0.971674589	0.988287391	0.991040261
R^2	0.970019748	0.944151507	0.976711967	0.9821608
$R^2_{adjusted}$	0.96844184	0.941212112	0.975486281	0.981221894
RSS	500.5099773	870.9715228	374.9938056	295.7349155

Graphical abstract of model fit presented as mean ± 1 SD of the fraction % of released carvedilol:

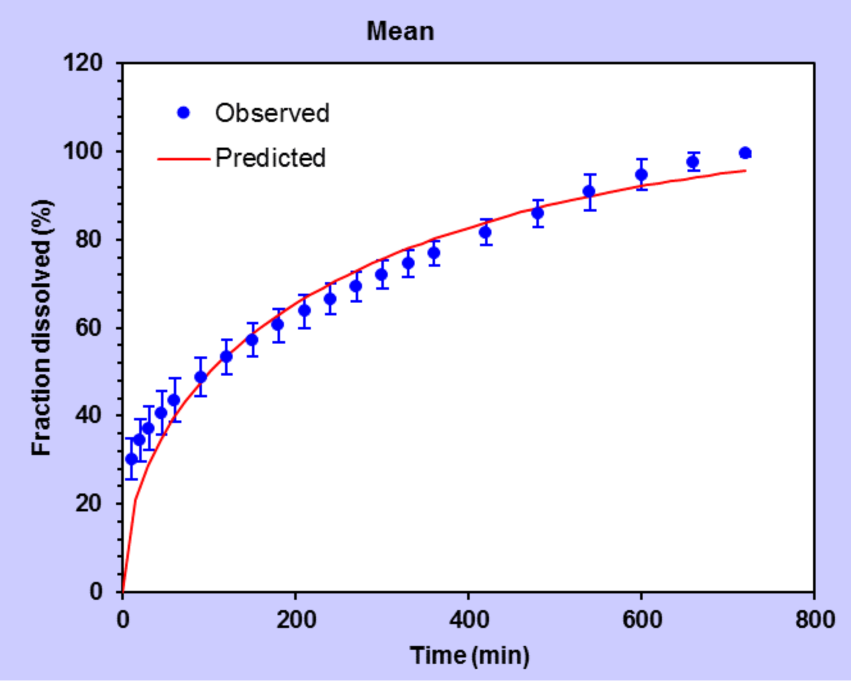

Supplementary materials – Model fitting summary – SuperTab® 11SD (modelling the entire relevant dissolution data)

Graphical abstract of model fit presented as the fraction % of released carvedilol per tested tablet:

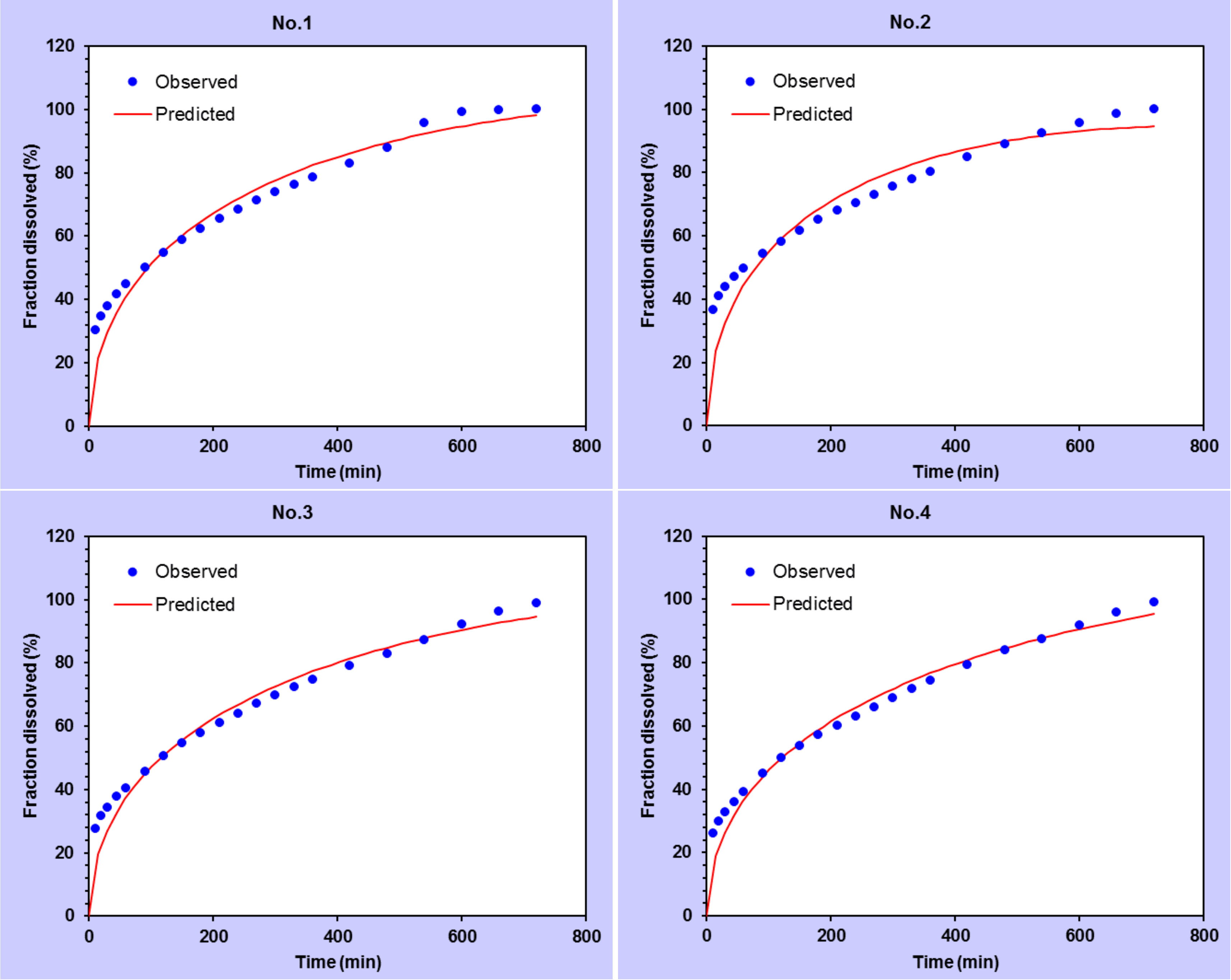

Supplementary materials – Model fitting summary – SuperTab® 11SD (modelling the entire relevant dissolution data)

Model: **Peppas–Sahlin_2 with T_{lag}**

Model equation: $F = k_1 \cdot (t - T_{lag})^{0.5} + k_2 \cdot (t - T_{lag})$

Fitted model parameters per tested tablet (N = 4) with statistics – mean, standard deviation (SD), and relative standard deviation expressed in % (RSD%) (output from DDSolver):

Parameter	No.1	No.2	No.3	No.4	Mean	SD	RSD(%)
k_1	6.117	6.815	5.528	5.347	5.952	0.662	11.129
k_2	-0.092	-0.123	-0.075	-0.067	-0.089	0.025	-27.748
T_{lag}	4.000	4.000	4.000	4.000	4.000	0.000	0.000

Number of dissolution data points (N), degrees of freedom (df), and selected goodness of fit criteria – Pearson correlation coefficient (R), coefficient of determination (R^2), adjusted coefficient of determination ($R^2_{adjusted}$), and residual sum of squares (RSS) (manual calculation in MS Excel):

Parameter	No.1	No.2	No.3	No.4
N	21	21	21	21
df	18	18	18	18
R	0.980619897	0.965640038	0.984386751	0.987609077
R^2	0.961615382	0.932460682	0.969017275	0.975371689
$R^2_{adjusted}$	0.957350424	0.924956313	0.96557475	0.97263521
RSS	708.2756666	1174.071109	544.8633948	444.2683606

Graphical abstract of model fit presented as mean ± 1 SD of the fraction % of released carvedilol:

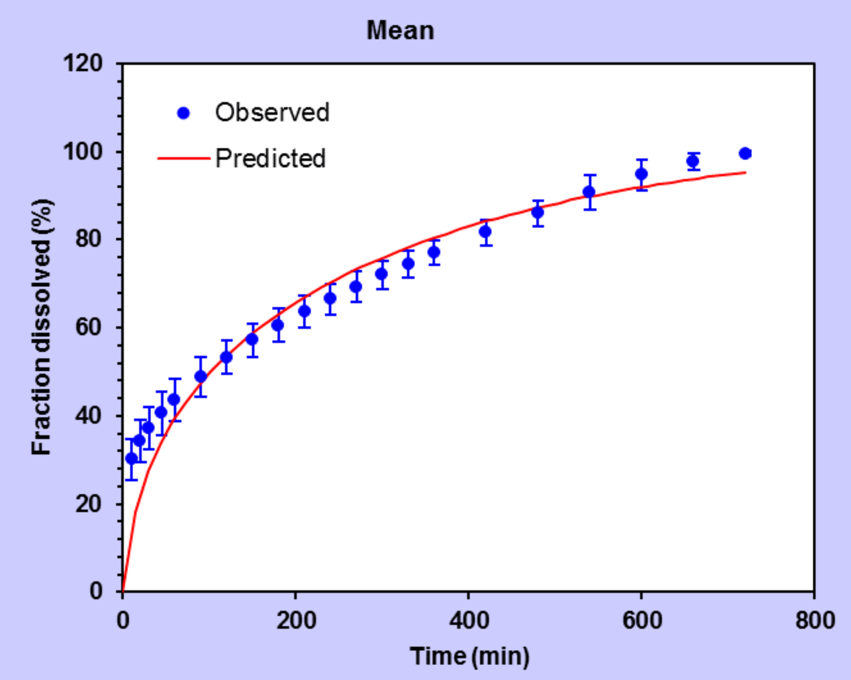

Supplementary materials – Model fitting summary – SuperTab® 11SD (modelling the entire relevant dissolution data)

Graphical abstract of model fit presented as the fraction % of released carvedilol per tested tablet:

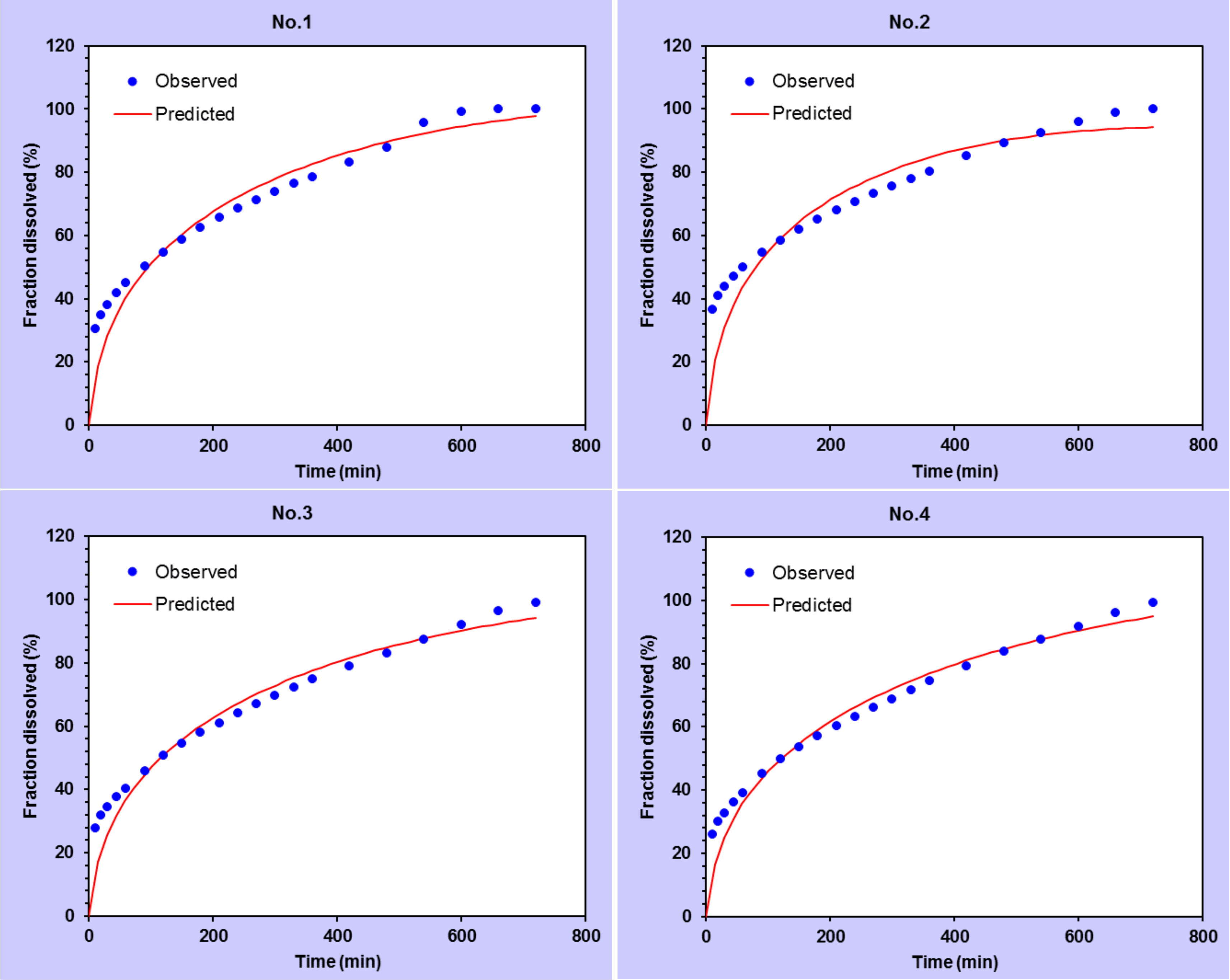

Supplementary materials – Model fitting summary – SuperTab® 11SD (modelling the entire relevant dissolution data)

Model: **Quadratic**

Model equation: $F = 100 \cdot (k_1 \cdot t^2 + k_2 \cdot t)$

Fitted model parameters per tested tablet (N = 4) with statistics – mean, standard deviation (SD), and relative standard deviation expressed in % (RSD%) (output from DDSolver):

Parameter	No.1	No.2	No.3	No.4	Mean	SD	RSD(%)
k1	-0.000003	-0.000004	-0.000003	-0.000003	-0.000003	0.000000	-10.322784
k2	0.003716	0.003898	0.003431	0.003383	0.003607	0.000243	6.744163

Number of dissolution data points (N), degrees of freedom (df), and selected goodness of fit criteria – Pearson correlation coefficient (R), coefficient of determination (R^2), adjusted coefficient of determination ($R^2_{adjusted}$), and residual sum of squares (RSS) (manual calculation in MS Excel):

Parameter	No.1	No.2	No.3	No.4
N	21	21	21	21
df	19	19	19	19
R	0.965421203	0.953932866	0.967615255	0.971082512
R^2	0.9320381	0.909987913	0.936279282	0.943001245
$R^2_{adjusted}$	0.928461158	0.905250435	0.93292556	0.940001311
RSS	4907.48562	6808.999981	4017.962458	3594.628101

Graphical abstract of model fit presented as mean ± 1 SD of the fraction % of released carvedilol:

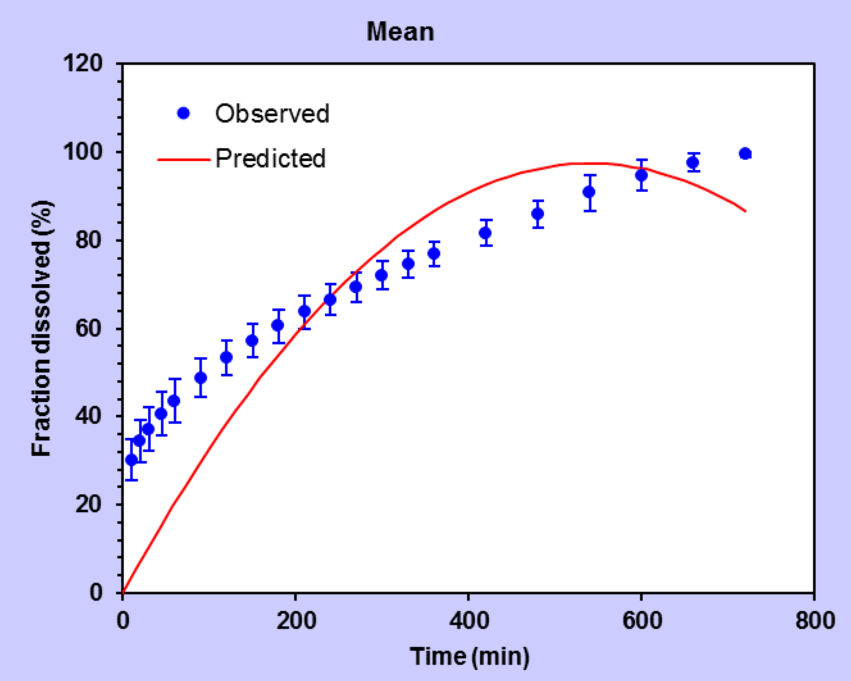

Supplementary materials – Model fitting summary – SuperTab® 11SD (modelling the entire relevant dissolution data)

Graphical abstract of model fit presented as the fraction % of released carvedilol per tested tablet:

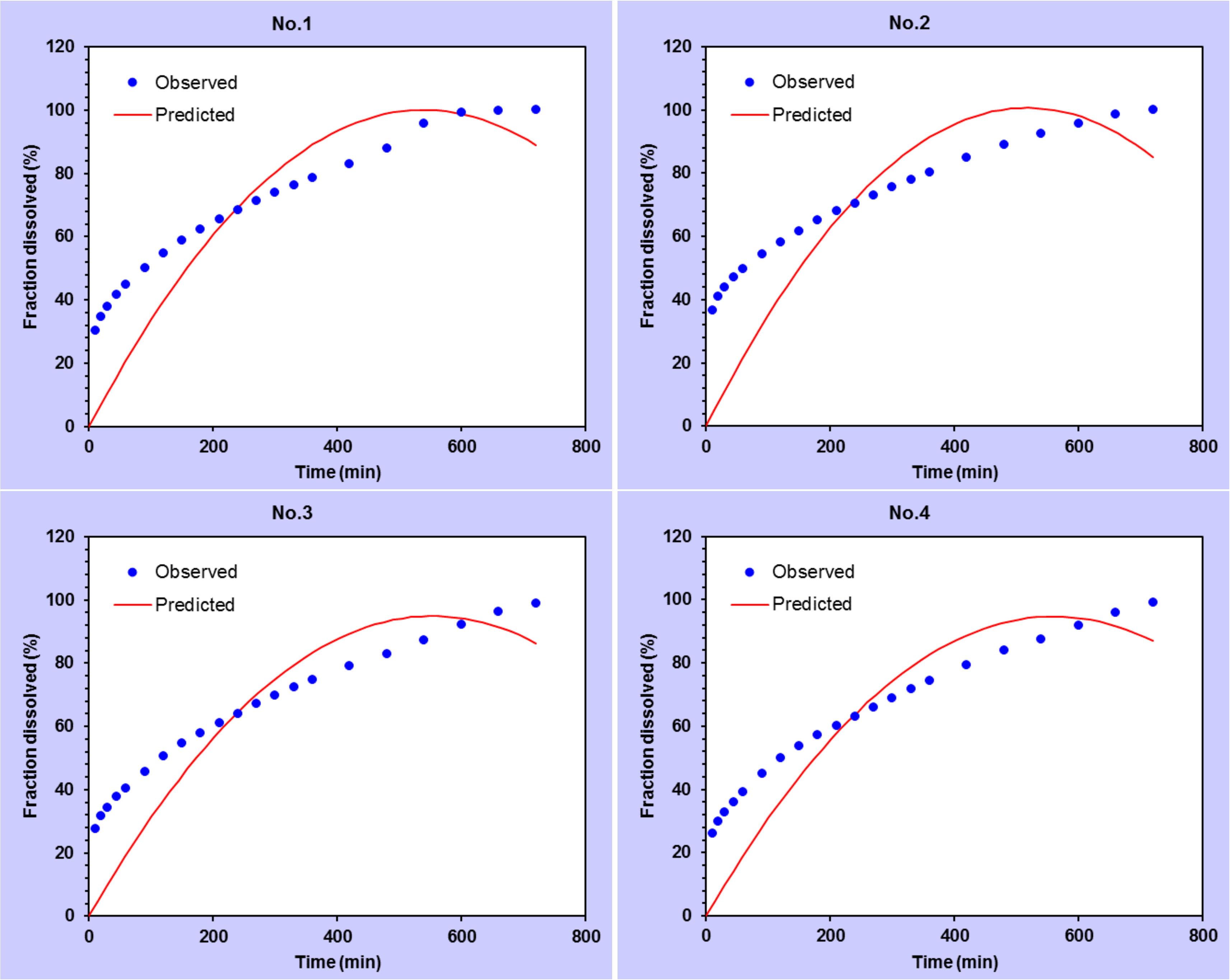

Supplementary materials – Model fitting summary – SuperTab® 11SD (modelling the entire relevant dissolution data)

Model: **Quadratic with T_{lag}**

Model equation: $F = 100 \cdot \left[k_1 \cdot (t - T_{lag})^2 + k_2 \cdot (t - T_{lag})\right]$

Fitted model parameters per tested tablet (N = 4) with statistics – mean, standard deviation (SD), and relative standard deviation expressed in % (RSD%) (output from DDSolver):

Parameter	No.1	No.2	No.3	No.4	Mean	SD	RSD(%)
k_1	-0.000004	-0.000004	-0.000003	-0.000003	-0.000003	0.000000	-10.158454
k_2	0.003752	0.003931	0.003466	0.003418	0.003642	0.000243	6.665152
T_{lag}	4.000000	4.000000	4.000000	4.000000	4.000000	0.000000	0.000000

Number of dissolution data points (N), degrees of freedom (df), and selected goodness of fit criteria – Pearson correlation coefficient (R), coefficient of determination (R^2), adjusted coefficient of determination ($R^2_{adjusted}$), and residual sum of squares (RSS) (manual calculation in MS Excel):

Parameter	No.1	No.2	No.3	No.4
N	21	21	21	21
df	18	18	18	18
R	0.965272834	0.954226414	0.967345737	0.970736568
R^2	0.931751645	0.910548049	0.935757775	0.942329484
$R^2_{adjusted}$	0.924168494	0.900608944	0.92861975	0.935921649
RSS	5352.673467	7360.071713	4390.353044	3941.966097

Graphical abstract of model fit presented as mean ± 1 SD of the fraction % of released carvedilol:

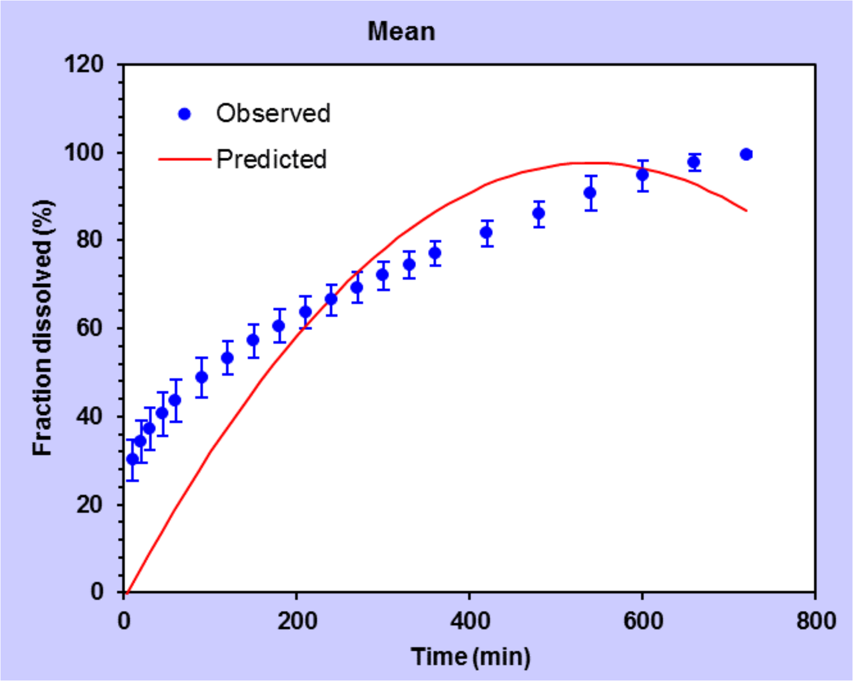

Supplementary materials – Model fitting summary – SuperTab® 11SD (modelling the entire relevant dissolution data)

Graphical abstract of model fit presented as the fraction % of released carvedilol per tested tablet:

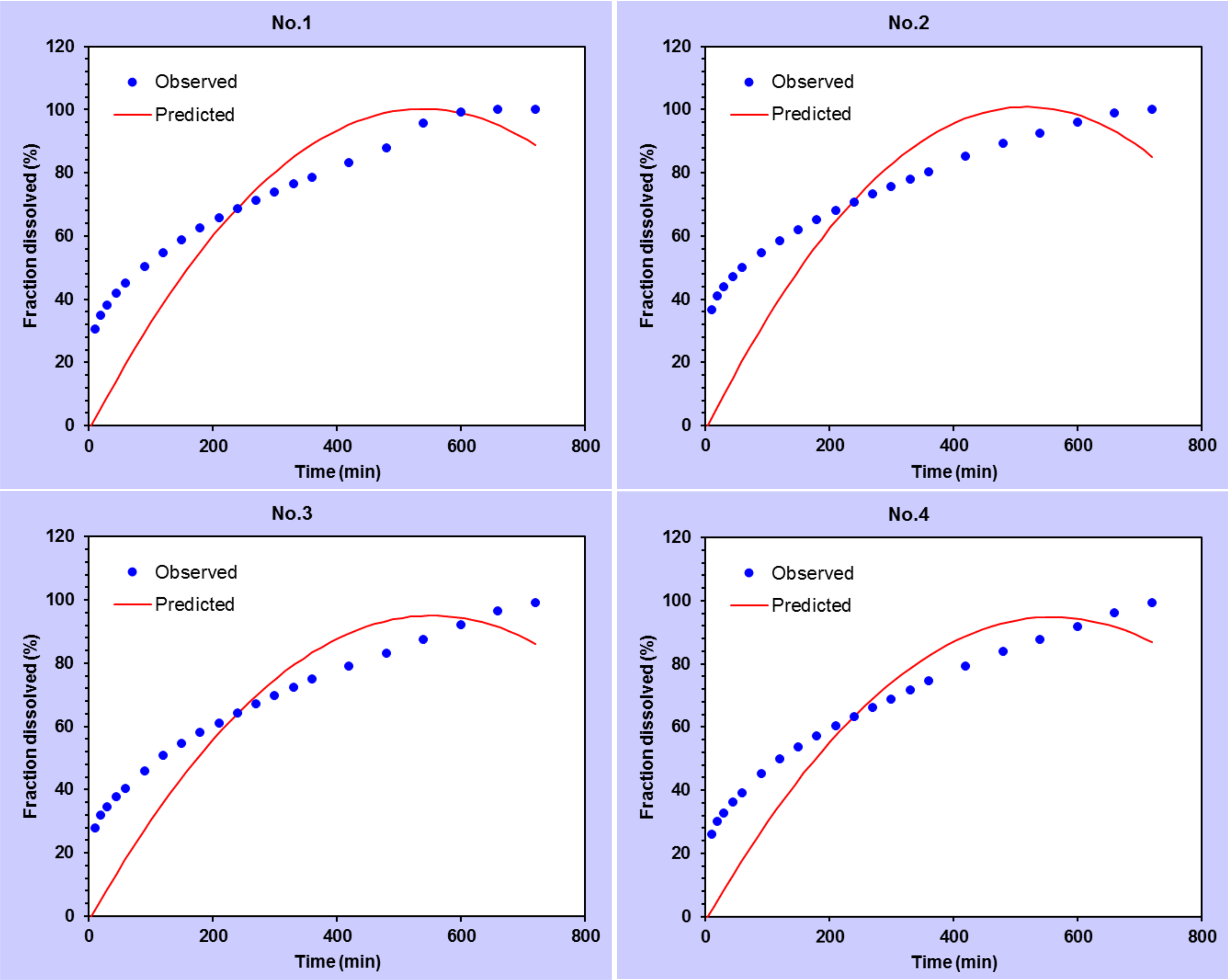

Supplementary materials – Model fitting summary – SuperTab® 11SD (modelling the entire relevant dissolution data)

Model: **Weibull_1**

Model equation: $F = 100 \cdot \left[1 - e^{-\frac{(t-T_i)^\beta}{\alpha}}\right]$

Fitted model parameters per tested tablet (N = 4) with statistics – mean, standard deviation (SD), and relative standard deviation expressed in % (RSD%) (output from DDSolver):

Parameter	No.1	No.2	No.3	No.4	Mean	SD	RSD(%)
α	12.563	9.056	12.541	14.084	12.061	2.130	17.657
β	0.537	0.489	0.504	0.522	0.513	0.021	4.144
T_i	4.000	4.000	4.000	4.000	4.000	0.000	0.000

Number of dissolution data points (N), degrees of freedom (df), and selected goodness of fit criteria – Pearson correlation coefficient (R), coefficient of determination (R^2), adjusted coefficient of determination ($R^2_{adjusted}$), and residual sum of squares (RSS) (manual calculation in MS Excel):

Parameter	No.1	No.2	No.3	No.4
N	21	21	21	21
df	18	18	18	18
R	0.949218621	0.943609387	0.958806826	0.961197388
R^2	0.901015991	0.890398674	0.919310529	0.923900419
$R^2_{adjusted}$	0.890017768	0.878220749	0.910345032	0.915444911
RSS	1131.943857	1067.921889	830.2793419	831.6579317

Graphical abstract of model fit presented as mean ± 1 SD of the fraction % of released carvedilol:

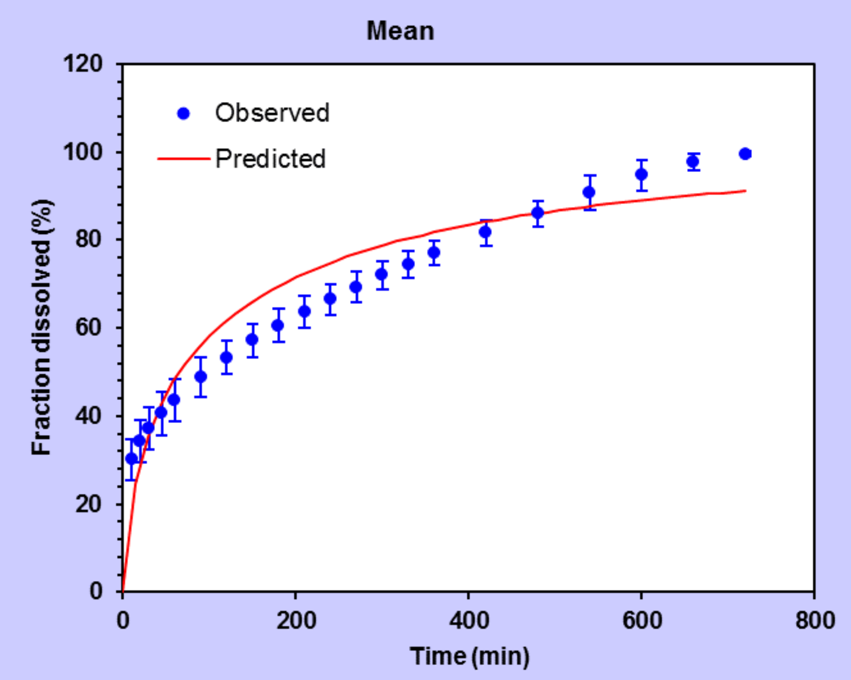

Supplementary materials – Model fitting summary – SuperTab® 11SD (modelling the entire relevant dissolution data)

Graphical abstract of model fit presented as the fraction % of released carvedilol per tested tablet:

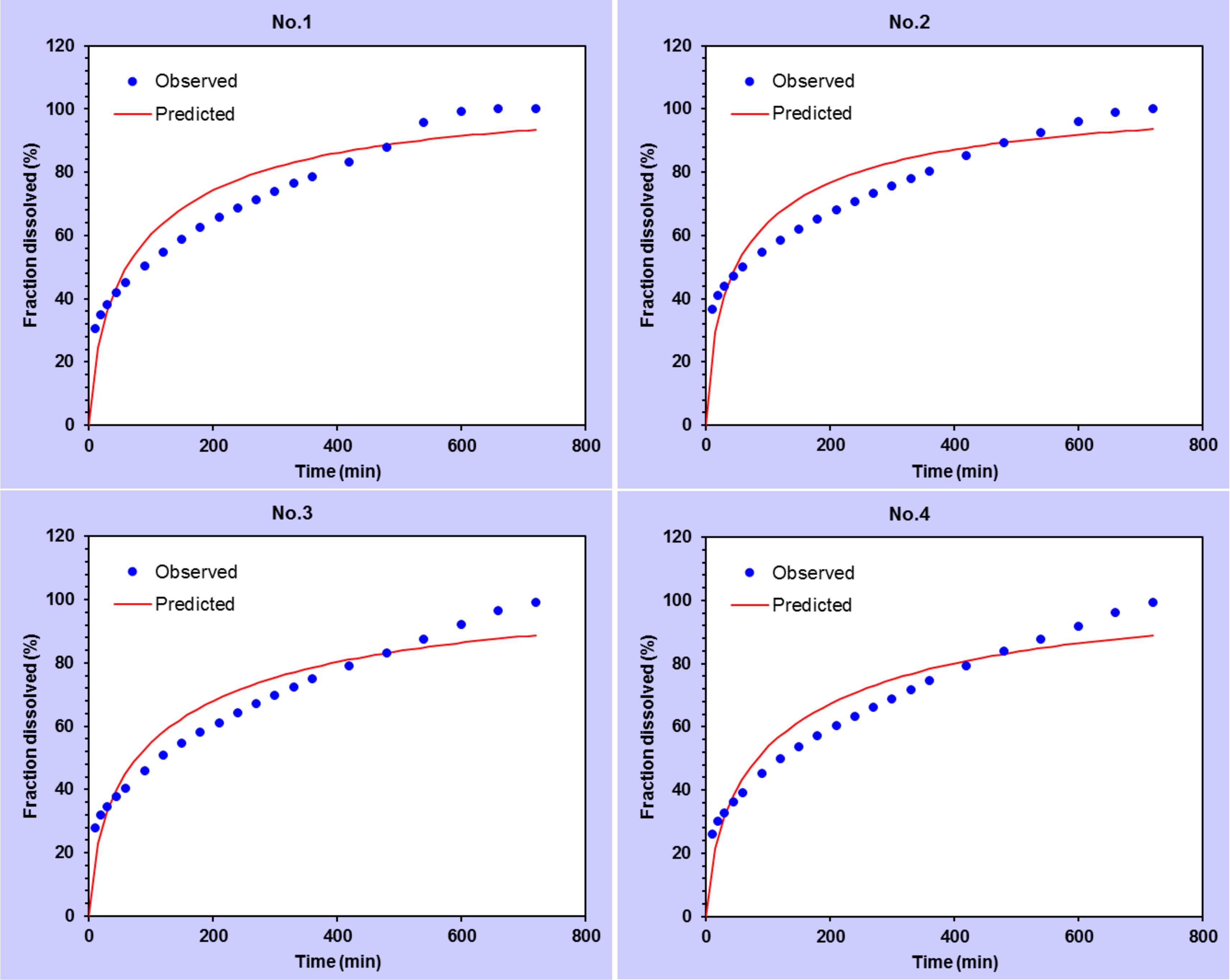

Supplementary materials – Model fitting summary – SuperTab® 11SD (modelling the entire relevant dissolution data)

Model: **Weibull_2**

Model equation: $F = 100 \cdot \left(1 - e^{-\frac{t^{\beta}}{\alpha}}\right)$

Fitted model parameters per tested tablet (N = 4) with statistics – mean, standard deviation (SD), and relative standard deviation expressed in % (RSD%) (output from DDSolver):

Parameter	No.1	No.2	No.3	No.4	Mean	SD	RSD(%)
α	17.101	11.932	16.543	18.755	16.083	2.922	18.169
β	0.591	0.536	0.552	0.572	0.563	0.024	4.245

Number of dissolution data points (N), degrees of freedom (df), and selected goodness of fit criteria – Pearson correlation coefficient (R), coefficient of determination (R^2), adjusted coefficient of determination ($R^2_{adjusted}$), and residual sum of squares (RSS) (manual calculation in MS Excel):

Parameter	No.1	No.2	No.3	No.4
N	21	21	21	21
df	19	19	19	19
R	0.956738329	0.952476684	0.966349059	0.968347782
R^2	0.915348229	0.907211834	0.933830504	0.937697427
$R^2_{adjusted}$	0.910892873	0.902328246	0.930347899	0.934418345
RSS	992.6811059	934.3213409	686.8459612	686.7560869

Graphical abstract of model fit presented as mean ± 1 SD of the fraction % of released carvedilol:

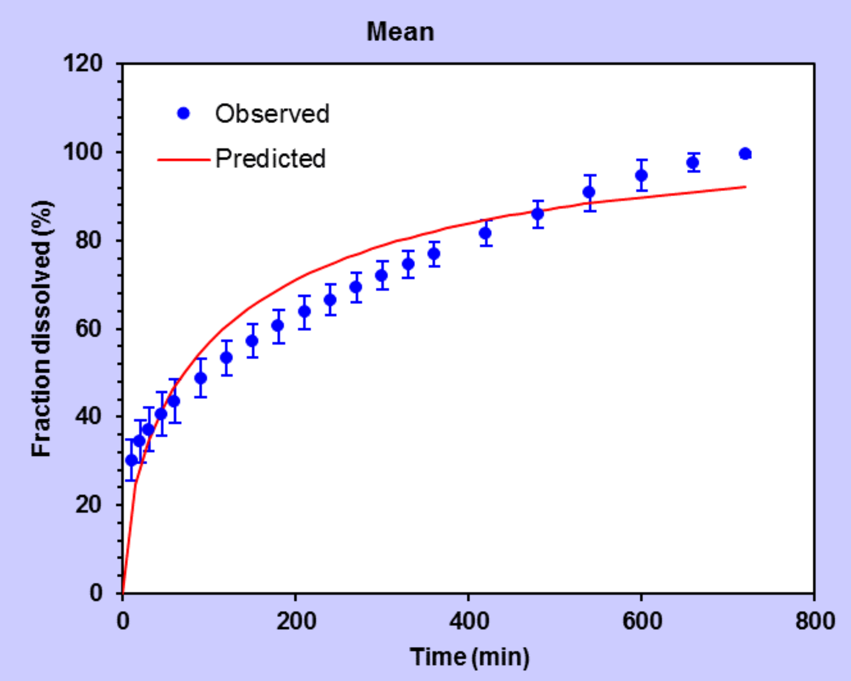

Supplementary materials – Model fitting summary – SuperTab® 11SD (modelling the entire relevant dissolution data)

Graphical abstract of model fit presented as the fraction % of released carvedilol per tested tablet:

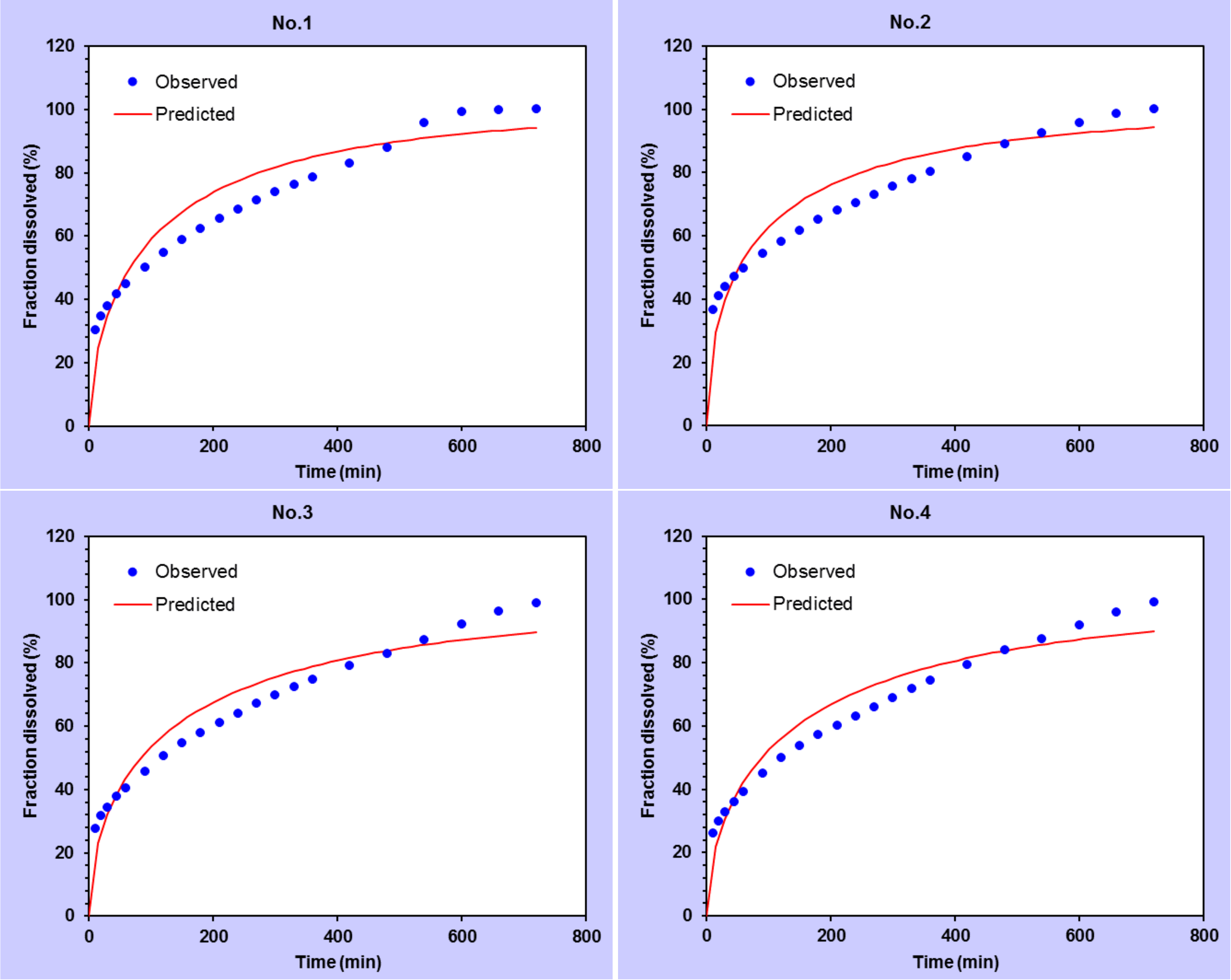

Supplementary materials – Model fitting summary – SuperTab® 11SD (modelling the entire relevant dissolution data)

Model: **Weibull_3**

Model equation: $F = F_{max} \cdot \left(1 - e^{-\frac{t^{\beta}}{\alpha}}\right)$

Fitted model parameters per tested tablet (N = 4) with statistics – mean, standard deviation (SD), and relative standard deviation expressed in % (RSD%) (output from DDSolver):

Parameter	No.1	No.2	No.3	No.4	Mean	SD	RSD(%)
α	17.955	9.034	16.557	18.585	15.533	4.415	28.421
β	0.522	0.450	0.447	0.462	0.470	0.035	7.427
F_{max}	113.462	104.976	129.351	129.653	119.360	12.213	10.232

Number of dissolution data points (N), degrees of freedom (df), and selected goodness of fit criteria – Pearson correlation coefficient (R), coefficient of determination (R^2), adjusted coefficient of determination ($R^2_{adjusted}$), and residual sum of squares (RSS) (manual calculation in MS Excel):

Parameter	No.1	No.2	No.3	No.4
N	21	21	21	21
df	18	18	18	18
R	0.979400611	0.964381428	0.98469668	0.986469711
R^2	0.959225557	0.93003154	0.969627552	0.973122492
$R^2_{adjusted}$	0.954695063	0.922257266	0.966252836	0.970136102
RSS	553.2576532	560.1905238	433.1353517	496.1336257

Graphical abstract of model fit presented as mean ± 1 SD of the fraction % of released carvedilol:

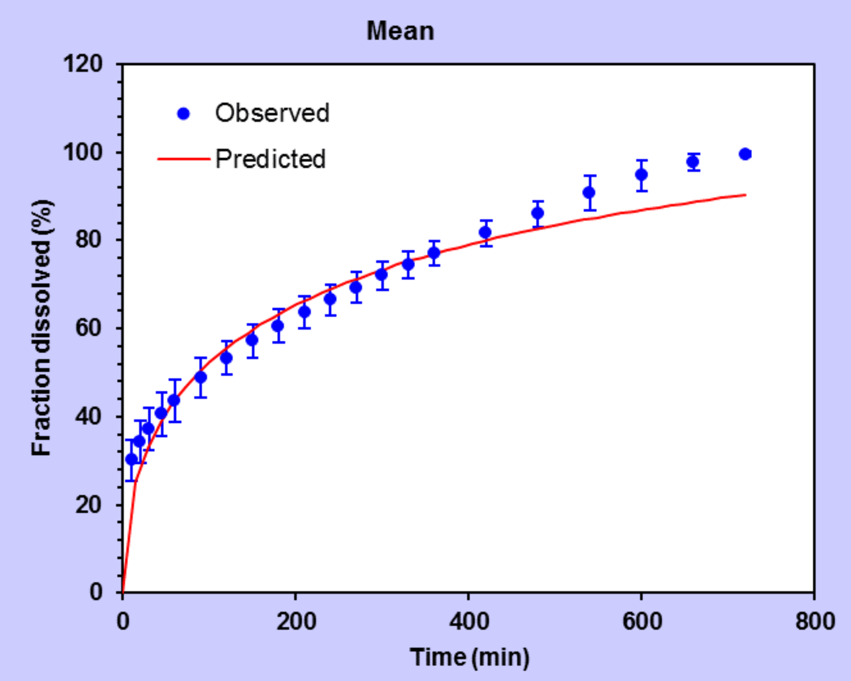

Supplementary materials – Model fitting summary – SuperTab® 11SD (modelling the entire relevant dissolution data)

Graphical abstract of model fit presented as the fraction % of released carvedilol per tested tablet:

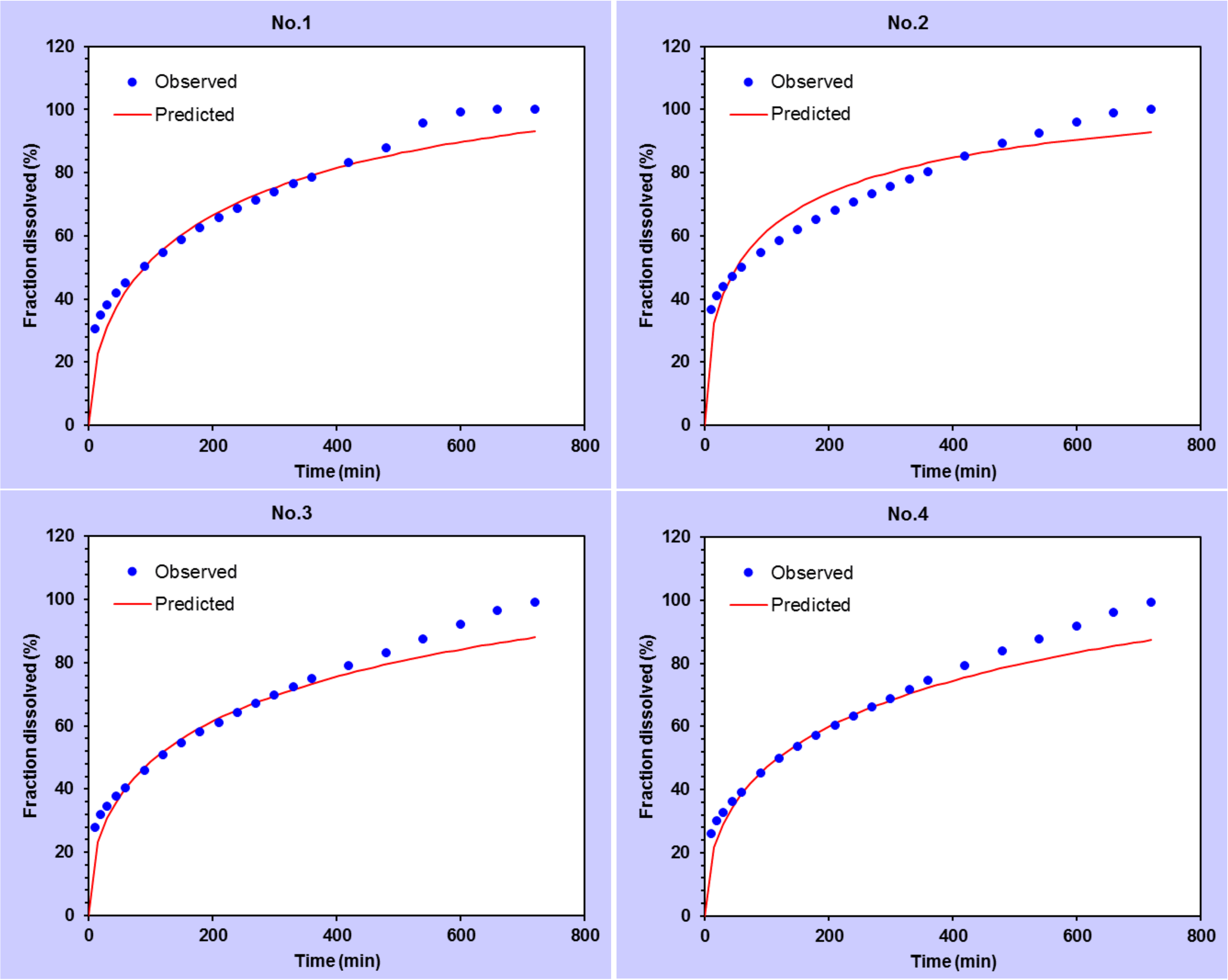

Supplementary materials – Model fitting summary – SuperTab® 11SD (modelling the entire relevant dissolution data)

Model: **Weibull_4**

Model equation: $F = F_{max} \cdot \left[1 - e^{-\frac{(t-T_i)^{\beta}}{\alpha}}\right]$

Fitted model parameters per tested tablet (N = 4) with statistics – mean, standard deviation (SD), and relative standard deviation expressed in % (RSD%) (output from DDSolver):

Parameter	No.1	No.2	No.3	No.4	Mean	SD	RSD(%)
α	10.710	7.214	11.571	12.890	10.596	2.427	22.901
β	0.477	0.412	0.470	0.486	0.461	0.034	7.274
T_i	4.000	4.000	4.000	4.000	4.000	0.000	0.000
F_{max}	105.109	104.976	103.833	104.076	104.498	0.638	0.610

Number of dissolution data points (N), degrees of freedom (df), and selected goodness of fit criteria – Pearson correlation coefficient (R), coefficient of determination (R^2), adjusted coefficient of determination ($R^2_{adjusted}$), and residual sum of squares (RSS) (manual calculation in MS Excel):

Parameter	No.1	No.2	No.3	No.4
N	21	21	21	21
df	17	17	17	17
R	0.959552665	0.955655602	0.963644607	0.966172629
R^2	0.920741317	0.91327763	0.928610928	0.933489549
$R^2_{adjusted}$	0.906754491	0.897973683	0.916012857	0.921752411
RSS	821.1667252	692.2661545	709.2627724	700.9108315

Graphical abstract of model fit presented as mean ± 1 SD of the fraction % of released carvedilol:

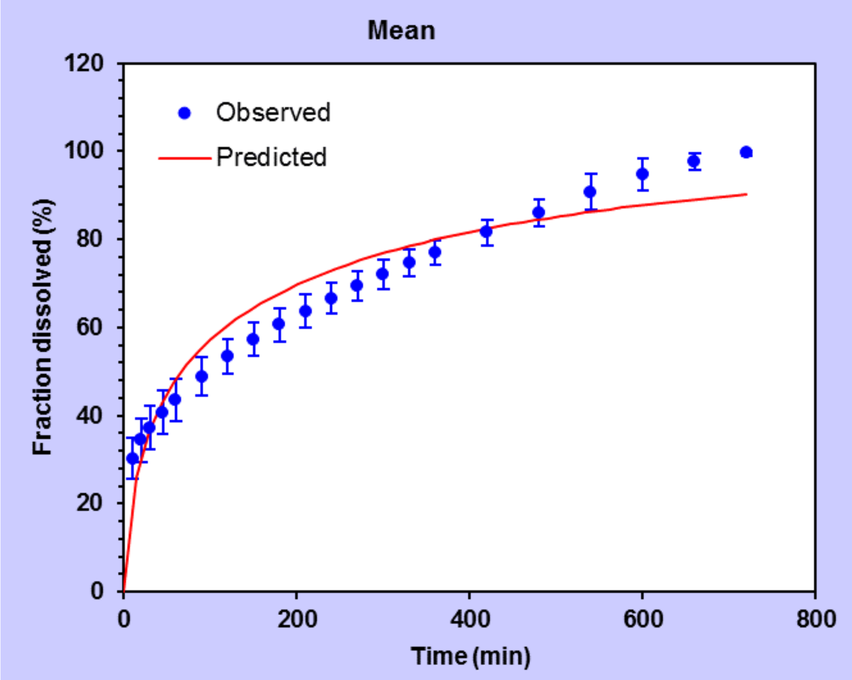

Supplementary materials – Model fitting summary – SuperTab® 11SD (modelling the entire relevant dissolution data)

Graphical abstract of model fit presented as the fraction % of released carvedilol per tested tablet:

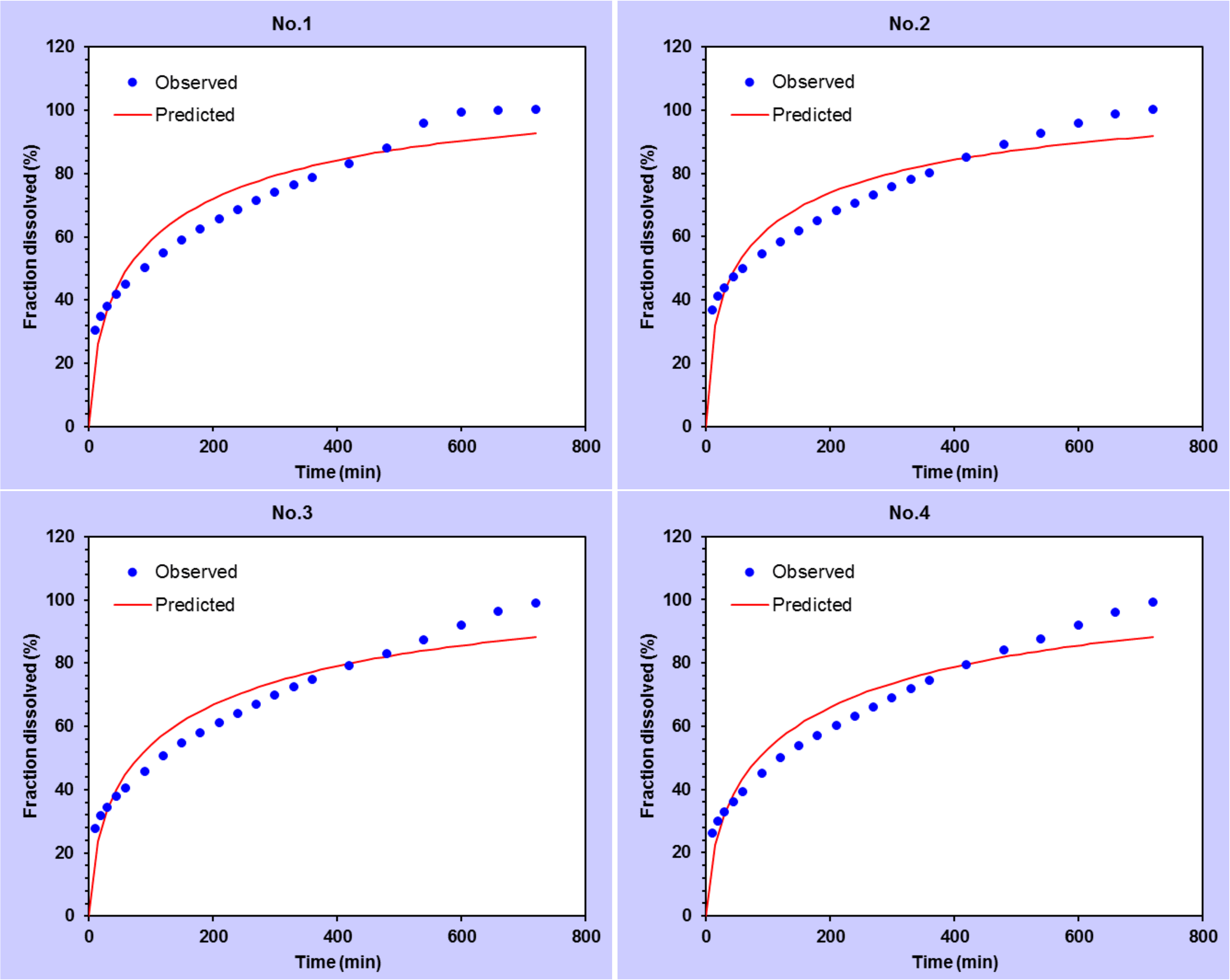

Supplementary materials – Model fitting summary – SuperTab® 11SD (modelling the entire relevant dissolution data)

Model: **Logistic_1**

Model equation: $F = 100 \cdot \frac{e^{\alpha+\beta \cdot \log(t)}}{1+e^{\alpha+\beta \cdot \log(t)}}$

Fitted model parameters per tested tablet (N = 4) with statistics – mean, standard deviation (SD), and relative standard deviation expressed in % (RSD%) (output from DDSolver):

Parameter	No.1	No.2	No.3	No.4	Mean	SD	RSD(%)
α	-6.281	-4.658	-4.131	-5.384	-5.114	0.933	-18.238
β	3.171	2.705	2.209	2.562	2.662	0.398	14.964

Number of dissolution data points (N), degrees of freedom (df), and selected goodness of fit criteria – Pearson correlation coefficient (R), coefficient of determination (R^2), adjusted coefficient of determination ($R^2_{adjusted}$), and residual sum of squares (RSS) (manual calculation in MS Excel):

Parameter	No.1	No.2	No.3	No.4
N	21	21	21	21
df	19	19	19	19
R	0.940019465	0.902202926	0.934559965	0.962572564
R^2	0.883636596	0.81397012	0.873402328	0.926545941
$R^2_{adjusted}$	0.877512206	0.804179074	0.866739292	0.922679938
RSS	2838.830607	2962.281183	1617.553807	1628.911153

Graphical abstract of model fit presented as mean ± 1 SD of the fraction % of released carvedilol:

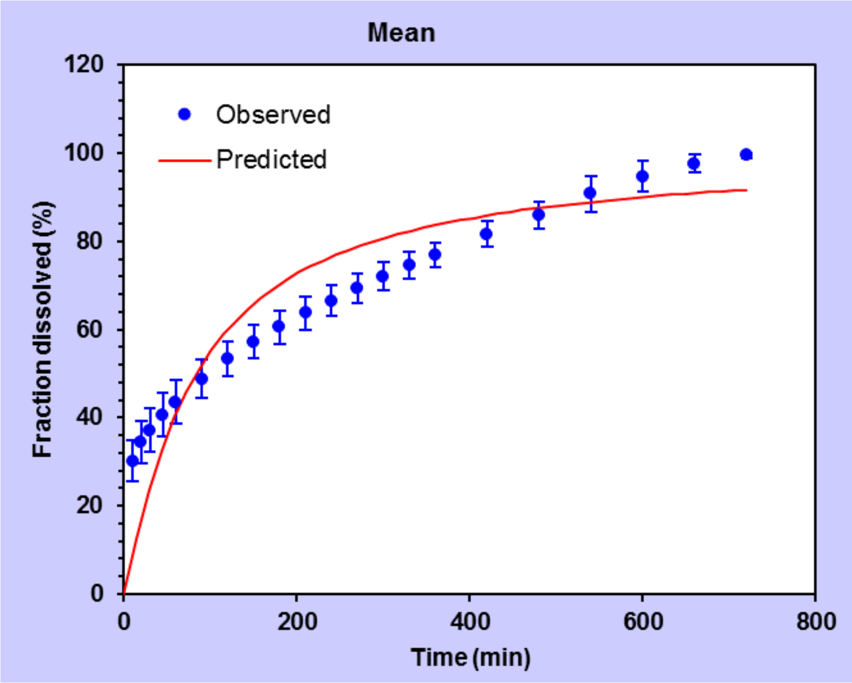

Supplementary materials – Model fitting summary – SuperTab® 11SD (modelling the entire relevant dissolution data)

Graphical abstract of model fit presented as the fraction % of released carvedilol per tested tablet:

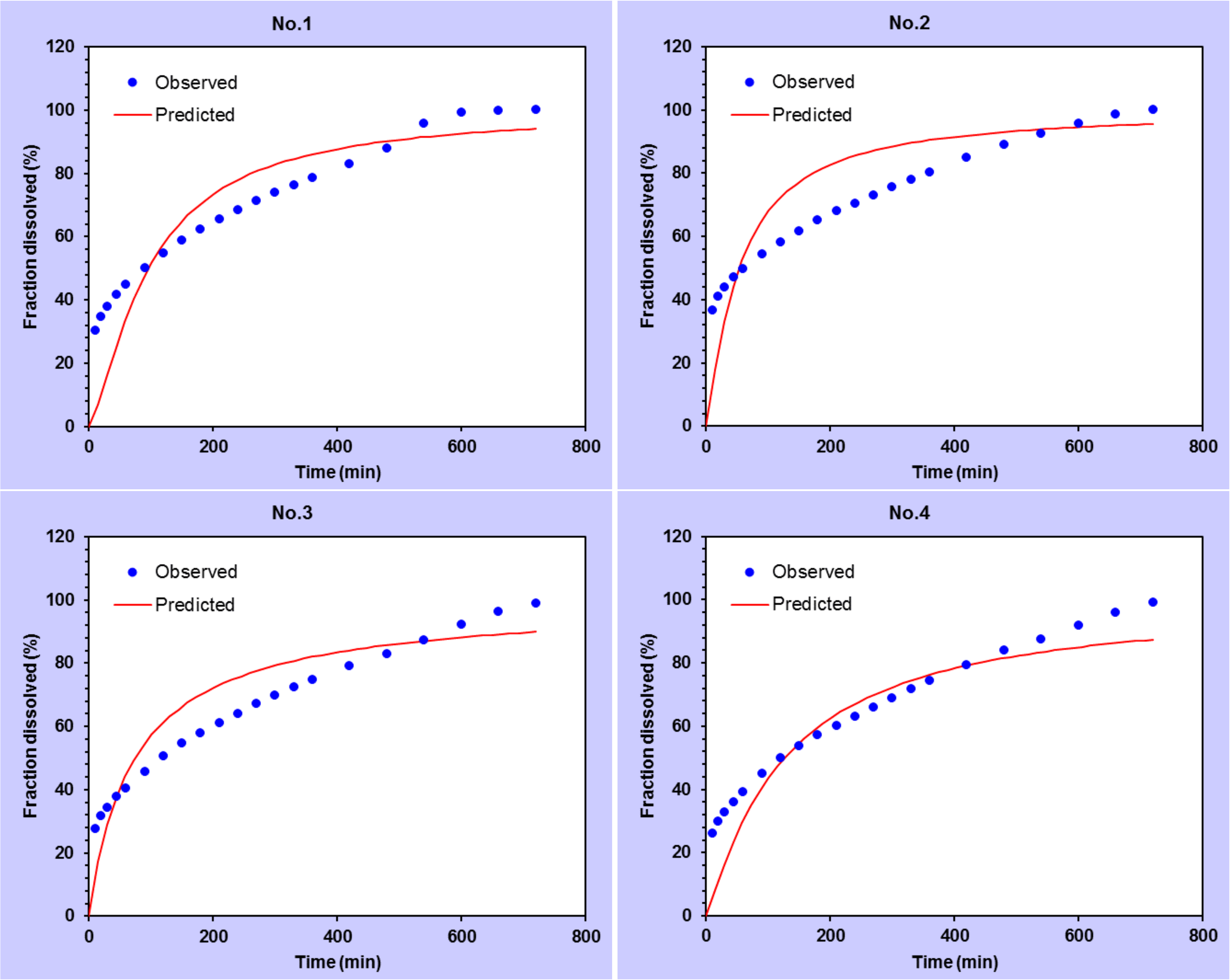

Supplementary materials – Model fitting summary – SuperTab® 11SD (modelling the entire relevant dissolution data)

Model: **Logistic_2**

Model equation: $F = F_{max} \cdot \frac{e^{\alpha+\beta\cdot\log(t)}}{1+e^{\alpha+\beta\cdot\log(t)}}$

Fitted model parameters per tested tablet (N = 4) with statistics – mean, standard deviation (SD), and relative standard deviation expressed in % (RSD%) (output from DDSolver):

Parameter	No.1	No.2	No.3	No.4	Mean	SD	RSD(%)
α	-3.742	-3.121	-3.638	-3.765	-3.567	0.302	-8.464
β	2.031	1.784	1.891	1.931	1.909	0.102	5.354
F_{max}	105.109	104.976	103.833	104.076	104.498	0.638	0.610

Number of dissolution data points (N), degrees of freedom (df), and selected goodness of fit criteria – Pearson correlation coefficient (R), coefficient of determination (R^2), adjusted coefficient of determination ($R^2_{adjusted}$), and residual sum of squares (RSS) (manual calculation in MS Excel):

Parameter	No.1	No.2	No.3	No.4
N	21	21	21	21
df	18	18	18	18
R	0.938118774	0.935796703	0.946159675	0.949007484
R^2	0.880066833	0.87571547	0.89521813	0.900615204
$R^2_{adjusted}$	0.866740926	0.861906077	0.8835757	0.889572449
RSS	1420.578854	1128.543105	1127.32367	1122.123812

Graphical abstract of model fit presented as mean ± 1 SD of the fraction % of released carvedilol:

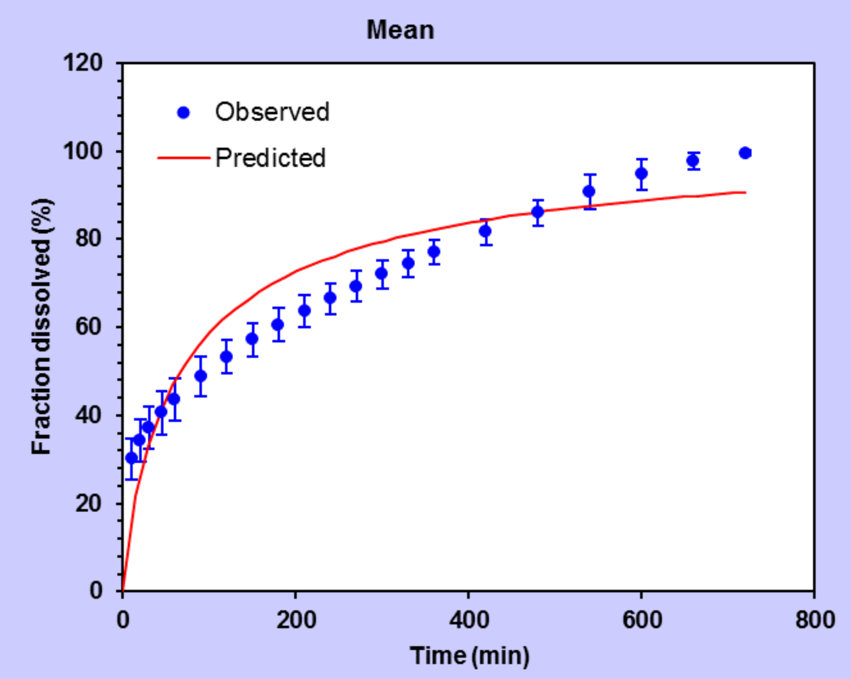

Supplementary materials – Model fitting summary – SuperTab® 11SD (modelling the entire relevant dissolution data)

Graphical abstract of model fit presented as the fraction % of released carvedilol per tested tablet:

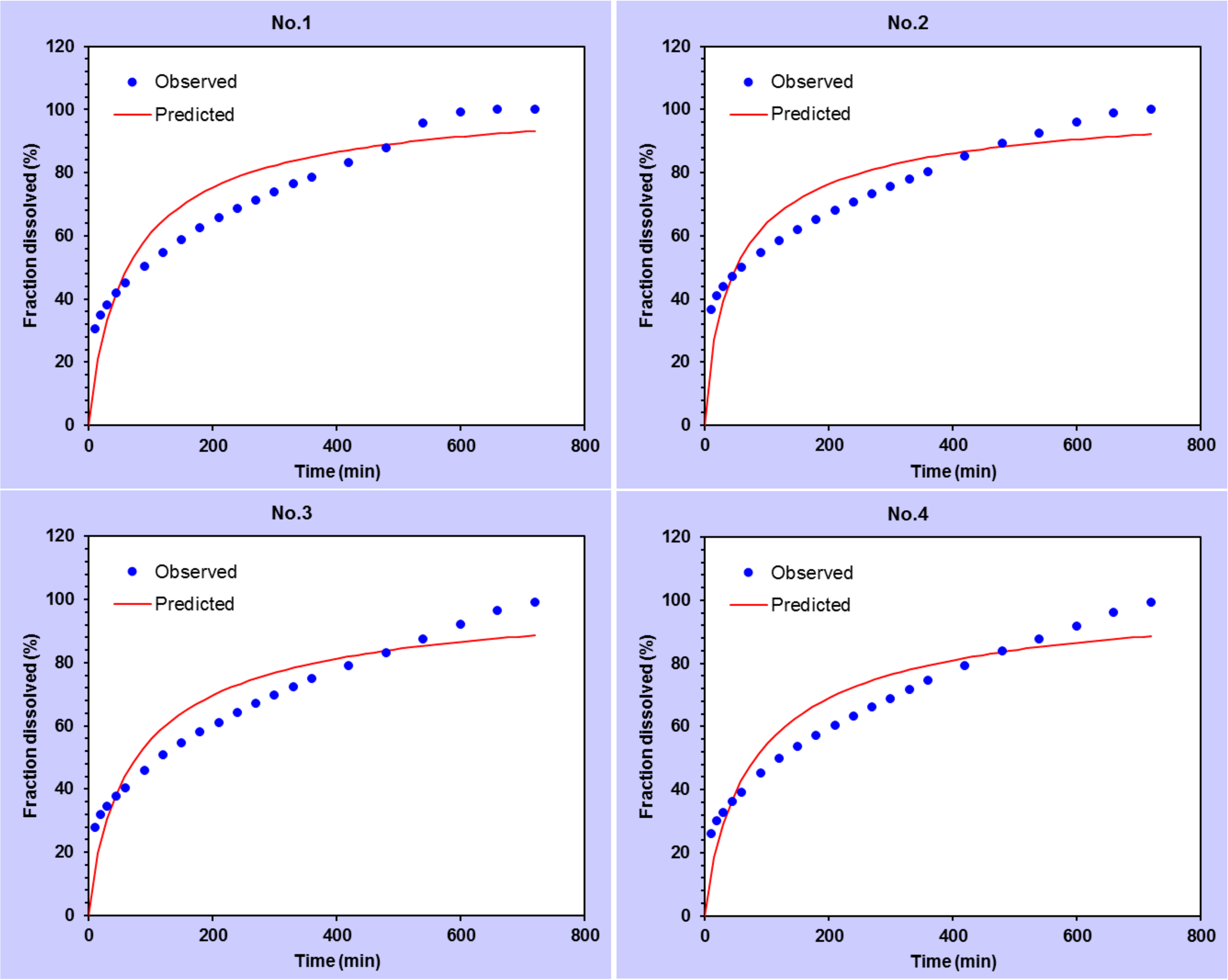

Supplementary materials – Model fitting summary – SuperTab® 11SD (modelling the entire relevant dissolution data)

Model: **Logistic_3**

Model equation: $F = F_{max} \cdot \frac{1}{1+e^{-k\cdot(t-\gamma)}}$

Fitted model parameters per tested tablet (N = 4) with statistics – mean, standard deviation (SD), and relative standard deviation expressed in % (RSD%) (output from DDSolver):

Parameter	No.1	No.2	No.3	No.4	Mean	SD	RSD(%)
k	0.005	0.005	0.005	0.005	0.005	0.000	5.452
γ	130.629	95.272	160.198	169.800	138.975	33.567	24.153
F_{max}	105.109	104.976	103.833	104.076	104.498	0.638	0.610

Number of dissolution data points (N), degrees of freedom (df), and selected goodness of fit criteria – Pearson correlation coefficient (R), coefficient of determination (R^2), adjusted coefficient of determination ($R^2_{adjusted}$), and residual sum of squares (RSS) (manual calculation in MS Excel):

Parameter	No.1	No.2	No.3	No.4
N	21	21	21	21
df	18	18	18	18
R	0.993015732	0.995066627	0.992936577	0.992746319
R^2	0.986080243	0.990157592	0.985923046	0.985545253
$R^2_{adjusted}$	0.984533603	0.989063991	0.98435894	0.98393917
RSS	139.5151092	76.38655414	136.0142759	147.0234333

Graphical abstract of model fit presented as mean ± 1 SD of the fraction % of released carvedilol:

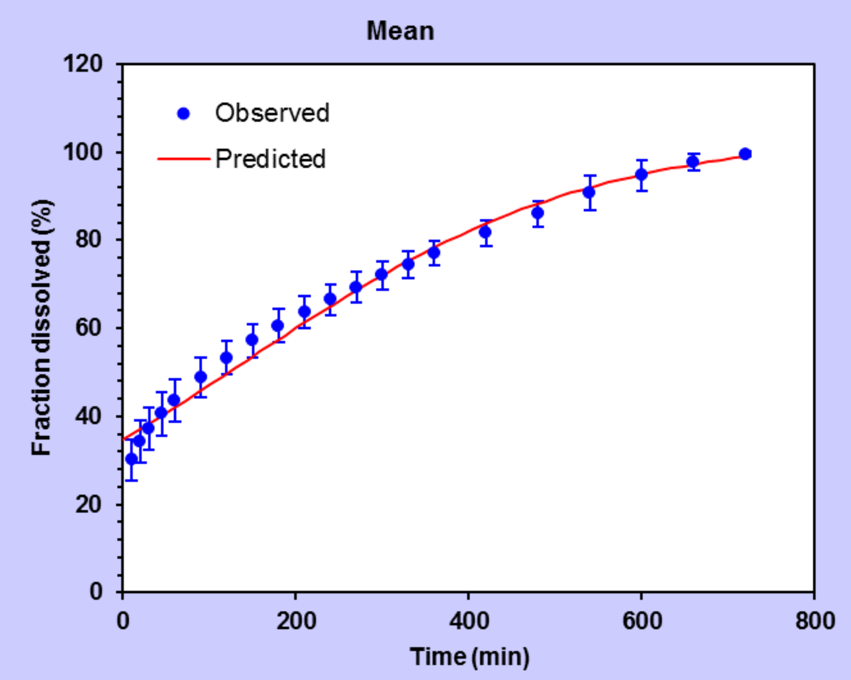

Supplementary materials – Model fitting summary – SuperTab® 11SD (modelling the entire relevant dissolution data)

Graphical abstract of model fit presented as the fraction % of released carvedilol per tested tablet:

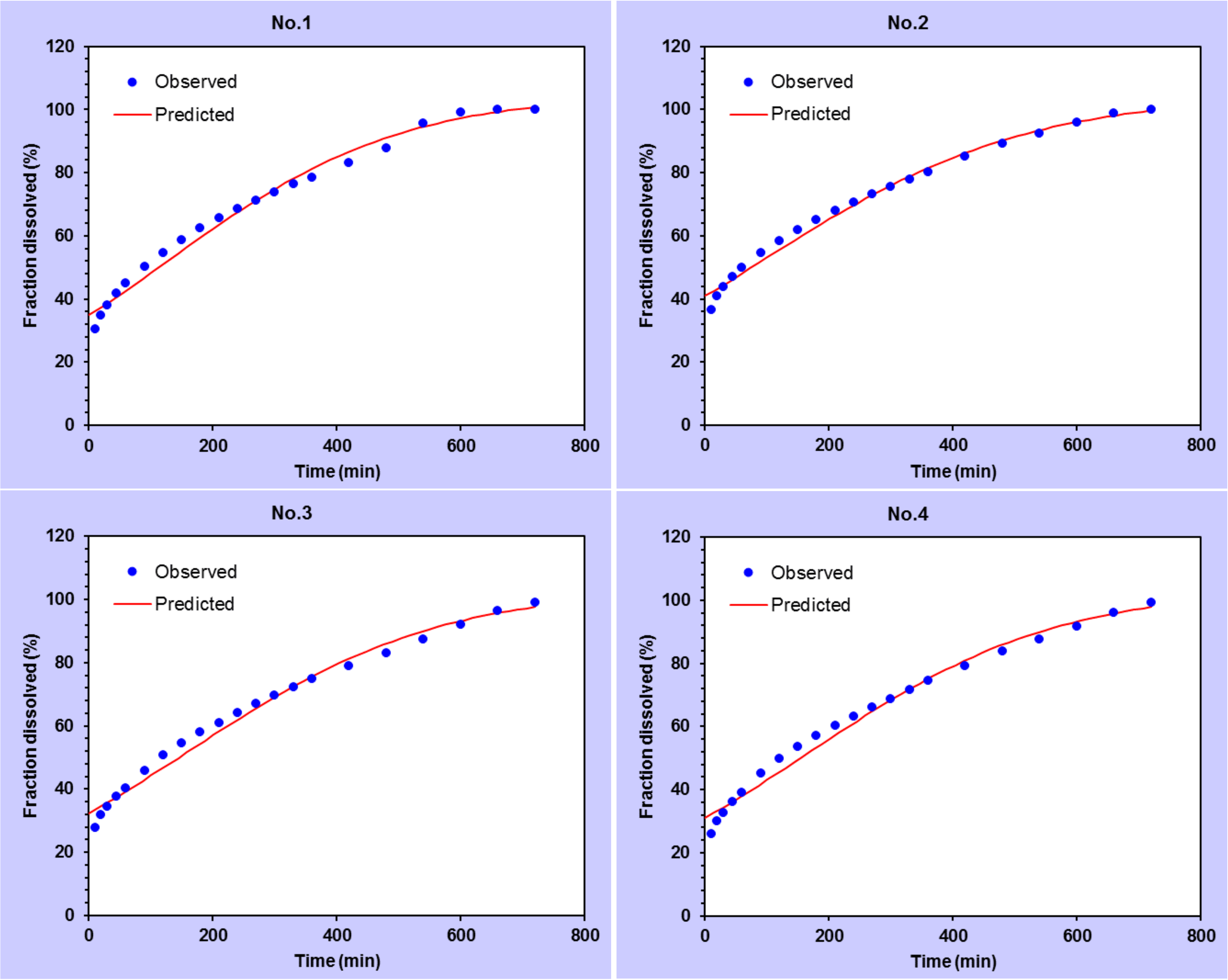

Supplementary materials – Model fitting summary – SuperTab® 11SD (modelling the entire relevant dissolution data)

Model: **Gompertz_1**

Model equation: $F = 100 \cdot e^{-\alpha \cdot e^{-\beta \cdot \log(t)}}$

Fitted model parameters per tested tablet (N = 4) with statistics – mean, standard deviation (SD), and relative standard deviation expressed in % (RSD%) (output from DDSolver):

Parameter	No.1	No.2	No.3	No.4	Mean	SD	RSD(%)
α	78.231	43.042	19.439	32.826	43.385	25.161	57.996
β	2.446	2.398	1.812	1.859	2.129	0.339	15.945

Number of dissolution data points (N), degrees of freedom (df), and selected goodness of fit criteria – Pearson correlation coefficient (R), coefficient of determination (R^2), adjusted coefficient of determination ($R^2_{adjusted}$), and residual sum of squares (RSS) (manual calculation in MS Excel):

Parameter	No.1	No.2	No.3	No.4
N	21	21	21	21
df	19	19	19	19
R	0.917366813	0.874930478	0.907280605	0.94409558
R^2	0.841561869	0.765503342	0.823158095	0.891316465
$R^2_{adjusted}$	0.83322302	0.753161413	0.813850627	0.885596279
RSS	3910.293034	4500.867242	2561.616324	2525.826123

Graphical abstract of model fit presented as mean ± 1 SD of the fraction % of released carvedilol:

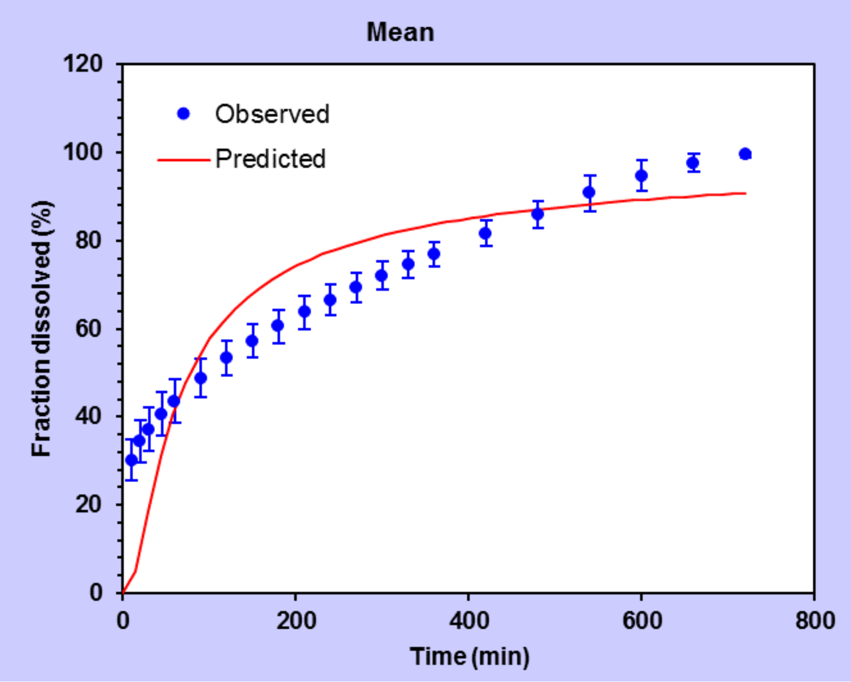

Supplementary materials – Model fitting summary – SuperTab® 11SD (modelling the entire relevant dissolution data)

Graphical abstract of model fit presented as the fraction % of released carvedilol per tested tablet:

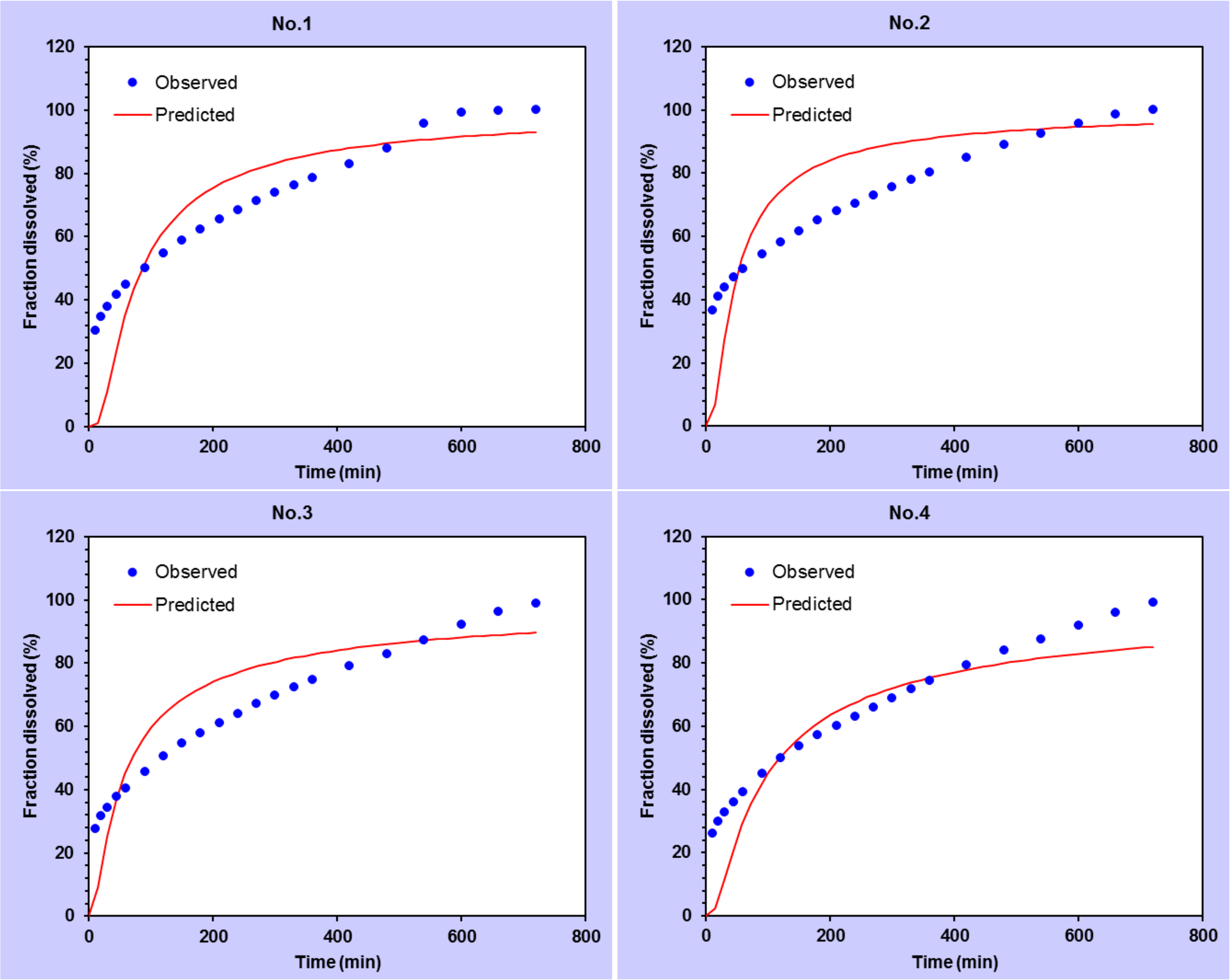

Supplementary materials – Model fitting summary – SuperTab® 11SD (modelling the entire relevant dissolution data)

Model: **Gompertz_2**

Model equation: $F = F_{max} \cdot e^{-\alpha \cdot e^{-\beta \cdot \log(t)}}$

Fitted model parameters per tested tablet (N = 4) with statistics – mean, standard deviation (SD), and relative standard deviation expressed in % (RSD%) (output from DDSolver):

Parameter	No.1	No.2	No.3	No.4	Mean	SD	RSD(%)
α	13.963	8.972	11.575	12.379	11.722	2.085	17.786
β	1.655	1.475	1.493	1.510	1.533	0.083	5.390
F_{max}	105.109	104.976	103.833	104.076	104.498	0.638	0.610

Number of dissolution data points (N), degrees of freedom (df), and selected goodness of fit criteria – Pearson correlation coefficient (R), coefficient of determination (R^2), adjusted coefficient of determination ($R^2_{adjusted}$), and residual sum of squares (RSS) (manual calculation in MS Excel):

Parameter	No.1	No.2	No.3	No.4
N	21	21	21	21
df	18	18	18	18
R	0.912099748	0.91058716	0.920568325	0.923633619
R^2	0.831925951	0.829168976	0.84744604	0.853099063
$R^2_{adjusted}$	0.813251056	0.810187751	0.8304956	0.836776737
RSS	2244.674905	1773.446974	1807.383855	1811.671631

Graphical abstract of model fit presented as mean ± 1 SD of the fraction % of released carvedilol:

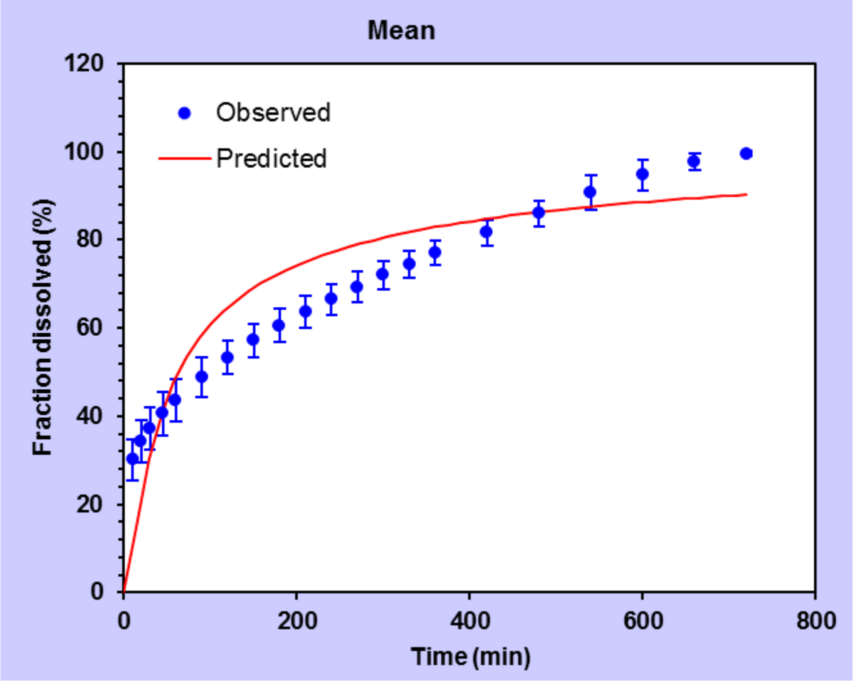

Supplementary materials – Model fitting summary – SuperTab® 11SD (modelling the entire relevant dissolution data)

Graphical abstract of model fit presented as the fraction % of released carvedilol per tested tablet:

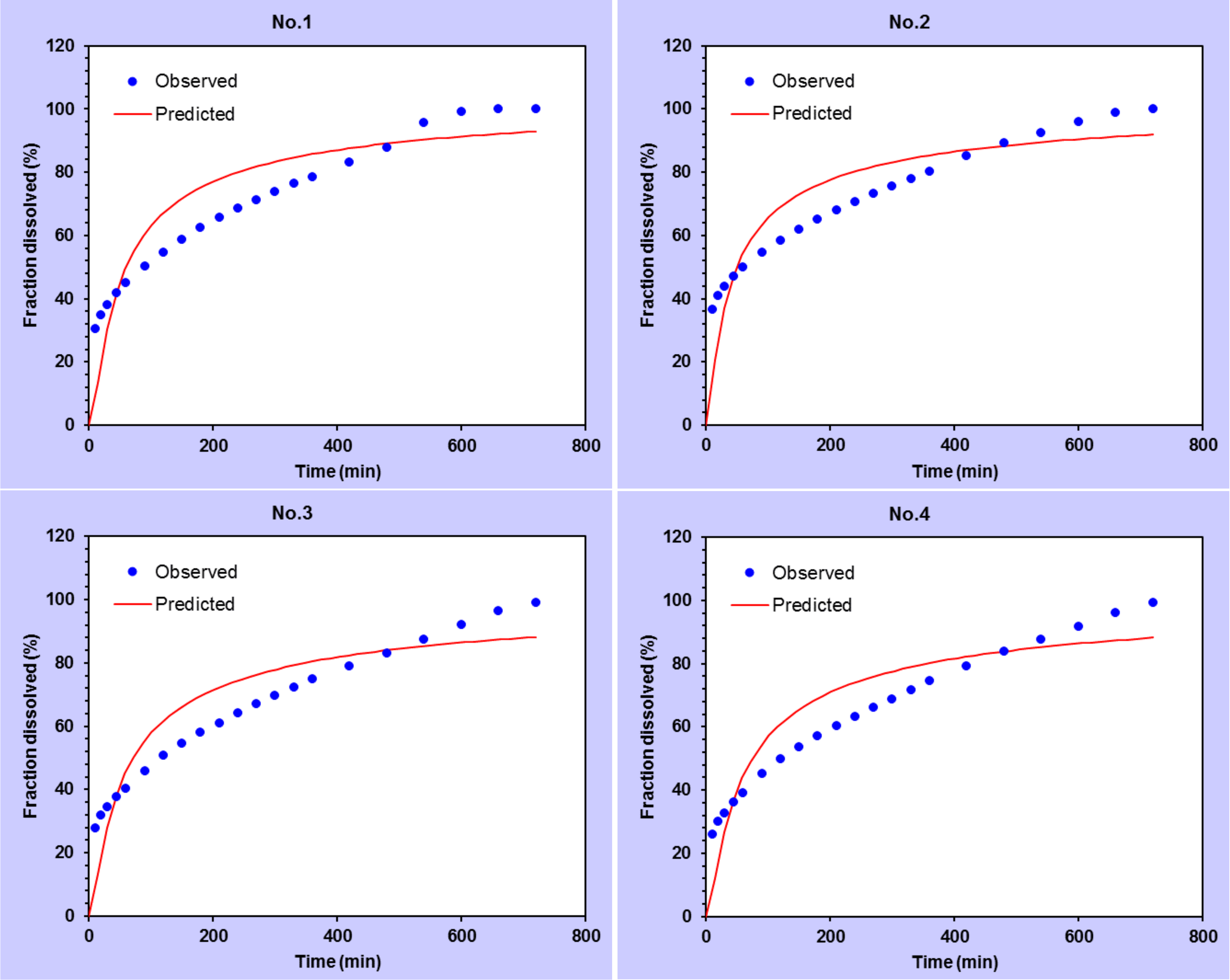

Supplementary materials – Model fitting summary – SuperTab® 11SD (modelling the entire relevant dissolution data)

Model: **Gompertz_3**

Model equation: $F = F_{max} \cdot e^{-e^{-k \cdot (t-\gamma)}}$

Fitted model parameters per tested tablet (N = 4) with statistics – mean, standard deviation (SD), and relative standard deviation expressed in % (RSD%) (output from DDSolver):

Parameter	No.1	No.2	No.3	No.4	Mean	SD	RSD(%)
k	0.005	0.004	0.004	0.004	0.004	0.000	5.788
γ	45.016	4.754	61.006	69.625	45.100	28.765	63.781
F_{max}	105.109	104.976	103.833	104.076	104.498	0.638	0.610

Number of dissolution data points (N), degrees of freedom (df), and selected goodness of fit criteria – Pearson correlation coefficient (R), coefficient of determination (R^2), adjusted coefficient of determination ($R^2_{adjusted}$), and residual sum of squares (RSS) (manual calculation in MS Excel):

Parameter	No.1	No.2	No.3	No.4
N	21	21	21	21
df	18	18	18	18
R	0.993960581	0.996496934	0.995735025	0.9958397
R^2	0.987957636	0.993006139	0.991488241	0.991696709
$R^2_{adjusted}$	0.986619596	0.992229043	0.99054249	0.990774121
RSS	146.7943563	63.58739937	97.92094792	97.4985752

Graphical abstract of model fit presented as mean ± 1 SD of the fraction % of released carvedilol:

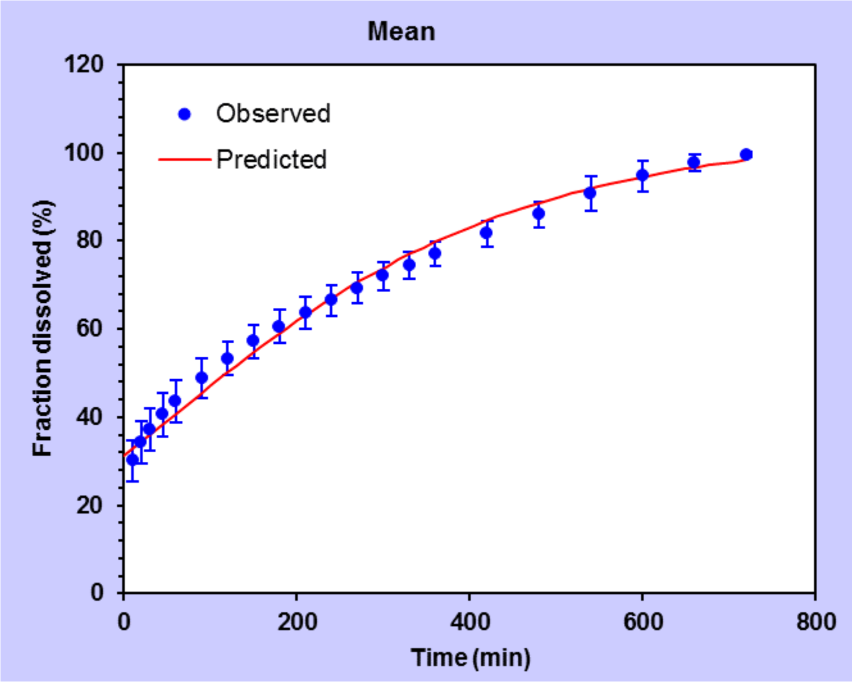

Supplementary materials – Model fitting summary – SuperTab® 11SD (modelling the entire relevant dissolution data)

Graphical abstract of model fit presented as the fraction % of released carvedilol per tested tablet:

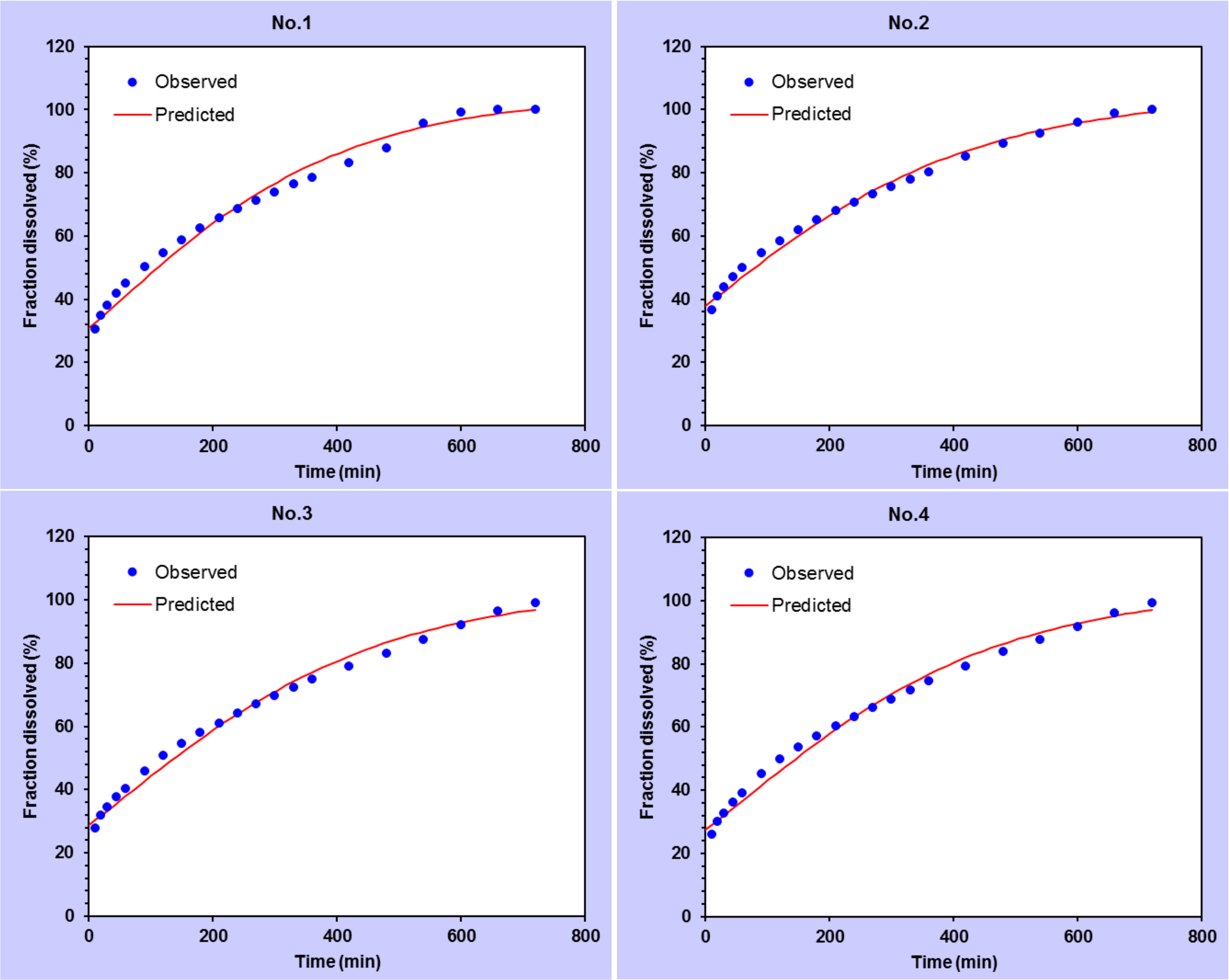

Supplementary materials – Model fitting summary – SuperTab® 11SD (modelling the entire relevant dissolution data)

Model: **Gompertz_4**

Model equation: $F = F_{max} \cdot e^{-\beta \cdot e^{-k \cdot t}}$

Fitted model parameters per tested tablet (N = 4) with statistics – mean, standard deviation (SD), and relative standard deviation expressed in % (RSD%) (output from DDSolver):

Parameter	No.1	No.2	No.3	No.4	Mean	SD	RSD(%)
k	0.005	0.004	0.004	0.004	0.004	0.000	5.788
β	1.227	1.019	1.281	1.330	1.214	0.137	11.261
F_{max}	105.109	104.976	103.833	104.076	104.498	0.638	0.610

Number of dissolution data points (N), degrees of freedom (df), and selected goodness of fit criteria – Pearson correlation coefficient (R), coefficient of determination (R^2), adjusted coefficient of determination ($R^2_{adjusted}$), and residual sum of squares (RSS) (manual calculation in MS Excel):

Parameter	No.1	No.2	No.3	No.4
N	21	21	21	21
df	18	18	18	18
R	0.993960581	0.996496934	0.995735025	0.9958397
R^2	0.987957636	0.993006139	0.991488241	0.991696709
$R^2_{adjusted}$	0.986619596	0.992229043	0.99054249	0.990774121
RSS	146.7943563	63.58739937	97.92094792	97.4985752

Graphical abstract of model fit presented as mean ± 1 SD of the fraction % of released carvedilol:

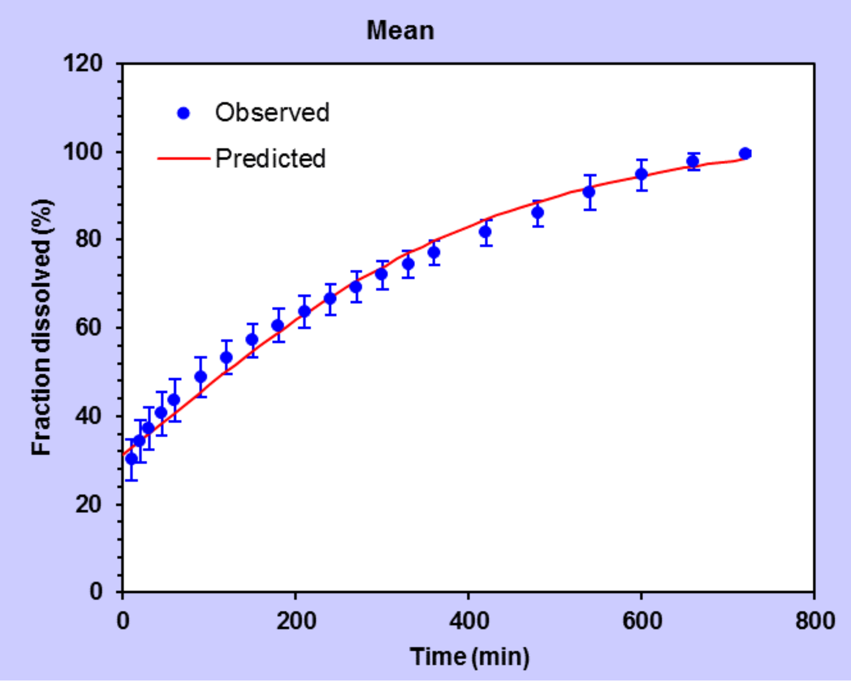

Supplementary materials – Model fitting summary – SuperTab® 11SD (modelling the entire relevant dissolution data)

Graphical abstract of model fit presented as the fraction % of released carvedilol per tested tablet:

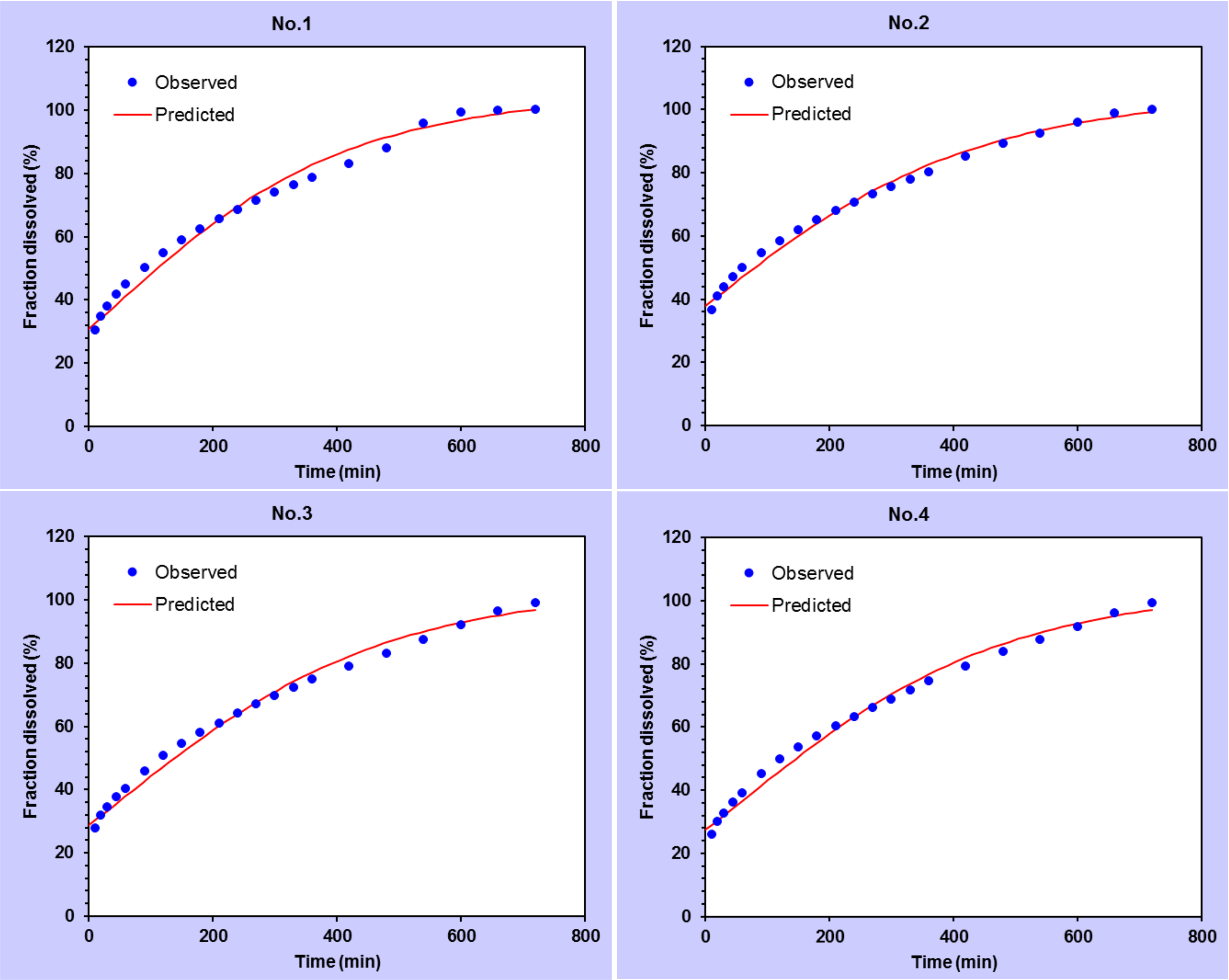

Supplementary materials – Model fitting summary – SuperTab® 11SD (modelling the entire relevant dissolution data)

Model: **Probit_1**

Model equation: $F = 100 \cdot \phi[\alpha + \beta \cdot \log(t)]$

Fitted model parameters per tested tablet (N = 4) with statistics – mean, standard deviation (SD), and relative standard deviation expressed in % (RSD%) (output from DDSolver):

Parameter	No.1	No.2	No.3	No.4	Mean	SD	RSD(%)
α	-2.645	-2.390	-2.382	-2.481	-2.475	0.122	-4.949
β	1.481	1.397	1.269	1.306	1.363	0.095	6.979

Number of dissolution data points (N), degrees of freedom (df), and selected goodness of fit criteria – Pearson correlation coefficient (R), coefficient of determination (R^2), adjusted coefficient of determination ($R^2_{adjusted}$), and residual sum of squares (RSS) (manual calculation in MS Excel):

Parameter	No.1	No.2	No.3	No.4
N	21	21	21	21
df	19	19	19	19
R	0.925838589	0.920814379	0.941563219	0.943687877
R^2	0.857177093	0.84789912	0.886541296	0.890546808
$R^2_{adjusted}$	0.849660098	0.839893811	0.880569785	0.884786114
RSS	2018.392253	1928.824885	1306.167589	1336.777929

Graphical abstract of model fit presented as mean ± 1 SD of the fraction % of released carvedilol:

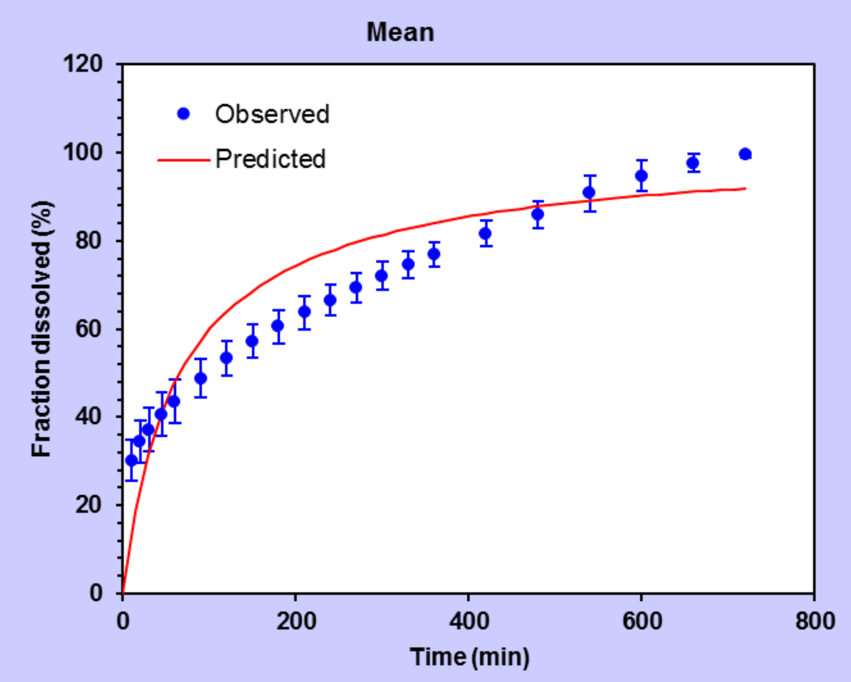

Supplementary materials – Model fitting summary – SuperTab® 11SD (modelling the entire relevant dissolution data)

Graphical abstract of model fit presented as the fraction % of released carvedilol per tested tablet:

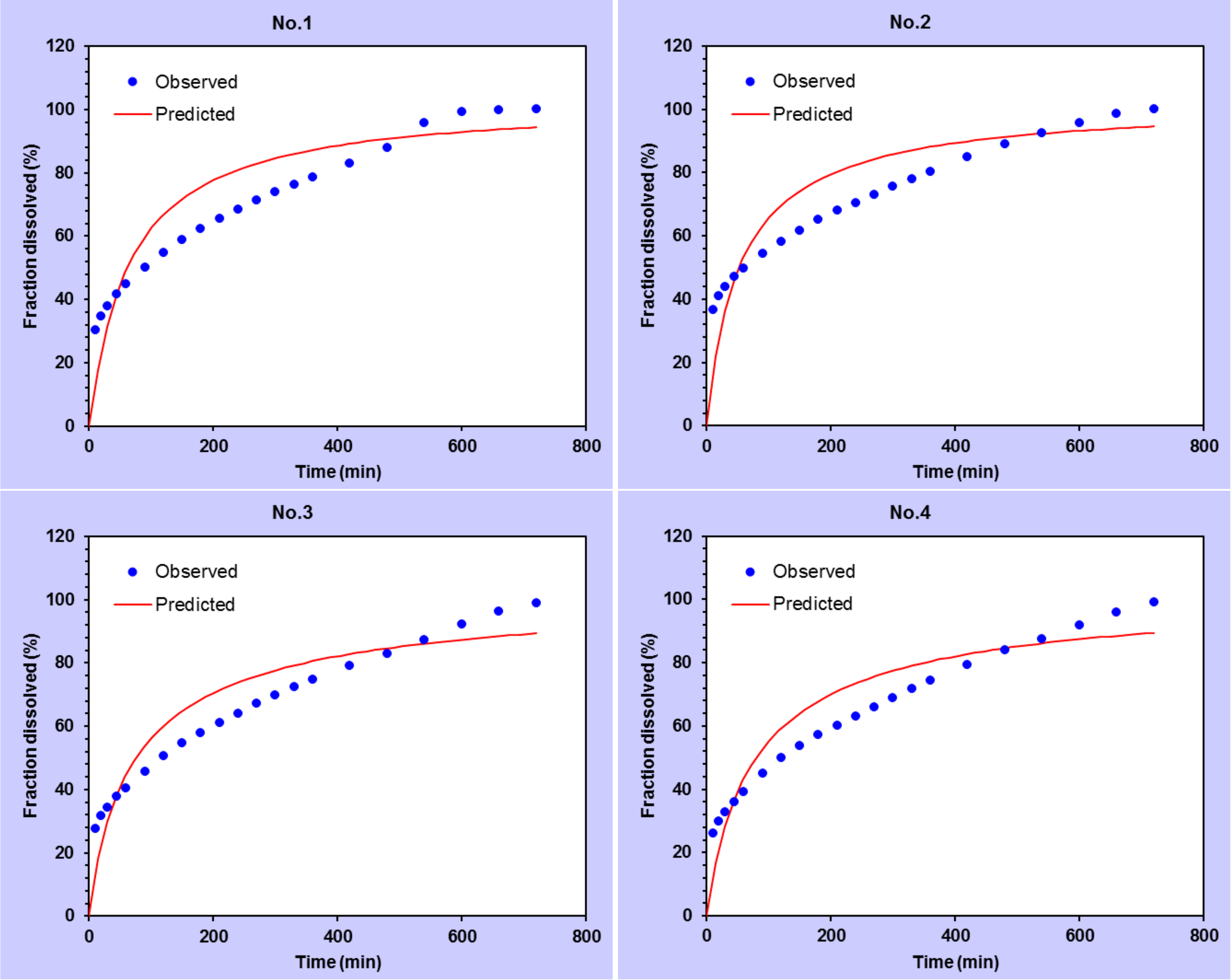

Supplementary materials – Model fitting summary – SuperTab® 11SD (modelling the entire relevant dissolution data)

Model: **Probit_2**

Model equation: $F = F_{max} \cdot \phi[\alpha + \beta \cdot \log(t)]$

Fitted model parameters per tested tablet (N = 4) with statistics – mean, standard deviation (SD), and relative standard deviation expressed in % (RSD%) (output from DDSolver):

Parameter	No.1	No.2	No.3	No.4	Mean	SD	RSD(%)
α	-2.199	-1.836	-2.173	-2.490	-2.174	0.268	-12.318
β	1.190	1.049	1.126	1.042	1.102	0.070	6.379
F_{max}	105.109	104.976	103.833	129.198	110.779	12.293	11.097

Number of dissolution data points (N), degrees of freedom (df), and selected goodness of fit criteria – Pearson correlation coefficient (R), coefficient of determination (R^2), adjusted coefficient of determination ($R^2_{adjusted}$), and residual sum of squares (RSS) (manual calculation in MS Excel):

Parameter	No.1	No.2	No.3	No.4
N	21	21	21	21
df	18	18	18	18
R	0.944091814	0.942261187	0.949671857	0.979102006
R^2	0.891309353	0.887856145	0.901876636	0.958640739
$R^2_{adjusted}$	0.879232615	0.875395716	0.89097404	0.954045266
RSS	1210.623949	964.727306	1009.119438	950.9777035

Graphical abstract of model fit presented as mean ± 1 SD of the fraction % of released carvedilol:

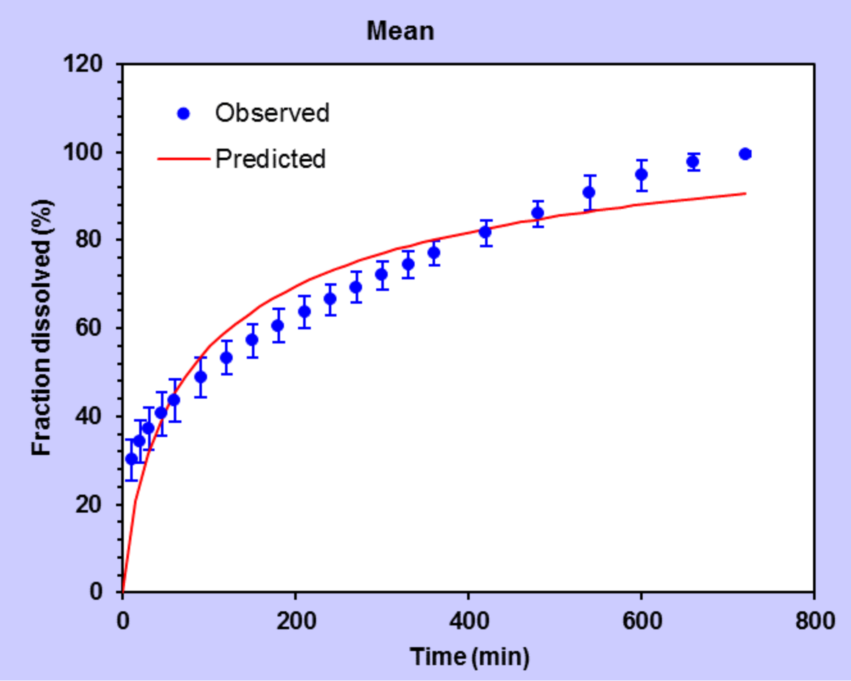

Supplementary materials – Model fitting summary – SuperTab® 11SD (modelling the entire relevant dissolution data)

Graphical abstract of model fit presented as the fraction % of released carvedilol per tested tablet:

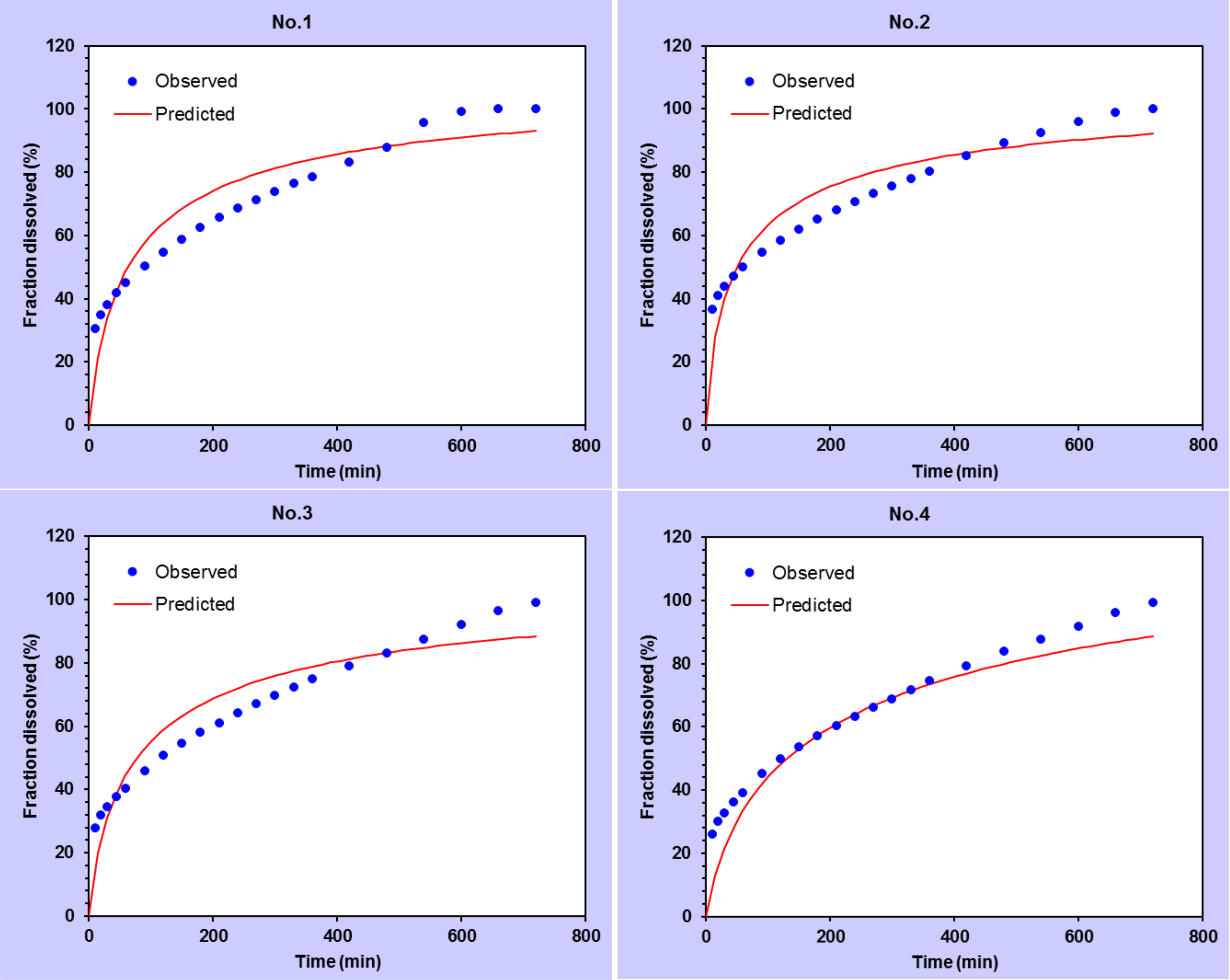

Supplementary materials – Model fitting summary – SuperTab® 11SD (modelling up to app. 60 % of carvedilol released)

Model: **Zero–order**

Model equation: $F = k_0 \cdot t$

Fitted model parameters per tested tablet (N = 4) with statistics – mean, standard deviation (SD), and relative standard deviation expressed in % (RSD%) (output from DDSolver):

Parameter	No.1	No.2	No.3	No.4	Mean	SD	RSD(%)
k_0	0.448	0.479	0.413	0.405	0.436	0.034	7.882

Number of dissolution data points (N), degrees of freedom (df), and selected goodness of fit criteria – Pearson correlation coefficient (R), coefficient of determination (R^2), adjusted coefficient of determination ($R^2_{adjusted}$), and residual sum of squares (RSS) (manual calculation in MS Excel):

Parameter	No.1	No.2	No.3	No.4
N	9	9	9	9
df	8	8	8	8
R	0.984851744	0.982371765	0.989657481	0.988750545
R^2	0.969932957	0.965054284	0.97942193	0.97762764
$R^2_{adjusted}$	0.969932957	0.965054284	0.97942193	0.97762764
RSS	3236.785956	4641.826216	2586.74967	2295.652692

Graphical abstract of model fit presented as mean ± 1 SD of the fraction % of released carvedilol:

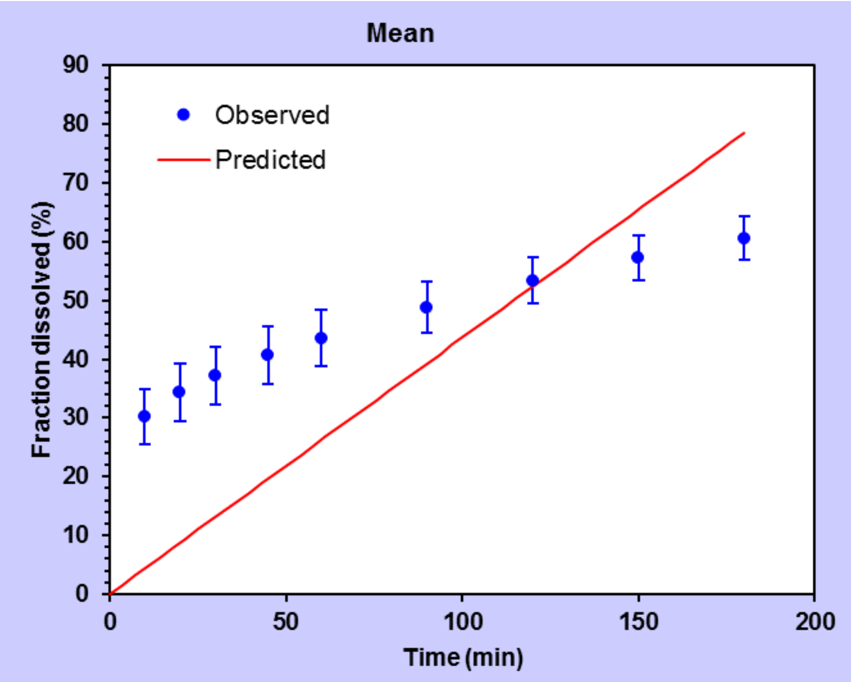

Supplementary materials – Model fitting summary – SuperTab® 11SD (modelling up to app. 60 % of carvedilol released)

Graphical abstract of model fit presented as the fraction % of released carvedilol per tested tablet:

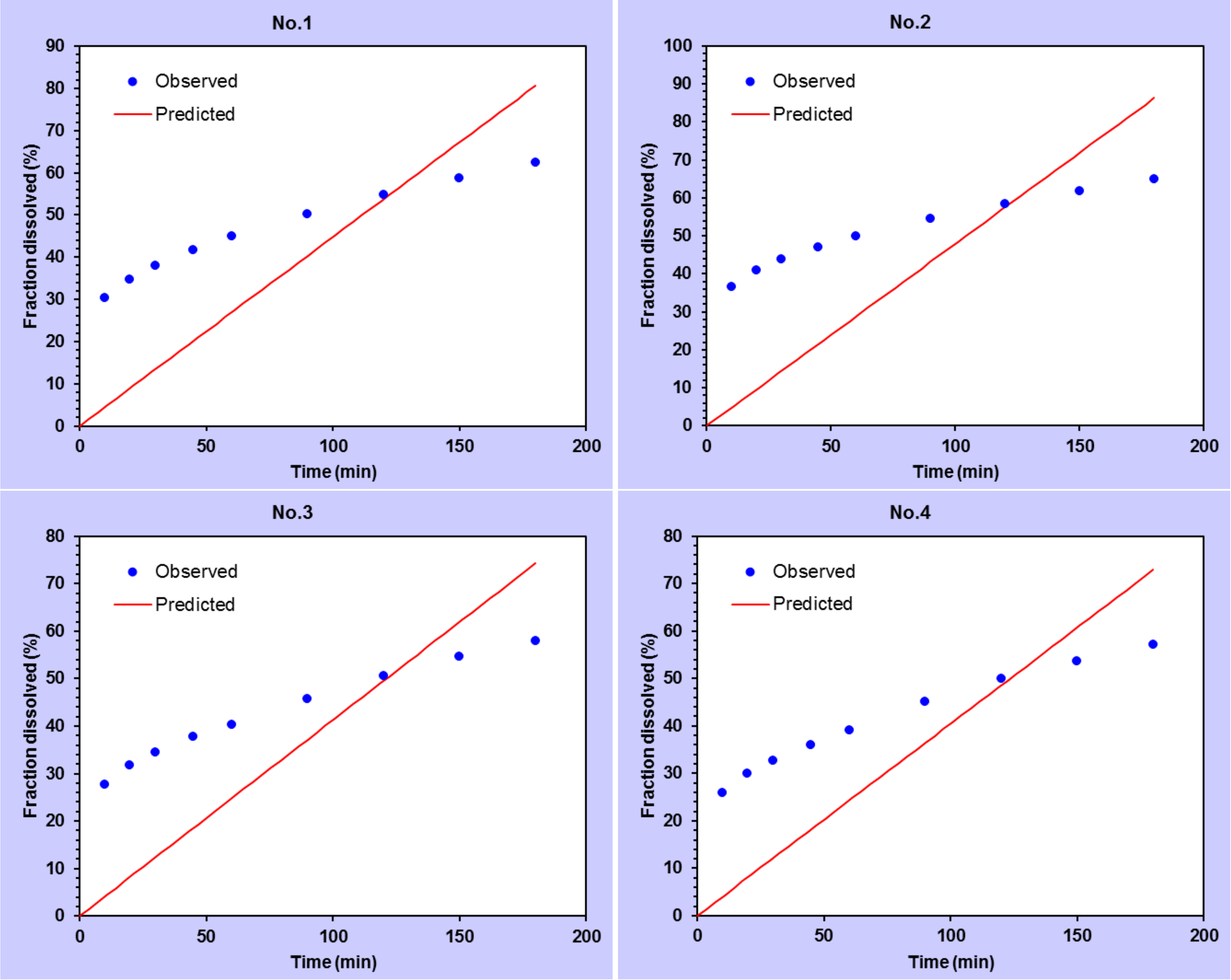

Supplementary materials – Model fitting summary – SuperTab® 11SD (modelling up to app. 60 % of carvedilol released)

Model: **Zero–order with T_{lag}**

Model equation: $F = k_0 \cdot (t - T_{lag})$

Fitted model parameters per tested tablet (N = 4) with statistics – mean, standard deviation (SD), and relative standard deviation expressed in % (RSD%) (output from DDSolver):

Parameter	No.1	No.2	No.3	No.4	Mean	SD	RSD(%)
k_0	0.180	0.158	0.173	0.179	0.173	0.010	5.944
T_{lag}	-178.155	-243.867	-166.072	-150.949	-184.761	40.946	-22.161

Number of dissolution data points (N), degrees of freedom (df), and selected goodness of fit criteria – Pearson correlation coefficient (R), coefficient of determination (R^2), adjusted coefficient of determination ($R^2_{adjusted}$), and residual sum of squares (RSS) (manual calculation in MS Excel):

Parameter	No.1	No.2	No.3	No.4
N	9	9	9	9
df	7	7	7	7
R	0.984851744	0.982371765	0.989657481	0.988750545
R^2	0.969932957	0.965054284	0.97942193	0.97762764
$R^2_{adjusted}$	0.965637665	0.960062039	0.976482206	0.974431589
RSS	29.38549782	26.36215568	18.35397737	21.42583201

Graphical abstract of model fit presented as mean ± 1 SD of the fraction % of released carvedilol:

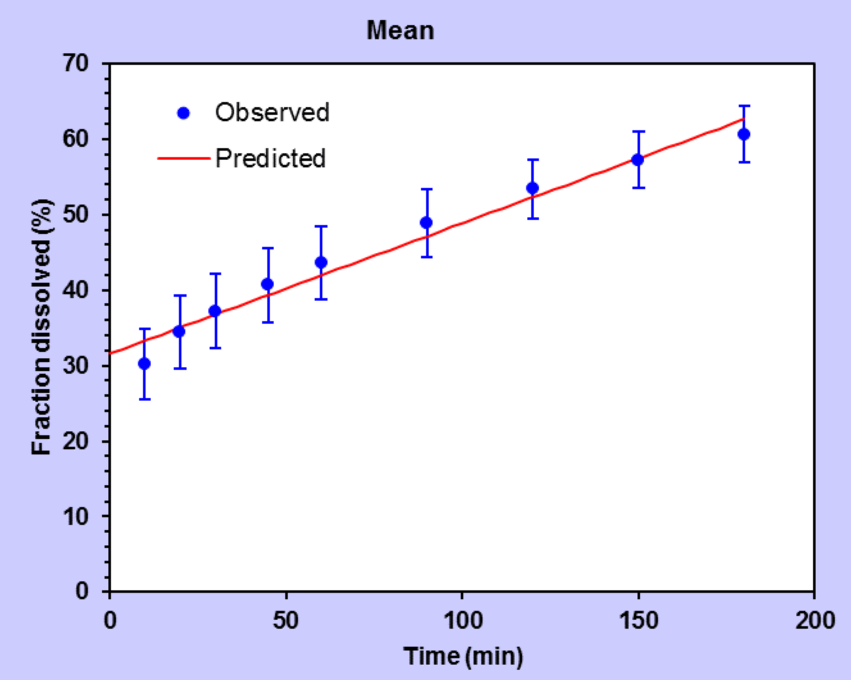

Supplementary materials – Model fitting summary – SuperTab® 11SD (modelling up to app. 60 % of carvedilol released)

Graphical abstract of model fit presented as the fraction % of released carvedilol per tested tablet:

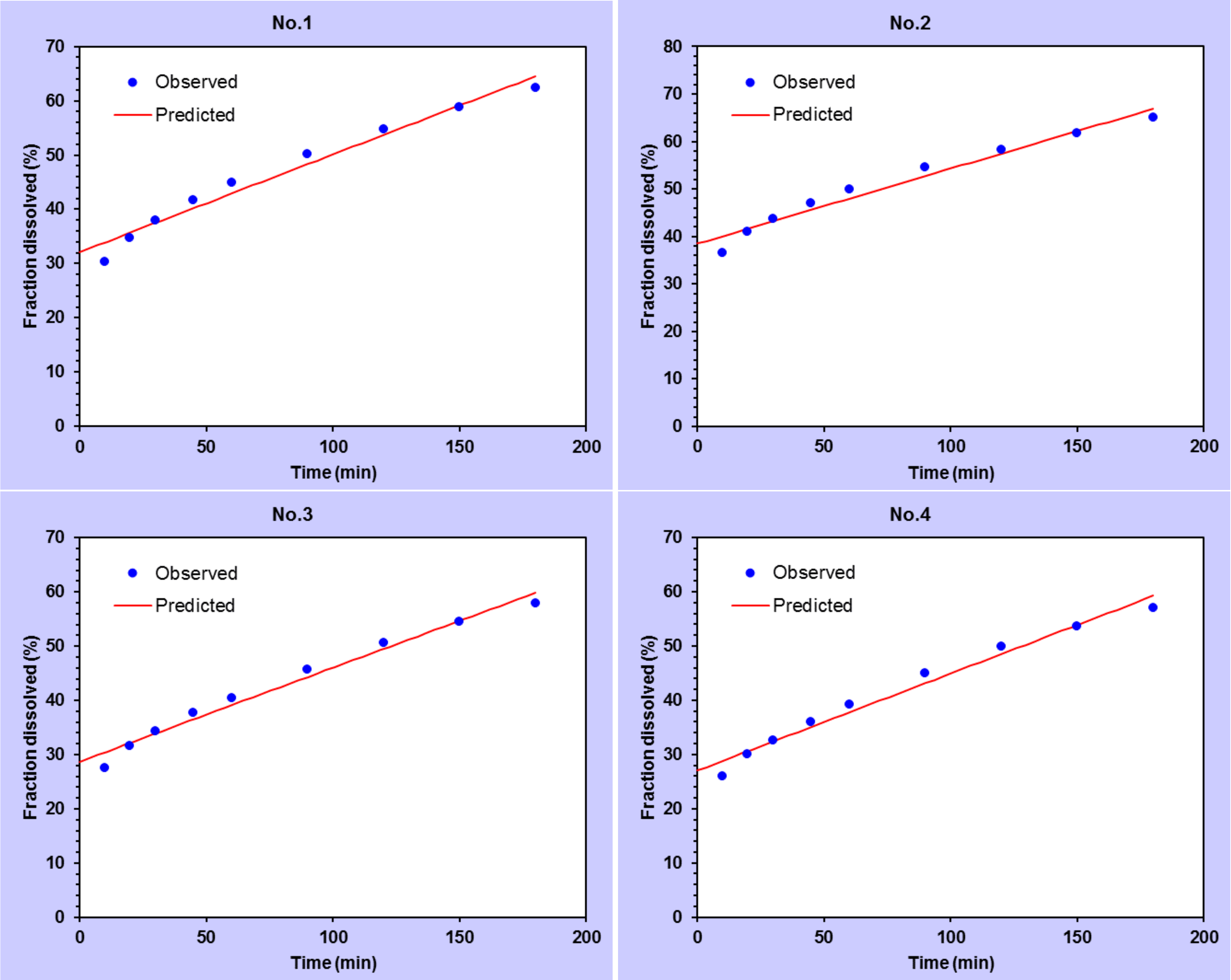

Supplementary materials – Model fitting summary – SuperTab® 11SD (modelling up to app. 60 % of carvedilol released)

Model: **Zero–order with F_0**

Model equation: $F = F_0 + k_0 \cdot t$

Fitted model parameters per tested tablet (N = 4) with statistics – mean, standard deviation (SD), and relative standard deviation expressed in % (RSD%) (output from DDSolver):

Parameter	No.1	No.2	No.3	No.4	Mean	SD	RSD(%)
k_0	0.180	0.158	0.173	0.179	0.173	0.010	5.944
F_0	32.100	38.506	28.725	27.030	31.590	5.070	16.048

Number of dissolution data points (N), degrees of freedom (df), and selected goodness of fit criteria – Pearson correlation coefficient (R), coefficient of determination (R^2), adjusted coefficient of determination ($R^2_{adjusted}$), and residual sum of squares (RSS) (manual calculation in MS Excel):

Parameter	No.1	No.2	No.3	No.4
N	9	9	9	9
df	7	7	7	7
R	0.984851744	0.982371765	0.989657481	0.988750545
R^2	0.969932957	0.965054284	0.97942193	0.97762764
$R^2_{adjusted}$	0.965637665	0.960062039	0.976482206	0.974431589
RSS	29.38549782	26.36215568	18.35397737	21.42583201

Graphical abstract of model fit presented as mean ± 1 SD of the fraction % of released carvedilol:

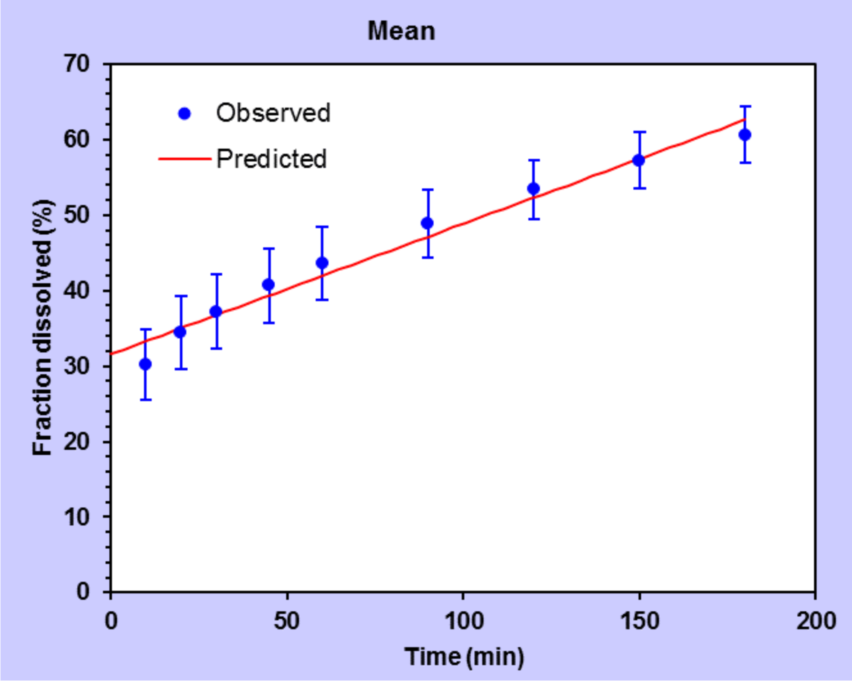

Supplementary materials – Model fitting summary – SuperTab® 11SD (modelling up to app. 60 % of carvedilol released)

Graphical abstract of model fit presented as the fraction % of released carvedilol per tested tablet:

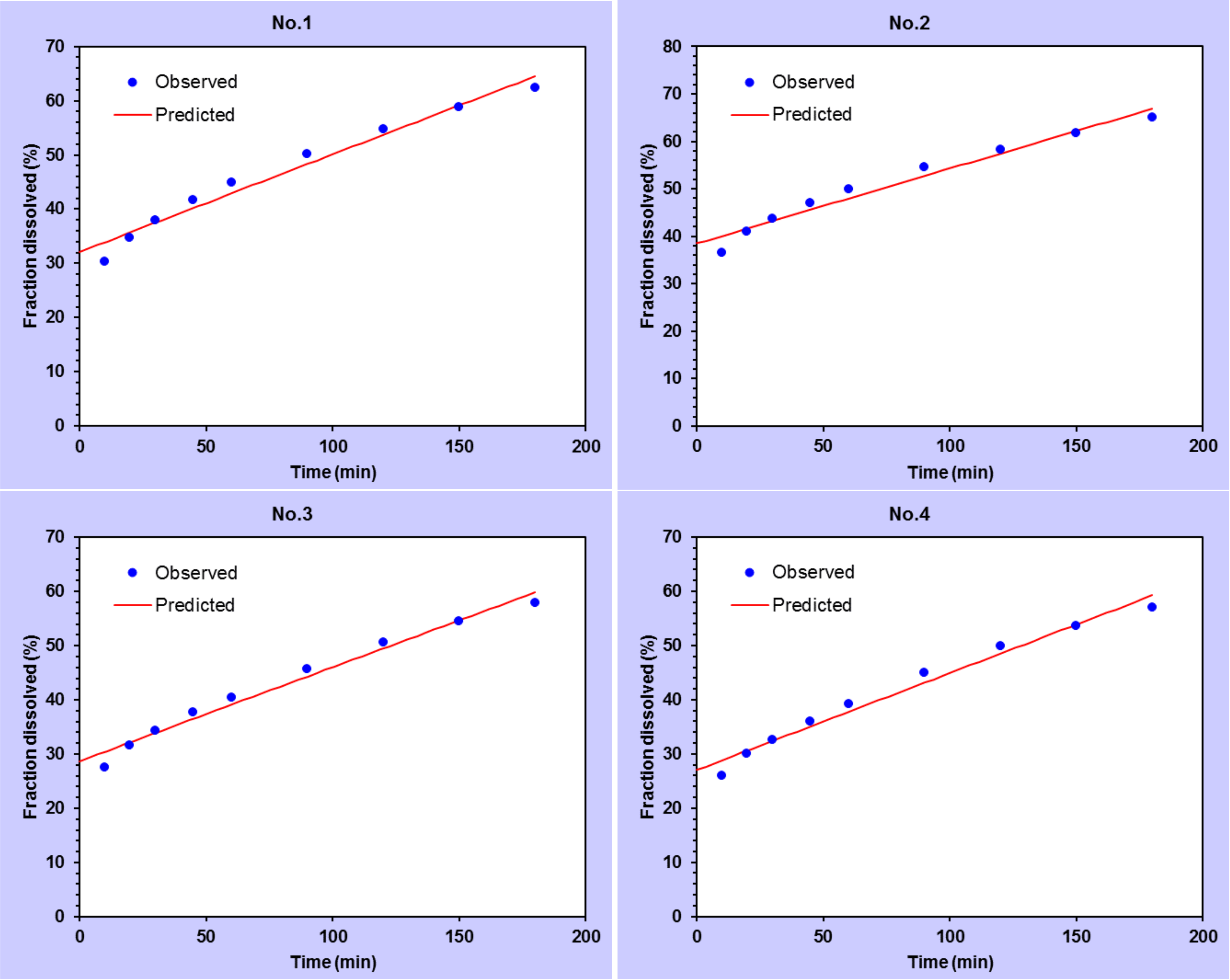

Supplementary materials – Model fitting summary – SuperTab® 11SD (modelling up to app. 60 % of carvedilol released)

Model: **First–order**

Model equation: $F = 100 \cdot (1 - e^{-k_1 \cdot t})$

Fitted model parameters per tested tablet (N = 4) with statistics – mean, standard deviation (SD), and relative standard deviation expressed in % (RSD%) (output from DDSolver):

Parameter	No.1	No.2	No.3	No.4	Mean	SD	RSD(%)
k_1	0.010	0.011	0.006	0.006	0.008	0.003	33.678

Number of dissolution data points (N), degrees of freedom (df), and selected goodness of fit criteria – Pearson correlation coefficient (R), coefficient of determination (R^2), adjusted coefficient of determination ($R^2_{adjusted}$), and residual sum of squares (RSS) (manual calculation in MS Excel):

Parameter	No.1	No.2	No.3	No.4
N	9	9	9	9
df	8	8	8	8
R	0.998109434	0.997076782	0.998883491	0.999231336
R^2	0.996222443	0.99416211	0.997768229	0.998463263
$R^2_{adjusted}$	0.996222443	0.99416211	0.997768229	0.998463263
RSS	1963.793682	2536.695507	1672.896522	1465.127241

Graphical abstract of model fit presented as mean ± 1 SD of the fraction % of released carvedilol:

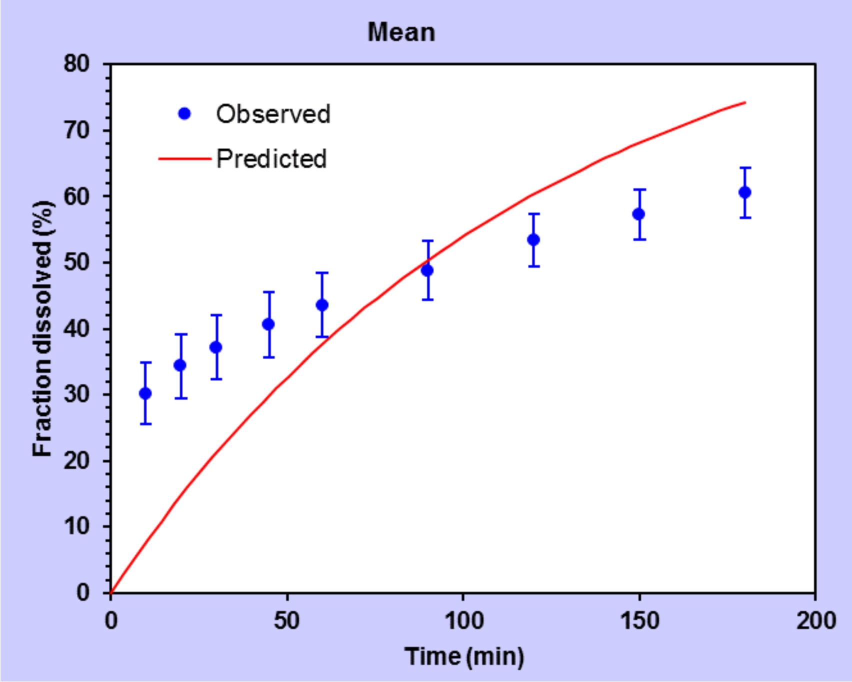

Supplementary materials – Model fitting summary – SuperTab® 11SD (modelling up to app. 60 % of carvedilol released)

Graphical abstract of model fit presented as the fraction % of released carvedilol per tested tablet:

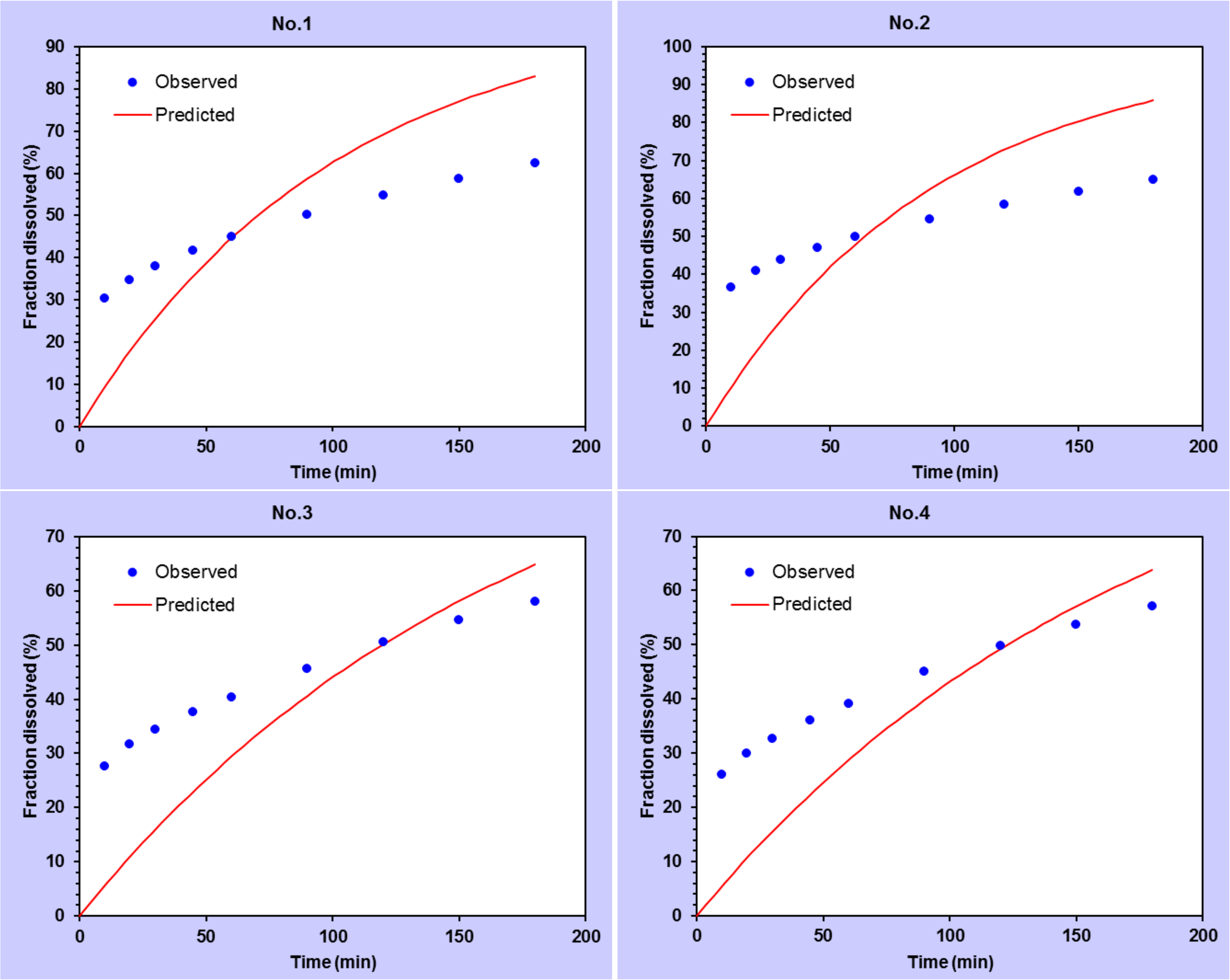

Supplementary materials – Model fitting summary – SuperTab® 11SD (modelling up to app. 60 % of carvedilol released)

Model: **First–order with T_{lag}**

Model equation: $F = 100 \cdot \left[1 - e^{-k_1 \cdot (t - T_{lag})}\right]$

Fitted model parameters per tested tablet (N = 4) with statistics – mean, standard deviation (SD), and relative standard deviation expressed in % (RSD%) (output from DDSolver):

Parameter	No.1	No.2	No.3	No.4	Mean	SD	RSD(%)
k_1	0.004	0.003	0.003	0.003	0.003	0.000	5.321
T_{lag}	-104.347	-139.471	-102.567	-93.775	-110.040	20.158	-18.318

Number of dissolution data points (N), degrees of freedom (df), and selected goodness of fit criteria – Pearson correlation coefficient (R), coefficient of determination (R^2), adjusted coefficient of determination ($R^2_{adjusted}$), and residual sum of squares (RSS) (manual calculation in MS Excel):

Parameter	No.1	No.2	No.3	No.4
N	9	9	9	9
df	7	7	7	7
R	0.994537517	0.992398951	0.996603262	0.996521394
R^2	0.989104873	0.984855679	0.993218062	0.993054889
$R^2_{adjusted}$	0.987548426	0.982692204	0.992249214	0.99206273
RSS	11.03251192	11.78136723	6.212718472	6.892428261

Graphical abstract of model fit presented as mean ± 1 SD of the fraction % of released carvedilol:

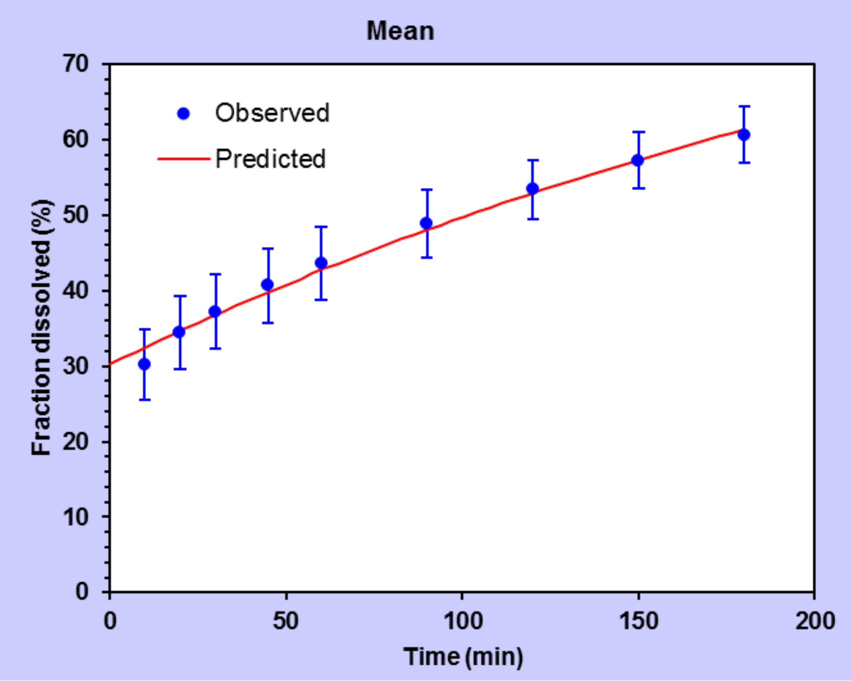

Supplementary materials – Model fitting summary – SuperTab® 11SD (modelling up to app. 60 % of carvedilol released)

Graphical abstract of model fit presented as the fraction % of released carvedilol per tested tablet:

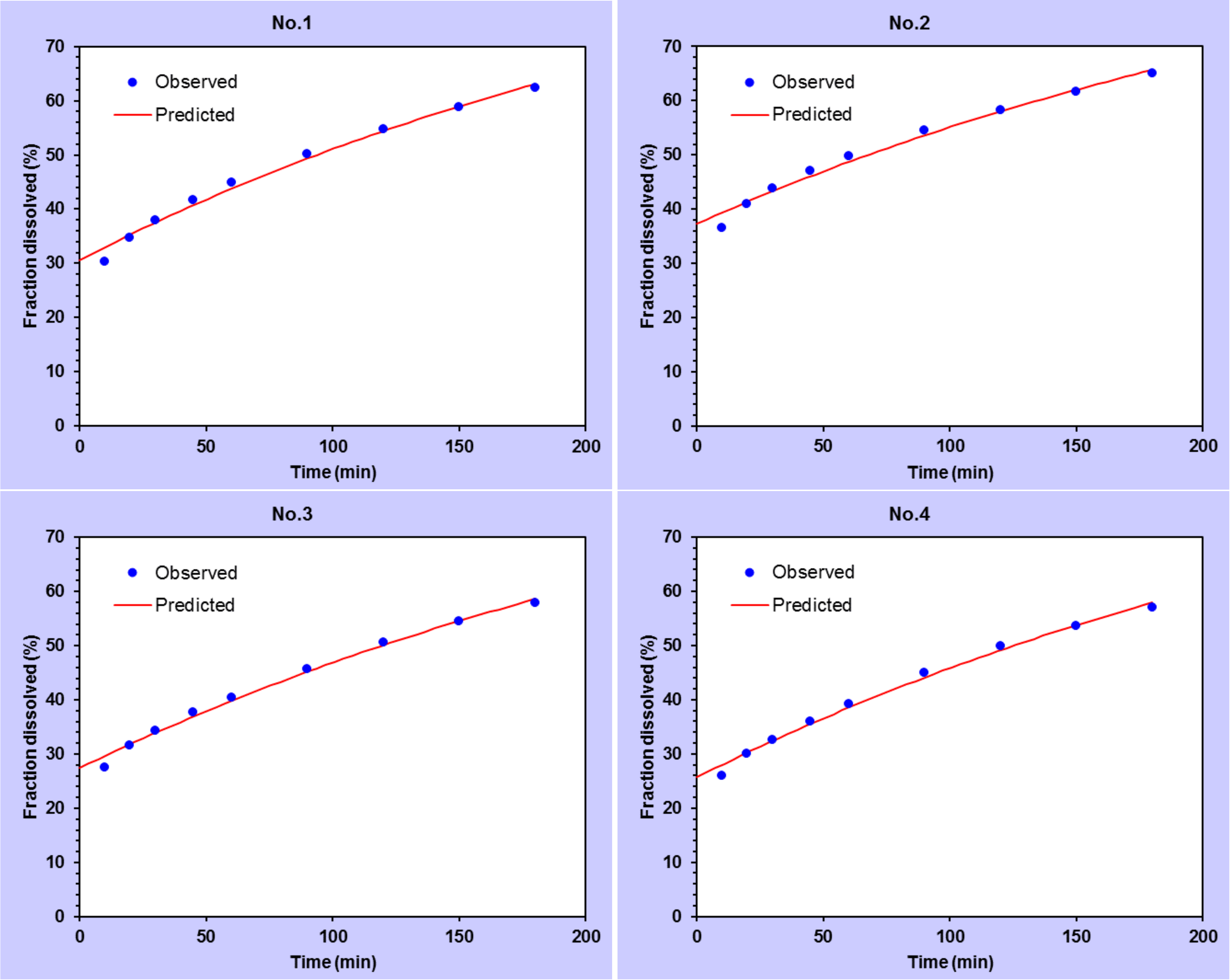

Supplementary materials – Model fitting summary – SuperTab® 11SD (modelling up to app. 60 % of carvedilol released)

Model: **First–order with F_{max}**

Model equation: $F = F_{max} \cdot (1 - e^{-k_1 \cdot t})$

Fitted model parameters per tested tablet (N = 4) with statistics – mean, standard deviation (SD), and relative standard deviation expressed in % (RSD%) (output from DDSolver):

Parameter	No.1	No.2	No.3	No.4	Mean	SD	RSD(%)
k_1	0.025	0.026	0.025	0.024	0.025	0.001	2.842
F_{max}	65.456	68.270	60.836	59.914	63.619	3.936	6.187

Number of dissolution data points (N), degrees of freedom (df), and selected goodness of fit criteria – Pearson correlation coefficient (R), coefficient of determination (R^2), adjusted coefficient of determination ($R^2_{adjusted}$), and residual sum of squares (RSS) (manual calculation in MS Excel):

Parameter	No.1	No.2	No.3	No.4
N	9	9	9	9
df	7	7	7	7
R	0.958454294	0.957715768	0.949703907	0.952268162
R^2	0.918634634	0.917219493	0.901937512	0.906814653
$R^2_{adjusted}$	0.90701101	0.905393706	0.887928585	0.89350246
RSS	545.1972332	812.395266	479.4050171	423.2217058

Graphical abstract of model fit presented as mean ± 1 SD of the fraction % of released carvedilol:

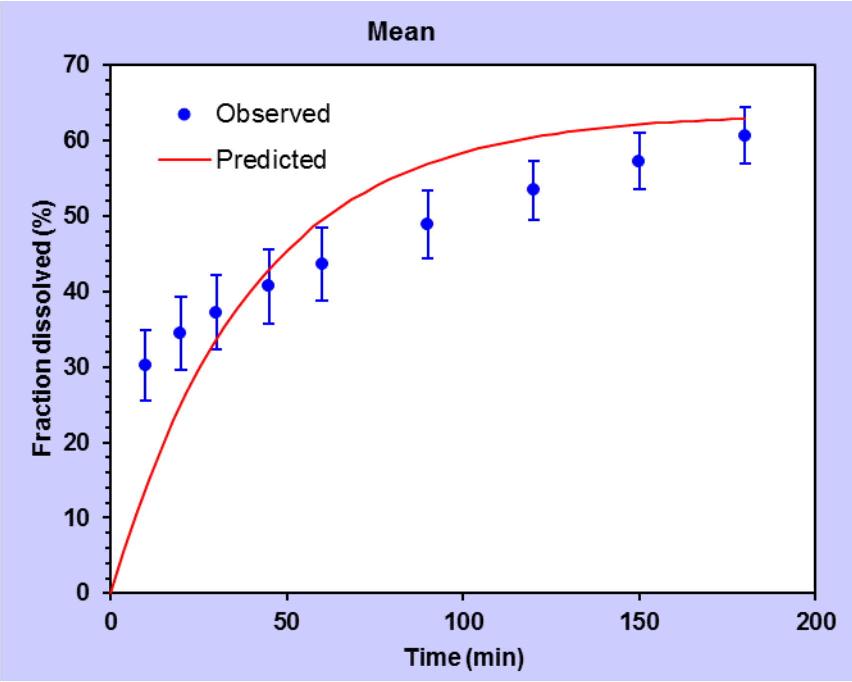

Supplementary materials – Model fitting summary – SuperTab® 11SD (modelling up to app. 60 % of carvedilol released)

Graphical abstract of model fit presented as the fraction % of released carvedilol per tested tablet:

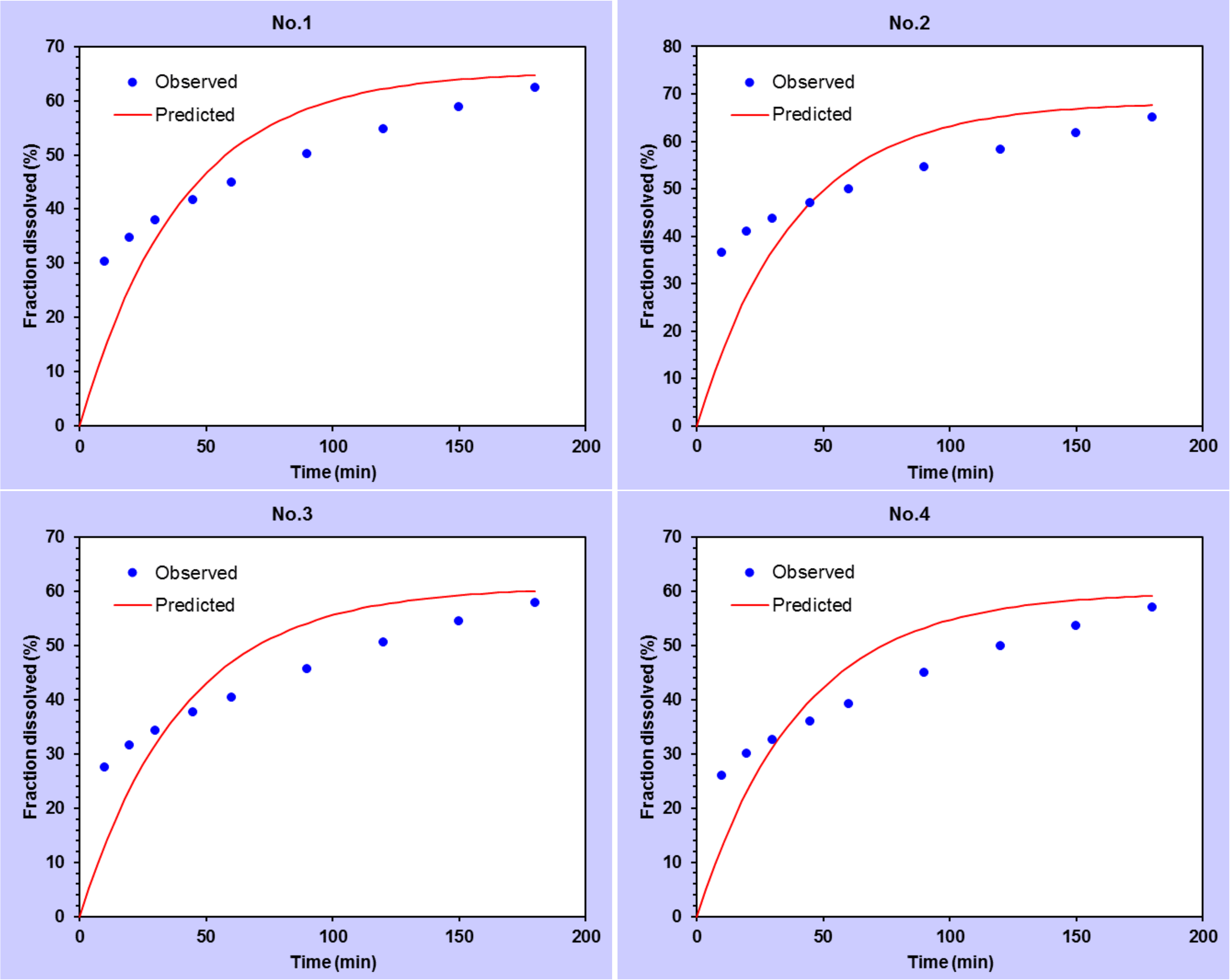

Supplementary materials – Model fitting summary – SuperTab® 11SD (modelling up to app. 60 % of carvedilol released)

Model: **First–order with T_{lag} and F_{max}**

Model equation: $F = F_{max} \cdot \left[1 - e^{-k_1 \cdot (t - T_{lag})}\right]$

Fitted model parameters per tested tablet (N = 4) with statistics – mean, standard deviation (SD), and relative standard deviation expressed in % (RSD%) (output from DDSolver):

Parameter	No.1	No.2	No.3	No.4	Mean	SD	RSD(%)
k_1	0.013	0.012	0.013	0.013	0.013	0.000	2.882
T_{lag}	-32.337	-49.464	-36.866	-31.712	-37.594	8.240	-21.917
F_{max}	65.456	68.270	57.667	56.794	62.047	5.690	9.170

Number of dissolution data points (N), degrees of freedom (df), and selected goodness of fit criteria – Pearson correlation coefficient (R), coefficient of determination (R^2), adjusted coefficient of determination ($R^2_{adjusted}$), and residual sum of squares (RSS) (manual calculation in MS Excel):

Parameter	No.1	No.2	No.3	No.4
N	9	9	9	9
df	6	6	6	6
R	0.994020379	0.995587652	0.991143065	0.992011218
R^2	0.988076513	0.991194772	0.982364576	0.984086256
$R^2_{adjusted}$	0.984102017	0.988259696	0.976486102	0.978781674
RSS	23.86737781	12.03132681	30.4030545	32.60503756

Graphical abstract of model fit presented as mean ± 1 SD of the fraction % of released carvedilol:

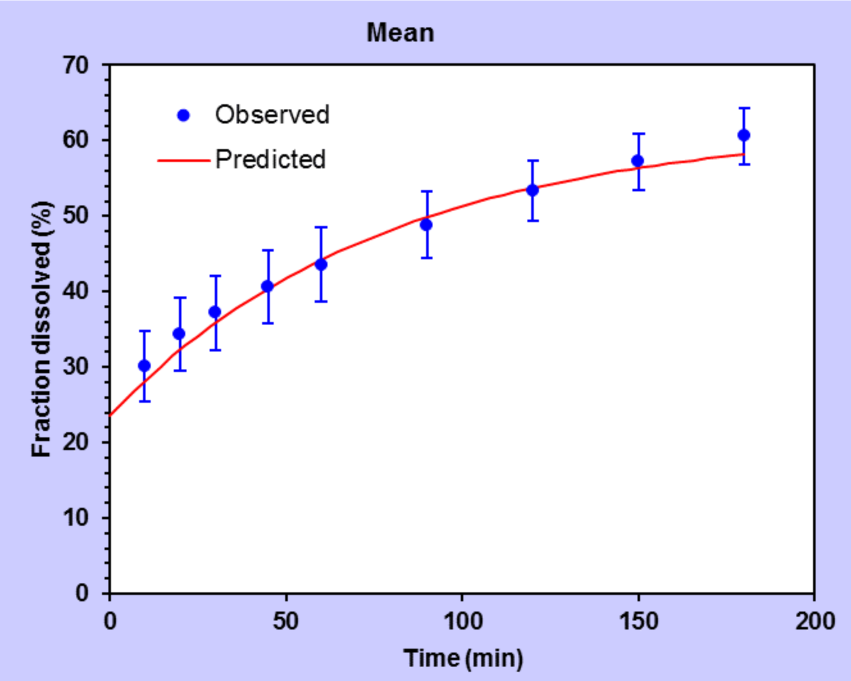

Supplementary materials – Model fitting summary – SuperTab® 11SD (modelling up to app. 60 % of carvedilol released)

Graphical abstract of model fit presented as the fraction % of released carvedilol per tested tablet:

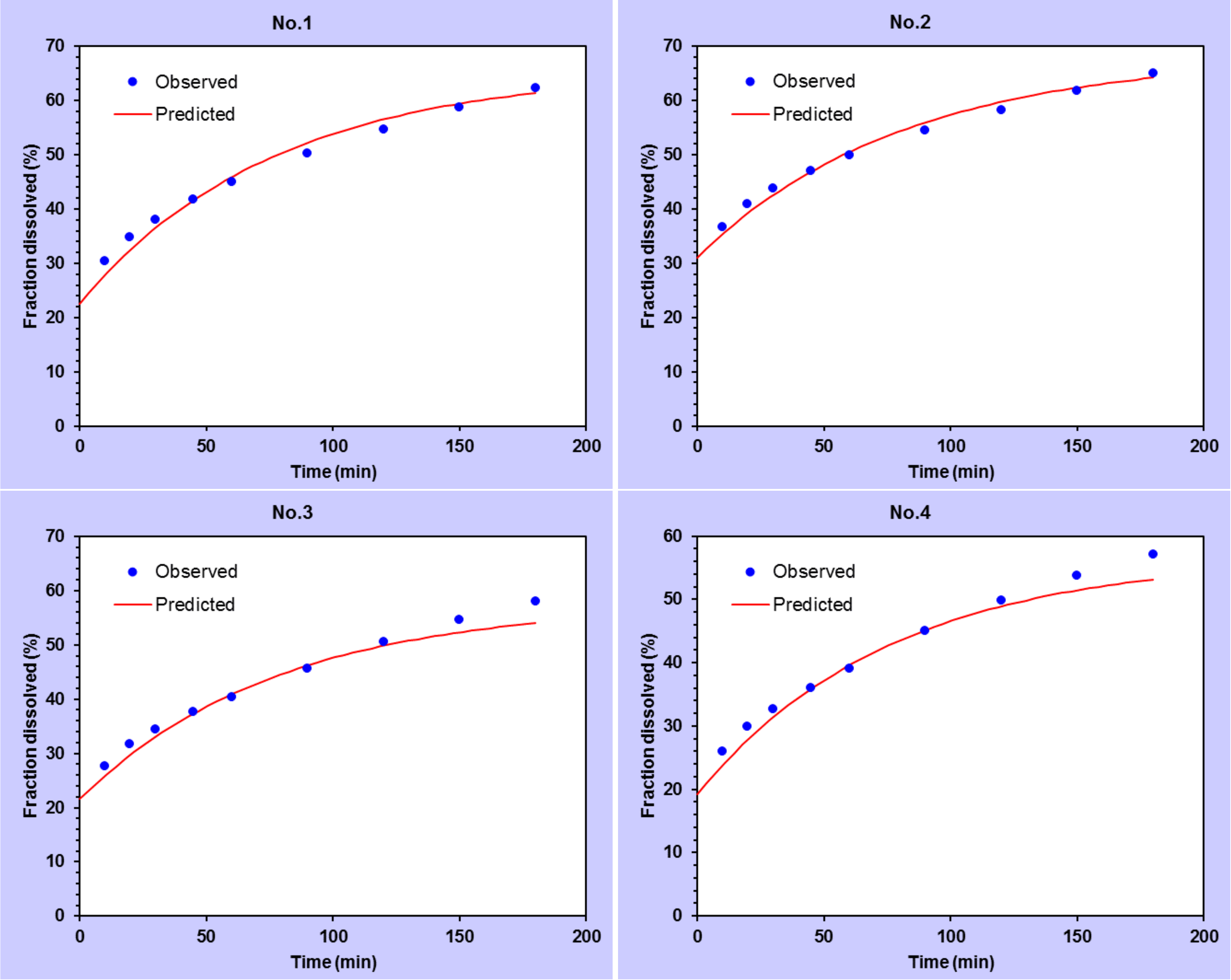

Supplementary materials – Model fitting summary – SuperTab® 11SD (modelling up to app. 60 % of carvedilol released)

Model: **Higuchi**

Model equation: $F = k_H \cdot t^{0.5}$

Fitted model parameters per tested tablet (N = 4) with statistics – mean, standard deviation (SD), and relative standard deviation expressed in % (RSD%) (output from DDSolver):

Parameter	No.1	No.2	No.3	No.4	Mean	SD	RSD(%)
k_H	5.277	5.710	4.845	4.733	5.141	0.446	8.666

Number of dissolution data points (N), degrees of freedom (df), and selected goodness of fit criteria – Pearson correlation coefficient (R), coefficient of determination (R^2), adjusted coefficient of determination ($R^2_{adjusted}$), and residual sum of squares (RSS) (manual calculation in MS Excel):

Parameter	No.1	No.2	No.3	No.4
N	9	9	9	9
df	8	8	8	8
R	0.999935022	0.999579194	0.999434605	0.999532585
R^2	0.999870048	0.999158564	0.99886953	0.999065388
$R^2_{adjusted}$	0.999870048	0.999158564	0.99886953	0.999065388
RSS	567.0982299	1066.602875	426.2836311	333.2657343

Graphical abstract of model fit presented as mean ± 1 SD of the fraction % of released carvedilol:

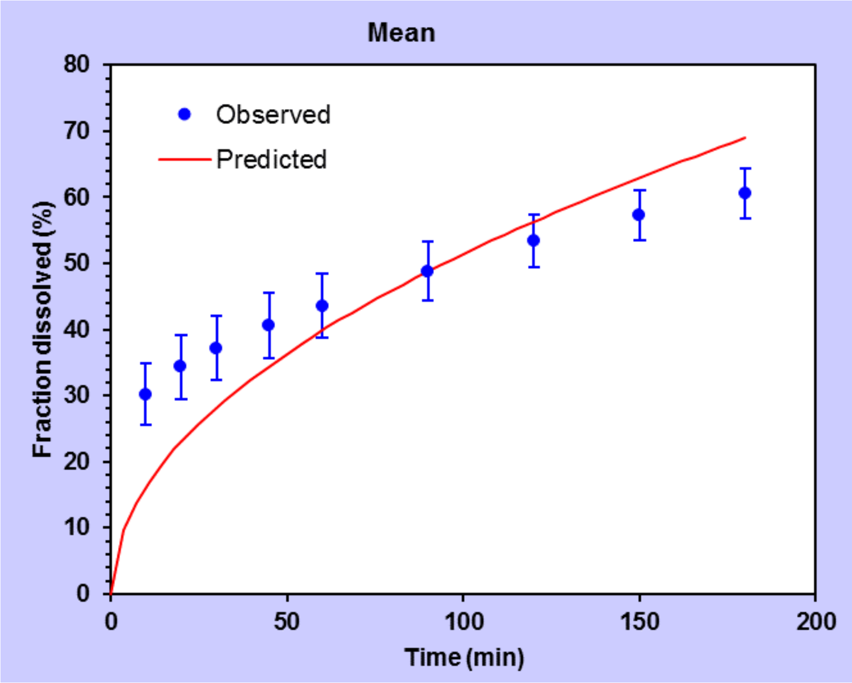

Supplementary materials – Model fitting summary – SuperTab® 11SD (modelling up to app. 60 % of carvedilol released)

Graphical abstract of model fit presented as the fraction % of released carvedilol per tested tablet:

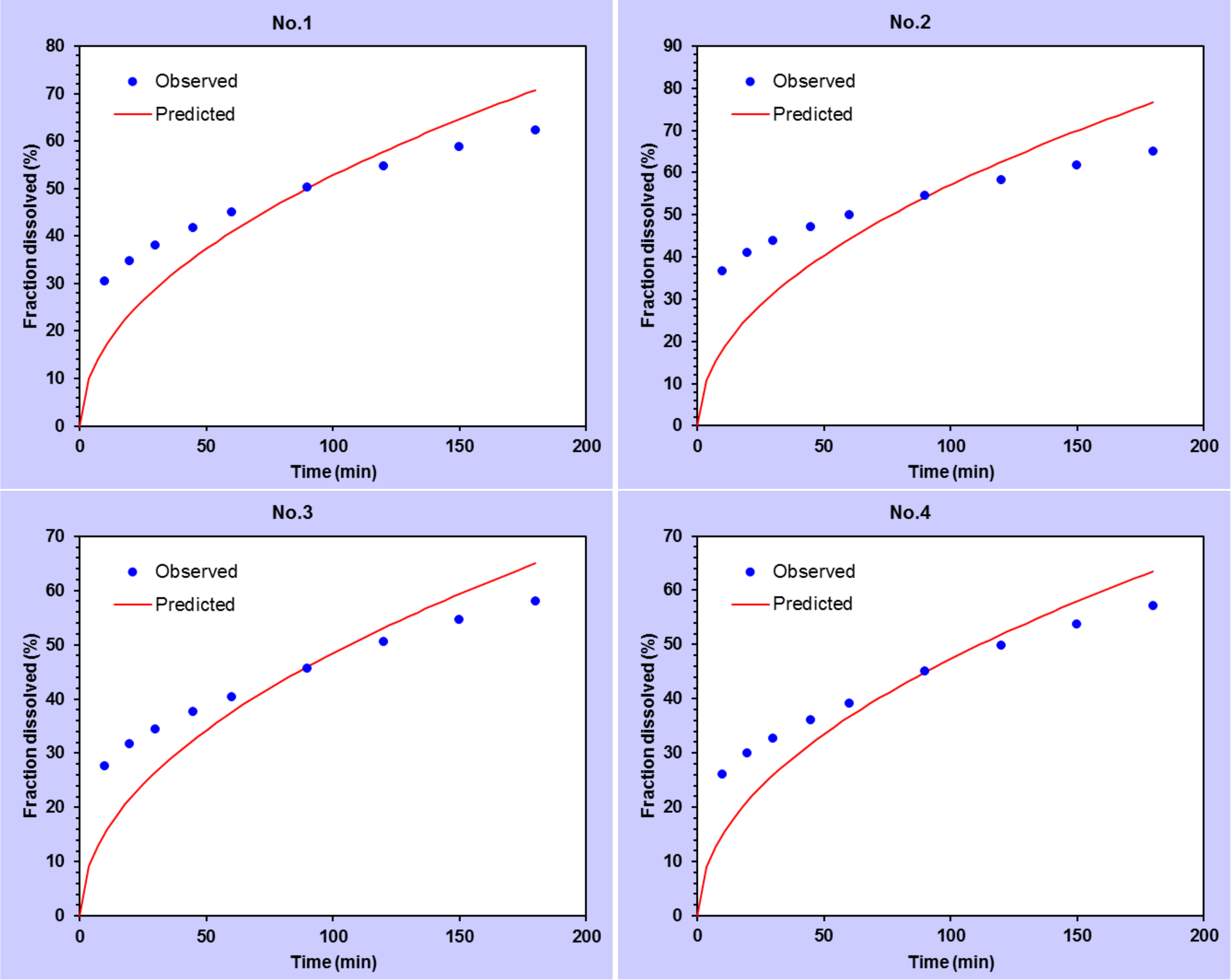

Supplementary materials – Model fitting summary – SuperTab® 11SD (modelling up to app. 60 % of carvedilol released)

Model: **Higuchi with T_{lag}**

Model equation: $F = k_H \cdot \left(t - T_{lag}\right)^{0.5}$

Fitted model parameters per tested tablet (N = 4) with statistics – mean, standard deviation (SD), and relative standard deviation expressed in % (RSD%) (output from DDSolver):

Parameter	No.1	No.2	No.3	No.4	Mean	SD	RSD(%)
k_H	4.132	4.045	3.885	3.896	3.990	0.120	3.000
T_{lag}	-53.106	-84.956	-46.614	-39.699	-56.094	20.005	-35.664

Number of dissolution data points (N), degrees of freedom (df), and selected goodness of fit criteria – Pearson correlation coefficient (R), coefficient of determination (R^2), adjusted coefficient of determination ($R^2_{adjusted}$), and residual sum of squares (RSS) (manual calculation in MS Excel):

Parameter	No.1	No.2	No.3	No.4
N	9	9	9	9
df	7	7	7	7
R	0.995506213	0.991955768	0.99802986	0.998263253
R^2	0.991032621	0.983976246	0.996063602	0.996529522
$R^2_{adjusted}$	0.989751567	0.981687139	0.995501259	0.996033739
RSS	9.276715443	12.50736081	3.689737903	3.576242117

Graphical abstract of model fit presented as mean ± 1 SD of the fraction % of released carvedilol:

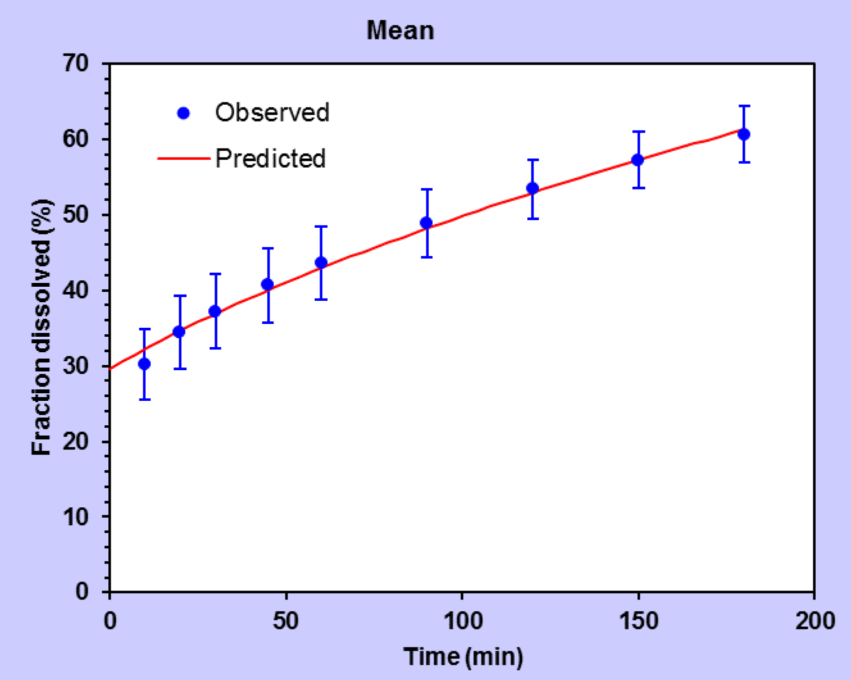

Supplementary materials – Model fitting summary – SuperTab® 11SD (modelling up to app. 60 % of carvedilol released)

Graphical abstract of model fit presented as the fraction % of released carvedilol per tested tablet:

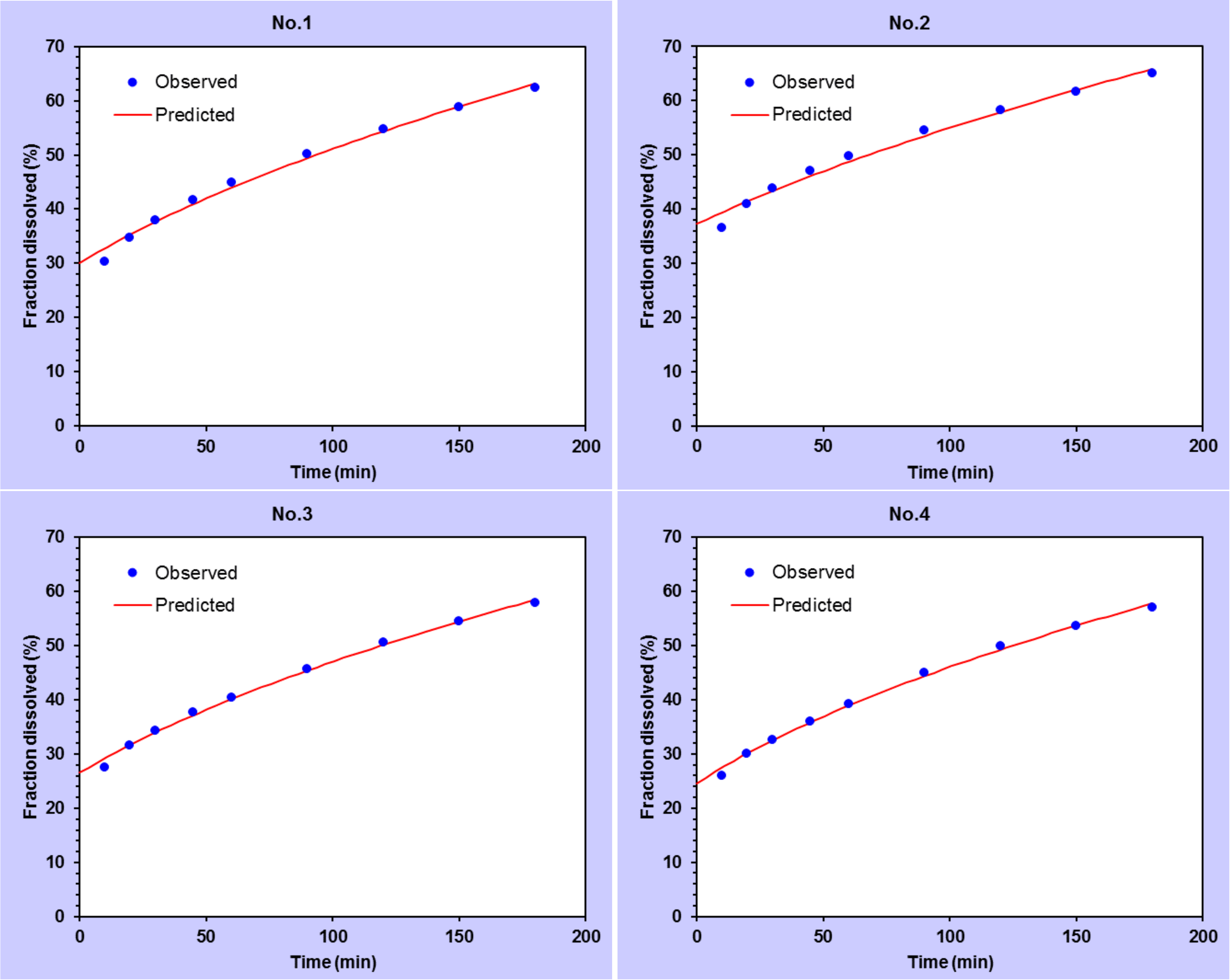

Supplementary materials – Model fitting summary – SuperTab® 11SD (modelling up to app. 60 % of carvedilol released)

Model: **Higuchi with F_0**

Model equation: $F = F_0 + k_H \cdot t^{0.5}$

Fitted model parameters per tested tablet (N = 4) with statistics – mean, standard deviation (SD), and relative standard deviation expressed in % (RSD%) (output from DDSolver):

Parameter	No.1	No.2	No.3	No.4	Mean	SD	RSD(%)
k_H	3.096	2.719	2.956	3.063	2.959	0.171	5.765
F_0	20.871	28.618	18.076	15.980	20.886	5.530	26.477

Number of dissolution data points (N), degrees of freedom (df), and selected goodness of fit criteria – Pearson correlation coefficient (R), coefficient of determination (R^2), adjusted coefficient of determination ($R^2_{adjusted}$), and residual sum of squares (RSS) (manual calculation in MS Excel):

Parameter	No.1	No.2	No.3	No.4
N	9	9	9	9
df	7	7	7	7
R	0.999935022	0.999579194	0.999434605	0.999532585
R^2	0.999870048	0.999158564	0.99886953	0.999065388
$R^2_{adjusted}$	0.999851484	0.999038359	0.998708034	0.998931872
RSS	0.127006063	0.634757707	1.008287971	0.895070143

Graphical abstract of model fit presented as mean ± 1 SD of the fraction % of released carvedilol:

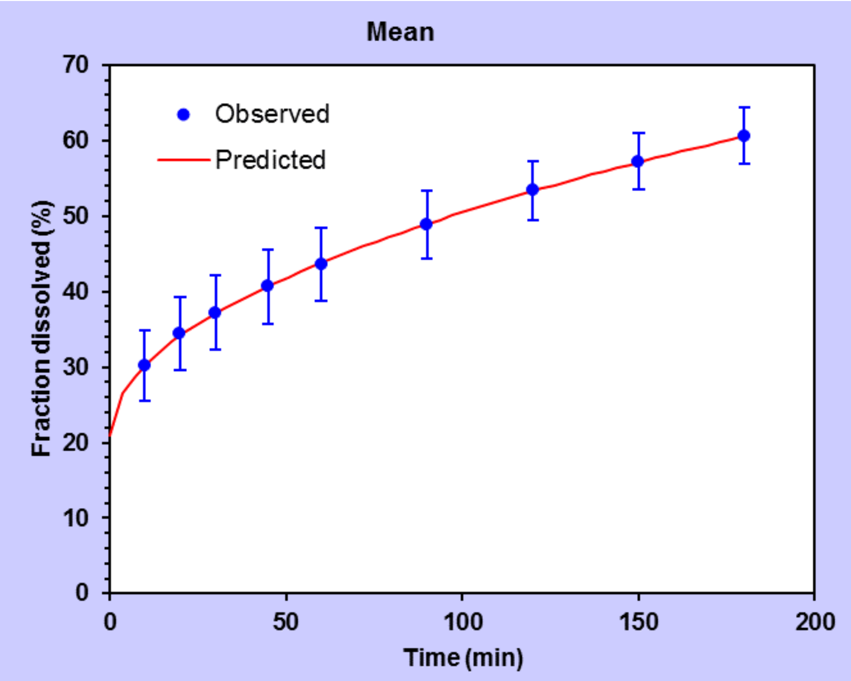

Supplementary materials – Model fitting summary – SuperTab® 11SD (modelling up to app. 60 % of carvedilol released)

Graphical abstract of model fit presented as the fraction % of released carvedilol per tested tablet:

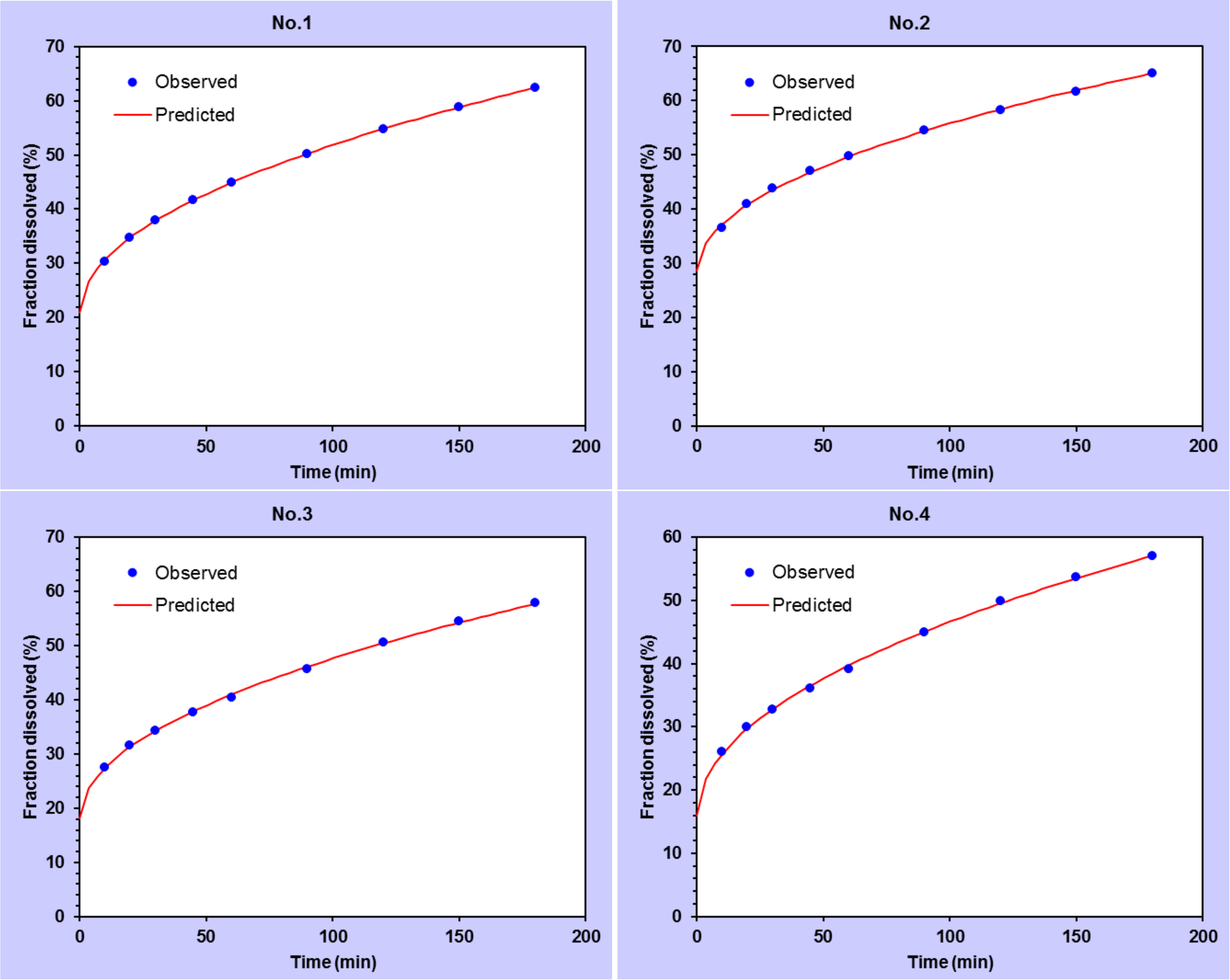

Supplementary materials – Model fitting summary – SuperTab® 11SD (modelling up to app. 60 % of carvedilol released)

Model: **Korsmeyer–Peppas**

Model equation: $F = k_{KP} \cdot t^n$

Fitted model parameters per tested tablet (N = 4) with statistics – mean, standard deviation (SD), and relative standard deviation expressed in % (RSD%) (output from DDSolver):

Parameter	No.1	No.2	No.3	No.4	Mean	SD	RSD(%)
k_{KP}	16.459	22.561	14.535	13.036	16.648	4.184	25.132
n	0.251	0.199	0.259	0.278	0.247	0.034	13.770

Number of dissolution data points (N), degrees of freedom (df), and selected goodness of fit criteria – Pearson correlation coefficient (R), coefficient of determination (R^2), adjusted coefficient of determination ($R^2_{adjusted}$), and residual sum of squares (RSS) (manual calculation in MS Excel):

Parameter	No.1	No.2	No.3	No.4
N	9	9	9	9
df	7	7	7	7
R	0.99615676	0.99549977	0.992995269	0.994163308
R^2	0.992328291	0.991019792	0.986039604	0.988360684
$R^2_{adjusted}$	0.991232333	0.989736906	0.984045262	0.986697924
RSS	8.217729204	7.159720245	13.66559124	12.47196315

Graphical abstract of model fit presented as mean ± 1 SD of the fraction % of released carvedilol:

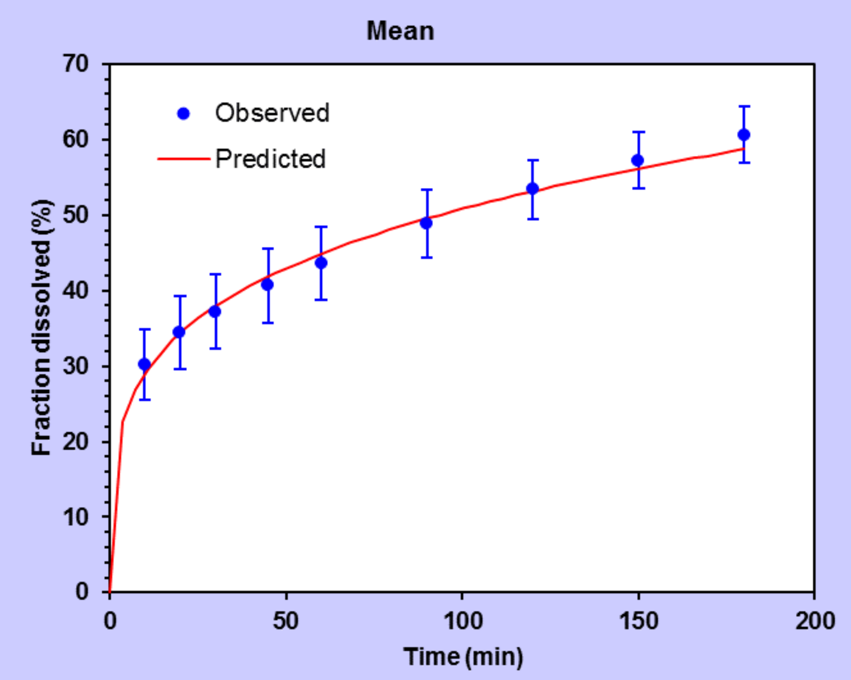

Supplementary materials – Model fitting summary – SuperTab® 11SD (modelling up to app. 60 % of carvedilol released)

Graphical abstract of model fit presented as the fraction % of released carvedilol per tested tablet:

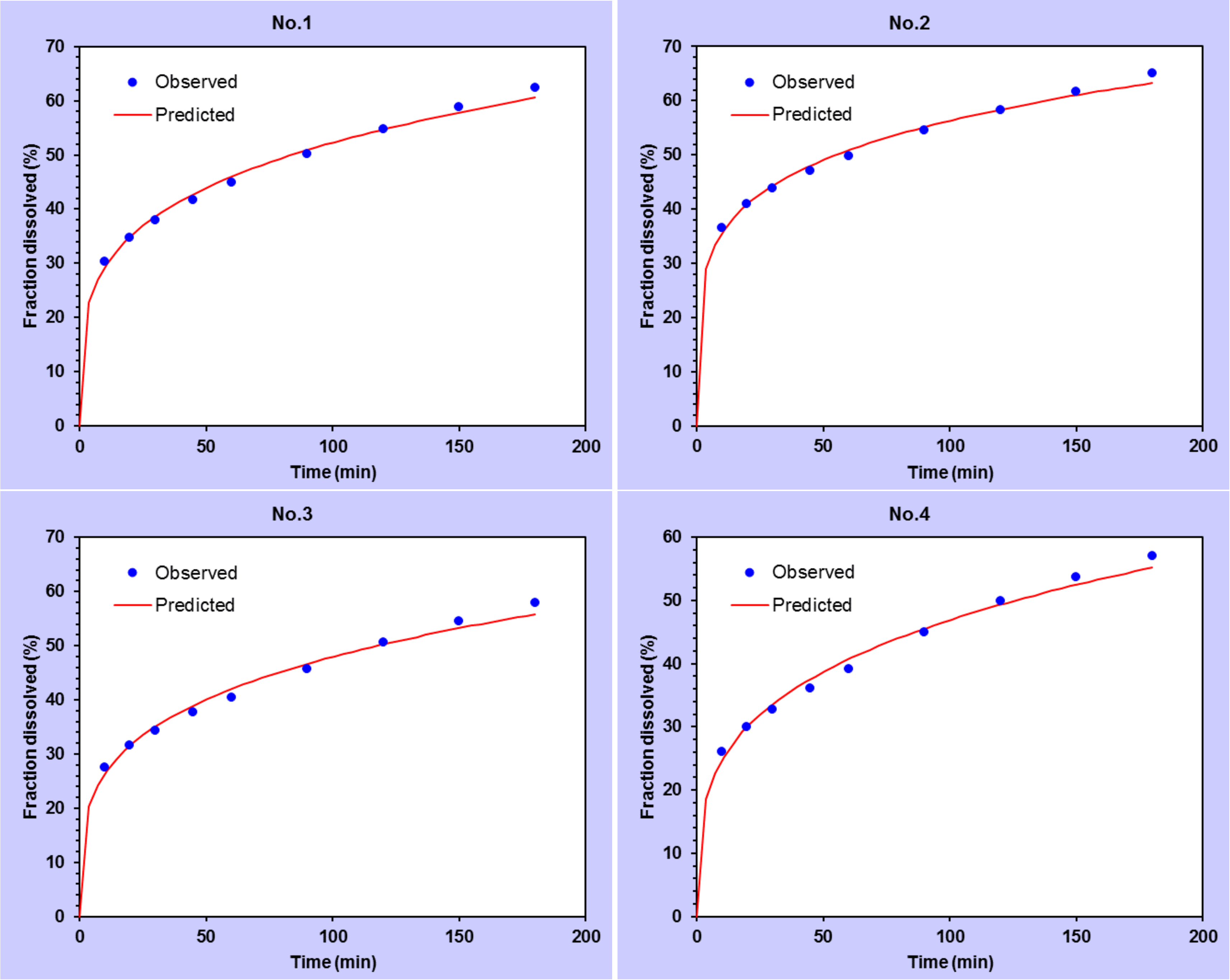

Supplementary materials – Model fitting summary – SuperTab® 11SD (modelling up to app. 60 % of carvedilol released)

Model: **Korsmeyer–Peppas with T_{lag}**

Model equation: $F = k_{KP} \cdot \left(t - T_{lag}\right)^n$

Fitted model parameters per tested tablet (N = 4) with statistics – mean, standard deviation (SD), and relative standard deviation expressed in % (RSD%) (output from DDSolver):

Parameter	No.1	No.2	No.3	No.4	Mean	SD	RSD(%)
k_{KP}	19.375	25.669	17.223	15.640	19.477	4.403	22.605
n	0.217	0.172	0.224	0.240	0.213	0.029	13.705
T_{lag}	4.000	4.000	4.000	4.000	4.000	0.000	0.000

Number of dissolution data points (N), degrees of freedom (df), and selected goodness of fit criteria – Pearson correlation coefficient (R), coefficient of determination (R^2), adjusted coefficient of determination ($R^2_{adjusted}$), and residual sum of squares (RSS) (manual calculation in MS Excel):

Parameter	No.1	No.2	No.3	No.4
N	9	9	9	9
df	6	6	6	6
R	0.990060671	0.989105461	0.985555993	0.987042825
R^2	0.980220133	0.978329614	0.971320615	0.974253539
$R^2_{adjusted}$	0.973626844	0.971106152	0.96176082	0.965671385
RSS	21.36471133	17.39806029	28.33653728	27.82793643

Graphical abstract of model fit presented as mean ± 1 SD of the fraction % of released carvedilol:

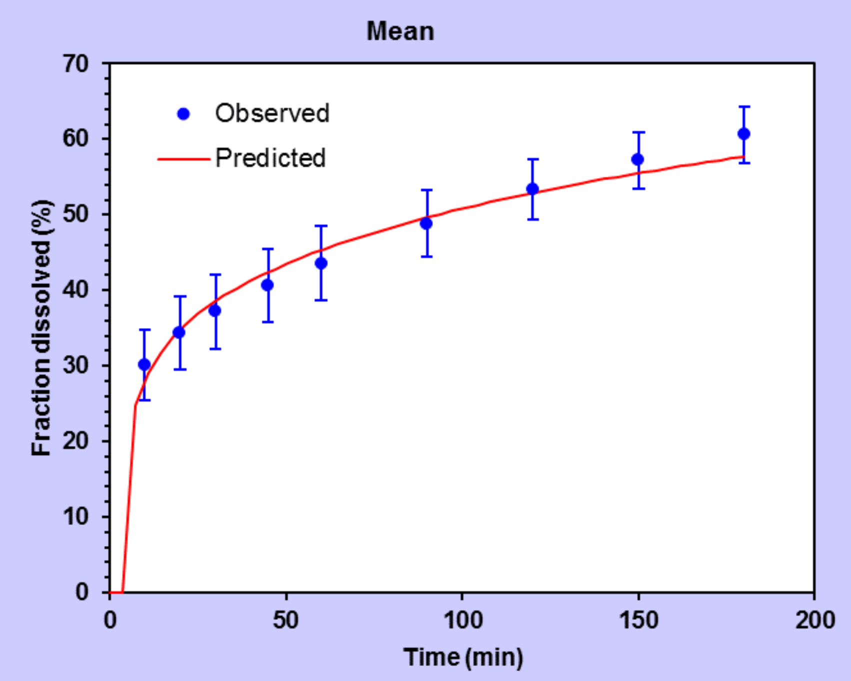

Supplementary materials – Model fitting summary – SuperTab® 11SD (modelling up to app. 60 % of carvedilol released)

Graphical abstract of model fit presented as the fraction % of released carvedilol per tested tablet:

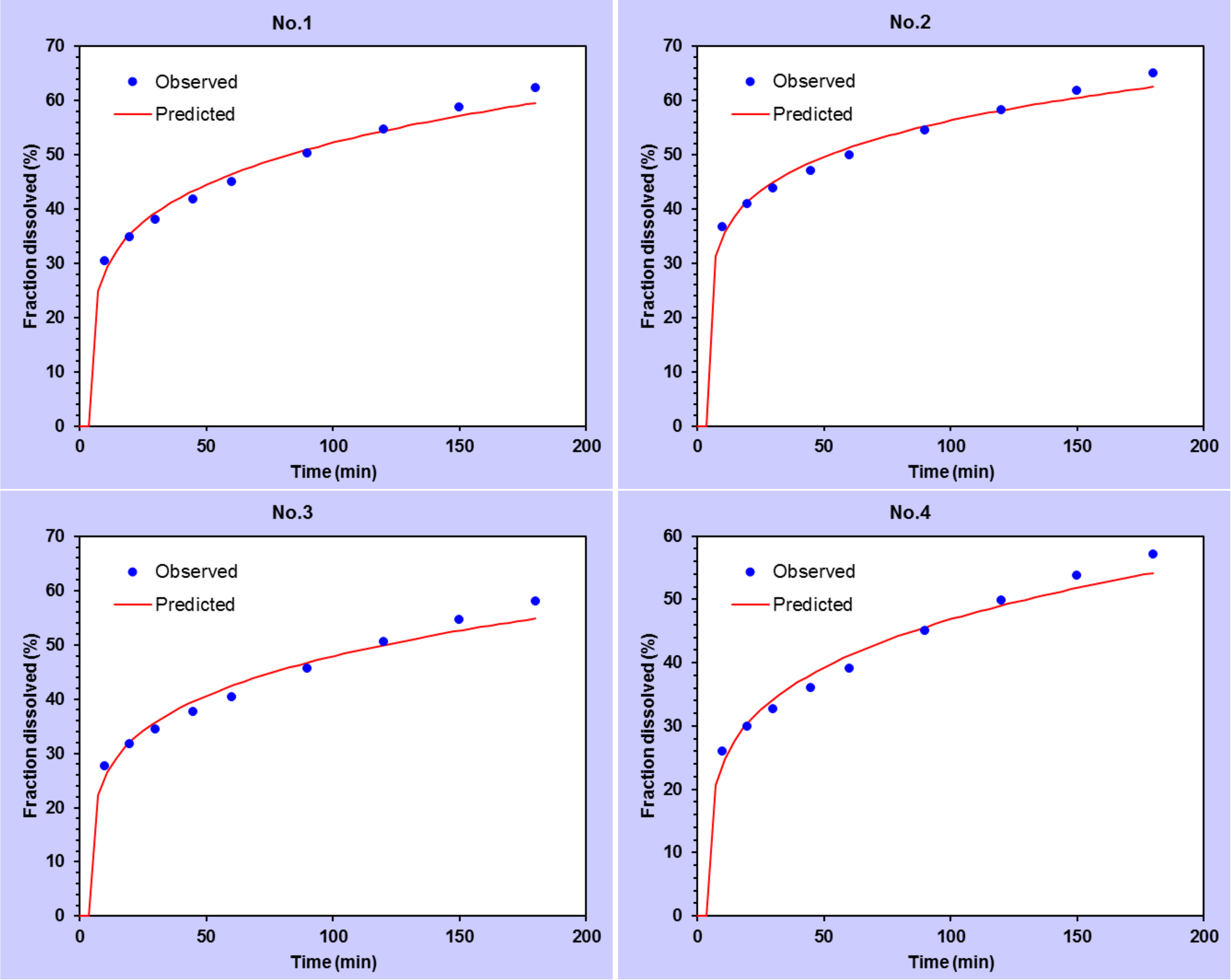

Supplementary materials – Model fitting summary – SuperTab® 11SD (modelling up to app. 60 % of carvedilol released)

Model: **Korsmeyer–Peppas with F_0**

Model equation: $F = F_0 + k_{KP} \cdot t^n$

Fitted model parameters per tested tablet (N = 4) with statistics – mean, standard deviation (SD), and relative standard deviation expressed in % (RSD%) (output from DDSolver):

Parameter	No.1	No.2	No.3	No.4	Mean	SD	RSD(%)
k_{KP}	7.901	11.129	6.929	5.897	7.964	2.263	28.420
n	0.352	0.286	0.362	0.400	0.350	0.047	13.511
F_0	12.157	14.638	11.038	10.199	12.008	1.928	16.054

Number of dissolution data points (N), degrees of freedom (df), and selected goodness of fit criteria – Pearson correlation coefficient (R), coefficient of determination (R^2), adjusted coefficient of determination ($R^2_{adjusted}$), and residual sum of squares (RSS) (manual calculation in MS Excel):

Parameter	No.1	No.2	No.3	No.4
N	9	9	9	9
df	6	6	6	6
R	0.998837064	0.998133457	0.996850907	0.998058892
R^2	0.99767548	0.996270399	0.993711731	0.996121553
$R^2_{adjusted}$	0.99690064	0.995027198	0.991615641	0.994828737
RSS	2.684377849	3.112493367	6.597942337	5.062089852

Graphical abstract of model fit presented as mean ± 1 SD of the fraction % of released carvedilol:

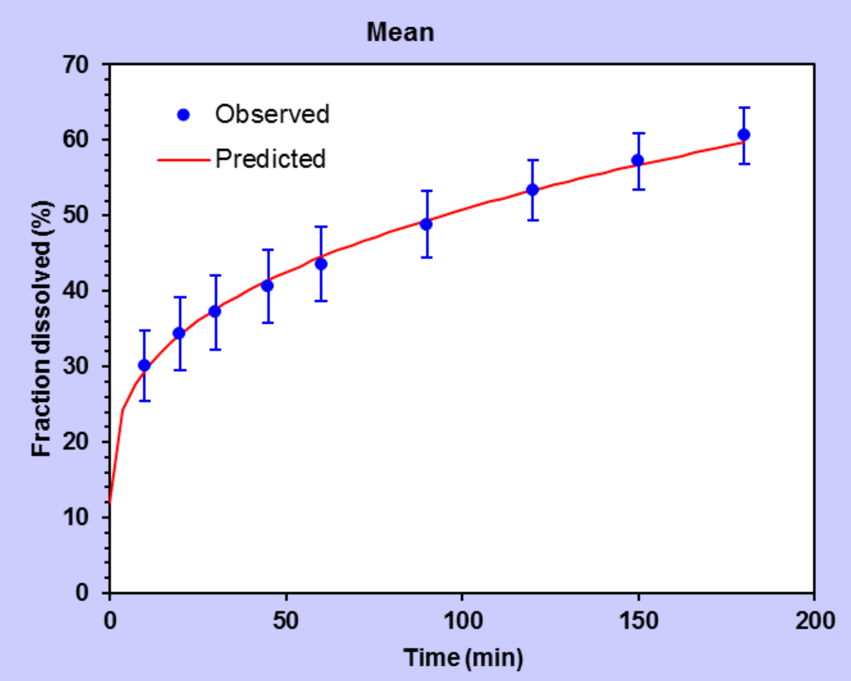

Supplementary materials – Model fitting summary – SuperTab® 11SD (modelling up to app. 60 % of carvedilol released)

Graphical abstract of model fit presented as the fraction % of released carvedilol per tested tablet:

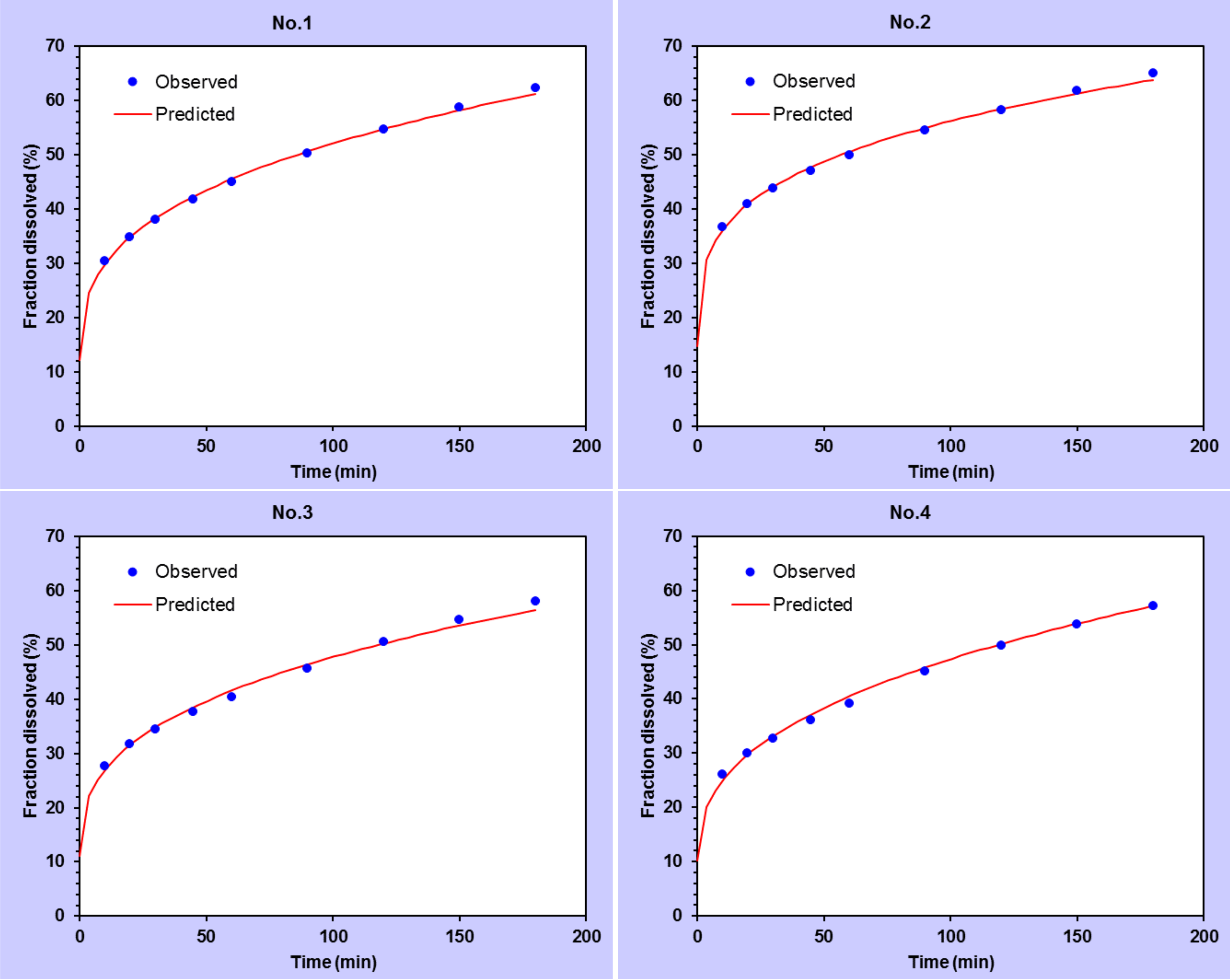

Supplementary materials – Model fitting summary – SuperTab® 11SD (modelling up to app. 60 % of carvedilol released)

Model: **Hixson–Crowell**

Model equation: $F = 100 \cdot [1 - (1 - k_{HC} \cdot t)^3]$

Fitted model parameters per tested tablet (N = 4) with statistics – mean, standard deviation (SD), and relative standard deviation expressed in % (RSD%) (output from DDSolver):

Parameter	No.1	No.2	No.3	No.4	Mean	SD	RSD(%)
k_{HC}	0.002	0.003	0.002	0.002	0.002	0.001	32.703

Number of dissolution data points (N), degrees of freedom (df), and selected goodness of fit criteria – Pearson correlation coefficient (R), coefficient of determination (R^2), adjusted coefficient of determination ($R^2_{adjusted}$), and residual sum of squares (RSS) (manual calculation in MS Excel):

Parameter	No.1	No.2	No.3	No.4
N	9	9	9	9
df	8	8	8	8
R	0.996255271	0.99732485	0.997701334	0.997648308
R^2	0.992524565	0.994656857	0.995407951	0.995302146
$R^2_{adjusted}$	0.992524565	0.994656857	0.995407951	0.995302146
RSS	2344.970979	3381.040898	1930.116344	1698.36931

Graphical abstract of model fit presented as mean ± 1 SD of the fraction % of released carvedilol:

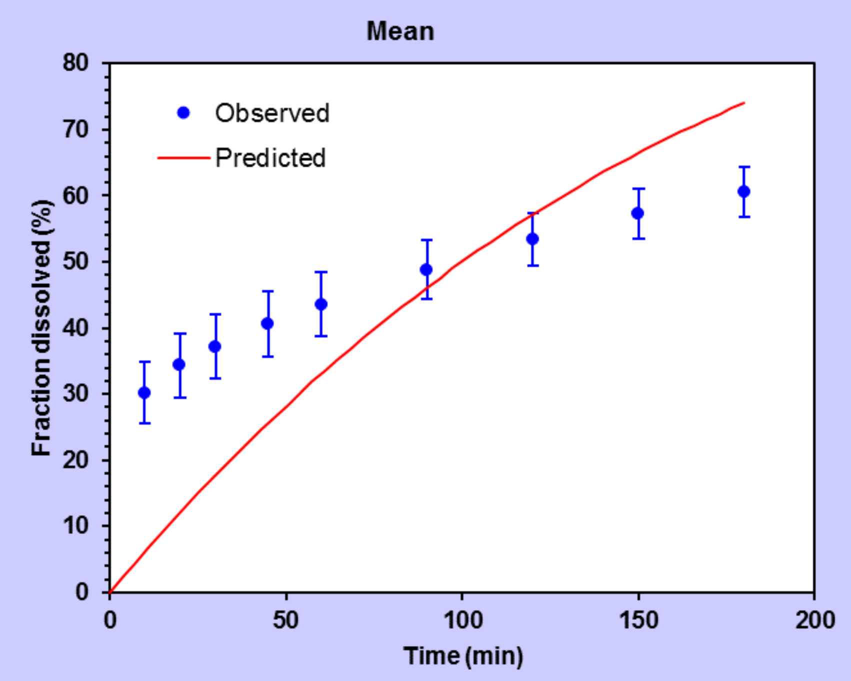

Supplementary materials – Model fitting summary – SuperTab® 11SD (modelling up to app. 60 % of carvedilol released)

Graphical abstract of model fit presented as the fraction % of released carvedilol per tested tablet:

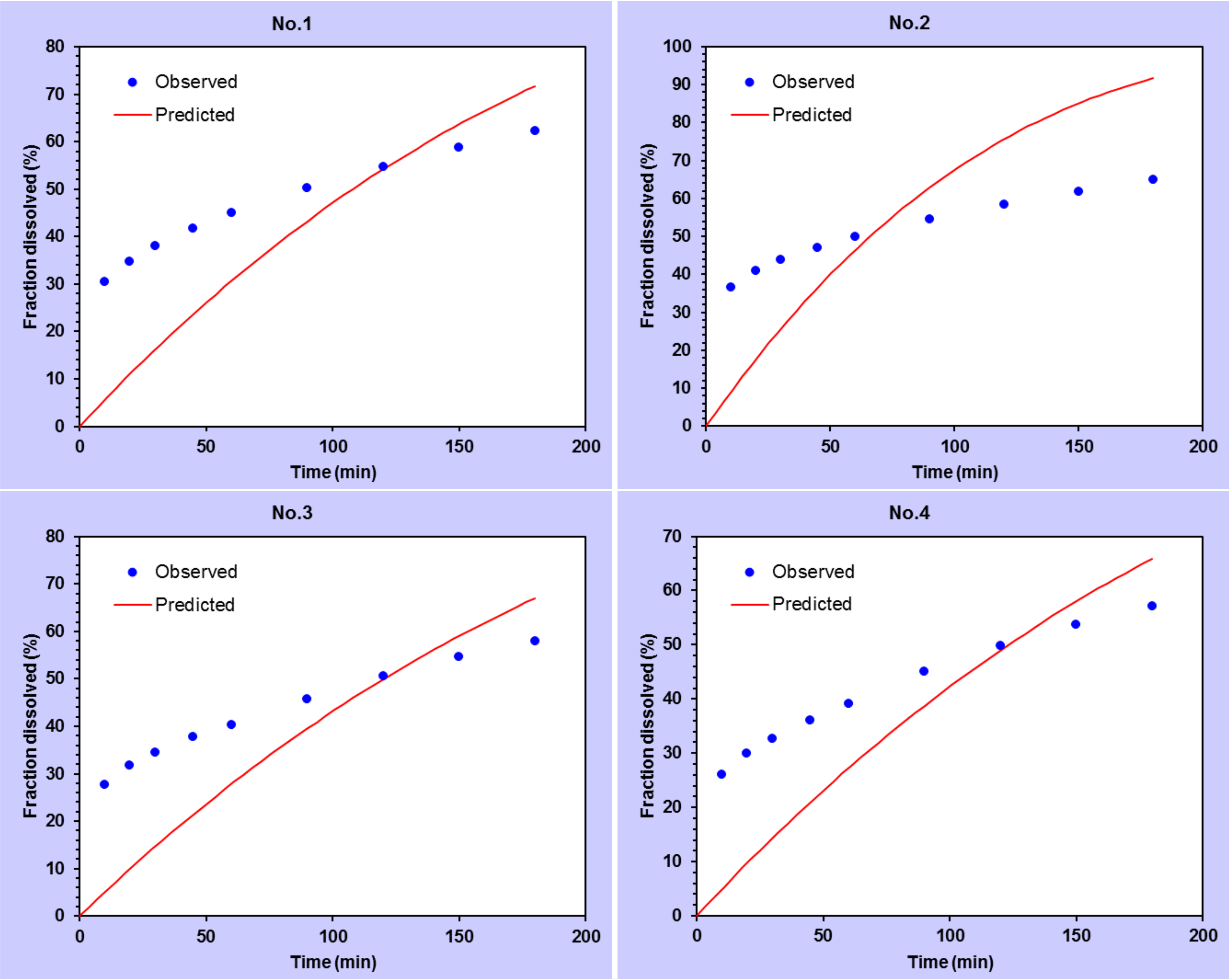

Supplementary materials – Model fitting summary – SuperTab® 11SD (modelling up to app. 60 % of carvedilol released)

Model: **Hixson–Crowell with T_{lag}**

Model equation: $F = 100 \cdot \left\{1 - \left[1 - k_{HC} \cdot \left(t - T_{lag}\right)\right]^3\right\}$

Fitted model parameters per tested tablet (N = 4) with statistics – mean, standard deviation (SD), and relative standard deviation expressed in % (RSD%) (output from DDSolver):

Parameter	No.1	No.2	No.3	No.4	Mean	SD	RSD(%)
k_{HC}	0.001	0.001	0.001	0.001	0.001	0.000	4.040
T_{lag}	-125.577	-168.781	-121.144	-110.597	-131.525	25.620	-19.479

Number of dissolution data points (N), degrees of freedom (df), and selected goodness of fit criteria – Pearson correlation coefficient (R), coefficient of determination (R^2), adjusted coefficient of determination ($R^2_{adjusted}$), and residual sum of squares (RSS) (manual calculation in MS Excel):

Parameter	No.1	No.2	No.3	No.4
N	9	9	9	9
df	7	7	7	7
R	0.991918601	0.989598139	0.994796496	0.994473428
R^2	0.983902512	0.979304477	0.989620069	0.9889774
$R^2_{adjusted}$	0.981602871	0.976347973	0.988137222	0.987402742
RSS	16.02142913	15.86028335	9.391081126	10.73938581

Graphical abstract of model fit presented as mean ± 1 SD of the fraction % of released carvedilol:

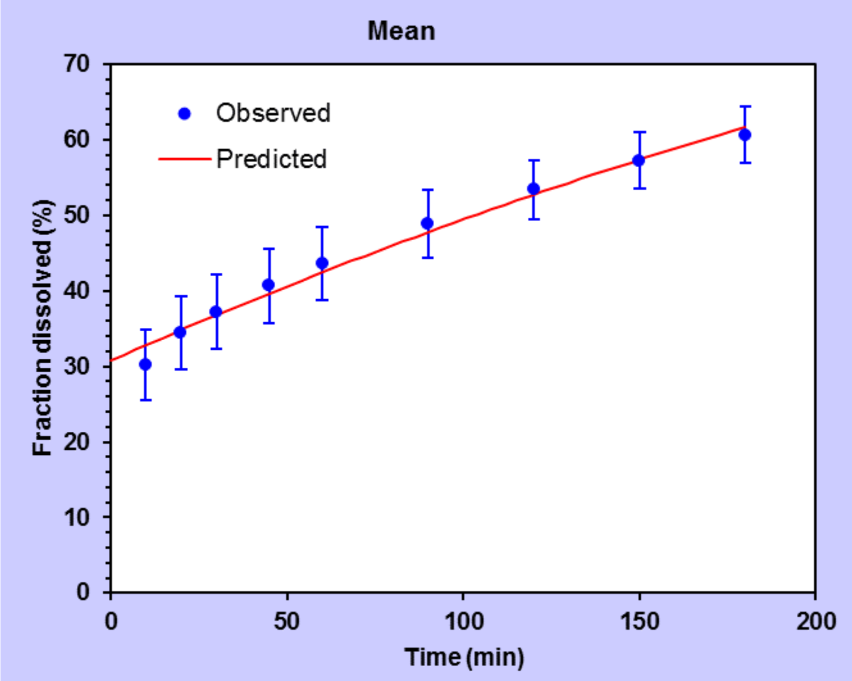

Supplementary materials – Model fitting summary – SuperTab® 11SD (modelling up to app. 60 % of carvedilol released)

Graphical abstract of model fit presented as the fraction % of released carvedilol per tested tablet:

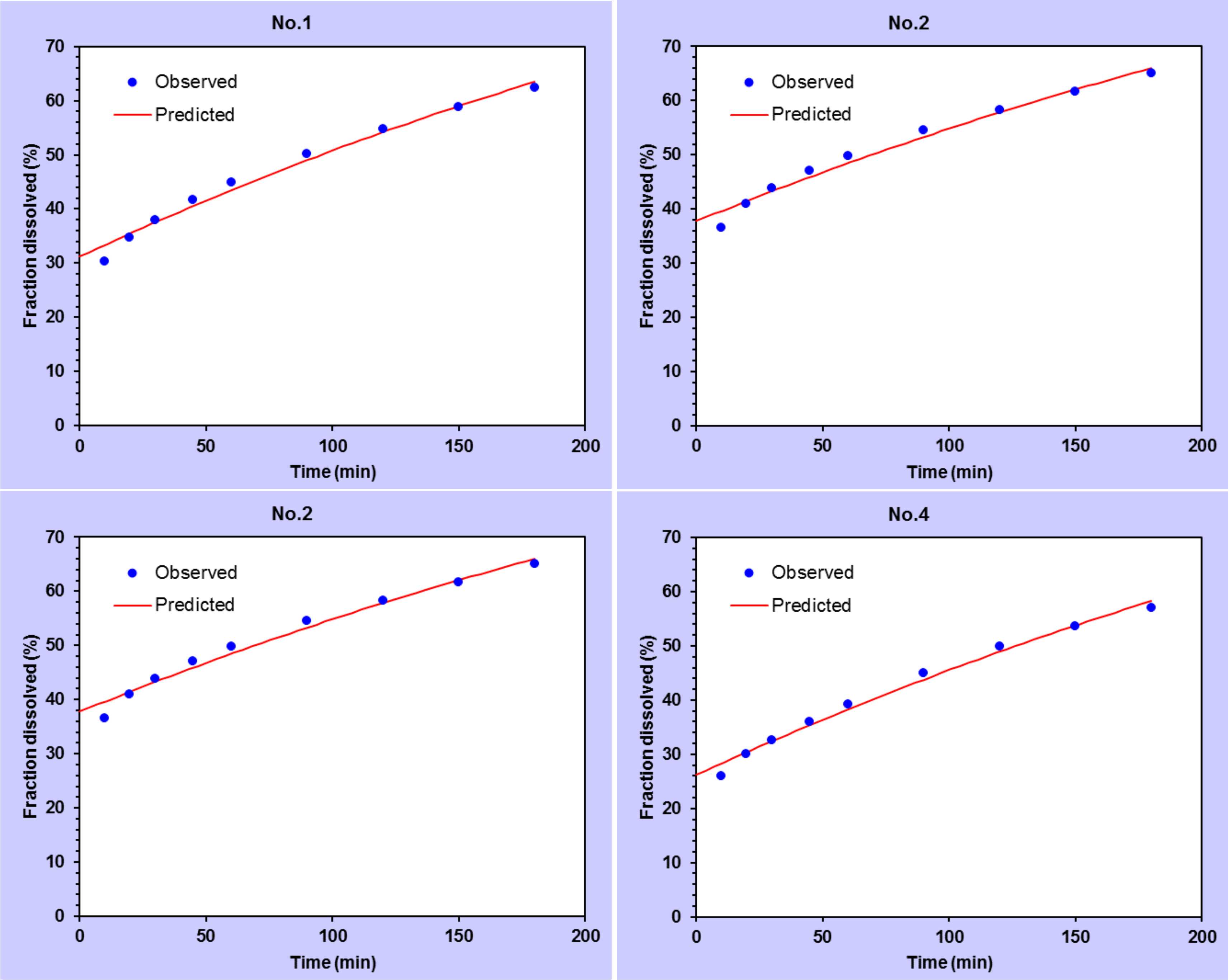

Supplementary materials – Model fitting summary – SuperTab® 11SD (modelling up to app. 60 % of carvedilol released)

Model: **Hopfenberg**

Model equation: $F = 100 \cdot [1 - (1 - k_{HB} \cdot t)^n]$

Fitted model parameters per tested tablet (N = 4) with statistics – mean, standard deviation (SD), and relative standard deviation expressed in % (RSD%) (output from DDSolver):

Parameter	No.1	No.2	No.3	No.4	Mean	SD	RSD(%)
k_{HB}	0.002	0.002	0.002	0.002	0.002	0.000	10.301
n	4.500	4.500	3.000	3.000	3.750	0.866	23.094

Number of dissolution data points (N), degrees of freedom (df), and selected goodness of fit criteria – Pearson correlation coefficient (R), coefficient of determination (R^2), adjusted coefficient of determination ($R^2_{adjusted}$), and residual sum of squares (RSS) (manual calculation in MS Excel):

Parameter	No.1	No.2	No.3	No.4
N	9	9	9	9
df	7	7	7	7
R	0.998502629	0.997611493	0.997701334	0.997648308
R^2	0.997007501	0.995228691	0.995407951	0.995302146
$R^2_{adjusted}$	0.996580001	0.994547075	0.994751944	0.994631024
RSS	2272.226423	2968.695007	1930.116344	1698.36931

Graphical abstract of model fit presented as mean ± 1 SD of the fraction % of released carvedilol:

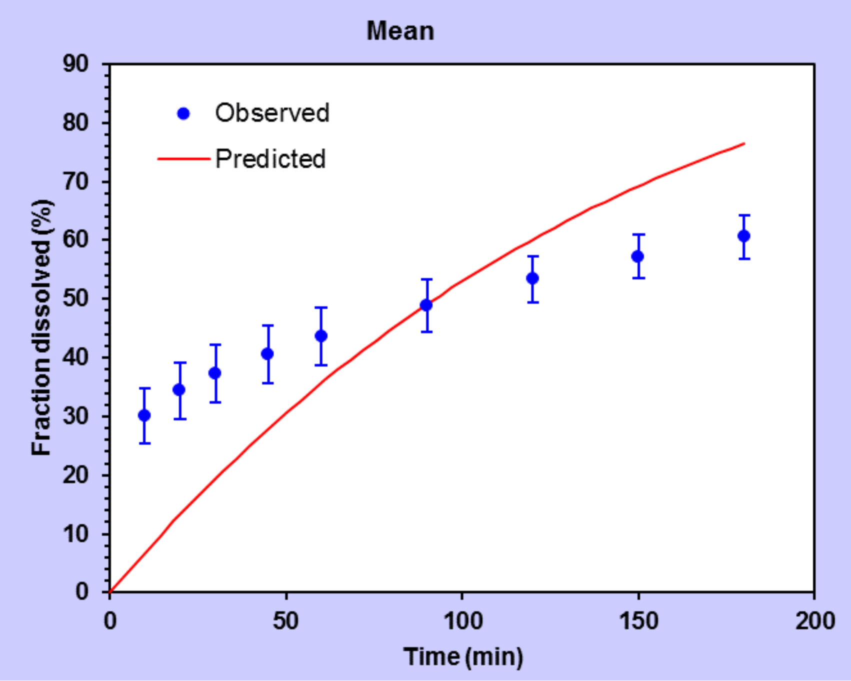

Supplementary materials – Model fitting summary – SuperTab® 11SD (modelling up to app. 60 % of carvedilol released)

Graphical abstract of model fit presented as the fraction % of released carvedilol per tested tablet:

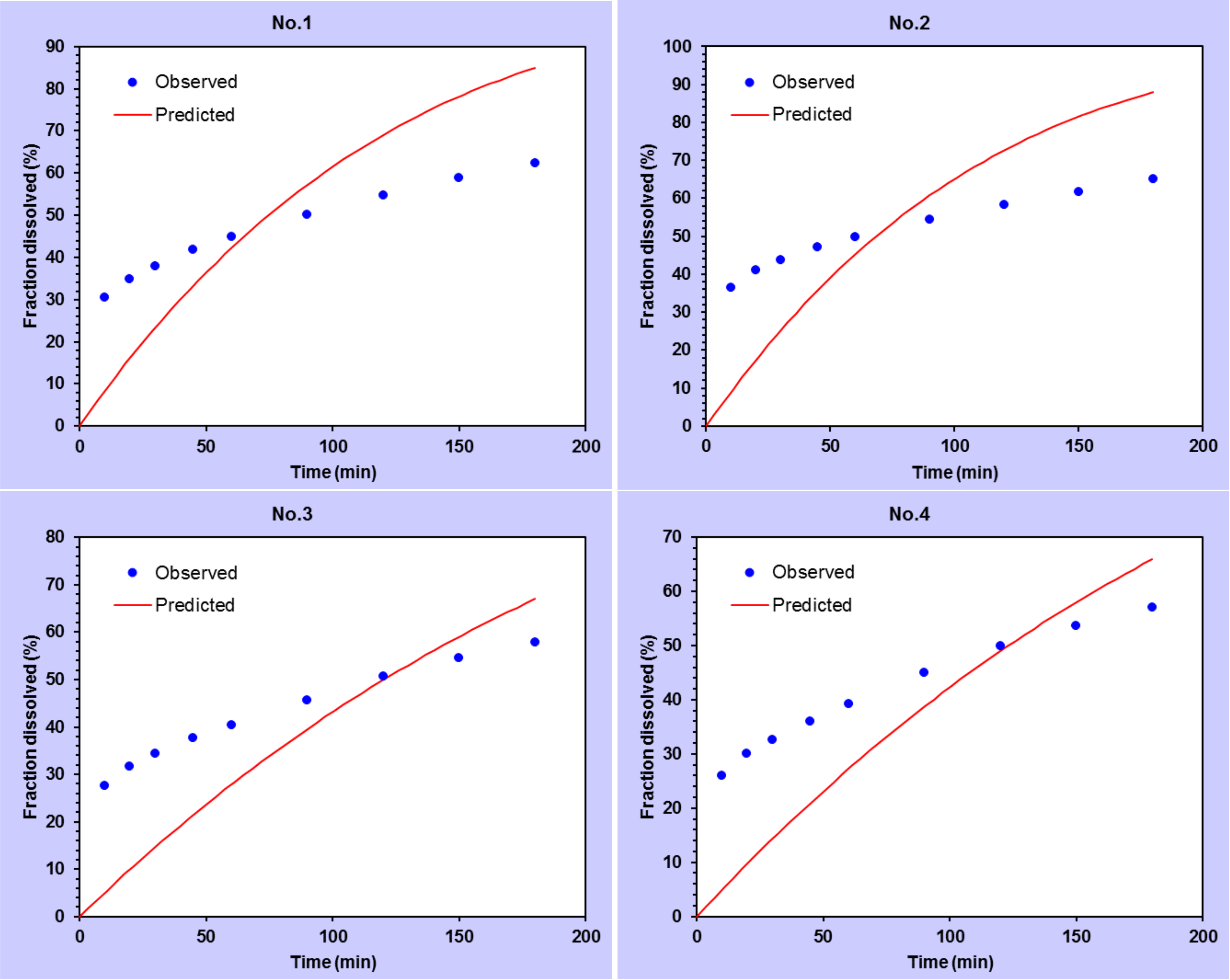

Supplementary materials – Model fitting summary – SuperTab® 11SD (modelling up to app. 60 % of carvedilol released)

Model: **Hopfenberg with T_{lag}**

Model equation: $F = 100 \cdot \{1 - [1 - k_{HB} \cdot (t - T_{lag})]^n\}$

Fitted model parameters per tested tablet (N = 4) with statistics – mean, standard deviation (SD), and relative standard deviation expressed in % (RSD%) (output from DDSolver):

Parameter	No.1	No.2	No.3	No.4	Mean	SD	RSD(%)
k_{HB}	0.001	0.001	0.001	0.001	0.001	0.000	4.040
n	3.000	3.000	3.000	3.000	3.000	0.000	0.000
T_{lag}	-125.577	-168.781	-121.144	-110.597	-131.525	25.620	-19.479

Number of dissolution data points (N), degrees of freedom (df), and selected goodness of fit criteria – Pearson correlation coefficient (R), coefficient of determination (R^2), adjusted coefficient of determination ($R^2_{adjusted}$), and residual sum of squares (RSS) (manual calculation in MS Excel):

Parameter	No.1	No.2	No.3	No.4
N	9	9	9	9
df	6	6	6	6
R	0.991918601	0.989598139	0.994796496	0.994473428
R^2	0.983902512	0.979304477	0.989620069	0.9889774
$R^2_{adjusted}$	0.978536682	0.972405969	0.986160092	0.9853032
RSS	16.02142913	15.86028335	9.391081126	10.73938581

Graphical abstract of model fit presented as mean ± 1 SD of the fraction % of released carvedilol:

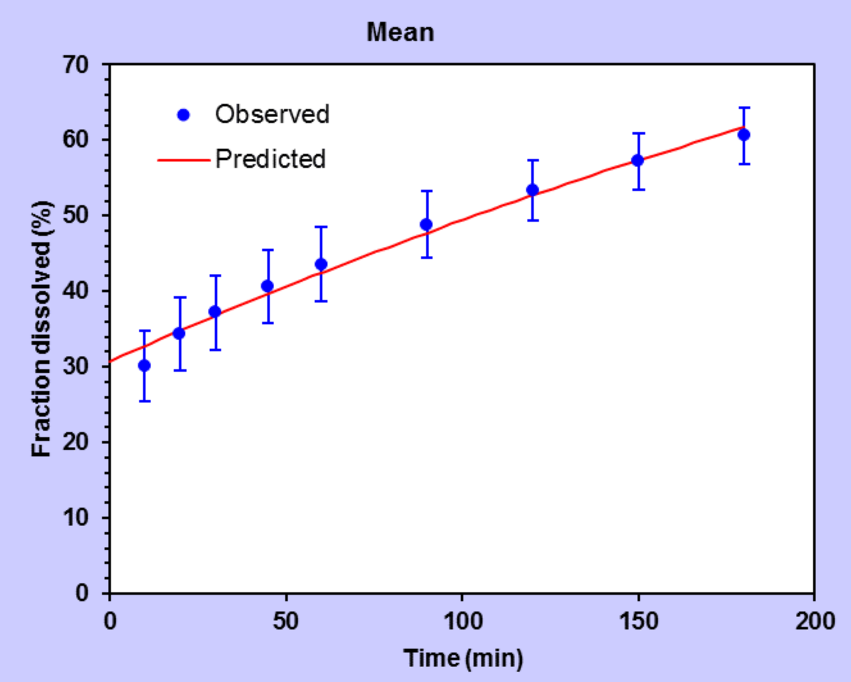

Supplementary materials – Model fitting summary – SuperTab® 11SD (modelling up to app. 60 % of carvedilol released)

Graphical abstract of model fit presented as the fraction % of released carvedilol per tested tablet:

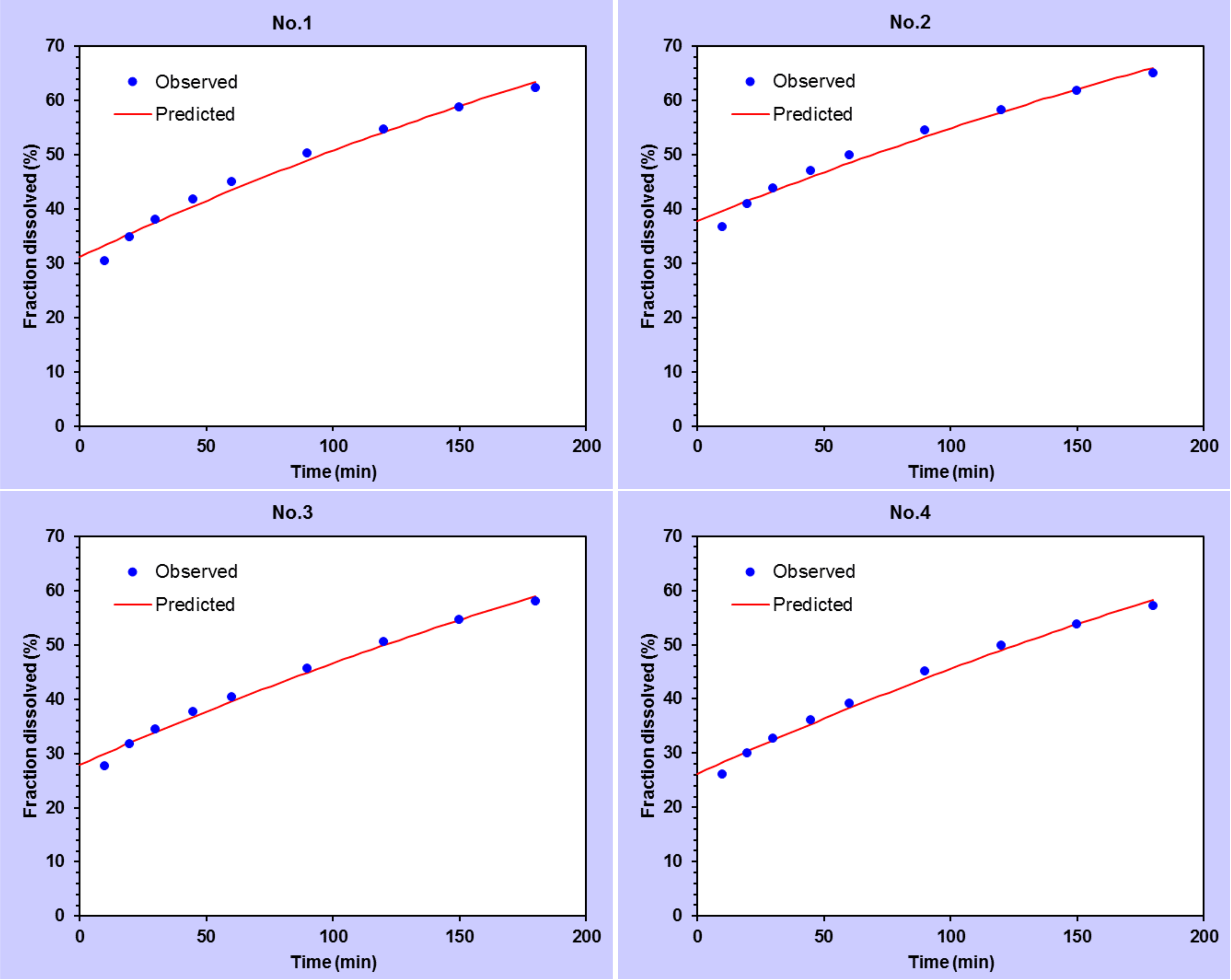

Supplementary materials – Model fitting summary – SuperTab® 11SD (modelling up to app. 60 % of carvedilol released)

Model: **Baker–Lonsdale**

Model equation: $\frac{3}{2}\cdot\left[1-\left(1-\frac{F}{100}\right)^{\frac{2}{3}}\right]-\frac{F}{100}=k_{BL}\cdot t$

Fitted model parameters per tested tablet (N = 4) with statistics – mean, standard deviation (SD), and relative standard deviation expressed in % (RSD%) (output from DDSolver):

Parameter	No.1	No.2	No.3	No.4	Mean	SD	RSD(%)
k_{BL}	0.001	0.001	0.001	0.001	0.001	0.000	10.170

Number of dissolution data points (N), degrees of freedom (df), and selected goodness of fit criteria – Pearson correlation coefficient (R), coefficient of determination (R^2), adjusted coefficient of determination ($R^2_{adjusted}$), and residual sum of squares (RSS) (manual calculation in MS Excel):

Parameter	No.1	No.2	No.3	No.4
N	9	9	9	9
df	8	8	8	8
R	0.998845803	0.999177633	0.997059675	0.997473099
R^2	0.997692938	0.998355941	0.994127995	0.994952582
$R^2_{adjusted}$	0.997692938	0.998355941	0.994127995	0.994952582
RSS	322.7532326	793.5261537	262.6696652	219.9361413

Graphical abstract of model fit presented as mean ± 1 SD of the fraction % of released carvedilol:

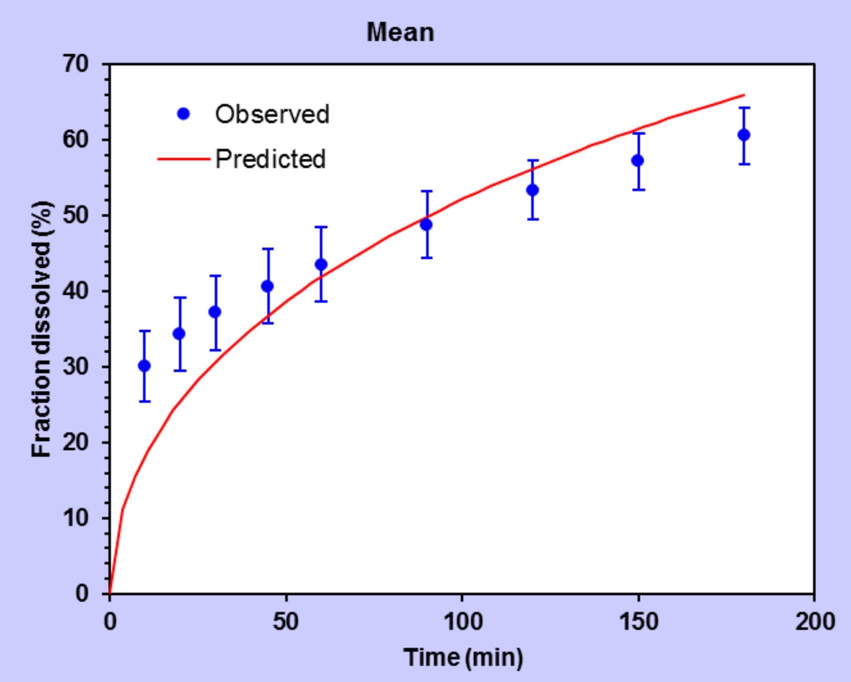

Supplementary materials – Model fitting summary – SuperTab® 11SD (modelling up to app. 60 % of carvedilol released)

Graphical abstract of model fit presented as the fraction % of released carvedilol per tested tablet:

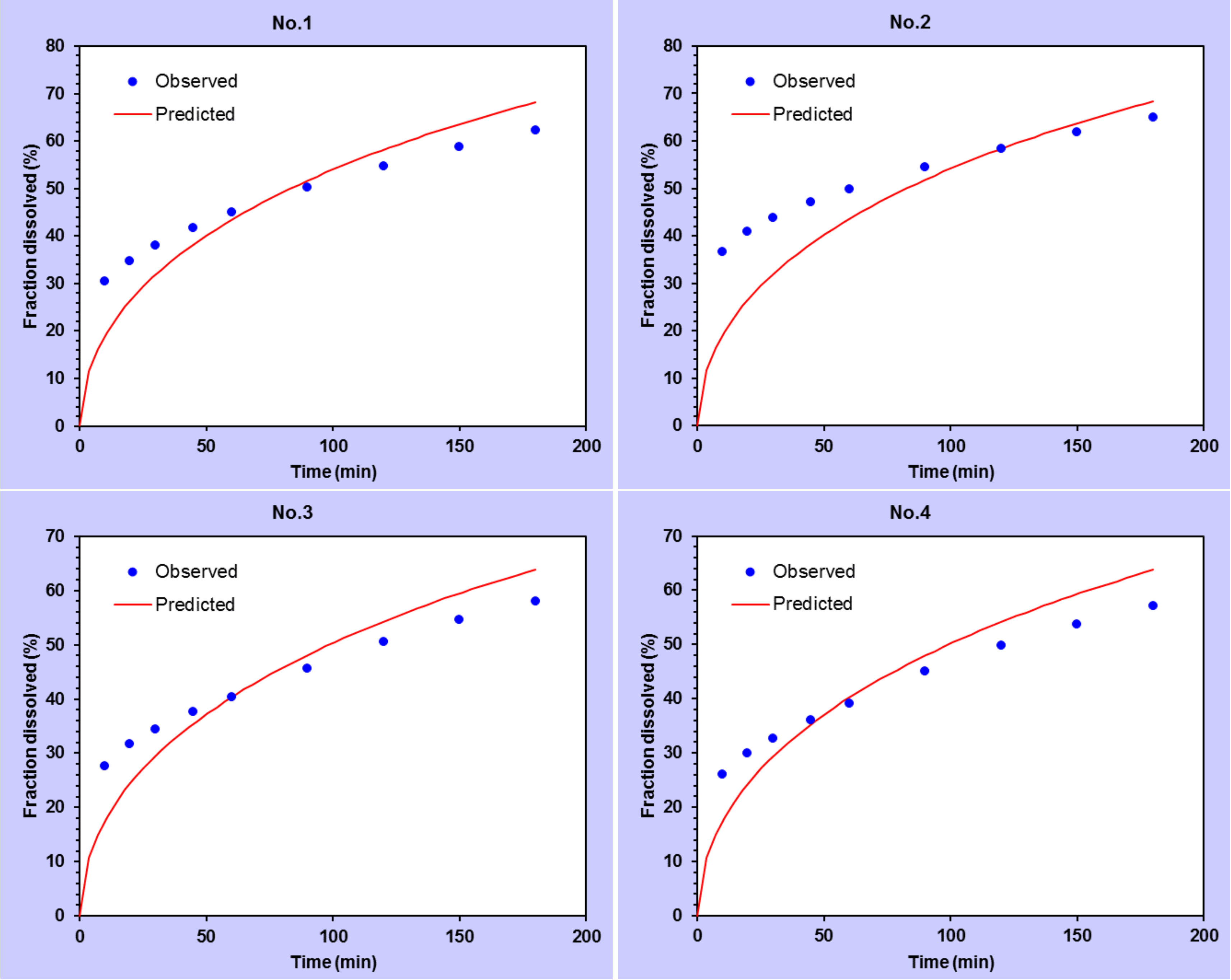

Supplementary materials – Model fitting summary – SuperTab® 11SD (modelling up to app. 60 % of carvedilol released)

Model: **Baker–Lonsdale with T_{lag}**

Model equation: $\frac{3}{2} \cdot \left[1 - \left(1 - \frac{F}{100}\right)^{\frac{2}{3}}\right] - \frac{F}{100} = k_{BL} \cdot (t - T_{lag})$

Fitted model parameters per tested tablet (N = 4) with statistics – mean, standard deviation (SD), and relative standard deviation expressed in % (RSD%) (output from DDSolver):

Parameter	No.1	No.2	No.3	No.4	Mean	SD	RSD(%)
k_{BL}	0.0004	0.0004	0.0004	0.0004	0.0004	0.0000	10.1701
T_{lag}	-35.0927	-59.0077	-31.3482	-26.0430	-37.8729	14.5708	-38.4729

Number of dissolution data points (N), degrees of freedom (df), and selected goodness of fit criteria – Pearson correlation coefficient (R), coefficient of determination (R^2), adjusted coefficient of determination ($R^2_{adjusted}$), and residual sum of squares (RSS) (manual calculation in MS Excel):

Parameter	No.1	No.2	No.3	No.4
N	9	9	9	9
df	7	7	7	7
R	0.99868055	0.996171633	0.999389684	0.999710696
R^2	0.997362842	0.992357923	0.99877974	0.999421475
$R^2_{adjusted}$	0.996986105	0.991266198	0.998605418	0.999338829
RSS	2.797103644	6.163910137	1.092539396	0.567403044

Graphical abstract of model fit presented as mean ± 1 SD of the fraction % of released carvedilol:

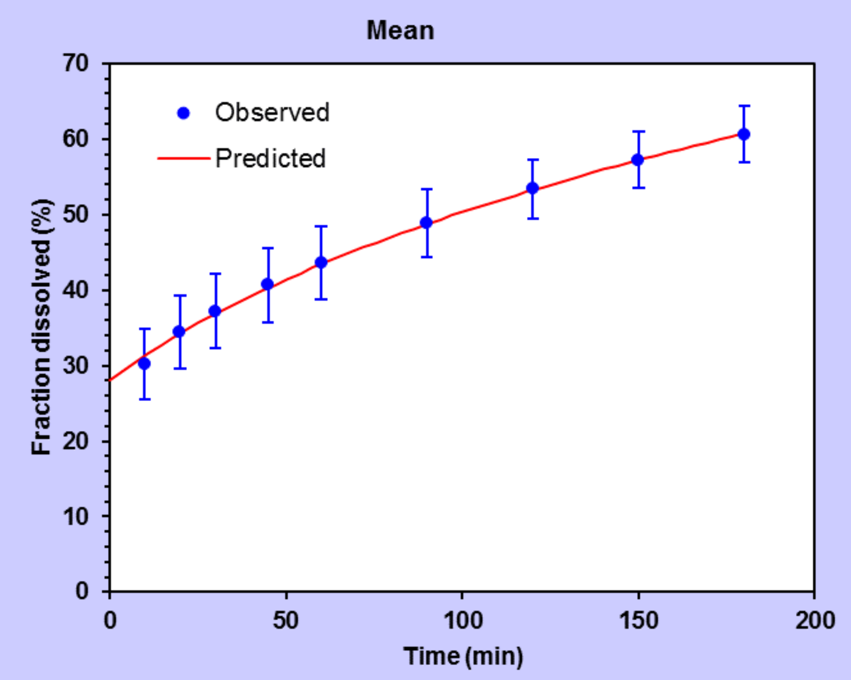

Supplementary materials – Model fitting summary – SuperTab® 11SD (modelling up to app. 60 % of carvedilol released)

Graphical abstract of model fit presented as the fraction % of released carvedilol per tested tablet:

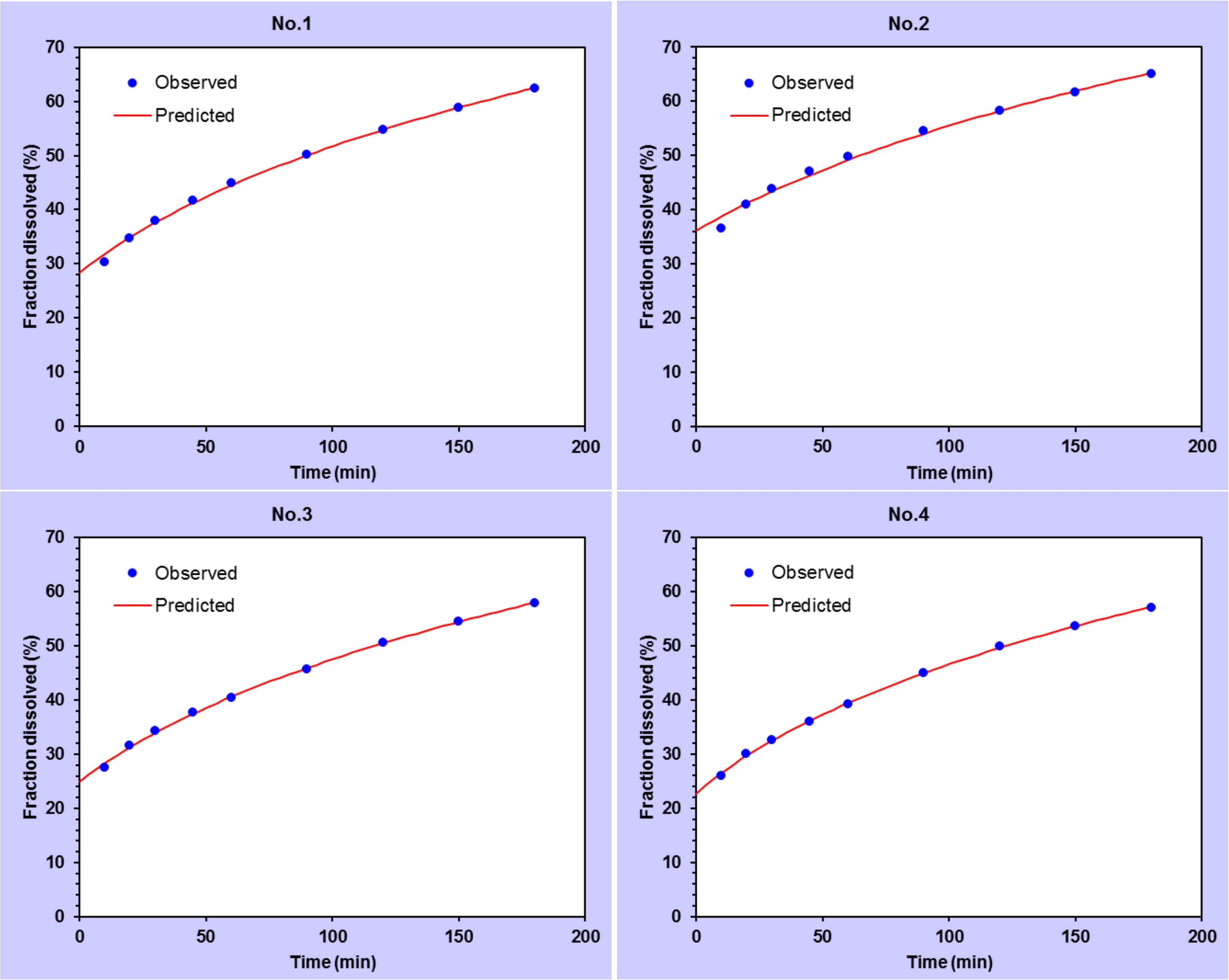

Supplementary materials – Model fitting summary – SuperTab® 11SD (modelling up to app. 60 % of carvedilol released)

Model: **Makoid–Banakar**

Model equation: $F = k_{MB} \cdot t^n \cdot e^{-k \cdot t}$

Fitted model parameters per tested tablet (N = 4) with statistics – mean, standard deviation (SD), and relative standard deviation expressed in % (RSD%) (output from DDSolver):

Parameter	No.1	No.2	No.3	No.4	Mean	SD	RSD(%)
k_{MB}	19.265	25.724	18.051	16.207	19.812	4.137	20.881
n	0.192	0.149	0.178	0.196	0.179	0.021	11.810
k	-0.001	-0.001	-0.001	-0.001	-0.001	0.000	-24.038

Number of dissolution data points (N), degrees of freedom (df), and selected goodness of fit criteria – Pearson correlation coefficient (R), coefficient of determination (R^2), adjusted coefficient of determination ($R^2_{adjusted}$), and residual sum of squares (RSS) (manual calculation in MS Excel):

Parameter	No.1	No.2	No.3	No.4
N	9	9	9	9
df	6	6	6	6
R	0.999868499	0.999961401	0.999524214	0.999039164
R^2	0.999737015	0.999922803	0.999048654	0.99807925
$R^2_{adjusted}$	0.999649353	0.999897071	0.998731539	0.997439
RSS	0.257140345	0.058241147	0.84912546	1.841802

Graphical abstract of model fit presented as mean ± 1 SD of the fraction % of released carvedilol:

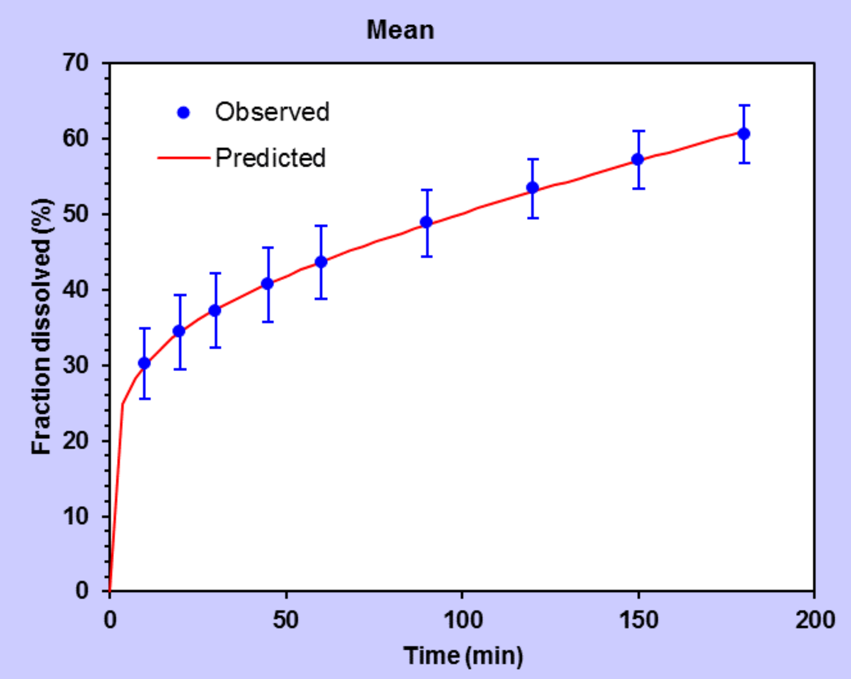

Supplementary materials – Model fitting summary – SuperTab® 11SD (modelling up to app. 60 % of carvedilol released)

Graphical abstract of model fit presented as the fraction % of released carvedilol per tested tablet:

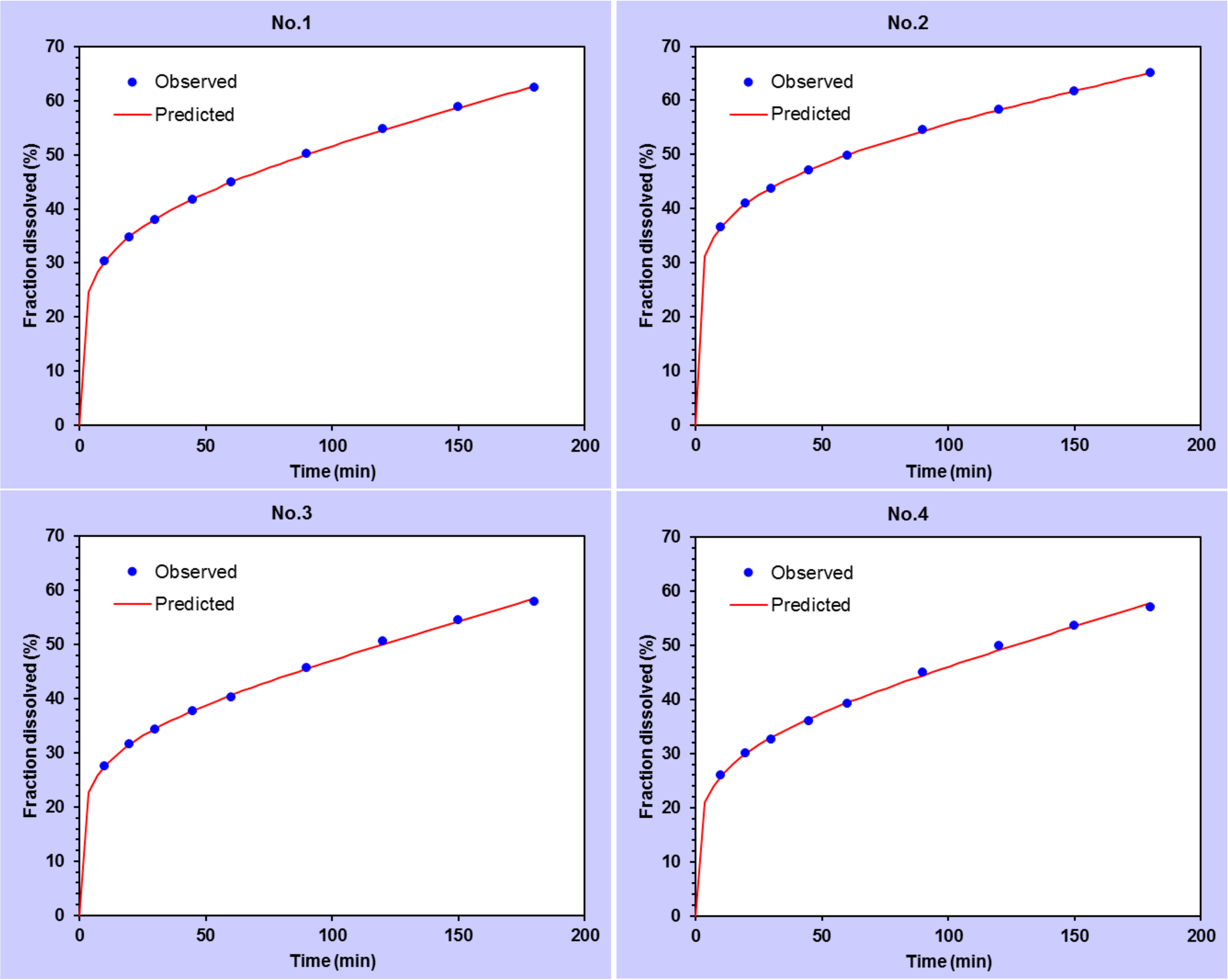

Supplementary materials – Model fitting summary – SuperTab® 11SD (modelling up to app. 60 % of carvedilol released)

Model: **Makoid–Banakar with T_{lag}**

Model equation: $F = k_{MB} \cdot (t - T_{lag})^n \cdot e^{-k\cdot(t-T_{lag})}$

Fitted model parameters per tested tablet (N = 4) with statistics – mean, standard deviation (SD), and relative standard deviation expressed in % (RSD%) (output from DDSolver):

Parameter	No.1	No.2	No.3	No.4	Mean	SD	RSD(%)
k_{MB}	22.999	29.509	21.301	19.483	23.323	4.367	18.723
n	0.145	0.113	0.135	0.148	0.135	0.016	11.578
k	-0.001	-0.001	-0.002	-0.002	-0.002	0.000	-20.185
T_{lag}	4.000	4.000	4.000	4.000	4.000	0.000	0.000

Number of dissolution data points (N), degrees of freedom (df), and selected goodness of fit criteria – Pearson correlation coefficient (R), coefficient of determination (R^2), adjusted coefficient of determination ($R^2_{adjusted}$), and residual sum of squares (RSS) (manual calculation in MS Excel):

Parameter	No.1	No.2	No.3	No.4
N	9	9	9	9
df	5	5	5	5
R	0.999366848	0.999688761	0.99909202	0.998247055
R^2	0.998734097	0.999377618	0.998184864	0.996497183
$R^2_{adjusted}$	0.997974556	0.999004189	0.997095782	0.994395493
RSS	1.239362879	0.469861376	1.623192009	3.366117159

Graphical abstract of model fit presented as mean ± 1 SD of the fraction % of released carvedilol:

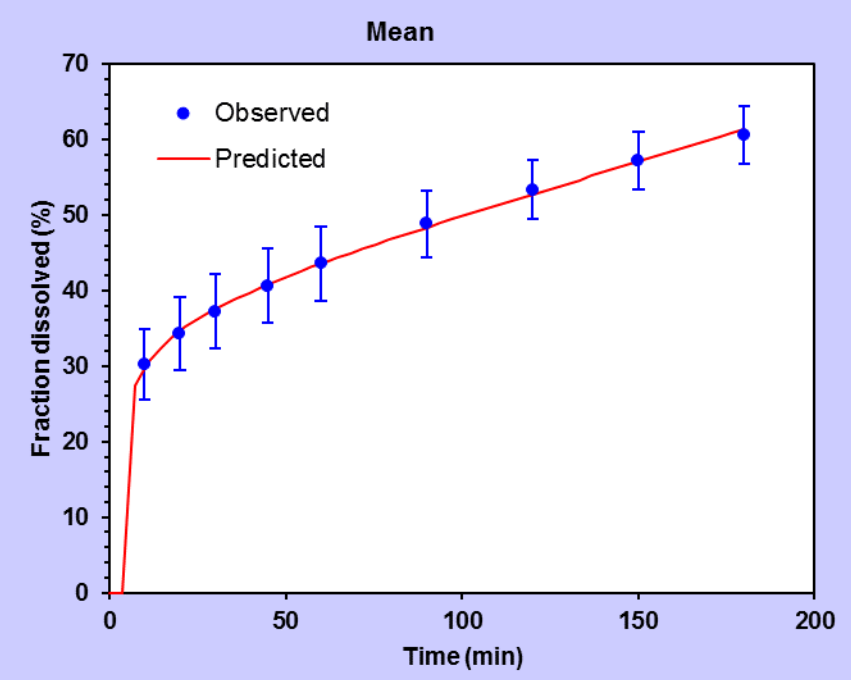

Supplementary materials – Model fitting summary – SuperTab® 11SD (modelling up to app. 60 % of carvedilol released)

Graphical abstract of model fit presented as the fraction % of released carvedilol per tested tablet:

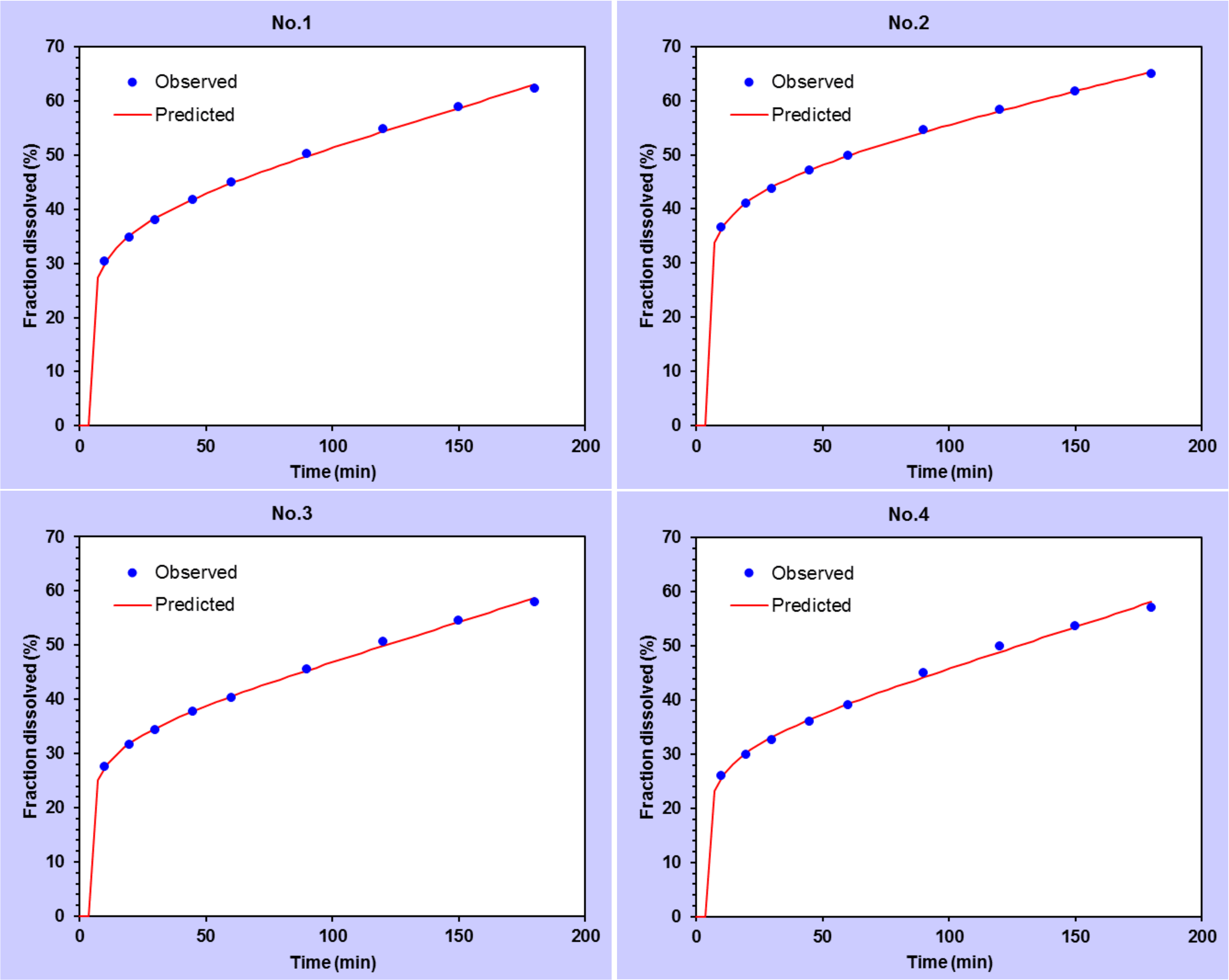

Supplementary materials – Model fitting summary – SuperTab® 11SD (modelling up to app. 60 % of carvedilol released)

Model: **Peppas–Sahlin_1**

Model equation: $F = k_1 \cdot t^m + k_2 \cdot t^{2m}$

Fitted model parameters per tested tablet (N = 4) with statistics – mean, standard deviation (SD), and relative standard deviation expressed in % (RSD%) (output from DDSolver):

Parameter	No.1	No.2	No.3	No.4	Mean	SD	RSD(%)
k_1	10.121	17.429	10.399	10.751	12.175	3.512	28.846
k_2	-0.419	-0.495	-0.334	-0.245	-0.373	0.108	-28.845
m	0.450	0.275	0.371	0.345	0.360	0.072	20.058

Number of dissolution data points (N), degrees of freedom (df), and selected goodness of fit criteria – Pearson correlation coefficient (R), coefficient of determination (R^2), adjusted coefficient of determination ($R^2_{adjusted}$), and residual sum of squares (RSS) (manual calculation in MS Excel):

Parameter	No.1	No.2	No.3	No.4
N	9	9	9	9
df	6	6	6	6
R	0.980198748	0.996228801	0.991280895	0.99383993
R^2	0.960789586	0.992471824	0.982637813	0.987717806
$R^2_{adjusted}$	0.947719448	0.989962432	0.976850417	0.983623741
RSS	52.47128756	55.71730709	47.764605	18.62038204

Graphical abstract of model fit presented as mean ± 1 SD of the fraction % of released carvedilol:

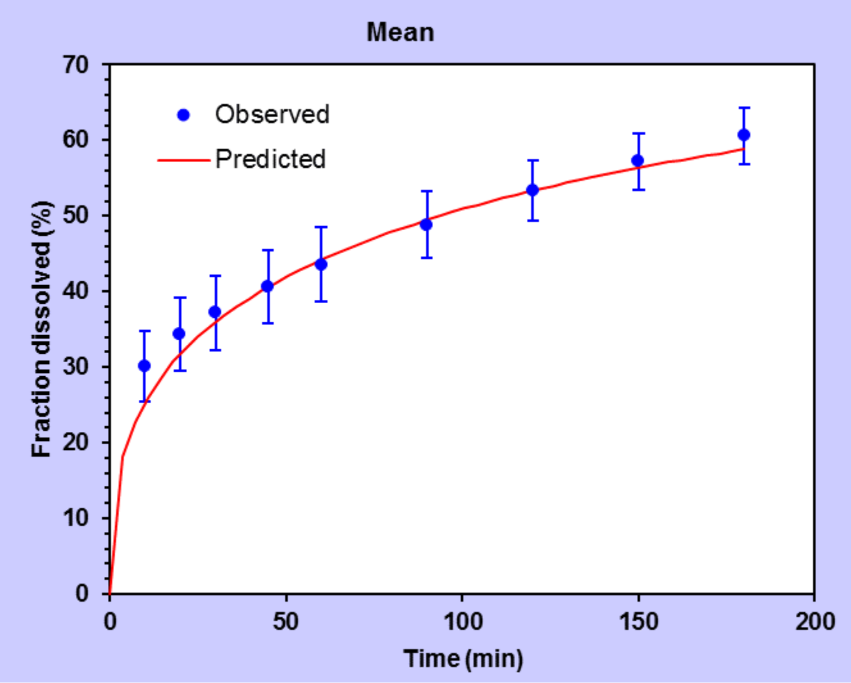

Supplementary materials – Model fitting summary – SuperTab® 11SD (modelling up to app. 60 % of carvedilol released)

Graphical abstract of model fit presented as the fraction % of released carvedilol per tested tablet:

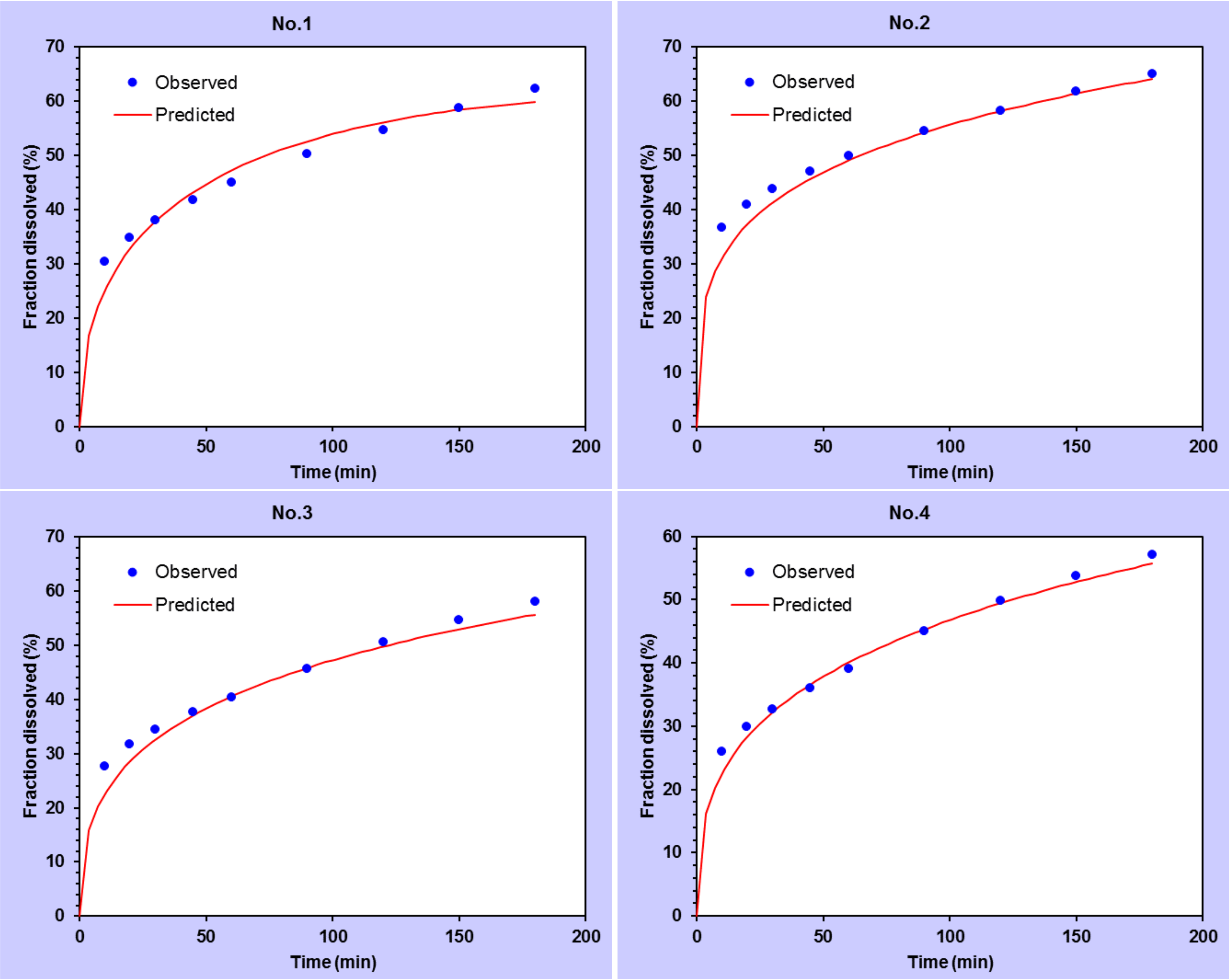

Supplementary materials – Model fitting summary – SuperTab® 11SD (modelling up to app. 60 % of carvedilol released)

Model: **Peppas–Sahlin_1 with T_{lag}**

Model equation: $F = k_1 \cdot (t - T_{lag})^m + k_2 \cdot (t - T_{lag})^{2m}$

Fitted model parameters per tested tablet (N = 4) with statistics – mean, standard deviation (SD), and relative standard deviation expressed in % (RSD%) (output from DDSolver):

Parameter	No.1	No.2	No.3	No.4	Mean	SD	RSD(%)
k_1	10.931	13.098	9.740	9.177	10.736	1.736	16.167
k_2	-0.504	-0.701	-0.427	-0.375	-0.502	0.143	-28.502
m	0.450	0.450	0.450	0.450	0.450	0.000	0.000
T_{lag}	4.000	4.000	4.000	4.000	4.000	0.000	0.000

Number of dissolution data points (N), degrees of freedom (df), and selected goodness of fit criteria – Pearson correlation coefficient (R), coefficient of determination (R^2), adjusted coefficient of determination ($R^2_{adjusted}$), and residual sum of squares (RSS) (manual calculation in MS Excel):

Parameter	No.1	No.2	No.3	No.4
N	9	9	9	9
df	5	5	5	5
R	0.961992845	0.930362909	0.960294061	0.968909219
R^2	0.925430234	0.865575143	0.922164684	0.938785075
$R^2_{adjusted}$	0.880688374	0.784920229	0.875463495	0.902056119
RSS	117.1679563	197.232756	107.1594888	86.750092

Graphical abstract of model fit presented as mean ± 1 SD of the fraction % of released carvedilol:

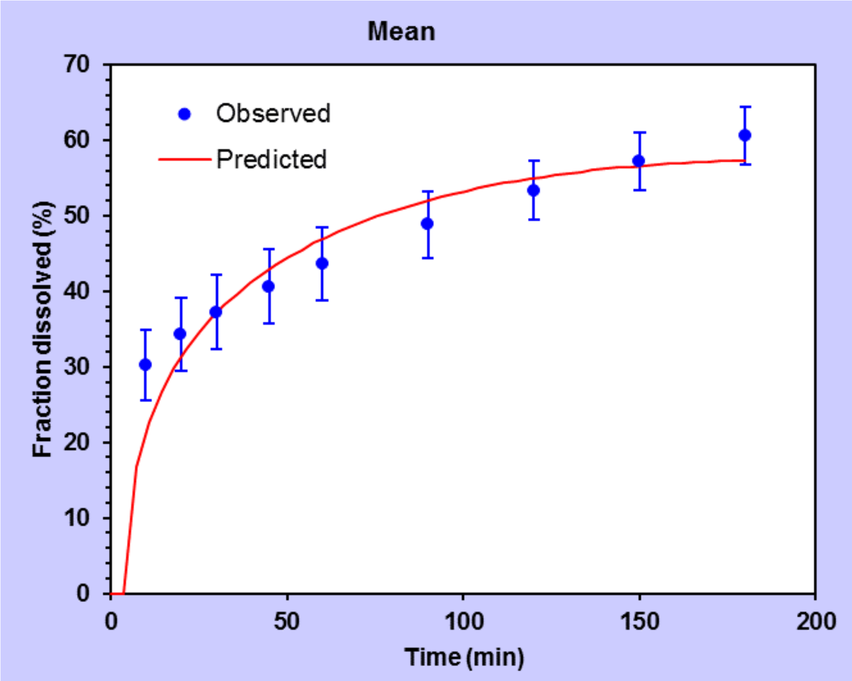

Supplementary materials – Model fitting summary – SuperTab® 11SD (modelling up to app. 60 % of carvedilol released)

Graphical abstract of model fit presented as the fraction % of released carvedilol per tested tablet:

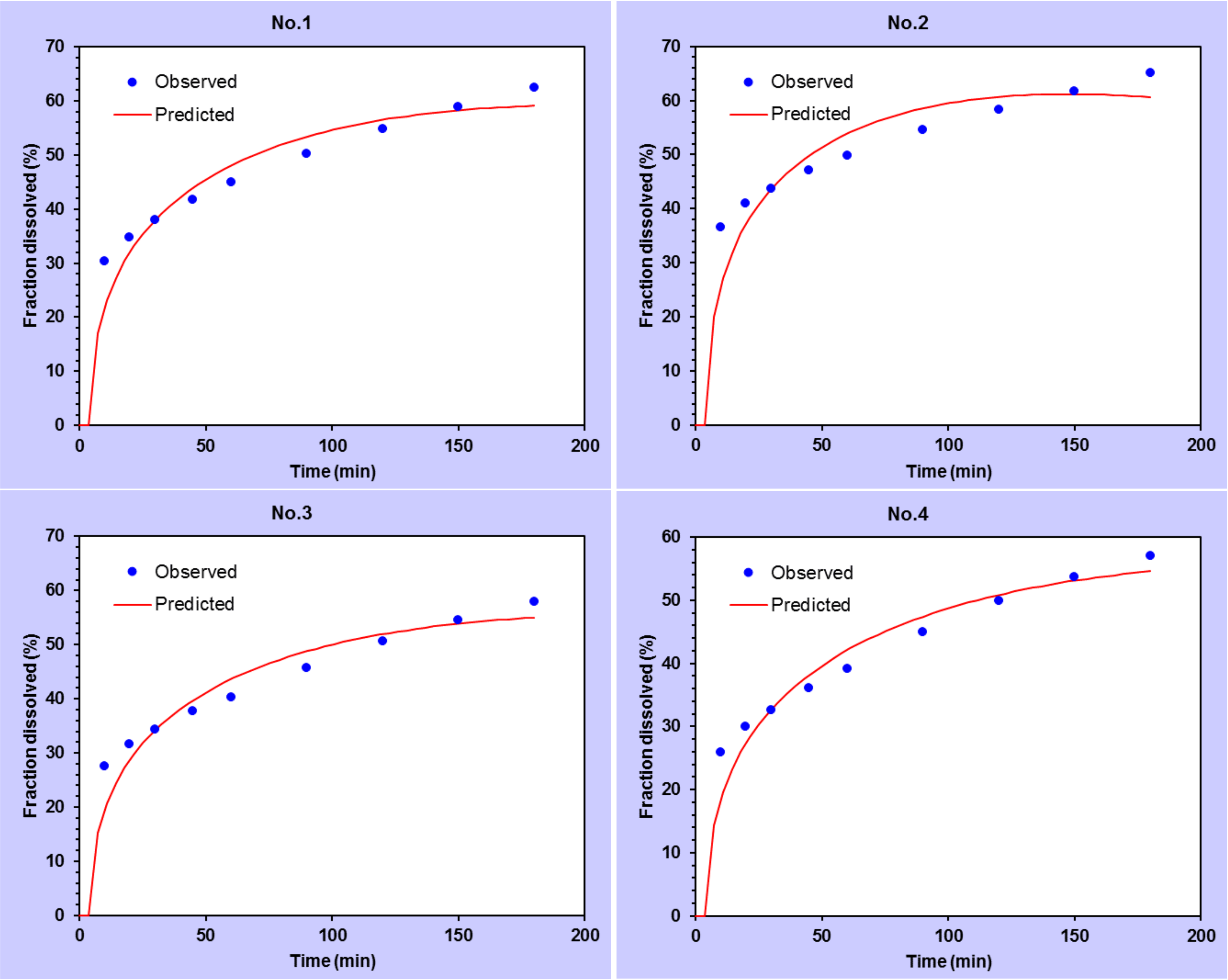

Supplementary materials – Model fitting summary – SuperTab® 11SD (modelling up to app. 60 % of carvedilol released)

Model: **Peppas–Sahlin_2**

Model equation: $F = k_1 \cdot t^{0.5} + k_2 \cdot t$

Fitted model parameters per tested tablet (N = 4) with statistics – mean, standard deviation (SD), and relative standard deviation expressed in % (RSD%) (output from DDSolver):

Parameter	No.1	No.2	No.3	No.4	Mean	SD	RSD(%)
k_1	8.503	10.147	7.585	7.160	8.349	1.323	15.847
k_2	-0.304	-0.419	-0.259	-0.229	-0.303	0.083	-27.533

Number of dissolution data points (N), degrees of freedom (df), and selected goodness of fit criteria – Pearson correlation coefficient (R), coefficient of determination (R^2), adjusted coefficient of determination ($R^2_{adjusted}$), and residual sum of squares (RSS) (manual calculation in MS Excel):

Parameter	No.1	No.2	No.3	No.4
N	9	9	9	9
df	7	7	7	7
R	0.972014956	0.944032997	0.97042177	0.978047131
R^2	0.944813074	0.8911983	0.941718412	0.95657619
$R^2_{adjusted}$	0.936929228	0.8756552	0.933392471	0.950372788
RSS	83.22550303	151.2505201	77.15021068	59.41621094

Graphical abstract of model fit presented as mean ± 1 SD of the fraction % of released carvedilol:

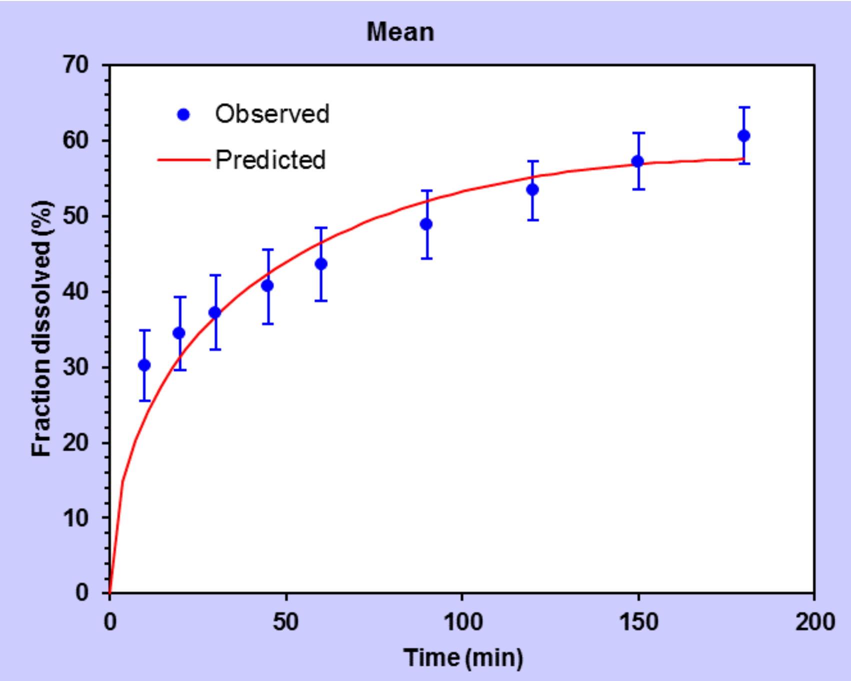

Supplementary materials – Model fitting summary – SuperTab® 11SD (modelling up to app. 60 % of carvedilol released)

Graphical abstract of model fit presented as the fraction % of released carvedilol per tested tablet:

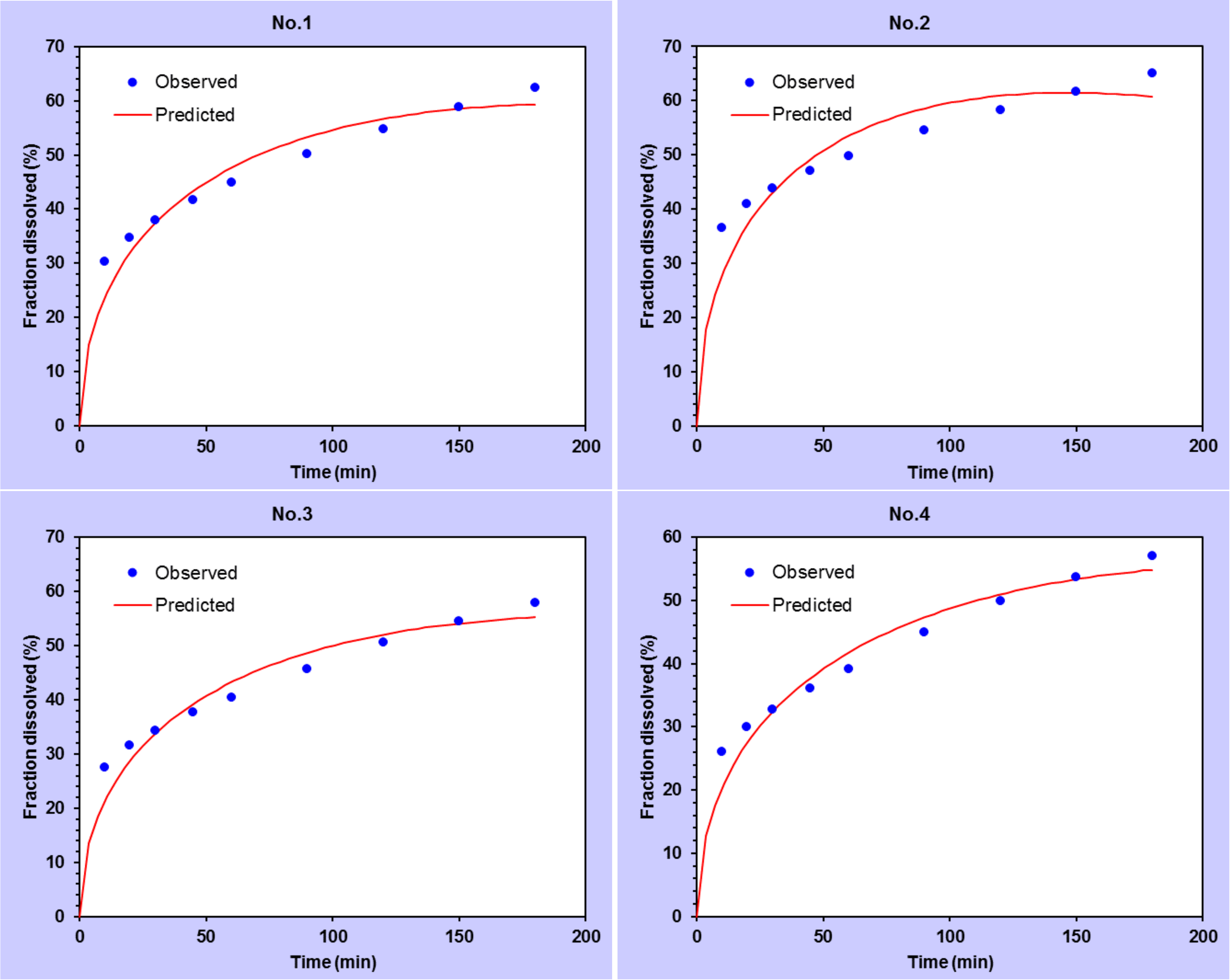

Supplementary materials – Model fitting summary – SuperTab® 11SD (modelling up to app. 60 % of carvedilol released)

Model: **Peppas–Sahlin_2 with T_{lag}**

Model equation: $F = k_1 \cdot (t - T_{lag})^{0.5} + k_2 \cdot (t - T_{lag})$

Fitted model parameters per tested tablet (N = 4) with statistics – mean, standard deviation (SD), and relative standard deviation expressed in % (RSD%) (output from DDSolver):

Parameter	No.1	No.2	No.3	No.4	Mean	SD	RSD(%)
k_1	9.134	10.829	8.157	7.720	8.960	1.379	15.389
k_2	-0.356	-0.476	-0.305	-0.274	-0.353	0.089	-25.156
T_{lag}	4.000	4.000	4.000	4.000	4.000	0.000	0.000

Number of dissolution data points (N), degrees of freedom (df), and selected goodness of fit criteria – Pearson correlation coefficient (R), coefficient of determination (R^2), adjusted coefficient of determination ($R^2_{adjusted}$), and residual sum of squares (RSS) (manual calculation in MS Excel):

Parameter	No.1	No.2	No.3	No.4
N	9	9	9	9
df	6	6	6	6
R	0.952191812	0.916593782	0.9509345	0.961074702
R^2	0.906669247	0.840144161	0.904276424	0.923664582
$R^2_{adjusted}$	0.875558996	0.786858881	0.872368565	0.898219443
RSS	171.1207941	284.8783993	152.0757728	123.6792021

Graphical abstract of model fit presented as mean ± 1 SD of the fraction % of released carvedilol:

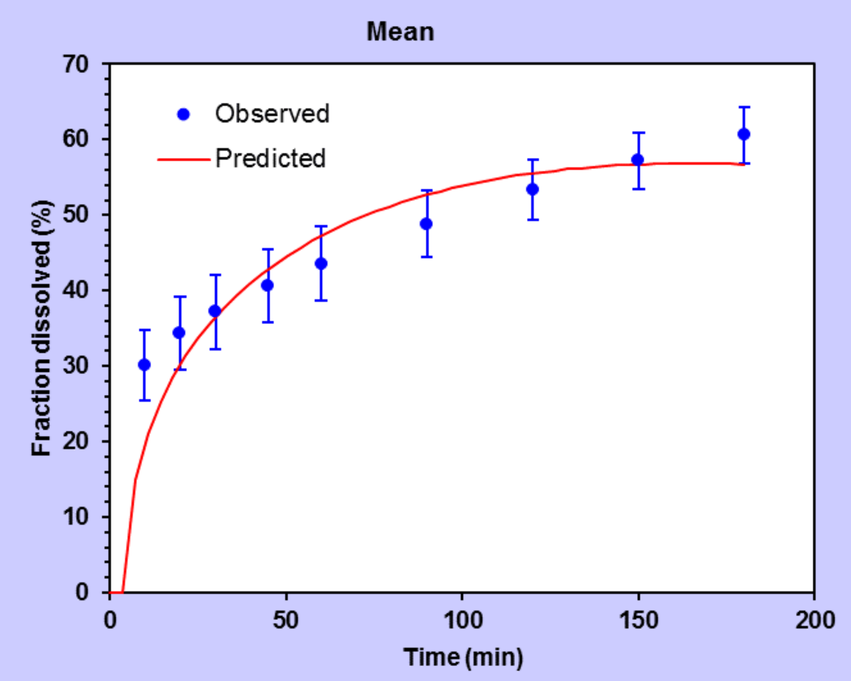

Supplementary materials – Model fitting summary – SuperTab® 11SD (modelling up to app. 60 % of carvedilol released)

Graphical abstract of model fit presented as the fraction % of released carvedilol per tested tablet:

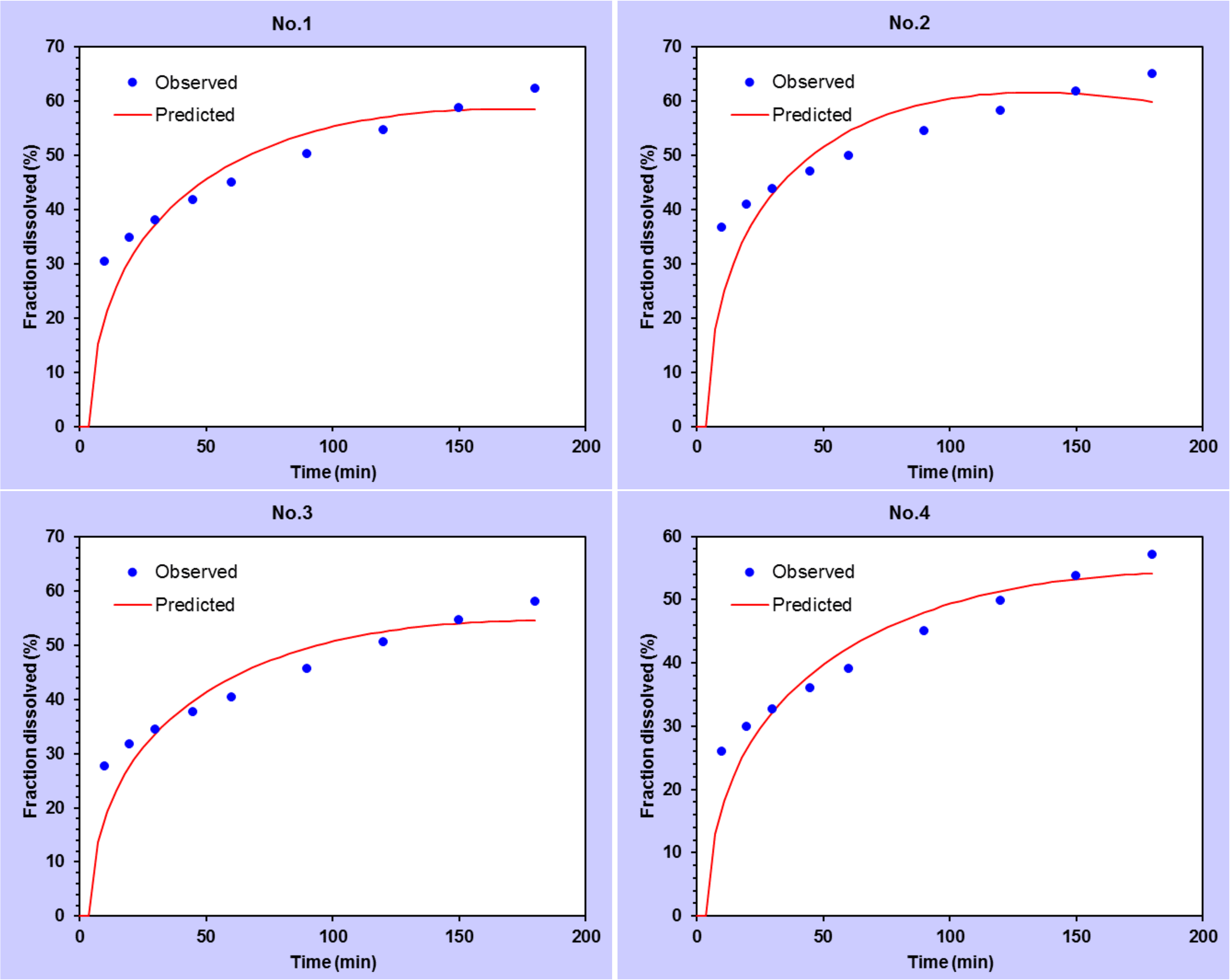

Supplementary materials – Model fitting summary – SuperTab® 11SD (modelling up to app. 60 % of carvedilol released)

Model: **Quadratic**

Model equation: $F = 100 \cdot (k_1 \cdot t^2 + k_2 \cdot t)$

Fitted model parameters per tested tablet (N = 4) with statistics – mean, standard deviation (SD), and relative standard deviation expressed in % (RSD%) (output from DDSolver):

Parameter	No.1	No.2	No.3	No.4	Mean	SD	RSD(%)
k_1	-0.00004	-0.00005	-0.00003	-0.00003	-0.00004	0.00001	-15.28000
k_2	0.01006	0.01138	0.00908	0.00877	0.00982	0.00118	11.97108

Number of dissolution data points (N), degrees of freedom (df), and selected goodness of fit criteria – Pearson correlation coefficient (R), coefficient of determination (R^2), adjusted coefficient of determination ($R^2_{adjusted}$), and residual sum of squares (RSS) (manual calculation in MS Excel):

Parameter	No.1	No.2	No.3	No.4
N	9	9	9	9
df	7	7	7	7
R	0.919881067	0.891099331	0.919856746	0.931997166
R^2	0.846181178	0.794058017	0.846136434	0.868618717
$R^2_{adjusted}$	0.824207061	0.764637734	0.824155924	0.849849963
RSS	1090.607919	1652.679807	895.4412318	761.2337445

Graphical abstract of model fit presented as mean ± 1 SD of the fraction % of released carvedilol:

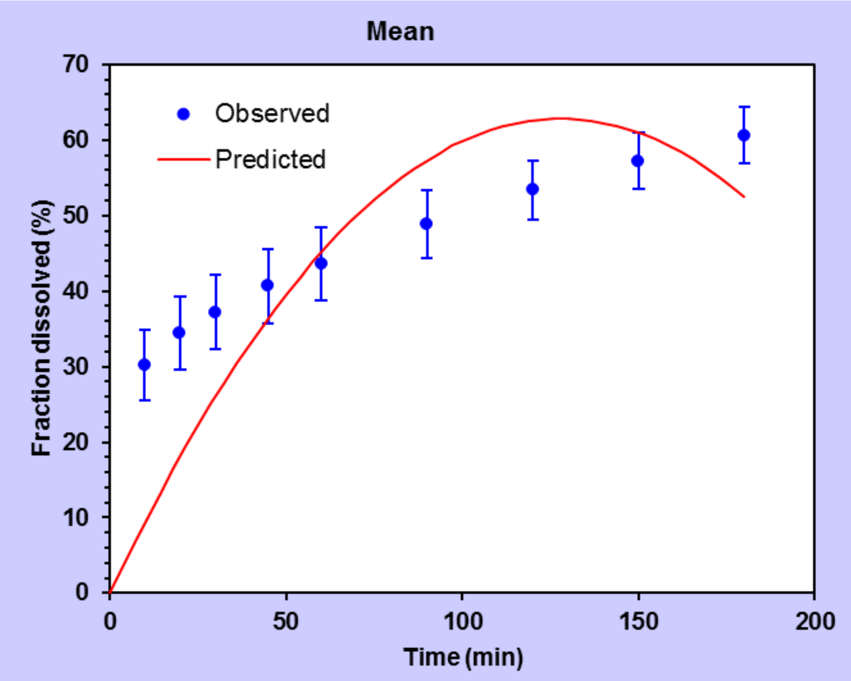

Supplementary materials – Model fitting summary – SuperTab® 11SD (modelling up to app. 60 % of carvedilol released)

Graphical abstract of model fit presented as the fraction % of released carvedilol per tested tablet:

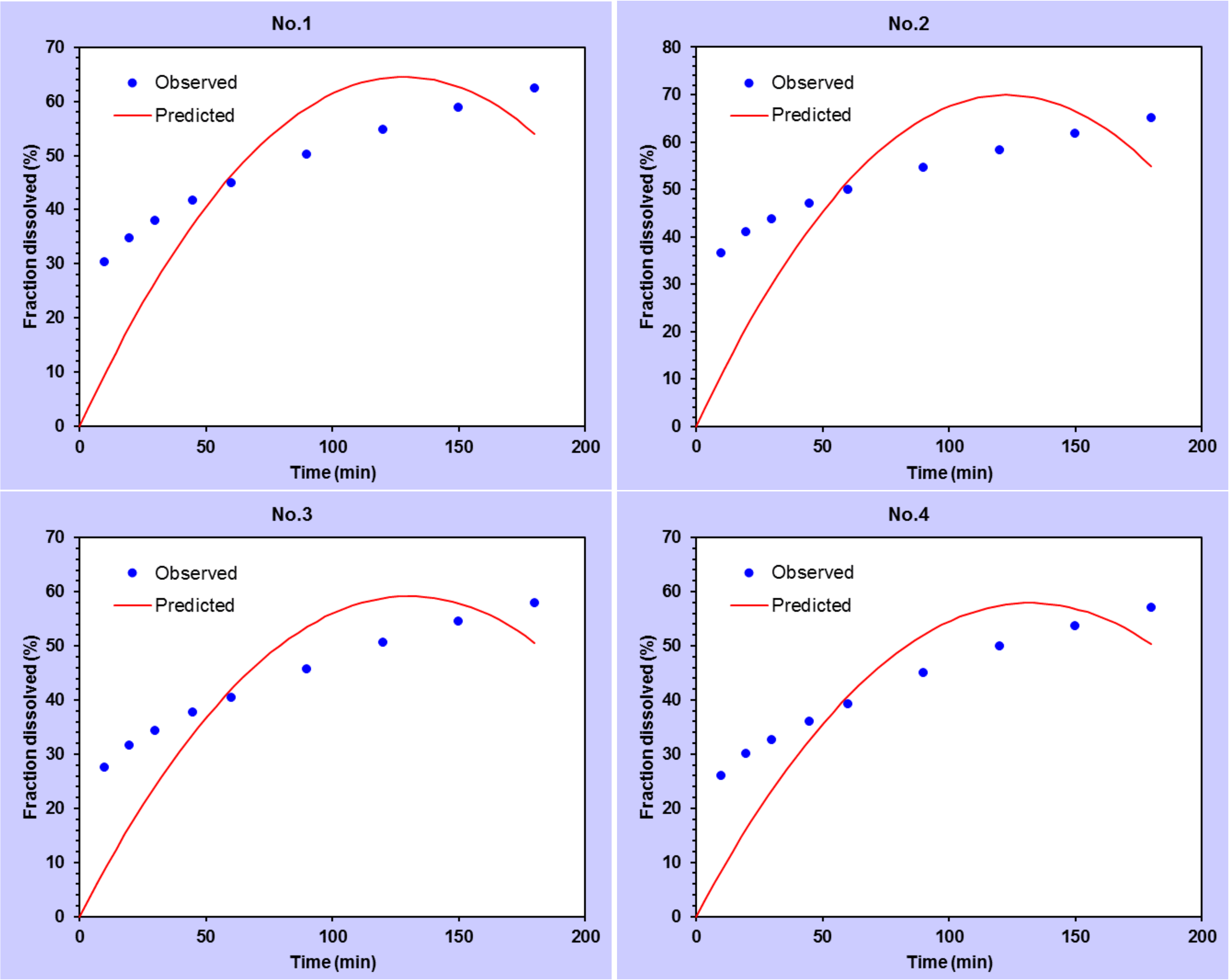

Supplementary materials – Model fitting summary – SuperTab® 11SD (modelling up to app. 60 % of carvedilol released)

Model: **Quadratic with T_{lag}**

Model equation: $F = 100 \cdot \left[k_1 \cdot (t - T_{lag})^2 + k_2 \cdot (t - T_{lag})\right]$

Fitted model parameters per tested tablet (N = 4) with statistics – mean, standard deviation (SD), and relative standard deviation expressed in % (RSD%) (output from DDSolver):

Parameter	No.1	No.2	No.3	No.4	Mean	SD	RSD(%)
k_1	-0.00004	-0.00005	-0.00004	-0.00004	-0.00004	0.00001	-14.81923
k_2	0.01048	0.01181	0.00946	0.00915	0.01022	0.00120	11.73151
T_{lag}	4.00000	4.00000	4.00000	4.00000	4.00000	0.00000	0.00000

Number of dissolution data points (N), degrees of freedom (df), and selected goodness of fit criteria – Pearson correlation coefficient (R), coefficient of determination (R^2), adjusted coefficient of determination ($R^2_{adjusted}$), and residual sum of squares (RSS) (manual calculation in MS Excel):

Parameter	No.1	No.2	No.3	No.4
N	9	9	9	9
df	6	6	6	6
R	0.919721959	0.894434584	0.919065786	0.930408677
R^2	0.845888482	0.800013226	0.844681919	0.865660307
$R^2_{adjusted}$	0.794517977	0.733350968	0.792909226	0.820880409
RSS	1441.364775	2137.496019	1182.427706	1017.792273

Graphical abstract of model fit presented as mean ± 1 SD of the fraction % of released carvedilol:

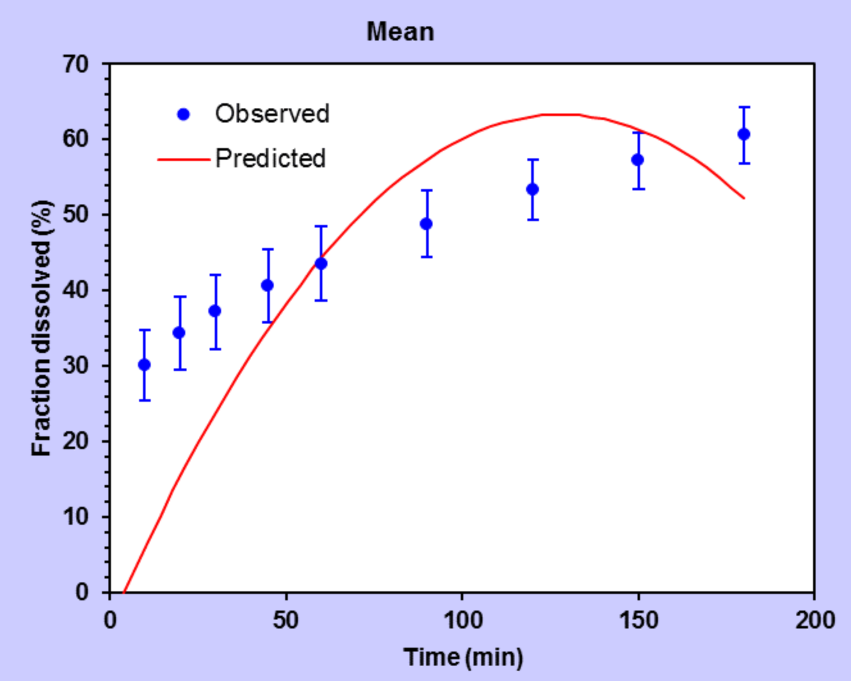

Supplementary materials – Model fitting summary – SuperTab® 11SD (modelling up to app. 60 % of carvedilol released)

Graphical abstract of model fit presented as the fraction % of released carvedilol per tested tablet:

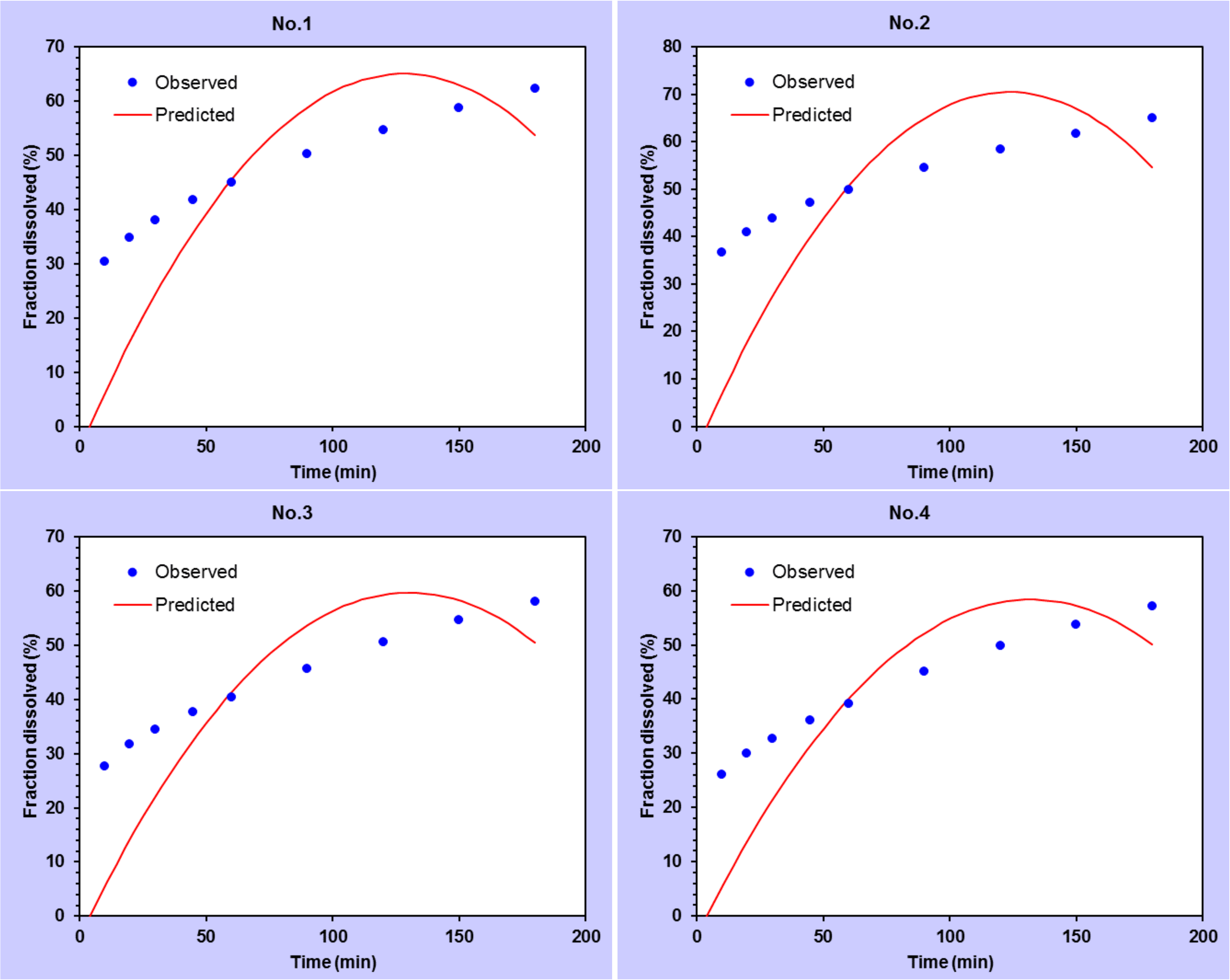

Supplementary materials – Model fitting summary – SuperTab® 11SD (modelling up to app. 60 % of carvedilol released)

Model: **Weibull_1**

Model equation: $F = 100 \cdot \left[1 - e^{-\frac{(t-T_i)^\beta}{\alpha}}\right]$

Fitted model parameters per tested tablet (N = 4) with statistics – mean, standard deviation (SD), and relative standard deviation expressed in % (RSD%) (output from DDSolver):

Parameter	No.1	No.2	No.3	No.4	Mean	SD	RSD(%)
α	5.218	3.723	5.874	6.563	5.344	1.212	22.685
β	0.298	0.248	0.296	0.314	0.289	0.028	9.794
T_i	4.000	4.000	4.000	4.000	4.000	0.000	0.000

Number of dissolution data points (N), degrees of freedom (df), and selected goodness of fit criteria – Pearson correlation coefficient (R), coefficient of determination (R^2), adjusted coefficient of determination ($R^2_{adjusted}$), and residual sum of squares (RSS) (manual calculation in MS Excel):

Parameter	No.1	No.2	No.3	No.4
N	9	9	9	9
df	6	6	6	6
R	0.982969724	0.981780467	0.978493785	0.980304753
R^2	0.966229479	0.963892885	0.957450088	0.960997409
$R^2_{adjusted}$	0.954972638	0.951857181	0.943266784	0.947996545
RSS	34.12947136	27.63279739	39.5858418	39.40279783

Graphical abstract of model fit presented as mean ± 1 SD of the fraction % of released carvedilol:

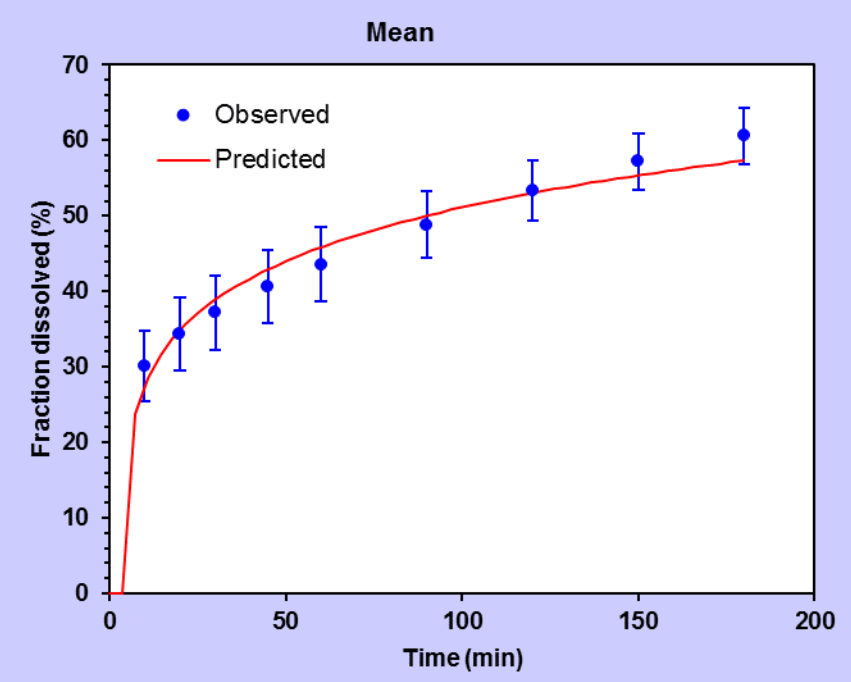

Supplementary materials – Model fitting summary – SuperTab® 11SD (modelling up to app. 60 % of carvedilol released)

Graphical abstract of model fit presented as the fraction % of released carvedilol per tested tablet:

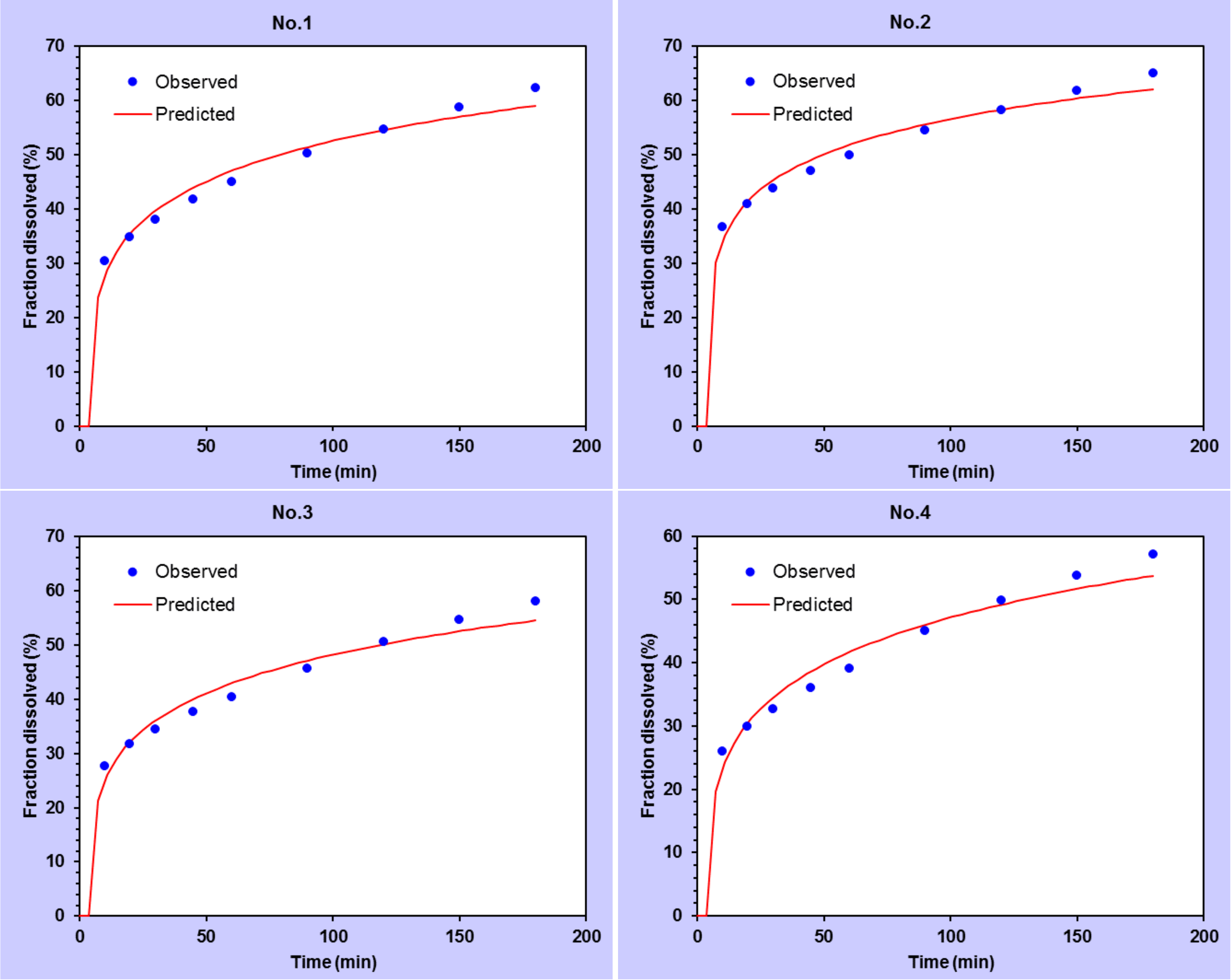

Supplementary materials – Model fitting summary – SuperTab® 11SD (modelling up to app. 60 % of carvedilol released)

Model: **Weibull_2**

Model equation: $F = 100 \cdot \left(1 - e^{-\frac{t^{\beta}}{\alpha}}\right)$

Fitted model parameters per tested tablet (N = 4) with statistics – mean, standard deviation (SD), and relative standard deviation expressed in % (RSD%) (output from DDSolver):

Parameter	No.1	No.2	No.3	No.4	Mean	SD	RSD(%)
α	6.547	4.498	7.372	8.350	6.692	1.638	24.473
β	0.345	0.287	0.343	0.364	0.335	0.033	9.839

Number of dissolution data points (N), degrees of freedom (df), and selected goodness of fit criteria – Pearson correlation coefficient (R), coefficient of determination (R^2), adjusted coefficient of determination ($R^2_{adjusted}$), and residual sum of squares (RSS) (manual calculation in MS Excel):

Parameter	No.1	No.2	No.3	No.4
N	9	9	9	9
df	7	7	7	7
R	0.991360392	0.990513876	0.987864862	0.989417546
R^2	0.982795428	0.981117739	0.975876985	0.978947081
$R^2_{adjusted}$	0.980337632	0.978420273	0.97243084	0.975939521
RSS	17.30549201	14.41445884	22.32361696	21.15157522

Graphical abstract of model fit presented as mean ± 1 SD of the fraction % of released carvedilol:

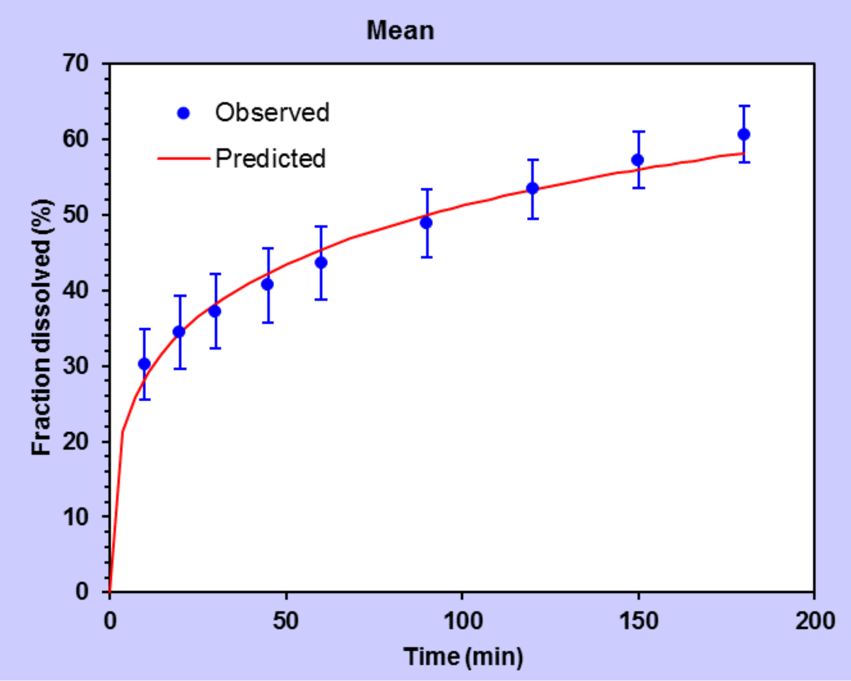

Supplementary materials – Model fitting summary – SuperTab® 11SD (modelling up to app. 60 % of carvedilol released)

Graphical abstract of model fit presented as the fraction % of released carvedilol per tested tablet:

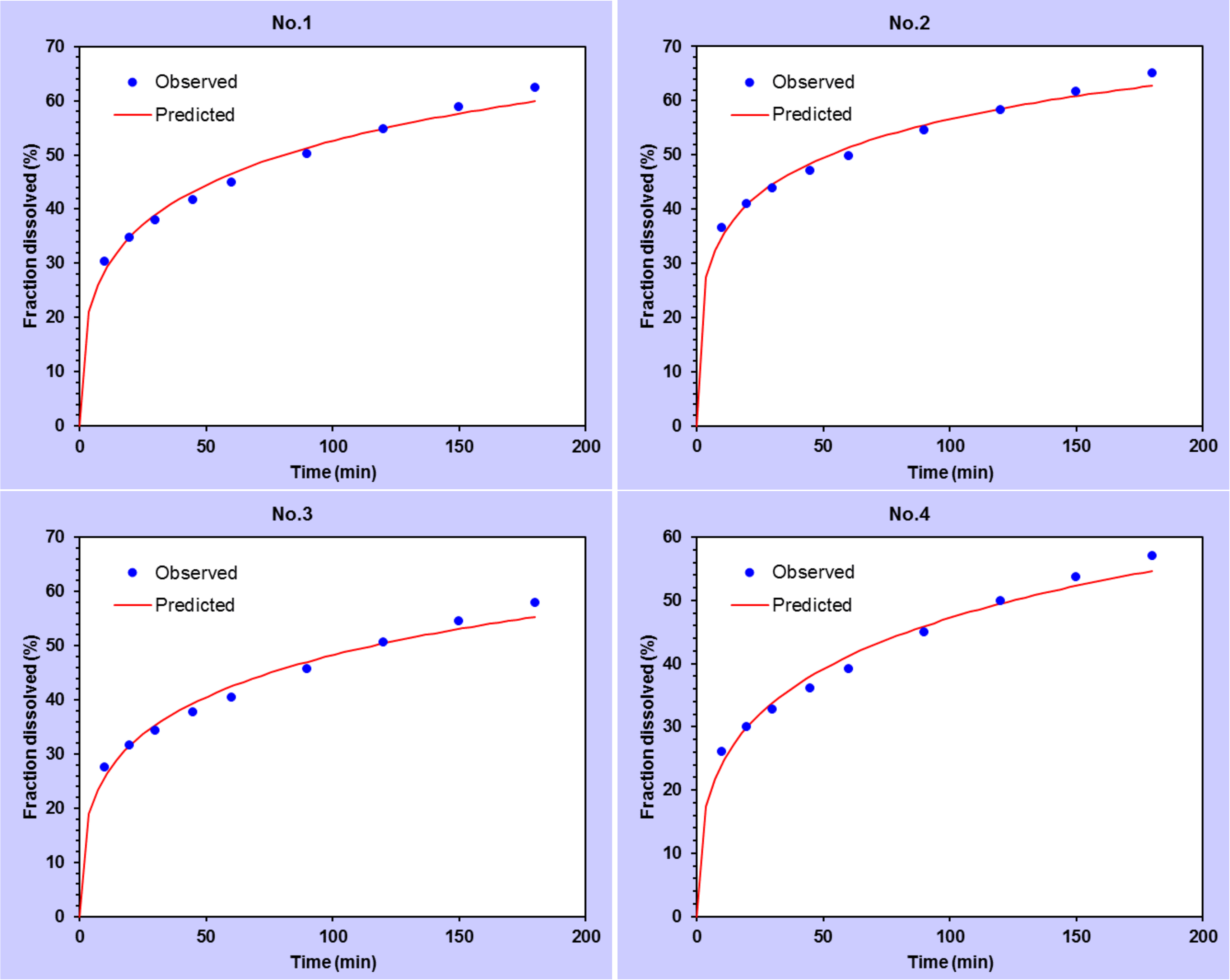

Supplementary materials – Model fitting summary – SuperTab® 11SD (modelling up to app. 60 % of carvedilol released)

Model: **Weibull_3**

Model equation: $F = F_{max} \cdot \left(1 - e^{-\frac{t^{\beta}}{\alpha}}\right)$

Fitted model parameters per tested tablet (N = 4) with statistics – mean, standard deviation (SD), and relative standard deviation expressed in % (RSD%) (output from DDSolver):

Parameter	No.1	No.2	No.3	No.4	Mean	SD	RSD(%)
α	6.361	4.265	6.829	7.813	6.317	1.496	23.681
β	0.522	0.450	0.532	0.558	0.516	0.046	8.962
F_{max}	65.456	68.270	60.836	59.914	63.619	3.936	6.187

Number of dissolution data points (N), degrees of freedom (df), and selected goodness of fit criteria – Pearson correlation coefficient (R), coefficient of determination (R^2), adjusted coefficient of determination ($R^2_{adjusted}$), and residual sum of squares (RSS) (manual calculation in MS Excel):

Parameter	No.1	No.2	No.3	No.4
N	9	9	9	9
df	6	6	6	6
R	0.973734694	0.972393602	0.96737167	0.969589485
R^2	0.948159254	0.945549317	0.935807949	0.94010377
$R^2_{adjusted}$	0.930879005	0.92739909	0.914410598	0.92013836
RSS	53.69986619	44.1250103	60.37352036	60.20232003

Graphical abstract of model fit presented as mean ± 1 SD of the fraction % of released carvedilol:

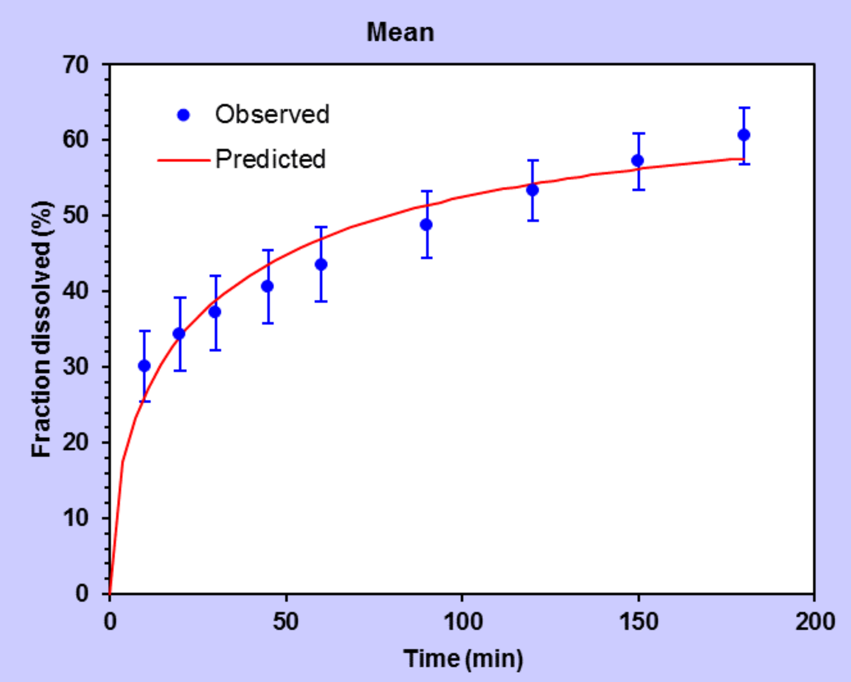

Supplementary materials – Model fitting summary – SuperTab® 11SD (modelling up to app. 60 % of carvedilol released)

Graphical abstract of model fit presented as the fraction % of released carvedilol per tested tablet:

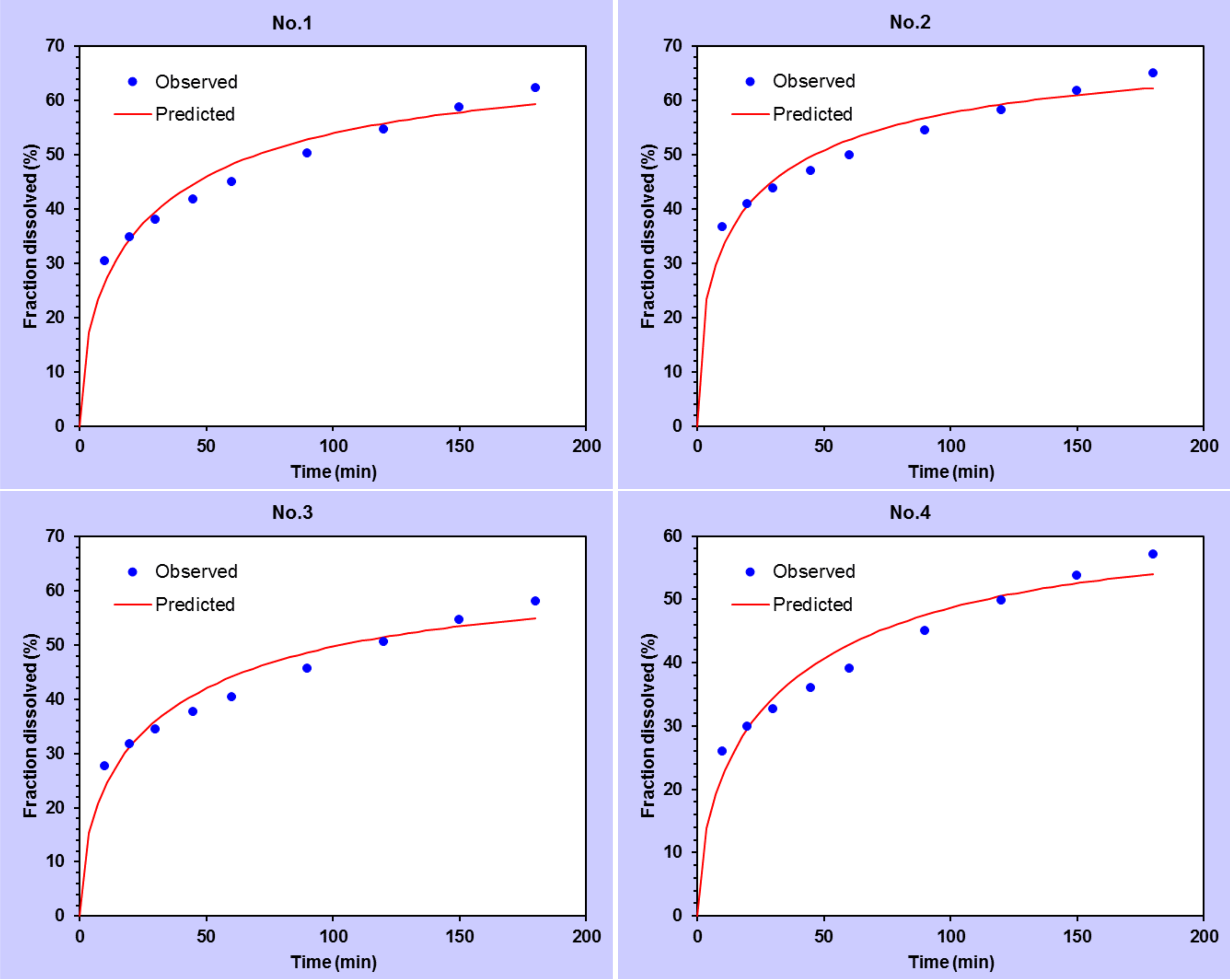

Supplementary materials – Model fitting summary – SuperTab® 11SD (modelling up to app. 60 % of carvedilol released)

Model: **Weibull_4**

Model equation: $F = F_{max} \cdot \left[1 - e^{-\frac{(t-T_i)^\beta}{\alpha}}\right]$

Fitted model parameters per tested tablet (N = 4) with statistics – mean, standard deviation (SD), and relative standard deviation expressed in % (RSD%) (output from DDSolver):

Parameter	No.1	No.2	No.3	No.4	Mean	SD	RSD(%)
α	4.464	3.143	4.750	5.337	4.424	0.928	20.977
β	0.448	0.387	0.456	0.479	0.442	0.039	8.908
T_i	4.000	4.000	4.000	4.000	4.000	0.000	0.000
F_{max}	65.456	68.270	60.836	59.914	63.619	3.936	6.187

Number of dissolution data points (N), degrees of freedom (df), and selected goodness of fit criteria – Pearson correlation coefficient (R), coefficient of determination (R^2), adjusted coefficient of determination ($R^2_{adjusted}$), and residual sum of squares (RSS) (manual calculation in MS Excel):

Parameter	No.1	No.2	No.3	No.4
N	9	9	9	9
df	5	5	5	5
R	0.961093308	0.959114067	0.95390305	0.956224839
R^2	0.923700347	0.919899793	0.909931029	0.914365943
$R^2_{adjusted}$	0.877920555	0.871839669	0.855889647	0.862985509
RSS	78.30594246	64.32911484	84.04967733	85.45576869

Graphical abstract of model fit presented as mean ± 1 SD of the fraction % of released carvedilol:

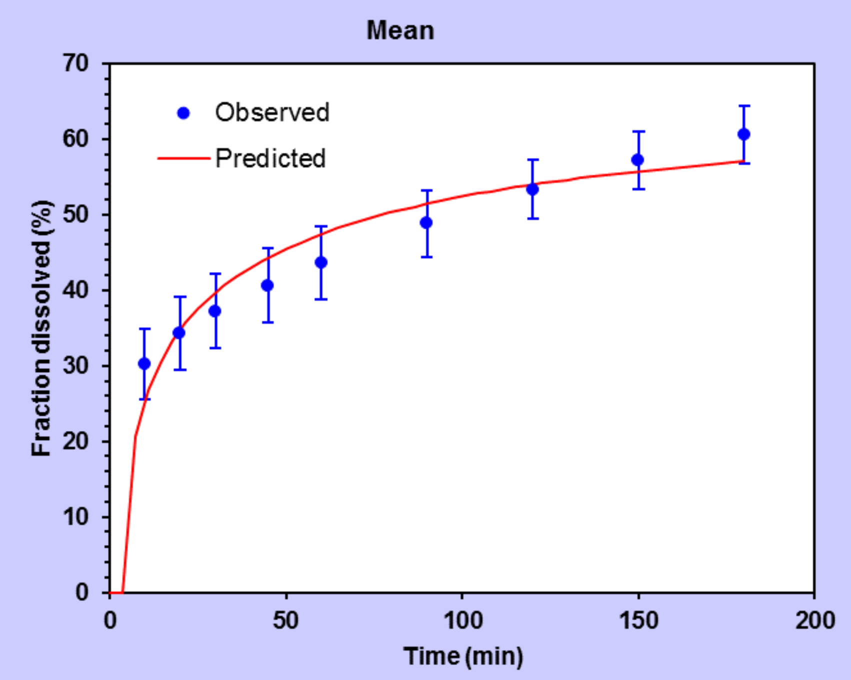

Supplementary materials – Model fitting summary – SuperTab® 11SD (modelling up to app. 60 % of carvedilol released)

Graphical abstract of model fit presented as the fraction % of released carvedilol per tested tablet:

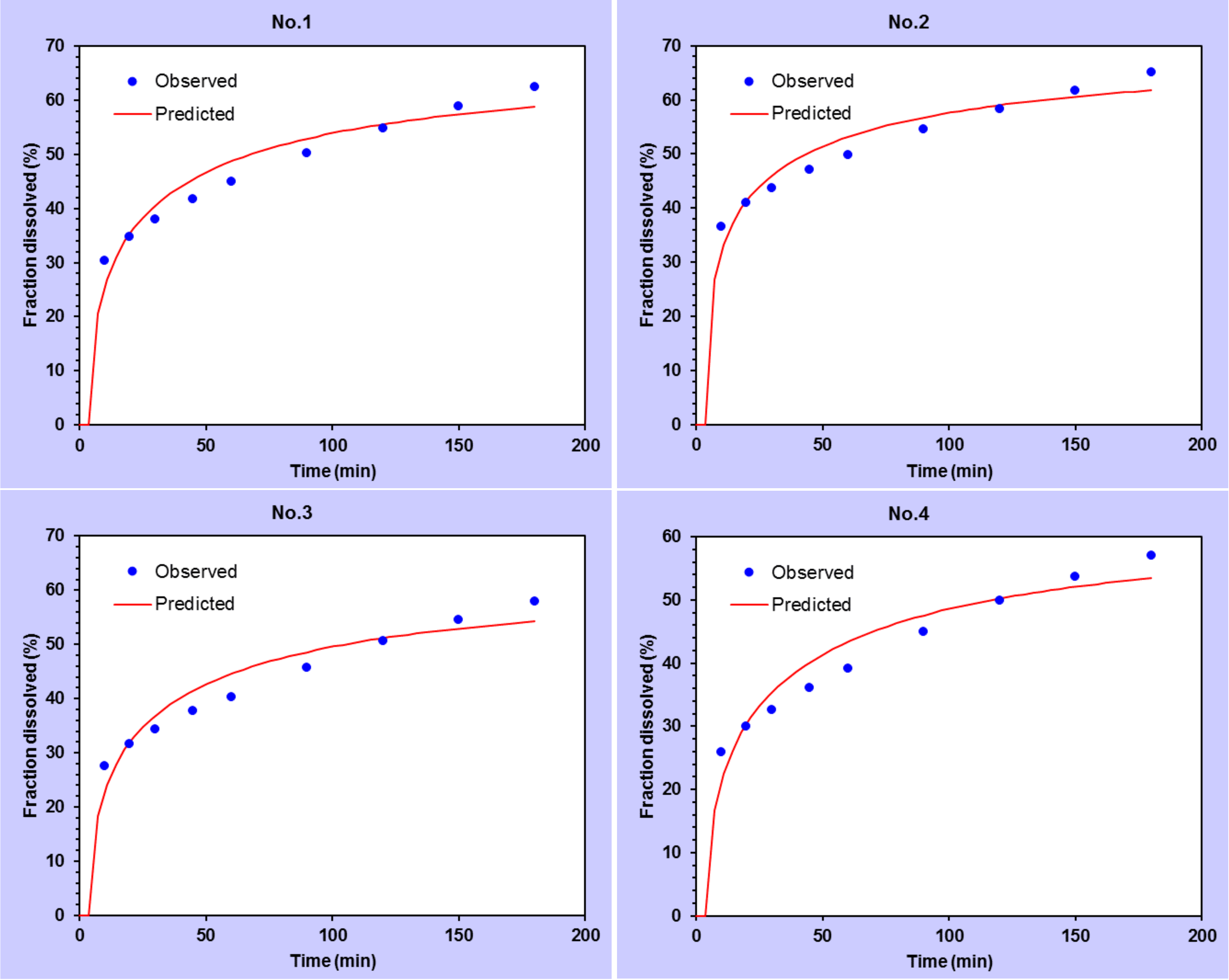

Supplementary materials – Model fitting summary – SuperTab® 11SD (modelling up to app. 60 % of carvedilol released)

Model: **Logistic_1**

Model equation: $F = 100 \cdot \frac{e^{\alpha+\beta \cdot \log(t)}}{1+e^{\alpha+\beta \cdot \log(t)}}$

Fitted model parameters per tested tablet (N = 4) with statistics – mean, standard deviation (SD), and relative standard deviation expressed in % (RSD%) (output from DDSolver):

Parameter	No.1	No.2	No.3	No.4	Mean	SD	RSD(%)
α	-2.006	-1.569	-2.109	-2.249	-1.983	0.293	-14.799
β	1.061	0.922	1.024	1.074	1.020	0.069	6.760

Number of dissolution data points (N), degrees of freedom (df), and selected goodness of fit criteria – Pearson correlation coefficient (R), coefficient of determination (R^2), adjusted coefficient of determination ($R^2_{adjusted}$), and residual sum of squares (RSS) (manual calculation in MS Excel):

Parameter	No.1	No.2	No.3	No.4
N	9	9	9	9
df	7	7	7	7
R	0.986056738	0.985242764	0.982498189	0.984269926
R^2	0.97230789	0.970703304	0.965302692	0.968787287
$R^2_{adjusted}$	0.968351874	0.966518061	0.960345934	0.964328327
RSS	27.2125852	22.12285638	31.28209608	30.38105193

Graphical abstract of model fit presented as mean ± 1 SD of the fraction % of released carvedilol:

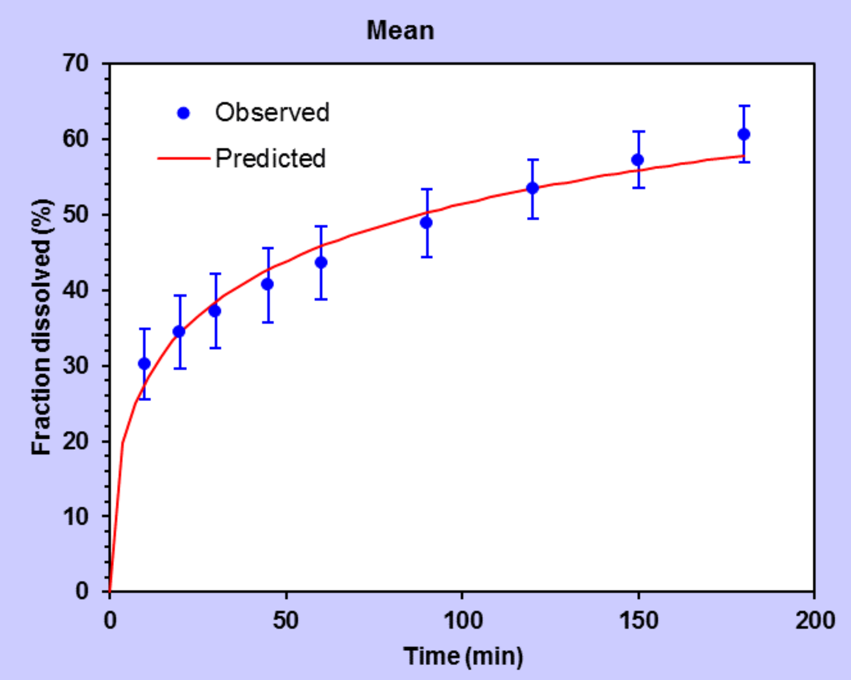

Supplementary materials – Model fitting summary – SuperTab® 11SD (modelling up to app. 60 % of carvedilol released)

Graphical abstract of model fit presented as the fraction % of released carvedilol per tested tablet:

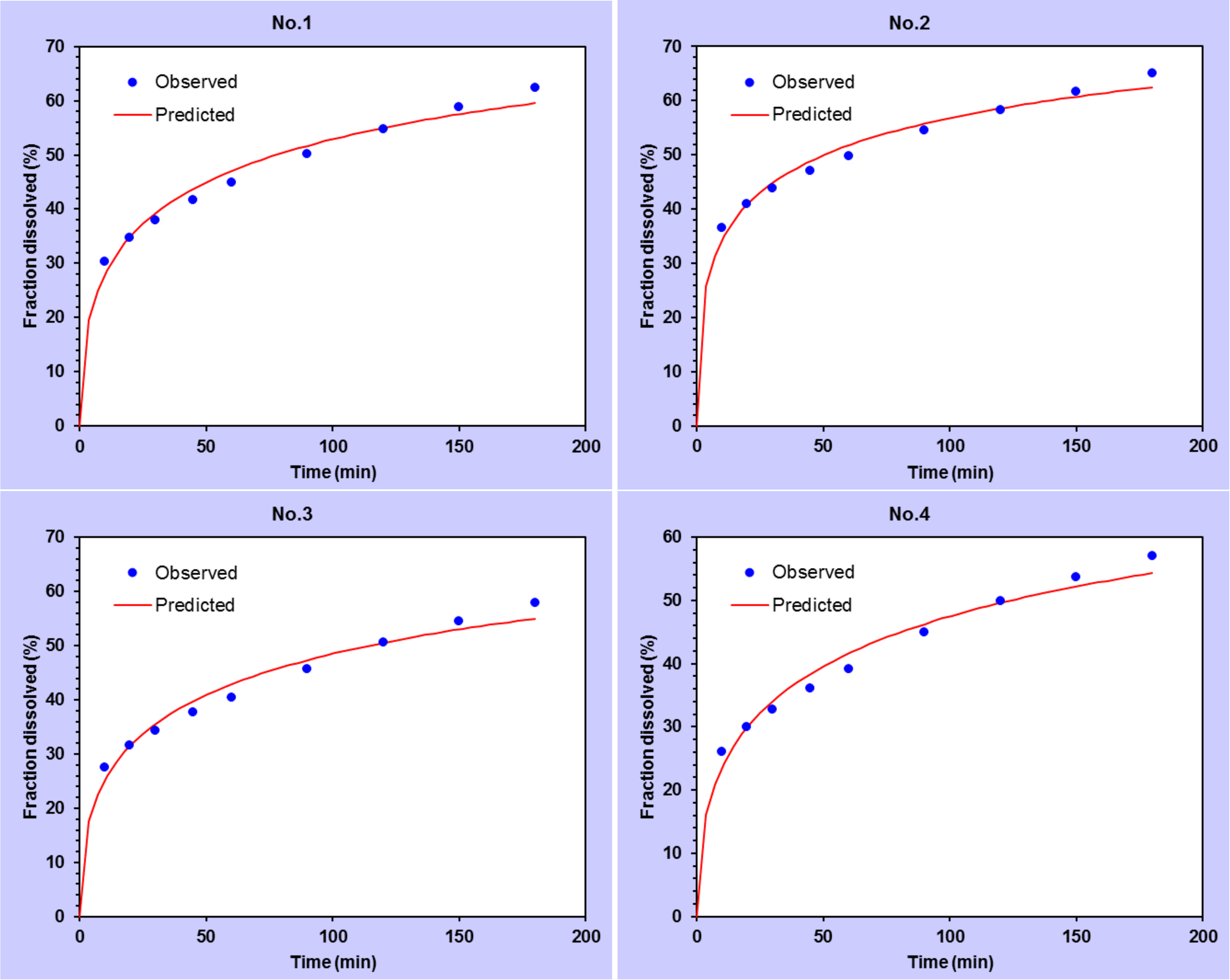

Supplementary materials – Model fitting summary – SuperTab® 11SD (modelling up to app. 60 % of carvedilol released)

Model: **Logistic_2**

Model equation: $F = F_{max} \cdot \frac{e^{\alpha+\beta \cdot \log(t)}}{1+e^{\alpha+\beta \cdot \log(t)}}$

Fitted model parameters per tested tablet (N = 4) with statistics – mean, standard deviation (SD), and relative standard deviation expressed in % (RSD%) (output from DDSolver):

Parameter	No.1	No.2	No.3	No.4	Mean	SD	RSD(%)
α	-2.851	-2.300	-2.944	-3.131	-2.807	0.357	-12.719
β	2.253	2.043	2.276	2.361	2.233	0.135	6.054
F_{max}	65.456	68.270	60.836	59.914	63.619	3.936	6.187

Number of dissolution data points (N), degrees of freedom (df), and selected goodness of fit criteria – Pearson correlation coefficient (R), coefficient of determination (R^2), adjusted coefficient of determination ($R^2_{adjusted}$), and residual sum of squares (RSS) (manual calculation in MS Excel):

Parameter	No.1	No.2	No.3	No.4
N	9	9	9	9
df	6	6	6	6
R	0.946377371	0.945471926	0.937761232	0.939910908
R^2	0.895630128	0.893917163	0.879396128	0.883432514
$R^2_{adjusted}$	0.860840171	0.858556218	0.839194837	0.844576686
RSS	129.2376591	104.7335693	134.7173276	137.8642848

Graphical abstract of model fit presented as mean ± 1 SD of the fraction % of released carvedilol:

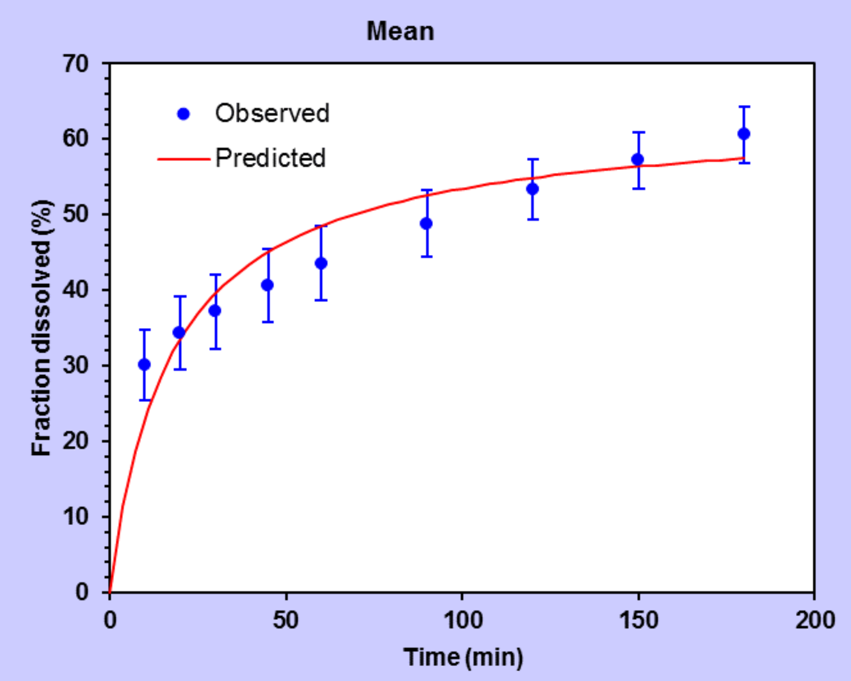

Supplementary materials – Model fitting summary – SuperTab® 11SD (modelling up to app. 60 % of carvedilol released)

Graphical abstract of model fit presented as the fraction % of released carvedilol per tested tablet:

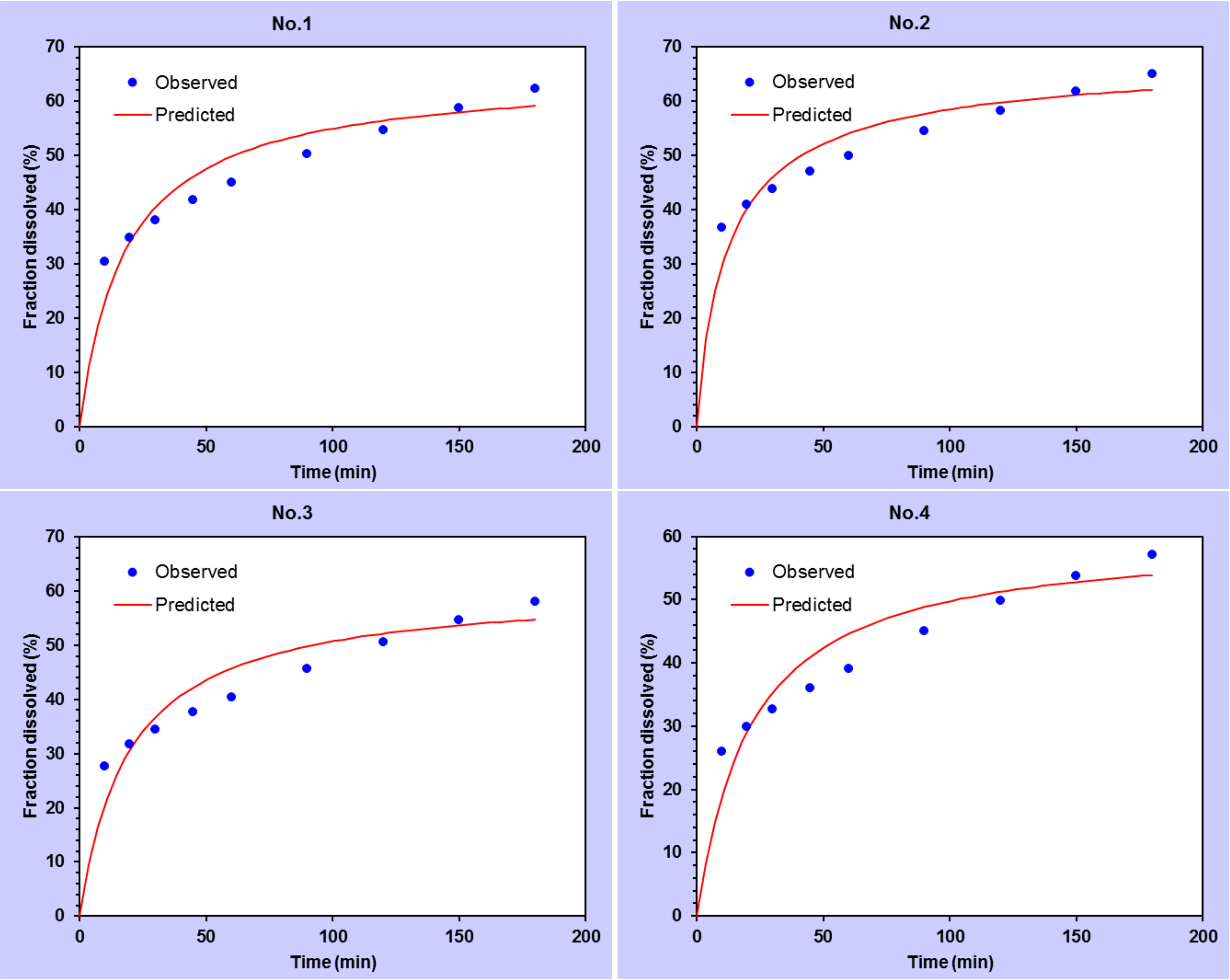

Supplementary materials – Model fitting summary – SuperTab® 11SD (modelling up to app. 60 % of carvedilol released)

Model: **Logistic_3**

Model equation: $F = F_{max} \cdot \frac{1}{1+e^{-k\cdot(t-\gamma)}}$

Fitted model parameters per tested tablet (N = 4) with statistics – mean, standard deviation (SD), and relative standard deviation expressed in % (RSD%) (output from DDSolver):

Parameter	No.1	No.2	No.3	No.4	Mean	SD	RSD(%)
k	0.017	0.015	0.017	0.018	0.017	0.001	6.413
γ	15.204	-3.443	19.406	23.557	13.681	11.914	87.086
F_{max}	65.456	68.270	60.836	59.914	63.619	3.936	6.187

Number of dissolution data points (N), degrees of freedom (df), and selected goodness of fit criteria – Pearson correlation coefficient (R), coefficient of determination (R^2), adjusted coefficient of determination ($R^2_{adjusted}$), and residual sum of squares (RSS) (manual calculation in MS Excel):

Parameter	No.1	No.2	No.3	No.4
N	9	9	9	9
df	6	6	6	6
R	0.996862263	0.996420119	0.996734975	0.997962502
R^2	0.993734371	0.992853053	0.993480611	0.995929155
$R^2_{adjusted}$	0.991645827	0.990470737	0.991307481	0.994572206
RSS	6.852089743	5.793839272	7.16037259	4.835436356

Graphical abstract of model fit presented as mean ± 1 SD of the fraction % of released carvedilol:

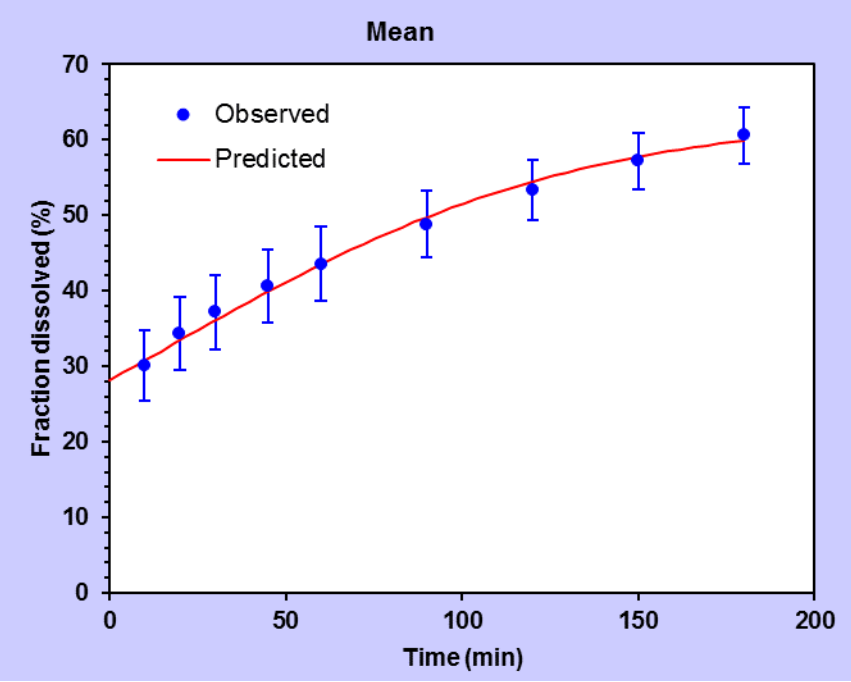

Supplementary materials – Model fitting summary – SuperTab® 11SD (modelling up to app. 60 % of carvedilol released)

Graphical abstract of model fit presented as the fraction % of released carvedilol per tested tablet:

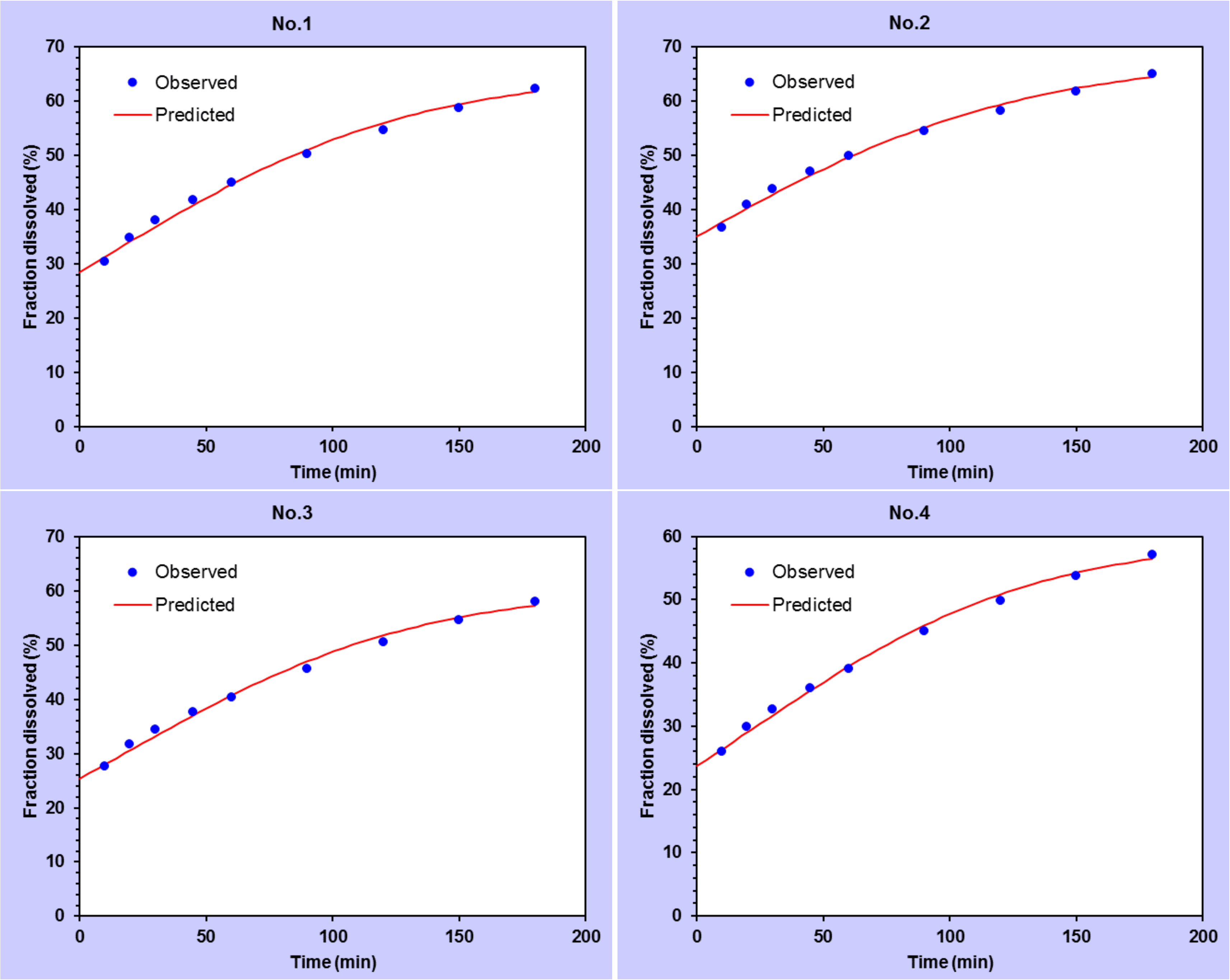

Supplementary materials – Model fitting summary – SuperTab® 11SD (modelling up to app. 60 % of carvedilol released)

Model: **Gompertz_1**

Model equation: $F = 100 \cdot e^{-\alpha \cdot e^{-\beta \cdot \log(t)}}$

Fitted model parameters per tested tablet (N = 4) with statistics – mean, standard deviation (SD), and relative standard deviation expressed in % (RSD%) (output from DDSolver):

Parameter	No.1	No.2	No.3	No.4	Mean	SD	RSD(%)
α	2.737	2.130	2.804	3.020	2.673	0.382	14.275
β	0.733	0.666	0.681	0.704	0.696	0.029	4.172

Number of dissolution data points (N), degrees of freedom (df), and selected goodness of fit criteria – Pearson correlation coefficient (R), coefficient of determination (R^2), adjusted coefficient of determination ($R^2_{adjusted}$), and residual sum of squares (RSS) (manual calculation in MS Excel):

Parameter	No.1	No.2	No.3	No.4
N	9	9	9	9
df	7	7	7	7
R	0.977623153	0.978329323	0.973219669	0.974671448
R^2	0.95574703	0.957128263	0.947156524	0.949984432
$R^2_{adjusted}$	0.949425177	0.95100373	0.939607456	0.942839351
RSS	43.48777285	32.68764213	47.22198491	47.96775675

Graphical abstract of model fit presented as mean ± 1 SD of the fraction % of released carvedilol:

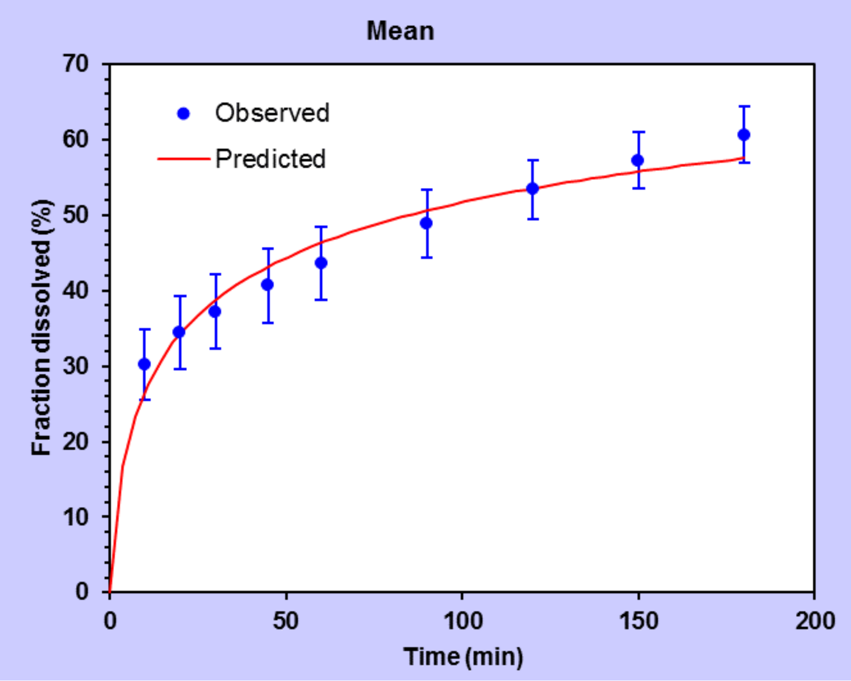

Supplementary materials – Model fitting summary – SuperTab® 11SD (modelling up to app. 60 % of carvedilol released)

Graphical abstract of model fit presented as the fraction % of released carvedilol per tested tablet:

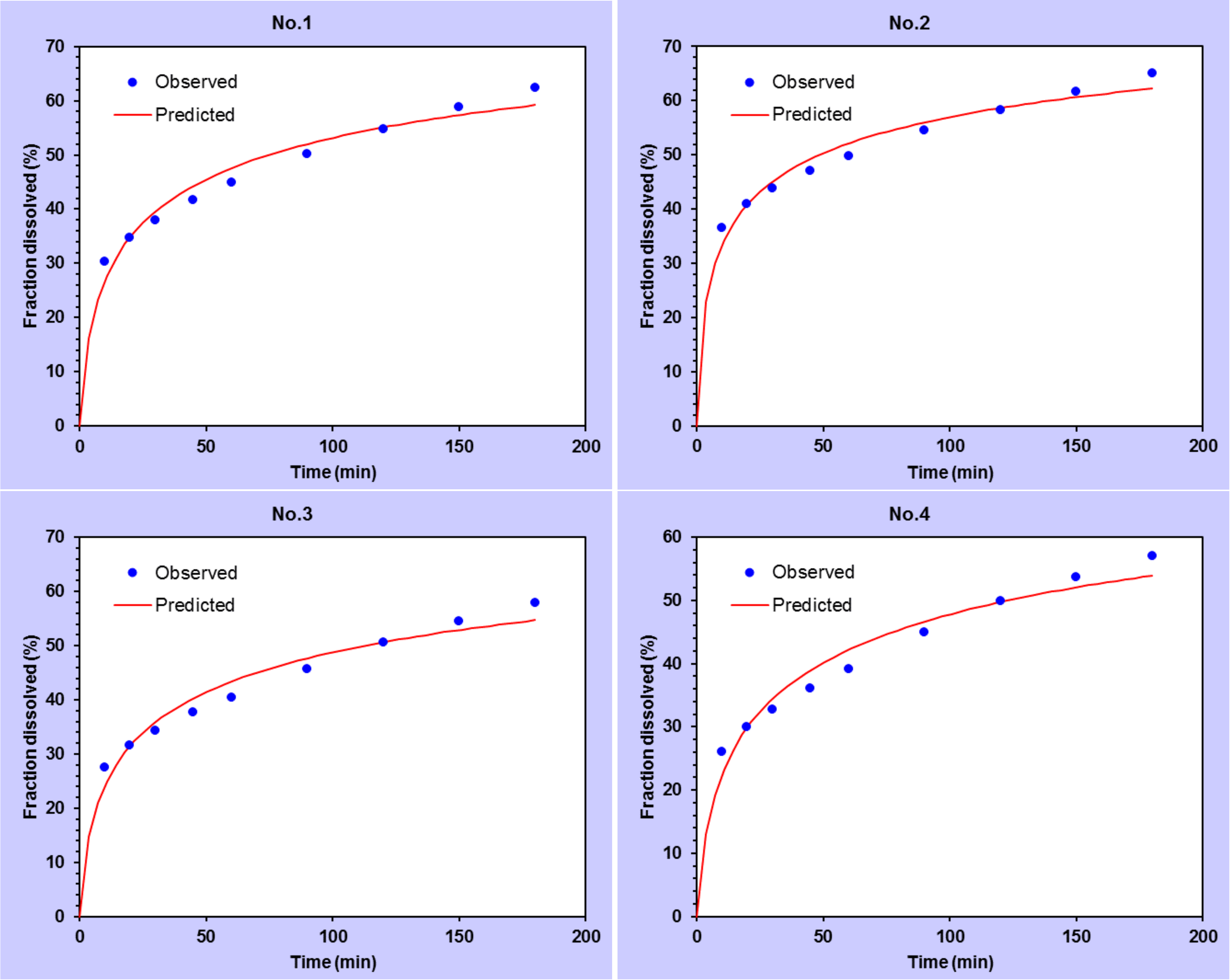

Supplementary materials – Model fitting summary – SuperTab® 11SD (modelling up to app. 60 % of carvedilol released)

Model: **Gompertz_2**

Model equation: $F = F_{max} \cdot e^{-\alpha \cdot e^{-\beta \cdot \log(t)}}$

Fitted model parameters per tested tablet (N = 4) with statistics – mean, standard deviation (SD), and relative standard deviation expressed in % (RSD%) (output from DDSolver):

Parameter	No.1	No.2	No.3	No.4	Mean	SD	RSD(%)
α	8.322	5.579	8.878	10.152	8.233	1.928	23.414
β	1.945	1.801	1.957	2.017	1.930	0.092	4.747
F_{max}	65.456	68.270	60.836	59.914	63.619	3.936	6.187

Number of dissolution data points (N), degrees of freedom (df), and selected goodness of fit criteria – Pearson correlation coefficient (R), coefficient of determination (R^2), adjusted coefficient of determination ($R^2_{adjusted}$), and residual sum of squares (RSS) (manual calculation in MS Excel):

Parameter	No.1	No.2	No.3	No.4
N	9	9	9	9
df	6	6	6	6
R	0.925274158	0.927890178	0.914895601	0.915795859
R^2	0.856132267	0.860980183	0.83703396	0.838682055
$R^2_{adjusted}$	0.808176356	0.814640243	0.782711947	0.784909407
RSS	207.2147092	158.2759966	211.3871691	221.3802459

Graphical abstract of model fit presented as mean ± 1 SD of the fraction % of released carvedilol:

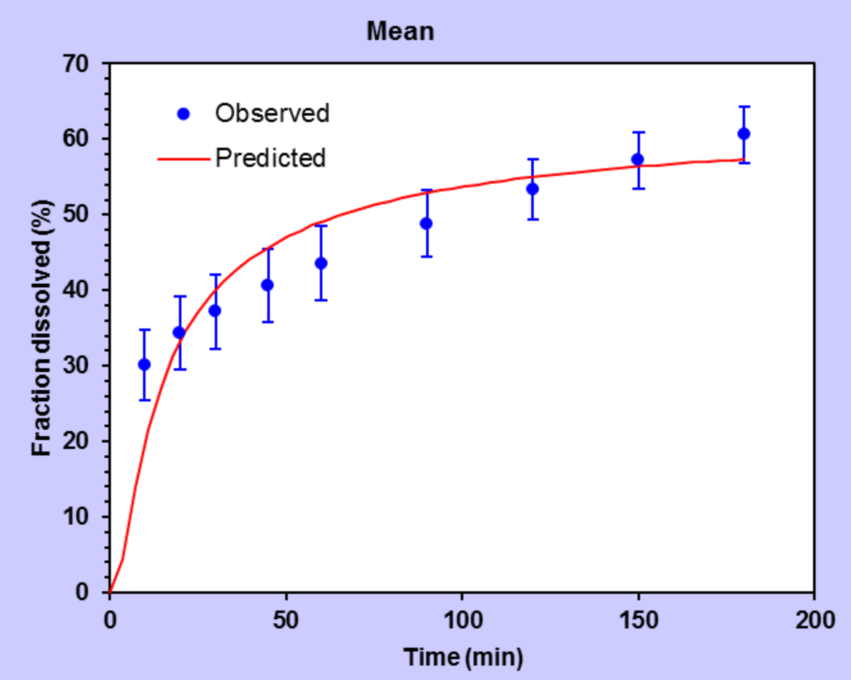

Supplementary materials – Model fitting summary – SuperTab® 11SD (modelling up to app. 60 % of carvedilol released)

Graphical abstract of model fit presented as the fraction % of released carvedilol per tested tablet:

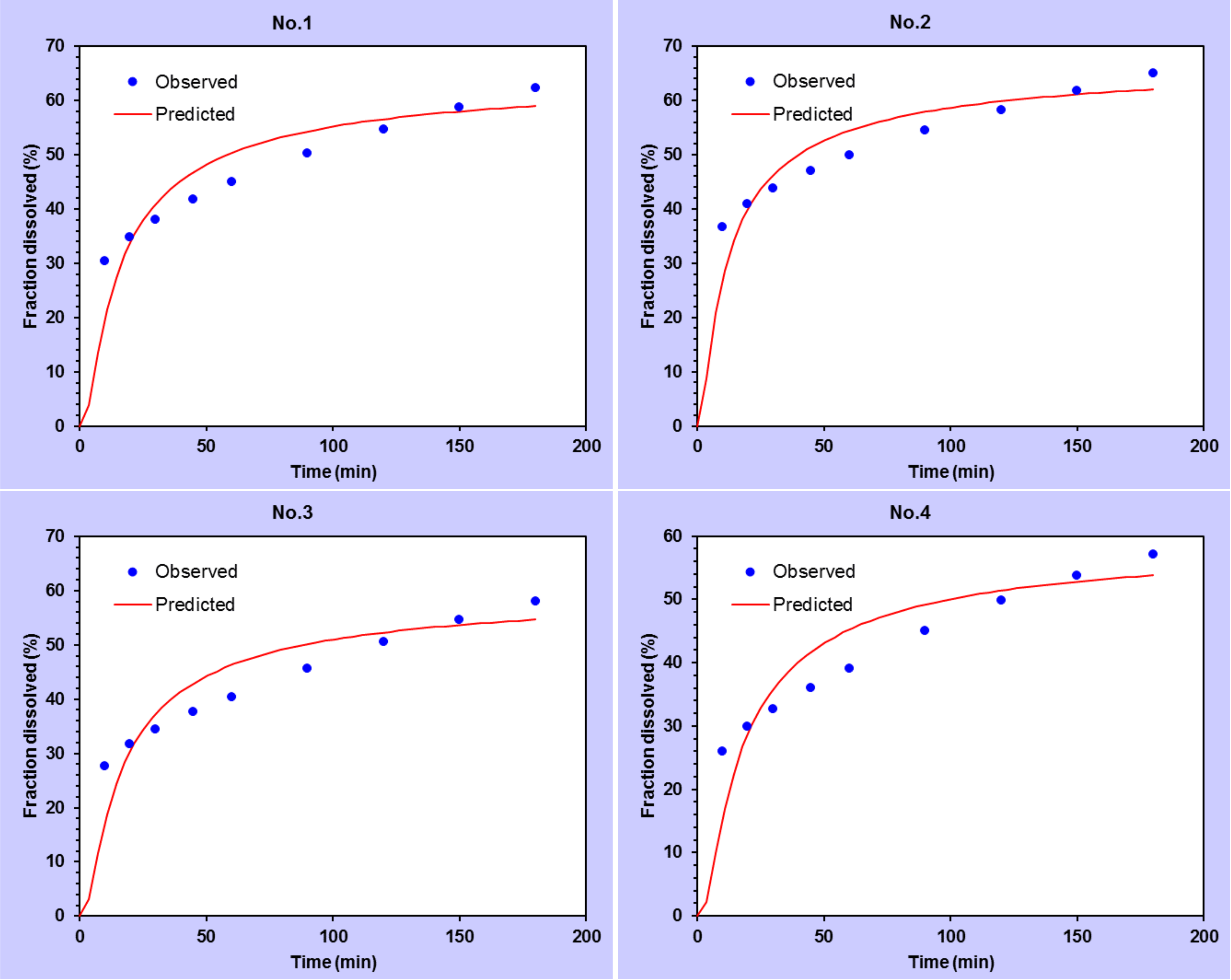

Supplementary materials – Model fitting summary – SuperTab® 11SD (modelling up to app. 60 % of carvedilol released)

Model: **Gompertz_3**

Model equation: $F = F_{max} \cdot e^{-e^{-k\cdot(t-\gamma)}}$

Fitted model parameters per tested tablet (N = 4) with statistics – mean, standard deviation (SD), and relative standard deviation expressed in % (RSD%) (output from DDSolver):

Parameter	No.1	No.2	No.3	No.4	Mean	SD	RSD(%)
k	0.015	0.014	0.015	0.016	0.015	0.001	5.278
γ	-6.682	-24.846	-3.875	1.551	-8.463	11.444	-135.228
F_{max}	65.456	68.270	60.836	59.914	63.619	3.936	6.187

Number of dissolution data points (N), degrees of freedom (df), and selected goodness of fit criteria – Pearson correlation coefficient (R), coefficient of determination (R^2), adjusted coefficient of determination ($R^2_{adjusted}$), and residual sum of squares (RSS) (manual calculation in MS Excel):

Parameter	No.1	No.2	No.3	No.4
N	9	9	9	9
df	6	6	6	6
R	0.996695732	0.996693453	0.994881967	0.996324129
R^2	0.993402382	0.993397839	0.989790128	0.99266177
$R^2_{adjusted}$	0.991203175	0.991197119	0.986386838	0.990215694
RSS	9.503331985	6.553236367	12.7557812	11.10279145

Graphical abstract of model fit presented as mean ± 1 SD of the fraction % of released carvedilol:

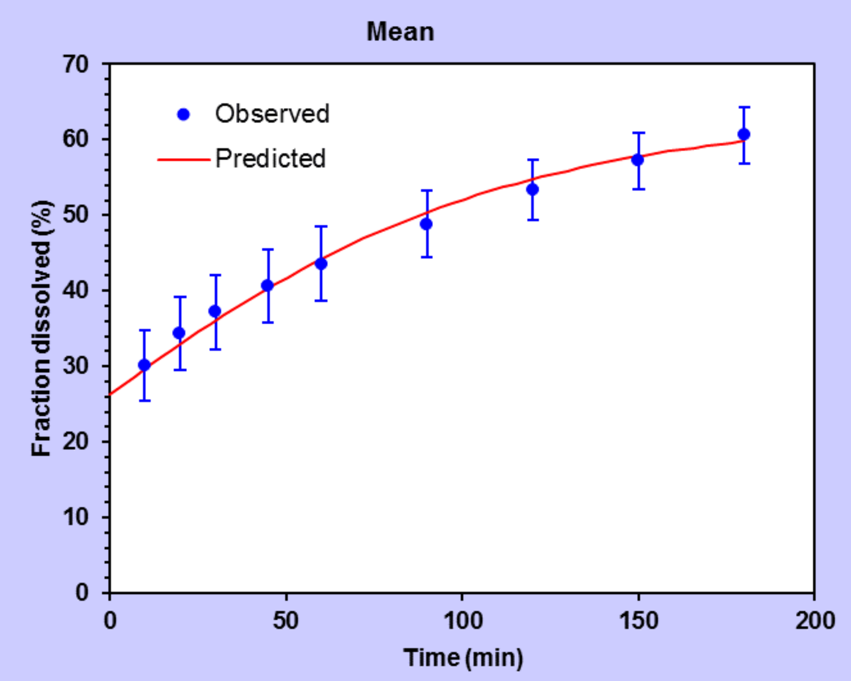

Supplementary materials – Model fitting summary – SuperTab® 11SD (modelling up to app. 60 % of carvedilol released)

Graphical abstract of model fit presented as the fraction % of released carvedilol per tested tablet:

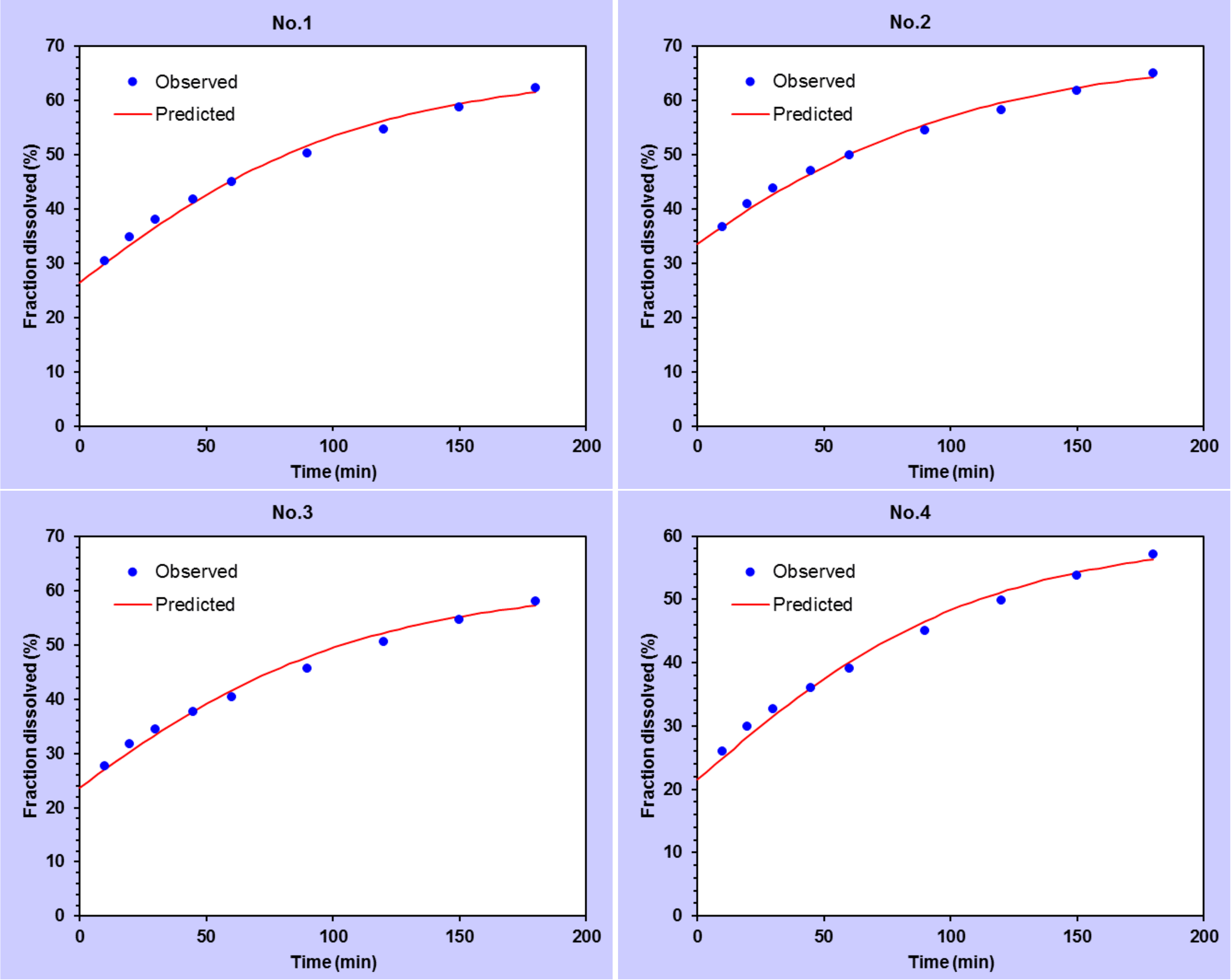

Supplementary materials – Model fitting summary – SuperTab® 11SD (modelling up to app. 60 % of carvedilol released)

Model: **Gompertz_4**

Model equation: $F = F_{max} \cdot e^{-\beta \cdot e^{-k \cdot t}}$

Fitted model parameters per tested tablet (N = 4) with statistics – mean, standard deviation (SD), and relative standard deviation expressed in % (RSD%) (output from DDSolver):

Parameter	No.1	No.2	No.3	No.4	Mean	SD	RSD(%)
k	0.015	0.014	0.015	0.016	0.015	0.001	5.278
β	0.905	0.711	0.962	1.024	0.901	0.136	15.053
F_{max}	65.456	68.270	60.836	59.914	63.619	3.936	6.187

Number of dissolution data points (N), degrees of freedom (df), and selected goodness of fit criteria – Pearson correlation coefficient (R), coefficient of determination (R^2), adjusted coefficient of determination ($R^2_{adjusted}$), and residual sum of squares (RSS) (manual calculation in MS Excel):

Parameter	No.1	No.2	No.3	No.4
N	9	9	9	9
df	6	6	6	6
R	0.996695732	0.996693453	0.994995113	0.996324129
R^2	0.993402382	0.993397839	0.990015275	0.99266177
$R^2_{adjusted}$	0.991203175	0.991197119	0.986687033	0.990215694
RSS	9.503331985	6.553236367	13.60163326	11.10279145

Graphical abstract of model fit presented as mean ± 1 SD of the fraction % of released carvedilol:

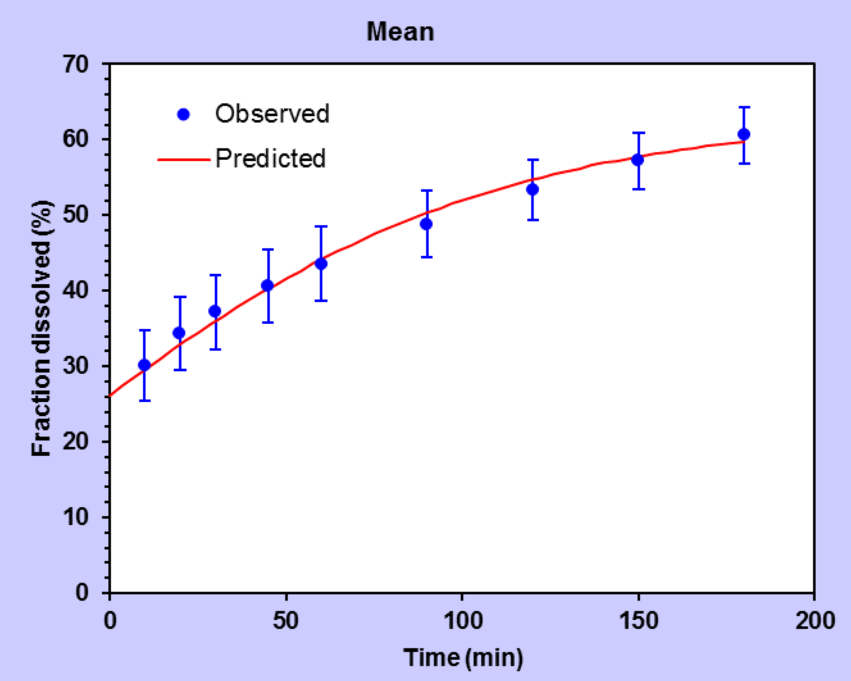

Supplementary materials – Model fitting summary – SuperTab® 11SD (modelling up to app. 60 % of carvedilol released)

Graphical abstract of model fit presented as the fraction % of released carvedilol per tested tablet:

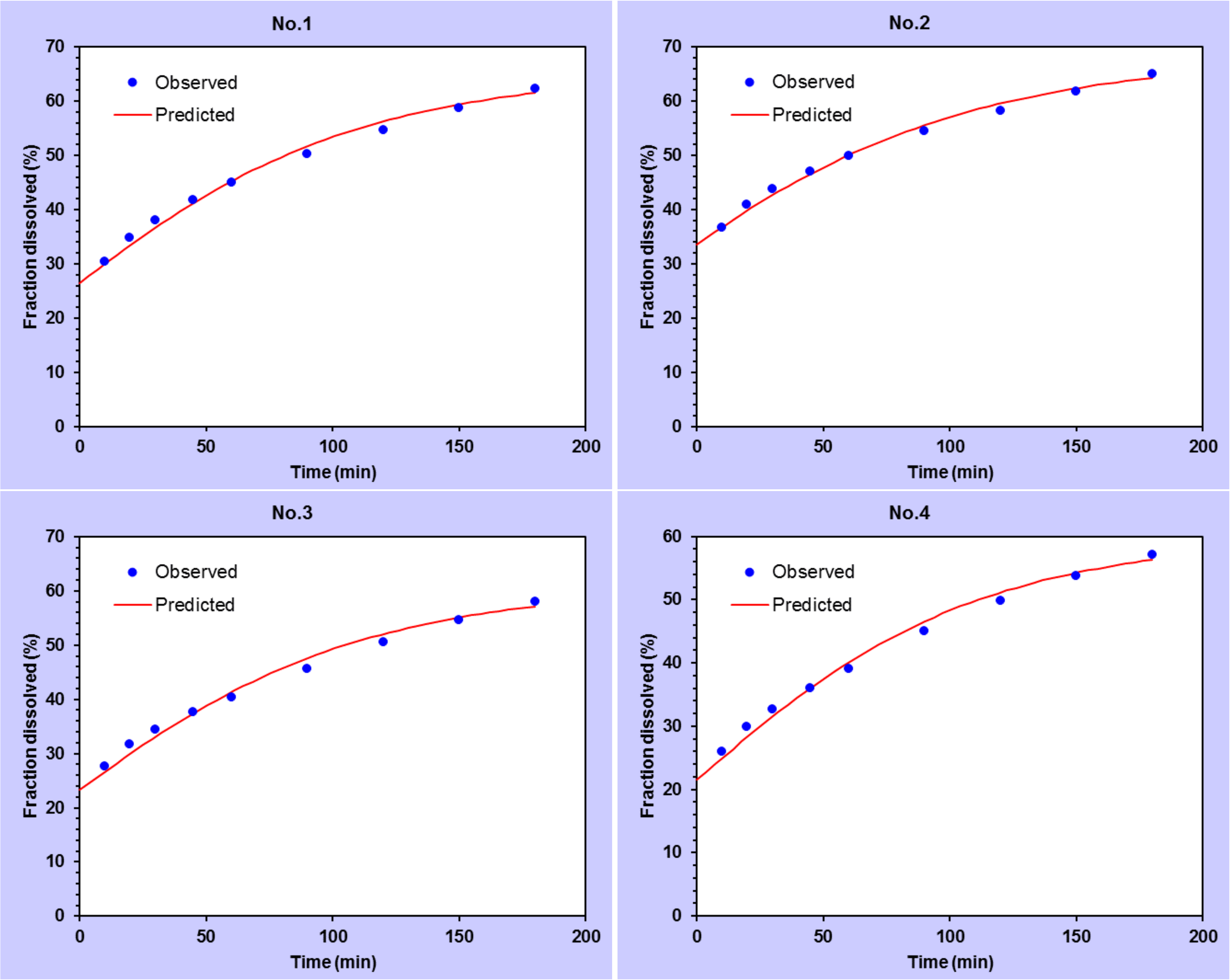

Supplementary materials – Model fitting summary – SuperTab® 11SD (modelling up to app. 60 % of carvedilol released)

Model: **Probit_1**

Model equation: $F = 100 \cdot \phi[\alpha + \beta \cdot \log(t)]$

Fitted model parameters per tested tablet (N = 4) with statistics – mean, standard deviation (SD), and relative standard deviation expressed in % (RSD%) (output from DDSolver):

Parameter	No.1	No.2	No.3	No.4	Mean	SD	RSD(%)
α	-1.248	-0.979	-1.307	-1.390	-1.231	0.178	-14.451
β	0.660	0.575	0.635	0.664	0.633	0.041	6.467

Number of dissolution data points (N), degrees of freedom (df), and selected goodness of fit criteria – Pearson correlation coefficient (R), coefficient of determination (R^2), adjusted coefficient of determination ($R^2_{adjusted}$), and residual sum of squares (RSS) (manual calculation in MS Excel):

Parameter	No.1	No.2	No.3	No.4
N	9	9	9	9
df	7	7	7	7
R	0.98527649	0.985125812	0.981148865	0.982700685
R^2	0.970769761	0.970472866	0.962653096	0.965700637
$R^2_{adjusted}$	0.966594013	0.966254703	0.957317824	0.960800728
RSS	28.67015294	22.28852073	33.54695003	33.20020644

Graphical abstract of model fit presented as mean ± 1 SD of the fraction % of released carvedilol:

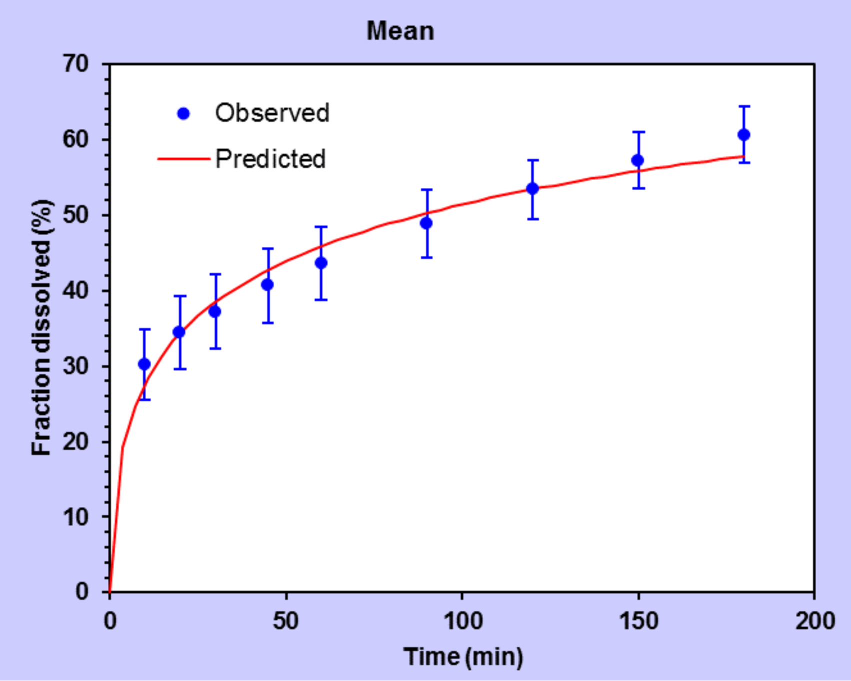

Supplementary materials – Model fitting summary – SuperTab® 11SD (modelling up to app. 60 % of carvedilol released)

Graphical abstract of model fit presented as the fraction % of released carvedilol per tested tablet:

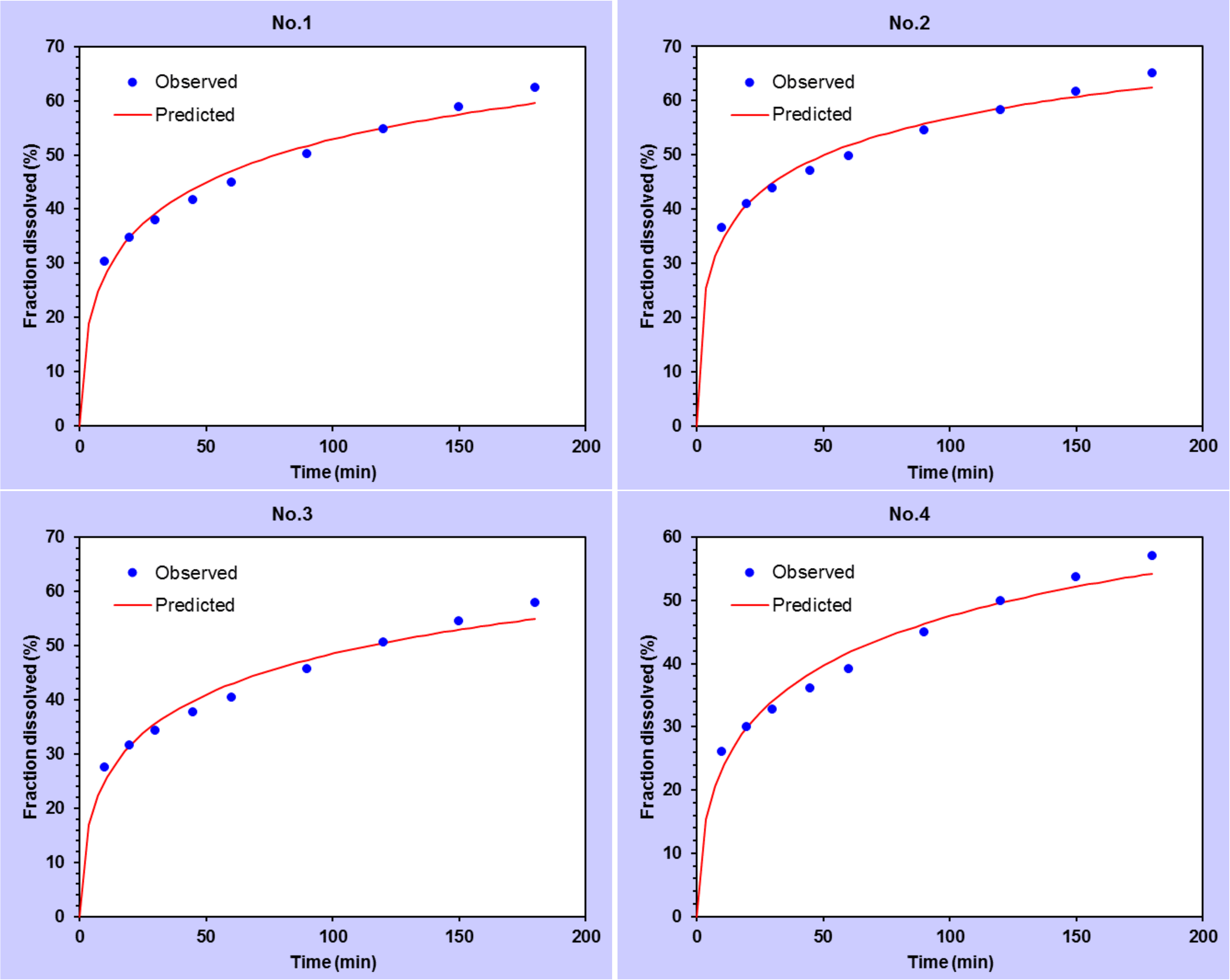

Supplementary materials – Model fitting summary – SuperTab® 11SD (modelling up to app. 60 % of carvedilol released)

Model: **Probit_2**

Model equation: $F = F_{max} \cdot \phi[\alpha + \beta \cdot \log(t)]$

Fitted model parameters per tested tablet (N = 4) with statistics – mean, standard deviation (SD), and relative standard deviation expressed in % (RSD%) (output from DDSolver):

Parameter	No.1	No.2	No.3	No.4	Mean	SD	RSD(%)
α	-1.626	-1.276	-1.686	-1.803	-1.598	0.227	-14.196
β	1.296	1.159	1.312	1.365	1.283	0.088	6.856
F_{max}	65.456	68.270	60.836	59.914	63.619	3.936	6.187

Number of dissolution data points (N), degrees of freedom (df), and selected goodness of fit criteria – Pearson correlation coefficient (R), coefficient of determination (R^2), adjusted coefficient of determination ($R^2_{adjusted}$), and residual sum of squares (RSS) (manual calculation in MS Excel):

Parameter	No.1	No.2	No.3	No.4
N	9	9	9	9
df	6	6	6	6
R	0.955459677	0.955713706	0.947208977	0.948935134
R^2	0.912903195	0.913388688	0.897204845	0.900477888
$R^2_{adjusted}$	0.883870927	0.884518251	0.862939794	0.86730385
RSS	100.0575634	78.36142193	106.8814724	110.0749897

Graphical abstract of model fit presented as mean ± 1 SD of the fraction % of released carvedilol:

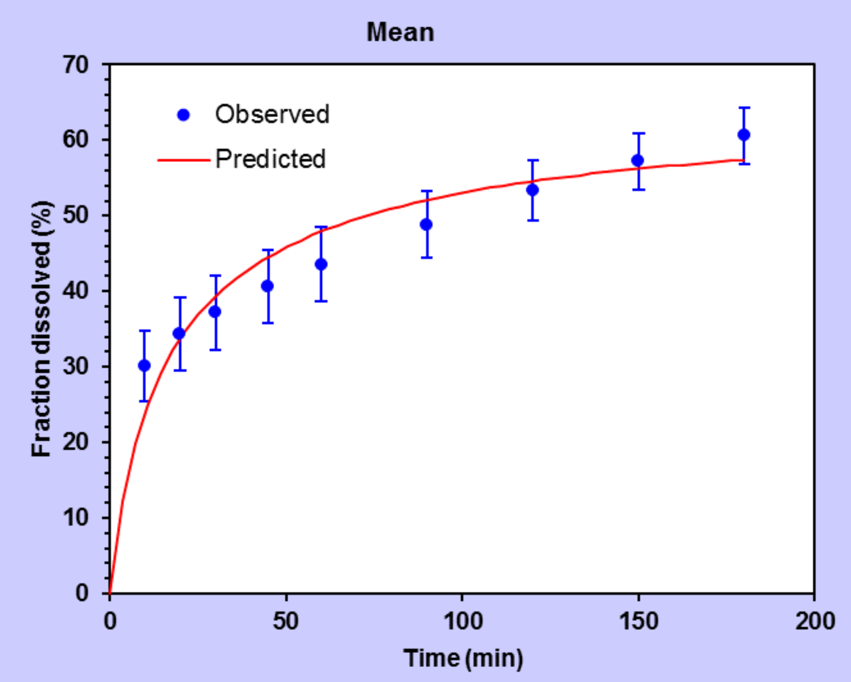

Supplementary materials – Model fitting summary – SuperTab® 11SD (modelling up to app. 60 % of carvedilol released)

Graphical abstract of model fit presented as the fraction % of released carvedilol per tested tablet:

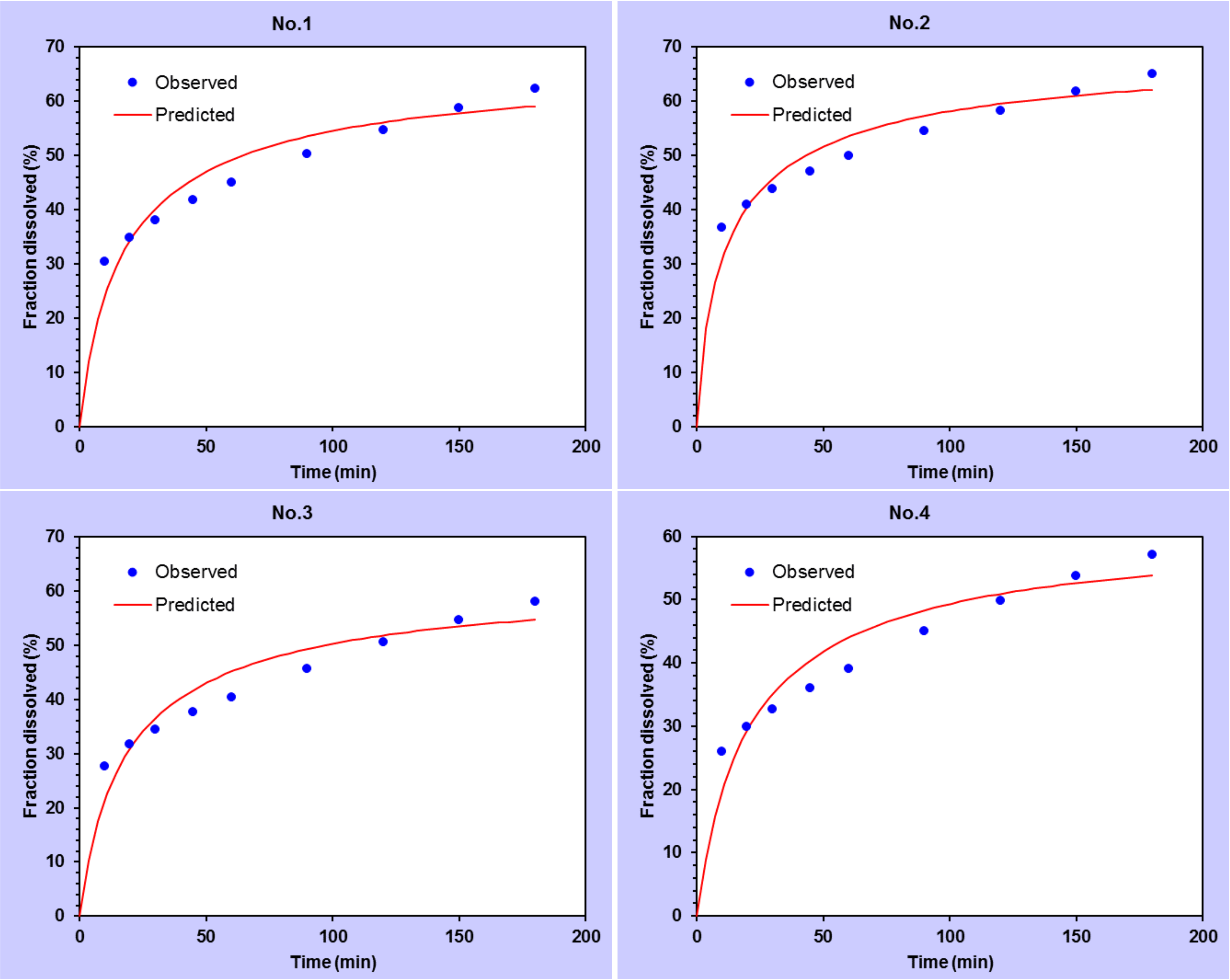

Supplement: Supplementary file 1 [file pharmaceutics-16-00498-s001.zip › Supplementary materials_Model fitting summary_SuperTab® 11SD.pdf]
