# Supplementary material for: Comparative Fitting of Mathematical Models to Carvedilol Release Profiles Obtained from Hypromellose Matrix Tablets
Source: Pharmaceutics. 2024 Apr 4;16(4):498. doi: 10.3390/pharmaceutics16040498 (PMC11053526; doi:10.3390/pharmaceutics16040498)

Supplementary materials – Model fitting summary – Tablettose® 70 (modelling the entire relevant dissolution data)

Model: **Zero–order**

Model equation: $F = k_0 \cdot t$

Fitted model parameters per tested tablet (N = 4) with statistics – mean, standard deviation (SD), and relative standard deviation expressed in % (RSD%) (output from DDSolver):

Parameter	No.1	No.2	No.3	No.4	Mean	SD	RSD(%)
k_0	0.197	0.195	0.194	0.199	0.196	0.002	1.126

Number of dissolution data points (N), degrees of freedom (df), and selected goodness of fit criteria – Pearson correlation coefficient (R), coefficient of determination (R^2), adjusted coefficient of determination ($R^2_{adjusted}$), and residual sum of squares (RSS) (manual calculation in MS Excel):

Parameter	No.1	No.2	No.3	No.4
N	20	20	20	20
df	19	19	19	19
R	0.969879509	0.963591241	0.975232582	0.976945702
R^2	0.940666261	0.92850808	0.951078588	0.954422904
$R^2_{adjusted}$	0.940666261	0.92850808	0.951078588	0.954422904
RSS	9754.338075	11593.15682	11751.01261	10653.05126

Graphical abstract of model fit presented as mean ± 1 SD of the fraction % of released carvedilol:

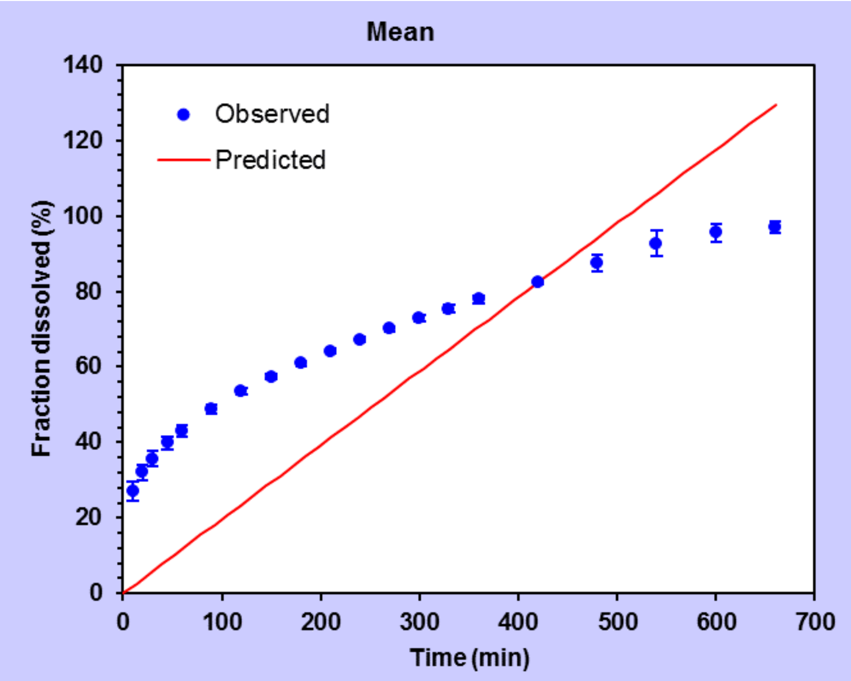

Supplementary materials – Model fitting summary – Tablettose® 70 (modelling the entire relevant dissolution data)

Graphical abstract of model fit presented as the fraction % of released carvedilol per tested tablet:

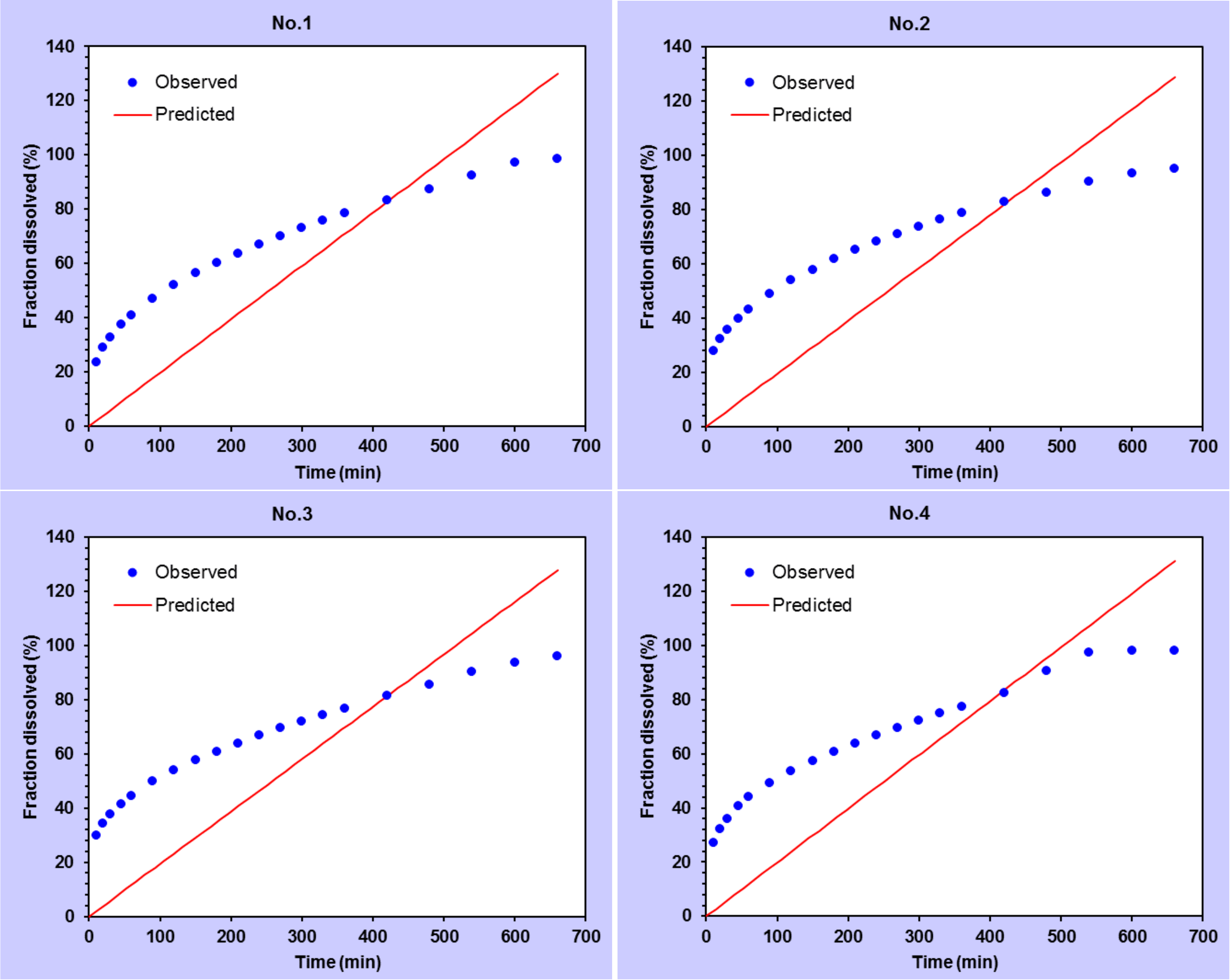

Supplementary materials – Model fitting summary – Tablettose® 70 (modelling the entire relevant dissolution data)

Model: **Zero–order with T_{lag}**

Model equation: $F = k_0 \cdot (t - T_{lag})$

Fitted model parameters per tested tablet (N = 4) with statistics – mean, standard deviation (SD), and relative standard deviation expressed in % (RSD%) (output from DDSolver):

Parameter	No.1	No.2	No.3	No.4	Mean	SD	RSD(%)
k_0	0.111	0.101	0.097	0.107	0.104	0.006	5.864
T_{lag}	-317.214	-382.504	-401.574	-345.184	-361.619	37.747	-10.438

Number of dissolution data points (N), degrees of freedom (df), and selected goodness of fit criteria – Pearson correlation coefficient (R), coefficient of determination (R^2), adjusted coefficient of determination ($R^2_{adjusted}$), and residual sum of squares (RSS) (manual calculation in MS Excel):

Parameter	No.1	No.2	No.3	No.4
N	20	20	20	20
df	18	18	18	18
R	0.969879509	0.963591241	0.975232582	0.976945702
R^2	0.940666261	0.92850808	0.951078588	0.954422904
$R^2_{adjusted}$	0.937369943	0.924536307	0.948360732	0.951890844
RSS	597.3972299	601.9958961	377.5674062	426.5631119

Graphical abstract of model fit presented as mean ± 1 SD of the fraction % of released carvedilol:

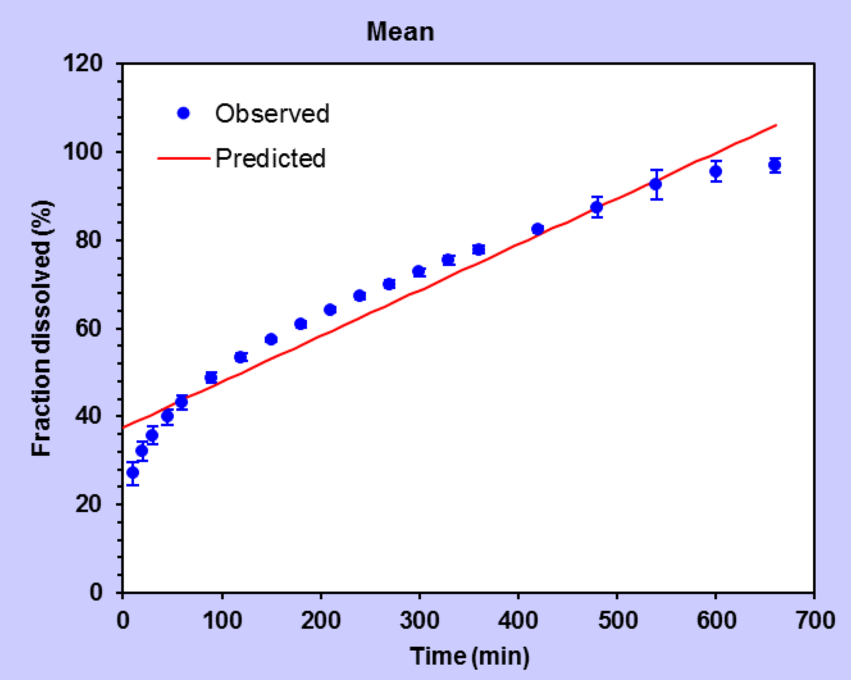

Supplementary materials – Model fitting summary – Tablettose® 70 (modelling the entire relevant dissolution data)

Graphical abstract of model fit presented as the fraction % of released carvedilol per tested tablet:

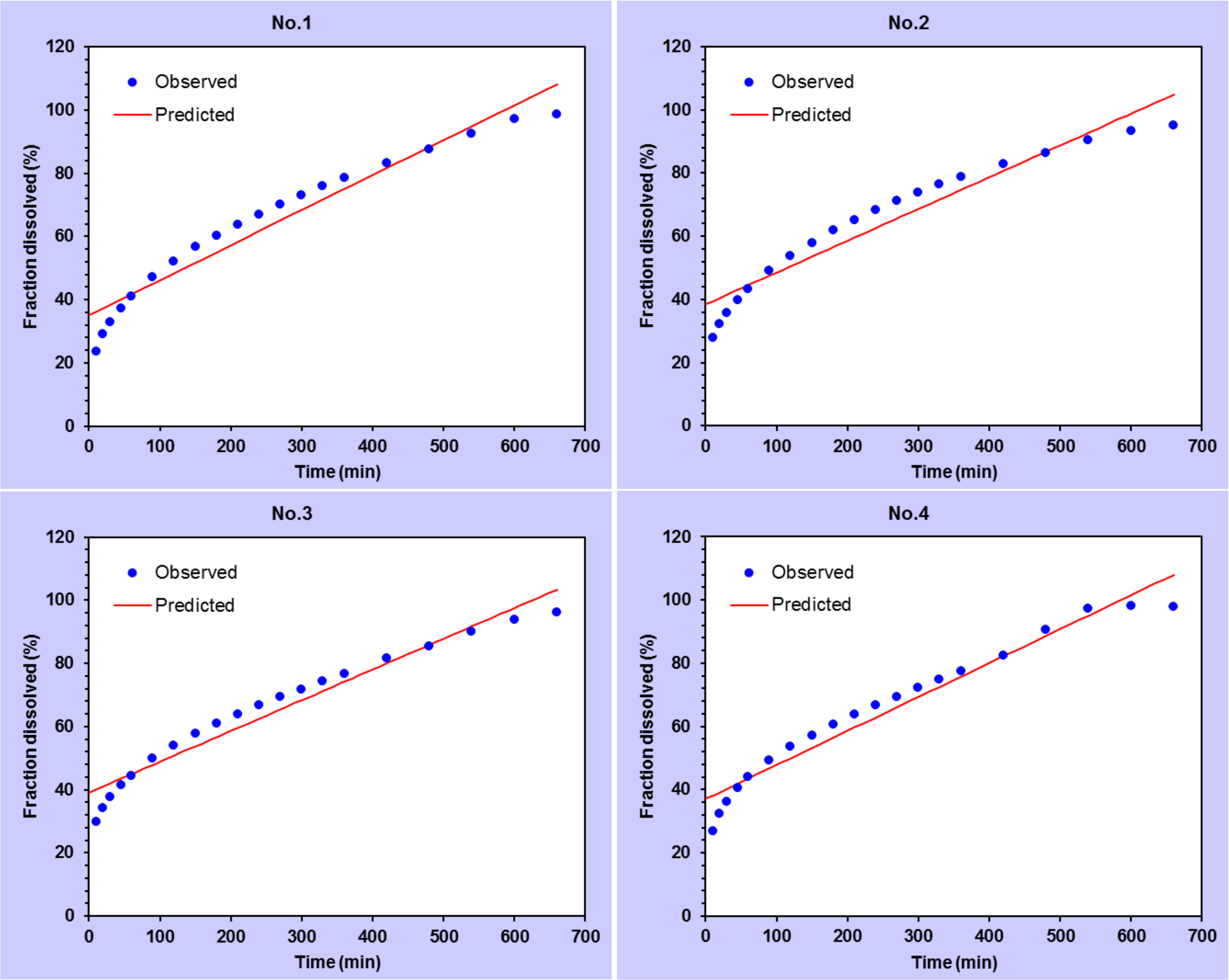

Supplementary materials – Model fitting summary – Tablettose® 70 (modelling the entire relevant dissolution data)

Model: **Zero–order with F_0**

Model equation: $F = F_0 + k_0 \cdot t$

Fitted model parameters per tested tablet (N = 4) with statistics – mean, standard deviation (SD), and relative standard deviation expressed in % (RSD%) (output from DDSolver):

Parameter	No.1	No.2	No.3	No.4	Mean	SD	RSD(%)
k_0	0.111	0.101	0.097	0.107	0.104	0.006	5.864
F_0	35.104	38.460	39.123	37.098	37.446	1.774	4.738

Number of dissolution data points (N), degrees of freedom (df), and selected goodness of fit criteria – Pearson correlation coefficient (R), coefficient of determination (R^2), adjusted coefficient of determination ($R^2_{adjusted}$), and residual sum of squares (RSS) (manual calculation in MS Excel):

Parameter	No.1	No.2	No.3	No.4
N	20	20	20	20
df	18	18	18	18
R	0.969879509	0.963591241	0.975232582	0.976945702
R^2	0.940666261	0.92850808	0.951078588	0.954422904
$R^2_{adjusted}$	0.937369943	0.924536307	0.948360732	0.951890844
RSS	597.3972299	601.9958961	377.5674062	426.5631119

Graphical abstract of model fit presented as mean ± 1 SD of the fraction % of released carvedilol:

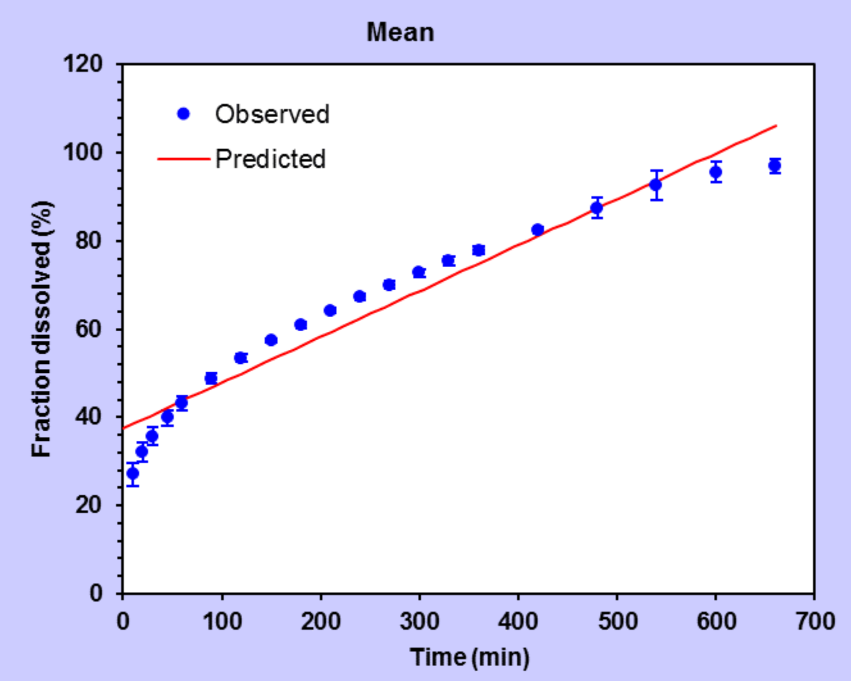

Supplementary materials – Model fitting summary – Tablettose® 70 (modelling the entire relevant dissolution data)

Graphical abstract of model fit presented as the fraction % of released carvedilol per tested tablet:

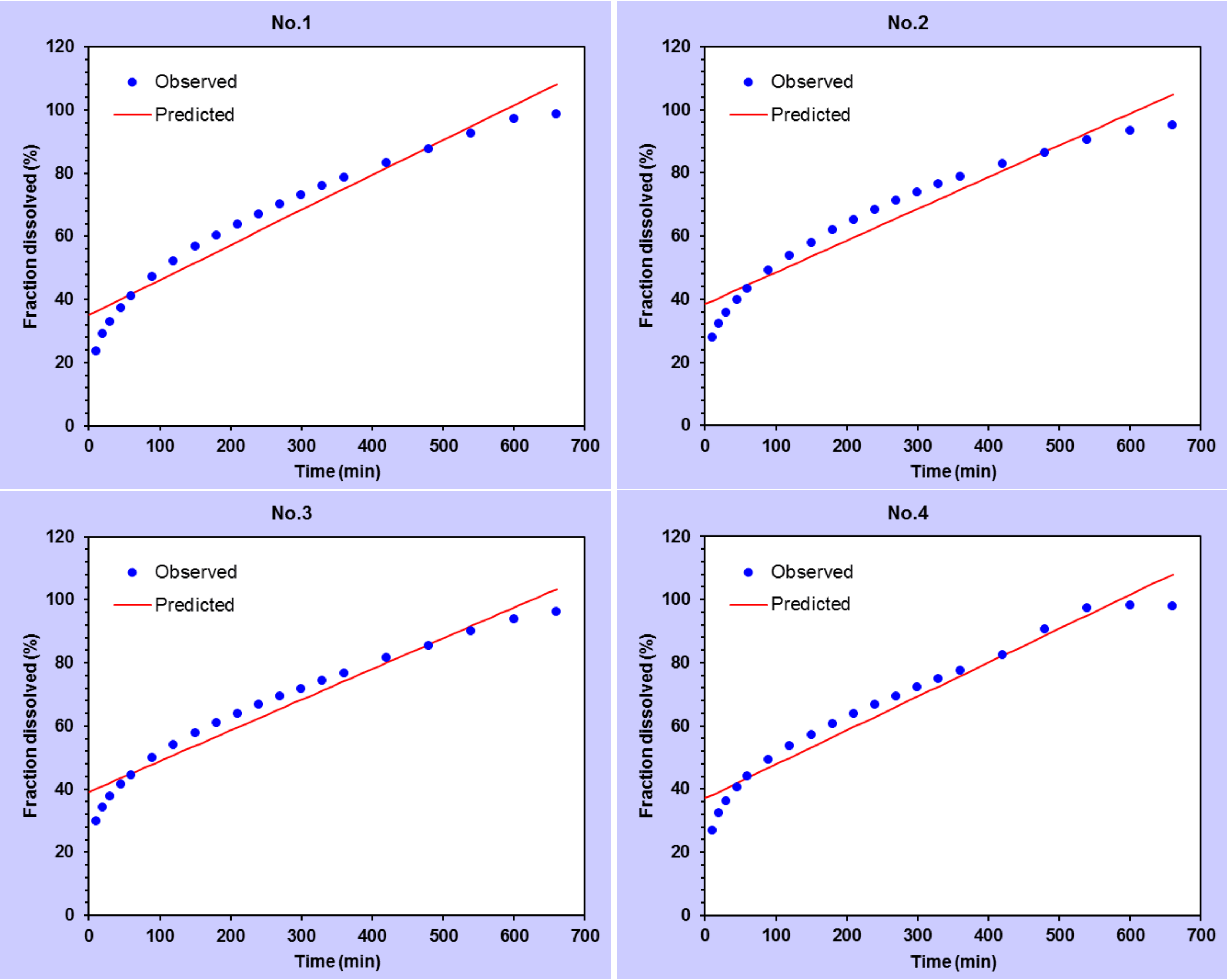

Supplementary materials – Model fitting summary – Tablettose® 70 (modelling the entire relevant dissolution data)

Model: **First–order**

Model equation: $F = 100 \cdot (1 - e^{-k_1 \cdot t})$

Fitted model parameters per tested tablet (N = 4) with statistics – mean, standard deviation (SD), and relative standard deviation expressed in % (RSD%) (output from DDSolver):

Parameter	No.1	No.2	No.3	No.4	Mean	SD	RSD(%)
k_1	0.005	0.007	0.007	0.006	0.006	0.001	13.519

Number of dissolution data points (N), degrees of freedom (df), and selected goodness of fit criteria – Pearson correlation coefficient (R), coefficient of determination (R^2), adjusted coefficient of determination ($R^2_{adjusted}$), and residual sum of squares (RSS) (manual calculation in MS Excel):

Parameter	No.1	No.2	No.3	No.4
N	20	20	20	20
df	19	19	19	19
R	0.985290762	0.976342519	0.963847951	0.970283483
R^2	0.970797886	0.953244715	0.929002873	0.941450037
$R^2_{adjusted}$	0.970797886	0.953244715	0.929002873	0.941450037
RSS	1874.219439	2768.365059	3428.003481	2552.258264

Graphical abstract of model fit presented as mean ± 1 SD of the fraction % of released carvedilol:

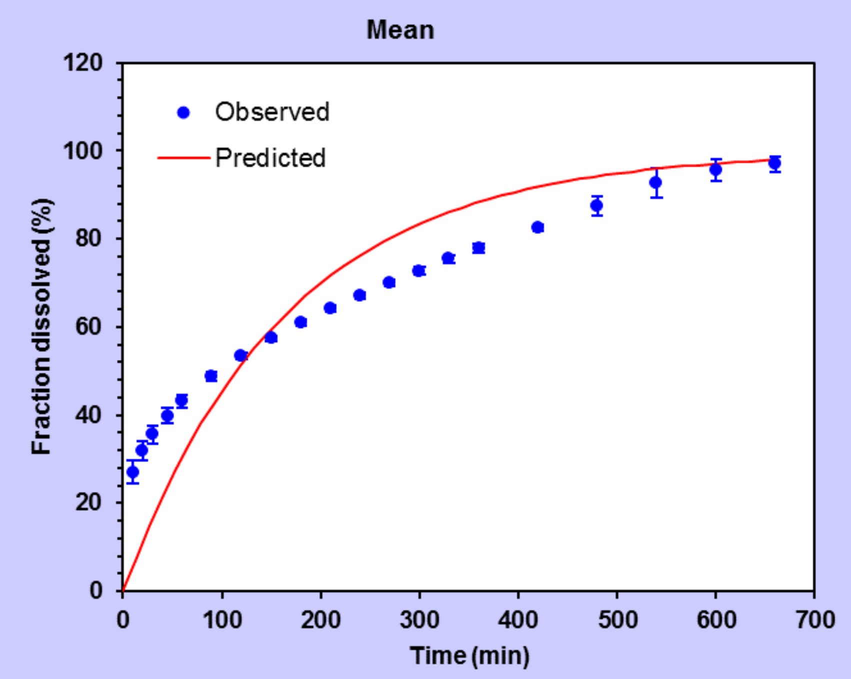

Supplementary materials – Model fitting summary – Tablettose® 70 (modelling the entire relevant dissolution data)

Graphical abstract of model fit presented as the fraction % of released carvedilol per tested tablet:

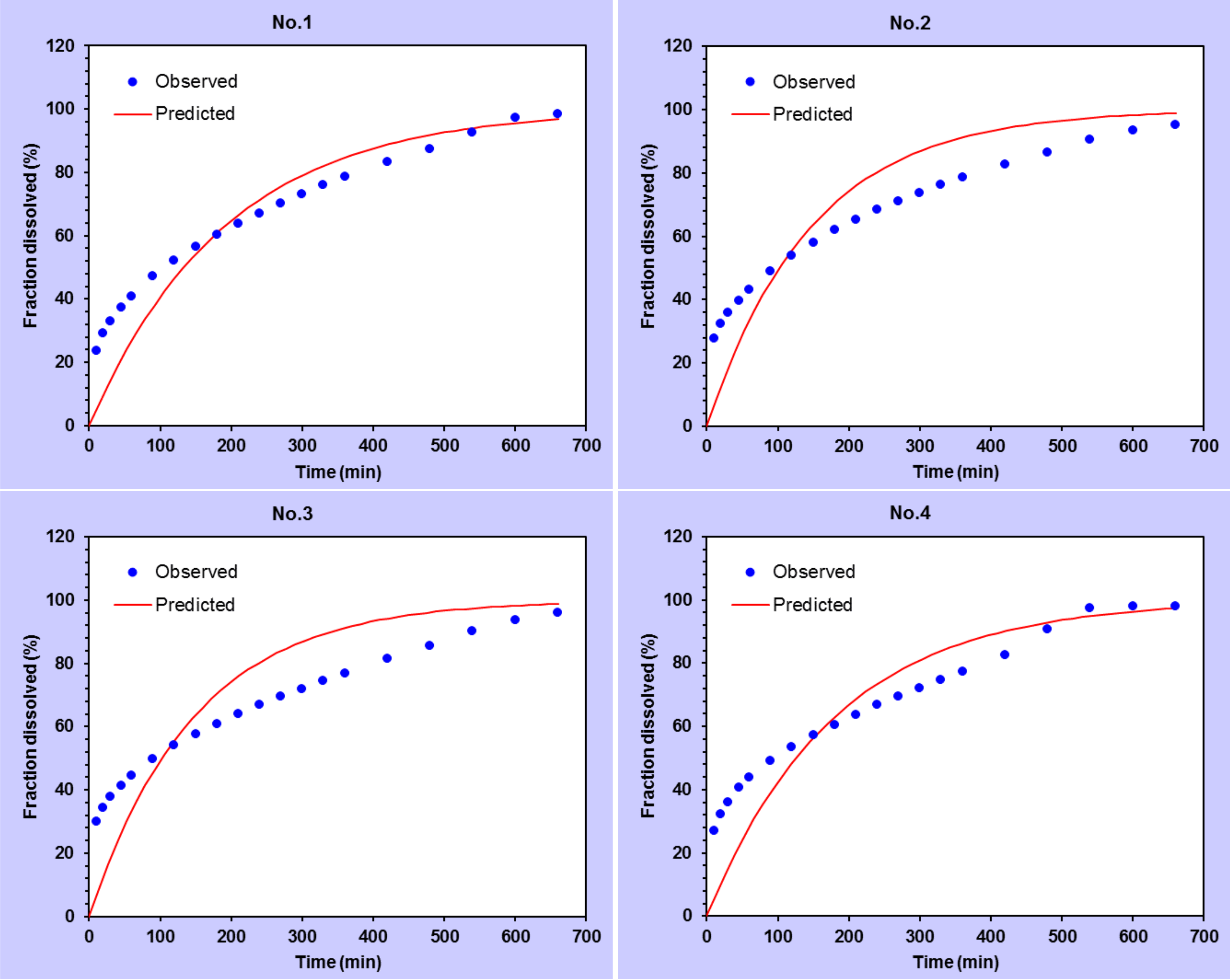

Supplementary materials – Model fitting summary – Tablettose® 70 (modelling the entire relevant dissolution data)

Model: **First–order with T_{lag}**

Model equation: $F = 100 \cdot \left[1 - e^{-k_1 \cdot (t - T_{lag})}\right]$

Fitted model parameters per tested tablet (N = 4) with statistics – mean, standard deviation (SD), and relative standard deviation expressed in % (RSD%) (output from DDSolver):

Parameter	No.1	No.2	No.3	No.4	Mean	SD	RSD(%)
k_1	0.005	0.003	0.003	0.005	0.004	0.001	25.290
T_{lag}	-16.214	-94.091	-87.793	-11.525	-52.406	44.613	-85.130

Number of dissolution data points (N), degrees of freedom (df), and selected goodness of fit criteria – Pearson correlation coefficient (R), coefficient of determination (R^2), adjusted coefficient of determination ($R^2_{adjusted}$), and residual sum of squares (RSS) (manual calculation in MS Excel):

Parameter	No.1	No.2	No.3	No.4
N	20	20	20	20
df	18	18	18	18
R	0.986450114	0.998323501	0.995131437	0.971493027
R^2	0.973083828	0.996649812	0.990286578	0.943798701
$R^2_{adjusted}$	0.971588485	0.99646369	0.989746943	0.940676407
RSS	944.611019	71.56811959	170.7400979	1712.507966

Graphical abstract of model fit presented as mean ± 1 SD of the fraction % of released carvedilol:

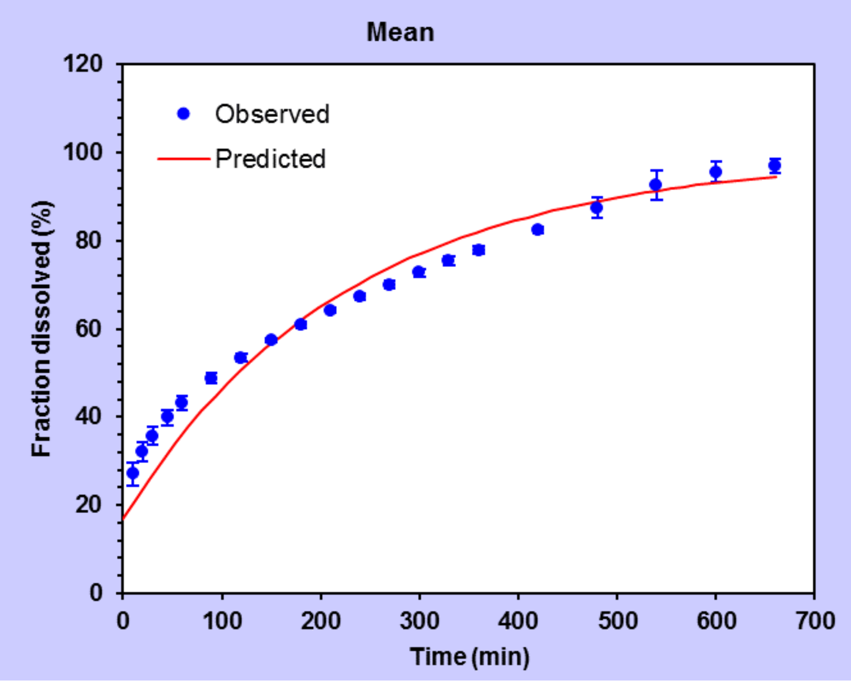

Supplementary materials – Model fitting summary – Tablettose® 70 (modelling the entire relevant dissolution data)

Graphical abstract of model fit presented as the fraction % of released carvedilol per tested tablet:

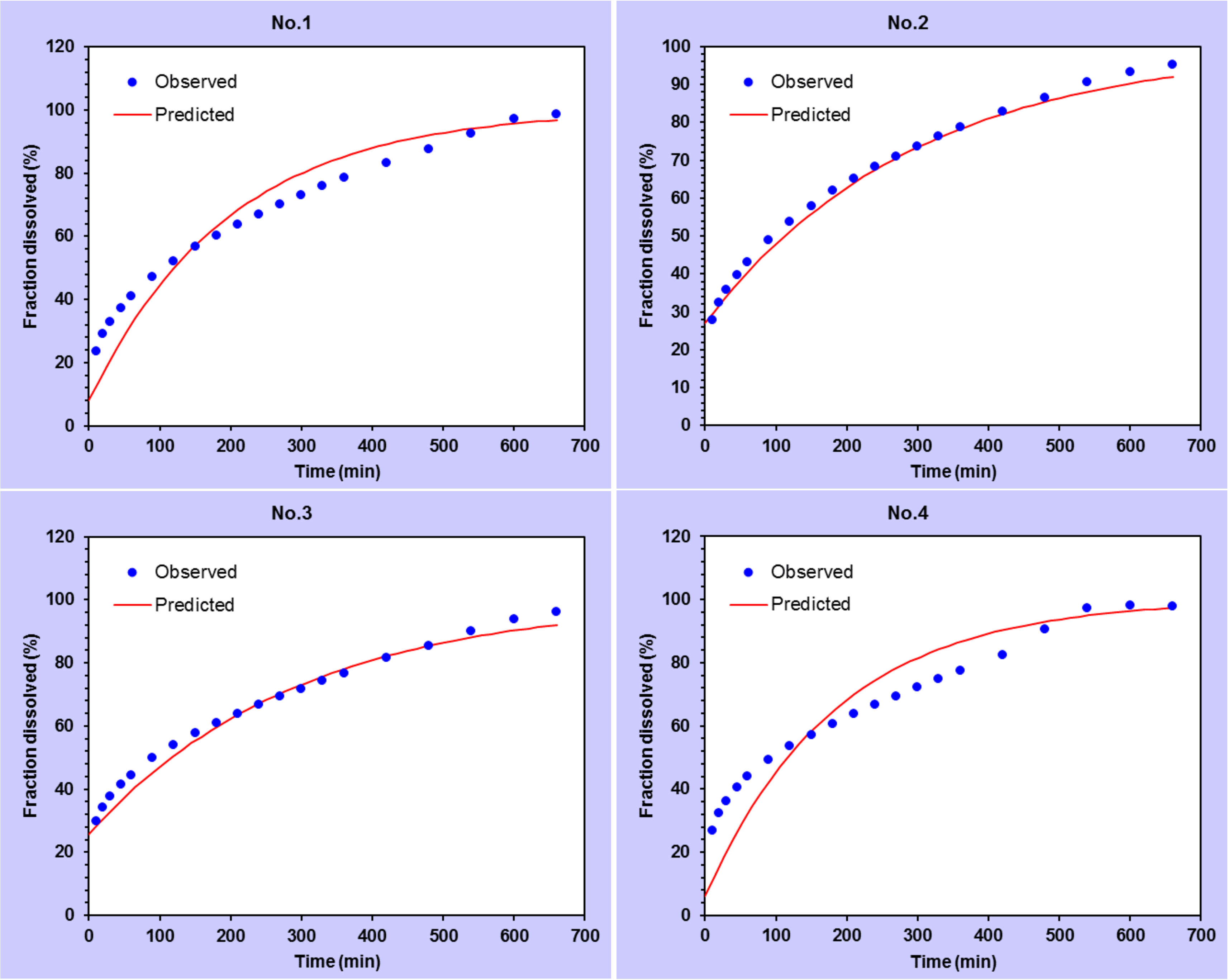

Supplementary materials – Model fitting summary – Tablettose® 70 (modelling the entire relevant dissolution data)

Model: **First–order with F_{max}**

Model equation: $F = F_{max} \cdot (1 - e^{-k_1 \cdot t})$

Fitted model parameters per tested tablet (N = 4) with statistics – mean, standard deviation (SD), and relative standard deviation expressed in % (RSD%) (output from DDSolver):

Parameter	No.1	No.2	No.3	No.4	Mean	SD	RSD(%)
k_1	0.004	0.007	0.007	0.005	0.006	0.001	22.698
F_{max}	103.515	99.877	100.914	102.988	101.823	1.716	1.685

Number of dissolution data points (N), degrees of freedom (df), and selected goodness of fit criteria – Pearson correlation coefficient (R), coefficient of determination (R^2), adjusted coefficient of determination ($R^2_{adjusted}$), and residual sum of squares (RSS) (manual calculation in MS Excel):

Parameter	No.1	No.2	No.3	No.4
N	20	20	20	20
df	18	18	18	18
R	0.991944016	0.976011463	0.966759374	0.980163522
R^2	0.98395293	0.952598377	0.934623687	0.960720529
$R^2_{adjusted}$	0.983061426	0.949964953	0.930991669	0.958538337
RSS	2266.166869	2762.310956	3471.934669	2860.999653

Graphical abstract of model fit presented as mean ± 1 SD of the fraction % of released carvedilol:

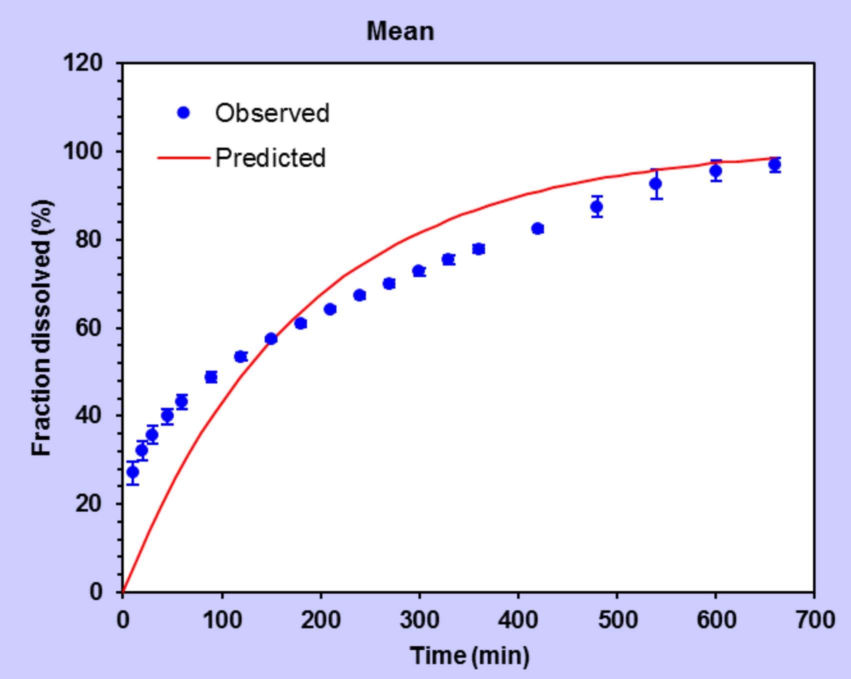

Supplementary materials – Model fitting summary – Tablettose® 70 (modelling the entire relevant dissolution data)

Graphical abstract of model fit presented as the fraction % of released carvedilol per tested tablet:

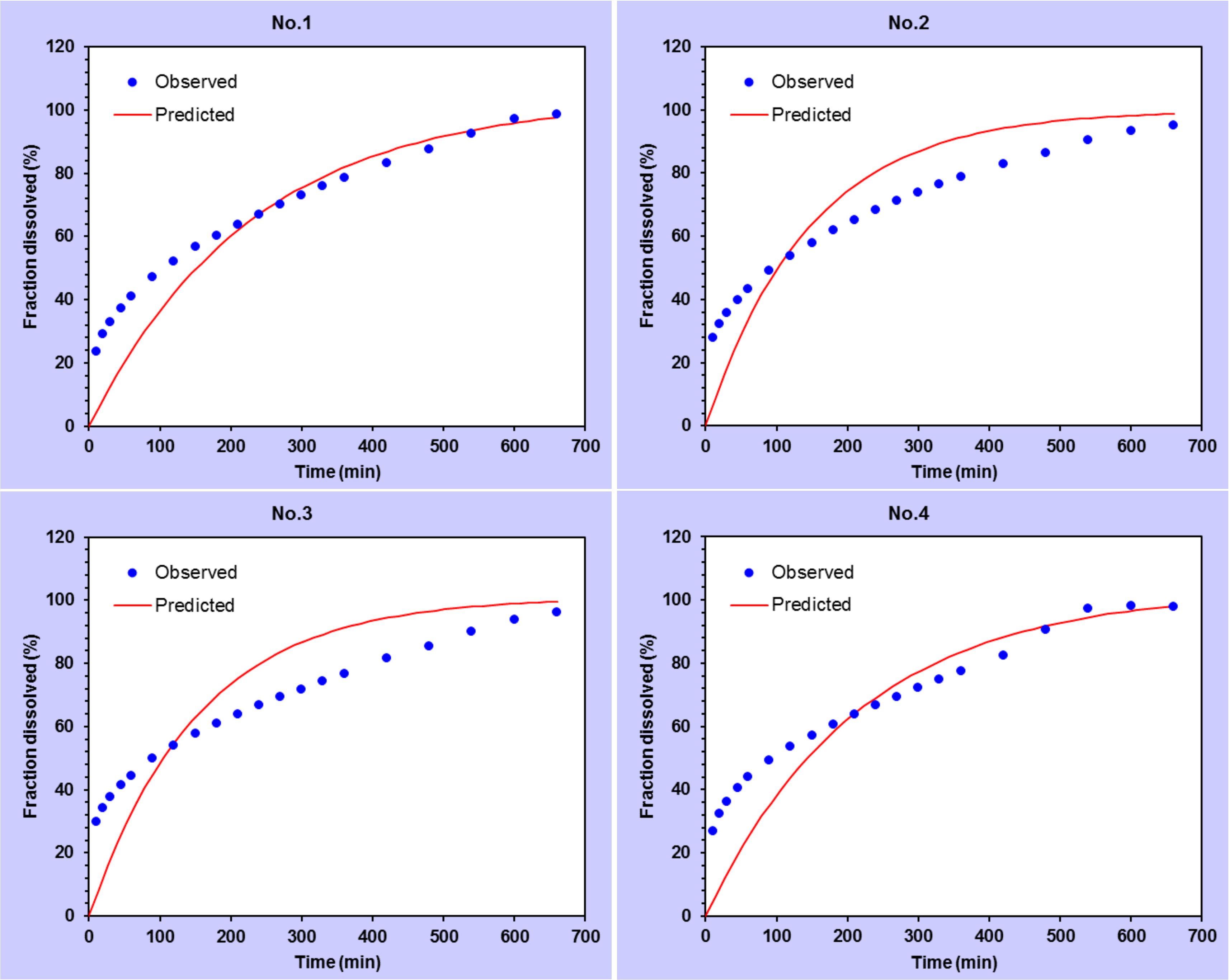

Supplementary materials – Model fitting summary – Tablettose® 70 (modelling the entire relevant dissolution data)

Model: **First–order with T_{lag} and F_{max}**

Model equation: $F = F_{max} \cdot \left[1 - e^{-k_1 \cdot (t - T_{lag})}\right]$

Fitted model parameters per tested tablet (N = 4) with statistics – mean, standard deviation (SD), and relative standard deviation expressed in % (RSD%) (output from DDSolver):

Parameter	No.1	No.2	No.3	No.4	Mean	SD	RSD(%)
k_1	0.004	0.004	0.003	0.004	0.004	0.000	8.041
T_{lag}	-47.461	-70.620	-98.300	-62.108	-69.622	21.378	-30.705
F_{max}	103.515	99.877	96.359	102.988	100.685	3.300	3.278

Number of dissolution data points (N), degrees of freedom (df), and selected goodness of fit criteria – Pearson correlation coefficient (R), coefficient of determination (R^2), adjusted coefficient of determination ($R^2_{adjusted}$), and residual sum of squares (RSS) (manual calculation in MS Excel):

Parameter	No.1	No.2	No.3	No.4
N	20	20	20	20
df	17	17	17	17
R	0.994520425	0.997395904	0.994854661	0.984323007
R^2	0.989070875	0.99479859	0.989735796	0.968891782
$R^2_{adjusted}$	0.987785096	0.994186659	0.988528242	0.965231992
RSS	252.7159367	92.40106341	185.7183589	500.0652515

Graphical abstract of model fit presented as mean ± 1 SD of the fraction % of released carvedilol:

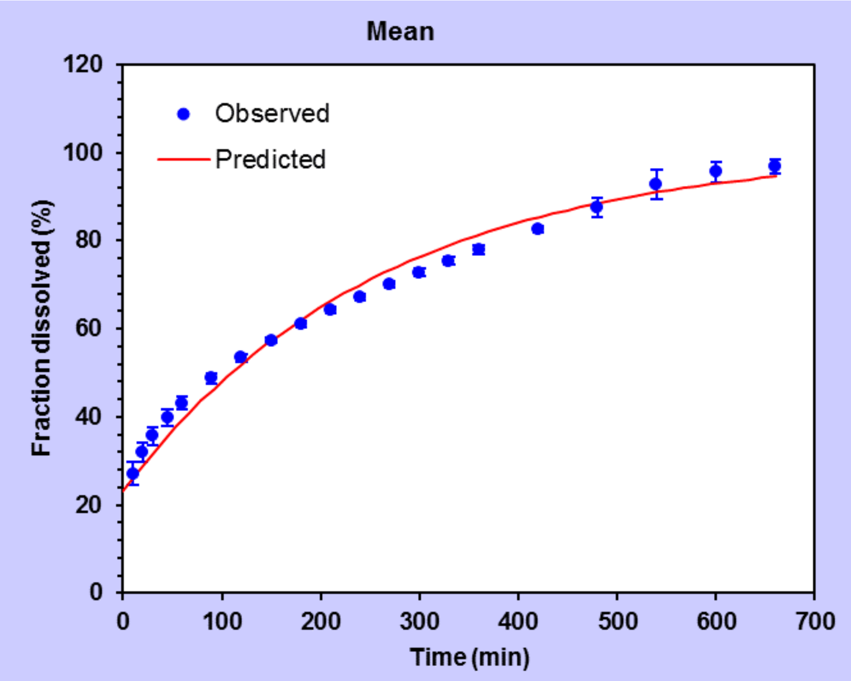

Supplementary materials – Model fitting summary – Tablettose® 70 (modelling the entire relevant dissolution data)

Graphical abstract of model fit presented as the fraction % of released carvedilol per tested tablet:

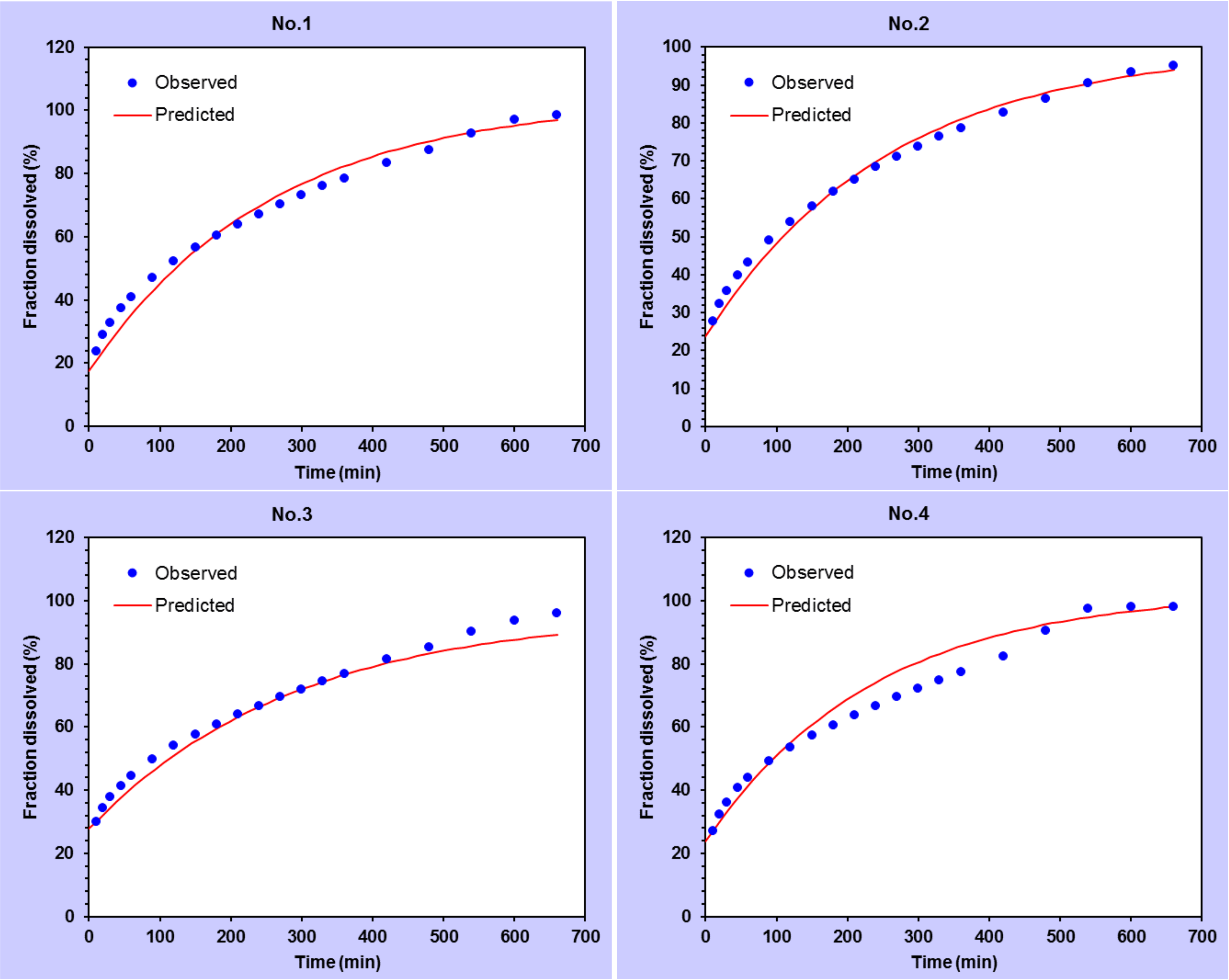

Supplementary materials – Model fitting summary – Tablettose® 70 (modelling the entire relevant dissolution data)

Model: **Higuchi**

Model equation: $F = k_H \cdot t^{0.5}$

Fitted model parameters per tested tablet (N = 4) with statistics – mean, standard deviation (SD), and relative standard deviation expressed in % (RSD%) (output from DDSolver):

Parameter	No.1	No.2	No.3	No.4	Mean	SD	RSD(%)
k_H	4.187	4.180	4.150	4.232	4.187	0.034	0.805

Number of dissolution data points (N), degrees of freedom (df), and selected goodness of fit criteria – Pearson correlation coefficient (R), coefficient of determination (R^2), adjusted coefficient of determination ($R^2_{adjusted}$), and residual sum of squares (RSS) (manual calculation in MS Excel):

Parameter	No.1	No.2	No.3	No.4
N	20	20	20	20
df	19	19	19	19
R	0.999390689	0.998431093	0.999799558	0.997173258
R^2	0.99878175	0.996864647	0.999599157	0.994354507
$R^2_{adjusted}$	0.99878175	0.996864647	0.999599157	0.994354507
RSS	788.026638	1406.952932	1630.253895	1176.418581

Graphical abstract of model fit presented as mean ± 1 SD of the fraction % of released carvedilol:

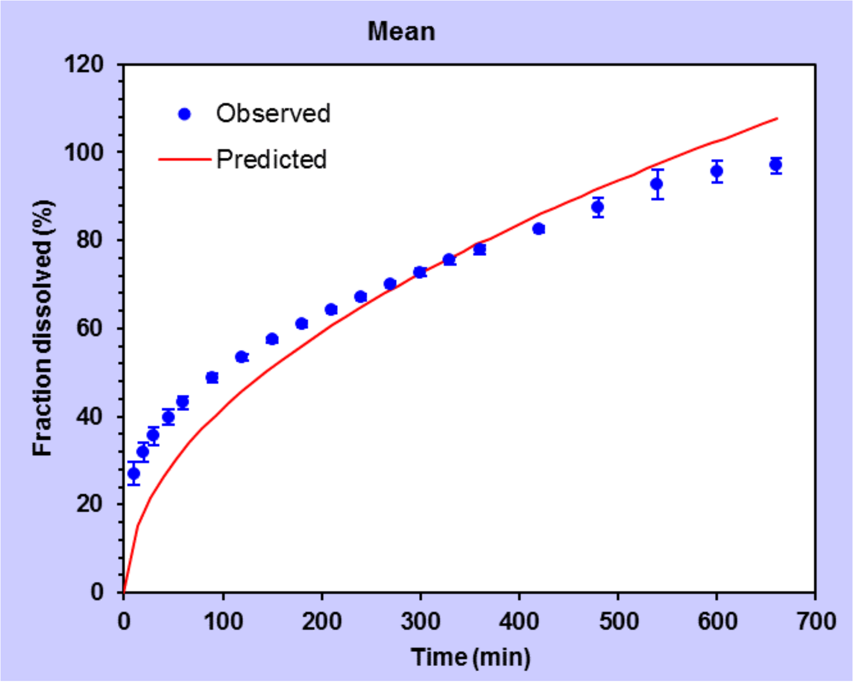

Supplementary materials – Model fitting summary – Tablettose® 70 (modelling the entire relevant dissolution data)

Graphical abstract of model fit presented as the fraction % of released carvedilol per tested tablet:

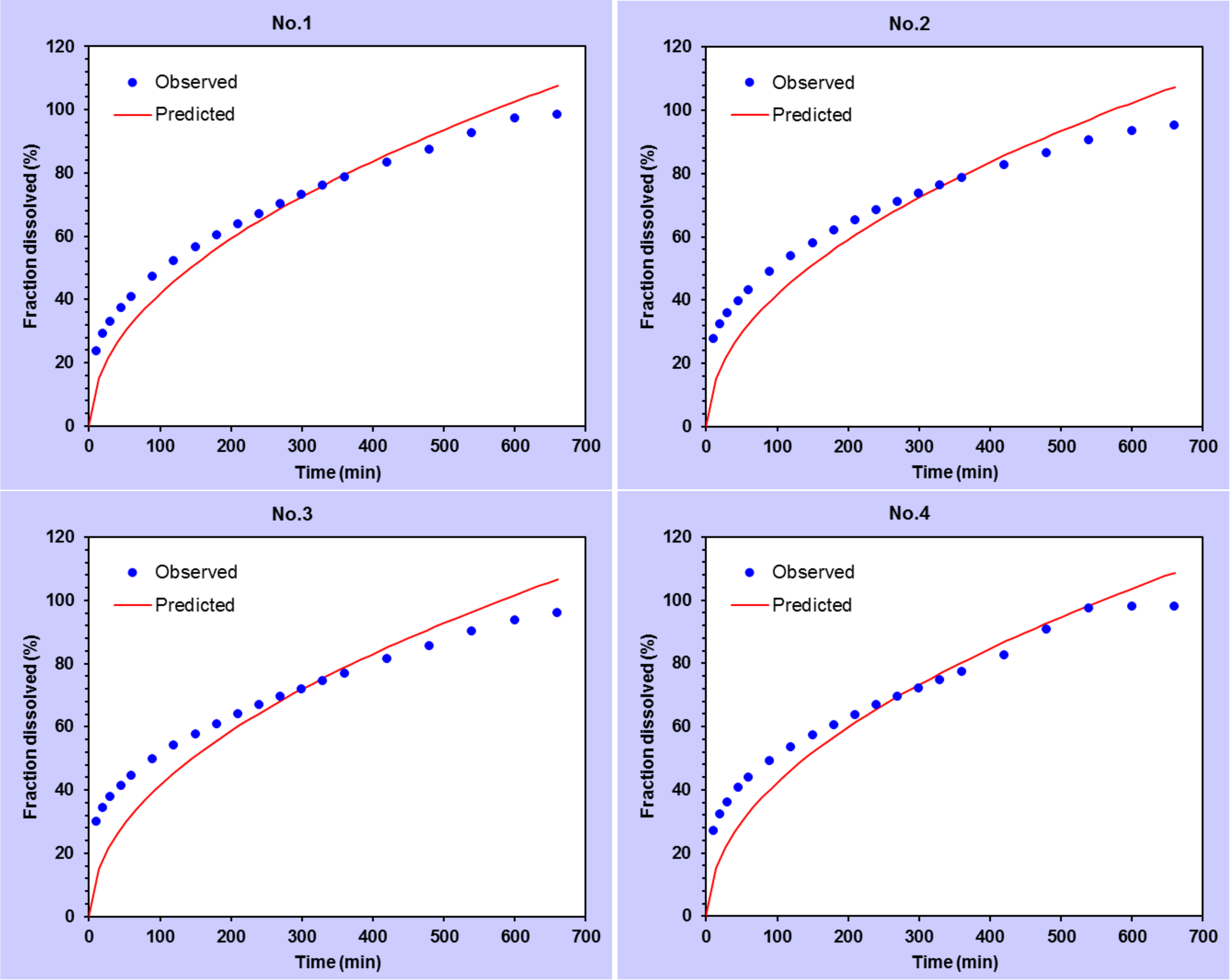

Supplementary materials – Model fitting summary – Tablettose® 70 (modelling the entire relevant dissolution data)

Model: **Higuchi with T_{lag}**

Model equation: $F = k_H \cdot (t - T_{lag})^{0.5}$

Fitted model parameters per tested tablet (N = 4) with statistics – mean, standard deviation (SD), and relative standard deviation expressed in % (RSD%) (output from DDSolver):

Parameter	No.1	No.2	No.3	No.4	Mean	SD	RSD(%)
k_H	3.778	3.605	3.571	3.783	3.684	0.112	3.048
T_{lag}	-61.193	-93.587	-96.193	-68.368	-79.835	17.661	-22.122

Number of dissolution data points (N), degrees of freedom (df), and selected goodness of fit criteria – Pearson correlation coefficient (R), coefficient of determination (R^2), adjusted coefficient of determination ($R^2_{adjusted}$), and residual sum of squares (RSS) (manual calculation in MS Excel):

Parameter	No.1	No.2	No.3	No.4
N	20	20	20	20
df	18	18	18	18
R	0.994513163	0.989866106	0.994725975	0.994665105
R^2	0.989056432	0.979834907	0.989479766	0.989358671
$R^2_{adjusted}$	0.988448456	0.978714624	0.988895309	0.988767486
RSS	134.2026147	202.8869997	92.34052483	105.5634926

Graphical abstract of model fit presented as mean ± 1 SD of the fraction % of released carvedilol:

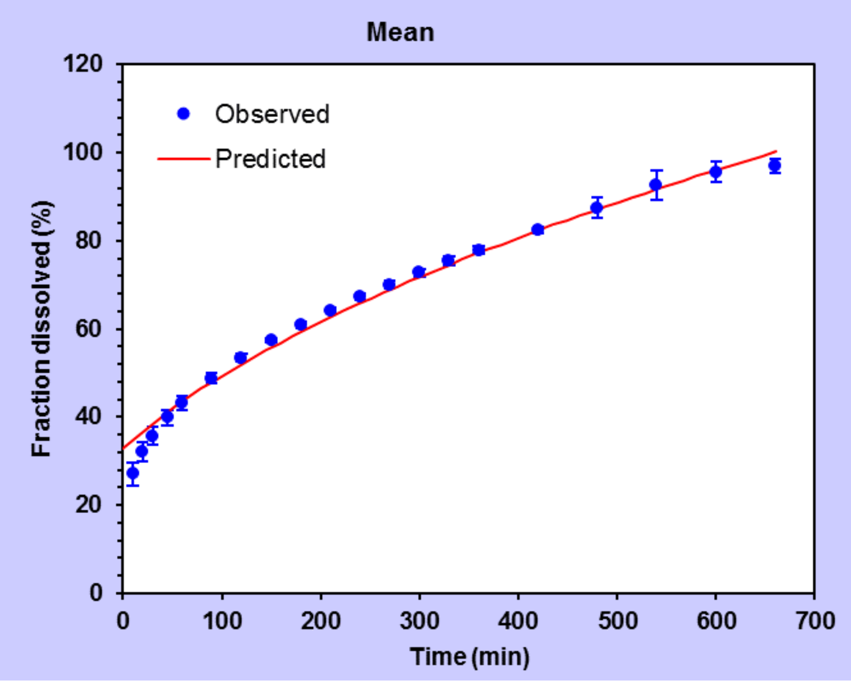

Supplementary materials – Model fitting summary – Tablettose® 70 (modelling the entire relevant dissolution data)

Graphical abstract of model fit presented as the fraction % of released carvedilol per tested tablet:

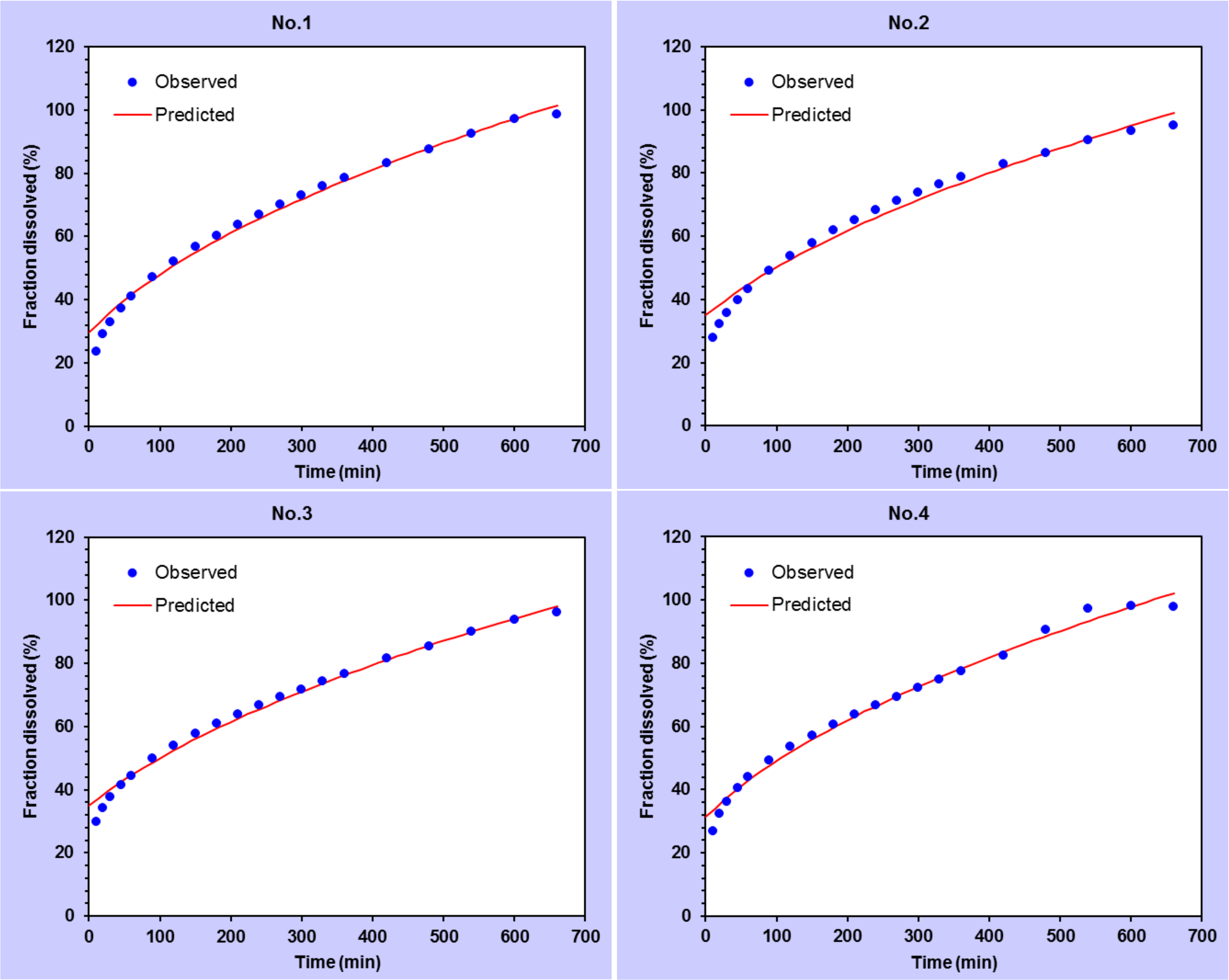

Supplementary materials – Model fitting summary – Tablettose® 70 (modelling the entire relevant dissolution data)

Model: **Higuchi with F_0**

Model equation: $F = F_0 + k_H \cdot t^{0.5}$

Fitted model parameters per tested tablet (N = 4) with statistics – mean, standard deviation (SD), and relative standard deviation expressed in % (RSD%) (output from DDSolver):

Parameter	No.1	No.2	No.3	No.4	Mean	SD	RSD(%)
k_H	3.344	3.055	2.929	3.217	3.136	0.182	5.799
F_0	14.854	19.816	21.513	17.876	18.515	2.857	15.431

Number of dissolution data points (N), degrees of freedom (df), and selected goodness of fit criteria – Pearson correlation coefficient (R), coefficient of determination (R^2), adjusted coefficient of determination ($R^2_{adjusted}$), and residual sum of squares (RSS) (manual calculation in MS Excel):

Parameter	No.1	No.2	No.3	No.4
N	20	20	20	20
df	18	18	18	18
R	0.999390689	0.998431093	0.999799558	0.997173258
R^2	0.99878175	0.996864647	0.999599157	0.994354507
$R^2_{adjusted}$	0.998714069	0.996690461	0.999576888	0.994040869
RSS	12.26585772	26.40116031	3.093639465	52.83704312

Graphical abstract of model fit presented as mean ± 1 SD of the fraction % of released carvedilol:

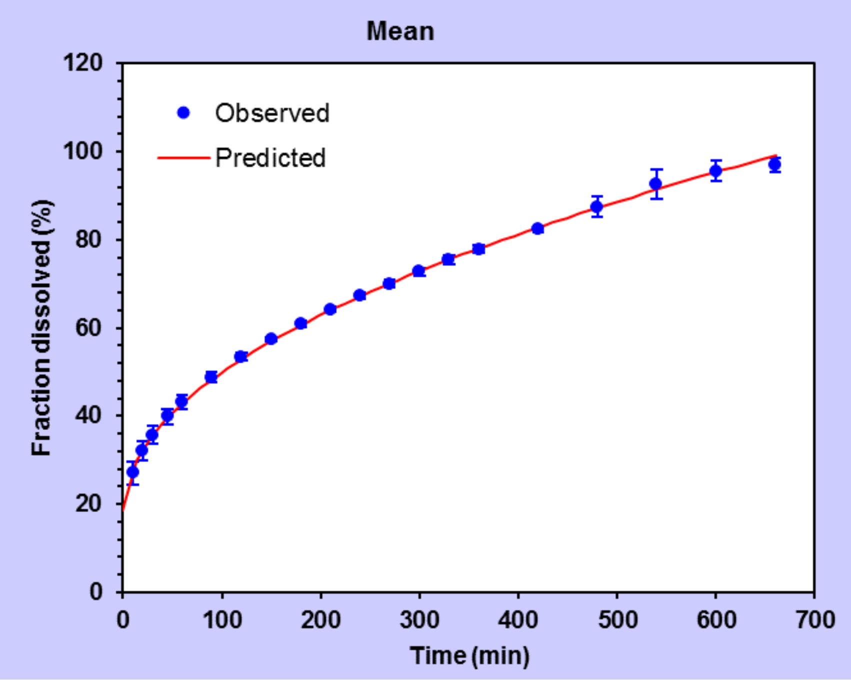

Supplementary materials – Model fitting summary – Tablettose® 70 (modelling the entire relevant dissolution data)

Graphical abstract of model fit presented as the fraction % of released carvedilol per tested tablet:

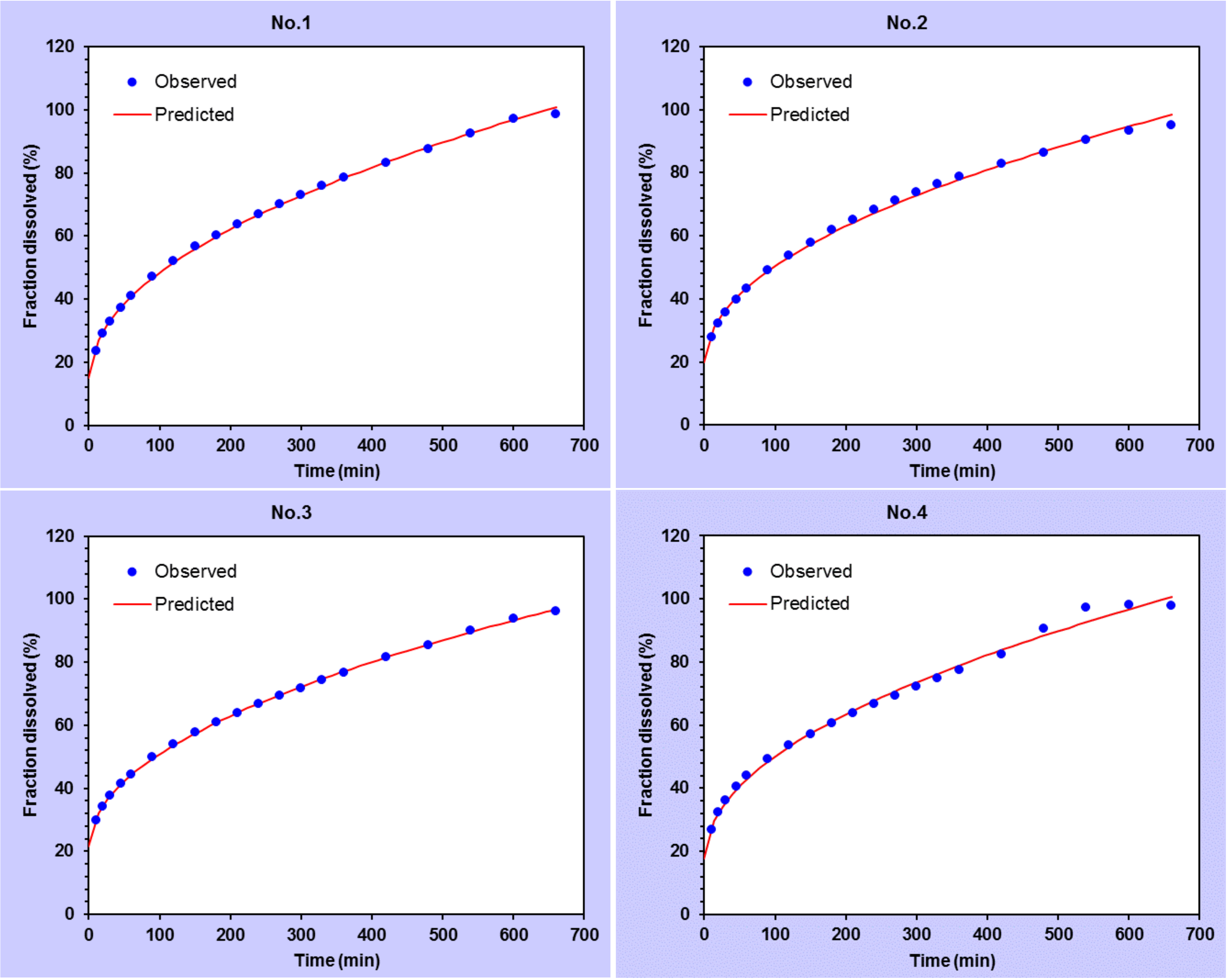

Supplementary materials – Model fitting summary – Tablettose® 70 (modelling the entire relevant dissolution data)

Model: **Korsmeyer–Peppas**

Model equation: $F = k_{KP} \cdot t^n$

Fitted model parameters per tested tablet (N = 4) with statistics – mean, standard deviation (SD), and relative standard deviation expressed in % (RSD%) (output from DDSolver):

Parameter	No.1	No.2	No.3	No.4	Mean	SD	RSD(%)
k_{KP}	10.059	12.661	14.208	12.231	12.290	1.713	13.935
n	0.349	0.309	0.287	0.316	0.315	0.026	8.138

Number of dissolution data points (N), degrees of freedom (df), and selected goodness of fit criteria – Pearson correlation coefficient (R), coefficient of determination (R^2), adjusted coefficient of determination ($R^2_{adjusted}$), and residual sum of squares (RSS) (manual calculation in MS Excel):

Parameter	No.1	No.2	No.3	No.4
N	20	20	20	20
df	18	18	18	18
R	0.998689777	0.998440807	0.994653693	0.991890735
R^2	0.997381271	0.996884046	0.989335968	0.98384723
$R^2_{adjusted}$	0.997235786	0.996710937	0.988743522	0.982949854
RSS	33.91470667	33.42469973	99.38927563	174.1218762

Graphical abstract of model fit presented as mean ± 1 SD of the fraction % of released carvedilol:

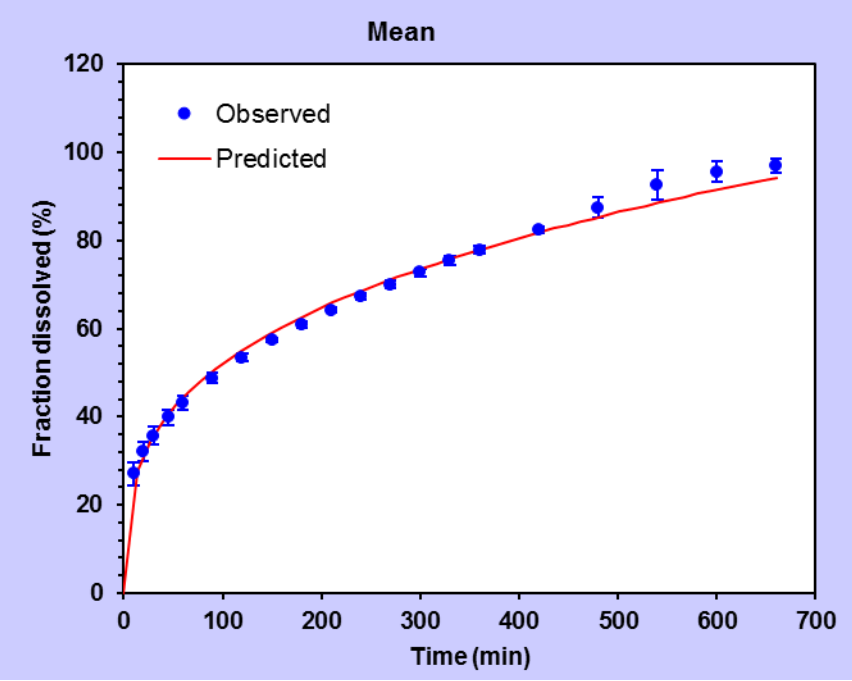

Supplementary materials – Model fitting summary – Tablettose® 70 (modelling the entire relevant dissolution data)

Graphical abstract of model fit presented as the fraction % of released carvedilol per tested tablet:

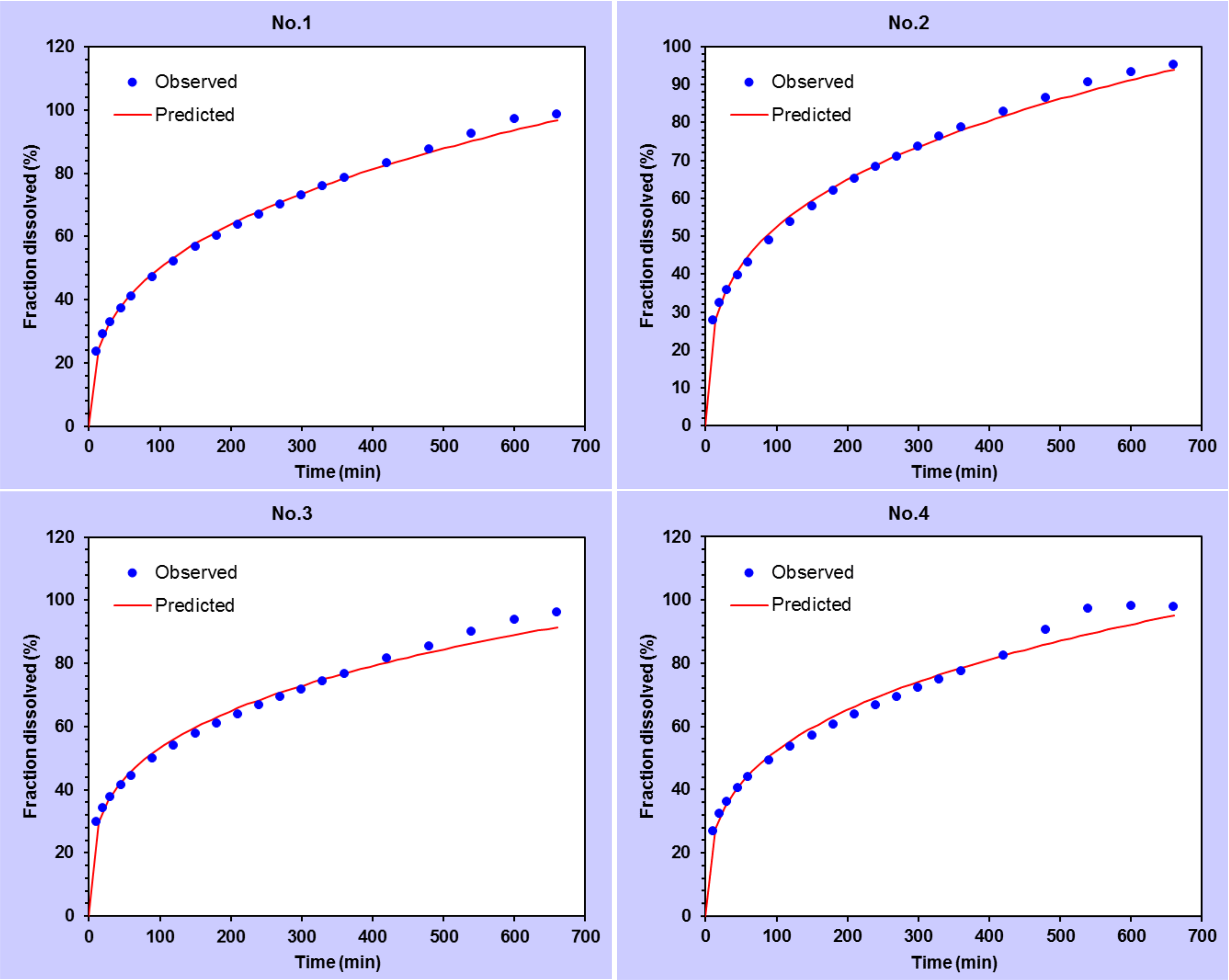

Supplementary materials – Model fitting summary – Tablettose® 70 (modelling the entire relevant dissolution data)

Model: **Korsmeyer–Peppas with T_{lag}**

Model equation: $F = k_{KP} \cdot (t - T_{lag})^n$

Fitted model parameters per tested tablet (N = 4) with statistics – mean, standard deviation (SD), and relative standard deviation expressed in % (RSD%) (output from DDSolver):

Parameter	No.1	No.2	No.3	No.4	Mean	SD	RSD(%)
k_{KP}	11.834	14.646	16.290	14.198	14.242	1.840	12.920
n	0.321	0.283	0.263	0.290	0.289	0.024	8.233
T_{lag}	4.000	4.000	4.000	4.000	4.000	0.000	0.000

Number of dissolution data points (N), degrees of freedom (df), and selected goodness of fit criteria – Pearson correlation coefficient (R), coefficient of determination (R^2), adjusted coefficient of determination ($R^2_{adjusted}$), and residual sum of squares (RSS) (manual calculation in MS Excel):

Parameter	No.1	No.2	No.3	No.4
N	20	20	20	20
df	17	17	17	17
R	0.996406642	0.995732385	0.990418936	0.987950548
R^2	0.992826197	0.991482982	0.980929669	0.976046286
$R^2_{adjusted}$	0.99198222	0.99048098	0.9786861	0.973228201
RSS	98.87144597	94.26707494	182.0889725	270.7812897

Graphical abstract of model fit presented as mean ± 1 SD of the fraction % of released carvedilol:

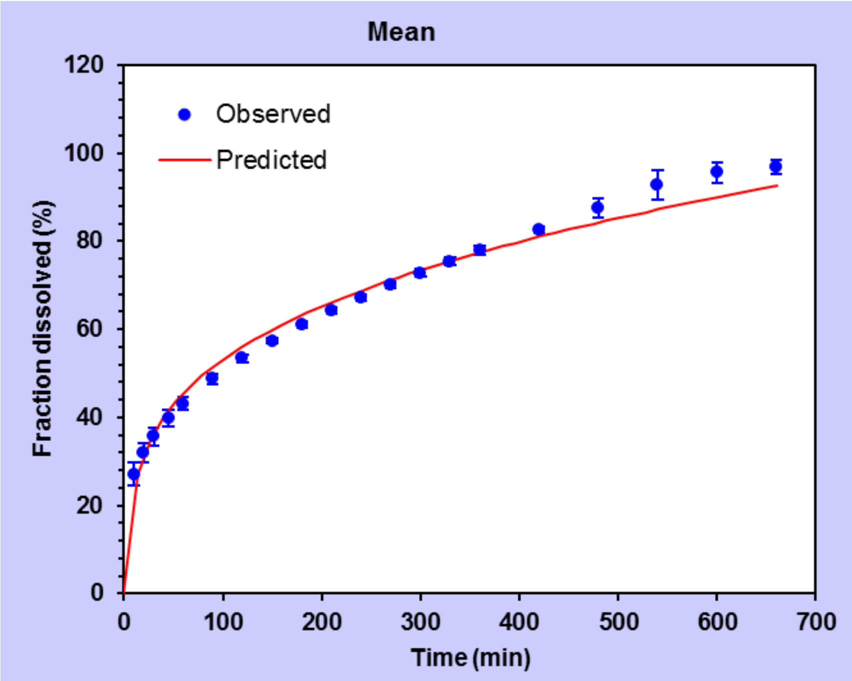

Supplementary materials – Model fitting summary – Tablettose® 70 (modelling the entire relevant dissolution data)

Graphical abstract of model fit presented as the fraction % of released carvedilol per tested tablet:

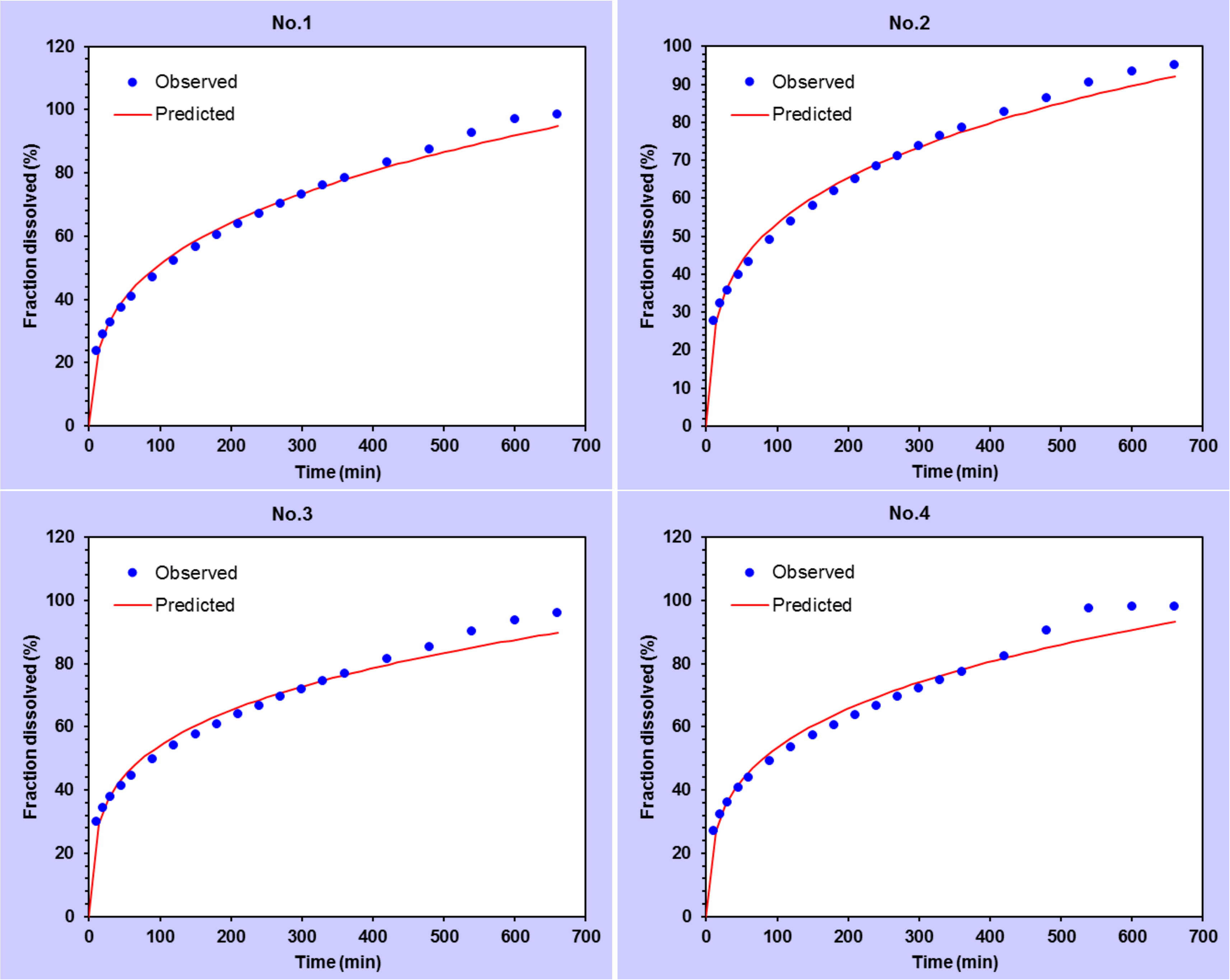

Supplementary materials – Model fitting summary – Tablettose® 70 (modelling the entire relevant dissolution data)

Model: **Korsmeyer–Peppas with F_0**

Model equation: $F = F_0 + k_{KP} \cdot t^n$

Fitted model parameters per tested tablet (N = 4) with statistics – mean, standard deviation (SD), and relative standard deviation expressed in % (RSD%) (output from DDSolver):

Parameter	No.1	No.2	No.3	No.4	Mean	SD	RSD(%)
k_{KP}	5.238	6.432	5.862	6.304	5.959	0.539	9.046
n	0.438	0.398	0.402	0.404	0.411	0.019	4.539
F_0	9.439	11.118	14.266	10.798	11.405	2.041	17.900

Number of dissolution data points (N), degrees of freedom (df), and selected goodness of fit criteria – Pearson correlation coefficient (R), coefficient of determination (R^2), adjusted coefficient of determination ($R^2_{adjusted}$), and residual sum of squares (RSS) (manual calculation in MS Excel):

Parameter	No.1	No.2	No.3	No.4
N	20	20	20	20
df	17	17	17	17
R	0.999823283	0.999682652	0.998975399	0.995528144
R^2	0.999646597	0.999365405	0.997951848	0.991076285
$R^2_{adjusted}$	0.99960502	0.999290747	0.997710889	0.990026437
RSS	3.560097788	5.733336716	20.00205526	92.62223063

Graphical abstract of model fit presented as mean ± 1 SD of the fraction % of released carvedilol:

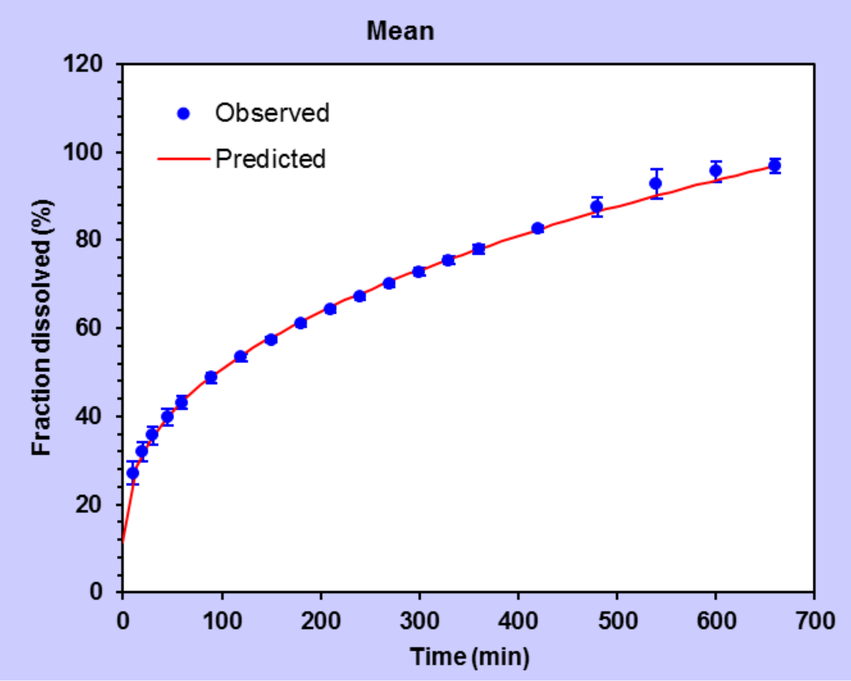

Supplementary materials – Model fitting summary – Tablettose® 70 (modelling the entire relevant dissolution data)

Graphical abstract of model fit presented as the fraction % of released carvedilol per tested tablet:

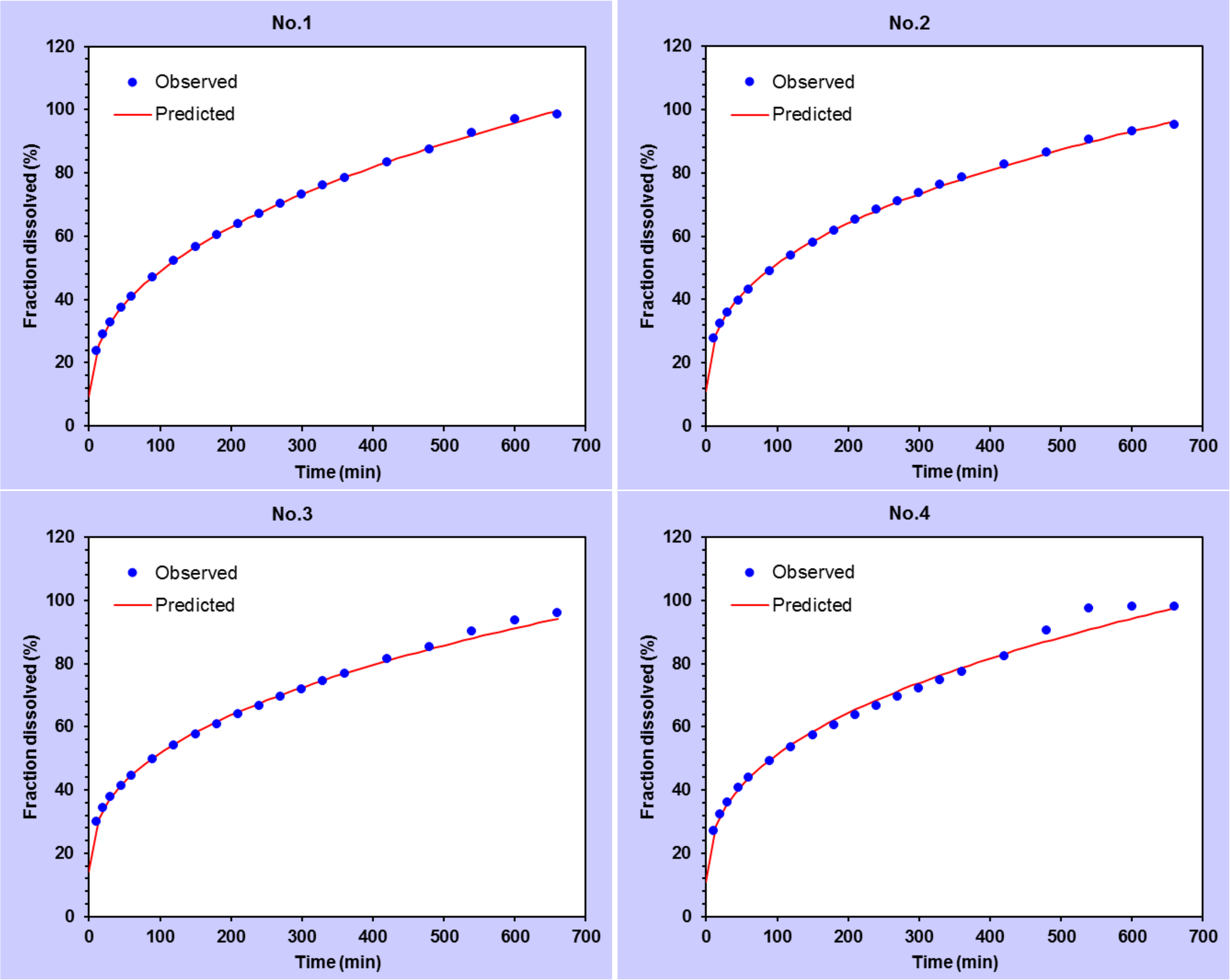

Supplementary materials – Model fitting summary – Tablettose® 70 (modelling the entire relevant dissolution data)

Model: **Hixson–Crowell**

Model equation: $F = 100 \cdot [1 - (1 - k_{HC} \cdot t)^3]$

Fitted model parameters per tested tablet (N = 4) with statistics – mean, standard deviation (SD), and relative standard deviation expressed in % (RSD%) (output from DDSolver):

Parameter	No.1	No.2	No.3	No.4	Mean	SD	RSD(%)
k_{HC}	0.002	0.002	0.002	0.001	0.002	0.000	15.402

Number of dissolution data points (N), degrees of freedom (df), and selected goodness of fit criteria – Pearson correlation coefficient (R), coefficient of determination (R^2), adjusted coefficient of determination ($R^2_{adjusted}$), and residual sum of squares (RSS) (manual calculation in MS Excel):

Parameter	No.1	No.2	No.3	No.4
N	20	20	20	20
df	19	19	19	19
R	0.972193741	0.983978304	0.973531547	0.987429115
R^2	0.945160671	0.968213303	0.947763674	0.975016257
$R^2_{adjusted}$	0.945160671	0.968213303	0.947763674	0.975016257
RSS	3265.133802	3660.93573	4370.3501	4086.758233

Graphical abstract of model fit presented as mean ± 1 SD of the fraction % of released carvedilol:

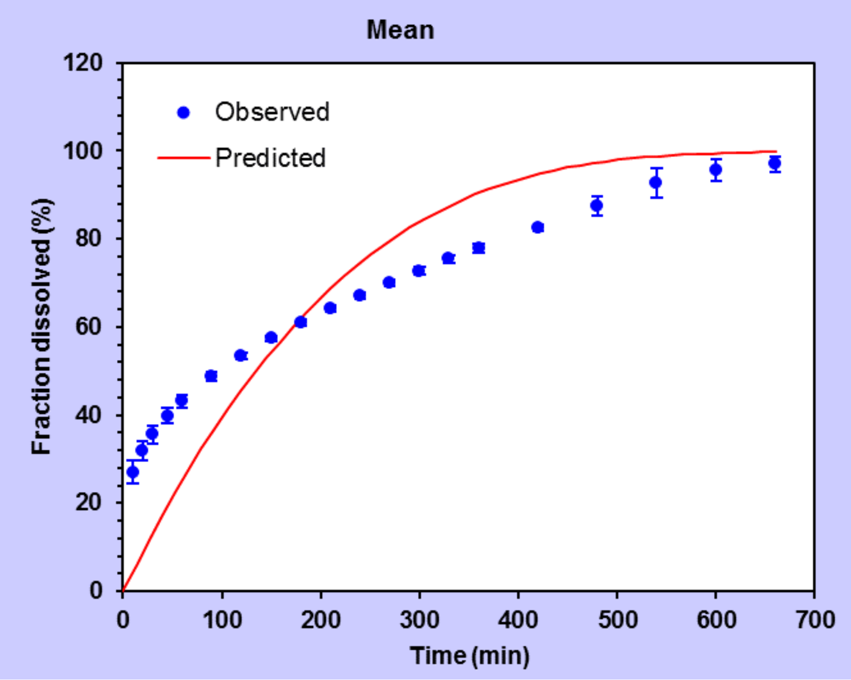

Supplementary materials – Model fitting summary – Tablettose® 70 (modelling the entire relevant dissolution data)

Graphical abstract of model fit presented as the fraction % of released carvedilol per tested tablet:

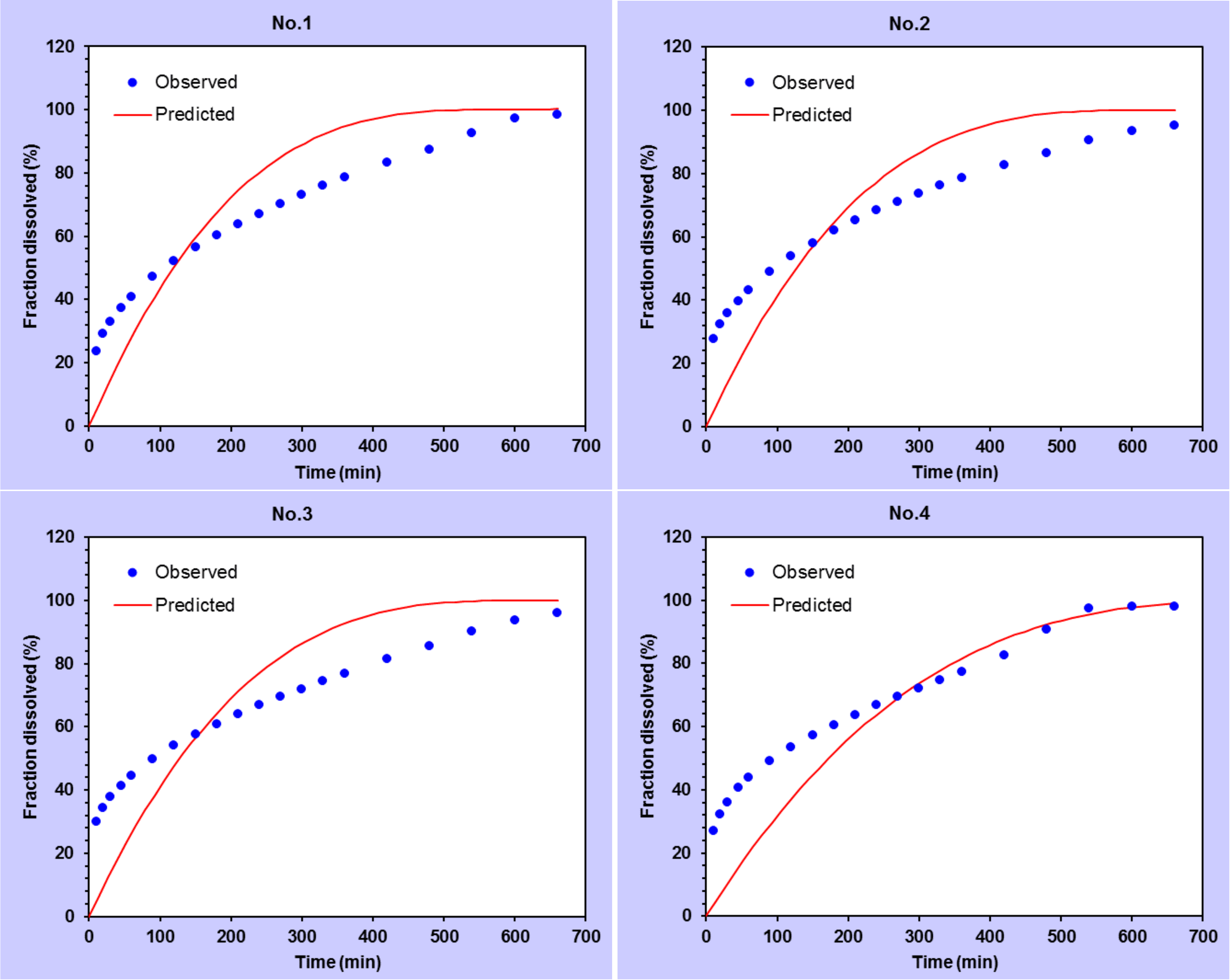

Supplementary materials – Model fitting summary – Tablettose® 70 (modelling the entire relevant dissolution data)

Model: **Hixson–Crowell with T_{lag}**

Model equation: $F = 100 \cdot \left\{1 - \left[1 - k_{HC} \cdot \left(t - T_{lag}\right)\right]^3\right\}$

Fitted model parameters per tested tablet (N = 4) with statistics – mean, standard deviation (SD), and relative standard deviation expressed in % (RSD%) (output from DDSolver):

Parameter	No.1	No.2	No.3	No.4	Mean	SD	RSD(%)
k_{HC}	0.001	0.001	0.001	0.001	0.001	0.000	4.634
T_{lag}	-116.750	-154.726	-155.810	-122.816	-137.526	20.641	-15.009

Number of dissolution data points (N), degrees of freedom (df), and selected goodness of fit criteria – Pearson correlation coefficient (R), coefficient of determination (R^2), adjusted coefficient of determination ($R^2_{adjusted}$), and residual sum of squares (RSS) (manual calculation in MS Excel):

Parameter	No.1	No.2	No.3	No.4
N	20	20	20	20
df	18	18	18	18
R	0.996125955	0.995890825	0.996311855	0.993504898
R^2	0.992266918	0.991798535	0.992637312	0.987051982
$R^2_{adjusted}$	0.991837302	0.991342898	0.992228273	0.986332648
RSS	86.37488388	74.78384603	58.33176525	202.9344582

Graphical abstract of model fit presented as mean ± 1 SD of the fraction % of released carvedilol:

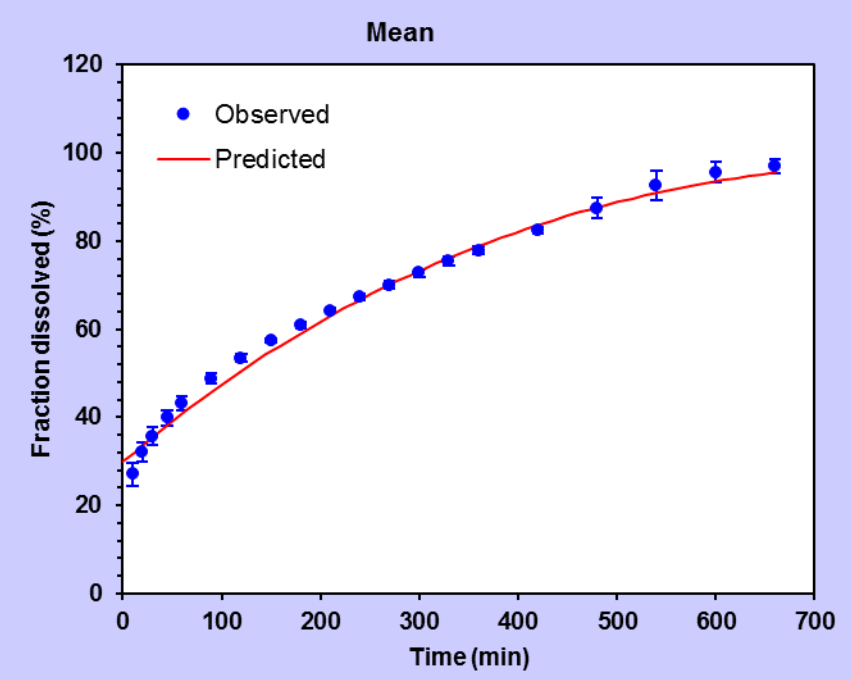

Supplementary materials – Model fitting summary – Tablettose® 70 (modelling the entire relevant dissolution data)

Graphical abstract of model fit presented as the fraction % of released carvedilol per tested tablet:

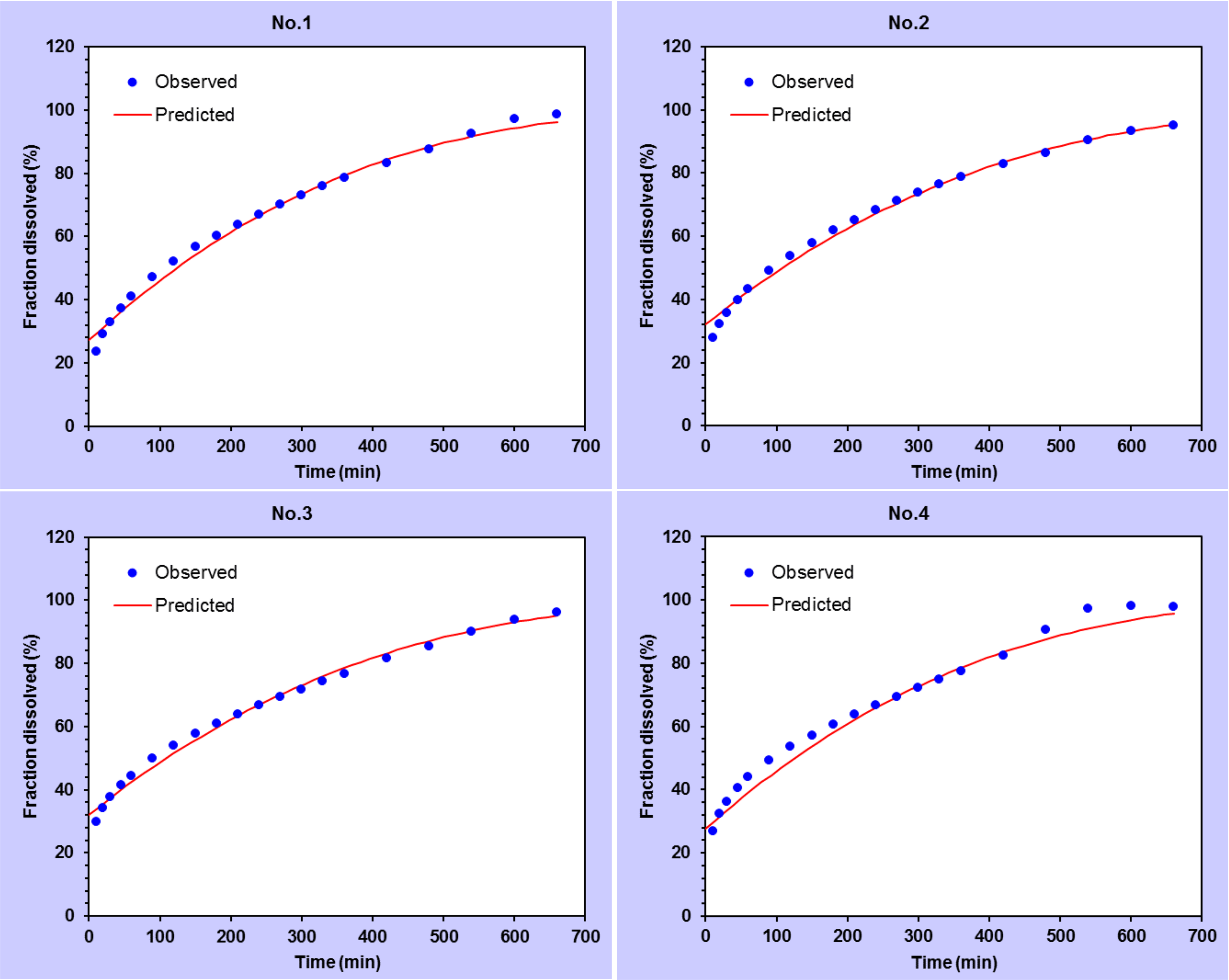

Supplementary materials – Model fitting summary – Tablettose® 70 (modelling the entire relevant dissolution data)

Model: **Hopfenberg**

Model equation: $F = 100 \cdot [1 - (1 - k_{HB} \cdot t)^n]$

Fitted model parameters per tested tablet (N = 4) with statistics – mean, standard deviation (SD), and relative standard deviation expressed in % (RSD%) (output from DDSolver):

Parameter	No.1	No.2	No.3	No.4	Mean	SD	RSD(%)
k_{HB}	0.001	0.001	0.001	0.001	0.001	0.000	5.168
n	4.500	4.500	4.500	4.500	4.500	0.000	0.000

Number of dissolution data points (N), degrees of freedom (df), and selected goodness of fit criteria – Pearson correlation coefficient (R), coefficient of determination (R^2), adjusted coefficient of determination ($R^2_{adjusted}$), and residual sum of squares (RSS) (manual calculation in MS Excel):

Parameter	No.1	No.2	No.3	No.4
N	20	20	20	20
df	18	18	18	18
R	0.97903191	0.988719439	0.979671604	0.963036303
R^2	0.958503482	0.977566128	0.959756451	0.92743892
$R^2_{adjusted}$	0.95619812	0.976319802	0.957520699	0.923407749
RSS	2597.197433	3149.239319	3787.046121	3371.231025

Graphical abstract of model fit presented as mean ± 1 SD of the fraction % of released carvedilol:

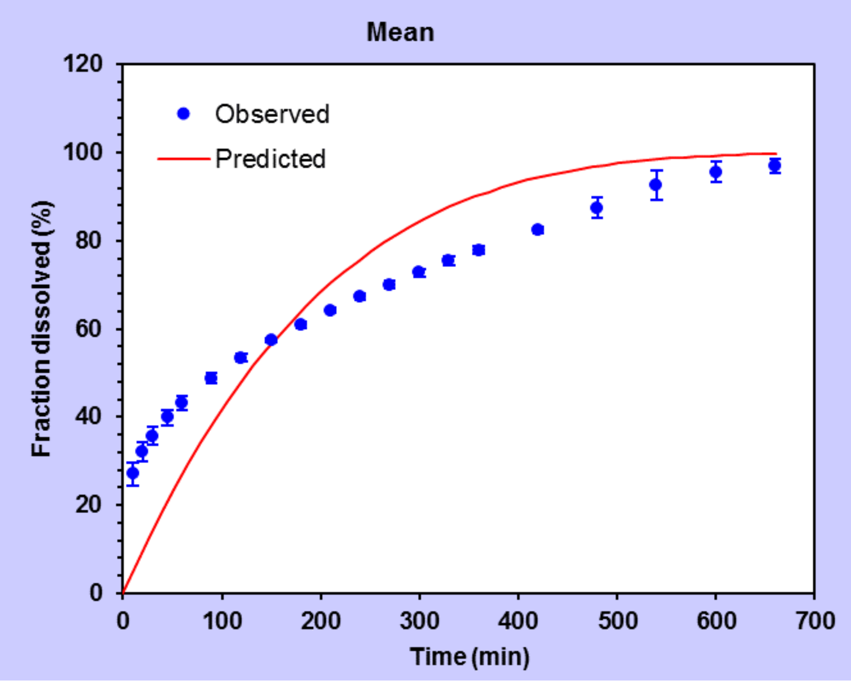

Supplementary materials – Model fitting summary – Tablettose® 70 (modelling the entire relevant dissolution data)

Graphical abstract of model fit presented as the fraction % of released carvedilol per tested tablet:

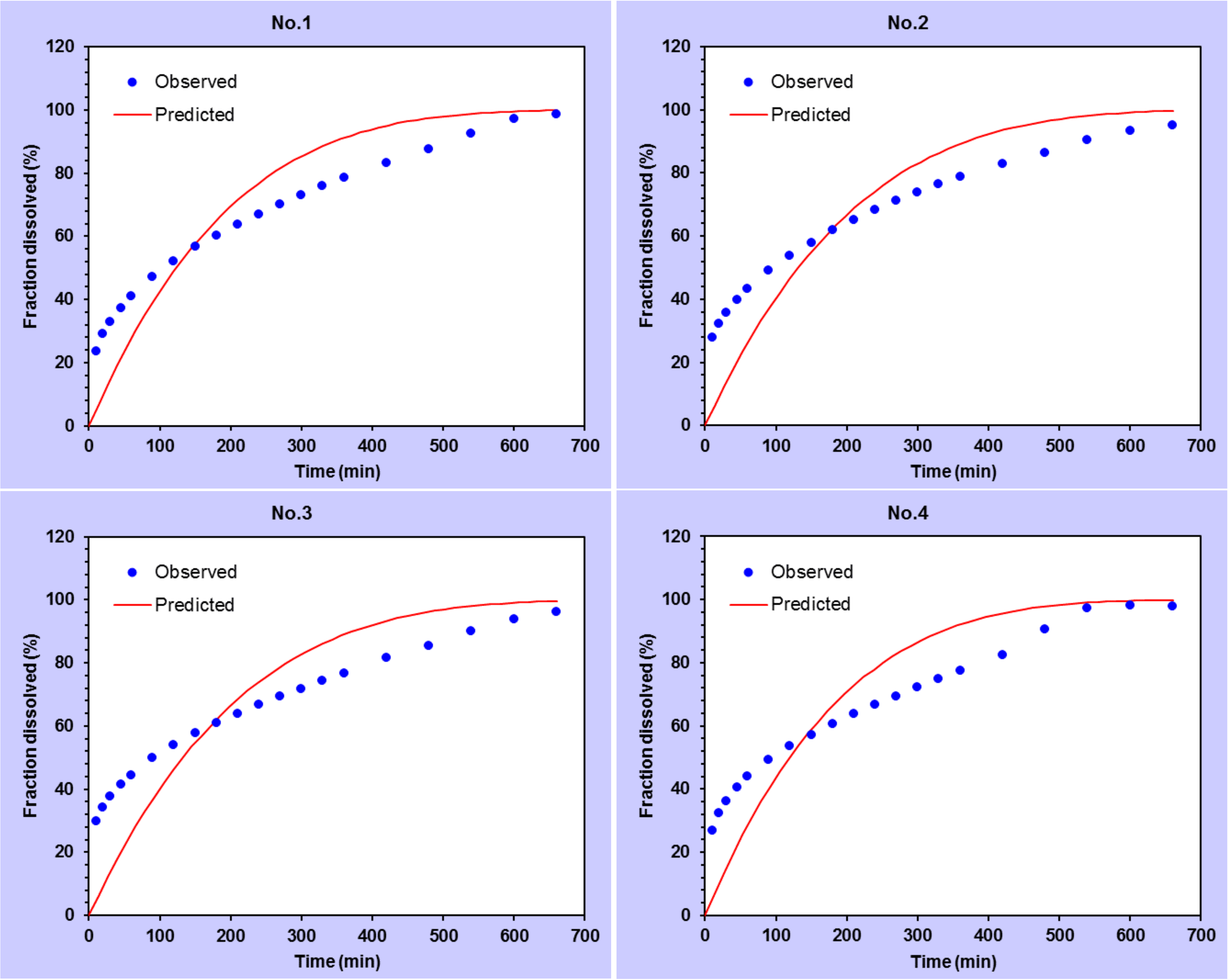

Supplementary materials – Model fitting summary – Tablettose® 70 (modelling the entire relevant dissolution data)

Model: **Hopfenberg with T_{lag}**

Model equation: $F = 100 \cdot \{1 - [1 - k_{HB} \cdot (t - T_{lag})]^n\}$

Fitted model parameters per tested tablet (N = 4) with statistics – mean, standard deviation (SD), and relative standard deviation expressed in % (RSD%) (output from DDSolver):

Parameter	No.1	No.2	No.3	No.4	Mean	SD	RSD(%)
k_{HB}	0.001	0.001	0.001	0.001	0.001	0.000	21.567
n	3.000	3.766	3.000	2.000	2.941	0.724	24.617
T_{lag}	-95.489	-138.475	-155.810	-149.985	-134.939	27.269	-20.208

Number of dissolution data points (N), degrees of freedom (df), and selected goodness of fit criteria – Pearson correlation coefficient (R), coefficient of determination (R^2), adjusted coefficient of determination ($R^2_{adjusted}$), and residual sum of squares (RSS) (manual calculation in MS Excel):

Parameter	No.1	No.2	No.3	No.4
N	20	20	20	20
df	17	17	17	17
R	0.995990932	0.996708873	0.996311855	0.993519032
R^2	0.991997936	0.993428578	0.992637312	0.987080068
$R^2_{adjusted}$	0.991056517	0.99265547	0.991771113	0.985560076
RSS	98.33167285	68.03176205	58.33176525	123.9082003

Graphical abstract of model fit presented as mean ± 1 SD of the fraction % of released carvedilol:

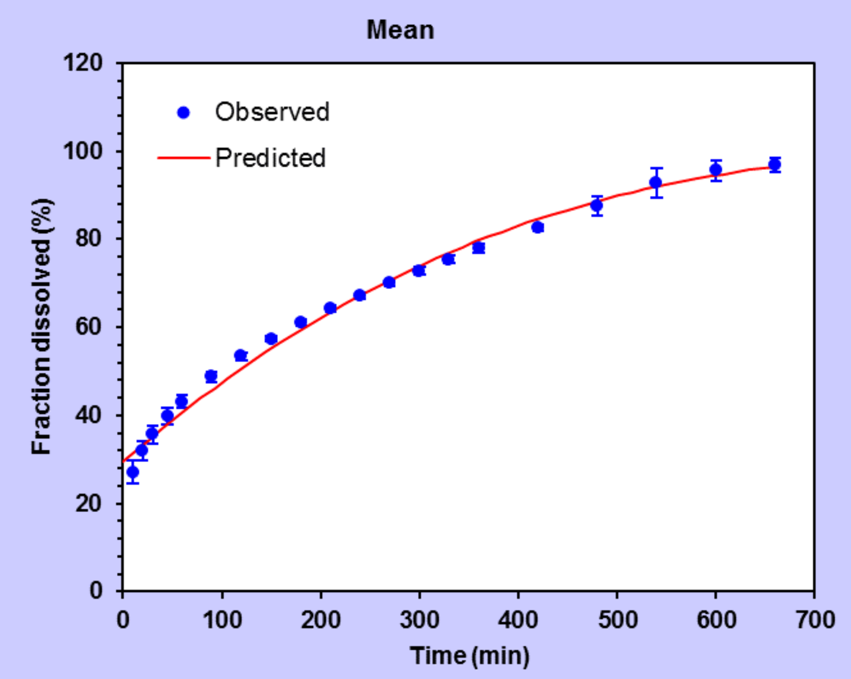

Supplementary materials – Model fitting summary – Tablettose® 70 (modelling the entire relevant dissolution data)

Graphical abstract of model fit presented as the fraction % of released carvedilol per tested tablet:

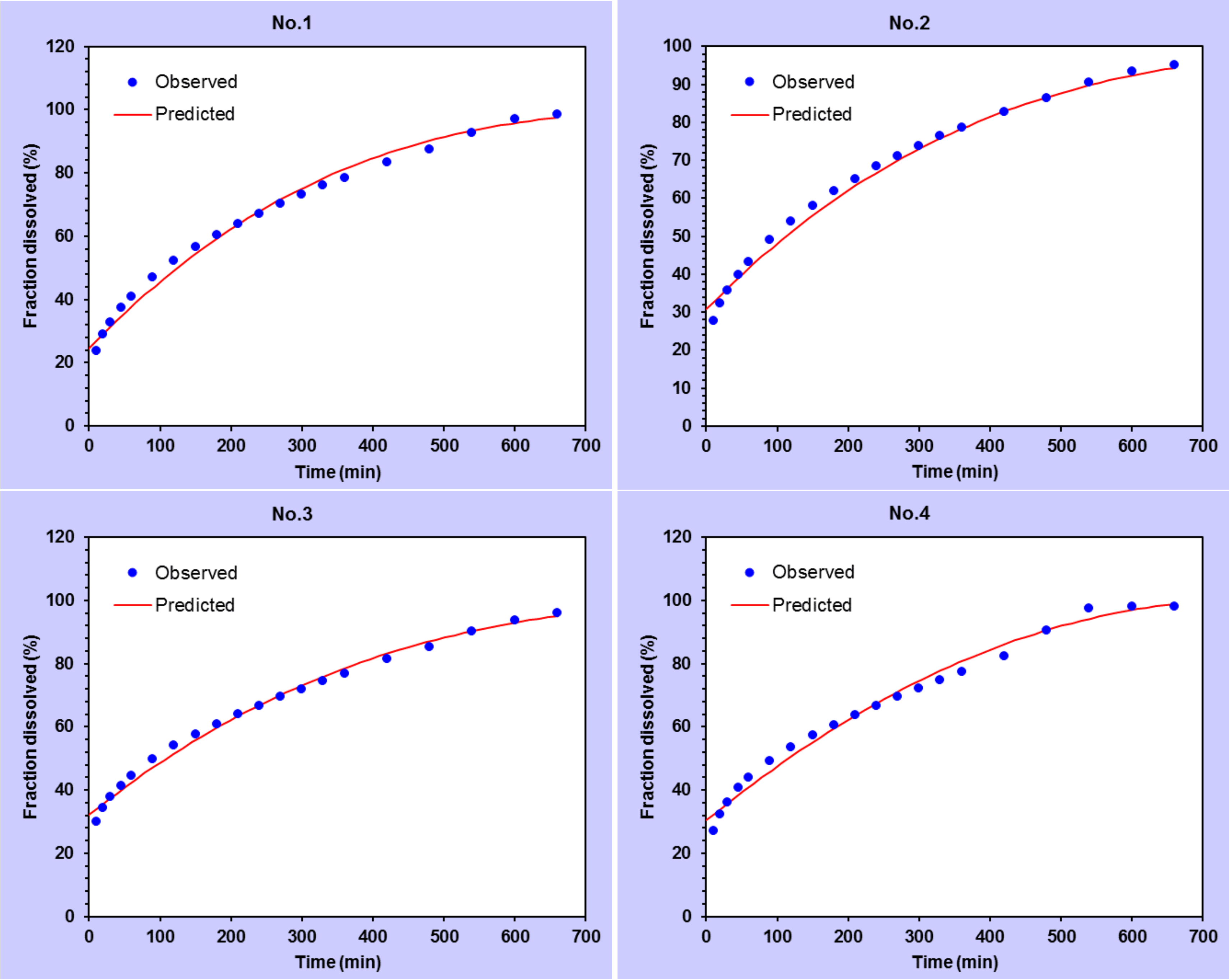

Supplementary materials – Model fitting summary – Tablettose® 70 (modelling the entire relevant dissolution data)

Model: **Baker–Lonsdale**

Model equation: $\frac{3}{2}\cdot\left[1-\left(1-\frac{F}{100}\right)^{\frac{2}{3}}\right]-\frac{F}{100}=k_{BL}\cdot t$

Fitted model parameters per tested tablet (N = 4) with statistics – mean, standard deviation (SD), and relative standard deviation expressed in % (RSD%) (output from DDSolver):

Parameter	No.1	No.2	No.3	No.4	Mean	SD	RSD(%)
k_{BL}	0.001	0.001	0.001	0.001	0.001	0.000	11.516

Number of dissolution data points (N), degrees of freedom (df), and selected goodness of fit criteria – Pearson correlation coefficient (R), coefficient of determination (R^2), adjusted coefficient of determination ($R^2_{adjusted}$), and residual sum of squares (RSS) (manual calculation in MS Excel):

Parameter	No.1	No.2	No.3	No.4
N	20	20	20	20
df	19	19	19	19
R	0.99114719	0.996499877	0.99132894	0.980709759
R^2	0.982372751	0.993012004	0.982733067	0.961791631
$R^2_{adjusted}$	0.982372751	0.993012004	0.982733067	0.961791631
RSS	348.4702016	340.5103398	541.9474708	634.3281038

Graphical abstract of model fit presented as mean ± 1 SD of the fraction % of released carvedilol:

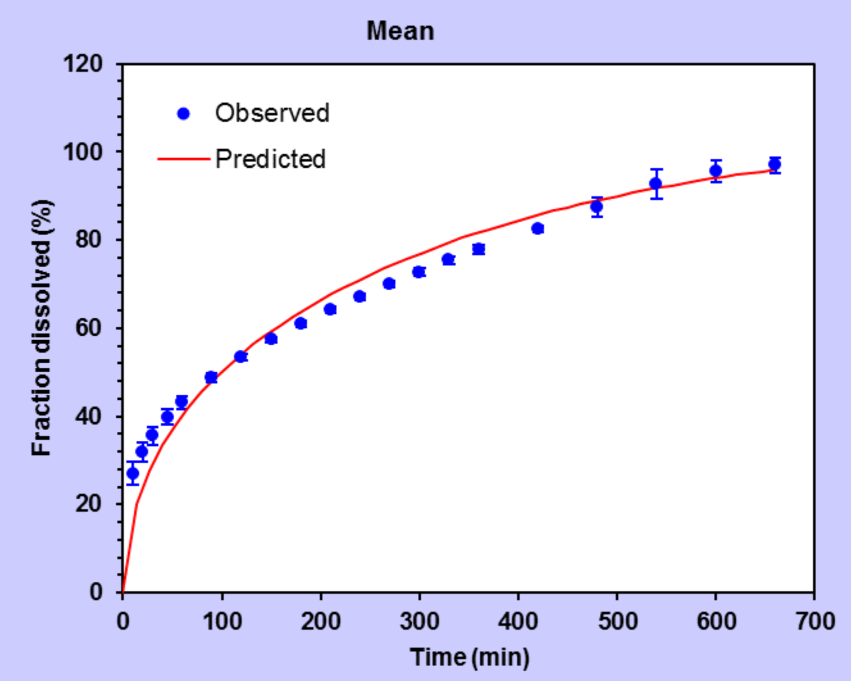

Supplementary materials – Model fitting summary – Tablettose® 70 (modelling the entire relevant dissolution data)

Graphical abstract of model fit presented as the fraction % of released carvedilol per tested tablet:

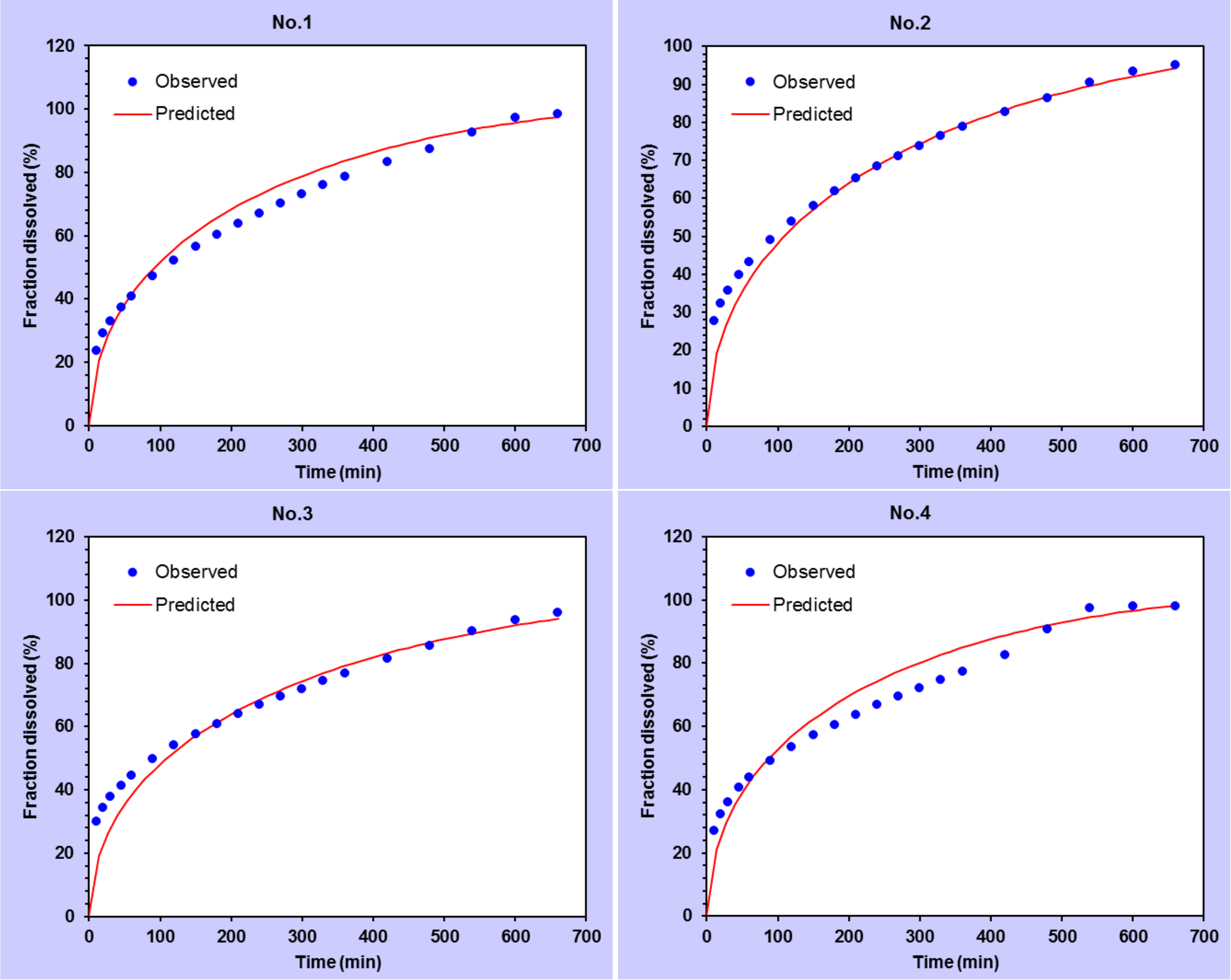

Supplementary materials – Model fitting summary – Tablettose® 70 (modelling the entire relevant dissolution data)

Model: **Baker–Lonsdale with T_{lag}**

Model equation: $\frac{3}{2} \cdot \left[1 - \left(1 - \frac{F}{100}\right)^{\frac{2}{3}}\right] - \frac{F}{100} = k_{BL} \cdot (t - T_{lag})$

Fitted model parameters per tested tablet (N = 4) with statistics – mean, standard deviation (SD), and relative standard deviation expressed in % (RSD%) (output from DDSolver):

Parameter	No.1	No.2	No.3	No.4	Mean	SD	RSD(%)
k_{BL}	0.001	0.001	0.001	0.001	0.001	0.000	11.516
T_{lag}	22.406	-13.420	-8.249	23.055	5.948	19.495	327.750

Number of dissolution data points (N), degrees of freedom (df), and selected goodness of fit criteria – Pearson correlation coefficient (R), coefficient of determination (R^2), adjusted coefficient of determination ($R^2_{adjusted}$), and residual sum of squares (RSS) (manual calculation in MS Excel):

Parameter	No.1	No.2	No.3	No.4
N	20	20	20	20
df	18	18	18	18
R	0.977890565	0.998347912	0.993235675	0.965413248
R^2	0.956269958	0.996698553	0.986517106	0.932022739
$R^2_{adjusted}$	0.953840511	0.996515139	0.985768057	0.928246224
RSS	1969.649829	48.85786984	254.1662386	2729.417557

Graphical abstract of model fit presented as mean ± 1 SD of the fraction % of released carvedilol:

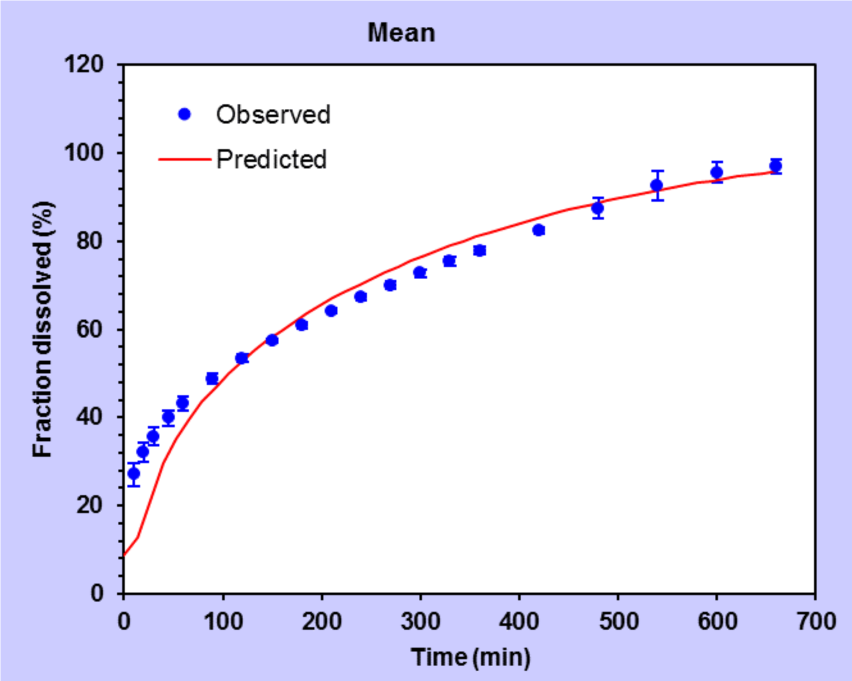

Supplementary materials – Model fitting summary – Tablettose® 70 (modelling the entire relevant dissolution data)

Graphical abstract of model fit presented as the fraction % of released carvedilol per tested tablet:

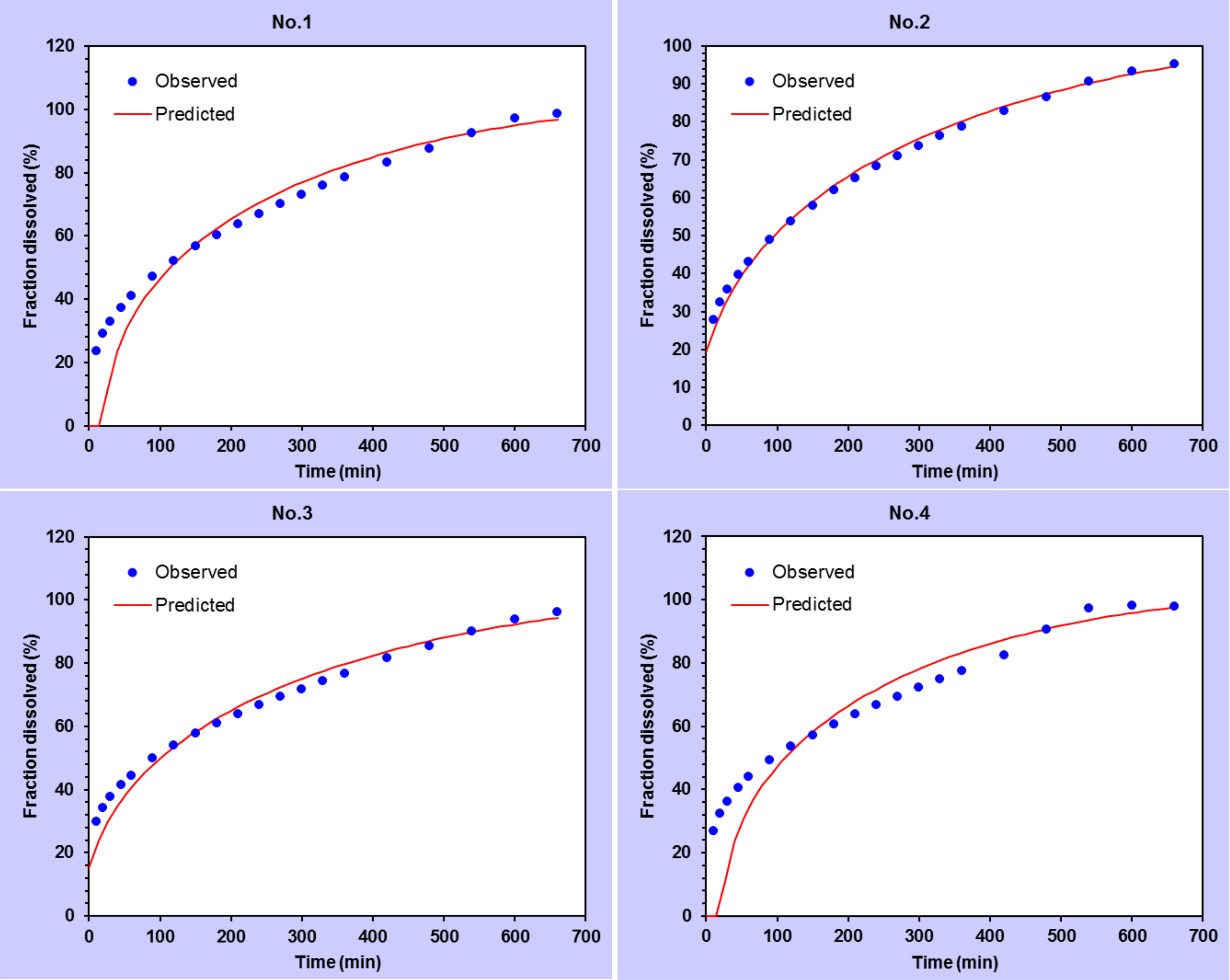

Supplementary materials – Model fitting summary – Tablettose® 70 (modelling the entire relevant dissolution data)

Model: **Makoid–Banakar**

Model equation: $F = k_{MB} \cdot t^{n} \cdot e^{-k \cdot t}$

Fitted model parameters per tested tablet (N = 4) with statistics – mean, standard deviation (SD), and relative standard deviation expressed in % (RSD%) (output from DDSolver):

Parameter	No.1	No.2	No.3	No.4	Mean	SD	RSD(%)
k_{MB}	11.1752	14.1303	17.0815	14.8391	14.3065	2.4373	17.0359
n	0.3173	0.2759	0.2319	0.2583	0.2708	0.0359	13.2441
k	-0.0002	-0.0002	-0.0004	-0.0004	-0.0003	0.0001	-31.7802

Number of dissolution data points (N), degrees of freedom (df), and selected goodness of fit criteria – Pearson correlation coefficient (R), coefficient of determination (R^2), adjusted coefficient of determination ($R^2_{adjusted}$), and residual sum of squares (RSS) (manual calculation in MS Excel):

Parameter	No.1	No.2	No.3	No.4
N	20	20	20	20
df	17	17	17	17
R	0.999647061	0.998848811	0.999547028	0.997366756
R^2	0.999294246	0.997698947	0.99909426	0.994740446
$R^2_{adjusted}$	0.999211216	0.997428235	0.998987703	0.994121675
RSS	7.108270094	19.38419513	7.001142108	49.22789606

Graphical abstract of model fit presented as mean ± 1 SD of the fraction % of released carvedilol:

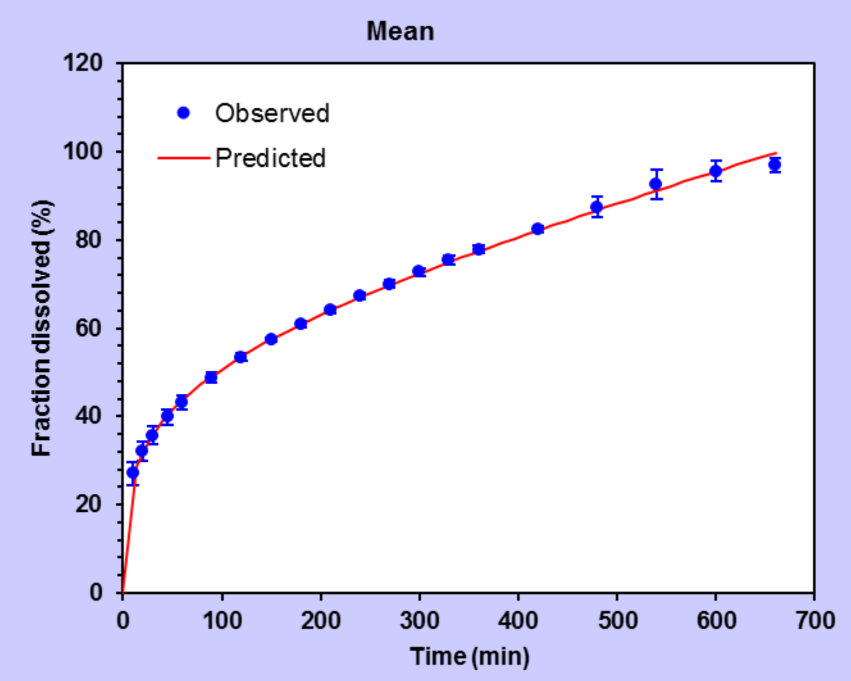

Supplementary materials – Model fitting summary – Tablettose® 70 (modelling the entire relevant dissolution data)

Graphical abstract of model fit presented as the fraction % of released carvedilol per tested tablet:

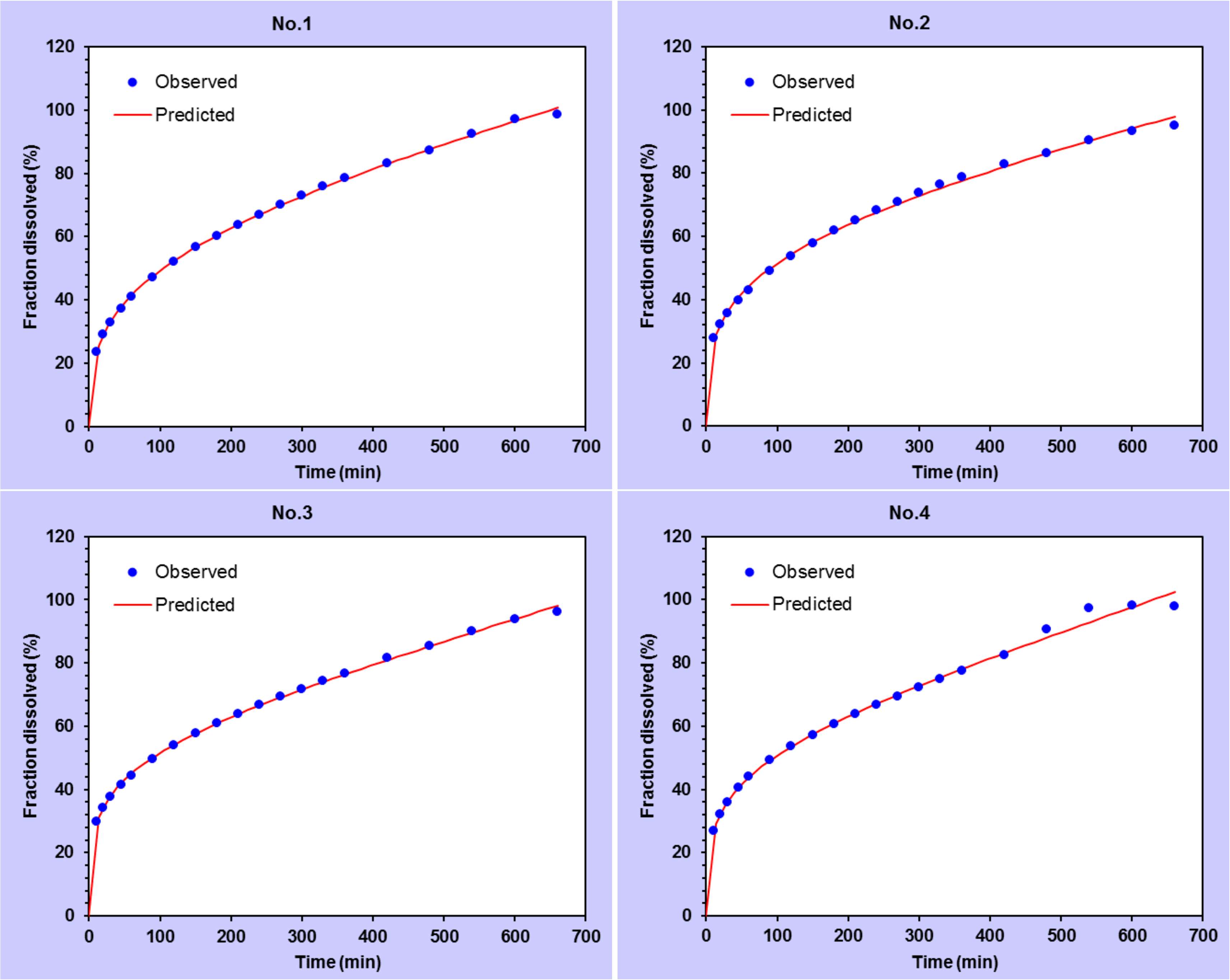

Supplementary materials – Model fitting summary – Tablettose® 70 (modelling the entire relevant dissolution data)

Model: **Makoid–Banakar with T_{lag}**

Model equation: $F = k_{MB} \cdot (t - T_{lag})^n \cdot e^{-k\cdot(t-T_{lag})}$

Fitted model parameters per tested tablet (N = 4) with statistics – mean, standard deviation (SD), and relative standard deviation expressed in % (RSD%) (output from DDSolver):

Parameter	No.1	No.2	No.3	No.4	Mean	SD	RSD(%)
k_{MB}	13.8055	17.0511	19.9816	17.6037	17.1105	2.5438	14.8669
n	0.2716	0.2350	0.1980	0.2216	0.2316	0.0307	13.2745
k	-0.0004	-0.0004	-0.0005	-0.0005	-0.0004	0.0001	-18.1815
T_{lag}	4.0000	4.0000	4.0000	4.0000	4.0000	0.0000	0.0000

Number of dissolution data points (N), degrees of freedom (df), and selected goodness of fit criteria – Pearson correlation coefficient (R), coefficient of determination (R^2), adjusted coefficient of determination ($R^2_{adjusted}$), and residual sum of squares (RSS) (manual calculation in MS Excel):

Parameter	No.1	No.2	No.3	No.4
N	20	20	20	20
df	16	16	16	16
R	0.998876348	0.997373575	0.998744357	0.996942619
R^2	0.997753959	0.994754047	0.99749029	0.993894586
$R^2_{adjusted}$	0.997332826	0.993770431	0.99701972	0.992749821
RSS	22.67887912	44.26876459	19.45910606	57.18395226

Graphical abstract of model fit presented as mean ± 1 SD of the fraction % of released carvedilol:

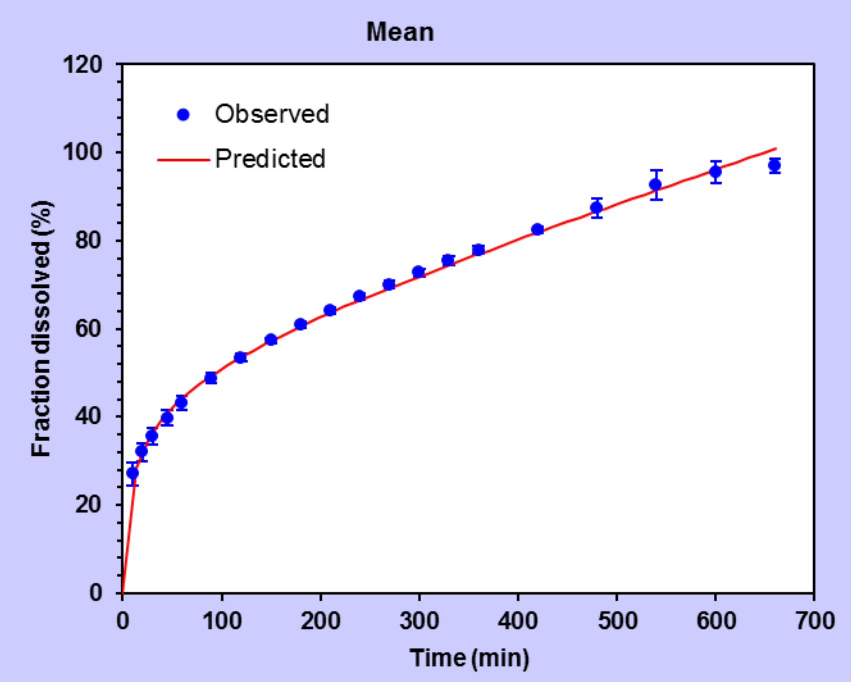

Supplementary materials – Model fitting summary – Tablettose® 70 (modelling the entire relevant dissolution data)

Graphical abstract of model fit presented as the fraction % of released carvedilol per tested tablet:

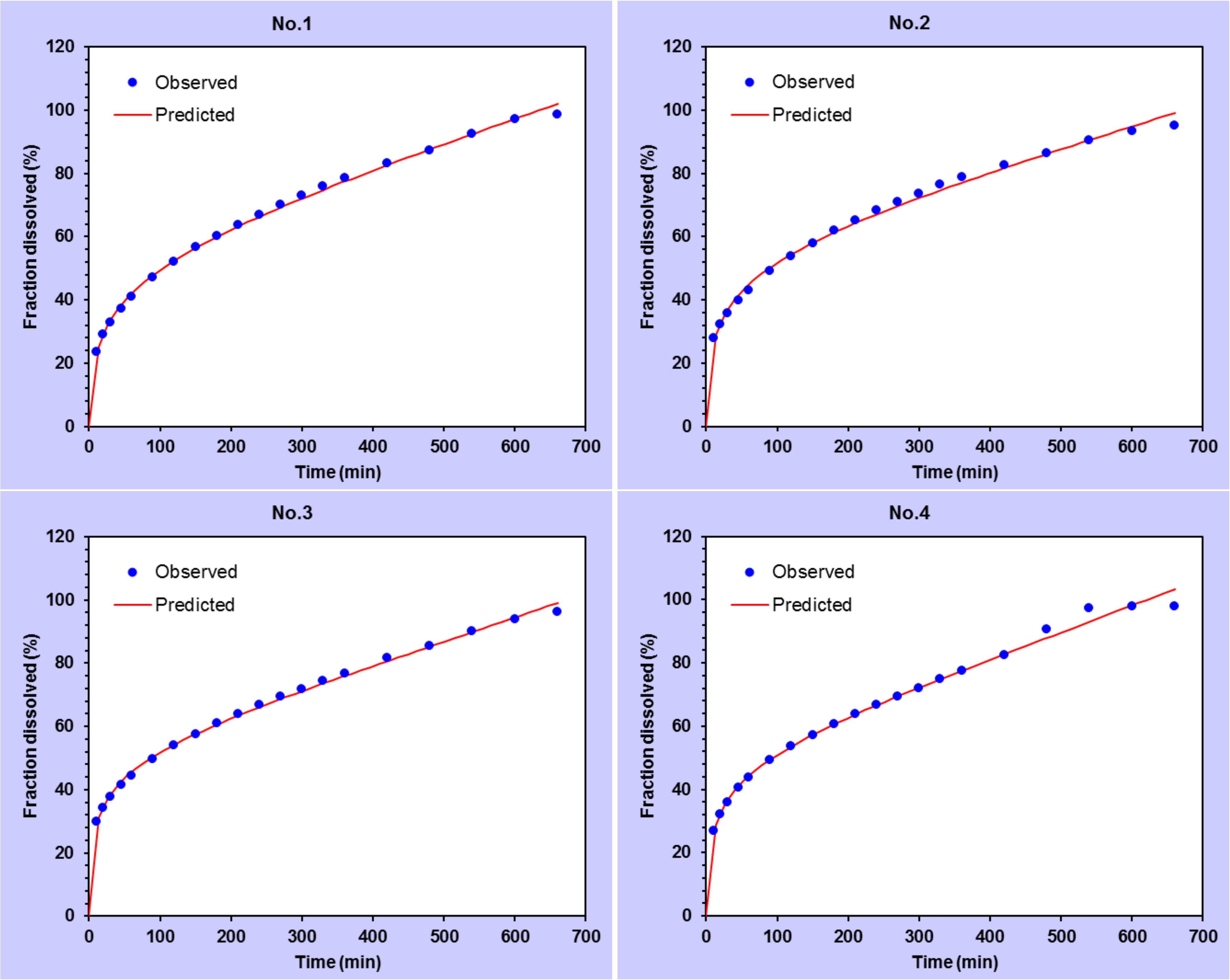

Supplementary materials – Model fitting summary – Tablettose® 70 (modelling the entire relevant dissolution data)

Model: **Peppas–Sahlin_1**

Model equation: $F = k_1 \cdot t^m + k_2 \cdot t^{2m}$

Fitted model parameters per tested tablet (N = 4) with statistics – mean, standard deviation (SD), and relative standard deviation expressed in % (RSD%) (output from DDSolver):

Parameter	No.1	No.2	No.3	No.4	Mean	SD	RSD(%)
k_1	6.872	7.568	7.592	7.156	7.297	0.347	4.758
k_2	-0.088	-0.137	-0.141	-0.103	-0.117	0.026	-22.175
m	0.450	0.450	0.450	0.450	0.450	0.000	0.000

Number of dissolution data points (N), degrees of freedom (df), and selected goodness of fit criteria – Pearson correlation coefficient (R), coefficient of determination (R^2), adjusted coefficient of determination ($R^2_{adjusted}$), and residual sum of squares (RSS) (manual calculation in MS Excel):

Parameter	No.1	No.2	No.3	No.4
N	20	20	20	20
df	17	17	17	17
R	0.997164874	0.994693088	0.988067613	0.988957732
R^2	0.994337786	0.98941434	0.976277608	0.978037396
$R^2_{adjusted}$	0.993671644	0.988168968	0.973486738	0.975453561
RSS	80.80592589	135.8946521	284.6333345	279.1045444

Graphical abstract of model fit presented as mean ± 1 SD of the fraction % of released carvedilol:

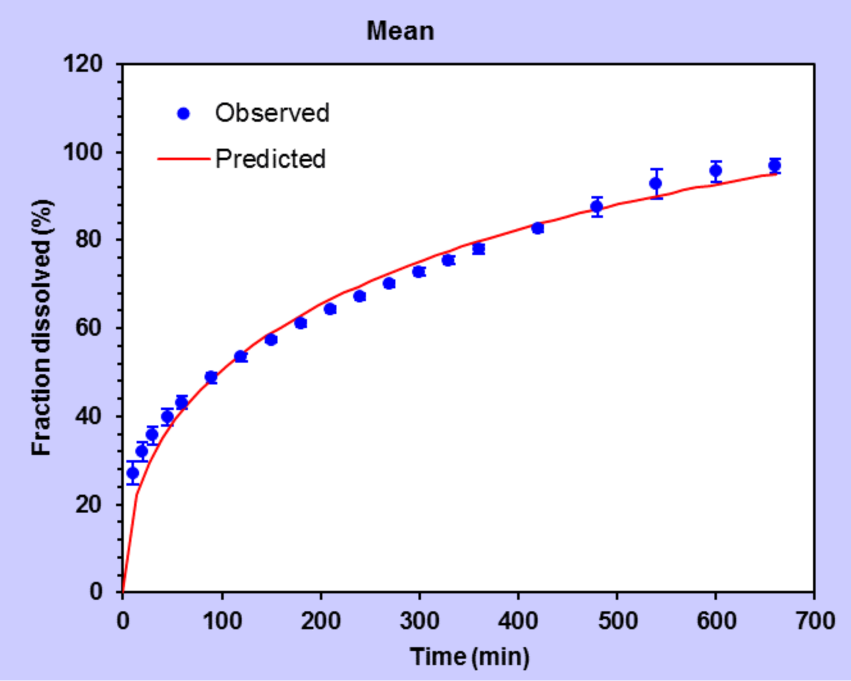

Supplementary materials – Model fitting summary – Tablettose® 70 (modelling the entire relevant dissolution data)

Graphical abstract of model fit presented as the fraction % of released carvedilol per tested tablet:

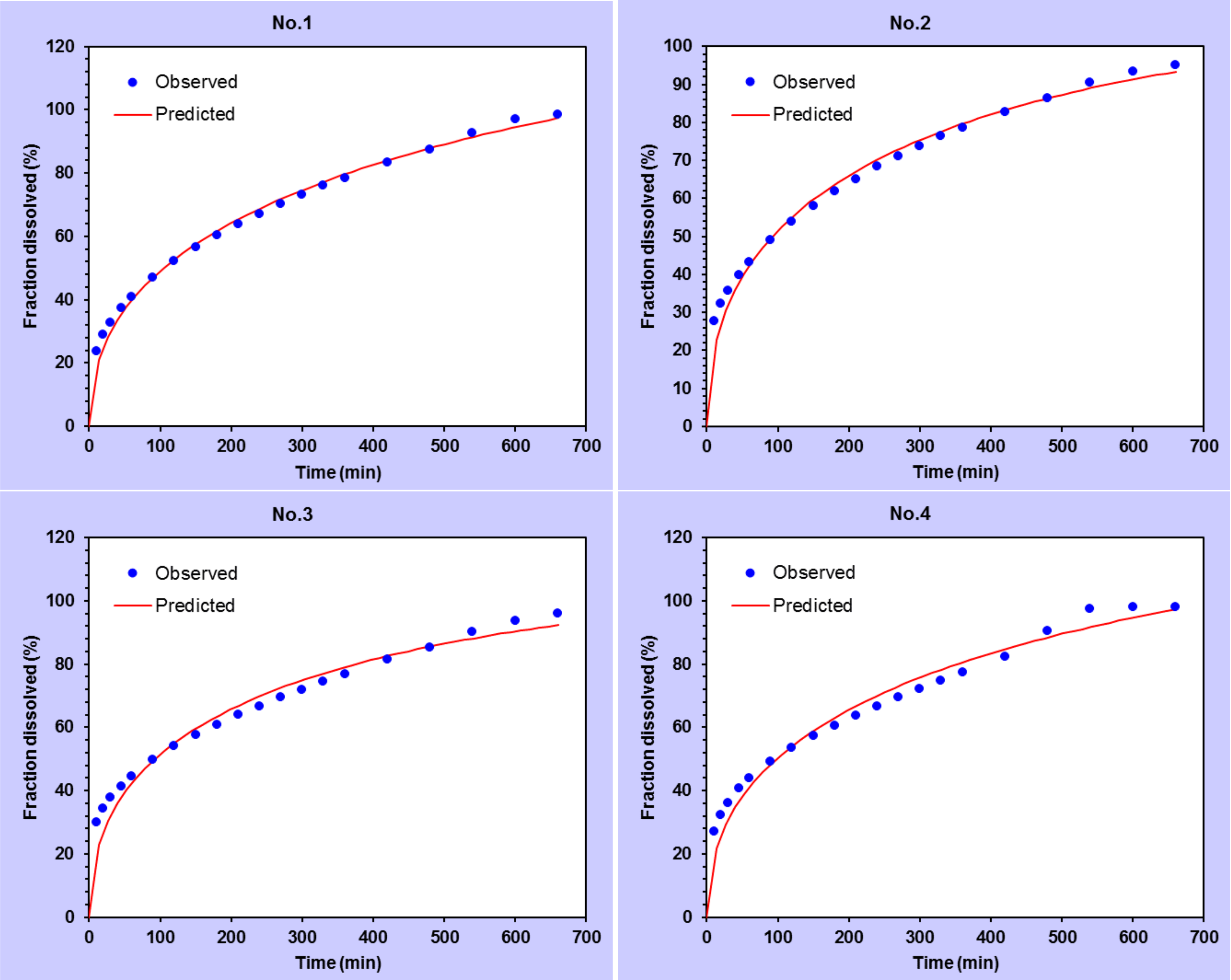

Supplementary materials – Model fitting summary – Tablettose® 70 (modelling the entire relevant dissolution data)

Model: **Peppas–Sahlin_1 with T_{lag}**

Model equation: $F = k_1 \cdot (t - T_{lag})^m + k_2 \cdot (t - T_{lag})^{2m}$

Fitted model parameters per tested tablet (N = 4) with statistics – mean, standard deviation (SD), and relative standard deviation expressed in % (RSD%) (output from DDSolver):

Parameter	No.1	No.2	No.3	No.4	Mean	SD	RSD(%)
k_1	7.104	7.798	7.810	7.378	7.523	0.344	4.571
k_2	-0.101	-0.151	-0.154	-0.116	-0.131	0.026	-19.827
m	0.450	0.450	0.450	0.450	0.450	0.000	0.000
T_{lag}	4.000	4.000	4.000	4.000	4.000	0.000	0.000

Number of dissolution data points (N), degrees of freedom (df), and selected goodness of fit criteria – Pearson correlation coefficient (R), coefficient of determination (R^2), adjusted coefficient of determination ($R^2_{adjusted}$), and residual sum of squares (RSS) (manual calculation in MS Excel):

Parameter	No.1	No.2	No.3	No.4
N	20	20	20	20
df	16	16	16	16
R	0.994731879	0.990944742	0.983174949	0.985217225
R^2	0.989491511	0.981971482	0.96663298	0.97065298
$R^2_{adjusted}$	0.987521169	0.978591135	0.960376663	0.965150413
RSS	163.4774992	252.6244127	439.0984412	410.2781949

Graphical abstract of model fit presented as mean ± 1 SD of the fraction % of released carvedilol:

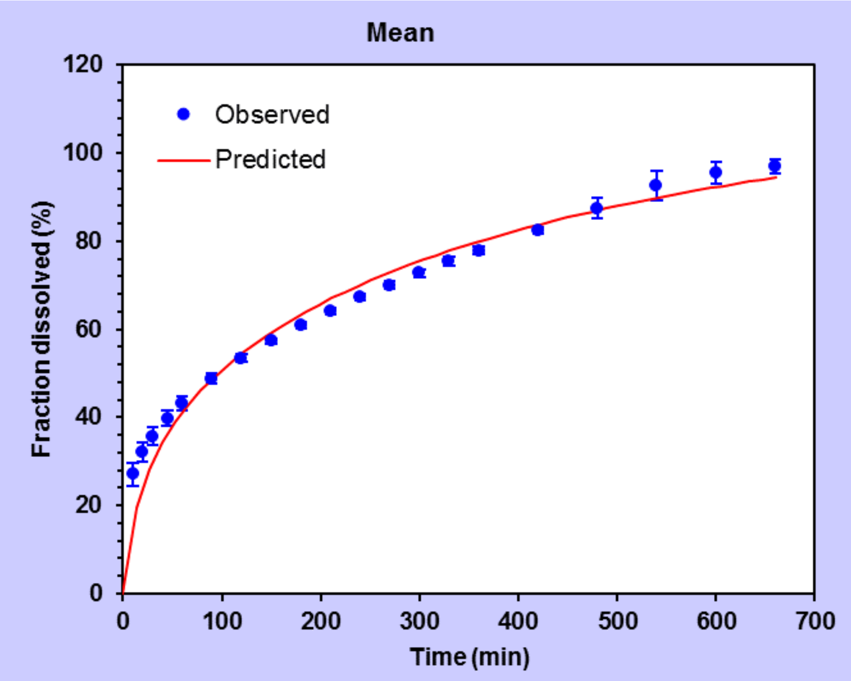

Supplementary materials – Model fitting summary – Tablettose® 70 (modelling the entire relevant dissolution data)

Graphical abstract of model fit presented as the fraction % of released carvedilol per tested tablet:

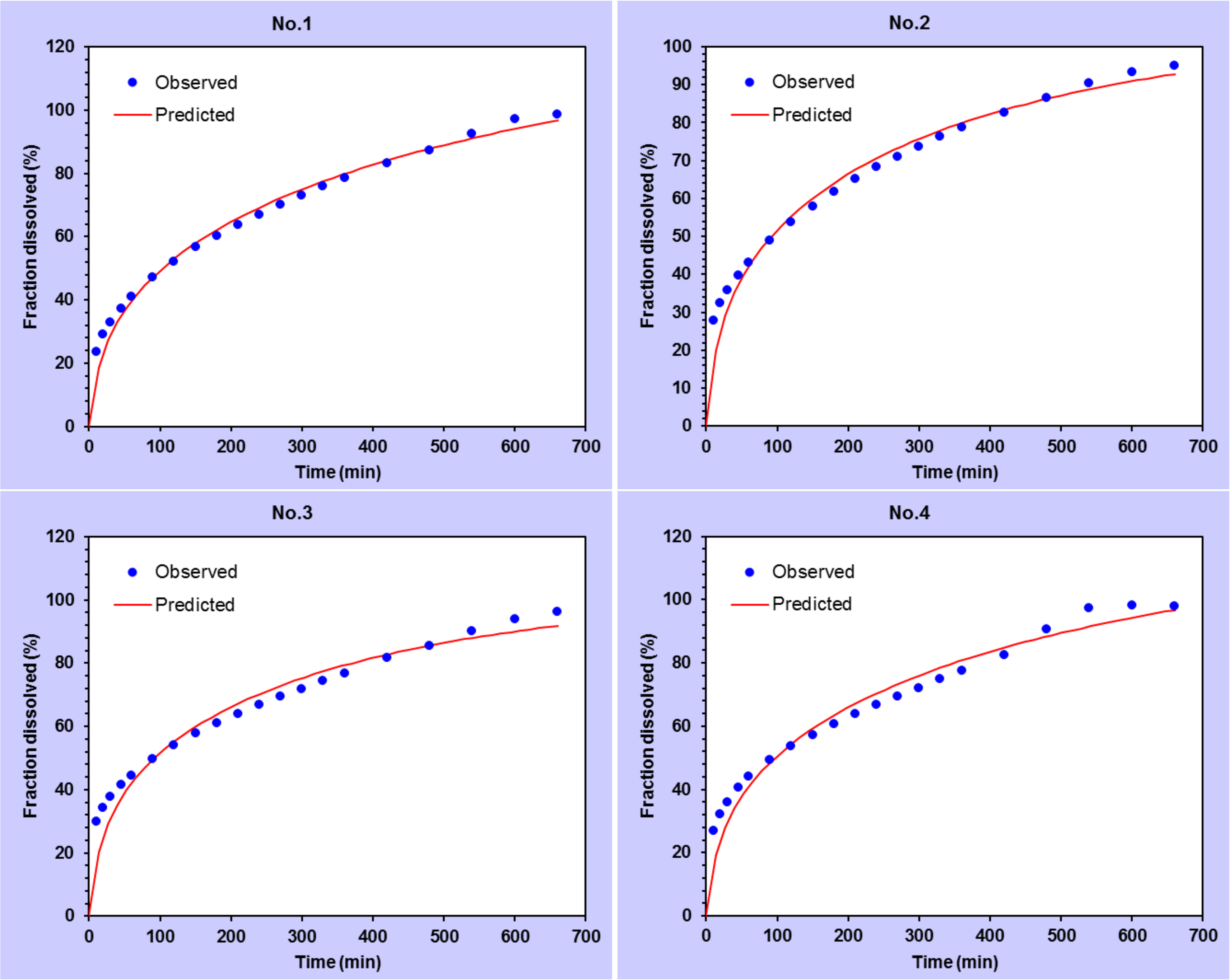

Supplementary materials – Model fitting summary – Tablettose® 70 (modelling the entire relevant dissolution data)

Model: **Peppas–Sahlin_2**

Model equation: $F = k_1 \cdot t^{0.5} + k_2 \cdot t$

Fitted model parameters per tested tablet (N = 4) with statistics – mean, standard deviation (SD), and relative standard deviation expressed in % (RSD%) (output from DDSolver):

Parameter	No.1	No.2	No.3	No.4	Mean	SD	RSD(%)
k_1	5.570	6.057	6.046	5.754	5.857	0.237	4.044
k_2	-0.071	-0.096	-0.097	-0.078	-0.085	0.013	-15.379

Number of dissolution data points (N), degrees of freedom (df), and selected goodness of fit criteria – Pearson correlation coefficient (R), coefficient of determination (R^2), adjusted coefficient of determination ($R^2_{adjusted}$), and residual sum of squares (RSS) (manual calculation in MS Excel):

Parameter	No.1	No.2	No.3	No.4
N	20	20	20	20
df	18	18	18	18
R	0.995133715	0.991741063	0.983781246	0.985650784
R^2	0.990291112	0.983550336	0.967825541	0.971507468
$R^2_{adjusted}$	0.989751729	0.982636465	0.966038071	0.96992455
RSS	158.4242157	247.5395782	447.781386	413.6471359

Graphical abstract of model fit presented as mean ± 1 SD of the fraction % of released carvedilol:

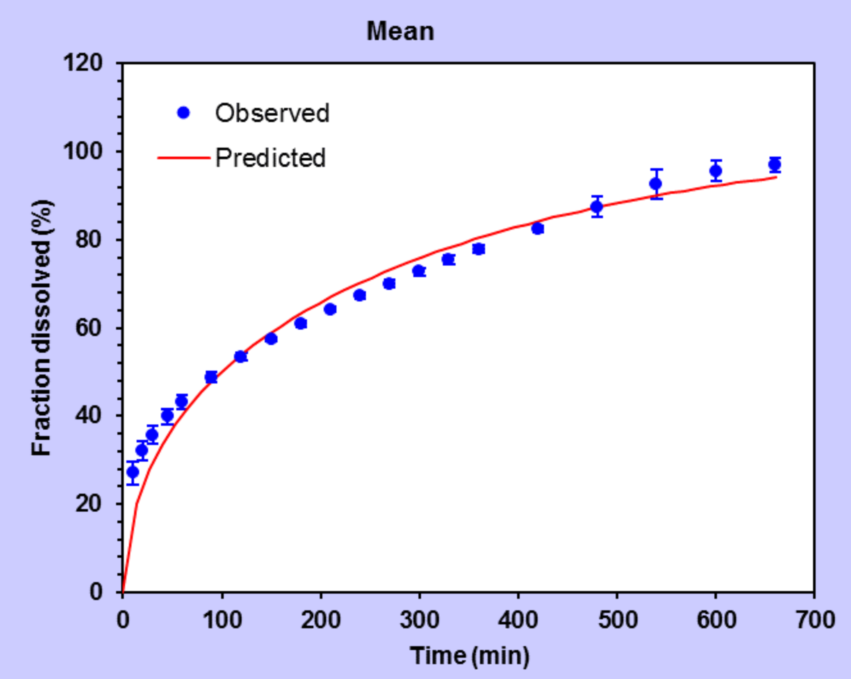

Supplementary materials – Model fitting summary – Tablettose® 70 (modelling the entire relevant dissolution data)

Graphical abstract of model fit presented as the fraction % of released carvedilol per tested tablet:

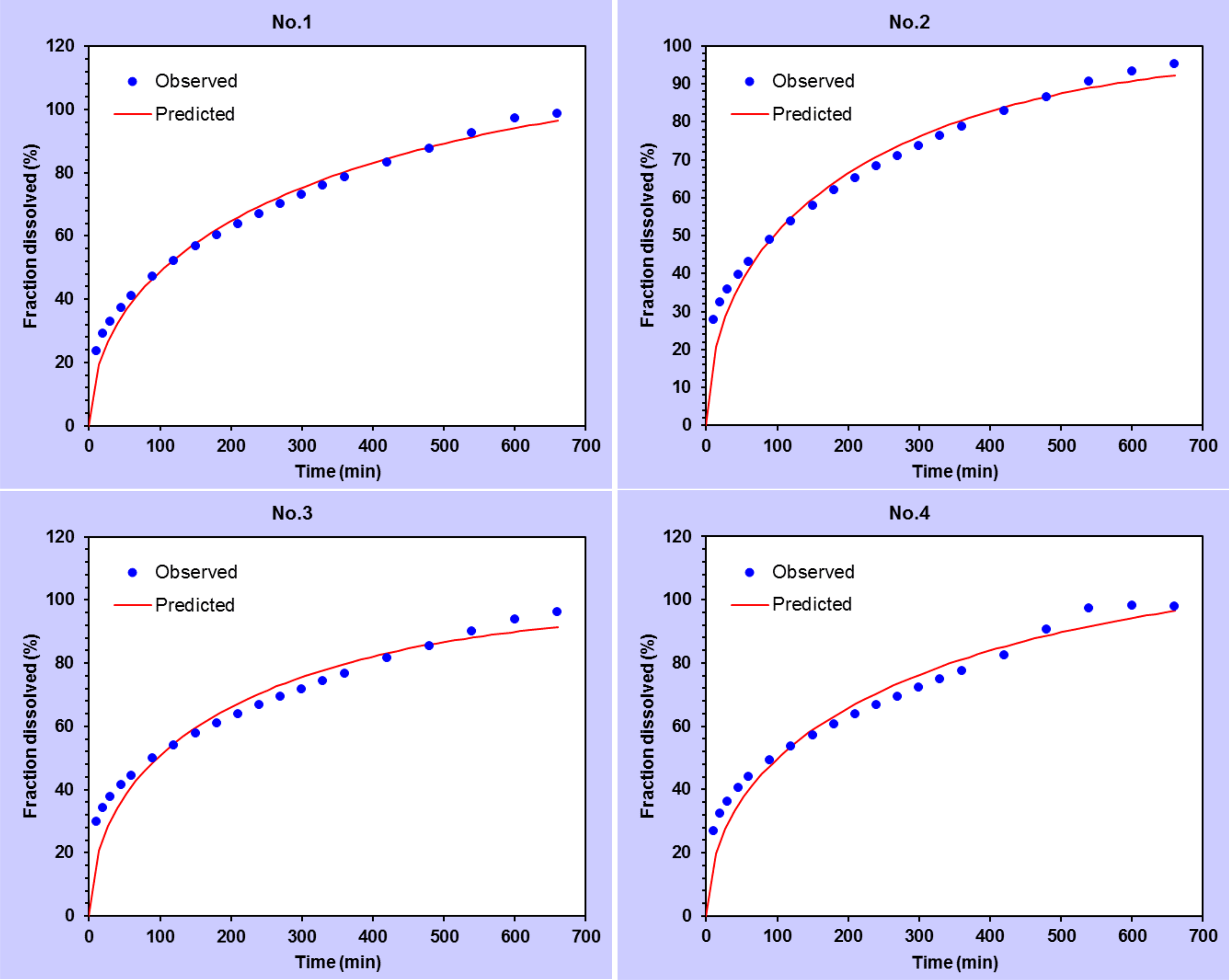

Supplementary materials – Model fitting summary – Tablettose® 70 (modelling the entire relevant dissolution data)

Model: **Peppas–Sahlin_2 with T_{lag}**

Model equation: $F = k_1 \cdot (t - T_{lag})^{0.5} + k_2 \cdot (t - T_{lag})$

Fitted model parameters per tested tablet (N = 4) with statistics – mean, standard deviation (SD), and relative standard deviation expressed in % (RSD%) (output from DDSolver):

Parameter	No.1	No.2	No.3	No.4	Mean	SD	RSD(%)
k_1	5.728	6.211	6.190	5.903	6.008	0.234	3.895
k_2	-0.077	-0.103	-0.103	-0.084	-0.092	0.013	-14.214
T_{lag}	4.000	4.000	4.000	4.000	4.000	0.000	0.000

Number of dissolution data points (N), degrees of freedom (df), and selected goodness of fit criteria – Pearson correlation coefficient (R), coefficient of determination (R^2), adjusted coefficient of determination ($R^2_{adjusted}$), and residual sum of squares (RSS) (manual calculation in MS Excel):

Parameter	No.1	No.2	No.3	No.4
N	20	20	20	20
df	17	17	17	17
R	0.992362778	0.987773246	0.978907975	0.98176832
R^2	0.984783884	0.975695986	0.958260824	0.963869035
$R^2_{adjusted}$	0.982993752	0.97283669	0.953350333	0.959618333
RSS	273.9547215	404.4361214	645.1424357	583.1764786

Graphical abstract of model fit presented as mean ± 1 SD of the fraction % of released carvedilol:

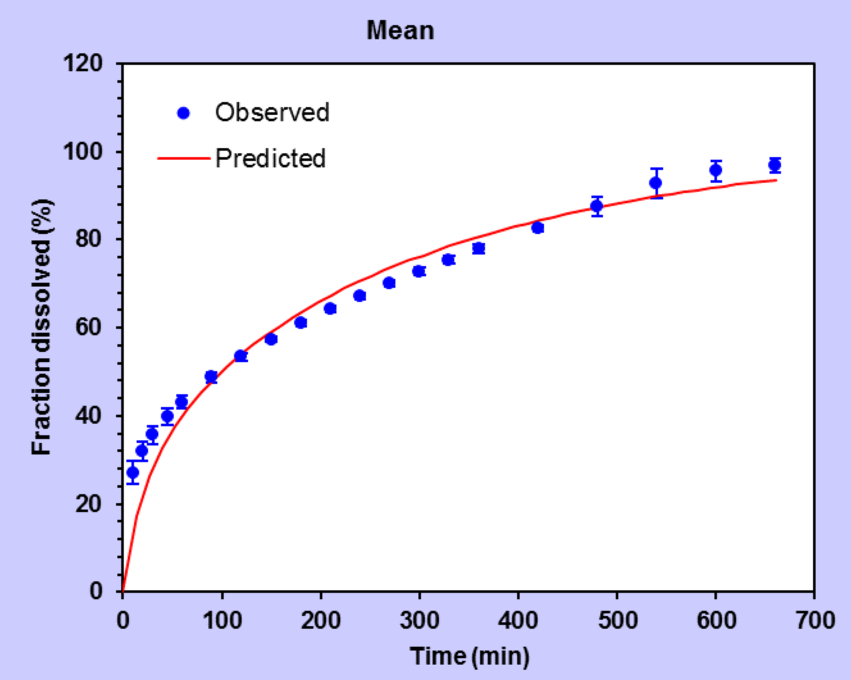

Supplementary materials – Model fitting summary – Tablettose® 70 (modelling the entire relevant dissolution data)

Graphical abstract of model fit presented as the fraction % of released carvedilol per tested tablet:

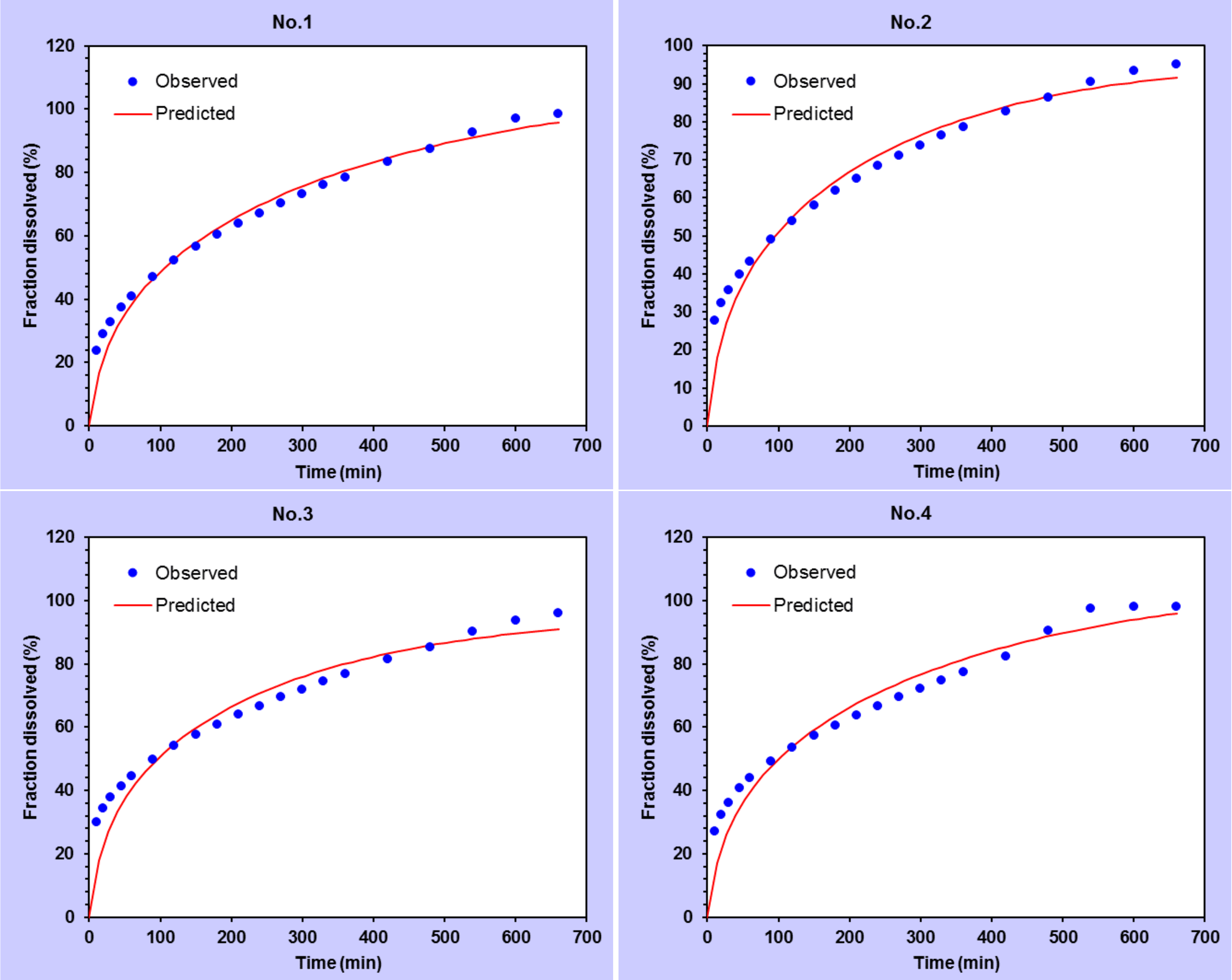

Supplementary materials – Model fitting summary – Tablettose® 70 (modelling the entire relevant dissolution data)

Model: **Quadratic**

Model equation: $F = 100 \cdot (k_1 \cdot t^2 + k_2 \cdot t)$

Fitted model parameters per tested tablet (N = 4) with statistics – mean, standard deviation (SD), and relative standard deviation expressed in % (RSD%) (output from DDSolver):

Parameter	No.1	No.2	No.3	No.4	Mean	SD	RSD(%)
k_1	-0.000004	-0.000004	-0.000004	-0.000004	-0.000004	0.000000	-3.868343
k_2	0.003766	0.003897	0.003818	0.003787	0.003817	0.000057	1.506312

Number of dissolution data points (N), degrees of freedom (df), and selected goodness of fit criteria – Pearson correlation coefficient (R), coefficient of determination (R^2), adjusted coefficient of determination ($R^2_{adjusted}$), and residual sum of squares (RSS) (manual calculation in MS Excel):

Parameter	No.1	No.2	No.3	No.4
N	20	20	20	20
df	18	18	18	18
R	0.973911012	0.971026288	0.961226631	0.964217511
R^2	0.94850266	0.942892052	0.923956637	0.929715408
$R^2_{adjusted}$	0.945641696	0.939719388	0.919732005	0.925810708
RSS	3216.659581	3920.448987	4575.701696	4088.231809

Graphical abstract of model fit presented as mean ± 1 SD of the fraction % of released carvedilol:

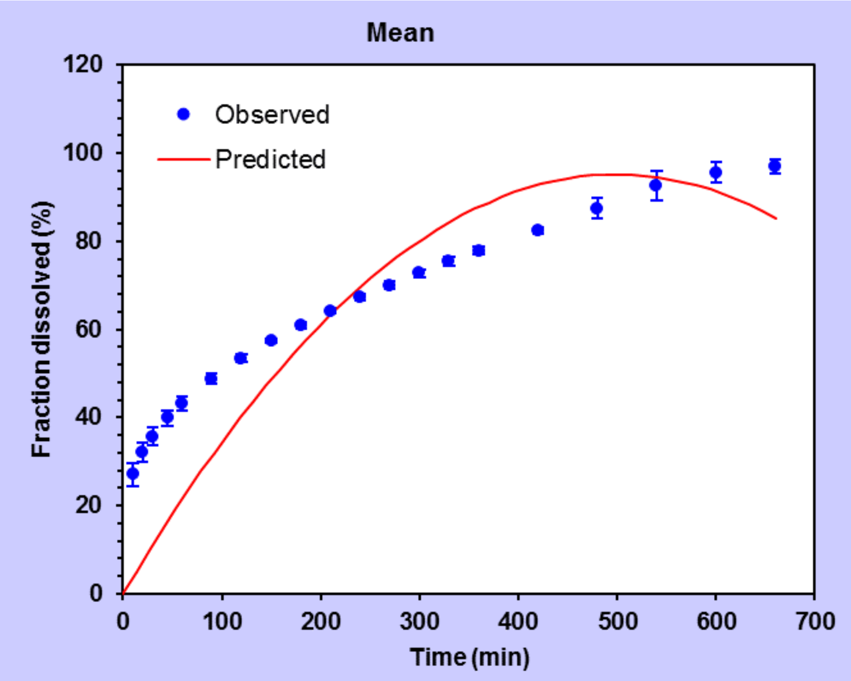

Supplementary materials – Model fitting summary – Tablettose® 70 (modelling the entire relevant dissolution data)

Graphical abstract of model fit presented as the fraction % of released carvedilol per tested tablet:

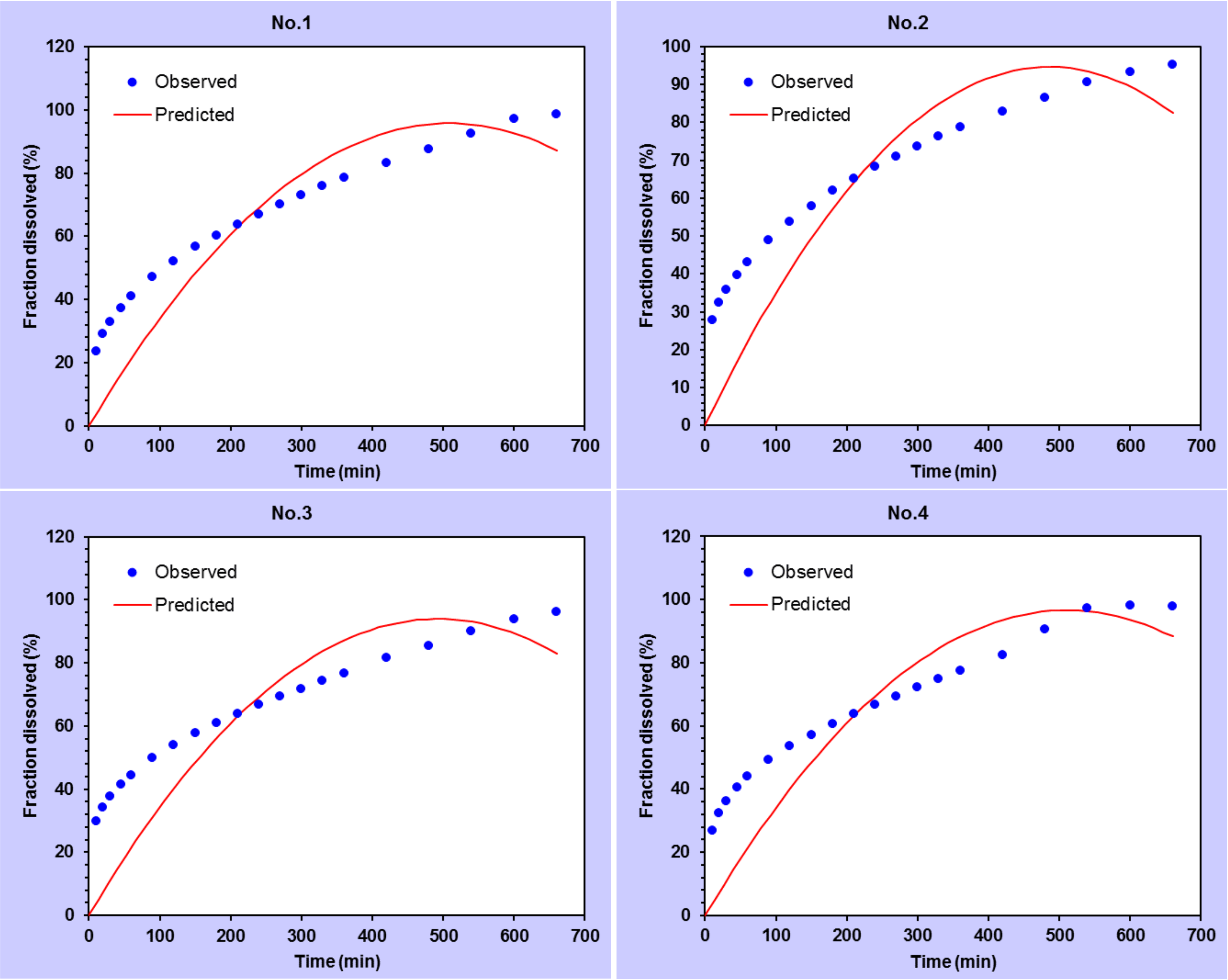

Supplementary materials – Model fitting summary – Tablettose® 70 (modelling the entire relevant dissolution data)

Model: **Quadratic with T_{lag}**

Model equation: $F = 100 \cdot \left[k_1 \cdot (t - T_{lag})^2 + k_2 \cdot (t - T_{lag})\right]$

Fitted model parameters per tested tablet (N = 4) with statistics – mean, standard deviation (SD), and relative standard deviation expressed in % (RSD%) (output from DDSolver):

Parameter	No.1	No.2	No.3	No.4	Mean	SD	RSD(%)
k_1	-0.000004	-0.000004	-0.000004	-0.000004	-0.000004	0.000000	-3.796541
k_2	0.003810	0.003939	0.003856	0.003828	0.003858	0.000057	1.480405
T_{lag}	4.000000	4.000000	4.000000	4.000000	4.000000	0.000000	0.000000

Number of dissolution data points (N), degrees of freedom (df), and selected goodness of fit criteria – Pearson correlation coefficient (R), coefficient of determination (R^2), adjusted coefficient of determination ($R^2_{adjusted}$), and residual sum of squares (RSS) (manual calculation in MS Excel):

Parameter	No.1	No.2	No.3	No.4
N	20	20	20	20
df	17	17	17	17
R	0.973555781	0.970913743	0.961202803	0.963956321
R^2	0.947810859	0.942673496	0.923910829	0.929211788
$R^2_{adjusted}$	0.94167096	0.935929201	0.914959162	0.920883764
RSS	3571.915418	4328.191159	5006.62771	4490.12623

Graphical abstract of model fit presented as mean ± 1 SD of the fraction % of released carvedilol:

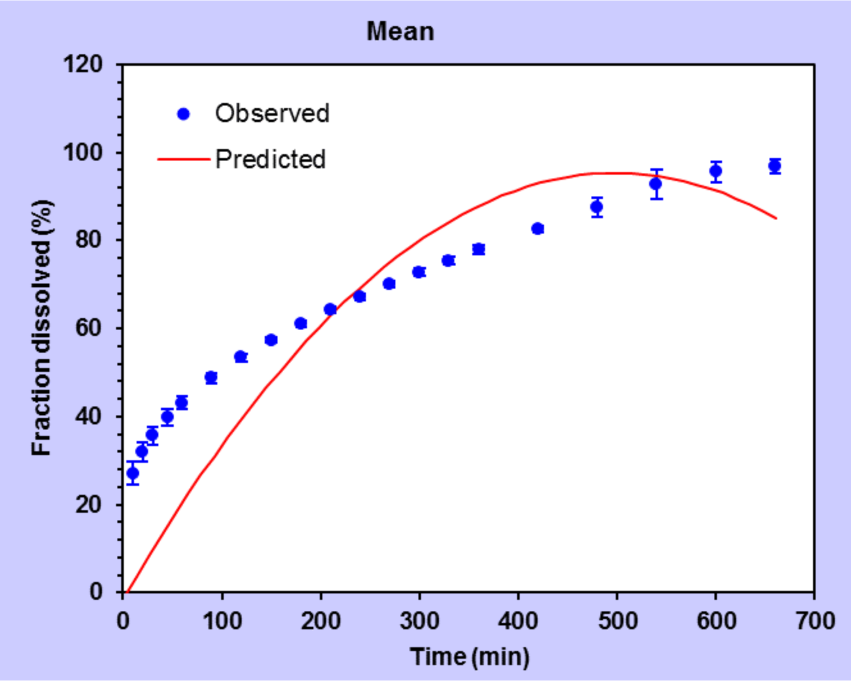

Supplementary materials – Model fitting summary – Tablettose® 70 (modelling the entire relevant dissolution data)

Graphical abstract of model fit presented as the fraction % of released carvedilol per tested tablet:

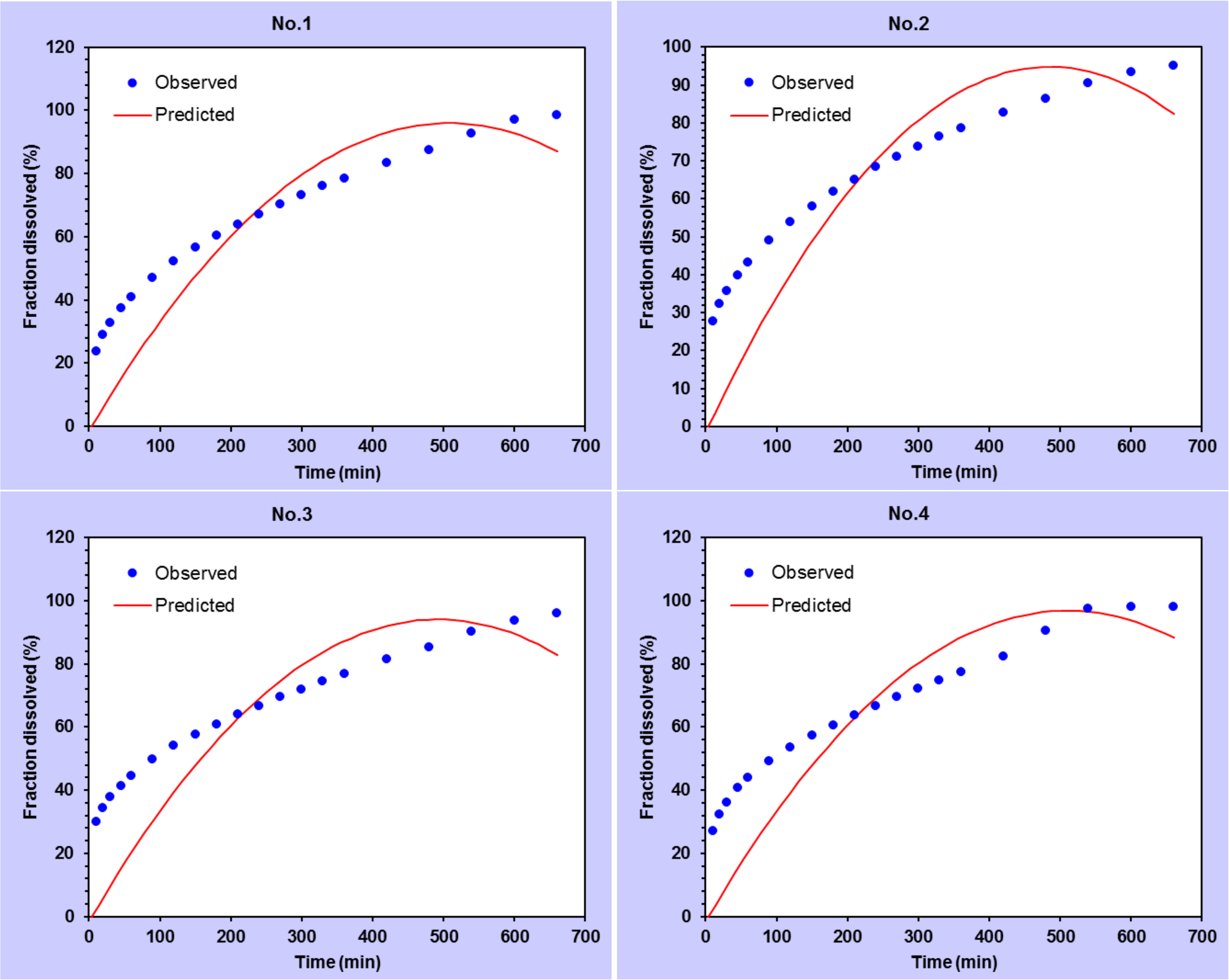

Supplementary materials – Model fitting summary – Tablettose® 70 (modelling the entire relevant dissolution data)

Model: **Weibull_1**

Model equation: $F = 100 \cdot \left[1 - e^{-\frac{(t-T_i)^\beta}{\alpha}}\right]$

Fitted model parameters per tested tablet (N = 4) with statistics – mean, standard deviation (SD), and relative standard deviation expressed in % (RSD%) (output from DDSolver):

Parameter	No.1	No.2	No.3	No.4	Mean	SD	RSD(%)
α	15.401	10.681	9.368	12.931	12.095	2.650	21.906
β	0.553	0.481	0.455	0.529	0.505	0.044	8.811
T_i	4.000	4.000	4.000	4.000	4.000	0.000	0.000

Number of dissolution data points (N), degrees of freedom (df), and selected goodness of fit criteria – Pearson correlation coefficient (R), coefficient of determination (R^2), adjusted coefficient of determination ($R^2_{adjusted}$), and residual sum of squares (RSS) (manual calculation in MS Excel):

Parameter	No.1	No.2	No.3	No.4
N	20	20	20	20
df	17	17	17	17
R	0.971463092	0.974353448	0.963847207	0.954718297
R^2	0.943740539	0.949364641	0.929001439	0.911487027
$R^2_{adjusted}$	0.937121779	0.94340754	0.920648667	0.901073737
RSS	622.6484333	444.6948982	569.6931577	919.406968

Graphical abstract of model fit presented as mean ± 1 SD of the fraction % of released carvedilol:

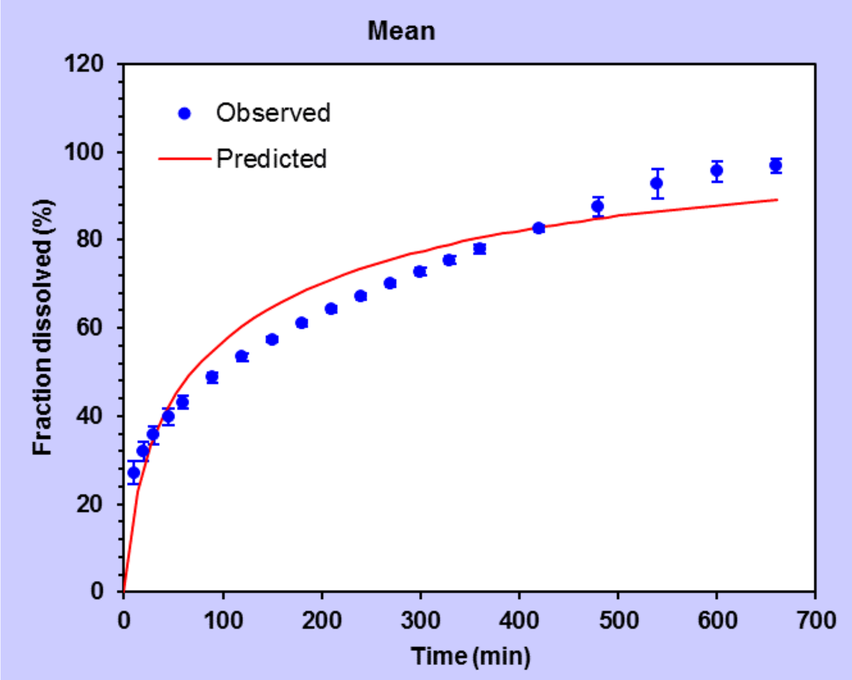

Supplementary materials – Model fitting summary – Tablettose® 70 (modelling the entire relevant dissolution data)

Graphical abstract of model fit presented as the fraction % of released carvedilol per tested tablet:

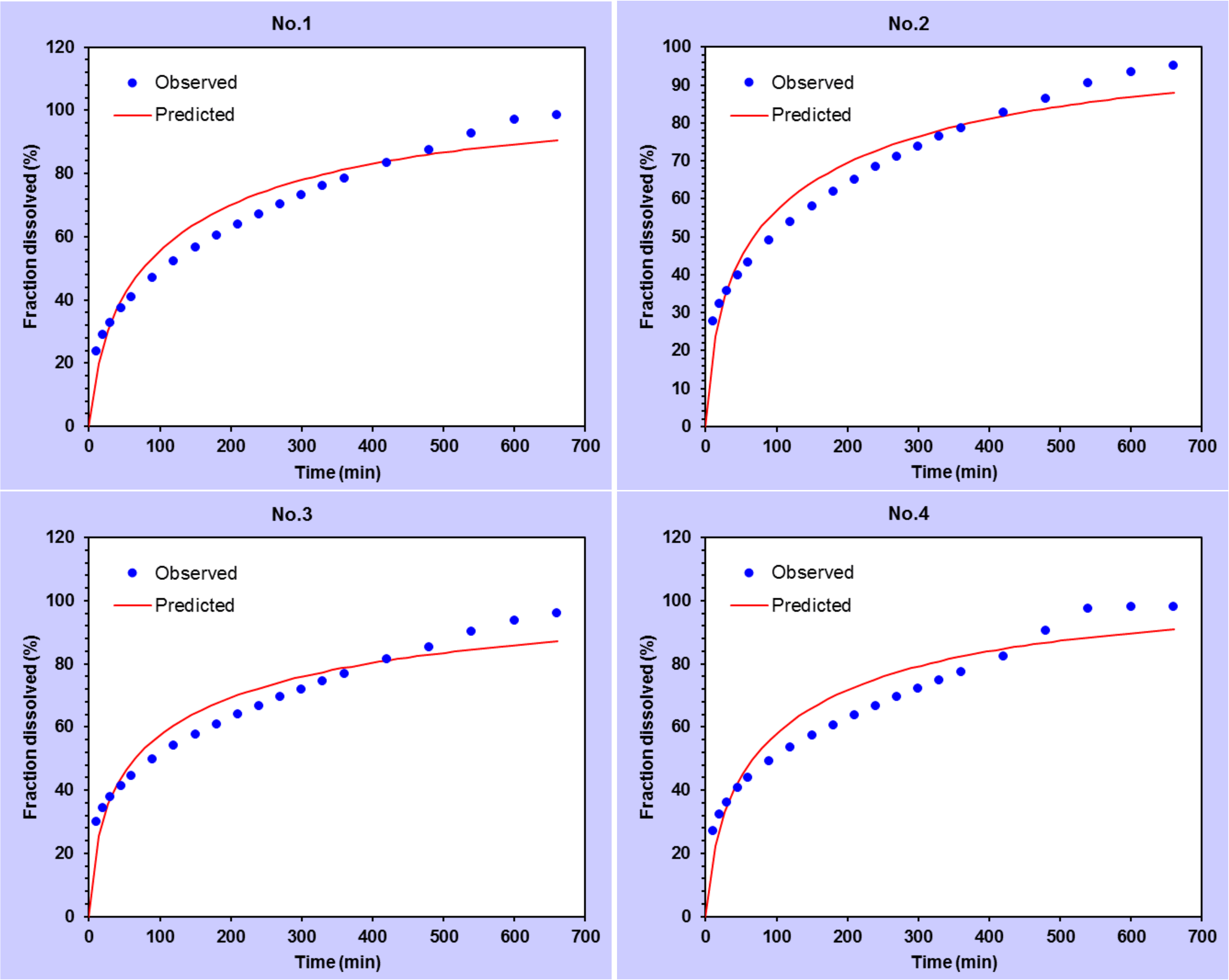

Supplementary materials – Model fitting summary – Tablettose® 70 (modelling the entire relevant dissolution data)

Model: **Weibull_2**

Model equation: $F = 100 \cdot \left(1 - e^{-\frac{t^{\beta}}{\alpha}}\right)$

Fitted model parameters per tested tablet (N = 4) with statistics – mean, standard deviation (SD), and relative standard deviation expressed in % (RSD%) (output from DDSolver):

Parameter	No.1	No.2	No.3	No.4	Mean	SD	RSD(%)
α	20.863	13.898	12.053	22.530	17.336	5.136	29.628
β	0.606	0.527	0.499	0.599	0.558	0.053	9.469

Number of dissolution data points (N), degrees of freedom (df), and selected goodness of fit criteria – Pearson correlation coefficient (R), coefficient of determination (R^2), adjusted coefficient of determination ($R^2_{adjusted}$), and residual sum of squares (RSS) (manual calculation in MS Excel):

Parameter	No.1	No.2	No.3	No.4
N	20	20	20	20
df	18	18	18	18
R	0.977253724	0.980963637	0.971289992	0.971482225
R^2	0.95502484	0.962289657	0.943404249	0.943777714
$R^2_{adjusted}$	0.95252622	0.960194638	0.940260041	0.940654254
RSS	502.9422207	330.7446	455.1715835	741.2276295

Graphical abstract of model fit presented as mean ± 1 SD of the fraction % of released carvedilol:

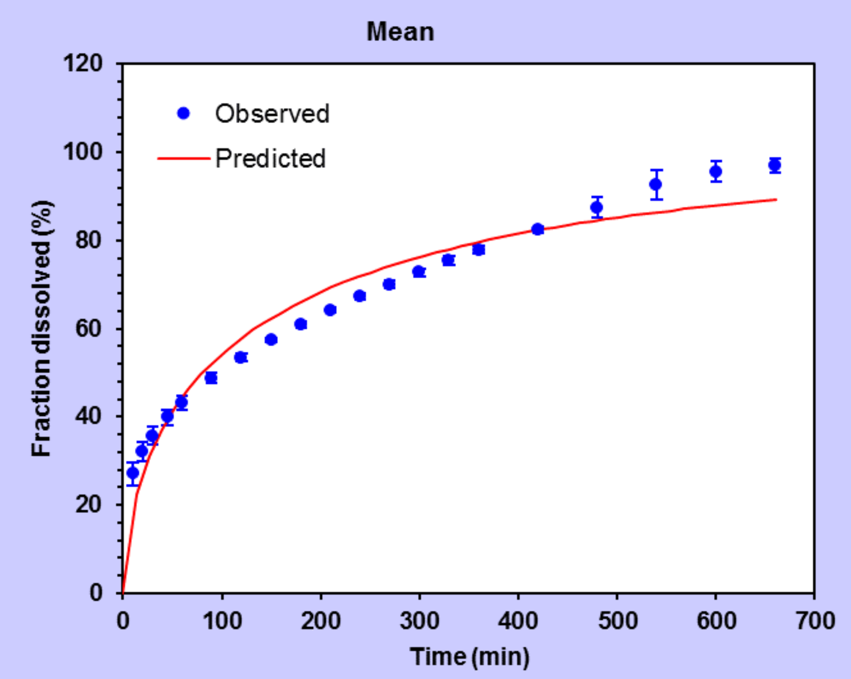

Supplementary materials – Model fitting summary – Tablettose® 70 (modelling the entire relevant dissolution data)

Graphical abstract of model fit presented as the fraction % of released carvedilol per tested tablet:

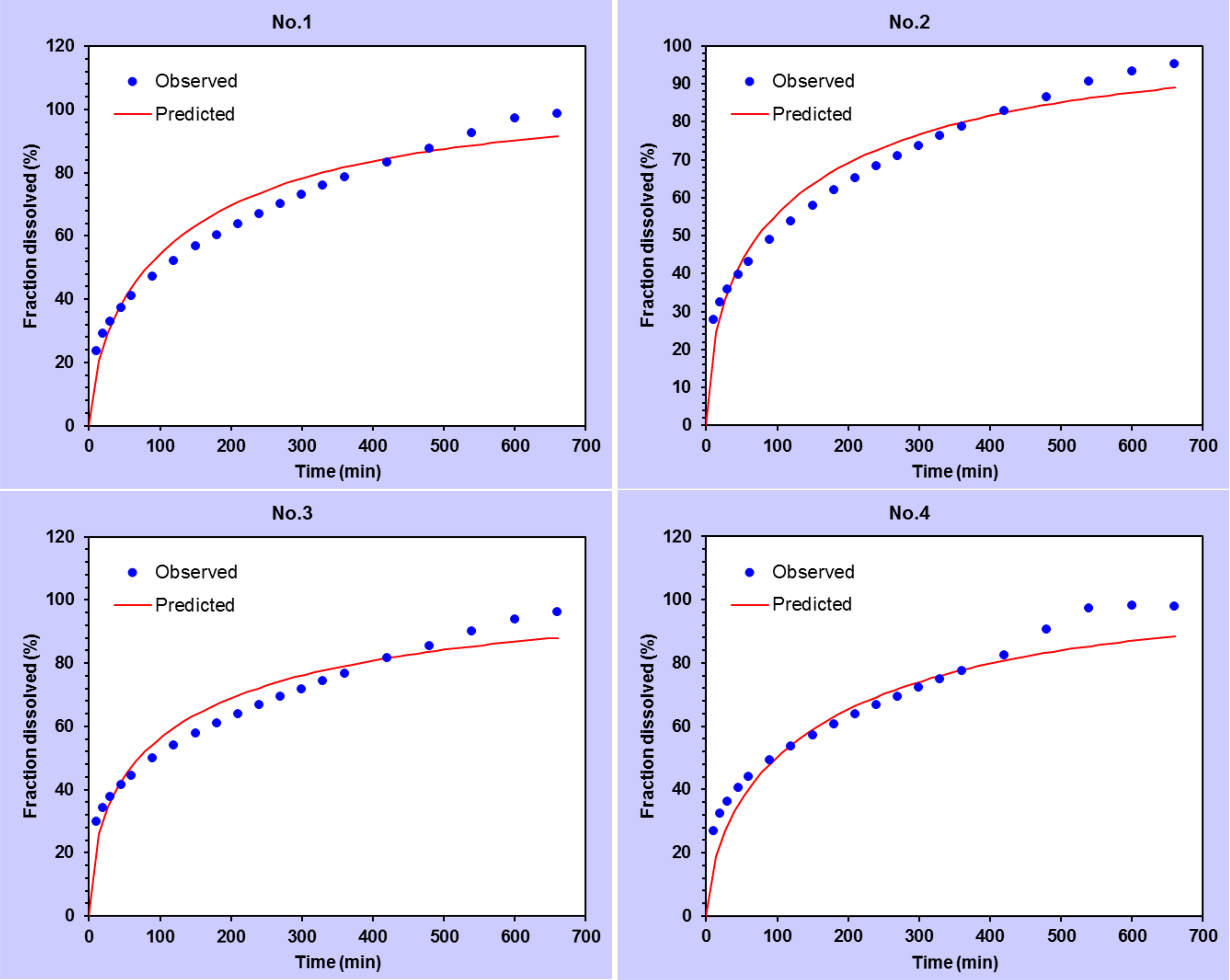

Supplementary materials – Model fitting summary – Tablettose® 70 (modelling the entire relevant dissolution data)

Model: **Weibull_3**

Model equation: $F = F_{max} \cdot \left(1 - e^{-\frac{t^{\beta}}{\alpha}}\right)$

Fitted model parameters per tested tablet (N = 4) with statistics – mean, standard deviation (SD), and relative standard deviation expressed in % (RSD%) (output from DDSolver):

Parameter	No.1	No.2	No.3	No.4	Mean	SD	RSD(%)
α	24.311	18.018	13.378	20.114	18.955	4.546	23.984
β	0.568	0.528	0.468	0.543	0.527	0.042	8.062
F_{max}	111.742	107.815	117.451	111.172	112.045	3.999	3.569

Number of dissolution data points (N), degrees of freedom (df), and selected goodness of fit criteria – Pearson correlation coefficient (R), coefficient of determination (R^2), adjusted coefficient of determination ($R^2_{adjusted}$), and residual sum of squares (RSS) (manual calculation in MS Excel):

Parameter	No.1	No.2	No.3	No.4
N	20	20	20	20
df	17	17	17	17
R	0.990094305	0.990082103	0.981743519	0.978204414
R^2	0.980286734	0.98026257	0.963820337	0.956883876
$R^2_{adjusted}$	0.977967526	0.97794052	0.959563906	0.951811391
RSS	372.2759101	329.7889829	408.6723909	541.7236222

Graphical abstract of model fit presented as mean ± 1 SD of the fraction % of released carvedilol:

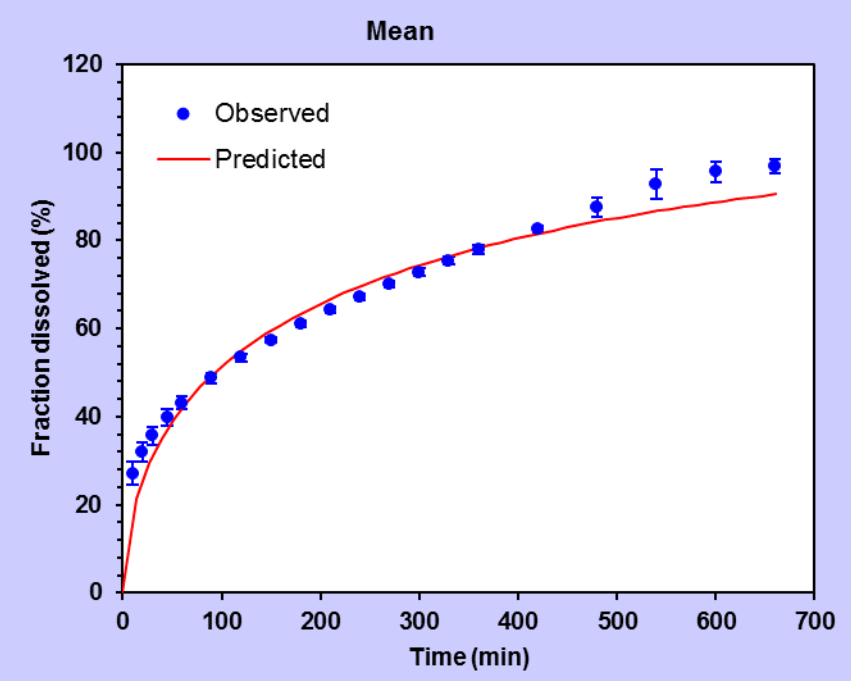

Supplementary materials – Model fitting summary – Tablettose® 70 (modelling the entire relevant dissolution data)

Graphical abstract of model fit presented as the fraction % of released carvedilol per tested tablet:

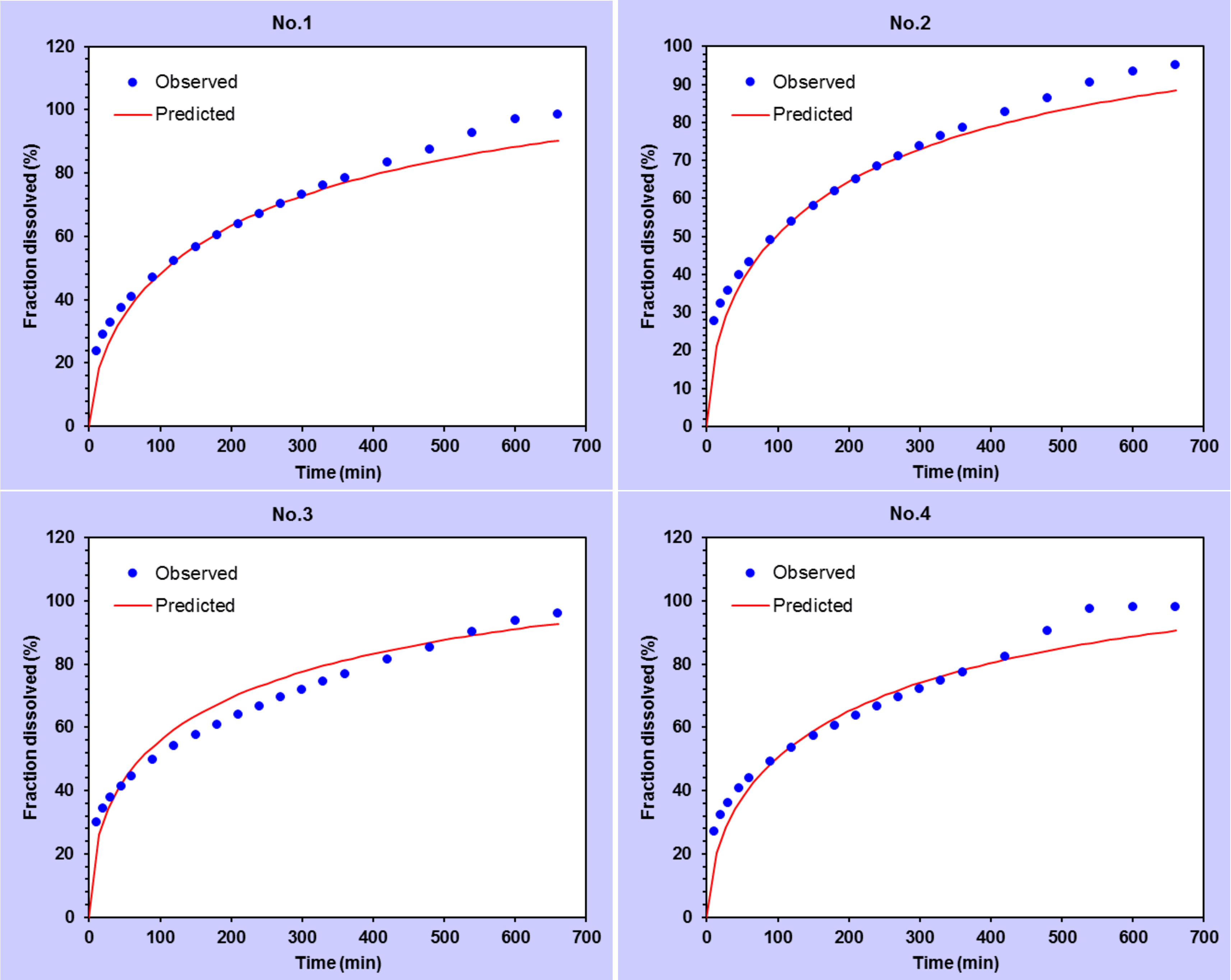

Supplementary materials – Model fitting summary – Tablettose® 70 (modelling the entire relevant dissolution data)

Model: **Weibull_4**

Model equation: $F = F_{max} \cdot \left[1 - e^{-\frac{(t-T_i)^{\beta}}{\alpha}}\right]$

Fitted model parameters per tested tablet (N = 4) with statistics – mean, standard deviation (SD), and relative standard deviation expressed in % (RSD%) (output from DDSolver):

Parameter	No.1	No.2	No.3	No.4	Mean	SD	RSD(%)
α	14.189	10.700	9.252	11.839	11.495	2.085	18.137
β	0.519	0.482	0.449	0.495	0.486	0.029	6.055
T_i	4.000	4.000	4.000	4.000	4.000	0.000	0.000
F_{max}	103.515	99.877	100.914	102.988	101.823	1.716	1.685

Number of dissolution data points (N), degrees of freedom (df), and selected goodness of fit criteria – Pearson correlation coefficient (R), coefficient of determination (R^2), adjusted coefficient of determination ($R^2_{adjusted}$), and residual sum of squares (RSS) (manual calculation in MS Excel):

Parameter	No.1	No.2	No.3	No.4
N	20	20	20	20
df	16	16	16	16
R	0.975662745	0.974223138	0.964908417	0.959734339
R^2	0.951917793	0.949110722	0.931048253	0.921090001
$R^2_{adjusted}$	0.942902379	0.939568983	0.9181198	0.906294376
RSS	510.2774592	447.1432427	550.5190379	775.4902839

Graphical abstract of model fit presented as mean ± 1 SD of the fraction % of released carvedilol:

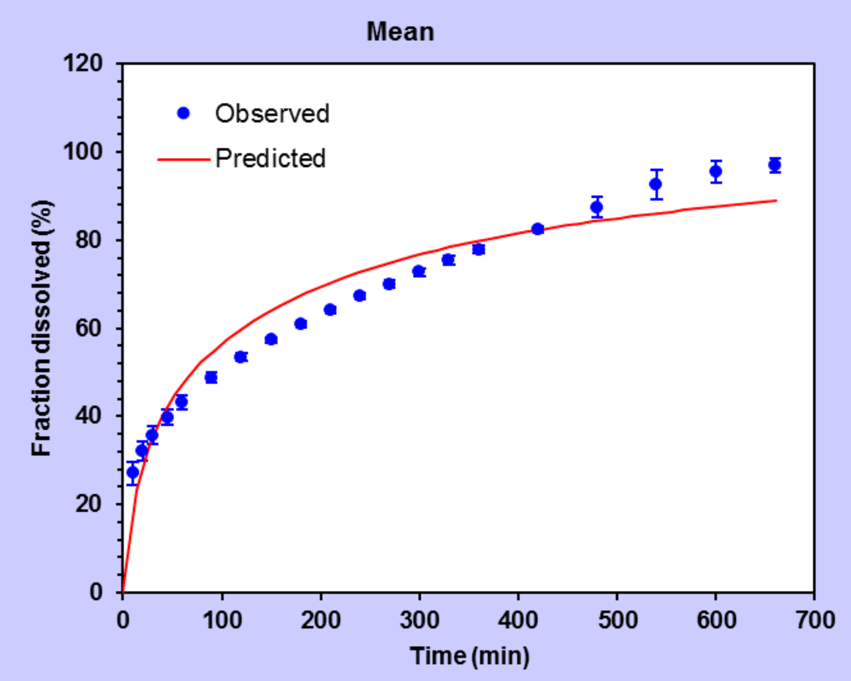

Supplementary materials – Model fitting summary – Tablettose® 70 (modelling the entire relevant dissolution data)

Graphical abstract of model fit presented as the fraction % of released carvedilol per tested tablet:

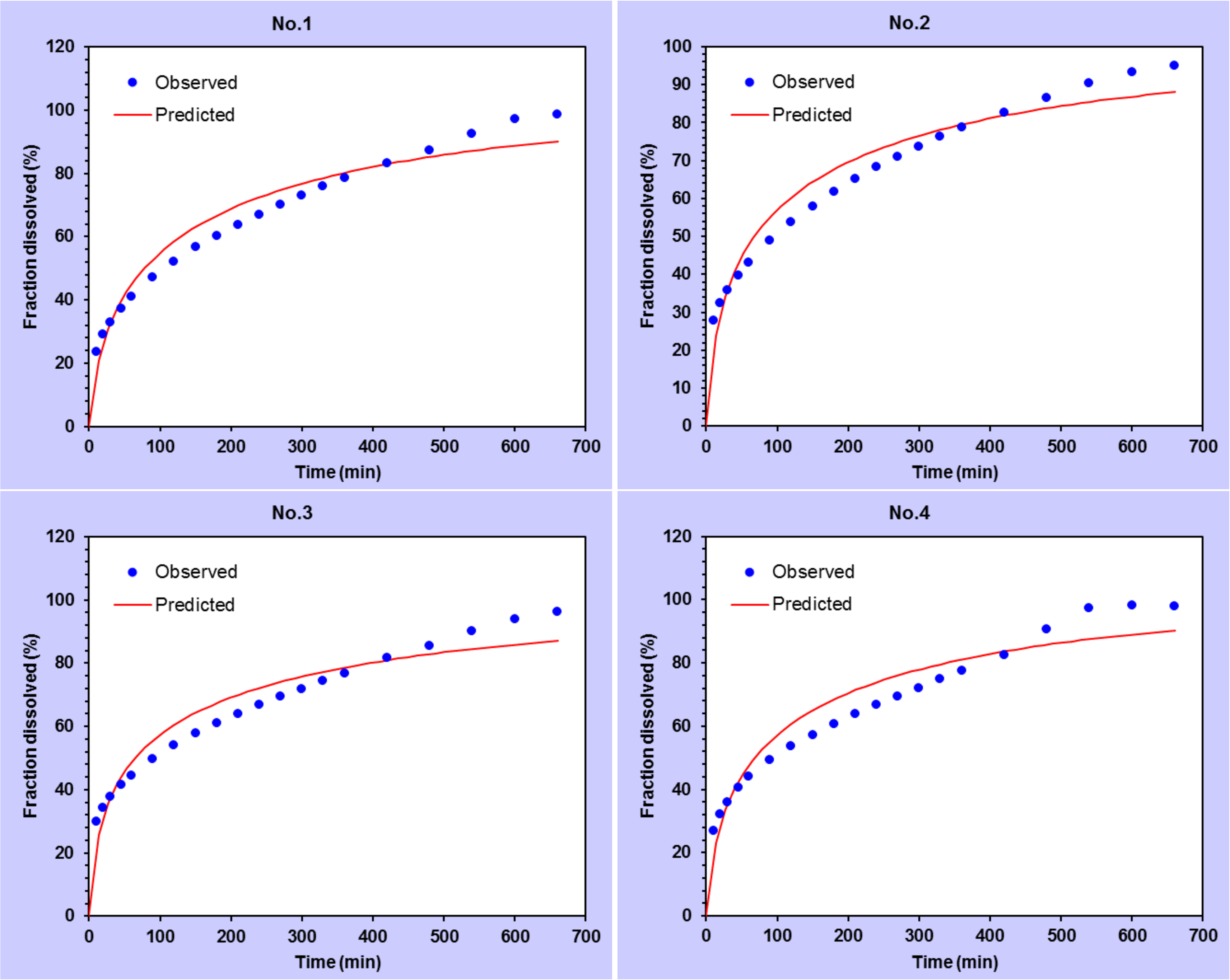

Supplementary materials – Model fitting summary – Tablettose® 70 (modelling the entire relevant dissolution data)

Model: **Logistic_1**

Model equation: $F = 100 \cdot \frac{e^{\alpha+\beta\cdot\log(t)}}{1+e^{\alpha+\beta\cdot\log(t)}}$

Fitted model parameters per tested tablet (N = 4) with statistics – mean, standard deviation (SD), and relative standard deviation expressed in % (RSD%) (output from DDSolver):

Parameter	No.1	No.2	No.3	No.4	Mean	SD	RSD(%)
α	-4.455	-3.634	-3.475	-4.369	-3.983	0.501	-12.567
β	2.399	1.995	1.921	2.407	2.181	0.259	11.870

Number of dissolution data points (N), degrees of freedom (df), and selected goodness of fit criteria – Pearson correlation coefficient (R), coefficient of determination (R^2), adjusted coefficient of determination ($R^2_{adjusted}$), and residual sum of squares (RSS) (manual calculation in MS Excel):

Parameter	No.1	No.2	No.3	No.4
N	20	20	20	20
df	18	18	18	18
R	0.949411655	0.958180097	0.945653712	0.927132741
R^2	0.901382491	0.918109097	0.894260944	0.85957512
$R^2_{adjusted}$	0.89590374	0.913559603	0.888386552	0.851773737
RSS	1348.463674	790.1504136	963.4760732	1876.300585

Graphical abstract of model fit presented as mean ± 1 SD of the fraction % of released carvedilol:

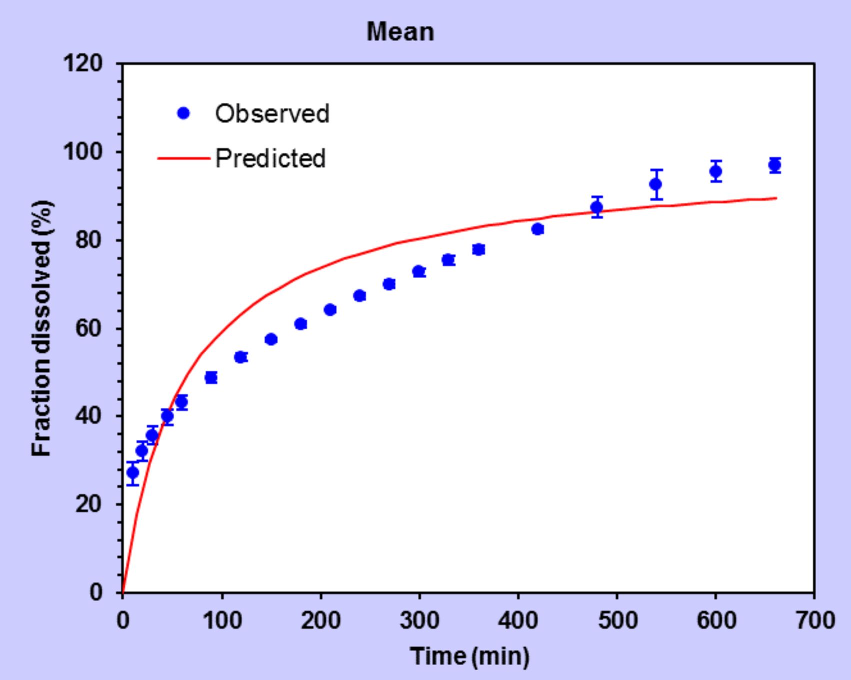

Supplementary materials – Model fitting summary – Tablettose® 70 (modelling the entire relevant dissolution data)

Graphical abstract of model fit presented as the fraction % of released carvedilol per tested tablet:

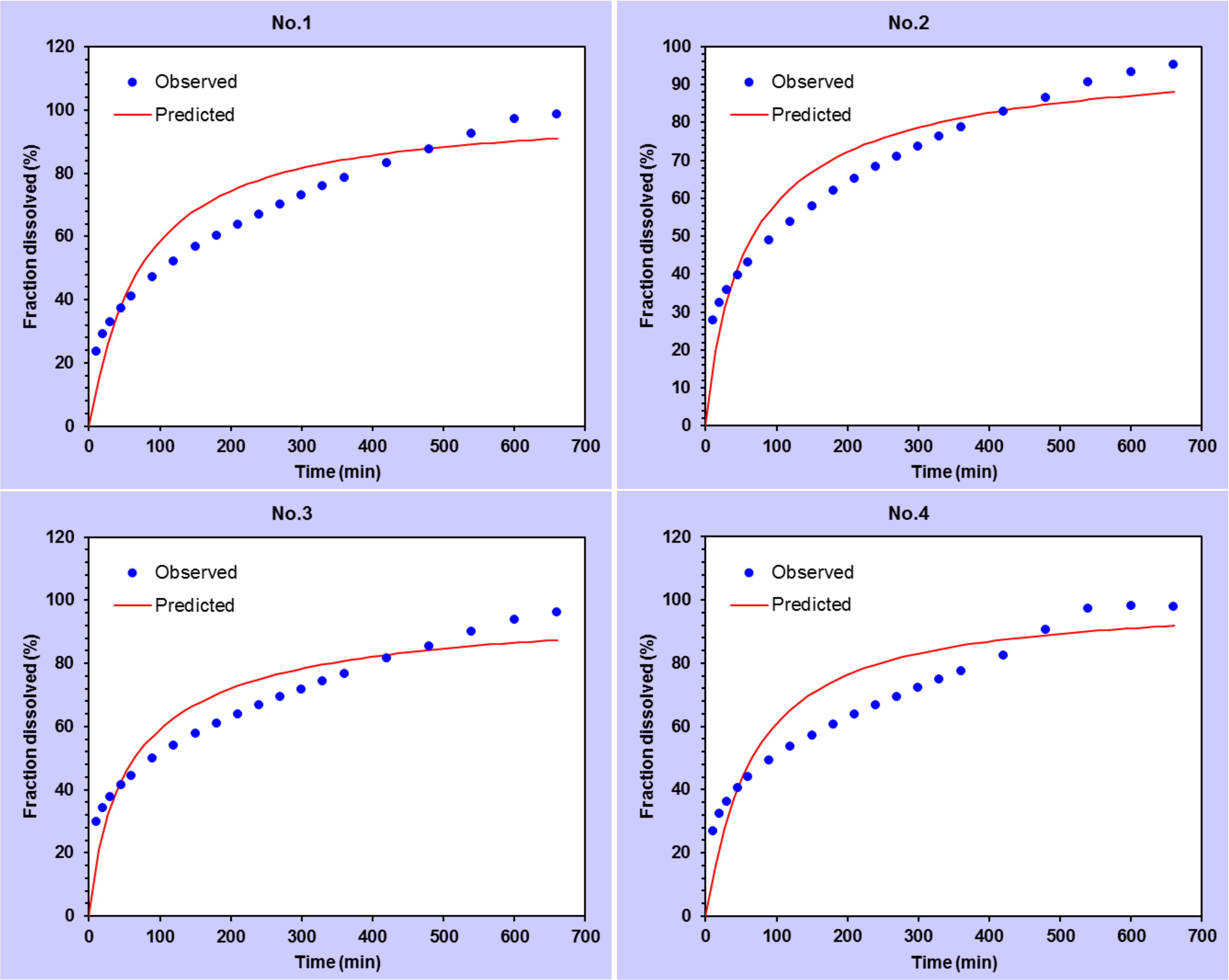

Supplementary materials – Model fitting summary – Tablettose® 70 (modelling the entire relevant dissolution data)

Model: **Logistic_2**

Model equation: $F = F_{max} \cdot \frac{e^{\alpha+\beta\cdot\log(t)}}{1+e^{\alpha+\beta\cdot\log(t)}}$

Fitted model parameters per tested tablet (N = 4) with statistics – mean, standard deviation (SD), and relative standard deviation expressed in % (RSD%) (output from DDSolver):

Parameter	No.1	No.2	No.3	No.4	Mean	SD	RSD(%)
α	-4.388	-3.646	-3.393	-3.857	-3.821	0.423	-11.076
β	1.885	2.002	1.864	2.081	1.958	0.102	5.209
F_{max}	128.502	99.877	100.914	102.988	108.070	13.682	12.660

Number of dissolution data points (N), degrees of freedom (df), and selected goodness of fit criteria – Pearson correlation coefficient (R), coefficient of determination (R^2), adjusted coefficient of determination ($R^2_{adjusted}$), and residual sum of squares (RSS) (manual calculation in MS Excel):

Parameter	No.1	No.2	No.3	No.4
N	20	20	20	20
df	17	17	17	17
R	0.989029805	0.957900783	0.947902881	0.939002192
R^2	0.978179955	0.917573909	0.898519872	0.881725117
$R^2_{adjusted}$	0.975612891	0.907876722	0.886581034	0.867810425
RSS	787.6879844	797.786404	900.4536728	1335.763409

Graphical abstract of model fit presented as mean ± 1 SD of the fraction % of released carvedilol:

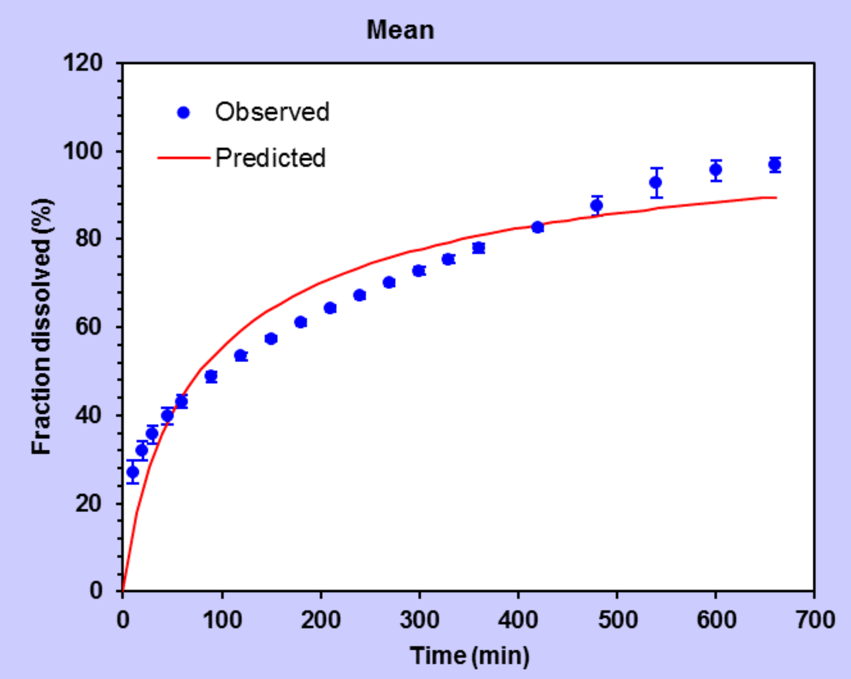

Supplementary materials – Model fitting summary – Tablettose® 70 (modelling the entire relevant dissolution data)

Graphical abstract of model fit presented as the fraction % of released carvedilol per tested tablet:

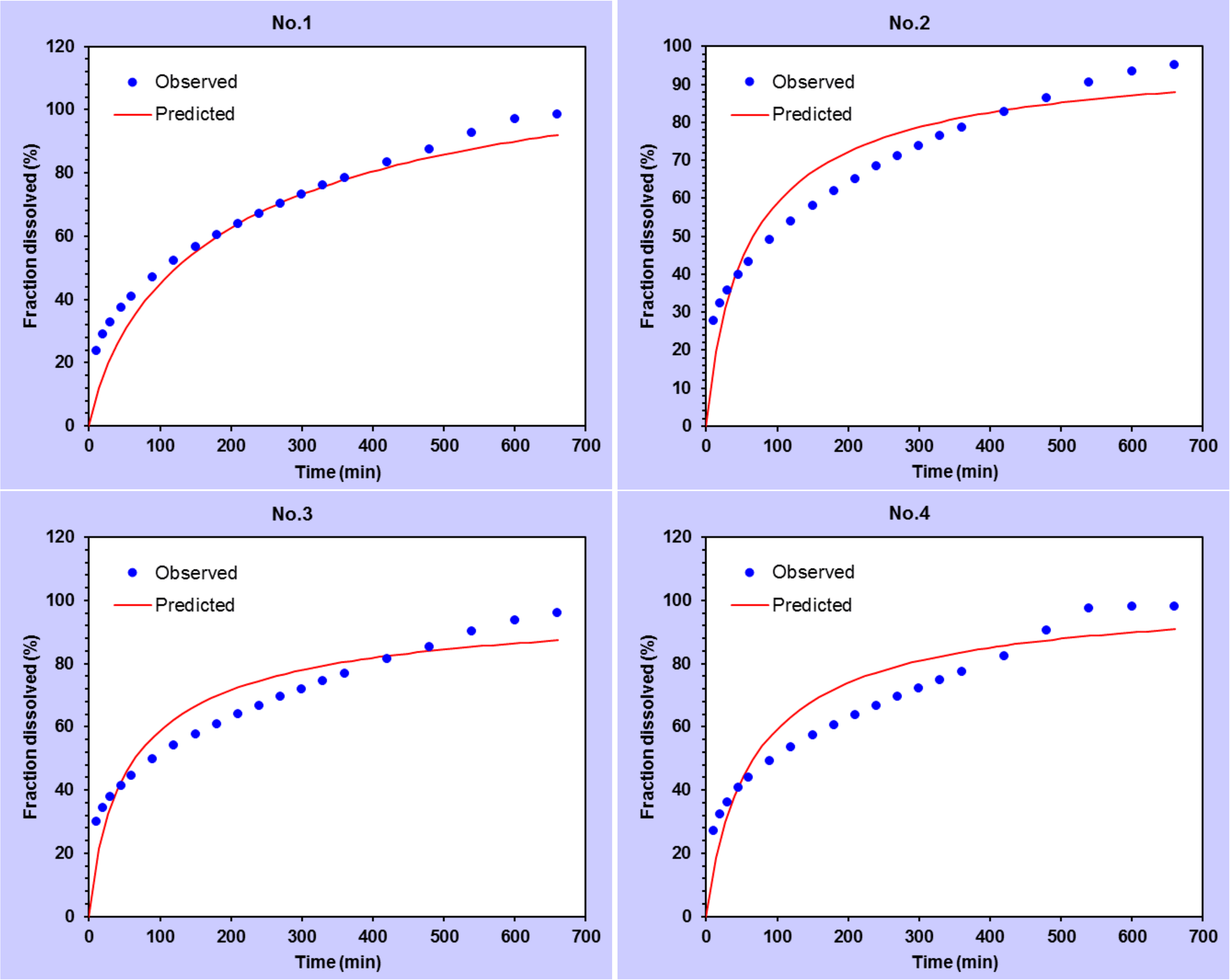

Supplementary materials – Model fitting summary – Tablettose® 70 (modelling the entire relevant dissolution data)

Model: **Logistic_3**

Model equation: $F = F_{max} \cdot \frac{1}{1+e^{-k\cdot(t-\gamma)}}$

Fitted model parameters per tested tablet (N = 4) with statistics – mean, standard deviation (SD), and relative standard deviation expressed in % (RSD%) (output from DDSolver):

Parameter	No.1	No.2	No.3	No.4	Mean	SD	RSD(%)
k	0.007	0.006	0.005	0.006	0.006	0.001	14.879
γ	121.805	118.631	121.238	134.875	124.137	7.290	5.873
F_{max}	97.405	99.877	100.914	102.988	100.296	2.321	2.314

Number of dissolution data points (N), degrees of freedom (df), and selected goodness of fit criteria – Pearson correlation coefficient (R), coefficient of determination (R^2), adjusted coefficient of determination ($R^2_{adjusted}$), and residual sum of squares (RSS) (manual calculation in MS Excel):

Parameter	No.1	No.2	No.3	No.4
N	20	20	20	20
df	17	17	17	17
R	0.990655918	0.991180929	0.99229416	0.989995368
R^2	0.981399148	0.982439634	0.984647699	0.980090828
$R^2_{adjusted}$	0.979210812	0.980373709	0.982841546	0.977748572
RSS	196.9879127	151.2731955	119.42848	195.4179184

Graphical abstract of model fit presented as mean ± 1 SD of the fraction % of released carvedilol:

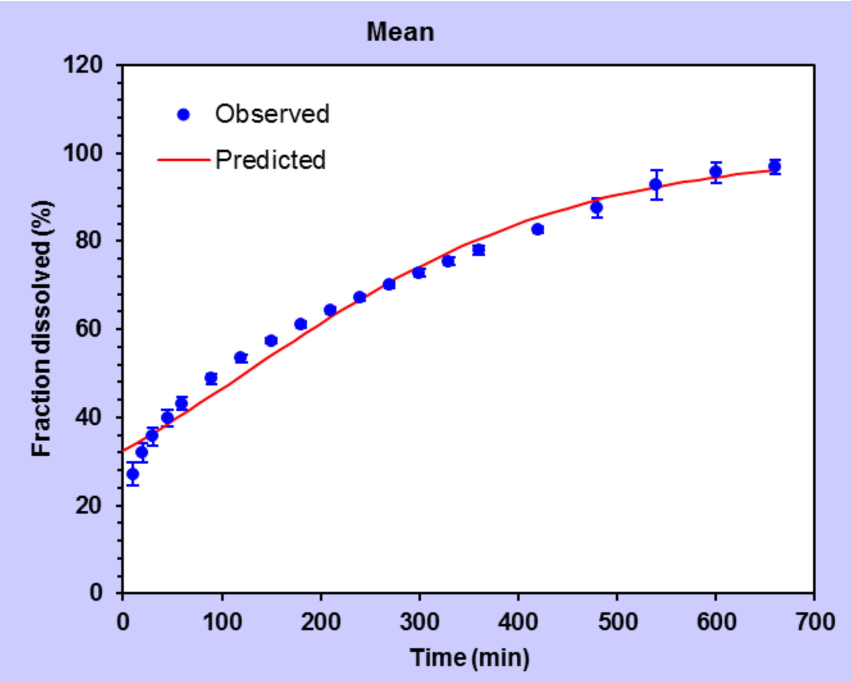

Supplementary materials – Model fitting summary – Tablettose® 70 (modelling the entire relevant dissolution data)

Graphical abstract of model fit presented as the fraction % of released carvedilol per tested tablet:

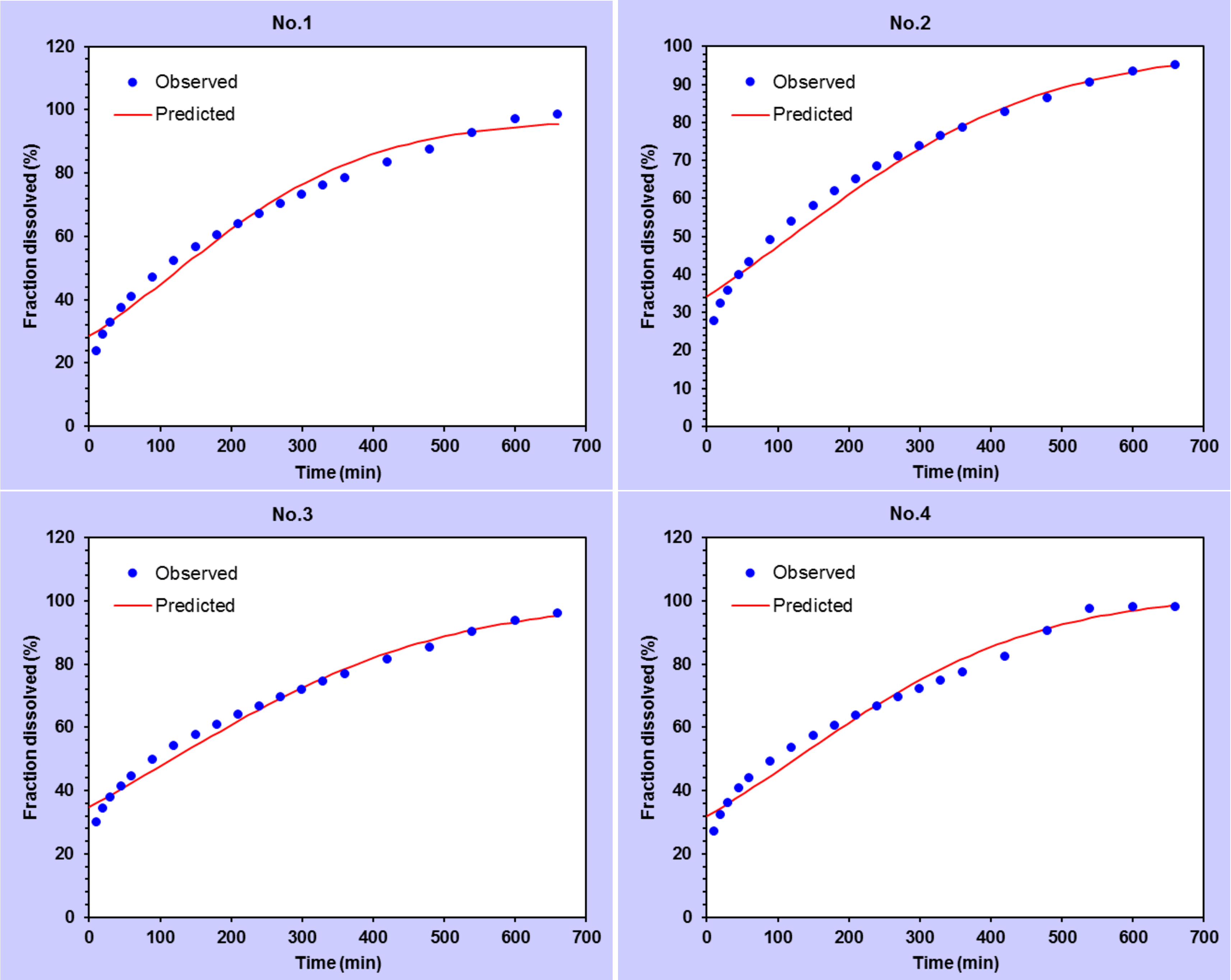

Supplementary materials – Model fitting summary – Tablettose® 70 (modelling the entire relevant dissolution data)

Model: **Gompertz_1**

Model equation: $F = 100 \cdot e^{-\alpha \cdot e^{-\beta \cdot \log(t)}}$

Fitted model parameters per tested tablet (N = 4) with statistics – mean, standard deviation (SD), and relative standard deviation expressed in % (RSD%) (output from DDSolver):

Parameter	No.1	No.2	No.3	No.4	Mean	SD	RSD(%)
α	24.274	12.230	11.150	37.554	21.302	12.360	58.022
β	1.953	1.602	1.558	2.006	1.780	0.232	13.050

Number of dissolution data points (N), degrees of freedom (df), and selected goodness of fit criteria – Pearson correlation coefficient (R), coefficient of determination (R^2), adjusted coefficient of determination ($R^2_{adjusted}$), and residual sum of squares (RSS) (manual calculation in MS Excel):

Parameter	No.1	No.2	No.3	No.4
N	20	20	20	20
df	18	18	18	18
R	0.925478953	0.935263201	0.921685648	0.933369489
R^2	0.856511293	0.874717256	0.849504435	0.871178604
$R^2_{adjusted}$	0.848539698	0.867757103	0.84114357	0.864021859
RSS	2189.982712	1345.694985	1549.128606	2793.236204

Graphical abstract of model fit presented as mean ± 1 SD of the fraction % of released carvedilol:

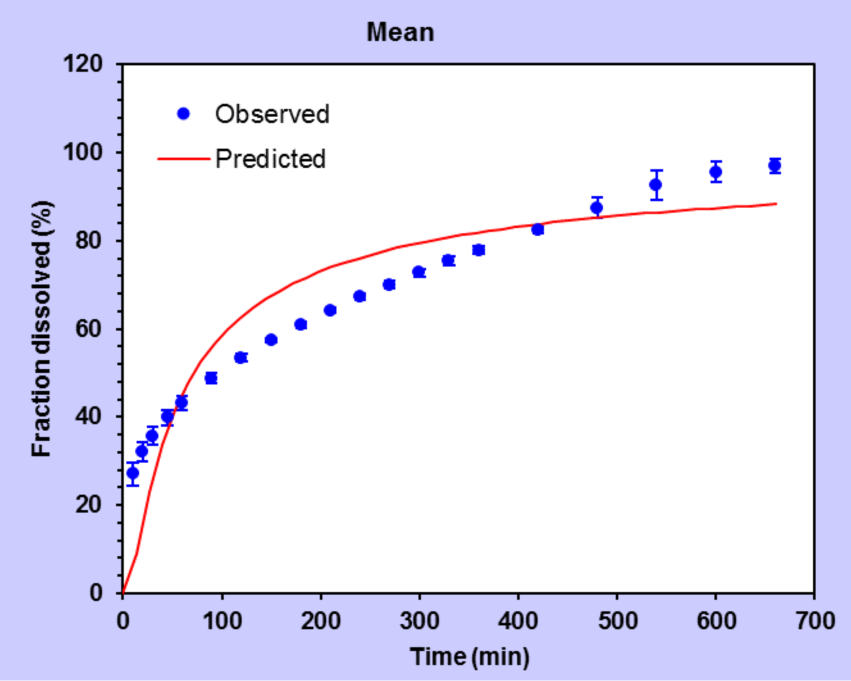

Supplementary materials – Model fitting summary – Tablettose® 70 (modelling the entire relevant dissolution data)

Graphical abstract of model fit presented as the fraction % of released carvedilol per tested tablet:

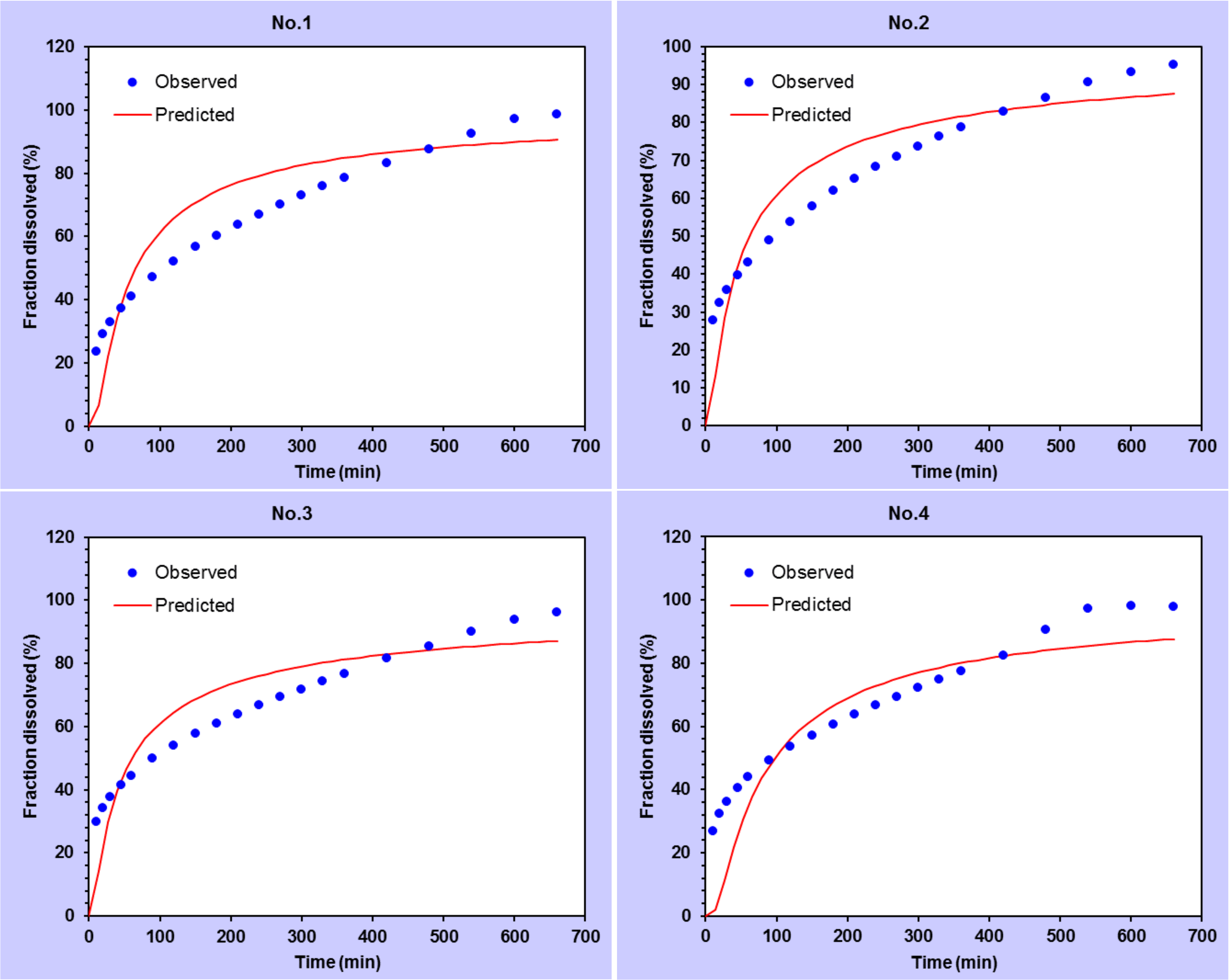

Supplementary materials – Model fitting summary – Tablettose® 70 (modelling the entire relevant dissolution data)

Model: **Gompertz_2**

Model equation: $F = F_{max} \cdot e^{-\alpha \cdot e^{-\beta \cdot \log(t)}}$

Fitted model parameters per tested tablet (N = 4) with statistics – mean, standard deviation (SD), and relative standard deviation expressed in % (RSD%) (output from DDSolver):

Parameter	No.1	No.2	No.3	No.4	Mean	SD	RSD(%)
α	14.551	12.377	10.210	14.708	12.961	2.121	16.362
β	1.634	1.610	1.500	1.678	1.606	0.076	4.714
F_{max}	103.515	99.877	100.914	102.988	101.823	1.716	1.685

Number of dissolution data points (N), degrees of freedom (df), and selected goodness of fit criteria – Pearson correlation coefficient (R), coefficient of determination (R^2), adjusted coefficient of determination ($R^2_{adjusted}$), and residual sum of squares (RSS) (manual calculation in MS Excel):

Parameter	No.1	No.2	No.3	No.4
N	20	20	20	20
df	17	17	17	17
R	0.936823371	0.934924085	0.924392714	0.914464063
R^2	0.877638029	0.874083045	0.85450189	0.836244523
$R^2_{adjusted}$	0.863242503	0.859269286	0.837384465	0.816979173
RSS	1551.684177	1357.968132	1447.424284	2073.180068

Graphical abstract of model fit presented as mean ± 1 SD of the fraction % of released carvedilol:

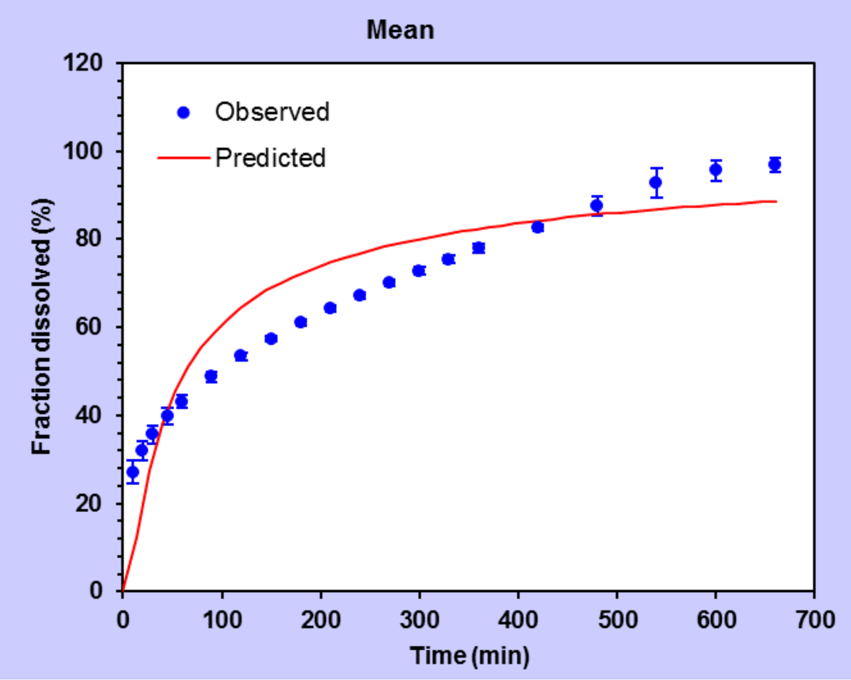

Supplementary materials – Model fitting summary – Tablettose® 70 (modelling the entire relevant dissolution data)

Graphical abstract of model fit presented as the fraction % of released carvedilol per tested tablet:

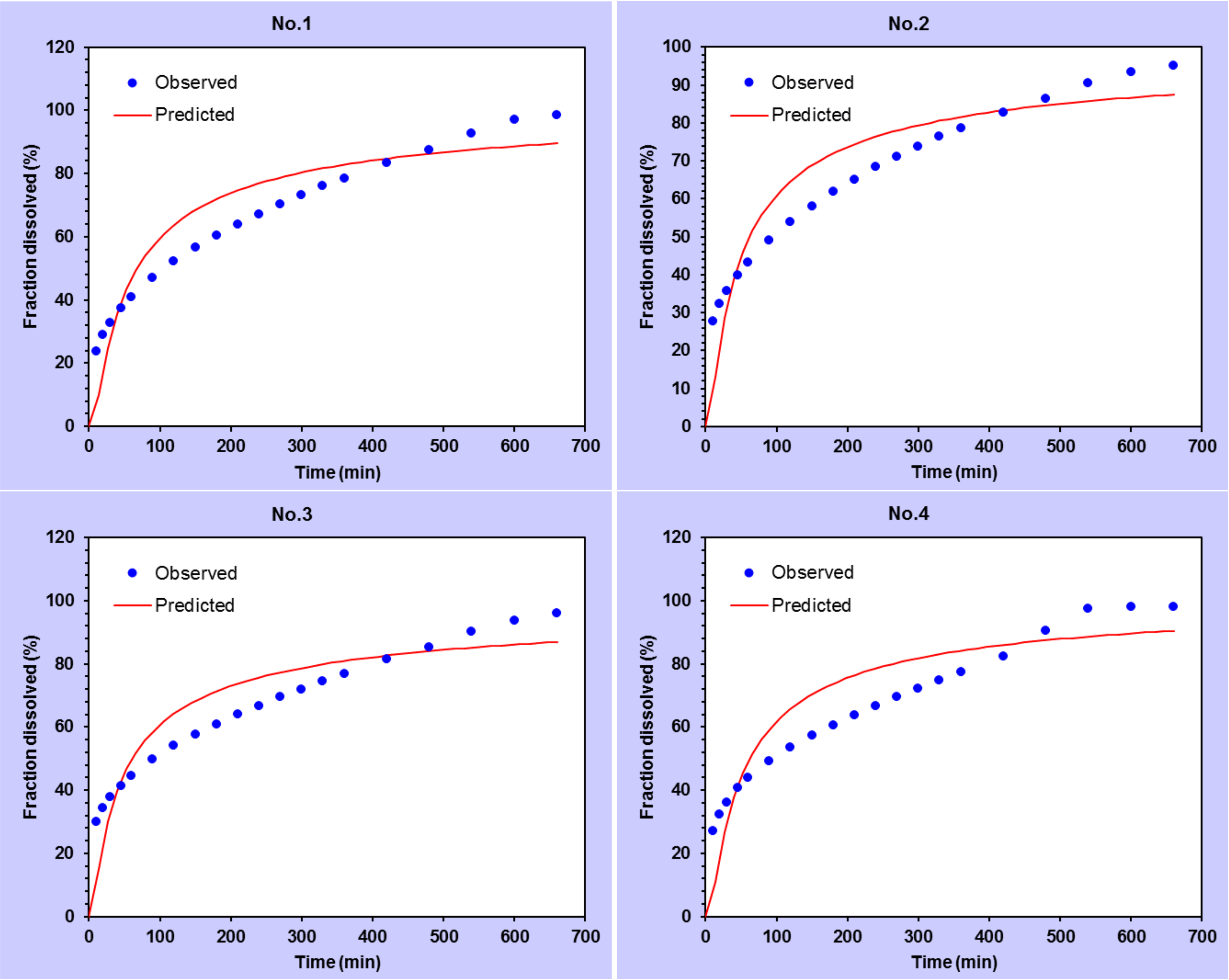

Supplementary materials – Model fitting summary – Tablettose® 70 (modelling the entire relevant dissolution data)

Model: **Gompertz_3**

Model equation: $F = F_{max} \cdot e^{-e^{-k\cdot(t-\gamma)}}$

Fitted model parameters per tested tablet (N = 4) with statistics – mean, standard deviation (SD), and relative standard deviation expressed in % (RSD%) (output from DDSolver):

Parameter	No.1	No.2	No.3	No.4	Mean	SD	RSD(%)
k	0.005	0.005	0.004	0.005	0.005	0.000	5.376
γ	62.080	33.902	33.453	55.300	46.184	14.705	31.840
F_{max}	103.515	99.877	100.914	102.988	101.823	1.716	1.685

Number of dissolution data points (N), degrees of freedom (df), and selected goodness of fit criteria – Pearson correlation coefficient (R), coefficient of determination (R^2), adjusted coefficient of determination ($R^2_{adjusted}$), and residual sum of squares (RSS) (manual calculation in MS Excel):

Parameter	No.1	No.2	No.3	No.4
N	20	20	20	20
df	17	17	17	17
R	0.99422406	0.995921187	0.994817193	0.989877579
R^2	0.988481482	0.991859011	0.989661247	0.979857622
$R^2_{adjusted}$	0.987126362	0.990901248	0.988444923	0.97748793
RSS	124.8069007	69.04204301	91.99802077	242.6636317

Graphical abstract of model fit presented as mean ± 1 SD of the fraction % of released carvedilol:

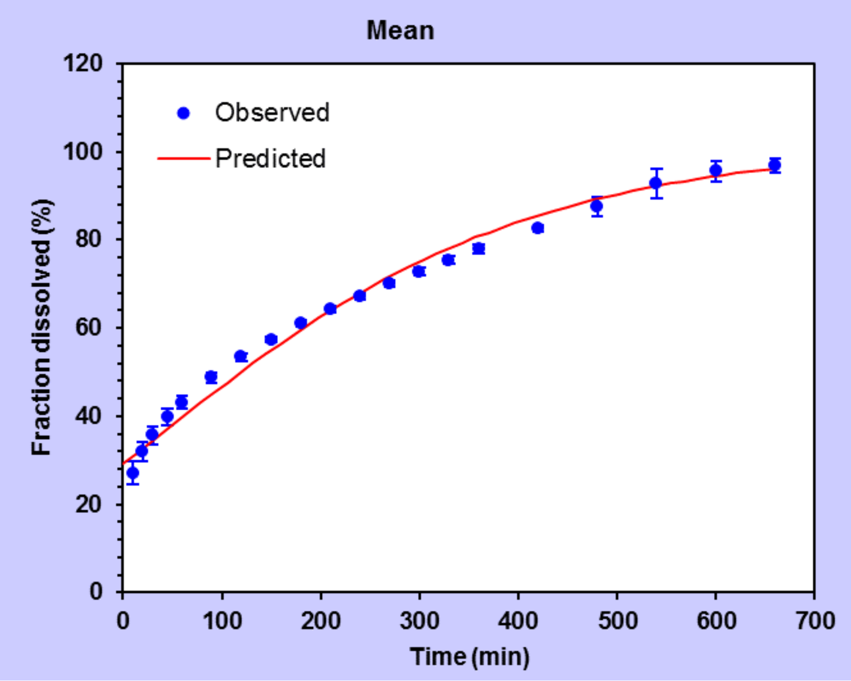

Supplementary materials – Model fitting summary – Tablettose® 70 (modelling the entire relevant dissolution data)

Graphical abstract of model fit presented as the fraction % of released carvedilol per tested tablet:

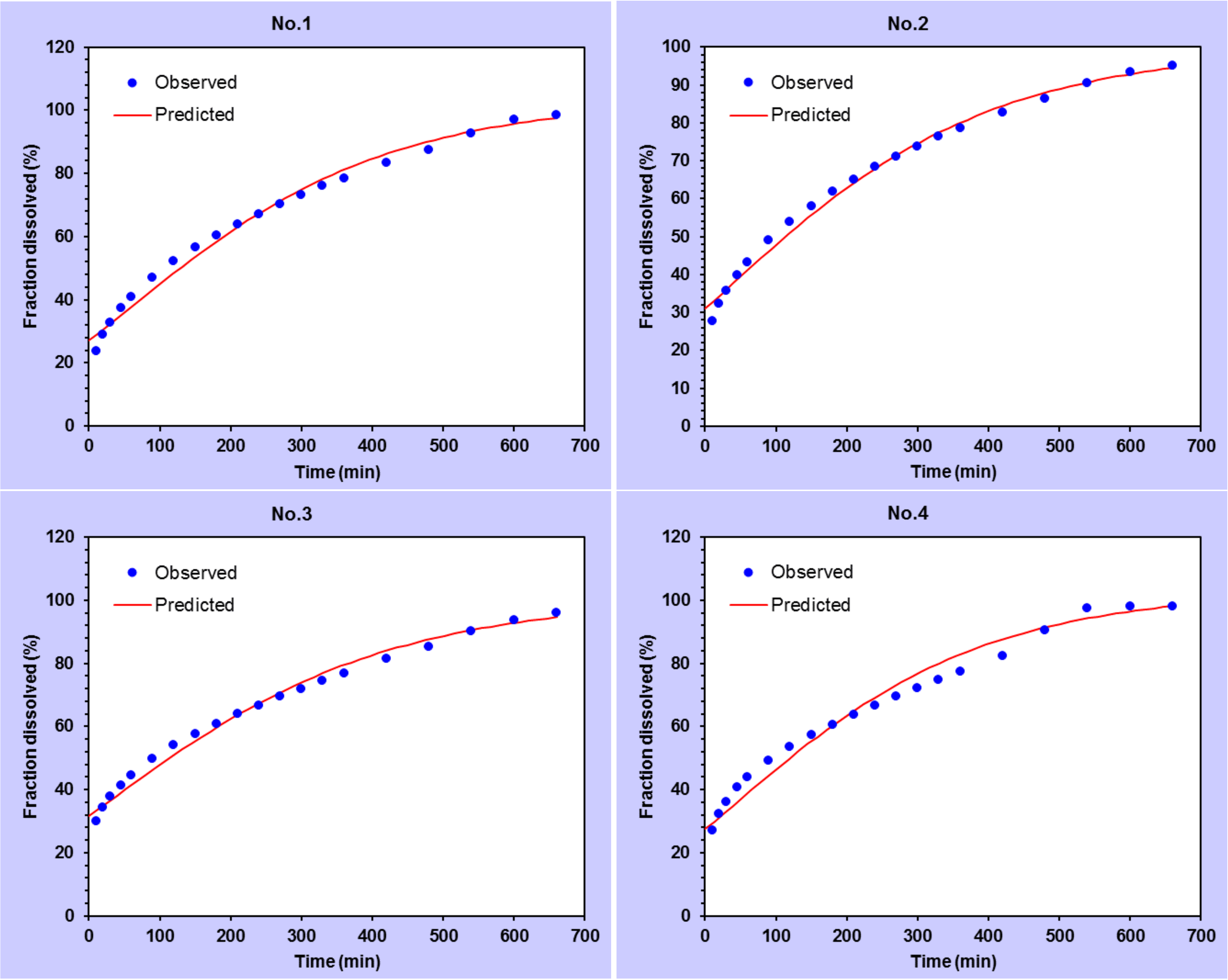

Supplementary materials – Model fitting summary – Tablettose® 70 (modelling the entire relevant dissolution data)

Model: **Gompertz_4**

Model equation: $F = F_{max} \cdot e^{-\beta \cdot e^{-k \cdot t}}$

Fitted model parameters per tested tablet (N = 4) with statistics – mean, standard deviation (SD), and relative standard deviation expressed in % (RSD%) (output from DDSolver):

Parameter	No.1	No.2	No.3	No.4	Mean	SD	RSD(%)
k	0.005	0.005	0.004	0.005	0.005	0.000	5.376
β	1.342	1.170	1.158	1.318	1.247	0.096	7.736
F_{max}	103.515	99.877	100.914	102.988	101.823	1.716	1.685

Number of dissolution data points (N), degrees of freedom (df), and selected goodness of fit criteria – Pearson correlation coefficient (R), coefficient of determination (R^2), adjusted coefficient of determination ($R^2_{adjusted}$), and residual sum of squares (RSS) (manual calculation in MS Excel):

Parameter	No.1	No.2	No.3	No.4
N	20	20	20	20
df	17	17	17	17
R	0.99422406	0.995921187	0.994817193	0.989877579
R^2	0.988481482	0.991859011	0.989661247	0.979857622
$R^2_{adjusted}$	0.987126362	0.990901248	0.988444923	0.97748793
RSS	124.8069007	69.04204301	91.99802077	242.6636317

Graphical abstract of model fit presented as mean ± 1 SD of the fraction % of released carvedilol:

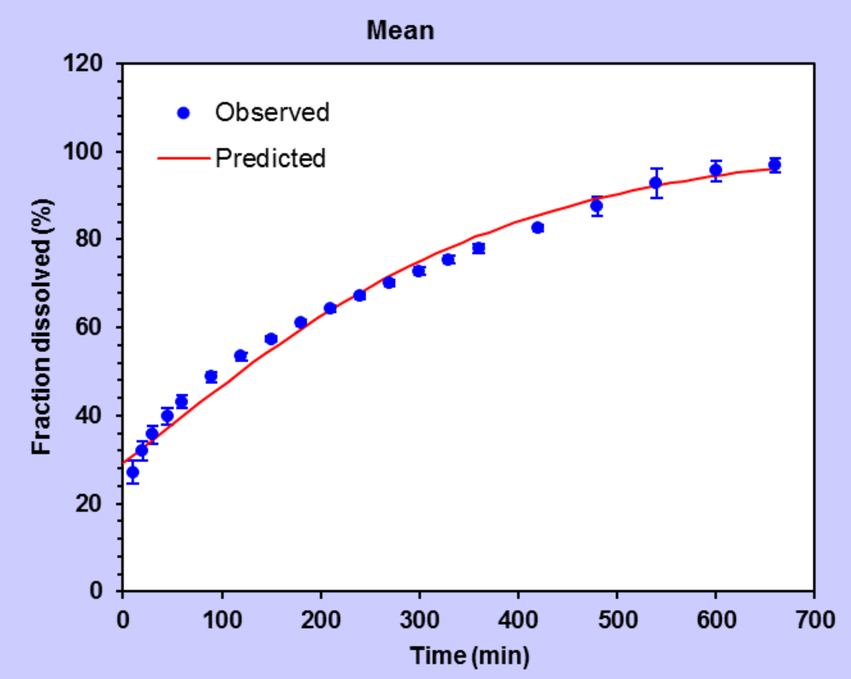

Supplementary materials – Model fitting summary – Tablettose® 70 (modelling the entire relevant dissolution data)

Graphical abstract of model fit presented as the fraction % of released carvedilol per tested tablet:

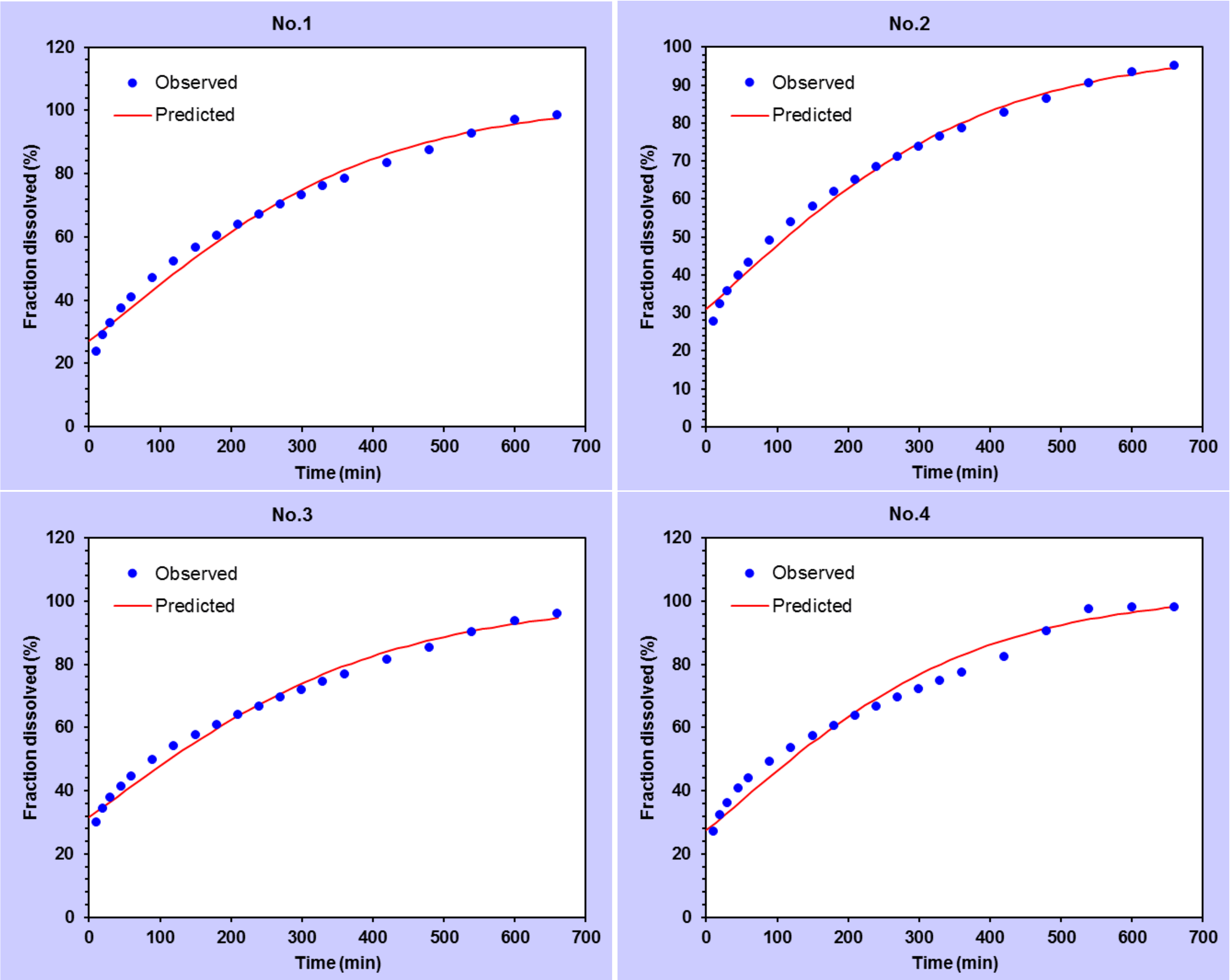

Supplementary materials – Model fitting summary – Tablettose® 70 (modelling the entire relevant dissolution data)

Model: **Probit_1**

Model equation: $F = 100 \cdot \phi[\alpha + \beta \cdot \log(t)]$

Fitted model parameters per tested tablet (N = 4) with statistics – mean, standard deviation (SD), and relative standard deviation expressed in % (RSD%) (output from DDSolver):

Parameter	No.1	No.2	No.3	No.4	Mean	SD	RSD(%)
α	-2.570	-2.158	-2.053	-2.486	-2.316	0.250	-10.801
β	1.379	1.182	1.133	1.365	1.264	0.126	9.930

Number of dissolution data points (N), degrees of freedom (df), and selected goodness of fit criteria – Pearson correlation coefficient (R), coefficient of determination (R^2), adjusted coefficient of determination ($R^2_{adjusted}$), and residual sum of squares (RSS) (manual calculation in MS Excel):

Parameter	No.1	No.2	No.3	No.4
N	20	20	20	20
df	18	18	18	18
R	0.955840639	0.962377092	0.950770913	0.936327216
R^2	0.913631327	0.926169668	0.903965329	0.876708656
$R^2_{adjusted}$	0.908833068	0.922067982	0.89863007	0.869859137
RSS	1065.03521	679.2510857	825.7060866	1471.620998

Graphical abstract of model fit presented as mean ± 1 SD of the fraction % of released carvedilol:

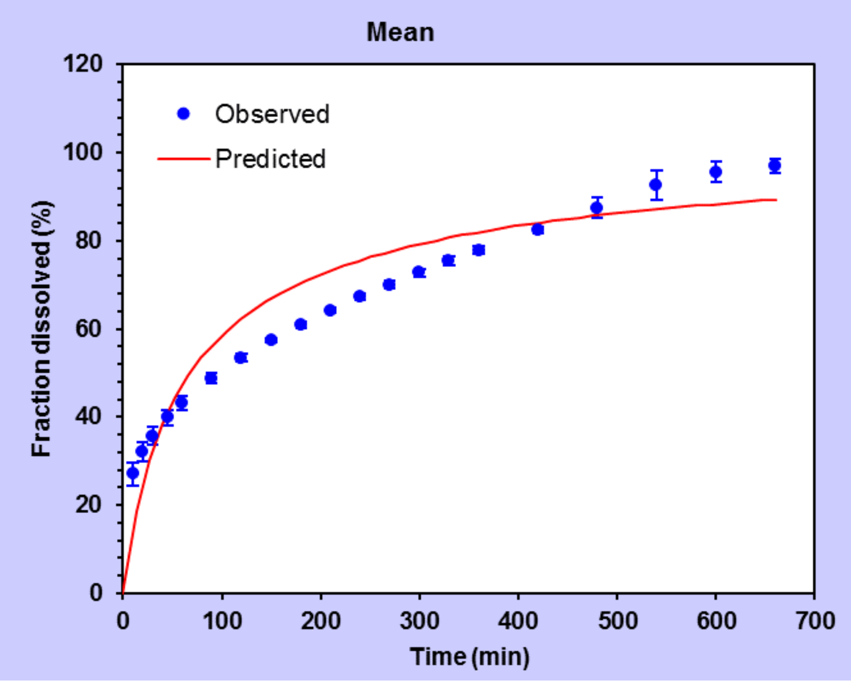

Supplementary materials – Model fitting summary – Tablettose® 70 (modelling the entire relevant dissolution data)

Graphical abstract of model fit presented as the fraction % of released carvedilol per tested tablet:

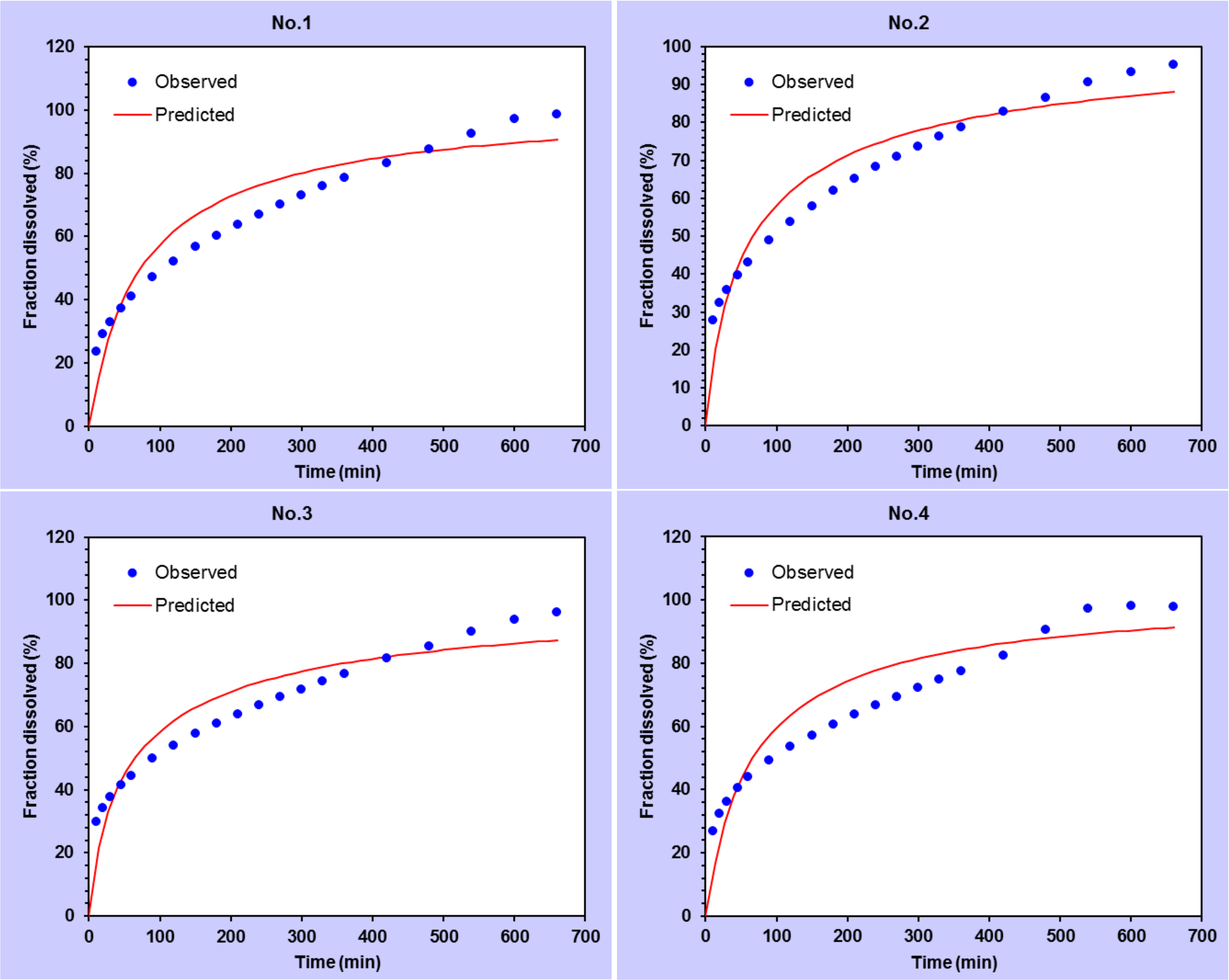

Supplementary materials – Model fitting summary – Tablettose® 70 (modelling the entire relevant dissolution data)

Model: **Probit_2**

Model equation: $F = F_{max} \cdot \phi[\alpha + \beta \cdot \log(t)]$

Fitted model parameters per tested tablet (N = 4) with statistics – mean, standard deviation (SD), and relative standard deviation expressed in % (RSD%) (output from DDSolver):

Parameter	No.1	No.2	No.3	No.4	Mean	SD	RSD(%)
α	-2.361	-2.163	-2.016	-2.270	-2.203	0.148	-6.738
β	1.236	1.185	1.105	1.221	1.187	0.058	4.913
F_{max}	103.515	99.877	100.914	102.988	101.823	1.716	1.685

Number of dissolution data points (N), degrees of freedom (df), and selected goodness of fit criteria – Pearson correlation coefficient (R), coefficient of determination (R^2), adjusted coefficient of determination ($R^2_{adjusted}$), and residual sum of squares (RSS) (manual calculation in MS Excel):

Parameter	No.1	No.2	No.3	No.4
N	20	20	20	20
df	17	17	17	17
R	0.962834555	0.962169937	0.952430299	0.944569949
R^2	0.92705038	0.925770988	0.907123475	0.892212388
$R^2_{adjusted}$	0.918468072	0.917038163	0.896196825	0.879531493
RSS	803.7410781	684.3705947	784.3838361	1140.792863

Graphical abstract of model fit presented as mean ± 1 SD of the fraction % of released carvedilol:

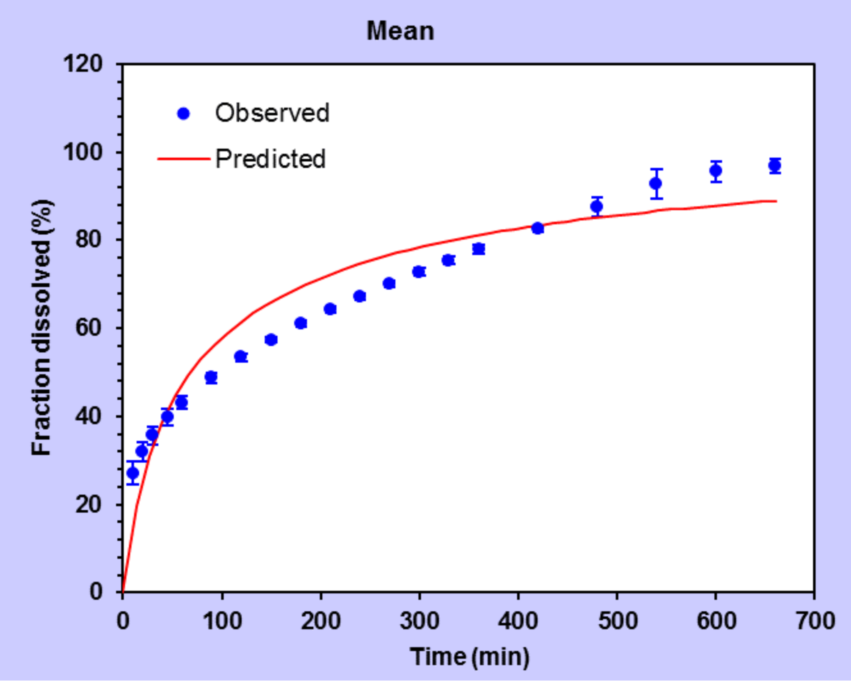

Supplementary materials – Model fitting summary – Tablettose® 70 (modelling the entire relevant dissolution data)

Graphical abstract of model fit presented as the fraction % of released carvedilol per tested tablet:

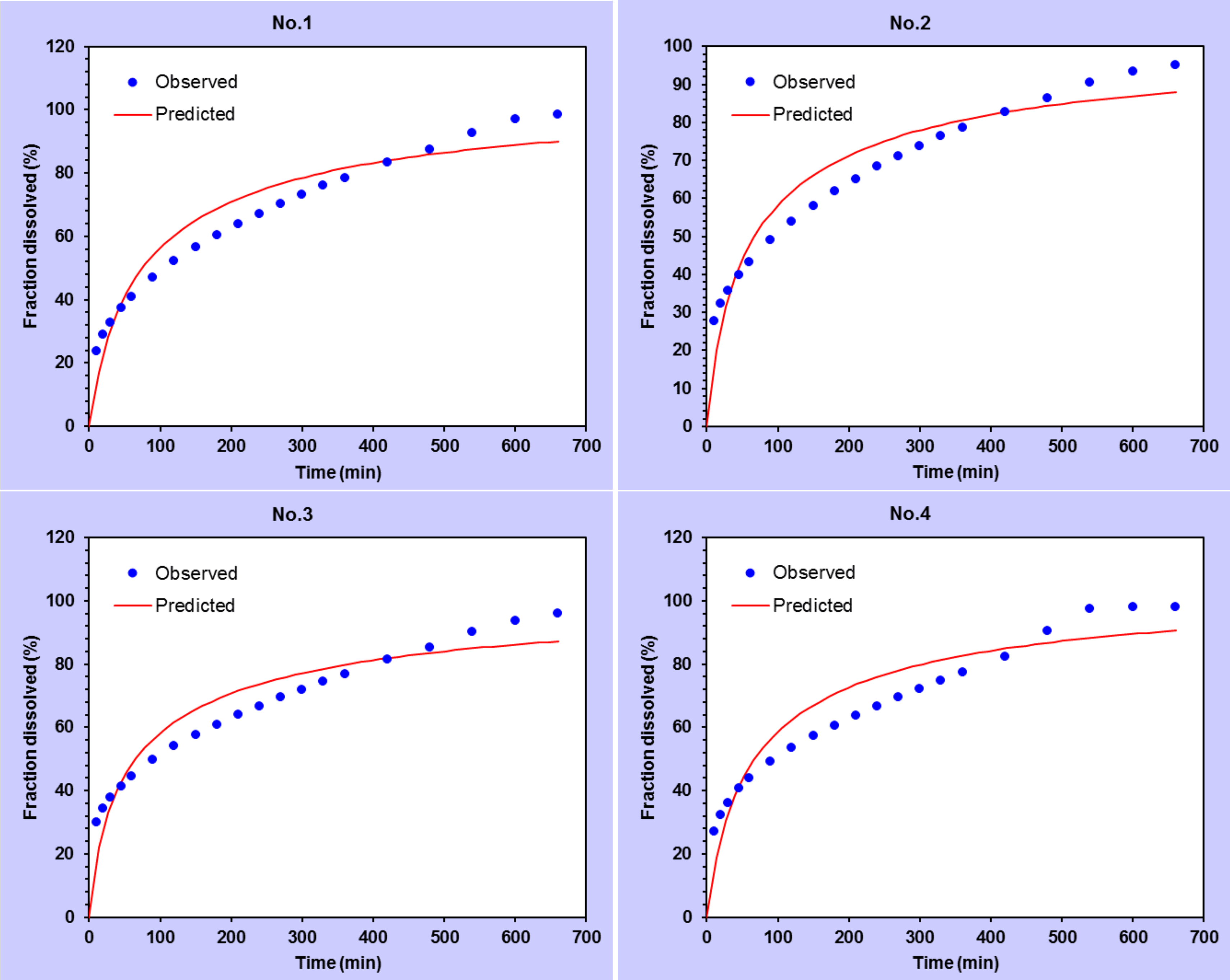

Supplementary materials – Model fitting summary – Tablettose® 70 (modelling up to app. 60 % of carvedilol released)

Model: **Zero–order**

Model equation: $F = k_0 \cdot t$

Fitted model parameters per tested tablet (N = 4) with statistics – mean, standard deviation (SD), and relative standard deviation expressed in % (RSD%) (output from DDSolver):

Parameter	No.1	No.2	No.3	No.4	Mean	SD	RSD(%)
k_0	0.424	0.439	0.441	0.436	0.435	0.008	1.760

Number of dissolution data points (N), degrees of freedom (df), and selected goodness of fit criteria – Pearson correlation coefficient (R), coefficient of determination (R^2), adjusted coefficient of determination ($R^2_{adjusted}$), and residual sum of squares (RSS) (manual calculation in MS Excel):

Parameter	No.1	No.2	No.3	No.4
N	9	9	9	9
df	8	8	8	8
R	0.980816576	0.985139038	0.980971287	0.973743624
R^2	0.962001156	0.970498923	0.962304666	0.948176645
$R^2_{adjusted}$	0.962001156	0.970498923	0.962304666	0.948176645
RSS	2167.658002	2746.687743	3205.164184	2843.25817

Graphical abstract of model fit presented as mean ± 1 SD of the fraction % of released carvedilol:

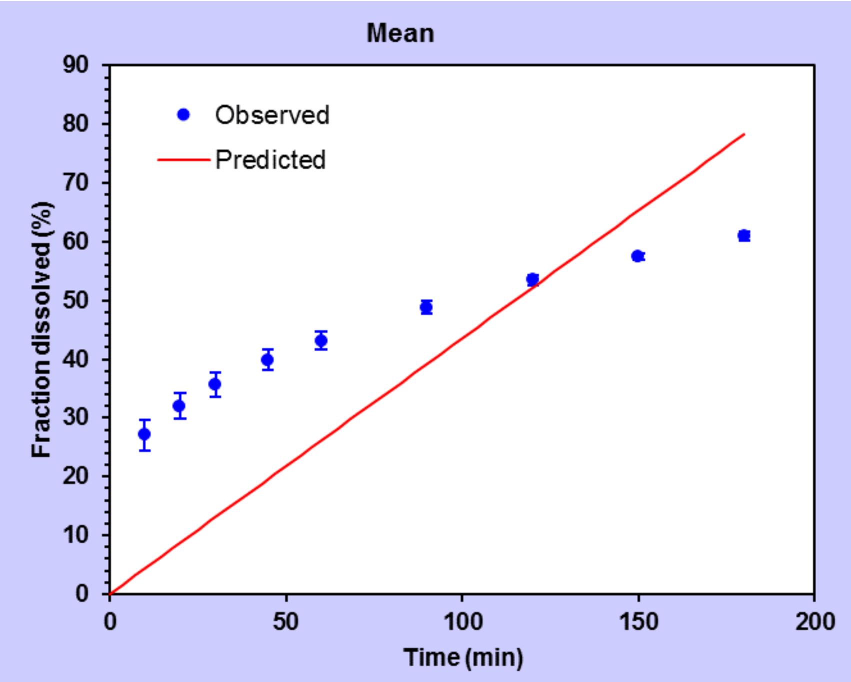

Supplementary materials – Model fitting summary – Tablettose® 70 (modelling up to app. 60 % of carvedilol released)

Graphical abstract of model fit presented as the fraction % of released carvedilol per tested tablet:

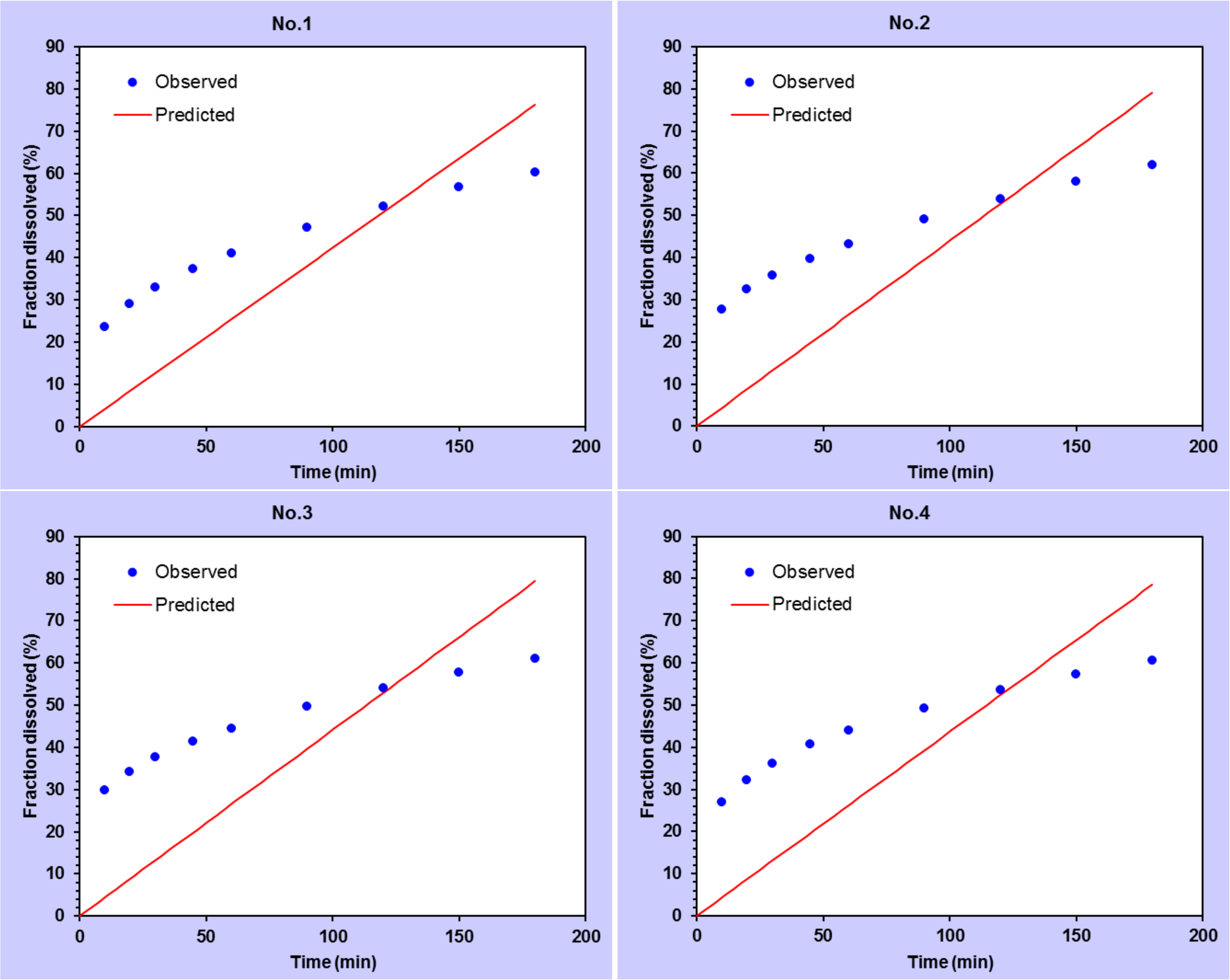

Supplementary materials – Model fitting summary – Tablettose® 70 (modelling up to app. 60 % of carvedilol released)

Model: **Zero–order with T_{lag}**

Model equation: $F = k_0 \cdot (t - T_{lag})$

Fitted model parameters per tested tablet (N = 4) with statistics – mean, standard deviation (SD), and relative standard deviation expressed in % (RSD%) (output from DDSolver):

Parameter	No.1	No.2	No.3	No.4	Mean	SD	RSD(%)
k_0	0.206	0.193	0.175	0.186	0.190	0.013	6.954
T_{lag}	-126.448	-153.062	-182.746	-160.574	-155.707	23.223	-14.914

Number of dissolution data points (N), degrees of freedom (df), and selected goodness of fit criteria – Pearson correlation coefficient (R), coefficient of determination (R^2), adjusted coefficient of determination ($R^2_{adjusted}$), and residual sum of squares (RSS) (manual calculation in MS Excel):

Parameter	No.1	No.2	No.3	No.4
N	9	9	9	9
df	7	7	7	7
R	0.980816576	0.985139038	0.980971287	0.973743624
R^2	0.962001156	0.970498923	0.962304666	0.948176645
$R^2_{adjusted}$	0.95657275	0.966284484	0.956919618	0.940773309
RSS	49.09572257	33.02875944	34.88236021	55.43451849

Graphical abstract of model fit presented as mean ± 1 SD of the fraction % of released carvedilol:

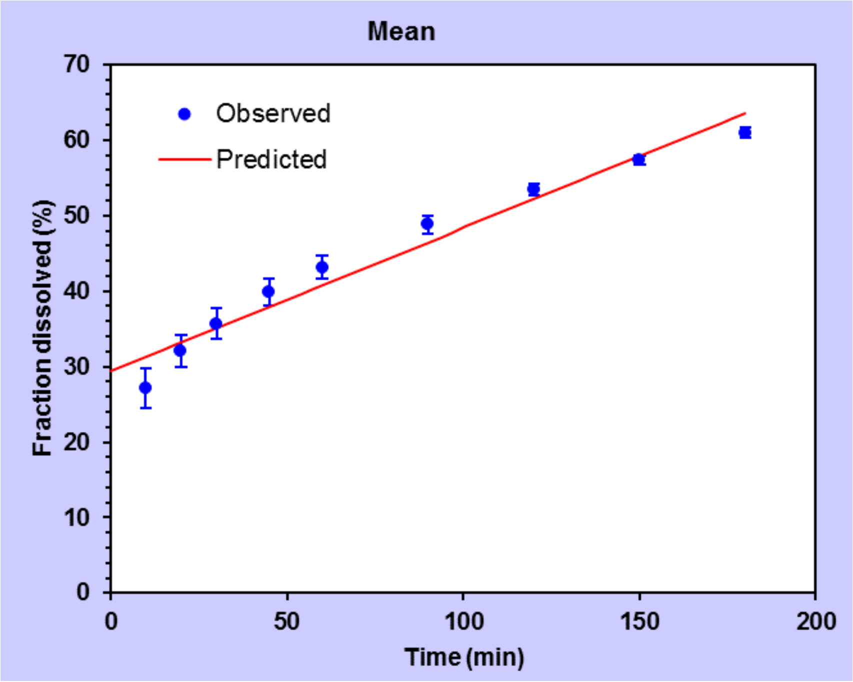

Supplementary materials – Model fitting summary – Tablettose® 70 (modelling up to app. 60 % of carvedilol released)

Graphical abstract of model fit presented as the fraction % of released carvedilol per tested tablet:

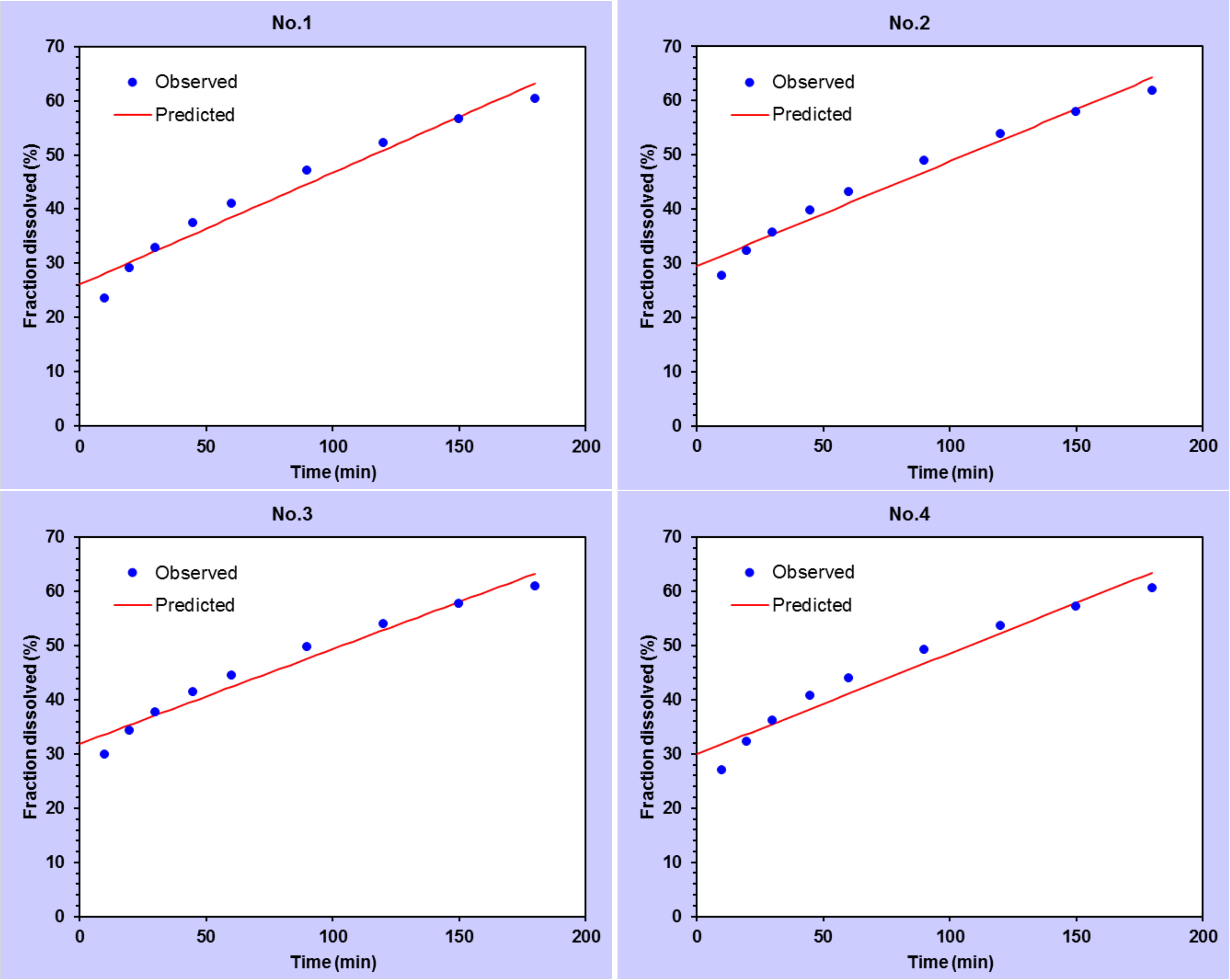

Supplementary materials – Model fitting summary – Tablettose® 70 (modelling up to app. 60 % of carvedilol released)

Model: **Zero–order with F_0**

Model equation: $F = F_0 + k_0 \cdot t$

Fitted model parameters per tested tablet (N = 4) with statistics – mean, standard deviation (SD), and relative standard deviation expressed in % (RSD%) (output from DDSolver):

Parameter	No.1	No.2	No.3	No.4	Mean	SD	RSD(%)
k_0	0.206	0.193	0.175	0.186	0.190	0.013	6.954
F_0	26.088	29.526	31.913	29.926	29.363	2.420	8.242

Number of dissolution data points (N), degrees of freedom (df), and selected goodness of fit criteria – Pearson correlation coefficient (R), coefficient of determination (R^2), adjusted coefficient of determination ($R^2_{adjusted}$), and residual sum of squares (RSS) (manual calculation in MS Excel):

Parameter	No.1	No.2	No.3	No.4
N	9	9	9	9
df	7	7	7	7
R	0.980816576	0.985139038	0.980971287	0.973743624
R^2	0.962001156	0.970498923	0.962304666	0.948176645
$R^2_{adjusted}$	0.95657275	0.966284484	0.956919618	0.940773309
RSS	49.09572257	33.02875944	34.88236021	55.43451849

Graphical abstract of model fit presented as mean ± 1 SD of the fraction % of released carvedilol:

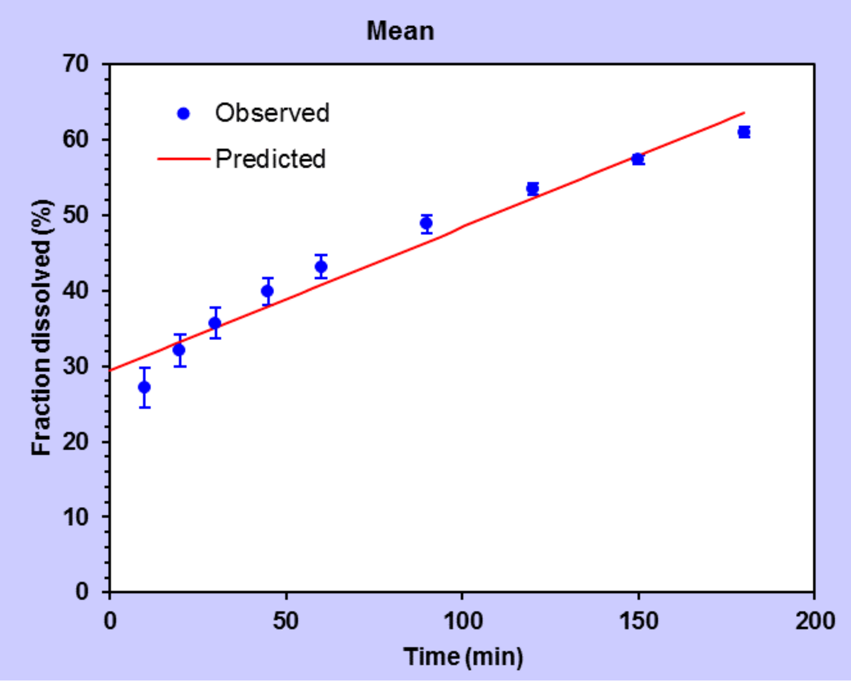

Supplementary materials – Model fitting summary – Tablettose® 70 (modelling up to app. 60 % of carvedilol released)

Graphical abstract of model fit presented as the fraction % of released carvedilol per tested tablet:

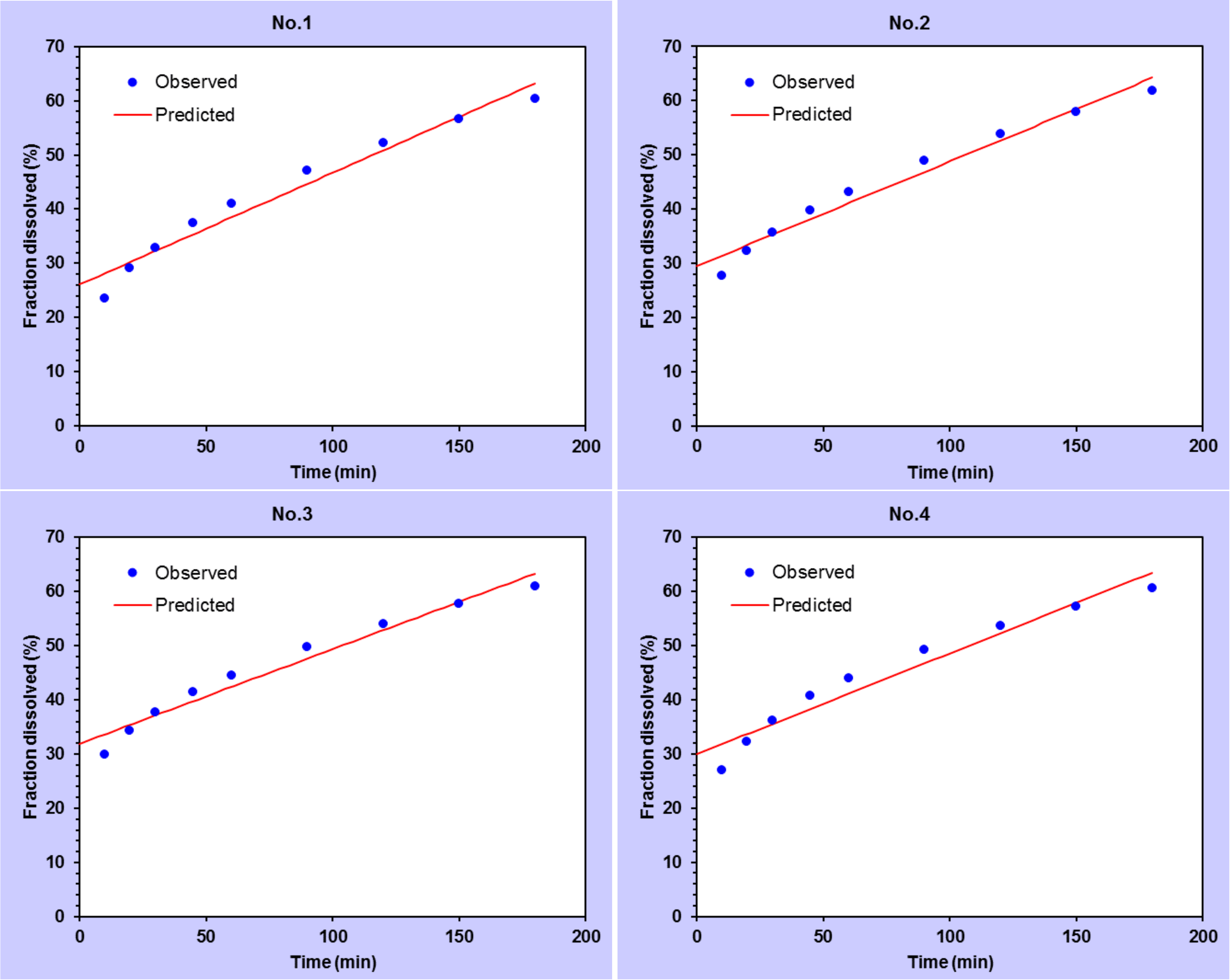

Supplementary materials – Model fitting summary – Tablettose® 70 (modelling up to app. 60 % of carvedilol released)

Model: **First–order**

Model equation: $F = 100 \cdot (1 - e^{-k_1 \cdot t})$

Fitted model parameters per tested tablet (N = 4) with statistics – mean, standard deviation (SD), and relative standard deviation expressed in % (RSD%) (output from DDSolver):

Parameter	No.1	No.2	No.3	No.4	Mean	SD	RSD(%)
k_1	0.006	0.006	0.010	0.006	0.007	0.002	23.629

Number of dissolution data points (N), degrees of freedom (df), and selected goodness of fit criteria – Pearson correlation coefficient (R), coefficient of determination (R^2), adjusted coefficient of determination ($R^2_{adjusted}$), and residual sum of squares (RSS) (manual calculation in MS Excel):

Parameter	No.1	No.2	No.3	No.4
N	9	9	9	9
df	8	8	8	8
R	0.996791324	0.998504875	0.998674978	0.994068762
R^2	0.993592944	0.997011986	0.997351712	0.988172703
$R^2_{adjusted}$	0.993592944	0.997011986	0.997351712	0.988172703
RSS	1253.832729	1652.408126	1981.854675	1727.560866

Graphical abstract of model fit presented as mean ± 1 SD of the fraction % of released carvedilol:

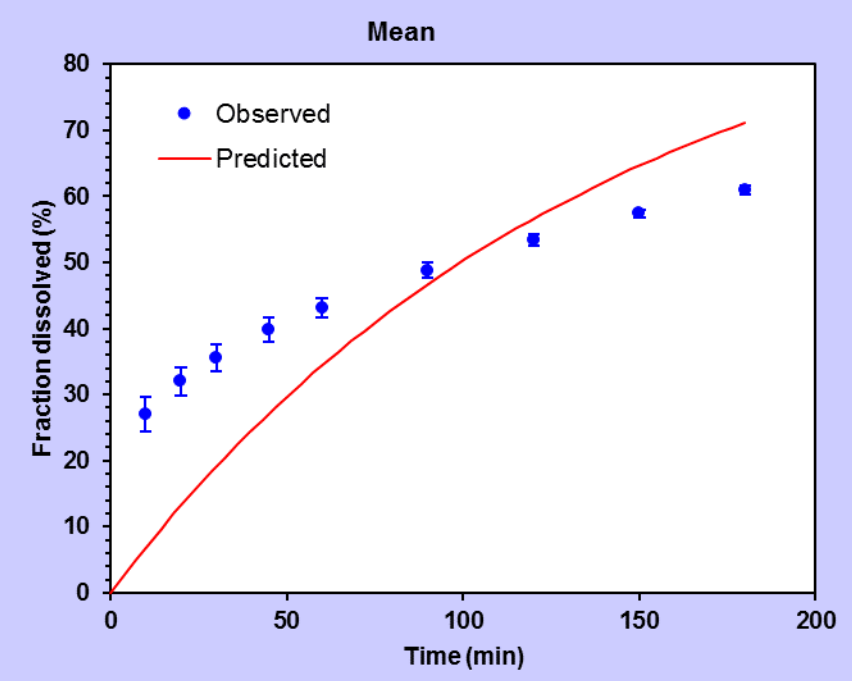

Supplementary materials – Model fitting summary – Tablettose® 70 (modelling up to app. 60 % of carvedilol released)

Graphical abstract of model fit presented as the fraction % of released carvedilol per tested tablet:

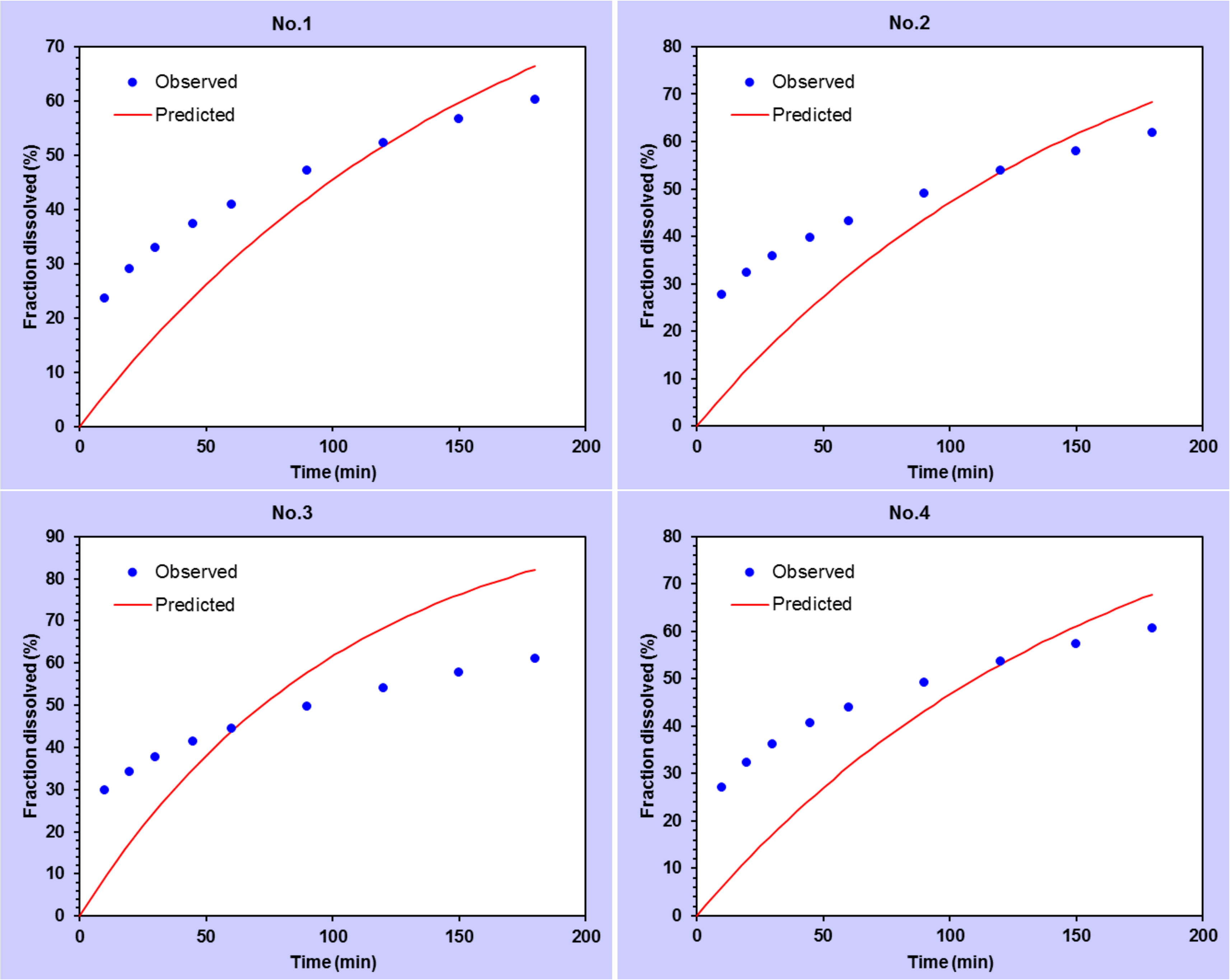

Supplementary materials – Model fitting summary – Tablettose® 70 (modelling up to app. 60 % of carvedilol released)

Model: **First–order with T_{lag}**

Model equation: $F = 100 \cdot \left[1 - e^{-k_1 \cdot (t - T_{lag})}\right]$

Fitted model parameters per tested tablet (N = 4) with statistics – mean, standard deviation (SD), and relative standard deviation expressed in % (RSD%) (output from DDSolver):

Parameter	No.1	No.2	No.3	No.4	Mean	SD	RSD(%)
k_1	0.004	0.004	0.003	0.003	0.004	0.000	5.055
T_{lag}	-74.541	-89.346	-109.572	-96.526	-92.496	14.608	-15.793

Number of dissolution data points (N), degrees of freedom (df), and selected goodness of fit criteria – Pearson correlation coefficient (R), coefficient of determination (R^2), adjusted coefficient of determination ($R^2_{adjusted}$), and residual sum of squares (RSS) (manual calculation in MS Excel):

Parameter	No.1	No.2	No.3	No.4
N	9	9	9	9
df	7	7	7	7
R	0.992674149	0.99510056	0.99183693	0.987211325
R^2	0.985401966	0.990225125	0.983740496	0.974586199
$R^2_{adjusted}$	0.983316532	0.988828714	0.98141771	0.970955656
RSS	19.68300723	11.38335497	15.59745141	28.26147803

Graphical abstract of model fit presented as mean ± 1 SD of the fraction % of released carvedilol:

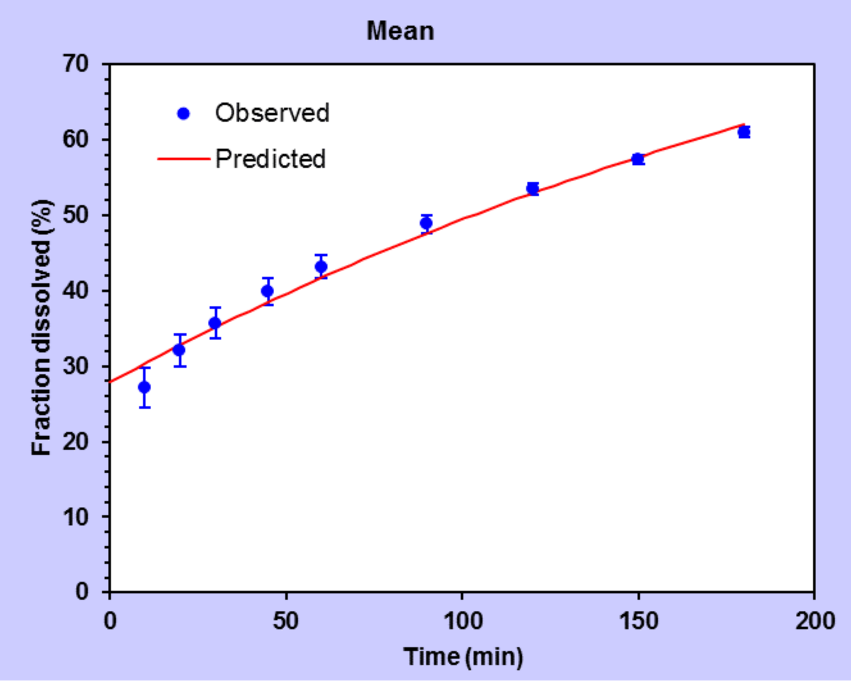

Supplementary materials – Model fitting summary – Tablettose® 70 (modelling up to app. 60 % of carvedilol released)

Graphical abstract of model fit presented as the fraction % of released carvedilol per tested tablet:

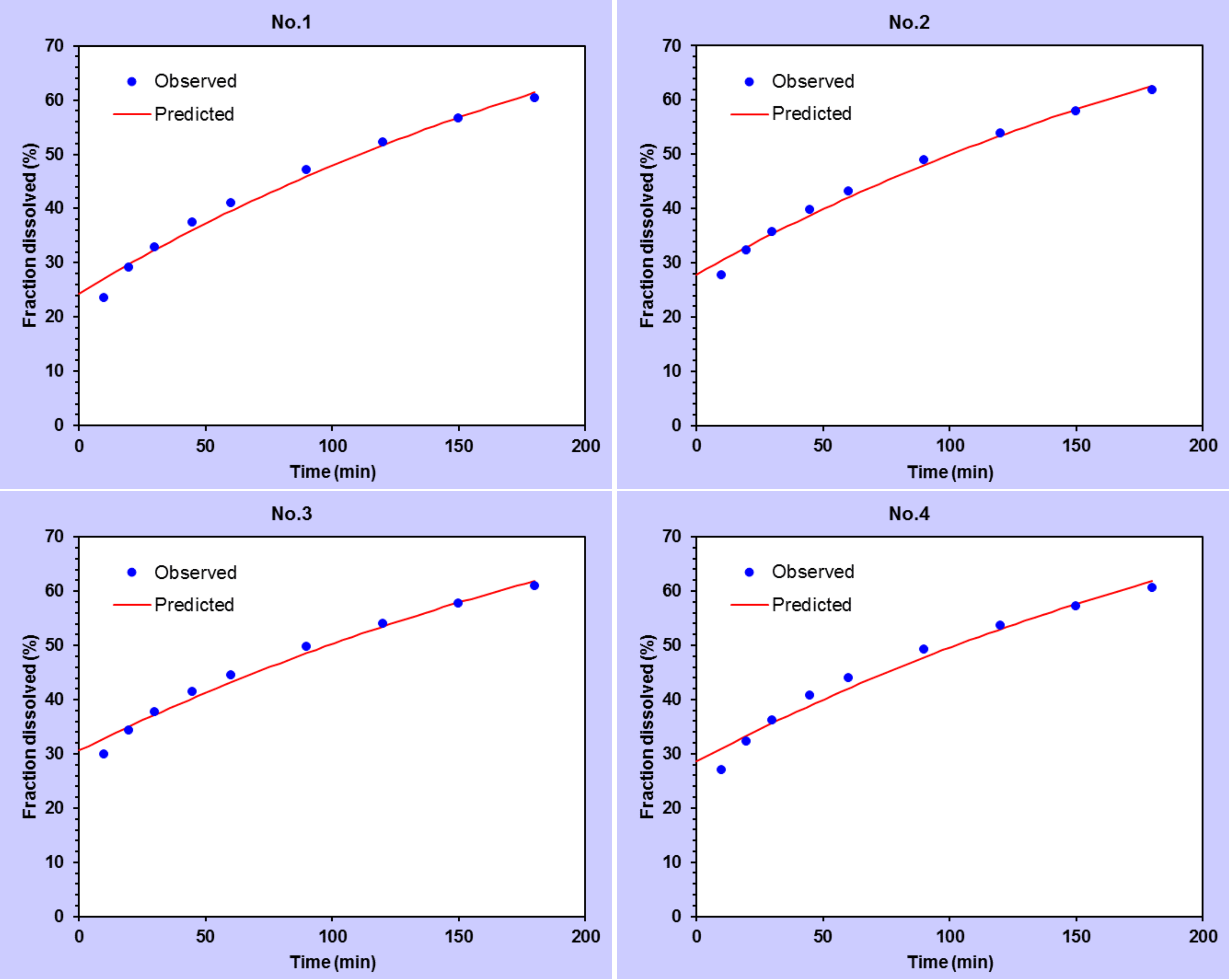

Supplementary materials – Model fitting summary – Tablettose® 70 (modelling up to app. 60 % of carvedilol released)

Model: **First–order with F_{max}**

Model equation: $F = F_{max} \cdot (1 - e^{-k_1 \cdot t})$

Fitted model parameters per tested tablet (N = 4) with statistics – mean, standard deviation (SD), and relative standard deviation expressed in % (RSD%) (output from DDSolver):

Parameter	No.1	No.2	No.3	No.4	Mean	SD	RSD(%)
k_1	0.024	0.024	0.025	0.025	0.025	0.001	2.171
F_{max}	63.349	65.024	63.992	63.663	64.007	0.727	1.136

Number of dissolution data points (N), degrees of freedom (df), and selected goodness of fit criteria – Pearson correlation coefficient (R), coefficient of determination (R^2), adjusted coefficient of determination ($R^2_{adjusted}$), and residual sum of squares (RSS) (manual calculation in MS Excel):

Parameter	No.1	No.2	No.3	No.4
N	9	9	9	9
df	7	7	7	7
R	0.966993011	0.960189323	0.962942518	0.972272557
R^2	0.935075484	0.921963537	0.927258292	0.945313926
$R^2_{adjusted}$	0.925800553	0.91081547	0.91686662	0.937501629
RSS	369.9132878	473.4289224	505.3667308	397.6244434

Graphical abstract of model fit presented as mean ± 1 SD of the fraction % of released carvedilol:

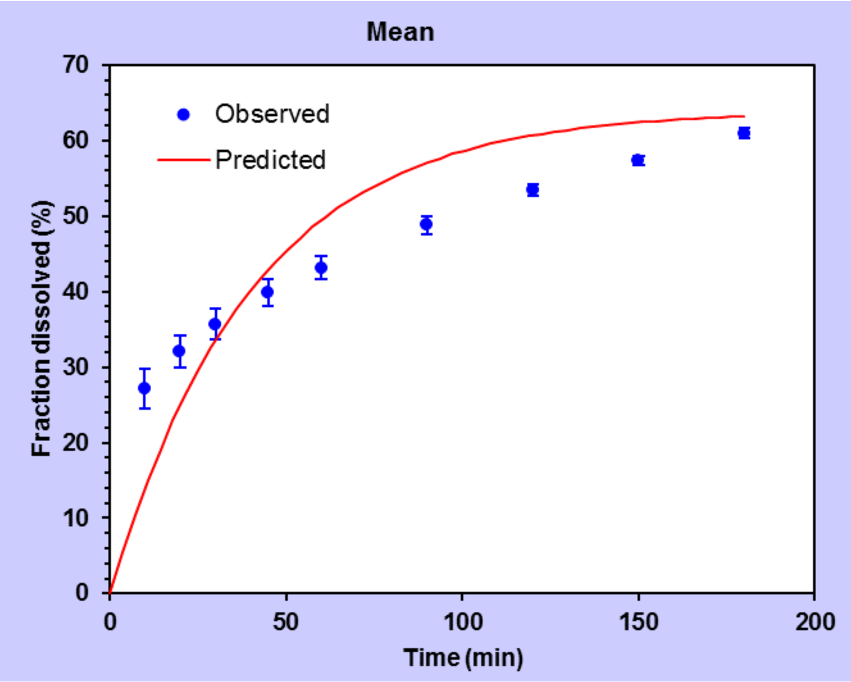

Supplementary materials – Model fitting summary – Tablettose® 70 (modelling up to app. 60 % of carvedilol released)

Graphical abstract of model fit presented as the fraction % of released carvedilol per tested tablet:

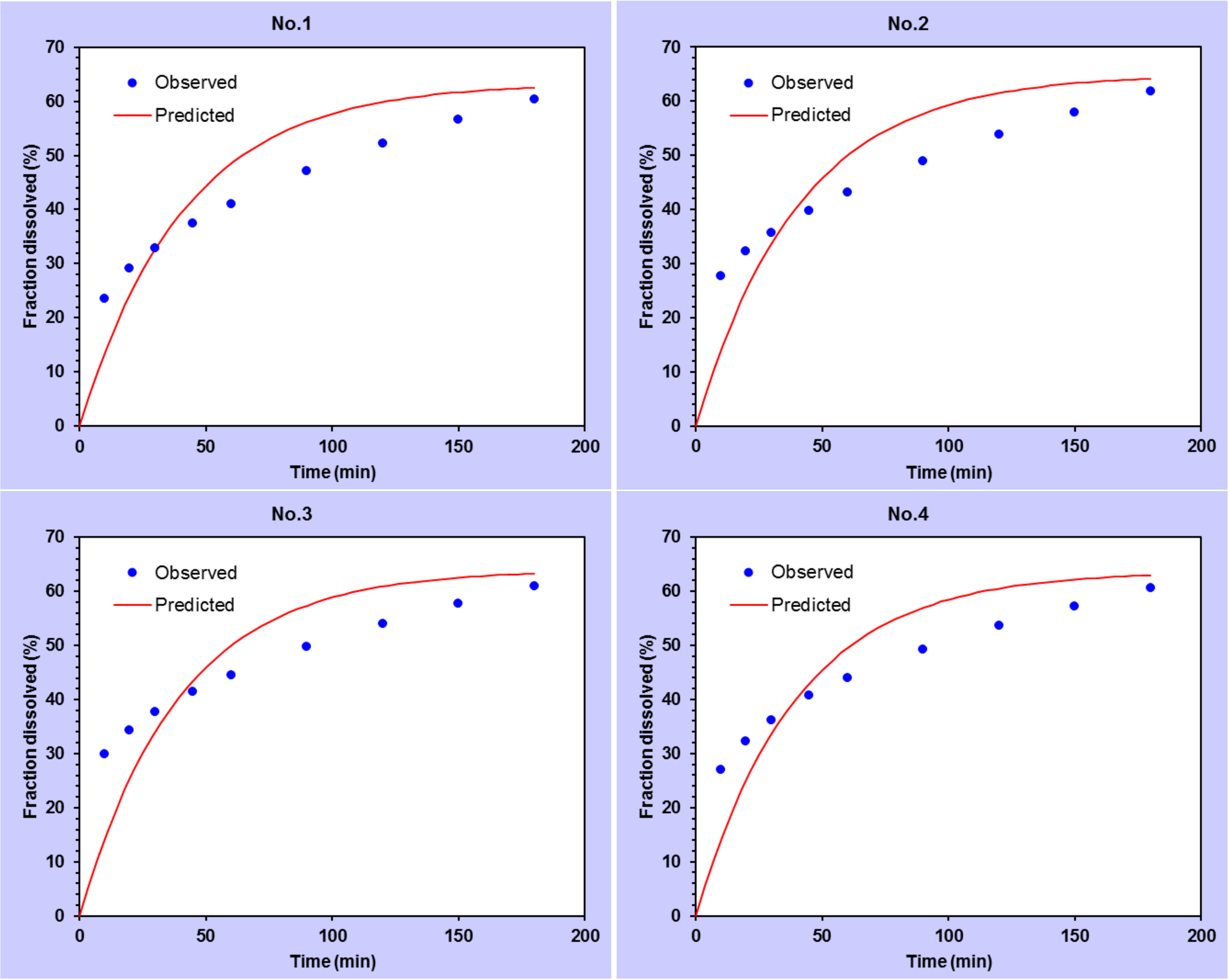

Supplementary materials – Model fitting summary – Tablettose® 70 (modelling up to app. 60 % of carvedilol released)

Model: **First–order with T_{lag} and F_{max}**

Model equation: $F = F_{max} \cdot \left[1 - e^{-k_1 \cdot (t - T_{lag})}\right]$

Fitted model parameters per tested tablet (N = 4) with statistics – mean, standard deviation (SD), and relative standard deviation expressed in % (RSD%) (output from DDSolver):

Parameter	No.1	No.2	No.3	No.4	Mean	SD	RSD(%)
k_1	0.014	0.014	0.013	0.013	0.014	0.000	2.584
T_{lag}	-18.395	-34.345	-33.774	-28.504	-28.754	7.390	-25.701
F_{max}	63.349	63.105	63.992	63.663	63.527	0.385	0.606

Number of dissolution data points (N), degrees of freedom (df), and selected goodness of fit criteria – Pearson correlation coefficient (R), coefficient of determination (R^2), adjusted coefficient of determination ($R^2_{adjusted}$), and residual sum of squares (RSS) (manual calculation in MS Excel):

Parameter	No.1	No.2	No.3	No.4
N	9	9	9	9
df	6	6	6	6
R	0.994502639	0.992840595	0.995769862	0.997028333
R^2	0.9890355	0.985732447	0.991557618	0.994065497
$R^2_{adjusted}$	0.985380666	0.980976596	0.988743491	0.99208733
RSS	31.07062865	29.16021548	15.82003073	12.50856454

Graphical abstract of model fit presented as mean ± 1 SD of the fraction % of released carvedilol:

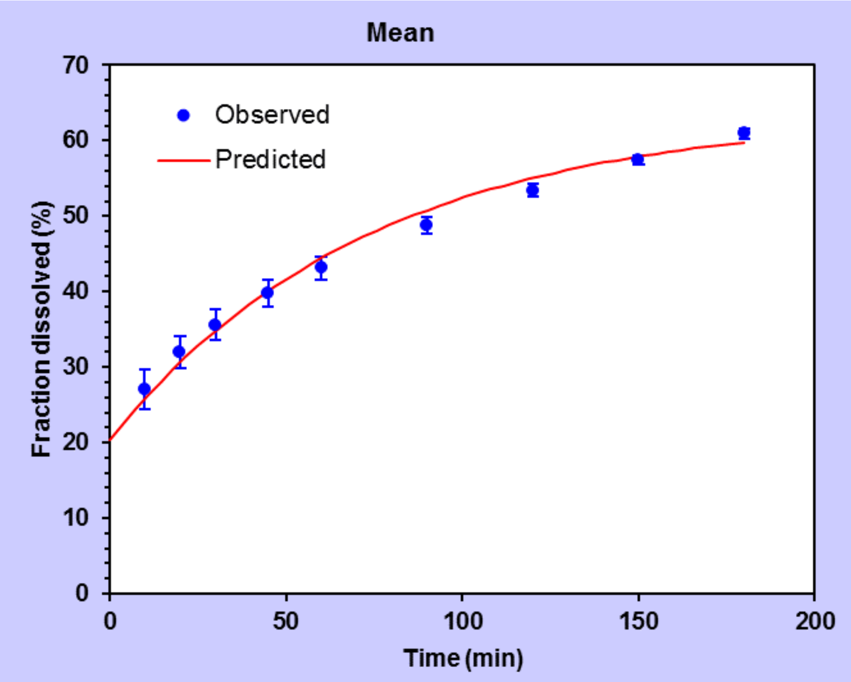

Supplementary materials – Model fitting summary – Tablettose® 70 (modelling up to app. 60 % of carvedilol released)

Graphical abstract of model fit presented as the fraction % of released carvedilol per tested tablet:

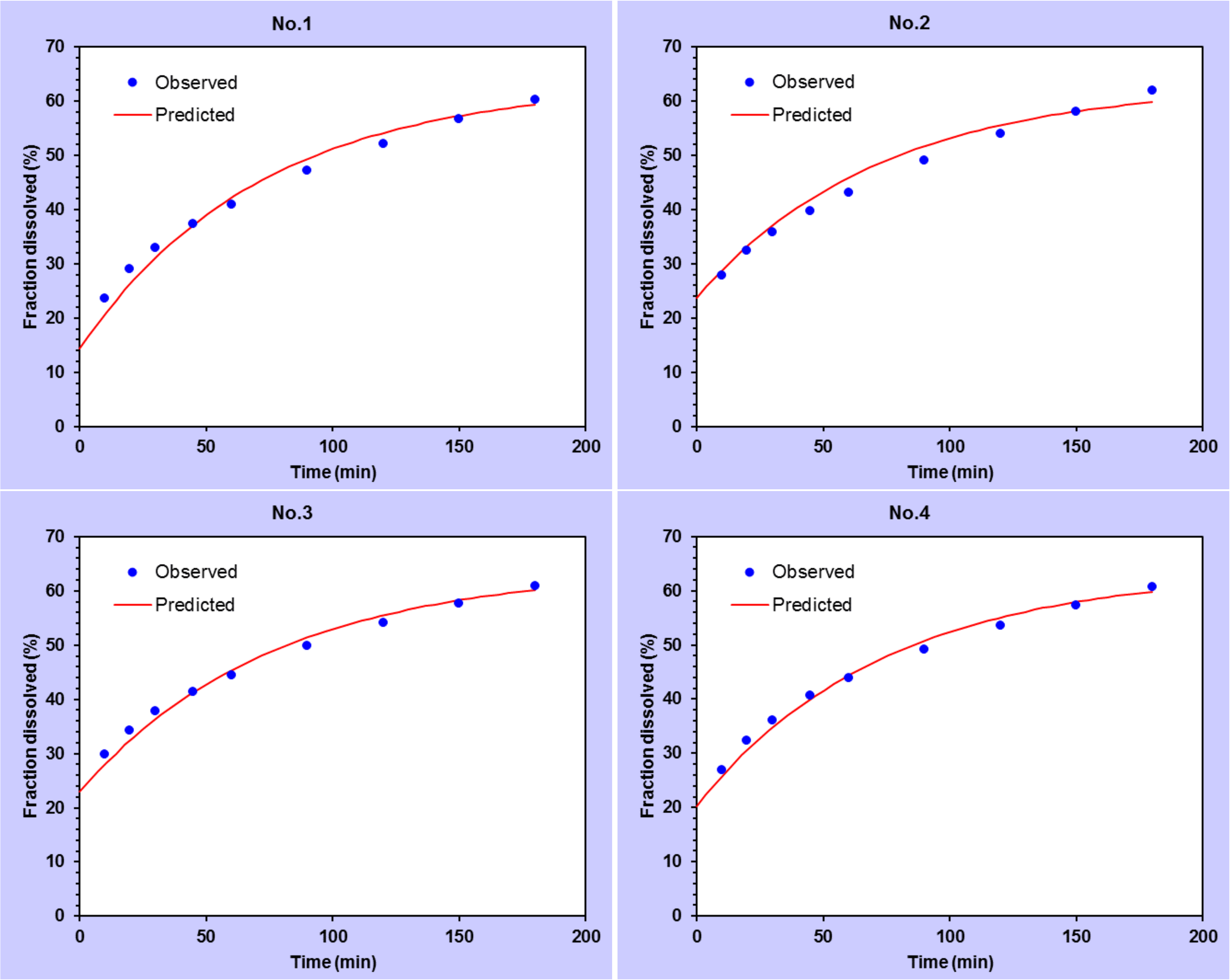

Supplementary materials – Model fitting summary – Tablettose® 70 (modelling up to app. 60 % of carvedilol released)

Model: **Higuchi**

Model equation: $F = k_H \cdot t^{0.5}$

Fitted model parameters per tested tablet (N = 4) with statistics – mean, standard deviation (SD), and relative standard deviation expressed in % (RSD%) (output from DDSolver):

Parameter	No.1	No.2	No.3	No.4	Mean	SD	RSD(%)
k_H	4.930	5.144	5.200	5.120	5.098	0.117	2.302

Number of dissolution data points (N), degrees of freedom (df), and selected goodness of fit criteria – Pearson correlation coefficient (R), coefficient of determination (R^2), adjusted coefficient of determination ($R^2_{adjusted}$), and residual sum of squares (RSS) (manual calculation in MS Excel):

Parameter	No.1	No.2	No.3	No.4
N	9	9	9	9
df	8	8	8	8
R	0.999510955	0.999959218	0.999538154	0.997912279
R^2	0.999022149	0.999918438	0.999076521	0.995828917
$R^2_{adjusted}$	0.999022149	0.999918438	0.999076521	0.995828917
RSS	225.4412236	399.2312001	571.7401285	429.2991876

Graphical abstract of model fit presented as mean ± 1 SD of the fraction % of released carvedilol:

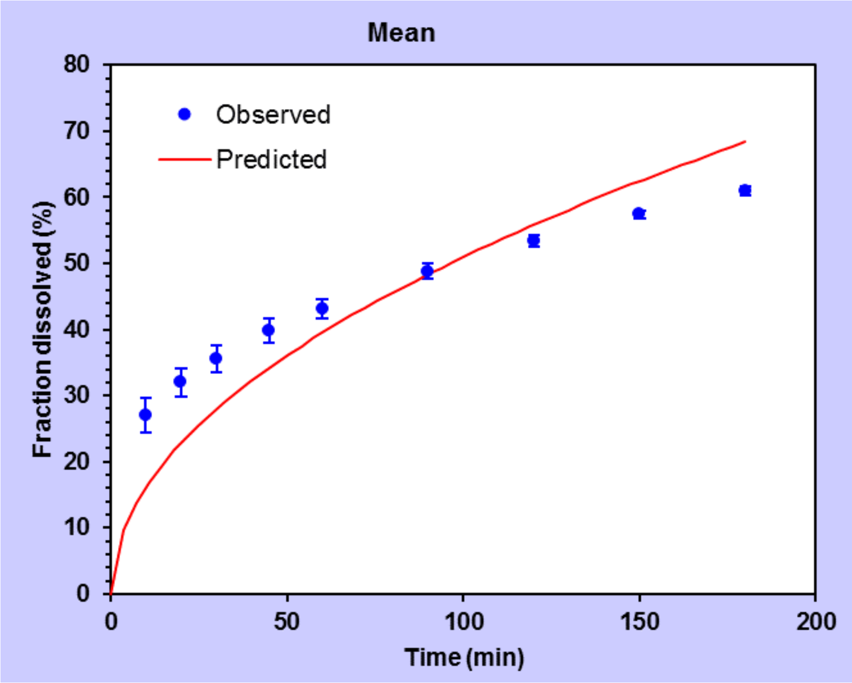

Supplementary materials – Model fitting summary – Tablettose® 70 (modelling up to app. 60 % of carvedilol released)

Graphical abstract of model fit presented as the fraction % of released carvedilol per tested tablet:

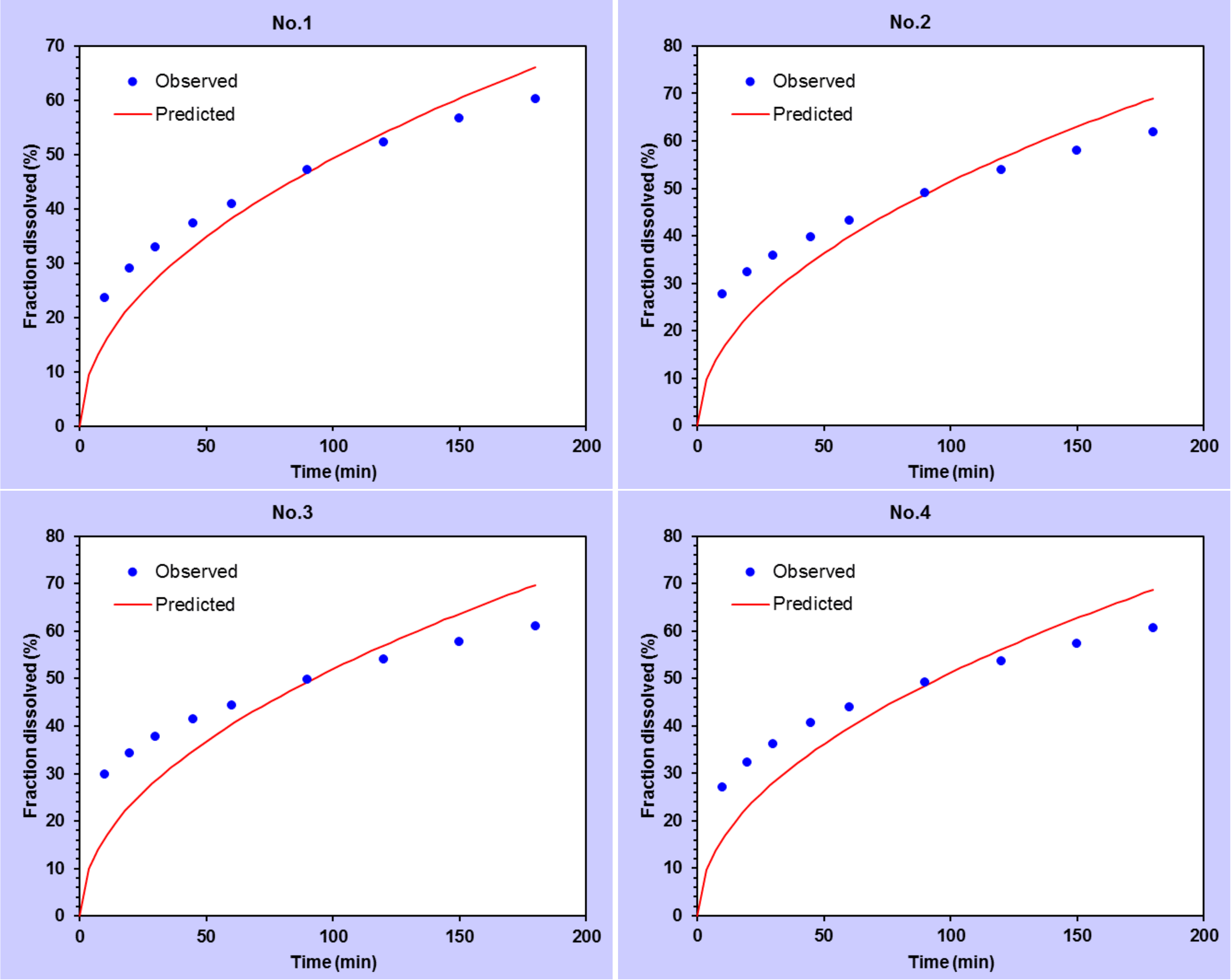

Supplementary materials – Model fitting summary – Tablettose® 70 (modelling up to app. 60 % of carvedilol released)

Model: **Higuchi with T_{lag}**

Model equation: $F = k_H \cdot \left(t - T_{lag}\right)^{0.5}$

Fitted model parameters per tested tablet (N = 4) with statistics – mean, standard deviation (SD), and relative standard deviation expressed in % (RSD%) (output from DDSolver):

Parameter	No.1	No.2	No.3	No.4	Mean	SD	RSD(%)
k_H	4.231	4.208	4.032	4.107	4.145	0.092	2.221
T_{lag}	-29.411	-41.215	-55.836	-46.221	-43.171	10.998	-25.475

Number of dissolution data points (N), degrees of freedom (df), and selected goodness of fit criteria – Pearson correlation coefficient (R), coefficient of determination (R^2), adjusted coefficient of determination ($R^2_{adjusted}$), and residual sum of squares (RSS) (manual calculation in MS Excel):

Parameter	No.1	No.2	No.3	No.4
N	9	9	9	9
df	7	7	7	7
R	0.995493019	0.996547449	0.993152401	0.989505335
R^2	0.99100635	0.993106818	0.986351692	0.979120809
$R^2_{adjusted}$	0.989721543	0.992122078	0.984401934	0.976138067
RSS	12.81559345	8.300523593	13.38486366	23.99121069

Graphical abstract of model fit presented as mean ± 1 SD of the fraction % of released carvedilol:

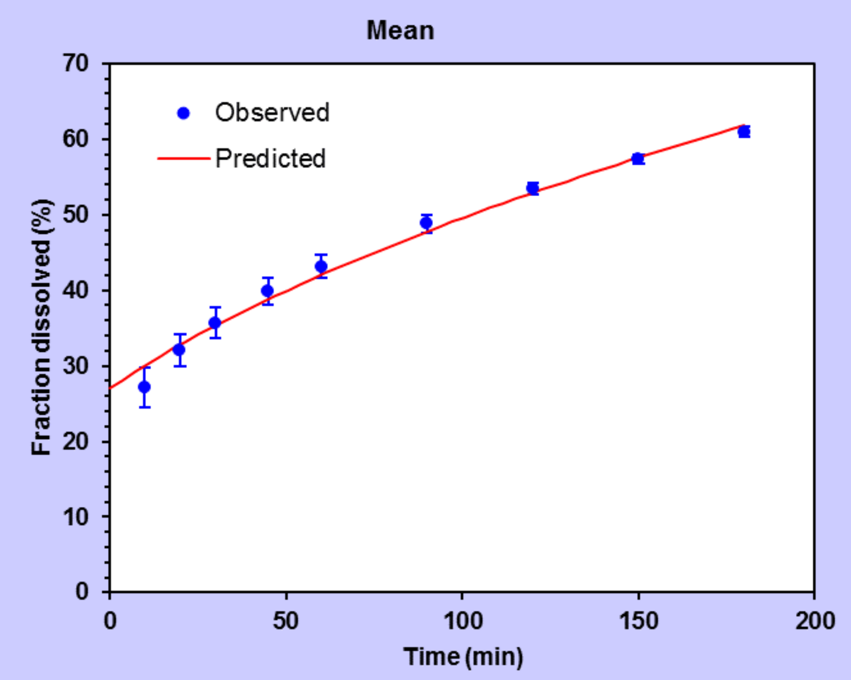

Supplementary materials – Model fitting summary – Tablettose® 70 (modelling up to app. 60 % of carvedilol released)

Graphical abstract of model fit presented as the fraction % of released carvedilol per tested tablet:

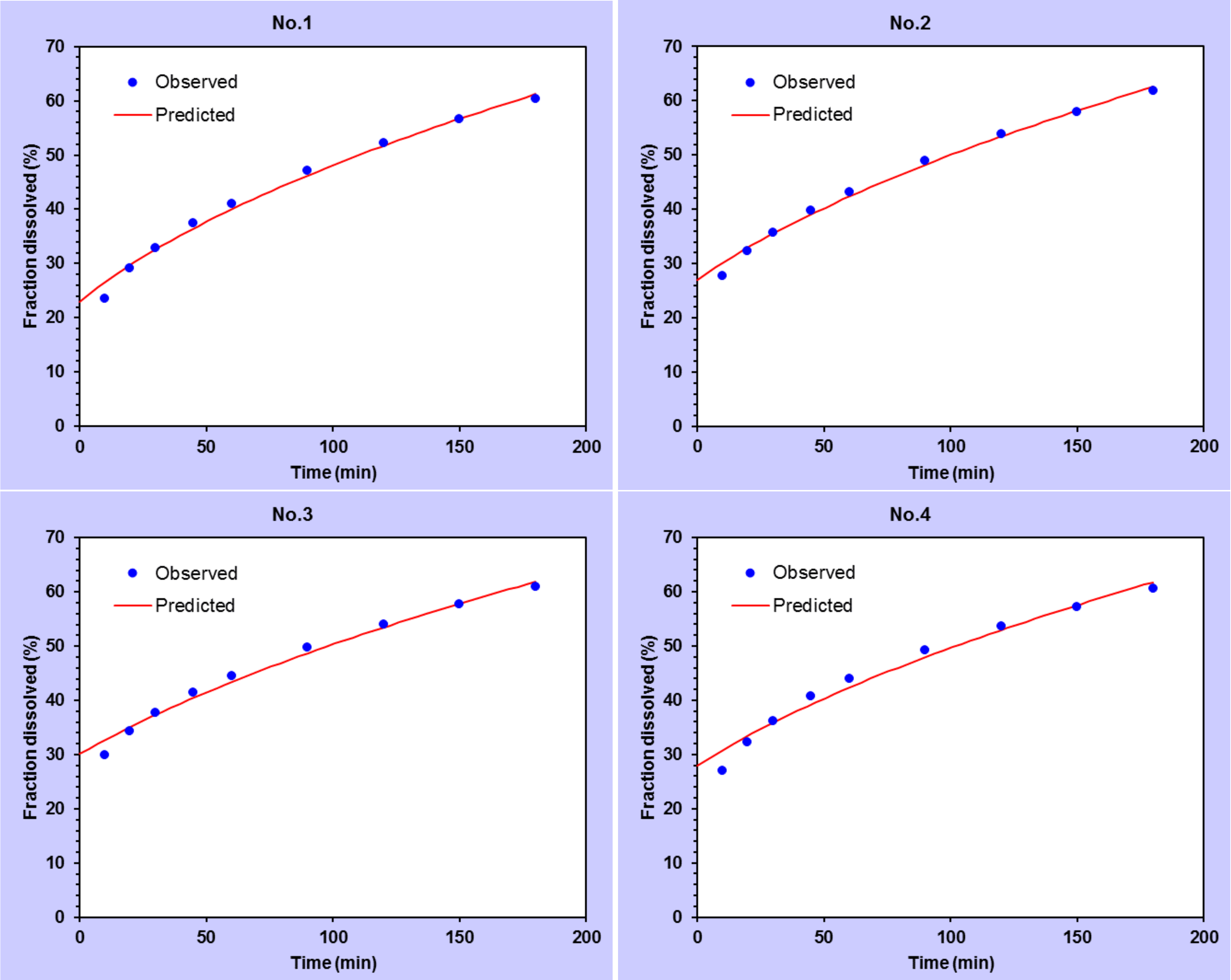

Supplementary materials – Model fitting summary – Tablettose® 70 (modelling up to app. 60 % of carvedilol released)

Model: **Higuchi with F_0**

Model equation: $F = F_0 + k_H \cdot t^{0.5}$

Fitted model parameters per tested tablet (N = 4) with statistics – mean, standard deviation (SD), and relative standard deviation expressed in % (RSD%) (output from DDSolver):

Parameter	No.1	No.2	No.3	No.4	Mean	SD	RSD(%)
k_H	3.558	3.314	3.011	3.232	3.279	0.226	6.886
F_0	13.124	17.512	20.943	18.067	17.411	3.230	18.550

Number of dissolution data points (N), degrees of freedom (df), and selected goodness of fit criteria – Pearson correlation coefficient (R), coefficient of determination (R^2), adjusted coefficient of determination ($R^2_{adjusted}$), and residual sum of squares (RSS) (manual calculation in MS Excel):

Parameter	No.1	No.2	No.3	No.4
N	9	9	9	9
df	7	7	7	7
R	0.999510955	0.999959218	0.999538154	0.997912279
R^2	0.999022149	0.999918438	0.999076521	0.995828917
$R^2_{adjusted}$	0.998882456	0.999906786	0.998944596	0.995233048
RSS	1.263415308	0.091315397	0.854565182	4.461732806

Graphical abstract of model fit presented as mean ± 1 SD of the fraction % of released carvedilol:

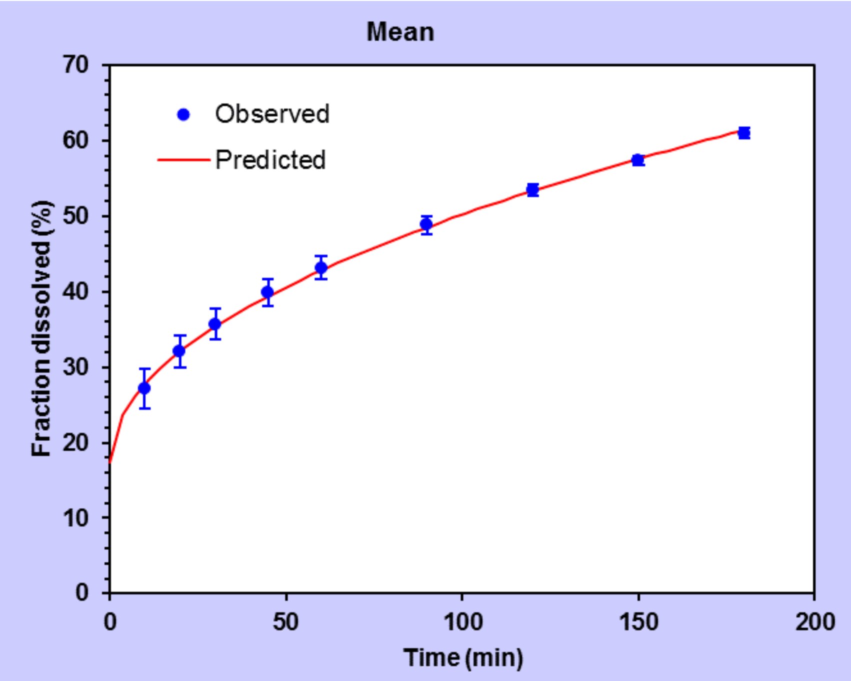

Supplementary materials – Model fitting summary – Tablettose® 70 (modelling up to app. 60 % of carvedilol released)

Graphical abstract of model fit presented as the fraction % of released carvedilol per tested tablet:

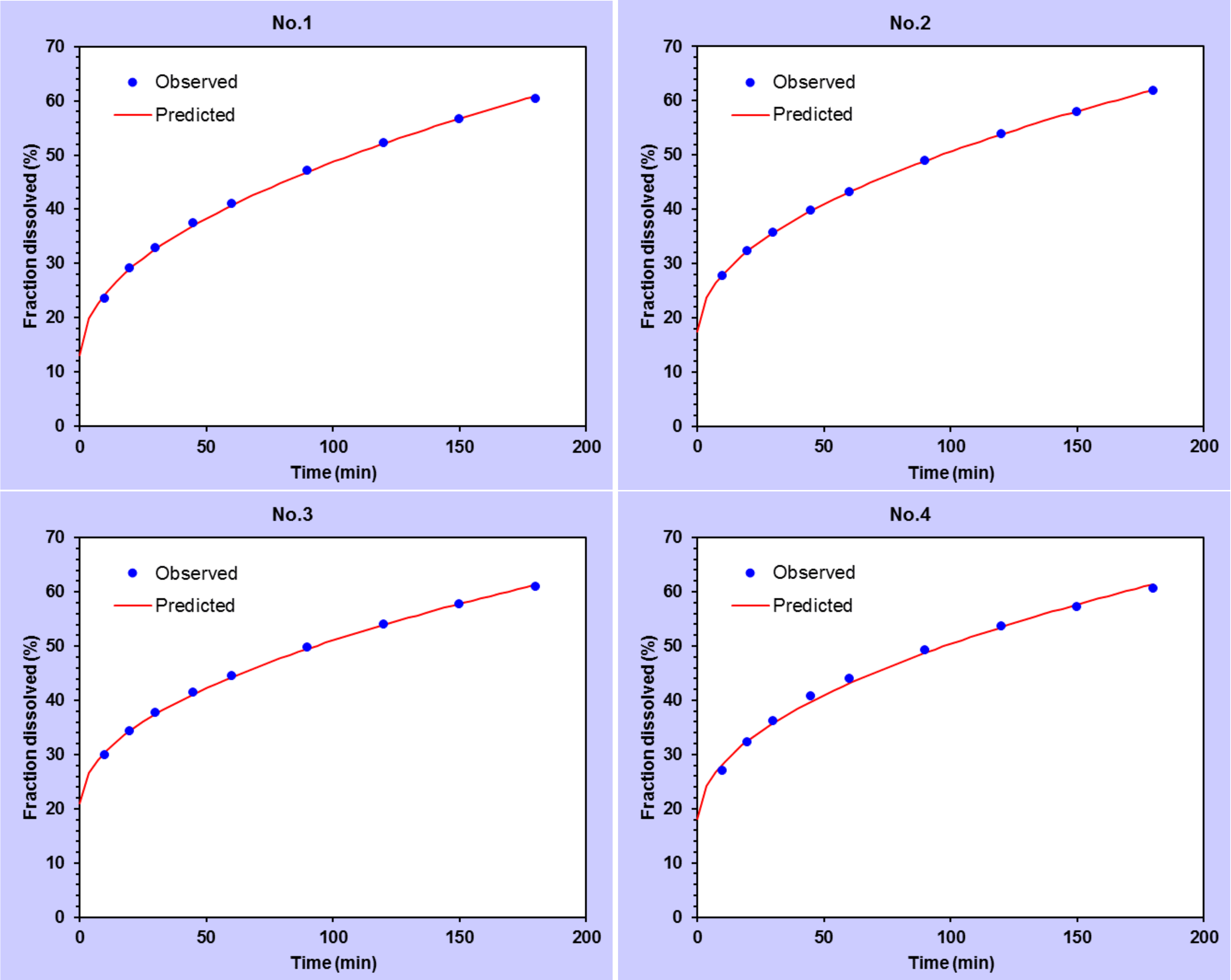

Supplementary materials – Model fitting summary – Tablettose® 70 (modelling up to app. 60 % of carvedilol released)

Model: **Korsmeyer–Peppas**

Model equation: $F = k_{KP} \cdot t^n$

Fitted model parameters per tested tablet (N = 4) with statistics – mean, standard deviation (SD), and relative standard deviation expressed in % (RSD%) (output from DDSolver):

Parameter	No.1	No.2	No.3	No.4	Mean	SD	RSD(%)
k_{KP}	10.941	14.054	16.356	13.986	13.834	2.221	16.056
n	0.326	0.280	0.249	0.281	0.284	0.032	11.155

Number of dissolution data points (N), degrees of freedom (df), and selected goodness of fit criteria – Pearson correlation coefficient (R), coefficient of determination (R^2), adjusted coefficient of determination ($R^2_{adjusted}$), and residual sum of squares (RSS) (manual calculation in MS Excel):

Parameter	No.1	No.2	No.3	No.4
N	9	9	9	9
df	7	7	7	7
R	0.999317615	0.99692494	0.99767074	0.999753347
R^2	0.998635696	0.993859335	0.995346905	0.999506754
$R^2_{adjusted}$	0.998440796	0.992982097	0.994682177	0.999436291
RSS	2.033654985	7.713477368	4.719362155	0.582291799

Graphical abstract of model fit presented as mean ± 1 SD of the fraction % of released carvedilol:

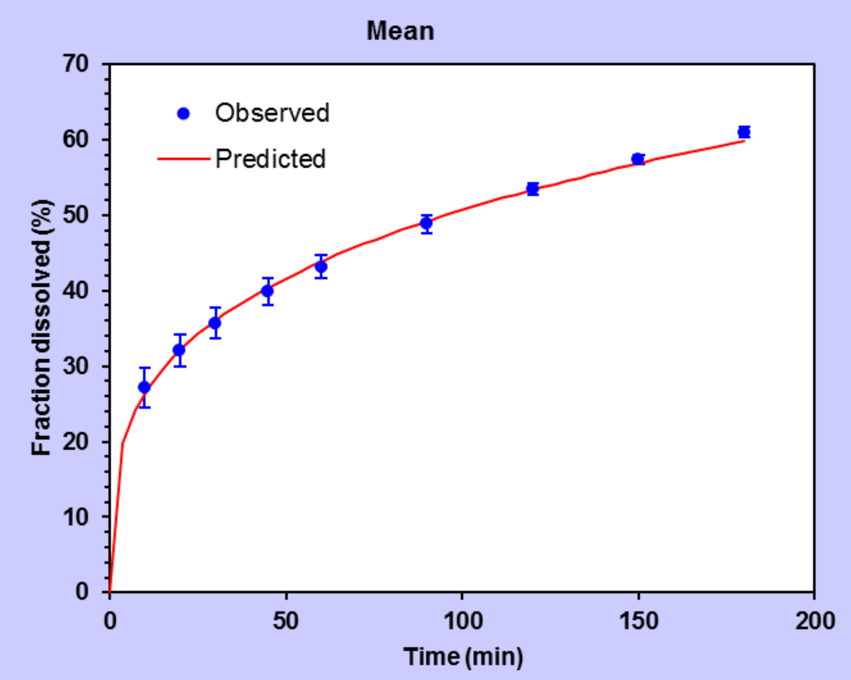

Supplementary materials – Model fitting summary – Tablettose® 70 (modelling up to app. 60 % of carvedilol released)

Graphical abstract of model fit presented as the fraction % of released carvedilol per tested tablet:

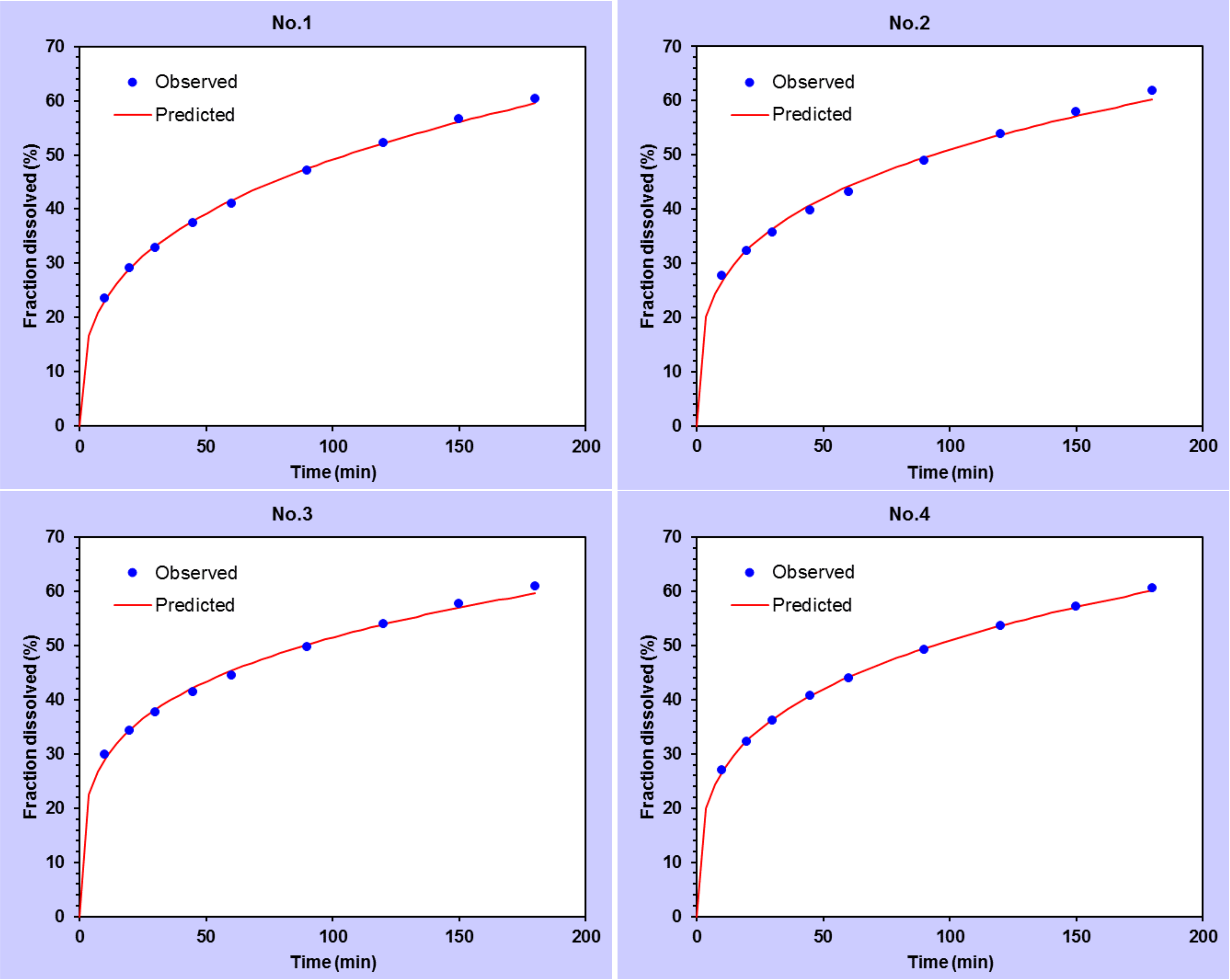

Supplementary materials – Model fitting summary – Tablettose® 70 (modelling up to app. 60 % of carvedilol released)

Model: **Korsmeyer–Peppas with T_{lag}**

Model equation: $F = k_{KP} \cdot (t - T_{lag})^n$

Fitted model parameters per tested tablet (N = 4) with statistics – mean, standard deviation (SD), and relative standard deviation expressed in % (RSD%) (output from DDSolver):

Parameter	No.1	No.2	No.3	No.4	Mean	SD	RSD(%)
k_{KP}	13.477	16.854	19.215	16.723	16.567	2.357	14.228
n	0.283	0.243	0.216	0.244	0.247	0.028	11.246
T_{lag}	4.000	4.000	4.000	4.000	4.000	0.000	0.000

Number of dissolution data points (N), degrees of freedom (df), and selected goodness of fit criteria – Pearson correlation coefficient (R), coefficient of determination (R^2), adjusted coefficient of determination ($R^2_{adjusted}$), and residual sum of squares (RSS) (manual calculation in MS Excel):

Parameter	No.1	No.2	No.3	No.4
N	9	9	9	9
df	6	6	6	6
R	0.996085715	0.991341416	0.992534997	0.997194434
R^2	0.992186752	0.982757802	0.985125721	0.994396739
$R^2_{adjusted}$	0.989582336	0.977010403	0.980167627	0.992528986
RSS	11.95261837	21.86905493	15.21763686	6.820373778

Graphical abstract of model fit presented as mean ± 1 SD of the fraction % of released carvedilol:

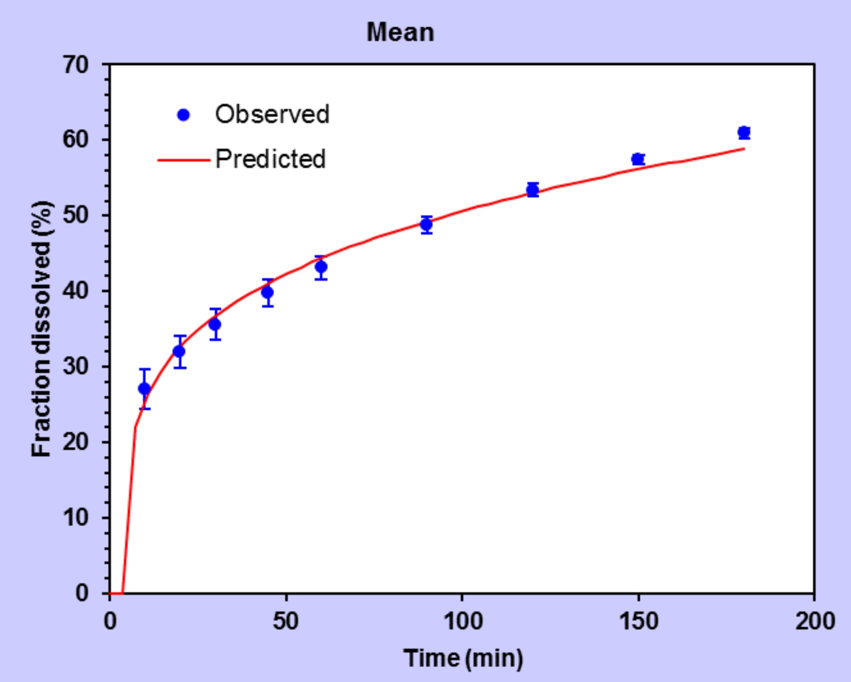

Supplementary materials – Model fitting summary – Tablettose® 70 (modelling up to app. 60 % of carvedilol released)

Graphical abstract of model fit presented as the fraction % of released carvedilol per tested tablet:

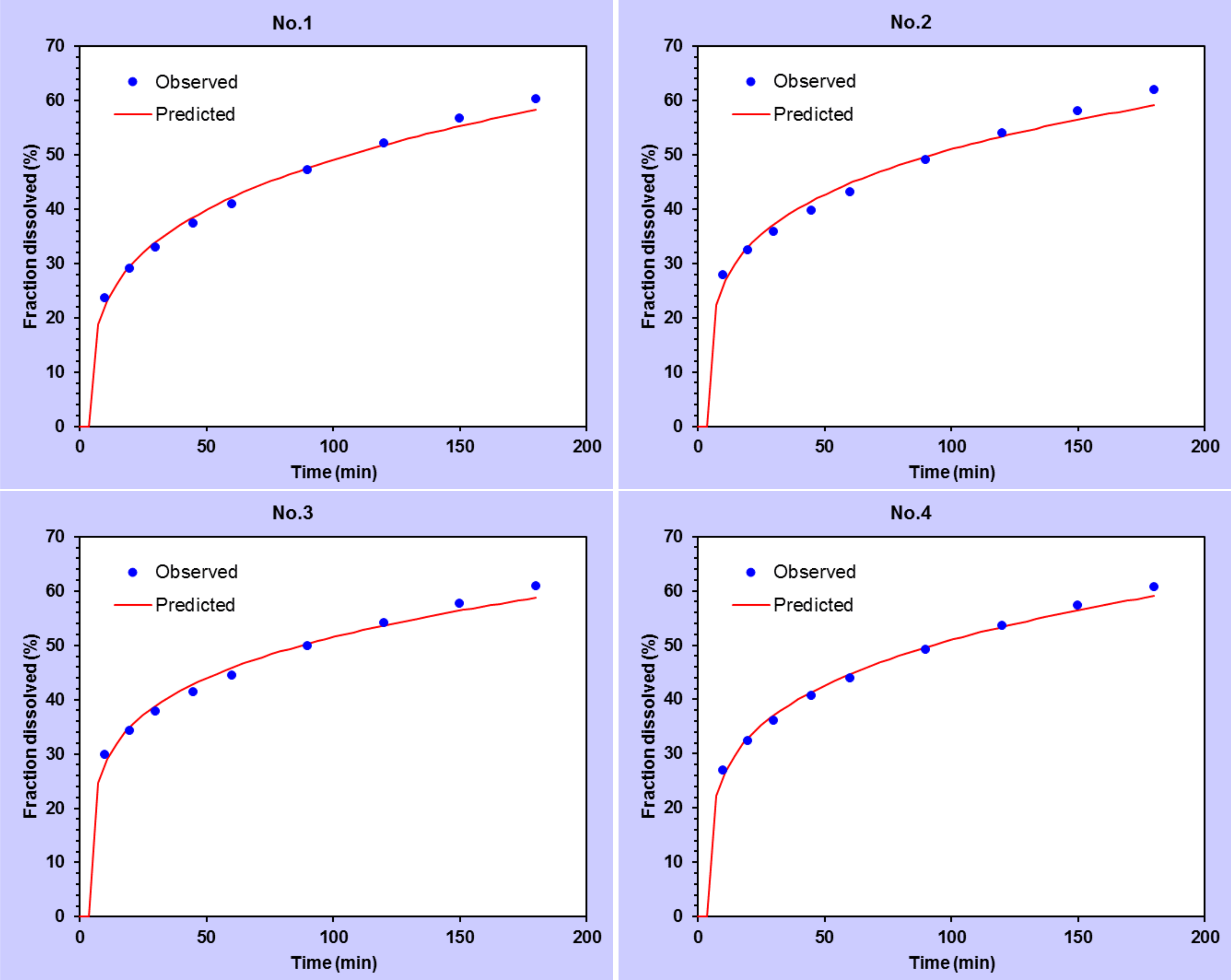

Supplementary materials – Model fitting summary – Tablettose® 70 (modelling up to app. 60 % of carvedilol released)

Model: **Korsmeyer–Peppas with F_0**

Model equation: $F = F_0 + k_{KP} \cdot t^n$

Fitted model parameters per tested tablet (N = 4) with statistics – mean, standard deviation (SD), and relative standard deviation expressed in % (RSD%) (output from DDSolver):

Parameter	No.1	No.2	No.3	No.4	Mean	SD	RSD(%)
k_{KP}	5.211	6.682	7.885	6.752	6.633	1.096	16.528
n	0.440	0.387	0.349	0.386	0.391	0.037	9.524
F_0	9.439	11.118	11.957	10.798	10.828	1.047	9.673

Number of dissolution data points (N), degrees of freedom (df), and selected goodness of fit criteria – Pearson correlation coefficient (R), coefficient of determination (R^2), adjusted coefficient of determination ($R^2_{adjusted}$), and residual sum of squares (RSS) (manual calculation in MS Excel):

Parameter	No.1	No.2	No.3	No.4
N	9	9	9	9
df	6	6	6	6
R	0.999936462	0.999320337	0.999568703	0.999735109
R^2	0.999872928	0.998641136	0.999137592	0.999470288
$R^2_{adjusted}$	0.99983057	0.998188182	0.998850123	0.999293718
RSS	0.201545894	1.866034427	0.937359029	0.689416387

Graphical abstract of model fit presented as mean ± 1 SD of the fraction % of released carvedilol:

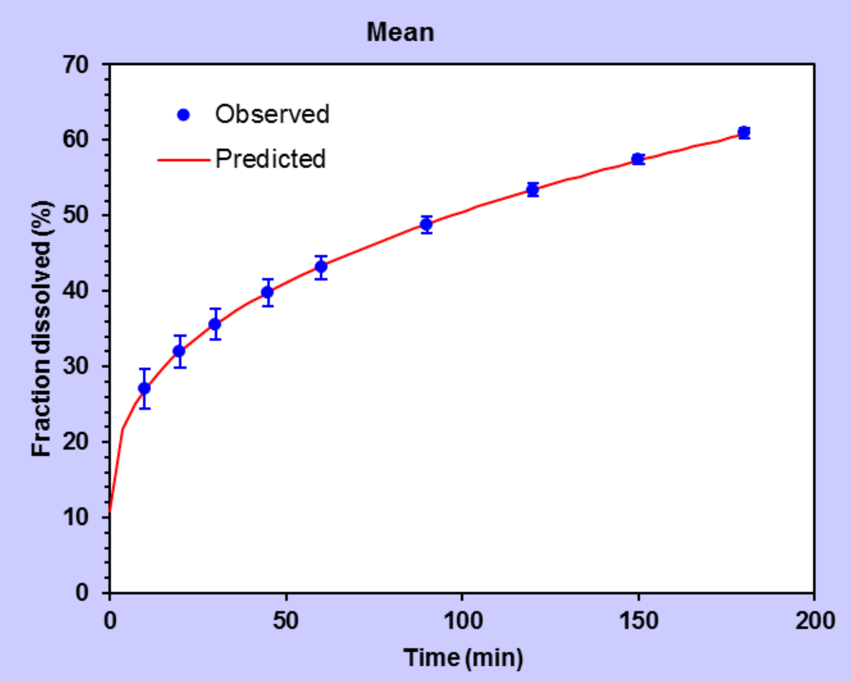

Supplementary materials – Model fitting summary – Tablettose® 70 (modelling up to app. 60 % of carvedilol released)

Graphical abstract of model fit presented as the fraction % of released carvedilol per tested tablet:

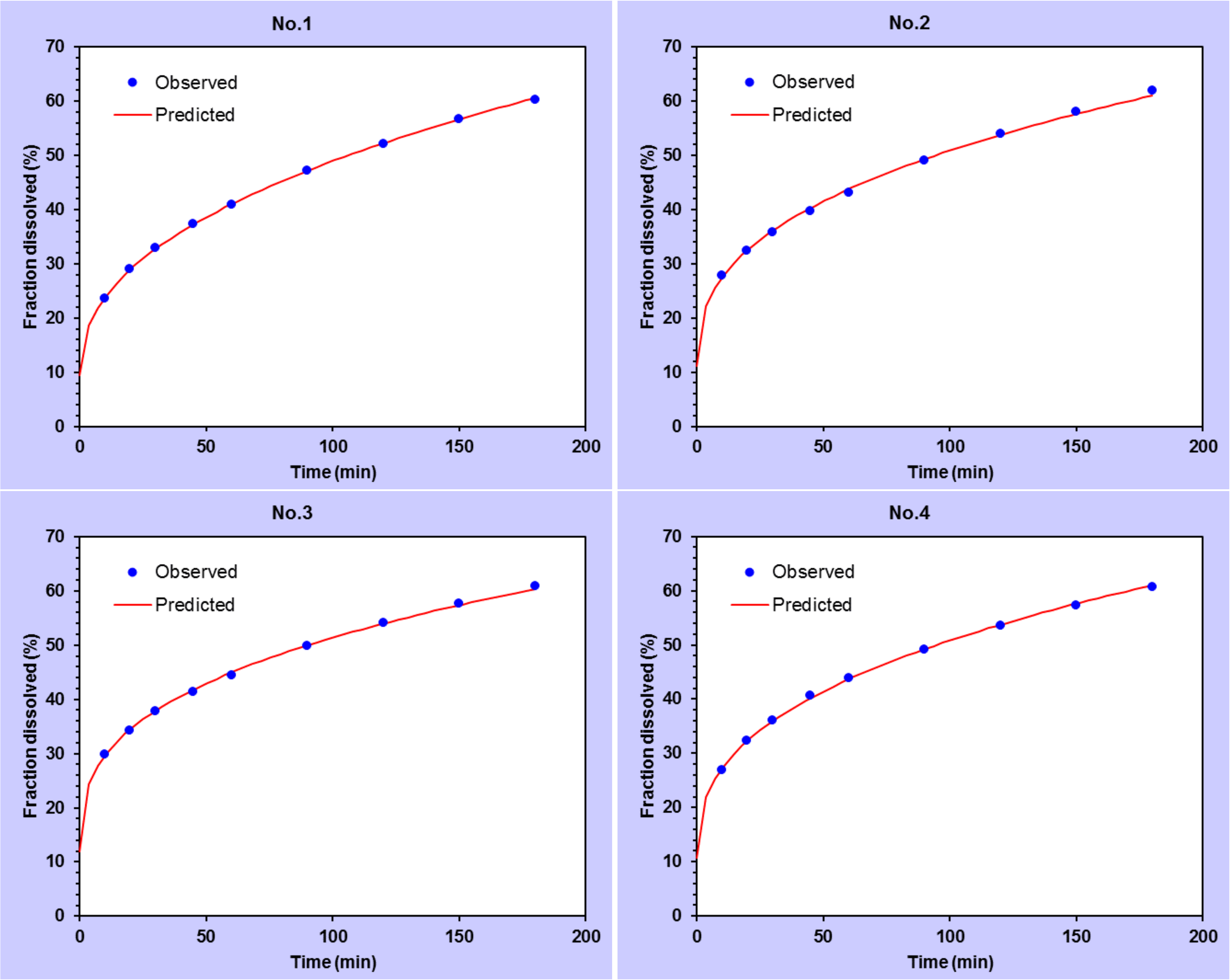

Supplementary materials – Model fitting summary – Tablettose® 70 (modelling up to app. 60 % of carvedilol released)

Model: **Hixson–Crowell**

Model equation: $F = 100 \cdot [1 - (1 - k_{HC} \cdot t)^3]$

Fitted model parameters per tested tablet (N = 4) with statistics – mean, standard deviation (SD), and relative standard deviation expressed in % (RSD%) (output from DDSolver):

Parameter	No.1	No.2	No.3	No.4	Mean	SD	RSD(%)
k_{HC}	0.002	0.002	0.002	0.002	0.002	0.000	2.159

Number of dissolution data points (N), degrees of freedom (df), and selected goodness of fit criteria – Pearson correlation coefficient (R), coefficient of determination (R^2), adjusted coefficient of determination ($R^2_{adjusted}$), and residual sum of squares (RSS) (manual calculation in MS Excel):

Parameter	No.1	No.2	No.3	No.4
N	9	9	9	9
df	8	8	8	8
R	0.993645308	0.996348733	0.994239065	0.989684066
R^2	0.987330998	0.992710798	0.988511319	0.97947455
$R^2_{adjusted}$	0.987330998	0.992710798	0.988511319	0.97947455
RSS	1506.177739	1955.950918	2343.451347	2037.380926

Graphical abstract of model fit presented as mean ± 1 SD of the fraction % of released carvedilol:

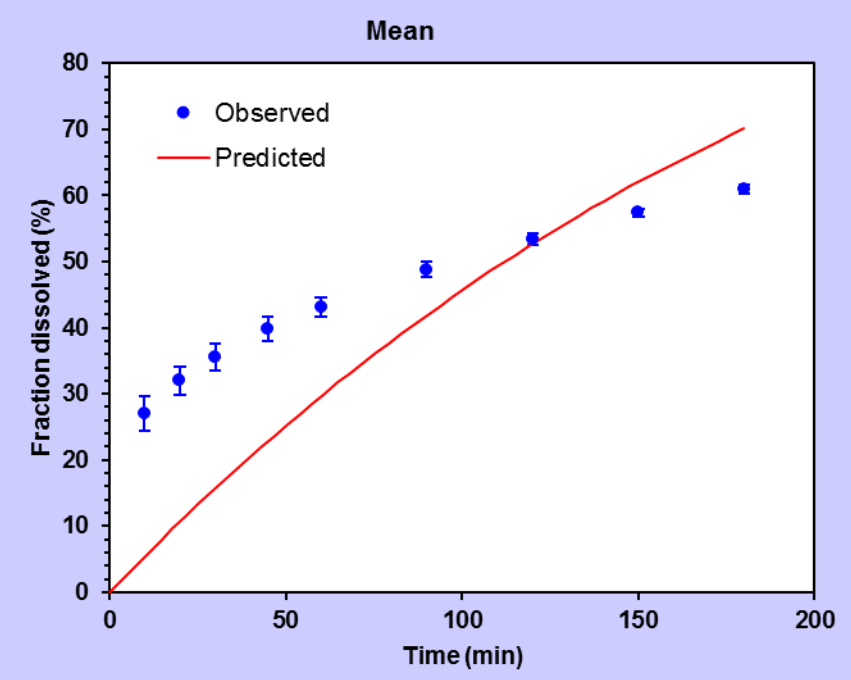

Supplementary materials – Model fitting summary – Tablettose® 70 (modelling up to app. 60 % of carvedilol released)

Graphical abstract of model fit presented as the fraction % of released carvedilol per tested tablet:

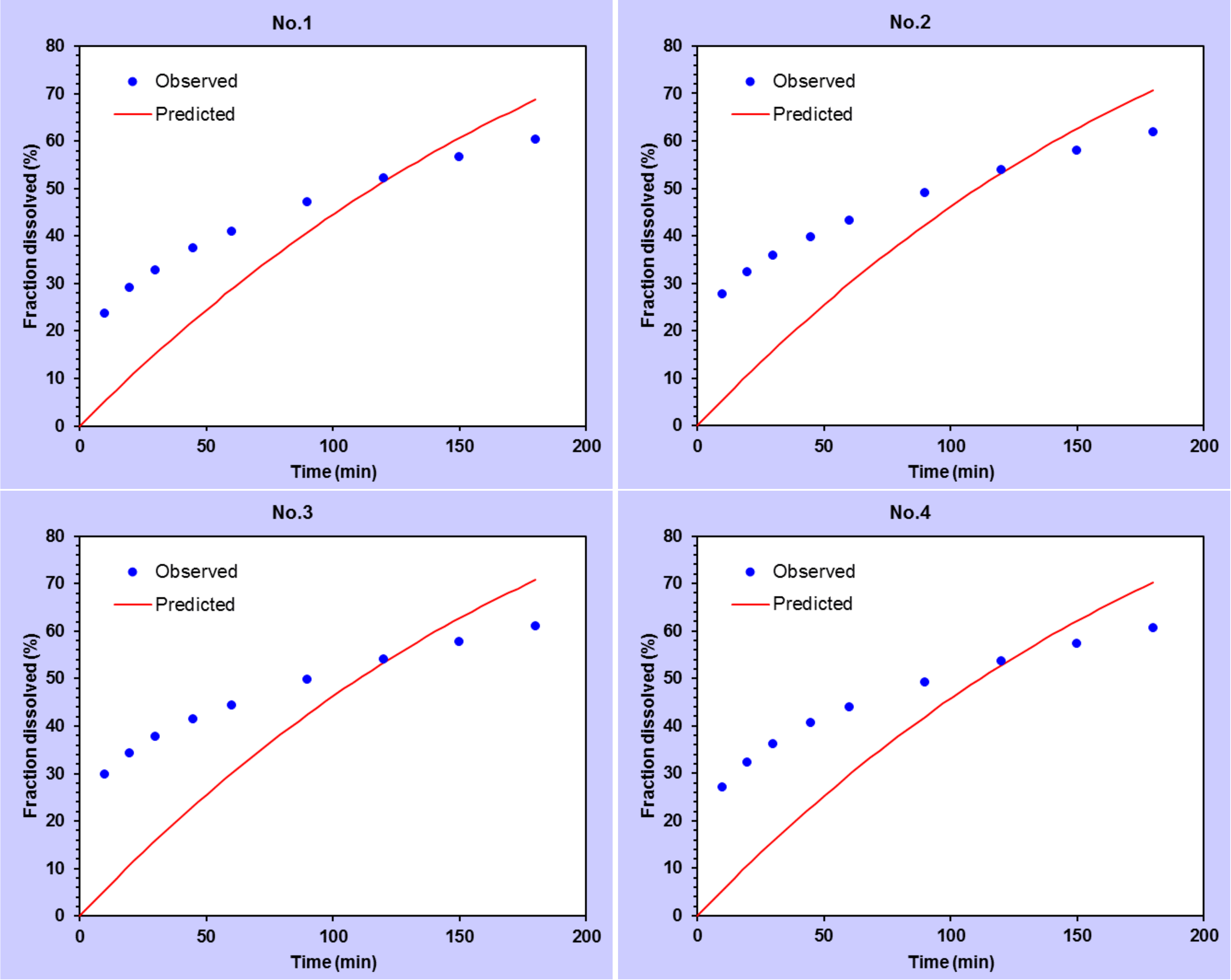

Supplementary materials – Model fitting summary – Tablettose® 70 (modelling up to app. 60 % of carvedilol released)

Model: **Hixson–Crowell with T_{lag}**

Model equation: $F = 100 \cdot \left\{1 - \left[1 - k_{HC} \cdot \left(t - T_{lag}\right)\right]^3\right\}$

Fitted model parameters per tested tablet (N = 4) with statistics – mean, standard deviation (SD), and relative standard deviation expressed in % (RSD%) (output from DDSolver):

Parameter	No.1	No.2	No.3	No.4	Mean	SD	RSD(%)
k_{HC}	0.001	0.001	0.001	0.001	0.001	0.000	5.622
T_{lag}	-89.792	-107.822	-130.699	-115.159	-110.868	16.982	-15.317

Number of dissolution data points (N), degrees of freedom (df), and selected goodness of fit criteria – Pearson correlation coefficient (R), coefficient of determination (R^2), adjusted coefficient of determination ($R^2_{adjusted}$), and residual sum of squares (RSS) (manual calculation in MS Excel):

Parameter	No.1	No.2	No.3	No.4
N	9	9	9	9
df	7	7	7	7
R	0.989419811	0.992447855	0.988776464	0.983316489
R^2	0.978951563	0.984952745	0.977678896	0.966911318
$R^2_{adjusted}$	0.975944643	0.982803137	0.974490166	0.962184363
RSS	27.7816026	17.19022593	21.02076238	36.06581805

Graphical abstract of model fit presented as mean ± 1 SD of the fraction % of released carvedilol:

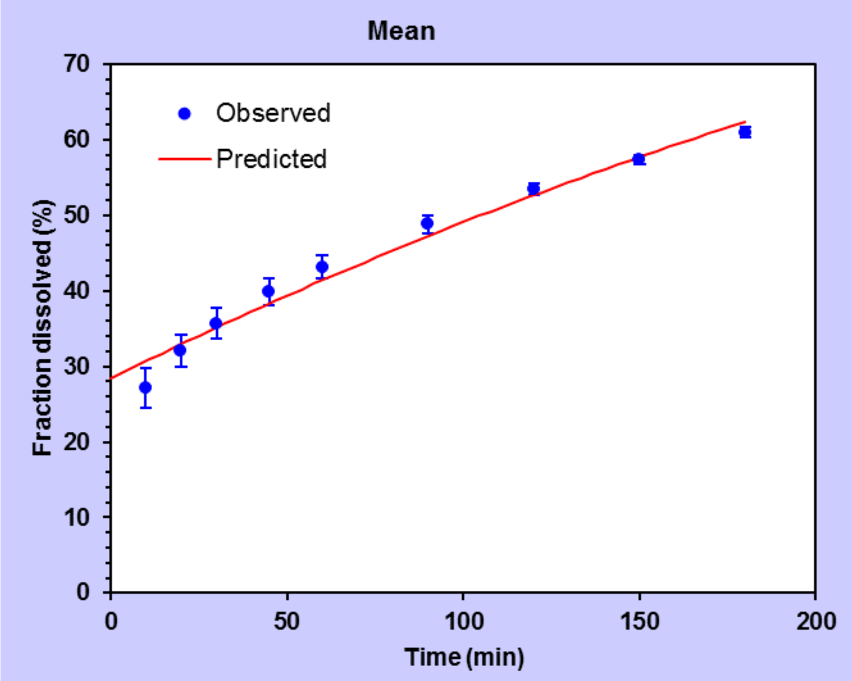

Supplementary materials – Model fitting summary – Tablettose® 70 (modelling up to app. 60 % of carvedilol released)

Graphical abstract of model fit presented as the fraction % of released carvedilol per tested tablet:

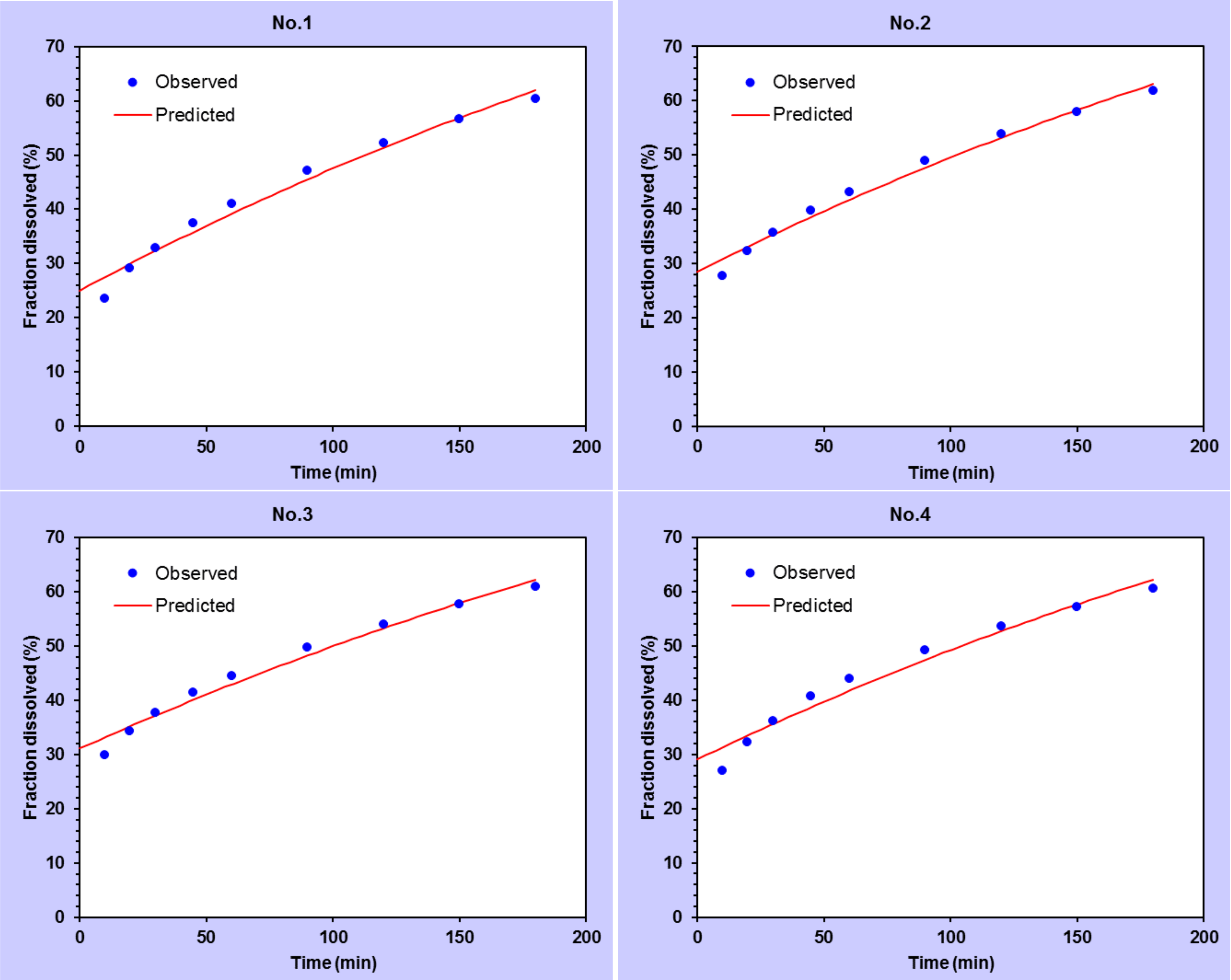

Supplementary materials – Model fitting summary – Tablettose® 70 (modelling up to app. 60 % of carvedilol released)

Model: **Hopfenberg**

Model equation: $F = 100 \cdot [1 - (1 - k_{HB} \cdot t)^n]$

Fitted model parameters per tested tablet (N = 4) with statistics – mean, standard deviation (SD), and relative standard deviation expressed in % (RSD%) (output from DDSolver):

Parameter	No.1	No.2	No.3	No.4	Mean	SD	RSD(%)
k_{HB}	0.002	0.002	0.002	0.002	0.002	0.000	2.159
n	3.000	3.000	4.500	3.000	3.375	0.750	22.222

Number of dissolution data points (N), degrees of freedom (df), and selected goodness of fit criteria – Pearson correlation coefficient (R), coefficient of determination (R^2), adjusted coefficient of determination ($R^2_{adjusted}$), and residual sum of squares (RSS) (manual calculation in MS Excel):

Parameter	No.1	No.2	No.3	No.4
N	9	9	9	9
df	7	7	7	7
R	0.993645308	0.996348733	0.998134439	0.989684066
R^2	0.987330998	0.992710798	0.996272359	0.97947455
$R^2_{adjusted}$	0.98552114	0.991669483	0.995739838	0.976542343
RSS	1506.177739	1955.950918	2289.677956	2037.380926

Graphical abstract of model fit presented as mean ± 1 SD of the fraction % of released carvedilol:

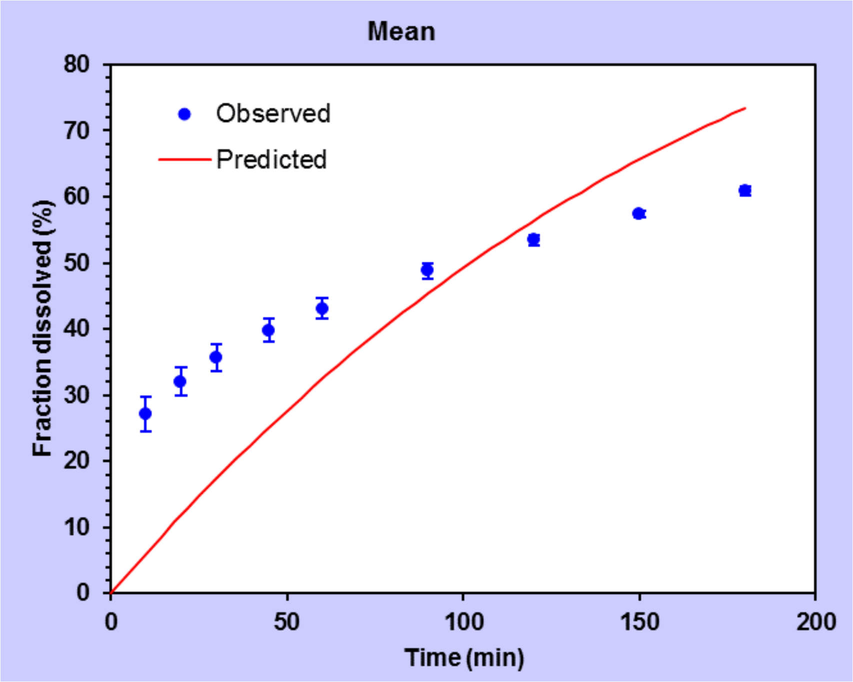

Supplementary materials – Model fitting summary – Tablettose® 70 (modelling up to app. 60 % of carvedilol released)

Graphical abstract of model fit presented as the fraction % of released carvedilol per tested tablet:

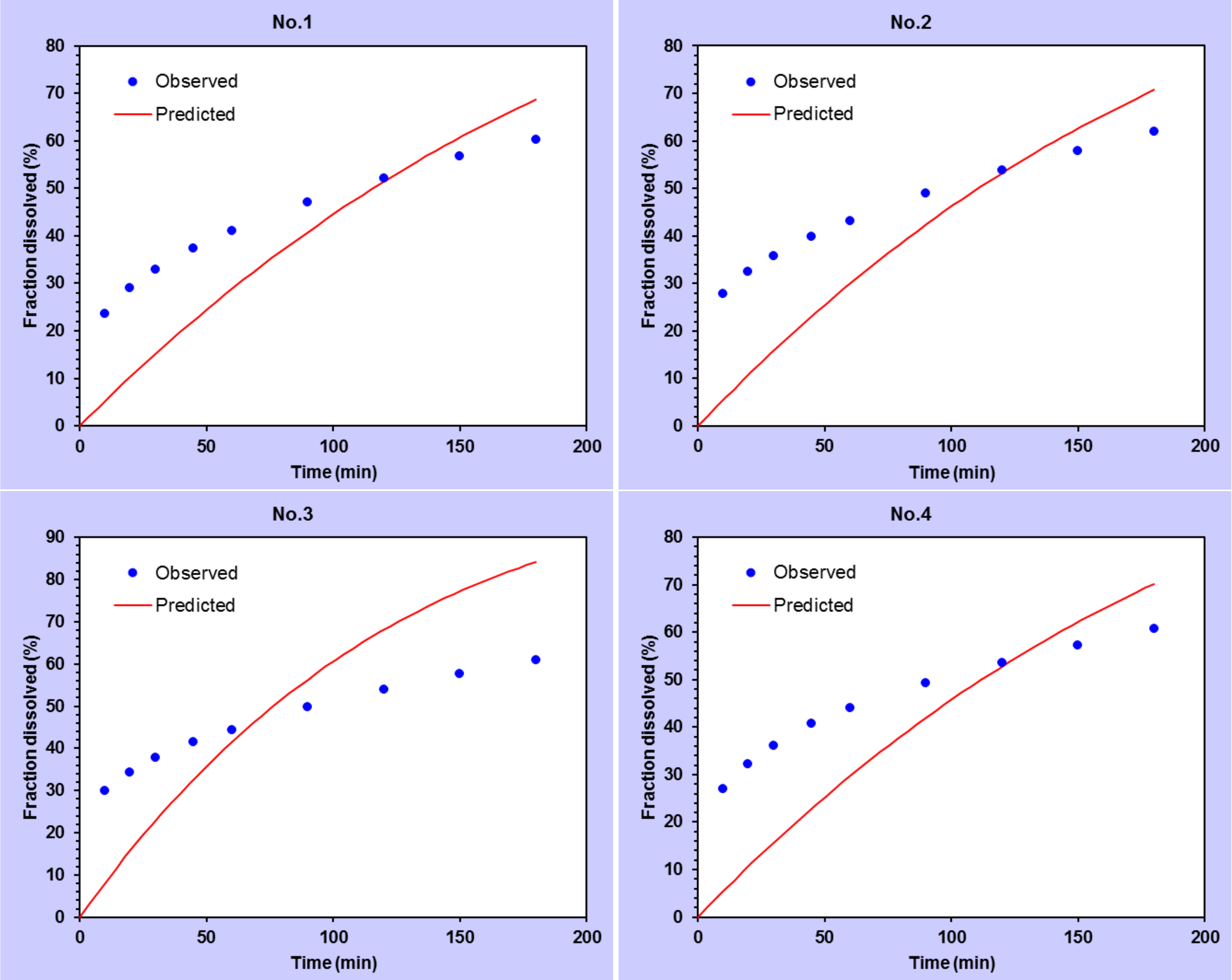

Supplementary materials – Model fitting summary – Tablettose® 70 (modelling up to app. 60 % of carvedilol released)

Model: **Hopfenberg with T_{lag}**

Model equation: $F = 100 \cdot \{1 - [1 - k_{HB} \cdot (t - T_{lag})]^n\}$

Fitted model parameters per tested tablet (N = 4) with statistics – mean, standard deviation (SD), and relative standard deviation expressed in % (RSD%) (output from DDSolver):

Parameter	No.1	No.2	No.3	No.4	Mean	SD	RSD(%)
k_{HB}	0.001	0.001	0.001	0.001	0.001	0.000	10.308
n	3.000	3.000	3.000	3.637	3.159	0.318	10.081
T_{lag}	-89.792	-107.822	-130.699	-111.708	-110.005	16.778	-15.252

Number of dissolution data points (N), degrees of freedom (df), and selected goodness of fit criteria – Pearson correlation coefficient (R), coefficient of determination (R^2), adjusted coefficient of determination ($R^2_{adjusted}$), and residual sum of squares (RSS) (manual calculation in MS Excel):

Parameter	No.1	No.2	No.3	No.4
N	9	9	9	9
df	6	6	6	6
R	0.989419811	0.992447855	0.988776464	0.984051305
R^2	0.978951563	0.984952745	0.977678896	0.968356971
$R^2_{adjusted}$	0.971935417	0.979936993	0.970238527	0.957809294
RSS	27.7816026	17.19022593	21.02076238	34.60456777

Graphical abstract of model fit presented as mean ± 1 SD of the fraction % of released carvedilol:

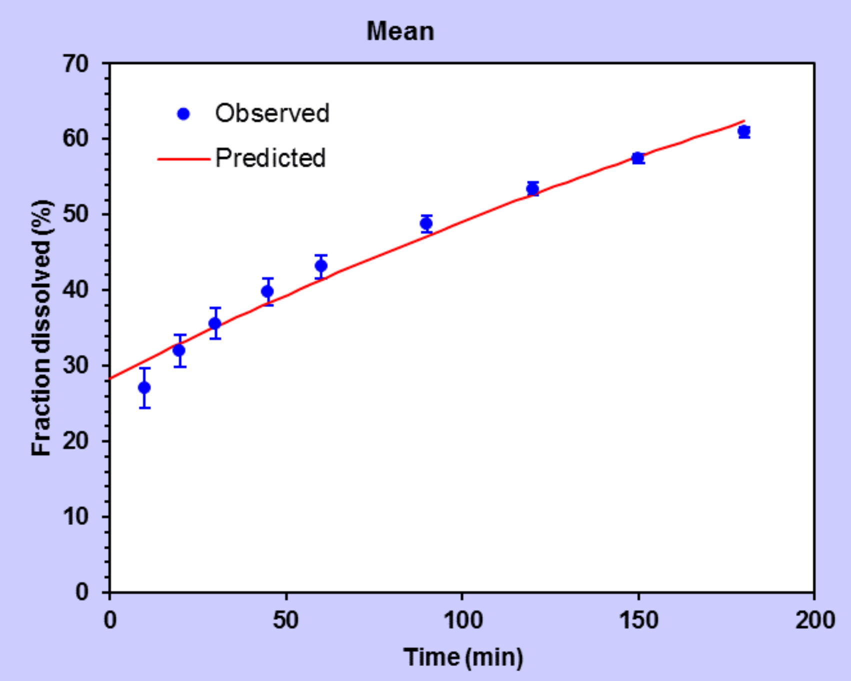

Supplementary materials – Model fitting summary – Tablettose® 70 (modelling up to app. 60 % of carvedilol released)

Graphical abstract of model fit presented as the fraction % of released carvedilol per tested tablet:

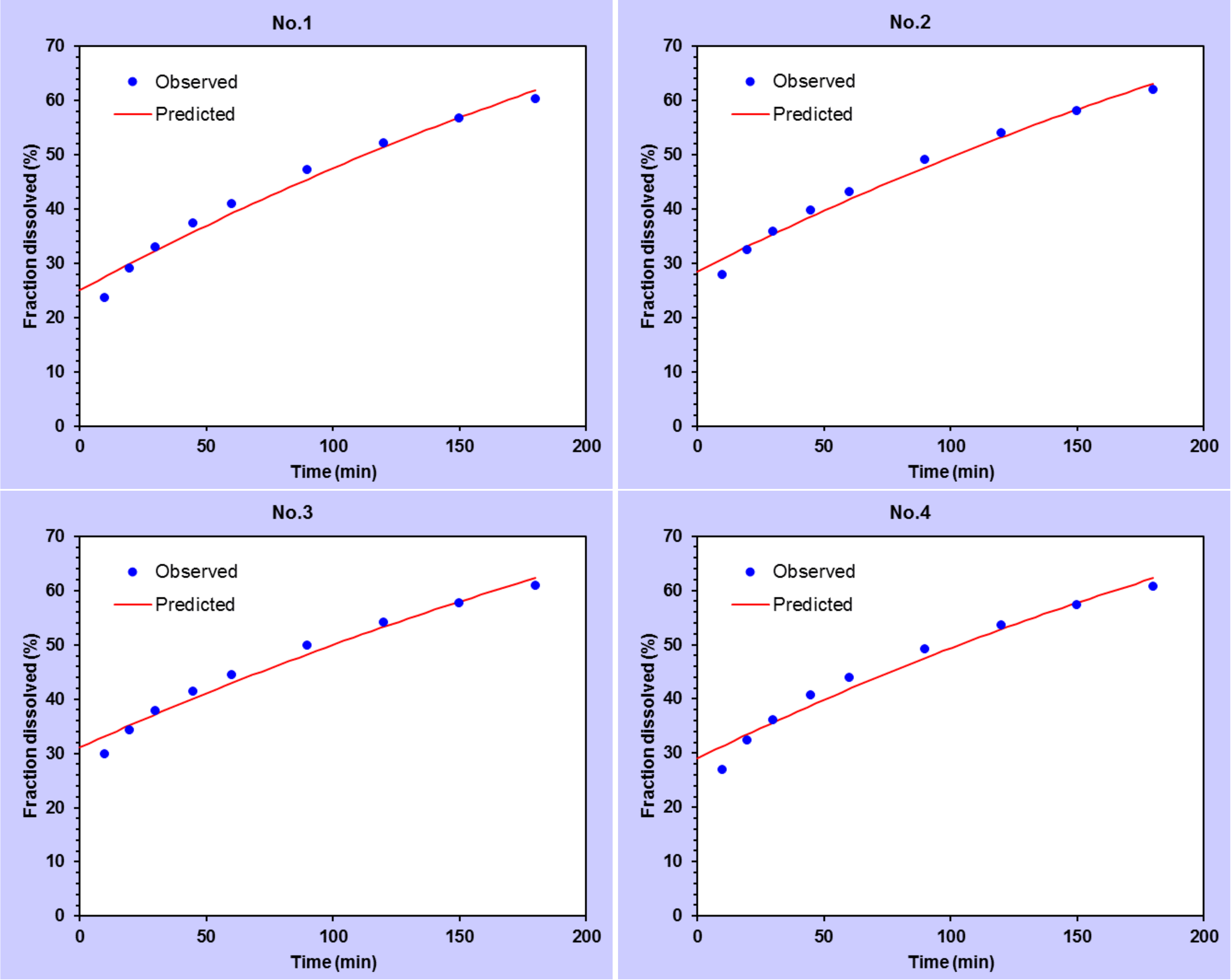

Supplementary materials – Model fitting summary – Tablettose® 70 (modelling up to app. 60 % of carvedilol released)

Model: **Baker–Lonsdale**

Model equation: $\frac{3}{2}\cdot\left[1-\left(1-\frac{F}{100}\right)^{\frac{2}{3}}\right]-\frac{F}{100}=k_{BL}\cdot t$

Fitted model parameters per tested tablet (N = 4) with statistics – mean, standard deviation (SD), and relative standard deviation expressed in % (RSD%) (output from DDSolver):

Parameter	No.1	No.2	No.3	No.4	Mean	SD	RSD(%)
k_{BL}	0.001	0.001	0.001	0.001	0.001	0.000	3.804

Number of dissolution data points (N), degrees of freedom (df), and selected goodness of fit criteria – Pearson correlation coefficient (R), coefficient of determination (R^2), adjusted coefficient of determination ($R^2_{adjusted}$), and residual sum of squares (RSS) (manual calculation in MS Excel):

Parameter	No.1	No.2	No.3	No.4
N	9	9	9	9
df	8	8	8	8
R	0.999607277	0.998742742	0.999657567	0.999817162
R^2	0.999214709	0.997487065	0.99931525	0.999634358
$R^2_{adjusted}$	0.999214709	0.997487065	0.99931525	0.999634358
RSS	212.256868	238.60413	332.6349347	232.8671474

Graphical abstract of model fit presented as mean ± 1 SD of the fraction % of released carvedilol:

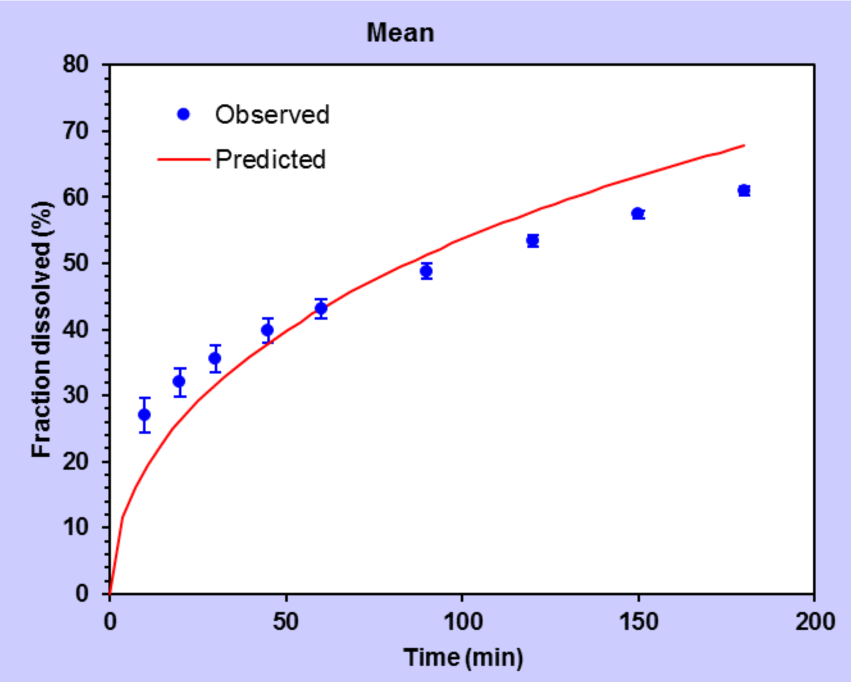

Supplementary materials – Model fitting summary – Tablettose® 70 (modelling up to app. 60 % of carvedilol released)

Graphical abstract of model fit presented as the fraction % of released carvedilol per tested tablet:

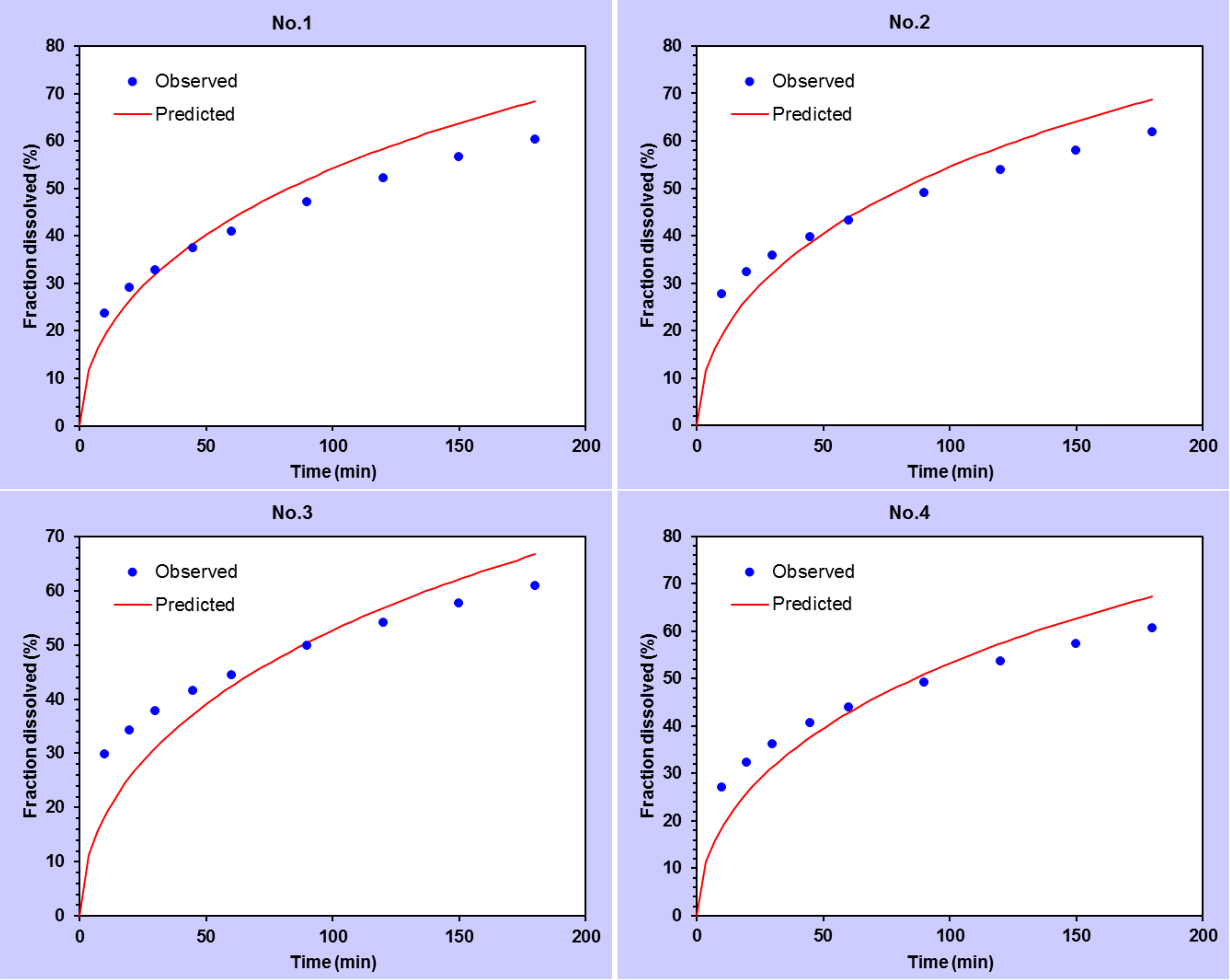

Supplementary materials – Model fitting summary – Tablettose® 70 (modelling up to app. 60 % of carvedilol released)

Model: **Baker–Lonsdale with T_{lag}**

Model equation: $\frac{3}{2}\cdot\left[1-\left(1-\frac{F}{100}\right)^{\frac{2}{3}}\right]-\frac{F}{100}=k_{BL}\cdot\left(t-T_{lag}\right)$

Fitted model parameters per tested tablet (N = 4) with statistics – mean, standard deviation (SD), and relative standard deviation expressed in % (RSD%) (output from DDSolver):

Parameter	No.1	No.2	No.3	No.4	Mean	SD	RSD(%)
k_{BL}	0.000	0.000	0.000	0.000	0.000	0.000	3.804
T_{lag}	-16.890	-25.763	-38.002	-30.619	-27.819	8.854	-31.829

Number of dissolution data points (N), degrees of freedom (df), and selected goodness of fit criteria – Pearson correlation coefficient (R), coefficient of determination (R^2), adjusted coefficient of determination ($R^2_{adjusted}$), and residual sum of squares (RSS) (manual calculation in MS Excel):

Parameter	No.1	No.2	No.3	No.4
N	9	9	9	9
df	7	7	7	7
R	0.998990156	0.99932942	0.997271847	0.995066177
R^2	0.997981331	0.99865929	0.994551138	0.990156696
$R^2_{adjusted}$	0.99769295	0.99846776	0.993772729	0.98875051
RSS	2.976577876	1.629583285	5.630869845	12.08364984

Graphical abstract of model fit presented as mean ± 1 SD of the fraction % of released carvedilol:

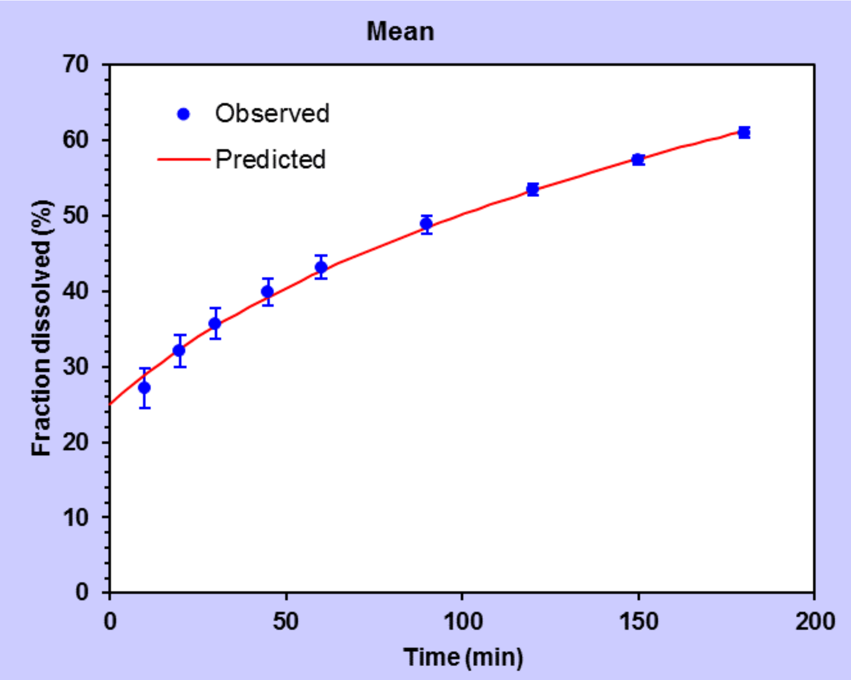

Supplementary materials – Model fitting summary – Tablettose® 70 (modelling up to app. 60 % of carvedilol released)

Graphical abstract of model fit presented as the fraction % of released carvedilol per tested tablet:

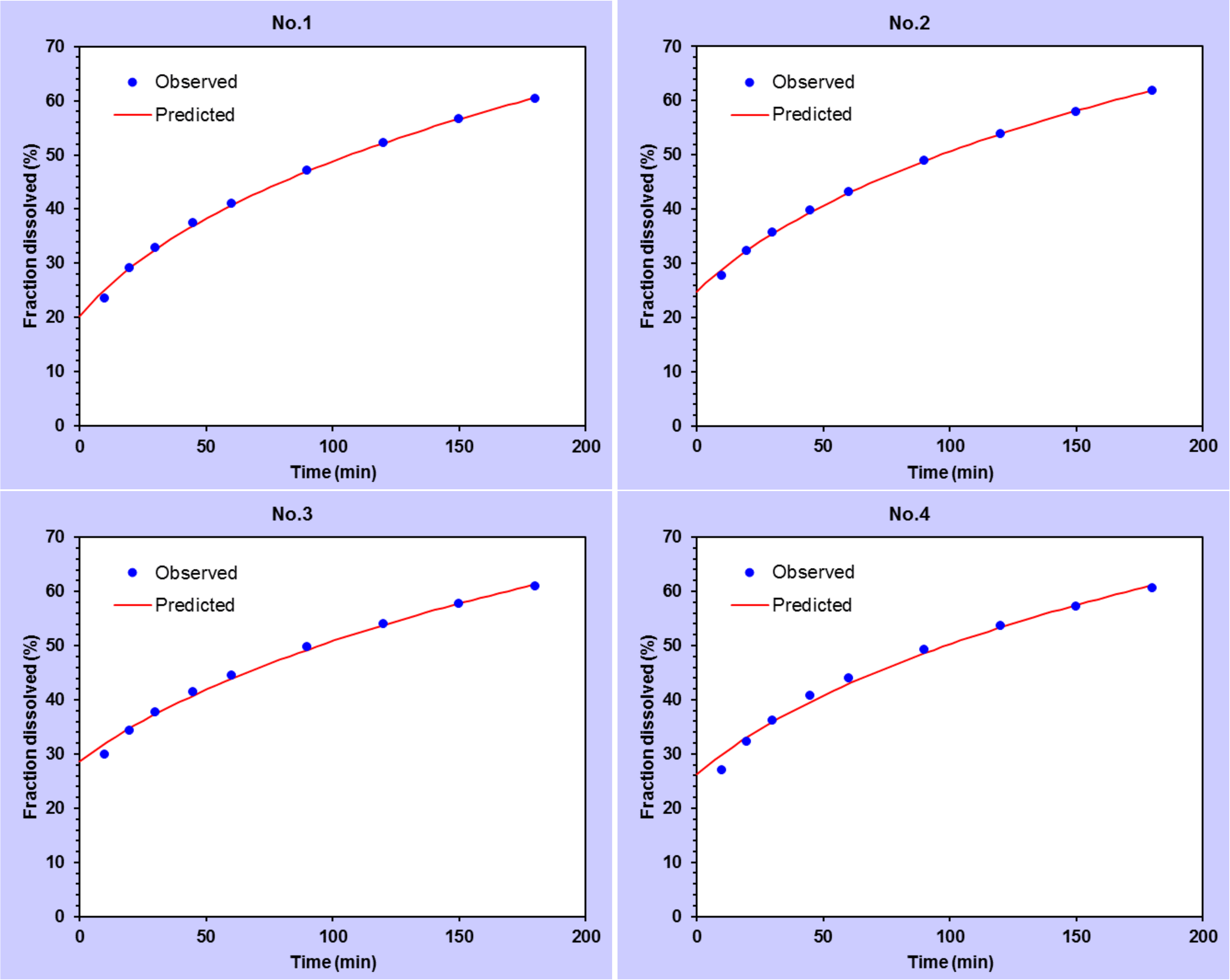

Supplementary materials – Model fitting summary – Tablettose® 70 (modelling up to app. 60 % of carvedilol released)

Model: **Makoid–Banakar**

Model equation: $F = k_{MB} \cdot t^n \cdot e^{-k \cdot t}$

Fitted model parameters per tested tablet (N = 4) with statistics – mean, standard deviation (SD), and relative standard deviation expressed in % (RSD%) (output from DDSolver):

Parameter	No.1	No.2	No.3	No.4	Mean	SD	RSD(%)
k_{MB}	11.8939	16.4720	18.4674	14.5669	15.3501	2.8009	18.2467
n	0.2947	0.2203	0.2035	0.2655	0.2460	0.0417	16.9486
k	-0.0005	-0.0010	-0.0008	-0.0003	-0.0007	0.0003	-50.0926

Number of dissolution data points (N), degrees of freedom (df), and selected goodness of fit criteria – Pearson correlation coefficient (R), coefficient of determination (R^2), adjusted coefficient of determination ($R^2_{adjusted}$), and residual sum of squares (RSS) (manual calculation in MS Excel):

Parameter	No.1	No.2	No.3	No.4
N	9	9	9	9
df	6	6	6	6
R	0.999942106	0.999799228	0.999825446	0.999959431
R^2	0.999884215	0.999598496	0.999650923	0.999918863
$R^2_{adjusted}$	0.99984562	0.999464661	0.999534564	0.999891817
RSS	0.149603451	0.449756765	0.323059621	0.086796429

Graphical abstract of model fit presented as mean ± 1 SD of the fraction % of released carvedilol:

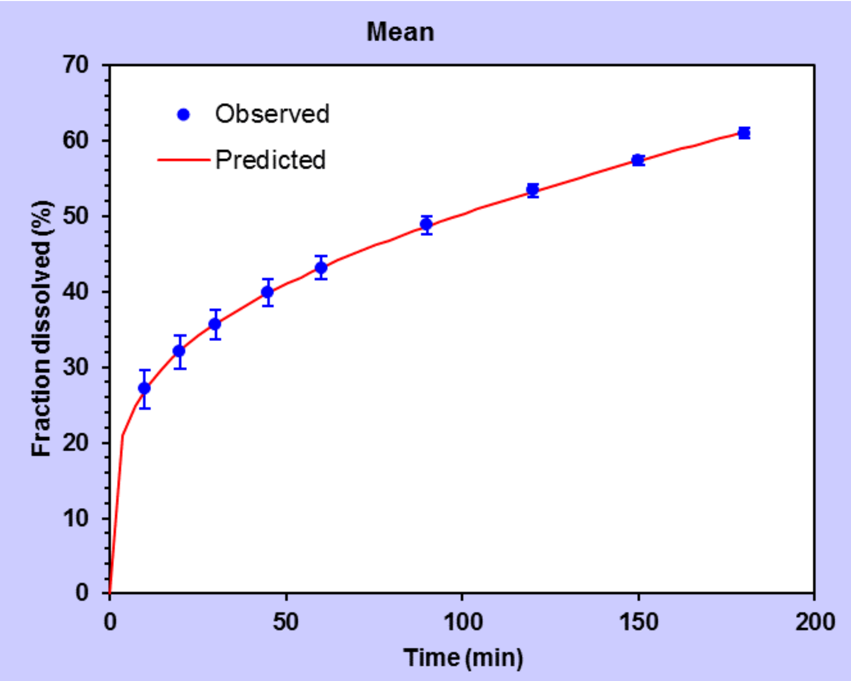

Supplementary materials – Model fitting summary – Tablettose® 70 (modelling up to app. 60 % of carvedilol released)

Graphical abstract of model fit presented as the fraction % of released carvedilol per tested tablet:

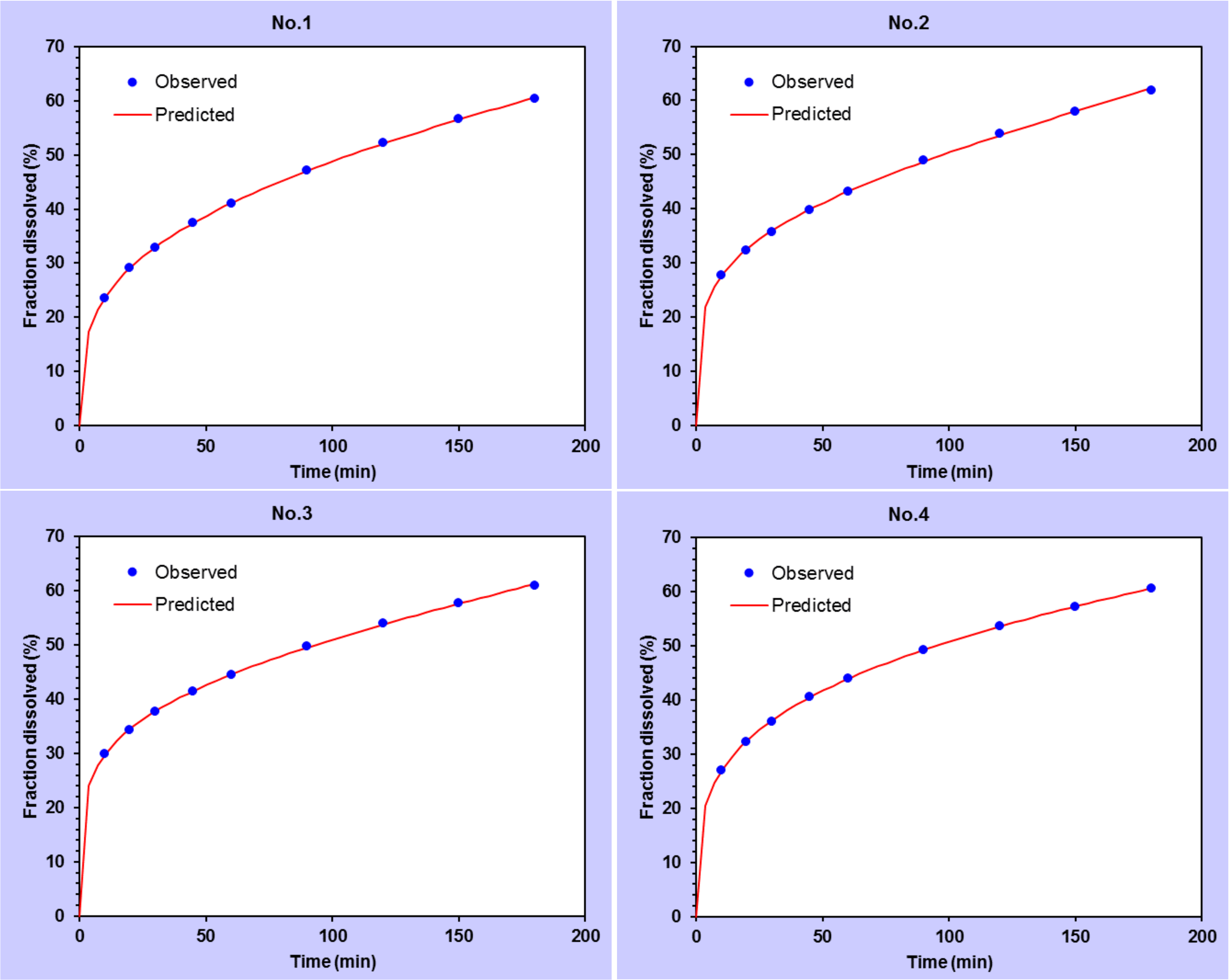

Supplementary materials – Model fitting summary – Tablettose® 70 (modelling up to app. 60 % of carvedilol released)

Model: **Makoid–Banakar with T_{lag}**

Model equation: $F = k_{MB} \cdot (t - T_{lag})^n \cdot e^{-k\cdot(t-T_{lag})}$

Fitted model parameters per tested tablet (N = 4) with statistics – mean, standard deviation (SD), and relative standard deviation expressed in % (RSD%) (output from DDSolver):

Parameter	No.1	No.2	No.3	No.4	Mean	SD	RSD(%)
k_{MB}	15.5267	20.1865	22.2651	18.5098	19.1220	2.8469	14.8878
n	0.2237	0.1666	0.1540	0.2013	0.1864	0.0319	17.1222
k	-0.0012	-0.0015	-0.0013	-0.0009	-0.0012	0.0003	-22.6775
T_{lag}	4.0000	4.0000	4.0000	4.0000	4.0000	0.0000	0.0000

Number of dissolution data points (N), degrees of freedom (df), and selected goodness of fit criteria – Pearson correlation coefficient (R), coefficient of determination (R^2), adjusted coefficient of determination ($R^2_{adjusted}$), and residual sum of squares (RSS) (manual calculation in MS Excel):

Parameter	No.1	No.2	No.3	No.4
N	9	9	9	9
df	5	5	5	5
R	0.999495718	0.999207808	0.999247496	0.999535194
R^2	0.99899169	0.998416244	0.998495557	0.999070605
$R^2_{adjusted}$	0.998386704	0.99746599	0.997592892	0.998512967
RSS	1.304162601	1.776975814	1.393453089	0.99429453

Graphical abstract of model fit presented as mean ± 1 SD of the fraction % of released carvedilol:

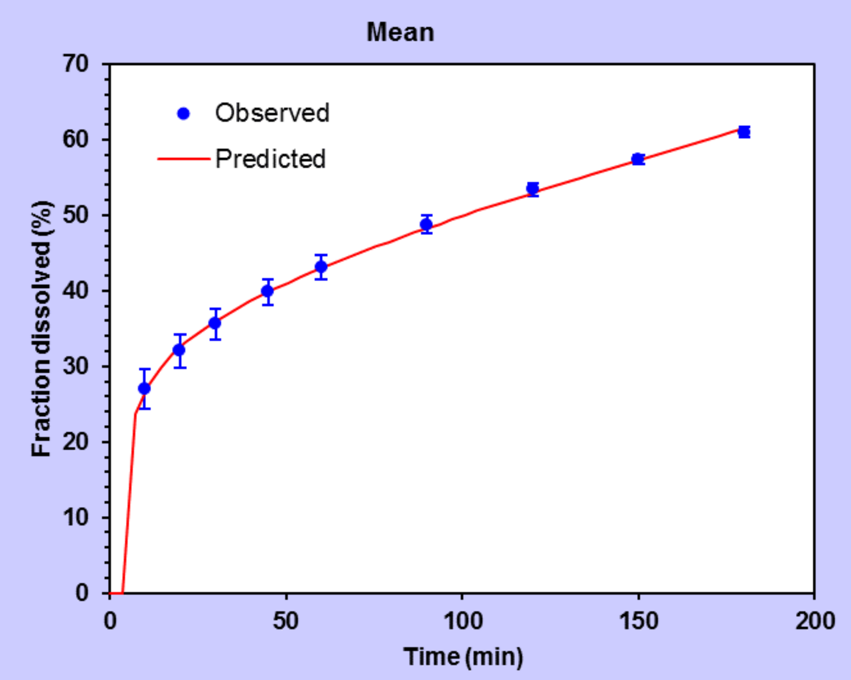

Supplementary materials – Model fitting summary – Tablettose® 70 (modelling up to app. 60 % of carvedilol released)

Graphical abstract of model fit presented as the fraction % of released carvedilol per tested tablet:

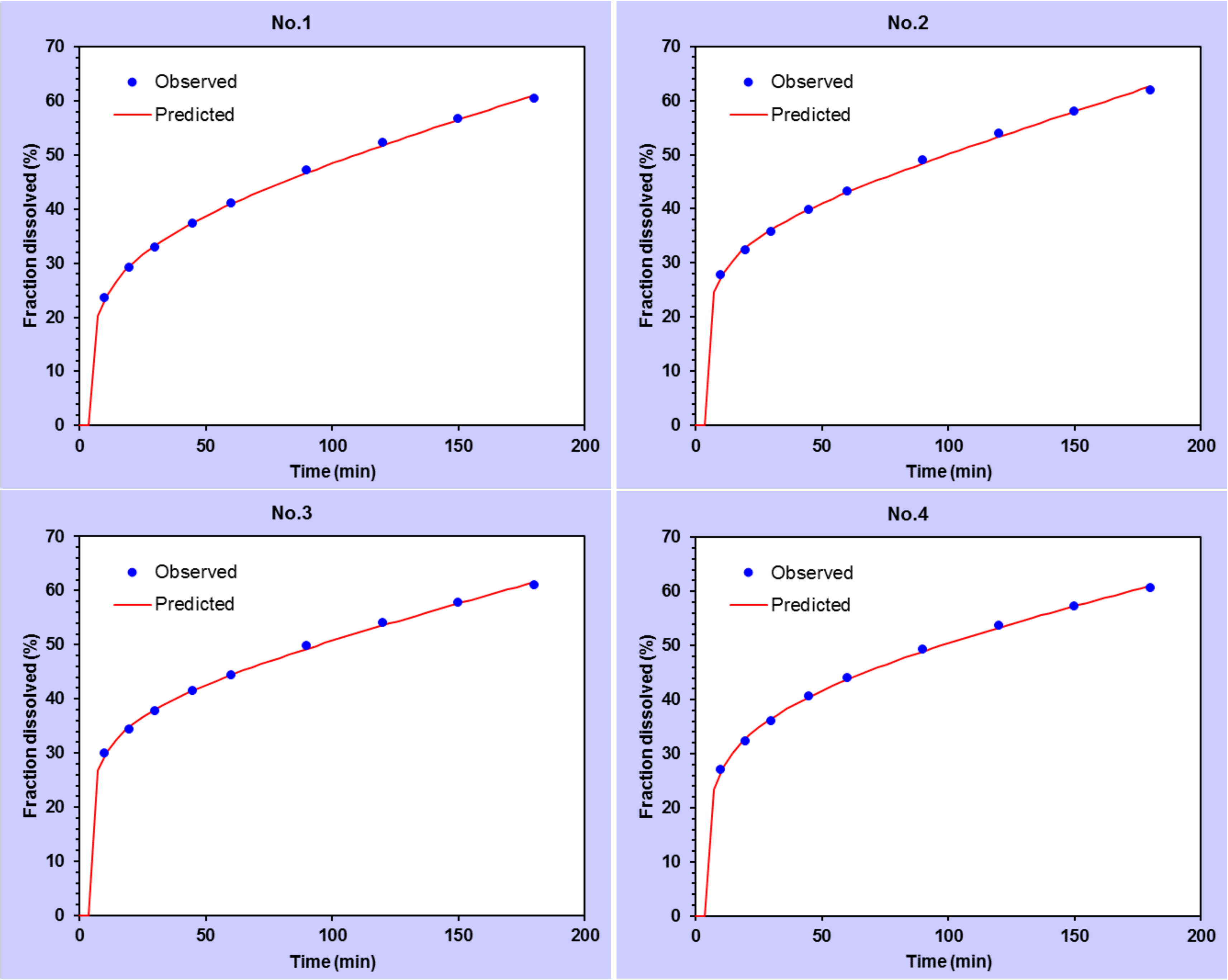

Supplementary materials – Model fitting summary – Tablettose® 70 (modelling up to app. 60 % of carvedilol released)

Model: **Peppas–Sahlin_1**

Model equation: $F = k_1 \cdot t^m + k_2 \cdot t^{2m}$

Fitted model parameters per tested tablet (N = 4) with statistics – mean, standard deviation (SD), and relative standard deviation expressed in % (RSD%) (output from DDSolver):

Parameter	No.1	No.2	No.3	No.4	Mean	SD	RSD(%)
k_1	8.189	11.791	11.452	10.994	10.606	1.644	15.503
k_2	-0.239	-0.275	-0.417	-0.374	-0.326	0.083	-25.539
m	0.450	0.345	0.374	0.377	0.387	0.045	11.538

Number of dissolution data points (N), degrees of freedom (df), and selected goodness of fit criteria – Pearson correlation coefficient (R), coefficient of determination (R^2), adjusted coefficient of determination ($R^2_{adjusted}$), and residual sum of squares (RSS) (manual calculation in MS Excel):

Parameter	No.1	No.2	No.3	No.4
N	9	9	9	9
df	6	6	6	6
R	0.996155406	0.996526832	0.995949664	0.9987627
R^2	0.992325593	0.993065726	0.991915733	0.99752693
$R^2_{adjusted}$	0.989767457	0.990754302	0.989220977	0.996702574
RSS	12.31523041	13.45898814	43.48299585	18.36242173

Graphical abstract of model fit presented as mean ± 1 SD of the fraction % of released carvedilol:

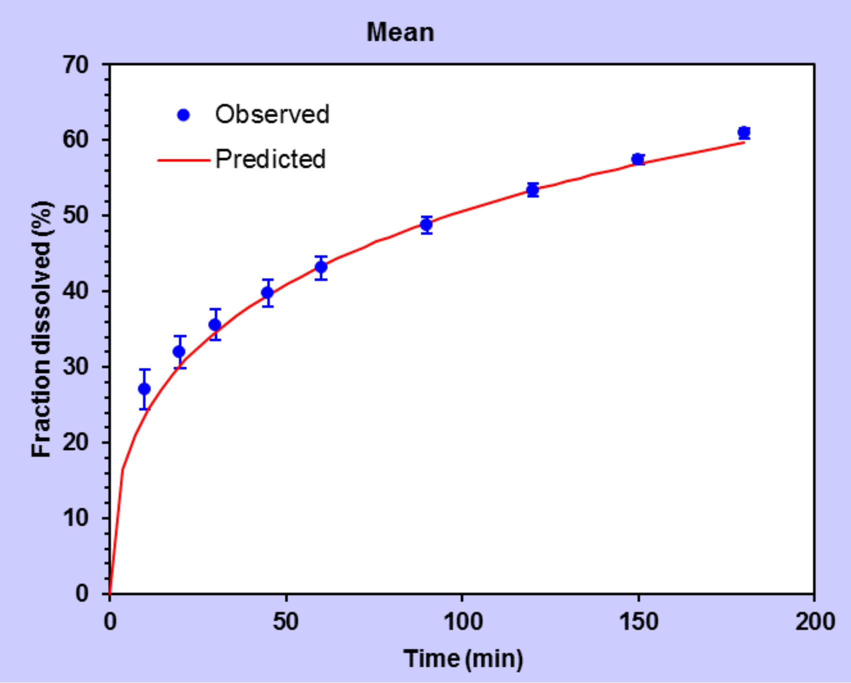

Supplementary materials – Model fitting summary – Tablettose® 70 (modelling up to app. 60 % of carvedilol released)

Graphical abstract of model fit presented as the fraction % of released carvedilol per tested tablet:

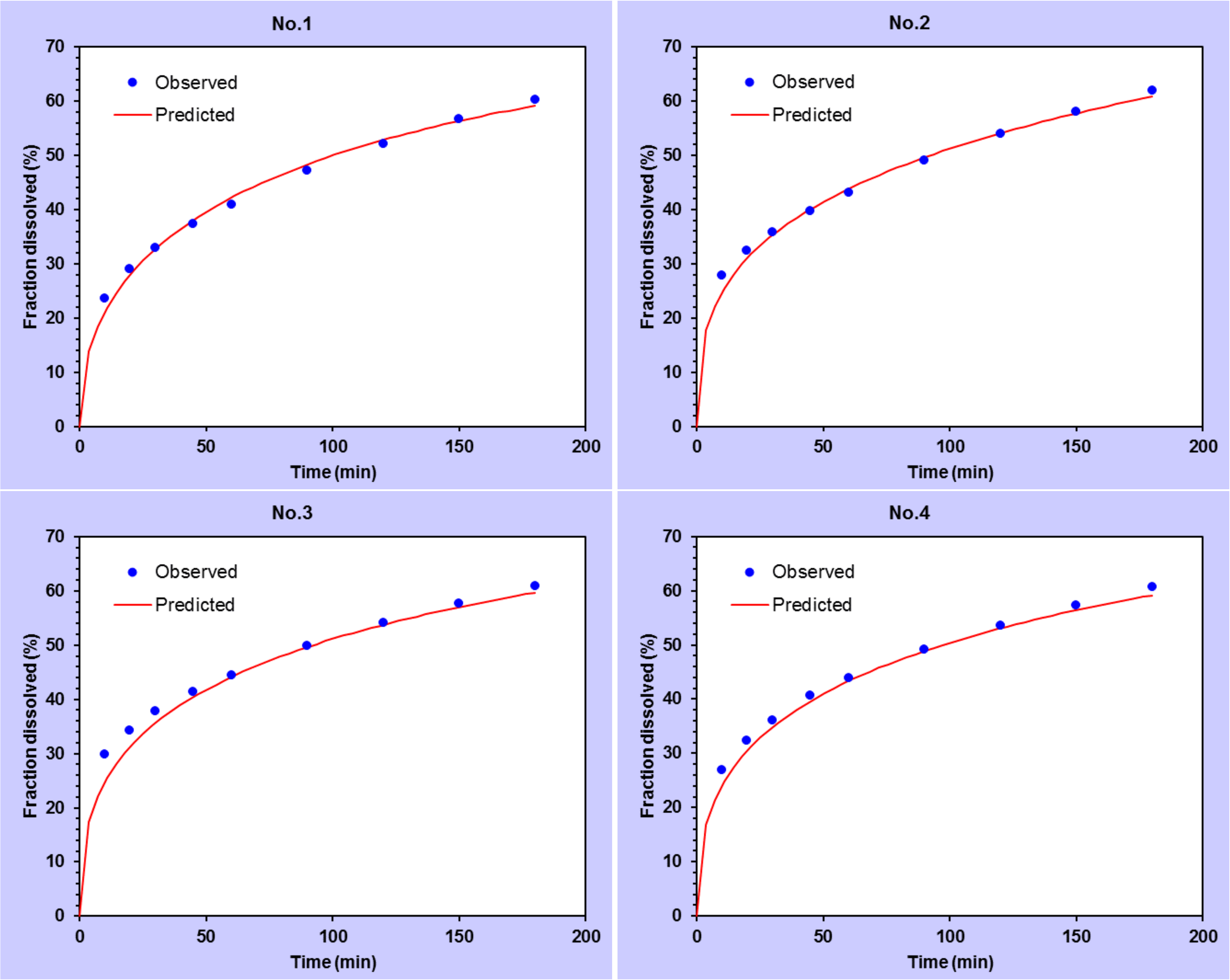

Supplementary materials – Model fitting summary – Tablettose® 70 (modelling up to app. 60 % of carvedilol released)

Model: **Peppas–Sahlin_1 with T_{lag}**

Model equation: $F = k_1 \cdot (t - T_{lag})^m + k_2 \cdot (t - T_{lag})^{2m}$

Fitted model parameters per tested tablet (N = 4) with statistics – mean, standard deviation (SD), and relative standard deviation expressed in % (RSD%) (output from DDSolver):

Parameter	No.1	No.2	No.3	No.4	Mean	SD	RSD(%)
k_1	8.958	10.067	10.897	10.293	10.054	0.810	8.056
k_2	-0.317	-0.419	-0.512	-0.451	-0.425	0.081	-19.149
m	0.450	0.450	0.450	0.450	0.450	0.000	0.000
T_{lag}	4.000	4.000	4.000	4.000	4.000	0.000	0.000

Number of dissolution data points (N), degrees of freedom (df), and selected goodness of fit criteria – Pearson correlation coefficient (R), coefficient of determination (R^2), adjusted coefficient of determination ($R^2_{adjusted}$), and residual sum of squares (RSS) (manual calculation in MS Excel):

Parameter	No.1	No.2	No.3	No.4
N	9	9	9	9
df	5	5	5	5
R	0.98805775	0.97357621	0.964601813	0.981071511
R^2	0.976258118	0.947850636	0.930456658	0.96250131
$R^2_{adjusted}$	0.962012989	0.916561018	0.888730653	0.940002096
RSS	43.02429227	87.55627204	105.1417697	61.94817206

Graphical abstract of model fit presented as mean ± 1 SD of the fraction % of released carvedilol:

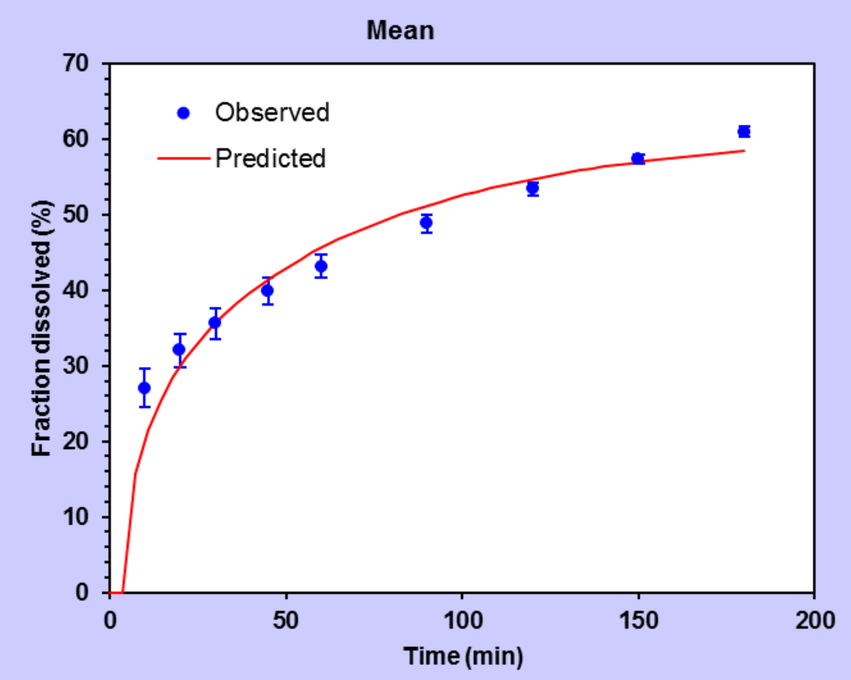

Supplementary materials – Model fitting summary – Tablettose® 70 (modelling up to app. 60 % of carvedilol released)

Graphical abstract of model fit presented as the fraction % of released carvedilol per tested tablet:

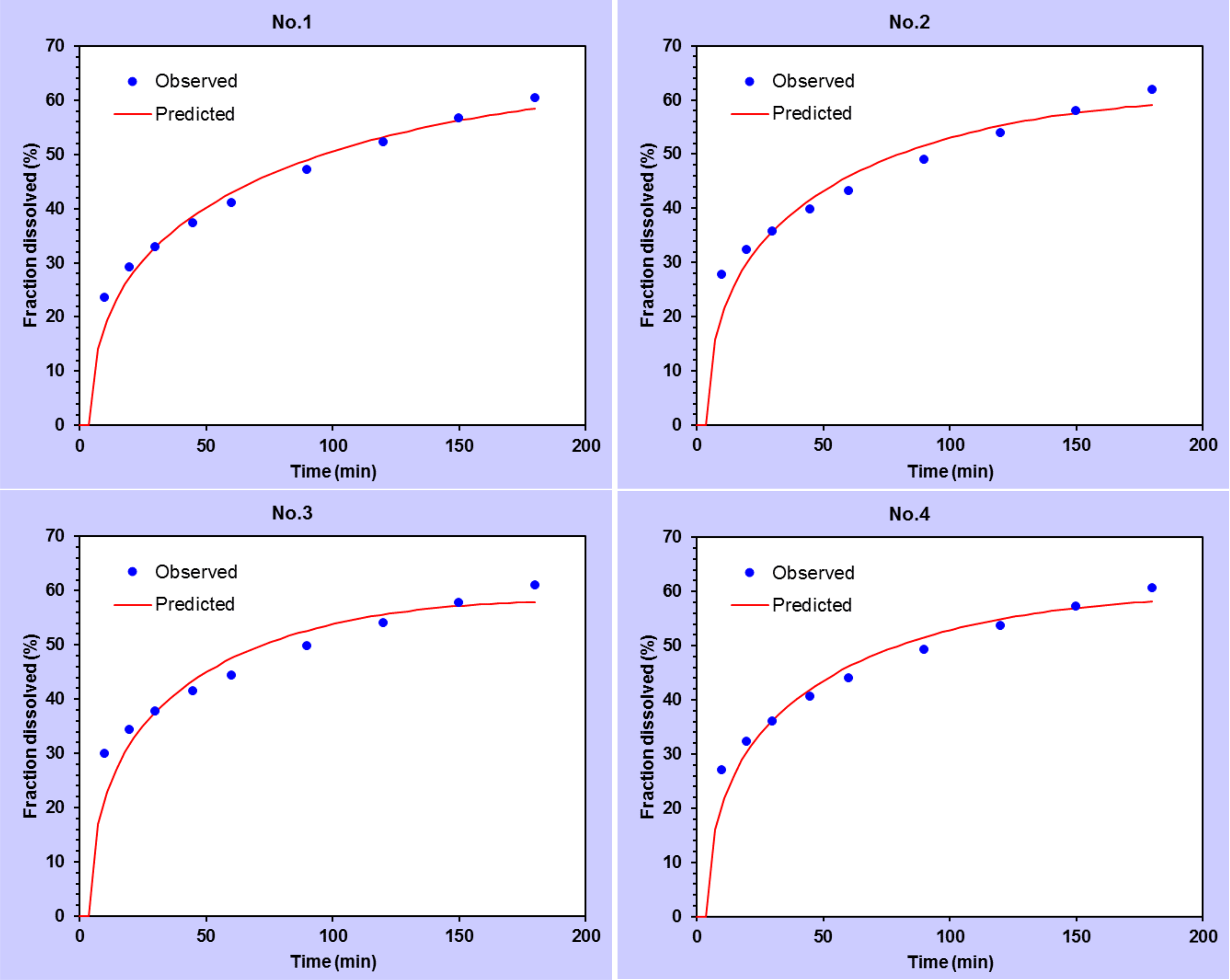

Supplementary materials – Model fitting summary – Tablettose® 70 (modelling up to app. 60 % of carvedilol released)

Model: **Peppas–Sahlin_2**

Model equation: $F = k_1 \cdot t^{0.5} + k_2 \cdot t$

Fitted model parameters per tested tablet (N = 4) with statistics – mean, standard deviation (SD), and relative standard deviation expressed in % (RSD%) (output from DDSolver):

Parameter	No.1	No.2	No.3	No.4	Mean	SD	RSD(%)
k_1	7.010	7.850	8.473	8.019	7.838	0.611	7.801
k_2	-0.196	-0.255	-0.309	-0.273	-0.258	0.047	-18.195

Number of dissolution data points (N), degrees of freedom (df), and selected goodness of fit criteria – Pearson correlation coefficient (R), coefficient of determination (R^2), adjusted coefficient of determination ($R^2_{adjusted}$), and residual sum of squares (RSS) (manual calculation in MS Excel):

Parameter	No.1	No.2	No.3	No.4
N	9	9	9	9
df	7	7	7	7
R	0.993088734	0.981755083	0.974324787	0.987720661
R^2	0.986225233	0.963843044	0.94930879	0.975592105
$R^2_{adjusted}$	0.984257409	0.958677764	0.942067189	0.972105263
RSS	24.16585148	58.5763799	73.51001829	38.73805391

Graphical abstract of model fit presented as mean ± 1 SD of the fraction % of released carvedilol:

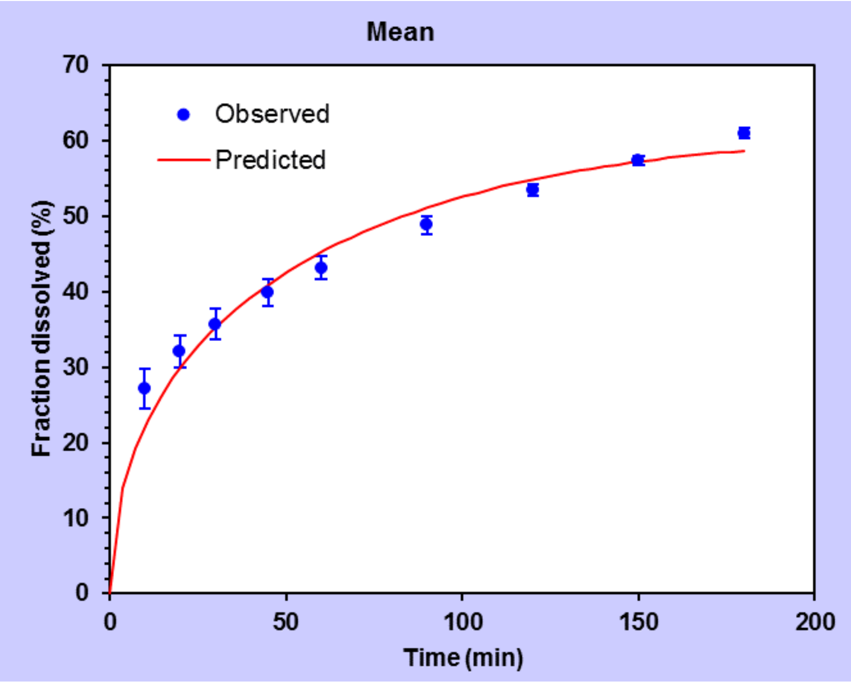

Supplementary materials – Model fitting summary – Tablettose® 70 (modelling up to app. 60 % of carvedilol released)

Graphical abstract of model fit presented as the fraction % of released carvedilol per tested tablet:

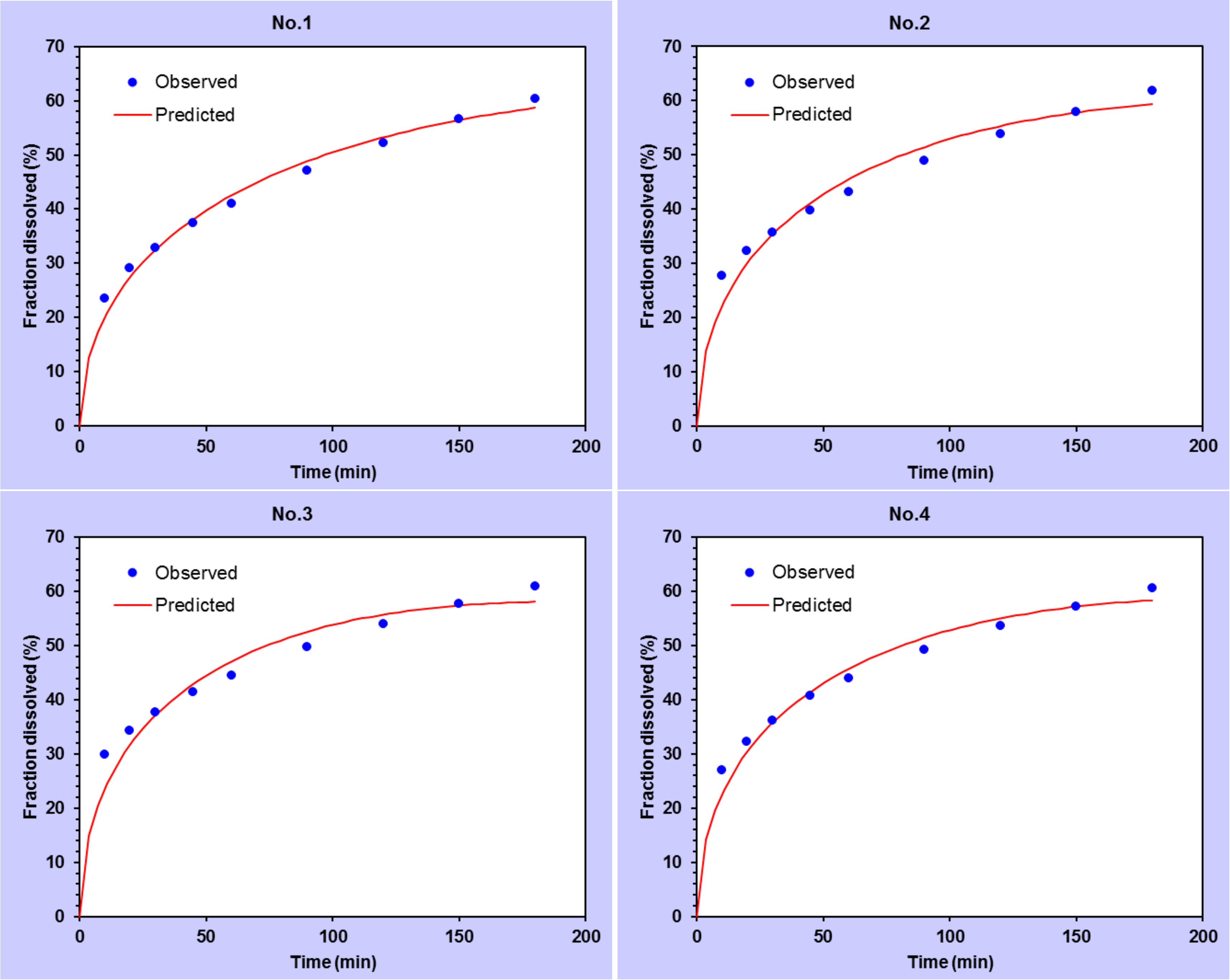

Supplementary materials – Model fitting summary – Tablettose® 70 (modelling up to app. 60 % of carvedilol released)

Model: **Peppas–Sahlin_2 with T_{lag}**

Model equation: $F = k_1 \cdot (t - T_{lag})^{0.5} + k_2 \cdot (t - T_{lag})$

Fitted model parameters per tested tablet (N = 4) with statistics – mean, standard deviation (SD), and relative standard deviation expressed in % (RSD%) (output from DDSolver):

Parameter	No.1	No.2	No.3	No.4	Mean	SD	RSD(%)
k_1	7.611	8.467	9.100	8.648	8.457	0.623	7.371
k_2	-0.244	-0.305	-0.360	-0.325	-0.309	0.049	-15.755
T_{lag}	4.000	4.000	4.000	4.000	4.000	0.000	0.000

Number of dissolution data points (N), degrees of freedom (df), and selected goodness of fit criteria – Pearson correlation coefficient (R), coefficient of determination (R^2), adjusted coefficient of determination ($R^2_{adjusted}$), and residual sum of squares (RSS) (manual calculation in MS Excel):

Parameter	No.1	No.2	No.3	No.4
N	9	9	9	9
df	6	6	6	6
R	0.983049237	0.965841581	0.954857373	0.973706268
R^2	0.966385803	0.932849959	0.911752603	0.948103896
$R^2_{adjusted}$	0.95518107	0.910466612	0.882336804	0.930805194
RSS	68.75856288	129.3758895	156.5232233	99.44534746

Graphical abstract of model fit presented as mean ± 1 SD of the fraction % of released carvedilol:

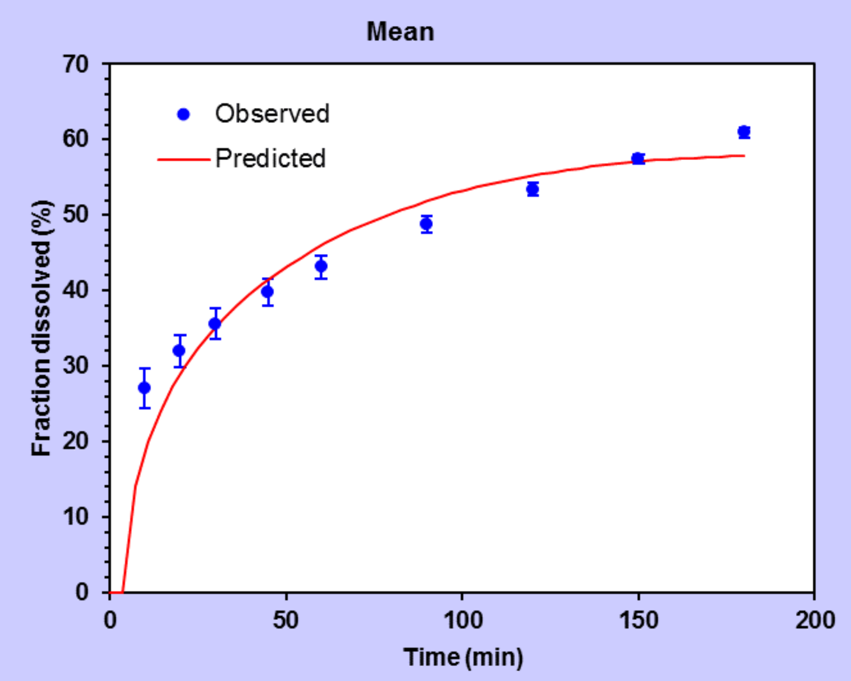

Supplementary materials – Model fitting summary – Tablettose® 70 (modelling up to app. 60 % of carvedilol released)

Graphical abstract of model fit presented as the fraction % of released carvedilol per tested tablet:

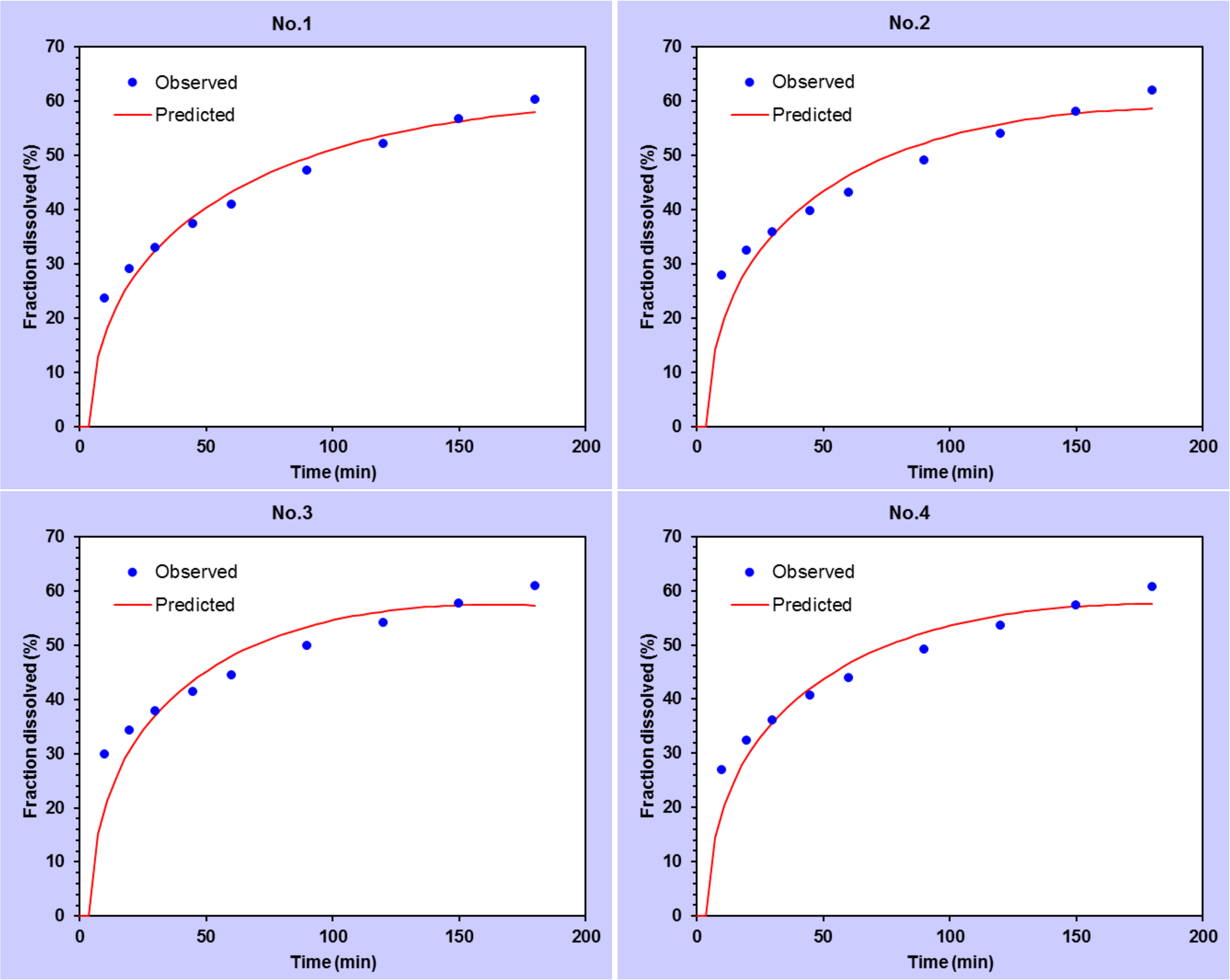

Supplementary materials – Model fitting summary – Tablettose® 70 (modelling up to app. 60 % of carvedilol released)

Model: **Quadratic**

Model equation: $F = 100 \cdot (k_1 \cdot t^2 + k_2 \cdot t)$

Fitted model parameters per tested tablet (N = 4) with statistics – mean, standard deviation (SD), and relative standard deviation expressed in % (RSD%) (output from DDSolver):

Parameter	No.1	No.2	No.3	No.4	Mean	SD	RSD(%)
k_1	-0.00003	-0.00004	-0.00004	-0.00004	-0.00004	0.00000	-7.02720
k_2	0.00897	0.00959	0.01000	0.00973	0.00957	0.00044	4.57339

Number of dissolution data points (N), degrees of freedom (df), and selected goodness of fit criteria – Pearson correlation coefficient (R), coefficient of determination (R^2), adjusted coefficient of determination ($R^2_{adjusted}$), and residual sum of squares (RSS) (manual calculation in MS Excel):

Parameter	No.1	No.2	No.3	No.4
N	9	9	9	9
df	7	7	7	7
R	0.951931642	0.933556127	0.922570082	0.93849515
R^2	0.906173851	0.871527043	0.851135556	0.880773146
$R^2_{adjusted}$	0.892770116	0.853173763	0.829869207	0.863740738
RSS	629.2530088	888.9270086	1053.553452	856.5906941

Graphical abstract of model fit presented as mean ± 1 SD of the fraction % of released carvedilol:

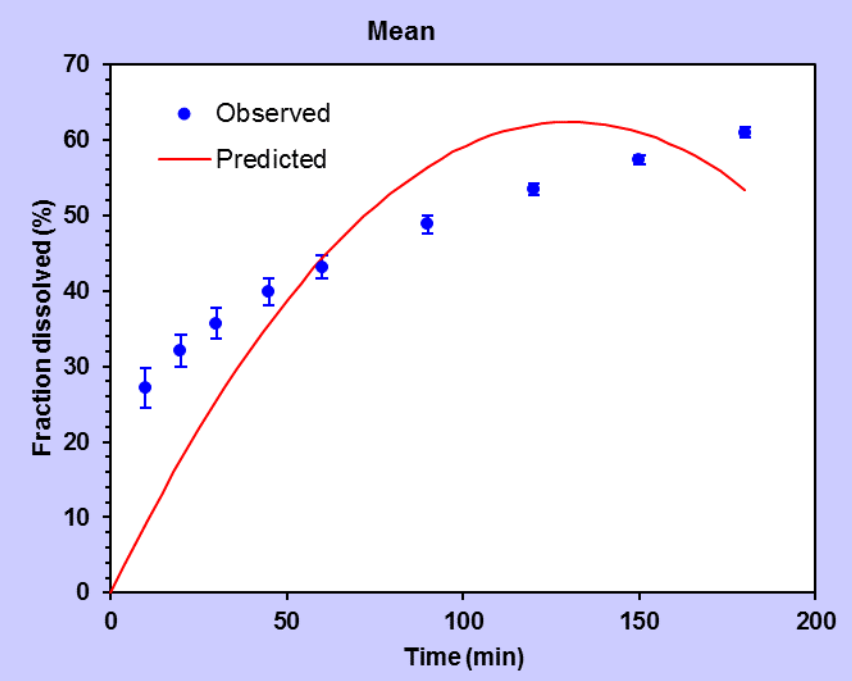

Supplementary materials – Model fitting summary – Tablettose® 70 (modelling up to app. 60 % of carvedilol released)

Graphical abstract of model fit presented as the fraction % of released carvedilol per tested tablet:

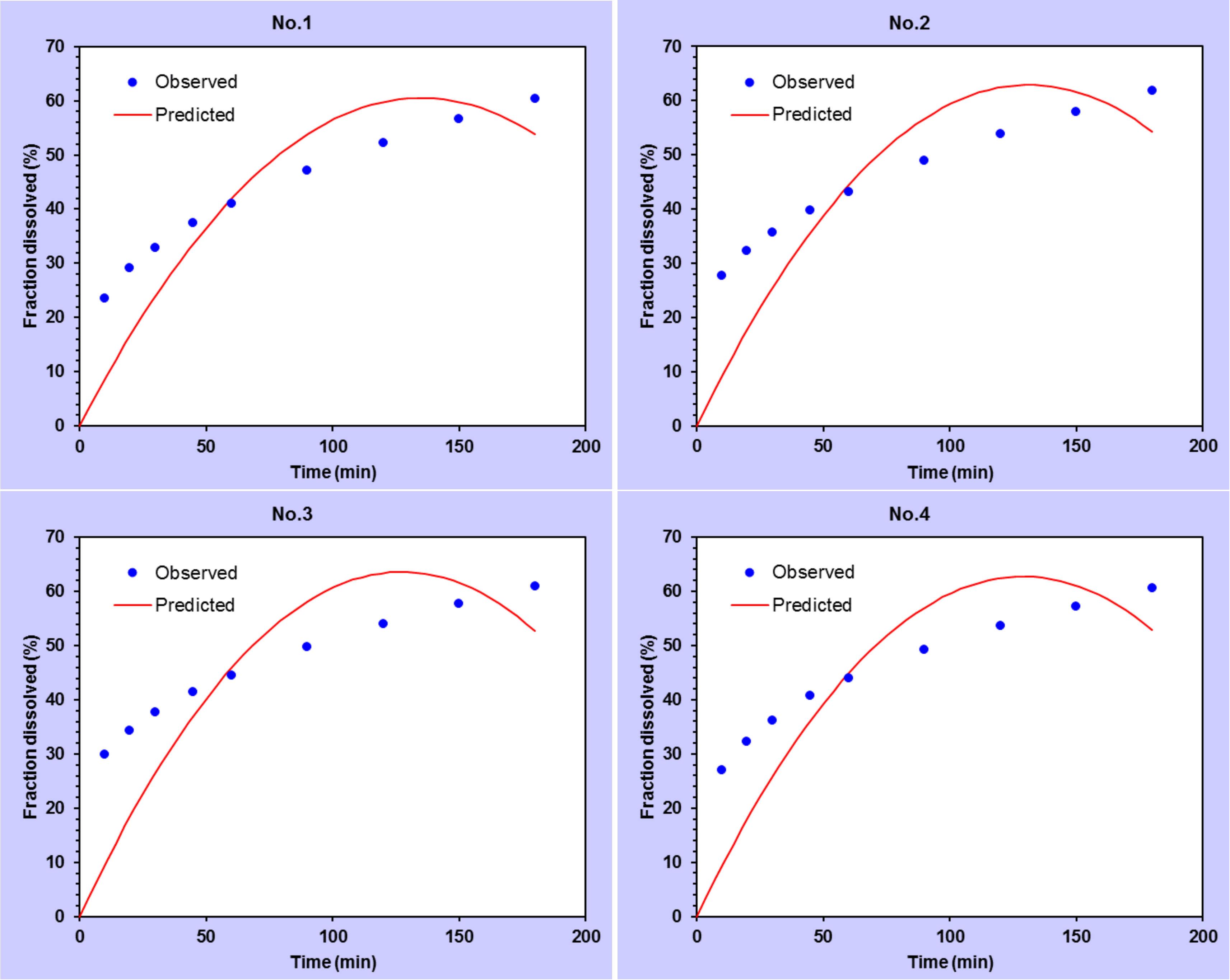

Supplementary materials – Model fitting summary – Tablettose® 70 (modelling up to app. 60 % of carvedilol released)

Model: **Quadratic with T_{lag}**

Model equation: $F = 100 \cdot \left[k_1 \cdot (t - T_{lag})^2 + k_2 \cdot (t - T_{lag})\right]$

Fitted model parameters per tested tablet (N = 4) with statistics – mean, standard deviation (SD), and relative standard deviation expressed in % (RSD%) (output from DDSolver):

Parameter	No.1	No.2	No.3	No.4	Mean	SD	RSD(%)
k_1	-0.00004	-0.00004	-0.00004	-0.00004	-0.00004	0.00000	-6.62046
k_2	0.00939	0.01000	0.01041	0.01016	0.00999	0.00044	4.36349
T_{lag}	4.00000	4.00000	4.00000	4.00000	4.00000	0.00000	0.00000

Number of dissolution data points (N), degrees of freedom (df), and selected goodness of fit criteria – Pearson correlation coefficient (R), coefficient of determination (R^2), adjusted coefficient of determination ($R^2_{adjusted}$), and residual sum of squares (RSS) (manual calculation in MS Excel):

Parameter	No.1	No.2	No.3	No.4
N	9	9	9	9
df	6	6	6	6
R	0.9492841	0.932084537	0.922633139	0.937425501
R^2	0.901140303	0.868781584	0.851251909	0.878766571
$R^2_{adjusted}$	0.868187071	0.825042112	0.801669212	0.838355428
RSS	869.4308286	1191.987081	1396.27828	1158.559871

Graphical abstract of model fit presented as mean ± 1 SD of the fraction % of released carvedilol:

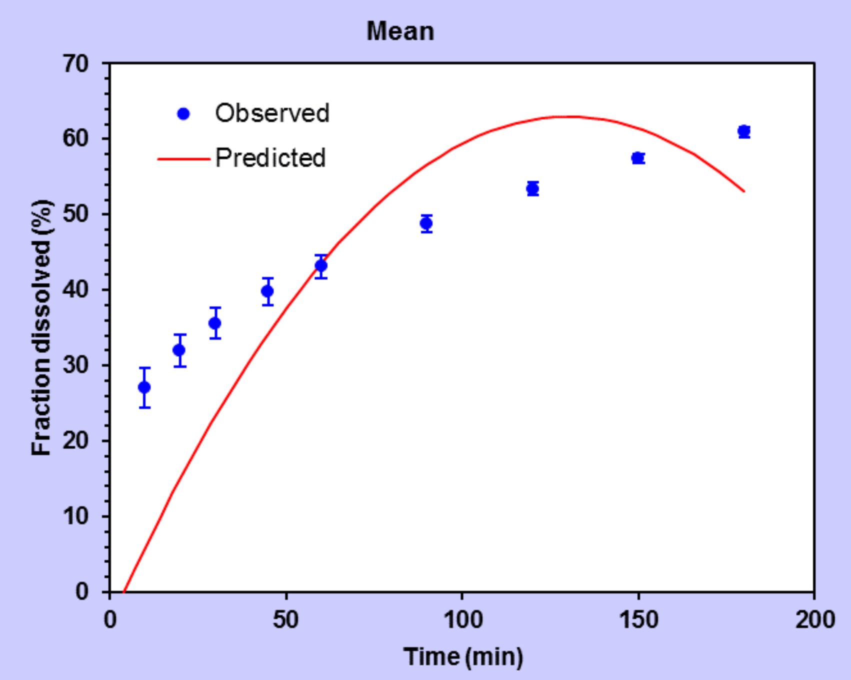

Supplementary materials – Model fitting summary – Tablettose® 70 (modelling up to app. 60 % of carvedilol released)

Graphical abstract of model fit presented as the fraction % of released carvedilol per tested tablet:

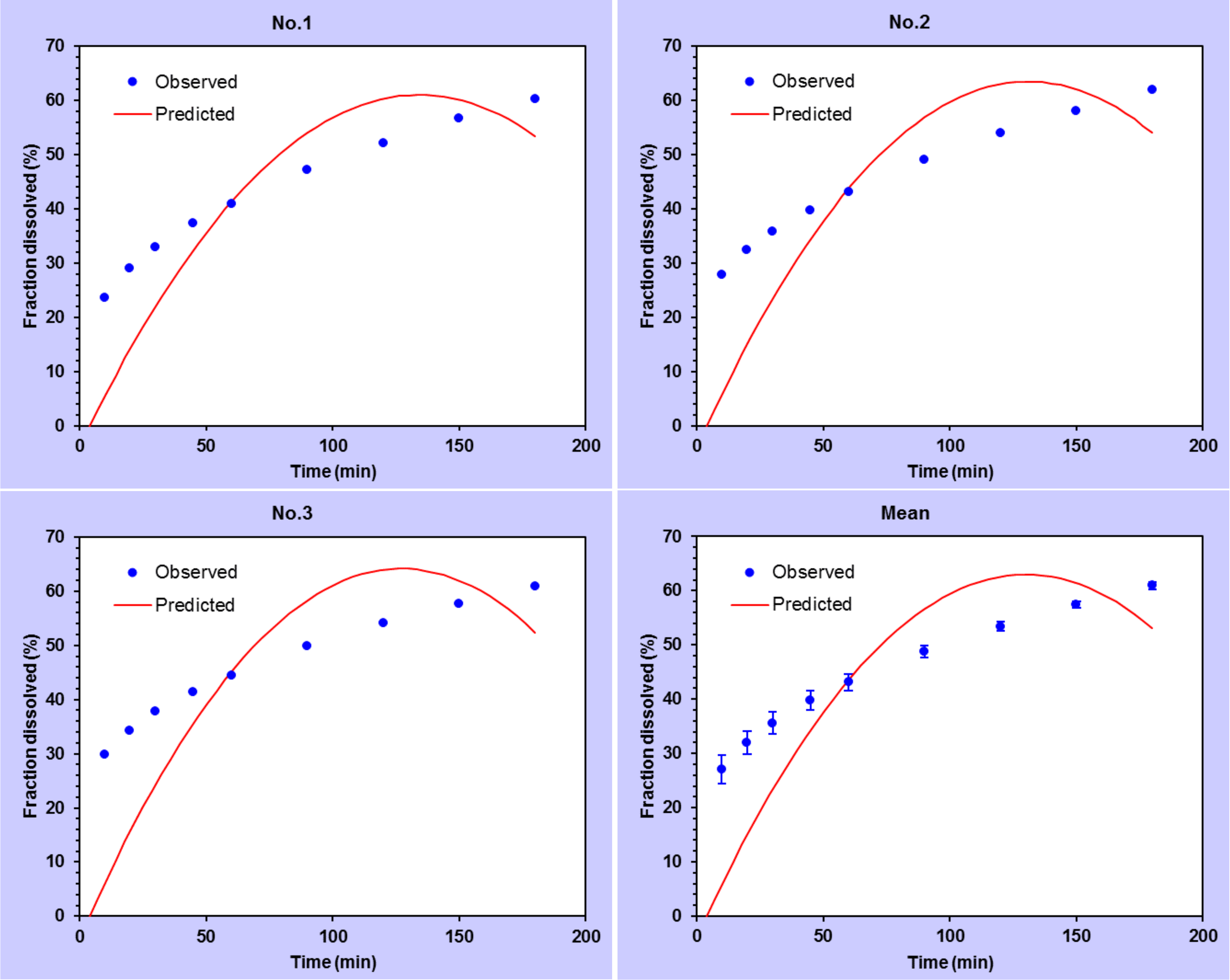

Supplementary materials – Model fitting summary – Tablettose® 70 (modelling up to app. 60 % of carvedilol released)

Model: **Weibull_1**

Model equation: $F = 100 \cdot \left[1 - e^{-\frac{(t-T_i)^{\beta}}{\alpha}}\right]$

Fitted model parameters per tested tablet (N = 4) with statistics – mean, standard deviation (SD), and relative standard deviation expressed in % (RSD%) (output from DDSolver):

Parameter	No.1	No.2	No.3	No.4	Mean	SD	RSD(%)
α	7.953	6.160	5.233	6.175	6.381	1.137	17.825
β	0.371	0.327	0.294	0.326	0.329	0.032	9.624
T_i	4.000	4.000	4.000	4.000	4.000	0.000	0.000

Number of dissolution data points (N), degrees of freedom (df), and selected goodness of fit criteria – Pearson correlation coefficient (R), coefficient of determination (R^2), adjusted coefficient of determination ($R^2_{adjusted}$), and residual sum of squares (RSS) (manual calculation in MS Excel):

Parameter	No.1	No.2	No.3	No.4
N	9	9	9	9
df	6	6	6	6
R	0.990908582	0.984455989	0.986486585	0.992850519
R^2	0.981899818	0.969153594	0.973155783	0.985752153
$R^2_{adjusted}$	0.975866424	0.958871459	0.96420771	0.98100287
RSS	25.11181661	36.16824336	25.73506404	16.01474547

Graphical abstract of model fit presented as mean ± 1 SD of the fraction % of released carvedilol:

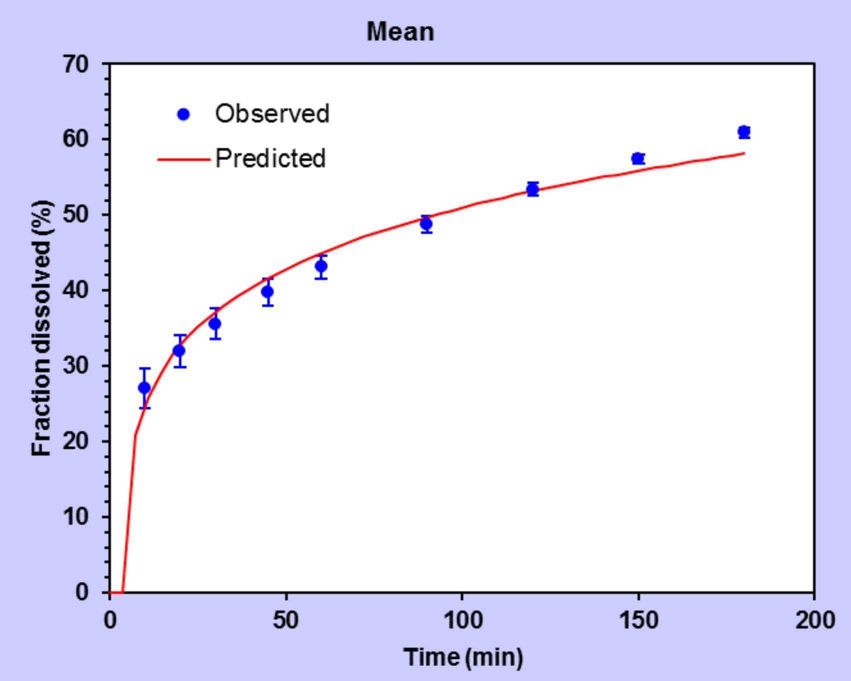

Supplementary materials – Model fitting summary – Tablettose® 70 (modelling up to app. 60 % of carvedilol released)

Graphical abstract of model fit presented as the fraction % of released carvedilol per tested tablet:

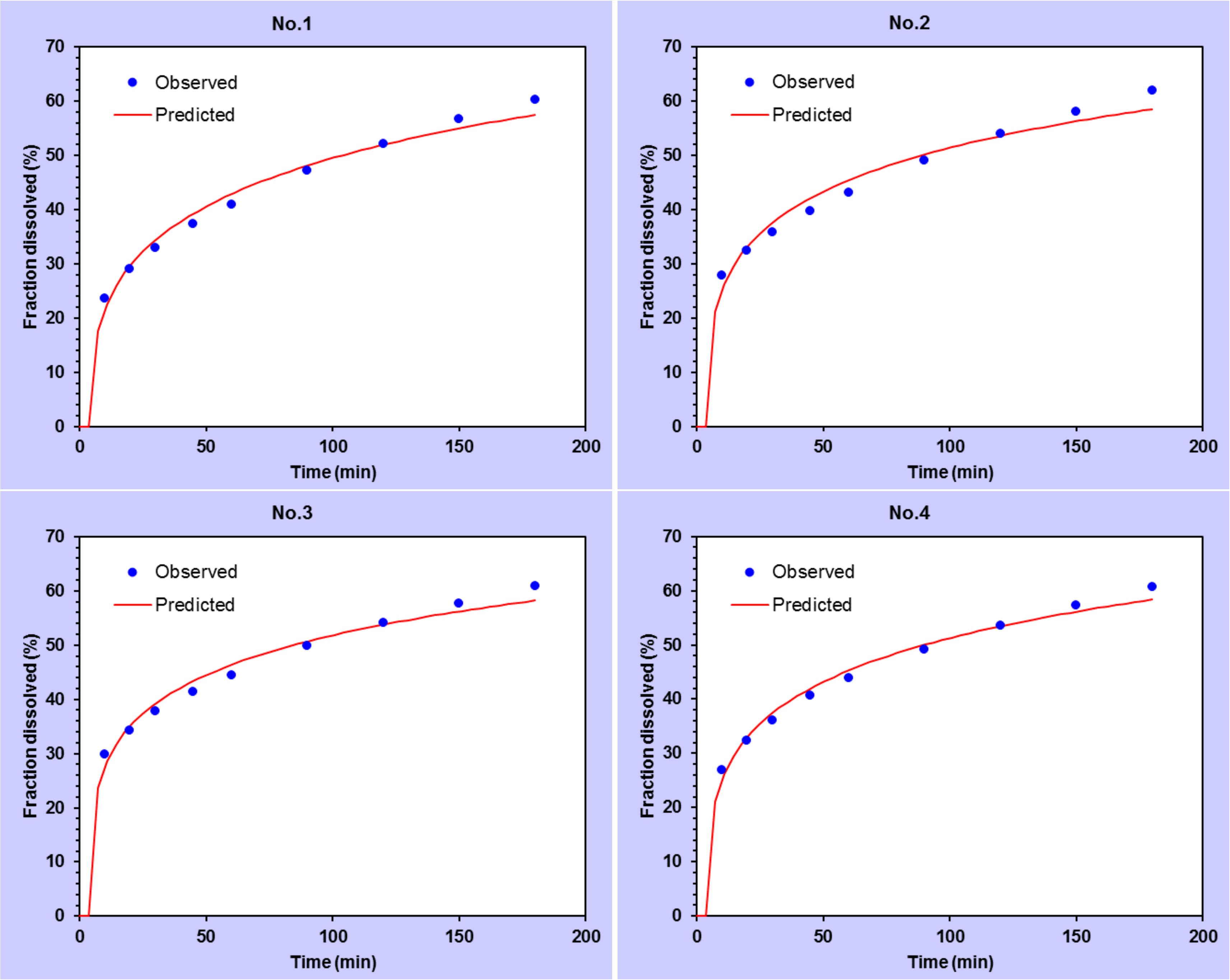

Supplementary materials – Model fitting summary – Tablettose® 70 (modelling up to app. 60 % of carvedilol released)

Model: **Weibull_2**

Model equation: $F = 100 \cdot \left(1 - e^{-\frac{t^{\beta}}{\alpha}}\right)$

Fitted model parameters per tested tablet (N = 4) with statistics – mean, standard deviation (SD), and relative standard deviation expressed in % (RSD%) (output from DDSolver):

Parameter	No.1	No.2	No.3	No.4	Mean	SD	RSD(%)
α	10.498	7.898	6.536	7.874	8.201	1.658	20.214
β	0.428	0.378	0.340	0.377	0.381	0.036	9.549

Number of dissolution data points (N), degrees of freedom (df), and selected goodness of fit criteria – Pearson correlation coefficient (R), coefficient of determination (R^2), adjusted coefficient of determination ($R^2_{adjusted}$), and residual sum of squares (RSS) (manual calculation in MS Excel):

Parameter	No.1	No.2	No.3	No.4
N	9	9	9	9
df	7	7	7	7
R	0.996483704	0.992365243	0.993890465	0.997785673
R^2	0.992979773	0.984788775	0.987818256	0.99557625
$R^2_{adjusted}$	0.991976883	0.982615743	0.986078007	0.994944285
RSS	9.614551288	17.7239774	11.62109851	4.925138176

Graphical abstract of model fit presented as mean ± 1 SD of the fraction % of released carvedilol:

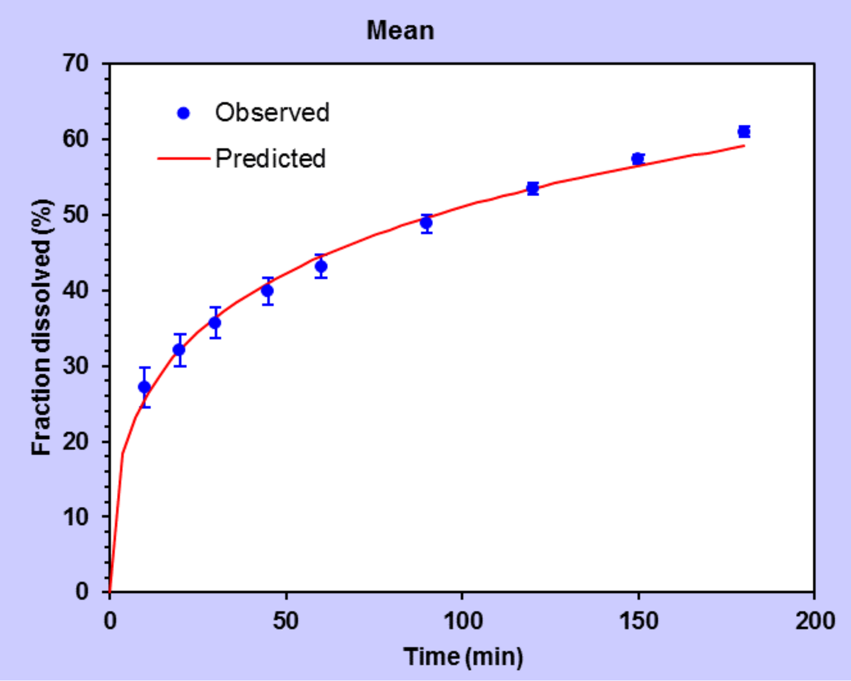

Supplementary materials – Model fitting summary – Tablettose® 70 (modelling up to app. 60 % of carvedilol released)

Graphical abstract of model fit presented as the fraction % of released carvedilol per tested tablet:

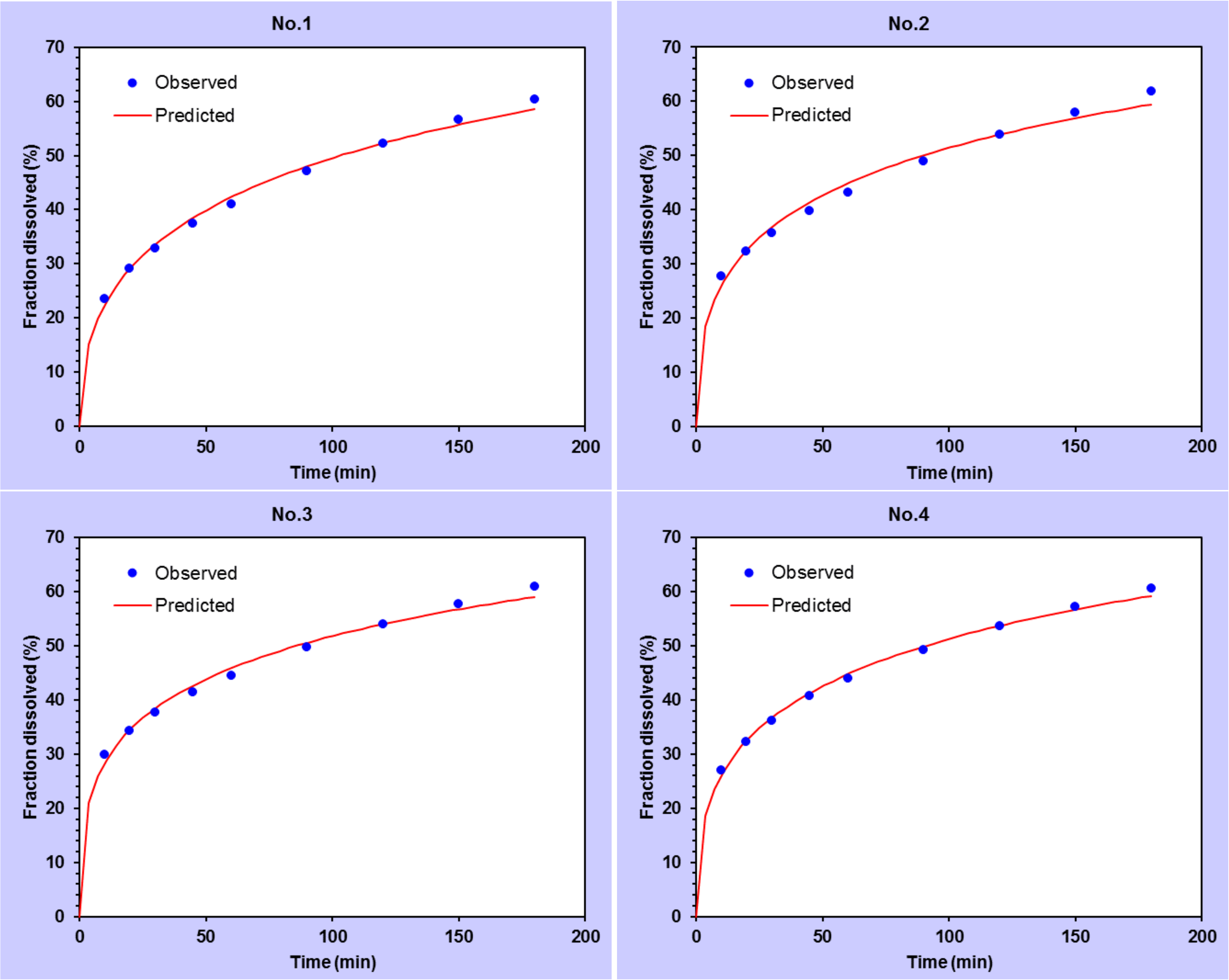

Supplementary materials – Model fitting summary – Tablettose® 70 (modelling up to app. 60 % of carvedilol released)

Model: **Weibull_3**

Model equation: $F = F_{max} \cdot \left(1 - e^{-\frac{t^{\beta}}{\alpha}}\right)$

Fitted model parameters per tested tablet (N = 4) with statistics – mean, standard deviation (SD), and relative standard deviation expressed in % (RSD%) (output from DDSolver):

Parameter	No.1	No.2	No.3	No.4	Mean	SD	RSD(%)
α	10.610	7.813	6.258	8.760	8.361	1.820	21.772
β	0.621	0.559	0.523	0.522	0.556	0.047	8.372
F_{max}	63.349	65.024	63.992	71.633	65.999	3.818	5.786

Number of dissolution data points (N), degrees of freedom (df), and selected goodness of fit criteria – Pearson correlation coefficient (R), coefficient of determination (R^2), adjusted coefficient of determination ($R^2_{adjusted}$), and residual sum of squares (RSS) (manual calculation in MS Excel):

Parameter	No.1	No.2	No.3	No.4
N	9	9	9	9
df	6	6	6	6
R	0.982848992	0.975802781	0.977235511	0.99399827
R^2	0.965992141	0.952191067	0.954989245	0.98803256
$R^2_{adjusted}$	0.954656188	0.936254755	0.939985659	0.984043414
RSS	46.3427085	56.4079191	44.24622917	29.87211236

Graphical abstract of model fit presented as mean ± 1 SD of the fraction % of released carvedilol:

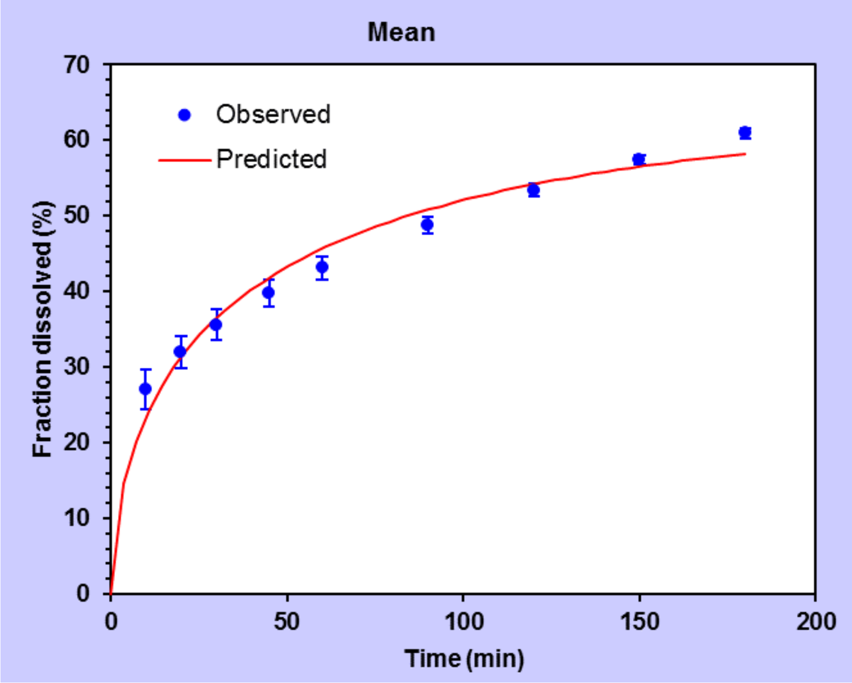

Supplementary materials – Model fitting summary – Tablettose® 70 (modelling up to app. 60 % of carvedilol released)

Graphical abstract of model fit presented as the fraction % of released carvedilol per tested tablet:

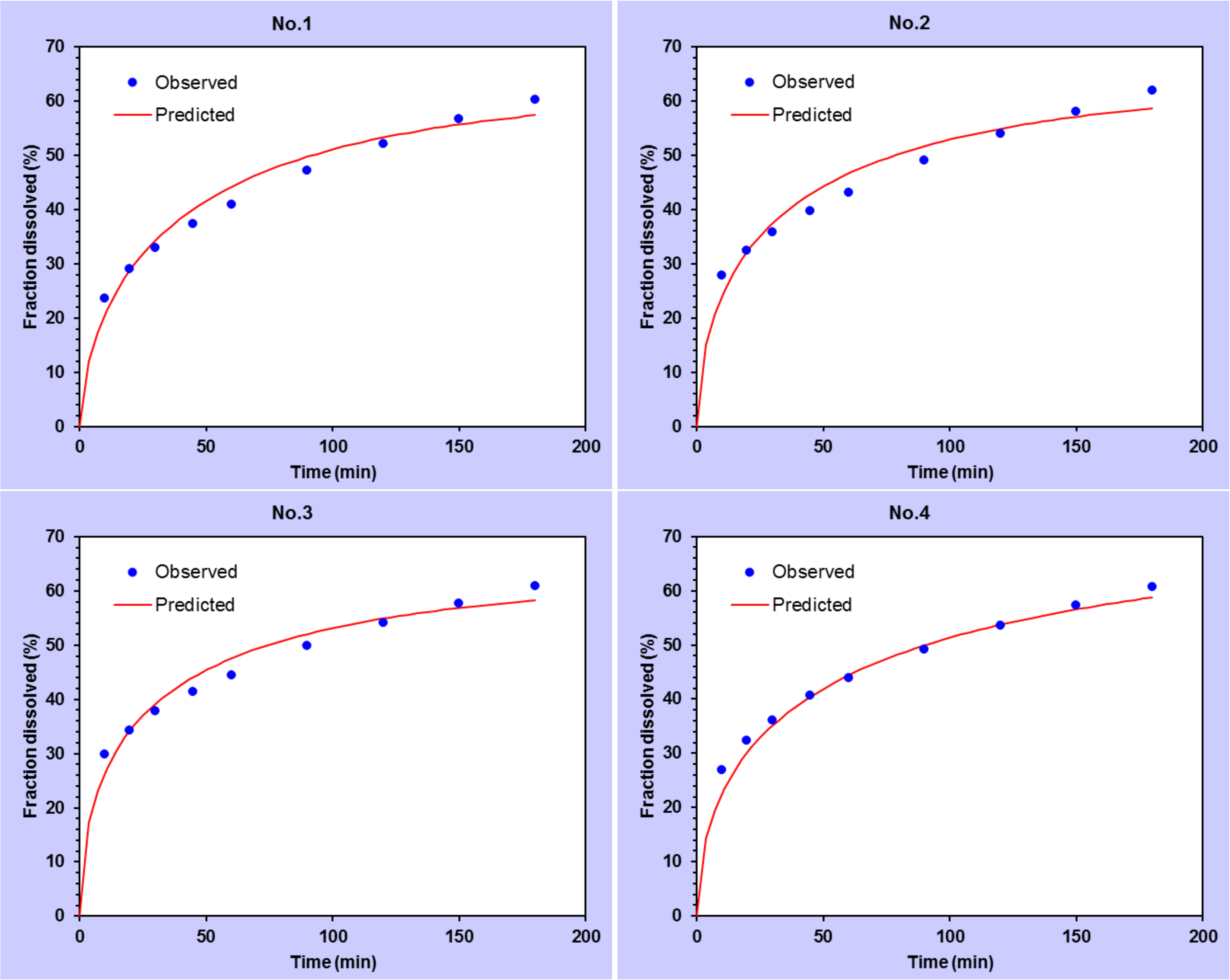

Supplementary materials – Model fitting summary – Tablettose® 70 (modelling up to app. 60 % of carvedilol released)

Model: **Weibull_4**

Model equation: $F = F_{max} \cdot \left[1 - e^{-\frac{(t-T_i)^{\beta}}{\alpha}}\right]$

Fitted model parameters per tested tablet (N = 4) with statistics – mean, standard deviation (SD), and relative standard deviation expressed in % (RSD%) (output from DDSolver):

Parameter	No.1	No.2	No.3	No.4	Mean	SD	RSD(%)
α	7.001	5.352	4.397	5.297	5.512	1.085	19.691
β	0.534	0.480	0.449	0.487	0.488	0.035	7.230
T_i	4.000	4.000	4.000	4.000	4.000	0.000	0.000
F_{max}	63.349	65.024	63.992	63.663	64.007	0.727	1.136

Number of dissolution data points (N), degrees of freedom (df), and selected goodness of fit criteria – Pearson correlation coefficient (R), coefficient of determination (R^2), adjusted coefficient of determination ($R^2_{adjusted}$), and residual sum of squares (RSS) (manual calculation in MS Excel):

Parameter	No.1	No.2	No.3	No.4
N	9	9	9	9
df	5	5	5	5
R	0.973154996	0.963835932	0.965186635	0.975502181
R^2	0.947030646	0.928979703	0.931585241	0.951604504
$R^2_{adjusted}$	0.915249034	0.886367526	0.890536386	0.922567207
RSS	71.52172616	83.04483144	66.60590497	54.40432922

Graphical abstract of model fit presented as mean ± 1 SD of the fraction % of released carvedilol:

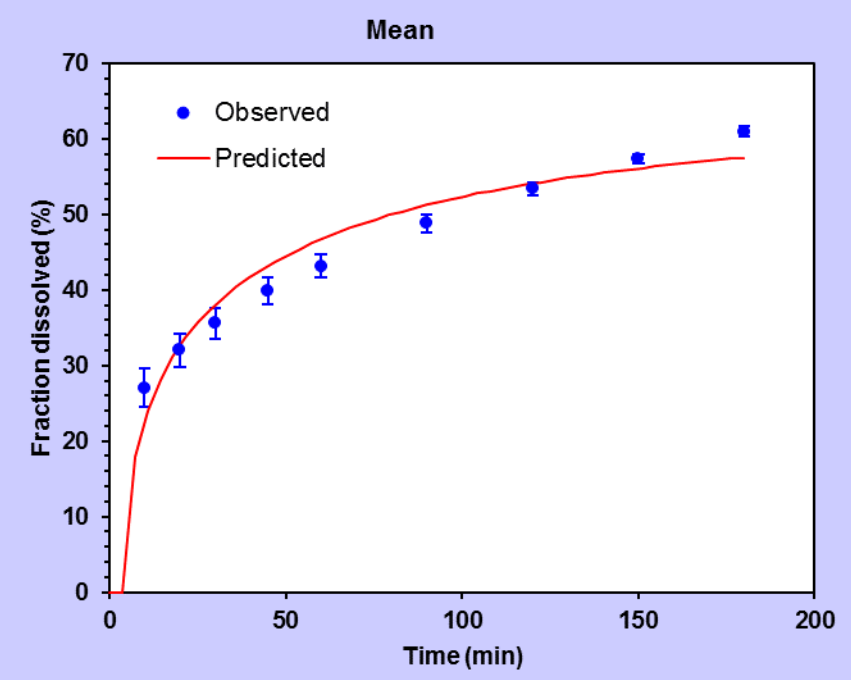

Supplementary materials – Model fitting summary – Tablettose® 70 (modelling up to app. 60 % of carvedilol released)

Graphical abstract of model fit presented as the fraction % of released carvedilol per tested tablet:

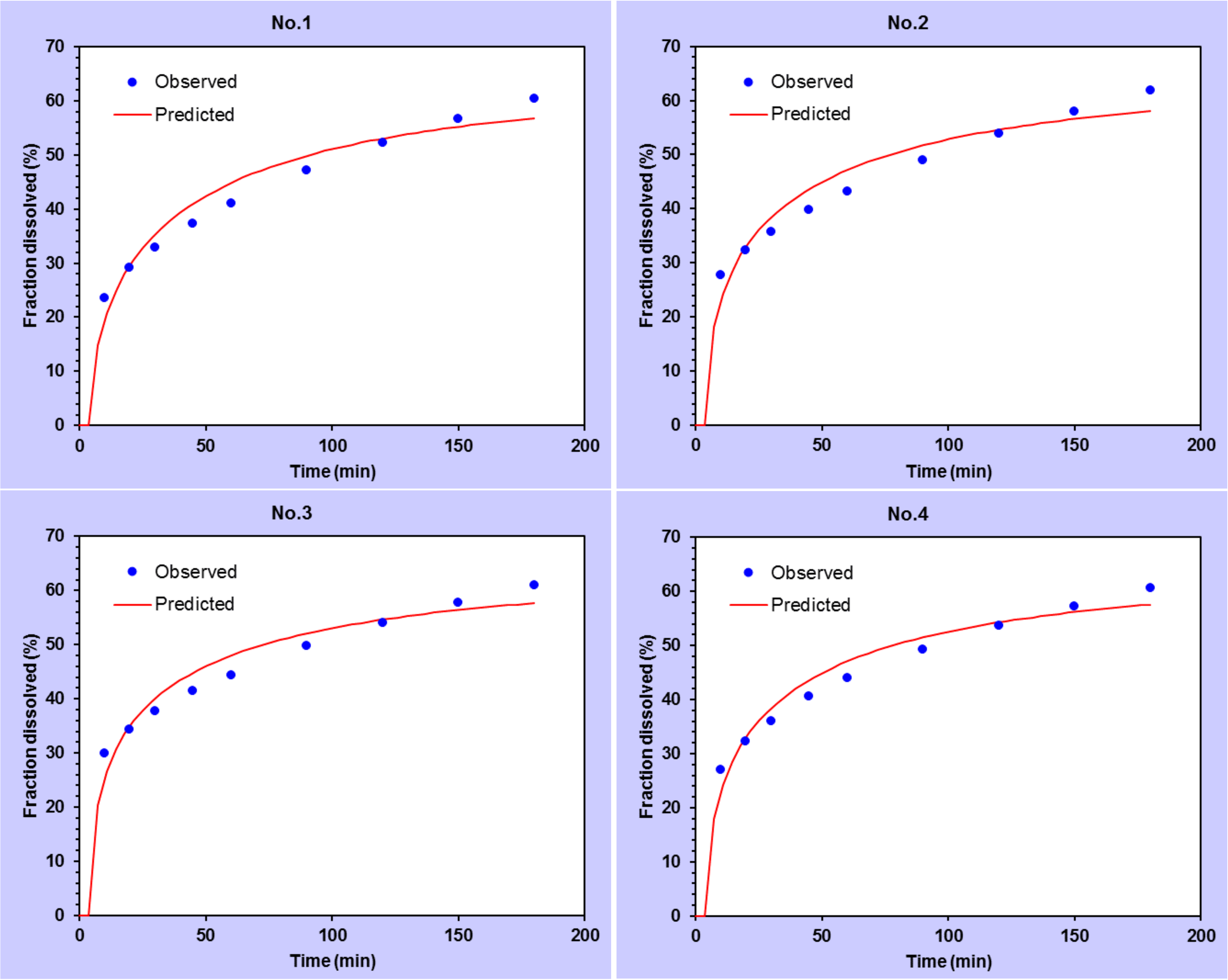

Supplementary materials – Model fitting summary – Tablettose® 70 (modelling up to app. 60 % of carvedilol released)

Model: **Logistic_1**

Model equation: $F = 100 \cdot \frac{e^{\alpha+\beta\cdot\log(t)}}{1+e^{\alpha+\beta\cdot\log(t)}}$

Fitted model parameters per tested tablet (N = 4) with statistics – mean, standard deviation (SD), and relative standard deviation expressed in % (RSD%) (output from DDSolver):

Parameter	No.1	No.2	No.3	No.4	Mean	SD	RSD(%)
α	-2.545	-2.226	-1.993	-2.211	-2.244	0.227	-10.133
β	1.270	1.148	1.038	1.137	1.148	0.095	8.269

Number of dissolution data points (N), degrees of freedom (df), and selected goodness of fit criteria – Pearson correlation coefficient (R), coefficient of determination (R^2), adjusted coefficient of determination ($R^2_{adjusted}$), and residual sum of squares (RSS) (manual calculation in MS Excel):

Parameter	No.1	No.2	No.3	No.4
N	9	9	9	9
df	7	7	7	7
R	0.992544958	0.987126365	0.989470395	0.99469093
R^2	0.985145494	0.97441846	0.979051662	0.989410045
$R^2_{adjusted}$	0.983023422	0.970763954	0.976059043	0.987897195
RSS	19.57411228	28.92420169	19.50983629	11.45015383

Graphical abstract of model fit presented as mean ± 1 SD of the fraction % of released carvedilol:

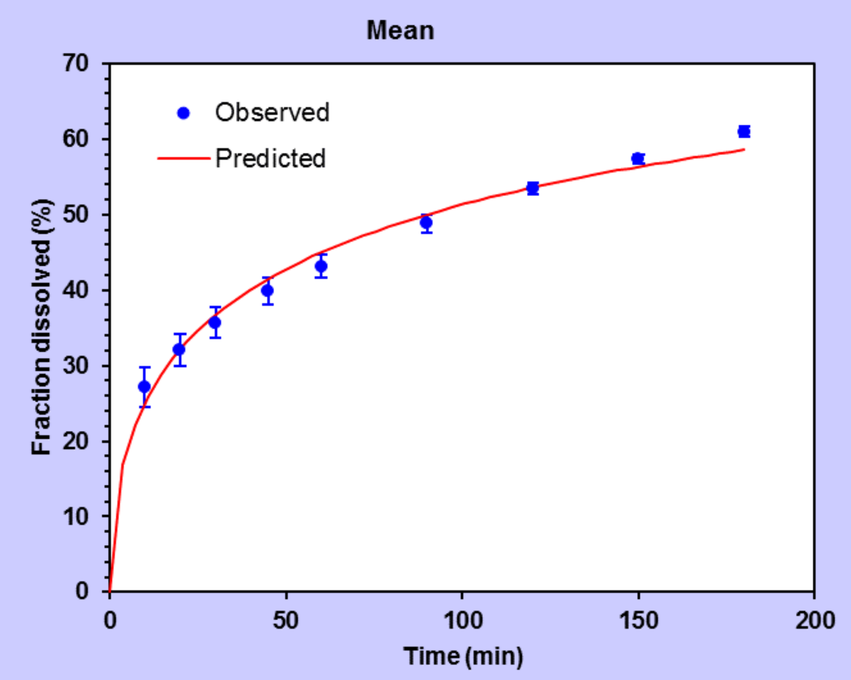

Supplementary materials – Model fitting summary – Tablettose® 70 (modelling up to app. 60 % of carvedilol released)

Graphical abstract of model fit presented as the fraction % of released carvedilol per tested tablet:

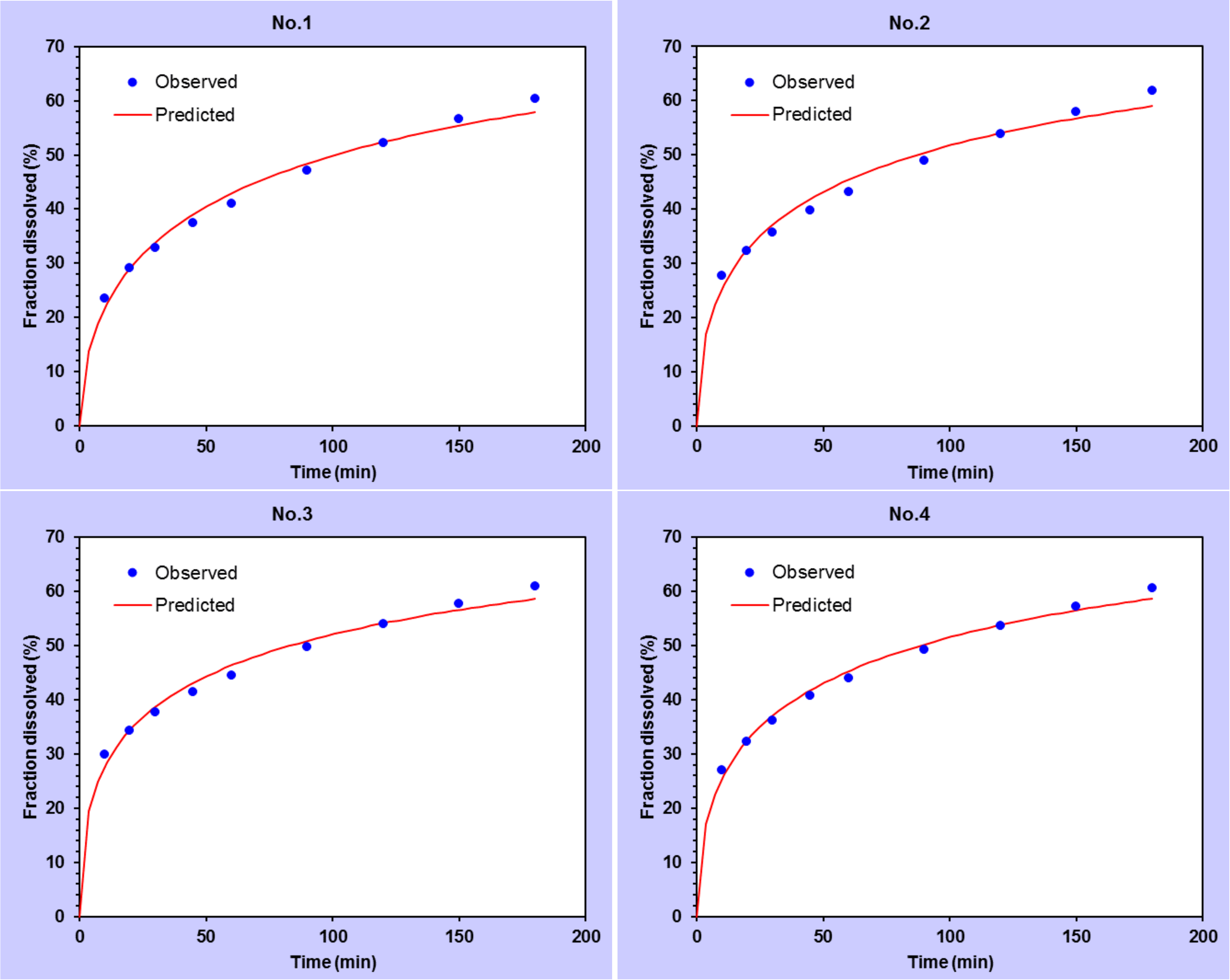

Supplementary materials – Model fitting summary – Tablettose® 70 (modelling up to app. 60 % of carvedilol released)

Model: **Logistic_2**

Model equation: $F = F_{max} \cdot \frac{e^{\alpha+\beta\cdot\log(t)}}{1+e^{\alpha+\beta\cdot\log(t)}}$

Fitted model parameters per tested tablet (N = 4) with statistics – mean, standard deviation (SD), and relative standard deviation expressed in % (RSD%) (output from DDSolver):

Parameter	No.1	No.2	No.3	No.4	Mean	SD	RSD(%)
α	-3.523	-3.116	-2.842	-3.124	-3.151	0.280	-8.896
β	2.540	2.353	2.268	2.397	2.389	0.114	4.762
F_{max}	63.349	65.024	63.992	63.663	64.007	0.727	1.136

Number of dissolution data points (N), degrees of freedom (df), and selected goodness of fit criteria – Pearson correlation coefficient (R), coefficient of determination (R^2), adjusted coefficient of determination ($R^2_{adjusted}$), and residual sum of squares (RSS) (manual calculation in MS Excel):

Parameter	No.1	No.2	No.3	No.4
N	9	9	9	9
df	6	6	6	6
R	0.959096565	0.949123922	0.950951013	0.962682752
R^2	0.919866221	0.90083622	0.904307829	0.926758081
$R^2_{adjusted}$	0.893154961	0.867781626	0.872410439	0.902344109
RSS	127.294183	138.1380171	112.7277118	99.09635371

Graphical abstract of model fit presented as mean ± 1 SD of the fraction % of released carvedilol:

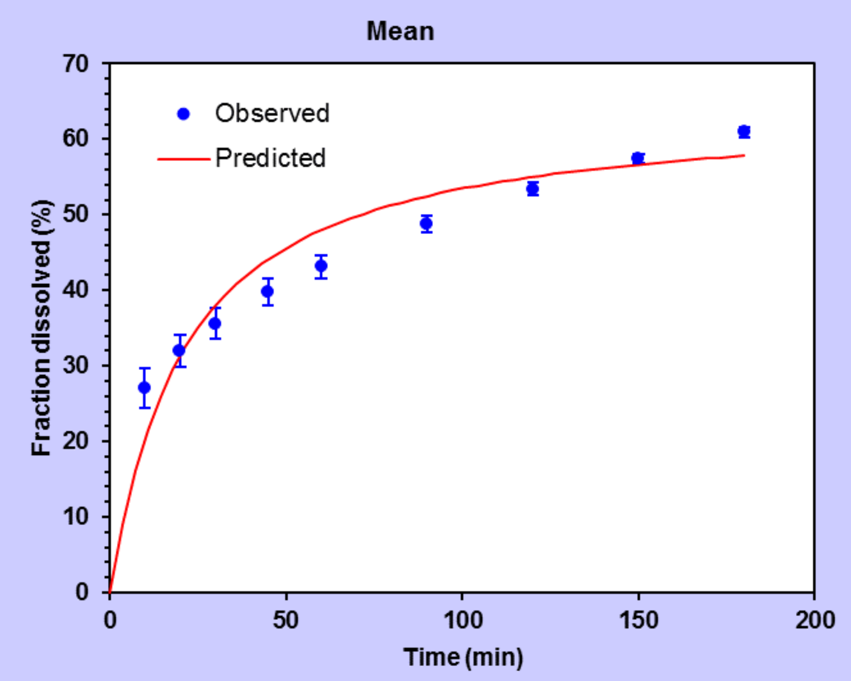

Supplementary materials – Model fitting summary – Tablettose® 70 (modelling up to app. 60 % of carvedilol released)

Graphical abstract of model fit presented as the fraction % of released carvedilol per tested tablet:

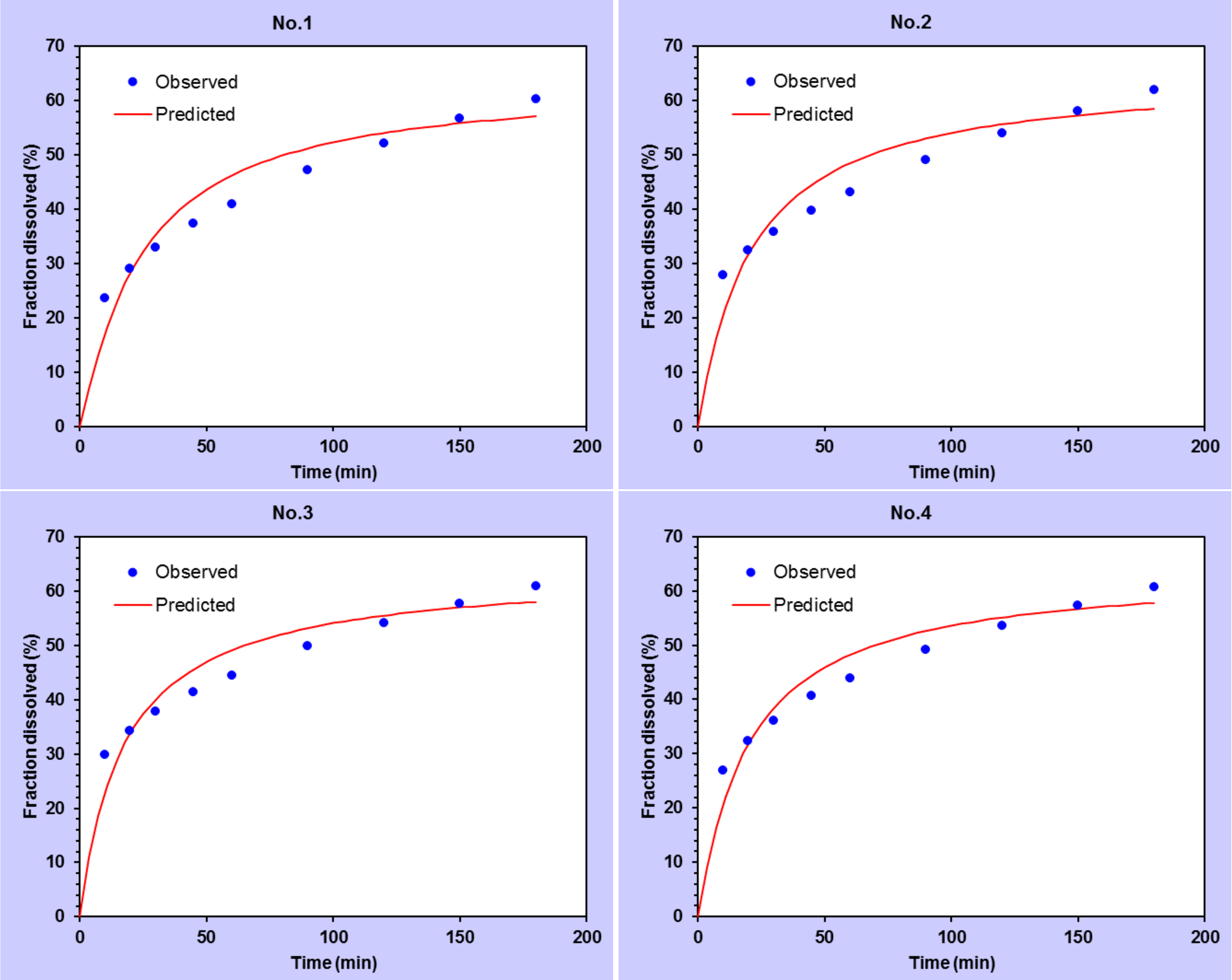

Supplementary materials – Model fitting summary – Tablettose® 70 (modelling up to app. 60 % of carvedilol released)

Model: **Logistic_3**

Model equation: $F = F_{max} \cdot \frac{1}{1+e^{-k\cdot(t-\gamma)}}$

Fitted model parameters per tested tablet (N = 4) with statistics – mean, standard deviation (SD), and relative standard deviation expressed in % (RSD%) (output from DDSolver):

Parameter	No.1	No.2	No.3	No.4	Mean	SD	RSD(%)
k	0.019	0.018	0.017	0.018	0.018	0.001	4.504
γ	30.772	22.915	13.161	19.155	21.501	7.371	34.285
F_{max}	63.349	65.024	63.992	63.663	64.007	0.727	1.136

Number of dissolution data points (N), degrees of freedom (df), and selected goodness of fit criteria – Pearson correlation coefficient (R), coefficient of determination (R^2), adjusted coefficient of determination ($R^2_{adjusted}$), and residual sum of squares (RSS) (manual calculation in MS Excel):

Parameter	No.1	No.2	No.3	No.4
N	9	9	9	9
df	6	6	6	6
R	0.99561973	0.996959589	0.996754353	0.99429628
R^2	0.991258646	0.993928421	0.99351924	0.988625092
$R^2_{adjusted}$	0.988344862	0.991904562	0.991358987	0.984833455
RSS	11.65777968	7.576082479	6.26655934	12.20135027

Graphical abstract of model fit presented as mean ± 1 SD of the fraction % of released carvedilol:

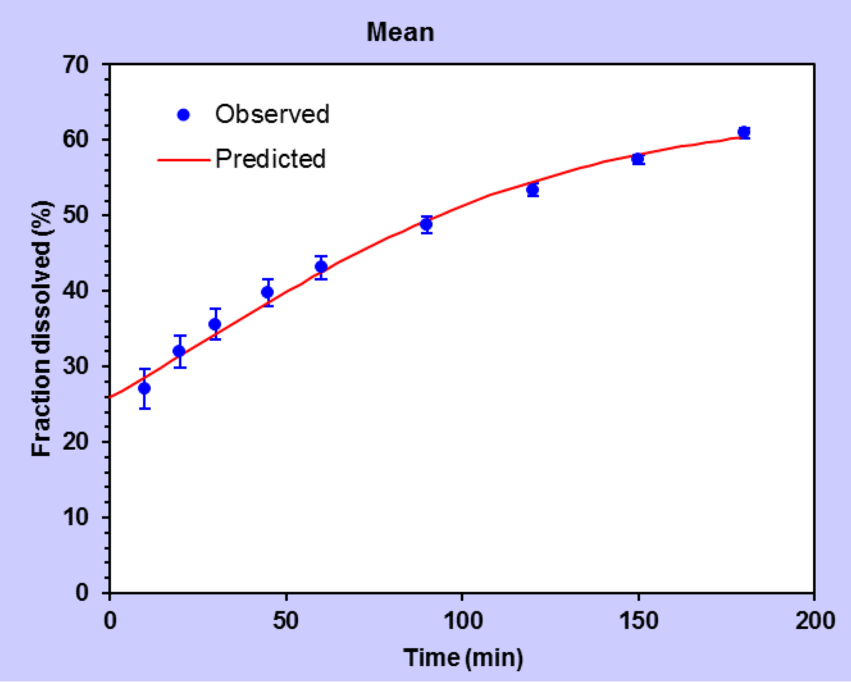

Supplementary materials – Model fitting summary – Tablettose® 70 (modelling up to app. 60 % of carvedilol released)

Graphical abstract of model fit presented as the fraction % of released carvedilol per tested tablet:

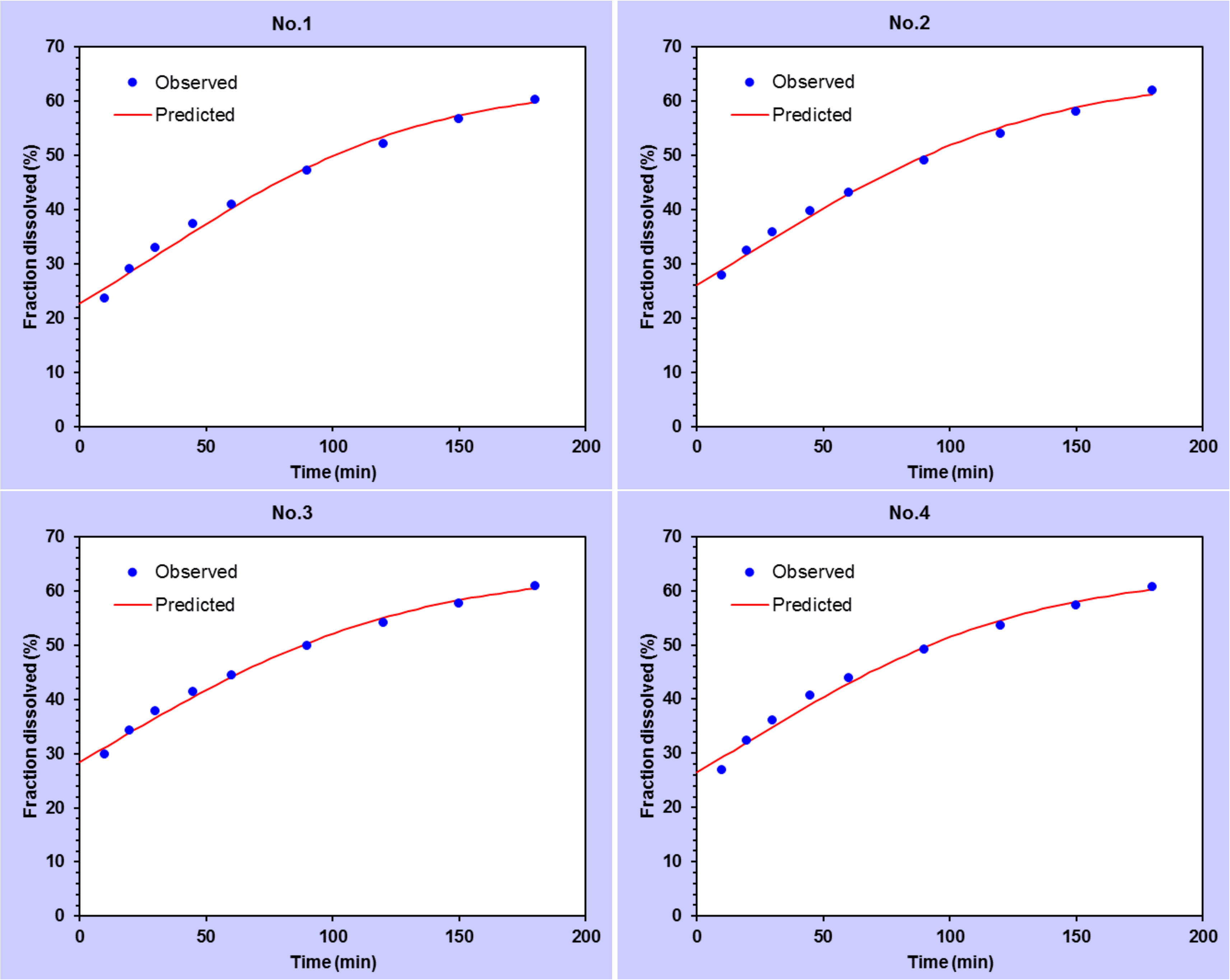

Supplementary materials – Model fitting summary – Tablettose® 70 (modelling up to app. 60 % of carvedilol released)

Model: **Gompertz_1**

Model equation: $F = 100 \cdot e^{-\alpha \cdot e^{-\beta \cdot \log(t)}}$

Fitted model parameters per tested tablet (N = 4) with statistics – mean, standard deviation (SD), and relative standard deviation expressed in % (RSD%) (output from DDSolver):

Parameter	No.1	No.2	No.3	No.4	Mean	SD	RSD(%)
α	3.663	3.103	2.687	3.045	3.124	0.403	12.913
β	0.835	0.779	0.711	0.766	0.773	0.051	6.590

Number of dissolution data points (N), degrees of freedom (df), and selected goodness of fit criteria – Pearson correlation coefficient (R), coefficient of determination (R^2), adjusted coefficient of determination ($R^2_{adjusted}$), and residual sum of squares (RSS) (manual calculation in MS Excel):

Parameter	No.1	No.2	No.3	No.4
N	9	9	9	9
df	7	7	7	7
R	0.984399091	0.978083342	0.982069246	0.988504455
R^2	0.96904157	0.956647024	0.964460003	0.977141058
$R^2_{adjusted}$	0.964618938	0.950453742	0.959382861	0.973875495
RSS	40.11300462	48.7422244	33.02810396	24.53704243

Graphical abstract of model fit presented as mean ± 1 SD of the fraction % of released carvedilol:

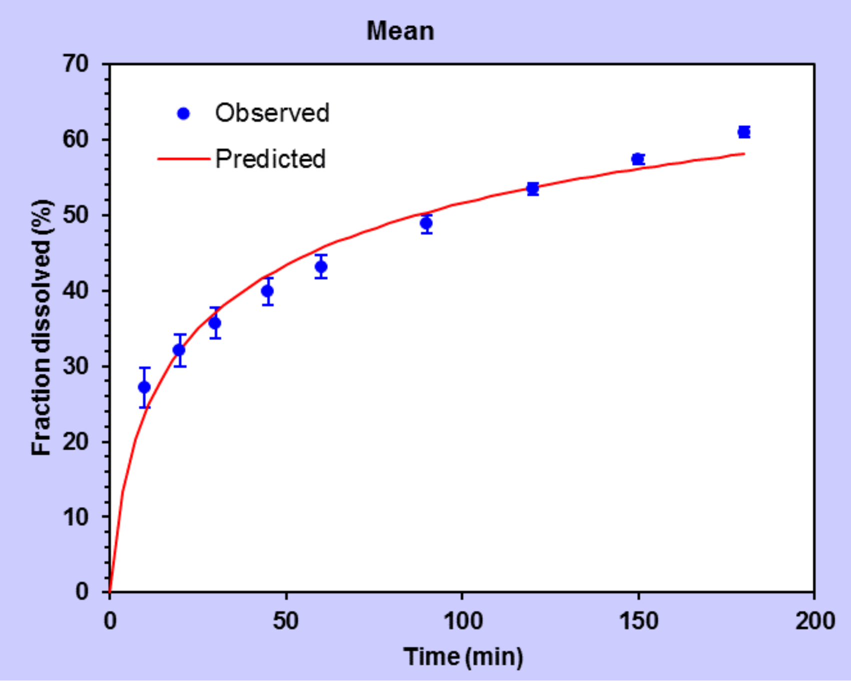

Supplementary materials – Model fitting summary – Tablettose® 70 (modelling up to app. 60 % of carvedilol released)

Graphical abstract of model fit presented as the fraction % of released carvedilol per tested tablet:

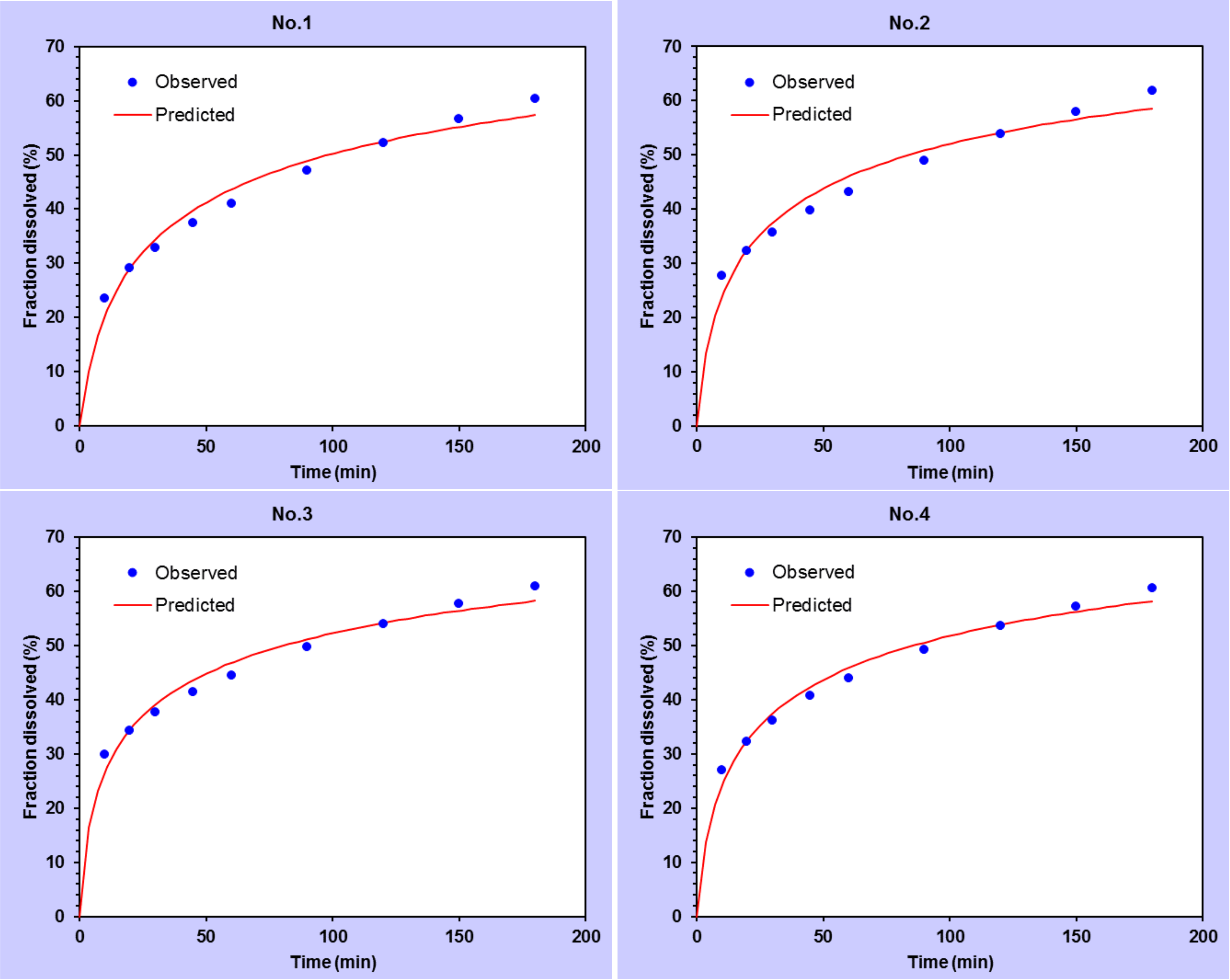

Supplementary materials – Model fitting summary – Tablettose® 70 (modelling up to app. 60 % of carvedilol released)

Model: **Gompertz_2**

Model equation: $F = F_{max} \cdot e^{-\alpha \cdot e^{-\beta \cdot \log(t)}}$

Fitted model parameters per tested tablet (N = 4) with statistics – mean, standard deviation (SD), and relative standard deviation expressed in % (RSD%) (output from DDSolver):

Parameter	No.1	No.2	No.3	No.4	Mean	SD	RSD(%)
α	13.174	9.959	8.323	10.134	10.398	2.023	19.452
β	2.133	2.006	1.962	2.050	2.038	0.073	3.582
F_{max}	63.349	65.024	63.992	63.663	64.007	0.727	1.136

Number of dissolution data points (N), degrees of freedom (df), and selected goodness of fit criteria – Pearson correlation coefficient (R), coefficient of determination (R^2), adjusted coefficient of determination ($R^2_{adjusted}$), and residual sum of squares (RSS) (manual calculation in MS Excel):

Parameter	No.1	No.2	No.3	No.4
N	9	9	9	9
df	6	6	6	6
R	0.937297099	0.92690924	0.930681517	0.943370996
R^2	0.878525851	0.859160738	0.866168086	0.889948837
$R^2_{adjusted}$	0.838034469	0.812214318	0.821557449	0.853265116
RSS	223.4928932	227.9068586	183.7325724	174.0029544

Graphical abstract of model fit presented as mean ± 1 SD of the fraction % of released carvedilol:

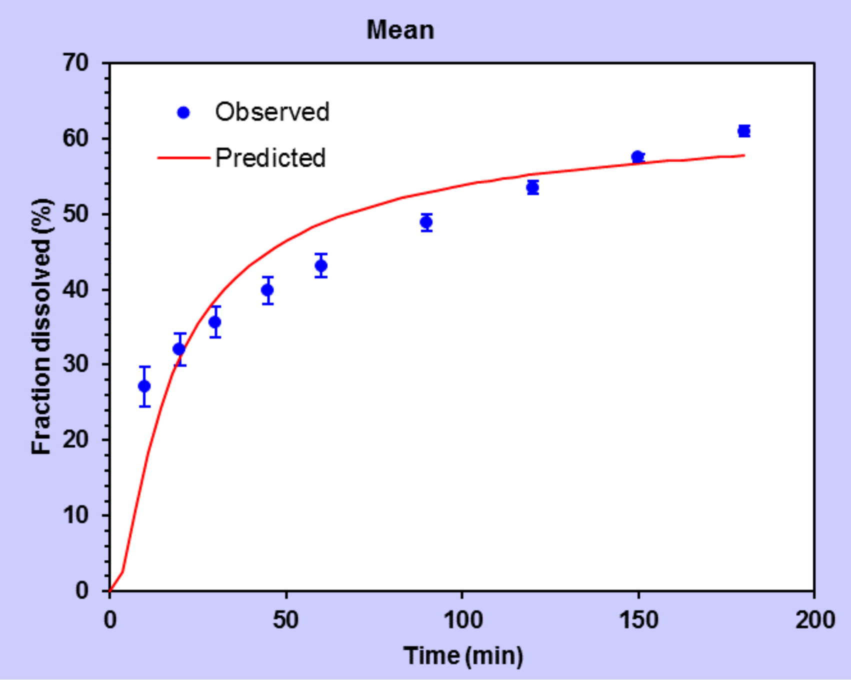

Supplementary materials – Model fitting summary – Tablettose® 70 (modelling up to app. 60 % of carvedilol released)

Graphical abstract of model fit presented as the fraction % of released carvedilol per tested tablet:

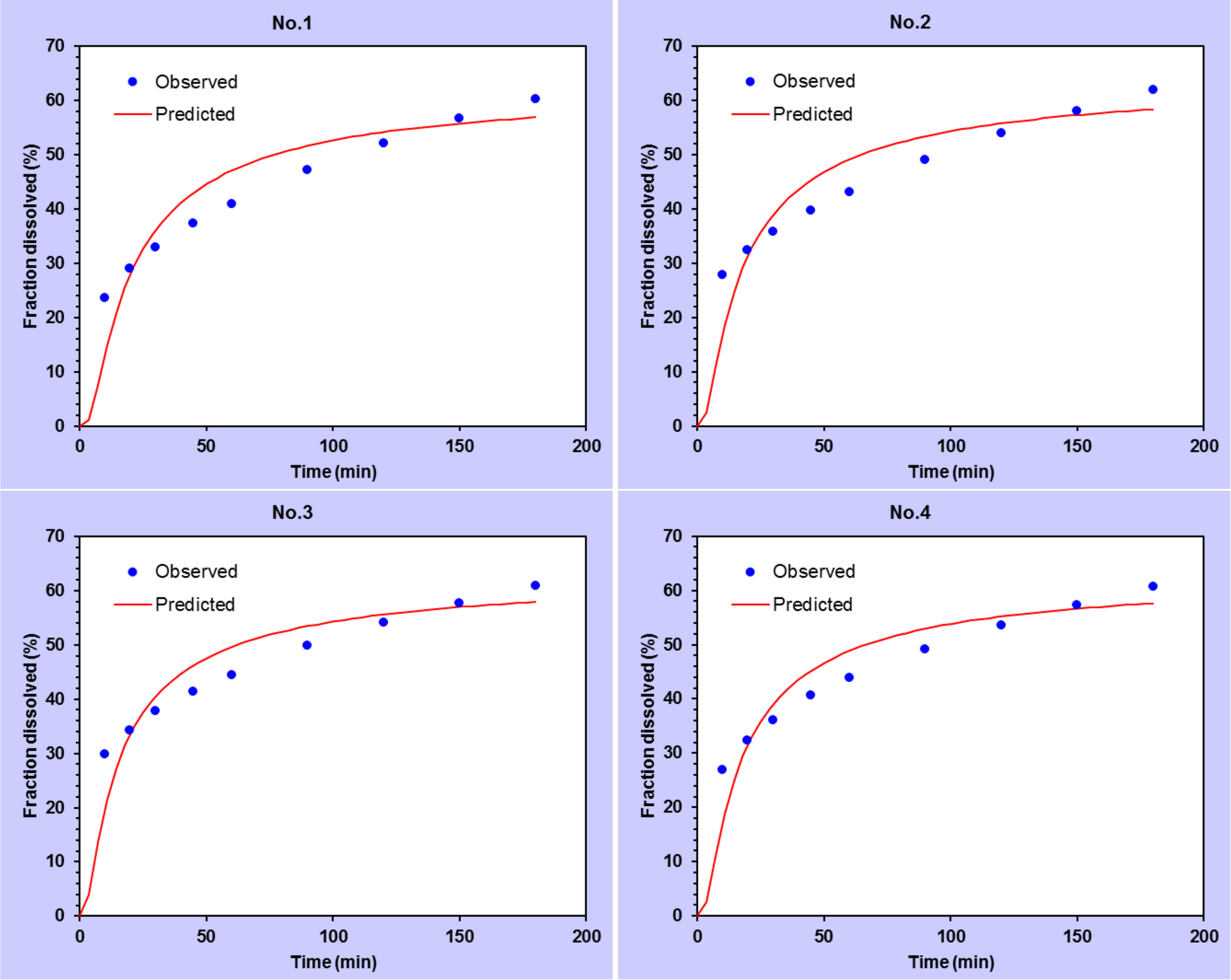

Supplementary materials – Model fitting summary – Tablettose® 70 (modelling up to app. 60 % of carvedilol released)

Model: **Gompertz_3**

Model equation: $F = F_{max} \cdot e^{-e^{-k\cdot(t-\gamma)}}$

Fitted model parameters per tested tablet (N = 4) with statistics – mean, standard deviation (SD), and relative standard deviation expressed in % (RSD%) (output from DDSolver):

Parameter	No.1	No.2	No.3	No.4	Mean	SD	RSD(%)
k	0.016	0.015	0.015	0.015	0.016	0.001	3.497
γ	8.390	0.642	-8.480	-4.089	-0.884	7.218	-816.288
F_{max}	63.349	65.024	63.992	63.663	64.007	0.727	1.136

Number of dissolution data points (N), degrees of freedom (df), and selected goodness of fit criteria – Pearson correlation coefficient (R), coefficient of determination (R^2), adjusted coefficient of determination ($R^2_{adjusted}$), and residual sum of squares (RSS) (manual calculation in MS Excel):

Parameter	No.1	No.2	No.3	No.4
N	9	9	9	9
df	6	6	6	6
R	0.996772756	0.996839228	0.997343967	0.996698647
R^2	0.993555928	0.993688447	0.994694989	0.993408193
$R^2_{adjusted}$	0.991407904	0.991584596	0.992926652	0.991210925
RSS	10.99246777	10.66652182	6.594054479	7.845613059

Graphical abstract of model fit presented as mean ± 1 SD of the fraction % of released carvedilol:

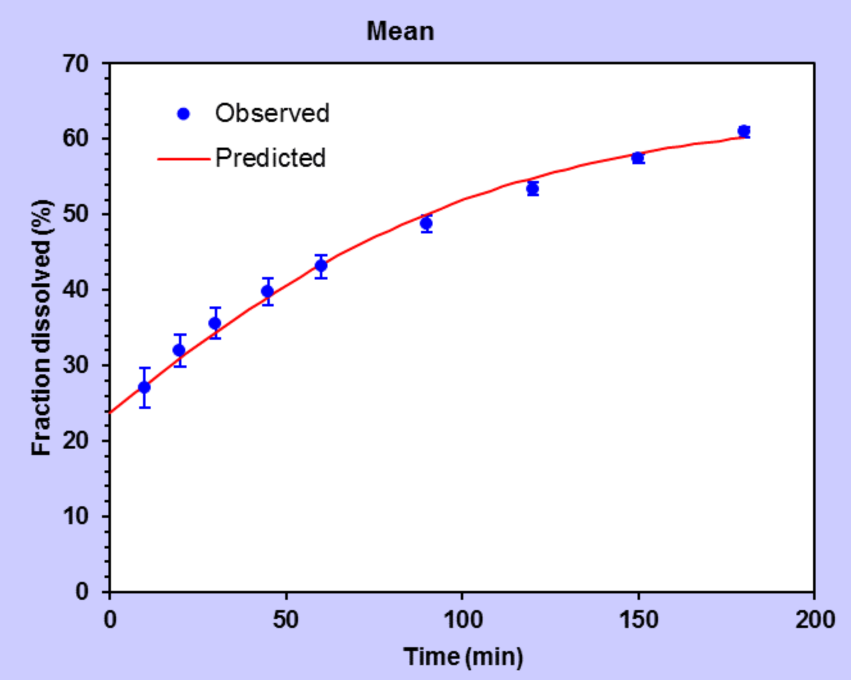

Supplementary materials – Model fitting summary – Tablettose® 70 (modelling up to app. 60 % of carvedilol released)

Graphical abstract of model fit presented as the fraction % of released carvedilol per tested tablet:

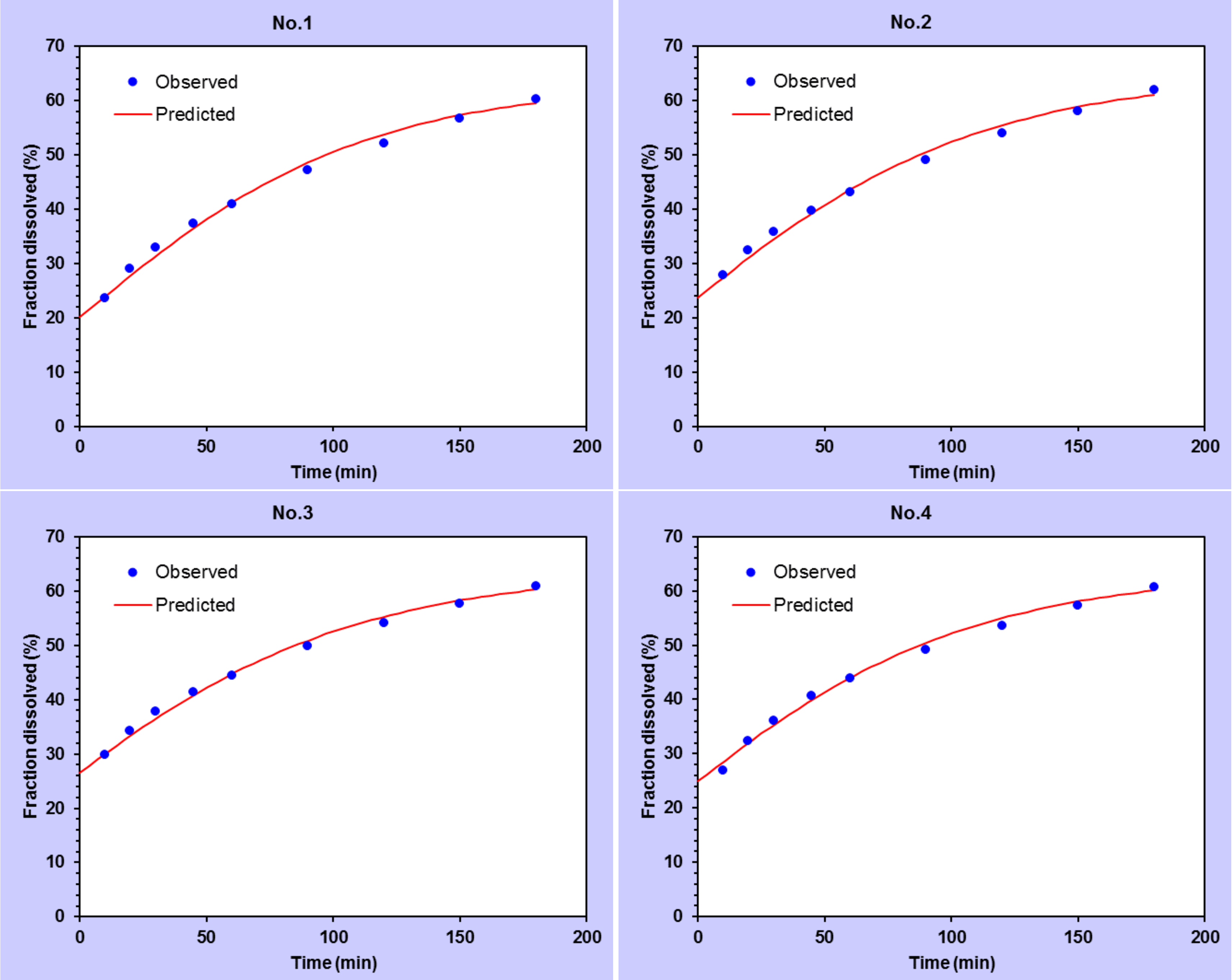

Supplementary materials – Model fitting summary – Tablettose® 70 (modelling up to app. 60 % of carvedilol released)

Model: **Gompertz_4**

Model equation: $F = F_{max} \cdot e^{-\beta \cdot e^{-k \cdot t}}$

Fitted model parameters per tested tablet (N = 4) with statistics – mean, standard deviation (SD), and relative standard deviation expressed in % (RSD%) (output from DDSolver):

Parameter	No.1	No.2	No.3	No.4	Mean	SD	RSD(%)
k	0.016	0.015	0.015	0.015	0.016	0.001	3.497
β	1.146	1.010	0.881	0.959	0.999	0.112	11.165
F_{max}	63.349	65.024	63.992	63.663	64.007	0.727	1.136

Number of dissolution data points (N), degrees of freedom (df), and selected goodness of fit criteria – Pearson correlation coefficient (R), coefficient of determination (R^2), adjusted coefficient of determination ($R^2_{adjusted}$), and residual sum of squares (RSS) (manual calculation in MS Excel):

Parameter	No.1	No.2	No.3	No.4
N	9	9	9	9
df	6	6	6	6
R	0.996772756	0.996839228	0.997343967	0.996649892
R^2	0.993555928	0.993688447	0.994694989	0.993311007
$R^2_{adjusted}$	0.991407904	0.991584596	0.992926652	0.991081343
RSS	10.99246777	10.66652182	6.594054479	7.926110689

Graphical abstract of model fit presented as mean ± 1 SD of the fraction % of released carvedilol:

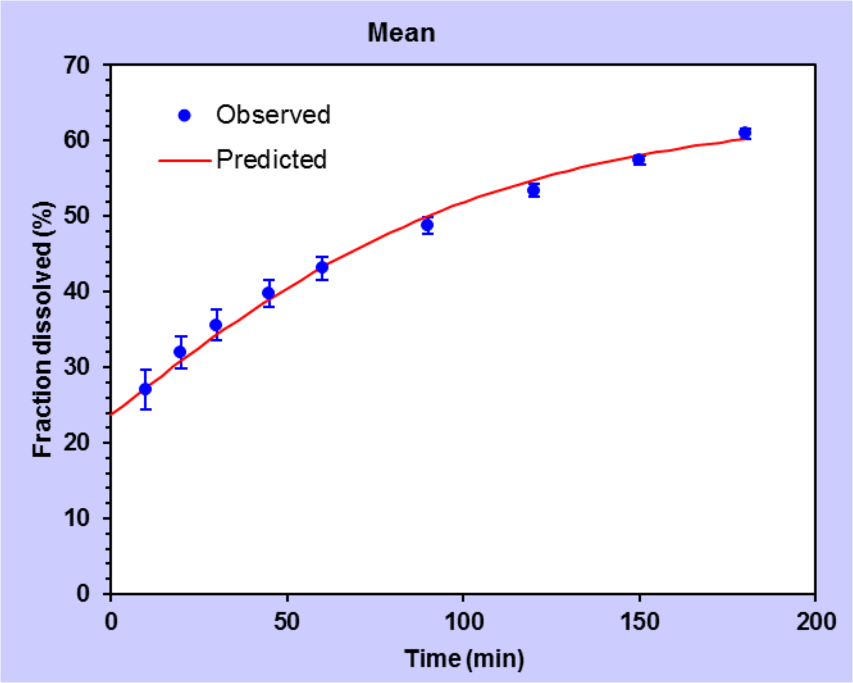

Supplementary materials – Model fitting summary – Tablettose® 70 (modelling up to app. 60 % of carvedilol released)

Graphical abstract of model fit presented as the fraction % of released carvedilol per tested tablet:

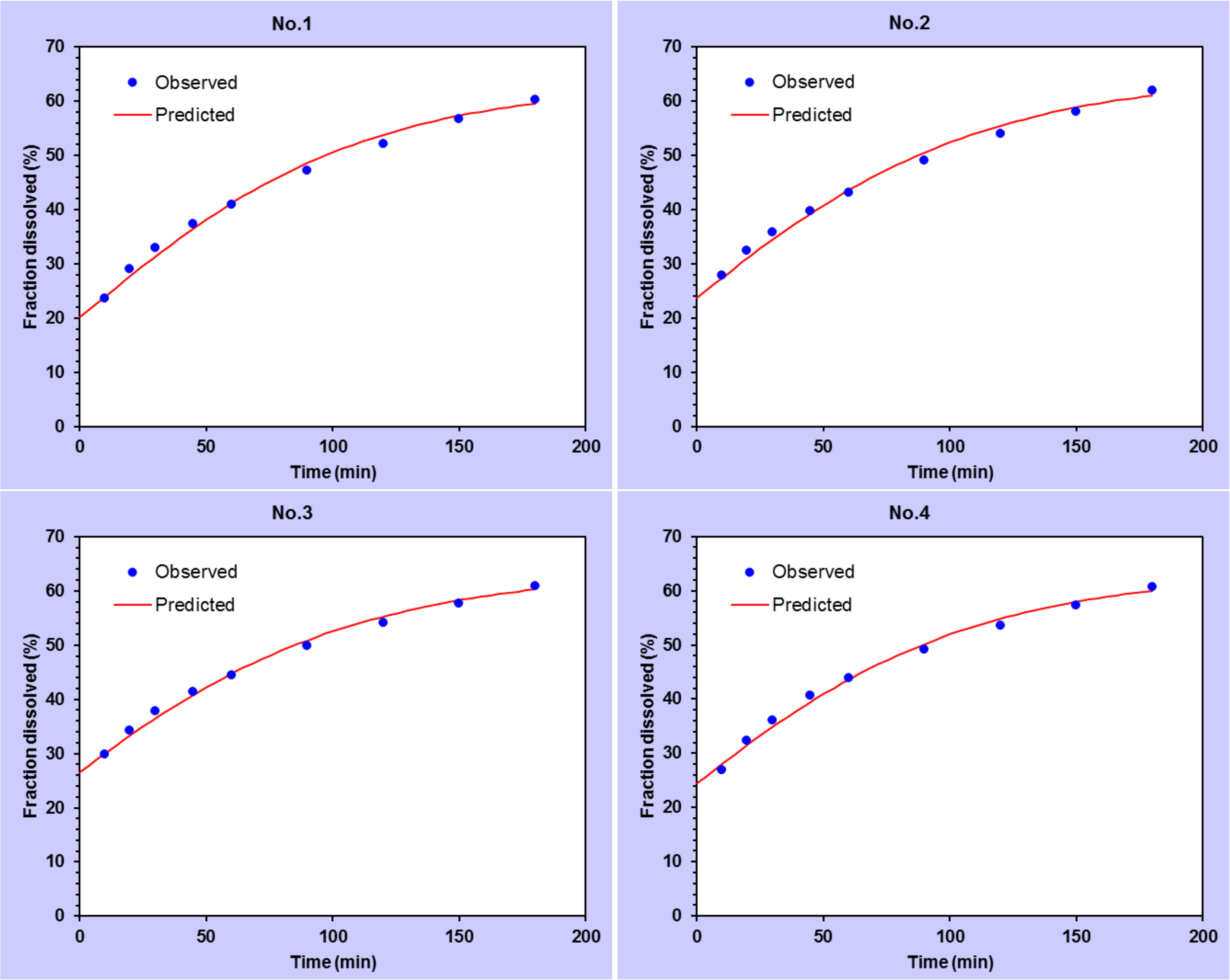

Supplementary materials – Model fitting summary – Tablettose® 70 (modelling up to app. 60 % of carvedilol released)

Model: **Probit_1**

Model equation: $F = 100 \cdot \phi[\alpha + \beta \cdot \log(t)]$

Fitted model parameters per tested tablet (N = 4) with statistics – mean, standard deviation (SD), and relative standard deviation expressed in % (RSD%) (output from DDSolver):

Parameter	No.1	No.2	No.3	No.4	Mean	SD	RSD(%)
α	-1.570	-1.381	-1.239	-1.371	-1.390	0.136	-9.812
β	0.783	0.712	0.645	0.705	0.711	0.057	7.949

Number of dissolution data points (N), degrees of freedom (df), and selected goodness of fit criteria – Pearson correlation coefficient (R), coefficient of determination (R^2), adjusted coefficient of determination ($R^2_{adjusted}$), and residual sum of squares (RSS) (manual calculation in MS Excel):

Parameter	No.1	No.2	No.3	No.4
N	9	9	9	9
df	7	7	7	7
R	0.991397729	0.986083803	0.988723793	0.994005333
R^2	0.982869457	0.972361267	0.977574738	0.988046603
$R^2_{adjusted}$	0.980422236	0.968412877	0.974371129	0.986338974
RSS	22.43398025	31.1468249	20.83920751	12.87890535

Graphical abstract of model fit presented as mean ± 1 SD of the fraction % of released carvedilol:

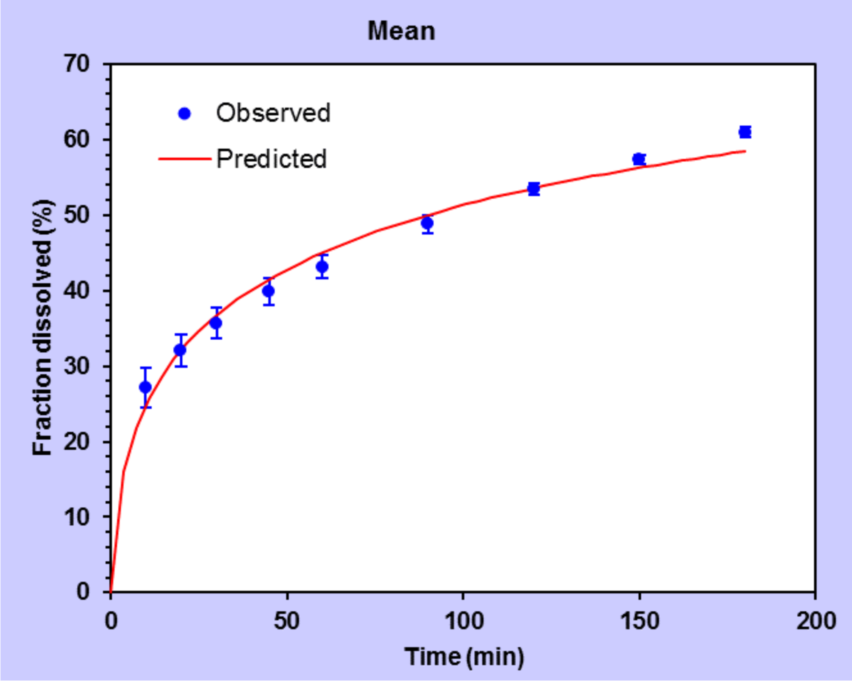

Supplementary materials – Model fitting summary – Tablettose® 70 (modelling up to app. 60 % of carvedilol released)

Graphical abstract of model fit presented as the fraction % of released carvedilol per tested tablet:

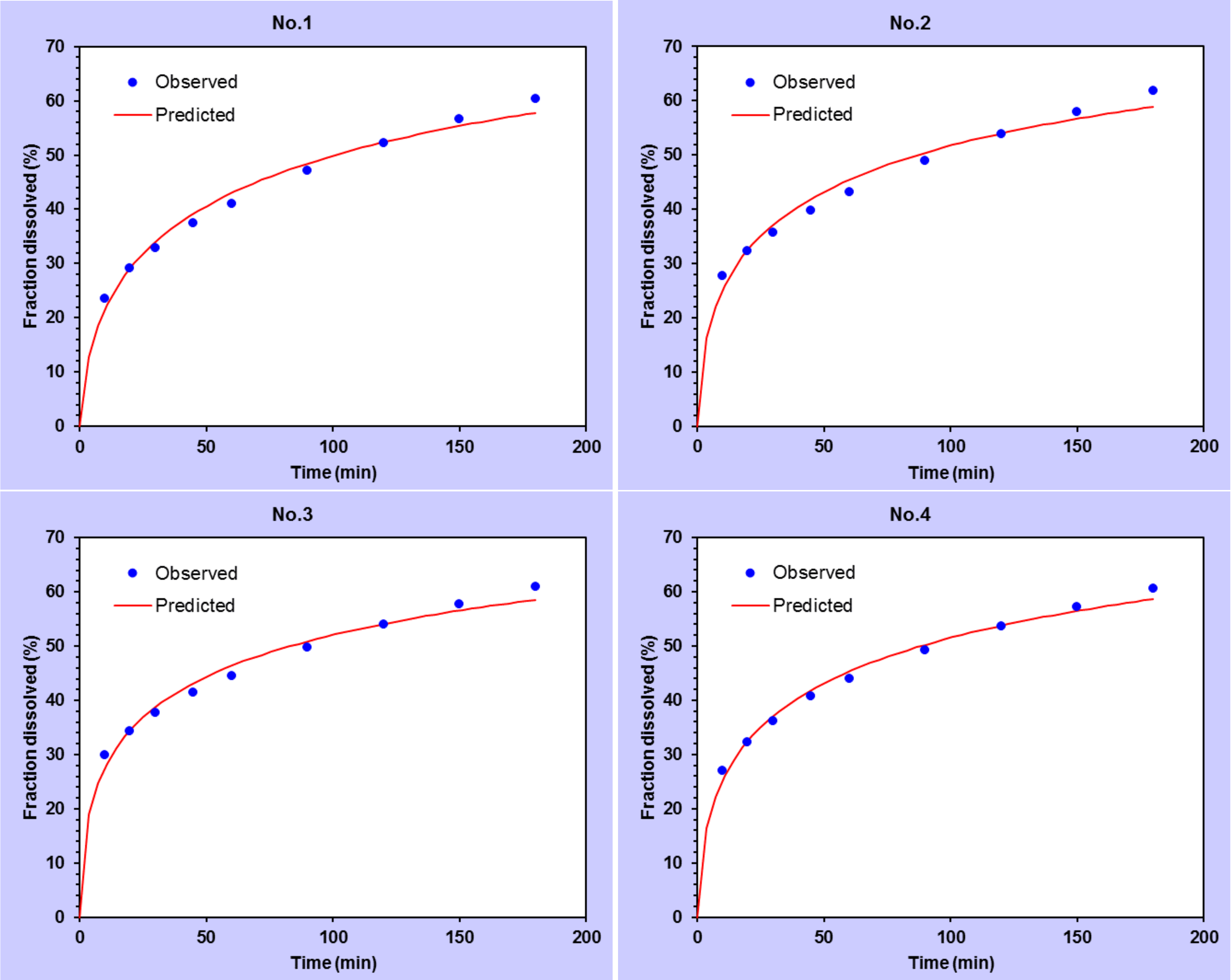

Supplementary materials – Model fitting summary – Tablettose® 70 (modelling up to app. 60 % of carvedilol released)

Model: **Probit_2**

Model equation: $F = F_{max} \cdot \phi[\alpha + \beta \cdot \log(t)]$

Fitted model parameters per tested tablet (N = 4) with statistics – mean, standard deviation (SD), and relative standard deviation expressed in % (RSD%) (output from DDSolver):

Parameter	No.1	No.2	No.3	No.4	Mean	SD	RSD(%)
α	-2.049	-1.796	-1.617	-1.795	-1.815	0.178	-9.794
β	1.479	1.362	1.303	1.385	1.382	0.073	5.285
F_{max}	63.349	65.024	63.992	63.663	64.007	0.727	1.136

Number of dissolution data points (N), degrees of freedom (df), and selected goodness of fit criteria – Pearson correlation coefficient (R), coefficient of determination (R^2), adjusted coefficient of determination ($R^2_{adjusted}$), and residual sum of squares (RSS) (manual calculation in MS Excel):

Parameter	No.1	No.2	No.3	No.4
N	9	9	9	9
df	6	6	6	6
R	0.965878807	0.957314675	0.959889547	0.97004797
R^2	0.93292187	0.916451387	0.921387942	0.940993065
$R^2_{adjusted}$	0.910562493	0.888601849	0.895183923	0.921324086
RSS	99.98329177	108.6407324	85.82138005	74.27657834

Graphical abstract of model fit presented as mean ± 1 SD of the fraction % of released carvedilol:

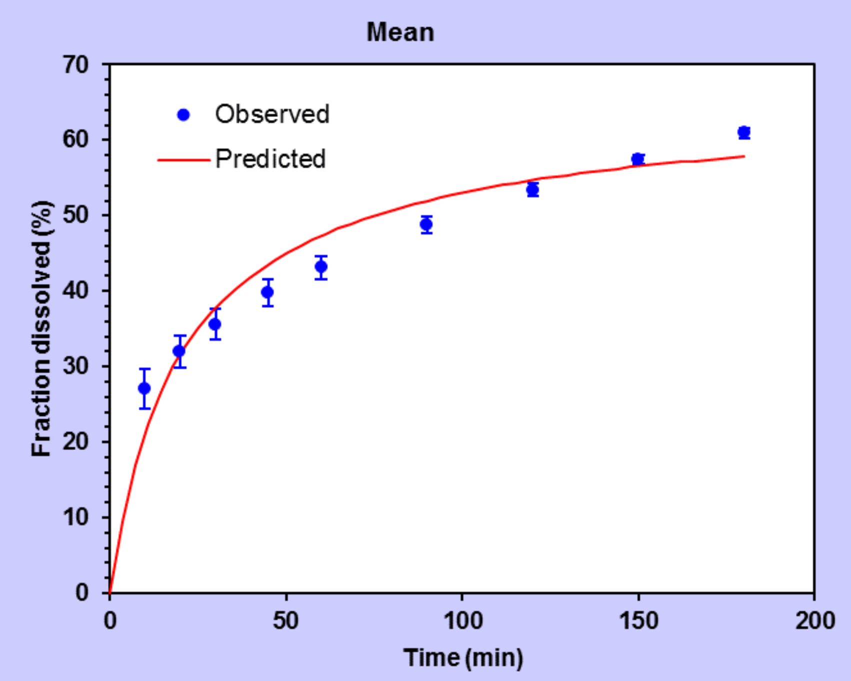

Supplementary materials – Model fitting summary – Tablettose® 70 (modelling up to app. 60 % of carvedilol released)

Graphical abstract of model fit presented as the fraction % of released carvedilol per tested tablet:

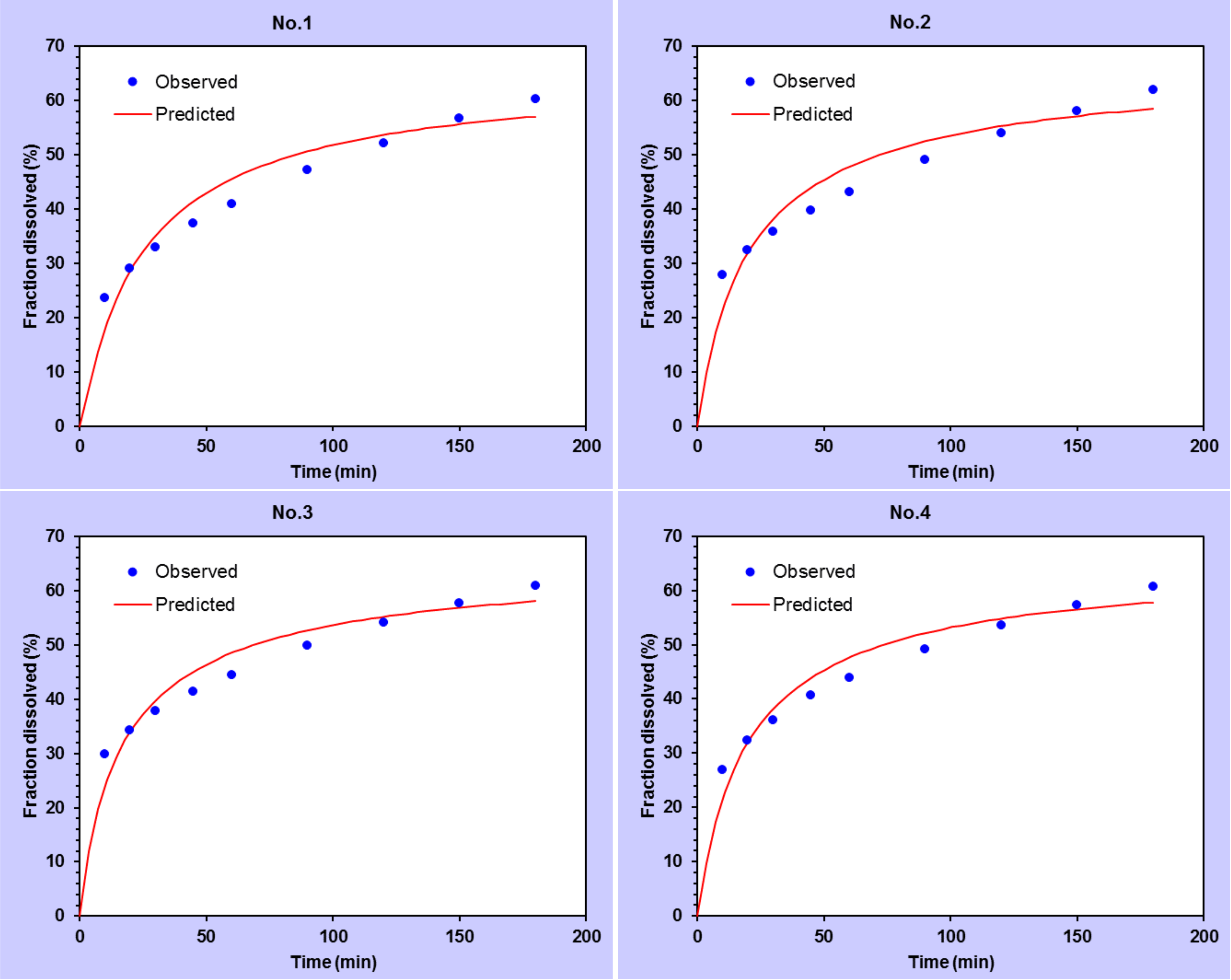

Supplement: Supplementary file 1 [file pharmaceutics-16-00498-s001.zip › Supplementary materials_Model fitting summary_Tablettose® 70.pdf]
