# Supplementary material for: Comparative Fitting of Mathematical Models to Carvedilol Release Profiles Obtained from Hypromellose Matrix Tablets
Source: Pharmaceutics. 2024 Apr 4;16(4):498. doi: 10.3390/pharmaceutics16040498 (PMC11053526; doi:10.3390/pharmaceutics16040498)
Supplement: Supplementary file 1 [file pharmaceutics-16-00498-s001.zip › Supplementary materials_Tables.pdf]

Tables S1, S2, S3, S4, S5, S6, S7, and S8 show experimental carvedilol dissolution results for individual tested tablets per formulation used for fitting mathematical models. In most cases results are presented with several decimal digits because dissolution media evaporation during the dissolution test was taken into account; initial results, obtained before considering dissolution media evaporation, contained a single decimal digit. For calculating % of carvedilol released, where dissolution media evaporation was taken into account ($F\%_{t,\ considering\ dissolution\ media\ evaporation}$) at each time point t, the below two equations were used:

$$F\%_{t,considering\ dissolution\ media\ evaporation} = F\%_t \cdot \frac{V_{dissolution\ media,end} - k_{evaporation} \cdot t}{V_{dissolution\ media,end}}$$

$$k_{evaporation} = \frac{V_{dissolution\ media,start} - V_{dissolution\ media,end}}{t_{end}}$$

$F\%_t$ …………………………………………………… experimentally measured % of carvedilol released at time t
$F\%_{t,considering\ dissolution\ media\ evaporation}$ ………………% of carvedilol released at time t where dissolution media evaporation was taken into account
$V_{dissolution\ media,start}$ …………………………………… volume of dissolution media at the start of dissolution testing (900 mL)
$V_{dissolution\ media,end}$ ……………………………………...volume of dissolution media at the end of dissolution testing
t ……………………………………………………………...time point at which % of carvedilol released was measured (from t = 10 min up to t = 1440 min)
t_{end} …………………………………………………………...final time point at which % of carvedilol release was measured (t = 1440 min)
$k_{evaporation}$ ……………………………………………….estimated rate of dissolution media evaporation in mL/min

Table S1. Experimental carvedilol dissolution results for individual tested tablets per formulation used for fitting mathematical models. Data is shown for the Polyglykol® 4000 P, the Polyglykol® 8000 P and the Polyox™ WSR N-80 (LEO NF Grade) formulations.

t (min]	Polyglykol® 4000 P_Tablet 1 – F% released	Polyglykol® 4000 P_Tablet 2 – F% released	Polyglykol® 4000 P_Tablet 3 – F% released	Polyglykol® 4000 P_Tablet 4 – F% released	Polyglykol® 8000 P_Tablet 1 – F% released	Polyglykol® 8000 P_Tablet 2 – F% released	Polyglykol® 8000 P_Tablet 3 – F% released	Polyglykol® 8000 P_Tablet 4 – F% released	Polyox™ WSR N-80 (LEO NF Grade)_Tablet 1 – F% released	Polyox ™ WSR N-80 (LEO NF Grade)_Tablet 2 – F% released	Polyox ™ WSR N-80 (LEO NF Grade)_Tablet 3 – F% released	Polyox ™ WSR N-80 (LEO NF Grade)_Tablet 4 – F% released
10	17.49594907	17.89723765	16.29748457	16.89608796	12.39808642	12.09813272	11.09743056	13.89785494	1.399675926	1.799722222	2.199660494	2.199490741
20	32.385	37.48842593	30.9904321	33.98425926	30.09070988	29.5908642	28.18694444	31.99012346	4.298009259	5.898179012	6.19808642	6.596944444
30	43.86951389	52.37574074	43.57981481	47.666875	45.9787037	44.77925926	43.07006944	47.57796296	7.994444444	10.99490741	11.19481481	11.89173611
45	58.23927083	72.04993056	57.85979167	66.530625	65.15472222	62.95625	61.83552083	66.25395833	15.2840625	19.58638889	19.48645833	20.27885417
60	70.00263889	86.71962963	67.83712963	79.98875	78.82694444	77.02861111	75.99430556	79.5262963	24.26625	28.07398148	28.17388889	28.46041667
90	84.92270833	97.16486111	81.38680556	95.60041667	92.77097222	95.36736111	95.20125	93.47	41.11416667	41.74194444	43.93888889	44.20770833
120	88.35388889	97.21962963	85.94055556	95.53388889	94.12537037	95.92203704	99.22361111	96.52092593	54.64777778	53.9	58.49148148	58.53694444
150	90.08611111	97.27430556	88.19537037	95.56701389	96.77546296	95.8775463	99.05486111	96.37638889	65.57152778	64.25092593	71.43425926	70.25520833
180	91.51708333	97.22916667	90.14888889	95.50041667	97.22916667	95.93277778	99.08541667	96.43138889	74.28916667	72.99666667	82.07138889	79.56708333
210	92.84645833	97.18402778	91.70185185	95.43381944	97.18402778	95.88824074	99.01631944	96.38662037	81.501875	80.63782407	90.00736111	86.875625
240	93.87555556	97.23851852	93.1537037	95.36722222	97.23851852	95.94333333	99.04666667	96.44148148	89.00277778	87.67407407	95.1462963	92.08555556
270	94.903125	97.19333333	94.50458333	95.4	97.29291667	95.89875	98.9775	96.49625	94.505625	92.91125	98.08958333	95.896875
300	95.33333333	97.24768519	95.45601852	95.33333333	97.24768519	95.85416667	99.00763889	96.45138889	96.92222222	96.55092593	98.24305556	97.12083333
330	95.76284722	97.30194444	96.2075463	95.36590278	97.30194444	95.90907407	99.03763889	96.50601852	97.54909722	98.99328704	98.19736111	97.05291667
360	95.795	97.25666667	96.85888889	95.29916667	97.25666667	95.86444444	98.96833333	96.46111111	97.48083333	99.74277778	98.25111111	96.985
420	95.75986111	97.26546296	97.06675926	95.16569444	97.26546296	95.87453704	98.92875	96.57	97.44333333	99.74925926	98.25898148	96.94819444
480	95.82333333	97.27407407	97.07555556	95.13111111	97.27407407	95.78518519	98.79	96.48	97.30666667	99.6562963	98.26666667	96.81222222
540	95.88625	97.2825	97.08416667	94.9975	97.2825	95.795	98.75	96.58833333	97.3675	99.6625	98.27416667	96.775
600	95.85	97.29074074	97.09259259	94.9625	97.19166667	95.80462963	98.61111111	96.59722222	97.23055556	99.66851852	98.28148148	96.7375
660	95.81347222	97.2987963	97.10083333	94.82875	97.2987963	95.81407407	98.57069444	96.60592593	97.19208333	99.57537037	98.28861111	96.69972222
720	95.67833333	97.20777778	97.01	94.695	97.30666667	95.72444444	98.53	96.61444444	97.15333333	99.58111111	98.29555556	96.56333333
780	95.73958333	97.21555556	97.01796296	94.56125	97.31435185	95.73361111	98.48902778	96.62277778	97.01611111	99.58666667	98.30231481	96.52513889
840	95.70222222	97.22314815	97.02574074	94.52555556	97.22314815	95.64388889	98.34972222	96.53222222	96.97694444	99.49333333	98.30888889	96.38861111
900	95.66458333	97.23055556	96.83611111	94.39166667	97.13194444	95.65277778	98.21041667	96.54027778	96.9375	99.49861111	98.21666667	96.25208333
960	95.62666667	97.13925926	96.94222222	94.35555556	97.13925926	95.56296296	98.16888889	96.54814815	96.8	99.40518519	98.22296296	96.21333333
1020	95.58847222	97.14638889	96.85111111	94.22152778	97.29291667	95.80462963	98.57069444	96.48	97.15333333	99.58666667	98.28148148	96.7375
1080	95.55	97.15333333	96.85833333	94.0875	97.25666667	95.81407407	98.48902778	96.49625	96.97694444	99.57537037	98.24305556	96.52513889
1140	95.51125	97.16009259	96.76712963	93.95347222	97.30194444	95.81407407	98.57069444	96.44148148	97.01611111	99.66851852	98.19736111	96.81222222
1200	95.47222222	97.16666667	96.77407407	93.81944444	97.13925926	95.88824074	98.53	96.60592593	96.9375	99.49861111	98.29555556	96.775
1260	95.33583333	97.075	96.68277778	93.7825	97.23851852	95.73361111	98.34972222	96.45138889	97.15333333	99.58666667	98.28148148	96.25208333
1320	95.39333333	97.17925926	96.68944444	93.64833333	97.25666667	95.88824074	98.16888889	96.61444444	97.19208333	99.49861111	98.19736111	97.12083333
1380	95.47222222	97.16666667	96.76712963	93.81944444	97.22916667	95.87453704	98.57069444	96.45138889	97.30666667	99.6625	98.22296296	96.69972222
1440	95.33583333	97.16009259	96.68277778	93.64833333	97.26546296	95.94333333	98.16888889	96.57	97.01611111	99.6562963	98.28861111	96.52513889

Table S2. Experimental carvedilol dissolution results for individual tested tablets per formulation used for fitting mathematical models. Data is shown for the Kollidon® 25, the Kollidon® 90 F and the C*Pharm Mannidex 16700 formulations.

t (min)	Kollidon® 25_Tablet 1 – F% released	Kollidon® 25_Tablet 2 – F% released	Kollidon® 25_Tablet 3 – F% released	Kollidon® 25_Tablet 4 – F% released	Kollidon® 90 F_Tablet 1 – F% released	Kollidon® 90 F_Tablet 2 – F% released	Kollidon® 90 F_Tablet 3 – F% released	Kollidon® 90 F_Tablet 4 – F% released	C*Pharm Mannidex 16700_Tablet 1 – F% released	C*Pharm Mannidex 16700_Tablet 2 – F% released	C*Pharm Mannidex 16700_Tablet 3 – F% released	C*Pharm Mannidex 16700_Tablet 4 – F% released
10	9.4	15.3	12.5	11.6	0.999768519	1.299799383	1.799722222	1.199907407	8.498032407	9.798487654	9.198580247	11.89724537
20	19.4	31.5	24.8	24.2	2.398888889	3.199012346	4.29867284	2.999537037	13.5937037	16.09503086	14.7954321	18.09162037
30	28.9	45.1	32.2	37.3	3.897291667	5.197592593	7.196666667	4.798888889	17.9875	21.39009259	19.59092593	23.48368056
45	40.3	64.3	38.1	55.9	6.493229167	8.294236111	11.691875	7.697326389	23.57541667	28.48020833	26.38166667	56.0415625
60	45.2	80	41.6	73.3	9.087361111	11.38944444	16.38481481	10.6950463	28.66013889	33.16925926	32.76962963	66.60736111
90	51.7	91.9	47.9	96.7	14.96875	18.77388889	26.06375	16.68840278	38.020625	41.54222222	45.43680556	75.741875
120	57.6	94.9	53.7	98.7	21.44027778	26.05166667	35.43425926	23.07861111	47.26833333	49.50814815	59.58944444	83.16833333
150	62.6	96.7	58.7	98.9	28.20173611	33.82152778	44.39699074	29.86539352	57.4	57.76597222	72.73125	90.48472222
180	69.7	98.2	63.5	98.9	34.85416667	41.285	52.055	37.14833333	66.02375	65.91638889	80.47583333	95.79916667
210	75	99.4	68.1	98.9	41.39777778	48.24314815	58.8087963	46.32481481	73.73979167	73.26180556	86.21967593	101.6036806
240	80	101.4	72.4	99	47.33555556	54.59703704	64.65962963	55.39722222	80.94777778	80.00259259	90.26444444	101.7316667
270	84.2	102.4	76.5	99.1	53.165625	60.3475	69.60875	63.56729167	86.05875	85.54208333	93.70791667	101.76
300	87.9	102.8	80.4	99	58.59027778	65.49537037	73.75694444	70.93541667	90.16944444	89.98148148	96.45138889	101.6888889
330	91.2	102.8	84	99	63.80881944	70.14097222	77.70226852	77.80138889	93.18270833	93.81976852	99.98819444	101.7170139
360	93.8	102.9	87.4	99.1	68.32583333	74.68277778	81.34555556	83.4675	95.29916667	96.85888889	101.0355556	101.6458333
420	98.4	102.9	92.5	99.1	77.53875	82.66074074	87.52898148	92.59893519	99.52291667	100.4447222	101.1401852	101.6025
480	101.6	103.2	96.3	99.2	85.73666667	89.03555556	92.01333333	98.23481481	100.4711111	100.8474074	101.2444444	101.5588889
540	103.6	103.3	98.9	99.3	92.33125	93.7125	96.78666667	100.67875	100.42875	100.9516667	101.2491667	101.515
600	105	103.5	100.4	99.4	96.7375	96.89444444	97.48888889	100.8310185	100.2875	100.6592593	101.1546296	101.2736111
660	105.2	103.6	101.3	99.4	99.55541667	97.89268519	97.69472222	100.8836111	100.2447222	100.6641667	101.0600926	101.1309722
720	105.4	103.7	101.3	99.5	101.775	97.80111111	97.50444444	100.9361111	100.2016667	100.6688889	101.0644444	101.0866667
780	105.4	103.9	101.4	99.5	102.0240278	98.00592593	97.51194444	100.8891204	100.0601389	100.4758333	101.0686111	100.8456944
840	105.5	104	101.5	99.6	101.9777778	97.91407407	97.51925926	100.9414815	100.1147222	100.4803704	101.0725926	100.8011111
900	105.8	104.3	101.6	99.7	102.0291667	98.01944444	97.52638889	100.99375	100.16875	100.4847222	101.0763889	100.75625
960	105.9	104.4	101.6	99.7	102.08	97.92740741	97.53333333	100.8474074	100.0266667	100.3903704	101.08	100.5155556
1020	106	104.6	101.7	99.8	102.1302778	98.03222222	97.54009259	100.8995833	100.42875	100.4447222	101.0725926	101.76
1080	106.2	104.8	101.8	99.9	102.18	98.03833333	97.54666667	100.9516667	100.16875	100.4847222	101.1401852	101.7170139
1140	106.3	104.9	101.8	100	102.1318056	98.04425926	97.55305556	100.904537	100.2016667	100.8474074	101.08	100.75625
1200	106.4	105.1	101.9	100	102.1805556	98.05	97.55925926	100.8574074	100.2447222	100.6688889	101.2444444	101.6025
1260	106.5	105.2	101.9	100.2	102.22875	98.15361111	97.56527778	100.9093056	100.16875	100.4758333	101.0763889	101.2736111
1320	106.3	104.9	101.7	100.1	102.2763889	98.15888889	97.57111111	100.8621296	100.16875	100.6688889	101.0644444	101.1309722
1380	106.5	105.2	101.8	100	102.4202778	98.16398148	97.47888889	100.8149537	100.16875	100.6688889	101.0686111	100.75625
1440	106.3	105.1	101.9	100.2	102.5633333	98.16888889	97.58222222	100.8666667	100.42875	100.6641667	101.0725926	101.76

Table S3. Experimental carvedilol dissolution results for individual tested tablets per formulation used for fitting mathematical models. Data is shown for the Pearlitol® 160C, the Parteck® M 100 and the Parteck® M 200 formulations.

t (min)	Pearlitol® 160C_Tablet 1 – F% released	Pearlitol ® 160C_Tablet 2 – F% released	Pearlitol ® 160C_Tablet 3 – F% released	Pearlitol ® 160C_Tablet 4 – F% released	Parteck® M 100_Tablet 1 – F% released	Parteck® M 100_Tablet 2 – F% released	Parteck® M 100_Tablet 3 – F% released	Parteck® M 100_Tablet 4 – F% released	Parteck® M 200_Tablet 1 – F% released	Parteck® M 200_Tablet 2 – F% released	Parteck® M 200_Tablet 3 – F% released	Parteck® M 200_Tablet 4 – F% released
10	10	11.9	10.6	9.6	6.198564815	4.699274691	5.199197531	4.998842593	6.199521605	6.999459877	6.8	6.19904321
20	15.7	18	16.7	15.6	17.59185185	18.59425926	14.39555556	18.89125	14.39777778	21.59666667	17	16.39493827
30	20.5	23	22.2	20.7	34.07631944	26.78759259	27.48726852	52.66340278	21.19509259	47.18907407	28.4	26.68763889
45	26.8	30.2	30.5	27.2	66.43072917	39.67243056	43.26993056	71.82510417	34.188125	72.47482639	44.7	40.67173611
60	32.5	37.5	46.7	32.9	77.89166667	64.24046296	55.54851852	82.88472222	52.27578704	85.36046296	63.1	50.55314815
90	45.2	52.3	77.5	42.8	90.21166667	78.19125	80.28833333	96.49854167	71.15055556	97.1325	86.5	64.51027778
120	57.9	65.3	91.2	51.6	96.0325	86.73907407	88.73537037	101.0186111	79.92592593	99.10814815	94.6	74.56166667
150	67.7	76.4	97.4	60	99.95173611	93.78240741	93.58287037	101.2472222	87.89814815	99.18506944	97.8	81.81018519
180	75.5	83.8	101.2	67.7	101.7741667	97.12944444	97.12944444	101.27625	92.87083333	99.26194444	99.3	87.35666667
210	81.4	90.3	103.7	74.3	102.7978472	99.77560185	100.872037	101.205625	98.44023148	99.23893519	99.9	92.69861111
240	86.3	96.2	104.9	80.1	102.8255556	102.1203704	102.1203704	101.135	99.51537037	99.31574074	99.9	97.23851852
270	90.4	99.6	105	85.5	102.853125	102.2720833	102.1725	101.064375	99.29270833	99.29270833	99.9	100.38
300	94.4	101.2	105	89.3	102.8805556	102.3240741	102.224537	101.0930556	99.36944444	99.26967593	100	100.6319444
330	97.7	102	105.1	92.3	103.0070833	102.3759722	102.2764815	101.0223611	99.44613426	99.34638889	100.1	100.7841204
360	99.9	102.3	105.1	94.8	103.0341667	102.2288889	102.2288889	100.9516667	99.52277778	99.42305556	100.1	100.8366667
420	103.2	102.4	105.3	100.5	103.1869444	102.3324074	102.2330556	100.9093056	99.47657407	99.37689815	100.2	100.8421296
480	103.4	102.7	105.5	103.3	103.24	102.4355556	102.3362963	100.8666667	99.62962963	99.53	100.3	100.9466667
540	103.4	102.9	105.6	104.1	103.2925	102.5383333	102.2408333	100.82375	99.48375	99.38416667	100.3	100.8525
600	103.5	103.1	105.8	104.4	103.3444444	102.4425926	102.2444444	100.7805556	99.63657407	99.4375	100.4	100.8574074
660	103.6	103.3	105.9	104.6	103.4943056	102.5448148	102.2478704	100.7370833	99.68972222	99.39125	100.5	100.8621296
720	103.7	103.5	106.1	104.7	103.545	102.4488889	102.2511111	100.4966667	99.64333333	99.44444444	100.4	100.8666667
780	103.8	103.7	106.3	105	103.5951389	102.5505556	102.2541667	100.4529167	99.69634259	99.4975463	100.6	100.8710185
840	103.8	103.9	106.5	105.2	103.6447222	102.4544444	102.1583333	100.3108333	99.74925926	99.4512037	100.6	100.8751852
900	103.9	104.2	106.7	105.4	103.5958333	102.4569444	102.0625	100.16875	99.60347222	99.40486111	100.6	100.7805556
960	103.9	104.3	106.8	105.6	103.6444444	102.4592593	102.0651852	100.1244444	99.6562963	99.45777778	100.7	100.7844444
1020	104.1	104.6	107	105.9	103.5958333	102.5448148	102.2408333	100.16875	99.70902778	99.51060185	100.7	100.7881481
1080	104.3	104.9	107.3	106.2	103.4943056	102.5383333	102.224537	100.3108333	99.76166667	99.56333333	100.7	100.7916667
1140	104.2	105	107.4	106.3	103.5951389	102.5448148	102.2288889	100.1244444	99.71509259	99.51685185	100.8	100.795
1200	104.3	105.3	107.6	106.6	103.5958333	102.4544444	102.0625	100.4529167	99.76759259	99.56944444	100.9	100.7981481
1260	104.4	105.5	107.8	106.8	103.6444444	102.4355556	102.1203704	100.1244444	99.82	99.52291667	100.9	100.8011111
1320	104.4	105.4	107.6	106.7	103.545	102.5383333	102.0651852	100.3108333	99.77333333	99.57537037	101	100.8038889
1380	104.3	105.3	107.8	106.6	103.6447222	102.4355556	102.1725	100.16875	99.82560185	99.5287963	101	100.7086111
1440	104.4	105.5	107.5	106.7	103.6447222	102.5448148	102.2408333	100.4966667	99.77888889	99.48222222	101	100.6133333

Table S4. Experimental carvedilol dissolution results for individual tested tablets per formulation used for fitting mathematical models. Data is shown for the Lactochem® Crystals, the Lactochem® Fine Powder and the SuperTab® 11SD formulations.

t (min)	Lactochem® Crystals_Tablet 1 – F% released	Lactochem® Crystals_Tablet 2 – F% released	Lactochem® Crystals_Tablet 3 – F% released	Lactochem® Crystals_Tablet 4 – F% released	Lactochem® Fine Powder_Tablet 1 – F% released	Lactochem® Fine Powder_Tablet 2 – F% released	Lactochem® Fine Powder_Tablet 3 – F% released	Lactochem® Fine Powder_Tablet 4 – F% released	SuperTab® 11SD_Tablet 1 – F% released	SuperTab® 11SD_Tablet 2 – F% released	SuperTab® 11SD_Tablet 3 – F% released	SuperTab® 11SD_Tablet 4 – F% released
10	19.2970216	23.49637346	19.19703704	23.99444444	6.698966049	7.399429012	8.399351852	6.399012346	30.39296296	36.59435185	27.59574074	25.99398148
20	24.19253086	27.89138889	24.79234568	28.98657407	9.896944444	10.5983642	11.59820988	9.497067901	34.78388889	40.98734568	31.69021605	29.98611111
30	27.38731481	31.08560185	28.88662037	32.47743056	12.59416667	13.19694444	14.19671296	11.99444444	37.97361111	43.77972222	34.38407407	32.67729167
45	31.17833333	34.77583333	33.37680556	36.66177083	16.68840278	16.69420139	17.69385417	15.289375	41.75645833	47.06729167	37.67381944	36.06239583
60	34.36814815	37.96481481	36.76592593	40.14416667	20.48101852	19.69087963	20.69041667	18.08324074	44.9375	49.8537963	40.36259259	39.14555556
90	40.14416667	43.53944444	42.44097222	46.00395833	26.56305556	25.08256944	25.78208333	23.06791667	50.19520833	54.52416667	45.63652778	45.00604167
120	45.01648148	48.31037037	47.41203704	50.75861111	31.6412963	29.77240741	30.07212963	27.44907407	54.7475	58.29185185	50.60611111	49.86111111
150	49.28564815	52.47824074	52.17893519	54.90868056	36.21597222	33.96064815	34.06053241	31.42708333	58.79513889	61.75671296	54.57337963	53.71284722
180	53.15194444	56.24333333	56.24333333	58.75416667	40.3875	38.04708333	37.94722222	35.20194444	62.33916667	65.01888889	57.93861111	57.06125
210	56.71560185	59.80555556	59.90523148	62.39520833	44.45546296	41.93194444	41.63243056	38.87361111	65.57965278	67.97898148	61.00166667	60.20590278
240	60.07666667	63.06555556	63.26481481	65.63333333	48.32037037	45.71518519	45.1162963	42.74111111	68.51722222	70.53777778	64.06185185	63.14722222
270	63.13583333	66.12333333	66.42208333	68.866875	51.88291667	49.19729167	48.49875	46.70458333	71.251875	73.09416667	67.01958333	65.985
300	66.09259259	68.97916667	69.37731481	71.89722222	55.44212963	52.67777778	51.77986111	50.46527778	73.88333333	75.54861111	69.77546296	68.81875
330	68.94708333	71.73282407	72.23027778	74.72479167	58.69953704	55.85740741	54.95969907	54.12296296	76.31256944	77.90125	72.32976852	71.64847222
360	71.6	74.285	74.88166667	77.6475	61.85444444	58.93583333	57.93861111	57.57833333	78.54	80.15222222	74.78222222	74.47416667
420	76.60027778	79.38212963	80.17694444	82.68819444	68.05601852	64.68967593	63.69291667	63.88324074	82.98527778	85.04518519	79.08407407	79.32125
480	81.09481481	84.07259259	85.2637037	87.22	73.74962963	70.03962963	69.04333333	69.97777778	87.91222222	89.13481481	82.98074074	83.85777778
540	85.28333333	88.25833333	89.44833333	91.0475	79.23416667	74.88666667	74.18958333	76.95333333	95.68875	92.42333333	87.26666667	87.59125
600	89.26574074	94.21944444	94.61574074	94.56805556	89.95925926	79.53009259	79.13194444	88.6712963	99.20277778	95.80462963	92.03981481	91.80694444
660	94.42833333	98.68453704	100.4662037	100.9340278	98.09064815	83.57222222	83.97018519	97.39777778	99.94930556	98.68453704	96.30898148	95.91194444
720	100.57	100.8666667	103.24	100.9883333	102.0533333	87.51111111	89.40055556	100.1744444	100.1033333	99.97666667	98.88888889	99.12
780	101.4637963	101.2662037	103.5385185	100.8456944	103.044537	91.64509259	96.8137963	100.5746296	100.0601389	100.4758333	99.68546296	99.86375
840	101.8622222	101.27	103.6388889	100.8011111	103.3427778	95.67583333	101.5375926	100.5790741	100.1147222	100.4803704	99.88814815	100.0166667
900	101.8652778	101.2736111	103.5416667	100.5604167	103.3444444	99.90138889	103.4763889	100.4847222	99.97291667	100.3861111	99.89305556	99.875
960	101.9666667	101.1785185	103.542963	100.6133333	103.3459259	102.7333333	104.3214815	100.5874074	99.92888889	100.3903704	99.89777778	99.92888889
1020	101.8708333	101.0834259	103.5440741	100.3727778	103.1503704	104.2728241	104.570463	100.3944444	99.88458333	100.4928704	99.80388889	99.78694444
1080	101.8733333	101.0866667	103.545	100.23	103.1516667	104.6208333	104.6208333	100.3983333	99.7425	100.4966667	99.80833333	99.7425
1140	101.9738889	100.9914815	103.5457407	100.1845833	103.1527778	104.6711111	104.6711111	100.402037	99.69777778	100.5002778	99.81259259	99.60041667
1200	101.9759259	101.0925926	103.6444444	100.0416667	103.1537037	104.7212963	104.6222222	100.4055556	99.65277778	100.5037037	99.81666667	99.65277778
1260	101.9777778	100.8991667	103.5466667	99.89875	103.0563889	104.6723611	104.5733333	100.3108333	99.51041667	100.4088889	99.82055556	99.51041667
1320	101.8814815	100.9998148	103.6448148	99.75583333	103.057037	104.5244444	104.6234259	100.2161111	99.27111111	100.3140741	99.53037037	99.27111111
1380	101.9809259	101.0022222	103.6447222	99.80652778	103.0575	104.5744907	104.7723611	100.3171296	99.22569444	100.3171296	99.53416667	99.3225
1440	102.1777778	101.1022222	103.84	99.85666667	103.2533333	104.8222222	105.02	100.5155556	99.08333333	100.2222222	99.53777778	99.37333333

Table S5. Experimental carvedilol dissolution results for individual tested tablets per formulation used for fitting mathematical models. Data is shown for the FlowLac® 100, the Tablettose® 70 and the Granulated sugar N°1 600 formulations.

t (min)	FlowLac® 100_Tablet 1 – F% released	FlowLac® 100_Tablet 2 – F% released	FlowLac® 100_Tablet 3 – F% released	FlowLac® 100_Tablet 4 – F% released	Tablettose® 70_Tablet 1 – F% released	Tablettose® 70_Tablet 2 – F% released	Tablettose® 70_Tablet 3 – F% released	Tablettose® 70_Tablet 4 – F% released	Granulated sugar N°1 600_Tablet 1 – F% released	Granulated sugar N°1 600_Tablet 2 – F% released	Granulated sugar N°1 600_Tablet 3 – F% released	Granulated sugar N°1 600_Tablet 4 – F% released
10	7.798796296	8.298719136	11.29738426	7.398858025	23.59635802	27.79570988	29.8930787	26.99583333	3.399737654	3.399737654	2.899776235	3.599722222
20	10.49675926	11.29651235	14.69319444	10.69669753	29.09101852	32.39	34.28412037	32.29003086	5.499151235	5.599135802	4.899243827	5.799104938
30	12.59416667	13.49375	17.088125	12.99398148	32.88476852	35.78342593	37.77375	36.08328704	7.198333333	7.398287037	6.598472222	7.598240741
45	15.289375	16.38861111	20.17895833	15.78902778	37.37402778	39.77236111	41.45677083	40.67173611	9.596666667	9.8965625	8.896909722	10.09649306
60	17.68361111	18.98240741	22.86819444	18.38296296	40.96203704	43.16	44.43819444	43.95925926	11.89449074	12.29430556	11.19481481	12.49421296
90	22.16916667	23.46736111	27.5425	23.06791667	47.13444444	49.03180556	49.79604167	49.23152778	16.28868056	16.78833333	15.38930556	17.18805556
120	26.45092593	27.54888889	31.71166667	27.34925926	52.20314815	53.9	54.04944444	53.60055556	20.58092593	21.08046296	19.48194444	21.67990741
150	30.62893519	31.2275463	35.57604167	31.42708333	56.66851852	57.96550926	57.69895833	57.26712963	24.67141204	25.07094907	23.37291667	25.96990741
180	34.50388889	34.80305556	39.33541667	35.40138889	60.33194444	61.9275	60.945	60.63111111	28.56027778	28.95972222	27.16222222	29.95833333
210	38.17587963	38.37523148	42.99	39.17263889	63.79259259	65.18805556	63.98743056	63.79259259	32.3475	32.54717593	30.75009259	33.74523148
240	41.64518519	41.74481481	46.44055556	42.74111111	67.05074074	68.34592593	66.82666667	66.75185185	36.03314815	35.93333333	34.23648148	37.53037037
270	45.01166667	45.11125	49.88625	46.30625	70.20625	71.1025	69.463125	69.40958333	39.5175	39.218125	37.52166667	41.014375
300	48.375	48.47453704	53.22777778	49.76851852	73.15972222	73.75694444	71.89722222	72.16435185	42.90046296	42.40162037	40.80532407	44.49675926
330	51.5362037	51.73518519	56.266875	53.02856481	76.01092593	76.40888889	74.42708333	74.81703704	46.08236111	45.68337963	43.98770833	47.87777778
360	54.69444444	54.89333333	59.5	56.28555556	78.56111111	78.76	76.755	77.46722222	49.16305556	48.76416667	46.96916667	51.05777778
420	60.60462963	60.70398148	65.55638889	62.29361111	83.25685185	82.76009259	81.59888889	82.46203704	54.92143519	54.52273148	52.52921296	57.11430556
480	66.10666667	66.70222222	71.10111111	67.89333333	87.44740741	86.45481481	85.44	90.6237037	60.37555556	59.97703704	57.88481481	62.76666667
540	71.59833333	72.09416667	76.33375	73.185	92.62166667	90.53916667	90.15875	97.38166667	65.32666667	65.1275	62.63791667	68.01541667
600	77.77314815	76.98055556	87.46805556	78.66481481	97.19166667	93.32777778	93.77916667	98.08333333	69.875	70.17361111	67.08796296	72.86111111
660	84.13425926	82.74851852	94.92722222	85.81694444	98.58555556	95.1212037	96.10888889	97.99166667	73.92162037	74.81703704	71.03638889	77.20481481
720	90.48333333	90.87888889	97.74333333	92.75777778	98.88888889	96.31777778	96.17	97.99888889	77.46722222	78.85944444	74.48388889	80.84833333
780	96.22759259	95.04203704	98.68541667	98.89509259	98.89509259	96.32638889	95.93597222	97.80833333	80.7112963	82.20226852	77.72935185	84.19023148
840	99.09851852	96.92703704	99.03611111	100.3816667	98.80240741	96.33481481	95.80027778	97.81537037	83.45555556	84.84648148	80.57435185	87.03222222
900	100.6819444	98.11805556	98.89583333	100.5833333	98.90694444	96.24444444	95.7625	97.82222222	85.60138889	86.99166667	82.92013889	89.17638889
960	101.08	98.51851852	98.85333333	100.5874074	98.81407407	96.15407407	95.62666667	97.63185185	87.54666667	88.63851852	84.86666667	91.41777778
1020	101.1818519	98.62277778	98.81055556	100.6897222	98.80240741	96.15407407	95.80027778	98.08333333	88.99402778	89.88694444	86.5137037	93.26018519
1080	100.9883333	98.62833333	98.67	100.6933333	98.88888889	96.24444444	96.10888889	97.81537037	90.34083333	90.63833333	87.86166667	94.50583333
1140	101.0897222	98.6337037	98.62680556	100.5985185	98.89509259	96.32638889	95.93597222	97.80833333	91.28986111	91.09162037	89.01009259	95.25467593
1200	101.0925926	98.54074074	98.48611111	100.6018519	98.88888889	96.33481481	96.17	98.08333333	92.23796296	91.24722222	90.05833333	95.70555556
1260	100.9972222	98.44777778	98.4425	100.5069444	98.80240741	96.24444444	95.80027778	97.99888889	92.98708333	91.20458333	90.9075	95.95791667
1320	100.9018519	98.25685185	98.20472222	100.412037	98.90694444	96.33481481	96.17	97.81537037	93.5375	91.26092593	91.45888889	96.21
1380	100.8064815	98.26185185	98.06402778	100.415	98.88888889	96.24444444	96.10888889	97.99166667	93.98842593	91.31717593	92.00972222	96.26393519
1440	100.8088889	98.16888889	97.92333333	100.32	98.81407407	96.31777778	95.62666667	97.82222222	94.24111111	91.17555556	92.36222222	96.31777778

Table S6. Experimental carvedilol dissolution results for individual tested tablets per formulation used for fitting mathematical models. Data is shown for the Glucidex® 19, the Di-Cafos® A12 and the Emcompress® Anhydrous formulations.

t (min)	Glucidex® 19_Tablet 1 – F% released	Glucidex® 19_Tablet 2 – F% released	Glucidex® 19_Tablet 3 – F% released	Glucidex® 19_Tablet 4 – F% released	Di-Cafos® A12_Tablet 1 – F% released	Di-Cafos® A12_Tablet 2 – F% released	Di-Cafos® A12_Tablet 3 – F% released	Di-Cafos® A12_Tablet 4 – F% released	Emcompress® Anhydrous_Tablet 1 – F% released	Emcompress® Anhydrous_Tablet 2 – F% released	Emcompress® Anhydrous_Tablet 3 – F% released	Emcompress® Anhydrous_Tablet 4 – F% released
10	4.499305556	3.299490741	3.999074074	2.899552469	3.899097222	3.699429012	3.698858025	3.898796296	6.797901235	7.597654321	6.898402778	6.698449074
20	6.597962963	5.198395062	5.797314815	4.798518519	5.797314815	5.498302469	5.59654321	5.796419753	8.994444444	10.1937037	9.395648148	9.095787037
30	8.296157407	6.696898148	7.294930556	6.397037037	7.394861111	6.896805556	6.993518519	7.293240741	10.59018519	11.88898148	11.19222222	10.7925
45	10.49270833	8.693958333	9.190416667	8.594027778	9.490104167	8.793888889	8.9875	9.386944444	12.68236111	14.08041667	13.18625	12.98645833
60	12.58833333	10.69009259	11.18444444	10.79	11.38416667	10.39037037	10.78	11.17925926	14.47314815	15.97037037	14.87930556	14.77944444
90	16.57694444	14.47986111	14.76916667	15.87791667	14.76916667	13.48125	13.96111111	14.45972222	17.45138889	19.24638889	17.86270833	17.9625
120	20.26240741	18.36592593	18.24916667	20.06277778	17.85027778	16.17	16.93703704	17.53481481	20.02555556	22.31703704	20.44305556	20.74222222
150	23.84467593	21.94907407	21.72430556	23.84467593	20.82743056	18.75648148	19.70833333	20.30555556	22.39583333	25.28240741	22.82048611	23.31875
180	27.32388889	25.42916667	25.095	27.52333333	23.70083333	21.14111111	22.375	23.07111111	24.76166667	28.04333333	25.095	25.6925
210	30.70018519	28.90601852	28.56048611	31.29824074	26.47069444	23.52351852	24.83796296	25.73212963	26.92435185	30.69972222	27.26680556	28.06291667
240	34.17296296	32.37962963	31.92166667	34.87037037	29.23666667	25.80407407	27.2962963	28.28888889	29.08296296	33.35111111	29.33611111	30.23111111
270	37.54291667	35.85	35.278125	38.43916667	31.8	27.98291667	29.65083333	30.74166667	31.03916667	35.89833333	31.4025	32.39625
300	40.81018519	39.21759259	38.53055556	41.80555556	34.35972222	30.15972222	32.00092593	33.18981481	32.99166667	38.34166667	33.36666667	34.55833333
330	43.97490741	42.58203704	41.77840278	45.26828704	36.91583333	32.33449074	34.24759259	35.43537037	34.94046296	40.78037037	35.32805556	36.618125
360	47.23611111	45.94333333	44.9225	48.62833333	39.36916667	34.30833333	36.49	37.77555556	36.78666667	43.11555556	37.1875	38.57583333
420	53.35194444	52.25907407	51.09833333	55.04092593	44.06736111	38.34981481	40.76462963	42.24518519	40.36981481	47.67388889	40.79944444	42.38388889
480	59.35703704	58.26518519	56.96	68.48888889	48.75222222	42.08592593	44.92444444	46.50074074	43.44666667	52.01777778	44.30222222	46.18111111
540	65.25166667	66.14416667	62.6075	74.27583333	53.22625	45.815	48.97	50.54333333	46.41333333	56.14833333	47.5975	49.86875
600	71.43240741	71.72962963	67.94305556	79.55648148	57.58888889	49.33888889	52.8037037	54.37407407	49.17222222	59.96851852	50.68611111	53.34861111
660	76.61166667	76.51268519	72.96791667	84.23324074	61.74208333	52.75712963	56.42666667	57.99407407	51.82240741	63.38203704	53.66736111	56.62152778
720	80.79222222	80.59444444	77.38833333	88.30777778	65.68666667	55.97111111	60.03555556	61.6	54.26666667	66.39111111	56.345	59.78666667
780	84.37203704	83.97685185	81.305	91.78175926	69.52166667	59.17898148	63.43518519	64.99666667	56.7012963	69.29074074	58.91666667	62.74625
840	87.35277778	86.85925926	84.81805556	94.75555556	73.14944444	62.18333333	66.72407407	68.28259259	58.93148148	72.3737037	61.48083333	65.59916667
900	89.83472222	89.34166667	87.83125	97.23055556	76.66875	65.18194444	70	71.55555556	61.15277778	75.44444444	63.93958333	68.24791667
960	92.0162963	91.5237037	90.44444444	99.30666667	79.98222222	67.87925926	73.16592593	74.91259259	63.46222222	78.30888889	66.19555556	70.79111111
1020	93.99675926	93.30777778	92.65930556	100.8865741	82.89541667	70.57138889	76.31925926	78.06259259	65.76240741	81.16185185	68.44486111	73.13152778
1080	95.48166667	94.89166667	94.575	101.9716667	85.605	73.16	79.17	81.2	68.15	83.61666667	70.395	75.27
1140	96.86537037	96.27592593	96.19277778	102.7598148	87.91708333	75.64537037	81.91277778	84.03537037	70.72092593	85.965	72.33930556	77.20736111
1200	97.95185185	97.46111111	97.61111111	103.1537037	90.02777778	78.02777778	84.35555556	86.66666667	72.99259259	87.91851852	74.18055556	79.13888889
1260	98.93805556	98.44777778	98.73375	103.3505556	91.93791667	80.20944444	86.59611111	89.095	74.77444444	89.86388889	76.01625	80.9675
1320	99.7262963	99.23648148	99.75583333	103.3509259	93.64833333	82.38685185	88.63555556	91.41740741	76.35703704	91.60925926	77.6525	82.69361111
1380	100.1201852	99.63037037	100.5439815	103.3505556	95.06305556	84.26638889	90.475	93.25148148	77.64574074	93.15574074	79.09013889	84.22083333
1440	100.317037	99.82722222	100.9378704	103.3509259	96.37666667	86.04444444	92.21111111	95.07777778	78.92888889	94.40888889	80.42666667	85.74333333

Table S7. Experimental carvedilol dissolution results for individual tested tablets per formulation used for fitting mathematical models. Data is shown for the Avicel® PH-102, the Avicel® PH-200 and the Ethocel™ Standard 20 Premium formulations.

t (min)	Avicel® PH-102_Tablet 1 – F% released	Avicel® PH-102_Tablet 2 – F% released	Avicel® PH-102_Tablet 3 – F% released	Avicel® PH-102_Tablet 4 – F% released	Avicel® PH-200_Tablet 1 – F% released	Avicel® PH-200_Tablet 2 – F% released	Avicel® PH-200_Tablet 3 – F% released	Avicel® PH-200_Tablet 4 – F% released	Ethocel™ Standard 20 Premium_Tablet 1 – F% released	Ethocel™ Standard 20 Premium_Tablet 2 – F% released	Ethocel™ Standard 20 Premium_Tablet 3 – F% released	Ethocel™ Standard 20 Premium_Tablet 4 – F% released
10	1.599753086	1.299699074	2.999189815	1.966213992	2.299645062	2.499614198	2.499228395	2.099351852	1.699868827	1.999691358	1.899560185	2.099675926
20	3.298981481	3.098564815	4.797407407	3.731651235	4.398641975	3.898796296	4.097469136	3.497839506	3.199506173	3.79882716	3.49837963	3.598888889
30	4.397962963	4.097152778	6.09505787	4.863391204	5.997222222	5.197592593	5.395	4.695648148	4.398981481	5.097638889	4.796666667	4.897731481
45	6.395555556	5.6940625	7.990277778	6.693298611	7.994444444	6.995138889	7.19	6.391111111	5.897951389	6.895208333	6.2934375	6.495486111
60	7.792777778	6.990277778	9.584444444	8.1225	9.790925926	8.592037037	8.783703704	7.88537037	7.29662037	8.392222222	7.689305556	7.892685185
90	10.38555556	9.58	12.36986111	10.77847222	12.98194444	11.58388889	11.6675	10.86972222	9.893125	11.38416667	10.478125	10.58527778
120	12.7762963	12.06638889	14.85171296	13.23146605	15.67092593	14.37333333	14.34666667	13.54962963	12.38851852	14.1737037	13.26305556	13.17555556
150	14.96527778	14.15069444	17.03072917	15.3822338	18.15787037	16.96064815	16.82175926	16.02546296	14.98263889	16.76111111	15.94444444	15.56388889
180	17.0525	16.23208333	19.10666667	17.46375	20.44305556	19.34611111	19.19277778	18.29777778	17.37583333	19.34611111	18.5225	17.95
210	18.93842593	18.21104167	20.98033565	19.37660108	22.62643519	21.72935185	21.46	20.56583333	19.86775463	21.72935185	20.99743056	20.13453704
240	20.82259259	20.08777778	22.85092593	21.25376543	24.70814815	24.01074074	23.72296296	22.63111111	22.2587037	24.01074074	23.36944444	22.41666667
270	22.705	21.961875	24.7184375	23.1284375	26.68833333	26.29	25.78333333	24.6925	24.54875	26.29	25.738125	24.59708333
300	24.48611111	23.73402778	26.58287037	24.93433642	28.66666667	28.46759259	27.93888889	26.75	26.83773148	28.46759259	28.00416667	26.77546296
330	26.26555556	25.60291667	28.44422454	26.77089892	30.54365741	30.54365741	29.89240741	28.60564815	29.12564815	30.54365741	30.26701389	28.85231481
360	27.94388889	27.46916667	30.20347222	28.53884259	32.41888889	32.61777778	31.94111111	30.55666667	31.31277778	32.61777778	32.4275	31.02666667
420	31.59388889	31.09472222	33.91094907	32.1998534	36.06472222	36.76018519	35.92814815	34.25018519	35.48462963	36.56148148	36.64027778	35.0712037
480	35.03851852	34.61111111	37.50740741	35.71901235	39.50518519	40.79555556	39.70296296	37.73259259	39.55296296	40.49777778	40.74222222	38.90962963
540	38.57583333	37.92	40.99333333	39.16305556	42.93916667	44.625	43.46333333	41.10333333	43.31875	44.12916667	44.73375	42.44333333
600	42.00740741	41.12083333	44.46759259	42.53194444	46.26759259	48.34814815	47.01296296	44.36296296	46.98148148	47.55555556	48.61527778	45.8712963
660	45.23453704	44.3125	47.93018519	45.82574074	49.39175926	52.06425926	50.45092593	47.51203704	50.34231481	50.7775	52.19027778	49.09481481
720	48.45555556	47.39666667	51.185	49.01240741	52.51	55.47666667	53.77777778	50.55111111	53.60055556	53.89444444	55.755	52.21333333
780	51.37407407	50.27555556	54.13511574	51.92824846	55.42472222	58.7837963	56.89648148	53.38314815	56.65694444	56.90666667	59.11305556	55.22712963
840	54.28703704	52.95	57.17291667	54.8033179	58.43259259	62.08462963	60.00296296	56.30148148	59.41240741	59.71574074	62.46138889	58.03777778
900	57.19444444	55.51875	60.00520833	57.57280093	61.53333333	65.18194444	62.90277778	59.01388889	62.06597222	62.42083333	65.60416667	60.64583333
960	60.0962963	57.98222222	62.73037037	60.26962963	64.62814815	68.17481481	65.79111111	61.71555556	64.51851852	64.9237037	68.54222222	63.24888889
1020	62.89416667	60.24319444	65.44613426	62.86116512	67.71703704	71.06351852	68.47425926	64.40648148	66.86953704	67.32333333	71.37402778	65.65009259
1080	65.49	62.4975	68.1525	65.38	70.70166667	73.84833333	71.24333333	66.99	69.02	69.62	74.1975	67.85
1140	68.08083333	64.93986111	70.655625	67.89210648	73.58231481	76.52953704	73.71185185	69.37018519	71.16842593	71.71574074	76.81791667	70.04564815
1200	70.37222222	67.375	72.95648148	70.2345679	76.35925926	78.81296296	76.17037037	71.64444444	73.31481481	73.70925926	79.23611111	72.13888889
1260	72.56111111	69.60875	75.24923611	72.47303241	78.93472222	80.99388889	78.42666667	73.81333333	75.16208333	75.60083333	81.55	74.03194444
1320	74.74574074	71.83583333	77.34101852	74.6408642	81.01537037	83.17055556	80.57777778	75.78148148	77.00759259	77.29277778	83.56611111	75.82333333
1380	76.92611111	73.95944444	79.23280093	76.70611883	82.99407407	85.34296296	82.81574074	77.64574074	78.85134259	79.07925926	85.57611111	77.70907407
1440	79.00444444	75.98	81.02166667	78.6687037	84.57777778	87.21777778	84.56666667	79.40666667	80.49555556	80.56888889	87.48333333	79.29777778

Table S8. Experimental carvedilol dissolution results for individual tested tablets per formulation used for fitting mathematical models. Data is shown for the Starch 1500® sample with smaller particle size (↓PS) and the Starch 1500® sample with larger particle size (↑PS) formulations.

t (min]	Starch 1500® sample with smaller particle size (↓PS)_Tablet 1 – F% released	Starch 1500® sample with smaller particle size (↓PS)_Tablet 2 – F% released	Starch 1500® sample with smaller particle size (↓PS)_Tablet 3 – F% released	Starch 1500® sample with smaller particle size (↓PS)_Tablet 4 – F% released	Starch 1500® sample with larger particle size (↑PS)_Tablet 1 – F% released	Starch 1500® sample with larger particle size (↑PS)_Tablet 2 – F% released	Starch 1500® sample with larger particle size (↑PS)_Tablet 3 – F% released	Starch 1500® sample with larger particle size (↑PS)_Tablet 4 – F% released
10	8.898626543	7.498263889	12.99699074	9.498533951	6.49849537	5.599135802	5.598703704	5.898861883
20	10.79666667	9.395648148	16.0925463	11.69638889	8.296157407	7.197777778	7.29662037	7.697029321
30	12.09439815	10.7925	17.48784722	13.09393519	9.693263889	8.496064815	8.594027778	8.994791667
45	13.69048611	12.48697917	19.27989583	14.88965278	11.388125	10.09298611	10.28927083	10.69071181
60	15.18592593	14.08041667	20.77111111	16.38481481	12.88208333	11.48935185	11.68375	12.08599537
90	17.67541667	16.66520833	23.25145833	18.97361111	15.46770833	13.88069444	14.37	14.57465278
120	19.76333333	18.74777778	25.52888889	21.26055556	17.65083333	15.97037037	16.55388889	17.06041667
150	21.74953704	20.92708333	27.50416667	23.24606481	19.53194444	17.85856481	18.53541667	19.24415509
180	23.53444444	22.705	29.2775	25.03027778	21.41041667	19.64527778	20.41458333	21.32569444
210	25.31768519	24.28138889	30.94881944	27.01217593	23.18673611	21.33064815	22.19159722	23.30520833
240	26.99962963	26.15388889	32.61777778	28.79296296	24.76166667	22.81518519	23.76722222	25.28240741
270	28.58041667	27.4275	34.085625	30.4725	26.334375	24.29833333	25.340625	27.05833333
300	30.06018519	29.09652778	35.75	32.15046296	27.80555556	25.78009259	26.91180556	28.83217593
330	31.53856481	30.66395833	37.21354167	33.72736111	29.27465278	27.16097222	28.38152778	30.50457176
360	33.01555556	31.93166667	38.675	35.30277778	30.74166667	28.54055556	29.94833333	32.175
420	35.86601852	34.75875	41.69069444	38.44916667	33.57041667	31.19648148	32.87722222	35.41076389
480	38.51259259	37.47888889	44.59888889	41.49037037	36.49	33.84740741	35.99555556	38.63888889
540	41.055	40.0925	47.4	44.52583333	39.3025	36.39416667	39.00625	41.76041667
600	43.69166667	42.79722222	50.19305556	47.55555556	42.00833333	38.93611111	42.10694444	44.77569444
660	46.22435185	45.49416667	52.97805556	50.57953704	44.70638889	41.47324074	45.19875	47.68506944
720	48.75222222	48.085	55.65666667	53.59777778	47.495	44.00555556	48.18333333	50.68611111
780	51.27527778	50.57013889	58.22930556	56.41268519	50.17736111	46.53305556	51.35569444	53.58148148
840	53.69481481	53.14611111	60.79444444	59.22222222	52.85194444	49.05574074	54.42083333	56.27314815
900	56.01111111	55.51875	63.35208333	62.02638889	55.51875	51.57361111	57.47708333	59.05659722
960	58.42148148	57.98222222	65.80444444	64.62814815	58.08	53.98814815	60.32888889	61.63703704
1020	60.82722222	60.43847222	68.34722222	67.12648148	60.63375	56.39805556	63.17236111	64.11319444
1080	63.13	62.6925	70.6875	69.52166667	63.0825	58.705	65.91	66.58333333
1140	65.42833333	64.93986111	73.02083333	71.91222222	65.52402778	61.10574074	68.54222222	68.85185185
1200	67.52592593	67.08333333	75.25	74.10185185	67.86111111	63.50185185	71.26388889	71.11481481
1260	69.7175	69.12333333	77.4725	76.28722222	70.09416667	65.69722222	73.88041667	73.37222222
1320	72.00277778	71.15722222	79.59138889	78.37037037	72.22361111	67.9862963	76.19833333	75.52662037
1380	74.18574074	73.185	81.60708333	80.54731481	74.24986111	70.07518519	78.4125	77.77303241
1440	76.16888889	75.11	83.52	82.52444444	76.17333333	72.06222222	80.52333333	79.72222222

Table S9. The RSS results for models fitted to carvedilol experimental dissolution data. Model fitting was performed on the entire relevant dissolution data range as determined from the paired t–tests. Data is presented for the Polyethylene Glycol & Polyethylene Oxide group, the Povidone group and the Mannitol group of formulations. Data is presented as average RSS ± one standard deviation.

Formulation / Model	Polyglykol® 4000 P	Polyglykol® 8000 P	Polyox™ WSR N-80 (LEO NF Grade)	Kollidon® 25	Kollidon® 90 F	C*Pharm Mannidex 16700	Pearlitol® 160C	Parteck® M 100	Parteck® M 200
Zero–order	1951.11 ± 602.7	1607.16 ± 285.22	800.26 ± 236.81	24657.36 ± 15433.32	1911.96 ± 1308.4	5754.43 ± 5732.45	6188.02 ± 4559.85	6518.02 ± 3379.67	3777.3 ± 3278.73
Zero–order with T_{lag}	653.97 ± 290.63	764.79 ± 91.06	668.83 ± 188.93	5252.33 ± 3665.25	1395.49 ± 757.75	2085.67 ± 2067.74	2598.8 ± 2415.06	3176.86 ± 1327.15	1923.55 ± 1479.62
Zero–order with F_0	653.97 ± 290.63	764.79 ± 91.06	668.83 ± 188.93	5252.33 ± 3665.25	1395.49 ± 757.75	2085.67 ± 2067.74	2598.8 ± 2415.06	3176.86 ± 1327.15	1923.55 ± 1479.62
First–order	121.19 ± 114.34	564.07 ± 708.12	1133.83 ± 1044.32	**567.76 ± 278.2**	2583.63 ± 2389.63	1042.91 ± 1288.09	1132.15 ± 1059.58	1722.04 ± 1117.56	987.99 ± 1225.82
First–order with T_{lag}	48.76 ± 21.41	535.19 ± 934.13	1124.41 ± 1438.75	/	/	/	/	/	1449.59 ± 1657.58
First–order with F_{max}	128.73 ± 88.52	384.45 ± 128.64	1294.9 ± 268.85	1426.46 ± 1035.98	1616.07 ± 809.39	296.12 ± 89.42	328.96 ± 437.03	559.75 ± 305.5	487.02 ± 286.52
First–order with T_{lag} and F_{max}	47.29 ± 17.88	64.85 ± 47.2	871.77 ± 135.35	4707.09 ± 5410.09	1916.07 ± 1262.62	1048.62 ± 1255.16	1035.86 ± 1718.4	1387.22 ± 1615.55	789.73 ± 1311.41
Higuchi	313.03 ± 156.11	552.98 ± 64.38	1725.2 ± 239.74	5996.77 ± 6009.63	2377.37 ± 865.67	1156.59 ± 1201.5	1154.93 ± 1311.06	1805.04 ± 863.54	1263.63 ± 787.81
Higuchi with T_{lag}	312.35 ± 235.75	268.56 ± 95.76	244.26 ± 86.09	4786.28 ± 4260.99	551.61 ± 201.8	1459.54 ± 2086.94	1759.46 ± 2500.84	2651.51 ± 1652.36	1372.95 ± 1676.89
Higuchi with F_0	237.6 ± 153.48	260.59 ± 60.4	162.8 ± 75.2	2996.85 ± 2971.32	508.09 ± 269.83	837.96 ± 1196.27	1021.96 ± 1407.11	1616.2 ± 1012.97	905.63 ± 1001.17
Korsmeyer–Peppas	530.76 ± 311.04	952.74 ± 82.79	1094.85 ± 191.67	15546.01 ± 25610.22	3777.83 ± 3051.48	11750.56 ± 21947.72	15766.42 ± 28907.6	31590.19 ± 32552.3	2829.59 ± 2436.05
Korsmeyer–Peppas with T_{lag}	350.35 ± 233.78	636.77 ± 73.77	1384.97 ± 181.94	3576.72 ± 3659.44	2133.06 ± 1021.9	1213.94 ± 1740.28	1468.64 ± 2078.72	4707.3 ± 2447.41	2336.43 ± 2230.37
Korsmeyer–Peppas with F_0	887.31 ± 448.19	1583.64 ± 110.15	3316.7 ± 407.43	5104.26 ± 4592.14	4371.91 ± 1736.48	2132.54 ± 2644.57	2592.39 ± 3078.52	7403.32 ± 3028.96	4113.58 ± 3012.55
Hixson–Crowell	142.78 ± 74.51	84.7 ± 25.04	287.86 ± 75.03	5773.04 ± 5014.83	629.58 ± 600.66	299.13 ± 338.82	277.87 ± 329.59	411.23 ± 276.19	368.48 ± 388.13
Hixson–Crowell with T_{lag}	139.85 ± 49.74	95.59 ± 75.31	89.26 ± 50.39	44205.11 ± 81313.46	577.06 ± 563.68	263.28 ± 251.86	1445.17 ± 2653.39	1231.81 ± 2132.13	698.08 ± 1133.46
Hopfenberg	135.86 ± 92.3	65.53 ± 31.72	147.74 ± 73.24	1795.99 ± 1736.65	**109.42 ± 38.74**	**200.74 ± 163.5**	**170.93 ± 134.74**	330.56 ± 161.97	264.48 ± 184.31
Hopfenberg with T_{lag}	139.85 ± 49.74	95.88 ± 74.87	**25.37 ± 13.15**	2499.47 ± 4003.14	**88.45 ± 67.04**	**140.46 ± 228.34**	**108.71 ± 166.09**	**157.45 ± 63.19**	698.08 ± 1133.46
Baker–Lonsdale	828.28 ± 332.95	31326.89 ± 60443.68	2930.11 ± 338.16	732782.83 ± 1136900.57	22571.38 ± 37743.84	/	548751.58 ± 719593.62	535145.72 ± 351844.44	/
Baker–Lonsdale with T_{lag}	247.79 ± 149.13	264.54 ± 13.48	594.83 ± 92.61	677668.16 ± 1173761	1078.55 ± 210.07	/	337550.55 ± 613927.88	331536.98 ± 336366.87	/
Makoid–Banakar	**3.09 ± 1.65**	**6.86 ± 1.52**	32.69 ± 27.97	1156.32 ± 1177.42	**114.84 ± 185.17**	**227.38 ± 261.85**	**177.59 ± 200.65**	937.04 ± 876.22	301.72 ± 443.55
Makoid–Banakar with T_{lag}	16.57 ± 10.63	**5.73 ± 4.38**	**23.59 ± 12.22**	887.73 ± 939.19	267.82 ± 347.23	267.83 ± 151.6	308.73 ± 247.91	564.14 ± 595.37	187.59 ± 241.46
Peppas–Sahlin_1	319.08 ± 171	440.42 ± 48.77	442.93 ± 138.74	931.8 ± 911.9	808.69 ± 427.19	737.03 ± 720.73	1061.48 ± 1181.7	1653.29 ± 610.54	1061.18 ± 784.65
Peppas–Sahlin_1 with T_{lag}	190.91 ± 111.29	288.08 ± 32.38	376.05 ± 126.61	**584.82 ± 622.08**	732.49 ± 399.45	545.42 ± 497.11	842.08 ± 963.05	1185.37 ± 391.67	771.01 ± 553.28
Peppas–Sahlin_2	299.67 ± 160.8	432.71 ± 43.12	488.13 ± 142.96	776.94 ± 790.57	855.32 ± 427.25	644.76 ± 584.9	945.01 ± 1038.74	1520.68 ± 511.03	1001.76 ± 697.09
Peppas–Sahlin_2 with T_{lag}	161.26 ± 95.18	259.85 ± 28.1	403.42 ± 128.79	**546.4 ± 580.32**	763.33 ± 401.6	455.27 ± 376.76	718.55 ± 813.14	1022.91 ± 295.11	685.19 ± 460.45
Quadratic	**12.88 ± 7.92**	28.77 ± 6.76	121.48 ± 53.75	6391.52 ± 4939.09	188.93 ± 215.17	618.93 ± 953.48	425.82 ± 480.95	656.83 ± 548.9	302.23 ± 372.75
Quadratic with T_{lag}	85.7 ± 27.55	**12.54 ± 13.49**	48.53 ± 31.64	6973.13 ± 5254.3	143.72 ± 184.42	774.84 ± 1093.3	555.35 ± 508	739.15 ± 657.58	320.04 ± 444.77
Weibull_1	29.5 ± 18.79	28.9 ± 20.91	32.49 ± 18.34	**414.94 ± 202.97**	224.91 ± 233.81	395.24 ± 55.88	454.09 ± 150.65	**89.28 ± 47.01**	**77.85 ± 90.57**
Weibull_2	**14.93 ± 3.5**	**16.31 ± 8.94**	30.5 ± 15.6	**417.32 ± 239.27**	155.81 ± 164.85	308.67 ± 29.29	293.2 ± 149.04	**129.39 ± 76.65**	**110.13 ± 147.75**
Weibull_3	**14.34 ± 10.58**	**13.14 ± 5.14**	**20.42 ± 17.06**	772.01 ± 703.57	**69.21 ± 109.33**	**254.95 ± 206.93**	**240.81 ± 267.33**	406.88 ± 348.04	192.7 ± 224.14
Weibull_4	35.47 ± 12.52	**16.68 ± 7.44**	**19.22 ± 9.17**	658.42 ± 542.94	141.17 ± 152.82	324.6 ± 103.54	310.88 ± 211	421.85 ± 409.88	181.71 ± 262.46
Logistic_1	108.23 ± 91.18	197.99 ± 177.07	415.73 ± 270.08	670.34 ± 472.16	909.94 ± 701.31	746.86 ± 267.08	910.73 ± 264.33	269.56 ± 162.35	233.91 ± 278.03
Logistic_2	127.36 ± 32.94	105.9 ± 35.24	170.9 ± 87.19	688 ± 270.49	488.17 ± 254.08	624.73 ± 261.03	524.16 ± 164.57	**131.66 ± 104.89**	**117.81 ± 28.85**
Logistic_3	88.28 ± 23.38	192.42 ± 9.11	484.89 ± 146.81	3973.6 ± 3792.61	845.81 ± 586.43	561.26 ± 527	442.7 ± 402.76	1336.09 ± 222.95	705.08 ± 264.56
Gompertz_1	278.64 ± 167.31	345.56 ± 76.08	493.09 ± 93.82	1145.43 ± 636.83	1363.53 ± 807.84	1233.44 ± 400.02	1330.19 ± 107.73	363.51 ± 99.09	356.2 ± 317.78
Gompertz_2	356.17 ± 62.67	325.74 ± 22.76	437.79 ± 86.25	971.88 ± 545.04	935.93 ± 418.07	920.95 ± 332.81	931.88 ± 171.65	294.49 ± 116.26	322.65 ± 52.01
Gompertz_3	59.71 ± 68.25	59 ± 17.7	76.56 ± 38.21	4301.28 ± 4613.04	120.51 ± 72.14	634.67 ± 1116.96	639.39 ± 1074.59	991.86 ± 1071.67	548.16 ± 891.81
Gompertz_4	49.53 ± 54.74	54.35 ± 26.01	76.9 ± 37.93	3469.62 ± 3584.67	138.46 ± 106.15	670.51 ± 1180.77	759.15 ± 1273.32	1329.03 ± 1033.09	681.42 ± 941.56
Probit_1	76.12 ± 56.15	126.75 ± 103.26	390.86 ± 187.13	**590.25 ± 320.92**	1011.19 ± 685.47	794.35 ± 319.53	939.4 ± 213.71	**162.54 ± 76**	182.96 ± 187.32
Probit_2	91.07 ± 24.94	75.78 ± 26.89	177.64 ± 99.01	694.84 ± 298.78	599.08 ± 359.28	410.35 ± 238.25	454.17 ± 198.5	**157.64 ± 122.01**	**136.03 ± 75.35**

Table S10. The RSS results for models fitted to carvedilol experimental dissolution data. Model fitting was performed on the entire relevant dissolution data range as determined from the paired t–tests. Data is presented for the Lactose Monohydrate group of formulations along with the Sucrose and Maltodextrin formulations. Data is presented as average RSS ± one standard deviation.

Formulation / Model	Lactochem® Crystals	Lactochem® Fine Powder	SuperTab® 11SD	FlowLac® 100	Tablettose® 70	Granulated sugar N°1 600	Glucidex® 19
Zero–order	8713.33 ± 1306.1	1947.37 ± 558.28	12747.51 ± 3066.48	2006.87 ± 656.59	10937.89 ± 926.01	3669.55 ± 360.78	4099 ± 894.41
Zero–order with T_{lag}	526.97 ± 78.98	316.75 ± 117.56	429.41 ± 55.31	219.62 ± 74.24	500.88 ± 115.86	1545.36 ± 136.83	2017.3 ± 675.79
Zero–order with F_0	526.97 ± 78.98	316.75 ± 117.56	429.41 ± 55.31	219.62 ± 74.24	500.88 ± 115.86	1545.36 ± 136.83	2017.3 ± 675.79
First–order	1812.24 ± 284.36	662.41 ± 395.44	2977.17 ± 753.71	1185.69 ± 1114.94	2655.71 ± 640.46	154.87 ± 66.8	2274.86 ± 1338.08
First–order with T_{lag}	/	/	/	/	724.86 ± 765.41	382.77 ± 406.25	130369.96 ± 10978.21
First–order with F_{max}	2199.43 ± 330.01	586.02 ± 236.88	3491.9 ± 836.03	1068.22 ± 288.63	2840.35 ± 495.01	509.74 ± 113.66	1040.98 ± 166.76
First–order with T_{lag} and F_{max}	329.63 ± 27.43	1921.47 ± 1395.36	295.23 ± 116.73	2227.82 ± 363.29	257.73 ± 174.42	768.49 ± 158.12	1393.14 ± 216.07
Higuchi	501.61 ± 235.25	614.22 ± 460.85	1708.21 ± 970.45	653.55 ± 136.47	1250.41 ± 359.66	916.89 ± 31.39	1752.42 ± 416.18
Higuchi with T_{lag}	52.53 ± 35.91	792.22 ± 376.17	86.48 ± 41.51	1052.47 ± 65.99	133.75 ± 49.29	380.53 ± 34.74	809.59 ± 174.56
Higuchi with F_0	**8.46 ± 1.3**	207.89 ± 192.91	**11.36 ± 14.46**	286.79 ± 26.4	**23.65 ± 21.69**	226.93 ± 37.16	559.31 ± 233.26
Korsmeyer–Peppas	118.4 ± 30.9	132.61 ± 162.81	157.96 ± 16.31	283.41 ± 104.32	85.21 ± 66.88	747.72 ± 23.74	1073.6 ± 877.34
Korsmeyer–Peppas with T_{lag}	192.66 ± 49.28	226.07 ± 183.24	266.21 ± 11.92	483.7 ± 142.09	161.5 ± 83.28	475.53 ± 26.45	777.44 ± 522.66
Korsmeyer–Peppas with F_0	**19.23 ± 15.29**	**64.35 ± 44.03**	**60.55 ± 20.44**	**95.49 ± 81.81**	**30.48 ± 42.07**	1348.97 ± 43.96	1578.79 ± 1038.41
Hixson–Crowell	2870.71 ± 365.31	498.55 ± 76.79	4427.33 ± 731.27	568.57 ± 173.36	3845.79 ± 484.62	153.57 ± 58.11	265.05 ± 141.38
Hixson–Crowell with T_{lag}	109.32 ± 25.96	345.8 ± 300.94	114.68 ± 56.63	700.88 ± 269.23	105.61 ± 65.9	**65.28 ± 37.46**	439.09 ± 318.81
Hopfenberg	2517.6 ± 258.97	440.4 ± 35.73	3781.75 ± 804.86	537.16 ± 203.83	3226.18 ± 495.7	153.57 ± 58.11	139.22 ± 83.54
Hopfenberg with T_{lag}	98.23 ± 27.16	103.42 ± 90	73.42 ± 29.01	143.25 ± 4.87	87.15 ± 29.85	**65.28 ± 37.46**	**90.64 ± 108.3**
Baker–Lonsdale	320.45 ± 95.74	2650.37 ± 893.39	705.38 ± 252.81	1851.18 ± 186.04	466.31 ± 145.67	3909.32 ± 726.52	/
Baker–Lonsdale with T_{lag}	1690.81 ± 775.7	1980.36 ± 523.59	2188.01 ± 1132.49	2783.4 ± 541.58	1250.52 ± 1309.07	1062.28 ± 142.73	/
Makoid–Banakar	**11.63 ± 7.57**	**38.05 ± 36.38**	**29.06 ± 16.6**	**67.3 ± 70.71**	**20.68 ± 19.9**	**98.76 ± 32.54**	495.91 ± 84.15
Makoid–Banakar with T_{lag}	29.23 ± 13.84	**60.38 ± 39.68**	**50.34 ± 17.41**	112.02 ± 78.79	35.9 ± 17.96	239.51 ± 45.99	800.6 ± 131.93
Peppas–Sahlin_1	92.09 ± 39.56	**39.49 ± 32.39**	335.15 ± 180.23	**44.69 ± 55.6**	195.11 ± 102.7	484.66 ± 48.84	853.05 ± 407.85
Peppas–Sahlin_1 with T_{lag}	168.47 ± 60.05	**41.06 ± 35.96**	501.8 ± 235.47	**59.3 ± 62.97**	316.37 ± 130.79	429.11 ± 45.8	799.75 ± 388.01
Peppas–Sahlin_2	161.33 ± 59.16	**37.66 ± 31.41**	510.55 ± 254.64	**46.29 ± 59.05**	316.85 ± 137.13	469.73 ± 45.3	855.9 ± 403.87
Peppas–Sahlin_2 with T_{lag}	264.34 ± 83.78	**40.46 ± 33.88**	717.87 ± 323.01	**62.91 ± 68.15**	476.68 ± 169.35	410.15 ± 42.47	795.81 ± 381.66
Quadratic	2881.19 ± 465.71	445.15 ± 95.22	4832.27 ± 1426.88	545.27 ± 205.35	3950.26 ± 562.52	**68.08 ± 9.96**	**73.05 ± 30.68**
Quadratic with T_{lag}	3184.46 ± 504.62	520.37 ± 105.43	5261.27 ± 1517.94	627.07 ± 225.13	4349.22 ± 593.49	**95.91 ± 13.2**	**89.65 ± 30.65**
Weibull_1	830.58 ± 68.74	1017.76 ± 542.26	965.45 ± 157.47	1328.3 ± 272.77	639.11 ± 201.21	333.09 ± 93.42	1154.08 ± 211.32
Weibull_2	665.34 ± 64.04	817.02 ± 463.32	825.15 ± 161.52	1129.25 ± 271.91	507.52 ± 171.88	203.94 ± 79.27	830.09 ± 254.63
Weibull_3	503.49 ± 43.98	658.19 ± 335.74	510.68 ± 59.13	942.75 ± 213.88	413.12 ± 91.6	203.79 ± 59.07	443.11 ± 47.34
Weibull_4	677.9 ± 58.09	853.26 ± 345.68	730.9 ± 60.58	1197.7 ± 194.47	570.86 ± 142.9	374.41 ± 67.69	800.11 ± 106.6
Logistic_1	1231.85 ± 192.65	1549.4 ± 635.78	2261.89 ± 739.2	2025.08 ± 187.71	1244.6 ± 481.45	979 ± 495.67	2390.96 ± 905.08
Logistic_2	979.94 ± 143.97	1476.57 ± 729.6	1199.64 ± 147.32	1763.58 ± 176.65	955.42 ± 258.63	794.54 ± 610.63	1681.01 ± 1042.86
Logistic_3	236.24 ± 56.8	423.57 ± 92.84	124.73 ± 32.56	319.91 ± 54	165.78 ± 37.47	848.68 ± 269.19	604.71 ± 441.18
Gompertz_1	1860.41 ± 319.01	2540.29 ± 843.49	3374.65 ± 989.41	2344.26 ± 436.84	1969.51 ± 656.51	1996.52 ± 250.65	3100.88 ± 645.38
Gompertz_2	1898.96 ± 140.96	1909.74 ± 500.45	1909.29 ± 224.24	2277.81 ± 298.62	1607.56 ± 320.35	1365.54 ± 200.94	2382.57 ± 90.48
Gompertz_3	149.25 ± 31.03	341.04 ± 203.23	101.45 ± 34.24	338.11 ± 71.6	132.13 ± 77.16	163.58 ± 23.31	103.64 ± 38.56
Gompertz_4	145.69 ± 35.13	341.04 ± 203.23	101.45 ± 34.24	338.11 ± 71.6	132.13 ± 77.16	163.58 ± 23.31	110.29 ± 26.63
Probit_1	1126.87 ± 120.61	1594.76 ± 639.85	1647.54 ± 378.49	2088.82 ± 110.64	1010.4 ± 346.16	1180.81 ± 315.27	2948.78 ± 441.29
Probit_2	1039.09 ± 72.54	1345.32 ± 798.74	1033.86 ± 120.42	1989.79 ± 275.07	853.32 ± 198.66	899.17 ± 508.88	1840.97 ± 882.97

Table S11. The RSS results for models fitted to carvedilol experimental dissolution data. Model fitting was performed on the entire relevant dissolution data range as determined from the paired t–tests. Data is presented for the Anhydrous Dibasic Calcium Phosphate group, the Microcrystalline Cellulose group, the Ethylcellulose formulation and for the Pregelatinized Starch group of formulations. Data is presented as average RSS ± one standard deviation.

Formulation / Model	Di-Cafos® A12	Emcompress® Anhydrous	Avicel® PH-102	Avicel® PH-200	Ethocel™ Standard 20 Premium	Starch 1500® sample with smaller particle size (↓PS)	Starch 1500® sample with larger particle size (↑PS)
Zero–order	2109.55 ± 328.98	3732.03 ± 474.42	931.04 ± 191.9	1273.22 ± 72.68	1362.09 ± 199.39	3350.97 ± 1171.96	1664.81 ± 273.92
Zero–order with T_{lag}	574.78 ± 167.02	625.03 ± 146.03	248.72 ± 20	359.34 ± 40.42	508.47 ± 66.33	184.56 ± 13.1	153.03 ± 56.19
Zero–order with F_0	574.78 ± 167.02	625.03 ± 146.03	248.72 ± 20	359.34 ± 40.42	508.47 ± 66.33	184.56 ± 13.1	153.03 ± 56.19
First–order	403.11 ± 253.28	561.63 ± 126.71	107 ± 44.85	189.23 ± 92.3	92.99 ± 123.18	1061.85 ± 472.23	417.06 ± 71.68
First–order with T_{lag}	1227.29 ± 858.87	224.94 ± 362.21	126.83 ± 51.84	257.38 ± 137.22	125.6 ± 190.9	122.14 ± 59.03	147.64 ± 97.74
First–order with F_{max}	829.93 ± 116.59	499.2 ± 41.97	793.97 ± 72.44	816.47 ± 110.02	696.38 ± 214.06	945.86 ± 348.76	717.58 ± 147.97
First–order with T_{lag} and F_{max}	1534.87 ± 196.87	756.59 ± 152.36	1335.64 ± 138.15	1422.01 ± 193.94	1130.42 ± 302	916.48 ± 128.51	1226.25 ± 279.54
Higuchi	1044.81 ± 157.21	177.43 ± 92.05	1072.54 ± 102.85	1085.52 ± 164.84	1125.2 ± 281.3	208.13 ± 30.86	477.35 ± 178.44
Higuchi with T_{lag}	625.96 ± 74.96	429.57 ± 112.76	521.32 ± 79.63	525.07 ± 86.21	394.09 ± 73.54	646.5 ± 203.38	724.69 ± 138.99
Higuchi with F_0	214.49 ± 30.78	71.52 ± 29.23	234.46 ± 27.03	209.07 ± 37.76	177.19 ± 63.39	170.02 ± 25.03	239.38 ± 81.46
Korsmeyer–Peppas	**49.46 ± 38.42**	91.07 ± 25.24	**22.43 ± 16.52**	**20.97 ± 10.75**	130.58 ± 57.95	445.11 ± 132.75	314.25 ± 91.74
Korsmeyer–Peppas with T_{lag}	126.78 ± 22.4	211.9 ± 48.7	55.4 ± 62.71	**35.65 ± 17.3**	62.92 ± 25.48	636.53 ± 158.22	492.51 ± 124.17
Korsmeyer–Peppas with F_0	99.78 ± 88.41	**27.03 ± 20.11**	88.25 ± 85.49	94.5 ± 70.96	349.37 ± 96.66	236.24 ± 102.6	129.93 ± 58.46
Hixson–Crowell	183.25 ± 37.68	1020.96 ± 261.39	110.26 ± 46.79	131.58 ± 61.01	112.83 ± 69.03	1539.67 ± 638.1	605.05 ± 129.85
Hixson–Crowell with T_{lag}	92.25 ± 61.07	**58.68 ± 30.21**	**15.32 ± 4.12**	30.67 ± 22.01	**26.66 ± 9.03**	**38.05 ± 6.12**	42.56 ± 24.82
Hopfenberg	177.7 ± 29.92	1020.96 ± 261.39	108.23 ± 48.81	131.11 ± 61.65	92.01 ± 54.71	1477.56 ± 678.64	576.73 ± 113.09
Hopfenberg with T_{lag}	**19.26 ± 9.35**	**58.68 ± 30.21**	**15.22 ± 4.02**	**24.87 ± 16.54**	**23.35 ± 9.71**	**35.67 ± 5.76**	**31.3 ± 6.87**
Baker–Lonsdale	5385.27 ± 1741.78	1537.61 ± 1646.01	4272.04 ± 284.7	4959.01 ± 812.09	4618.12 ± 1269.76	626.15 ± 108.77	1946.83 ± 1385.96
Baker–Lonsdale with T_{lag}	1884.95 ± 401.2	1436.07 ± 455.13	1144.11 ± 166.2	1348.98 ± 285.32	965.77 ± 279.37	1677.71 ± 400.61	1434.28 ± 211.92
Makoid–Banakar	**78.22 ± 50.79**	90.72 ± 54.73	**13 ± 13.28**	**15.77 ± 10.91**	**20.73 ± 8.81**	**44.42 ± 22.47**	**36.07 ± 18.81**
Makoid–Banakar with T_{lag}	170.97 ± 80.76	159.59 ± 83.62	35.57 ± 30.13	50.64 ± 39.37	81.8 ± 25.31	**73.44 ± 30.28**	67.21 ± 30.2
Peppas–Sahlin_1	80.44 ± 62.3	**36.62 ± 26.25**	**24.87 ± 6.56**	**39.94 ± 19.4**	109.52 ± 25.88	**82.59 ± 65.87**	**21.9 ± 10.29**
Peppas–Sahlin_1 with T_{lag}	**70.52 ± 57.63**	**39.09 ± 24.56**	**20.02 ± 4.7**	**31.31 ± 17.51**	94.16 ± 23.97	110.18 ± 80.5	**31.65 ± 12.27**
Peppas–Sahlin_2	85.51 ± 63.7	**33.81 ± 23.53**	**31.39 ± 8.25**	**46.8 ± 22.94**	120.71 ± 26.58	105.31 ± 84.99	**23.5 ± 9.96**
Peppas–Sahlin_2 with T_{lag}	**74.08 ± 58.43**	**39.35 ± 21.64**	**24.79 ± 5.75**	**36.18 ± 20.4**	102.4 ± 24.74	137.62 ± 101.88	**34.61 ± 12.22**
Quadratic	207.58 ± 33.46	699.43 ± 52.28	104.18 ± 47.38	137.19 ± 66.39	**59.24 ± 25.91**	1199.92 ± 534.98	506.11 ± 73.95
Quadratic with T_{lag}	241.7 ± 34.49	765.61 ± 53.39	122.13 ± 52.61	159.44 ± 70.81	74.67 ± 29.2	1276.05 ± 558.72	548.58 ± 77
Weibull_1	842.68 ± 159.39	724.18 ± 245.68	400.69 ± 160.28	466.45 ± 120.42	283.73 ± 117.29	1017.33 ± 221.04	878.4 ± 170
Weibull_2	645.98 ± 145.7	543.55 ± 211.24	269.41 ± 128.83	322.83 ± 101.4	158.21 ± 79.16	822.48 ± 198.82	688.68 ± 147.1
Weibull_3	661.37 ± 75.67	564.85 ± 73.17	446.88 ± 187.2	502.65 ± 91.03	264.48 ± 52.67	1011.04 ± 182.45	883.7 ± 140.26
Weibull_4	889.29 ± 78.77	881.35 ± 157.77	592.49 ± 174.69	638.38 ± 103.87	454.15 ± 94.71	1194.89 ± 208.27	1056.4 ± 158.15
Logistic_1	1761.4 ± 578.68	1153.64 ± 614.6	678.9 ± 196.68	825.64 ± 202.26	636.36 ± 240.72	1197.9 ± 269.08	1075.07 ± 220.03
Logistic_2	1087.96 ± 103.91	1722.66 ± 335.46	862.35 ± 127.06	860.04 ± 196.99	746.62 ± 152.64	1805.24 ± 276.37	1675.11 ± 224.12
Logistic_3	635.82 ± 60.22	412.53 ± 65.65	433.74 ± 16.54	531.34 ± 38.06	664.77 ± 98.89	202.12 ± 23.39	259.39 ± 27.96
Gompertz_1	2786.18 ± 349.09	2082.88 ± 589.75	1860.73 ± 286.98	2300.45 ± 413.23	1989.07 ± 507.59	2005.29 ± 397.65	1942.16 ± 374.43
Gompertz_2	1873.03 ± 567.54	1610.37 ± 241.92	1613.99 ± 438.63	1478.43 ± 151.74	1258.69 ± 215.97	2538.84 ± 373.01	2248.73 ± 285.57
Gompertz_3	222.5 ± 39.48	155.38 ± 25.13	202.6 ± 9.93	250.84 ± 52.8	188.74 ± 25.01	168 ± 45.94	219.21 ± 40.46
Gompertz_4	222.5 ± 39.48	155.38 ± 25.13	202.6 ± 9.93	250.84 ± 52.8	188.74 ± 25.01	195.29 ± 8.84	221.89 ± 36.45
Probit_1	1921.3 ± 507.12	1277.63 ± 540.63	1014.14 ± 196.4	1187.85 ± 211.23	933.17 ± 262.09	1344.07 ± 247.84	1288.9 ± 244.02
Probit_2	1356.32 ± 121.44	1700.62 ± 335.99	1036.62 ± 163.48	1047.21 ± 220.96	897.65 ± 171.46	1785.48 ± 254.32	1722.27 ± 240.06

Table S12. Summary of model performance for fitting the entire relevant carvedilol dissolution data. The table depicts the number of times each model performed as a first–choice model, a near first–choice model, a second–choice model and a third–choice model. Summary statistics depict the total number of times each model was chosen as a candidate for carvedilol release modelling from all studied formulations (N = 23) expressed as a count and as %.

Model	Number of parameters in the model	First–choice model	Near first–choice model	Second–choice model	Third–choice model	SUM	% of all formulations (N = 23)
Zero–order	1					0	0 %
Zero–order with T_{lag}	2					0	0 %
Zero–order with F_0	2					0	0 %
First–order	1			1		1	4.3 %
First–order with T_{lag}	2					0	0 %
First–order with F_{max}	2					0	0 %
First–order with T_{lag} and F_{max}	3					0	0 %
Higuchi	1					0	0 %
Higuchi with T_{lag}	2					0	0 %
Higuchi with F_0	2	3				3	13 %
Korsmeyer–Peppas	2			2	1	3	13 %
Korsmeyer–Peppas with T_{lag}	3				1	1	4.3 %
**Korsmeyer–Peppas with F_0**	**3**	**1**		**1**	**4**	**6**	**26.1 %**
Hixson–Crowell	1					0	0 %
**Hixson–Crowell with T_{lag}**	**2**		**1**	**3**	**1**	**5**	**21.7 %**
Hopfenberg	2			2		2	8.7 %
**Hopfenberg with T_{lag}**	**3**	**4**		**6**	**4**	**14**	**60.9 %**
Baker–Lonsdale	1					0	0 %
Baker–Lonsdale with T_{lag}	2					0	0 %
**Makoid–Banakar**	**3**	**4**	**2**	**5**	**6**	**17**	**73.9 %**
Makoid–Banakar with T_{lag}	4	1	1			2	8.7 %
**Peppas–Sahlin_1**	**3**	**3**		**1**	**3**	**7**	**30.4 %**
Peppas–Sahlin_1 with T_{lag}	4				3	3	13 %
Peppas–Sahlin_2	2					0	0 %
Peppas–Sahlin_2 with T_{lag}	3					0	0 %
**Quadratic**	**2**	**2**			**2**	**4**	**17.4 %**
Quadratic with T_{lag}	3				1	1	4.3 %
Weibull_1	3	3				3	13 %
**Weibull_2**	**2**		**1**	**4**		**5**	**21.7 %**
**Weibull_3**	**3**	**1**	**1**	**2**	**2**	**6**	**26.1 %**
Weibull_4	4	1		1		2	8.7 %
Logistic_1	2					0	0 %
Logistic_2	3			2		2	8.7 %
Logistic_3	3					0	0 %
Gompertz_1	2					0	0 %
Gompertz_2	3					0	0 %
Gompertz_3	3					0	0 %
Gompertz_4	3					0	0 %
Probit_1	2			1	1	2	8.7 %
Probit_2	3				2	2	8.7 %

Table S13. The RSS results for models fitted to carvedilol experimental dissolution data for up to and including t = 60 min. Model fitting was performed on the entire relevant dissolution data range as determined from the paired t–tests. Data is presented for the Polyethylene Glycol & Polyethylene Oxide group, the Povidone group and the Mannitol group of formulations. Data is presented as average RSS ± one standard deviation.

Formulation / Model	Polyglykol® 4000 P	Polyglykol® 8000 P	Polyox™ WSR N-80 (LEO NF Grade)	Kollidon® 25	Kollidon® 90 F	C*Pharm Mannidex 16700	Pearlitol® 160C	Parteck® M 100	Parteck® M 200
Zero–order	1298.12 ± 393.03	1003.65 ± 218.89	38.57 ± 10.2	5445.47 ± 3246.34	11.15 ± 14.86	1423.65 ± 1726.92	815.2 ± 210.44	2937.55 ± 2139.19	1768.34 ± 2017.91
Zero–order with T_{lag}	457.17 ± 215.28	522.87 ± 86.18	127.96 ± 21.79	2836.23 ± 2163.57	268.82 ± 242.95	806.09 ± 1108.25	730.89 ± 753.84	1741.37 ± 1084.06	1012.99 ± 1057.77
Zero–order with F_0	457.17 ± 215.28	522.87 ± 86.18	127.96 ± 21.79	2836.23 ± 2163.57	268.82 ± 242.95	806.09 ± 1108.25	730.89 ± 753.84	1741.37 ± 1084.06	1012.99 ± 1057.77
First–order	67.06 ± 64.16	495.09 ± 753.05	675.19 ± 690.8	374.7 ± 223.37	372.24 ± 295.26	457.31 ± 590.95	622.39 ± 1060.14	1598.07 ± 1203.76	901.47 ± 1247.9
First–order with T_{lag}	39.7 ± 21.5	529.02 ± 937.81	899.95 ± 1301.08	/	/	/	/	/	1437.65 ± 1654.9
First–order with F_{max}	120.62 ± 82.86	376.06 ± 126.53	859.07 ± 216.04	707.03 ± 420.5	259.04 ± 97.02	126.51 ± 132.09	261.32 ± 416.09	521.46 ± 283.61	417.63 ± 223.01
First–order with T_{lag} and F_{max}	40.07 ± 12.99	57.54 ± 47.01	620.89 ± 155.93	4486.84 ± 5178.81	1479.89 ± 921.66	926.99 ± 1244.95	920.4 ± 1598.21	1300.11 ± 1561.96	733.1 ± 1264.06
Higuchi	244.05 ± 115.61	472.82 ± 50.49	1401.12 ± 195.4	1080.35 ± 1088.62	1085.08 ± 143.72	445.04 ± 278.33	319.82 ± 134.86	1078.36 ± 394.45	849.22 ± 390.71
Higuchi with T_{lag}	245.44 ± 205.69	193.11 ± 98.96	168.46 ± 48.59	3761.92 ± 3352.13	175.71 ± 57	1074.98 ± 1677.97	1189.2 ± 1792.39	2164.58 ± 1460.83	1058.55 ± 1451.64
Higuchi with F_0	130.36 ± 97.7	134.57 ± 48.2	76.24 ± 31.53	1328.98 ± 1325.76	131.45 ± 53.93	303.05 ± 548.22	169.91 ± 265.21	830.58 ± 727.85	438.37 ± 653.26
Korsmeyer–Peppas	140.27 ± 104.71	223.84 ± 36.71	47.28 ± 12.42	1503.56 ± 2326.63	2.14 ± 2.73	149.12 ± 294.48	453.21 ± 888.2	4293.69 ± 7111.62	628.61 ± 886.25
Korsmeyer–Peppas with T_{lag}	91.56 ± 70.54	139.43 ± 30.28	48.07 ± 16.34	436.68 ± 412.77	**0.69 ± 1.03**	199.1 ± 383.07	**26.37 ± 29.77**	1099.69 ± 919.49	582.67 ± 724.39
Korsmeyer–Peppas with F_0	211.85 ± 120.11	318.28 ± 41.5	79.89 ± 18.02	620.84 ± 522.89	3.84 ± 4.99	269.82 ± 523.21	**26.27 ± 40.32**	1558.96 ± 1165.64	840.53 ± 996.93
Hixson–Crowell	95.49 ± 84.47	60.6 ± 22.25	148.61 ± 75.51	2117.67 ± 1589.87	50.25 ± 9.61	145.8 ± 173.01	78.48 ± 39.42	324.99 ± 197.08	235.28 ± 198.01
Hixson–Crowell with T_{lag}	120.55 ± 53.59	80.55 ± 69.59	65.61 ± 36.98	6006.61 ± 6901.17	371.03 ± 353.28	172.14 ± 185.79	370.29 ± 585.18	923.08 ± 1611.66	638.61 ± 1090.03
Hopfenberg	116.33 ± 84.58	49.64 ± 20.55	133.18 ± 68.36	626 ± 721.76	31.01 ± 13.78	146.03 ± 169.44	65.78 ± 47.47	301.89 ± 169.6	235.28 ± 198.01
Hopfenberg with T_{lag}	120.55 ± 53.59	81.37 ± 68.37	**14.15 ± 7.93**	1556.62 ± 2333	36.62 ± 53.65	**117.32 ± 217.5**	**24.94 ± 37.55**	**125.91 ± 82.15**	638.61 ± 1090.03
Baker–Lonsdale	449.75 ± 184.96	1374.44 ± 1431.53	1997.33 ± 325.6	1913.94 ± 2081.28	1154.03 ± 672.28	/	2948.97 ± 2431.29	3876.41 ± 393.53	/
Baker–Lonsdale with T_{lag}	232.71 ± 143.7	250.93 ± 12.73	254.99 ± 63.94	4526.27 ± 4398.62	267.48 ± 145.76	/	1464.68 ± 1230.15	3444.56 ± 3378	/
Makoid–Banakar	**1.26 ± 0.71**	**4.75 ± 2.46**	**11.99 ± 16.83**	238.61 ± 189.14	**0.22 ± 0.16**	**107.86 ± 186.8**	**25.32 ± 30.75**	199.34 ± 203.28	**53.68 ± 78.45**
Makoid–Banakar with T_{lag}	**7.19 ± 5.51**	**1.03 ± 0.91**	**1.94 ± 1.4**	165.27 ± 140.21	1.84 ± 0.41	**97.2 ± 171.79**	**44.36 ± 49.53**	155.29 ± 147.04	**46.51 ± 52.61**
Peppas–Sahlin_1	224.93 ± 131.41	306.78 ± 49.4	136.22 ± 25.13	623.49 ± 604.32	202.73 ± 161.54	403.81 ± 584.63	480.74 ± 631.47	1115.41 ± 631.24	649.52 ± 645.15
Peppas–Sahlin_1 with T_{lag}	119.57 ± 80.64	182.43 ± 32.22	80.63 ± 17.74	293.01 ± 316.64	148.23 ± 122.15	256.58 ± 396.89	329.13 ± 482.51	734.06 ± 439.02	412.42 ± 440.27
Peppas–Sahlin_2	216.16 ± 126.28	309.27 ± 44.66	175.18 ± 31.23	442.5 ± 425.25	243.82 ± 159.91	374.8 ± 515.91	475.72 ± 617.68	1078.31 ± 566.19	640.14 ± 594.32
Peppas–Sahlin_2 with T_{lag}	100.07 ± 69.21	164.51 ± 27.11	107.09 ± 23.27	195.33 ± 192.35	177.08 ± 117.75	225.5 ± 331.24	313.92 ± 455.18	667.37 ± 369.72	380.15 ± 378.39
Quadratic	**7.65 ± 5.33**	26.11 ± 7.16	92.68 ± 38.29	2640.93 ± 1623.22	41.53 ± 17.97	351.86 ± 497.29	93.51 ± 54.35	395.25 ± 349.04	176.15 ± 216.6
Quadratic with T_{lag}	66.64 ± 25.93	**6.58 ± 6.43**	24.34 ± 20.61	3014.39 ± 1797.29	16.38 ± 9.78	471.43 ± 586.57	176.18 ± 75.47	410.19 ± 411.8	157.32 ± 238.89
Weibull_1	20.05 ± 15.19	20.56 ± 22	**4.35 ± 2.75**	**58.87 ± 52.79**	2.85 ± 1.03	118.25 ± 96.96	161.8 ± 104.89	**68.2 ± 52.81**	**64.48 ± 81.14**
Weibull_2	**5.71 ± 3.76**	**8.22 ± 11.31**	**4.45 ± 5.85**	**92.3 ± 76.59**	**0.88 ± 0.77**	**99.05 ± 109.97**	94.13 ± 111.4	**105.94 ± 82.84**	99.44 ± 150.66
Weibull_3	**7.63 ± 7.79**	**5.25 ± 4.21**	**5.37 ± 6.34**	245.44 ± 208.53	**0.41 ± 0.41**	123.2 ± 192.28	**57.43 ± 49.13**	341.78 ± 323.98	134.96 ± 204.6
Weibull_4	25.45 ± 7.3	**7.66 ± 2.15**	**3.77 ± 3.48**	160.51 ± 102.32	2.41 ± 0.98	128.51 ± 145.99	103.6 ± 56.2	263.76 ± 340.05	131.37 ± 215.65
Logistic_1	101.19 ± 89.08	191.86 ± 178.9	113.57 ± 50.55	103.7 ± 62.83	48.99 ± 36.44	346.11 ± 237.77	382.66 ± 231.27	198.97 ± 119.52	211.82 ± 280.79
Logistic_2	119.69 ± 29.85	80.65 ± 27.73	59.06 ± 26.71	218.92 ± 198.97	32.99 ± 26.03	179.97 ± 50.86	230.13 ± 68.61	**113.08 ± 81.09**	**82.18 ± 44.52**
Logistic_3	76.63 ± 27.74	168.97 ± 12.84	167.27 ± 28.77	3277.38 ± 3403.78	130.9 ± 54.26	368.02 ± 510.87	158.26 ± 136.29	986.75 ± 431.34	554.8 ± 295.01
Gompertz_1	249.29 ± 146.16	307.89 ± 83.81	94 ± 39.38	382.34 ± 157.09	68.31 ± 26.83	475.33 ± 81.98	530.94 ± 83.11	245.25 ± 132.08	304.93 ± 327.88
Gompertz_2	331.59 ± 43.43	275.79 ± 27.18	121.51 ± 66.48	173.49 ± 109.03	106.11 ± 48.03	407.71 ± 76.82	438.29 ± 45.87	218.01 ± 104.15	189.78 ± 35.03
Gompertz_3	52.81 ± 63.52	43.99 ± 12.6	36.99 ± 23.01	3943.81 ± 4275.04	12.56 ± 6.62	520.26 ± 954.61	331.6 ± 536.76	802.05 ± 994.14	446.53 ± 783.67
Gompertz_4	34.34 ± 33.49	46.43 ± 24.77	41.7 ± 19.27	2361.61 ± 2462.2	25.26 ± 31.23	573.22 ± 1048.74	547.21 ± 949.29	1112.88 ± 989.02	570.58 ± 879.13
Probit_1	67.84 ± 53.19	119.27 ± 105.28	89.81 ± 51.2	**73.34 ± 35.18**	47.53 ± 27.44	351.47 ± 218.95	502.81 ± 278.97	**129.14 ± 78.93**	160.05 ± 190.13
Probit_2	82.34 ± 21.17	66.68 ± 22.97	36.53 ± 19.45	230.08 ± 211.44	49.54 ± 24.52	176.71 ± 65.48	339.72 ± 137.41	**128.58 ± 93.74**	87.8 ± 68.75

Table S14. The RSS results for models fitted to carvedilol experimental dissolution data for up to and including t = 60 min. Model fitting was performed on the entire relevant dissolution data range as determined from the paired t–tests. Data is presented for the Lactose Monohydrate group of formulations along with the Sucrose and Maltodextrin formulations. Data is presented as average RSS ± one standard deviation.

Formulation / Model	Lactochem® Crystals	Lactochem® Fine Powder	SuperTab® 11SD	FlowLac® 100	Tablettose® 70	Granulated sugar N°1 600	Glucidex® 19
Zero–order	2914.17 ± 525.02	390.77 ± 93.44	4941.51 ± 1503.16	493.14 ± 203.87	4265.75 ± 574.49	103.16 ± 15.4	98.58 ± 39.38
Zero–order with T_{lag}	237.23 ± 37.65	126.18 ± 52.47	182.39 ± 18.83	80.52 ± 10.54	219.34 ± 43.52	407.79 ± 33.9	371.2 ± 101.41
Zero–order with F_0	237.23 ± 37.65	126.18 ± 52.47	182.39 ± 18.83	80.52 ± 10.54	219.34 ± 43.52	407.79 ± 33.9	371.2 ± 101.41
First–order	1437.55 ± 265.66	133.55 ± 44.43	2222.8 ± 653.18	256.33 ± 204.48	1665.03 ± 246.71	6.24 ± 1	33.72 ± 30.16
First–order with T_{lag}	/	/	/	/	463.06 ± 516.33	264.48 ± 307.63	/
First–order with F_{max}	1696.45 ± 306.68	99.74 ± 44.15	2671.18 ± 419.19	134.56 ± 70.95	1861.76 ± 389.94	3.37 ± 0.73	12.29 ± 3.75
First–order with T_{lag} and F_{max}	**15.88 ± 12.52**	1221.52 ± 916	148.63 ± 90.89	1435.5 ± 248.98	66.19 ± 49.1	552.28 ± 100.04	957.43 ± 125.35
Higuchi	337.18 ± 147.32	109.74 ± 62.54	1138.56 ± 633.83	67.2 ± 39.34	797.46 ± 253.23	286.14 ± 12.08	347.84 ± 104.45
Higuchi with T_{lag}	40.14 ± 28.04	599.02 ± 197.14	58.78 ± 22.89	832.15 ± 52.57	94.35 ± 38.66	140.25 ± 14.99	282 ± 21.51
Higuchi with F_0	4023.46 ± 664.77	49.43 ± 42.14	**0.73 ± 0.46**	79.89 ± 7.95	**3.59 ± 1.73**	29.22 ± 1.62	85.36 ± 17.19
Korsmeyer–Peppas	**5.35 ± 3.8**	**0.58 ± 0.21**	17.29 ± 2.5	3.96 ± 1.76	6.19 ± 3.32	**0.84 ± 0.3**	**2.92 ± 2.61**
Korsmeyer–Peppas with T_{lag}	18.28 ± 7.87	6.05 ± 3.26	37.16 ± 2.72	15.41 ± 4.93	19.59 ± 6.33	3.05 ± 0.8	4.82 ± 0.75
Korsmeyer–Peppas with F_0	**1.08 ± 0.76**	**0.96 ± 0.92**	20.76 ± 28.18	**0.5 ± 0.28**	**0.84 ± 0.55**	**0.48 ± 0.03**	**0.29 ± 0.1**
Hixson–Crowell	1773.14 ± 299.52	183.65 ± 83.46	2955.51 ± 766.91	270.73 ± 146.09	2323.49 ± 689.24	30.11 ± 4.72	20.77 ± 17.19
Hixson–Crowell with T_{lag}	28.83 ± 8.62	95.99 ± 97.25	39.32 ± 29.51	301.6 ± 144.54	44.43 ± 14.69	29.68 ± 17.63	244.83 ± 154.22
Hopfenberg	1226.83 ± 261.7	205.3 ± 51.64	2458.84 ± 905.41	292.91 ± 139.94	2073.83 ± 486.42	30.11 ± 4.72	33.23 ± 27.02
Hopfenberg with T_{lag}	30.22 ± 6.94	19.19 ± 11.78	24.3 ± 3.29	50.75 ± 20.23	32.87 ± 8.04	29.68 ± 17.63	20.41 ± 33.63
Baker–Lonsdale	80.11 ± 71.83	280.25 ± 217.92	435.5 ± 348.96	152.22 ± 60.08	252.66 ± 190.62	649.15 ± 356.01	/
Baker–Lonsdale with T_{lag}	1531.29 ± 745.69	997.66 ± 127.6	2022.21 ± 1084.34	1093.62 ± 319.33	1136.93 ± 1218.33	329.18 ± 35.54	/
Makoid–Banakar	**1.27 ± 0.9**	**0.26 ± 0.19**	**3.92 ± 1.69**	**0.9 ± 0.44**	**1.45 ± 1.63**	**1.18 ± 1.08**	**3.28 ± 3.23**
Makoid–Banakar with T_{lag}	**5.03 ± 2.49**	2.03 ± 0.86	**9.45 ± 2.62**	**3.43 ± 0.88**	5.27 ± 3.72	3.22 ± 0.83	4.59 ± 0.45
Peppas–Sahlin_1	61.69 ± 30.27	3.71 ± 2.48	231.52 ± 139.11	8.8 ± 8.46	125.68 ± 60.59	95.94 ± 6.79	80.61 ± 48.96
Peppas–Sahlin_1 with T_{lag}	125.6 ± 48.64	5.1 ± 4.64	382.32 ± 180.27	21.06 ± 15.01	227.29 ± 85.79	58.57 ± 4.53	50.52 ± 35.72
Peppas–Sahlin_2	113.7 ± 46.92	3.05 ± 1.49	377.3 ± 192.75	10.69 ± 11.45	218.2 ± 88.87	96.68 ± 6.13	87.13 ± 52.22
Peppas–Sahlin_2 with T_{lag}	202.4 ± 69.59	5.55 ± 4.53	561.76 ± 255.31	25.28 ± 19.74	356.55 ± 118.87	57.25 ± 4.05	53.83 ± 37.47
Quadratic	1880.5 ± 364.04	214.38 ± 54.05	3390.04 ± 1106.17	310.97 ± 138.12	2776.04 ± 448.87	26.71 ± 5.51	31.06 ± 22.75
Quadratic with T_{lag}	2110.78 ± 396.64	264.53 ± 60.66	3728.28 ± 1183.76	367.71 ± 154.4	3096.15 ± 477.61	41.57 ± 7.52	45.71 ± 27.79
Weibull_1	69.72 ± 24.22	22.18 ± 5.17	180.44 ± 49.95	47.9 ± 22.54	101.68 ± 9.12	7.5 ± 1.93	15.14 ± 4.58
Weibull_2	42.67 ± 18.95	6.95 ± 2.42	139 ± 46.87	23.79 ± 13.83	120.71 ± 114.95	2.75 ± 1.05	13.43 ± 7.95
Weibull_3	104.03 ± 77.53	14.61 ± 15.26	135.37 ± 79.34	33.03 ± 6.53	150.39 ± 60.99	2.97 ± 0.63	5.8 ± 2.25
Weibull_4	73.14 ± 20.47	23.65 ± 3.79	128.36 ± 15.05	42.86 ± 17	90.06 ± 12.99	6.41 ± 0.67	11.66 ± 4.16
Logistic_1	145.61 ± 63.19	58.47 ± 60.64	1162.1 ± 714.54	295.36 ± 310.64	260.76 ± 66.05	59.38 ± 31.46	158.47 ± 101.14
Logistic_2	468.84 ± 271.49	72.09 ± 83.95	269.31 ± 55.09	127.12 ± 166.47	318.05 ± 206.04	77.37 ± 42.29	61.38 ± 47.37
Logistic_3	89.89 ± 22.81	75.08 ± 19.09	46.74 ± 6.11	48.93 ± 6.88	64.49 ± 12.03	134.41 ± 54.95	153.58 ± 136.61
Gompertz_1	423.48 ± 150.54	207.81 ± 194.94	1965.71 ± 818.05	568.61 ± 186.36	1000.28 ± 772.19	188.77 ± 24.14	230.49 ± 58.45
Gompertz_2	471.97 ± 96.03	347.79 ± 140.65	682.2 ± 116.62	498.5 ± 200.69	553.77 ± 63.88	214.35 ± 35.73	220.4 ± 34.44
Gompertz_3	55.97 ± 25.66	34.8 ± 9.54	22.39 ± 6.39	29.57 ± 14.67	43.08 ± 16.25	56.31 ± 6.19	39.98 ± 13.48
Gompertz_4	48.98 ± 13.79	34.8 ± 9.54	22.39 ± 6.39	29.57 ± 14.67	43.08 ± 16.25	56.31 ± 6.19	32.75 ± 13.12
Probit_1	145.97 ± 52.64	40.91 ± 8.01	440.2 ± 163.25	240.36 ± 339.21	228.7 ± 46.05	61.48 ± 48.33	182.74 ± 112
Probit_2	163.12 ± 36.19	112.17 ± 83.65	359.15 ± 195.3	77.67 ± 30.35	187.76 ± 20.91	97.75 ± 50.33	82.1 ± 58.26

Table S15. The RSS results for models fitted to carvedilol experimental dissolution data for up to and including t = 60 min. Model fitting was performed on the entire relevant dissolution data range as determined from the paired t–tests. Data is presented for the Anhydrous Dibasic Calcium Phosphate group, the Microcrystalline Cellulose group, the Ethylcellulose formulation and for the Pregelatinized Starch group of formulations. Data is presented as average RSS ± one standard deviation.

Formulation / Model	Di-Cafos® A12	Emcompress® Anhydrous	Avicel® PH-102	Avicel® PH-200	Ethocel™ Standard 20 Premium	Starch 1500® sample with smaller particle size (↓PS)	Starch 1500® sample with larger particle size (↑PS)
Zero–order	126.55 ± 6.42	402.63 ± 33.96	55.17 ± 27.97	62.37 ± 18.01	41.94 ± 10.03	668.77 ± 330.4	254.6 ± 37.94
Zero–order with T_{lag}	178.55 ± 29.02	220.89 ± 29.86	88.74 ± 6.03	128.74 ± 5.64	142.85 ± 24.42	89.54 ± 5.35	72.35 ± 16.9
Zero–order with F_0	178.55 ± 29.02	220.89 ± 29.86	88.74 ± 6.03	128.74 ± 5.64	142.85 ± 24.42	89.54 ± 5.35	72.35 ± 16.9
First–order	45.34 ± 3.06	242.44 ± 33.79	19.9 ± 14.65	16.79 ± 8.68	10 ± 4.5	519.72 ± 269.88	176.39 ± 34.29
First–order with T_{lag}	712.11 ± 564.91	120.18 ± 213.63	39.57 ± 18.35	97.03 ± 67.15	62.44 ± 100.21	25.66 ± 20.91	24.66 ± 23.73
First–order with F_{max}	27.11 ± 6.29	189.28 ± 14.64	6.25 ± 8.21	5.61 ± 4.54	1.8 ± 0.71	431.55 ± 253.86	123.8 ± 26.49
First–order with T_{lag} and F_{max}	889.71 ± 136.67	412.56 ± 113.9	731.3 ± 66.94	776.01 ± 102.85	691.39 ± 177.9	409.53 ± 105.98	594.71 ± 144.01
Higuchi	147.46 ± 28.07	9.08 ± 7.58	141.45 ± 27.55	165.46 ± 26.25	198.51 ± 32.84	49.53 ± 71.16	12.52 ± 8.34
Higuchi with T_{lag}	301.48 ± 21.43	387.69 ± 94.3	155.22 ± 49.65	178.58 ± 32.49	141.58 ± 17.43	533.91 ± 180.64	431.5 ± 50.09
Higuchi with F_0	90.88 ± 10.83	29.1 ± 11.81	89.14 ± 6.16	84.21 ± 17.84	82 ± 28.14	51.43 ± 10.6	72.31 ± 21.65
Korsmeyer–Peppas	**0.38 ± 0.02**	1.98 ± 0.89	**0.3 ± 0.18**	**0.28 ± 0.28**	**0.07 ± 0.03**	8 ± 4.47	**2.44 ± 0.39**
Korsmeyer–Peppas with T_{lag}	4.03 ± 1.18	5.25 ± 1.81	**0.23 ± 0.25**	**0.55 ± 0.37**	**0.49 ± 0.16**	12.35 ± 5.42	5.67 ± 2.12
Korsmeyer–Peppas with F_0	**0.63 ± 0.46**	**0.22 ± 0.16**	0.96 ± 0.44	0.75 ± 0.58	**0.41 ± 0.27**	**2.15 ± 1.79**	**0.36 ± 0.09**
Hixson–Crowell	63.79 ± 7.99	307.11 ± 18.73	32.25 ± 19.9	32.46 ± 12.41	19.3 ± 7.89	579.41 ± 295.45	206.54 ± 35.31
Hixson–Crowell with T_{lag}	21.42 ± 18.28	38.17 ± 25.42	4.6 ± 1.45	5.53 ± 4.14	13.85 ± 9.41	12.58 ± 4.51	7.93 ± 1.65
Hopfenberg	62.84 ± 6.42	307.11 ± 18.73	31.88 ± 20.16	32.02 ± 12.83	17.91 ± 7.11	573.56 ± 299.01	203.75 ± 33.08
Hopfenberg with T_{lag}	5.94 ± 2.59	38.17 ± 25.42	7.03 ± 4.22	12.11 ± 9.08	11.14 ± 2.39	18.88 ± 5.34	13.22 ± 6.16
Baker–Lonsdale	517.1 ± 208.04	104.01 ± 136.74	429.88 ± 35.78	537.33 ± 81.41	570.67 ± 118.06	28.55 ± 41.92	60.56 ± 80.43
Baker–Lonsdale with T_{lag}	301.48 ± 21.43	663.36 ± 64.84	155.22 ± 49.65	178.58 ± 32.49	141.58 ± 17.43	950.19 ± 406.76	431.5 ± 50.09
Makoid–Banakar	**0.28 ± 0.04**	**0.74 ± 0.55**	**0.31 ± 0.24**	**0.16 ± 0.25**	**0.21 ± 0.19**	**0.71 ± 0.21**	**0.39 ± 0.23**
Makoid–Banakar with T_{lag}	1.63 ± 0.2	2.63 ± 1.18	**0.12 ± 0.1**	**0.5 ± 0.36**	**0.47 ± 0.13**	**2.5 ± 0.65**	**1.79 ± 0.53**
Peppas–Sahlin_1	7.44 ± 4.04	**0.93 ± 0.46**	4.12 ± 2.91	8.75 ± 3.11	18.04 ± 4.93	55.07 ± 45.56	10.26 ± 4.27
Peppas–Sahlin_1 with T_{lag}	2.58 ± 1.56	5.68 ± 1.06	**0.8 ± 1.08**	2.67 ± 2.02	8.35 ± 2.95	78.58 ± 58.36	18.14 ± 5.58
Peppas–Sahlin_2	8.39 ± 4.45	2.52 ± 0.92	5.9 ± 3.94	10.91 ± 3.85	21.74 ± 4.68	73.05 ± 60.19	12.34 ± 4.9
Peppas–Sahlin_2 with T_{lag}	3.06 ± 1.71	10.2 ± 1.81	1.45 ± 1.62	3.6 ± 2.51	10.29 ± 2.95	100.92 ± 75.09	21.58 ± 6.46
Quadratic	70.2 ± 3.57	272.36 ± 20.81	30.4 ± 19.13	31.93 ± 12.8	16.53 ± 5.26	525.88 ± 274.06	189.57 ± 29.47
Quadratic with T_{lag}	86.14 ± 3.94	305.15 ± 24.06	38.04 ± 21.94	40.85 ± 14.38	23.26 ± 6.3	565.63 ± 287.9	210.28 ± 31.35
Weibull_1	9.74 ± 1.72	24.33 ± 8.56	2.62 ± 1.49	3.1 ± 0.74	2.31 ± 0.44	29.13 ± 11.56	16.3 ± 2.38
Weibull_2	4.23 ± 1.16	10.97 ± 5.64	1.16 ± 0.45	1.05 ± 0.54	0.9 ± 0.69	21.89 ± 12.69	7.38 ± 1.34
Weibull_3	6.13 ± 3.24	14.99 ± 3.23	2.31 ± 0.76	1.89 ± 0.75	1.61 ± 0.4	50.9 ± 21.13	22.86 ± 13.91
Weibull_4	10.29 ± 0.77	29.92 ± 5.42	3.62 ± 2.35	4.16 ± 0.84	3.26 ± 0.41	47.74 ± 12.79	22.06 ± 2.85
Logistic_1	47.6 ± 32.49	56.85 ± 50.57	12.72 ± 16.47	18.94 ± 18.32	2.91 ± 0.85	77.85 ± 70.7	16.27 ± 3.2
Logistic_2	103.14 ± 9.16	50.71 ± 8.33	55.06 ± 16.79	60.63 ± 13.6	49.54 ± 6.44	85.04 ± 39.93	34.54 ± 4.59
Logistic_3	430.39 ± 222.73	116.03 ± 14.1	261.69 ± 124.7	408.91 ± 22.69	168.99 ± 162.43	52.34 ± 4.97	56.19 ± 7.31
Gompertz_1	176.85 ± 84.85	230.35 ± 185.56	28.61 ± 11.45	27.86 ± 6.73	23.38 ± 5.89	154.32 ± 74.48	69.64 ± 11.46
Gompertz_2	222.36 ± 13.62	430.82 ± 51.46	110.93 ± 35.66	124.35 ± 22	103.46 ± 15.65	606.74 ± 237.24	278.8 ± 46.62
Gompertz_3	26.6 ± 2.29	30.09 ± 1.19	18.95 ± 0.78	24.36 ± 2.39	25.88 ± 4.84	20.62 ± 20.3	26.45 ± 33.1
Gompertz_4	26.6 ± 2.29	30.09 ± 1.19	18.95 ± 0.78	24.36 ± 2.39	25.88 ± 4.84	11.73 ± 2.72	10.37 ± 1.13
Probit_1	65.88 ± 40.78	79.86 ± 63.1	7.24 ± 3.54	21.07 ± 24.96	30.25 ± 17.97	106.8 ± 88.9	28.84 ± 4.82
Probit_2	135.25 ± 11.49	68.16 ± 11.42	72.53 ± 21.75	80.55 ± 16.13	65.95 ± 7.63	105.91 ± 43.47	48.72 ± 5.75

Table S16. Summary of model performance up to and including t = 60 minutes for fitting the entire relevant carvedilol dissolution data. The table depicts the number of times each model performed as a first–choice model, a near first–choice model, a second–choice model and a third–choice model. Summary statistics depict the total number of times each model was chosen as a candidate for carvedilol release modelling from all studied formulations (N = 23) expressed as a count and as %.

Model	Number of parameters in the model	First–choice model	Near first–choice model	Second–choice model	Third–choice model	SUM	% of all formulations
Zero–order	1					0	0 %
Zero–order with T_{lag}	2					0	0 %
Zero–order with F_0	2					0	0 %
First–order	1					0	0 %
First–order with T_{lag}	2					0	0 %
First–order with F_{max}	2					0	0 %
First–order with T_{lag} and F_{max}	3				1	1	4.3 %
Higuchi	1					0	0 %
Higuchi with T_{lag}	2					0	0 %
Higuchi with F_0	2	1			1	2	8.7 %
**Korsmeyer–Peppas**	**2**	**1**		**7**	**1**	**9**	**39.1 %**
**Korsmeyer–Peppas with T_{lag}**	**3**		**1**	**1**	**3**	**5**	**21.7 %**
**Korsmeyer–Peppas with F_0**	**3**	**7**	**1**	**1**	**3**	**12**	**52.2 %**
Hixson–Crowell	1					0	0 %
Hixson–Crowell with T_{lag}	2					0	0 %
Hopfenberg	2					0	0 %
**Hopfenberg with T_{lag}**	**3**		**1**		**3**	**4**	**17.4 %**
Baker–Lonsdale	1					0	0 %
Baker–Lonsdale with T_{lag}	2					0	0 %
**Makoid–Banakar**	**3**	**7**	**2**	**9**	**3**	**21**	**91.3 %**
**Makoid–Banakar with T_{lag}**	**4**	**5**		**3**	**6**	**14**	**60.9 %**
Peppas–Sahlin_1	3				1	1	4.3 %
Peppas–Sahlin_1 with T_{lag}	4				1	1	4.3 %
Peppas–Sahlin_2	2					0	0 %
Peppas–Sahlin_2 with T_{lag}	3					0	0 %
Quadratic	2				1	1	4.3 %
Quadratic with T_{lag}	3				1	1	4.3 %
**Weibull_1**	**3**	**2**		**1**	**1**	**4**	**17.4 %**
**Weibull_2**	**2**		**1**	**3**	**3**	**7**	**30.4 %**
**Weibull_3**	**3**			**3**	**2**	**5**	**21.7 %**
Weibull_4	4			1	1	2	8.7 %
Logistic_1	2					0	0 %
Logistic_2	3				2	2	8.7 %
Logistic_3	3					0	0 %
Gompertz_1	2					0	0 %
Gompertz_2	3					0	0 %
Gompertz_3	3					0	0 %
Gompertz_4	3					0	0 %
Probit_1	2			1	1	2	8.7 %
Probit_2	3				1	1	4.3 %

Table S17. The RSS results for models fitted to carvedilol experimental dissolution data. Model fitting was performed for up to app. 60 % of carvedilol released. Data is presented for the Polyethylene Glycol & Polyethylene Oxide group and the Povidone group of formulations. For Polyglykol® 4000 P and Polyglykol® 8000 P formulations, model fitting was also performed for up to 75 % of carvedilol released. Data is presented as average RSS ± one standard deviation.

Formulation / Model	Polyglykol® 4000 P (up to app. 60 % of carvedilol released)	Polyglykol® 4000 P (up to app. 75 % of carvedilol released)	Polyglykol® 8000 P (up to app. 60 % of carvedilol released)	Polyglykol® 8000 P (up to app. 75 % of carvedilol released)	Polyox™ WSR N-80 (LEO NF Grade)	Kollidon® 25	Kollidon® 90 F
Zero–order	38.1 ± 14.74	127.28 ± 21.69	11.14 ± 1.1	53.04 ± 16.68	42.76 ± 28.35	75.76 ± 89.61	44.5 ± 26.48
Zero–order with T_{lag}	10.47 ± 3.1	36.15 ± 9.14	10.08 ± 1.85	42.04 ± 7.22	5.88 ± 4.66	25.59 ± 22.85	24.77 ± 23.31
Zero–order with F_0	10.47 ± 3.1	36.15 ± 9.14	10.08 ± 1.85	42.04 ± 7.22	5.88 ± 4.66	25.59 ± 22.85	24.77 ± 23.31
First–order	21.98 ± 26.31	45.38 ± 52.15	76.52 ± 9.65	120.77 ± 11.81	160.59 ± 52.7	86.77 ± 53.06	203.06 ± 97.59
First–order with T_{lag}	4.29 ± 4.94	27.65 ± 37.2	5.02 ± 1.73	18.59 ± 0.87	38.16 ± 21.28	45.17 ± 25.17	103.68 ± 85.35
First–order with F_{max}	247.21 ± 73.87	358.51 ± 132	532.33 ± 27.15	558.62 ± 92.45	845.39 ± 130.08	355.31 ± 332.27	897.14 ± 202.4
First–order with T_{lag} and F_{max}	230.62 ± 76.51	324.87 ± 119.68	350.2 ± 122.47	457.37 ± 25.02	630 ± 85.22	326.59 ± 334.45	1004.88 ± 266.34
Higuchi	163.62 ± 70.65	238.6 ± 108.06	310.1 ± 17.94	454.71 ± 33.45	651.64 ± 84.72	255.46 ± 220.44	973.61 ± 152.58
Higuchi with T_{lag}	22.15 ± 14.1	67.68 ± 61.51	168.32 ± 27.34	169.72 ± 26.2	135.36 ± 20.9	119.36 ± 119.57	259.65 ± 88.31
Higuchi with F_0	**0.76 ± 0.94**	**1.34 ± 1.14**	1.6 ± 0.62	2.12 ± 0.68	70.08 ± 28.6	15.82 ± 10.54	185.62 ± 91.57
Korsmeyer–Peppas	6.74 ± 2.71	24.04 ± 13.55	19.31 ± 3.06	76.58 ± 4.85	68.09 ± 45.18	22.09 ± 16.63	52.57 ± 74.56
Korsmeyer–Peppas with T_{lag}	**0.3 ± 0.28**	4.19 ± 3.1	3.6 ± 0.36	24.01 ± 2.24	19.61 ± 14.96	10.78 ± 10.2	34.01 ± 30.83
Korsmeyer–Peppas with F_0	27.76 ± 16.1	80.54 ± 34.94	74.66 ± 4.25	191.99 ± 10.55	230.05 ± 65.77	47.46 ± 22.69	138.64 ± 177.25
Hixson–Crowell	6.41 ± 4.38	16.31 ± 10.96	36.27 ± 7.28	50.41 ± 11.41	107.85 ± 44.02	65 ± 47.86	120.64 ± 77.69
Hixson–Crowell with T_{lag}	**1.12 ± 0.21**	4.09 ± 2.7	**0.15 ± 0.17**	**0.22 ± 0.18**	17.71 ± 13.52	23.37 ± 14.73	53.59 ± 58.16
Hopfenberg	2.51 ± 1.31	6.95 ± 4.92	11.14 ± 1.1	24.78 ± 7.59	42.76 ± 28.35	39.4 ± 58.83	40.11 ± 26.18
Hopfenberg with T_{lag}	**0.99 ± 0.41**	**2.64 ± 2.71**	**0.07 ± 0.06**	**0.22 ± 0.18**	4.55 ± 4.57	16.02 ± 20.09	13.37 ± 15.22
Baker–Lonsdale	844.02 ± 363.68	1294.24 ± 637.79	1316.42 ± 63.98	1949.71 ± 83.97	1914.23 ± 249.44	1074.44 ± 1100.14	2526.8 ± 487.82
Baker–Lonsdale with T_{lag}	202.8 ± 149.37	326.95 ± 41.62	187.76 ± 27.8	187.28 ± 23.38	167.54 ± 36.06	161.21 ± 129.13	321.22 ± 73.62
Makoid–Banakar	**0.49 ± 0.52**	**0.71 ± 0.71**	**0.26 ± 0.18**	**1.3 ± 0.73**	**2.15 ± 1.44**	**1.04 ± 0.92**	**2.86 ± 2.93**
Makoid–Banakar with T_{lag}	/	**0.77 ± 0.66**	/	**0.26 ± 0.26**	5.86 ± 7.75	**1.78 ± 1.79**	14.78 ± 9.51
Peppas–Sahlin_1	5.07 ± 2.17	17.15 ± 6.32	7.68 ± 1.04	28.83 ± 4.07	4.54 ± 3.42	14.33 ± 13.01	18.71 ± 16.88
Peppas–Sahlin_1 with T_{lag}	/	6.25 ± 3.34	/	15.79 ± 2.47	4.17 ± 3.25	8.1 ± 7.87	18.55 ± 15.92
Peppas–Sahlin_2	6.27 ± 2.67	19.59 ± 7.4	10.34 ± 1.08	34.78 ± 4.11	6.46 ± 4.06	15.34 ± 12.04	18.36 ± 20.94
Peppas–Sahlin_2 with T_{lag}	**1.18 ± 1.01**	6.37 ± 3.59	4.44 ± 0.51	18.4 ± 2.2	5.14 ± 3.39	7.5 ± 7.23	16.95 ± 17.56
Quadratic	**0.94 ± 1.09**	**1.61 ± 1.22**	9.46 ± 1.37	16.25 ± 2.82	19.07 ± 5.66	**2.92 ± 2.3**	**12.33 ± 10.21**
Quadratic with T_{lag}	14.51 ± 4.25	24.51 ± 9.37	**0.32 ± 0.53**	**0.59 ± 0.82**	9.36 ± 5.67	10.48 ± 7.61	**6.46 ± 3.87**
Weibull_1	6.17 ± 3.8	15.91 ± 11.74	1.96 ± 1	4.92 ± 1.12	**4.13 ± 2.46**	23.28 ± 15.95	58.85 ± 62.04
Weibull_2	**0.81 ± 0.78**	**3.4 ± 3.44**	**1.24 ± 0.71**	**1.18 ± 0.74**	8.98 ± 11.3	15.32 ± 7.64	18.81 ± 32.44
Weibull_3	22.52 ± 5.65	26.32 ± 8.18	16.44 ± 1.88	16.87 ± 1.05	14.08 ± 1.36	24.06 ± 25.27	79.79 ± 62.04
Weibull_4	/	50.55 ± 12.4	/	39.86 ± 0.69	29.52 ± 10.97	44.42 ± 43.98	120.06 ± 70.69
Logistic_1	6.66 ± 5.64	27.31 ± 26.11	2.6 ± 1.19	13.48 ± 2.27	**3.84 ± 2.72**	29.55 ± 20.58	67.17 ± 63.72
Logistic_2	100.06 ± 26.12	129.99 ± 34.05	87.4 ± 7.5	122.91 ± 1.57	112.99 ± 46.04	98.51 ± 91.2	243.91 ± 112.51
Logistic_3	15.36 ± 6.36	15.44 ± 7.46	14.1 ± 1.01	22.86 ± 2.77	38.4 ± 8.88	19.62 ± 15.62	66.66 ± 19.73
Gompertz_1	26.44 ± 16.33	85.98 ± 64.51	26.1 ± 4.18	77.49 ± 6.64	82.92 ± 26.21	68.9 ± 73.35	304.16 ± 107.67
Gompertz_2	193.05 ± 51.33	269.46 ± 63.97	152.32 ± 18.44	204.33 ± 19.63	104.51 ± 24.25	154.53 ± 120.65	358.84 ± 79.14
Gompertz_3	29.94 ± 26.76	41.14 ± 15.26	41.04 ± 12.94	38.67 ± 3.55	53.43 ± 13.32	34.09 ± 45.26	91.9 ± 73.5
Gompertz_4	49.4 ± 16.48	41.45 ± 15.54	50.47 ± 3.18	38.67 ± 3.55	53.43 ± 13.32	45.98 ± 49.09	97.02 ± 67.71
Probit_1	8.75 ± 6.2	28.36 ± 23.27	5.65 ± 1.54	18.1 ± 1.79	23.52 ± 12.47	32.24 ± 27.46	144.96 ± 92.43
Probit_2	74.61 ± 20.78	102.21 ± 29.11	77.62 ± 4.79	96.7 ± 1.43	121.76 ± 23.87	77.8 ± 72.25	279.77 ± 120.38

Table S18. The RSS results for models fitted to carvedilol experimental dissolution data. Model fitting was performed for up to app. 60 % of carvedilol released. Data is presented for the Mannitol group and the Lactose Monohydrate group of formulations. For Parteck® M 100 formulation, model fitting was also performed for up to 70 % of carvedilol released. Data is presented as average RSS ± one standard deviation.

Formulation / Model	C*Pharm Mannidex 16700	Pearlitol® 160C	Parteck® M 100 (up to app. 60 % of carvedilol released)	Parteck® M 100 (up to app. 70 % of carvedilol released)	Parteck® M 200	Lactochem® Crystals	Lactochem® Fine Powder	SuperTab® 11SD	FlowLac® 100	Tablettose® 70
Zero–order	338.95 ± 394.8	88.72 ± 15.23	138.68 ± 135.25	164.76 ± 134.65	64.05 ± 76.42	2099.85 ± 406.6	568.76 ± 124.01	3190.25 ± 1044.63	741.74 ± 289.46	2740.69 ± 429.98
Zero–order with T_{lag}	177.18 ± 326.11	20.12 ± 31.9	39.1 ± 46.78	100.64 ± 117.62	35.75 ± 53.93	48.48 ± 11.86	63.03 ± 25.99	23.88 ± 4.93	43.93 ± 7.01	43.11 ± 10.91
Zero–order with F_0	177.18 ± 326.11	20.12 ± 31.9	39.1 ± 46.78	100.64 ± 117.62	35.75 ± 53.93	48.48 ± 11.86	63.03 ± 25.99	23.88 ± 4.93	43.93 ± 7.01	43.11 ± 10.91
First–order	120.61 ± 148.78	118.86 ± 178.59	317.35 ± 319.61	437.8 ± 290.7	239.52 ± 258.14	1237.35 ± 249.71	192.06 ± 65.74	1909.63 ± 465.39	287.34 ± 145.78	1653.91 ± 301.68
First–order with T_{lag}	**72.82 ± 123.82**	121.27 ± 235.74	95.94 ± 105.14	132.69 ± 95.65	65.14 ± 59.64	17.99 ± 5.89	8.63 ± 1.84	8.98 ± 2.83	**6.62 ± 2.21**	18.73 ± 7.2
First–order with F_{max}	303.13 ± 285.98	437.58 ± 661.18	915.34 ± 658.75	1244.83 ± 523.63	860.56 ± 525.35	419.88 ± 80.42	154.05 ± 23.85	565.05 ± 172.26	226.97 ± 47.11	436.58 ± 63.38
First–order with T_{lag} and F_{max}	284.28 ± 171.97	572.86 ± 699.65	475.29 ± 335.41	803.41 ± 350.29	674.82 ± 278.18	45.98 ± 5.76	301.85 ± 50.72	24.73 ± 9.24	361.92 ± 30.39	22.14 ± 9.34
Higuchi	281.42 ± 360.46	245.82 ± 373.3	594.97 ± 444.85	900.52 ± 425.2	599.68 ± 411.3	205.22 ± 90.39	87.03 ± 38.61	598.31 ± 326.66	78.94 ± 37.09	406.43 ± 142.2
Higuchi with T_{lag}	163.81 ± 182.16	198.16 ± 206.04	122.22 ± 95.88	169.09 ± 84.22	92.75 ± 34.94	9.02 ± 4.1	136.13 ± 38.16	7.26 ± 4.39	213.3 ± 53.18	14.62 ± 6.65
Higuchi with F_0	104.06 ± 177.61	72.42 ± 125.69	63.9 ± 64.02	96.98 ± 57.96	45.9 ± 28.59	**0.77 ± 0.87**	17.83 ± 11.03	**0.67 ± 0.39**	36.86 ± 5.77	**1.67 ± 1.92**
Korsmeyer–Peppas	166.52 ± 322.14	45.24 ± 82.01	98.83 ± 167.67	236.99 ± 284.79	116.44 ± 206.34	5.88 ± 3.41	**7.29 ± 5.38**	10.38 ± 3.17	38.56 ± 9.48	3.76 ± 3.14
Korsmeyer–Peppas with T_{lag}	132.25 ± 234.79	83.63 ± 138.26	66.55 ± 93.53	208.55 ± 313.26	66.78 ± 109.29	19.42 ± 6.56	29.58 ± 14.66	23.73 ± 5.28	86.4 ± 12.6	13.96 ± 6.3
Korsmeyer–Peppas with F_0	244.31 ± 466.55	24.47 ± 44.82	186.5 ± 285.77	576.12 ± 745.48	227.57 ± 377.98	**1.3 ± 0.99**	**7 ± 10.3**	**4.36 ± 1.81**	**5.34 ± 1.78**	**0.92 ± 0.7**
Hixson–Crowell	129.36 ± 130.42	106.1 ± 123.48	247.81 ± 243.34	334.8 ± 235.31	179.5 ± 212.32	1477.38 ± 292.32	282.6 ± 79.95	2338.62 ± 744.64	394.42 ± 183.86	1960.74 ± 345.92
Hixson–Crowell with T_{lag}	92.44 ± 166.58	65.72 ± 126.59	60.39 ± 63.81	79.55 ± 51.99	31.82 ± 24.35	26.16 ± 7.58	18.11 ± 7.5	13 ± 3.44	7.9 ± 3.07	25.51 ± 8.29
Hopfenberg	129.36 ± 130.42	51.08 ± 14.37	138.68 ± 135.25	164.76 ± 134.65	64.05 ± 76.42	1477.38 ± 292.32	282.6 ± 79.95	2217.35 ± 553.59	383.21 ± 162.03	1947.3 ± 326.6
Hopfenberg with T_{lag}	90.74 ± 167.75	**18.29 ± 32.91**	35.01 ± 42.63	**60.61 ± 49.07**	**15.62 ± 19.84**	26.16 ± 7.58	17.69 ± 7.49	13 ± 3.44	7.9 ± 3.07	25.15 ± 7.68
Baker–Lonsdale	784.39 ± 883.41	1003.43 ± 1679.84	2057.92 ± 1504.1	2908.44 ± 1231.17	2117.12 ± 1430.75	178.46 ± 99.85	196.11 ± 43.25	399.72 ± 265.9	191.59 ± 32.04	254.09 ± 53.57
Baker–Lonsdale with T_{lag}	236.7 ± 196.7	311.42 ± 362.63	178.09 ± 167.53	238.64 ± 112.71	154.21 ± 82.94	1.72 ± 0.9	291.13 ± 41.09	**2.66 ± 2.53**	445.65 ± 24.78	5.58 ± 4.64
Makoid–Banakar	**79.74 ± 158.36**	**2.38 ± 4.5**	**23.65 ± 40.02**	**55.24 ± 35.61**	**6.9 ± 10.61**	**0.38 ± 0.16**	**0.57 ± 0.18**	**0.75 ± 0.8**	**3.17 ± 1.65**	**0.25 ± 0.17**
Makoid–Banakar with T_{lag}	106.44 ± 211.75	**2.9 ± 5.27**	/	67.62 ± 45.88	**13.48 ± 24.54**	1.45 ± 0.74	**5.41 ± 2.67**	**1.67 ± 1.23**	12.17 ± 5.1	**1.37 ± 0.32**
Peppas–Sahlin_1	132.3 ± 258.2	**19.8 ± 36.7**	37.22 ± 49.09	101.13 ± 118.56	35.28 ± 54.65	16.77 ± 11.02	**2.17 ± 2.96**	43.64 ± 17	**5.71 ± 4.91**	21.9 ± 14.62
Peppas–Sahlin_1 with T_{lag}	117.35 ± 227.85	25.39 ± 43.31	/	94.82 ± 107.63	32.36 ± 47.47	49.14 ± 20.82	**3.27 ± 1.31**	127.08 ± 48.45	14.95 ± 9.71	74.42 ± 27.43
Peppas–Sahlin_2	134.46 ± 263.39	**15.94 ± 29.31**	37.79 ± 53.65	109.55 ± 130.58	39.2 ± 63.63	31.39 ± 15.62	**2.08 ± 2.59**	92.76 ± 40.28	7.64 ± 7.29	48.75 ± 21.71
Peppas–Sahlin_2 with T_{lag}	115.79 ± 225.69	21.5 ± 35.38	36.34 ± 51.75	101.24 ± 117.91	34.78 ± 54.98	75.87 ± 29.15	**4.03 ± 2.2**	182.94 ± 70.7	19.18 ± 13.64	113.53 ± 37.87
Quadratic	**81.14 ± 119.01**	22.87 ± 10.96	52.21 ± 88.27	132.71 ± 146.59	47.58 ± 82.91	668.38 ± 155.77	136.5 ± 37.53	1099.99 ± 392.49	244.97 ± 118.56	857.08 ± 174.66
Quadratic with T_{lag}	91.52 ± 76.24	53.99 ± 15.99	39.01 ± 71.55	89.14 ± 92.77	27.3 ± 47.48	883.82 ± 194.2	183.84 ± 44.67	1444.77 ± 493.63	305.34 ± 137.94	1154.06 ± 216.9
Weibull_1	89.72 ± 119.97	105.29 ± 164.36	39.85 ± 36.42	**61.43 ± 46.39**	**24.3 ± 19.85**	36.02 ± 6.97	73.64 ± 19	35.19 ± 5.64	151 ± 16.07	25.76 ± 8.24
Weibull_2	**74.21 ± 122.59**	67.65 ± 116.1	37.75 ± 40.05	68.93 ± 72.87	**16.36 ± 10.64**	16.78 ± 4.73	35.32 ± 14.16	18.8 ± 3.62	93.75 ± 13.69	10.97 ± 5.3
Weibull_3	**81.63 ± 80.46**	99.73 ± 128.06	**31.63 ± 31.9**	**60.45 ± 45.85**	30.09 ± 28.84	60.31 ± 6.11	112.74 ± 23.22	54.6 ± 7.64	181.74 ± 14.54	44.22 ± 10.94
Weibull_4	108.65 ± 89.91	131.24 ± 151.05	/	65.18 ± 39.34	47.34 ± 28.35	88.46 ± 7.76	160.47 ± 18.57	78.04 ± 9.65	236.78 ± 18.86	68.89 ± 11.87
Logistic_1	**72.02 ± 97.77**	93.48 ± 155.77	**32.88 ± 28.84**	**49.8 ± 33.14**	**22.17 ± 16.22**	29.84 ± 5.36	74.57 ± 18.82	27.75 ± 4.14	151.11 ± 17.72	19.86 ± 7.14
Logistic_2	147.22 ± 71.51	225.02 ± 243.45	112.17 ± 117.18	120.7 ± 89.75	104.75 ± 52.03	151.18 ± 7.98	299.96 ± 14.97	126.64 ± 15.03	373.79 ± 21.1	119.31 ± 17.03
Logistic_3	92.28 ± 140.44	53.07 ± 75.52	**27.26 ± 32.08**	82.16 ± 86.15	30.43 ± 24.18	16.17 ± 6.03	63.12 ± 14.29	6.16 ± 1.06	54.08 ± 4.72	9.43 ± 2.95
Gompertz_1	133.82 ± 154.59	157.84 ± 243.21	76.62 ± 81.01	127.17 ± 81.39	93.44 ± 69.58	57.15 ± 4.99	193.9 ± 16.94	42.84 ± 7.05	294.5 ± 19.04	36.61 ± 10.3
Gompertz_2	229.55 ± 93.52	250.46 ± 177.06	94.92 ± 79.72	131.66 ± 82.81	101.6 ± 44.39	260.2 ± 8.32	451.48 ± 70.59	199.56 ± 28.16	628.89 ± 27.34	202.28 ± 27.39
Gompertz_3	**77.97 ± 75.46**	135.75 ± 178.24	80.2 ± 90.8	92.17 ± 84.44	65.51 ± 57.17	17.11 ± 3.32	57.82 ± 11.4	9.98 ± 2.64	65.92 ± 5	9.02 ± 2.15
Gompertz_4	**80.73 ± 72.98**	127.18 ± 158.83	55.27 ± 32.67	92.17 ± 84.44	67.37 ± 54.97	17.11 ± 3.32	57.82 ± 11.4	10.19 ± 2.95	65.92 ± 5	9.04 ± 2.14
Probit_1	85.73 ± 108.51	108.26 ± 172.41	41.77 ± 43.05	63.64 ± 41.72	32.39 ± 22.78	34.24 ± 5.04	109.91 ± 16.8	29.43 ± 5.25	191.18 ± 14.11	21.82 ± 7.49
Probit_2	141.83 ± 79.19	156.96 ± 143.18	107.58 ± 101.99	107.79 ± 56.24	96.33 ± 42.39	123.31 ± 6.16	254.71 ± 63.17	98.84 ± 14.28	360.1 ± 16.56	92.18 ± 15.2

Table S19. The RSS results for models fitted to carvedilol experimental dissolution data. Model fitting was performed for up to app. 60 % of carvedilol released. Data is presented for the Sucrose and Maltodextrin formulations, the Anhydrous Dibasic Calcium Phosphate group, the Microcrystalline Cellulose group, the Ethylcellulose formulation and the Pregelatinized Starch group of formulations. Data is presented as average RSS ± one standard deviation.

Formulation / Model	Granulated sugar N°1 600	Glucidex® 19	Di-Cafos® A12	Emcompress® Anhydrous	Avicel® PH-102	Avicel® PH-200	Ethocel™ Standard 20 Premium	Starch 1500® sample with smaller particle size (↓PS)	Starch 1500® sample with larger particle size (↑PS)
Zero–order	182.23 ± 29.15	119.74 ± 51.32	427.01 ± 24.81	1083.13 ± 61.02	352.23 ± 101.73	372.56 ± 97.13	266.19 ± 68.69	1958.91 ± 777.2	1046.51 ± 145.89
Zero–order with T_{lag}	43.97 ± 4.85	15.13 ± 9.55	66.82 ± 6.47	95.24 ± 10	70.03 ± 3.8	83.95 ± 19.99	73.62 ± 17.41	80.06 ± 7.75	77.62 ± 17.32
Zero–order with F_0	43.97 ± 4.85	15.13 ± 9.55	66.82 ± 6.47	95.24 ± 10	70.03 ± 3.8	83.95 ± 19.99	73.62 ± 17.41	80.06 ± 7.75	77.62 ± 17.32
First–order	15.04 ± 4.27	54.4 ± 37.56	83.77 ± 14.7	432.78 ± 65.25	53.05 ± 32.33	58.45 ± 36.92	20.54 ± 10.87	1029.72 ± 462.15	407.84 ± 89.04
First–order with T_{lag}	**4.97 ± 1.96**	46.93 ± 51.6	**9.19 ± 7.67**	**14.69 ± 6.62**	**14.14 ± 6.06**	**9.87 ± 4.75**	**6.17 ± 5.06**	**19.77 ± 2.76**	**23.21 ± 10.88**
First–order with F_{max}	313.08 ± 21.2	419.09 ± 117.57	332.42 ± 69.92	268.1 ± 59.05	447.58 ± 9.98	352.26 ± 85.88	456.25 ± 82.78	547.33 ± 271.68	369.73 ± 59.98
First–order with T_{lag} and F_{max}	572.19 ± 36.01	744.5 ± 154.34	630.01 ± 121.34	398.57 ± 140.77	744.65 ± 26.04	610.7 ± 127.71	728.71 ± 102.41	317.5 ± 78.03	571.35 ± 130.37
Higuchi	388.67 ± 26.08	454.02 ± 157.31	320.53 ± 77.03	62.28 ± 41.64	517.13 ± 54.46	411.99 ± 106.09	559.11 ± 101.29	90.31 ± 76.42	167.48 ± 71.61
Higuchi with T_{lag}	220.83 ± 17.64	312.58 ± 45.09	306.29 ± 63.52	241.08 ± 116.1	304.91 ± 44.76	259.37 ± 35.91	257.92 ± 17.08	149.22 ± 102.66	419.18 ± 82.55
Higuchi with F_0	68.92 ± 6.67	133.11 ± 48.18	84.67 ± 22.01	39.04 ± 21.61	127.2 ± 6.52	86.38 ± 31.85	117.19 ± 28.67	49.54 ± 16.88	100.16 ± 34.97
Korsmeyer–Peppas	**2.16 ± 1.29**	41.49 ± 10.47	22.12 ± 6.29	57.19 ± 27.15	**12.39 ± 15.85**	**2.98 ± 2**	**3.28 ± 1.46**	149.36 ± 52.78	142.42 ± 37.83
Korsmeyer–Peppas with T_{lag}	25.34 ± 6.81	98.75 ± 13.98	75.42 ± 16.14	115.18 ± 46.28	39.37 ± 42.59	23.52 ± 21.65	20.78 ± 10.09	222.83 ± 64.84	233.67 ± 51.87
Korsmeyer–Peppas with F_0	15.68 ± 6.28	**10.6 ± 11.42**	**0.5 ± 0.27**	**12.73 ± 9.68**	29.69 ± 28.12	26.54 ± 18.42	24.69 ± 18.73	76.41 ± 38.97	55.47 ± 23.08
Hixson–Crowell	35.03 ± 8.96	48.32 ± 21.86	146.05 ± 22.14	588.07 ± 57.41	100.44 ± 47.44	115.31 ± 58.1	52.35 ± 30.21	1272.48 ± 545.61	558.57 ± 104.52
Hixson–Crowell with T_{lag}	**1.66 ± 1.09**	19.9 ± 29.97	**8.74 ± 2.64**	27.81 ± 13	**10.89 ± 1.7**	**16.23 ± 12.15**	**6.46 ± 5.97**	**30.07 ± 6.28**	**24.18 ± 4.75**
Hopfenberg	35.03 ± 8.96	45.97 ± 19.98	146.05 ± 22.14	575.86 ± 75.27	100.44 ± 47.44	115.31 ± 58.1	52.05 ± 30.55	1255.62 ± 554.35	558.57 ± 104.52
Hopfenberg with T_{lag}	**1.66 ± 1.09**	**8.24 ± 13.39**	**8.53 ± 2.64**	27.23 ± 13.26	**10.89 ± 1.7**	**16.23 ± 12.15**	**6.46 ± 5.97**	**30.07 ± 6.28**	**24.18 ± 4.75**
Baker–Lonsdale	1056.26 ± 283.6	1393.75 ± 634.44	920.1 ± 92.71	194.43 ± 66.27	1471.43 ± 346.86	1194.29 ± 412.49	1534.97 ± 572.02	185.69 ± 33.4	342.39 ± 110.85
Baker–Lonsdale with T_{lag}	327.68 ± 28.58	446.01 ± 94.27	457.14 ± 66.41	482.53 ± 169.03	497.2 ± 82.46	396.36 ± 94.22	415.14 ± 60.44	519.24 ± 174.07	646.66 ± 105.88
Makoid–Banakar	**4.59 ± 2.53**	**10.66 ± 6.13**	**4.17 ± 1.15**	**7.34 ± 5.97**	**4.39 ± 2.42**	**2.43 ± 3.22**	**2.94 ± 2.09**	**4.24 ± 1.7**	**5.26 ± 5.86**
Makoid–Banakar with T_{lag}	22.66 ± 6.18	22.62 ± 3.73	18.52 ± 3.73	18.95 ± 11.83	**4.28 ± 3.83**	**7.84 ± 6.21**	16.96 ± 6.45	**10.97 ± 3.22**	**14.82 ± 11.76**
Peppas–Sahlin_1	**4.09 ± 0.39**	**8.64 ± 13.09**	**0.55 ± 0.37**	**5.8 ± 2.63**	**2.77 ± 1.62**	**3.7 ± 2.14**	**7.09 ± 2.46**	54.07 ± 43.49	**15.35 ± 7.53**
Peppas–Sahlin_1 with T_{lag}	**2.77 ± 0.62**	11.41 ± 12.9	**1.17 ± 0.3**	**13.47 ± 4.34**	**1.96 ± 1.09**	**1.54 ± 1.21**	**5.07 ± 1.94**	76.64 ± 55.88	**23.68 ± 8.71**
Peppas–Sahlin_2	6.58 ± 0.55	**6.93 ± 11.81**	**0.96 ± 0.5**	**8.35 ± 2.92**	**4.03 ± 2.83**	**5.26 ± 2.35**	**10.63 ± 2.86**	72.58 ± 58.11	**17.77 ± 7.76**
Peppas–Sahlin_2 with T_{lag}	**4.15 ± 0.74**	**9.06 ± 11.4**	**1.29 ± 0.31**	18.25 ± 4.62	**2.41 ± 1.5**	**2.31 ± 1.54**	**7.25 ± 2.31**	99.9 ± 72.84	**27.64 ± 9.22**
Quadratic	20.67 ± 5.39	42.39 ± 15.67	82.48 ± 1.85	301.15 ± 19.61	62.5 ± 35.04	54.04 ± 22.87	23.11 ± 9.31	736.17 ± 358.55	342.49 ± 51.17
Quadratic with T_{lag}	34.5 ± 7.48	57.98 ± 18.51	103.23 ± 2.93	346.87 ± 22.99	75.49 ± 39.6	67.64 ± 25.75	32.28 ± 11.23	801 ± 381.53	378.19 ± 54.16
Weibull_1	87.83 ± 14.66	181.01 ± 29.55	177.41 ± 41.3	199.19 ± 78.22	147.68 ± 72.91	105.36 ± 55.09	110.61 ± 32.37	303.67 ± 84.68	359.02 ± 74.59
Weibull_2	35.03 ± 13.44	111.87 ± 25.46	101.44 ± 30.15	131.38 ± 58.22	84.9 ± 53.77	52.93 ± 33.29	42.63 ± 13.07	209.77 ± 71.32	260.96 ± 59.8
Weibull_3	125.85 ± 11.71	198.43 ± 21.12	227.69 ± 35.76	250.73 ± 70.21	242.36 ± 59.97	186.6 ± 39.38	185.99 ± 19.4	358.85 ± 76.52	399.99 ± 64.76
Weibull_4	171.08 ± 15.98	254.24 ± 24	291.81 ± 45.55	314.24 ± 85.92	305.36 ± 71.22	241.88 ± 50.73	246.76 ± 25.45	431.87 ± 86.33	494.73 ± 77.29
Logistic_1	93.63 ± 17.02	183.26 ± 42.53	197.75 ± 52.97	207.13 ± 88.45	194.99 ± 73.87	139.61 ± 49.24	137.21 ± 21.47	305.43 ± 89.28	372.23 ± 87.6
Logistic_2	255.39 ± 13.16	286.14 ± 105.3	274.13 ± 48.26	513.64 ± 129.51	328.37 ± 54.99	252.5 ± 58.76	280.23 ± 49.26	568.29 ± 123.99	762.79 ± 110.23
Logistic_3	99.11 ± 6.47	104.19 ± 72.82	126.6 ± 16.44	77.9 ± 9.21	207.6 ± 19.11	195.79 ± 10.69	196.84 ± 21.62	79.87 ± 9.05	135.17 ± 11.27
Gompertz_1	304.06 ± 32.47	403 ± 85.42	481.73 ± 106.77	406.19 ± 149.19	605.83 ± 80.37	466.97 ± 101.6	520.67 ± 66.91	502.7 ± 108.33	689.03 ± 138
Gompertz_2	589.41 ± 39.74	690.52 ± 108.09	612.96 ± 247.1	826.11 ± 161.92	915.3 ± 213.33	764.17 ± 205.15	580.67 ± 60.22	1007.24 ± 150.69	1156.32 ± 138.73
Gompertz_3	52.43 ± 10.34	112.41 ± 53.77	79.92 ± 12.86	63.63 ± 15.71	110.67 ± 10.41	91.12 ± 9.32	76.03 ± 7.3	67.87 ± 23.71	115.91 ± 23.01
Gompertz_4	64.77 ± 4.22	112.41 ± 53.77	79.94 ± 12.9	63.63 ± 15.71	110.67 ± 10.41	91.12 ± 9.32	77.62 ± 9.95	81.92 ± 14.16	115.91 ± 23.01
Probit_1	167.46 ± 20.42	253.15 ± 63.14	291.47 ± 73.84	267.57 ± 102.87	346.81 ± 71.46	259.05 ± 70.1	284.01 ± 43.05	365.63 ± 86.17	469.11 ± 91.55
Probit_2	254.42 ± 28.15	330.33 ± 88.58	341.68 ± 59.89	498.6 ± 129.67	418.38 ± 81.9	319.59 ± 67.09	341.98 ± 97.62	615.23 ± 103.01	770.05 ± 116.65

Table S20. Summary of model performance for fitting carvedilol release data up to app. 60 % of carvedilol released[1]. The table depicts the number of times each model performed as a first–choice model, a near first–choice model, a second–choice model and a third–choice model. Summary statistics depict the total number of times each model was chosen as a candidate for carvedilol release modelling from all studied formulations (N = 23) expressed as a count and as %.

Model	Number of parameters in the model	First–choice model	Near first–choice model	Second–choice model	Third–choice model	SUM	% of all formulations
Zero–order	1					0	0 %
Zero–order with T_{lag}	2					0	0 %
Zero–order with F_0	2					0	0 %
First–order	1					0	0 %
**First–order with T_{lag}**	**2**			**2**	**8**	**10**	**43.5 %**
First–order with F_{max}	2					0	0 %
First–order with T_{lag} and F_{max}	3					0	0 %
Higuchi	1					0	0 %
Higuchi with T_{lag}	2					0	0 %
**Higuchi with F_0**	**2**	**1**		**2**	**1**	**4**	**17.4 %**
**Korsmeyer–Peppas**	**2**		**1**	**2**	**2**	**5**	**21.7 %**
Korsmeyer–Peppas with T_{lag}	3					0	0 %
**Korsmeyer–Peppas with F_0**	**3**	**1**		**3**	**4**	**8**	**34.8 %**
Hixson–Crowell	1					0	0 %
**Hixson–Crowell with T_{lag}**	**2**	**2**			**6**	**8**	**34.8 %**
Hopfenberg	2					0	0 %
**Hopfenberg with T_{lag}**	**3**	**3**		**2**	**8**	**13**	**56.5 %**
Baker–Lonsdale	1					0	0 %
Baker–Lonsdale with T_{lag}	2				1	1	4.3 %
**Makoid–Banakar**	**3**	**13**	**1**	**6**	**3**	**23**	**100 %**
**Makoid–Banakar with T_{lag}**	**4**		**2**	**3**		**5**	**21.7 %**
**Peppas–Sahlin_1**	**3**	**1**	**3**	**5**	**2**	**11**	**47.8 %**
**Peppas–Sahlin_1 with T_{lag}**	**4**	**2**		**1**		**3**	**13 %**
Peppas–Sahlin_2	2					0	0 %
Peppas–Sahlin_2 with T_{lag}	3					0	0 %
**Quadratic**	**2**			**1**	**3**	**4**	**17.4 %**
Quadratic with T_{lag}	3			2		2	8.7 %
**Weibull_1**	**3**				**3**	**3**	**13 %**
**Weibull_2**	**2**			**2**	**2**	**4**	**17.4 %**
Weibull_3	3				2	2	8.7 %
Weibull_4	4					0	0 %
**Logistic_1**	**2**	**2**		**1**	**1**	**4**	**17.4 %**
Logistic_2	3					0	0 %
Logistic_3	3					0	0 %
Gompertz_1	2					0	0 %
Gompertz_2	3					0	0 %
Gompertz_3	3				1	1	4.3 %
Gompertz_4	3				1	1	4.3 %
Probit_1	2					0	0 %
Probit_2	3					0	0 %

[1] *For the Polyglykol® 4000 P and the Polyglykol® 8000 P formulations, dissolution data up to app. 75 % of carvedilol released was used in order to include enough dissolution analysis data points to fit all the available models in DDSolver; for the Parteck® M 100 formulation, dissolution data up to app. 70 % of carvedilol released was used due to the same reason.*

Table S21. The RSS results for models fitted to carvedilol experimental dissolution data for up to and including t = 60 min. Model fitting was performed for up to app. 60 % of carvedilol released. Data is presented for the Polyethylene Glycol & Polyethylene Oxide group and the Povidone group of formulations. For Polyglykol® 4000 P and Polyglykol® 8000 P formulations, model fitting was also performed for up to 75 % of carvedilol released. Data is presented as average RSS ± one standard deviation.

Formulation / Model	Polyglykol® 4000 P (up to app. 60 % of carvedilol released)	Polyglykol® 4000 P (up to app. 75 % of carvedilol released)	Polyglykol® 8000 P (up to app. 60 % of carvedilol released)	Polyglykol® 8000 P (up to app. 75 % of carvedilol released)	Polyox™ WSR N-80 (LEO NF Grade)	Kollidon® 25	Kollidon® 90 F
Zero–order	/	127.28 ± 21.69	/	53.04 ± 16.68	37.45 ± 22.95	75.76 ± 89.61	12.62 ± 6.23
Zero–order with T_{lag}	/	36.15 ± 9.14	/	42.04 ± 7.22	4.22 ± 3.81	25.59 ± 22.85	4.42 ± 2.46
Zero–order with F_0	/	36.15 ± 9.14	/	42.04 ± 7.22	4.22 ± 3.81	25.59 ± 22.85	4.42 ± 2.46
First–order	/	45.38 ± 52.15	/	120.77 ± 11.81	144.29 ± 46.77	86.77 ± 53.06	83.31 ± 16.93
First–order with T_{lag}	/	27.65 ± 37.2	/	18.59 ± 0.87	34.78 ± 20.27	45.17 ± 25.17	46.82 ± 26.75
First–order with F_{max}	/	358.51 ± 132	/	558.62 ± 92.45	804.9 ± 125.78	355.31 ± 332.27	311.92 ± 80.84
First–order with T_{lag} and F_{max}	/	324.87 ± 119.68	/	457.37 ± 25.02	292.41 ± 84.18	326.59 ± 334.45	691.02 ± 132.95
Higuchi	/	238.6 ± 108.06	/	454.71 ± 33.45	414.85 ± 41.81	255.46 ± 220.44	539.16 ± 106.12
Higuchi with T_{lag}	/	67.68 ± 61.51	/	169.72 ± 26.2	112.53 ± 25.02	119.36 ± 119.57	108.63 ± 72.25
Higuchi with F_0	/	**1.34 ± 1.14**	/	2.12 ± 0.68	56.35 ± 23.15	15.82 ± 10.54	78.33 ± 10.94
Korsmeyer–Peppas	/	24.04 ± 13.55	/	76.58 ± 4.85	31.35 ± 33.86	22.09 ± 16.63	**0.3 ± 0.43**
Korsmeyer–Peppas with T_{lag}	/	4.19 ± 3.1	/	24.01 ± 2.24	9.57 ± 14.31	10.78 ± 10.2	0.61 ± 0.23
Korsmeyer–Peppas with F_0	/	80.54 ± 34.94	/	191.99 ± 10.55	17.51 ± 7.23	47.46 ± 22.69	3.85 ± 3.08
Hixson–Crowell	/	16.31 ± 10.96	/	50.41 ± 11.41	95.75 ± 38.08	65 ± 47.86	48.35 ± 6.17
Hixson–Crowell with T_{lag}	/	4.09 ± 2.7	/	**0.22 ± 0.18**	16.34 ± 12.95	23.37 ± 14.73	20.35 ± 15.23
Hopfenberg	/	6.95 ± 4.92	/	24.78 ± 7.59	37.45 ± 22.95	39.4 ± 58.83	18.9 ± 7.05
Hopfenberg with T_{lag}	/	**2.64 ± 2.71**	/	**0.22 ± 0.18**	3.63 ± 3.81	16.02 ± 20.09	3.68 ± 2.28
Baker–Lonsdale	/	1294.24 ± 637.79	/	1949.71 ± 83.97	1830.53 ± 238.95	1074.44 ± 1100.14	1509 ± 310.76
Baker–Lonsdale with T_{lag}	/	326.95 ± 41.62	/	187.28 ± 23.38	142.52 ± 43.64	161.21 ± 129.13	138.56 ± 53.68
Makoid–Banakar	/	**0.71 ± 0.71**	/	**1.3 ± 0.73**	**0.48 ± 0.35**	**1.04 ± 0.92**	**0.08 ± 0.07**
Makoid–Banakar with T_{lag}	/	**0.77 ± 0.66**	/	**0.26 ± 0.26**	**1.65 ± 1.25**	**1.78 ± 1.79**	**0.22 ± 0.17**
Peppas–Sahlin_1	/	17.15 ± 6.32	/	28.83 ± 4.07	2.97 ± 2.39	14.33 ± 13.01	4.91 ± 3.61
Peppas–Sahlin_1 with T_{lag}	/	6.25 ± 3.34	/	15.79 ± 2.47	2.95 ± 2.34	8.1 ± 7.87	5.48 ± 3.89
Peppas–Sahlin_2	/	19.59 ± 7.4	/	34.78 ± 4.11	3.77 ± 3.08	15.34 ± 12.04	4.65 ± 5.5
Peppas–Sahlin_2 with T_{lag}	/	6.37 ± 3.59	/	18.4 ± 2.2	3 ± 2.41	7.5 ± 7.23	2.94 ± 2.54
Quadratic	/	**1.61 ± 1.22**	/	16.25 ± 2.82	13.53 ± 3.58	**2.92 ± 2.3**	6.67 ± 6.72
Quadratic with T_{lag}	/	24.51 ± 9.37	/	**0.59 ± 0.82**	6.15 ± 2.79	10.48 ± 7.61	1.84 ± 0.99
Weibull_1	/	15.91 ± 11.74	/	4.92 ± 1.12	**2.03 ± 1.74**	23.28 ± 15.95	3.36 ± 0.6
Weibull_2	/	**3.4 ± 3.44**	/	**1.18 ± 0.74**	**2.15 ± 2.65**	15.32 ± 7.64	**0.33 ± 0.28**
Weibull_3	/	26.32 ± 8.18	/	16.87 ± 1.05	3.57 ± 1.36	24.06 ± 25.27	1.82 ± 0.9
Weibull_4	/	50.55 ± 12.4	/	39.86 ± 0.69	12.62 ± 7.81	44.42 ± 43.98	6.33 ± 2.13
Logistic_1	/	27.31 ± 26.11	/	13.48 ± 2.27	**1.21 ± 0.28**	29.55 ± 20.58	2.39 ± 1.13
Logistic_2	/	129.99 ± 34.05	/	122.91 ± 1.57	74.24 ± 39.67	98.51 ± 91.2	28.41 ± 18.64
Logistic_3	/	15.44 ± 7.46	/	22.86 ± 2.77	21.44 ± 7.7	19.62 ± 15.62	28.91 ± 19.13
Gompertz_1	/	85.98 ± 64.51	/	77.49 ± 6.64	39.99 ± 13.01	68.9 ± 73.35	44.07 ± 19.43
Gompertz_2	/	269.46 ± 63.97	/	204.33 ± 19.63	35.23 ± 8.82	154.53 ± 120.65	26.98 ± 10.01
Gompertz_3	/	41.14 ± 15.26	/	38.67 ± 3.55	25.82 ± 8.53	34.09 ± 45.26	11.43 ± 5.02
Gompertz_4	/	41.45 ± 15.54	/	38.67 ± 3.55	25.82 ± 8.53	45.98 ± 49.09	10.15 ± 3.33
Probit_1	/	28.36 ± 23.27	/	18.1 ± 1.79	12.79 ± 7.84	32.24 ± 27.46	11.26 ± 2.3
Probit_2	/	102.21 ± 29.11	/	96.7 ± 1.43	73.33 ± 39.61	77.8 ± 72.25	33.31 ± 8.57

Table S22. The RSS results for models fitted to carvedilol experimental dissolution data for up to and including t = 60 min. Model fitting was performed for up to app. 60 % of carvedilol released. Data is presented for the Mannitol group and the Lactose Monohydrate group of formulations. For Parteck® M 100 formulation, model fitting was also performed for up to 70 % of carvedilol released. Data is presented as average RSS ± one standard deviation.

Formulation / Model	C*Pharm Mannidex 16700	Pearlitol® 160C	Parteck® M 100 (up to app. 60 % of carvedilol released)	Parteck® M 100 (up to app. 70 % of carvedilol released)	Parteck® M 200	Lactochem® Crystals	Lactochem® Fine Powder	SuperTab® 11SD	FlowLac® 100	Tablettose® 70
Zero–order	248.99 ± 272.97	68.43 ± 14.07	/	164.76 ± 134.65	64.05 ± 76.42	1670.58 ± 335.03	274.88 ± 65.51	2700.06 ± 902.2	383.92 ± 169.36	2280.67 ± 375.5
Zero–order with T_{lag}	144.54 ± 265.29	15.87 ± 24.85	/	100.64 ± 117.62	35.75 ± 53.93	30.3 ± 9.42	26.73 ± 8.25	15.33 ± 4.53	19.01 ± 4.43	28.9 ± 8.31
Zero–order with F_0	144.54 ± 265.29	15.87 ± 24.85	/	100.64 ± 117.62	35.75 ± 53.93	30.3 ± 9.42	26.73 ± 8.25	15.33 ± 4.53	19.01 ± 4.43	28.9 ± 8.31
First–order	115.73 ± 150.61	33.69 ± 11.36	/	437.8 ± 290.7	239.52 ± 258.14	1144.42 ± 236.34	149.08 ± 51.72	1342.7 ± 294.32	237.74 ± 118.96	1324.39 ± 334.05
First–order with T_{lag}	69.95 ± 123.93	115.32 ± 224.89	/	132.69 ± 95.65	65.14 ± 59.64	15.45 ± 5.77	5.56 ± 1.65	7.53 ± 2.91	3.26 ± 1.26	15.88 ± 6.26
First–order with F_{max}	258.25 ± 314.62	372.9 ± 683.92	/	1244.83 ± 523.63	860.56 ± 525.35	290.65 ± 53.67	25.63 ± 17.29	420.09 ± 178.37	87.78 ± 68.28	284.55 ± 71.97
First–order with T_{lag} and F_{max}	264.47 ± 158.4	220.71 ± 107.46	/	803.41 ± 350.29	674.82 ± 278.18	16.03 ± 6.22	205.7 ± 31.21	10.68 ± 3.59	248.04 ± 24.88	13.24 ± 6.08
Higuchi	239.86 ± 357.22	120.31 ± 175.71	/	900.52 ± 425.2	599.68 ± 411.3	165.54 ± 72.78	40.85 ± 23.69	479.19 ± 256.66	19.24 ± 12.52	317.46 ± 112.44
Higuchi with T_{lag}	155.06 ± 176.28	187.28 ± 188.84	/	169.09 ± 84.22	92.75 ± 34.94	8.05 ± 3.77	119.41 ± 27.62	6 ± 3.95	187.34 ± 47.01	12.31 ± 5.79
Higuchi with F_0	87.67 ± 156.34	48.32 ± 84.31	/	96.98 ± 57.96	45.9 ± 28.59	**0.69 ± 0.84**	5.61 ± 1.84	**0.52 ± 0.27**	10.65 ± 3.07	**1.24 ± 1.48**
Korsmeyer–Peppas	101.23 ± 198.41	**9.68 ± 17.21**	/	236.99 ± 284.79	116.44 ± 206.34	2.06 ± 1.56	**0.42 ± 0.16**	5.06 ± 1.86	**2.59 ± 0.96**	1.81 ± 1.57
Korsmeyer–Peppas with T_{lag}	100.03 ± 180.53	15.98 ± 25.4	/	208.55 ± 313.26	66.78 ± 109.29	7.73 ± 3.55	3.8 ± 0.88	11.88 ± 2.76	8.4 ± 2.08	7.05 ± 3.16
Korsmeyer–Peppas with F_0	248.99 ± 272.97	**5.68 ± 9.55**	/	576.12 ± 745.48	227.57 ± 377.98	**0.75 ± 0.67**	**1.26 ± 1.32**	**2.29 ± 1.56**	**1.88 ± 2.51**	**0.45 ± 0.27**
Hixson–Crowell	119.59 ± 125.6	89.58 ± 98.14	/	334.8 ± 235.31	179.5 ± 212.32	1321.45 ± 269.81	190.73 ± 56.65	1821.88 ± 260.81	287.83 ± 137.17	1807.01 ± 324.46
Hixson–Crowell with T_{lag}	87.97 ± 161.66	59.96 ± 115.78	/	79.55 ± 51.99	31.82 ± 24.35	20.44 ± 7.06	11.61 ± 4.07	10.04 ± 3.47	6.06 ± 2.3	20.2 ± 6.96
Hopfenberg	119.59 ± 125.6	42.93 ± 5.59	/	164.76 ± 134.65	64.05 ± 76.42	1321.45 ± 269.81	190.73 ± 56.65	1571.18 ± 314.11	283.19 ± 128.08	1546.45 ± 356.28
Hopfenberg with T_{lag}	87.34 ± 162.1	14.49 ± 25.62	/	**60.61 ± 49.07**	**15.62 ± 19.84**	20.44 ± 7.06	10.57 ± 4.19	10.04 ± 3.47	6.06 ± 2.3	19.99 ± 6.6
Baker–Lonsdale	706.59 ± 916.82	907.44 ± 1741.79	/	2908.44 ± 1231.17	2117.12 ± 1430.75	53.89 ± 31.32	65.12 ± 37.62	330.65 ± 298.64	35.34 ± 25.67	142.61 ± 98.14
Baker–Lonsdale with T_{lag}	229.85 ± 197.88	301.64 ± 348.56	/	238.64 ± 112.71	154.21 ± 82.94	**1.48 ± 0.82**	247.83 ± 26.33	2.5 ± 2.41	383.61 ± 32.88	5.22 ± 4.3
Makoid–Banakar	70.41 ± 140.54	**2.18 ± 4.17**	/	**55.24 ± 35.61**	**6.9 ± 10.61**	**0.1 ± 0.05**	**0.14 ± 0.16**	**0.12 ± 0.11**	**0.57 ± 0.84**	**0.07 ± 0.04**
Makoid–Banakar with T_{lag}	87.75 ± 174.6	**2.46 ± 4.55**	/	67.62 ± 45.88	**13.48 ± 24.54**	**0.34 ± 0.17**	**0.89 ± 0.52**	**0.31 ± 0.18**	**1.42 ± 0.88**	**0.46 ± 0.13**
Peppas–Sahlin_1	114.13 ± 223.44	16.33 ± 30.39	/	101.13 ± 118.56	35.28 ± 54.65	10.53 ± 8.79	**0.98 ± 1.66**	36.92 ± 16	3.32 ± 2.78	19.34 ± 14.85
Peppas–Sahlin_1 with T_{lag}	104.45 ± 204.22	21.19 ± 36.29	/	94.82 ± 107.63	32.36 ± 47.47	37.09 ± 16.92	2.01 ± 0.71	102.06 ± 36.32	10.27 ± 6.38	59.82 ± 22.39
Peppas–Sahlin_2	117.54 ± 230.75	13.56 ± 25.03	/	109.55 ± 130.58	39.2 ± 63.63	21 ± 11.74	**1.03 ± 1.59**	69.17 ± 27.69	4.87 ± 4.54	35.62 ± 16.68
Peppas–Sahlin_2 with T_{lag}	104.12 ± 204.14	18.51 ± 30.63	/	101.24 ± 117.91	34.78 ± 54.98	57.1 ± 23.52	2.35 ± 1.92	146.47 ± 53.3	14 ± 9.5	90.51 ± 30.76
Quadratic	75.81 ± 120.94	21.98 ± 10.84	/	132.71 ± 146.59	47.58 ± 82.91	511.63 ± 124.2	100.08 ± 31.38	856.36 ± 301.5	190.25 ± 92.52	653.79 ± 139.18
Quadratic with T_{lag}	81.84 ± 77.7	52.51 ± 15.8	/	89.14 ± 92.77	27.3 ± 47.48	714.16 ± 161.85	141.25 ± 38.34	1183.04 ± 399.62	243.46 ± 110.14	930.75 ± 180.89
Weibull_1	80.92 ± 123.14	32.61 ± 51.86	/	**61.43 ± 46.39**	**24.3 ± 19.85**	15.94 ± 4.97	9.31 ± 1.51	19.67 ± 3.09	15.29 ± 3.55	14.33 ± 4.65
Weibull_2	**64.89 ± 121.45**	23.36 ± 40.99	/	68.93 ± 72.87	**16.36 ± 10.64**	6.33 ± 3.06	2.35 ± 0.91	10.08 ± 2.27	6.46 ± 1.95	5.64 ± 3.01
Weibull_3	**61.18 ± 89.77**	55.9 ± 80.55	/	**60.45 ± 45.85**	30.09 ± 28.84	29.74 ± 6.73	12.07 ± 3.52	37.12 ± 5.6	14.67 ± 3.56	30.47 ± 5.1
Weibull_4	85.21 ± 97.09	71.17 ± 61.64	/	65.18 ± 39.34	47.34 ± 28.35	50.27 ± 8.6	24.09 ± 4.37	55.95 ± 6.91	31.22 ± 3.52	48.63 ± 8.36
Logistic_1	**59.97 ± 102.85**	48.02 ± 79.4	/	**49.8 ± 33.14**	**22.17 ± 16.22**	12.88 ± 4.35	6.56 ± 1.18	16.24 ± 2.61	12.36 ± 3.5	11.29 ± 4.4
Logistic_2	120.95 ± 76.34	195.54 ± 207.85	/	120.7 ± 89.75	104.75 ± 52.03	95.18 ± 11.49	36.18 ± 5.19	99.77 ± 11.37	41.86 ± 9.22	92.58 ± 12.6
Logistic_3	**68.87 ± 112.74**	46.56 ± 76.78	/	82.16 ± 86.15	30.43 ± 24.18	11.58 ± 5.78	24.7 ± 4.75	3.17 ± 0.59	17.31 ± 2.66	6.97 ± 3.2
Gompertz_1	110.74 ± 161.02	100.07 ± 164.06	/	127.17 ± 81.39	93.44 ± 69.58	30.89 ± 5.43	32.59 ± 3.23	28.06 ± 4.89	44.18 ± 8.09	23.97 ± 6.85
Gompertz_2	185.32 ± 105.46	171.14 ± 84.84	/	131.66 ± 82.81	101.6 ± 44.39	192.67 ± 13.1	194.07 ± 23.9	169.27 ± 23.66	125.15 ± 21.61	171.41 ± 21.41
Gompertz_3	**61.24 ± 80.78**	130.66 ± 177.41	/	92.17 ± 84.44	65.51 ± 57.17	7.87 ± 2.33	17.55 ± 1.48	4.45 ± 1.31	18.15 ± 2.54	4.17 ± 0.96
Gompertz_4	**65.33 ± 77.32**	118.19 ± 142.81	/	92.17 ± 84.44	67.37 ± 54.97	7.87 ± 2.33	17.55 ± 1.48	5.02 ± 1.81	18.15 ± 2.54	4.45 ± 0.9
Probit_1	72.1 ± 113.21	53.15 ± 88.69	/	63.64 ± 41.72	32.39 ± 22.78	15.77 ± 4.39	12.86 ± 1.46	17.56 ± 3.46	20.56 ± 4.14	12.79 ± 4.63
Probit_2	120.41 ± 79.52	92.51 ± 46.13	/	107.79 ± 56.24	96.33 ± 42.39	76.58 ± 8.9	54.79 ± 23.01	75.69 ± 11.19	49.83 ± 8.61	69.7 ± 11.15

Table S23. The RSS results for models fitted to carvedilol experimental dissolution data for up to and including t = 60 min. Model fitting was performed for up to app. 60 % of carvedilol released. Data is presented for the Sucrose and Maltodextrin formulations, the Anhydrous Dibasic Calcium Phosphate group, the Microcrystalline Cellulose group, the Ethylcellulose formulation and the Pregelatinized Starch group of formulations. Data is presented as average RSS ± one standard deviation.

Formulation / Model	Granulated sugar N°1 600	Glucidex® 19	Di-Cafos® A12	Emcompress® Anhydrous	Avicel® PH-102	Avicel® PH-200	Ethocel™ Standard 20 Premium	Starch 1500® sample with smaller particle size (↓PS)	Starch 1500® sample with larger particle size (↑PS)
Zero–order	45.24 ± 8.54	48.99 ± 26.99	92.67 ± 3.99	323.43 ± 25.98	55.17 ± 27.97	47.66 ± 15.82	29.06 ± 7.77	613.64 ± 309.12	236.43 ± 35.55
Zero–order with T_{lag}	12.86 ± 1.94	3.81 ± 1.74	26.81 ± 2.03	40.78 ± 2.51	88.74 ± 6.03	35.4 ± 11.07	25.3 ± 7.84	41.33 ± 4.64	39.66 ± 5.88
Zero–order with F_0	12.86 ± 1.94	3.81 ± 1.74	26.81 ± 2.03	40.78 ± 2.51	88.74 ± 6.03	35.4 ± 11.07	25.3 ± 7.84	41.33 ± 4.64	39.66 ± 5.88
First–order	12.02 ± 3.1	18.85 ± 15.74	50.97 ± 4.8	239.92 ± 14.69	19.9 ± 14.65	23.63 ± 11.12	10.96 ± 4.92	517.09 ± 268.66	182.91 ± 31.92
First–order with T_{lag}	1.75 ± 0.86	13.26 ± 12.48	2.86 ± 2.09	9.8 ± 5.33	39.57 ± 18.35	3.71 ± 3.6	2.08 ± 1	10.83 ± 3.44	5.26 ± 2.48
First–order with F_{max}	14.79 ± 2.45	12.08 ± 8.87	9.88 ± 1.34	117.19 ± 9.65	6.25 ± 8.21	2.55 ± 1.69	2.95 ± 1.64	360.45 ± 221.2	105.65 ± 23.6
First–order with T_{lag} and F_{max}	412.04 ± 27.41	497.87 ± 73.31	418.73 ± 81.28	259.82 ± 97.11	731.3 ± 66.94	388.54 ± 88.63	489.75 ± 71.83	142.21 ± 74.21	308.82 ± 75.01
Higuchi	163.66 ± 9.28	142.38 ± 56.2	71.68 ± 15.91	**3.1 ± 2.8**	141.45 ± 27.55	98.69 ± 18.14	129.23 ± 18.01	57.86 ± 73.53	5.13 ± 3.74
Higuchi with T_{lag}	151.45 ± 12.07	185.88 ± 20.69	232.29 ± 43.77	217.94 ± 102.75	155.22 ± 49.65	170.96 ± 20.94	141.58 ± 17.43	122.48 ± 98.98	339.98 ± 48.26
Higuchi with F_0	26.14 ± 2.85	35.89 ± 7.92	26.94 ± 6.62	11.43 ± 6.91	89.14 ± 6.16	28.81 ± 11.22	42.22 ± 10	12.19 ± 4.19	26.64 ± 8.84
Korsmeyer–Peppas	**0.36 ± 0.22**	**1.21 ± 0.43**	**0.34 ± 0.03**	**1.29 ± 0.77**	**0.3 ± 0.18**	**0.22 ± 0.25**	**0.1 ± 0.07**	**3.79 ± 1.66**	**1.56 ± 0.36**
Korsmeyer–Peppas with T_{lag}	3.39 ± 1.44	5.62 ± 3.13	2.05 ± 0.19	4.16 ± 1.62	**0.23 ± 0.25**	**0.61 ± 0.42**	**0.98 ± 0.59**	7.43 ± 2.23	**3.87 ± 0.68**
Korsmeyer–Peppas with F_0	**0.4 ± 0.08**	**0.2 ± 0.2**	**0.17 ± 0.09**	**0.28 ± 0.11**	**0.96 ± 0.44**	**0.93 ± 1.17**	**0.52 ± 0.41**	**0.92 ± 0.72**	**0.17 ± 0.08**
Hixson–Crowell	21.6 ± 4.76	27.6 ± 20.3	65.17 ± 3.47	269.87 ± 16.95	32.25 ± 19.9	31.67 ± 12.85	16.7 ± 6.01	552.71 ± 284.07	202.46 ± 33.11
Hixson–Crowell with T_{lag}	**1.05 ± 0.6**	2.66 ± 2.6	5.56 ± 2.09	18 ± 6.75	4.6 ± 1.45	10.19 ± 8.03	4.06 ± 3.72	19.7 ± 4.46	12.87 ± 4.94
Hopfenberg	21.6 ± 4.76	28.38 ± 19.23	65.17 ± 3.47	267.48 ± 14.78	31.88 ± 20.16	31.67 ± 12.85	15.85 ± 6.95	550.35 ± 284.79	202.46 ± 33.11
Hopfenberg with T_{lag}	**1.05 ± 0.6**	**1.3 ± 1.18**	4.67 ± 2.47	16.02 ± 8.3	7.03 ± 4.22	10.19 ± 8.03	4.06 ± 3.72	19.7 ± 4.46	12.87 ± 4.94
Baker–Lonsdale	289.85 ± 216.67	381.55 ± 261.42	40.37 ± 15.96	8.5 ± 10.29	429.88 ± 35.78	127.29 ± 137.94	230.63 ± 174.11	33.38 ± 42.44	14.2 ± 11.47
Baker–Lonsdale with T_{lag}	201.14 ± 22.12	245.32 ± 23.42	301.48 ± 21.43	417.34 ± 132.7	155.22 ± 49.65	178.58 ± 32.49	141.58 ± 17.43	454.7 ± 151.55	431.5 ± 50.09
Makoid–Banakar	**0.36 ± 0.39**	**0.27 ± 0.05**	**0.1 ± 0.01**	**0.27 ± 0.33**	**0.31 ± 0.24**	**0.13 ± 0.22**	**0.07 ± 0.03**	**0.34 ± 0.14**	**0.25 ± 0.14**
Makoid–Banakar with T_{lag}	**0.97 ± 0.27**	**1.07 ± 0.24**	0.8 ± 0.08	**0.97 ± 0.44**	**0.12 ± 0.1**	**0.42 ± 0.28**	**0.25 ± 0.09**	**1.17 ± 0.43**	**1.64 ± 0.78**
Peppas–Sahlin_1	**1.01 ± 0.18**	**1.07 ± 0.65**	**0.04 ± 0.04**	**3.7 ± 1.79**	4.12 ± 2.91	2.11 ± 1.52	1.58 ± 1.15	35.07 ± 28.93	7.23 ± 2.38
Peppas–Sahlin_1 with T_{lag}	**0.65 ± 0.16**	3 ± 1.22	0.73 ± 0.21	10.03 ± 3.23	**0.8 ± 1.08**	**0.27 ± 0.26**	**0.3 ± 0.08**	54.07 ± 39.63	13.84 ± 3.37
Peppas–Sahlin_2	2.15 ± 0.21	**0.47 ± 0.47**	**0.13 ± 0.09**	5.91 ± 2.1	5.9 ± 3.94	2.99 ± 1.56	3.01 ± 1.27	49.93 ± 40.68	9.56 ± 3.07
Peppas–Sahlin_2 with T_{lag}	**1.06 ± 0.13**	2.1 ± 1.3	0.69 ± 0.12	14.4 ± 3.61	1.45 ± 1.62	**0.53 ± 0.43**	**0.47 ± 0.26**	73.59 ± 53.77	17.66 ± 4.34
Quadratic	14.69 ± 3.29	25.52 ± 13.96	48.3 ± 1.91	197 ± 18.4	30.4 ± 19.13	21.8 ± 8.27	11.88 ± 3.45	439.42 ± 235.25	164.01 ± 25.76
Quadratic with T_{lag}	25.92 ± 4.95	38.05 ± 17.82	63.01 ± 2.74	231.22 ± 21.67	38.04 ± 21.94	29.9 ± 9.89	17.84 ± 4.39	484.19 ± 252.21	185.63 ± 27.92
Weibull_1	5.08 ± 1.21	6.84 ± 1.22	4.85 ± 0.57	7.27 ± 2.85	2.62 ± 1.49	1.54 ± 0.69	1.45 ± 0.29	13.42 ± 4.56	8.42 ± 3.24
Weibull_2	1.35 ± 0.56	2.6 ± 0.6	1.35 ± 0.33	**3.22 ± 1.7**	1.16 ± 0.45	**0.55 ± 0.51**	**0.5 ± 0.18**	9.91 ± 2.97	**3.46 ± 0.77**
Weibull_3	3.53 ± 0.9	5.05 ± 1.39	2.8 ± 0.19	8.14 ± 2.33	2.31 ± 0.76	**0.97 ± 0.2**	**0.96 ± 0.12**	16.99 ± 7.27	8.71 ± 2.7
Weibull_4	8.94 ± 1.74	11.19 ± 2.79	7.92 ± 0.56	20.21 ± 4.33	3.62 ± 2.35	3.1 ± 0.55	2.73 ± 0.3	24.49 ± 6.18	16.77 ± 2.24
Logistic_1	5.13 ± 2.43	4.67 ± 0.8	2.43 ± 0.48	6.28 ± 3.16	12.72 ± 16.47	5.07 ± 5.17	7 ± 0.59	16.05 ± 4.96	8.99 ± 3.92
Logistic_2	47.78 ± 3.83	33.89 ± 17.15	53.88 ± 4.87	25.06 ± 5.18	55.06 ± 16.79	31.2 ± 16.3	26.24 ± 10.22	77.62 ± 79.91	22.44 ± 3.06
Logistic_3	16.03 ± 1.13	11.22 ± 2.09	21.34 ± 1.94	22 ± 0.61	261.69 ± 124.7	59.51 ± 69.82	22.44 ± 3.02	19.54 ± 1.91	27.07 ± 1.73
Gompertz_1	22.16 ± 3.95	23.32 ± 2.05	16.24 ± 2.9	27.97 ± 10.9	28.61 ± 11.45	8.08 ± 1.67	7.93 ± 0.85	43.06 ± 16.19	29.22 ± 5.41
Gompertz_2	89.89 ± 9.76	86.27 ± 31.37	132.13 ± 9.46	265.47 ± 33.89	110.93 ± 35.66	86.02 ± 16.98	67.77 ± 6.48	141.84 ± 50.46	234.52 ± 24.93
Gompertz_3	11.63 ± 0.76	11.71 ± 1.18	8.69 ± 1.28	11.56 ± 1.81	18.95 ± 0.78	8.53 ± 3.14	11.39 ± 9.37	15.85 ± 8.28	7.43 ± 0.61
Gompertz_4	10.46 ± 0.49	11.71 ± 1.18	8.53 ± 0.99	11.56 ± 1.81	18.95 ± 0.78	8.53 ± 3.14	6.57 ± 1.1	10.06 ± 3.72	7.43 ± 0.61
Probit_1	8.81 ± 1.9	12.64 ± 2.35	7.9 ± 2.37	15.58 ± 7.18	7.24 ± 3.54	2.62 ± 0.81	2.71 ± 0.4	26.69 ± 16.2	21.07 ± 10.63
Probit_2	50.94 ± 2.39	43.76 ± 16.37	66.58 ± 6.17	32.33 ± 6.88	72.53 ± 21.75	39.33 ± 18.13	28.96 ± 9.72	54.56 ± 19.32	30.68 ± 3.86

Table S24. Summary of model performance up to and including t = 60 minutes for fitting carvedilol release data up to app. 60 % of carvedilol released[1]. The table depicts the number of times each model performed as a first–choice model, a near first–choice model, a second–choice model and a third–choice model. Summary statistics depict the total number of times each model was chosen as a candidate for carvedilol release modelling from all studied formulations (N = 23) expressed as a count and as %.

Model	Number of parameters in the model	First–choice model	Near first–choice model	Second–choice model	Third–choice model	SUM	% of all formulations
Zero–order	1					0	0 %
Zero–order with T_{lag}	2					0	0 %
Zero–order with F_0	2					0	0 %
First–order	1					0	0 %
First–order with T_{lag}	2					0	0 %
First–order with F_{max}	2					0	0 %
First–order with T_{lag} and F_{max}	3					0	0 %
Higuchi	1				1	1	4.3 %
Higuchi with T_{lag}	2					0	0 %
**Higuchi with F_0**	**2**			**3**	**1**	**4**	**17.4 %**
**Korsmeyer–Peppas**	**2**	**1**	**2**	**4**	**6**	**13**	**56.5 %**
**Korsmeyer–Peppas with T_{lag}**	**3**		**1**	**1**	**2**	**4**	**17.4 %**
**Korsmeyer–Peppas with F_0**	**3**	**2**	**2**	**7**	**4**	**15**	**65.2 %**
Hixson–Crowell	1					0	0 %
Hixson–Crowell with T_{lag}	2	1			1	2	8.7 %
Hopfenberg	2					0	0 %
**Hopfenberg with T_{lag}**	**3**	**1**		**1**	**4**	**6**	**26.1 %**
Baker–Lonsdale	1					0	0 %
Baker–Lonsdale with T_{lag}	2				1	1	4.3 %
**Makoid–Banakar**	**3**	**15**	**3**	**3**	**1**	**22**	**95.7 %**
**Makoid–Banakar with T_{lag}**	**4**	**1**	**5**	**11**	**3**	**20**	**87 %**
**Peppas–Sahlin_1**	**3**	**1**			**4**	**5**	**21.7 %**
**Peppas–Sahlin_1 with T_{lag}**	**4**		**1**	**2**	**1**	**4**	**17.4 %**
Peppas–Sahlin_2	2			2	1	3	13 %
Peppas–Sahlin_2 with T_{lag}	3			2	1	3	13 %
Quadratic	2			1	1	2	8.7 %
Quadratic with T_{lag}	3			1		1	4.3 %
Weibull_1	3				3	3	13 %
**Weibull_2**	**2**			**4**	**6**	**10**	**43.5 %**
**Weibull_3**	**3**		**1**		**3**	**4**	**17.4 %**
Weibull_4	4					0	0 %
**Logistic_1**	**2**	**2**		**1**	**1**	**4**	**17.4 %**
Logistic_2	3					0	0 %
Logistic_3	3				1	1	4.3 %
Gompertz_1	2					0	0 %
Gompertz_2	3					0	0 %
Gompertz_3	3		1			1	4.3 %
Gompertz_4	3			1		1	4.3 %
Probit_1	2					0	0 %
Probit_2	3					0	0 %

[1] For the Polyglykol® 4000 P and the Polyglykol® 8000 P formulations, dissolution data up to app. 75 % of carvedilol released was used in order to include enough dissolution analysis data points to fit all the available models in DDSolver; for the Parteck® M 100 formulation, dissolution data up to app. 70 % of carvedilol released was used due to the same reason

Table S25. LOESS analysis of burst release (positive $F_{0,LOESS}$) and lag time (negative F_0 and positive T_{lag}) from obtained carvedilol release data. Estimated % of carvedilol released at t = 0, namely $F_{0,LOESS}$, was used as a burst release indicator/estimator to identify the presence and estimate the extent of burst release (in the case $F_{0,LOESS}$ was significantly > 0 %, i.e., subtracting one standard deviation from mean $F_{0,LOESS}$ was still above 0 % of carvedilol release) and as a lag time indicator to estimate the presence of lag time (in the case estimated $F_{0,LOESS}$ was significantly < 0 %, i.e., adding one standard deviation to mean $F_{0,LOESS}$ was still below 0 % of carvedilol release).

Formulation	$F_{0, LOESS}$ – AVERAGE (%)	$F_{0, LOESS}$ – SD (%)	$F_{0, LOESS}$ – AVERAGE-SD (%)	$F_{0, LOESS}$ – AVERAGE+SD (%)	$T_{lag time, LOESS}$ – AVERAGE (min)	$T_{lag time, LOESS}$ – SD (min)	$T_{lag time, LOESS}$ – AVERAGE-SD (min)
Polyglykol® 4000 P	4.388506384	1.506701533	2.881804851	5.895207917			
Polyglykol® 8000 P	-2.08518878	1.001865215	-3.087053994	-1.083323565	1.330626751	0.653942898	0.676683853
Polyox™ WSR N-80 (LEO NF Grade)	-3.030636854	0.324486112	-3.355122966	-2.706150742	6.739358614	0.627245617	6.112112997
Kollidon® 25	1.779131162	3.016956179	-1.237825017	4.796087341			
Kollidon® 90 F	-0.760212877	0.227966706	-0.988179583	-0.53224617	3.760348644	0.188813073	3.571535572
C*Pharm Mannidex 16700	3.28497305	2.321722858	0.963250193	5.606695908			
Pearlitol® 160C	5.479450476	0.952262586	4.527187891	6.431713062			
Parteck® M 100	-10.11405361	6.111961019	-16.22601463	-4.002092591	6.472398715	1.937385655	4.535013061
Parteck® M 200	-5.810027554	5.4492429	-11.25927045	-0.360784654	4.171165078	2.158482421	2.012682658
Lactochem® Crystals	18.15700244	2.769288841	15.3877136	20.92629128			
Lactochem® Fine Powder	4.641241905	0.904157242	3.737084664	5.545399147			
SuperTab® 11SD	27.36710224	4.63147733	22.73562491	31.99857958			
FlowLac® 100	6.489029804	1.669475503	4.819554301	8.158505307			
Tablettose® 70	23.63442999	2.838989096	20.7954409	26.47341909			
Granulated sugar N°1 600	1.586463573	0.249112955	1.337350618	1.835576528			
Glucidex® 19	2.138605282	0.696054488	1.442550794	2.83465977			
Di-Cafos® A12	2.331383133	0.053619544	2.277763589	2.385002677			
Emcompress® Anhydrous	5.33350584	0.384671836	4.948834004	5.718177676			
Avicel® PH-102	0.725801106	0.662017584	0.063783523	1.38781869			
Avicel® PH-200	1.023795503	0.234104804	0.789690699	1.257900307			
Ethocel™ Standard 20 Premium	0.697870291	0.133586479	0.564283812	0.831456771			
Starch 1500® ↓PS	8.338863654	2.255641443	6.083222211	10.5945051			
Starch 1500® ↑PS	4.614592269	0.387606861	4.226985407	5.00219913			

Data in Table S25 was taken from:

Ojsteršek, T., Hudovornik, G., & Vrečer, F. (2023). Comparative Study of Selected Excipients' Influence on Carvedilol Release from Hypromellose Matrix Tablets. Pharmaceutics, 15(5), 1525.
